# Supplementary material for: Macroevolutionary dynamics of gene family gain and loss along multicellular eukaryotic lineages
Source: Nat Commun. 2024 Mar 26;15:2663. doi: 10.1038/s41467-024-47017-w (PMC10966110; doi:10.1038/s41467-024-47017-w)
Supplement: Supplementary file 1 — Supplementary Information [file 41467_2024_47017_MOESM1_ESM.pdf]

Supplementary Information

Macroevolutionary dynamics of gene family gain and loss along multicellular eukaryotic lineages

Mirjana Domazet-Lošo[1*], Tin Široki[1], Korina Šimičević[1], Tomislav Domazet-Lošo[2,3*]

[1]Department of Applied Computing, Faculty of Electrical Engineering and Computing, University of Zagreb, Unska 3, HR-10000 Zagreb, Croatia

[2]Laboratory of Evolutionary Genetics, Ruđer Bošković Institute, Bijenička cesta 54, HR-10000 Zagreb, Croatia

[3]School of Medicine, Catholic University of Croatia, Ilica 242, HR-10000 Zagreb, Croatia

[*]Corresponding authors E-mail: mirjana.domazet-loso@fer.hr, tdomazet@irb.hr

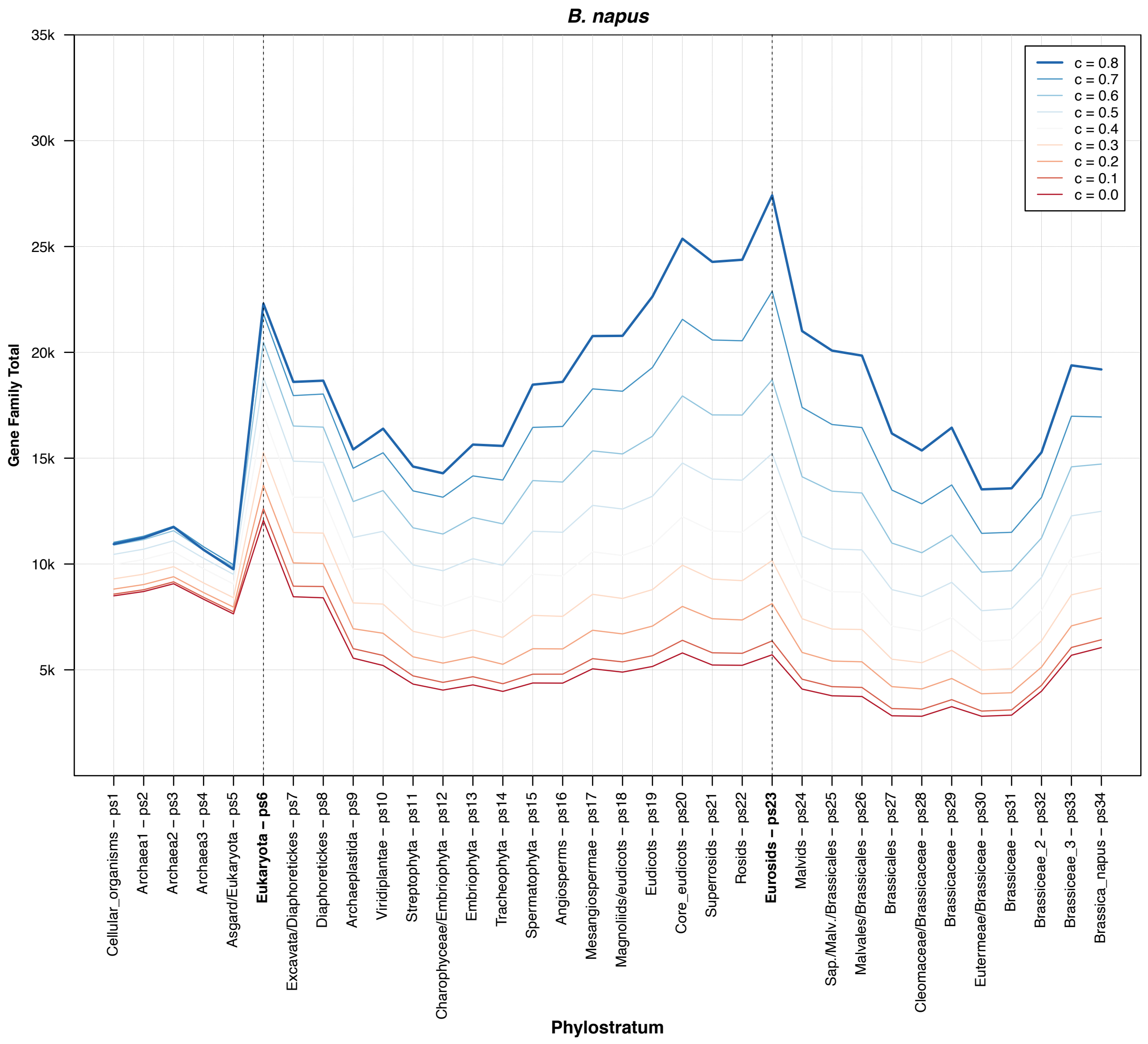

Supplementary Figure 1. The total number of gene families in *Brassica napus*. The total number of estimated gene families across phylostrata (ps) is depicted for *Brassica napus* as a focal species. The first phylostratum (ps1) represents the ancestor of cellular organisms and the last one corresponds to a focal species. The colored lines in each plot correspond to different c-values of the MMseqs *cluster* algorithm. This parameter determines the minimal percentage of protein sequence alignment overlap in a cluster. The darkest blue graph corresponds to c-value = 0.8 which forces at least 80% of sequence length alignment overlap with the cluster's representative sequence. The darkest red graph corresponds to c-value = 0, which allows clustering without restrictions on the alignment overlap length. The dashed vertical lines mark the evolutionary range with the highest number of gene families. In comparison to other plant lineages, *Brassica napus* has an increase of gene families in the most recent phylostrata,

probably related to the hybridization events (allopolyploidy). The source data of this figure are provided in the Source Data file.

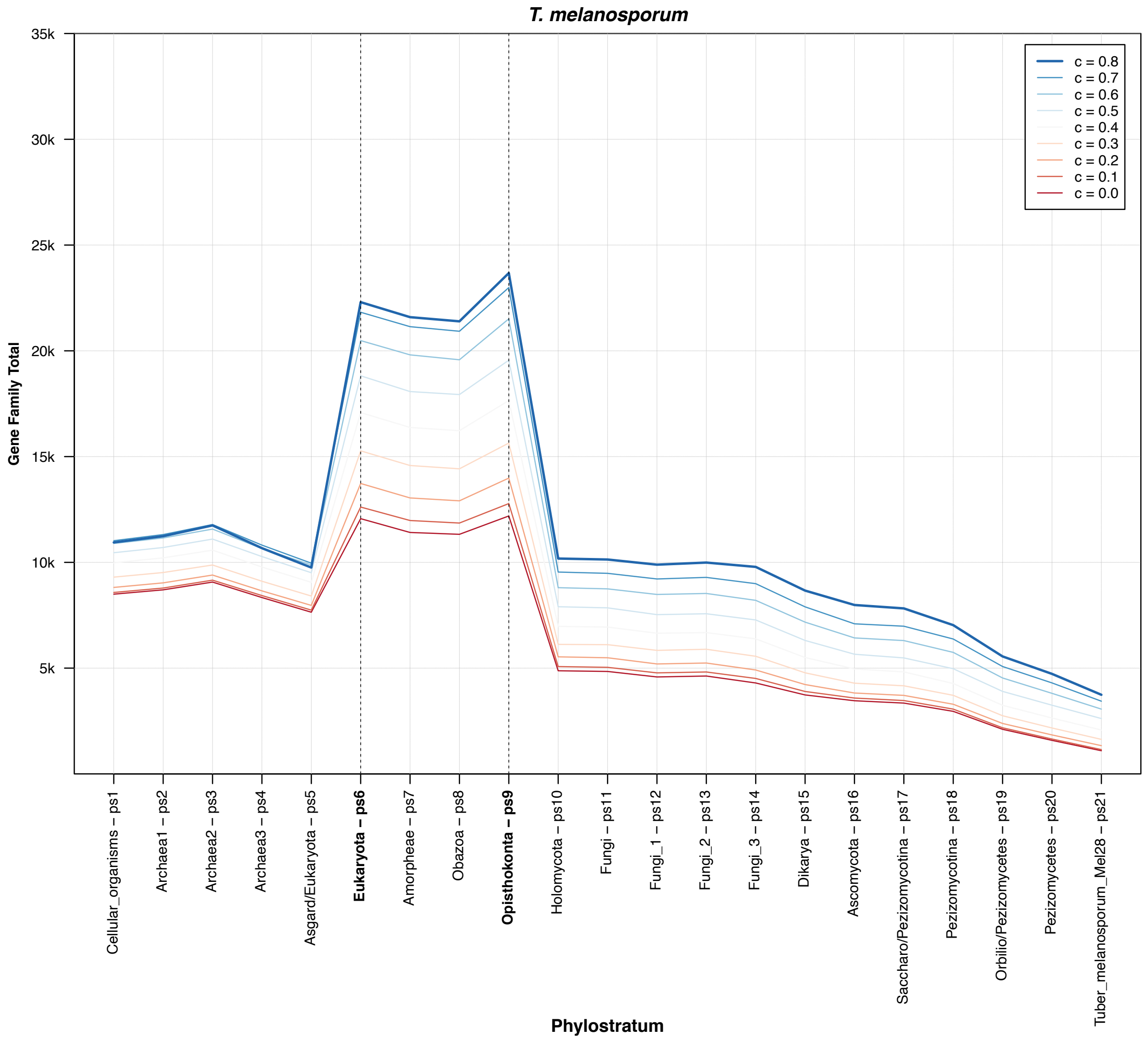

Supplementary Figure 2. The total number of gene families in *Tuber melanosporum*. The total number of estimated gene families across phylostrata (ps) is depicted for *Tuber melanosporum* as a focal species. The first phylostratum (ps1) represents the ancestor of cellular organisms and the last one corresponds to the focal species. The colored lines in each plot correspond to different c-values of the MMseqs2 *cluster* algorithm. This parameter determines the minimal percentage of protein sequence alignment overlap in a cluster. The darkest blue graph corresponds to c-value = 0.8 which forces at least 80% of sequence length alignment overlap with the cluster's representative sequence. The darkest red graph corresponds to c-value = 0 which allows clustering without restrictions on the alignment overlap length. The dashed

vertical lines mark the evolutionary range with the highest number of gene families. All fungal lineages show similar changes in the total number of gene families across evolutionary time. The source data of this figure are provided in the Source Data file.

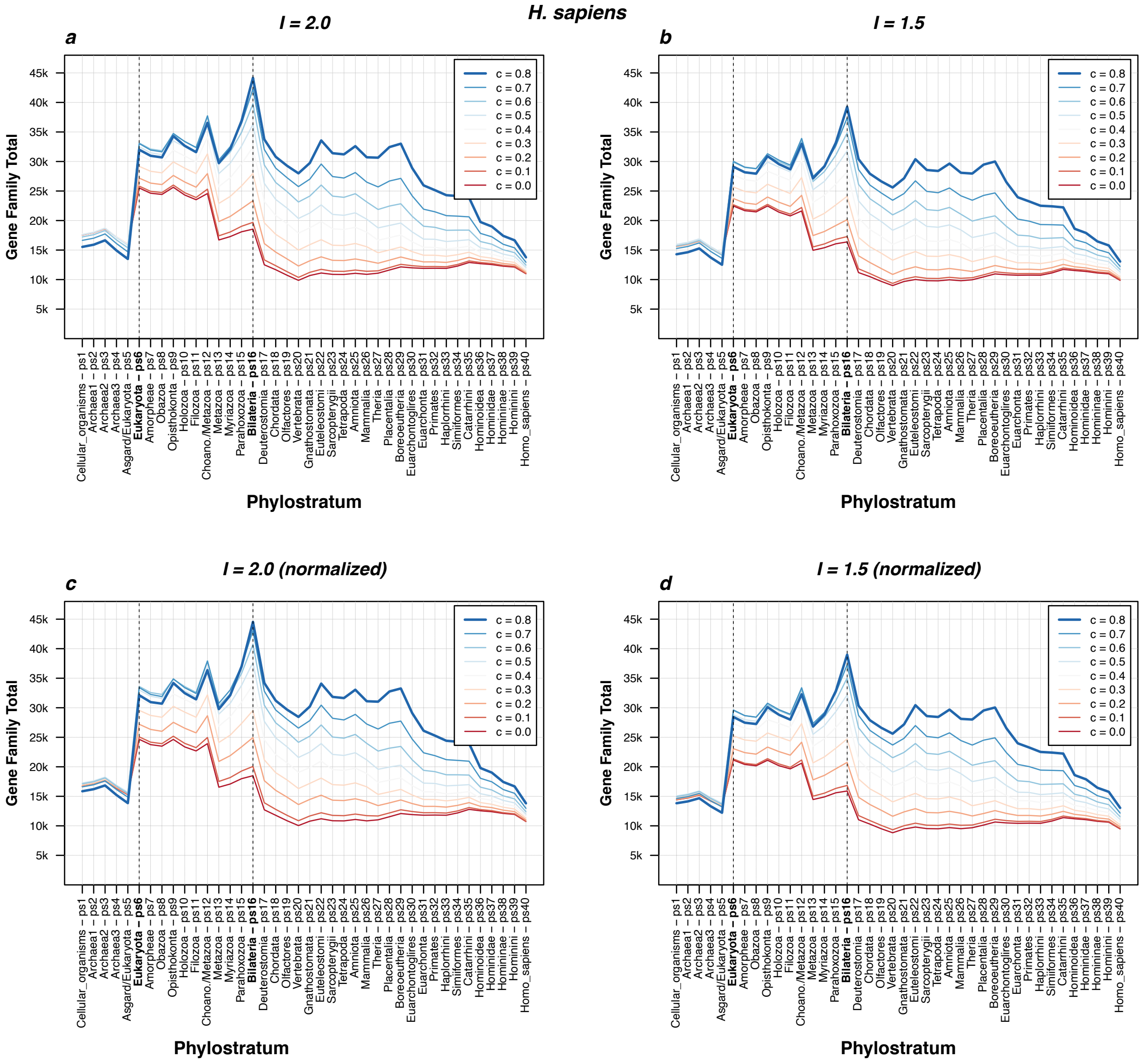

Supplementary Figure 3. The total number of gene families estimated by the MCL algorithm along the human lineage. The total number of estimated gene families across phylostrata (ps) is depicted for *H. sapiens* as a focal species. The first phylostratum (ps1) represents the ancestor of cellular organisms and the last one corresponds to the focal species (ps40 - *H. sapiens*). Colored lines in each plot correspond to the different c-values of the MMseqs2 *search* algorithm used in all-against-all comparisons of reference genomes. This parameter determines the minimal percentage of protein sequence alignment overlap. In this

analysis we clustered the obtained sequence similarity scores using the MCL algorithm. We first performed clustering without any normalization using two values of the MCL inflation parameter **a**, I=2.0 and **b**, I=1.5. In the next test, we included gene length and phylogenetic distance normalization of bit scores as described in the OrthoFinder paper and repeated the analysis **c**, I=2.0 (normalized) and **d**, I=1.5 (normalized). The dashed vertical lines on each plot mark the evolutionary range with the highest number of gene families. This analysis shows that the pattern of change in the total number of gene families along phylostrata is not dependent on the alternative clustering algorithms — MMseqs2 *cluster* (Fig.1) vs. MCL (here). This analysis also reveals that the normalization by the gene length and phylogenetic distance, as described in the OrthoFinder paper, does not change recovered profiles. Direct comparison between mmseqs cluster and MCL with or without OrthoFinder-style normalization for the two most extreme c-values (0.8 and 0) is shown in Supplementary Fig. 4. The source data of this figure are provided in the Source Data file.

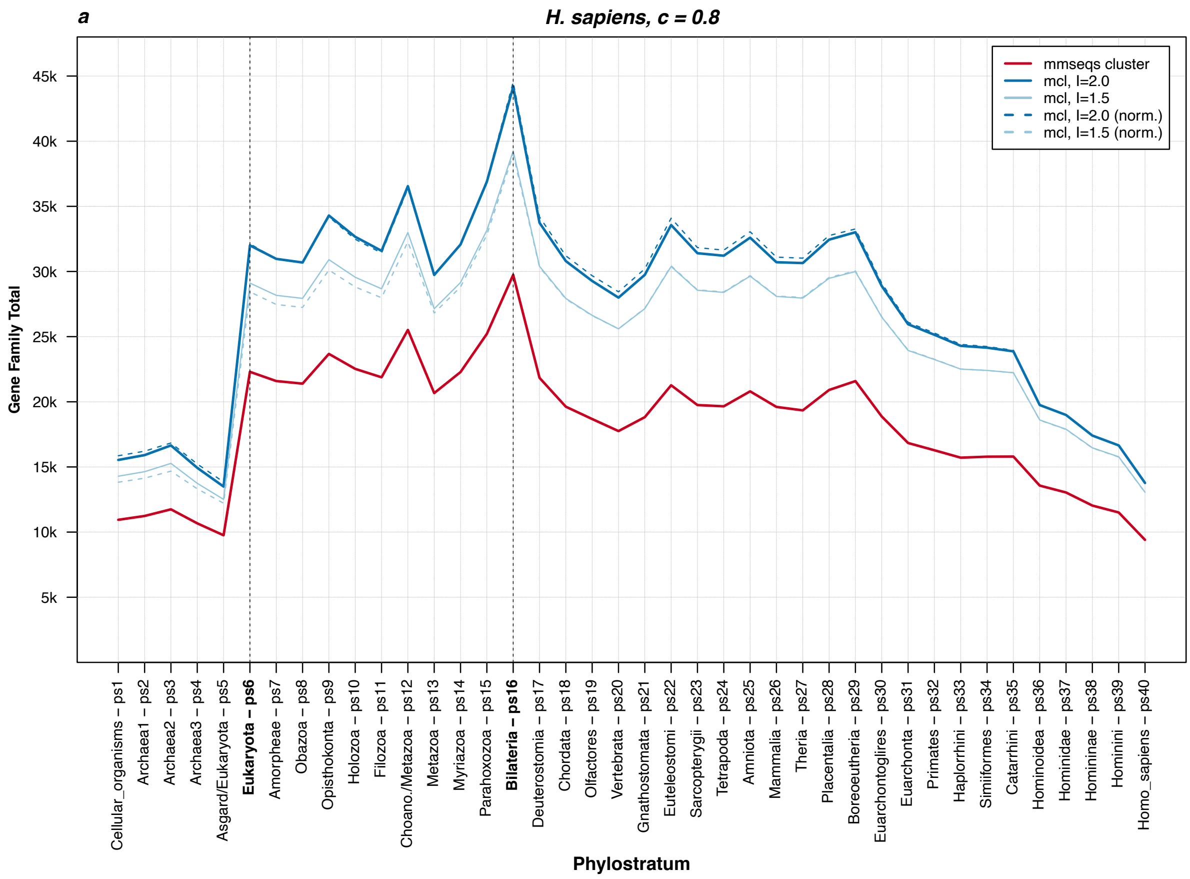
a
H. sapiens, c = 0.8
mmseqs cluster
mcl, I=2.0
mcl, I=1.5
mcl, I=2.0 (norm.)
mcl, I=1.5 (norm.)
Gene Family Total
45k
40k
35k
30k
25k
20k
15k
10k
5k
Cellular_organisms – ps1
Archaea1 – ps2
Archaea2 – ps3
Archaea3 – ps4
Asgard/Eukaryota – ps5
Eukaryota – ps6
Amorpheae – ps7
Obazoa – ps8
Opisthokonta – ps9
Holozoa – ps10
Filozoa – ps11
Choano./Metazoa – ps12
Metazoa – ps13
Myriazoa – ps14
Parahoxozoa – ps15
Bilateria – ps16
Deuterostomia – ps17
Chordata – ps18
Olfactores – ps19
Vertebrata – ps20
Gnathostomata – ps21
Euteleostomi – ps22
Sarcopterygii – ps23
Tetrapoda – ps24
Amniota – ps25
Mammalia – ps26
Theria – ps27
Placentalia – ps28
Boreoeutheria – ps29
Euarchontoglires – ps30
Euarchonta – ps31
Primates – ps32
Haplorrhini – ps33
Simiiformes – ps34
Catarrhini – ps35
Hominoidea – ps36
Hominidae – ps37
Homininae – ps38
Hominini – ps39
Homo_sapiens – ps40
Phylostratum

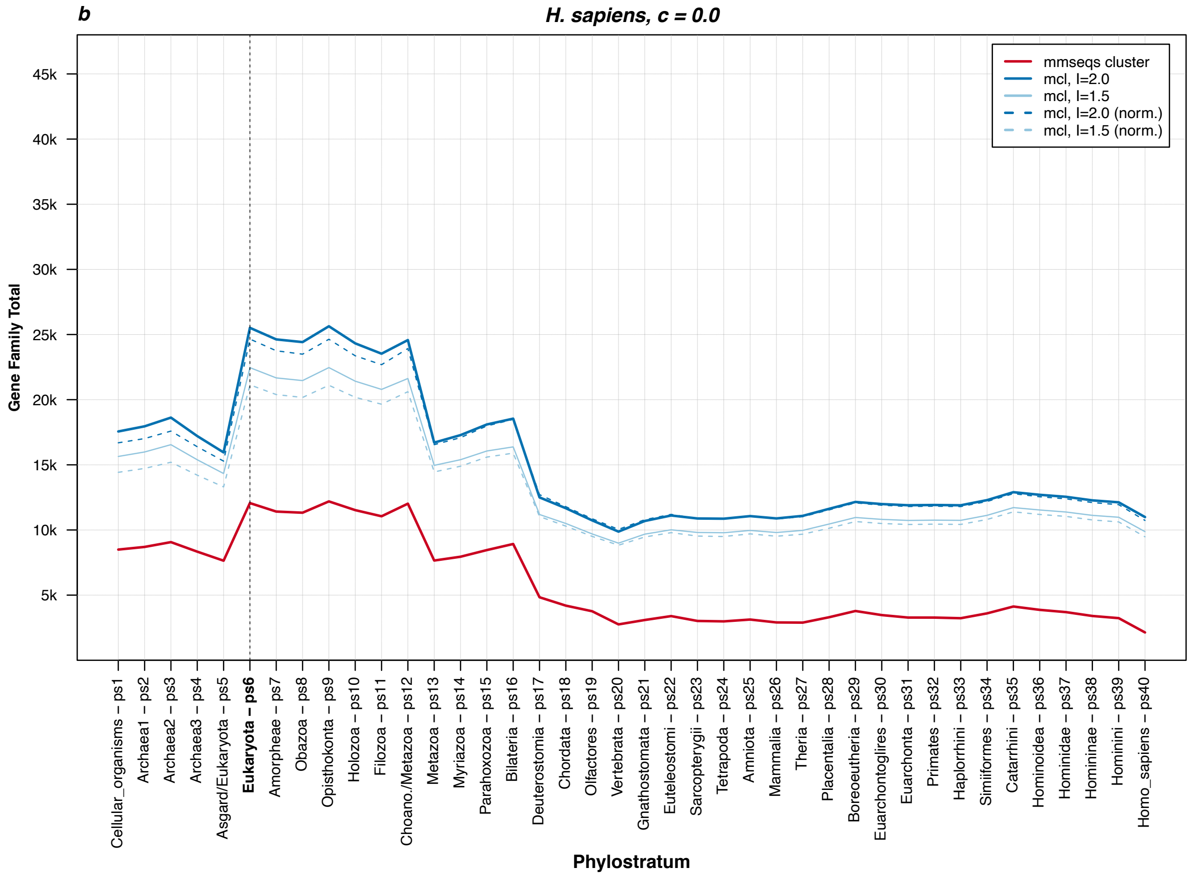
b
H. sapiens, c = 0.0
mmseqs cluster
mcl, I=2.0
mcl, I=1.5
mcl, I=2.0 (norm.)
mcl, I=1.5 (norm.)
Gene Family Total
45k
40k
35k
30k
25k
20k
15k
10k
5k
Cellular_organisms – ps1
Archaea1 – ps2
Archaea2 – ps3
Archaea3 – ps4
Asgard/Eukaryota – ps5
Eukaryota – ps6
Amorpheae – ps7
Obazoa – ps8
Opisthokonta – ps9
Holozoa – ps10
Filozoa – ps11
Choano./Metazoa – ps12
Metazoa – ps13
Myriazoa – ps14
Parahoxozoa – ps15
Bilateria – ps16
Deuterostomia – ps17
Chordata – ps18
Olfactores – ps19
Vertebrata – ps20
Gnathostomata – ps21
Euteleostomi – ps22
Sarcopterygii – ps23
Tetrapoda – ps24
Amniota – ps25
Mammalia – ps26
Theria – ps27
Placentalia – ps28
Boreoeutheria – ps29
Euarchontoglires – ps30
Euarchonta – ps31
Primates – ps32
Haplorrhini – ps33
Simiiformes – ps34
Catarrhini – ps35
Hominoidea – ps36
Hominidae – ps37
Homininae – ps38
Hominini – ps39
Homo_sapiens – ps40
Phylostratum

Supplementary Figure 4. The total number of gene families estimated by the MMseqs2 *cluster* and the MCL algorithm along the human lineage. The total number of estimated gene families across phylostrata (ps) is depicted for *H. sapiens* as a focal species. The first phylostratum (ps1) represents the ancestor of cellular organisms and the last one corresponds to a focal species (ps40 - *H. sapiens*). We compared trajectories obtained by MMseqs2 *cluster* and MCL clustering algorithms. We made the MCL analysis with two different inflation parameters; first without any normalization (I=1.5 and I=2.0) and then with the gene length and phylogenetic distance normalization of bit scores as described in the OrthoFinder paper (I=1.5 norm. and I=2.0 norm.). We showed comparisons for the two most extreme c-values **a,** 0.8 and **b,** 0. This parameter determines the minimal percentage of protein sequence alignment overlap. The dashed vertical lines on each plot mark the phylostrata with the highest number of gene families. These analyses show that the overall pattern of change in the total number of gene families is not dependent on the clustering algorithms and normalization procedure. However, it is evident that MMseqs2 *cluster* generally returns less clusters than MCL. See also Supplementary Fig. 3. The source data of this figure are provided in the Source Data file.

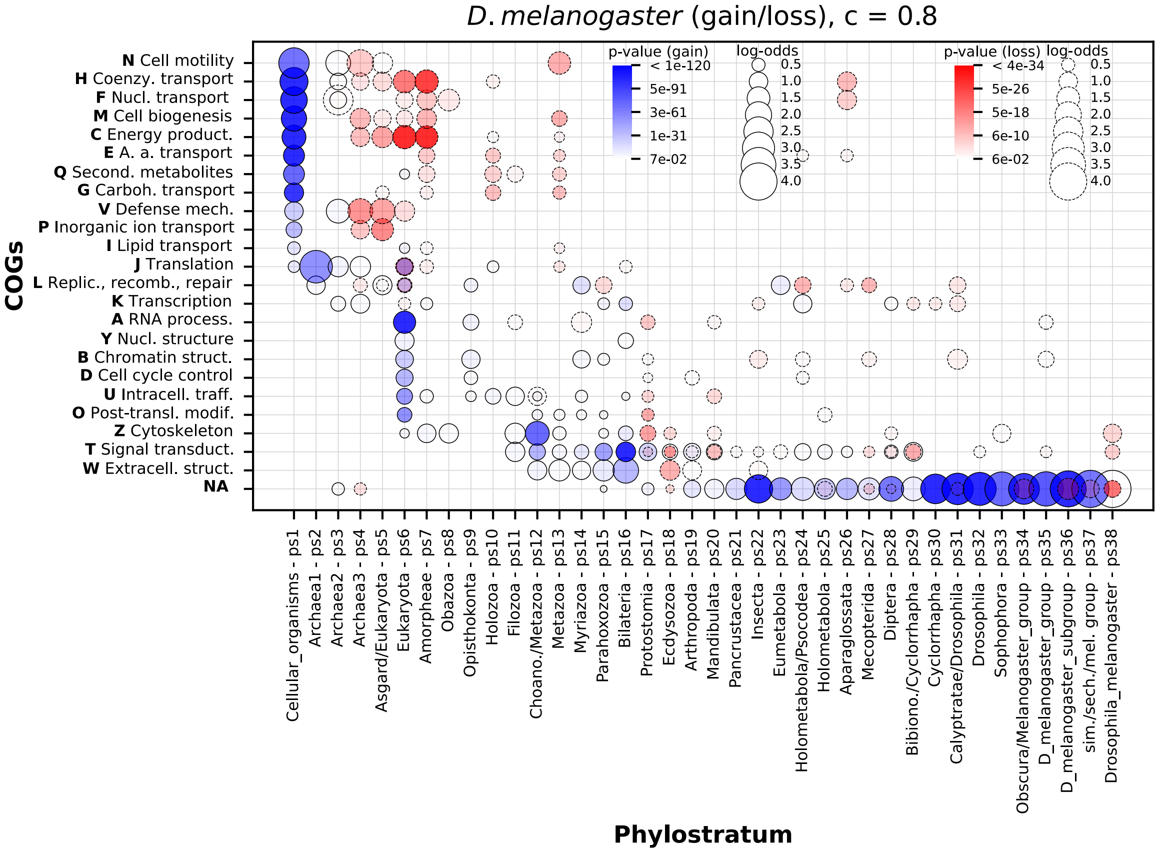

Supplementary Figure 5. The enrichment of COG functional categories in gained and lost gene families along the *D. melanogaster* lineage, c-value = 0.8. The abbreviated names of COG functional categories and corresponding one-letter symbols are depicted at y-axis. The protein families without COG annotation are annotated with NA. The names and symbols of phylostrata are shown on the x-axis. The gene families are reconstructed with the MMseqs *cluster* program using a c-value of 0.8. The figures with c-values in the range between 0 and 0.8 for all four species are in Supplementary Data 5. Functional categories significantly enriched among gained gene families across phylostrata are depicted by solid circles painted in shades of blue that reflect the underlying p-values. The size of circles is proportional to the enrichment values estimated by log-odds. Functional categories significantly enriched among lost gene families across phylostrata are depicted by dashed circles painted in shades of red that reflect p-values. The size of circles is proportional to enrichment values estimated by log-odds. The significance of enrichment was estimated by two-tailed hypergeometric test corrected for multiple testing. The source data of this figure are provided in the Source Data file.

Table 1. GO functions are more often enriched among gene family gain than loss events along four focal lineages.

Number (%) of Enriched GO Functions			
Focal species, c-value	Gain	Loss	Gain/Loss Ratio
H. sap. c=0.8	8,958 (29.94)	2,593 (9.01)	3.32
D. mel. c=0.8	8,222 (28.01)	2,444 (8.63)	3.25
S. cer. c=0.8	4,020 (14.21)	1,252 (4.66)	3.05
A. thal. c=0.8	6,336 (22.54)	2,348 (8.99)	2.51
**Number (%) of Enriched GO Functions (adjusted)**			
Focal species, c-value	Gain	Loss	Gain/Loss Ratio
H. sap. c=0.8	7,535 (26.17)	2,593 (9.01)	2.91
D. mel. c=0.8	7,297 (25.77)	2,444 (8.63)	2.99
S. cer. c=0.8	3,491 (12.99)	1,252 (4.66)	2.79
A. thal. c=0.8	5,857 (22.43)	2,348 (8.99)	2.49

The source data of this Table are provided in the Source Data file.

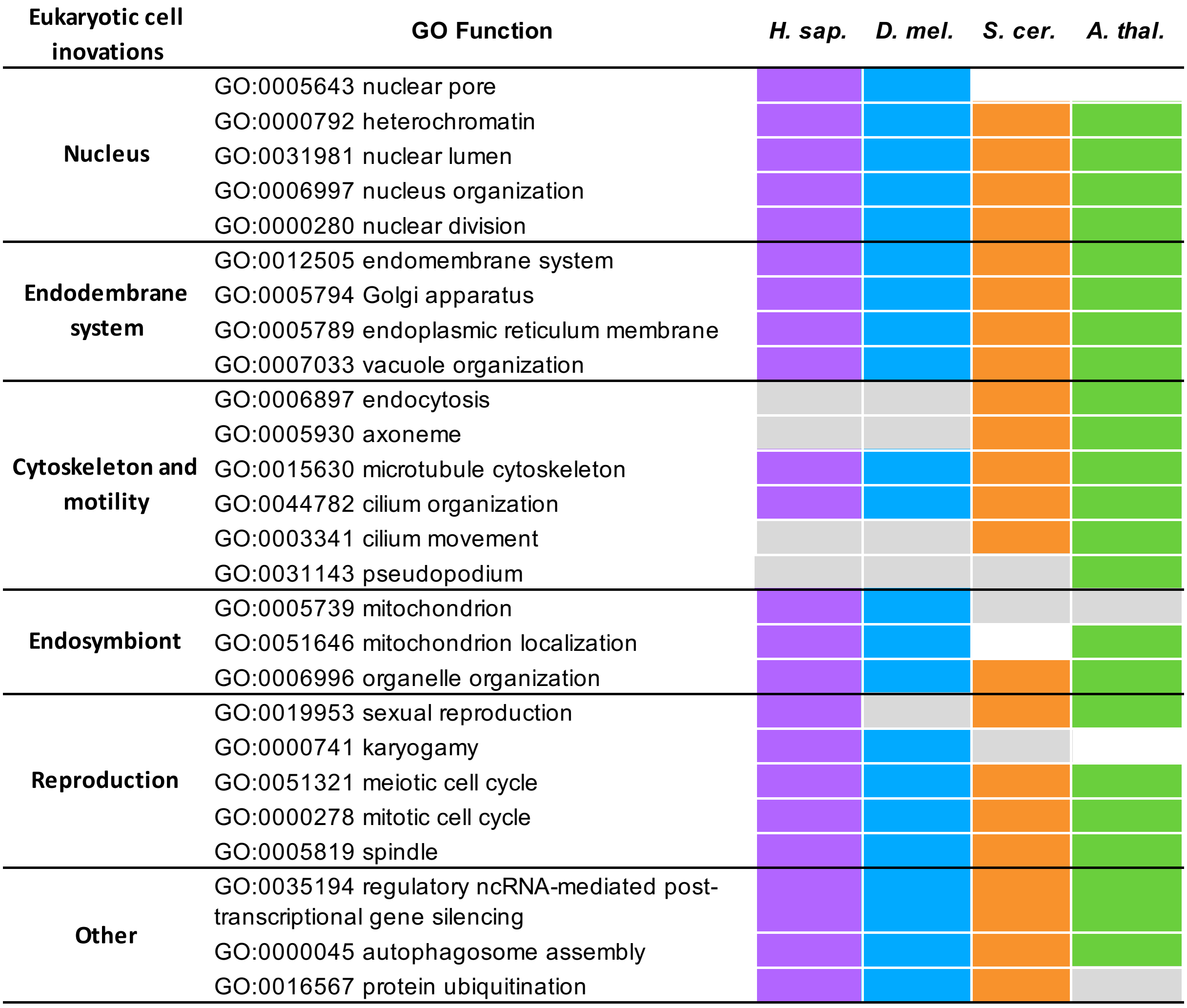

Supplementary Figure 6. An overview of eukaryogenesis-related GO terms that show significant enrichment signals at Eukaryota (ps6). The colored fields mark the presence of the enrichment signal for a particular GO term at Eukaryota (ps6); gray fields mark that enrichment signal is present but not at ps6, and an empty field denotes complete absence of enrichment signals in a particular species. We performed an independent enrichment analysis for every focal species (*H. sapiens*, *D. melanogaster*, *S. cerevisiae*, and *A. thaliana*). The full enrichment profiles in the form of charts for each term and focal species are accessible in Supplementary Data 15.

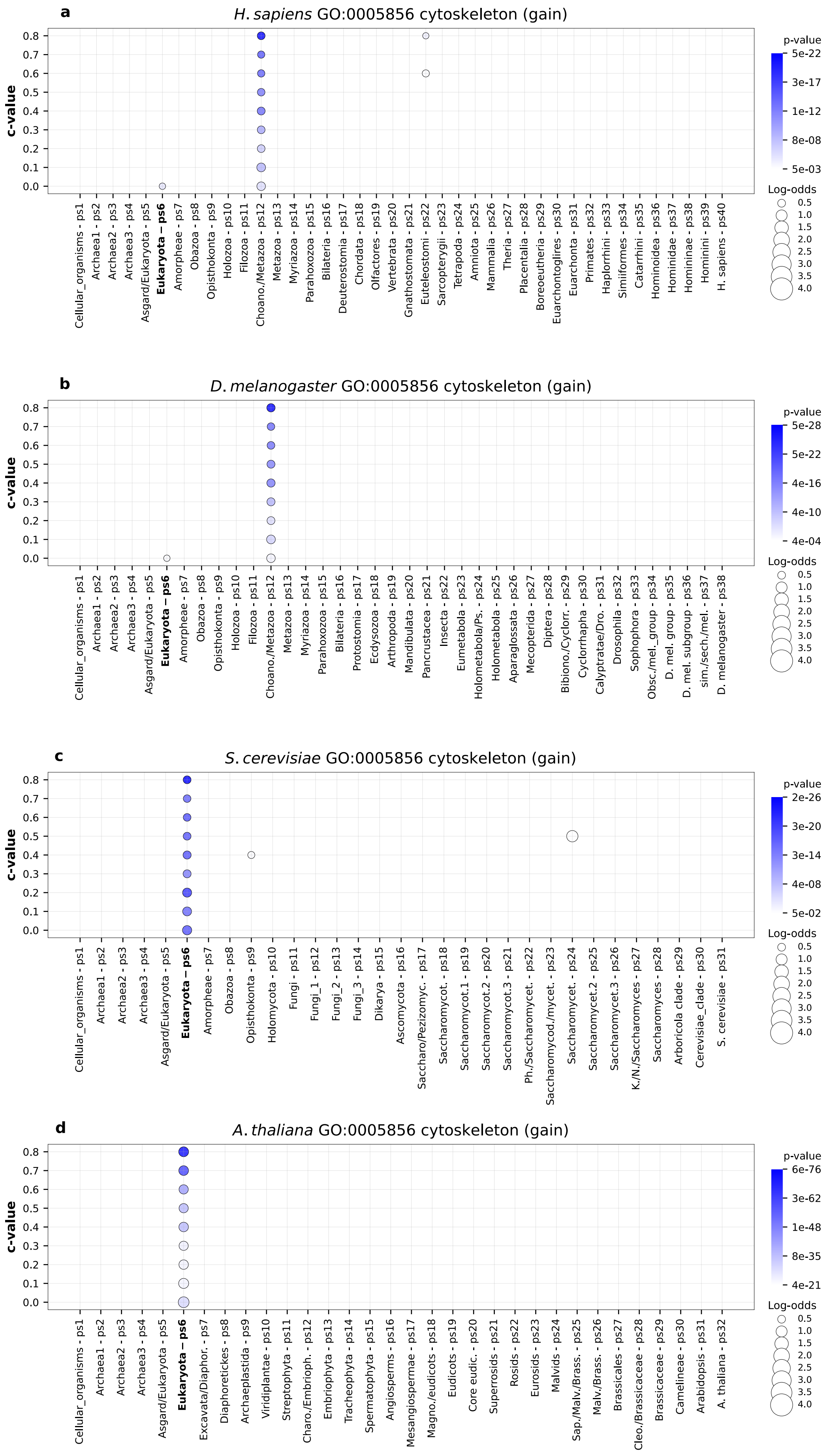
a
H. sapiens GO:0005856 cytoskeleton (gain)
c-value
p-value
5e-22
3e-17
1e-12
8e-08
5e-03
Log-odds
0.5 1.0 1.5 2.0 2.5 3.0 3.5 4.0
Cellular_organisms - ps1
Archaea1 - ps2
Archaea2 - ps3
Archaea3 - ps4
Asgard/Eukaryota - ps5
Eukaryota – ps6
Amorpheae - ps7
Obazoa - ps8
Opisthokonta - ps9
Holozoa - ps10
Filozoa - ps11
Choano./Metazoa - ps12
Metazoa - ps13
Myriazoa - ps14
Parahoxozoa - ps15
Bilateria - ps16
Deuterostomia - ps17
Chordata - ps18
Olfactores - ps19
Vertebrata - ps20
Gnathostomata - ps21
Euteleostomi - ps22
Sarcopterygii - ps23
Tetrapoda - ps24
Amniota - ps25
Mammalia - ps26
Theria - ps27
Placentalia - ps28
Boreoeutheria - ps29
Euarchontoglires - ps30
Euarchonta - ps31
Primates - ps32
Haplorrhini - ps33
Simiiformes - ps34
Catarrhini - ps35
Hominoidea - ps36
Hominidae - ps37
Homininae - ps38
Hominini - ps39
H. sapiens - ps40
b
D. melanogaster GO:0005856 cytoskeleton (gain)
c-value
p-value
5e-28
5e-22
4e-16
4e-10
4e-04
Log-odds
0.5 1.0 1.5 2.0 2.5 3.0 3.5 4.0
Cellular_organisms - ps1
Archaea1 - ps2
Archaea2 - ps3
Archaea3 - ps4
Asgard/Eukaryota - ps5
Eukaryota – ps6
Amorpheae - ps7
Obazoa - ps8
Opisthokonta - ps9
Holozoa - ps10
Filozoa - ps11
Choano./Metazoa - ps12
Metazoa - ps13
Myriazoa - ps14
Parahoxozoa - ps15
Bilateria - ps16
Protostomia - ps17
Ecdysozoa - ps18
Arthropoda - ps19
Mandibulata - ps20
Pancrustacea - ps21
Insecta - ps22
Eumetabola - ps23
Holometabola/Ps. - ps24
Holometabola - ps25
Aparaglossata - ps26
Mecopterida - ps27
Diptera - ps28
Bibiono./Cyclorr. - ps29
Cyclorrhapha - ps30
Calyptratae/Dro. - ps31
Drosophila - ps32
Sophophora - ps33
Obsc./mel._group - ps34
D. mel. group - ps35
D. mel. subgroup - ps36
sim./sech./mel. - ps37
D. melanogaster - ps38
c
S. cerevisiae GO:0005856 cytoskeleton (gain)
c-value
p-value
2e-26
3e-20
3e-14
4e-08
5e-02
Log-odds
0.5 1.0 1.5 2.0 2.5 3.0 3.5 4.0
Cellular_organisms - ps1
Archaea1 - ps2
Archaea2 - ps3
Archaea3 - ps4
Asgard/Eukaryota - ps5
Eukaryota – ps6
Amorpheae - ps7
Obazoa - ps8
Opisthokonta - ps9
Holomycota - ps10
Fungi - ps11
Fungi_1 - ps12
Fungi_2 - ps13
Fungi_3 - ps14
Dikarya - ps15
Ascomycota - ps16
Saccharo/Pezizomyc. - ps17
Saccharomycot. - ps18
Saccharomycot.1 - ps19
Saccharomycot.2 - ps20
Saccharomycot.3 - ps21
Ph./Saccharomycet. - ps22
Saccharomycod./mycet. - ps23
Saccharomycet. - ps24
Saccharomycet.2 - ps25
Saccharomycet.3 - ps26
K./N./Saccharomyces - ps27
Saccharomyces - ps28
Arboricola clade - ps29
Cerevisiae_clade - ps30
S. cerevisiae - ps31
d
A. thaliana GO:0005856 cytoskeleton (gain)
c-value
p-value
6e-76
3e-62
1e-48
8e-35
4e-21
Log-odds
0.5 1.0 1.5 2.0 2.5 3.0 3.5 4.0
Cellular_organisms - ps1
Archaea1 - ps2
Archaea2 - ps3
Archaea3 - ps4
Asgard/Eukaryota - ps5
Eukaryota – ps6
Excavata/Diaphor. - ps7
Diaphoretickes - ps8
Archaeplastida - ps9
Viridiplantae - ps10
Streptophyta - ps11
Charo./Embrioph. - ps12
Embriophyta - ps13
Tracheophyta - ps14
Spermatophyta - ps15
Angiosperms - ps16
Mesangiospermae - ps17
Magno./eudicots - ps18
Eudicots - ps19
Core eudic. - ps20
Superrosids - ps21
Rosids - ps22
Eurosids - ps23
Malvids - ps24
Sap./Malv./Brass. - ps25
Malv./Brass. - ps26
Brassicales - ps27
Cleo./Brassicaceae - ps28
Brassicaceae - ps29
Camelineae - ps30
Arabidopsis - ps31
A. thaliana - ps32

Supplementary Figure 7. The enrichment of GO functional categories related to cytoskeleton in four focal species. The enrichment profiles are shown for the GO term GO:0005856 cytoskeleton. The functional enrichments were calculated using the sets of gained gene families along **a**, *H. sapiens*, **b**, *D. melanogaster* **c**, *S. cerevisiae* and **d**, *A. thaliana* lineage (x-axis). The gene families are reconstructed with MMseqs2 *cluster* using a range of c-values (0 to 0.8, y-axis). Solid circles depict significant enrichments of the GO term in gained gene families at a particular phylostratum. The size of circles is proportional to the enrichment values estimated by log-odds, while the shades of blue (gain) correspond to p-values. The significance of enrichments was estimated by two-tailed hypergeometric test corrected for multiple comparisons. Only the enrichments with p-value < 0.05 are shown. This GO term shows the significant enrichments signals for some c-values at the origin of Eukaryota (ps6) in all four focal species (**a-d**), with additional signals at Choanozoa (ps12) in *H. sapiens* (**a**) and *D. melanogaster* (**b**) lineages. The source data of this figure are provided in the Source Data file.

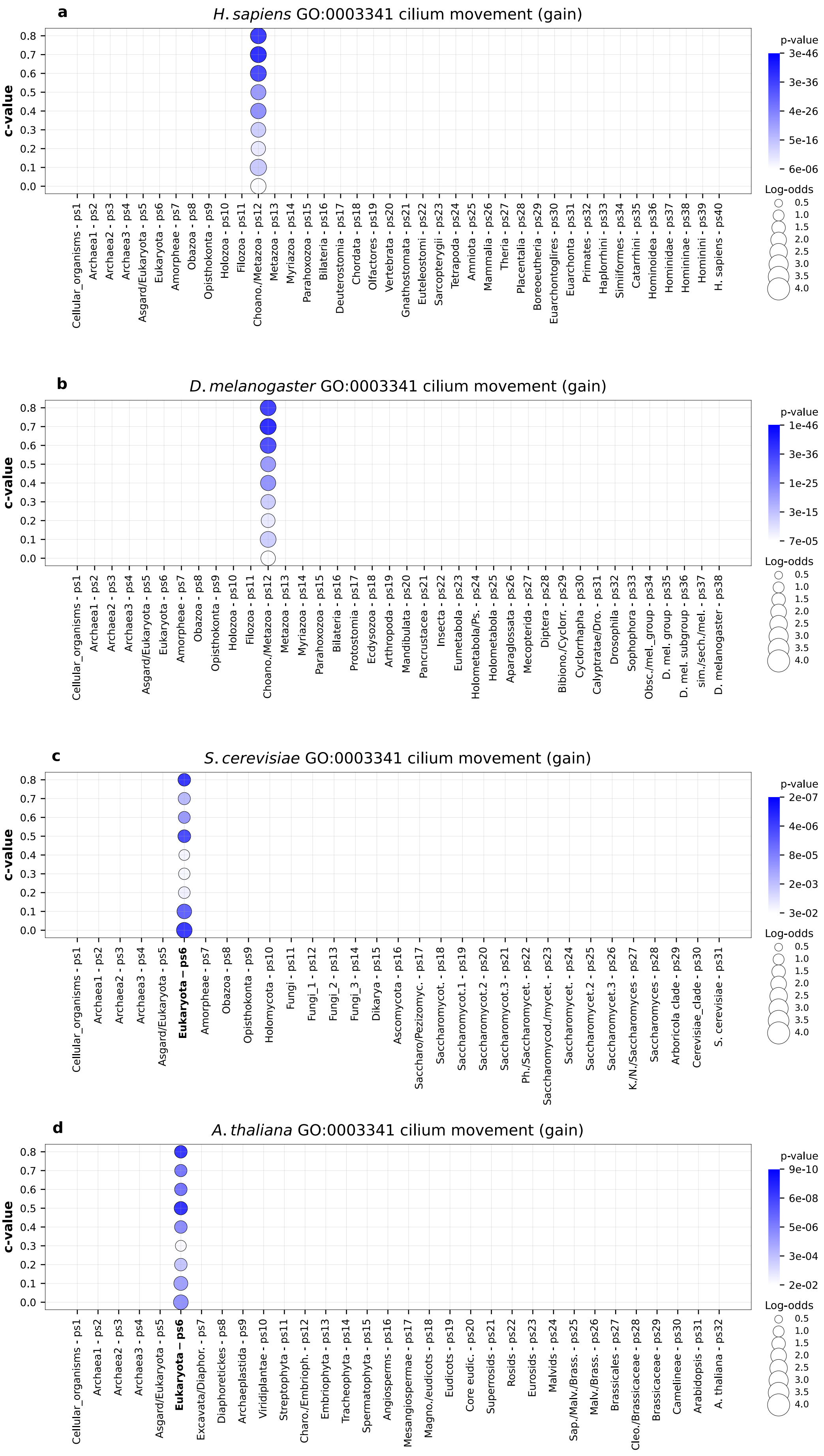
a
H. sapiens GO:0003341 cilium movement (gain)
c-value
0.0
0.1
0.2
0.3
0.4
0.5
0.6
0.7
0.8
p-value
3e-46
3e-36
4e-26
5e-16
6e-06
Log-odds
0.5
1.0
1.5
2.0
2.5
3.0
3.5
4.0
Cellular_organisms - ps1
Archaea1 - ps2
Archaea2 - ps3
Archaea3 - ps4
Asgard/Eukaryota - ps5
Eukaryota - ps6
Amorpheae - ps7
Obazoa - ps8
Opisthokonta - ps9
Holozoa - ps10
Filozoa - ps11
Choano./Metazoa - ps12
Metazoa - ps13
Myriazoa - ps14
Parahoxozoa - ps15
Bilateria - ps16
Deuterostomia - ps17
Chordata - ps18
Olfactores - ps19
Vertebrata - ps20
Gnathostomata - ps21
Euteleostomi - ps22
Sarcopterygii - ps23
Tetrapoda - ps24
Amniota - ps25
Mammalia - ps26
Theria - ps27
Placentalia - ps28
Boreoeutheria - ps29
Euarchontoglires - ps30
Euarchonta - ps31
Primates - ps32
Haplorrhini - ps33
Simiiformes - ps34
Catarrhini - ps35
Hominoidea - ps36
Hominidae - ps37
Homininae - ps38
Hominini - ps39
H. sapiens - ps40
b
D. melanogaster GO:0003341 cilium movement (gain)
c-value
p-value
1e-46
3e-36
1e-25
3e-15
7e-05
Log-odds
Cellular_organisms - ps1
Archaea1 - ps2
Archaea2 - ps3
Archaea3 - ps4
Asgard/Eukaryota - ps5
Eukaryota - ps6
Amorpheae - ps7
Obazoa - ps8
Opisthokonta - ps9
Holozoa - ps10
Filozoa - ps11
Choano./Metazoa - ps12
Metazoa - ps13
Myriazoa - ps14
Parahoxozoa - ps15
Bilateria - ps16
Protostomia - ps17
Ecdysozoa - ps18
Arthropoda - ps19
Mandibulata - ps20
Pancrustacea - ps21
Insecta - ps22
Eumetabola - ps23
Holometabola/Ps. - ps24
Holometabola - ps25
Aparaglossata - ps26
Mecopterida - ps27
Diptera - ps28
Bibiono./Cyclorr. - ps29
Cyclorrhapha - ps30
Calyptratae/Dro. - ps31
Drosophila - ps32
Sophophora - ps33
Obsc./mel._group - ps34
D. mel. group - ps35
D. mel. subgroup - ps36
sim./sech./mel. - ps37
D. melanogaster - ps38
c
S. cerevisiae GO:0003341 cilium movement (gain)
c-value
p-value
2e-07
4e-06
8e-05
2e-03
3e-02
Log-odds
Cellular_organisms - ps1
Archaea1 - ps2
Archaea2 - ps3
Archaea3 - ps4
Asgard/Eukaryota - ps5
Eukaryota – ps6
Amorpheae - ps7
Obazoa - ps8
Opisthokonta - ps9
Holomycota - ps10
Fungi - ps11
Fungi_1 - ps12
Fungi_2 - ps13
Fungi_3 - ps14
Dikarya - ps15
Ascomycota - ps16
Saccharo/Pezizomyc. - ps17
Saccharomycot. - ps18
Saccharomycot.1 - ps19
Saccharomycot.2 - ps20
Saccharomycot.3 - ps21
Ph./Saccharomycet. - ps22
Saccharomycod./mycet. - ps23
Saccharomycet. - ps24
Saccharomycet.2 - ps25
Saccharomycet.3 - ps26
K./N./Saccharomyces - ps27
Saccharomyces - ps28
Arboricola clade - ps29
Cerevisiae_clade - ps30
S. cerevisiae - ps31
d
A. thaliana GO:0003341 cilium movement (gain)
c-value
p-value
9e-10
6e-08
5e-06
3e-04
2e-02
Log-odds
Cellular_organisms - ps1
Archaea1 - ps2
Archaea2 - ps3
Archaea3 - ps4
Asgard/Eukaryota - ps5
Eukaryota – ps6
Excavata/Diaphor. - ps7
Diaphoretickes - ps8
Archaeplastida - ps9
Viridiplantae - ps10
Streptophyta - ps11
Charo./Embrioph. - ps12
Embriophyta - ps13
Tracheophyta - ps14
Spermatophyta - ps15
Angiosperms - ps16
Mesangiospermae - ps17
Magno./eudicots - ps18
Eudicots - ps19
Core eudic. - ps20
Superrosids - ps21
Rosids - ps22
Eurosids - ps23
Malvids - ps24
Sap./Malv./Brass. - ps25
Malv./Brass. - ps26
Brassicales - ps27
Cleo./Brassicaceae - ps28
Brassicaceae - ps29
Camelineae - ps30
Arabidopsis - ps31
A. thaliana - ps32

Supplementary Figure 8. The enrichment of GO functional categories related to cilium movement in four focal species. The enrichment profiles are shown for the GO term GO:0003341 (cilium movement). The functional enrichments were calculated using the sets of gained gene families along **a**, *H. sapiens*, **b**, *D. melanogaster* **c**, *S. cerevisiae* and **d**, *A. thaliana* lineage (x-axis). The gene families are reconstructed with MMseqs2 *cluster* using a range of c-values (0 to 0.8, y-axis). Solid circles depict significant enrichments of the GO term in gained gene families at a particular phylostratum. The size of circles is proportional to the enrichment values estimated by log-odds, while the shades of blue (gain) correspond to p-values. The significance of enrichments was estimated by two-tailed hypergeometric test corrected for multiple comparisons. Only enrichments with p-value < 0.05 are shown. This GO term shows the significant enrichments signals at Choanozoa (ps12) in *H. sapiens* (**a**) and *D. melanogaster* (**b**) lineages and at the origin of Eukaryota (ps6) in *S. cerevisiae* (**c**) and *A. thaliana* (**d**) lineages. The source data of this figure are provided in the Source Data file.

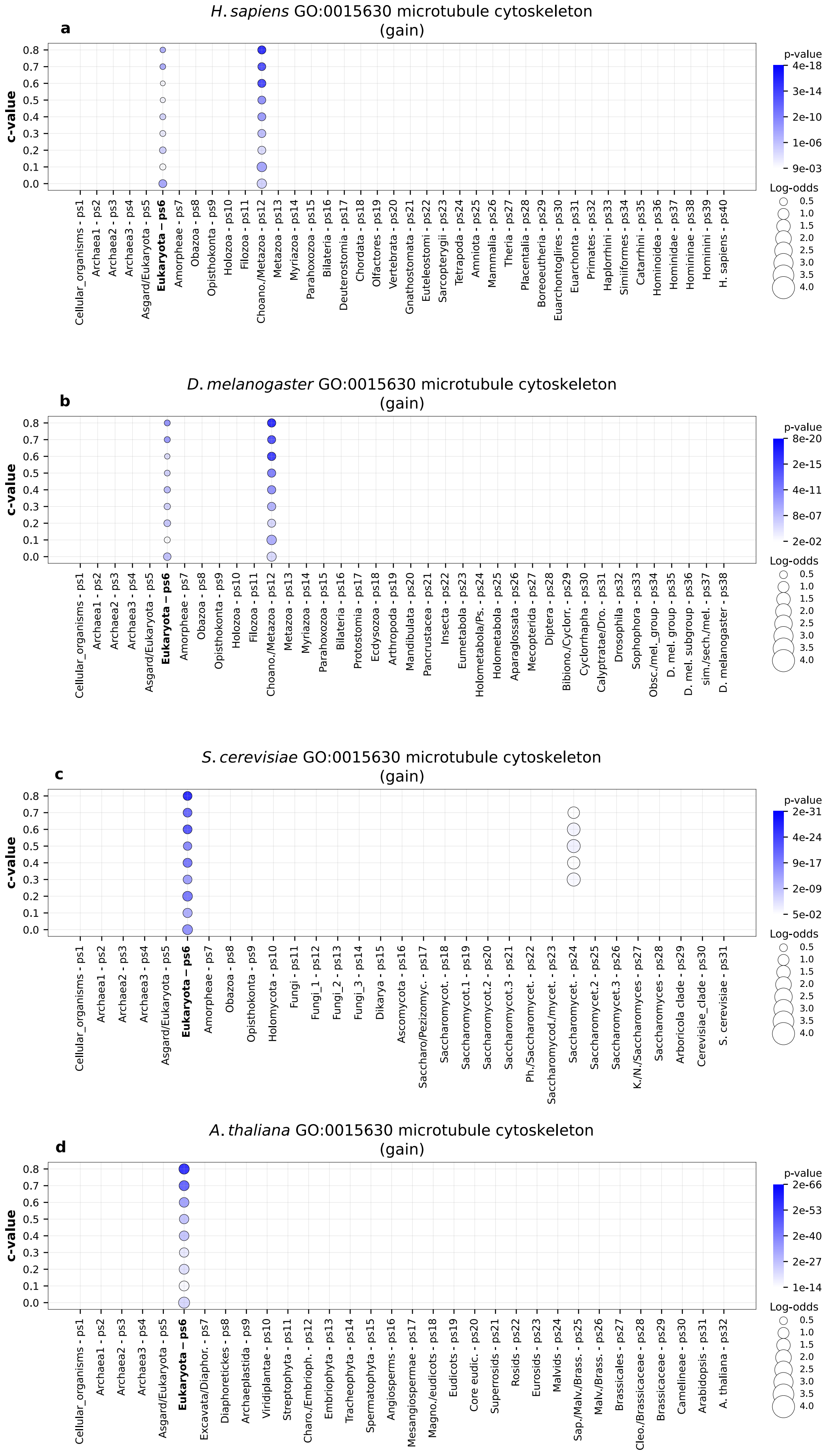

a
H. sapiens GO:0015630 microtubule cytoskeleton (gain)
c-value
0.0 0.1 0.2 0.3 0.4 0.5 0.6 0.7 0.8
p-value
4e-18 3e-14 2e-10 1e-06 9e-03
Log-odds
0.5 1.0 1.5 2.0 2.5 3.0 3.5 4.0
Cellular_organisms - ps1
Archaea1 - ps2
Archaea2 - ps3
Archaea3 - ps4
Asgard/Eukaryota - ps5
Eukaryota – ps6
Amorpheae - ps7
Obazoa - ps8
Opisthokonta - ps9
Holozoa - ps10
Filozoa - ps11
Choano./Metazoa - ps12
Metazoa - ps13
Myriazoa - ps14
Parahoxozoa - ps15
Bilateria - ps16
Deuterostomia - ps17
Chordata - ps18
Olfactores - ps19
Vertebrata - ps20
Gnathostomata - ps21
Euteleostomi - ps22
Sarcopterygii - ps23
Tetrapoda - ps24
Amniota - ps25
Mammalia - ps26
Theria - ps27
Placentalia - ps28
Boreoeutheria - ps29
Euarchontoglires - ps30
Euarchonta - ps31
Primates - ps32
Haplorrhini - ps33
Simiiformes - ps34
Catarrhini - ps35
Hominoidea - ps36
Hominidae - ps37
Homininae - ps38
Hominini - ps39
H. sapiens - ps40
b
D. melanogaster GO:0015630 microtubule cytoskeleton (gain)
c-value
0.0 0.1 0.2 0.3 0.4 0.5 0.6 0.7 0.8
p-value
8e-20 2e-15 4e-11 8e-07 2e-02
Log-odds
0.5 1.0 1.5 2.0 2.5 3.0 3.5 4.0
Cellular_organisms - ps1
Archaea1 - ps2
Archaea2 - ps3
Archaea3 - ps4
Asgard/Eukaryota - ps5
Eukaryota – ps6
Amorpheae - ps7
Obazoa - ps8
Opisthokonta - ps9
Holozoa - ps10
Filozoa - ps11
Choano./Metazoa - ps12
Metazoa - ps13
Myriazoa - ps14
Parahoxozoa - ps15
Bilateria - ps16
Protostomia - ps17
Ecdysozoa - ps18
Arthropoda - ps19
Mandibulata - ps20
Pancrustacea - ps21
Insecta - ps22
Eumetabola - ps23
Holometabola/Ps. - ps24
Holometabola - ps25
Aparaglossata - ps26
Mecopterida - ps27
Diptera - ps28
Bibiono./Cyclorr. - ps29
Cyclorrhapha - ps30
Calyptratae/Dro. - ps31
Drosophila - ps32
Sophophora - ps33
Obsc./mel._group - ps34
D. mel. group - ps35
D. mel. subgroup - ps36
sim./sech./mel. - ps37
D. melanogaster - ps38
c
S. cerevisiae GO:0015630 microtubule cytoskeleton (gain)
c-value
0.0 0.1 0.2 0.3 0.4 0.5 0.6 0.7 0.8
p-value
2e-31 4e-24 9e-17 2e-09 5e-02
Log-odds
0.5 1.0 1.5 2.0 2.5 3.0 3.5 4.0
Cellular_organisms - ps1
Archaea1 - ps2
Archaea2 - ps3
Archaea3 - ps4
Asgard/Eukaryota - ps5
Eukaryota – ps6
Amorpheae - ps7
Obazoa - ps8
Opisthokonta - ps9
Holomycota - ps10
Fungi - ps11
Fungi_1 - ps12
Fungi_2 - ps13
Fungi_3 - ps14
Dikarya - ps15
Ascomycota - ps16
Saccharo/Pezizomyc. - ps17
Saccharomycot. - ps18
Saccharomycot.1 - ps19
Saccharomycot.2 - ps20
Saccharomycot.3 - ps21
Ph./Saccharomycet. - ps22
Saccharomycod./mycet. - ps23
Saccharomycet. - ps24
Saccharomycet.2 - ps25
Saccharomycet.3 - ps26
K./N./Saccharomyces - ps27
Saccharomyces - ps28
Arboricola clade - ps29
Cerevisiae_clade - ps30
S. cerevisiae - ps31
d
A. thaliana GO:0015630 microtubule cytoskeleton (gain)
c-value
0.0 0.1 0.2 0.3 0.4 0.5 0.6 0.7 0.8
p-value
2e-66 2e-53 2e-40 2e-27 1e-14
Log-odds
0.5 1.0 1.5 2.0 2.5 3.0 3.5 4.0
Cellular_organisms - ps1
Archaea1 - ps2
Archaea2 - ps3
Archaea3 - ps4
Asgard/Eukaryota - ps5
Eukaryota – ps6
Excavata/Diaphor. - ps7
Diaphoretickes - ps8
Archaeplastida - ps9
Viridiplantae - ps10
Streptophyta - ps11
Charo./Embrioph. - ps12
Embriophyta - ps13
Tracheophyta - ps14
Spermatophyta - ps15
Angiosperms - ps16
Mesangiospermae - ps17
Magno./eudicots - ps18
Eudicots - ps19
Core eudic. - ps20
Superrosids - ps21
Rosids - ps22
Eurosids - ps23
Malvids - ps24
Sap./Malv./Brass. - ps25
Malv./Brass. - ps26
Brassicales - ps27
Cleo./Brassicaceae - ps28
Brassicaceae - ps29
Camelineae - ps30
Arabidopsis - ps31
A. thaliana - ps32

Supplementary Figure 9. The enrichment of GO functional categories related to microtubule cytoskeleton in four focal species. The enrichment profiles are shown for the GO term GO:0015630 (microtubule cytoskeleton). The functional enrichments were calculated using the sets of gained gene families along **a**, *H. sapiens*, **b**, *D. melanogaster* **c**, *S. cerevisiae* and **d**, *A. thaliana* lineage (x-axis). The gene families are reconstructed with MMseqs2 *cluster* using a range of c-values (0 to 0.8, y-axis). Solid circles depict significant enrichments of the GO term in gained gene families at a particular phylostratum. The size of circles is proportional to the enrichment values estimated by log-odds, while the shades of blue (gain) correspond to p-values. The significance of enrichments was estimated by two-tailed hypergeometric test corrected for multiple comparisons. Only enrichments with p-value < 0.05 are shown. This GO term shows the significant enrichments signals at higher c-values at the origin of Eukaryota (ps6) in all four focal species (**a-d**), with additional signals at Choanozoa (ps12) in *H. sapiens* (**a**) and *D. melanogaster* (**b**) analysis. The source data of this figure are provided in the Source Data file.

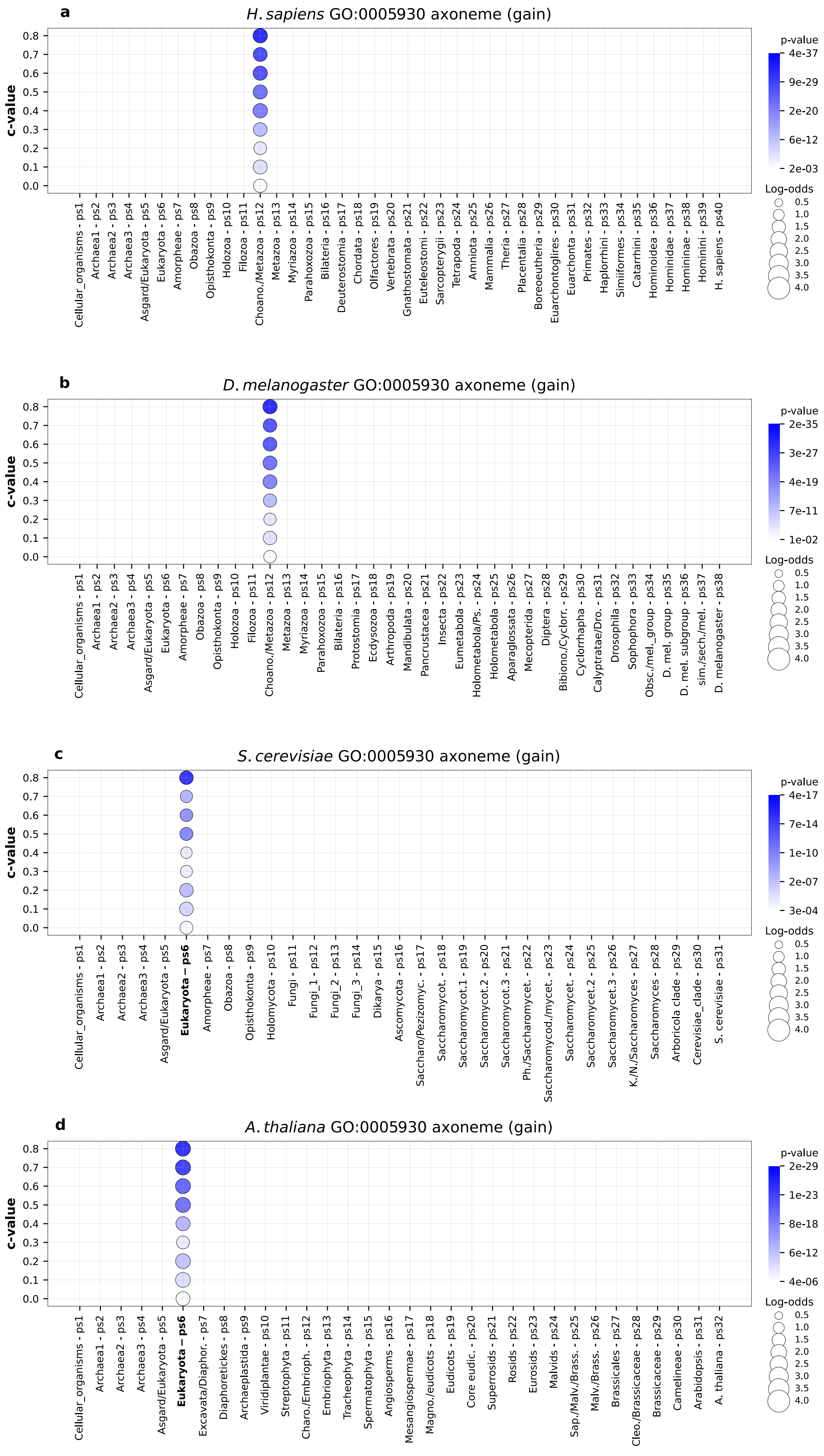
a
H. sapiens GO:0005930 axoneme (gain)
c-value
0.0
0.1
0.2
0.3
0.4
0.5
0.6
0.7
0.8
p-value
4e-37
9e-29
2e-20
6e-12
2e-03
Log-odds
0.5
1.0
1.5
2.0
2.5
3.0
3.5
4.0
Cellular_organisms - ps1
Archaea1 - ps2
Archaea2 - ps3
Archaea3 - ps4
Asgard/Eukaryota - ps5
Eukaryota - ps6
Amorpheae - ps7
Obazoa - ps8
Opisthokonta - ps9
Holozoa - ps10
Filozoa - ps11
Choano./Metazoa - ps12
Metazoa - ps13
Myriazoa - ps14
Parahoxozoa - ps15
Bilateria - ps16
Deuterostomia - ps17
Chordata - ps18
Olfactores - ps19
Vertebrata - ps20
Gnathostomata - ps21
Euteleostomi - ps22
Sarcopterygii - ps23
Tetrapoda - ps24
Amniota - ps25
Mammalia - ps26
Theria - ps27
Placentalia - ps28
Boreoeutheria - ps29
Euarchontoglires - ps30
Euarchonta - ps31
Primates - ps32
Haplorrhini - ps33
Simiiformes - ps34
Catarrhini - ps35
Hominoidea - ps36
Hominidae - ps37
Homininae - ps38
Hominini - ps39
H. sapiens - ps40
b
D. melanogaster GO:0005930 axoneme (gain)
c-value
p-value
2e-35
3e-27
4e-19
7e-11
1e-02
Log-odds
Cellular_organisms - ps1
Archaea1 - ps2
Archaea2 - ps3
Archaea3 - ps4
Asgard/Eukaryota - ps5
Eukaryota - ps6
Amorpheae - ps7
Obazoa - ps8
Opisthokonta - ps9
Holozoa - ps10
Filozoa - ps11
Choano./Metazoa - ps12
Metazoa - ps13
Myriazoa - ps14
Parahoxozoa - ps15
Bilateria - ps16
Protostomia - ps17
Ecdysozoa - ps18
Arthropoda - ps19
Mandibulata - ps20
Pancrustacea - ps21
Insecta - ps22
Eumetabola - ps23
Holometabola/Ps. - ps24
Holometabola - ps25
Aparaglossata - ps26
Mecopterida - ps27
Diptera - ps28
Bibiono./Cyclorr. - ps29
Cyclorrhapha - ps30
Calyptratae/Dro. - ps31
Drosophila - ps32
Sophophora - ps33
Obsc./mel._group - ps34
D. mel. group - ps35
D. mel. subgroup - ps36
sim./sech./mel. - ps37
D. melanogaster - ps38
c
S. cerevisiae GO:0005930 axoneme (gain)
c-value
p-value
4e-17
7e-14
1e-10
2e-07
3e-04
Log-odds
Cellular_organisms - ps1
Archaea1 - ps2
Archaea2 - ps3
Archaea3 - ps4
Asgard/Eukaryota - ps5
Eukaryota – ps6
Amorpheae - ps7
Obazoa - ps8
Opisthokonta - ps9
Holomycota - ps10
Fungi - ps11
Fungi_1 - ps12
Fungi_2 - ps13
Fungi_3 - ps14
Dikarya - ps15
Ascomycota - ps16
Saccharo/Pezizomyc. - ps17
Saccharomycot. - ps18
Saccharomycot.1 - ps19
Saccharomycot.2 - ps20
Saccharomycot.3 - ps21
Ph./Saccharomycet. - ps22
Saccharomycod./mycet. - ps23
Saccharomycet. - ps24
Saccharomycet.2 - ps25
Saccharomycet.3 - ps26
K./N./Saccharomyces - ps27
Saccharomyces - ps28
Arboricola clade - ps29
Cerevisiae_clade - ps30
S. cerevisiae - ps31
d
A. thaliana GO:0005930 axoneme (gain)
c-value
p-value
2e-29
1e-23
8e-18
6e-12
4e-06
Log-odds
Cellular_organisms - ps1
Archaea1 - ps2
Archaea2 - ps3
Archaea3 - ps4
Asgard/Eukaryota - ps5
Eukaryota – ps6
Excavata/Diaphor. - ps7
Diaphoretickes - ps8
Archaeplastida - ps9
Viridiplantae - ps10
Streptophyta - ps11
Charo./Embrioph. - ps12
Embriophyta - ps13
Tracheophyta - ps14
Spermatophyta - ps15
Angiosperms - ps16
Mesangiospermae - ps17
Magno./eudicots - ps18
Eudicots - ps19
Core eudic. - ps20
Superrosids - ps21
Rosids - ps22
Eurosids - ps23
Malvids - ps24
Sap./Malv./Brass. - ps25
Malv./Brass. - ps26
Brassicales - ps27
Cleo./Brassicaceae - ps28
Brassicaceae - ps29
Camelineae - ps30
Arabidopsis - ps31
A. thaliana - ps32

Supplementary Figure 10. The enrichment of GO functional categories related to axoneme in four focal species. The enrichment profiles are shown for the GO term GO:0005930 (axoneme). The functional enrichments were calculated using the sets of gained gene families along **a**, *H. sapiens*, **b**, *D. melanogaster* **c**, *S. cerevisiae* and **d**, *A. thaliana* lineage (x-axis). The gene families are reconstructed with MMseqs2 *cluster* using a range of c-values (0 to 0.8, y-axis). Solid circles depict significant enrichments of the GO term in gained gene families at a particular phylostratum. The size of circles is proportional to the enrichment values estimated by log-odds, while the shades of blue (gain) correspond to p-values. The significance of enrichments was estimated by two-tailed hypergeometric test corrected for multiple comparisons. Only enrichments with p-value < 0.05 are shown. This GO term shows the significant enrichments signals at higher c-values at the origin of Eukaryota (ps6) in all four focal species (**a-d**), with additional signals at Choanozoa (ps12) in *H. sapiens* (**a**) and *D. melanogaster* (**b**) analysis. The source data of this figure are provided in the Source Data file.

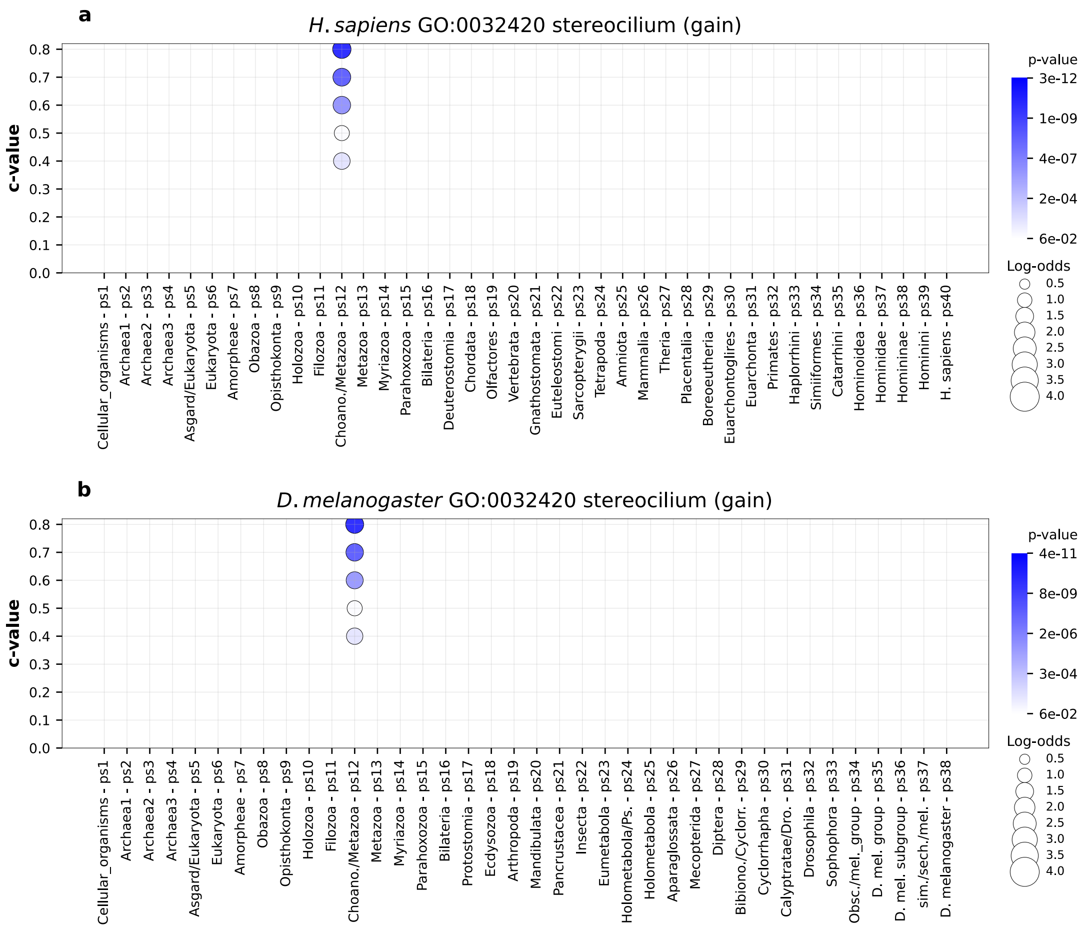

Supplementary Figure 11. The enrichment of GO functional categories related to stereocilium in four focal species. The enrichment profiles are shown for the GO term GO:0032420 (stereocilium). The functional enrichments were calculated using the sets of gained gene families along **a**, *H. sapiens* and **b**, *D. melanogaster* lineage (x-axis). The gene families are reconstructed with MMseqs2 *cluster* using a range of c-values (0 to 0.8, y-axis). Solid circles depict significant enrichments of the GO term in gained gene families at a particular phylostratum. The size of circles is proportional to the enrichment values estimated by log-odds, while the shades of blue (gain) correspond to p-values. The significance of enrichments was estimated by two-tailed hypergeometric test corrected for multiple comparisons. Only enrichments with p-value < 0.05 are shown. This GO term shows the significant enrichments signals at higher c-values at the origin of Choanozoa (ps12) in the two focal species (**a, b**). The source data of this figure are provided in the Source Data file.

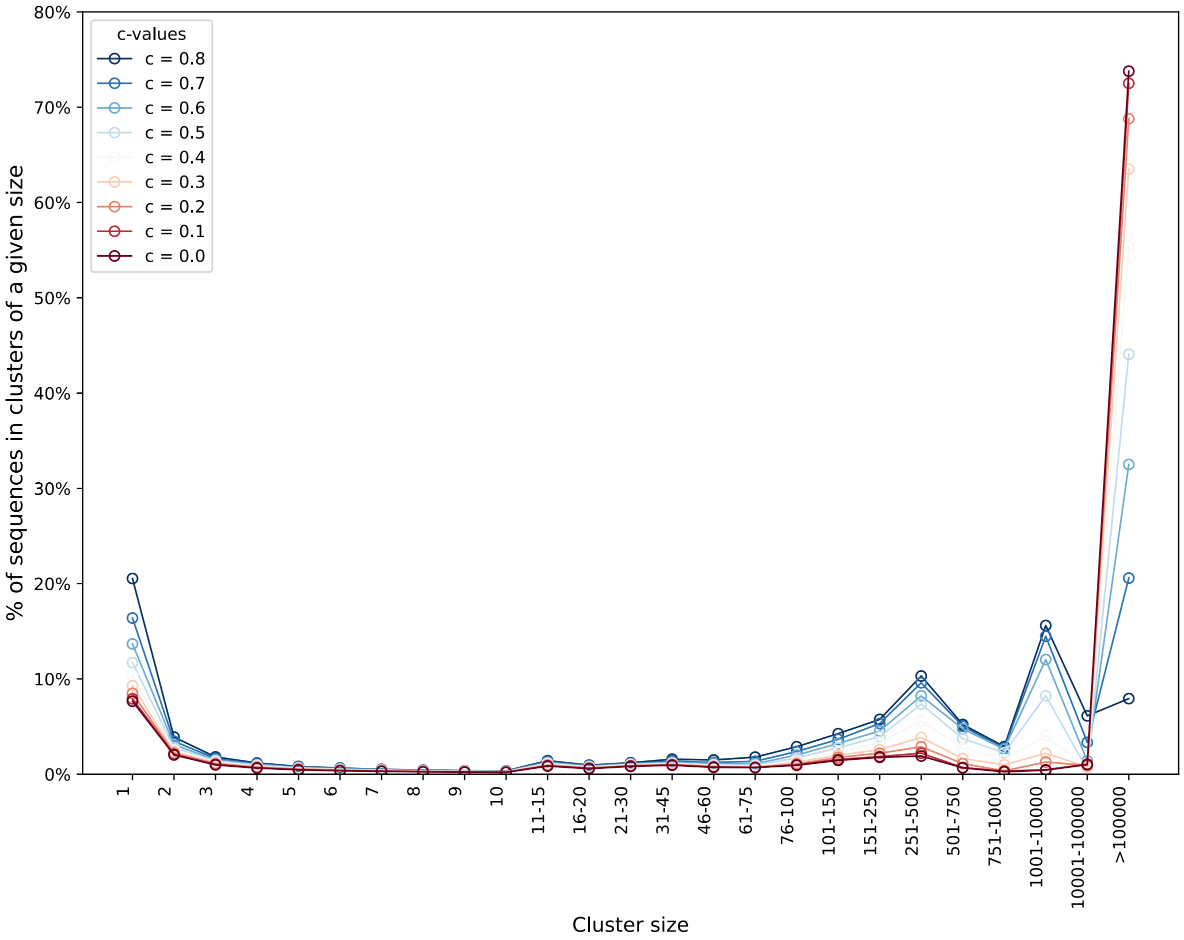

Supplementary Figure 12. Distribution of sequences by cluster size. The x-axis shows the number of sequences in clusters sorted in bins, while the y-axis shows the percentage of all sequences allocated to clusters of a given size. Clustering was performed using the MMseqs2 *cluster* algorithm and the full set of reference genomes (667 genomes, Supplementary Data 1). The colored lines in each plot correspond to different c-values of the MMseqs2 *cluster* algorithm. This parameter determines the minimal percentage of protein sequence alignment overlap in a cluster. The darkest blue graph corresponds to c-value = 0.8 which forces at least 80% of sequence length alignment overlap with the cluster's representative sequence. The darkest red graph corresponds to c-value = 0 which allows clustering without restrictions on the alignment overlap length. It is evident that c-value = 0 pushes sequences to the largest clusters at the expense of the middle to large-sized clusters. The source data of this figure are provided in the Source Data file.

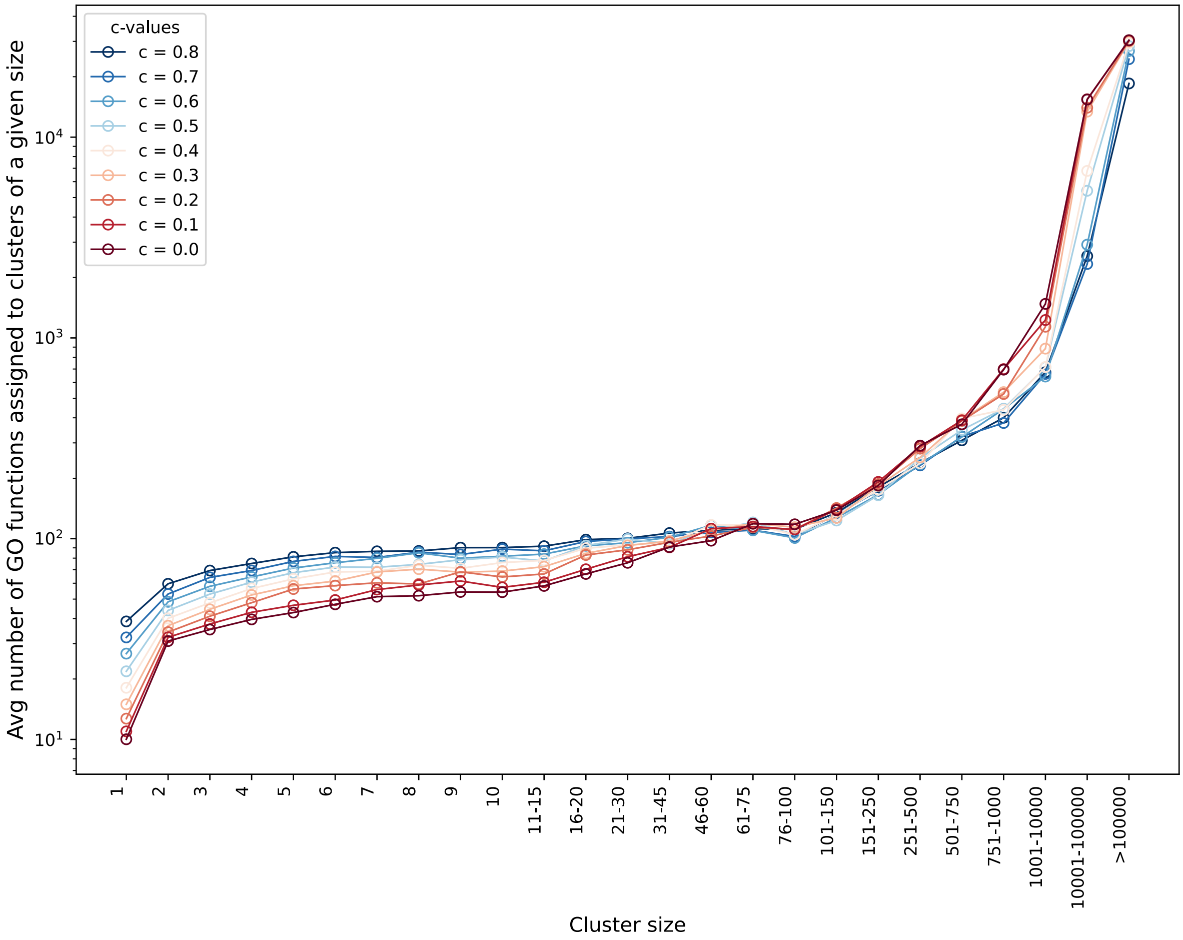

Supplementary Figure 13. Average number of GO functions per cluster size. The x-axis shows the number of sequences in clusters sorted in bins, while the y-axis shows the average number of GO annotations per cluster. Clustering was performed using the MMseqs2 *cluster* algorithm and the full set of reference genomes (667 genomes, Supplementary Data 1). The colored lines in each plot correspond to different c-values of the MMseqs2 *cluster* algorithm. This parameter determines the minimal percentage of protein sequence alignment overlap in a cluster. The darkest blue graph corresponds to c-value = 0.8 which forces at least 80% of sequence length alignment overlap with the cluster's representative sequence. The darkest red graph corresponds to c-value = 0 which allows clustering without restrictions on the alignment overlap length. It is evident that c-value = 0 clustering decreases the percentage of GO annotations in small to middle size clusters. The source data of this figure are provided in the Source Data file.

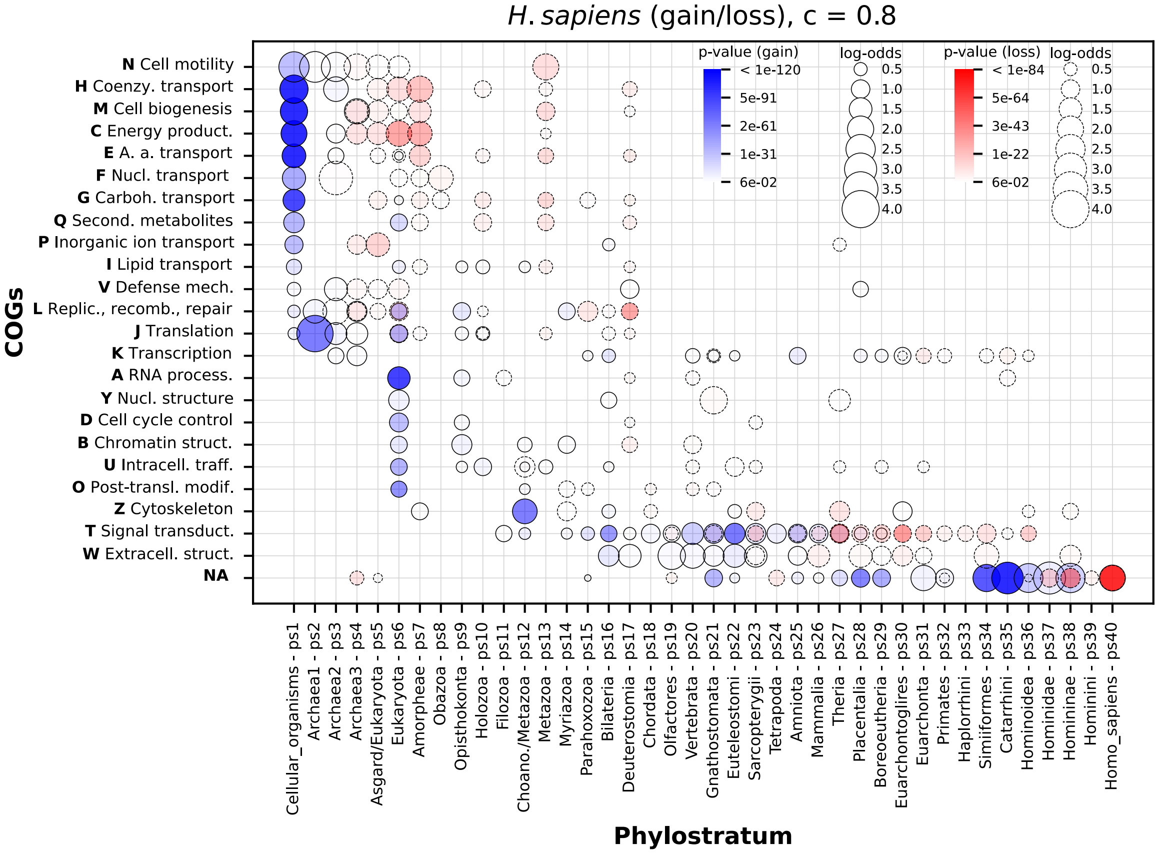

Supplementary Figure 14. The enrichment of COG functional categories in gained and lost gene families along the *H. sapiens* lineage - stringent annotation criteria. The abbreviated names of COG functional categories and corresponding one-letter symbols are depicted at y-axis. The protein families without COG annotation are annotated with NA. The names and symbols of phylostrata are shown on the x-axis. The gene families are reconstructed with the MMseqs *cluster* program using a c-value of 0.8. Functional categories significantly enriched among gained gene families across phylostrata are depicted by solid circles painted in shades of blue that reflect the underlying p-values. The size of circles is proportional to the enrichment values estimated by log-odds. Functional categories significantly enriched among lost gene families across phylostrata are depicted by dashed circles painted in shades of red that reflect p-values. The size of circles is proportional to the enrichment values estimated by log-odds. The significance of enrichment was estimated by two-tailed hypergeometric test corrected for multiple testing. The source data of this figure are provided in the Source Data file.

Supplementary References

The phylogentic literature that we used to construct the consensus phylogeny[1-62].

1. Shao, Y. *et al.* Phylogenomic analyses provide insights into primate evolution. *Science* **380**, 913–924 (2023).
2. D'Elía, G., Fabre, P.-H. & Lessa, E. P. Rodent systematics in an age of discovery: recent advances and prospects. *Journal of Mammalogy* **100**, 852–871 (2019).
3. Fabre, P.-H., Hautier, L., Dimitrov, D. & P Douzery, E. J. A glimpse on the pattern of rodent diversification: a phylogenetic approach. *BMC Evol Biol* **12**, 88 (2012).
4. Foley, N. M. *et al.* A genomic timescale for placental mammal evolution. *Science* **380**, eabl8189 (2023).
5. Álvarez-Carretero, S. *et al.* A species-level timeline of mammal evolution integrating phylogenomic data. *Nature* **602**, 263–267 (2022).
6. Dornburg, A. & Near, T. J. The Emerging Phylogenetic Perspective on the Evolution of Actinopterygian Fishes. *Annu. Rev. Ecol. Evol. Syst.* **52**, 427–452 (2021).
7. Ghezelayagh, A. *et al.* Prolonged morphological expansion of spiny-rayed fishes following the end-Cretaceous. *Nat Ecol Evol* **6**, 1211–1220 (2022).
8. Prum, R. O. *et al.* A comprehensive phylogeny of birds (Aves) using targeted next-generation DNA sequencing. *Nature* **526**, 569–573 (2015).
9. Wiegmann, B. M. *et al.* Episodic radiations in the fly tree of life. *Proc. Natl. Acad. Sci. U.S.A.* **108**, 5690–5695 (2011).
10. Narayanan Kutty, S., Wong, W. H., Meusemann, K., Meier, R. & Cranston, P. S. A phylogenomic analysis of Culicomorpha (Diptera) resolves the relationships among the eight constituent families. *Systematic Entomology* **43**, 434–446 (2018).
11. Li, F. *et al.* Phylogenomic analyses of the genus *Drosophila* reveals genomic signals of climate adaptation. *Molecular Ecology Resources* **22**, 1559–1581 (2022).

12. Suvorov, A. *et al.* Widespread introgression across a phylogeny of 155 Drosophila genomes. *Current Biology* **32**, 111-123.e5 (2022).
13. Kawahara, A. Y. *et al.* A global phylogeny of butterflies reveals their evolutionary history, ancestral hosts and biogeographic origins. *Nat Ecol Evol* **7**, 903–913 (2023).
14. Kawahara, A. Y. *et al.* Phylogenomics reveals the evolutionary timing and pattern of butterflies and moths. *Proc. Natl. Acad. Sci. U.S.A.* **116**, 22657–22663 (2019).
15. McKenna, D. D. *et al.* The evolution and genomic basis of beetle diversity. *Proc. Natl. Acad. Sci. U.S.A.* **116**, 24729–24737 (2019).
16. Cai, C. *et al.* Integrated phylogenomics and fossil data illuminate the evolution of beetles. *R. Soc. open sci.* **9**, 211771 (2022).
17. Misof, B. *et al.* Phylogenomics resolves the timing and pattern of insect evolution. *Science* **346**, 763–767 (2014).
18. Johnson, K. P. *et al.* Phylogenomics and the evolution of hemipteroid insects. *Proc. Natl. Acad. Sci. U.S.A.* **115**, 12775–12780 (2018).
19. Bernot, J. P. *et al.* Major Revisions in Pancrustacean Phylogeny and Evidence of Sensitivity to Taxon Sampling. *Molecular Biology and Evolution* **40**, msad175 (2023).
20. Giribet, G. & Edgecombe, G. D. The Phylogeny and Evolutionary History of Arthropods. *Current Biology* **29**, R592–R602 (2019).
21. Smythe, A. B., Holovachov, O. & Kocot, K. M. Improved phylogenomic sampling of free-living nematodes enhances resolution of higher-level nematode phylogeny. *BMC Evol Biol* **19**, 121 (2019).
22. Marlétaz, F., Peijnenburg, K. T. C. A., Goto, T., Satoh, N. & Rokhsar, D. S. A New Spiralian Phylogeny Places the Enigmatic Arrow Worms among Gnathiferans. *Current Biology* **29**, 312-318.e3 (2019).

23. Khalturin, K. *et al.* Polyzoa is back: The effect of complete gene sets on the placement of Ectoprocta and Entoprocta. *Sci. Adv.* **8**, eabo4400 (2022).
24. Laumer, C. E. *et al.* Spiralian Phylogeny Informs the Evolution of Microscopic Lineages. *Current Biology* **25**, 2000–2006 (2015).
25. Steenwyk, J. L., Li, Y., Zhou, X., Shen, X.-X. & Rokas, A. Incongruence in the phylogenomics era. *Nat Rev Genet* **24**, 834–850 (2023).
26. Schultz, D. T. *et al.* Ancient gene linkages support ctenophores as sister to other animals. *Nature* **618**, 110–117 (2023).
27. Laumer, C. E. *et al.* Revisiting metazoan phylogeny with genomic sampling of all phyla. *Proc. R. Soc. B.* **286**, 20190831 (2019).
28. Laumer, C. E. *et al.* Support for a clade of Placozoa and Cnidaria in genes with minimal compositional bias. *eLife* **7**, e36278 (2018).
29. Carr, M. *et al.* A six-gene phylogeny provides new insights into choanoflagellate evolution. *Molecular Phylogenetics and Evolution* **107**, 166–178 (2017).
30. Richter, D. J., Fozouni, P., Eisen, M. B. & King, N. Gene family innovation, conservation and loss on the animal stem lineage. *eLife* **7**, e34226 (2018).
31. Shen, X.-X. *et al.* Reconstructing the Backbone of the Saccharomycotina Yeast Phylogeny Using Genome-Scale Data. *G3 Genes|Genomes|Genetics* **6**, 3927–3939 (2016).
32. Shen, X.-X. *et al.* Genome-scale phylogeny and contrasting modes of genome evolution in the fungal phylum Ascomycota. *Sci. Adv.* **6**, eabd0079 (2020).
33. Li, Y. *et al.* A genome-scale phylogeny of the kingdom Fungi. *Current Biology* **31**, 1653-1665.e5 (2021).
34. Strassert, J. F. H. & Monaghan, M. T. Phylogenomic insights into the early diversification of fungi. *Current Biology* **32**, 3628-3635.e3 (2022).

35. Chang, Y. *et al.* Genome-scale phylogenetic analyses confirm Olpidium as the closest living zoosporic fungus to the non-flagellated, terrestrial fungi. *Sci Rep* **11**, 3217 (2021).
36. Wadi, L. & Reinke, A. W. Evolution of microsporidia: An extremely successful group of eukaryotic intracellular parasites. *PLoS Pathog* **16**, e1008276 (2020).
37. Tekle, Y. I., Wang, F., Wood, F. C., Anderson, O. R. & Smirnov, A. New insights on the evolutionary relationships between the major lineages of Amoebozoa. *Sci Rep* **12**, 11173 (2022).
38. Liu, L., Du, X., Guo, C. & Li, D. Resolving robust phylogenetic relationships of core Brassicaceae using genome skimming data. *J of Sytematics Evolution* **59**, 442–453 (2021).
39. Hendriks, K. P. *et al. Global Phylogeny of the Brassicaceae Provides Important Insights into Gene Discordance*. http://biorxiv.org/lookup/doi/10.1101/2022.09.01.506188 (2022) doi:10.1101/2022.09.01.506188.
40. Nikolov, L. A. *et al.* Resolving the backbone of the Brassicaceae phylogeny for investigating trait diversity. *New Phytologist* **222**, 1638–1651 (2019).
41. Zhao, T. *et al.* Whole-genome microsynteny-based phylogeny of angiosperms. *Nat Commun* **12**, 3498 (2021).
42. Richardson, J. E., Whitlock, B. A., Meerow, A. W. & Madriñán, S. The age of chocolate: a diversification history of Theobroma and Malvaceae. *Front. Ecol. Evol.* **3**, (2015).
43. Ramírez-Barahona, S., Sauquet, H. & Magallón, S. The delayed and geographically heterogeneous diversification of flowering plant families. *Nat Ecol Evol* **4**, 1232–1238 (2020).
44. Bredeson, J. V. *et al.* Sequencing wild and cultivated cassava and related species reveals extensive interspecific hybridization and genetic diversity. *Nat Biotechnol* **34**, 562–570 (2016).

45. Li, H.-T. *et al.* Plastid phylogenomic insights into relationships of all flowering plant families. *BMC Biol* **19**, 232 (2021).
46. Li, H.-T. *et al.* Origin of angiosperms and the puzzle of the Jurassic gap. *Nat. Plants* **5**, 461–470 (2019).
47. Zhao, Y. *et al.* Nuclear phylotranscriptomics and phylogenomics support numerous polyploidization events and hypotheses for the evolution of rhizobial nitrogen-fixing symbiosis in Fabaceae. *Molecular Plant* **14**, 748–773 (2021).
48. Yang, Y.-Y., Qu, X.-J., Zhang, R., Stull, G. W. & Yi, T.-S. Plastid phylogenomic analyses of Fagales reveal signatures of conflict and ancient chloroplast capture. *Molecular Phylogenetics and Evolution* **163**, 107232 (2021).
49. Guo, J. *et al.* Phylotranscriptomics in Cucurbitaceae Reveal Multiple Whole-Genome Duplications and Key Morphological and Molecular Innovations. *Molecular Plant* **13**, 1117–1133 (2020).
50. Zhang, S. *et al.* Diversification of Rosaceae since the Late Cretaceous based on plastid phylogenomics. *New Phytologist* **214**, 1355–1367 (2017).
51. One Thousand Plant Transcriptomes Initiative. One thousand plant transcriptomes and the phylogenomics of green plants. *Nature* **574**, 679–685 (2019).
52. Guo, X. *et al.* Chloranthus genome provides insights into the early diversification of angiosperms. *Nat Commun* **12**, 6930 (2021).
53. Lv, Q. *et al.* The *Chimonanthus salicifolius* genome provides insight into magnoliid evolution and flavonoid biosynthesis. *The Plant Journal* **103**, 1910–1923 (2020).
54. Su, D. *et al.* Large-Scale Phylogenomic Analyses Reveal the Monophyly of Bryophytes and Neoproterozoic Origin of Land Plants. *Molecular Biology and Evolution* **38**, 3332–3344 (2021).

55. Puttick, M. N. *et al.* The Interrelationships of Land Plants and the Nature of the Ancestral Embryophyte. *Current Biology* **28**, 733-745.e2 (2018).

56. Sánchez-Baracaldo, P., Raven, J. A., Pisani, D. & Knoll, A. H. Early photosynthetic eukaryotes inhabited low-salinity habitats. *Proc. Natl. Acad. Sci. U.S.A.* **114**, (2017).

57. Craig, R. J., Hasan, A. R., Ness, R. W. & Keightley, P. D. Comparative genomics of *Chlamydomonas*. *The Plant Cell* **33**, 1016–1041 (2021).

58. Martínez-Alberola, F. *et al.* Dynamic evolution of mitochondrial genomes in Trebouxiophyceae, including the first completely assembled mtDNA from a lichen-symbiont microalga (Trebouxia sp. TR9). *Sci Rep* **9**, 8209 (2019).

59. Li, L. *et al.* The genome of Prasinoderma coloniale unveils the existence of a third phylum within green plants. *Nat Ecol Evol* **4**, 1220–1231 (2020).

60. Bowles, A. M. C., Williamson, C. J., Williams, T. A., Lenton, T. M. & Donoghue, P. C. J. The origin and early evolution of plants. *Trends in Plant Science* **28**, 312–329 (2023).

61. Strassert, J. F. H., Irisarri, I., Williams, T. A. & Burki, F. A molecular timescale for eukaryote evolution with implications for the origin of red algal-derived plastids. *Nat Commun* **12**, 1879 (2021).

62. Seeleuthner, Y. *et al.* Single-cell genomics of multiple uncultured stramenopiles reveals underestimated functional diversity across oceans. *Nat Commun* **9**, 310 (2018).
