# Supplementary material for: Macroevolutionary dynamics of gene family gain and loss along multicellular eukaryotic lineages
Source: Nat Commun. 2024 Mar 26;15:2663. doi: 10.1038/s41467-024-47017-w (PMC10966110; doi:10.1038/s41467-024-47017-w)
Supplement: Supplementary file 3 — Reporting Summary [file 41467_2024_47017_MOESM3_ESM.pdf]

nature portfolio

Corresponding author(s): Tomislav Domazet-Lošo

Last updated by author(s): Feb 14, 2024

Reporting Summary

Nature Portfolio wishes to improve the reproducibility of the work that we publish. This form provides structure for consistency and transparency in reporting. For further information on Nature Portfolio policies, see our Editorial Policies and the Editorial Policy Checklist.

Statistics

For all statistical analyses, confirm that the following items are present in the figure legend, table legend, main text, or Methods section.

n/a	Confirmed	
☒	☐	The exact sample size (n) for each experimental group/condition, given as a discrete number and unit of measurement
☒	☐	A statement on whether measurements were taken from distinct samples or whether the same sample was measured repeatedly
☐	☒	The statistical test(s) used AND whether they are one- or two-sided<br>*Only common tests should be described solely by name; describe more complex techniques in the Methods section.*
☒	☐	A description of all covariates tested
☒	☐	A description of any assumptions or corrections, such as tests of normality and adjustment for multiple comparisons
☒	☐	A full description of the statistical parameters including central tendency (e.g. means) or other basic estimates (e.g. regression coefficient) AND variation (e.g. standard deviation) or associated estimates of uncertainty (e.g. confidence intervals)
☐	☒	For null hypothesis testing, the test statistic (e.g. F, t, r) with confidence intervals, effect sizes, degrees of freedom and P value noted<br>*Give P values as exact values whenever suitable.*
☒	☐	For Bayesian analysis, information on the choice of priors and Markov chain Monte Carlo settings
☒	☐	For hierarchical and complex designs, identification of the appropriate level for tests and full reporting of outcomes
☒	☐	Estimates of effect sizes (e.g. Cohen's d, Pearson's r), indicating how they were calculated

Our web collection on statistics for biologists contains articles on many of the points above.

Software and code

Policy information about availability of computer code

Data collection	No specific software was used for data collection.
Data analysis	The completeness of protein content in genomes was tested using BUSCO v5.4.3. Clustering and sequence similarity searches were performed using the MMseqs2 package (version 14-7e284). The control clustering was performed using the MCL algorithm (version 22-282). The functional annotation of clusters was performed using the EggNOG package (emapper version 2.1.9, the database version 5.0.2 and the diamond search algorithm version 2.0.15). Gene family gain-and-loss patterns were calculated using the custom made scripts written in Python. The figures in the manuscript were generated using Python and R. All custom made scripts are available in GitHub at https://github.com/PhyLoss/PhyLoss.

For manuscripts utilizing custom algorithms or software that are central to the research but not yet described in published literature, software must be made available to editors and reviewers. We strongly encourage code deposition in a community repository (e.g. GitHub). See the Nature Portfolio guidelines for submitting code & software for further information.

Data

Policy information about availability of data

All manuscripts must include a data availability statement. This statement should provide the following information, where applicable:

- Accession codes, unique identifiers, or web links for publicly available datasets
- A description of any restrictions on data availability
- For clinical datasets or third party data, please ensure that the statement adheres to our policy

Publicly available genomes are listed in Supplementary Data 16. Supplementary Data 6 to 13 are also available in Figshare at https://figshare.com/s/0e723feb5add189d9bb6 (doi:10.6084/m9.figshare.20522103) . The scripts used in this study are available in GitHub at https://github.com/PhyLoss/PhyLoss.

Human research participants

Policy information about studies involving human research participants and Sex and Gender in Research.

Reporting on sex and gender	This study did not involve human participants.
Population characteristics	This study did not involve human participants.
Recruitment	This study did not involve human participants.
Ethics oversight	This study did not involve human participants.

Note that full information on the approval of the study protocol must also be provided in the manuscript.

Field-specific reporting

Please select the one below that is the best fit for your research. If you are not sure, read the appropriate sections before making your selection.

☒ Life sciences ☐ Behavioural & social sciences ☐ Ecological, evolutionary & environmental sciences

For a reference copy of the document with all sections, see nature.com/documents/nr-reporting-summary-flat.pdf

Life sciences study design

All studies must disclose on these points even when the disclosure is negative.

Sample size	For the calculation of gain-and-loss patters, we retrieved 667 reference genomes from public databases, which is so far the richest collection of taxa used in this type of analysis. The choice of taxa was dictated by phylogenetic nodes that were present along lineages of focal species and by the availability of reference genomes.
Data exclusions	No data were excluded from the analyses.
Replication	This is not experimental or simulation study, thus experimental replication is not applicable.
Randomization	No randomization was required in the analysis.
Blinding	No blinding was required.

Reporting for specific materials, systems and methods

We require information from authors about some types of materials, experimental systems and methods used in many studies. Here, indicate whether each material, system or method listed is relevant to your study. If you are not sure if a list item applies to your research, read the appropriate section before selecting a response.

Materials & experimental systems

n/a	Involved in the study	
☒	☐	Antibodies
☒	☐	Eukaryotic cell lines
☒	☐	Palaeontology and archaeology
☒	☐	Animals and other organisms
☒	☐	Clinical data
☒	☐	Dual use research of concern

Methods

n/a	Involved in the study	
☒	☐	ChIP-seq
☒	☐	Flow cytometry
☒	☐	MRI-based neuroimaging
