# Supplementary material for: Macroevolutionary dynamics of gene family gain and loss along multicellular eukaryotic lineages
Source: Nat Commun. 2024 Mar 26;15:2663. doi: 10.1038/s41467-024-47017-w (PMC10966110; doi:10.1038/s41467-024-47017-w)
Supplement: Supplementary file 5 — Description of Additional Supplementary Files [file 41467_2024_47017_MOESM5_ESM.docx]

**Description of Additional Supplementary Files**

Supplementary Data 1

Description: The consensus phylogeny of 667 species used to estimate gene family gain and loss patterns.

Supplementary Data 2

Description: The number of total, gained and lost gene families per phylogenetic node depending on the c-value in the four focal species.

Supplementary Data 3

Description: The number of total gene families along all 352 eukaryotic lineages (charts).

Supplementary Data 4

Description: Evolutionary dynamics of gene family gain and loss ratios (all c-values).

Supplementary Data 5

Description: The enrichment of COG functional categories in gained and lost gene families in the four focal species (all c-values).

Supplementary Data 6

Description: The enrichment of GO functional categories in gained gene families along the *H. sapiens* lineage.

Supplementary Data 7

Description: The enrichment of GO functional categories in gained gene families along the *D. melanogaster* lineage.

Supplementary Data 8

Description: The enrichment of GO functional categories in gained gene families along the *S. cerevisiae* lineage.

Supplementary Data 9

Description: The enrichment of GO functional categories in gained gene families along the *A. thaliana* lineage.

Supplementary Data 10

Description: The enrichment of GO functional categories in lost gene families along the *H. sapiens* lineage.

Supplementary Data 11

Description: The enrichment of GO functional categories in lost gene families along the *D. melanogaster* lineage.

Supplementary Data 12

Description: The enrichment of GO functional categories in lost gene families along the *S. cerevisiae* lineage.

Supplementary Data 13

Description: The enrichment of GO functional categories in lost gene families along the *A. thaliana* lineage.
