# Supplementary figures and images for: Macroevolutionary dynamics of gene family gain and loss along multicellular eukaryotic lineages
Source: Nat Commun. 2024 Mar 26;15:2663. doi: 10.1038/s41467-024-47017-w (PMC10966110; doi:10.1038/s41467-024-47017-w)

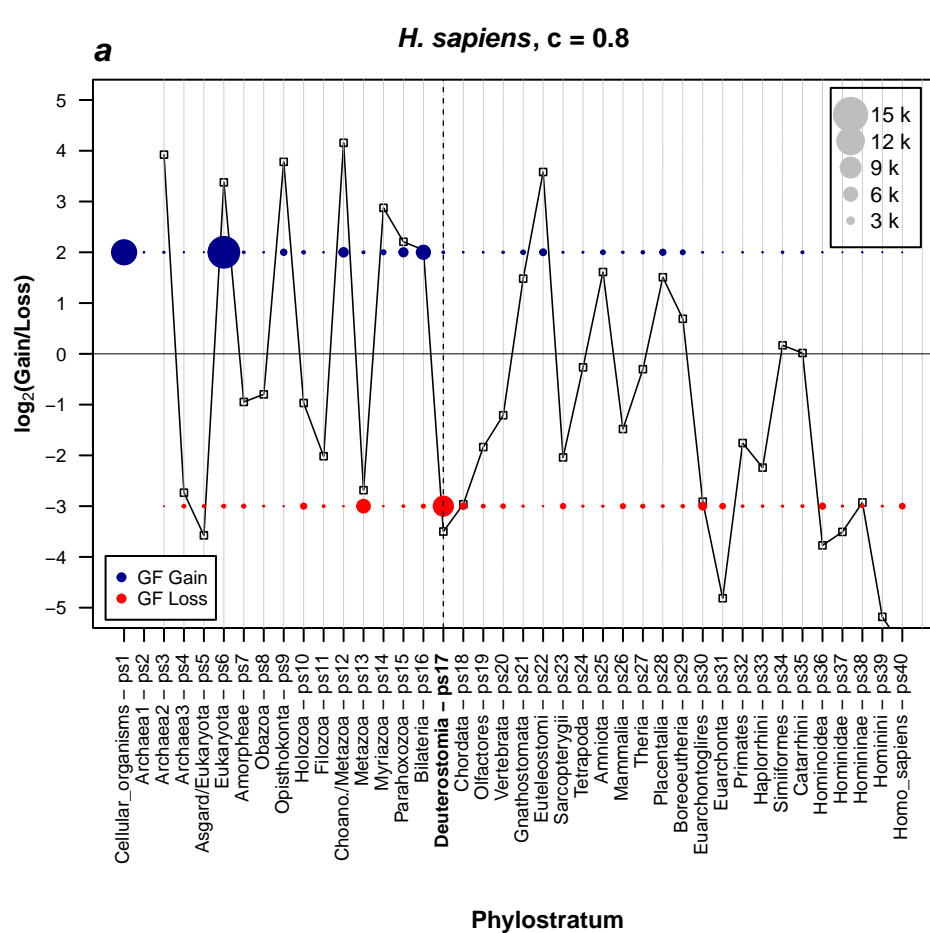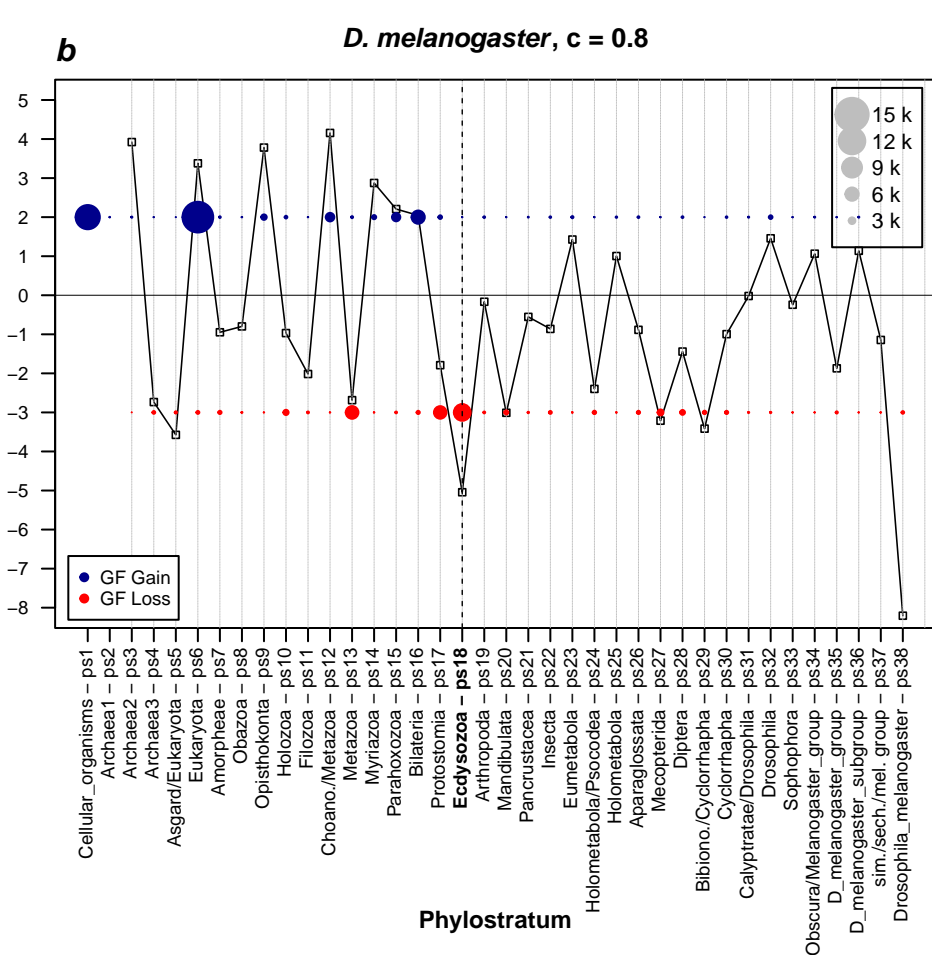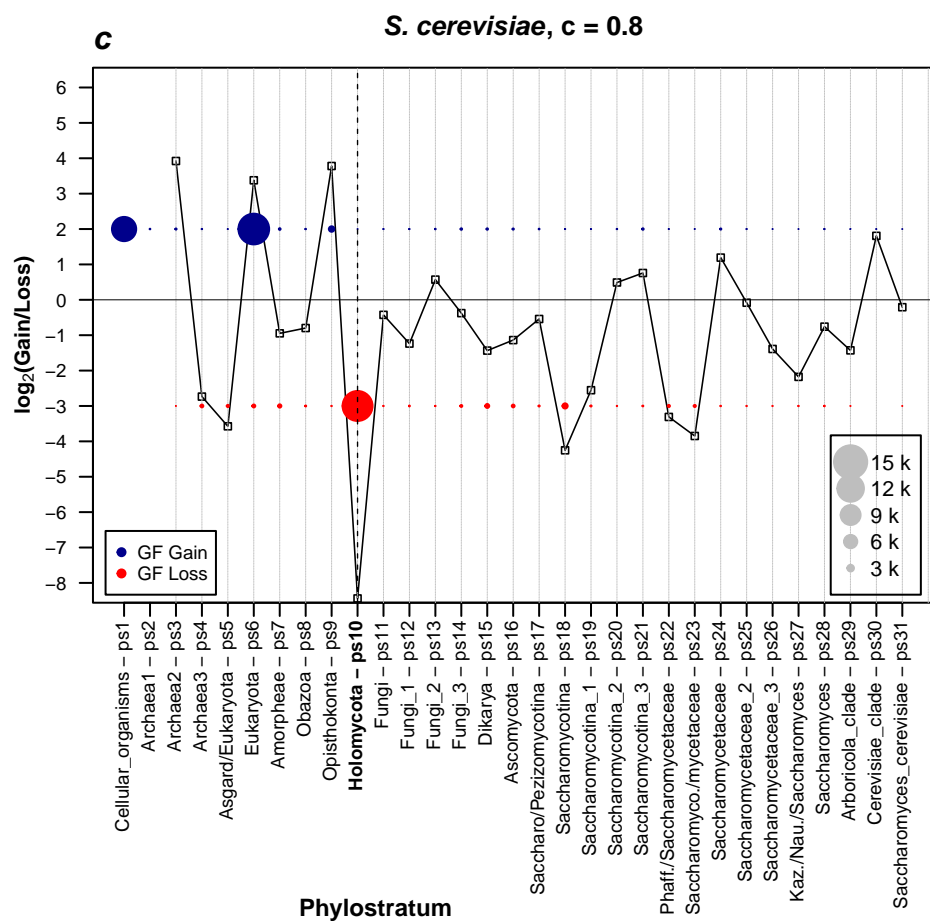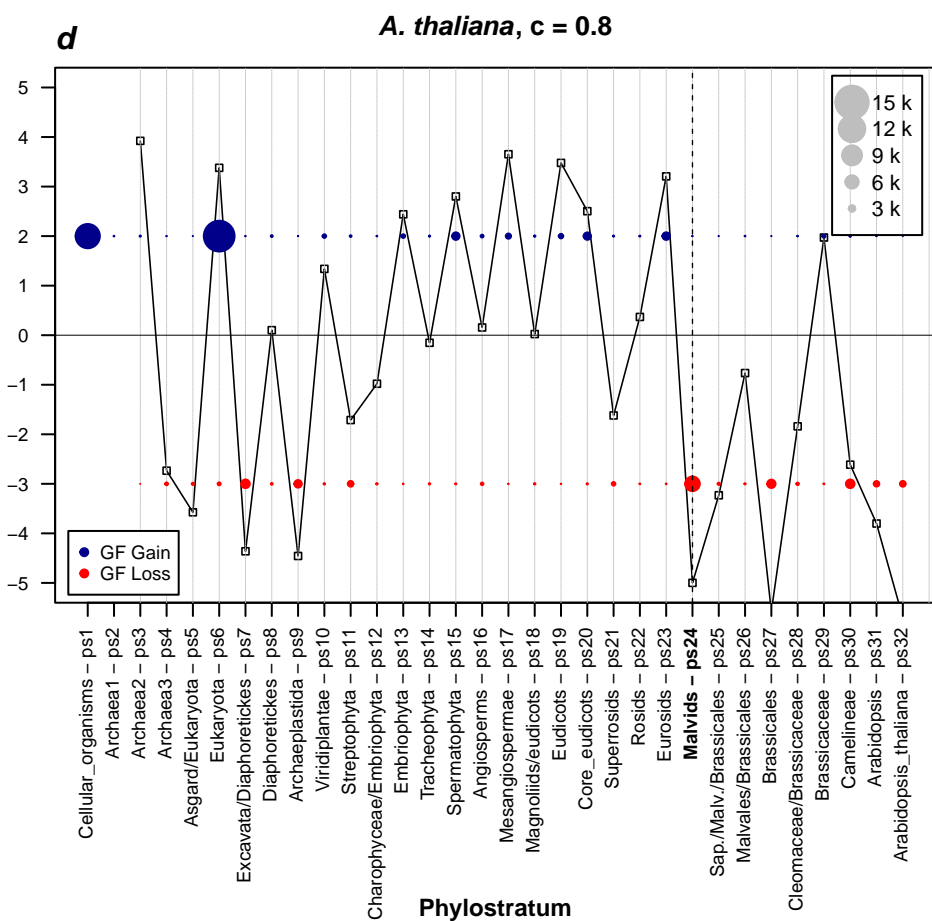

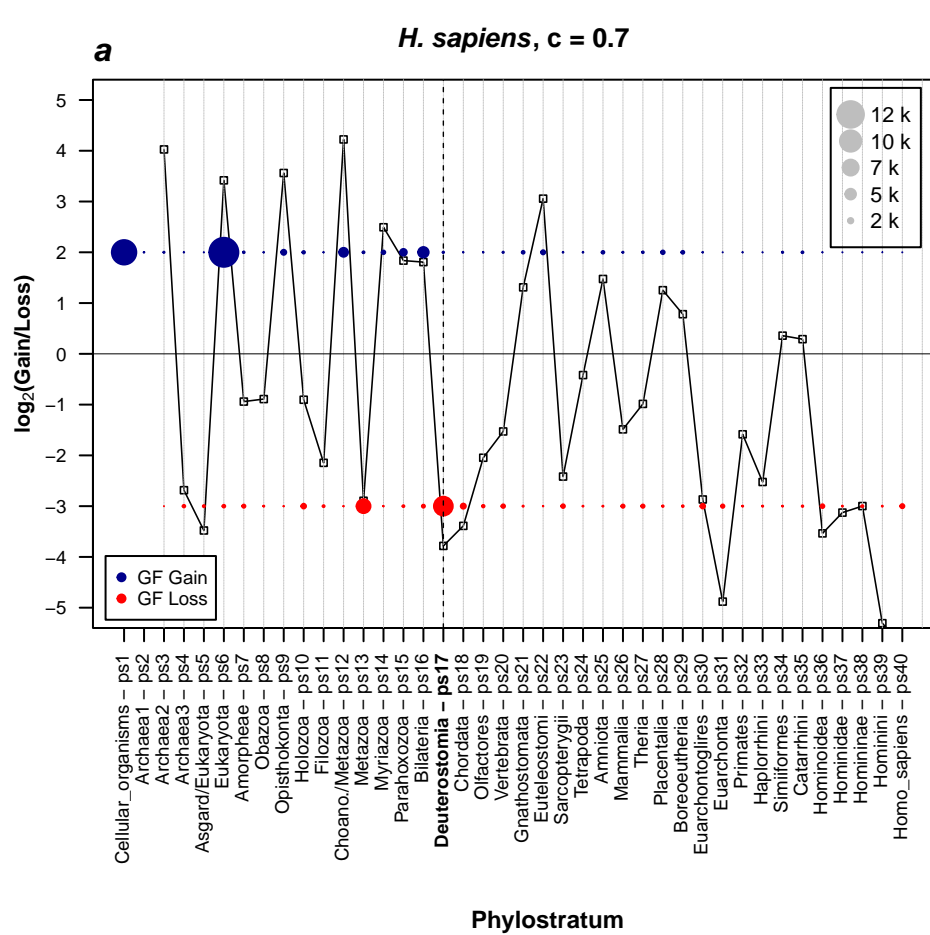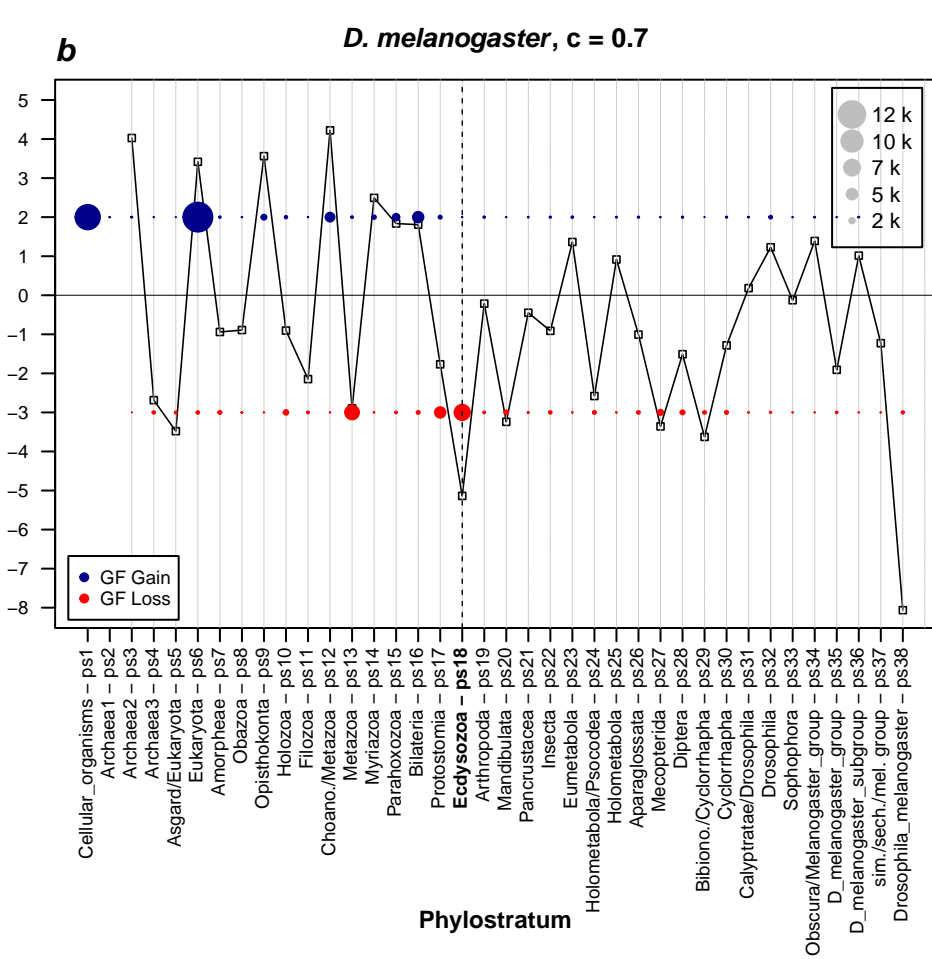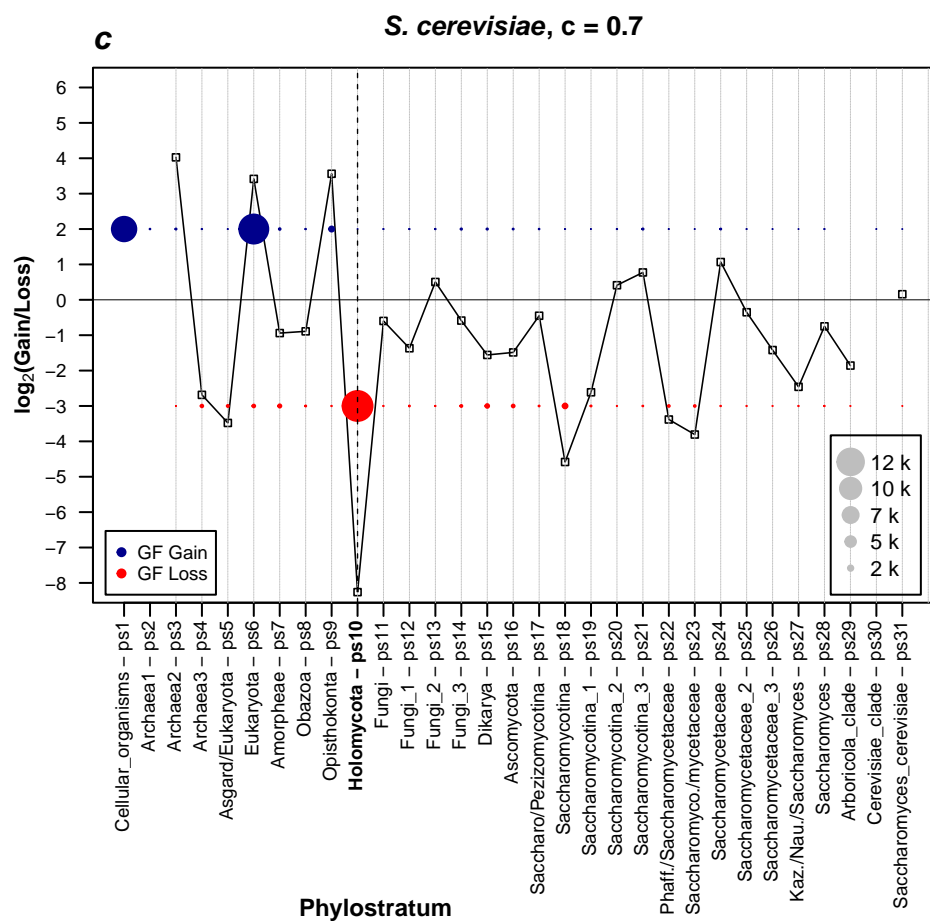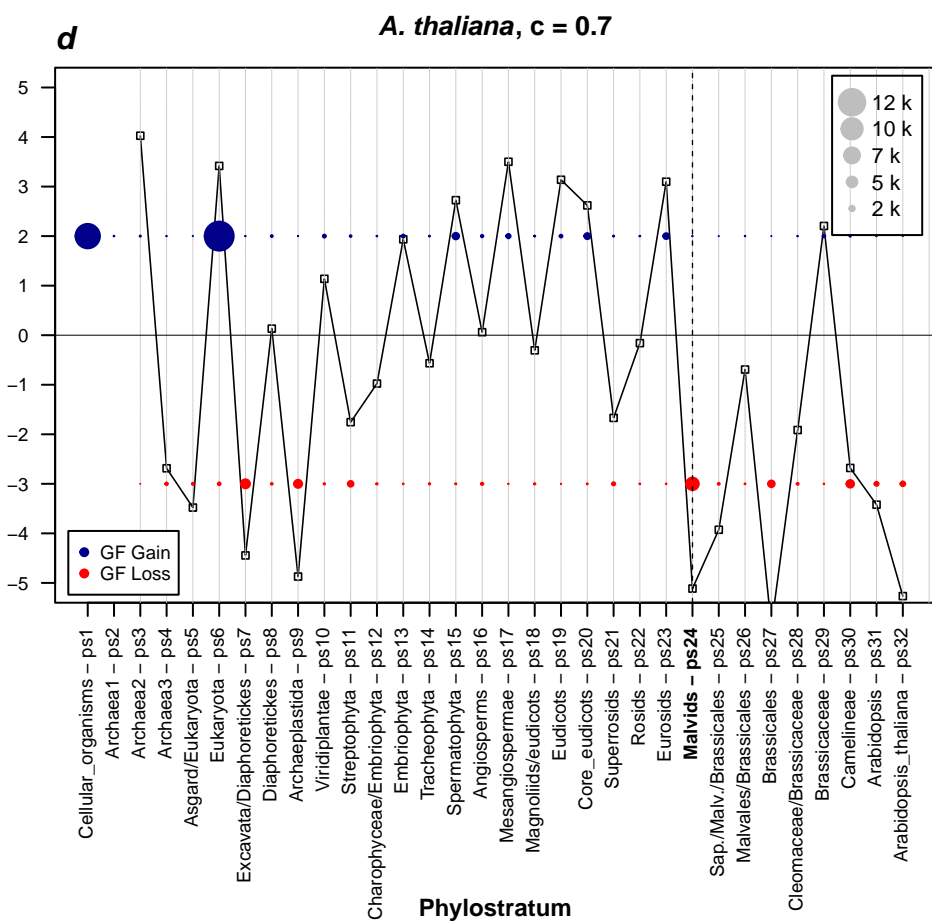

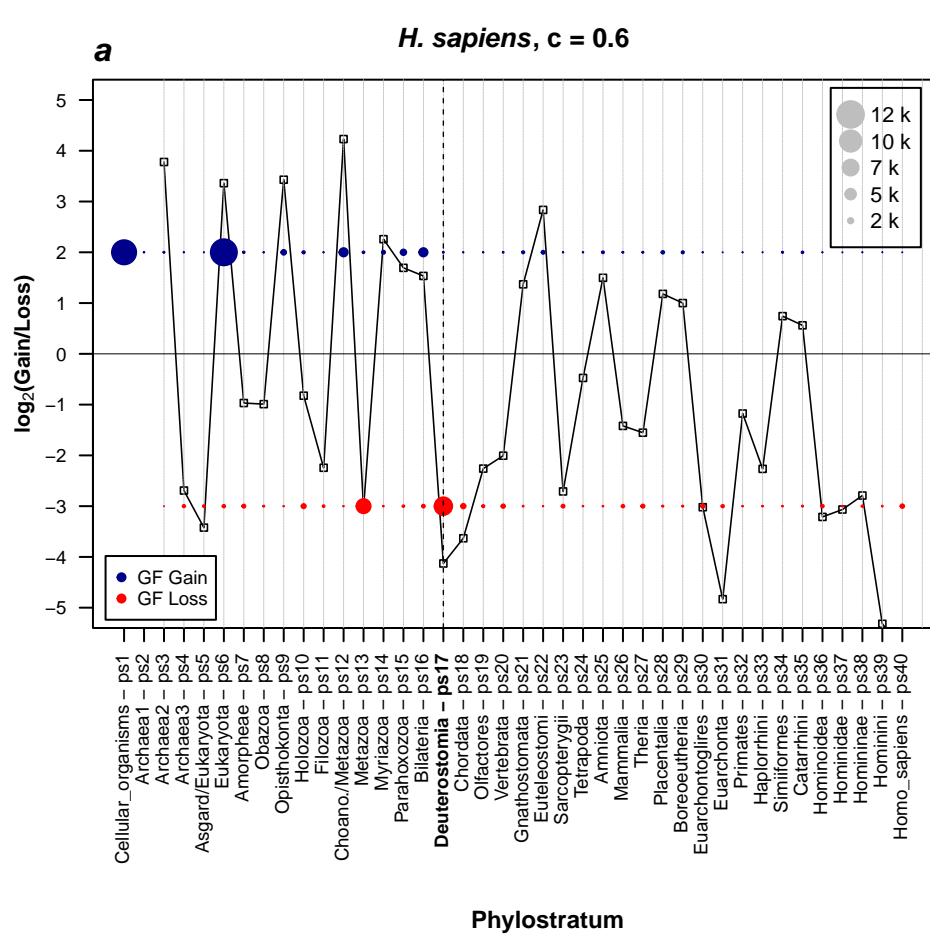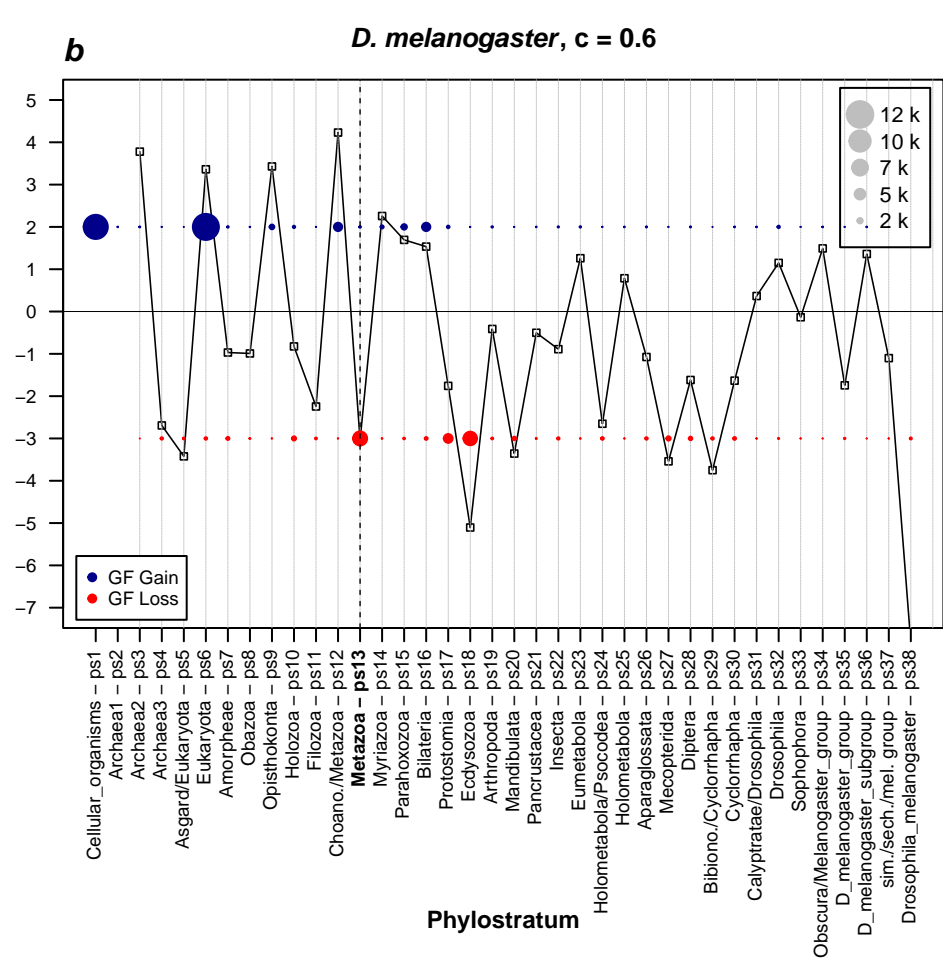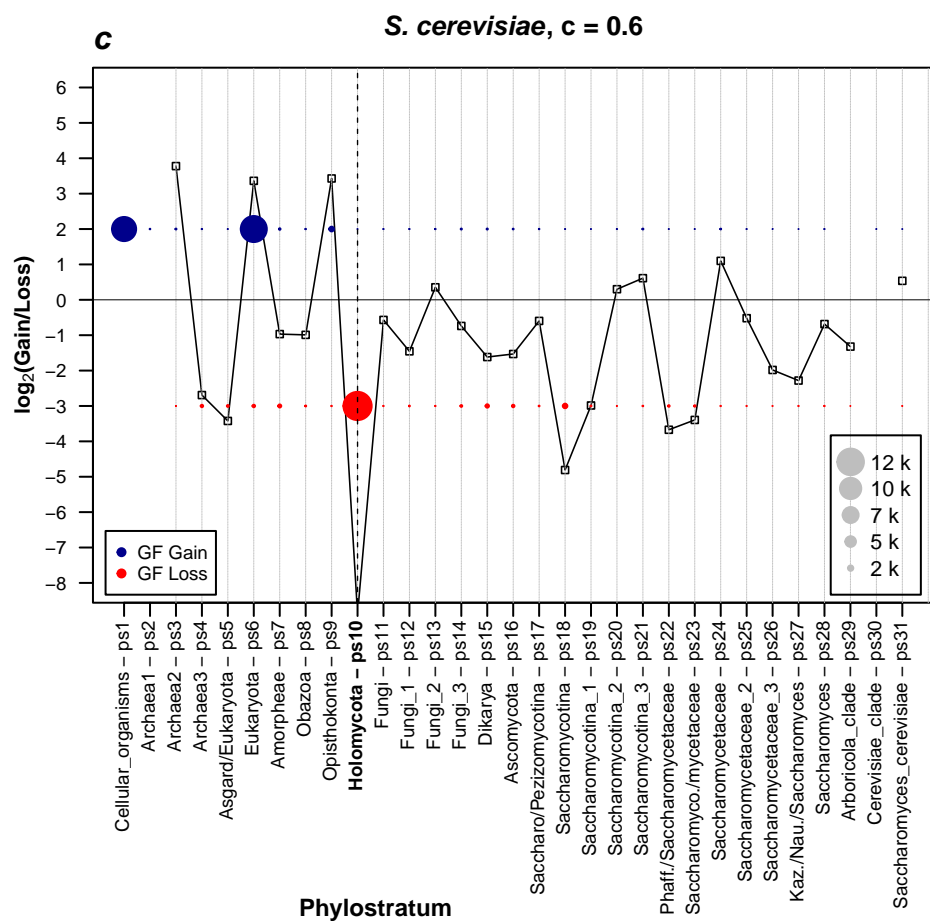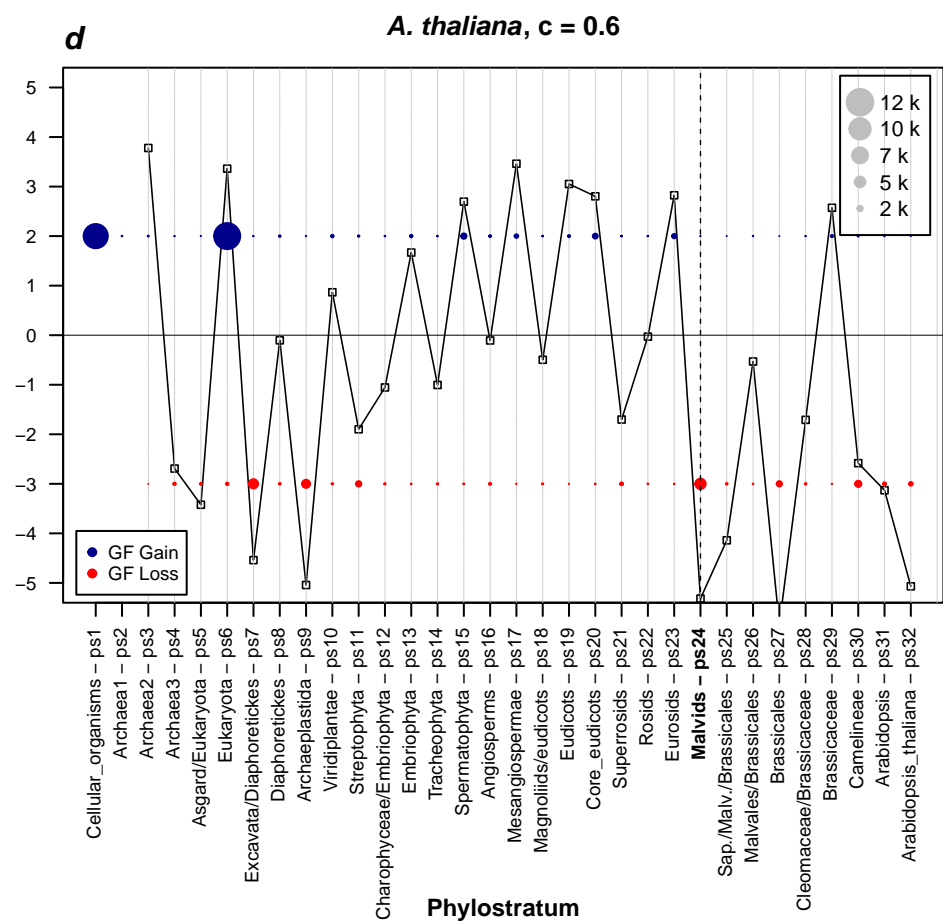

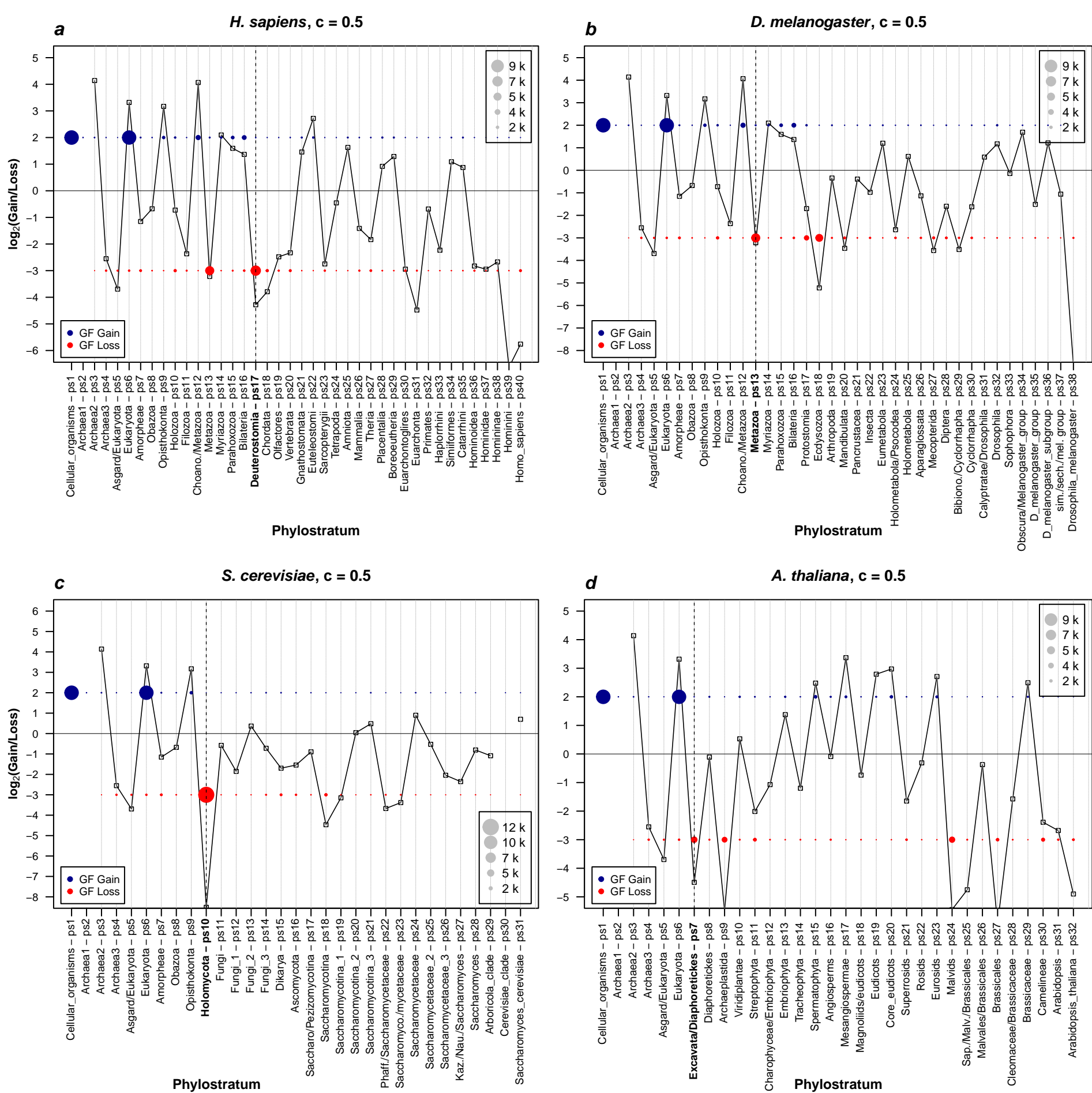

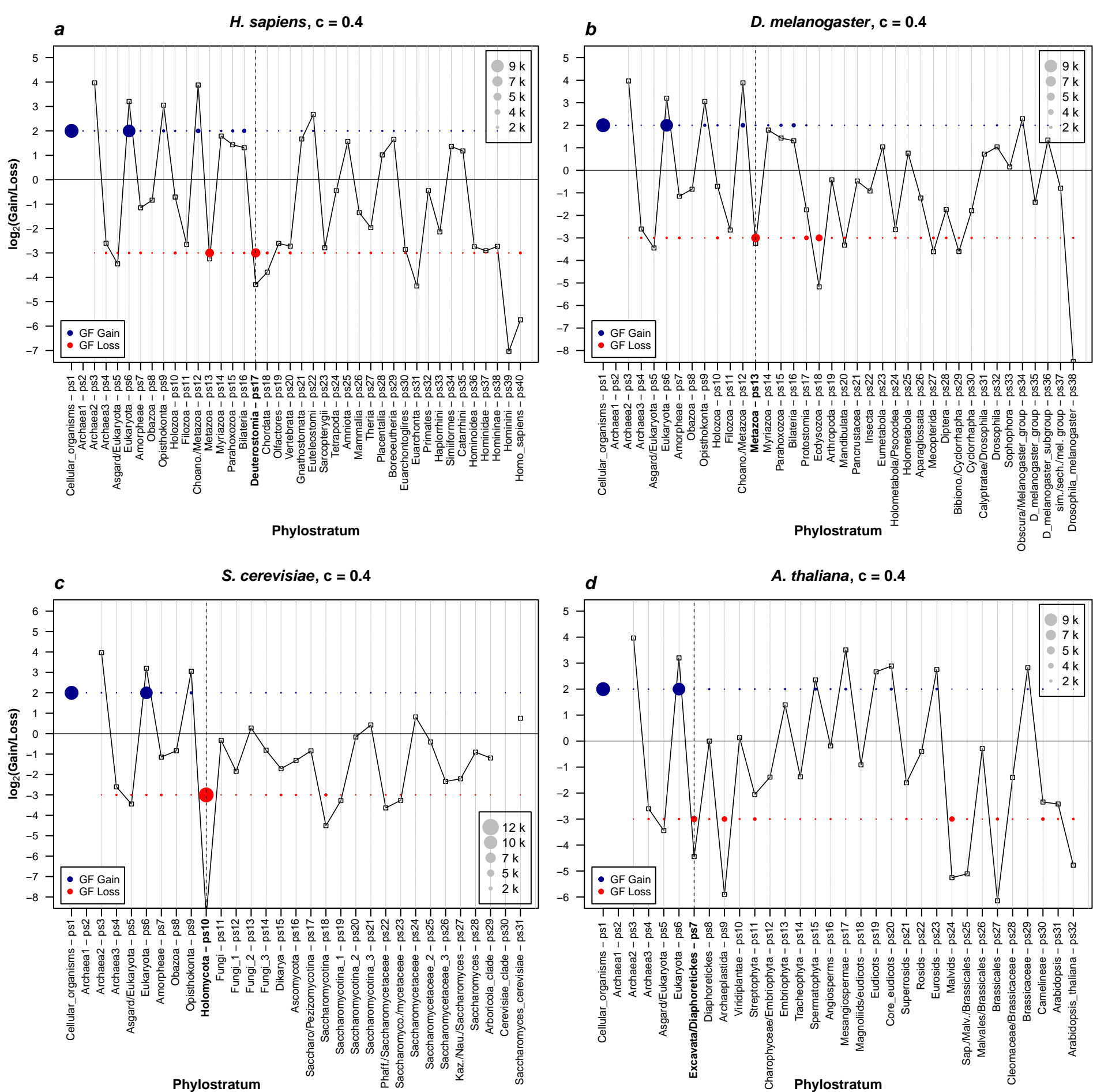

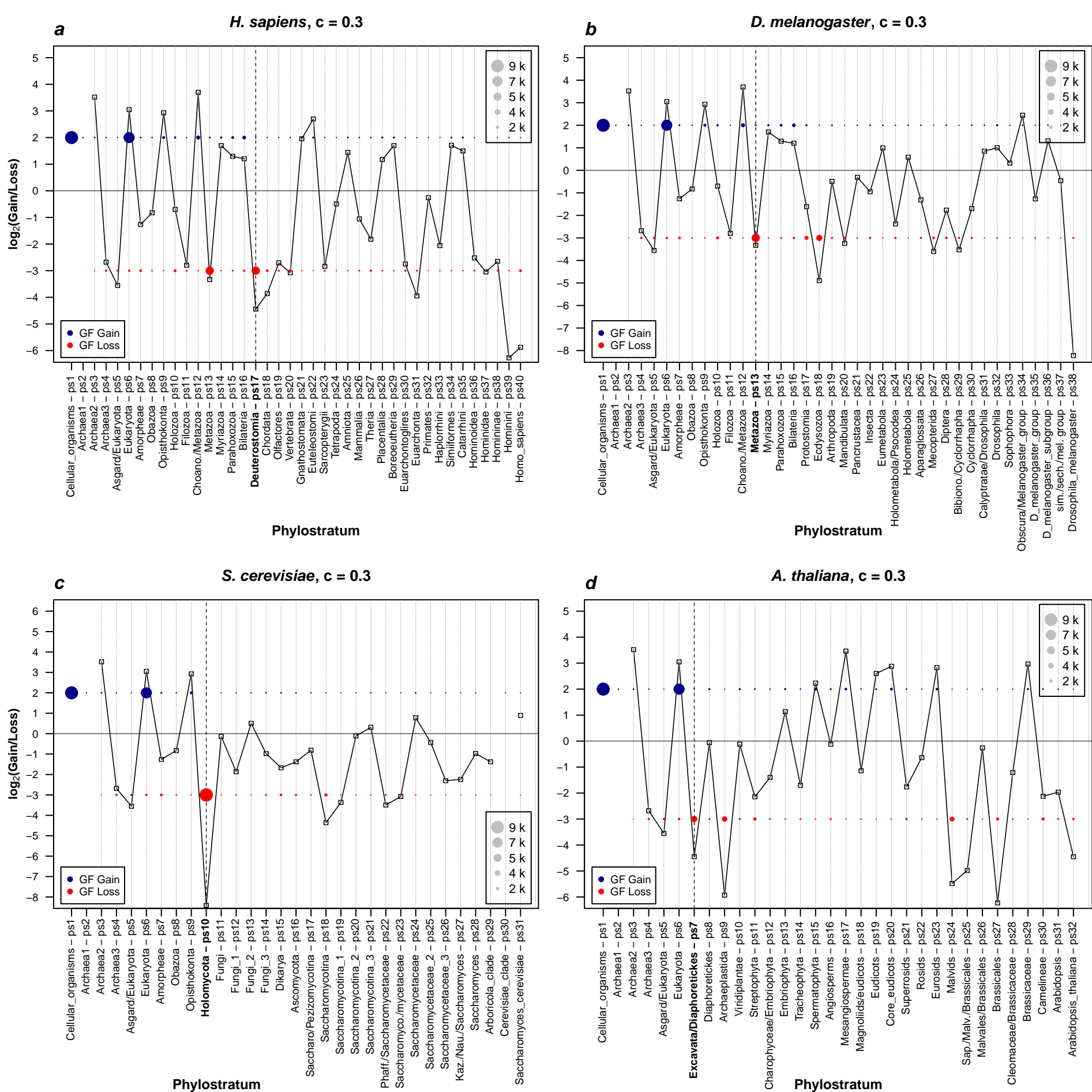

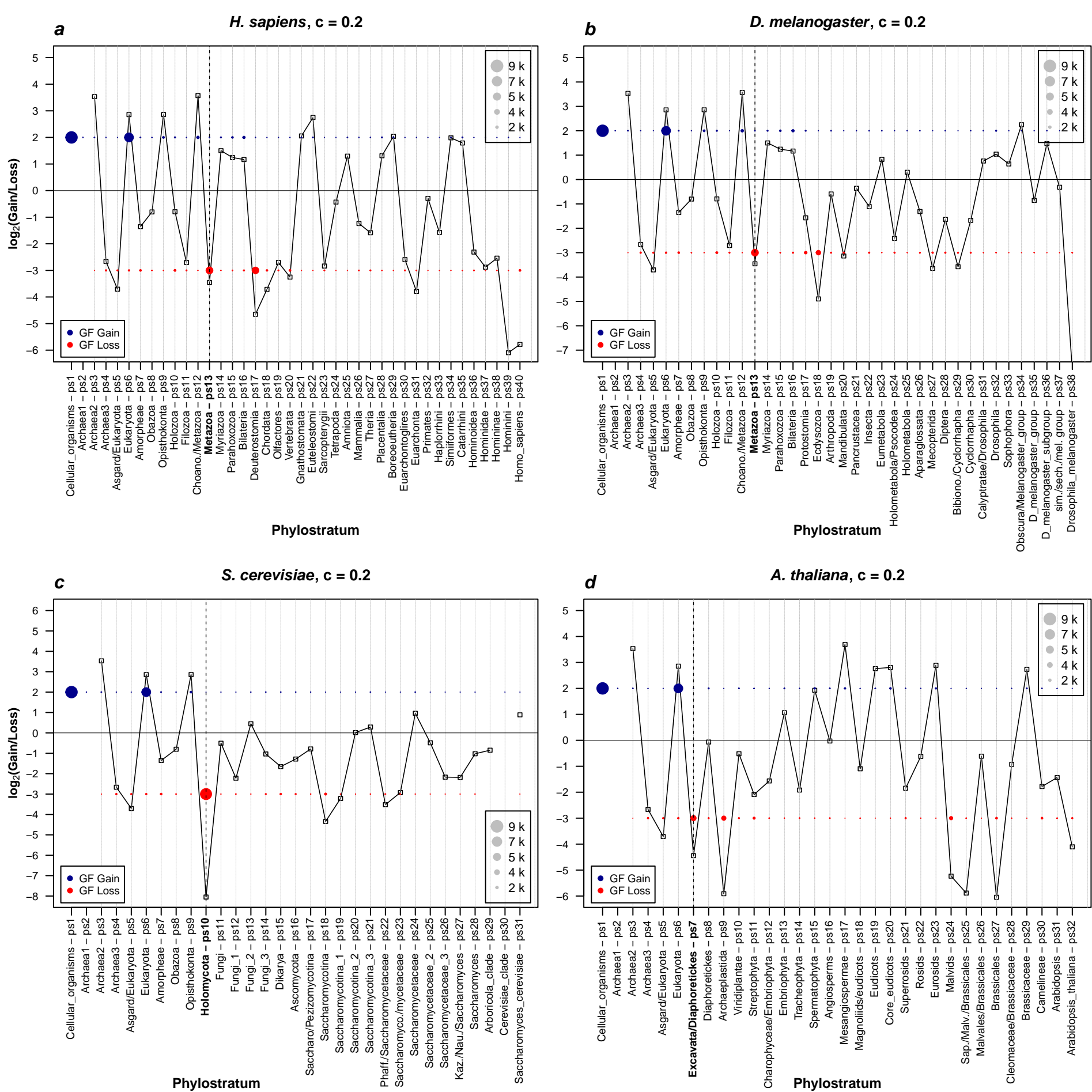

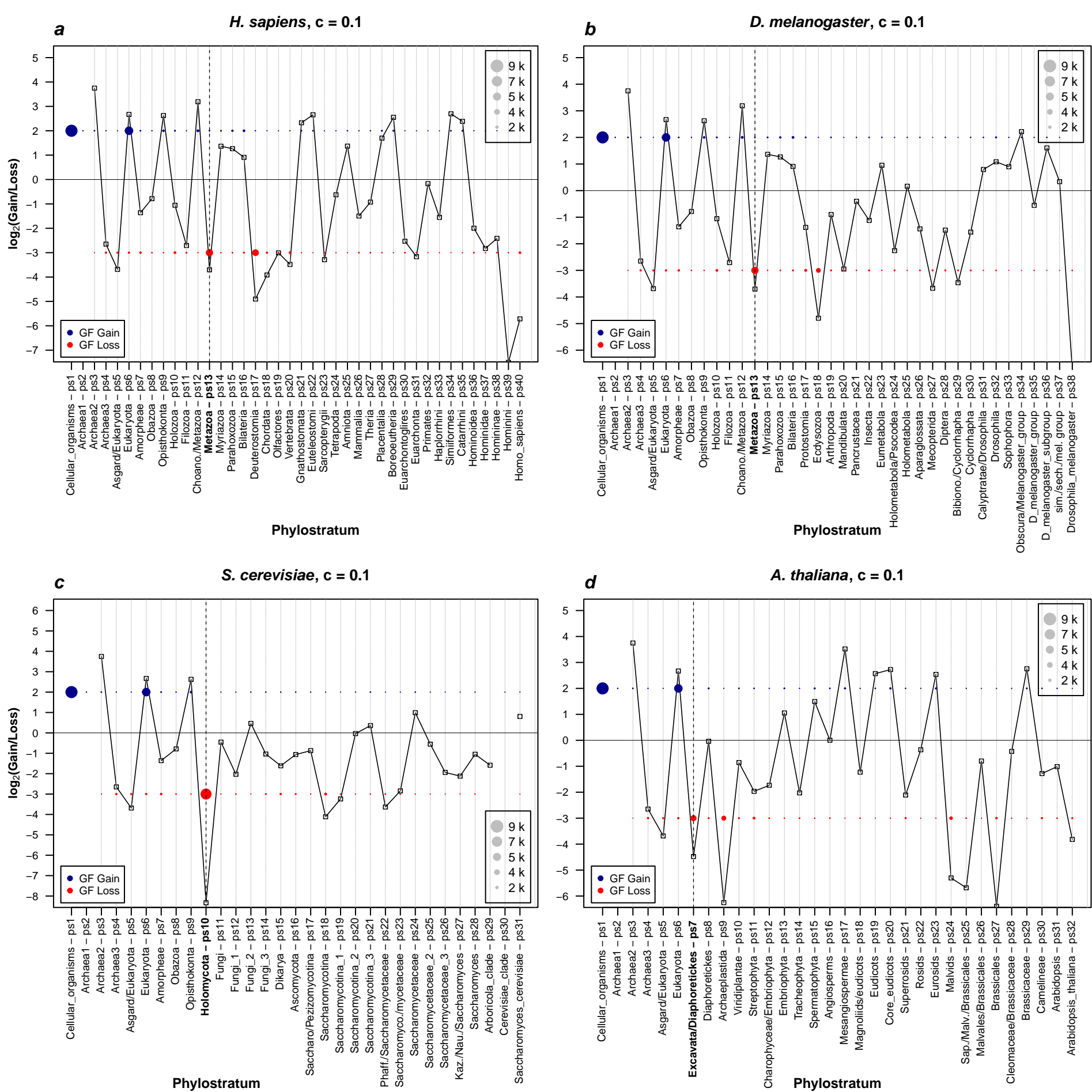

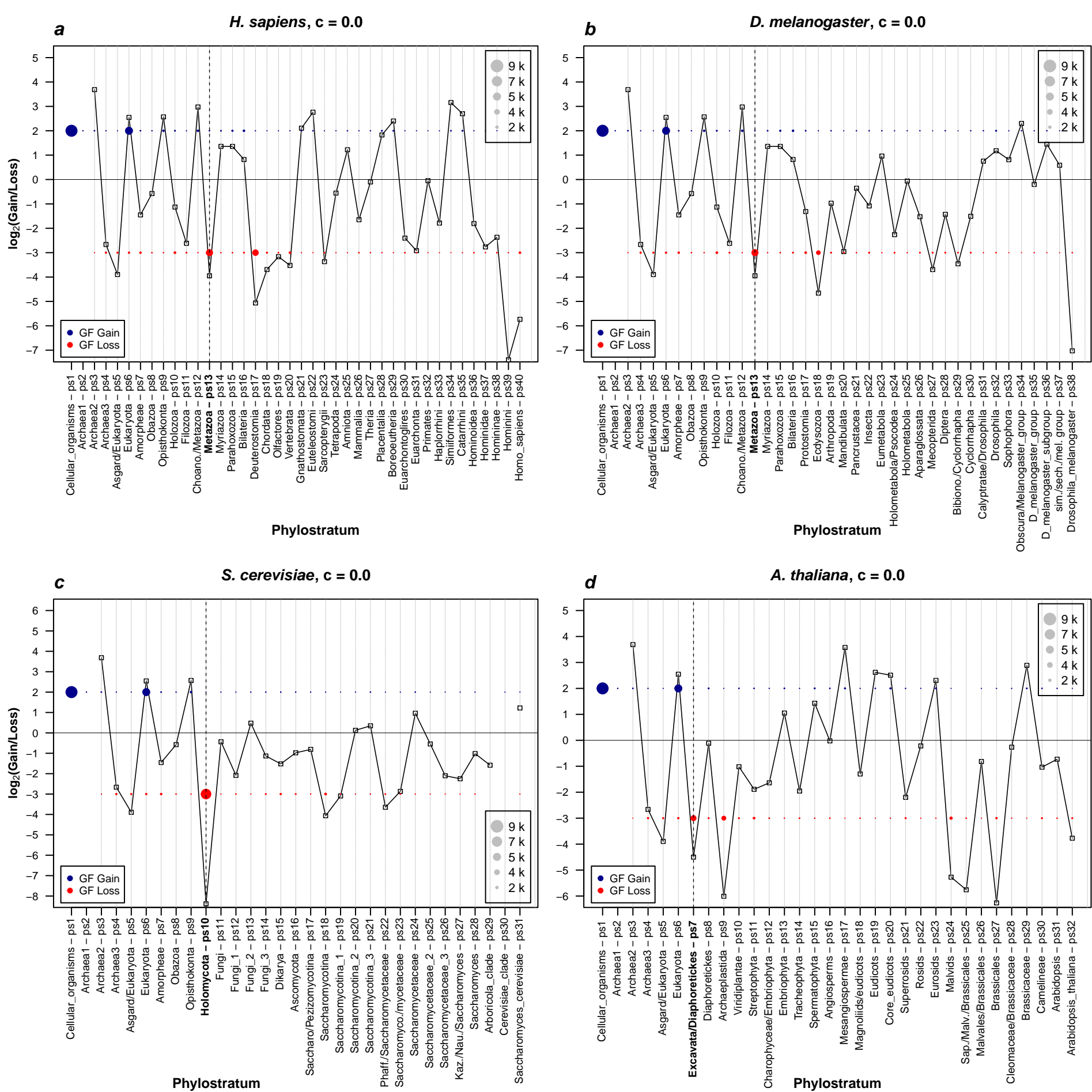

Supplement: Supplementary file 9 — Supplementary Dataset 4 [file 41467_2024_47017_MOESM9_ESM.pdf]
