# Supplementary material for: Macroevolutionary dynamics of gene family gain and loss along multicellular eukaryotic lineages
Source: Nat Commun. 2024 Mar 26;15:2663. doi: 10.1038/s41467-024-47017-w (PMC10966110; doi:10.1038/s41467-024-47017-w)
Supplement: Supplementary file 10 — Supplementary Dataset 5 [file 41467_2024_47017_MOESM10_ESM.pdf]

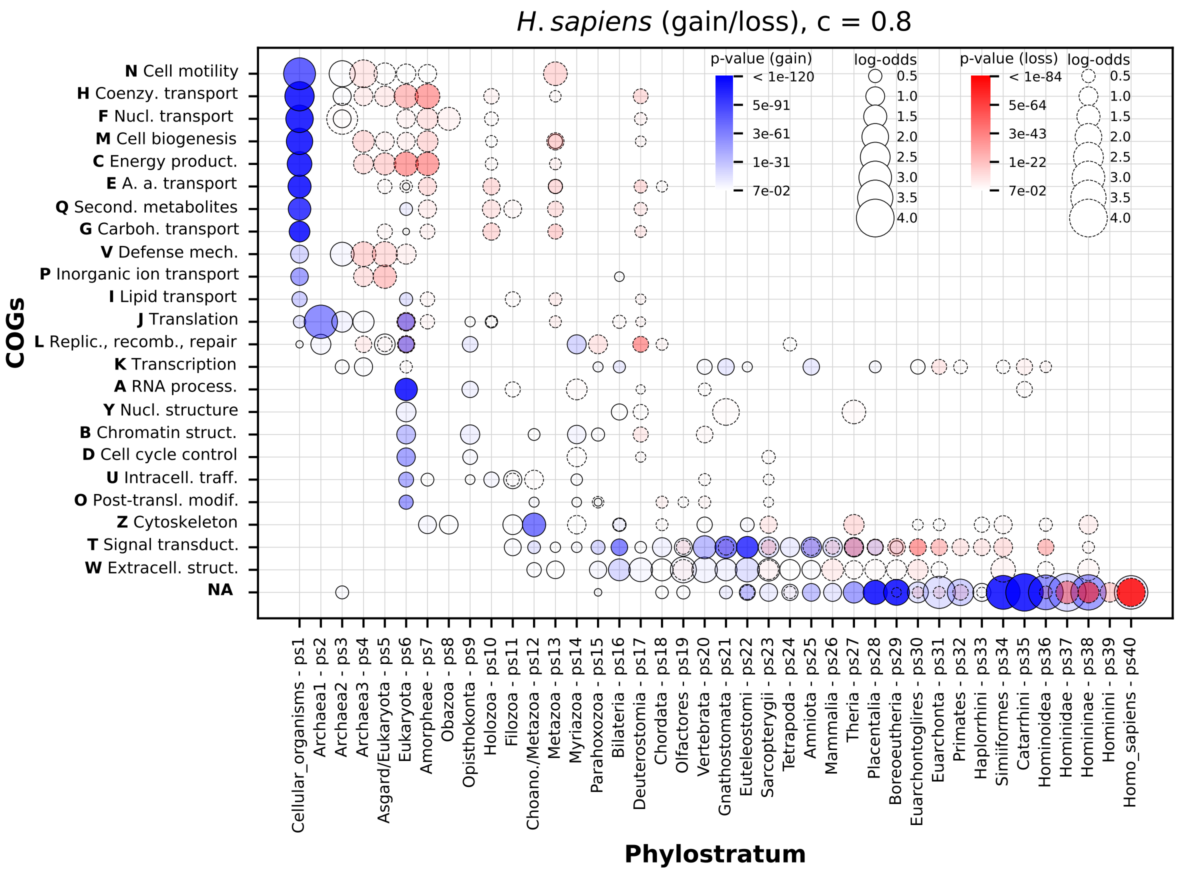
H. sapiens (gain/loss), c = 0.8
p-value (gain)
< 1e-120
5e-91
3e-61
1e-31
7e-02
log-odds
0.5
1.0
1.5
2.0
2.5
3.0
3.5
4.0
p-value (loss)
< 1e-84
5e-64
3e-43
1e-22
7e-02
log-odds
COGs
N Cell motility
H Coenzy. transport
F Nucl. transport
M Cell biogenesis
C Energy product.
E A. a. transport
Q Second. metabolites
G Carboh. transport
V Defense mech.
P Inorganic ion transport
I Lipid transport
J Translation
L Replic., recomb., repair
K Transcription
A RNA process.
Y Nucl. structure
B Chromatin struct.
D Cell cycle control
U Intracell. traff.
O Post-transl. modif.
Z Cytoskeleton
T Signal transduct.
W Extracell. struct.
NA
Cellular_organisms - ps1
Archaea1 - ps2
Archaea2 - ps3
Archaea3 - ps4
Asgard/Eukaryota - ps5
Eukaryota - ps6
Amorpheae - ps7
Obazoa - ps8
Opisthokonta - ps9
Holozoa - ps10
Filozoa - ps11
Choano./Metazoa - ps12
Metazoa - ps13
Myriazoa - ps14
Parahoxozoa - ps15
Bilateria - ps16
Deuterostomia - ps17
Chordata - ps18
Olfactores - ps19
Vertebrata - ps20
Gnathostomata - ps21
Euteleostomi - ps22
Sarcopterygii - ps23
Tetrapoda - ps24
Amniota - ps25
Mammalia - ps26
Theria - ps27
Placentalia - ps28
Boreoeutheria - ps29
Euarchontoglires - ps30
Euarchonta - ps31
Primates - ps32
Haplorrhini - ps33
Simiiformes - ps34
Catarrhini - ps35
Hominoidea - ps36
Hominidae - ps37
Homininae - ps38
Hominini - ps39
Homo_sapiens - ps40
Phylostratum

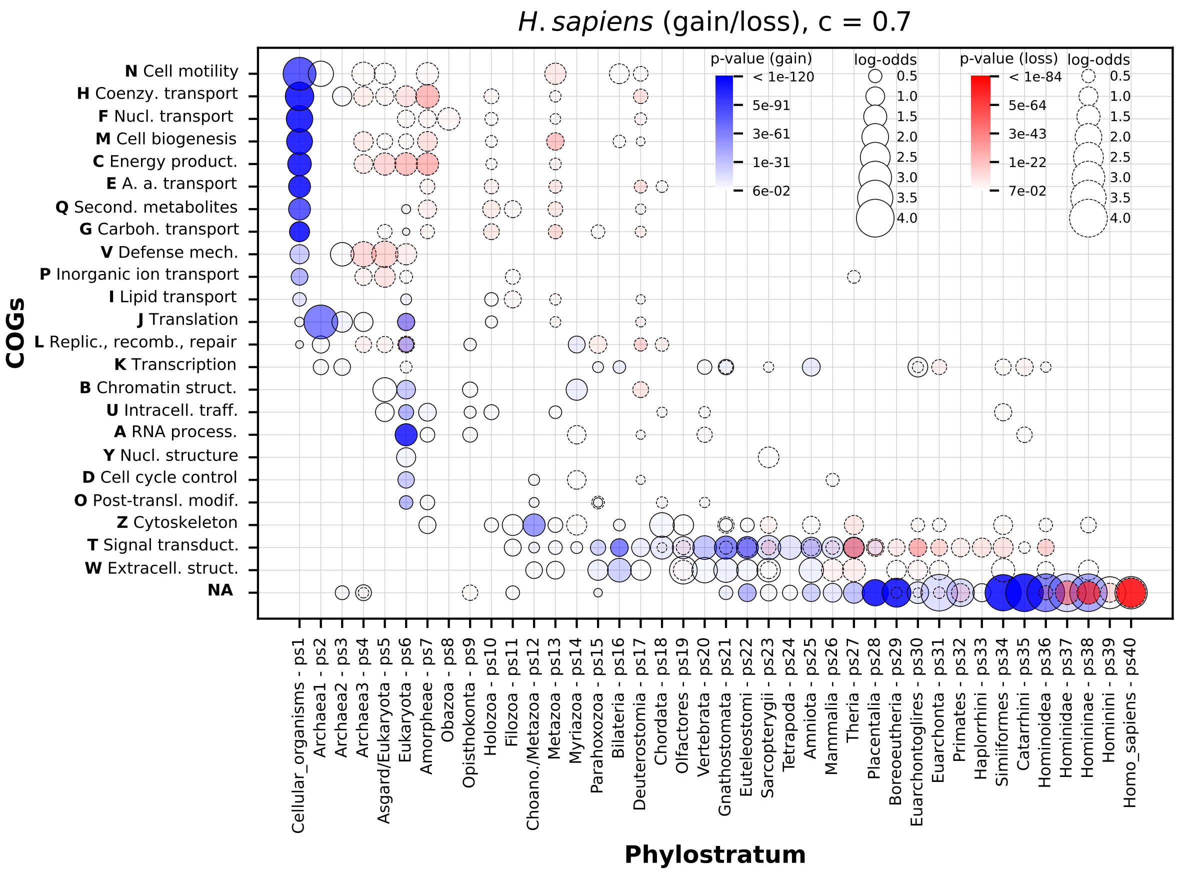

H. sapiens (gain/loss), c = 0.7
p-value (gain)
< 1e-120
5e-91
3e-61
1e-31
6e-02
log-odds
0.5
1.0
1.5
2.0
2.5
3.0
3.5
4.0
p-value (loss)
< 1e-84
5e-64
3e-43
1e-22
7e-02
log-odds
0.5
1.0
1.5
2.0
2.5
3.0
3.5
4.0
COGs
N Cell motility
H Coenzy. transport
F Nucl. transport
M Cell biogenesis
C Energy product.
E A. a. transport
Q Second. metabolites
G Carboh. transport
V Defense mech.
P Inorganic ion transport
I Lipid transport
J Translation
L Replic., recomb., repair
K Transcription
B Chromatin struct.
U Intracell. traff.
A RNA process.
Y Nucl. structure
D Cell cycle control
O Post-transl. modif.
Z Cytoskeleton
T Signal transduct.
W Extracell. struct.
NA
Phylostratum
Cellular_organisms - ps1
Archaea1 - ps2
Archaea2 - ps3
Archaea3 - ps4
Asgard/Eukaryota - ps5
Eukaryota - ps6
Amorpheae - ps7
Obazoa - ps8
Opisthokonta - ps9
Holozoa - ps10
Filozoa - ps11
Choano./Metazoa - ps12
Metazoa - ps13
Myriazoa - ps14
Parahoxozoa - ps15
Bilateria - ps16
Deuterostomia - ps17
Chordata - ps18
Olfactores - ps19
Vertebrata - ps20
Gnathostomata - ps21
Euteleostomi - ps22
Sarcopterygii - ps23
Tetrapoda - ps24
Amniota - ps25
Mammalia - ps26
Theria - ps27
Placentalia - ps28
Boreoeutheria - ps29
Euarchontoglires - ps30
Euarchonta - ps31
Primates - ps32
Haplorrhini - ps33
Simiiformes - ps34
Catarrhini - ps35
Hominoidea - ps36
Hominidae - ps37
Homininae - ps38
Hominini - ps39
Homo_sapiens - ps40

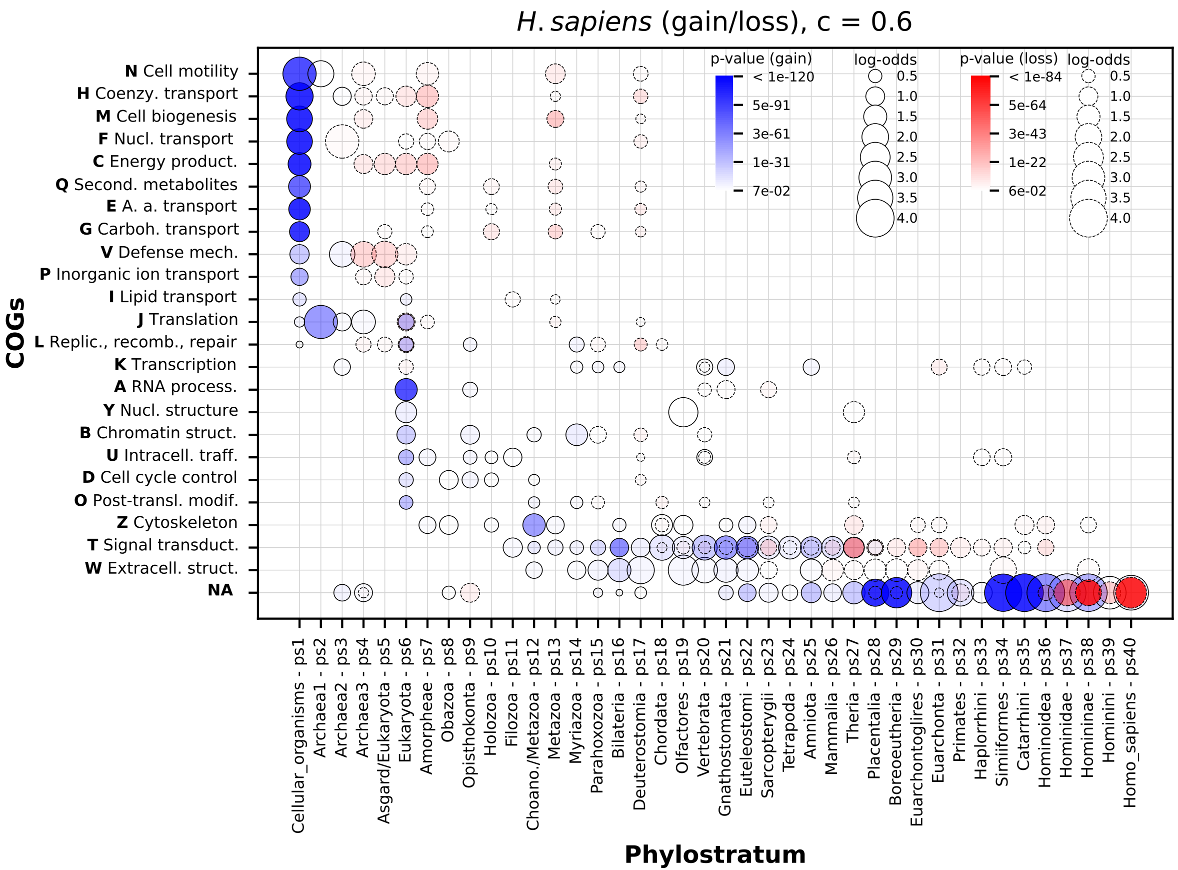

H. sapiens (gain/loss), c = 0.6
p-value (gain)
< 1e-120
5e-91
3e-61
1e-31
7e-02
log-odds
0.5
1.0
1.5
2.0
2.5
3.0
3.5
4.0
p-value (loss)
< 1e-84
5e-64
3e-43
1e-22
6e-02
COGs
N Cell motility
H Coenzy. transport
M Cell biogenesis
F Nucl. transport
C Energy product.
Q Second. metabolites
E A. a. transport
G Carboh. transport
V Defense mech.
P Inorganic ion transport
I Lipid transport
J Translation
L Replic., recomb., repair
K Transcription
A RNA process.
Y Nucl. structure
B Chromatin struct.
U Intracell. traff.
D Cell cycle control
O Post-transl. modif.
Z Cytoskeleton
T Signal transduct.
W Extracell. struct.
NA
Cellular_organisms - ps1
Archaea1 - ps2
Archaea2 - ps3
Archaea3 - ps4
Asgard/Eukaryota - ps5
Eukaryota - ps6
Amorpheae - ps7
Obazoa - ps8
Opisthokonta - ps9
Holozoa - ps10
Filozoa - ps11
Choano./Metazoa - ps12
Metazoa - ps13
Myriazoa - ps14
Parahoxozoa - ps15
Bilateria - ps16
Deuterostomia - ps17
Chordata - ps18
Olfactores - ps19
Vertebrata - ps20
Gnathostomata - ps21
Euteleostomi - ps22
Sarcopterygii - ps23
Tetrapoda - ps24
Amniota - ps25
Mammalia - ps26
Theria - ps27
Placentalia - ps28
Boreoeutheria - ps29
Euarchontoglires - ps30
Euarchonta - ps31
Primates - ps32
Haplorrhini - ps33
Simiiformes - ps34
Catarrhini - ps35
Hominoidea - ps36
Hominidae - ps37
Homininae - ps38
Hominini - ps39
Homo_sapiens - ps40
Phylostratum

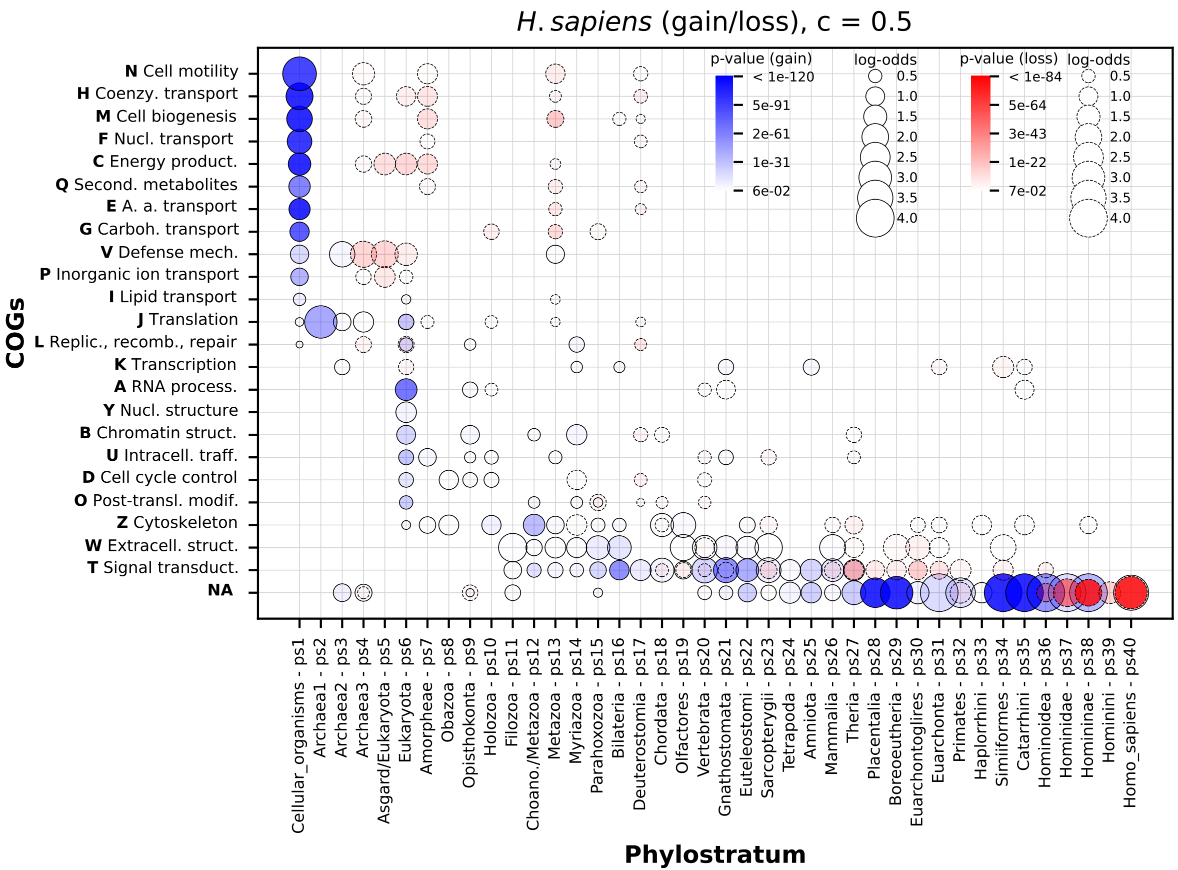
H. sapiens (gain/loss), c = 0.5
COGs
N Cell motility
H Coenzy. transport
M Cell biogenesis
F Nucl. transport
C Energy product.
Q Second. metabolites
E A. a. transport
G Carboh. transport
V Defense mech.
P Inorganic ion transport
I Lipid transport
J Translation
L Replic., recomb., repair
K Transcription
A RNA process.
Y Nucl. structure
B Chromatin struct.
U Intracell. traff.
D Cell cycle control
O Post-transl. modif.
Z Cytoskeleton
W Extracell. struct.
T Signal transduct.
NA
p-value (gain)
< 1e-120
5e-91
2e-61
1e-31
6e-02
log-odds
0.5
1.0
1.5
2.0
2.5
3.0
3.5
4.0
p-value (loss)
< 1e-84
5e-64
3e-43
1e-22
7e-02
log-odds
0.5
1.0
1.5
2.0
2.5
3.0
3.5
4.0
Cellular_organisms - ps1
Archaea1 - ps2
Archaea2 - ps3
Archaea3 - ps4
Asgard/Eukaryota - ps5
Eukaryota - ps6
Amorpheae - ps7
Obazoa - ps8
Opisthokonta - ps9
Holozoa - ps10
Filozoa - ps11
Choano./Metazoa - ps12
Metazoa - ps13
Myriazoa - ps14
Parahoxozoa - ps15
Bilateria - ps16
Deuterostomia - ps17
Chordata - ps18
Olfactores - ps19
Vertebrata - ps20
Gnathostomata - ps21
Euteleostomi - ps22
Sarcopterygii - ps23
Tetrapoda - ps24
Amniota - ps25
Mammalia - ps26
Theria - ps27
Placentalia - ps28
Boreoeutheria - ps29
Euarchontoglires - ps30
Euarchonta - ps31
Primates - ps32
Haplorrhini - ps33
Simiiformes - ps34
Catarrhini - ps35
Hominoidea - ps36
Hominidae - ps37
Homininae - ps38
Hominini - ps39
Homo_sapiens - ps40
Phylostratum

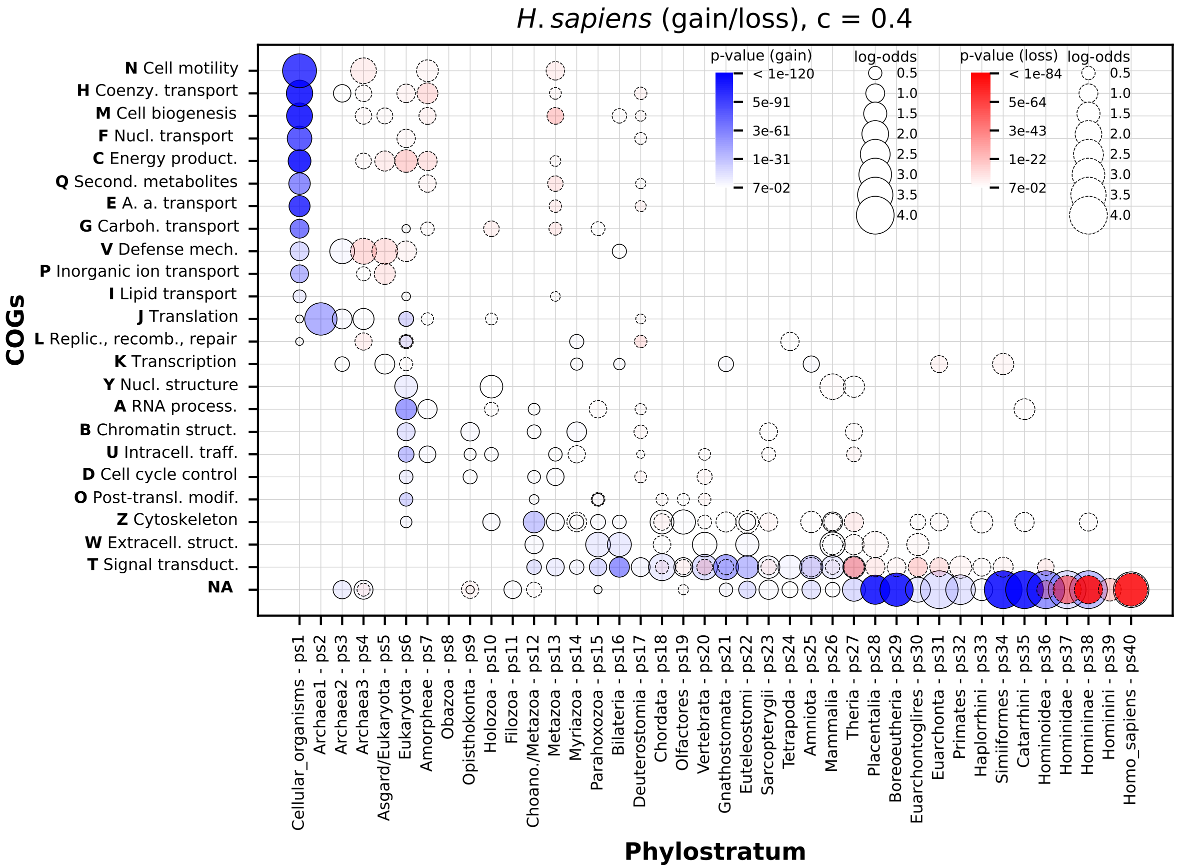

H. sapiens (gain/loss), c = 0.4
p-value (gain)
< 1e-120
5e-91
3e-61
1e-31
7e-02
log-odds
0.5
1.0
1.5
2.0
2.5
3.0
3.5
4.0
p-value (loss)
< 1e-84
5e-64
3e-43
1e-22
7e-02
log-odds
0.5
1.0
1.5
2.0
2.5
3.0
3.5
4.0
COGs
N Cell motility
H Coenzy. transport
M Cell biogenesis
F Nucl. transport
C Energy product.
Q Second. metabolites
E A. a. transport
G Carboh. transport
V Defense mech.
P Inorganic ion transport
I Lipid transport
J Translation
L Replic., recomb., repair
K Transcription
Y Nucl. structure
A RNA process.
B Chromatin struct.
U Intracell. traff.
D Cell cycle control
O Post-transl. modif.
Z Cytoskeleton
W Extracell. struct.
T Signal transduct.
NA
Phylostratum
Cellular_organisms - ps1
Archaea1 - ps2
Archaea2 - ps3
Archaea3 - ps4
Asgard/Eukaryota - ps5
Eukaryota - ps6
Amorpheae - ps7
Obazoa - ps8
Opisthokonta - ps9
Holozoa - ps10
Filozoa - ps11
Choano./Metazoa - ps12
Metazoa - ps13
Myriazoa - ps14
Parahoxozoa - ps15
Bilateria - ps16
Deuterostomia - ps17
Chordata - ps18
Olfactores - ps19
Vertebrata - ps20
Gnathostomata - ps21
Euteleostomi - ps22
Sarcopterygii - ps23
Tetrapoda - ps24
Amniota - ps25
Mammalia - ps26
Theria - ps27
Placentalia - ps28
Boreoeutheria - ps29
Euarchontoglires - ps30
Euarchonta - ps31
Primates - ps32
Haplorrhini - ps33
Simiiformes - ps34
Catarrhini - ps35
Hominoidea - ps36
Hominidae - ps37
Homininae - ps38
Hominini - ps39
Homo_sapiens - ps40

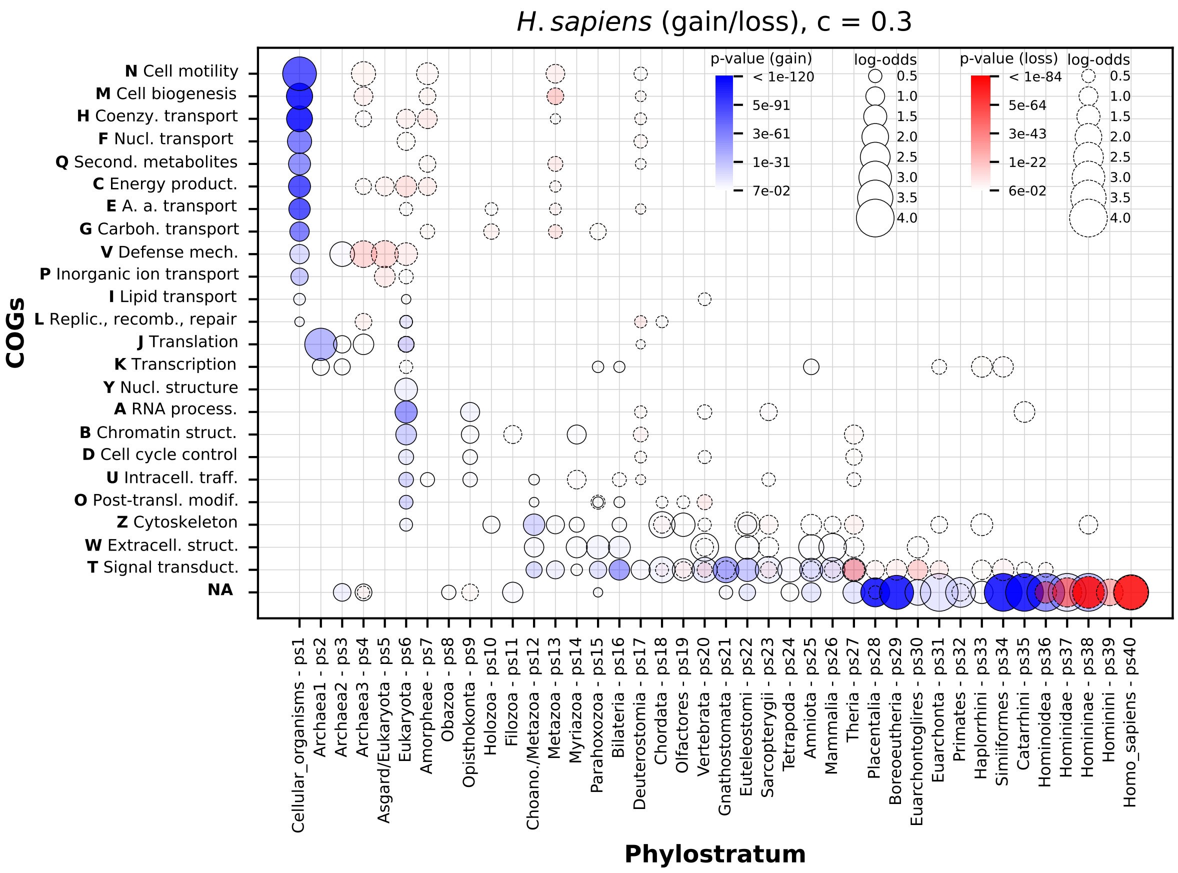

H. sapiens (gain/loss), c = 0.3
p-value (gain)
< 1e-120
5e-91
3e-61
1e-31
7e-02
log-odds
0.5
1.0
1.5
2.0
2.5
3.0
3.5
4.0
p-value (loss)
< 1e-84
5e-64
3e-43
1e-22
6e-02
COGs
N Cell motility
M Cell biogenesis
H Coenzy. transport
F Nucl. transport
Q Second. metabolites
C Energy product.
E A. a. transport
G Carboh. transport
V Defense mech.
P Inorganic ion transport
I Lipid transport
L Replic., recomb., repair
J Translation
K Transcription
Y Nucl. structure
A RNA process.
B Chromatin struct.
D Cell cycle control
U Intracell. traff.
O Post-transl. modif.
Z Cytoskeleton
W Extracell. struct.
T Signal transduct.
NA
Phylostratum
Cellular_organisms - ps1
Archaea1 - ps2
Archaea2 - ps3
Archaea3 - ps4
Asgard/Eukaryota - ps5
Eukaryota - ps6
Amorpheae - ps7
Obazoa - ps8
Opisthokonta - ps9
Holozoa - ps10
Filozoa - ps11
Choano./Metazoa - ps12
Metazoa - ps13
Myriazoa - ps14
Parahoxozoa - ps15
Bilateria - ps16
Deuterostomia - ps17
Chordata - ps18
Olfactores - ps19
Vertebrata - ps20
Gnathostomata - ps21
Euteleostomi - ps22
Sarcopterygii - ps23
Tetrapoda - ps24
Amniota - ps25
Mammalia - ps26
Theria - ps27
Placentalia - ps28
Boreoeutheria - ps29
Euarchontoglires - ps30
Euarchonta - ps31
Primates - ps32
Haplorrhini - ps33
Simiiformes - ps34
Catarrhini - ps35
Hominoidea - ps36
Hominidae - ps37
Homininae - ps38
Hominini - ps39
Homo_sapiens - ps40

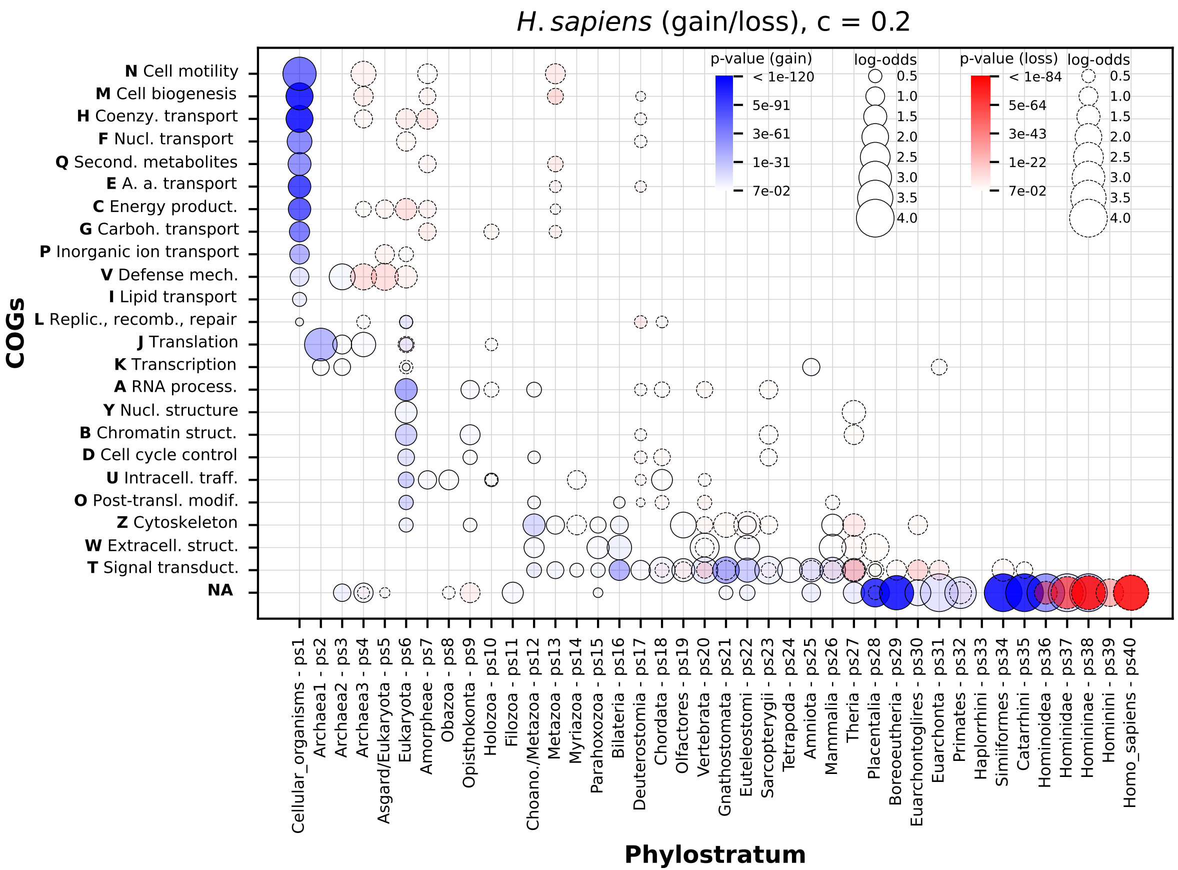

H. sapiens (gain/loss), c = 0.2
COGs
N Cell motility
M Cell biogenesis
H Coenzy. transport
F Nucl. transport
Q Second. metabolites
E A. a. transport
C Energy product.
G Carboh. transport
P Inorganic ion transport
V Defense mech.
I Lipid transport
L Replic., recomb., repair
J Translation
K Transcription
A RNA process.
Y Nucl. structure
B Chromatin struct.
D Cell cycle control
U Intracell. traff.
O Post-transl. modif.
Z Cytoskeleton
W Extracell. struct.
T Signal transduct.
NA
p-value (gain)
< 1e-120
5e-91
3e-61
1e-31
7e-02
log-odds
0.5
1.0
1.5
2.0
2.5
3.0
3.5
4.0
p-value (loss)
< 1e-84
5e-64
3e-43
1e-22
7e-02
log-odds
0.5
1.0
1.5
2.0
2.5
3.0
3.5
4.0
Cellular_organisms - ps1
Archaea1 - ps2
Archaea2 - ps3
Archaea3 - ps4
Asgard/Eukaryota - ps5
Eukaryota - ps6
Amorpheae - ps7
Obazoa - ps8
Opisthokonta - ps9
Holozoa - ps10
Filozoa - ps11
Choano./Metazoa - ps12
Metazoa - ps13
Myriazoa - ps14
Parahoxozoa - ps15
Bilateria - ps16
Deuterostomia - ps17
Chordata - ps18
Olfactores - ps19
Vertebrata - ps20
Gnathostomata - ps21
Euteleostomi - ps22
Sarcopterygii - ps23
Tetrapoda - ps24
Amniota - ps25
Mammalia - ps26
Theria - ps27
Placentalia - ps28
Boreoeutheria - ps29
Euarchontoglires - ps30
Euarchonta - ps31
Primates - ps32
Haplorrhini - ps33
Simiiformes - ps34
Catarrhini - ps35
Hominoidea - ps36
Hominidae - ps37
Homininae - ps38
Hominini - ps39
Homo_sapiens - ps40
Phylostratum

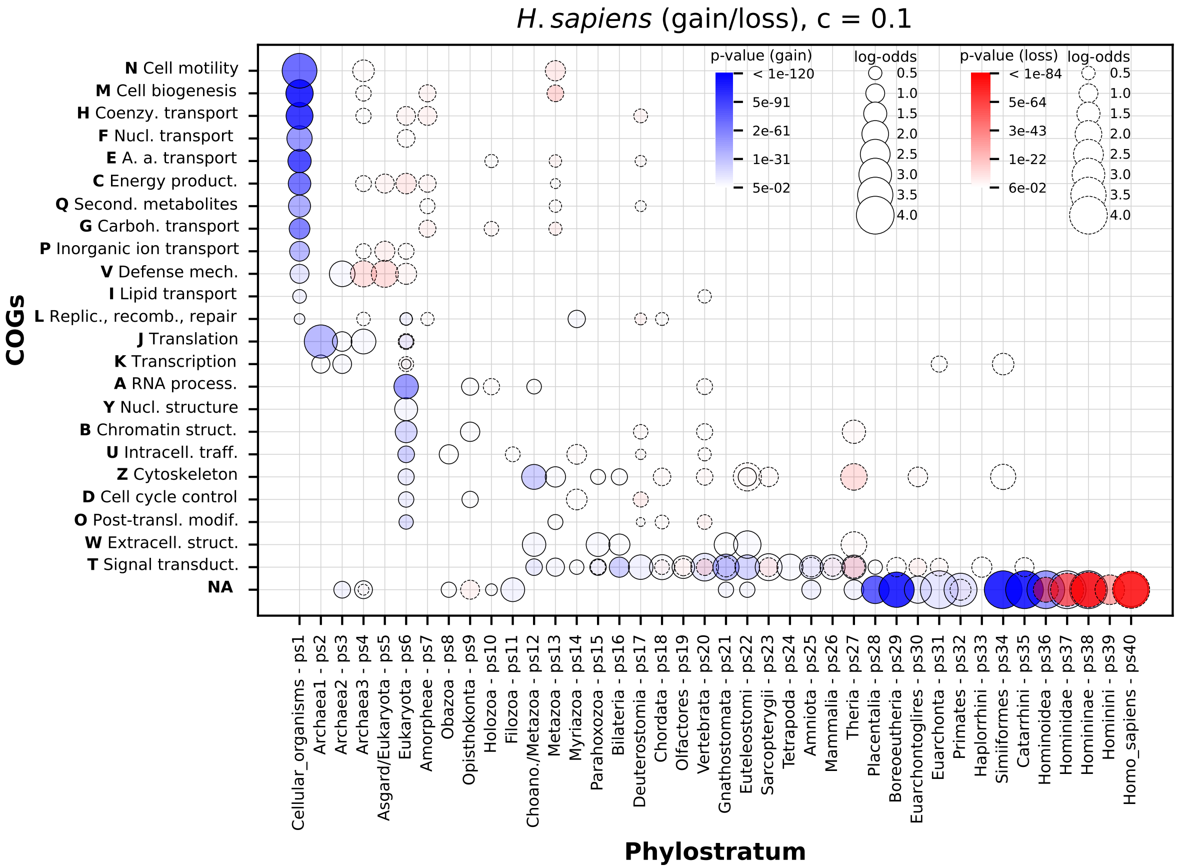

H. sapiens (gain/loss), c = 0.1
COGs
N Cell motility
M Cell biogenesis
H Coenzy. transport
F Nucl. transport
E A. a. transport
C Energy product.
Q Second. metabolites
G Carboh. transport
P Inorganic ion transport
V Defense mech.
I Lipid transport
L Replic., recomb., repair
J Translation
K Transcription
A RNA process.
Y Nucl. structure
B Chromatin struct.
U Intracell. traff.
Z Cytoskeleton
D Cell cycle control
O Post-transl. modif.
W Extracell. struct.
T Signal transduct.
NA
p-value (gain)
< 1e-120
5e-91
2e-61
1e-31
5e-02
log-odds
0.5
1.0
1.5
2.0
2.5
3.0
3.5
4.0
p-value (loss)
< 1e-84
5e-64
3e-43
1e-22
6e-02
log-odds
0.5
1.0
1.5
2.0
2.5
3.0
3.5
4.0
Cellular_organisms - ps1
Archaea1 - ps2
Archaea2 - ps3
Archaea3 - ps4
Asgard/Eukaryota - ps5
Eukaryota - ps6
Amorpheae - ps7
Obazoa - ps8
Opisthokonta - ps9
Holozoa - ps10
Filozoa - ps11
Choano./Metazoa - ps12
Metazoa - ps13
Myriazoa - ps14
Parahoxozoa - ps15
Bilateria - ps16
Deuterostomia - ps17
Chordata - ps18
Olfactores - ps19
Vertebrata - ps20
Gnathostomata - ps21
Euteleostomi - ps22
Sarcopterygii - ps23
Tetrapoda - ps24
Amniota - ps25
Mammalia - ps26
Theria - ps27
Placentalia - ps28
Boreoeutheria - ps29
Euarchontoglires - ps30
Euarchonta - ps31
Primates - ps32
Haplorrhini - ps33
Simiiformes - ps34
Catarrhini - ps35
Hominoidea - ps36
Hominidae - ps37
Homininae - ps38
Hominini - ps39
Homo_sapiens - ps40
Phylostratum

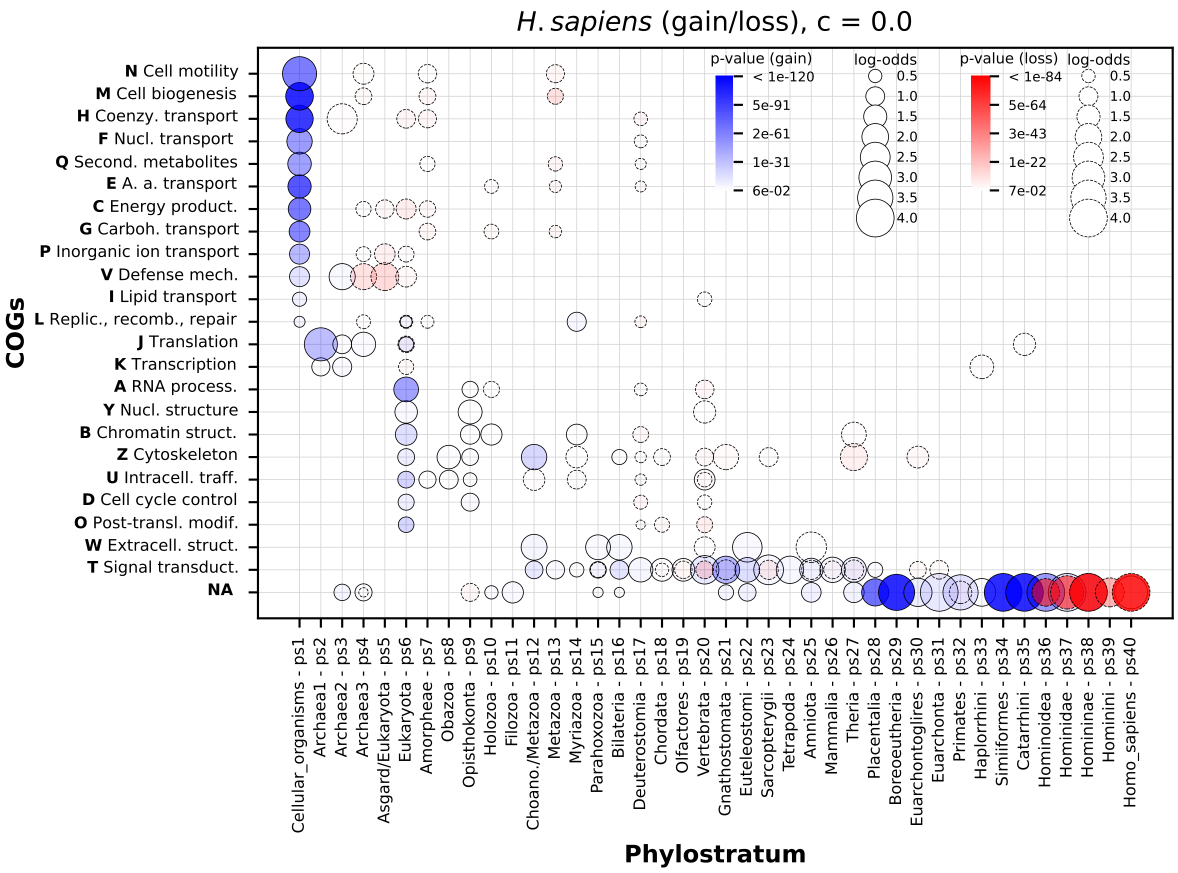
H. sapiens (gain/loss), c = 0.0
p-value (gain)
< 1e-120
5e-91
2e-61
1e-31
6e-02
log-odds
0.5
1.0
1.5
2.0
2.5
3.0
3.5
4.0
p-value (loss)
< 1e-84
5e-64
3e-43
1e-22
7e-02
log-odds
0.5
1.0
1.5
2.0
2.5
3.0
3.5
4.0
COGs
N Cell motility
M Cell biogenesis
H Coenzy. transport
F Nucl. transport
Q Second. metabolites
E A. a. transport
C Energy product.
G Carboh. transport
P Inorganic ion transport
V Defense mech.
I Lipid transport
L Replic., recomb., repair
J Translation
K Transcription
A RNA process.
Y Nucl. structure
B Chromatin struct.
Z Cytoskeleton
U Intracell. traff.
D Cell cycle control
O Post-transl. modif.
W Extracell. struct.
T Signal transduct.
NA
Phylostratum
Cellular_organisms - ps1
Archaea1 - ps2
Archaea2 - ps3
Archaea3 - ps4
Asgard/Eukaryota - ps5
Eukaryota - ps6
Amorpheae - ps7
Obazoa - ps8
Opisthokonta - ps9
Holozoa - ps10
Filozoa - ps11
Choano./Metazoa - ps12
Metazoa - ps13
Myriazoa - ps14
Parahoxozoa - ps15
Bilateria - ps16
Deuterostomia - ps17
Chordata - ps18
Olfactores - ps19
Vertebrata - ps20
Gnathostomata - ps21
Euteleostomi - ps22
Sarcopterygii - ps23
Tetrapoda - ps24
Amniota - ps25
Mammalia - ps26
Theria - ps27
Placentalia - ps28
Boreoeutheria - ps29
Euarchontoglires - ps30
Euarchonta - ps31
Primates - ps32
Haplorrhini - ps33
Simiiformes - ps34
Catarrhini - ps35
Hominoidea - ps36
Hominidae - ps37
Homininae - ps38
Hominini - ps39
Homo_sapiens - ps40

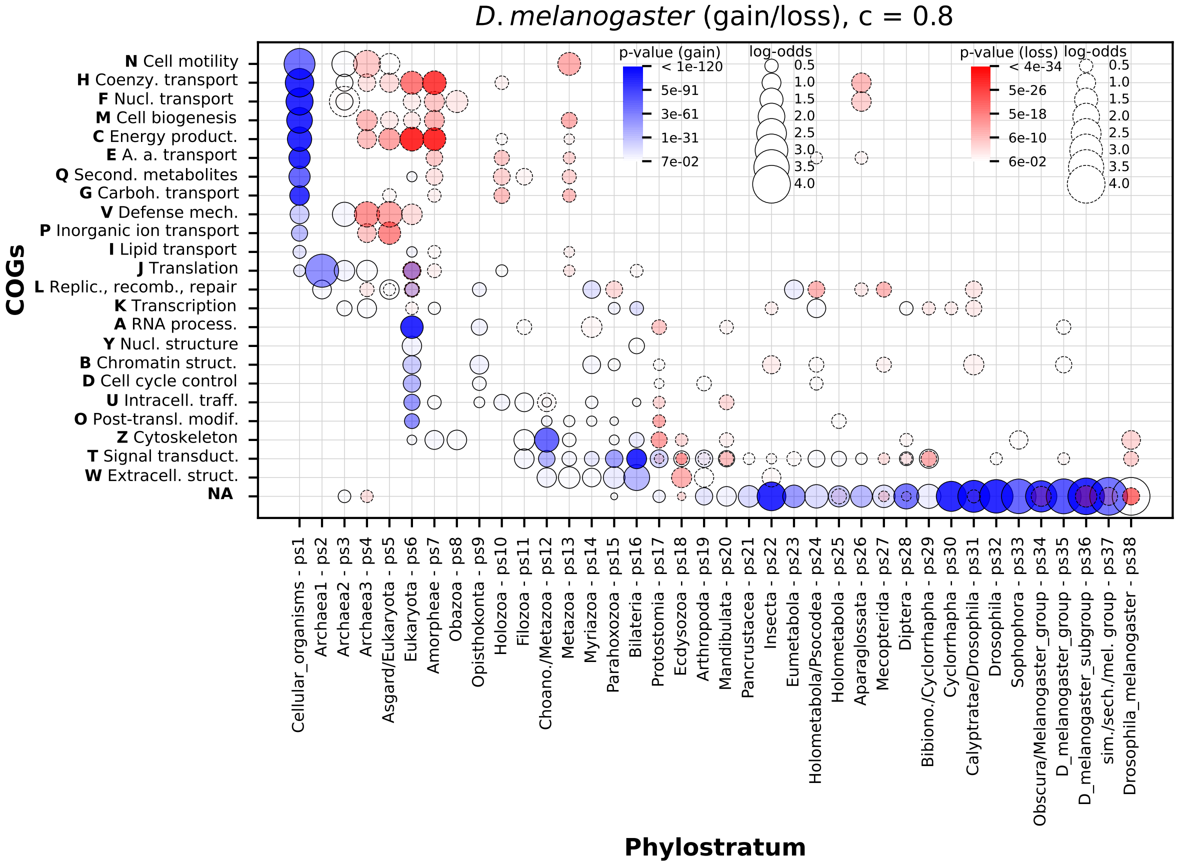

D. melanogaster (gain/loss), c = 0.8
COGs
N Cell motility
H Coenzy. transport
F Nucl. transport
M Cell biogenesis
C Energy product.
E A. a. transport
Q Second. metabolites
G Carboh. transport
V Defense mech.
P Inorganic ion transport
I Lipid transport
J Translation
L Replic., recomb., repair
K Transcription
A RNA process.
Y Nucl. structure
B Chromatin struct.
D Cell cycle control
U Intracell. traff.
O Post-transl. modif.
Z Cytoskeleton
T Signal transduct.
W Extracell. struct.
NA
p-value (gain)
< 1e-120
5e-91
3e-61
1e-31
7e-02
log-odds
0.5
1.0
1.5
2.0
2.5
3.0
3.5
4.0
p-value (loss)
< 4e-34
5e-26
5e-18
6e-10
6e-02
log-odds
0.5
1.0
1.5
2.0
2.5
3.0
3.5
4.0
Cellular_organisms - ps1
Archaea1 - ps2
Archaea2 - ps3
Archaea3 - ps4
Asgard/Eukaryota - ps5
Eukaryota - ps6
Amorpheae - ps7
Obazoa - ps8
Opisthokonta - ps9
Holozoa - ps10
Filozoa - ps11
Choano./Metazoa - ps12
Metazoa - ps13
Myriazoa - ps14
Parahoxozoa - ps15
Bilateria - ps16
Protostomia - ps17
Ecdysozoa - ps18
Arthropoda - ps19
Mandibulata - ps20
Pancrustacea - ps21
Insecta - ps22
Eumetabola - ps23
Holometabola/Psocodea - ps24
Holometabola - ps25
Aparaglossata - ps26
Mecopterida - ps27
Diptera - ps28
Bibiono./Cyclorrhapha - ps29
Cyclorrhapha - ps30
Calyptratae/Drosophila - ps31
Drosophila - ps32
Sophophora - ps33
Obscura/Melanogaster_group - ps34
D_melanogaster_group - ps35
D_melanogaster_subgroup - ps36
sim./sech./mel. group - ps37
Drosophila_melanogaster - ps38
Phylostratum

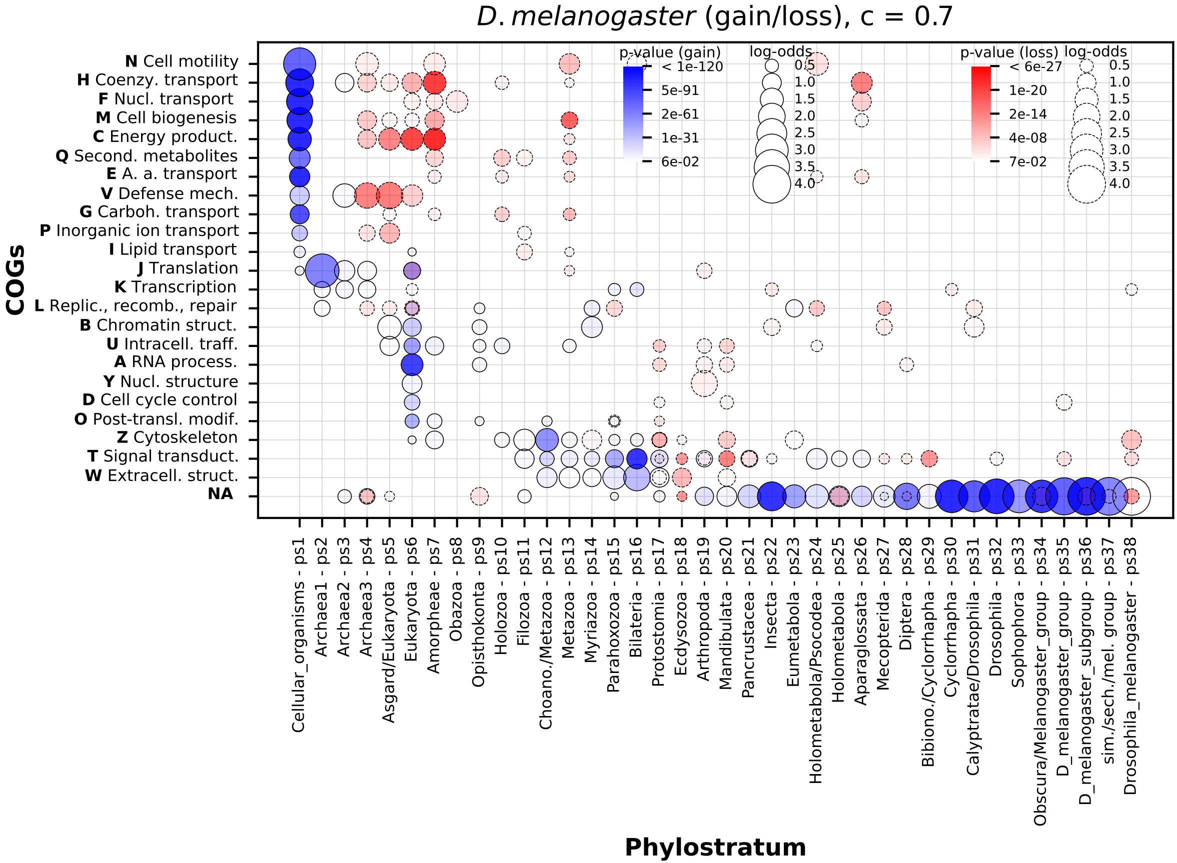

D. melanogaster (gain/loss), c = 0.7
COGs
N Cell motility
H Coenzy. transport
F Nucl. transport
M Cell biogenesis
C Energy product.
Q Second. metabolites
E A. a. transport
V Defense mech.
G Carboh. transport
P Inorganic ion transport
I Lipid transport
J Translation
K Transcription
L Replic., recomb., repair
B Chromatin struct.
U Intracell. traff.
A RNA process.
Y Nucl. structure
D Cell cycle control
O Post-transl. modif.
Z Cytoskeleton
T Signal transduct.
W Extracell. struct.
NA
p-value (gain)
< 1e-120
5e-91
2e-61
1e-31
6e-02
log-odds
0.5
1.0
1.5
2.0
2.5
3.0
3.5
4.0
p-value (loss)
< 6e-27
1e-20
2e-14
4e-08
7e-02
log-odds
0.5
1.0
1.5
2.0
2.5
3.0
3.5
4.0
Cellular_organisms - ps1
Archaea1 - ps2
Archaea2 - ps3
Archaea3 - ps4
Asgard/Eukaryota - ps5
Eukaryota - ps6
Amorpheae - ps7
Obazoa - ps8
Opisthokonta - ps9
Holozoa - ps10
Filozoa - ps11
Choano./Metazoa - ps12
Metazoa - ps13
Myriazoa - ps14
Parahoxozoa - ps15
Bilateria - ps16
Protostomia - ps17
Ecdysozoa - ps18
Arthropoda - ps19
Mandibulata - ps20
Pancrustacea - ps21
Insecta - ps22
Eumetabola - ps23
Holometabola/Psocodea - ps24
Holometabola - ps25
Aparaglossata - ps26
Mecopterida - ps27
Diptera - ps28
Bibiono./Cyclorrhapha - ps29
Cyclorrhapha - ps30
Calyptratae/Drosophila - ps31
Drosophila - ps32
Sophophora - ps33
Obscura/Melanogaster_group - ps34
D_melanogaster_group - ps35
D_melanogaster_subgroup - ps36
sim./sech./mel. group - ps37
Drosophila_melanogaster - ps38
Phylostratum

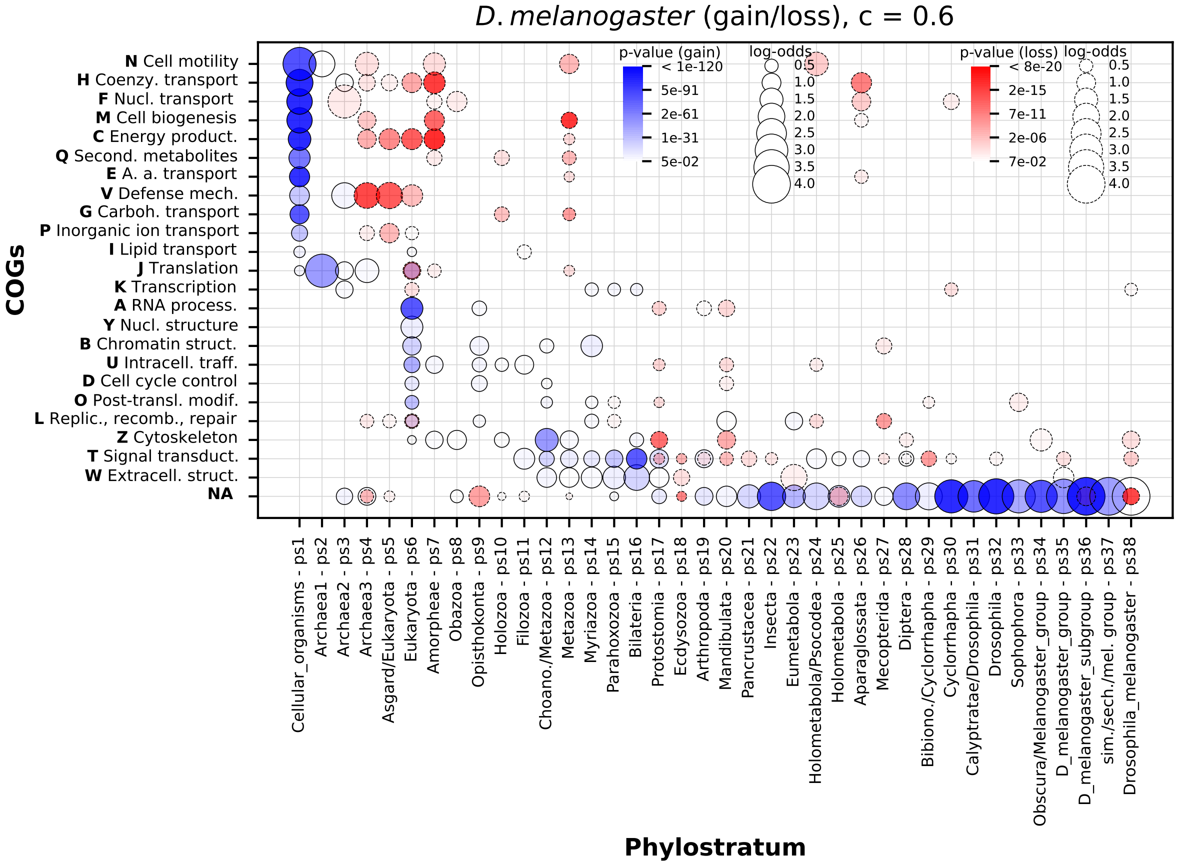

D. melanogaster (gain/loss), c = 0.6
COGs
N Cell motility
H Coenzy. transport
F Nucl. transport
M Cell biogenesis
C Energy product.
Q Second. metabolites
E A. a. transport
V Defense mech.
G Carboh. transport
P Inorganic ion transport
I Lipid transport
J Translation
K Transcription
A RNA process.
Y Nucl. structure
B Chromatin struct.
U Intracell. traff.
D Cell cycle control
O Post-transl. modif.
L Replic., recomb., repair
Z Cytoskeleton
T Signal transduct.
W Extracell. struct.
NA
p-value (gain)
< 1e-120
5e-91
2e-61
1e-31
5e-02
log-odds
0.5
1.0
1.5
2.0
2.5
3.0
3.5
4.0
p-value (loss)
< 8e-20
2e-15
7e-11
2e-06
7e-02
log-odds
0.5
1.0
1.5
2.0
2.5
3.0
3.5
4.0
Cellular_organisms - ps1
Archaea1 - ps2
Archaea2 - ps3
Archaea3 - ps4
Asgard/Eukaryota - ps5
Eukaryota - ps6
Amorpheae - ps7
Obazoa - ps8
Opisthokonta - ps9
Holozoa - ps10
Filozoa - ps11
Choano./Metazoa - ps12
Metazoa - ps13
Myriazoa - ps14
Parahoxozoa - ps15
Bilateria - ps16
Protostomia - ps17
Ecdysozoa - ps18
Arthropoda - ps19
Mandibulata - ps20
Pancrustacea - ps21
Insecta - ps22
Eumetabola - ps23
Holometabola/Psocodea - ps24
Holometabola - ps25
Aparaglossata - ps26
Mecopterida - ps27
Diptera - ps28
Bibiono./Cyclorrhapha - ps29
Cyclorrhapha - ps30
Calyptratae/Drosophila - ps31
Drosophila - ps32
Sophophora - ps33
Obscura/Melanogaster_group - ps34
D_melanogaster_group - ps35
D_melanogaster_subgroup - ps36
sim./sech./mel. group - ps37
Drosophila_melanogaster - ps38
Phylostratum

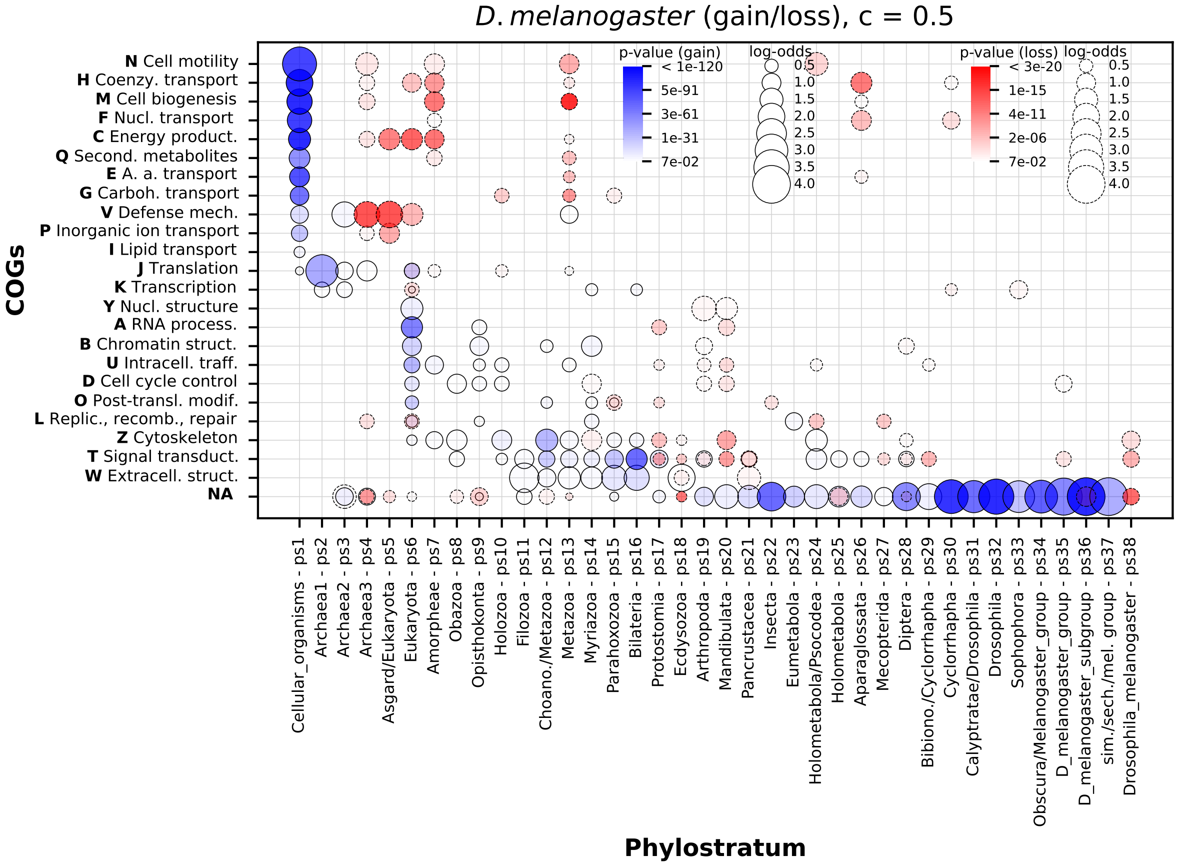

D. melanogaster (gain/loss), c = 0.5
COGs
N Cell motility
H Coenzy. transport
M Cell biogenesis
F Nucl. transport
C Energy product.
Q Second. metabolites
E A. a. transport
G Carboh. transport
V Defense mech.
P Inorganic ion transport
I Lipid transport
J Translation
K Transcription
Y Nucl. structure
A RNA process.
B Chromatin struct.
U Intracell. traff.
D Cell cycle control
O Post-transl. modif.
L Replic., recomb., repair
Z Cytoskeleton
T Signal transduct.
W Extracell. struct.
NA
p-value (gain)
< 1e-120
5e-91
3e-61
1e-31
7e-02
log-odds
0.5
1.0
1.5
2.0
2.5
3.0
3.5
4.0
p-value (loss)
< 3e-20
1e-15
4e-11
2e-06
7e-02
log-odds
0.5
1.0
1.5
2.0
2.5
3.0
3.5
4.0
Cellular_organisms - ps1
Archaea1 - ps2
Archaea2 - ps3
Archaea3 - ps4
Asgard/Eukaryota - ps5
Eukaryota - ps6
Amorpheae - ps7
Obazoa - ps8
Opisthokonta - ps9
Holozoa - ps10
Filozoa - ps11
Choano./Metazoa - ps12
Metazoa - ps13
Myriazoa - ps14
Parahoxozoa - ps15
Bilateria - ps16
Protostomia - ps17
Ecdysozoa - ps18
Arthropoda - ps19
Mandibulata - ps20
Pancrustacea - ps21
Insecta - ps22
Eumetabola - ps23
Holometabola/Psocodea - ps24
Holometabola - ps25
Aparaglossata - ps26
Mecopterida - ps27
Diptera - ps28
Bibiono./Cyclorrhapha - ps29
Cyclorrhapha - ps30
Calyptratae/Drosophila - ps31
Drosophila - ps32
Sophophora - ps33
Obscura/Melanogaster_group - ps34
D_melanogaster_group - ps35
D_melanogaster_subgroup - ps36
sim./sech./mel. group - ps37
Drosophila_melanogaster - ps38
Phylostratum

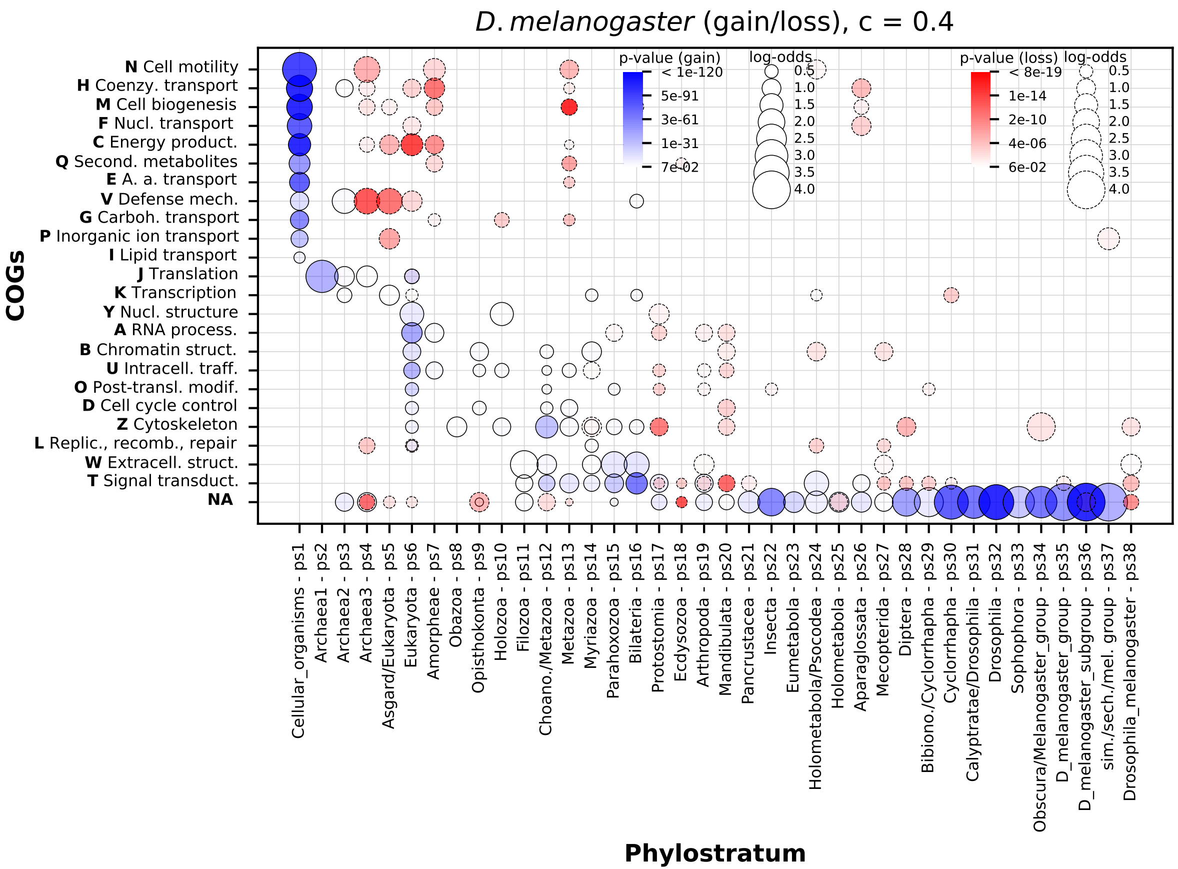

D. melanogaster (gain/loss), c = 0.4
COGs
N Cell motility
H Coenzy. transport
M Cell biogenesis
F Nucl. transport
C Energy product.
Q Second. metabolites
E A. a. transport
V Defense mech.
G Carboh. transport
P Inorganic ion transport
I Lipid transport
J Translation
K Transcription
Y Nucl. structure
A RNA process.
B Chromatin struct.
U Intracell. traff.
O Post-transl. modif.
D Cell cycle control
Z Cytoskeleton
L Replic., recomb., repair
W Extracell. struct.
T Signal transduct.
NA
p-value (gain)
< 1e-120
5e-91
3e-61
1e-31
7e-02
log-odds
0.5
1.0
1.5
2.0
2.5
3.0
3.5
4.0
p-value (loss)
< 8e-19
1e-14
2e-10
4e-06
6e-02
log-odds
0.5
1.0
1.5
2.0
2.5
3.0
3.5
4.0
Cellular_organisms - ps1
Archaea1 - ps2
Archaea2 - ps3
Archaea3 - ps4
Asgard/Eukaryota - ps5
Eukaryota - ps6
Amorpheae - ps7
Obazoa - ps8
Opisthokonta - ps9
Holozoa - ps10
Filozoa - ps11
Choano./Metazoa - ps12
Metazoa - ps13
Myriazoa - ps14
Parahoxozoa - ps15
Bilateria - ps16
Protostomia - ps17
Ecdysozoa - ps18
Arthropoda - ps19
Mandibulata - ps20
Pancrustacea - ps21
Insecta - ps22
Eumetabola - ps23
Holometabola/Psocodea - ps24
Holometabola - ps25
Aparaglossata - ps26
Mecopterida - ps27
Diptera - ps28
Bibiono./Cyclorrhapha - ps29
Cyclorrhapha - ps30
Calyptratae/Drosophila - ps31
Drosophila - ps32
Sophophora - ps33
Obscura/Melanogaster_group - ps34
D_melanogaster_group - ps35
D_melanogaster_subgroup - ps36
sim./sech./mel. group - ps37
Drosophila_melanogaster - ps38
Phylostratum

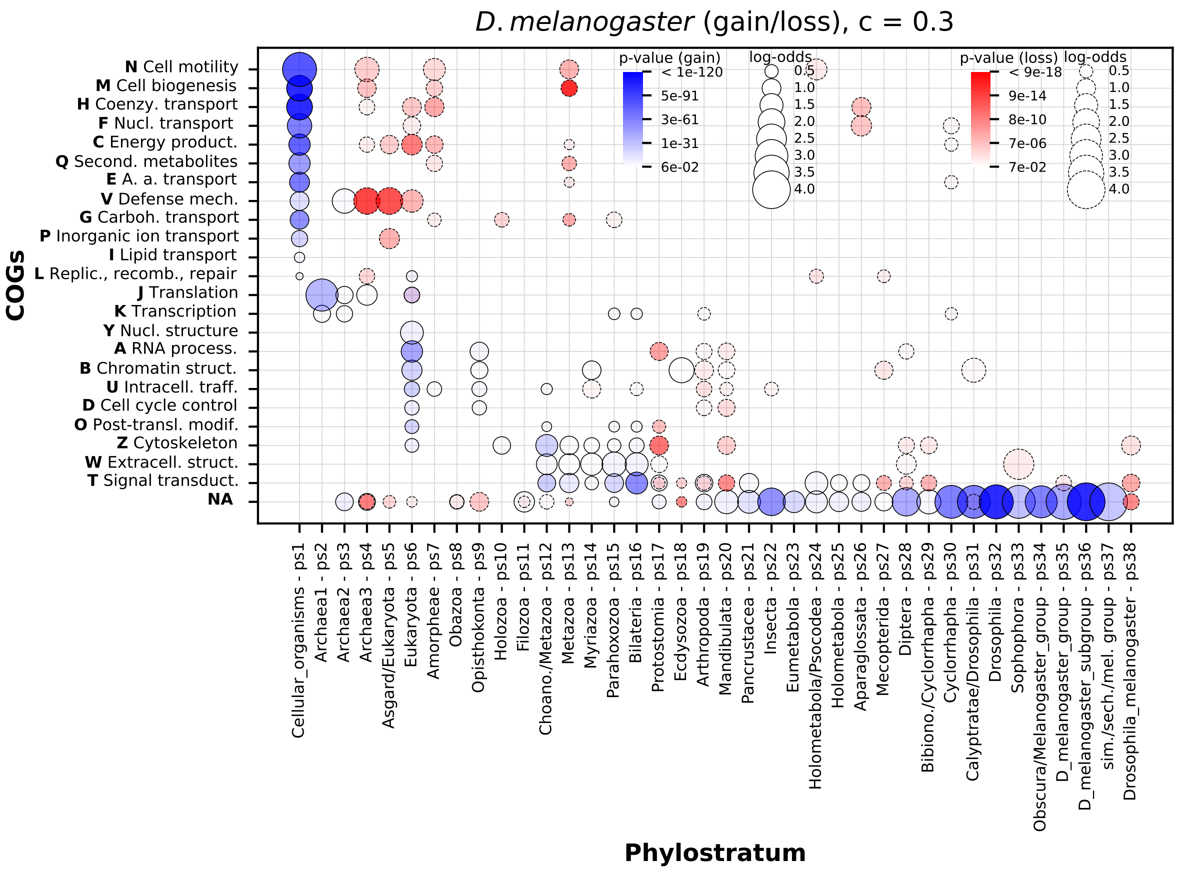

D. melanogaster (gain/loss), c = 0.3
p-value (gain)
< 1e-120
5e-91
3e-61
1e-31
6e-02
log-odds
0.5
1.0
1.5
2.0
2.5
3.0
3.5
4.0
p-value (loss)
< 9e-18
9e-14
8e-10
7e-06
7e-02
log-odds
0.5
1.0
1.5
2.0
2.5
3.0
3.5
4.0
COGs
N Cell motility
M Cell biogenesis
H Coenzy. transport
F Nucl. transport
C Energy product.
Q Second. metabolites
E A. a. transport
V Defense mech.
G Carboh. transport
P Inorganic ion transport
I Lipid transport
L Replic., recomb., repair
J Translation
K Transcription
Y Nucl. structure
A RNA process.
B Chromatin struct.
U Intracell. traff.
D Cell cycle control
O Post-transl. modif.
Z Cytoskeleton
W Extracell. struct.
T Signal transduct.
NA
Cellular_organisms - ps1
Archaea1 - ps2
Archaea2 - ps3
Archaea3 - ps4
Asgard/Eukaryota - ps5
Eukaryota - ps6
Amorpheae - ps7
Obazoa - ps8
Opisthokonta - ps9
Holozoa - ps10
Filozoa - ps11
Choano./Metazoa - ps12
Metazoa - ps13
Myriazoa - ps14
Parahoxozoa - ps15
Bilateria - ps16
Protostomia - ps17
Ecdysozoa - ps18
Arthropoda - ps19
Mandibulata - ps20
Pancrustacea - ps21
Insecta - ps22
Eumetabola - ps23
Holometabola/Psocodea - ps24
Holometabola - ps25
Aparaglossata - ps26
Mecopterida - ps27
Diptera - ps28
Bibiono./Cyclorrhapha - ps29
Cyclorrhapha - ps30
Calyptratae/Drosophila - ps31
Drosophila - ps32
Sophophora - ps33
Obscura/Melanogaster_group - ps34
D_melanogaster_group - ps35
D_melanogaster_subgroup - ps36
sim./sech./mel. group - ps37
Drosophila_melanogaster - ps38
Phylostratum

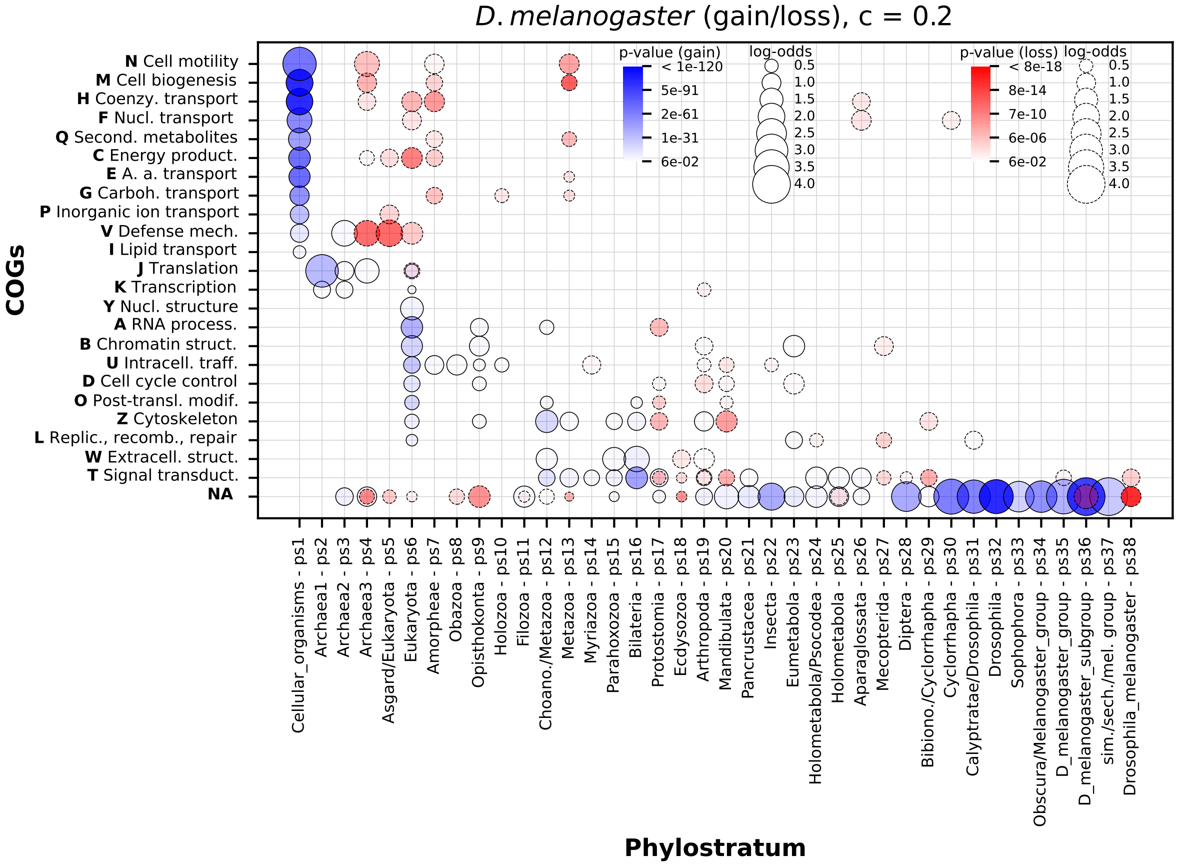

D. melanogaster (gain/loss), c = 0.2
COGs
N Cell motility
M Cell biogenesis
H Coenzy. transport
F Nucl. transport
Q Second. metabolites
C Energy product.
E A. a. transport
G Carboh. transport
P Inorganic ion transport
V Defense mech.
I Lipid transport
J Translation
K Transcription
Y Nucl. structure
A RNA process.
B Chromatin struct.
U Intracell. traff.
D Cell cycle control
O Post-transl. modif.
Z Cytoskeleton
L Replic., recomb., repair
W Extracell. struct.
T Signal transduct.
NA
p-value (gain)
< 1e-120
5e-91
2e-61
1e-31
6e-02
log-odds
0.5
1.0
1.5
2.0
2.5
3.0
3.5
4.0
p-value (loss)
< 8e-18
8e-14
7e-10
6e-06
6e-02
log-odds
0.5
1.0
1.5
2.0
2.5
3.0
3.5
4.0
Cellular_organisms - ps1
Archaea1 - ps2
Archaea2 - ps3
Archaea3 - ps4
Asgard/Eukaryota - ps5
Eukaryota - ps6
Amorpheae - ps7
Obazoa - ps8
Opisthokonta - ps9
Holozoa - ps10
Filozoa - ps11
Choano./Metazoa - ps12
Metazoa - ps13
Myriazoa - ps14
Parahoxozoa - ps15
Bilateria - ps16
Protostomia - ps17
Ecdysozoa - ps18
Arthropoda - ps19
Mandibulata - ps20
Pancrustacea - ps21
Insecta - ps22
Eumetabola - ps23
Holometabola/Psocodea - ps24
Holometabola - ps25
Aparaglossata - ps26
Mecopterida - ps27
Diptera - ps28
Bibiono./Cyclorrhapha - ps29
Cyclorrhapha - ps30
Calyptratae/Drosophila - ps31
Drosophila - ps32
Sophophora - ps33
Obscura/Melanogaster_group - ps34
D_melanogaster_group - ps35
D_melanogaster_subgroup - ps36
sim./sech./mel. group - ps37
Drosophila_melanogaster - ps38
Phylostratum

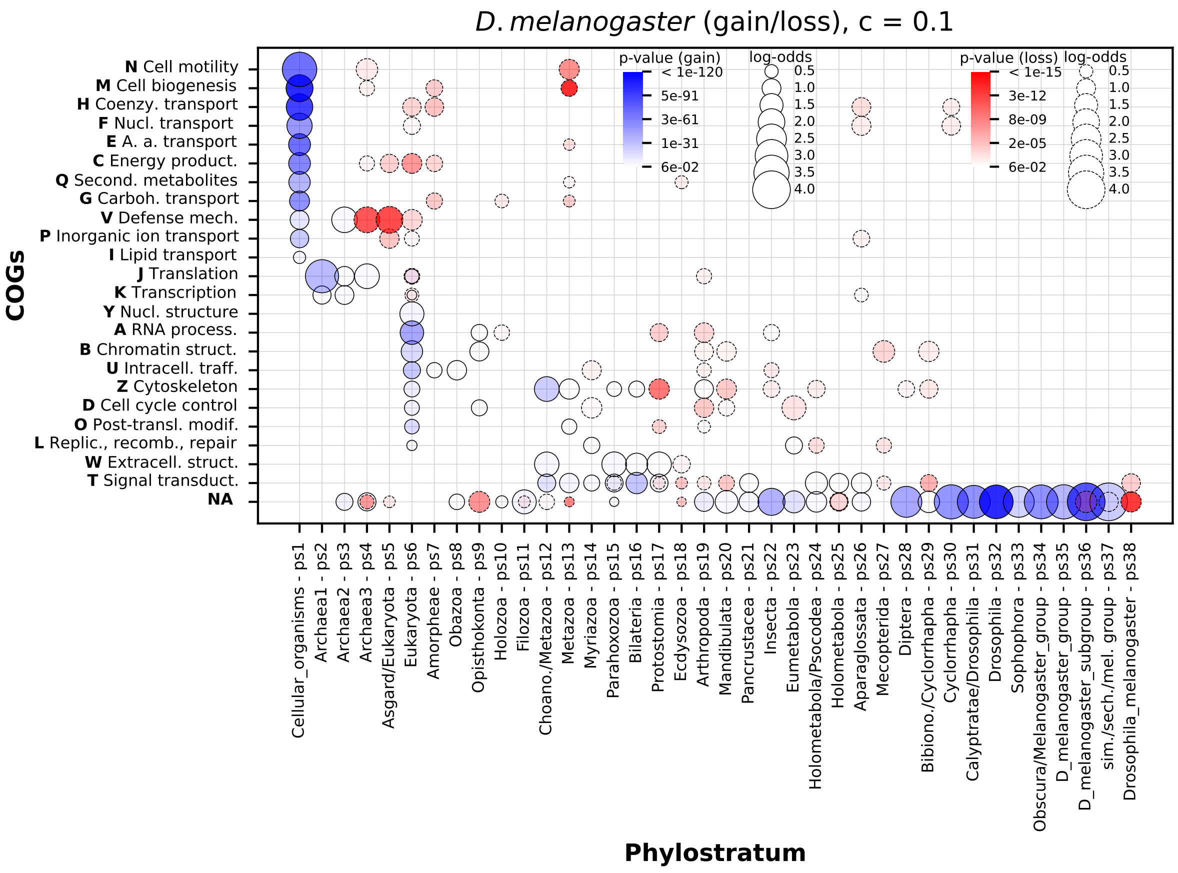

D. melanogaster (gain/loss), c = 0.1
COGs
N Cell motility
M Cell biogenesis
H Coenzy. transport
F Nucl. transport
E A. a. transport
C Energy product.
Q Second. metabolites
G Carboh. transport
V Defense mech.
P Inorganic ion transport
I Lipid transport
J Translation
K Transcription
Y Nucl. structure
A RNA process.
B Chromatin struct.
U Intracell. traff.
Z Cytoskeleton
D Cell cycle control
O Post-transl. modif.
L Replic., recomb., repair
W Extracell. struct.
T Signal transduct.
NA
p-value (gain)
< 1e-120
5e-91
3e-61
1e-31
6e-02
log-odds
0.5
1.0
1.5
2.0
2.5
3.0
3.5
4.0
p-value (loss)
< 1e-15
3e-12
8e-09
2e-05
6e-02
log-odds
0.5
1.0
1.5
2.0
2.5
3.0
3.5
4.0
Cellular_organisms - ps1
Archaea1 - ps2
Archaea2 - ps3
Archaea3 - ps4
Asgard/Eukaryota - ps5
Eukaryota - ps6
Amorpheae - ps7
Obazoa - ps8
Opisthokonta - ps9
Holozoa - ps10
Filozoa - ps11
Choano./Metazoa - ps12
Metazoa - ps13
Myriazoa - ps14
Parahoxozoa - ps15
Bilateria - ps16
Protostomia - ps17
Ecdysozoa - ps18
Arthropoda - ps19
Mandibulata - ps20
Pancrustacea - ps21
Insecta - ps22
Eumetabola - ps23
Holometabola/Psocodea - ps24
Holometabola - ps25
Aparaglossata - ps26
Mecopterida - ps27
Diptera - ps28
Bibiono./Cyclorrhapha - ps29
Cyclorrhapha - ps30
Calyptratae/Drosophila - ps31
Drosophila - ps32
Sophophora - ps33
Obscura/Melanogaster_group - ps34
D_melanogaster_group - ps35
D_melanogaster_subgroup - ps36
sim./sech./mel. group - ps37
Drosophila_melanogaster - ps38
Phylostratum

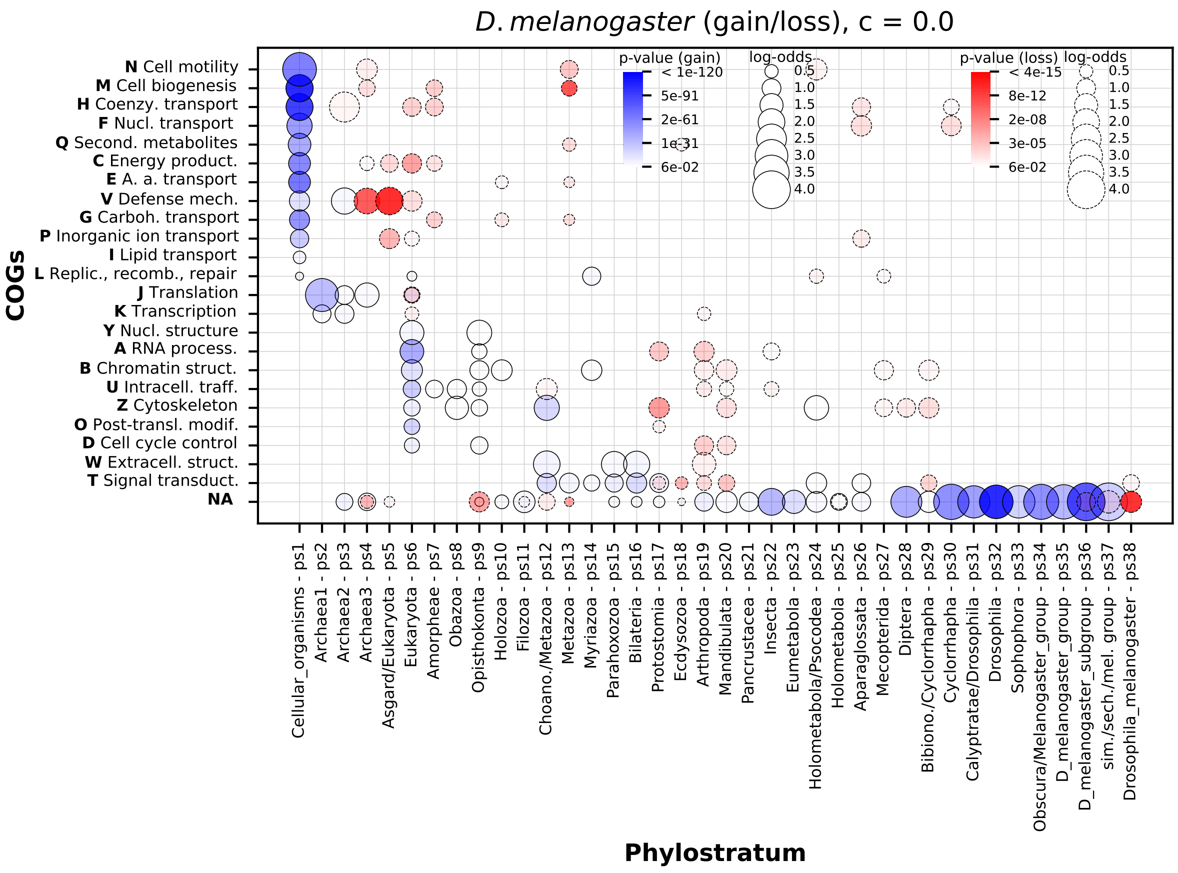

D. melanogaster (gain/loss), c = 0.0
p-value (gain)
< 1e-120
5e-91
2e-61
1e-31
6e-02
log-odds
0.5
1.0
1.5
2.0
2.5
3.0
3.5
4.0
p-value (loss)
< 4e-15
8e-12
2e-08
3e-05
6e-02
COGs
N Cell motility
M Cell biogenesis
H Coenzy. transport
F Nucl. transport
Q Second. metabolites
C Energy product.
E A. a. transport
V Defense mech.
G Carboh. transport
P Inorganic ion transport
I Lipid transport
L Replic., recomb., repair
J Translation
K Transcription
Y Nucl. structure
A RNA process.
B Chromatin struct.
U Intracell. traff.
Z Cytoskeleton
O Post-transl. modif.
D Cell cycle control
W Extracell. struct.
T Signal transduct.
NA
Cellular_organisms - ps1
Archaea1 - ps2
Archaea2 - ps3
Archaea3 - ps4
Asgard/Eukaryota - ps5
Eukaryota - ps6
Amorpheae - ps7
Obazoa - ps8
Opisthokonta - ps9
Holozoa - ps10
Filozoa - ps11
Choano./Metazoa - ps12
Metazoa - ps13
Myriazoa - ps14
Parahoxozoa - ps15
Bilateria - ps16
Protostomia - ps17
Ecdysozoa - ps18
Arthropoda - ps19
Mandibulata - ps20
Pancrustacea - ps21
Insecta - ps22
Eumetabola - ps23
Holometabola/Psocodea - ps24
Holometabola - ps25
Aparaglossata - ps26
Mecopterida - ps27
Diptera - ps28
Bibiono./Cyclorrhapha - ps29
Cyclorrhapha - ps30
Calyptratae/Drosophila - ps31
Drosophila - ps32
Sophophora - ps33
Obscura/Melanogaster_group - ps34
D_melanogaster_group - ps35
D_melanogaster_subgroup - ps36
sim./sech./mel. group - ps37
Drosophila_melanogaster - ps38
Phylostratum

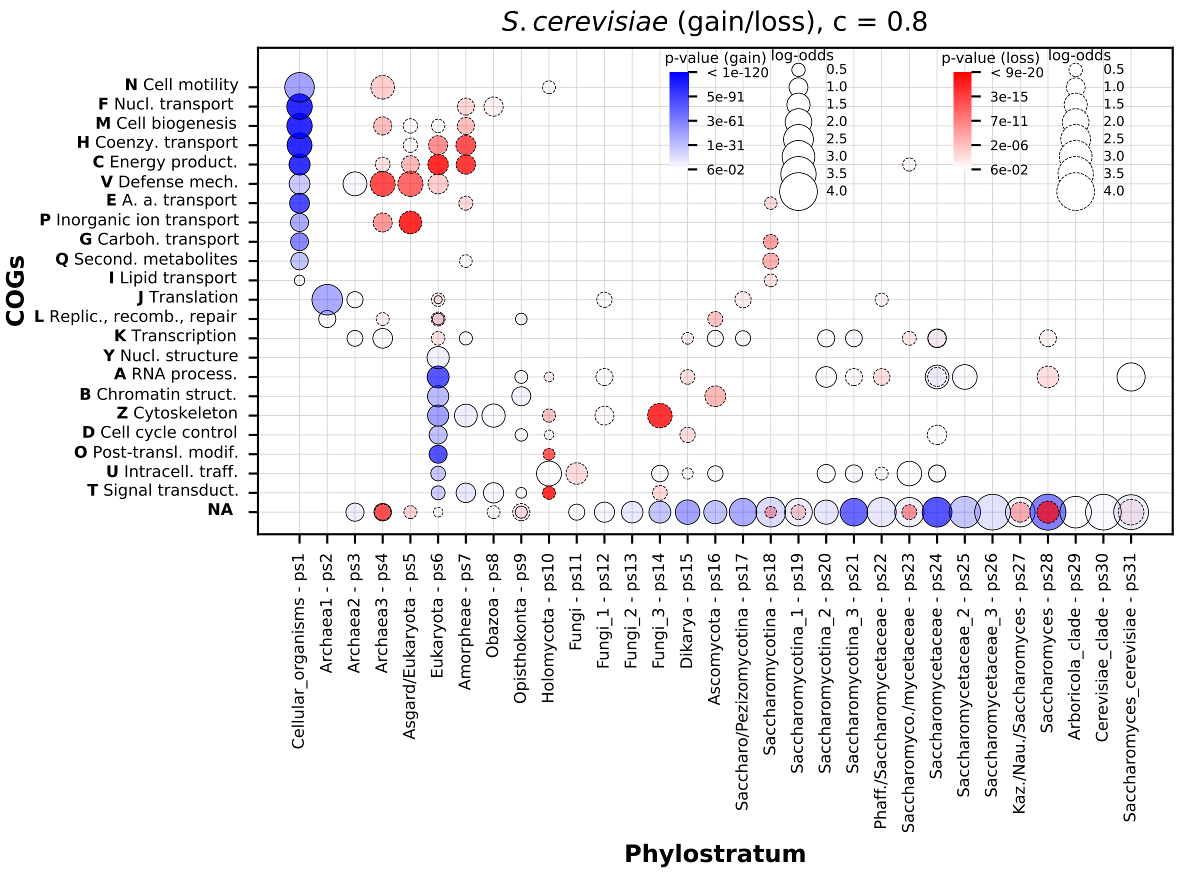
S. cerevisiae (gain/loss), c = 0.8
p-value (gain)
< 1e-120
5e-91
3e-61
1e-31
6e-02
log-odds
0.5
1.0
1.5
2.0
2.5
3.0
3.5
4.0
p-value (loss)
< 9e-20
3e-15
7e-11
2e-06
6e-02
COGs
N Cell motility
F Nucl. transport
M Cell biogenesis
H Coenzy. transport
C Energy product.
V Defense mech.
E A. a. transport
P Inorganic ion transport
G Carboh. transport
Q Second. metabolites
I Lipid transport
J Translation
L Replic., recomb., repair
K Transcription
Y Nucl. structure
A RNA process.
B Chromatin struct.
Z Cytoskeleton
D Cell cycle control
O Post-transl. modif.
U Intracell. traff.
T Signal transduct.
NA
Cellular_organisms - ps1
Archaea1 - ps2
Archaea2 - ps3
Archaea3 - ps4
Asgard/Eukaryota - ps5
Eukaryota - ps6
Amorpheae - ps7
Obazoa - ps8
Opisthokonta - ps9
Holomycota - ps10
Fungi - ps11
Fungi_1 - ps12
Fungi_2 - ps13
Fungi_3 - ps14
Dikarya - ps15
Ascomycota - ps16
Saccharo/Pezizomycotina - ps17
Saccharomycotina - ps18
Saccharomycotina_1 - ps19
Saccharomycotina_2 - ps20
Saccharomycotina_3 - ps21
Phaff./Saccharomycetaceae - ps22
Saccharomyco./mycetaceae - ps23
Saccharomycetaceae - ps24
Saccharomycetaceae_2 - ps25
Saccharomycetaceae_3 - ps26
Kaz./Nau./Saccharomyces - ps27
Saccharomyces - ps28
Arboricola_clade - ps29
Cerevisiae_clade - ps30
Saccharomyces_cerevisiae - ps31
Phylostratum

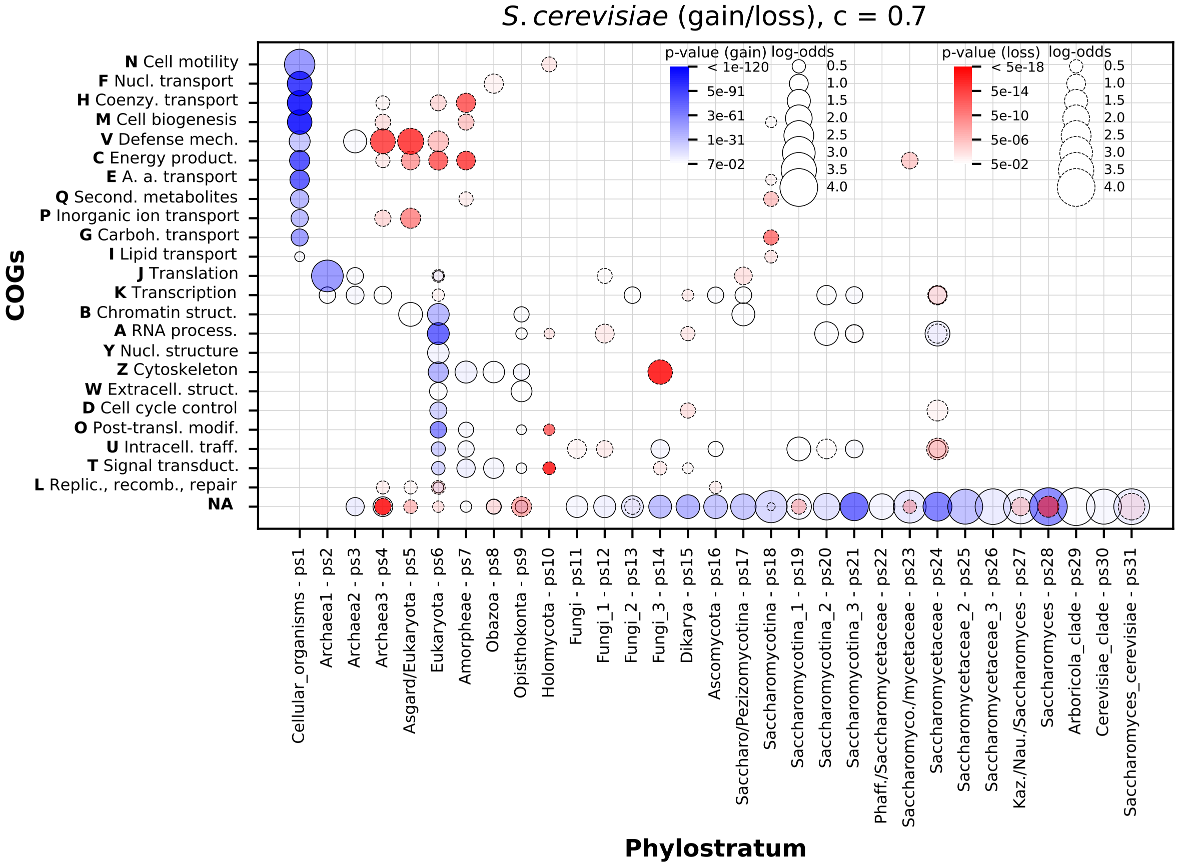

S. cerevisiae (gain/loss), c = 0.7
COGs
N Cell motility
F Nucl. transport
H Coenzy. transport
M Cell biogenesis
V Defense mech.
C Energy product.
E A. a. transport
Q Second. metabolites
P Inorganic ion transport
G Carboh. transport
I Lipid transport
J Translation
K Transcription
B Chromatin struct.
A RNA process.
Y Nucl. structure
Z Cytoskeleton
W Extracell. struct.
D Cell cycle control
O Post-transl. modif.
U Intracell. traff.
T Signal transduct.
L Replic., recomb., repair
NA
p-value (gain)
< 1e-120
5e-91
3e-61
1e-31
7e-02
log-odds
0.5
1.0
1.5
2.0
2.5
3.0
3.5
4.0
p-value (loss)
< 5e-18
5e-14
5e-10
5e-06
5e-02
Cellular_organisms - ps1
Archaea1 - ps2
Archaea2 - ps3
Archaea3 - ps4
Asgard/Eukaryota - ps5
Eukaryota - ps6
Amorpheae - ps7
Obazoa - ps8
Opisthokonta - ps9
Holomycota - ps10
Fungi - ps11
Fungi_1 - ps12
Fungi_2 - ps13
Fungi_3 - ps14
Dikarya - ps15
Ascomycota - ps16
Saccharo/Pezizomycotina - ps17
Saccharomycotina - ps18
Saccharomycotina_1 - ps19
Saccharomycotina_2 - ps20
Saccharomycotina_3 - ps21
Phaff./Saccharomycetaceae - ps22
Saccharomyco./mycetaceae - ps23
Saccharomycetaceae - ps24
Saccharomycetaceae_2 - ps25
Saccharomycetaceae_3 - ps26
Kaz./Nau./Saccharomyces - ps27
Saccharomyces - ps28
Arboricola_clade - ps29
Cerevisiae_clade - ps30
Saccharomyces_cerevisiae - ps31
Phylostratum

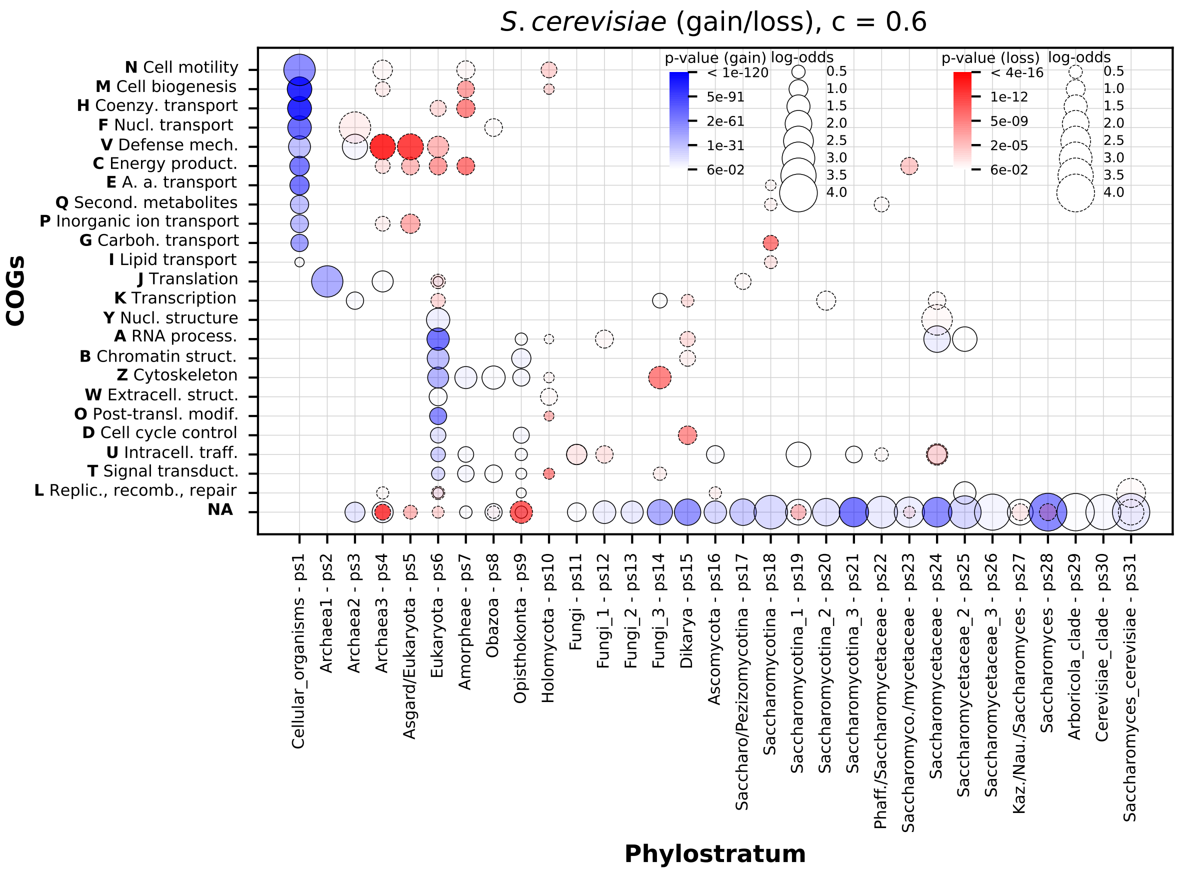

S. cerevisiae (gain/loss), c = 0.6
p-value (gain)
log-odds
< 1e-120
5e-91
2e-61
1e-31
6e-02
0.5
1.0
1.5
2.0
2.5
3.0
3.5
4.0
p-value (loss)
log-odds
< 4e-16
1e-12
5e-09
2e-05
6e-02
COGs
N Cell motility
M Cell biogenesis
H Coenzy. transport
F Nucl. transport
V Defense mech.
C Energy product.
E A. a. transport
Q Second. metabolites
P Inorganic ion transport
G Carboh. transport
I Lipid transport
J Translation
K Transcription
Y Nucl. structure
A RNA process.
B Chromatin struct.
Z Cytoskeleton
W Extracell. struct.
O Post-transl. modif.
D Cell cycle control
U Intracell. traff.
T Signal transduct.
L Replic., recomb., repair
NA
Cellular_organisms - ps1
Archaea1 - ps2
Archaea2 - ps3
Archaea3 - ps4
Asgard/Eukaryota - ps5
Eukaryota - ps6
Amorpheae - ps7
Obazoa - ps8
Opisthokonta - ps9
Holomycota - ps10
Fungi - ps11
Fungi_1 - ps12
Fungi_2 - ps13
Fungi_3 - ps14
Dikarya - ps15
Ascomycota - ps16
Saccharo/Pezizomycotina - ps17
Saccharomycotina - ps18
Saccharomycotina_1 - ps19
Saccharomycotina_2 - ps20
Saccharomycotina_3 - ps21
Phaff./Saccharomycetaceae - ps22
Saccharomyco./mycetaceae - ps23
Saccharomycetaceae - ps24
Saccharomycetaceae_2 - ps25
Saccharomycetaceae_3 - ps26
Kaz./Nau./Saccharomyces - ps27
Saccharomyces - ps28
Arboricola_clade - ps29
Cerevisiae_clade - ps30
Saccharomyces_cerevisiae - ps31
Phylostratum

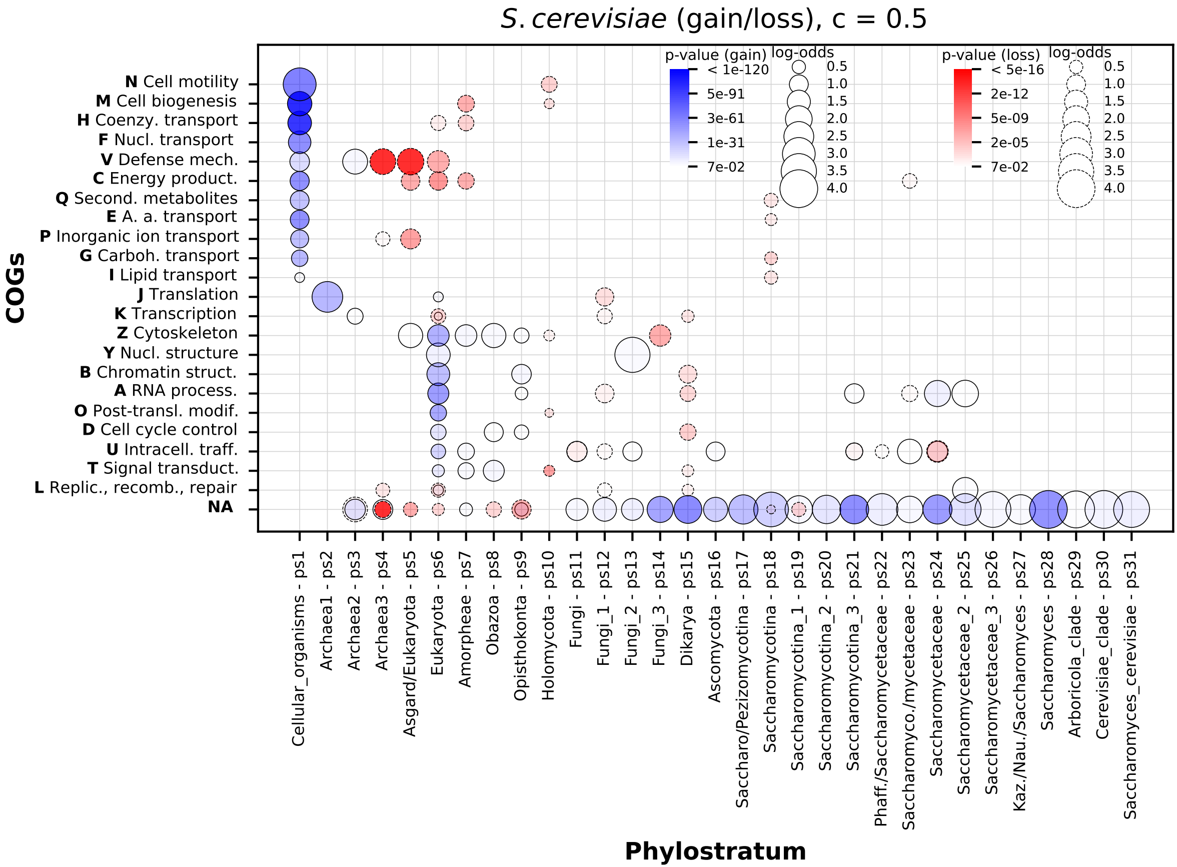

S. cerevisiae (gain/loss), c = 0.5
p-value (gain)
< 1e-120
5e-91
3e-61
1e-31
7e-02
log-odds
0.5
1.0
1.5
2.0
2.5
3.0
3.5
4.0
p-value (loss)
< 5e-16
2e-12
5e-09
2e-05
7e-02
log-odds
0.5
1.0
1.5
2.0
2.5
3.0
3.5
4.0
COGs
N Cell motility
M Cell biogenesis
H Coenzy. transport
F Nucl. transport
V Defense mech.
C Energy product.
Q Second. metabolites
E A. a. transport
P Inorganic ion transport
G Carboh. transport
I Lipid transport
J Translation
K Transcription
Z Cytoskeleton
Y Nucl. structure
B Chromatin struct.
A RNA process.
O Post-transl. modif.
D Cell cycle control
U Intracell. traff.
T Signal transduct.
L Replic., recomb., repair
NA
Cellular_organisms - ps1
Archaea1 - ps2
Archaea2 - ps3
Archaea3 - ps4
Asgard/Eukaryota - ps5
Eukaryota - ps6
Amorpheae - ps7
Obazoa - ps8
Opisthokonta - ps9
Holomycota - ps10
Fungi - ps11
Fungi_1 - ps12
Fungi_2 - ps13
Fungi_3 - ps14
Dikarya - ps15
Ascomycota - ps16
Saccharo/Pezizomycotina - ps17
Saccharomycotina - ps18
Saccharomycotina_1 - ps19
Saccharomycotina_2 - ps20
Saccharomycotina_3 - ps21
Phaff./Saccharomycetaceae - ps22
Saccharomyco./mycetaceae - ps23
Saccharomycetaceae - ps24
Saccharomycetaceae_2 - ps25
Saccharomycetaceae_3 - ps26
Kaz./Nau./Saccharomyces - ps27
Saccharomyces - ps28
Arboricola_clade - ps29
Cerevisiae_clade - ps30
Saccharomyces_cerevisiae - ps31
Phylostratum

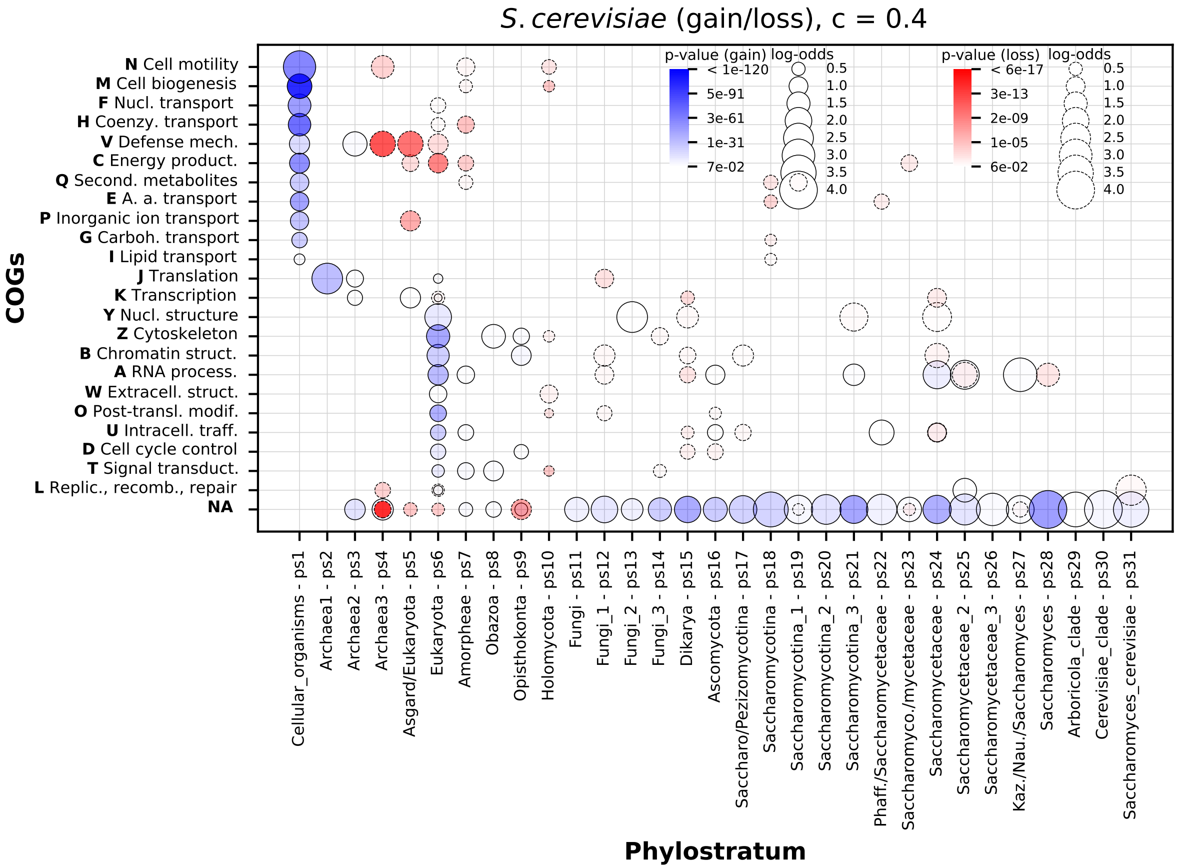
S. cerevisiae (gain/loss), c = 0.4
p-value (gain)
log-odds
< 1e-120
5e-91
3e-61
1e-31
7e-02
0.5
1.0
1.5
2.0
2.5
3.0
3.5
4.0
p-value (loss)
log-odds
< 6e-17
3e-13
2e-09
1e-05
6e-02
0.5
1.0
1.5
2.0
2.5
3.0
3.5
4.0
COGs
N Cell motility
M Cell biogenesis
F Nucl. transport
H Coenzy. transport
V Defense mech.
C Energy product.
Q Second. metabolites
E A. a. transport
P Inorganic ion transport
G Carboh. transport
I Lipid transport
J Translation
K Transcription
Y Nucl. structure
Z Cytoskeleton
B Chromatin struct.
A RNA process.
W Extracell. struct.
O Post-transl. modif.
U Intracell. traff.
D Cell cycle control
T Signal transduct.
L Replic., recomb., repair
NA
Cellular_organisms - ps1
Archaea1 - ps2
Archaea2 - ps3
Archaea3 - ps4
Asgard/Eukaryota - ps5
Eukaryota - ps6
Amorpheae - ps7
Obazoa - ps8
Opisthokonta - ps9
Holomycota - ps10
Fungi - ps11
Fungi_1 - ps12
Fungi_2 - ps13
Fungi_3 - ps14
Dikarya - ps15
Ascomycota - ps16
Saccharo/Pezizomycotina - ps17
Saccharomycotina - ps18
Saccharomycotina_1 - ps19
Saccharomycotina_2 - ps20
Saccharomycotina_3 - ps21
Phaff./Saccharomycetaceae - ps22
Saccharomyco./mycetaceae - ps23
Saccharomycetaceae - ps24
Saccharomycetaceae_2 - ps25
Saccharomycetaceae_3 - ps26
Kaz./Nau./Saccharomyces - ps27
Saccharomyces - ps28
Arboricola_clade - ps29
Cerevisiae_clade - ps30
Saccharomyces_cerevisiae - ps31
Phylostratum

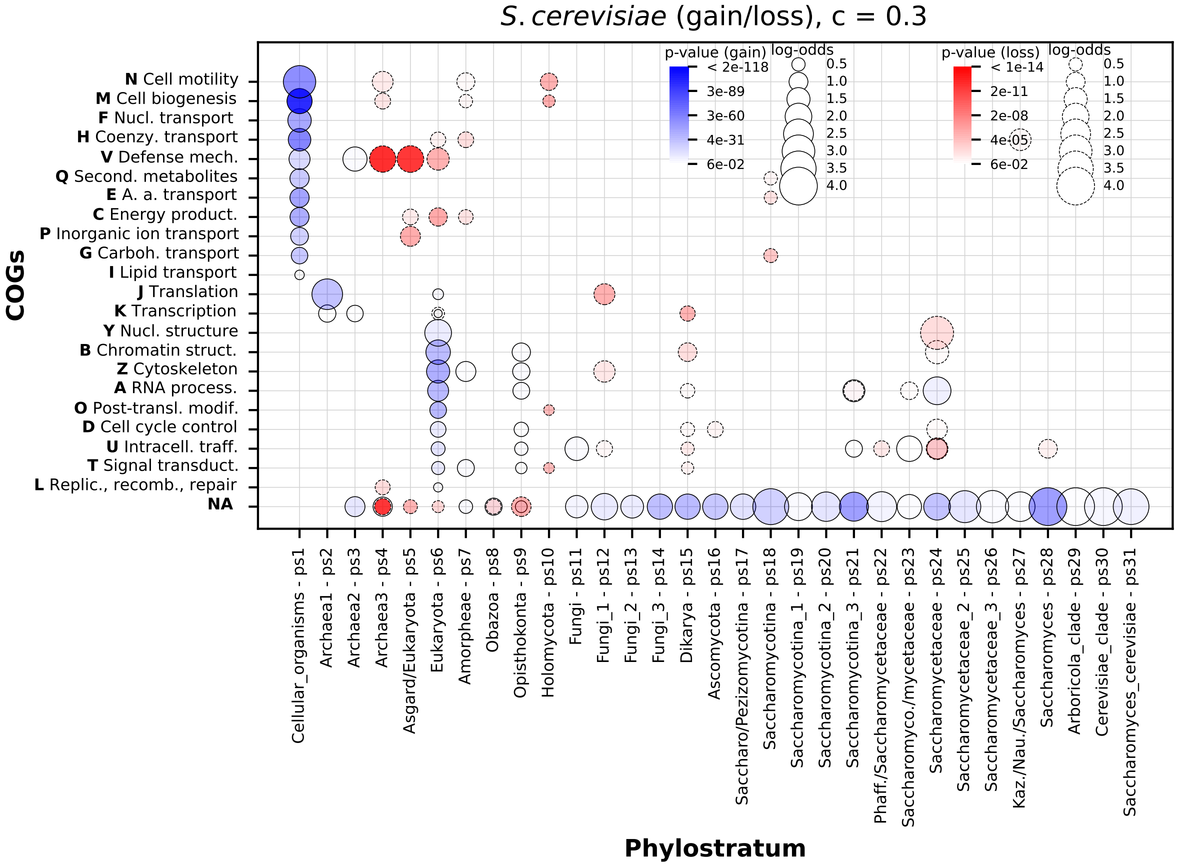

S. cerevisiae (gain/loss), c = 0.3
p-value (gain)
< 2e-118
3e-89
3e-60
4e-31
6e-02
log-odds
0.5
1.0
1.5
2.0
2.5
3.0
3.5
4.0
p-value (loss)
< 1e-14
2e-11
2e-08
4e-05
6e-02
log-odds
0.5
1.0
1.5
2.0
2.5
3.0
3.5
4.0
COGs
N Cell motility
M Cell biogenesis
F Nucl. transport
H Coenzy. transport
V Defense mech.
Q Second. metabolites
E A. a. transport
C Energy product.
P Inorganic ion transport
G Carboh. transport
I Lipid transport
J Translation
K Transcription
Y Nucl. structure
B Chromatin struct.
Z Cytoskeleton
A RNA process.
O Post-transl. modif.
D Cell cycle control
U Intracell. traff.
T Signal transduct.
L Replic., recomb., repair
NA
Cellular_organisms - ps1
Archaea1 - ps2
Archaea2 - ps3
Archaea3 - ps4
Asgard/Eukaryota - ps5
Eukaryota - ps6
Amorpheae - ps7
Obazoa - ps8
Opisthokonta - ps9
Holomycota - ps10
Fungi - ps11
Fungi_1 - ps12
Fungi_2 - ps13
Fungi_3 - ps14
Dikarya - ps15
Ascomycota - ps16
Saccharo/Pezizomycotina - ps17
Saccharomycotina - ps18
Saccharomycotina_1 - ps19
Saccharomycotina_2 - ps20
Saccharomycotina_3 - ps21
Phaff./Saccharomycetaceae - ps22
Saccharomyco./mycetaceae - ps23
Saccharomycetaceae - ps24
Saccharomycetaceae_2 - ps25
Saccharomycetaceae_3 - ps26
Kaz./Nau./Saccharomyces - ps27
Saccharomyces - ps28
Arboricola_clade - ps29
Cerevisiae_clade - ps30
Saccharomyces_cerevisiae - ps31
Phylostratum

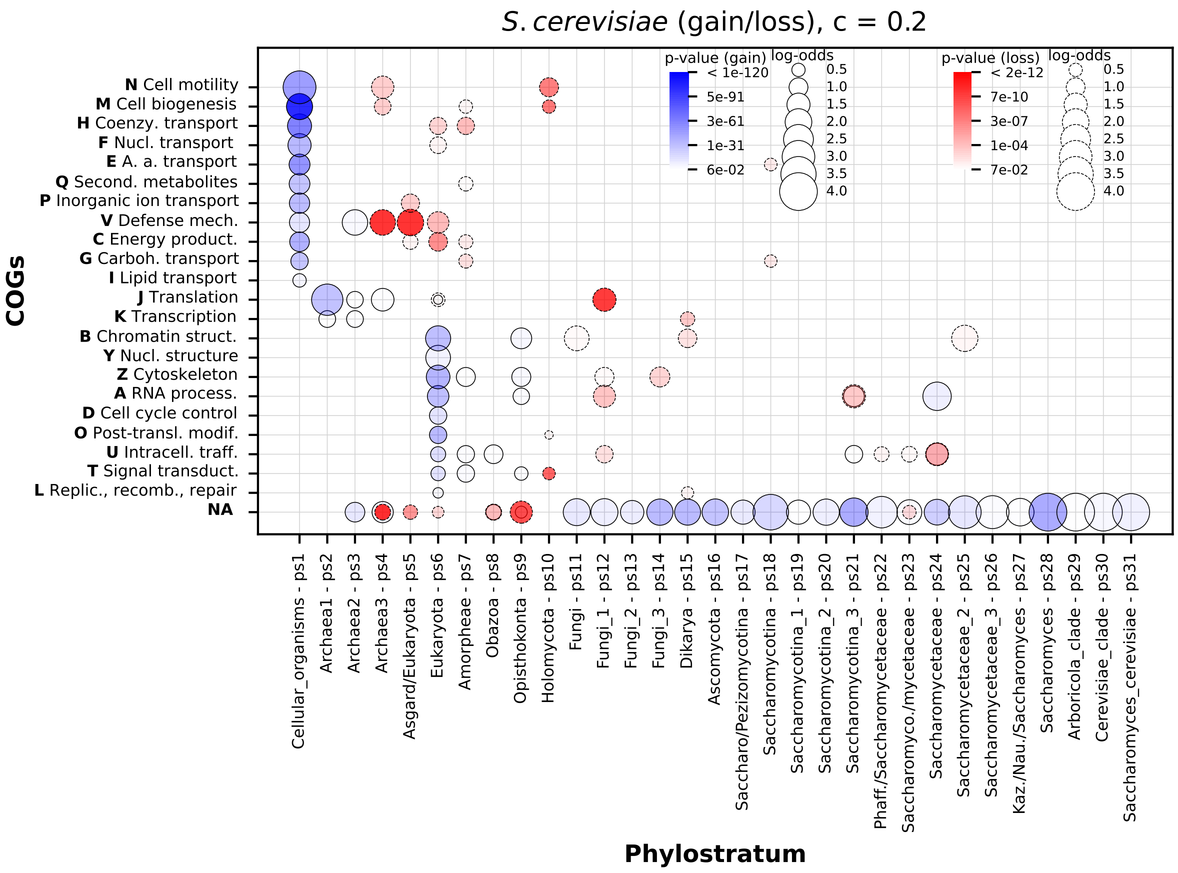

S. cerevisiae (gain/loss), c = 0.2
p-value (gain)
< 1e-120
5e-91
3e-61
1e-31
6e-02
log-odds
0.5
1.0
1.5
2.0
2.5
3.0
3.5
4.0
p-value (loss)
< 2e-12
7e-10
3e-07
1e-04
7e-02
COGs
N Cell motility
M Cell biogenesis
H Coenzy. transport
F Nucl. transport
E A. a. transport
Q Second. metabolites
P Inorganic ion transport
V Defense mech.
C Energy product.
G Carboh. transport
I Lipid transport
J Translation
K Transcription
B Chromatin struct.
Y Nucl. structure
Z Cytoskeleton
A RNA process.
D Cell cycle control
O Post-transl. modif.
U Intracell. traff.
T Signal transduct.
L Replic., recomb., repair
NA
Cellular_organisms - ps1
Archaea1 - ps2
Archaea2 - ps3
Archaea3 - ps4
Asgard/Eukaryota - ps5
Eukaryota - ps6
Amorpheae - ps7
Obazoa - ps8
Opisthokonta - ps9
Holomycota - ps10
Fungi - ps11
Fungi_1 - ps12
Fungi_2 - ps13
Fungi_3 - ps14
Dikarya - ps15
Ascomycota - ps16
Saccharo/Pezizomycotina - ps17
Saccharomycotina - ps18
Saccharomycotina_1 - ps19
Saccharomycotina_2 - ps20
Saccharomycotina_3 - ps21
Phaff./Saccharomycetaceae - ps22
Saccharomyco./mycetaceae - ps23
Saccharomycetaceae - ps24
Saccharomycetaceae_2 - ps25
Saccharomycetaceae_3 - ps26
Kaz./Nau./Saccharomyces - ps27
Saccharomyces - ps28
Arboricola_clade - ps29
Cerevisiae_clade - ps30
Saccharomyces_cerevisiae - ps31
Phylostratum

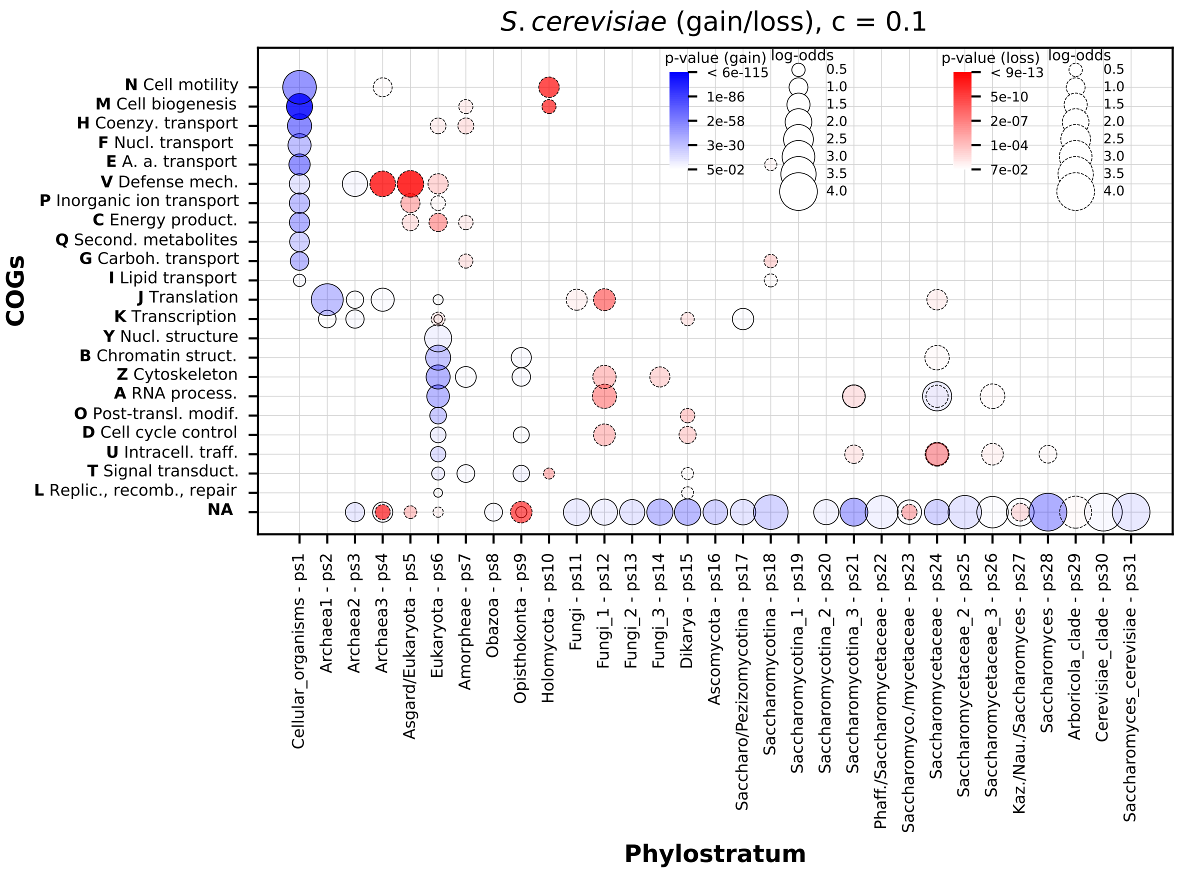

S. cerevisiae (gain/loss), c = 0.1
p-value (gain)
< 6e-115
1e-86
2e-58
3e-30
5e-02
log-odds
0.5
1.0
1.5
2.0
2.5
3.0
3.5
4.0
p-value (loss)
< 9e-13
5e-10
2e-07
1e-04
7e-02
COGs
N Cell motility
M Cell biogenesis
H Coenzy. transport
F Nucl. transport
E A. a. transport
V Defense mech.
P Inorganic ion transport
C Energy product.
Q Second. metabolites
G Carboh. transport
I Lipid transport
J Translation
K Transcription
Y Nucl. structure
B Chromatin struct.
Z Cytoskeleton
A RNA process.
O Post-transl. modif.
D Cell cycle control
U Intracell. traff.
T Signal transduct.
L Replic., recomb., repair
NA
Cellular_organisms - ps1
Archaea1 - ps2
Archaea2 - ps3
Archaea3 - ps4
Asgard/Eukaryota - ps5
Eukaryota - ps6
Amorpheae - ps7
Obazoa - ps8
Opisthokonta - ps9
Holomycota - ps10
Fungi - ps11
Fungi_1 - ps12
Fungi_2 - ps13
Fungi_3 - ps14
Dikarya - ps15
Ascomycota - ps16
Saccharo/Pezizomycotina - ps17
Saccharomycotina - ps18
Saccharomycotina_1 - ps19
Saccharomycotina_2 - ps20
Saccharomycotina_3 - ps21
Phaff./Saccharomycetaceae - ps22
Saccharomyco./mycetaceae - ps23
Saccharomycetaceae - ps24
Saccharomycetaceae_2 - ps25
Saccharomycetaceae_3 - ps26
Kaz./Nau./Saccharomyces - ps27
Saccharomyces - ps28
Arboricola_clade - ps29
Cerevisiae_clade - ps30
Saccharomyces_cerevisiae - ps31
Phylostratum

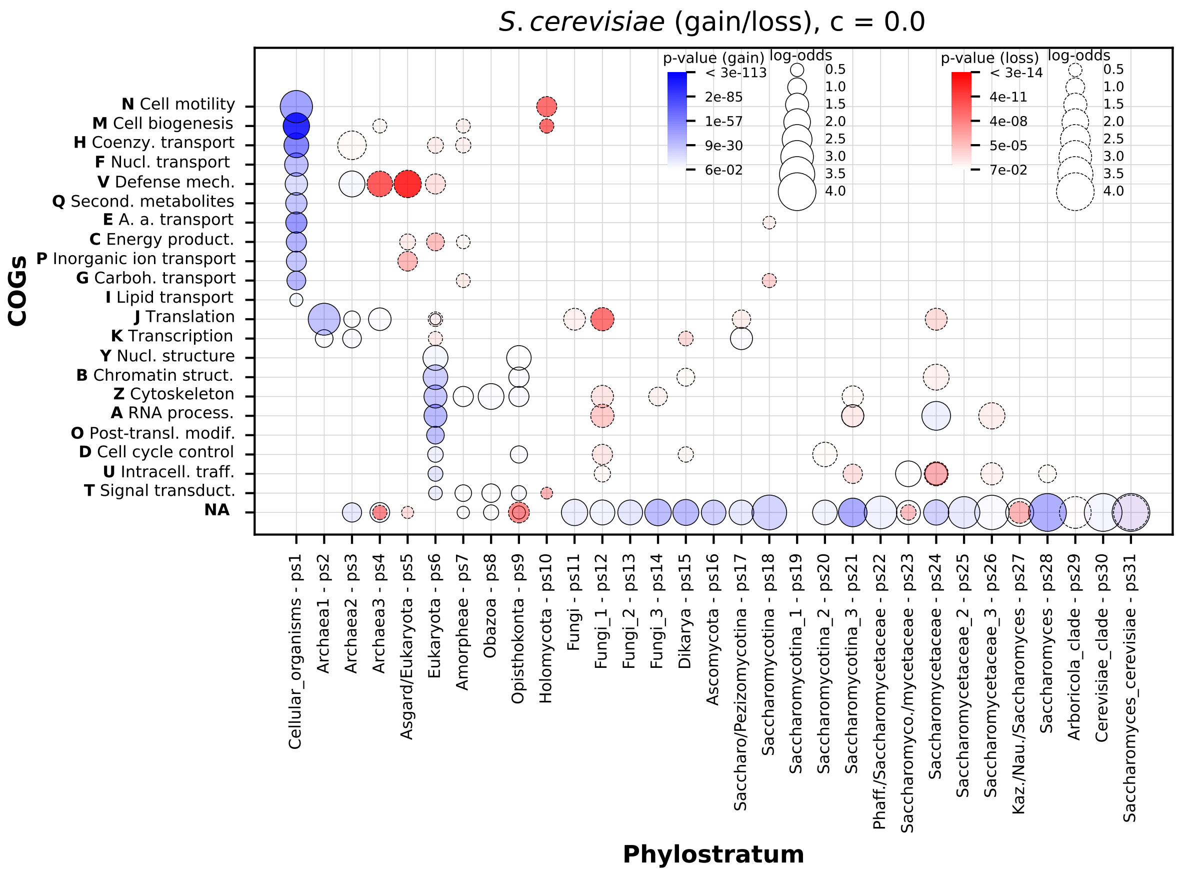
S. cerevisiae (gain/loss), c = 0.0
p-value (gain)
< 3e-113
2e-85
1e-57
9e-30
6e-02
log-odds
0.5
1.0
1.5
2.0
2.5
3.0
3.5
4.0
p-value (loss)
< 3e-14
4e-11
4e-08
5e-05
7e-02
COGs
N Cell motility
M Cell biogenesis
H Coenzy. transport
F Nucl. transport
V Defense mech.
Q Second. metabolites
E A. a. transport
C Energy product.
P Inorganic ion transport
G Carboh. transport
I Lipid transport
J Translation
K Transcription
Y Nucl. structure
B Chromatin struct.
Z Cytoskeleton
A RNA process.
O Post-transl. modif.
D Cell cycle control
U Intracell. traff.
T Signal transduct.
NA
Cellular_organisms - ps1
Archaea1 - ps2
Archaea2 - ps3
Archaea3 - ps4
Asgard/Eukaryota - ps5
Eukaryota - ps6
Amorpheae - ps7
Obazoa - ps8
Opisthokonta - ps9
Holomycota - ps10
Fungi - ps11
Fungi_1 - ps12
Fungi_2 - ps13
Fungi_3 - ps14
Dikarya - ps15
Ascomycota - ps16
Saccharo/Pezizomycotina - ps17
Saccharomycotina - ps18
Saccharomycotina_1 - ps19
Saccharomycotina_2 - ps20
Saccharomycotina_3 - ps21
Phaff./Saccharomycetaceae - ps22
Saccharomyco./mycetaceae - ps23
Saccharomycetaceae - ps24
Saccharomycetaceae_2 - ps25
Saccharomycetaceae_3 - ps26
Kaz./Nau./Saccharomyces - ps27
Saccharomyces - ps28
Arboricola_clade - ps29
Cerevisiae_clade - ps30
Saccharomyces_cerevisiae - ps31
Phylostratum

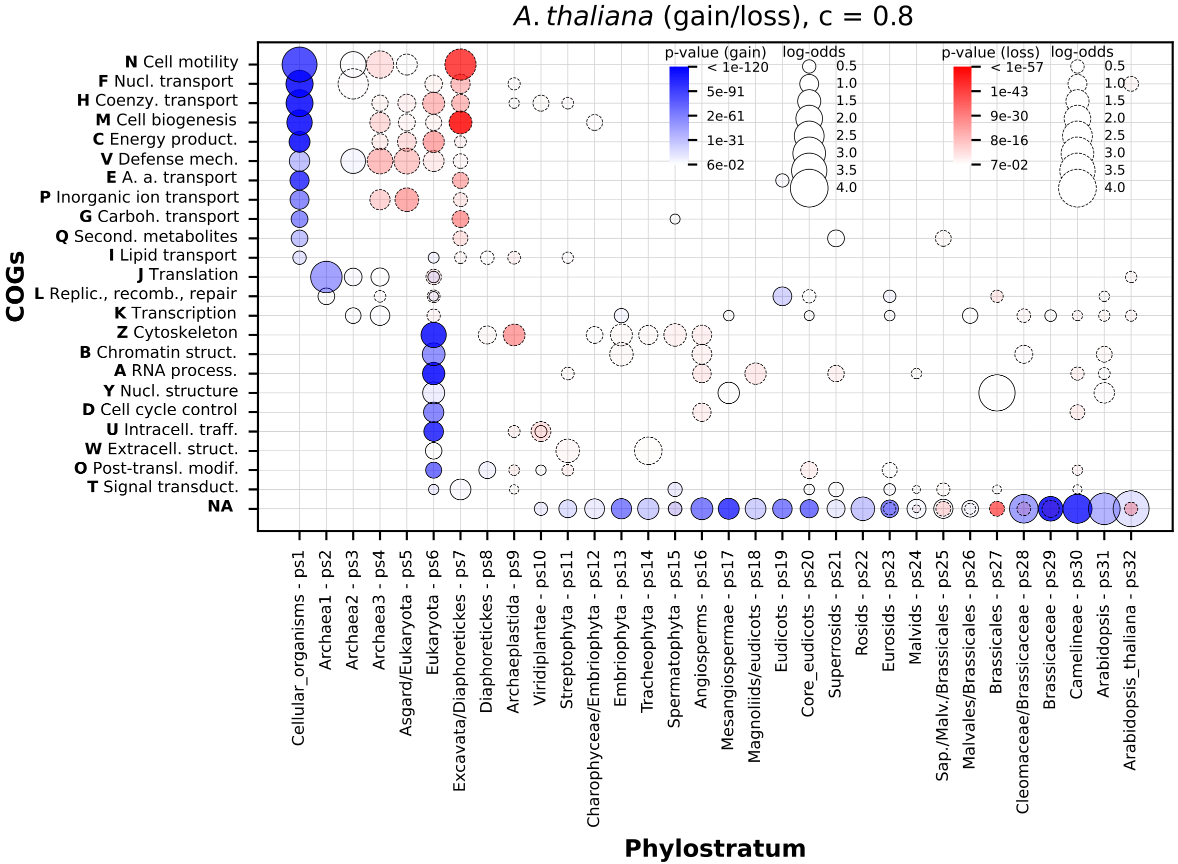

A. thaliana (gain/loss), c = 0.8
COGs
N Cell motility
F Nucl. transport
H Coenzy. transport
M Cell biogenesis
C Energy product.
V Defense mech.
E A. a. transport
P Inorganic ion transport
G Carboh. transport
Q Second. metabolites
I Lipid transport
J Translation
L Replic., recomb., repair
K Transcription
Z Cytoskeleton
B Chromatin struct.
A RNA process.
Y Nucl. structure
D Cell cycle control
U Intracell. traff.
W Extracell. struct.
O Post-transl. modif.
T Signal transduct.
NA
p-value (gain)
< 1e-120
5e-91
2e-61
1e-31
6e-02
log-odds
0.5
1.0
1.5
2.0
2.5
3.0
3.5
4.0
p-value (loss)
< 1e-57
1e-43
9e-30
8e-16
7e-02
log-odds
0.5
1.0
1.5
2.0
2.5
3.0
3.5
4.0
Cellular_organisms - ps1
Archaea1 - ps2
Archaea2 - ps3
Archaea3 - ps4
Asgard/Eukaryota - ps5
Eukaryota - ps6
Excavata/Diaphoretickes - ps7
Diaphoretickes - ps8
Archaeplastida - ps9
Viridiplantae - ps10
Streptophyta - ps11
Charophyceae/Embriophyta - ps12
Embriophyta - ps13
Tracheophyta - ps14
Spermatophyta - ps15
Angiosperms - ps16
Mesangiospermae - ps17
Magnoliids/eudicots - ps18
Eudicots - ps19
Core_eudicots - ps20
Superrosids - ps21
Rosids - ps22
Eurosids - ps23
Malvids - ps24
Sap./Malv./Brassicales - ps25
Malvales/Brassicales - ps26
Brassicales - ps27
Cleomaceae/Brassicaceae - ps28
Brassicaceae - ps29
Camelineae - ps30
Arabidopsis - ps31
Arabidopsis_thaliana - ps32
Phylostratum

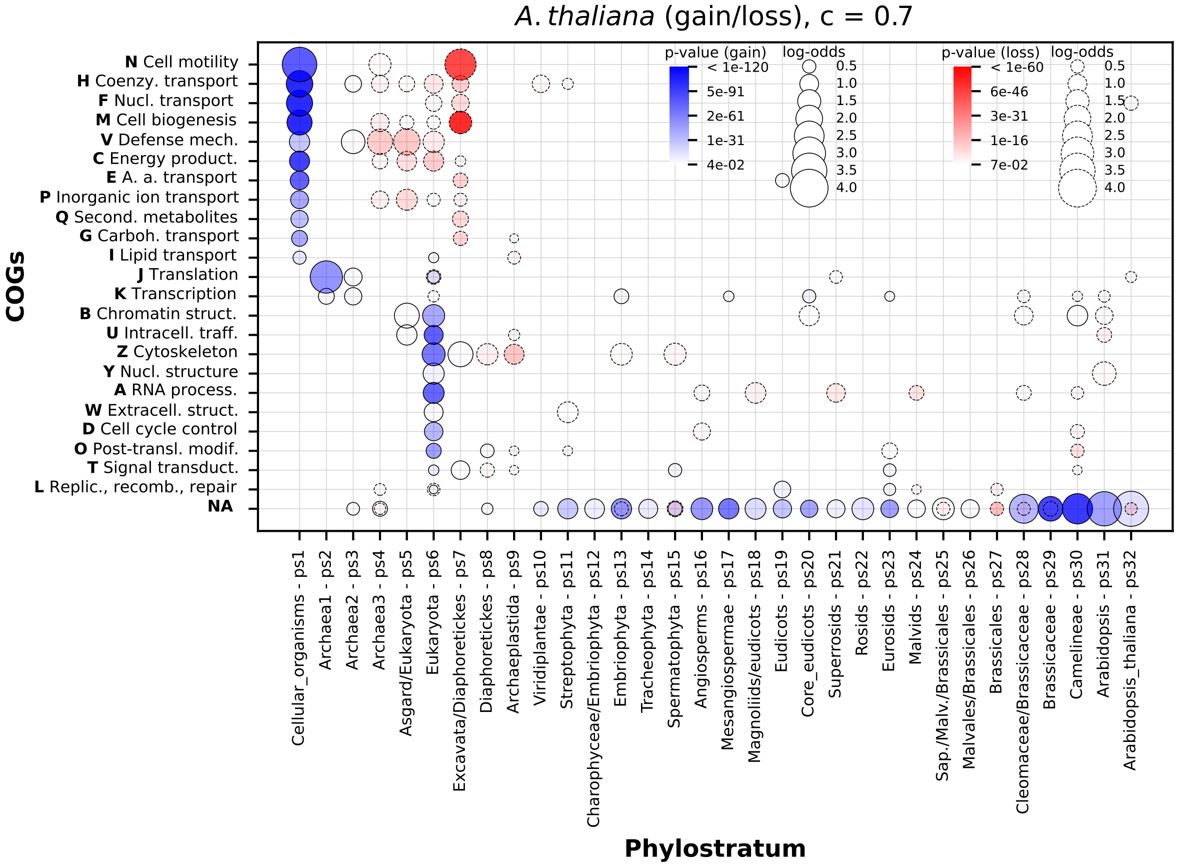

A. thaliana (gain/loss), c = 0.7
COGs
N Cell motility
H Coenzy. transport
F Nucl. transport
M Cell biogenesis
V Defense mech.
C Energy product.
E A. a. transport
P Inorganic ion transport
Q Second. metabolites
G Carboh. transport
I Lipid transport
J Translation
K Transcription
B Chromatin struct.
U Intracell. traff.
Z Cytoskeleton
Y Nucl. structure
A RNA process.
W Extracell. struct.
D Cell cycle control
O Post-transl. modif.
T Signal transduct.
L Replic., recomb., repair
NA
p-value (gain)
< 1e-120
5e-91
2e-61
1e-31
4e-02
log-odds
0.5
1.0
1.5
2.0
2.5
3.0
3.5
4.0
p-value (loss)
< 1e-60
6e-46
3e-31
1e-16
7e-02
log-odds
0.5
1.0
1.5
2.0
2.5
3.0
3.5
4.0
Cellular_organisms - ps1
Archaea1 - ps2
Archaea2 - ps3
Archaea3 - ps4
Asgard/Eukaryota - ps5
Eukaryota - ps6
Excavata/Diaphoretickes - ps7
Diaphoretickes - ps8
Archaeplastida - ps9
Viridiplantae - ps10
Streptophyta - ps11
Charophyceae/Embriophyta - ps12
Embriophyta - ps13
Tracheophyta - ps14
Spermatophyta - ps15
Angiosperms - ps16
Mesangiospermae - ps17
Magnoliids/eudicots - ps18
Eudicots - ps19
Core_eudicots - ps20
Superrosids - ps21
Rosids - ps22
Eurosids - ps23
Malvids - ps24
Sap./Malv./Brassicales - ps25
Malvales/Brassicales - ps26
Brassicales - ps27
Cleomaceae/Brassicaceae - ps28
Brassicaceae - ps29
Camelineae - ps30
Arabidopsis - ps31
Arabidopsis_thaliana - ps32
Phylostratum

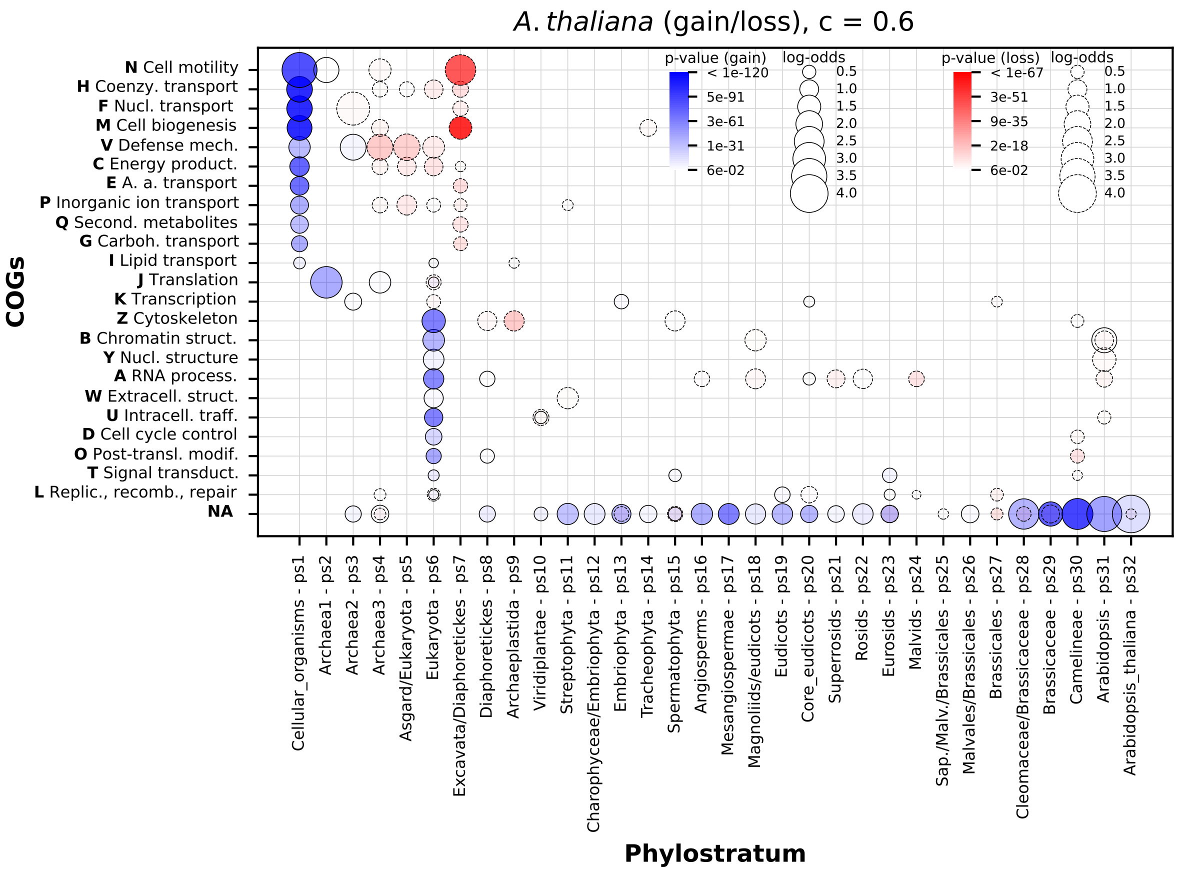

A. thaliana (gain/loss), c = 0.6
COGs
Phylostratum
p-value (gain)
< 1e-120
5e-91
3e-61
1e-31
6e-02
log-odds
0.5
1.0
1.5
2.0
2.5
3.0
3.5
4.0
p-value (loss)
< 1e-67
3e-51
9e-35
2e-18
6e-02
N Cell motility
H Coenzy. transport
F Nucl. transport
M Cell biogenesis
V Defense mech.
C Energy product.
E A. a. transport
P Inorganic ion transport
Q Second. metabolites
G Carboh. transport
I Lipid transport
J Translation
K Transcription
Z Cytoskeleton
B Chromatin struct.
Y Nucl. structure
A RNA process.
W Extracell. struct.
U Intracell. traff.
D Cell cycle control
O Post-transl. modif.
T Signal transduct.
L Replic., recomb., repair
NA
Cellular_organisms - ps1
Archaea1 - ps2
Archaea2 - ps3
Archaea3 - ps4
Asgard/Eukaryota - ps5
Eukaryota - ps6
Excavata/Diaphoretickes - ps7
Diaphoretickes - ps8
Archaeplastida - ps9
Viridiplantae - ps10
Streptophyta - ps11
Charophyceae/Embriophyta - ps12
Embriophyta - ps13
Tracheophyta - ps14
Spermatophyta - ps15
Angiosperms - ps16
Mesangiospermae - ps17
Magnoliids/eudicots - ps18
Eudicots - ps19
Core_eudicots - ps20
Superrosids - ps21
Rosids - ps22
Eurosids - ps23
Malvids - ps24
Sap./Malv./Brassicales - ps25
Malvales/Brassicales - ps26
Brassicales - ps27
Cleomaceae/Brassicaceae - ps28
Brassicaceae - ps29
Camelineae - ps30
Arabidopsis - ps31
Arabidopsis_thaliana - ps32

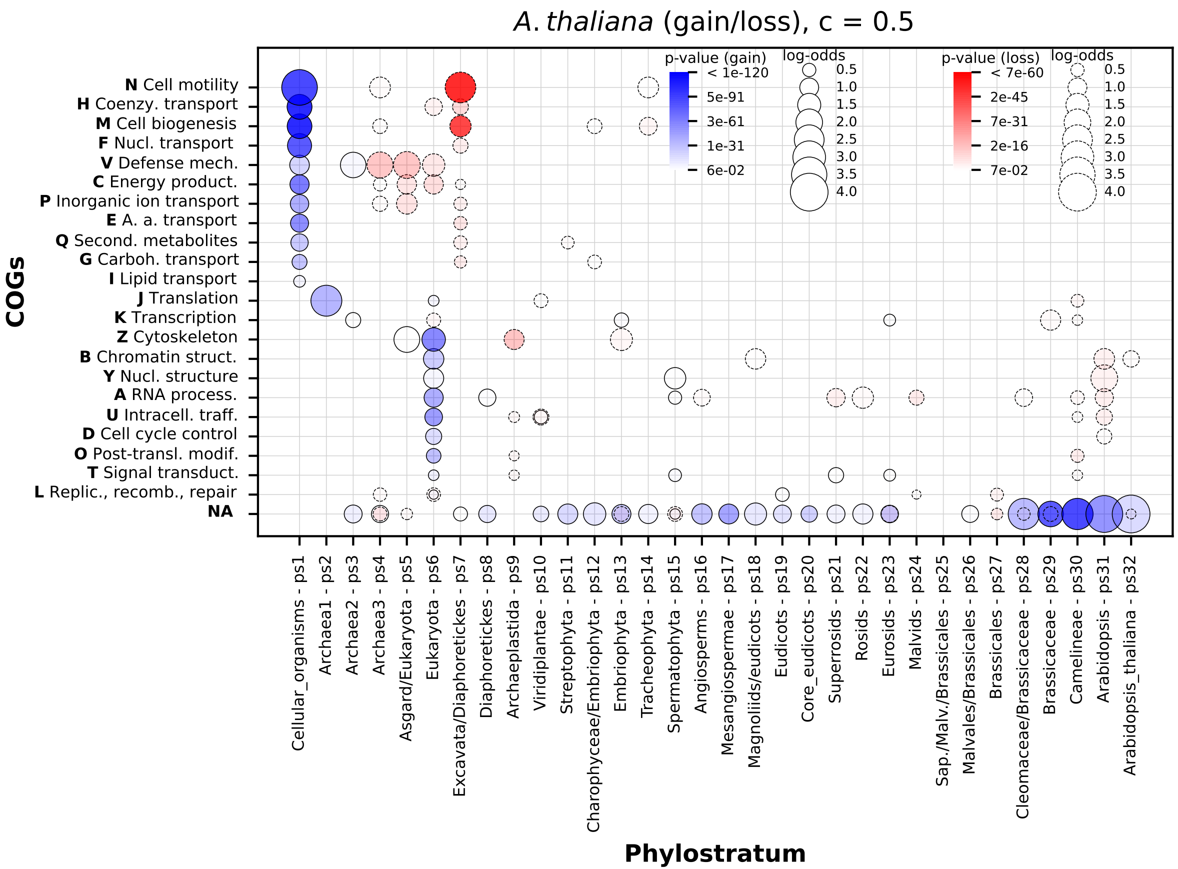

A. thaliana (gain/loss), c = 0.5
COGs
N Cell motility
H Coenzy. transport
M Cell biogenesis
F Nucl. transport
V Defense mech.
C Energy product.
P Inorganic ion transport
E A. a. transport
Q Second. metabolites
G Carboh. transport
I Lipid transport
J Translation
K Transcription
Z Cytoskeleton
B Chromatin struct.
Y Nucl. structure
A RNA process.
U Intracell. traff.
D Cell cycle control
O Post-transl. modif.
T Signal transduct.
L Replic., recomb., repair
NA
p-value (gain)
< 1e-120
5e-91
3e-61
1e-31
6e-02
log-odds
0.5
1.0
1.5
2.0
2.5
3.0
3.5
4.0
p-value (loss)
< 7e-60
2e-45
7e-31
2e-16
7e-02
log-odds
0.5
1.0
1.5
2.0
2.5
3.0
3.5
4.0
Cellular_organisms - ps1
Archaea1 - ps2
Archaea2 - ps3
Archaea3 - ps4
Asgard/Eukaryota - ps5
Eukaryota - ps6
Excavata/Diaphoretickes - ps7
Diaphoretickes - ps8
Archaeplastida - ps9
Viridiplantae - ps10
Streptophyta - ps11
Charophyceae/Embriophyta - ps12
Embriophyta - ps13
Tracheophyta - ps14
Spermatophyta - ps15
Angiosperms - ps16
Mesangiospermae - ps17
Magnoliids/eudicots - ps18
Eudicots - ps19
Core_eudicots - ps20
Superrosids - ps21
Rosids - ps22
Eurosids - ps23
Malvids - ps24
Sap./Malv./Brassicales - ps25
Malvales/Brassicales - ps26
Brassicales - ps27
Cleomaceae/Brassicaceae - ps28
Brassicaceae - ps29
Camelineae - ps30
Arabidopsis - ps31
Arabidopsis_thaliana - ps32
Phylostratum

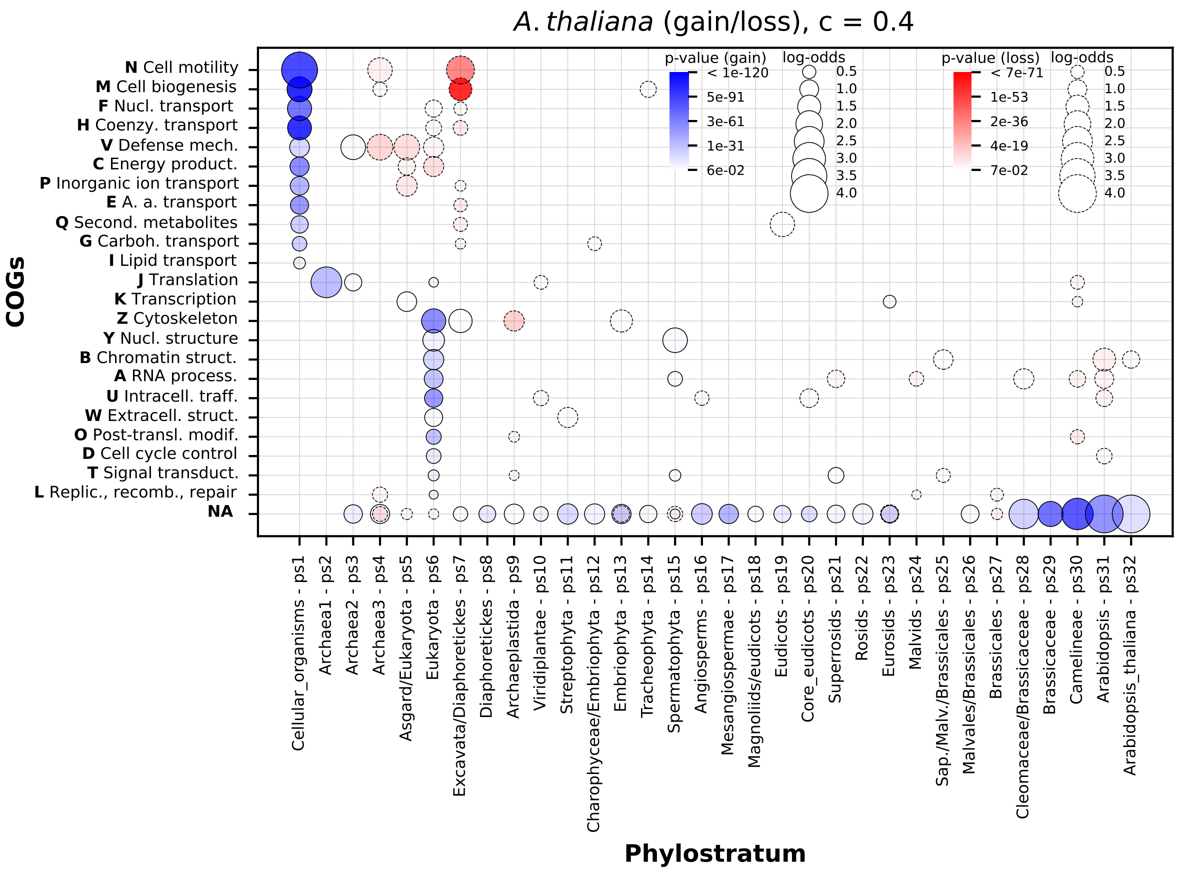
A. thaliana (gain/loss), c = 0.4
COGs
N Cell motility
M Cell biogenesis
F Nucl. transport
H Coenzy. transport
V Defense mech.
C Energy product.
P Inorganic ion transport
E A. a. transport
Q Second. metabolites
G Carboh. transport
I Lipid transport
J Translation
K Transcription
Z Cytoskeleton
Y Nucl. structure
B Chromatin struct.
A RNA process.
U Intracell. traff.
W Extracell. struct.
O Post-transl. modif.
D Cell cycle control
T Signal transduct.
L Replic., recomb., repair
NA
p-value (gain)
< 1e-120
5e-91
3e-61
1e-31
6e-02
log-odds
0.5
1.0
1.5
2.0
2.5
3.0
3.5
4.0
p-value (loss)
< 7e-71
1e-53
2e-36
4e-19
7e-02
log-odds
0.5
1.0
1.5
2.0
2.5
3.0
3.5
4.0
Cellular_organisms - ps1
Archaea1 - ps2
Archaea2 - ps3
Archaea3 - ps4
Asgard/Eukaryota - ps5
Eukaryota - ps6
Excavata/Diaphoretickes - ps7
Diaphoretickes - ps8
Archaeplastida - ps9
Viridiplantae - ps10
Streptophyta - ps11
Charophyceae/Embriophyta - ps12
Embriophyta - ps13
Tracheophyta - ps14
Spermatophyta - ps15
Angiosperms - ps16
Mesangiospermae - ps17
Magnoliids/eudicots - ps18
Eudicots - ps19
Core_eudicots - ps20
Superrosids - ps21
Rosids - ps22
Eurosids - ps23
Malvids - ps24
Sap./Malv./Brassicales - ps25
Malvales/Brassicales - ps26
Brassicales - ps27
Cleomaceae/Brassicaceae - ps28
Brassicaceae - ps29
Camelineae - ps30
Arabidopsis - ps31
Arabidopsis_thaliana - ps32
Phylostratum

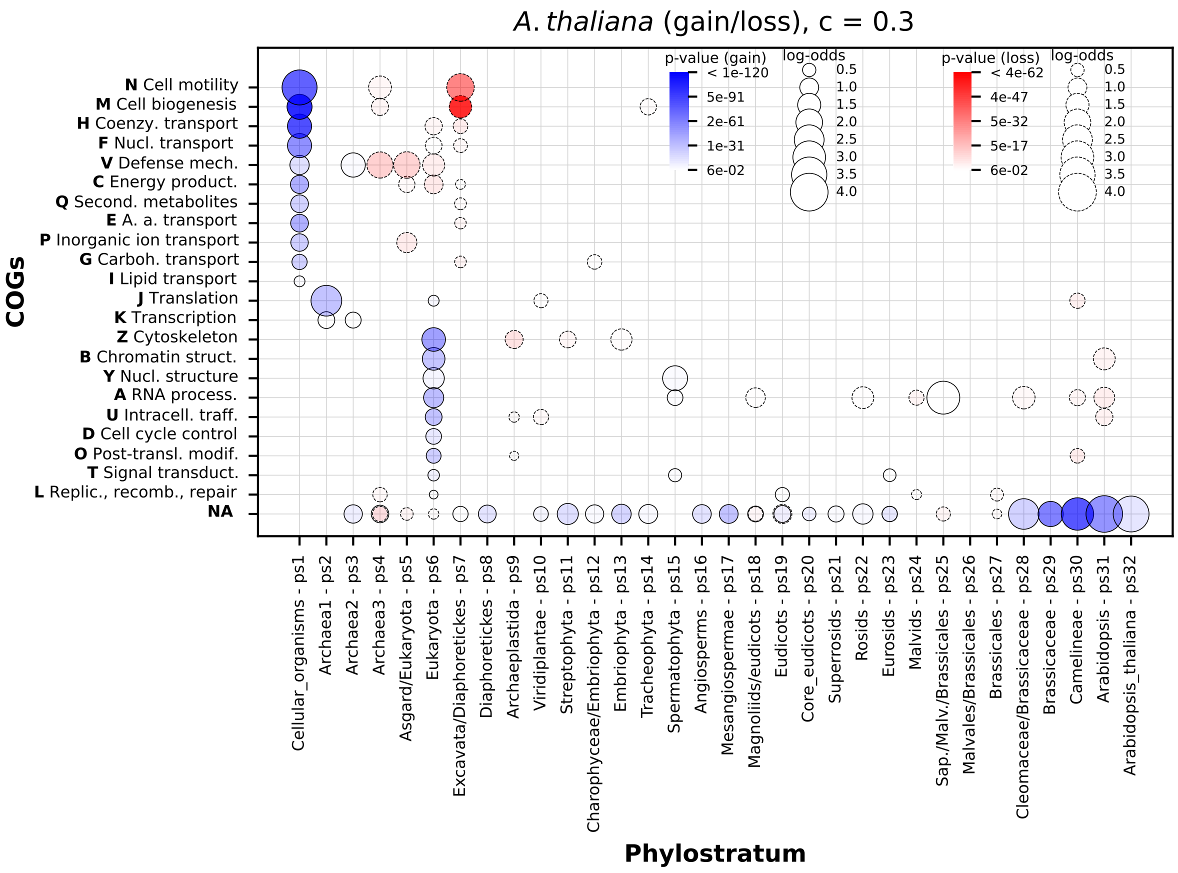

A. thaliana (gain/loss), c = 0.3
p-value (gain)
< 1e-120
5e-91
2e-61
1e-31
6e-02
log-odds
0.5
1.0
1.5
2.0
2.5
3.0
3.5
4.0
p-value (loss)
< 4e-62
4e-47
5e-32
5e-17
6e-02
COGs
N Cell motility
M Cell biogenesis
H Coenzy. transport
F Nucl. transport
V Defense mech.
C Energy product.
Q Second. metabolites
E A. a. transport
P Inorganic ion transport
G Carboh. transport
I Lipid transport
J Translation
K Transcription
Z Cytoskeleton
B Chromatin struct.
Y Nucl. structure
A RNA process.
U Intracell. traff.
D Cell cycle control
O Post-transl. modif.
T Signal transduct.
L Replic., recomb., repair
NA
Cellular_organisms - ps1
Archaea1 - ps2
Archaea2 - ps3
Archaea3 - ps4
Asgard/Eukaryota - ps5
Eukaryota - ps6
Excavata/Diaphoretickes - ps7
Diaphoretickes - ps8
Archaeplastida - ps9
Viridiplantae - ps10
Streptophyta - ps11
Charophyceae/Embriophyta - ps12
Embriophyta - ps13
Tracheophyta - ps14
Spermatophyta - ps15
Angiosperms - ps16
Mesangiospermae - ps17
Magnoliids/eudicots - ps18
Eudicots - ps19
Core_eudicots - ps20
Superrosids - ps21
Rosids - ps22
Eurosids - ps23
Malvids - ps24
Sap./Malv./Brassicales - ps25
Malvales/Brassicales - ps26
Brassicales - ps27
Cleomaceae/Brassicaceae - ps28
Brassicaceae - ps29
Camelineae - ps30
Arabidopsis - ps31
Arabidopsis_thaliana - ps32
Phylostratum

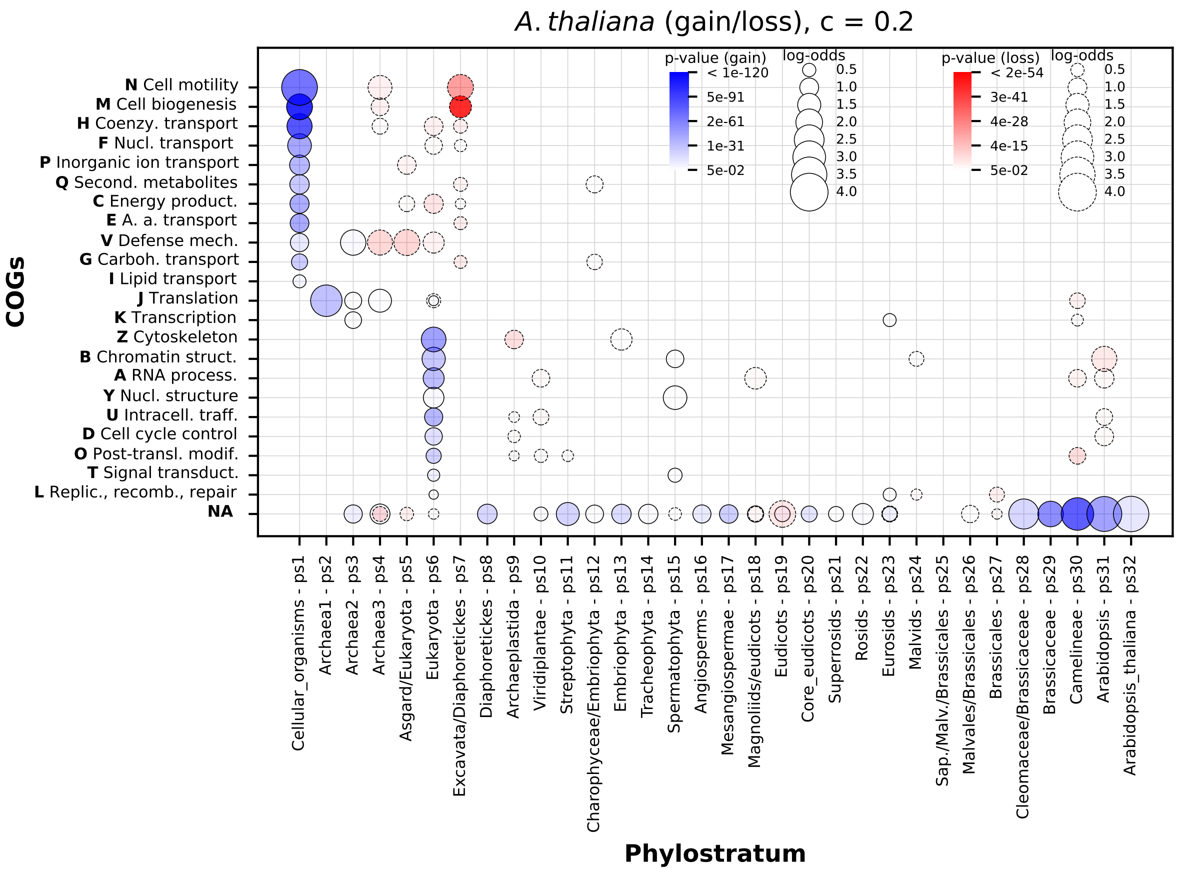
A. thaliana (gain/loss), c = 0.2
COGs
N Cell motility
M Cell biogenesis
H Coenzy. transport
F Nucl. transport
P Inorganic ion transport
Q Second. metabolites
C Energy product.
E A. a. transport
V Defense mech.
G Carboh. transport
I Lipid transport
J Translation
K Transcription
Z Cytoskeleton
B Chromatin struct.
A RNA process.
Y Nucl. structure
U Intracell. traff.
D Cell cycle control
O Post-transl. modif.
T Signal transduct.
L Replic., recomb., repair
NA
p-value (gain)
< 1e-120
5e-91
2e-61
1e-31
5e-02
log-odds
0.5
1.0
1.5
2.0
2.5
3.0
3.5
4.0
p-value (loss)
< 2e-54
3e-41
4e-28
4e-15
5e-02
log-odds
0.5
1.0
1.5
2.0
2.5
3.0
3.5
4.0
Cellular_organisms - ps1
Archaea1 - ps2
Archaea2 - ps3
Archaea3 - ps4
Asgard/Eukaryota - ps5
Eukaryota - ps6
Excavata/Diaphoretickes - ps7
Diaphoretickes - ps8
Archaeplastida - ps9
Viridiplantae - ps10
Streptophyta - ps11
Charophyceae/Embriophyta - ps12
Embriophyta - ps13
Tracheophyta - ps14
Spermatophyta - ps15
Angiosperms - ps16
Mesangiospermae - ps17
Magnoliids/eudicots - ps18
Eudicots - ps19
Core_eudicots - ps20
Superrosids - ps21
Rosids - ps22
Eurosids - ps23
Malvids - ps24
Sap./Malv./Brassicales - ps25
Malvales/Brassicales - ps26
Brassicales - ps27
Cleomaceae/Brassicaceae - ps28
Brassicaceae - ps29
Camelineae - ps30
Arabidopsis - ps31
Arabidopsis_thaliana - ps32
Phylostratum

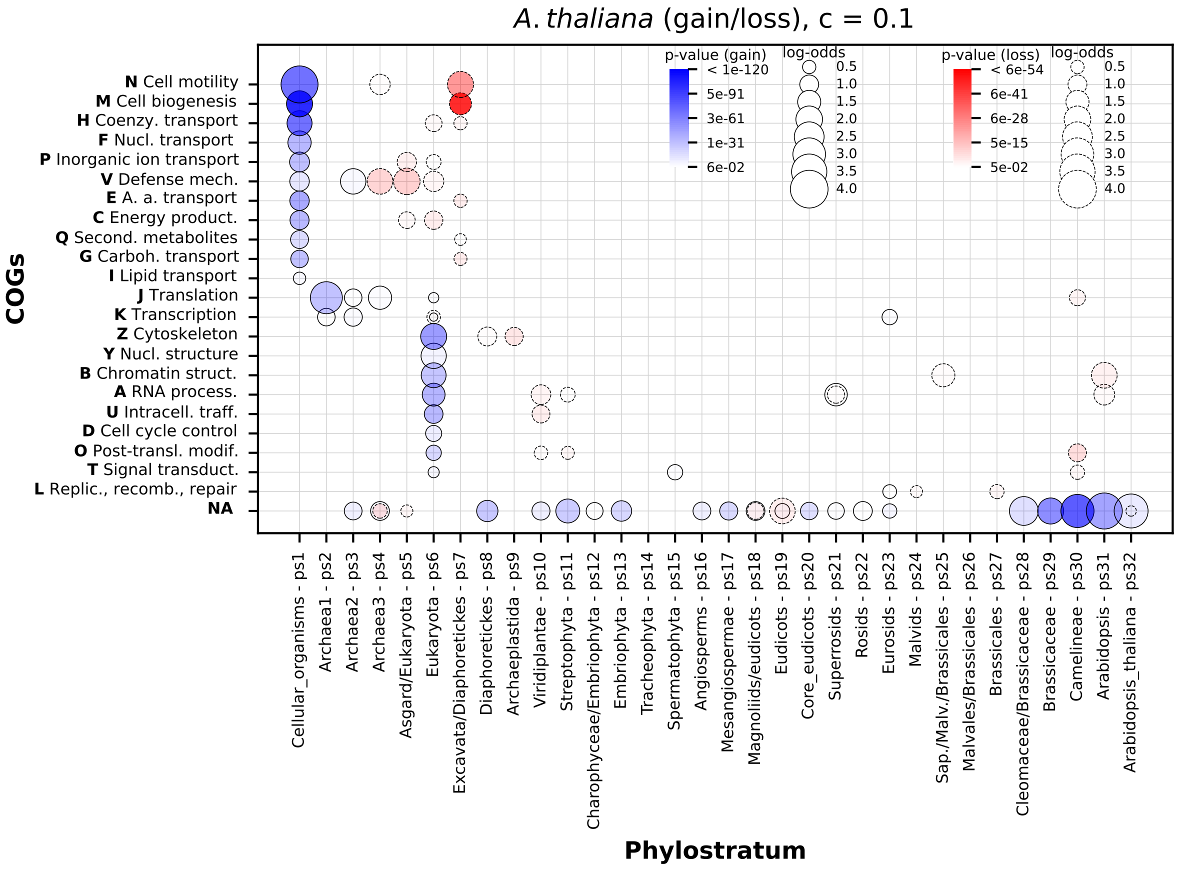
A. thaliana (gain/loss), c = 0.1
p-value (gain)
< 1e-120
5e-91
3e-61
1e-31
6e-02
log-odds
0.5
1.0
1.5
2.0
2.5
3.0
3.5
4.0
p-value (loss)
< 6e-54
6e-41
6e-28
5e-15
5e-02
log-odds
0.5
1.0
1.5
2.0
2.5
3.0
3.5
4.0
COGs
N Cell motility
M Cell biogenesis
H Coenzy. transport
F Nucl. transport
P Inorganic ion transport
V Defense mech.
E A. a. transport
C Energy product.
Q Second. metabolites
G Carboh. transport
I Lipid transport
J Translation
K Transcription
Z Cytoskeleton
Y Nucl. structure
B Chromatin struct.
A RNA process.
U Intracell. traff.
D Cell cycle control
O Post-transl. modif.
T Signal transduct.
L Replic., recomb., repair
NA
Cellular_organisms - ps1
Archaea1 - ps2
Archaea2 - ps3
Archaea3 - ps4
Asgard/Eukaryota - ps5
Eukaryota - ps6
Excavata/Diaphoretickes - ps7
Diaphoretickes - ps8
Archaeplastida - ps9
Viridiplantae - ps10
Streptophyta - ps11
Charophyceae/Embriophyta - ps12
Embriophyta - ps13
Tracheophyta - ps14
Spermatophyta - ps15
Angiosperms - ps16
Mesangiospermae - ps17
Magnoliids/eudicots - ps18
Eudicots - ps19
Core_eudicots - ps20
Superrosids - ps21
Rosids - ps22
Eurosids - ps23
Malvids - ps24
Sap./Malv./Brassicales - ps25
Malvales/Brassicales - ps26
Brassicales - ps27
Cleomaceae/Brassicaceae - ps28
Brassicaceae - ps29
Camelineae - ps30
Arabidopsis - ps31
Arabidopsis_thaliana - ps32
Phylostratum

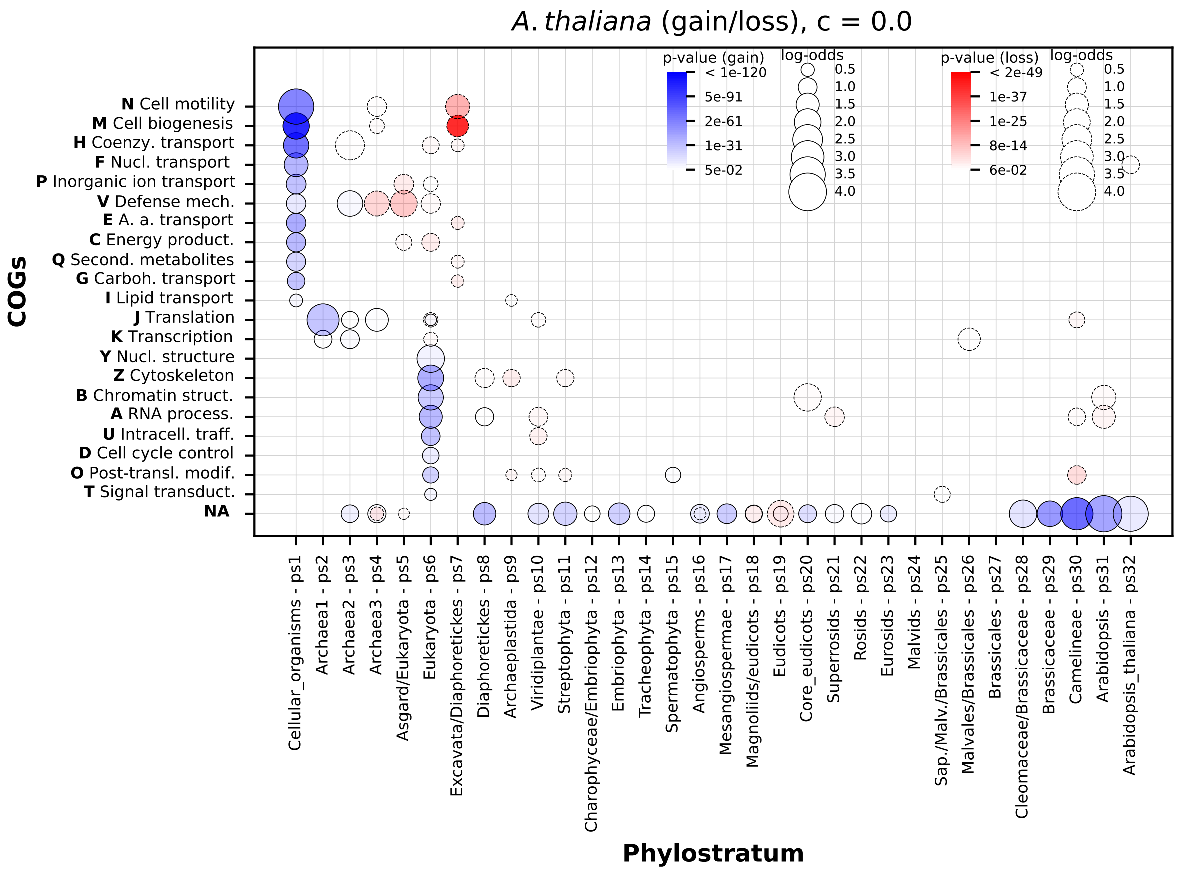

A. thaliana (gain/loss), c = 0.0
p-value (gain)
< 1e-120
5e-91
2e-61
1e-31
5e-02
log-odds
0.5
1.0
1.5
2.0
2.5
3.0
3.5
4.0
p-value (loss)
< 2e-49
1e-37
1e-25
8e-14
6e-02
COGs
N Cell motility
M Cell biogenesis
H Coenzy. transport
F Nucl. transport
P Inorganic ion transport
V Defense mech.
E A. a. transport
C Energy product.
Q Second. metabolites
G Carboh. transport
I Lipid transport
J Translation
K Transcription
Y Nucl. structure
Z Cytoskeleton
B Chromatin struct.
A RNA process.
U Intracell. traff.
D Cell cycle control
O Post-transl. modif.
T Signal transduct.
NA
Cellular_organisms - ps1
Archaea1 - ps2
Archaea2 - ps3
Archaea3 - ps4
Asgard/Eukaryota - ps5
Eukaryota - ps6
Excavata/Diaphoretickes - ps7
Diaphoretickes - ps8
Archaeplastida - ps9
Viridiplantae - ps10
Streptophyta - ps11
Charophyceae/Embriophyta - ps12
Embriophyta - ps13
Tracheophyta - ps14
Spermatophyta - ps15
Angiosperms - ps16
Mesangiospermae - ps17
Magnoliids/eudicots - ps18
Eudicots - ps19
Core_eudicots - ps20
Superrosids - ps21
Rosids - ps22
Eurosids - ps23
Malvids - ps24
Sap./Malv./Brassicales - ps25
Malvales/Brassicales - ps26
Brassicales - ps27
Cleomaceae/Brassicaceae - ps28
Brassicaceae - ps29
Camelineae - ps30
Arabidopsis - ps31
Arabidopsis_thaliana - ps32
Phylostratum
