# Supplementary material for: Macroevolutionary dynamics of gene family gain and loss along multicellular eukaryotic lineages
Source: Nat Commun. 2024 Mar 26;15:2663. doi: 10.1038/s41467-024-47017-w (PMC10966110; doi:10.1038/s41467-024-47017-w)
Supplement: Supplementary file 20 — Supplementary Dataset 15 [file 41467_2024_47017_MOESM20_ESM.pdf]

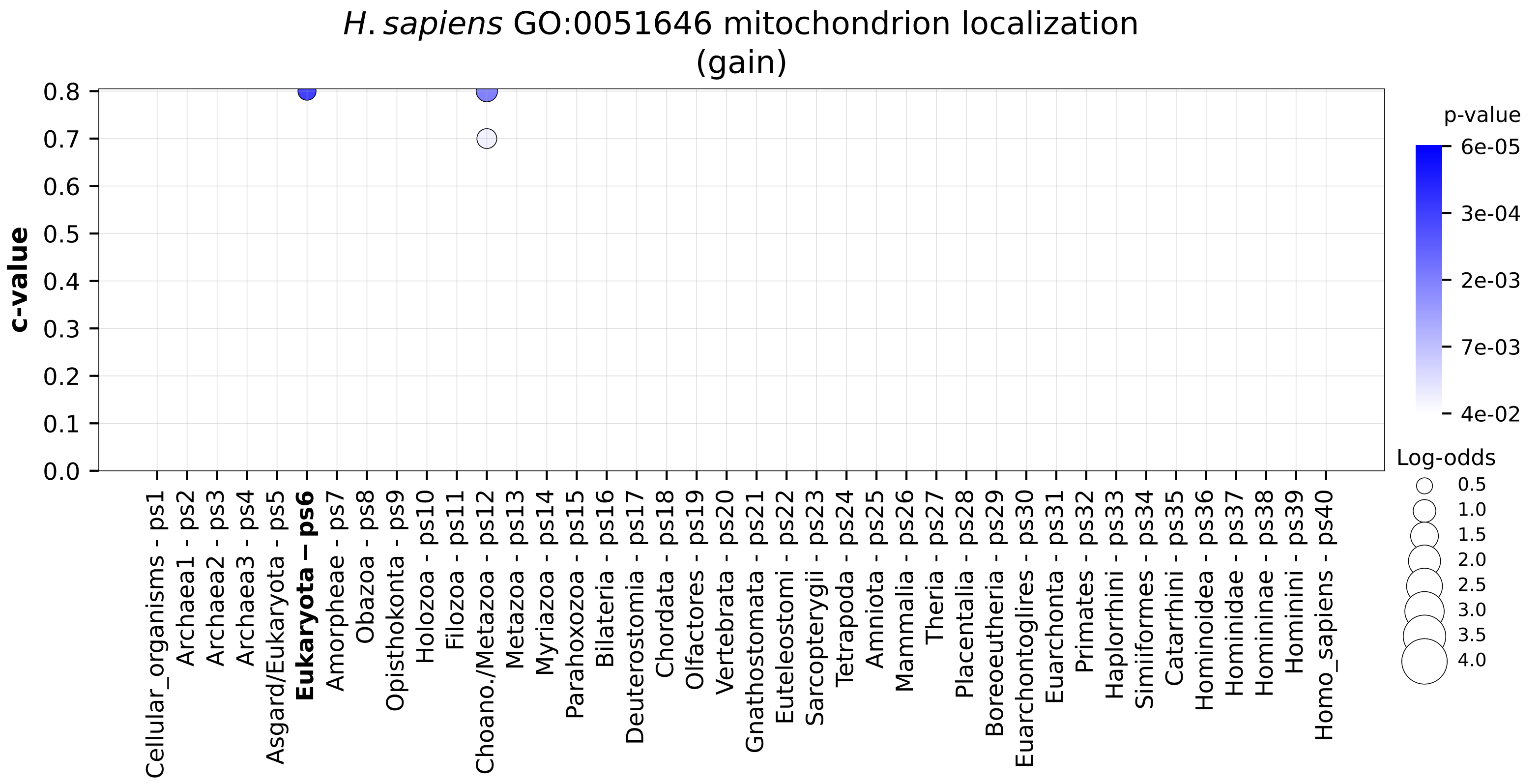

H. sapiens GO:0051646 mitochondrion localization
(gain)
c-value
p-value
6e-05
3e-04
2e-03
7e-03
4e-02
Log-odds
0.5
1.0
1.5
2.0
2.5
3.0
3.5
4.0
Cellular_organisms - ps1
Archaea1 - ps2
Archaea2 - ps3
Archaea3 - ps4
Asgard/Eukaryota - ps5
Eukaryota – ps6
Amorpheae - ps7
Obazoa - ps8
Opisthokonta - ps9
Holozoa - ps10
Filozoa - ps11
Choano./Metazoa - ps12
Metazoa - ps13
Myriazoa - ps14
Parahoxozoa - ps15
Bilateria - ps16
Deuterostomia - ps17
Chordata - ps18
Olfactores - ps19
Vertebrata - ps20
Gnathostomata - ps21
Euteleostomi - ps22
Sarcopterygii - ps23
Tetrapoda - ps24
Amniota - ps25
Mammalia - ps26
Theria - ps27
Placentalia - ps28
Boreoeutheria - ps29
Euarchontoglires - ps30
Euarchonta - ps31
Primates - ps32
Haplorrhini - ps33
Simiiformes - ps34
Catarrhini - ps35
Hominoidea - ps36
Hominidae - ps37
Homininae - ps38
Hominini - ps39
Homo_sapiens - ps40

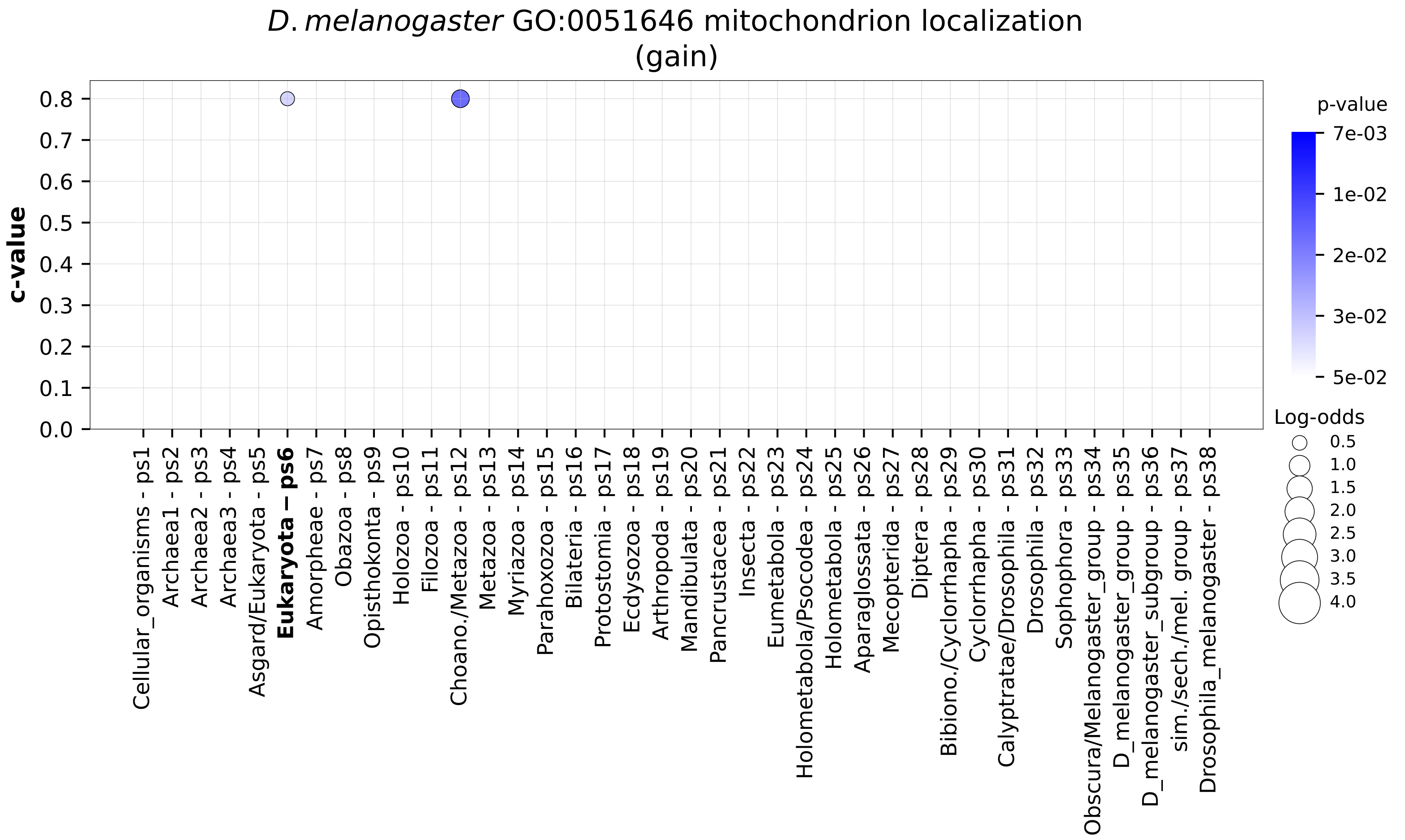

D. melanogaster GO:0051646 mitochondrion localization
(gain)
c-value
p-value
7e-03
1e-02
2e-02
3e-02
5e-02
Log-odds
Cellular_organisms - ps1
Archaea1 - ps2
Archaea2 - ps3
Archaea3 - ps4
Asgard/Eukaryota - ps5
Eukaryota – ps6
Amorpheae - ps7
Obazoa - ps8
Opisthokonta - ps9
Holozoa - ps10
Filozoa - ps11
Choano./Metazoa - ps12
Metazoa - ps13
Myriazoa - ps14
Parahoxozoa - ps15
Bilateria - ps16
Protostomia - ps17
Ecdysozoa - ps18
Arthropoda - ps19
Mandibulata - ps20
Pancrustacea - ps21
Insecta - ps22
Eumetabola - ps23
Holometabola/Psocodea - ps24
Holometabola - ps25
Aparaglossata - ps26
Mecopterida - ps27
Diptera - ps28
Bibiono./Cyclorrhapha - ps29
Cyclorrhapha - ps30
Calyptratae/Drosophila - ps31
Drosophila - ps32
Sophophora - ps33
Obscura/Melanogaster_group - ps34
D_melanogaster_group - ps35
D_melanogaster_subgroup - ps36
sim./sech./mel. group - ps37
Drosophila_melanogaster - ps38

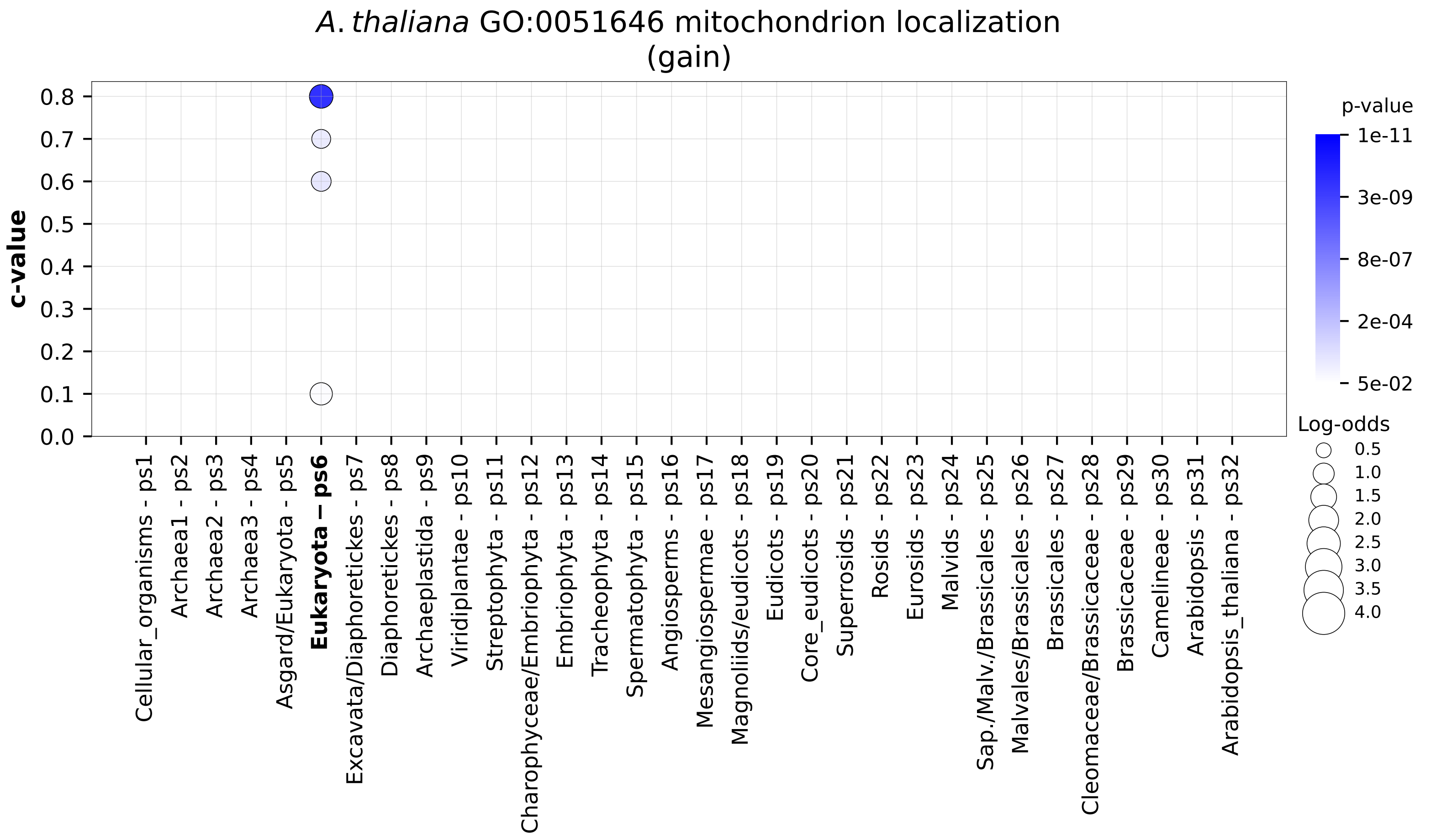

A. thaliana GO:0051646 mitochondrion localization
(gain)
c-value
p-value
1e-11
3e-09
8e-07
2e-04
5e-02
Log-odds
Cellular_organisms - ps1
Archaea1 - ps2
Archaea2 - ps3
Archaea3 - ps4
Asgard/Eukaryota - ps5
Eukaryota – ps6
Excavata/Diaphoretickes - ps7
Diaphoretickes - ps8
Archaeplastida - ps9
Viridiplantae - ps10
Streptophyta - ps11
Charophyceae/Embriophyta - ps12
Embriophyta - ps13
Tracheophyta - ps14
Spermatophyta - ps15
Angiosperms - ps16
Mesangiospermae - ps17
Magnoliids/eudicots - ps18
Eudicots - ps19
Core_eudicots - ps20
Superrosids - ps21
Rosids - ps22
Eurosids - ps23
Malvids - ps24
Sap./Malv./Brassicales - ps25
Malvales/Brassicales - ps26
Brassicales - ps27
Cleomaceae/Brassicaceae - ps28
Brassicaceae - ps29
Camelineae - ps30
Arabidopsis - ps31
Arabidopsis_thaliana - ps32

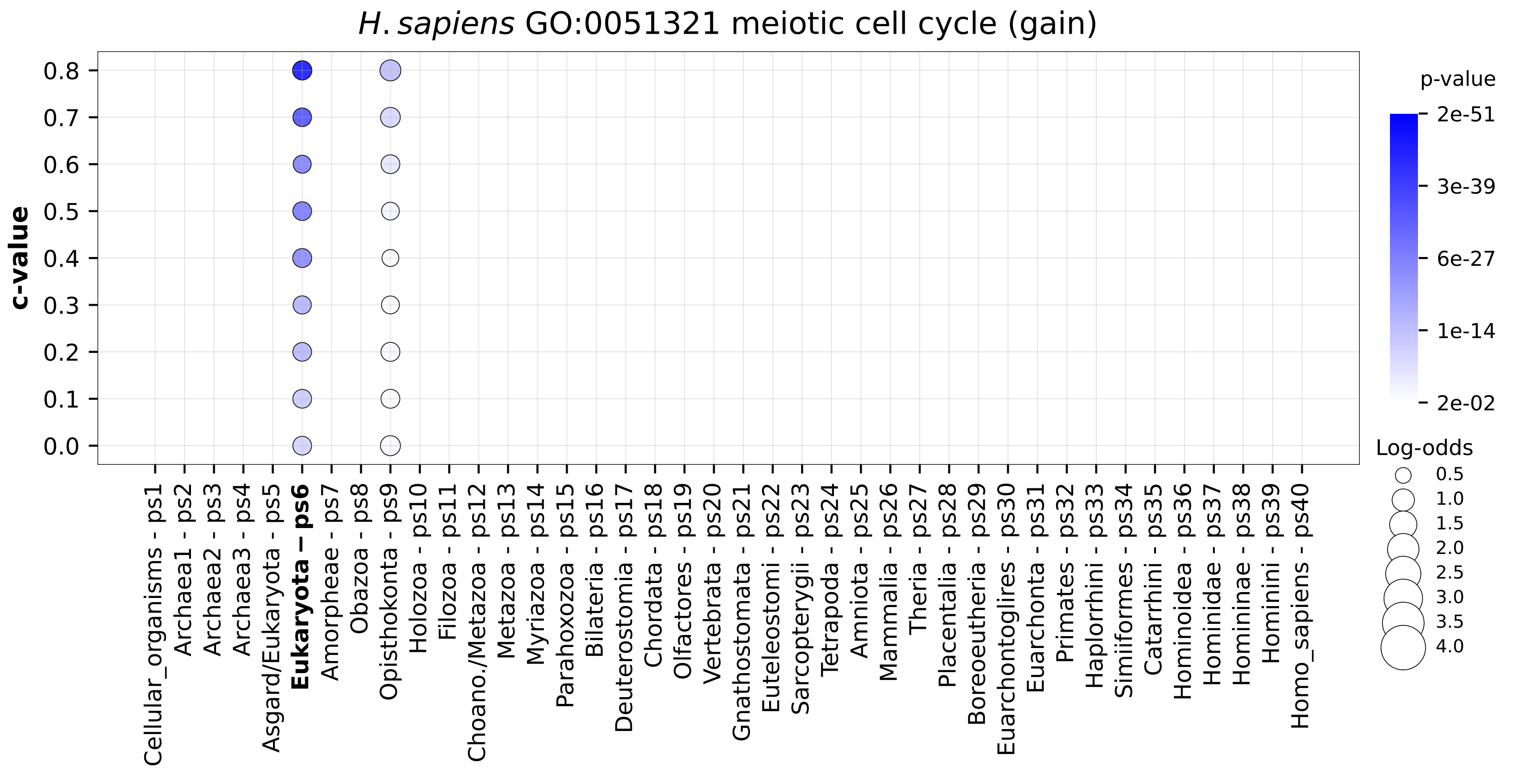
H. sapiens GO:0051321 meiotic cell cycle (gain)
c-value
0.0
0.1
0.2
0.3
0.4
0.5
0.6
0.7
0.8
p-value
2e-51
3e-39
6e-27
1e-14
2e-02
Log-odds
0.5
1.0
1.5
2.0
2.5
3.0
3.5
4.0
Cellular_organisms - ps1
Archaea1 - ps2
Archaea2 - ps3
Archaea3 - ps4
Asgard/Eukaryota - ps5
Eukaryota – ps6
Amorpheae - ps7
Obazoa - ps8
Opisthokonta - ps9
Holozoa - ps10
Filozoa - ps11
Choano./Metazoa - ps12
Metazoa - ps13
Myriazoa - ps14
Parahoxozoa - ps15
Bilateria - ps16
Deuterostomia - ps17
Chordata - ps18
Olfactores - ps19
Vertebrata - ps20
Gnathostomata - ps21
Euteleostomi - ps22
Sarcopterygii - ps23
Tetrapoda - ps24
Amniota - ps25
Mammalia - ps26
Theria - ps27
Placentalia - ps28
Boreoeutheria - ps29
Euarchontoglires - ps30
Euarchonta - ps31
Primates - ps32
Haplorrhini - ps33
Simiiformes - ps34
Catarrhini - ps35
Hominoidea - ps36
Hominidae - ps37
Homininae - ps38
Hominini - ps39
Homo_sapiens - ps40

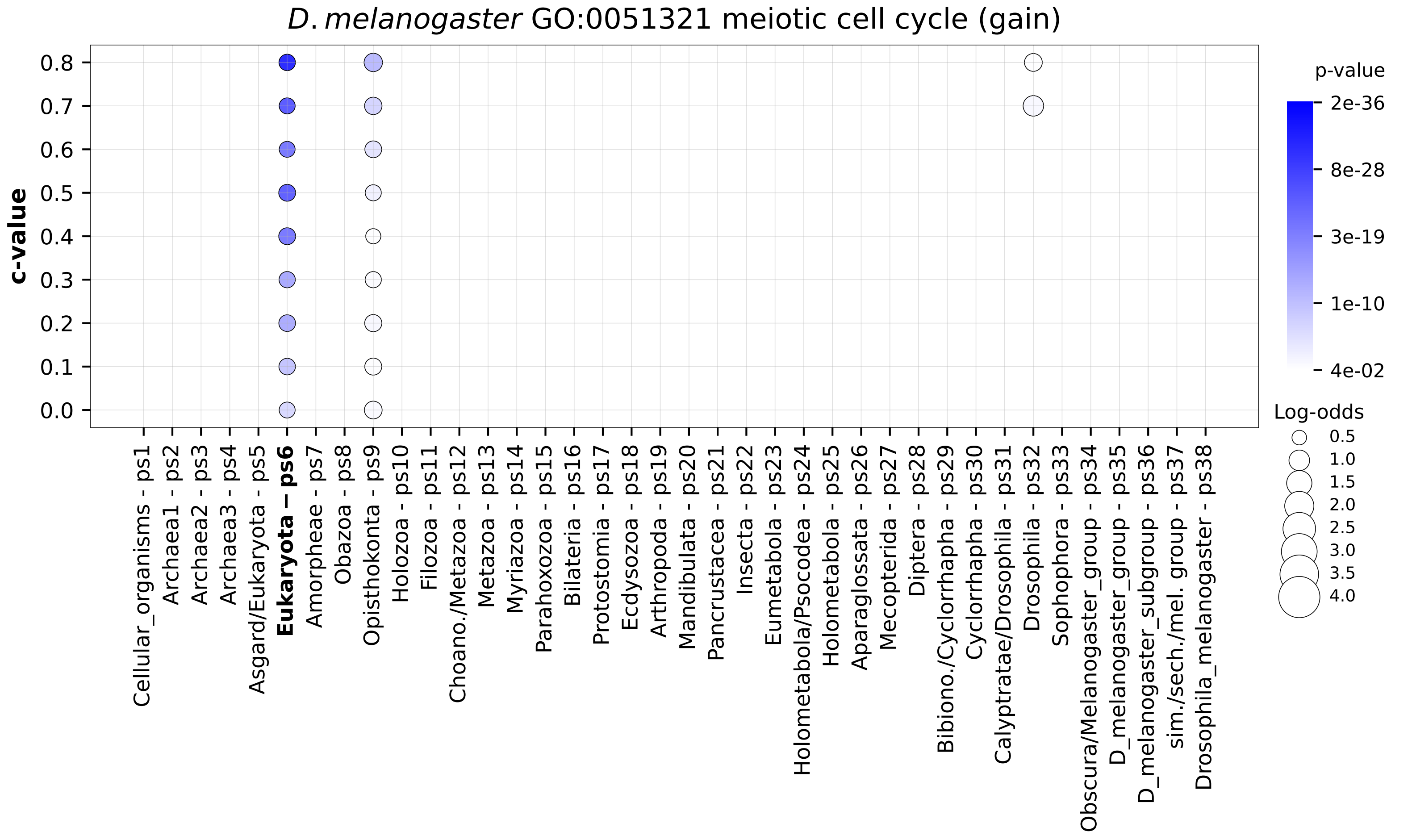
D. melanogaster GO:0051321 meiotic cell cycle (gain)
c-value
0.0
0.1
0.2
0.3
0.4
0.5
0.6
0.7
0.8
p-value
2e-36
8e-28
3e-19
1e-10
4e-02
Log-odds
0.5
1.0
1.5
2.0
2.5
3.0
3.5
4.0
Cellular_organisms - ps1
Archaea1 - ps2
Archaea2 - ps3
Archaea3 - ps4
Asgard/Eukaryota - ps5
Eukaryota – ps6
Amorpheae - ps7
Obazoa - ps8
Opisthokonta - ps9
Holozoa - ps10
Filozoa - ps11
Choano./Metazoa - ps12
Metazoa - ps13
Myriazoa - ps14
Parahoxozoa - ps15
Bilateria - ps16
Protostomia - ps17
Ecdysozoa - ps18
Arthropoda - ps19
Mandibulata - ps20
Pancrustacea - ps21
Insecta - ps22
Eumetabola - ps23
Holometabola/Psocodea - ps24
Holometabola - ps25
Aparaglossata - ps26
Mecopterida - ps27
Diptera - ps28
Bibiono./Cyclorrhapha - ps29
Cyclorrhapha - ps30
Calyptratae/Drosophila - ps31
Drosophila - ps32
Sophophora - ps33
Obscura/Melanogaster_group - ps34
D_melanogaster_group - ps35
D_melanogaster_subgroup - ps36
sim./sech./mel. group - ps37
Drosophila_melanogaster - ps38

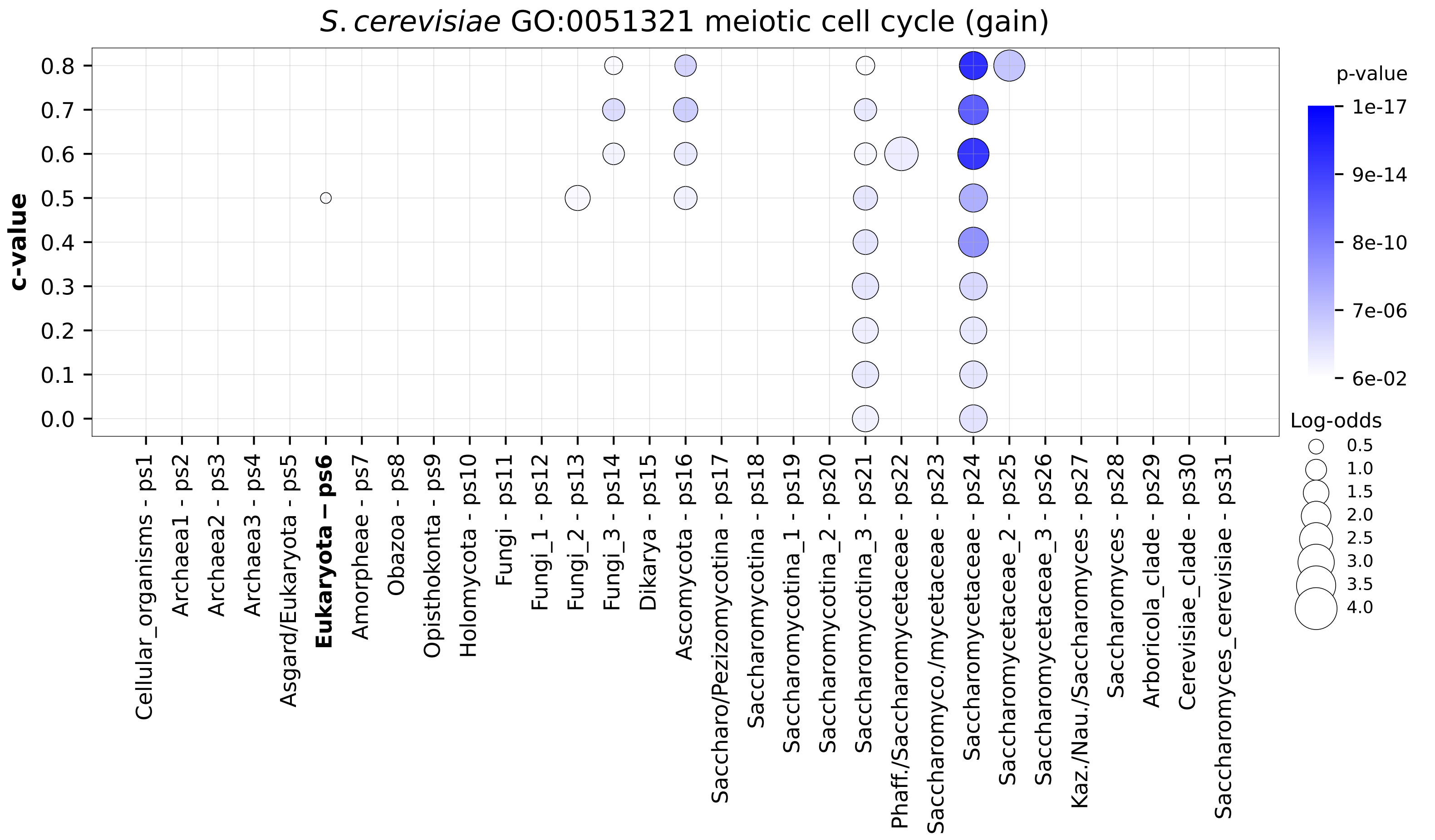
S. cerevisiae GO:0051321 meiotic cell cycle (gain)
c-value
0.0
0.1
0.2
0.3
0.4
0.5
0.6
0.7
0.8
p-value
1e-17
9e-14
8e-10
7e-06
6e-02
Log-odds
0.5
1.0
1.5
2.0
2.5
3.0
3.5
4.0
Cellular_organisms - ps1
Archaea1 - ps2
Archaea2 - ps3
Archaea3 - ps4
Asgard/Eukaryota - ps5
Eukaryota – ps6
Amorpheae - ps7
Obazoa - ps8
Opisthokonta - ps9
Holomycota - ps10
Fungi - ps11
Fungi_1 - ps12
Fungi_2 - ps13
Fungi_3 - ps14
Dikarya - ps15
Ascomycota - ps16
Saccharo/Pezizomycotina - ps17
Saccharomycotina - ps18
Saccharomycotina_1 - ps19
Saccharomycotina_2 - ps20
Saccharomycotina_3 - ps21
Phaff./Saccharomycetaceae - ps22
Saccharomyco./mycetaceae - ps23
Saccharomycetaceae - ps24
Saccharomycetaceae_2 - ps25
Saccharomycetaceae_3 - ps26
Kaz./Nau./Saccharomyces - ps27
Saccharomyces - ps28
Arboricola_clade - ps29
Cerevisiae_clade - ps30
Saccharomyces_cerevisiae - ps31

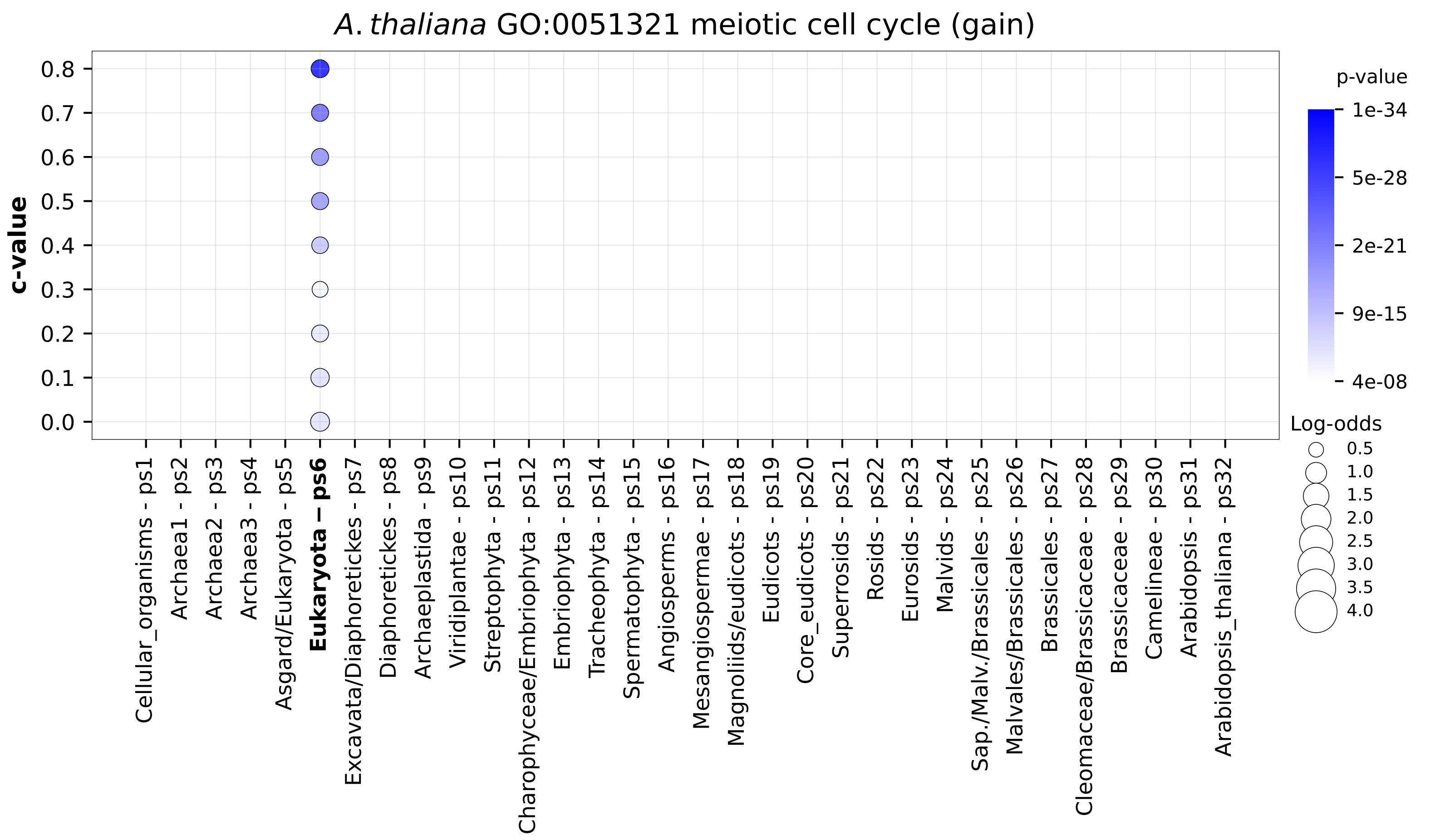
A. thaliana GO:0051321 meiotic cell cycle (gain)
c-value
0.0
0.1
0.2
0.3
0.4
0.5
0.6
0.7
0.8
p-value
1e-34
5e-28
2e-21
9e-15
4e-08
Log-odds
0.5
1.0
1.5
2.0
2.5
3.0
3.5
4.0
Cellular_organisms - ps1
Archaea1 - ps2
Archaea2 - ps3
Archaea3 - ps4
Asgard/Eukaryota - ps5
Eukaryota – ps6
Excavata/Diaphoretickes - ps7
Diaphoretickes - ps8
Archaeplastida - ps9
Viridiplantae - ps10
Streptophyta - ps11
Charophyceae/Embriophyta - ps12
Embriophyta - ps13
Tracheophyta - ps14
Spermatophyta - ps15
Angiosperms - ps16
Mesangiospermae - ps17
Magnoliids/eudicots - ps18
Eudicots - ps19
Core_eudicots - ps20
Superrosids - ps21
Rosids - ps22
Eurosids - ps23
Malvids - ps24
Sap./Malv./Brassicales - ps25
Malvales/Brassicales - ps26
Brassicales - ps27
Cleomaceae/Brassicaceae - ps28
Brassicaceae - ps29
Camelineae - ps30
Arabidopsis - ps31
Arabidopsis_thaliana - ps32

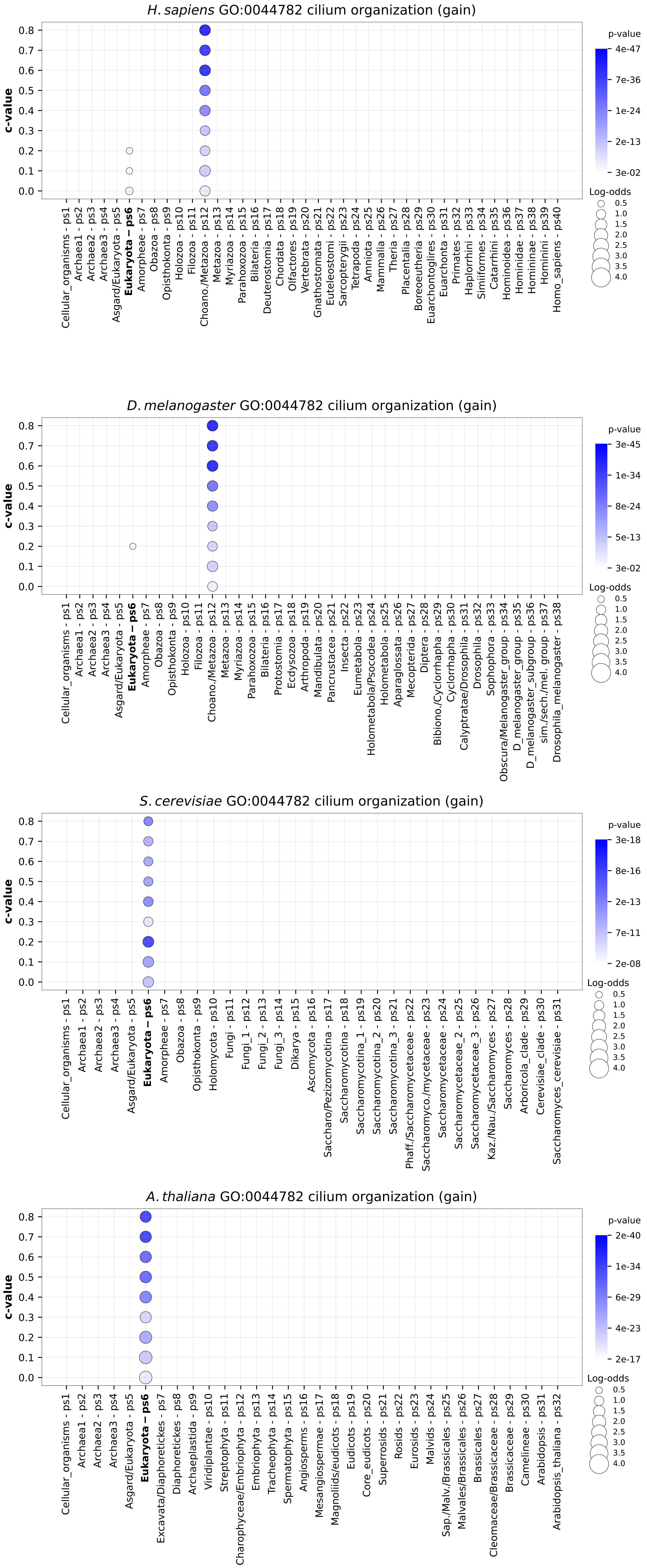

H. sapiens GO:0044782 cilium organization (gain)
c-value
p-value
4e-47
7e-36
1e-24
2e-13
3e-02
Log-odds
0.5
1.0
1.5
2.0
2.5
3.0
3.5
4.0
Cellular_organisms - ps1
Archaea1 - ps2
Archaea2 - ps3
Archaea3 - ps4
Asgard/Eukaryota - ps5
Eukaryota – ps6
Amorpheae - ps7
Obazoa - ps8
Opisthokonta - ps9
Holozoa - ps10
Filozoa - ps11
Choano./Metazoa - ps12
Metazoa - ps13
Myriazoa - ps14
Parahoxozoa - ps15
Bilateria - ps16
Deuterostomia - ps17
Chordata - ps18
Olfactores - ps19
Vertebrata - ps20
Gnathostomata - ps21
Euteleostomi - ps22
Sarcopterygii - ps23
Tetrapoda - ps24
Amniota - ps25
Mammalia - ps26
Theria - ps27
Placentalia - ps28
Boreoeutheria - ps29
Euarchontoglires - ps30
Euarchonta - ps31
Primates - ps32
Haplorrhini - ps33
Simiiformes - ps34
Catarrhini - ps35
Hominoidea - ps36
Hominidae - ps37
Homininae - ps38
Hominini - ps39
Homo_sapiens - ps40
D. melanogaster GO:0044782 cilium organization (gain)
c-value
p-value
3e-45
1e-34
8e-24
5e-13
3e-02
Log-odds
Cellular_organisms - ps1
Archaea1 - ps2
Archaea2 - ps3
Archaea3 - ps4
Asgard/Eukaryota - ps5
Eukaryota – ps6
Amorpheae - ps7
Obazoa - ps8
Opisthokonta - ps9
Holozoa - ps10
Filozoa - ps11
Choano./Metazoa - ps12
Metazoa - ps13
Myriazoa - ps14
Parahoxozoa - ps15
Bilateria - ps16
Protostomia - ps17
Ecdysozoa - ps18
Arthropoda - ps19
Mandibulata - ps20
Pancrustacea - ps21
Insecta - ps22
Eumetabola - ps23
Holometabola/Psocodea - ps24
Holometabola - ps25
Aparaglossata - ps26
Mecopterida - ps27
Diptera - ps28
Bibiono./Cyclorrhapha - ps29
Cyclorrhapha - ps30
Calyptratae/Drosophila - ps31
Drosophila - ps32
Sophophora - ps33
Obscura/Melanogaster_group - ps34
D_melanogaster_group - ps35
D_melanogaster_subgroup - ps36
sim./sech./mel. group - ps37
Drosophila_melanogaster - ps38
S. cerevisiae GO:0044782 cilium organization (gain)
c-value
p-value
3e-18
8e-16
2e-13
7e-11
2e-08
Log-odds
Cellular_organisms - ps1
Archaea1 - ps2
Archaea2 - ps3
Archaea3 - ps4
Asgard/Eukaryota - ps5
Eukaryota – ps6
Amorpheae - ps7
Obazoa - ps8
Opisthokonta - ps9
Holomycota - ps10
Fungi - ps11
Fungi_1 - ps12
Fungi_2 - ps13
Fungi_3 - ps14
Dikarya - ps15
Ascomycota - ps16
Saccharo/Pezizomycotina - ps17
Saccharomycotina - ps18
Saccharomycotina_1 - ps19
Saccharomycotina_2 - ps20
Saccharomycotina_3 - ps21
Phaff./Saccharomycetaceae - ps22
Saccharomyco./mycetaceae - ps23
Saccharomycetaceae - ps24
Saccharomycetaceae_2 - ps25
Saccharomycetaceae_3 - ps26
Kaz./Nau./Saccharomyces - ps27
Saccharomyces - ps28
Arboricola_clade - ps29
Cerevisiae_clade - ps30
Saccharomyces_cerevisiae - ps31
A. thaliana GO:0044782 cilium organization (gain)
c-value
p-value
2e-40
1e-34
6e-29
4e-23
2e-17
Log-odds
Cellular_organisms - ps1
Archaea1 - ps2
Archaea2 - ps3
Archaea3 - ps4
Asgard/Eukaryota - ps5
Eukaryota – ps6
Excavata/Diaphoretickes - ps7
Diaphoretickes - ps8
Archaeplastida - ps9
Viridiplantae - ps10
Streptophyta - ps11
Charophyceae/Embriophyta - ps12
Embriophyta - ps13
Tracheophyta - ps14
Spermatophyta - ps15
Angiospermae - ps16
Mesangiospermae - ps17
Magnoliids/eudicots - ps18
Eudicots - ps19
Core_eudicots - ps20
Superrosids - ps21
Rosids - ps22
Eurosids - ps23
Malvids - ps24
Sap./Malv./Brassicales - ps25
Malvales/Brassicales - ps26
Brassicales - ps27
Cleomaceae/Brassicaceae - ps28
Brassicaceae - ps29
Camelineae - ps30
Arabidopsis - ps31
Arabidopsis_thaliana - ps32

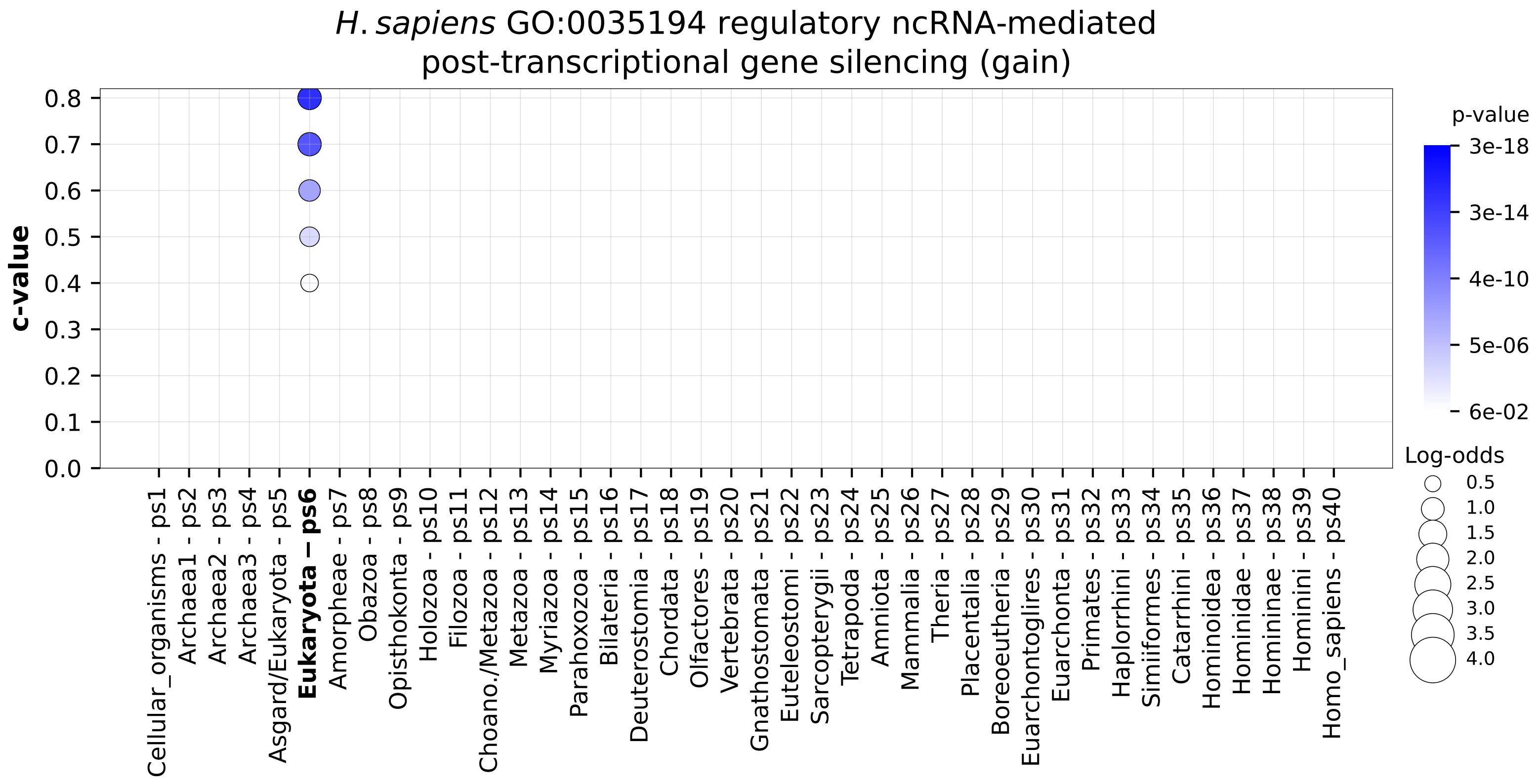
H. sapiens GO:0035194 regulatory ncRNA-mediated post-transcriptional gene silencing (gain)
c-value
p-value
3e-18
3e-14
4e-10
5e-06
6e-02
Log-odds
0.5
1.0
1.5
2.0
2.5
3.0
3.5
4.0
Cellular_organisms - ps1
Archaea1 - ps2
Archaea2 - ps3
Archaea3 - ps4
Asgard/Eukaryota - ps5
Eukaryota – ps6
Amorpheae - ps7
Obazoa - ps8
Opisthokonta - ps9
Holozoa - ps10
Filozoa - ps11
Choano./Metazoa - ps12
Metazoa - ps13
Myriazoa - ps14
Parahoxozoa - ps15
Bilateria - ps16
Deuterostomia - ps17
Chordata - ps18
Olfactores - ps19
Vertebrata - ps20
Gnathostomata - ps21
Euteleostomi - ps22
Sarcopterygii - ps23
Tetrapoda - ps24
Amniota - ps25
Mammalia - ps26
Theria - ps27
Placentalia - ps28
Boreoeutheria - ps29
Euarchontoglires - ps30
Euarchonta - ps31
Primates - ps32
Haplorrhini - ps33
Simiiformes - ps34
Catarrhini - ps35
Hominoidea - ps36
Hominidae - ps37
Homininae - ps38
Hominini - ps39
Homo_sapiens - ps40

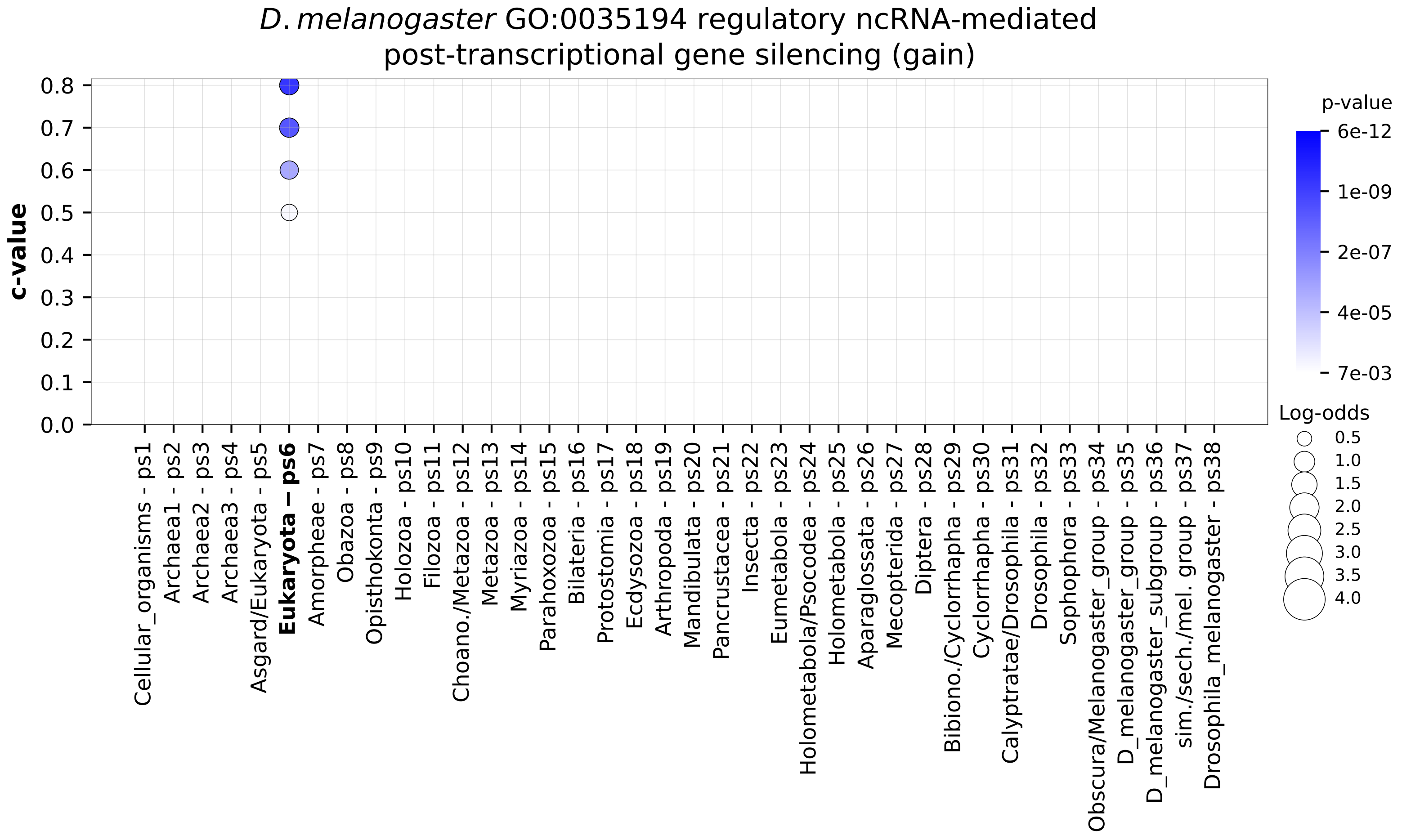
D. melanogaster GO:0035194 regulatory ncRNA-mediated post-transcriptional gene silencing (gain)
c-value
p-value
6e-12
1e-09
2e-07
4e-05
7e-03
Log-odds
Cellular_organisms - ps1
Archaea1 - ps2
Archaea2 - ps3
Archaea3 - ps4
Asgard/Eukaryota - ps5
Eukaryota – ps6
Amorpheae - ps7
Obazoa - ps8
Opisthokonta - ps9
Holozoa - ps10
Filozoa - ps11
Choano./Metazoa - ps12
Metazoa - ps13
Myriazoa - ps14
Parahoxozoa - ps15
Bilateria - ps16
Protostomia - ps17
Ecdysozoa - ps18
Arthropoda - ps19
Mandibulata - ps20
Pancrustacea - ps21
Insecta - ps22
Eumetabola - ps23
Holometabola/Psocodea - ps24
Holometabola - ps25
Aparaglossata - ps26
Mecopterida - ps27
Diptera - ps28
Bibiono./Cyclorrhapha - ps29
Cyclorrhapha - ps30
Calyptratae/Drosophila - ps31
Drosophila - ps32
Sophophora - ps33
Obscura/Melanogaster_group - ps34
D_melanogaster_group - ps35
D_melanogaster_subgroup - ps36
sim./sech./mel. group - ps37
Drosophila_melanogaster - ps38

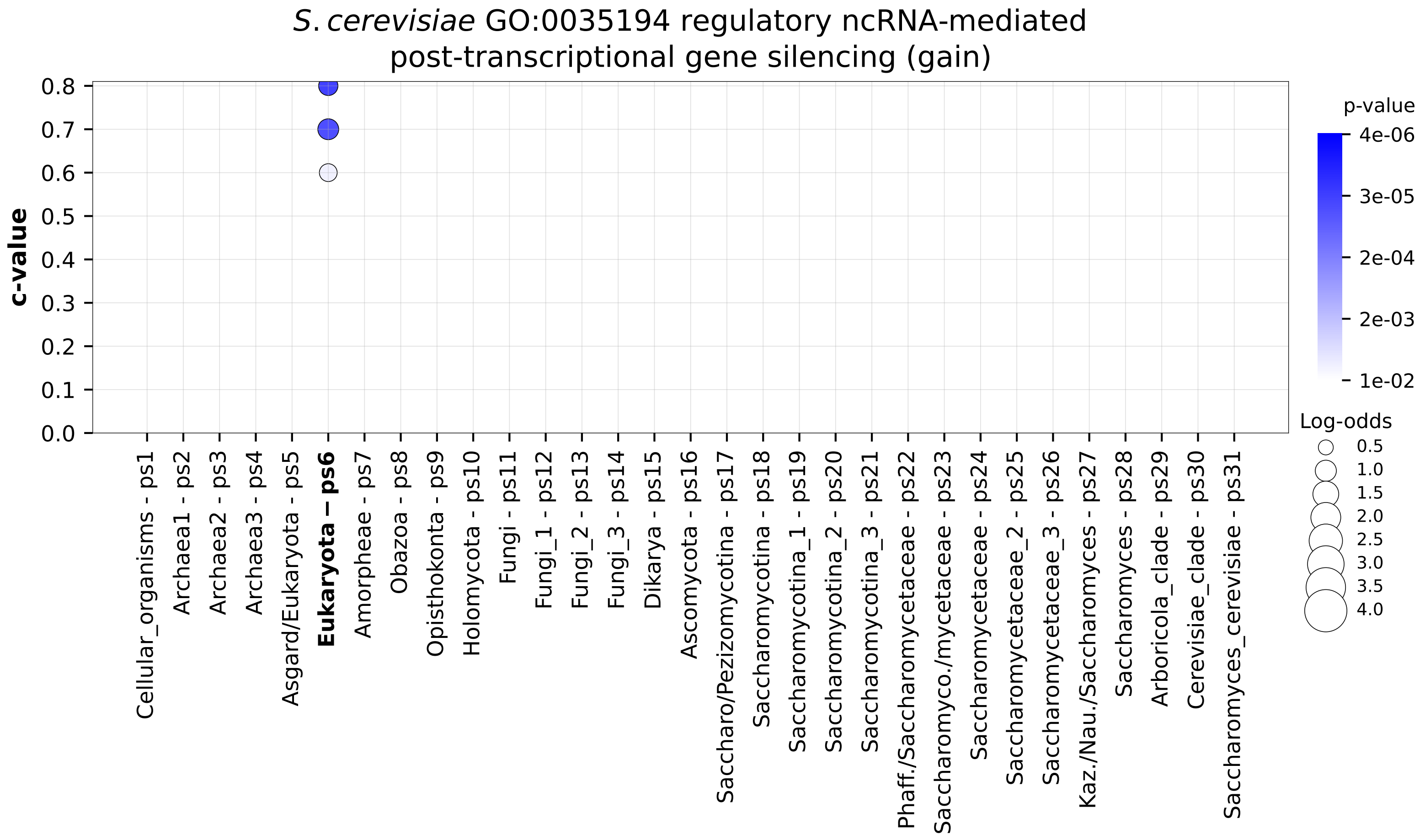
S. cerevisiae GO:0035194 regulatory ncRNA-mediated post-transcriptional gene silencing (gain)
c-value
p-value
4e-06
3e-05
2e-04
2e-03
1e-02
Log-odds
Cellular_organisms - ps1
Archaea1 - ps2
Archaea2 - ps3
Archaea3 - ps4
Asgard/Eukaryota - ps5
Eukaryota – ps6
Amorpheae - ps7
Obazoa - ps8
Opisthokonta - ps9
Holomycota - ps10
Fungi - ps11
Fungi_1 - ps12
Fungi_2 - ps13
Fungi_3 - ps14
Dikarya - ps15
Ascomycota - ps16
Saccharo/Pezizomycotina - ps17
Saccharomycotina - ps18
Saccharomycotina_1 - ps19
Saccharomycotina_2 - ps20
Saccharomycotina_3 - ps21
Phaff./Saccharomycetaceae - ps22
Saccharomyco./mycetaceae - ps23
Saccharomycetaceae - ps24
Saccharomycetaceae_2 - ps25
Saccharomycetaceae_3 - ps26
Kaz./Nau./Saccharomyces - ps27
Saccharomyces - ps28
Arboricola_clade - ps29
Cerevisiae_clade - ps30
Saccharomyces_cerevisiae - ps31

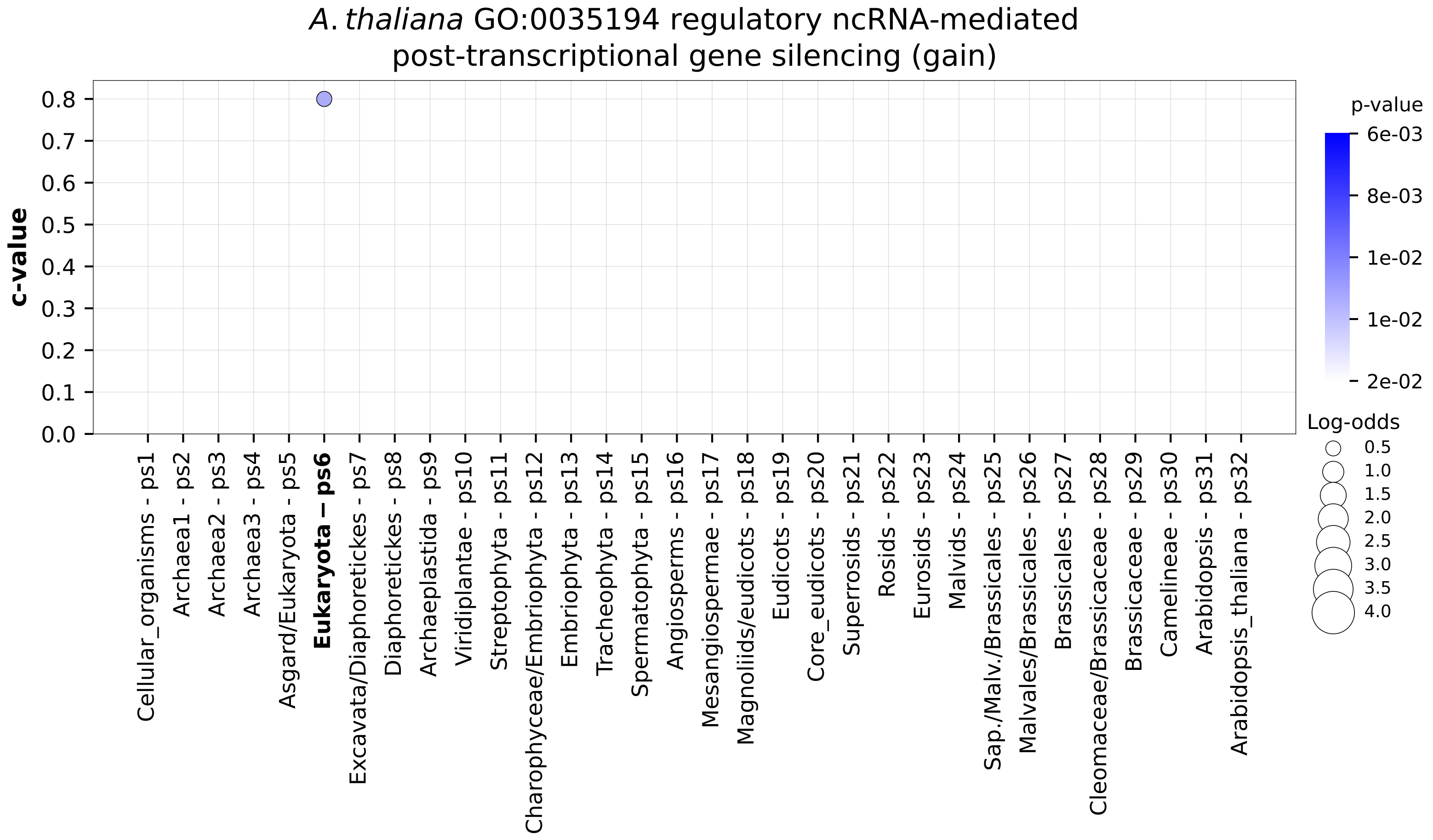
A. thaliana GO:0035194 regulatory ncRNA-mediated post-transcriptional gene silencing (gain)
c-value
p-value
6e-03
8e-03
1e-02
1e-02
2e-02
Log-odds
Cellular_organisms - ps1
Archaea1 - ps2
Archaea2 - ps3
Archaea3 - ps4
Asgard/Eukaryota - ps5
Eukaryota – ps6
Excavata/Diaphoretickes - ps7
Diaphoretickes - ps8
Archaeplastida - ps9
Viridiplantae - ps10
Streptophyta - ps11
Charophyceae/Embriophyta - ps12
Embriophyta - ps13
Tracheophyta - ps14
Spermatophyta - ps15
Angiosperms - ps16
Mesangiospermae - ps17
Magnoliids/eudicots - ps18
Eudicots - ps19
Core_eudicots - ps20
Superrosids - ps21
Rosids - ps22
Eurosids - ps23
Malvids - ps24
Sap./Malv./Brassicales - ps25
Malvales/Brassicales - ps26
Brassicales - ps27
Cleomaceae/Brassicaceae - ps28
Brassicaceae - ps29
Camelineae - ps30
Arabidopsis - ps31
Arabidopsis_thaliana - ps32

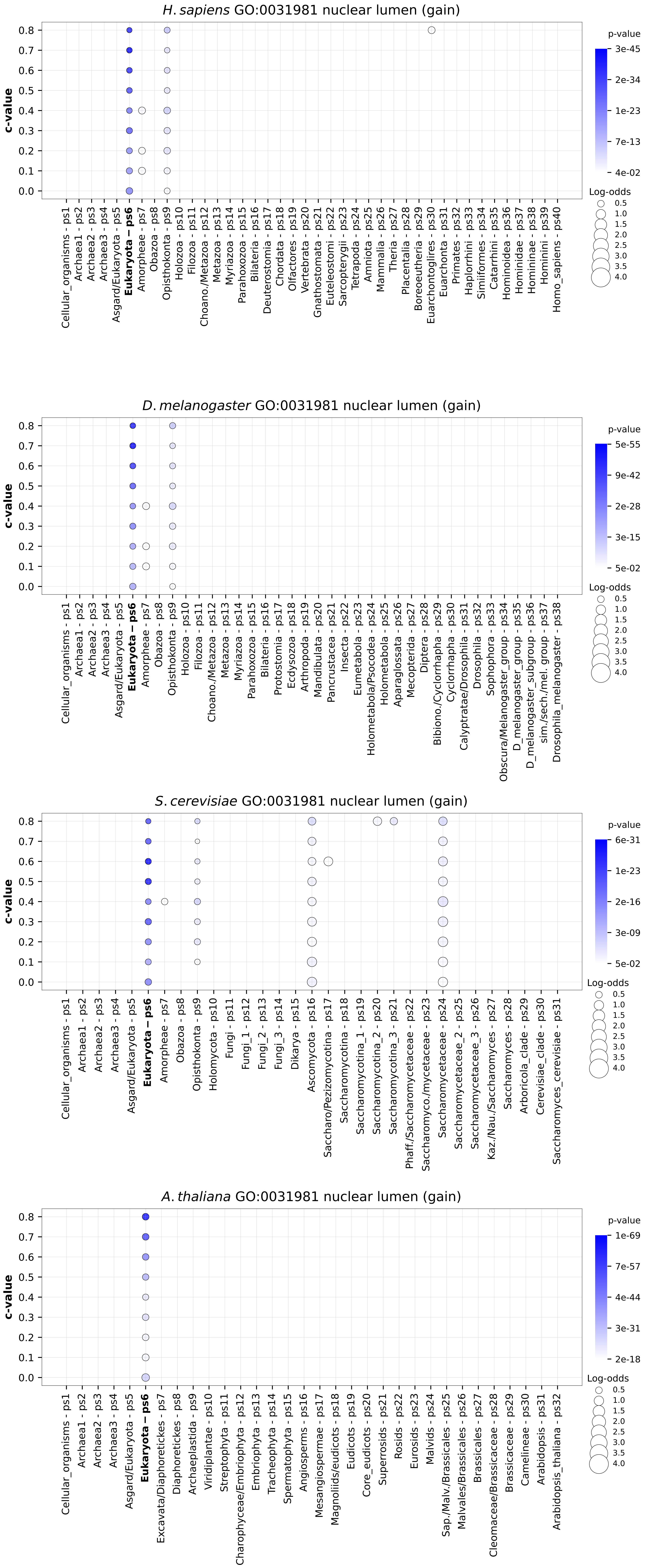
H. sapiens GO:0031981 nuclear lumen (gain)
c-value
0.0
0.1
0.2
0.3
0.4
0.5
0.6
0.7
0.8
p-value
3e-45
2e-34
1e-23
7e-13
4e-02
Log-odds
0.5
1.0
1.5
2.0
2.5
3.0
3.5
4.0
Cellular_organisms - ps1
Archaea1 - ps2
Archaea2 - ps3
Archaea3 - ps4
Asgard/Eukaryota - ps5
Eukaryota – ps6
Amorpheae - ps7
Obazoa - ps8
Opisthokonta - ps9
Holozoa - ps10
Filozoa - ps11
Choano./Metazoa - ps12
Metazoa - ps13
Myriazoa - ps14
Parahoxozoa - ps15
Bilateria - ps16
Deuterostomia - ps17
Chordata - ps18
Olfactores - ps19
Vertebrata - ps20
Gnathostomata - ps21
Euteleostomi - ps22
Sarcopterygii - ps23
Tetrapoda - ps24
Amniota - ps25
Mammalia - ps26
Theria - ps27
Placentalia - ps28
Boreoeutheria - ps29
Euarchontoglires - ps30
Euarchonta - ps31
Primates - ps32
Haplorrhini - ps33
Simiiformes - ps34
Catarrhini - ps35
Hominoidea - ps36
Hominidae - ps37
Homininae - ps38
Hominini - ps39
Homo_sapiens - ps40
D. melanogaster GO:0031981 nuclear lumen (gain)
c-value
p-value
5e-55
9e-42
2e-28
3e-15
5e-02
Log-odds
Cellular_organisms - ps1
Archaea1 - ps2
Archaea2 - ps3
Archaea3 - ps4
Asgard/Eukaryota - ps5
Eukaryota – ps6
Amorpheae - ps7
Obazoa - ps8
Opisthokonta - ps9
Holozoa - ps10
Filozoa - ps11
Choano./Metazoa - ps12
Metazoa - ps13
Myriazoa - ps14
Parahoxozoa - ps15
Bilateria - ps16
Protostomia - ps17
Ecdysozoa - ps18
Arthropoda - ps19
Mandibulata - ps20
Pancrustacea - ps21
Insecta - ps22
Eumetabola - ps23
Holometabola/Psocodea - ps24
Holometabola - ps25
Aparaglossata - ps26
Mecopterida - ps27
Diptera - ps28
Bibiono./Cyclorrhapha - ps29
Cyclorrhapha - ps30
Calyptratae/Drosophila - ps31
Drosophila - ps32
Sophophora - ps33
Obscura/Melanogaster_group - ps34
D_melanogaster_group - ps35
D_melanogaster_subgroup - ps36
sim./sech./mel. group - ps37
Drosophila_melanogaster - ps38
S. cerevisiae GO:0031981 nuclear lumen (gain)
c-value
p-value
6e-31
1e-23
2e-16
3e-09
5e-02
Log-odds
Cellular_organisms - ps1
Archaea1 - ps2
Archaea2 - ps3
Archaea3 - ps4
Asgard/Eukaryota - ps5
Eukaryota – ps6
Amorpheae - ps7
Obazoa - ps8
Opisthokonta - ps9
Holomycota - ps10
Fungi - ps11
Fungi_1 - ps12
Fungi_2 - ps13
Fungi_3 - ps14
Dikarya - ps15
Ascomycota - ps16
Saccharo/Pezizomycotina - ps17
Saccharomycotina - ps18
Saccharomycotina_1 - ps19
Saccharomycotina_2 - ps20
Saccharomycotina_3 - ps21
Phaff./Saccharomycetaceae - ps22
Saccharomyco./mycetaceae - ps23
Saccharomycetaceae - ps24
Saccharomycetaceae_2 - ps25
Saccharomycetaceae_3 - ps26
Kaz./Nau./Saccharomyces - ps27
Saccharomyces - ps28
Arboricola_clade - ps29
Cerevisiae_clade - ps30
Saccharomyces_cerevisiae - ps31
A. thaliana GO:0031981 nuclear lumen (gain)
c-value
p-value
1e-69
7e-57
4e-44
3e-31
2e-18
Log-odds
Cellular_organisms - ps1
Archaea1 - ps2
Archaea2 - ps3
Archaea3 - ps4
Asgard/Eukaryota - ps5
Eukaryota – ps6
Excavata/Diaphoretickes - ps7
Diaphoretickes - ps8
Archaeplastida - ps9
Viridiplantae - ps10
Streptophyta - ps11
Charophyceae/Embriophyta - ps12
Embriophyta - ps13
Tracheophyta - ps14
Spermatophyta - ps15
Angiosperms - ps16
Mesangiospermae - ps17
Magnoliids/eudicots - ps18
Eudicots - ps19
Core_eudicots - ps20
Superrosids - ps21
Rosids - ps22
Eurosids - ps23
Malvids - ps24
Sap./Malv./Brassicales - ps25
Malvales/Brassicales - ps26
Brassicales - ps27
Cleomaceae/Brassicaceae - ps28
Brassicaceae - ps29
Camelineae - ps30
Arabidopsis - ps31
Arabidopsis_thaliana - ps32

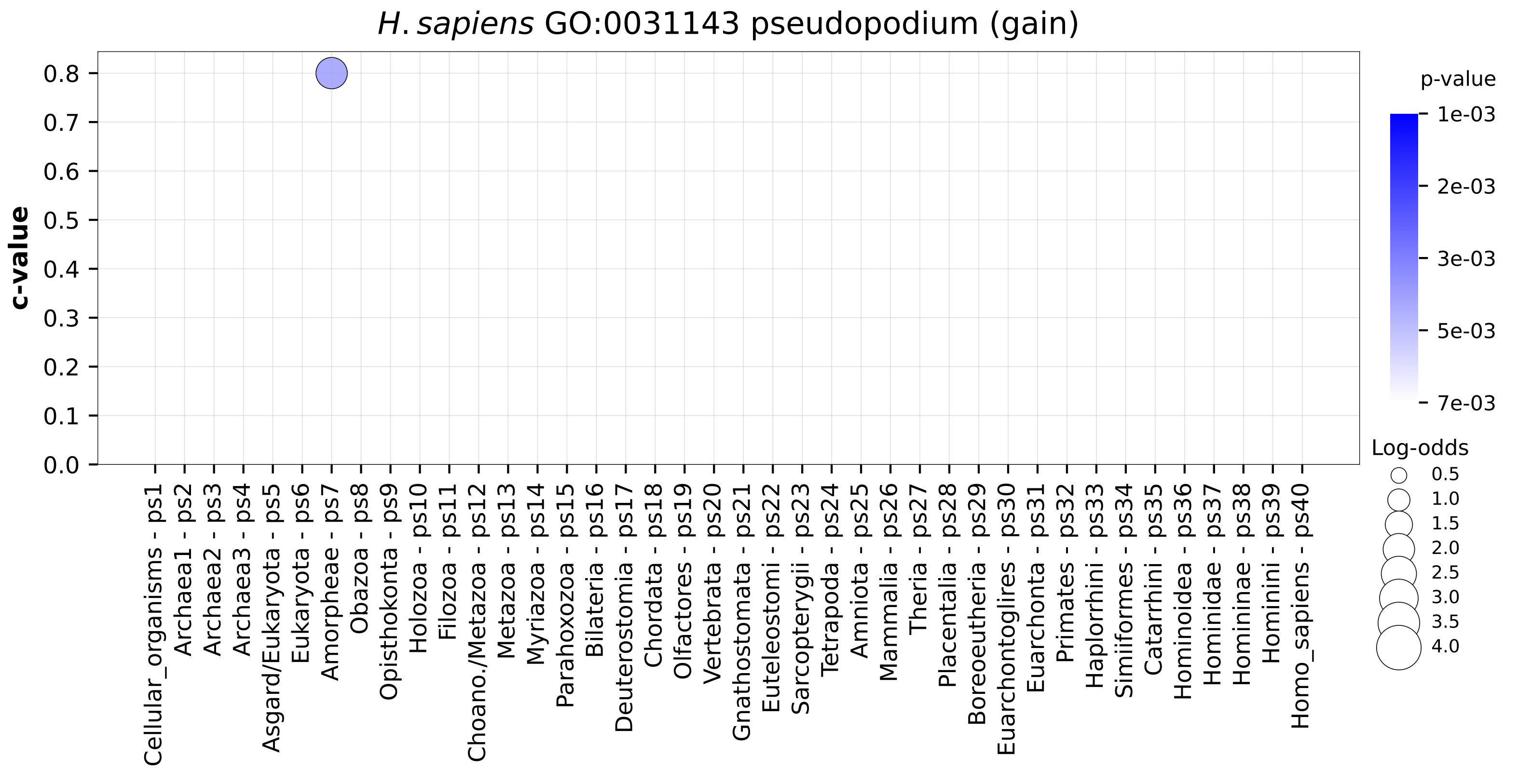
H. sapiens GO:0031143 pseudopodium (gain)
c-value
p-value
Log-odds

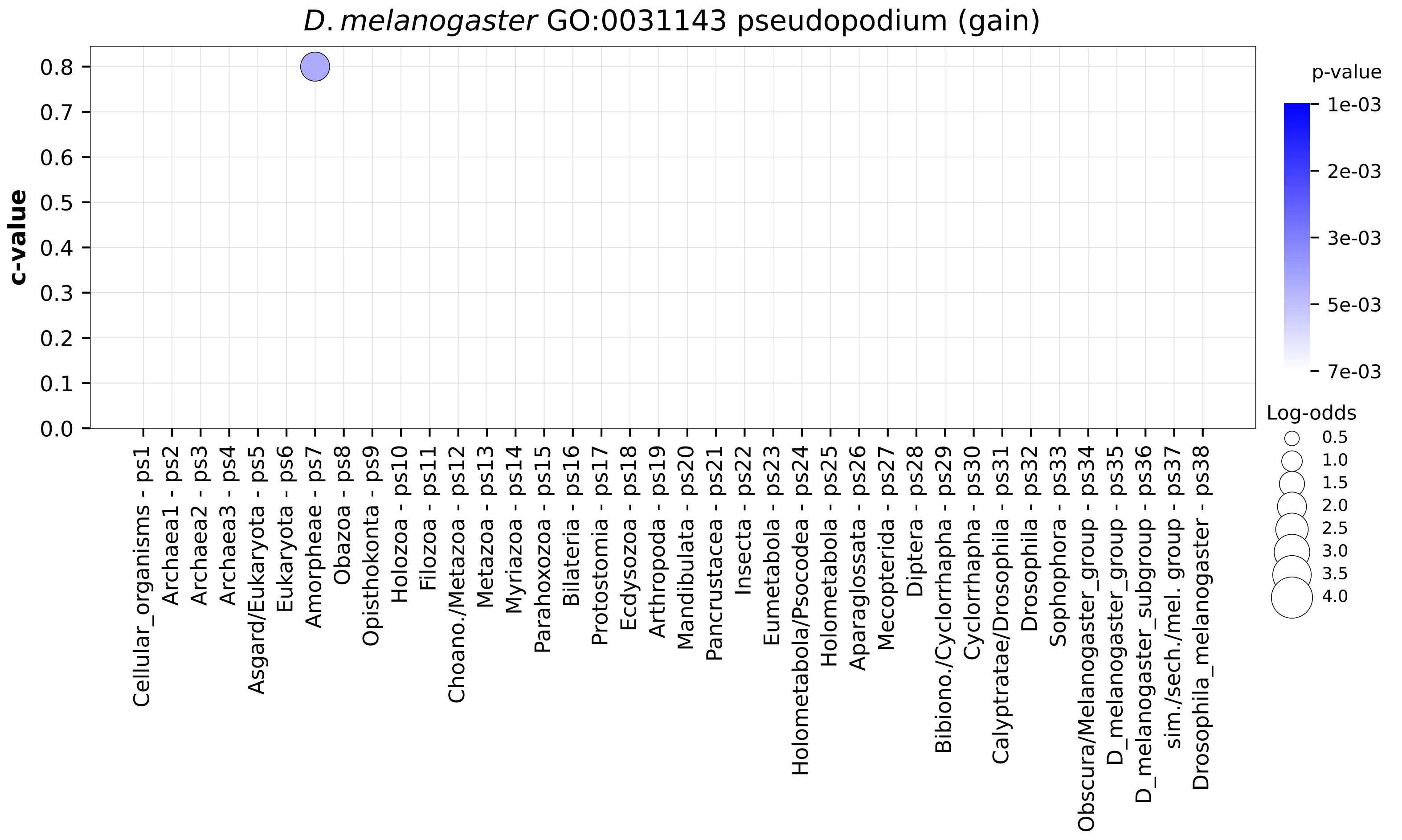
D. melanogaster GO:0031143 pseudopodium (gain)
c-value
p-value
Log-odds

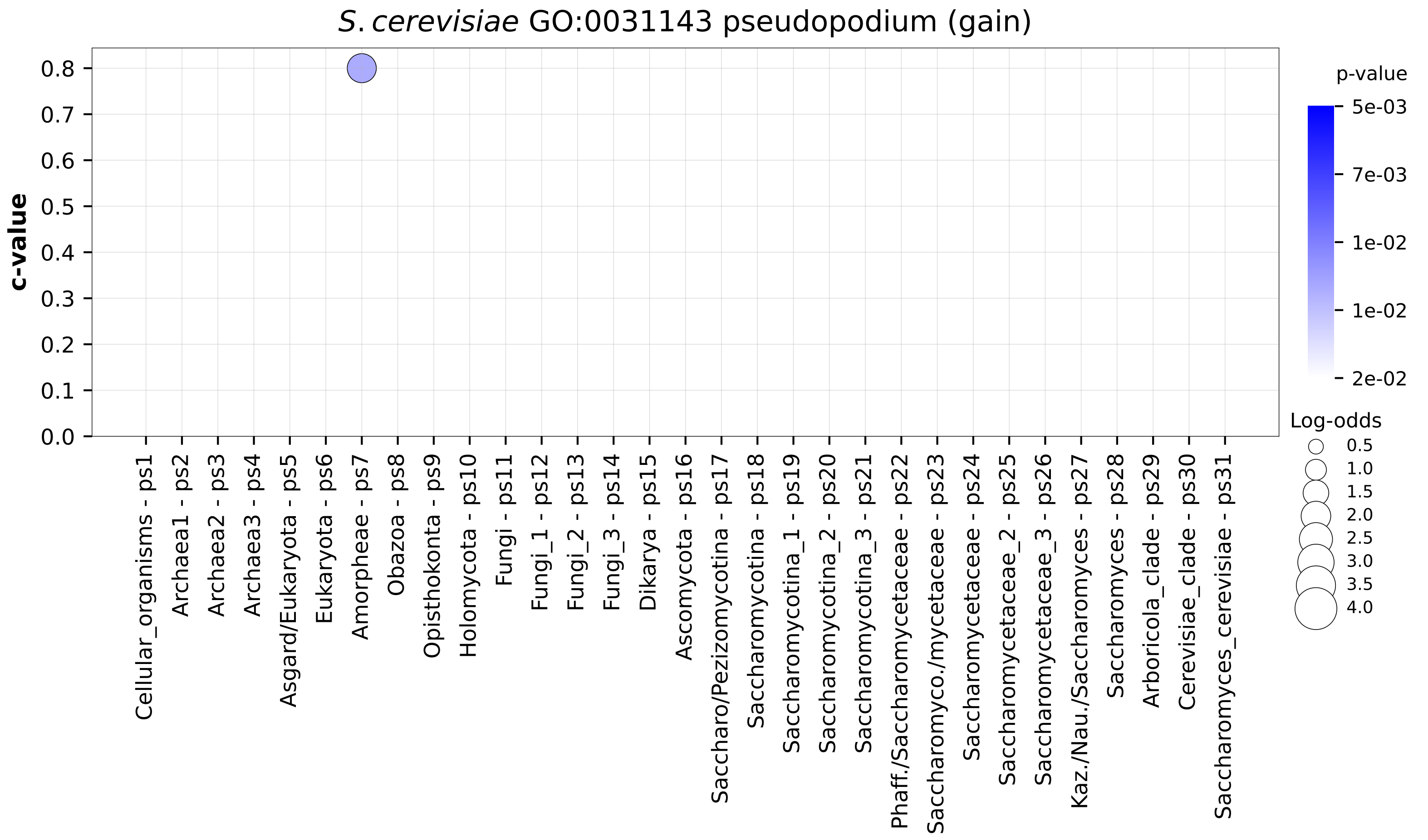
S. cerevisiae GO:0031143 pseudopodium (gain)
c-value
p-value
Log-odds

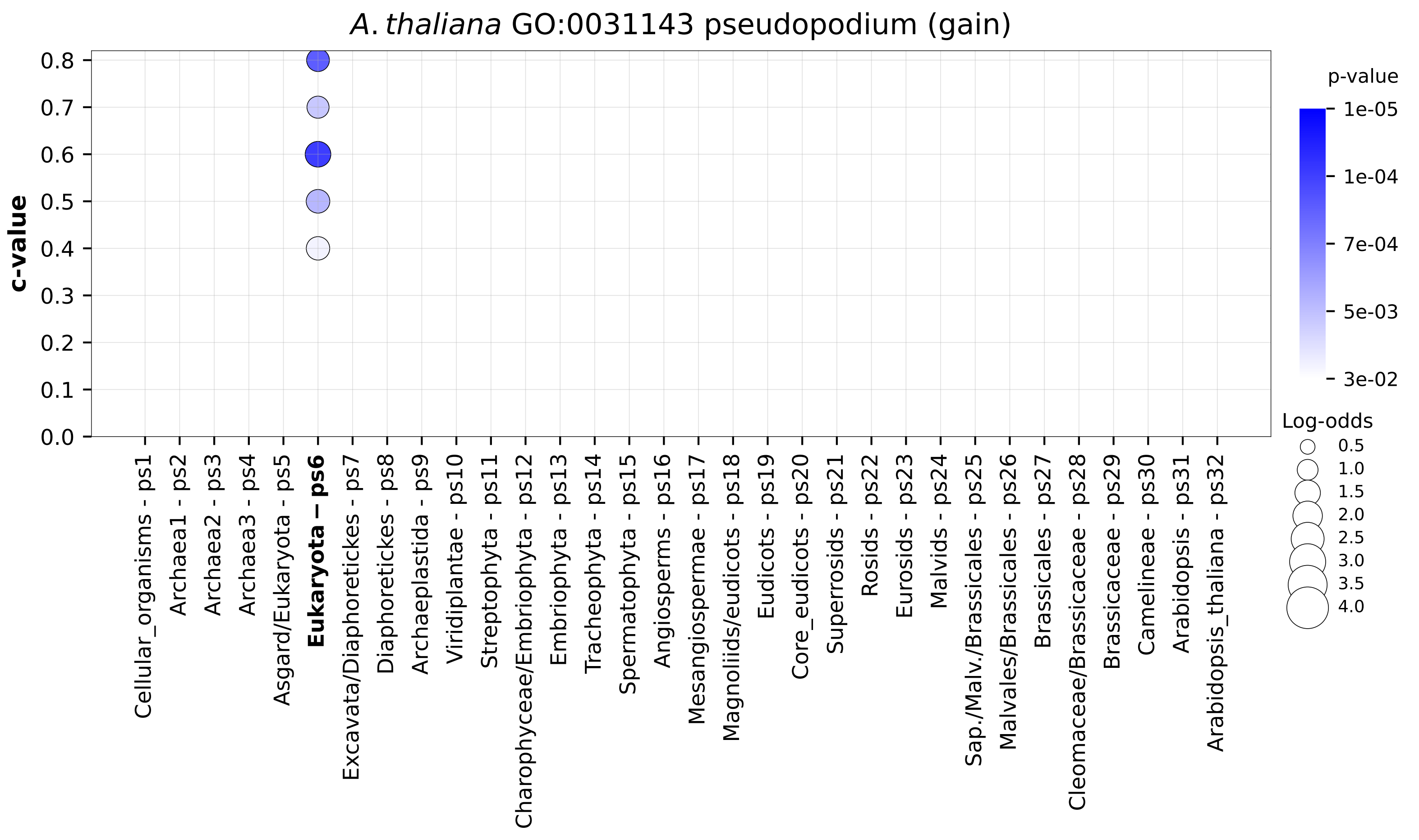
A. thaliana GO:0031143 pseudopodium (gain)
c-value
p-value
Log-odds

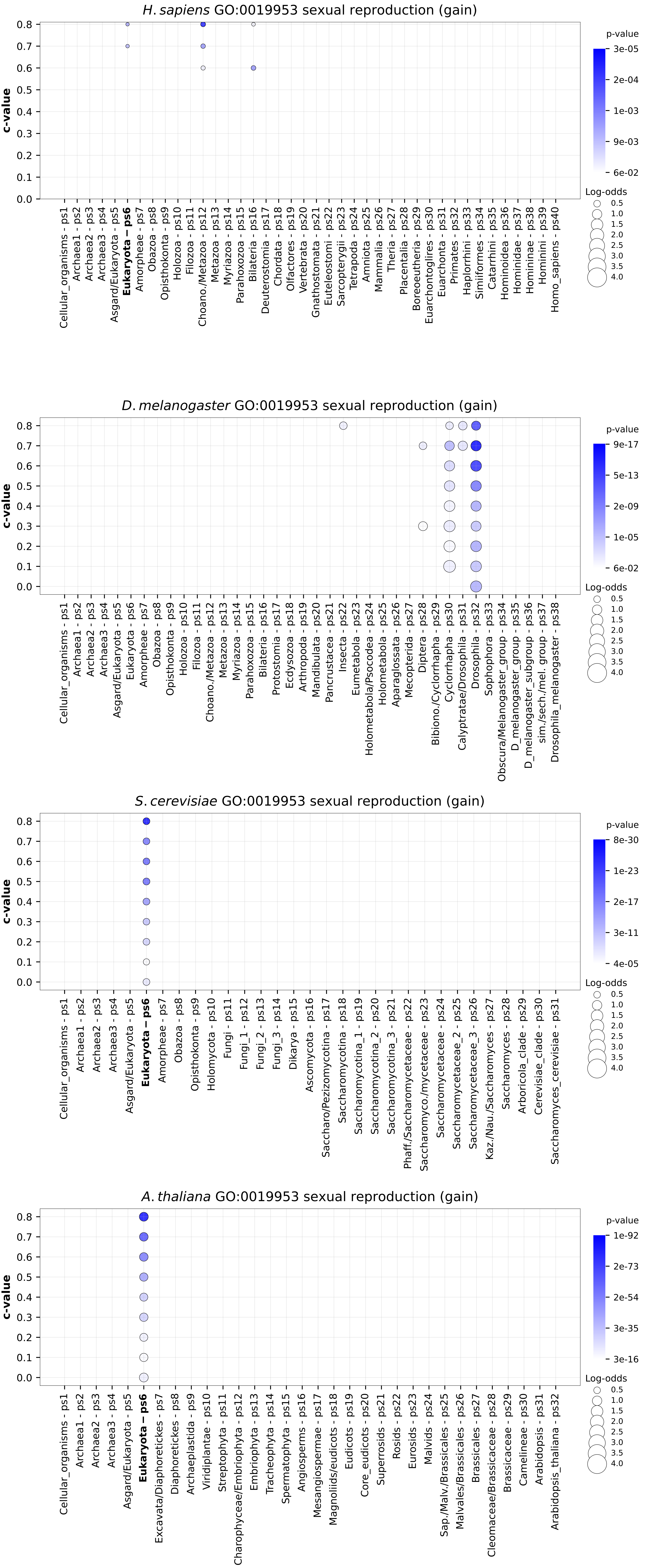

H. sapiens GO:0019953 sexual reproduction (gain)
c-value
p-value
3e-05
2e-04
1e-03
9e-03
6e-02
Log-odds
0.5 1.0 1.5 2.0 2.5 3.0 3.5 4.0
Cellular_organisms - ps1
Archaea1 - ps2
Archaea2 - ps3
Archaea3 - ps4
Asgard/Eukaryota - ps5
Eukaryota – ps6
Amorpheae - ps7
Obazoa - ps8
Opisthokonta - ps9
Holozoa - ps10
Filozoa - ps11
Choano./Metazoa - ps12
Metazoa - ps13
Myriazoa - ps14
Parahoxozoa - ps15
Bilateria - ps16
Deuterostomia - ps17
Chordata - ps18
Olfactores - ps19
Vertebrata - ps20
Gnathostomata - ps21
Euteleostomi - ps22
Sarcopterygii - ps23
Tetrapoda - ps24
Amniota - ps25
Mammalia - ps26
Theria - ps27
Placentalia - ps28
Boreoeutheria - ps29
Euarchontoglires - ps30
Euarchonta - ps31
Primates - ps32
Haplorrhini - ps33
Simiiformes - ps34
Catarrhini - ps35
Hominoidea - ps36
Hominidae - ps37
Homininae - ps38
Hominini - ps39
Homo_sapiens - ps40
D. melanogaster GO:0019953 sexual reproduction (gain)
c-value
p-value
9e-17
5e-13
2e-09
1e-05
6e-02
Log-odds
0.5 1.0 1.5 2.0 2.5 3.0 3.5 4.0
Cellular_organisms - ps1
Archaea1 - ps2
Archaea2 - ps3
Archaea3 - ps4
Asgard/Eukaryota - ps5
Eukaryota - ps6
Amorpheae - ps7
Obazoa - ps8
Opisthokonta - ps9
Holozoa - ps10
Filozoa - ps11
Choano./Metazoa - ps12
Metazoa - ps13
Myriazoa - ps14
Parahoxozoa - ps15
Bilateria - ps16
Protostomia - ps17
Ecdysozoa - ps18
Arthropoda - ps19
Mandibulata - ps20
Pancrustacea - ps21
Insecta - ps22
Eumetabola - ps23
Holometabola/Psocodea - ps24
Holometabola - ps25
Aparaglossata - ps26
Mecopterida - ps27
Diptera - ps28
Bibiono./Cyclorrhapha - ps29
Cyclorrhapha - ps30
Calyptratae/Drosophila - ps31
Drosophila - ps32
Sophophora - ps33
Obscura/Melanogaster_group - ps34
D_melanogaster_group - ps35
D_melanogaster_subgroup - ps36
sim./sech./mel. group - ps37
Drosophila_melanogaster - ps38
S. cerevisiae GO:0019953 sexual reproduction (gain)
c-value
p-value
8e-30
1e-23
2e-17
3e-11
4e-05
Log-odds
0.5 1.0 1.5 2.0 2.5 3.0 3.5 4.0
Cellular_organisms - ps1
Archaea1 - ps2
Archaea2 - ps3
Archaea3 - ps4
Asgard/Eukaryota - ps5
Eukaryota – ps6
Amorpheae - ps7
Obazoa - ps8
Opisthokonta - ps9
Holomycota - ps10
Fungi - ps11
Fungi_1 - ps12
Fungi_2 - ps13
Fungi_3 - ps14
Dikarya - ps15
Ascomycota - ps16
Saccharo/Pezizomycotina - ps17
Saccharomycotina - ps18
Saccharomycotina_1 - ps19
Saccharomycotina_2 - ps20
Saccharomycotina_3 - ps21
Phaff./Saccharomycetaceae - ps22
Saccharomyco./mycetaceae - ps23
Saccharomycetaceae - ps24
Saccharomycetaceae_2 - ps25
Saccharomycetaceae_3 - ps26
Kaz./Nau./Saccharomyces - ps27
Saccharomyces - ps28
Arboricola_clade - ps29
Cerevisiae_clade - ps30
Saccharomyces_cerevisiae - ps31
A. thaliana GO:0019953 sexual reproduction (gain)
c-value
p-value
1e-92
2e-73
2e-54
3e-35
3e-16
Log-odds
0.5 1.0 1.5 2.0 2.5 3.0 3.5 4.0
Cellular_organisms - ps1
Archaea1 - ps2
Archaea2 - ps3
Archaea3 - ps4
Asgard/Eukaryota - ps5
Eukaryota – ps6
Excavata/Diaphoretickes - ps7
Diaphoretickes - ps8
Archaeplastida - ps9
Viridiplantae - ps10
Streptophyta - ps11
Charophyceae/Embriophyta - ps12
Embriophyta - ps13
Tracheophyta - ps14
Spermatophyta - ps15
Angiospermae - ps16
Mesangiospermae - ps17
Magnoliids/eudicots - ps18
Eudicots - ps19
Core_eudicots - ps20
Superrosids - ps21
Rosids - ps22
Eurosids - ps23
Malvids - ps24
Sap./Malv./Brassicales - ps25
Malvales/Brassicales - ps26
Brassicales - ps27
Cleomaceae/Brassicaceae - ps28
Brassicaceae - ps29
Camelineae - ps30
Arabidopsis - ps31
Arabidopsis_thaliana - ps32

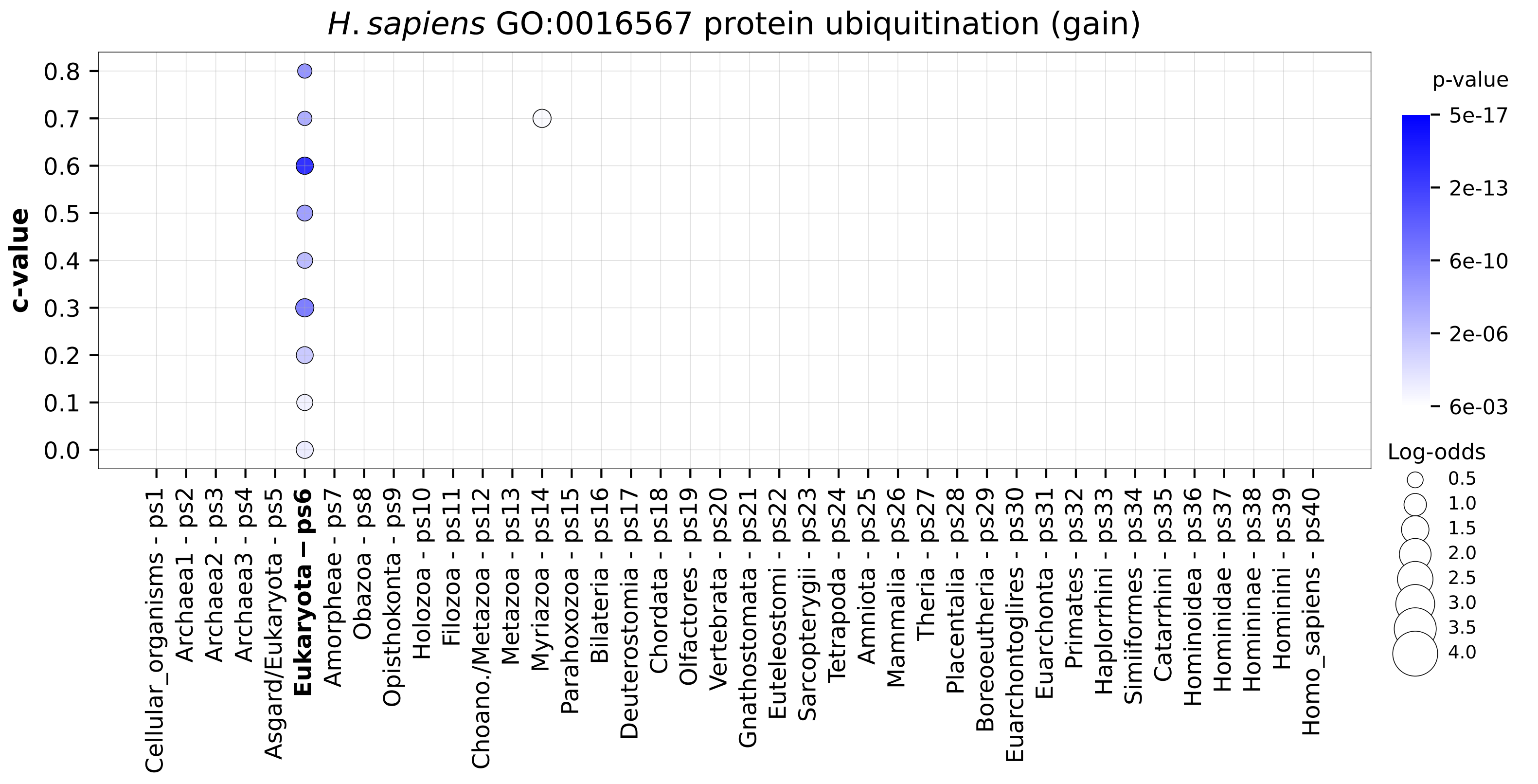
H. sapiens GO:0016567 protein ubiquitination (gain)
c-value
0.8
0.7
0.6
0.5
0.4
0.3
0.2
0.1
0.0
p-value
5e-17
2e-13
6e-10
2e-06
6e-03
Log-odds
0.5
1.0
1.5
2.0
2.5
3.0
3.5
4.0
Cellular_organisms - ps1
Archaea1 - ps2
Archaea2 - ps3
Archaea3 - ps4
Asgard/Eukaryota - ps5
Eukaryota – ps6
Amorpheae - ps7
Obazoa - ps8
Opisthokonta - ps9
Holozoa - ps10
Filozoa - ps11
Choano./Metazoa - ps12
Metazoa - ps13
Myriazoa - ps14
Parahoxozoa - ps15
Bilateria - ps16
Deuterostomia - ps17
Chordata - ps18
Olfactores - ps19
Vertebrata - ps20
Gnathostomata - ps21
Euteleostomi - ps22
Sarcopterygii - ps23
Tetrapoda - ps24
Amniota - ps25
Mammalia - ps26
Theria - ps27
Placentalia - ps28
Boreoeutheria - ps29
Euarchontoglires - ps30
Euarchonta - ps31
Primates - ps32
Haplorrhini - ps33
Simiiformes - ps34
Catarrhini - ps35
Hominoidea - ps36
Hominidae - ps37
Homininae - ps38
Hominini - ps39
Homo_sapiens - ps40

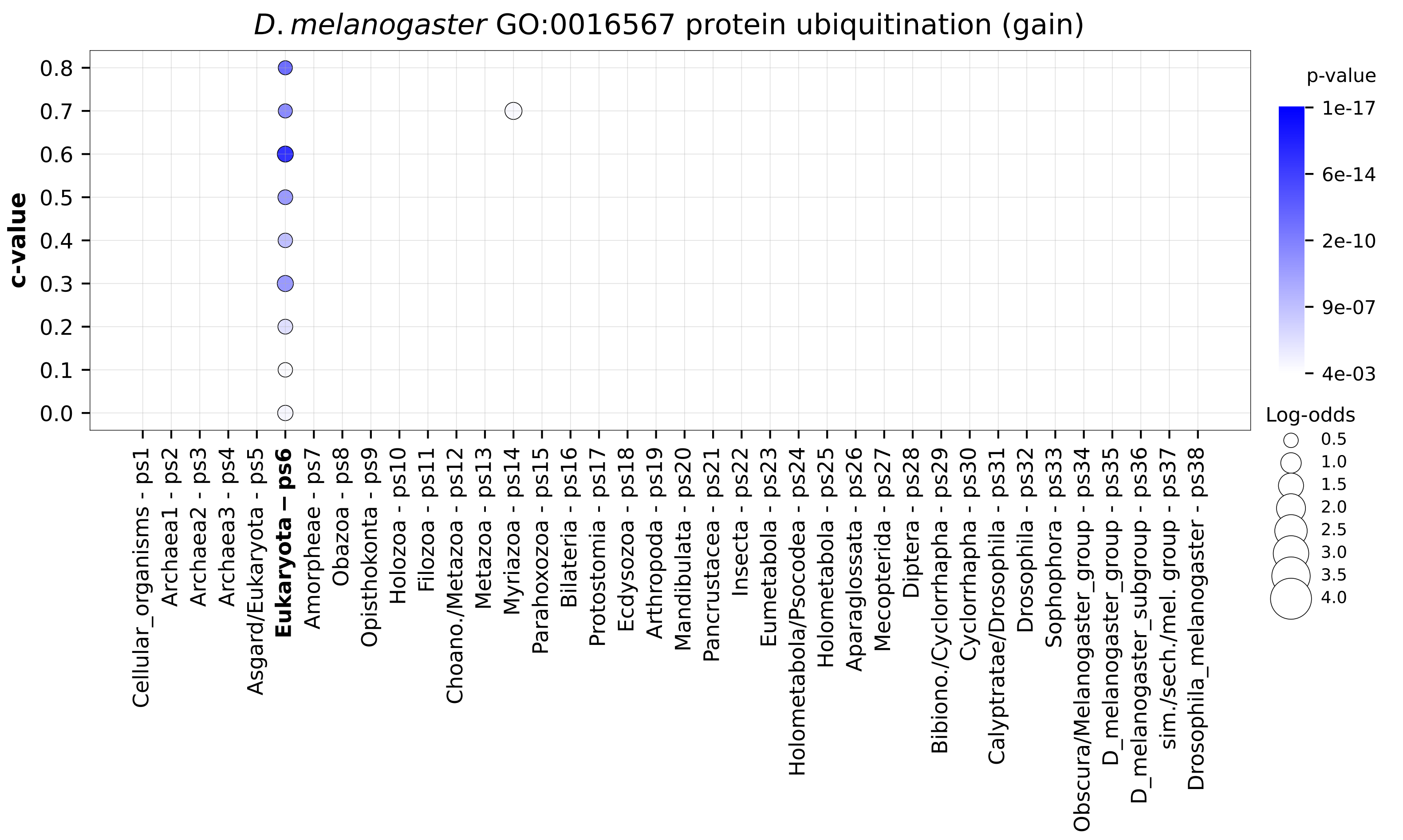
D. melanogaster GO:0016567 protein ubiquitination (gain)
c-value
0.8
0.7
0.6
0.5
0.4
0.3
0.2
0.1
0.0
p-value
1e-17
6e-14
2e-10
9e-07
4e-03
Log-odds
0.5
1.0
1.5
2.0
2.5
3.0
3.5
4.0
Cellular_organisms - ps1
Archaea1 - ps2
Archaea2 - ps3
Archaea3 - ps4
Asgard/Eukaryota - ps5
Eukaryota – ps6
Amorpheae - ps7
Obazoa - ps8
Opisthokonta - ps9
Holozoa - ps10
Filozoa - ps11
Choano./Metazoa - ps12
Metazoa - ps13
Myriazoa - ps14
Parahoxozoa - ps15
Bilateria - ps16
Protostomia - ps17
Ecdysozoa - ps18
Arthropoda - ps19
Mandibulata - ps20
Pancrustacea - ps21
Insecta - ps22
Eumetabola - ps23
Holometabola/Psocodea - ps24
Holometabola - ps25
Aparaglossata - ps26
Mecopterida - ps27
Diptera - ps28
Bibiono./Cyclorrhapha - ps29
Cyclorrhapha - ps30
Calyptratae/Drosophila - ps31
Drosophila - ps32
Sophophora - ps33
Obscura/Melanogaster_group - ps34
D_melanogaster_group - ps35
D_melanogaster_subgroup - ps36
sim./sech./mel. group - ps37
Drosophila_melanogaster - ps38

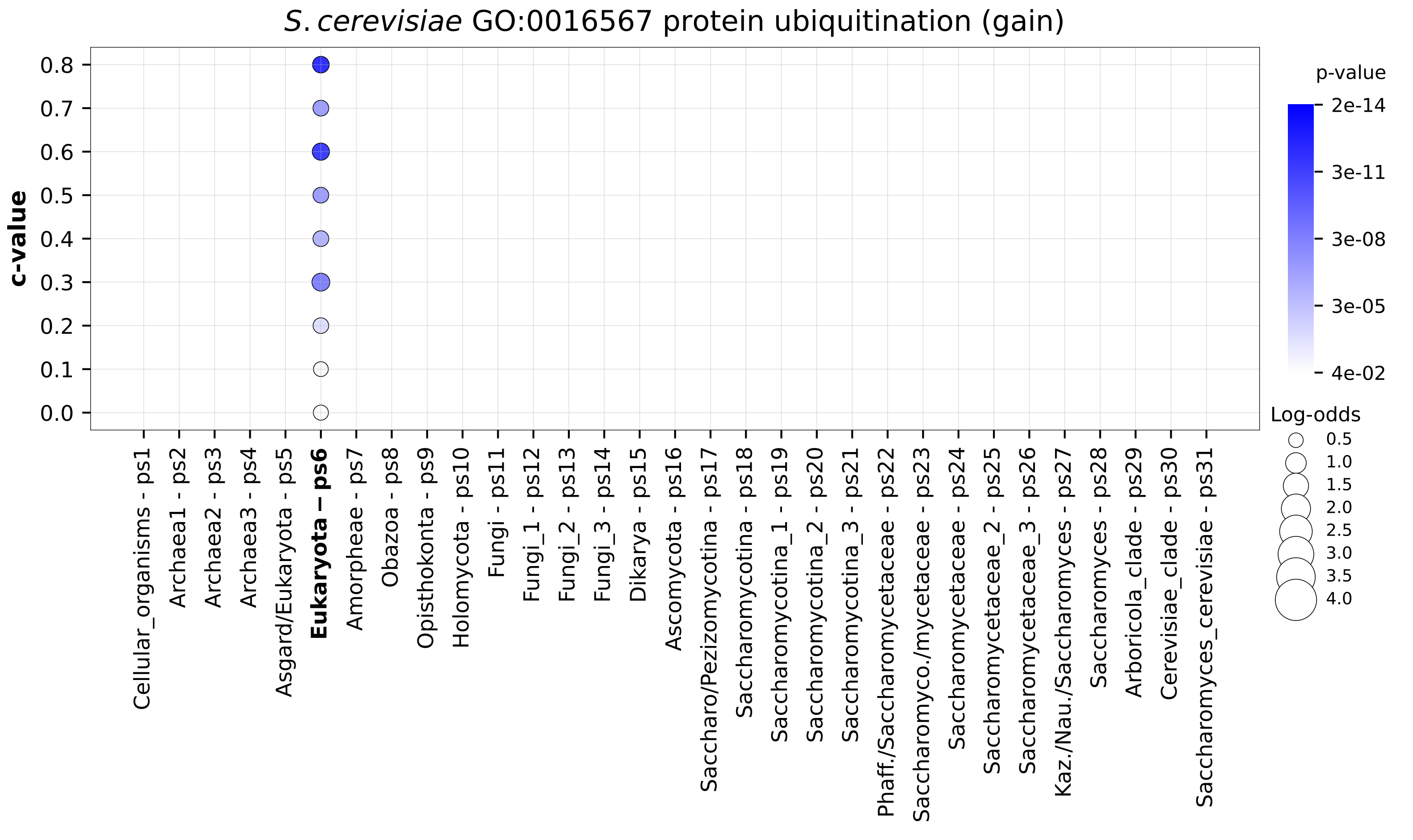
S. cerevisiae GO:0016567 protein ubiquitination (gain)
c-value
0.8
0.7
0.6
0.5
0.4
0.3
0.2
0.1
0.0
p-value
2e-14
3e-11
3e-08
3e-05
4e-02
Log-odds
0.5
1.0
1.5
2.0
2.5
3.0
3.5
4.0
Cellular_organisms - ps1
Archaea1 - ps2
Archaea2 - ps3
Archaea3 - ps4
Asgard/Eukaryota - ps5
Eukaryota – ps6
Amorpheae - ps7
Obazoa - ps8
Opisthokonta - ps9
Holomycota - ps10
Fungi - ps11
Fungi_1 - ps12
Fungi_2 - ps13
Fungi_3 - ps14
Dikarya - ps15
Ascomycota - ps16
Saccharo/Pezizomycotina - ps17
Saccharomycotina - ps18
Saccharomycotina_1 - ps19
Saccharomycotina_2 - ps20
Saccharomycotina_3 - ps21
Phaff./Saccharomycetaceae - ps22
Saccharomyco./mycetaceae - ps23
Saccharomycetaceae - ps24
Saccharomycetaceae_2 - ps25
Saccharomycetaceae_3 - ps26
Kaz./Nau./Saccharomyces - ps27
Saccharomyces - ps28
Arboricola_clade - ps29
Cerevisiae_clade - ps30
Saccharomyces_cerevisiae - ps31

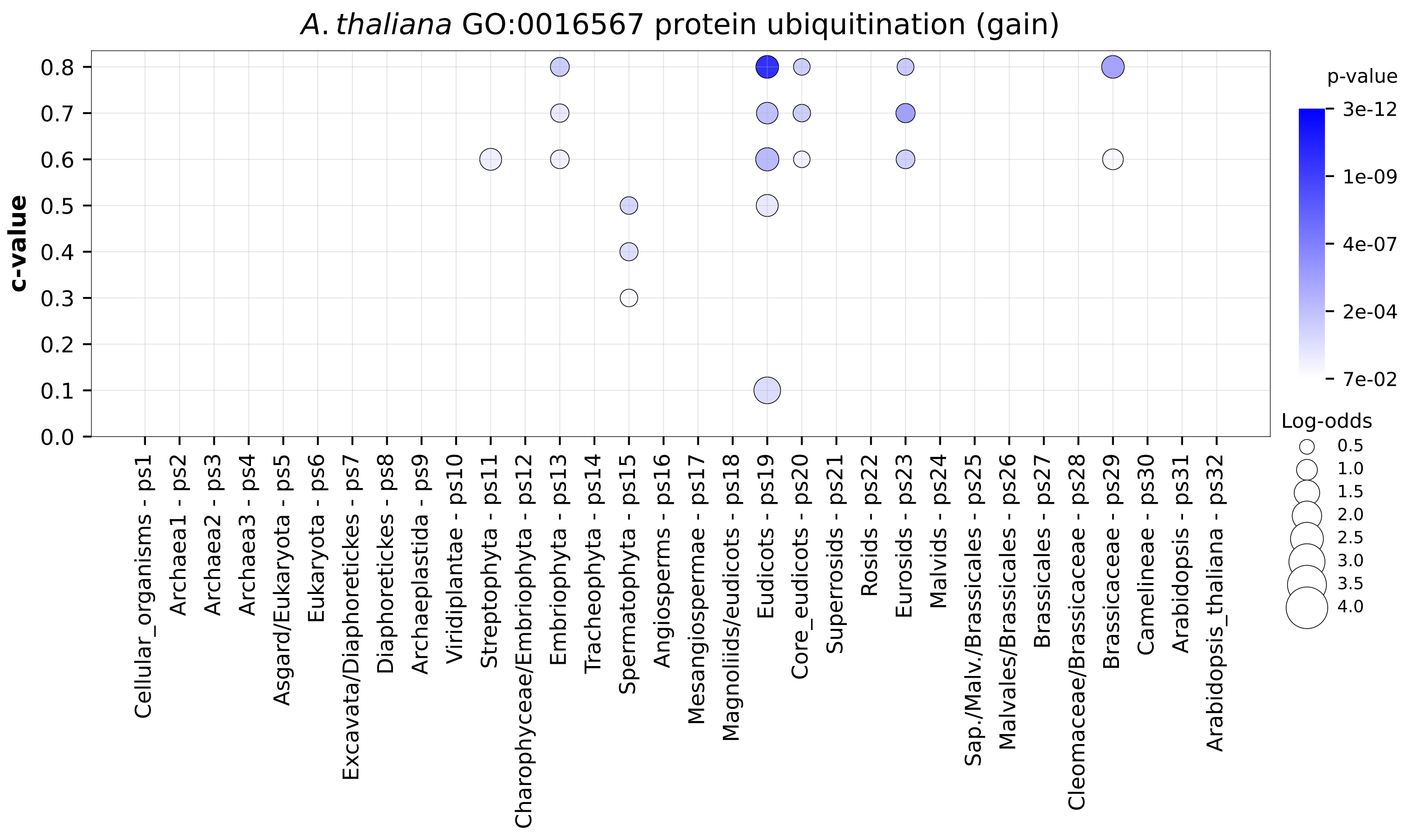
A. thaliana GO:0016567 protein ubiquitination (gain)
c-value
0.8
0.7
0.6
0.5
0.4
0.3
0.2
0.1
0.0
p-value
3e-12
1e-09
4e-07
2e-04
7e-02
Log-odds
0.5
1.0
1.5
2.0
2.5
3.0
3.5
4.0
Cellular_organisms - ps1
Archaea1 - ps2
Archaea2 - ps3
Archaea3 - ps4
Asgard/Eukaryota - ps5
Eukaryota - ps6
Excavata/Diaphoretickes - ps7
Diaphoretickes - ps8
Archaeplastida - ps9
Viridiplantae - ps10
Streptophyta - ps11
Charophyceae/Embriophyta - ps12
Embriophyta - ps13
Tracheophyta - ps14
Spermatophyta - ps15
Angiosperms - ps16
Mesangiospermae - ps17
Magnoliids/eudicots - ps18
Eudicots - ps19
Core_eudicots - ps20
Superrosids - ps21
Rosids - ps22
Eurosids - ps23
Malvids - ps24
Sap./Malv./Brassicales - ps25
Malvales/Brassicales - ps26
Brassicales - ps27
Cleomaceae/Brassicaceae - ps28
Brassicaceae - ps29
Camelineae - ps30
Arabidopsis - ps31
Arabidopsis_thaliana - ps32

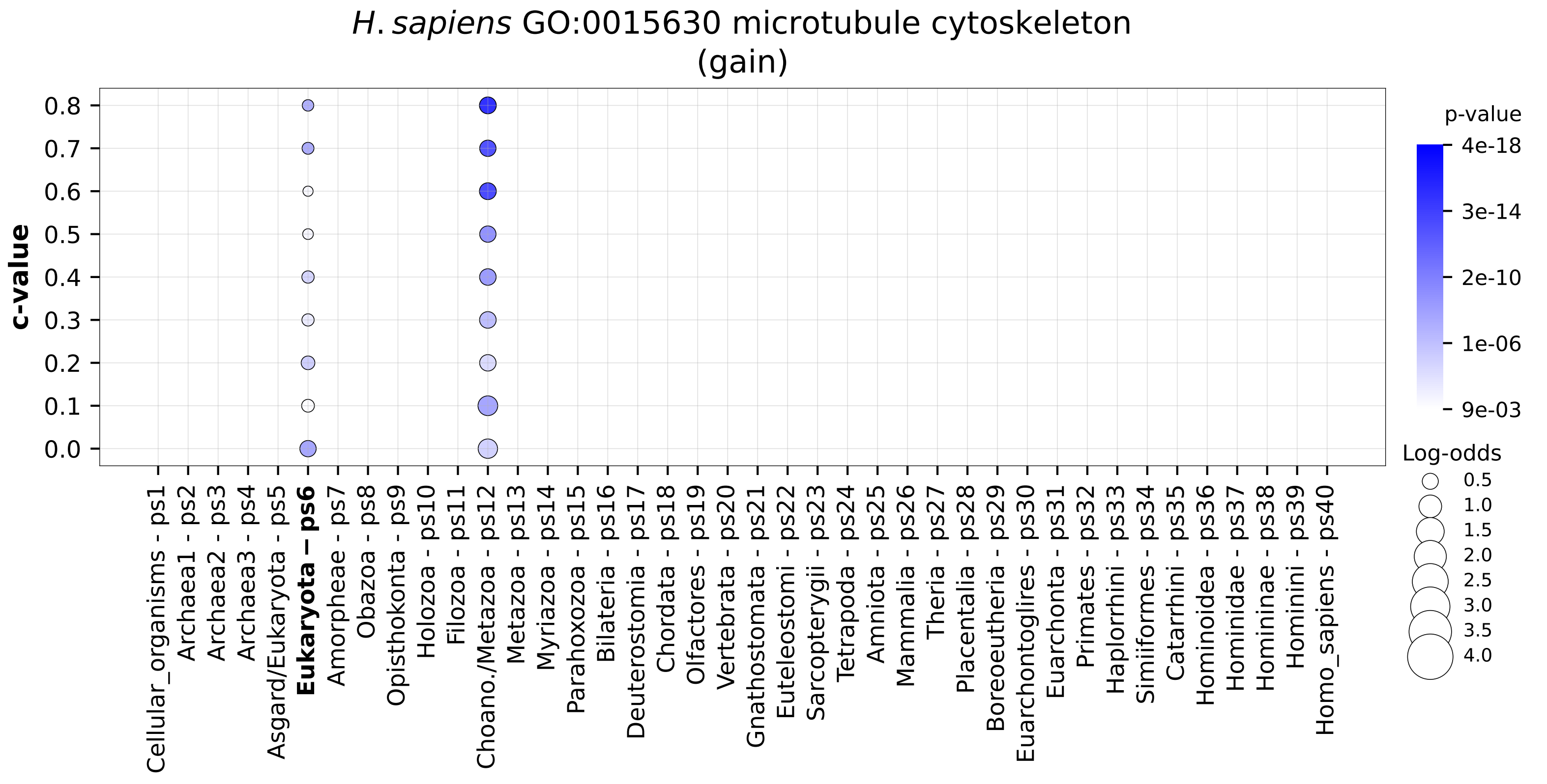
H. sapiens GO:0015630 microtubule cytoskeleton
(gain)
c-value
p-value
4e-18
3e-14
2e-10
1e-06
9e-03
Log-odds
0.5
1.0
1.5
2.0
2.5
3.0
4.0
Cellular_organisms - ps1
Archaea1 - ps2
Archaea2 - ps3
Archaea3 - ps4
Asgard/Eukaryota - ps5
Eukaryota – ps6
Amorpheae - ps7
Obazoa - ps8
Opisthokonta - ps9
Holozoa - ps10
Filozoa - ps11
Choano./Metazoa - ps12
Metazoa - ps13
Myriazoa - ps14
Parahoxozoa - ps15
Bilateria - ps16
Deuterostomia - ps17
Chordata - ps18
Olfactores - ps19
Vertebrata - ps20
Gnathostomata - ps21
Euteleostomi - ps22
Sarcopterygii - ps23
Tetrapoda - ps24
Amniota - ps25
Mammalia - ps26
Theria - ps27
Placentalia - ps28
Boreoeutheria - ps29
Euarchontoglires - ps30
Euarchonta - ps31
Primates - ps32
Haplorrhini - ps33
Simiiformes - ps34
Catarrhini - ps35
Hominoidea - ps36
Hominidae - ps37
Homininae - ps38
Hominini - ps39
Homo_sapiens - ps40

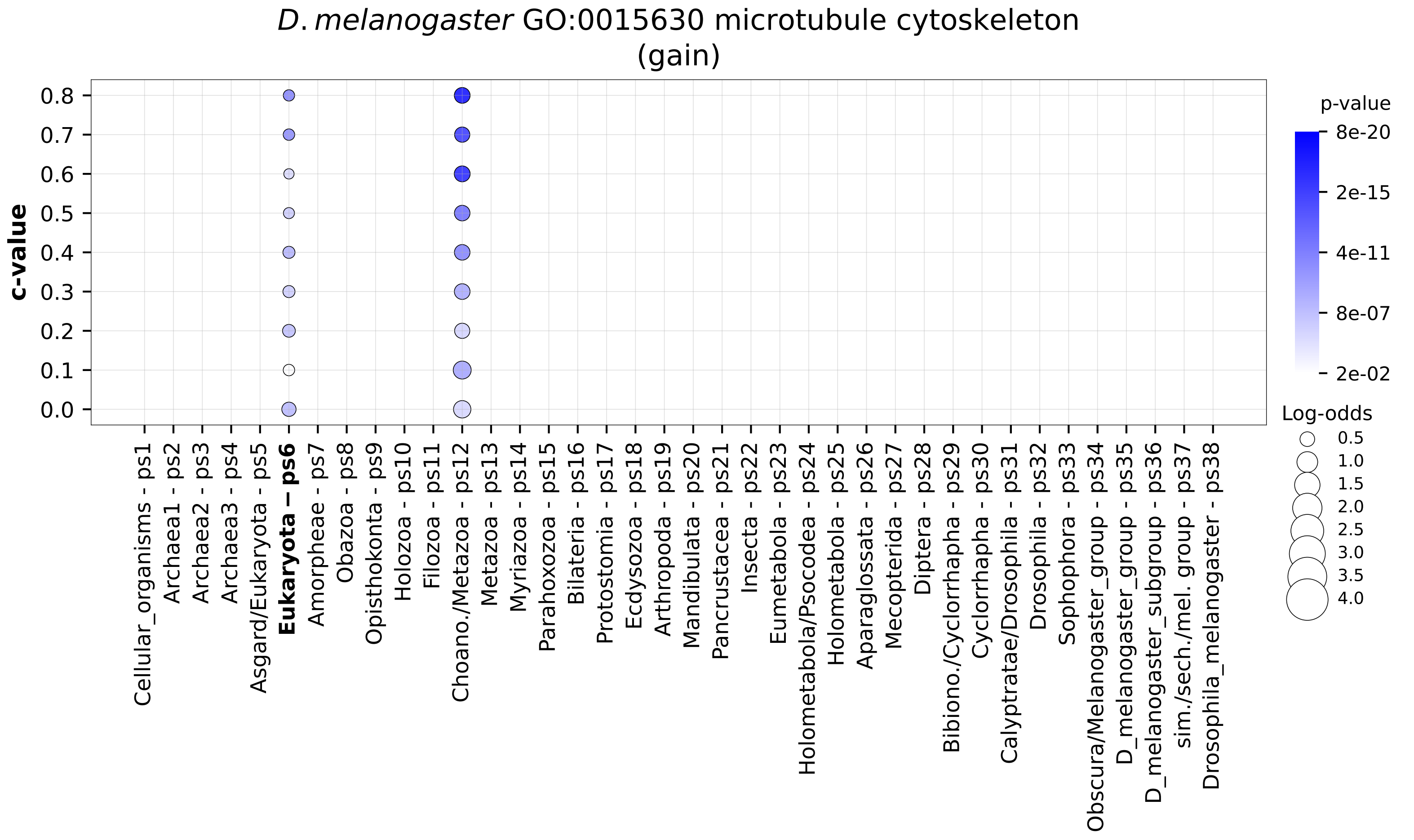
D. melanogaster GO:0015630 microtubule cytoskeleton
(gain)
c-value
p-value
8e-20
2e-15
4e-11
8e-07
2e-02
Log-odds
0.5
1.0
1.5
2.0
2.5
3.0
3.5
4.0
Cellular_organisms - ps1
Archaea1 - ps2
Archaea2 - ps3
Archaea3 - ps4
Asgard/Eukaryota - ps5
Eukaryota – ps6
Amorpheae - ps7
Obazoa - ps8
Opisthokonta - ps9
Holozoa - ps10
Filozoa - ps11
Choano./Metazoa - ps12
Metazoa - ps13
Myriazoa - ps14
Parahoxozoa - ps15
Bilateria - ps16
Protostomia - ps17
Ecdysozoa - ps18
Arthropoda - ps19
Mandibulata - ps20
Pancrustacea - ps21
Insecta - ps22
Eumetabola - ps23
Holometabola/Psocodea - ps24
Holometabola - ps25
Aparaglossata - ps26
Mecopterida - ps27
Diptera - ps28
Bibiono./Cyclorrhapha - ps29
Cyclorrhapha - ps30
Calyptratae/Drosophila - ps31
Drosophila - ps32
Sophophora - ps33
Obscura/Melanogaster_group - ps34
D_melanogaster_group - ps35
D_melanogaster_subgroup - ps36
sim./sech./mel. group - ps37
Drosophila_melanogaster - ps38

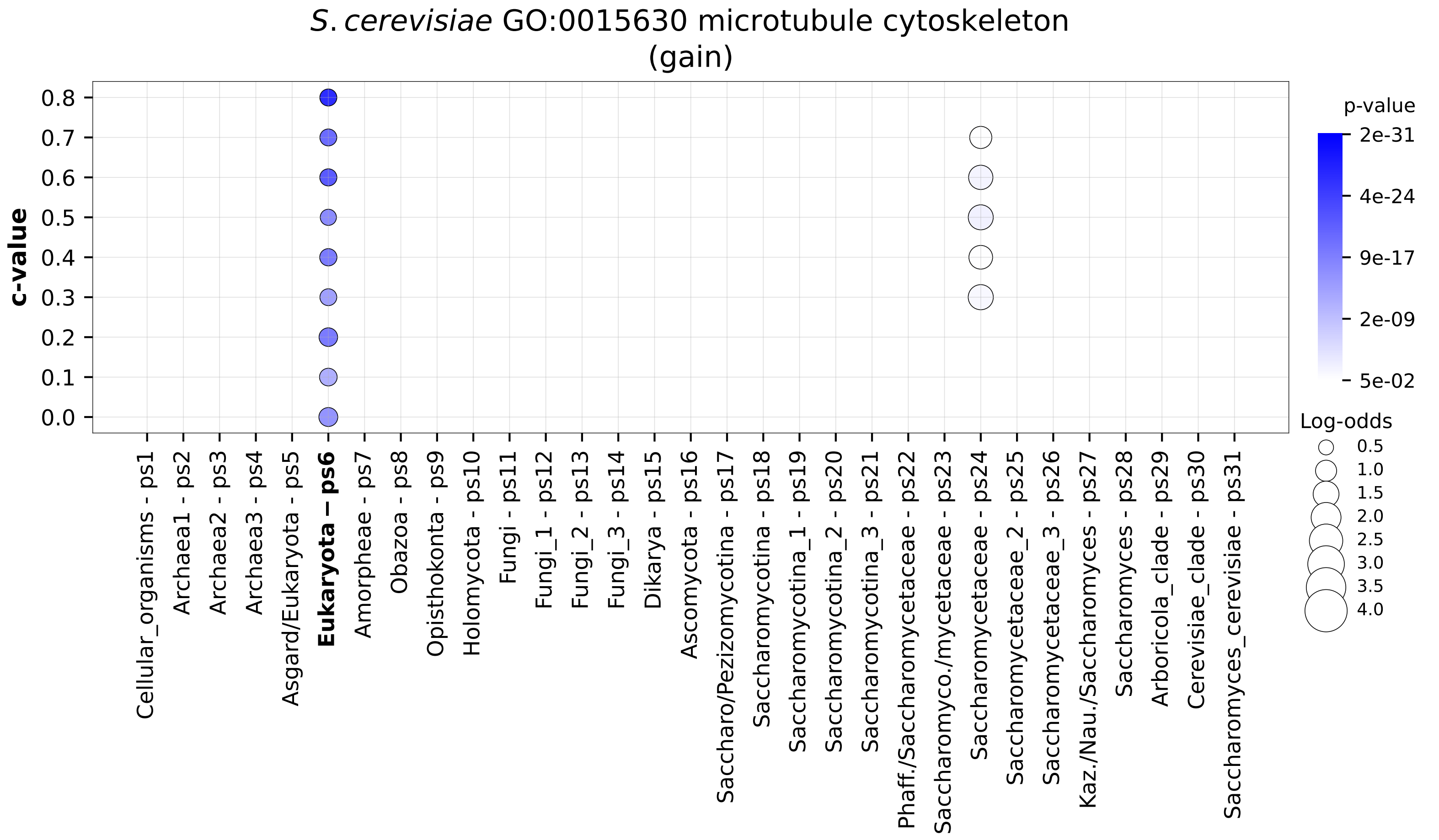
S. cerevisiae GO:0015630 microtubule cytoskeleton
(gain)
c-value
p-value
2e-31
4e-24
9e-17
2e-09
5e-02
Log-odds
0.5
1.0
1.5
2.0
2.5
3.0
3.5
4.0
Cellular_organisms - ps1
Archaea1 - ps2
Archaea2 - ps3
Archaea3 - ps4
Asgard/Eukaryota - ps5
Eukaryota – ps6
Amorpheae - ps7
Obazoa - ps8
Opisthokonta - ps9
Holomycota - ps10
Fungi - ps11
Fungi_1 - ps12
Fungi_2 - ps13
Fungi_3 - ps14
Dikarya - ps15
Ascomycota - ps16
Saccharo/Pezizomycotina - ps17
Saccharomycotina - ps18
Saccharomycotina_1 - ps19
Saccharomycotina_2 - ps20
Saccharomycotina_3 - ps21
Phaff./Saccharomycetaceae - ps22
Saccharomyco./mycetaceae - ps23
Saccharomycetaceae - ps24
Saccharomycetaceae_2 - ps25
Saccharomycetaceae_3 - ps26
Kaz./Nau./Saccharomyces - ps27
Saccharomyces - ps28
Arboricola_clade - ps29
Cerevisiae_clade - ps30
Saccharomyces_cerevisiae - ps31

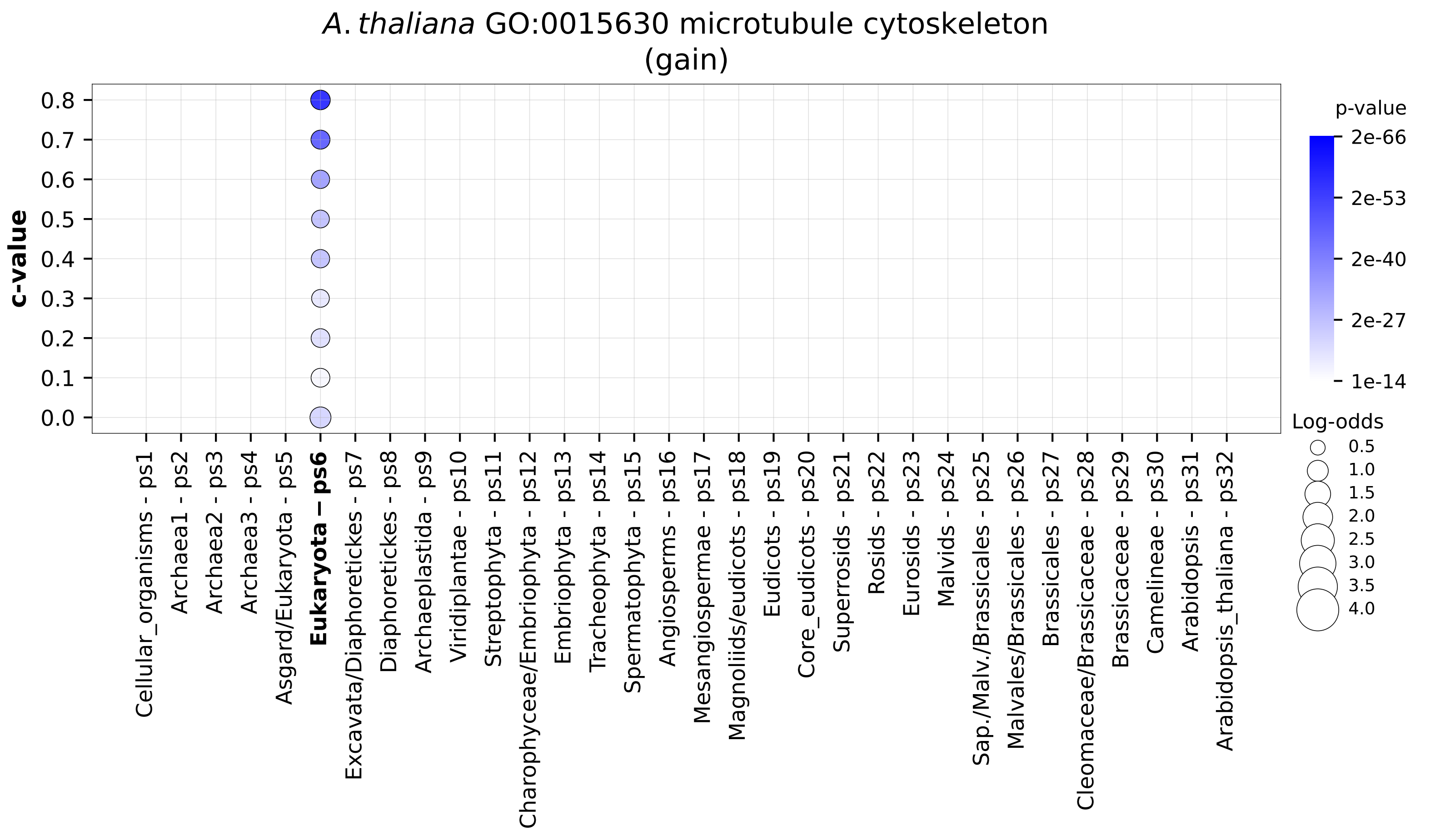
A. thaliana GO:0015630 microtubule cytoskeleton
(gain)
c-value
p-value
2e-66
2e-53
2e-40
2e-27
1e-14
Log-odds
0.5
1.0
1.5
2.0
2.5
3.0
3.5
4.0
Cellular_organisms - ps1
Archaea1 - ps2
Archaea2 - ps3
Archaea3 - ps4
Asgard/Eukaryota - ps5
Eukaryota – ps6
Excavata/Diaphoretickes - ps7
Diaphoretickes - ps8
Archaeplastida - ps9
Viridiplantae - ps10
Streptophyta - ps11
Charophyceae/Embriophyta - ps12
Embriophyta - ps13
Tracheophyta - ps14
Spermatophyta - ps15
Angiosperms - ps16
Mesangiospermae - ps17
Magnoliids/eudicots - ps18
Eudicots - ps19
Core_eudicots - ps20
Superrosids - ps21
Rosids - ps22
Eurosids - ps23
Malvids - ps24
Sap./Malv./Brassicales - ps25
Malvales/Brassicales - ps26
Brassicales - ps27
Cleomaceae/Brassicaceae - ps28
Brassicaceae - ps29
Camelineae - ps30
Arabidopsis - ps31
Arabidopsis_thaliana - ps32

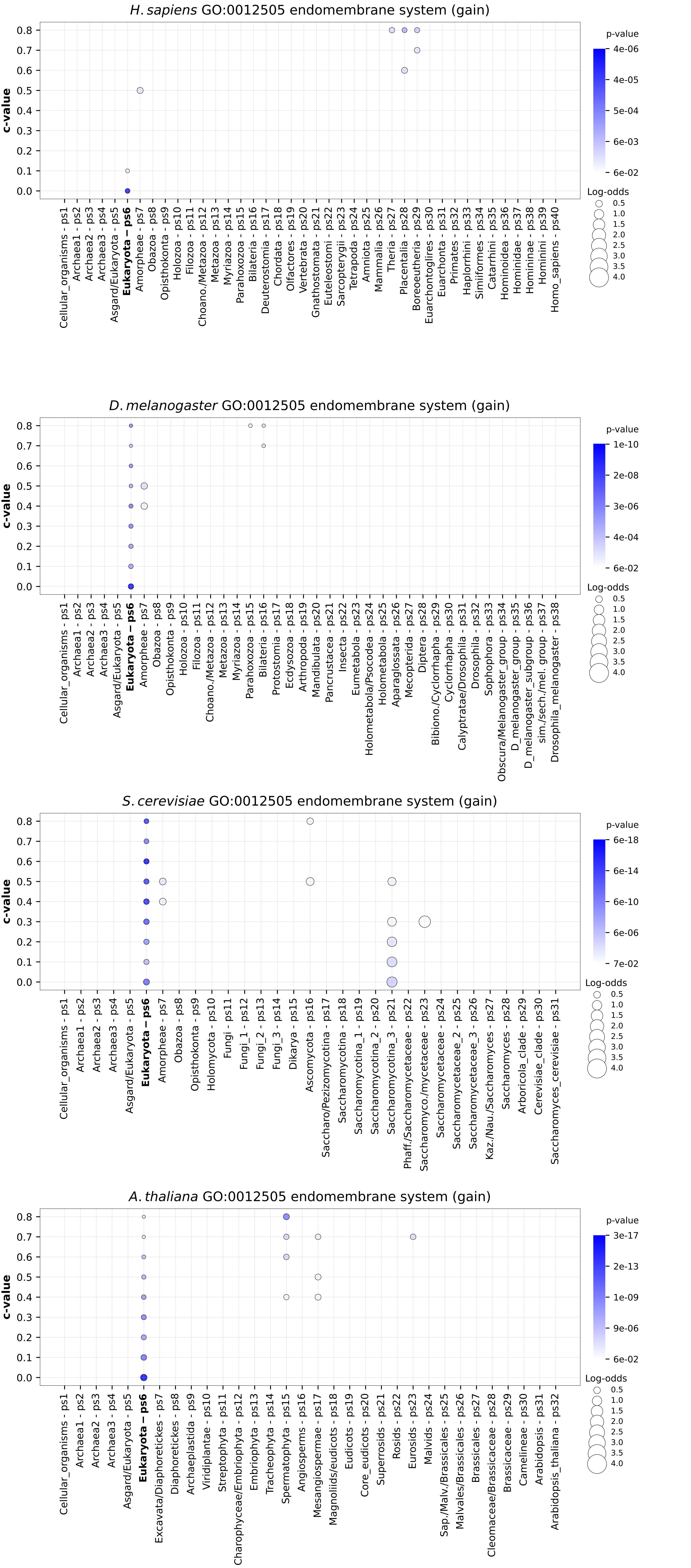

H. sapiens GO:0012505 endomembrane system (gain)
c-value
0.0
0.1
0.2
0.3
0.4
0.5
0.6
0.7
0.8
p-value
4e-06
4e-05
5e-04
6e-03
6e-02
Log-odds
0.5
1.0
1.5
2.0
2.5
3.0
3.5
4.0
Cellular_organisms - ps1
Archaea1 - ps2
Archaea2 - ps3
Archaea3 - ps4
Asgard/Eukaryota - ps5
Eukaryota – ps6
Amorpheae - ps7
Obazoa - ps8
Opisthokonta - ps9
Holozoa - ps10
Filozoa - ps11
Choano./Metazoa - ps12
Metazoa - ps13
Myriazoa - ps14
Parahoxozoa - ps15
Bilateria - ps16
Deuterostomia - ps17
Chordata - ps18
Olfactores - ps19
Vertebrata - ps20
Gnathostomata - ps21
Euteleostomi - ps22
Sarcopterygii - ps23
Tetrapoda - ps24
Amniota - ps25
Mammalia - ps26
Theria - ps27
Placentalia - ps28
Boreoeutheria - ps29
Euarchontoglires - ps30
Euarchonta - ps31
Primates - ps32
Haplorrhini - ps33
Simiiformes - ps34
Catarrhini - ps35
Hominoidea - ps36
Hominidae - ps37
Homininae - ps38
Hominini - ps39
Homo_sapiens - ps40
D. melanogaster GO:0012505 endomembrane system (gain)
c-value
p-value
1e-10
2e-08
3e-06
4e-04
6e-02
Log-odds
Cellular_organisms - ps1
Archaea1 - ps2
Archaea2 - ps3
Archaea3 - ps4
Asgard/Eukaryota - ps5
Eukaryota – ps6
Amorpheae - ps7
Obazoa - ps8
Opisthokonta - ps9
Holozoa - ps10
Filozoa - ps11
Choano./Metazoa - ps12
Metazoa - ps13
Myriazoa - ps14
Parahoxozoa - ps15
Bilateria - ps16
Protostomia - ps17
Ecdysozoa - ps18
Arthropoda - ps19
Mandibulata - ps20
Pancrustacea - ps21
Insecta - ps22
Eumetabola - ps23
Holometabola/Psocodea - ps24
Holometabola - ps25
Aparaglossata - ps26
Mecopterida - ps27
Diptera - ps28
Bibiono./Cyclorrhapha - ps29
Cyclorrhapha - ps30
Calyptratae/Drosophila - ps31
Drosophila - ps32
Sophophora - ps33
Obscura/Melanogaster_group - ps34
D_melanogaster_group - ps35
D_melanogaster_subgroup - ps36
sim./sech./mel. group - ps37
Drosophila_melanogaster - ps38
S. cerevisiae GO:0012505 endomembrane system (gain)
c-value
p-value
6e-18
6e-14
6e-10
6e-06
7e-02
Log-odds
Cellular_organisms - ps1
Archaea1 - ps2
Archaea2 - ps3
Archaea3 - ps4
Asgard/Eukaryota - ps5
Eukaryota – ps6
Amorpheae - ps7
Obazoa - ps8
Opisthokonta - ps9
Holomycota - ps10
Fungi - ps11
Fungi_1 - ps12
Fungi_2 - ps13
Fungi_3 - ps14
Dikarya - ps15
Ascomycota - ps16
Saccharo/Pezizomycotina - ps17
Saccharomycotina - ps18
Saccharomycotina_1 - ps19
Saccharomycotina_2 - ps20
Saccharomycotina_3 - ps21
Phaff./Saccharomycetaceae - ps22
Saccharomyco./mycetaceae - ps23
Saccharomycetaceae - ps24
Saccharomycetaceae_2 - ps25
Saccharomycetaceae_3 - ps26
Kaz./Nau./Saccharomyces - ps27
Saccharomyces - ps28
Arboricola_clade - ps29
Cerevisiae_clade - ps30
Saccharomyces_cerevisiae - ps31
A. thaliana GO:0012505 endomembrane system (gain)
c-value
p-value
3e-17
2e-13
1e-09
9e-06
6e-02
Log-odds
Cellular_organisms - ps1
Archaea1 - ps2
Archaea2 - ps3
Archaea3 - ps4
Asgard/Eukaryota - ps5
Eukaryota – ps6
Excavata/Diaphoretickes - ps7
Diaphoretickes - ps8
Archaeplastida - ps9
Viridiplantae - ps10
Streptophyta - ps11
Charophyceae/Embriophyta - ps12
Embriophyta - ps13
Tracheophyta - ps14
Spermatophyta - ps15
Angiosperms - ps16
Mesangiospermae - ps17
Magnoliids/eudicots - ps18
Eudicots - ps19
Core_eudicots - ps20
Superrosids - ps21
Rosids - ps22
Eurosids - ps23
Malvids - ps24
Sap./Malv./Brassicales - ps25
Malvales/Brassicales - ps26
Brassicales - ps27
Cleomaceae/Brassicaceae - ps28
Brassicaceae - ps29
Camelineae - ps30
Arabidopsis - ps31
Arabidopsis_thaliana - ps32

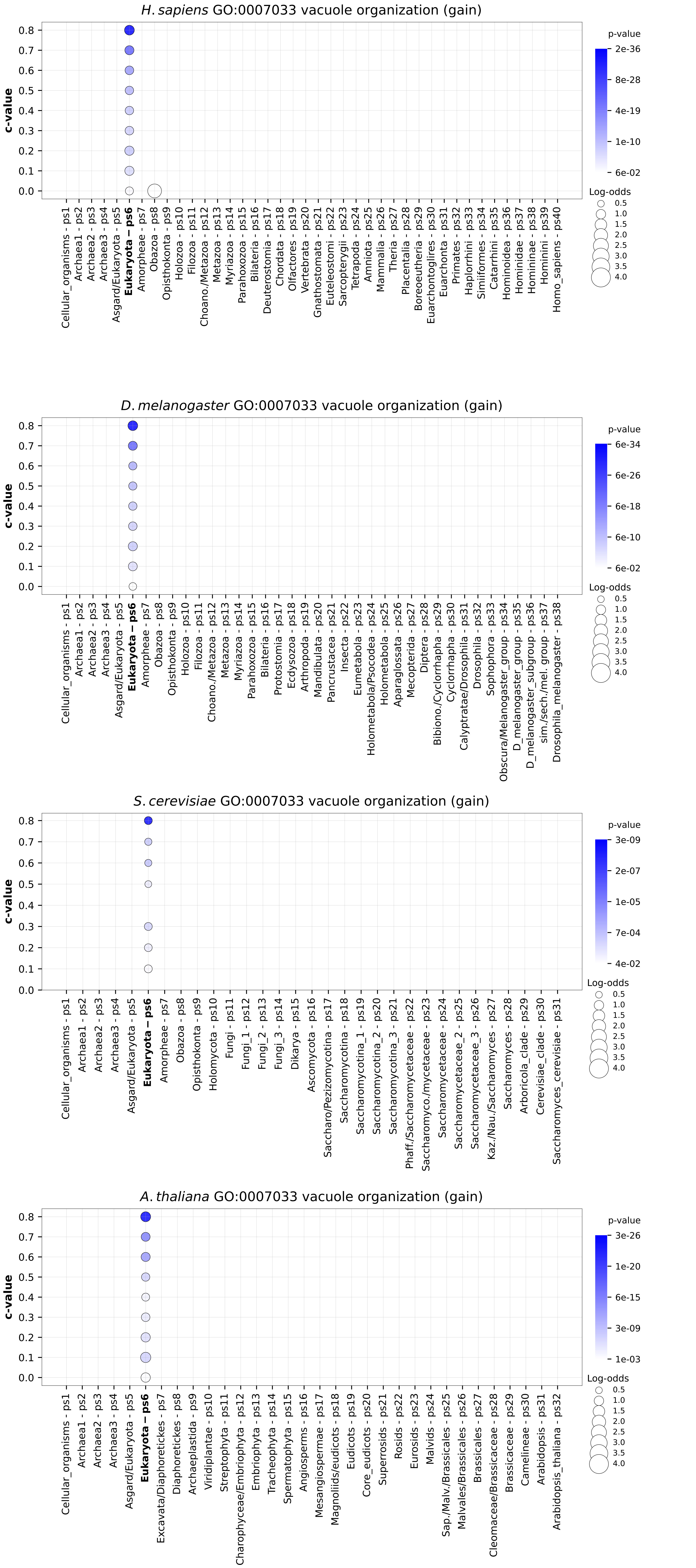
H. sapiens GO:0007033 vacuole organization (gain)
c-value
p-value
Log-odds
Cellular_organisms - ps1
Archaea1 - ps2
Archaea2 - ps3
Archaea3 - ps4
Asgard/Eukaryota - ps5
Eukaryota – ps6
Amorpheae - ps7
Obazoa - ps8
Opisthokonta - ps9
Holozoa - ps10
Filozoa - ps11
Choano./Metazoa - ps12
Metazoa - ps13
Myriazoa - ps14
Parahoxozoa - ps15
Bilateria - ps16
Deuterostomia - ps17
Chordata - ps18
Olfactores - ps19
Vertebrata - ps20
Gnathostomata - ps21
Euteleostomi - ps22
Sarcopterygii - ps23
Tetrapoda - ps24
Amniota - ps25
Mammalia - ps26
Theria - ps27
Placentalia - ps28
Boreoeutheria - ps29
Euarchontoglires - ps30
Euarchonta - ps31
Primates - ps32
Haplorrhini - ps33
Simiiformes - ps34
Catarrhini - ps35
Hominoidea - ps36
Hominidae - ps37
Homininae - ps38
Hominini - ps39
Homo_sapiens - ps40
D. melanogaster GO:0007033 vacuole organization (gain)
Protostomia - ps17
Ecdysozoa - ps18
Arthropoda - ps19
Mandibulata - ps20
Pancrustacea - ps21
Insecta - ps22
Eumetabola - ps23
Holometabola/Psocodea - ps24
Holometabola - ps25
Aparaglossata - ps26
Mecopterida - ps27
Diptera - ps28
Bibiono./Cyclorrhapha - ps29
Cyclorrhapha - ps30
Calyptratae/Drosophila - ps31
Drosophila - ps32
Sophophora - ps33
Obscura/Melanogaster_group - ps34
D_melanogaster_group - ps35
D_melanogaster_subgroup - ps36
sim./sech./mel. group - ps37
Drosophila_melanogaster - ps38
S. cerevisiae GO:0007033 vacuole organization (gain)
Holomycota - ps10
Fungi - ps11
Fungi_1 - ps12
Fungi_2 - ps13
Fungi_3 - ps14
Dikarya - ps15
Ascomycota - ps16
Saccharo/Pezizomycotina - ps17
Saccharomycotina - ps18
Saccharomycotina_1 - ps19
Saccharomycotina_2 - ps20
Saccharomycotina_3 - ps21
Phaff./Saccharomycetaceae - ps22
Saccharomyco./mycetaceae - ps23
Saccharomycetaceae - ps24
Saccharomycetaceae_2 - ps25
Saccharomycetaceae_3 - ps26
Kaz./Nau./Saccharomyces - ps27
Saccharomyces - ps28
Arboricola_clade - ps29
Cerevisiae_clade - ps30
Saccharomyces_cerevisiae - ps31
A. thaliana GO:0007033 vacuole organization (gain)
Excavata/Diaphoretickes - ps7
Diaphoretickes - ps8
Archaeplastida - ps9
Viridiplantae - ps10
Streptophyta - ps11
Charophyceae/Embriophyta - ps12
Embriophyta - ps13
Tracheophyta - ps14
Spermatophyta - ps15
Angiospermae - ps16
Mesangiospermae - ps17
Magnoliids/eudicots - ps18
Eudicots - ps19
Core_eudicots - ps20
Superrosids - ps21
Rosids - ps22
Eurosids - ps23
Malvids - ps24
Sap./Malv./Brassicales - ps25
Malvales/Brassicales - ps26
Brassicales - ps27
Cleomaceae/Brassicaceae - ps28
Brassicaceae - ps29
Camelineae - ps30
Arabidopsis - ps31
Arabidopsis_thaliana - ps32

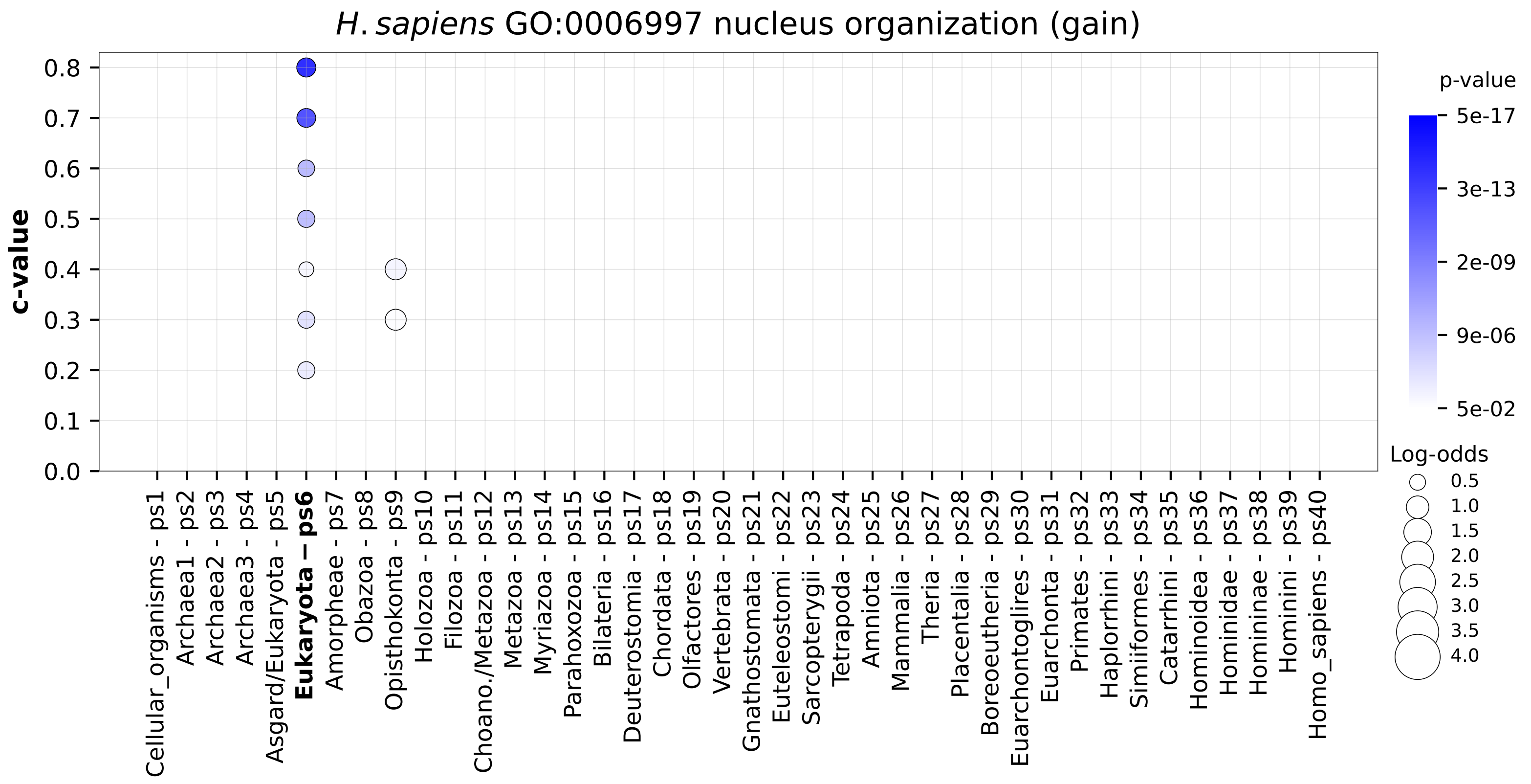
H. sapiens GO:0006997 nucleus organization (gain)
c-value
p-value
Log-odds
Cellular_organisms - ps1
Archaea1 - ps2
Archaea2 - ps3
Archaea3 - ps4
Asgard/Eukaryota - ps5
Eukaryota – ps6
Amorpheae - ps7
Obazoa - ps8
Opisthokonta - ps9
Holozoa - ps10
Filozoa - ps11
Choano./Metazoa - ps12
Metazoa - ps13
Myriazoa - ps14
Parahoxozoa - ps15
Bilateria - ps16
Deuterostomia - ps17
Chordata - ps18
Olfactores - ps19
Vertebrata - ps20
Gnathostomata - ps21
Euteleostomi - ps22
Sarcopterygii - ps23
Tetrapoda - ps24
Amniota - ps25
Mammalia - ps26
Theria - ps27
Placentalia - ps28
Boreoeutheria - ps29
Euarchontoglires - ps30
Euarchonta - ps31
Primates - ps32
Haplorrhini - ps33
Simiiformes - ps34
Catarrhini - ps35
Hominoidea - ps36
Hominidae - ps37
Homininae - ps38
Hominini - ps39
Homo_sapiens - ps40

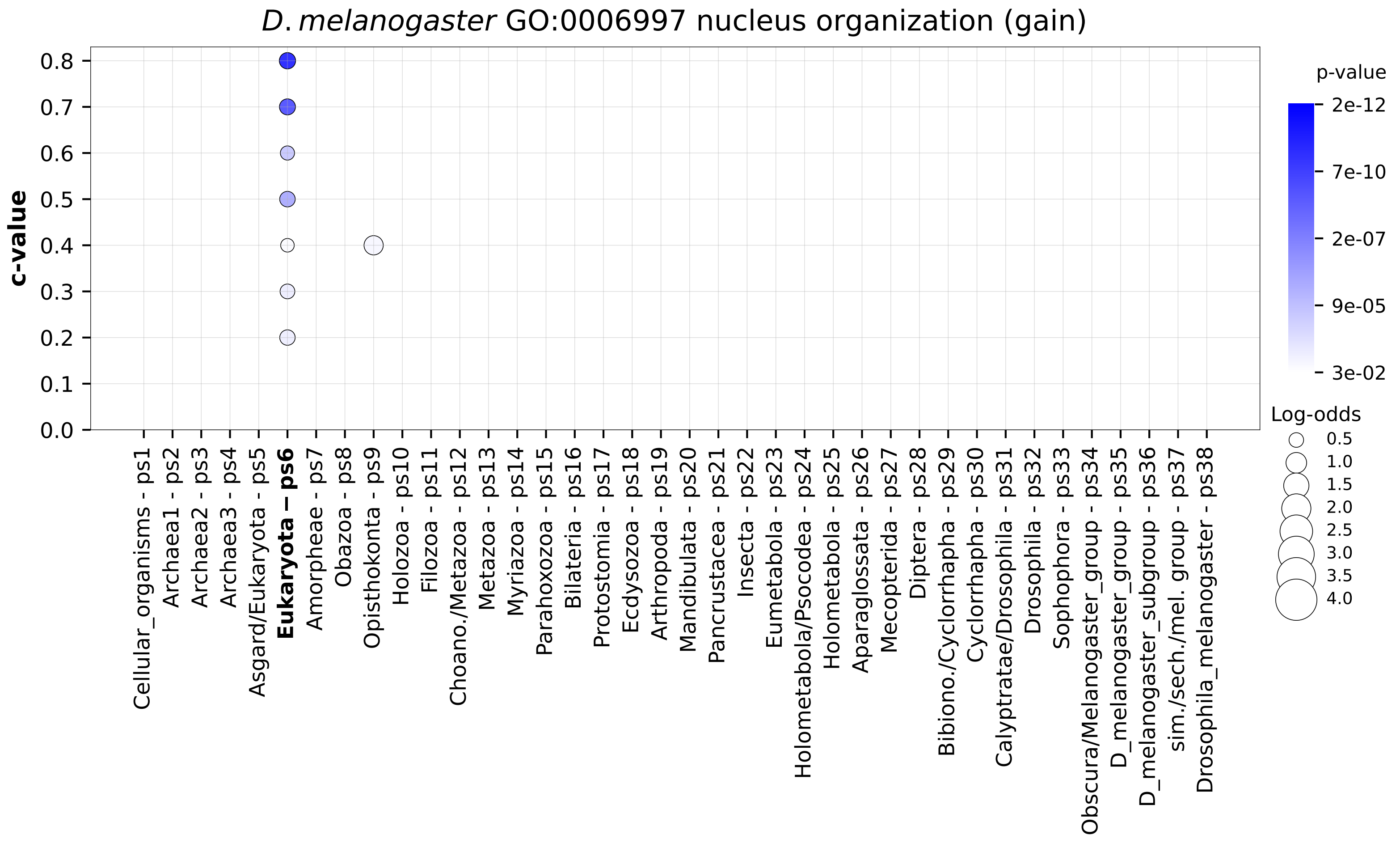
D. melanogaster GO:0006997 nucleus organization (gain)
c-value
p-value
Log-odds
Cellular_organisms - ps1
Archaea1 - ps2
Archaea2 - ps3
Archaea3 - ps4
Asgard/Eukaryota - ps5
Eukaryota – ps6
Amorpheae - ps7
Obazoa - ps8
Opisthokonta - ps9
Holozoa - ps10
Filozoa - ps11
Choano./Metazoa - ps12
Metazoa - ps13
Myriazoa - ps14
Parahoxozoa - ps15
Bilateria - ps16
Protostomia - ps17
Ecdysozoa - ps18
Arthropoda - ps19
Mandibulata - ps20
Pancrustacea - ps21
Insecta - ps22
Eumetabola - ps23
Holometabola/Psocodea - ps24
Holometabola - ps25
Aparaglossata - ps26
Mecopterida - ps27
Diptera - ps28
Bibiono./Cyclorrhapha - ps29
Cyclorrhapha - ps30
Calyptratae/Drosophila - ps31
Drosophila - ps32
Sophophora - ps33
Obscura/Melanogaster_group - ps34
D_melanogaster_group - ps35
D_melanogaster_subgroup - ps36
sim./sech./mel. group - ps37
Drosophila_melanogaster - ps38

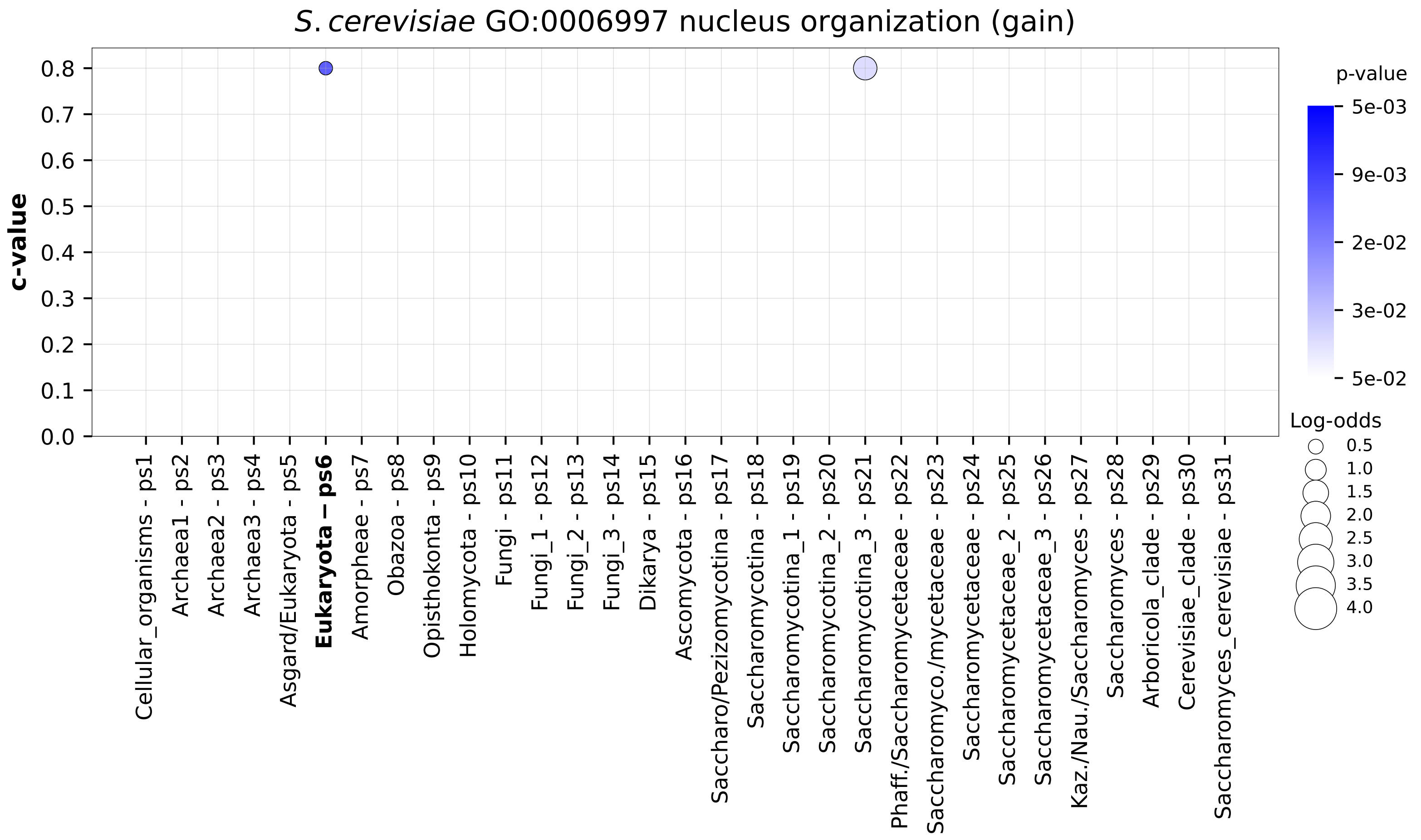
S. cerevisiae GO:0006997 nucleus organization (gain)
c-value
p-value
Log-odds
Cellular_organisms - ps1
Archaea1 - ps2
Archaea2 - ps3
Archaea3 - ps4
Asgard/Eukaryota - ps5
Eukaryota – ps6
Amorpheae - ps7
Obazoa - ps8
Opisthokonta - ps9
Holomycota - ps10
Fungi - ps11
Fungi_1 - ps12
Fungi_2 - ps13
Fungi_3 - ps14
Dikarya - ps15
Ascomycota - ps16
Saccharo/Pezizomycotina - ps17
Saccharomycotina - ps18
Saccharomycotina_1 - ps19
Saccharomycotina_2 - ps20
Saccharomycotina_3 - ps21
Phaff./Saccharomycetaceae - ps22
Saccharomyco./mycetaceae - ps23
Saccharomycetaceae - ps24
Saccharomycetaceae_2 - ps25
Saccharomycetaceae_3 - ps26
Kaz./Nau./Saccharomyces - ps27
Saccharomyces - ps28
Arboricola_clade - ps29
Cerevisiae_clade - ps30
Saccharomyces_cerevisiae - ps31

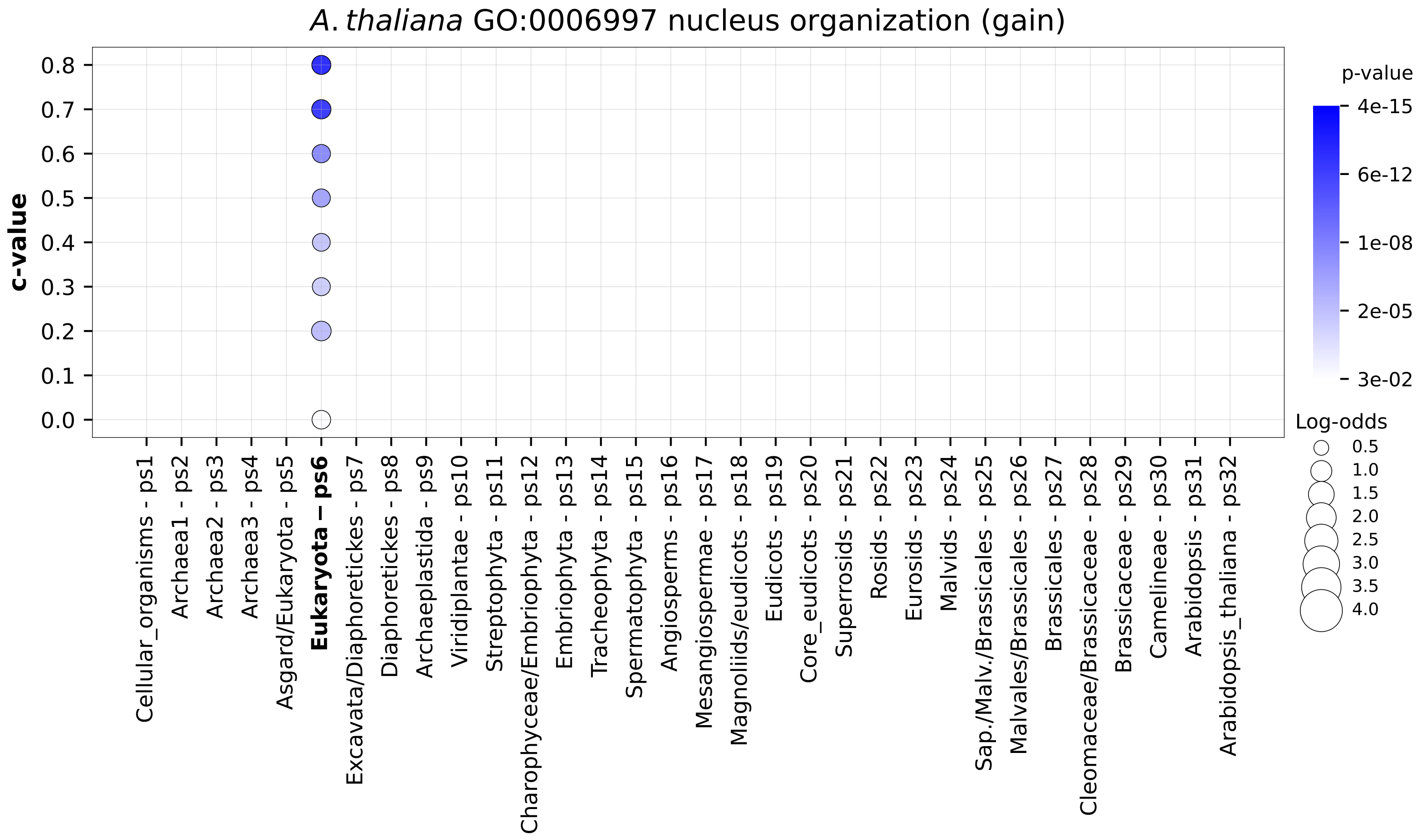
A. thaliana GO:0006997 nucleus organization (gain)
c-value
p-value
Log-odds
Cellular_organisms - ps1
Archaea1 - ps2
Archaea2 - ps3
Archaea3 - ps4
Asgard/Eukaryota - ps5
Eukaryota – ps6
Excavata/Diaphoretickes - ps7
Diaphoretickes - ps8
Archaeplastida - ps9
Viridiplantae - ps10
Streptophyta - ps11
Charophyceae/Embriophyta - ps12
Embriophyta - ps13
Tracheophyta - ps14
Spermatophyta - ps15
Angiospermae - ps16
Mesangiospermae - ps17
Magnoliids/eudicots - ps18
Eudicots - ps19
Core_eudicots - ps20
Superrosids - ps21
Rosids - ps22
Eurosids - ps23
Malvids - ps24
Sap./Malv./Brassicales - ps25
Malvales/Brassicales - ps26
Brassicales - ps27
Cleomaceae/Brassicaceae - ps28
Brassicaceae - ps29
Camelineae - ps30
Arabidopsis - ps31
Arabidopsis_thaliana - ps32

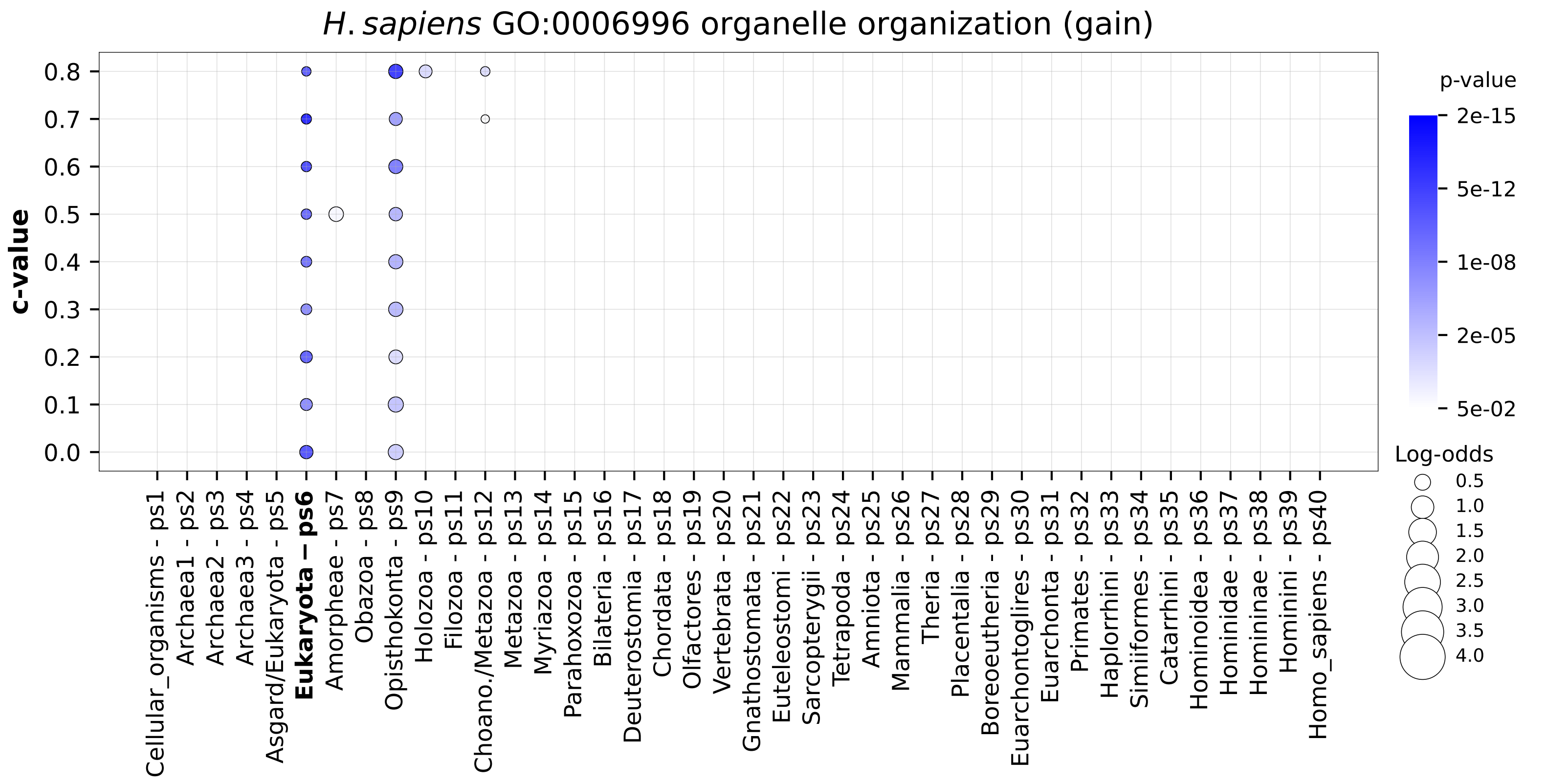
H. sapiens GO:0006996 organelle organization (gain)
c-value
p-value
2e-15
5e-12
1e-08
2e-05
5e-02
Log-odds
0.5
1.0
1.5
2.0
2.5
3.0
3.5
4.0
Cellular_organisms - ps1
Archaea1 - ps2
Archaea2 - ps3
Archaea3 - ps4
Asgard/Eukaryota - ps5
Eukaryota – ps6
Amorpheae - ps7
Obazoa - ps8
Opisthokonta - ps9
Holozoa - ps10
Filozoa - ps11
Choano./Metazoa - ps12
Metazoa - ps13
Myriazoa - ps14
Parahoxozoa - ps15
Bilateria - ps16
Deuterostomia - ps17
Chordata - ps18
Olfactores - ps19
Vertebrata - ps20
Gnathostomata - ps21
Euteleostomi - ps22
Sarcopterygii - ps23
Tetrapoda - ps24
Amniota - ps25
Mammalia - ps26
Theria - ps27
Placentalia - ps28
Boreoeutheria - ps29
Euarchontoglires - ps30
Euarchonta - ps31
Primates - ps32
Haplorrhini - ps33
Simiiformes - ps34
Catarrhini - ps35
Hominoidea - ps36
Hominidae - ps37
Homininae - ps38
Hominini - ps39
Homo_sapiens - ps40

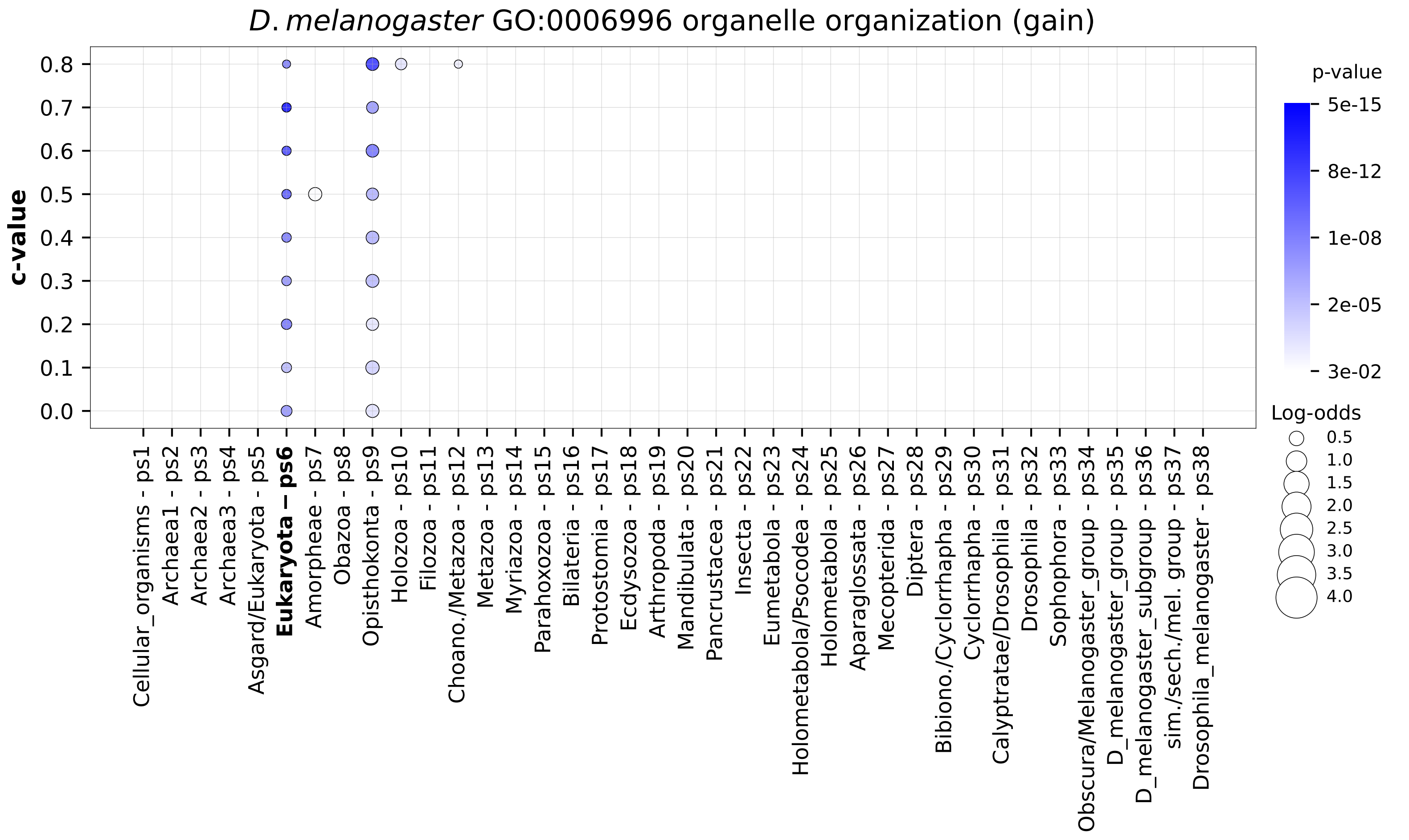
D. melanogaster GO:0006996 organelle organization (gain)
c-value
p-value
5e-15
8e-12
1e-08
2e-05
3e-02
Log-odds
0.5
1.0
1.5
2.0
2.5
3.0
3.5
4.0
Cellular_organisms - ps1
Archaea1 - ps2
Archaea2 - ps3
Archaea3 - ps4
Asgard/Eukaryota - ps5
Eukaryota – ps6
Amorpheae - ps7
Obazoa - ps8
Opisthokonta - ps9
Holozoa - ps10
Filozoa - ps11
Choano./Metazoa - ps12
Metazoa - ps13
Myriazoa - ps14
Parahoxozoa - ps15
Bilateria - ps16
Protostomia - ps17
Ecdysozoa - ps18
Arthropoda - ps19
Mandibulata - ps20
Pancrustacea - ps21
Insecta - ps22
Eumetabola - ps23
Holometabola/Psocodea - ps24
Holometabola - ps25
Aparaglossata - ps26
Mecopterida - ps27
Diptera - ps28
Bibiono./Cyclorrhapha - ps29
Cyclorrhapha - ps30
Calyptratae/Drosophila - ps31
Drosophila - ps32
Sophophora - ps33
Obscura/Melanogaster_group - ps34
D_melanogaster_group - ps35
D_melanogaster_subgroup - ps36
sim./sech./mel. group - ps37
Drosophila_melanogaster - ps38

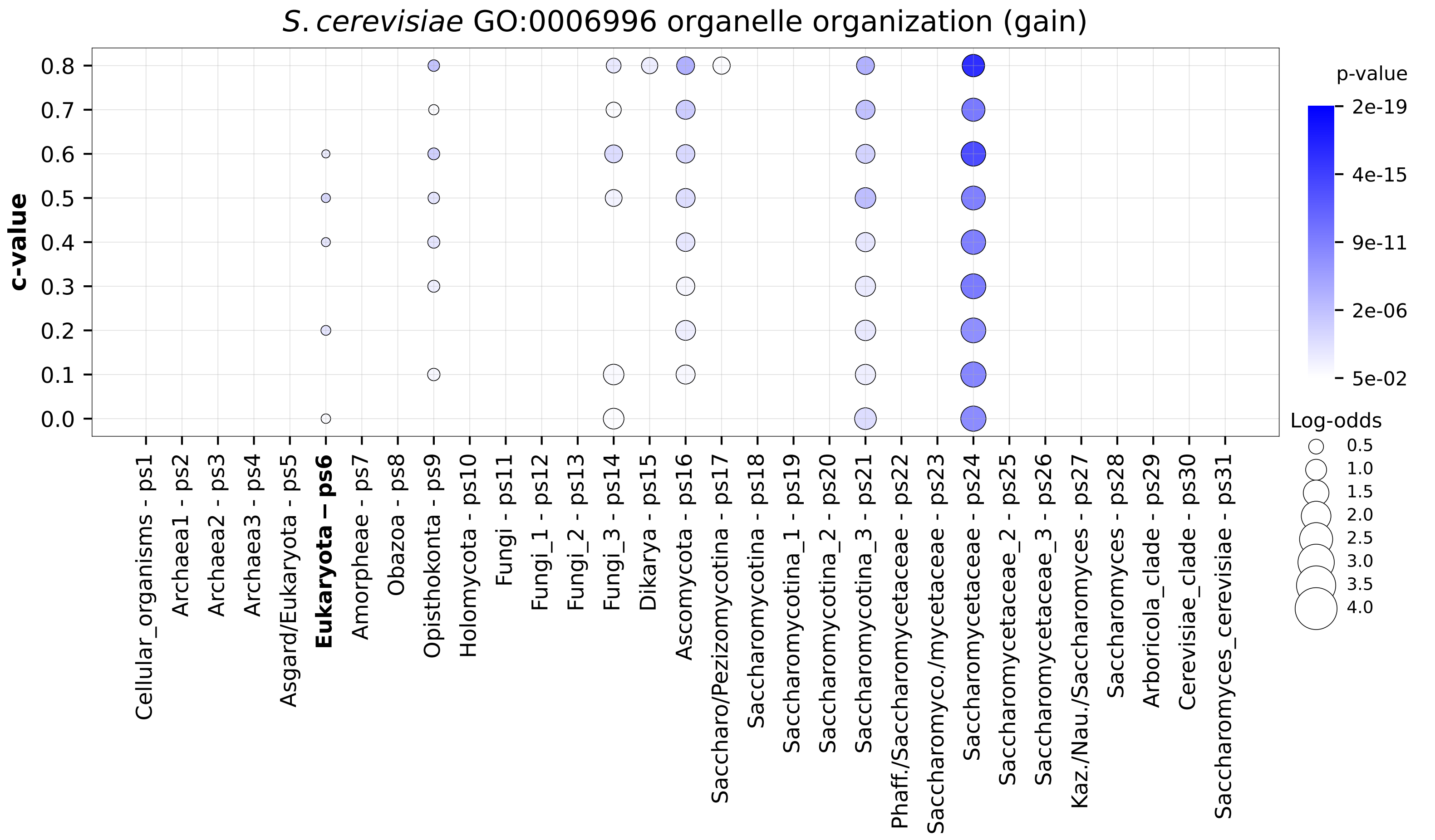
S. cerevisiae GO:0006996 organelle organization (gain)
c-value
p-value
2e-19
4e-15
9e-11
2e-06
5e-02
Log-odds
0.5
1.0
1.5
2.0
2.5
3.0
3.5
4.0
Cellular_organisms - ps1
Archaea1 - ps2
Archaea2 - ps3
Archaea3 - ps4
Asgard/Eukaryota - ps5
Eukaryota – ps6
Amorpheae - ps7
Obazoa - ps8
Opisthokonta - ps9
Holomycota - ps10
Fungi - ps11
Fungi_1 - ps12
Fungi_2 - ps13
Fungi_3 - ps14
Dikarya - ps15
Ascomycota - ps16
Saccharo/Pezizomycotina - ps17
Saccharomycotina - ps18
Saccharomycotina_1 - ps19
Saccharomycotina_2 - ps20
Saccharomycotina_3 - ps21
Phaff./Saccharomycetaceae - ps22
Saccharomyco./mycetaceae - ps23
Saccharomycetaceae - ps24
Saccharomycetaceae_2 - ps25
Saccharomycetaceae_3 - ps26
Kaz./Nau./Saccharomyces - ps27
Saccharomyces - ps28
Arboricola_clade - ps29
Cerevisiae_clade - ps30
Saccharomyces_cerevisiae - ps31

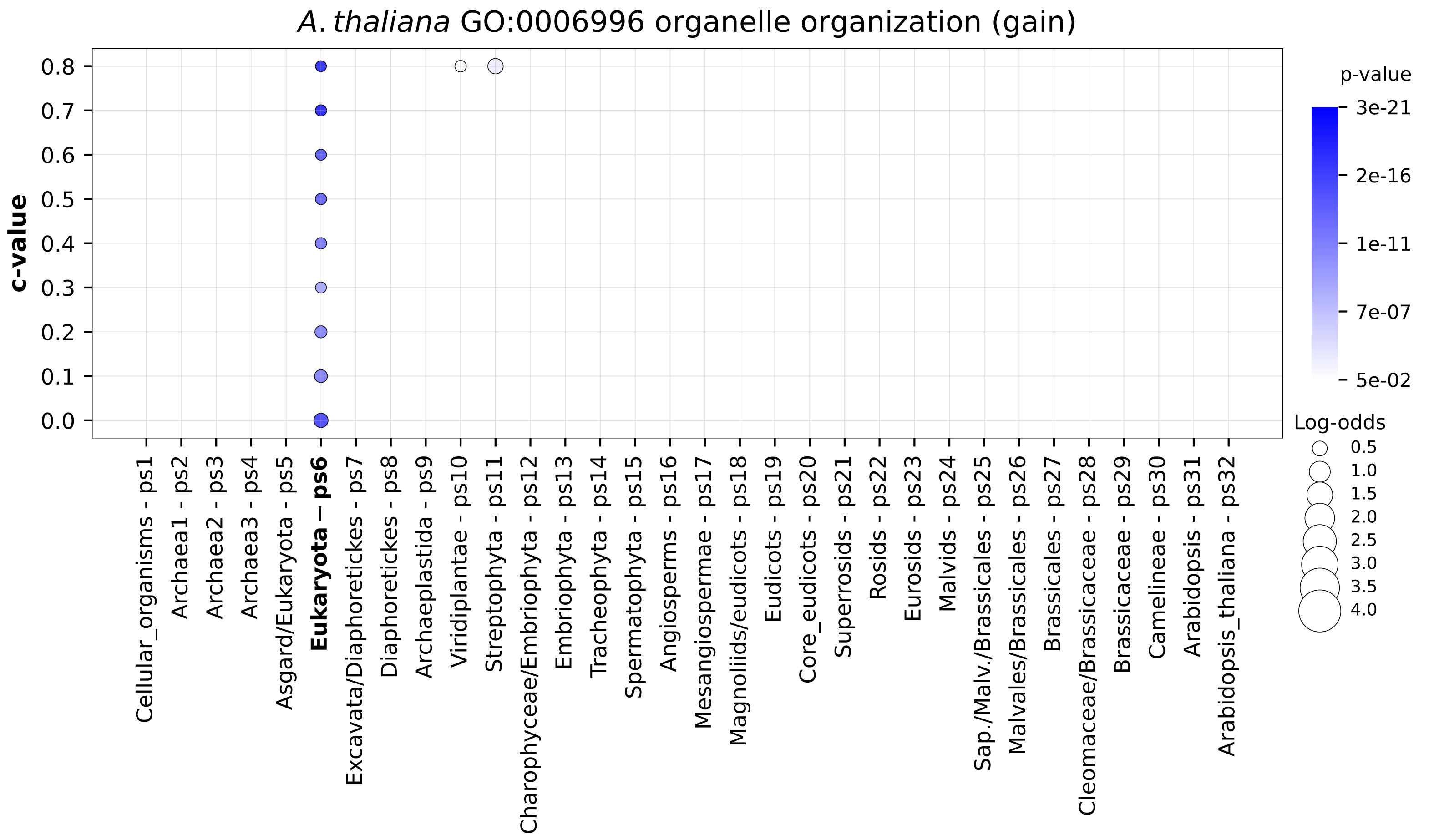
A. thaliana GO:0006996 organelle organization (gain)
c-value
p-value
3e-21
2e-16
1e-11
7e-07
5e-02
Log-odds
0.5
1.0
1.5
2.0
2.5
3.0
3.5
4.0
Cellular_organisms - ps1
Archaea1 - ps2
Archaea2 - ps3
Archaea3 - ps4
Asgard/Eukaryota - ps5
Eukaryota – ps6
Excavata/Diaphoretickes - ps7
Diaphoretickes - ps8
Archaeplastida - ps9
Viridiplantae - ps10
Streptophyta - ps11
Charophyceae/Embriophyta - ps12
Embriophyta - ps13
Tracheophyta - ps14
Spermatophyta - ps15
Angiosperms - ps16
Mesangiospermae - ps17
Magnoliids/eudicots - ps18
Eudicots - ps19
Core_eudicots - ps20
Superrosids - ps21
Rosids - ps22
Eurosids - ps23
Malvids - ps24
Sap./Malv./Brassicales - ps25
Malvales/Brassicales - ps26
Brassicales - ps27
Cleomaceae/Brassicaceae - ps28
Brassicaceae - ps29
Camelineae - ps30
Arabidopsis - ps31
Arabidopsis_thaliana - ps32

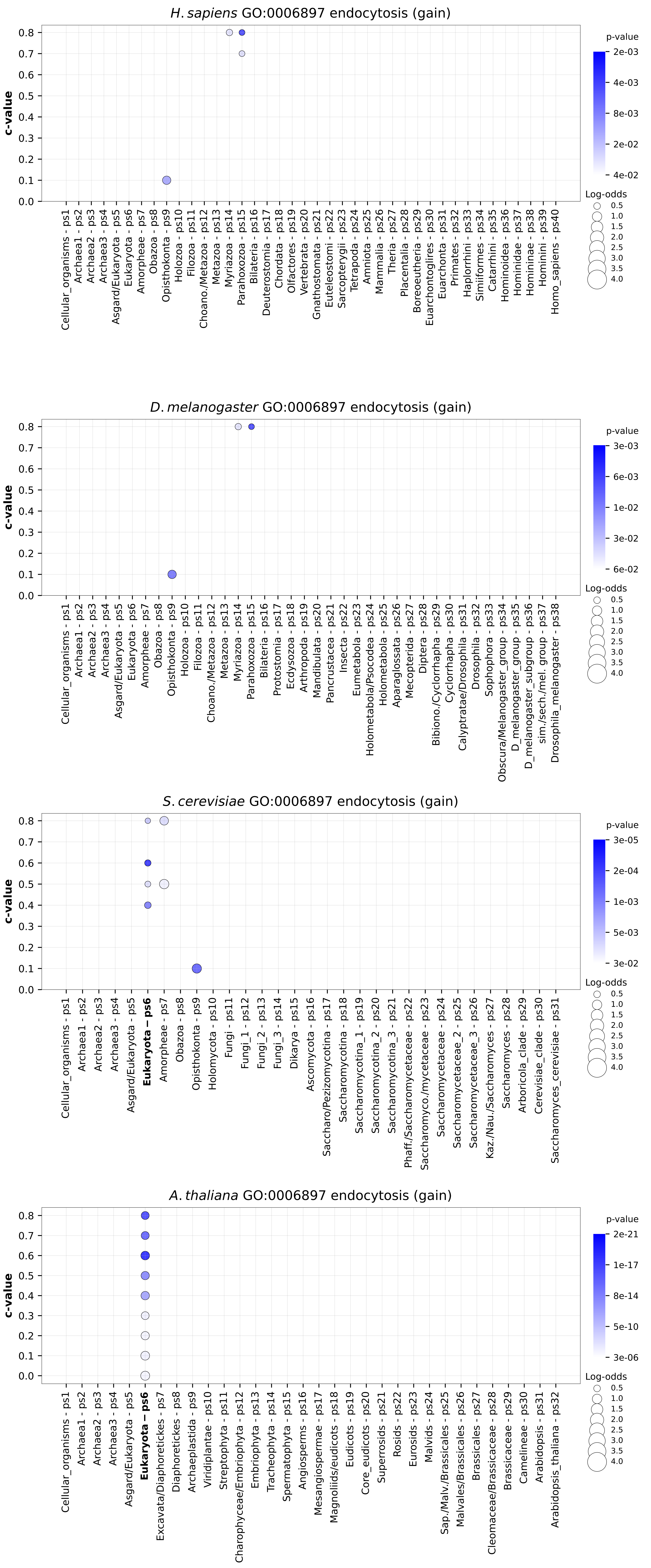

H. sapiens GO:0006897 endocytosis (gain)
c-value
p-value
2e-03
4e-03
8e-03
2e-02
4e-02
Log-odds
0.5
1.0
1.5
2.0
2.5
3.0
3.5
4.0
Cellular_organisms - ps1
Archaea1 - ps2
Archaea2 - ps3
Archaea3 - ps4
Asgard/Eukaryota - ps5
Eukaryota - ps6
Amorpheae - ps7
Obazoa - ps8
Opisthokonta - ps9
Holozoa - ps10
Filozoa - ps11
Choano./Metazoa - ps12
Metazoa - ps13
Myriazoa - ps14
Parahoxozoa - ps15
Bilateria - ps16
Deuterostomia - ps17
Chordata - ps18
Olfactores - ps19
Vertebrata - ps20
Gnathostomata - ps21
Euteleostomi - ps22
Sarcopterygii - ps23
Tetrapoda - ps24
Amniota - ps25
Mammalia - ps26
Theria - ps27
Placentalia - ps28
Boreoeutheria - ps29
Euarchontoglires - ps30
Euarchonta - ps31
Primates - ps32
Haplorrhini - ps33
Simiiformes - ps34
Catarrhini - ps35
Hominoidea - ps36
Hominidae - ps37
Homininae - ps38
Hominini - ps39
Homo_sapiens - ps40
D. melanogaster GO:0006897 endocytosis (gain)
c-value
p-value
3e-03
6e-03
1e-02
3e-02
6e-02
Log-odds
Cellular_organisms - ps1
Archaea1 - ps2
Archaea2 - ps3
Archaea3 - ps4
Asgard/Eukaryota - ps5
Eukaryota - ps6
Amorpheae - ps7
Obazoa - ps8
Opisthokonta - ps9
Holozoa - ps10
Filozoa - ps11
Choano./Metazoa - ps12
Metazoa - ps13
Myriazoa - ps14
Parahoxozoa - ps15
Bilateria - ps16
Protostomia - ps17
Ecdysozoa - ps18
Arthropoda - ps19
Mandibulata - ps20
Pancrustacea - ps21
Insecta - ps22
Eumetabola - ps23
Holometabola/Psocodea - ps24
Holometabola - ps25
Aparaglossata - ps26
Mecopterida - ps27
Diptera - ps28
Bibiono./Cyclorrhapha - ps29
Cyclorrhapha - ps30
Calyptratae/Drosophila - ps31
Drosophila - ps32
Sophophora - ps33
Obscura/Melanogaster_group - ps34
D_melanogaster_group - ps35
D_melanogaster_subgroup - ps36
sim./sech./mel. group - ps37
Drosophila_melanogaster - ps38
S. cerevisiae GO:0006897 endocytosis (gain)
c-value
p-value
3e-05
2e-04
1e-03
5e-03
3e-02
Log-odds
Cellular_organisms - ps1
Archaea1 - ps2
Archaea2 - ps3
Archaea3 - ps4
Asgard/Eukaryota - ps5
Eukaryota – ps6
Amorpheae - ps7
Obazoa - ps8
Opisthokonta - ps9
Holomycota - ps10
Fungi - ps11
Fungi_1 - ps12
Fungi_2 - ps13
Fungi_3 - ps14
Dikarya - ps15
Ascomycota - ps16
Saccharo/Pezizomycotina - ps17
Saccharomycotina - ps18
Saccharomycotina_1 - ps19
Saccharomycotina_2 - ps20
Saccharomycotina_3 - ps21
Phaff./Saccharomycetaceae - ps22
Saccharomyco./mycetaceae - ps23
Saccharomycetaceae - ps24
Saccharomycetaceae_2 - ps25
Saccharomycetaceae_3 - ps26
Kaz./Nau./Saccharomyces - ps27
Saccharomyces - ps28
Arboricola_clade - ps29
Cerevisiae_clade - ps30
Saccharomyces_cerevisiae - ps31
A. thaliana GO:0006897 endocytosis (gain)
c-value
p-value
2e-21
1e-17
8e-14
5e-10
3e-06
Log-odds
Cellular_organisms - ps1
Archaea1 - ps2
Archaea2 - ps3
Archaea3 - ps4
Asgard/Eukaryota - ps5
Eukaryota – ps6
Excavata/Diaphoretickes - ps7
Diaphoretickes - ps8
Archaeplastida - ps9
Viridiplantae - ps10
Streptophyta - ps11
Charophyceae/Embriophyta - ps12
Embriophyta - ps13
Tracheophyta - ps14
Spermatophyta - ps15
Angiospermae - ps16
Mesangiospermae - ps17
Magnoliids/eudicots - ps18
Eudicots - ps19
Core_eudicots - ps20
Superrosids - ps21
Rosids - ps22
Eurosids - ps23
Malvids - ps24
Sap./Malv./Brassicales - ps25
Malvales/Brassicales - ps26
Brassicales - ps27
Cleomaceae/Brassicaceae - ps28
Brassicaceae - ps29
Camelineae - ps30
Arabidopsis - ps31
Arabidopsis_thaliana - ps32

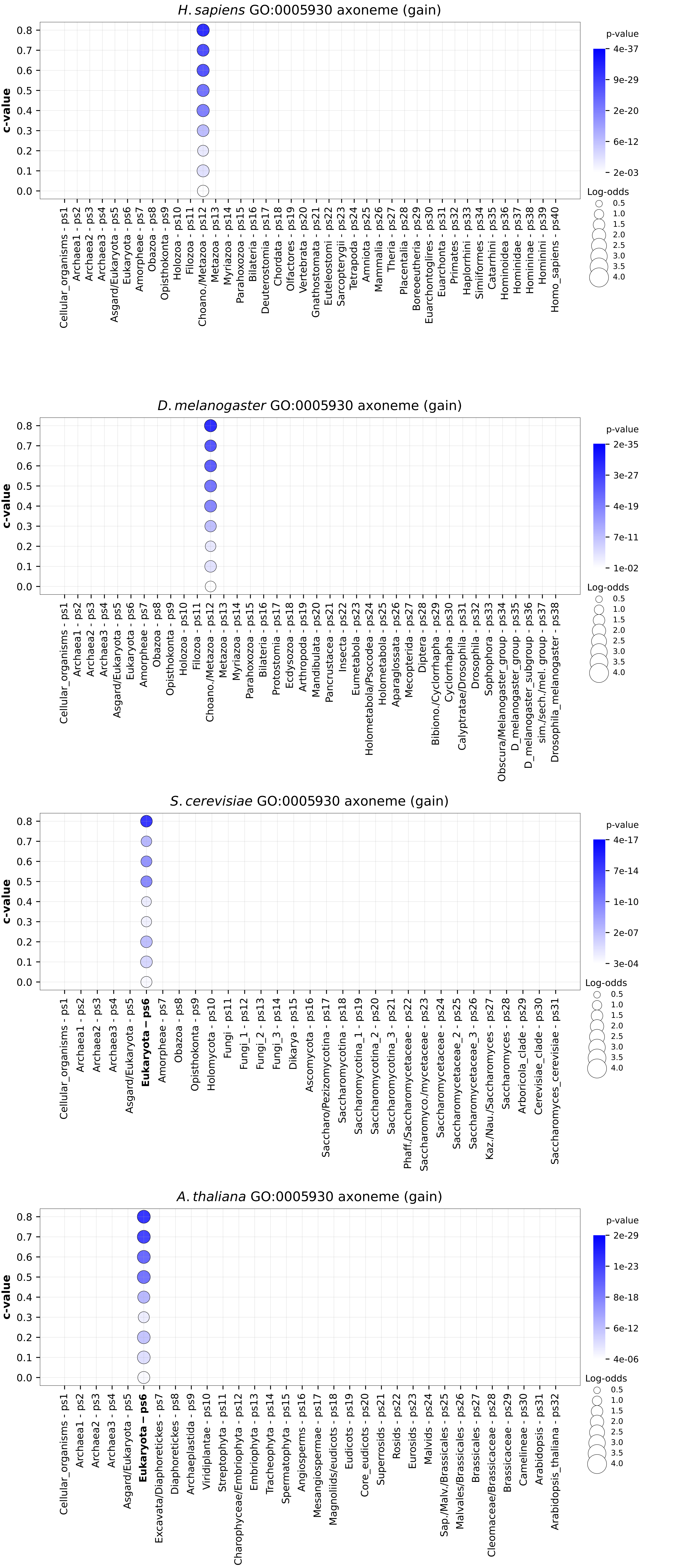

H. sapiens GO:0005930 axoneme (gain)
c-value
0.0
0.1
0.2
0.3
0.4
0.5
0.6
0.7
0.8
p-value
4e-37
9e-29
2e-20
6e-12
2e-03
Log-odds
0.5
1.0
1.5
2.0
2.5
3.0
3.5
4.0
Cellular_organisms - ps1
Archaea1 - ps2
Archaea2 - ps3
Archaea3 - ps4
Asgard/Eukaryota - ps5
Eukaryota - ps6
Amorpheae - ps7
Obazoa - ps8
Opisthokonta - ps9
Holozoa - ps10
Filozoa - ps11
Choano./Metazoa - ps12
Metazoa - ps13
Eumetazoa - ps14
Parahoxozoa - ps15
Bilateria - ps16
Deuterostomia - ps17
Chordata - ps18
Olfactores - ps19
Vertebrata - ps20
Gnathostomata - ps21
Euteleostomi - ps22
Sarcopterygii - ps23
Tetrapoda - ps24
Amniota - ps25
Mammalia - ps26
Theria - ps27
Placentalia - ps28
Boreoeutheria - ps29
Euarchontoglires - ps30
Euarchonta - ps31
Primates - ps32
Haplorrhini - ps33
Simiiformes - ps34
Catarrhini - ps35
Hominoidea - ps36
Hominidae - ps37
Homininae - ps38
Hominini - ps39
Homo_sapiens - ps40
D. melanogaster GO:0005930 axoneme (gain)
c-value
p-value
2e-35
3e-27
4e-19
7e-11
1e-02
Log-odds
Cellular_organisms - ps1
Archaea1 - ps2
Archaea2 - ps3
Archaea3 - ps4
Asgard/Eukaryota - ps5
Eukaryota - ps6
Amorpheae - ps7
Obazoa - ps8
Opisthokonta - ps9
Holozoa - ps10
Filozoa - ps11
Choano./Metazoa - ps12
Metazoa - ps13
Myriazoa - ps14
Parahoxozoa - ps15
Bilateria - ps16
Protostomia - ps17
Ecdysozoa - ps18
Arthropoda - ps19
Mandibulata - ps20
Pancrustacea - ps21
Insecta - ps22
Eumetabola - ps23
Holometabola/Psocodea - ps24
Holometabola - ps25
Aparaglossata - ps26
Mecopterida - ps27
Diptera - ps28
Bibiono./Cyclorrhapha - ps29
Cyclorrhapha - ps30
Calyptratae/Drosophila - ps31
Drosophila - ps32
Sophophora - ps33
Obscura/Melanogaster_group - ps34
D_melanogaster_group - ps35
D_melanogaster_subgroup - ps36
sim./sech./mel. group - ps37
Drosophila_melanogaster - ps38
S. cerevisiae GO:0005930 axoneme (gain)
c-value
p-value
4e-17
7e-14
1e-10
2e-07
3e-04
Log-odds
Cellular_organisms - ps1
Archaea1 - ps2
Archaea2 - ps3
Archaea3 - ps4
Asgard/Eukaryota - ps5
Eukaryota – ps6
Amorpheae - ps7
Obazoa - ps8
Opisthokonta - ps9
Holomycota - ps10
Fungi - ps11
Fungi_1 - ps12
Fungi_2 - ps13
Fungi_3 - ps14
Dikarya - ps15
Ascomycota - ps16
Saccharo/Pezizomycotina - ps17
Saccharomycotina - ps18
Saccharomycotina_1 - ps19
Saccharomycotina_2 - ps20
Saccharomycotina_3 - ps21
Phaff./Saccharomycetaceae - ps22
Saccharomyco./mycetaceae - ps23
Saccharomycetaceae - ps24
Saccharomycetaceae_2 - ps25
Saccharomycetaceae_3 - ps26
Kaz./Nau./Saccharomyces - ps27
Saccharomyces - ps28
Arboricola_clade - ps29
Cerevisiae_clade - ps30
Saccharomyces_cerevisiae - ps31
A. thaliana GO:0005930 axoneme (gain)
c-value
p-value
2e-29
1e-23
8e-18
6e-12
4e-06
Log-odds
Cellular_organisms - ps1
Archaea1 - ps2
Archaea2 - ps3
Archaea3 - ps4
Asgard/Eukaryota - ps5
Eukaryota – ps6
Excavata/Diaphoretickes - ps7
Diaphoretickes - ps8
Archaeplastida - ps9
Viridiplantae - ps10
Streptophyta - ps11
Charophyceae/Embriophyta - ps12
Embriophyta - ps13
Tracheophyta - ps14
Spermatophyta - ps15
Angiosperms - ps16
Mesangiospermae - ps17
Magnoliids/eudicots - ps18
Eudicots - ps19
Core_eudicots - ps20
Superrosids - ps21
Rosids - ps22
Eurosids - ps23
Malvids - ps24
Sap./Malv./Brassicales - ps25
Malvales/Brassicales - ps26
Brassicales - ps27
Cleomaceae/Brassicaceae - ps28
Brassicaceae - ps29
Camelineae - ps30
Arabidopsis - ps31
Arabidopsis_thaliana - ps32

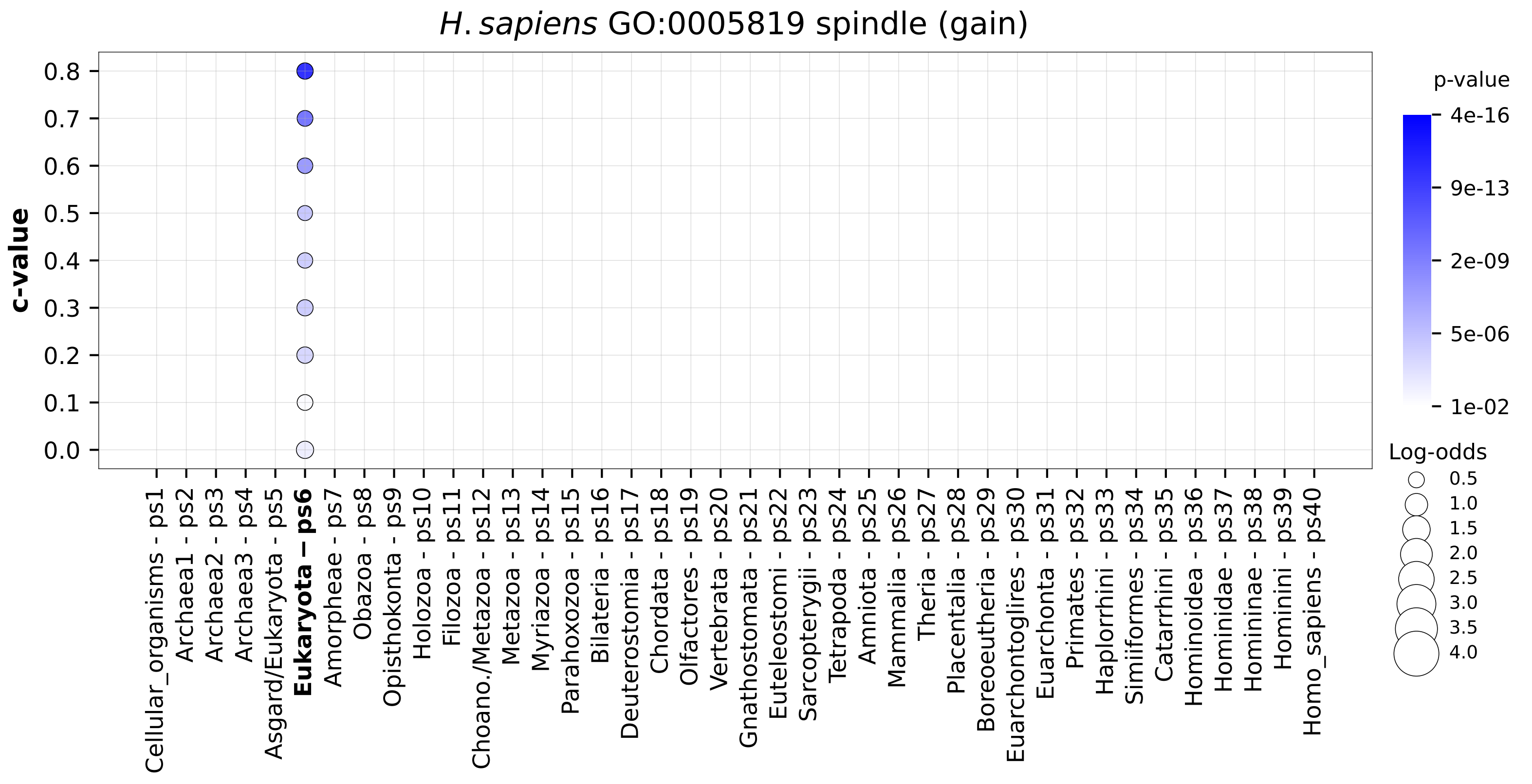

H. sapiens GO:0005819 spindle (gain)
c-value
0.0 0.1 0.2 0.3 0.4 0.5 0.6 0.7 0.8
p-value
4e-16
9e-13
2e-09
5e-06
1e-02
Log-odds
0.5 1.0 1.5 2.0 2.5 3.0 3.5 4.0
Cellular_organisms - ps1
Archaea1 - ps2
Archaea2 - ps3
Archaea3 - ps4
Asgard/Eukaryota - ps5
Eukaryota – ps6
Amorpheae - ps7
Obazoa - ps8
Opisthokonta - ps9
Holozoa - ps10
Filozoa - ps11
Choano./Metazoa - ps12
Metazoa - ps13
Myriazoa - ps14
Parahoxozoa - ps15
Bilateria - ps16
Deuterostomia - ps17
Chordata - ps18
Olfactores - ps19
Vertebrata - ps20
Gnathostomata - ps21
Euteleostomi - ps22
Sarcopterygii - ps23
Tetrapoda - ps24
Amniota - ps25
Mammalia - ps26
Theria - ps27
Placentalia - ps28
Boreoeutheria - ps29
Euarchontoglires - ps30
Euarchonta - ps31
Primates - ps32
Haplorrhini - ps33
Simiiformes - ps34
Catarrhini - ps35
Hominoidea - ps36
Hominidae - ps37
Homininae - ps38
Hominini - ps39
Homo_sapiens - ps40

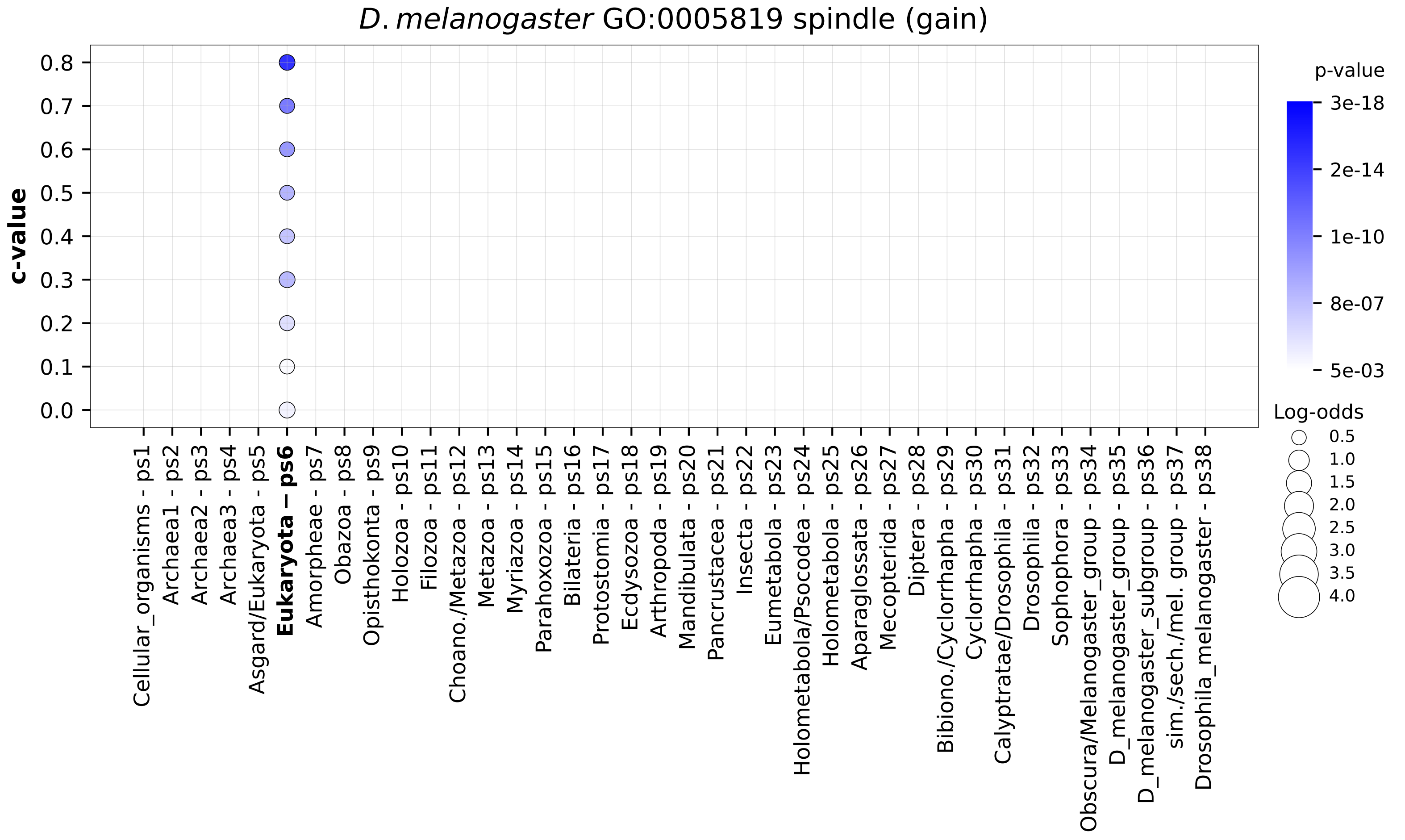

D. melanogaster GO:0005819 spindle (gain)
c-value
0.0 0.1 0.2 0.3 0.4 0.5 0.6 0.7 0.8
p-value
3e-18
2e-14
1e-10
8e-07
5e-03
Log-odds
0.5 1.0 1.5 2.0 2.5 3.0 3.5 4.0
Cellular_organisms - ps1
Archaea1 - ps2
Archaea2 - ps3
Archaea3 - ps4
Asgard/Eukaryota - ps5
Eukaryota – ps6
Amorpheae - ps7
Obazoa - ps8
Opisthokonta - ps9
Holozoa - ps10
Filozoa - ps11
Choano./Metazoa - ps12
Metazoa - ps13
Myriazoa - ps14
Parahoxozoa - ps15
Bilateria - ps16
Protostomia - ps17
Ecdysozoa - ps18
Arthropoda - ps19
Mandibulata - ps20
Pancrustacea - ps21
Insecta - ps22
Eumetabola - ps23
Holometabola/Psocodea - ps24
Holometabola - ps25
Aparaglossata - ps26
Mecopterida - ps27
Diptera - ps28
Bibiono./Cyclorrhapha - ps29
Cyclorrhapha - ps30
Calyptratae/Drosophila - ps31
Drosophila - ps32
Sophophora - ps33
Obscura/Melanogaster_group - ps34
D_melanogaster_group - ps35
D_melanogaster_subgroup - ps36
sim./sech./mel. group - ps37
Drosophila_melanogaster - ps38

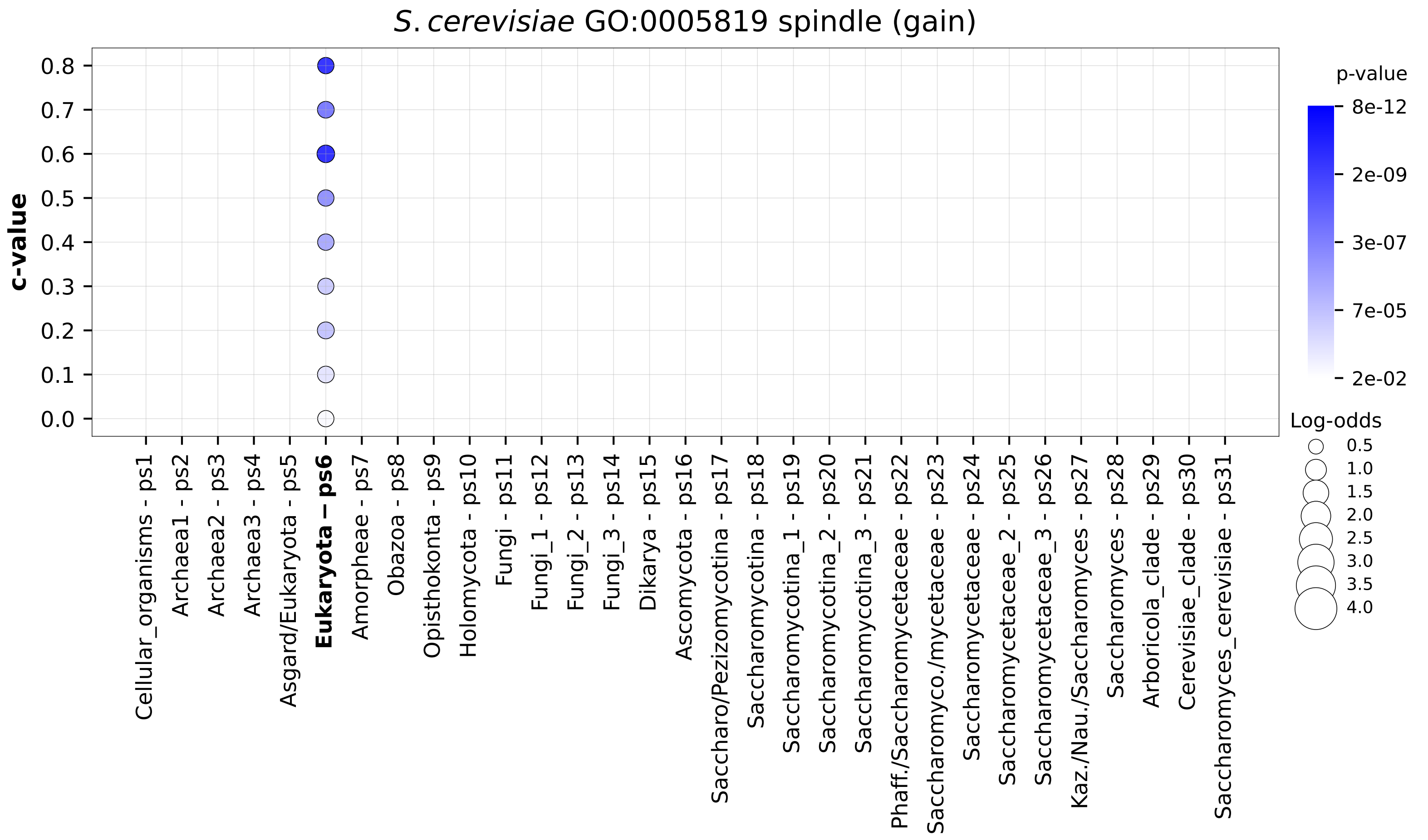

S. cerevisiae GO:0005819 spindle (gain)
c-value
0.0 0.1 0.2 0.3 0.4 0.5 0.6 0.7 0.8
p-value
8e-12
2e-09
3e-07
7e-05
2e-02
Log-odds
0.5 1.0 1.5 2.0 2.5 3.0 3.5 4.0
Cellular_organisms - ps1
Archaea1 - ps2
Archaea2 - ps3
Archaea3 - ps4
Asgard/Eukaryota - ps5
Eukaryota – ps6
Amorpheae - ps7
Obazoa - ps8
Opisthokonta - ps9
Holomycota - ps10
Fungi - ps11
Fungi_1 - ps12
Fungi_2 - ps13
Fungi_3 - ps14
Dikarya - ps15
Ascomycota - ps16
Saccharo/Pezizomycotina - ps17
Saccharomycotina - ps18
Saccharomycotina_1 - ps19
Saccharomycotina_2 - ps20
Saccharomycotina_3 - ps21
Phaff./Saccharomycetaceae - ps22
Saccharomyco./mycetaceae - ps23
Saccharomycetaceae - ps24
Saccharomycetaceae_2 - ps25
Saccharomycetaceae_3 - ps26
Kaz./Nau./Saccharomyces - ps27
Saccharomyces - ps28
Arboricola_clade - ps29
Cerevisiae_clade - ps30
Saccharomyces_cerevisiae - ps31

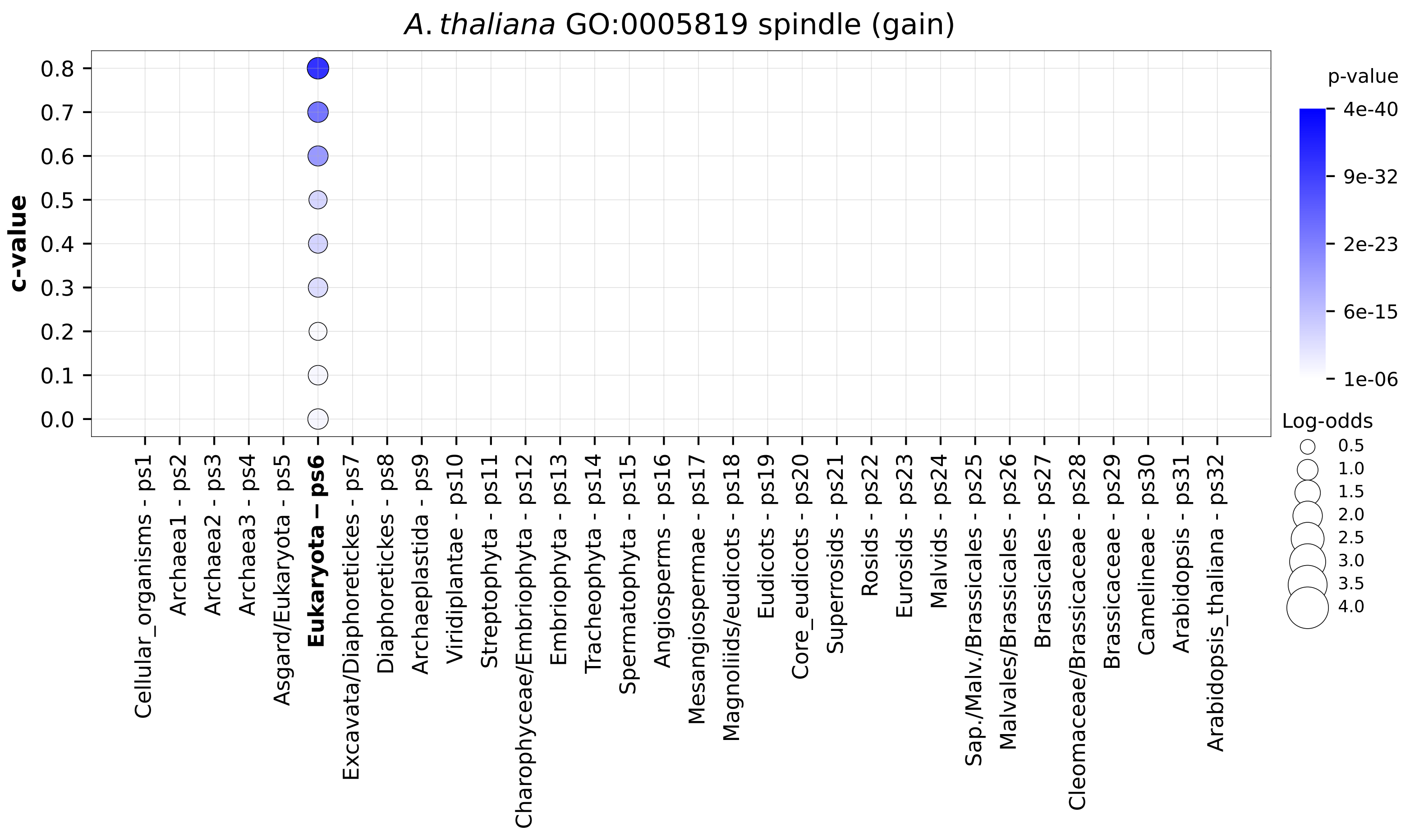

A. thaliana GO:0005819 spindle (gain)
c-value
0.0 0.1 0.2 0.3 0.4 0.5 0.6 0.7 0.8
p-value
4e-40
9e-32
2e-23
6e-15
1e-06
Log-odds
0.5 1.0 1.5 2.0 2.5 3.0 3.5 4.0
Cellular_organisms - ps1
Archaea1 - ps2
Archaea2 - ps3
Archaea3 - ps4
Asgard/Eukaryota - ps5
Eukaryota – ps6
Excavata/Diaphoretickes - ps7
Diaphoretickes - ps8
Archaeplastida - ps9
Viridiplantae - ps10
Streptophyta - ps11
Charophyceae/Embriophyta - ps12
Embriophyta - ps13
Tracheophyta - ps14
Spermatophyta - ps15
Angiosperms - ps16
Mesangiospermae - ps17
Magnoliids/eudicots - ps18
Eudicots - ps19
Core_eudicots - ps20
Superrosids - ps21
Rosids - ps22
Eurosids - ps23
Malvids - ps24
Sap./Malv./Brassicales - ps25
Malvales/Brassicales - ps26
Brassicales - ps27
Cleomaceae/Brassicaceae - ps28
Brassicaceae - ps29
Camelineae - ps30
Arabidopsis - ps31
Arabidopsis_thaliana - ps32

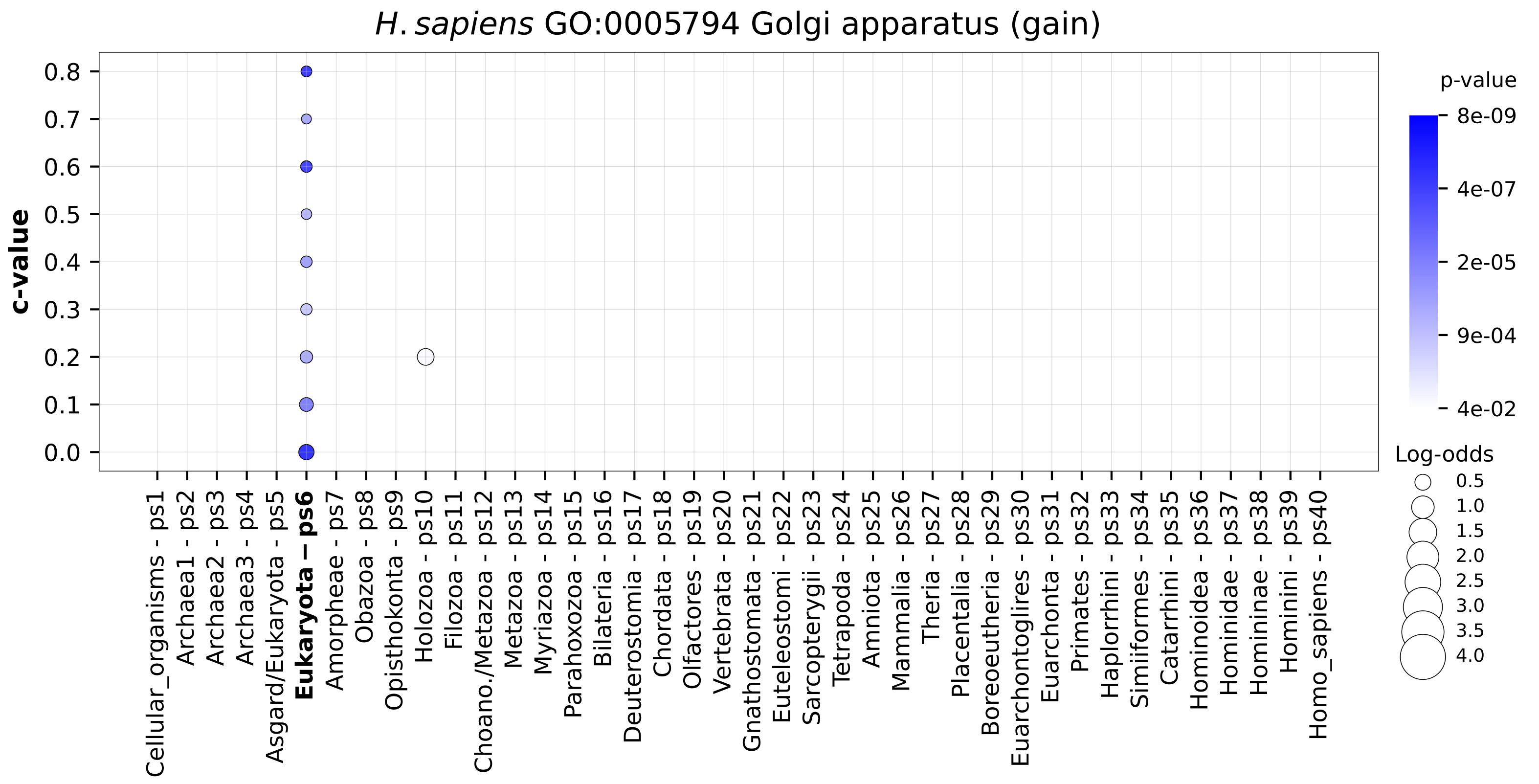
H. sapiens GO:0005794 Golgi apparatus (gain)
c-value
p-value
Log-odds

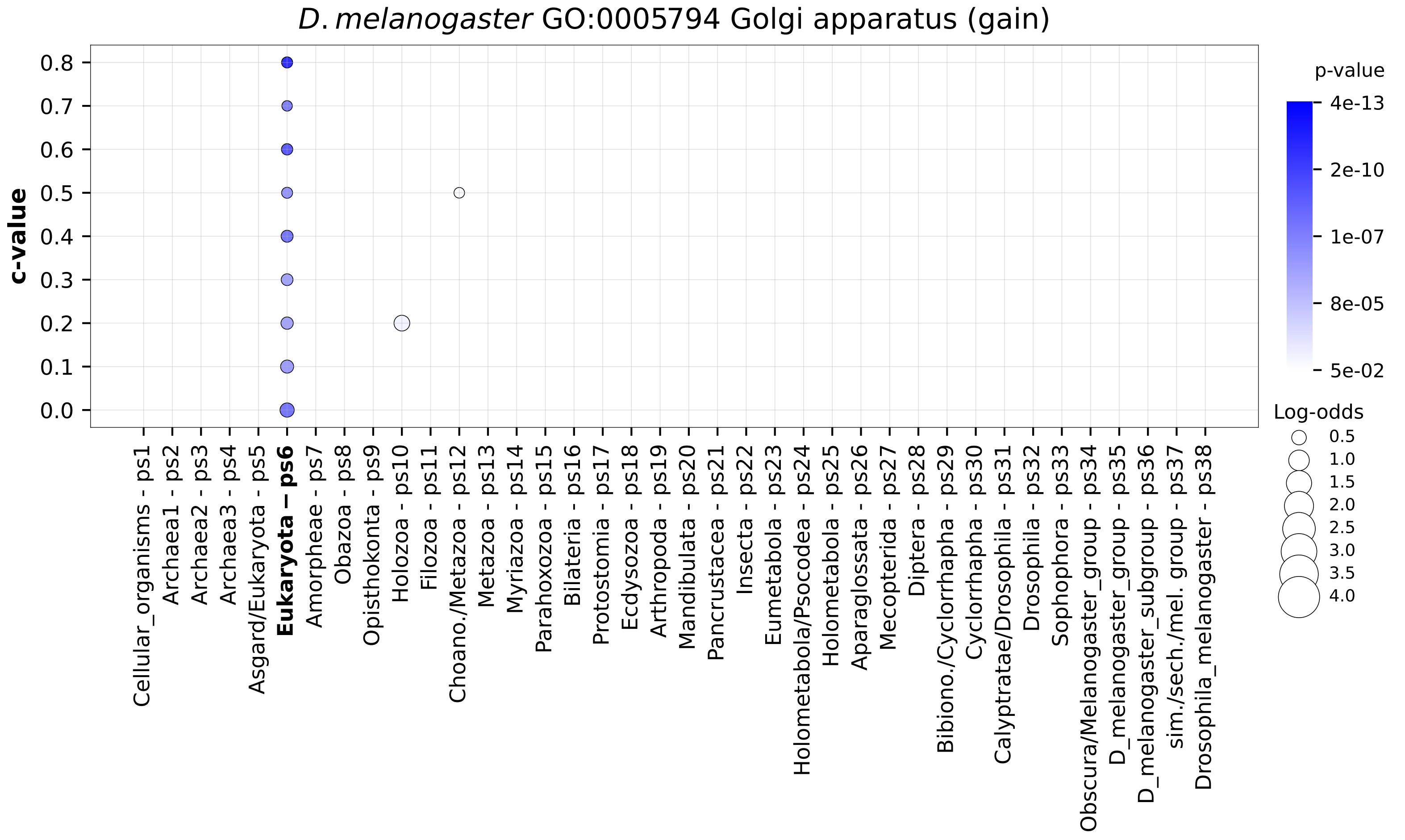
D. melanogaster GO:0005794 Golgi apparatus (gain)
c-value
p-value
Log-odds

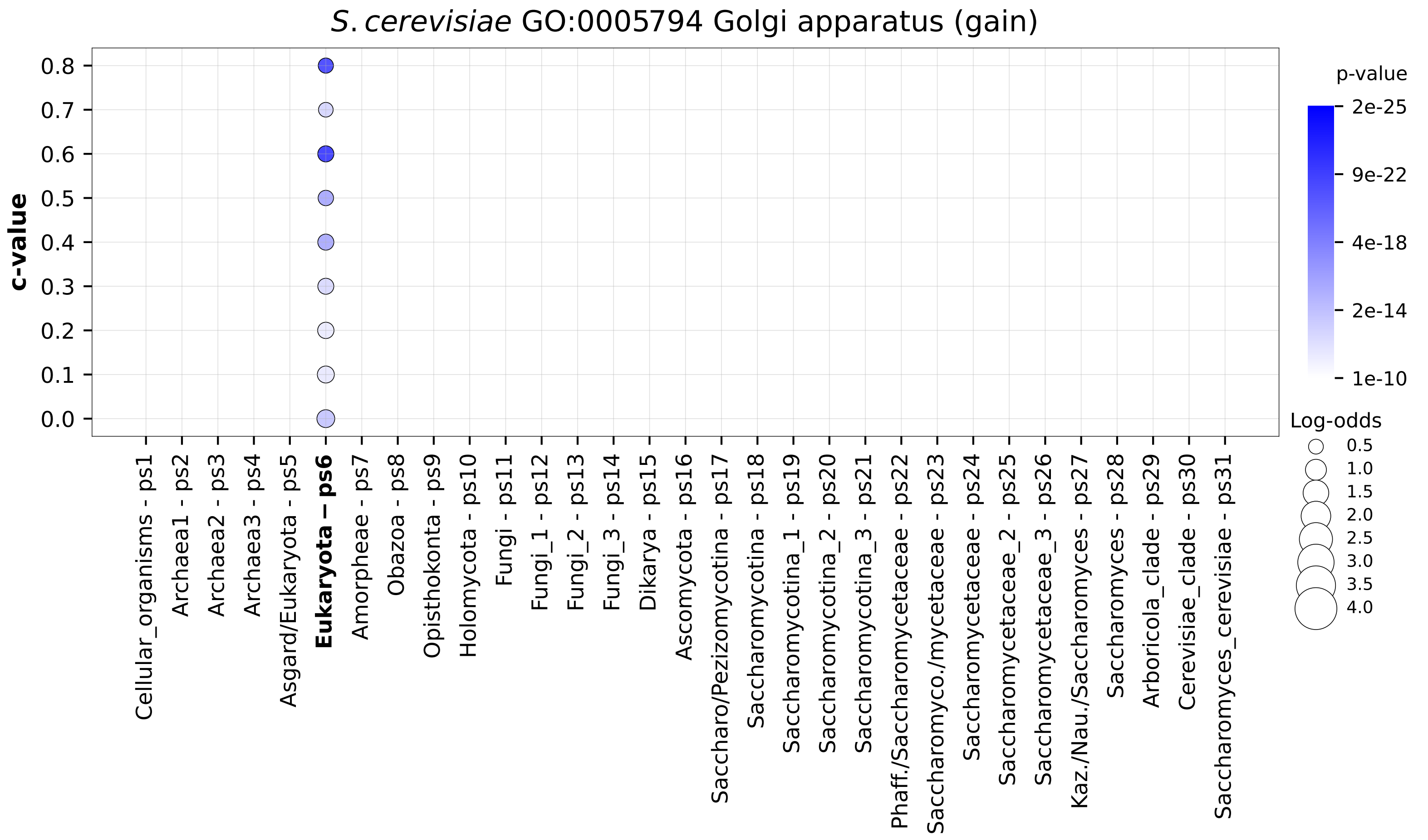
S. cerevisiae GO:0005794 Golgi apparatus (gain)
c-value
p-value
Log-odds

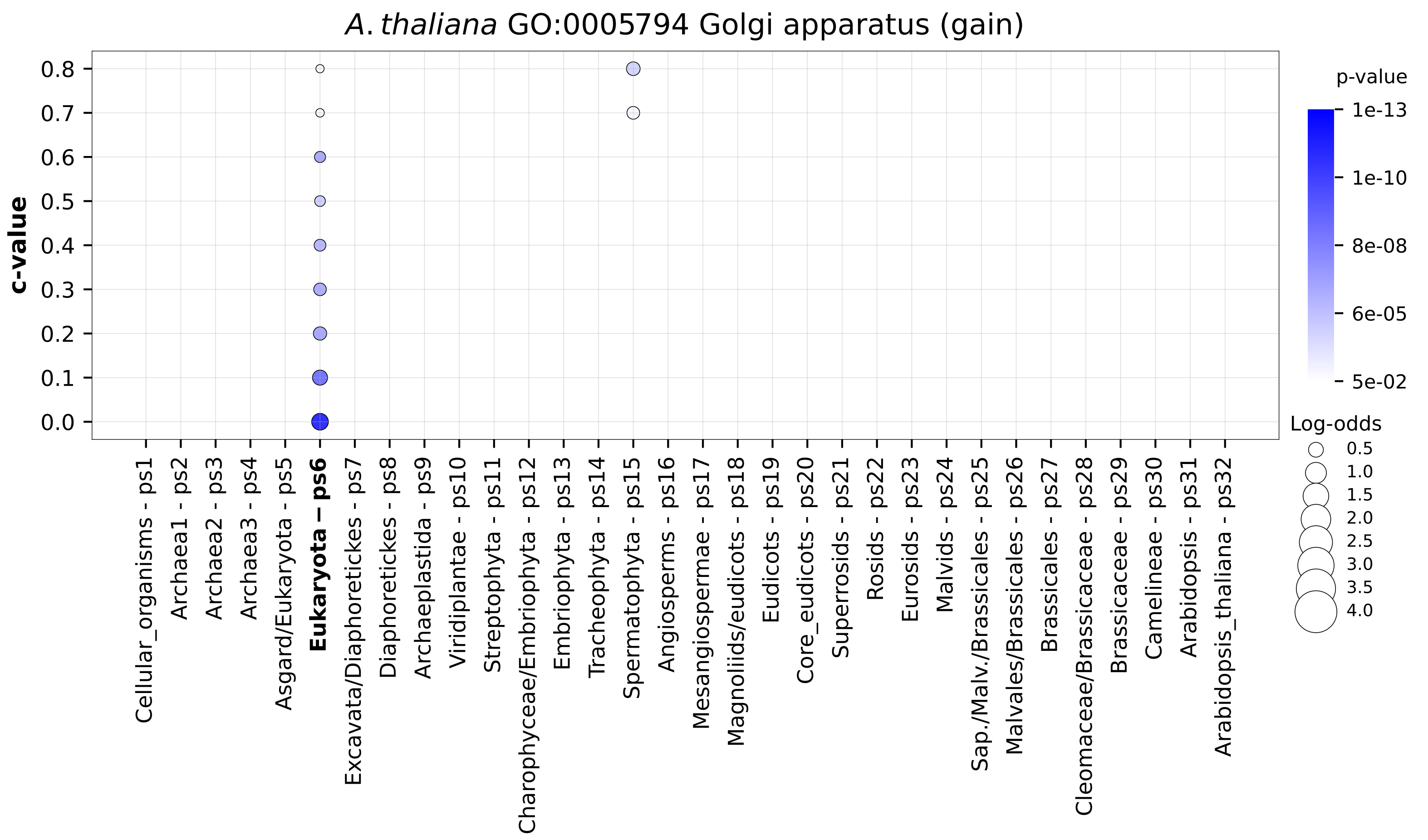
A. thaliana GO:0005794 Golgi apparatus (gain)
c-value
p-value
Log-odds

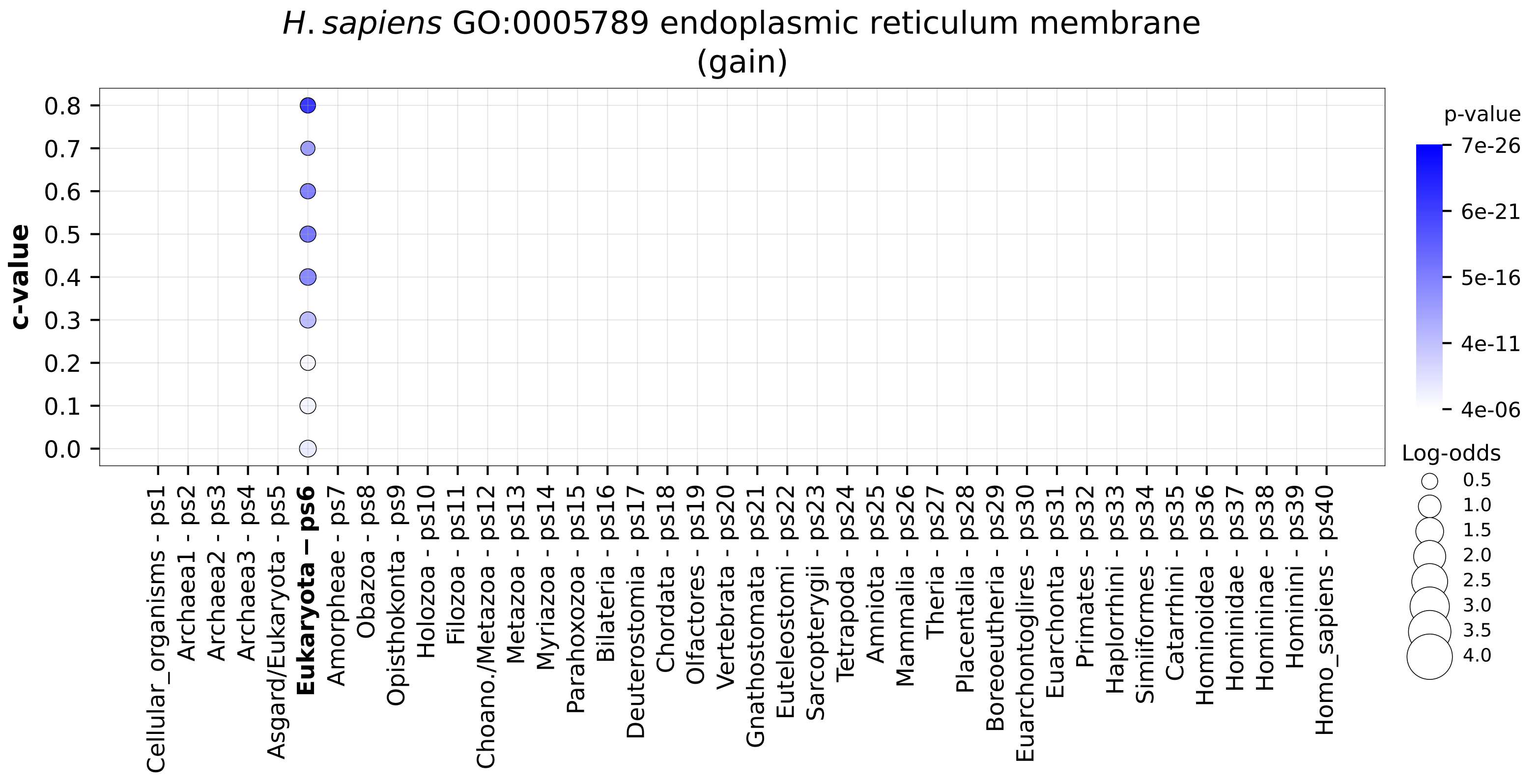
H. sapiens GO:0005789 endoplasmic reticulum membrane (gain)
c-value
p-value
7e-26
6e-21
5e-16
4e-11
4e-06
Log-odds
0.5
1.0
1.5
2.0
2.5
3.0
3.5
4.0
Cellular_organisms - ps1
Archaea1 - ps2
Archaea2 - ps3
Archaea3 - ps4
Asgard/Eukaryota - ps5
Eukaryota – ps6
Amorpheae - ps7
Obazoa - ps8
Opisthokonta - ps9
Holozoa - ps10
Filozoa - ps11
Choano./Metazoa - ps12
Metazoa - ps13
Myriazoa - ps14
Parahoxozoa - ps15
Bilateria - ps16
Deuterostomia - ps17
Chordata - ps18
Olfactores - ps19
Vertebrata - ps20
Gnathostomata - ps21
Euteleostomi - ps22
Sarcopterygii - ps23
Tetrapoda - ps24
Amniota - ps25
Mammalia - ps26
Theria - ps27
Placentalia - ps28
Boreoeutheria - ps29
Euarchontoglires - ps30
Euarchonta - ps31
Primates - ps32
Haplorrhini - ps33
Simiiformes - ps34
Catarrhini - ps35
Hominoidea - ps36
Hominidae - ps37
Homininae - ps38
Hominini - ps39
Homo_sapiens - ps40

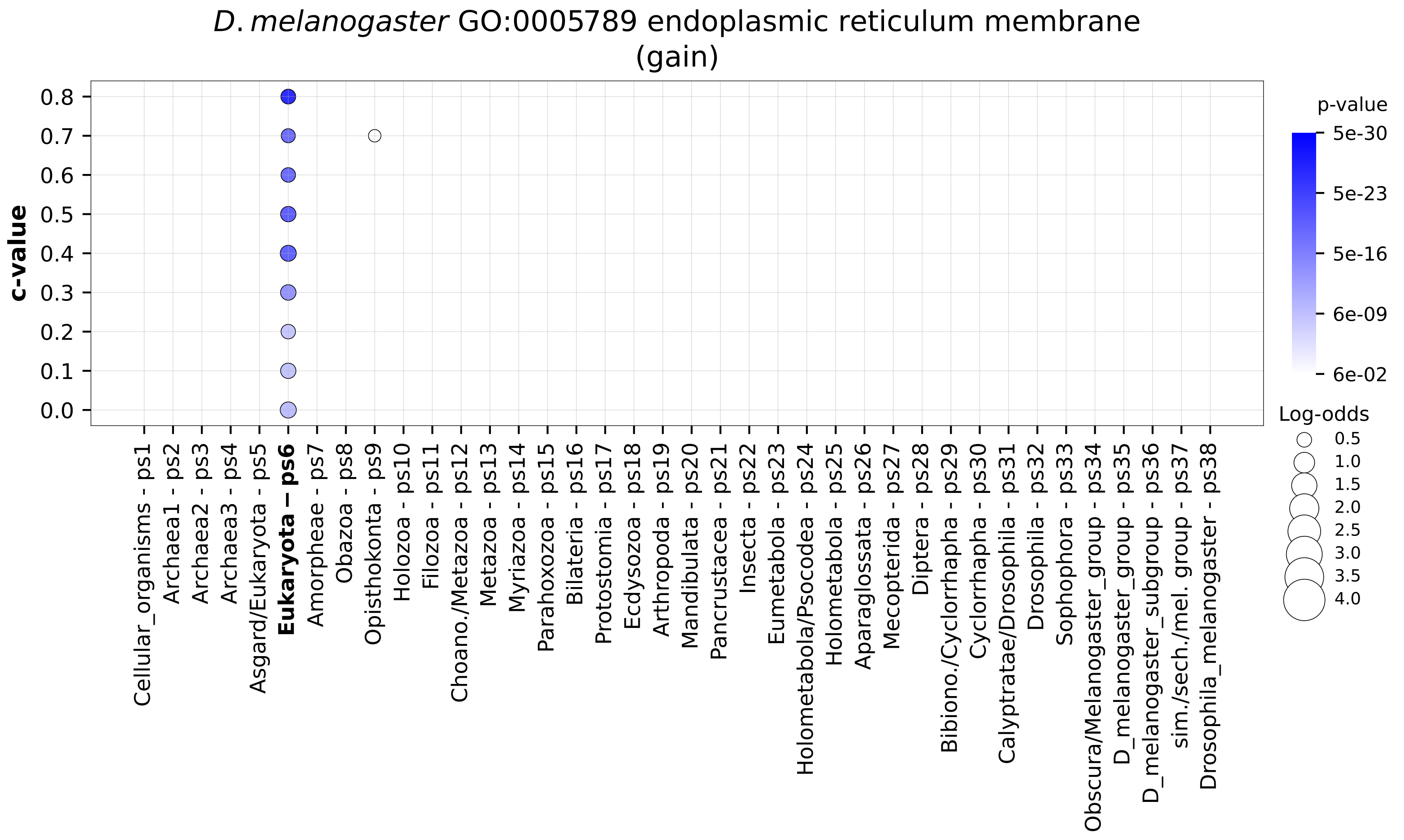
D. melanogaster GO:0005789 endoplasmic reticulum membrane (gain)
c-value
p-value
5e-30
5e-23
5e-16
6e-09
6e-02
Log-odds
0.5
1.0
1.5
2.0
2.5
3.0
3.5
4.0
Cellular_organisms - ps1
Archaea1 - ps2
Archaea2 - ps3
Archaea3 - ps4
Asgard/Eukaryota - ps5
Eukaryota – ps6
Amorpheae - ps7
Obazoa - ps8
Opisthokonta - ps9
Holozoa - ps10
Filozoa - ps11
Choano./Metazoa - ps12
Metazoa - ps13
Myriazoa - ps14
Parahoxozoa - ps15
Bilateria - ps16
Protostomia - ps17
Ecdysozoa - ps18
Arthropoda - ps19
Mandibulata - ps20
Pancrustacea - ps21
Insecta - ps22
Eumetabola - ps23
Holometabola/Psocodea - ps24
Holometabola - ps25
Aparaglossata - ps26
Mecopterida - ps27
Diptera - ps28
Bibiono./Cyclorrhapha - ps29
Cyclorrhapha - ps30
Calyptratae/Drosophila - ps31
Drosophila - ps32
Sophophora - ps33
Obscura/Melanogaster_group - ps34
D_melanogaster_group - ps35
D_melanogaster_subgroup - ps36
sim./sech./mel. group - ps37
Drosophila_melanogaster - ps38

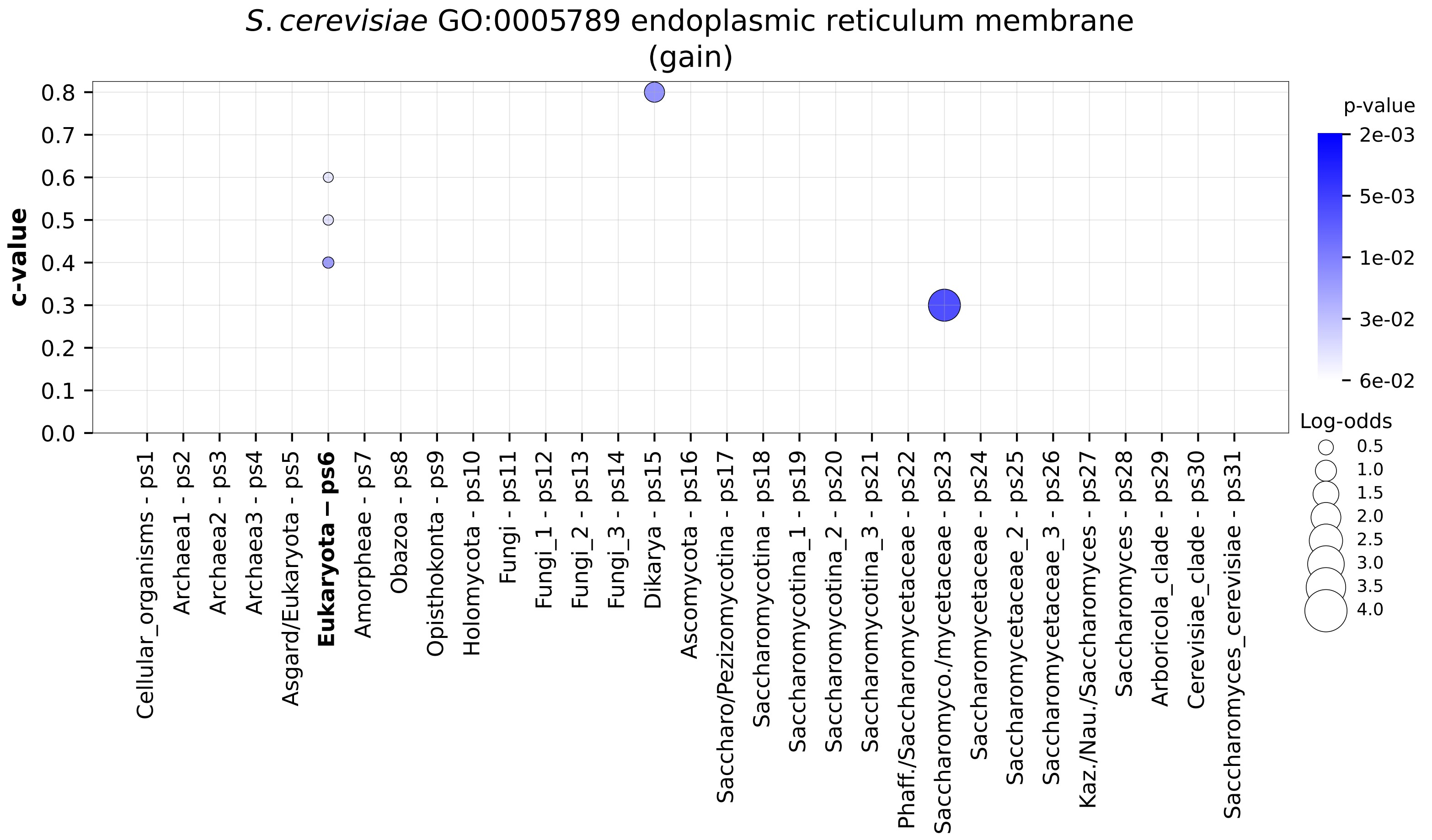
S. cerevisiae GO:0005789 endoplasmic reticulum membrane (gain)
c-value
p-value
2e-03
5e-03
1e-02
3e-02
6e-02
Log-odds
0.5
1.0
1.5
2.0
2.5
3.0
3.5
4.0
Cellular_organisms - ps1
Archaea1 - ps2
Archaea2 - ps3
Archaea3 - ps4
Asgard/Eukaryota - ps5
Eukaryota – ps6
Amorpheae - ps7
Obazoa - ps8
Opisthokonta - ps9
Holomycota - ps10
Fungi - ps11
Fungi_1 - ps12
Fungi_2 - ps13
Fungi_3 - ps14
Dikarya - ps15
Ascomycota - ps16
Saccharo/Pezizomycotina - ps17
Saccharomycotina - ps18
Saccharomycotina_1 - ps19
Saccharomycotina_2 - ps20
Saccharomycotina_3 - ps21
Phaff./Saccharomycetaceae - ps22
Saccharomyco./mycetaceae - ps23
Saccharomycetaceae - ps24
Saccharomycetaceae_2 - ps25
Saccharomycetaceae_3 - ps26
Kaz./Nau./Saccharomyces - ps27
Saccharomyces - ps28
Arboricola_clade - ps29
Cerevisiae_clade - ps30
Saccharomyces_cerevisiae - ps31

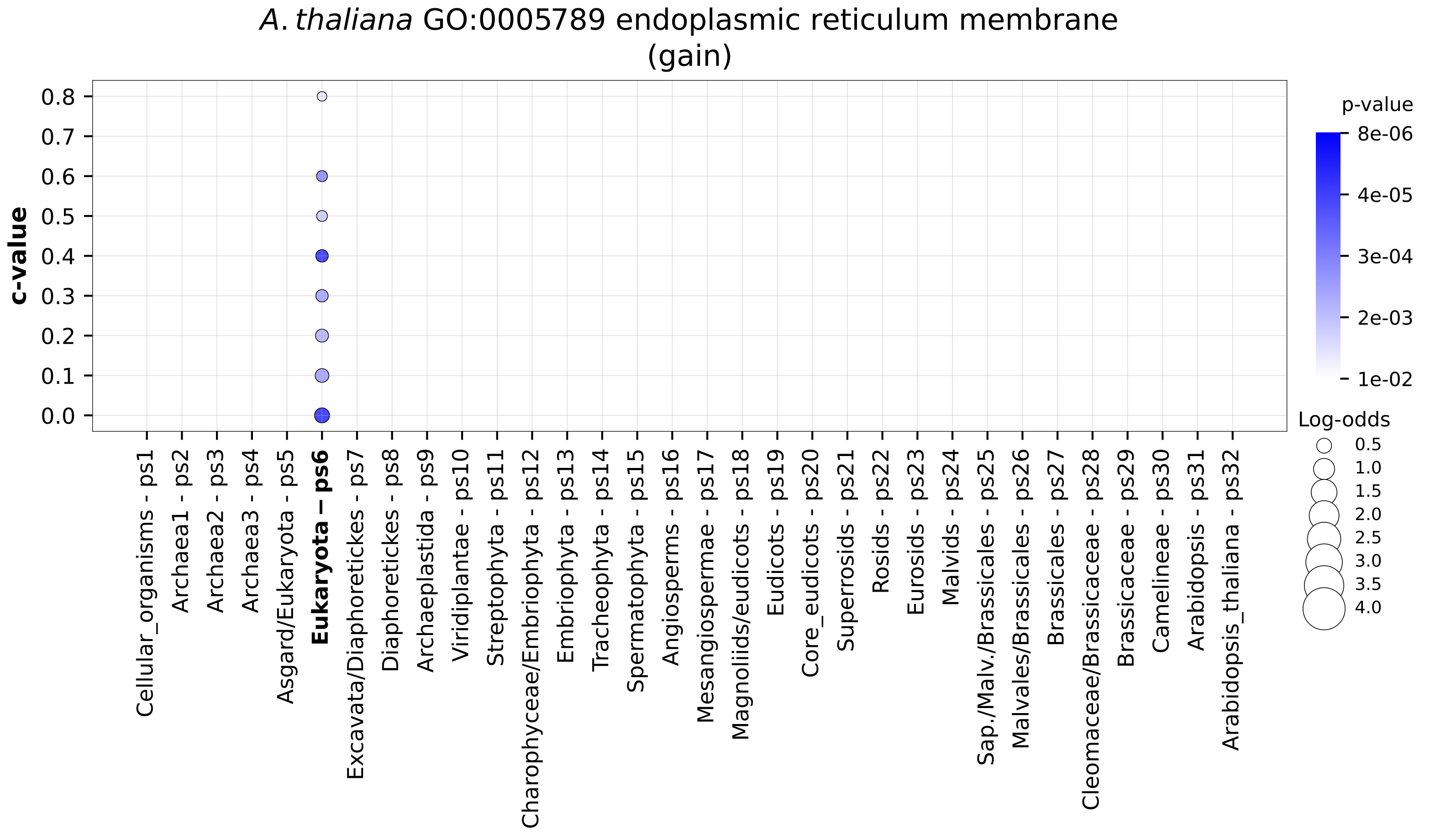
A. thaliana GO:0005789 endoplasmic reticulum membrane (gain)
c-value
p-value
8e-06
4e-05
3e-04
2e-03
1e-02
Log-odds
0.5
1.0
1.5
2.0
2.5
3.0
3.5
4.0
Cellular_organisms - ps1
Archaea1 - ps2
Archaea2 - ps3
Archaea3 - ps4
Asgard/Eukaryota - ps5
Eukaryota – ps6
Excavata/Diaphoretickes - ps7
Diaphoretickes - ps8
Archaeplastida - ps9
Viridiplantae - ps10
Streptophyta - ps11
Charophyceae/Embriophyta - ps12
Embriophyta - ps13
Tracheophyta - ps14
Spermatophyta - ps15
Angiosperms - ps16
Mesangiospermae - ps17
Magnoliids/eudicots - ps18
Eudicots - ps19
Core_eudicots - ps20
Superrosids - ps21
Rosids - ps22
Eurosids - ps23
Malvids - ps24
Sap./Malv./Brassicales - ps25
Malvales/Brassicales - ps26
Brassicales - ps27
Cleomaceae/Brassicaceae - ps28
Brassicaceae - ps29
Camelineae - ps30
Arabidopsis - ps31
Arabidopsis_thaliana - ps32

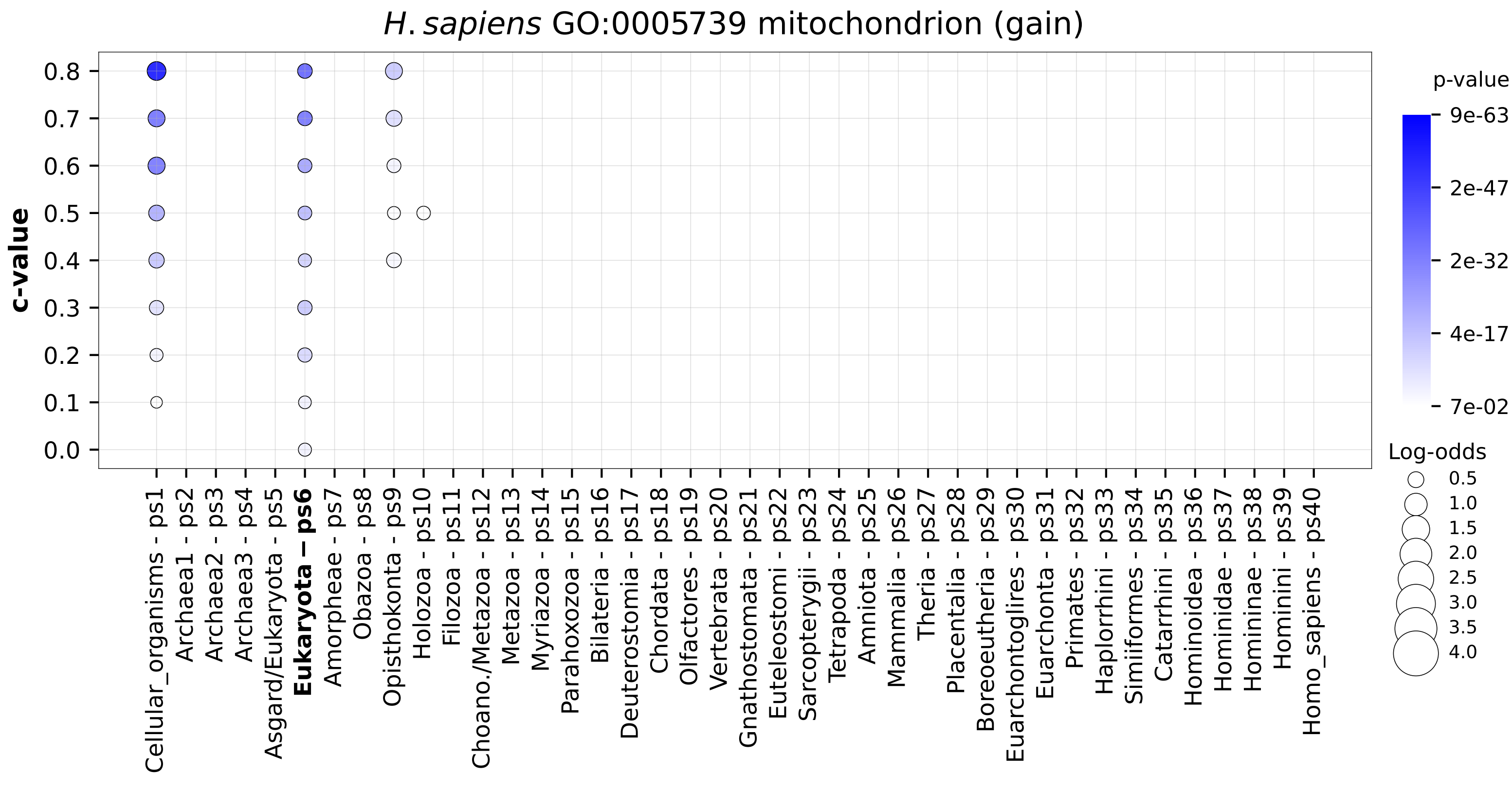
H. sapiens GO:0005739 mitochondrion (gain)
c-value
p-value
9e-63
2e-47
2e-32
4e-17
7e-02
Log-odds
0.5
1.0
1.5
2.0
2.5
3.0
3.5
4.0
Cellular_organisms - ps1
Archaea1 - ps2
Archaea2 - ps3
Archaea3 - ps4
Asgard/Eukaryota - ps5
Eukaryota – ps6
Amorpheae - ps7
Obazoa - ps8
Opisthokonta - ps9
Holozoa - ps10
Filozoa - ps11
Choano./Metazoa - ps12
Metazoa - ps13
Myriazoa - ps14
Parahoxozoa - ps15
Bilateria - ps16
Deuterostomia - ps17
Chordata - ps18
Olfactores - ps19
Vertebrata - ps20
Gnathostomata - ps21
Euteleostomi - ps22
Sarcopterygii - ps23
Tetrapoda - ps24
Amniota - ps25
Mammalia - ps26
Theria - ps27
Placentalia - ps28
Boreoeutheria - ps29
Euarchontoglires - ps30
Euarchonta - ps31
Primates - ps32
Haplorrhini - ps33
Simiiformes - ps34
Catarrhini - ps35
Hominoidea - ps36
Hominidae - ps37
Homininae - ps38
Hominini - ps39
Homo_sapiens - ps40

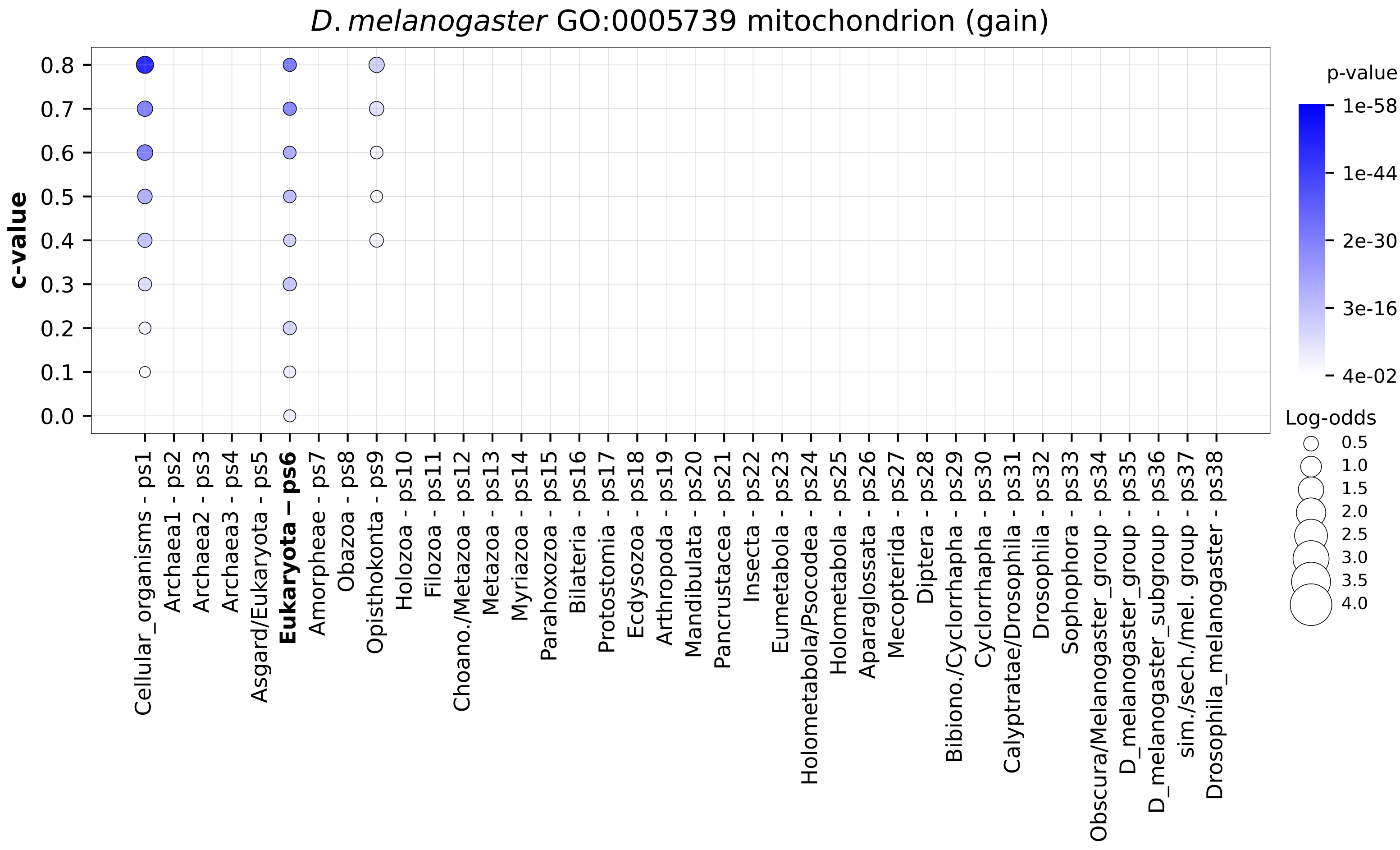
D. melanogaster GO:0005739 mitochondrion (gain)
c-value
p-value
1e-58
1e-44
2e-30
3e-16
4e-02
Log-odds
0.5
1.0
1.5
2.0
2.5
3.0
3.5
4.0
Cellular_organisms - ps1
Archaea1 - ps2
Archaea2 - ps3
Archaea3 - ps4
Asgard/Eukaryota - ps5
Eukaryota – ps6
Amorpheae - ps7
Obazoa - ps8
Opisthokonta - ps9
Holozoa - ps10
Filozoa - ps11
Choano./Metazoa - ps12
Metazoa - ps13
Myriazoa - ps14
Parahoxozoa - ps15
Bilateria - ps16
Protostomia - ps17
Ecdysozoa - ps18
Arthropoda - ps19
Mandibulata - ps20
Pancrustacea - ps21
Insecta - ps22
Eumetabola - ps23
Holometabola/Psocodea - ps24
Holometabola - ps25
Aparaglossata - ps26
Mecopterida - ps27
Diptera - ps28
Bibiono./Cyclorrhapha - ps29
Cyclorrhapha - ps30
Calyptratae/Drosophila - ps31
Drosophila - ps32
Sophophora - ps33
Obscura/Melanogaster_group - ps34
D_melanogaster_group - ps35
D_melanogaster_subgroup - ps36
sim./sech./mel. group - ps37
Drosophila_melanogaster - ps38

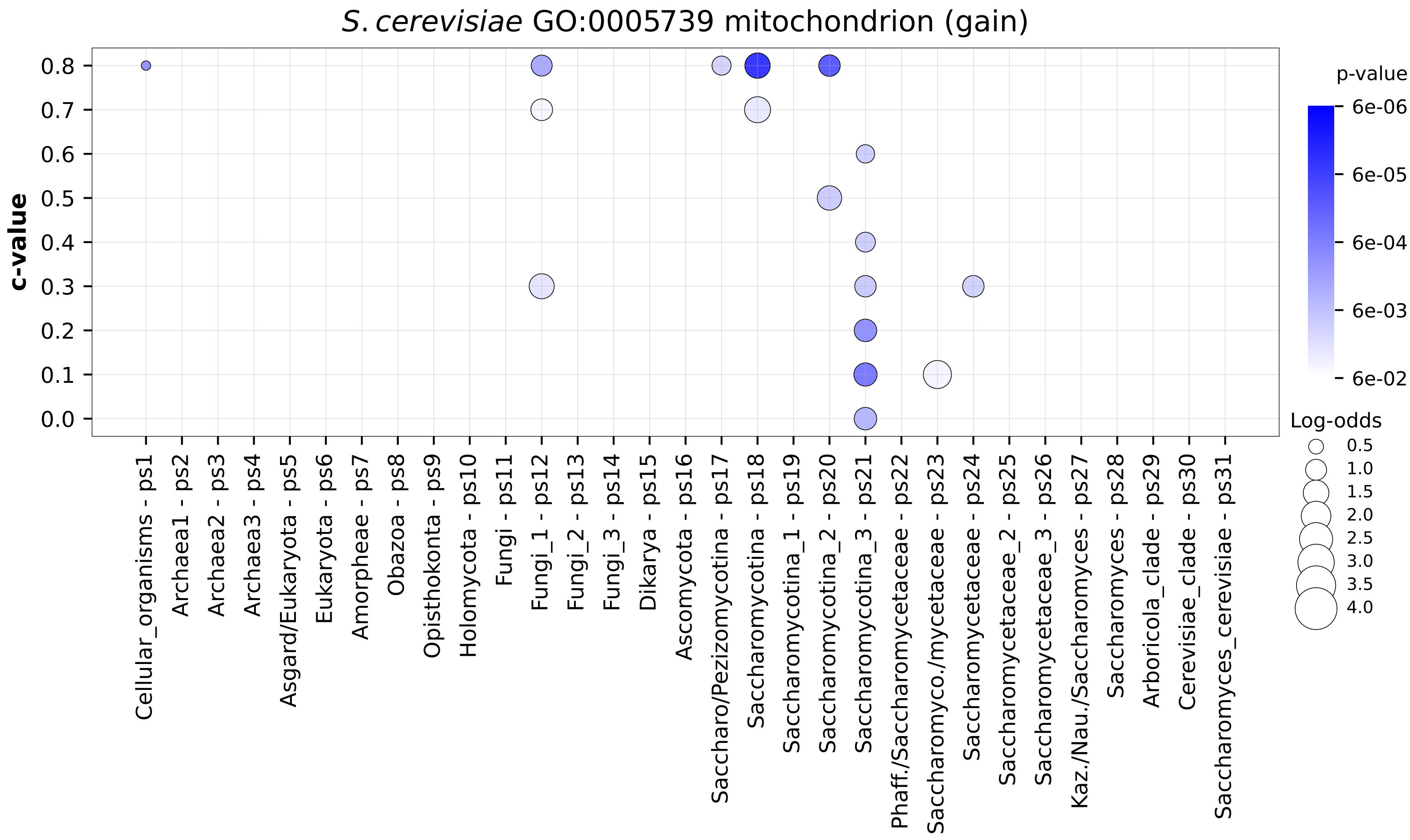
S. cerevisiae GO:0005739 mitochondrion (gain)
c-value
p-value
6e-06
6e-05
6e-04
6e-03
6e-02
Log-odds
0.5
1.0
1.5
2.0
2.5
3.0
3.5
4.0
Cellular_organisms - ps1
Archaea1 - ps2
Archaea2 - ps3
Archaea3 - ps4
Asgard/Eukaryota - ps5
Eukaryota - ps6
Amorpheae - ps7
Obazoa - ps8
Opisthokonta - ps9
Holomycota - ps10
Fungi - ps11
Fungi_1 - ps12
Fungi_2 - ps13
Fungi_3 - ps14
Dikarya - ps15
Ascomycota - ps16
Saccharo/Pezizomycotina - ps17
Saccharomycotina - ps18
Saccharomycotina_1 - ps19
Saccharomycotina_2 - ps20
Saccharomycotina_3 - ps21
Phaff./Saccharomycetaceae - ps22
Saccharomyco./mycetaceae - ps23
Saccharomycetaceae - ps24
Saccharomycetaceae_2 - ps25
Saccharomycetaceae_3 - ps26
Kaz./Nau./Saccharomyces - ps27
Saccharomyces - ps28
Arboricola_clade - ps29
Cerevisiae_clade - ps30
Saccharomyces_cerevisiae - ps31

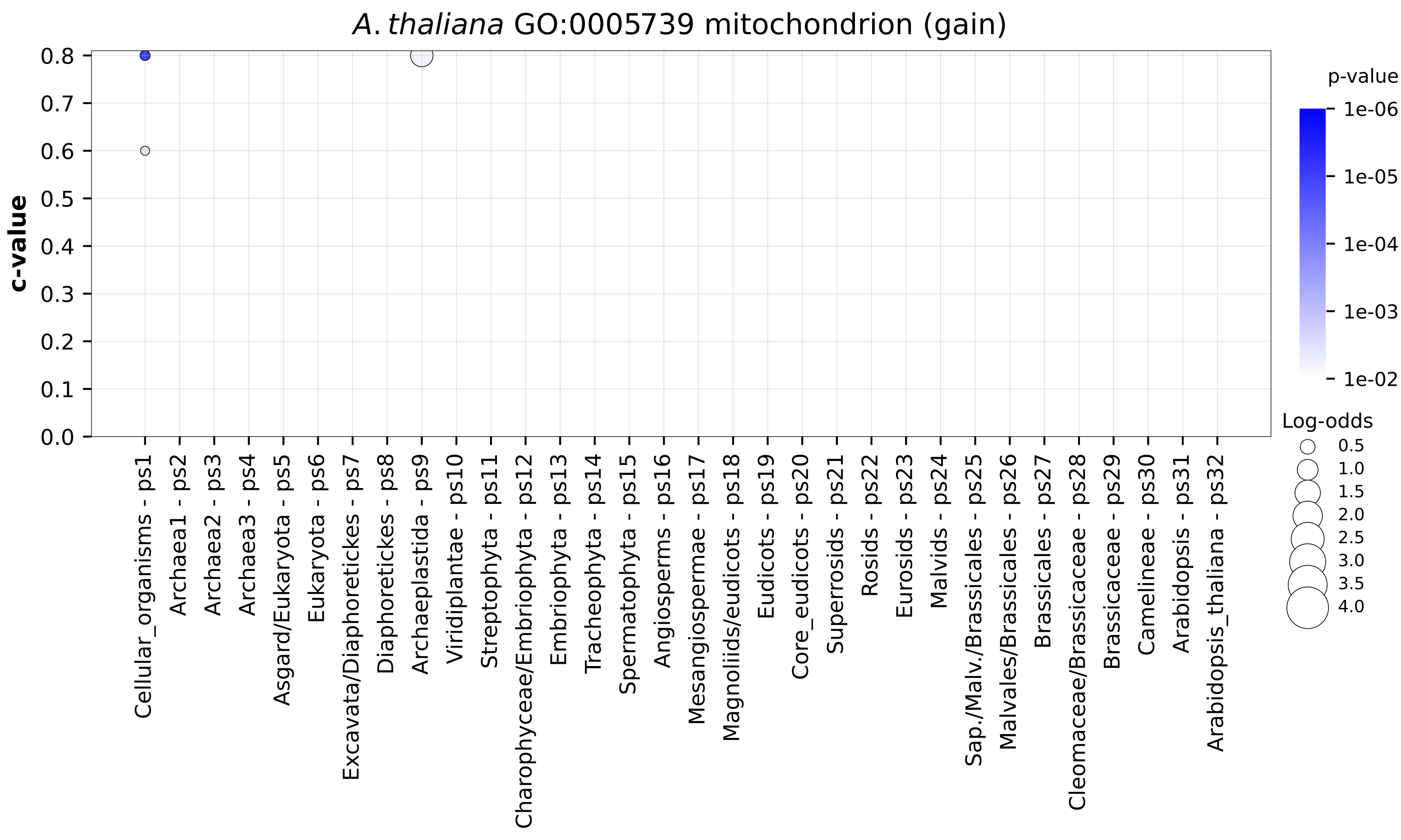
A. thaliana GO:0005739 mitochondrion (gain)
c-value
p-value
1e-06
1e-05
1e-04
1e-03
1e-02
Log-odds
0.5
1.0
1.5
2.0
2.5
3.0
3.5
4.0
Cellular_organisms - ps1
Archaea1 - ps2
Archaea2 - ps3
Archaea3 - ps4
Asgard/Eukaryota - ps5
Eukaryota - ps6
Excavata/Diaphoretickes - ps7
Diaphoretickes - ps8
Archaeplastida - ps9
Viridiplantae - ps10
Streptophyta - ps11
Charophyceae/Embriophyta - ps12
Embriophyta - ps13
Tracheophyta - ps14
Spermatophyta - ps15
Angiospermae - ps16
Mesangiospermae - ps17
Magnoliids/eudicots - ps18
Eudicots - ps19
Core_eudicots - ps20
Superrosids - ps21
Rosids - ps22
Eurosids - ps23
Malvids - ps24
Sap./Malv./Brassicales - ps25
Malvales/Brassicales - ps26
Brassicales - ps27
Cleomaceae/Brassicaceae - ps28
Brassicaceae - ps29
Camelineae - ps30
Arabidopsis - ps31
Arabidopsis_thaliana - ps32

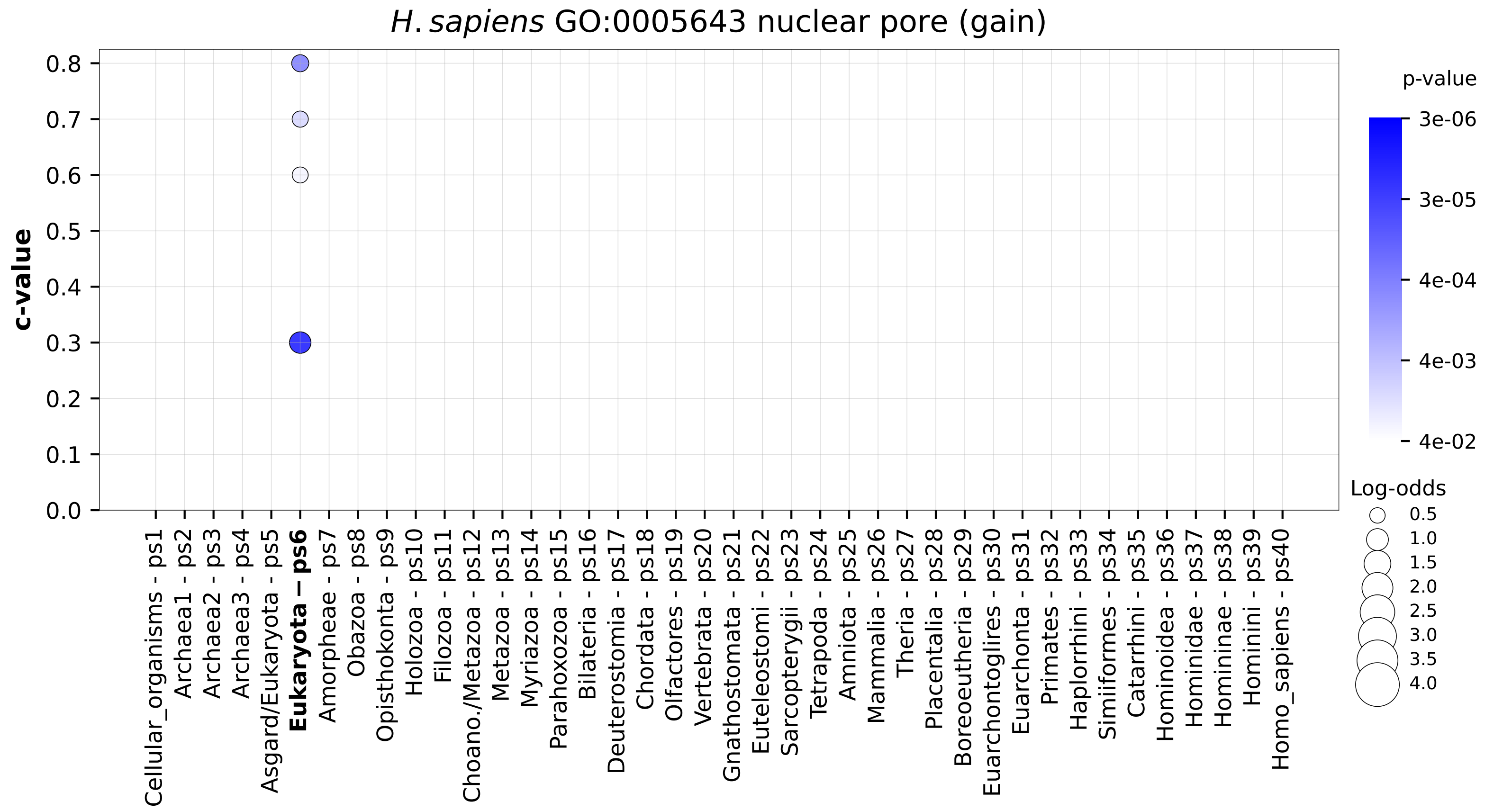
H. sapiens GO:0005643 nuclear pore (gain)
c-value
0.0
0.1
0.2
0.3
0.4
0.5
0.6
0.7
0.8
p-value
3e-06
3e-05
4e-04
4e-03
4e-02
Log-odds
0.5
1.0
1.5
2.0
2.5
3.0
3.5
4.0
Cellular_organisms - ps1
Archaea1 - ps2
Archaea2 - ps3
Archaea3 - ps4
Asgard/Eukaryota - ps5
Eukaryota – ps6
Amorpheae - ps7
Obazoa - ps8
Opisthokonta - ps9
Holozoa - ps10
Filozoa - ps11
Choano./Metazoa - ps12
Metazoa - ps13
Myriazoa - ps14
Parahoxozoa - ps15
Bilateria - ps16
Deuterostomia - ps17
Chordata - ps18
Olfactores - ps19
Vertebrata - ps20
Gnathostomata - ps21
Euteleostomi - ps22
Sarcopterygii - ps23
Tetrapoda - ps24
Amniota - ps25
Mammalia - ps26
Theria - ps27
Placentalia - ps28
Boreoeutheria - ps29
Euarchontoglires - ps30
Euarchonta - ps31
Primates - ps32
Haplorrhini - ps33
Simiiformes - ps34
Catarrhini - ps35
Hominoidea - ps36
Hominidae - ps37
Homininae - ps38
Hominini - ps39
Homo_sapiens - ps40

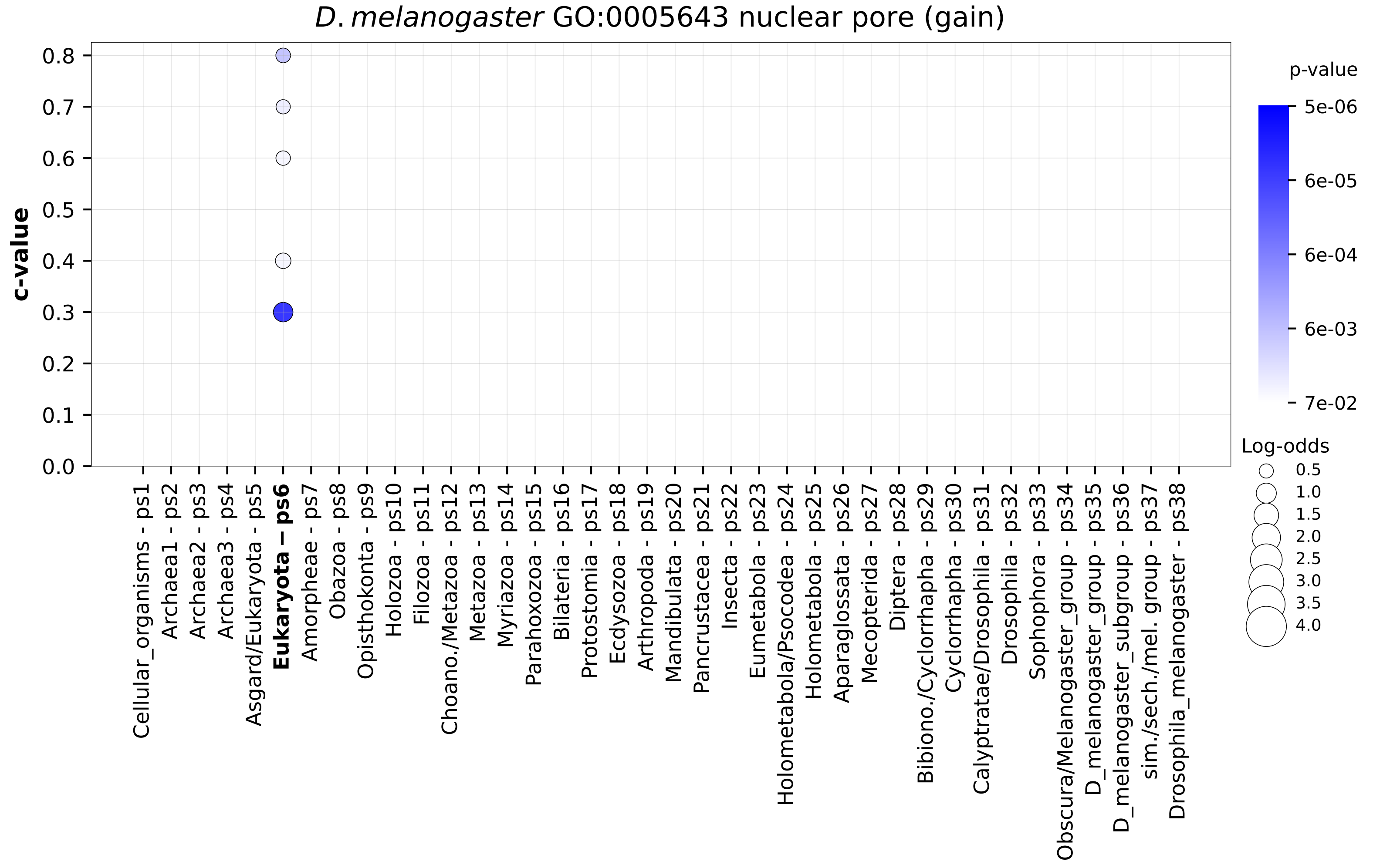
D. melanogaster GO:0005643 nuclear pore (gain)
c-value
0.0
0.1
0.2
0.3
0.4
0.5
0.6
0.7
0.8
p-value
5e-06
6e-05
6e-04
6e-03
7e-02
Log-odds
0.5
1.0
1.5
2.0
2.5
3.0
3.5
4.0
Cellular_organisms - ps1
Archaea1 - ps2
Archaea2 - ps3
Archaea3 - ps4
Asgard/Eukaryota - ps5
Eukaryota – ps6
Amorpheae - ps7
Obazoa - ps8
Opisthokonta - ps9
Holozoa - ps10
Filozoa - ps11
Choano./Metazoa - ps12
Metazoa - ps13
Myriazoa - ps14
Parahoxozoa - ps15
Bilateria - ps16
Protostomia - ps17
Ecdysozoa - ps18
Arthropoda - ps19
Mandibulata - ps20
Pancrustacea - ps21
Insecta - ps22
Eumetabola - ps23
Holometabola/Psocodea - ps24
Holometabola - ps25
Aparaglossata - ps26
Mecopterida - ps27
Diptera - ps28
Bibiono./Cyclorrhapha - ps29
Cyclorrhapha - ps30
Calyptratae/Drosophila - ps31
Drosophila - ps32
Sophophora - ps33
Obscura/Melanogaster_group - ps34
D_melanogaster_group - ps35
D_melanogaster_subgroup - ps36
sim./sech./mel. group - ps37
Drosophila_melanogaster - ps38

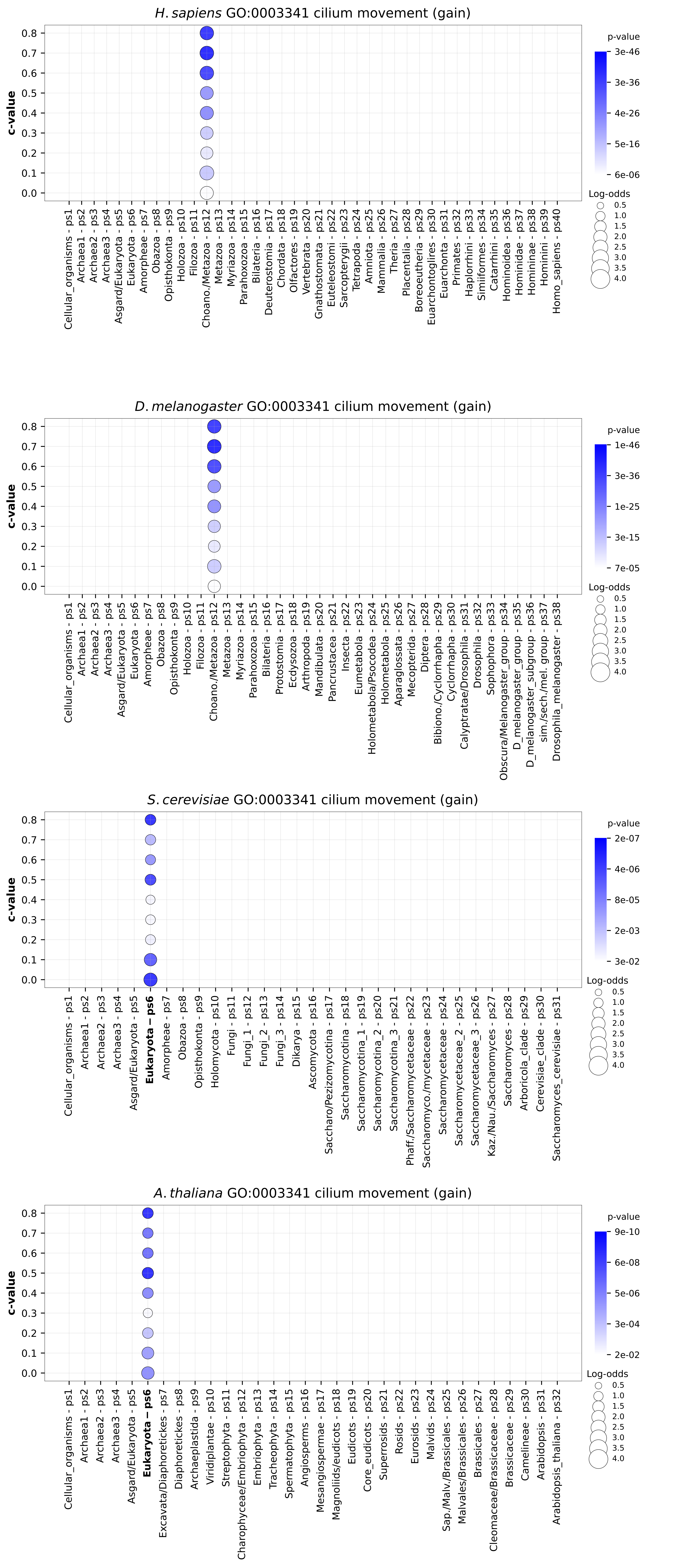

H. sapiens GO:0003341 cilium movement (gain)
D. melanogaster GO:0003341 cilium movement (gain)
S. cerevisiae GO:0003341 cilium movement (gain)
A. thaliana GO:0003341 cilium movement (gain)
c-value
p-value
Log-odds
Cellular_organisms - ps1
Archaea1 - ps2
Archaea2 - ps3
Archaea3 - ps4
Asgard/Eukaryota - ps5
Eukaryota - ps6
Choano./Metazoa - ps12
Homo_sapiens - ps40
Drosophila_melanogaster - ps38
Saccharomyces_cerevisiae - ps31
Arabidopsis_thaliana - ps32

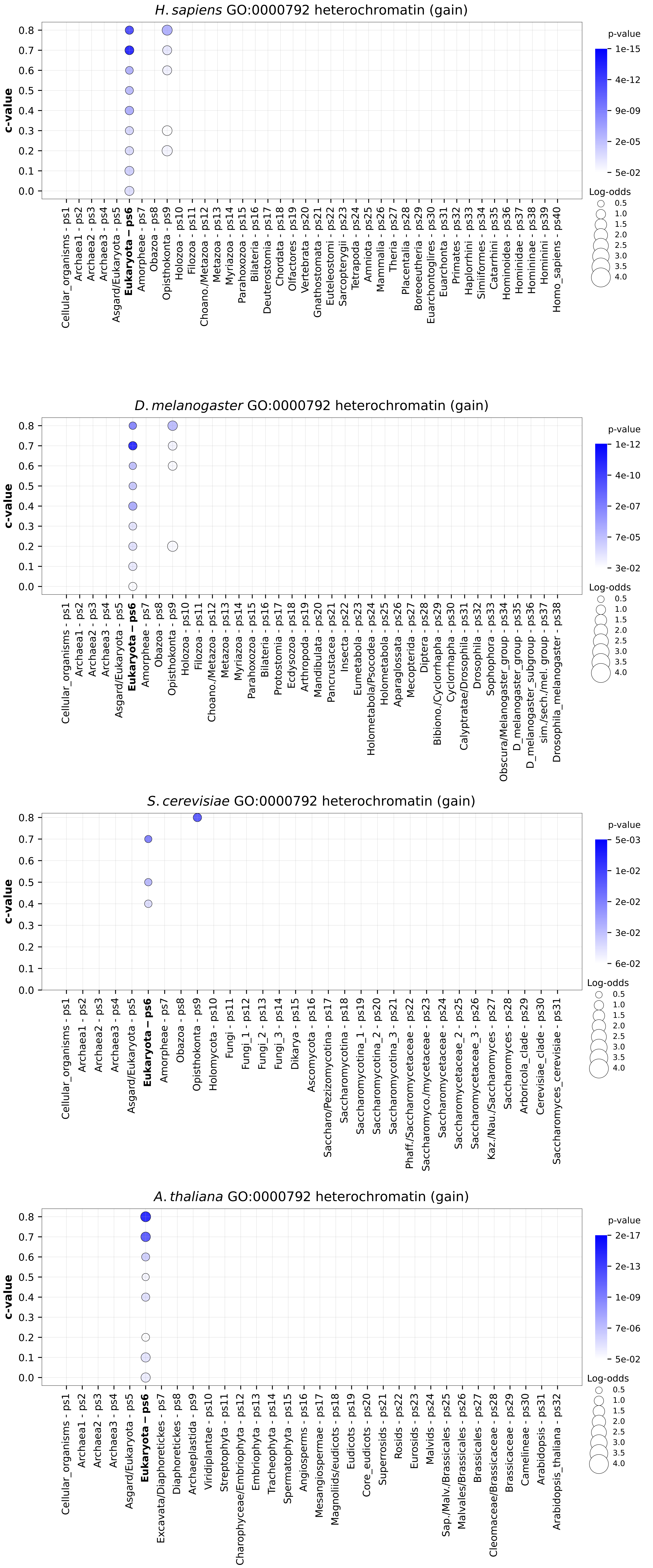
H. sapiens GO:0000792 heterochromatin (gain)
c-value
p-value
1e-15
4e-12
9e-09
2e-05
5e-02
Log-odds
0.5
1.0
1.5
2.0
2.5
3.0
3.5
4.0
Cellular_organisms - ps1
Archaea1 - ps2
Archaea2 - ps3
Archaea3 - ps4
Asgard/Eukaryota - ps5
Eukaryota – ps6
Amorpheae - ps7
Obazoa - ps8
Opisthokonta - ps9
Holozoa - ps10
Filozoa - ps11
Choano./Metazoa - ps12
Metazoa - ps13
Myriazoa - ps14
Parahoxozoa - ps15
Bilateria - ps16
Deuterostomia - ps17
Chordata - ps18
Olfactores - ps19
Vertebrata - ps20
Gnathostomata - ps21
Euteleostomi - ps22
Sarcopterygii - ps23
Tetrapoda - ps24
Amniota - ps25
Mammalia - ps26
Theria - ps27
Placentalia - ps28
Boreoeutheria - ps29
Euarchontoglires - ps30
Euarchonta - ps31
Primates - ps32
Haplorrhini - ps33
Simiiformes - ps34
Catarrhini - ps35
Hominoidea - ps36
Hominidae - ps37
Homininae - ps38
Hominini - ps39
Homo_sapiens - ps40
D. melanogaster GO:0000792 heterochromatin (gain)
c-value
p-value
1e-12
4e-10
2e-07
7e-05
3e-02
Log-odds
Cellular_organisms - ps1
Archaea1 - ps2
Archaea2 - ps3
Archaea3 - ps4
Asgard/Eukaryota - ps5
Eukaryota – ps6
Amorpheae - ps7
Obazoa - ps8
Opisthokonta - ps9
Holozoa - ps10
Filozoa - ps11
Choano./Metazoa - ps12
Metazoa - ps13
Myriazoa - ps14
Parahoxozoa - ps15
Bilateria - ps16
Protostomia - ps17
Ecdysozoa - ps18
Arthropoda - ps19
Mandibulata - ps20
Pancrustacea - ps21
Insecta - ps22
Eumetabola - ps23
Holometabola/Psocodea - ps24
Holometabola - ps25
Aparaglossata - ps26
Mecopterida - ps27
Diptera - ps28
Bibiono./Cyclorrhapha - ps29
Cyclorrhapha - ps30
Calyptratae/Drosophila - ps31
Drosophila - ps32
Sophophora - ps33
Obscura/Melanogaster_group - ps34
D_melanogaster_group - ps35
D_melanogaster_subgroup - ps36
sim./sech./mel. group - ps37
Drosophila_melanogaster - ps38
S. cerevisiae GO:0000792 heterochromatin (gain)
c-value
p-value
5e-03
1e-02
2e-02
3e-02
6e-02
Log-odds
Cellular_organisms - ps1
Archaea1 - ps2
Archaea2 - ps3
Archaea3 - ps4
Asgard/Eukaryota - ps5
Eukaryota – ps6
Amorpheae - ps7
Obazoa - ps8
Opisthokonta - ps9
Holomycota - ps10
Fungi - ps11
Fungi_1 - ps12
Fungi_2 - ps13
Fungi_3 - ps14
Dikarya - ps15
Ascomycota - ps16
Saccharo/Pezizomycotina - ps17
Saccharomycotina - ps18
Saccharomycotina_1 - ps19
Saccharomycotina_2 - ps20
Saccharomycotina_3 - ps21
Phaff./Saccharomycetaceae - ps22
Saccharomyco./mycetaceae - ps23
Saccharomycetaceae - ps24
Saccharomycetaceae_2 - ps25
Saccharomycetaceae_3 - ps26
Kaz./Nau./Saccharomyces - ps27
Saccharomyces - ps28
Arboricola_clade - ps29
Cerevisiae_clade - ps30
Saccharomyces_cerevisiae - ps31
A. thaliana GO:0000792 heterochromatin (gain)
c-value
p-value
2e-17
2e-13
1e-09
7e-06
5e-02
Log-odds
Cellular_organisms - ps1
Archaea1 - ps2
Archaea2 - ps3
Archaea3 - ps4
Asgard/Eukaryota - ps5
Eukaryota – ps6
Excavata/Diaphoretickes - ps7
Diaphoretickes - ps8
Archaeplastida - ps9
Viridiplantae - ps10
Streptophyta - ps11
Charophyceae/Embriophyta - ps12
Embriophyta - ps13
Tracheophyta - ps14
Spermatophyta - ps15
Angiosperms - ps16
Mesangiospermae - ps17
Magnoliids/eudicots - ps18
Eudicots - ps19
Core_eudicots - ps20
Superrosids - ps21
Rosids - ps22
Eurosids - ps23
Malvids - ps24
Sap./Malv./Brassicales - ps25
Malvales/Brassicales - ps26
Brassicales - ps27
Cleomaceae/Brassicaceae - ps28
Brassicaceae - ps29
Camelineae - ps30
Arabidopsis - ps31
Arabidopsis_thaliana - ps32

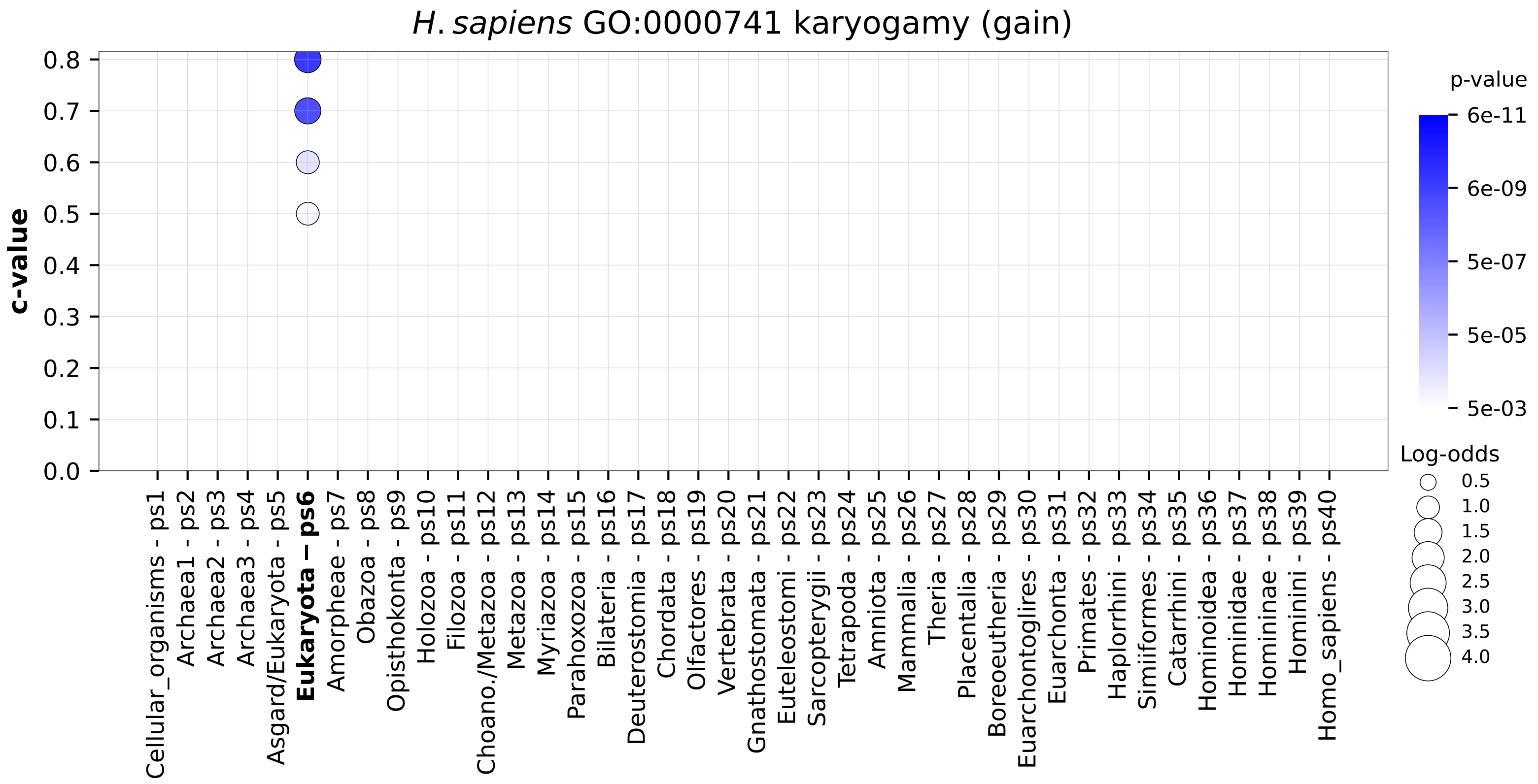

H. sapiens GO:0000741 karyogamy (gain)
c-value
p-value
Log-odds
Cellular_organisms - ps1
Archaea1 - ps2
Archaea2 - ps3
Archaea3 - ps4
Asgard/Eukaryota - ps5
Eukaryota – ps6
Amorpheae - ps7
Obazoa - ps8
Opisthokonta - ps9
Holozoa - ps10
Filozoa - ps11
Choano./Metazoa - ps12
Metazoa - ps13
Myriazoa - ps14
Parahoxozoa - ps15
Bilateria - ps16
Deuterostomia - ps17
Chordata - ps18
Olfactores - ps19
Vertebrata - ps20
Gnathostomata - ps21
Euteleostomi - ps22
Sarcopterygii - ps23
Tetrapoda - ps24
Amniota - ps25
Mammalia - ps26
Theria - ps27
Placentalia - ps28
Boreoeutheria - ps29
Euarchontoglires - ps30
Euarchonta - ps31
Primates - ps32
Haplorrhini - ps33
Simiiformes - ps34
Catarrhini - ps35
Hominoidea - ps36
Hominidae - ps37
Homininae - ps38
Hominini - ps39
Homo_sapiens - ps40

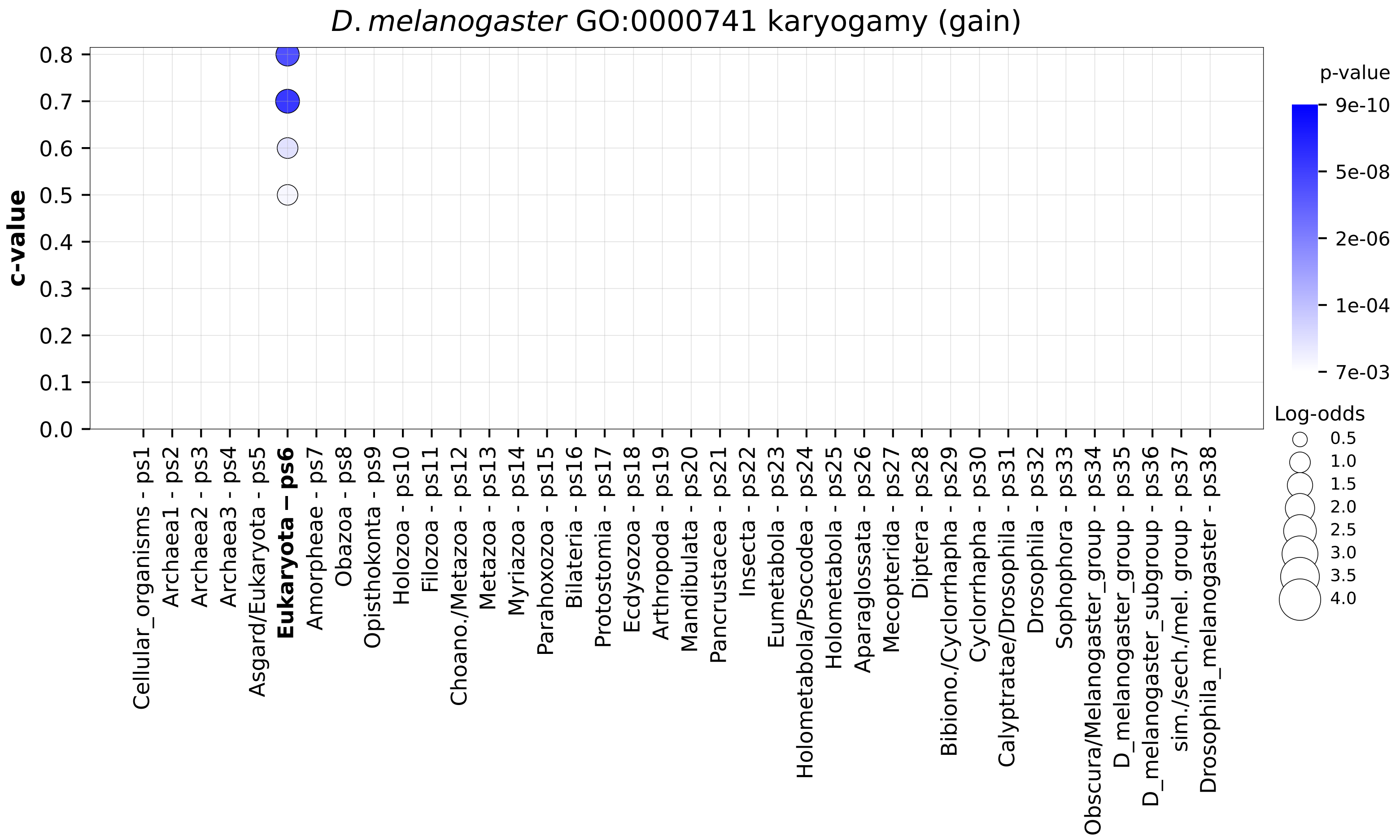

D. melanogaster GO:0000741 karyogamy (gain)
c-value
p-value
Log-odds
Cellular_organisms - ps1
Archaea1 - ps2
Archaea2 - ps3
Archaea3 - ps4
Asgard/Eukaryota - ps5
Eukaryota – ps6
Amorpheae - ps7
Obazoa - ps8
Opisthokonta - ps9
Holozoa - ps10
Filozoa - ps11
Choano./Metazoa - ps12
Metazoa - ps13
Myriazoa - ps14
Parahoxozoa - ps15
Bilateria - ps16
Protostomia - ps17
Ecdysozoa - ps18
Arthropoda - ps19
Mandibulata - ps20
Pancrustacea - ps21
Insecta - ps22
Eumetabola - ps23
Holometabola/Psocodea - ps24
Holometabola - ps25
Aparaglossata - ps26
Mecopterida - ps27
Diptera - ps28
Bibiono./Cyclorrhapha - ps29
Cyclorrhapha - ps30
Calyptratae/Drosophila - ps31
Drosophila - ps32
Sophophora - ps33
Obscura/Melanogaster_group - ps34
D_melanogaster_group - ps35
D_melanogaster_subgroup - ps36
sim./sech./mel. group - ps37
Drosophila_melanogaster - ps38

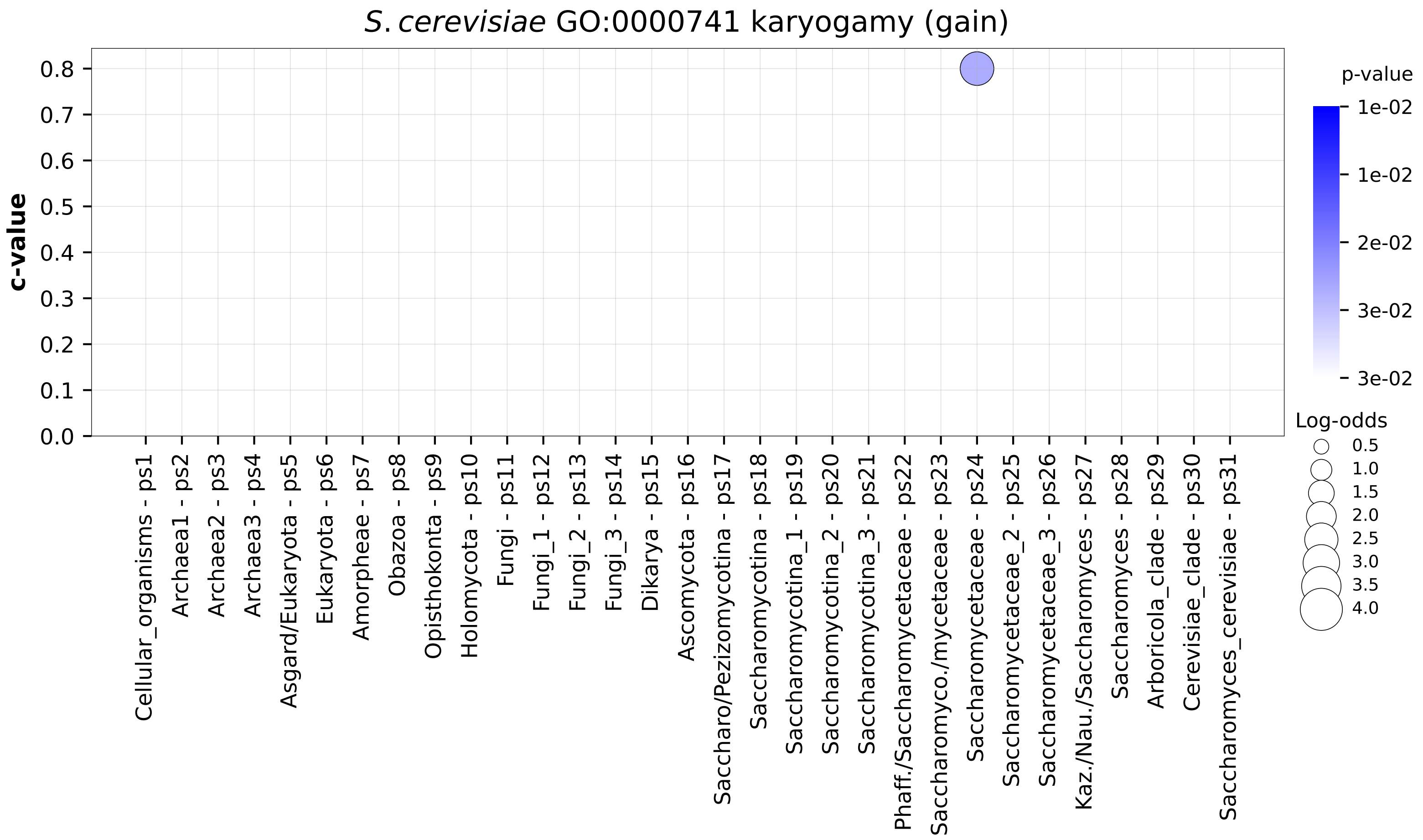

S. cerevisiae GO:0000741 karyogamy (gain)
c-value
p-value
Log-odds
Cellular_organisms - ps1
Archaea1 - ps2
Archaea2 - ps3
Archaea3 - ps4
Asgard/Eukaryota - ps5
Eukaryota - ps6
Amorpheae - ps7
Obazoa - ps8
Opisthokonta - ps9
Holomycota - ps10
Fungi - ps11
Fungi_1 - ps12
Fungi_2 - ps13
Fungi_3 - ps14
Dikarya - ps15
Ascomycota - ps16
Saccharo/Pezizomycotina - ps17
Saccharomycotina - ps18
Saccharomycotina_1 - ps19
Saccharomycotina_2 - ps20
Saccharomycotina_3 - ps21
Phaff./Saccharomycetaceae - ps22
Saccharomyco./mycetaceae - ps23
Saccharomycetaceae - ps24
Saccharomycetaceae_2 - ps25
Saccharomycetaceae_3 - ps26
Kaz./Nau./Saccharomyces - ps27
Saccharomyces - ps28
Arboricola_clade - ps29
Cerevisiae_clade - ps30
Saccharomyces_cerevisiae - ps31

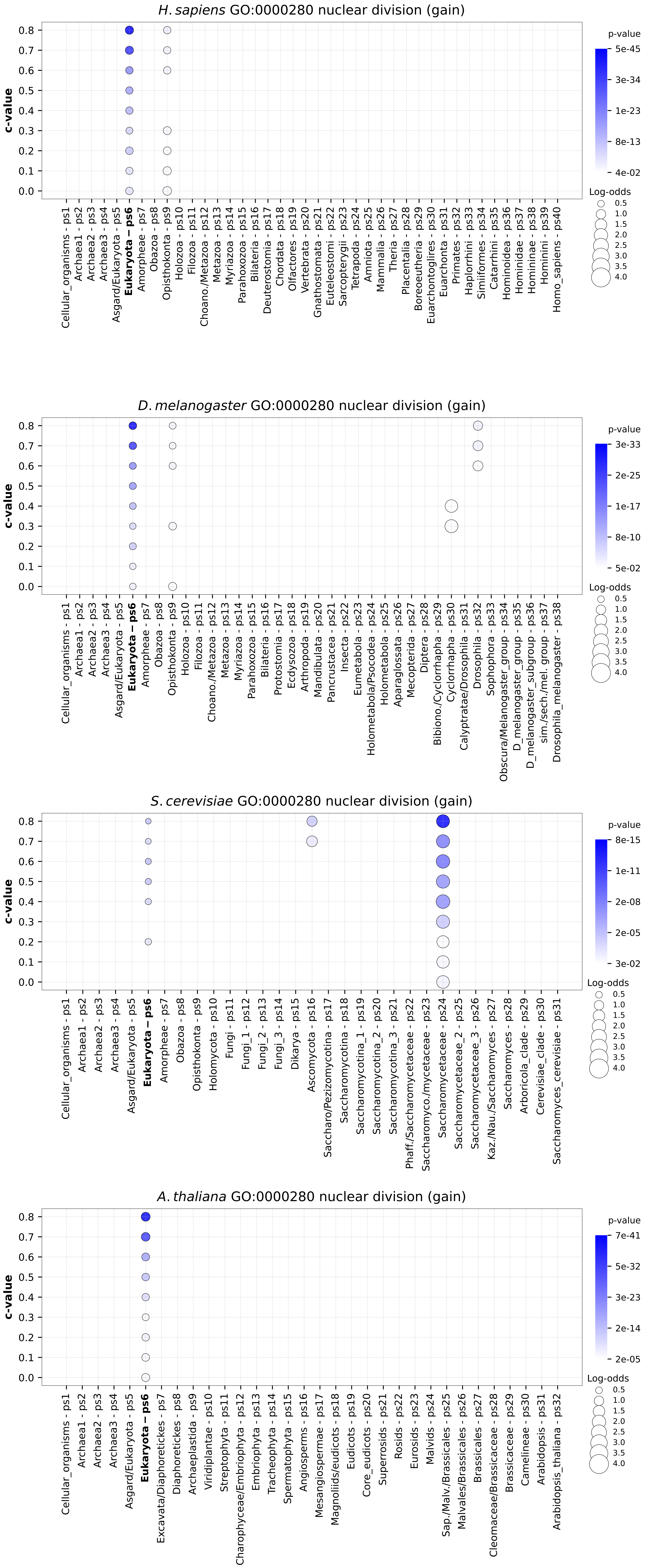
H. sapiens GO:0000280 nuclear division (gain)
c-value
p-value
5e-45
3e-34
1e-23
8e-13
4e-02
Log-odds
0.5 1.0 1.5 2.0 2.5 3.0 3.5 4.0
Cellular_organisms - ps1
Archaea1 - ps2
Archaea2 - ps3
Archaea3 - ps4
Asgard/Eukaryota - ps5
Eukaryota – ps6
Amorpheae - ps7
Obazoa - ps8
Opisthokonta - ps9
Holozoa - ps10
Filozoa - ps11
Choano./Metazoa - ps12
Metazoa - ps13
Myriazoa - ps14
Parahoxozoa - ps15
Bilateria - ps16
Deuterostomia - ps17
Chordata - ps18
Olfactores - ps19
Vertebrata - ps20
Gnathostomata - ps21
Euteleostomi - ps22
Sarcopterygii - ps23
Tetrapoda - ps24
Amniota - ps25
Mammalia - ps26
Theria - ps27
Placentalia - ps28
Boreoeutheria - ps29
Euarchontoglires - ps30
Euarchonta - ps31
Primates - ps32
Haplorrhini - ps33
Simiiformes - ps34
Catarrhini - ps35
Hominoidea - ps36
Hominidae - ps37
Homininae - ps38
Hominini - ps39
Homo_sapiens - ps40
D. melanogaster GO:0000280 nuclear division (gain)
c-value
p-value
3e-33
2e-25
1e-17
8e-10
5e-02
Log-odds
0.5 1.0 1.5 2.0 2.5 3.0 3.5 4.0
Cellular_organisms - ps1
Archaea1 - ps2
Archaea2 - ps3
Archaea3 - ps4
Asgard/Eukaryota - ps5
Eukaryota – ps6
Amorpheae - ps7
Obazoa - ps8
Opisthokonta - ps9
Holozoa - ps10
Filozoa - ps11
Choano./Metazoa - ps12
Metazoa - ps13
Myriazoa - ps14
Parahoxozoa - ps15
Bilateria - ps16
Protostomia - ps17
Ecdysozoa - ps18
Arthropoda - ps19
Mandibulata - ps20
Pancrustacea - ps21
Insecta - ps22
Eumetabola - ps23
Holometabola/Psocodea - ps24
Holometabola - ps25
Aparaglossata - ps26
Mecopterida - ps27
Diptera - ps28
Bibiono./Cyclorrhapha - ps29
Cyclorrhapha - ps30
Calyptratae/Drosophila - ps31
Drosophila - ps32
Sophophora - ps33
Obscura/Melanogaster_group - ps34
D_melanogaster_group - ps35
D_melanogaster_subgroup - ps36
sim./sech./mel. group - ps37
Drosophila_melanogaster - ps38
S. cerevisiae GO:0000280 nuclear division (gain)
c-value
p-value
8e-15
1e-11
2e-08
2e-05
3e-02
Log-odds
0.5 1.0 1.5 2.0 2.5 3.0 3.5 4.0
Cellular_organisms - ps1
Archaea1 - ps2
Archaea2 - ps3
Archaea3 - ps4
Asgard/Eukaryota - ps5
Eukaryota – ps6
Amorpheae - ps7
Obazoa - ps8
Opisthokonta - ps9
Holomycota - ps10
Fungi - ps11
Fungi_1 - ps12
Fungi_2 - ps13
Fungi_3 - ps14
Dikarya - ps15
Ascomycota - ps16
Saccharo/Pezizomycotina - ps17
Saccharomycotina - ps18
Saccharomycotina_1 - ps19
Saccharomycotina_2 - ps20
Saccharomycotina_3 - ps21
Phaff./Saccharomycetaceae - ps22
Saccharomyco./mycetaceae - ps23
Saccharomycetaceae - ps24
Saccharomycetaceae_2 - ps25
Saccharomycetaceae_3 - ps26
Kaz./Nau./Saccharomyces - ps27
Saccharomyces - ps28
Arboricola_clade - ps29
Cerevisiae_clade - ps30
Saccharomyces_cerevisiae - ps31
A. thaliana GO:0000280 nuclear division (gain)
c-value
p-value
7e-41
5e-32
3e-23
2e-14
2e-05
Log-odds
0.5 1.0 1.5 2.0 2.5 3.0 3.5 4.0
Cellular_organisms - ps1
Archaea1 - ps2
Archaea2 - ps3
Archaea3 - ps4
Asgard/Eukaryota - ps5
Eukaryota – ps6
Excavata/Diaphoretickes - ps7
Diaphoretickes - ps8
Archaeplastida - ps9
Viridiplantae - ps10
Streptophyta - ps11
Charophyceae/Embriophyta - ps12
Embriophyta - ps13
Tracheophyta - ps14
Spermatophyta - ps15
Angiosperms - ps16
Mesangiospermae - ps17
Magnoliids/eudicots - ps18
Eudicots - ps19
Core_eudicots - ps20
Superrosids - ps21
Rosids - ps22
Eurosids - ps23
Malvids - ps24
Sap./Malv./Brassicales - ps25
Malvales/Brassicales - ps26
Brassicales - ps27
Cleomaceae/Brassicaceae - ps28
Brassicaceae - ps29
Camelineae - ps30
Arabidopsis - ps31
Arabidopsis_thaliana - ps32

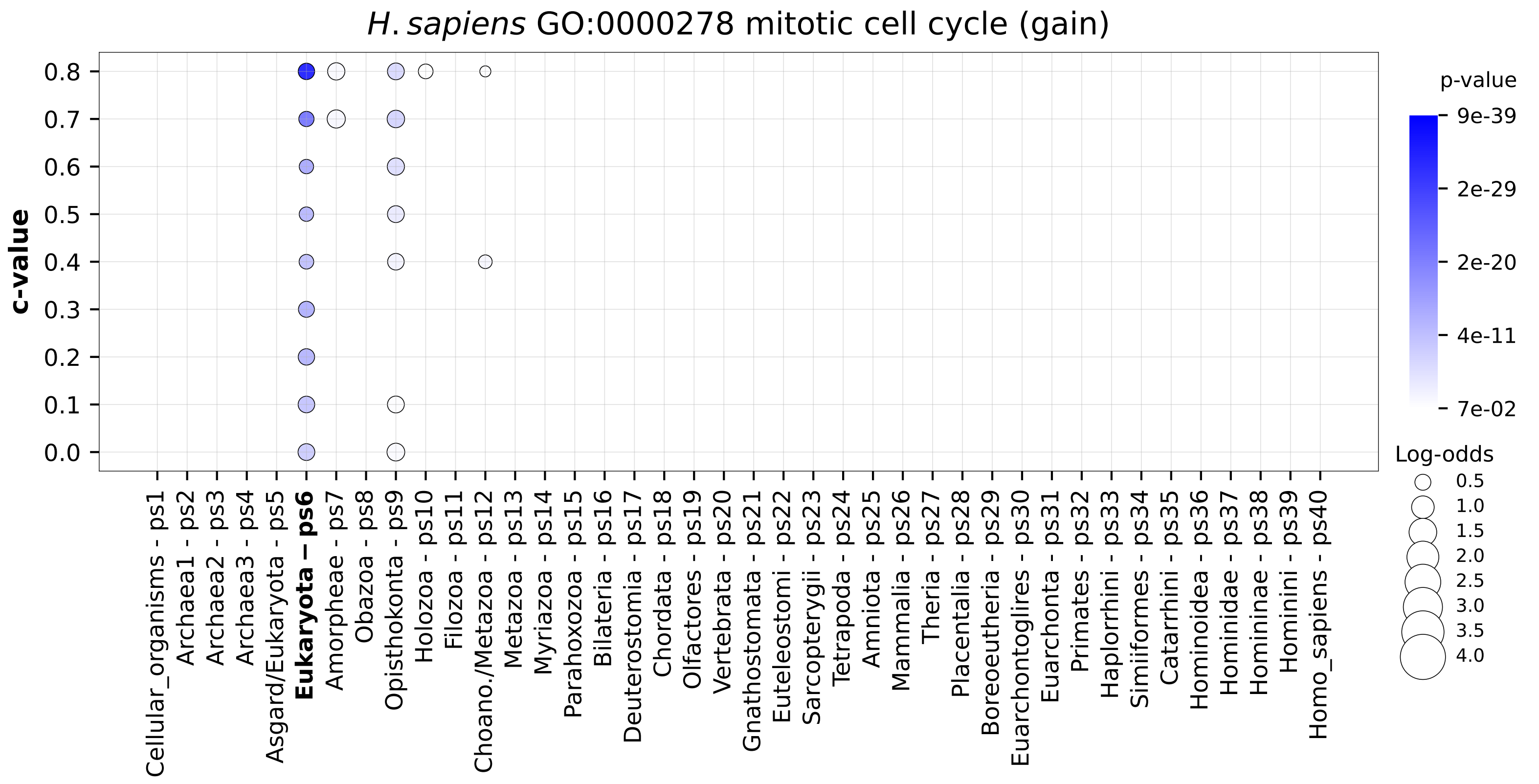
H. sapiens GO:0000278 mitotic cell cycle (gain)
c-value
0.0 0.1 0.2 0.3 0.4 0.5 0.6 0.7 0.8
p-value
9e-39
2e-29
2e-20
4e-11
7e-02
Log-odds
0.5 1.0 1.5 2.0 2.5 3.0 3.5 4.0
Cellular_organisms - ps1
Archaea1 - ps2
Archaea2 - ps3
Archaea3 - ps4
Asgard/Eukaryota - ps5
Eukaryota – ps6
Amorpheae - ps7
Obazoa - ps8
Opisthokonta - ps9
Holozoa - ps10
Filozoa - ps11
Choano./Metazoa - ps12
Metazoa - ps13
Myriazoa - ps14
Parahoxozoa - ps15
Bilateria - ps16
Deuterostomia - ps17
Chordata - ps18
Olfactores - ps19
Vertebrata - ps20
Gnathostomata - ps21
Euteleostomi - ps22
Sarcopterygii - ps23
Tetrapoda - ps24
Amniota - ps25
Mammalia - ps26
Theria - ps27
Placentalia - ps28
Boreoeutheria - ps29
Euarchontoglires - ps30
Euarchonta - ps31
Primates - ps32
Haplorrhini - ps33
Simiiformes - ps34
Catarrhini - ps35
Hominoidea - ps36
Hominidae - ps37
Homininae - ps38
Hominini - ps39
Homo_sapiens - ps40

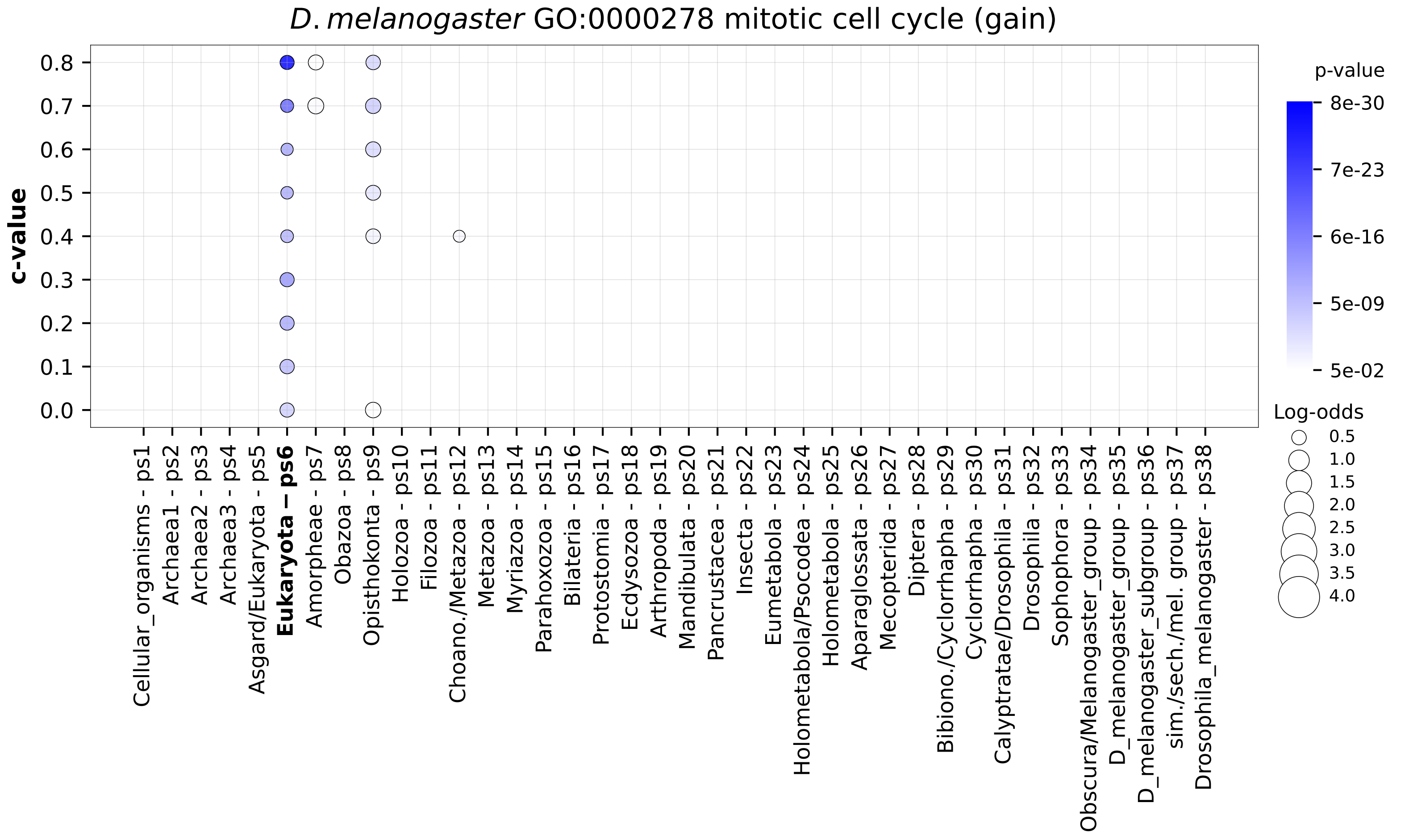
D. melanogaster GO:0000278 mitotic cell cycle (gain)
c-value
0.0 0.1 0.2 0.3 0.4 0.5 0.6 0.7 0.8
p-value
8e-30
7e-23
6e-16
5e-09
5e-02
Log-odds
0.5 1.0 1.5 2.0 2.5 3.0 3.5 4.0
Cellular_organisms - ps1
Archaea1 - ps2
Archaea2 - ps3
Archaea3 - ps4
Asgard/Eukaryota - ps5
Eukaryota – ps6
Amorpheae - ps7
Obazoa - ps8
Opisthokonta - ps9
Holozoa - ps10
Filozoa - ps11
Choano./Metazoa - ps12
Metazoa - ps13
Myriazoa - ps14
Parahoxozoa - ps15
Bilateria - ps16
Protostomia - ps17
Ecdysozoa - ps18
Arthropoda - ps19
Mandibulata - ps20
Pancrustacea - ps21
Insecta - ps22
Eumetabola - ps23
Holometabola/Psocodea - ps24
Holometabola - ps25
Aparaglossata - ps26
Mecopterida - ps27
Diptera - ps28
Bibiono./Cyclorrhapha - ps29
Cyclorrhapha - ps30
Calyptratae/Drosophila - ps31
Drosophila - ps32
Sophophora - ps33
Obscura/Melanogaster_group - ps34
D_melanogaster_group - ps35
D_melanogaster_subgroup - ps36
sim./sech./mel. group - ps37
Drosophila_melanogaster - ps38

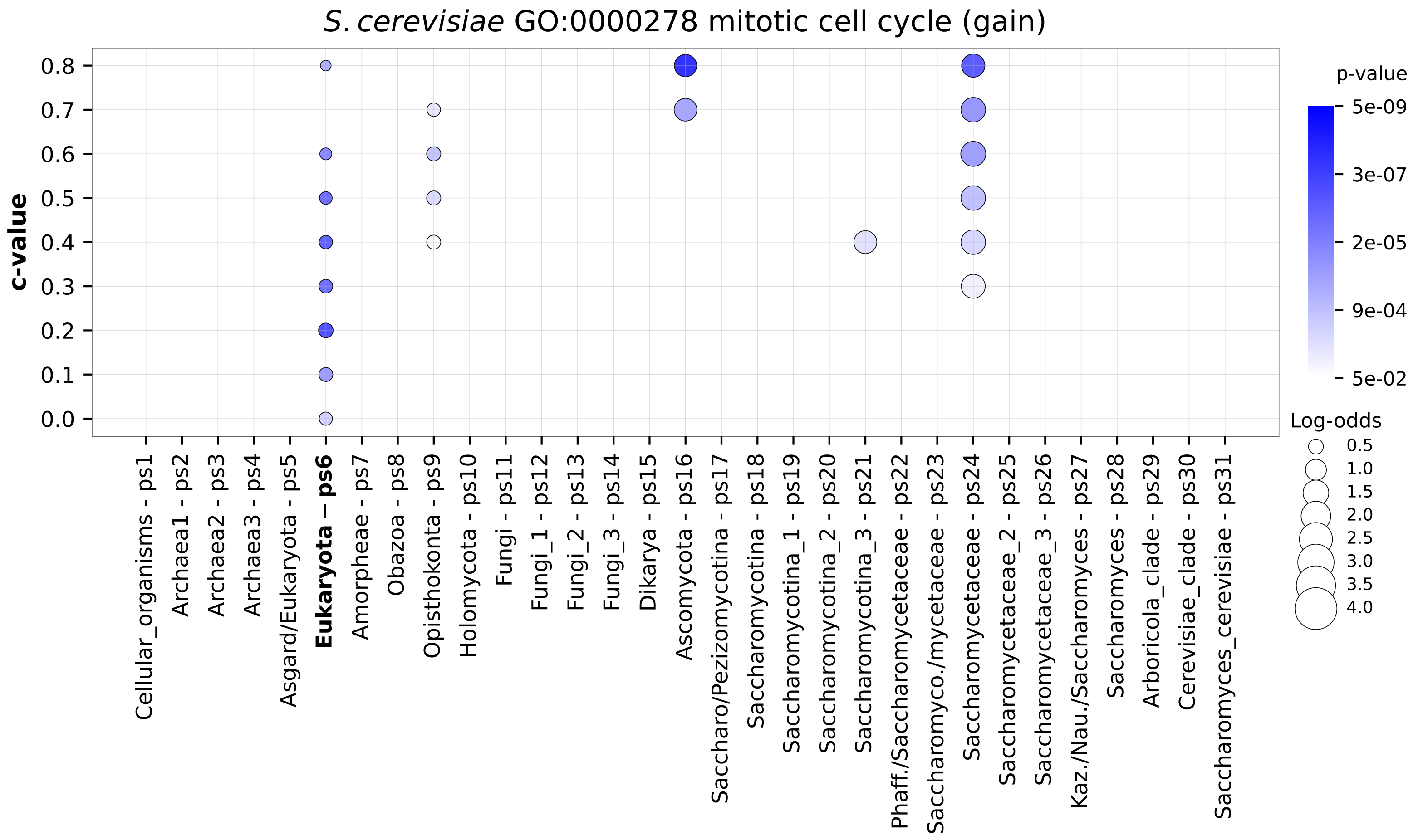
S. cerevisiae GO:0000278 mitotic cell cycle (gain)
c-value
0.0 0.1 0.2 0.3 0.4 0.5 0.6 0.7 0.8
p-value
5e-09
3e-07
2e-05
9e-04
5e-02
Log-odds
0.5 1.0 1.5 2.0 2.5 3.0 3.5 4.0
Cellular_organisms - ps1
Archaea1 - ps2
Archaea2 - ps3
Archaea3 - ps4
Asgard/Eukaryota - ps5
Eukaryota – ps6
Amorpheae - ps7
Obazoa - ps8
Opisthokonta - ps9
Holomycota - ps10
Fungi - ps11
Fungi_1 - ps12
Fungi_2 - ps13
Fungi_3 - ps14
Dikarya - ps15
Ascomycota - ps16
Saccharo/Pezizomycotina - ps17
Saccharomycotina - ps18
Saccharomycotina_1 - ps19
Saccharomycotina_2 - ps20
Saccharomycotina_3 - ps21
Phaff./Saccharomycetaceae - ps22
Saccharomyco./mycetaceae - ps23
Saccharomycetaceae - ps24
Saccharomycetaceae_2 - ps25
Saccharomycetaceae_3 - ps26
Kaz./Nau./Saccharomyces - ps27
Saccharomyces - ps28
Arboricola_clade - ps29
Cerevisiae_clade - ps30
Saccharomyces_cerevisiae - ps31

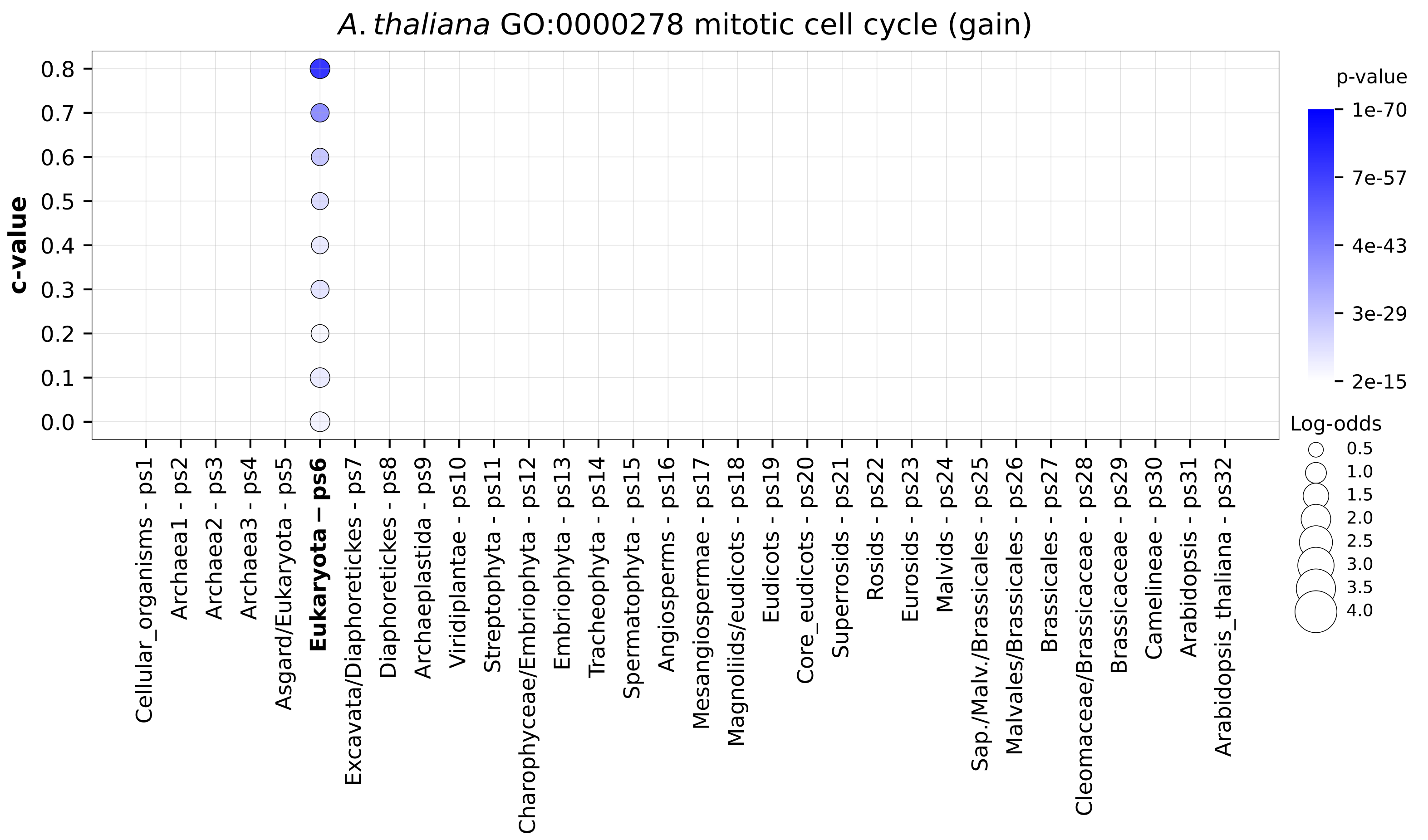
A. thaliana GO:0000278 mitotic cell cycle (gain)
c-value
0.0 0.1 0.2 0.3 0.4 0.5 0.6 0.7 0.8
p-value
1e-70
7e-57
4e-43
3e-29
2e-15
Log-odds
0.5 1.0 1.5 2.0 2.5 3.0 3.5 4.0
Cellular_organisms - ps1
Archaea1 - ps2
Archaea2 - ps3
Archaea3 - ps4
Asgard/Eukaryota - ps5
Eukaryota – ps6
Excavata/Diaphoretickes - ps7
Diaphoretickes - ps8
Archaeplastida - ps9
Viridiplantae - ps10
Streptophyta - ps11
Charophyceae/Embriophyta - ps12
Embriophyta - ps13
Tracheophyta - ps14
Spermatophyta - ps15
Angiosperms - ps16
Mesangiospermae - ps17
Magnoliids/eudicots - ps18
Eudicots - ps19
Core_eudicots - ps20
Superrosids - ps21
Rosids - ps22
Eurosids - ps23
Malvids - ps24
Sap./Malv./Brassicales - ps25
Malvales/Brassicales - ps26
Brassicales - ps27
Cleomaceae/Brassicaceae - ps28
Brassicaceae - ps29
Camelineae - ps30
Arabidopsis - ps31
Arabidopsis_thaliana - ps32

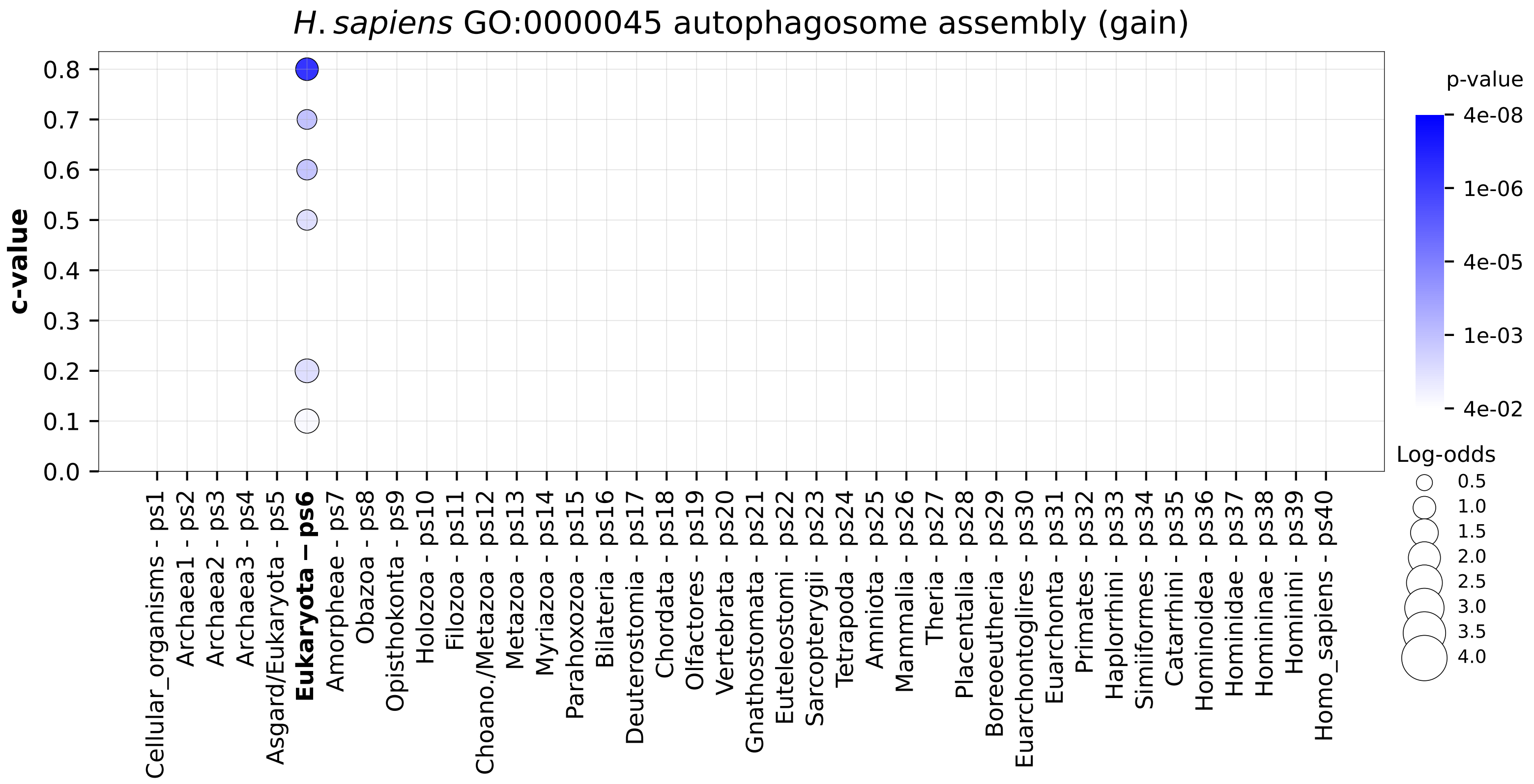

H. sapiens GO:0000045 autophagosome assembly (gain)
c-value
0.0
0.1
0.2
0.3
0.4
0.5
0.6
0.7
0.8
p-value
4e-08
1e-06
4e-05
1e-03
4e-02
Log-odds
0.5
1.0
1.5
2.0
2.5
3.0
3.5
4.0
Cellular_organisms - ps1
Archaea1 - ps2
Archaea2 - ps3
Archaea3 - ps4
Asgard/Eukaryota - ps5
Eukaryota – ps6
Amorpheae - ps7
Obazoa - ps8
Opisthokonta - ps9
Holozoa - ps10
Filozoa - ps11
Choano./Metazoa - ps12
Metazoa - ps13
Myriazoa - ps14
Parahoxozoa - ps15
Bilateria - ps16
Deuterostomia - ps17
Chordata - ps18
Olfactores - ps19
Vertebrata - ps20
Gnathostomata - ps21
Euteleostomi - ps22
Sarcopterygii - ps23
Tetrapoda - ps24
Amniota - ps25
Mammalia - ps26
Theria - ps27
Placentalia - ps28
Boreoeutheria - ps29
Euarchontoglires - ps30
Euarchonta - ps31
Primates - ps32
Haplorrhini - ps33
Simiiformes - ps34
Catarrhini - ps35
Hominoidea - ps36
Hominidae - ps37
Homininae - ps38
Hominini - ps39
Homo_sapiens - ps40

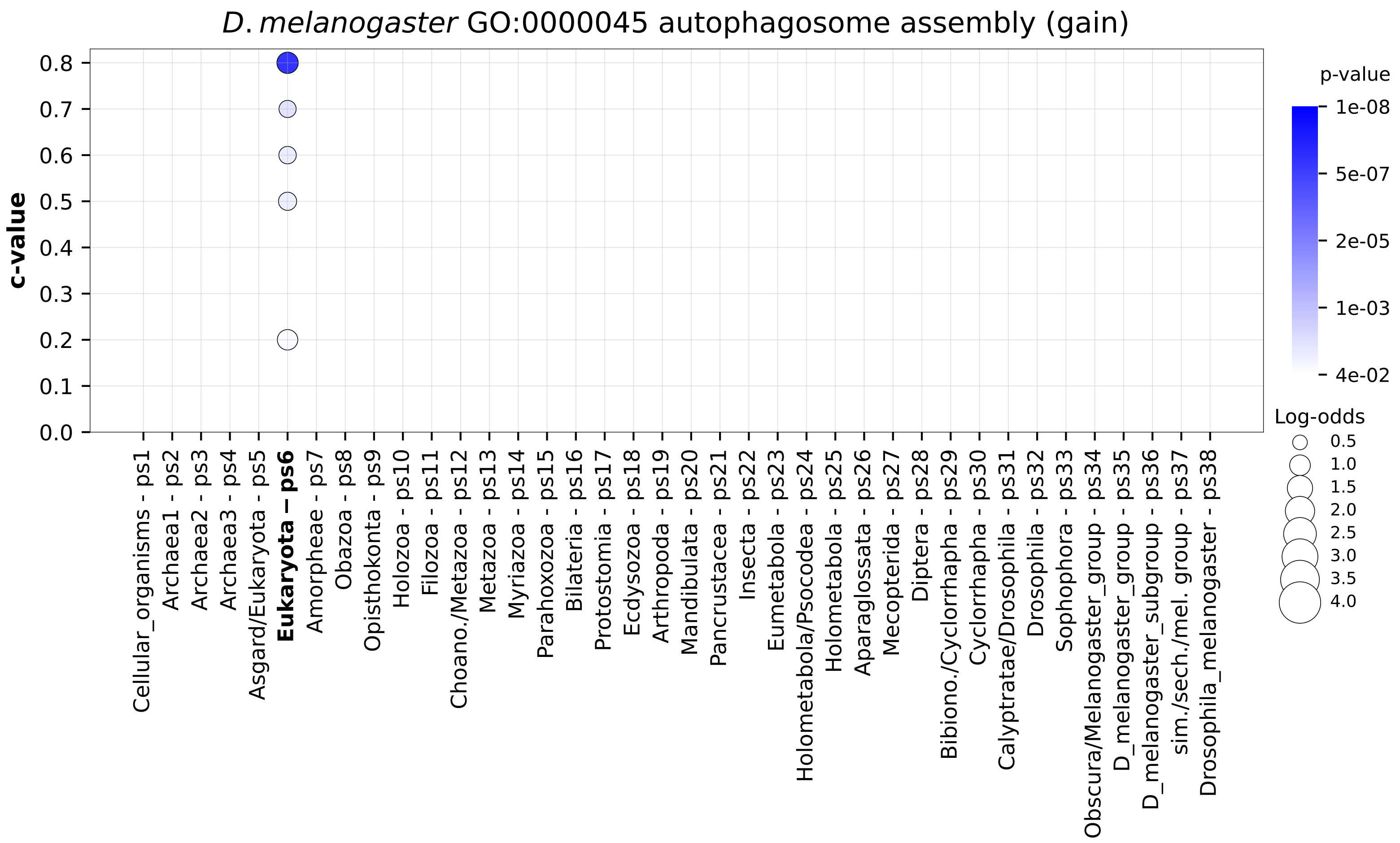

D. melanogaster GO:0000045 autophagosome assembly (gain)
c-value
0.0
0.1
0.2
0.3
0.4
0.5
0.6
0.7
0.8
p-value
1e-08
5e-07
2e-05
1e-03
4e-02
Log-odds
0.5
1.0
1.5
2.0
2.5
3.0
3.5
4.0
Cellular_organisms - ps1
Archaea1 - ps2
Archaea2 - ps3
Archaea3 - ps4
Asgard/Eukaryota - ps5
Eukaryota – ps6
Amorpheae - ps7
Obazoa - ps8
Opisthokonta - ps9
Holozoa - ps10
Filozoa - ps11
Choano./Metazoa - ps12
Metazoa - ps13
Myriazoa - ps14
Parahoxozoa - ps15
Bilateria - ps16
Protostomia - ps17
Ecdysozoa - ps18
Arthropoda - ps19
Mandibulata - ps20
Pancrustacea - ps21
Insecta - ps22
Eumetabola - ps23
Holometabola/Psocodea - ps24
Holometabola - ps25
Aparaglossata - ps26
Mecopterida - ps27
Diptera - ps28
Bibiono./Cyclorrhapha - ps29
Cyclorrhapha - ps30
Calyptratae/Drosophila - ps31
Drosophila - ps32
Sophophora - ps33
Obscura/Melanogaster_group - ps34
D_melanogaster_group - ps35
D_melanogaster_subgroup - ps36
sim./sech./mel. group - ps37
Drosophila_melanogaster - ps38

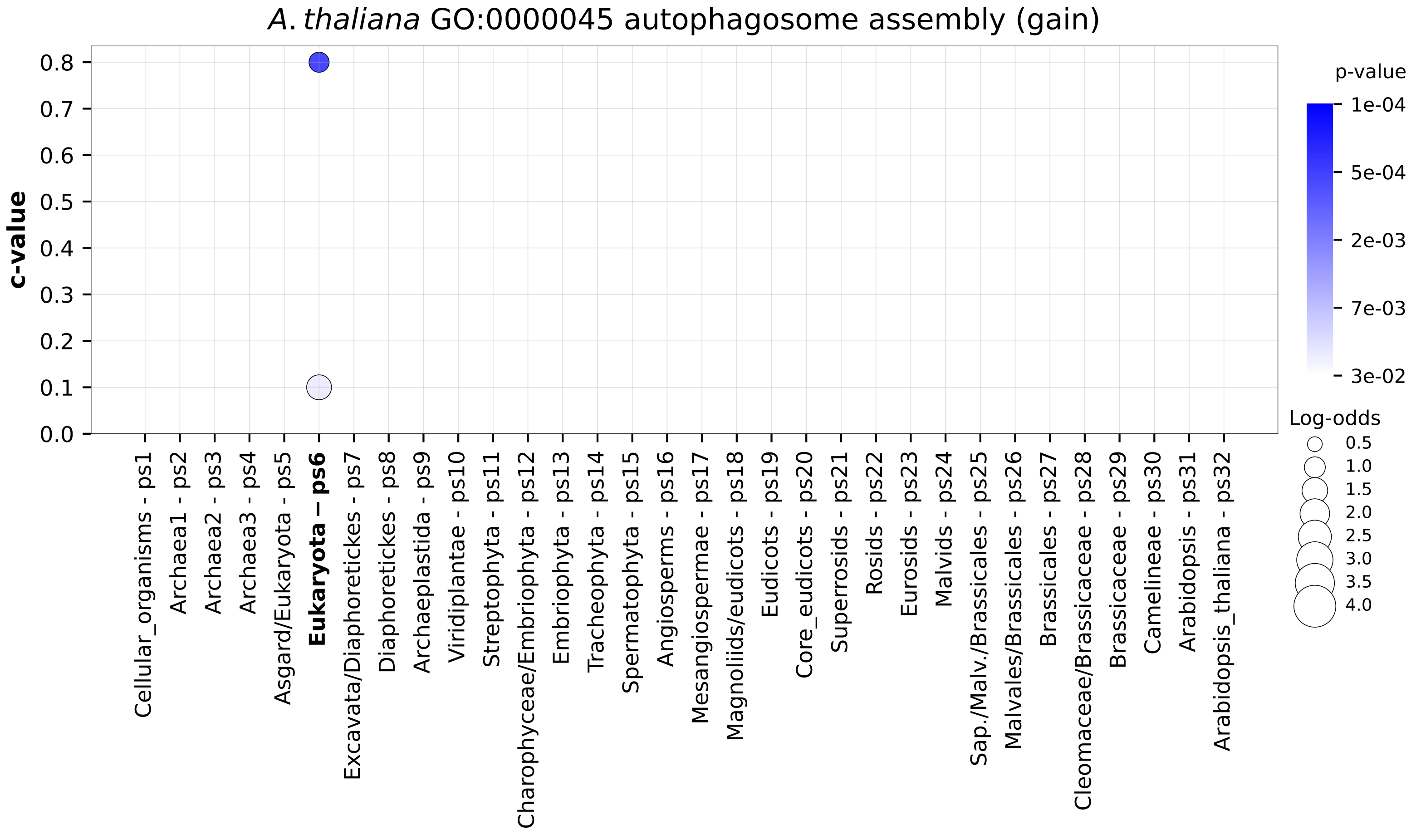

A. thaliana GO:0000045 autophagosome assembly (gain)
c-value
0.0
0.1
0.2
0.3
0.4
0.5
0.6
0.7
0.8
p-value
1e-04
5e-04
2e-03
7e-03
3e-02
Log-odds
0.5
1.0
1.5
2.0
2.5
3.0
3.5
4.0
Cellular_organisms - ps1
Archaea1 - ps2
Archaea2 - ps3
Archaea3 - ps4
Asgard/Eukaryota - ps5
Eukaryota – ps6
Excavata/Diaphoretickes - ps7
Diaphoretickes - ps8
Archaeplastida - ps9
Viridiplantae - ps10
Streptophyta - ps11
Charophyceae/Embriophyta - ps12
Embriophyta - ps13
Tracheophyta - ps14
Spermatophyta - ps15
Angiosperms - ps16
Mesangiospermae - ps17
Magnoliids/eudicots - ps18
Eudicots - ps19
Core_eudicots - ps20
Superrosids - ps21
Rosids - ps22
Eurosids - ps23
Malvids - ps24
Sap./Malv./Brassicales - ps25
Malvales/Brassicales - ps26
Brassicales - ps27
Cleomaceae/Brassicaceae - ps28
Brassicaceae - ps29
Camelineae - ps30
Arabidopsis - ps31
Arabidopsis_thaliana - ps32
