# Supplementary material for: Macroevolutionary dynamics of gene family gain and loss along multicellular eukaryotic lineages
Source: Nat Commun. 2024 Mar 26;15:2663. doi: 10.1038/s41467-024-47017-w (PMC10966110; doi:10.1038/s41467-024-47017-w)

Supplementary Data 1. The consensus phylogeny of 667 species used to estimate gene family gain and loss patterns.
The number of total, gained and lost gene families at a particular node (*total/+gain/-loss*) are calculated using c-value=0.8.
Paths to four functionally analysed focal species are colored (**A.thailana**, **S. cerevisiae**, **D. melanogaster**, **H.sapiens**).

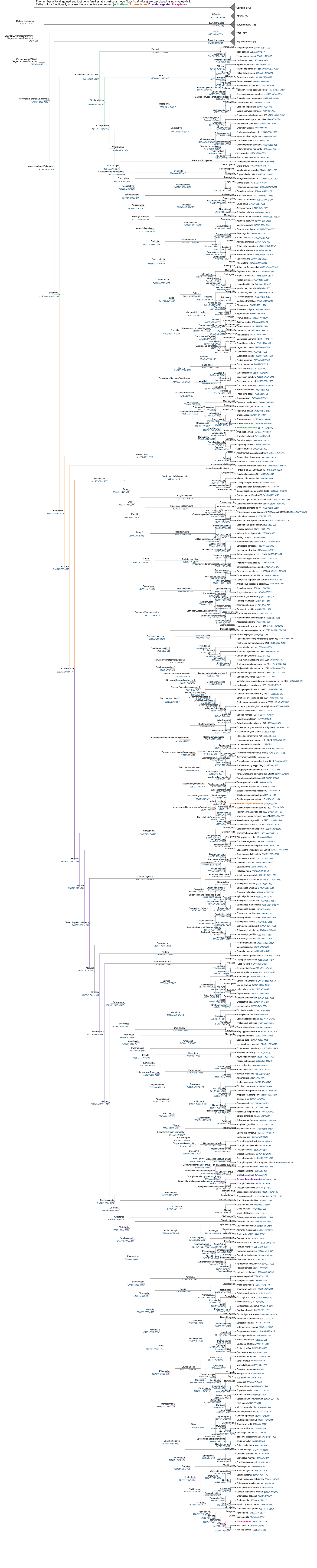

Supplement: Supplementary file 6 — Supplementary Dataset 1 [file 41467_2024_47017_MOESM6_ESM.pdf]
