# Supplementary material for: Macroevolutionary dynamics of gene family gain and loss along multicellular eukaryotic lineages
Source: Nat Commun. 2024 Mar 26;15:2663. doi: 10.1038/s41467-024-47017-w (PMC10966110; doi:10.1038/s41467-024-47017-w)
Supplement: Supplementary file 8 — Supplementary Dataset 3 [file 41467_2024_47017_MOESM8_ESM.pdf]

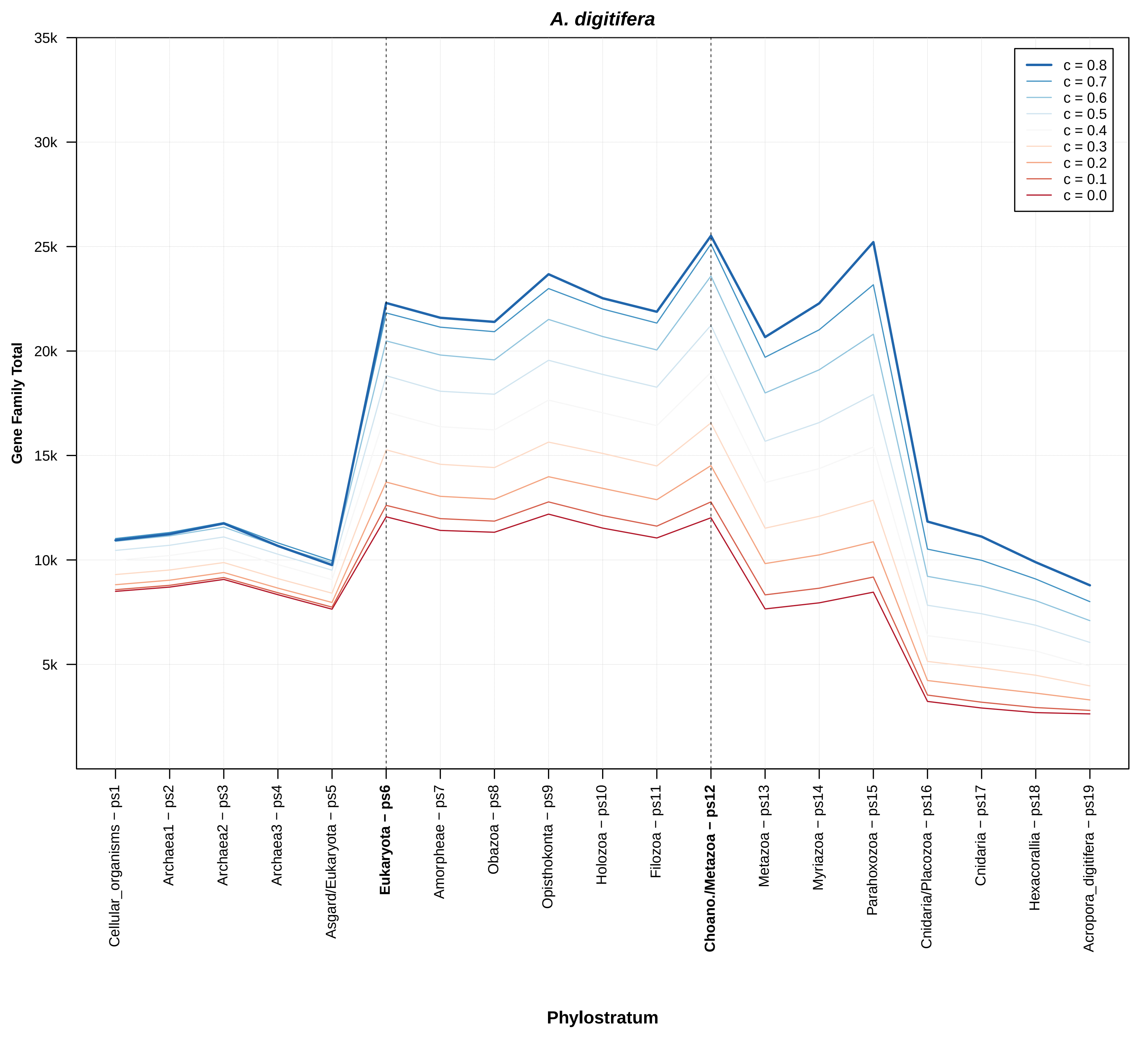
A. digitifera
Gene Family Total
35k
30k
25k
20k
15k
10k
5k
c = 0.8
c = 0.7
c = 0.6
c = 0.5
c = 0.4
c = 0.3
c = 0.2
c = 0.1
c = 0.0
Cellular_organisms – ps1
Archaea1 – ps2
Archaea2 – ps3
Archaea3 – ps4
Asgard/Eukaryota – ps5
Eukaryota – ps6
Amorpheae – ps7
Obazoa – ps8
Opisthokonta – ps9
Holozoa – ps10
Filozoa – ps11
Choano./Metazoa – ps12
Metazoa – ps13
Myriazoa – ps14
Parahoxozoa – ps15
Cnidaria/Placozoa – ps16
Cnidaria – ps17
Hexacorallia – ps18
Acropora_digitifera – ps19
Phylostratum

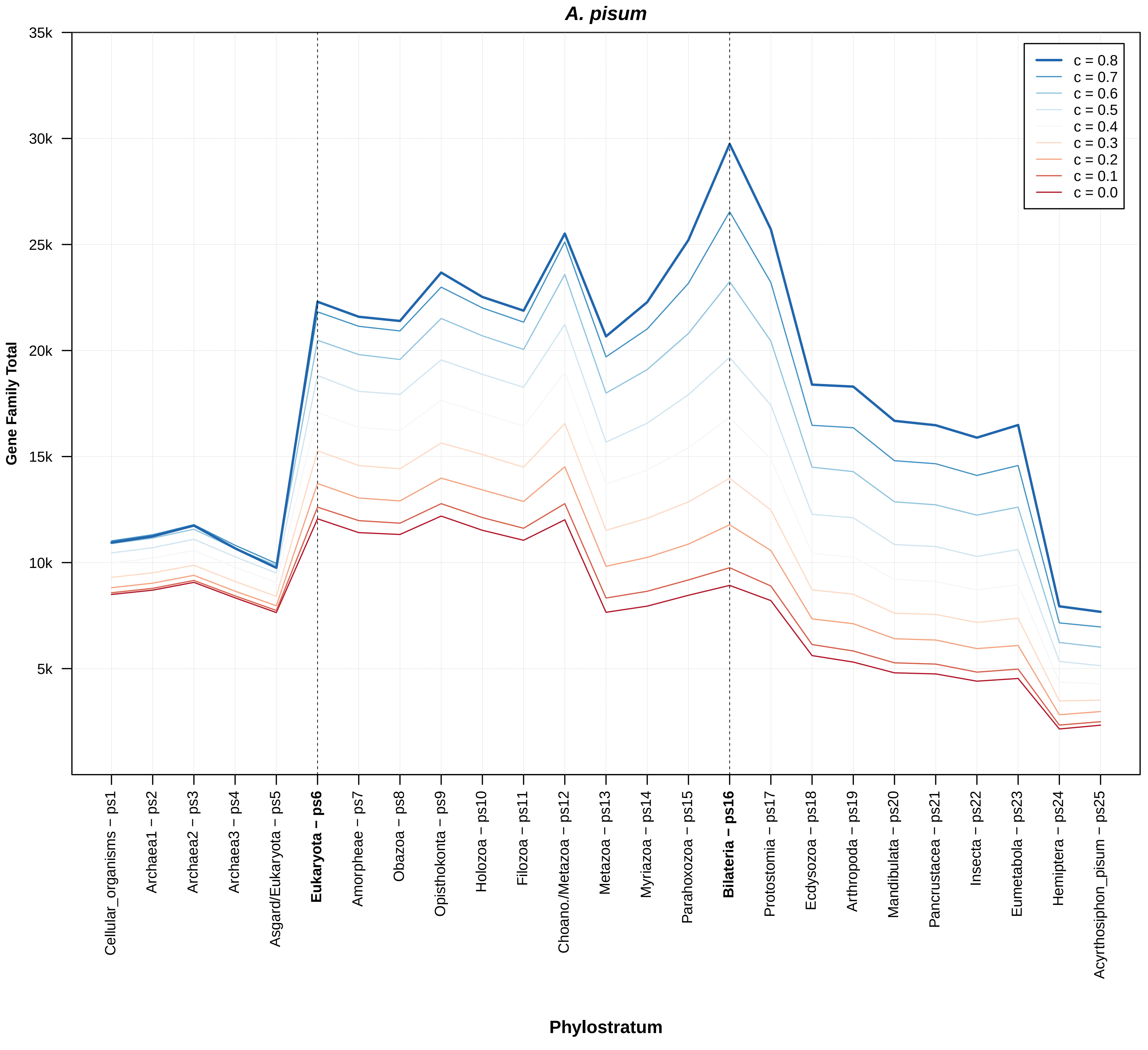
A. pisum
Gene Family Total
35k
30k
25k
20k
15k
10k
5k
c = 0.8
c = 0.7
c = 0.6
c = 0.5
c = 0.4
c = 0.3
c = 0.2
c = 0.1
c = 0.0
Cellular_organisms – ps1
Archaea1 – ps2
Archaea2 – ps3
Archaea3 – ps4
Asgard/Eukaryota – ps5
Eukaryota – ps6
Amorpheae – ps7
Obazoa – ps8
Opisthokonta – ps9
Holozoa – ps10
Filozoa – ps11
Choano./Metazoa – ps12
Metazoa – ps13
Myriazoa – ps14
Parahoxozoa – ps15
Bilateria – ps16
Protostomia – ps17
Ecdysozoa – ps18
Arthropoda – ps19
Mandibulata – ps20
Pancrustacea – ps21
Insecta – ps22
Eumetabola – ps23
Hemiptera – ps24
Acyrthosiphon_pisum – ps25
Phylostratum

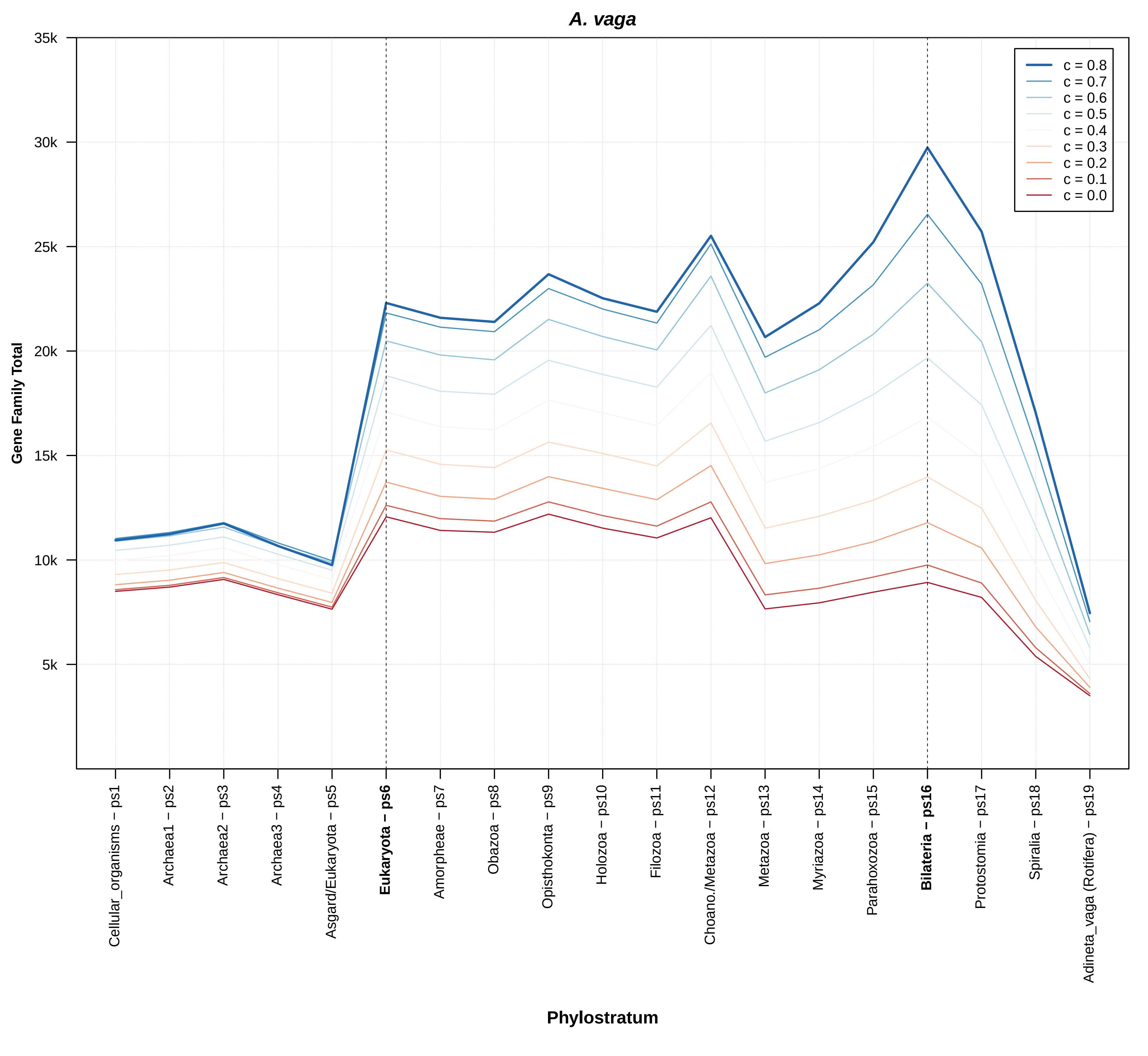

A. vaga
Gene Family Total
35k
30k
25k
20k
15k
10k
5k
c = 0.8
c = 0.7
c = 0.6
c = 0.5
c = 0.4
c = 0.3
c = 0.2
c = 0.1
c = 0.0
Cellular_organisms – ps1
Archaea1 – ps2
Archaea2 – ps3
Archaea3 – ps4
Asgard/Eukaryota – ps5
Eukaryota – ps6
Amorpheae – ps7
Obazoa – ps8
Opisthokonta – ps9
Holozoa – ps10
Filozoa – ps11
Choano./Metazoa – ps12
Metazoa – ps13
Myriazoa – ps14
Parahoxozoa – ps15
Bilateria – ps16
Protostomia – ps17
Spiralia – ps18
Adineta_vaga (Rotifera) – ps19
Phylostratum

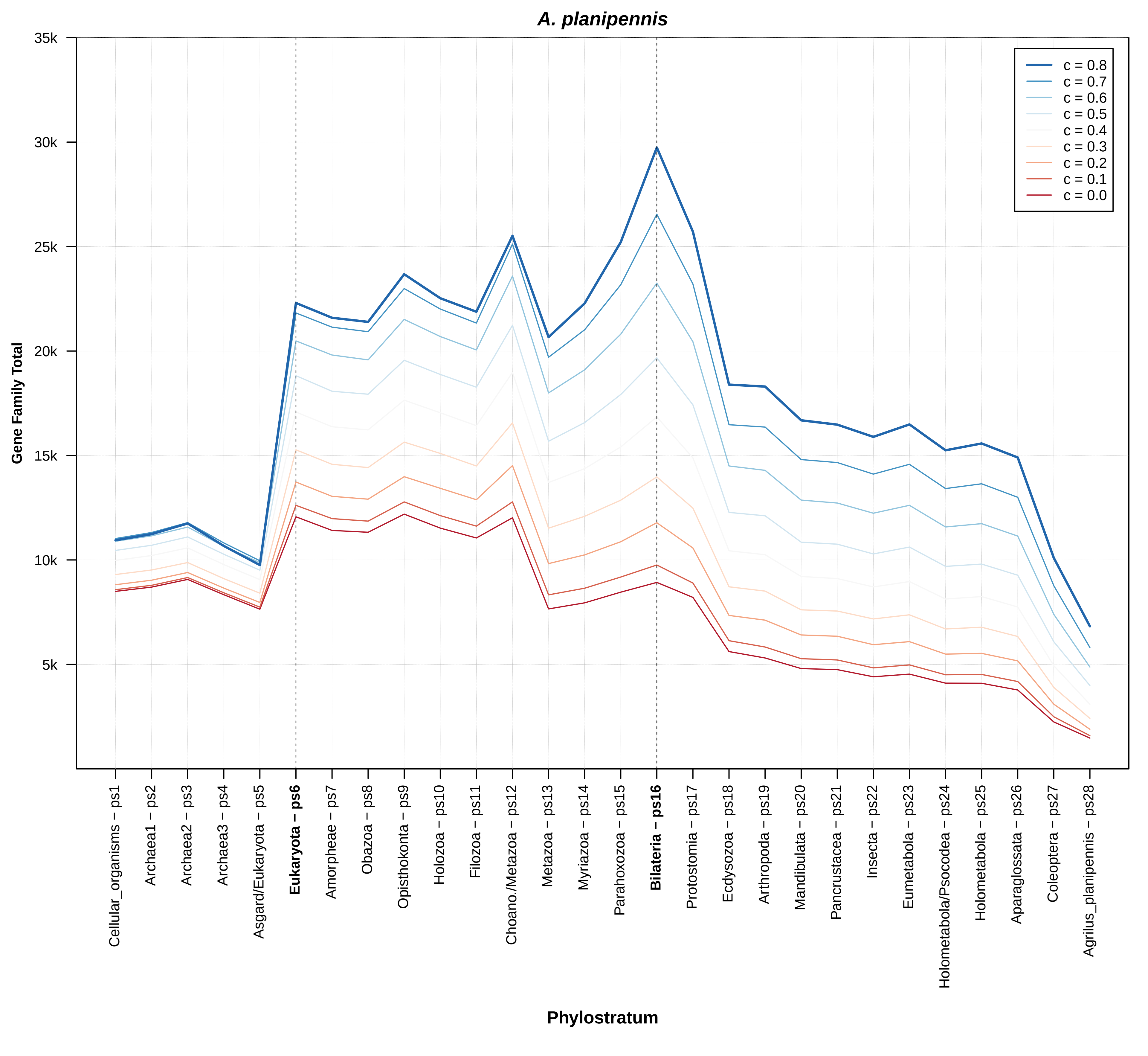
A. planipennis
Gene Family Total
35k
30k
25k
20k
15k
10k
5k
c = 0.8
c = 0.7
c = 0.6
c = 0.5
c = 0.4
c = 0.3
c = 0.2
c = 0.1
c = 0.0
Cellular_organisms – ps1
Archaea1 – ps2
Archaea2 – ps3
Archaea3 – ps4
Asgard/Eukaryota – ps5
Eukaryota – ps6
Amorpheae – ps7
Obazoa – ps8
Opisthokonta – ps9
Holozoa – ps10
Filozoa – ps11
Choano./Metazoa – ps12
Metazoa – ps13
Myriazoa – ps14
Parahoxozoa – ps15
Bilateria – ps16
Protostomia – ps17
Ecdysozoa – ps18
Arthropoda – ps19
Mandibulata – ps20
Pancrustacea – ps21
Insecta – ps22
Eumetabola – ps23
Holometabola/Psocodea – ps24
Holometabola – ps25
Aparaglossata – ps26
Coleoptera – ps27
Agrilus_planipennis – ps28
Phylostratum

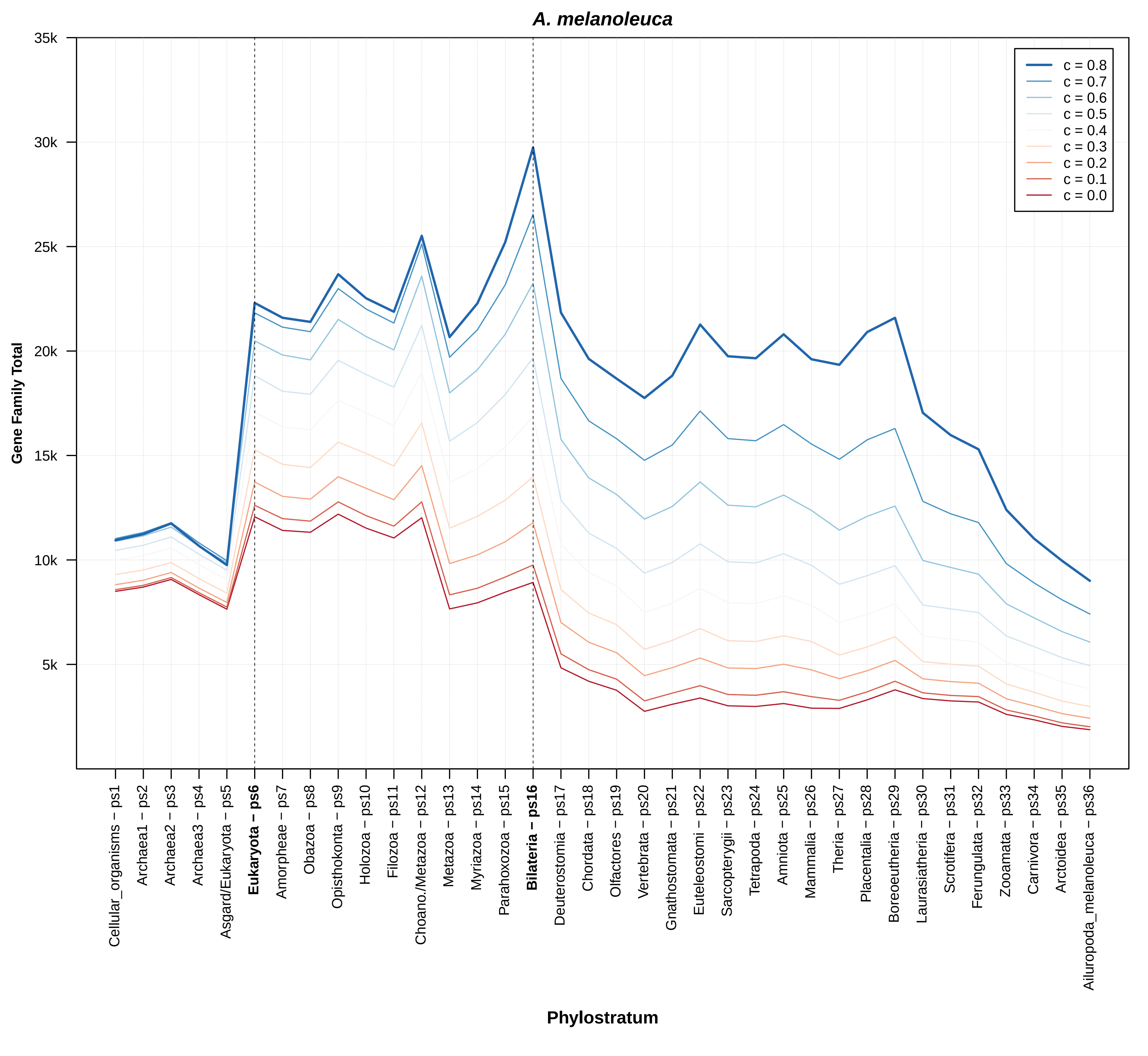

A. melanoleuca
35k
30k
25k
20k
15k
10k
5k
Gene Family Total
c = 0.8
c = 0.7
c = 0.6
c = 0.5
c = 0.4
c = 0.3
c = 0.2
c = 0.1
c = 0.0
Cellular_organisms – ps1
Archaea1 – ps2
Archaea2 – ps3
Archaea3 – ps4
Asgard/Eukaryota – ps5
Eukaryota – ps6
Amorpheae – ps7
Obazoa – ps8
Opisthokonta – ps9
Holozoa – ps10
Filozoa – ps11
Choano./Metazoa – ps12
Metazoa – ps13
Myriazoa – ps14
Parahoxozoa – ps15
Bilateria – ps16
Deuterostomia – ps17
Chordata – ps18
Olfactores – ps19
Vertebrata – ps20
Gnathostomata – ps21
Euteleostomi – ps22
Sarcopterygii – ps23
Tetrapoda – ps24
Amniota – ps25
Mammalia – ps26
Theria – ps27
Placentalia – ps28
Boreoeutheria – ps29
Laurasiatheria – ps30
Scrotifera – ps31
Ferungulata – ps32
Zooamata – ps33
Carnivora – ps34
Arctoidea – ps35
Ailuropoda_melanoleuca – ps36
Phylostratum

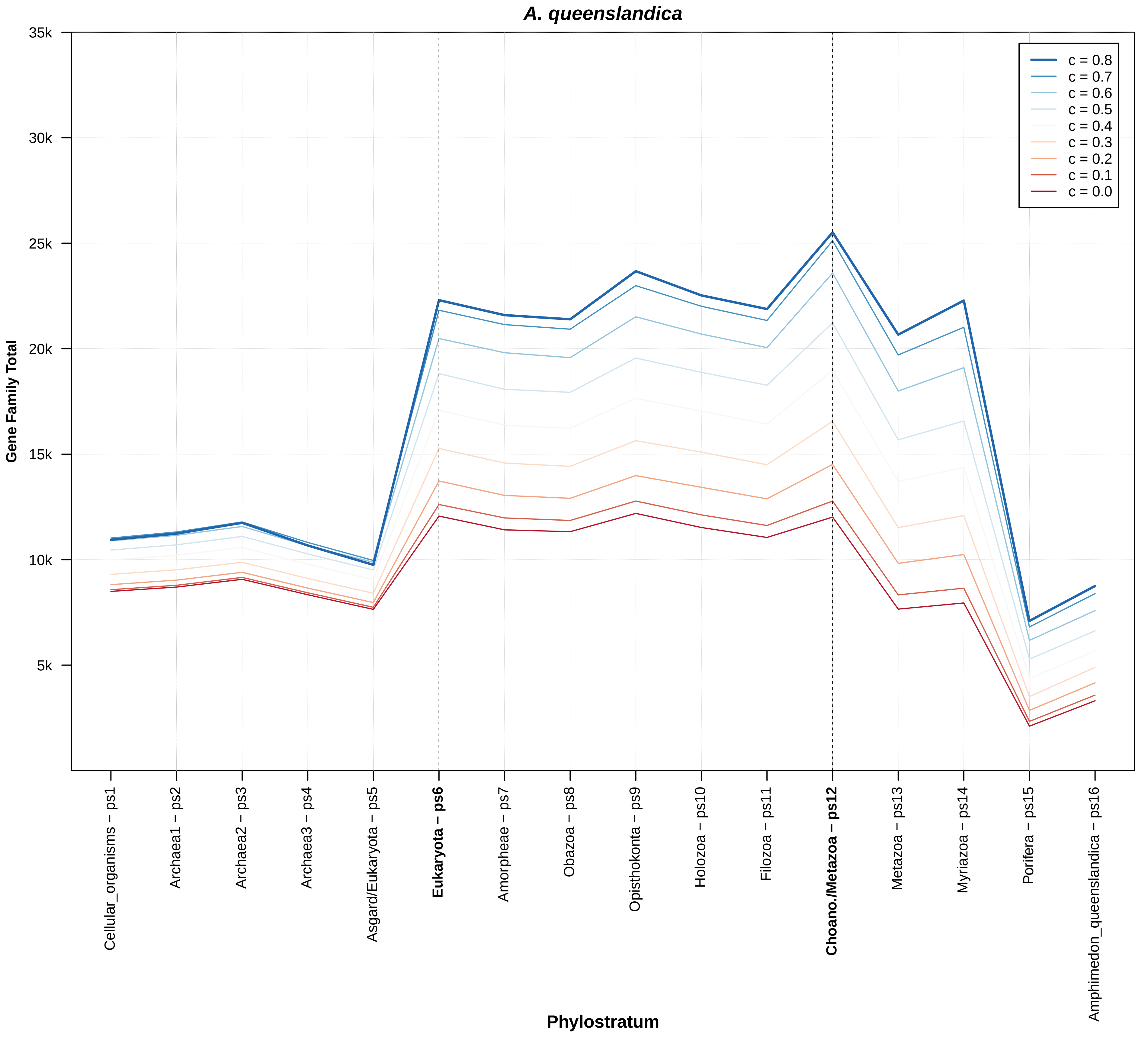
A. queenslandica
Gene Family Total
35k
30k
25k
20k
15k
10k
5k
c = 0.8
c = 0.7
c = 0.6
c = 0.5
c = 0.4
c = 0.3
c = 0.2
c = 0.1
c = 0.0
Cellular_organisms – ps1
Archaea1 – ps2
Archaea2 – ps3
Archaea3 – ps4
Asgard/Eukaryota – ps5
Eukaryota – ps6
Amorpheae – ps7
Obazoa – ps8
Opisthokonta – ps9
Holozoa – ps10
Filozoa – ps11
Choano./Metazoa – ps12
Metazoa – ps13
Myriazoa – ps14
Porifera – ps15
Amphimedon_queenslandica – ps16
Phylostratum

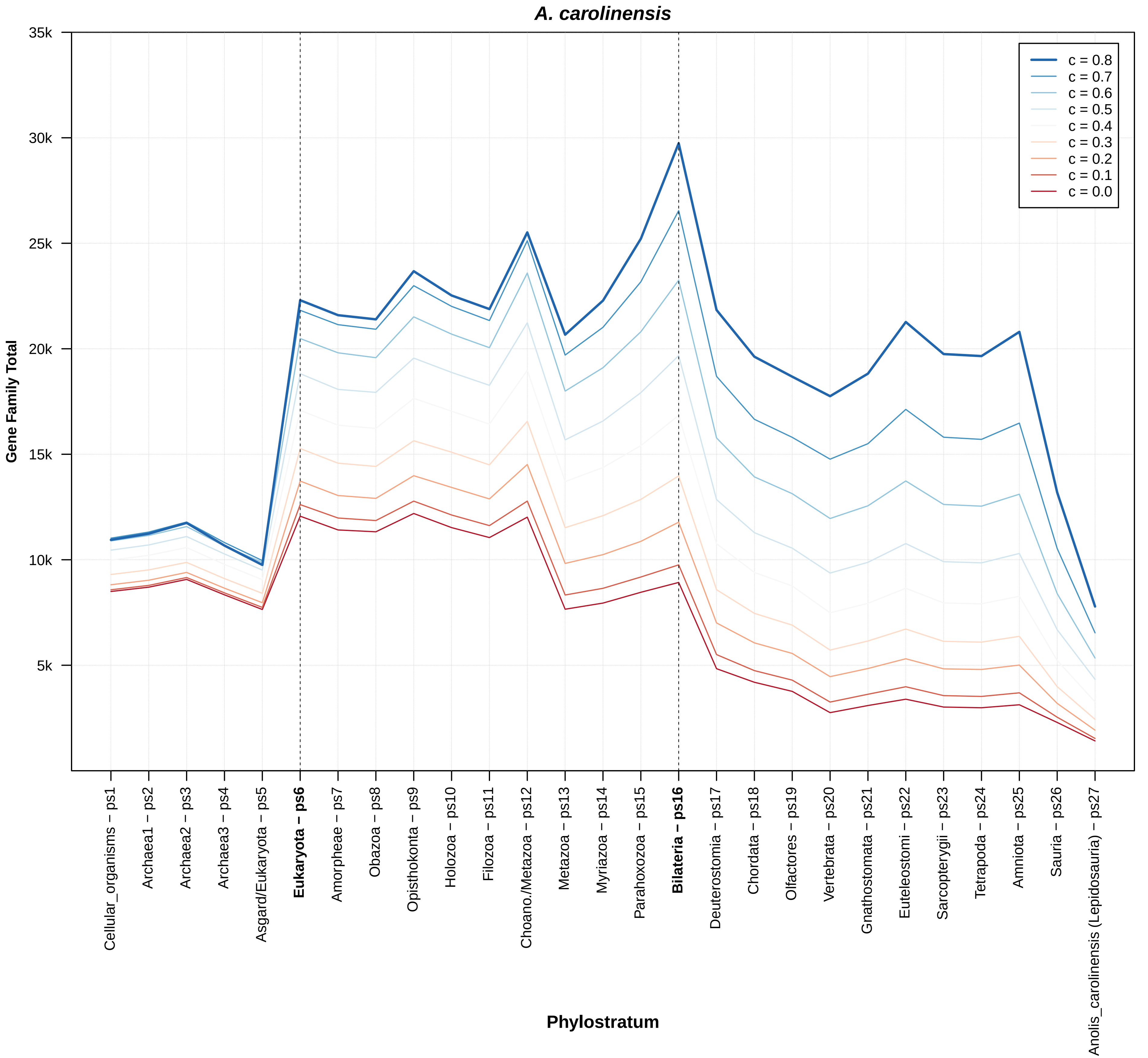

A. carolinensis
35k
30k
25k
20k
15k
10k
5k
Gene Family Total
c = 0.8
c = 0.7
c = 0.6
c = 0.5
c = 0.4
c = 0.3
c = 0.2
c = 0.1
c = 0.0
Cellular_organisms – ps1
Archaea1 – ps2
Archaea2 – ps3
Archaea3 – ps4
Asgard/Eukaryota – ps5
Eukaryota – ps6
Amorpheae – ps7
Obazoa – ps8
Opisthokonta – ps9
Holozoa – ps10
Filozoa – ps11
Choano./Metazoa – ps12
Metazoa – ps13
Myriazoa – ps14
Parahoxozoa – ps15
Bilateria – ps16
Deuterostomia – ps17
Chordata – ps18
Olfactores – ps19
Vertebrata – ps20
Gnathostomata – ps21
Euteleostomi – ps22
Sarcopterygii – ps23
Tetrapoda – ps24
Amniota – ps25
Sauria – ps26
Anolis_carolinensis (Lepidosauria) – ps27
Phylostratum

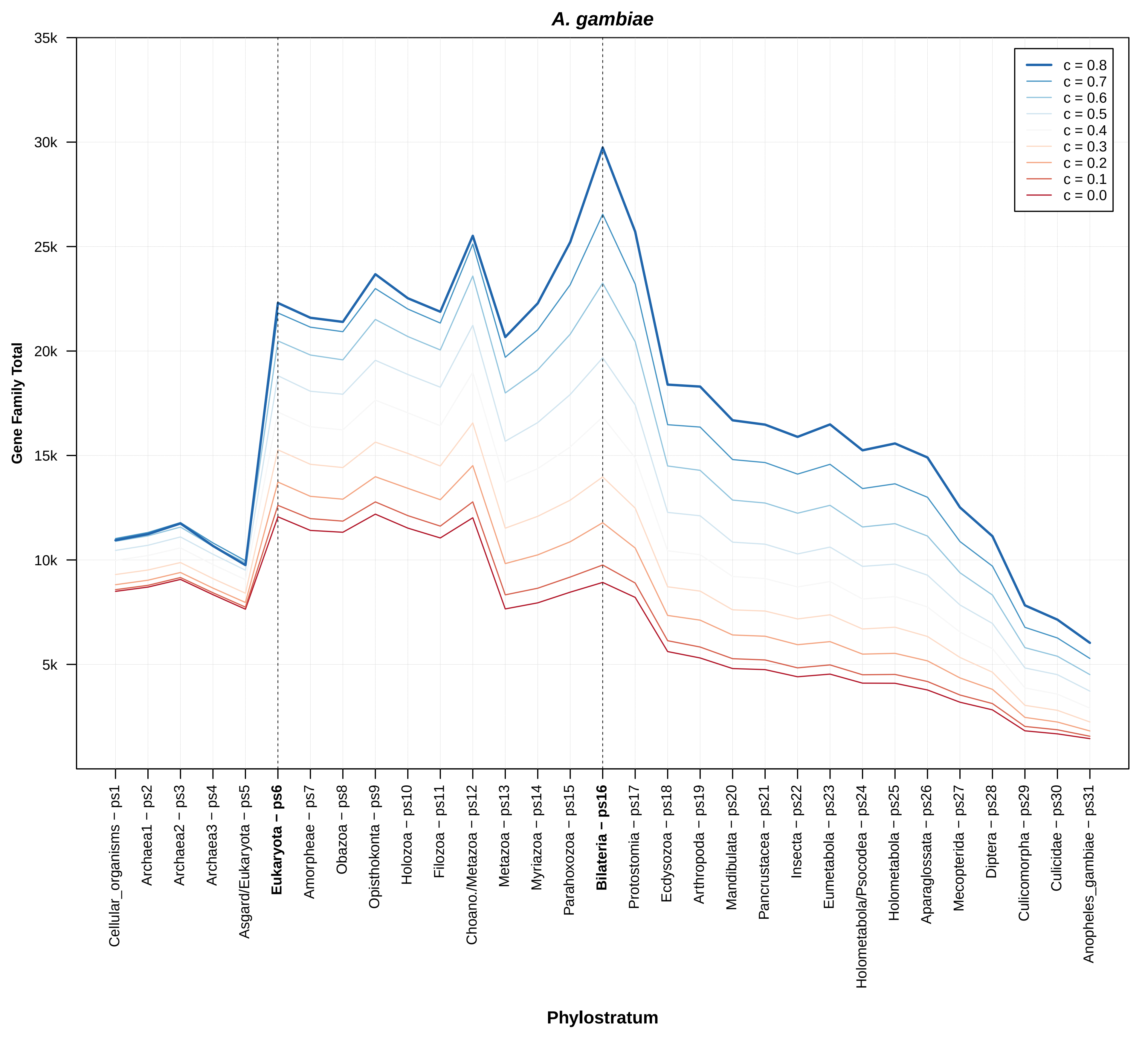
A. gambiae
Gene Family Total
35k
30k
25k
20k
15k
10k
5k
c = 0.8
c = 0.7
c = 0.6
c = 0.5
c = 0.4
c = 0.3
c = 0.2
c = 0.1
c = 0.0
Cellular_organisms – ps1
Archaea1 – ps2
Archaea2 – ps3
Archaea3 – ps4
Asgard/Eukaryota – ps5
Eukaryota – ps6
Amorpheae – ps7
Obazoa – ps8
Opisthokonta – ps9
Holozoa – ps10
Filozoa – ps11
Choano./Metazoa – ps12
Metazoa – ps13
Myriazoa – ps14
Parahoxozoa – ps15
Bilateria – ps16
Protostomia – ps17
Ecdysozoa – ps18
Arthropoda – ps19
Mandibulata – ps20
Pancrustacea – ps21
Insecta – ps22
Eumetabola – ps23
Holometabola/Psocodea – ps24
Holometabola – ps25
Aparaglossata – ps26
Mecopterida – ps27
Diptera – ps28
Culicomorpha – ps29
Culicidae – ps30
Anopheles_gambiae – ps31
Phylostratum

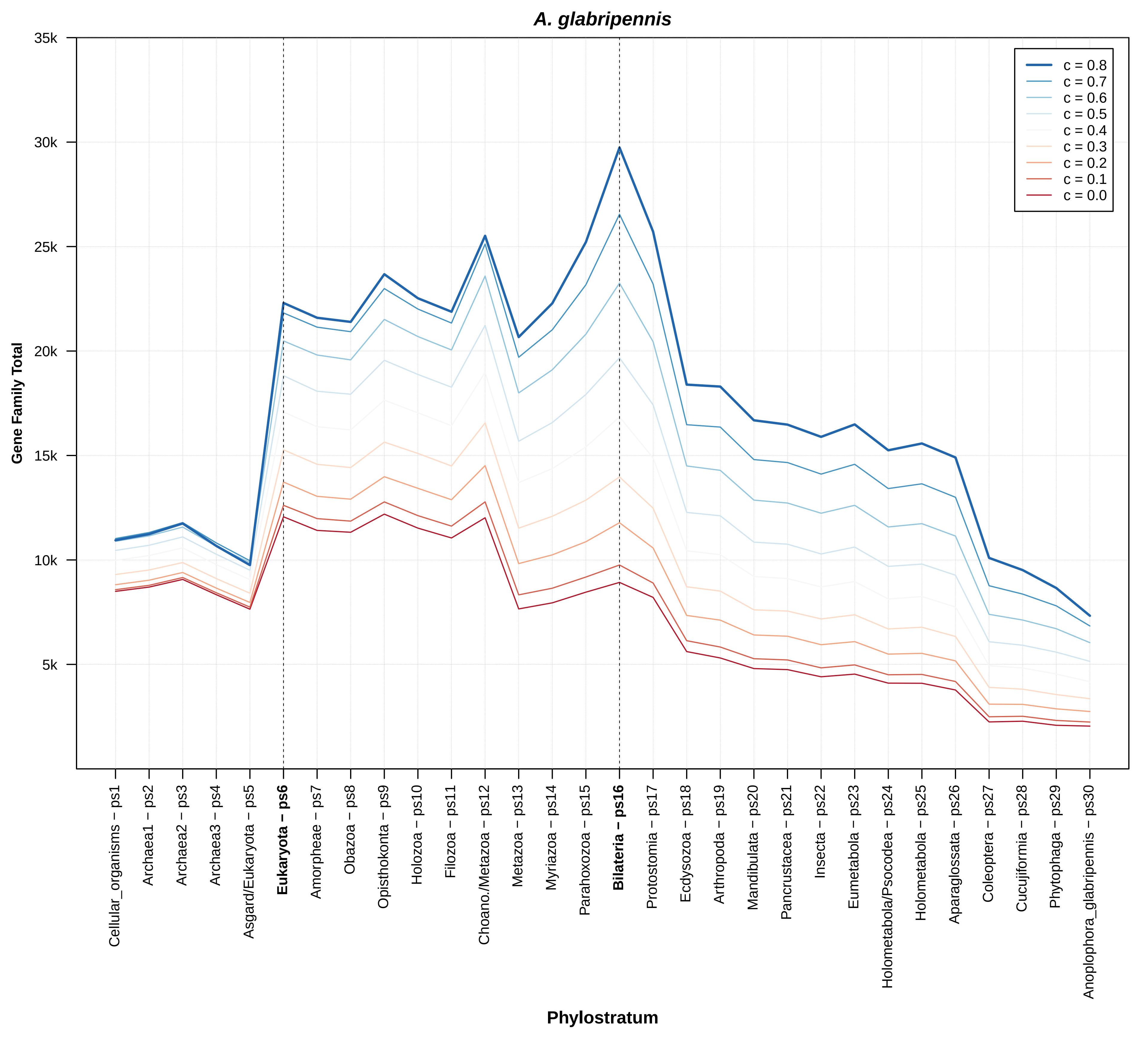
A. glabripennis
Gene Family Total
35k
30k
25k
20k
15k
10k
5k
c = 0.8
c = 0.7
c = 0.6
c = 0.5
c = 0.4
c = 0.3
c = 0.2
c = 0.1
c = 0.0
Cellular_organisms – ps1
Archaea1 – ps2
Archaea2 – ps3
Archaea3 – ps4
Asgard/Eukaryota – ps5
Eukaryota – ps6
Amorpheae – ps7
Obazoa – ps8
Opisthokonta – ps9
Holozoa – ps10
Filozoa – ps11
Choano./Metazoa – ps12
Metazoa – ps13
Myriazoa – ps14
Parahoxozoa – ps15
Bilateria – ps16
Protostomia – ps17
Ecdysozoa – ps18
Arthropoda – ps19
Mandibulata – ps20
Pancrustacea – ps21
Insecta – ps22
Eumetabola – ps23
Holometabola/Psocodea – ps24
Holometabola – ps25
Aparaglossata – ps26
Coleoptera – ps27
Cucujiformia – ps28
Phytophaga – ps29
Anoplophora_glabripennis – ps30
Phylostratum

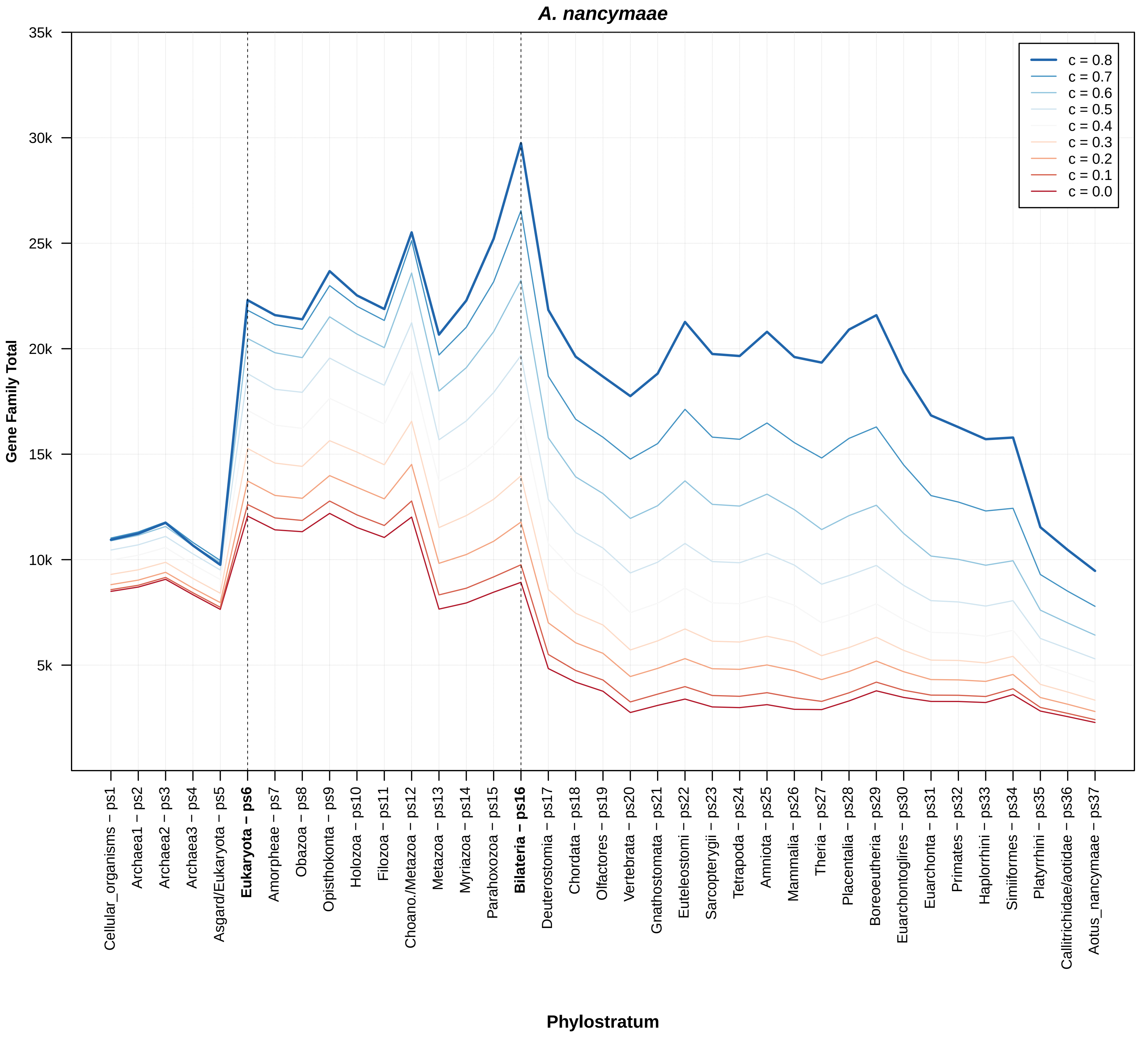

A. nancymaae
Gene Family Total
35k
30k
25k
20k
15k
10k
5k
c = 0.8
c = 0.7
c = 0.6
c = 0.5
c = 0.4
c = 0.3
c = 0.2
c = 0.1
c = 0.0
Cellular_organisms – ps1
Archaea1 – ps2
Archaea2 – ps3
Archaea3 – ps4
Asgard/Eukaryota – ps5
Eukaryota – ps6
Amorpheae – ps7
Obazoa – ps8
Opisthokonta – ps9
Holozoa – ps10
Filozoa – ps11
Choano./Metazoa – ps12
Metazoa – ps13
Myriazoa – ps14
Parahoxozoa – ps15
Bilateria – ps16
Deuterostomia – ps17
Chordata – ps18
Olfactores – ps19
Vertebrata – ps20
Gnathostomata – ps21
Euteleostomi – ps22
Sarcopterygii – ps23
Tetrapoda – ps24
Amniota – ps25
Mammalia – ps26
Theria – ps27
Placentalia – ps28
Boreoeutheria – ps29
Euarchontoglires – ps30
Euarchonta – ps31
Primates – ps32
Haplorrhini – ps33
Simiiformes – ps34
Platyrrhini – ps35
Callitrichidae/aotidae – ps36
Aotus_nancymaae – ps37
Phylostratum

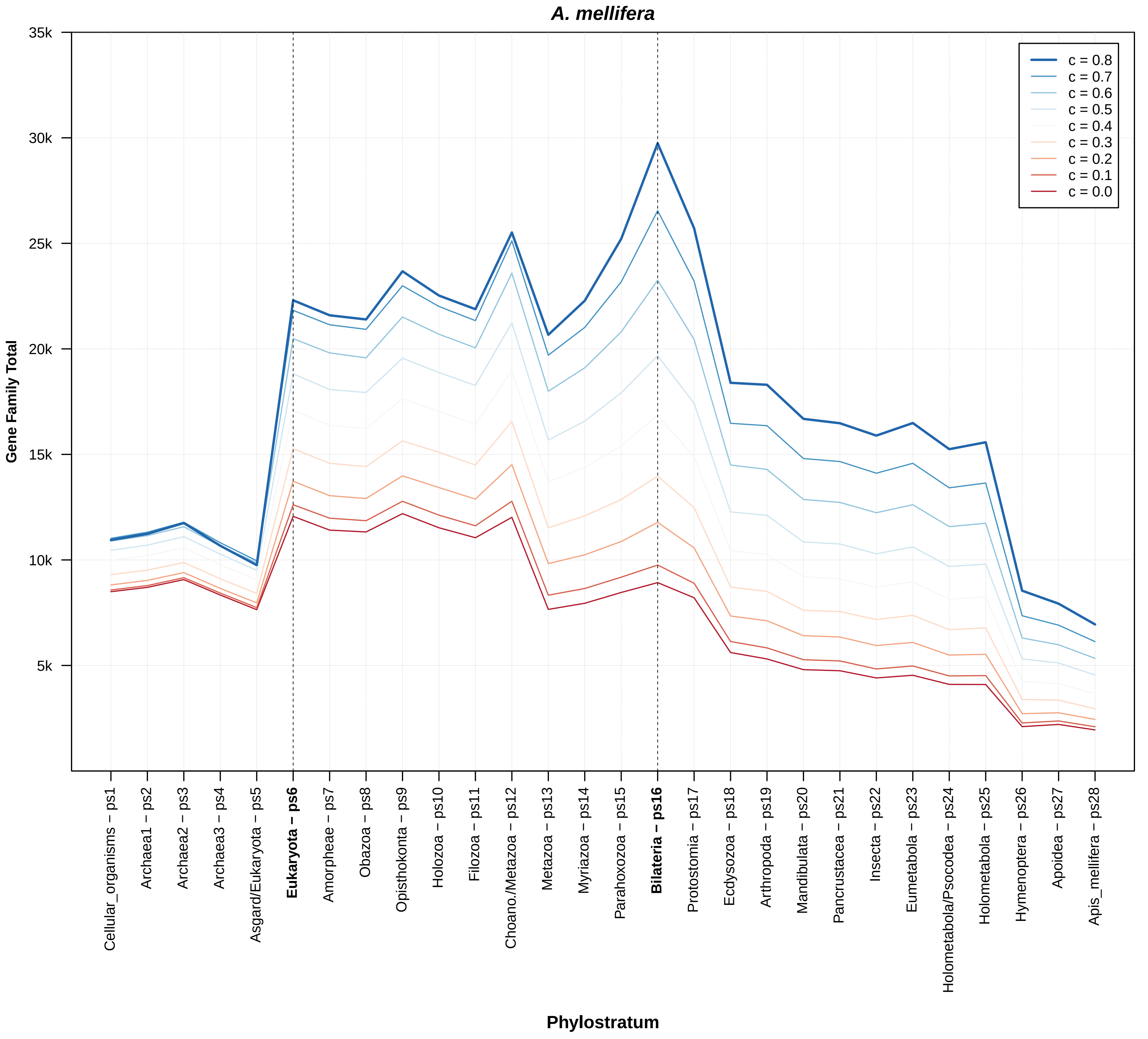

A. mellifera
Gene Family Total
Phylostratum
35k
30k
25k
20k
15k
10k
5k
c = 0.8
c = 0.7
c = 0.6
c = 0.5
c = 0.4
c = 0.3
c = 0.2
c = 0.1
c = 0.0
Cellular_organisms – ps1
Archaea1 – ps2
Archaea2 – ps3
Archaea3 – ps4
Asgard/Eukaryota – ps5
Eukaryota – ps6
Amorpheae – ps7
Obazoa – ps8
Opisthokonta – ps9
Holozoa – ps10
Filozoa – ps11
Choano./Metazoa – ps12
Metazoa – ps13
Myriazoa – ps14
Parahoxozoa – ps15
Bilateria – ps16
Protostomia – ps17
Ecdysozoa – ps18
Arthropoda – ps19
Mandibulata – ps20
Pancrustacea – ps21
Insecta – ps22
Eumetabola – ps23
Holometabola/Psocodea – ps24
Holometabola – ps25
Hymenoptera – ps26
Apoidea – ps27
Apis_mellifera – ps28

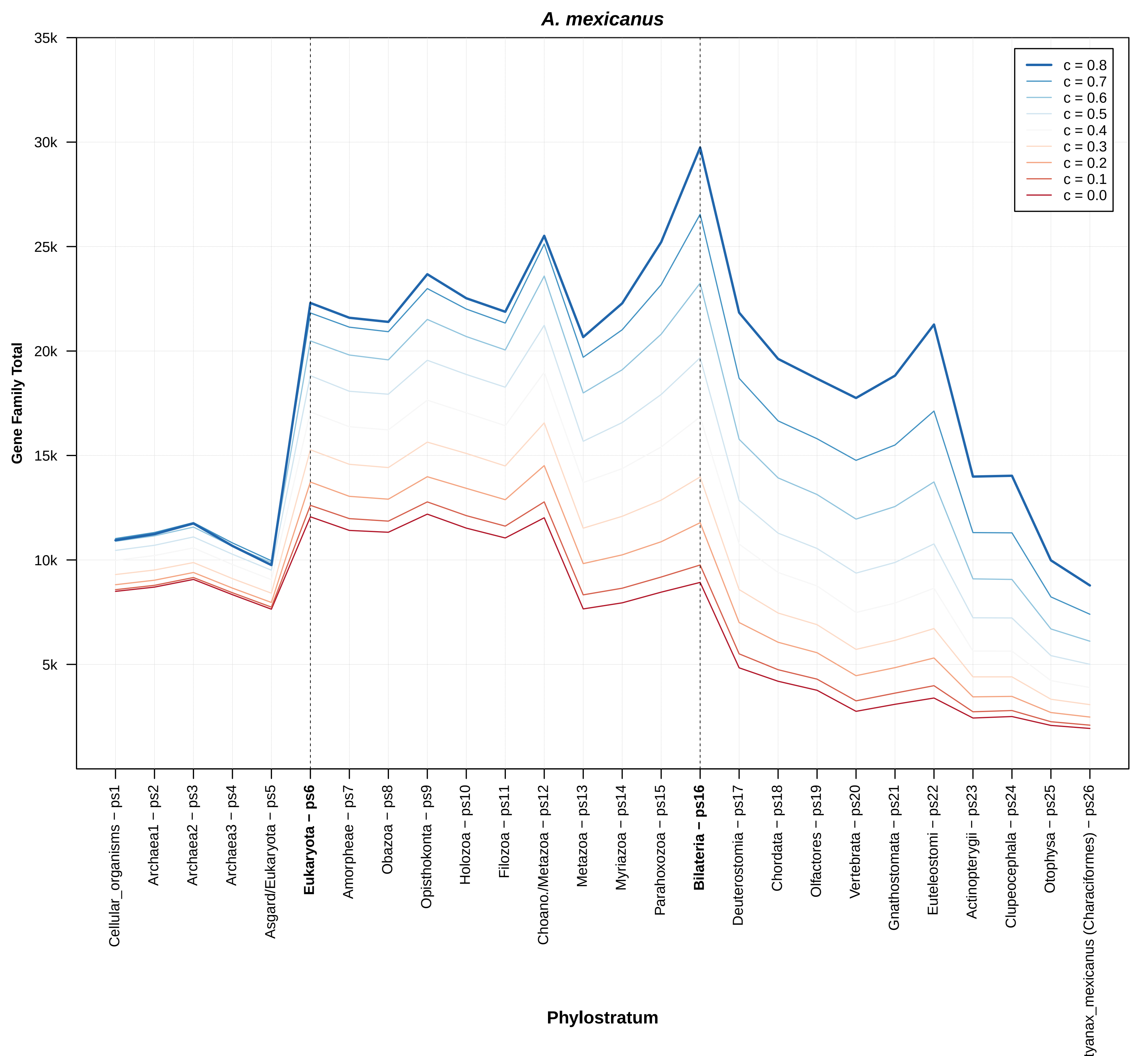

A. mexicanus
Gene Family Total
35k
30k
25k
20k
15k
10k
5k
Phylostratum
c = 0.8
c = 0.7
c = 0.6
c = 0.5
c = 0.4
c = 0.3
c = 0.2
c = 0.1
c = 0.0
Cellular_organisms – ps1
Archaea1 – ps2
Archaea2 – ps3
Archaea3 – ps4
Asgard/Eukaryota – ps5
Eukaryota – ps6
Amorpheae – ps7
Obazoa – ps8
Opisthokonta – ps9
Holozoa – ps10
Filozoa – ps11
Choano./Metazoa – ps12
Metazoa – ps13
Myriazoa – ps14
Parahoxozoa – ps15
Bilateria – ps16
Deuterostomia – ps17
Chordata – ps18
Olfactores – ps19
Vertebrata – ps20
Gnathostomata – ps21
Euteleostomi – ps22
Actinopterygii – ps23
Clupeocephala – ps24
Otophysa – ps25
tyanax_mexicanus (Characiformes) – ps26

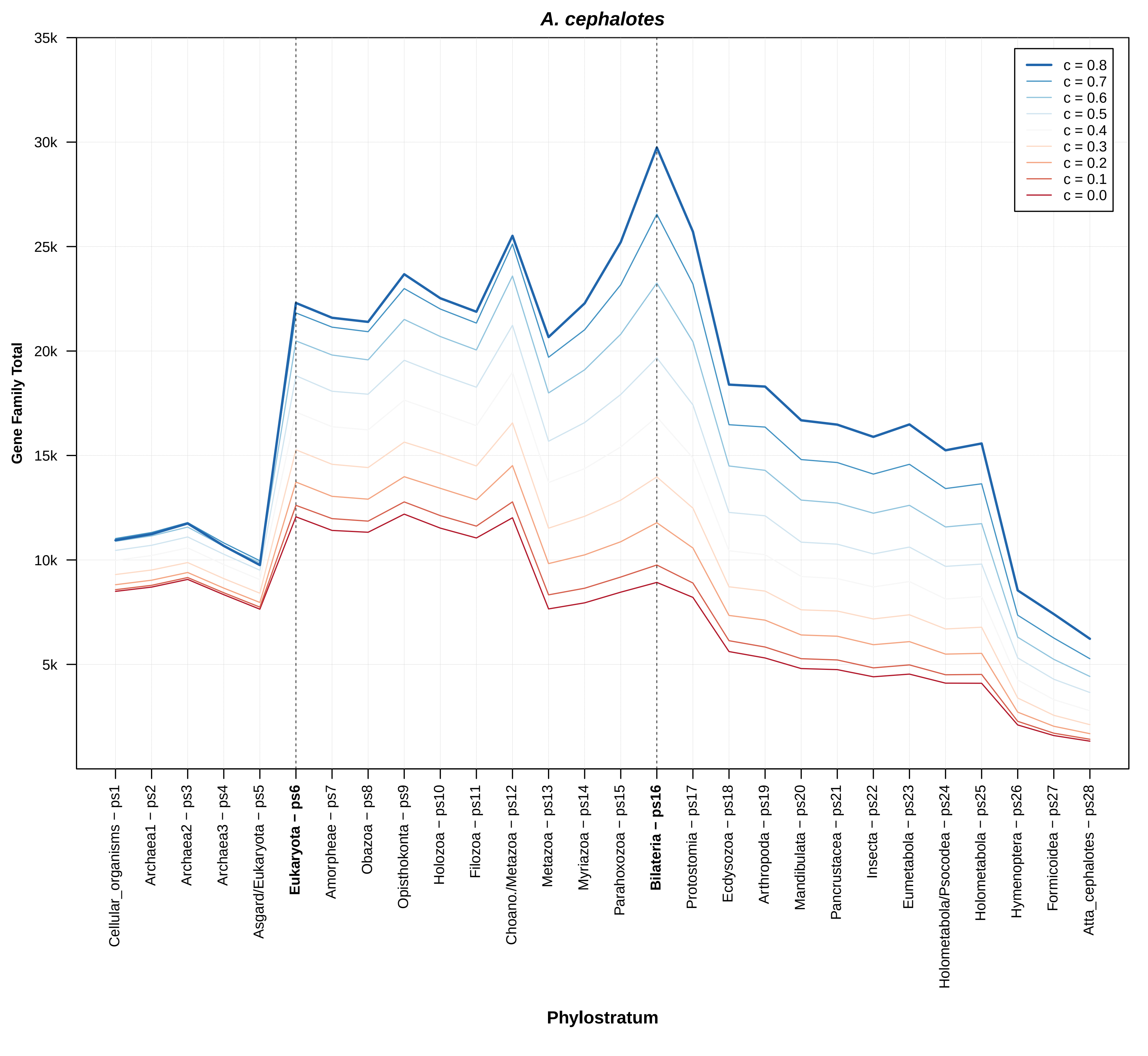

A. cephalotes
Gene Family Total
35k
30k
25k
20k
15k
10k
5k
c = 0.8
c = 0.7
c = 0.6
c = 0.5
c = 0.4
c = 0.3
c = 0.2
c = 0.1
c = 0.0
Cellular_organisms – ps1
Archaea1 – ps2
Archaea2 – ps3
Archaea3 – ps4
Asgard/Eukaryota – ps5
Eukaryota – ps6
Amorpheae – ps7
Obazoa – ps8
Opisthokonta – ps9
Holozoa – ps10
Filozoa – ps11
Choano./Metazoa – ps12
Metazoa – ps13
Myriazoa – ps14
Parahoxozoa – ps15
Bilateria – ps16
Protostomia – ps17
Ecdysozoa – ps18
Arthropoda – ps19
Mandibulata – ps20
Pancrustacea – ps21
Insecta – ps22
Eumetabola – ps23
Holometabola/Psocodea – ps24
Holometabola – ps25
Hymenoptera – ps26
Formicoidea – ps27
Atta_cephalotes – ps28
Phylostratum

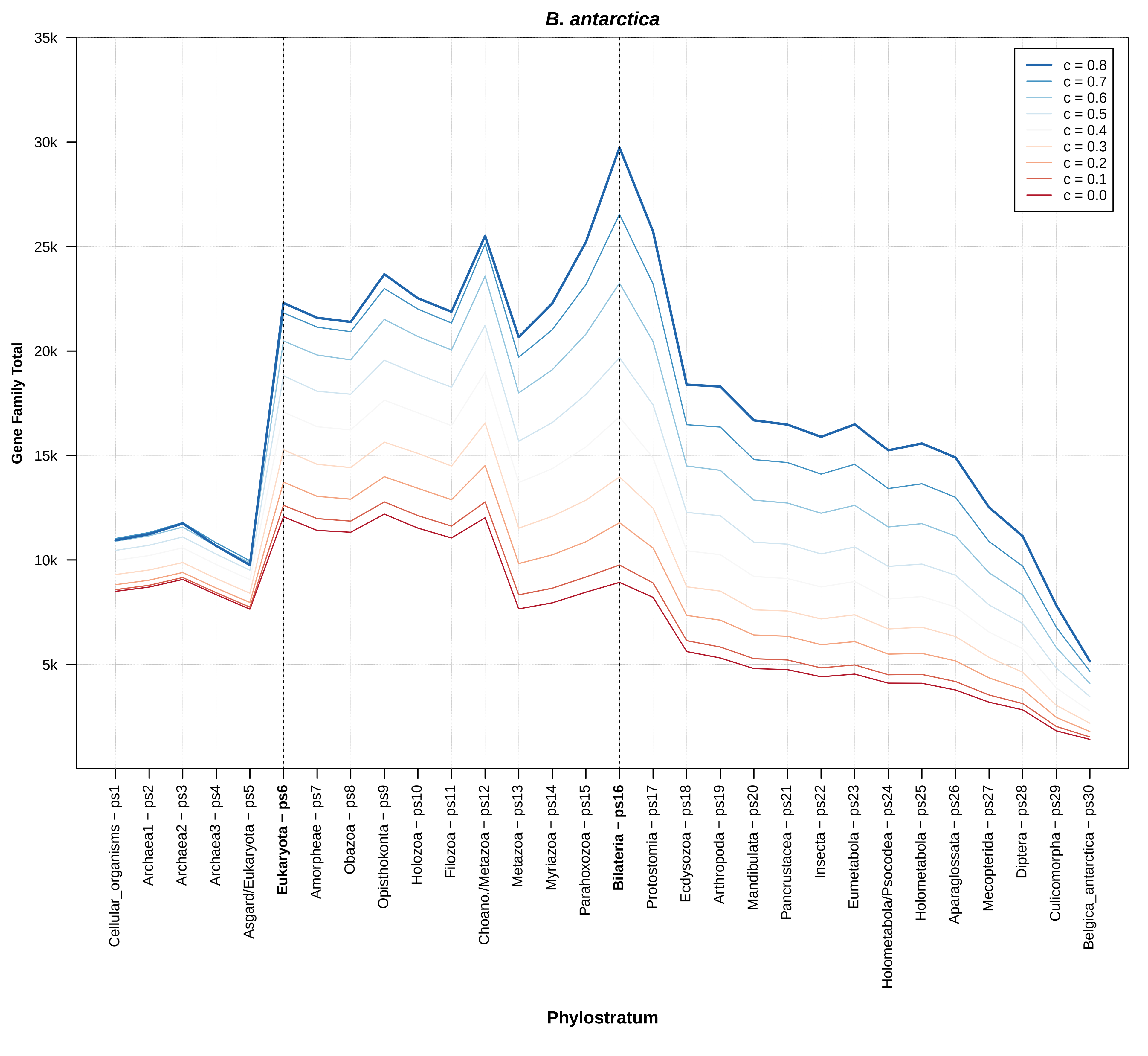
B. antarctica
Gene Family Total
Phylostratum
35k
30k
25k
20k
15k
10k
5k
c = 0.8
c = 0.7
c = 0.6
c = 0.5
c = 0.4
c = 0.3
c = 0.2
c = 0.1
c = 0.0
Cellular_organisms – ps1
Archaea1 – ps2
Archaea2 – ps3
Archaea3 – ps4
Asgard/Eukaryota – ps5
Eukaryota – ps6
Amorpheae – ps7
Obazoa – ps8
Opisthokonta – ps9
Holozoa – ps10
Filozoa – ps11
Choano./Metazoa – ps12
Metazoa – ps13
Myriazoa – ps14
Parahoxozoa – ps15
Bilateria – ps16
Protostomia – ps17
Ecdysozoa – ps18
Arthropoda – ps19
Mandibulata – ps20
Pancrustacea – ps21
Insecta – ps22
Eumetabola – ps23
Holometabola/Psocodea – ps24
Holometabola – ps25
Aparaglossata – ps26
Mecopterida – ps27
Diptera – ps28
Culicomorpha – ps29
Belgica_antarctica – ps30

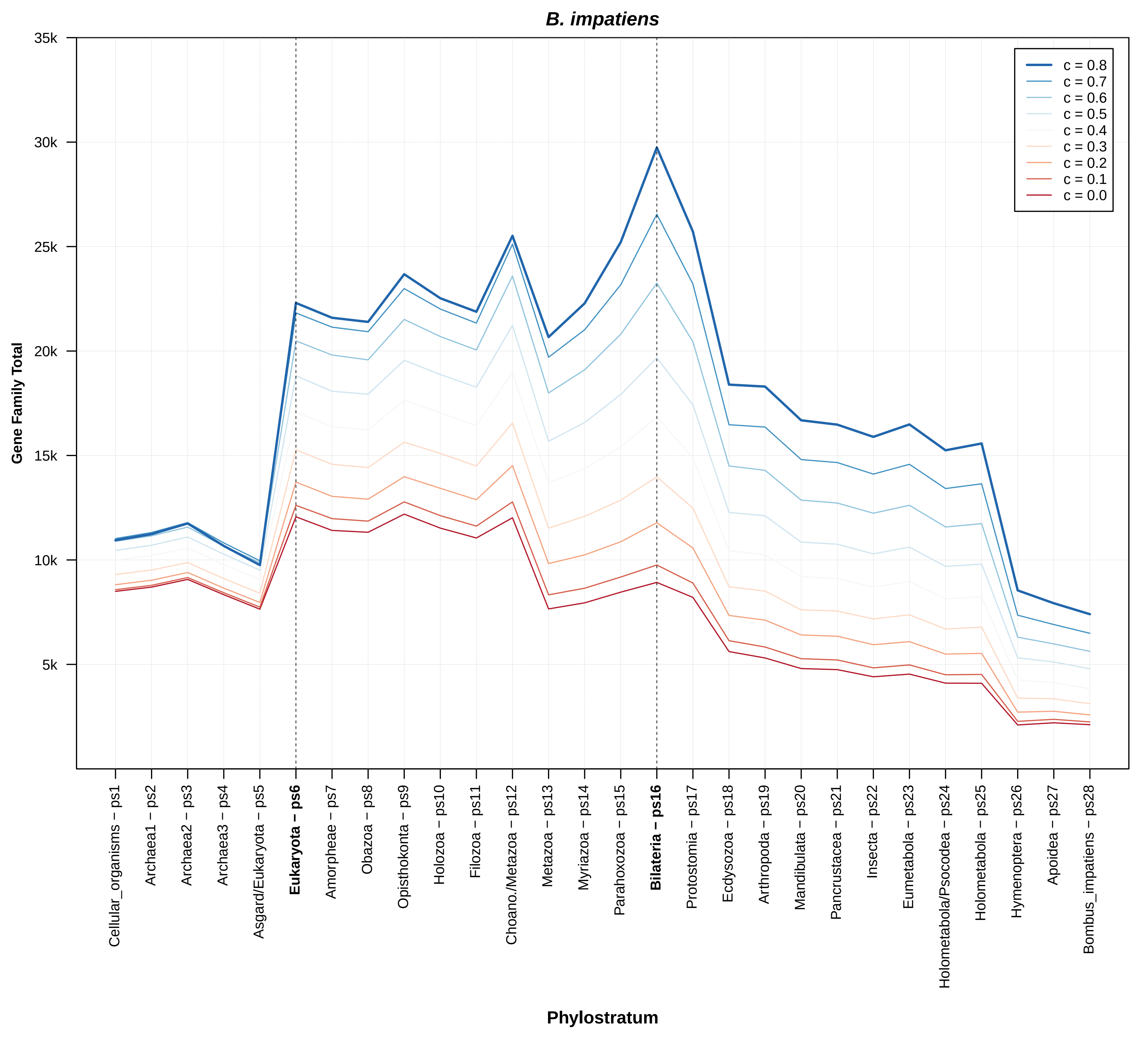

B. impatiens
35k
30k
25k
20k
15k
10k
5k
Gene Family Total
c = 0.8
c = 0.7
c = 0.6
c = 0.5
c = 0.4
c = 0.3
c = 0.2
c = 0.1
c = 0.0
Cellular_organisms – ps1
Archaea1 – ps2
Archaea2 – ps3
Archaea3 – ps4
Asgard/Eukaryota – ps5
Eukaryota – ps6
Amorpheae – ps7
Obazoa – ps8
Opisthokonta – ps9
Holozoa – ps10
Filozoa – ps11
Choano./Metazoa – ps12
Metazoa – ps13
Myriazoa – ps14
Parahoxozoa – ps15
Bilateria – ps16
Protostomia – ps17
Ecdysozoa – ps18
Arthropoda – ps19
Mandibulata – ps20
Pancrustacea – ps21
Insecta – ps22
Eumetabola – ps23
Holometabola/Psocodea – ps24
Holometabola – ps25
Hymenoptera – ps26
Apoidea – ps27
Bombus_impatiens – ps28
Phylostratum

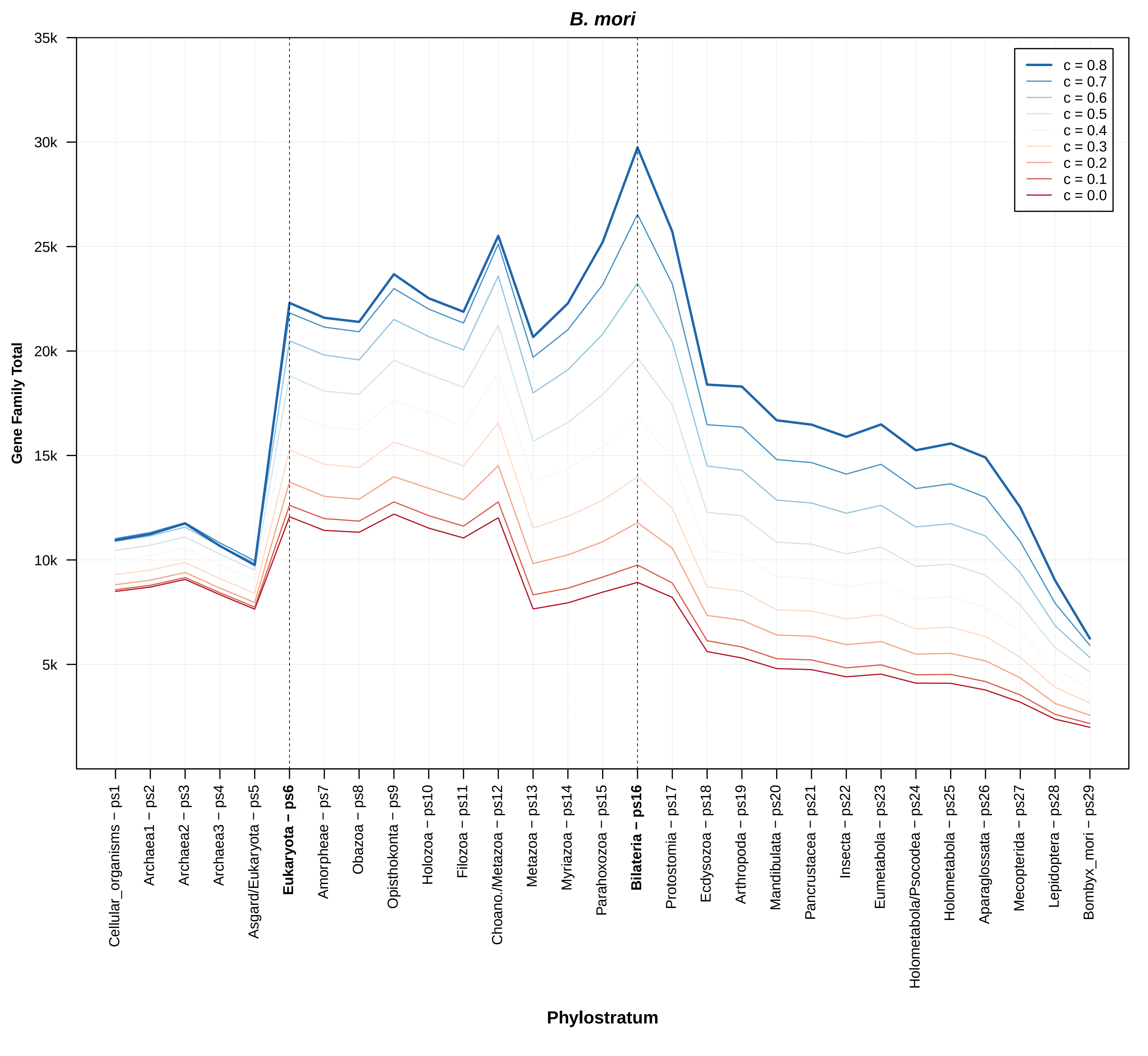
B. mori
Gene Family Total
35k
30k
25k
20k
15k
10k
5k
c = 0.8
c = 0.7
c = 0.6
c = 0.5
c = 0.4
c = 0.3
c = 0.2
c = 0.1
c = 0.0
Cellular_organisms – ps1
Archaea1 – ps2
Archaea2 – ps3
Archaea3 – ps4
Asgard/Eukaryota – ps5
Eukaryota – ps6
Amorpheae – ps7
Obazoa – ps8
Opisthokonta – ps9
Holozoa – ps10
Filozoa – ps11
Choano./Metazoa – ps12
Metazoa – ps13
Myriazoa – ps14
Parahoxozoa – ps15
Bilateria – ps16
Protostomia – ps17
Ecdysozoa – ps18
Arthropoda – ps19
Mandibulata – ps20
Pancrustacea – ps21
Insecta – ps22
Eumetabola – ps23
Holometabola/Psocodea – ps24
Holometabola – ps25
Aparaglossata – ps26
Mecopterida – ps27
Lepidoptera – ps28
Bombyx_mori – ps29
Phylostratum

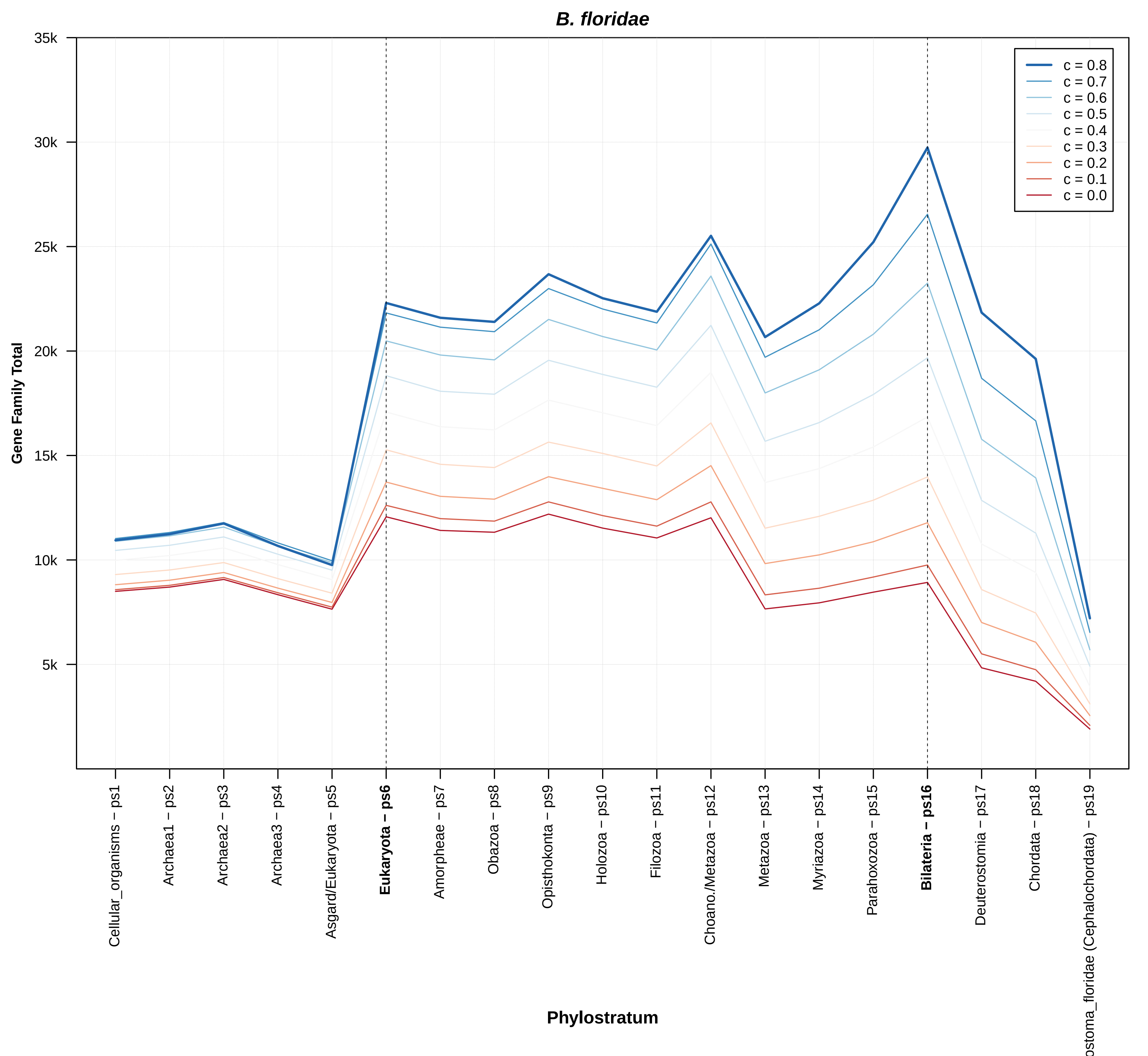
B. floridae
35k
30k
25k
20k
15k
10k
5k
Gene Family Total
c = 0.8
c = 0.7
c = 0.6
c = 0.5
c = 0.4
c = 0.3
c = 0.2
c = 0.1
c = 0.0
Cellular_organisms – ps1
Archaea1 – ps2
Archaea2 – ps3
Archaea3 – ps4
Asgard/Eukaryota – ps5
Eukaryota – ps6
Amorpheae – ps7
Obazoa – ps8
Opisthokonta – ps9
Holozoa – ps10
Filozoa – ps11
Choano./Metazoa – ps12
Metazoa – ps13
Myriazoa – ps14
Parahoxozoa – ps15
Bilateria – ps16
Deuterostomia – ps17
Chordata – ps18
ostoma_floridae (Cephalochordata) – ps19
Phylostratum

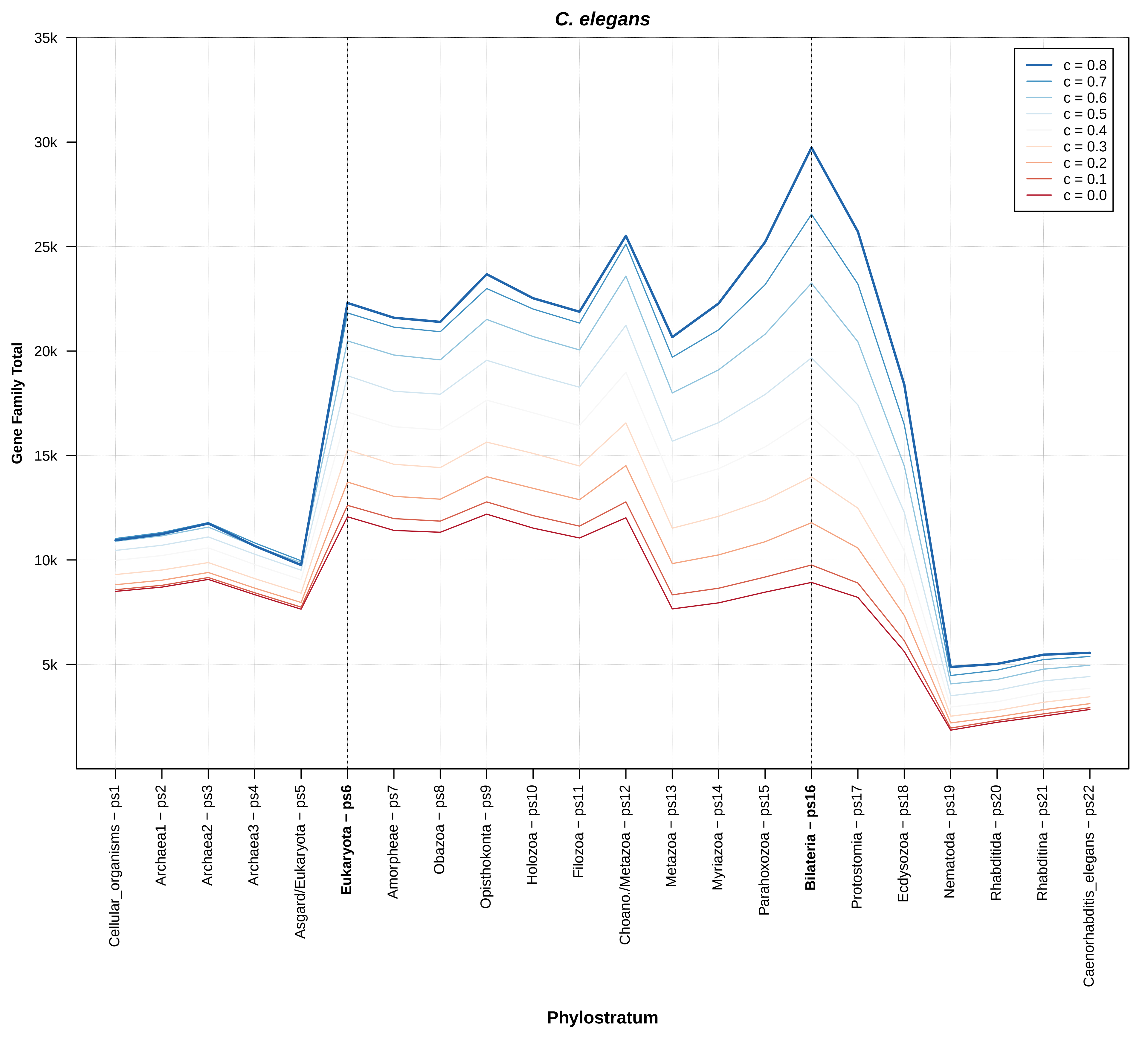

C. elegans
Gene Family Total
35k
30k
25k
20k
15k
10k
5k
c = 0.8
c = 0.7
c = 0.6
c = 0.5
c = 0.4
c = 0.3
c = 0.2
c = 0.1
c = 0.0
Cellular_organisms – ps1
Archaea1 – ps2
Archaea2 – ps3
Archaea3 – ps4
Asgard/Eukaryota – ps5
Eukaryota – ps6
Amorpheae – ps7
Obazoa – ps8
Opisthokonta – ps9
Holozoa – ps10
Filozoa – ps11
Choano./Metazoa – ps12
Metazoa – ps13
Myriazoa – ps14
Parahoxozoa – ps15
Bilateria – ps16
Protostomia – ps17
Ecdysozoa – ps18
Nematoda – ps19
Rhabditida – ps20
Rhabditina – ps21
Caenorhabditis_elegans – ps22
Phylostratum

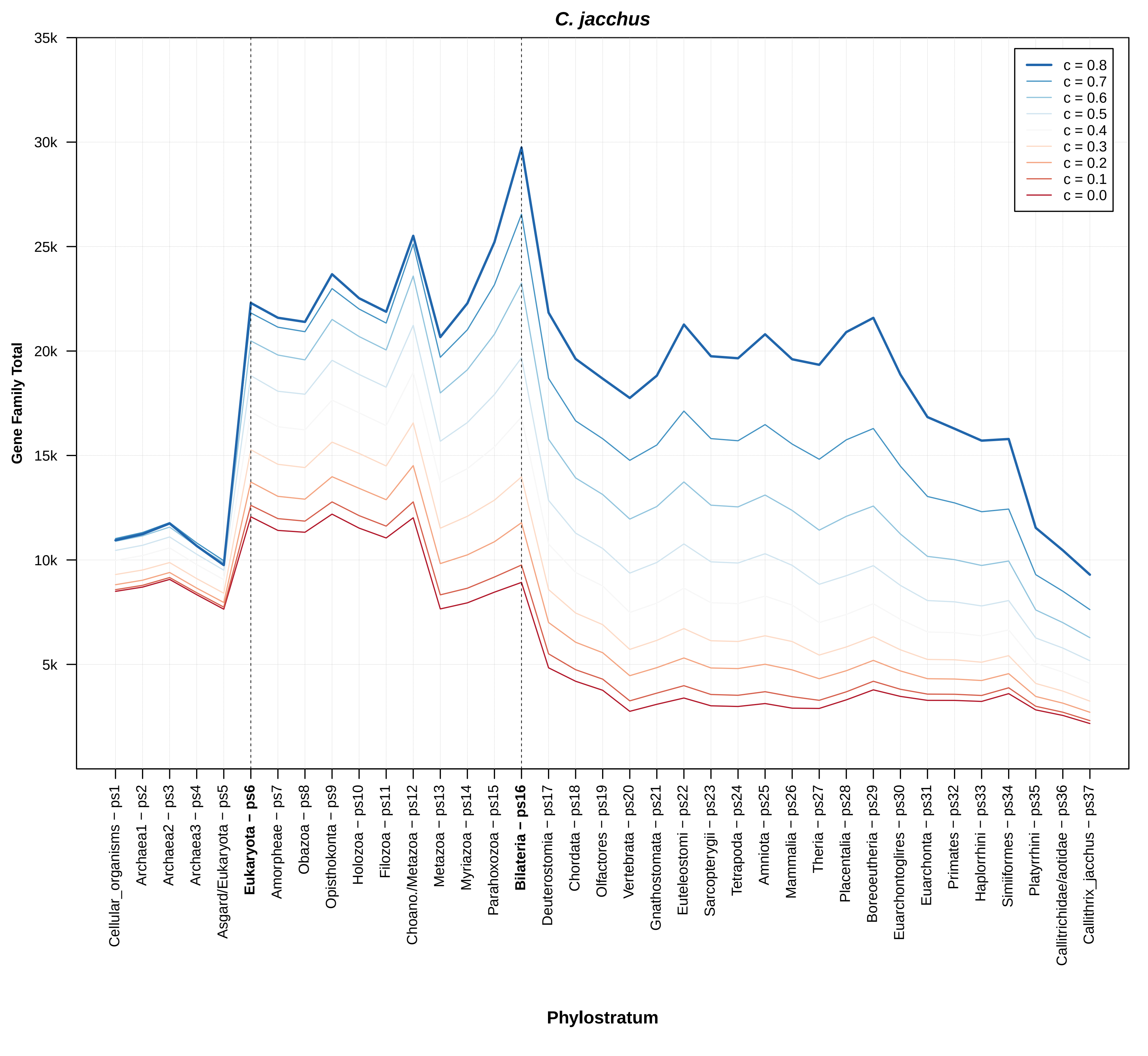

C. jacchus
Gene Family Total
35k
30k
25k
20k
15k
10k
5k
c = 0.8
c = 0.7
c = 0.6
c = 0.5
c = 0.4
c = 0.3
c = 0.2
c = 0.1
c = 0.0
Cellular_organisms – ps1
Archaea1 – ps2
Archaea2 – ps3
Archaea3 – ps4
Asgard/Eukaryota – ps5
Eukaryota – ps6
Amorpheae – ps7
Obazoa – ps8
Opisthokonta – ps9
Holozoa – ps10
Filozoa – ps11
Choano./Metazoa – ps12
Metazoa – ps13
Myriazoa – ps14
Parahoxozoa – ps15
Bilateria – ps16
Deuterostomia – ps17
Chordata – ps18
Olfactores – ps19
Vertebrata – ps20
Gnathostomata – ps21
Euteleostomi – ps22
Sarcopterygii – ps23
Tetrapoda – ps24
Amniota – ps25
Mammalia – ps26
Theria – ps27
Placentalia – ps28
Boreoeutheria – ps29
Euarchontoglires – ps30
Euarchonta – ps31
Primates – ps32
Haplorrhini – ps33
Simiiformes – ps34
Platyrrhini – ps35
Callitrichidae/aotidae – ps36
Callithrix_jacchus – ps37
Phylostratum

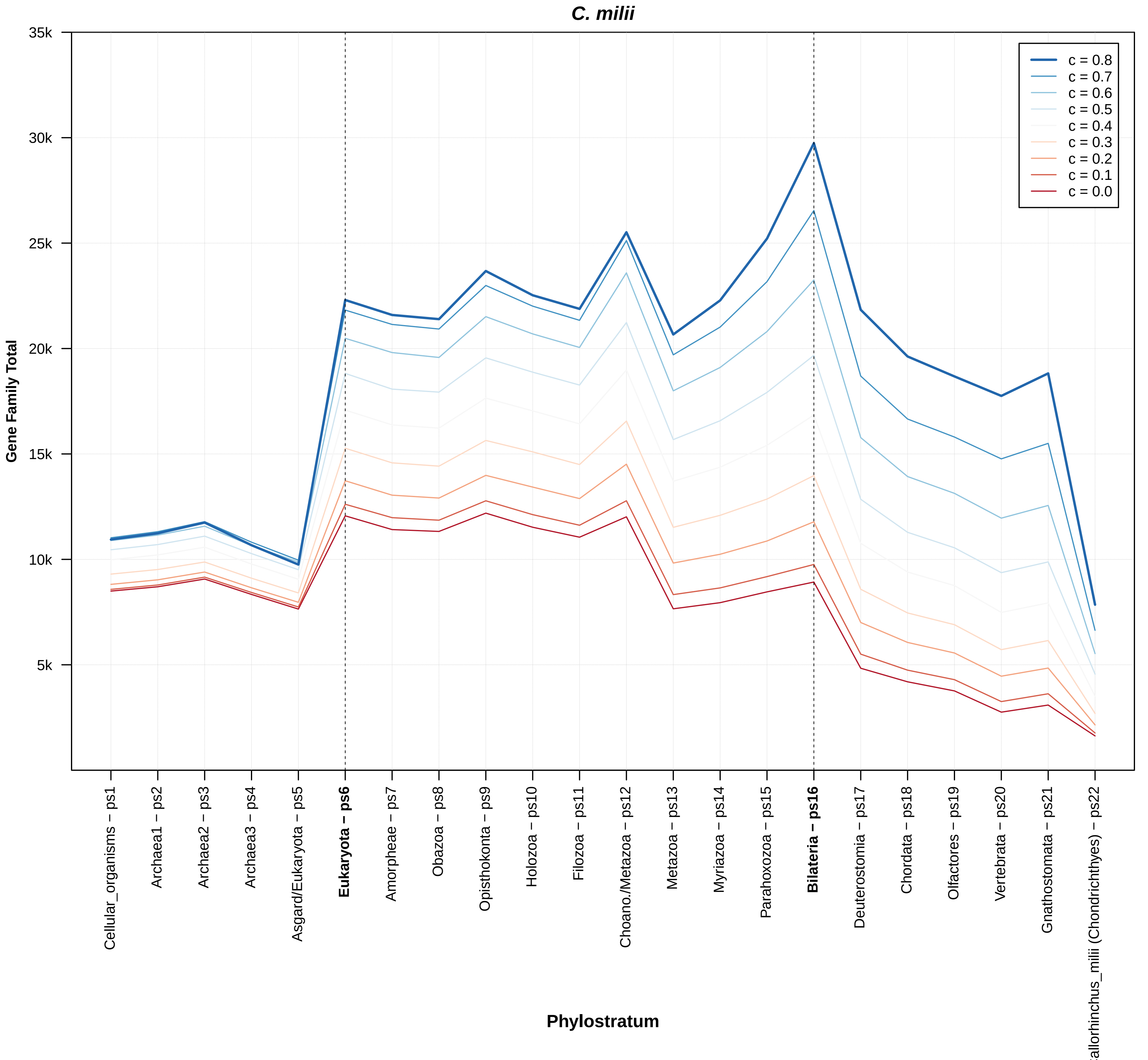

C. milii
Gene Family Total
Phylostratum
35k
30k
25k
20k
15k
10k
5k
c = 0.8
c = 0.7
c = 0.6
c = 0.5
c = 0.4
c = 0.3
c = 0.2
c = 0.1
c = 0.0
Cellular_organisms – ps1
Archaea1 – ps2
Archaea2 – ps3
Archaea3 – ps4
Asgard/Eukaryota – ps5
Eukaryota – ps6
Amorpheae – ps7
Obazoa – ps8
Opisthokonta – ps9
Holozoa – ps10
Filozoa – ps11
Choano./Metazoa – ps12
Metazoa – ps13
Myriazoa – ps14
Parahoxozoa – ps15
Bilateria – ps16
Deuterostomia – ps17
Chordata – ps18
Olfactores – ps19
Vertebrata – ps20
Gnathostomata – ps21
allorhinchus_milii (Chondrichthyes) – ps22

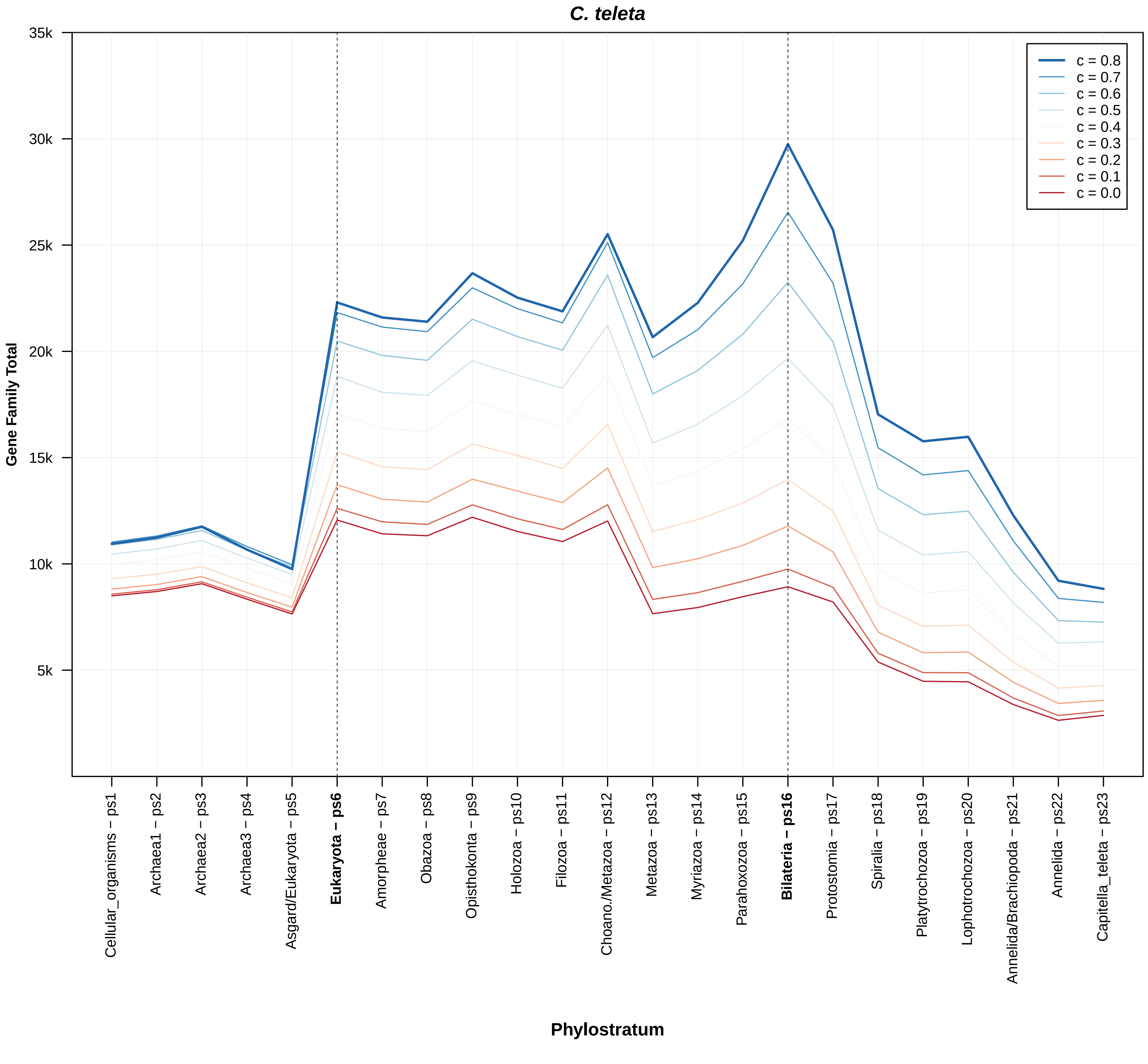

C. teleta
Gene Family Total
35k
30k
25k
20k
15k
10k
5k
c = 0.8
c = 0.7
c = 0.6
c = 0.5
c = 0.4
c = 0.3
c = 0.2
c = 0.1
c = 0.0
Cellular_organisms – ps1
Archaea1 – ps2
Archaea2 – ps3
Archaea3 – ps4
Asgard/Eukaryota – ps5
Eukaryota – ps6
Amorpheae – ps7
Obazoa – ps8
Opisthokonta – ps9
Holozoa – ps10
Filozoa – ps11
Choano./Metazoa – ps12
Metazoa – ps13
Myriazoa – ps14
Parahoxozoa – ps15
Bilateria – ps16
Protostomia – ps17
Spiralia – ps18
Platytrochozoa – ps19
Lophotrochozoa – ps20
Annelida/Brachiopoda – ps21
Annelida – ps22
Capitella_teleta – ps23
Phylostratum

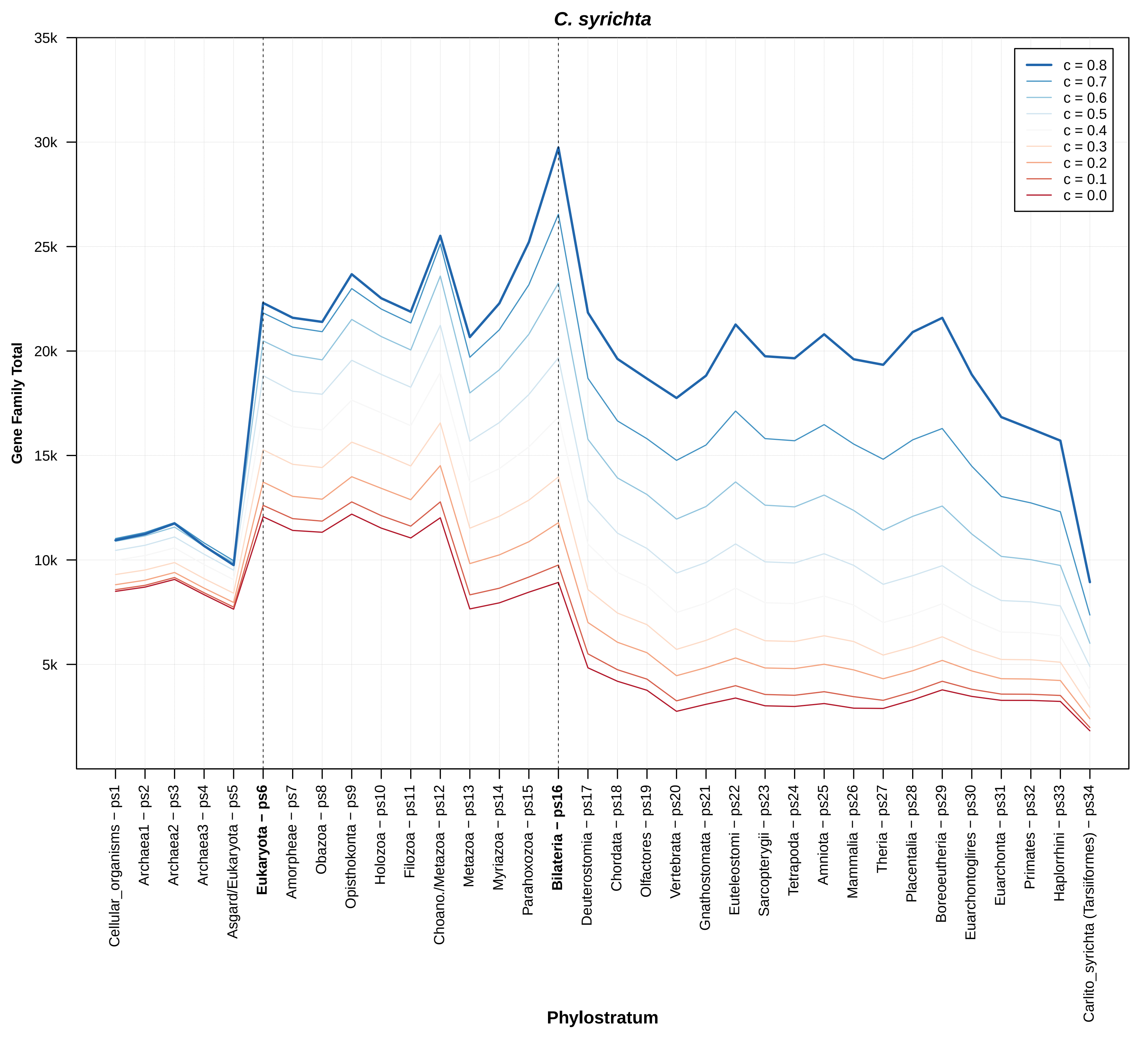
C. syrichta
Gene Family Total
Phylostratum
35k
30k
25k
20k
15k
10k
5k
c = 0.8
c = 0.7
c = 0.6
c = 0.5
c = 0.4
c = 0.3
c = 0.2
c = 0.1
c = 0.0
Cellular_organisms – ps1
Archaea1 – ps2
Archaea2 – ps3
Archaea3 – ps4
Asgard/Eukaryota – ps5
Eukaryota – ps6
Amorpheae – ps7
Obazoa – ps8
Opisthokonta – ps9
Holozoa – ps10
Filozoa – ps11
Choano./Metazoa – ps12
Metazoa – ps13
Myriazoa – ps14
Parahoxozoa – ps15
Bilateria – ps16
Deuterostomia – ps17
Chordata – ps18
Olfactores – ps19
Vertebrata – ps20
Gnathostomata – ps21
Euteleostomi – ps22
Sarcopterygii – ps23
Tetrapoda – ps24
Amniota – ps25
Mammalia – ps26
Theria – ps27
Placentalia – ps28
Boreoeutheria – ps29
Euarchontoglires – ps30
Euarchonta – ps31
Primates – ps32
Haplorrhini – ps33
Carlito_syrichta (Tarsiiformes) – ps34

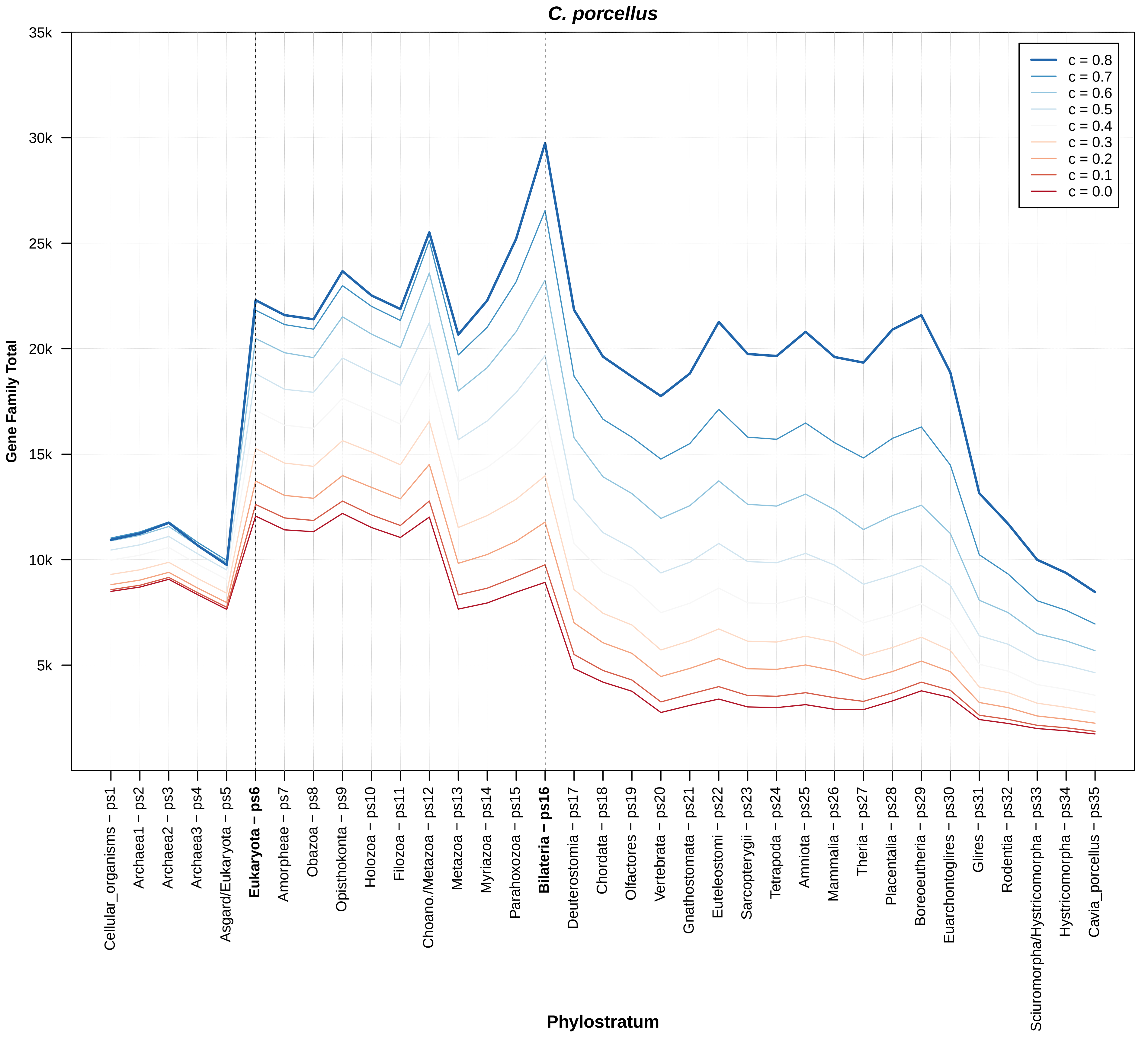
C. porcellus
Gene Family Total
35k
30k
25k
20k
15k
10k
5k
c = 0.8
c = 0.7
c = 0.6
c = 0.5
c = 0.4
c = 0.3
c = 0.2
c = 0.1
c = 0.0
Cellular_organisms – ps1
Archaea1 – ps2
Archaea2 – ps3
Archaea3 – ps4
Asgard/Eukaryota – ps5
Eukaryota – ps6
Amorpheae – ps7
Obazoa – ps8
Opisthokonta – ps9
Holozoa – ps10
Filozoa – ps11
Choano./Metazoa – ps12
Metazoa – ps13
Myriazoa – ps14
Parahoxozoa – ps15
Bilateria – ps16
Deuterostomia – ps17
Chordata – ps18
Olfactores – ps19
Vertebrata – ps20
Gnathostomata – ps21
Euteleostomi – ps22
Sarcopterygii – ps23
Tetrapoda – ps24
Amniota – ps25
Mammalia – ps26
Theria – ps27
Placentalia – ps28
Boreoeutheria – ps29
Euarchontoglires – ps30
Glires – ps31
Rodentia – ps32
Sciuromorpha/Hystricomorpha – ps33
Hystricomorpha – ps34
Cavia_porcellus – ps35
Phylostratum

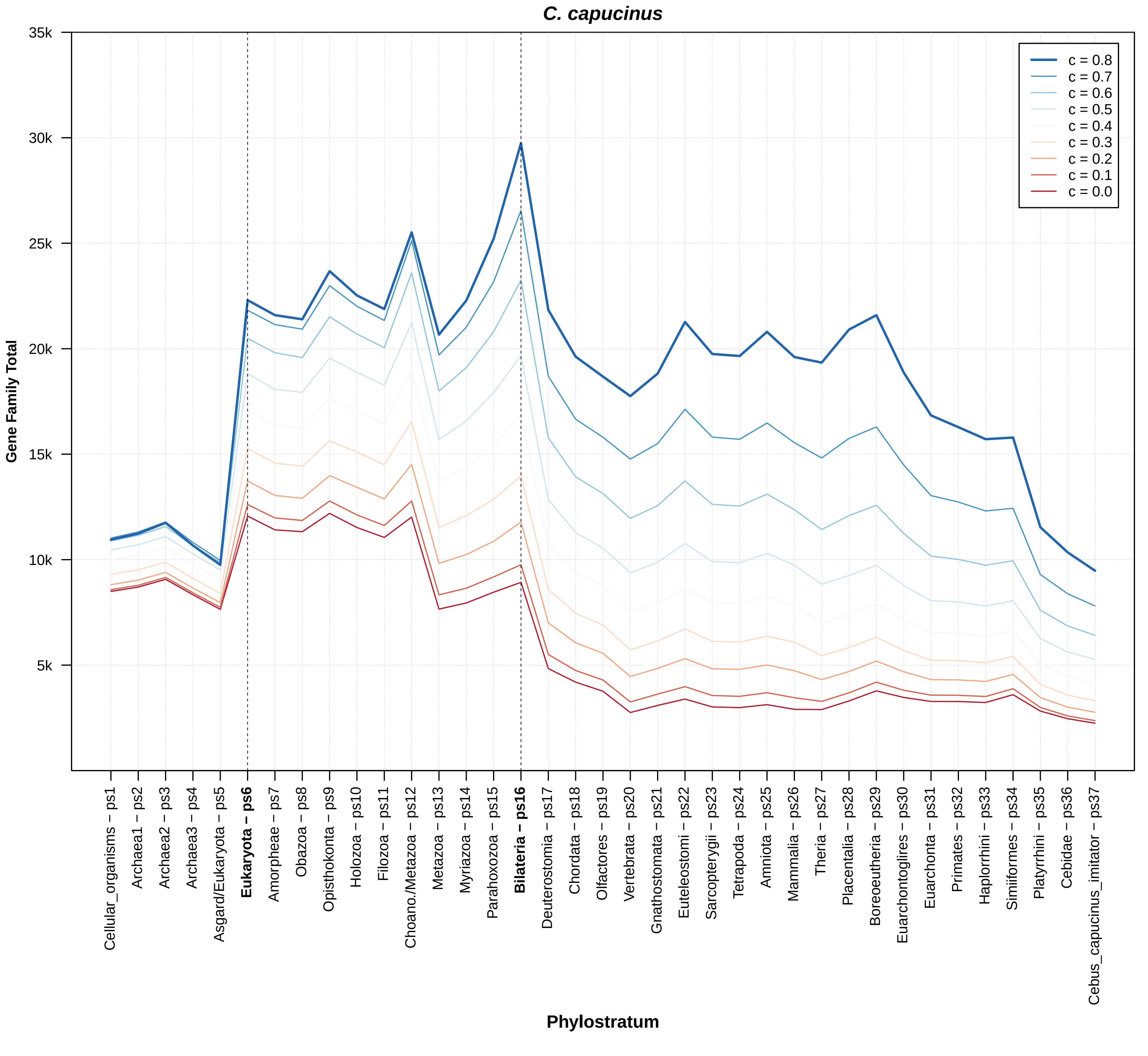
C. capucinus
Gene Family Total
35k
30k
25k
20k
15k
10k
5k
c = 0.8
c = 0.7
c = 0.6
c = 0.5
c = 0.4
c = 0.3
c = 0.2
c = 0.1
c = 0.0
Cellular_organisms – ps1
Archaea1 – ps2
Archaea2 – ps3
Archaea3 – ps4
Asgard/Eukaryota – ps5
Eukaryota – ps6
Amorpheae – ps7
Obazoa – ps8
Opisthokonta – ps9
Holozoa – ps10
Filozoa – ps11
Choano./Metazoa – ps12
Metazoa – ps13
Myriazoa – ps14
Parahoxozoa – ps15
Bilateria – ps16
Deuterostomia – ps17
Chordata – ps18
Olfactores – ps19
Vertebrata – ps20
Gnathostomata – ps21
Euteleostomi – ps22
Sarcopterygii – ps23
Tetrapoda – ps24
Amniota – ps25
Mammalia – ps26
Theria – ps27
Placentalia – ps28
Boreoeutheria – ps29
Euarchontoglires – ps30
Euarchonta – ps31
Primates – ps32
Haplorrhini – ps33
Simiiformes – ps34
Platyrrhini – ps35
Cebidae – ps36
Cebus_capucinus_imitator – ps37
Phylostratum

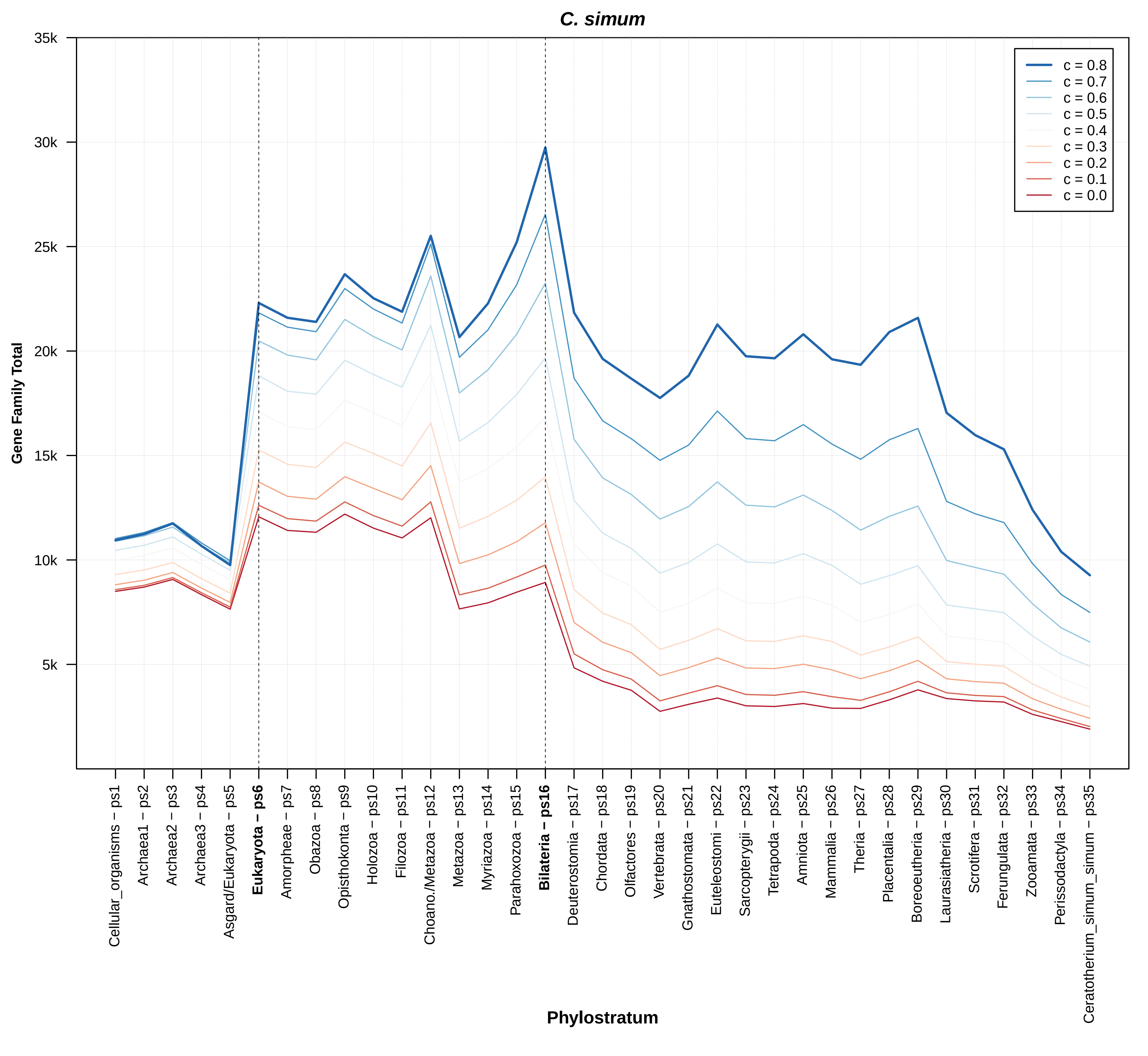
C. simum
Gene Family Total
35k
30k
25k
20k
15k
10k
5k
c = 0.8
c = 0.7
c = 0.6
c = 0.5
c = 0.4
c = 0.3
c = 0.2
c = 0.1
c = 0.0
Cellular_organisms – ps1
Archaea1 – ps2
Archaea2 – ps3
Archaea3 – ps4
Asgard/Eukaryota – ps5
Eukaryota – ps6
Amorpheae – ps7
Obazoa – ps8
Opisthokonta – ps9
Holozoa – ps10
Filozoa – ps11
Choano./Metazoa – ps12
Metazoa – ps13
Myriazoa – ps14
Parahoxozoa – ps15
Bilateria – ps16
Deuterostomia – ps17
Chordata – ps18
Olfactores – ps19
Vertebrata – ps20
Gnathostomata – ps21
Euteleostomi – ps22
Sarcopterygii – ps23
Tetrapoda – ps24
Amniota – ps25
Mammalia – ps26
Theria – ps27
Placentalia – ps28
Boreoeutheria – ps29
Laurasiatheria – ps30
Scrotifera – ps31
Ferungulata – ps32
Zooamata – ps33
Perissodactyla – ps34
Ceratotherium_simum_simum – ps35
Phylostratum

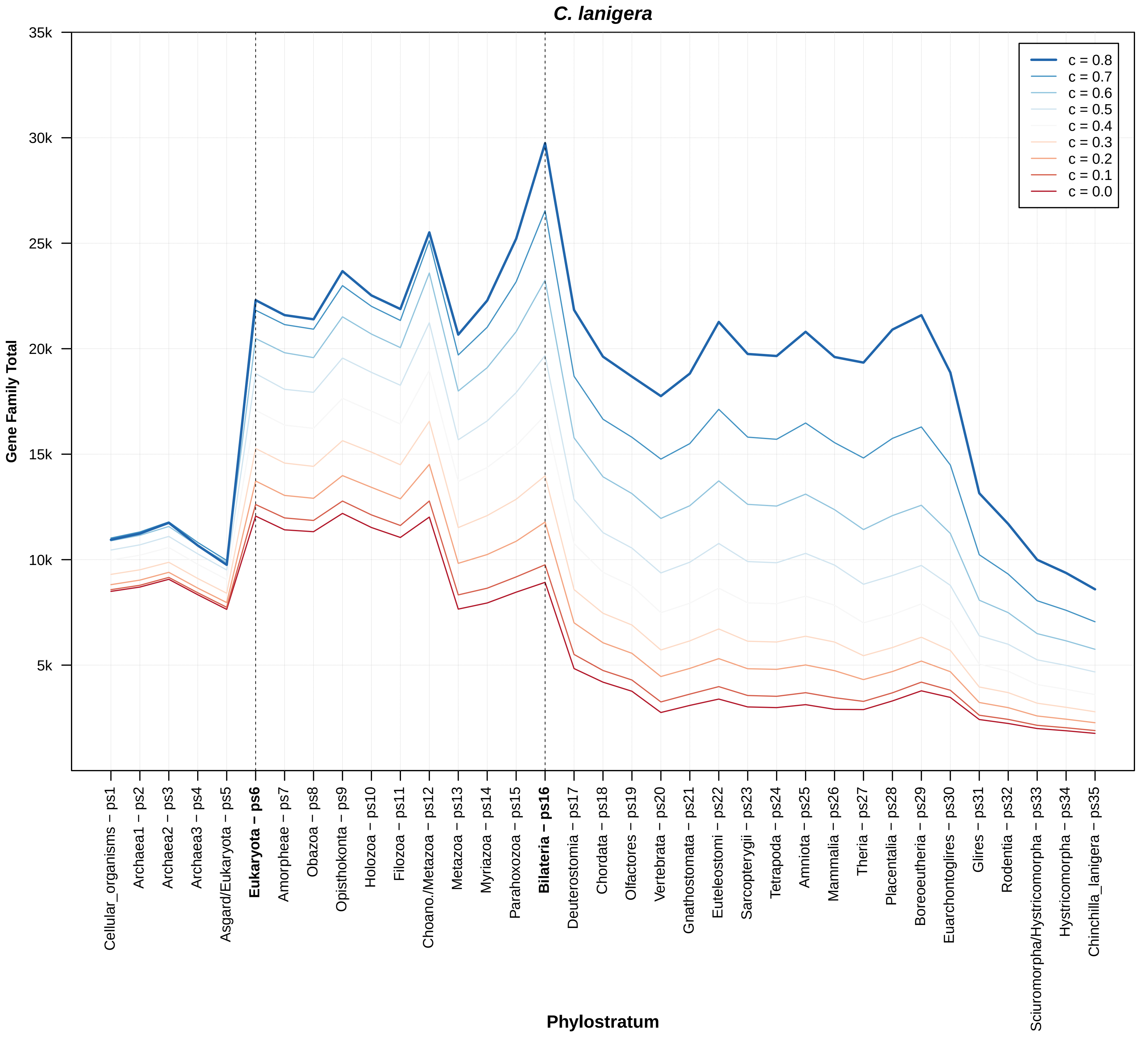

C. lanigera
Gene Family Total
Phylostratum
c = 0.8
c = 0.7
c = 0.6
c = 0.5
c = 0.4
c = 0.3
c = 0.2
c = 0.1
c = 0.0
35k
30k
25k
20k
15k
10k
5k
Cellular_organisms – ps1
Archaea1 – ps2
Archaea2 – ps3
Archaea3 – ps4
Asgard/Eukaryota – ps5
Eukaryota – ps6
Amorpheae – ps7
Obazoa – ps8
Opisthokonta – ps9
Holozoa – ps10
Filozoa – ps11
Choano./Metazoa – ps12
Metazoa – ps13
Myriazoa – ps14
Parahoxozoa – ps15
Bilateria – ps16
Deuterostomia – ps17
Chordata – ps18
Olfactores – ps19
Vertebrata – ps20
Gnathostomata – ps21
Euteleostomi – ps22
Sarcopterygii – ps23
Tetrapoda – ps24
Amniota – ps25
Mammalia – ps26
Theria – ps27
Placentalia – ps28
Boreoeutheria – ps29
Euarchontoglires – ps30
Glires – ps31
Rodentia – ps32
Sciuromorpha/Hystricomorpha – ps33
Hystricomorpha – ps34
Chinchilla_lanigera – ps35

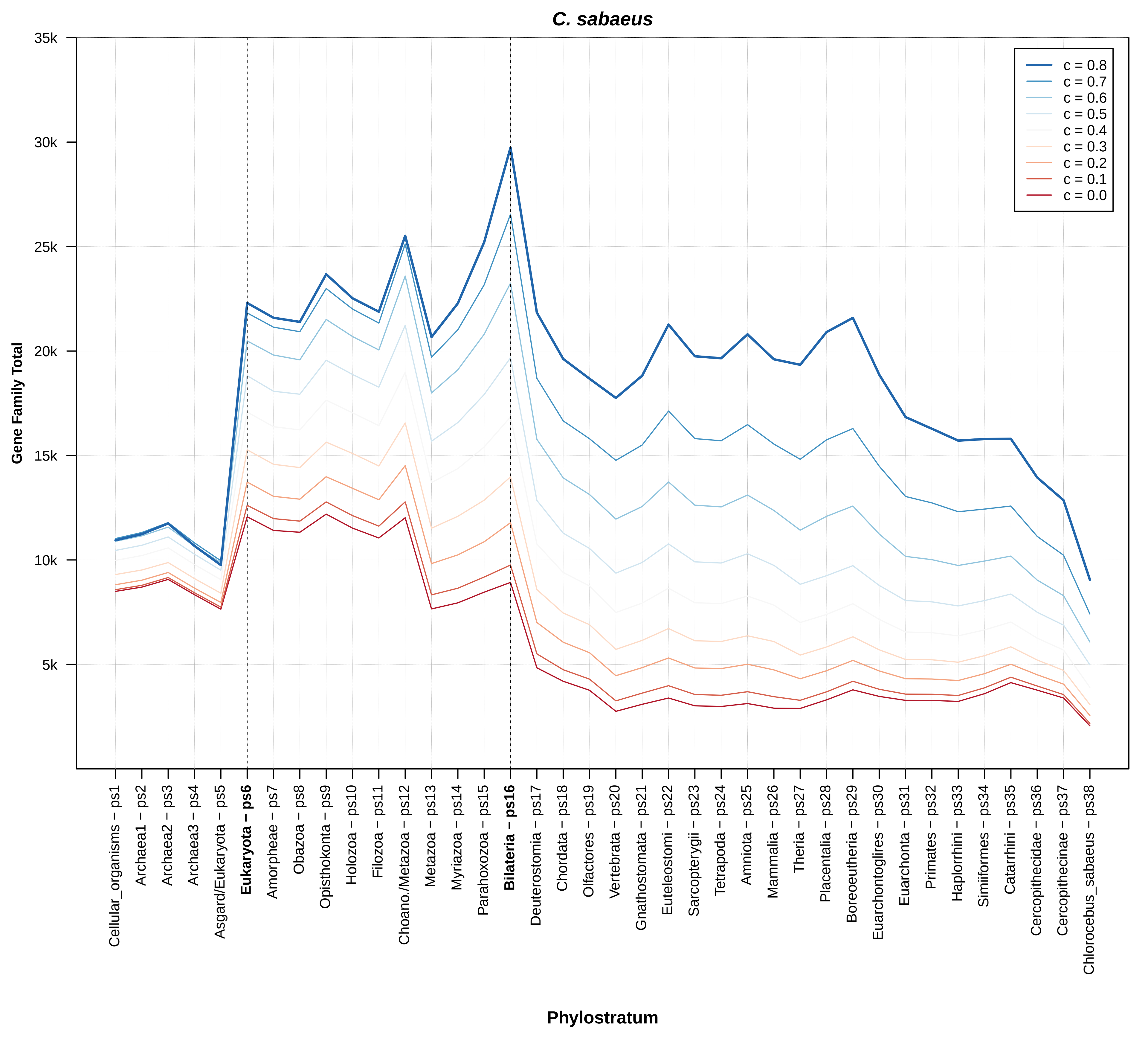
C. sabaeus
Gene Family Total
35k
30k
25k
20k
15k
10k
5k
c = 0.8
c = 0.7
c = 0.6
c = 0.5
c = 0.4
c = 0.3
c = 0.2
c = 0.1
c = 0.0
Cellular_organisms – ps1
Archaea1 – ps2
Archaea2 – ps3
Archaea3 – ps4
Asgard/Eukaryota – ps5
Eukaryota – ps6
Amorpheae – ps7
Obazoa – ps8
Opisthokonta – ps9
Holozoa – ps10
Filozoa – ps11
Choano./Metazoa – ps12
Metazoa – ps13
Myriazoa – ps14
Parahoxozoa – ps15
Bilateria – ps16
Deuterostomia – ps17
Chordata – ps18
Olfactores – ps19
Vertebrata – ps20
Gnathostomata – ps21
Euteleostomi – ps22
Sarcopterygii – ps23
Tetrapoda – ps24
Amniota – ps25
Mammalia – ps26
Theria – ps27
Placentalia – ps28
Boreoeutheria – ps29
Euarchontoglires – ps30
Euarchonta – ps31
Primates – ps32
Haplorrhini – ps33
Simiiformes – ps34
Catarrhini – ps35
Cercopithecidae – ps36
Cercopithecinae – ps37
Chlorocebus_sabaeus – ps38
Phylostratum

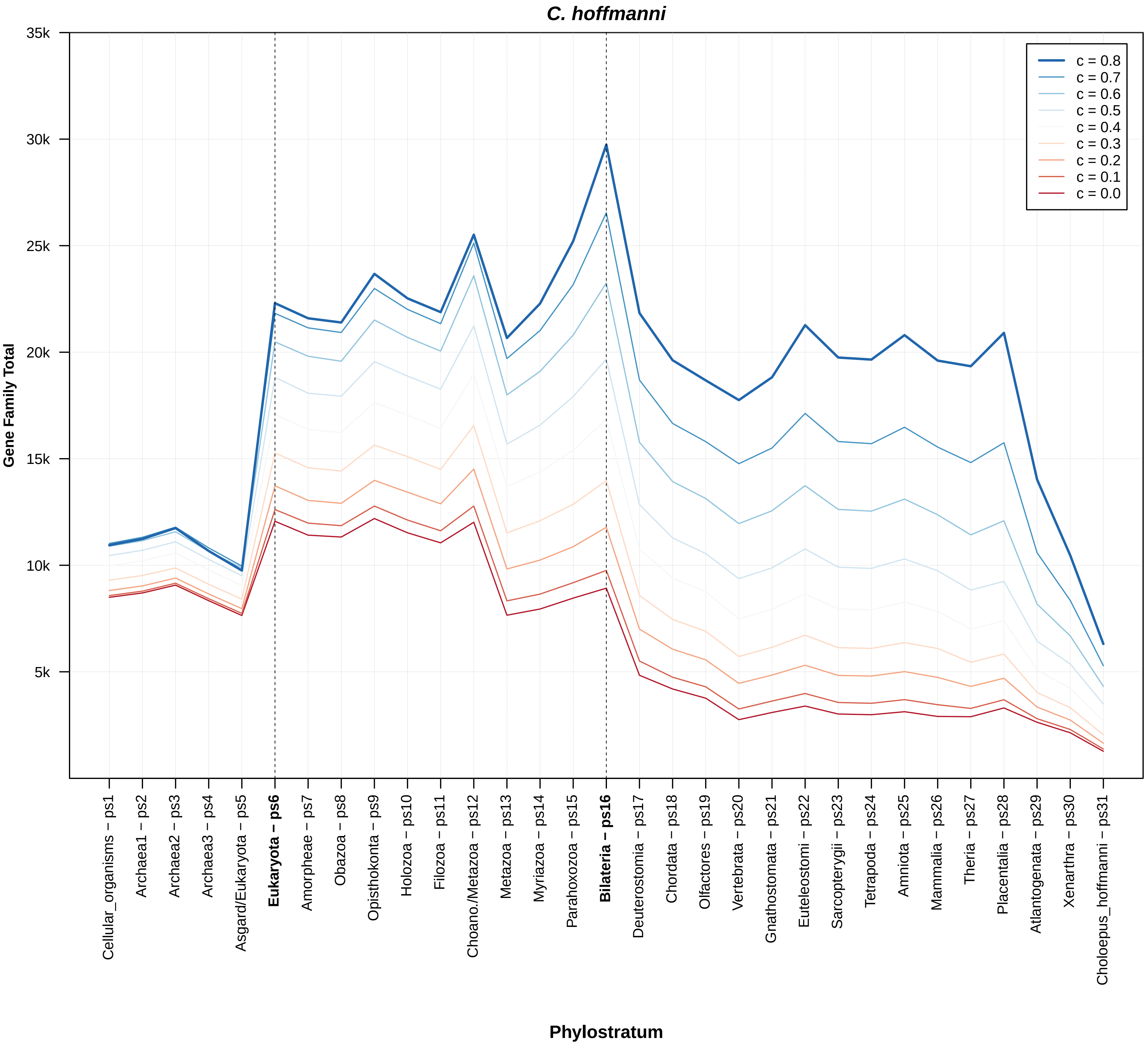

C. hoffmanni
Gene Family Total
Phylostratum
c = 0.8
c = 0.7
c = 0.6
c = 0.5
c = 0.4
c = 0.3
c = 0.2
c = 0.1
c = 0.0
35k
30k
25k
20k
15k
10k
5k
Cellular_organisms – ps1
Archaea1 – ps2
Archaea2 – ps3
Archaea3 – ps4
Asgard/Eukaryota – ps5
Eukaryota – ps6
Amorpheae – ps7
Obazoa – ps8
Opisthokonta – ps9
Holozoa – ps10
Filozoa – ps11
Choano./Metazoa – ps12
Metazoa – ps13
Myriazoa – ps14
Parahoxozoa – ps15
Bilateria – ps16
Deuterostomia – ps17
Chordata – ps18
Olfactores – ps19
Vertebrata – ps20
Gnathostomata – ps21
Euteleostomi – ps22
Sarcopterygii – ps23
Tetrapoda – ps24
Amniota – ps25
Mammalia – ps26
Theria – ps27
Placentalia – ps28
Atlantogenata – ps29
Xenarthra – ps30
Choloepus_hoffmanni – ps31

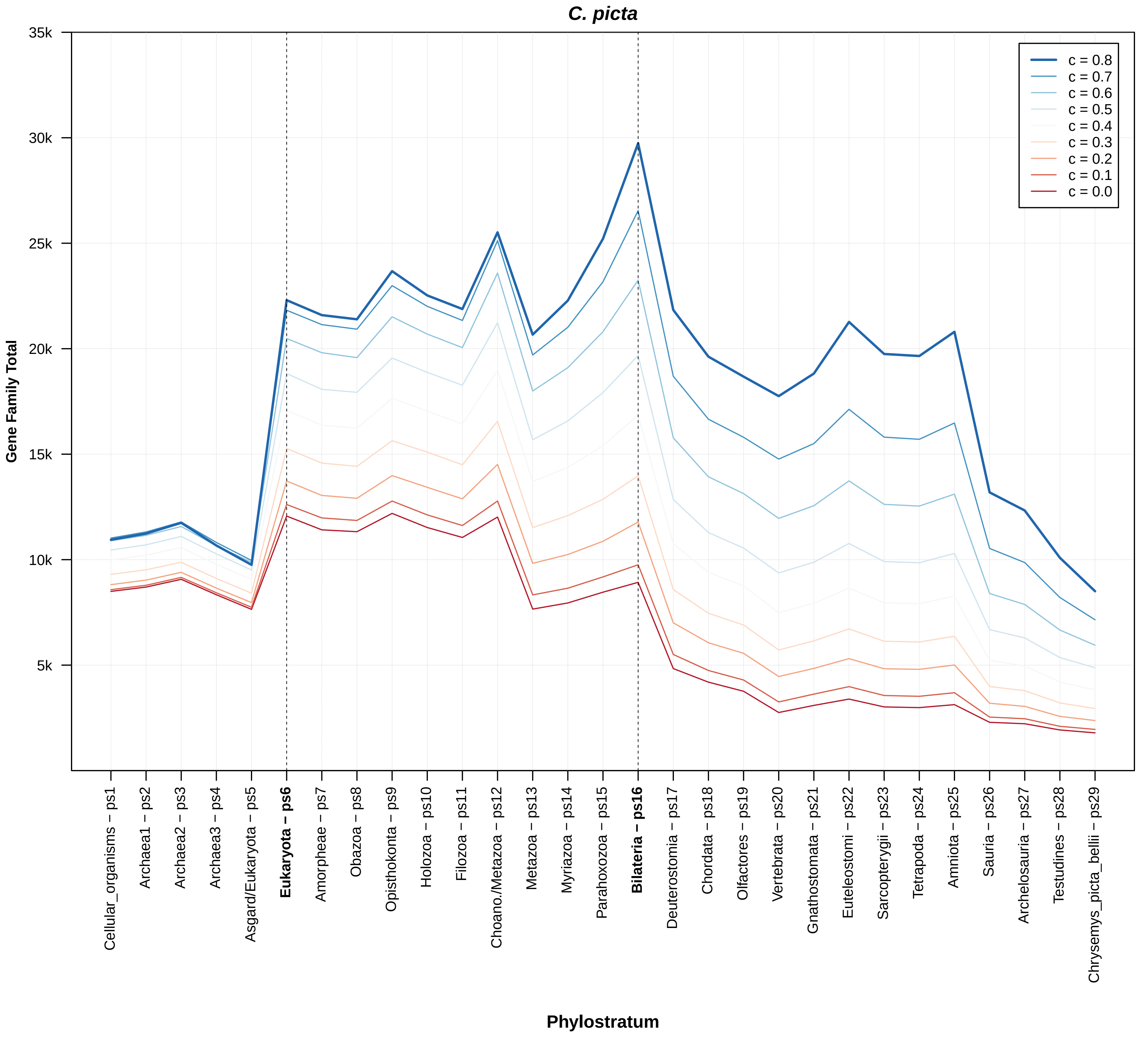

C. picta
35k
30k
25k
20k
15k
10k
5k
Gene Family Total
c = 0.8
c = 0.7
c = 0.6
c = 0.5
c = 0.4
c = 0.3
c = 0.2
c = 0.1
c = 0.0
Cellular_organisms – ps1
Archaea1 – ps2
Archaea2 – ps3
Archaea3 – ps4
Asgard/Eukaryota – ps5
Eukaryota – ps6
Amorpheae – ps7
Obazoa – ps8
Opisthokonta – ps9
Holozoa – ps10
Filozoa – ps11
Choano./Metazoa – ps12
Metazoa – ps13
Myriazoa – ps14
Parahoxozoa – ps15
Bilateria – ps16
Deuterostomia – ps17
Chordata – ps18
Olfactores – ps19
Vertebrata – ps20
Gnathostomata – ps21
Euteleostomi – ps22
Sarcopterygii – ps23
Tetrapoda – ps24
Amniota – ps25
Sauria – ps26
Archelosauria – ps27
Testudines – ps28
Chrysemys_picta_bellii – ps29
Phylostratum

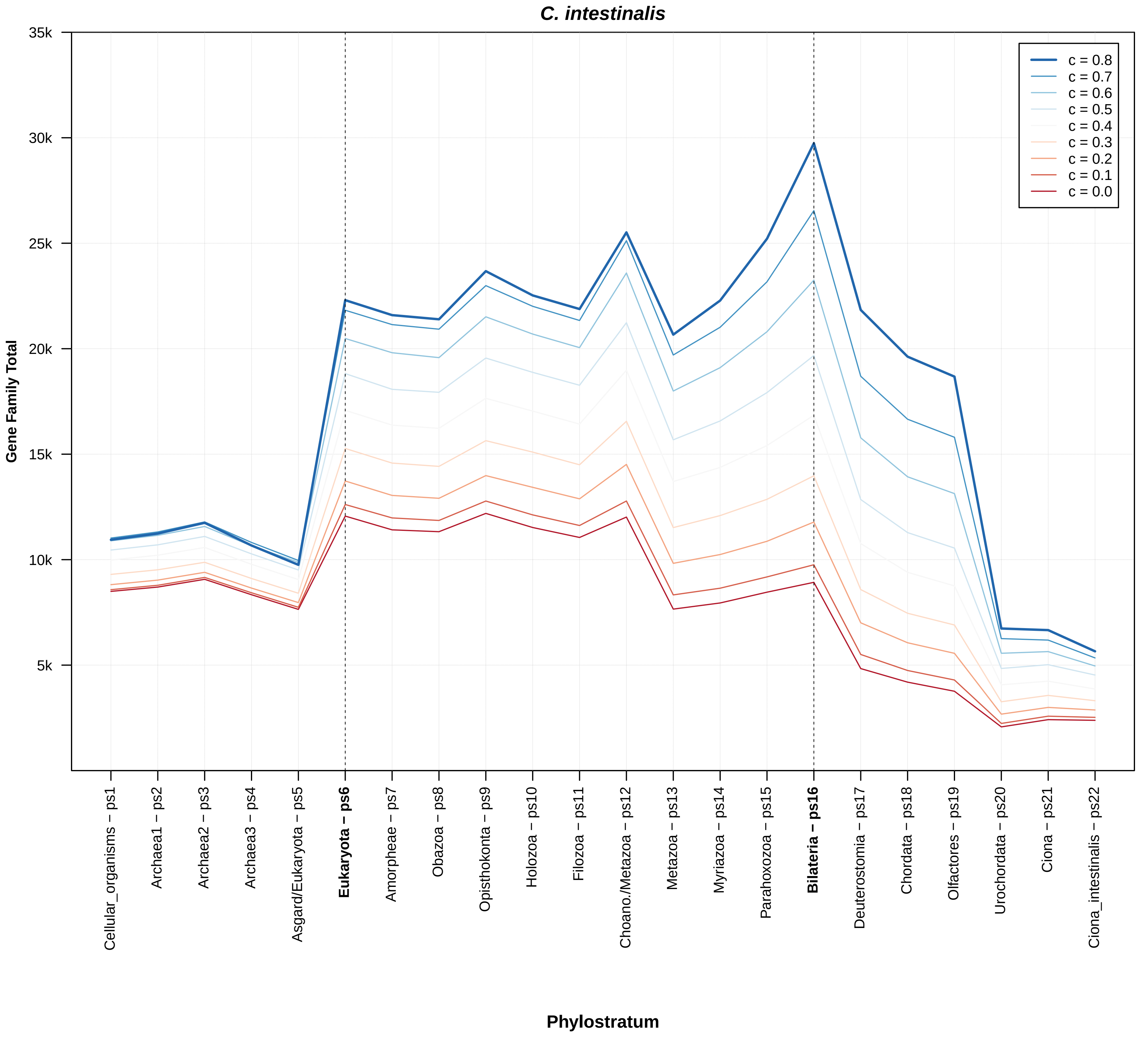
C. intestinalis
Gene Family Total
35k
30k
25k
20k
15k
10k
5k
c = 0.8
c = 0.7
c = 0.6
c = 0.5
c = 0.4
c = 0.3
c = 0.2
c = 0.1
c = 0.0
Cellular_organisms – ps1
Archaea1 – ps2
Archaea2 – ps3
Archaea3 – ps4
Asgard/Eukaryota – ps5
Eukaryota – ps6
Amorpheae – ps7
Obazoa – ps8
Opisthokonta – ps9
Holozoa – ps10
Filozoa – ps11
Choano./Metazoa – ps12
Metazoa – ps13
Myriazoa – ps14
Parahoxozoa – ps15
Bilateria – ps16
Deuterostomia – ps17
Chordata – ps18
Olfactores – ps19
Urochordata – ps20
Ciona – ps21
Ciona_intestinalis – ps22
Phylostratum

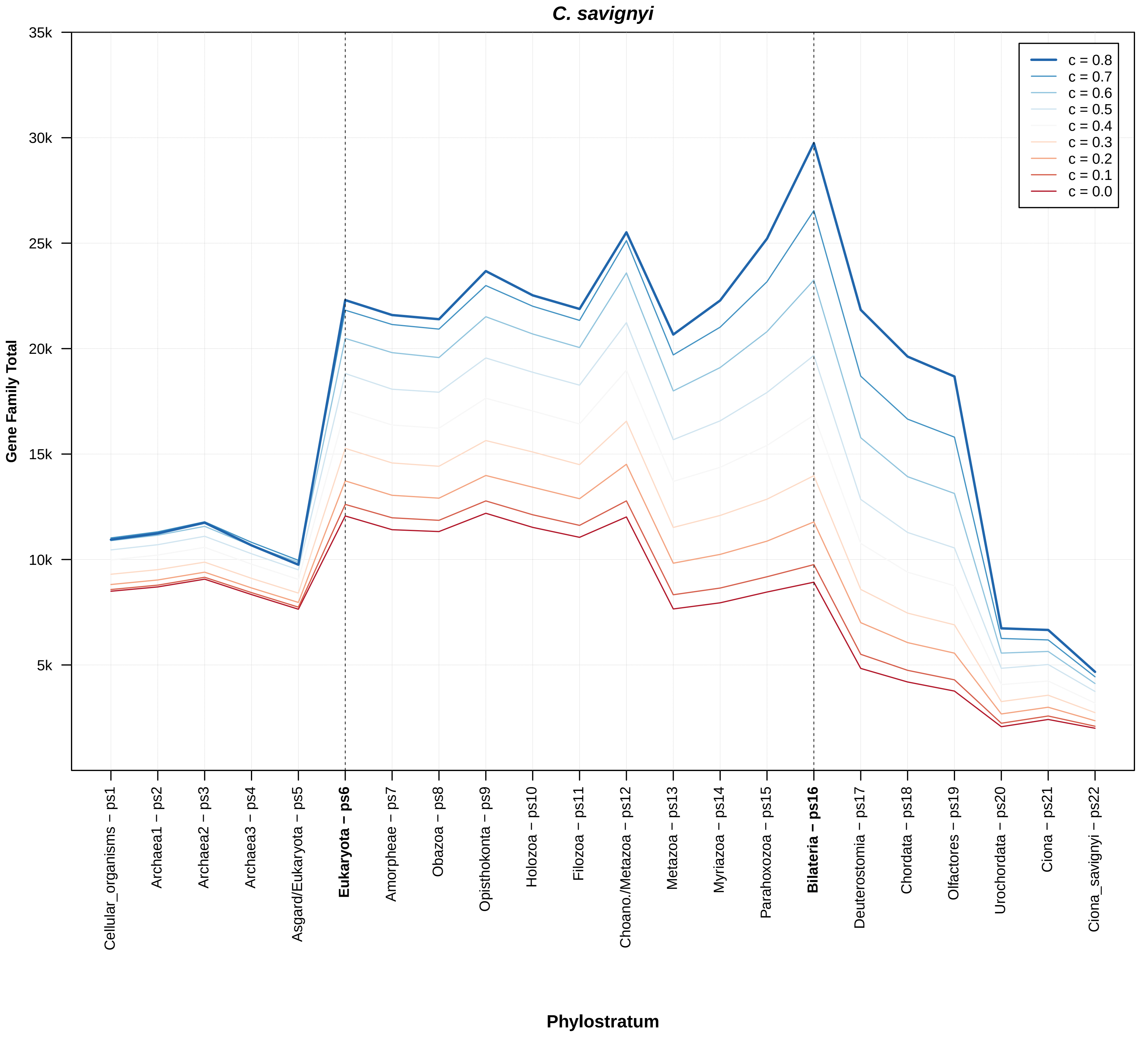

C. savignyi
Gene Family Total
35k
30k
25k
20k
15k
10k
5k
c = 0.8
c = 0.7
c = 0.6
c = 0.5
c = 0.4
c = 0.3
c = 0.2
c = 0.1
c = 0.0
Cellular_organisms – ps1
Archaea1 – ps2
Archaea2 – ps3
Archaea3 – ps4
Asgard/Eukaryota – ps5
Eukaryota – ps6
Amorpheae – ps7
Obazoa – ps8
Opisthokonta – ps9
Holozoa – ps10
Filozoa – ps11
Choano./Metazoa – ps12
Metazoa – ps13
Myriazoa – ps14
Parahoxozoa – ps15
Bilateria – ps16
Deuterostomia – ps17
Chordata – ps18
Olfactores – ps19
Urochordata – ps20
Ciona – ps21
Ciona_savignyi – ps22
Phylostratum

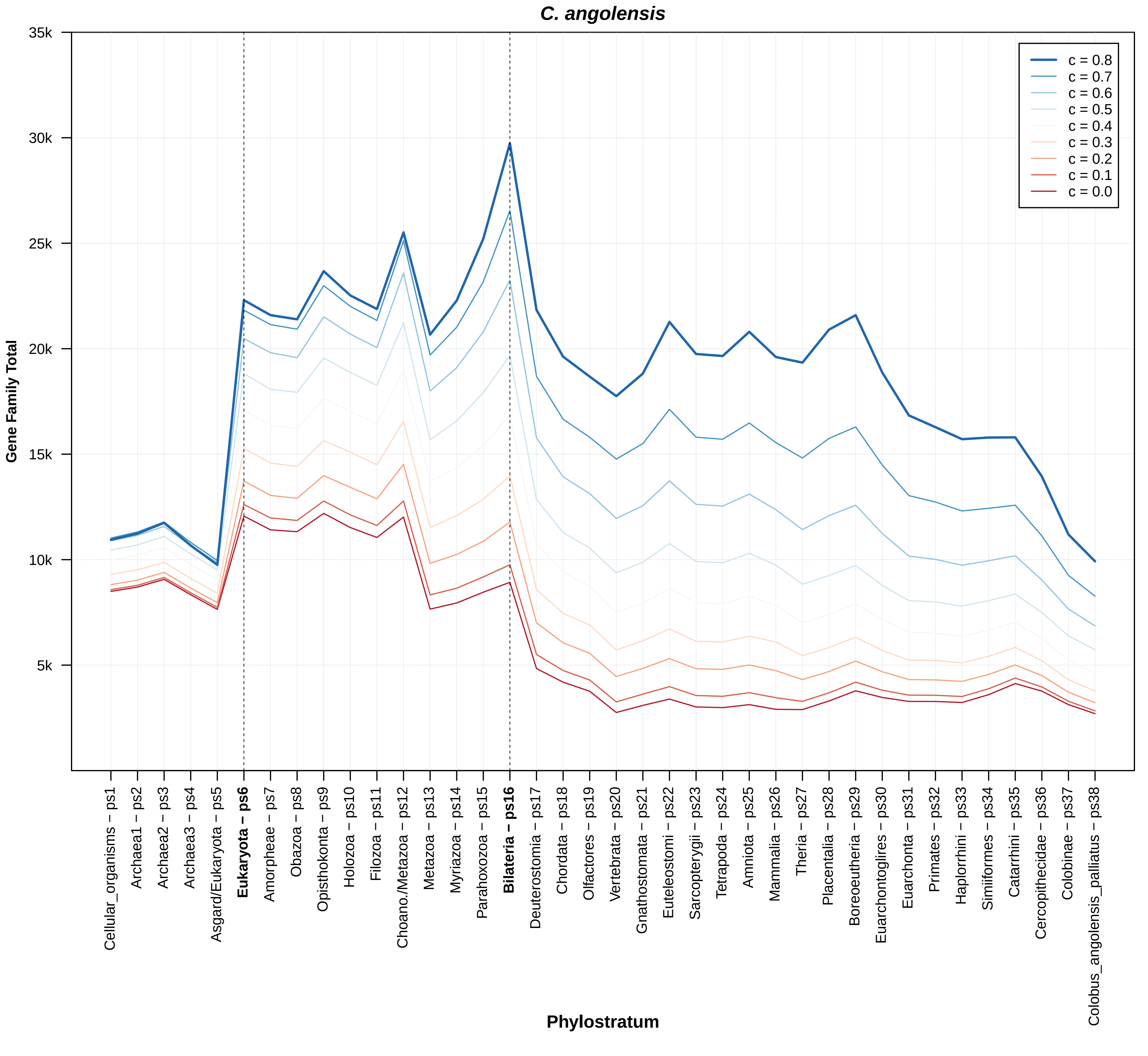

C. angolensis
Gene Family Total
35k
30k
25k
20k
15k
10k
5k
c = 0.8
c = 0.7
c = 0.6
c = 0.5
c = 0.4
c = 0.3
c = 0.2
c = 0.1
c = 0.0
Cellular_organisms – ps1
Archaea1 – ps2
Archaea2 – ps3
Archaea3 – ps4
Asgard/Eukaryota – ps5
Eukaryota – ps6
Amorpheae – ps7
Obazoa – ps8
Opisthokonta – ps9
Holozoa – ps10
Filozoa – ps11
Choano./Metazoa – ps12
Metazoa – ps13
Myriazoa – ps14
Parahoxozoa – ps15
Bilateria – ps16
Deuterostomia – ps17
Chordata – ps18
Olfactores – ps19
Vertebrata – ps20
Gnathostomata – ps21
Euteleostomi – ps22
Sarcopterygii – ps23
Tetrapoda – ps24
Amniota – ps25
Mammalia – ps26
Theria – ps27
Placentalia – ps28
Boreoeutheria – ps29
Euarchontoglires – ps30
Euarchonta – ps31
Primates – ps32
Haplorrhini – ps33
Simiiformes – ps34
Catarrhini – ps35
Cercopithecidae – ps36
Colobinae – ps37
Colobus_angolensis_palliatus – ps38
Phylostratum

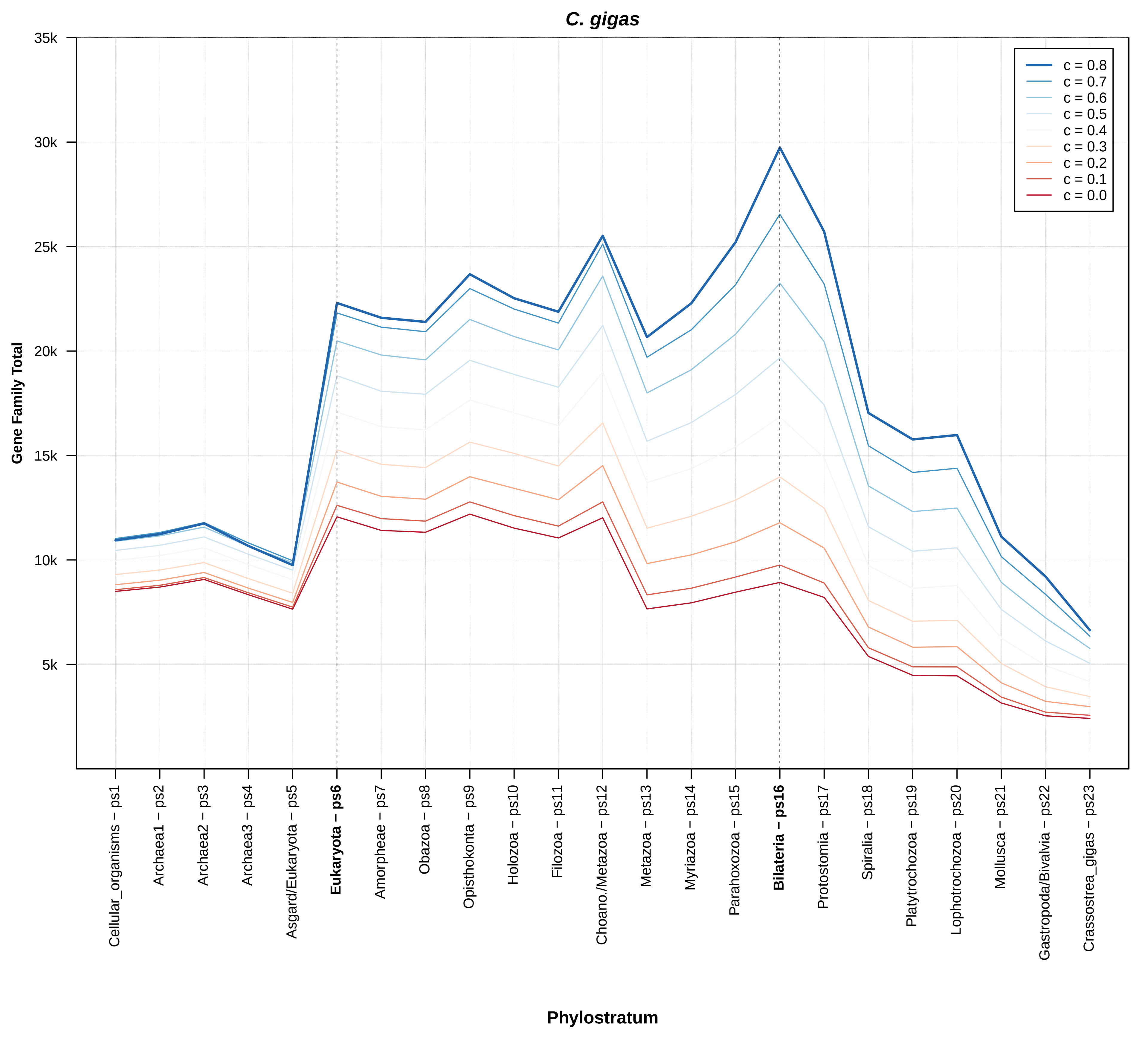
C. gigas
Gene Family Total
35k
30k
25k
20k
15k
10k
5k
c = 0.8
c = 0.7
c = 0.6
c = 0.5
c = 0.4
c = 0.3
c = 0.2
c = 0.1
c = 0.0
Cellular_organisms – ps1
Archaea1 – ps2
Archaea2 – ps3
Archaea3 – ps4
Asgard/Eukaryota – ps5
Eukaryota – ps6
Amorpheae – ps7
Obazoa – ps8
Opisthokonta – ps9
Holozoa – ps10
Filozoa – ps11
Choano./Metazoa – ps12
Metazoa – ps13
Myriazoa – ps14
Parahoxozoa – ps15
Bilateria – ps16
Protostomia – ps17
Spiralia – ps18
Platytrochozoa – ps19
Lophotrochozoa – ps20
Mollusca – ps21
Gastropoda/Bivalvia – ps22
Crassostrea_gigas – ps23
Phylostratum

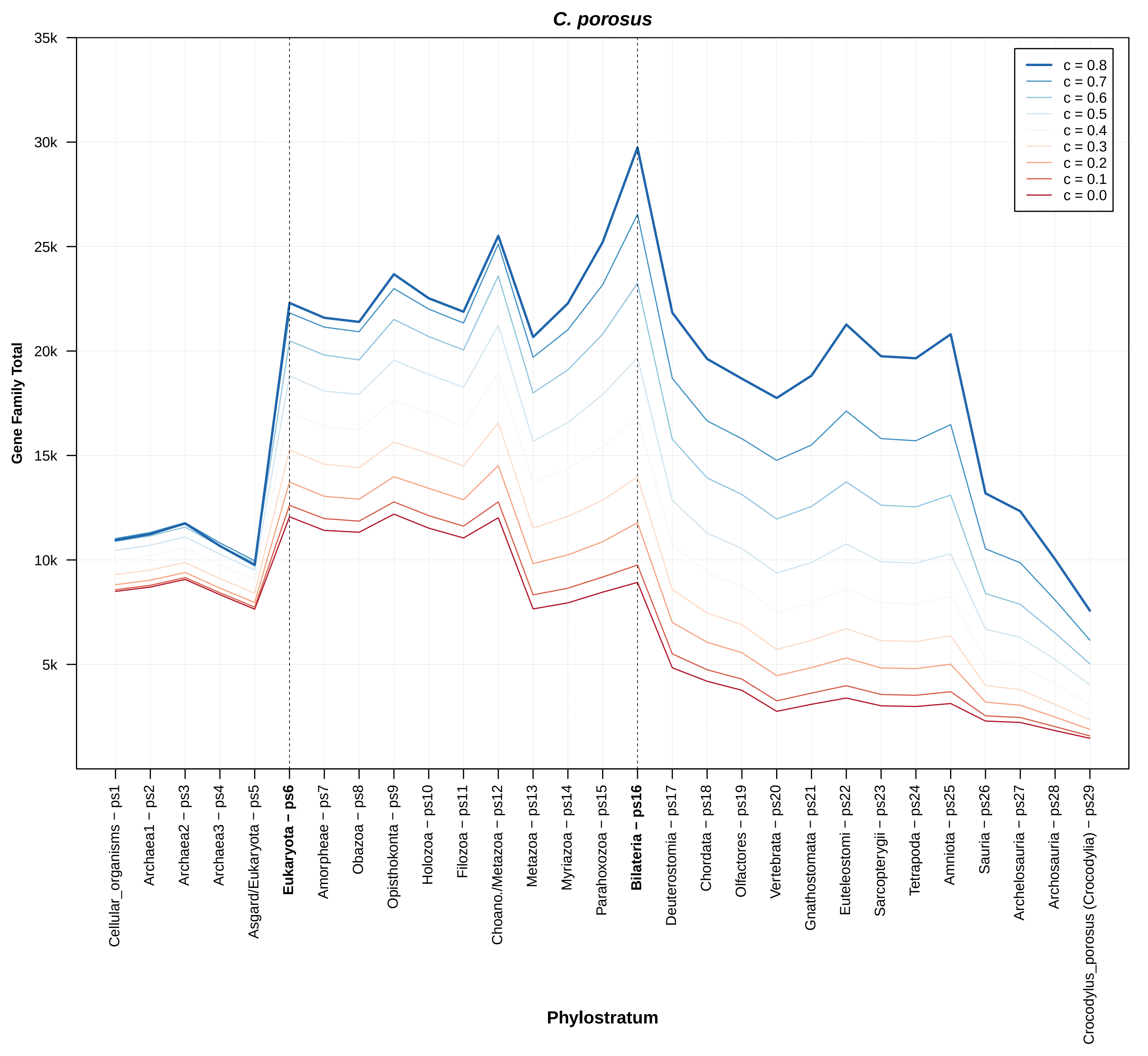
C. porosus
Gene Family Total
35k
30k
25k
20k
15k
10k
5k
c = 0.8
c = 0.7
c = 0.6
c = 0.5
c = 0.4
c = 0.3
c = 0.2
c = 0.1
c = 0.0
Cellular_organisms – ps1
Archaea1 – ps2
Archaea2 – ps3
Archaea3 – ps4
Asgard/Eukaryota – ps5
Eukaryota – ps6
Amorpheae – ps7
Obazoa – ps8
Opisthokonta – ps9
Holozoa – ps10
Filozoa – ps11
Choano./Metazoa – ps12
Metazoa – ps13
Myriazoa – ps14
Parahoxozoa – ps15
Bilateria – ps16
Deuterostomia – ps17
Chordata – ps18
Olfactores – ps19
Vertebrata – ps20
Gnathostomata – ps21
Euteleostomi – ps22
Sarcopterygii – ps23
Tetrapoda – ps24
Amniota – ps25
Sauria – ps26
Archelosauria – ps27
Archosauria – ps28
Crocodylus_porosus (Crocodylia) – ps29
Phylostratum

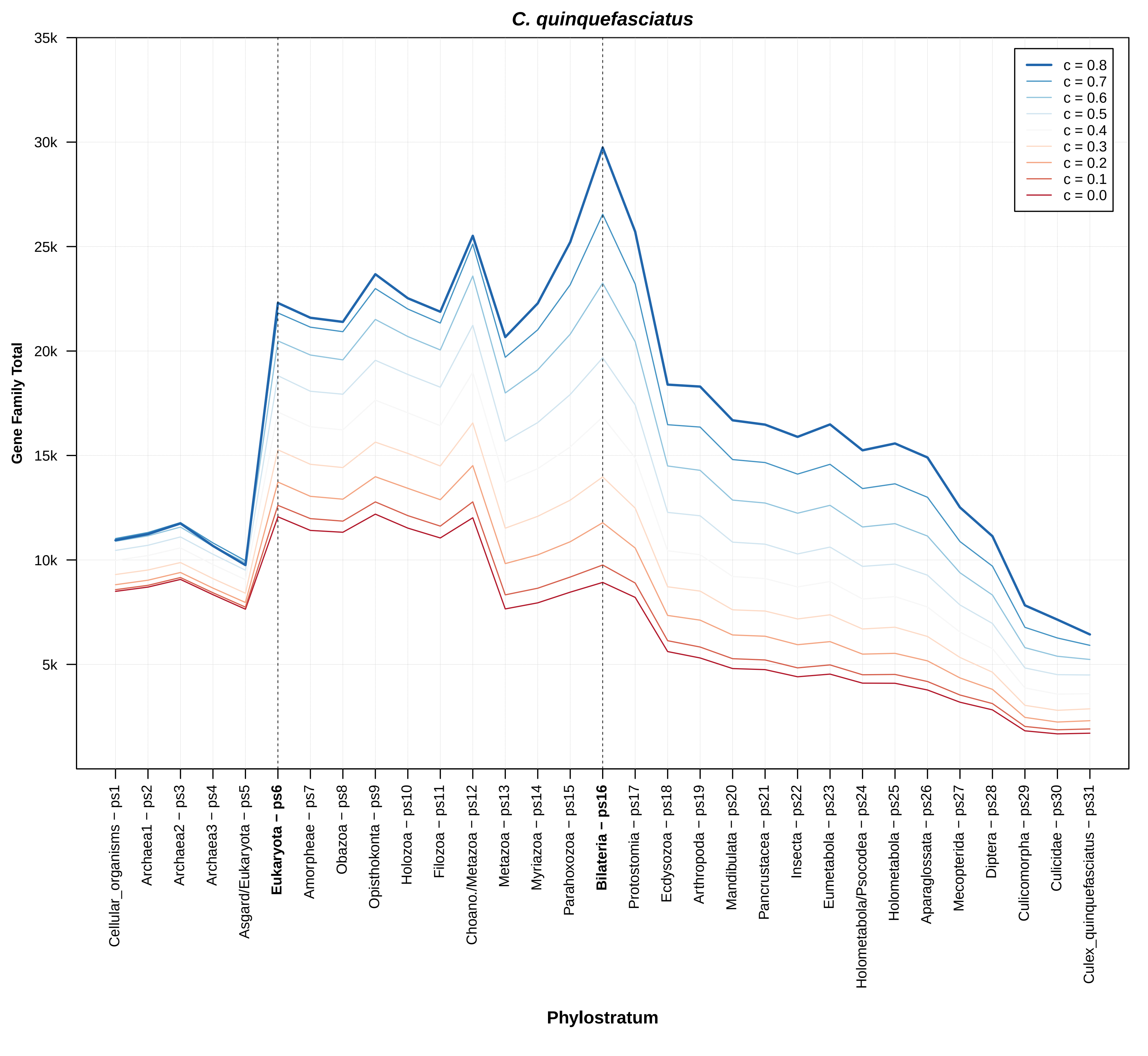

C. quinquefasciatus
Gene Family Total
Phylostratum
35k
30k
25k
20k
15k
10k
5k
c = 0.8
c = 0.7
c = 0.6
c = 0.5
c = 0.4
c = 0.3
c = 0.2
c = 0.1
c = 0.0
Cellular_organisms – ps1
Archaea1 – ps2
Archaea2 – ps3
Archaea3 – ps4
Asgard/Eukaryota – ps5
Eukaryota – ps6
Amorpheae – ps7
Obazoa – ps8
Opisthokonta – ps9
Holozoa – ps10
Filozoa – ps11
Choano./Metazoa – ps12
Metazoa – ps13
Myriazoa – ps14
Parahoxozoa – ps15
Bilateria – ps16
Protostomia – ps17
Ecdysozoa – ps18
Arthropoda – ps19
Mandibulata – ps20
Pancrustacea – ps21
Insecta – ps22
Eumetabola – ps23
Holometabola/Psocodea – ps24
Holometabola – ps25
Aparaglossata – ps26
Mecopterida – ps27
Diptera – ps28
Culicomorpha – ps29
Culicidae – ps30
Culex_quinquefasciatus – ps31

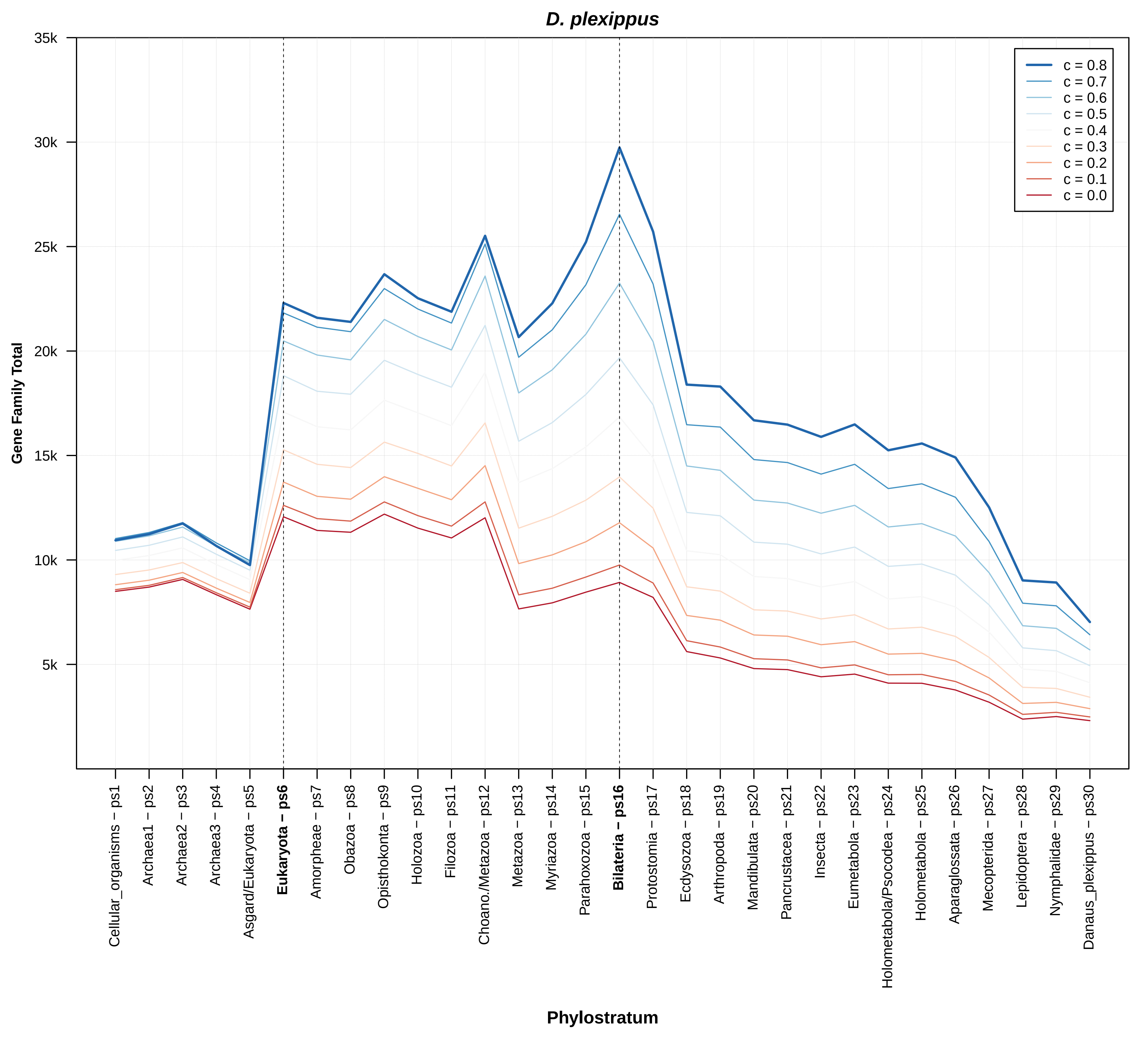

D. plexippus
Gene Family Total
35k
30k
25k
20k
15k
10k
5k
c = 0.8
c = 0.7
c = 0.6
c = 0.5
c = 0.4
c = 0.3
c = 0.2
c = 0.1
c = 0.0
Cellular_organisms – ps1
Archaea1 – ps2
Archaea2 – ps3
Archaea3 – ps4
Asgard/Eukaryota – ps5
Eukaryota – ps6
Amorpheae – ps7
Obazoa – ps8
Opisthokonta – ps9
Holozoa – ps10
Filozoa – ps11
Choano./Metazoa – ps12
Metazoa – ps13
Myriazoa – ps14
Parahoxozoa – ps15
Bilateria – ps16
Protostomia – ps17
Ecdysozoa – ps18
Arthropoda – ps19
Mandibulata – ps20
Pancrustacea – ps21
Insecta – ps22
Eumetabola – ps23
Holometabola/Psocodea – ps24
Holometabola – ps25
Aparaglossata – ps26
Mecopterida – ps27
Lepidoptera – ps28
Nymphalidae – ps29
Danaus_plexippus – ps30
Phylostratum

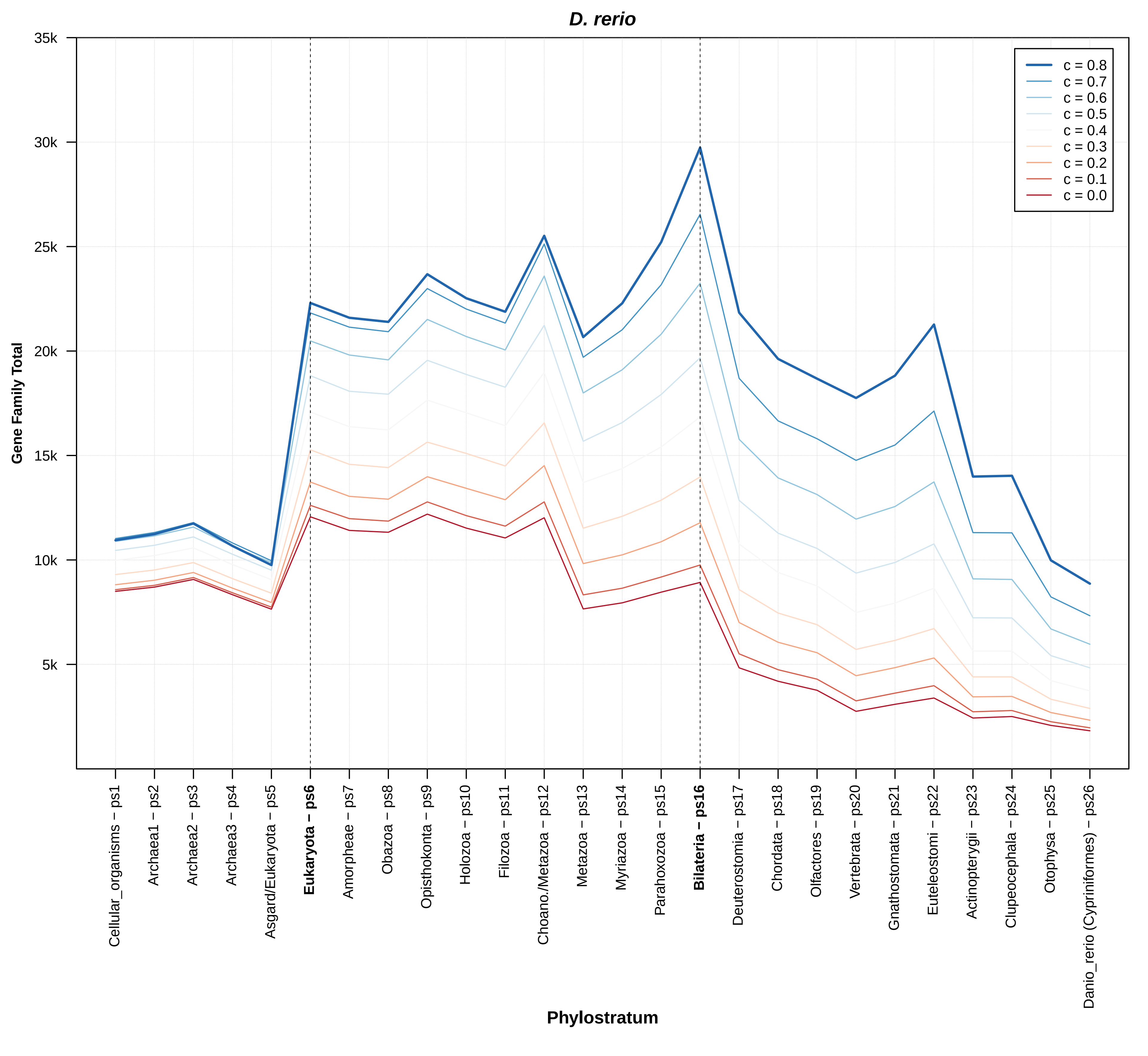

D. rerio
Gene Family Total
35k
30k
25k
20k
15k
10k
5k
c = 0.8
c = 0.7
c = 0.6
c = 0.5
c = 0.4
c = 0.3
c = 0.2
c = 0.1
c = 0.0
Cellular_organisms – ps1
Archaea1 – ps2
Archaea2 – ps3
Archaea3 – ps4
Asgard/Eukaryota – ps5
Eukaryota – ps6
Amorpheae – ps7
Obazoa – ps8
Opisthokonta – ps9
Holozoa – ps10
Filozoa – ps11
Choano./Metazoa – ps12
Metazoa – ps13
Myriazoa – ps14
Parahoxozoa – ps15
Bilateria – ps16
Deuterostomia – ps17
Chordata – ps18
Olfactores – ps19
Vertebrata – ps20
Gnathostomata – ps21
Euteleostomi – ps22
Actinopterygii – ps23
Clupeocephala – ps24
Otophysa – ps25
Danio_rerio (Cypriniformes) – ps26
Phylostratum

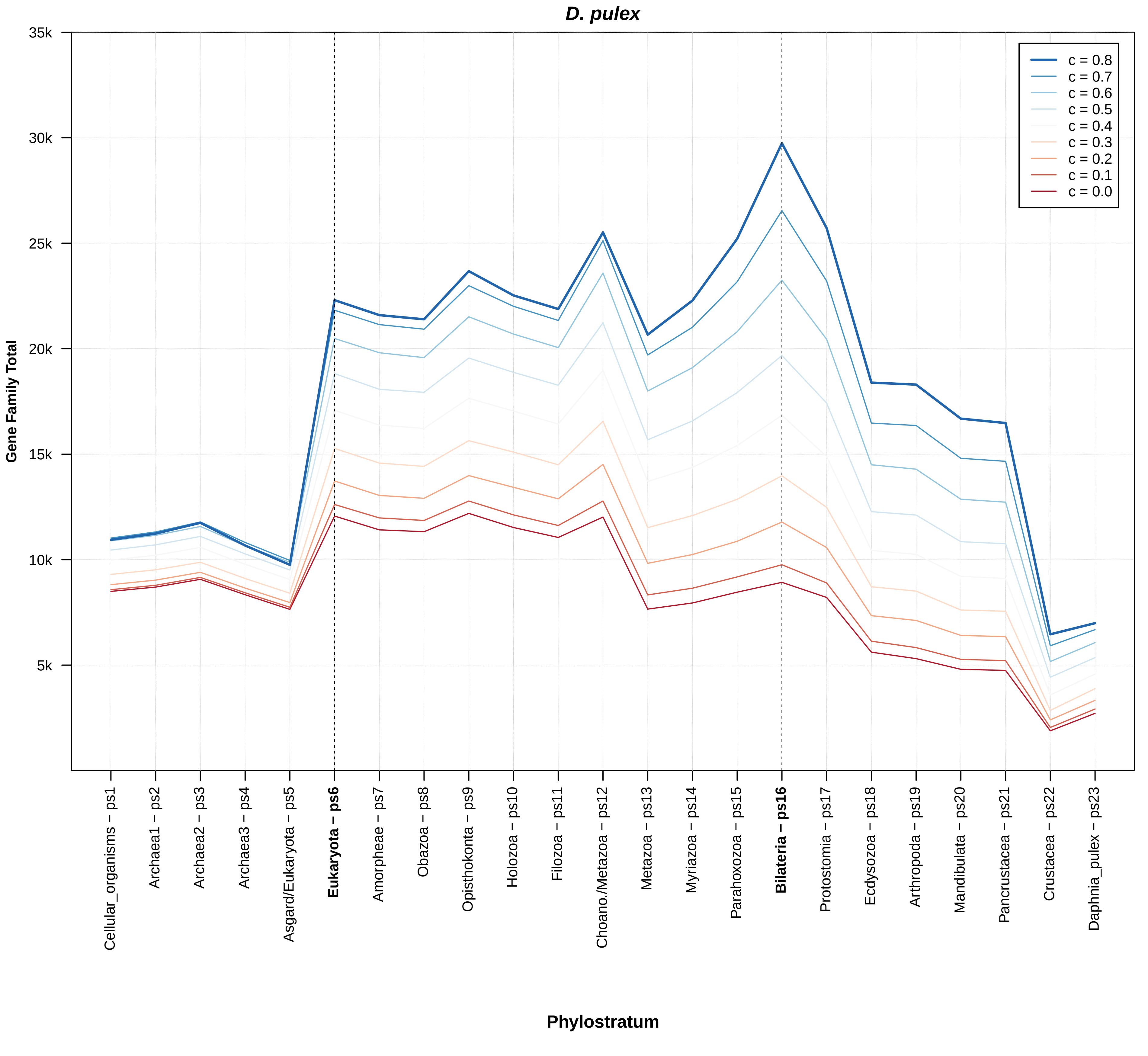

D. pulex
Gene Family Total
35k
30k
25k
20k
15k
10k
5k
c = 0.8
c = 0.7
c = 0.6
c = 0.5
c = 0.4
c = 0.3
c = 0.2
c = 0.1
c = 0.0
Cellular_organisms – ps1
Archaea1 – ps2
Archaea2 – ps3
Archaea3 – ps4
Asgard/Eukaryota – ps5
Eukaryota – ps6
Amorpheae – ps7
Obazoa – ps8
Opisthokonta – ps9
Holozoa – ps10
Filozoa – ps11
Choano./Metazoa – ps12
Metazoa – ps13
Myriazoa – ps14
Parahoxozoa – ps15
Bilateria – ps16
Protostomia – ps17
Ecdysozoa – ps18
Arthropoda – ps19
Mandibulata – ps20
Pancrustacea – ps21
Crustacea – ps22
Daphnia_pulex – ps23
Phylostratum

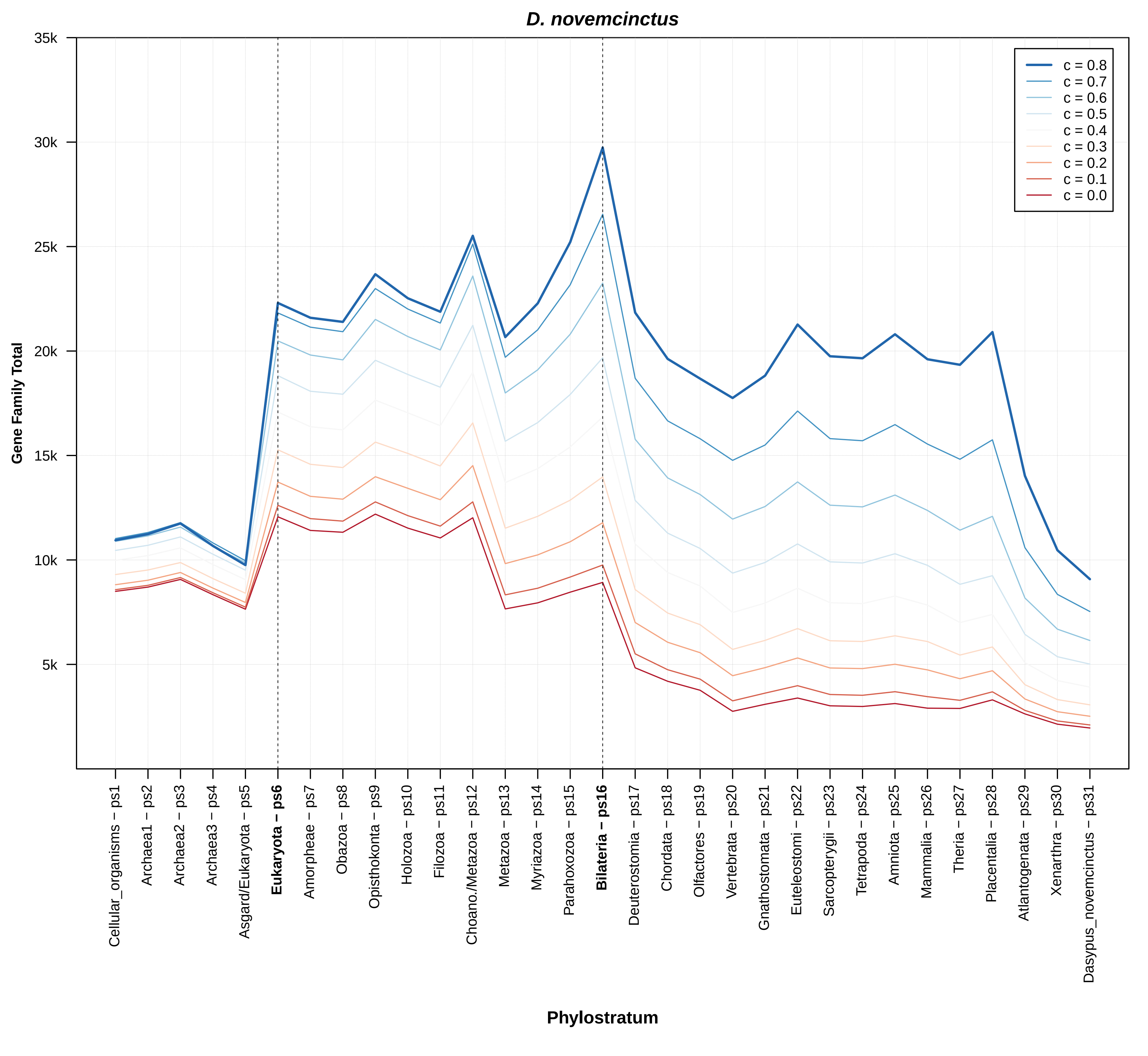

D. novemcinctus
Gene Family Total
35k
30k
25k
20k
15k
10k
5k
c = 0.8
c = 0.7
c = 0.6
c = 0.5
c = 0.4
c = 0.3
c = 0.2
c = 0.1
c = 0.0
Cellular_organisms – ps1
Archaea1 – ps2
Archaea2 – ps3
Archaea3 – ps4
Asgard/Eukaryota – ps5
Eukaryota – ps6
Amorpheae – ps7
Obazoa – ps8
Opisthokonta – ps9
Holozoa – ps10
Filozoa – ps11
Choano./Metazoa – ps12
Metazoa – ps13
Myriazoa – ps14
Parahoxozoa – ps15
Bilateria – ps16
Deuterostomia – ps17
Chordata – ps18
Olfactores – ps19
Vertebrata – ps20
Gnathostomata – ps21
Euteleostomi – ps22
Sarcopterygii – ps23
Tetrapoda – ps24
Amniota – ps25
Mammalia – ps26
Theria – ps27
Placentalia – ps28
Atlantogenata – ps29
Xenarthra – ps30
Dasypus_novemcinctus – ps31
Phylostratum

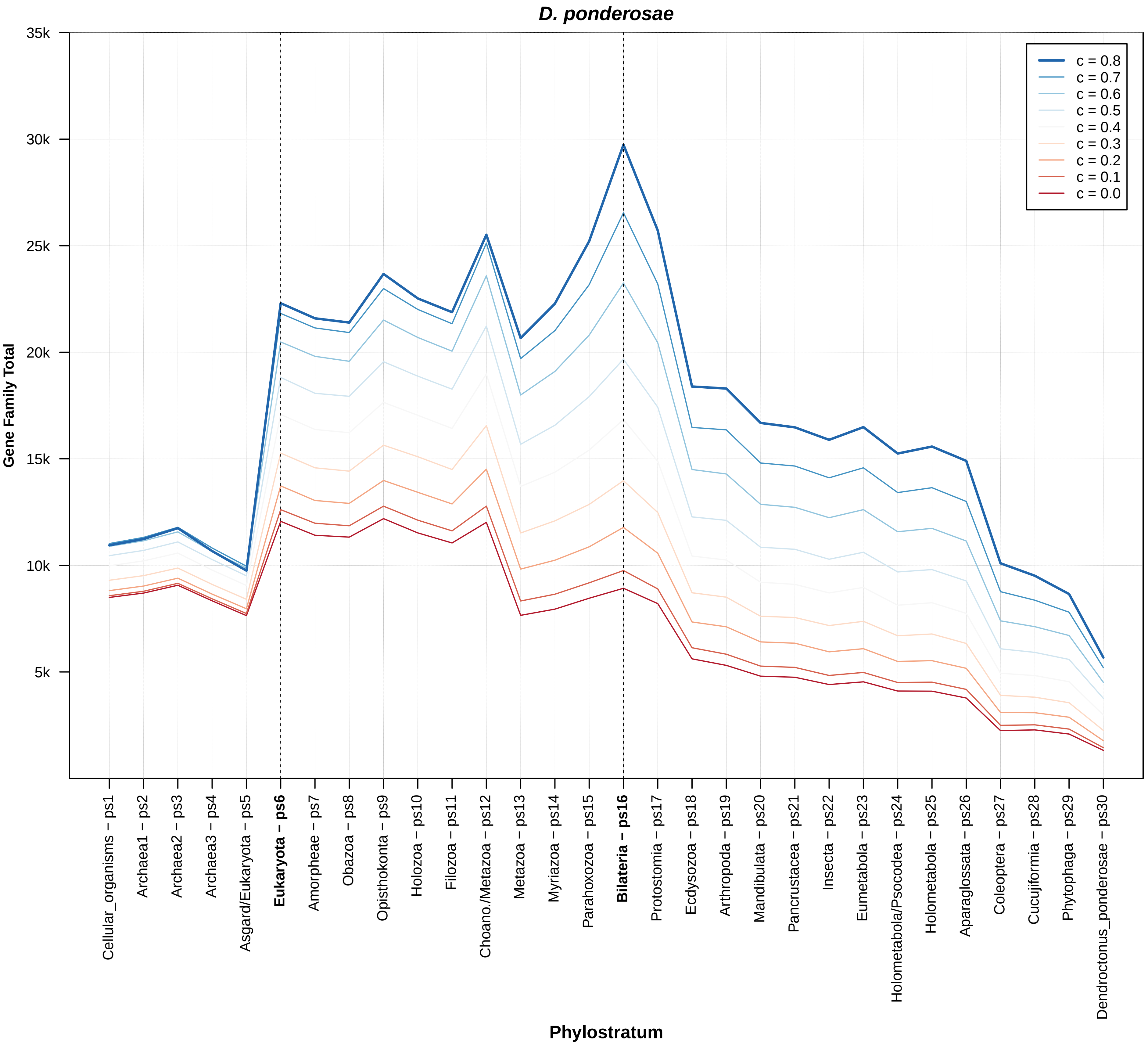
D. ponderosae
Gene Family Total
Phylostratum
35k
30k
25k
20k
15k
10k
5k
c = 0.8
c = 0.7
c = 0.6
c = 0.5
c = 0.4
c = 0.3
c = 0.2
c = 0.1
c = 0.0
Cellular_organisms – ps1
Archaea1 – ps2
Archaea2 – ps3
Archaea3 – ps4
Asgard/Eukaryota – ps5
Eukaryota – ps6
Amorpheae – ps7
Obazoa – ps8
Opisthokonta – ps9
Holozoa – ps10
Filozoa – ps11
Choano./Metazoa – ps12
Metazoa – ps13
Myriazoa – ps14
Parahoxozoa – ps15
Bilateria – ps16
Protostomia – ps17
Ecdysozoa – ps18
Arthropoda – ps19
Mandibulata – ps20
Pancrustacea – ps21
Insecta – ps22
Eumetabola – ps23
Holometabola/Psocodea – ps24
Holometabola – ps25
Aparaglossata – ps26
Coleoptera – ps27
Cucujiformia – ps28
Phytophaga – ps29
Dendroctonus_ponderosae – ps30

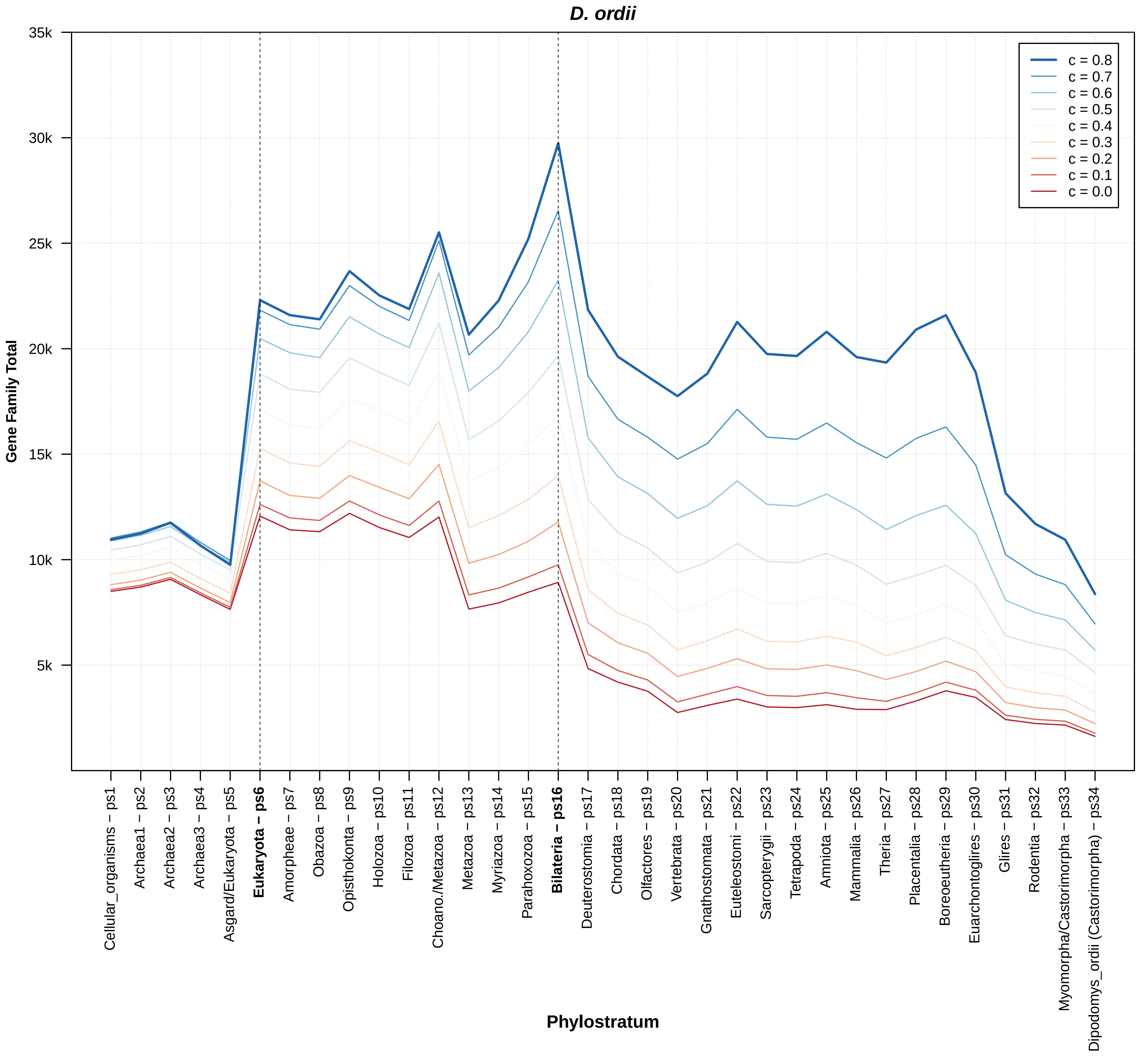

D. ordii
Gene Family Total
Phylostratum
35k
30k
25k
20k
15k
10k
5k
c = 0.8
c = 0.7
c = 0.6
c = 0.5
c = 0.4
c = 0.3
c = 0.2
c = 0.1
c = 0.0
Cellular_organisms – ps1
Archaea1 – ps2
Archaea2 – ps3
Archaea3 – ps4
Asgard/Eukaryota – ps5
Eukaryota – ps6
Amorpheae – ps7
Obazoa – ps8
Opisthokonta – ps9
Holozoa – ps10
Filozoa – ps11
Choano./Metazoa – ps12
Metazoa – ps13
Myriazoa – ps14
Parahoxozoa – ps15
Bilateria – ps16
Deuterostomia – ps17
Chordata – ps18
Olfactores – ps19
Vertebrata – ps20
Gnathostomata – ps21
Euteleostomi – ps22
Sarcopterygii – ps23
Tetrapoda – ps24
Amniota – ps25
Mammalia – ps26
Theria – ps27
Placentalia – ps28
Boreoeutheria – ps29
Euarchontoglires – ps30
Glires – ps31
Rodentia – ps32
Myomorpha/Castorimorpha – ps33
Dipodomys_ordii (Castorimorpha) – ps34

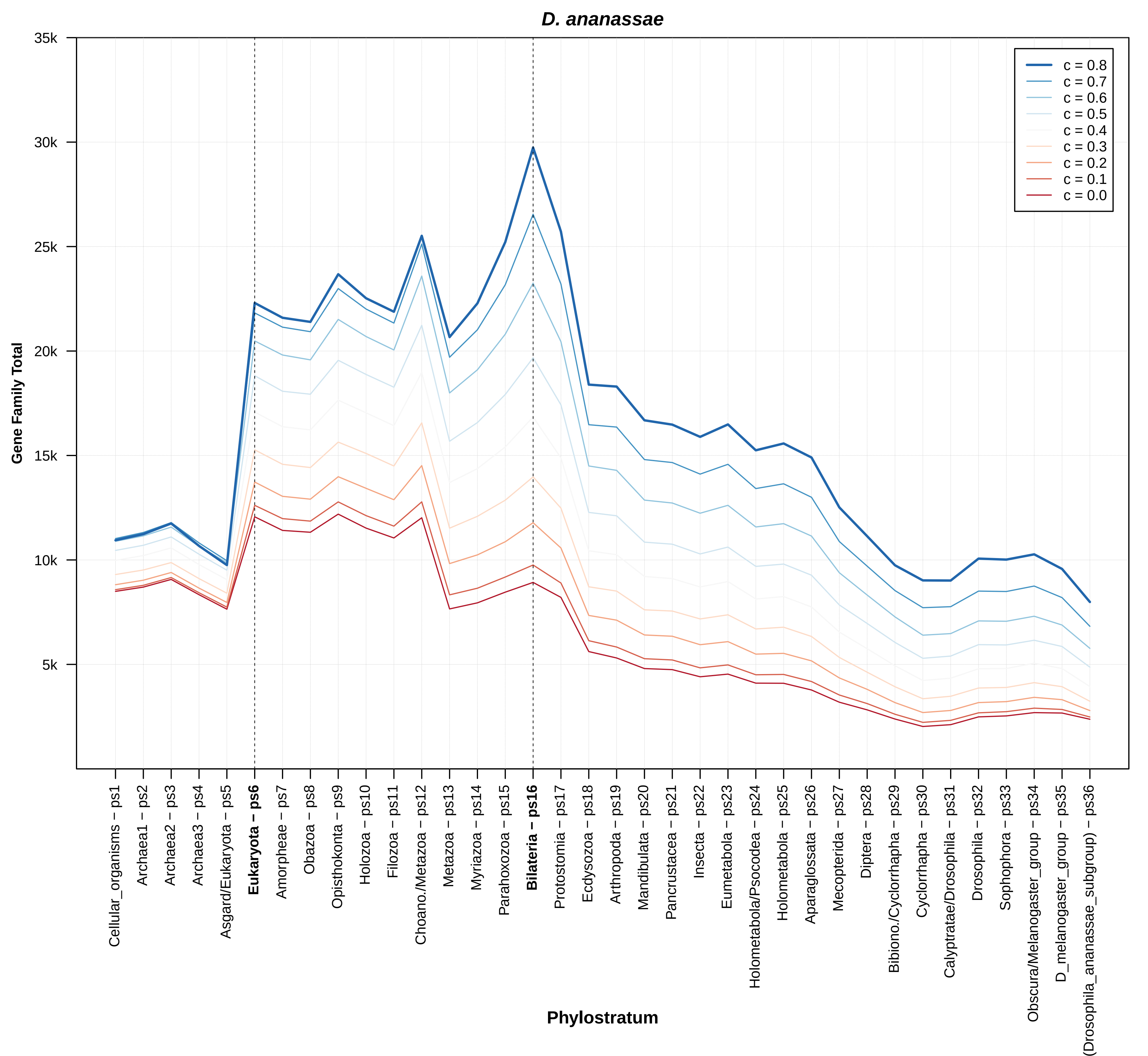
D. ananassae
Gene Family Total
35k
30k
25k
20k
15k
10k
5k
c = 0.8
c = 0.7
c = 0.6
c = 0.5
c = 0.4
c = 0.3
c = 0.2
c = 0.1
c = 0.0
Cellular_organisms – ps1
Archaea1 – ps2
Archaea2 – ps3
Archaea3 – ps4
Asgard/Eukaryota – ps5
Eukaryota – ps6
Amorpheae – ps7
Obazoa – ps8
Opisthokonta – ps9
Holozoa – ps10
Filozoa – ps11
Choano./Metazoa – ps12
Metazoa – ps13
Myriazoa – ps14
Parahoxozoa – ps15
Bilateria – ps16
Protostomia – ps17
Ecdysozoa – ps18
Arthropoda – ps19
Mandibulata – ps20
Pancrustacea – ps21
Insecta – ps22
Eumetabola – ps23
Holometabola/Psocodea – ps24
Holometabola – ps25
Aparaglossata – ps26
Mecopterida – ps27
Diptera – ps28
Bibiono./Cyclorrhapha – ps29
Cyclorrhapha – ps30
Calyptratae/Drosophila – ps31
Drosophila – ps32
Sophophora – ps33
Obscura/Melanogaster_group – ps34
D_melanogaster_group – ps35
(Drosophila_ananassae_subgroup) – ps36
Phylostratum

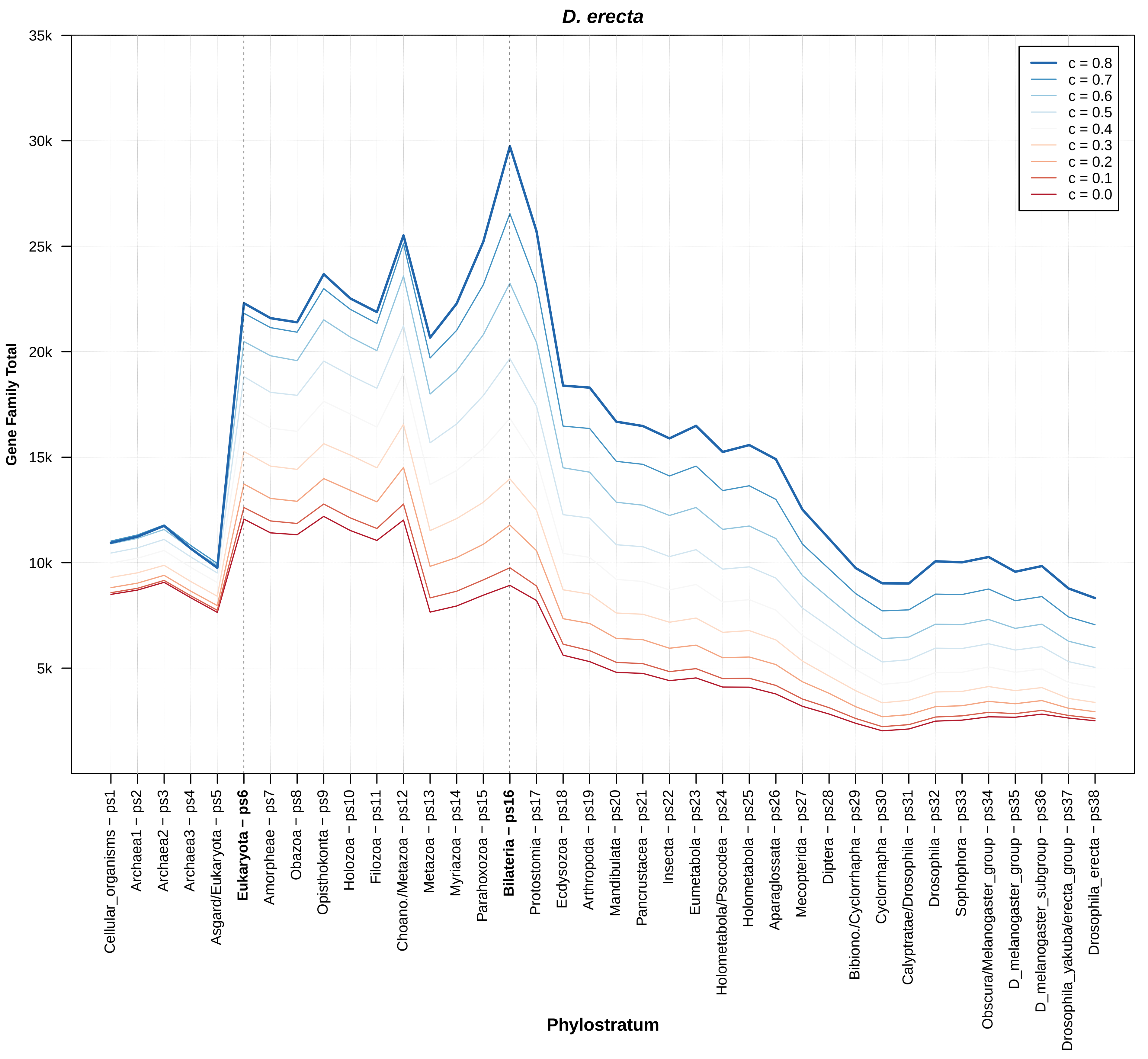
D. erecta
Gene Family Total
Phylostratum
35k
30k
25k
20k
15k
10k
5k
c = 0.8
c = 0.7
c = 0.6
c = 0.5
c = 0.4
c = 0.3
c = 0.2
c = 0.1
c = 0.0
Cellular_organisms – ps1
Archaea1 – ps2
Archaea2 – ps3
Archaea3 – ps4
Asgard/Eukaryota – ps5
Eukaryota – ps6
Amorpheae – ps7
Obazoa – ps8
Opisthokonta – ps9
Holozoa – ps10
Filozoa – ps11
Choano./Metazoa – ps12
Metazoa – ps13
Myriazoa – ps14
Parahoxozoa – ps15
Bilateria – ps16
Protostomia – ps17
Ecdysozoa – ps18
Arthropoda – ps19
Mandibulata – ps20
Pancrustacea – ps21
Insecta – ps22
Eumetabola – ps23
Holometabola/Psocodea – ps24
Holometabola – ps25
Aparaglossata – ps26
Mecopterida – ps27
Diptera – ps28
Bibiono./Cyclorrhapha – ps29
Cyclorrhapha – ps30
Calyptratae/Drosophila – ps31
Drosophila – ps32
Sophophora – ps33
Obscura/Melanogaster_group – ps34
D_melanogaster_group – ps35
D_melanogaster_subgroup – ps36
Drosophila_yakuba/erecta_group – ps37
Drosophila_erecta – ps38

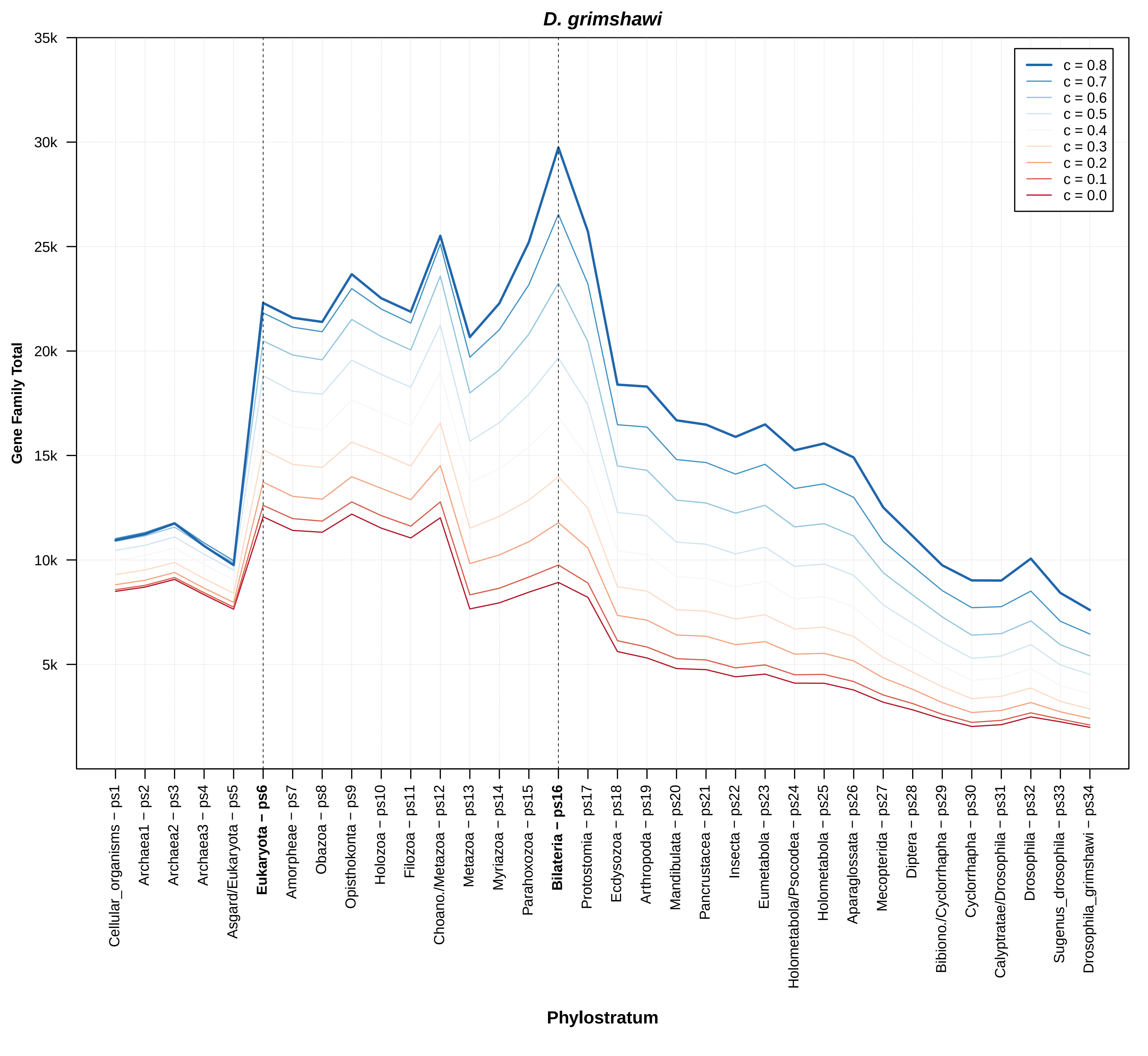

D. grimshawi
c = 0.8
c = 0.7
c = 0.6
c = 0.5
c = 0.4
c = 0.3
c = 0.2
c = 0.1
c = 0.0
Gene Family Total
35k
30k
25k
20k
15k
10k
5k
Phylostratum
Cellular_organisms – ps1
Archaea1 – ps2
Archaea2 – ps3
Archaea3 – ps4
Asgard/Eukaryota – ps5
Eukaryota – ps6
Amorpheae – ps7
Obazoa – ps8
Opisthokonta – ps9
Holozoa – ps10
Filozoa – ps11
Choano./Metazoa – ps12
Metazoa – ps13
Myriazoa – ps14
Parahoxozoa – ps15
Bilateria – ps16
Protostomia – ps17
Ecdysozoa – ps18
Arthropoda – ps19
Mandibulata – ps20
Pancrustacea – ps21
Insecta – ps22
Eumetabola – ps23
Holometabola/Psocodea – ps24
Holometabola – ps25
Aparaglossata – ps26
Mecopterida – ps27
Diptera – ps28
Bibiono./Cyclorrhapha – ps29
Cyclorrhapha – ps30
Calyptratae/Drosophila – ps31
Drosophila – ps32
Sugenus_drosophila – ps33
Drosophila_grimshawi – ps34

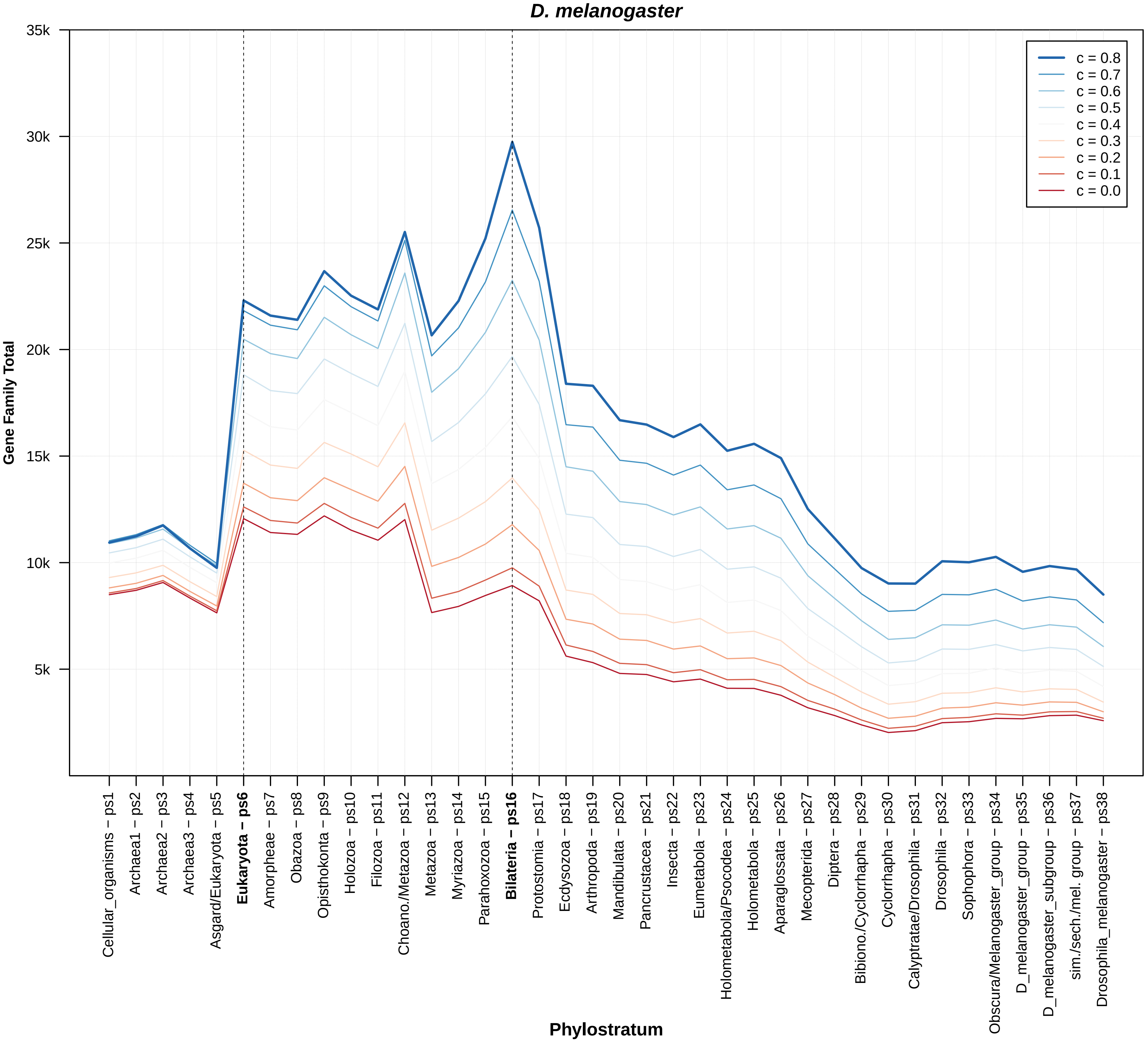

D. melanogaster
Gene Family Total
35k
30k
25k
20k
15k
10k
5k
c = 0.8
c = 0.7
c = 0.6
c = 0.5
c = 0.4
c = 0.3
c = 0.2
c = 0.1
c = 0.0
Cellular_organisms – ps1
Archaea1 – ps2
Archaea2 – ps3
Archaea3 – ps4
Asgard/Eukaryota – ps5
Eukaryota – ps6
Amorpheae – ps7
Obazoa – ps8
Opisthokonta – ps9
Holozoa – ps10
Filozoa – ps11
Choano./Metazoa – ps12
Metazoa – ps13
Myriazoa – ps14
Parahoxozoa – ps15
Bilateria – ps16
Protostomia – ps17
Ecdysozoa – ps18
Arthropoda – ps19
Mandibulata – ps20
Pancrustacea – ps21
Insecta – ps22
Eumetabola – ps23
Holometabola/Psocodea – ps24
Holometabola – ps25
Aparaglossata – ps26
Mecopterida – ps27
Diptera – ps28
Bibiono./Cyclorrhapha – ps29
Cyclorrhapha – ps30
Calyptratae/Drosophila – ps31
Drosophila – ps32
Sophophora – ps33
Obscura/Melanogaster_group – ps34
D_melanogaster_group – ps35
D_melanogaster_subgroup – ps36
sim./sech./mel. group – ps37
Drosophila_melanogaster – ps38
Phylostratum

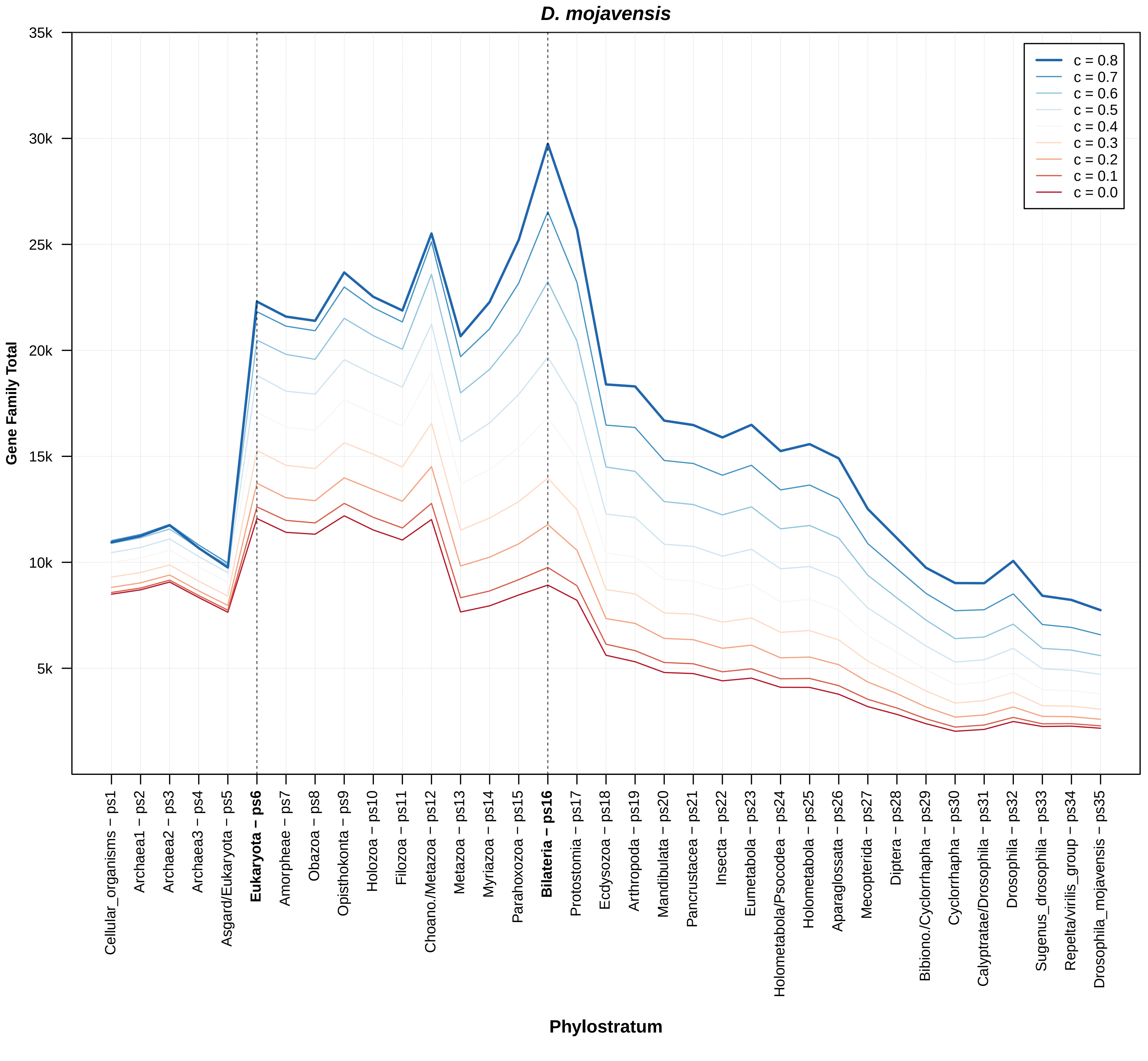

D. mojavensis
Gene Family Total
35k
30k
25k
20k
15k
10k
5k
c = 0.8
c = 0.7
c = 0.6
c = 0.5
c = 0.4
c = 0.3
c = 0.2
c = 0.1
c = 0.0
Cellular_organisms – ps1
Archaea1 – ps2
Archaea2 – ps3
Archaea3 – ps4
Asgard/Eukaryota – ps5
Eukaryota – ps6
Amorpheae – ps7
Obazoa – ps8
Opisthokonta – ps9
Holozoa – ps10
Filozoa – ps11
Choano./Metazoa – ps12
Metazoa – ps13
Myriazoa – ps14
Parahoxozoa – ps15
Bilateria – ps16
Protostomia – ps17
Ecdysozoa – ps18
Arthropoda – ps19
Mandibulata – ps20
Pancrustacea – ps21
Insecta – ps22
Eumetabola – ps23
Holometabola/Psocodea – ps24
Holometabola – ps25
Aparaglossata – ps26
Mecopterida – ps27
Diptera – ps28
Bibiono./Cyclorrhapha – ps29
Cyclorrhapha – ps30
Calyptratae/Drosophila – ps31
Drosophila – ps32
Sugenus_drosophila – ps33
Repelta/virilis_group – ps34
Drosophila_mojavensis – ps35
Phylostratum

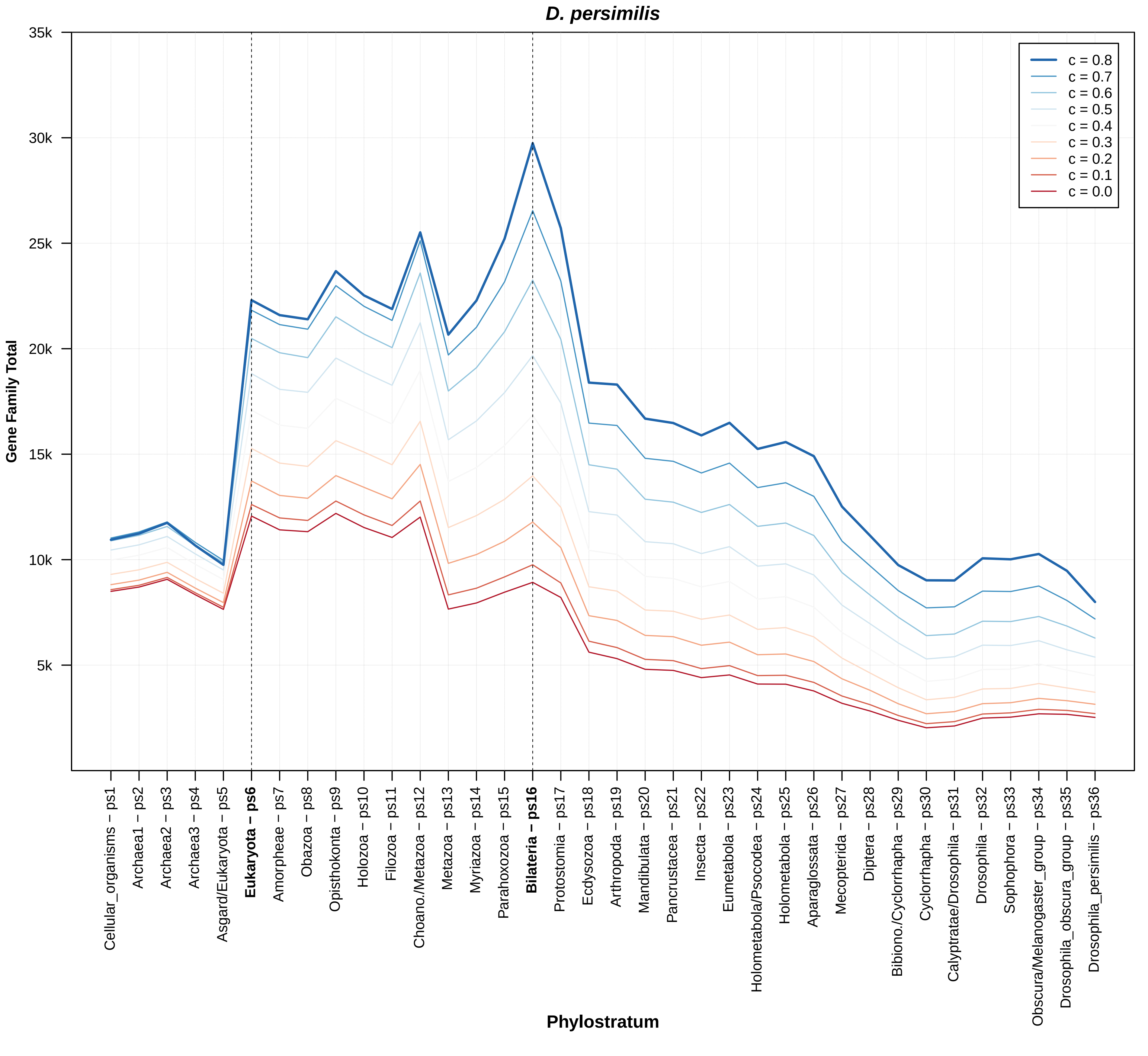

D. persimilis
Gene Family Total
35k
30k
25k
20k
15k
10k
5k
c = 0.8
c = 0.7
c = 0.6
c = 0.5
c = 0.4
c = 0.3
c = 0.2
c = 0.1
c = 0.0
Cellular_organisms – ps1
Archaea1 – ps2
Archaea2 – ps3
Archaea3 – ps4
Asgard/Eukaryota – ps5
Eukaryota – ps6
Amorpheae – ps7
Obazoa – ps8
Opisthokonta – ps9
Holozoa – ps10
Filozoa – ps11
Choano./Metazoa – ps12
Metazoa – ps13
Myriazoa – ps14
Parahoxozoa – ps15
Bilateria – ps16
Protostomia – ps17
Ecdysozoa – ps18
Arthropoda – ps19
Mandibulata – ps20
Pancrustacea – ps21
Insecta – ps22
Eumetabola – ps23
Holometabola/Psocodea – ps24
Holometabola – ps25
Aparaglossata – ps26
Mecopterida – ps27
Diptera – ps28
Bibiono./Cyclorrhapha – ps29
Cyclorrhapha – ps30
Calyptratae/Drosophila – ps31
Drosophila – ps32
Sophophora – ps33
Obscura/Melanogaster_group – ps34
Drosophila_obscura_group – ps35
Drosophila_persimilis – ps36
Phylostratum

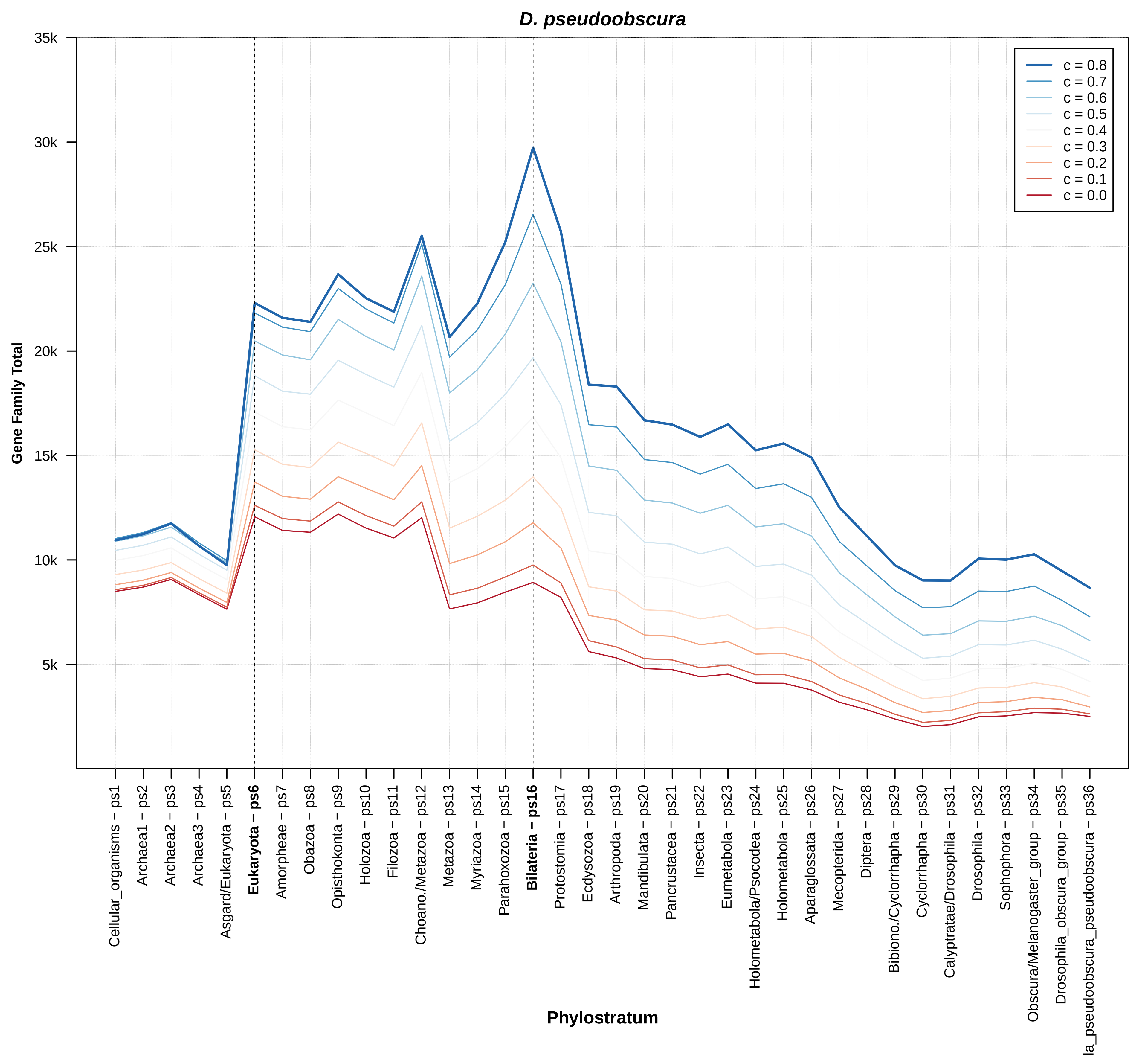

D. pseudoobscura
Gene Family Total
Phylostratum
35k
30k
25k
20k
15k
10k
5k
c = 0.8
c = 0.7
c = 0.6
c = 0.5
c = 0.4
c = 0.3
c = 0.2
c = 0.1
c = 0.0
Cellular_organisms – ps1
Archaea1 – ps2
Archaea2 – ps3
Archaea3 – ps4
Asgard/Eukaryota – ps5
Eukaryota – ps6
Amorpheae – ps7
Obazoa – ps8
Opisthokonta – ps9
Holozoa – ps10
Filozoa – ps11
Choano./Metazoa – ps12
Metazoa – ps13
Myriazoa – ps14
Parahoxozoa – ps15
Bilateria – ps16
Protostomia – ps17
Ecdysozoa – ps18
Arthropoda – ps19
Mandibulata – ps20
Pancrustacea – ps21
Insecta – ps22
Eumetabola – ps23
Holometabola/Psocodea – ps24
Holometabola – ps25
Aparaglossata – ps26
Mecopterida – ps27
Diptera – ps28
Bibiono./Cyclorrhapha – ps29
Cyclorrhapha – ps30
Calyptratae/Drosophila – ps31
Drosophila – ps32
Sophophora – ps33
Obscura/Melanogaster_group – ps34
Drosophila_obscura_group – ps35
la_pseudoobscura_pseudoobscura – ps36

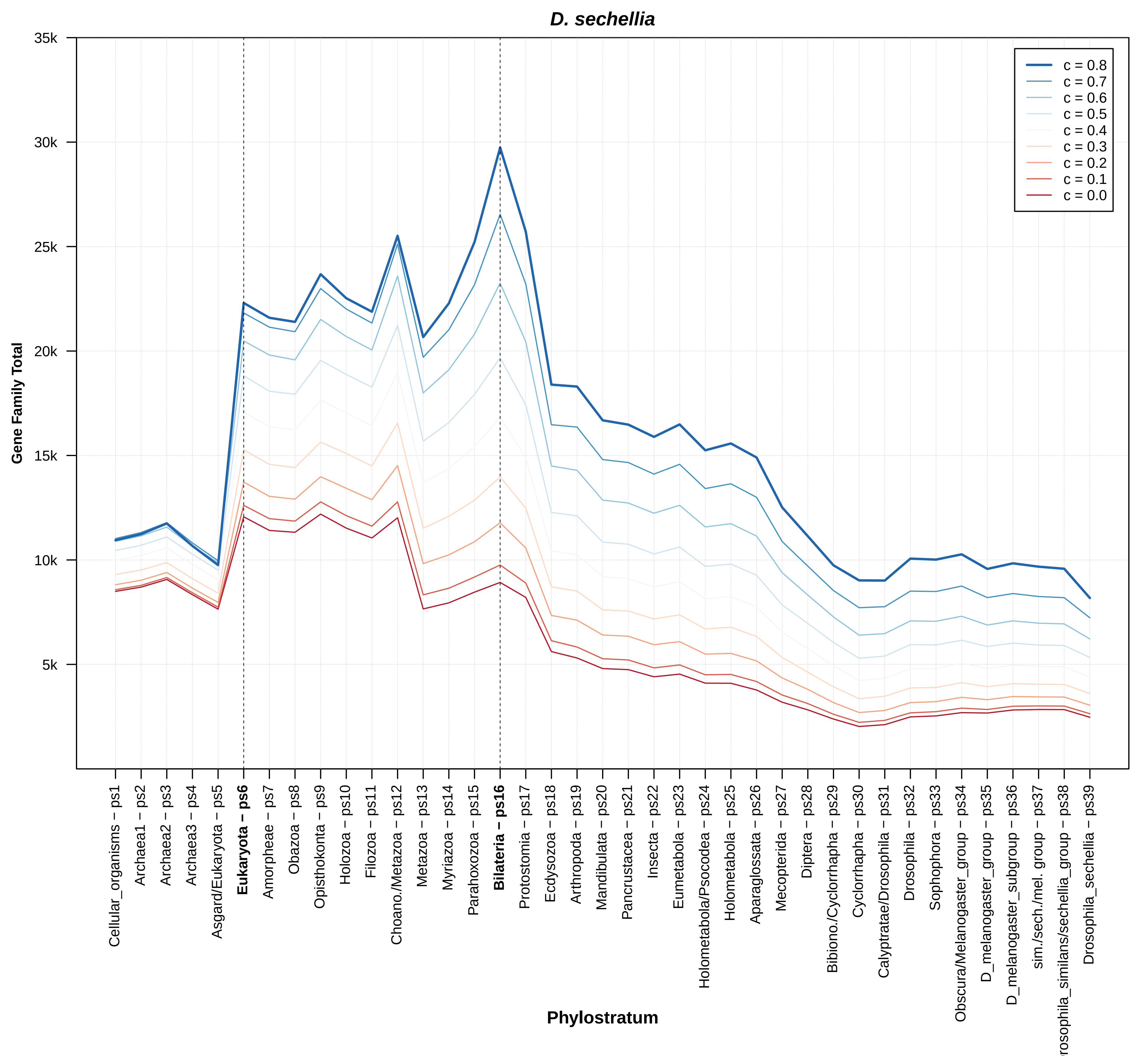

D. sechellia
35k
30k
25k
20k
15k
10k
5k
Gene Family Total
c = 0.8
c = 0.7
c = 0.6
c = 0.5
c = 0.4
c = 0.3
c = 0.2
c = 0.1
c = 0.0
Cellular_organisms – ps1
Archaea1 – ps2
Archaea2 – ps3
Archaea3 – ps4
Asgard/Eukaryota – ps5
Eukaryota – ps6
Amorpheae – ps7
Obazoa – ps8
Opisthokonta – ps9
Holozoa – ps10
Filozoa – ps11
Choano./Metazoa – ps12
Metazoa – ps13
Myriazoa – ps14
Parahoxozoa – ps15
Bilateria – ps16
Protostomia – ps17
Ecdysozoa – ps18
Arthropoda – ps19
Mandibulata – ps20
Pancrustacea – ps21
Insecta – ps22
Eumetabola – ps23
Holometabola/Psocodea – ps24
Holometabola – ps25
Aparaglossata – ps26
Mecopterida – ps27
Diptera – ps28
Bibiono./Cyclorrhapha – ps29
Cyclorrhapha – ps30
Calyptratae/Drosophila – ps31
Drosophila – ps32
Sophophora – ps33
Obscura/Melanogaster_group – ps34
D_melanogaster_group – ps35
D_melanogaster_subgroup – ps36
sim./sech./mel. group – ps37
rosophila_similans/sechellia_group – ps38
Drosophila_sechellia – ps39
Phylostratum

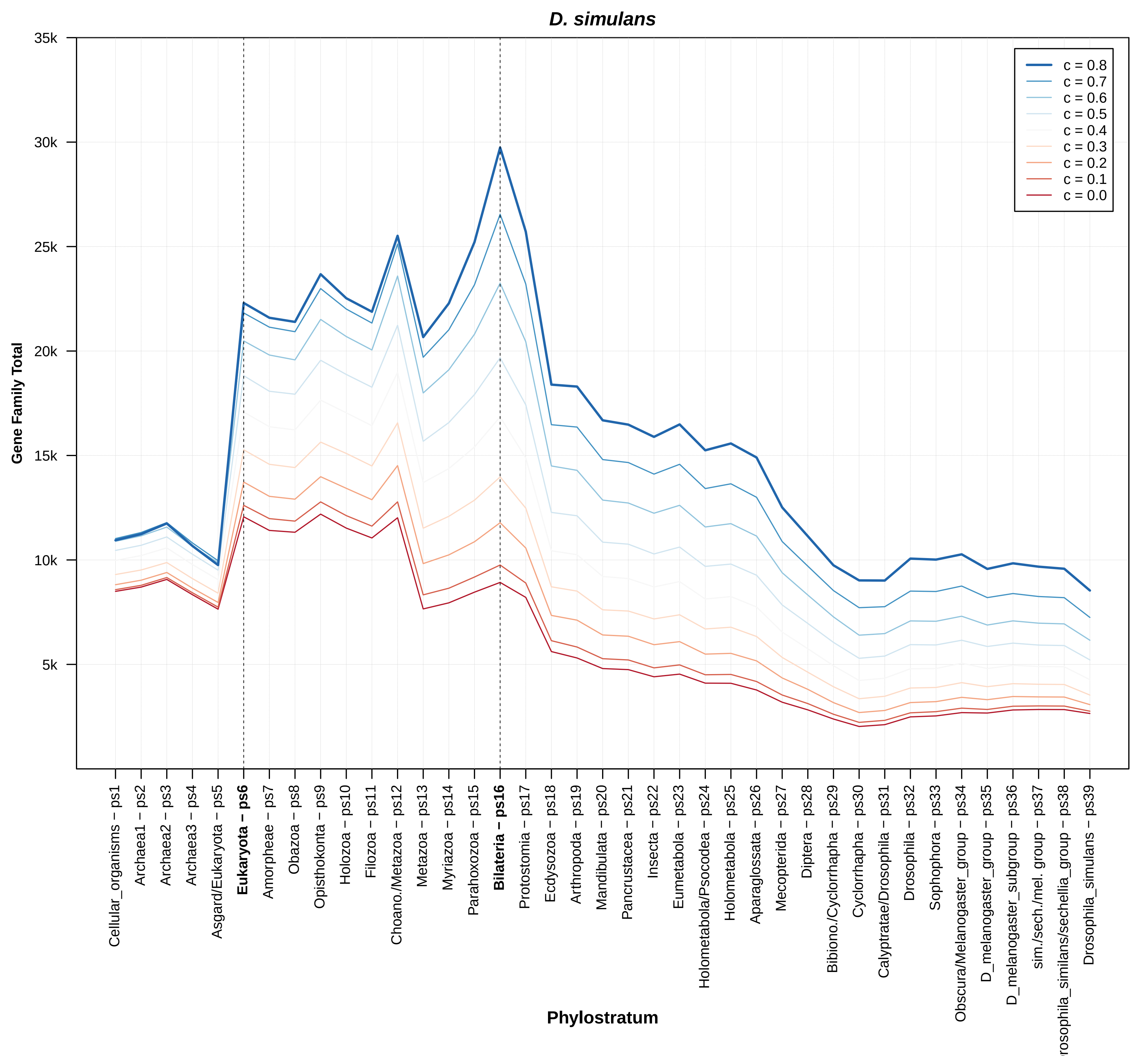
D. simulans
Gene Family Total
Phylostratum
35k
30k
25k
20k
15k
10k
5k
c = 0.8
c = 0.7
c = 0.6
c = 0.5
c = 0.4
c = 0.3
c = 0.2
c = 0.1
c = 0.0
Cellular_organisms – ps1
Archaea1 – ps2
Archaea2 – ps3
Archaea3 – ps4
Asgard/Eukaryota – ps5
Eukaryota – ps6
Amorpheae – ps7
Obazoa – ps8
Opisthokonta – ps9
Holozoa – ps10
Filozoa – ps11
Choano./Metazoa – ps12
Metazoa – ps13
Myriazoa – ps14
Parahoxozoa – ps15
Bilateria – ps16
Protostomia – ps17
Ecdysozoa – ps18
Arthropoda – ps19
Mandibulata – ps20
Pancrustacea – ps21
Insecta – ps22
Eumetabola – ps23
Holometabola/Psocodea – ps24
Holometabola – ps25
Aparaglossata – ps26
Mecopterida – ps27
Diptera – ps28
Bibiono./Cyclorrhapha – ps29
Cyclorrhapha – ps30
Calyptratae/Drosophila – ps31
Drosophila – ps32
Sophophora – ps33
Obscura/Melanogaster_group – ps34
D_melanogaster_group – ps35
D_melanogaster_subgroup – ps36
sim./sech./mel. group – ps37
rosophila_similans/sechellia_group – ps38
Drosophila_simulans – ps39

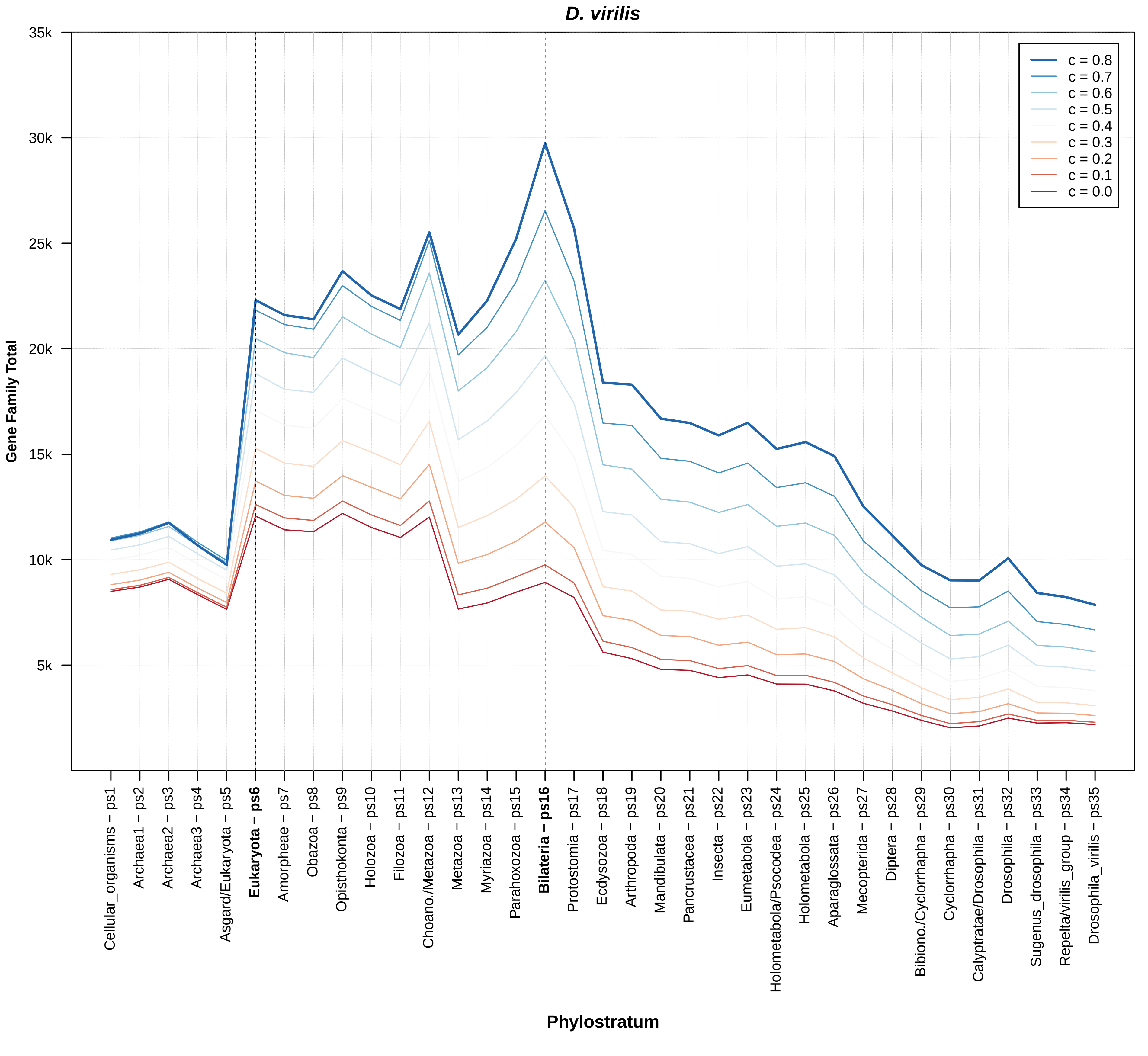
D. virilis
Gene Family Total
35k
30k
25k
20k
15k
10k
5k
c = 0.8
c = 0.7
c = 0.6
c = 0.5
c = 0.4
c = 0.3
c = 0.2
c = 0.1
c = 0.0
Cellular_organisms – ps1
Archaea1 – ps2
Archaea2 – ps3
Archaea3 – ps4
Asgard/Eukaryota – ps5
Eukaryota – ps6
Amorpheae – ps7
Obazoa – ps8
Opisthokonta – ps9
Holozoa – ps10
Filozoa – ps11
Choano./Metazoa – ps12
Metazoa – ps13
Myriazoa – ps14
Parahoxozoa – ps15
Bilateria – ps16
Protostomia – ps17
Ecdysozoa – ps18
Arthropoda – ps19
Mandibulata – ps20
Pancrustacea – ps21
Insecta – ps22
Eumetabola – ps23
Holometabola/Psocodea – ps24
Holometabola – ps25
Aparaglossata – ps26
Mecopterida – ps27
Diptera – ps28
Bibiono./Cyclorrhapha – ps29
Cyclorrhapha – ps30
Calyptratae/Drosophila – ps31
Drosophila – ps32
Sugenus_drosophila – ps33
Repelta/virilis_group – ps34
Drosophila_virilis – ps35
Phylostratum

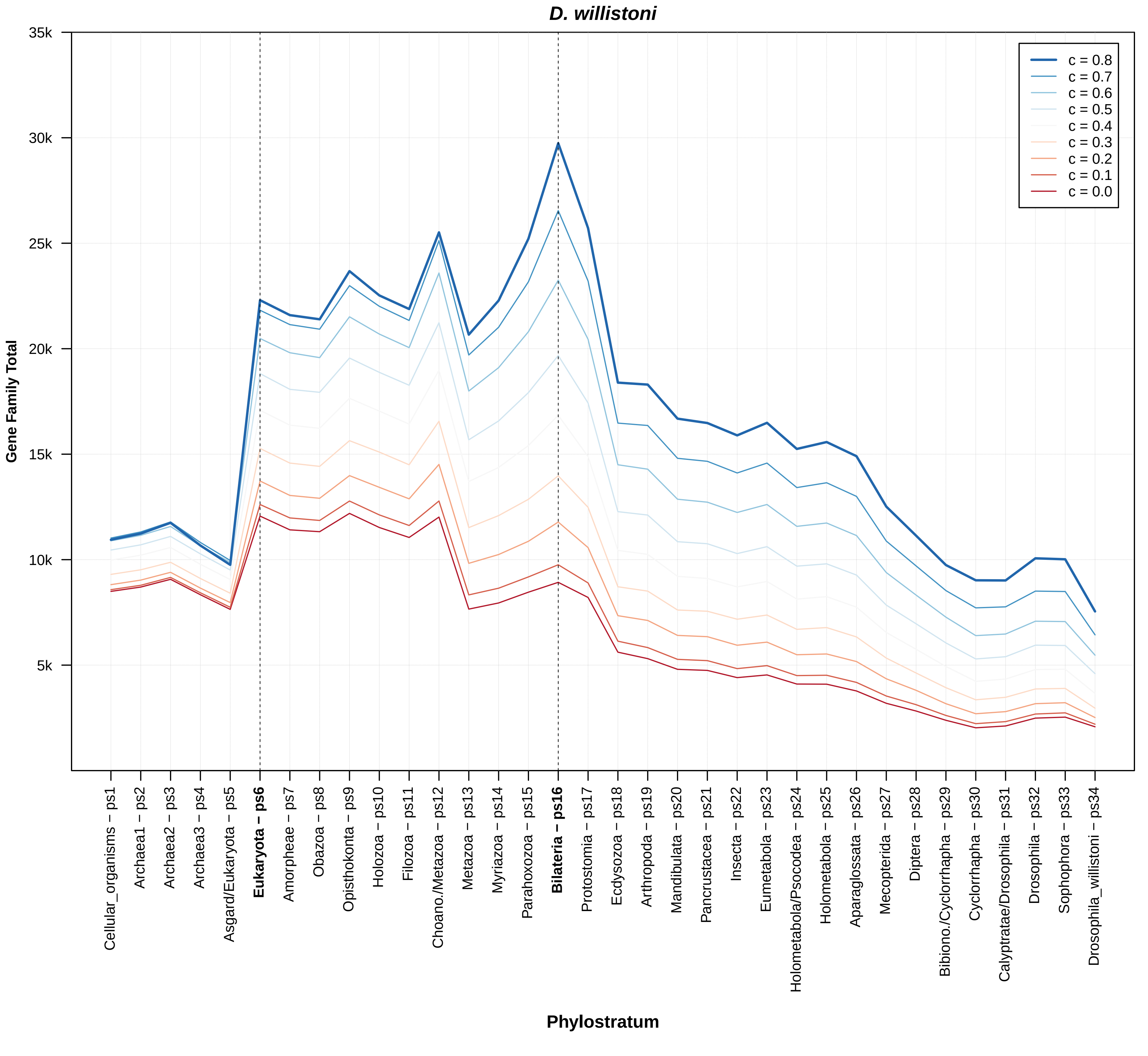
D. willistoni
Gene Family Total
35k
30k
25k
20k
15k
10k
5k
c = 0.8
c = 0.7
c = 0.6
c = 0.5
c = 0.4
c = 0.3
c = 0.2
c = 0.1
c = 0.0
Cellular_organisms – ps1
Archaea1 – ps2
Archaea2 – ps3
Archaea3 – ps4
Asgard/Eukaryota – ps5
Eukaryota – ps6
Amorpheae – ps7
Obazoa – ps8
Opisthokonta – ps9
Holozoa – ps10
Filozoa – ps11
Choano./Metazoa – ps12
Metazoa – ps13
Myriazoa – ps14
Parahoxozoa – ps15
Bilateria – ps16
Protostomia – ps17
Ecdysozoa – ps18
Arthropoda – ps19
Mandibulata – ps20
Pancrustacea – ps21
Insecta – ps22
Eumetabola – ps23
Holometabola/Psocodea – ps24
Holometabola – ps25
Aparaglossata – ps26
Mecopterida – ps27
Diptera – ps28
Bibiono./Cyclorrhapha – ps29
Cyclorrhapha – ps30
Calyptratae/Drosophila – ps31
Drosophila – ps32
Sophophora – ps33
Drosophila_willistoni – ps34
Phylostratum

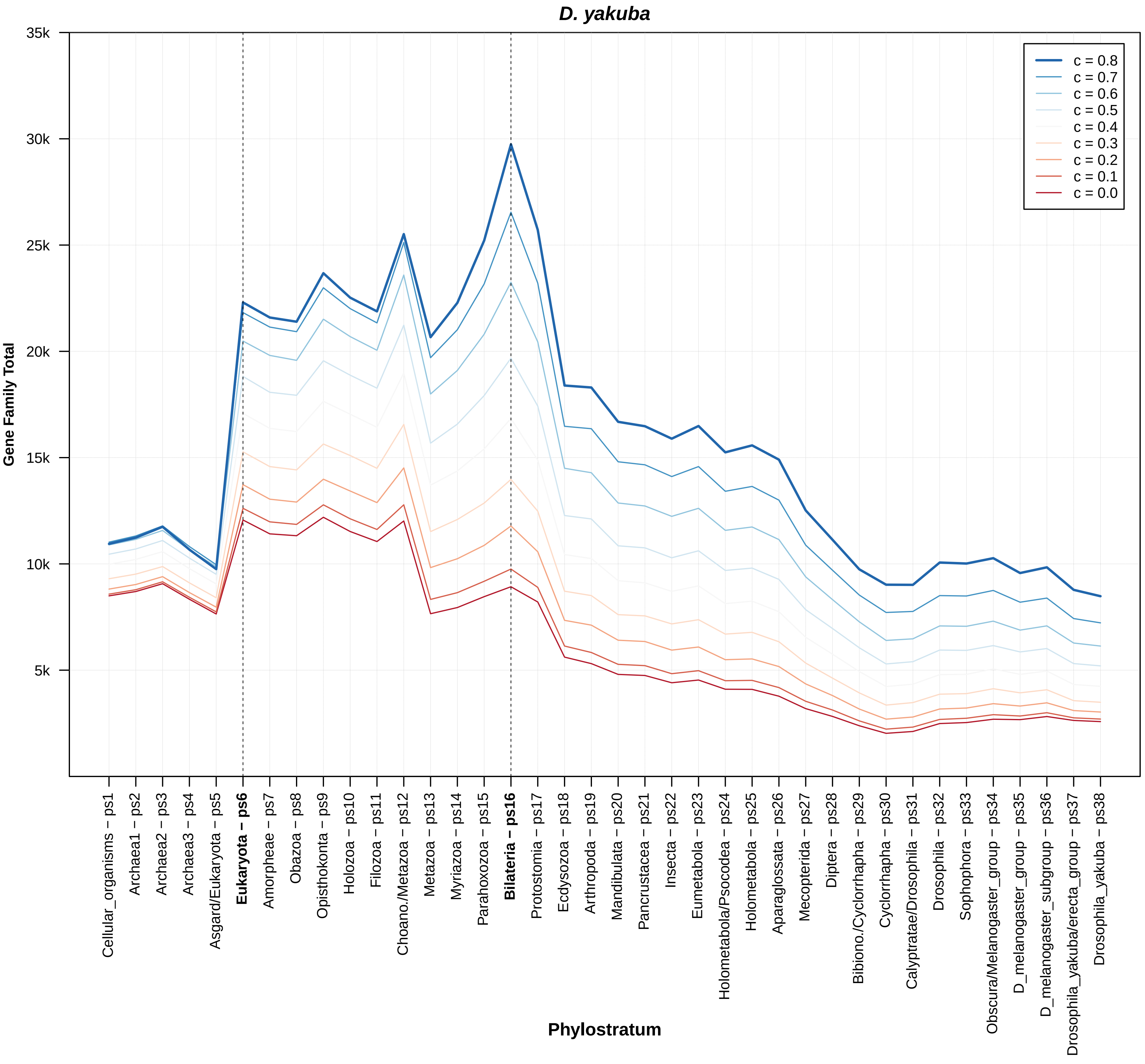
D. yakuba
Gene Family Total
Phylostratum
35k
30k
25k
20k
15k
10k
5k
c = 0.8
c = 0.7
c = 0.6
c = 0.5
c = 0.4
c = 0.3
c = 0.2
c = 0.1
c = 0.0
Cellular_organisms – ps1
Archaea1 – ps2
Archaea2 – ps3
Archaea3 – ps4
Asgard/Eukaryota – ps5
Eukaryota – ps6
Amorpheae – ps7
Obazoa – ps8
Opisthokonta – ps9
Holozoa – ps10
Filozoa – ps11
Choano./Metazoa – ps12
Metazoa – ps13
Myriazoa – ps14
Parahoxozoa – ps15
Bilateria – ps16
Protostomia – ps17
Ecdysozoa – ps18
Arthropoda – ps19
Mandibulata – ps20
Pancrustacea – ps21
Insecta – ps22
Eumetabola – ps23
Holometabola/Psocodea – ps24
Holometabola – ps25
Aparaglossata – ps26
Mecopterida – ps27
Diptera – ps28
Bibiono./Cyclorrhapha – ps29
Cyclorrhapha – ps30
Calyptratae/Drosophila – ps31
Drosophila – ps32
Sophophora – ps33
Obscura/Melanogaster_group – ps34
D_melanogaster_group – ps35
D_melanogaster_subgroup – ps36
Drosophila_yakuba/erecta_group – ps37
Drosophila_yakuba – ps38

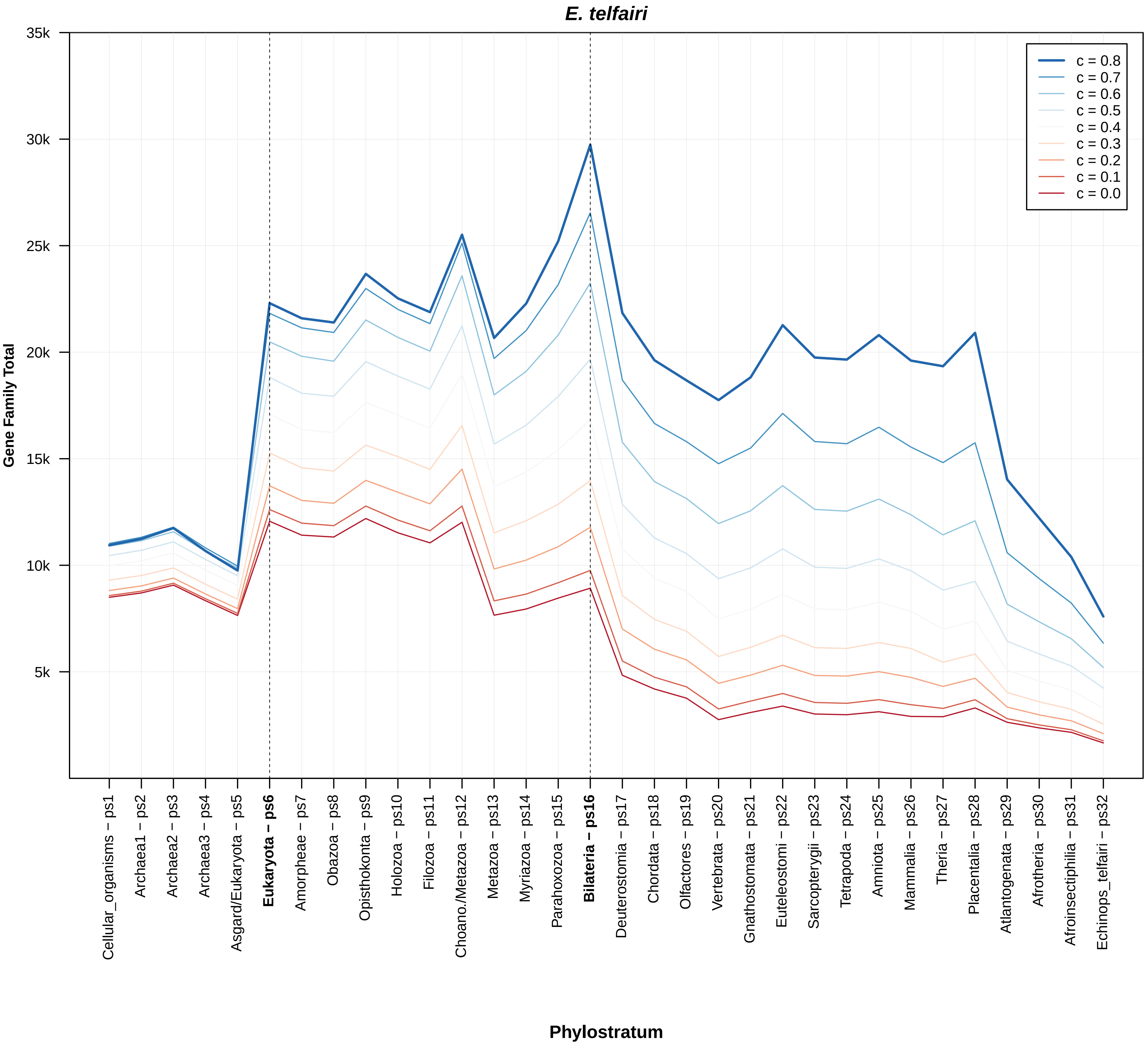

E. telfairi
Gene Family Total
Phylostratum
c = 0.8
c = 0.7
c = 0.6
c = 0.5
c = 0.4
c = 0.3
c = 0.2
c = 0.1
c = 0.0
35k
30k
25k
20k
15k
10k
5k
Cellular_organisms – ps1
Archaea1 – ps2
Archaea2 – ps3
Archaea3 – ps4
Asgard/Eukaryota – ps5
Eukaryota – ps6
Amorpheae – ps7
Obazoa – ps8
Opisthokonta – ps9
Holozoa – ps10
Filozoa – ps11
Choano./Metazoa – ps12
Metazoa – ps13
Myriazoa – ps14
Parahoxozoa – ps15
Bilateria – ps16
Deuterostomia – ps17
Chordata – ps18
Olfactores – ps19
Vertebrata – ps20
Gnathostomata – ps21
Euteleostomi – ps22
Sarcopterygii – ps23
Tetrapoda – ps24
Amniota – ps25
Mammalia – ps26
Theria – ps27
Placentalia – ps28
Atlantogenata – ps29
Afrotheria – ps30
Afroinsectiphilia – ps31
Echinops_telfairi – ps32

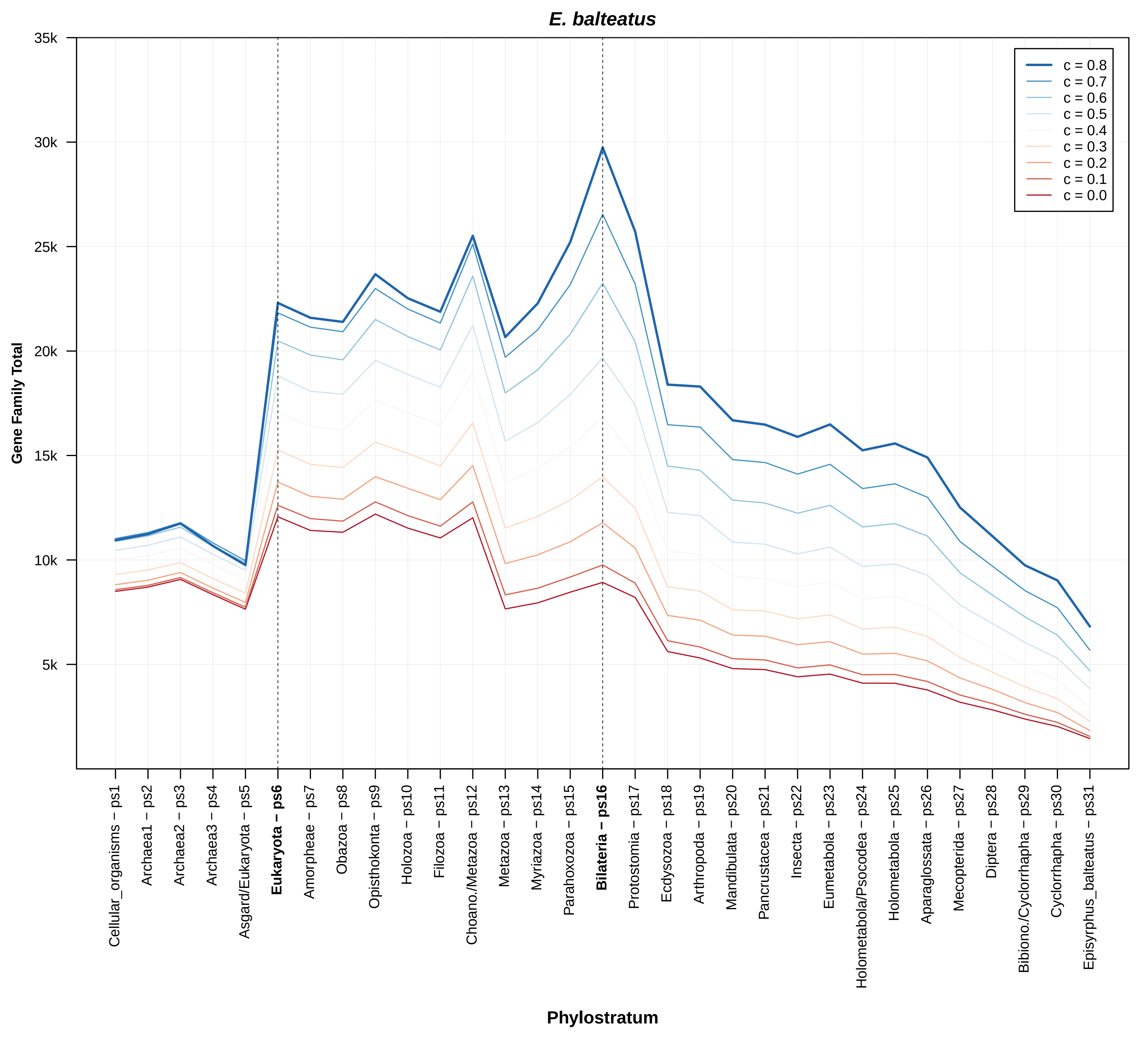

E. balteatus
Gene Family Total
Phylostratum
35k
30k
25k
20k
15k
10k
5k
c = 0.8
c = 0.7
c = 0.6
c = 0.5
c = 0.4
c = 0.3
c = 0.2
c = 0.1
c = 0.0
Cellular_organisms – ps1
Archaea1 – ps2
Archaea2 – ps3
Archaea3 – ps4
Asgard/Eukaryota – ps5
Eukaryota – ps6
Amorpheae – ps7
Obazoa – ps8
Opisthokonta – ps9
Holozoa – ps10
Filozoa – ps11
Choano./Metazoa – ps12
Metazoa – ps13
Myriazoa – ps14
Parahoxozoa – ps15
Bilateria – ps16
Protostomia – ps17
Ecdysozoa – ps18
Arthropoda – ps19
Mandibulata – ps20
Pancrustacea – ps21
Insecta – ps22
Eumetabola – ps23
Holometabola/Psocodea – ps24
Holometabola – ps25
Aparaglossata – ps26
Mecopterida – ps27
Diptera – ps28
Bibiono./Cyclorrhapha – ps29
Cyclorrhapha – ps30
Episyrphus_balteatus – ps31

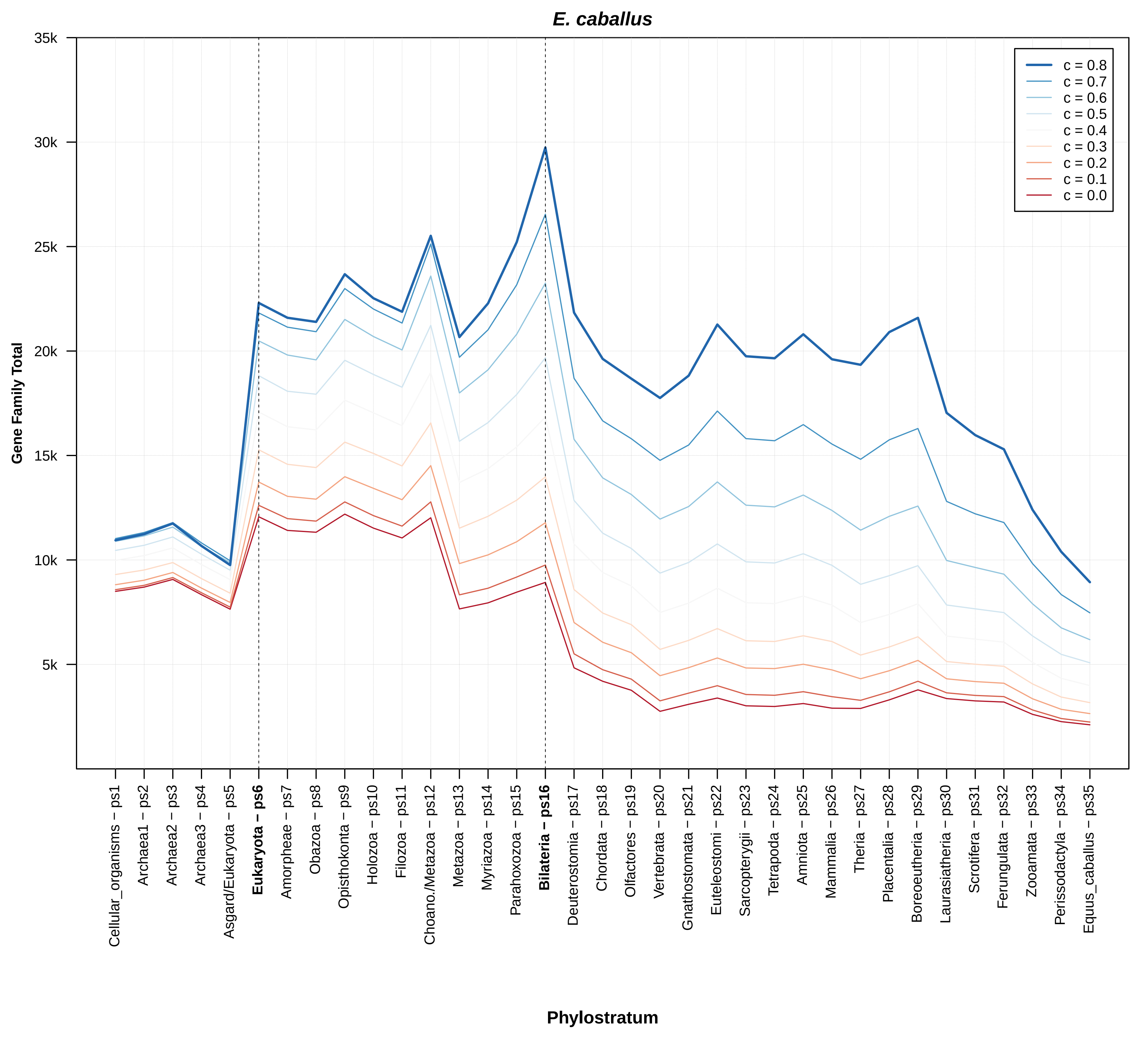
E. caballus
Gene Family Total
Phylostratum
35k
30k
25k
20k
15k
10k
5k
c = 0.8
c = 0.7
c = 0.6
c = 0.5
c = 0.4
c = 0.3
c = 0.2
c = 0.1
c = 0.0
Cellular_organisms – ps1
Archaea1 – ps2
Archaea2 – ps3
Archaea3 – ps4
Asgard/Eukaryota – ps5
Eukaryota – ps6
Amorpheae – ps7
Obazoa – ps8
Opisthokonta – ps9
Holozoa – ps10
Filozoa – ps11
Choano./Metazoa – ps12
Metazoa – ps13
Myriazoa – ps14
Parahoxozoa – ps15
Bilateria – ps16
Deuterostomia – ps17
Chordata – ps18
Olfactores – ps19
Vertebrata – ps20
Gnathostomata – ps21
Euteleostomi – ps22
Sarcopterygii – ps23
Tetrapoda – ps24
Amniota – ps25
Mammalia – ps26
Theria – ps27
Placentalia – ps28
Boreoeutheria – ps29
Laurasiatheria – ps30
Scrotifera – ps31
Ferungulata – ps32
Zooamata – ps33
Perissodactyla – ps34
Equus_caballus – ps35

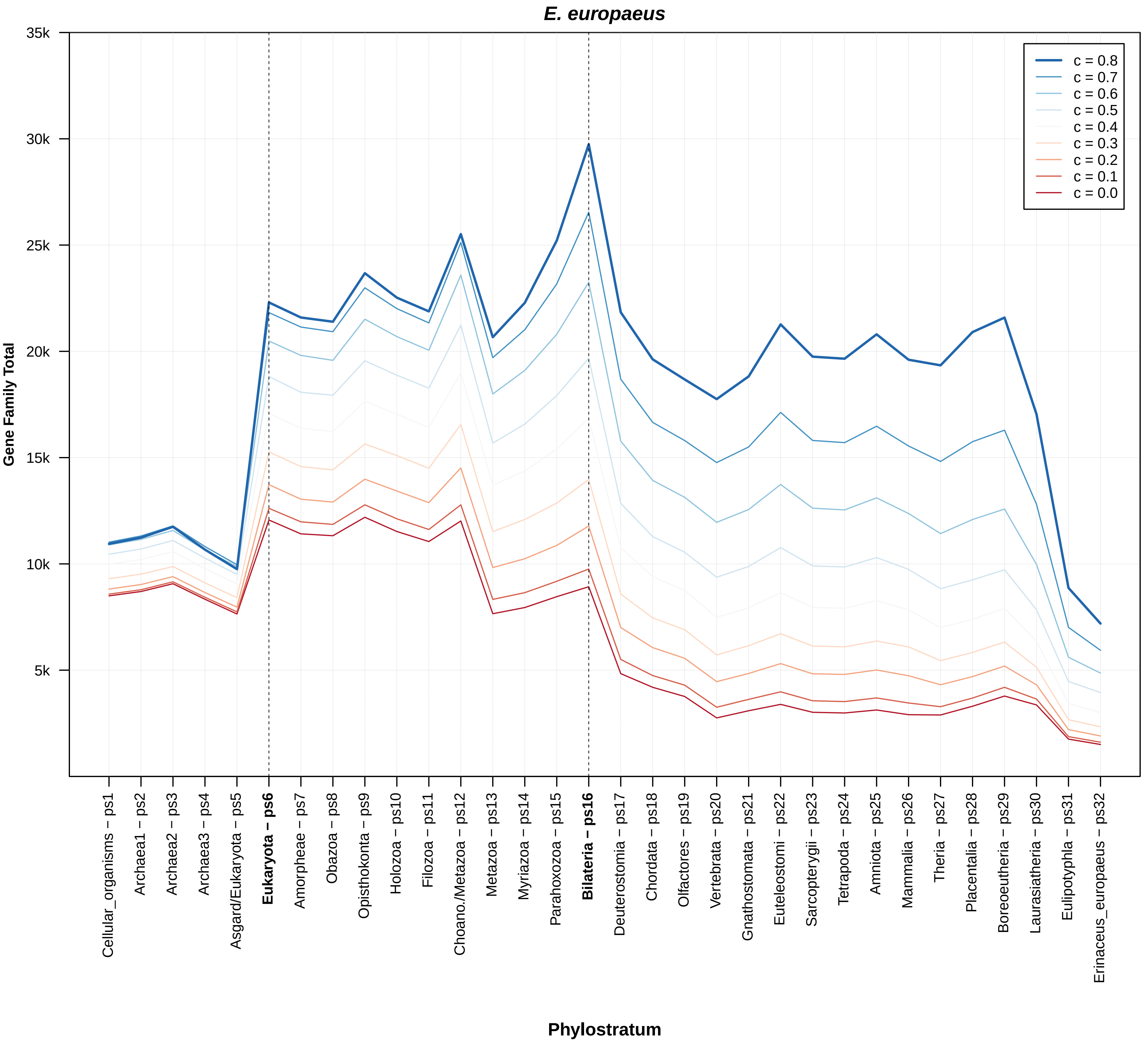
E. europaeus
Gene Family Total
Phylostratum
35k
30k
25k
20k
15k
10k
5k
c = 0.8
c = 0.7
c = 0.6
c = 0.5
c = 0.4
c = 0.3
c = 0.2
c = 0.1
c = 0.0
Cellular_organisms – ps1
Archaea1 – ps2
Archaea2 – ps3
Archaea3 – ps4
Asgard/Eukaryota – ps5
Eukaryota – ps6
Amorpheae – ps7
Obazoa – ps8
Opisthokonta – ps9
Holozoa – ps10
Filozoa – ps11
Choano./Metazoa – ps12
Metazoa – ps13
Myriazoa – ps14
Parahoxozoa – ps15
Bilateria – ps16
Deuterostomia – ps17
Chordata – ps18
Olfactores – ps19
Vertebrata – ps20
Gnathostomata – ps21
Euteleostomi – ps22
Sarcopterygii – ps23
Tetrapoda – ps24
Amniota – ps25
Mammalia – ps26
Theria – ps27
Placentalia – ps28
Boreoeutheria – ps29
Laurasiatheria – ps30
Eulipotyphla – ps31
Erinaceus_europaeus – ps32

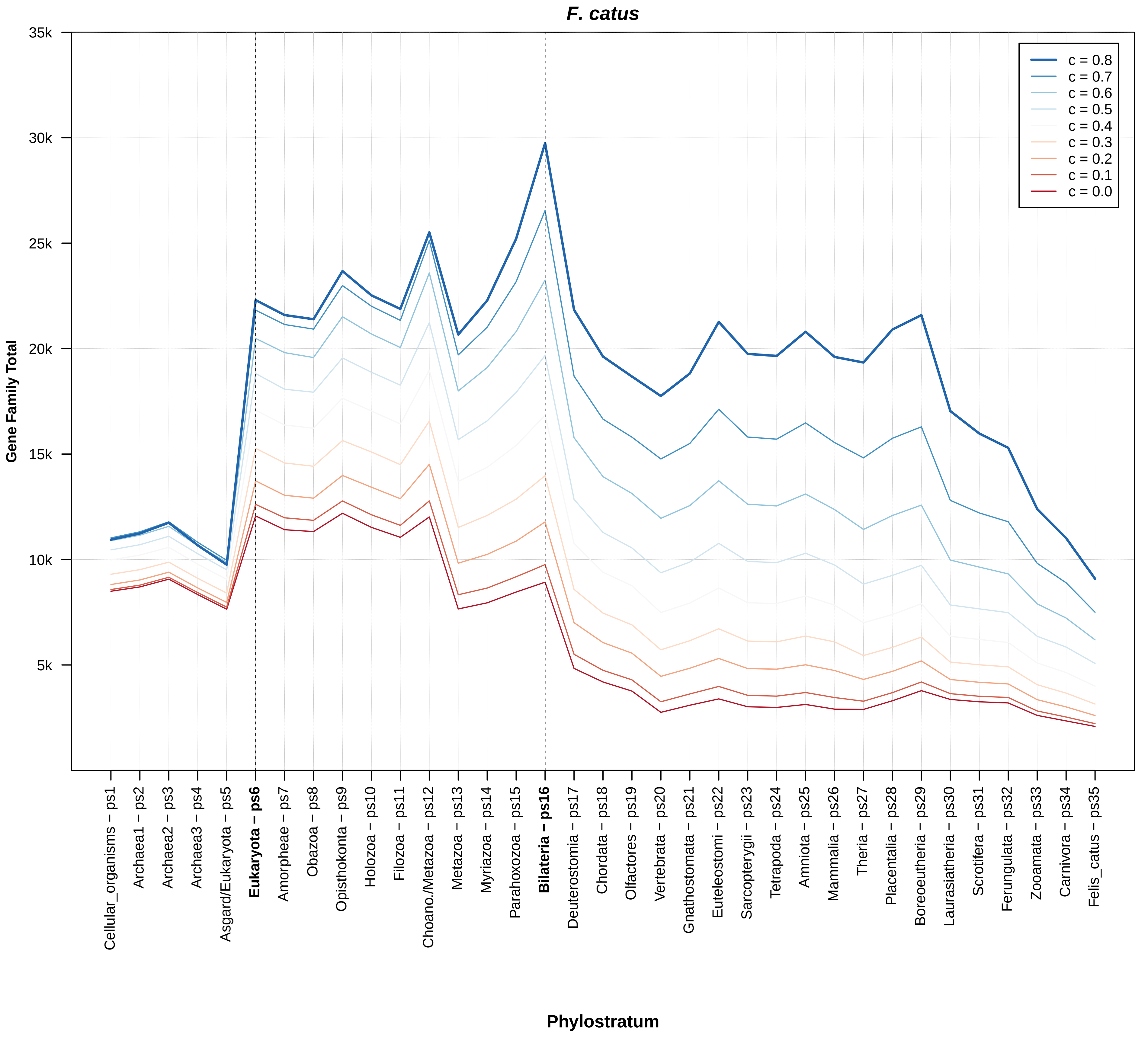
F. catus
35k
30k
25k
20k
15k
10k
5k
Gene Family Total
c = 0.8
c = 0.7
c = 0.6
c = 0.5
c = 0.4
c = 0.3
c = 0.2
c = 0.1
c = 0.0
Cellular_organisms – ps1
Archaea1 – ps2
Archaea2 – ps3
Archaea3 – ps4
Asgard/Eukaryota – ps5
Eukaryota – ps6
Amorpheae – ps7
Obazoa – ps8
Opisthokonta – ps9
Holozoa – ps10
Filozoa – ps11
Choano./Metazoa – ps12
Metazoa – ps13
Myriazoa – ps14
Parahoxozoa – ps15
Bilateria – ps16
Deuterostomia – ps17
Chordata – ps18
Olfactores – ps19
Vertebrata – ps20
Gnathostomata – ps21
Euteleostomi – ps22
Sarcopterygii – ps23
Tetrapoda – ps24
Amniota – ps25
Mammalia – ps26
Theria – ps27
Placentalia – ps28
Boreoeutheria – ps29
Laurasiatheria – ps30
Scrotifera – ps31
Ferungulata – ps32
Zooamata – ps33
Carnivora – ps34
Felis_catus – ps35
Phylostratum

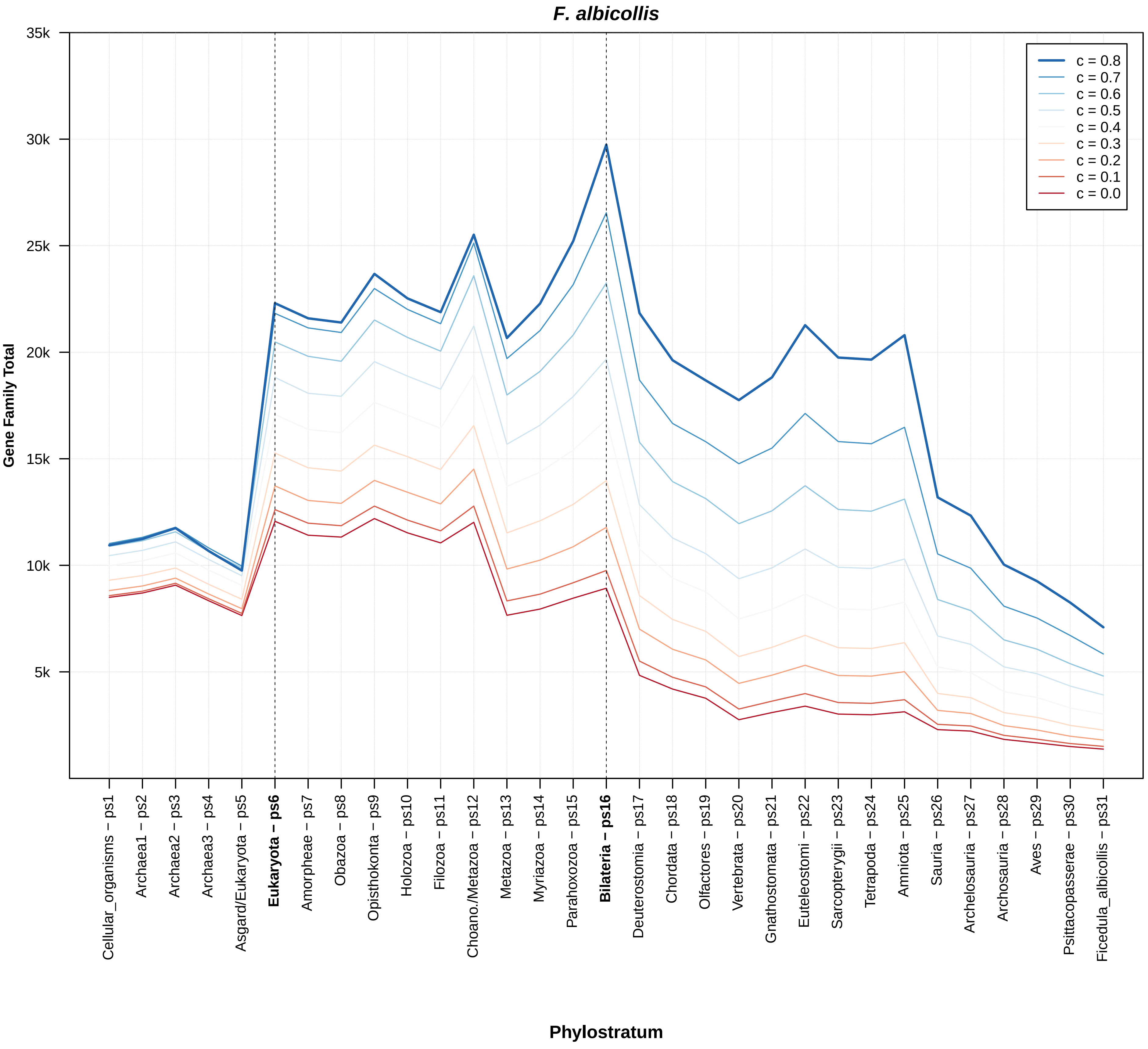

F. albicollis
35k
30k
25k
20k
15k
10k
5k
Gene Family Total
c = 0.8
c = 0.7
c = 0.6
c = 0.5
c = 0.4
c = 0.3
c = 0.2
c = 0.1
c = 0.0
Cellular_organisms – ps1
Archaea1 – ps2
Archaea2 – ps3
Archaea3 – ps4
Asgard/Eukaryota – ps5
Eukaryota – ps6
Amorpheae – ps7
Obazoa – ps8
Opisthokonta – ps9
Holozoa – ps10
Filozoa – ps11
Choano./Metazoa – ps12
Metazoa – ps13
Myriazoa – ps14
Parahoxozoa – ps15
Bilateria – ps16
Deuterostomia – ps17
Chordata – ps18
Olfactores – ps19
Vertebrata – ps20
Gnathostomata – ps21
Euteleostomi – ps22
Sarcopterygii – ps23
Tetrapoda – ps24
Amniota – ps25
Sauria – ps26
Archelosauria – ps27
Archosauria – ps28
Aves – ps29
Psittacopasserae – ps30
Ficedula_albicollis – ps31
Phylostratum

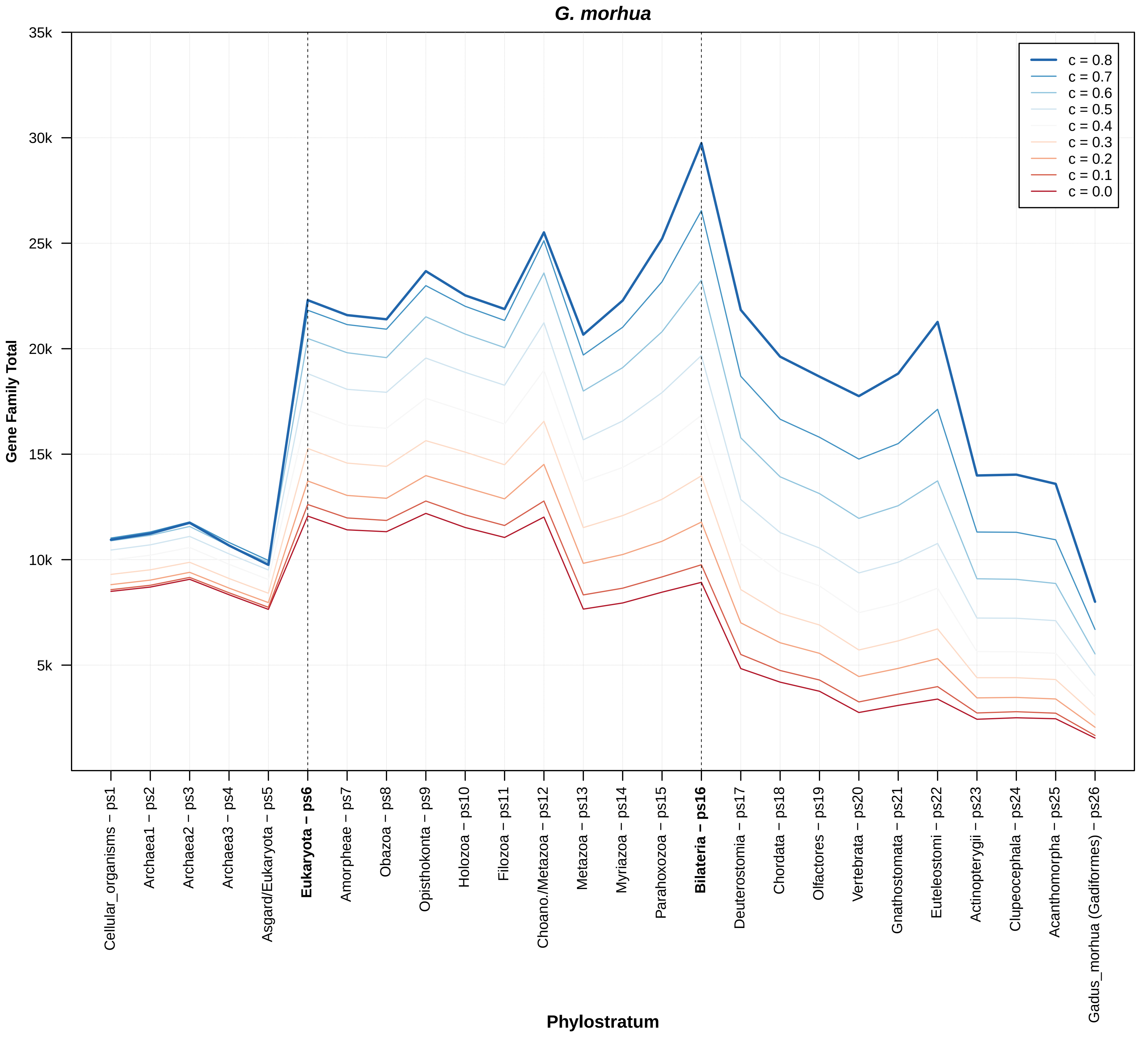
G. morhua
Gene Family Total
35k
30k
25k
20k
15k
10k
5k
c = 0.8
c = 0.7
c = 0.6
c = 0.5
c = 0.4
c = 0.3
c = 0.2
c = 0.1
c = 0.0
Cellular_organisms – ps1
Archaea1 – ps2
Archaea2 – ps3
Archaea3 – ps4
Asgard/Eukaryota – ps5
Eukaryota – ps6
Amorpheae – ps7
Obazoa – ps8
Opisthokonta – ps9
Holozoa – ps10
Filozoa – ps11
Choano./Metazoa – ps12
Metazoa – ps13
Myriazoa – ps14
Parahoxozoa – ps15
Bilateria – ps16
Deuterostomia – ps17
Chordata – ps18
Olfactores – ps19
Vertebrata – ps20
Gnathostomata – ps21
Euteleostomi – ps22
Actinopterygii – ps23
Clupeocephala – ps24
Acanthomorpha – ps25
Gadus_morhua (Gadiformes) – ps26
Phylostratum

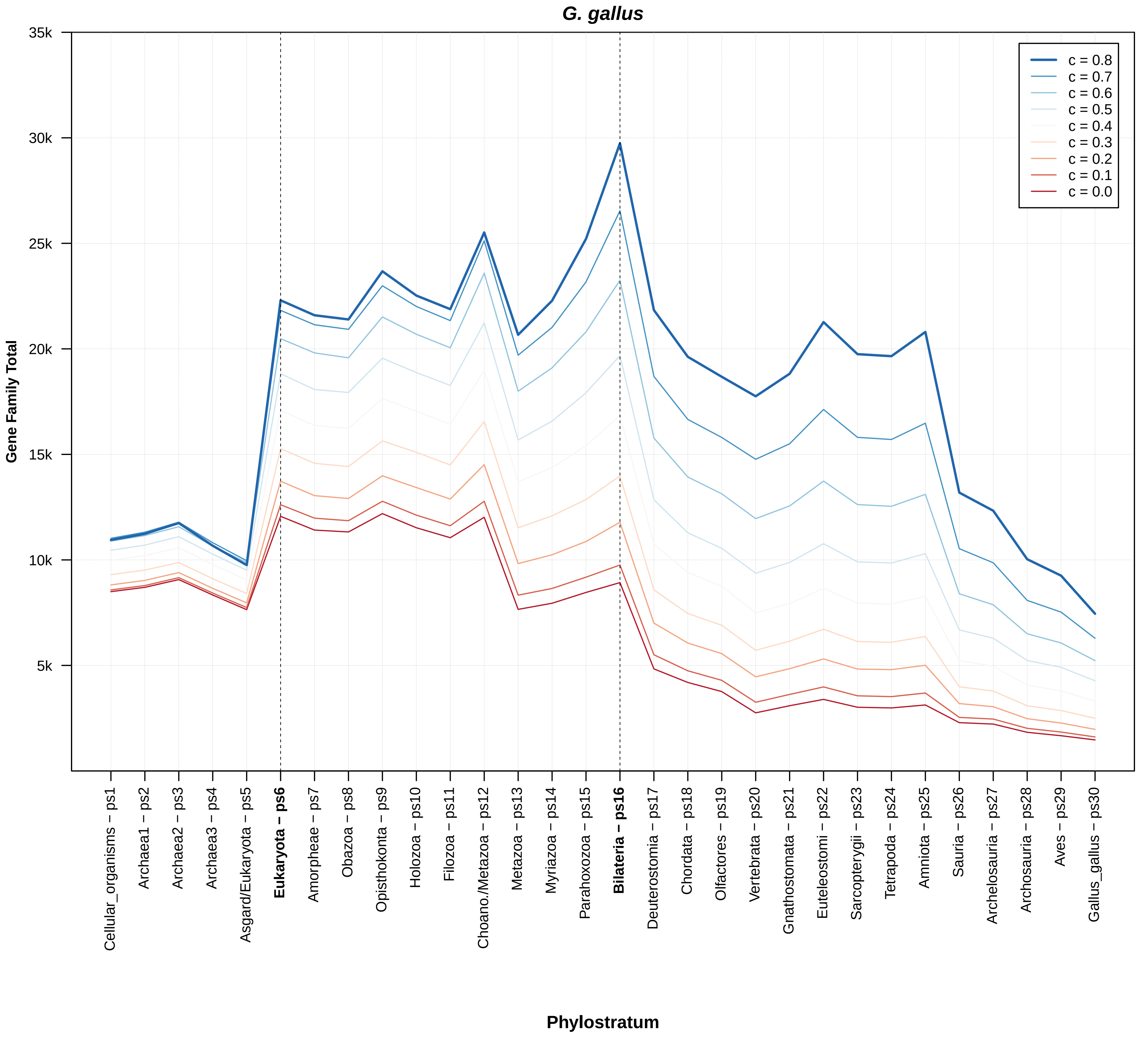

G. gallus
Gene Family Total
35k
30k
25k
20k
15k
10k
5k
c = 0.8
c = 0.7
c = 0.6
c = 0.5
c = 0.4
c = 0.3
c = 0.2
c = 0.1
c = 0.0
Cellular_organisms – ps1
Archaea1 – ps2
Archaea2 – ps3
Archaea3 – ps4
Asgard/Eukaryota – ps5
Eukaryota – ps6
Amorpheae – ps7
Obazoa – ps8
Opisthokonta – ps9
Holozoa – ps10
Filozoa – ps11
Choano./Metazoa – ps12
Metazoa – ps13
Myriazoa – ps14
Parahoxozoa – ps15
Bilateria – ps16
Deuterostomia – ps17
Chordata – ps18
Olfactores – ps19
Vertebrata – ps20
Gnathostomata – ps21
Euteleostomi – ps22
Sarcopterygii – ps23
Tetrapoda – ps24
Amniota – ps25
Sauria – ps26
Archelosauria – ps27
Archosauria – ps28
Aves – ps29
Gallus_gallus – ps30
Phylostratum

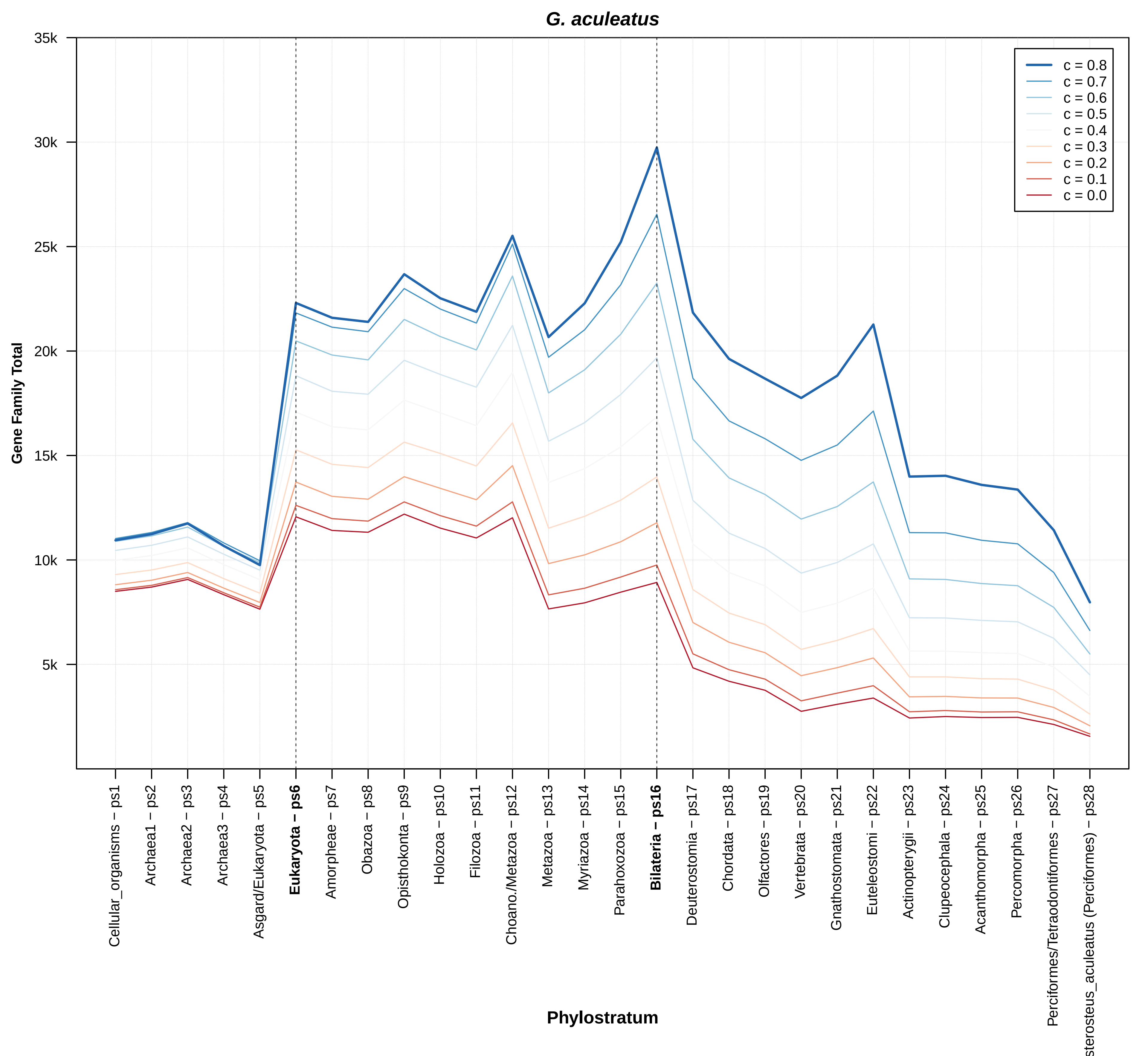

G. aculeatus
35k
30k
25k
20k
15k
10k
5k
Gene Family Total
c = 0.8
c = 0.7
c = 0.6
c = 0.5
c = 0.4
c = 0.3
c = 0.2
c = 0.1
c = 0.0
Cellular_organisms – ps1
Archaea1 – ps2
Archaea2 – ps3
Archaea3 – ps4
Asgard/Eukaryota – ps5
Eukaryota – ps6
Amorpheae – ps7
Obazoa – ps8
Opisthokonta – ps9
Holozoa – ps10
Filozoa – ps11
Choano./Metazoa – ps12
Metazoa – ps13
Myriazoa – ps14
Parahoxozoa – ps15
Bilateria – ps16
Deuterostomia – ps17
Chordata – ps18
Olfactores – ps19
Vertebrata – ps20
Gnathostomata – ps21
Euteleostomi – ps22
Actinopterygii – ps23
Clupeocephala – ps24
Acanthomorpha – ps25
Percomorpha – ps26
Perciformes/Tetraodontiformes – ps27
sterosteus_aculeatus (Perciformes) – ps28
Phylostratum

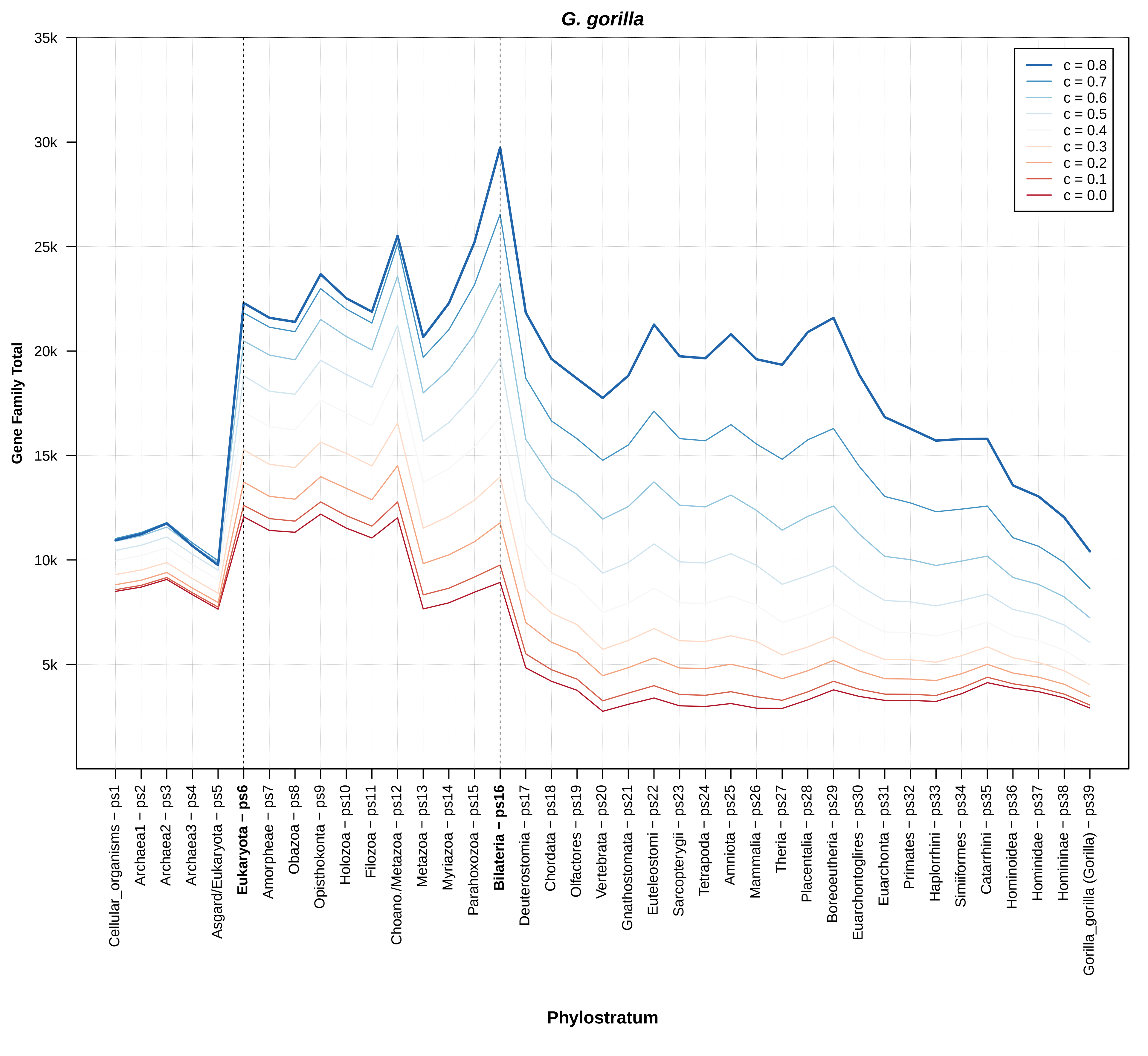

G. gorilla
Gene Family Total
35k
30k
25k
20k
15k
10k
5k
c = 0.8
c = 0.7
c = 0.6
c = 0.5
c = 0.4
c = 0.3
c = 0.2
c = 0.1
c = 0.0
Cellular_organisms – ps1
Archaea1 – ps2
Archaea2 – ps3
Archaea3 – ps4
Asgard/Eukaryota – ps5
Eukaryota – ps6
Amorpheae – ps7
Obazoa – ps8
Opisthokonta – ps9
Holozoa – ps10
Filozoa – ps11
Choano./Metazoa – ps12
Metazoa – ps13
Myriazoa – ps14
Parahoxozoa – ps15
Bilateria – ps16
Deuterostomia – ps17
Chordata – ps18
Olfactores – ps19
Vertebrata – ps20
Gnathostomata – ps21
Euteleostomi – ps22
Sarcopterygii – ps23
Tetrapoda – ps24
Amniota – ps25
Mammalia – ps26
Theria – ps27
Placentalia – ps28
Boreoeutheria – ps29
Euarchontoglires – ps30
Euarchonta – ps31
Primates – ps32
Haplorrhini – ps33
Simiiformes – ps34
Catarrhini – ps35
Hominoidea – ps36
Hominidae – ps37
Homininae – ps38
Gorilla_gorilla (Gorilla) – ps39
Phylostratum

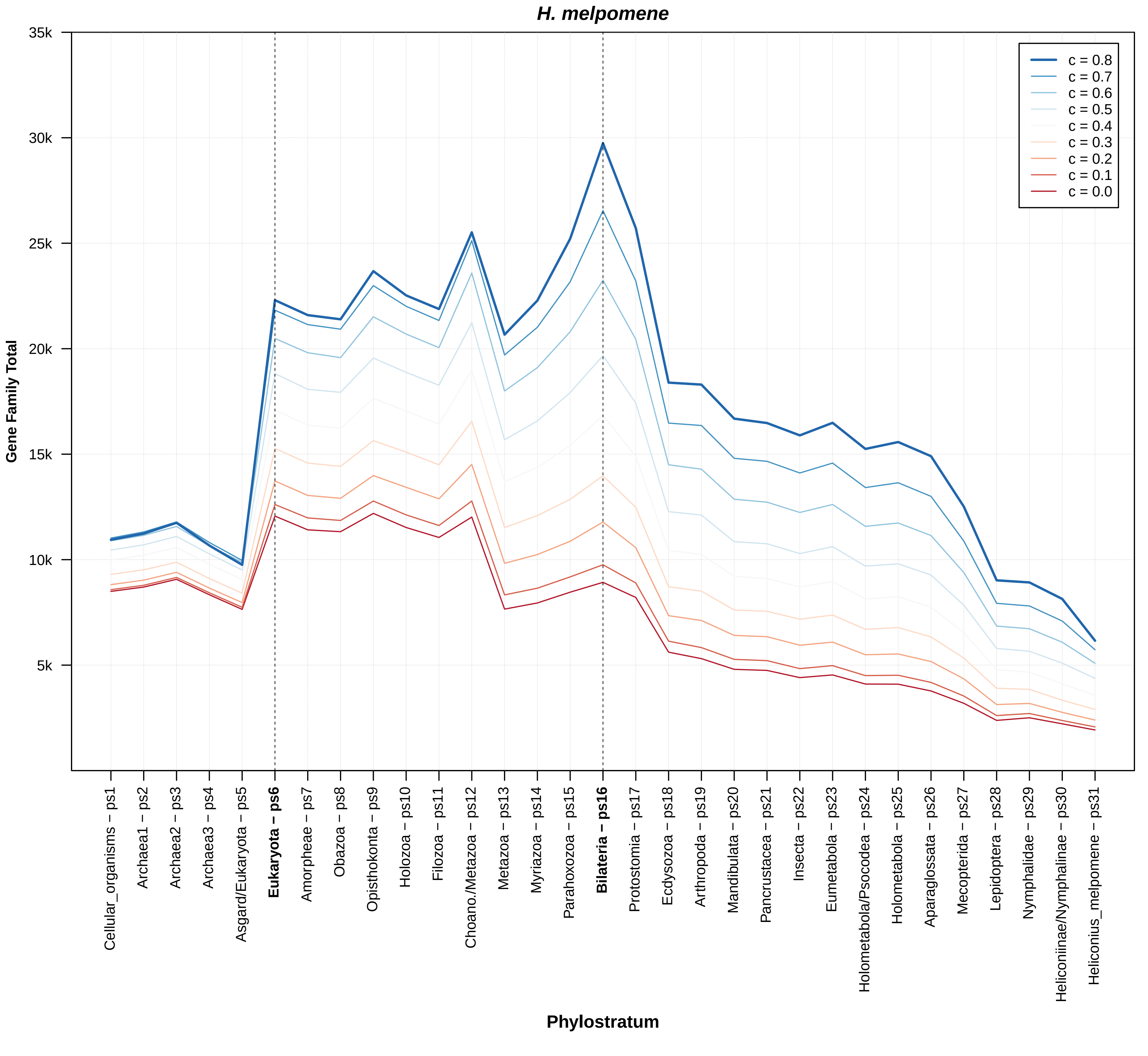

H. melpomene
Gene Family Total
35k
30k
25k
20k
15k
10k
5k
c = 0.8
c = 0.7
c = 0.6
c = 0.5
c = 0.4
c = 0.3
c = 0.2
c = 0.1
c = 0.0
Cellular_organisms – ps1
Archaea1 – ps2
Archaea2 – ps3
Archaea3 – ps4
Asgard/Eukaryota – ps5
Eukaryota – ps6
Amorpheae – ps7
Obazoa – ps8
Opisthokonta – ps9
Holozoa – ps10
Filozoa – ps11
Choano./Metazoa – ps12
Metazoa – ps13
Myriazoa – ps14
Parahoxozoa – ps15
Bilateria – ps16
Protostomia – ps17
Ecdysozoa – ps18
Arthropoda – ps19
Mandibulata – ps20
Pancrustacea – ps21
Insecta – ps22
Eumetabola – ps23
Holometabola/Psocodea – ps24
Holometabola – ps25
Aparaglossata – ps26
Mecopterida – ps27
Lepidoptera – ps28
Nymphalidae – ps29
Heliconiinae/Nymphalinae – ps30
Heliconius_melpomene – ps31
Phylostratum

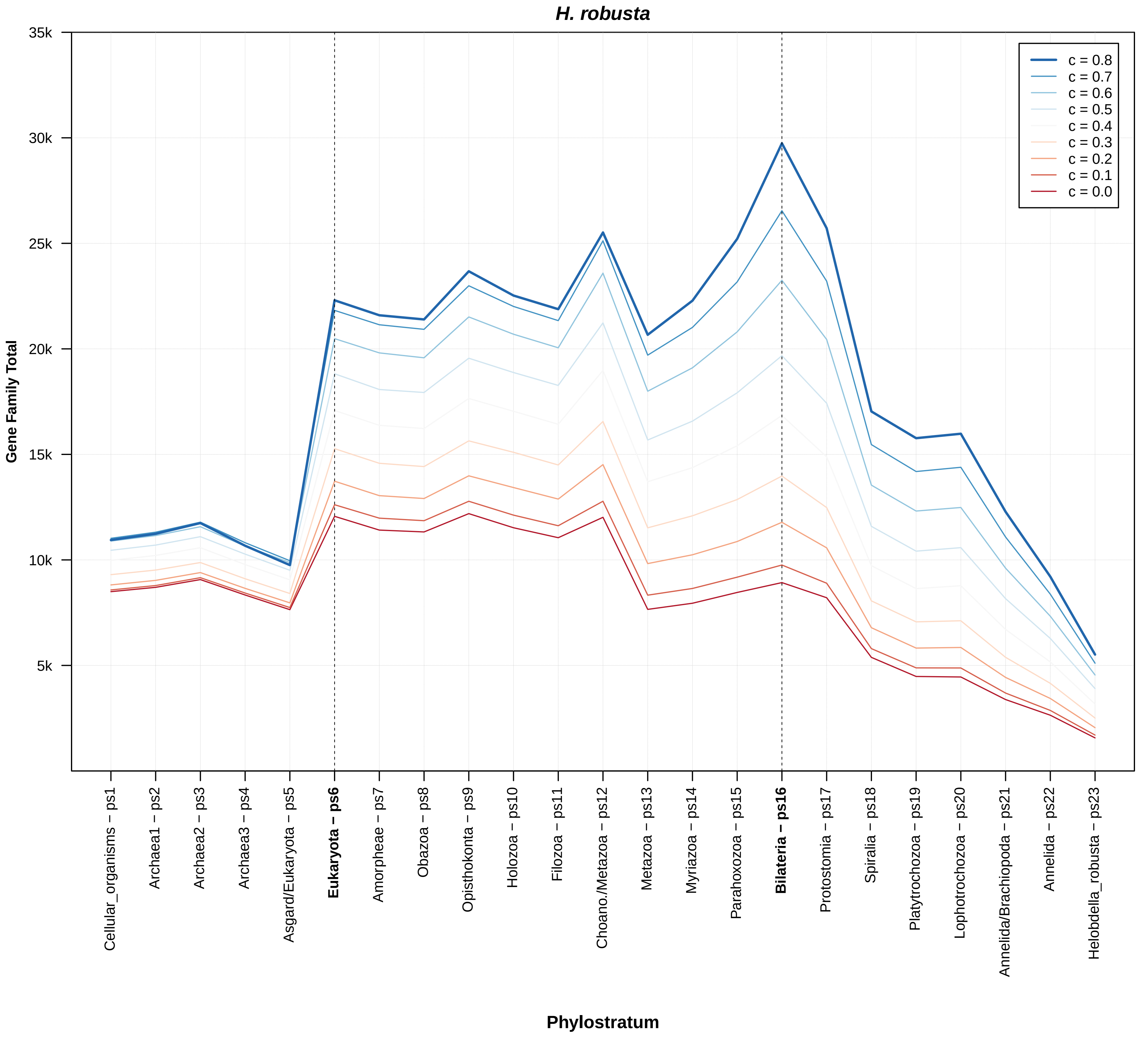

H. robusta
Gene Family Total
35k
30k
25k
20k
15k
10k
5k
c = 0.8
c = 0.7
c = 0.6
c = 0.5
c = 0.4
c = 0.3
c = 0.2
c = 0.1
c = 0.0
Cellular_organisms – ps1
Archaea1 – ps2
Archaea2 – ps3
Archaea3 – ps4
Asgard/Eukaryota – ps5
Eukaryota – ps6
Amorpheae – ps7
Obazoa – ps8
Opisthokonta – ps9
Holozoa – ps10
Filozoa – ps11
Choano./Metazoa – ps12
Metazoa – ps13
Myriazoa – ps14
Parahoxozoa – ps15
Bilateria – ps16
Protostomia – ps17
Spiralia – ps18
Platytrochozoa – ps19
Lophotrochozoa – ps20
Annelida/Brachiopoda – ps21
Annelida – ps22
Helobdella_robusta – ps23
Phylostratum

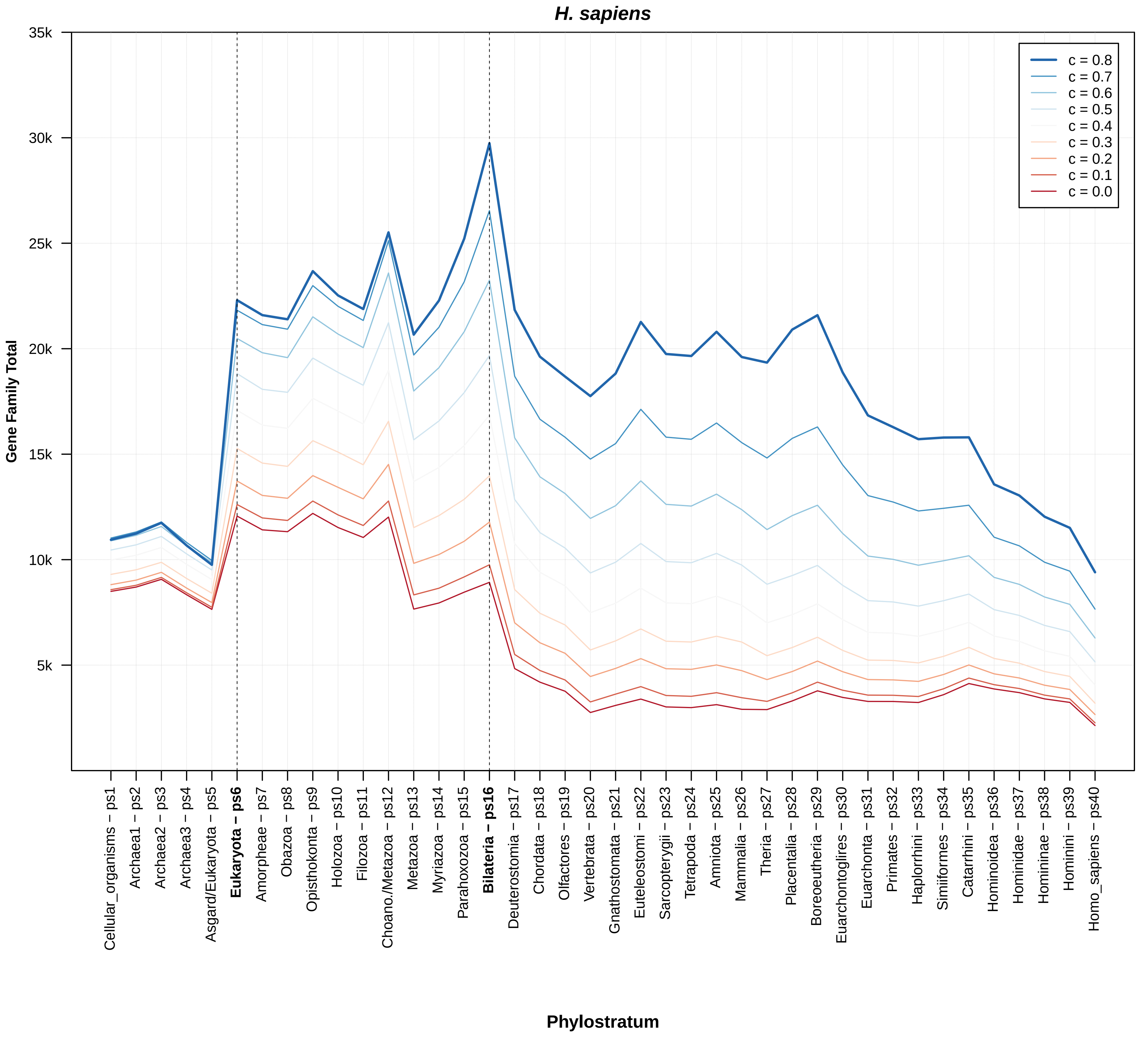

H. sapiens
Gene Family Total
Phylostratum
35k
30k
25k
20k
15k
10k
5k
c = 0.8
c = 0.7
c = 0.6
c = 0.5
c = 0.4
c = 0.3
c = 0.2
c = 0.1
c = 0.0
Cellular_organisms – ps1
Archaea1 – ps2
Archaea2 – ps3
Archaea3 – ps4
Asgard/Eukaryota – ps5
Eukaryota – ps6
Amorpheae – ps7
Obazoa – ps8
Opisthokonta – ps9
Holozoa – ps10
Filozoa – ps11
Choano./Metazoa – ps12
Metazoa – ps13
Myriazoa – ps14
Parahoxozoa – ps15
Bilateria – ps16
Deuterostomia – ps17
Chordata – ps18
Olfactores – ps19
Vertebrata – ps20
Gnathostomata – ps21
Euteleostomi – ps22
Sarcopterygii – ps23
Tetrapoda – ps24
Amniota – ps25
Mammalia – ps26
Theria – ps27
Placentalia – ps28
Boreoeutheria – ps29
Euarchontoglires – ps30
Euarchonta – ps31
Primates – ps32
Haplorrhini – ps33
Simiiformes – ps34
Catarrhini – ps35
Hominoidea – ps36
Hominidae – ps37
Homininae – ps38
Hominini – ps39
Homo_sapiens – ps40

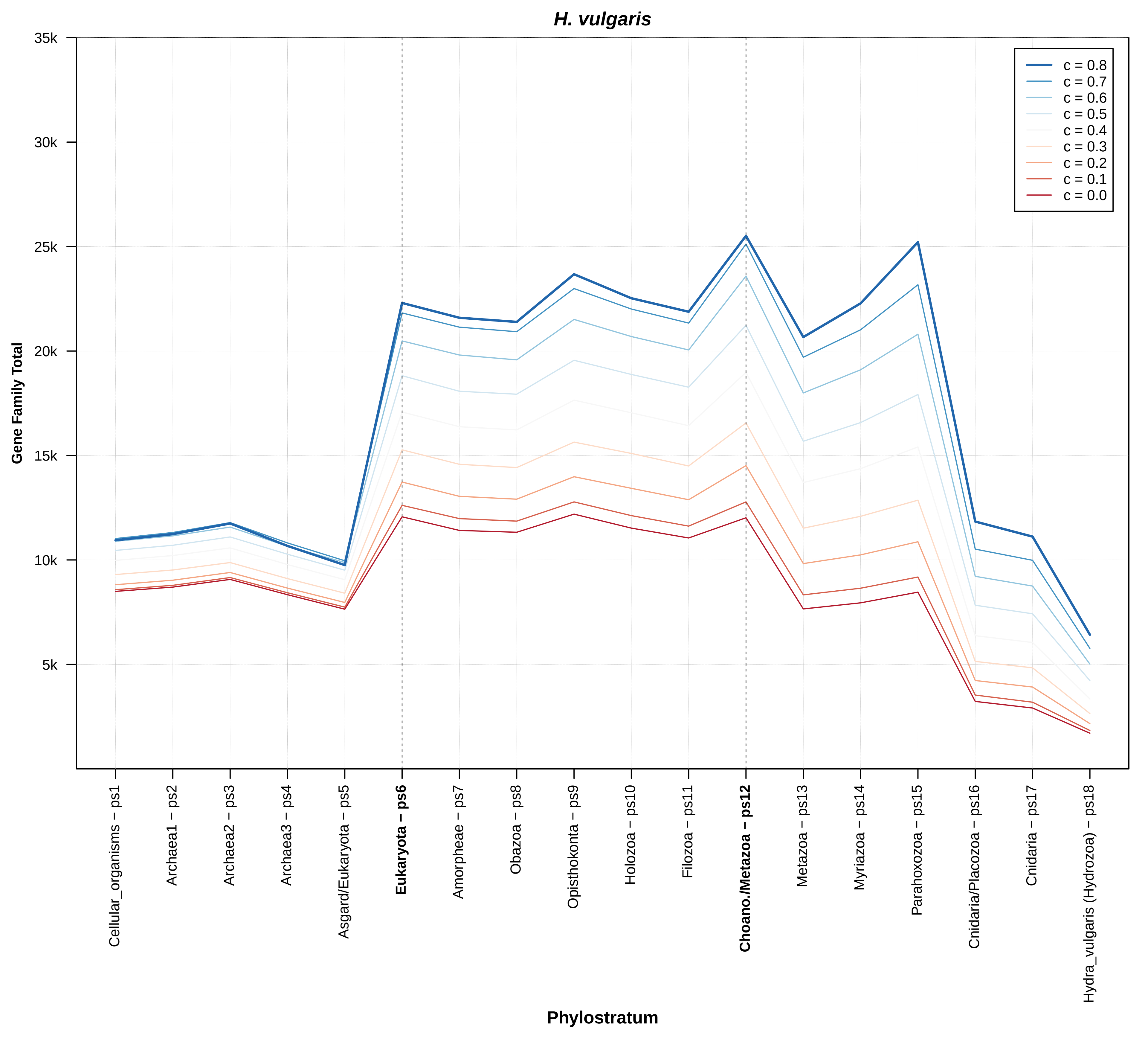
H. vulgaris
35k
30k
25k
20k
15k
10k
5k
Gene Family Total
c = 0.8
c = 0.7
c = 0.6
c = 0.5
c = 0.4
c = 0.3
c = 0.2
c = 0.1
c = 0.0
Cellular_organisms – ps1
Archaea1 – ps2
Archaea2 – ps3
Archaea3 – ps4
Asgard/Eukaryota – ps5
Eukaryota – ps6
Amorpheae – ps7
Obazoa – ps8
Opisthokonta – ps9
Holozoa – ps10
Filozoa – ps11
Choano./Metazoa – ps12
Metazoa – ps13
Myriazoa – ps14
Parahoxozoa – ps15
Cnidaria/Placozoa – ps16
Cnidaria – ps17
Hydra_vulgaris (Hydrozoa) – ps18
Phylostratum

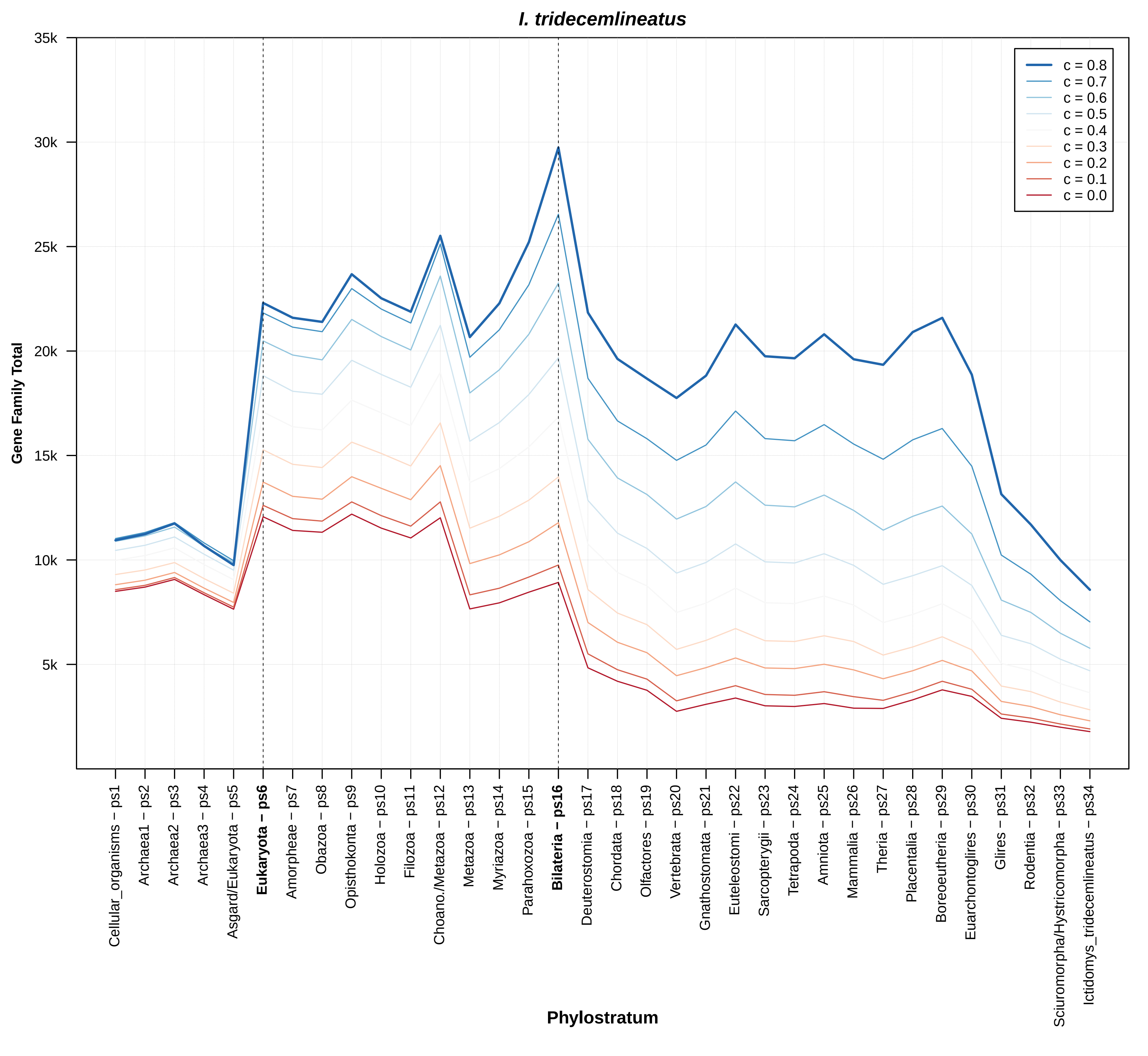
I. tridecemlineatus
Gene Family Total
Phylostratum
35k
30k
25k
20k
15k
10k
5k
c = 0.8
c = 0.7
c = 0.6
c = 0.5
c = 0.4
c = 0.3
c = 0.2
c = 0.1
c = 0.0
Cellular_organisms – ps1
Archaea1 – ps2
Archaea2 – ps3
Archaea3 – ps4
Asgard/Eukaryota – ps5
Eukaryota – ps6
Amorpheae – ps7
Obazoa – ps8
Opisthokonta – ps9
Holozoa – ps10
Filozoa – ps11
Choano./Metazoa – ps12
Metazoa – ps13
Myriazoa – ps14
Parahoxozoa – ps15
Bilateria – ps16
Deuterostomia – ps17
Chordata – ps18
Olfactores – ps19
Vertebrata – ps20
Gnathostomata – ps21
Euteleostomi – ps22
Sarcopterygii – ps23
Tetrapoda – ps24
Amniota – ps25
Mammalia – ps26
Theria – ps27
Placentalia – ps28
Boreoeutheria – ps29
Euarchontoglires – ps30
Glires – ps31
Rodentia – ps32
Sciuromorpha/Hystricomorpha – ps33
Ictidomys_tridecemlineatus – ps34

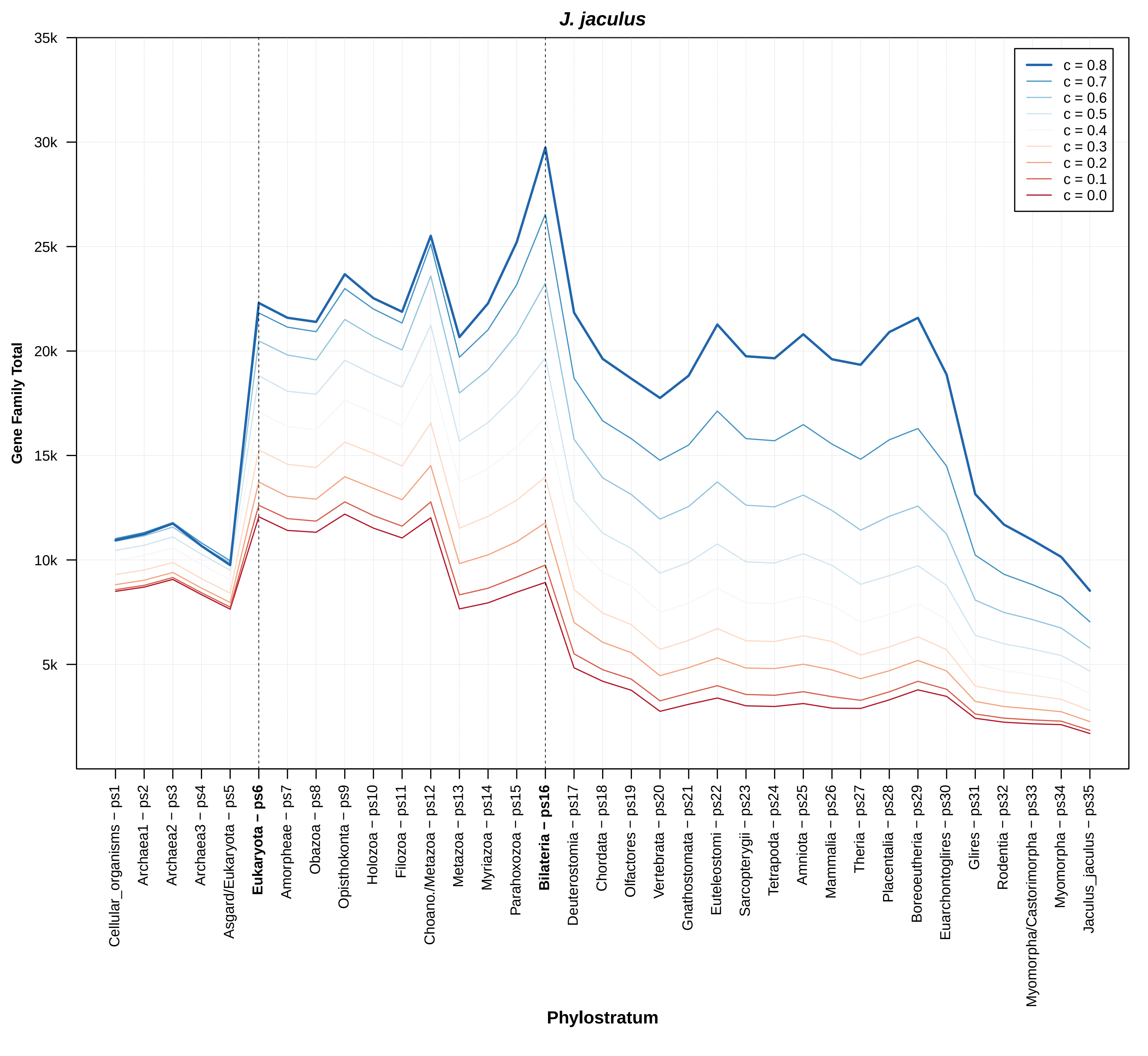

J. jaculus
Gene Family Total
35k
30k
25k
20k
15k
10k
5k
c = 0.8
c = 0.7
c = 0.6
c = 0.5
c = 0.4
c = 0.3
c = 0.2
c = 0.1
c = 0.0
Cellular_organisms – ps1
Archaea1 – ps2
Archaea2 – ps3
Archaea3 – ps4
Asgard/Eukaryota – ps5
Eukaryota – ps6
Amorpheae – ps7
Obazoa – ps8
Opisthokonta – ps9
Holozoa – ps10
Filozoa – ps11
Choano./Metazoa – ps12
Metazoa – ps13
Myriazoa – ps14
Parahoxozoa – ps15
Bilateria – ps16
Deuterostomia – ps17
Chordata – ps18
Olfactores – ps19
Vertebrata – ps20
Gnathostomata – ps21
Euteleostomi – ps22
Sarcopterygii – ps23
Tetrapoda – ps24
Amniota – ps25
Mammalia – ps26
Theria – ps27
Placentalia – ps28
Boreoeutheria – ps29
Euarchontoglires – ps30
Glires – ps31
Rodentia – ps32
Myomorpha/Castorimorpha – ps33
Myomorpha – ps34
Jaculus_jaculus – ps35
Phylostratum

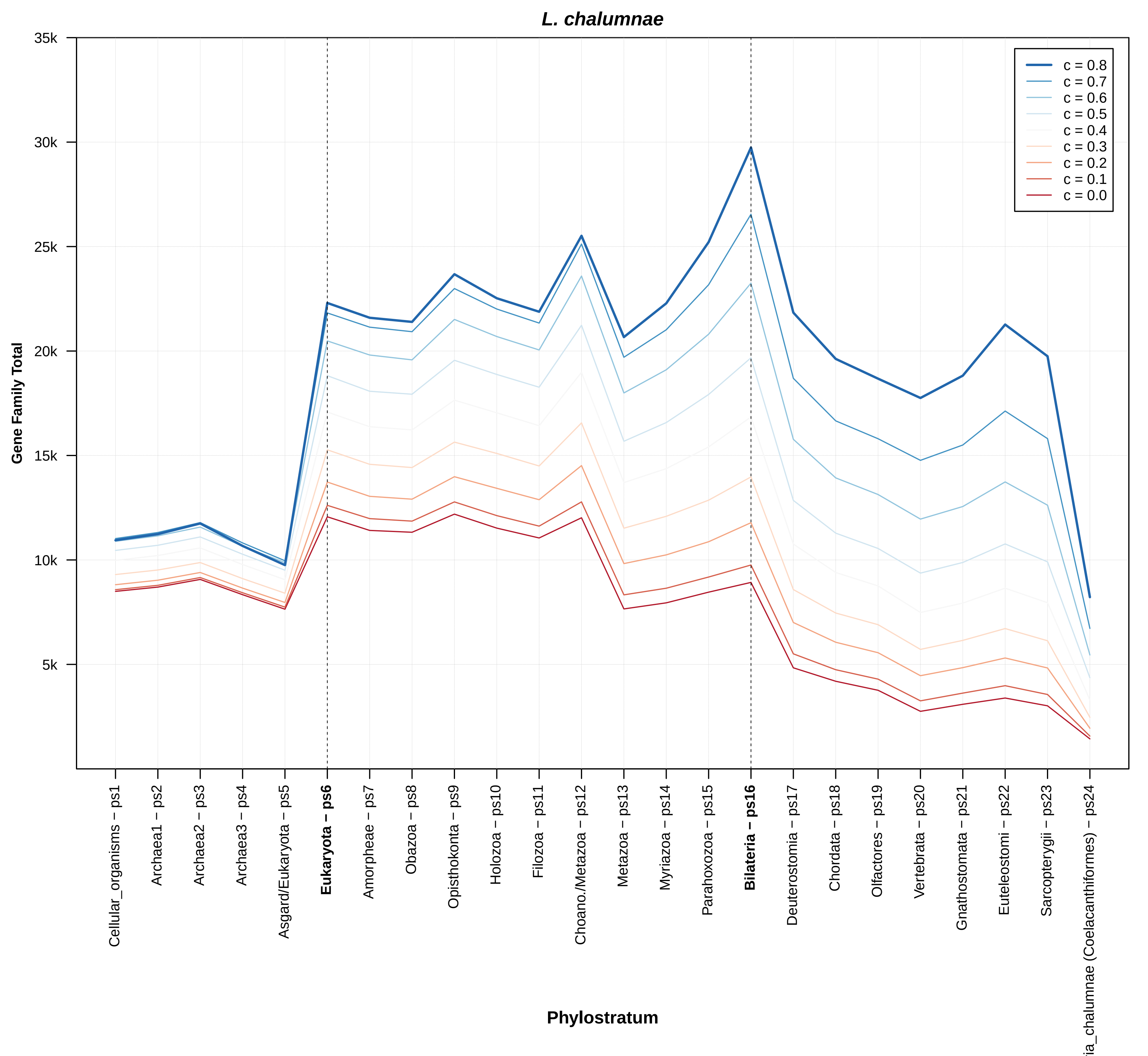

L. chalumnae
Gene Family Total
Phylostratum
35k
30k
25k
20k
15k
10k
5k
c = 0.8
c = 0.7
c = 0.6
c = 0.5
c = 0.4
c = 0.3
c = 0.2
c = 0.1
c = 0.0
Cellular_organisms – ps1
Archaea1 – ps2
Archaea2 – ps3
Archaea3 – ps4
Asgard/Eukaryota – ps5
Eukaryota – ps6
Amorpheae – ps7
Obazoa – ps8
Opisthokonta – ps9
Holozoa – ps10
Filozoa – ps11
Choano./Metazoa – ps12
Metazoa – ps13
Myriazoa – ps14
Parahoxozoa – ps15
Bilateria – ps16
Deuterostomia – ps17
Chordata – ps18
Olfactores – ps19
Vertebrata – ps20
Gnathostomata – ps21
Euteleostomi – ps22
Sarcopterygii – ps23
ia_chalumnae (Coelacanthiformes) – ps24

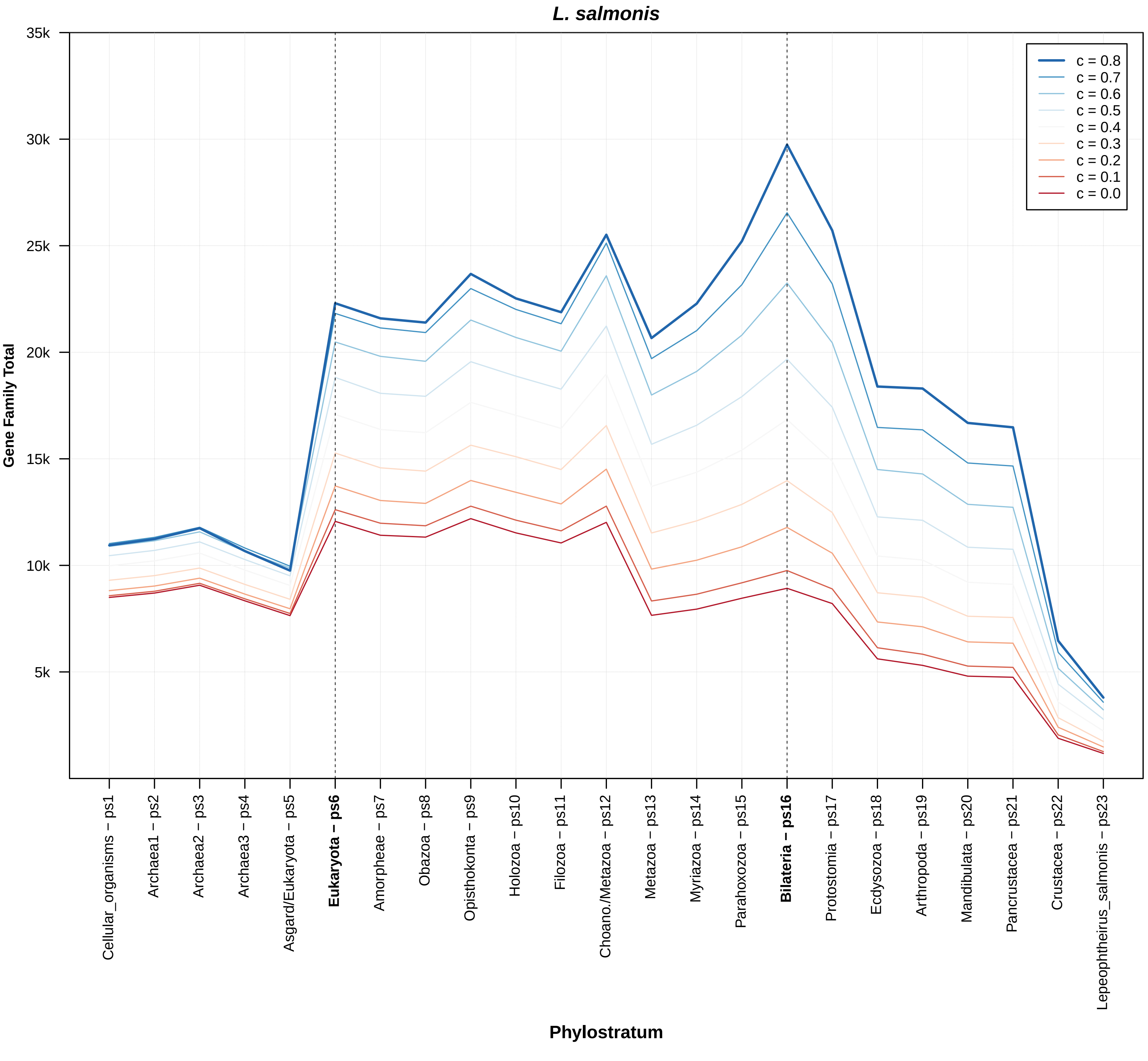

L. salmonis
Gene Family Total
35k
30k
25k
20k
15k
10k
5k
c = 0.8
c = 0.7
c = 0.6
c = 0.5
c = 0.4
c = 0.3
c = 0.2
c = 0.1
c = 0.0
Cellular_organisms – ps1
Archaea1 – ps2
Archaea2 – ps3
Archaea3 – ps4
Asgard/Eukaryota – ps5
Eukaryota – ps6
Amorpheae – ps7
Obazoa – ps8
Opisthokonta – ps9
Holozoa – ps10
Filozoa – ps11
Choano./Metazoa – ps12
Metazoa – ps13
Myriazoa – ps14
Parahoxozoa – ps15
Bilateria – ps16
Protostomia – ps17
Ecdysozoa – ps18
Arthropoda – ps19
Mandibulata – ps20
Pancrustacea – ps21
Crustacea – ps22
Lepeophtheirus_salmonis – ps23
Phylostratum

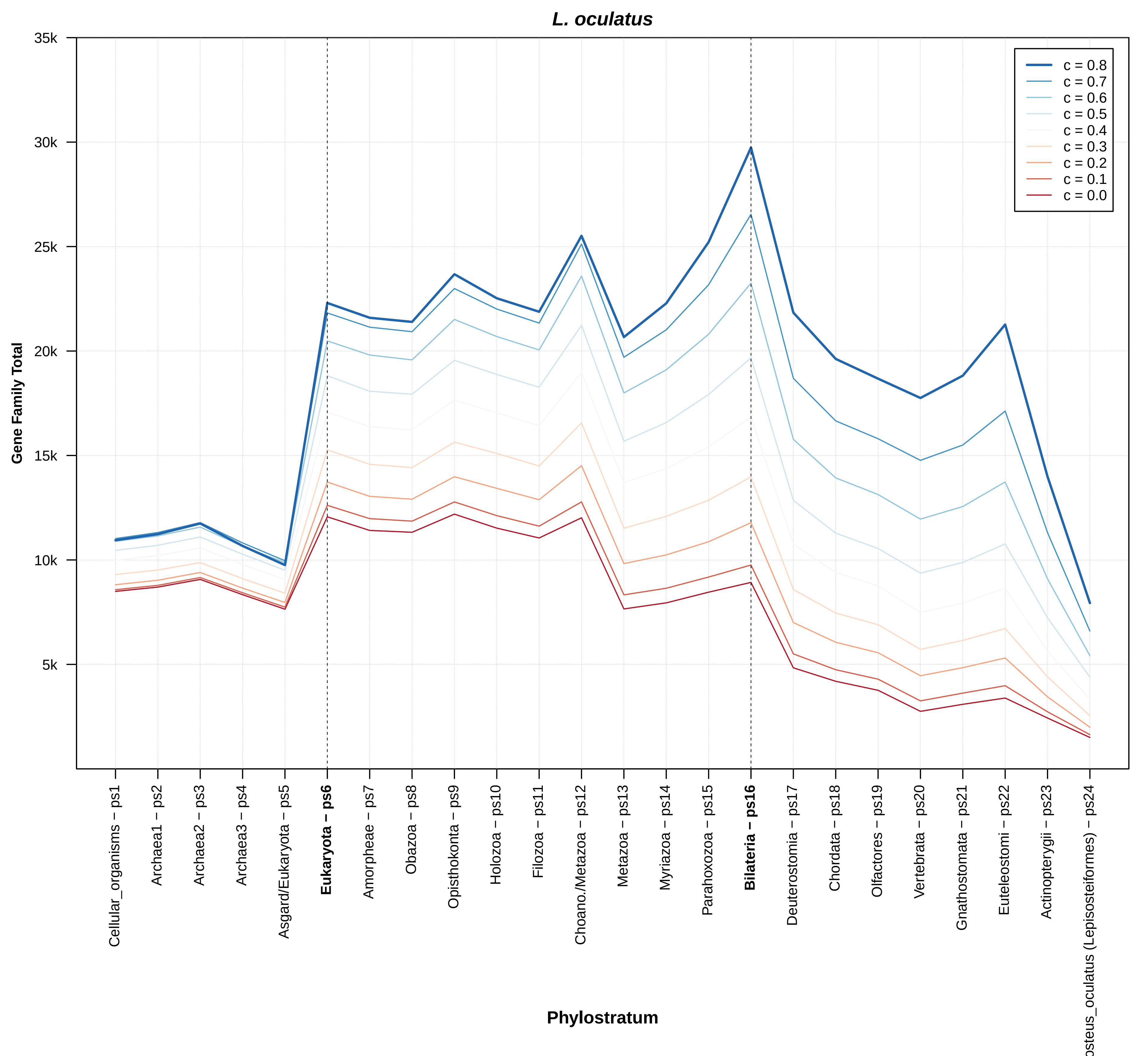
L. oculatus
Gene Family Total
35k
30k
25k
20k
15k
10k
5k
c = 0.8
c = 0.7
c = 0.6
c = 0.5
c = 0.4
c = 0.3
c = 0.2
c = 0.1
c = 0.0
Cellular_organisms – ps1
Archaea1 – ps2
Archaea2 – ps3
Archaea3 – ps4
Asgard/Eukaryota – ps5
Eukaryota – ps6
Amorpheae – ps7
Obazoa – ps8
Opisthokonta – ps9
Holozoa – ps10
Filozoa – ps11
Choano./Metazoa – ps12
Metazoa – ps13
Myriazoa – ps14
Parahoxozoa – ps15
Bilateria – ps16
Deuterostomia – ps17
Chordata – ps18
Olfactores – ps19
Vertebrata – ps20
Gnathostomata – ps21
Euteleostomi – ps22
Actinopterygii – ps23
osteus_oculatus (Lepisosteiformes) – ps24
Phylostratum

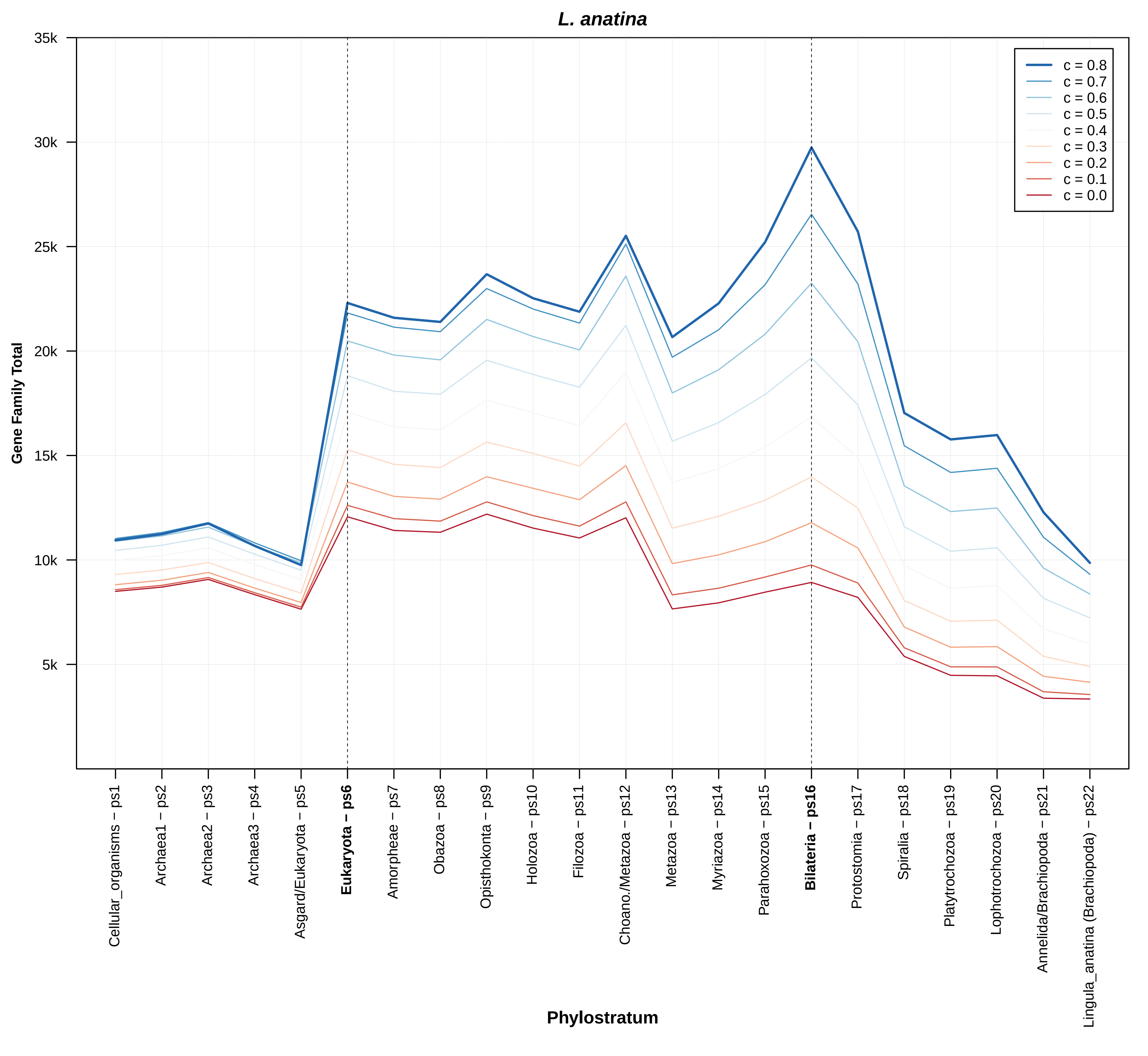

L. anatina
Gene Family Total
Phylostratum
35k
30k
25k
20k
15k
10k
5k
c = 0.8
c = 0.7
c = 0.6
c = 0.5
c = 0.4
c = 0.3
c = 0.2
c = 0.1
c = 0.0
Cellular_organisms – ps1
Archaea1 – ps2
Archaea2 – ps3
Archaea3 – ps4
Asgard/Eukaryota – ps5
Eukaryota – ps6
Amorpheae – ps7
Obazoa – ps8
Opisthokonta – ps9
Holozoa – ps10
Filozoa – ps11
Choano./Metazoa – ps12
Metazoa – ps13
Myriazoa – ps14
Parahoxozoa – ps15
Bilateria – ps16
Protostomia – ps17
Spiralia – ps18
Platytrochozoa – ps19
Lophotrochozoa – ps20
Annelida/Brachiopoda – ps21
Lingula_anatina (Brachiopoda) – ps22

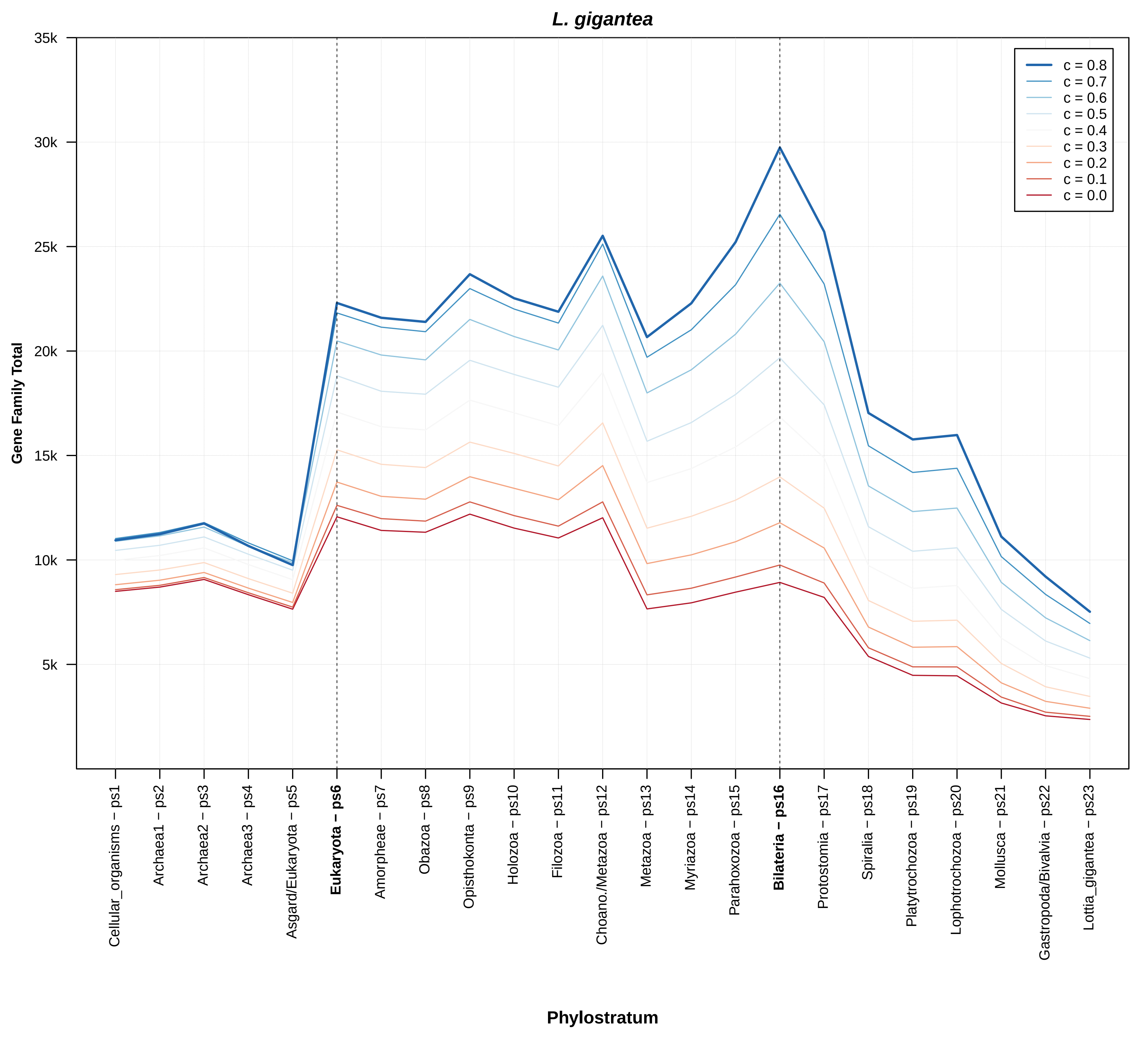

L. gigantea
35k
30k
25k
20k
15k
10k
5k
Gene Family Total
c = 0.8
c = 0.7
c = 0.6
c = 0.5
c = 0.4
c = 0.3
c = 0.2
c = 0.1
c = 0.0
Cellular_organisms – ps1
Archaea1 – ps2
Archaea2 – ps3
Archaea3 – ps4
Asgard/Eukaryota – ps5
Eukaryota – ps6
Amorpheae – ps7
Obazoa – ps8
Opisthokonta – ps9
Holozoa – ps10
Filozoa – ps11
Choano./Metazoa – ps12
Metazoa – ps13
Myriazoa – ps14
Parahoxozoa – ps15
Bilateria – ps16
Protostomia – ps17
Spiralia – ps18
Platytrochozoa – ps19
Lophotrochozoa – ps20
Mollusca – ps21
Gastropoda/Bivalvia – ps22
Lottia_gigantea – ps23
Phylostratum

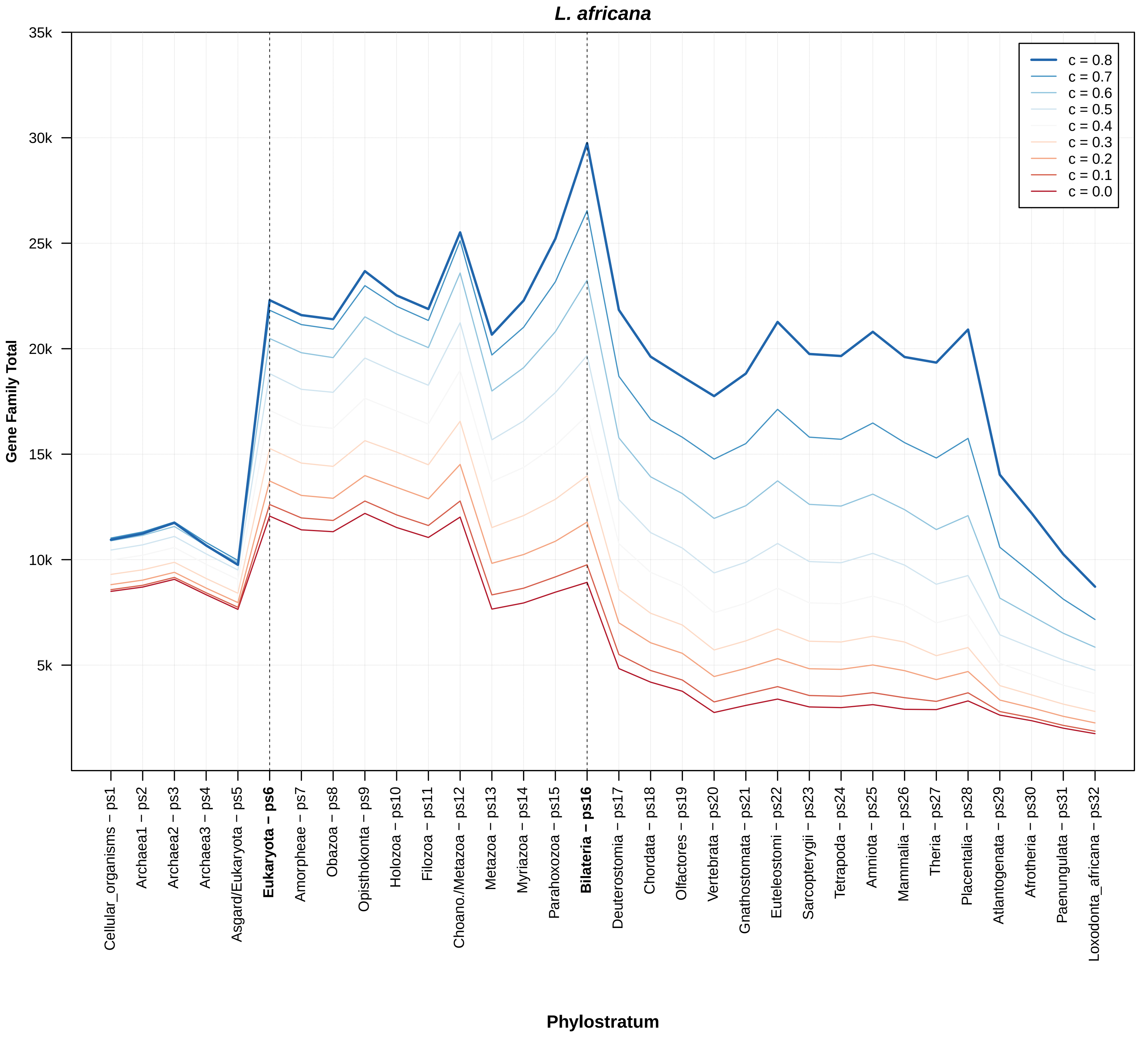

L. africana
35k
30k
25k
20k
15k
10k
5k
Gene Family Total
c = 0.8
c = 0.7
c = 0.6
c = 0.5
c = 0.4
c = 0.3
c = 0.2
c = 0.1
c = 0.0
Cellular_organisms – ps1
Archaea1 – ps2
Archaea2 – ps3
Archaea3 – ps4
Asgard/Eukaryota – ps5
Eukaryota – ps6
Amorpheae – ps7
Obazoa – ps8
Opisthokonta – ps9
Holozoa – ps10
Filozoa – ps11
Choano./Metazoa – ps12
Metazoa – ps13
Myriazoa – ps14
Parahoxozoa – ps15
Bilateria – ps16
Deuterostomia – ps17
Chordata – ps18
Olfactores – ps19
Vertebrata – ps20
Gnathostomata – ps21
Euteleostomi – ps22
Sarcopterygii – ps23
Tetrapoda – ps24
Amniota – ps25
Mammalia – ps26
Theria – ps27
Placentalia – ps28
Atlantogenata – ps29
Afrotheria – ps30
Paenungulata – ps31
Loxodonta_africana – ps32
Phylostratum

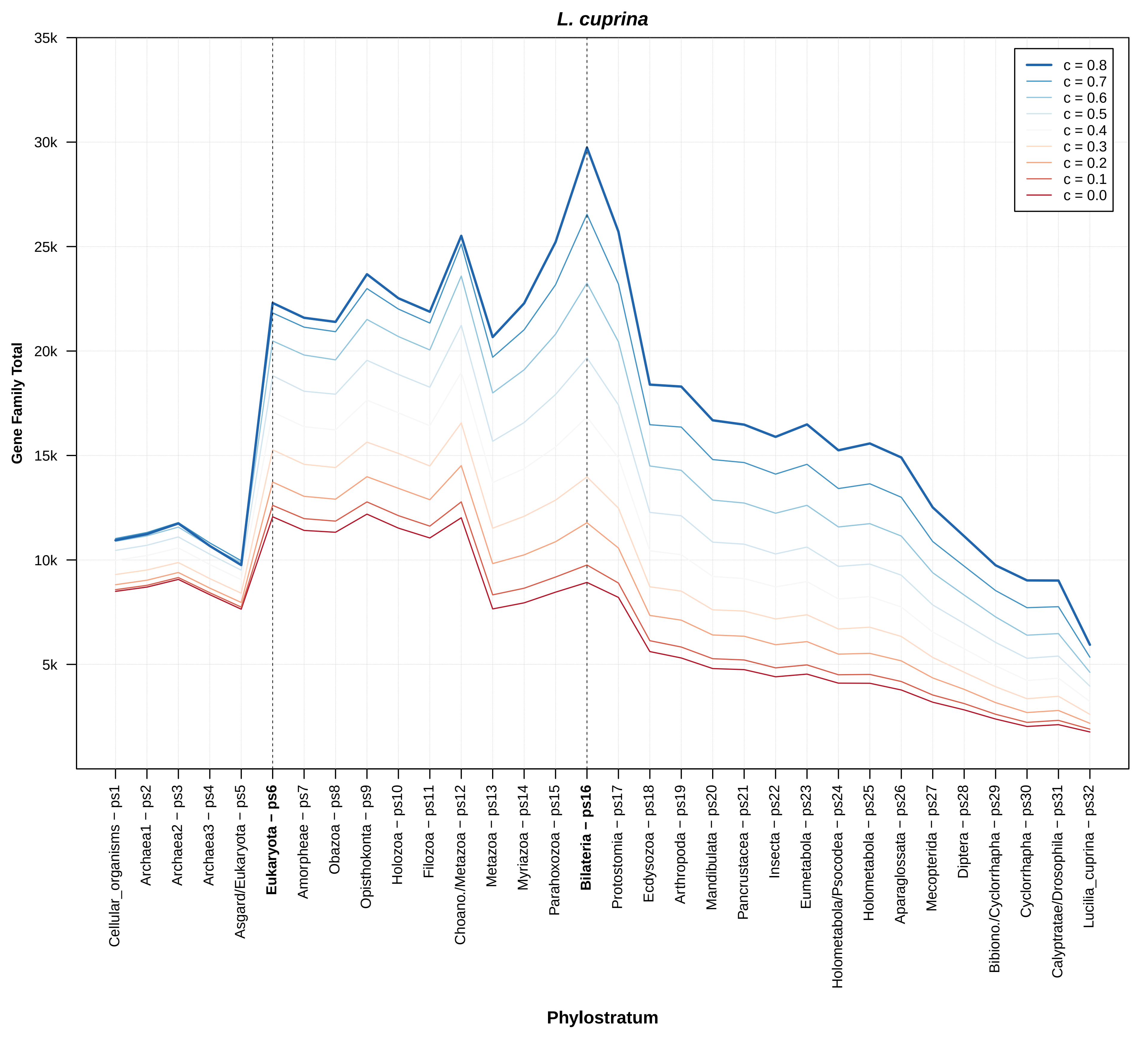
L. cuprina
c = 0.8
c = 0.7
c = 0.6
c = 0.5
c = 0.4
c = 0.3
c = 0.2
c = 0.1
c = 0.0
Gene Family Total
35k
30k
25k
20k
15k
10k
5k
Phylostratum
Cellular_organisms – ps1
Archaea1 – ps2
Archaea2 – ps3
Archaea3 – ps4
Asgard/Eukaryota – ps5
Eukaryota – ps6
Amorpheae – ps7
Obazoa – ps8
Opisthokonta – ps9
Holozoa – ps10
Filozoa – ps11
Choano./Metazoa – ps12
Metazoa – ps13
Myriazoa – ps14
Parahoxozoa – ps15
Bilateria – ps16
Protostomia – ps17
Ecdysozoa – ps18
Arthropoda – ps19
Mandibulata – ps20
Pancrustacea – ps21
Insecta – ps22
Eumetabola – ps23
Holometabola/Psocodea – ps24
Holometabola – ps25
Aparaglossata – ps26
Mecopterida – ps27
Diptera – ps28
Bibiono./Cyclorrhapha – ps29
Cyclorrhapha – ps30
Calyptratae/Drosophila – ps31
Lucilia_cuprina – ps32

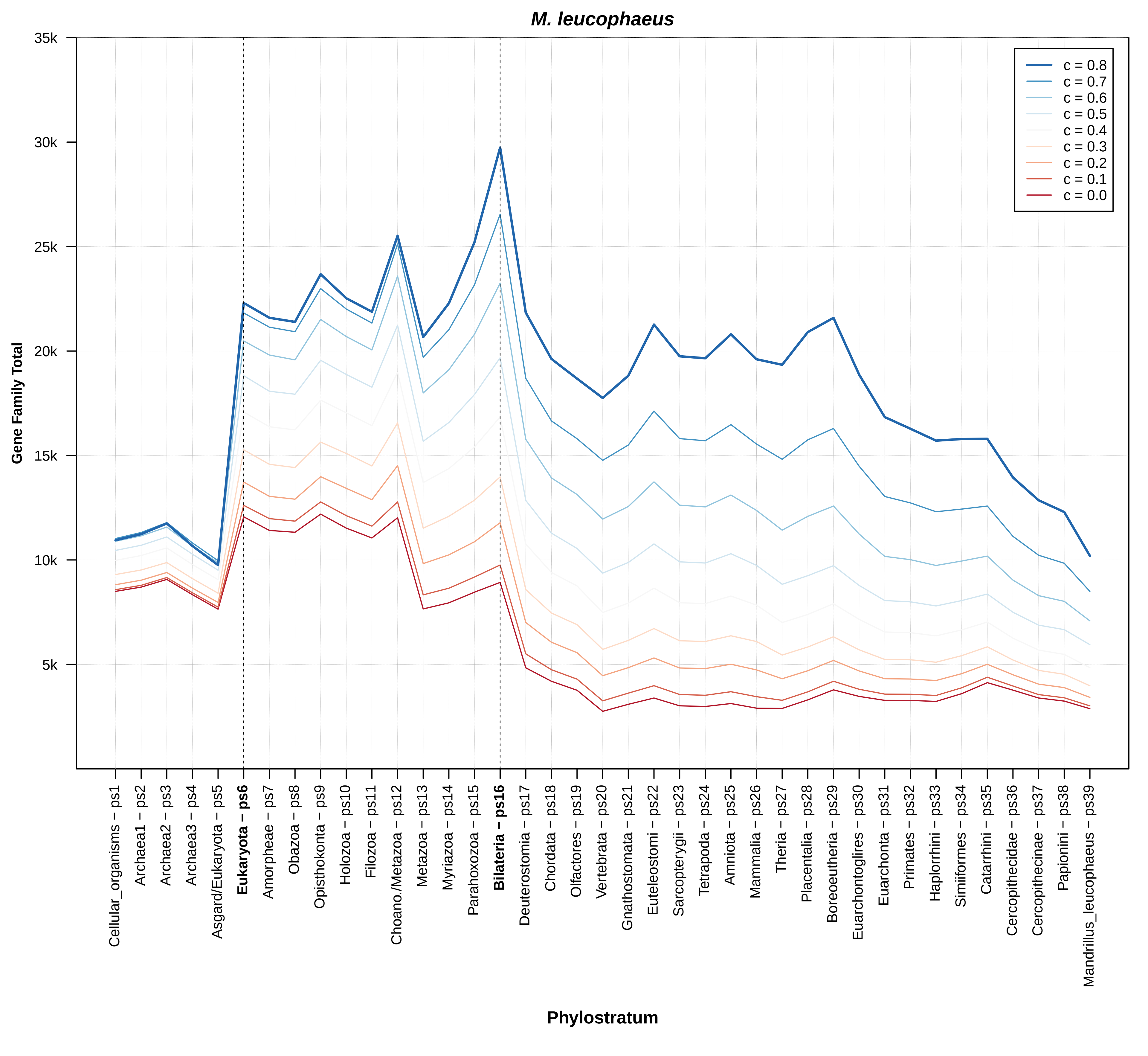
M. leucophaeus
Gene Family Total
Phylostratum
35k
30k
25k
20k
15k
10k
5k
c = 0.8
c = 0.7
c = 0.6
c = 0.5
c = 0.4
c = 0.3
c = 0.2
c = 0.1
c = 0.0
Cellular_organisms – ps1
Archaea1 – ps2
Archaea2 – ps3
Archaea3 – ps4
Asgard/Eukaryota – ps5
Eukaryota – ps6
Amorpheae – ps7
Obazoa – ps8
Opisthokonta – ps9
Holozoa – ps10
Filozoa – ps11
Choano./Metazoa – ps12
Metazoa – ps13
Myriazoa – ps14
Parahoxozoa – ps15
Bilateria – ps16
Deuterostomia – ps17
Chordata – ps18
Olfactores – ps19
Vertebrata – ps20
Gnathostomata – ps21
Euteleostomi – ps22
Sarcopterygii – ps23
Tetrapoda – ps24
Amniota – ps25
Mammalia – ps26
Theria – ps27
Placentalia – ps28
Boreoeutheria – ps29
Euarchontoglires – ps30
Euarchonta – ps31
Primates – ps32
Haplorrhini – ps33
Simiiformes – ps34
Catarrhini – ps35
Cercopithecidae – ps36
Cercopithecinae – ps37
Papionini – ps38
Mandrillus_leucophaeus – ps39

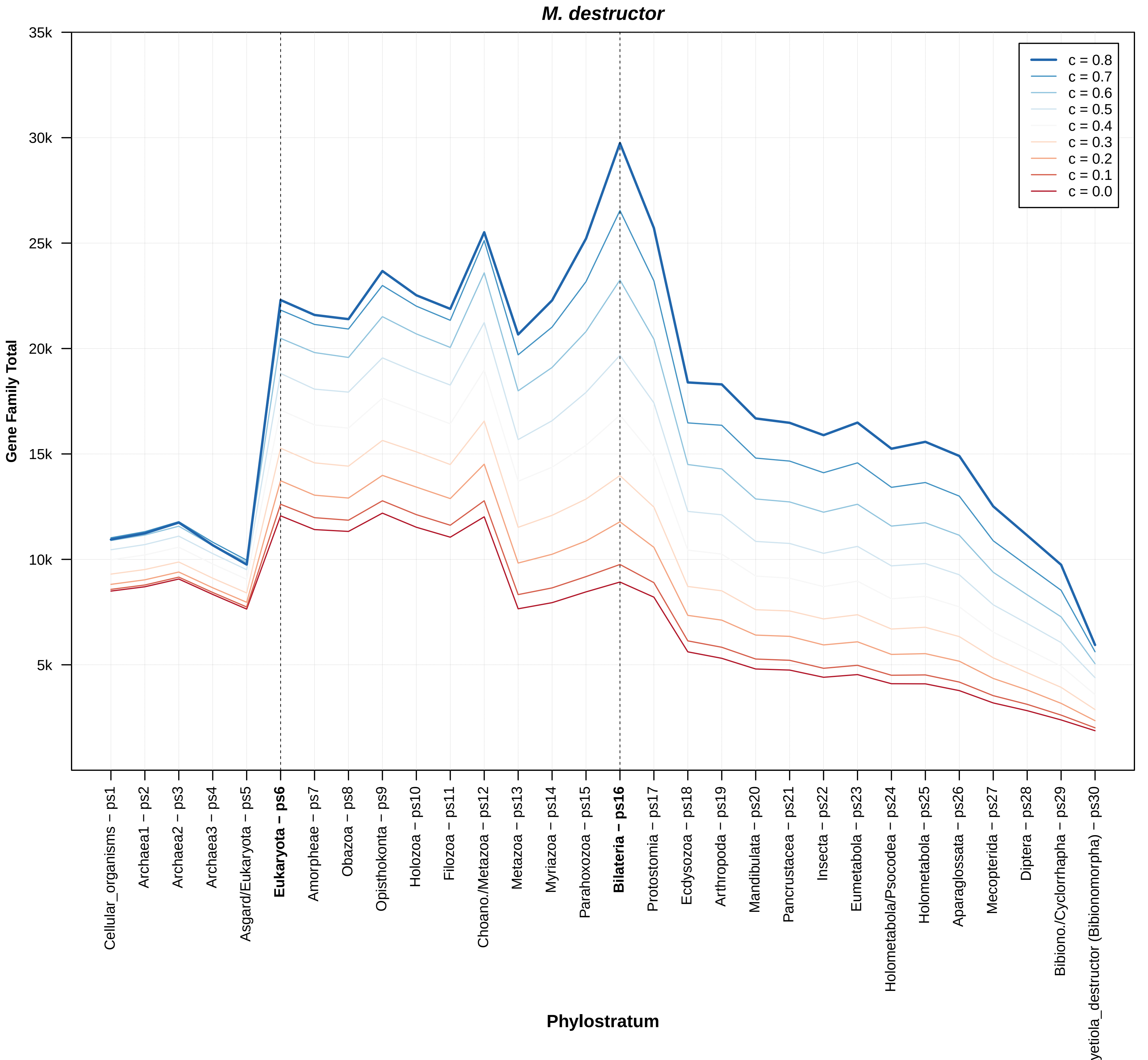
M. destructor
Gene Family Total
35k
30k
25k
20k
15k
10k
5k
c = 0.8
c = 0.7
c = 0.6
c = 0.5
c = 0.4
c = 0.3
c = 0.2
c = 0.1
c = 0.0
Cellular_organisms – ps1
Archaea1 – ps2
Archaea2 – ps3
Archaea3 – ps4
Asgard/Eukaryota – ps5
Eukaryota – ps6
Amorpheae – ps7
Obazoa – ps8
Opisthokonta – ps9
Holozoa – ps10
Filozoa – ps11
Choano./Metazoa – ps12
Metazoa – ps13
Myriazoa – ps14
Parahoxozoa – ps15
Bilateria – ps16
Protostomia – ps17
Ecdysozoa – ps18
Arthropoda – ps19
Mandibulata – ps20
Pancrustacea – ps21
Insecta – ps22
Eumetabola – ps23
Holometabola/Psocodea – ps24
Holometabola – ps25
Aparaglossata – ps26
Mecopterida – ps27
Diptera – ps28
Bibiono./Cyclorrhapha – ps29
Phylostratum

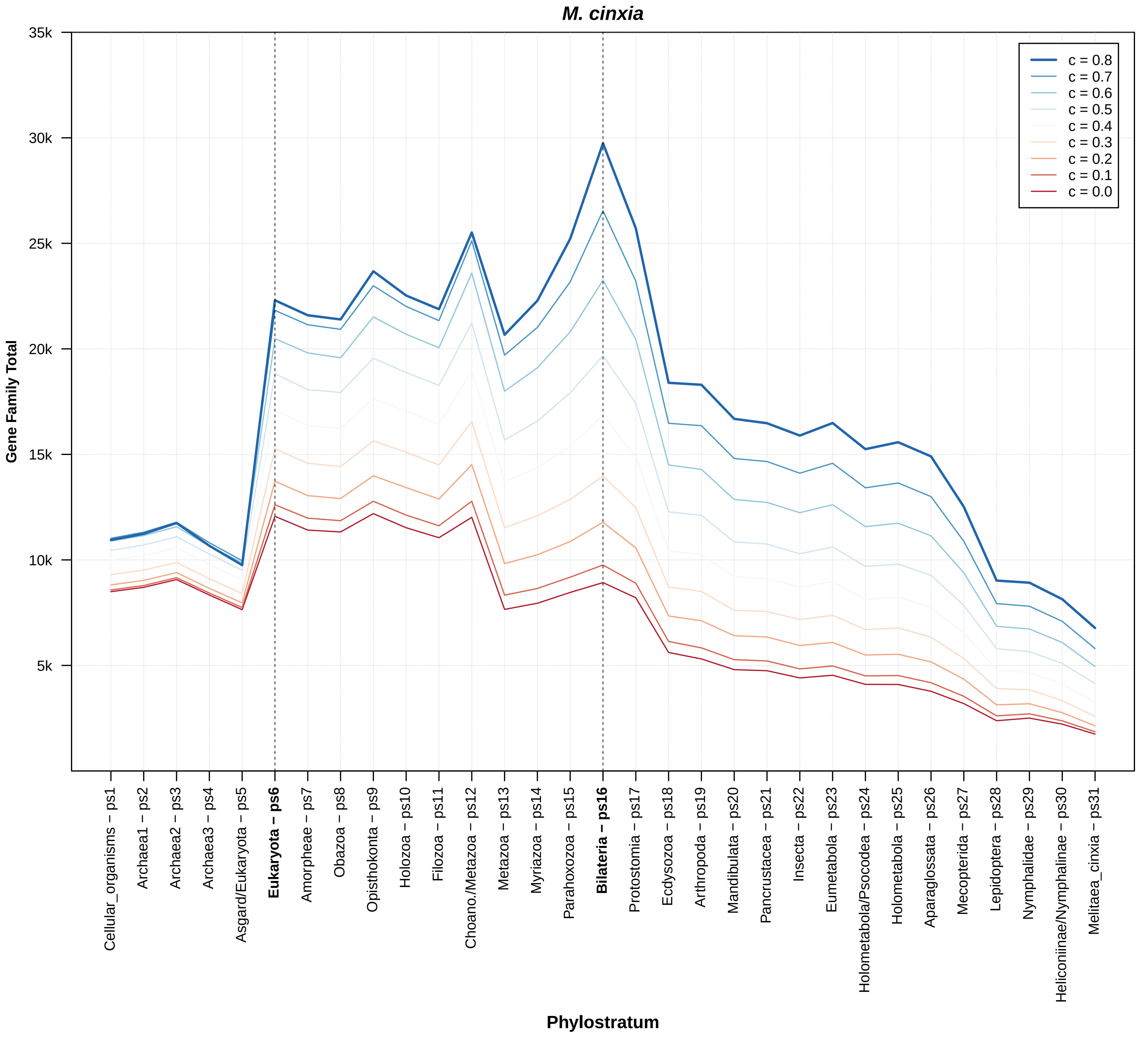
M. cinxia
Gene Family Total
35k
30k
25k
20k
15k
10k
5k
c = 0.8
c = 0.7
c = 0.6
c = 0.5
c = 0.4
c = 0.3
c = 0.2
c = 0.1
c = 0.0
Cellular_organisms – ps1
Archaea1 – ps2
Archaea2 – ps3
Archaea3 – ps4
Asgard/Eukaryota – ps5
Eukaryota – ps6
Amorpheae – ps7
Obazoa – ps8
Opisthokonta – ps9
Holozoa – ps10
Filozoa – ps11
Choano./Metazoa – ps12
Metazoa – ps13
Myriazoa – ps14
Parahoxozoa – ps15
Bilateria – ps16
Protostomia – ps17
Ecdysozoa – ps18
Arthropoda – ps19
Mandibulata – ps20
Pancrustacea – ps21
Insecta – ps22
Eumetabola – ps23
Holometabola/Psocodea – ps24
Holometabola – ps25
Aparaglossata – ps26
Mecopterida – ps27
Lepidoptera – ps28
Nymphalidae – ps29
Heliconiinae/Nymphalinae – ps30
Melitaea_cinxia – ps31
Phylostratum

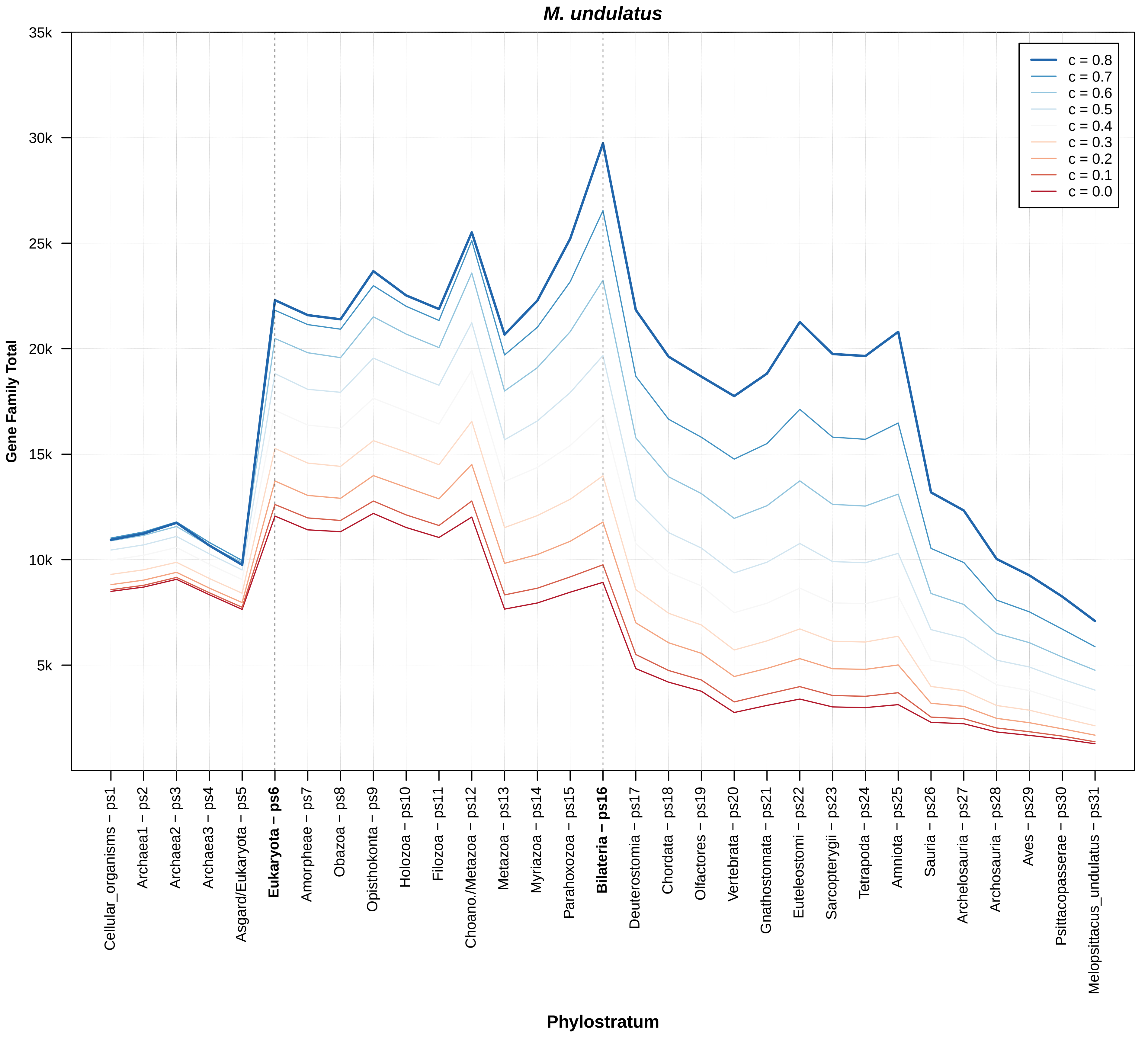

M. undulatus
35k
30k
25k
20k
15k
10k
5k
Gene Family Total
c = 0.8
c = 0.7
c = 0.6
c = 0.5
c = 0.4
c = 0.3
c = 0.2
c = 0.1
c = 0.0
Cellular_organisms – ps1
Archaea1 – ps2
Archaea2 – ps3
Archaea3 – ps4
Asgard/Eukaryota – ps5
Eukaryota – ps6
Amorpheae – ps7
Obazoa – ps8
Opisthokonta – ps9
Holozoa – ps10
Filozoa – ps11
Choano./Metazoa – ps12
Metazoa – ps13
Myriazoa – ps14
Parahoxozoa – ps15
Bilateria – ps16
Deuterostomia – ps17
Chordata – ps18
Olfactores – ps19
Vertebrata – ps20
Gnathostomata – ps21
Euteleostomi – ps22
Sarcopterygii – ps23
Tetrapoda – ps24
Amniota – ps25
Sauria – ps26
Archelosauria – ps27
Archosauria – ps28
Aves – ps29
Psittacopasserae – ps30
Melopsittacus_undulatus – ps31
Phylostratum

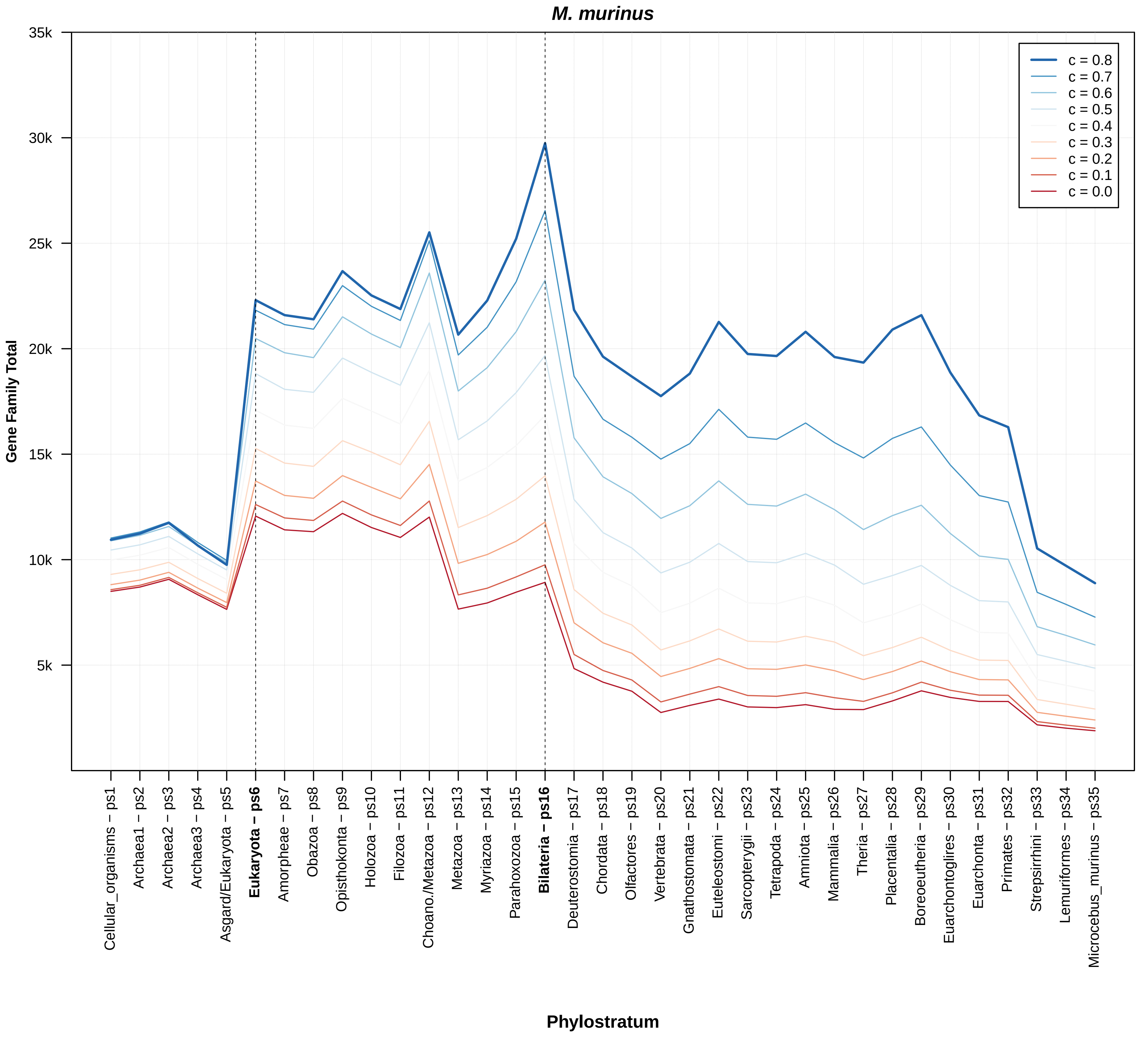

M. murinus
Gene Family Total
35k
30k
25k
20k
15k
10k
5k
c = 0.8
c = 0.7
c = 0.6
c = 0.5
c = 0.4
c = 0.3
c = 0.2
c = 0.1
c = 0.0
Cellular_organisms – ps1
Archaea1 – ps2
Archaea2 – ps3
Archaea3 – ps4
Asgard/Eukaryota – ps5
Eukaryota – ps6
Amorpheae – ps7
Obazoa – ps8
Opisthokonta – ps9
Holozoa – ps10
Filozoa – ps11
Choano./Metazoa – ps12
Metazoa – ps13
Myriazoa – ps14
Parahoxozoa – ps15
Bilateria – ps16
Deuterostomia – ps17
Chordata – ps18
Olfactores – ps19
Vertebrata – ps20
Gnathostomata – ps21
Euteleostomi – ps22
Sarcopterygii – ps23
Tetrapoda – ps24
Amniota – ps25
Mammalia – ps26
Theria – ps27
Placentalia – ps28
Boreoeutheria – ps29
Euarchontoglires – ps30
Euarchonta – ps31
Primates – ps32
Strepsirrhini – ps33
Lemuriformes – ps34
Microcebus_murinus – ps35
Phylostratum

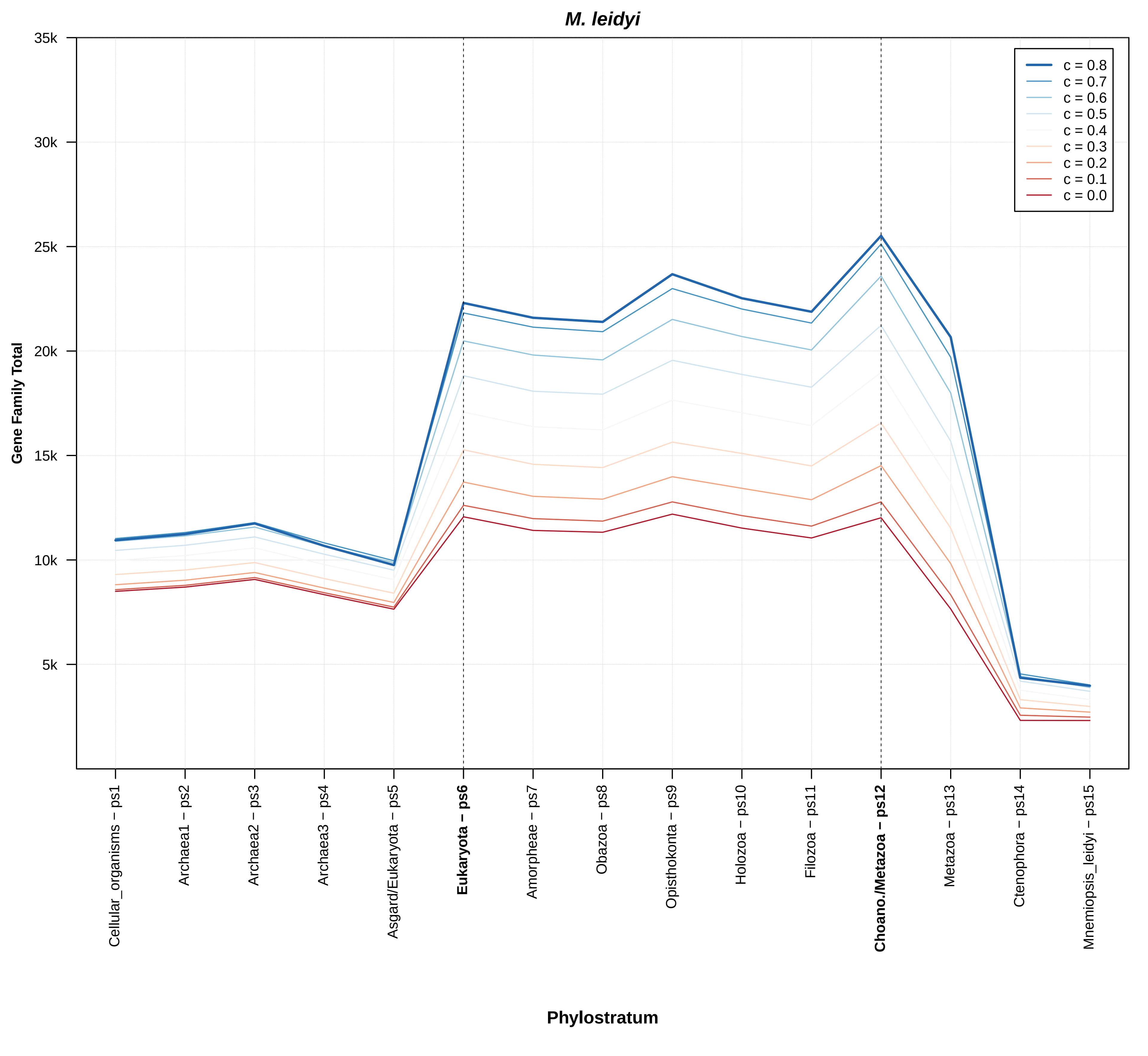

M. leidyi
Gene Family Total
35k
30k
25k
20k
15k
10k
5k
c = 0.8
c = 0.7
c = 0.6
c = 0.5
c = 0.4
c = 0.3
c = 0.2
c = 0.1
c = 0.0
Cellular_organisms – ps1
Archaea1 – ps2
Archaea2 – ps3
Archaea3 – ps4
Asgard/Eukaryota – ps5
Eukaryota – ps6
Amorpheae – ps7
Obazoa – ps8
Opisthokonta – ps9
Holozoa – ps10
Filozoa – ps11
Choano./Metazoa – ps12
Metazoa – ps13
Ctenophora – ps14
Mnemiopsis_leidyi – ps15
Phylostratum

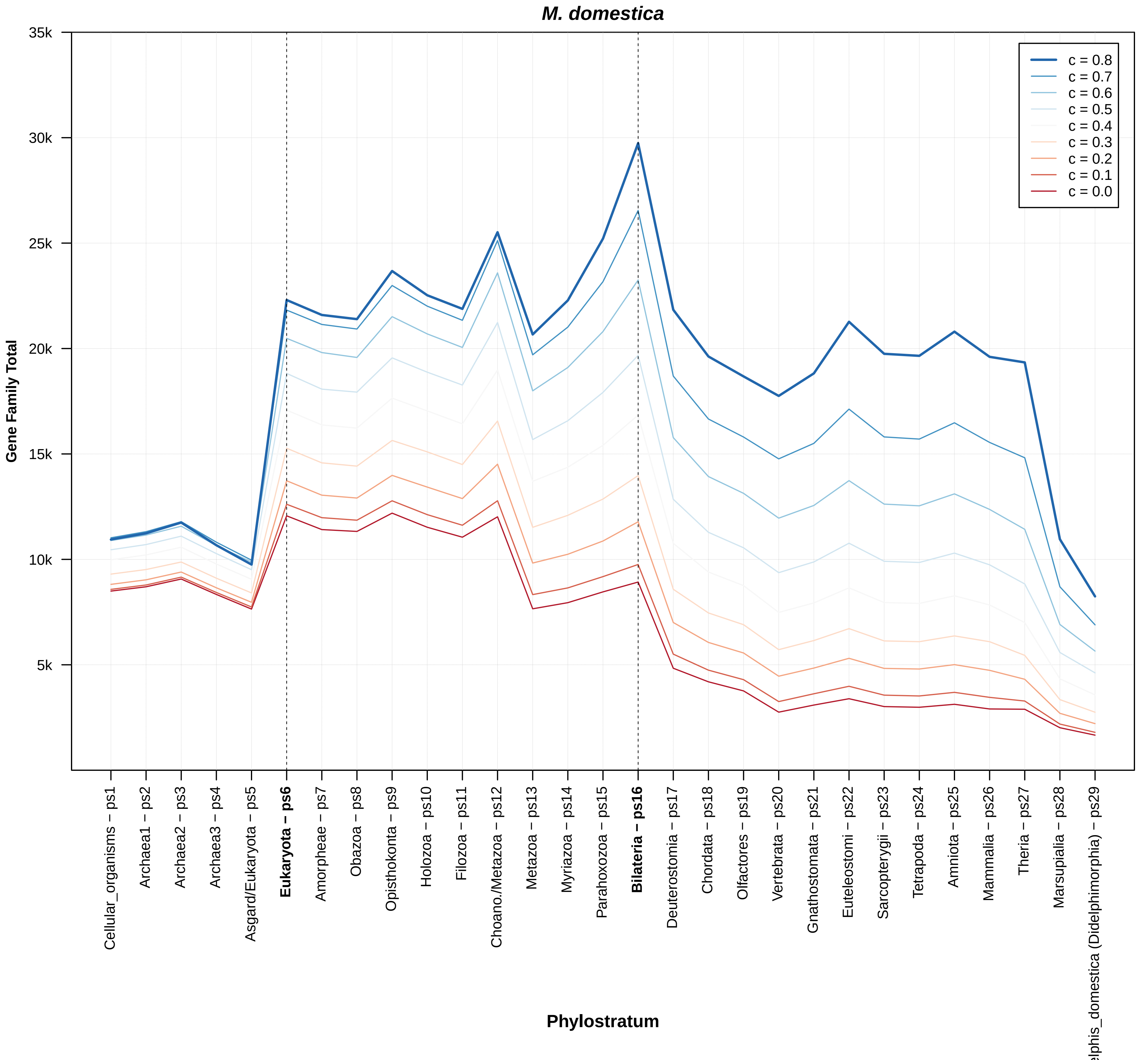

M. domestica
Gene Family Total
35k
30k
25k
20k
15k
10k
5k
c = 0.8
c = 0.7
c = 0.6
c = 0.5
c = 0.4
c = 0.3
c = 0.2
c = 0.1
c = 0.0
Cellular_organisms – ps1
Archaea1 – ps2
Archaea2 – ps3
Archaea3 – ps4
Asgard/Eukaryota – ps5
Eukaryota – ps6
Amorpheae – ps7
Obazoa – ps8
Opisthokonta – ps9
Holozoa – ps10
Filozoa – ps11
Choano./Metazoa – ps12
Metazoa – ps13
Myriazoa – ps14
Parahoxozoa – ps15
Bilateria – ps16
Deuterostomia – ps17
Chordata – ps18
Olfactores – ps19
Vertebrata – ps20
Gnathostomata – ps21
Euteleostomi – ps22
Sarcopterygii – ps23
Tetrapoda – ps24
Amniota – ps25
Mammalia – ps26
Theria – ps27
Marsupialia – ps28
elphis_domestica (Didelphimorphia) – ps29
Phylostratum

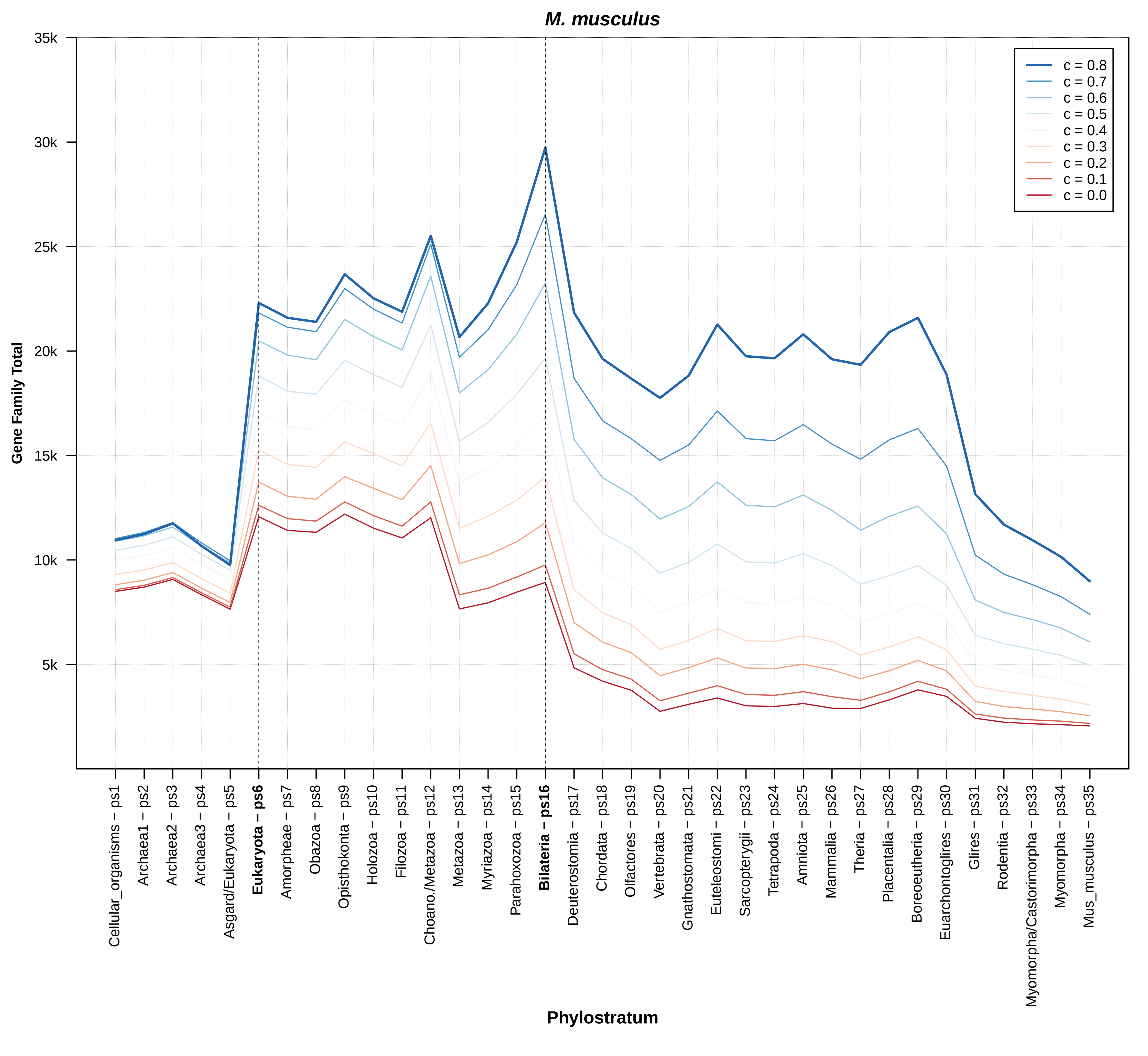

M. musculus
Gene Family Total
35k
30k
25k
20k
15k
10k
5k
c = 0.8
c = 0.7
c = 0.6
c = 0.5
c = 0.4
c = 0.3
c = 0.2
c = 0.1
c = 0.0
Cellular_organisms – ps1
Archaea1 – ps2
Archaea2 – ps3
Archaea3 – ps4
Asgard/Eukaryota – ps5
Eukaryota – ps6
Amorpheae – ps7
Obazoa – ps8
Opisthokonta – ps9
Holozoa – ps10
Filozoa – ps11
Choano./Metazoa – ps12
Metazoa – ps13
Myriazoa – ps14
Parahoxozoa – ps15
Bilateria – ps16
Deuterostomia – ps17
Chordata – ps18
Olfactores – ps19
Vertebrata – ps20
Gnathostomata – ps21
Euteleostomi – ps22
Sarcopterygii – ps23
Tetrapoda – ps24
Amniota – ps25
Mammalia – ps26
Theria – ps27
Placentalia – ps28
Boreoeutheria – ps29
Euarchontoglires – ps30
Glires – ps31
Rodentia – ps32
Myomorpha/Castorimorpha – ps33
Myomorpha – ps34
Mus_musculus – ps35
Phylostratum

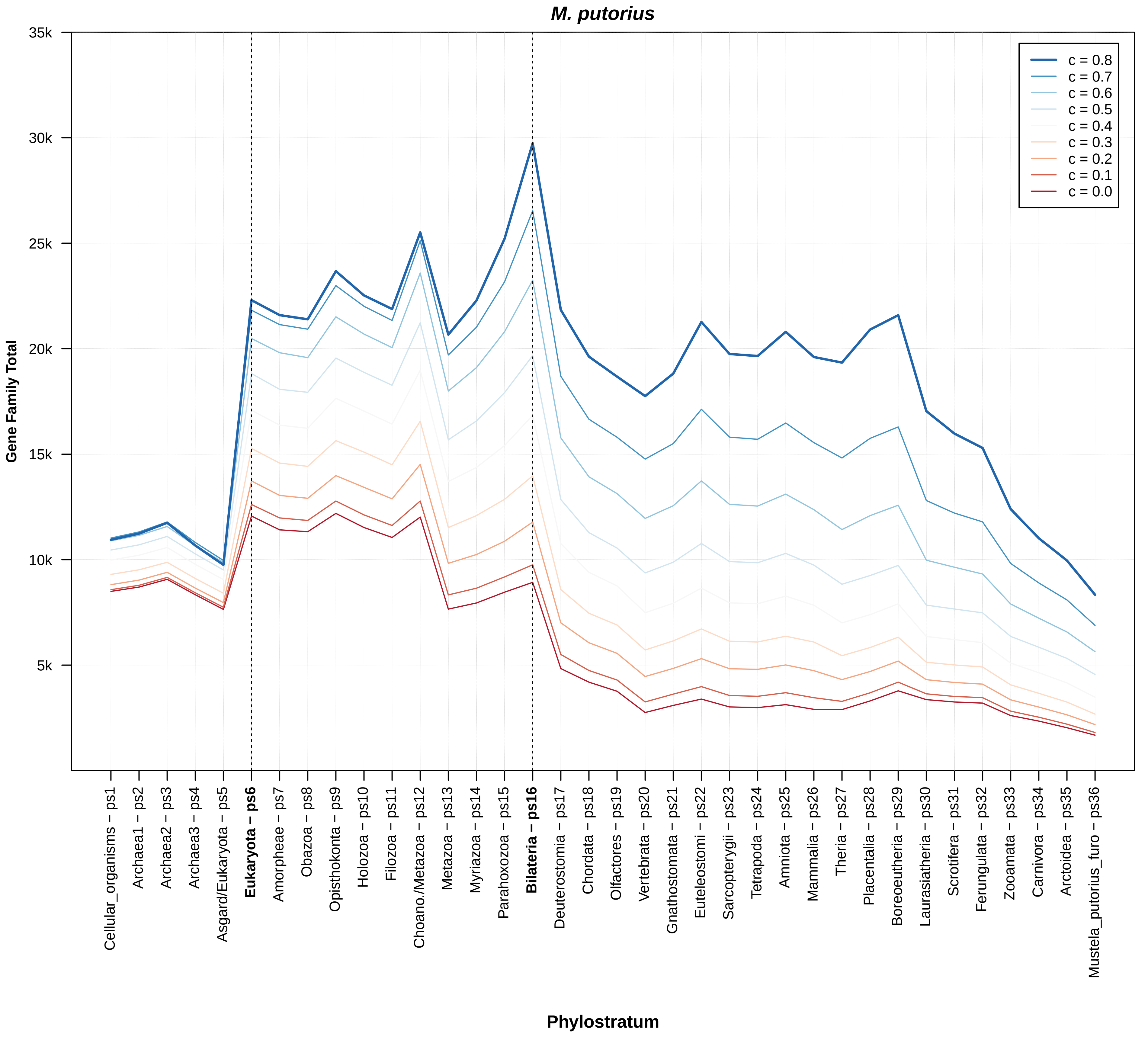
M. putorius
Gene Family Total
35k
30k
25k
20k
15k
10k
5k
c = 0.8
c = 0.7
c = 0.6
c = 0.5
c = 0.4
c = 0.3
c = 0.2
c = 0.1
c = 0.0
Cellular_organisms – ps1
Archaea1 – ps2
Archaea2 – ps3
Archaea3 – ps4
Asgard/Eukaryota – ps5
Eukaryota – ps6
Amorpheae – ps7
Obazoa – ps8
Opisthokonta – ps9
Holozoa – ps10
Filozoa – ps11
Choano./Metazoa – ps12
Metazoa – ps13
Myriazoa – ps14
Parahoxozoa – ps15
Bilateria – ps16
Deuterostomia – ps17
Chordata – ps18
Olfactores – ps19
Vertebrata – ps20
Gnathostomata – ps21
Euteleostomi – ps22
Sarcopterygii – ps23
Tetrapoda – ps24
Amniota – ps25
Mammalia – ps26
Theria – ps27
Placentalia – ps28
Boreoeutheria – ps29
Laurasiatheria – ps30
Scrotifera – ps31
Ferungulata – ps32
Zooamata – ps33
Carnivora – ps34
Arctoidea – ps35
Mustela_putorius_furo – ps36
Phylostratum

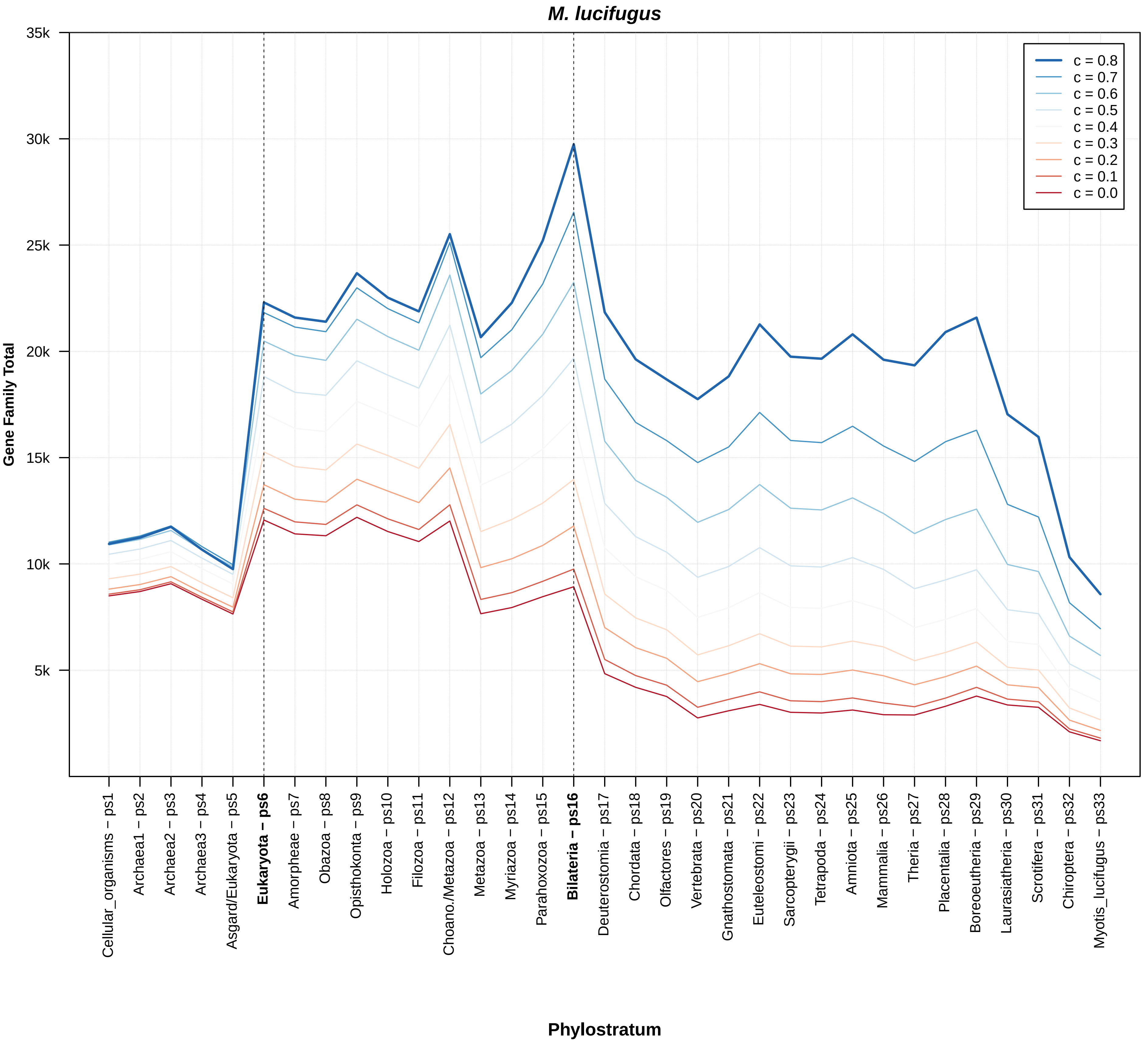
M. lucifugus
Gene Family Total
Phylostratum
35k
30k
25k
20k
15k
10k
5k
c = 0.8
c = 0.7
c = 0.6
c = 0.5
c = 0.4
c = 0.3
c = 0.2
c = 0.1
c = 0.0
Cellular_organisms – ps1
Archaea1 – ps2
Archaea2 – ps3
Archaea3 – ps4
Asgard/Eukaryota – ps5
Eukaryota – ps6
Amorpheae – ps7
Obazoa – ps8
Opisthokonta – ps9
Holozoa – ps10
Filozoa – ps11
Choano./Metazoa – ps12
Metazoa – ps13
Myriazoa – ps14
Parahoxozoa – ps15
Bilateria – ps16
Deuterostomia – ps17
Chordata – ps18
Olfactores – ps19
Vertebrata – ps20
Gnathostomata – ps21
Euteleostomi – ps22
Sarcopterygii – ps23
Tetrapoda – ps24
Amniota – ps25
Mammalia – ps26
Theria – ps27
Placentalia – ps28
Boreoeutheria – ps29
Laurasiatheria – ps30
Scrotifera – ps31
Chiroptera – ps32
Myotis_lucifugus – ps33

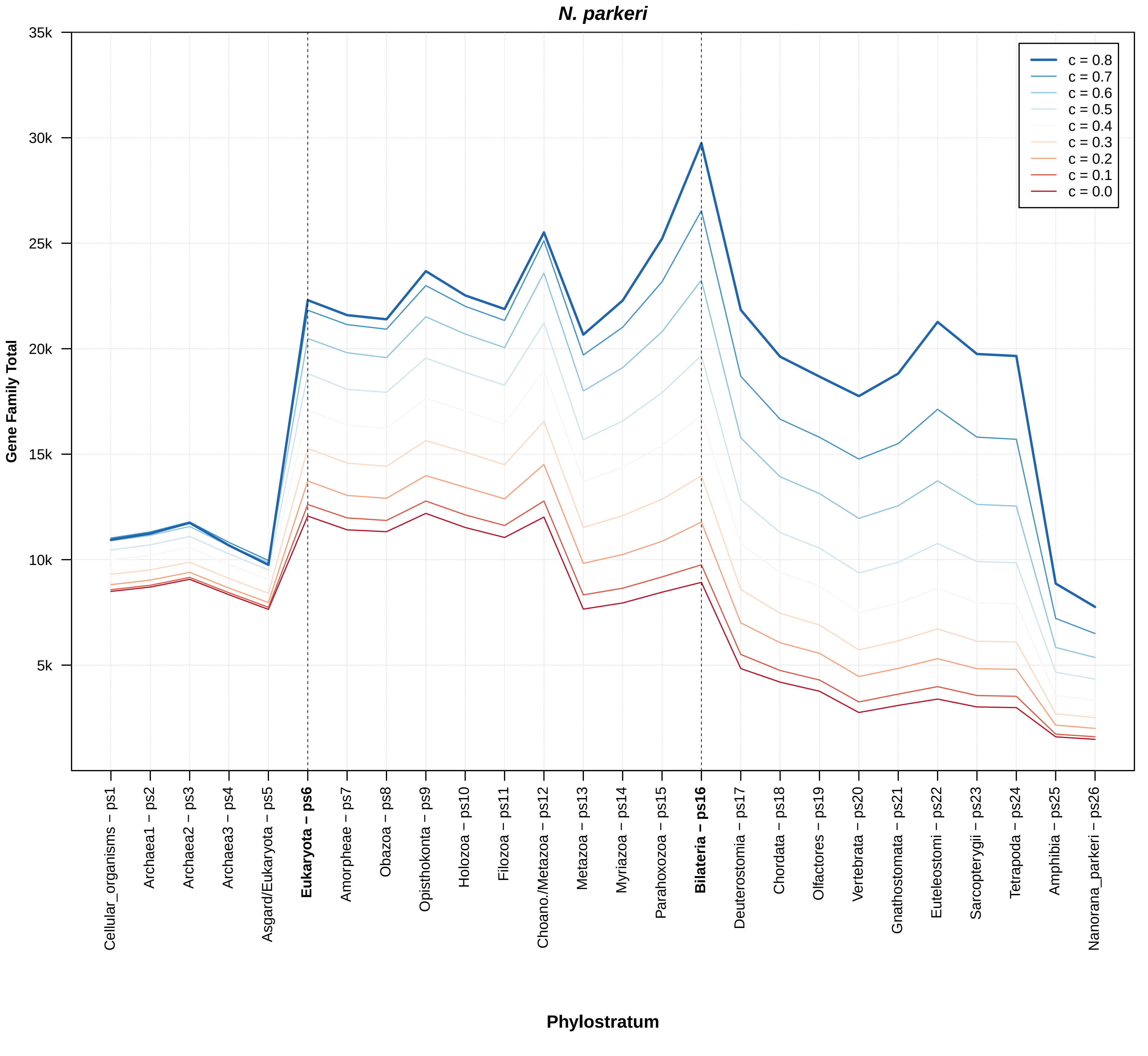
N. parkeri
Gene Family Total
35k
30k
25k
20k
15k
10k
5k
c = 0.8
c = 0.7
c = 0.6
c = 0.5
c = 0.4
c = 0.3
c = 0.2
c = 0.1
c = 0.0
Cellular_organisms – ps1
Archaea1 – ps2
Archaea2 – ps3
Archaea3 – ps4
Asgard/Eukaryota – ps5
Eukaryota – ps6
Amorpheae – ps7
Obazoa – ps8
Opisthokonta – ps9
Holozoa – ps10
Filozoa – ps11
Choano./Metazoa – ps12
Metazoa – ps13
Myriazoa – ps14
Parahoxozoa – ps15
Bilateria – ps16
Deuterostomia – ps17
Chordata – ps18
Olfactores – ps19
Vertebrata – ps20
Gnathostomata – ps21
Euteleostomi – ps22
Sarcopterygii – ps23
Tetrapoda – ps24
Amphibia – ps25
Nanorana_parkeri – ps26
Phylostratum

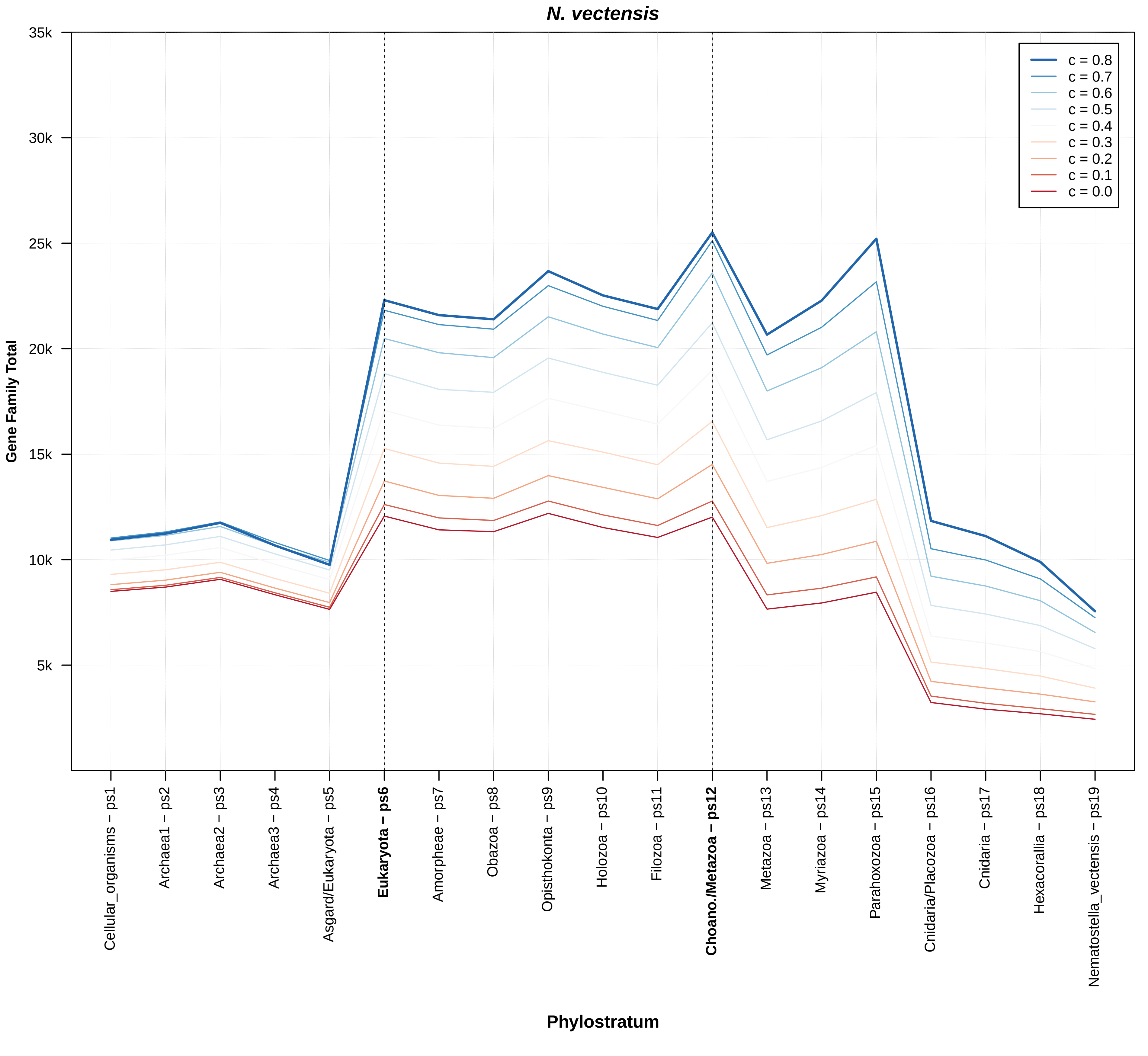
N. vectensis
Gene Family Total
35k
30k
25k
20k
15k
10k
5k
c = 0.8
c = 0.7
c = 0.6
c = 0.5
c = 0.4
c = 0.3
c = 0.2
c = 0.1
c = 0.0
Cellular_organisms – ps1
Archaea1 – ps2
Archaea2 – ps3
Archaea3 – ps4
Asgard/Eukaryota – ps5
Eukaryota – ps6
Amorpheae – ps7
Obazoa – ps8
Opisthokonta – ps9
Holozoa – ps10
Filozoa – ps11
Choano./Metazoa – ps12
Metazoa – ps13
Myriazoa – ps14
Parahoxozoa – ps15
Cnidaria/Placozoa – ps16
Cnidaria – ps17
Hexacorallia – ps18
Nematostella_vectensis – ps19
Phylostratum

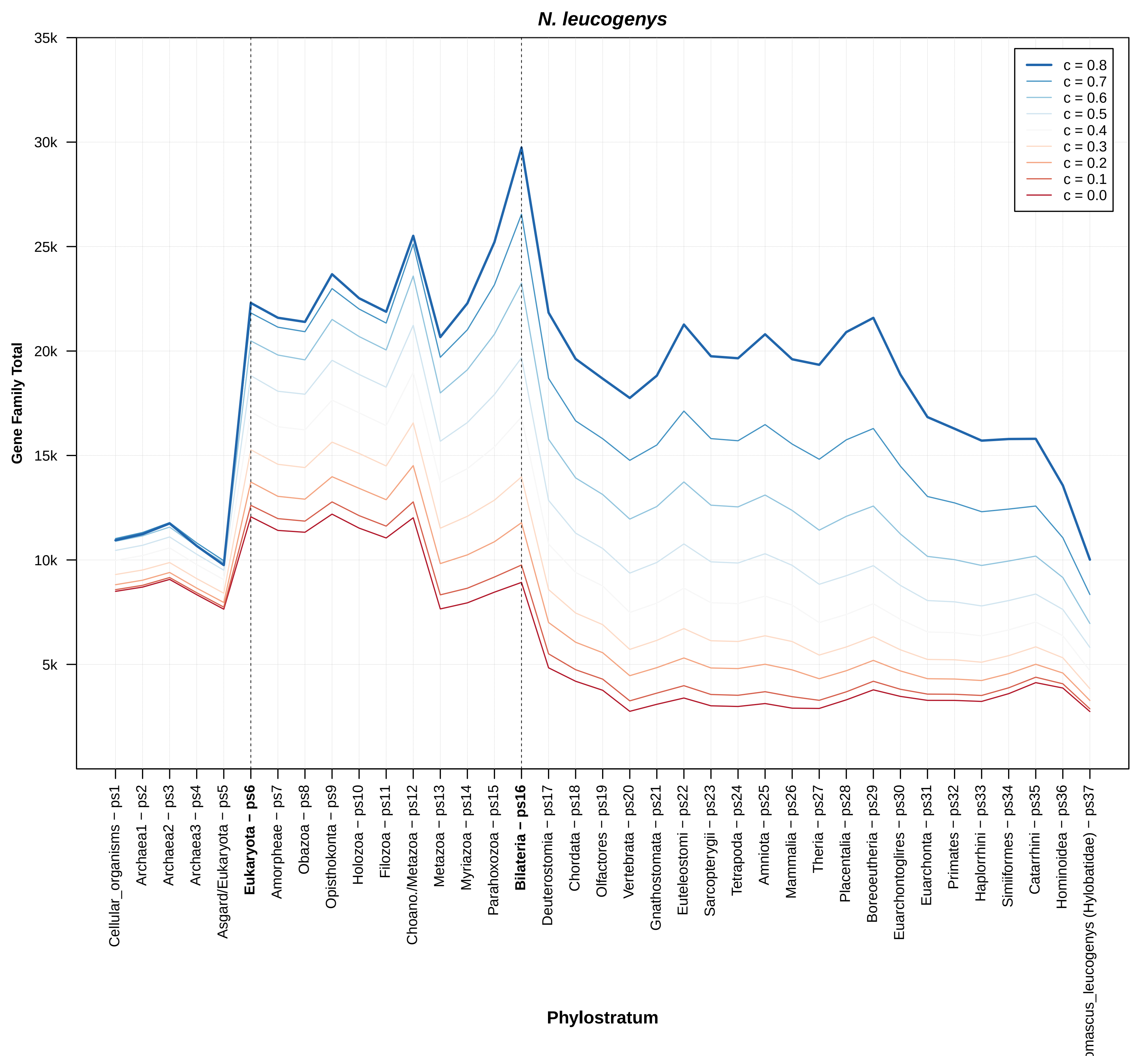

N. leucogenys
Gene Family Total
Phylostratum
35k
30k
25k
20k
15k
10k
5k
c = 0.8
c = 0.7
c = 0.6
c = 0.5
c = 0.4
c = 0.3
c = 0.2
c = 0.1
c = 0.0
Cellular_organisms – ps1
Archaea1 – ps2
Archaea2 – ps3
Archaea3 – ps4
Asgard/Eukaryota – ps5
Eukaryota – ps6
Amorpheae – ps7
Obazoa – ps8
Opisthokonta – ps9
Holozoa – ps10
Filozoa – ps11
Choano./Metazoa – ps12
Metazoa – ps13
Myriazoa – ps14
Parahoxozoa – ps15
Bilateria – ps16
Deuterostomia – ps17
Chordata – ps18
Olfactores – ps19
Vertebrata – ps20
Gnathostomata – ps21
Euteleostomi – ps22
Sarcopterygii – ps23
Tetrapoda – ps24
Amniota – ps25
Mammalia – ps26
Theria – ps27
Placentalia – ps28
Boreoeutheria – ps29
Euarchontoglires – ps30
Euarchonta – ps31
Primates – ps32
Haplorrhini – ps33
Simiiformes – ps34
Catarrhini – ps35
Hominoidea – ps36

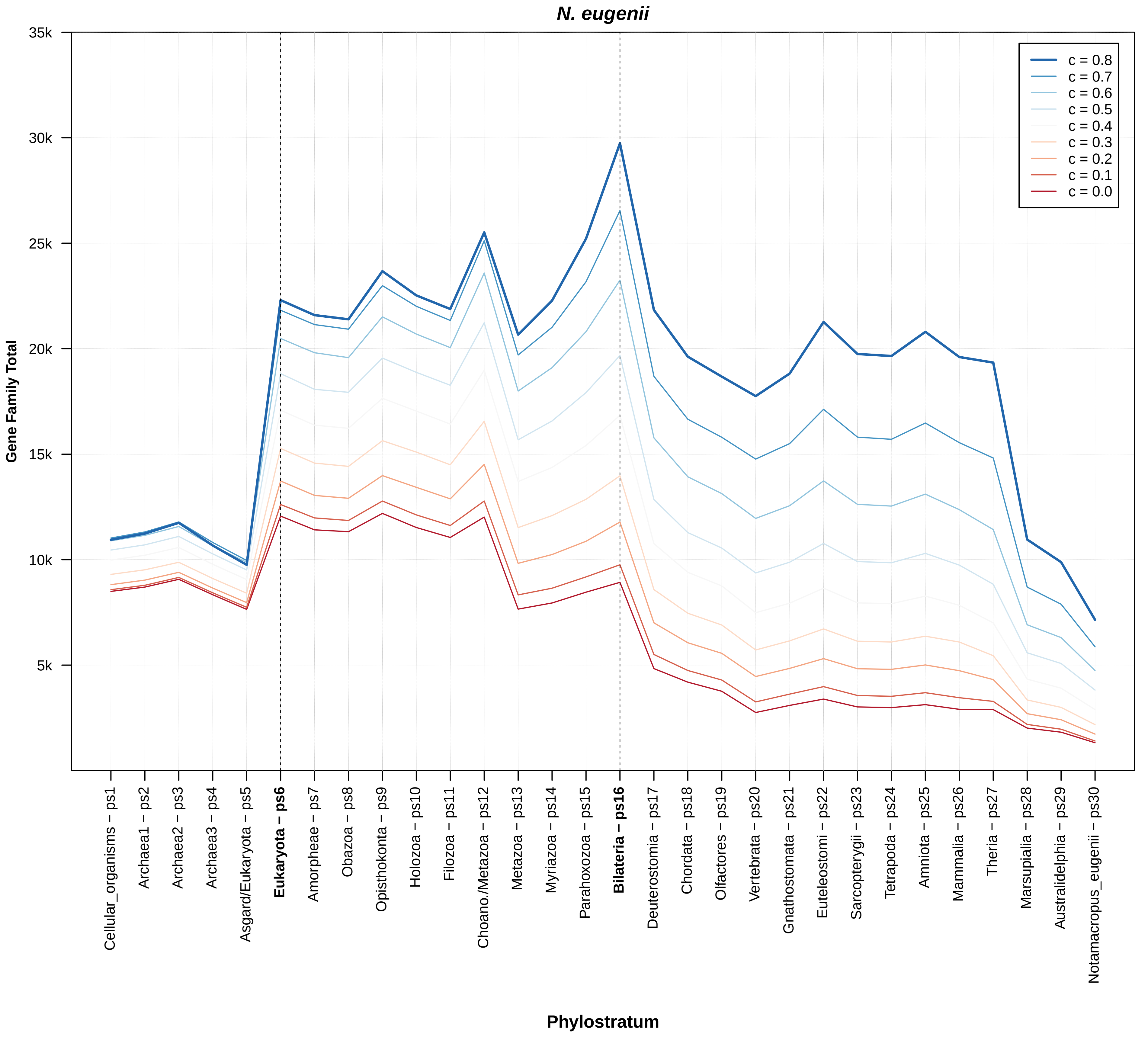

N. eugenii
Gene Family Total
Phylostratum
35k
30k
25k
20k
15k
10k
5k
c = 0.8
c = 0.7
c = 0.6
c = 0.5
c = 0.4
c = 0.3
c = 0.2
c = 0.1
c = 0.0
Cellular_organisms – ps1
Archaea1 – ps2
Archaea2 – ps3
Archaea3 – ps4
Asgard/Eukaryota – ps5
Eukaryota – ps6
Amorpheae – ps7
Obazoa – ps8
Opisthokonta – ps9
Holozoa – ps10
Filozoa – ps11
Choano./Metazoa – ps12
Metazoa – ps13
Myriazoa – ps14
Parahoxozoa – ps15
Bilateria – ps16
Deuterostomia – ps17
Chordata – ps18
Olfactores – ps19
Vertebrata – ps20
Gnathostomata – ps21
Euteleostomi – ps22
Sarcopterygii – ps23
Tetrapoda – ps24
Amniota – ps25
Mammalia – ps26
Theria – ps27
Marsupialia – ps28
Australidelphia – ps29
Notamacropus_eugenii – ps30

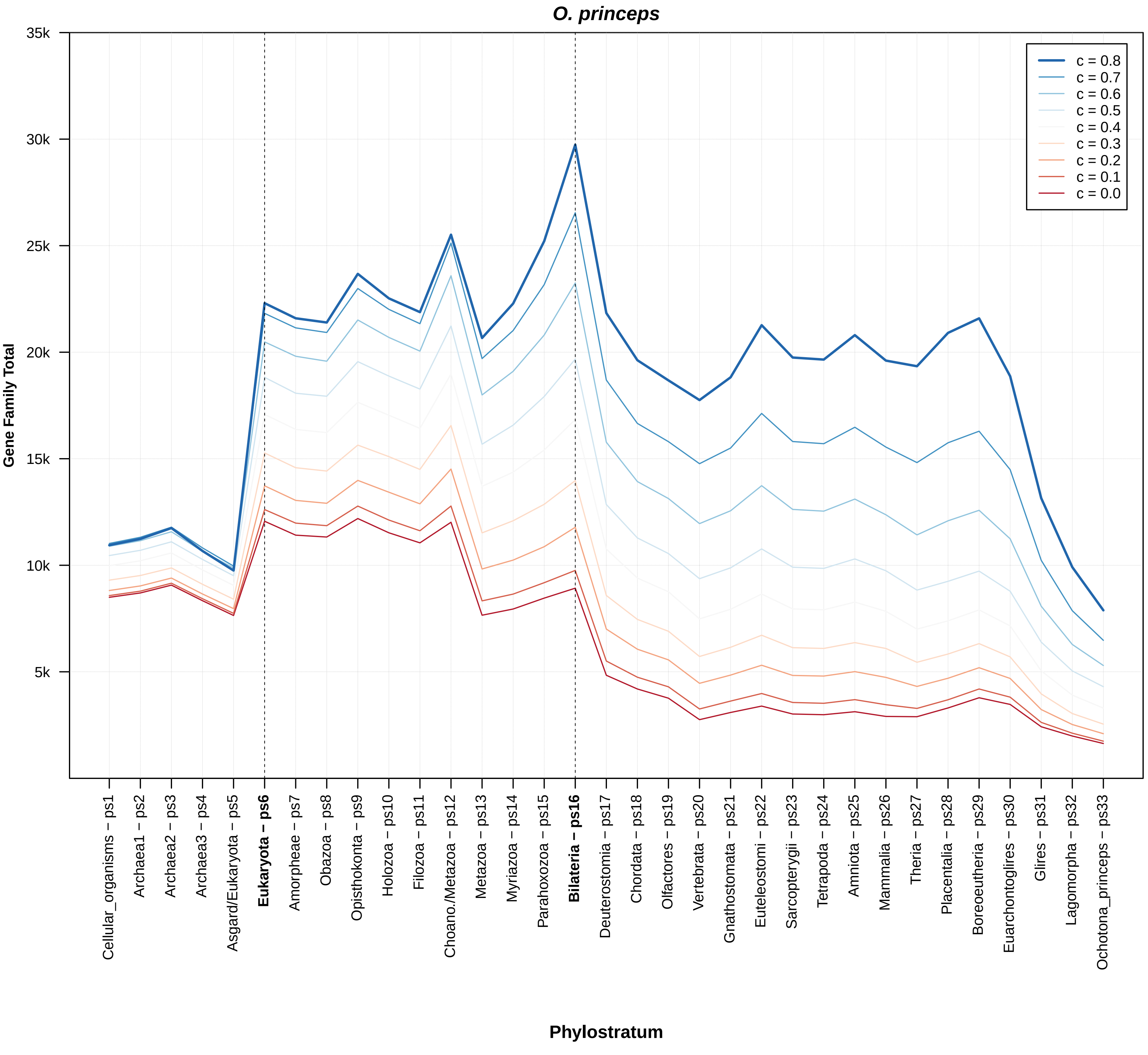
O. princeps
Gene Family Total
35k
30k
25k
20k
15k
10k
5k
c = 0.8
c = 0.7
c = 0.6
c = 0.5
c = 0.4
c = 0.3
c = 0.2
c = 0.1
c = 0.0
Cellular_organisms – ps1
Archaea1 – ps2
Archaea2 – ps3
Archaea3 – ps4
Asgard/Eukaryota – ps5
Eukaryota – ps6
Amorpheae – ps7
Obazoa – ps8
Opisthokonta – ps9
Holozoa – ps10
Filozoa – ps11
Choano./Metazoa – ps12
Metazoa – ps13
Myriazoa – ps14
Parahoxozoa – ps15
Bilateria – ps16
Deuterostomia – ps17
Chordata – ps18
Olfactores – ps19
Vertebrata – ps20
Gnathostomata – ps21
Euteleostomi – ps22
Sarcopterygii – ps23
Tetrapoda – ps24
Amniota – ps25
Mammalia – ps26
Theria – ps27
Placentalia – ps28
Boreoeutheria – ps29
Euarchontoglires – ps30
Glires – ps31
Lagomorpha – ps32
Ochotona_princeps – ps33
Phylostratum

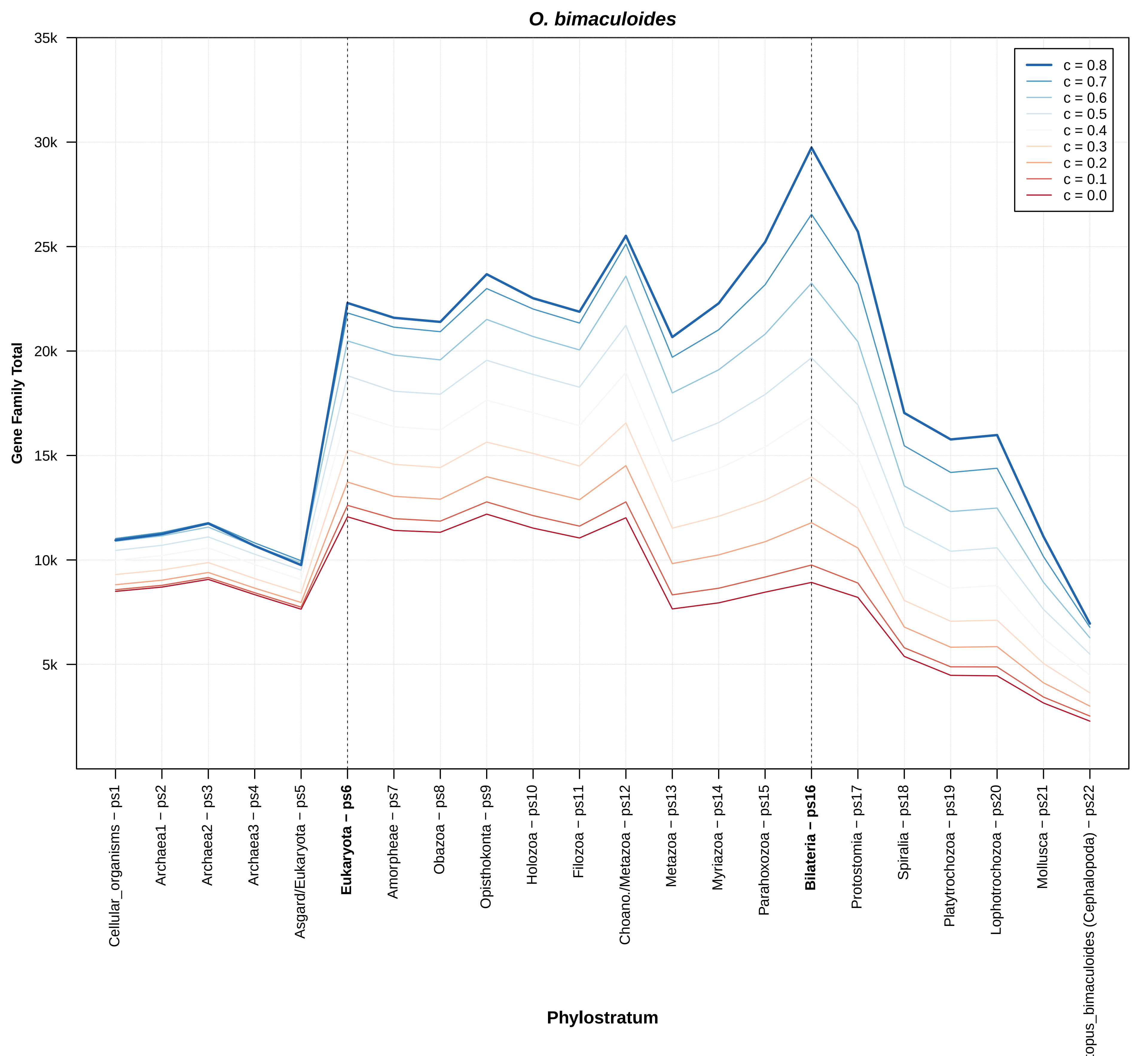

O. bimaculoides
Gene Family Total
35k
30k
25k
20k
15k
10k
5k
c = 0.8
c = 0.7
c = 0.6
c = 0.5
c = 0.4
c = 0.3
c = 0.2
c = 0.1
c = 0.0
Cellular_organisms – ps1
Archaea1 – ps2
Archaea2 – ps3
Archaea3 – ps4
Asgard/Eukaryota – ps5
Eukaryota – ps6
Amorpheae – ps7
Obazoa – ps8
Opisthokonta – ps9
Holozoa – ps10
Filozoa – ps11
Choano./Metazoa – ps12
Metazoa – ps13
Myriazoa – ps14
Parahoxozoa – ps15
Bilateria – ps16
Protostomia – ps17
Spiralia – ps18
Platytrochozoa – ps19
Lophotrochozoa – ps20
Mollusca – ps21
Phylostratum

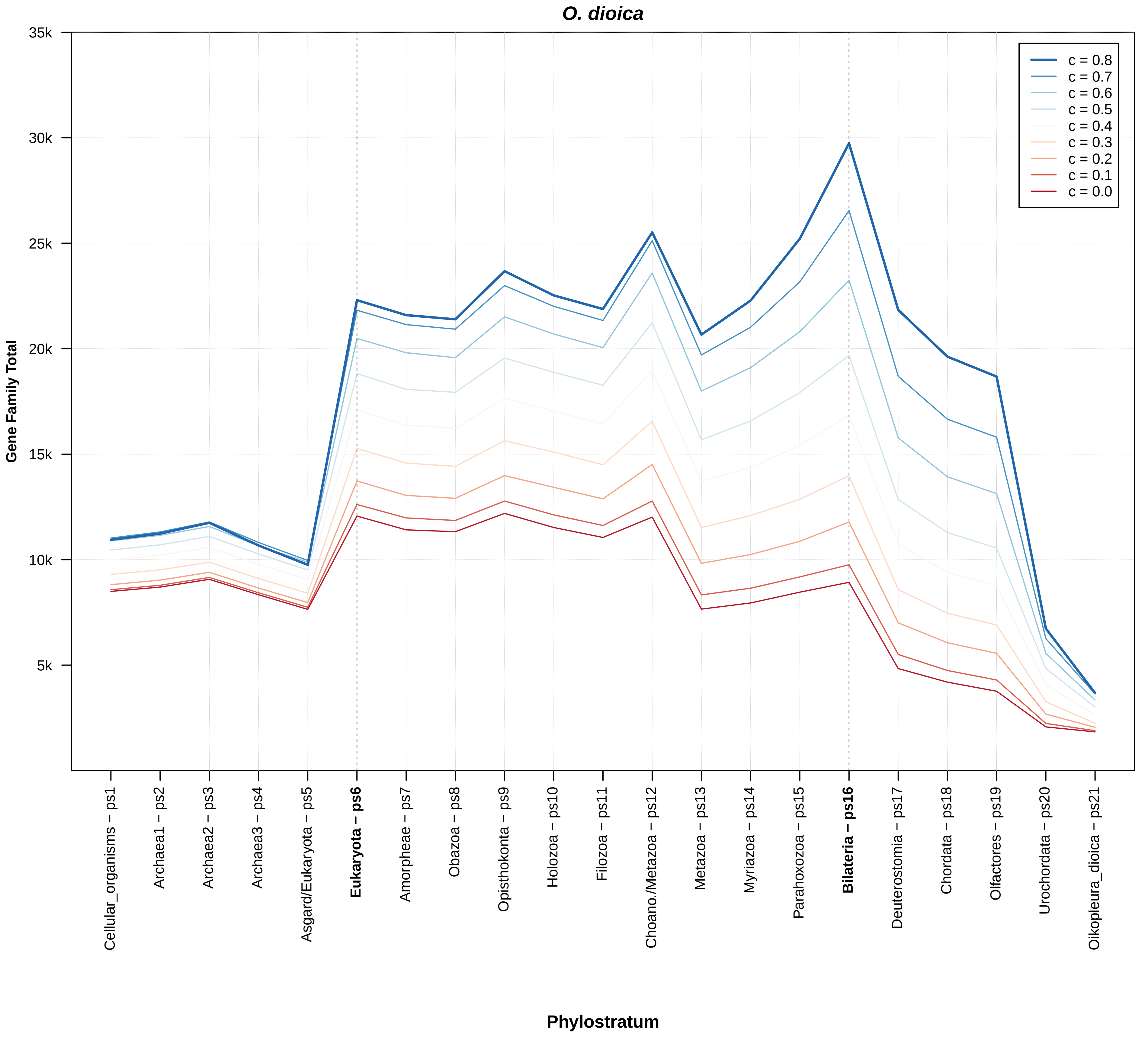

O. dioica
Gene Family Total
35k
30k
25k
20k
15k
10k
5k
c = 0.8
c = 0.7
c = 0.6
c = 0.5
c = 0.4
c = 0.3
c = 0.2
c = 0.1
c = 0.0
Cellular_organisms – ps1
Archaea1 – ps2
Archaea2 – ps3
Archaea3 – ps4
Asgard/Eukaryota – ps5
Eukaryota – ps6
Amorpheae – ps7
Obazoa – ps8
Opisthokonta – ps9
Holozoa – ps10
Filozoa – ps11
Choano./Metazoa – ps12
Metazoa – ps13
Myriazoa – ps14
Parahoxozoa – ps15
Bilateria – ps16
Deuterostomia – ps17
Chordata – ps18
Olfactores – ps19
Urochordata – ps20
Oikopleura_dioica – ps21
Phylostratum

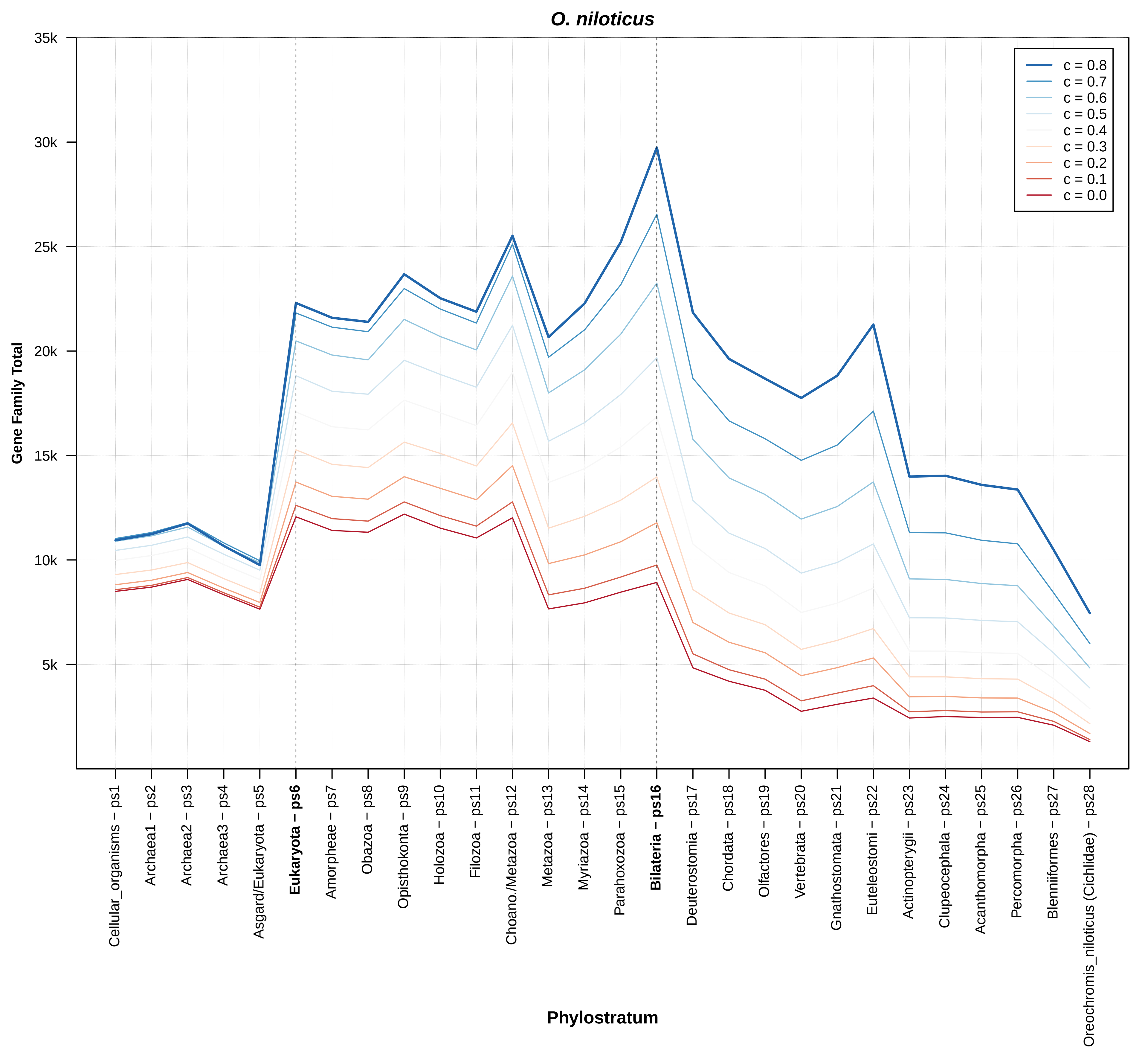

O. niloticus
Gene Family Total
Phylostratum
35k
30k
25k
20k
15k
10k
5k
c = 0.8
c = 0.7
c = 0.6
c = 0.5
c = 0.4
c = 0.3
c = 0.2
c = 0.1
c = 0.0
Cellular_organisms – ps1
Archaea1 – ps2
Archaea2 – ps3
Archaea3 – ps4
Asgard/Eukaryota – ps5
Eukaryota – ps6
Amorpheae – ps7
Obazoa – ps8
Opisthokonta – ps9
Holozoa – ps10
Filozoa – ps11
Choano./Metazoa – ps12
Metazoa – ps13
Myriazoa – ps14
Parahoxozoa – ps15
Bilateria – ps16
Deuterostomia – ps17
Chordata – ps18
Olfactores – ps19
Vertebrata – ps20
Gnathostomata – ps21
Euteleostomi – ps22
Actinopterygii – ps23
Clupeocephala – ps24
Acanthomorpha – ps25
Percomorpha – ps26
Blenniiformes – ps27
Oreochromis_niloticus (Cichlidae) – ps28

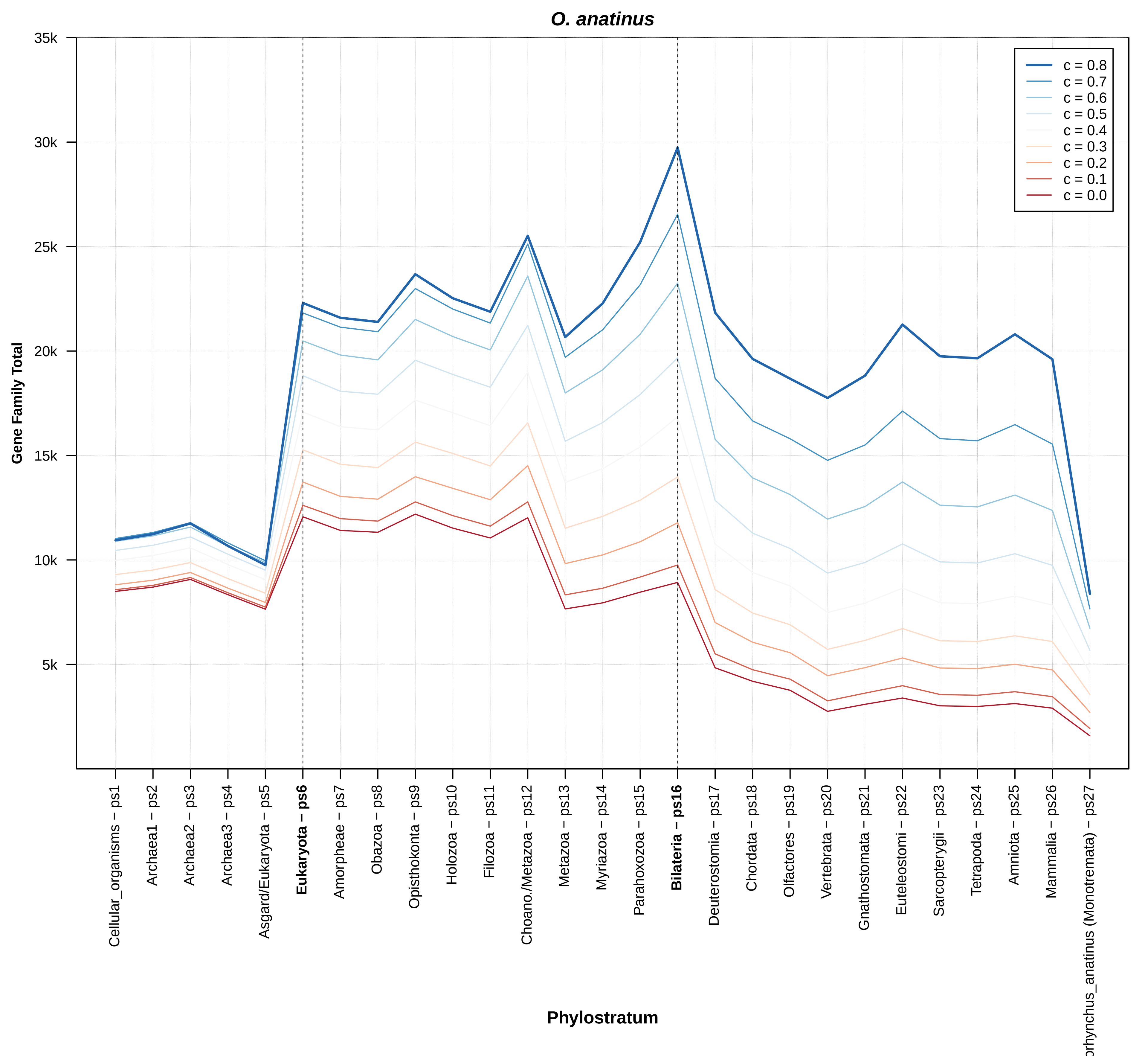
O. anatinus
Gene Family Total
35k
30k
25k
20k
15k
10k
5k
c = 0.8
c = 0.7
c = 0.6
c = 0.5
c = 0.4
c = 0.3
c = 0.2
c = 0.1
c = 0.0
Cellular_organisms – ps1
Archaea1 – ps2
Archaea2 – ps3
Archaea3 – ps4
Asgard/Eukaryota – ps5
Eukaryota – ps6
Amorpheae – ps7
Obazoa – ps8
Opisthokonta – ps9
Holozoa – ps10
Filozoa – ps11
Choano./Metazoa – ps12
Metazoa – ps13
Myriazoa – ps14
Parahoxozoa – ps15
Bilateria – ps16
Deuterostomia – ps17
Chordata – ps18
Olfactores – ps19
Vertebrata – ps20
Gnathostomata – ps21
Euteleostomi – ps22
Sarcopterygii – ps23
Tetrapoda – ps24
Amniota – ps25
Mammalia – ps26
orhynchus_anatinus (Monotremata) – ps27
Phylostratum

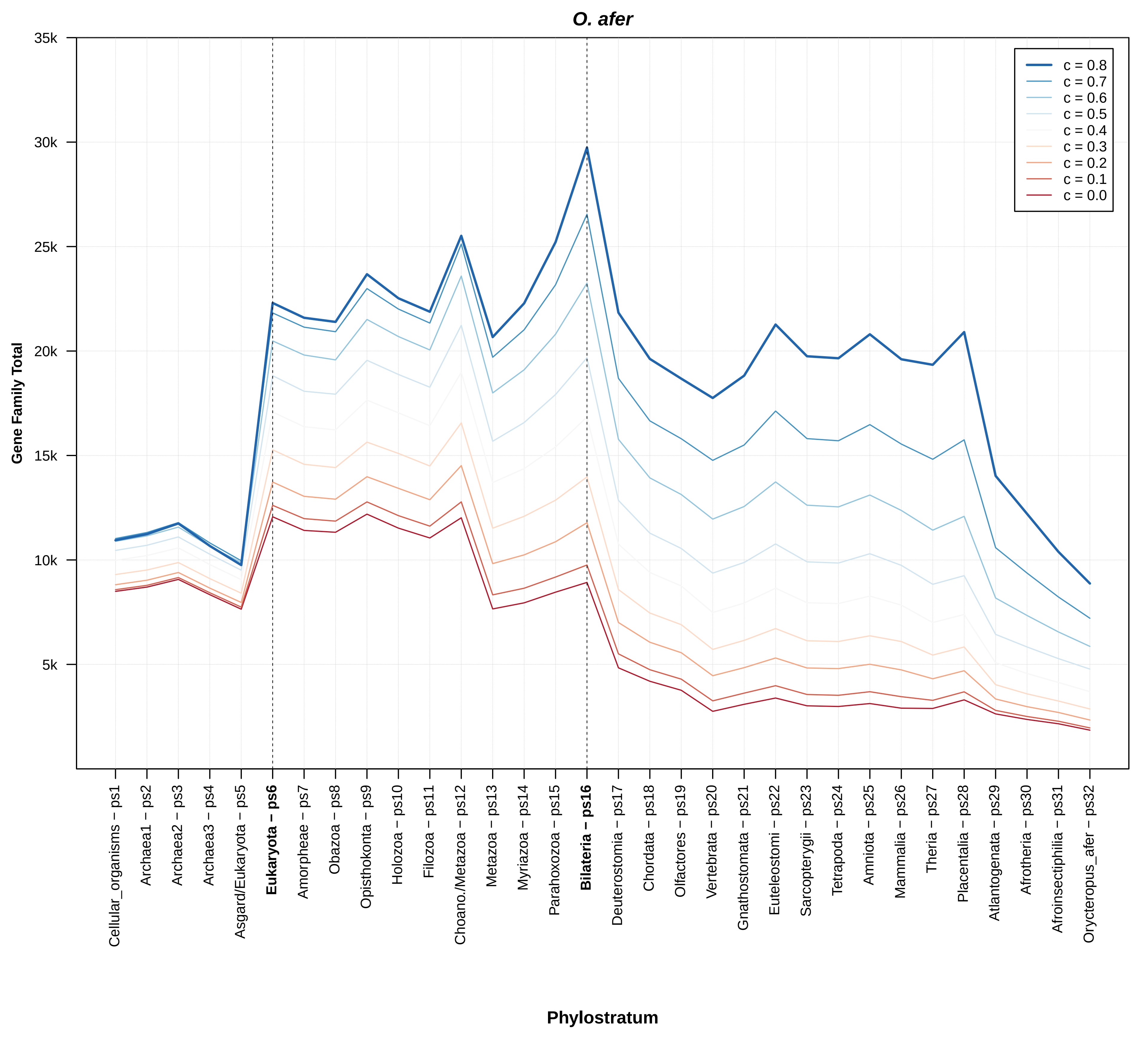

O. afer
35k
30k
25k
20k
15k
10k
5k
Gene Family Total
c = 0.8
c = 0.7
c = 0.6
c = 0.5
c = 0.4
c = 0.3
c = 0.2
c = 0.1
c = 0.0
Cellular_organisms – ps1
Archaea1 – ps2
Archaea2 – ps3
Archaea3 – ps4
Asgard/Eukaryota – ps5
Eukaryota – ps6
Amorpheae – ps7
Obazoa – ps8
Opisthokonta – ps9
Holozoa – ps10
Filozoa – ps11
Choano./Metazoa – ps12
Metazoa – ps13
Myriazoa – ps14
Parahoxozoa – ps15
Bilateria – ps16
Deuterostomia – ps17
Chordata – ps18
Olfactores – ps19
Vertebrata – ps20
Gnathostomata – ps21
Euteleostomi – ps22
Sarcopterygii – ps23
Tetrapoda – ps24
Amniota – ps25
Mammalia – ps26
Theria – ps27
Placentalia – ps28
Atlantogenata – ps29
Afrotheria – ps30
Afroinsectiphilia – ps31
Orycteropus_afer – ps32
Phylostratum

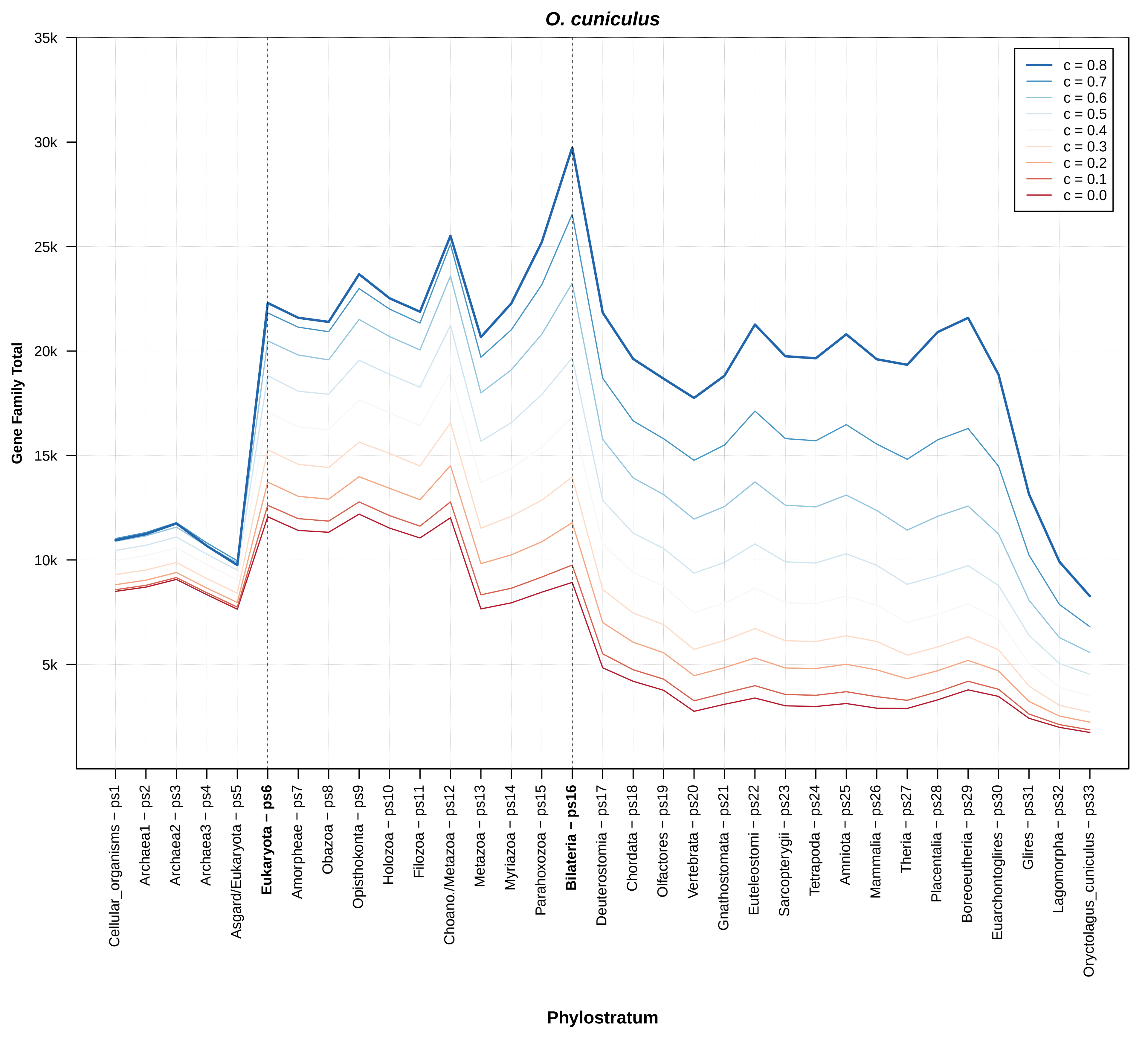

O. cuniculus
Gene Family Total
35k
30k
25k
20k
15k
10k
5k
c = 0.8
c = 0.7
c = 0.6
c = 0.5
c = 0.4
c = 0.3
c = 0.2
c = 0.1
c = 0.0
Cellular_organisms – ps1
Archaea1 – ps2
Archaea2 – ps3
Archaea3 – ps4
Asgard/Eukaryota – ps5
Eukaryota – ps6
Amorpheae – ps7
Obazoa – ps8
Opisthokonta – ps9
Holozoa – ps10
Filozoa – ps11
Choano./Metazoa – ps12
Metazoa – ps13
Myriazoa – ps14
Parahoxozoa – ps15
Bilateria – ps16
Deuterostomia – ps17
Chordata – ps18
Olfactores – ps19
Vertebrata – ps20
Gnathostomata – ps21
Euteleostomi – ps22
Sarcopterygii – ps23
Tetrapoda – ps24
Amniota – ps25
Mammalia – ps26
Theria – ps27
Placentalia – ps28
Boreoeutheria – ps29
Euarchontoglires – ps30
Glires – ps31
Lagomorpha – ps32
Oryctolagus_cuniculus – ps33
Phylostratum

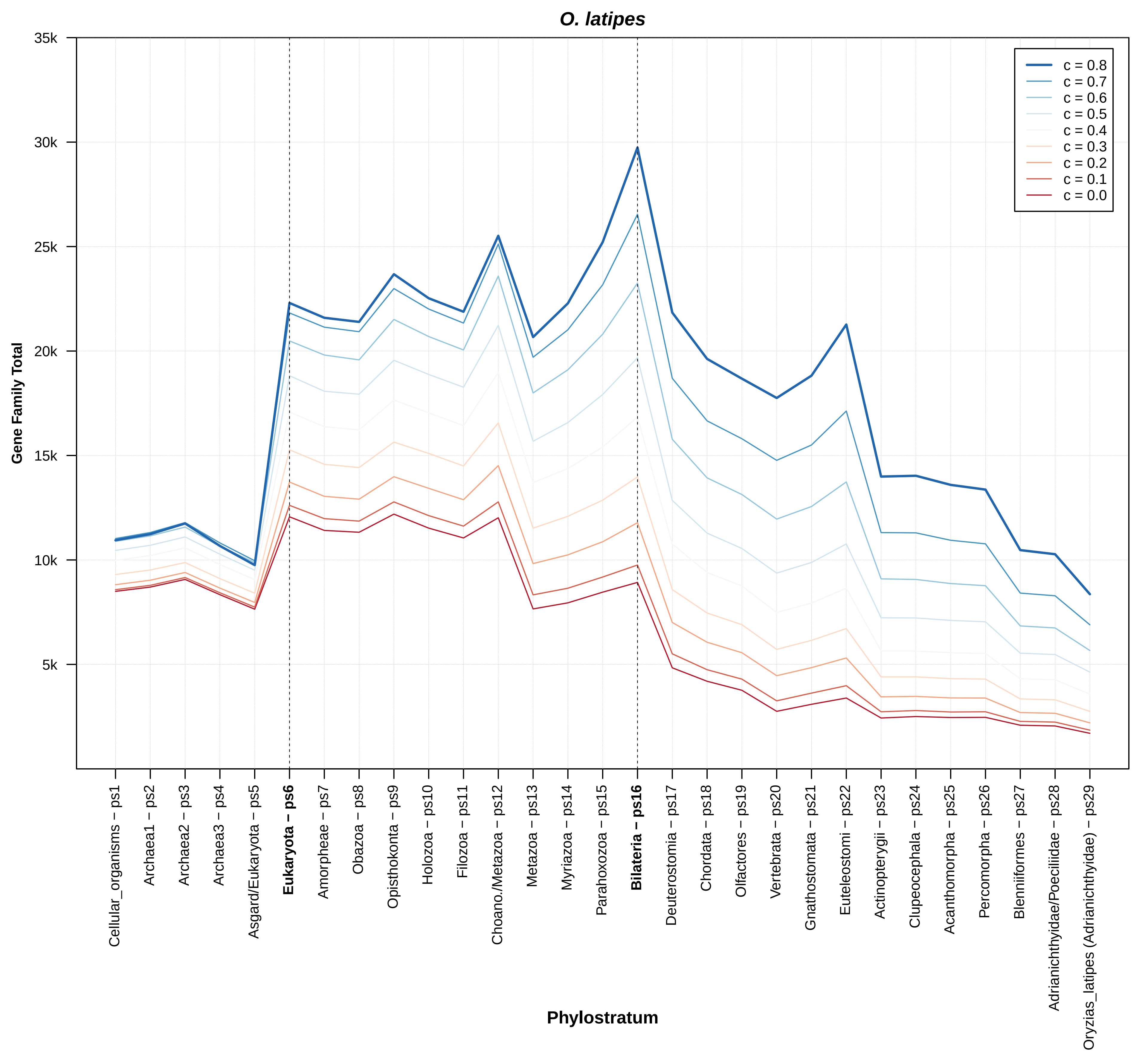

O. latipes
Gene Family Total
35k
30k
25k
20k
15k
10k
5k
c = 0.8
c = 0.7
c = 0.6
c = 0.5
c = 0.4
c = 0.3
c = 0.2
c = 0.1
c = 0.0
Cellular_organisms – ps1
Archaea1 – ps2
Archaea2 – ps3
Archaea3 – ps4
Asgard/Eukaryota – ps5
Eukaryota – ps6
Amorpheae – ps7
Obazoa – ps8
Opisthokonta – ps9
Holozoa – ps10
Filozoa – ps11
Choano./Metazoa – ps12
Metazoa – ps13
Myriazoa – ps14
Parahoxozoa – ps15
Bilateria – ps16
Deuterostomia – ps17
Chordata – ps18
Olfactores – ps19
Vertebrata – ps20
Gnathostomata – ps21
Euteleostomi – ps22
Actinopterygii – ps23
Clupeocephala – ps24
Acanthomorpha – ps25
Percomorpha – ps26
Blenniiformes – ps27
Adrianichthyidae/Poeciliidae – ps28
Oryzias_latipes (Adrianichthyidae) – ps29
Phylostratum

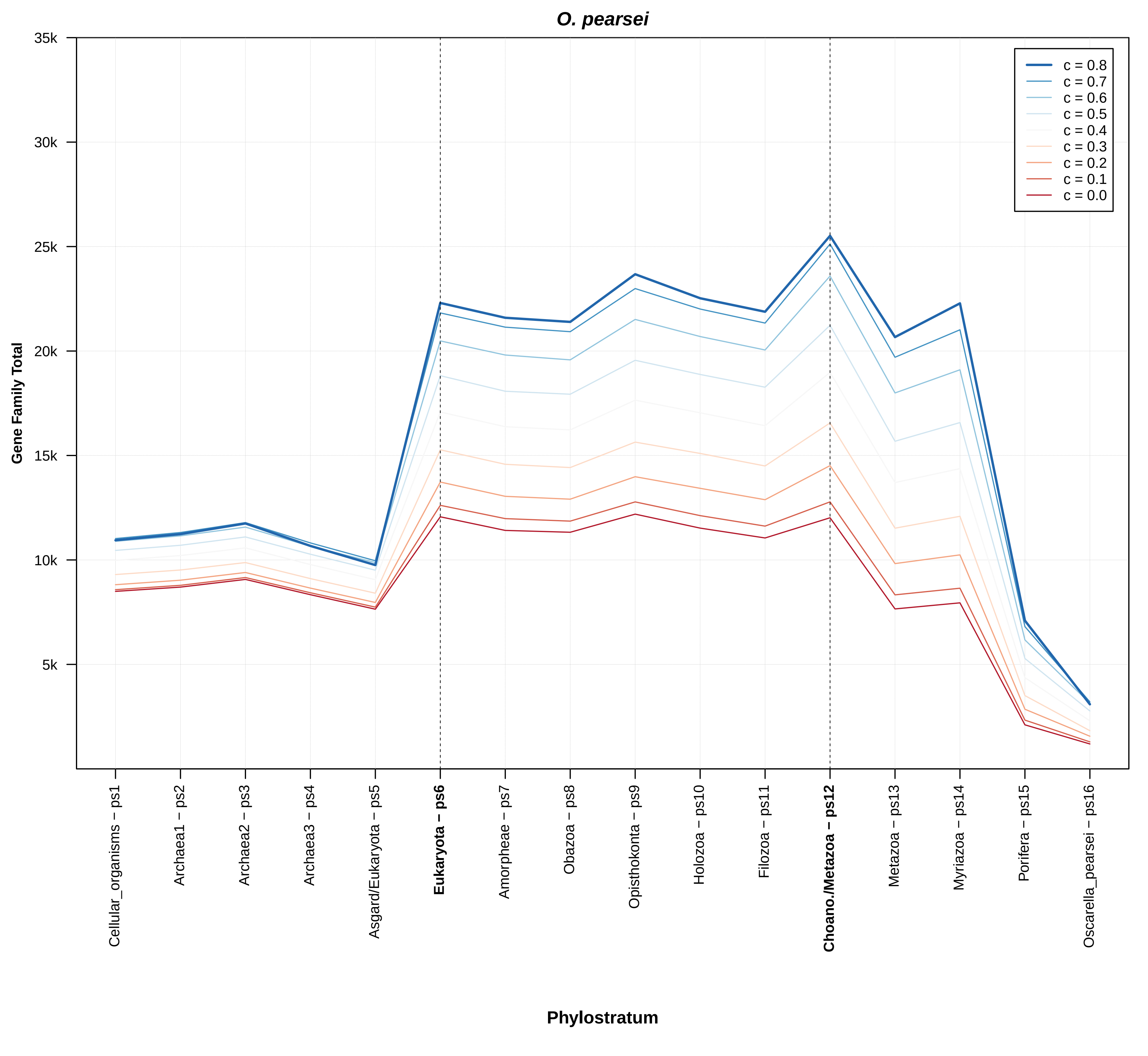

O. pearsei
35k
30k
25k
20k
15k
10k
5k
Gene Family Total
c = 0.8
c = 0.7
c = 0.6
c = 0.5
c = 0.4
c = 0.3
c = 0.2
c = 0.1
c = 0.0
Cellular_organisms – ps1
Archaea1 – ps2
Archaea2 – ps3
Archaea3 – ps4
Asgard/Eukaryota – ps5
Eukaryota – ps6
Amorpheae – ps7
Obazoa – ps8
Opisthokonta – ps9
Holozoa – ps10
Filozoa – ps11
Choano./Metazoa – ps12
Metazoa – ps13
Myriazoa – ps14
Porifera – ps15
Oscarella_pearsei – ps16
Phylostratum

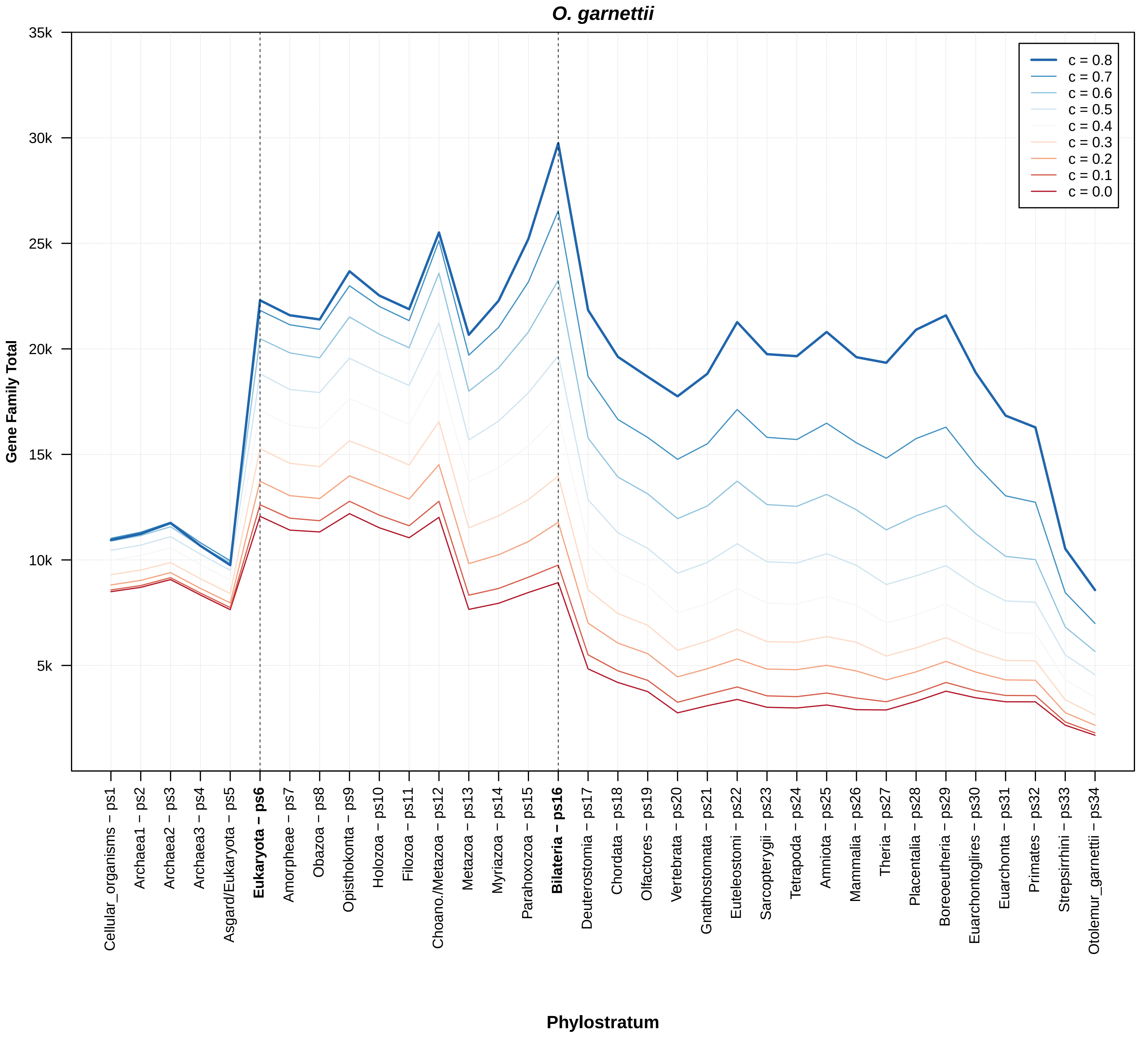
O. garnettii
Gene Family Total
Phylostratum
35k
30k
25k
20k
15k
10k
5k
c = 0.8
c = 0.7
c = 0.6
c = 0.5
c = 0.4
c = 0.3
c = 0.2
c = 0.1
c = 0.0
Cellular_organisms – ps1
Archaea1 – ps2
Archaea2 – ps3
Archaea3 – ps4
Asgard/Eukaryota – ps5
Eukaryota – ps6
Amorpheae – ps7
Obazoa – ps8
Opisthokonta – ps9
Holozoa – ps10
Filozoa – ps11
Choano./Metazoa – ps12
Metazoa – ps13
Myriazoa – ps14
Parahoxozoa – ps15
Bilateria – ps16
Deuterostomia – ps17
Chordata – ps18
Olfactores – ps19
Vertebrata – ps20
Gnathostomata – ps21
Euteleostomi – ps22
Sarcopterygii – ps23
Tetrapoda – ps24
Amniota – ps25
Mammalia – ps26
Theria – ps27
Placentalia – ps28
Boreoeutheria – ps29
Euarchontoglires – ps30
Euarchonta – ps31
Primates – ps32
Strepsirrhini – ps33
Otolemur_garnettii – ps34

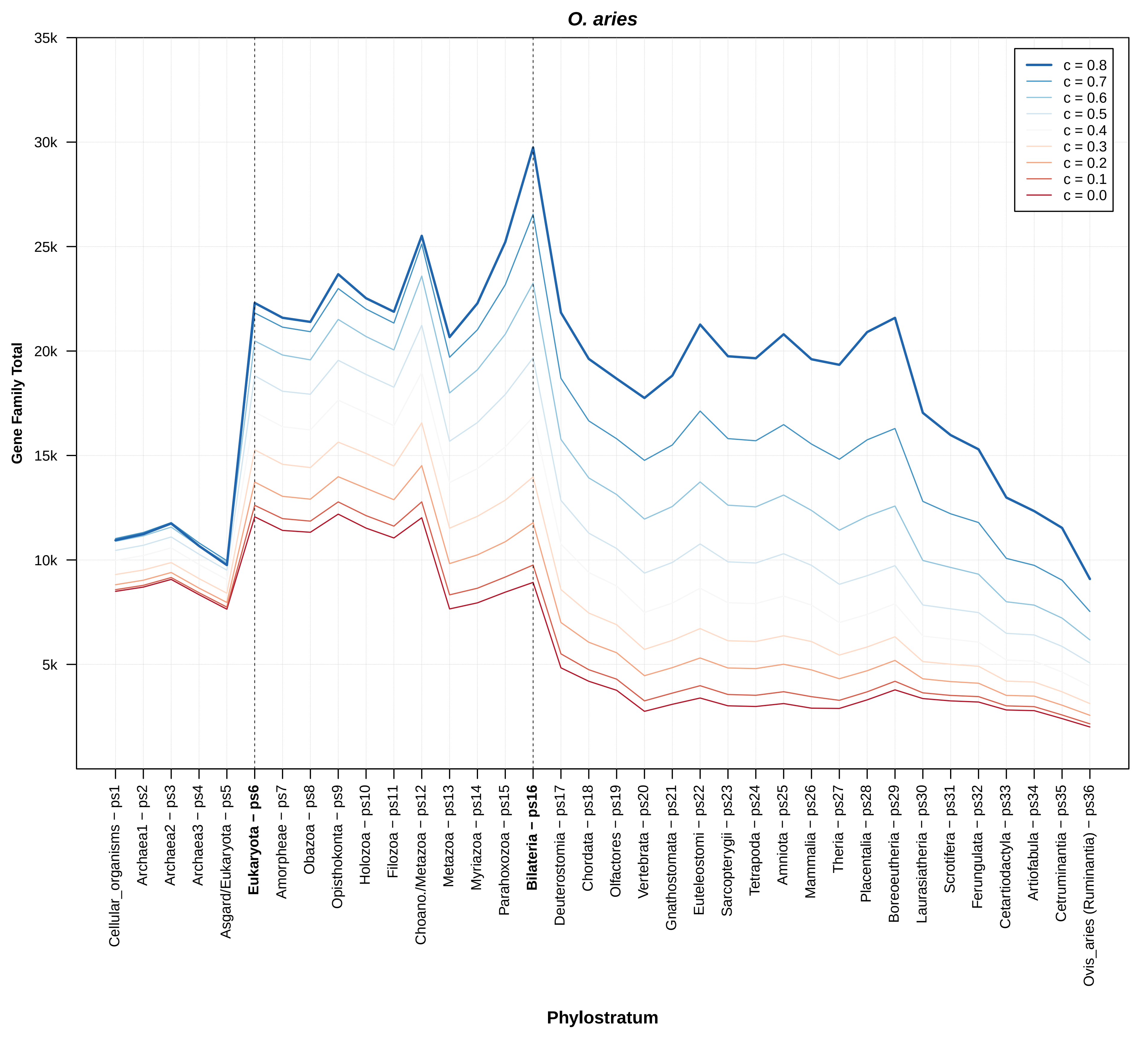
O. aries
Gene Family Total
35k
30k
25k
20k
15k
10k
5k
c = 0.8
c = 0.7
c = 0.6
c = 0.5
c = 0.4
c = 0.3
c = 0.2
c = 0.1
c = 0.0
Cellular_organisms – ps1
Archaea1 – ps2
Archaea2 – ps3
Archaea3 – ps4
Asgard/Eukaryota – ps5
Eukaryota – ps6
Amorpheae – ps7
Obazoa – ps8
Opisthokonta – ps9
Holozoa – ps10
Filozoa – ps11
Choano./Metazoa – ps12
Metazoa – ps13
Myriazoa – ps14
Parahoxozoa – ps15
Bilateria – ps16
Deuterostomia – ps17
Chordata – ps18
Olfactores – ps19
Vertebrata – ps20
Gnathostomata – ps21
Euteleostomi – ps22
Sarcopterygii – ps23
Tetrapoda – ps24
Amniota – ps25
Mammalia – ps26
Theria – ps27
Placentalia – ps28
Boreoeutheria – ps29
Laurasiatheria – ps30
Scrotifera – ps31
Ferungulata – ps32
Cetartiodactyla – ps33
Artiofabula – ps34
Cetruminantia – ps35
Ovis_aries (Ruminantia) – ps36
Phylostratum

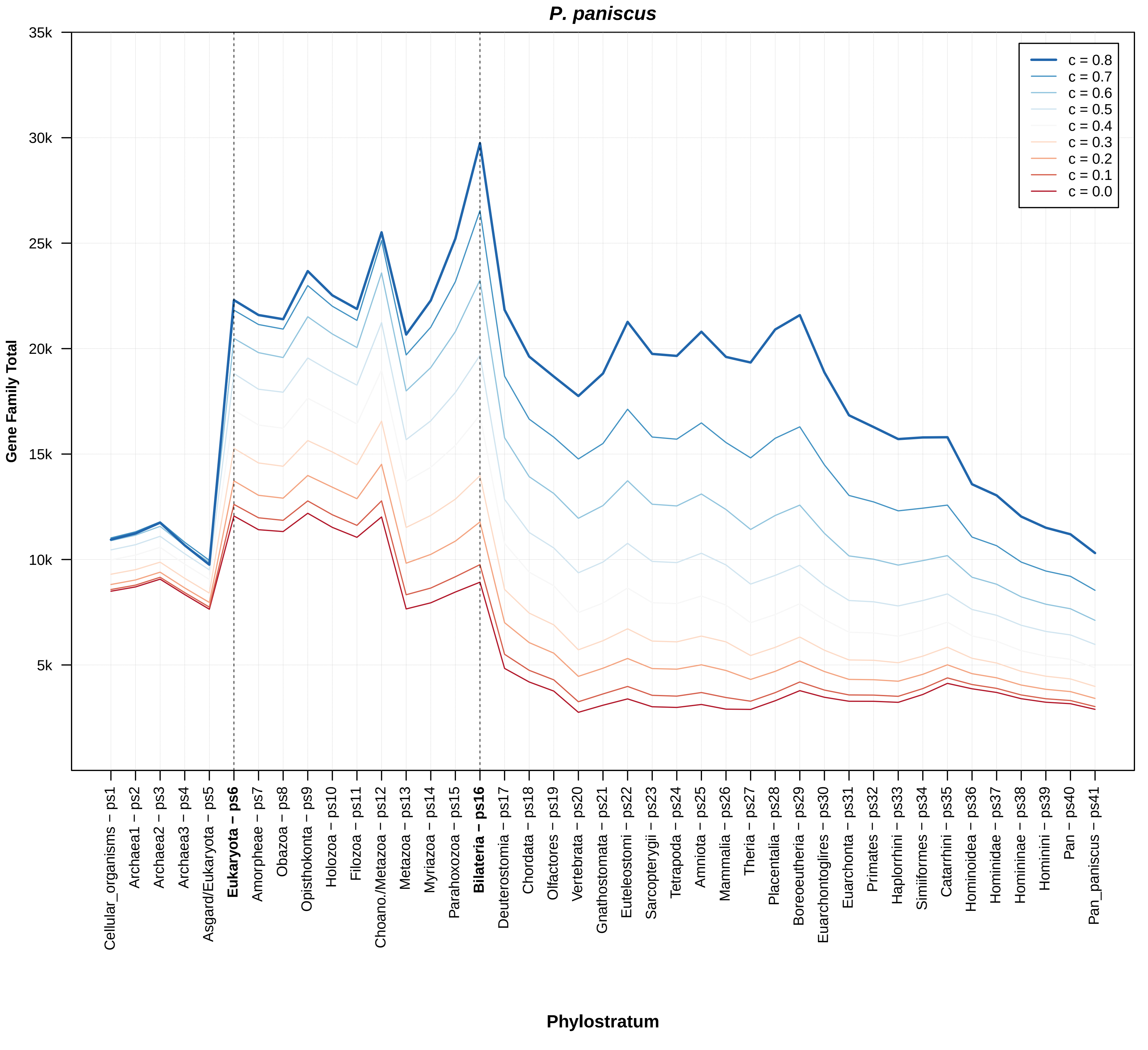

P. paniscus
Gene Family Total
35k
30k
25k
20k
15k
10k
5k
c = 0.8
c = 0.7
c = 0.6
c = 0.5
c = 0.4
c = 0.3
c = 0.2
c = 0.1
c = 0.0
Cellular_organisms – ps1
Archaea1 – ps2
Archaea2 – ps3
Archaea3 – ps4
Asgard/Eukaryota – ps5
Eukaryota – ps6
Amorpheae – ps7
Obazoa – ps8
Opisthokonta – ps9
Holozoa – ps10
Filozoa – ps11
Choano./Metazoa – ps12
Metazoa – ps13
Myriazoa – ps14
Parahoxozoa – ps15
Bilateria – ps16
Deuterostomia – ps17
Chordata – ps18
Olfactores – ps19
Vertebrata – ps20
Gnathostomata – ps21
Euteleostomi – ps22
Sarcopterygii – ps23
Tetrapoda – ps24
Amniota – ps25
Mammalia – ps26
Theria – ps27
Placentalia – ps28
Boreoeutheria – ps29
Euarchontoglires – ps30
Euarchonta – ps31
Primates – ps32
Haplorrhini – ps33
Simiiformes – ps34
Catarrhini – ps35
Hominoidea – ps36
Hominidae – ps37
Homininae – ps38
Hominini – ps39
Pan – ps40
Pan_paniscus – ps41
Phylostratum

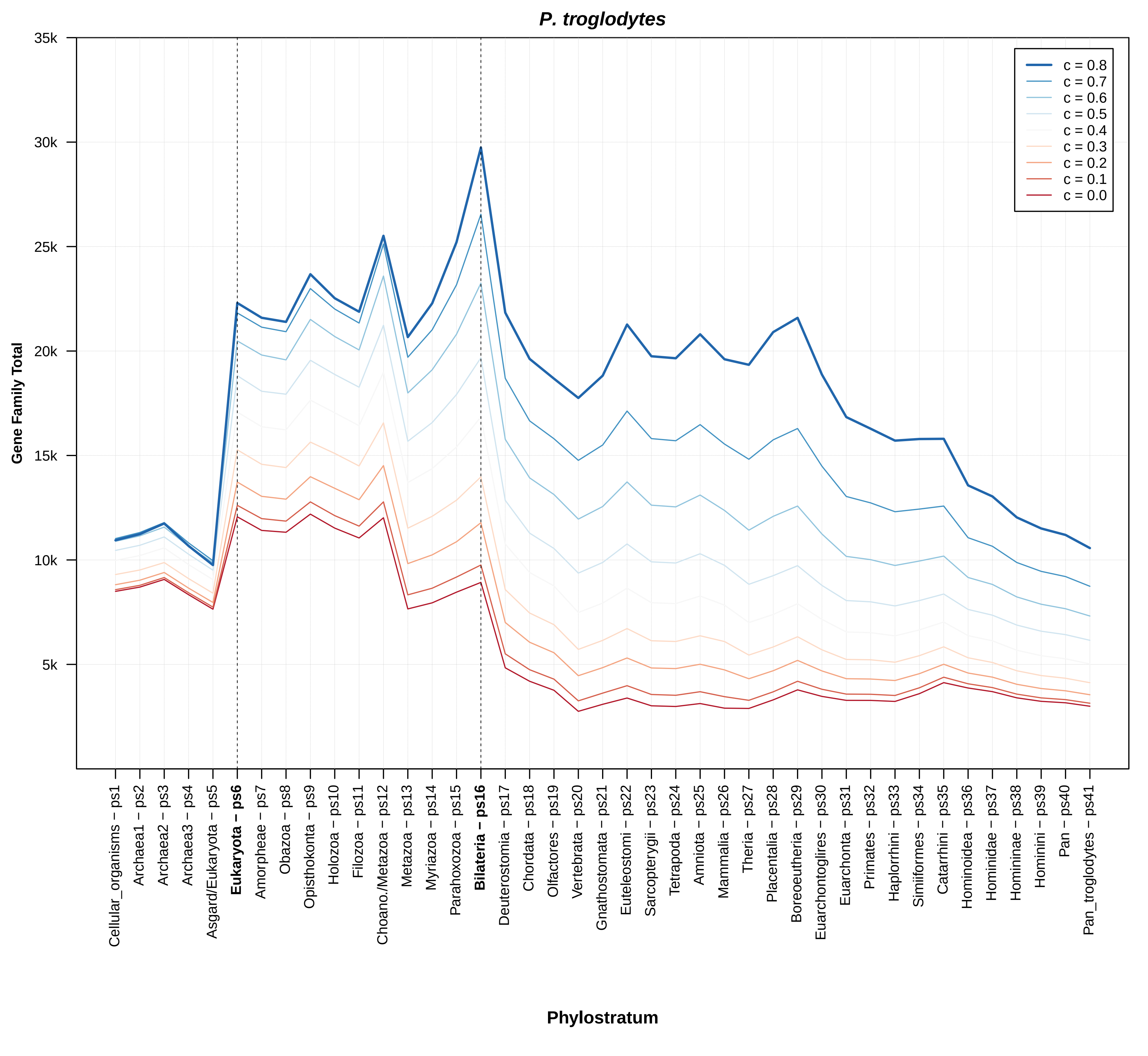

P. troglodytes
Gene Family Total
35k
30k
25k
20k
15k
10k
5k
c = 0.8
c = 0.7
c = 0.6
c = 0.5
c = 0.4
c = 0.3
c = 0.2
c = 0.1
c = 0.0
Cellular_organisms – ps1
Archaea1 – ps2
Archaea2 – ps3
Archaea3 – ps4
Asgard/Eukaryota – ps5
Eukaryota – ps6
Amorpheae – ps7
Obazoa – ps8
Opisthokonta – ps9
Holozoa – ps10
Filozoa – ps11
Choano./Metazoa – ps12
Metazoa – ps13
Myriazoa – ps14
Parahoxozoa – ps15
Bilateria – ps16
Deuterostomia – ps17
Chordata – ps18
Olfactores – ps19
Vertebrata – ps20
Gnathostomata – ps21
Euteleostomi – ps22
Sarcopterygii – ps23
Tetrapoda – ps24
Amniota – ps25
Mammalia – ps26
Theria – ps27
Placentalia – ps28
Boreoeutheria – ps29
Euarchontoglires – ps30
Euarchonta – ps31
Primates – ps32
Haplorrhini – ps33
Simiiformes – ps34
Catarrhini – ps35
Hominoidea – ps36
Hominidae – ps37
Homininae – ps38
Hominini – ps39
Pan – ps40
Pan_troglodytes – ps41
Phylostratum

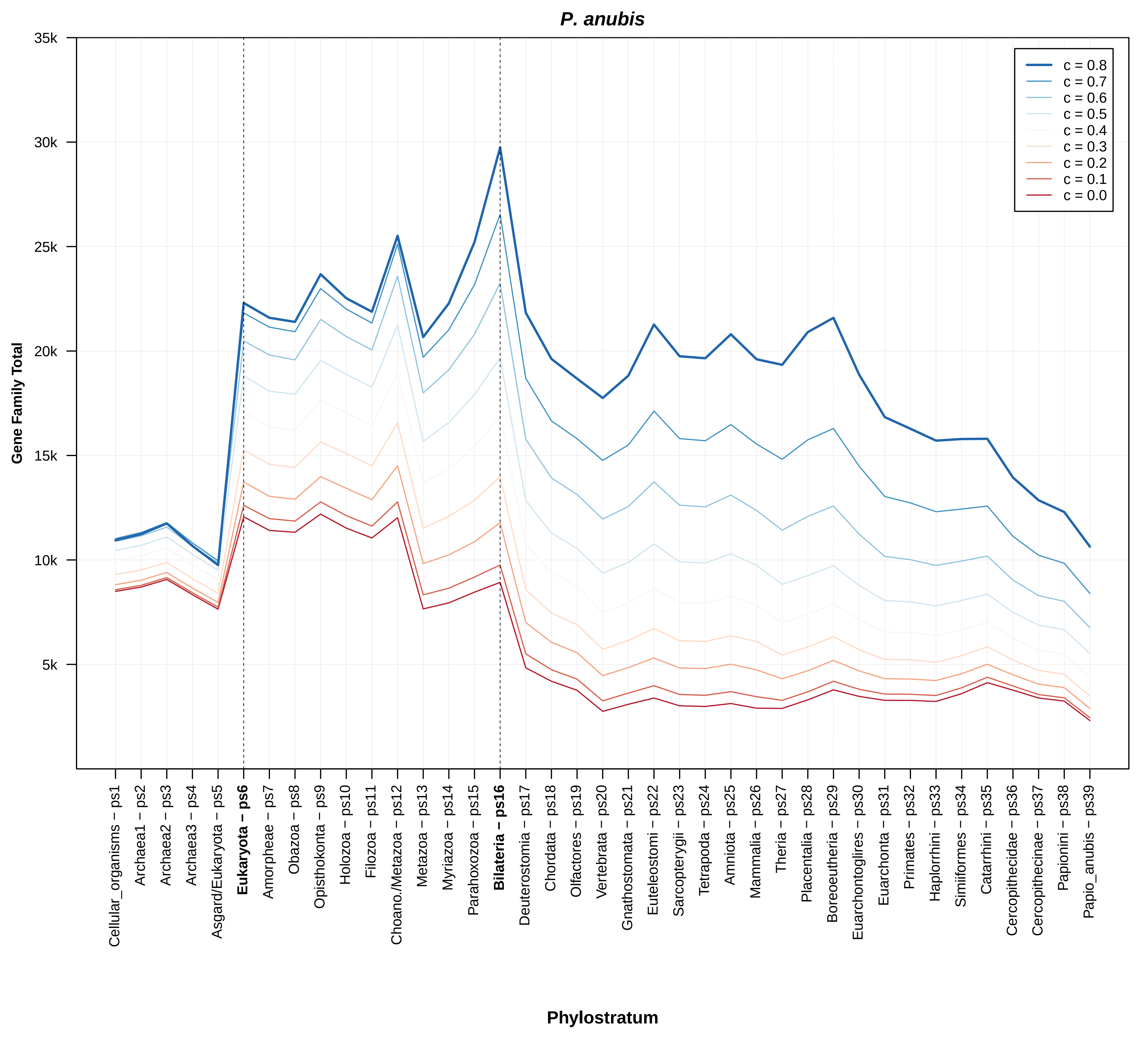

P. anubis
Gene Family Total
Phylostratum
35k
30k
25k
20k
15k
10k
5k
c = 0.8
c = 0.7
c = 0.6
c = 0.5
c = 0.4
c = 0.3
c = 0.2
c = 0.1
c = 0.0
Cellular_organisms – ps1
Archaea1 – ps2
Archaea2 – ps3
Archaea3 – ps4
Asgard/Eukaryota – ps5
Eukaryota – ps6
Amorpheae – ps7
Obazoa – ps8
Opisthokonta – ps9
Holozoa – ps10
Filozoa – ps11
Choano./Metazoa – ps12
Metazoa – ps13
Myriazoa – ps14
Parahoxozoa – ps15
Bilateria – ps16
Deuterostomia – ps17
Chordata – ps18
Olfactores – ps19
Vertebrata – ps20
Gnathostomata – ps21
Euteleostomi – ps22
Sarcopterygii – ps23
Tetrapoda – ps24
Amniota – ps25
Mammalia – ps26
Theria – ps27
Placentalia – ps28
Boreoeutheria – ps29
Euarchontoglires – ps30
Euarchonta – ps31
Primates – ps32
Haplorrhini – ps33
Simiiformes – ps34
Catarrhini – ps35
Cercopithecidae – ps36
Cercopithecinae – ps37
Papionini – ps38
Papio_anubis – ps39

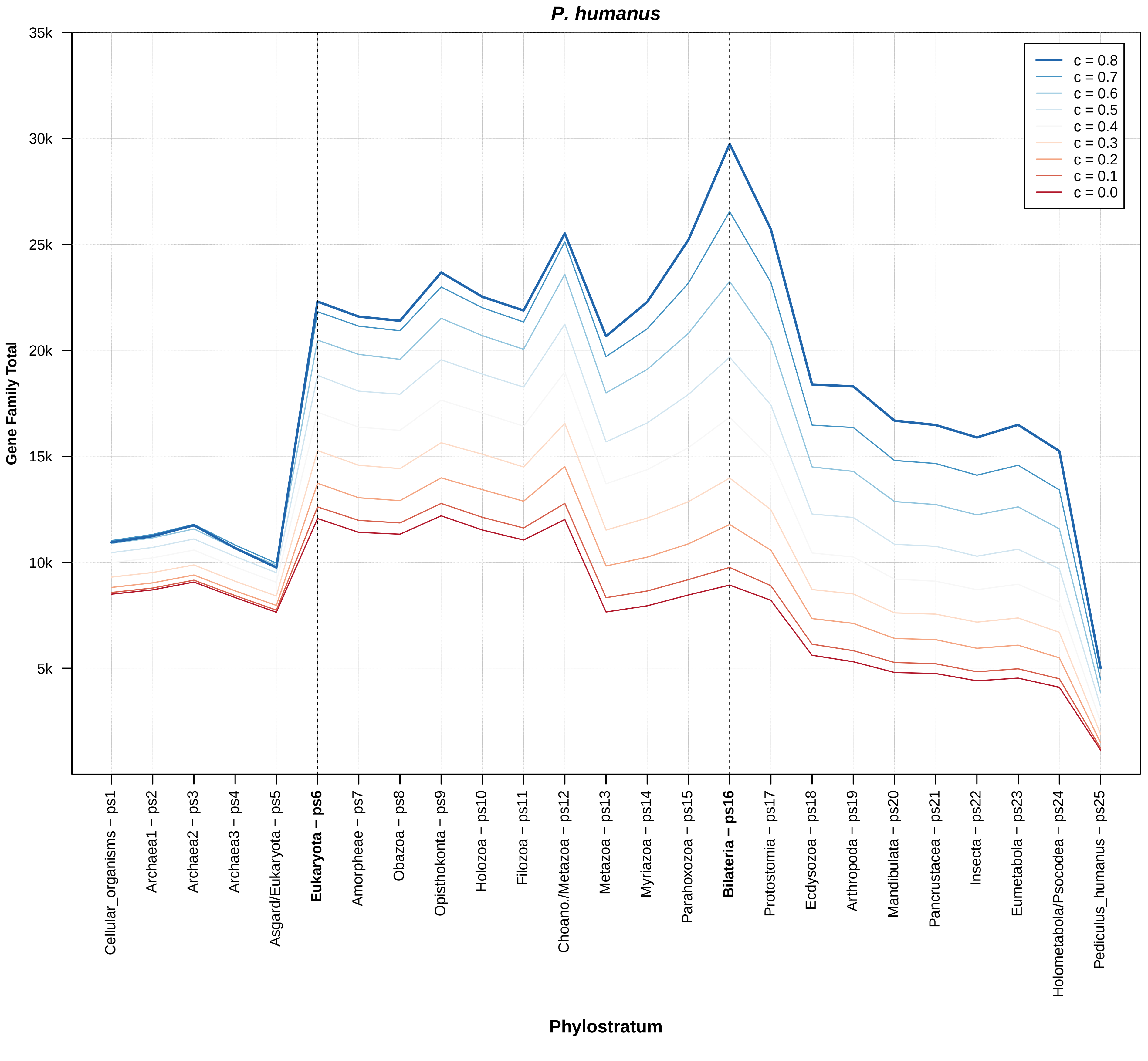

P. humanus
Gene Family Total
35k
30k
25k
20k
15k
10k
5k
c = 0.8
c = 0.7
c = 0.6
c = 0.5
c = 0.4
c = 0.3
c = 0.2
c = 0.1
c = 0.0
Cellular_organisms – ps1
Archaea1 – ps2
Archaea2 – ps3
Archaea3 – ps4
Asgard/Eukaryota – ps5
Eukaryota – ps6
Amorpheae – ps7
Obazoa – ps8
Opisthokonta – ps9
Holozoa – ps10
Filozoa – ps11
Choano./Metazoa – ps12
Metazoa – ps13
Myriazoa – ps14
Parahoxozoa – ps15
Bilateria – ps16
Protostomia – ps17
Ecdysozoa – ps18
Arthropoda – ps19
Mandibulata – ps20
Pancrustacea – ps21
Insecta – ps22
Eumetabola – ps23
Holometabola/Psocodea – ps24
Pediculus_humanus – ps25
Phylostratum

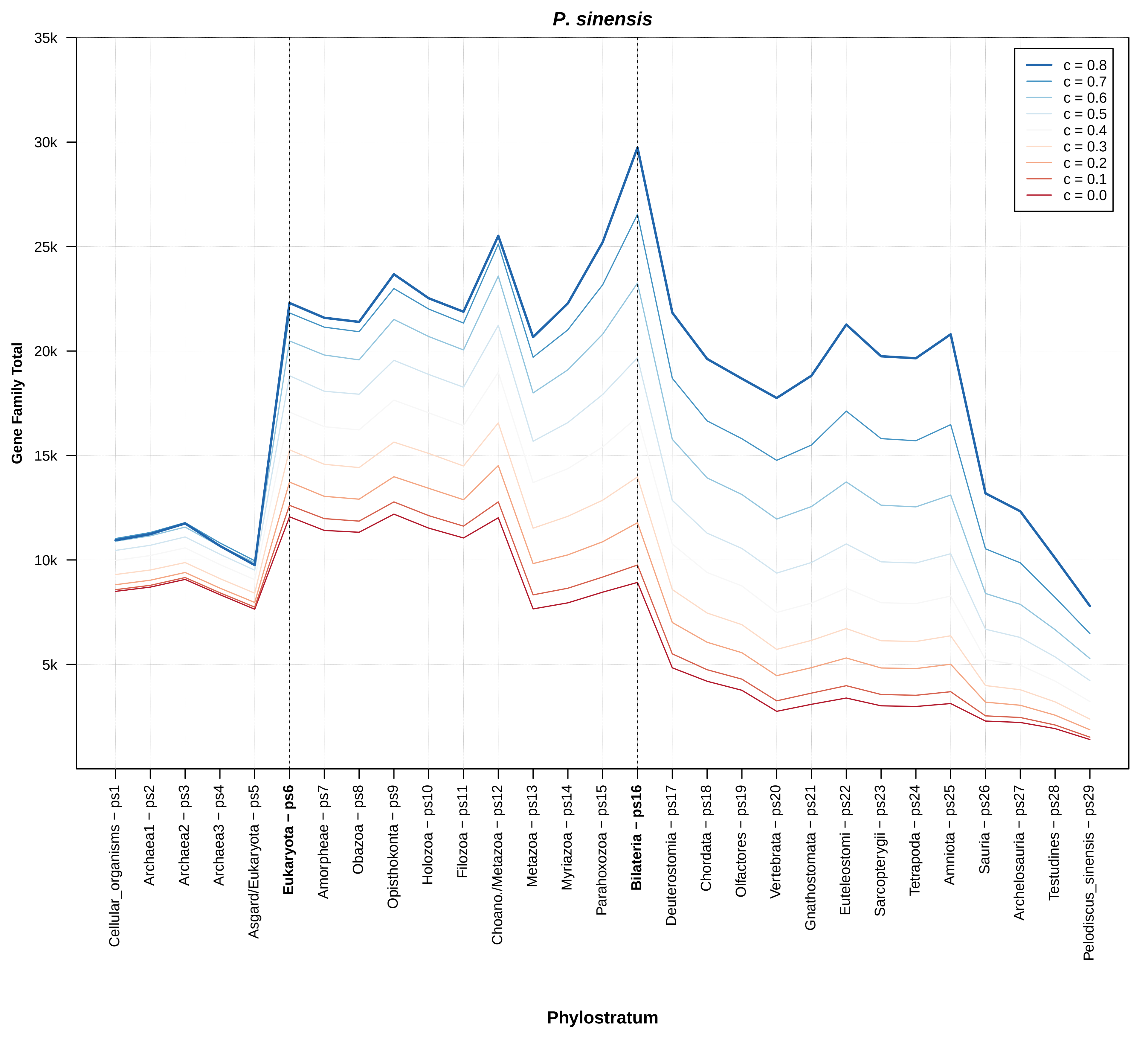

P. sinensis
Gene Family Total
35k
30k
25k
20k
15k
10k
5k
c = 0.8
c = 0.7
c = 0.6
c = 0.5
c = 0.4
c = 0.3
c = 0.2
c = 0.1
c = 0.0
Cellular_organisms – ps1
Archaea1 – ps2
Archaea2 – ps3
Archaea3 – ps4
Asgard/Eukaryota – ps5
Eukaryota – ps6
Amorpheae – ps7
Obazoa – ps8
Opisthokonta – ps9
Holozoa – ps10
Filozoa – ps11
Choano./Metazoa – ps12
Metazoa – ps13
Myriazoa – ps14
Parahoxozoa – ps15
Bilateria – ps16
Deuterostomia – ps17
Chordata – ps18
Olfactores – ps19
Vertebrata – ps20
Gnathostomata – ps21
Euteleostomi – ps22
Sarcopterygii – ps23
Tetrapoda – ps24
Amniota – ps25
Sauria – ps26
Archelosauria – ps27
Testudines – ps28
Pelodiscus_sinensis – ps29
Phylostratum

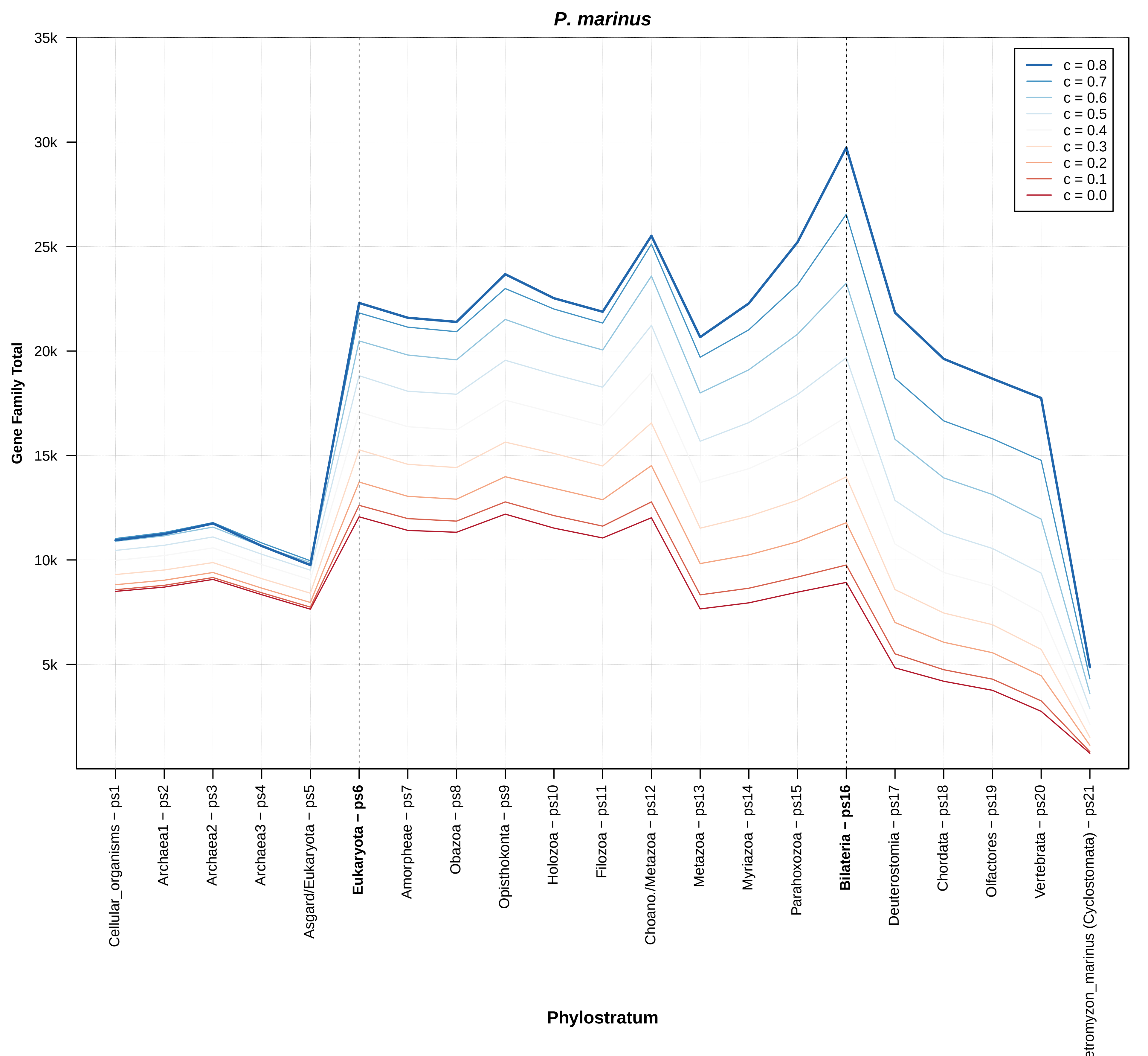

P. marinus
35k
30k
25k
20k
15k
10k
5k
Gene Family Total
c = 0.8
c = 0.7
c = 0.6
c = 0.5
c = 0.4
c = 0.3
c = 0.2
c = 0.1
c = 0.0
Cellular_organisms – ps1
Archaea1 – ps2
Archaea2 – ps3
Archaea3 – ps4
Asgard/Eukaryota – ps5
Eukaryota – ps6
Amorpheae – ps7
Obazoa – ps8
Opisthokonta – ps9
Holozoa – ps10
Filozoa – ps11
Choano./Metazoa – ps12
Metazoa – ps13
Myriazoa – ps14
Parahoxozoa – ps15
Bilateria – ps16
Deuterostomia – ps17
Chordata – ps18
Olfactores – ps19
Vertebrata – ps20
etromyzon_marinus (Cyclostomata) – ps21
Phylostratum

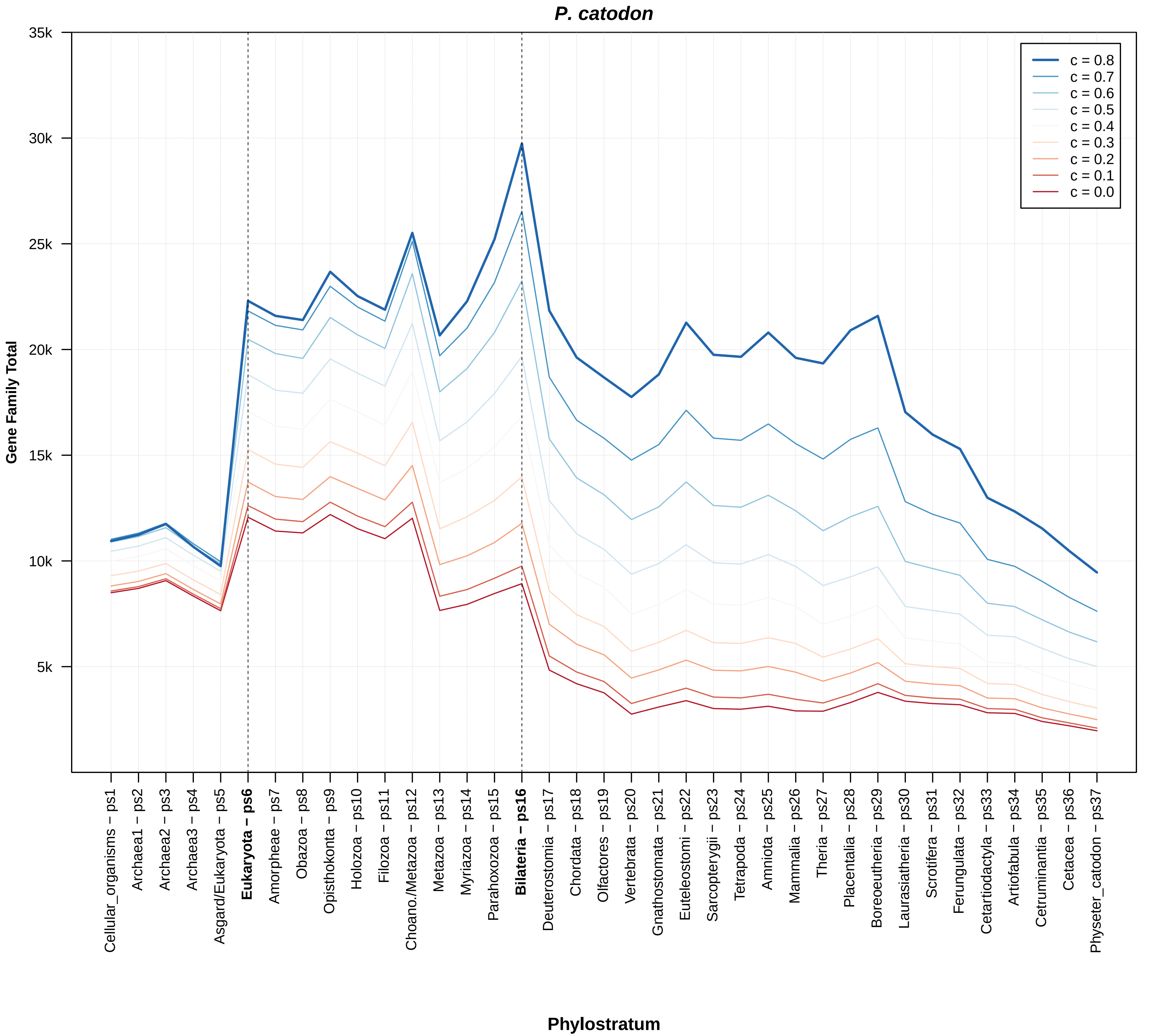

P. catodon
Gene Family Total
35k
30k
25k
20k
15k
10k
5k
c = 0.8
c = 0.7
c = 0.6
c = 0.5
c = 0.4
c = 0.3
c = 0.2
c = 0.1
c = 0.0
Cellular_organisms – ps1
Archaea1 – ps2
Archaea2 – ps3
Archaea3 – ps4
Asgard/Eukaryota – ps5
Eukaryota – ps6
Amorpheae – ps7
Obazoa – ps8
Opisthokonta – ps9
Holozoa – ps10
Filozoa – ps11
Choano./Metazoa – ps12
Metazoa – ps13
Myriazoa – ps14
Parahoxozoa – ps15
Bilateria – ps16
Deuterostomia – ps17
Chordata – ps18
Olfactores – ps19
Vertebrata – ps20
Gnathostomata – ps21
Euteleostomi – ps22
Sarcopterygii – ps23
Tetrapoda – ps24
Amniota – ps25
Mammalia – ps26
Theria – ps27
Placentalia – ps28
Boreoeutheria – ps29
Laurasiatheria – ps30
Scrotifera – ps31
Ferungulata – ps32
Cetartiodactyla – ps33
Artiofabula – ps34
Cetruminantia – ps35
Cetacea – ps36
Physeter_catodon – ps37
Phylostratum

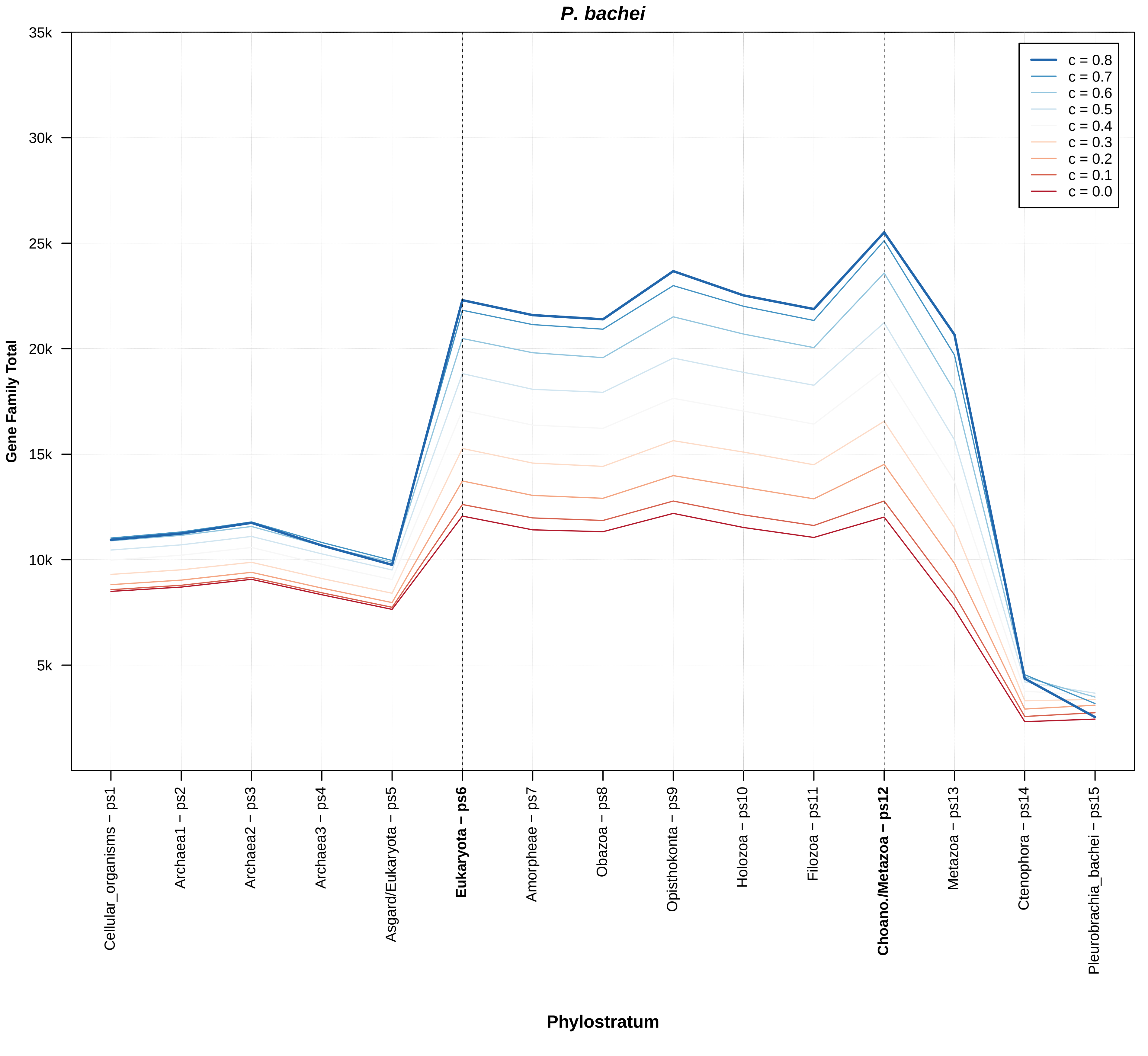

P. bachei
35k
30k
25k
20k
15k
10k
5k
Gene Family Total
c = 0.8
c = 0.7
c = 0.6
c = 0.5
c = 0.4
c = 0.3
c = 0.2
c = 0.1
c = 0.0
Cellular_organisms – ps1
Archaea1 – ps2
Archaea2 – ps3
Archaea3 – ps4
Asgard/Eukaryota – ps5
Eukaryota – ps6
Amorpheae – ps7
Obazoa – ps8
Opisthokonta – ps9
Holozoa – ps10
Filozoa – ps11
Choano./Metazoa – ps12
Metazoa – ps13
Ctenophora – ps14
Pleurobrachia_bachei – ps15
Phylostratum

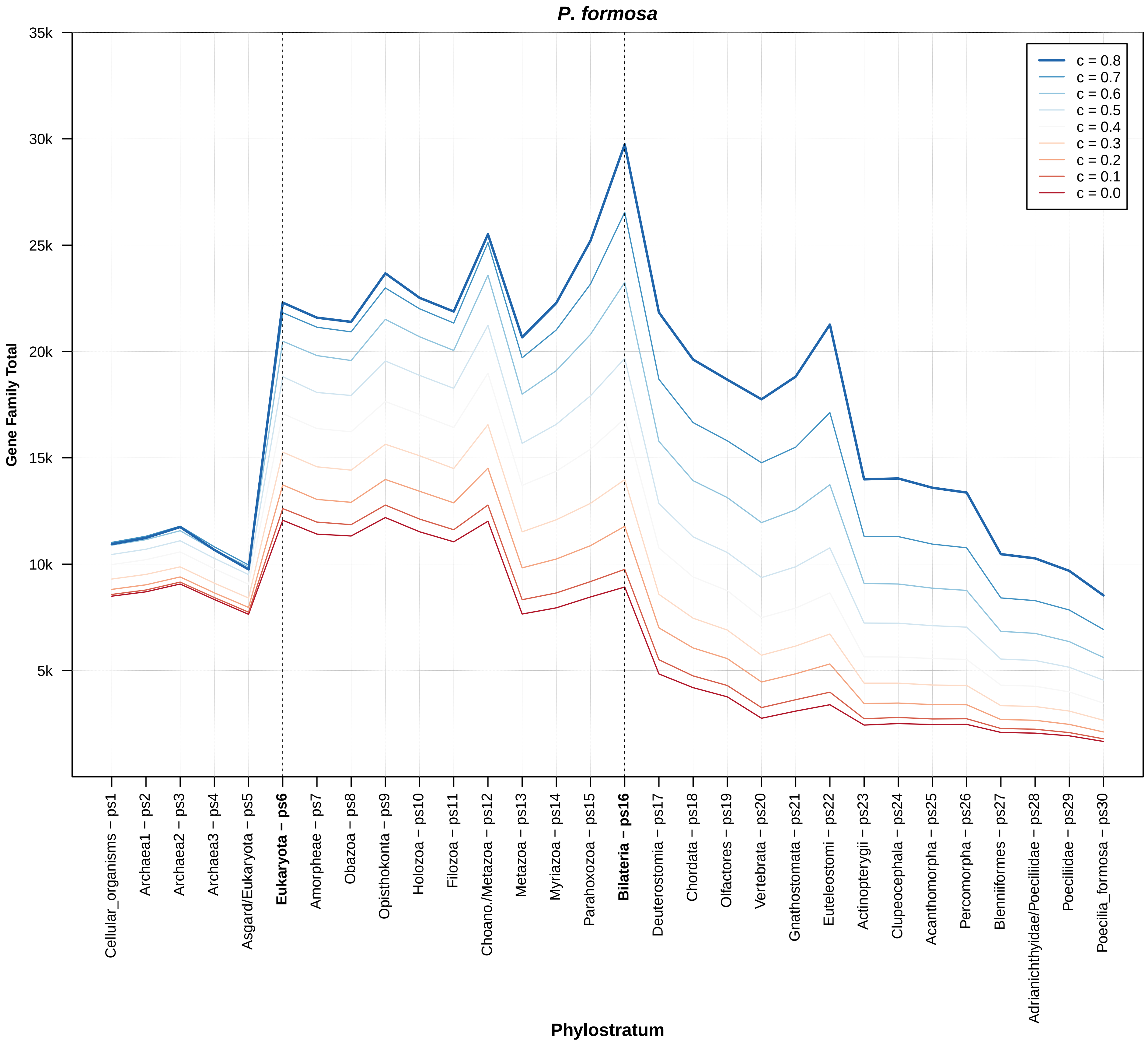
P. formosa
Gene Family Total
35k
30k
25k
20k
15k
10k
5k
c = 0.8
c = 0.7
c = 0.6
c = 0.5
c = 0.4
c = 0.3
c = 0.2
c = 0.1
c = 0.0
Cellular_organisms – ps1
Archaea1 – ps2
Archaea2 – ps3
Archaea3 – ps4
Asgard/Eukaryota – ps5
Eukaryota – ps6
Amorpheae – ps7
Obazoa – ps8
Opisthokonta – ps9
Holozoa – ps10
Filozoa – ps11
Choano./Metazoa – ps12
Metazoa – ps13
Myriazoa – ps14
Parahoxozoa – ps15
Bilateria – ps16
Deuterostomia – ps17
Chordata – ps18
Olfactores – ps19
Vertebrata – ps20
Gnathostomata – ps21
Euteleostomi – ps22
Actinopterygii – ps23
Clupeocephala – ps24
Acanthomorpha – ps25
Percomorpha – ps26
Blenniiformes – ps27
Adrianichthyidae/Poeciliidae – ps28
Poeciliidae – ps29
Poecilia_formosa – ps30
Phylostratum

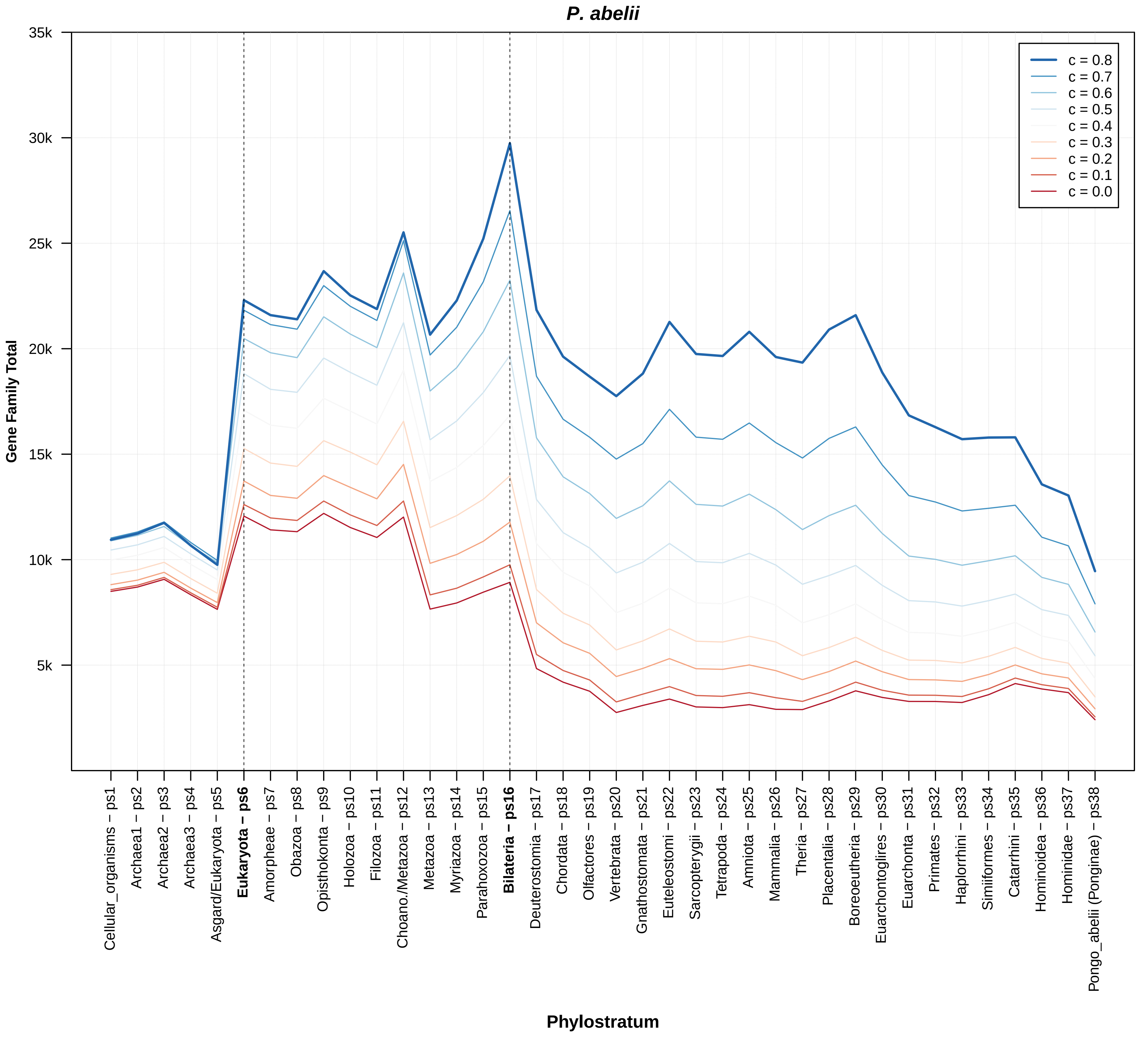

P. abelii
Gene Family Total
Phylostratum
35k
30k
25k
20k
15k
10k
5k
c = 0.8
c = 0.7
c = 0.6
c = 0.5
c = 0.4
c = 0.3
c = 0.2
c = 0.1
c = 0.0
Cellular_organisms – ps1
Archaea1 – ps2
Archaea2 – ps3
Archaea3 – ps4
Asgard/Eukaryota – ps5
Eukaryota – ps6
Amorpheae – ps7
Obazoa – ps8
Opisthokonta – ps9
Holozoa – ps10
Filozoa – ps11
Choano./Metazoa – ps12
Metazoa – ps13
Myriazoa – ps14
Parahoxozoa – ps15
Bilateria – ps16
Deuterostomia – ps17
Chordata – ps18
Olfactores – ps19
Vertebrata – ps20
Gnathostomata – ps21
Euteleostomi – ps22
Sarcopterygii – ps23
Tetrapoda – ps24
Amniota – ps25
Mammalia – ps26
Theria – ps27
Placentalia – ps28
Boreoeutheria – ps29
Euarchontoglires – ps30
Euarchonta – ps31
Primates – ps32
Haplorrhini – ps33
Simiiformes – ps34
Catarrhini – ps35
Hominoidea – ps36
Hominidae – ps37
Pongo_abelii (Ponginae) – ps38

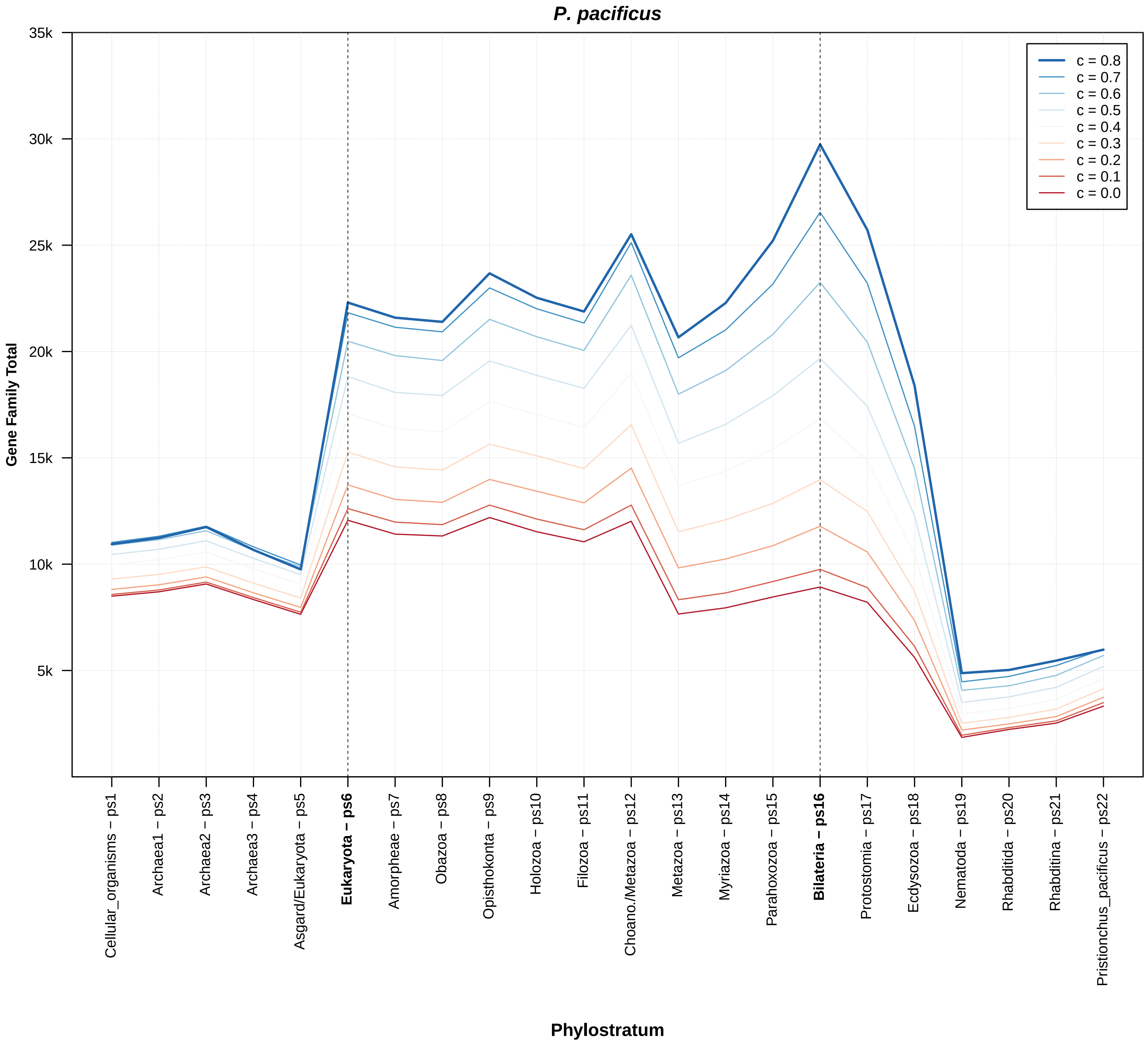
P. pacificus
Gene Family Total
35k
30k
25k
20k
15k
10k
5k
c = 0.8
c = 0.7
c = 0.6
c = 0.5
c = 0.4
c = 0.3
c = 0.2
c = 0.1
c = 0.0
Cellular_organisms – ps1
Archaea1 – ps2
Archaea2 – ps3
Archaea3 – ps4
Asgard/Eukaryota – ps5
Eukaryota – ps6
Amorpheae – ps7
Obazoa – ps8
Opisthokonta – ps9
Holozoa – ps10
Filozoa – ps11
Choano./Metazoa – ps12
Metazoa – ps13
Myriazoa – ps14
Parahoxozoa – ps15
Bilateria – ps16
Protostomia – ps17
Ecdysozoa – ps18
Nematoda – ps19
Rhabditida – ps20
Rhabditina – ps21
Pristionchus_pacificus – ps22
Phylostratum

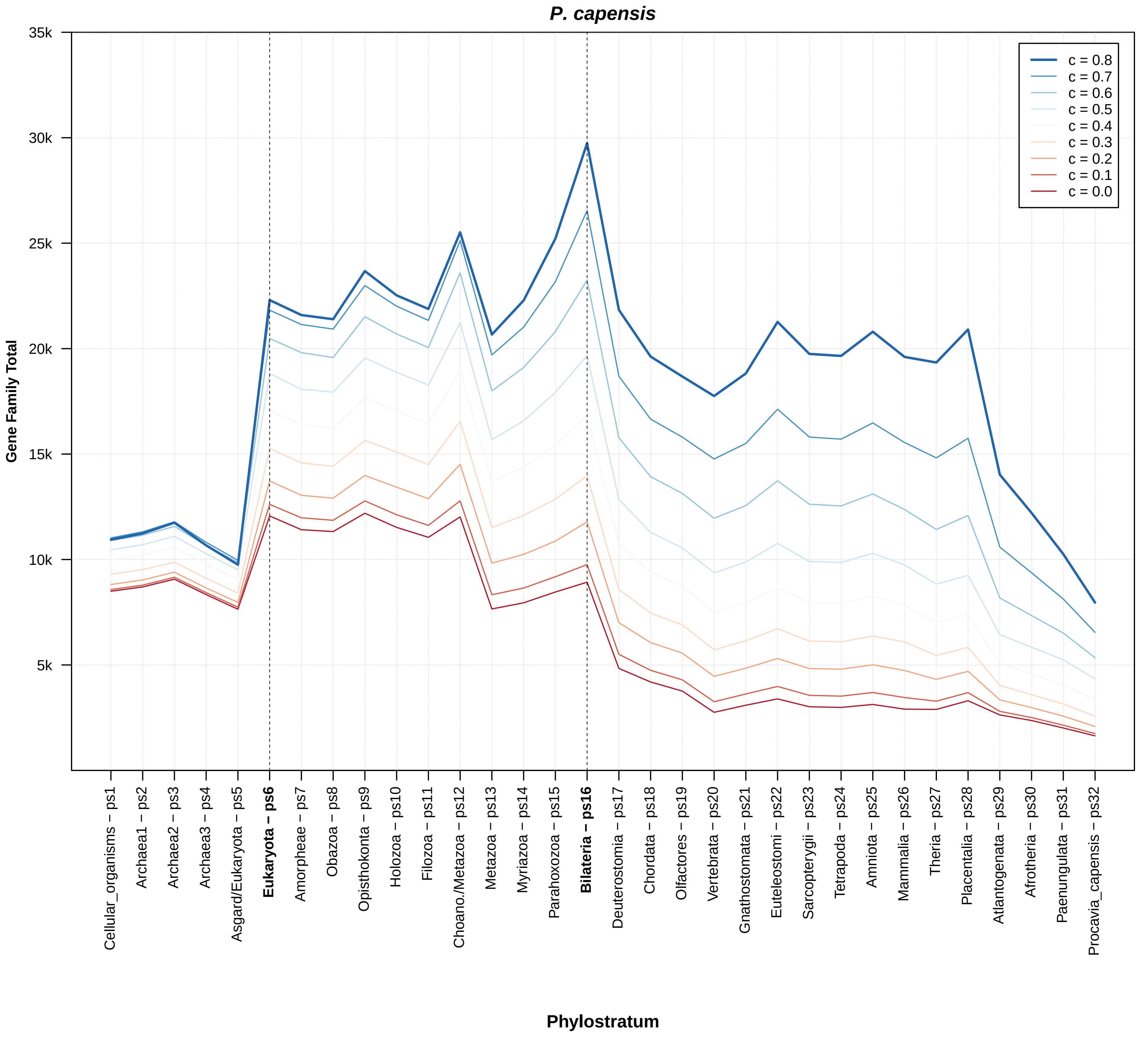
P. capensis
Gene Family Total
35k
30k
25k
20k
15k
10k
5k
c = 0.8
c = 0.7
c = 0.6
c = 0.5
c = 0.4
c = 0.3
c = 0.2
c = 0.1
c = 0.0
Cellular_organisms – ps1
Archaea1 – ps2
Archaea2 – ps3
Archaea3 – ps4
Asgard/Eukaryota – ps5
Eukaryota – ps6
Amorpheae – ps7
Obazoa – ps8
Opisthokonta – ps9
Holozoa – ps10
Filozoa – ps11
Choano./Metazoa – ps12
Metazoa – ps13
Myriazoa – ps14
Parahoxozoa – ps15
Bilateria – ps16
Deuterostomia – ps17
Chordata – ps18
Olfactores – ps19
Vertebrata – ps20
Gnathostomata – ps21
Euteleostomi – ps22
Sarcopterygii – ps23
Tetrapoda – ps24
Amniota – ps25
Mammalia – ps26
Theria – ps27
Placentalia – ps28
Atlantogenata – ps29
Afrotheria – ps30
Paenungulata – ps31
Procavia_capensis – ps32
Phylostratum

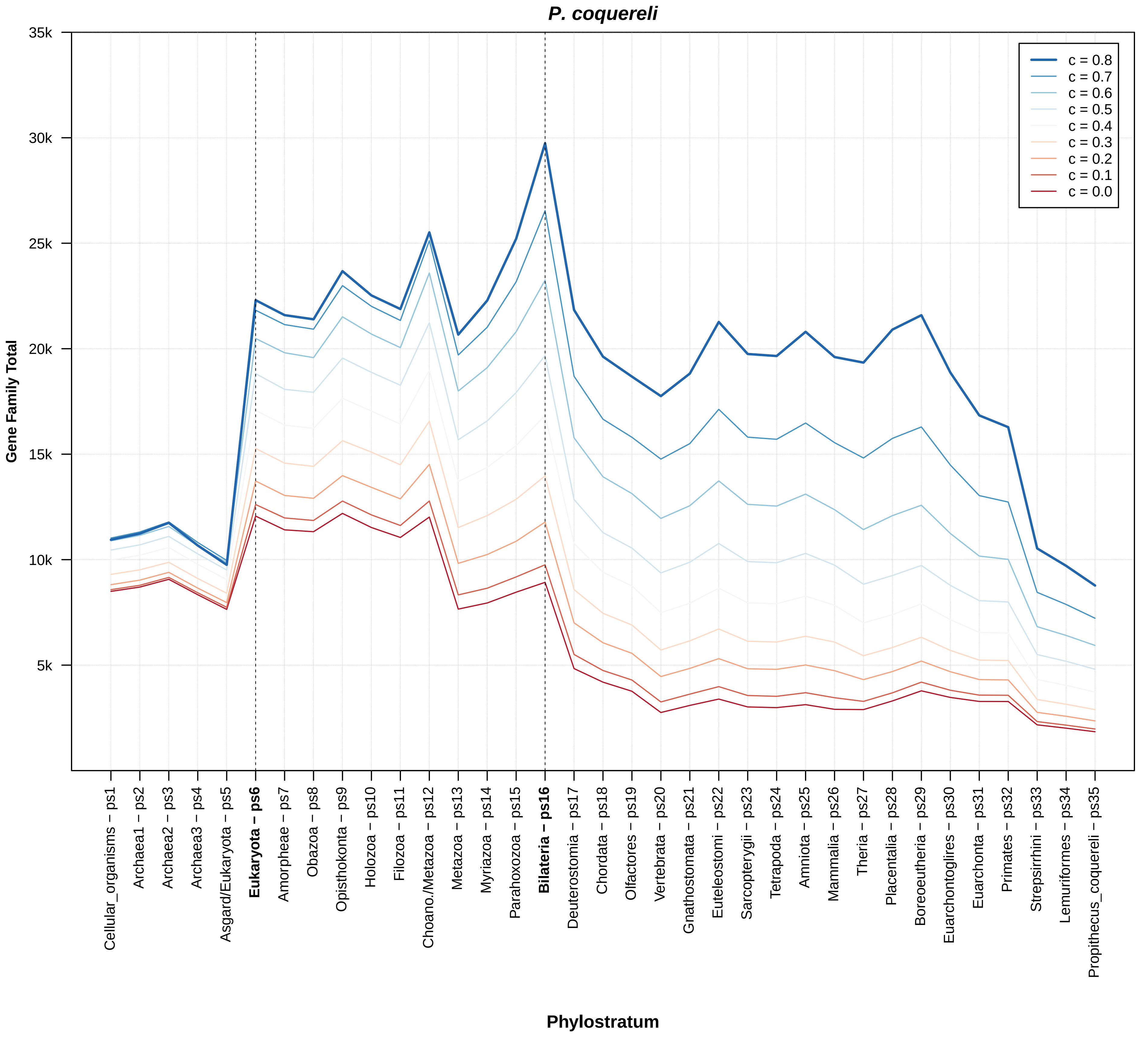

P. coquereli
Gene Family Total
Phylostratum
35k
30k
25k
20k
15k
10k
5k
c = 0.8
c = 0.7
c = 0.6
c = 0.5
c = 0.4
c = 0.3
c = 0.2
c = 0.1
c = 0.0
Cellular_organisms – ps1
Archaea1 – ps2
Archaea2 – ps3
Archaea3 – ps4
Asgard/Eukaryota – ps5
Eukaryota – ps6
Amorpheae – ps7
Obazoa – ps8
Opisthokonta – ps9
Holozoa – ps10
Filozoa – ps11
Choano./Metazoa – ps12
Metazoa – ps13
Myriazoa – ps14
Parahoxozoa – ps15
Bilateria – ps16
Deuterostomia – ps17
Chordata – ps18
Olfactores – ps19
Vertebrata – ps20
Gnathostomata – ps21
Euteleostomi – ps22
Sarcopterygii – ps23
Tetrapoda – ps24
Amniota – ps25
Mammalia – ps26
Theria – ps27
Placentalia – ps28
Boreoeutheria – ps29
Euarchontoglires – ps30
Euarchonta – ps31
Primates – ps32
Strepsirrhini – ps33
Lemuriformes – ps34
Propithecus_coquereli – ps35

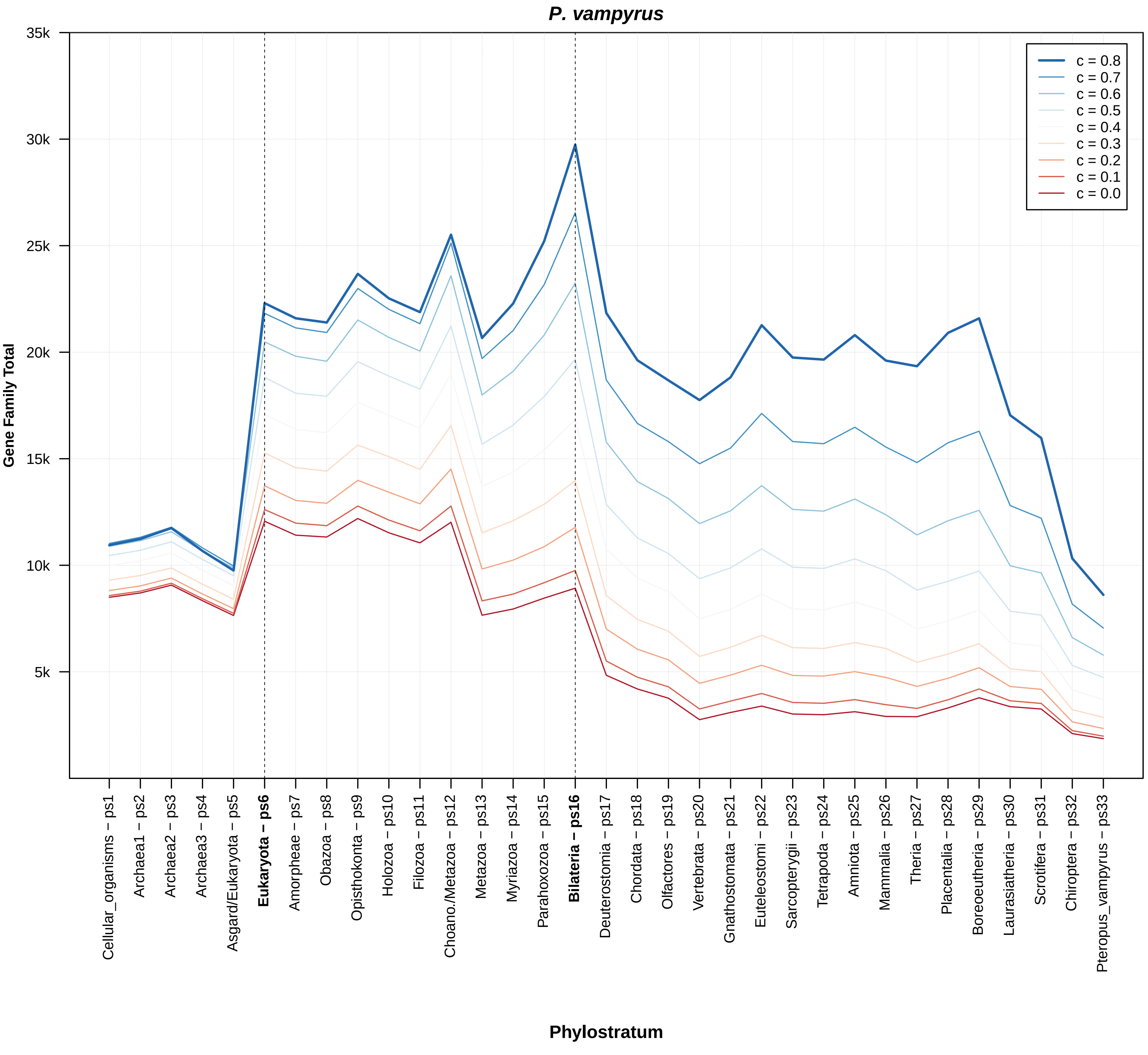

P. vampyrus
Gene Family Total
35k
30k
25k
20k
15k
10k
5k
c = 0.8
c = 0.7
c = 0.6
c = 0.5
c = 0.4
c = 0.3
c = 0.2
c = 0.1
c = 0.0
Cellular_organisms – ps1
Archaea1 – ps2
Archaea2 – ps3
Archaea3 – ps4
Asgard/Eukaryota – ps5
Eukaryota – ps6
Amorpheae – ps7
Obazoa – ps8
Opisthokonta – ps9
Holozoa – ps10
Filozoa – ps11
Choano./Metazoa – ps12
Metazoa – ps13
Myriazoa – ps14
Parahoxozoa – ps15
Bilateria – ps16
Deuterostomia – ps17
Chordata – ps18
Olfactores – ps19
Vertebrata – ps20
Gnathostomata – ps21
Euteleostomi – ps22
Sarcopterygii – ps23
Tetrapoda – ps24
Amniota – ps25
Mammalia – ps26
Theria – ps27
Placentalia – ps28
Boreoeutheria – ps29
Laurasiatheria – ps30
Scrotifera – ps31
Chiroptera – ps32
Pteropus_vampyrus – ps33
Phylostratum

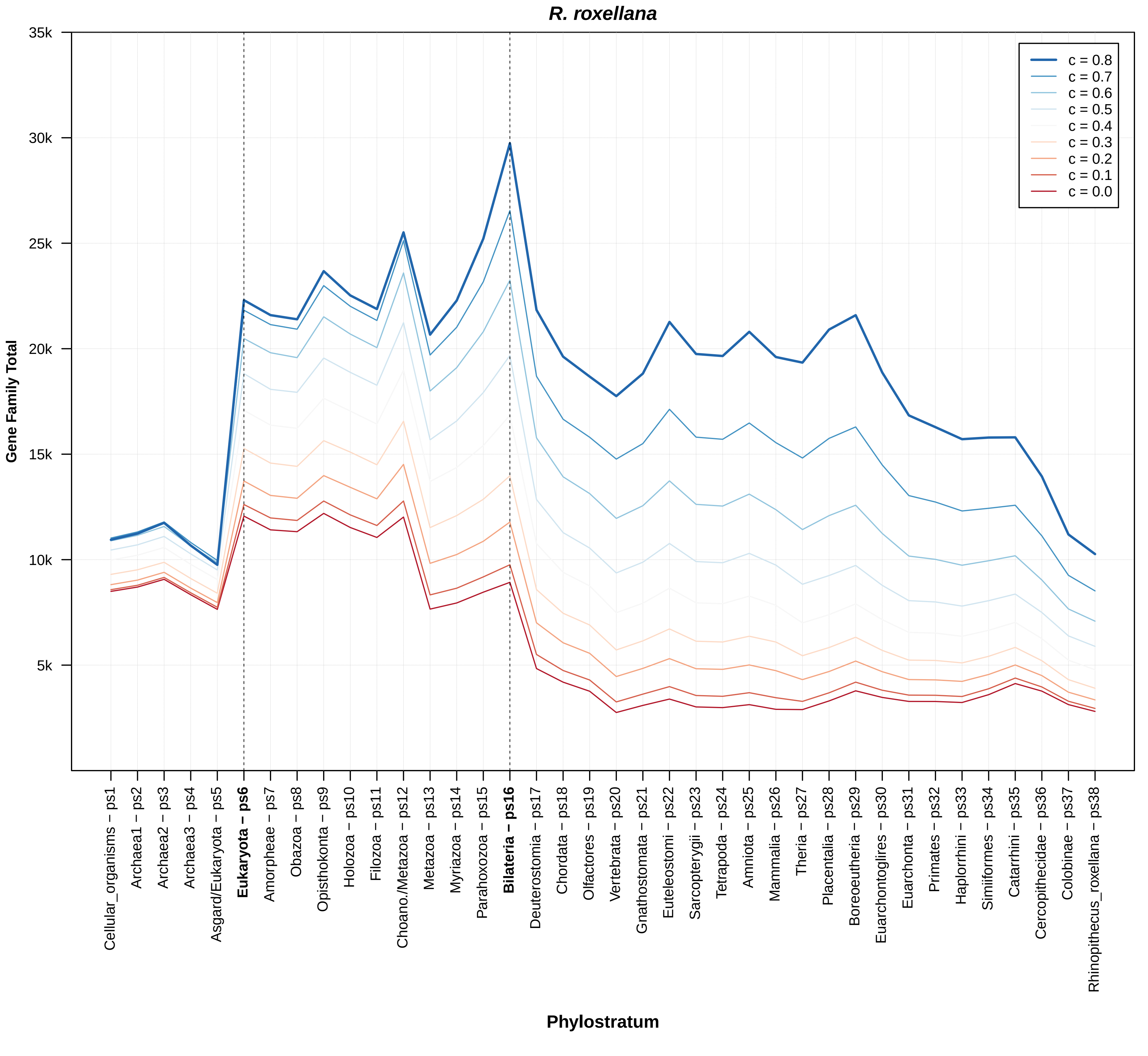
R. roxellana
Gene Family Total
35k
30k
25k
20k
15k
10k
5k
c = 0.8
c = 0.7
c = 0.6
c = 0.5
c = 0.4
c = 0.3
c = 0.2
c = 0.1
c = 0.0
Cellular_organisms – ps1
Archaea1 – ps2
Archaea2 – ps3
Archaea3 – ps4
Asgard/Eukaryota – ps5
Eukaryota – ps6
Amorpheae – ps7
Obazoa – ps8
Opisthokonta – ps9
Holozoa – ps10
Filozoa – ps11
Choano./Metazoa – ps12
Metazoa – ps13
Myriazoa – ps14
Parahoxozoa – ps15
Bilateria – ps16
Deuterostomia – ps17
Chordata – ps18
Olfactores – ps19
Vertebrata – ps20
Gnathostomata – ps21
Euteleostomi – ps22
Sarcopterygii – ps23
Tetrapoda – ps24
Amniota – ps25
Mammalia – ps26
Theria – ps27
Placentalia – ps28
Boreoeutheria – ps29
Euarchontoglires – ps30
Euarchonta – ps31
Primates – ps32
Haplorrhini – ps33
Simiiformes – ps34
Catarrhini – ps35
Cercopithecidae – ps36
Colobinae – ps37
Rhinopithecus_roxellana – ps38
Phylostratum

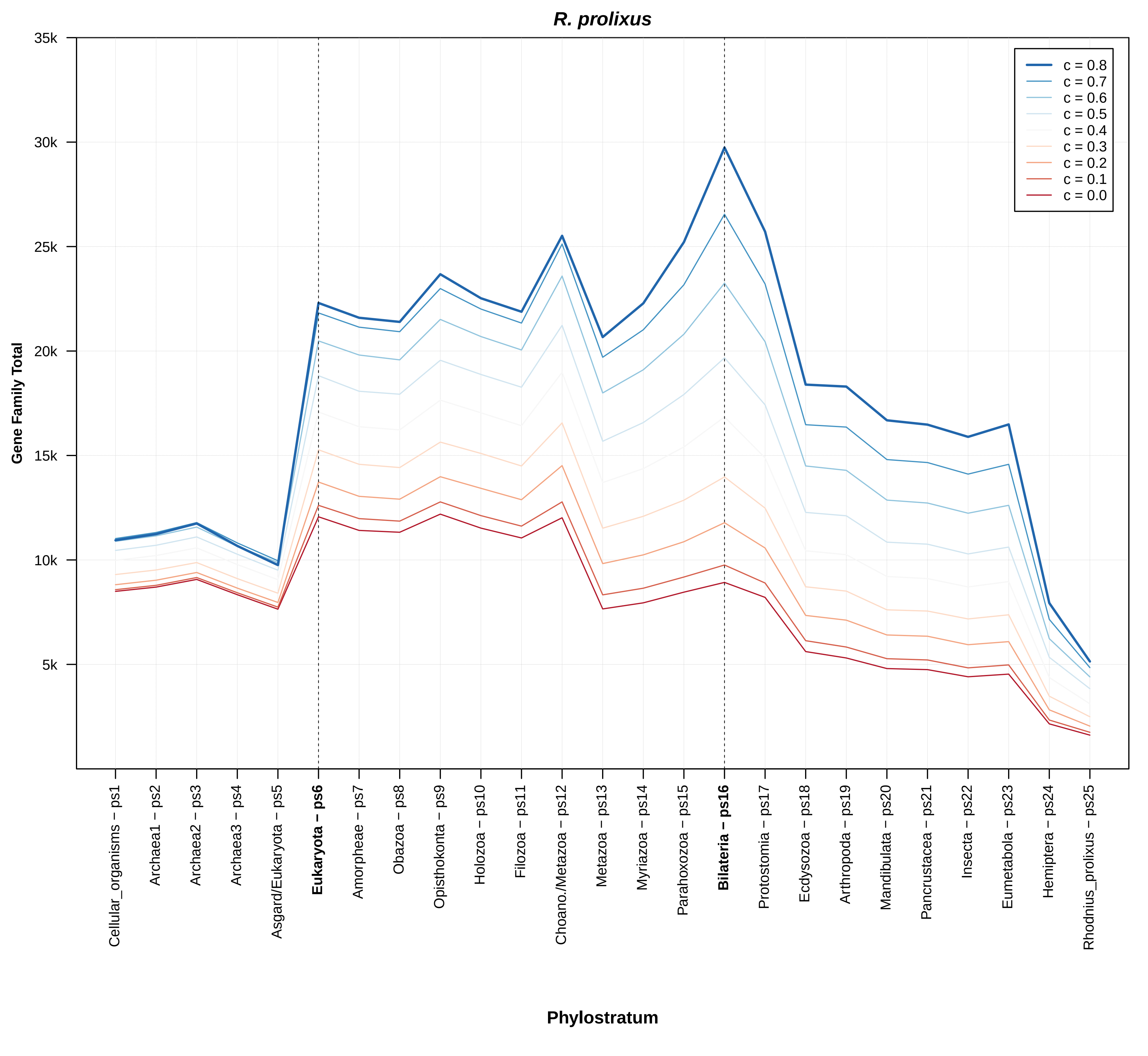
R. prolixus
Gene Family Total
Phylostratum
35k
30k
25k
20k
15k
10k
5k
c = 0.8
c = 0.7
c = 0.6
c = 0.5
c = 0.4
c = 0.3
c = 0.2
c = 0.1
c = 0.0
Cellular_organisms – ps1
Archaea1 – ps2
Archaea2 – ps3
Archaea3 – ps4
Asgard/Eukaryota – ps5
Eukaryota – ps6
Amorpheae – ps7
Obazoa – ps8
Opisthokonta – ps9
Holozoa – ps10
Filozoa – ps11
Choano./Metazoa – ps12
Metazoa – ps13
Myriazoa – ps14
Parahoxozoa – ps15
Bilateria – ps16
Protostomia – ps17
Ecdysozoa – ps18
Arthropoda – ps19
Mandibulata – ps20
Pancrustacea – ps21
Insecta – ps22
Eumetabola – ps23
Hemiptera – ps24
Rhodnius_prolixus – ps25

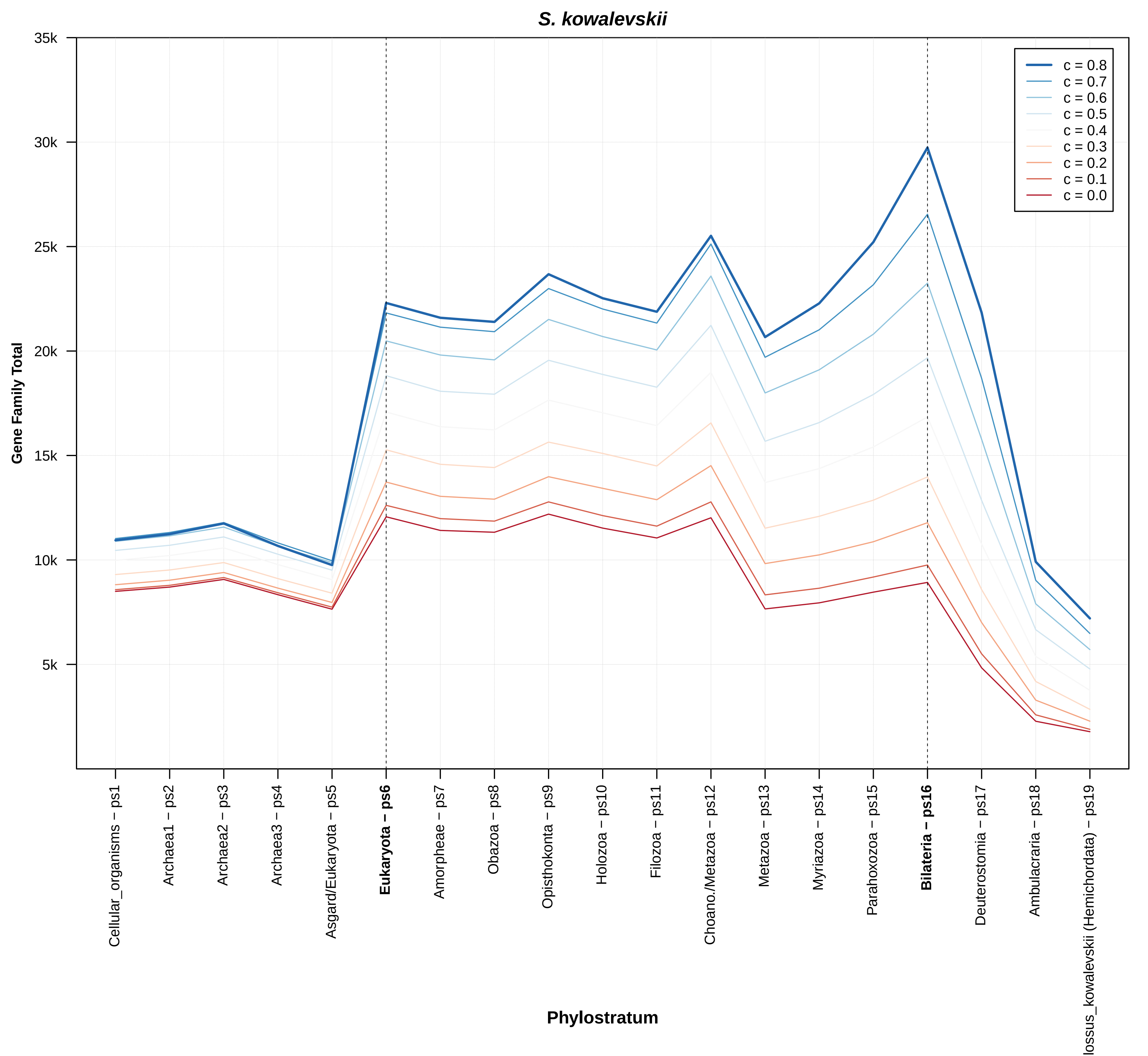
S. kowalevskii
Gene Family Total
35k
30k
25k
20k
15k
10k
5k
c = 0.8
c = 0.7
c = 0.6
c = 0.5
c = 0.4
c = 0.3
c = 0.2
c = 0.1
c = 0.0
Cellular_organisms – ps1
Archaea1 – ps2
Archaea2 – ps3
Archaea3 – ps4
Asgard/Eukaryota – ps5
Eukaryota – ps6
Amorpheae – ps7
Obazoa – ps8
Opisthokonta – ps9
Holozoa – ps10
Filozoa – ps11
Choano./Metazoa – ps12
Metazoa – ps13
Myriazoa – ps14
Parahoxozoa – ps15
Bilateria – ps16
Deuterostomia – ps17
Ambulacraria – ps18
lossus_kowalevskii (Hemichordata) – ps19
Phylostratum

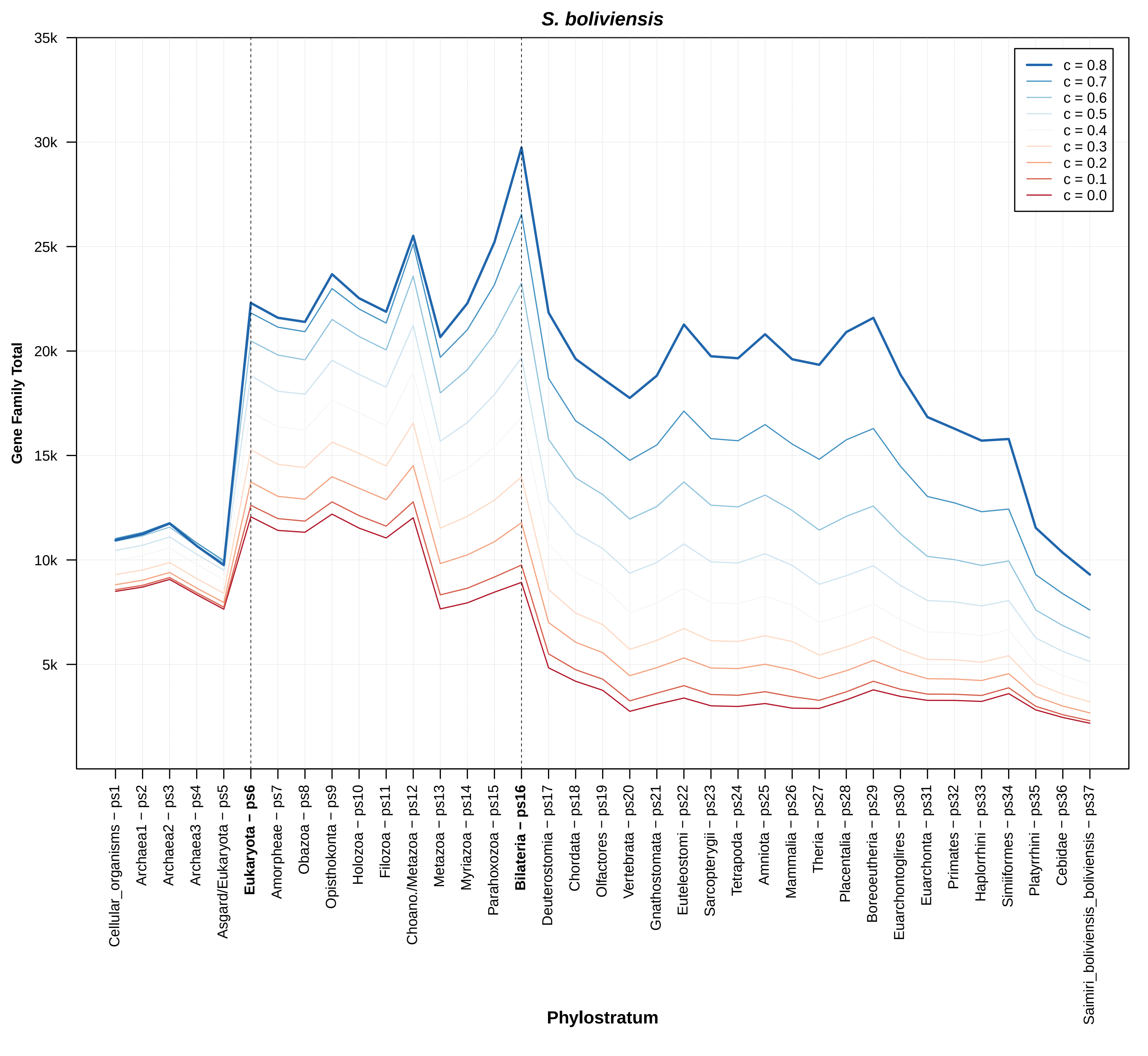

S. boliviensis
Gene Family Total
35k
30k
25k
20k
15k
10k
5k
c = 0.8
c = 0.7
c = 0.6
c = 0.5
c = 0.4
c = 0.3
c = 0.2
c = 0.1
c = 0.0
Cellular_organisms – ps1
Archaea1 – ps2
Archaea2 – ps3
Archaea3 – ps4
Asgard/Eukaryota – ps5
Eukaryota – ps6
Amorpheae – ps7
Obazoa – ps8
Opisthokonta – ps9
Holozoa – ps10
Filozoa – ps11
Choano./Metazoa – ps12
Metazoa – ps13
Myriazoa – ps14
Parahoxozoa – ps15
Bilateria – ps16
Deuterostomia – ps17
Chordata – ps18
Olfactores – ps19
Vertebrata – ps20
Gnathostomata – ps21
Euteleostomi – ps22
Sarcopterygii – ps23
Tetrapoda – ps24
Amniota – ps25
Mammalia – ps26
Theria – ps27
Placentalia – ps28
Boreoeutheria – ps29
Euarchontoglires – ps30
Euarchonta – ps31
Primates – ps32
Haplorrhini – ps33
Simiiformes – ps34
Platyrrhini – ps35
Cebidae – ps36
Saimiri_boliviensis_boliviensis – ps37
Phylostratum

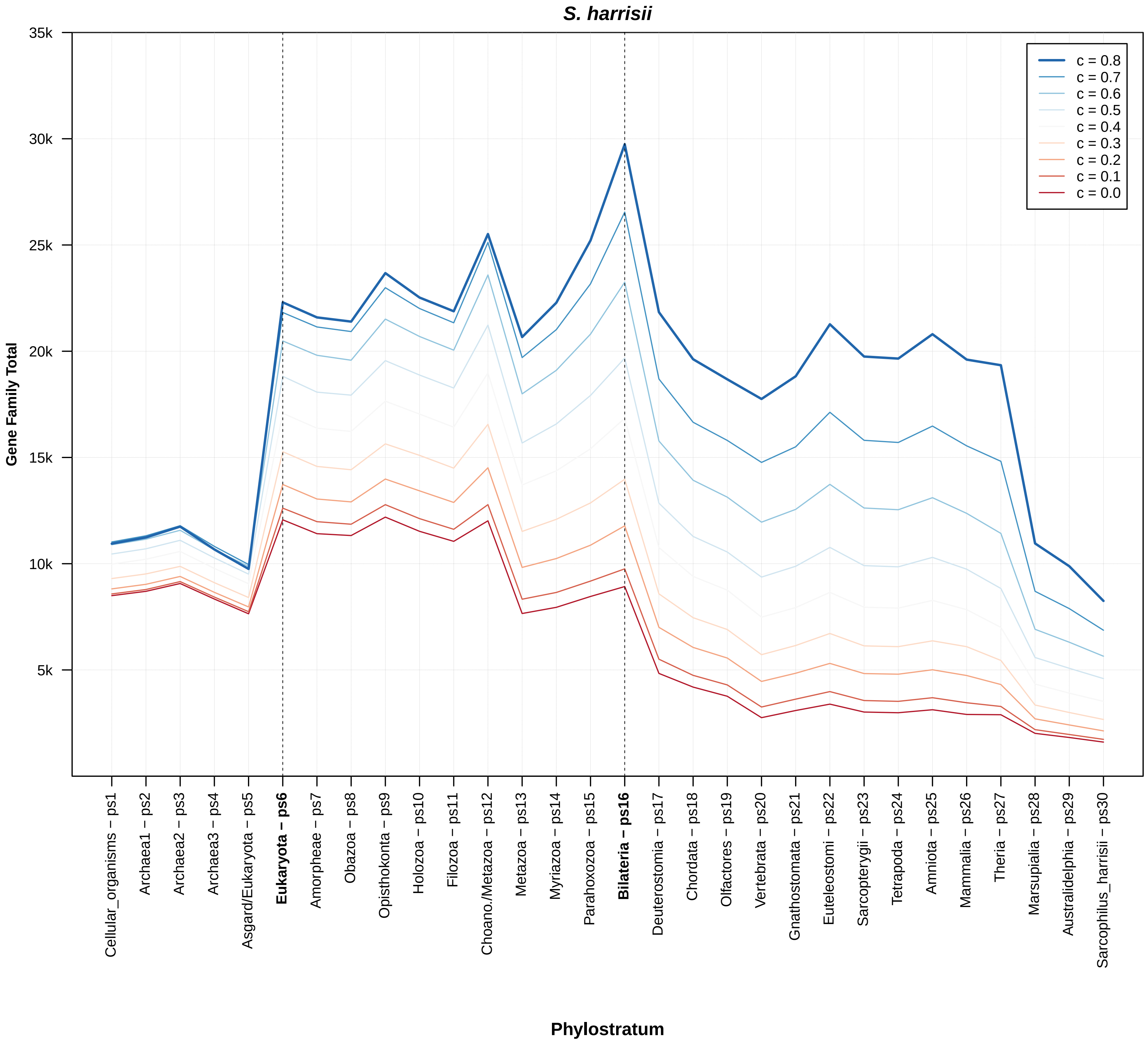

S. harrisii
Gene Family Total
35k
30k
25k
20k
15k
10k
5k
c = 0.8
c = 0.7
c = 0.6
c = 0.5
c = 0.4
c = 0.3
c = 0.2
c = 0.1
c = 0.0
Cellular_organisms – ps1
Archaea1 – ps2
Archaea2 – ps3
Archaea3 – ps4
Asgard/Eukaryota – ps5
Eukaryota – ps6
Amorpheae – ps7
Obazoa – ps8
Opisthokonta – ps9
Holozoa – ps10
Filozoa – ps11
Choano./Metazoa – ps12
Metazoa – ps13
Myriazoa – ps14
Parahoxozoa – ps15
Bilateria – ps16
Deuterostomia – ps17
Chordata – ps18
Olfactores – ps19
Vertebrata – ps20
Gnathostomata – ps21
Euteleostomi – ps22
Sarcopterygii – ps23
Tetrapoda – ps24
Amniota – ps25
Mammalia – ps26
Theria – ps27
Marsupialia – ps28
Australidelphia – ps29
Sarcophilus_harrisii – ps30
Phylostratum

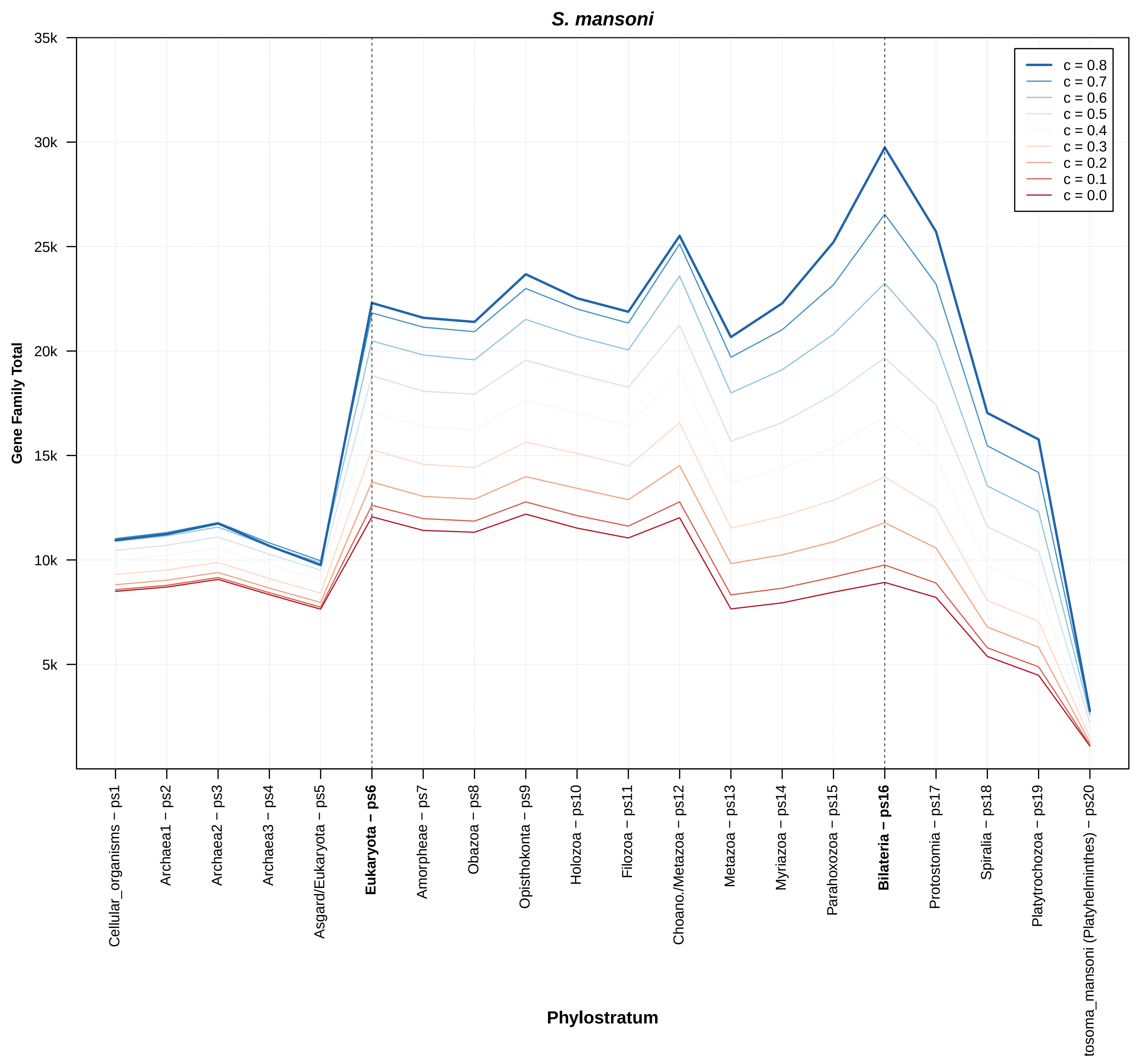
S. mansoni
35k
30k
25k
20k
15k
10k
5k
Gene Family Total
c = 0.8
c = 0.7
c = 0.6
c = 0.5
c = 0.4
c = 0.3
c = 0.2
c = 0.1
c = 0.0
Cellular_organisms – ps1
Archaea1 – ps2
Archaea2 – ps3
Archaea3 – ps4
Asgard/Eukaryota – ps5
Eukaryota – ps6
Amorpheae – ps7
Obazoa – ps8
Opisthokonta – ps9
Holozoa – ps10
Filozoa – ps11
Choano./Metazoa – ps12
Metazoa – ps13
Myriazoa – ps14
Parahoxozoa – ps15
Bilateria – ps16
Protostomia – ps17
Spiralia – ps18
Platytrochozoa – ps19
tosoma_mansoni (Platyhelminthes) – ps20
Phylostratum

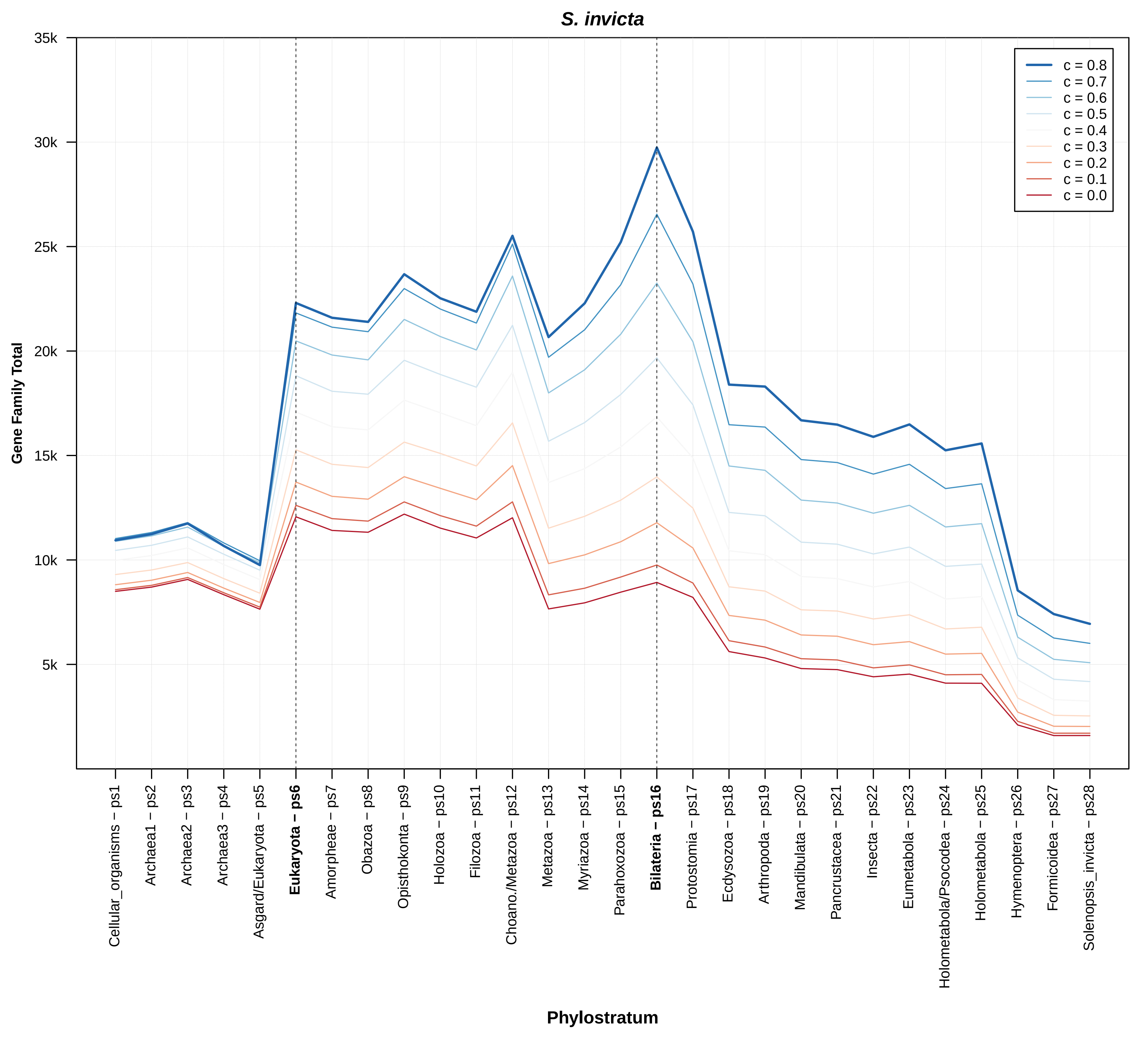

S. invicta
Gene Family Total
35k
30k
25k
20k
15k
10k
5k
c = 0.8
c = 0.7
c = 0.6
c = 0.5
c = 0.4
c = 0.3
c = 0.2
c = 0.1
c = 0.0
Cellular_organisms – ps1
Archaea1 – ps2
Archaea2 – ps3
Archaea3 – ps4
Asgard/Eukaryota – ps5
Eukaryota – ps6
Amorpheae – ps7
Obazoa – ps8
Opisthokonta – ps9
Holozoa – ps10
Filozoa – ps11
Choano./Metazoa – ps12
Metazoa – ps13
Myriazoa – ps14
Parahoxozoa – ps15
Bilateria – ps16
Protostomia – ps17
Ecdysozoa – ps18
Arthropoda – ps19
Mandibulata – ps20
Pancrustacea – ps21
Insecta – ps22
Eumetabola – ps23
Holometabola/Psocodea – ps24
Holometabola – ps25
Hymenoptera – ps26
Formicoidea – ps27
Solenopsis_invicta – ps28
Phylostratum

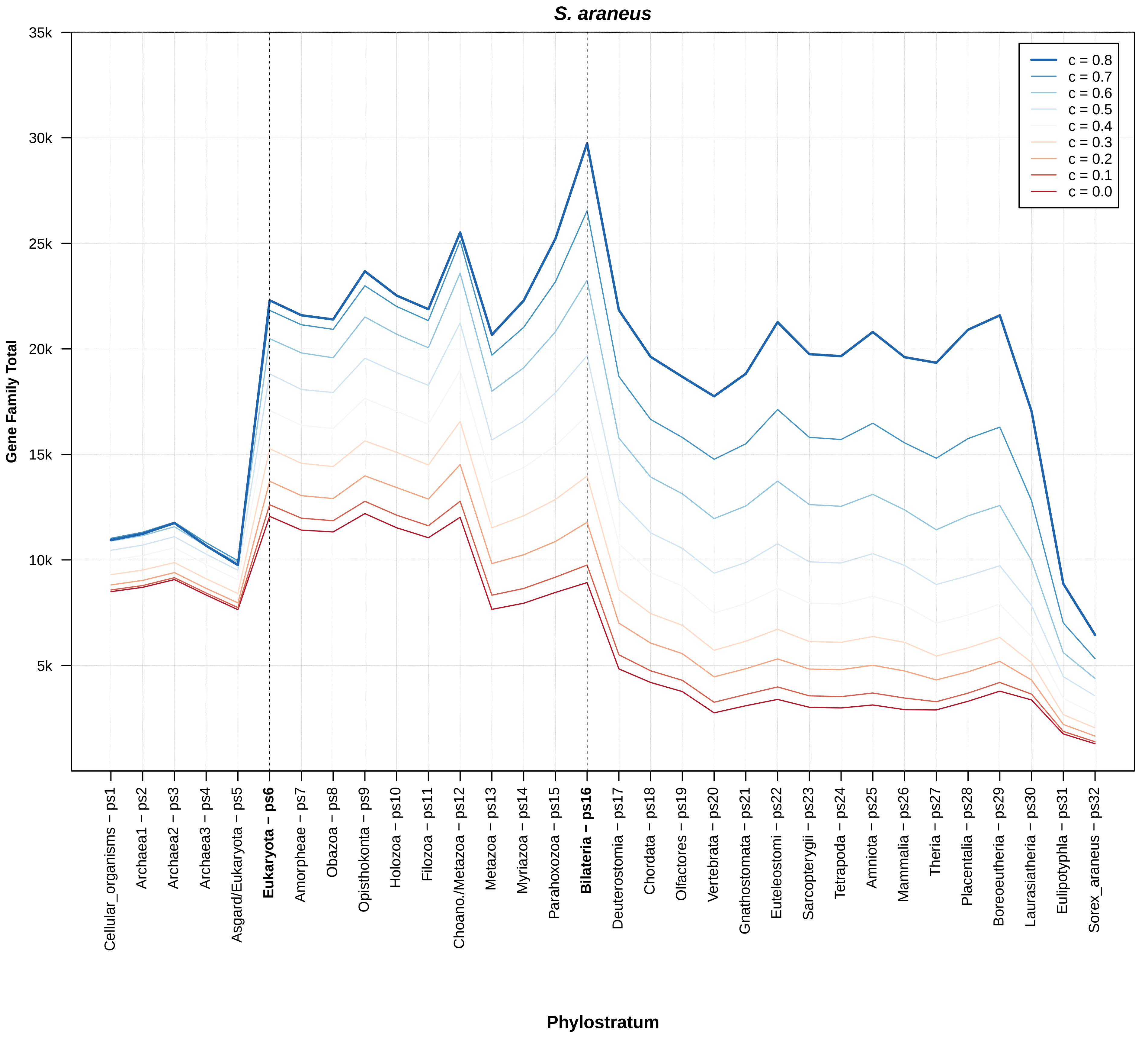

S. araneus
Gene Family Total
35k
30k
25k
20k
15k
10k
5k
c = 0.8
c = 0.7
c = 0.6
c = 0.5
c = 0.4
c = 0.3
c = 0.2
c = 0.1
c = 0.0
Cellular_organisms – ps1
Archaea1 – ps2
Archaea2 – ps3
Archaea3 – ps4
Asgard/Eukaryota – ps5
Eukaryota – ps6
Amorpheae – ps7
Obazoa – ps8
Opisthokonta – ps9
Holozoa – ps10
Filozoa – ps11
Choano./Metazoa – ps12
Metazoa – ps13
Myriazoa – ps14
Parahoxozoa – ps15
Bilateria – ps16
Deuterostomia – ps17
Chordata – ps18
Olfactores – ps19
Vertebrata – ps20
Gnathostomata – ps21
Euteleostomi – ps22
Sarcopterygii – ps23
Tetrapoda – ps24
Amniota – ps25
Mammalia – ps26
Theria – ps27
Placentalia – ps28
Boreoeutheria – ps29
Laurasiatheria – ps30
Eulipotyphla – ps31
Sorex_araneus – ps32
Phylostratum

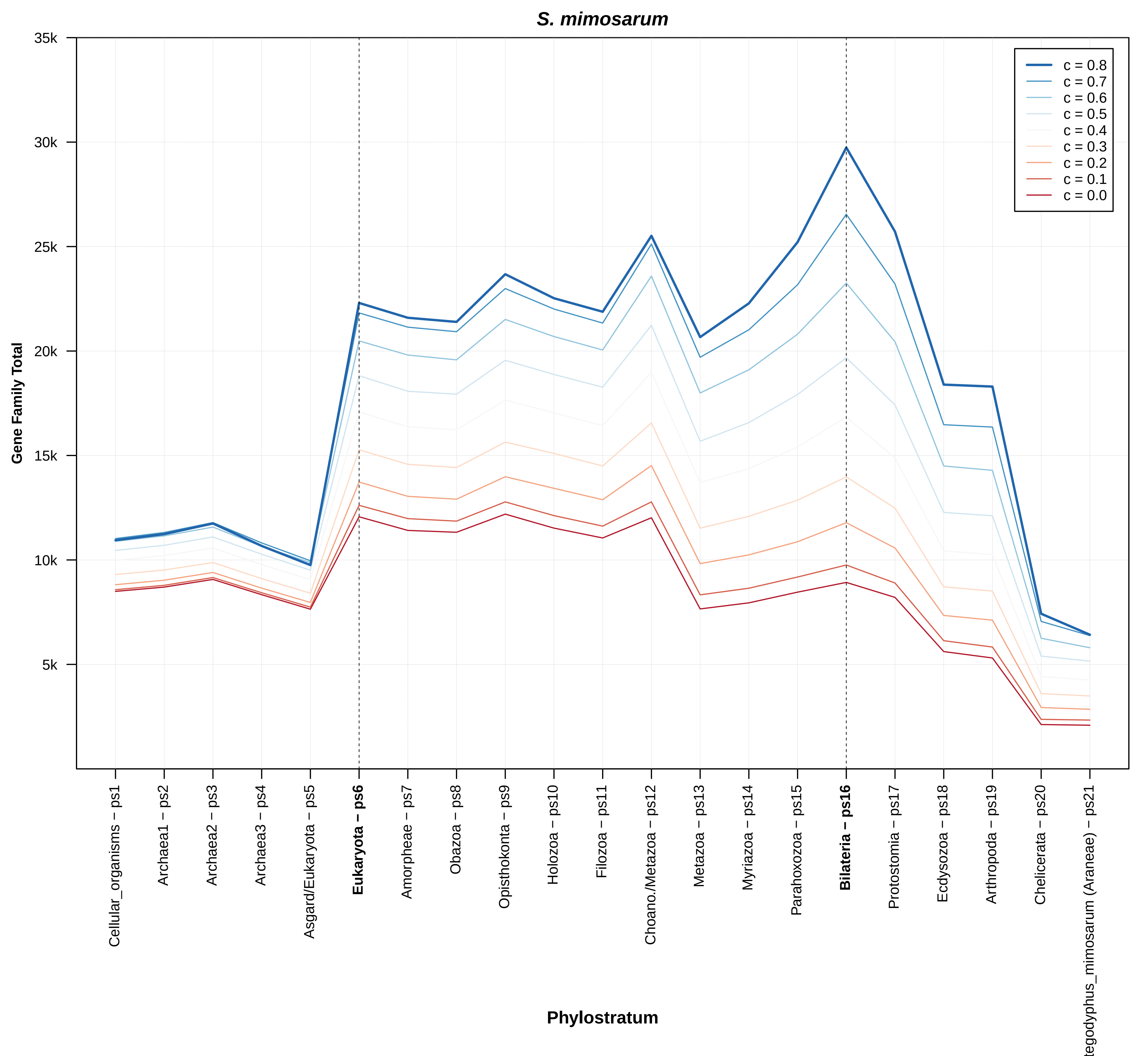
S. mimosarum
Gene Family Total
35k
30k
25k
20k
15k
10k
5k
c = 0.8
c = 0.7
c = 0.6
c = 0.5
c = 0.4
c = 0.3
c = 0.2
c = 0.1
c = 0.0
Cellular_organisms – ps1
Archaea1 – ps2
Archaea2 – ps3
Archaea3 – ps4
Asgard/Eukaryota – ps5
Eukaryota – ps6
Amorpheae – ps7
Obazoa – ps8
Opisthokonta – ps9
Holozoa – ps10
Filozoa – ps11
Choano./Metazoa – ps12
Metazoa – ps13
Myriazoa – ps14
Parahoxozoa – ps15
Bilateria – ps16
Protostomia – ps17
Ecdysozoa – ps18
Arthropoda – ps19
Chelicerata – ps20
tegodyphus_mimosarum (Araneae) – ps21
Phylostratum

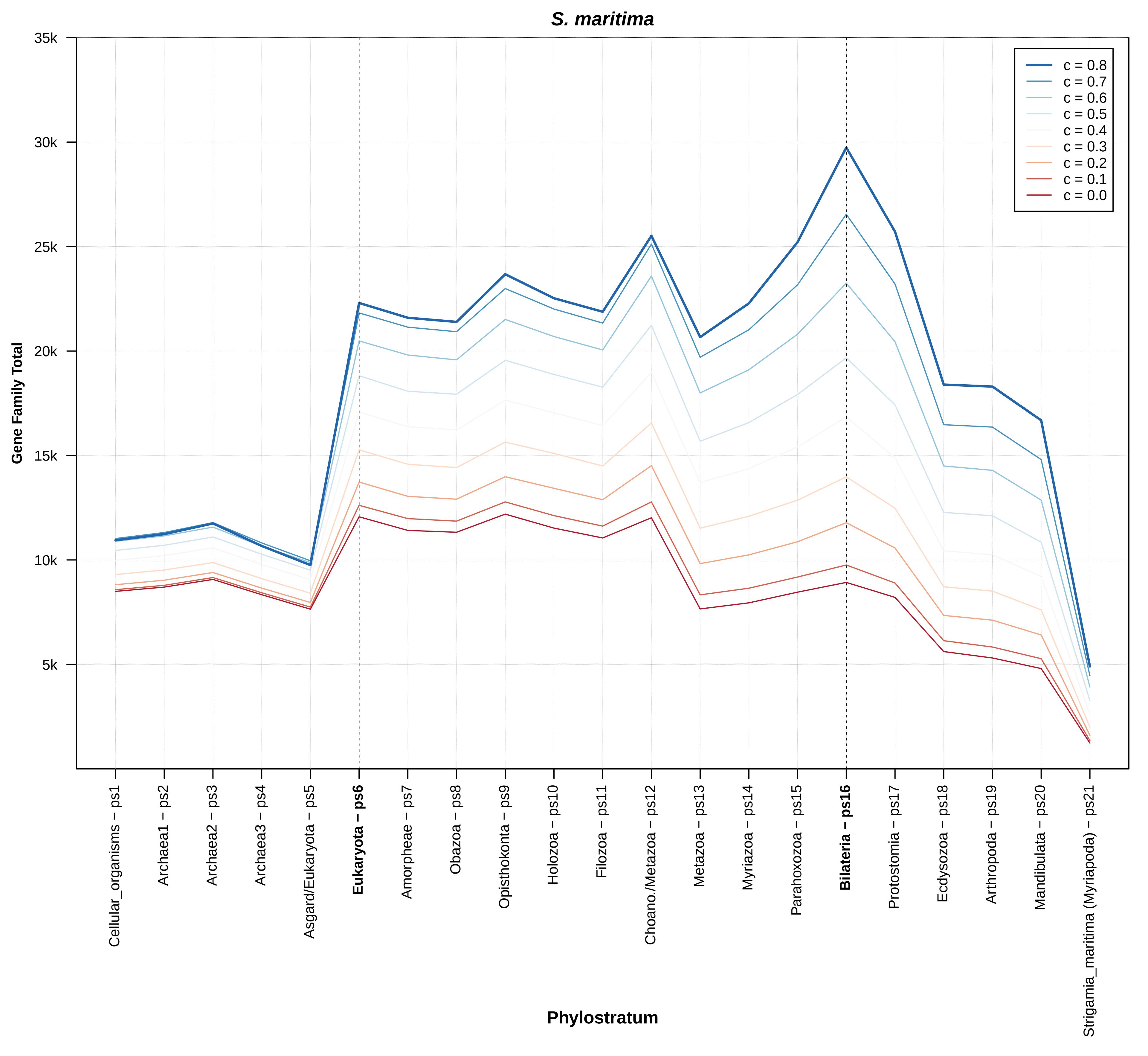

S. maritima
35k
30k
25k
20k
15k
10k
5k
Gene Family Total
c = 0.8
c = 0.7
c = 0.6
c = 0.5
c = 0.4
c = 0.3
c = 0.2
c = 0.1
c = 0.0
Cellular_organisms – ps1
Archaea1 – ps2
Archaea2 – ps3
Archaea3 – ps4
Asgard/Eukaryota – ps5
Eukaryota – ps6
Amorpheae – ps7
Obazoa – ps8
Opisthokonta – ps9
Holozoa – ps10
Filozoa – ps11
Choano./Metazoa – ps12
Metazoa – ps13
Myriazoa – ps14
Parahoxozoa – ps15
Bilateria – ps16
Protostomia – ps17
Ecdysozoa – ps18
Arthropoda – ps19
Mandibulata – ps20
Strigamia_maritima (Myriapoda) – ps21
Phylostratum

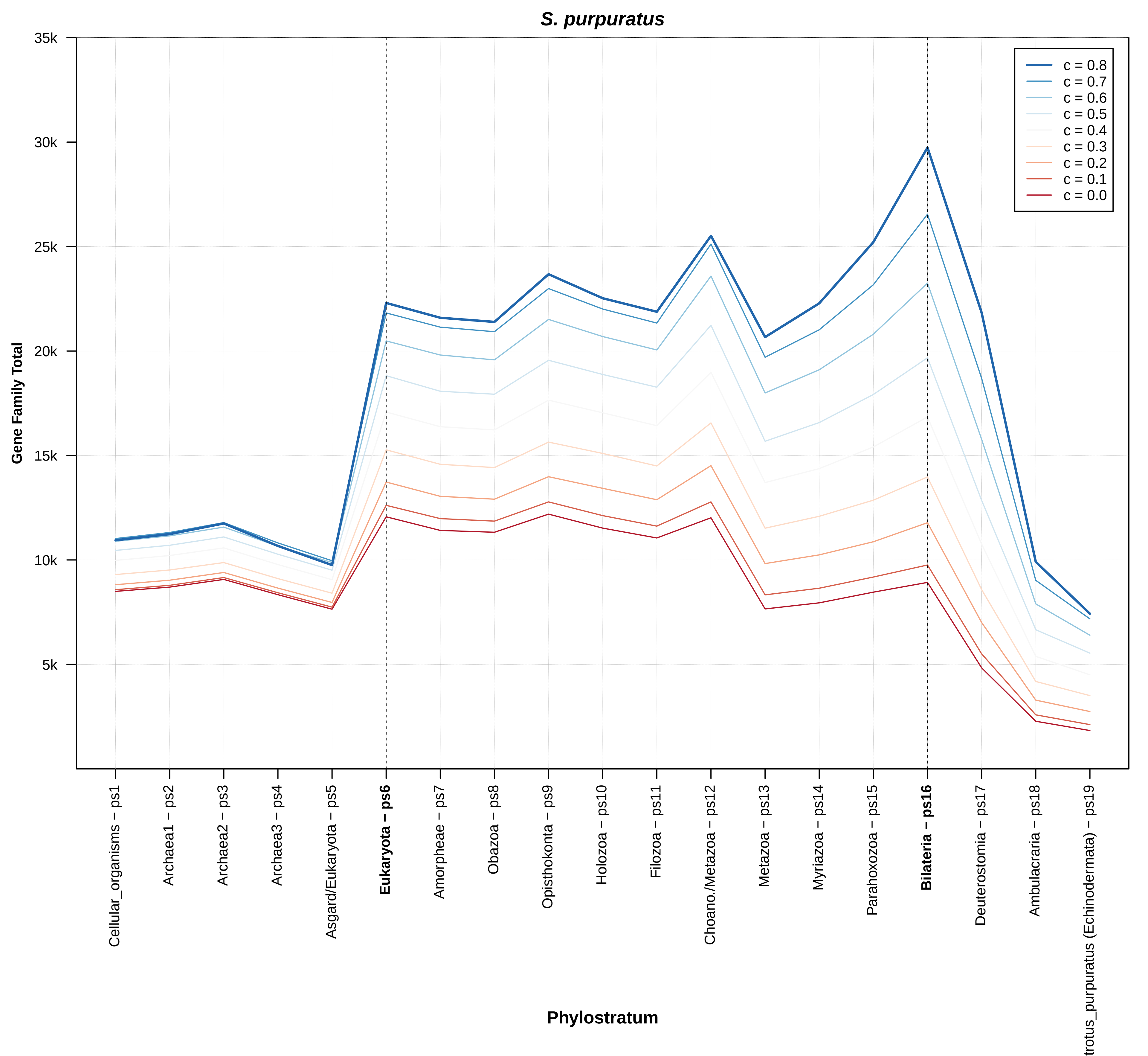

S. purpuratus
Gene Family Total
35k
30k
25k
20k
15k
10k
5k
c = 0.8
c = 0.7
c = 0.6
c = 0.5
c = 0.4
c = 0.3
c = 0.2
c = 0.1
c = 0.0
Cellular_organisms – ps1
Archaea1 – ps2
Archaea2 – ps3
Archaea3 – ps4
Asgard/Eukaryota – ps5
Eukaryota – ps6
Amorpheae – ps7
Obazoa – ps8
Opisthokonta – ps9
Holozoa – ps10
Filozoa – ps11
Choano./Metazoa – ps12
Metazoa – ps13
Myriazoa – ps14
Parahoxozoa – ps15
Bilateria – ps16
Deuterostomia – ps17
Ambulacraria – ps18
trotus_purpuratus (Echinodermata) – ps19
Phylostratum

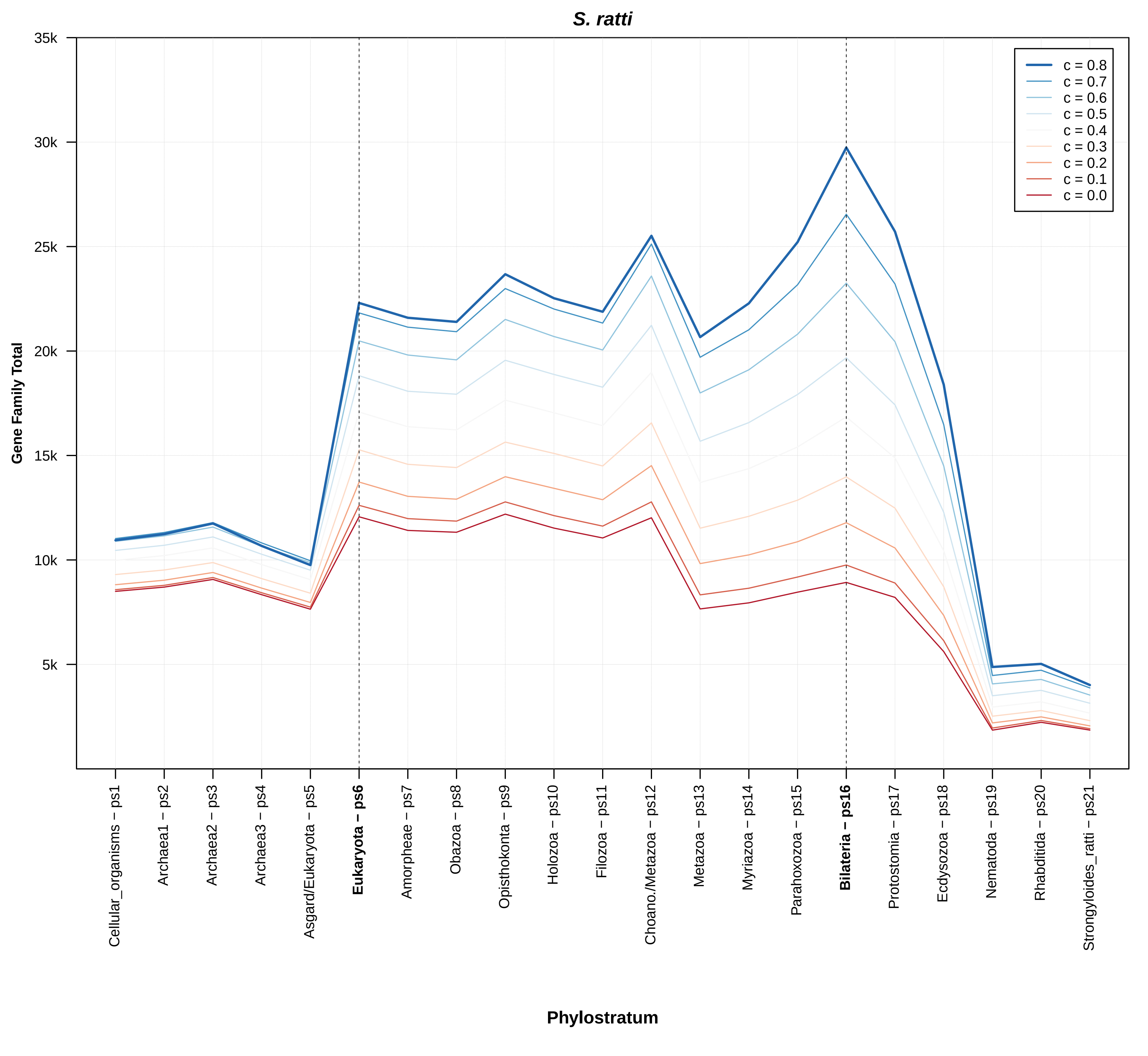

S. ratti
Gene Family Total
35k
30k
25k
20k
15k
10k
5k
c = 0.8
c = 0.7
c = 0.6
c = 0.5
c = 0.4
c = 0.3
c = 0.2
c = 0.1
c = 0.0
Cellular_organisms – ps1
Archaea1 – ps2
Archaea2 – ps3
Archaea3 – ps4
Asgard/Eukaryota – ps5
Eukaryota – ps6
Amorpheae – ps7
Obazoa – ps8
Opisthokonta – ps9
Holozoa – ps10
Filozoa – ps11
Choano./Metazoa – ps12
Metazoa – ps13
Myriazoa – ps14
Parahoxozoa – ps15
Bilateria – ps16
Protostomia – ps17
Ecdysozoa – ps18
Nematoda – ps19
Rhabditida – ps20
Strongyloides_ratti – ps21
Phylostratum

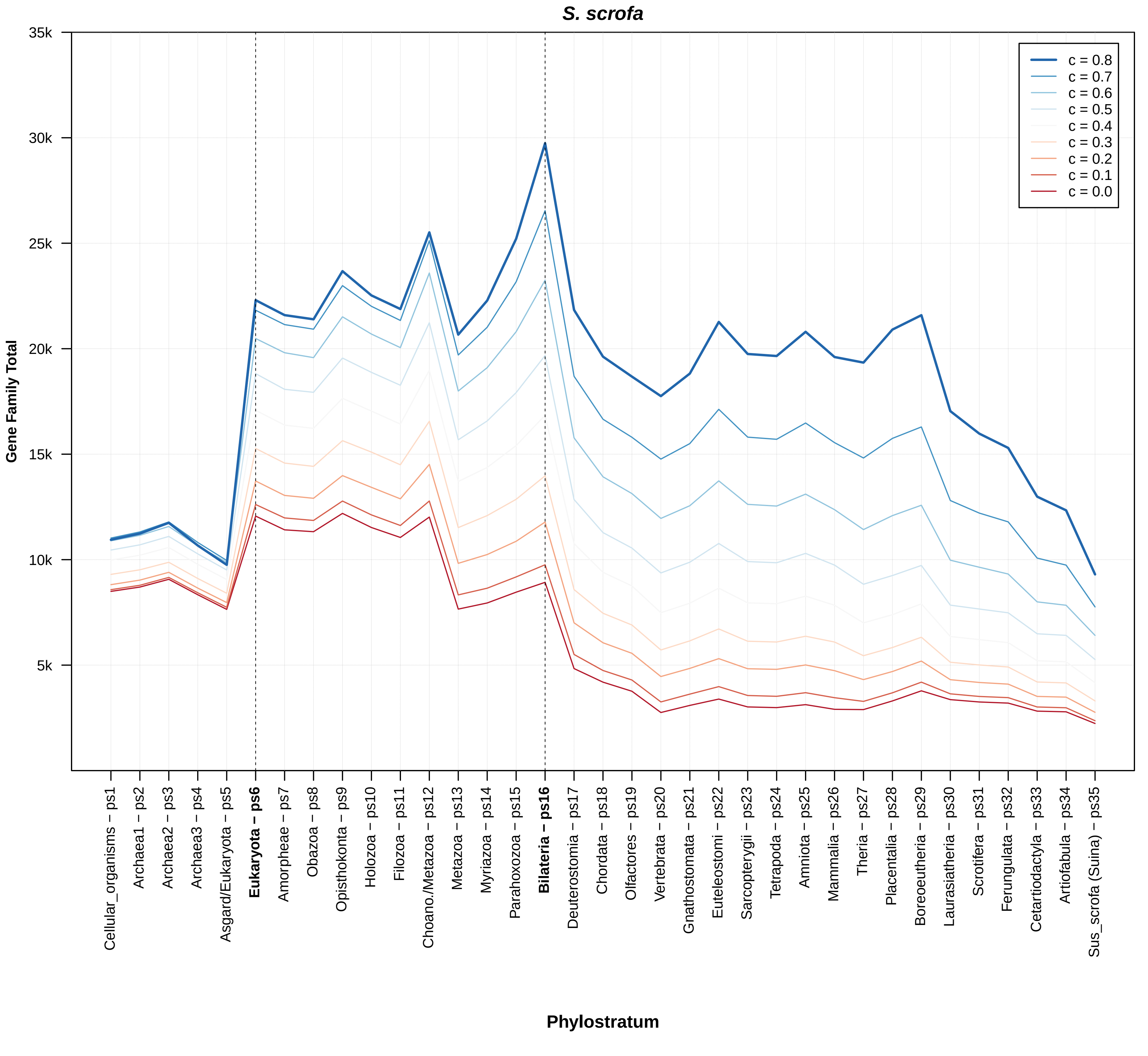
S. scrofa
c = 0.8
c = 0.7
c = 0.6
c = 0.5
c = 0.4
c = 0.3
c = 0.2
c = 0.1
c = 0.0
Gene Family Total
35k
30k
25k
20k
15k
10k
5k
Cellular_organisms – ps1
Archaea1 – ps2
Archaea2 – ps3
Archaea3 – ps4
Asgard/Eukaryota – ps5
Eukaryota – ps6
Amorpheae – ps7
Obazoa – ps8
Opisthokonta – ps9
Holozoa – ps10
Filozoa – ps11
Choano./Metazoa – ps12
Metazoa – ps13
Myriazoa – ps14
Parahoxozoa – ps15
Bilateria – ps16
Deuterostomia – ps17
Chordata – ps18
Olfactores – ps19
Vertebrata – ps20
Gnathostomata – ps21
Euteleostomi – ps22
Sarcopterygii – ps23
Tetrapoda – ps24
Amniota – ps25
Mammalia – ps26
Theria – ps27
Placentalia – ps28
Boreoeutheria – ps29
Laurasiatheria – ps30
Scrotifera – ps31
Ferungulata – ps32
Cetartiodactyla – ps33
Artiofabula – ps34
Sus_scrofa (Suina) – ps35
Phylostratum

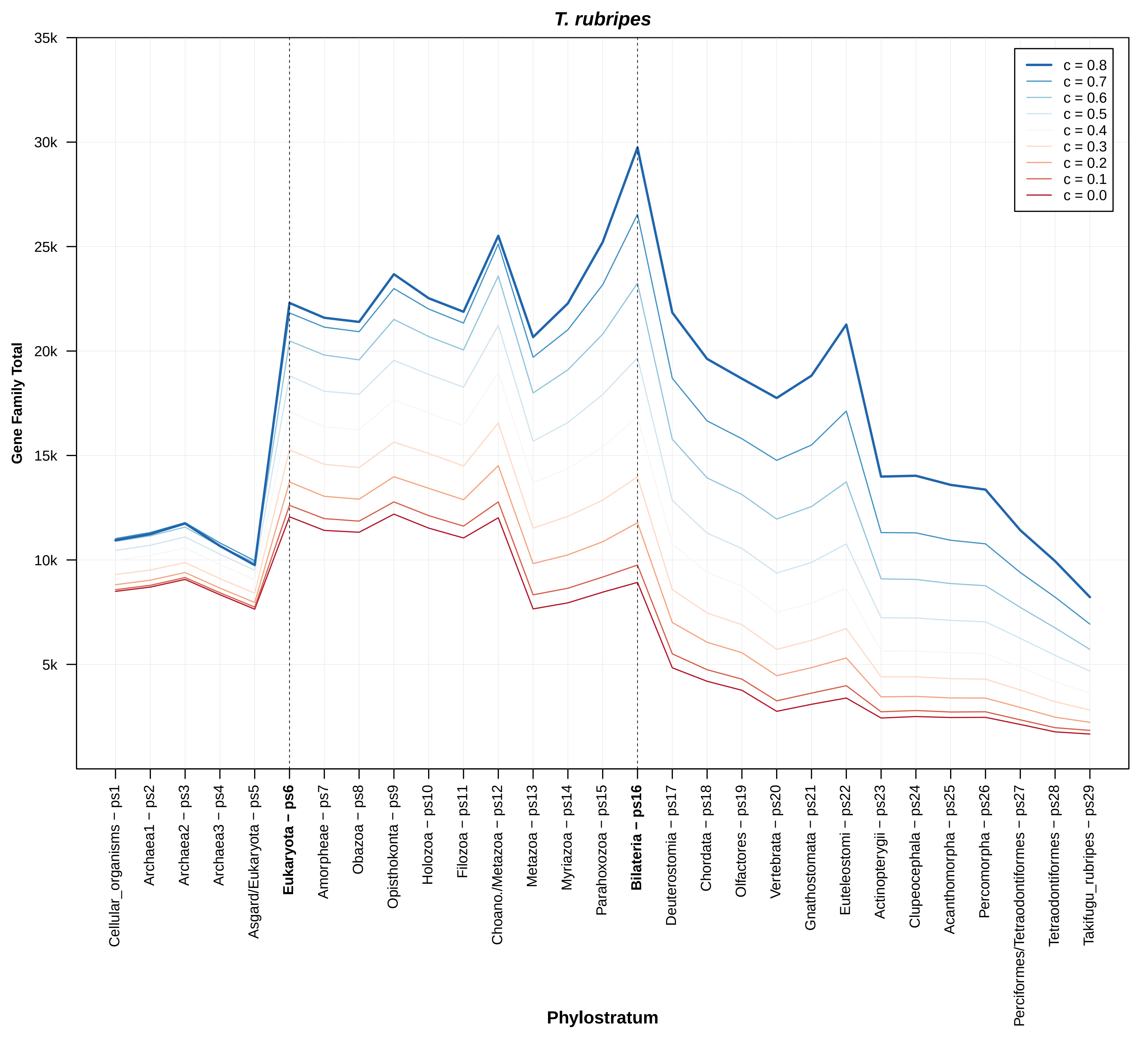

T. rubripes
Gene Family Total
35k
30k
25k
20k
15k
10k
5k
c = 0.8
c = 0.7
c = 0.6
c = 0.5
c = 0.4
c = 0.3
c = 0.2
c = 0.1
c = 0.0
Cellular_organisms – ps1
Archaea1 – ps2
Archaea2 – ps3
Archaea3 – ps4
Asgard/Eukaryota – ps5
Eukaryota – ps6
Amorpheae – ps7
Obazoa – ps8
Opisthokonta – ps9
Holozoa – ps10
Filozoa – ps11
Choano./Metazoa – ps12
Metazoa – ps13
Myriazoa – ps14
Parahoxozoa – ps15
Bilateria – ps16
Deuterostomia – ps17
Chordata – ps18
Olfactores – ps19
Vertebrata – ps20
Gnathostomata – ps21
Euteleostomi – ps22
Actinopterygii – ps23
Clupeocephala – ps24
Acanthomorpha – ps25
Percomorpha – ps26
Perciformes/Tetraodontiformes – ps27
Tetraodontiformes – ps28
Takifugu_rubripes – ps29
Phylostratum

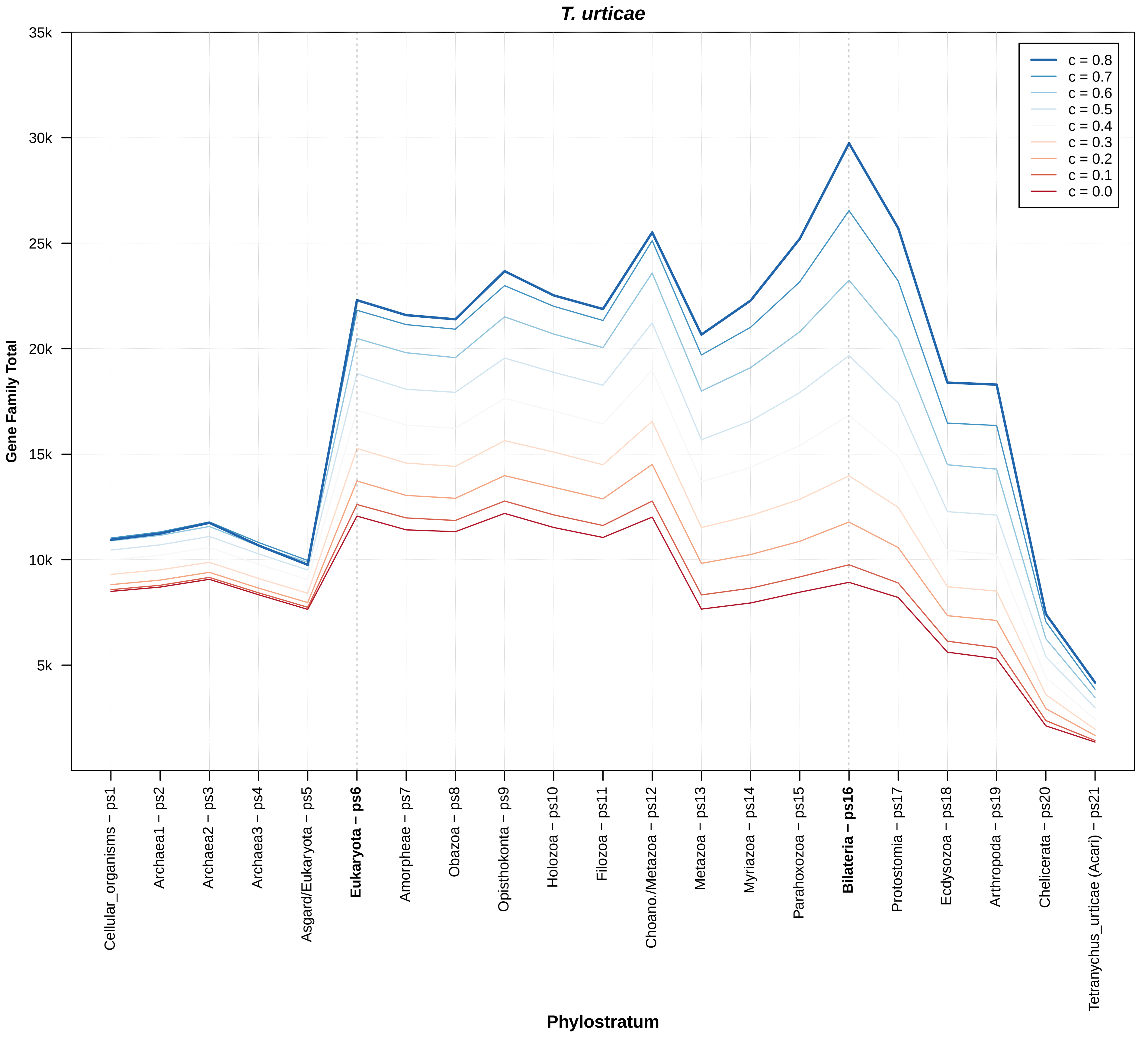
T. urticae
Gene Family Total
35k
30k
25k
20k
15k
10k
5k
c = 0.8
c = 0.7
c = 0.6
c = 0.5
c = 0.4
c = 0.3
c = 0.2
c = 0.1
c = 0.0
Cellular_organisms – ps1
Archaea1 – ps2
Archaea2 – ps3
Archaea3 – ps4
Asgard/Eukaryota – ps5
Eukaryota – ps6
Amorpheae – ps7
Obazoa – ps8
Opisthokonta – ps9
Holozoa – ps10
Filozoa – ps11
Choano./Metazoa – ps12
Metazoa – ps13
Myriazoa – ps14
Parahoxozoa – ps15
Bilateria – ps16
Protostomia – ps17
Ecdysozoa – ps18
Arthropoda – ps19
Chelicerata – ps20
Tetranychus_urticae (Acari) – ps21
Phylostratum

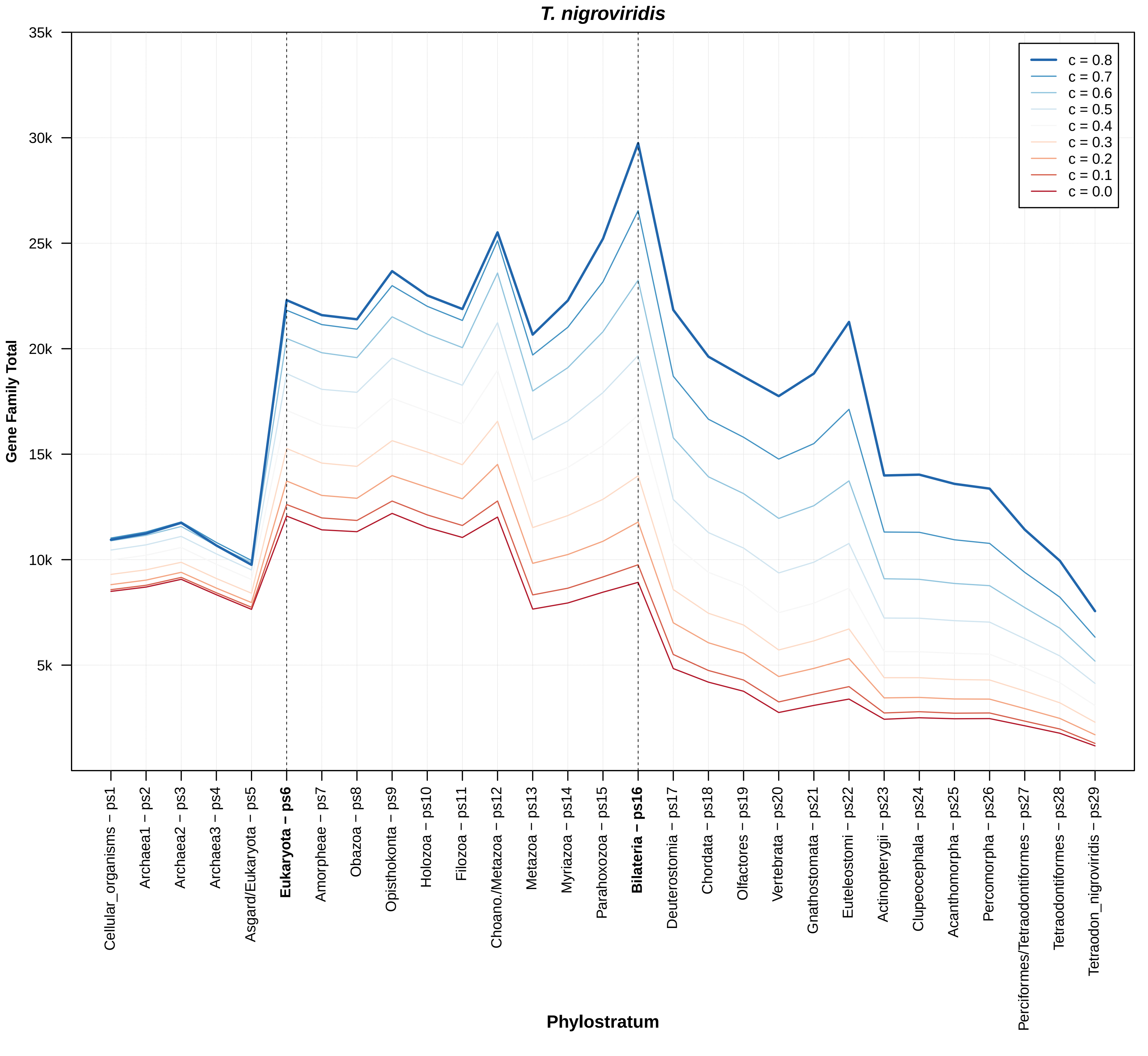

T. nigroviridis
Gene Family Total
Phylostratum
35k
30k
25k
20k
15k
10k
5k
c = 0.8
c = 0.7
c = 0.6
c = 0.5
c = 0.4
c = 0.3
c = 0.2
c = 0.1
c = 0.0
Cellular_organisms – ps1
Archaea1 – ps2
Archaea2 – ps3
Archaea3 – ps4
Asgard/Eukaryota – ps5
Eukaryota – ps6
Amorpheae – ps7
Obazoa – ps8
Opisthokonta – ps9
Holozoa – ps10
Filozoa – ps11
Choano./Metazoa – ps12
Metazoa – ps13
Myriazoa – ps14
Parahoxozoa – ps15
Bilateria – ps16
Deuterostomia – ps17
Chordata – ps18
Olfactores – ps19
Vertebrata – ps20
Gnathostomata – ps21
Euteleostomi – ps22
Actinopterygii – ps23
Clupeocephala – ps24
Acanthomorpha – ps25
Percomorpha – ps26
Perciformes/Tetraodontiformes – ps27
Tetraodontiformes – ps28
Tetraodon_nigroviridis – ps29

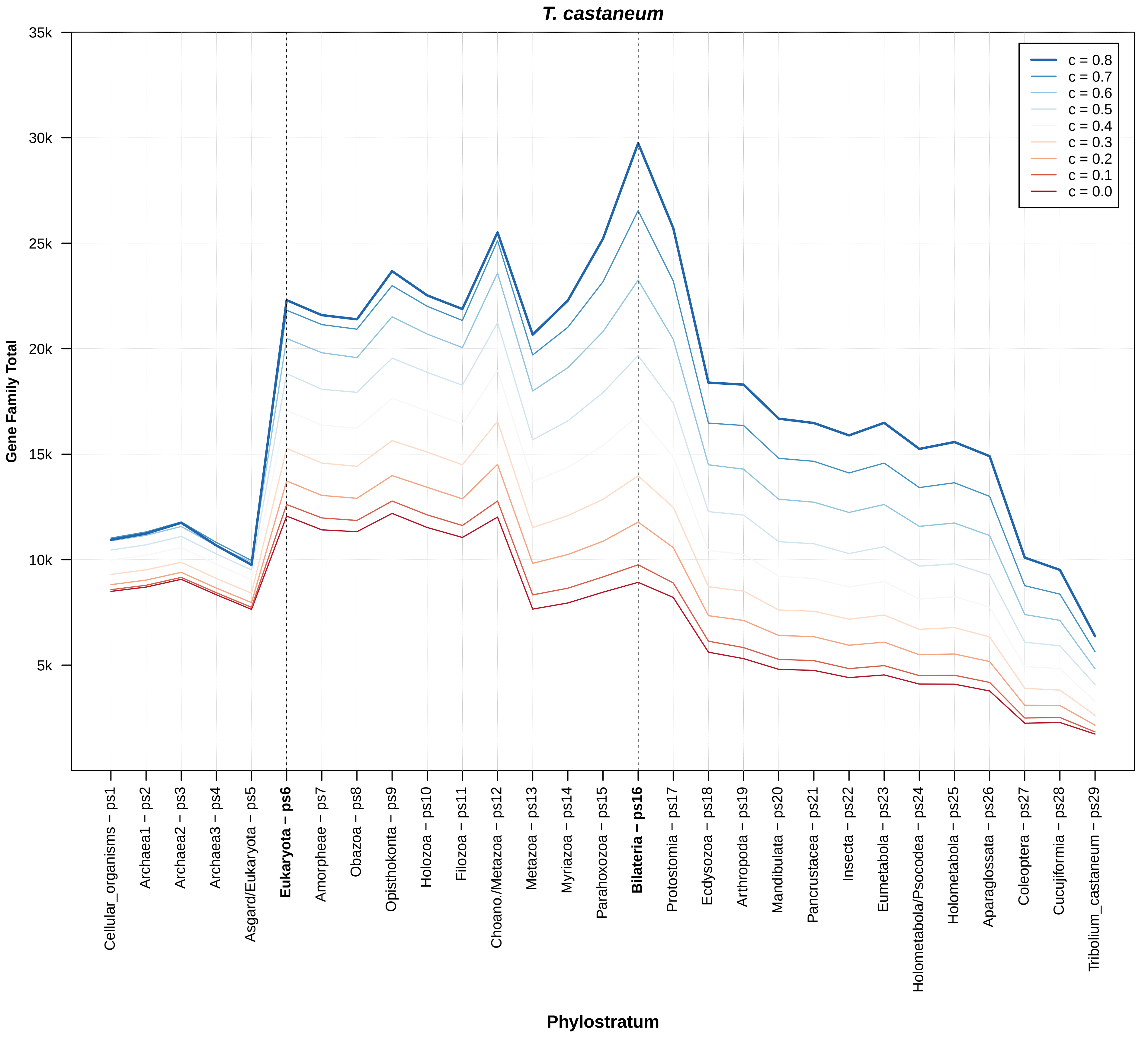

T. castaneum
Gene Family Total
35k
30k
25k
20k
15k
10k
5k
c = 0.8
c = 0.7
c = 0.6
c = 0.5
c = 0.4
c = 0.3
c = 0.2
c = 0.1
c = 0.0
Cellular_organisms – ps1
Archaea1 – ps2
Archaea2 – ps3
Archaea3 – ps4
Asgard/Eukaryota – ps5
Eukaryota – ps6
Amorpheae – ps7
Obazoa – ps8
Opisthokonta – ps9
Holozoa – ps10
Filozoa – ps11
Choano./Metazoa – ps12
Metazoa – ps13
Myriazoa – ps14
Parahoxozoa – ps15
Bilateria – ps16
Protostomia – ps17
Ecdysozoa – ps18
Arthropoda – ps19
Mandibulata – ps20
Pancrustacea – ps21
Insecta – ps22
Eumetabola – ps23
Holometabola/Psocodea – ps24
Holometabola – ps25
Aparaglossata – ps26
Coleoptera – ps27
Cucujiformia – ps28
Tribolium_castaneum – ps29
Phylostratum

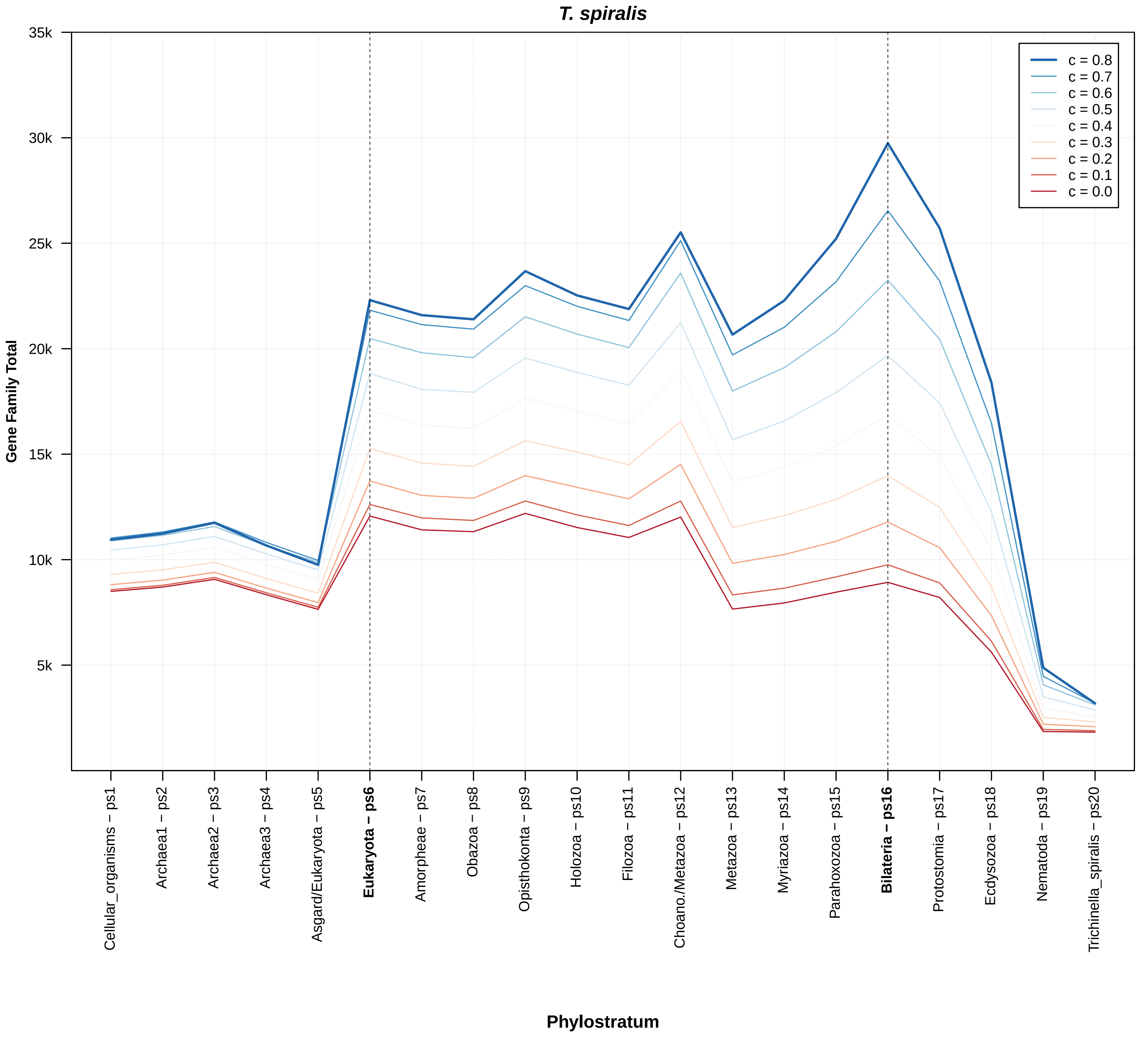

T. spiralis
Gene Family Total
35k
30k
25k
20k
15k
10k
5k
c = 0.8
c = 0.7
c = 0.6
c = 0.5
c = 0.4
c = 0.3
c = 0.2
c = 0.1
c = 0.0
Cellular_organisms – ps1
Archaea1 – ps2
Archaea2 – ps3
Archaea3 – ps4
Asgard/Eukaryota – ps5
Eukaryota – ps6
Amorpheae – ps7
Obazoa – ps8
Opisthokonta – ps9
Holozoa – ps10
Filozoa – ps11
Choano./Metazoa – ps12
Metazoa – ps13
Myriazoa – ps14
Parahoxozoa – ps15
Bilateria – ps16
Protostomia – ps17
Ecdysozoa – ps18
Nematoda – ps19
Trichinella_spiralis – ps20
Phylostratum

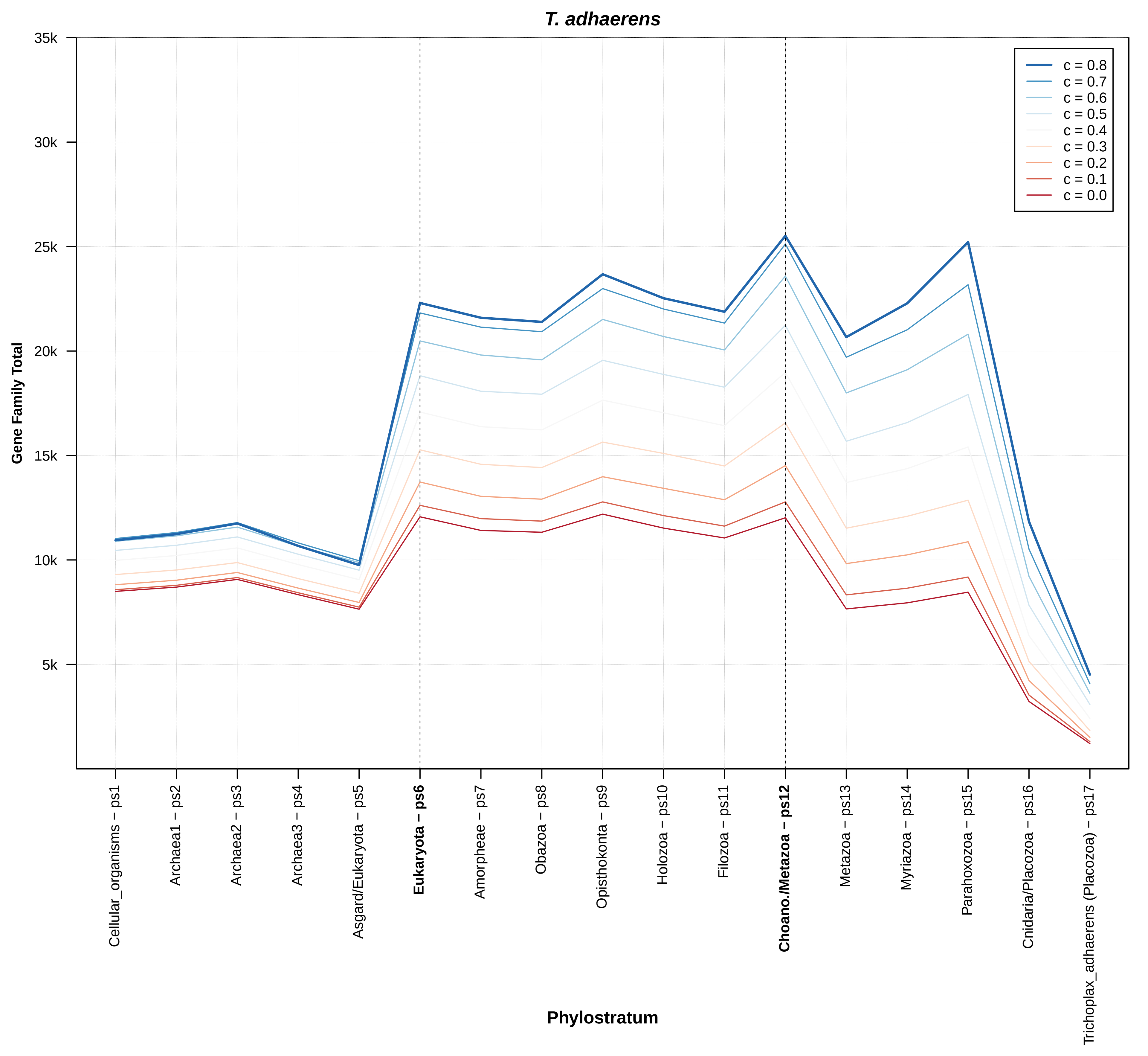

T. adhaerens
Gene Family Total
35k
30k
25k
20k
15k
10k
5k
c = 0.8
c = 0.7
c = 0.6
c = 0.5
c = 0.4
c = 0.3
c = 0.2
c = 0.1
c = 0.0
Cellular_organisms – ps1
Archaea1 – ps2
Archaea2 – ps3
Archaea3 – ps4
Asgard/Eukaryota – ps5
Eukaryota – ps6
Amorpheae – ps7
Obazoa – ps8
Opisthokonta – ps9
Holozoa – ps10
Filozoa – ps11
Choano./Metazoa – ps12
Metazoa – ps13
Myriazoa – ps14
Parahoxozoa – ps15
Cnidaria/Placozoa – ps16
Trichoplax_adhaerens (Placozoa) – ps17
Phylostratum

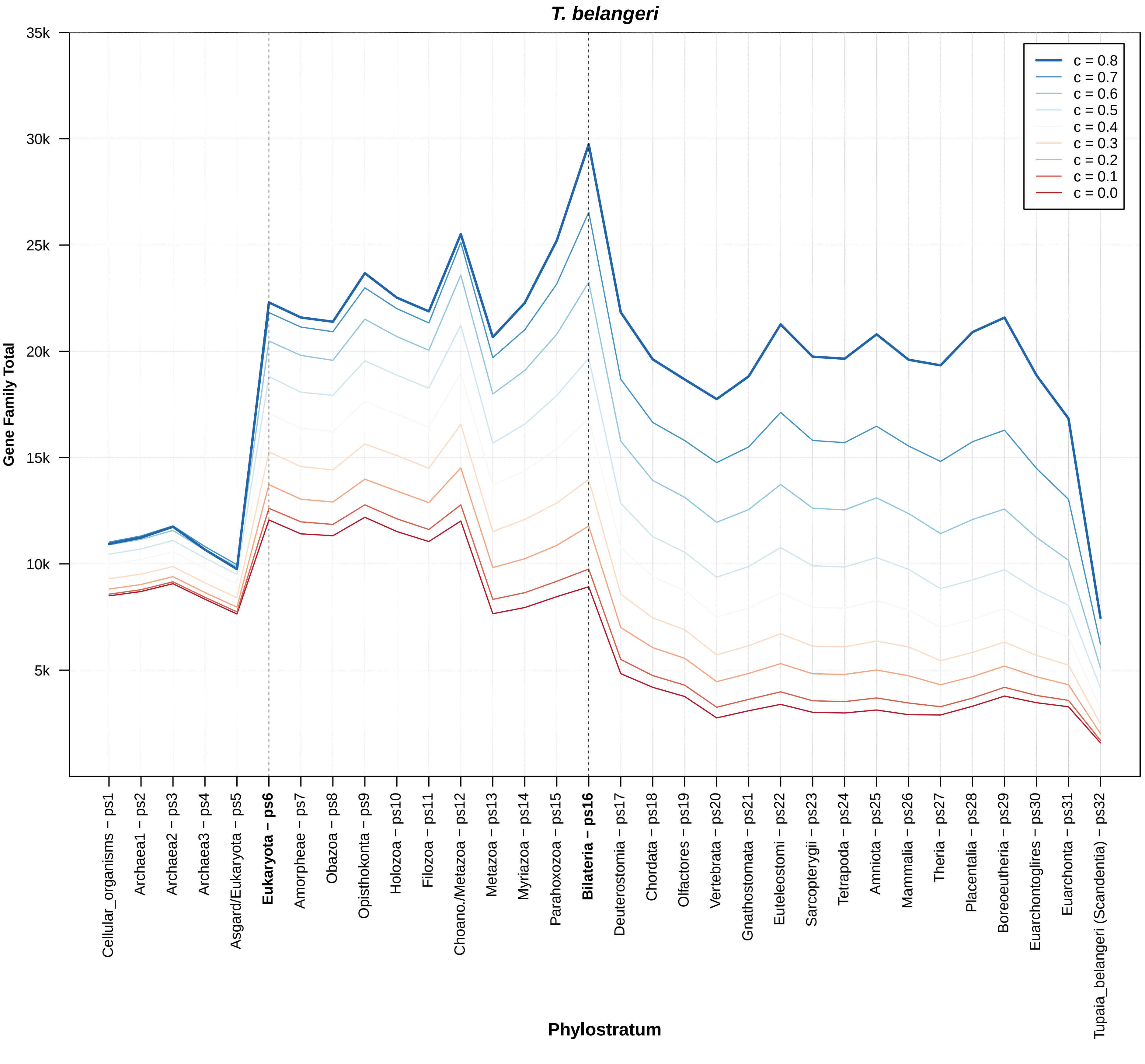

T. belangeri
35k
30k
25k
20k
15k
10k
5k
Gene Family Total
c = 0.8
c = 0.7
c = 0.6
c = 0.5
c = 0.4
c = 0.3
c = 0.2
c = 0.1
c = 0.0
Cellular_organisms – ps1
Archaea1 – ps2
Archaea2 – ps3
Archaea3 – ps4
Asgard/Eukaryota – ps5
Eukaryota – ps6
Amorpheae – ps7
Obazoa – ps8
Opisthokonta – ps9
Holozoa – ps10
Filozoa – ps11
Choano./Metazoa – ps12
Metazoa – ps13
Myriazoa – ps14
Parahoxozoa – ps15
Bilateria – ps16
Deuterostomia – ps17
Chordata – ps18
Olfactores – ps19
Vertebrata – ps20
Gnathostomata – ps21
Euteleostomi – ps22
Sarcopterygii – ps23
Tetrapoda – ps24
Amniota – ps25
Mammalia – ps26
Theria – ps27
Placentalia – ps28
Boreoeutheria – ps29
Euarchontoglires – ps30
Euarchonta – ps31
Tupaia_belangeri (Scandentia) – ps32
Phylostratum

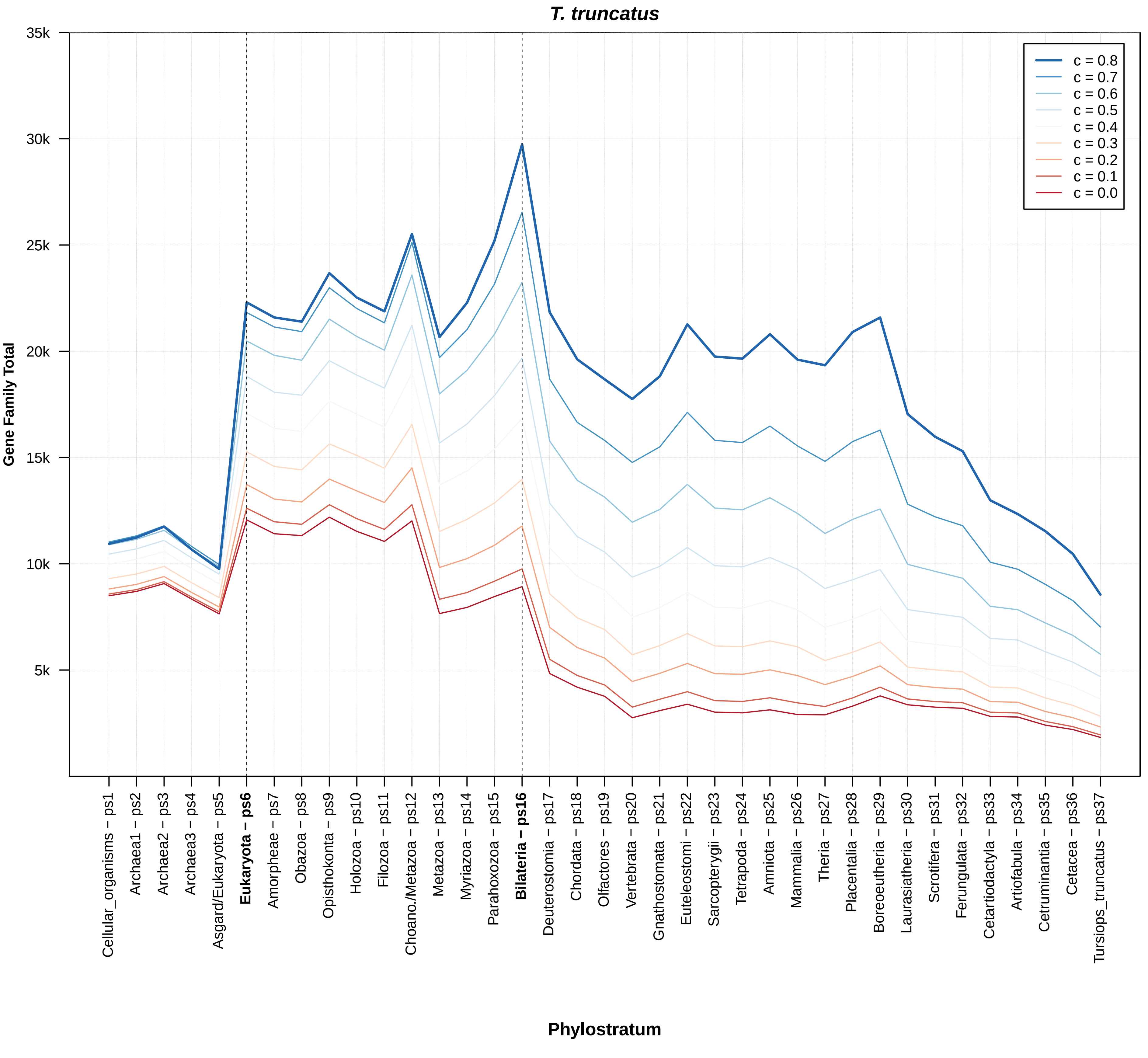
T. truncatus
Gene Family Total
35k
30k
25k
20k
15k
10k
5k
c = 0.8
c = 0.7
c = 0.6
c = 0.5
c = 0.4
c = 0.3
c = 0.2
c = 0.1
c = 0.0
Cellular_organisms – ps1
Archaea1 – ps2
Archaea2 – ps3
Archaea3 – ps4
Asgard/Eukaryota – ps5
Eukaryota – ps6
Amorpheae – ps7
Obazoa – ps8
Opisthokonta – ps9
Holozoa – ps10
Filozoa – ps11
Choano./Metazoa – ps12
Metazoa – ps13
Myriazoa – ps14
Parahoxozoa – ps15
Bilateria – ps16
Deuterostomia – ps17
Chordata – ps18
Olfactores – ps19
Vertebrata – ps20
Gnathostomata – ps21
Euteleostomi – ps22
Sarcopterygii – ps23
Tetrapoda – ps24
Amniota – ps25
Mammalia – ps26
Theria – ps27
Placentalia – ps28
Boreoeutheria – ps29
Laurasiatheria – ps30
Scrotifera – ps31
Ferungulata – ps32
Cetartiodactyla – ps33
Artiofabula – ps34
Cetruminantia – ps35
Cetacea – ps36
Tursiops_truncatus – ps37
Phylostratum

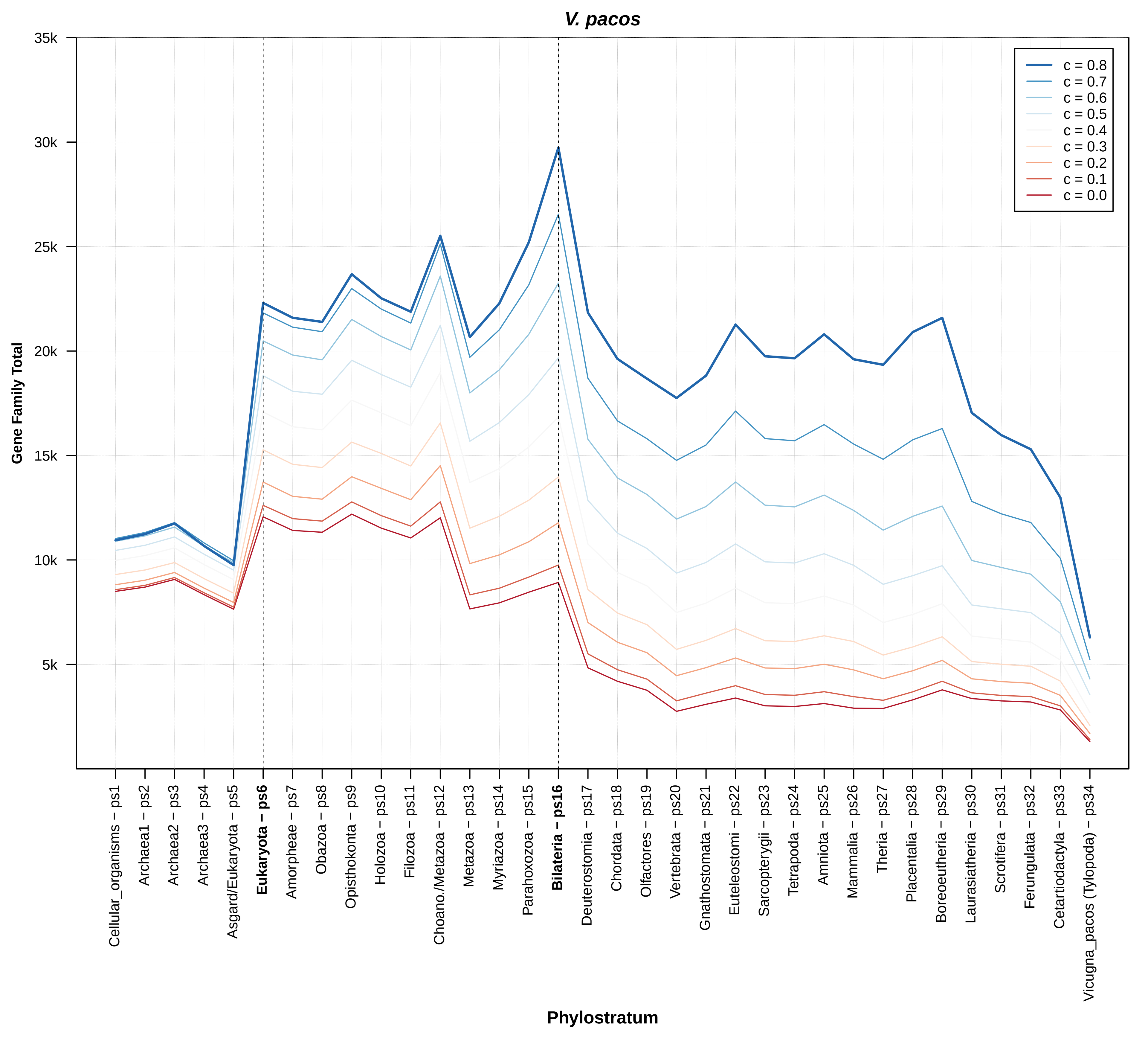

V. pacos
Gene Family Total
Phylostratum
35k
30k
25k
20k
15k
10k
5k
c = 0.8
c = 0.7
c = 0.6
c = 0.5
c = 0.4
c = 0.3
c = 0.2
c = 0.1
c = 0.0
Cellular_organisms – ps1
Archaea1 – ps2
Archaea2 – ps3
Archaea3 – ps4
Asgard/Eukaryota – ps5
Eukaryota – ps6
Amorpheae – ps7
Obazoa – ps8
Opisthokonta – ps9
Holozoa – ps10
Filozoa – ps11
Choano./Metazoa – ps12
Metazoa – ps13
Myriazoa – ps14
Parahoxozoa – ps15
Bilateria – ps16
Deuterostomia – ps17
Chordata – ps18
Olfactores – ps19
Vertebrata – ps20
Gnathostomata – ps21
Euteleostomi – ps22
Sarcopterygii – ps23
Tetrapoda – ps24
Amniota – ps25
Mammalia – ps26
Theria – ps27
Placentalia – ps28
Boreoeutheria – ps29
Laurasiatheria – ps30
Scrotifera – ps31
Ferungulata – ps32
Cetartiodactyla – ps33
Vicugna_pacos (Tylopoda) – ps34

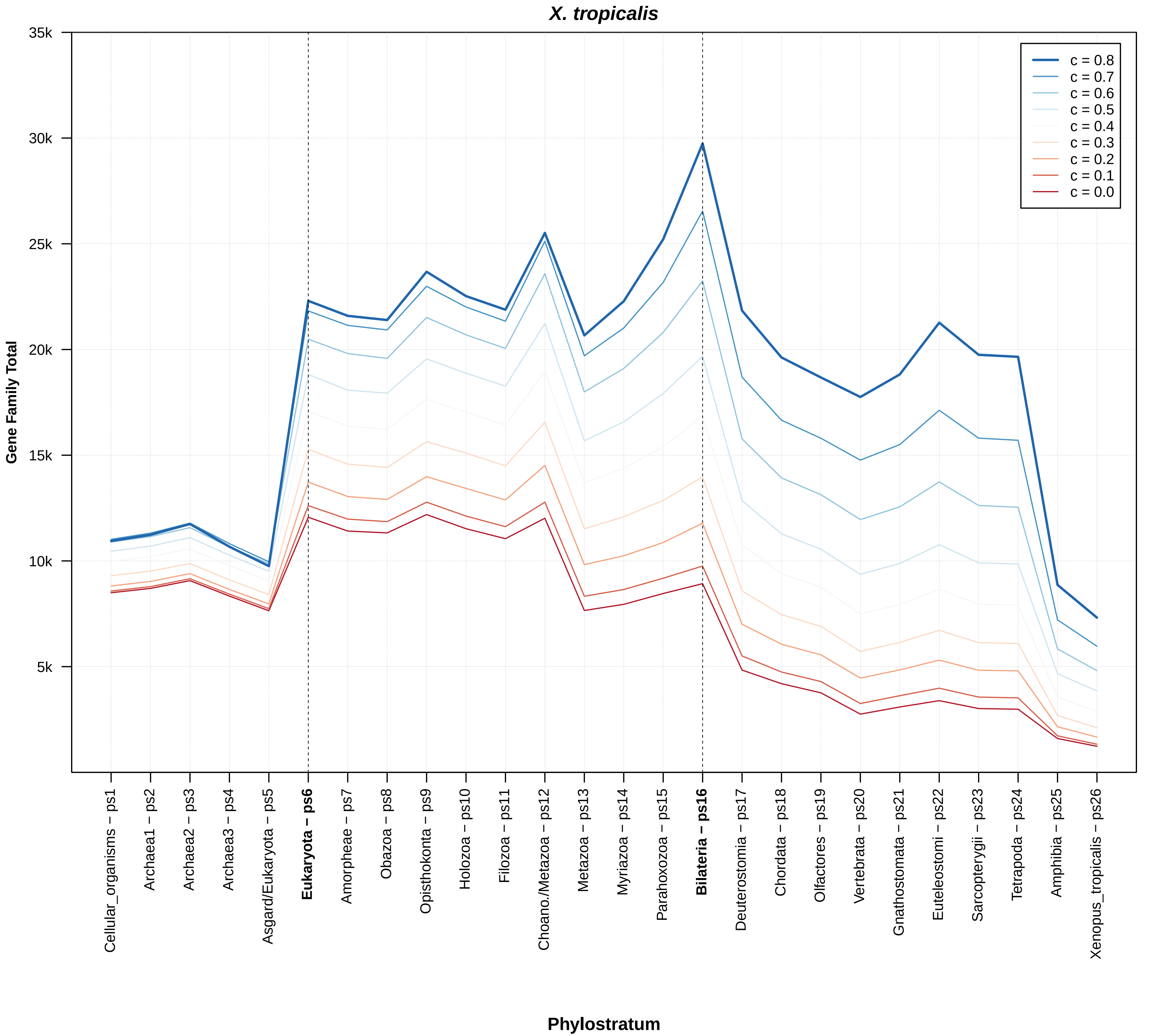

X. tropicalis
Gene Family Total
35k
30k
25k
20k
15k
10k
5k
c = 0.8
c = 0.7
c = 0.6
c = 0.5
c = 0.4
c = 0.3
c = 0.2
c = 0.1
c = 0.0
Cellular_organisms – ps1
Archaea1 – ps2
Archaea2 – ps3
Archaea3 – ps4
Asgard/Eukaryota – ps5
Eukaryota – ps6
Amorpheae – ps7
Obazoa – ps8
Opisthokonta – ps9
Holozoa – ps10
Filozoa – ps11
Choano./Metazoa – ps12
Metazoa – ps13
Myriazoa – ps14
Parahoxozoa – ps15
Bilateria – ps16
Deuterostomia – ps17
Chordata – ps18
Olfactores – ps19
Vertebrata – ps20
Gnathostomata – ps21
Euteleostomi – ps22
Sarcopterygii – ps23
Tetrapoda – ps24
Amphibia – ps25
Xenopus_tropicalis – ps26
Phylostratum

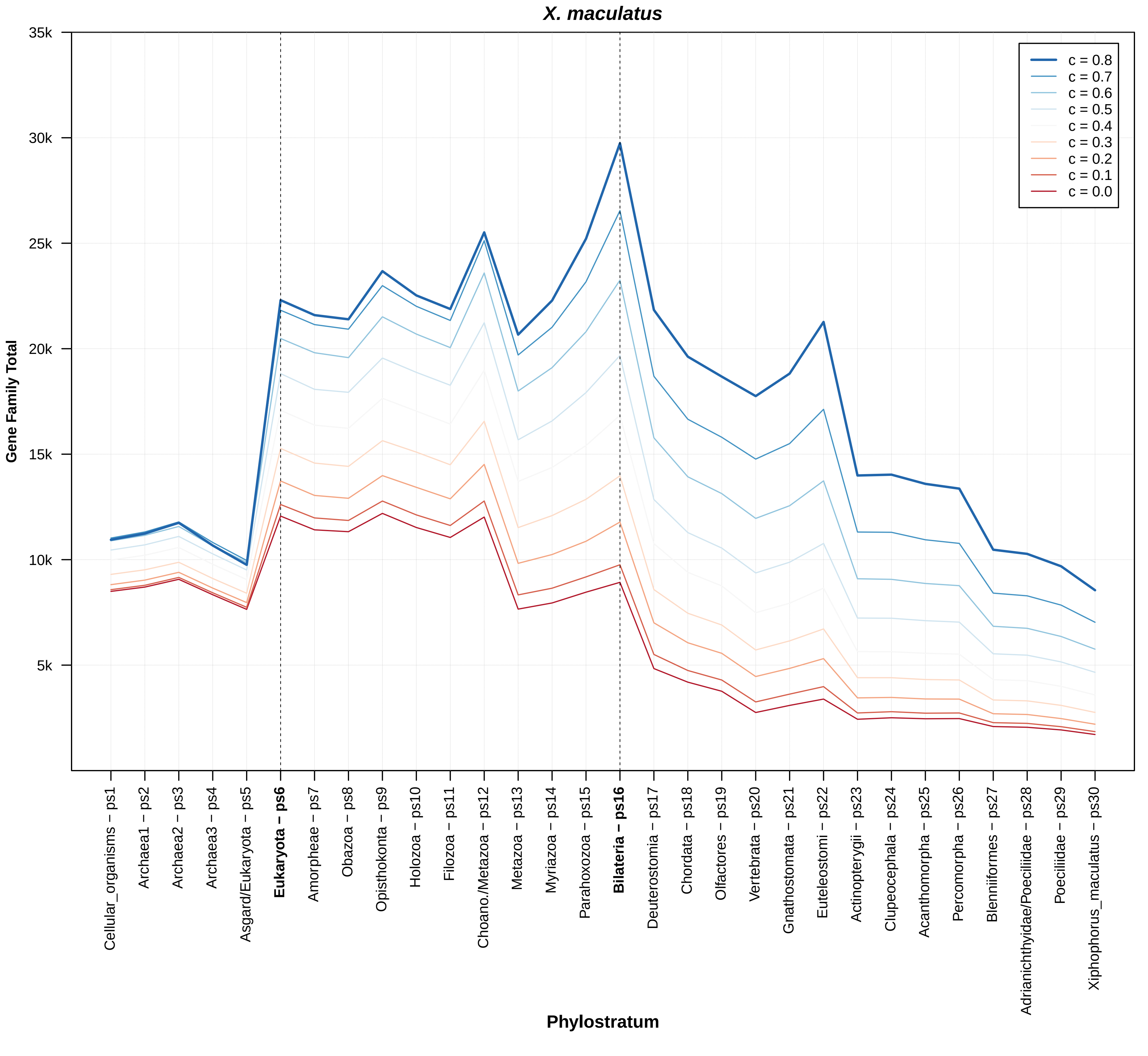

X. maculatus
Gene Family Total
Phylostratum
35k
30k
25k
20k
15k
10k
5k
c = 0.8
c = 0.7
c = 0.6
c = 0.5
c = 0.4
c = 0.3
c = 0.2
c = 0.1
c = 0.0
Cellular_organisms – ps1
Archaea1 – ps2
Archaea2 – ps3
Archaea3 – ps4
Asgard/Eukaryota – ps5
Eukaryota – ps6
Amorpheae – ps7
Obazoa – ps8
Opisthokonta – ps9
Holozoa – ps10
Filozoa – ps11
Choano./Metazoa – ps12
Metazoa – ps13
Myriazoa – ps14
Parahoxozoa – ps15
Bilateria – ps16
Deuterostomia – ps17
Chordata – ps18
Olfactores – ps19
Vertebrata – ps20
Gnathostomata – ps21
Euteleostomi – ps22
Actinopterygii – ps23
Clupeocephala – ps24
Acanthomorpha – ps25
Percomorpha – ps26
Blenniiformes – ps27
Adrianichthyidae/Poeciliidae – ps28
Poeciliidae – ps29
Xiphophorus_maculatus – ps30

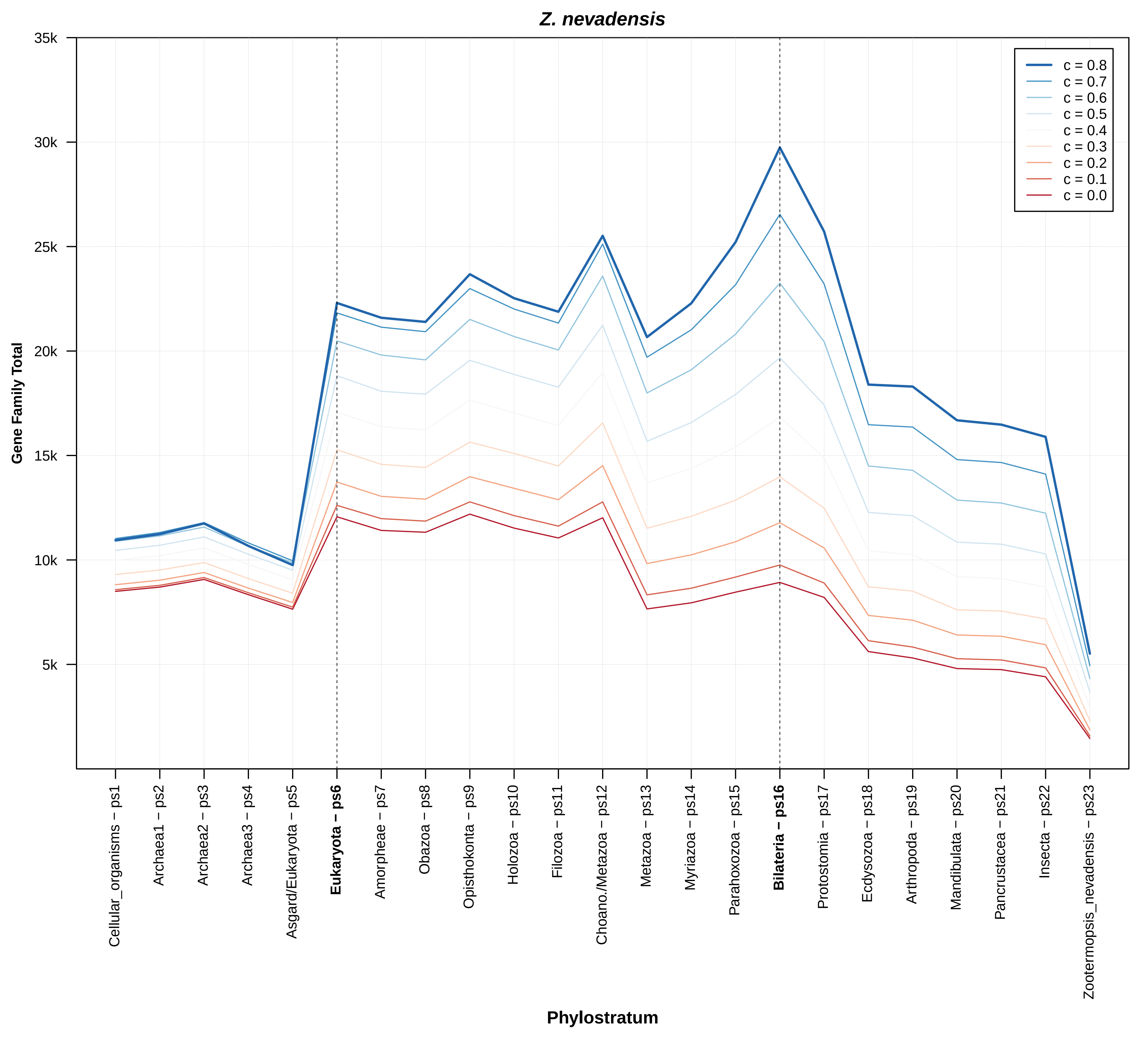

Z. nevadensis
Gene Family Total
Phylostratum
35k
30k
25k
20k
15k
10k
5k
c = 0.8
c = 0.7
c = 0.6
c = 0.5
c = 0.4
c = 0.3
c = 0.2
c = 0.1
c = 0.0
Cellular_organisms – ps1
Archaea1 – ps2
Archaea2 – ps3
Archaea3 – ps4
Asgard/Eukaryota – ps5
Eukaryota – ps6
Amorpheae – ps7
Obazoa – ps8
Opisthokonta – ps9
Holozoa – ps10
Filozoa – ps11
Choano./Metazoa – ps12
Metazoa – ps13
Myriazoa – ps14
Parahoxozoa – ps15
Bilateria – ps16
Protostomia – ps17
Ecdysozoa – ps18
Arthropoda – ps19
Mandibulata – ps20
Pancrustacea – ps21
Insecta – ps22
Zootermopsis_nevadensis – ps23

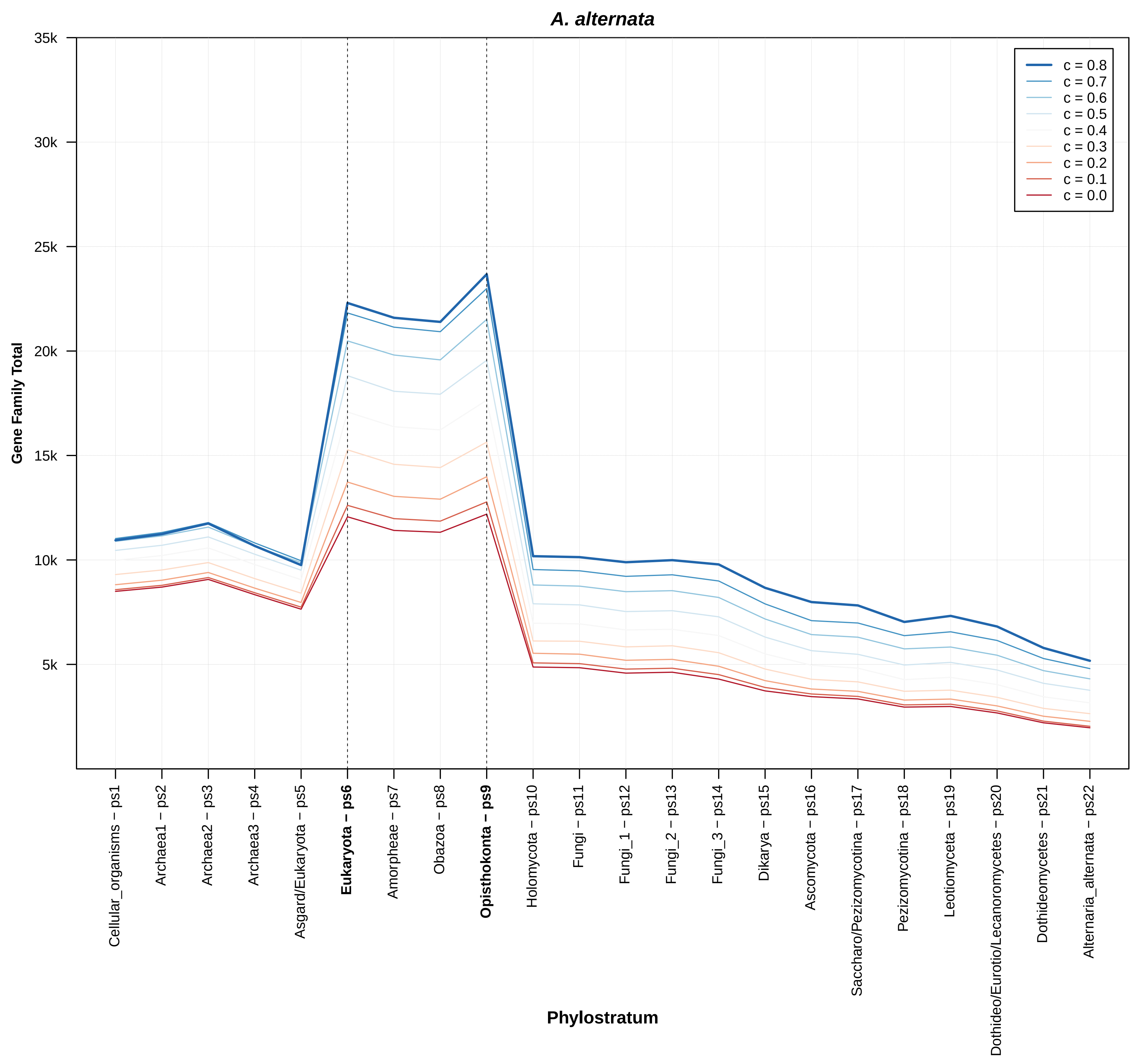
A. alternata
Gene Family Total
35k
30k
25k
20k
15k
10k
5k
c = 0.8
c = 0.7
c = 0.6
c = 0.5
c = 0.4
c = 0.3
c = 0.2
c = 0.1
c = 0.0
Cellular_organisms – ps1
Archaea1 – ps2
Archaea2 – ps3
Archaea3 – ps4
Asgard/Eukaryota – ps5
Eukaryota – ps6
Amorpheae – ps7
Obazoa – ps8
Opisthokonta – ps9
Holomycota – ps10
Fungi – ps11
Fungi_1 – ps12
Fungi_2 – ps13
Fungi_3 – ps14
Dikarya – ps15
Ascomycota – ps16
Saccharo/Pezizomycotina – ps17
Pezizomycotina – ps18
Leotiomyceta – ps19
Dothideo/Eurotio/Lecanoromycetes – ps20
Dothideomycetes – ps21
Alternaria_alternata – ps22
Phylostratum

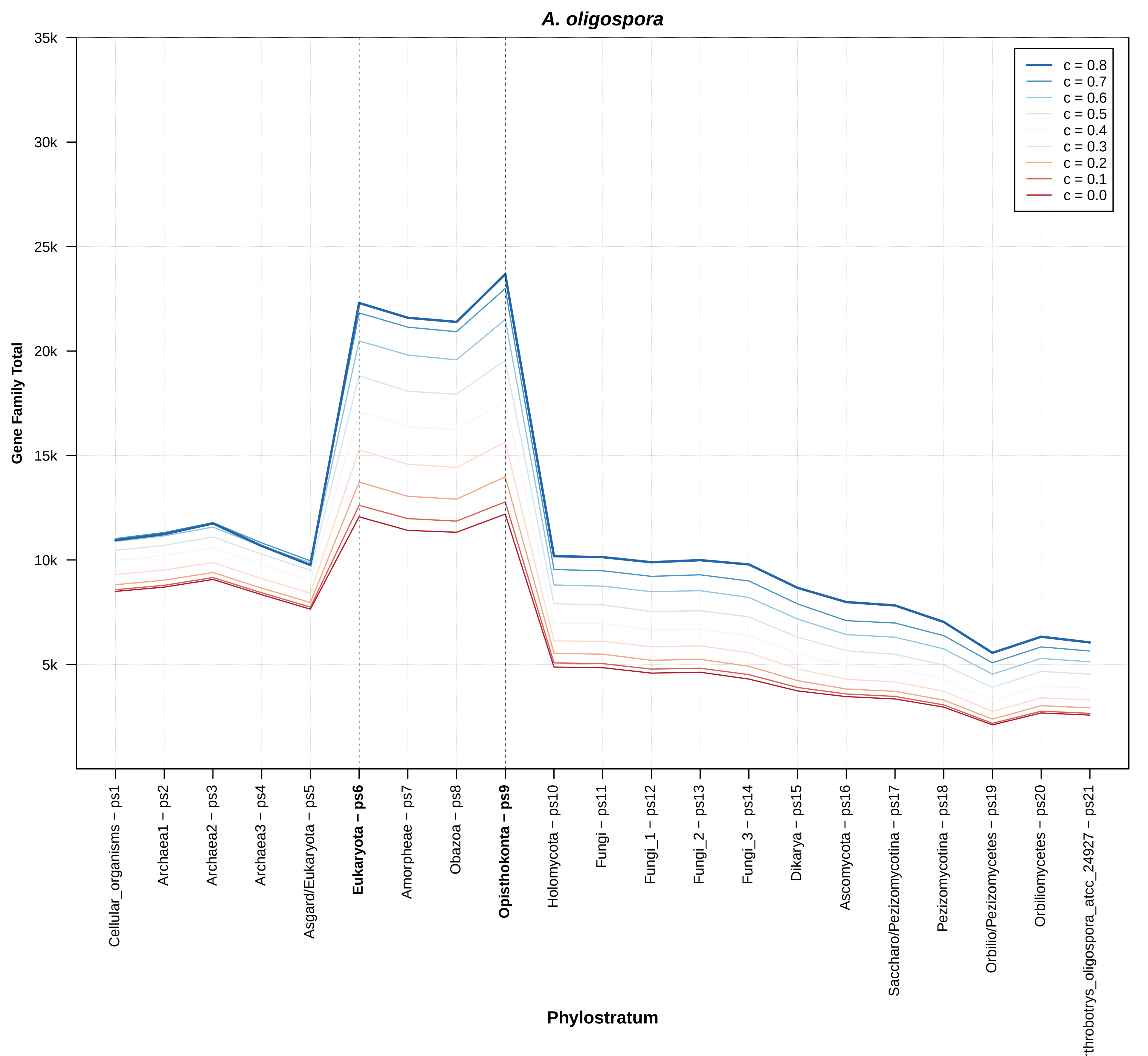

A. oligospora
35k
30k
25k
20k
15k
10k
5k
Gene Family Total
c = 0.8
c = 0.7
c = 0.6
c = 0.5
c = 0.4
c = 0.3
c = 0.2
c = 0.1
c = 0.0
Cellular_organisms – ps1
Archaea1 – ps2
Archaea2 – ps3
Archaea3 – ps4
Asgard/Eukaryota – ps5
Eukaryota – ps6
Amorpheae – ps7
Obazoa – ps8
Opisthokonta – ps9
Holomycota – ps10
Fungi – ps11
Fungi_1 – ps12
Fungi_2 – ps13
Fungi_3 – ps14
Dikarya – ps15
Ascomycota – ps16
Saccharo/Pezizomycotina – ps17
Pezizomycotina – ps18
Orbilio/Pezizomycetes – ps19
Orbiliomycetes – ps20
rthrobotrys_oligospora_atcc_24927 – ps21
Phylostratum

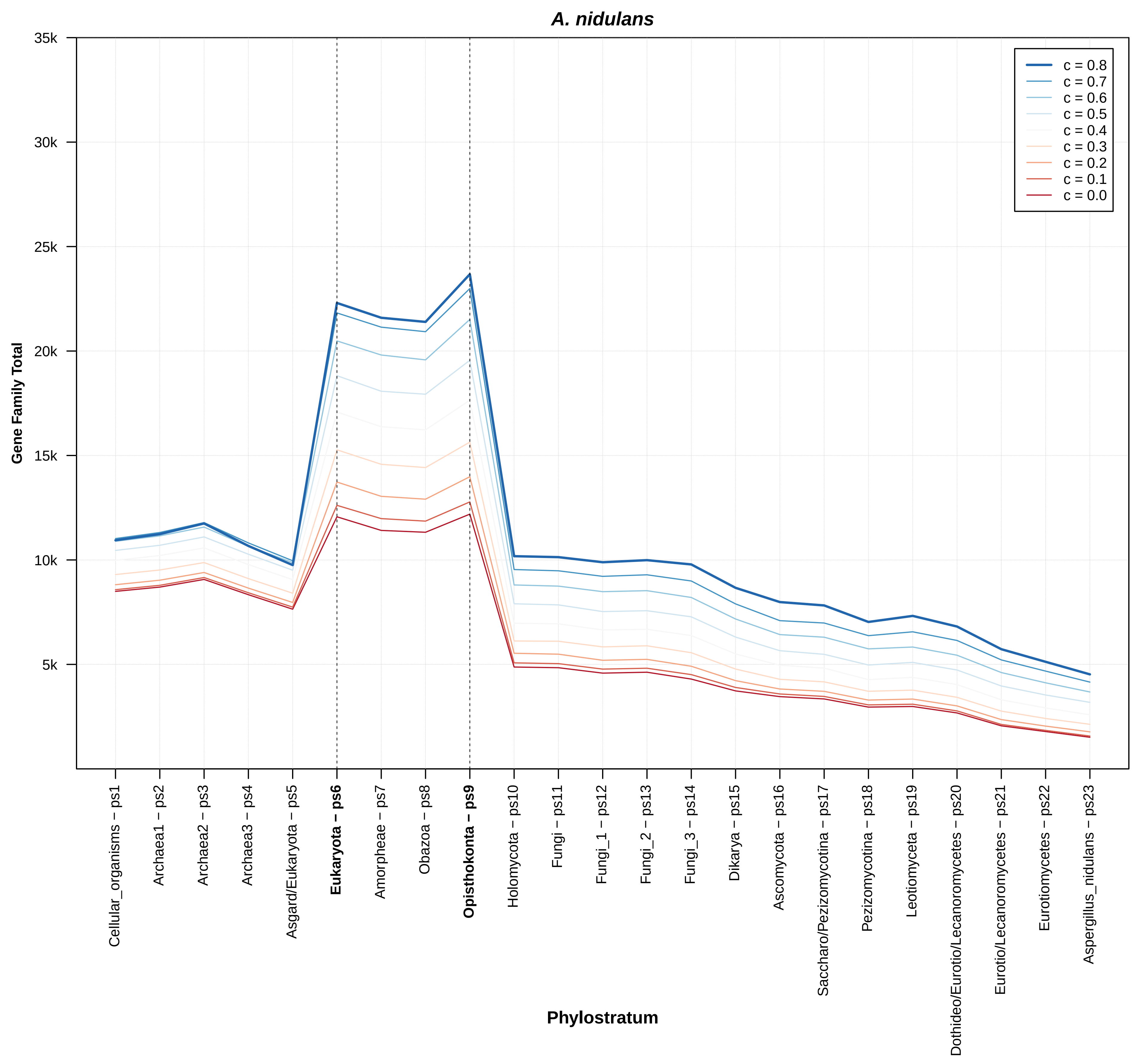
A. nidulans
Gene Family Total
35k
30k
25k
20k
15k
10k
5k
c = 0.8
c = 0.7
c = 0.6
c = 0.5
c = 0.4
c = 0.3
c = 0.2
c = 0.1
c = 0.0
Cellular_organisms – ps1
Archaea1 – ps2
Archaea2 – ps3
Archaea3 – ps4
Asgard/Eukaryota – ps5
Eukaryota – ps6
Amorpheae – ps7
Obazoa – ps8
Opisthokonta – ps9
Holomycota – ps10
Fungi – ps11
Fungi_1 – ps12
Fungi_2 – ps13
Fungi_3 – ps14
Dikarya – ps15
Ascomycota – ps16
Saccharo/Pezizomycotina – ps17
Pezizomycotina – ps18
Leotiomyceta – ps19
Dothideo/Eurotio/Lecanoromycetes – ps20
Eurotio/Lecanoromycetes – ps21
Eurotiomycetes – ps22
Aspergillus_nidulans – ps23
Phylostratum

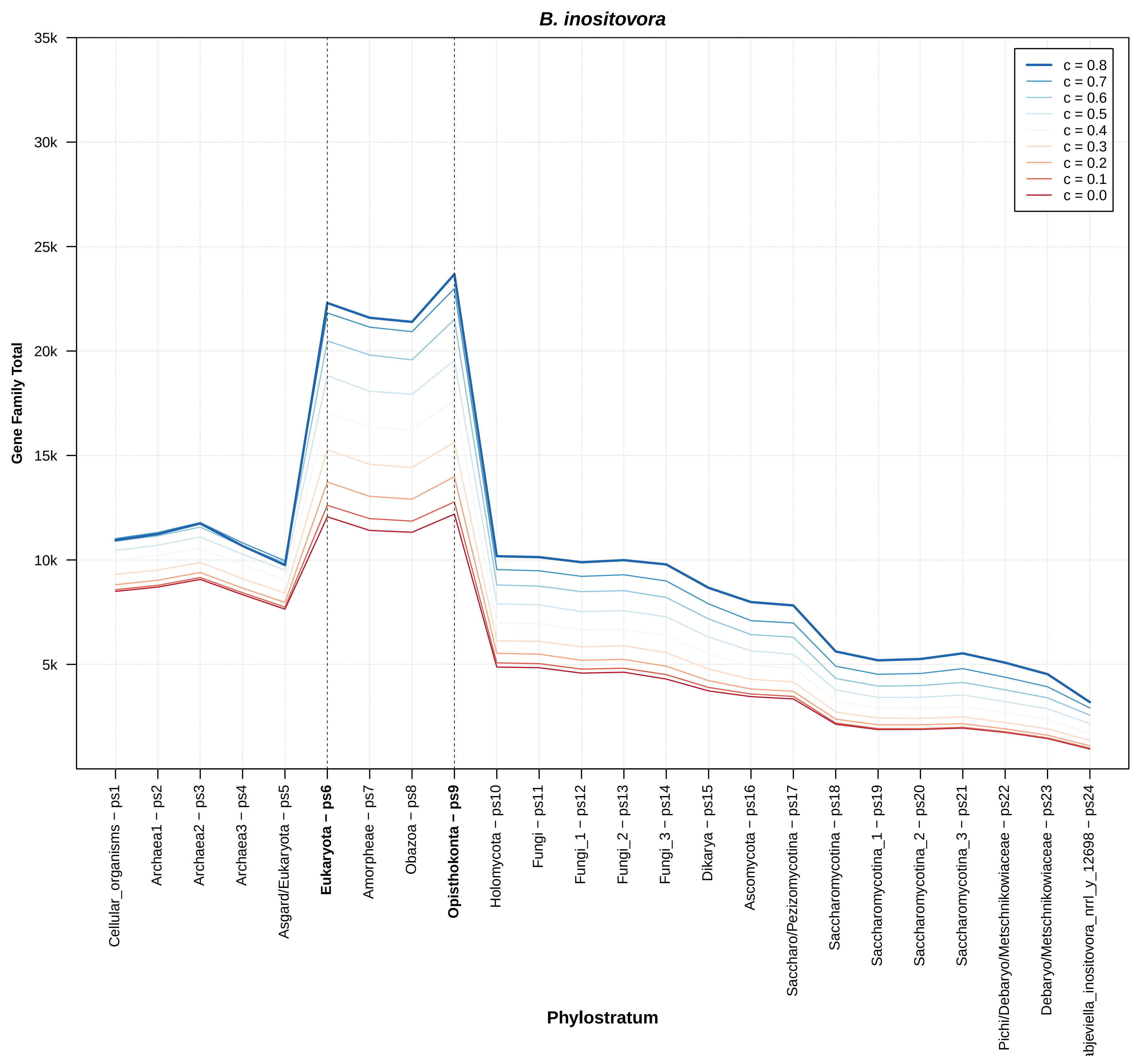
B. inositovora
Gene Family Total
35k
30k
25k
20k
15k
10k
5k
c = 0.8
c = 0.7
c = 0.6
c = 0.5
c = 0.4
c = 0.3
c = 0.2
c = 0.1
c = 0.0
Cellular_organisms – ps1
Archaea1 – ps2
Archaea2 – ps3
Archaea3 – ps4
Asgard/Eukaryota – ps5
Eukaryota – ps6
Amorpheae – ps7
Obazoa – ps8
Opisthokonta – ps9
Holomycota – ps10
Fungi – ps11
Fungi_1 – ps12
Fungi_2 – ps13
Fungi_3 – ps14
Dikarya – ps15
Ascomycota – ps16
Saccharo/Pezizomycotina – ps17
Saccharomycotina – ps18
Saccharomycotina_1 – ps19
Saccharomycotina_2 – ps20
Saccharomycotina_3 – ps21
Pichi/Debaryo/Metschnikowiaceae – ps22
Debaryo/Metschnikowiaceae – ps23
abjeviella_inositovora_nrrl_y_12698 – ps24
Phylostratum

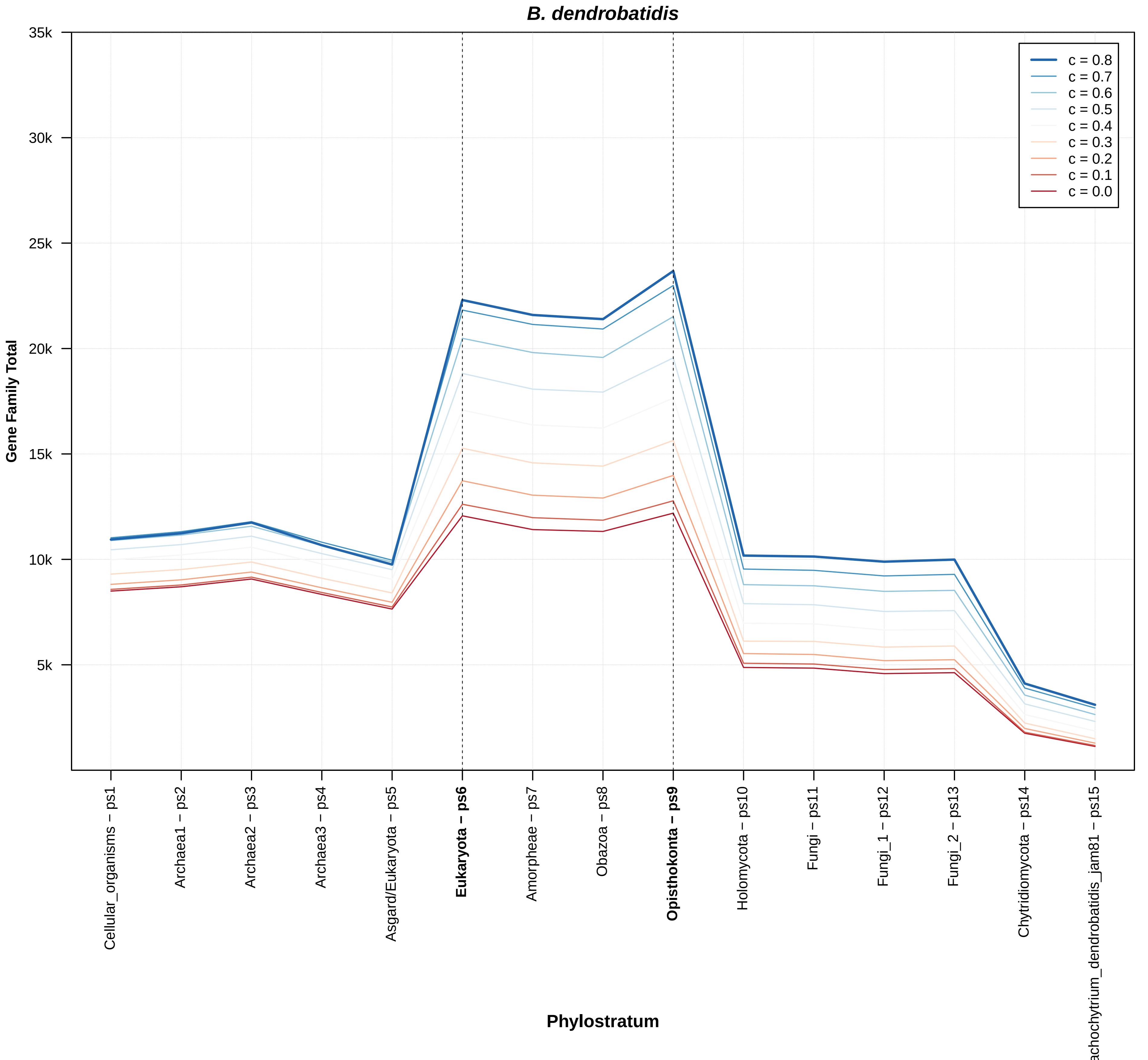

B. dendrobatidis
Gene Family Total
35k
30k
25k
20k
15k
10k
5k
c = 0.8
c = 0.7
c = 0.6
c = 0.5
c = 0.4
c = 0.3
c = 0.2
c = 0.1
c = 0.0
Cellular_organisms – ps1
Archaea1 – ps2
Archaea2 – ps3
Archaea3 – ps4
Asgard/Eukaryota – ps5
Eukaryota – ps6
Amorpheae – ps7
Obazoa – ps8
Opisthokonta – ps9
Holomycota – ps10
Fungi – ps11
Fungi_1 – ps12
Fungi_2 – ps13
Chytridiomycota – ps14
achochytrium_dendrobatidis_jam81 – ps15
Phylostratum

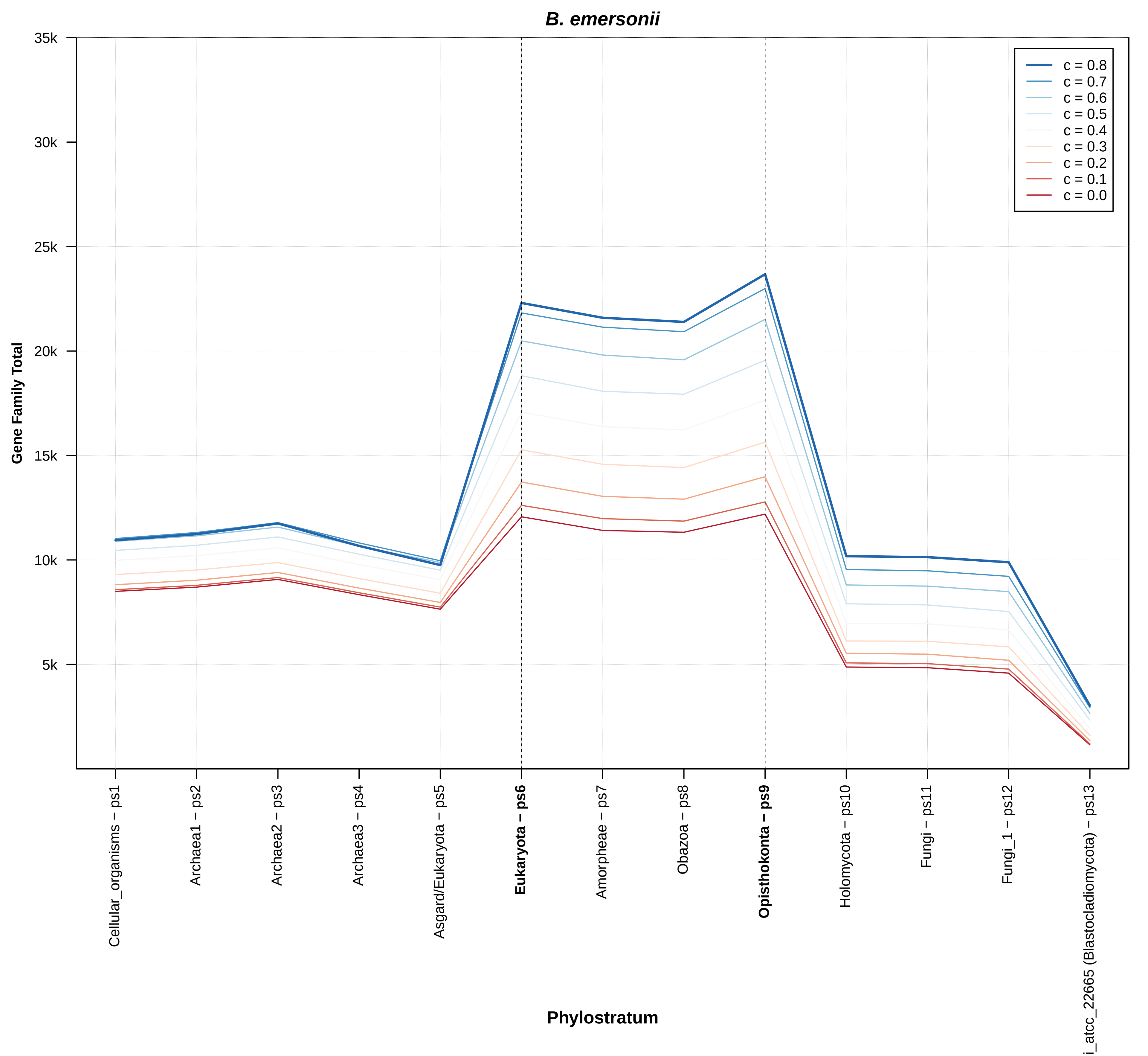
B. emersonii
35k
30k
25k
20k
15k
10k
5k
Gene Family Total
c = 0.8
c = 0.7
c = 0.6
c = 0.5
c = 0.4
c = 0.3
c = 0.2
c = 0.1
c = 0.0
Cellular_organisms – ps1
Archaea1 – ps2
Archaea2 – ps3
Archaea3 – ps4
Asgard/Eukaryota – ps5
Eukaryota – ps6
Amorpheae – ps7
Obazoa – ps8
Opisthokonta – ps9
Holomycota – ps10
Fungi – ps11
Fungi_1 – ps12
i_atcc_22665 (Blastocladiomycota) – ps13
Phylostratum

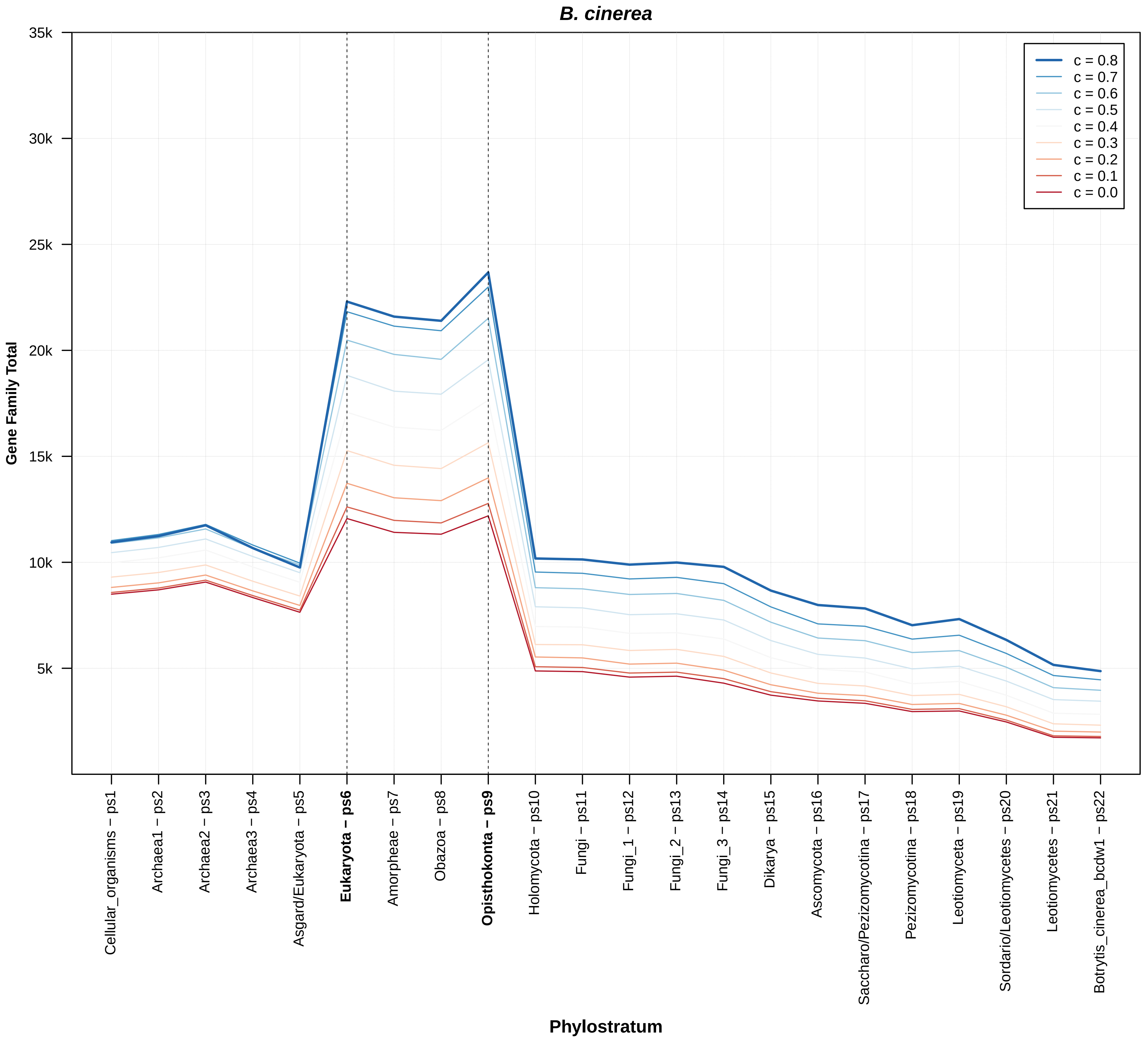

B. cinerea
35k
30k
25k
20k
15k
10k
5k
Gene Family Total
c = 0.8
c = 0.7
c = 0.6
c = 0.5
c = 0.4
c = 0.3
c = 0.2
c = 0.1
c = 0.0
Cellular_organisms – ps1
Archaea1 – ps2
Archaea2 – ps3
Archaea3 – ps4
Asgard/Eukaryota – ps5
Eukaryota – ps6
Amorpheae – ps7
Obazoa – ps8
Opisthokonta – ps9
Holomycota – ps10
Fungi – ps11
Fungi_1 – ps12
Fungi_2 – ps13
Fungi_3 – ps14
Dikarya – ps15
Ascomycota – ps16
Saccharo/Pezizomycotina – ps17
Pezizomycotina – ps18
Leotiomyceta – ps19
Sordario/Leotiomycetes – ps20
Leotiomycetes – ps21
Botrytis_cinerea_bcdw1 – ps22
Phylostratum

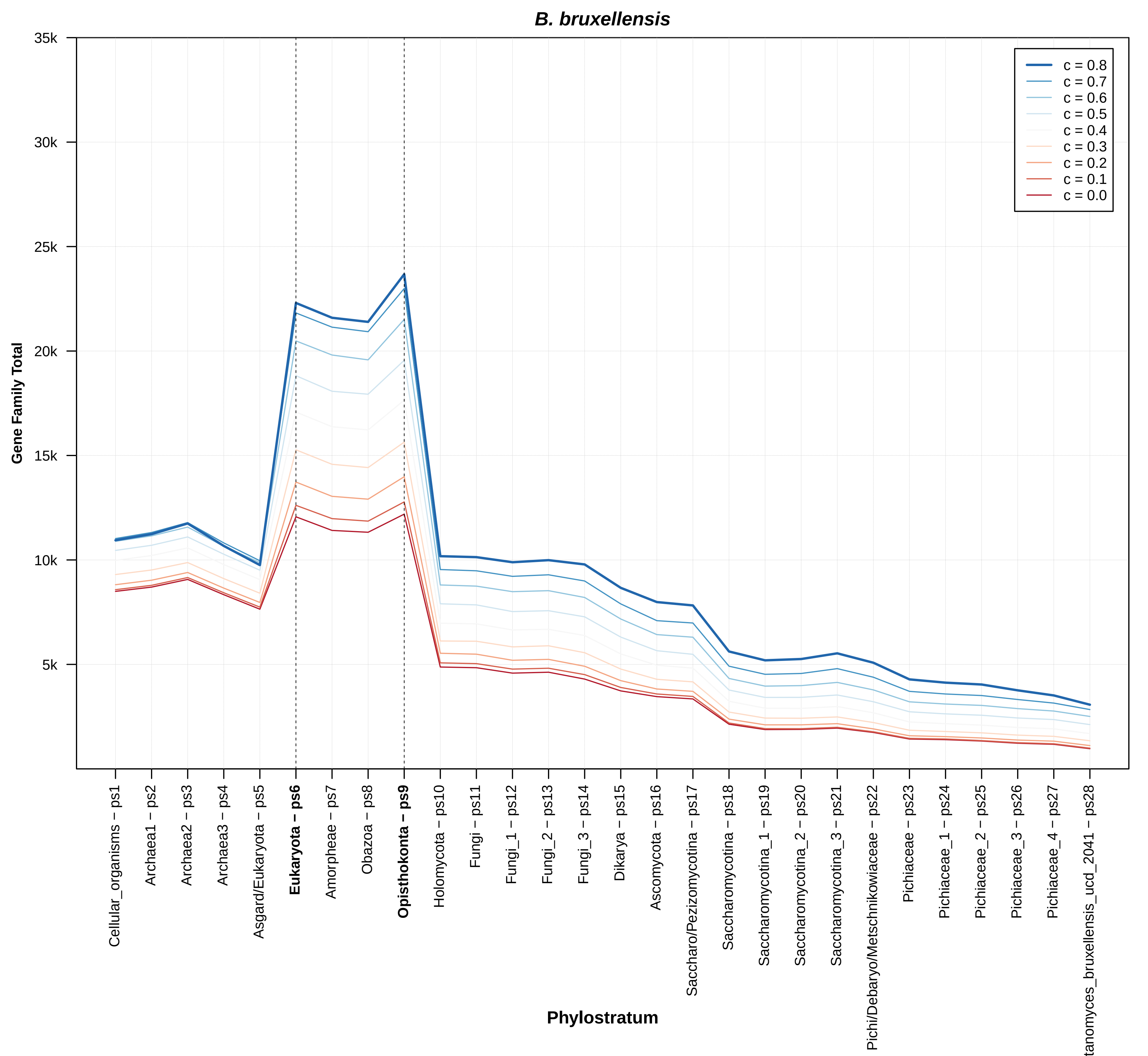

B. bruxellensis
Gene Family Total
35k
30k
25k
20k
15k
10k
5k
c = 0.8
c = 0.7
c = 0.6
c = 0.5
c = 0.4
c = 0.3
c = 0.2
c = 0.1
c = 0.0
Cellular_organisms – ps1
Archaea1 – ps2
Archaea2 – ps3
Archaea3 – ps4
Asgard/Eukaryota – ps5
Eukaryota – ps6
Amorpheae – ps7
Obazoa – ps8
Opisthokonta – ps9
Holomycota – ps10
Fungi – ps11
Fungi_1 – ps12
Fungi_2 – ps13
Fungi_3 – ps14
Dikarya – ps15
Ascomycota – ps16
Saccharo/Pezizomycotina – ps17
Saccharomycotina – ps18
Saccharomycotina_1 – ps19
Saccharomycotina_2 – ps20
Saccharomycotina_3 – ps21
Pichi/Debaryo/Metschnikowiaceae – ps22
Pichiaceae – ps23
Pichiaceae_1 – ps24
Pichiaceae_2 – ps25
Pichiaceae_3 – ps26
Pichiaceae_4 – ps27
tanomyces_bruxellensis_ucd_2041 – ps28
Phylostratum

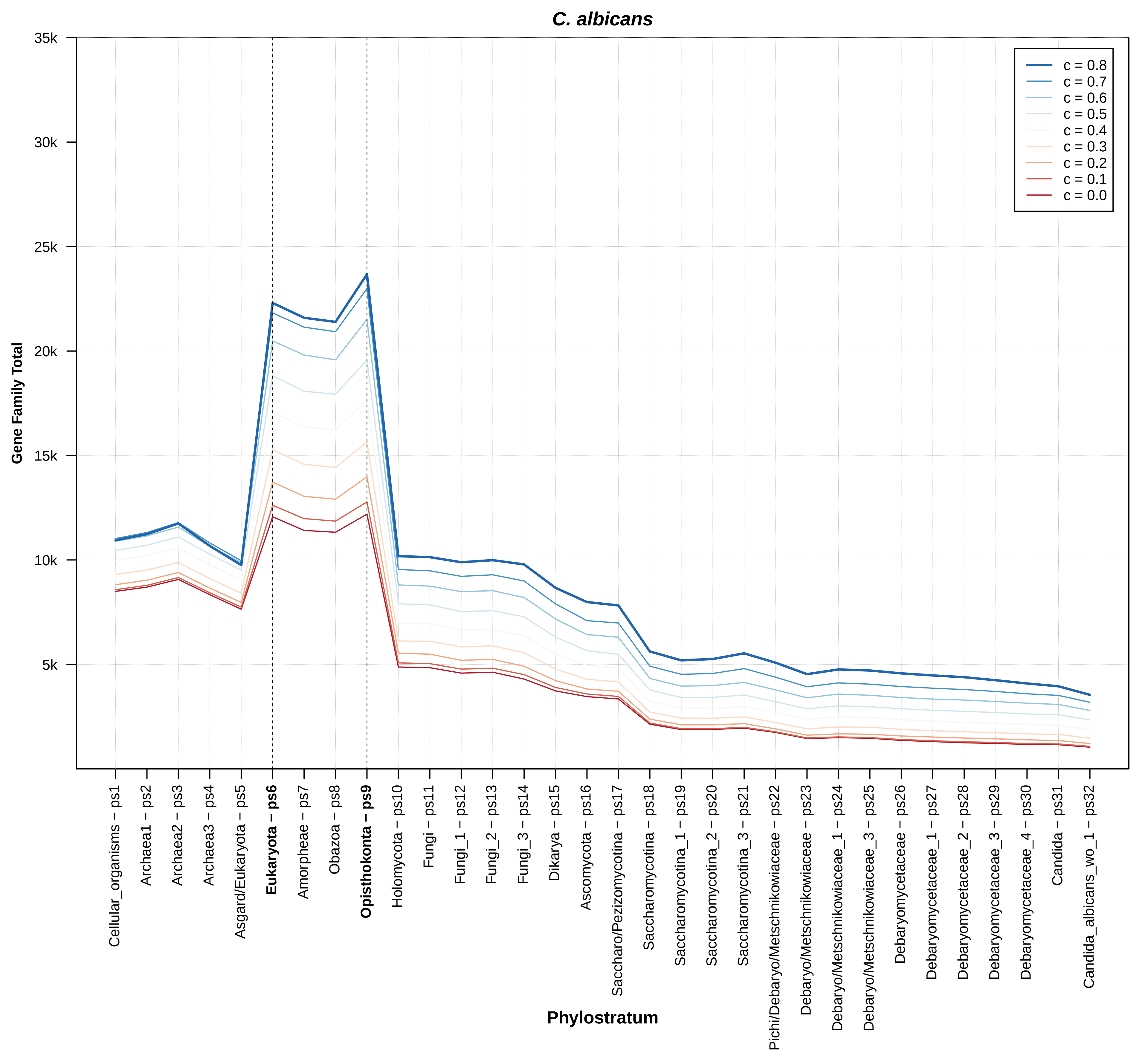

C. albicans
Gene Family Total
35k
30k
25k
20k
15k
10k
5k
c = 0.8
c = 0.7
c = 0.6
c = 0.5
c = 0.4
c = 0.3
c = 0.2
c = 0.1
c = 0.0
Cellular_organisms – ps1
Archaea1 – ps2
Archaea2 – ps3
Archaea3 – ps4
Asgard/Eukaryota – ps5
Eukaryota – ps6
Amorpheae – ps7
Obazoa – ps8
Opisthokonta – ps9
Holomycota – ps10
Fungi – ps11
Fungi_1 – ps12
Fungi_2 – ps13
Fungi_3 – ps14
Dikarya – ps15
Ascomycota – ps16
Saccharo/Pezizomycotina – ps17
Saccharomycotina – ps18
Saccharomycotina_1 – ps19
Saccharomycotina_2 – ps20
Saccharomycotina_3 – ps21
Pichi/Debaryo/Metschnikowiaceae – ps22
Debaryo/Metschnikowiaceae – ps23
Debaryo/Metschnikowiaceae_1 – ps24
Debaryo/Metschnikowiaceae_3 – ps25
Debaryomycetaceae – ps26
Debaryomycetaceae_1 – ps27
Debaryomycetaceae_2 – ps28
Debaryomycetaceae_3 – ps29
Debaryomycetaceae_4 – ps30
Candida – ps31
Candida_albicans_wo_1 – ps32
Phylostratum

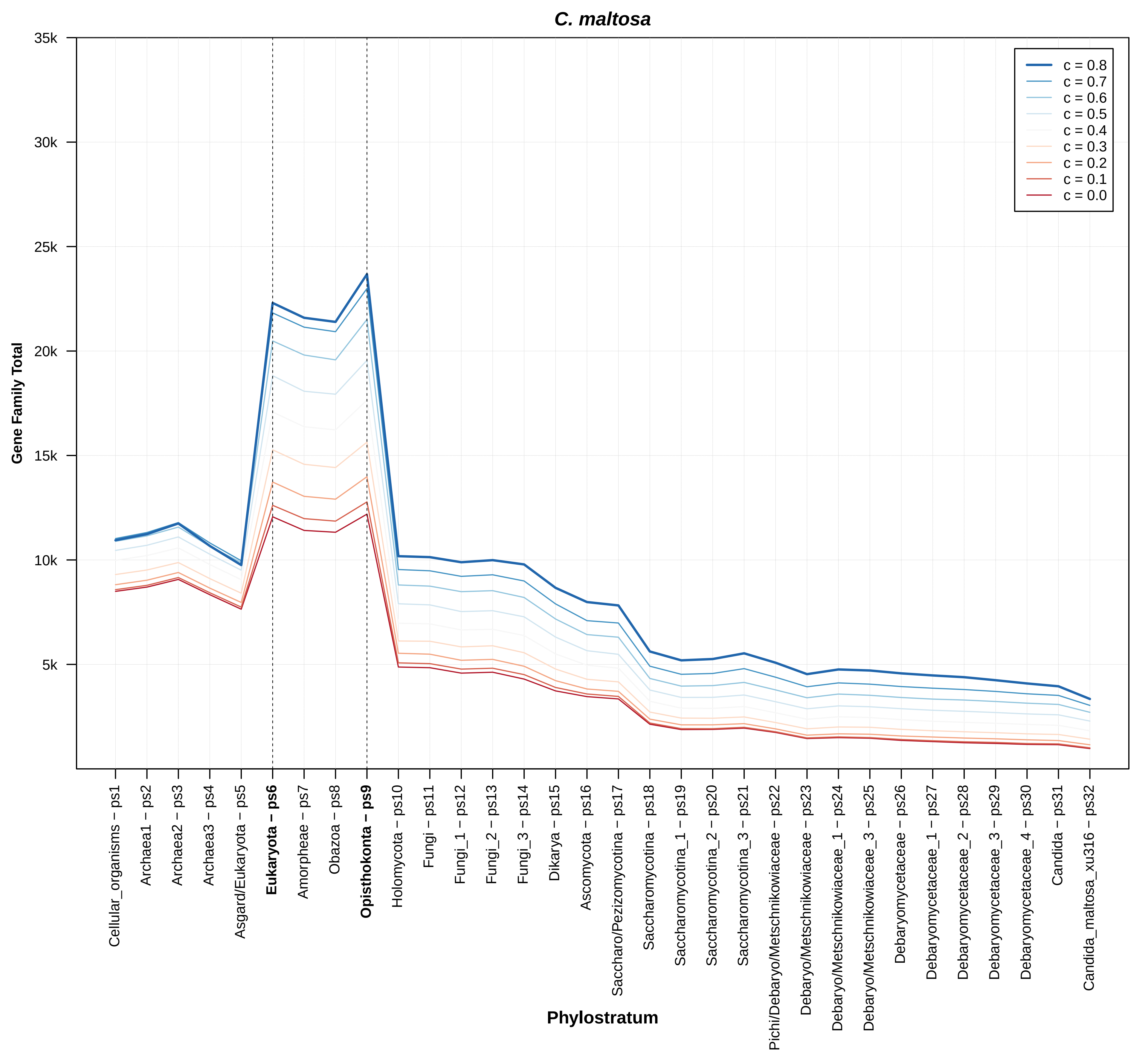

C. maltosa
Gene Family Total
35k
30k
25k
20k
15k
10k
5k
c = 0.8
c = 0.7
c = 0.6
c = 0.5
c = 0.4
c = 0.3
c = 0.2
c = 0.1
c = 0.0
Cellular_organisms – ps1
Archaea1 – ps2
Archaea2 – ps3
Archaea3 – ps4
Asgard/Eukaryota – ps5
Eukaryota – ps6
Amorpheae – ps7
Obazoa – ps8
Opisthokonta – ps9
Holomycota – ps10
Fungi – ps11
Fungi_1 – ps12
Fungi_2 – ps13
Fungi_3 – ps14
Dikarya – ps15
Ascomycota – ps16
Saccharo/Pezizomycotina – ps17
Saccharomycotina – ps18
Saccharomycotina_1 – ps19
Saccharomycotina_2 – ps20
Saccharomycotina_3 – ps21
Pichi/Debaryo/Metschnikowiaceae – ps22
Debaryo/Metschnikowiaceae – ps23
Debaryo/Metschnikowiaceae_1 – ps24
Debaryo/Metschnikowiaceae_3 – ps25
Debaryomycetaceae – ps26
Debaryomycetaceae_1 – ps27
Debaryomycetaceae_2 – ps28
Debaryomycetaceae_3 – ps29
Debaryomycetaceae_4 – ps30
Candida – ps31
Candida_maltosa_xu316 – ps32
Phylostratum

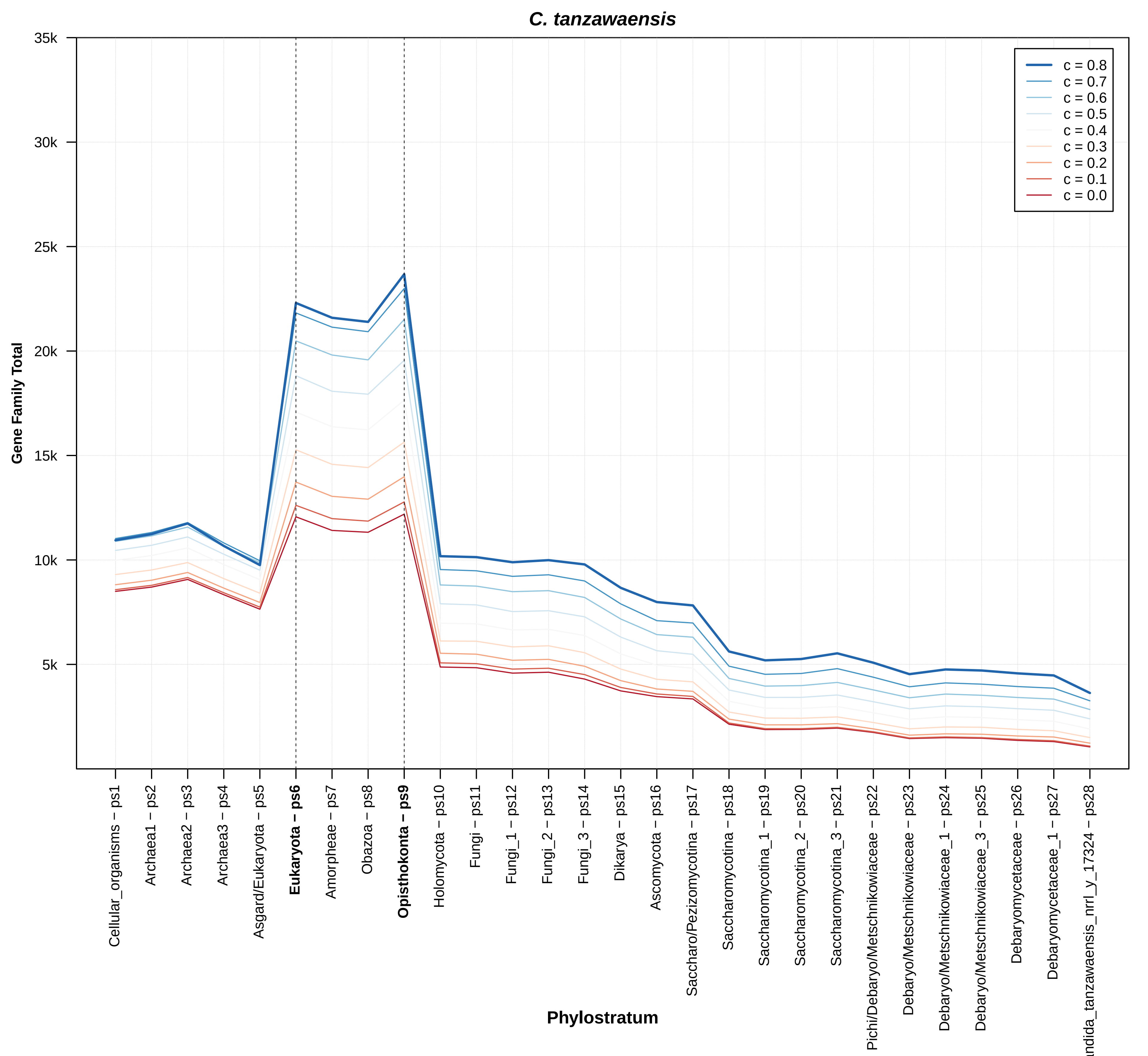

C. tanzawaensis
Gene Family Total
Phylostratum
35k
30k
25k
20k
15k
10k
5k
c = 0.8
c = 0.7
c = 0.6
c = 0.5
c = 0.4
c = 0.3
c = 0.2
c = 0.1
c = 0.0
Cellular_organisms – ps1
Archaea1 – ps2
Archaea2 – ps3
Archaea3 – ps4
Asgard/Eukaryota – ps5
Eukaryota – ps6
Amorpheae – ps7
Obazoa – ps8
Opisthokonta – ps9
Holomycota – ps10
Fungi – ps11
Fungi_1 – ps12
Fungi_2 – ps13
Fungi_3 – ps14
Dikarya – ps15
Ascomycota – ps16
Saccharo/Pezizomycotina – ps17
Saccharomycotina – ps18
Saccharomycotina_1 – ps19
Saccharomycotina_2 – ps20
Saccharomycotina_3 – ps21
Pichi/Debaryo/Metschnikowiaceae – ps22
Debaryo/Metschnikowiaceae – ps23
Debaryo/Metschnikowiaceae_1 – ps24
Debaryo/Metschnikowiaceae_3 – ps25
Debaryomycetaceae – ps26
Debaryomycetaceae_1 – ps27
ndida_tanzawaensis_nrrl_y_17324 – ps28

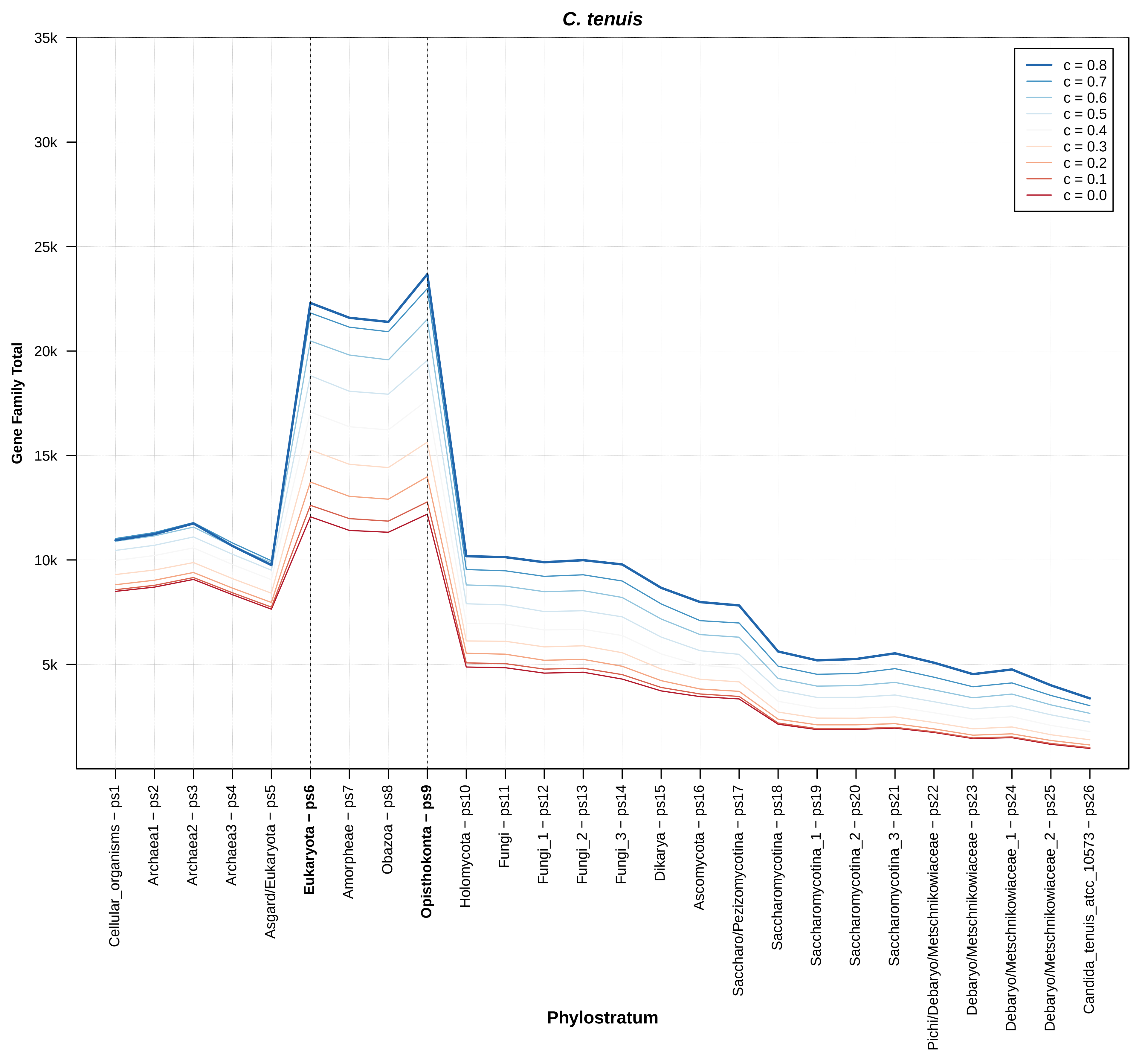
C. tenuis
Gene Family Total
35k
30k
25k
20k
15k
10k
5k
c = 0.8
c = 0.7
c = 0.6
c = 0.5
c = 0.4
c = 0.3
c = 0.2
c = 0.1
c = 0.0
Cellular_organisms – ps1
Archaea1 – ps2
Archaea2 – ps3
Archaea3 – ps4
Asgard/Eukaryota – ps5
Eukaryota – ps6
Amorpheae – ps7
Obazoa – ps8
Opisthokonta – ps9
Holomycota – ps10
Fungi – ps11
Fungi_1 – ps12
Fungi_2 – ps13
Fungi_3 – ps14
Dikarya – ps15
Ascomycota – ps16
Saccharo/Pezizomycotina – ps17
Saccharomycotina – ps18
Saccharomycotina_1 – ps19
Saccharomycotina_2 – ps20
Saccharomycotina_3 – ps21
Pichi/Debaryo/Metschnikowiaceae – ps22
Debaryo/Metschnikowiaceae – ps23
Debaryo/Metschnikowiaceae_1 – ps24
Debaryo/Metschnikowiaceae_2 – ps25
Candida_tenuis_atcc_10573 – ps26
Phylostratum

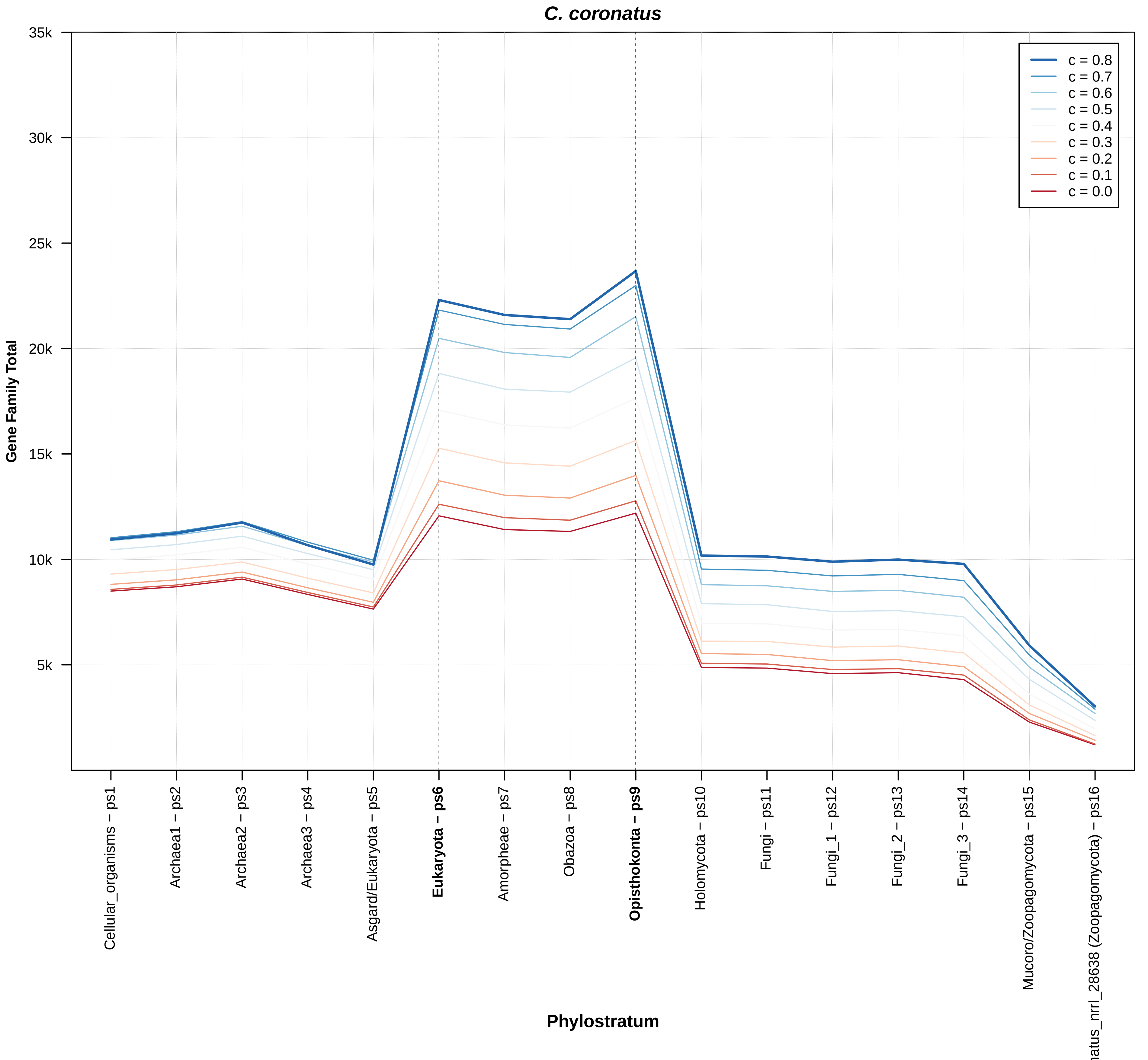
C. coronatus
Gene Family Total
35k
30k
25k
20k
15k
10k
5k
c = 0.8
c = 0.7
c = 0.6
c = 0.5
c = 0.4
c = 0.3
c = 0.2
c = 0.1
c = 0.0
Cellular_organisms – ps1
Archaea1 – ps2
Archaea2 – ps3
Archaea3 – ps4
Asgard/Eukaryota – ps5
Eukaryota – ps6
Amorpheae – ps7
Obazoa – ps8
Opisthokonta – ps9
Holomycota – ps10
Fungi – ps11
Fungi_1 – ps12
Fungi_2 – ps13
Fungi_3 – ps14
Mucoro/Zoopagomycota – ps15
atus_nrrl_28638 (Zoopagomycota) – ps16
Phylostratum

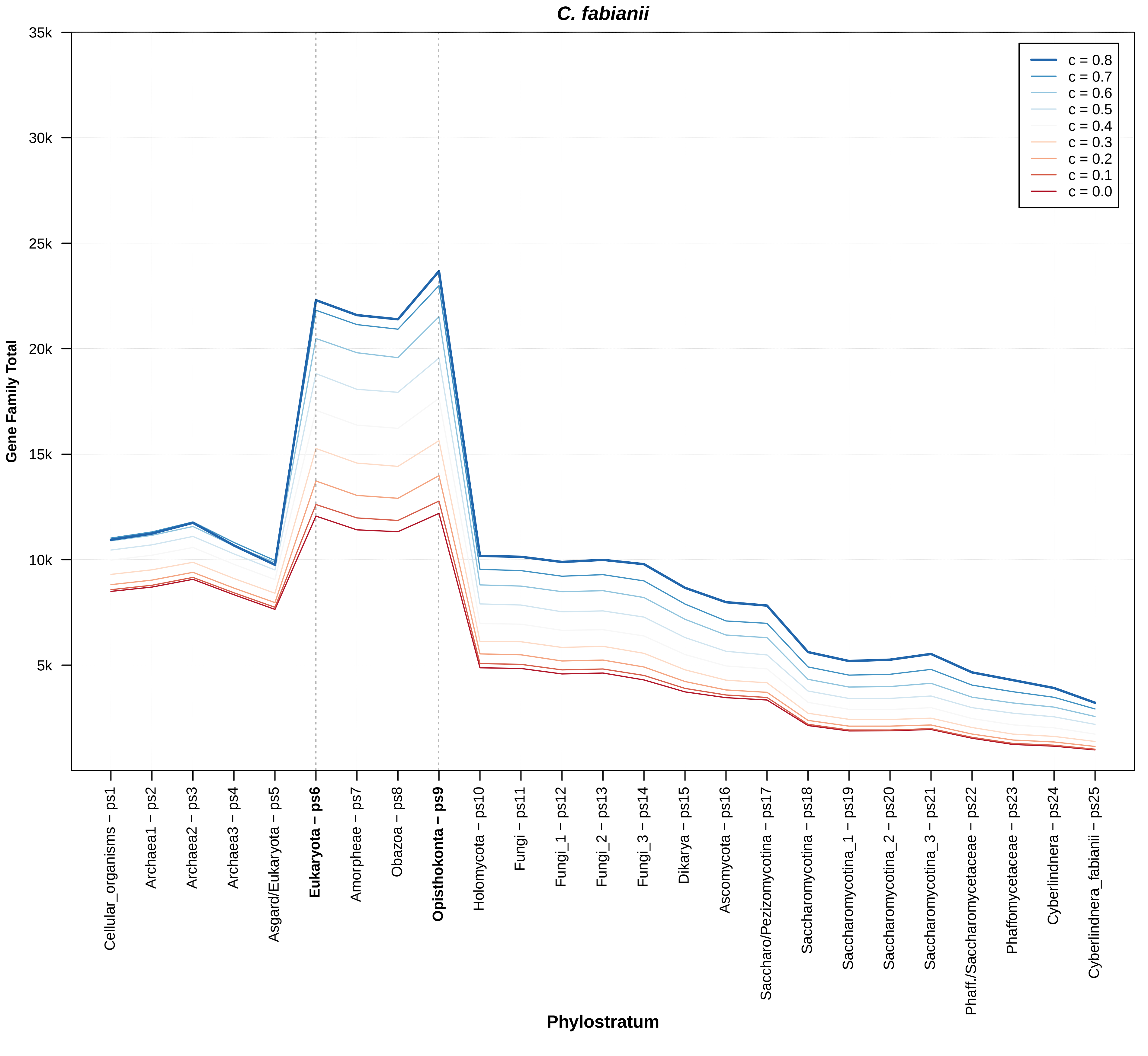

C. fabianii
35k
30k
25k
20k
15k
10k
5k
Gene Family Total
c = 0.8
c = 0.7
c = 0.6
c = 0.5
c = 0.4
c = 0.3
c = 0.2
c = 0.1
c = 0.0
Cellular_organisms – ps1
Archaea1 – ps2
Archaea2 – ps3
Archaea3 – ps4
Asgard/Eukaryota – ps5
Eukaryota – ps6
Amorpheae – ps7
Obazoa – ps8
Opisthokonta – ps9
Holomycota – ps10
Fungi – ps11
Fungi_1 – ps12
Fungi_2 – ps13
Fungi_3 – ps14
Dikarya – ps15
Ascomycota – ps16
Saccharo/Pezizomycotina – ps17
Saccharomycotina – ps18
Saccharomycotina_1 – ps19
Saccharomycotina_2 – ps20
Saccharomycotina_3 – ps21
Phaff./Saccharomycetaceae – ps22
Phaffomycetaceae – ps23
Cyberlindnera – ps24
Cyberlindnera_fabianii – ps25
Phylostratum

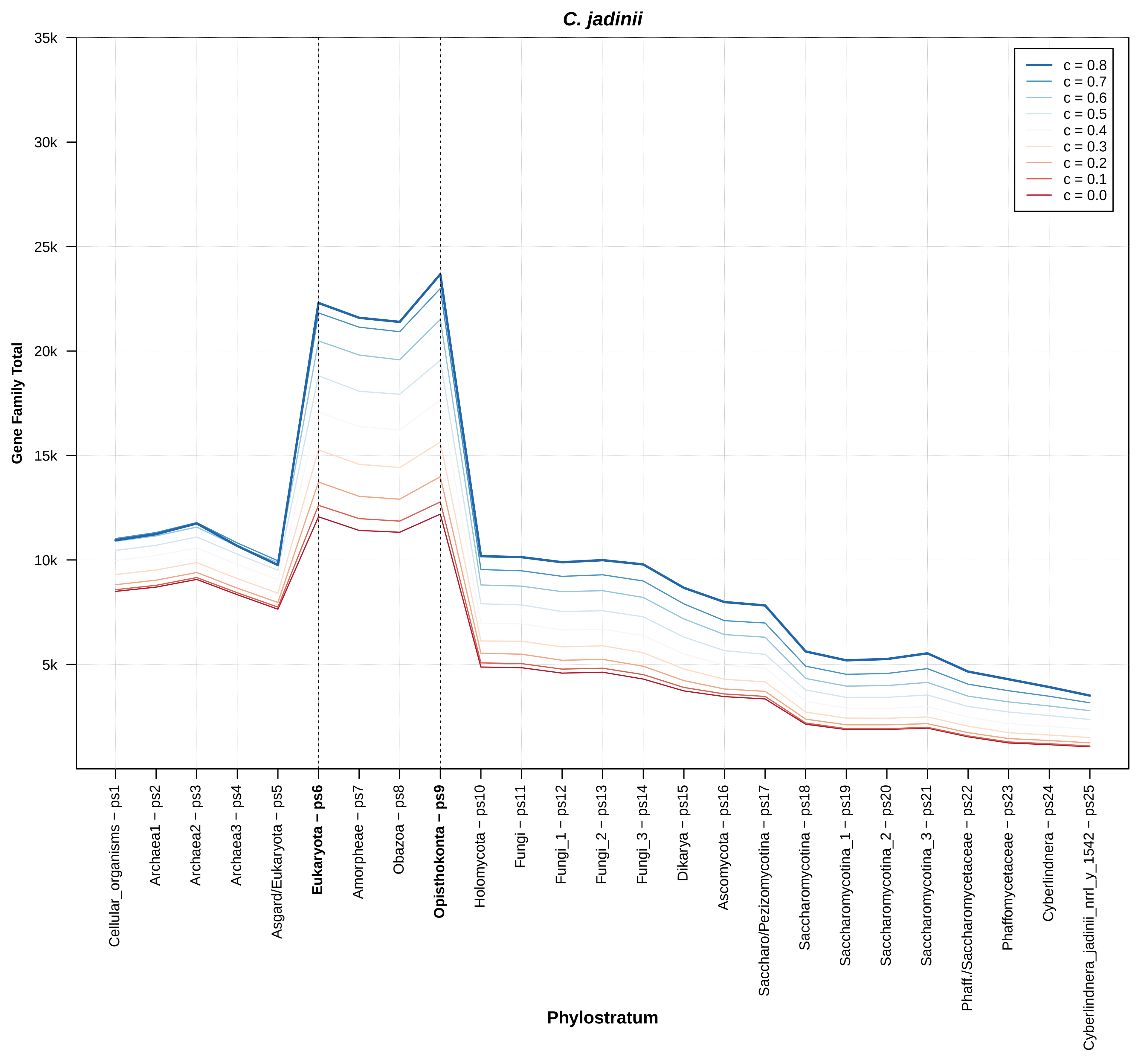
C. jadinii
Gene Family Total
35k
30k
25k
20k
15k
10k
5k
c = 0.8
c = 0.7
c = 0.6
c = 0.5
c = 0.4
c = 0.3
c = 0.2
c = 0.1
c = 0.0
Cellular_organisms – ps1
Archaea1 – ps2
Archaea2 – ps3
Archaea3 – ps4
Asgard/Eukaryota – ps5
Eukaryota – ps6
Amorpheae – ps7
Obazoa – ps8
Opisthokonta – ps9
Holomycota – ps10
Fungi – ps11
Fungi_1 – ps12
Fungi_2 – ps13
Fungi_3 – ps14
Dikarya – ps15
Ascomycota – ps16
Saccharo/Pezizomycotina – ps17
Saccharomycotina – ps18
Saccharomycotina_1 – ps19
Saccharomycotina_2 – ps20
Saccharomycotina_3 – ps21
Phaff./Saccharomycetaceae – ps22
Phaffomycetaceae – ps23
Cyberlindnera – ps24
Cyberlindnera_jadinii_nrrl_y_1542 – ps25
Phylostratum

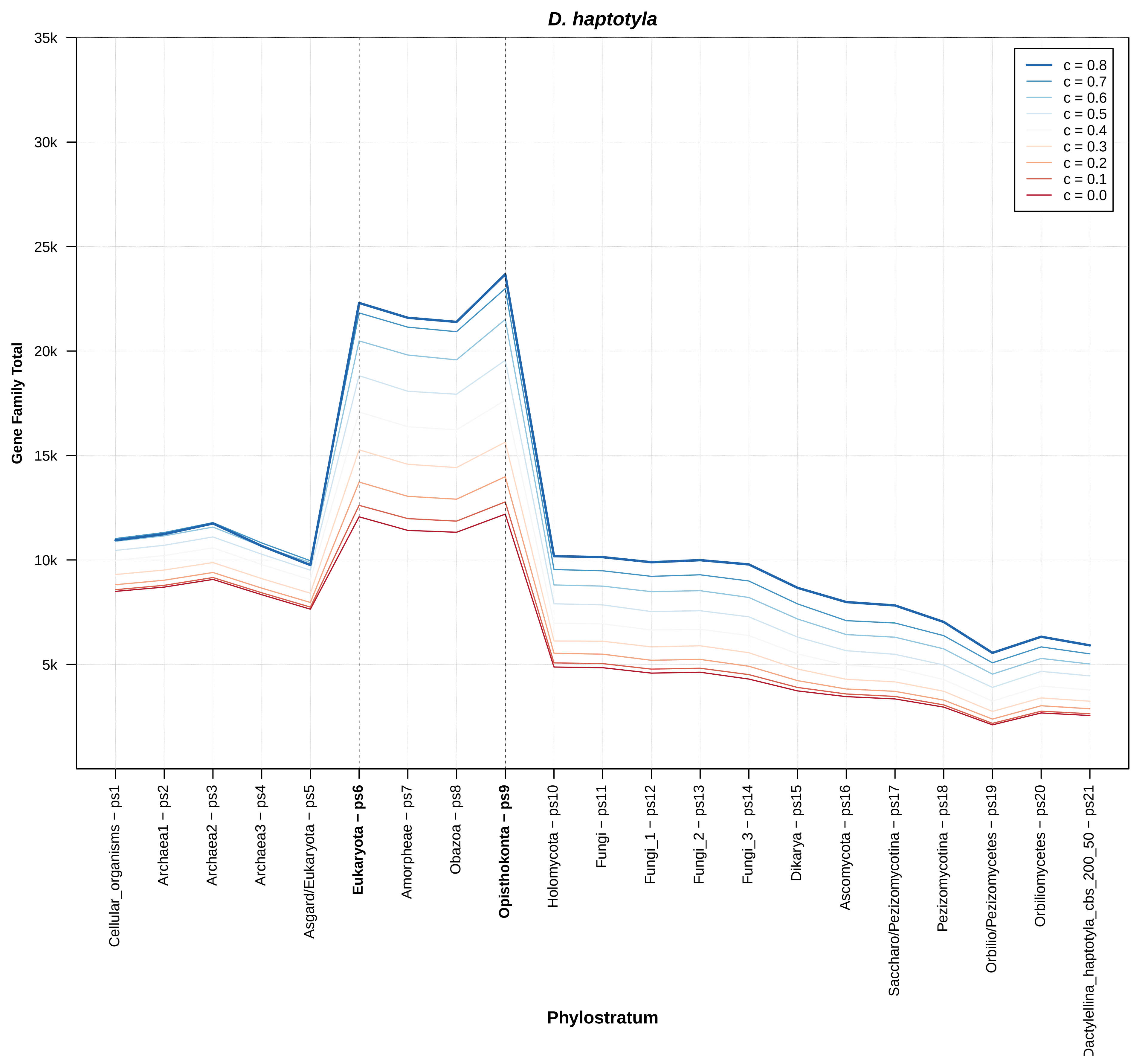

D. haptotyla
35k
30k
25k
20k
15k
10k
5k
Gene Family Total
c = 0.8
c = 0.7
c = 0.6
c = 0.5
c = 0.4
c = 0.3
c = 0.2
c = 0.1
c = 0.0
Cellular_organisms – ps1
Archaea1 – ps2
Archaea2 – ps3
Archaea3 – ps4
Asgard/Eukaryota – ps5
Eukaryota – ps6
Amorpheae – ps7
Obazoa – ps8
Opisthokonta – ps9
Holomycota – ps10
Fungi – ps11
Fungi_1 – ps12
Fungi_2 – ps13
Fungi_3 – ps14
Dikarya – ps15
Ascomycota – ps16
Saccharo/Pezizomycotina – ps17
Pezizomycotina – ps18
Orbilio/Pezizomycetes – ps19
Orbiliomycetes – ps20
Dactylellina_haptotyla_cbs_200_50 – ps21
Phylostratum

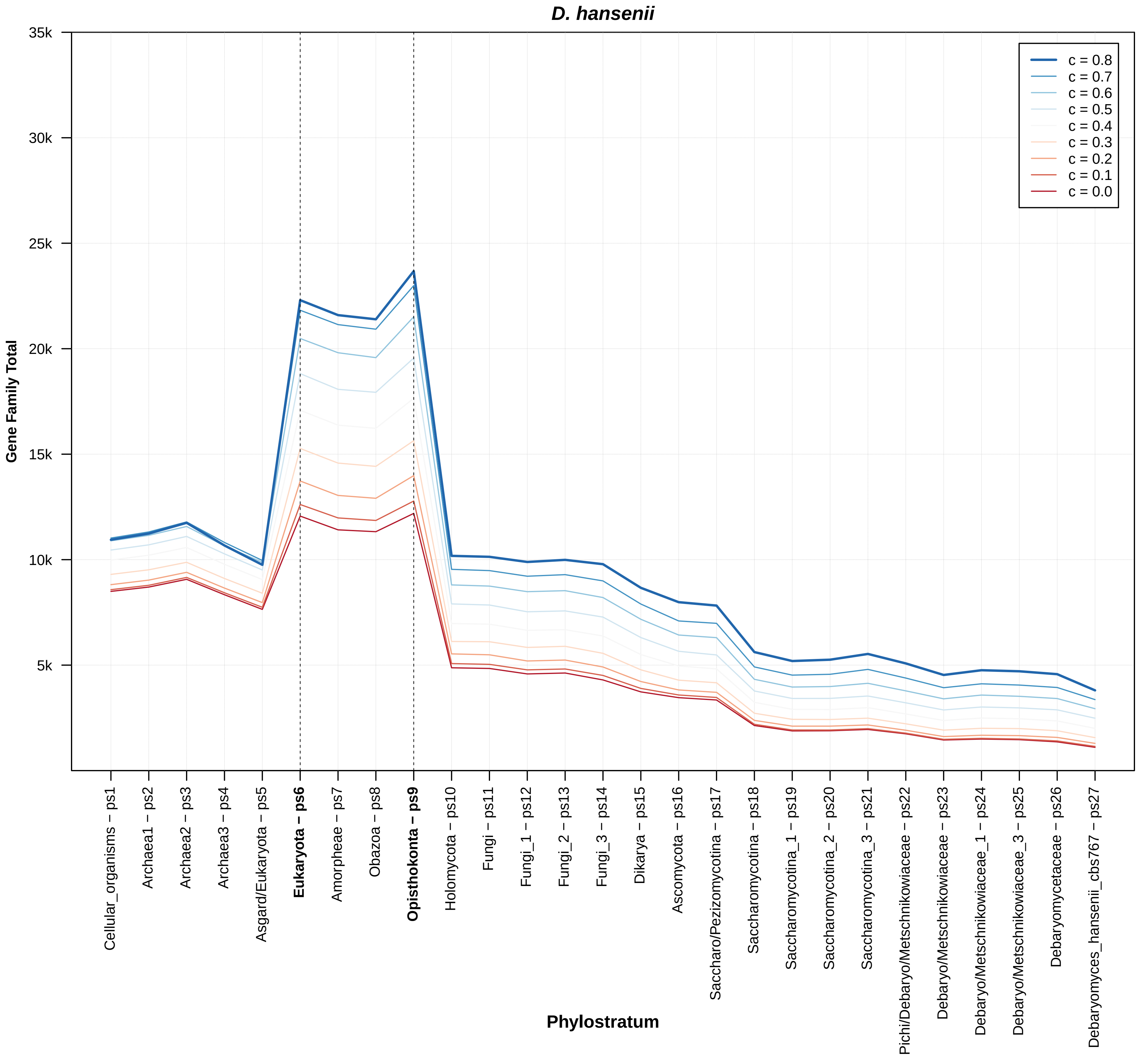

D. hansenii
Gene Family Total
35k
30k
25k
20k
15k
10k
5k
c = 0.8
c = 0.7
c = 0.6
c = 0.5
c = 0.4
c = 0.3
c = 0.2
c = 0.1
c = 0.0
Cellular_organisms – ps1
Archaea1 – ps2
Archaea2 – ps3
Archaea3 – ps4
Asgard/Eukaryota – ps5
Eukaryota – ps6
Amorpheae – ps7
Obazoa – ps8
Opisthokonta – ps9
Holomycota – ps10
Fungi – ps11
Fungi_1 – ps12
Fungi_2 – ps13
Fungi_3 – ps14
Dikarya – ps15
Ascomycota – ps16
Saccharo/Pezizomycotina – ps17
Saccharomycotina – ps18
Saccharomycotina_1 – ps19
Saccharomycotina_2 – ps20
Saccharomycotina_3 – ps21
Pichi/Debaryo/Metschnikowiaceae – ps22
Debaryo/Metschnikowiaceae – ps23
Debaryo/Metschnikowiaceae_1 – ps24
Debaryo/Metschnikowiaceae_3 – ps25
Debaryomycetaceae – ps26
Debaryomyces_hansenii_cbs767 – ps27
Phylostratum

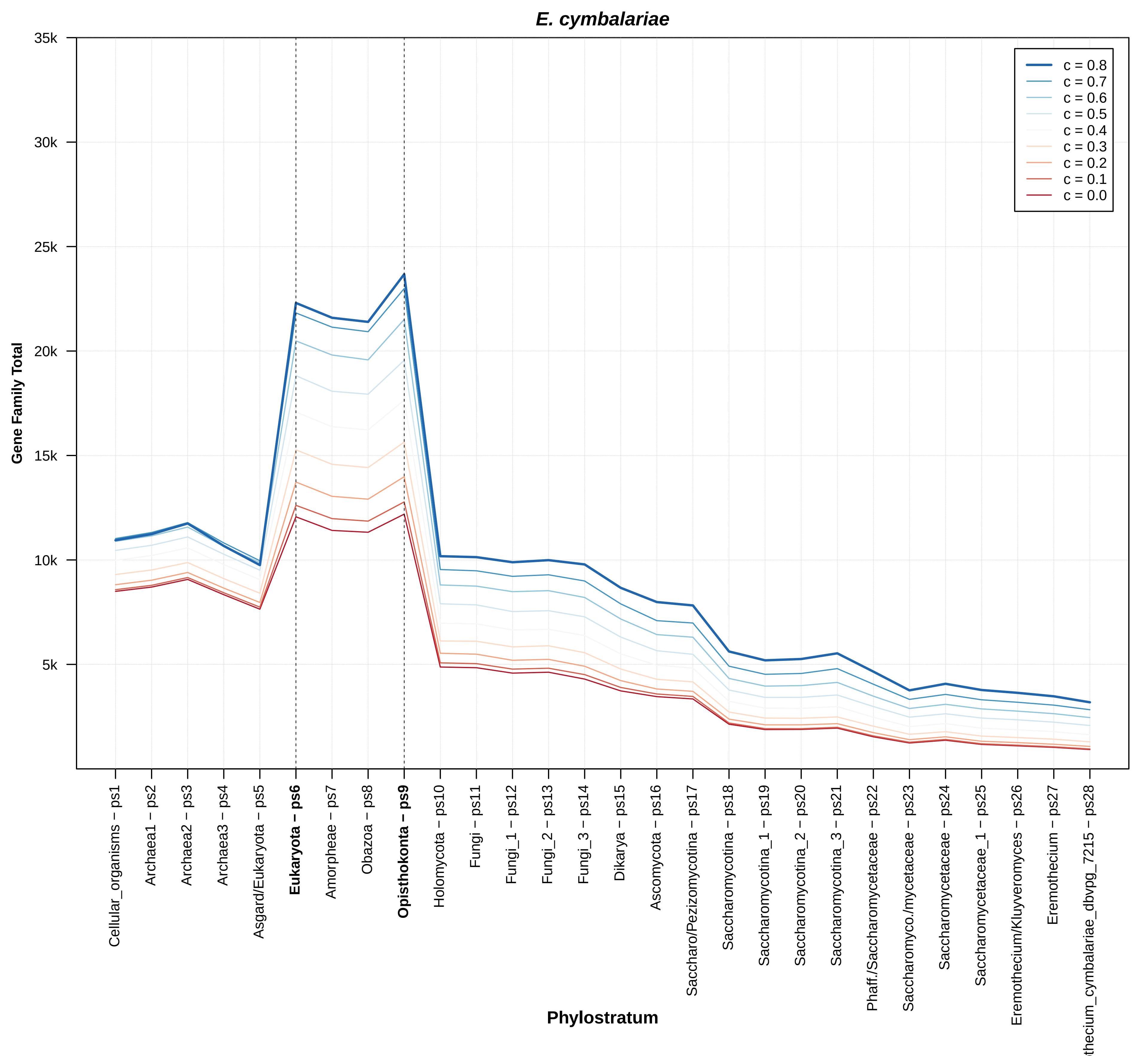
E. cymbalariae
Gene Family Total
Phylostratum
35k
30k
25k
20k
15k
10k
5k
c = 0.8
c = 0.7
c = 0.6
c = 0.5
c = 0.4
c = 0.3
c = 0.2
c = 0.1
c = 0.0
Cellular_organisms – ps1
Archaea1 – ps2
Archaea2 – ps3
Archaea3 – ps4
Asgard/Eukaryota – ps5
Eukaryota – ps6
Amorpheae – ps7
Obazoa – ps8
Opisthokonta – ps9
Holomycota – ps10
Fungi – ps11
Fungi_1 – ps12
Fungi_2 – ps13
Fungi_3 – ps14
Dikarya – ps15
Ascomycota – ps16
Saccharo/Pezizomycotina – ps17
Saccharomycotina – ps18
Saccharomycotina_1 – ps19
Saccharomycotina_2 – ps20
Saccharomycotina_3 – ps21
Phaff./Saccharomycetaceae – ps22
Saccharomyco./mycetaceae – ps23
Saccharomycetaceae – ps24
Saccharomycetaceae_1 – ps25
Eremothecium/Kluyveromyces – ps26
Eremothecium – ps27
thecium_cymbalariae_dbvpg_7215 – ps28

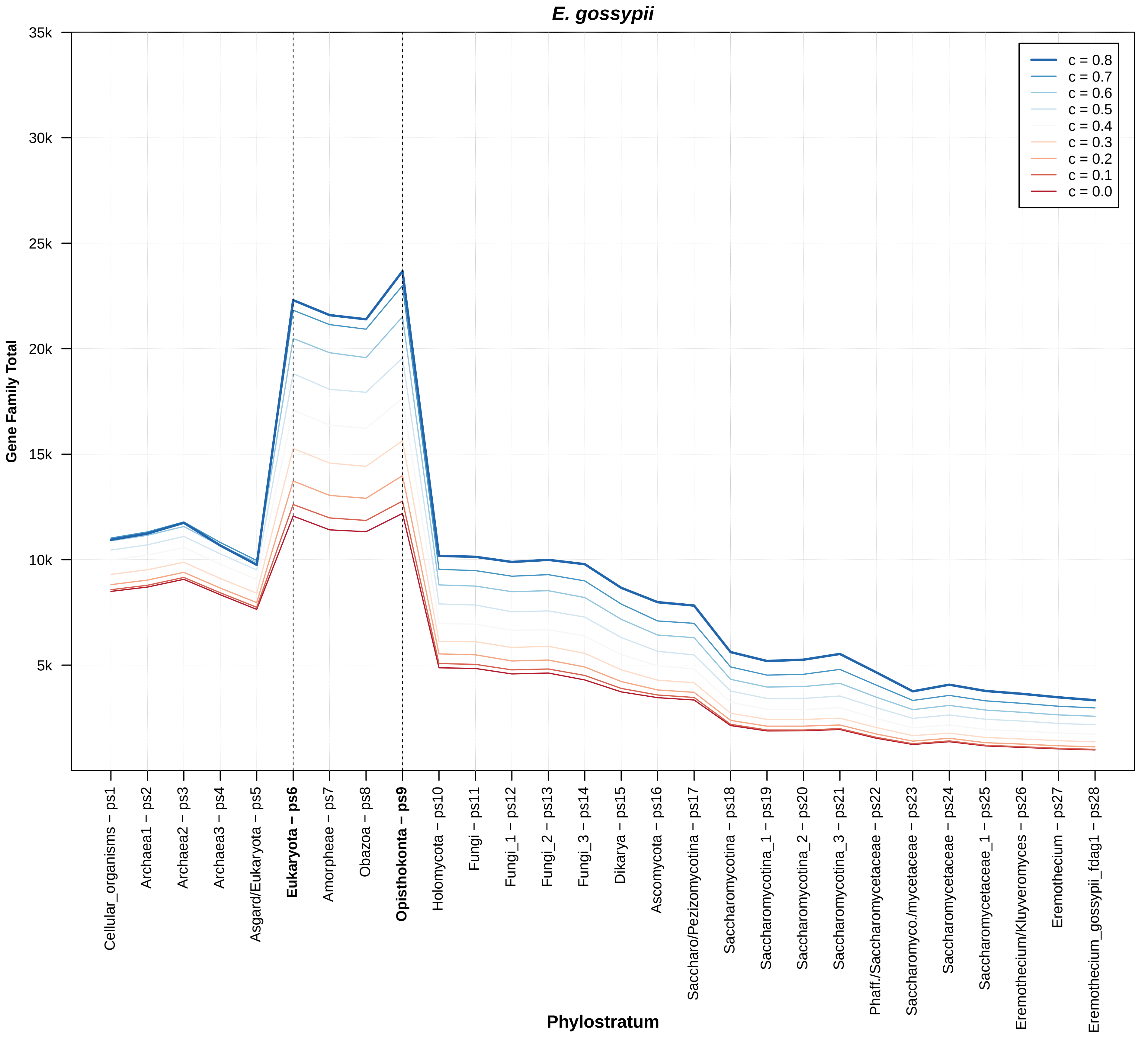

E. gossypii
Gene Family Total
35k
30k
25k
20k
15k
10k
5k
c = 0.8
c = 0.7
c = 0.6
c = 0.5
c = 0.4
c = 0.3
c = 0.2
c = 0.1
c = 0.0
Cellular_organisms – ps1
Archaea1 – ps2
Archaea2 – ps3
Archaea3 – ps4
Asgard/Eukaryota – ps5
Eukaryota – ps6
Amorpheae – ps7
Obazoa – ps8
Opisthokonta – ps9
Holomycota – ps10
Fungi – ps11
Fungi_1 – ps12
Fungi_2 – ps13
Fungi_3 – ps14
Dikarya – ps15
Ascomycota – ps16
Saccharo/Pezizomycotina – ps17
Saccharomycotina – ps18
Saccharomycotina_1 – ps19
Saccharomycotina_2 – ps20
Saccharomycotina_3 – ps21
Phaff./Saccharomycetaceae – ps22
Saccharomyco./mycetaceae – ps23
Saccharomycetaceae – ps24
Saccharomycetaceae_1 – ps25
Eremothecium/Kluyveromyces – ps26
Eremothecium – ps27
Eremothecium_gossypii_fdag1 – ps28
Phylostratum

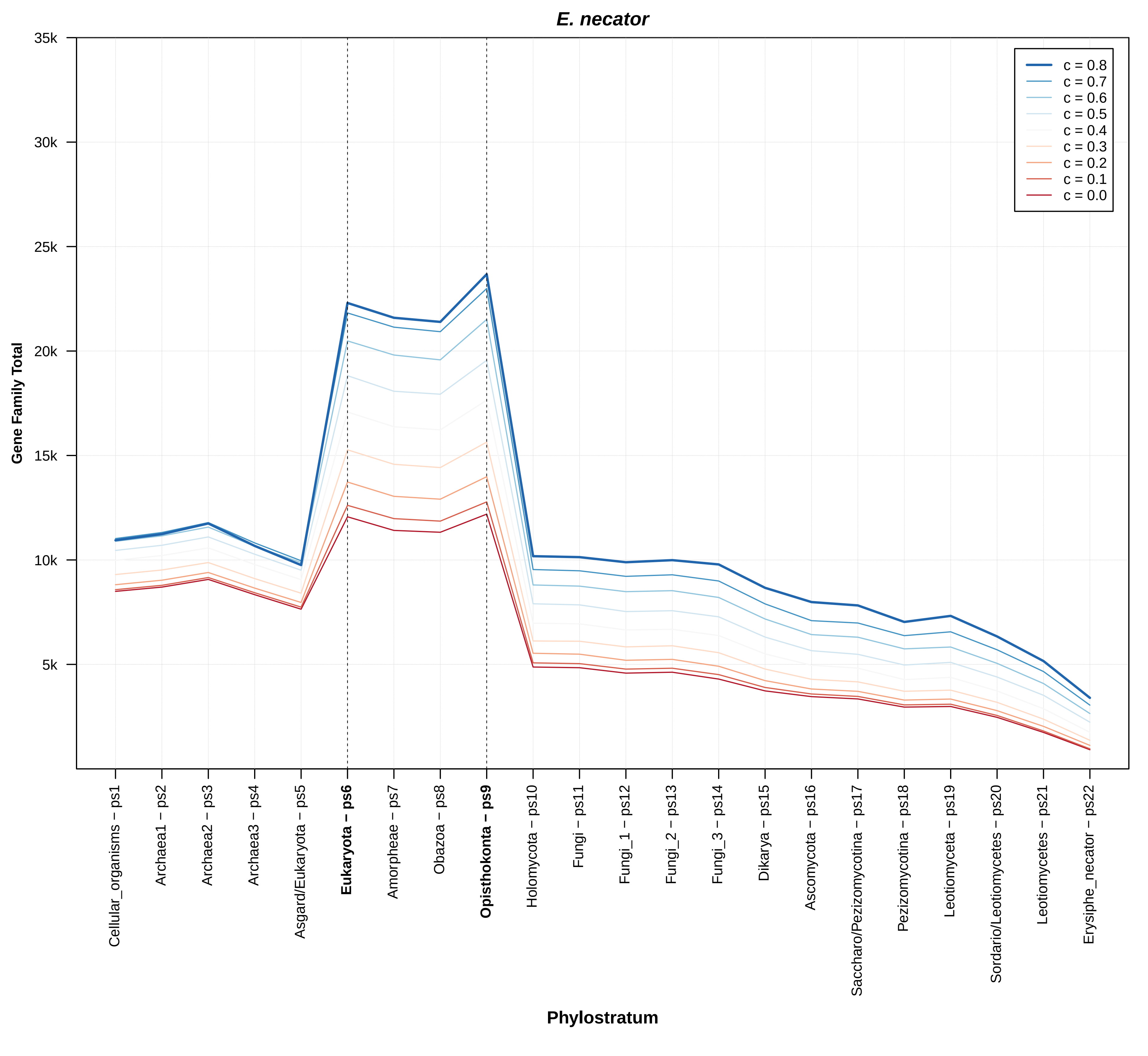
E. necator
Gene Family Total
Phylostratum
35k
30k
25k
20k
15k
10k
5k
c = 0.8
c = 0.7
c = 0.6
c = 0.5
c = 0.4
c = 0.3
c = 0.2
c = 0.1
c = 0.0
Cellular_organisms – ps1
Archaea1 – ps2
Archaea2 – ps3
Archaea3 – ps4
Asgard/Eukaryota – ps5
Eukaryota – ps6
Amorpheae – ps7
Obazoa – ps8
Opisthokonta – ps9
Holomycota – ps10
Fungi – ps11
Fungi_1 – ps12
Fungi_2 – ps13
Fungi_3 – ps14
Dikarya – ps15
Ascomycota – ps16
Saccharo/Pezizomycotina – ps17
Pezizomycotina – ps18
Leotiomyceta – ps19
Sordario/Leotiomycetes – ps20
Leotiomycetes – ps21
Erysiphe_necator – ps22

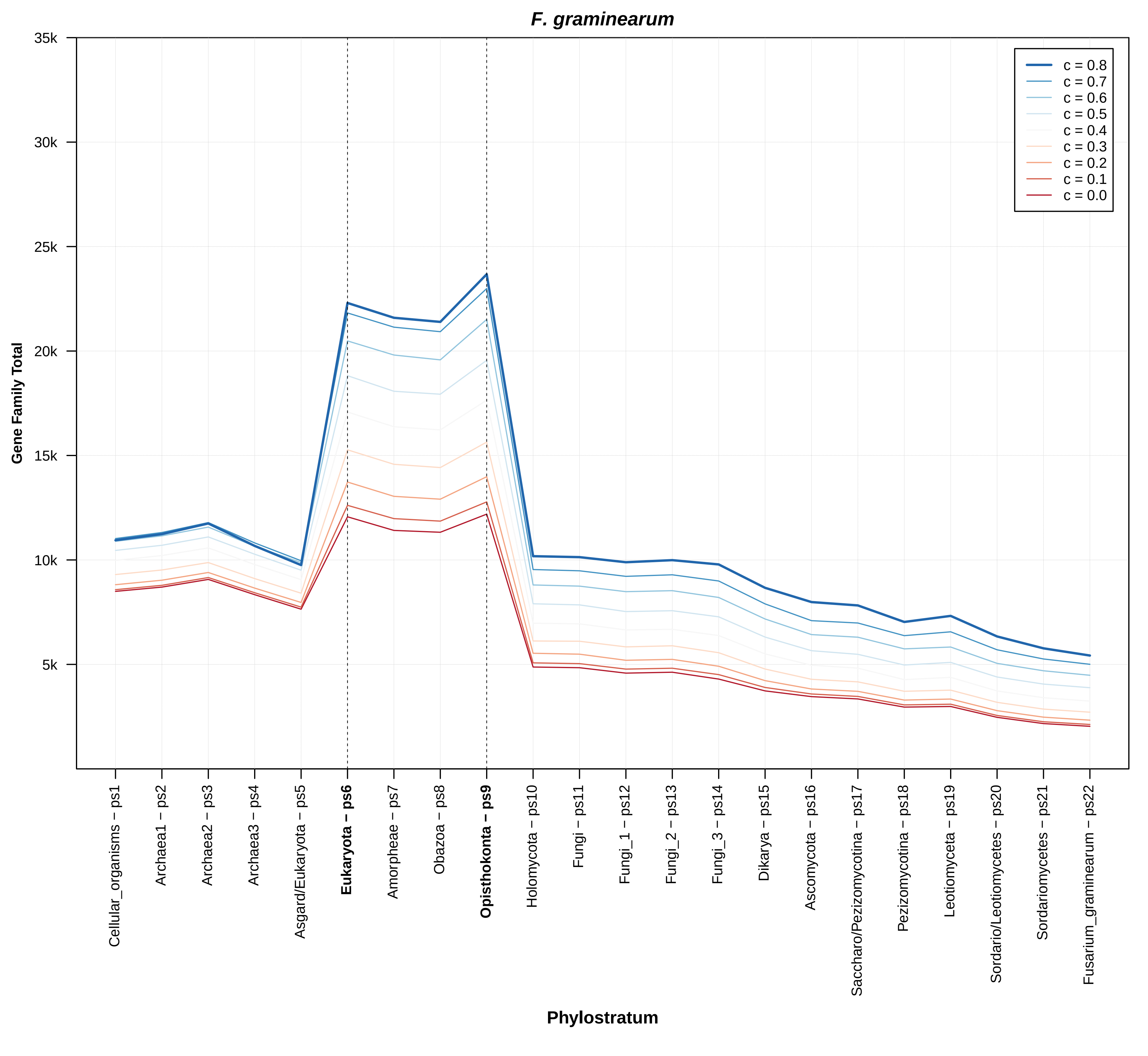

F. graminearum
Gene Family Total
35k
30k
25k
20k
15k
10k
5k
c = 0.8
c = 0.7
c = 0.6
c = 0.5
c = 0.4
c = 0.3
c = 0.2
c = 0.1
c = 0.0
Cellular_organisms – ps1
Archaea1 – ps2
Archaea2 – ps3
Archaea3 – ps4
Asgard/Eukaryota – ps5
Eukaryota – ps6
Amorpheae – ps7
Obazoa – ps8
Opisthokonta – ps9
Holomycota – ps10
Fungi – ps11
Fungi_1 – ps12
Fungi_2 – ps13
Fungi_3 – ps14
Dikarya – ps15
Ascomycota – ps16
Saccharo/Pezizomycotina – ps17
Pezizomycotina – ps18
Leotiomyceta – ps19
Sordario/Leotiomycetes – ps20
Sordariomycetes – ps21
Fusarium_graminearum – ps22
Phylostratum

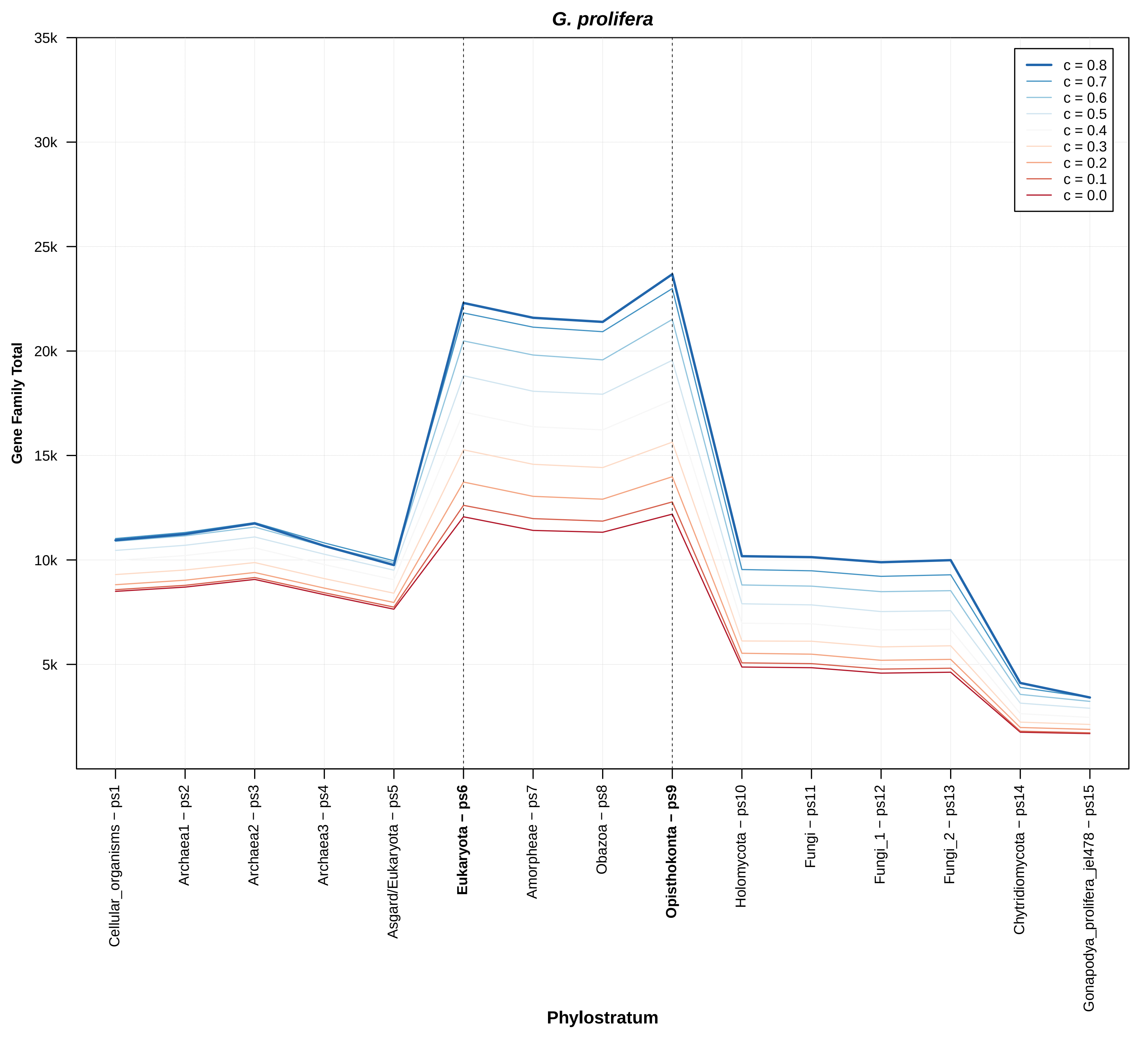

G. prolifera
Gene Family Total
35k
30k
25k
20k
15k
10k
5k
c = 0.8
c = 0.7
c = 0.6
c = 0.5
c = 0.4
c = 0.3
c = 0.2
c = 0.1
c = 0.0
Cellular_organisms – ps1
Archaea1 – ps2
Archaea2 – ps3
Archaea3 – ps4
Asgard/Eukaryota – ps5
Eukaryota – ps6
Amorpheae – ps7
Obazoa – ps8
Opisthokonta – ps9
Holomycota – ps10
Fungi – ps11
Fungi_1 – ps12
Fungi_2 – ps13
Chytridiomycota – ps14
Gonapodya_prolifera_jel478 – ps15
Phylostratum

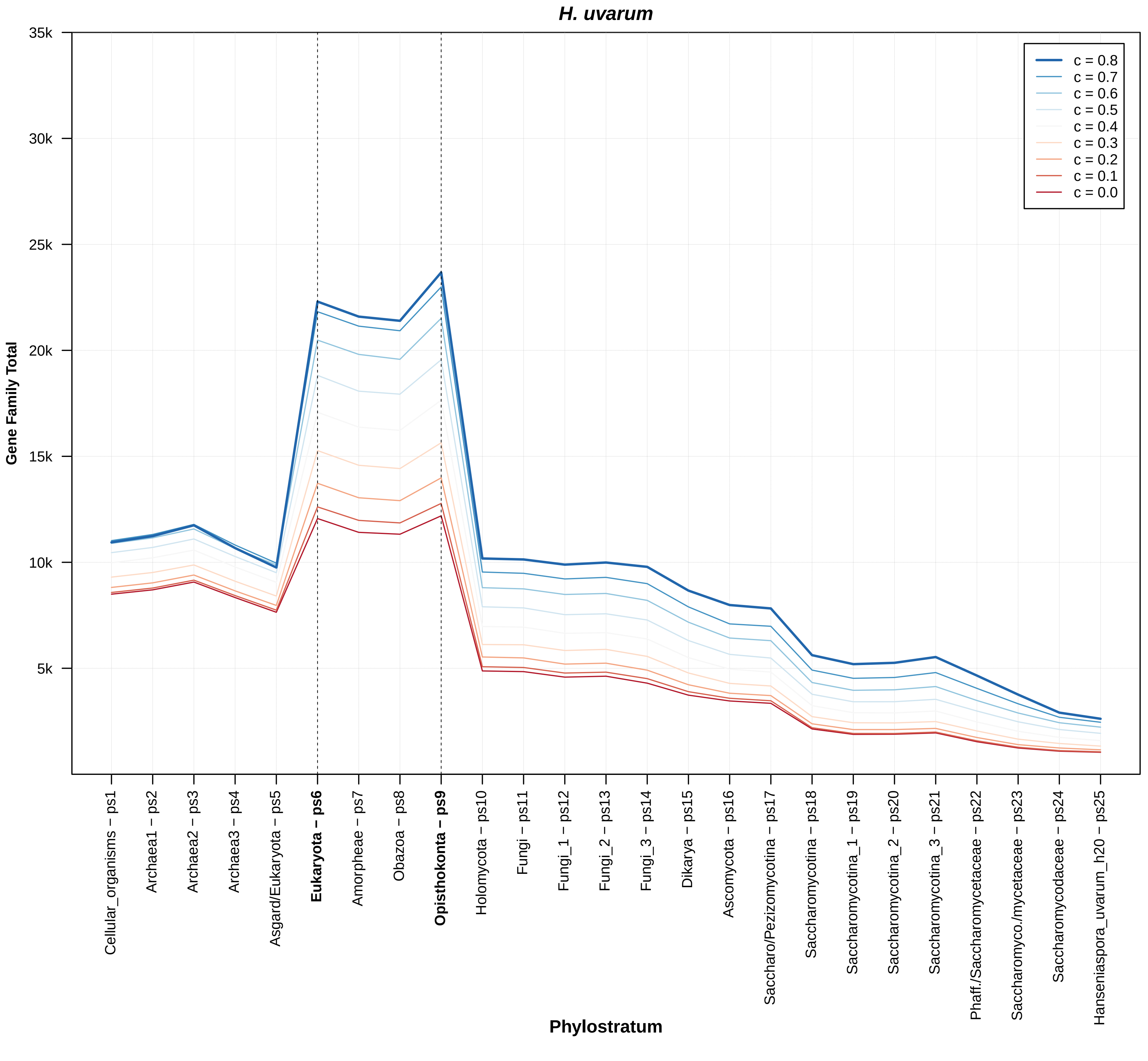
H. uvarum
Gene Family Total
35k
30k
25k
20k
15k
10k
5k
c = 0.8
c = 0.7
c = 0.6
c = 0.5
c = 0.4
c = 0.3
c = 0.2
c = 0.1
c = 0.0
Cellular_organisms – ps1
Archaea1 – ps2
Archaea2 – ps3
Archaea3 – ps4
Asgard/Eukaryota – ps5
Eukaryota – ps6
Amorpheae – ps7
Obazoa – ps8
Opisthokonta – ps9
Holomycota – ps10
Fungi – ps11
Fungi_1 – ps12
Fungi_2 – ps13
Fungi_3 – ps14
Dikarya – ps15
Ascomycota – ps16
Saccharo/Pezizomycotina – ps17
Saccharomycotina – ps18
Saccharomycotina_1 – ps19
Saccharomycotina_2 – ps20
Saccharomycotina_3 – ps21
Phaff./Saccharomycetaceae – ps22
Saccharomyco./mycetaceae – ps23
Saccharomycodaceae – ps24
Hanseniaspora_uvarum_h20 – ps25
Phylostratum

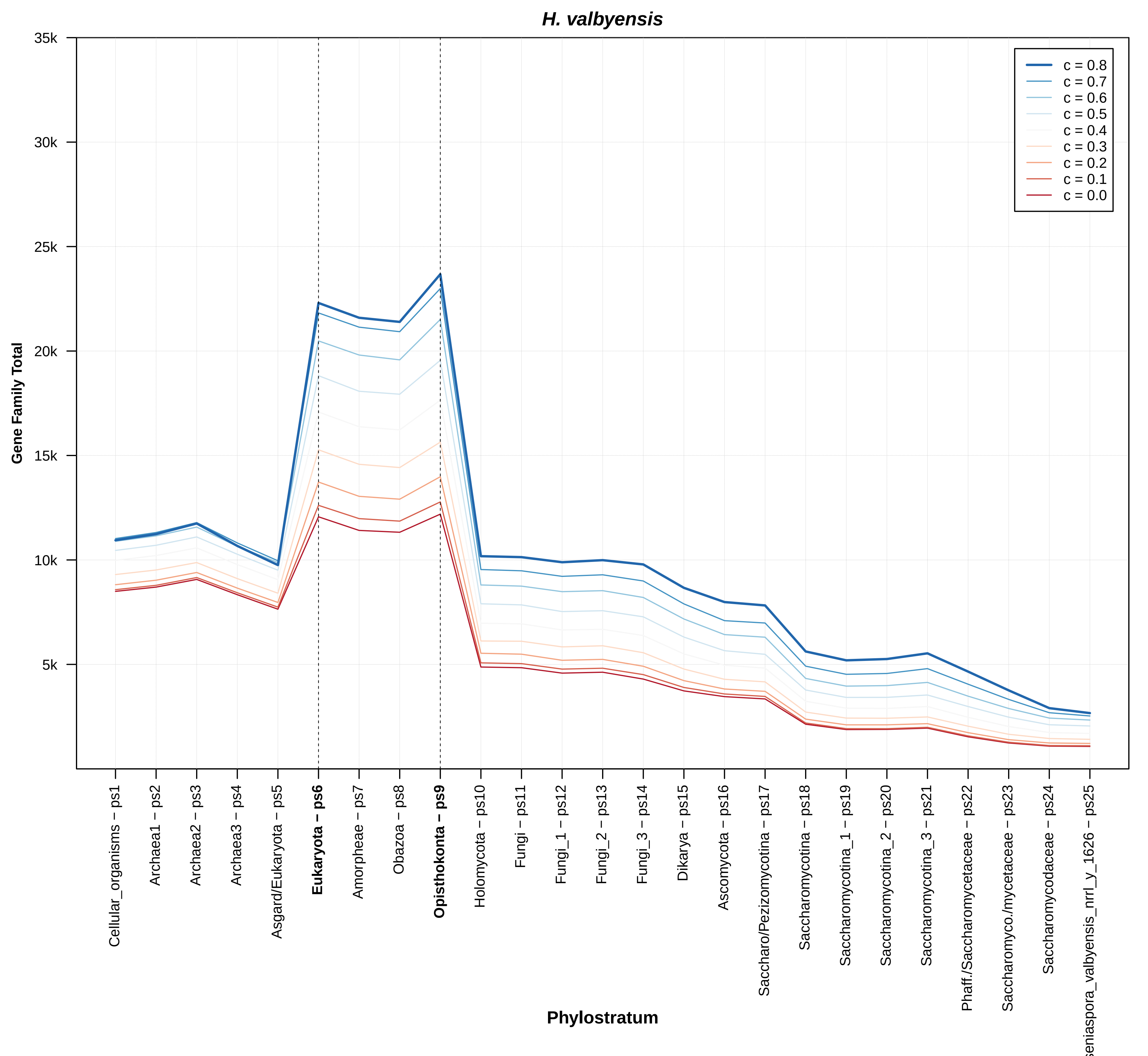

H. valbyensis
Gene Family Total
35k
30k
25k
20k
15k
10k
5k
c = 0.8
c = 0.7
c = 0.6
c = 0.5
c = 0.4
c = 0.3
c = 0.2
c = 0.1
c = 0.0
Cellular_organisms – ps1
Archaea1 – ps2
Archaea2 – ps3
Archaea3 – ps4
Asgard/Eukaryota – ps5
Eukaryota – ps6
Amorpheae – ps7
Obazoa – ps8
Opisthokonta – ps9
Holomycota – ps10
Fungi – ps11
Fungi_1 – ps12
Fungi_2 – ps13
Fungi_3 – ps14
Dikarya – ps15
Ascomycota – ps16
Saccharo/Pezizomycotina – ps17
Saccharomycotina – ps18
Saccharomycotina_1 – ps19
Saccharomycotina_2 – ps20
Saccharomycotina_3 – ps21
Phaff./Saccharomycetaceae – ps22
Saccharomyco./mycetaceae – ps23
Saccharomycodaceae – ps24
Phylostratum

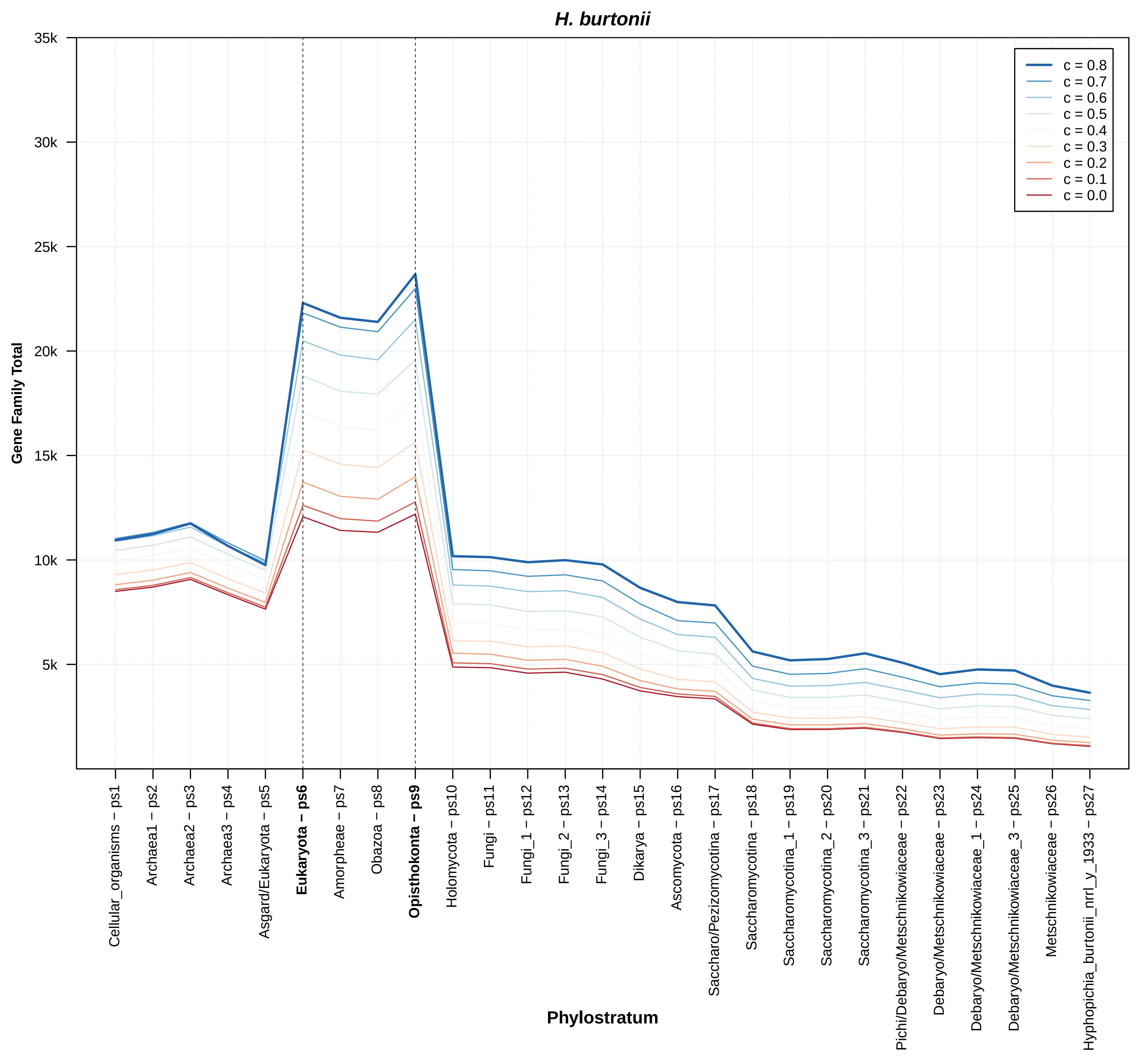

H. burtonii
Gene Family Total
35k
30k
25k
20k
15k
10k
5k
c = 0.8
c = 0.7
c = 0.6
c = 0.5
c = 0.4
c = 0.3
c = 0.2
c = 0.1
c = 0.0
Cellular_organisms – ps1
Archaea1 – ps2
Archaea2 – ps3
Archaea3 – ps4
Asgard/Eukaryota – ps5
Eukaryota – ps6
Amorpheae – ps7
Obazoa – ps8
Opisthokonta – ps9
Holomycota – ps10
Fungi – ps11
Fungi_1 – ps12
Fungi_2 – ps13
Fungi_3 – ps14
Dikarya – ps15
Ascomycota – ps16
Saccharo/Pezizomycotina – ps17
Saccharomycotina – ps18
Saccharomycotina_1 – ps19
Saccharomycotina_2 – ps20
Saccharomycotina_3 – ps21
Pichi/Debaryo/Metschnikowiaceae – ps22
Debaryo/Metschnikowiaceae – ps23
Debaryo/Metschnikowiaceae_1 – ps24
Debaryo/Metschnikowiaceae_3 – ps25
Metschnikowiaceae – ps26
Hyphopichia_burtonii_nrrl_y_1933 – ps27
Phylostratum

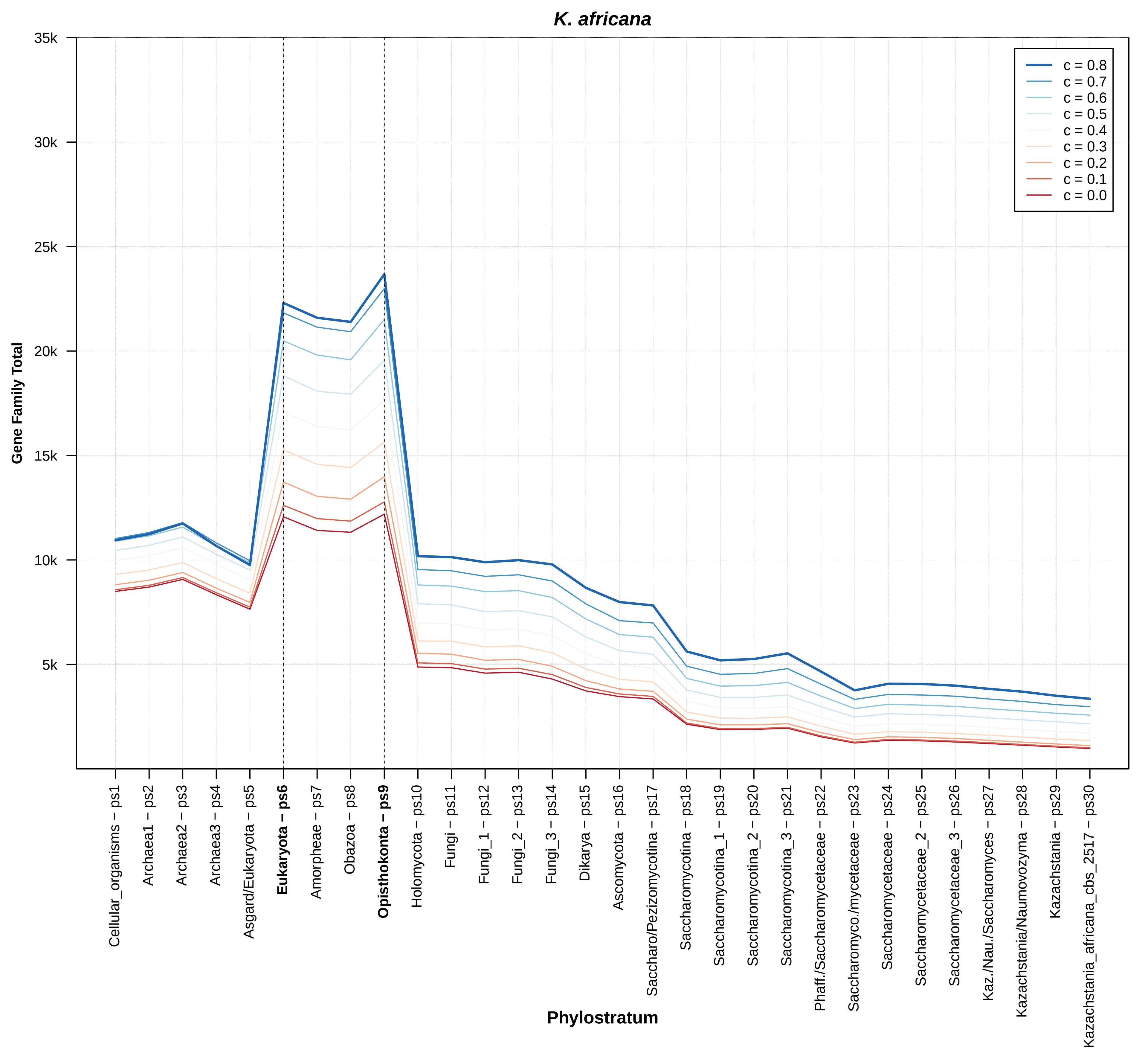
K. africana
Gene Family Total
Phylostratum
35k
30k
25k
20k
15k
10k
5k
c = 0.8
c = 0.7
c = 0.6
c = 0.5
c = 0.4
c = 0.3
c = 0.2
c = 0.1
c = 0.0
Cellular_organisms – ps1
Archaea1 – ps2
Archaea2 – ps3
Archaea3 – ps4
Asgard/Eukaryota – ps5
Eukaryota – ps6
Amorpheae – ps7
Obazoa – ps8
Opisthokonta – ps9
Holomycota – ps10
Fungi – ps11
Fungi_1 – ps12
Fungi_2 – ps13
Fungi_3 – ps14
Dikarya – ps15
Ascomycota – ps16
Saccharo/Pezizomycotina – ps17
Saccharomycotina – ps18
Saccharomycotina_1 – ps19
Saccharomycotina_2 – ps20
Saccharomycotina_3 – ps21
Phaff./Saccharomycetaceae – ps22
Saccharomyco./mycetaceae – ps23
Saccharomycetaceae – ps24
Saccharomycetaceae_2 – ps25
Saccharomycetaceae_3 – ps26
Kaz./Nau./Saccharomyces – ps27
Kazachstania/Naumovozyma – ps28
Kazachstania – ps29
Kazachstania_africana_cbs_2517 – ps30

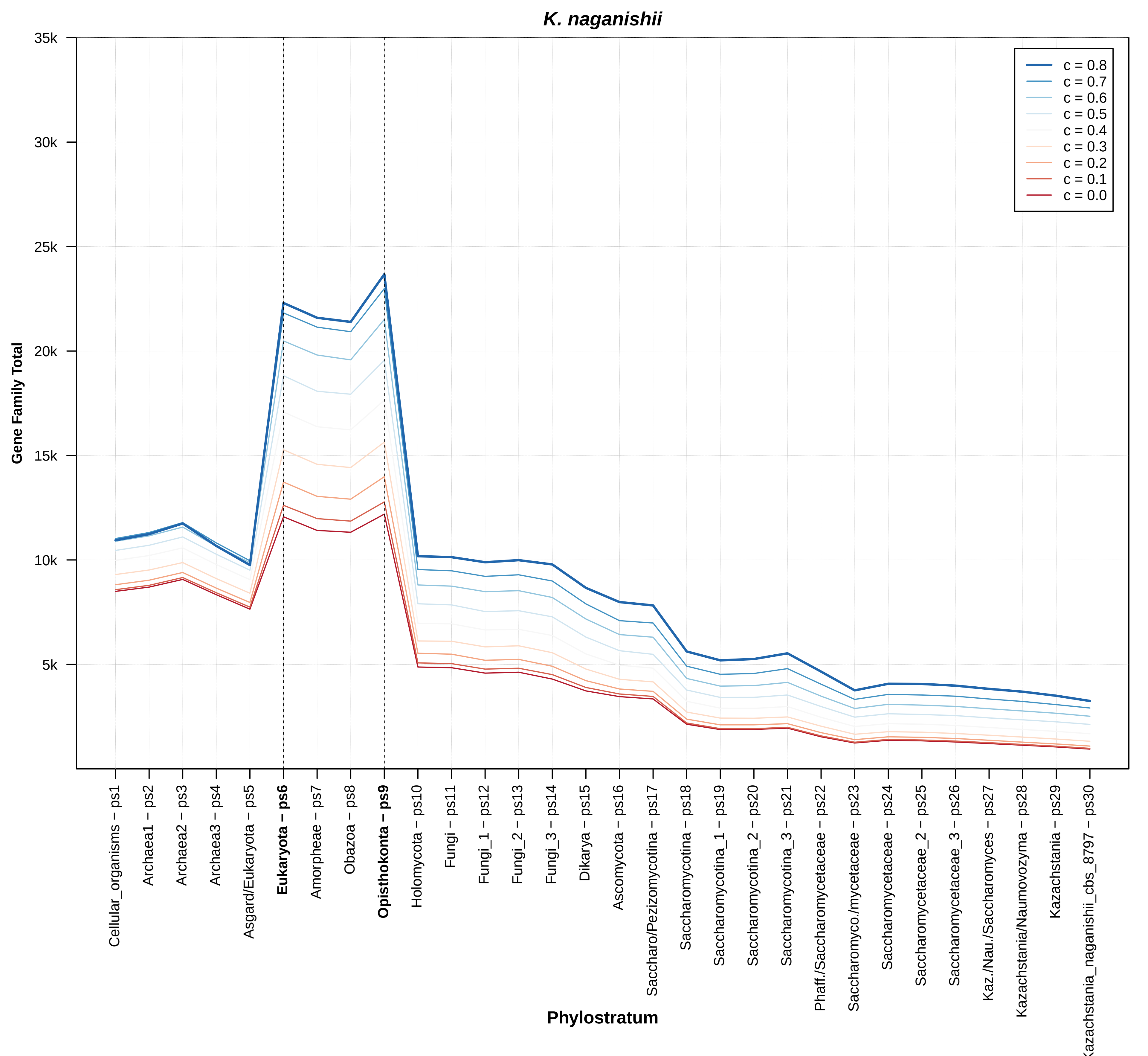
K. naganishii
Gene Family Total
35k
30k
25k
20k
15k
10k
5k
c = 0.8
c = 0.7
c = 0.6
c = 0.5
c = 0.4
c = 0.3
c = 0.2
c = 0.1
c = 0.0
Cellular_organisms – ps1
Archaea1 – ps2
Archaea2 – ps3
Archaea3 – ps4
Asgard/Eukaryota – ps5
Eukaryota – ps6
Amorpheae – ps7
Obazoa – ps8
Opisthokonta – ps9
Holomycota – ps10
Fungi – ps11
Fungi_1 – ps12
Fungi_2 – ps13
Fungi_3 – ps14
Dikarya – ps15
Ascomycota – ps16
Saccharo/Pezizomycotina – ps17
Saccharomycotina – ps18
Saccharomycotina_1 – ps19
Saccharomycotina_2 – ps20
Saccharomycotina_3 – ps21
Phaff./Saccharomycetaceae – ps22
Saccharomyco./mycetaceae – ps23
Saccharomycetaceae – ps24
Saccharomycetaceae_2 – ps25
Saccharomycetaceae_3 – ps26
Kaz./Nau./Saccharomyces – ps27
Kazachstania/Naumovozyma – ps28
Kazachstania – ps29
Kazachstania_naganishii_cbs_8797 – ps30
Phylostratum

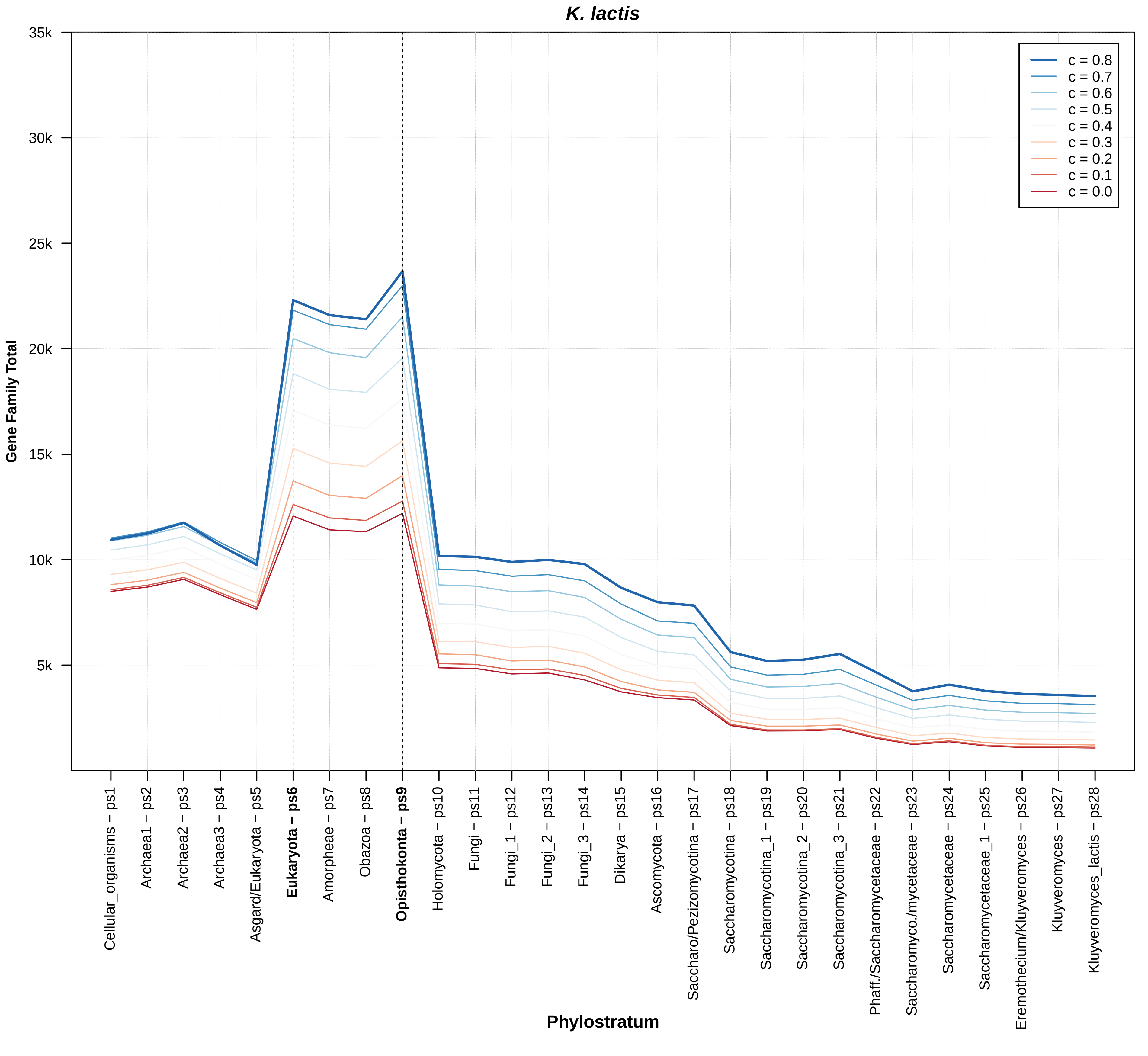

K. lactis
Gene Family Total
35k
30k
25k
20k
15k
10k
5k
c = 0.8
c = 0.7
c = 0.6
c = 0.5
c = 0.4
c = 0.3
c = 0.2
c = 0.1
c = 0.0
Cellular_organisms – ps1
Archaea1 – ps2
Archaea2 – ps3
Archaea3 – ps4
Asgard/Eukaryota – ps5
Eukaryota – ps6
Amorpheae – ps7
Obazoa – ps8
Opisthokonta – ps9
Holomycota – ps10
Fungi – ps11
Fungi_1 – ps12
Fungi_2 – ps13
Fungi_3 – ps14
Dikarya – ps15
Ascomycota – ps16
Saccharo/Pezizomycotina – ps17
Saccharomycotina – ps18
Saccharomycotina_1 – ps19
Saccharomycotina_2 – ps20
Saccharomycotina_3 – ps21
Phaff./Saccharomycetaceae – ps22
Saccharomyco./mycetaceae – ps23
Saccharomycetaceae – ps24
Saccharomycetaceae_1 – ps25
Eremothecium/Kluyveromyces – ps26
Kluyveromyces – ps27
Kluyveromyces_lactis – ps28
Phylostratum

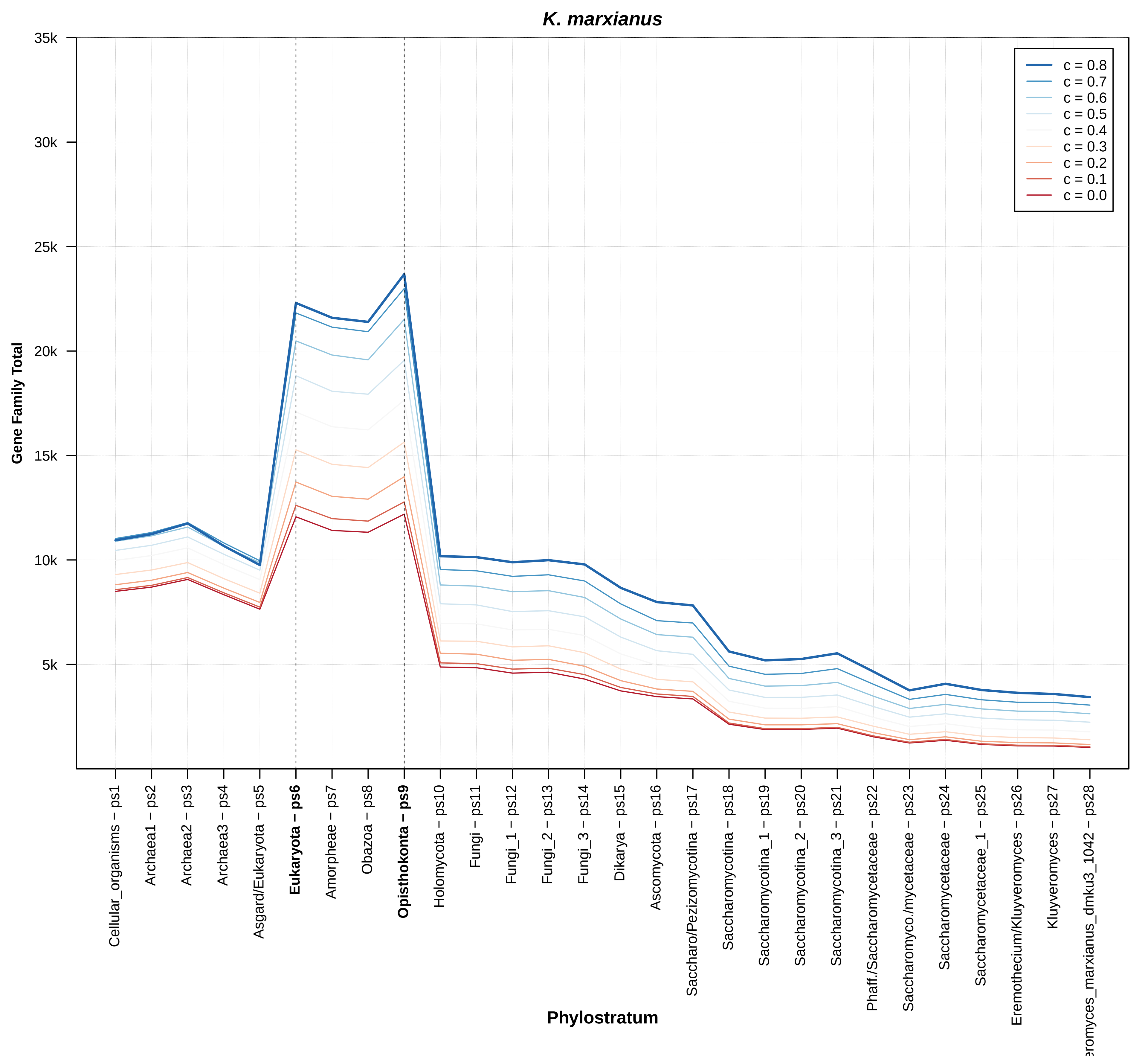

K. marxianus
Gene Family Total
35k
30k
25k
20k
15k
10k
5k
c = 0.8
c = 0.7
c = 0.6
c = 0.5
c = 0.4
c = 0.3
c = 0.2
c = 0.1
c = 0.0
Cellular_organisms – ps1
Archaea1 – ps2
Archaea2 – ps3
Archaea3 – ps4
Asgard/Eukaryota – ps5
Eukaryota – ps6
Amorpheae – ps7
Obazoa – ps8
Opisthokonta – ps9
Holomycota – ps10
Fungi – ps11
Fungi_1 – ps12
Fungi_2 – ps13
Fungi_3 – ps14
Dikarya – ps15
Ascomycota – ps16
Saccharo/Pezizomycotina – ps17
Saccharomycotina – ps18
Saccharomycotina_1 – ps19
Saccharomycotina_2 – ps20
Saccharomycotina_3 – ps21
Phaff./Saccharomycetaceae – ps22
Saccharomyco./mycetaceae – ps23
Saccharomycetaceae – ps24
Saccharomycetaceae_1 – ps25
Eremothecium/Kluyveromyces – ps26
Kluyveromyces – ps27
Phylostratum

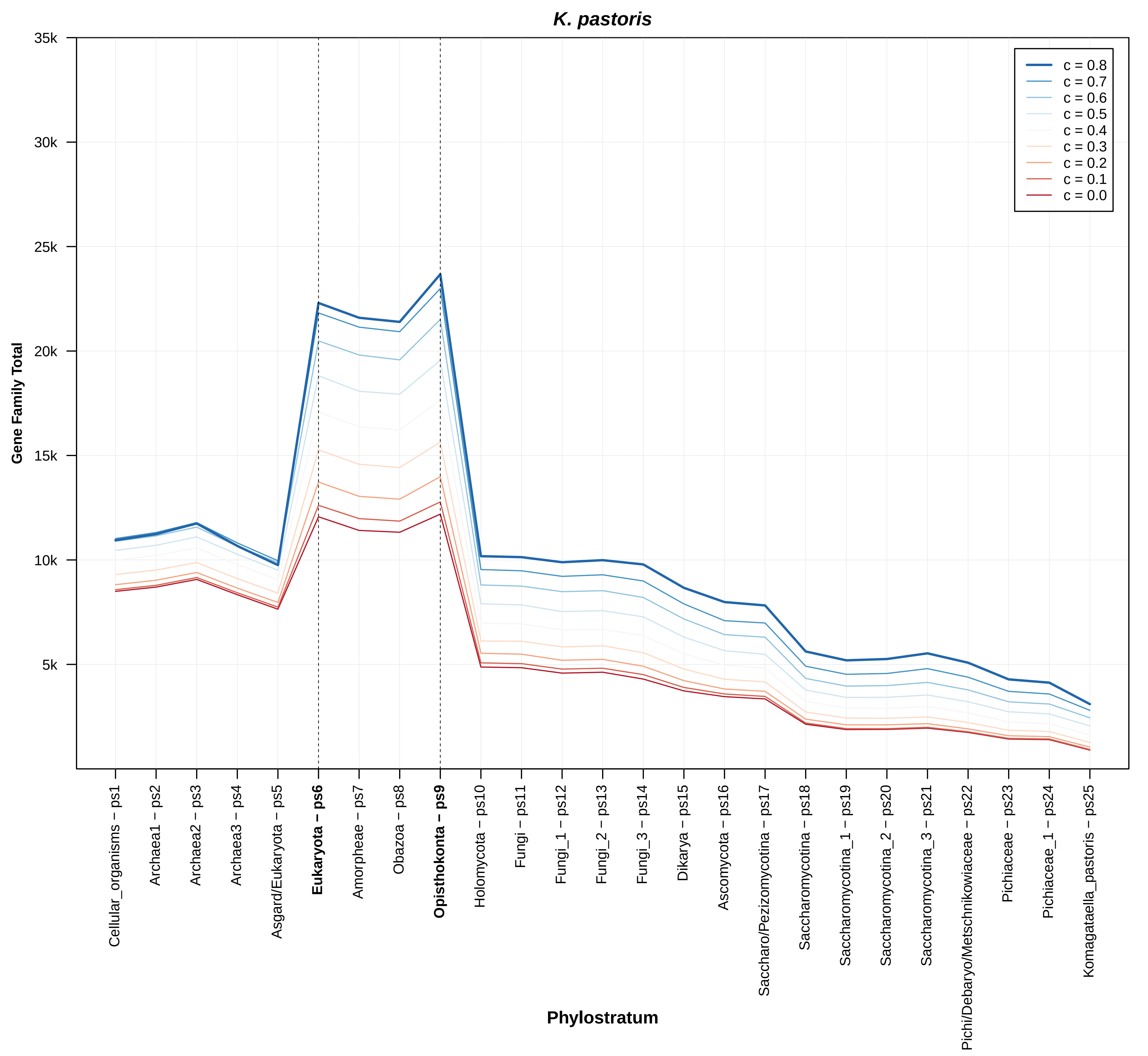
K. pastoris
Gene Family Total
35k
30k
25k
20k
15k
10k
5k
c = 0.8
c = 0.7
c = 0.6
c = 0.5
c = 0.4
c = 0.3
c = 0.2
c = 0.1
c = 0.0
Cellular_organisms – ps1
Archaea1 – ps2
Archaea2 – ps3
Archaea3 – ps4
Asgard/Eukaryota – ps5
Eukaryota – ps6
Amorpheae – ps7
Obazoa – ps8
Opisthokonta – ps9
Holomycota – ps10
Fungi – ps11
Fungi_1 – ps12
Fungi_2 – ps13
Fungi_3 – ps14
Dikarya – ps15
Ascomycota – ps16
Saccharo/Pezizomycotina – ps17
Saccharomycotina – ps18
Saccharomycotina_1 – ps19
Saccharomycotina_2 – ps20
Saccharomycotina_3 – ps21
Pichi/Debaryo/Metschnikowiaceae – ps22
Pichiaceae – ps23
Pichiaceae_1 – ps24
Komagataella_pastoris – ps25
Phylostratum

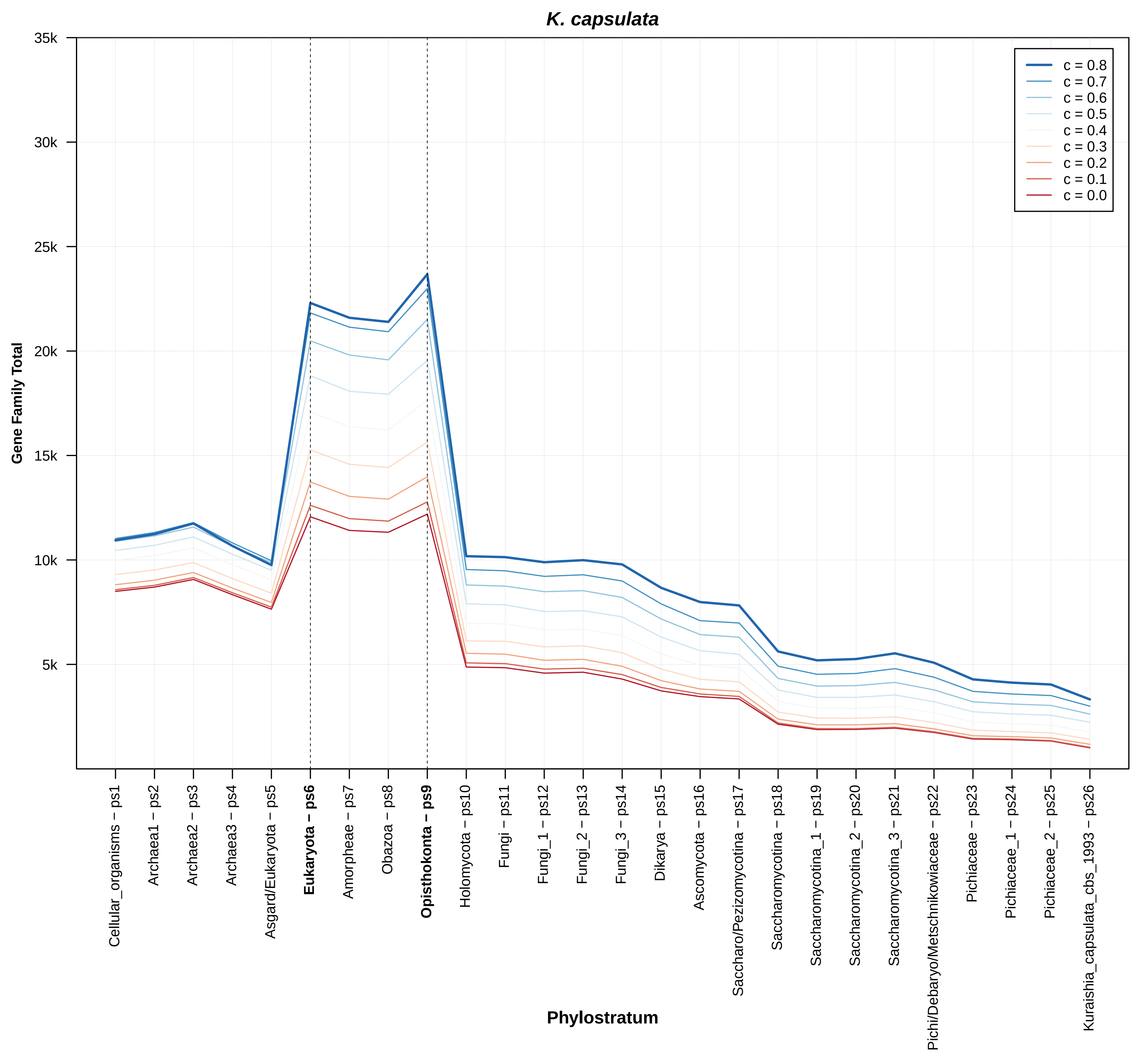

K. capsulata
Gene Family Total
35k
30k
25k
20k
15k
10k
5k
c = 0.8
c = 0.7
c = 0.6
c = 0.5
c = 0.4
c = 0.3
c = 0.2
c = 0.1
c = 0.0
Cellular_organisms – ps1
Archaea1 – ps2
Archaea2 – ps3
Archaea3 – ps4
Asgard/Eukaryota – ps5
Eukaryota – ps6
Amorpheae – ps7
Obazoa – ps8
Opisthokonta – ps9
Holomycota – ps10
Fungi – ps11
Fungi_1 – ps12
Fungi_2 – ps13
Fungi_3 – ps14
Dikarya – ps15
Ascomycota – ps16
Saccharo/Pezizomycotina – ps17
Saccharomycotina – ps18
Saccharomycotina_1 – ps19
Saccharomycotina_2 – ps20
Saccharomycotina_3 – ps21
Pichi/Debaryo/Metschnikowiaceae – ps22
Pichiaceae – ps23
Pichiaceae_1 – ps24
Pichiaceae_2 – ps25
Kuraishia_capsulata_cbs_1993 – ps26
Phylostratum

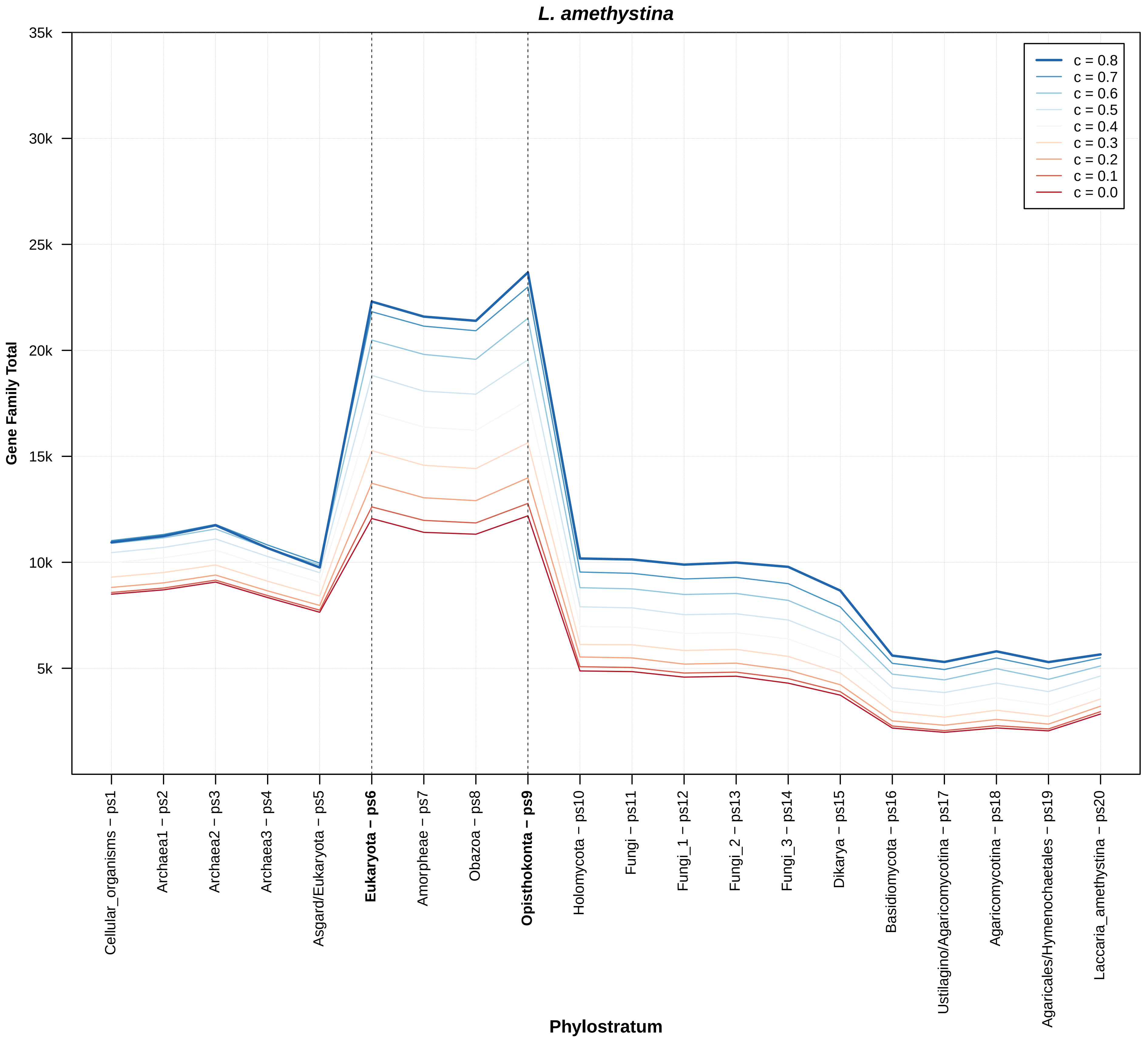
L. amethystina
Gene Family Total
35k
30k
25k
20k
15k
10k
5k
c = 0.8
c = 0.7
c = 0.6
c = 0.5
c = 0.4
c = 0.3
c = 0.2
c = 0.1
c = 0.0
Cellular_organisms – ps1
Archaea1 – ps2
Archaea2 – ps3
Archaea3 – ps4
Asgard/Eukaryota – ps5
Eukaryota – ps6
Amorpheae – ps7
Obazoa – ps8
Opisthokonta – ps9
Holomycota – ps10
Fungi – ps11
Fungi_1 – ps12
Fungi_2 – ps13
Fungi_3 – ps14
Dikarya – ps15
Basidiomycota – ps16
Ustilagino/Agaricomycotina – ps17
Agaricomycotina – ps18
Agaricales/Hymenochaetales – ps19
Laccaria_amethystina – ps20
Phylostratum

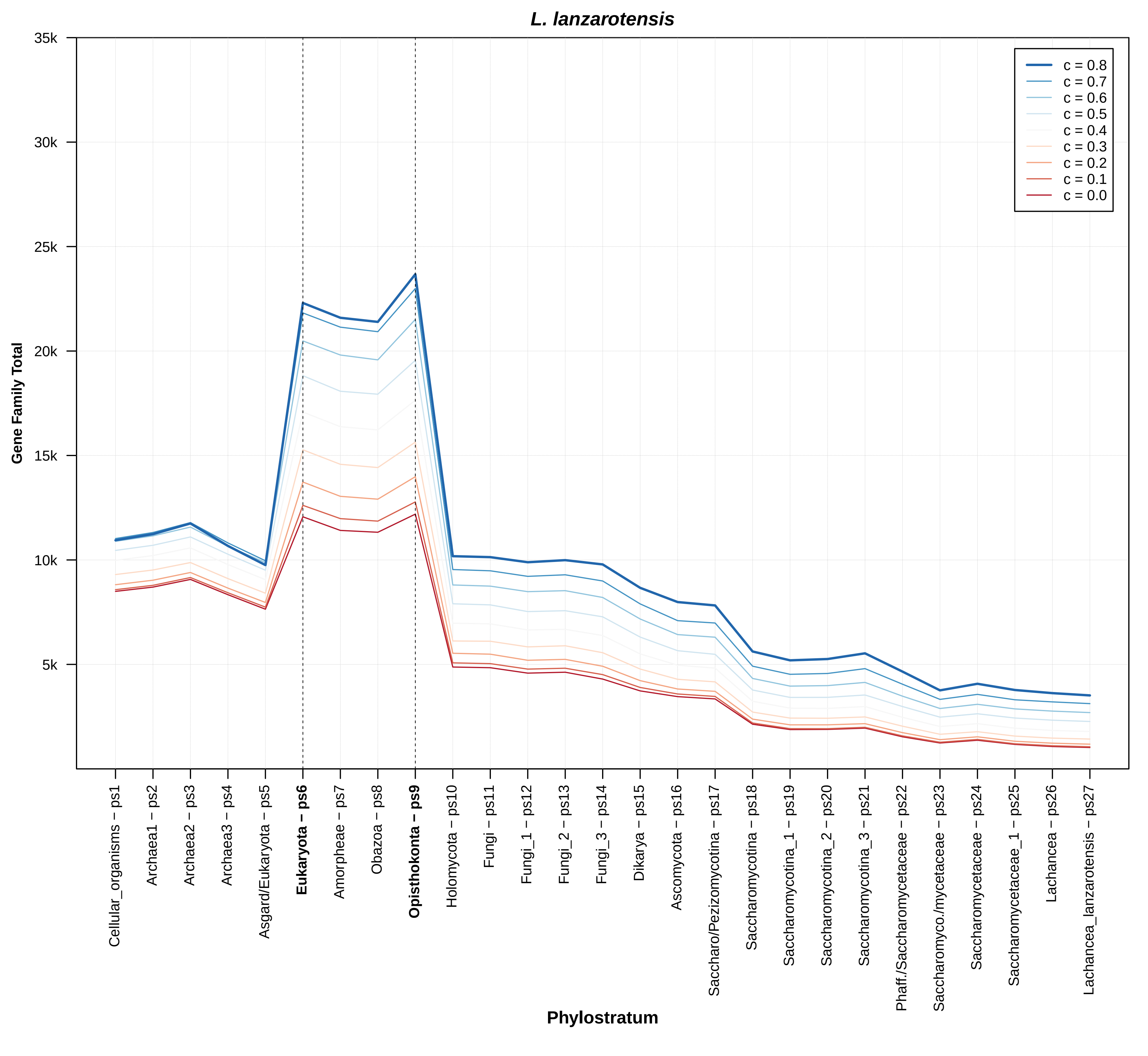

L. lanzarotensis
Gene Family Total
35k
30k
25k
20k
15k
10k
5k
c = 0.8
c = 0.7
c = 0.6
c = 0.5
c = 0.4
c = 0.3
c = 0.2
c = 0.1
c = 0.0
Cellular_organisms – ps1
Archaea1 – ps2
Archaea2 – ps3
Archaea3 – ps4
Asgard/Eukaryota – ps5
Eukaryota – ps6
Amorpheae – ps7
Obazoa – ps8
Opisthokonta – ps9
Holomycota – ps10
Fungi – ps11
Fungi_1 – ps12
Fungi_2 – ps13
Fungi_3 – ps14
Dikarya – ps15
Ascomycota – ps16
Saccharo/Pezizomycotina – ps17
Saccharomycotina – ps18
Saccharomycotina_1 – ps19
Saccharomycotina_2 – ps20
Saccharomycotina_3 – ps21
Phaff./Saccharomycetaceae – ps22
Saccharomyco./mycetaceae – ps23
Saccharomycetaceae – ps24
Saccharomycetaceae_1 – ps25
Lachancea – ps26
Lachancea_lanzarotensis – ps27
Phylostratum

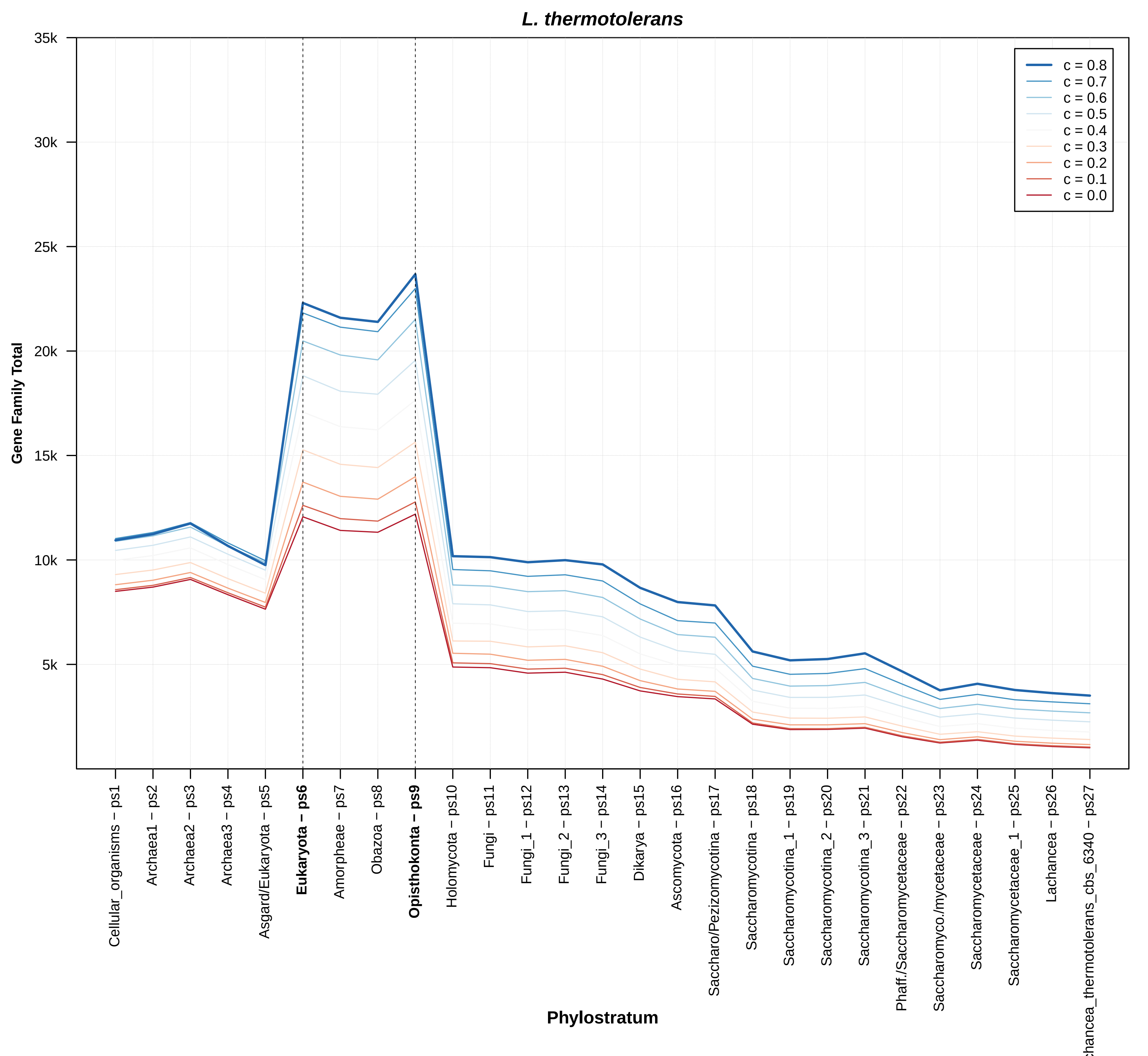

L. thermotolerans
Gene Family Total
35k
30k
25k
20k
15k
10k
5k
c = 0.8
c = 0.7
c = 0.6
c = 0.5
c = 0.4
c = 0.3
c = 0.2
c = 0.1
c = 0.0
Cellular_organisms – ps1
Archaea1 – ps2
Archaea2 – ps3
Archaea3 – ps4
Asgard/Eukaryota – ps5
Eukaryota – ps6
Amorpheae – ps7
Obazoa – ps8
Opisthokonta – ps9
Holomycota – ps10
Fungi – ps11
Fungi_1 – ps12
Fungi_2 – ps13
Fungi_3 – ps14
Dikarya – ps15
Ascomycota – ps16
Saccharo/Pezizomycotina – ps17
Saccharomycotina – ps18
Saccharomycotina_1 – ps19
Saccharomycotina_2 – ps20
Saccharomycotina_3 – ps21
Phaff./Saccharomycetaceae – ps22
Saccharomyco./mycetaceae – ps23
Saccharomycetaceae – ps24
Saccharomycetaceae_1 – ps25
Lachancea – ps26
chancea_thermotolerans_cbs_6340 – ps27
Phylostratum

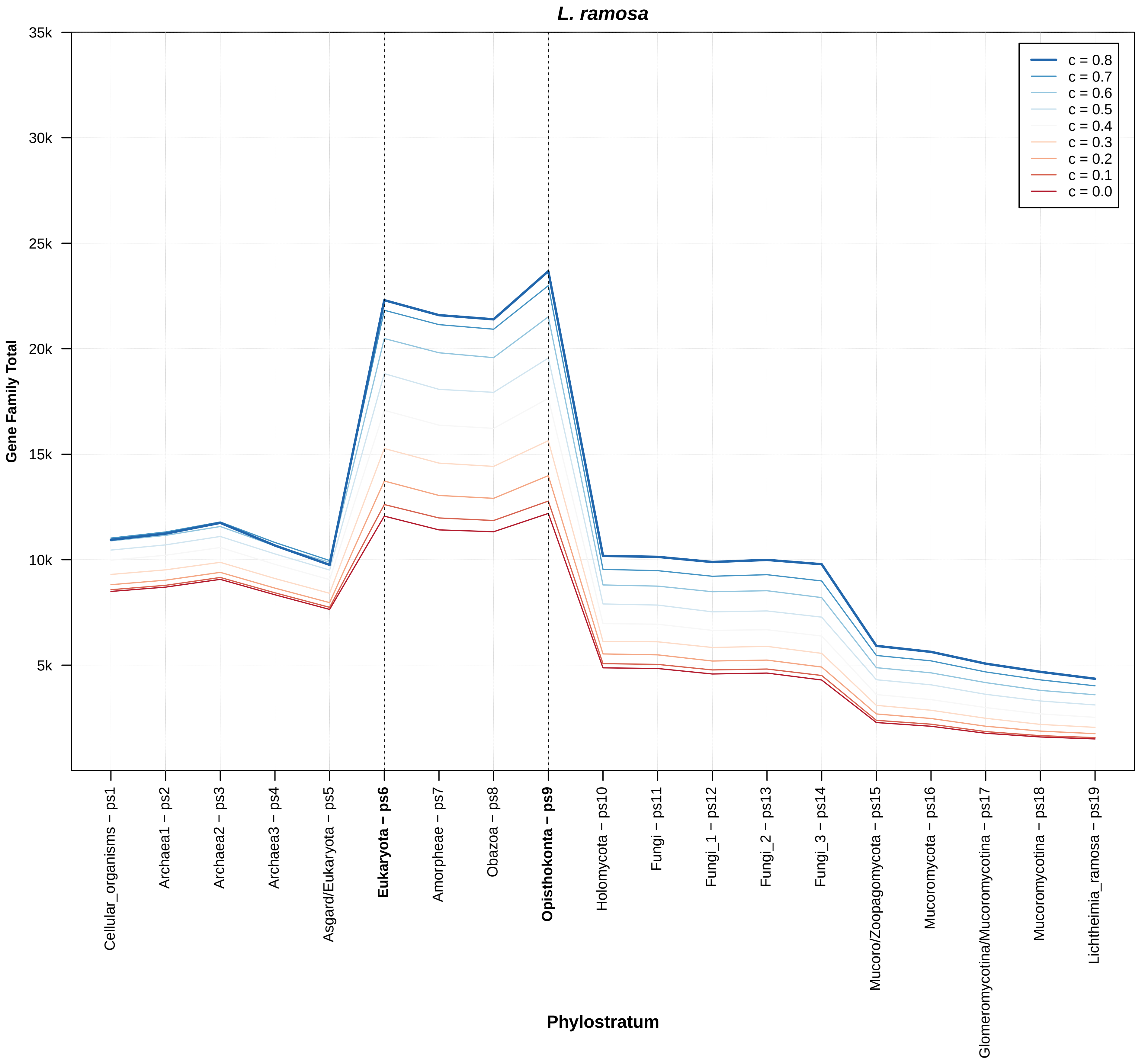
L. ramosa
Gene Family Total
35k
30k
25k
20k
15k
10k
5k
c = 0.8
c = 0.7
c = 0.6
c = 0.5
c = 0.4
c = 0.3
c = 0.2
c = 0.1
c = 0.0
Cellular_organisms – ps1
Archaea1 – ps2
Archaea2 – ps3
Archaea3 – ps4
Asgard/Eukaryota – ps5
Eukaryota – ps6
Amorpheae – ps7
Obazoa – ps8
Opisthokonta – ps9
Holomycota – ps10
Fungi – ps11
Fungi_1 – ps12
Fungi_2 – ps13
Fungi_3 – ps14
Mucoro/Zoopagomycota – ps15
Mucoromycota – ps16
Glomeromycotina/Mucoromycotina – ps17
Mucoromycotina – ps18
Lichtheimia_ramosa – ps19
Phylostratum

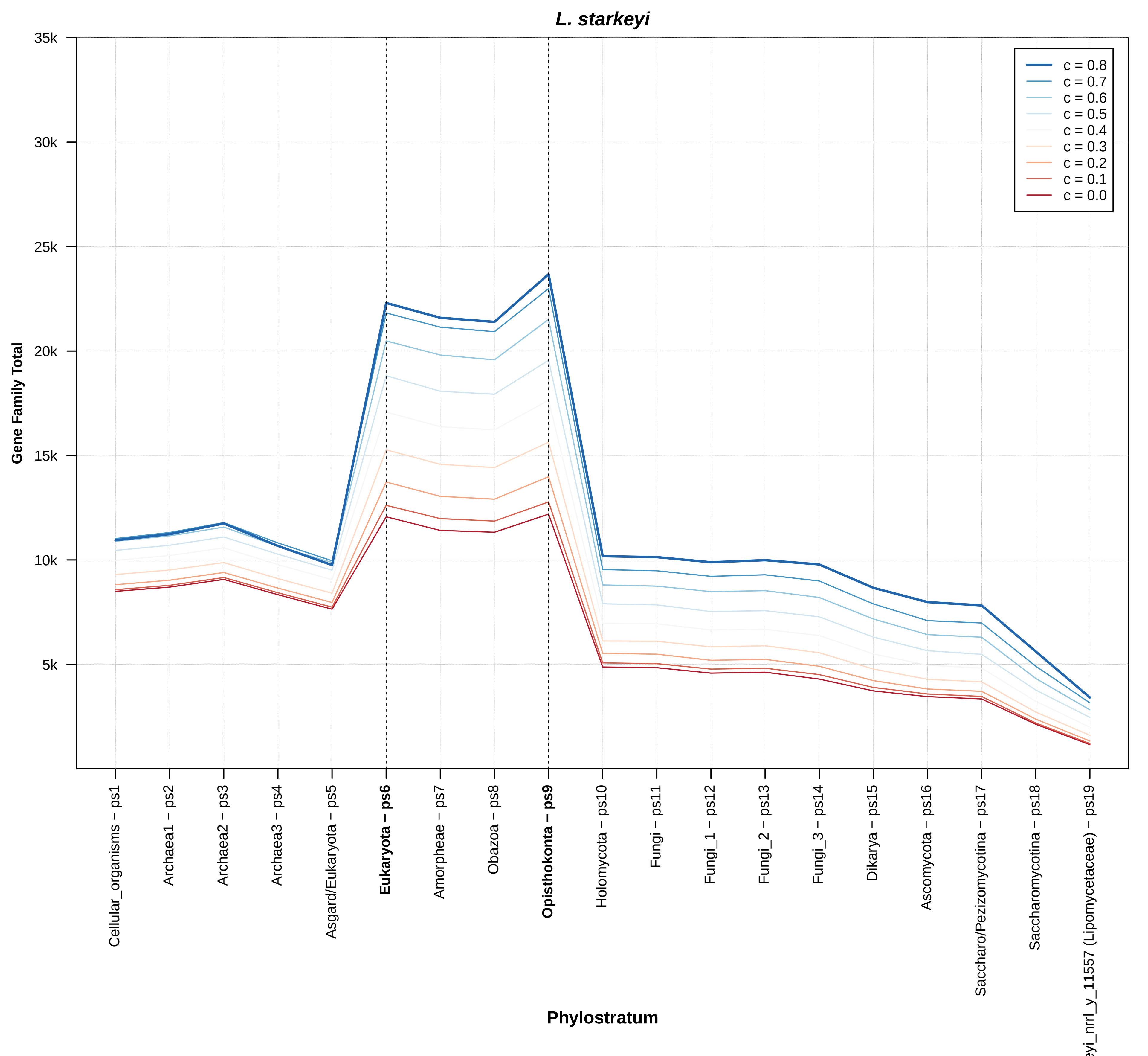
L. starkeyi
Gene Family Total
35k
30k
25k
20k
15k
10k
5k
c = 0.8
c = 0.7
c = 0.6
c = 0.5
c = 0.4
c = 0.3
c = 0.2
c = 0.1
c = 0.0
Cellular_organisms – ps1
Archaea1 – ps2
Archaea2 – ps3
Archaea3 – ps4
Asgard/Eukaryota – ps5
Eukaryota – ps6
Amorpheae – ps7
Obazoa – ps8
Opisthokonta – ps9
Holomycota – ps10
Fungi – ps11
Fungi_1 – ps12
Fungi_2 – ps13
Fungi_3 – ps14
Dikarya – ps15
Ascomycota – ps16
Saccharo/Pezizomycotina – ps17
Saccharomycotina – ps18
yi_nrrl_y_11557 (Lipomycetaceae) – ps19
Phylostratum

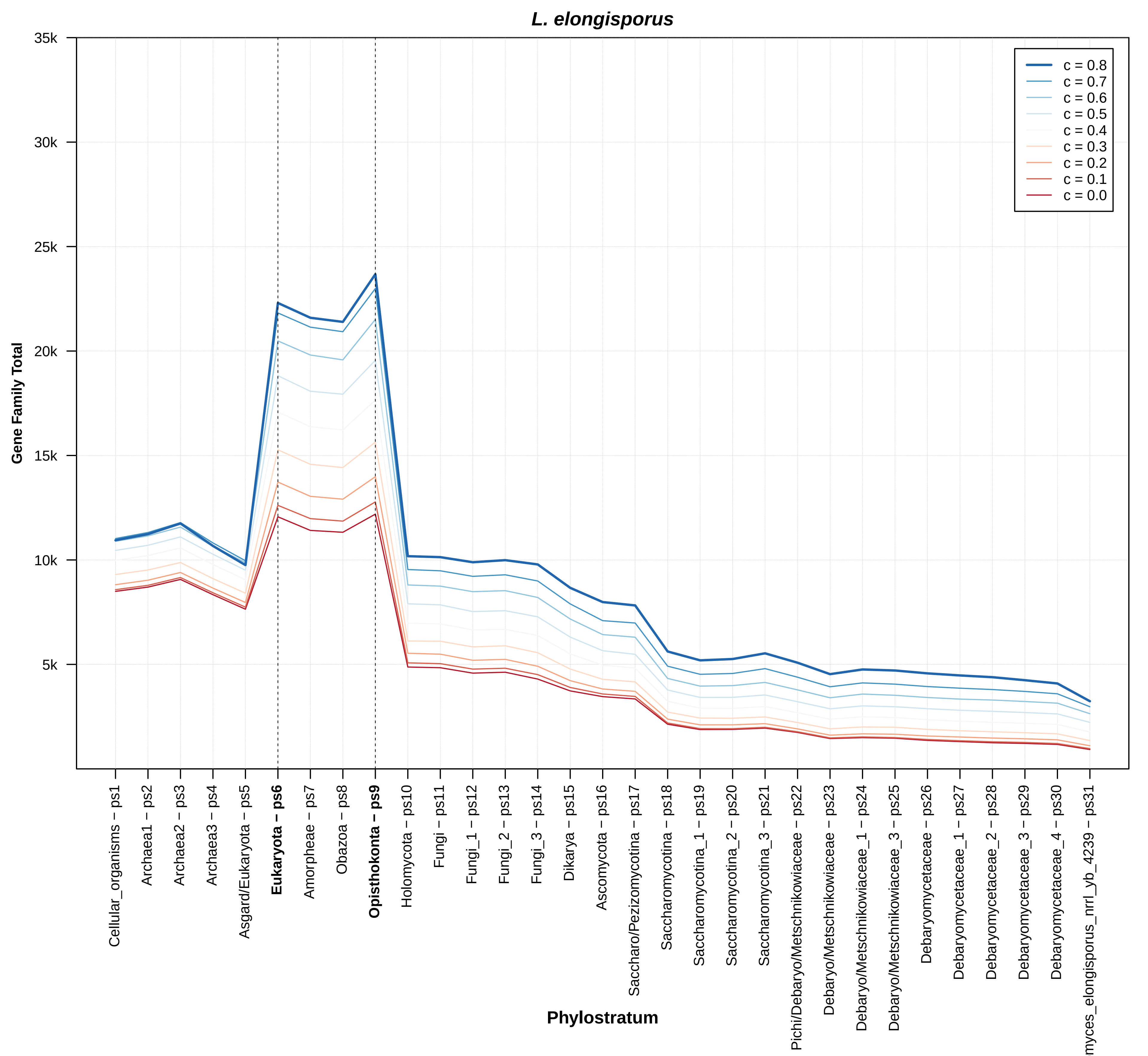
L. elongisporus
Gene Family Total
Phylostratum
35k
30k
25k
20k
15k
10k
5k
c = 0.8
c = 0.7
c = 0.6
c = 0.5
c = 0.4
c = 0.3
c = 0.2
c = 0.1
c = 0.0
Cellular_organisms – ps1
Archaea1 – ps2
Archaea2 – ps3
Archaea3 – ps4
Asgard/Eukaryota – ps5
Eukaryota – ps6
Amorpheae – ps7
Obazoa – ps8
Opisthokonta – ps9
Holomycota – ps10
Fungi – ps11
Fungi_1 – ps12
Fungi_2 – ps13
Fungi_3 – ps14
Dikarya – ps15
Ascomycota – ps16
Saccharo/Pezizomycotina – ps17
Saccharomycotina – ps18
Saccharomycotina_1 – ps19
Saccharomycotina_2 – ps20
Saccharomycotina_3 – ps21
Pichi/Debaryo/Metschnikowiaceae – ps22
Debaryo/Metschnikowiaceae – ps23
Debaryo/Metschnikowiaceae_1 – ps24
Debaryo/Metschnikowiaceae_3 – ps25
Debaryomycetaceae – ps26
Debaryomycetaceae_1 – ps27
Debaryomycetaceae_2 – ps28
Debaryomycetaceae_3 – ps29
Debaryomycetaceae_4 – ps30
myces_elongisporus_nrrl_yb_4239 – ps31

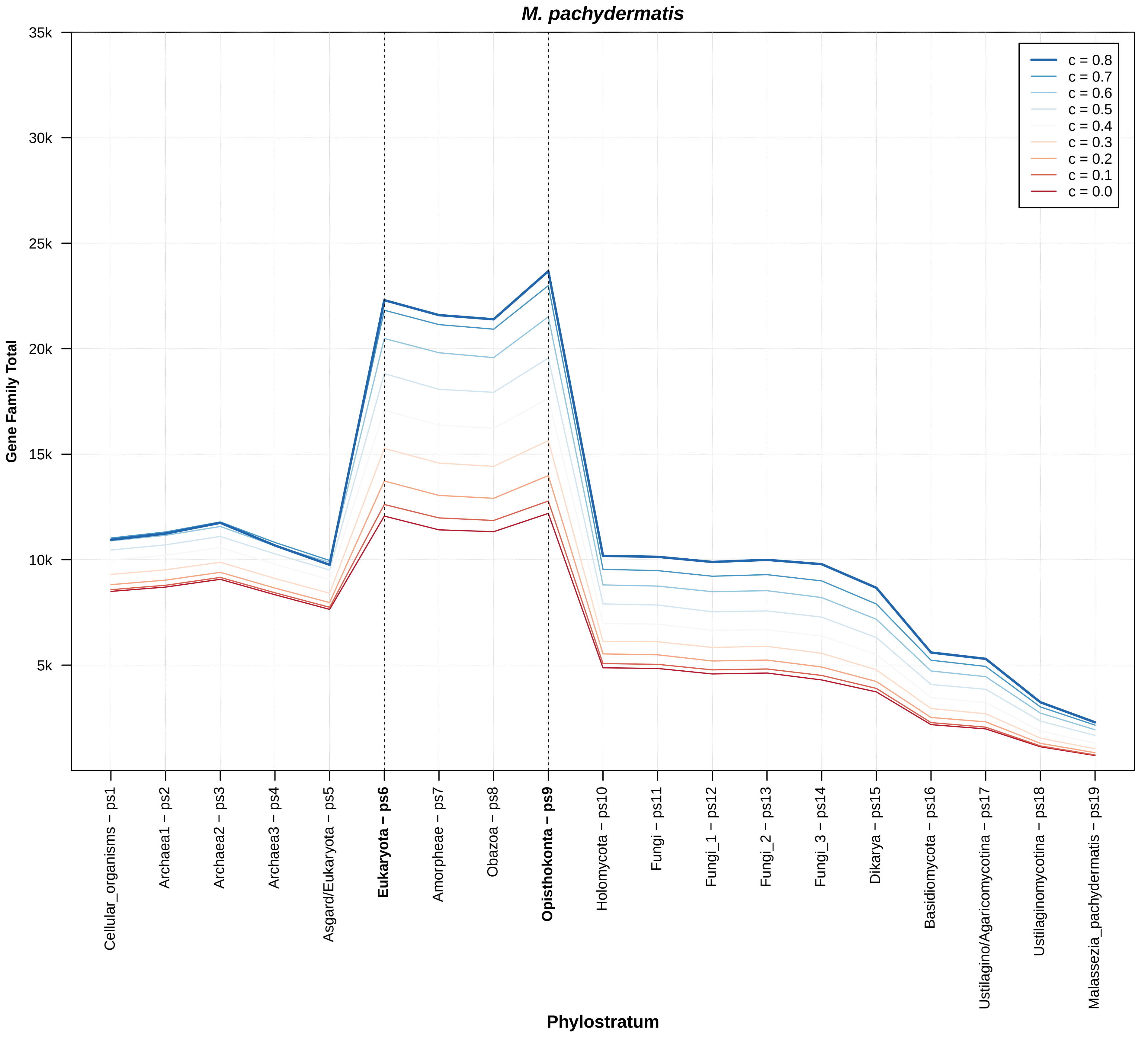
M. pachydermatis
Gene Family Total
Phylostratum
35k
30k
25k
20k
15k
10k
5k
c = 0.8
c = 0.7
c = 0.6
c = 0.5
c = 0.4
c = 0.3
c = 0.2
c = 0.1
c = 0.0
Cellular_organisms – ps1
Archaea1 – ps2
Archaea2 – ps3
Archaea3 – ps4
Asgard/Eukaryota – ps5
Eukaryota – ps6
Amorpheae – ps7
Obazoa – ps8
Opisthokonta – ps9
Holomycota – ps10
Fungi – ps11
Fungi_1 – ps12
Fungi_2 – ps13
Fungi_3 – ps14
Dikarya – ps15
Basidiomycota – ps16
Ustilagino/Agaricomycotina – ps17
Ustilaginomycotina – ps18
Malassezia_pachydermatis – ps19

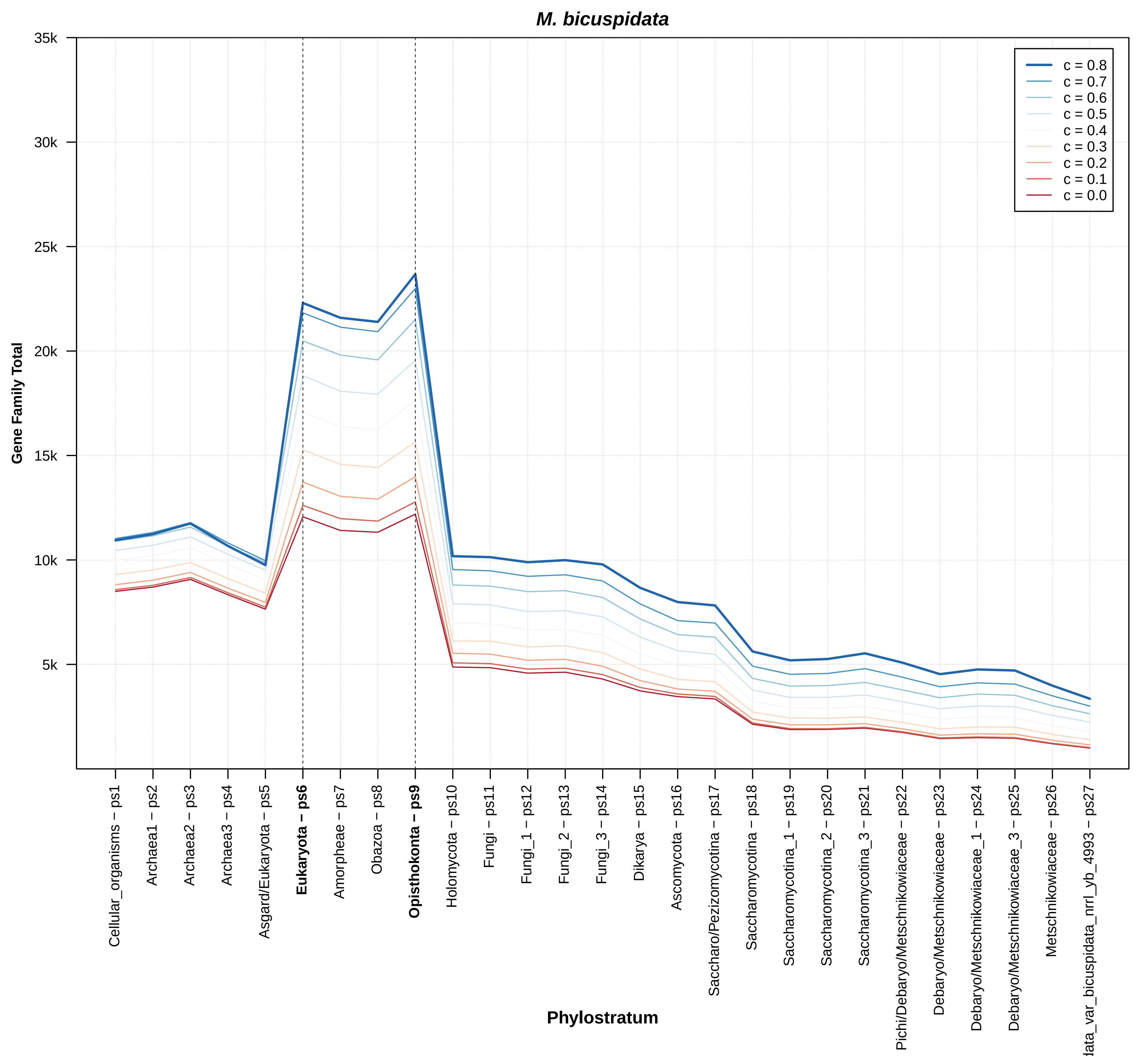

M. bicuspidata
Gene Family Total
35k
30k
25k
20k
15k
10k
5k
c = 0.8
c = 0.7
c = 0.6
c = 0.5
c = 0.4
c = 0.3
c = 0.2
c = 0.1
c = 0.0
Cellular_organisms – ps1
Archaea1 – ps2
Archaea2 – ps3
Archaea3 – ps4
Asgard/Eukaryota – ps5
Eukaryota – ps6
Amorpheae – ps7
Obazoa – ps8
Opisthokonta – ps9
Holomycota – ps10
Fungi – ps11
Fungi_1 – ps12
Fungi_2 – ps13
Fungi_3 – ps14
Dikarya – ps15
Ascomycota – ps16
Saccharo/Pezizomycotina – ps17
Saccharomycotina – ps18
Saccharomycotina_1 – ps19
Saccharomycotina_2 – ps20
Saccharomycotina_3 – ps21
Pichi/Debaryo/Metschnikowiaceae – ps22
Debaryo/Metschnikowiaceae – ps23
Debaryo/Metschnikowiaceae_1 – ps24
Debaryo/Metschnikowiaceae_3 – ps25
Metschnikowiaceae – ps26
data_var_bicuspidata_nrrl_yb_4993 – ps27
Phylostratum

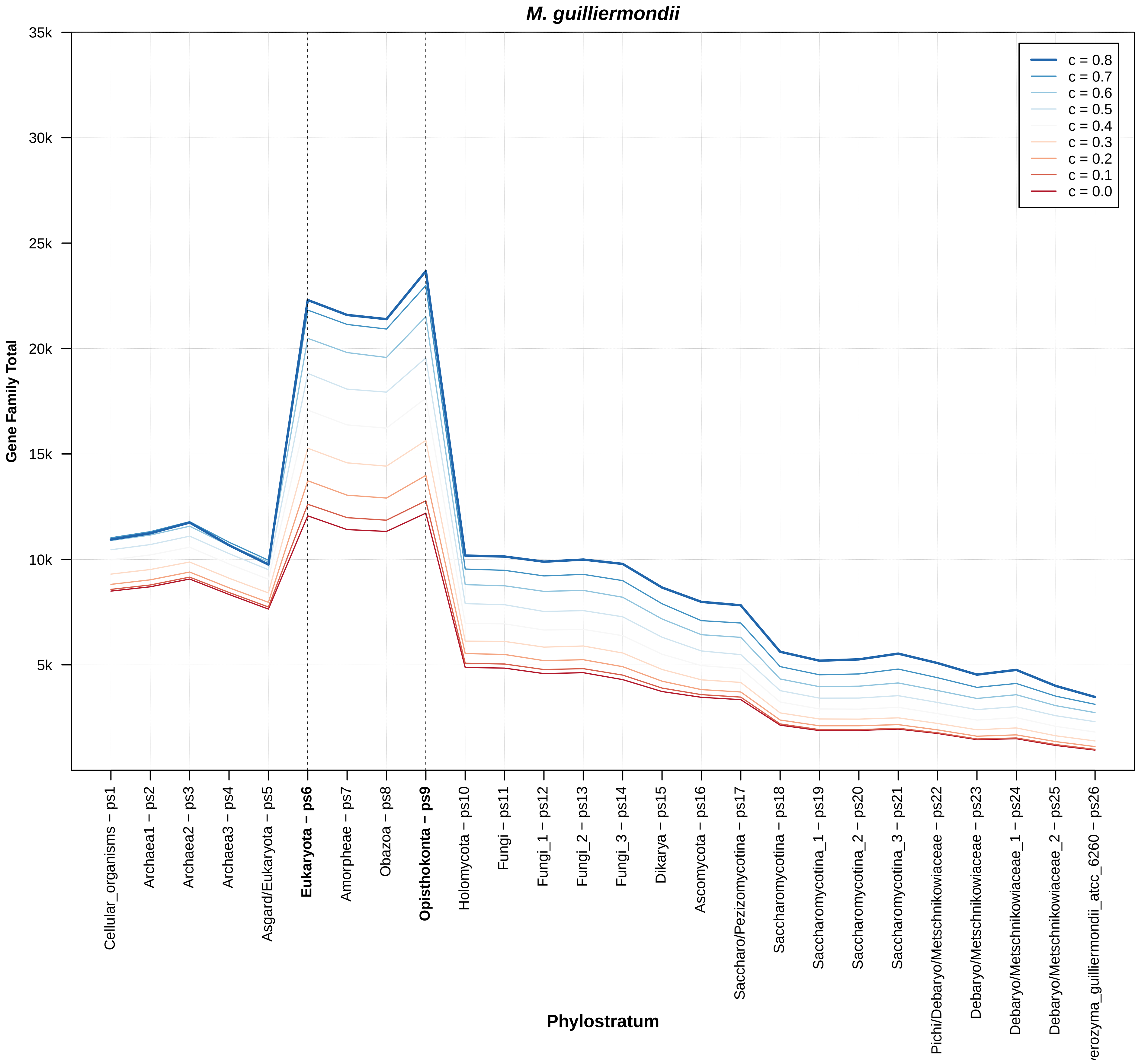

M. guilliermondii
Gene Family Total
35k
30k
25k
20k
15k
10k
5k
c = 0.8
c = 0.7
c = 0.6
c = 0.5
c = 0.4
c = 0.3
c = 0.2
c = 0.1
c = 0.0
Cellular_organisms – ps1
Archaea1 – ps2
Archaea2 – ps3
Archaea3 – ps4
Asgard/Eukaryota – ps5
Eukaryota – ps6
Amorpheae – ps7
Obazoa – ps8
Opisthokonta – ps9
Holomycota – ps10
Fungi – ps11
Fungi_1 – ps12
Fungi_2 – ps13
Fungi_3 – ps14
Dikarya – ps15
Ascomycota – ps16
Saccharo/Pezizomycotina – ps17
Saccharomycotina – ps18
Saccharomycotina_1 – ps19
Saccharomycotina_2 – ps20
Saccharomycotina_3 – ps21
Pichi/Debaryo/Metschnikowiaceae – ps22
Debaryo/Metschnikowiaceae – ps23
Debaryo/Metschnikowiaceae_1 – ps24
Debaryo/Metschnikowiaceae_2 – ps25
erozyma_guilliermondii_atcc_6260 – ps26
Phylostratum

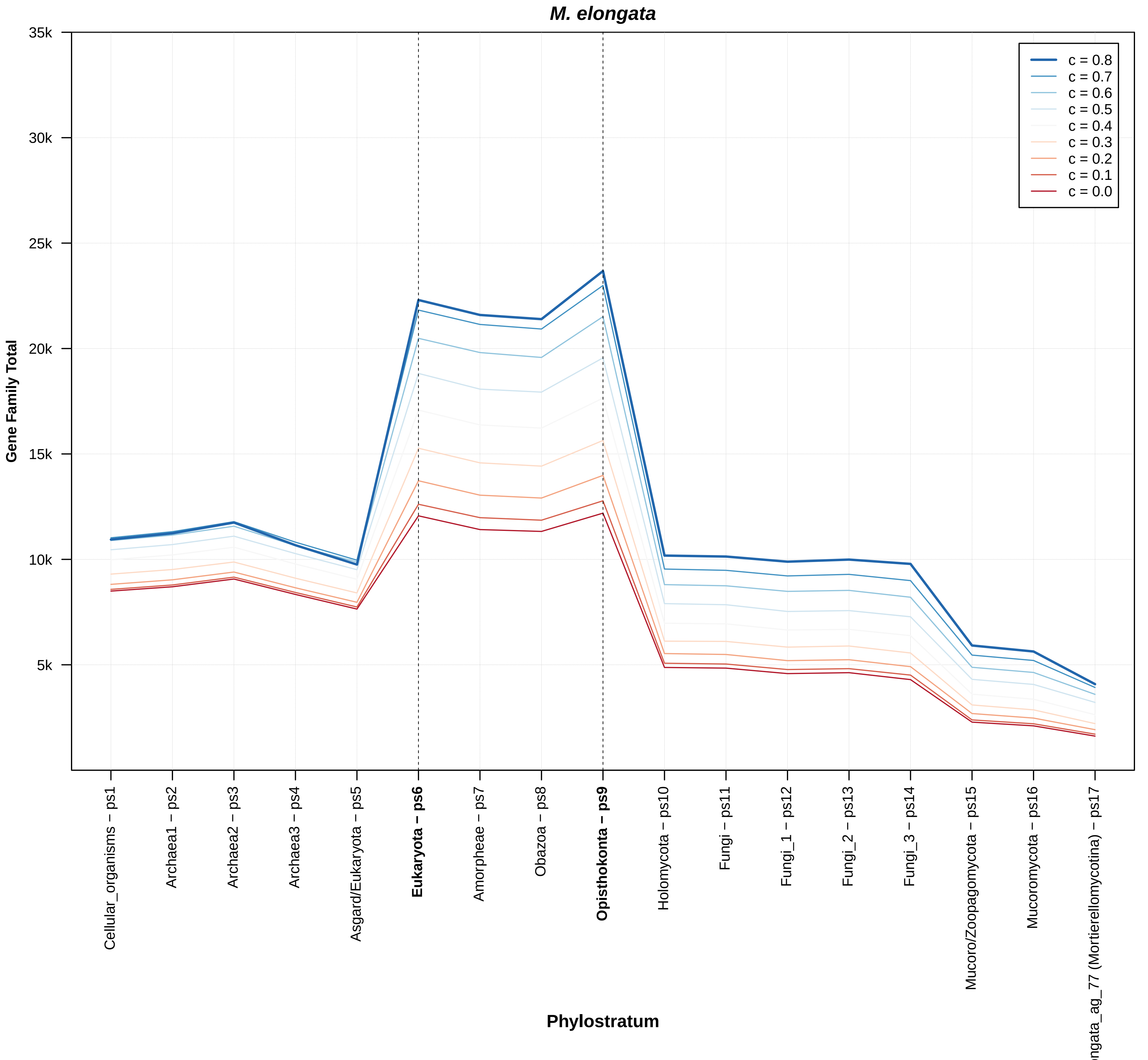

M. elongata
Gene Family Total
35k
30k
25k
20k
15k
10k
5k
c = 0.8
c = 0.7
c = 0.6
c = 0.5
c = 0.4
c = 0.3
c = 0.2
c = 0.1
c = 0.0
Cellular_organisms – ps1
Archaea1 – ps2
Archaea2 – ps3
Archaea3 – ps4
Asgard/Eukaryota – ps5
Eukaryota – ps6
Amorpheae – ps7
Obazoa – ps8
Opisthokonta – ps9
Holomycota – ps10
Fungi – ps11
Fungi_1 – ps12
Fungi_2 – ps13
Fungi_3 – ps14
Mucoro/Zoopagomycota – ps15
Mucoromycota – ps16
ngata_ag_77 (Mortierellomycotina) – ps17
Phylostratum

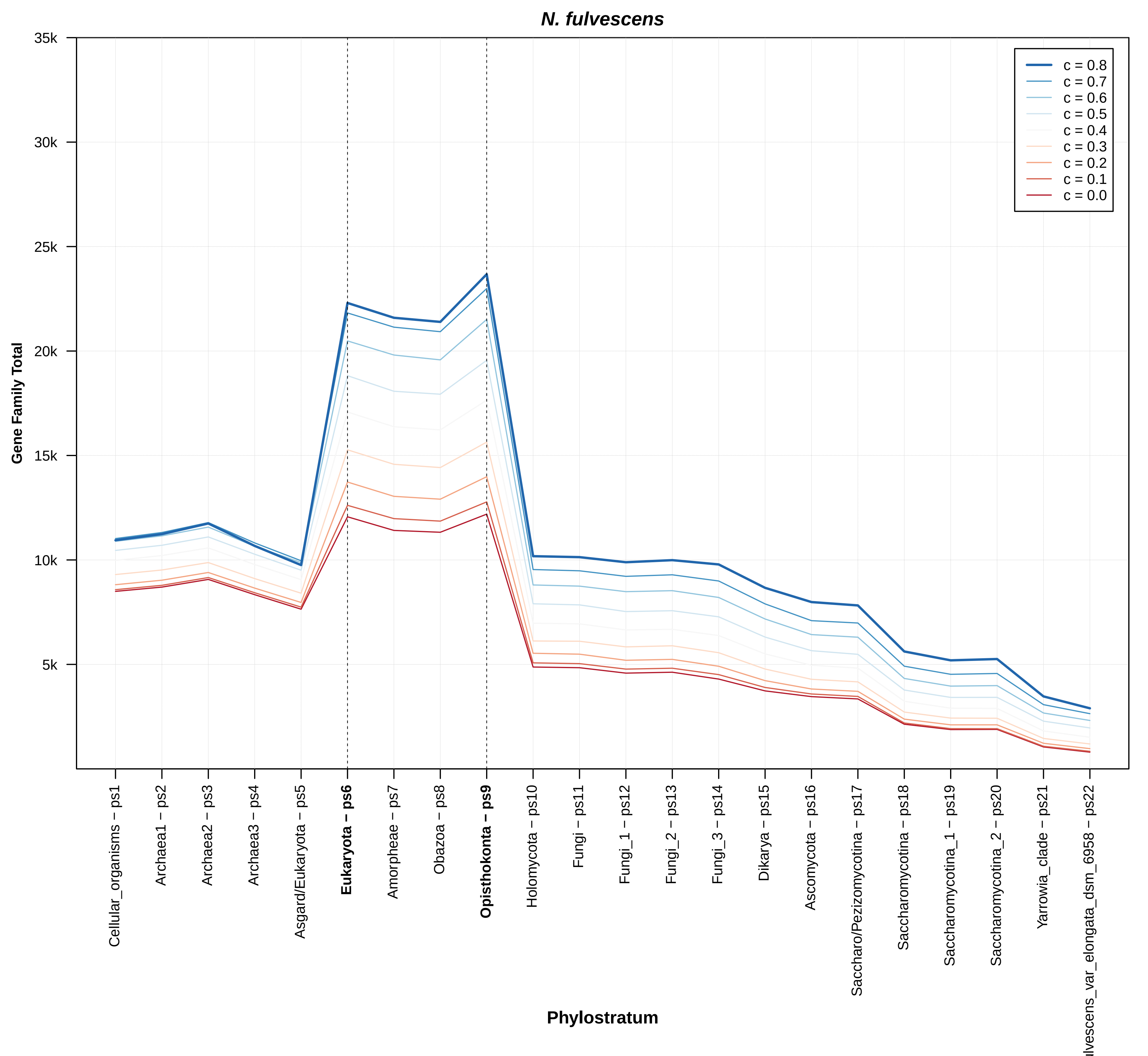

N. fulvescens
Gene Family Total
35k
30k
25k
20k
15k
10k
5k
c = 0.8
c = 0.7
c = 0.6
c = 0.5
c = 0.4
c = 0.3
c = 0.2
c = 0.1
c = 0.0
Cellular_organisms – ps1
Archaea1 – ps2
Archaea2 – ps3
Archaea3 – ps4
Asgard/Eukaryota – ps5
Eukaryota – ps6
Amorpheae – ps7
Obazoa – ps8
Opisthokonta – ps9
Holomycota – ps10
Fungi – ps11
Fungi_1 – ps12
Fungi_2 – ps13
Fungi_3 – ps14
Dikarya – ps15
Ascomycota – ps16
Saccharo/Pezizomycotina – ps17
Saccharomycotina – ps18
Saccharomycotina_1 – ps19
Saccharomycotina_2 – ps20
Yarrowia_clade – ps21
lvescens_var_elongata_dsm_6958 – ps22
Phylostratum

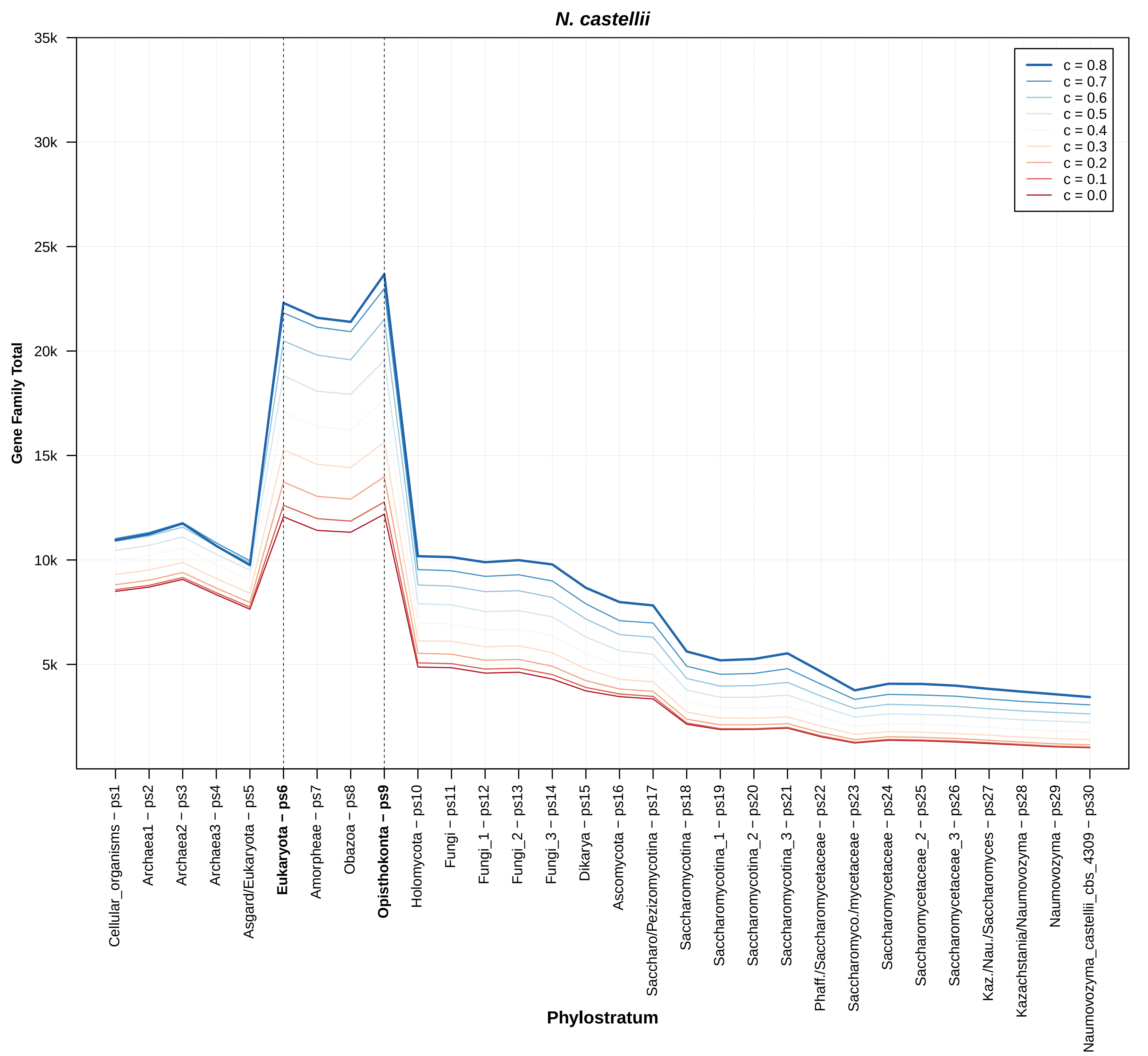

N. castellii
Gene Family Total
35k
30k
25k
20k
15k
10k
5k
c = 0.8
c = 0.7
c = 0.6
c = 0.5
c = 0.4
c = 0.3
c = 0.2
c = 0.1
c = 0.0
Cellular_organisms – ps1
Archaea1 – ps2
Archaea2 – ps3
Archaea3 – ps4
Asgard/Eukaryota – ps5
Eukaryota – ps6
Amorpheae – ps7
Obazoa – ps8
Opisthokonta – ps9
Holomycota – ps10
Fungi – ps11
Fungi_1 – ps12
Fungi_2 – ps13
Fungi_3 – ps14
Dikarya – ps15
Ascomycota – ps16
Saccharo/Pezizomycotina – ps17
Saccharomycotina – ps18
Saccharomycotina_1 – ps19
Saccharomycotina_2 – ps20
Saccharomycotina_3 – ps21
Phaff./Saccharomycetaceae – ps22
Saccharomyco./mycetaceae – ps23
Saccharomycetaceae – ps24
Saccharomycetaceae_2 – ps25
Saccharomycetaceae_3 – ps26
Kaz./Nau./Saccharomyces – ps27
Kazachstania/Naumovozyma – ps28
Naumovozyma – ps29
Naumovozyma_castellii_cbs_4309 – ps30
Phylostratum

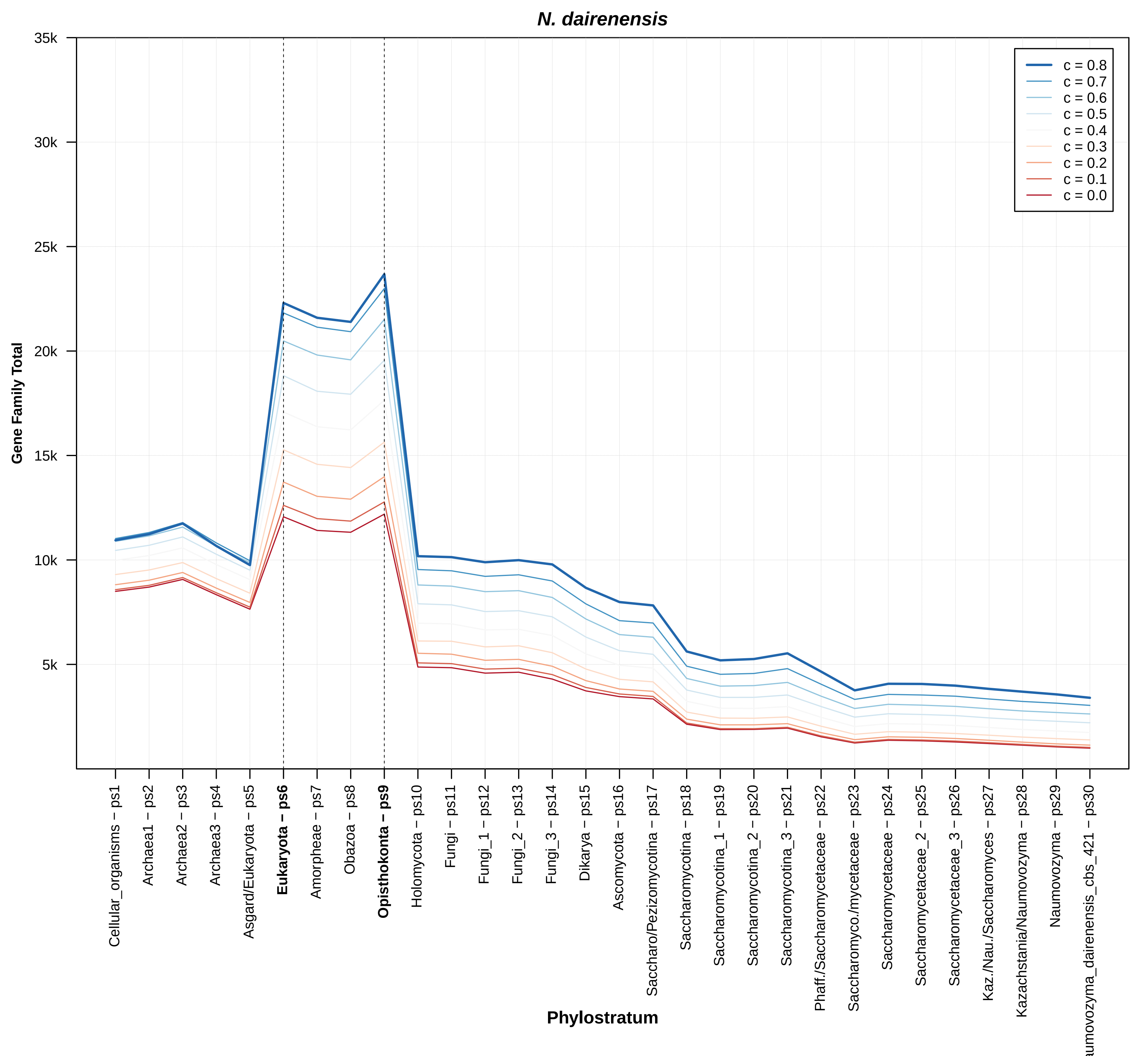

N. dairenensis
Gene Family Total
35k
30k
25k
20k
15k
10k
5k
c = 0.8
c = 0.7
c = 0.6
c = 0.5
c = 0.4
c = 0.3
c = 0.2
c = 0.1
c = 0.0
Cellular_organisms – ps1
Archaea1 – ps2
Archaea2 – ps3
Archaea3 – ps4
Asgard/Eukaryota – ps5
Eukaryota – ps6
Amorpheae – ps7
Obazoa – ps8
Opisthokonta – ps9
Holomycota – ps10
Fungi – ps11
Fungi_1 – ps12
Fungi_2 – ps13
Fungi_3 – ps14
Dikarya – ps15
Ascomycota – ps16
Saccharo/Pezizomycotina – ps17
Saccharomycotina – ps18
Saccharomycotina_1 – ps19
Saccharomycotina_2 – ps20
Saccharomycotina_3 – ps21
Phaff./Saccharomycetaceae – ps22
Saccharomyco./mycetaceae – ps23
Saccharomycetaceae – ps24
Saccharomycetaceae_2 – ps25
Saccharomycetaceae_3 – ps26
Kaz./Nau./Saccharomyces – ps27
Kazachstania/Naumovozyma – ps28
Naumovozyma – ps29
aumovozyma_dairenensis_cbs_421 – ps30
Phylostratum

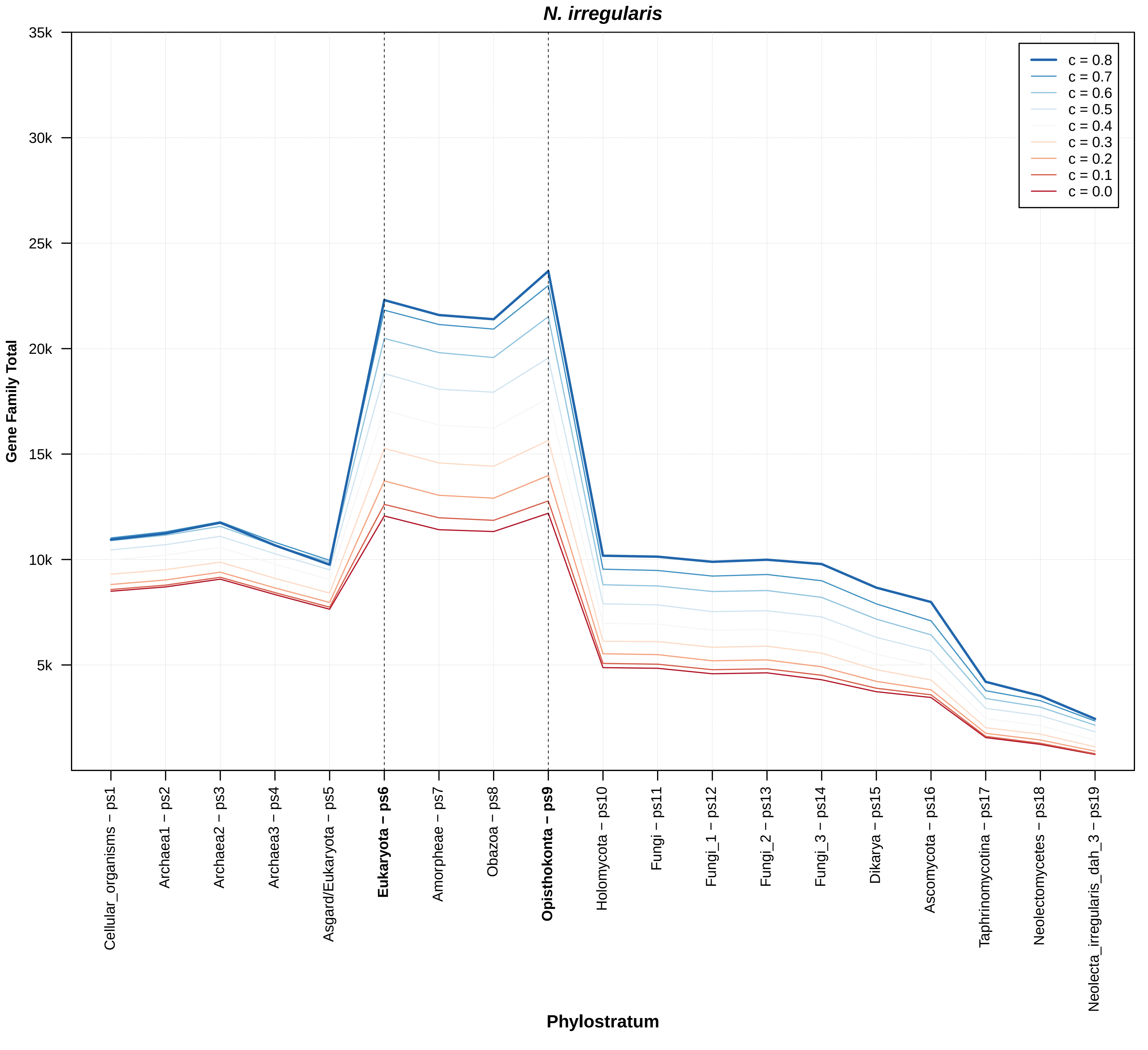
N. irregularis
Gene Family Total
35k
30k
25k
20k
15k
10k
5k
c = 0.8
c = 0.7
c = 0.6
c = 0.5
c = 0.4
c = 0.3
c = 0.2
c = 0.1
c = 0.0
Cellular_organisms – ps1
Archaea1 – ps2
Archaea2 – ps3
Archaea3 – ps4
Asgard/Eukaryota – ps5
Eukaryota – ps6
Amorpheae – ps7
Obazoa – ps8
Opisthokonta – ps9
Holomycota – ps10
Fungi – ps11
Fungi_1 – ps12
Fungi_2 – ps13
Fungi_3 – ps14
Dikarya – ps15
Ascomycota – ps16
Taphrinomycotina – ps17
Neolectomycetes – ps18
Neolecta_irregularis_dah_3 – ps19
Phylostratum

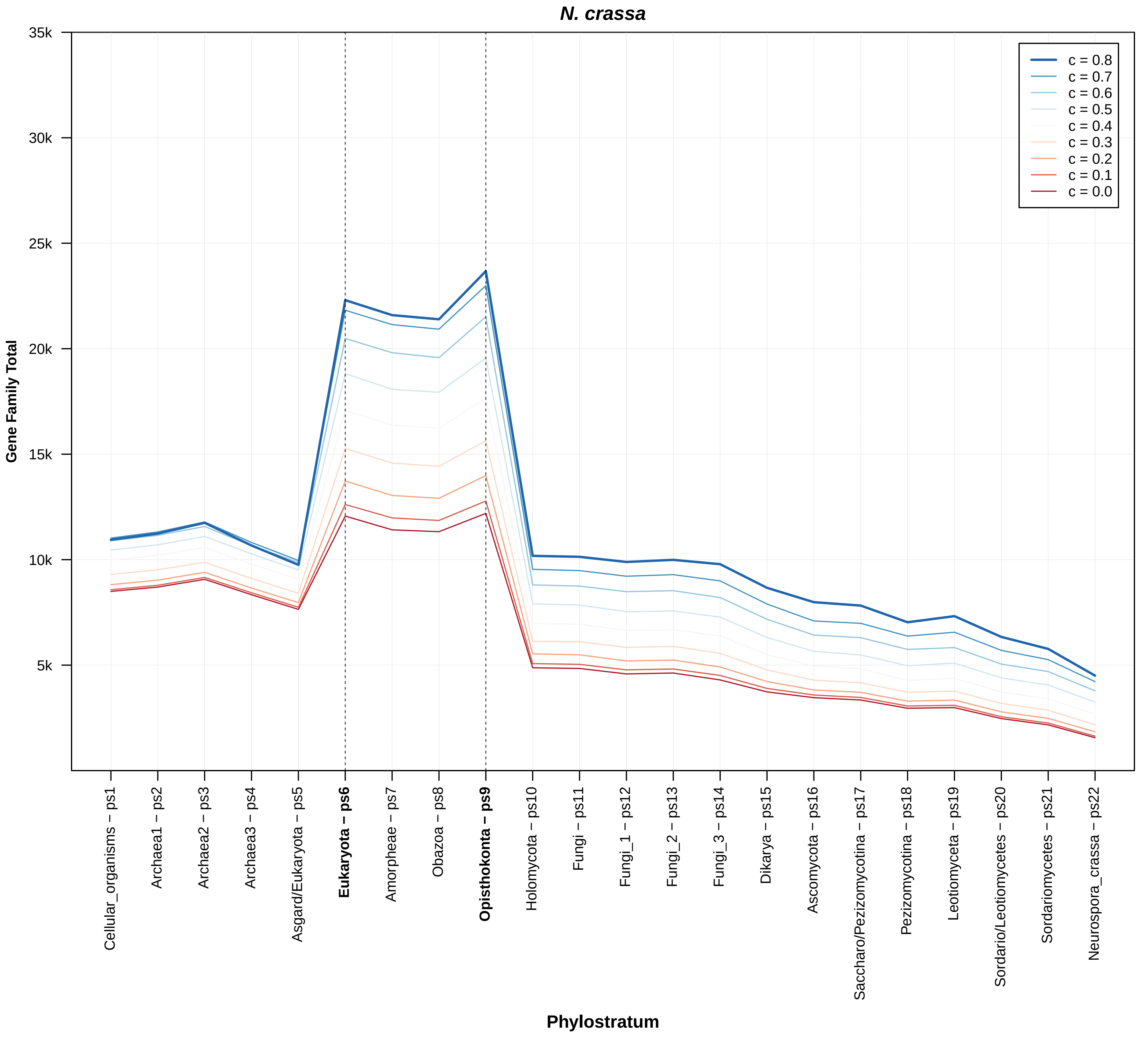

N. crassa
Gene Family Total
35k
30k
25k
20k
15k
10k
5k
c = 0.8
c = 0.7
c = 0.6
c = 0.5
c = 0.4
c = 0.3
c = 0.2
c = 0.1
c = 0.0
Cellular_organisms – ps1
Archaea1 – ps2
Archaea2 – ps3
Archaea3 – ps4
Asgard/Eukaryota – ps5
Eukaryota – ps6
Amorpheae – ps7
Obazoa – ps8
Opisthokonta – ps9
Holomycota – ps10
Fungi – ps11
Fungi_1 – ps12
Fungi_2 – ps13
Fungi_3 – ps14
Dikarya – ps15
Ascomycota – ps16
Saccharo/Pezizomycotina – ps17
Pezizomycotina – ps18
Leotiomyceta – ps19
Sordario/Leotiomycetes – ps20
Sordariomycetes – ps21
Neurospora_crassa – ps22
Phylostratum

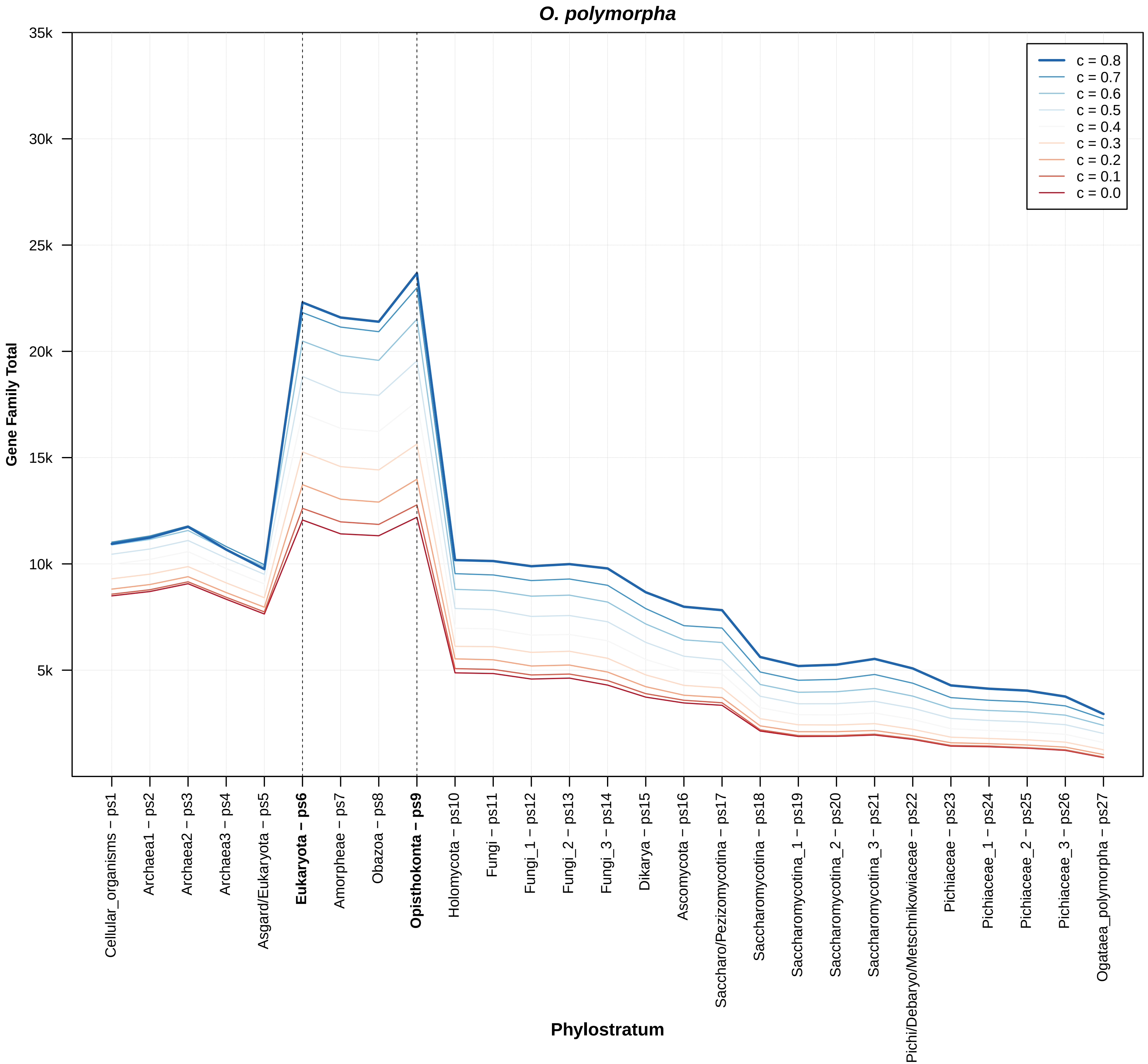
O. polymorpha
Gene Family Total
Phylostratum
35k
30k
25k
20k
15k
10k
5k
c = 0.8
c = 0.7
c = 0.6
c = 0.5
c = 0.4
c = 0.3
c = 0.2
c = 0.1
c = 0.0
Cellular_organisms – ps1
Archaea1 – ps2
Archaea2 – ps3
Archaea3 – ps4
Asgard/Eukaryota – ps5
Eukaryota – ps6
Amorpheae – ps7
Obazoa – ps8
Opisthokonta – ps9
Holomycota – ps10
Fungi – ps11
Fungi_1 – ps12
Fungi_2 – ps13
Fungi_3 – ps14
Dikarya – ps15
Ascomycota – ps16
Saccharo/Pezizomycotina – ps17
Saccharomycotina – ps18
Saccharomycotina_1 – ps19
Saccharomycotina_2 – ps20
Saccharomycotina_3 – ps21
Pichi/Debaryo/Metschnikowiaceae – ps22
Pichiaceae – ps23
Pichiaceae_1 – ps24
Pichiaceae_2 – ps25
Pichiaceae_3 – ps26
Ogataea_polymorpha – ps27

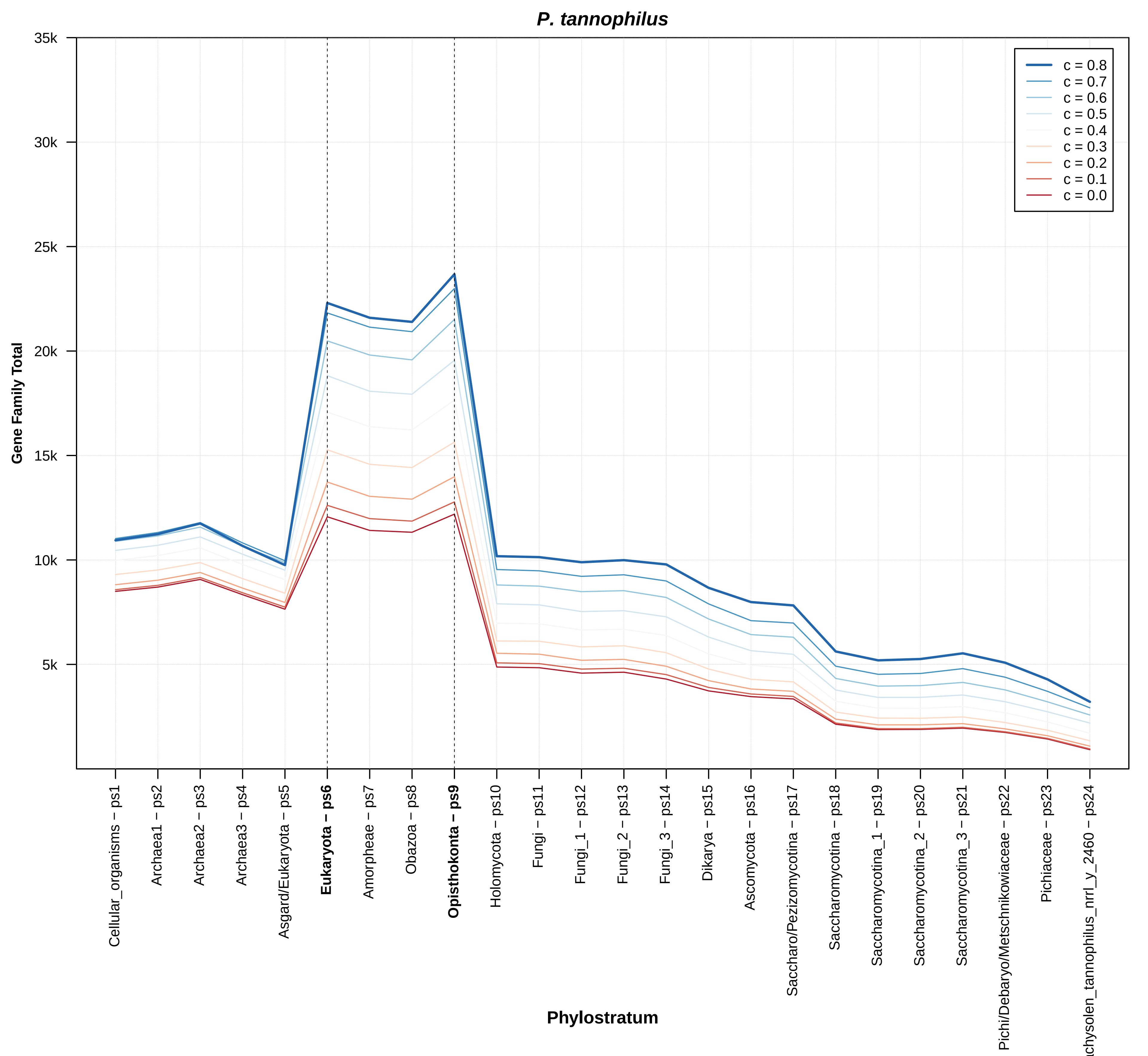

P. tannophilus
Gene Family Total
35k
30k
25k
20k
15k
10k
5k
c = 0.8
c = 0.7
c = 0.6
c = 0.5
c = 0.4
c = 0.3
c = 0.2
c = 0.1
c = 0.0
Cellular_organisms – ps1
Archaea1 – ps2
Archaea2 – ps3
Archaea3 – ps4
Asgard/Eukaryota – ps5
Eukaryota – ps6
Amorpheae – ps7
Obazoa – ps8
Opisthokonta – ps9
Holomycota – ps10
Fungi – ps11
Fungi_1 – ps12
Fungi_2 – ps13
Fungi_3 – ps14
Dikarya – ps15
Ascomycota – ps16
Saccharo/Pezizomycotina – ps17
Saccharomycotina – ps18
Saccharomycotina_1 – ps19
Saccharomycotina_2 – ps20
Saccharomycotina_3 – ps21
Pichi/Debaryo/Metschnikowiaceae – ps22
Pichiaceae – ps23
achysolen_tannophilus_nrrl_y_2460 – ps24
Phylostratum

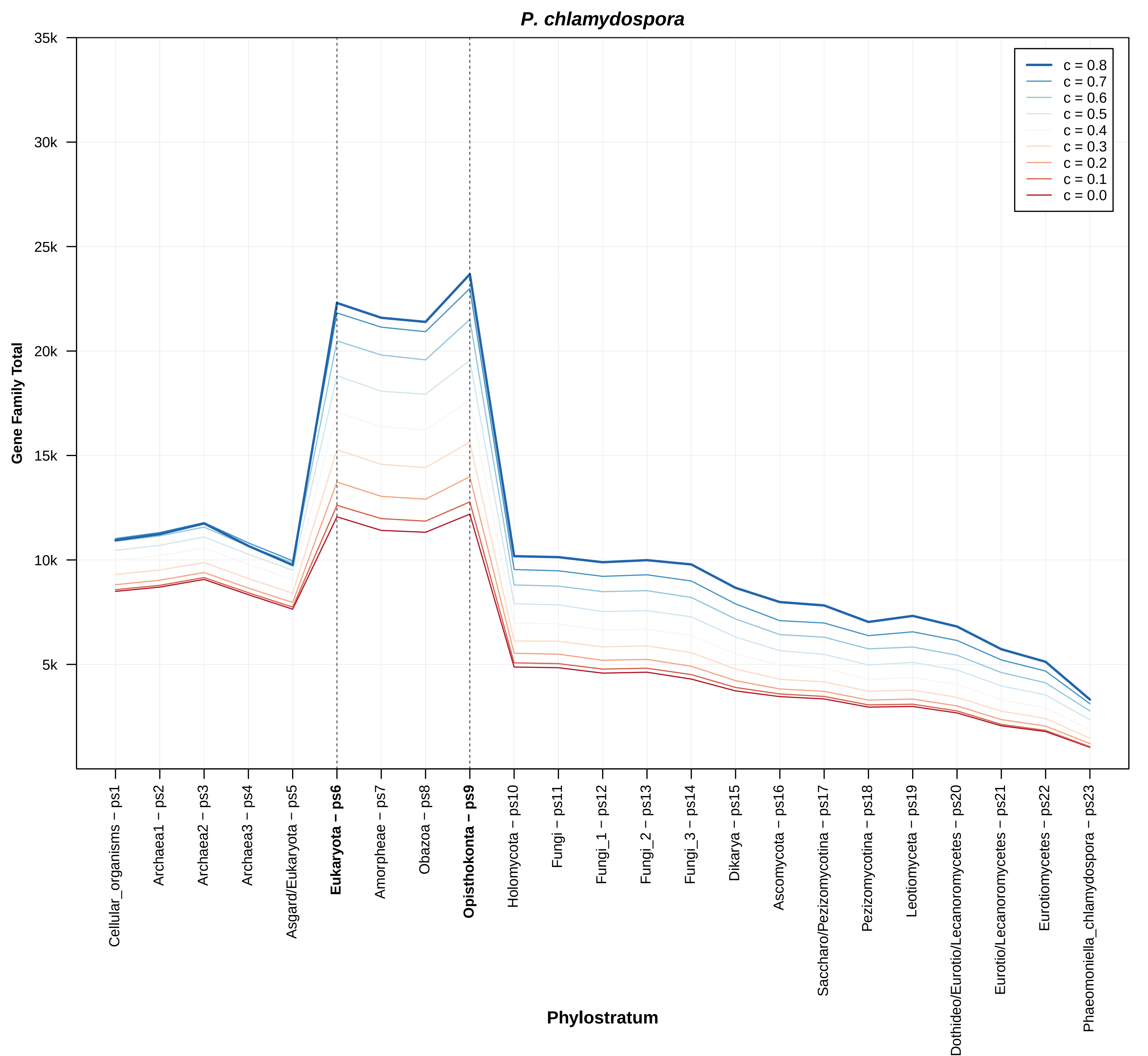
P. chlamydospora
Gene Family Total
35k
30k
25k
20k
15k
10k
5k
c = 0.8
c = 0.7
c = 0.6
c = 0.5
c = 0.4
c = 0.3
c = 0.2
c = 0.1
c = 0.0
Cellular_organisms – ps1
Archaea1 – ps2
Archaea2 – ps3
Archaea3 – ps4
Asgard/Eukaryota – ps5
Eukaryota – ps6
Amorpheae – ps7
Obazoa – ps8
Opisthokonta – ps9
Holomycota – ps10
Fungi – ps11
Fungi_1 – ps12
Fungi_2 – ps13
Fungi_3 – ps14
Dikarya – ps15
Ascomycota – ps16
Saccharo/Pezizomycotina – ps17
Pezizomycotina – ps18
Leotiomyceta – ps19
Dothideo/Eurotio/Lecanoromycetes – ps20
Eurotio/Lecanoromycetes – ps21
Eurotiomycetes – ps22
Phaeomoniella_chlamydospora – ps23
Phylostratum

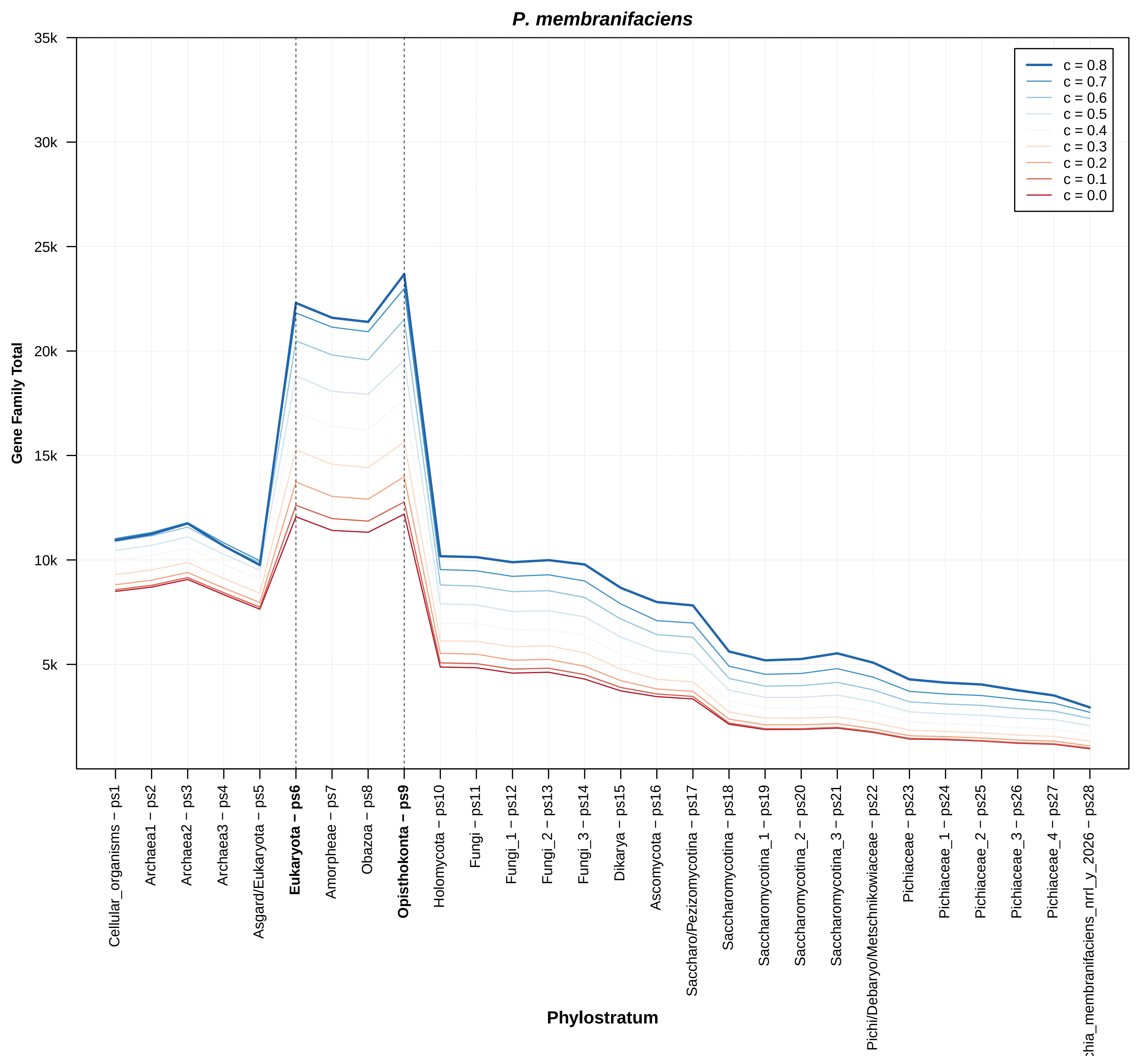
P. membranifaciens
Gene Family Total
35k
30k
25k
20k
15k
10k
5k
c = 0.8
c = 0.7
c = 0.6
c = 0.5
c = 0.4
c = 0.3
c = 0.2
c = 0.1
c = 0.0
Cellular_organisms – ps1
Archaea1 – ps2
Archaea2 – ps3
Archaea3 – ps4
Asgard/Eukaryota – ps5
Eukaryota – ps6
Amorpheae – ps7
Obazoa – ps8
Opisthokonta – ps9
Holomycota – ps10
Fungi – ps11
Fungi_1 – ps12
Fungi_2 – ps13
Fungi_3 – ps14
Dikarya – ps15
Ascomycota – ps16
Saccharo/Pezizomycotina – ps17
Saccharomycotina – ps18
Saccharomycotina_1 – ps19
Saccharomycotina_2 – ps20
Saccharomycotina_3 – ps21
Pichi/Debaryo/Metschnikowiaceae – ps22
Pichiaceae – ps23
Pichiaceae_1 – ps24
Pichiaceae_2 – ps25
Pichiaceae_3 – ps26
Pichiaceae_4 – ps27
chia_membranifaciens_nrrl_y_2026 – ps28
Phylostratum

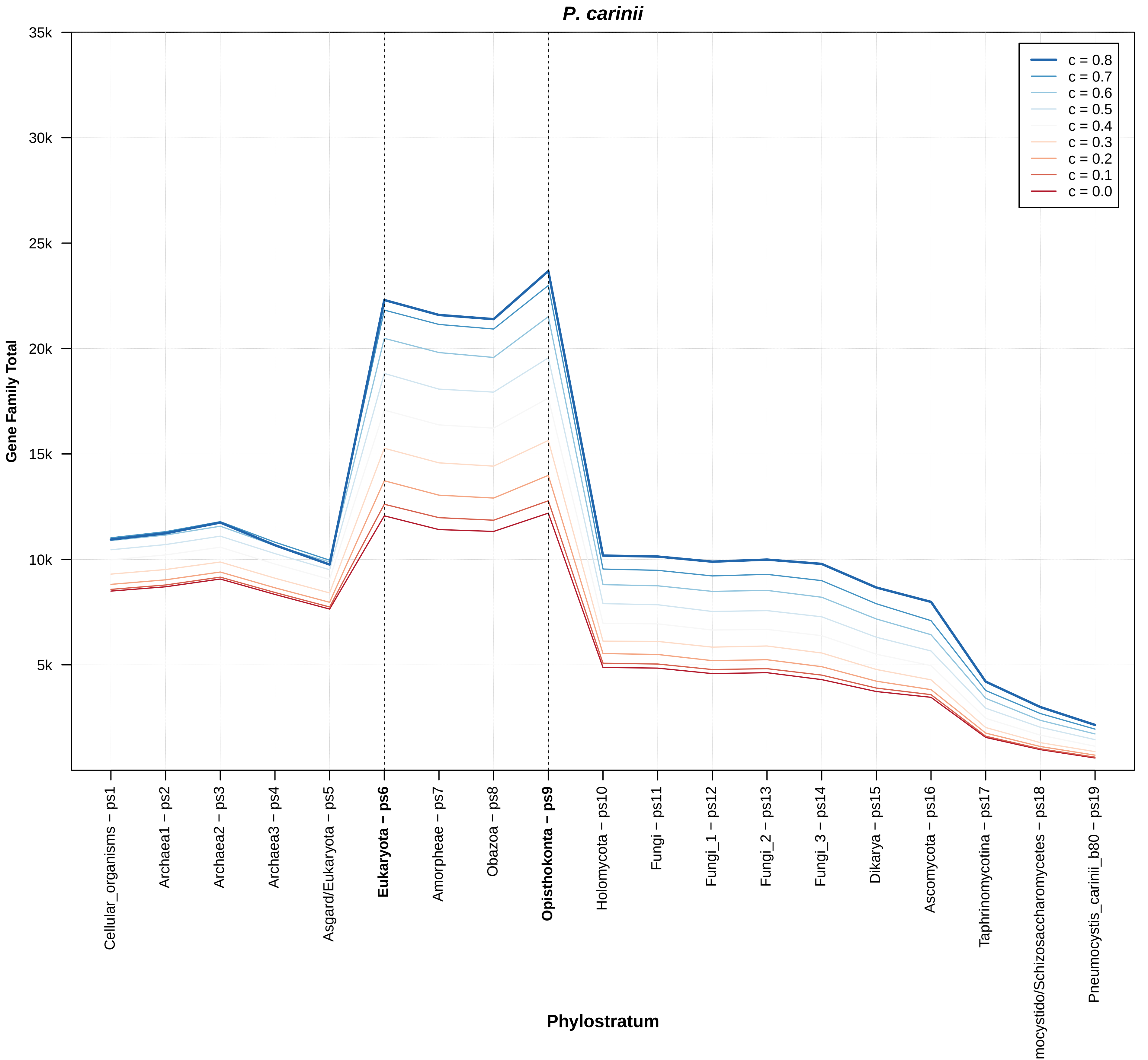

P. carinii
Gene Family Total
35k
30k
25k
20k
15k
10k
5k
c = 0.8
c = 0.7
c = 0.6
c = 0.5
c = 0.4
c = 0.3
c = 0.2
c = 0.1
c = 0.0
Cellular_organisms – ps1
Archaea1 – ps2
Archaea2 – ps3
Archaea3 – ps4
Asgard/Eukaryota – ps5
Eukaryota – ps6
Amorpheae – ps7
Obazoa – ps8
Opisthokonta – ps9
Holomycota – ps10
Fungi – ps11
Fungi_1 – ps12
Fungi_2 – ps13
Fungi_3 – ps14
Dikarya – ps15
Ascomycota – ps16
Taphrinomycotina – ps17
mocystido/Schizosaccharomycetes – ps18
Pneumocystis_carinii_b80 – ps19
Phylostratum

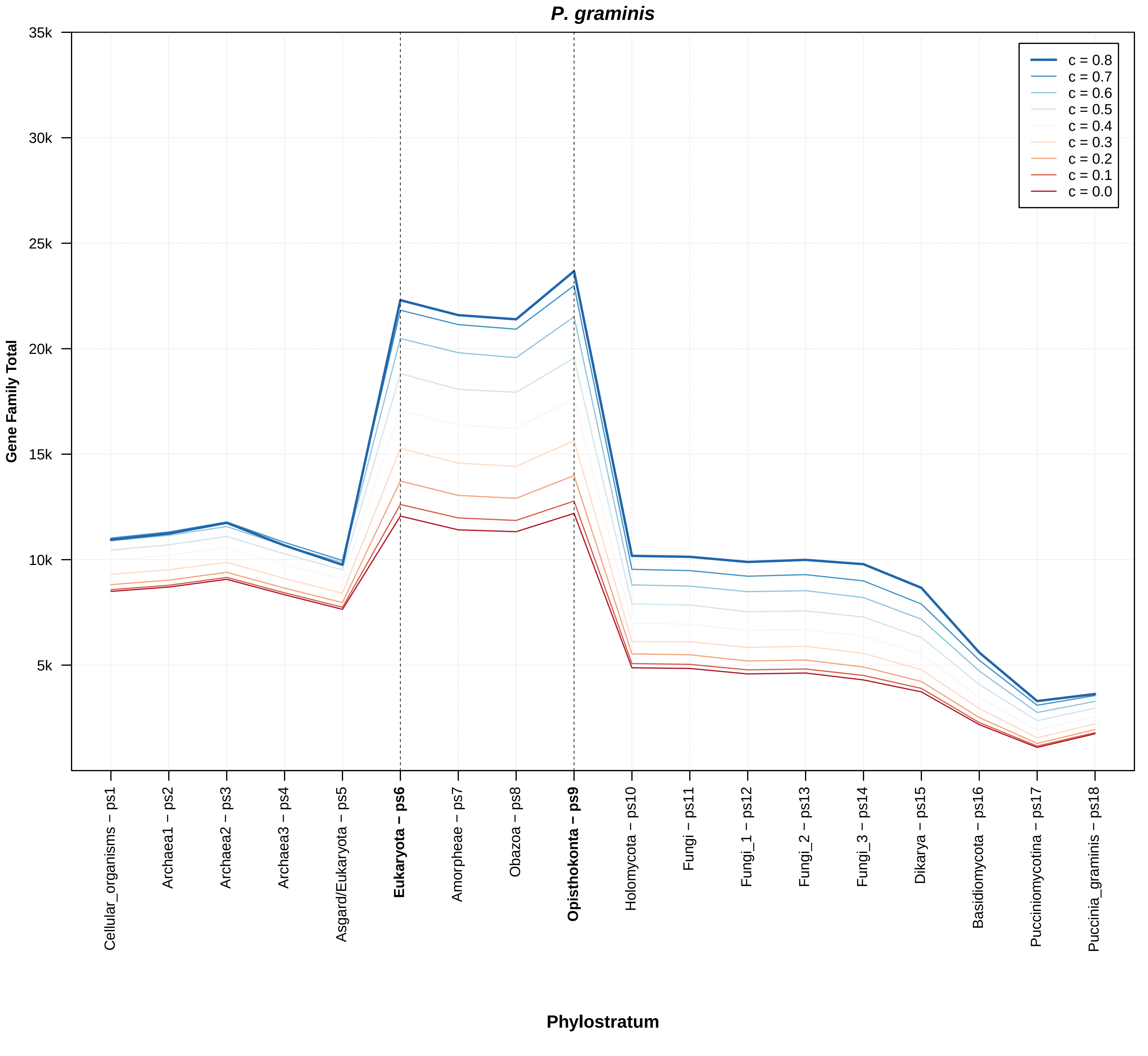
P. graminis
Gene Family Total
35k
30k
25k
20k
15k
10k
5k
c = 0.8
c = 0.7
c = 0.6
c = 0.5
c = 0.4
c = 0.3
c = 0.2
c = 0.1
c = 0.0
Cellular_organisms – ps1
Archaea1 – ps2
Archaea2 – ps3
Archaea3 – ps4
Asgard/Eukaryota – ps5
Eukaryota – ps6
Amorpheae – ps7
Obazoa – ps8
Opisthokonta – ps9
Holomycota – ps10
Fungi – ps11
Fungi_1 – ps12
Fungi_2 – ps13
Fungi_3 – ps14
Dikarya – ps15
Basidiomycota – ps16
Pucciniomycotina – ps17
Puccinia_graminis – ps18
Phylostratum

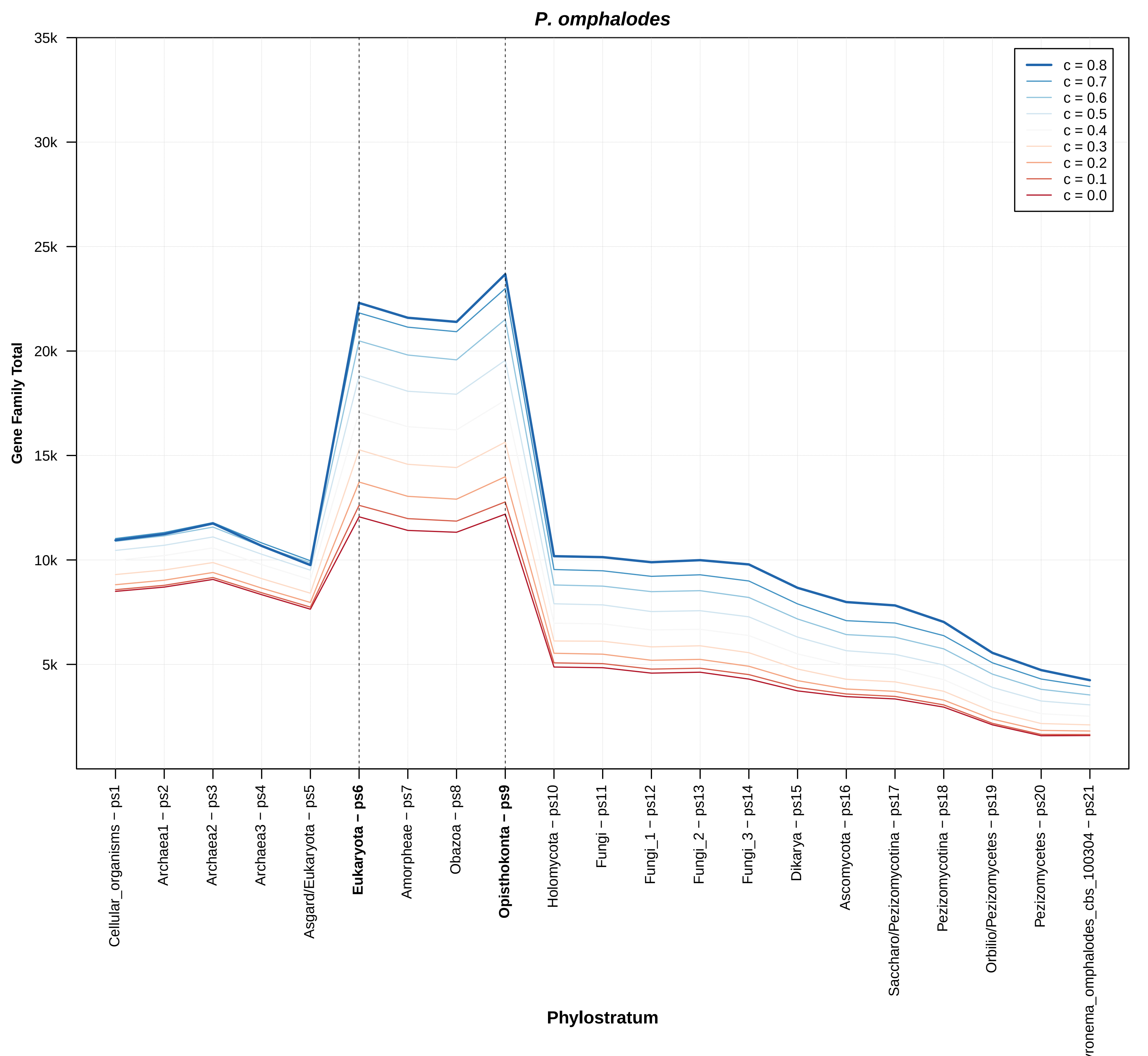
P. omphalodes
Gene Family Total
35k
30k
25k
20k
15k
10k
5k
c = 0.8
c = 0.7
c = 0.6
c = 0.5
c = 0.4
c = 0.3
c = 0.2
c = 0.1
c = 0.0
Cellular_organisms – ps1
Archaea1 – ps2
Archaea2 – ps3
Archaea3 – ps4
Asgard/Eukaryota – ps5
Eukaryota – ps6
Amorpheae – ps7
Obazoa – ps8
Opisthokonta – ps9
Holomycota – ps10
Fungi – ps11
Fungi_1 – ps12
Fungi_2 – ps13
Fungi_3 – ps14
Dikarya – ps15
Ascomycota – ps16
Saccharo/Pezizomycotina – ps17
Pezizomycotina – ps18
Orbilio/Pezizomycetes – ps19
Pezizomycetes – ps20
ronema_omphalodes_cbs_100304 – ps21
Phylostratum

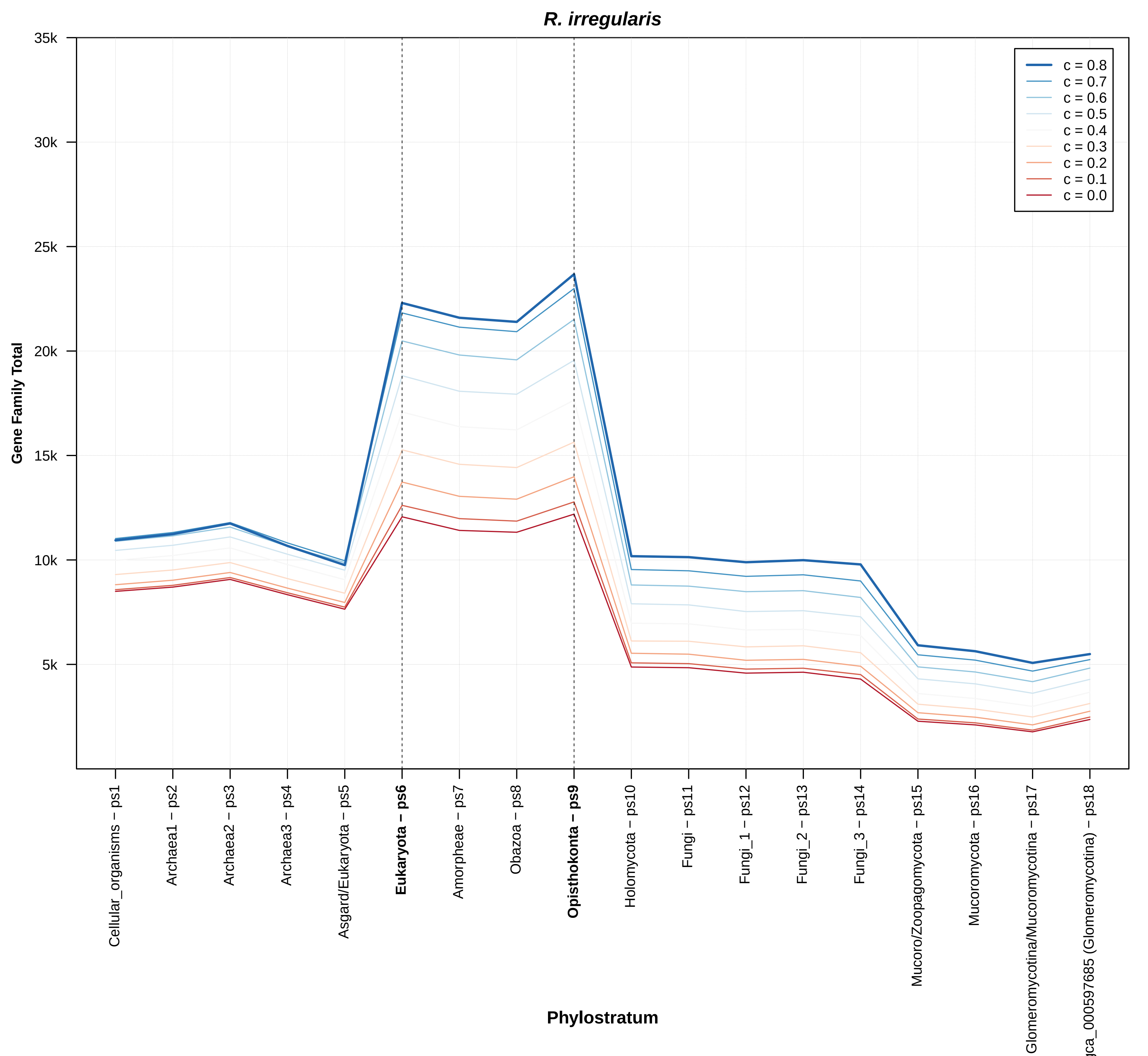

R. irregularis
Gene Family Total
35k
30k
25k
20k
15k
10k
5k
c = 0.8
c = 0.7
c = 0.6
c = 0.5
c = 0.4
c = 0.3
c = 0.2
c = 0.1
c = 0.0
Cellular_organisms – ps1
Archaea1 – ps2
Archaea2 – ps3
Archaea3 – ps4
Asgard/Eukaryota – ps5
Eukaryota – ps6
Amorpheae – ps7
Obazoa – ps8
Opisthokonta – ps9
Holomycota – ps10
Fungi – ps11
Fungi_1 – ps12
Fungi_2 – ps13
Fungi_3 – ps14
Mucoro/Zoopagomycota – ps15
Mucoromycota – ps16
Glomeromycotina/Mucoromycotina – ps17
gca_000597685 (Glomeromycotina) – ps18
Phylostratum

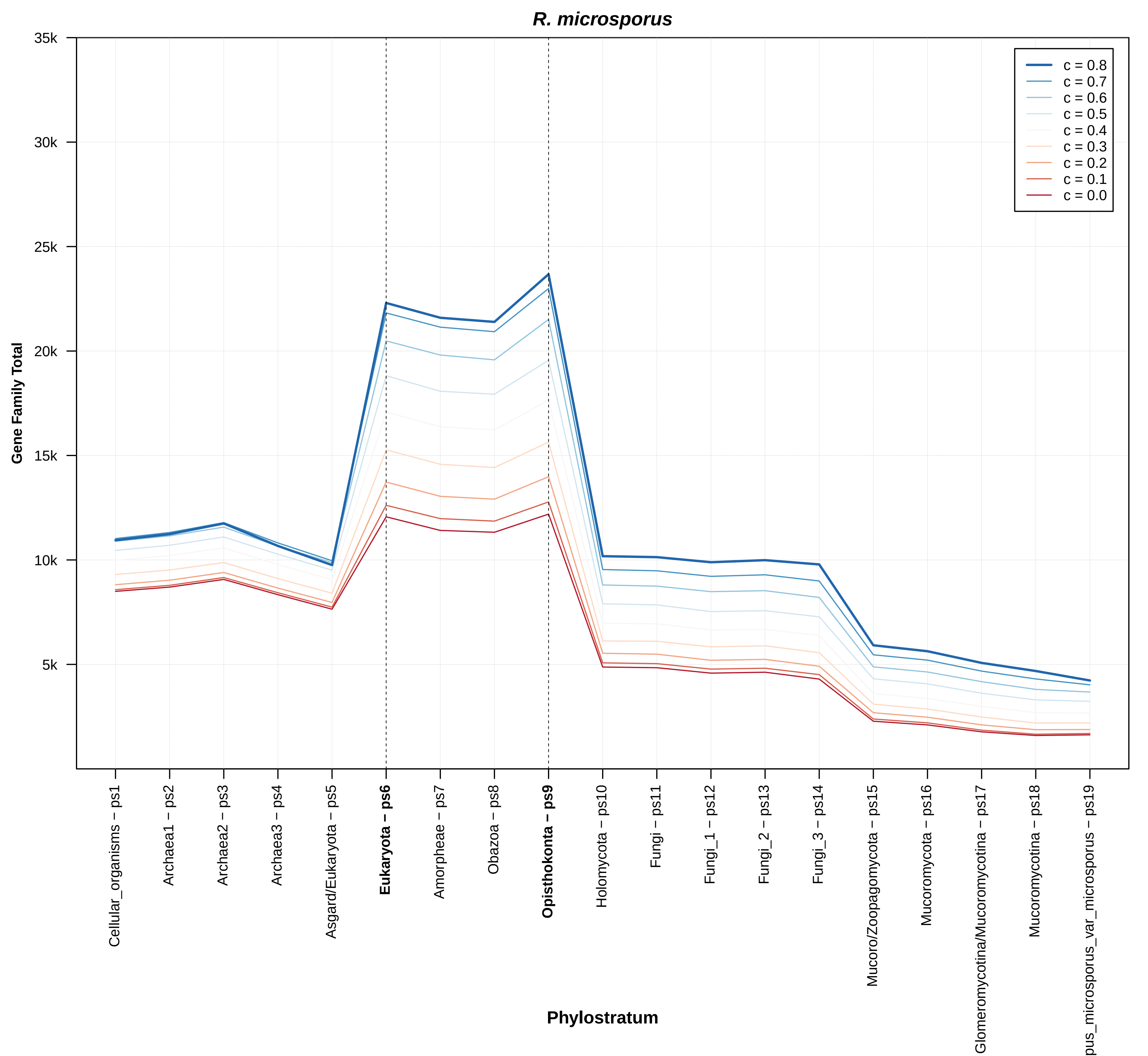

R. microsporus
Gene Family Total
35k
30k
25k
20k
15k
10k
5k
c = 0.8
c = 0.7
c = 0.6
c = 0.5
c = 0.4
c = 0.3
c = 0.2
c = 0.1
c = 0.0
Cellular_organisms – ps1
Archaea1 – ps2
Archaea2 – ps3
Archaea3 – ps4
Asgard/Eukaryota – ps5
Eukaryota – ps6
Amorpheae – ps7
Obazoa – ps8
Opisthokonta – ps9
Holomycota – ps10
Fungi – ps11
Fungi_1 – ps12
Fungi_2 – ps13
Fungi_3 – ps14
Mucoro/Zoopagomycota – ps15
Mucoromycota – ps16
Glomeromycotina/Mucoromycotina – ps17
Mucoromycotina – ps18
pus_microsporus_var_microsporus – ps19
Phylostratum

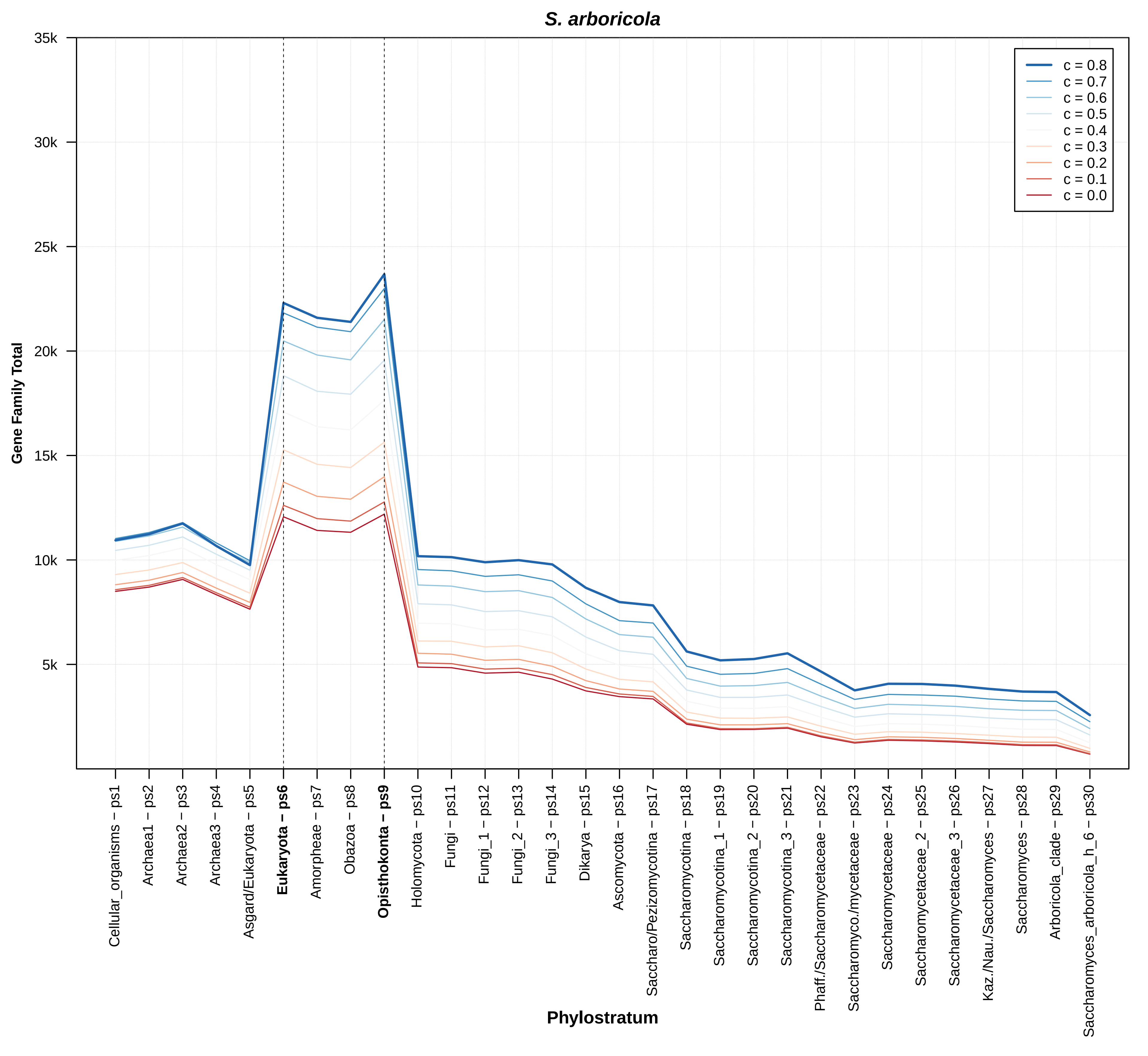
S. arboricola
Gene Family Total
35k
30k
25k
20k
15k
10k
5k
c = 0.8
c = 0.7
c = 0.6
c = 0.5
c = 0.4
c = 0.3
c = 0.2
c = 0.1
c = 0.0
Cellular_organisms – ps1
Archaea1 – ps2
Archaea2 – ps3
Archaea3 – ps4
Asgard/Eukaryota – ps5
Eukaryota – ps6
Amorpheae – ps7
Obazoa – ps8
Opisthokonta – ps9
Holomycota – ps10
Fungi – ps11
Fungi_1 – ps12
Fungi_2 – ps13
Fungi_3 – ps14
Dikarya – ps15
Ascomycota – ps16
Saccharo/Pezizomycotina – ps17
Saccharomycotina – ps18
Saccharomycotina_1 – ps19
Saccharomycotina_2 – ps20
Saccharomycotina_3 – ps21
Phaff./Saccharomycetaceae – ps22
Saccharomyco./mycetaceae – ps23
Saccharomycetaceae – ps24
Saccharomycetaceae_2 – ps25
Saccharomycetaceae_3 – ps26
Kaz./Nau./Saccharomyces – ps27
Saccharomyces – ps28
Arboricola_clade – ps29
Saccharomyces_arboricola_h_6 – ps30
Phylostratum

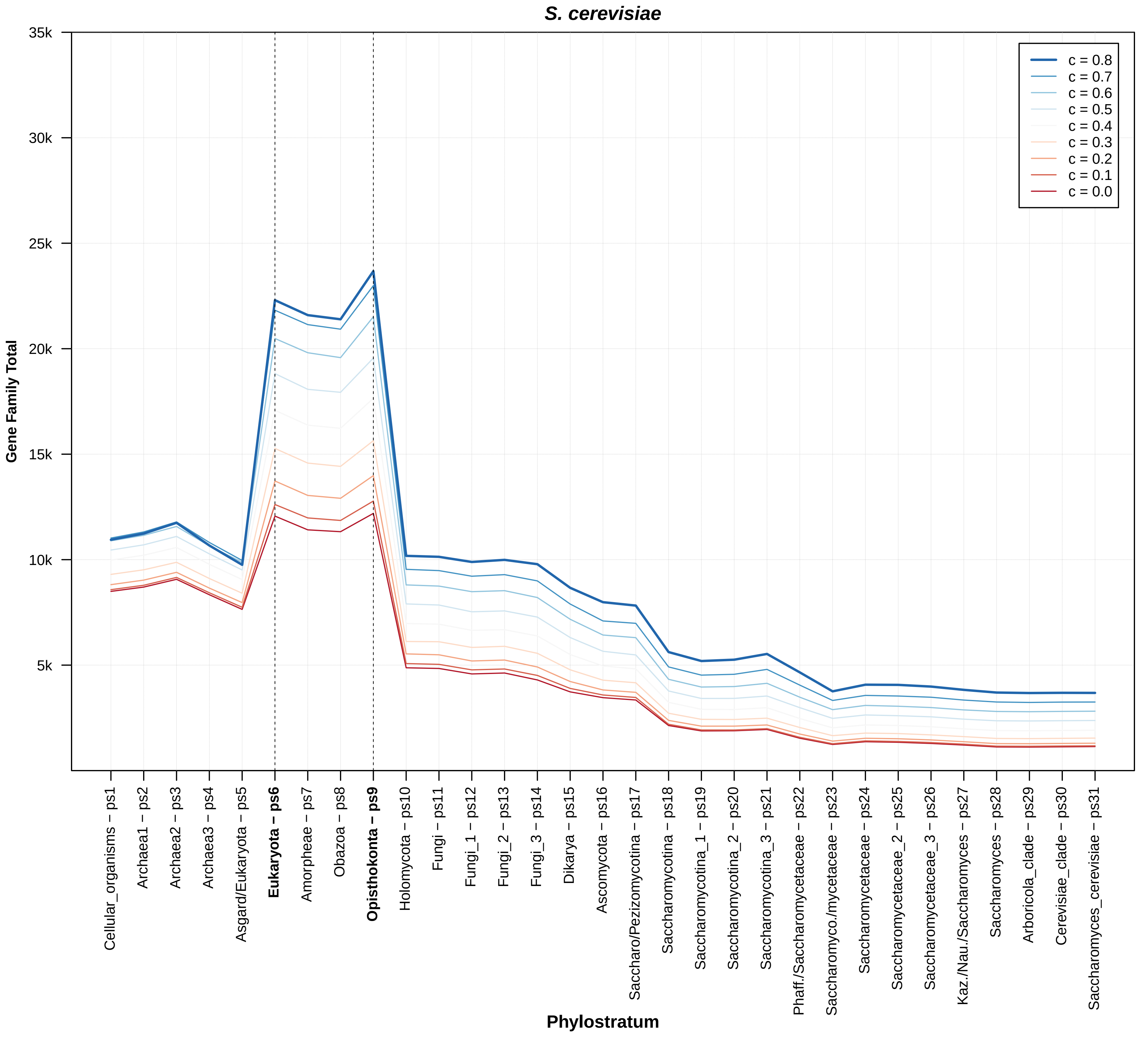

S. cerevisiae
Gene Family Total
35k
30k
25k
20k
15k
10k
5k
c = 0.8
c = 0.7
c = 0.6
c = 0.5
c = 0.4
c = 0.3
c = 0.2
c = 0.1
c = 0.0
Cellular_organisms – ps1
Archaea1 – ps2
Archaea2 – ps3
Archaea3 – ps4
Asgard/Eukaryota – ps5
Eukaryota – ps6
Amorpheae – ps7
Obazoa – ps8
Opisthokonta – ps9
Holomycota – ps10
Fungi – ps11
Fungi_1 – ps12
Fungi_2 – ps13
Fungi_3 – ps14
Dikarya – ps15
Ascomycota – ps16
Saccharo/Pezizomycotina – ps17
Saccharomycotina – ps18
Saccharomycotina_1 – ps19
Saccharomycotina_2 – ps20
Saccharomycotina_3 – ps21
Phaff./Saccharomycetaceae – ps22
Saccharomyco./mycetaceae – ps23
Saccharomycetaceae – ps24
Saccharomycetaceae_2 – ps25
Saccharomycetaceae_3 – ps26
Kaz./Nau./Saccharomyces – ps27
Saccharomyces – ps28
Arboricola_clade – ps29
Cerevisiae_clade – ps30
Saccharomyces_cerevisiae – ps31
Phylostratum

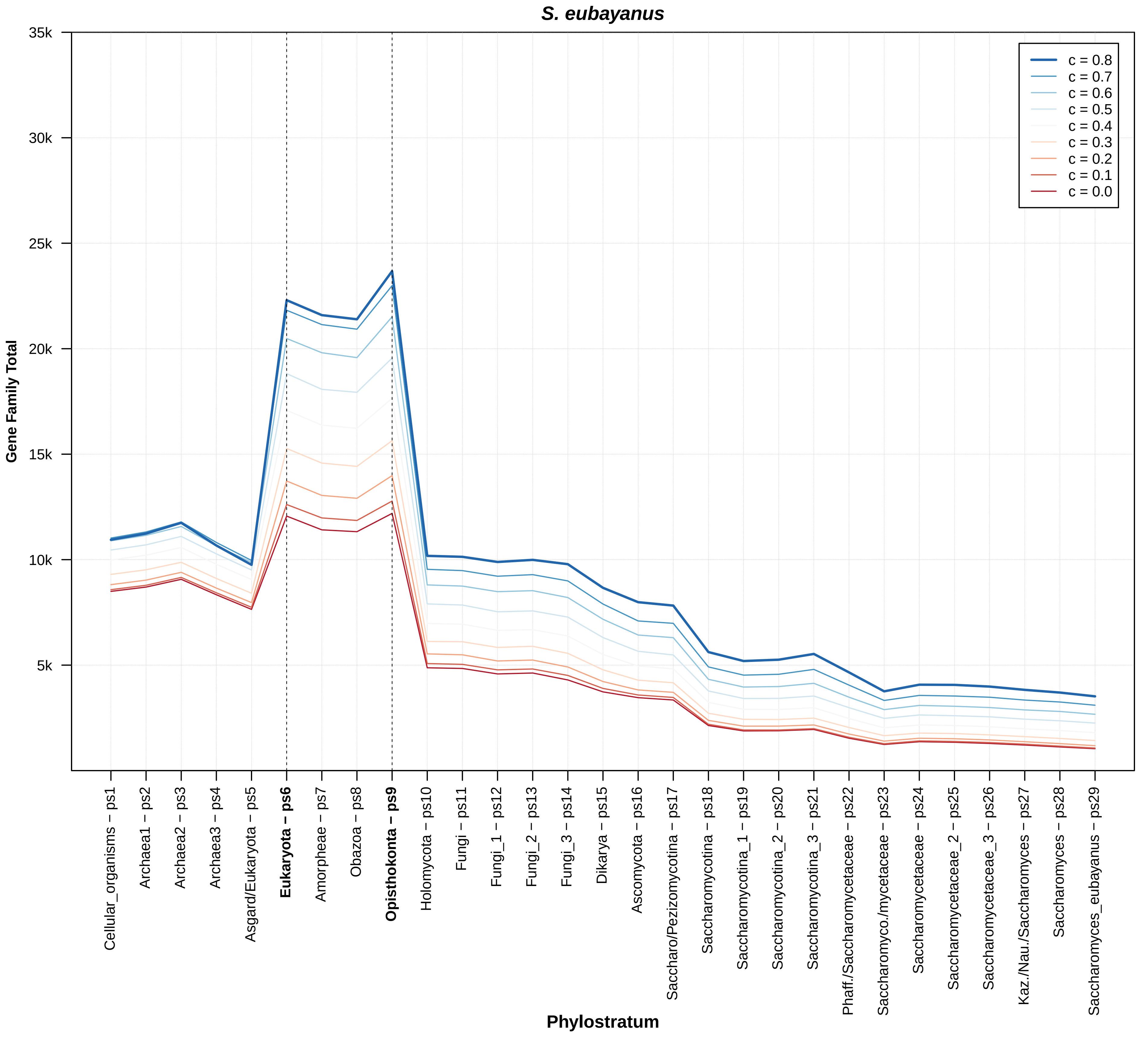
S. eubayanus
Gene Family Total
35k
30k
25k
20k
15k
10k
5k
c = 0.8
c = 0.7
c = 0.6
c = 0.5
c = 0.4
c = 0.3
c = 0.2
c = 0.1
c = 0.0
Cellular_organisms – ps1
Archaea1 – ps2
Archaea2 – ps3
Archaea3 – ps4
Asgard/Eukaryota – ps5
Eukaryota – ps6
Amorpheae – ps7
Obazoa – ps8
Opisthokonta – ps9
Holomycota – ps10
Fungi – ps11
Fungi_1 – ps12
Fungi_2 – ps13
Fungi_3 – ps14
Dikarya – ps15
Ascomycota – ps16
Saccharo/Pezizomycotina – ps17
Saccharomycotina – ps18
Saccharomycotina_1 – ps19
Saccharomycotina_2 – ps20
Saccharomycotina_3 – ps21
Phaff./Saccharomycetaceae – ps22
Saccharomyco./mycetaceae – ps23
Saccharomycetaceae – ps24
Saccharomycetaceae_2 – ps25
Saccharomycetaceae_3 – ps26
Kaz./Nau./Saccharomyces – ps27
Saccharomyces – ps28
Saccharomyces_eubayanus – ps29
Phylostratum

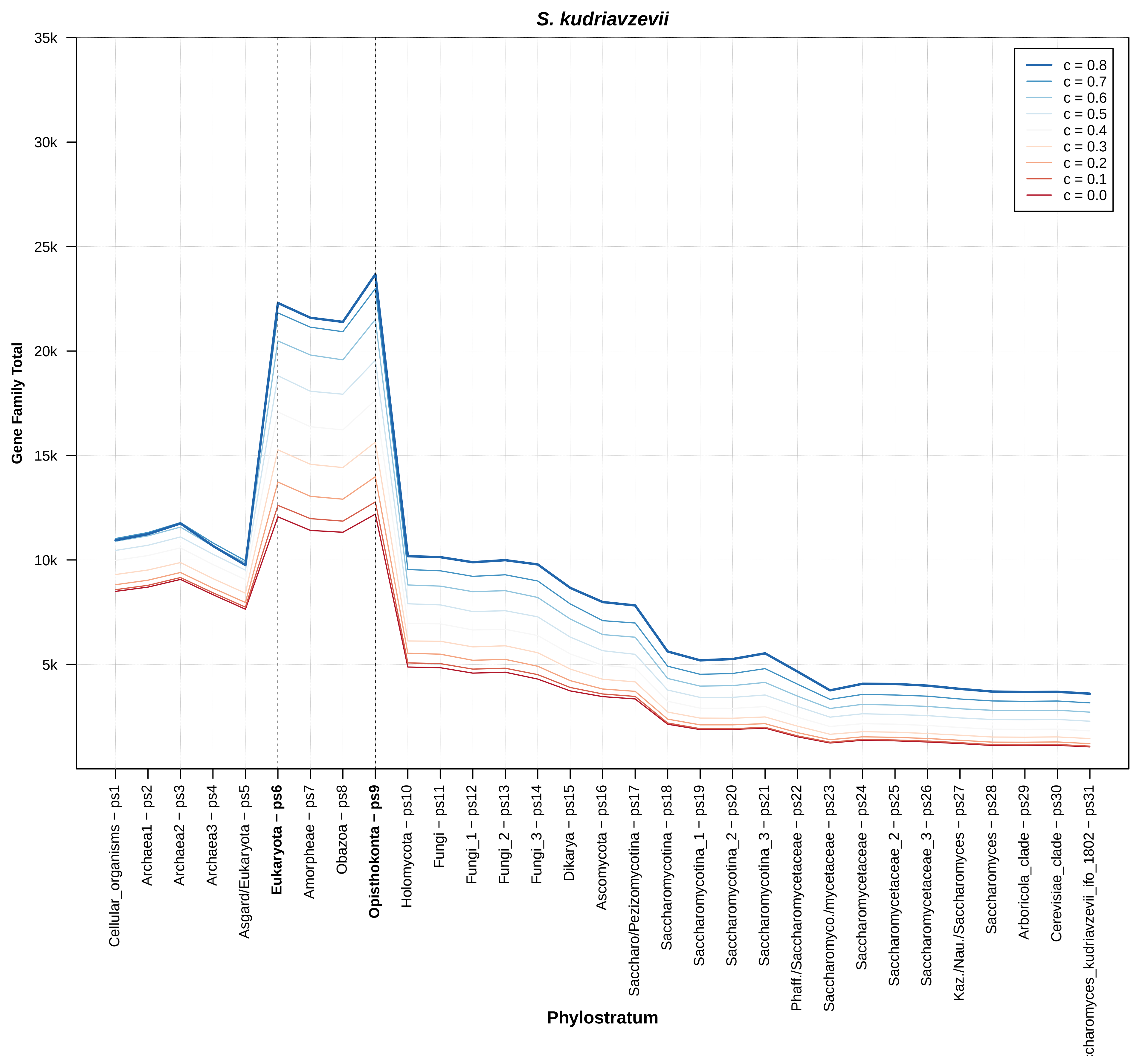

S. kudriavzevii
Gene Family Total
35k
30k
25k
20k
15k
10k
5k
c = 0.8
c = 0.7
c = 0.6
c = 0.5
c = 0.4
c = 0.3
c = 0.2
c = 0.1
c = 0.0
Cellular_organisms – ps1
Archaea1 – ps2
Archaea2 – ps3
Archaea3 – ps4
Asgard/Eukaryota – ps5
Eukaryota – ps6
Amorpheae – ps7
Obazoa – ps8
Opisthokonta – ps9
Holomycota – ps10
Fungi – ps11
Fungi_1 – ps12
Fungi_2 – ps13
Fungi_3 – ps14
Dikarya – ps15
Ascomycota – ps16
Saccharo/Pezizomycotina – ps17
Saccharomycotina – ps18
Saccharomycotina_1 – ps19
Saccharomycotina_2 – ps20
Saccharomycotina_3 – ps21
Phaff./Saccharomycetaceae – ps22
Saccharomyco./mycetaceae – ps23
Saccharomycetaceae – ps24
Saccharomycetaceae_2 – ps25
Saccharomycetaceae_3 – ps26
Kaz./Nau./Saccharomyces – ps27
Saccharomyces – ps28
Arboricola_clade – ps29
Cerevisiae_clade – ps30
charomyces_kudriavzevii_ifo_1802 – ps31
Phylostratum

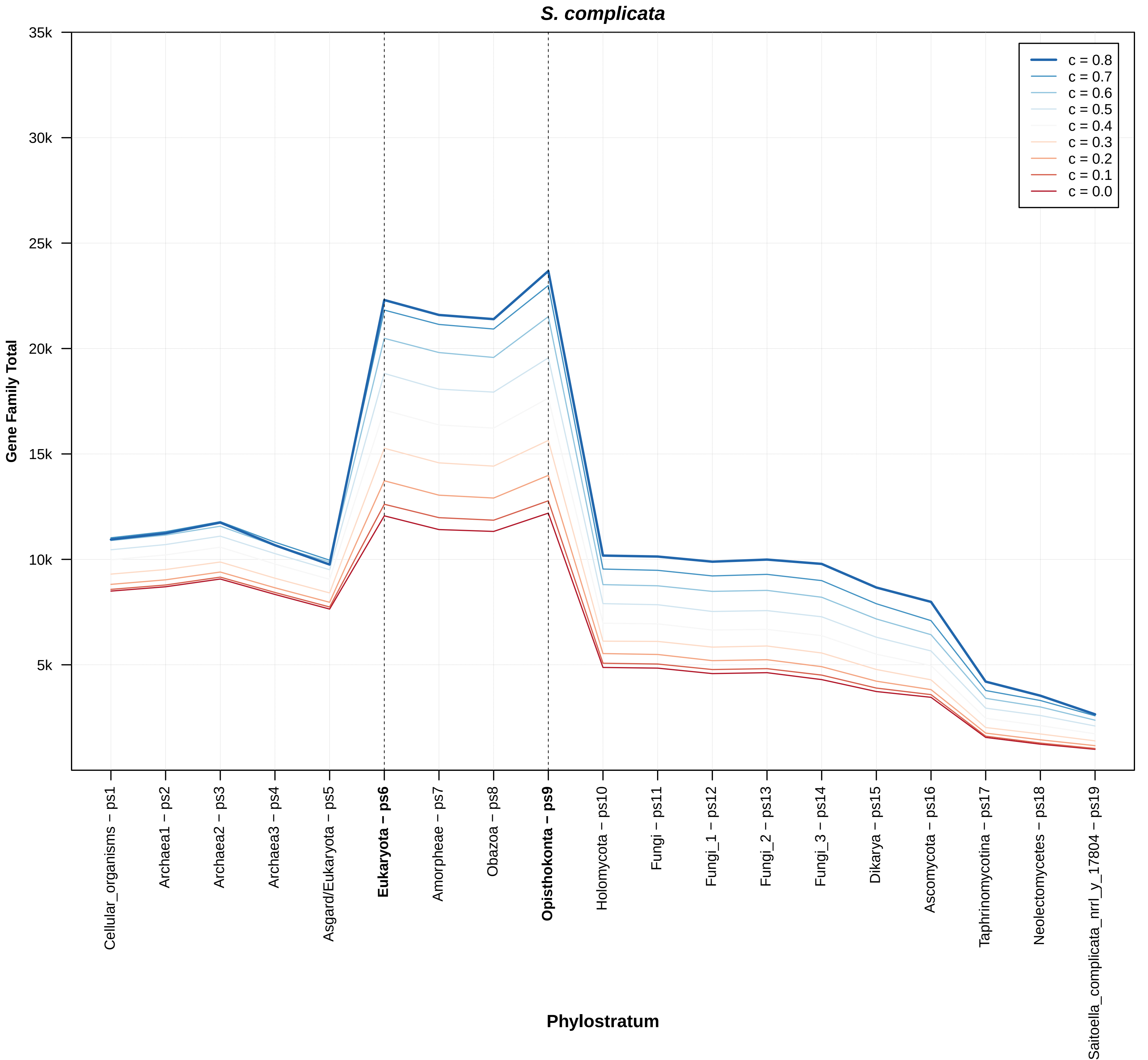
S. complicata
Gene Family Total
35k
30k
25k
20k
15k
10k
5k
c = 0.8
c = 0.7
c = 0.6
c = 0.5
c = 0.4
c = 0.3
c = 0.2
c = 0.1
c = 0.0
Cellular_organisms – ps1
Archaea1 – ps2
Archaea2 – ps3
Archaea3 – ps4
Asgard/Eukaryota – ps5
Eukaryota – ps6
Amorpheae – ps7
Obazoa – ps8
Opisthokonta – ps9
Holomycota – ps10
Fungi – ps11
Fungi_1 – ps12
Fungi_2 – ps13
Fungi_3 – ps14
Dikarya – ps15
Ascomycota – ps16
Taphrinomycotina – ps17
Neolectomycetes – ps18
Saitoella_complicata_nrrl_y_17804 – ps19
Phylostratum

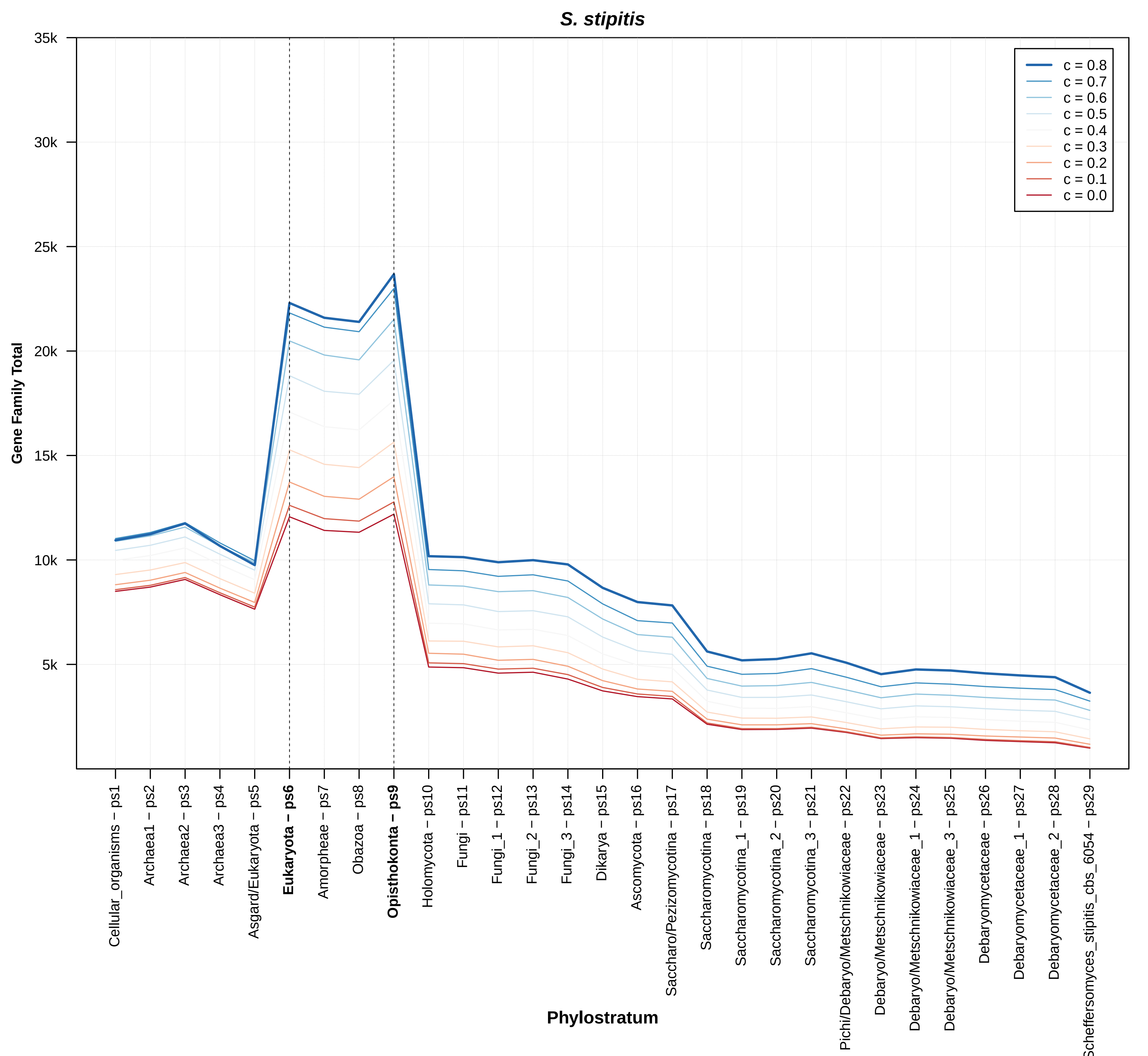

S. stipitis
Gene Family Total
35k
30k
25k
20k
15k
10k
5k
c = 0.8
c = 0.7
c = 0.6
c = 0.5
c = 0.4
c = 0.3
c = 0.2
c = 0.1
c = 0.0
Cellular_organisms – ps1
Archaea1 – ps2
Archaea2 – ps3
Archaea3 – ps4
Asgard/Eukaryota – ps5
Eukaryota – ps6
Amorpheae – ps7
Obazoa – ps8
Opisthokonta – ps9
Holomycota – ps10
Fungi – ps11
Fungi_1 – ps12
Fungi_2 – ps13
Fungi_3 – ps14
Dikarya – ps15
Ascomycota – ps16
Saccharo/Pezizomycotina – ps17
Saccharomycotina – ps18
Saccharomycotina_1 – ps19
Saccharomycotina_2 – ps20
Saccharomycotina_3 – ps21
Pichi/Debaryo/Metschnikowiaceae – ps22
Debaryo/Metschnikowiaceae – ps23
Debaryo/Metschnikowiaceae_1 – ps24
Debaryo/Metschnikowiaceae_3 – ps25
Debaryomycetaceae – ps26
Debaryomycetaceae_1 – ps27
Debaryomycetaceae_2 – ps28
Scheffersomyces_stipitis_cbs_6054 – ps29
Phylostratum

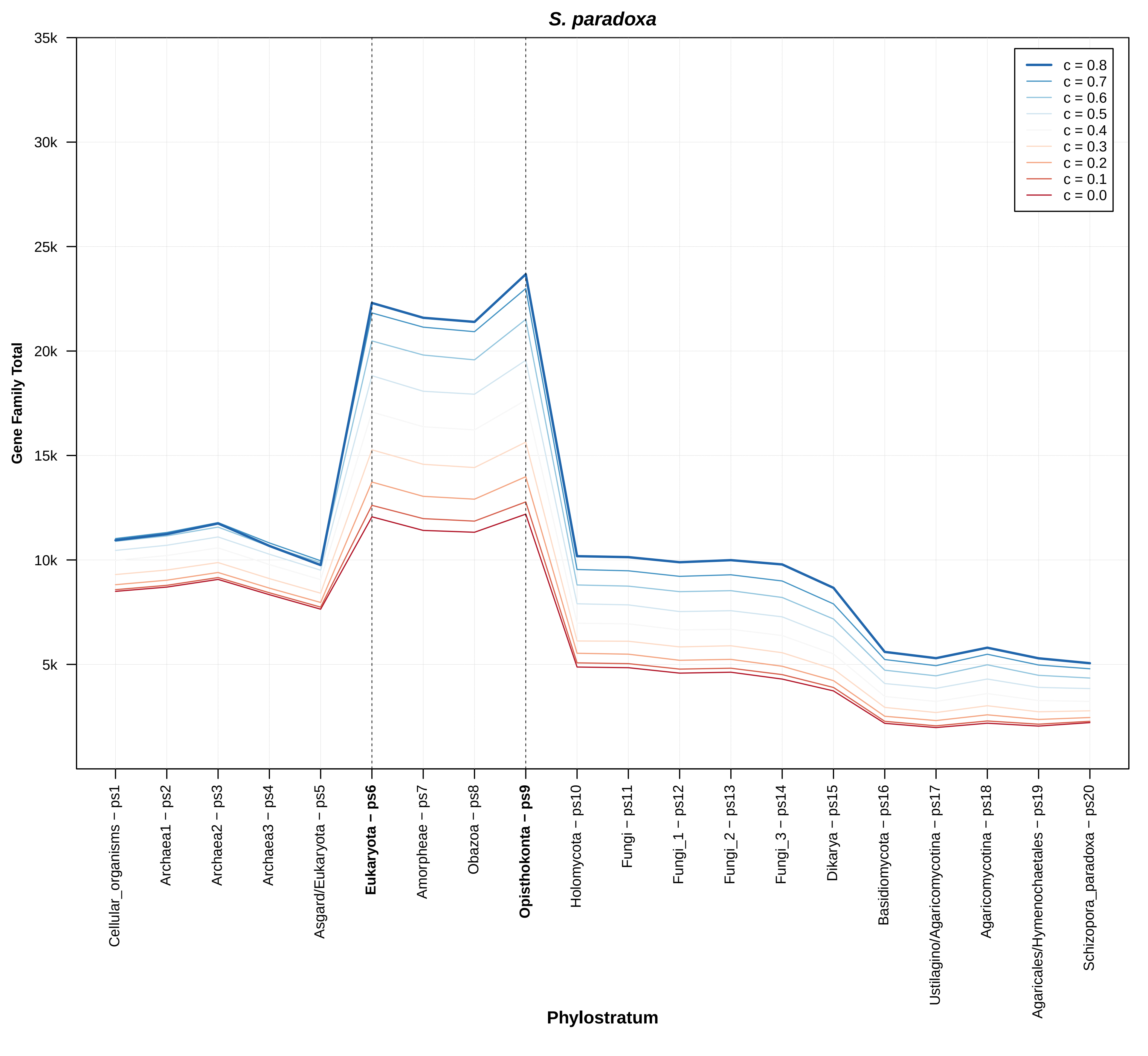

S. paradoxa
Gene Family Total
35k
30k
25k
20k
15k
10k
5k
c = 0.8
c = 0.7
c = 0.6
c = 0.5
c = 0.4
c = 0.3
c = 0.2
c = 0.1
c = 0.0
Cellular_organisms – ps1
Archaea1 – ps2
Archaea2 – ps3
Archaea3 – ps4
Asgard/Eukaryota – ps5
Eukaryota – ps6
Amorpheae – ps7
Obazoa – ps8
Opisthokonta – ps9
Holomycota – ps10
Fungi – ps11
Fungi_1 – ps12
Fungi_2 – ps13
Fungi_3 – ps14
Dikarya – ps15
Basidiomycota – ps16
Ustilagino/Agaricomycotina – ps17
Agaricomycotina – ps18
Agaricales/Hymenochaetales – ps19
Schizopora_paradoxa – ps20
Phylostratum

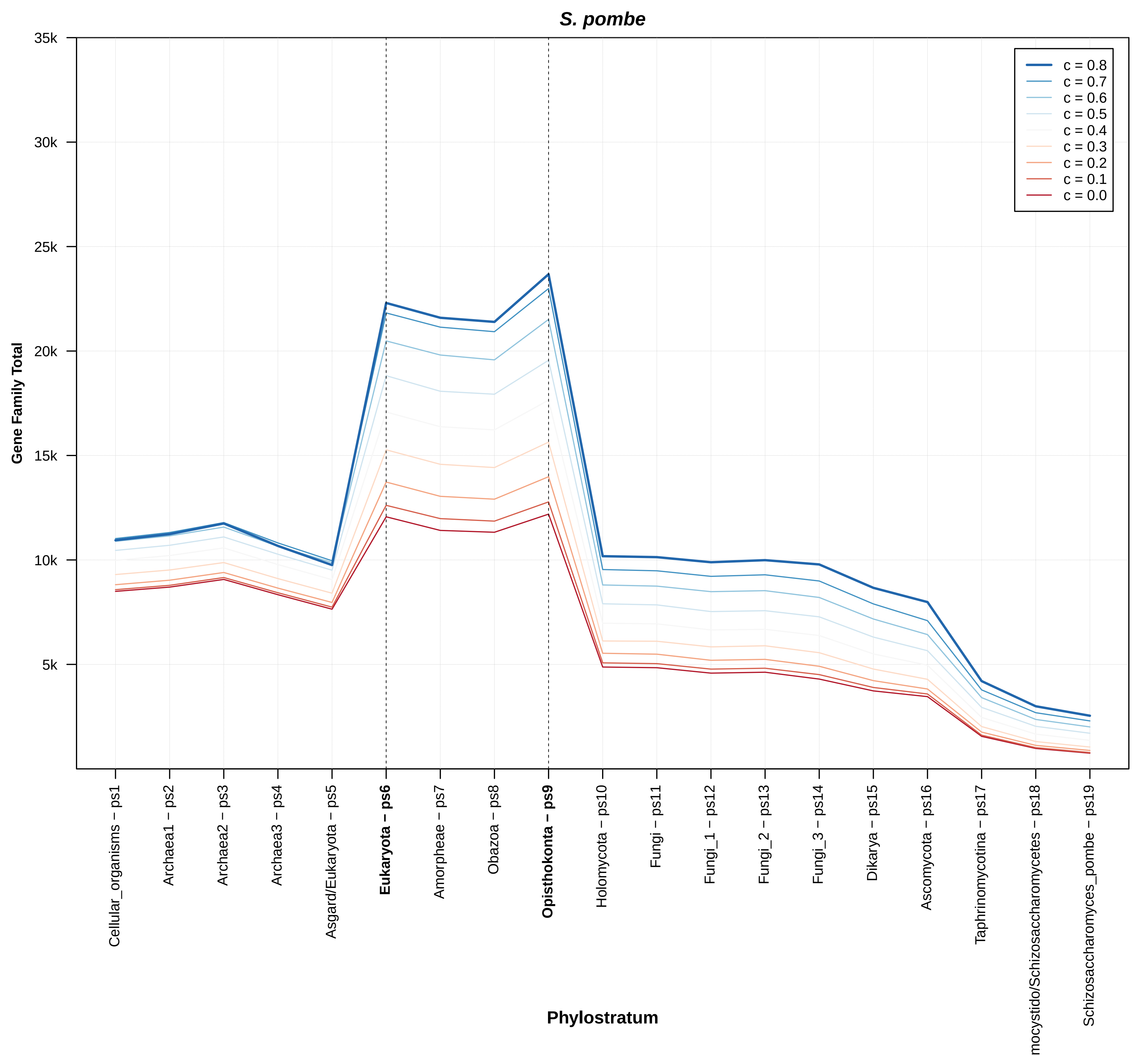
S. pombe
35k
30k
25k
20k
15k
10k
5k
Gene Family Total
c = 0.8
c = 0.7
c = 0.6
c = 0.5
c = 0.4
c = 0.3
c = 0.2
c = 0.1
c = 0.0
Cellular_organisms – ps1
Archaea1 – ps2
Archaea2 – ps3
Archaea3 – ps4
Asgard/Eukaryota – ps5
Eukaryota – ps6
Amorpheae – ps7
Obazoa – ps8
Opisthokonta – ps9
Holomycota – ps10
Fungi – ps11
Fungi_1 – ps12
Fungi_2 – ps13
Fungi_3 – ps14
Dikarya – ps15
Ascomycota – ps16
Taphrinomycotina – ps17
mocystido/Schizosaccharomycetes – ps18
Schizosaccharomyces_pombe – ps19
Phylostratum

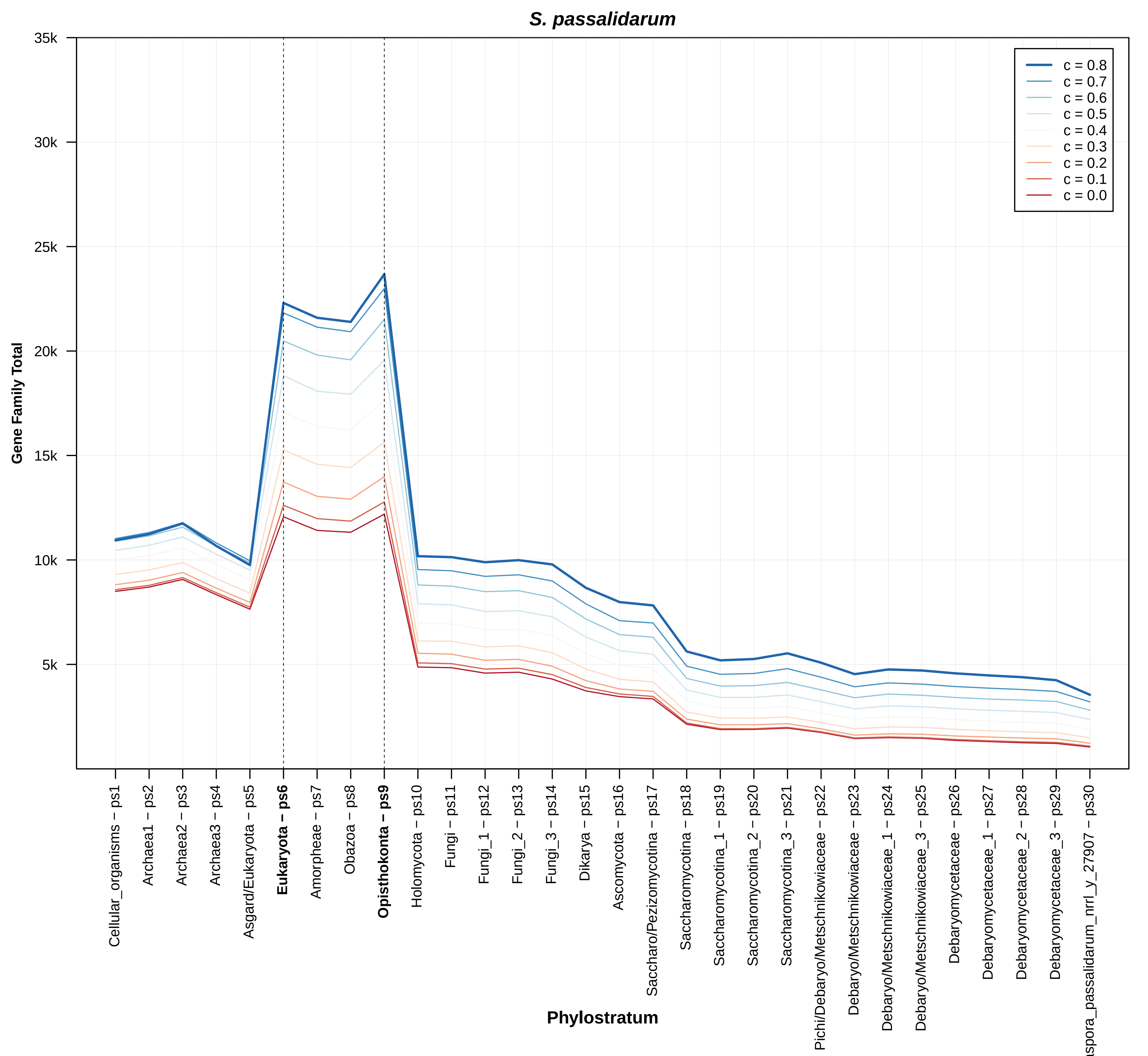

S. passalidarum
Gene Family Total
Phylostratum
35k
30k
25k
20k
15k
10k
5k
c = 0.8
c = 0.7
c = 0.6
c = 0.5
c = 0.4
c = 0.3
c = 0.2
c = 0.1
c = 0.0
Cellular_organisms – ps1
Archaea1 – ps2
Archaea2 – ps3
Archaea3 – ps4
Asgard/Eukaryota – ps5
Eukaryota – ps6
Amorpheae – ps7
Obazoa – ps8
Opisthokonta – ps9
Holomycota – ps10
Fungi – ps11
Fungi_1 – ps12
Fungi_2 – ps13
Fungi_3 – ps14
Dikarya – ps15
Ascomycota – ps16
Saccharo/Pezizomycotina – ps17
Saccharomycotina – ps18
Saccharomycotina_1 – ps19
Saccharomycotina_2 – ps20
Saccharomycotina_3 – ps21
Pichi/Debaryo/Metschnikowiaceae – ps22
Debaryo/Metschnikowiaceae – ps23
Debaryo/Metschnikowiaceae_1 – ps24
Debaryo/Metschnikowiaceae_3 – ps25
Debaryomycetaceae – ps26
Debaryomycetaceae_1 – ps27
Debaryomycetaceae_2 – ps28
Debaryomycetaceae_3 – ps29
spora_passalidarum_nrrl_y_27907 – ps30

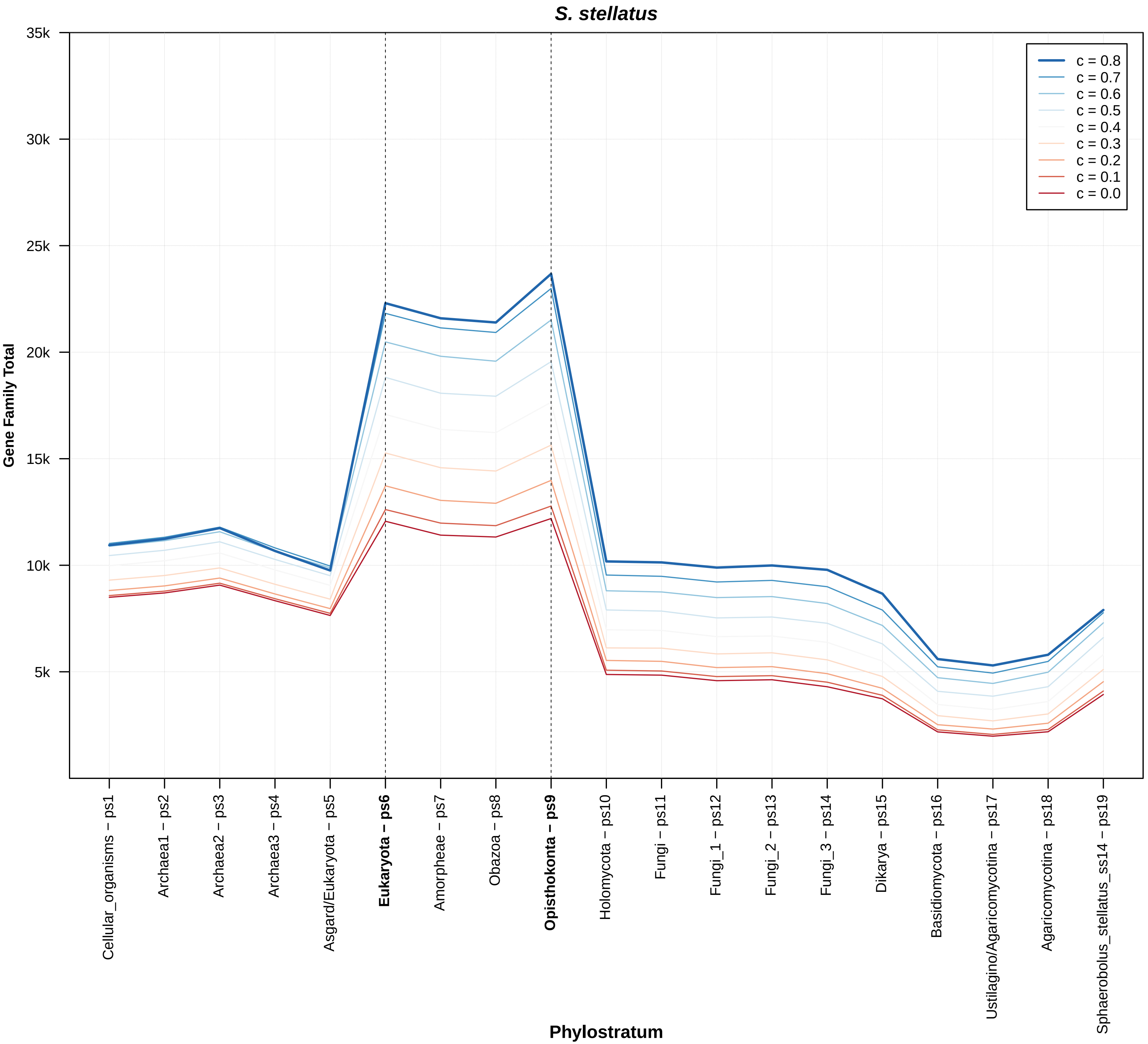

S. stellatus
c = 0.8
c = 0.7
c = 0.6
c = 0.5
c = 0.4
c = 0.3
c = 0.2
c = 0.1
c = 0.0
Gene Family Total
35k
30k
25k
20k
15k
10k
5k
Phylostratum
Cellular_organisms – ps1
Archaea1 – ps2
Archaea2 – ps3
Archaea3 – ps4
Asgard/Eukaryota – ps5
Eukaryota – ps6
Amorpheae – ps7
Obazoa – ps8
Opisthokonta – ps9
Holomycota – ps10
Fungi – ps11
Fungi_1 – ps12
Fungi_2 – ps13
Fungi_3 – ps14
Dikarya – ps15
Basidiomycota – ps16
Ustilagino/Agaricomycotina – ps17
Agaricomycotina – ps18
Sphaerobolus_stellatus_ss14 – ps19

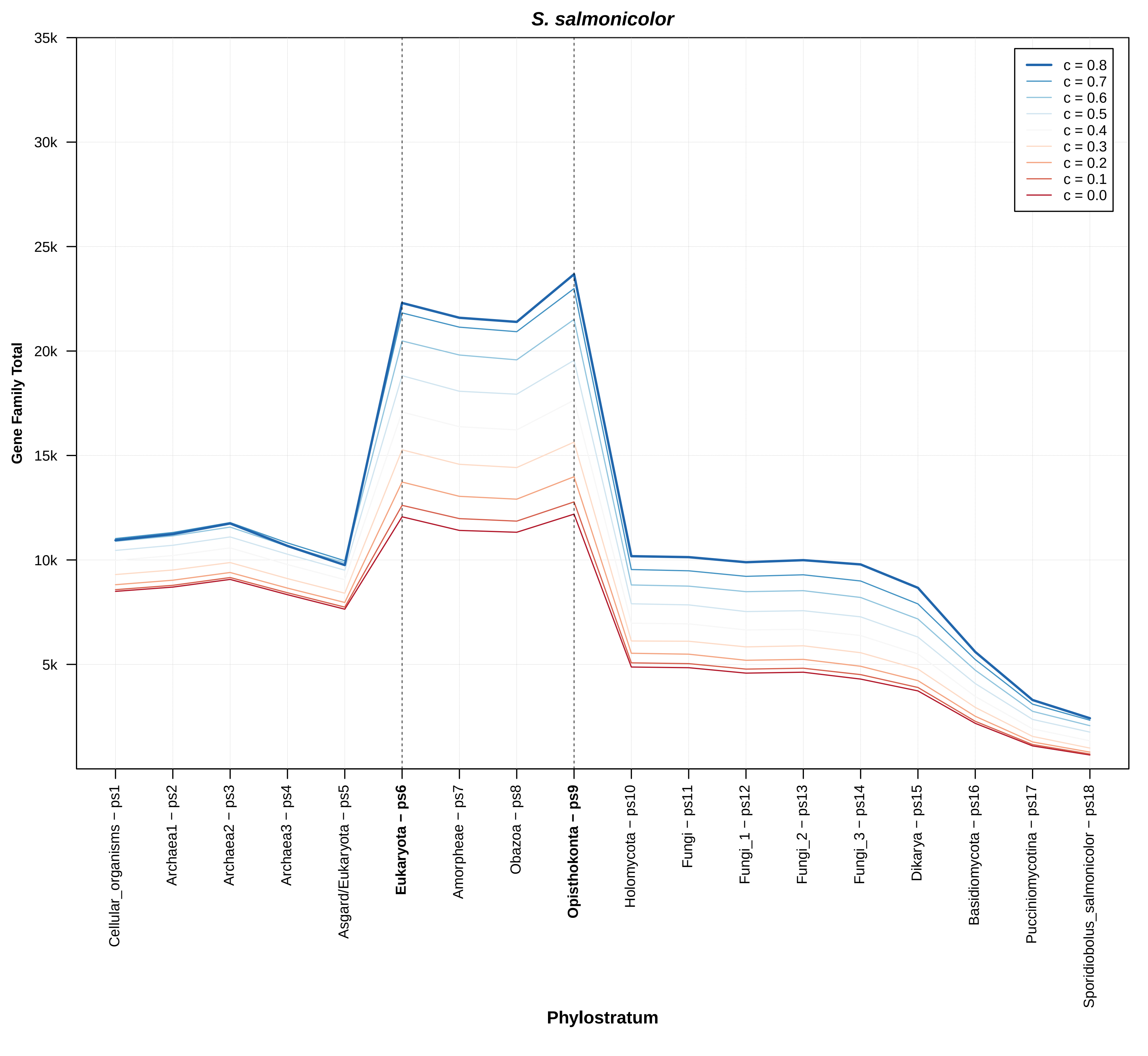
S. salmonicolor
Gene Family Total
Phylostratum
35k
30k
25k
20k
15k
10k
5k
c = 0.8
c = 0.7
c = 0.6
c = 0.5
c = 0.4
c = 0.3
c = 0.2
c = 0.1
c = 0.0
Cellular_organisms – ps1
Archaea1 – ps2
Archaea2 – ps3
Archaea3 – ps4
Asgard/Eukaryota – ps5
Eukaryota – ps6
Amorpheae – ps7
Obazoa – ps8
Opisthokonta – ps9
Holomycota – ps10
Fungi – ps11
Fungi_1 – ps12
Fungi_2 – ps13
Fungi_3 – ps14
Dikarya – ps15
Basidiomycota – ps16
Pucciniomycotina – ps17
Sporidiobolus_salmonicolor – ps18

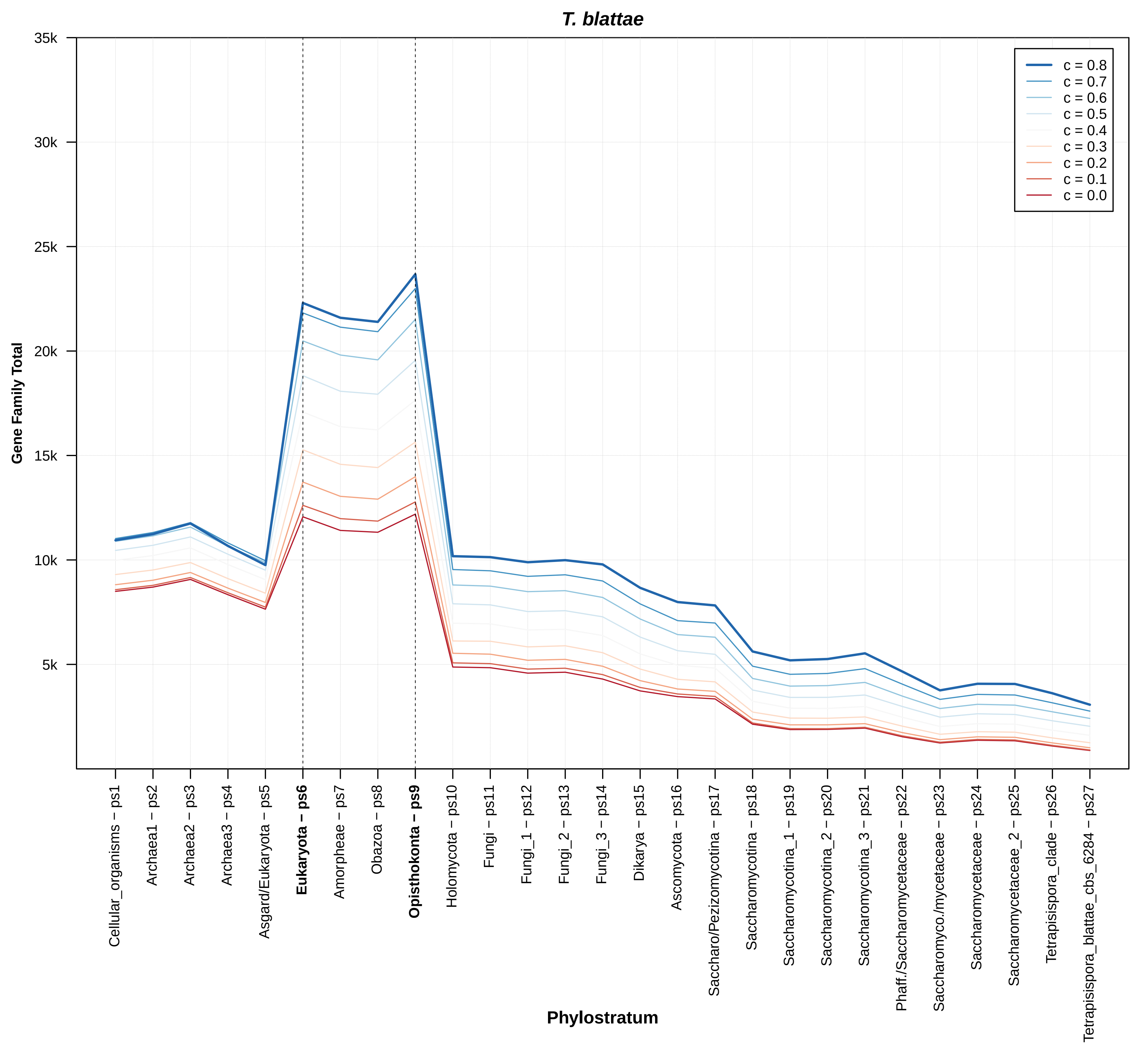
T. blattae
c = 0.8
c = 0.7
c = 0.6
c = 0.5
c = 0.4
c = 0.3
c = 0.2
c = 0.1
c = 0.0
35k
30k
25k
20k
15k
10k
5k
Gene Family Total
Cellular_organisms – ps1
Archaea1 – ps2
Archaea2 – ps3
Archaea3 – ps4
Asgard/Eukaryota – ps5
Eukaryota – ps6
Amorpheae – ps7
Obazoa – ps8
Opisthokonta – ps9
Holomycota – ps10
Fungi – ps11
Fungi_1 – ps12
Fungi_2 – ps13
Fungi_3 – ps14
Dikarya – ps15
Ascomycota – ps16
Saccharo/Pezizomycotina – ps17
Saccharomycotina – ps18
Saccharomycotina_1 – ps19
Saccharomycotina_2 – ps20
Saccharomycotina_3 – ps21
Phaff./Saccharomycetaceae – ps22
Saccharomyco./mycetaceae – ps23
Saccharomycetaceae – ps24
Saccharomycetaceae_2 – ps25
Tetrapisispora_clade – ps26
Tetrapisispora_blattae_cbs_6284 – ps27
Phylostratum

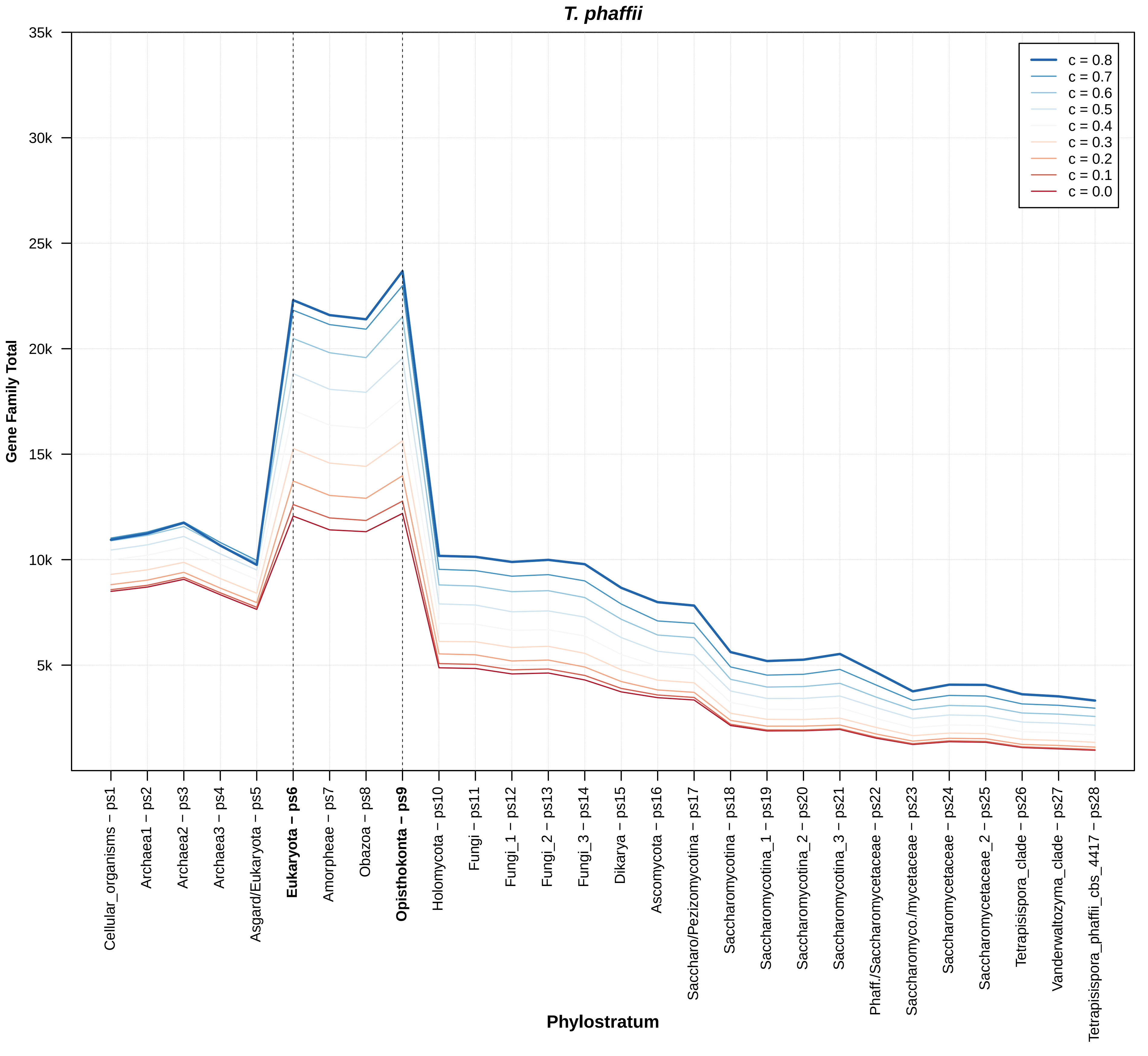
T. phaffii
Gene Family Total
35k
30k
25k
20k
15k
10k
5k
c = 0.8
c = 0.7
c = 0.6
c = 0.5
c = 0.4
c = 0.3
c = 0.2
c = 0.1
c = 0.0
Cellular_organisms – ps1
Archaea1 – ps2
Archaea2 – ps3
Archaea3 – ps4
Asgard/Eukaryota – ps5
Eukaryota – ps6
Amorpheae – ps7
Obazoa – ps8
Opisthokonta – ps9
Holomycota – ps10
Fungi – ps11
Fungi_1 – ps12
Fungi_2 – ps13
Fungi_3 – ps14
Dikarya – ps15
Ascomycota – ps16
Saccharo/Pezizomycotina – ps17
Saccharomycotina – ps18
Saccharomycotina_1 – ps19
Saccharomycotina_2 – ps20
Saccharomycotina_3 – ps21
Phaff./Saccharomycetaceae – ps22
Saccharomyco./mycetaceae – ps23
Saccharomycetaceae – ps24
Saccharomycetaceae_2 – ps25
Tetrapisispora_clade – ps26
Vanderwaltozyma_clade – ps27
Tetrapisispora_phaffii_cbs_4417 – ps28
Phylostratum

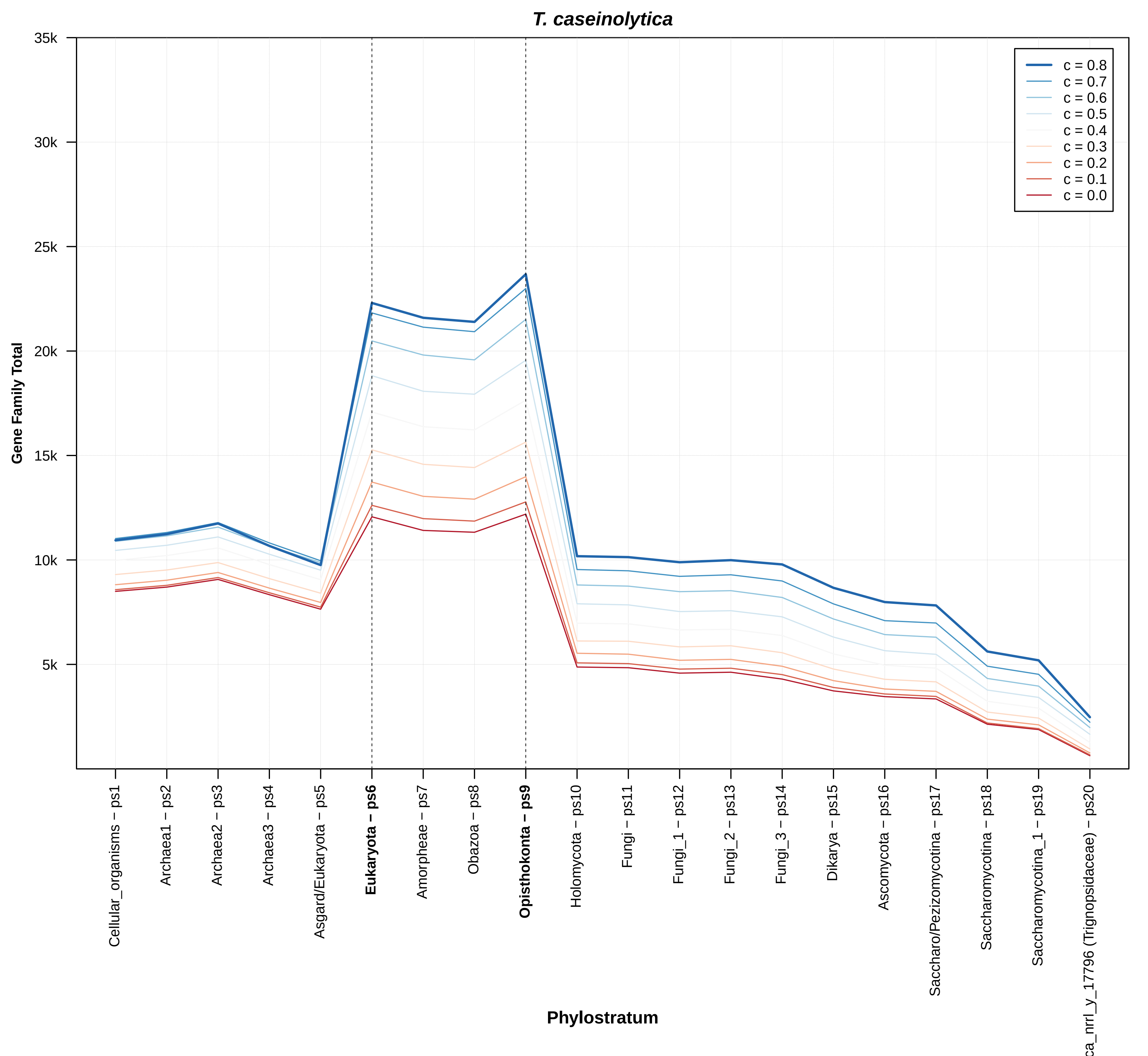
T. caseinolytica
Gene Family Total
35k
30k
25k
20k
15k
10k
5k
c = 0.8
c = 0.7
c = 0.6
c = 0.5
c = 0.4
c = 0.3
c = 0.2
c = 0.1
c = 0.0
Cellular_organisms – ps1
Archaea1 – ps2
Archaea2 – ps3
Archaea3 – ps4
Asgard/Eukaryota – ps5
Eukaryota – ps6
Amorpheae – ps7
Obazoa – ps8
Opisthokonta – ps9
Holomycota – ps10
Fungi – ps11
Fungi_1 – ps12
Fungi_2 – ps13
Fungi_3 – ps14
Dikarya – ps15
Ascomycota – ps16
Saccharo/Pezizomycotina – ps17
Saccharomycotina – ps18
Saccharomycotina_1 – ps19
ca_nrrl_y_17796 (Trignopsidaceae) – ps20
Phylostratum

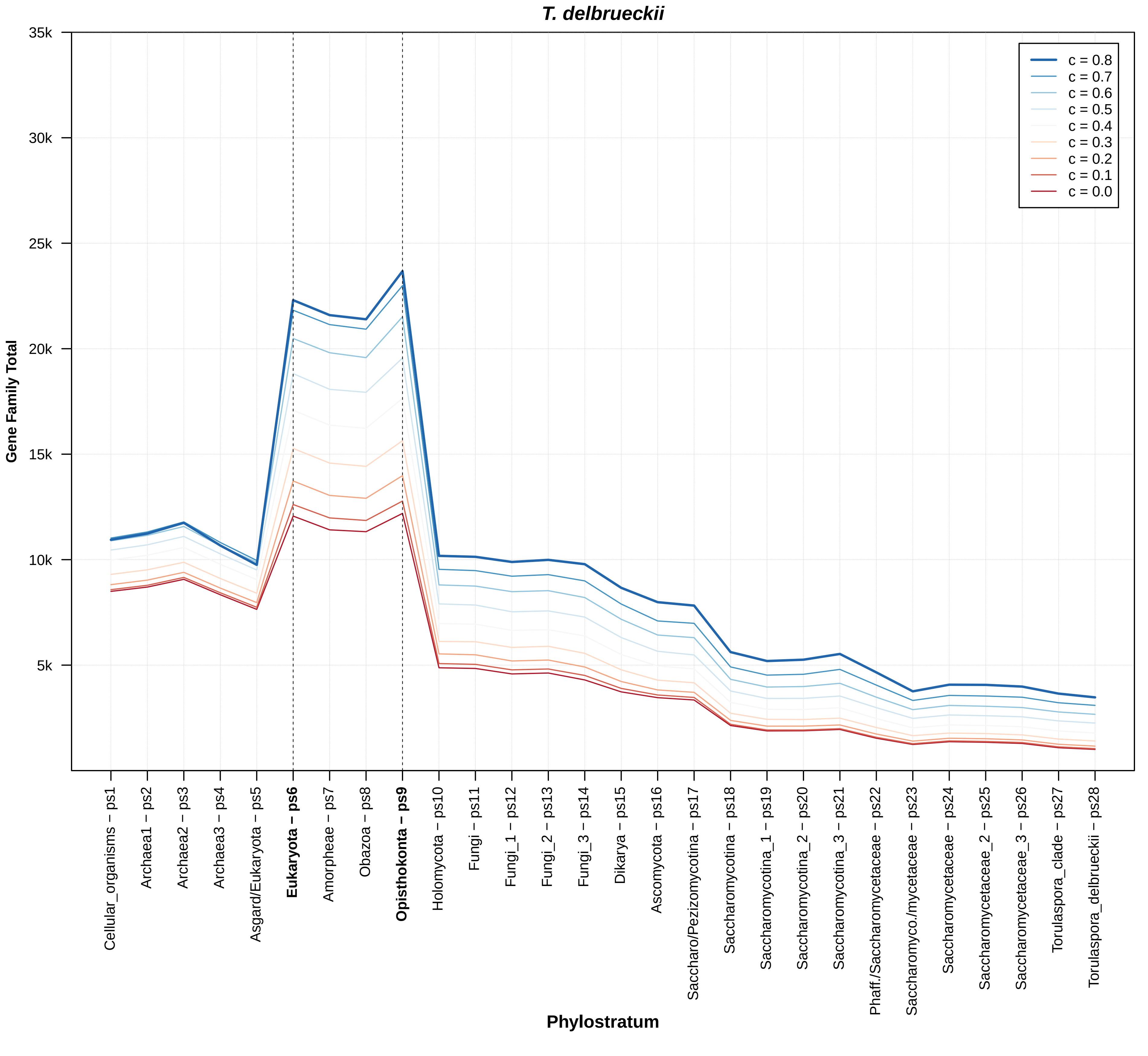
T. delbrueckii
35k
30k
25k
20k
15k
10k
5k
Gene Family Total
c = 0.8
c = 0.7
c = 0.6
c = 0.5
c = 0.4
c = 0.3
c = 0.2
c = 0.1
c = 0.0
Cellular_organisms – ps1
Archaea1 – ps2
Archaea2 – ps3
Archaea3 – ps4
Asgard/Eukaryota – ps5
Eukaryota – ps6
Amorpheae – ps7
Obazoa – ps8
Opisthokonta – ps9
Holomycota – ps10
Fungi – ps11
Fungi_1 – ps12
Fungi_2 – ps13
Fungi_3 – ps14
Dikarya – ps15
Ascomycota – ps16
Saccharo/Pezizomycotina – ps17
Saccharomycotina – ps18
Saccharomycotina_1 – ps19
Saccharomycotina_2 – ps20
Saccharomycotina_3 – ps21
Phaff./Saccharomycetaceae – ps22
Saccharomyco./mycetaceae – ps23
Saccharomycetaceae – ps24
Saccharomycetaceae_2 – ps25
Saccharomycetaceae_3 – ps26
Torulaspora_clade – ps27
Torulaspora_delbrueckii – ps28
Phylostratum

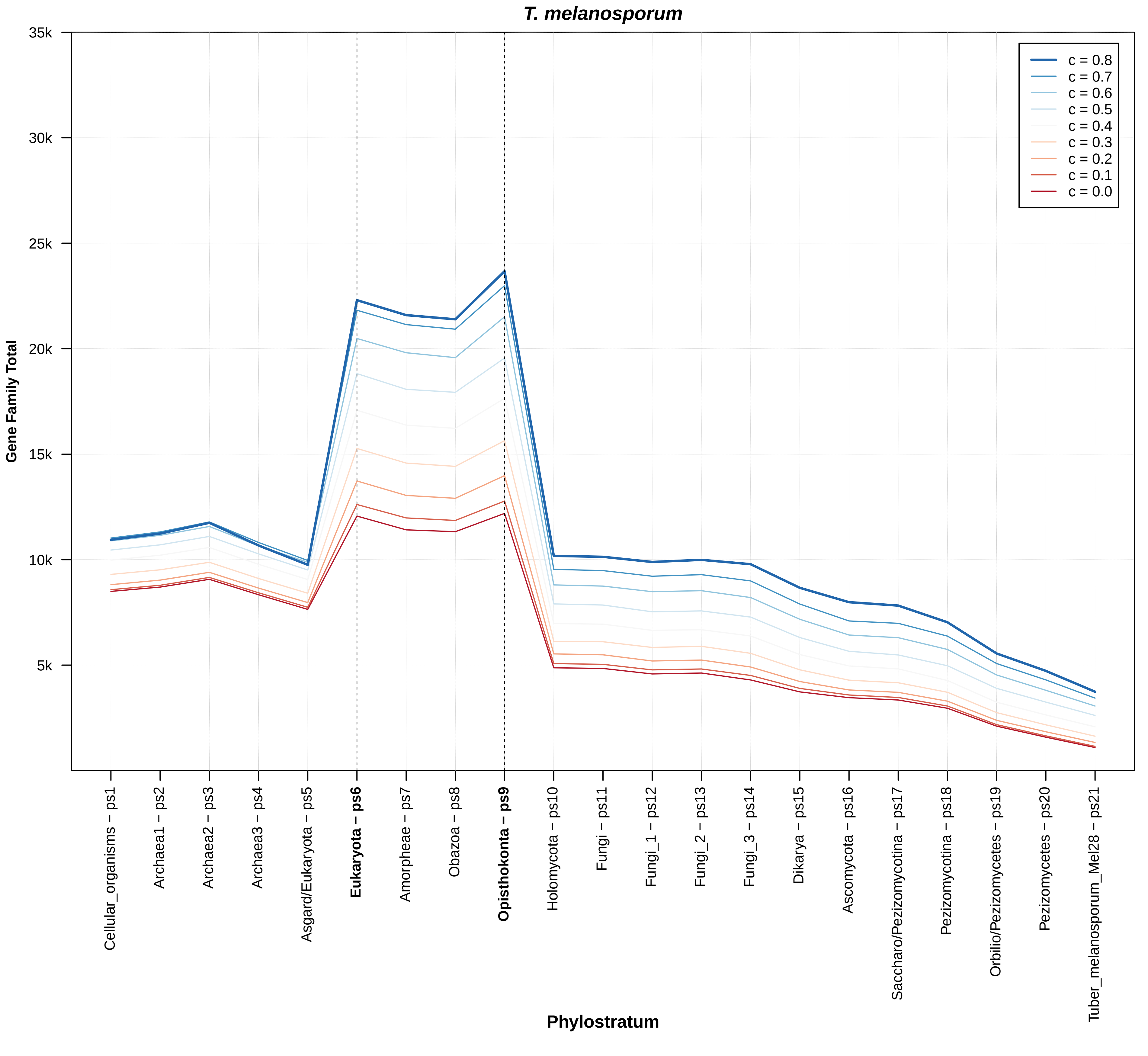
T. melanosporum
Gene Family Total
35k
30k
25k
20k
15k
10k
5k
c = 0.8
c = 0.7
c = 0.6
c = 0.5
c = 0.4
c = 0.3
c = 0.2
c = 0.1
c = 0.0
Cellular_organisms – ps1
Archaea1 – ps2
Archaea2 – ps3
Archaea3 – ps4
Asgard/Eukaryota – ps5
Eukaryota – ps6
Amorpheae – ps7
Obazoa – ps8
Opisthokonta – ps9
Holomycota – ps10
Fungi – ps11
Fungi_1 – ps12
Fungi_2 – ps13
Fungi_3 – ps14
Dikarya – ps15
Ascomycota – ps16
Saccharo/Pezizomycotina – ps17
Pezizomycotina – ps18
Orbilio/Pezizomycetes – ps19
Pezizomycetes – ps20
Tuber_melanosporum_Mel28 – ps21
Phylostratum

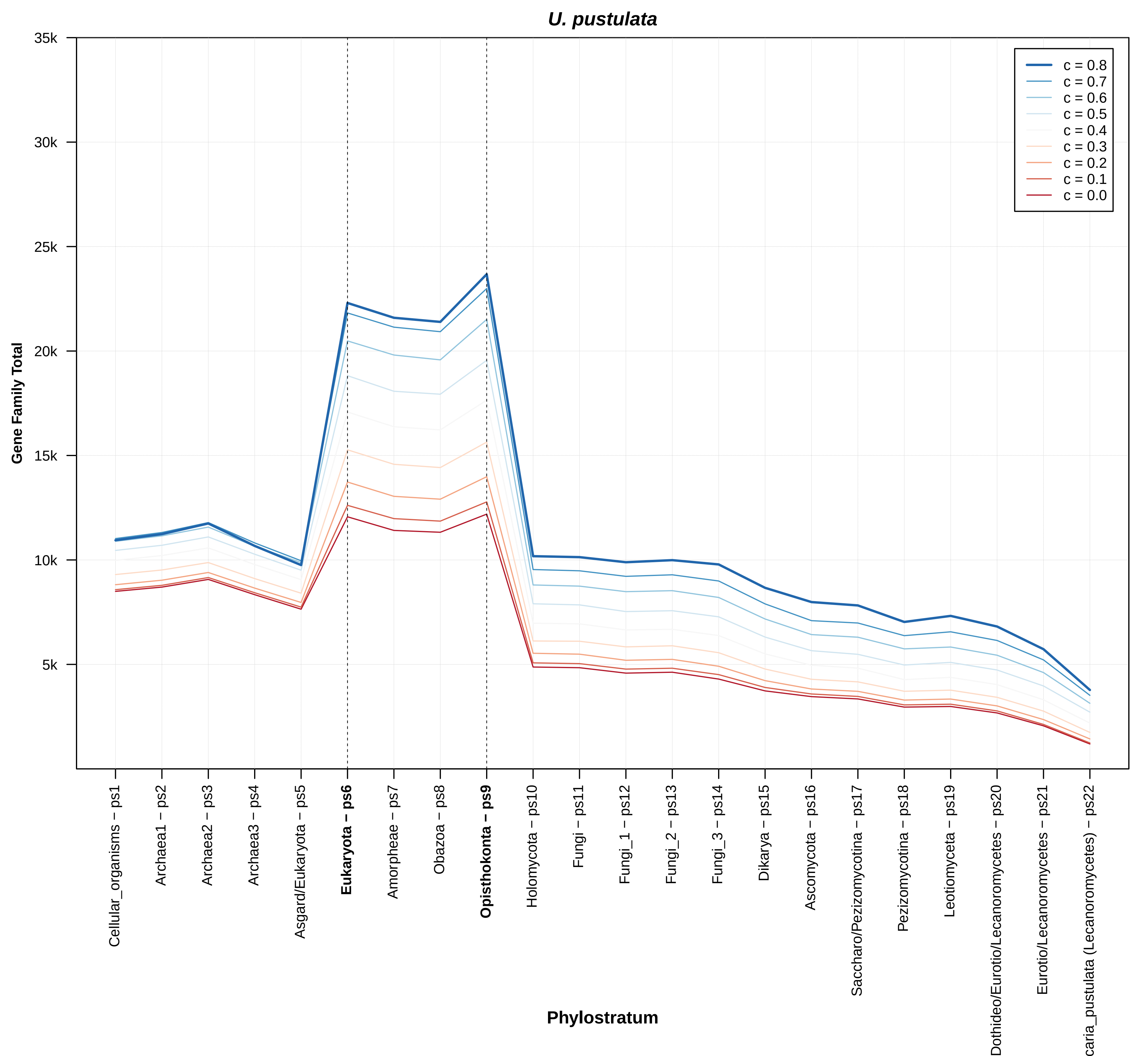
U. pustulata
Gene Family Total
35k
30k
25k
20k
15k
10k
5k
c = 0.8
c = 0.7
c = 0.6
c = 0.5
c = 0.4
c = 0.3
c = 0.2
c = 0.1
c = 0.0
Cellular_organisms – ps1
Archaea1 – ps2
Archaea2 – ps3
Archaea3 – ps4
Asgard/Eukaryota – ps5
Eukaryota – ps6
Amorpheae – ps7
Obazoa – ps8
Opisthokonta – ps9
Holomycota – ps10
Fungi – ps11
Fungi_1 – ps12
Fungi_2 – ps13
Fungi_3 – ps14
Dikarya – ps15
Ascomycota – ps16
Saccharo/Pezizomycotina – ps17
Pezizomycotina – ps18
Leotiomyceta – ps19
Dothideo/Eurotio/Lecanoromycetes – ps20
Eurotio/Lecanoromycetes – ps21
caria_pustulata (Lecanoromycetes) – ps22
Phylostratum

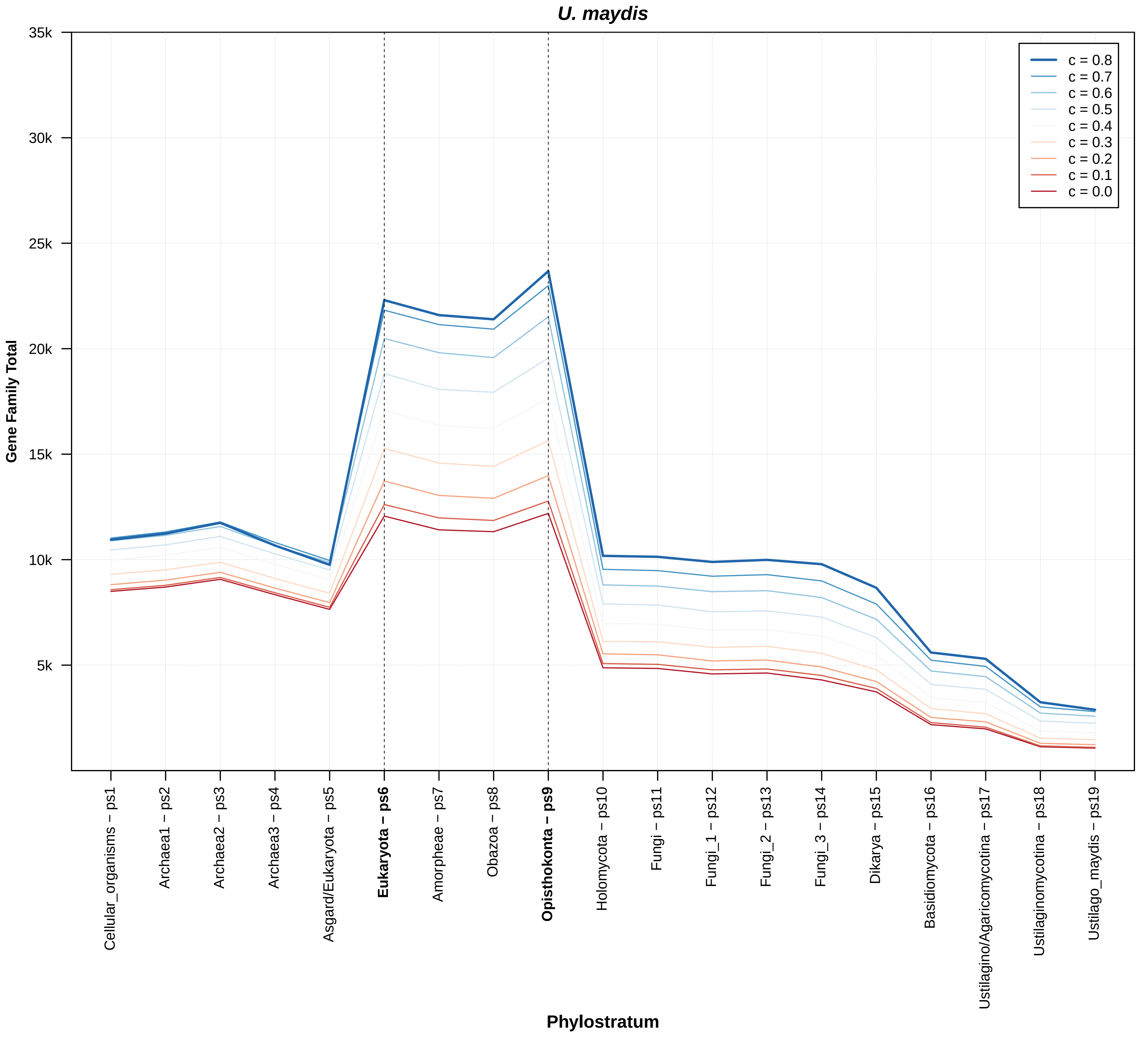
U. maydis
Gene Family Total
35k
30k
25k
20k
15k
10k
5k
c = 0.8
c = 0.7
c = 0.6
c = 0.5
c = 0.4
c = 0.3
c = 0.2
c = 0.1
c = 0.0
Cellular_organisms – ps1
Archaea1 – ps2
Archaea2 – ps3
Archaea3 – ps4
Asgard/Eukaryota – ps5
Eukaryota – ps6
Amorpheae – ps7
Obazoa – ps8
Opisthokonta – ps9
Holomycota – ps10
Fungi – ps11
Fungi_1 – ps12
Fungi_2 – ps13
Fungi_3 – ps14
Dikarya – ps15
Basidiomycota – ps16
Ustilagino/Agaricomycotina – ps17
Ustilaginomycotina – ps18
Ustilago_maydis – ps19
Phylostratum

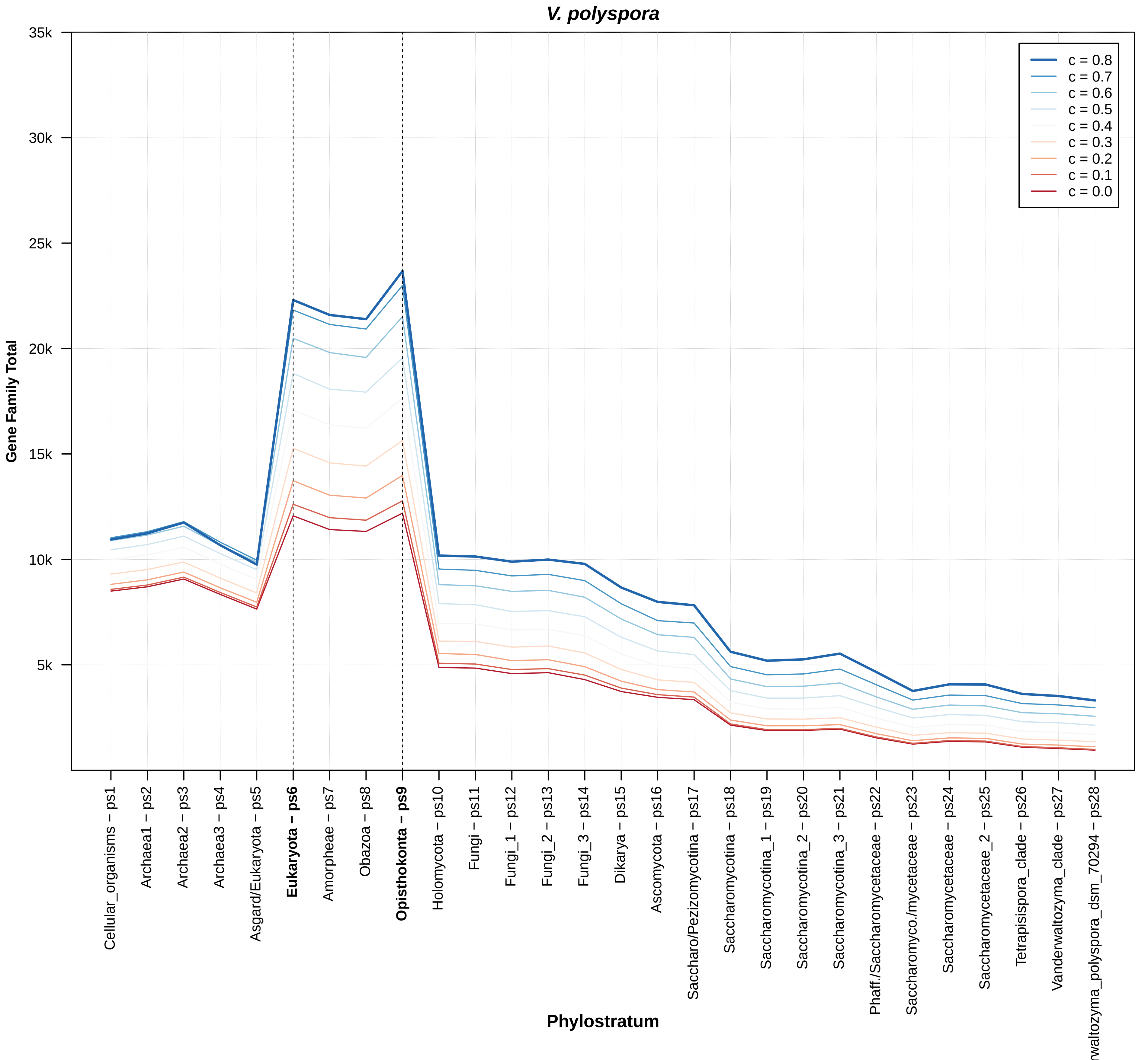

V. polyspora
Gene Family Total
35k
30k
25k
20k
15k
10k
5k
c = 0.8
c = 0.7
c = 0.6
c = 0.5
c = 0.4
c = 0.3
c = 0.2
c = 0.1
c = 0.0
Cellular_organisms – ps1
Archaea1 – ps2
Archaea2 – ps3
Archaea3 – ps4
Asgard/Eukaryota – ps5
Eukaryota – ps6
Amorpheae – ps7
Obazoa – ps8
Opisthokonta – ps9
Holomycota – ps10
Fungi – ps11
Fungi_1 – ps12
Fungi_2 – ps13
Fungi_3 – ps14
Dikarya – ps15
Ascomycota – ps16
Saccharo/Pezizomycotina – ps17
Saccharomycotina – ps18
Saccharomycotina_1 – ps19
Saccharomycotina_2 – ps20
Saccharomycotina_3 – ps21
Phaff./Saccharomycetaceae – ps22
Saccharomyco./mycetaceae – ps23
Saccharomycetaceae – ps24
Saccharomycetaceae_2 – ps25
Tetrapisispora_clade – ps26
Vanderwaltozyma_clade – ps27
rwaltozyma_polyspora_dsm_70294 – ps28
Phylostratum

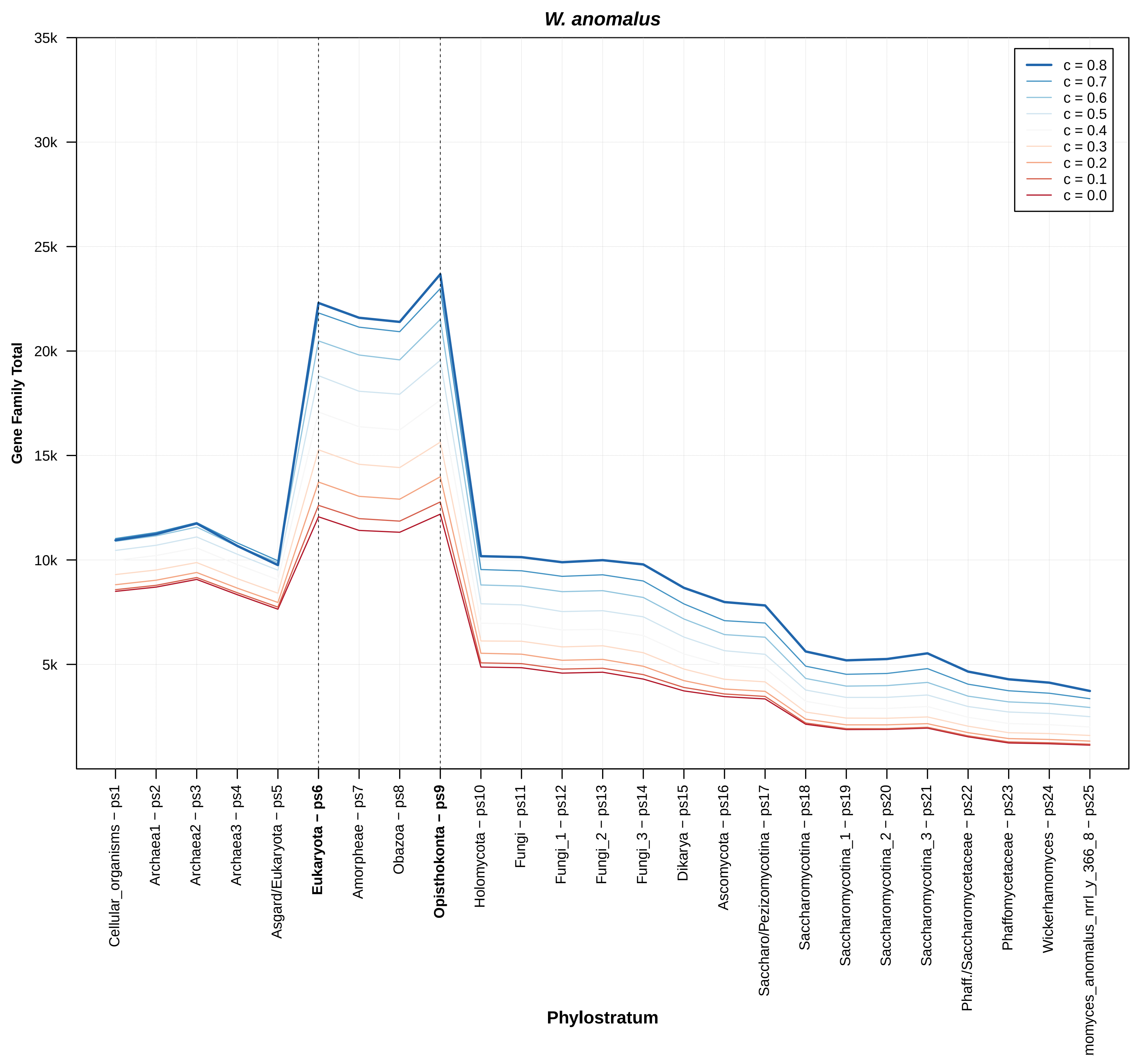

W. anomalus
35k
30k
25k
20k
15k
10k
5k
Gene Family Total
c = 0.8
c = 0.7
c = 0.6
c = 0.5
c = 0.4
c = 0.3
c = 0.2
c = 0.1
c = 0.0
Cellular_organisms – ps1
Archaea1 – ps2
Archaea2 – ps3
Archaea3 – ps4
Asgard/Eukaryota – ps5
Eukaryota – ps6
Amorpheae – ps7
Obazoa – ps8
Opisthokonta – ps9
Holomycota – ps10
Fungi – ps11
Fungi_1 – ps12
Fungi_2 – ps13
Fungi_3 – ps14
Dikarya – ps15
Ascomycota – ps16
Saccharo/Pezizomycotina – ps17
Saccharomycotina – ps18
Saccharomycotina_1 – ps19
Saccharomycotina_2 – ps20
Saccharomycotina_3 – ps21
Phaff./Saccharomycetaceae – ps22
Phaffomycetaceae – ps23
Wickerhamomyces – ps24
momyces_anomalus_nrrl_y_366_8 – ps25
Phylostratum

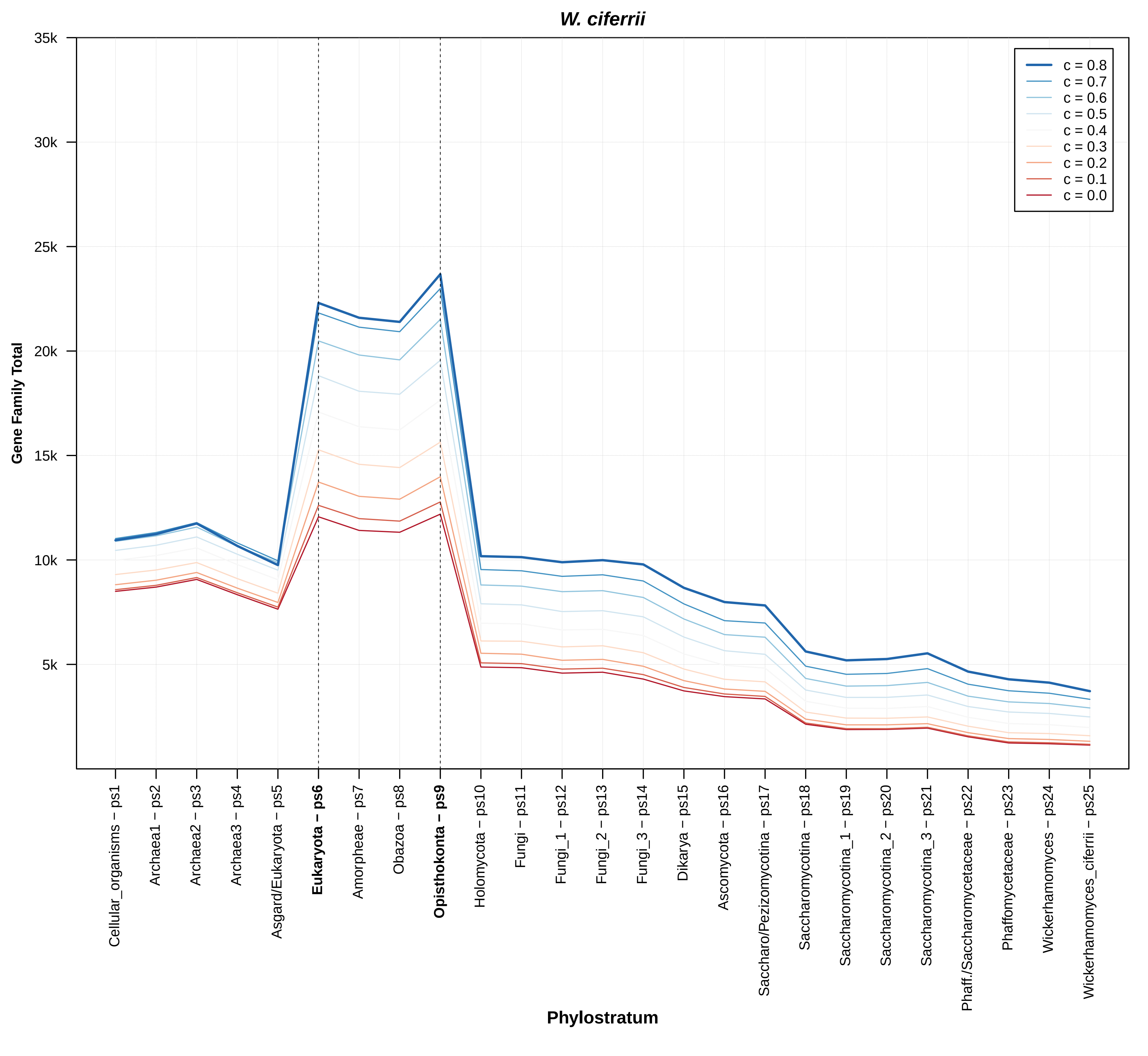

W. ciferrii
35k
30k
25k
20k
15k
10k
5k
Gene Family Total
c = 0.8
c = 0.7
c = 0.6
c = 0.5
c = 0.4
c = 0.3
c = 0.2
c = 0.1
c = 0.0
Cellular_organisms – ps1
Archaea1 – ps2
Archaea2 – ps3
Archaea3 – ps4
Asgard/Eukaryota – ps5
Eukaryota – ps6
Amorpheae – ps7
Obazoa – ps8
Opisthokonta – ps9
Holomycota – ps10
Fungi – ps11
Fungi_1 – ps12
Fungi_2 – ps13
Fungi_3 – ps14
Dikarya – ps15
Ascomycota – ps16
Saccharo/Pezizomycotina – ps17
Saccharomycotina – ps18
Saccharomycotina_1 – ps19
Saccharomycotina_2 – ps20
Saccharomycotina_3 – ps21
Phaff./Saccharomycetaceae – ps22
Phaffomycetaceae – ps23
Wickerhamomyces – ps24
Wickerhamomyces_ciferrii – ps25
Phylostratum

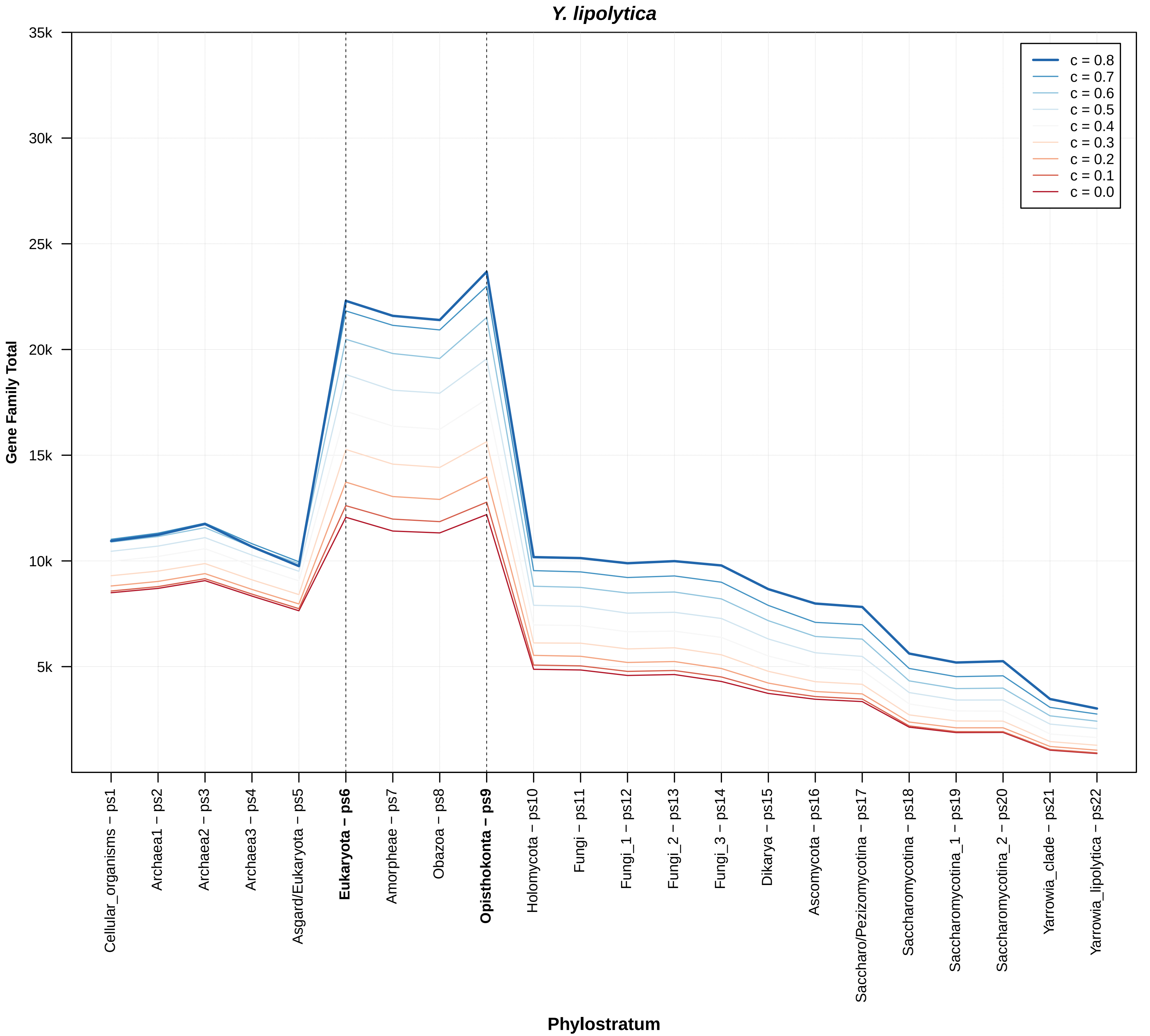

Y. lipolytica
Gene Family Total
35k
30k
25k
20k
15k
10k
5k
c = 0.8
c = 0.7
c = 0.6
c = 0.5
c = 0.4
c = 0.3
c = 0.2
c = 0.1
c = 0.0
Cellular_organisms – ps1
Archaea1 – ps2
Archaea2 – ps3
Archaea3 – ps4
Asgard/Eukaryota – ps5
Eukaryota – ps6
Amorpheae – ps7
Obazoa – ps8
Opisthokonta – ps9
Holomycota – ps10
Fungi – ps11
Fungi_1 – ps12
Fungi_2 – ps13
Fungi_3 – ps14
Dikarya – ps15
Ascomycota – ps16
Saccharo/Pezizomycotina – ps17
Saccharomycotina – ps18
Saccharomycotina_1 – ps19
Saccharomycotina_2 – ps20
Yarrowia_clade – ps21
Yarrowia_lipolytica – ps22
Phylostratum

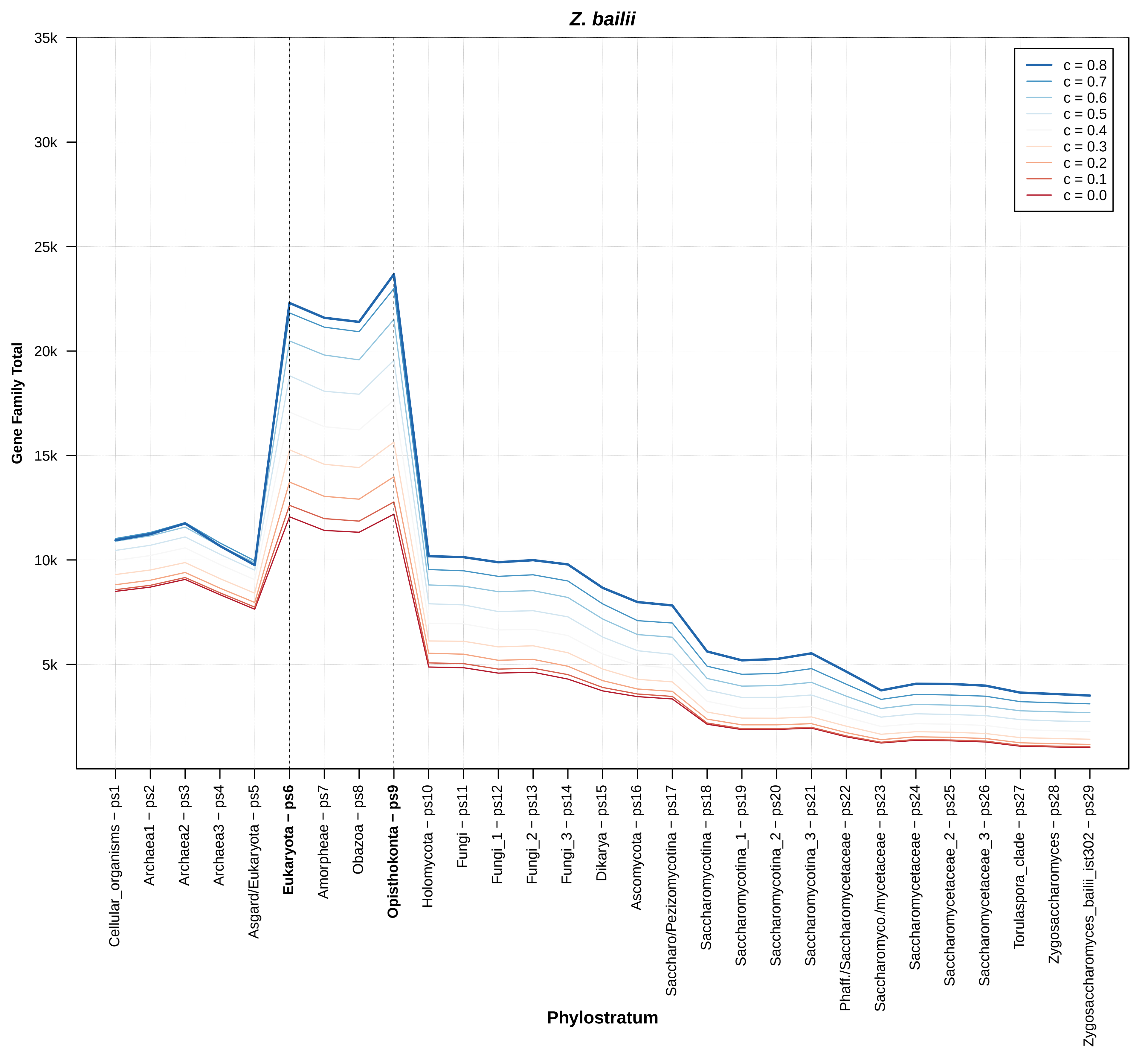

Z. bailii
Gene Family Total
35k
30k
25k
20k
15k
10k
5k
c = 0.8
c = 0.7
c = 0.6
c = 0.5
c = 0.4
c = 0.3
c = 0.2
c = 0.1
c = 0.0
Cellular_organisms – ps1
Archaea1 – ps2
Archaea2 – ps3
Archaea3 – ps4
Asgard/Eukaryota – ps5
Eukaryota – ps6
Amorpheae – ps7
Obazoa – ps8
Opisthokonta – ps9
Holomycota – ps10
Fungi – ps11
Fungi_1 – ps12
Fungi_2 – ps13
Fungi_3 – ps14
Dikarya – ps15
Ascomycota – ps16
Saccharo/Pezizomycotina – ps17
Saccharomycotina – ps18
Saccharomycotina_1 – ps19
Saccharomycotina_2 – ps20
Saccharomycotina_3 – ps21
Phaff./Saccharomycetaceae – ps22
Saccharomyco./mycetaceae – ps23
Saccharomycetaceae – ps24
Saccharomycetaceae_2 – ps25
Saccharomycetaceae_3 – ps26
Torulaspora_clade – ps27
Zygosaccharomyces – ps28
Zygosaccharomyces_bailii_ist302 – ps29
Phylostratum

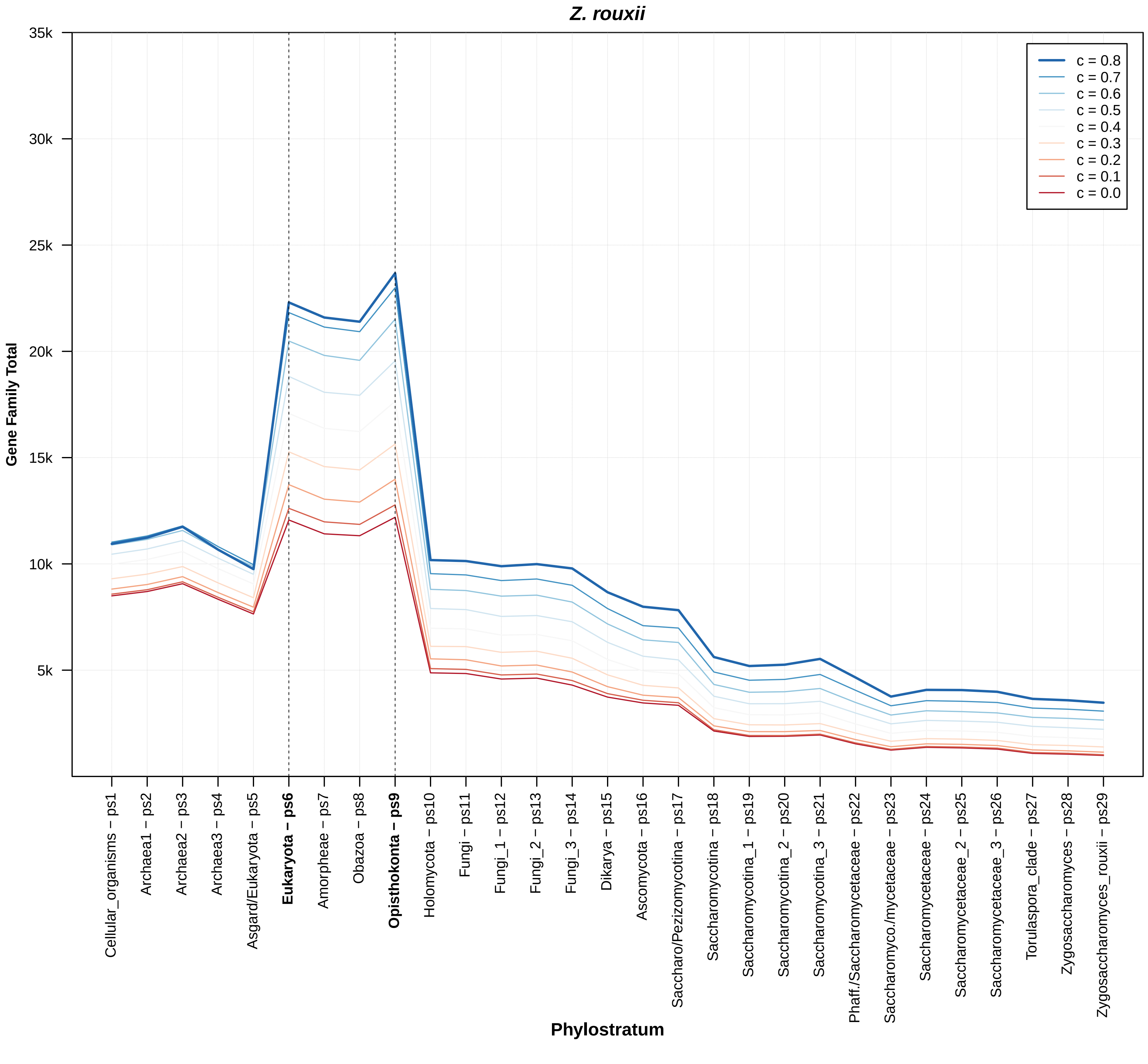
Z. rouxii
Gene Family Total
35k
30k
25k
20k
15k
10k
5k
c = 0.8
c = 0.7
c = 0.6
c = 0.5
c = 0.4
c = 0.3
c = 0.2
c = 0.1
c = 0.0
Cellular_organisms – ps1
Archaea1 – ps2
Archaea2 – ps3
Archaea3 – ps4
Asgard/Eukaryota – ps5
Eukaryota – ps6
Amorpheae – ps7
Obazoa – ps8
Opisthokonta – ps9
Holomycota – ps10
Fungi – ps11
Fungi_1 – ps12
Fungi_2 – ps13
Fungi_3 – ps14
Dikarya – ps15
Ascomycota – ps16
Saccharo/Pezizomycotina – ps17
Saccharomycotina – ps18
Saccharomycotina_1 – ps19
Saccharomycotina_2 – ps20
Saccharomycotina_3 – ps21
Phaff./Saccharomycetaceae – ps22
Saccharomyco./mycetaceae – ps23
Saccharomycetaceae – ps24
Saccharomycetaceae_2 – ps25
Saccharomycetaceae_3 – ps26
Torulaspora_clade – ps27
Zygosaccharomyces – ps28
Zygosaccharomyces_rouxii – ps29
Phylostratum

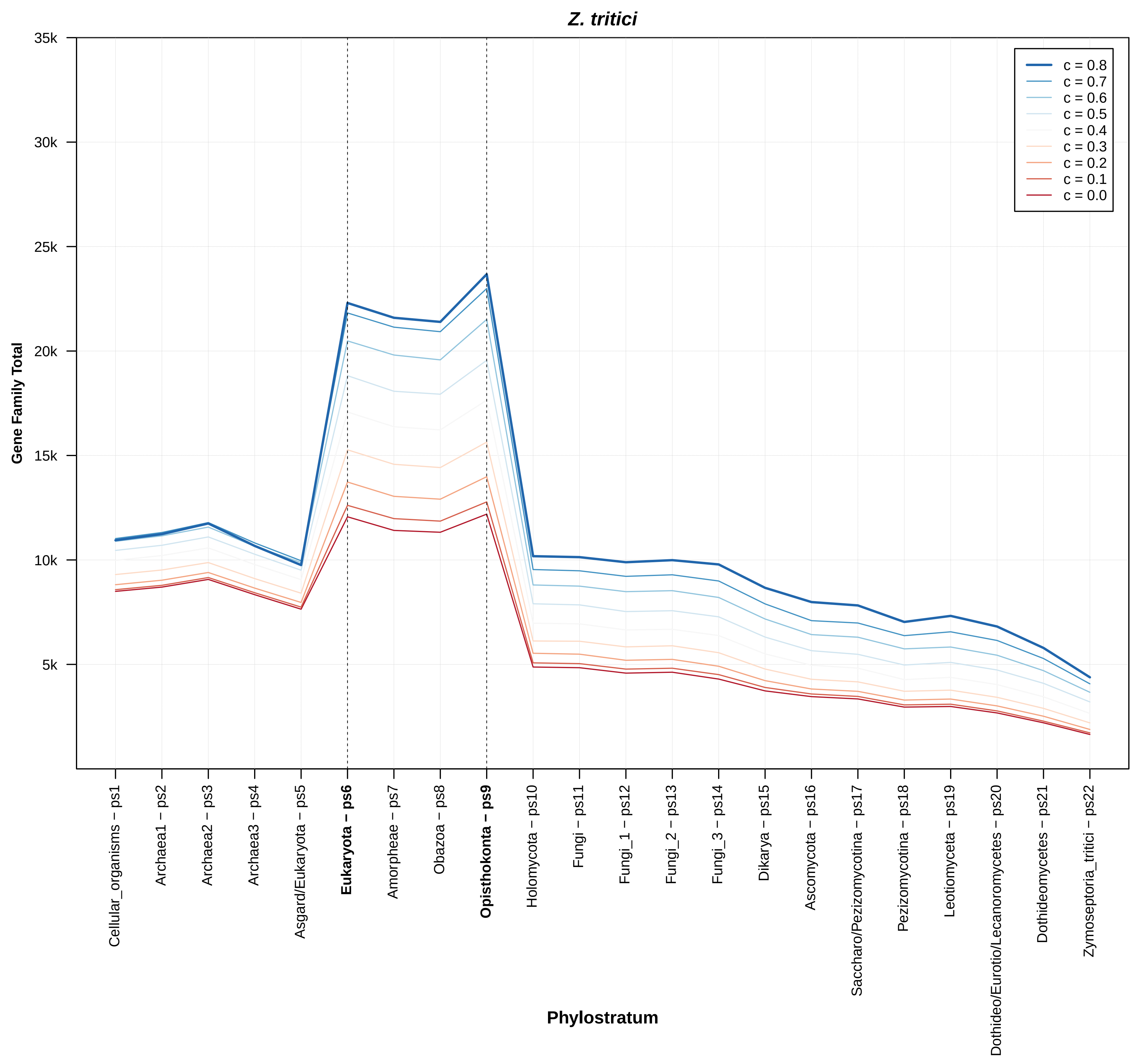

Z. tritici
Gene Family Total
35k
30k
25k
20k
15k
10k
5k
c = 0.8
c = 0.7
c = 0.6
c = 0.5
c = 0.4
c = 0.3
c = 0.2
c = 0.1
c = 0.0
Cellular_organisms – ps1
Archaea1 – ps2
Archaea2 – ps3
Archaea3 – ps4
Asgard/Eukaryota – ps5
Eukaryota – ps6
Amorpheae – ps7
Obazoa – ps8
Opisthokonta – ps9
Holomycota – ps10
Fungi – ps11
Fungi_1 – ps12
Fungi_2 – ps13
Fungi_3 – ps14
Dikarya – ps15
Ascomycota – ps16
Saccharo/Pezizomycotina – ps17
Pezizomycotina – ps18
Leotiomyceta – ps19
Dothideo/Eurotio/Lecanoromycetes – ps20
Dothideomycetes – ps21
Zymoseptoria_tritici – ps22
Phylostratum

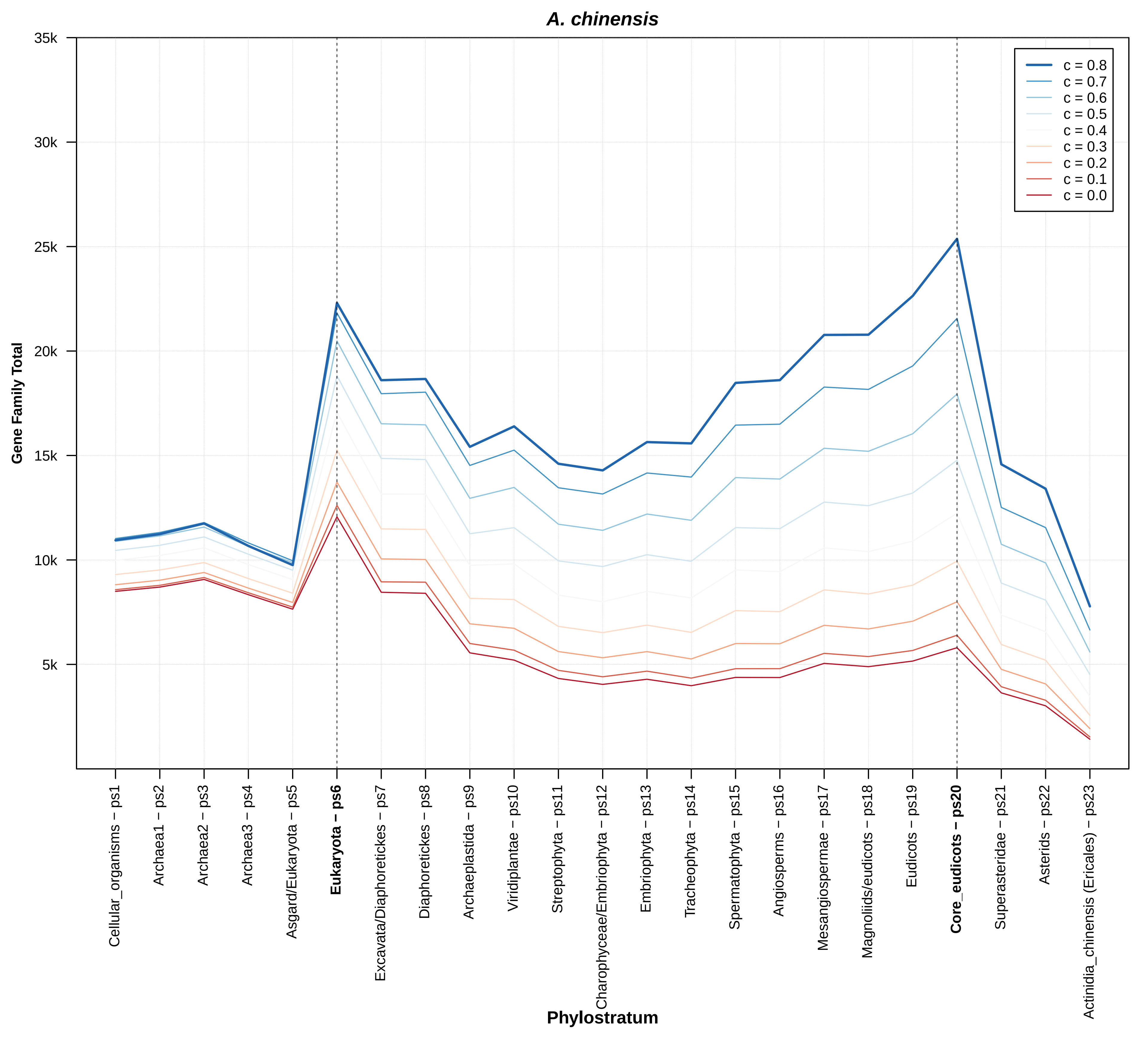
A. chinensis
Gene Family Total
35k
30k
25k
20k
15k
10k
5k
c = 0.8
c = 0.7
c = 0.6
c = 0.5
c = 0.4
c = 0.3
c = 0.2
c = 0.1
c = 0.0
Cellular_organisms – ps1
Archaea1 – ps2
Archaea2 – ps3
Archaea3 – ps4
Asgard/Eukaryota – ps5
Eukaryota – ps6
Excavata/Diaphoretickes – ps7
Diaphoretickes – ps8
Archaeplastida – ps9
Viridiplantae – ps10
Streptophyta – ps11
Charophyceae/Embriophyta – ps12
Embriophyta – ps13
Tracheophyta – ps14
Spermatophyta – ps15
Angiosperms – ps16
Mesangiospermae – ps17
Magnoliids/eudicots – ps18
Eudicots – ps19
Core_eudicots – ps20
Superasteridae – ps21
Asterids – ps22
Actinidia_chinensis (Ericales) – ps23
Phylostratum

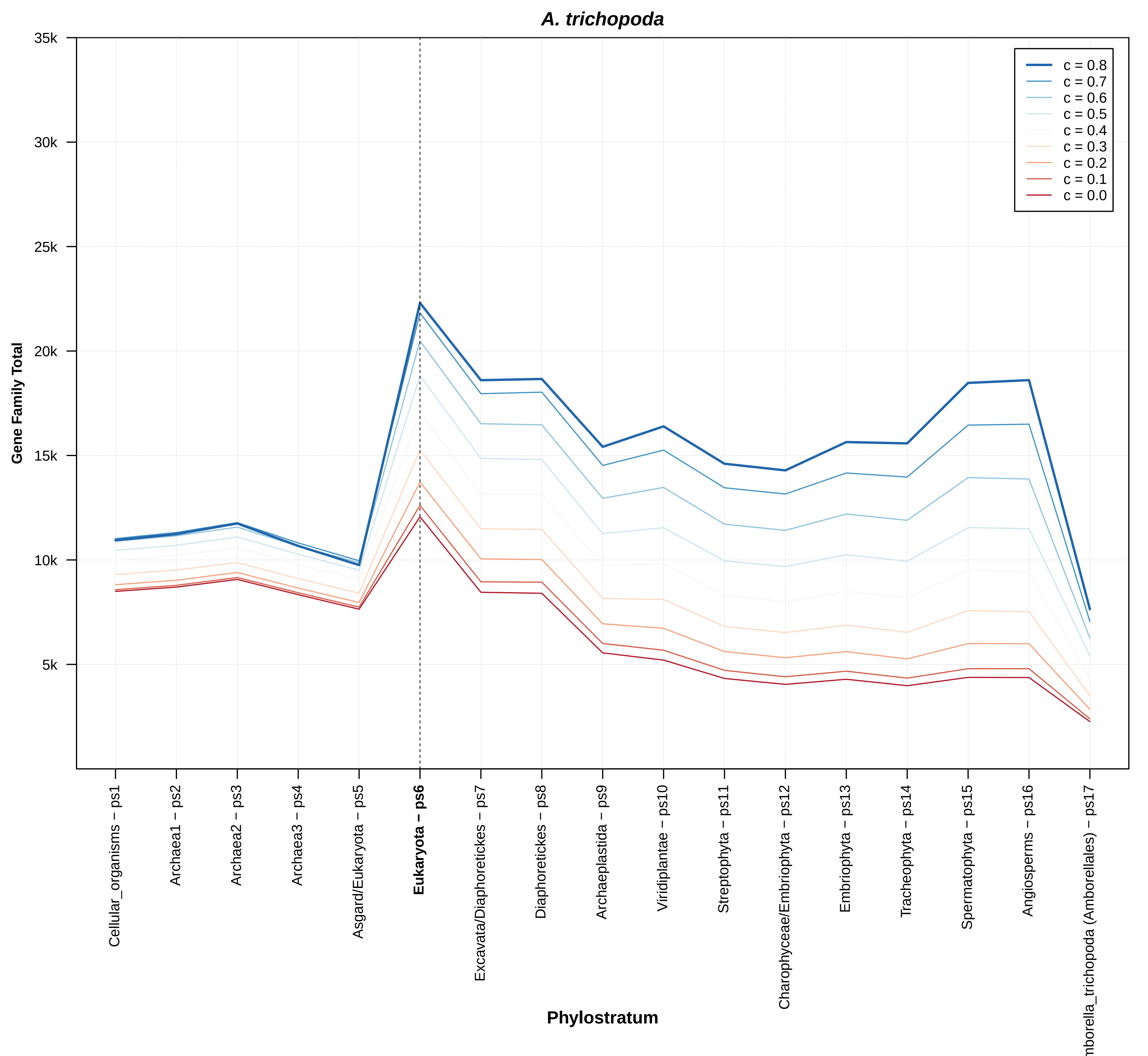

A. trichopoda
Gene Family Total
35k
30k
25k
20k
15k
10k
5k
c = 0.8
c = 0.7
c = 0.6
c = 0.5
c = 0.4
c = 0.3
c = 0.2
c = 0.1
c = 0.0
Cellular_organisms – ps1
Archaea1 – ps2
Archaea2 – ps3
Archaea3 – ps4
Asgard/Eukaryota – ps5
Eukaryota – ps6
Excavata/Diaphoretickes – ps7
Diaphoretickes – ps8
Archaeplastida – ps9
Viridiplantae – ps10
Streptophyta – ps11
Charophyceae/Embriophyta – ps12
Embriophyta – ps13
Tracheophyta – ps14
Spermatophyta – ps15
Angiosperms – ps16
mborella_trichopoda (Amborellales) – ps17
Phylostratum

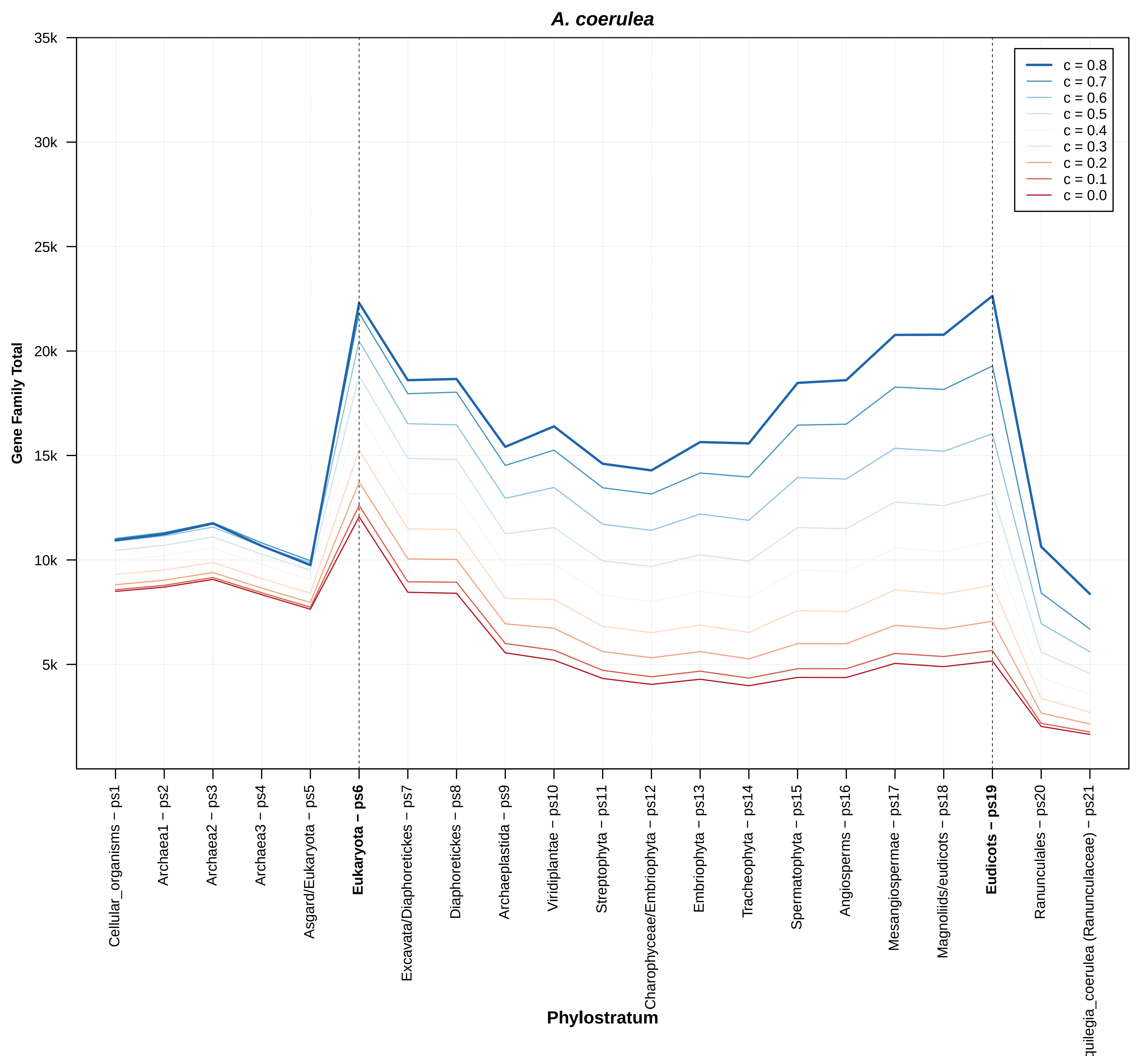

A. coerulea
35k
30k
25k
20k
15k
10k
5k
Gene Family Total
c = 0.8
c = 0.7
c = 0.6
c = 0.5
c = 0.4
c = 0.3
c = 0.2
c = 0.1
c = 0.0
Cellular_organisms – ps1
Archaea1 – ps2
Archaea2 – ps3
Archaea3 – ps4
Asgard/Eukaryota – ps5
Eukaryota – ps6
Excavata/Diaphoretickes – ps7
Diaphoretickes – ps8
Archaeplastida – ps9
Viridiplantae – ps10
Streptophyta – ps11
Charophyceae/Embriophyta – ps12
Embriophyta – ps13
Tracheophyta – ps14
Spermatophyta – ps15
Angiosperms – ps16
Mesangiospermae – ps17
Magnoliids/eudicots – ps18
Eudicots – ps19
Ranunculales – ps20
quilegia_coerulea (Ranunculaceae) – ps21
Phylostratum

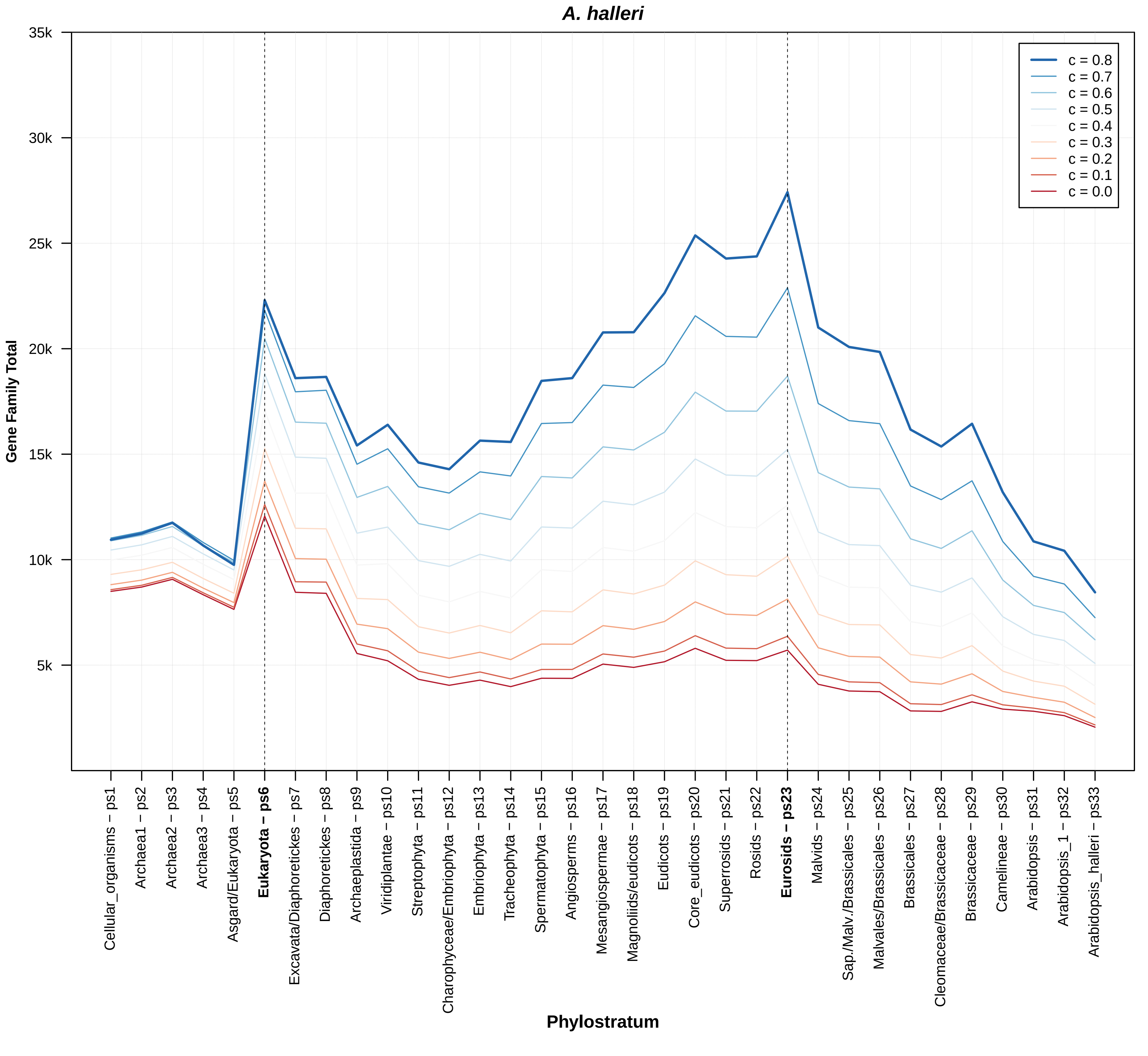
A. halleri
Gene Family Total
35k
30k
25k
20k
15k
10k
5k
c = 0.8
c = 0.7
c = 0.6
c = 0.5
c = 0.4
c = 0.3
c = 0.2
c = 0.1
c = 0.0
Cellular_organisms – ps1
Archaea1 – ps2
Archaea2 – ps3
Archaea3 – ps4
Asgard/Eukaryota – ps5
Eukaryota – ps6
Excavata/Diaphoretickes – ps7
Diaphoretickes – ps8
Archaeplastida – ps9
Viridiplantae – ps10
Streptophyta – ps11
Charophyceae/Embriophyta – ps12
Embriophyta – ps13
Tracheophyta – ps14
Spermatophyta – ps15
Angiosperms – ps16
Mesangiospermae – ps17
Magnoliids/eudicots – ps18
Eudicots – ps19
Core_eudicots – ps20
Superrosids – ps21
Rosids – ps22
Eurosids – ps23
Malvids – ps24
Sap./Malv./Brassicales – ps25
Malvales/Brassicales – ps26
Brassicales – ps27
Cleomaceae/Brassicaceae – ps28
Brassicaceae – ps29
Camelineae – ps30
Arabidopsis – ps31
Arabidopsis_1 – ps32
Arabidopsis_halleri – ps33
Phylostratum

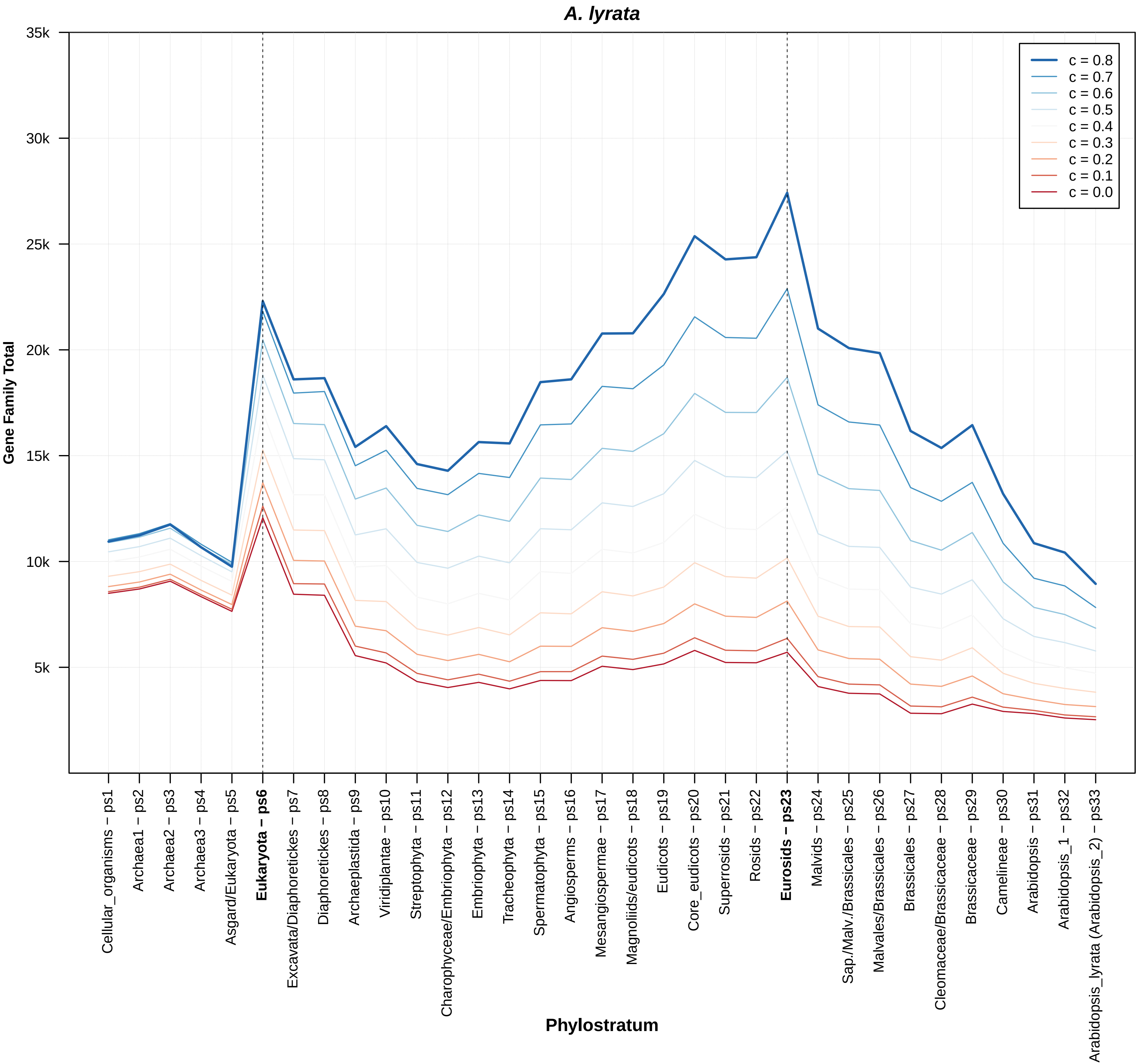
A. lyrata
Gene Family Total
Phylostratum
35k
30k
25k
20k
15k
10k
5k
c = 0.8
c = 0.7
c = 0.6
c = 0.5
c = 0.4
c = 0.3
c = 0.2
c = 0.1
c = 0.0
Cellular_organisms – ps1
Archaea1 – ps2
Archaea2 – ps3
Archaea3 – ps4
Asgard/Eukaryota – ps5
Eukaryota – ps6
Excavata/Diaphoretickes – ps7
Diaphoretickes – ps8
Archaeplastida – ps9
Viridiplantae – ps10
Streptophyta – ps11
Charophyceae/Embriophyta – ps12
Embriophyta – ps13
Tracheophyta – ps14
Spermatophyta – ps15
Angiosperms – ps16
Mesangiospermae – ps17
Magnoliids/eudicots – ps18
Eudicots – ps19
Core_eudicots – ps20
Superrosids – ps21
Rosids – ps22
Eurosids – ps23
Malvids – ps24
Sap./Malv./Brassicales – ps25
Malvales/Brassicales – ps26
Brassicales – ps27
Cleomaceae/Brassicaceae – ps28
Brassicaceae – ps29
Camelineae – ps30
Arabidopsis – ps31
Arabidopsis_1 – ps32
Arabidopsis_lyrata (Arabidopsis_2) – ps33

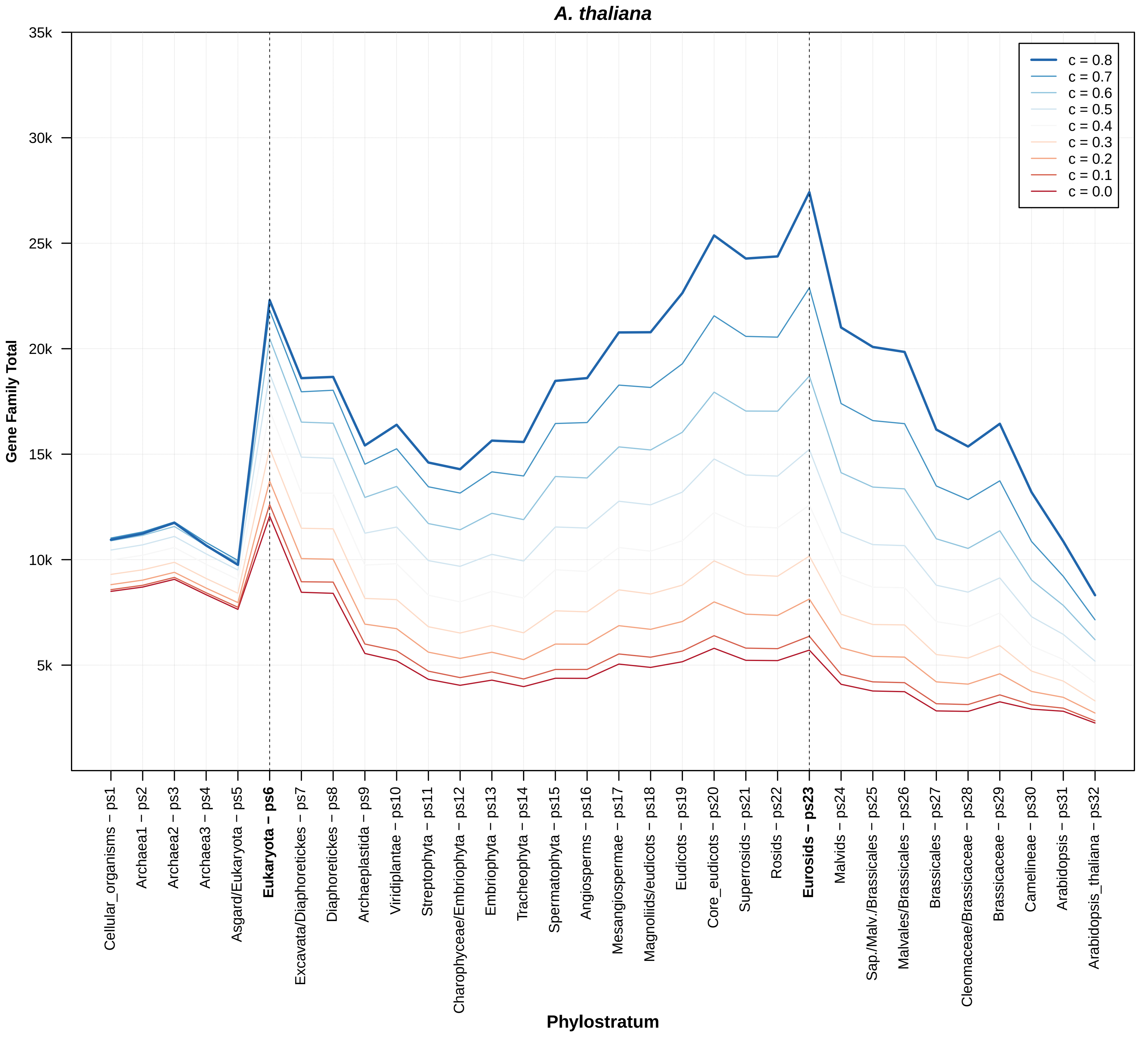
A. thaliana
Gene Family Total
35k
30k
25k
20k
15k
10k
5k
c = 0.8
c = 0.7
c = 0.6
c = 0.5
c = 0.4
c = 0.3
c = 0.2
c = 0.1
c = 0.0
Cellular_organisms – ps1
Archaea1 – ps2
Archaea2 – ps3
Archaea3 – ps4
Asgard/Eukaryota – ps5
Eukaryota – ps6
Excavata/Diaphoretickes – ps7
Diaphoretickes – ps8
Archaeplastida – ps9
Viridiplantae – ps10
Streptophyta – ps11
Charophyceae/Embriophyta – ps12
Embriophyta – ps13
Tracheophyta – ps14
Spermatophyta – ps15
Angiosperms – ps16
Mesangiospermae – ps17
Magnoliids/eudicots – ps18
Eudicots – ps19
Core_eudicots – ps20
Superrosids – ps21
Rosids – ps22
Eurosids – ps23
Malvids – ps24
Sap./Malv./Brassicales – ps25
Malvales/Brassicales – ps26
Brassicales – ps27
Cleomaceae/Brassicaceae – ps28
Brassicaceae – ps29
Camelineae – ps30
Arabidopsis – ps31
Arabidopsis_thaliana – ps32
Phylostratum

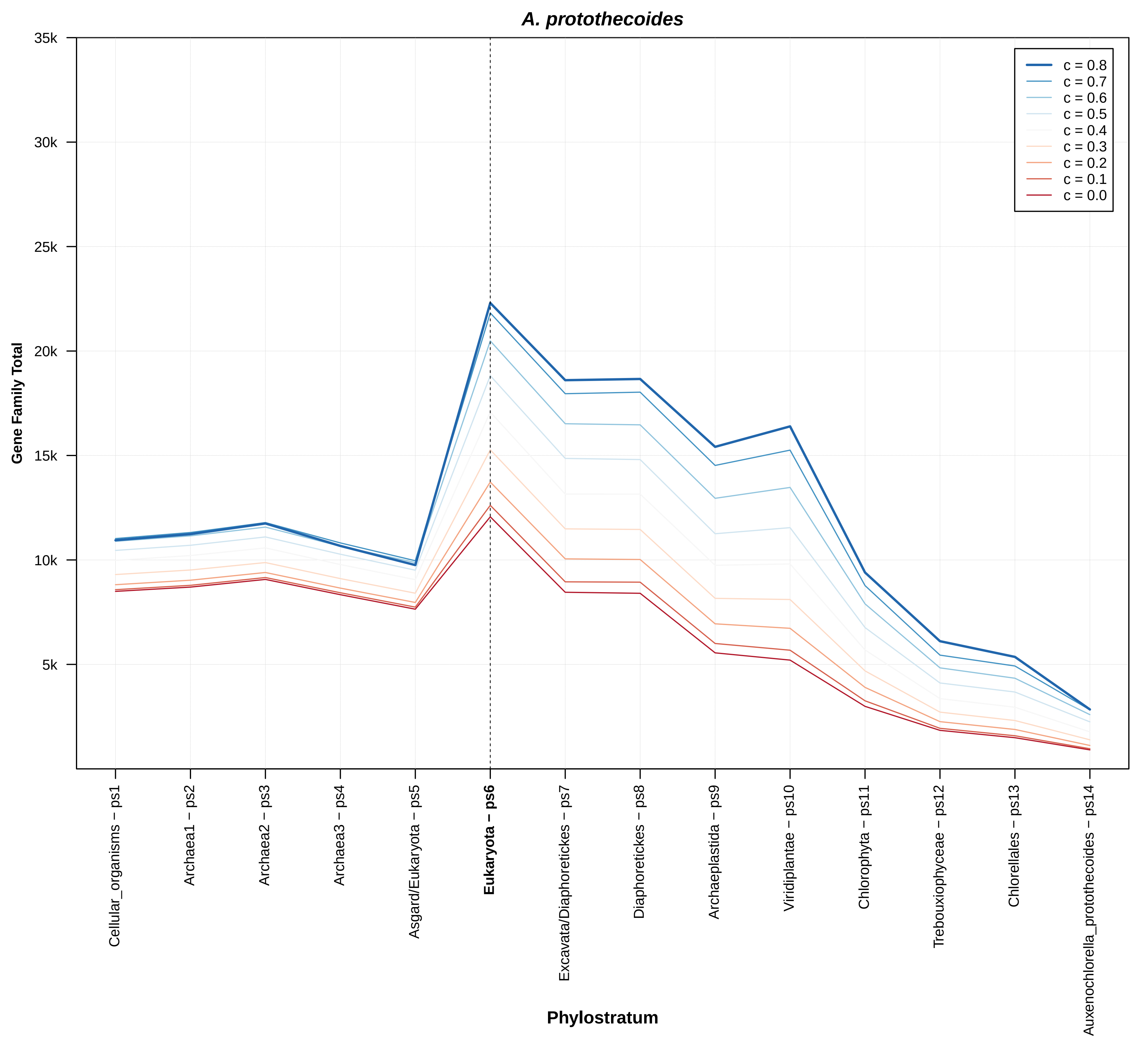
A. protothecoides
Gene Family Total
35k
30k
25k
20k
15k
10k
5k
c = 0.8
c = 0.7
c = 0.6
c = 0.5
c = 0.4
c = 0.3
c = 0.2
c = 0.1
c = 0.0
Cellular_organisms – ps1
Archaea1 – ps2
Archaea2 – ps3
Archaea3 – ps4
Asgard/Eukaryota – ps5
Eukaryota – ps6
Excavata/Diaphoretickes – ps7
Diaphoretickes – ps8
Archaeplastida – ps9
Viridiplantae – ps10
Chlorophyta – ps11
Trebouxiophyceae – ps12
Chlorellales – ps13
Auxenochlorella_protothecoides – ps14
Phylostratum

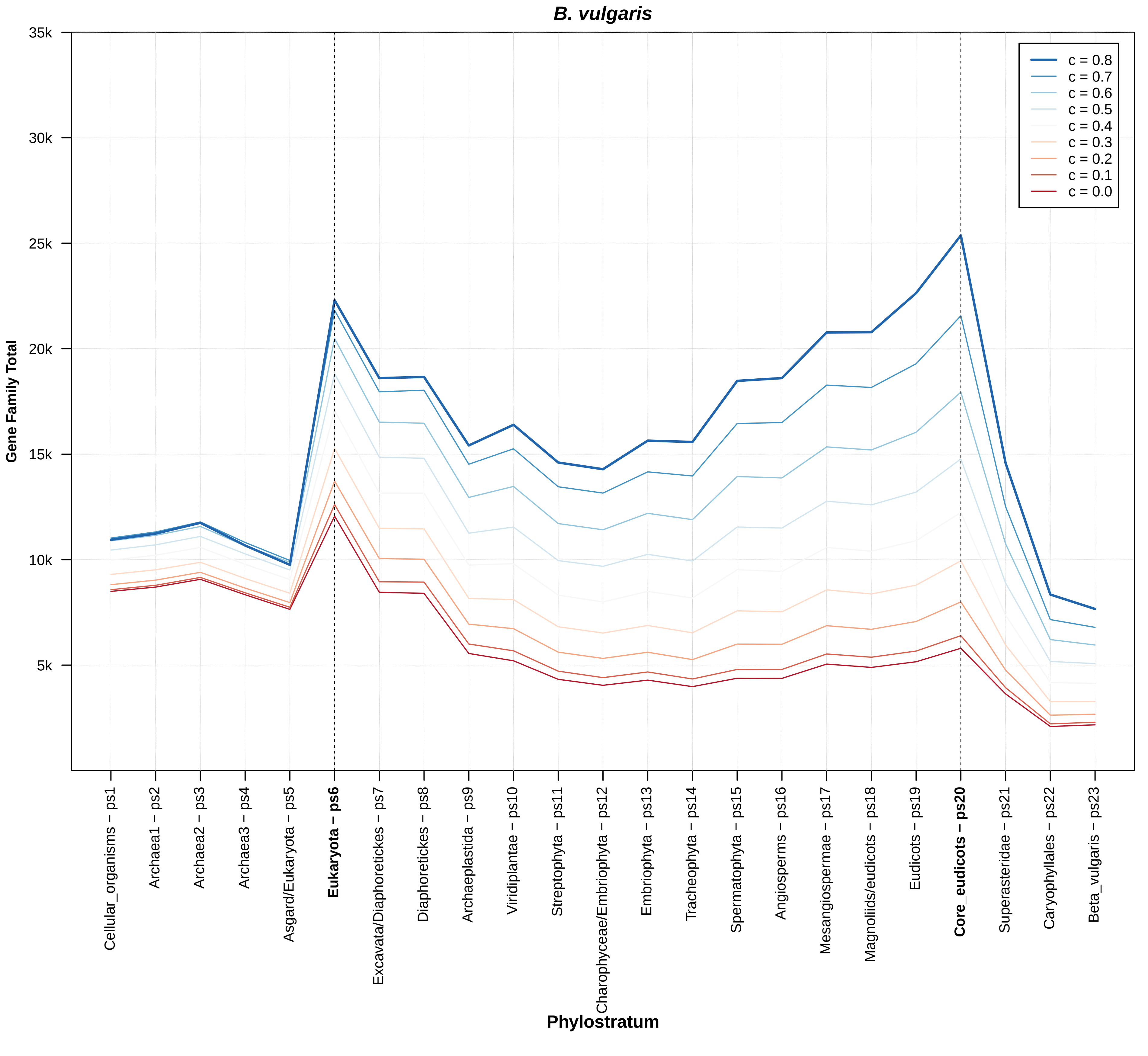

B. vulgaris
Gene Family Total
35k
30k
25k
20k
15k
10k
5k
c = 0.8
c = 0.7
c = 0.6
c = 0.5
c = 0.4
c = 0.3
c = 0.2
c = 0.1
c = 0.0
Cellular_organisms – ps1
Archaea1 – ps2
Archaea2 – ps3
Archaea3 – ps4
Asgard/Eukaryota – ps5
Eukaryota – ps6
Excavata/Diaphoretickes – ps7
Diaphoretickes – ps8
Archaeplastida – ps9
Viridiplantae – ps10
Streptophyta – ps11
Charophyceae/Embriophyta – ps12
Embriophyta – ps13
Tracheophyta – ps14
Spermatophyta – ps15
Angiosperms – ps16
Mesangiospermae – ps17
Magnoliids/eudicots – ps18
Eudicots – ps19
Core_eudicots – ps20
Superasteridae – ps21
Caryophyllales – ps22
Beta_vulgaris – ps23
Phylostratum

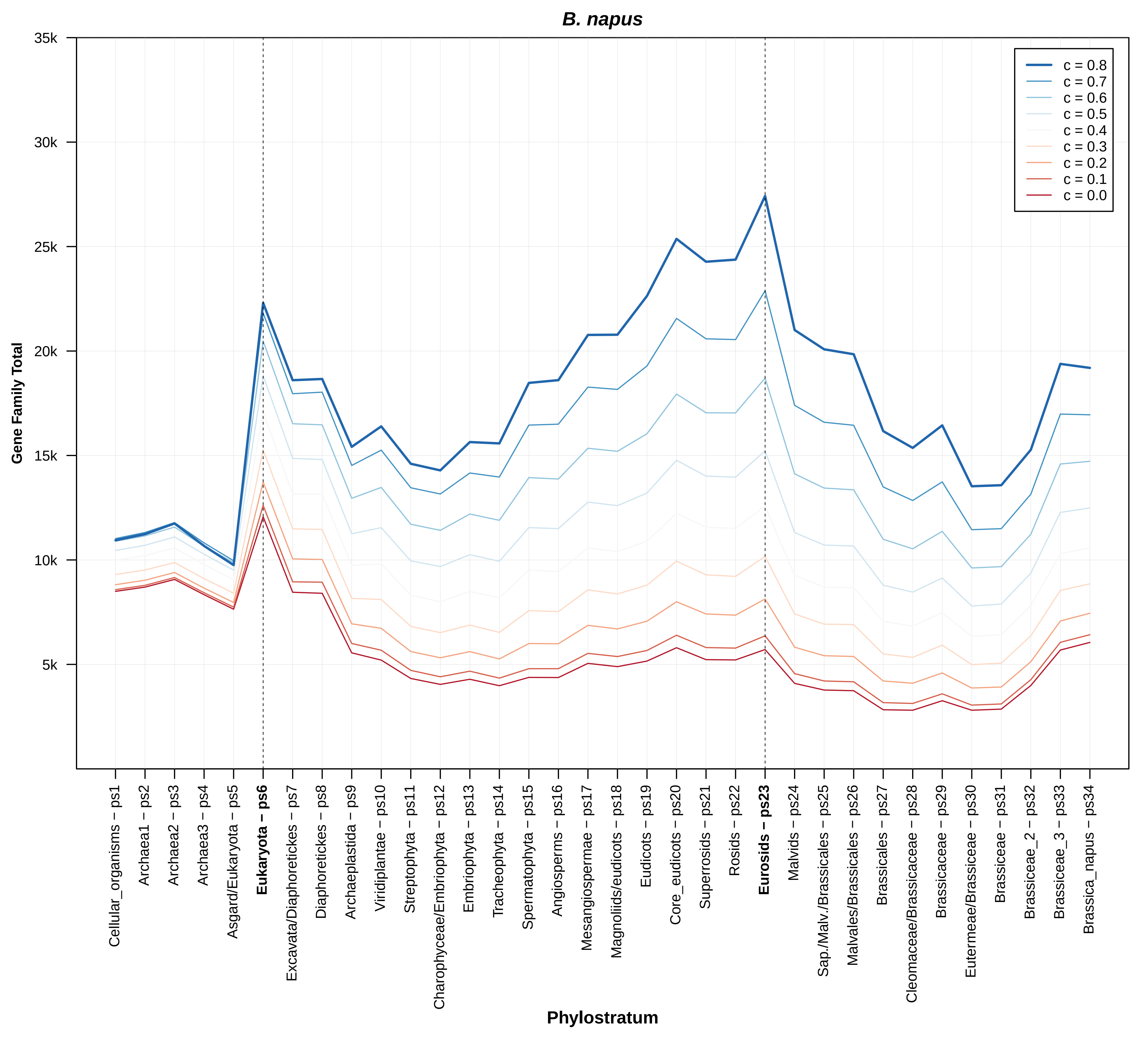

B. napus
Gene Family Total
35k
30k
25k
20k
15k
10k
5k
c = 0.8
c = 0.7
c = 0.6
c = 0.5
c = 0.4
c = 0.3
c = 0.2
c = 0.1
c = 0.0
Cellular_organisms – ps1
Archaea1 – ps2
Archaea2 – ps3
Archaea3 – ps4
Asgard/Eukaryota – ps5
Eukaryota – ps6
Excavata/Diaphoretickes – ps7
Diaphoretickes – ps8
Archaeplastida – ps9
Viridiplantae – ps10
Streptophyta – ps11
Charophyceae/Embriophyta – ps12
Embriophyta – ps13
Tracheophyta – ps14
Spermatophyta – ps15
Angiosperms – ps16
Mesangiospermae – ps17
Magnoliids/eudicots – ps18
Eudicots – ps19
Core_eudicots – ps20
Superrosids – ps21
Rosids – ps22
Eurosids – ps23
Malvids – ps24
Sap./Malv./Brassicales – ps25
Malvales/Brassicales – ps26
Brassicales – ps27
Cleomaceae/Brassicaceae – ps28
Brassicaceae – ps29
Eutermeae/Brassiceae – ps30
Brassiceae – ps31
Brassiceae_2 – ps32
Brassiceae_3 – ps33
Brassica_napus – ps34
Phylostratum

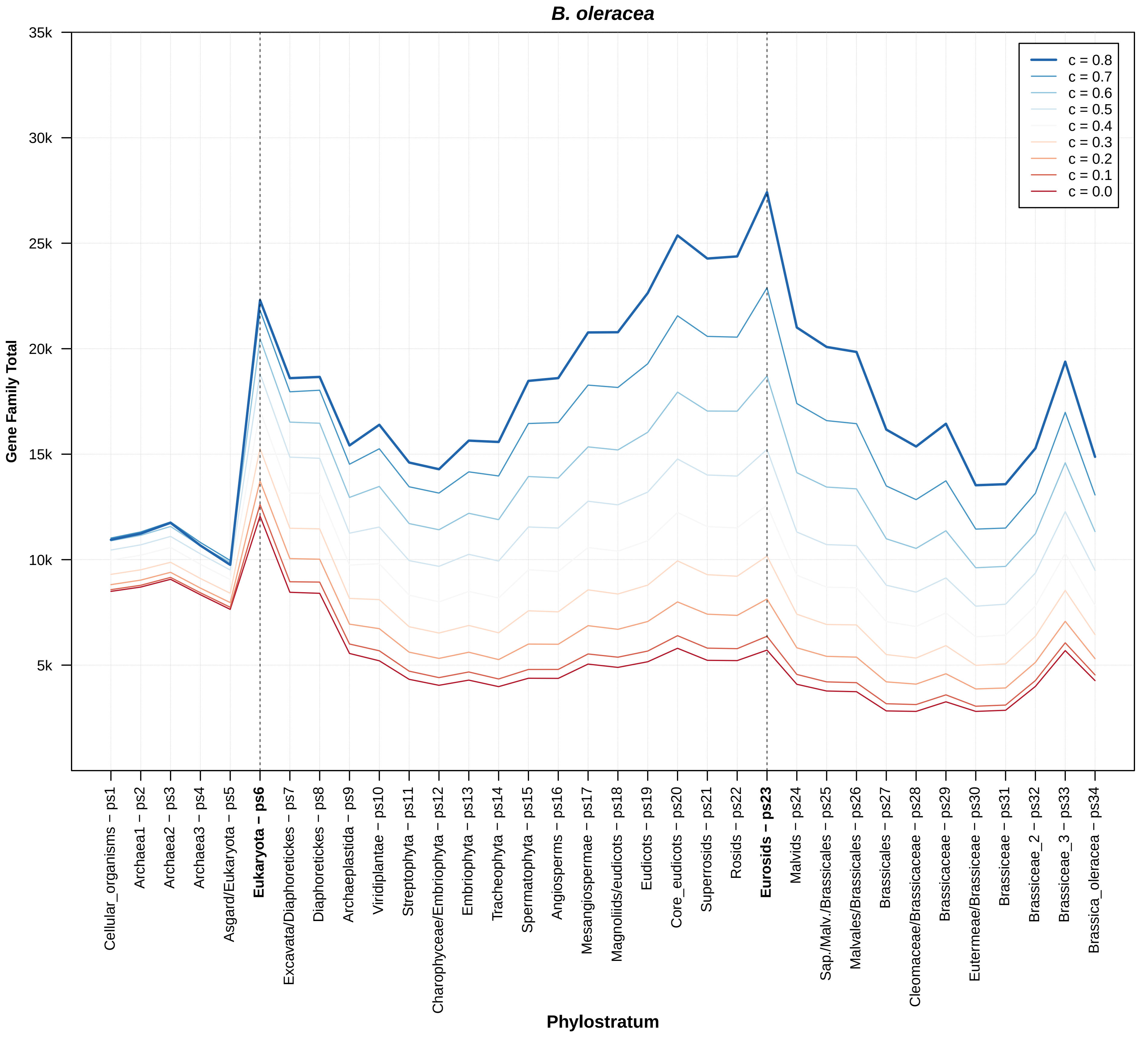

B. oleracea
Gene Family Total
Phylostratum
35k
30k
25k
20k
15k
10k
5k
c = 0.8
c = 0.7
c = 0.6
c = 0.5
c = 0.4
c = 0.3
c = 0.2
c = 0.1
c = 0.0
Cellular_organisms – ps1
Archaea1 – ps2
Archaea2 – ps3
Archaea3 – ps4
Asgard/Eukaryota – ps5
Eukaryota – ps6
Excavata/Diaphoretickes – ps7
Diaphoretickes – ps8
Archaeplastida – ps9
Viridiplantae – ps10
Streptophyta – ps11
Charophyceae/Embriophyta – ps12
Embriophyta – ps13
Tracheophyta – ps14
Spermatophyta – ps15
Angiosperms – ps16
Mesangiospermae – ps17
Magnoliids/eudicots – ps18
Eudicots – ps19
Core_eudicots – ps20
Superrosids – ps21
Rosids – ps22
Eurosids – ps23
Malvids – ps24
Sap./Malv./Brassicales – ps25
Malvales/Brassicales – ps26
Brassicales – ps27
Cleomaceae/Brassicaceae – ps28
Brassicaceae – ps29
Eutermeae/Brassiceae – ps30
Brassiceae – ps31
Brassiceae_2 – ps32
Brassiceae_3 – ps33
Brassica_oleracea – ps34

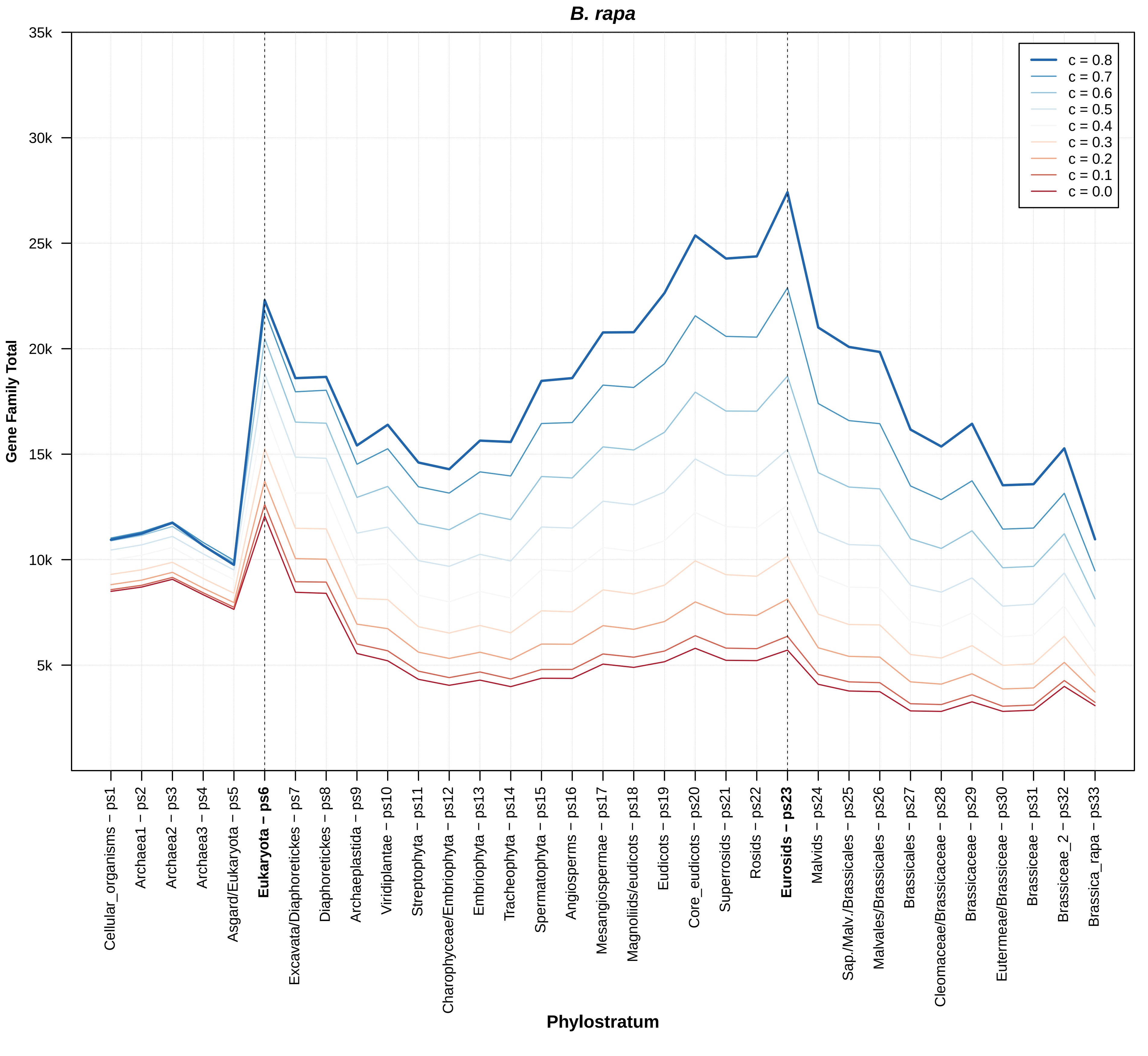
B. rapa
Gene Family Total
35k
30k
25k
20k
15k
10k
5k
c = 0.8
c = 0.7
c = 0.6
c = 0.5
c = 0.4
c = 0.3
c = 0.2
c = 0.1
c = 0.0
Cellular_organisms – ps1
Archaea1 – ps2
Archaea2 – ps3
Archaea3 – ps4
Asgard/Eukaryota – ps5
Eukaryota – ps6
Excavata/Diaphoretickes – ps7
Diaphoretickes – ps8
Archaeplastida – ps9
Viridiplantae – ps10
Streptophyta – ps11
Charophyceae/Embriophyta – ps12
Embriophyta – ps13
Tracheophyta – ps14
Spermatophyta – ps15
Angiosperms – ps16
Mesangiospermae – ps17
Magnoliids/eudicots – ps18
Eudicots – ps19
Core_eudicots – ps20
Superrosids – ps21
Rosids – ps22
Eurosids – ps23
Malvids – ps24
Sap./Malv./Brassicales – ps25
Malvales/Brassicales – ps26
Brassicales – ps27
Cleomaceae/Brassicaceae – ps28
Brassicaceae – ps29
Eutermeae/Brassiceae – ps30
Brassiceae – ps31
Brassiceae_2 – ps32
Brassica_rapa – ps33
Phylostratum

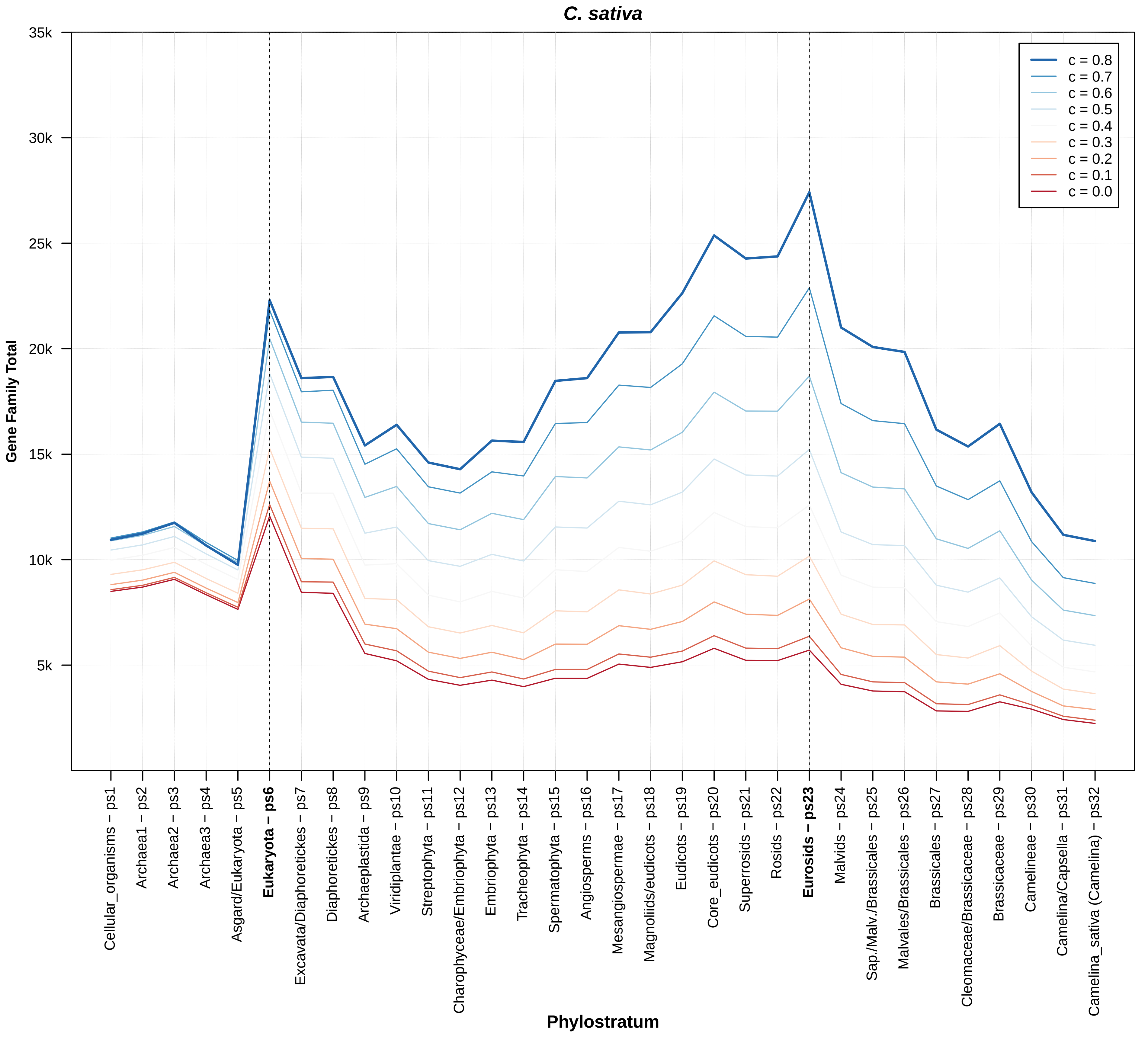

C. sativa
Gene Family Total
35k
30k
25k
20k
15k
10k
5k
c = 0.8
c = 0.7
c = 0.6
c = 0.5
c = 0.4
c = 0.3
c = 0.2
c = 0.1
c = 0.0
Cellular_organisms – ps1
Archaea1 – ps2
Archaea2 – ps3
Archaea3 – ps4
Asgard/Eukaryota – ps5
Eukaryota – ps6
Excavata/Diaphoretickes – ps7
Diaphoretickes – ps8
Archaeplastida – ps9
Viridiplantae – ps10
Streptophyta – ps11
Charophyceae/Embriophyta – ps12
Embriophyta – ps13
Tracheophyta – ps14
Spermatophyta – ps15
Angiosperms – ps16
Mesangiospermae – ps17
Magnoliids/eudicots – ps18
Eudicots – ps19
Core_eudicots – ps20
Superrosids – ps21
Rosids – ps22
Eurosids – ps23
Malvids – ps24
Sap./Malv./Brassicales – ps25
Malvales/Brassicales – ps26
Brassicales – ps27
Cleomaceae/Brassicaceae – ps28
Brassicaceae – ps29
Camelineae – ps30
Camelina/Capsella – ps31
Camelina_sativa (Camelina) – ps32
Phylostratum

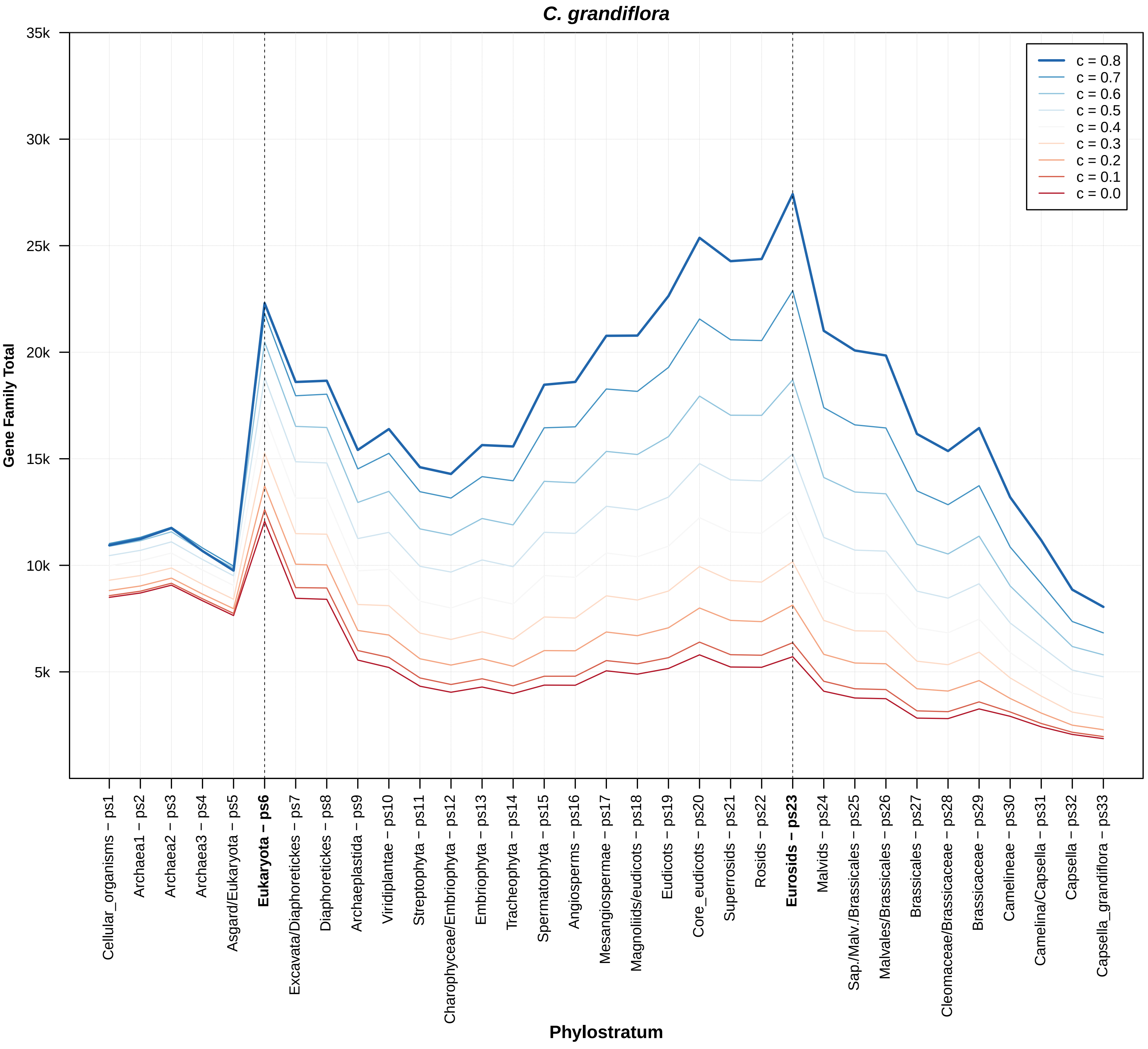

C. grandiflora
Gene Family Total
35k
30k
25k
20k
15k
10k
5k
c = 0.8
c = 0.7
c = 0.6
c = 0.5
c = 0.4
c = 0.3
c = 0.2
c = 0.1
c = 0.0
Cellular_organisms – ps1
Archaea1 – ps2
Archaea2 – ps3
Archaea3 – ps4
Asgard/Eukaryota – ps5
Eukaryota – ps6
Excavata/Diaphoretickes – ps7
Diaphoretickes – ps8
Archaeplastida – ps9
Viridiplantae – ps10
Streptophyta – ps11
Charophyceae/Embriophyta – ps12
Embriophyta – ps13
Tracheophyta – ps14
Spermatophyta – ps15
Angiosperms – ps16
Mesangiospermae – ps17
Magnoliids/eudicots – ps18
Eudicots – ps19
Core_eudicots – ps20
Superrosids – ps21
Rosids – ps22
Eurosids – ps23
Malvids – ps24
Sap./Malv./Brassicales – ps25
Malvales/Brassicales – ps26
Brassicales – ps27
Cleomaceae/Brassicaceae – ps28
Brassicaceae – ps29
Camelineae – ps30
Camelina/Capsella – ps31
Capsella – ps32
Capsella_grandiflora – ps33
Phylostratum

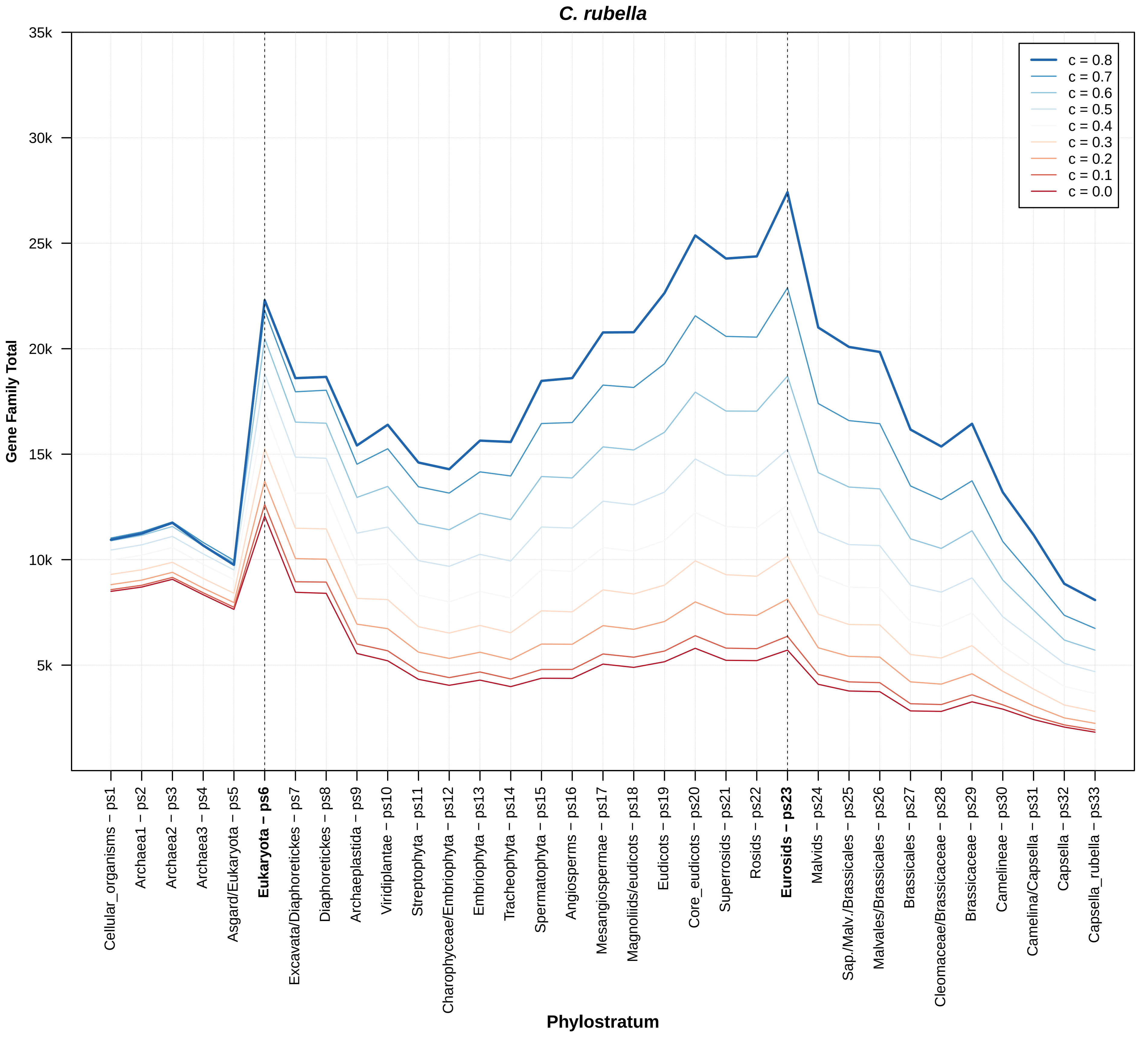

C. rubella
Gene Family Total
35k
30k
25k
20k
15k
10k
5k
c = 0.8
c = 0.7
c = 0.6
c = 0.5
c = 0.4
c = 0.3
c = 0.2
c = 0.1
c = 0.0
Cellular_organisms – ps1
Archaea1 – ps2
Archaea2 – ps3
Archaea3 – ps4
Asgard/Eukaryota – ps5
Eukaryota – ps6
Excavata/Diaphoretickes – ps7
Diaphoretickes – ps8
Archaeplastida – ps9
Viridiplantae – ps10
Streptophyta – ps11
Charophyceae/Embriophyta – ps12
Embriophyta – ps13
Tracheophyta – ps14
Spermatophyta – ps15
Angiosperms – ps16
Mesangiospermae – ps17
Magnoliids/eudicots – ps18
Eudicots – ps19
Core_eudicots – ps20
Superrosids – ps21
Rosids – ps22
Eurosids – ps23
Malvids – ps24
Sap./Malv./Brassicales – ps25
Malvales/Brassicales – ps26
Brassicales – ps27
Cleomaceae/Brassicaceae – ps28
Brassicaceae – ps29
Camelineae – ps30
Camelina/Capsella – ps31
Capsella – ps32
Capsella_rubella – ps33
Phylostratum

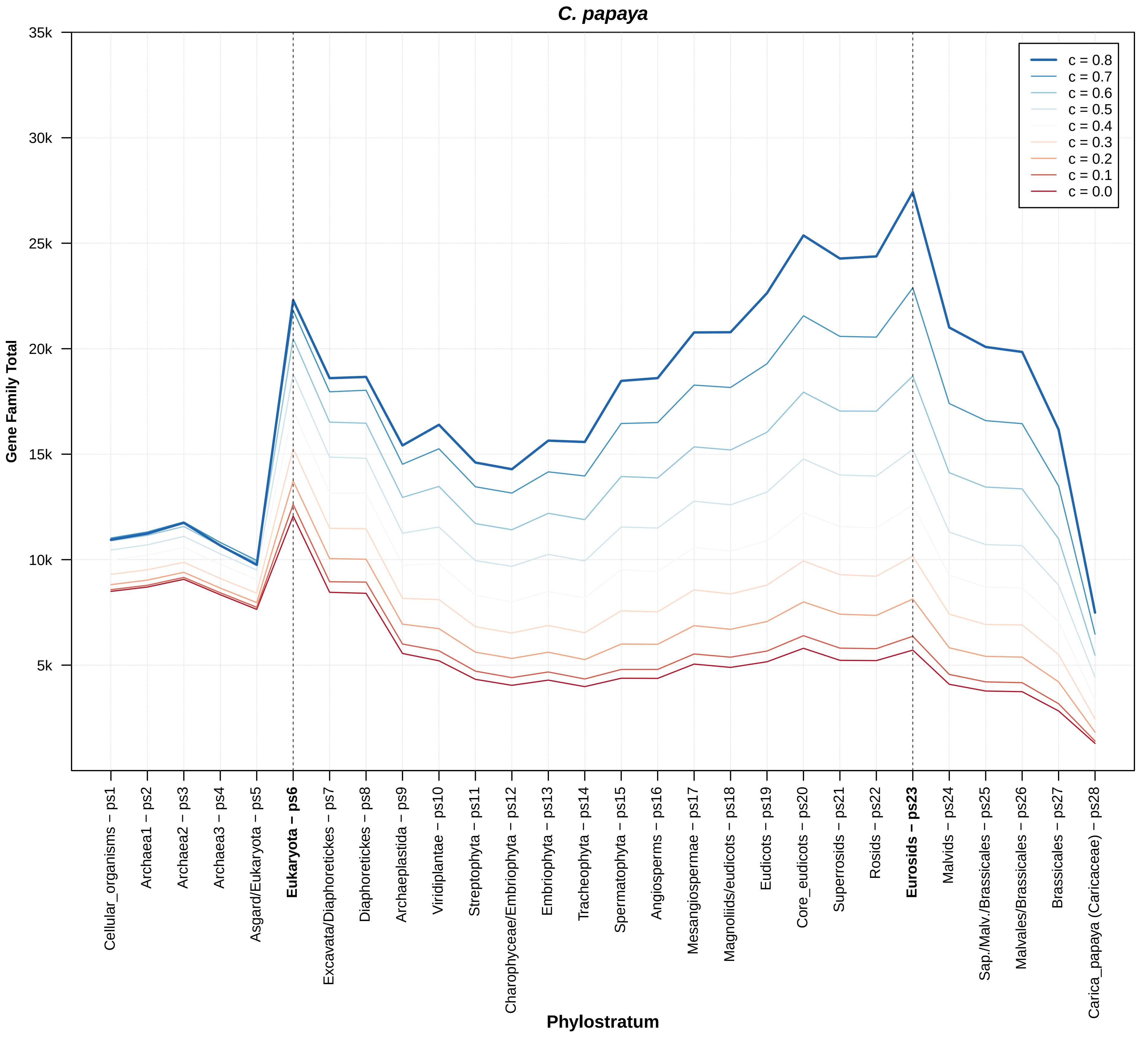
C. papaya
Gene Family Total
Phylostratum
35k
30k
25k
20k
15k
10k
5k
c = 0.8
c = 0.7
c = 0.6
c = 0.5
c = 0.4
c = 0.3
c = 0.2
c = 0.1
c = 0.0
Cellular_organisms – ps1
Archaea1 – ps2
Archaea2 – ps3
Archaea3 – ps4
Asgard/Eukaryota – ps5
Eukaryota – ps6
Excavata/Diaphoretickes – ps7
Diaphoretickes – ps8
Archaeplastida – ps9
Viridiplantae – ps10
Streptophyta – ps11
Charophyceae/Embriophyta – ps12
Embriophyta – ps13
Tracheophyta – ps14
Spermatophyta – ps15
Angiosperms – ps16
Mesangiospermae – ps17
Magnoliids/eudicots – ps18
Eudicots – ps19
Core_eudicots – ps20
Superrosids – ps21
Rosids – ps22
Eurosids – ps23
Malvids – ps24
Sap./Malv./Brassicales – ps25
Malvales/Brassicales – ps26
Brassicales – ps27
Carica_papaya (Caricaceae) – ps28

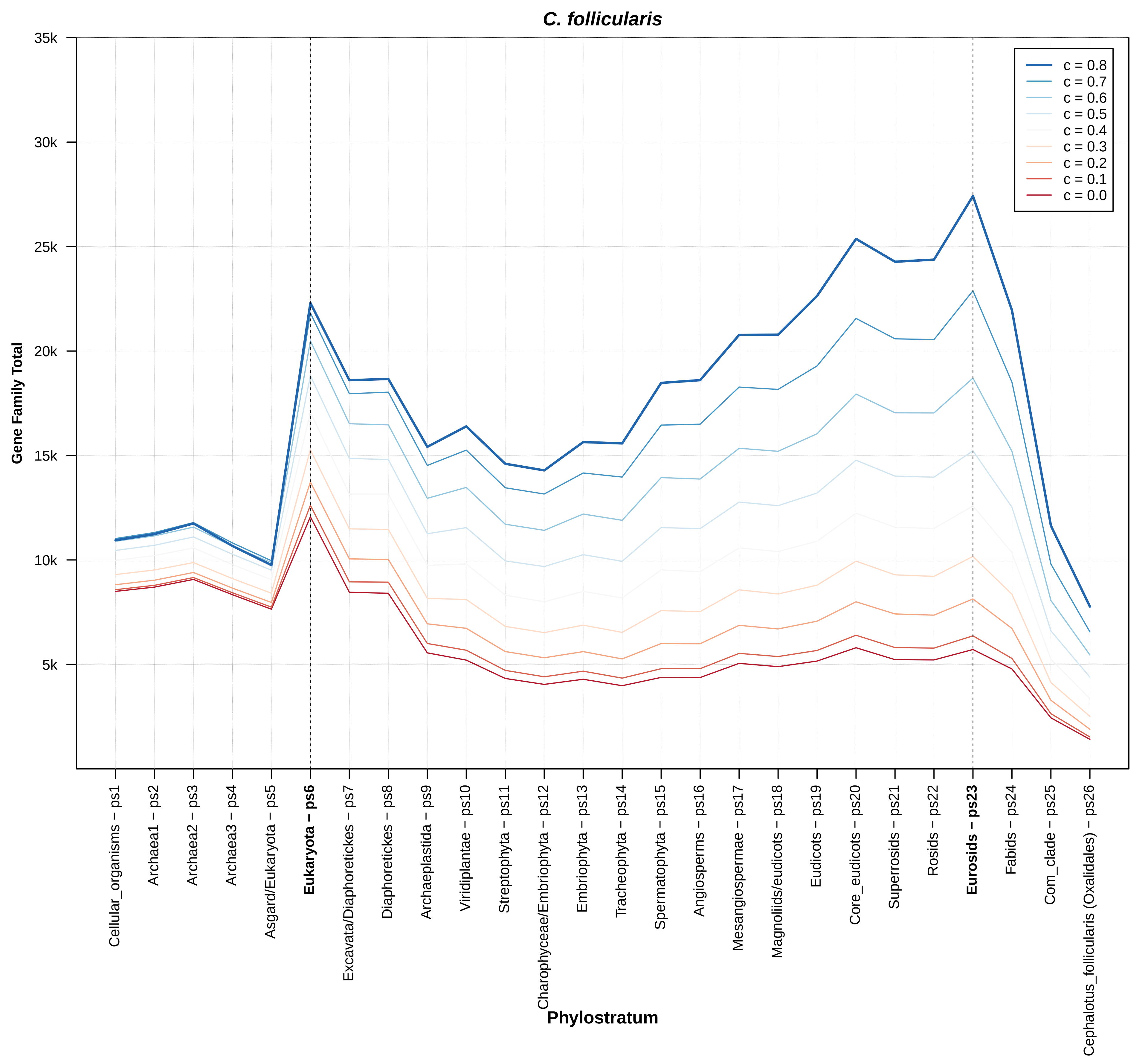

C. follicularis
Gene Family Total
35k
30k
25k
20k
15k
10k
5k
c = 0.8
c = 0.7
c = 0.6
c = 0.5
c = 0.4
c = 0.3
c = 0.2
c = 0.1
c = 0.0
Cellular_organisms – ps1
Archaea1 – ps2
Archaea2 – ps3
Archaea3 – ps4
Asgard/Eukaryota – ps5
Eukaryota – ps6
Excavata/Diaphoretickes – ps7
Diaphoretickes – ps8
Archaeplastida – ps9
Viridiplantae – ps10
Streptophyta – ps11
Charophyceae/Embriophyta – ps12
Embriophyta – ps13
Tracheophyta – ps14
Spermatophyta – ps15
Angiosperms – ps16
Mesangiospermae – ps17
Magnoliids/eudicots – ps18
Eudicots – ps19
Core_eudicots – ps20
Superrosids – ps21
Rosids – ps22
Eurosids – ps23
Fabids – ps24
Com_clade – ps25
Cephalotus_follicularis (Oxalidales) – ps26
Phylostratum

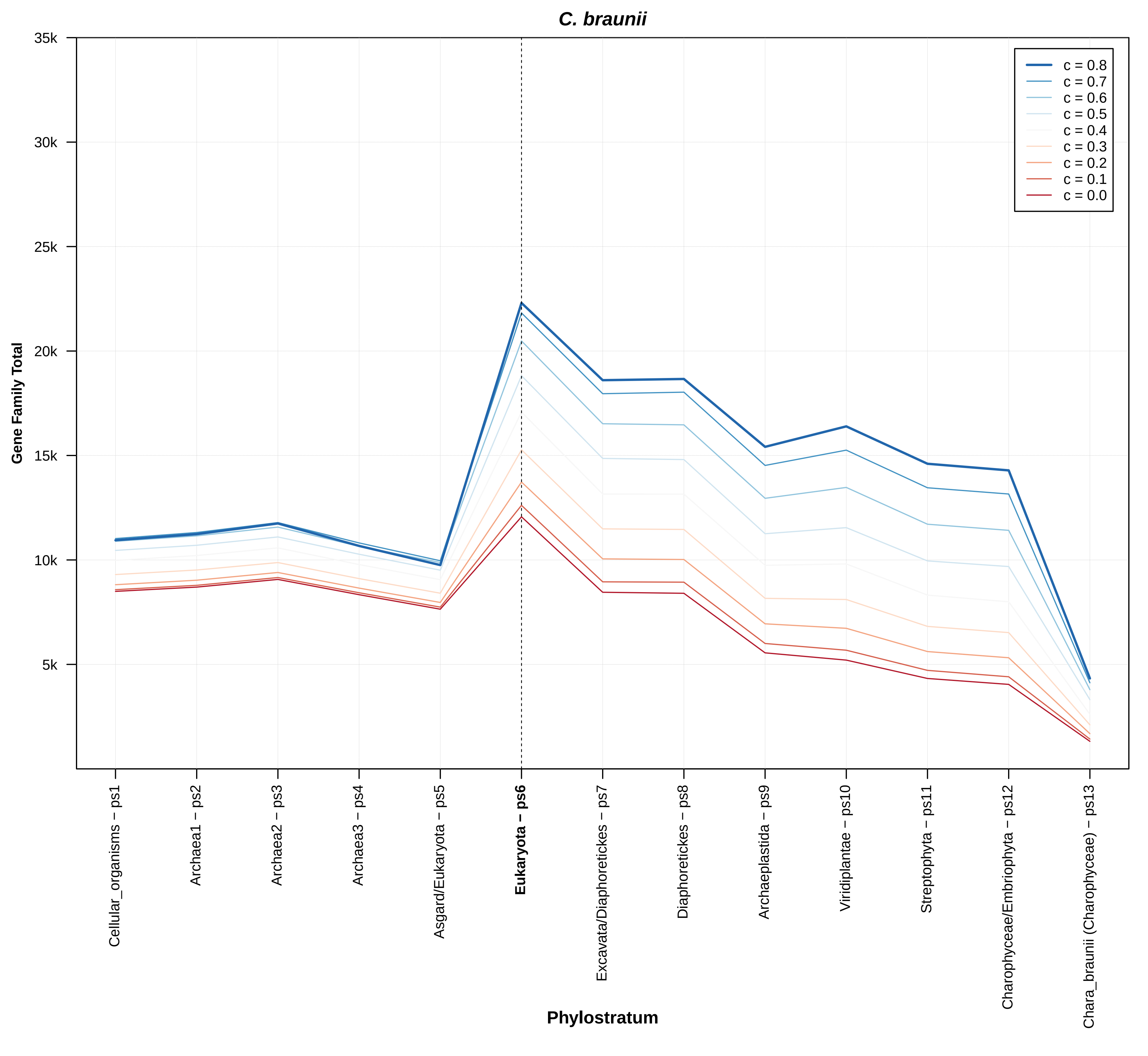
C. braunii
Gene Family Total
35k
30k
25k
20k
15k
10k
5k
c = 0.8
c = 0.7
c = 0.6
c = 0.5
c = 0.4
c = 0.3
c = 0.2
c = 0.1
c = 0.0
Cellular_organisms – ps1
Archaea1 – ps2
Archaea2 – ps3
Archaea3 – ps4
Asgard/Eukaryota – ps5
Eukaryota – ps6
Excavata/Diaphoretickes – ps7
Diaphoretickes – ps8
Archaeplastida – ps9
Viridiplantae – ps10
Streptophyta – ps11
Charophyceae/Embriophyta – ps12
Chara_braunii (Charophyceae) – ps13
Phylostratum

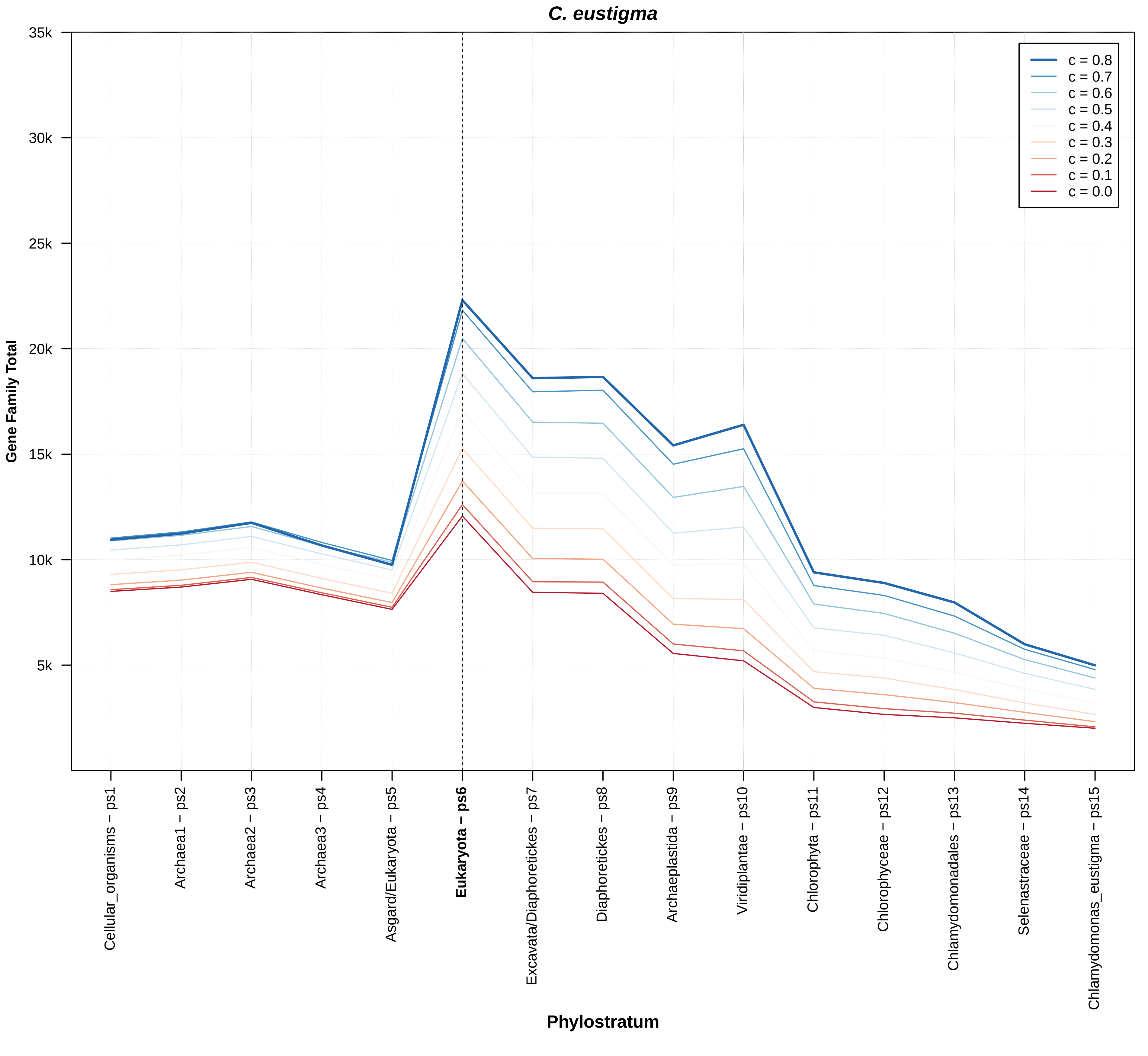

C. eustigma
Gene Family Total
35k
30k
25k
20k
15k
10k
5k
c = 0.8
c = 0.7
c = 0.6
c = 0.5
c = 0.4
c = 0.3
c = 0.2
c = 0.1
c = 0.0
Cellular_organisms – ps1
Archaea1 – ps2
Archaea2 – ps3
Archaea3 – ps4
Asgard/Eukaryota – ps5
Eukaryota – ps6
Excavata/Diaphoretickes – ps7
Diaphoretickes – ps8
Archaeplastida – ps9
Viridiplantae – ps10
Chlorophyta – ps11
Chlorophyceae – ps12
Chlamydomonadales – ps13
Selenastraceae – ps14
Chlamydomonas_eustigma – ps15
Phylostratum

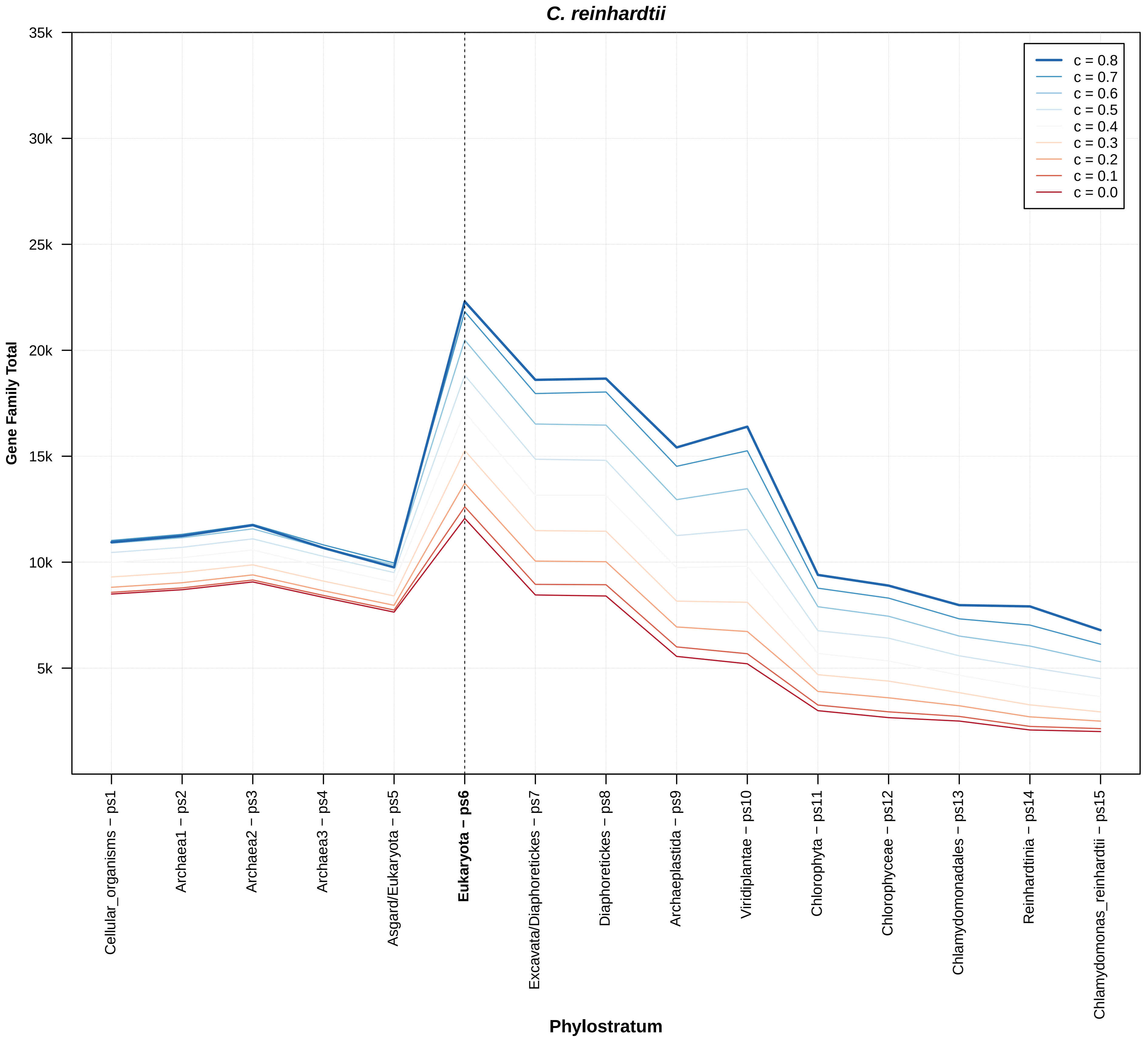

C. reinhardtii
Gene Family Total
35k
30k
25k
20k
15k
10k
5k
c = 0.8
c = 0.7
c = 0.6
c = 0.5
c = 0.4
c = 0.3
c = 0.2
c = 0.1
c = 0.0
Cellular_organisms – ps1
Archaea1 – ps2
Archaea2 – ps3
Archaea3 – ps4
Asgard/Eukaryota – ps5
Eukaryota – ps6
Excavata/Diaphoretickes – ps7
Diaphoretickes – ps8
Archaeplastida – ps9
Viridiplantae – ps10
Chlorophyta – ps11
Chlorophyceae – ps12
Chlamydomonadales – ps13
Reinhardtinia – ps14
Chlamydomonas_reinhardtii – ps15
Phylostratum

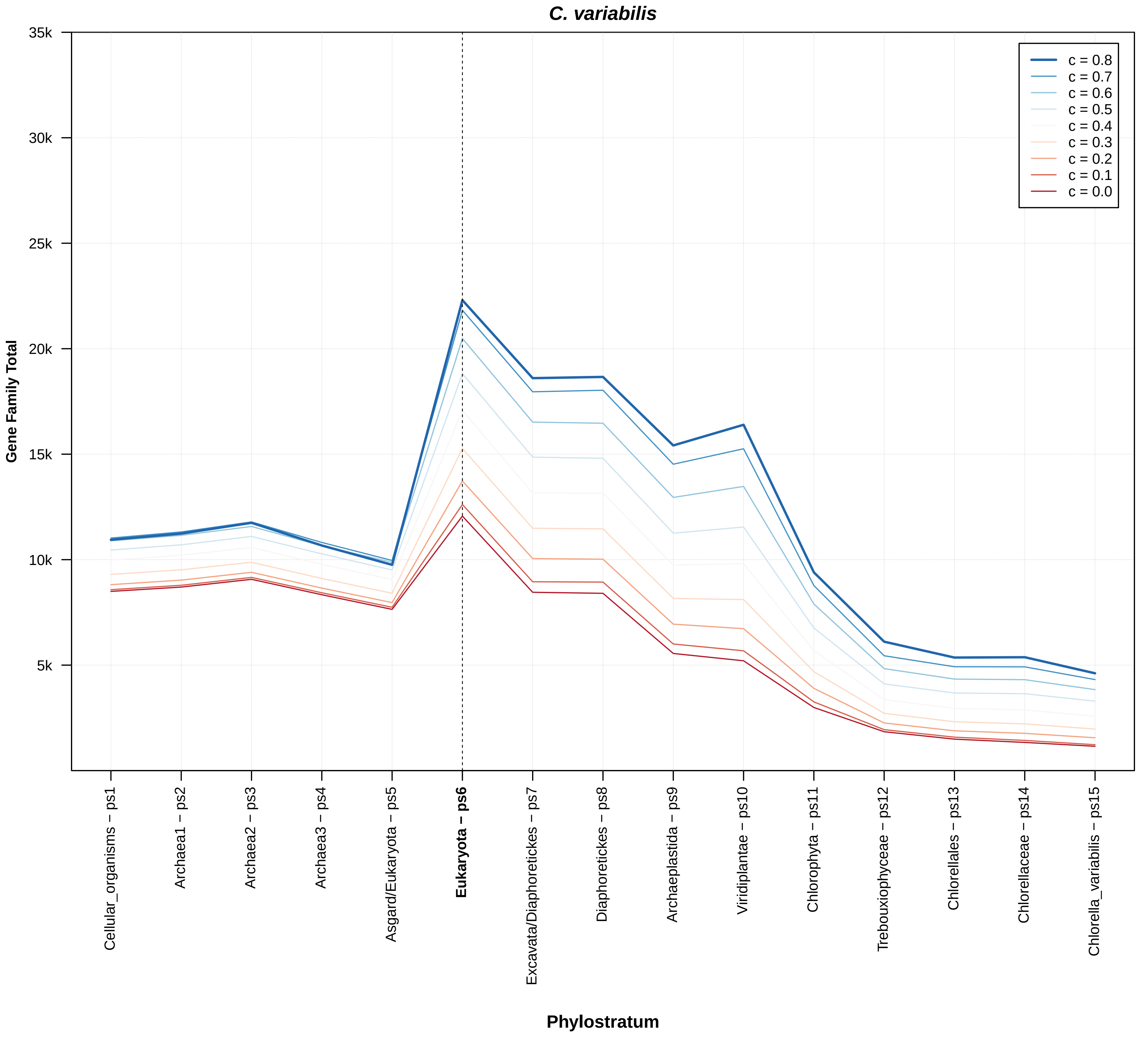
C. variabilis
35k
30k
25k
20k
15k
10k
5k
Gene Family Total
c = 0.8
c = 0.7
c = 0.6
c = 0.5
c = 0.4
c = 0.3
c = 0.2
c = 0.1
c = 0.0
Cellular_organisms – ps1
Archaea1 – ps2
Archaea2 – ps3
Archaea3 – ps4
Asgard/Eukaryota – ps5
Eukaryota – ps6
Excavata/Diaphoretickes – ps7
Diaphoretickes – ps8
Archaeplastida – ps9
Viridiplantae – ps10
Chlorophyta – ps11
Trebouxiophyceae – ps12
Chlorellales – ps13
Chlorellaceae – ps14
Chlorella_variabilis – ps15
Phylostratum

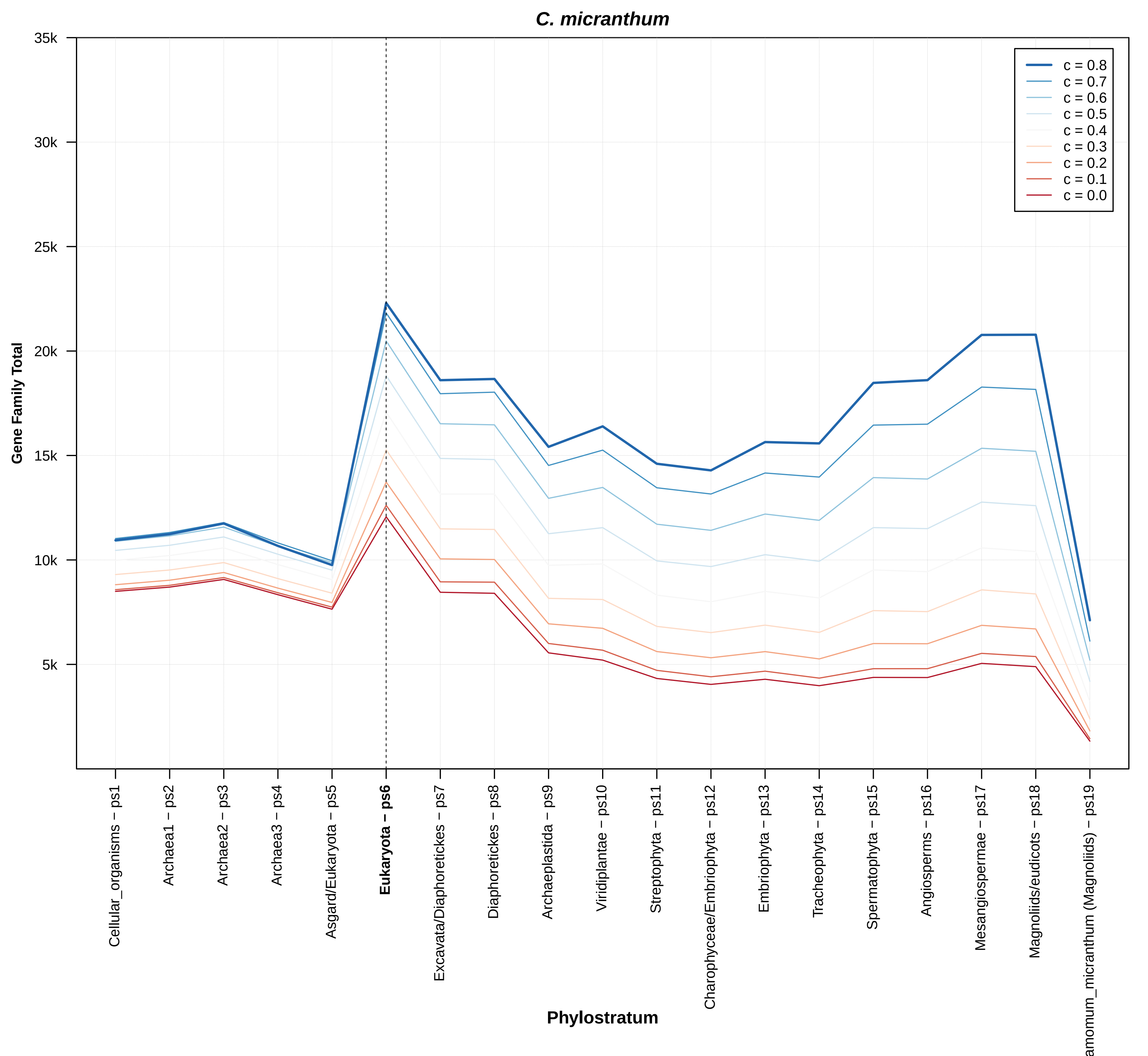
C. micranthum
Gene Family Total
35k
30k
25k
20k
15k
10k
5k
c = 0.8
c = 0.7
c = 0.6
c = 0.5
c = 0.4
c = 0.3
c = 0.2
c = 0.1
c = 0.0
Cellular_organisms – ps1
Archaea1 – ps2
Archaea2 – ps3
Archaea3 – ps4
Asgard/Eukaryota – ps5
Eukaryota – ps6
Excavata/Diaphoretickes – ps7
Diaphoretickes – ps8
Archaeplastida – ps9
Viridiplantae – ps10
Streptophyta – ps11
Charophyceae/Embriophyta – ps12
Embriophyta – ps13
Tracheophyta – ps14
Spermatophyta – ps15
Angiosperms – ps16
Mesangiospermae – ps17
Magnoliids/eudicots – ps18
amomum_micranthum (Magnoliids) – ps19
Phylostratum

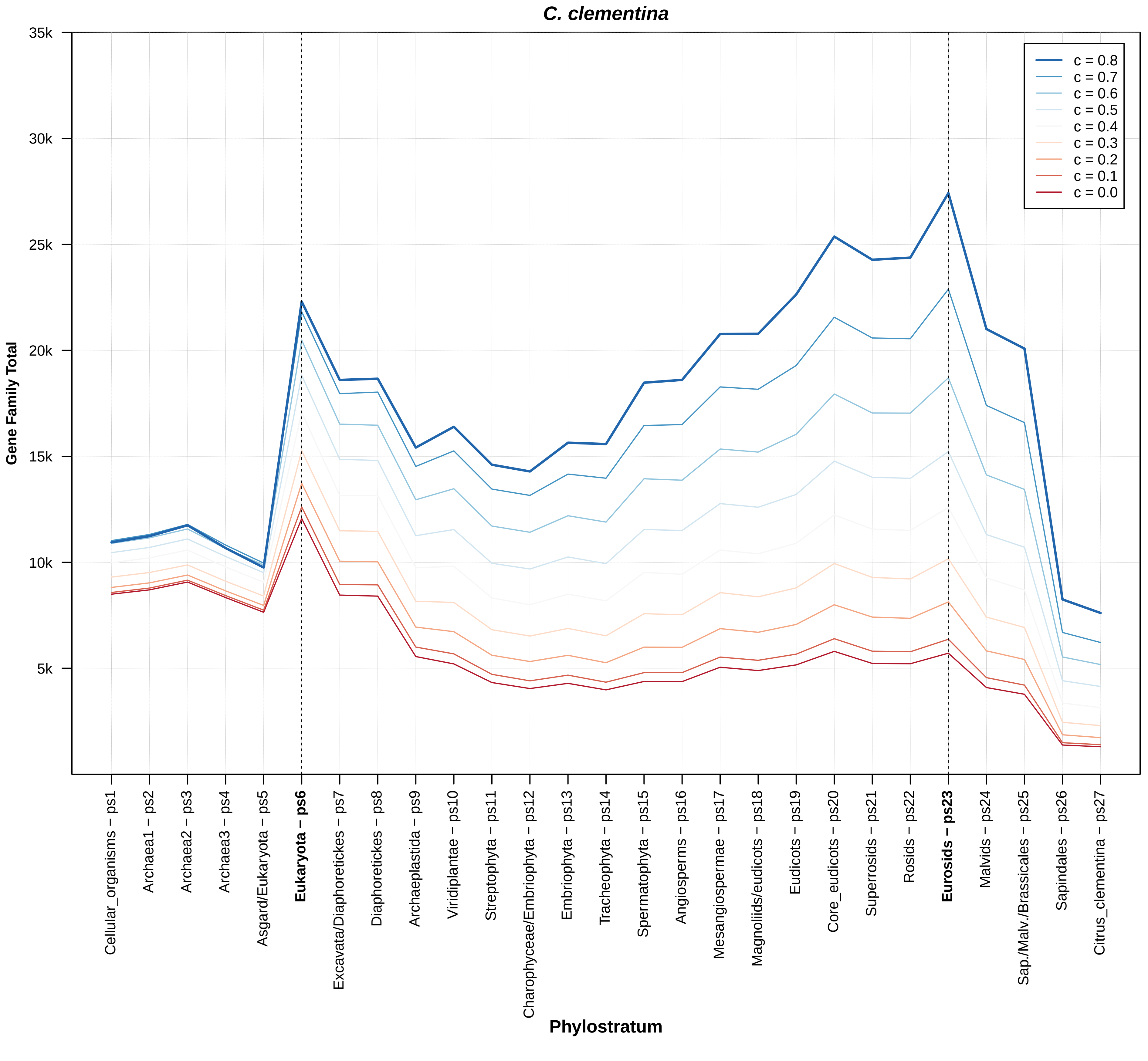

C. clementina
Gene Family Total
35k
30k
25k
20k
15k
10k
5k
c = 0.8
c = 0.7
c = 0.6
c = 0.5
c = 0.4
c = 0.3
c = 0.2
c = 0.1
c = 0.0
Cellular_organisms – ps1
Archaea1 – ps2
Archaea2 – ps3
Archaea3 – ps4
Asgard/Eukaryota – ps5
Eukaryota – ps6
Excavata/Diaphoretickes – ps7
Diaphoretickes – ps8
Archaeplastida – ps9
Viridiplantae – ps10
Streptophyta – ps11
Charophyceae/Embriophyta – ps12
Embriophyta – ps13
Tracheophyta – ps14
Spermatophyta – ps15
Angiosperms – ps16
Mesangiospermae – ps17
Magnoliids/eudicots – ps18
Eudicots – ps19
Core_eudicots – ps20
Superrosids – ps21
Rosids – ps22
Eurosids – ps23
Malvids – ps24
Sap./Malv./Brassicales – ps25
Sapindales – ps26
Citrus_clementina – ps27
Phylostratum

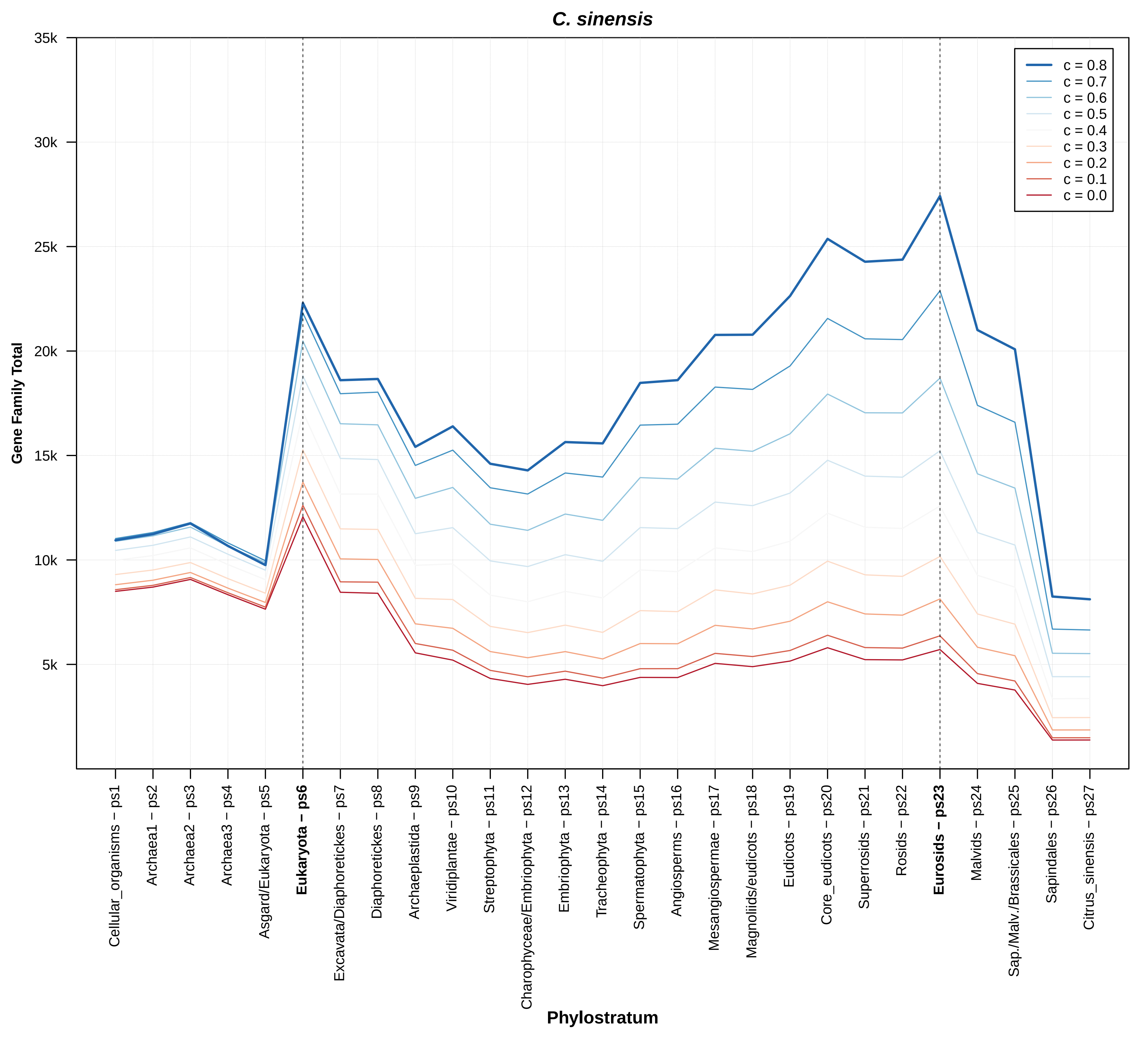
C. sinensis
Gene Family Total
35k
30k
25k
20k
15k
10k
5k
c = 0.8
c = 0.7
c = 0.6
c = 0.5
c = 0.4
c = 0.3
c = 0.2
c = 0.1
c = 0.0
Cellular_organisms – ps1
Archaea1 – ps2
Archaea2 – ps3
Archaea3 – ps4
Asgard/Eukaryota – ps5
Eukaryota – ps6
Excavata/Diaphoretickes – ps7
Diaphoretickes – ps8
Archaeplastida – ps9
Viridiplantae – ps10
Streptophyta – ps11
Charophyceae/Embriophyta – ps12
Embriophyta – ps13
Tracheophyta – ps14
Spermatophyta – ps15
Angiosperms – ps16
Mesangiospermae – ps17
Magnoliids/eudicots – ps18
Eudicots – ps19
Core_eudicots – ps20
Superrosids – ps21
Rosids – ps22
Eurosids – ps23
Malvids – ps24
Sap./Malv./Brassicales – ps25
Sapindales – ps26
Citrus_sinensis – ps27
Phylostratum

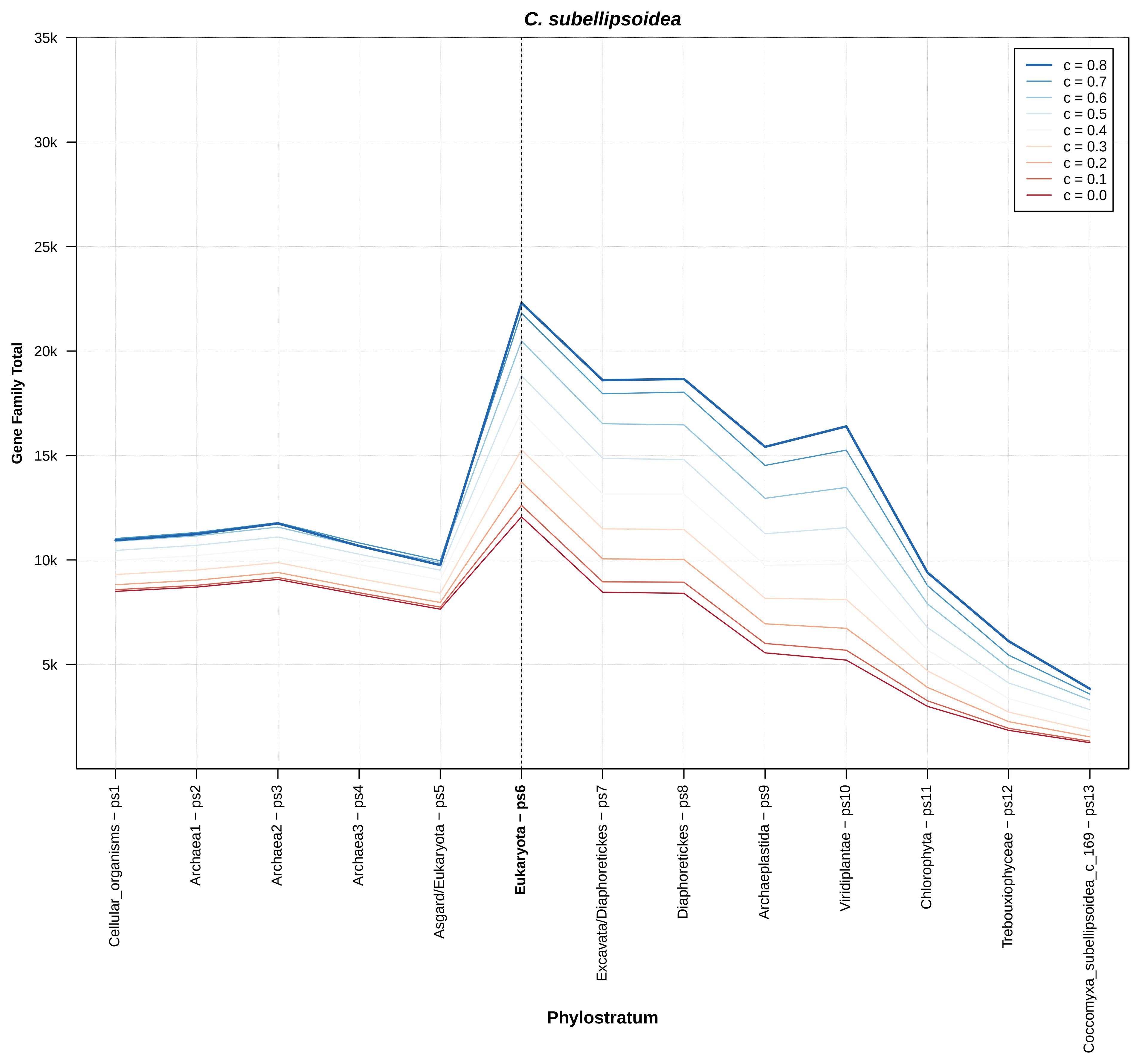

C. subellipsoidea
Gene Family Total
35k
30k
25k
20k
15k
10k
5k
c = 0.8
c = 0.7
c = 0.6
c = 0.5
c = 0.4
c = 0.3
c = 0.2
c = 0.1
c = 0.0
Cellular_organisms – ps1
Archaea1 – ps2
Archaea2 – ps3
Archaea3 – ps4
Asgard/Eukaryota – ps5
Eukaryota – ps6
Excavata/Diaphoretickes – ps7
Diaphoretickes – ps8
Archaeplastida – ps9
Viridiplantae – ps10
Chlorophyta – ps11
Trebouxiophyceae – ps12
Coccomyxa_subellipsoidea_c_169 – ps13
Phylostratum

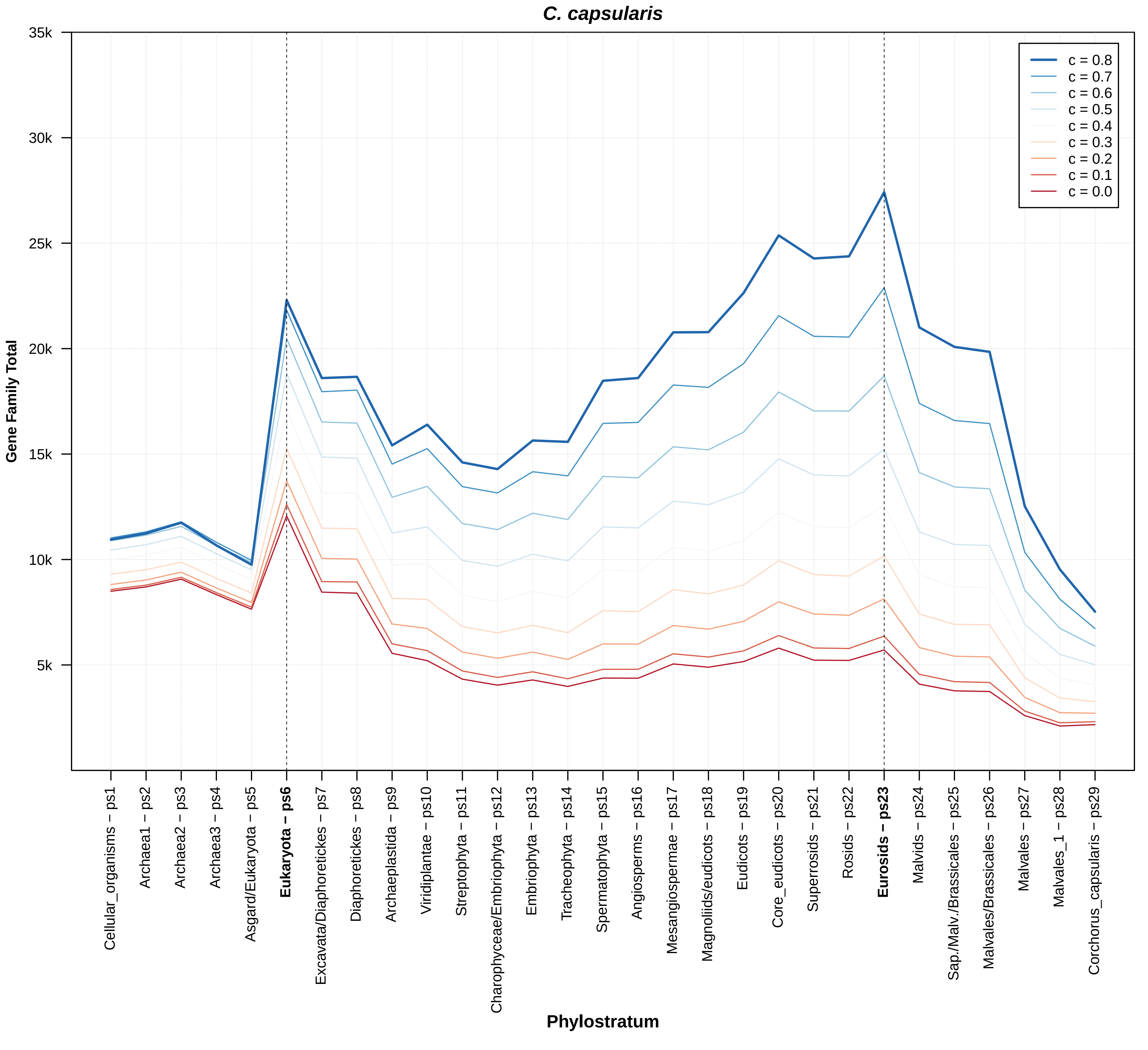

C. capsularis
Gene Family Total
35k
30k
25k
20k
15k
10k
5k
c = 0.8
c = 0.7
c = 0.6
c = 0.5
c = 0.4
c = 0.3
c = 0.2
c = 0.1
c = 0.0
Cellular_organisms – ps1
Archaea1 – ps2
Archaea2 – ps3
Archaea3 – ps4
Asgard/Eukaryota – ps5
Eukaryota – ps6
Excavata/Diaphoretickes – ps7
Diaphoretickes – ps8
Archaeplastida – ps9
Viridiplantae – ps10
Streptophyta – ps11
Charophyceae/Embriophyta – ps12
Embriophyta – ps13
Tracheophyta – ps14
Spermatophyta – ps15
Angiosperms – ps16
Mesangiospermae – ps17
Magnoliids/eudicots – ps18
Eudicots – ps19
Core_eudicots – ps20
Superrosids – ps21
Rosids – ps22
Eurosids – ps23
Malvids – ps24
Sap./Malv./Brassicales – ps25
Malvales/Brassicales – ps26
Malvales – ps27
Malvales_1 – ps28
Corchorus_capsularis – ps29
Phylostratum

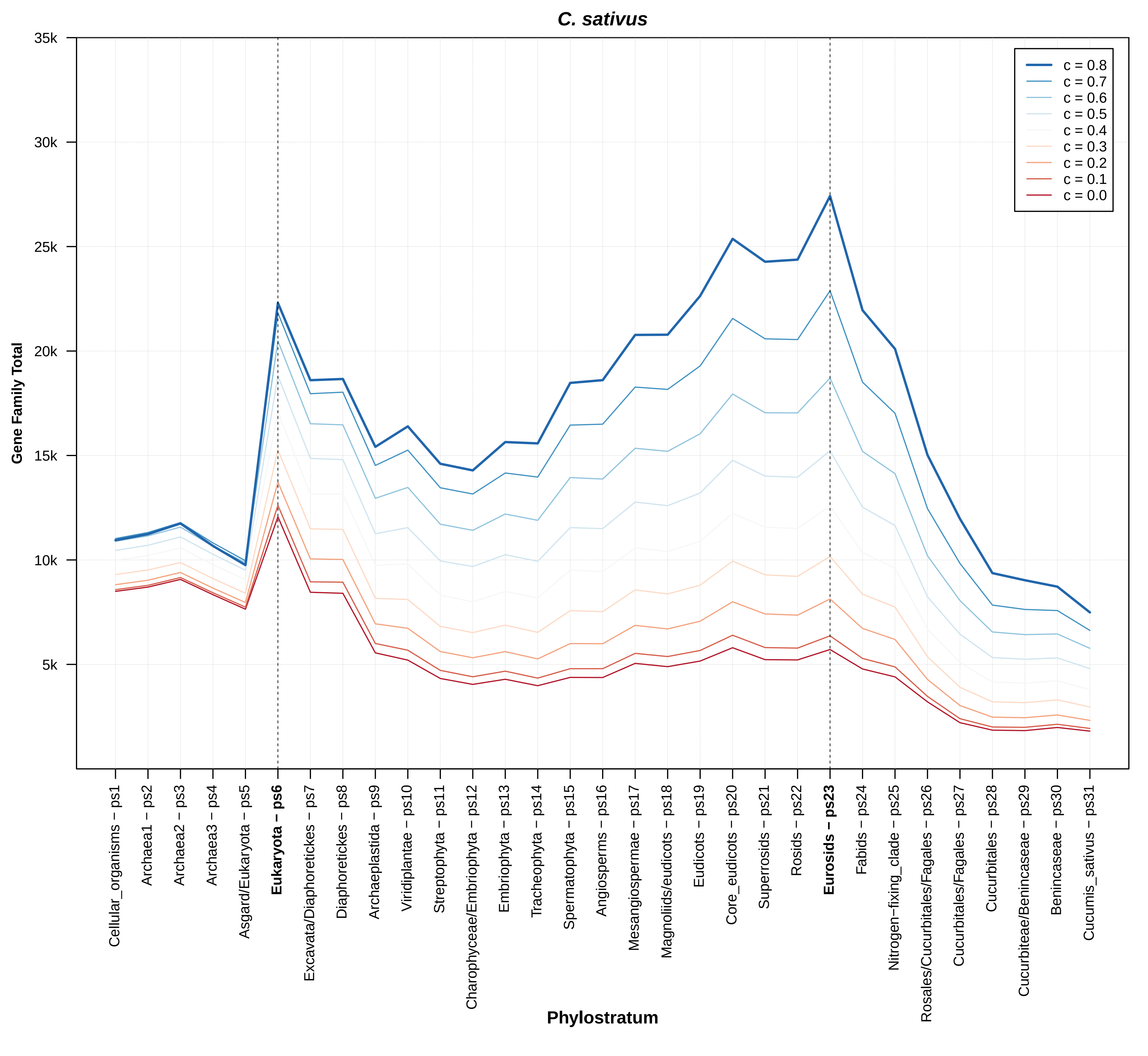
C. sativus
Gene Family Total
35k
30k
25k
20k
15k
10k
5k
c = 0.8
c = 0.7
c = 0.6
c = 0.5
c = 0.4
c = 0.3
c = 0.2
c = 0.1
c = 0.0
Cellular_organisms – ps1
Archaea1 – ps2
Archaea2 – ps3
Archaea3 – ps4
Asgard/Eukaryota – ps5
Eukaryota – ps6
Excavata/Diaphoretickes – ps7
Diaphoretickes – ps8
Archaeplastida – ps9
Viridiplantae – ps10
Streptophyta – ps11
Charophyceae/Embriophyta – ps12
Embriophyta – ps13
Tracheophyta – ps14
Spermatophyta – ps15
Angiosperms – ps16
Mesangiospermae – ps17
Magnoliids/eudicots – ps18
Eudicots – ps19
Core_eudicots – ps20
Superrosids – ps21
Rosids – ps22
Eurosids – ps23
Fabids – ps24
Nitrogen–fixing_clade – ps25
Rosales/Cucurbitales/Fagales – ps26
Cucurbitales/Fagales – ps27
Cucurbitales – ps28
Cucurbiteae/Benincaseae – ps29
Benincaseae – ps30
Cucumis_sativus – ps31
Phylostratum

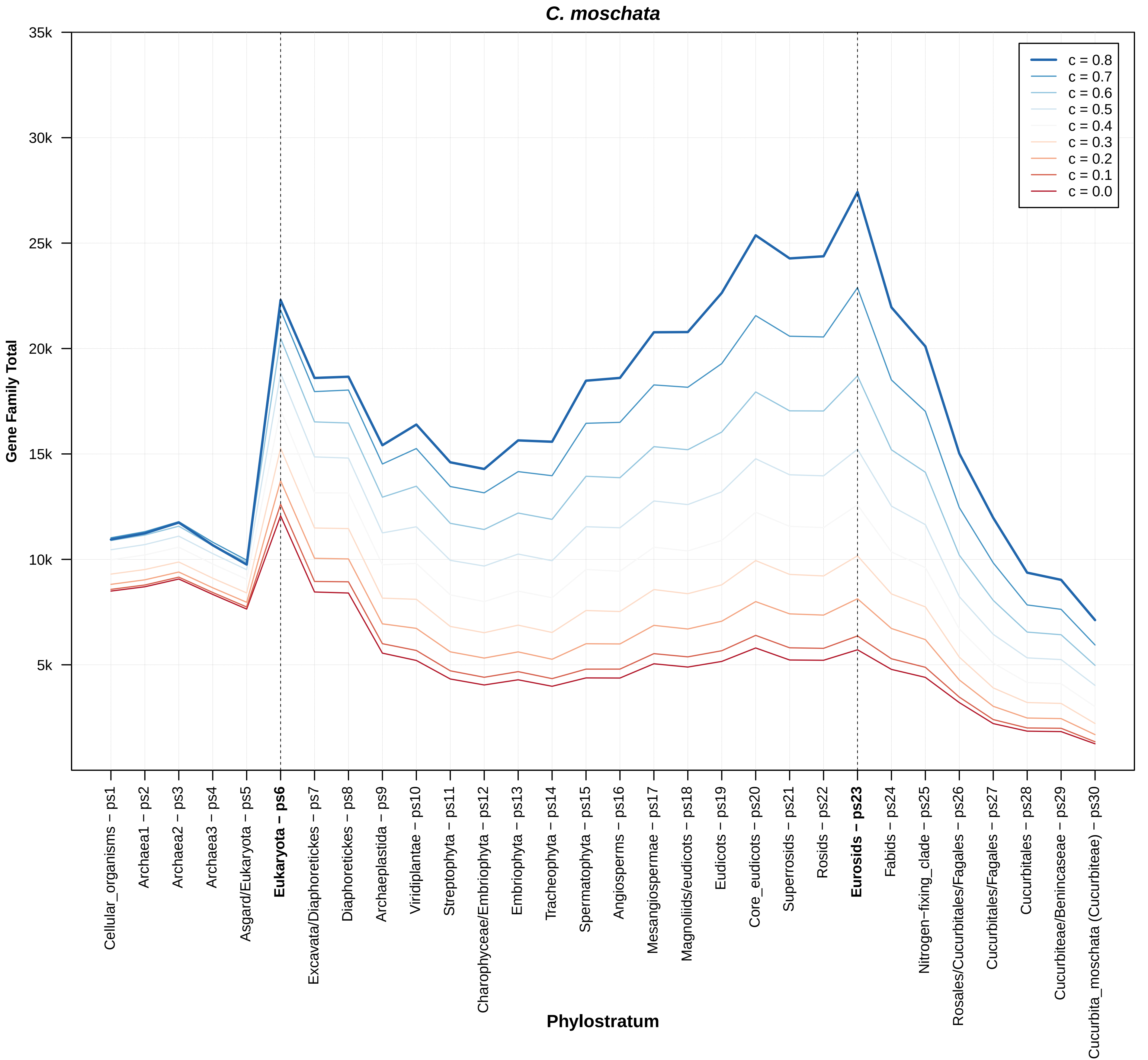

C. moschata
35k
30k
25k
20k
15k
10k
5k
Gene Family Total
c = 0.8
c = 0.7
c = 0.6
c = 0.5
c = 0.4
c = 0.3
c = 0.2
c = 0.1
c = 0.0
Cellular_organisms – ps1
Archaea1 – ps2
Archaea2 – ps3
Archaea3 – ps4
Asgard/Eukaryota – ps5
Eukaryota – ps6
Excavata/Diaphoretickes – ps7
Diaphoretickes – ps8
Archaeplastida – ps9
Viridiplantae – ps10
Streptophyta – ps11
Charophyceae/Embriophyta – ps12
Embriophyta – ps13
Tracheophyta – ps14
Spermatophyta – ps15
Angiosperms – ps16
Mesangiospermae – ps17
Magnoliids/eudicots – ps18
Eudicots – ps19
Core_eudicots – ps20
Superrosids – ps21
Rosids – ps22
Eurosids – ps23
Fabids – ps24
Nitrogen–fixing_clade – ps25
Rosales/Cucurbitales/Fagales – ps26
Cucurbitales/Fagales – ps27
Cucurbitales – ps28
Cucurbiteae/Benincaseae – ps29
Cucurbita_moschata (Cucurbiteae) – ps30
Phylostratum

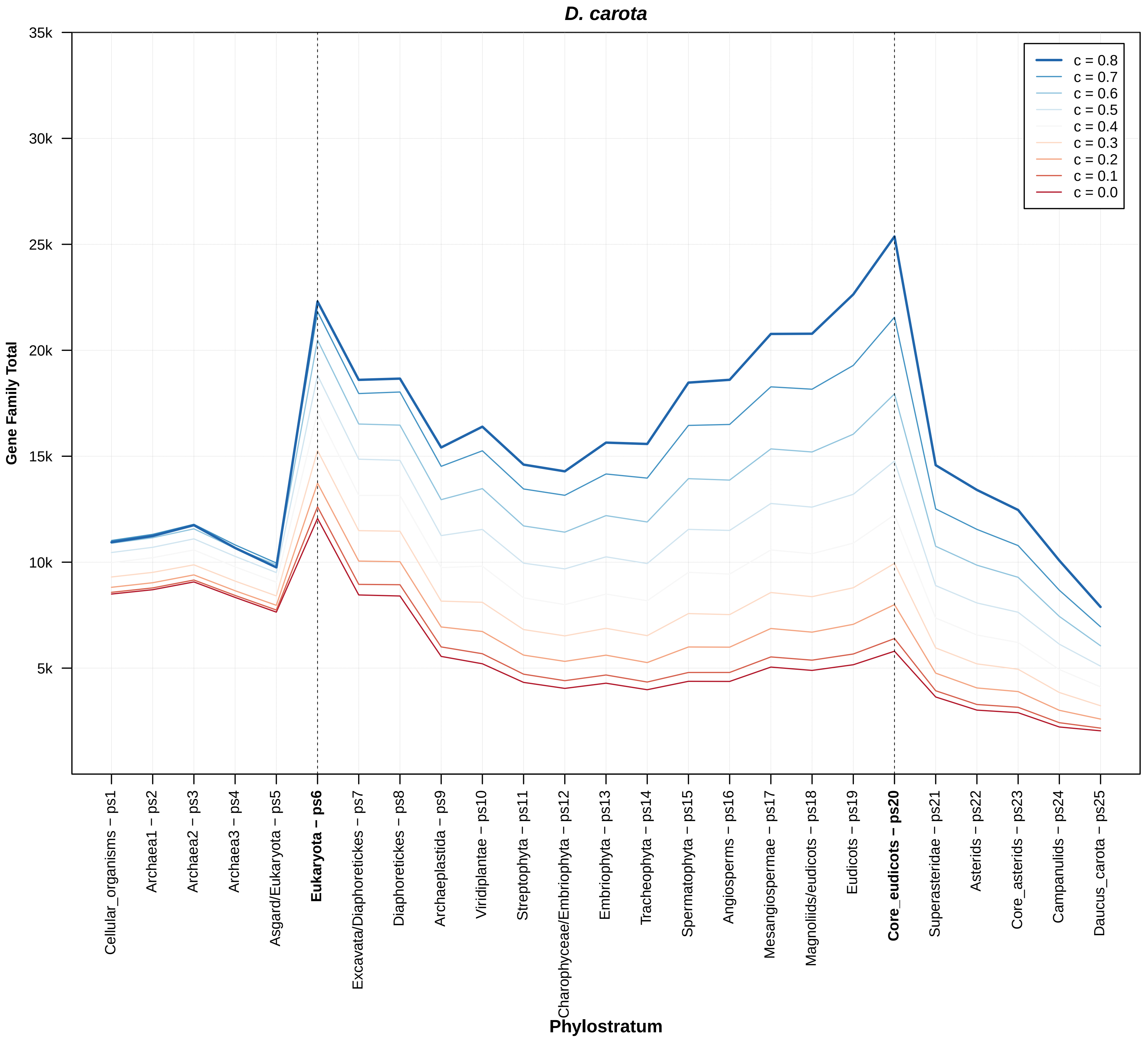
D. carota
35k
30k
25k
20k
15k
10k
5k
Gene Family Total
c = 0.8
c = 0.7
c = 0.6
c = 0.5
c = 0.4
c = 0.3
c = 0.2
c = 0.1
c = 0.0
Cellular_organisms – ps1
Archaea1 – ps2
Archaea2 – ps3
Archaea3 – ps4
Asgard/Eukaryota – ps5
Eukaryota – ps6
Excavata/Diaphoretickes – ps7
Diaphoretickes – ps8
Archaeplastida – ps9
Viridiplantae – ps10
Streptophyta – ps11
Charophyceae/Embriophyta – ps12
Embriophyta – ps13
Tracheophyta – ps14
Spermatophyta – ps15
Angiosperms – ps16
Mesangiospermae – ps17
Magnoliids/eudicots – ps18
Eudicots – ps19
Core_eudicots – ps20
Superasteridae – ps21
Asterids – ps22
Core_asterids – ps23
Campanulids – ps24
Daucus_carota – ps25
Phylostratum

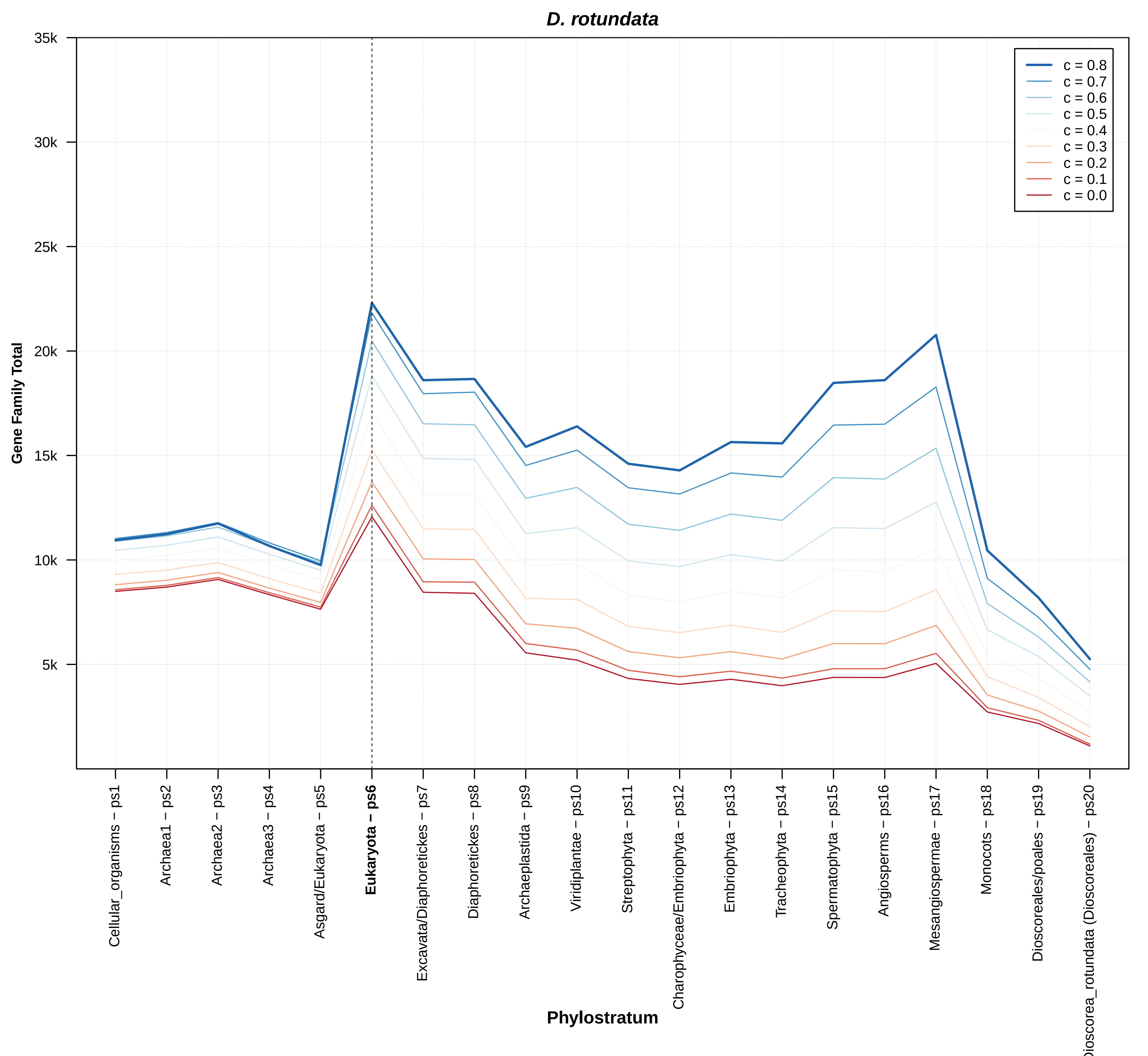

D. rotundata
Gene Family Total
Phylostratum
35k
30k
25k
20k
15k
10k
5k
c = 0.8
c = 0.7
c = 0.6
c = 0.5
c = 0.4
c = 0.3
c = 0.2
c = 0.1
c = 0.0
Cellular_organisms – ps1
Archaea1 – ps2
Archaea2 – ps3
Archaea3 – ps4
Asgard/Eukaryota – ps5
Eukaryota – ps6
Excavata/Diaphoretickes – ps7
Diaphoretickes – ps8
Archaeplastida – ps9
Viridiplantae – ps10
Streptophyta – ps11
Charophyceae/Embriophyta – ps12
Embriophyta – ps13
Tracheophyta – ps14
Spermatophyta – ps15
Angiosperms – ps16
Mesangiospermae – ps17
Monocots – ps18
Dioscoreales/poales – ps19

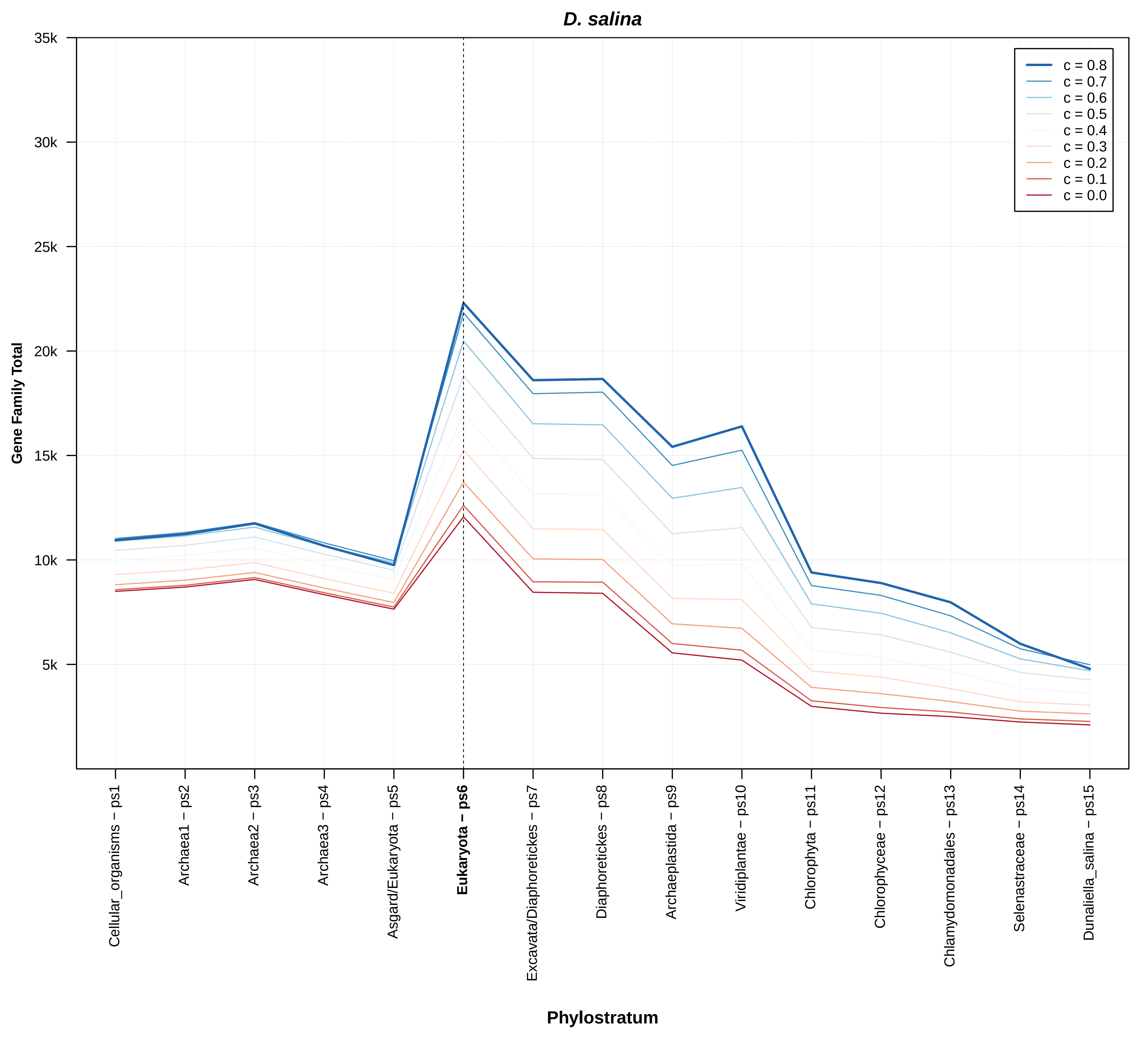
D. salina
35k
30k
25k
20k
15k
10k
5k
Gene Family Total
c = 0.8
c = 0.7
c = 0.6
c = 0.5
c = 0.4
c = 0.3
c = 0.2
c = 0.1
c = 0.0
Cellular_organisms – ps1
Archaea1 – ps2
Archaea2 – ps3
Archaea3 – ps4
Asgard/Eukaryota – ps5
Eukaryota – ps6
Excavata/Diaphoretickes – ps7
Diaphoretickes – ps8
Archaeplastida – ps9
Viridiplantae – ps10
Chlorophyta – ps11
Chlorophyceae – ps12
Chlamydomonadales – ps13
Selenastraceae – ps14
Dunaliella_salina – ps15
Phylostratum

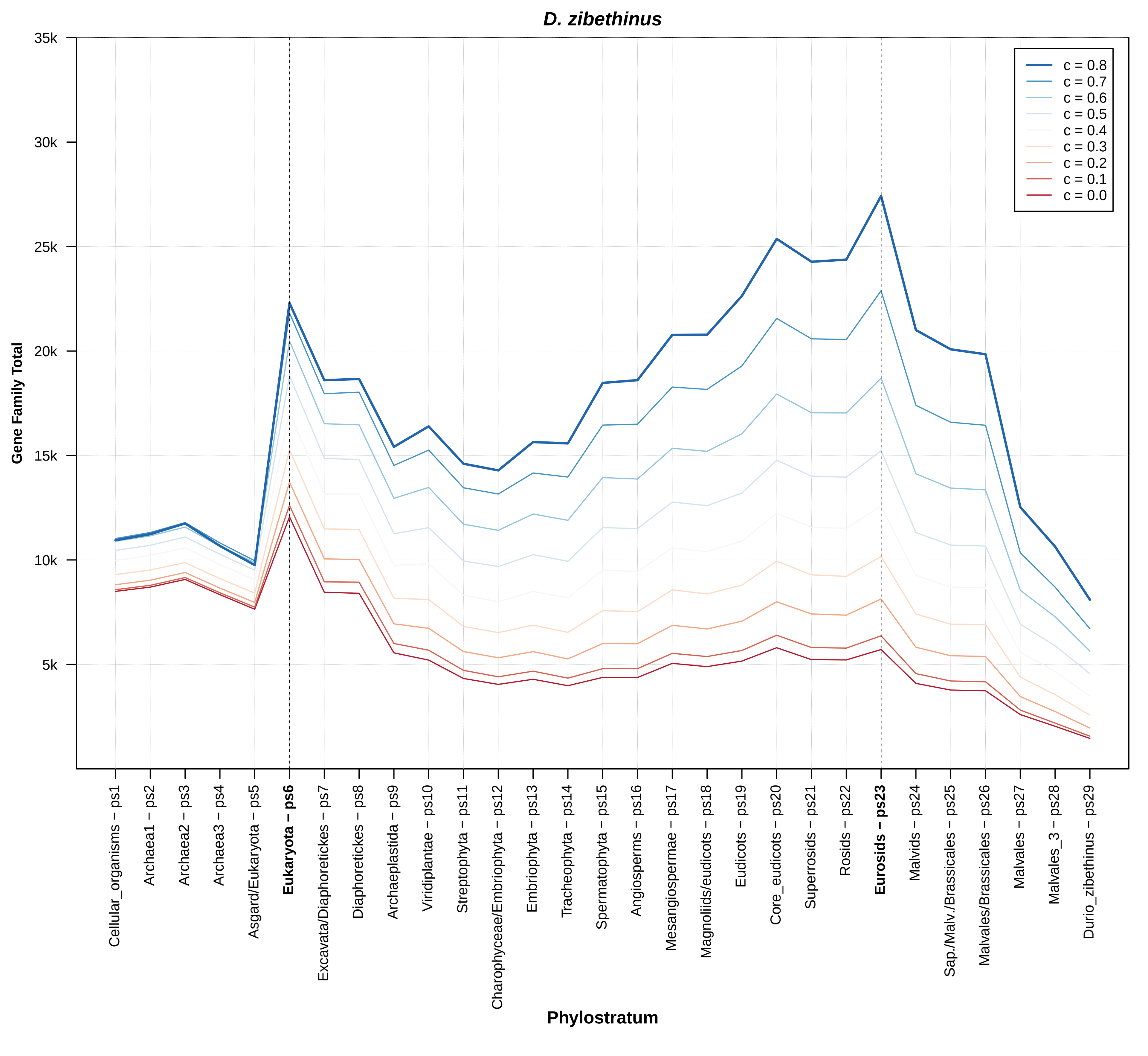
D. zibethinus
Gene Family Total
35k
30k
25k
20k
15k
10k
5k
c = 0.8
c = 0.7
c = 0.6
c = 0.5
c = 0.4
c = 0.3
c = 0.2
c = 0.1
c = 0.0
Cellular_organisms – ps1
Archaea1 – ps2
Archaea2 – ps3
Archaea3 – ps4
Asgard/Eukaryota – ps5
Eukaryota – ps6
Excavata/Diaphoretickes – ps7
Diaphoretickes – ps8
Archaeplastida – ps9
Viridiplantae – ps10
Streptophyta – ps11
Charophyceae/Embriophyta – ps12
Embriophyta – ps13
Tracheophyta – ps14
Spermatophyta – ps15
Angiosperms – ps16
Mesangiospermae – ps17
Magnoliids/eudicots – ps18
Eudicots – ps19
Core_eudicots – ps20
Superrosids – ps21
Rosids – ps22
Eurosids – ps23
Malvids – ps24
Sap./Malv./Brassicales – ps25
Malvales/Brassicales – ps26
Malvales – ps27
Malvales_3 – ps28
Durio_zibethinus – ps29
Phylostratum

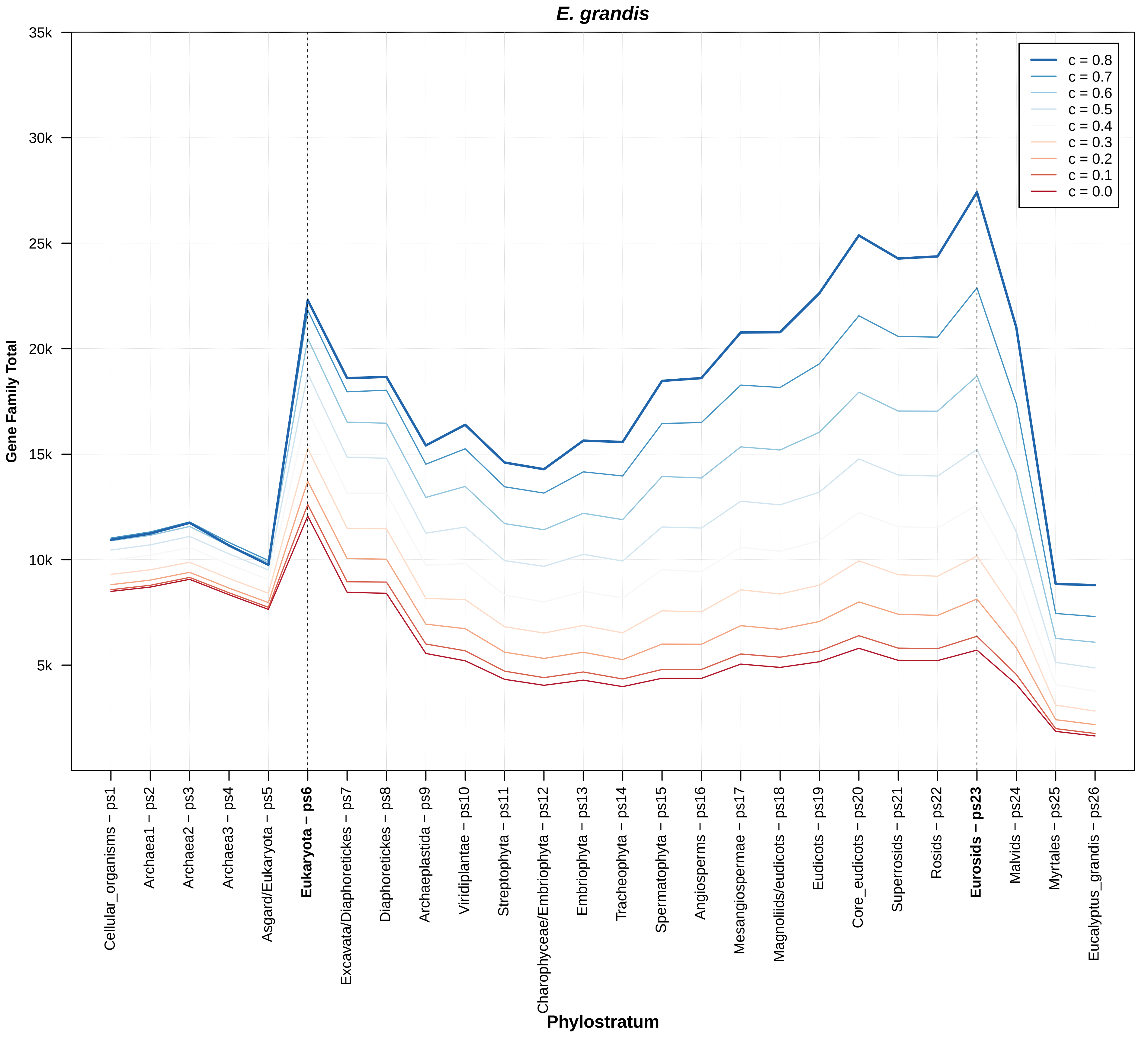

E. grandis
35k
30k
25k
20k
15k
10k
5k
Gene Family Total
c = 0.8
c = 0.7
c = 0.6
c = 0.5
c = 0.4
c = 0.3
c = 0.2
c = 0.1
c = 0.0
Cellular_organisms – ps1
Archaea1 – ps2
Archaea2 – ps3
Archaea3 – ps4
Asgard/Eukaryota – ps5
Eukaryota – ps6
Excavata/Diaphoretickes – ps7
Diaphoretickes – ps8
Archaeplastida – ps9
Viridiplantae – ps10
Streptophyta – ps11
Charophyceae/Embriophyta – ps12
Embriophyta – ps13
Tracheophyta – ps14
Spermatophyta – ps15
Angiosperms – ps16
Mesangiospermae – ps17
Magnoliids/eudicots – ps18
Eudicots – ps19
Core_eudicots – ps20
Superrosids – ps21
Rosids – ps22
Eurosids – ps23
Malvids – ps24
Myrtales – ps25
Eucalyptus_grandis – ps26
Phylostratum

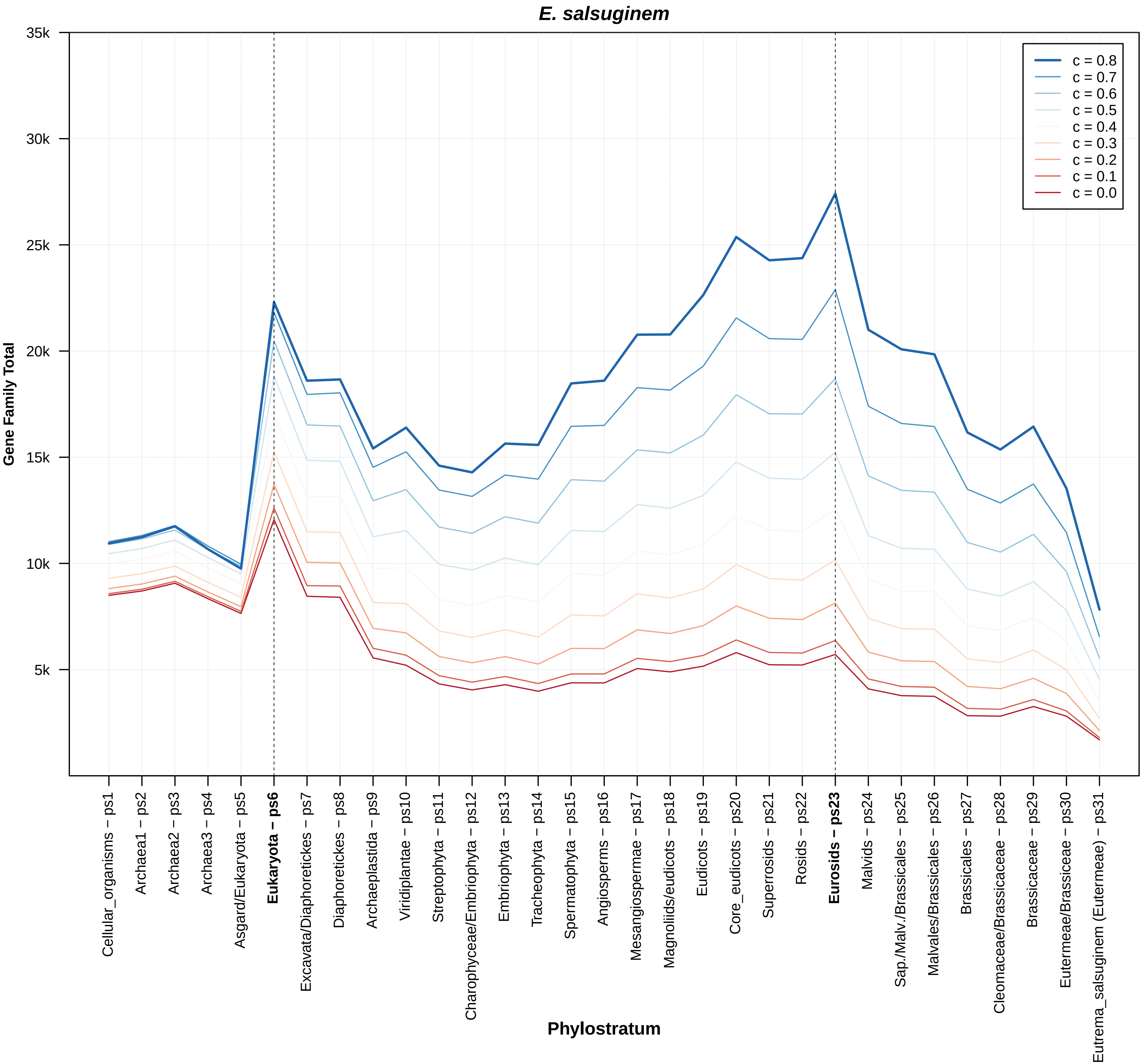

E. salsuginem
Gene Family Total
35k
30k
25k
20k
15k
10k
5k
c = 0.8
c = 0.7
c = 0.6
c = 0.5
c = 0.4
c = 0.3
c = 0.2
c = 0.1
c = 0.0
Cellular_organisms – ps1
Archaea1 – ps2
Archaea2 – ps3
Archaea3 – ps4
Asgard/Eukaryota – ps5
Eukaryota – ps6
Excavata/Diaphoretickes – ps7
Diaphoretickes – ps8
Archaeplastida – ps9
Viridiplantae – ps10
Streptophyta – ps11
Charophyceae/Embriophyta – ps12
Embriophyta – ps13
Tracheophyta – ps14
Spermatophyta – ps15
Angiosperms – ps16
Mesangiospermae – ps17
Magnoliids/eudicots – ps18
Eudicots – ps19
Core_eudicots – ps20
Superrosids – ps21
Rosids – ps22
Eurosids – ps23
Malvids – ps24
Sap./Malv./Brassicales – ps25
Malvales/Brassicales – ps26
Brassicales – ps27
Cleomaceae/Brassicaceae – ps28
Brassicaceae – ps29
Eutermeae/Brassiceae – ps30
Eutrema_salsuginem (Eutermeae) – ps31
Phylostratum

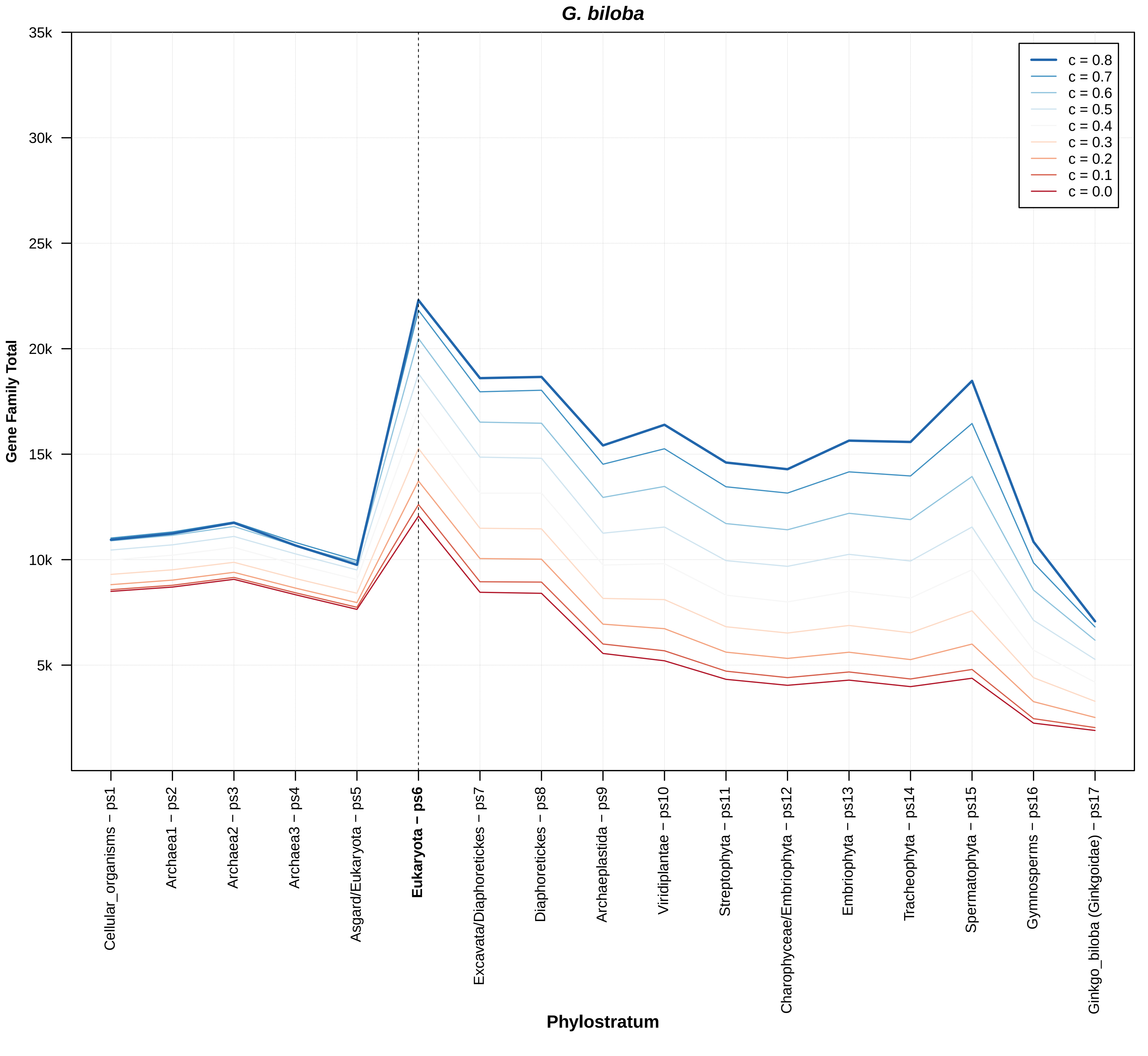
G. biloba
Gene Family Total
35k
30k
25k
20k
15k
10k
5k
c = 0.8
c = 0.7
c = 0.6
c = 0.5
c = 0.4
c = 0.3
c = 0.2
c = 0.1
c = 0.0
Cellular_organisms – ps1
Archaea1 – ps2
Archaea2 – ps3
Archaea3 – ps4
Asgard/Eukaryota – ps5
Eukaryota – ps6
Excavata/Diaphoretickes – ps7
Diaphoretickes – ps8
Archaeplastida – ps9
Viridiplantae – ps10
Streptophyta – ps11
Charophyceae/Embriophyta – ps12
Embriophyta – ps13
Tracheophyta – ps14
Spermatophyta – ps15
Gymnosperms – ps16
Ginkgo_biloba (Ginkgoidae) – ps17
Phylostratum

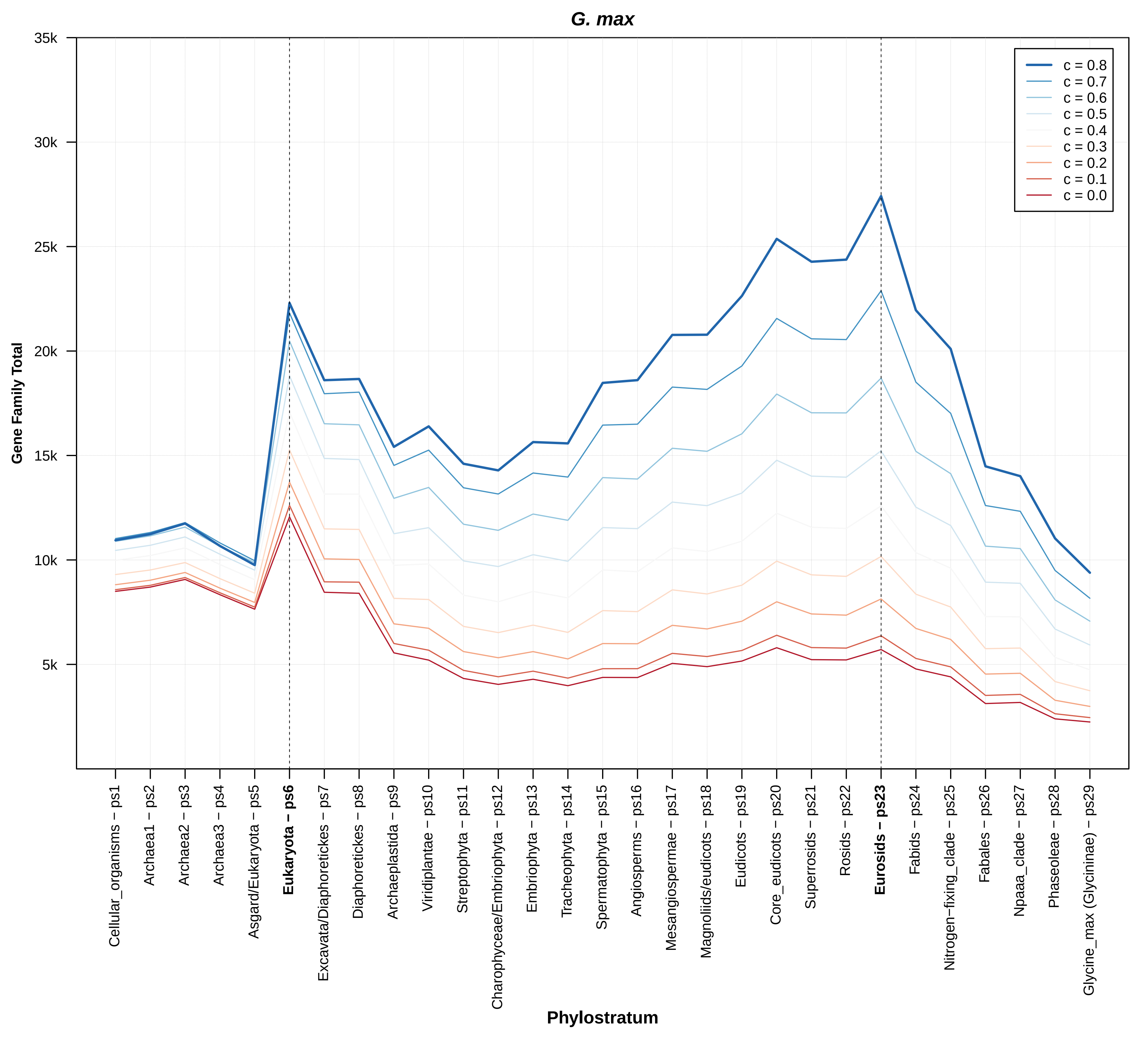
G. max
Gene Family Total
35k
30k
25k
20k
15k
10k
5k
c = 0.8
c = 0.7
c = 0.6
c = 0.5
c = 0.4
c = 0.3
c = 0.2
c = 0.1
c = 0.0
Cellular_organisms – ps1
Archaea1 – ps2
Archaea2 – ps3
Archaea3 – ps4
Asgard/Eukaryota – ps5
Eukaryota – ps6
Excavata/Diaphoretickes – ps7
Diaphoretickes – ps8
Archaeplastida – ps9
Viridiplantae – ps10
Streptophyta – ps11
Charophyceae/Embriophyta – ps12
Embriophyta – ps13
Tracheophyta – ps14
Spermatophyta – ps15
Angiosperms – ps16
Mesangiospermae – ps17
Magnoliids/eudicots – ps18
Eudicots – ps19
Core_eudicots – ps20
Superrosids – ps21
Rosids – ps22
Eurosids – ps23
Fabids – ps24
Nitrogen−fixing_clade – ps25
Fabales – ps26
Npaaa_clade – ps27
Phaseoleae – ps28
Glycine_max (Glycininae) – ps29
Phylostratum

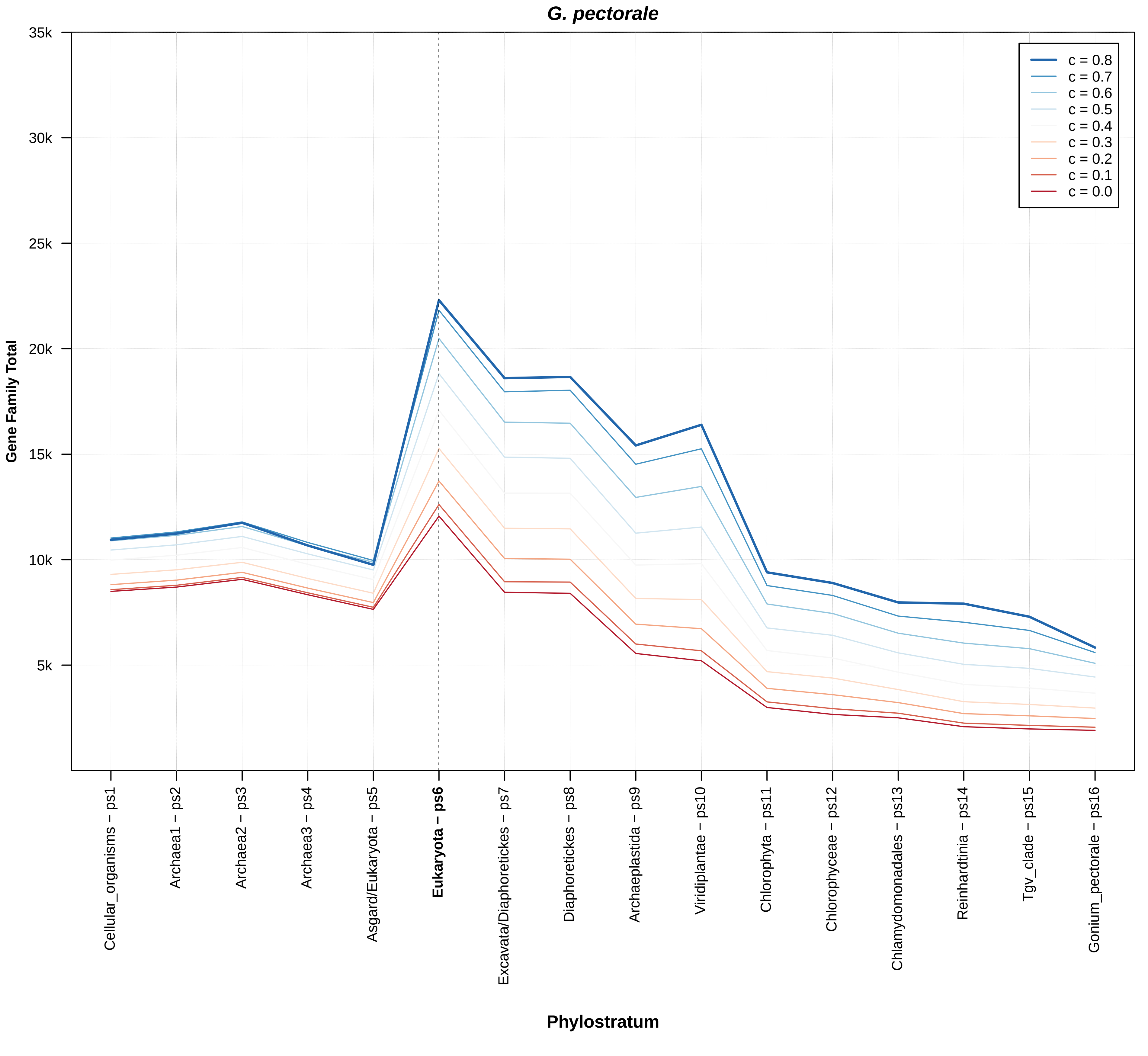

G. pectorale
Gene Family Total
35k
30k
25k
20k
15k
10k
5k
c = 0.8
c = 0.7
c = 0.6
c = 0.5
c = 0.4
c = 0.3
c = 0.2
c = 0.1
c = 0.0
Cellular_organisms – ps1
Archaea1 – ps2
Archaea2 – ps3
Archaea3 – ps4
Asgard/Eukaryota – ps5
Eukaryota – ps6
Excavata/Diaphoretickes – ps7
Diaphoretickes – ps8
Archaeplastida – ps9
Viridiplantae – ps10
Chlorophyta – ps11
Chlorophyceae – ps12
Chlamydomonadales – ps13
Reinhardtinia – ps14
Tgv_clade – ps15
Gonium_pectorale – ps16
Phylostratum

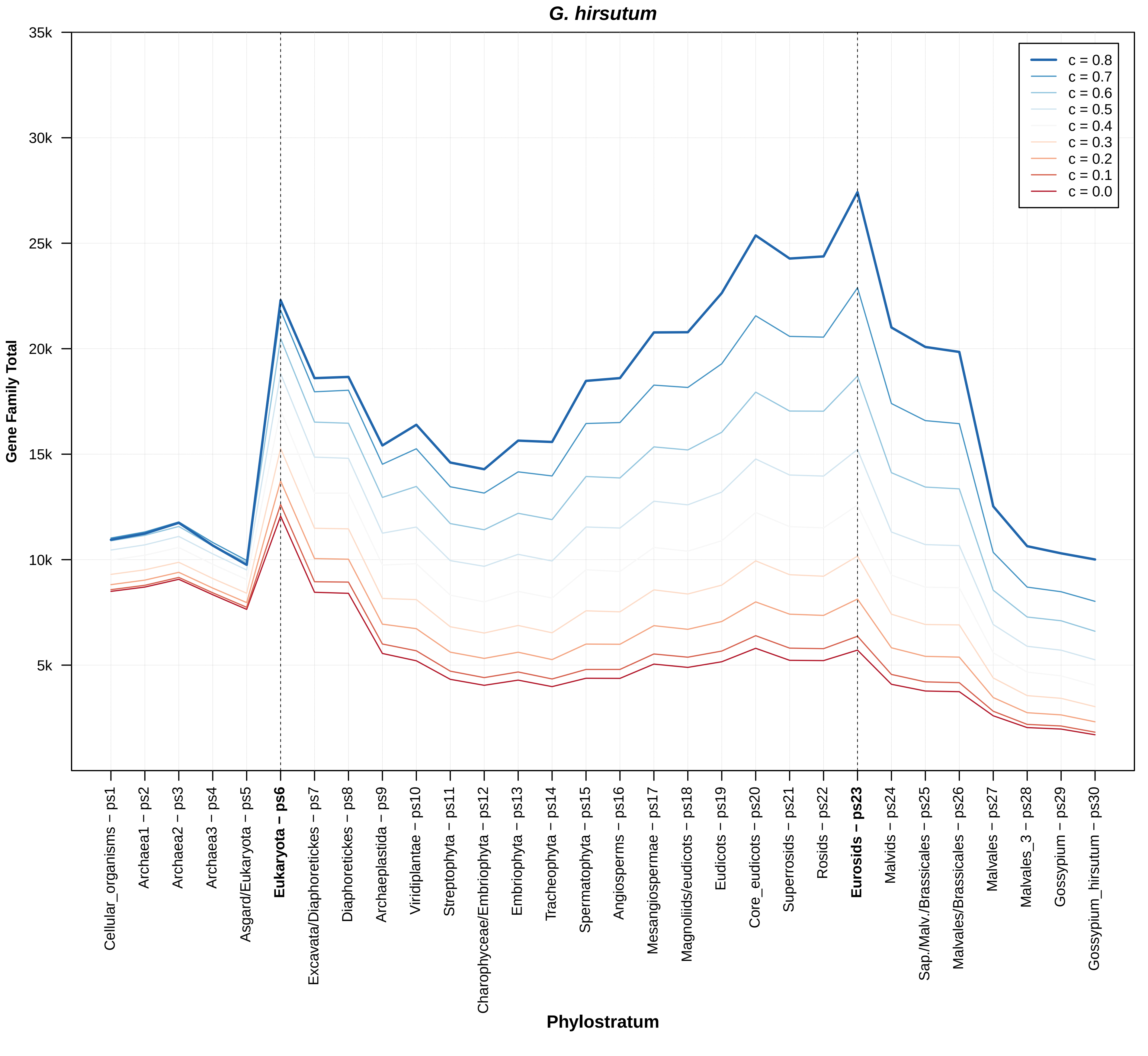

G. hirsutum
Gene Family Total
35k
30k
25k
20k
15k
10k
5k
c = 0.8
c = 0.7
c = 0.6
c = 0.5
c = 0.4
c = 0.3
c = 0.2
c = 0.1
c = 0.0
Cellular_organisms – ps1
Archaea1 – ps2
Archaea2 – ps3
Archaea3 – ps4
Asgard/Eukaryota – ps5
Eukaryota – ps6
Excavata/Diaphoretickes – ps7
Diaphoretickes – ps8
Archaeplastida – ps9
Viridiplantae – ps10
Streptophyta – ps11
Charophyceae/Embriophyta – ps12
Embriophyta – ps13
Tracheophyta – ps14
Spermatophyta – ps15
Angiosperms – ps16
Mesangiospermae – ps17
Magnoliids/eudicots – ps18
Eudicots – ps19
Core_eudicots – ps20
Superrosids – ps21
Rosids – ps22
Eurosids – ps23
Malvids – ps24
Sap./Malv./Brassicales – ps25
Malvales/Brassicales – ps26
Malvales – ps27
Malvales_3 – ps28
Gossypium – ps29
Gossypium_hirsutum – ps30
Phylostratum

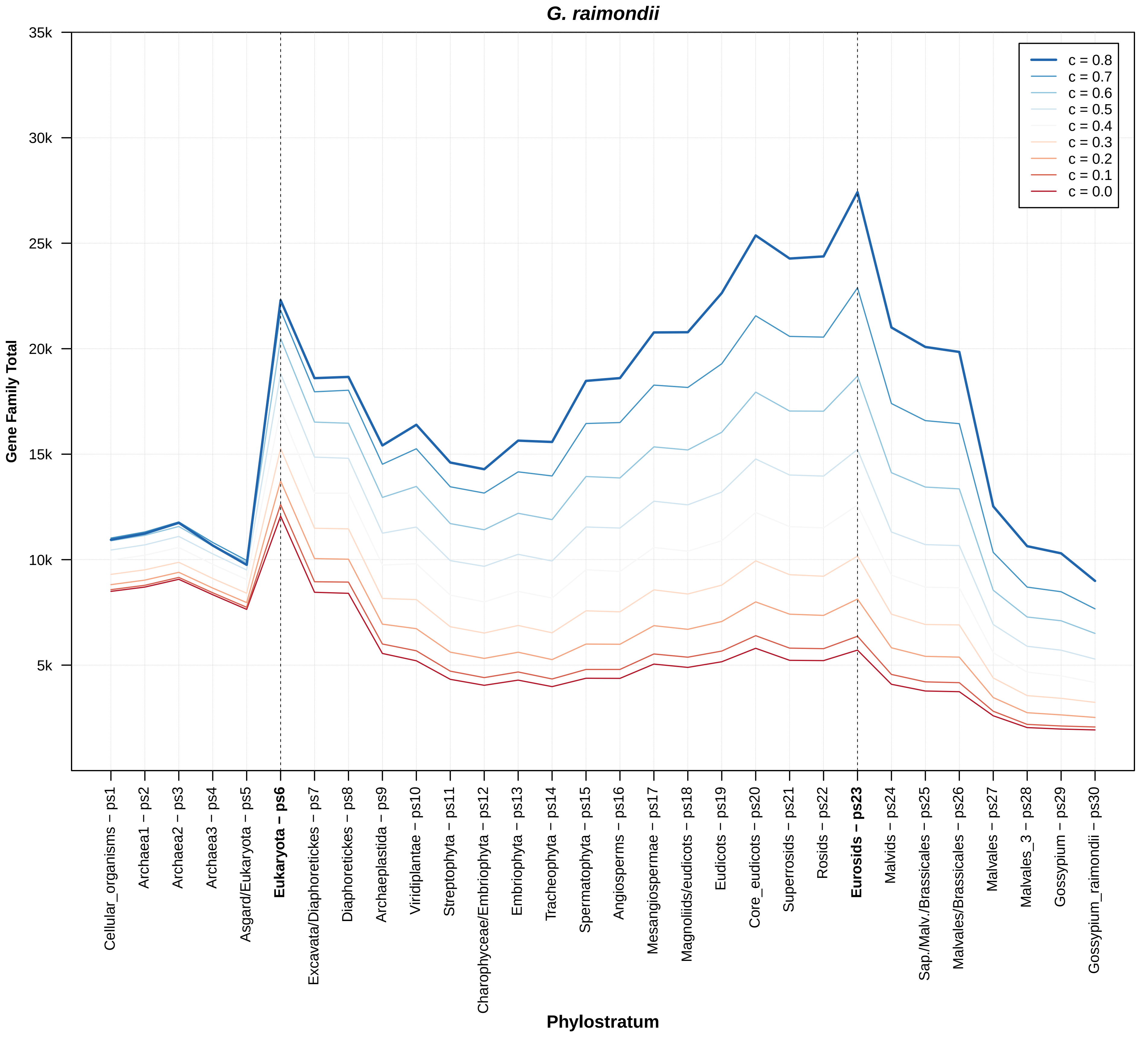

G. raimondii
Gene Family Total
35k
30k
25k
20k
15k
10k
5k
c = 0.8
c = 0.7
c = 0.6
c = 0.5
c = 0.4
c = 0.3
c = 0.2
c = 0.1
c = 0.0
Cellular_organisms – ps1
Archaea1 – ps2
Archaea2 – ps3
Archaea3 – ps4
Asgard/Eukaryota – ps5
Eukaryota – ps6
Excavata/Diaphoretickes – ps7
Diaphoretickes – ps8
Archaeplastida – ps9
Viridiplantae – ps10
Streptophyta – ps11
Charophyceae/Embriophyta – ps12
Embriophyta – ps13
Tracheophyta – ps14
Spermatophyta – ps15
Angiosperms – ps16
Mesangiospermae – ps17
Magnoliids/eudicots – ps18
Eudicots – ps19
Core_eudicots – ps20
Superrosids – ps21
Rosids – ps22
Eurosids – ps23
Malvids – ps24
Sap./Malv./Brassicales – ps25
Malvales/Brassicales – ps26
Malvales – ps27
Malvales_3 – ps28
Gossypium – ps29
Gossypium_raimondii – ps30
Phylostratum

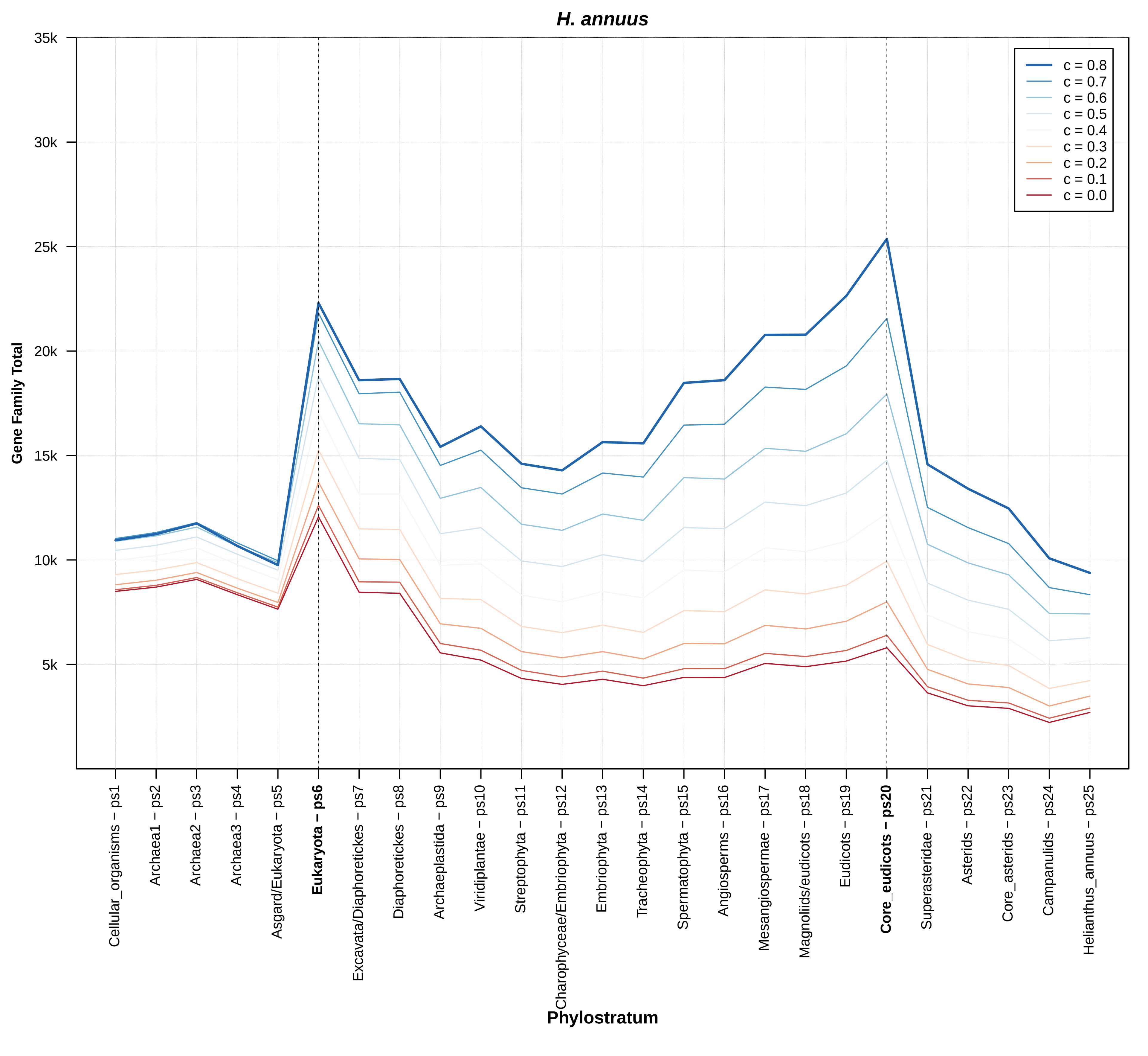

H. annuus
35k
30k
25k
20k
15k
10k
5k
Gene Family Total
c = 0.8
c = 0.7
c = 0.6
c = 0.5
c = 0.4
c = 0.3
c = 0.2
c = 0.1
c = 0.0
Cellular_organisms – ps1
Archaea1 – ps2
Archaea2 – ps3
Archaea3 – ps4
Asgard/Eukaryota – ps5
Eukaryota – ps6
Excavata/Diaphoretickes – ps7
Diaphoretickes – ps8
Archaeplastida – ps9
Viridiplantae – ps10
Streptophyta – ps11
Charophyceae/Embriophyta – ps12
Embriophyta – ps13
Tracheophyta – ps14
Spermatophyta – ps15
Angiosperms – ps16
Mesangiospermae – ps17
Magnoliids/eudicots – ps18
Eudicots – ps19
Core_eudicots – ps20
Superasteridae – ps21
Asterids – ps22
Core_asterids – ps23
Campanulids – ps24
Helianthus_annuus – ps25
Phylostratum

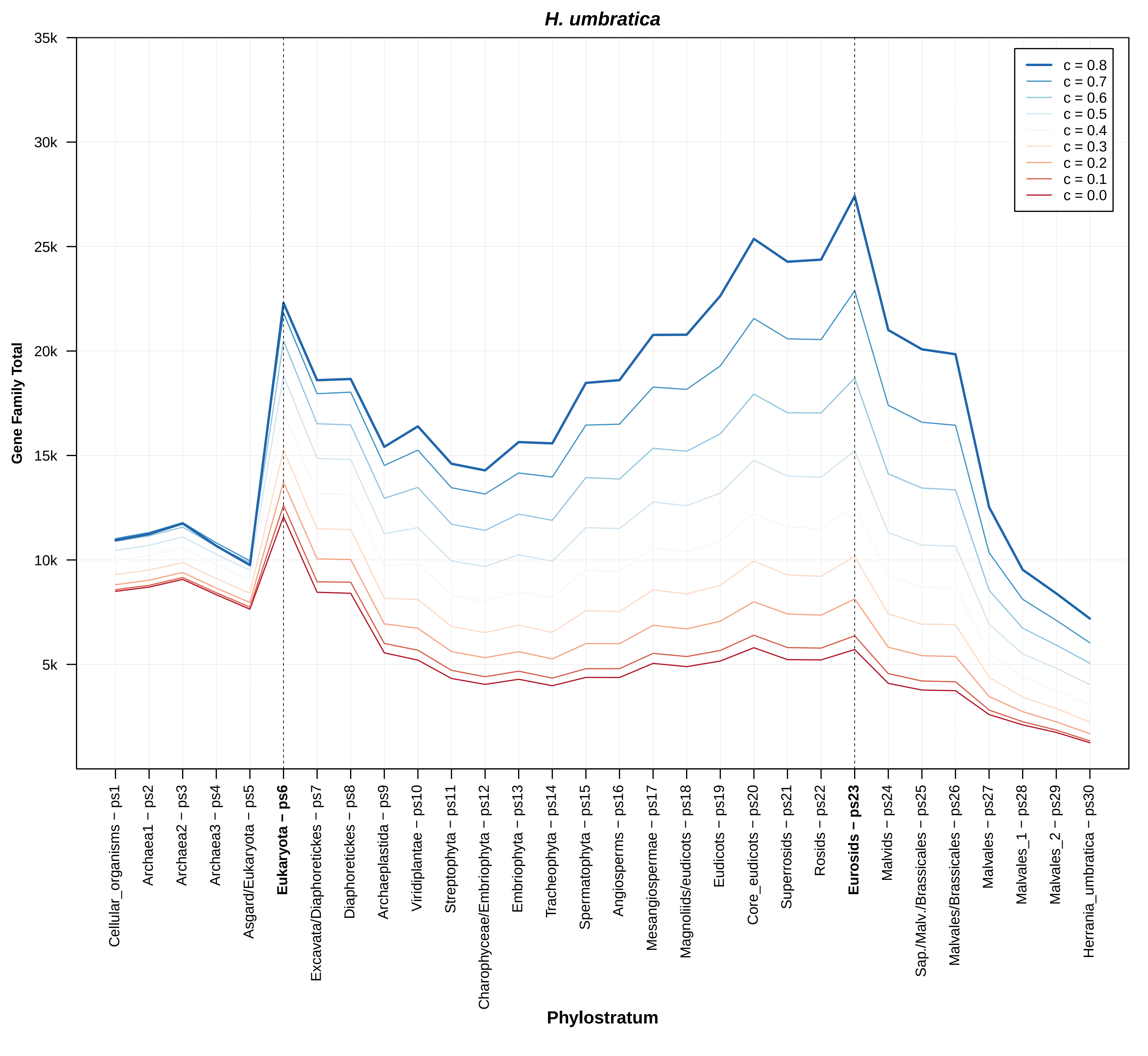

H. umbratica
Gene Family Total
35k
30k
25k
20k
15k
10k
5k
c = 0.8
c = 0.7
c = 0.6
c = 0.5
c = 0.4
c = 0.3
c = 0.2
c = 0.1
c = 0.0
Cellular_organisms – ps1
Archaea1 – ps2
Archaea2 – ps3
Archaea3 – ps4
Asgard/Eukaryota – ps5
Eukaryota – ps6
Excavata/Diaphoretickes – ps7
Diaphoretickes – ps8
Archaeplastida – ps9
Viridiplantae – ps10
Streptophyta – ps11
Charophyceae/Embriophyta – ps12
Embriophyta – ps13
Tracheophyta – ps14
Spermatophyta – ps15
Angiosperms – ps16
Mesangiospermae – ps17
Magnoliids/eudicots – ps18
Eudicots – ps19
Core_eudicots – ps20
Superrosids – ps21
Rosids – ps22
Eurosids – ps23
Malvids – ps24
Sap./Malv./Brassicales – ps25
Malvales/Brassicales – ps26
Malvales – ps27
Malvales_1 – ps28
Malvales_2 – ps29
Herrania_umbratica – ps30
Phylostratum

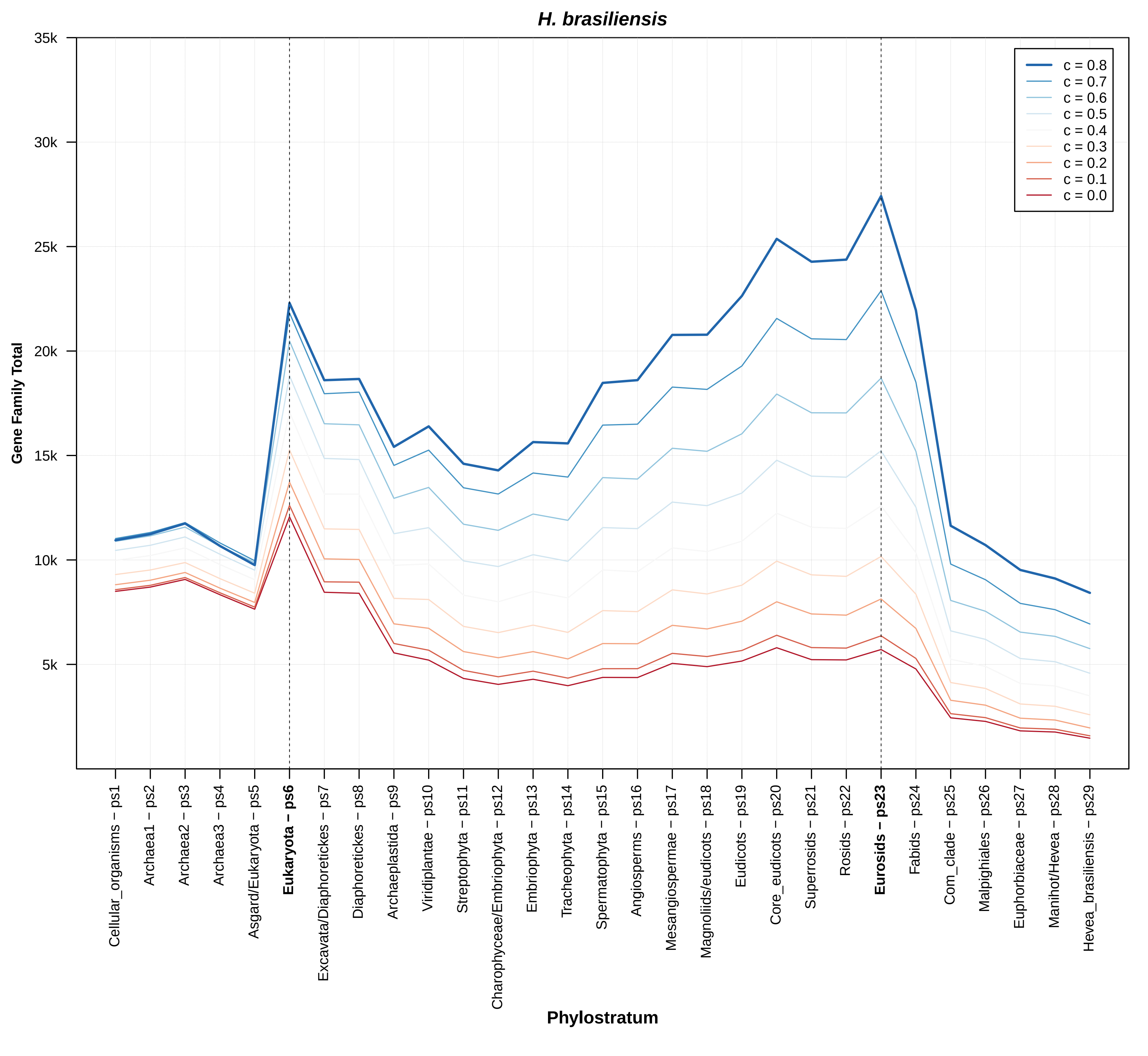
H. brasiliensis
Gene Family Total
35k
30k
25k
20k
15k
10k
5k
c = 0.8
c = 0.7
c = 0.6
c = 0.5
c = 0.4
c = 0.3
c = 0.2
c = 0.1
c = 0.0
Cellular_organisms – ps1
Archaea1 – ps2
Archaea2 – ps3
Archaea3 – ps4
Asgard/Eukaryota – ps5
Eukaryota – ps6
Excavata/Diaphoretickes – ps7
Diaphoretickes – ps8
Archaeplastida – ps9
Viridiplantae – ps10
Streptophyta – ps11
Charophyceae/Embriophyta – ps12
Embriophyta – ps13
Tracheophyta – ps14
Spermatophyta – ps15
Angiosperms – ps16
Mesangiospermae – ps17
Magnoliids/eudicots – ps18
Eudicots – ps19
Core_eudicots – ps20
Superrosids – ps21
Rosids – ps22
Eurosids – ps23
Fabids – ps24
Com_clade – ps25
Malpighiales – ps26
Euphorbiaceae – ps27
Manihot/Hevea – ps28
Hevea_brasiliensis – ps29
Phylostratum

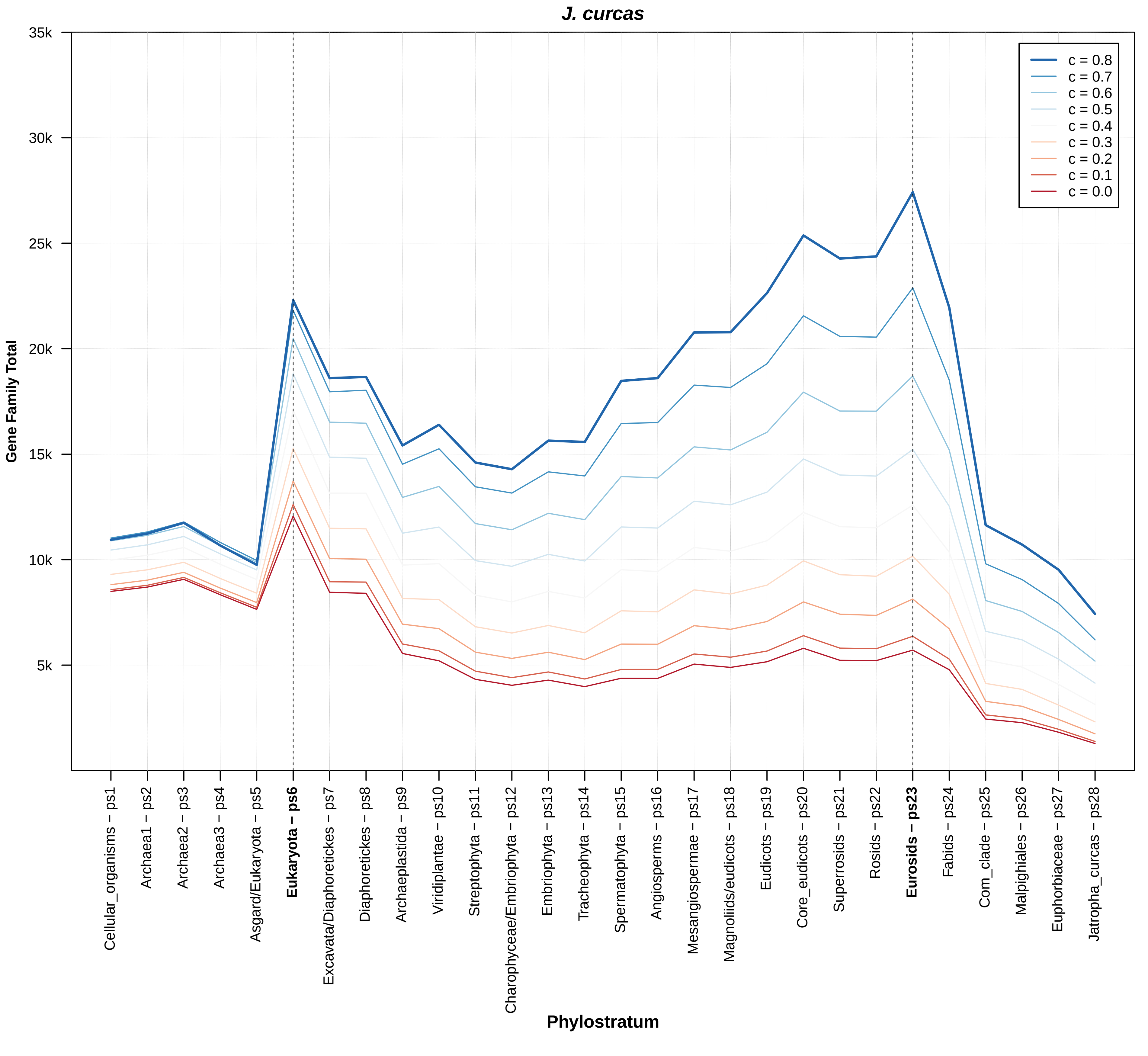

J. curcas
Gene Family Total
35k
30k
25k
20k
15k
10k
5k
c = 0.8
c = 0.7
c = 0.6
c = 0.5
c = 0.4
c = 0.3
c = 0.2
c = 0.1
c = 0.0
Cellular_organisms – ps1
Archaea1 – ps2
Archaea2 – ps3
Archaea3 – ps4
Asgard/Eukaryota – ps5
Eukaryota – ps6
Excavata/Diaphoretickes – ps7
Diaphoretickes – ps8
Archaeplastida – ps9
Viridiplantae – ps10
Streptophyta – ps11
Charophyceae/Embriophyta – ps12
Embriophyta – ps13
Tracheophyta – ps14
Spermatophyta – ps15
Angiosperms – ps16
Mesangiospermae – ps17
Magnoliids/eudicots – ps18
Eudicots – ps19
Core_eudicots – ps20
Superrosids – ps21
Rosids – ps22
Eurosids – ps23
Fabids – ps24
Com_clade – ps25
Malpighiales – ps26
Euphorbiaceae – ps27
Jatropha_curcas – ps28
Phylostratum

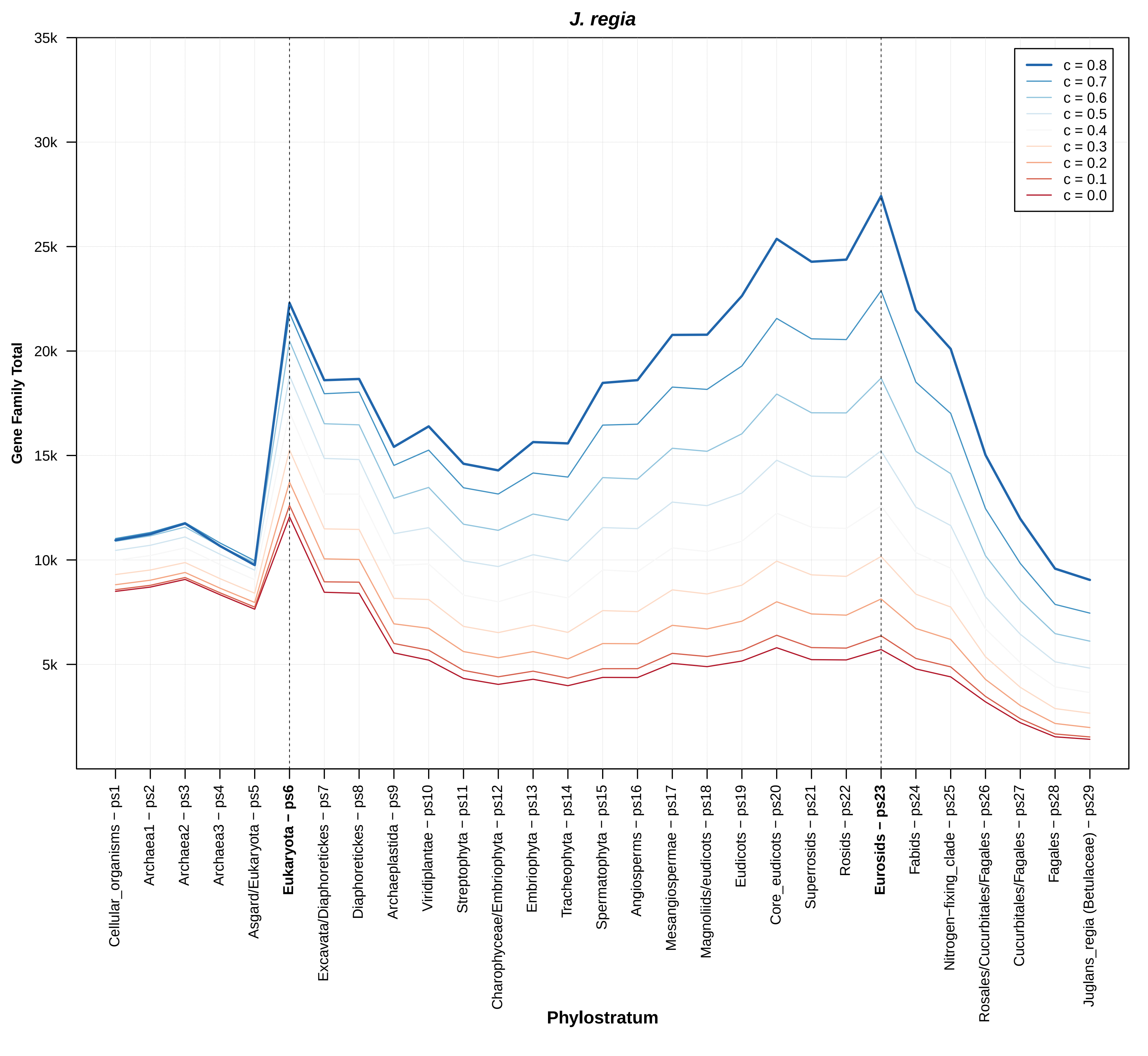

J. regia
Gene Family Total
35k
30k
25k
20k
15k
10k
5k
c = 0.8
c = 0.7
c = 0.6
c = 0.5
c = 0.4
c = 0.3
c = 0.2
c = 0.1
c = 0.0
Cellular_organisms – ps1
Archaea1 – ps2
Archaea2 – ps3
Archaea3 – ps4
Asgard/Eukaryota – ps5
Eukaryota – ps6
Excavata/Diaphoretickes – ps7
Diaphoretickes – ps8
Archaeplastida – ps9
Viridiplantae – ps10
Streptophyta – ps11
Charophyceae/Embriophyta – ps12
Embriophyta – ps13
Tracheophyta – ps14
Spermatophyta – ps15
Angiosperms – ps16
Mesangiospermae – ps17
Magnoliids/eudicots – ps18
Eudicots – ps19
Core_eudicots – ps20
Superrosids – ps21
Rosids – ps22
Eurosids – ps23
Fabids – ps24
Nitrogen–fixing_clade – ps25
Rosales/Cucurbitales/Fagales – ps26
Cucurbitales/Fagales – ps27
Fagales – ps28
Juglans_regia (Betulaceae) – ps29
Phylostratum

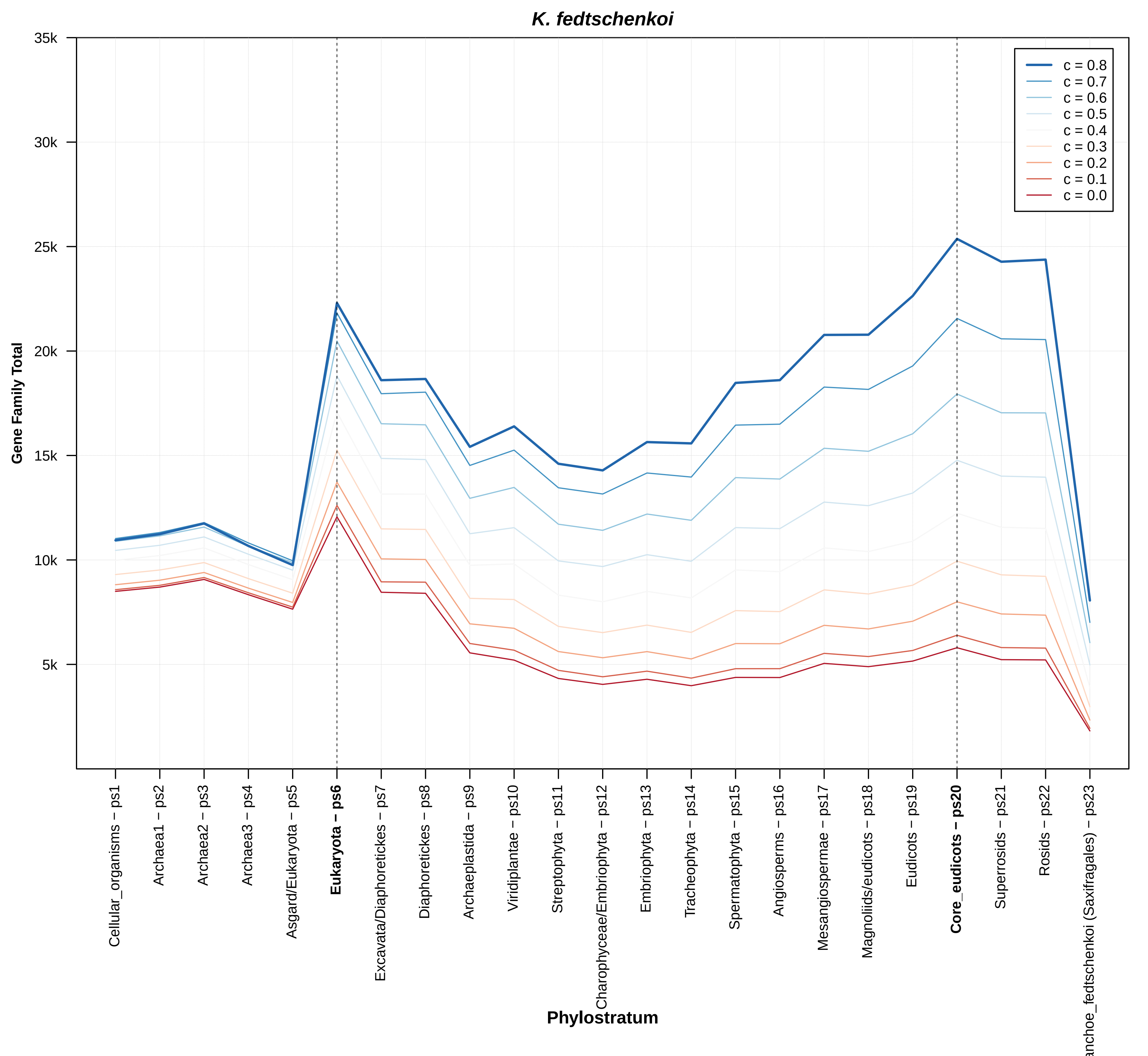

K. fedtschenkoi
Gene Family Total
Phylostratum
35k
30k
25k
20k
15k
10k
5k
c = 0.8
c = 0.7
c = 0.6
c = 0.5
c = 0.4
c = 0.3
c = 0.2
c = 0.1
c = 0.0
Cellular_organisms – ps1
Archaea1 – ps2
Archaea2 – ps3
Archaea3 – ps4
Asgard/Eukaryota – ps5
Eukaryota – ps6
Excavata/Diaphoretickes – ps7
Diaphoretickes – ps8
Archaeplastida – ps9
Viridiplantae – ps10
Streptophyta – ps11
Charophyceae/Embriophyta – ps12
Embriophyta – ps13
Tracheophyta – ps14
Spermatophyta – ps15
Angiosperms – ps16
Mesangiospermae – ps17
Magnoliids/eudicots – ps18
Eudicots – ps19
Core_eudicots – ps20
Superrosids – ps21
Rosids – ps22

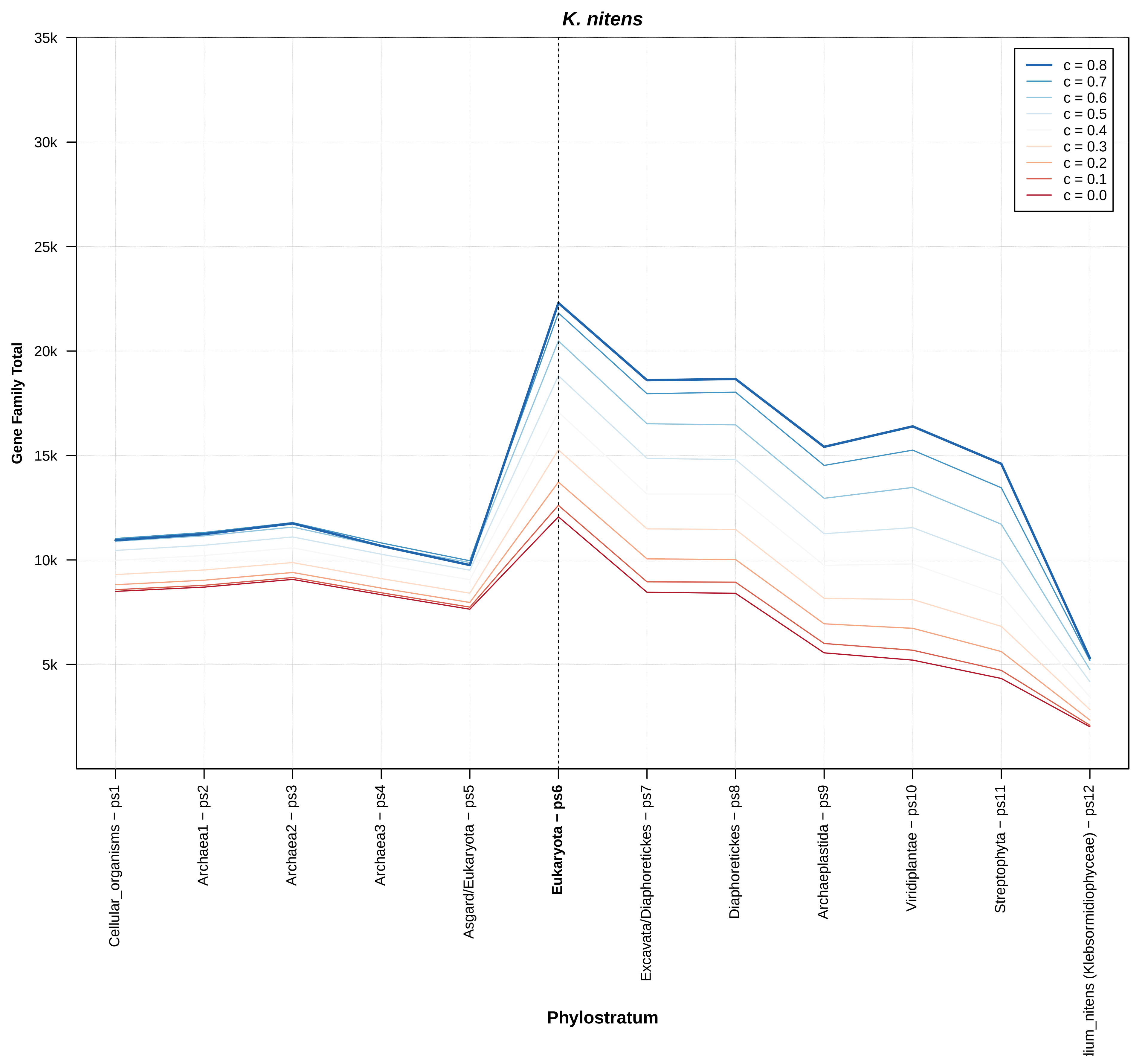
K. nitens
35k
30k
25k
20k
15k
10k
5k
Gene Family Total
c = 0.8
c = 0.7
c = 0.6
c = 0.5
c = 0.4
c = 0.3
c = 0.2
c = 0.1
c = 0.0
Cellular_organisms – ps1
Archaea1 – ps2
Archaea2 – ps3
Archaea3 – ps4
Asgard/Eukaryota – ps5
Eukaryota – ps6
Excavata/Diaphoretickes – ps7
Diaphoretickes – ps8
Archaeplastida – ps9
Viridiplantae – ps10
Streptophyta – ps11
dium_nitens (Klebsormidiophyceae) – ps12
Phylostratum

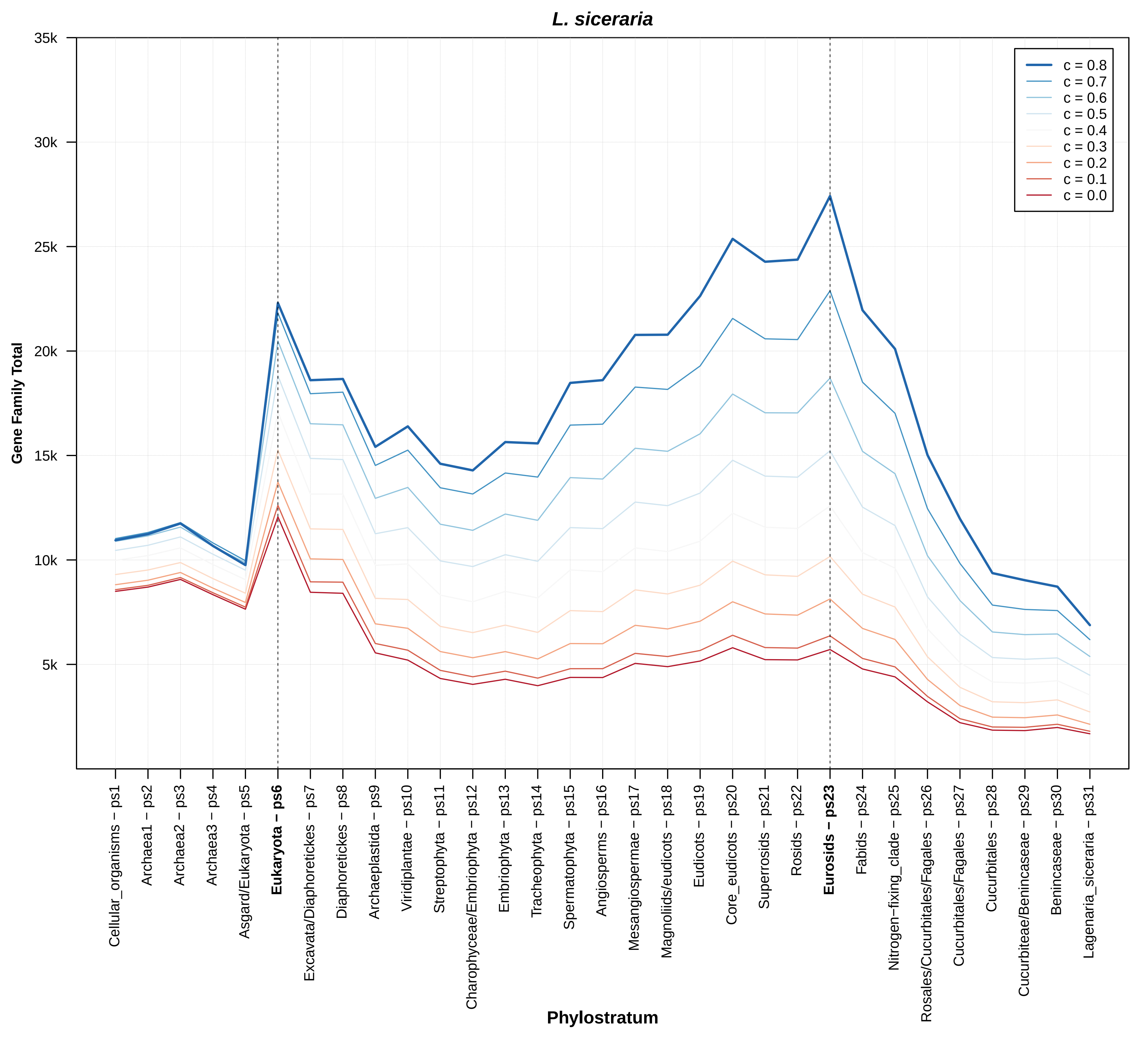
L. siceraria
Gene Family Total
35k
30k
25k
20k
15k
10k
5k
c = 0.8
c = 0.7
c = 0.6
c = 0.5
c = 0.4
c = 0.3
c = 0.2
c = 0.1
c = 0.0
Cellular_organisms – ps1
Archaea1 – ps2
Archaea2 – ps3
Archaea3 – ps4
Asgard/Eukaryota – ps5
Eukaryota – ps6
Excavata/Diaphoretickes – ps7
Diaphoretickes – ps8
Archaeplastida – ps9
Viridiplantae – ps10
Streptophyta – ps11
Charophyceae/Embriophyta – ps12
Embriophyta – ps13
Tracheophyta – ps14
Spermatophyta – ps15
Angiosperms – ps16
Mesangiospermae – ps17
Magnoliids/eudicots – ps18
Eudicots – ps19
Core_eudicots – ps20
Superrosids – ps21
Rosids – ps22
Eurosids – ps23
Fabids – ps24
Nitrogen–fixing_clade – ps25
Rosales/Cucurbitales/Fagales – ps26
Cucurbitales/Fagales – ps27
Cucurbitales – ps28
Cucurbiteae/Benincaseae – ps29
Benincaseae – ps30
Lagenaria_siceraria – ps31
Phylostratum

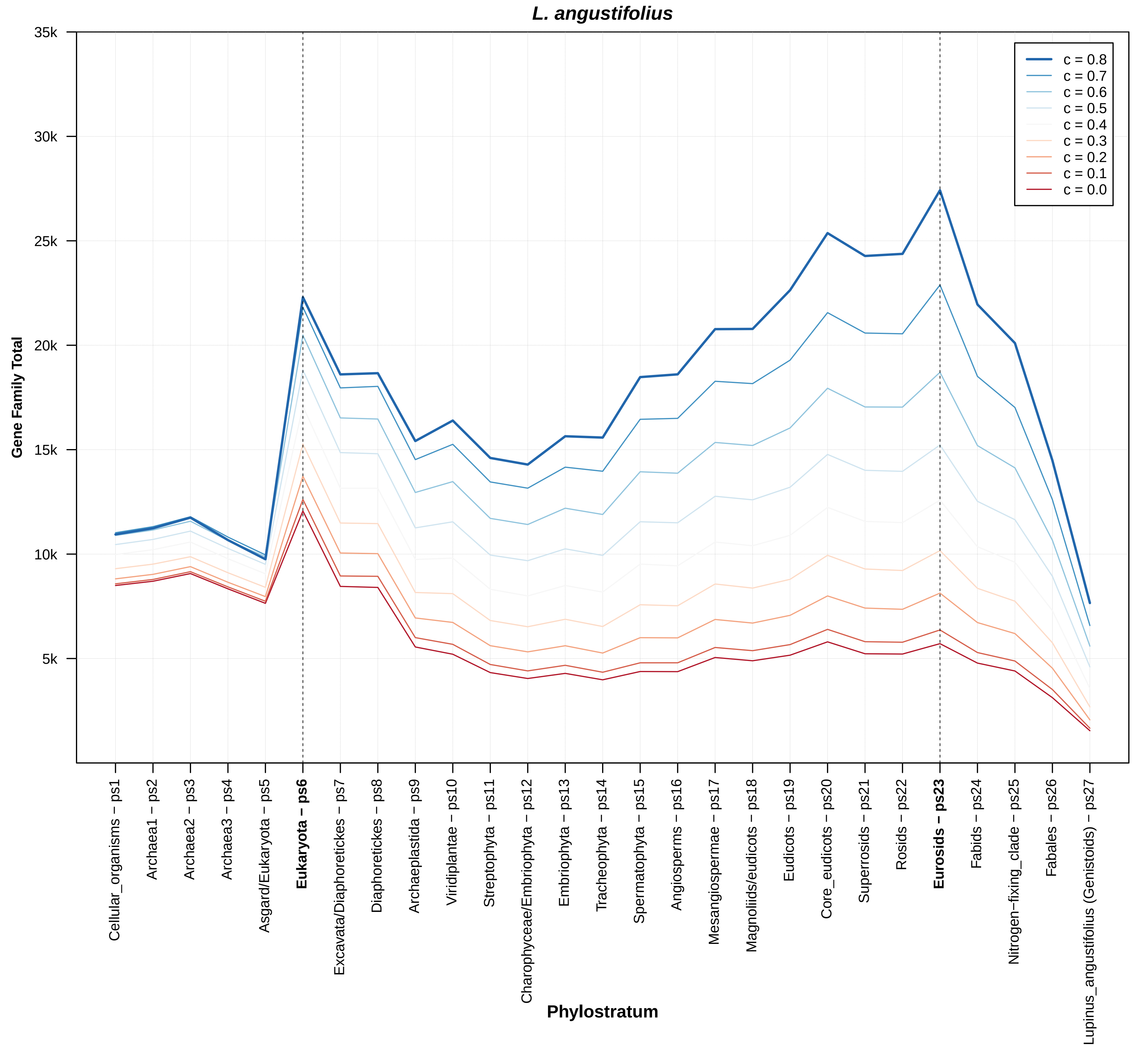

L. angustifolius
35k
30k
25k
20k
15k
10k
5k
Gene Family Total
c = 0.8
c = 0.7
c = 0.6
c = 0.5
c = 0.4
c = 0.3
c = 0.2
c = 0.1
c = 0.0
Cellular_organisms – ps1
Archaea1 – ps2
Archaea2 – ps3
Archaea3 – ps4
Asgard/Eukaryota – ps5
Eukaryota – ps6
Excavata/Diaphoretickes – ps7
Diaphoretickes – ps8
Archaeplastida – ps9
Viridiplantae – ps10
Streptophyta – ps11
Charophyceae/Embriophyta – ps12
Embriophyta – ps13
Tracheophyta – ps14
Spermatophyta – ps15
Angiosperms – ps16
Mesangiospermae – ps17
Magnoliids/eudicots – ps18
Eudicots – ps19
Core_eudicots – ps20
Superrosids – ps21
Rosids – ps22
Eurosids – ps23
Fabids – ps24
Nitrogen–fixing_clade – ps25
Fabales – ps26
Lupinus_angustifolius (Genistoids) – ps27
Phylostratum

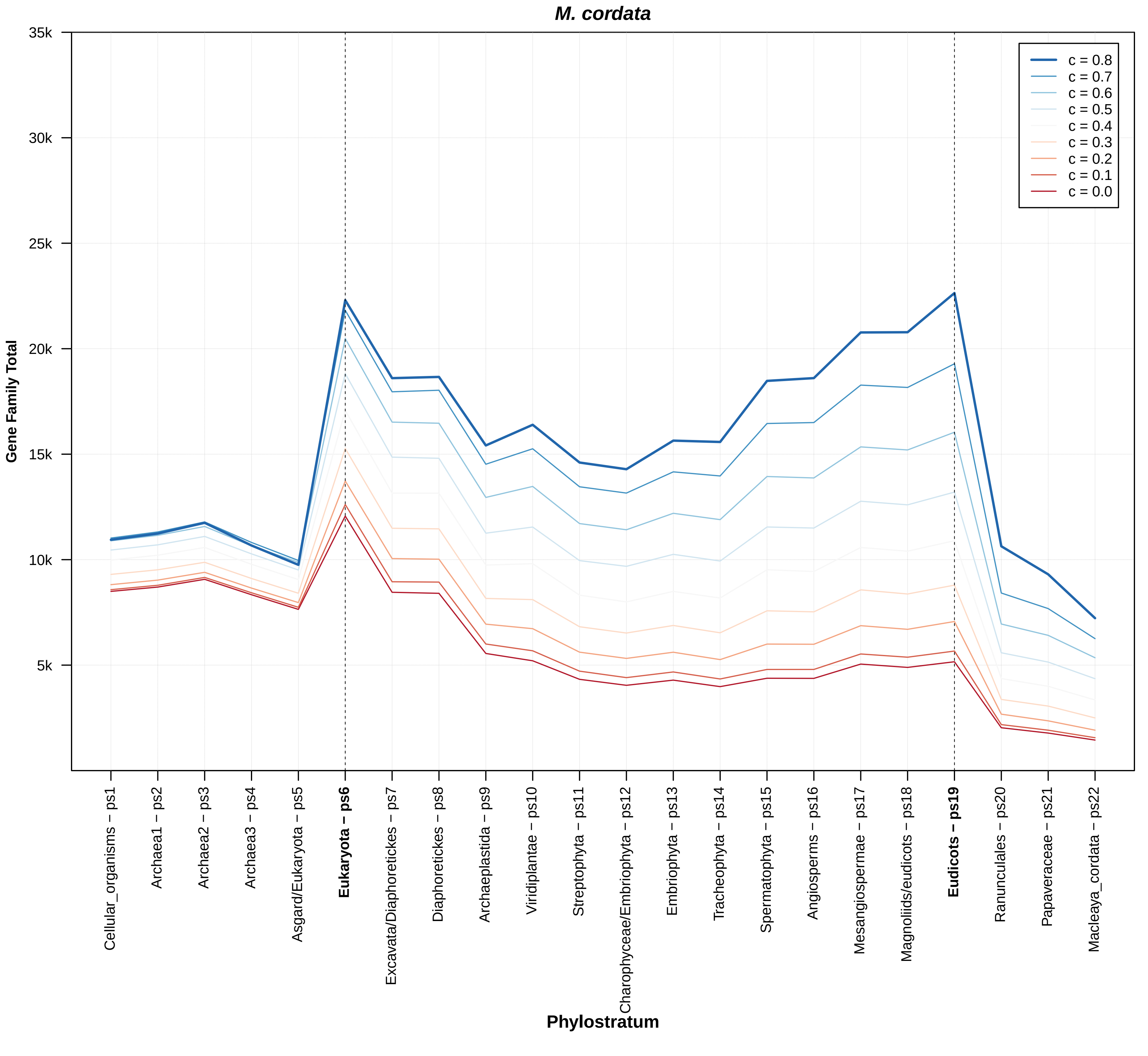
M. cordata
Gene Family Total
35k
30k
25k
20k
15k
10k
5k
c = 0.8
c = 0.7
c = 0.6
c = 0.5
c = 0.4
c = 0.3
c = 0.2
c = 0.1
c = 0.0
Cellular_organisms – ps1
Archaea1 – ps2
Archaea2 – ps3
Archaea3 – ps4
Asgard/Eukaryota – ps5
Eukaryota – ps6
Excavata/Diaphoretickes – ps7
Diaphoretickes – ps8
Archaeplastida – ps9
Viridiplantae – ps10
Streptophyta – ps11
Charophyceae/Embriophyta – ps12
Embriophyta – ps13
Tracheophyta – ps14
Spermatophyta – ps15
Angiosperms – ps16
Mesangiospermae – ps17
Magnoliids/eudicots – ps18
Eudicots – ps19
Ranunculales – ps20
Papaveraceae – ps21
Macleaya_cordata – ps22
Phylostratum

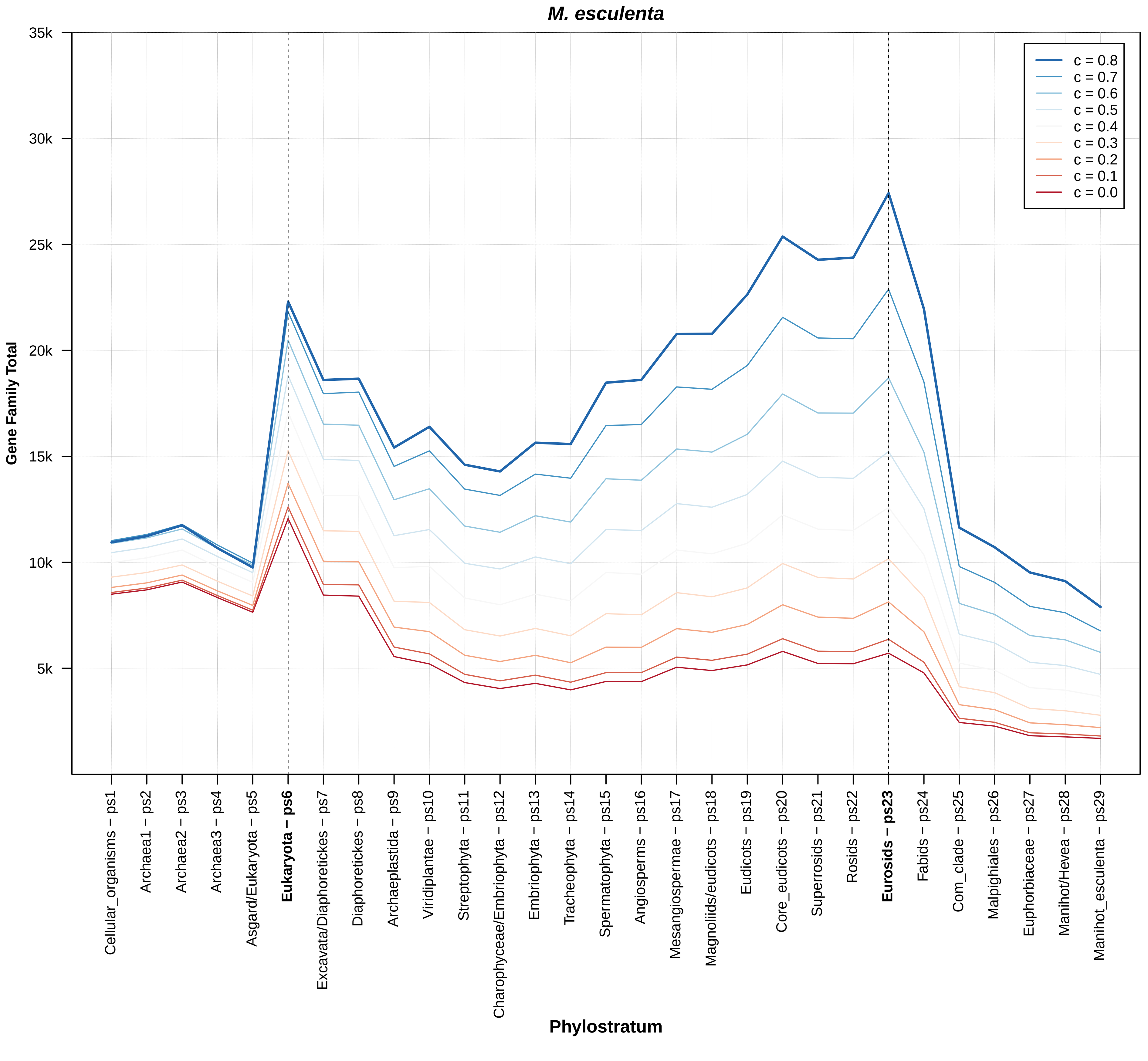

M. esculenta
Gene Family Total
35k
30k
25k
20k
15k
10k
5k
c = 0.8
c = 0.7
c = 0.6
c = 0.5
c = 0.4
c = 0.3
c = 0.2
c = 0.1
c = 0.0
Cellular_organisms – ps1
Archaea1 – ps2
Archaea2 – ps3
Archaea3 – ps4
Asgard/Eukaryota – ps5
Eukaryota – ps6
Excavata/Diaphoretickes – ps7
Diaphoretickes – ps8
Archaeplastida – ps9
Viridiplantae – ps10
Streptophyta – ps11
Charophyceae/Embriophyta – ps12
Embriophyta – ps13
Tracheophyta – ps14
Spermatophyta – ps15
Angiosperms – ps16
Mesangiospermae – ps17
Magnoliids/eudicots – ps18
Eudicots – ps19
Core_eudicots – ps20
Superrosids – ps21
Rosids – ps22
Eurosids – ps23
Fabids – ps24
Com_clade – ps25
Malpighiales – ps26
Euphorbiaceae – ps27
Manihot/Hevea – ps28
Manihot_esculenta – ps29
Phylostratum

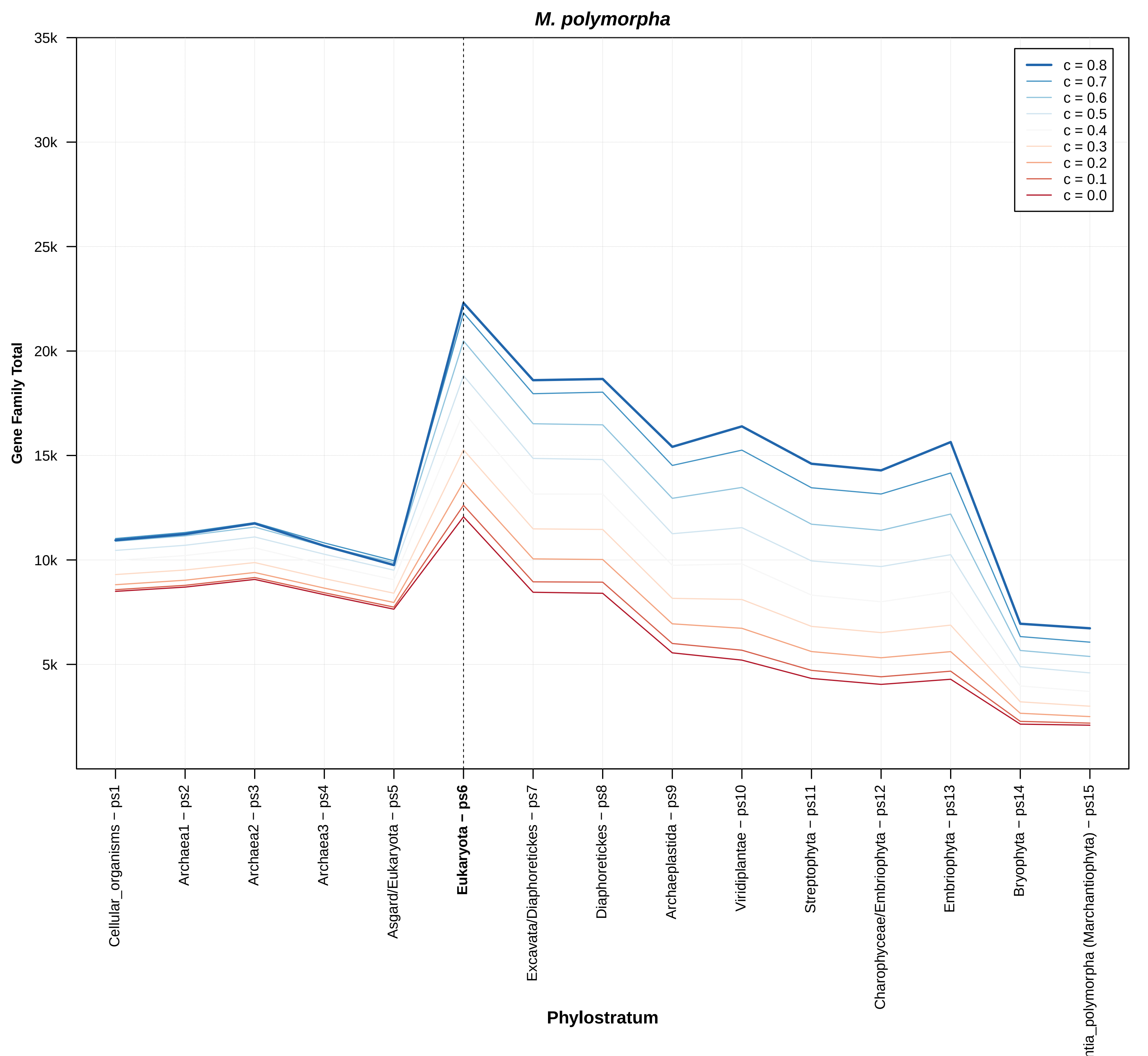

M. polymorpha
35k
30k
25k
20k
15k
10k
5k
Gene Family Total
c = 0.8
c = 0.7
c = 0.6
c = 0.5
c = 0.4
c = 0.3
c = 0.2
c = 0.1
c = 0.0
Cellular_organisms – ps1
Archaea1 – ps2
Archaea2 – ps3
Archaea3 – ps4
Asgard/Eukaryota – ps5
Eukaryota – ps6
Excavata/Diaphoretickes – ps7
Diaphoretickes – ps8
Archaeplastida – ps9
Viridiplantae – ps10
Streptophyta – ps11
Charophyceae/Embriophyta – ps12
Embriophyta – ps13
Bryophyta – ps14
ntia_polymorpha (Marchantiophyta) – ps15
Phylostratum

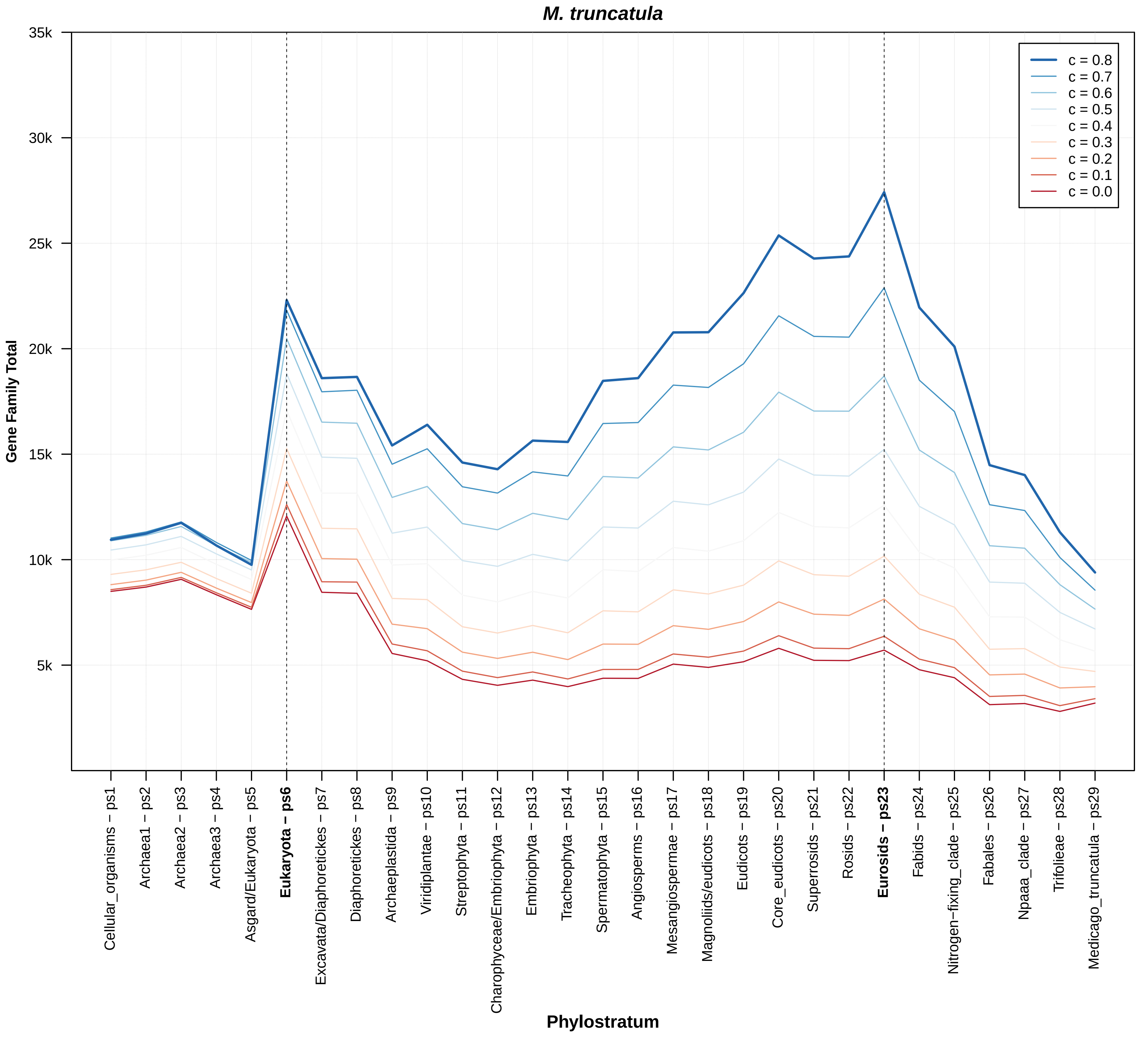

M. truncatula
Gene Family Total
35k
30k
25k
20k
15k
10k
5k
c = 0.8
c = 0.7
c = 0.6
c = 0.5
c = 0.4
c = 0.3
c = 0.2
c = 0.1
c = 0.0
Cellular_organisms – ps1
Archaea1 – ps2
Archaea2 – ps3
Archaea3 – ps4
Asgard/Eukaryota – ps5
Eukaryota – ps6
Excavata/Diaphoretickes – ps7
Diaphoretickes – ps8
Archaeplastida – ps9
Viridiplantae – ps10
Streptophyta – ps11
Charophyceae/Embriophyta – ps12
Embriophyta – ps13
Tracheophyta – ps14
Spermatophyta – ps15
Angiosperms – ps16
Mesangiospermae – ps17
Magnoliids/eudicots – ps18
Eudicots – ps19
Core_eudicots – ps20
Superrosids – ps21
Rosids – ps22
Eurosids – ps23
Fabids – ps24
Nitrogen–fixing_clade – ps25
Fabales – ps26
Npaaa_clade – ps27
Trifolieae – ps28
Medicago_truncatula – ps29
Phylostratum

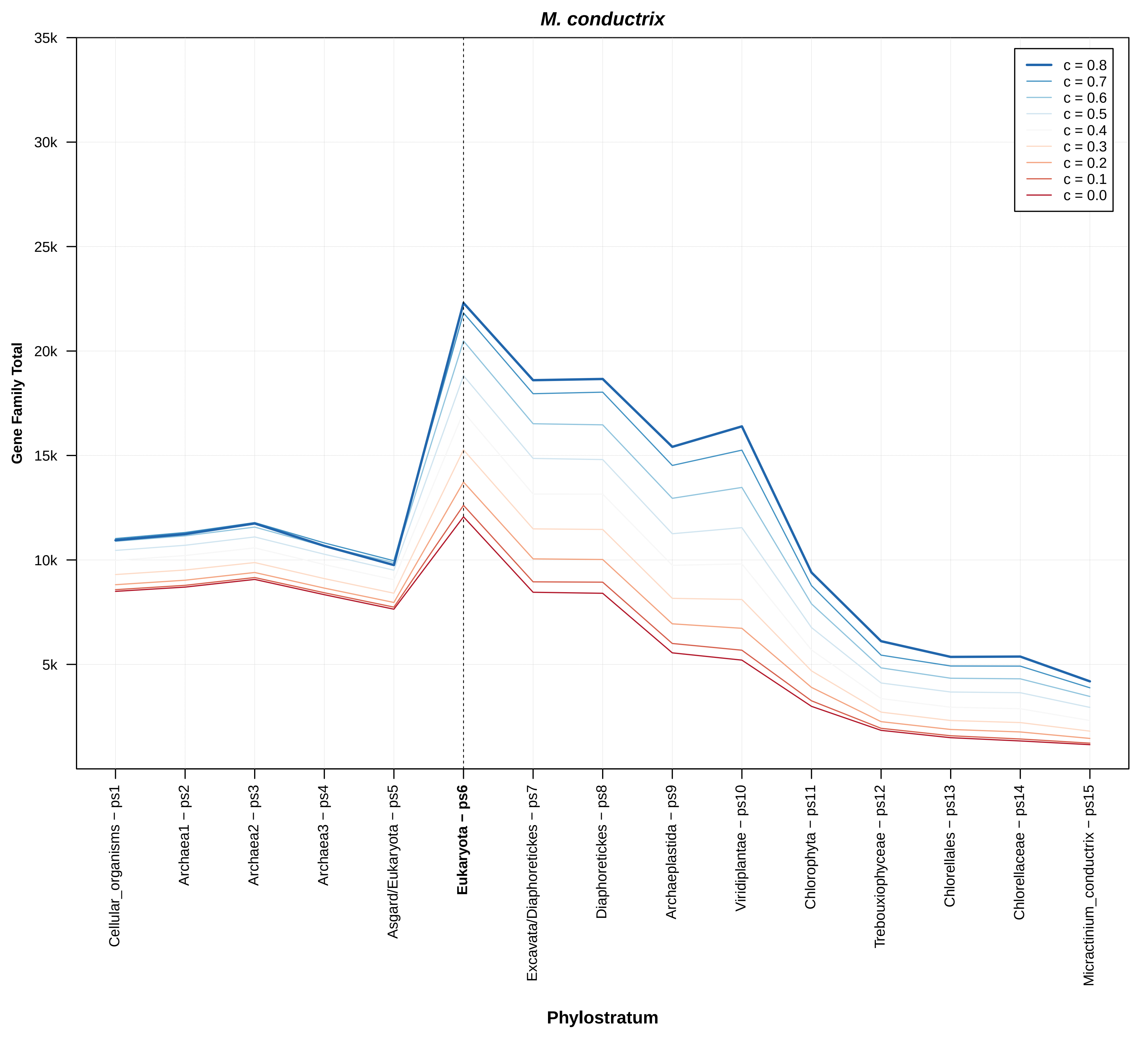
M. conductrix
Gene Family Total
35k
30k
25k
20k
15k
10k
5k
c = 0.8
c = 0.7
c = 0.6
c = 0.5
c = 0.4
c = 0.3
c = 0.2
c = 0.1
c = 0.0
Cellular_organisms – ps1
Archaea1 – ps2
Archaea2 – ps3
Archaea3 – ps4
Asgard/Eukaryota – ps5
Eukaryota – ps6
Excavata/Diaphoretickes – ps7
Diaphoretickes – ps8
Archaeplastida – ps9
Viridiplantae – ps10
Chlorophyta – ps11
Trebouxiophyceae – ps12
Chlorellales – ps13
Chlorellaceae – ps14
Micractinium_conductrix – ps15
Phylostratum

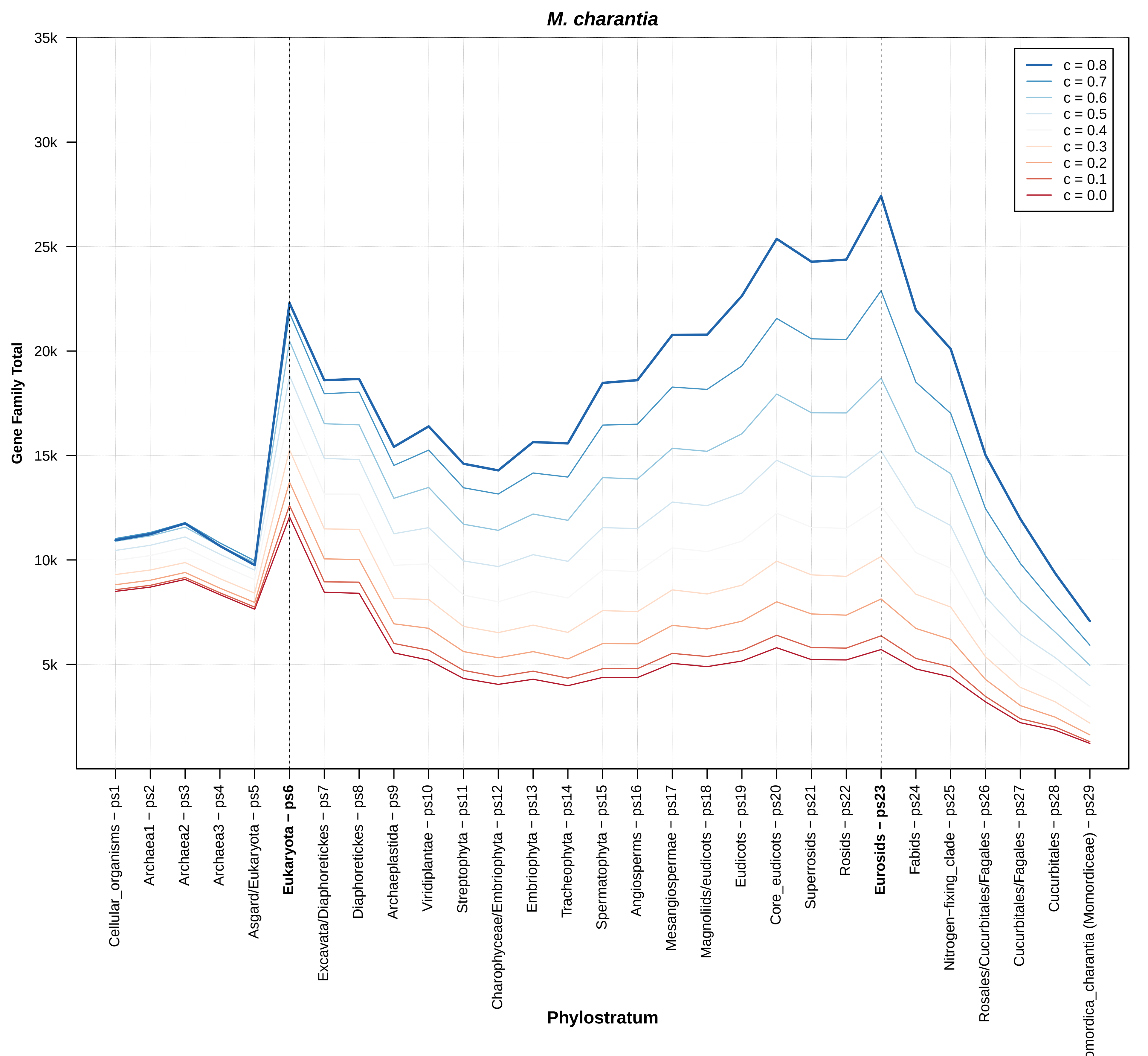
M. charantia
Gene Family Total
35k
30k
25k
20k
15k
10k
5k
c = 0.8
c = 0.7
c = 0.6
c = 0.5
c = 0.4
c = 0.3
c = 0.2
c = 0.1
c = 0.0
Cellular_organisms – ps1
Archaea1 – ps2
Archaea2 – ps3
Archaea3 – ps4
Asgard/Eukaryota – ps5
Eukaryota – ps6
Excavata/Diaphoretickes – ps7
Diaphoretickes – ps8
Archaeplastida – ps9
Viridiplantae – ps10
Streptophyta – ps11
Charophyceae/Embriophyta – ps12
Embriophyta – ps13
Tracheophyta – ps14
Spermatophyta – ps15
Angiosperms – ps16
Mesangiospermae – ps17
Magnoliids/eudicots – ps18
Eudicots – ps19
Core_eudicots – ps20
Superrosids – ps21
Rosids – ps22
Eurosids – ps23
Fabids – ps24
Nitrogen–fixing_clade – ps25
Rosales/Cucurbitales/Fagales – ps26
Cucurbitales/Fagales – ps27
Cucurbitales – ps28
omordica_charantia (Momordiceae) – ps29
Phylostratum

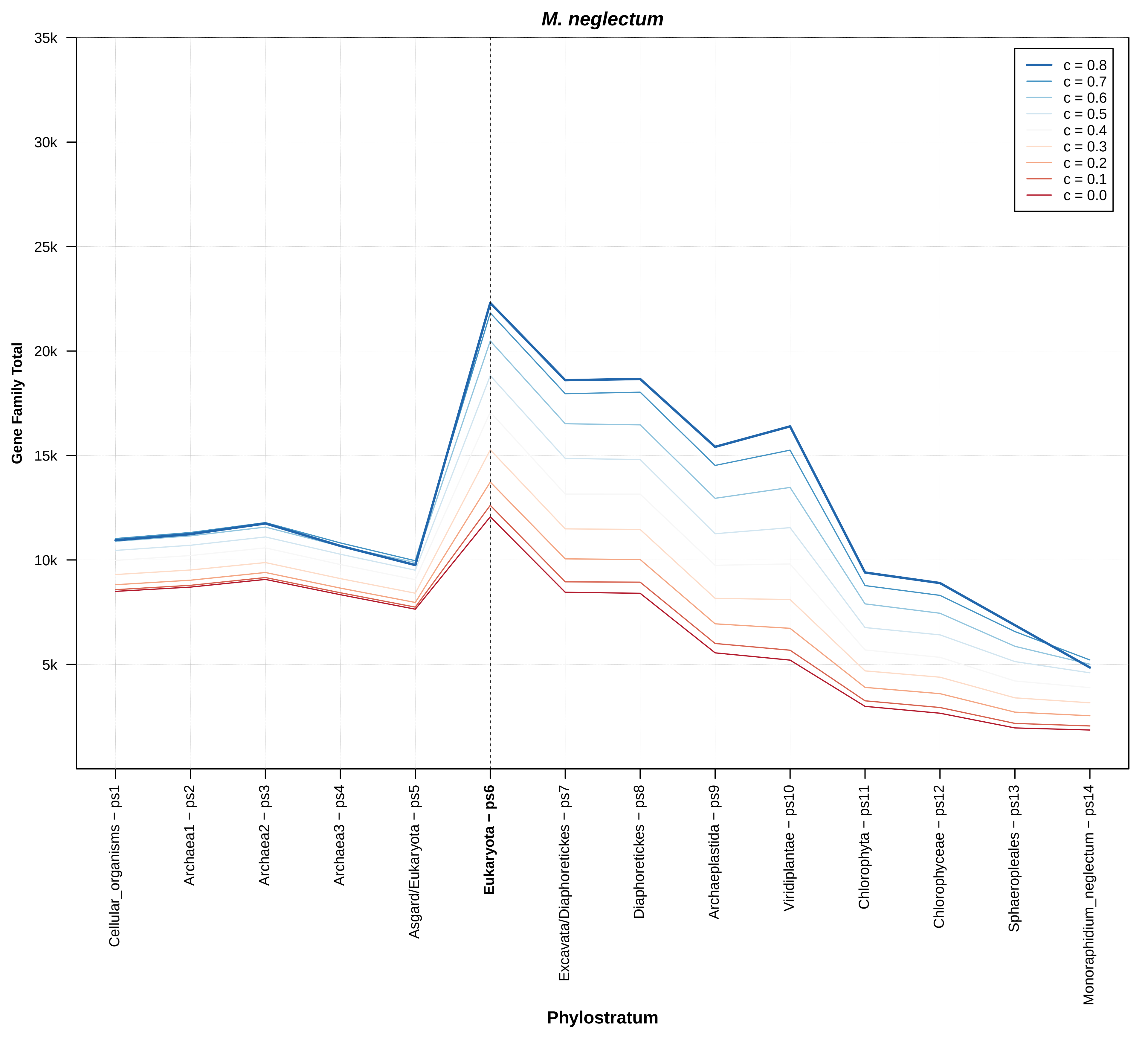

M. neglectum
Gene Family Total
35k
30k
25k
20k
15k
10k
5k
c = 0.8
c = 0.7
c = 0.6
c = 0.5
c = 0.4
c = 0.3
c = 0.2
c = 0.1
c = 0.0
Cellular_organisms – ps1
Archaea1 – ps2
Archaea2 – ps3
Archaea3 – ps4
Asgard/Eukaryota – ps5
Eukaryota – ps6
Excavata/Diaphoretickes – ps7
Diaphoretickes – ps8
Archaeplastida – ps9
Viridiplantae – ps10
Chlorophyta – ps11
Chlorophyceae – ps12
Sphaeropleales – ps13
Monoraphidium_neglectum – ps14
Phylostratum

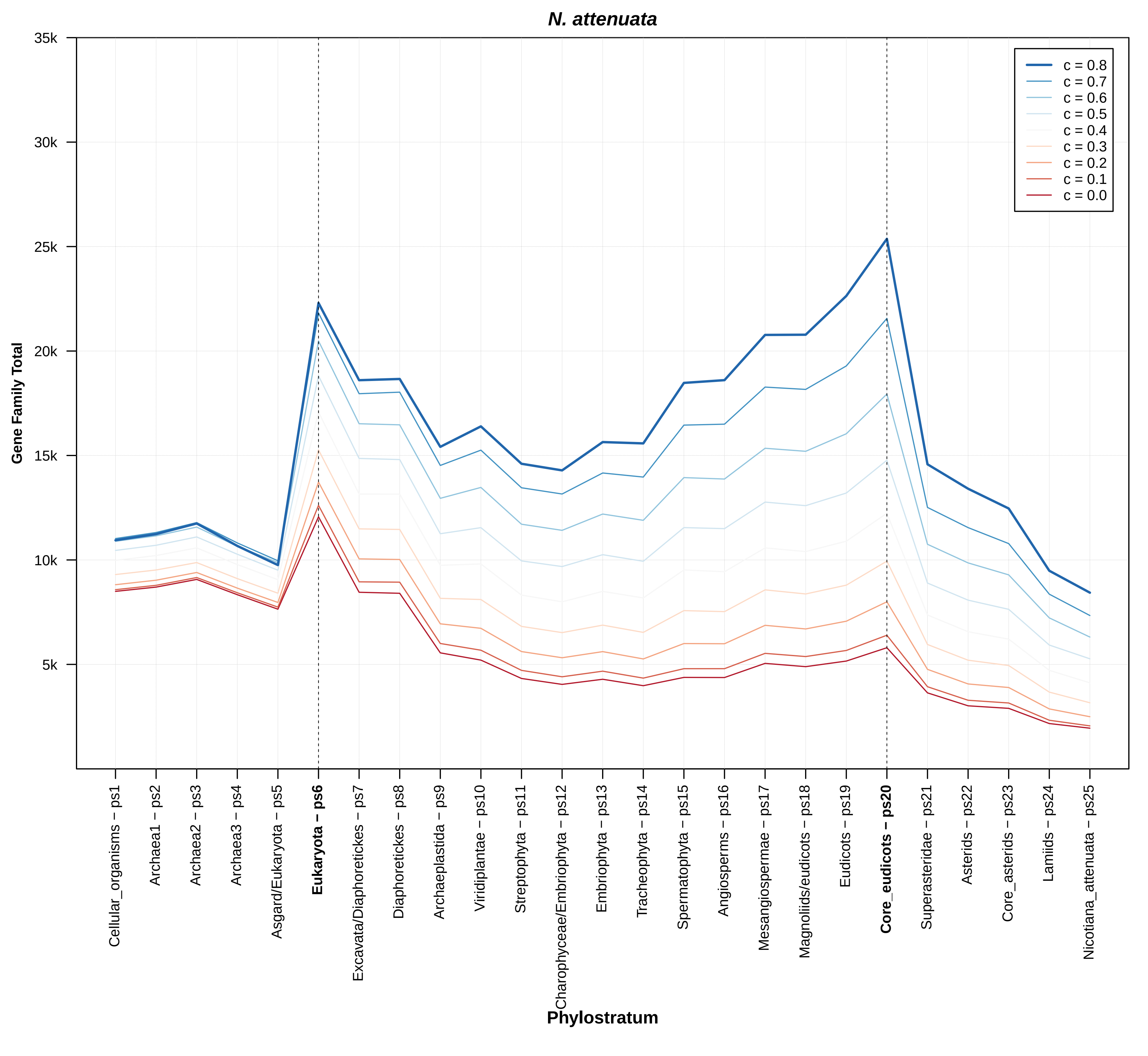

N. attenuata
Gene Family Total
35k
30k
25k
20k
15k
10k
5k
c = 0.8
c = 0.7
c = 0.6
c = 0.5
c = 0.4
c = 0.3
c = 0.2
c = 0.1
c = 0.0
Cellular_organisms – ps1
Archaea1 – ps2
Archaea2 – ps3
Archaea3 – ps4
Asgard/Eukaryota – ps5
Eukaryota – ps6
Excavata/Diaphoretickes – ps7
Diaphoretickes – ps8
Archaeplastida – ps9
Viridiplantae – ps10
Streptophyta – ps11
Charophyceae/Embriophyta – ps12
Embriophyta – ps13
Tracheophyta – ps14
Spermatophyta – ps15
Angiosperms – ps16
Mesangiospermae – ps17
Magnoliids/eudicots – ps18
Eudicots – ps19
Core_eudicots – ps20
Superasteridae – ps21
Asterids – ps22
Core_asterids – ps23
Lamiids – ps24
Nicotiana_attenuata – ps25
Phylostratum

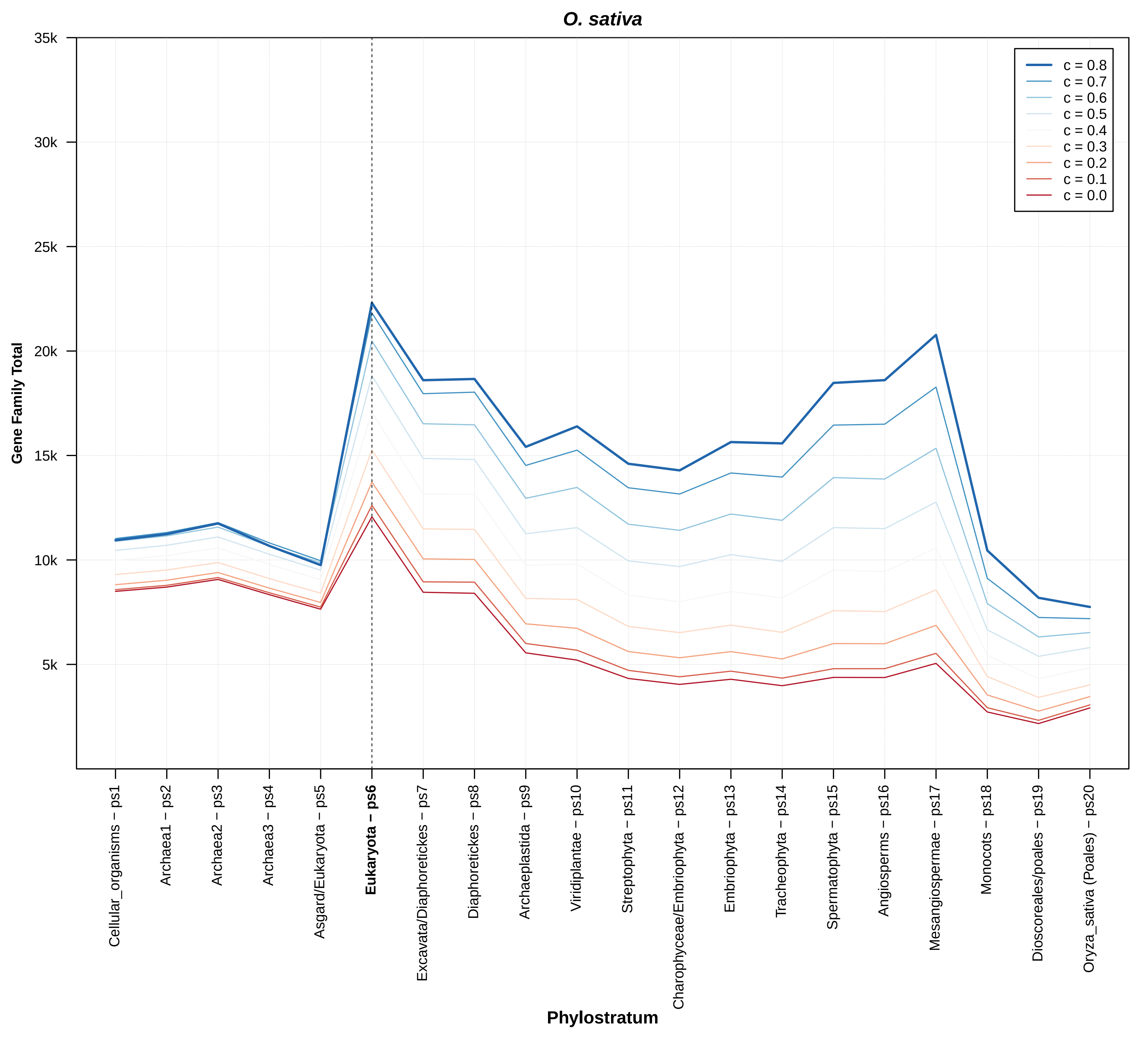
O. sativa
35k
30k
25k
20k
15k
10k
5k
Gene Family Total
c = 0.8
c = 0.7
c = 0.6
c = 0.5
c = 0.4
c = 0.3
c = 0.2
c = 0.1
c = 0.0
Cellular_organisms – ps1
Archaea1 – ps2
Archaea2 – ps3
Archaea3 – ps4
Asgard/Eukaryota – ps5
Eukaryota – ps6
Excavata/Diaphoretickes – ps7
Diaphoretickes – ps8
Archaeplastida – ps9
Viridiplantae – ps10
Streptophyta – ps11
Charophyceae/Embriophyta – ps12
Embriophyta – ps13
Tracheophyta – ps14
Spermatophyta – ps15
Angiosperms – ps16
Mesangiospermae – ps17
Monocots – ps18
Dioscoreales/poales – ps19
Oryza_sativa (Poales) – ps20
Phylostratum

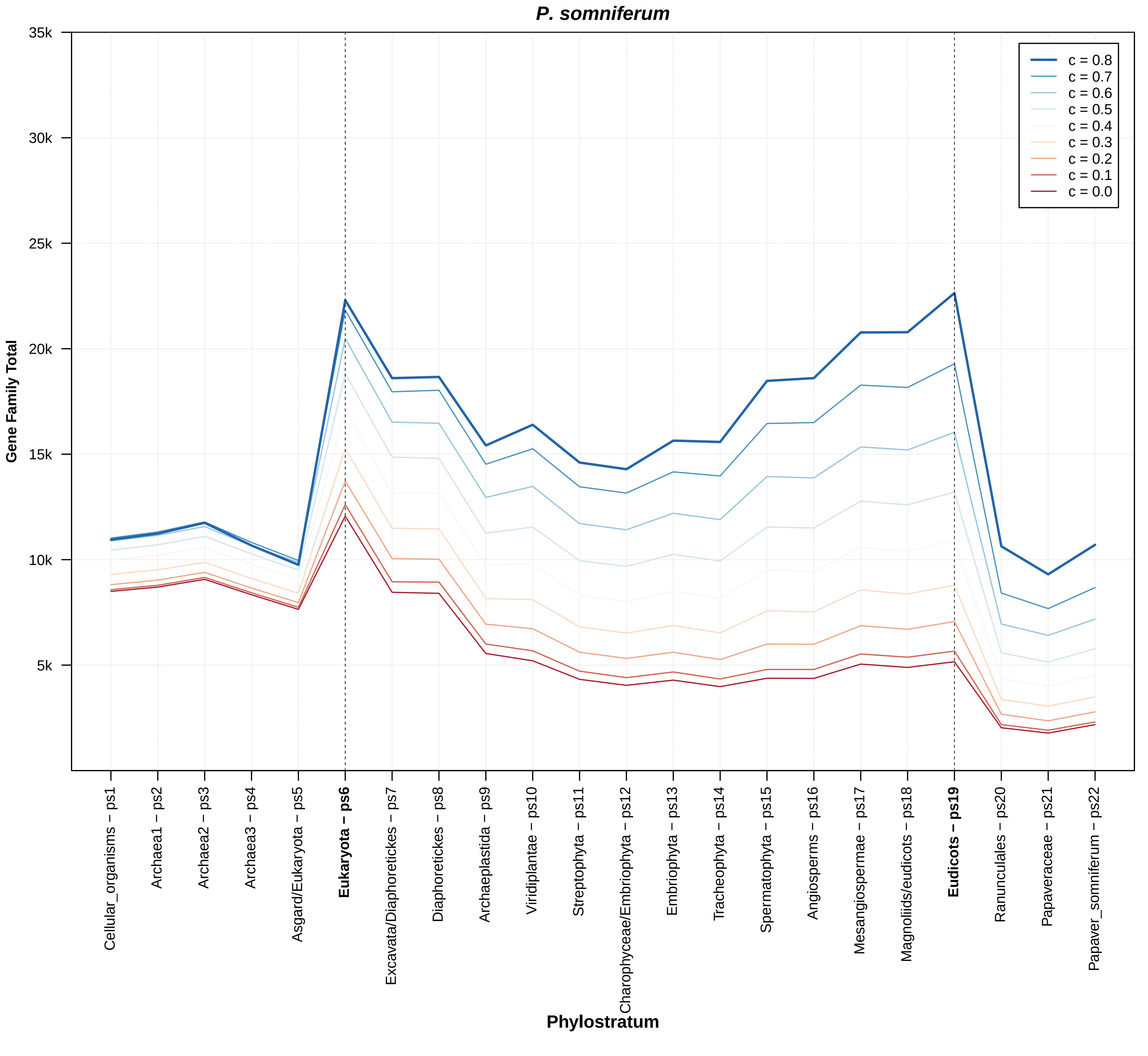

P. somniferum
Gene Family Total
35k
30k
25k
20k
15k
10k
5k
c = 0.8
c = 0.7
c = 0.6
c = 0.5
c = 0.4
c = 0.3
c = 0.2
c = 0.1
c = 0.0
Cellular_organisms – ps1
Archaea1 – ps2
Archaea2 – ps3
Archaea3 – ps4
Asgard/Eukaryota – ps5
Eukaryota – ps6
Excavata/Diaphoretickes – ps7
Diaphoretickes – ps8
Archaeplastida – ps9
Viridiplantae – ps10
Streptophyta – ps11
Charophyceae/Embriophyta – ps12
Embriophyta – ps13
Tracheophyta – ps14
Spermatophyta – ps15
Angiosperms – ps16
Mesangiospermae – ps17
Magnoliids/eudicots – ps18
Eudicots – ps19
Ranunculales – ps20
Papaveraceae – ps21
Papaver_somniferum – ps22
Phylostratum

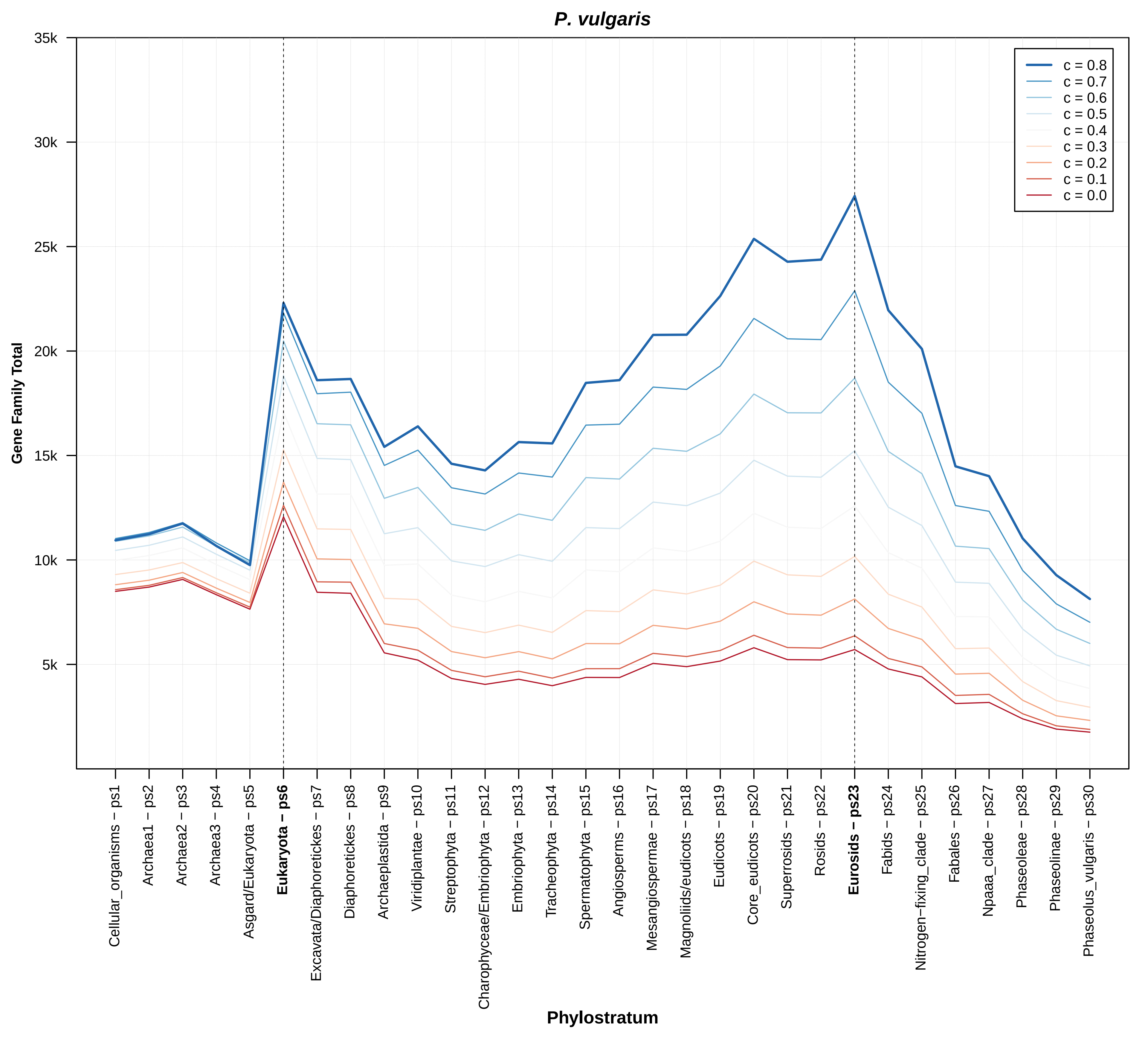
P. vulgaris
Gene Family Total
35k
30k
25k
20k
15k
10k
5k
c = 0.8
c = 0.7
c = 0.6
c = 0.5
c = 0.4
c = 0.3
c = 0.2
c = 0.1
c = 0.0
Cellular_organisms – ps1
Archaea1 – ps2
Archaea2 – ps3
Archaea3 – ps4
Asgard/Eukaryota – ps5
Eukaryota – ps6
Excavata/Diaphoretickes – ps7
Diaphoretickes – ps8
Archaeplastida – ps9
Viridiplantae – ps10
Streptophyta – ps11
Charophyceae/Embriophyta – ps12
Embriophyta – ps13
Tracheophyta – ps14
Spermatophyta – ps15
Angiosperms – ps16
Mesangiospermae – ps17
Magnoliids/eudicots – ps18
Eudicots – ps19
Core_eudicots – ps20
Superrosids – ps21
Rosids – ps22
Eurosids – ps23
Fabids – ps24
Nitrogen–fixing_clade – ps25
Fabales – ps26
Npaaa_clade – ps27
Phaseoleae – ps28
Phaseolinae – ps29
Phaseolus_vulgaris – ps30
Phylostratum

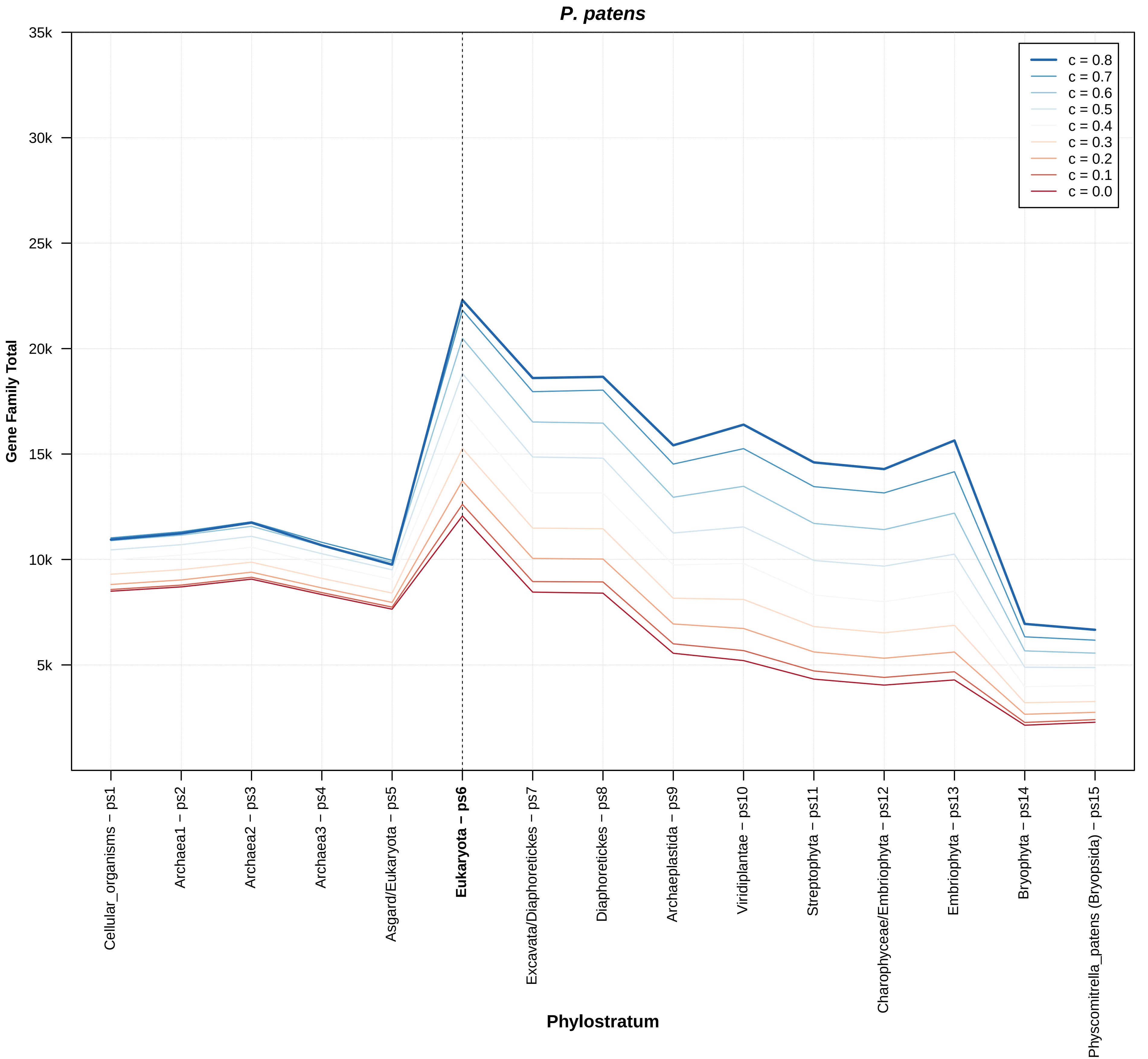
P. patens
35k
30k
25k
20k
15k
10k
5k
Gene Family Total
c = 0.8
c = 0.7
c = 0.6
c = 0.5
c = 0.4
c = 0.3
c = 0.2
c = 0.1
c = 0.0
Cellular_organisms – ps1
Archaea1 – ps2
Archaea2 – ps3
Archaea3 – ps4
Asgard/Eukaryota – ps5
Eukaryota – ps6
Excavata/Diaphoretickes – ps7
Diaphoretickes – ps8
Archaeplastida – ps9
Viridiplantae – ps10
Streptophyta – ps11
Charophyceae/Embriophyta – ps12
Embriophyta – ps13
Bryophyta – ps14
Physcomitrella_patens (Bryopsida) – ps15
Phylostratum

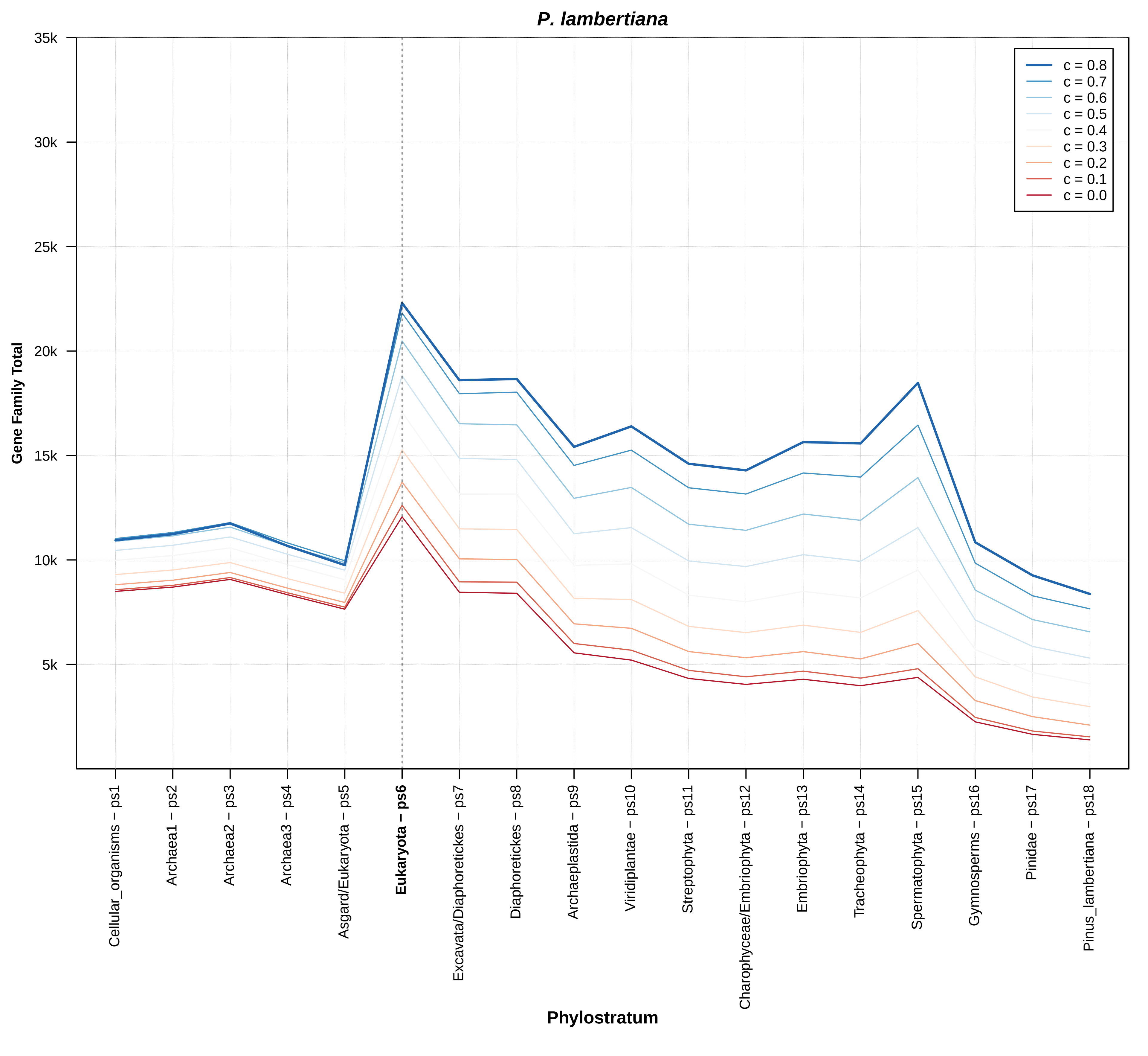

P. lambertiana
Gene Family Total
35k
30k
25k
20k
15k
10k
5k
c = 0.8
c = 0.7
c = 0.6
c = 0.5
c = 0.4
c = 0.3
c = 0.2
c = 0.1
c = 0.0
Cellular_organisms – ps1
Archaea1 – ps2
Archaea2 – ps3
Archaea3 – ps4
Asgard/Eukaryota – ps5
Eukaryota – ps6
Excavata/Diaphoretickes – ps7
Diaphoretickes – ps8
Archaeplastida – ps9
Viridiplantae – ps10
Streptophyta – ps11
Charophyceae/Embriophyta – ps12
Embriophyta – ps13
Tracheophyta – ps14
Spermatophyta – ps15
Gymnosperms – ps16
Pinidae – ps17
Pinus_lambertiana – ps18
Phylostratum

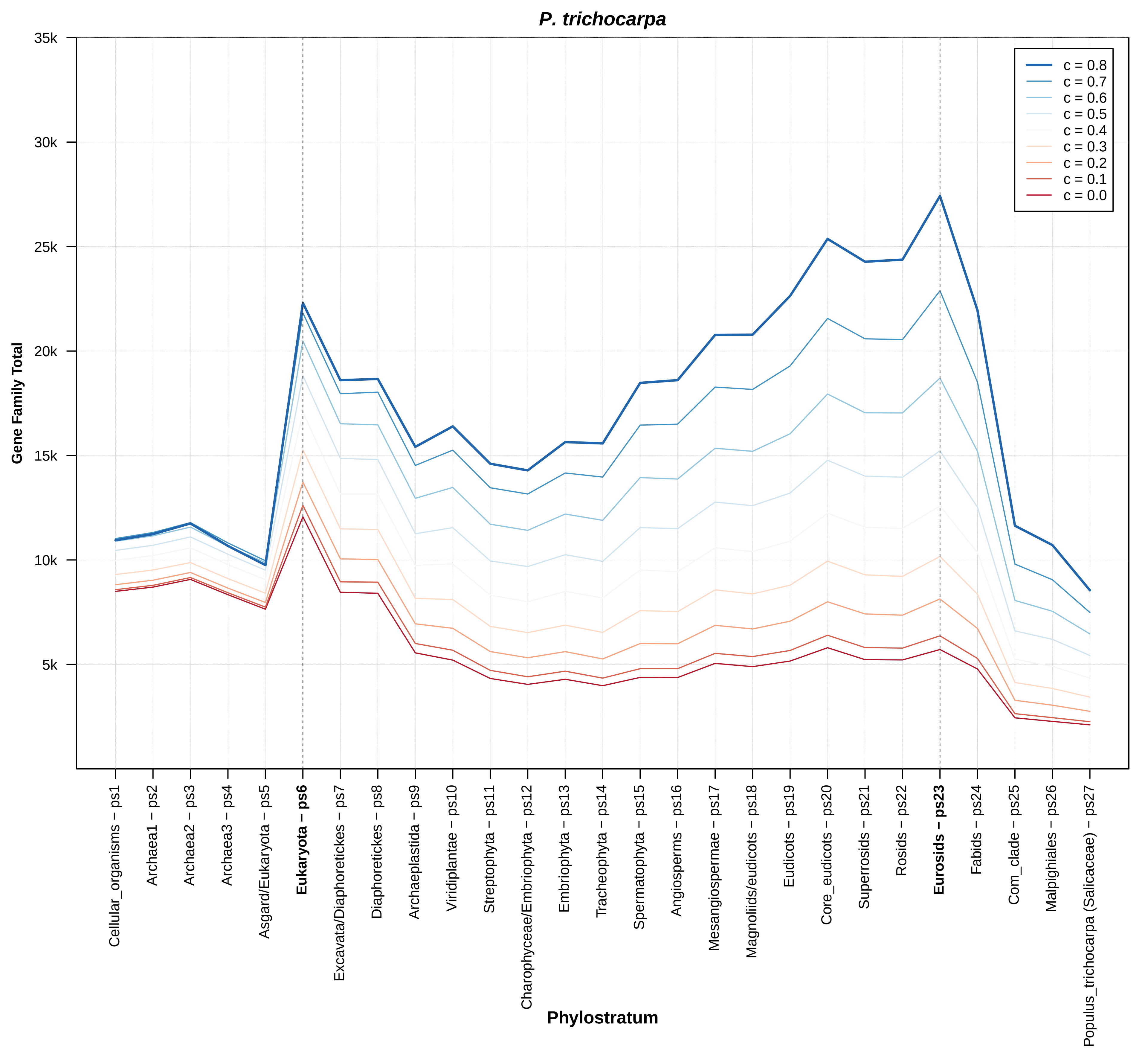
P. trichocarpa
Gene Family Total
Phylostratum
35k
30k
25k
20k
15k
10k
5k
c = 0.8
c = 0.7
c = 0.6
c = 0.5
c = 0.4
c = 0.3
c = 0.2
c = 0.1
c = 0.0
Cellular_organisms – ps1
Archaea1 – ps2
Archaea2 – ps3
Archaea3 – ps4
Asgard/Eukaryota – ps5
Eukaryota – ps6
Excavata/Diaphoretickes – ps7
Diaphoretickes – ps8
Archaeplastida – ps9
Viridiplantae – ps10
Streptophyta – ps11
Charophyceae/Embriophyta – ps12
Embriophyta – ps13
Tracheophyta – ps14
Spermatophyta – ps15
Angiosperms – ps16
Mesangiospermae – ps17
Magnoliids/eudicots – ps18
Eudicots – ps19
Core_eudicots – ps20
Superrosids – ps21
Rosids – ps22
Eurosids – ps23
Fabids – ps24
Com_clade – ps25
Malpighiales – ps26
Populus_trichocarpa (Salicaceae) – ps27

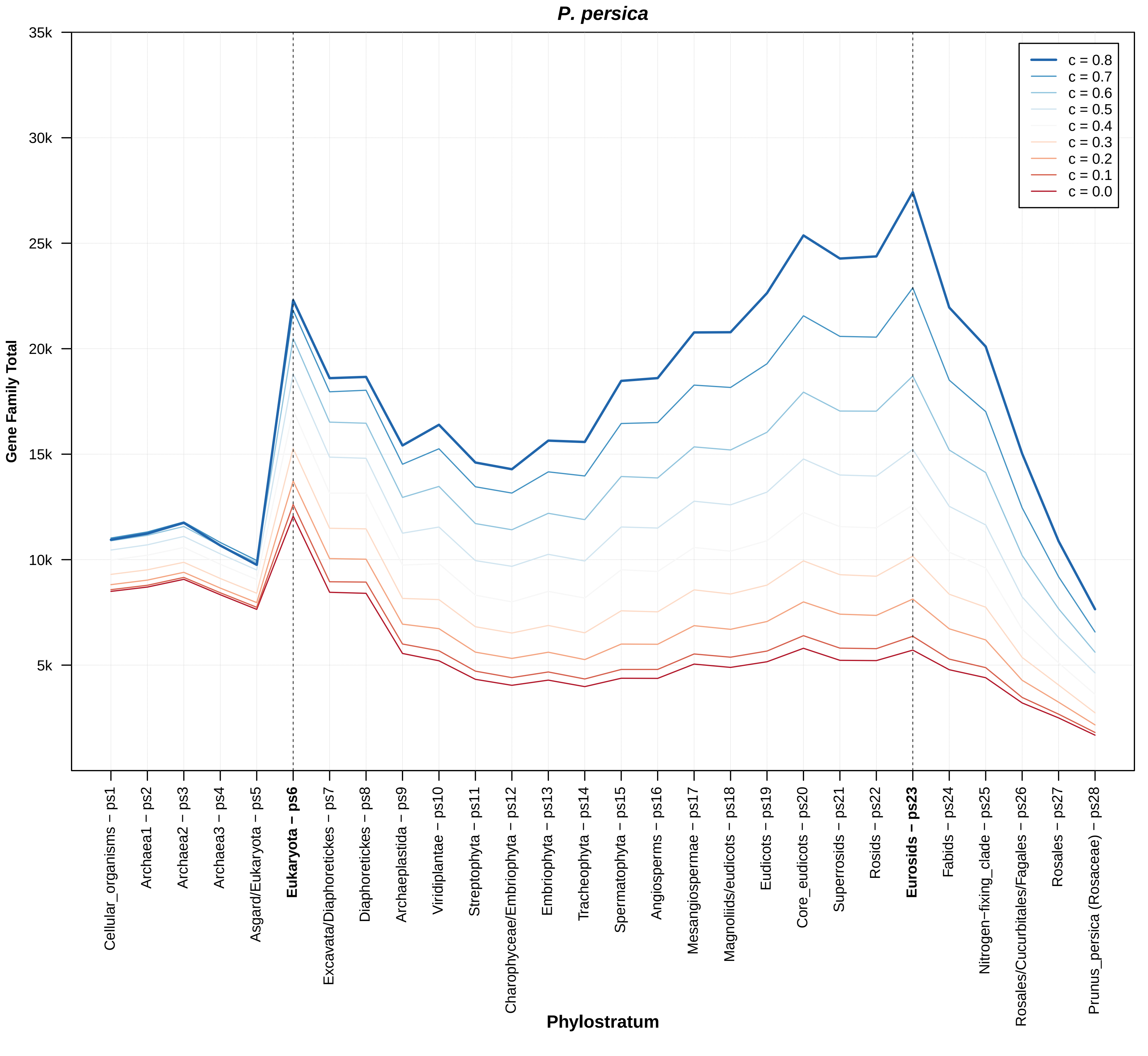
P. persica
Gene Family Total
35k
30k
25k
20k
15k
10k
5k
c = 0.8
c = 0.7
c = 0.6
c = 0.5
c = 0.4
c = 0.3
c = 0.2
c = 0.1
c = 0.0
Cellular_organisms – ps1
Archaea1 – ps2
Archaea2 – ps3
Archaea3 – ps4
Asgard/Eukaryota – ps5
Eukaryota – ps6
Excavata/Diaphoretickes – ps7
Diaphoretickes – ps8
Archaeplastida – ps9
Viridiplantae – ps10
Streptophyta – ps11
Charophyceae/Embriophyta – ps12
Embriophyta – ps13
Tracheophyta – ps14
Spermatophyta – ps15
Angiosperms – ps16
Mesangiospermae – ps17
Magnoliids/eudicots – ps18
Eudicots – ps19
Core_eudicots – ps20
Superrosids – ps21
Rosids – ps22
Eurosids – ps23
Fabids – ps24
Nitrogen−fixing_clade – ps25
Rosales/Cucurbitales/Fagales – ps26
Rosales – ps27
Prunus_persica (Rosaceae) – ps28
Phylostratum

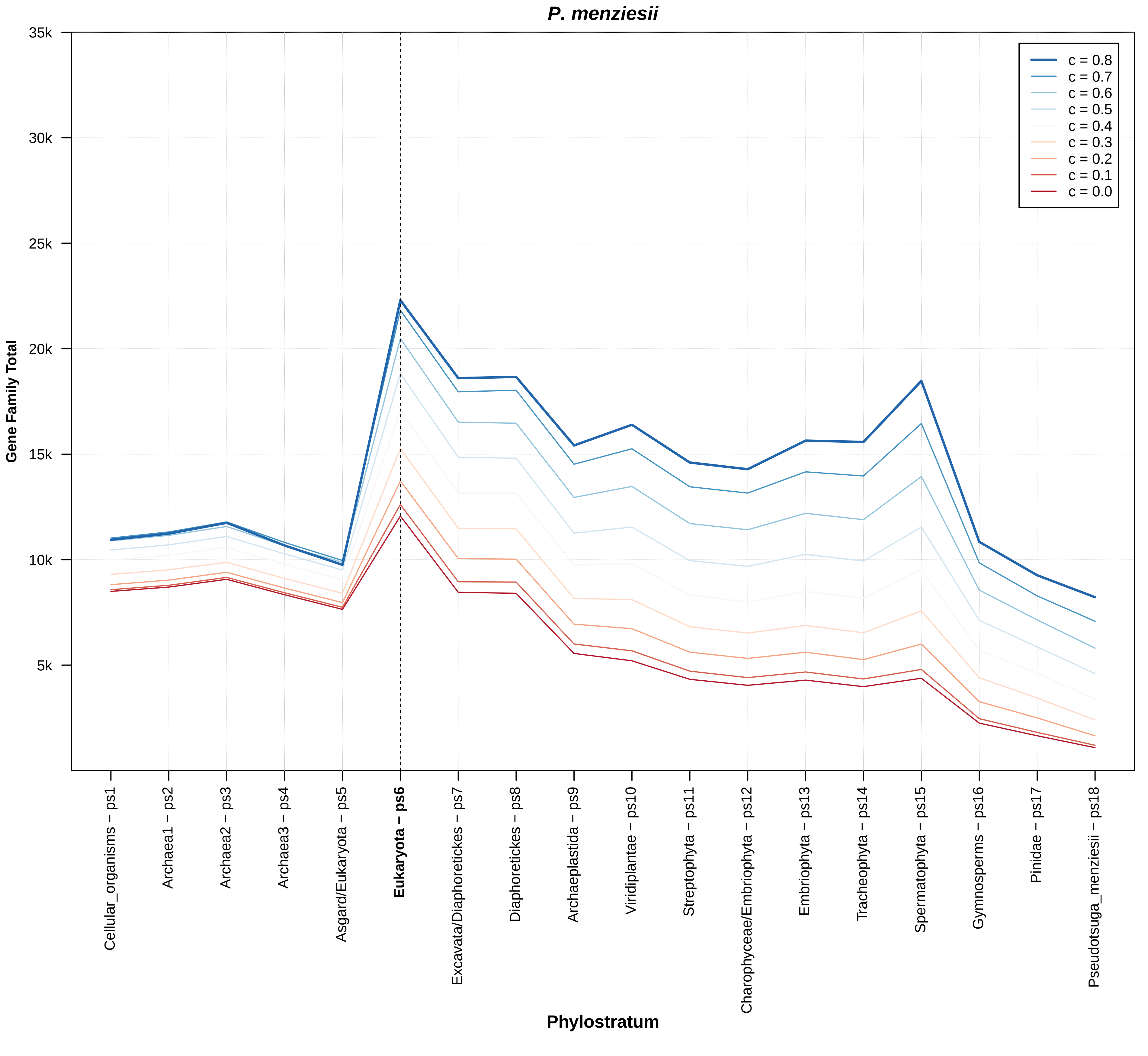

P. menziesii
Gene Family Total
35k
30k
25k
20k
15k
10k
5k
c = 0.8
c = 0.7
c = 0.6
c = 0.5
c = 0.4
c = 0.3
c = 0.2
c = 0.1
c = 0.0
Cellular_organisms – ps1
Archaea1 – ps2
Archaea2 – ps3
Archaea3 – ps4
Asgard/Eukaryota – ps5
Eukaryota – ps6
Excavata/Diaphoretickes – ps7
Diaphoretickes – ps8
Archaeplastida – ps9
Viridiplantae – ps10
Streptophyta – ps11
Charophyceae/Embriophyta – ps12
Embriophyta – ps13
Tracheophyta – ps14
Spermatophyta – ps15
Gymnosperms – ps16
Pinidae – ps17
Pseudotsuga_menziesii – ps18
Phylostratum

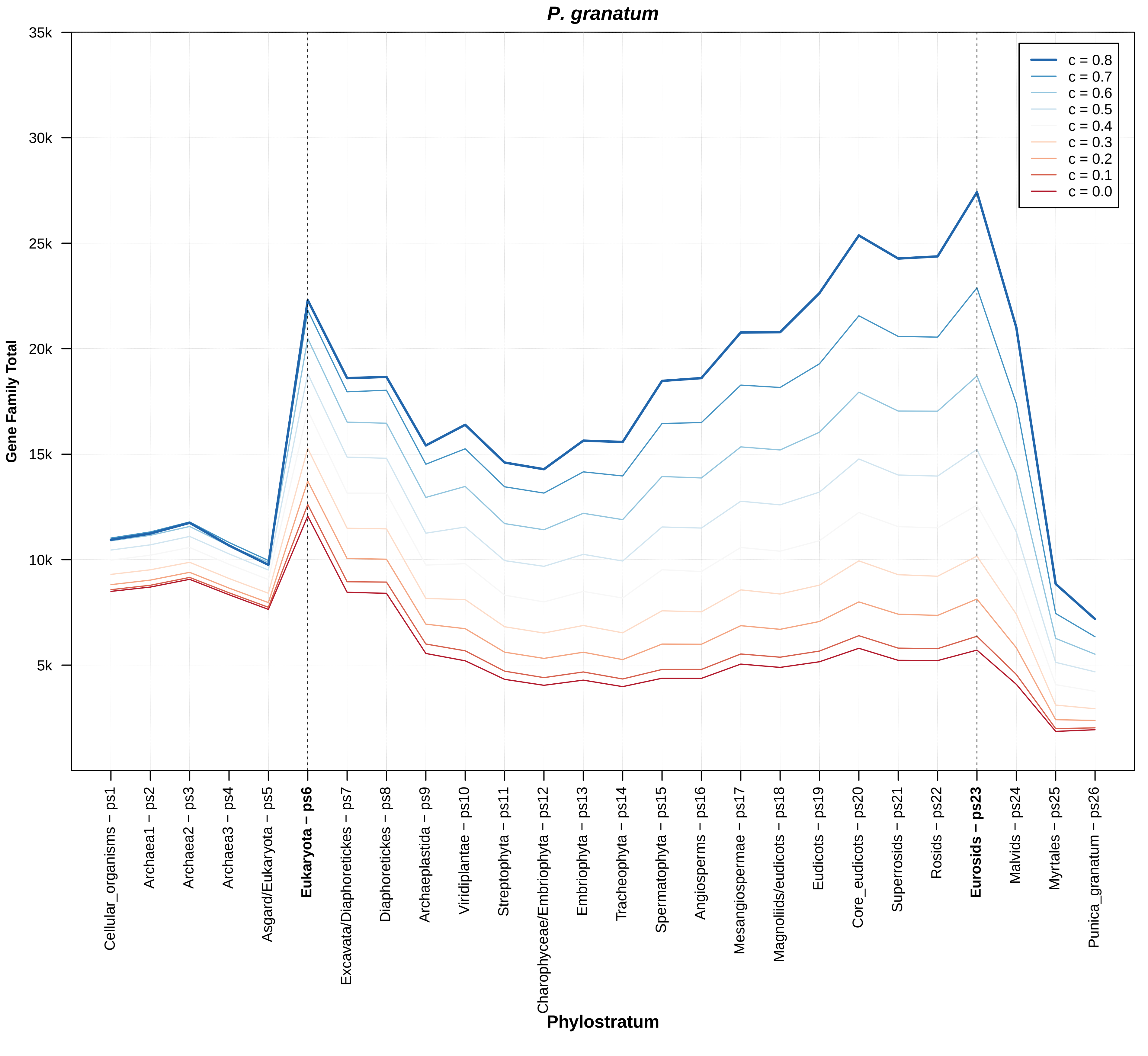
P. granatum
Gene Family Total
35k
30k
25k
20k
15k
10k
5k
c = 0.8
c = 0.7
c = 0.6
c = 0.5
c = 0.4
c = 0.3
c = 0.2
c = 0.1
c = 0.0
Cellular_organisms – ps1
Archaea1 – ps2
Archaea2 – ps3
Archaea3 – ps4
Asgard/Eukaryota – ps5
Eukaryota – ps6
Excavata/Diaphoretickes – ps7
Diaphoretickes – ps8
Archaeplastida – ps9
Viridiplantae – ps10
Streptophyta – ps11
Charophyceae/Embriophyta – ps12
Embriophyta – ps13
Tracheophyta – ps14
Spermatophyta – ps15
Angiosperms – ps16
Mesangiospermae – ps17
Magnoliids/eudicots – ps18
Eudicots – ps19
Core_eudicots – ps20
Superrosids – ps21
Rosids – ps22
Eurosids – ps23
Malvids – ps24
Myrtales – ps25
Punica_granatum – ps26
Phylostratum

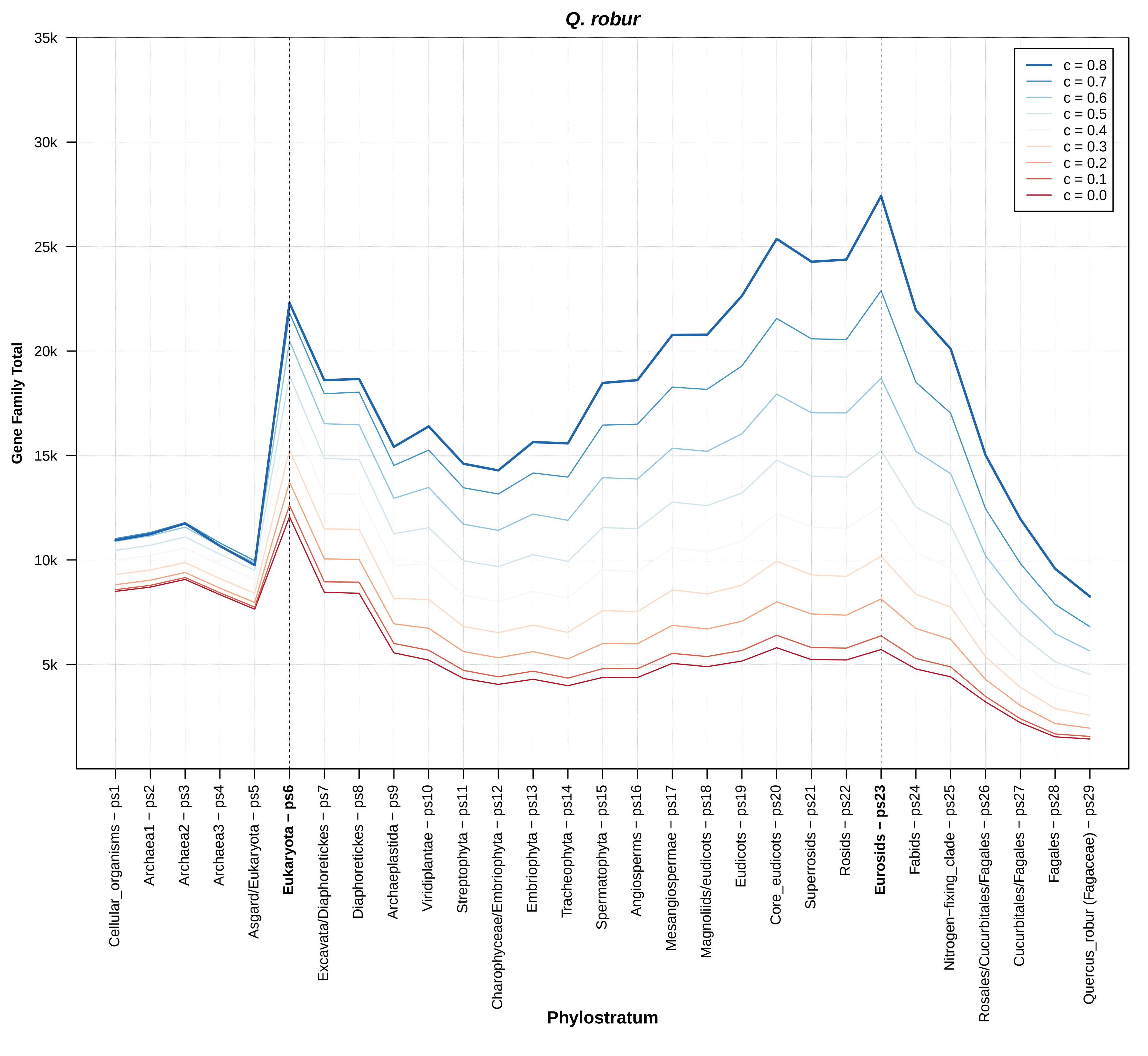

Q. robur
Gene Family Total
Phylostratum
35k
30k
25k
20k
15k
10k
5k
c = 0.8
c = 0.7
c = 0.6
c = 0.5
c = 0.4
c = 0.3
c = 0.2
c = 0.1
c = 0.0
Cellular_organisms – ps1
Archaea1 – ps2
Archaea2 – ps3
Archaea3 – ps4
Asgard/Eukaryota – ps5
Eukaryota – ps6
Excavata/Diaphoretickes – ps7
Diaphoretickes – ps8
Archaeplastida – ps9
Viridiplantae – ps10
Streptophyta – ps11
Charophyceae/Embriophyta – ps12
Embriophyta – ps13
Tracheophyta – ps14
Spermatophyta – ps15
Angiosperms – ps16
Mesangiospermae – ps17
Magnoliids/eudicots – ps18
Eudicots – ps19
Core_eudicots – ps20
Superrosids – ps21
Rosids – ps22
Eurosids – ps23
Fabids – ps24
Nitrogen–fixing_clade – ps25
Rosales/Cucurbitales/Fagales – ps26
Cucurbitales/Fagales – ps27
Fagales – ps28
Quercus_robur (Fagaceae) – ps29

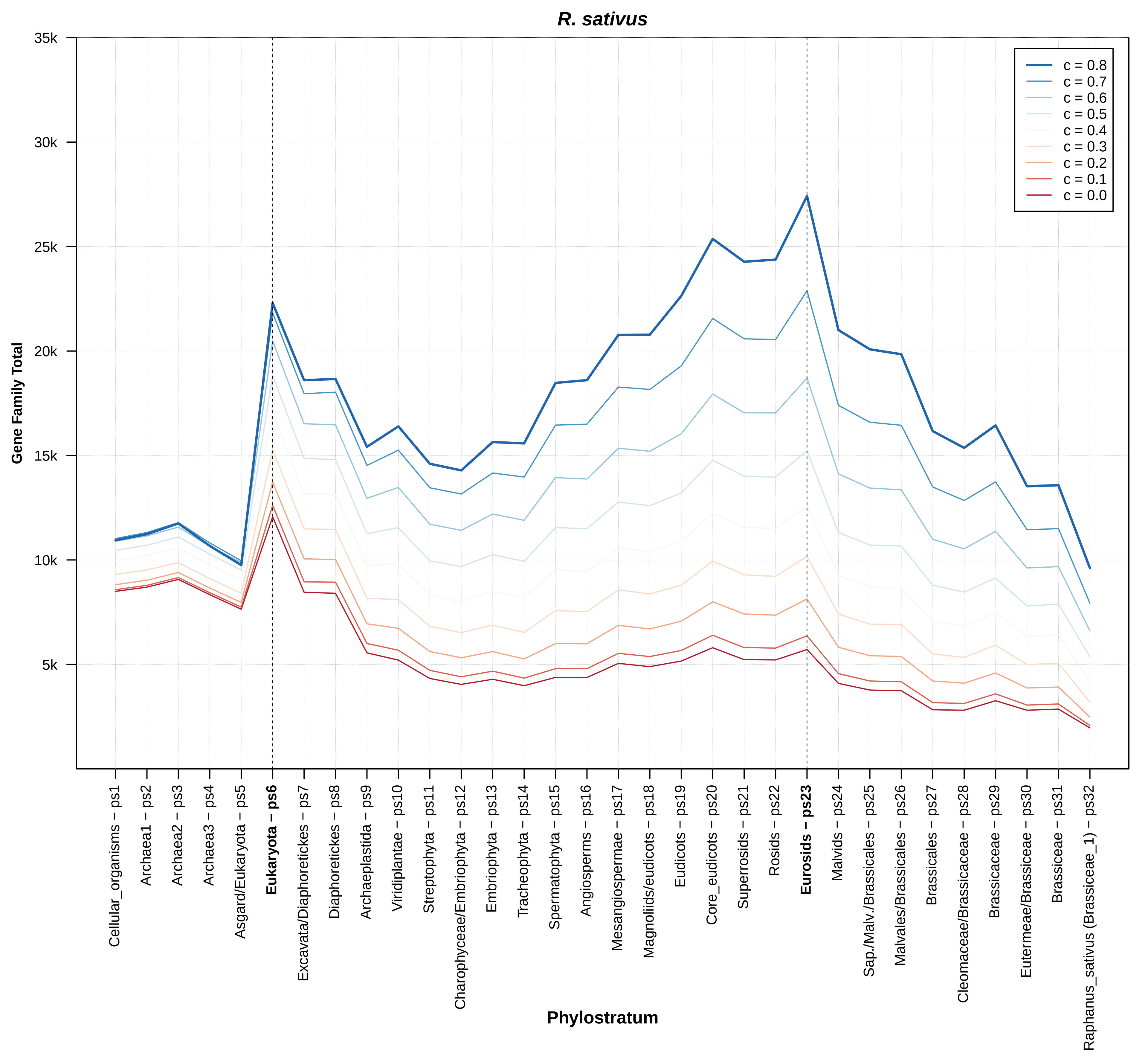

R. sativus
Gene Family Total
Phylostratum
35k
30k
25k
20k
15k
10k
5k
c = 0.8
c = 0.7
c = 0.6
c = 0.5
c = 0.4
c = 0.3
c = 0.2
c = 0.1
c = 0.0
Cellular_organisms – ps1
Archaea1 – ps2
Archaea2 – ps3
Archaea3 – ps4
Asgard/Eukaryota – ps5
Eukaryota – ps6
Excavata/Diaphoretickes – ps7
Diaphoretickes – ps8
Archaeplastida – ps9
Viridiplantae – ps10
Streptophyta – ps11
Charophyceae/Embriophyta – ps12
Embriophyta – ps13
Tracheophyta – ps14
Spermatophyta – ps15
Angiosperms – ps16
Mesangiospermae – ps17
Magnoliids/eudicots – ps18
Eudicots – ps19
Core_eudicots – ps20
Superrosids – ps21
Rosids – ps22
Eurosids – ps23
Malvids – ps24
Sap./Malv./Brassicales – ps25
Malvales/Brassicales – ps26
Brassicales – ps27
Cleomaceae/Brassicaceae – ps28
Brassicaceae – ps29
Eutermeae/Brassiceae – ps30
Brassiceae – ps31
Raphanus_sativus (Brassiceae_1) – ps32

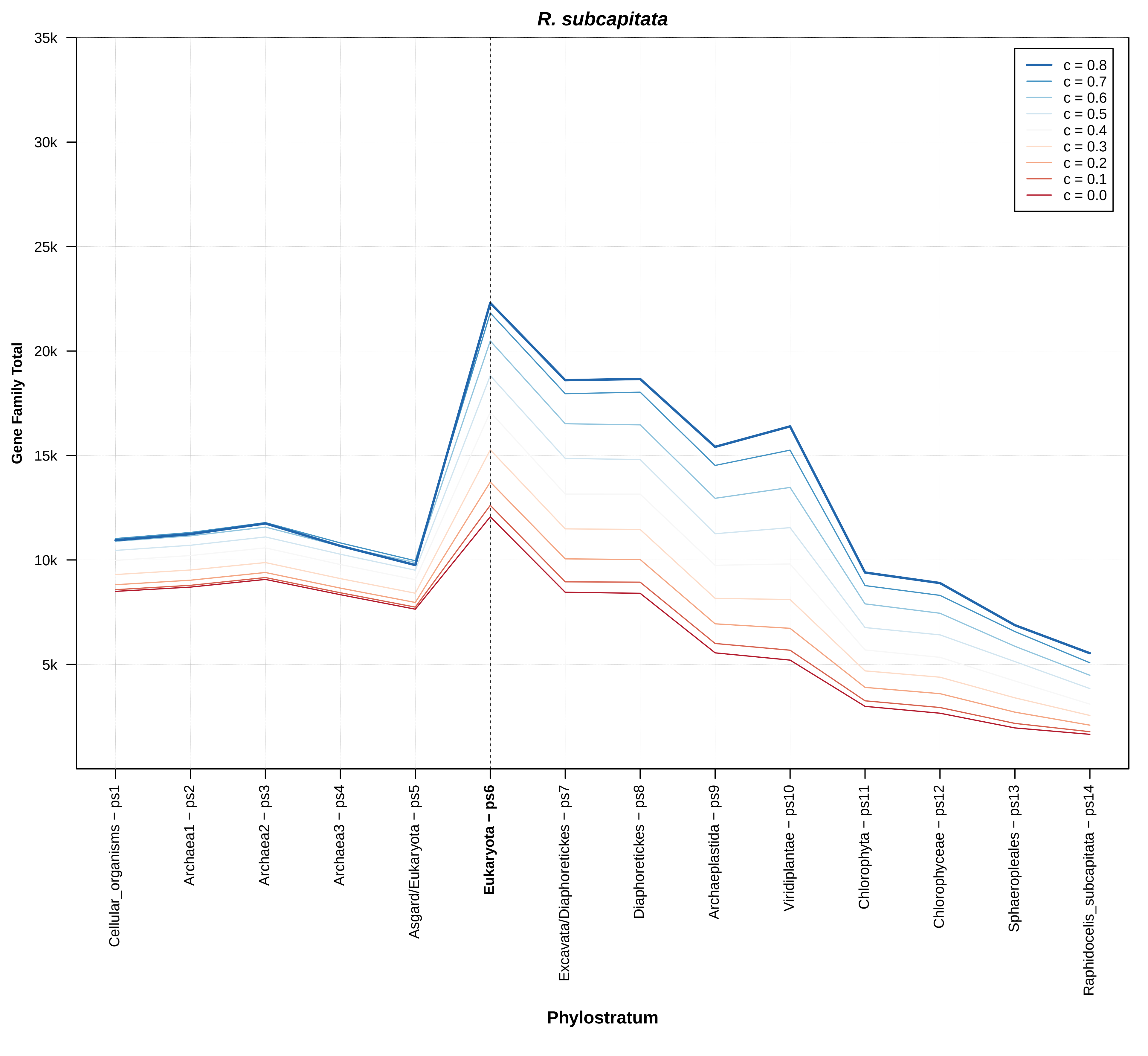
R. subcapitata
Gene Family Total
35k
30k
25k
20k
15k
10k
5k
c = 0.8
c = 0.7
c = 0.6
c = 0.5
c = 0.4
c = 0.3
c = 0.2
c = 0.1
c = 0.0
Cellular_organisms – ps1
Archaea1 – ps2
Archaea2 – ps3
Archaea3 – ps4
Asgard/Eukaryota – ps5
Eukaryota – ps6
Excavata/Diaphoretickes – ps7
Diaphoretickes – ps8
Archaeplastida – ps9
Viridiplantae – ps10
Chlorophyta – ps11
Chlorophyceae – ps12
Sphaeropleales – ps13
Raphidocelis_subcapitata – ps14
Phylostratum

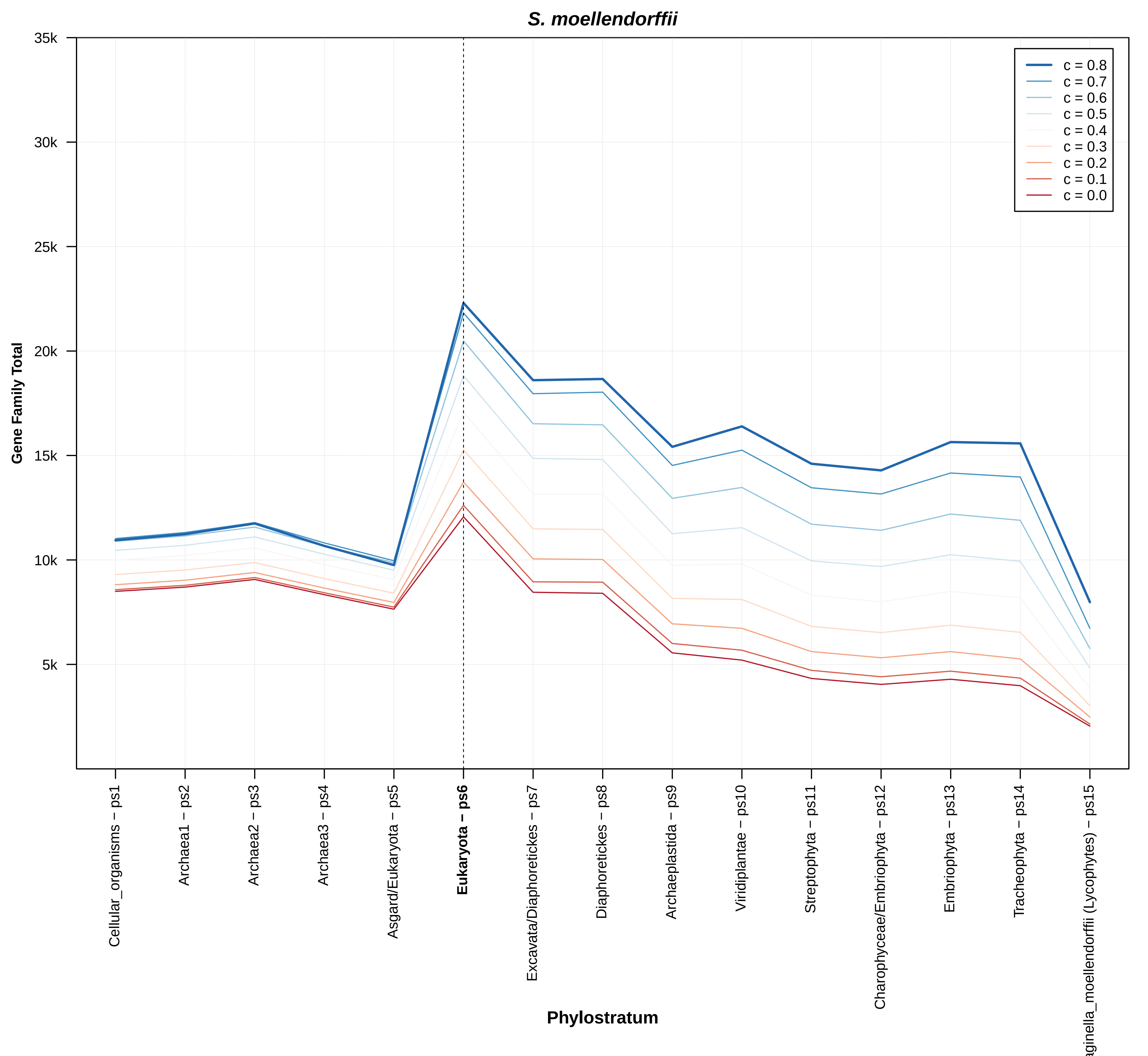

S. moellendorffii
Gene Family Total
35k
30k
25k
20k
15k
10k
5k
c = 0.8
c = 0.7
c = 0.6
c = 0.5
c = 0.4
c = 0.3
c = 0.2
c = 0.1
c = 0.0
Cellular_organisms – ps1
Archaea1 – ps2
Archaea2 – ps3
Archaea3 – ps4
Asgard/Eukaryota – ps5
Eukaryota – ps6
Excavata/Diaphoretickes – ps7
Diaphoretickes – ps8
Archaeplastida – ps9
Viridiplantae – ps10
Streptophyta – ps11
Charophyceae/Embriophyta – ps12
Embriophyta – ps13
Tracheophyta – ps14
Phylostratum

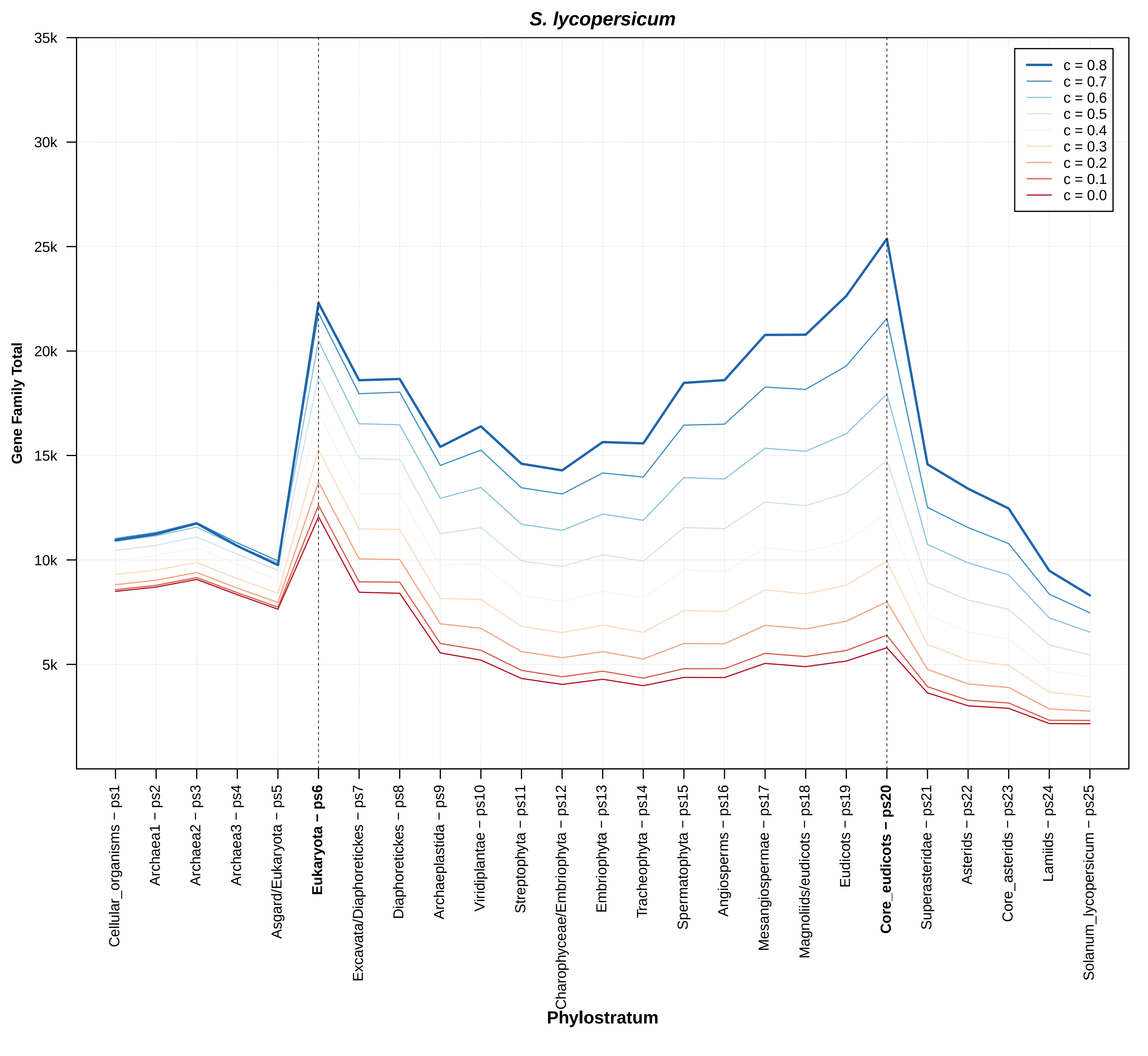
S. lycopersicum
Gene Family Total
Phylostratum
35k
30k
25k
20k
15k
10k
5k
c = 0.8
c = 0.7
c = 0.6
c = 0.5
c = 0.4
c = 0.3
c = 0.2
c = 0.1
c = 0.0
Cellular_organisms – ps1
Archaea1 – ps2
Archaea2 – ps3
Archaea3 – ps4
Asgard/Eukaryota – ps5
Eukaryota – ps6
Excavata/Diaphoretickes – ps7
Diaphoretickes – ps8
Archaeplastida – ps9
Viridiplantae – ps10
Streptophyta – ps11
Charophyceae/Embriophyta – ps12
Embriophyta – ps13
Tracheophyta – ps14
Spermatophyta – ps15
Angiosperms – ps16
Mesangiospermae – ps17
Magnoliids/eudicots – ps18
Eudicots – ps19
Core_eudicots – ps20
Superasteridae – ps21
Asterids – ps22
Core_asterids – ps23
Lamiids – ps24
Solanum_lycopersicum – ps25

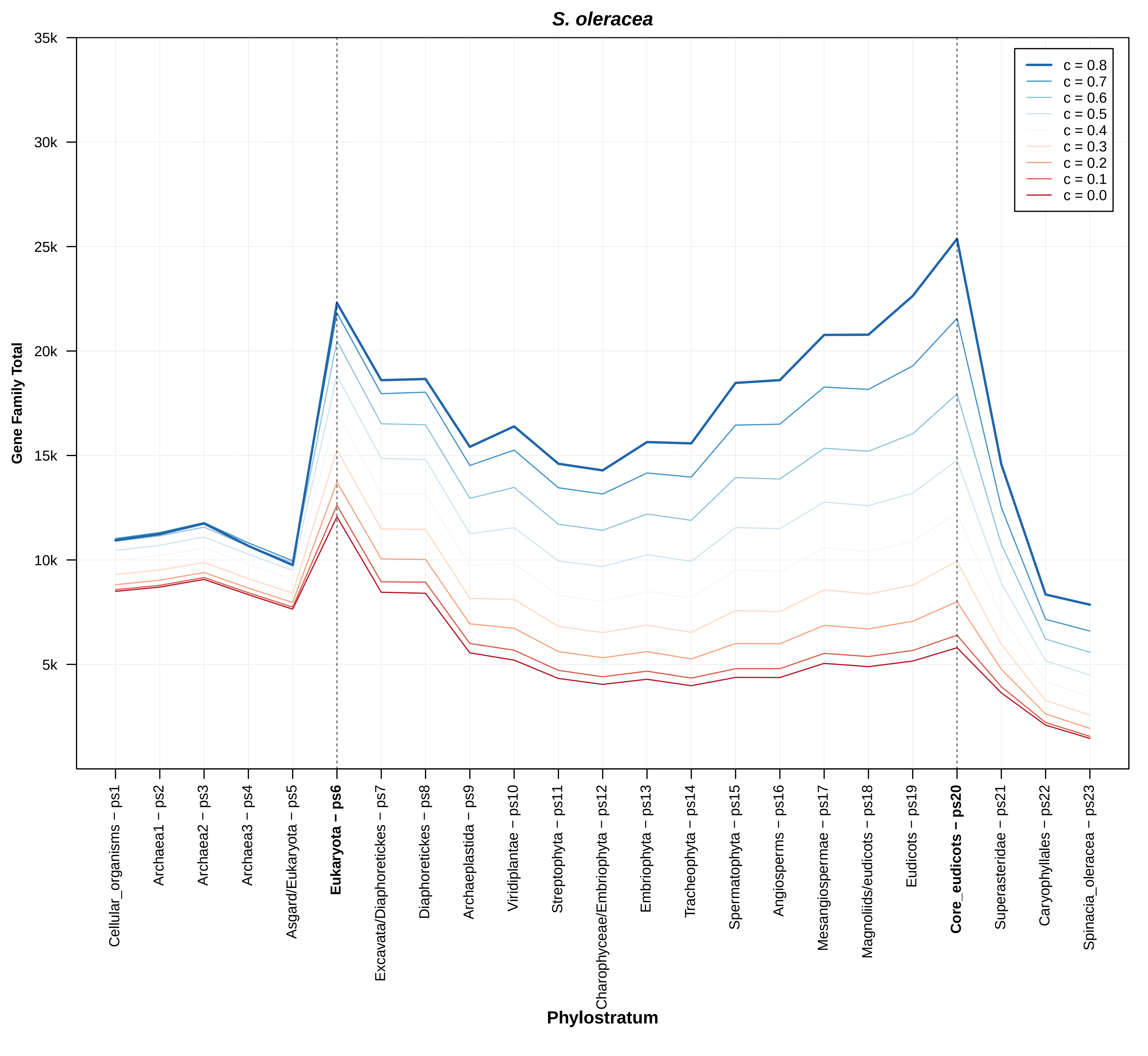

S. oleracea
Gene Family Total
35k
30k
25k
20k
15k
10k
5k
c = 0.8
c = 0.7
c = 0.6
c = 0.5
c = 0.4
c = 0.3
c = 0.2
c = 0.1
c = 0.0
Cellular_organisms – ps1
Archaea1 – ps2
Archaea2 – ps3
Archaea3 – ps4
Asgard/Eukaryota – ps5
Eukaryota – ps6
Excavata/Diaphoretickes – ps7
Diaphoretickes – ps8
Archaeplastida – ps9
Viridiplantae – ps10
Streptophyta – ps11
Charophyceae/Embriophyta – ps12
Embriophyta – ps13
Tracheophyta – ps14
Spermatophyta – ps15
Angiosperms – ps16
Mesangiospermae – ps17
Magnoliids/eudicots – ps18
Eudicots – ps19
Core_eudicots – ps20
Superasteridae – ps21
Caryophyllales – ps22
Spinacia_oleracea – ps23
Phylostratum

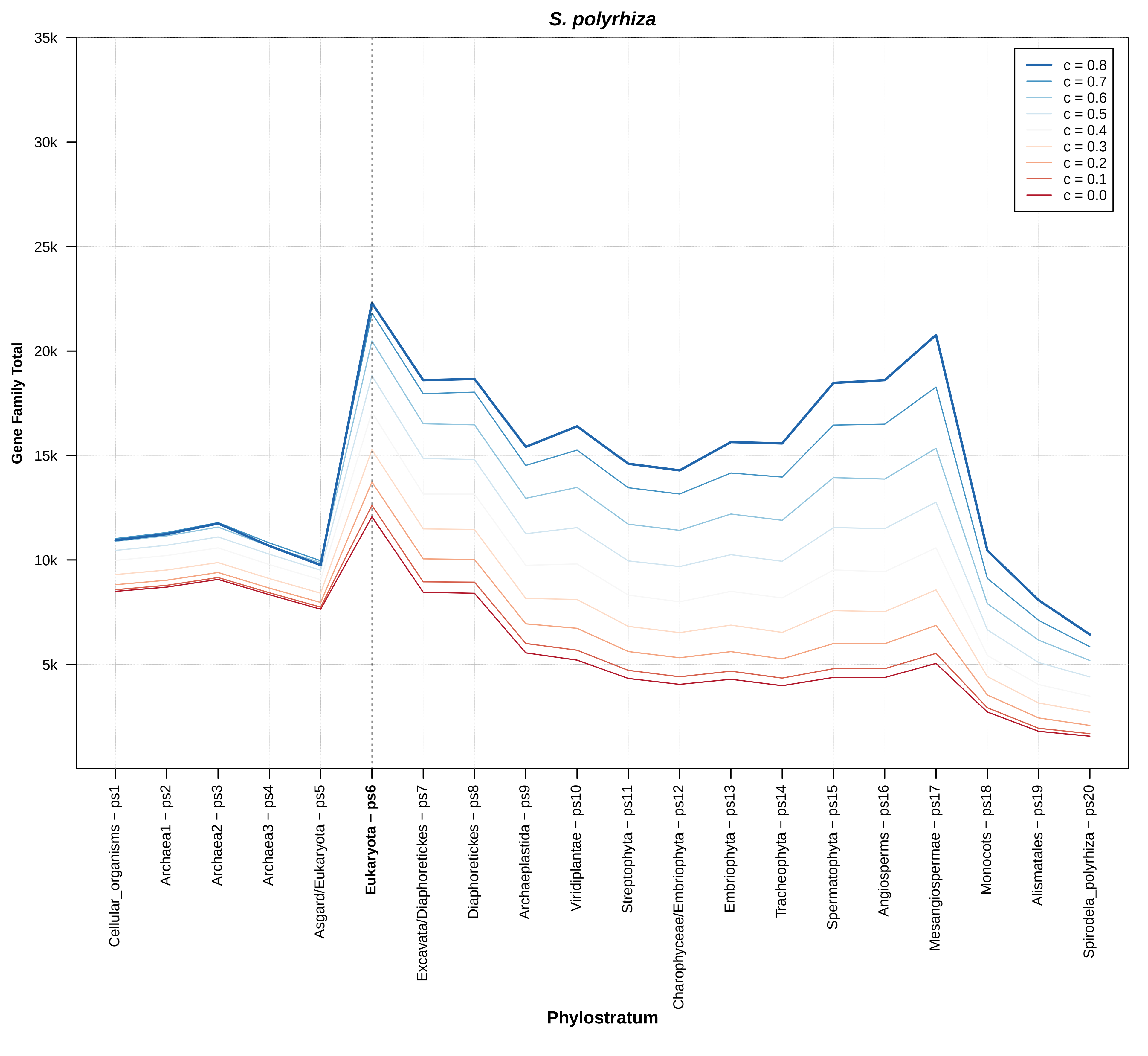
S. polyrhiza
35k
30k
25k
20k
15k
10k
5k
Gene Family Total
c = 0.8
c = 0.7
c = 0.6
c = 0.5
c = 0.4
c = 0.3
c = 0.2
c = 0.1
c = 0.0
Cellular_organisms – ps1
Archaea1 – ps2
Archaea2 – ps3
Archaea3 – ps4
Asgard/Eukaryota – ps5
Eukaryota – ps6
Excavata/Diaphoretickes – ps7
Diaphoretickes – ps8
Archaeplastida – ps9
Viridiplantae – ps10
Streptophyta – ps11
Charophyceae/Embriophyta – ps12
Embriophyta – ps13
Tracheophyta – ps14
Spermatophyta – ps15
Angiosperms – ps16
Mesangiospermae – ps17
Monocots – ps18
Alismatales – ps19
Spirodela_polyrhiza – ps20
Phylostratum

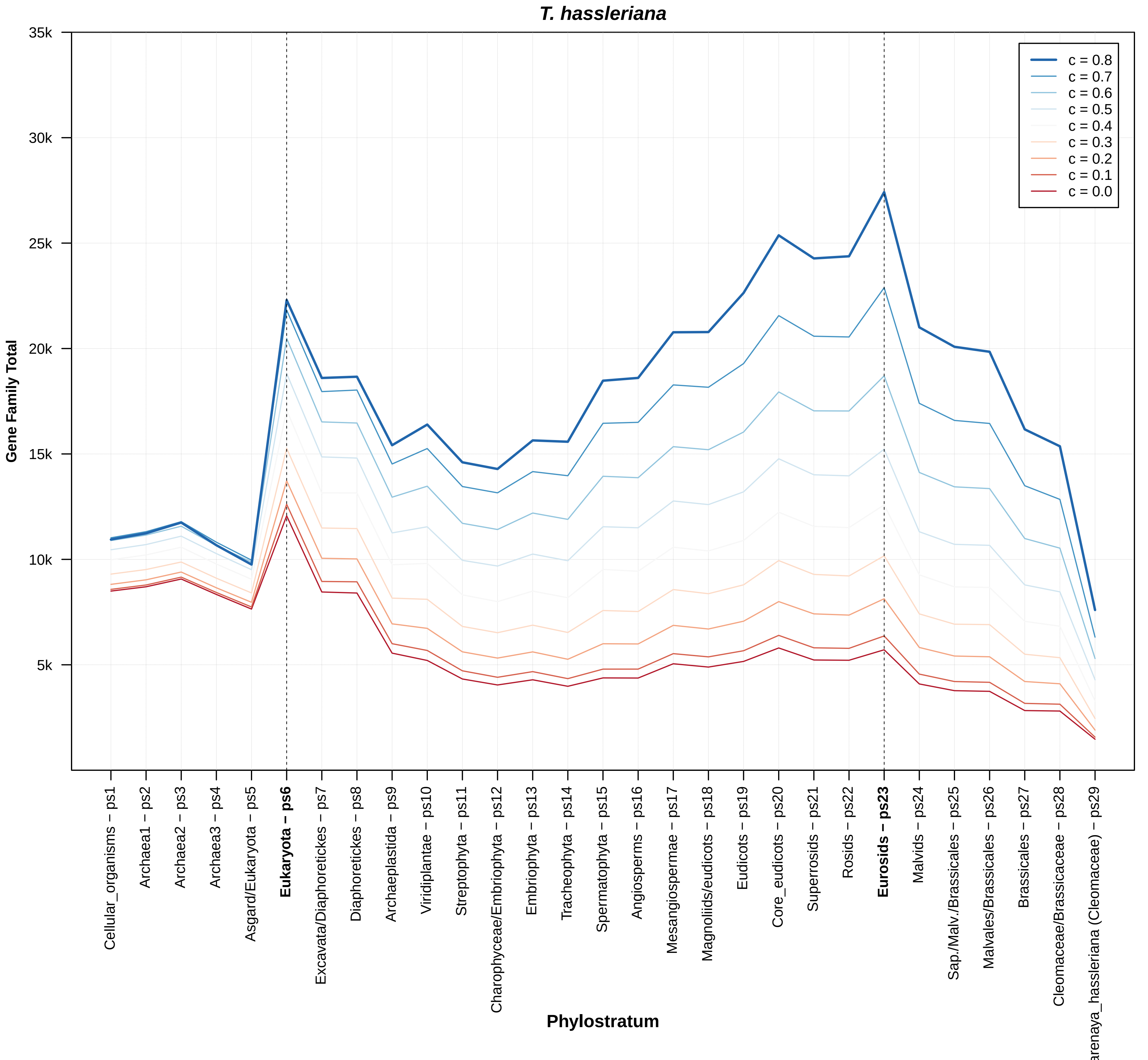

T. hassleriana
Gene Family Total
35k
30k
25k
20k
15k
10k
5k
c = 0.8
c = 0.7
c = 0.6
c = 0.5
c = 0.4
c = 0.3
c = 0.2
c = 0.1
c = 0.0
Cellular_organisms – ps1
Archaea1 – ps2
Archaea2 – ps3
Archaea3 – ps4
Asgard/Eukaryota – ps5
Eukaryota – ps6
Excavata/Diaphoretickes – ps7
Diaphoretickes – ps8
Archaeplastida – ps9
Viridiplantae – ps10
Streptophyta – ps11
Charophyceae/Embriophyta – ps12
Embriophyta – ps13
Tracheophyta – ps14
Spermatophyta – ps15
Angiosperms – ps16
Mesangiospermae – ps17
Magnoliids/eudicots – ps18
Eudicots – ps19
Core_eudicots – ps20
Superrosids – ps21
Rosids – ps22
Eurosids – ps23
Malvids – ps24
Sap./Malv./Brassicales – ps25
Malvales/Brassicales – ps26
Brassicales – ps27
Cleomaceae/Brassicaceae – ps28
arenaya_hassleriana (Cleomaceae) – ps29
Phylostratum

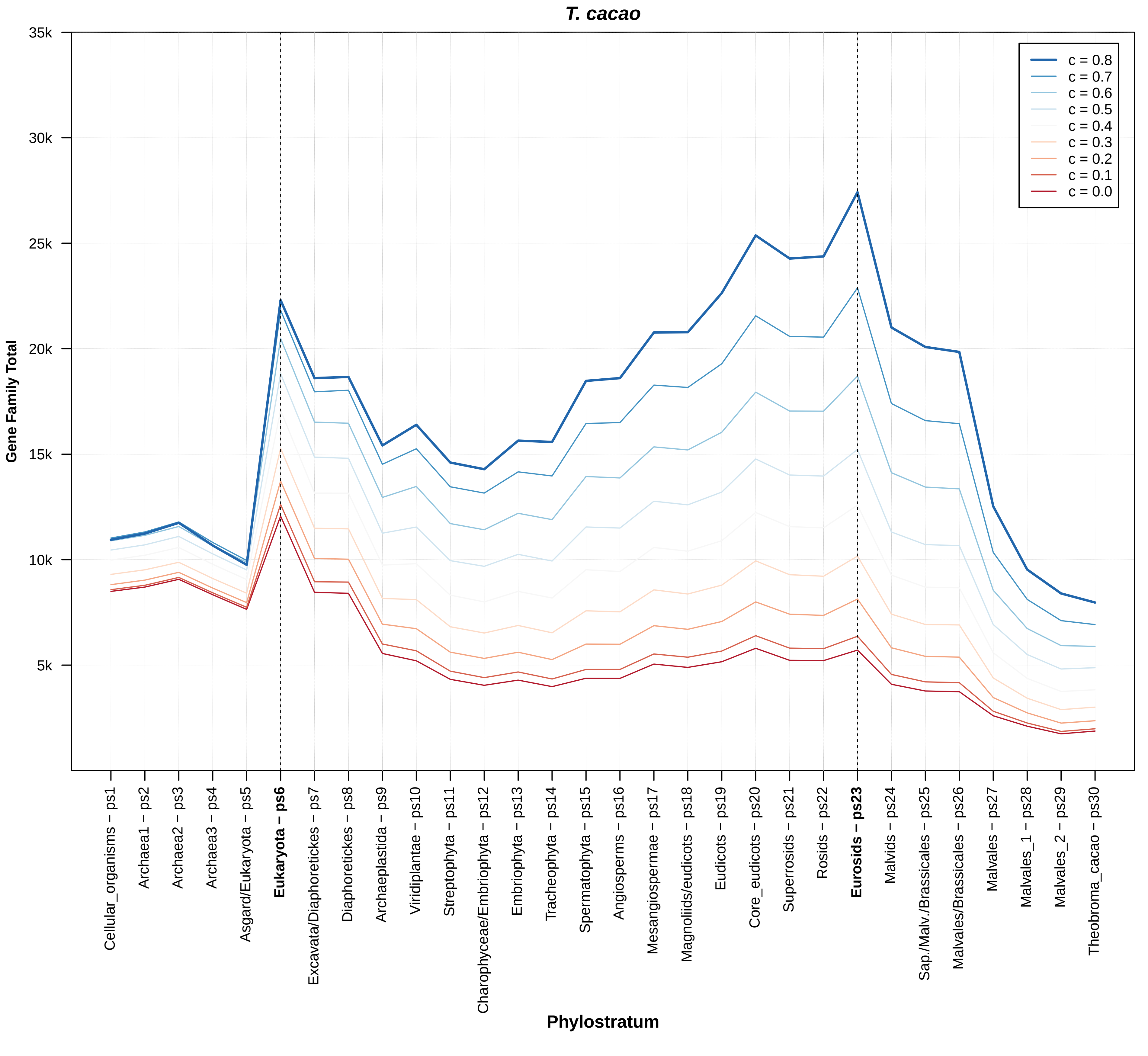

T. cacao
Gene Family Total
35k
30k
25k
20k
15k
10k
5k
c = 0.8
c = 0.7
c = 0.6
c = 0.5
c = 0.4
c = 0.3
c = 0.2
c = 0.1
c = 0.0
Cellular_organisms – ps1
Archaea1 – ps2
Archaea2 – ps3
Archaea3 – ps4
Asgard/Eukaryota – ps5
Eukaryota – ps6
Excavata/Diaphoretickes – ps7
Diaphoretickes – ps8
Archaeplastida – ps9
Viridiplantae – ps10
Streptophyta – ps11
Charophyceae/Embriophyta – ps12
Embriophyta – ps13
Tracheophyta – ps14
Spermatophyta – ps15
Angiosperms – ps16
Mesangiospermae – ps17
Magnoliids/eudicots – ps18
Eudicots – ps19
Core_eudicots – ps20
Superrosids – ps21
Rosids – ps22
Eurosids – ps23
Malvids – ps24
Sap./Malv./Brassicales – ps25
Malvales/Brassicales – ps26
Malvales – ps27
Malvales_1 – ps28
Malvales_2 – ps29
Theobroma_cacao – ps30
Phylostratum

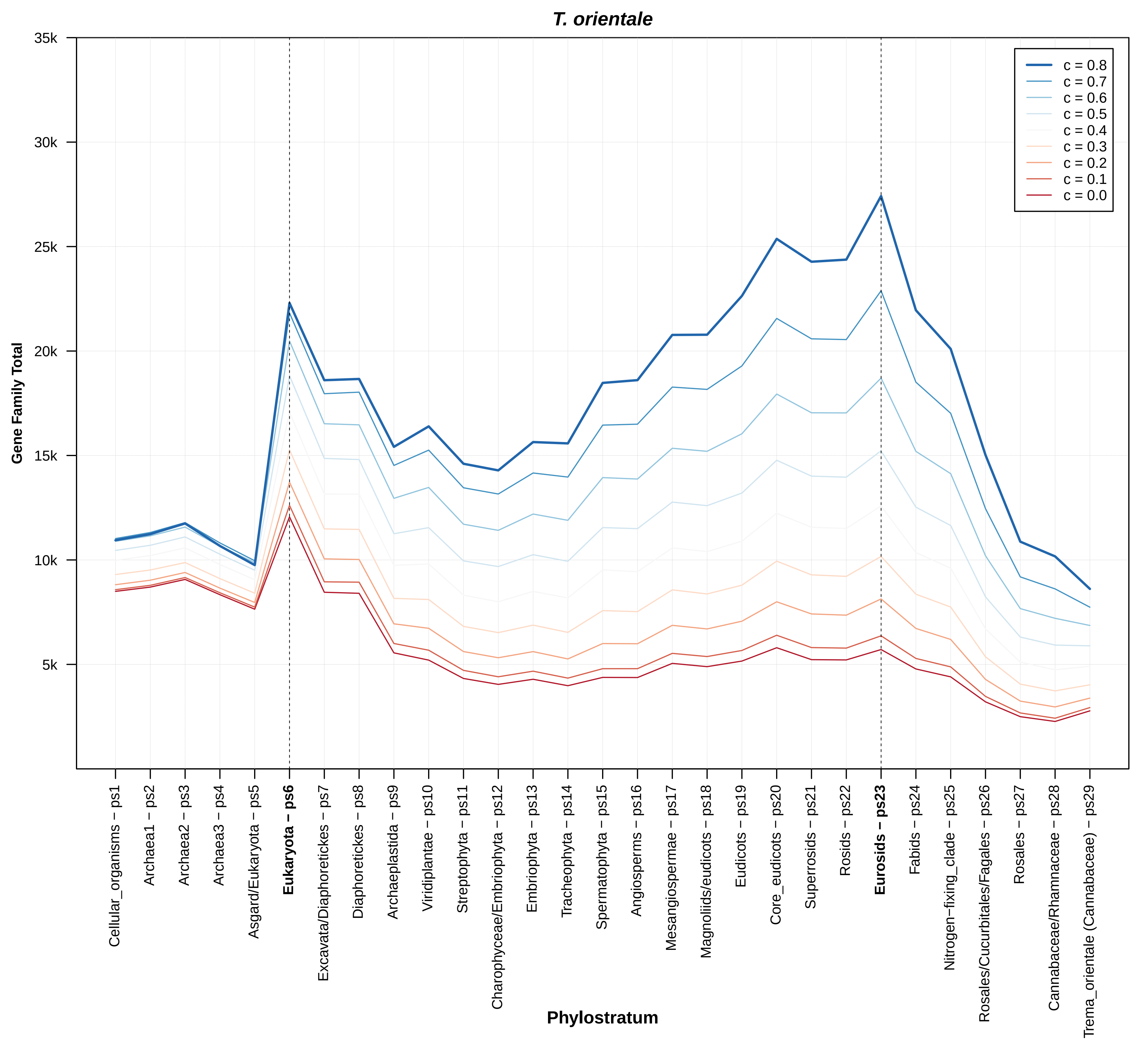
T. orientale
Gene Family Total
35k
30k
25k
20k
15k
10k
5k
c = 0.8
c = 0.7
c = 0.6
c = 0.5
c = 0.4
c = 0.3
c = 0.2
c = 0.1
c = 0.0
Cellular_organisms – ps1
Archaea1 – ps2
Archaea2 – ps3
Archaea3 – ps4
Asgard/Eukaryota – ps5
Eukaryota – ps6
Excavata/Diaphoretickes – ps7
Diaphoretickes – ps8
Archaeplastida – ps9
Viridiplantae – ps10
Streptophyta – ps11
Charophyceae/Embriophyta – ps12
Embriophyta – ps13
Tracheophyta – ps14
Spermatophyta – ps15
Angiosperms – ps16
Mesangiospermae – ps17
Magnoliids/eudicots – ps18
Eudicots – ps19
Core_eudicots – ps20
Superrosids – ps21
Rosids – ps22
Eurosids – ps23
Fabids – ps24
Nitrogen–fixing_clade – ps25
Rosales/Cucurbitales/Fagales – ps26
Rosales – ps27
Cannabaceae/Rhamnaceae – ps28
Trema_orientale (Cannabaceae) – ps29
Phylostratum

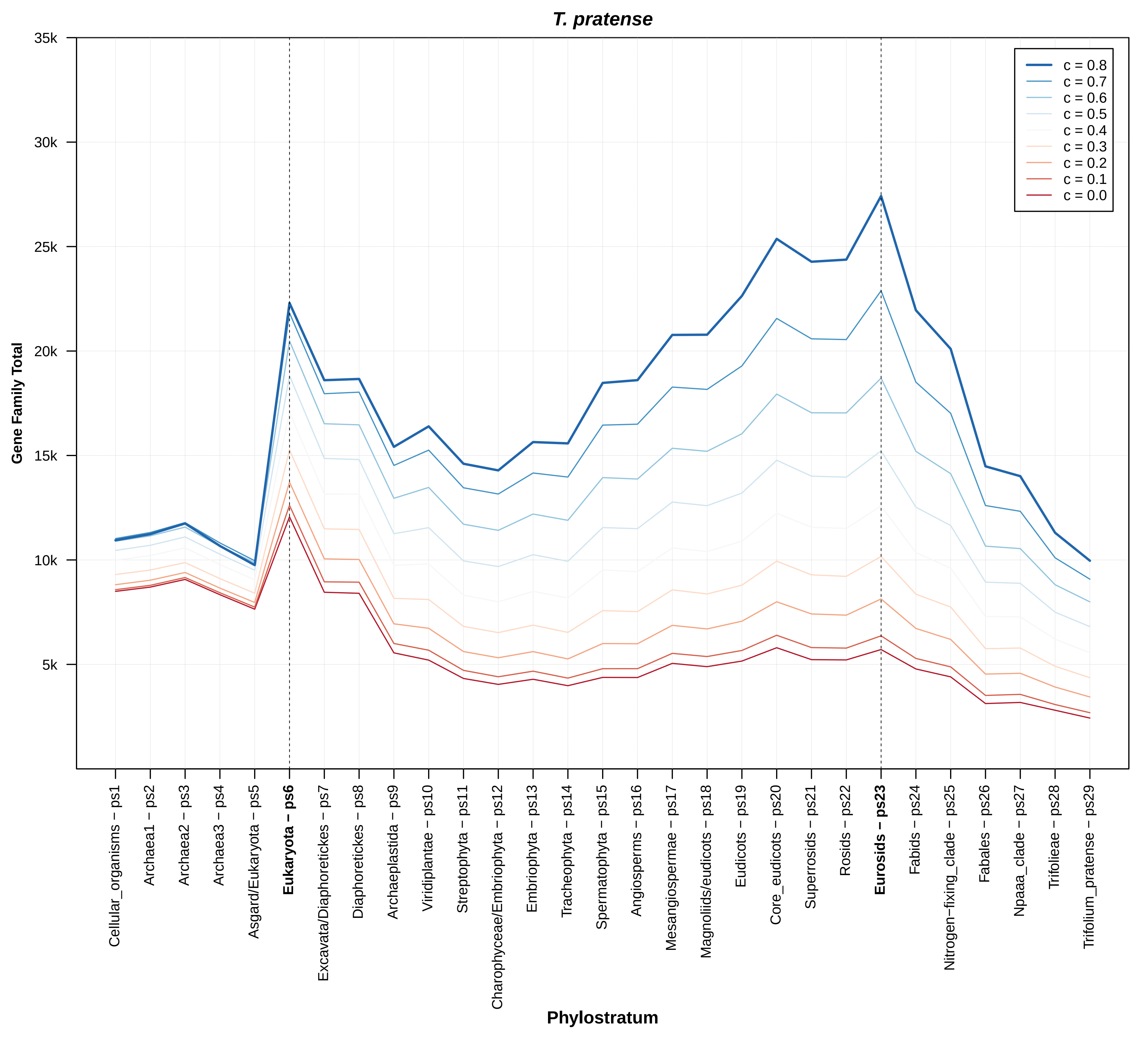
T. pratense
Gene Family Total
35k
30k
25k
20k
15k
10k
5k
c = 0.8
c = 0.7
c = 0.6
c = 0.5
c = 0.4
c = 0.3
c = 0.2
c = 0.1
c = 0.0
Cellular_organisms – ps1
Archaea1 – ps2
Archaea2 – ps3
Archaea3 – ps4
Asgard/Eukaryota – ps5
Eukaryota – ps6
Excavata/Diaphoretickes – ps7
Diaphoretickes – ps8
Archaeplastida – ps9
Viridiplantae – ps10
Streptophyta – ps11
Charophyceae/Embriophyta – ps12
Embriophyta – ps13
Tracheophyta – ps14
Spermatophyta – ps15
Angiosperms – ps16
Mesangiospermae – ps17
Magnoliids/eudicots – ps18
Eudicots – ps19
Core_eudicots – ps20
Superrosids – ps21
Rosids – ps22
Eurosids – ps23
Fabids – ps24
Nitrogen–fixing_clade – ps25
Fabales – ps26
Npaaa_clade – ps27
Trifolieae – ps28
Trifolium_pratense – ps29
Phylostratum

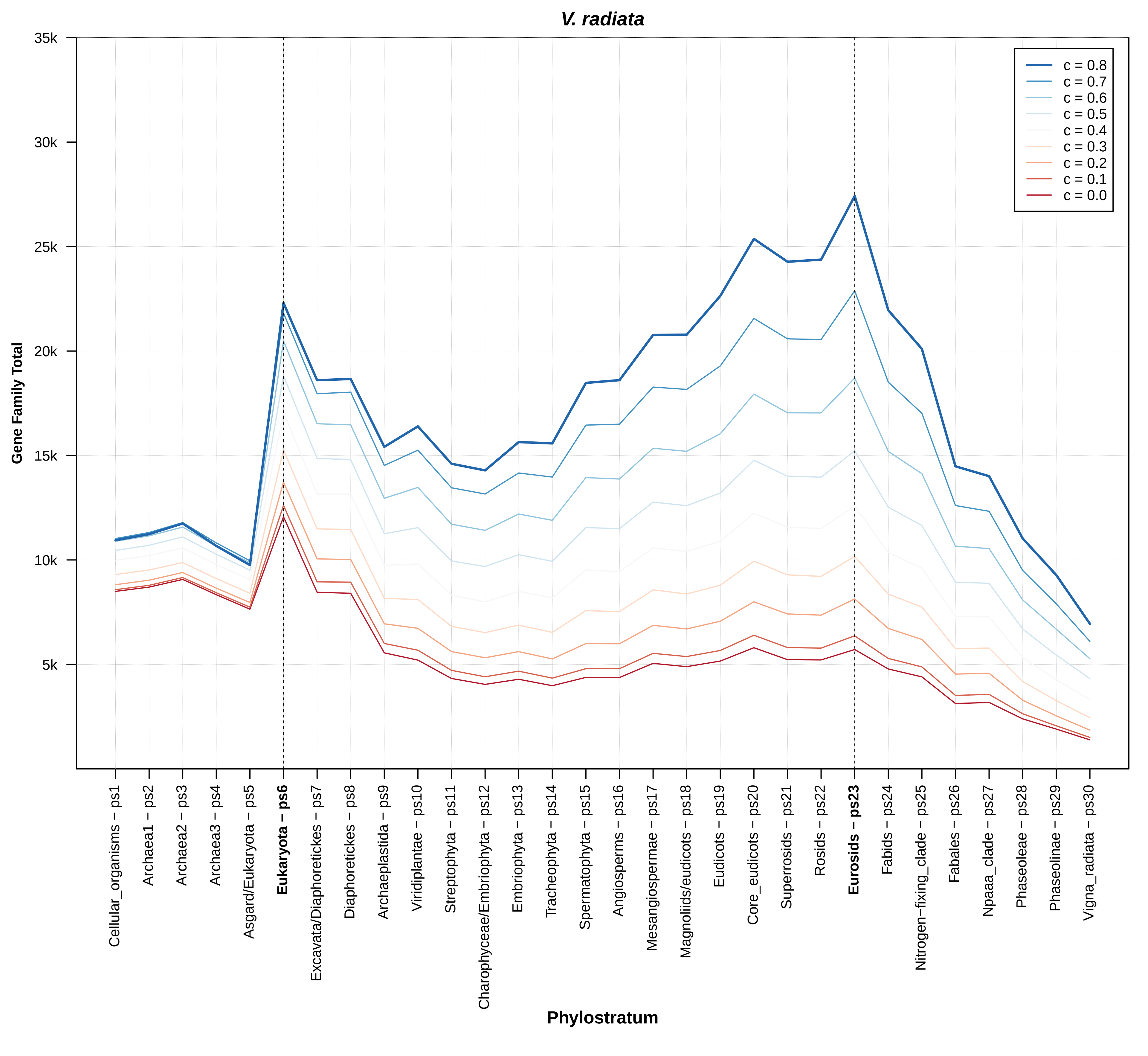

V. radiata
Gene Family Total
35k
30k
25k
20k
15k
10k
5k
c = 0.8
c = 0.7
c = 0.6
c = 0.5
c = 0.4
c = 0.3
c = 0.2
c = 0.1
c = 0.0
Cellular_organisms – ps1
Archaea1 – ps2
Archaea2 – ps3
Archaea3 – ps4
Asgard/Eukaryota – ps5
Eukaryota – ps6
Excavata/Diaphoretickes – ps7
Diaphoretickes – ps8
Archaeplastida – ps9
Viridiplantae – ps10
Streptophyta – ps11
Charophyceae/Embriophyta – ps12
Embriophyta – ps13
Tracheophyta – ps14
Spermatophyta – ps15
Angiosperms – ps16
Mesangiospermae – ps17
Magnoliids/eudicots – ps18
Eudicots – ps19
Core_eudicots – ps20
Superrosids – ps21
Rosids – ps22
Eurosids – ps23
Fabids – ps24
Nitrogen–fixing_clade – ps25
Fabales – ps26
Npaaa_clade – ps27
Phaseoleae – ps28
Phaseolinae – ps29
Vigna_radiata – ps30
Phylostratum

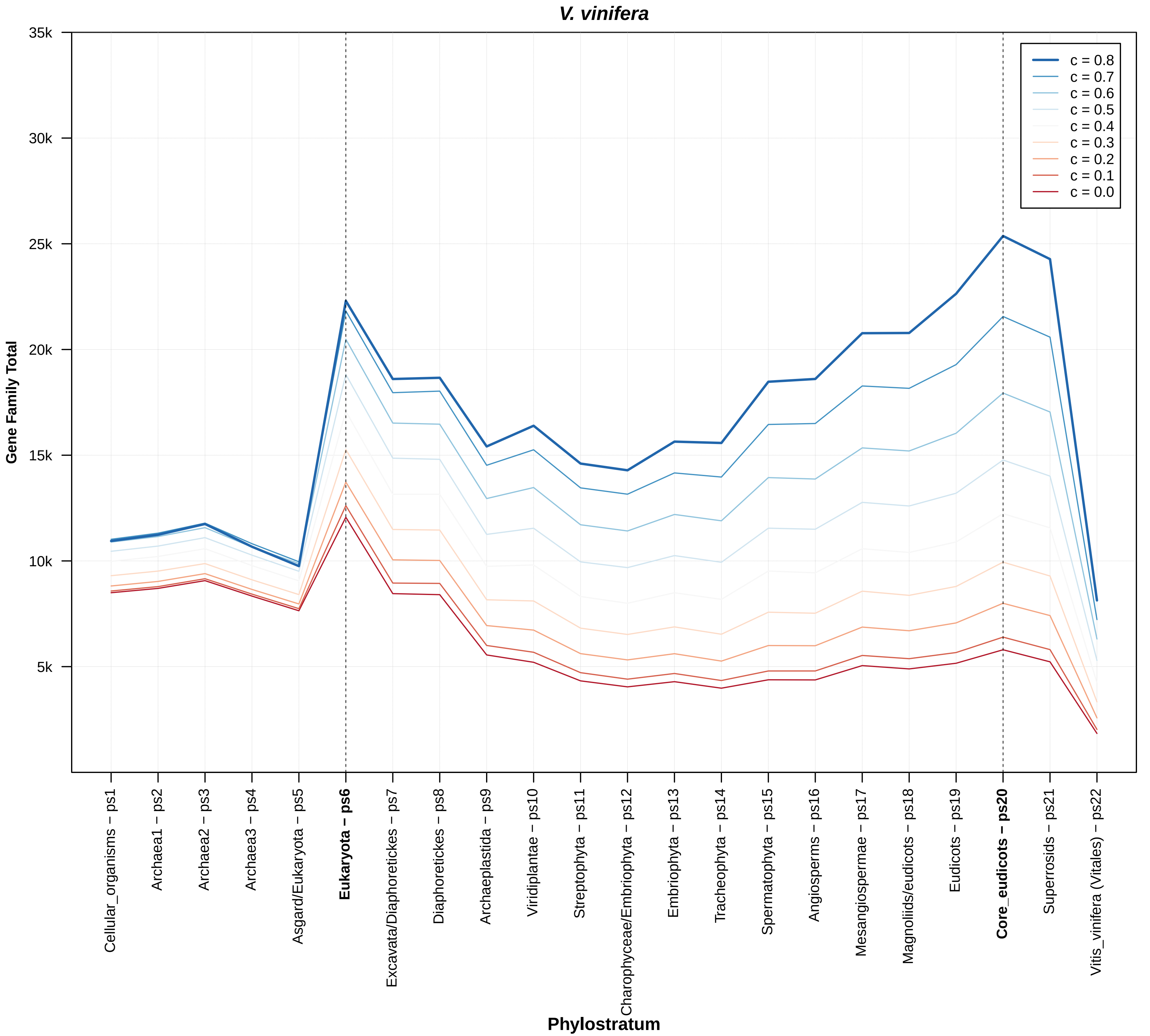

V. vinifera
Gene Family Total
Phylostratum
35k
30k
25k
20k
15k
10k
5k
c = 0.8
c = 0.7
c = 0.6
c = 0.5
c = 0.4
c = 0.3
c = 0.2
c = 0.1
c = 0.0
Cellular_organisms – ps1
Archaea1 – ps2
Archaea2 – ps3
Archaea3 – ps4
Asgard/Eukaryota – ps5
Eukaryota – ps6
Excavata/Diaphoretickes – ps7
Diaphoretickes – ps8
Archaeplastida – ps9
Viridiplantae – ps10
Streptophyta – ps11
Charophyceae/Embriophyta – ps12
Embriophyta – ps13
Tracheophyta – ps14
Spermatophyta – ps15
Angiosperms – ps16
Mesangiospermae – ps17
Magnoliids/eudicots – ps18
Eudicots – ps19
Core_eudicots – ps20
Superrosids – ps21
Vitis_vinifera (Vitales) – ps22

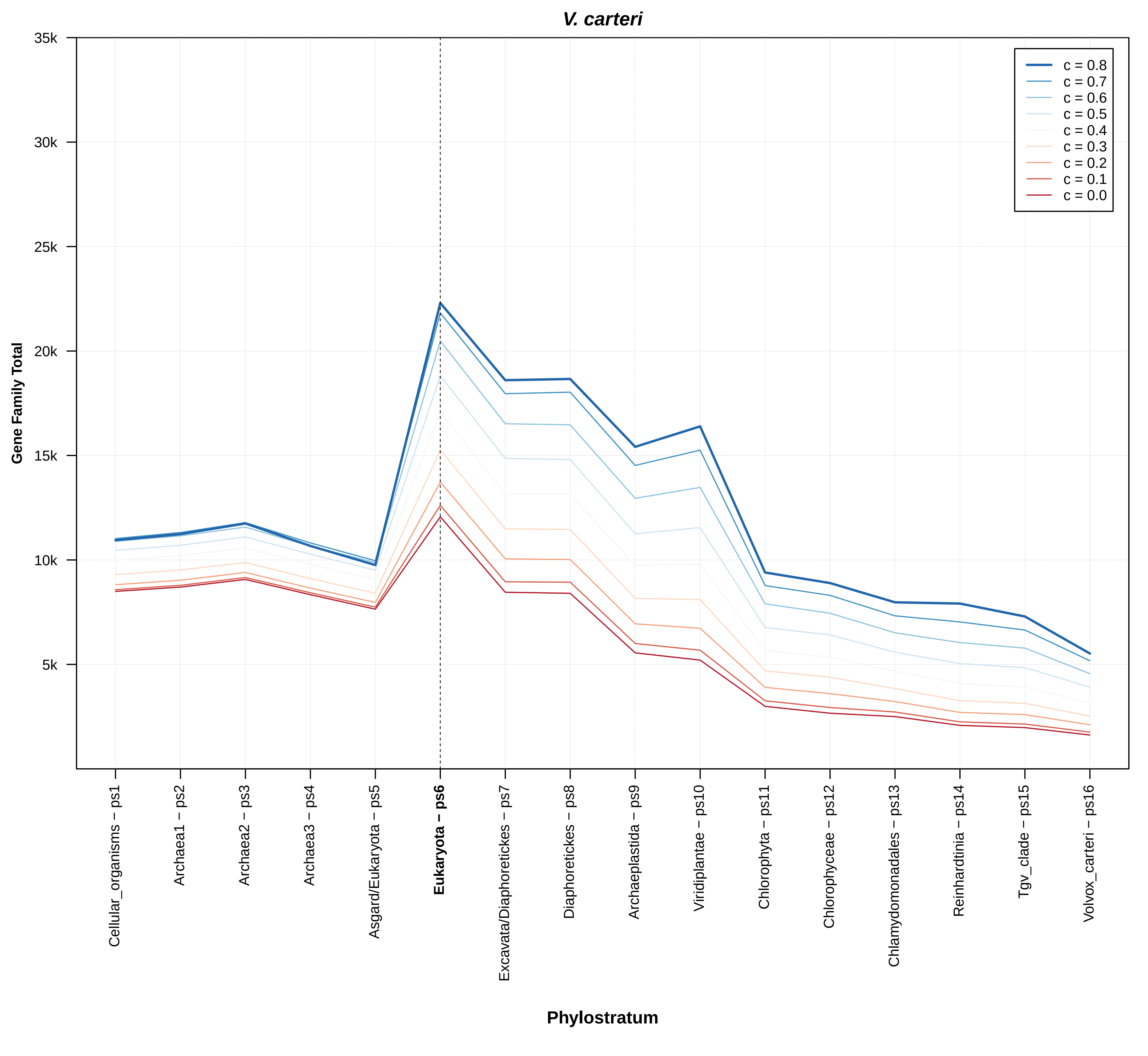
V. carteri
35k
30k
25k
20k
15k
10k
5k
Gene Family Total
c = 0.8
c = 0.7
c = 0.6
c = 0.5
c = 0.4
c = 0.3
c = 0.2
c = 0.1
c = 0.0
Cellular_organisms – ps1
Archaea1 – ps2
Archaea2 – ps3
Archaea3 – ps4
Asgard/Eukaryota – ps5
Eukaryota – ps6
Excavata/Diaphoretickes – ps7
Diaphoretickes – ps8
Archaeplastida – ps9
Viridiplantae – ps10
Chlorophyta – ps11
Chlorophyceae – ps12
Chlamydomonadales – ps13
Reinhardtinia – ps14
Tgv_clade – ps15
Volvox_carteri – ps16
Phylostratum

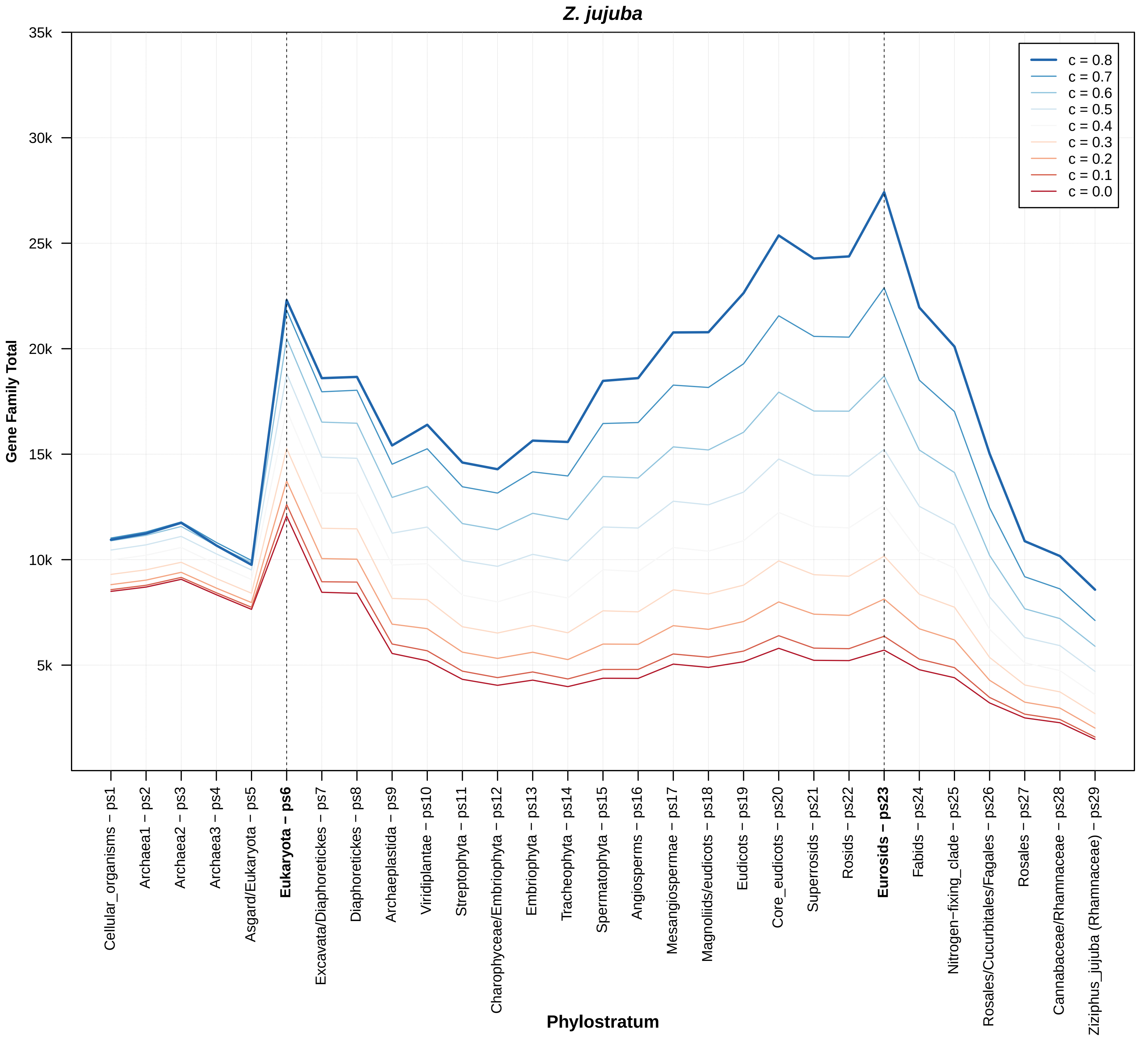

Z. jujuba
Gene Family Total
35k
30k
25k
20k
15k
10k
5k
c = 0.8
c = 0.7
c = 0.6
c = 0.5
c = 0.4
c = 0.3
c = 0.2
c = 0.1
c = 0.0
Cellular_organisms – ps1
Archaea1 – ps2
Archaea2 – ps3
Archaea3 – ps4
Asgard/Eukaryota – ps5
Eukaryota – ps6
Excavata/Diaphoretickes – ps7
Diaphoretickes – ps8
Archaeplastida – ps9
Viridiplantae – ps10
Streptophyta – ps11
Charophyceae/Embriophyta – ps12
Embriophyta – ps13
Tracheophyta – ps14
Spermatophyta – ps15
Angiosperms – ps16
Mesangiospermae – ps17
Magnoliids/eudicots – ps18
Eudicots – ps19
Core_eudicots – ps20
Superrosids – ps21
Rosids – ps22
Eurosids – ps23
Fabids – ps24
Nitrogen–fixing_clade – ps25
Rosales/Cucurbitales/Fagales – ps26
Rosales – ps27
Cannabaceae/Rhamnaceae – ps28
Ziziphus_jujuba (Rhamnaceae) – ps29
Phylostratum

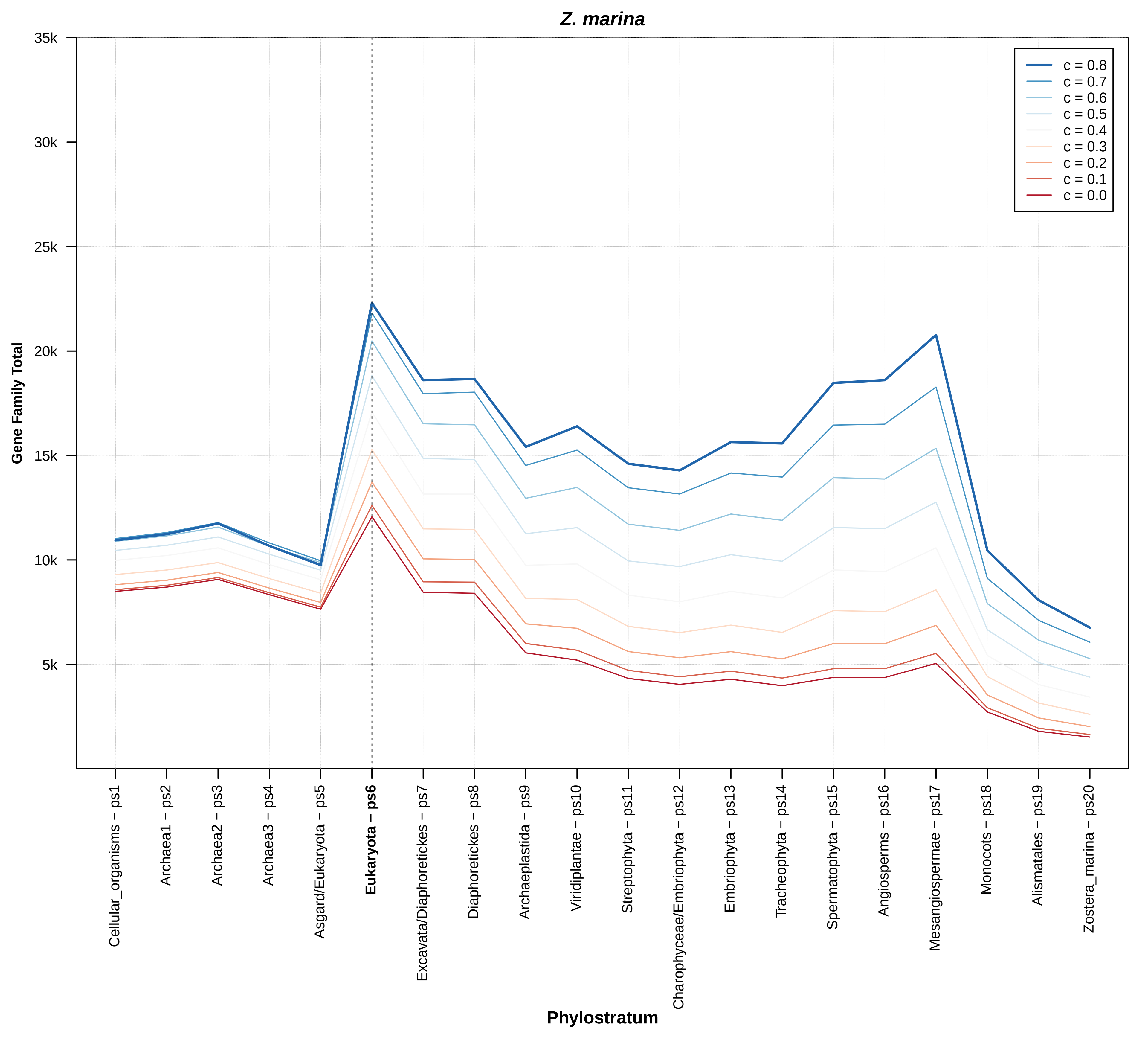

Z. marina
Gene Family Total
Phylostratum
35k
30k
25k
20k
15k
10k
5k
c = 0.8
c = 0.7
c = 0.6
c = 0.5
c = 0.4
c = 0.3
c = 0.2
c = 0.1
c = 0.0
Cellular_organisms – ps1
Archaea1 – ps2
Archaea2 – ps3
Archaea3 – ps4
Asgard/Eukaryota – ps5
Eukaryota – ps6
Excavata/Diaphoretickes – ps7
Diaphoretickes – ps8
Archaeplastida – ps9
Viridiplantae – ps10
Streptophyta – ps11
Charophyceae/Embriophyta – ps12
Embriophyta – ps13
Tracheophyta – ps14
Spermatophyta – ps15
Angiosperms – ps16
Mesangiospermae – ps17
Monocots – ps18
Alismatales – ps19
Zostera_marina – ps20

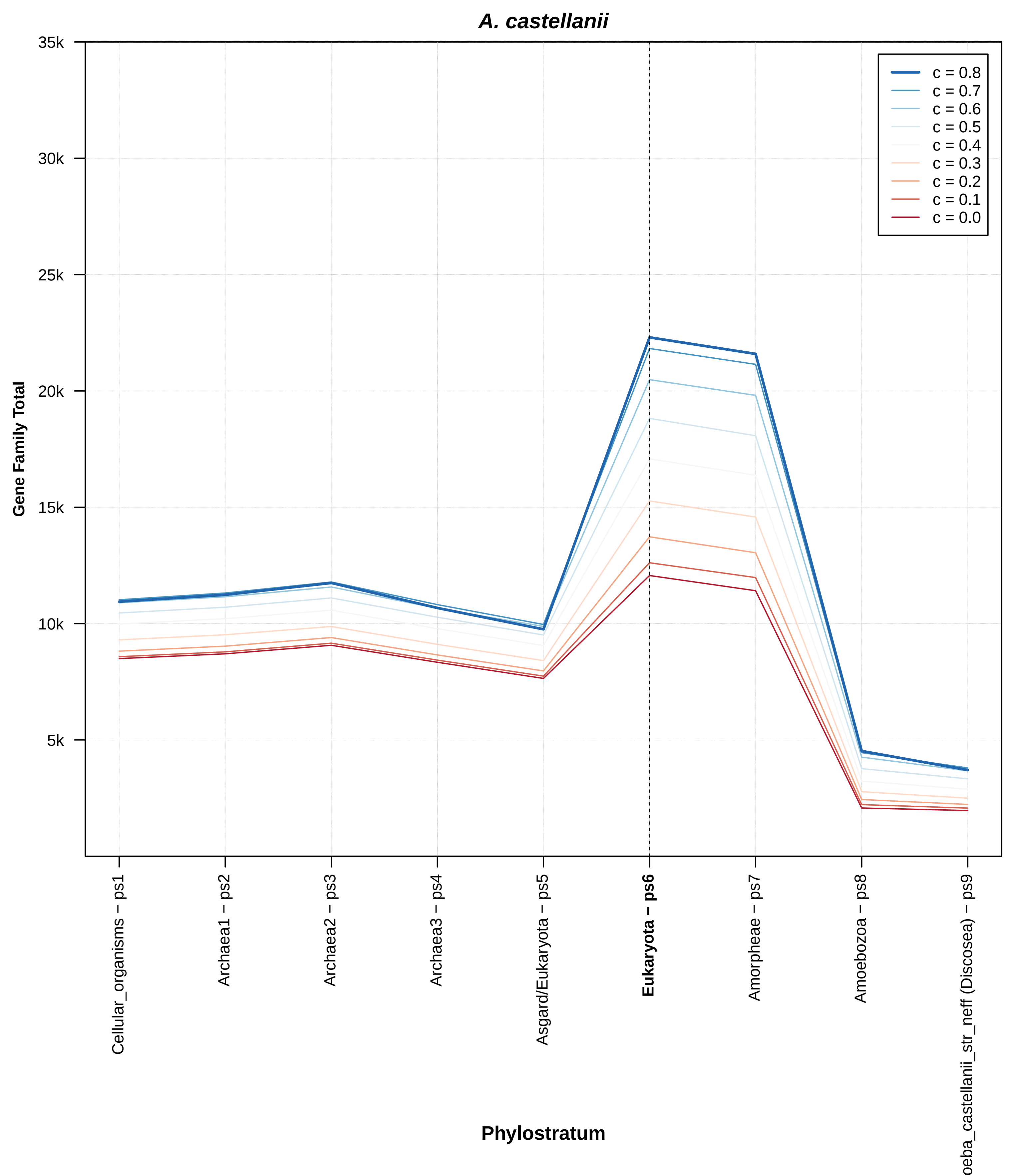

A. castellanii
Gene Family Total
35k
30k
25k
20k
15k
10k
5k
c = 0.8
c = 0.7
c = 0.6
c = 0.5
c = 0.4
c = 0.3
c = 0.2
c = 0.1
c = 0.0
Cellular_organisms – ps1
Archaea1 – ps2
Archaea2 – ps3
Archaea3 – ps4
Asgard/Eukaryota – ps5
Eukaryota – ps6
Amorpheae – ps7
Amoebozoa – ps8
oeba_castellanii_str_neff (Discosea) – ps9
Phylostratum

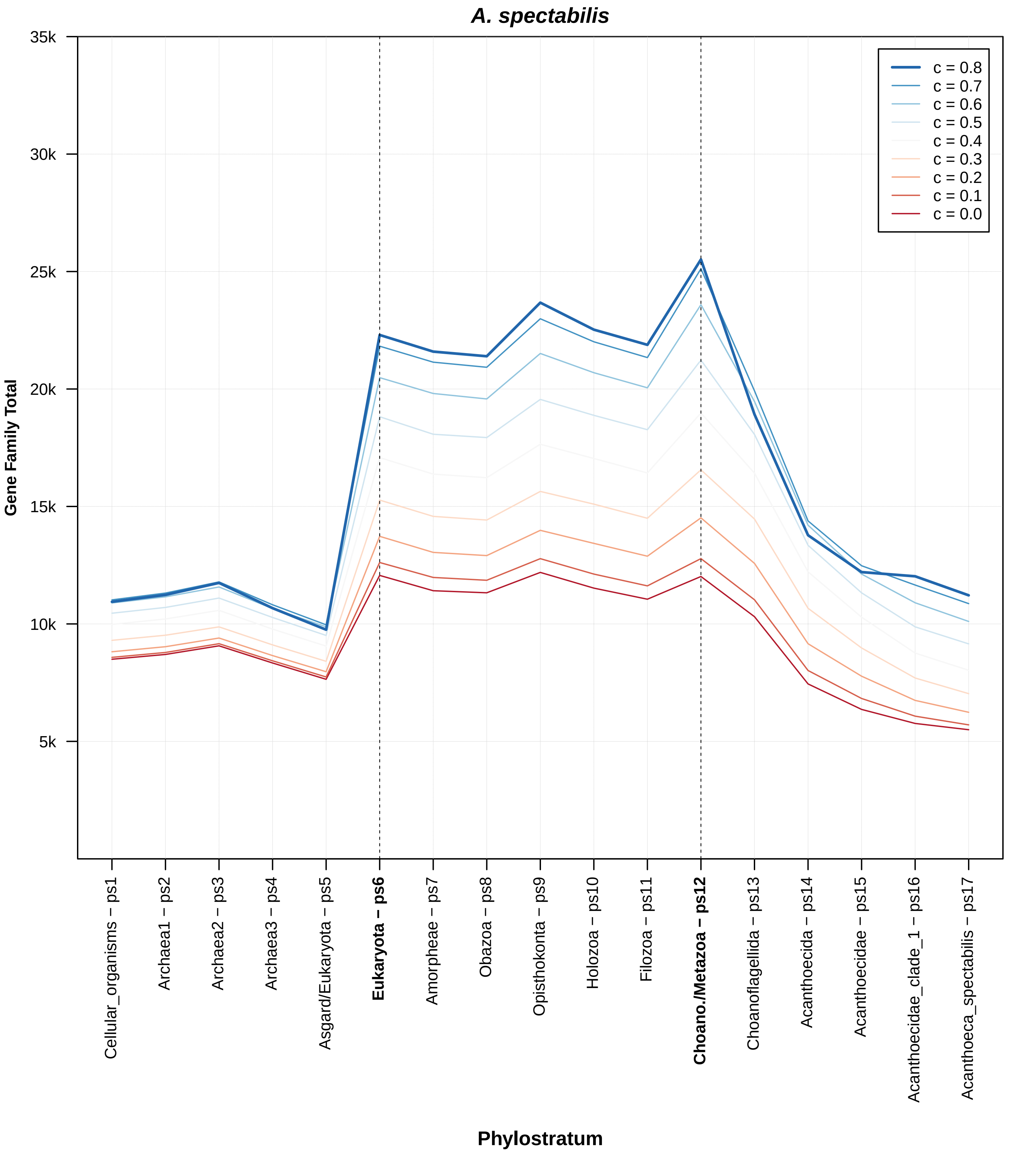
A. spectabilis
Gene Family Total
35k
30k
25k
20k
15k
10k
5k
c = 0.8
c = 0.7
c = 0.6
c = 0.5
c = 0.4
c = 0.3
c = 0.2
c = 0.1
c = 0.0
Cellular_organisms – ps1
Archaea1 – ps2
Archaea2 – ps3
Archaea3 – ps4
Asgard/Eukaryota – ps5
Eukaryota – ps6
Amorpheae – ps7
Obazoa – ps8
Opisthokonta – ps9
Holozoa – ps10
Filozoa – ps11
Choano./Metazoa – ps12
Choanoflagellida – ps13
Acanthoecida – ps14
Acanthoecidae – ps15
Acanthoecidae_clade_1 – ps16
Acanthoeca_spectabilis – ps17
Phylostratum

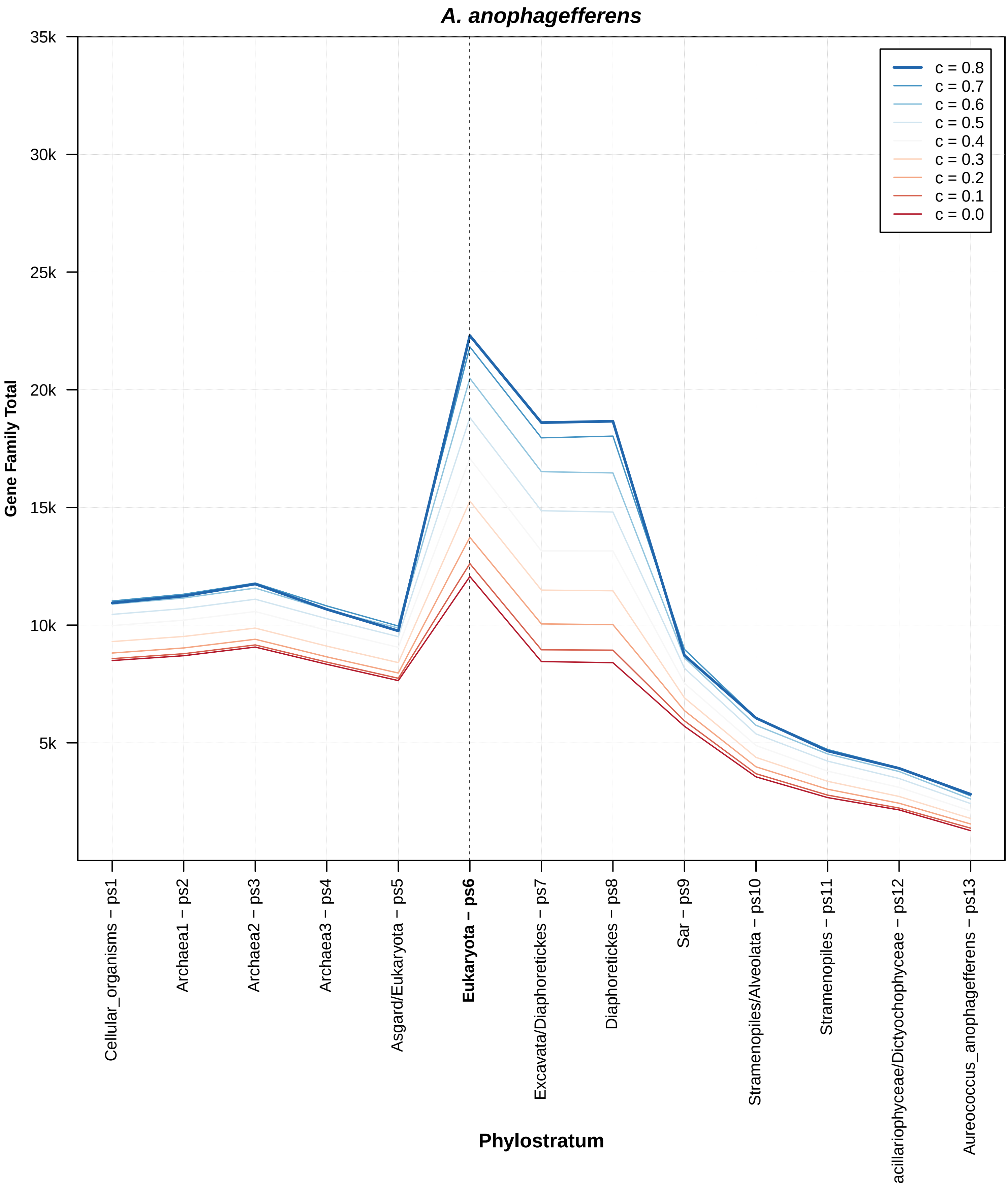

A. anophagefferens
c = 0.8
c = 0.7
c = 0.6
c = 0.5
c = 0.4
c = 0.3
c = 0.2
c = 0.1
c = 0.0
35k
30k
25k
20k
15k
10k
5k
Gene Family Total
Cellular_organisms – ps1
Archaea1 – ps2
Archaea2 – ps3
Archaea3 – ps4
Asgard/Eukaryota – ps5
Eukaryota – ps6
Excavata/Diaphoretickes – ps7
Diaphoretickes – ps8
Sar – ps9
Stramenopiles/Alveolata – ps10
Stramenopiles – ps11
acillariophyceae/Dictyochophyceae – ps12
Aureococcus_anophagefferens – ps13
Phylostratum

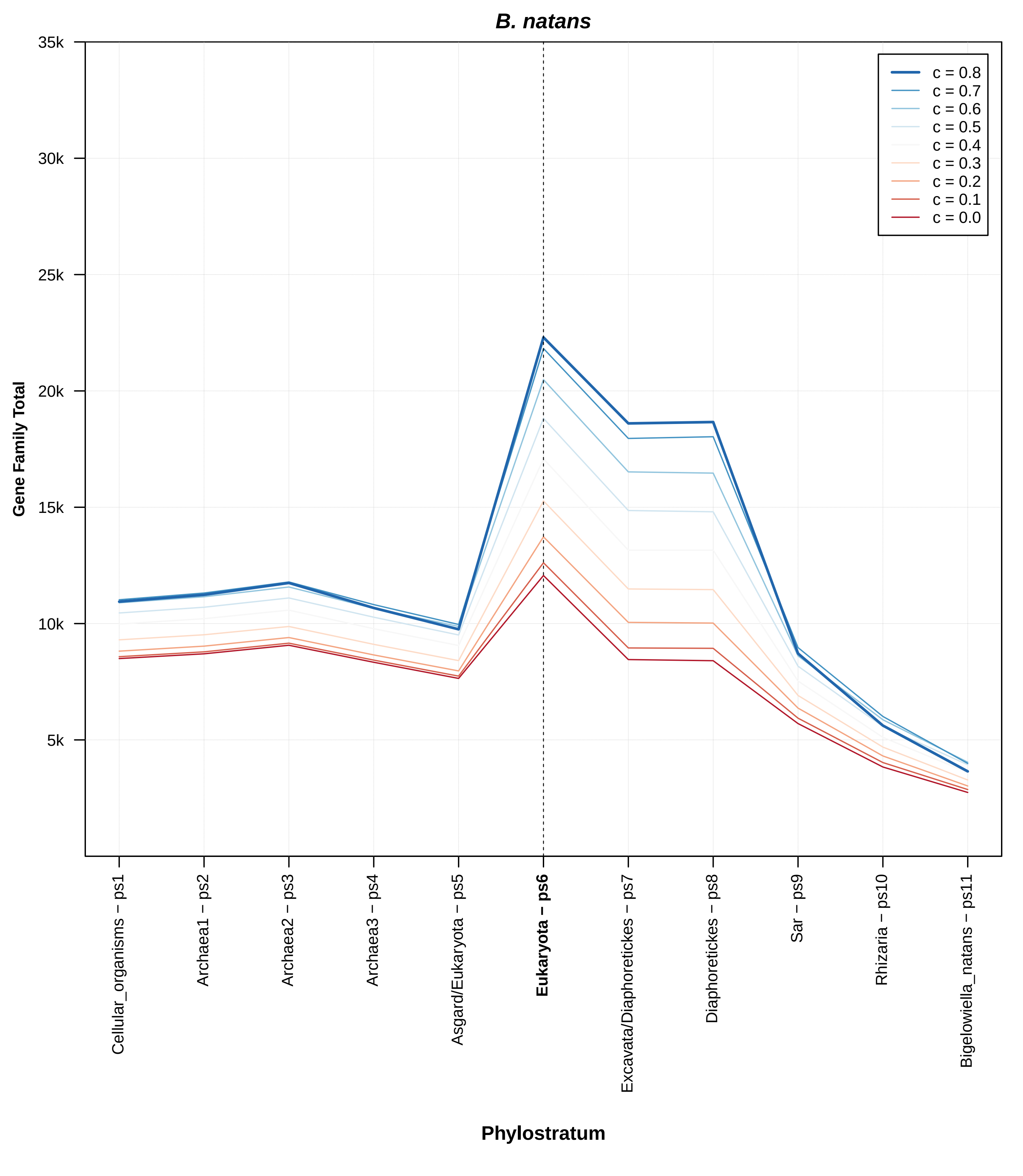

B. natans
35k
30k
25k
20k
15k
10k
5k
Gene Family Total
c = 0.8
c = 0.7
c = 0.6
c = 0.5
c = 0.4
c = 0.3
c = 0.2
c = 0.1
c = 0.0
Cellular_organisms – ps1
Archaea1 – ps2
Archaea2 – ps3
Archaea3 – ps4
Asgard/Eukaryota – ps5
Eukaryota – ps6
Excavata/Diaphoretickes – ps7
Diaphoretickes – ps8
Sar – ps9
Rhizaria – ps10
Bigelowiella_natans – ps11
Phylostratum

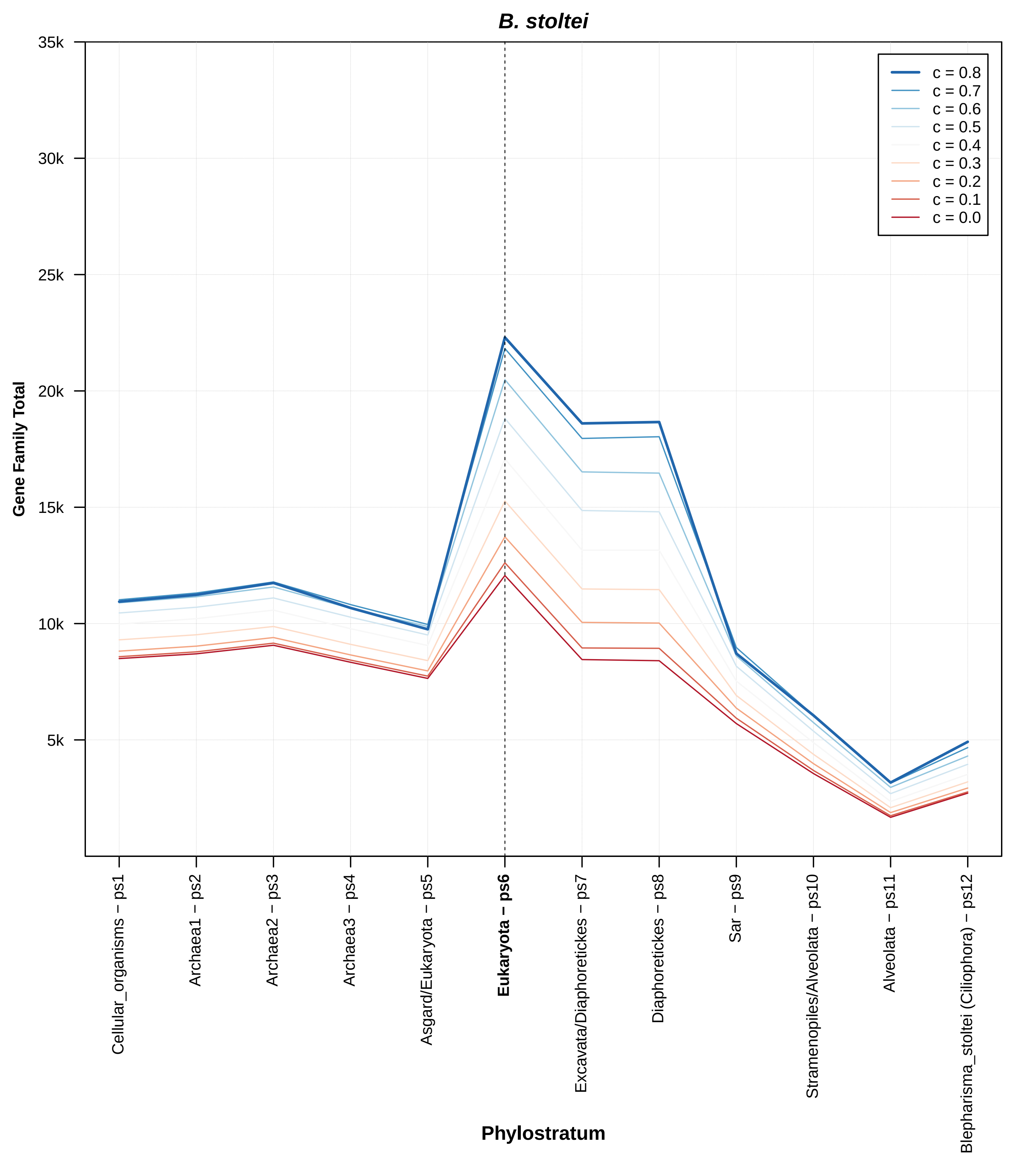
B. stoltei
35k
30k
25k
20k
15k
10k
5k
Gene Family Total
c = 0.8
c = 0.7
c = 0.6
c = 0.5
c = 0.4
c = 0.3
c = 0.2
c = 0.1
c = 0.0
Cellular_organisms – ps1
Archaea1 – ps2
Archaea2 – ps3
Archaea3 – ps4
Asgard/Eukaryota – ps5
Eukaryota – ps6
Excavata/Diaphoretickes – ps7
Diaphoretickes – ps8
Sar – ps9
Stramenopiles/Alveolata – ps10
Alveolata – ps11
Blepharisma_stoltei (Ciliophora) – ps12
Phylostratum

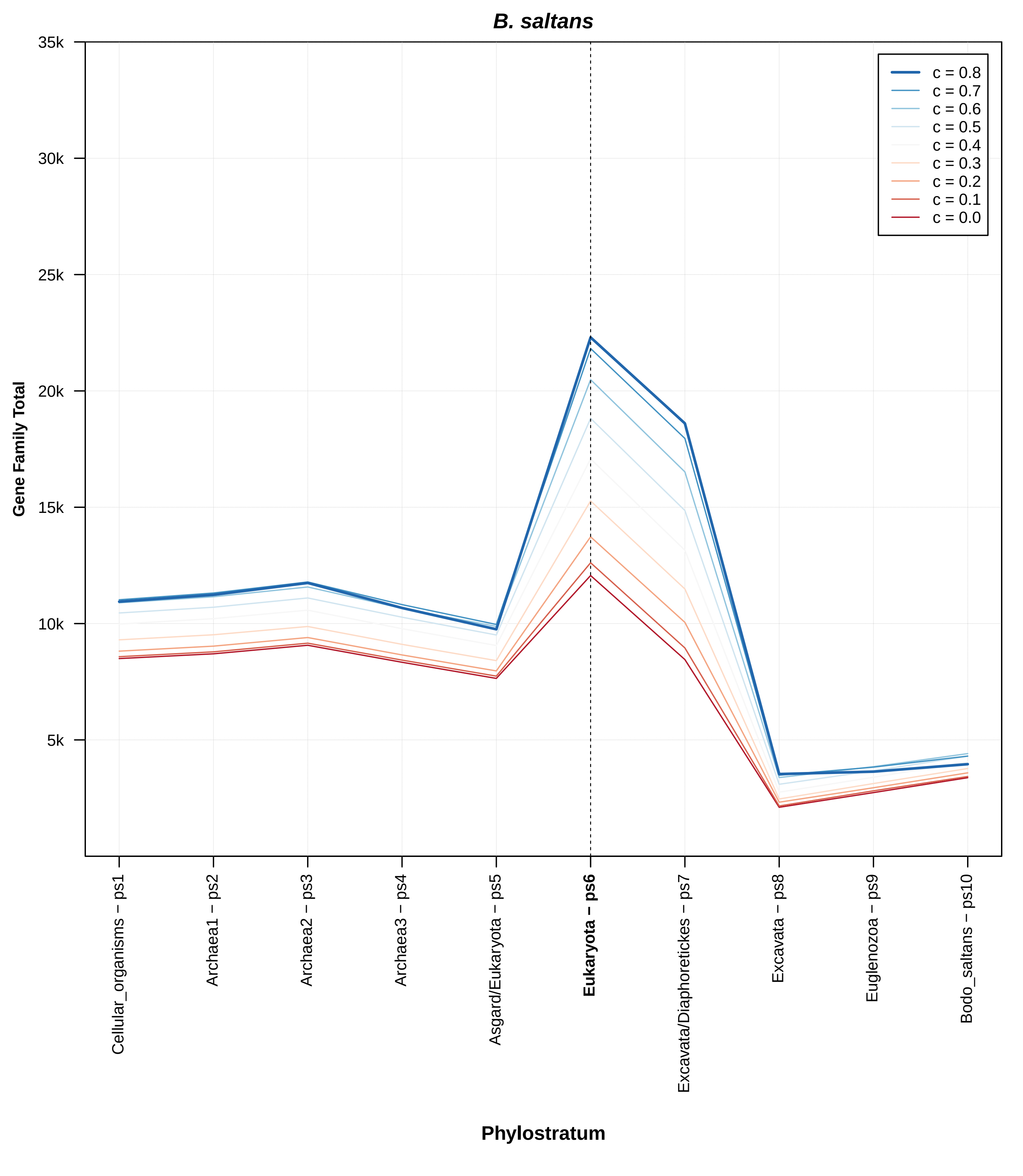

B. saltans
Gene Family Total
35k
30k
25k
20k
15k
10k
5k
c = 0.8
c = 0.7
c = 0.6
c = 0.5
c = 0.4
c = 0.3
c = 0.2
c = 0.1
c = 0.0
Cellular_organisms – ps1
Archaea1 – ps2
Archaea2 – ps3
Archaea3 – ps4
Asgard/Eukaryota – ps5
Eukaryota – ps6
Excavata/Diaphoretickes – ps7
Excavata – ps8
Euglenozoa – ps9
Bodo_saltans – ps10
Phylostratum

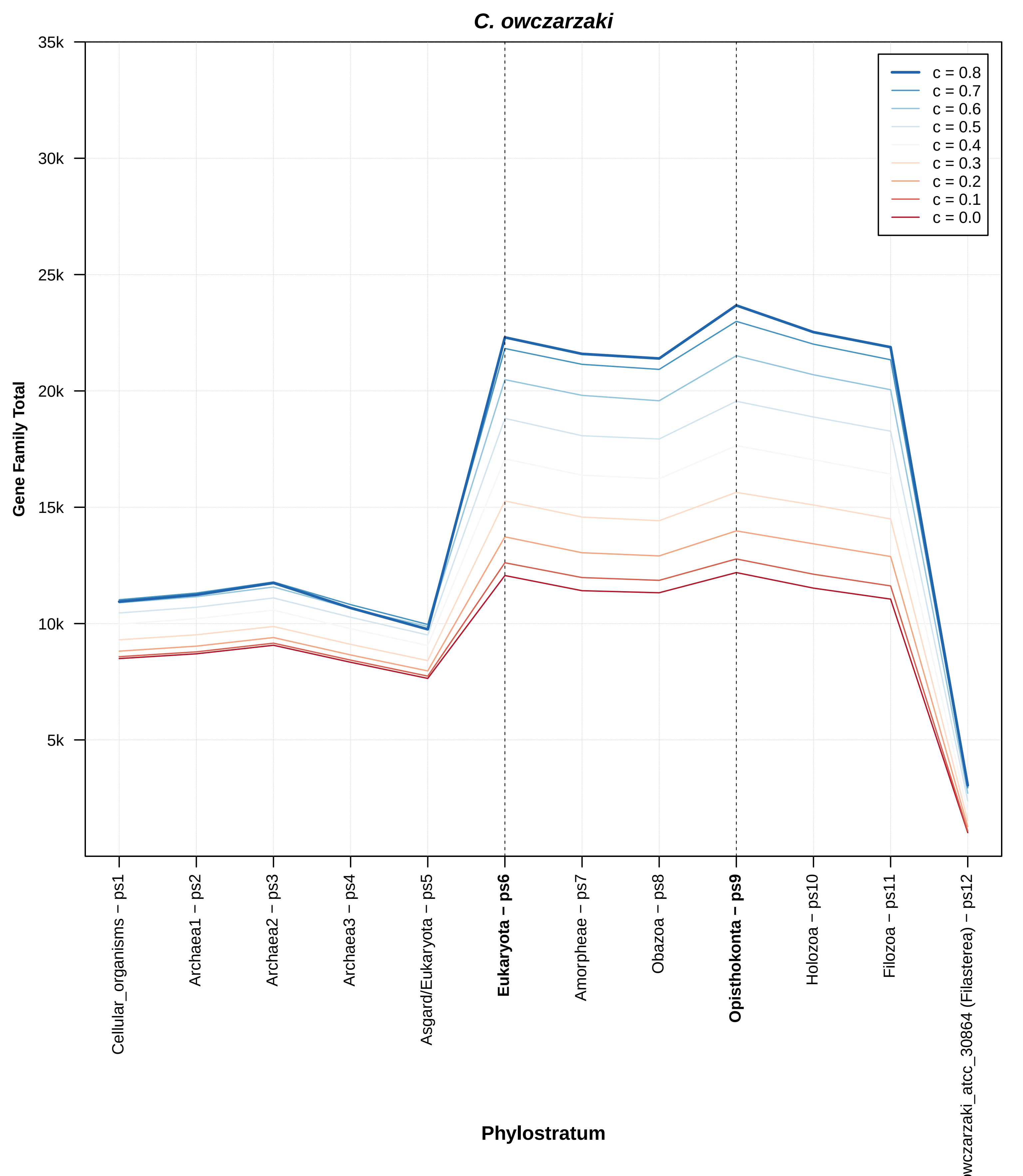
C. owczarzaki
Gene Family Total
35k
30k
25k
20k
15k
10k
5k
c = 0.8
c = 0.7
c = 0.6
c = 0.5
c = 0.4
c = 0.3
c = 0.2
c = 0.1
c = 0.0
Cellular_organisms – ps1
Archaea1 – ps2
Archaea2 – ps3
Archaea3 – ps4
Asgard/Eukaryota – ps5
Eukaryota – ps6
Amorpheae – ps7
Obazoa – ps8
Opisthokonta – ps9
Holozoa – ps10
Filozoa – ps11
wczarzaki_atcc_30864 (Filasterea) – ps12
Phylostratum

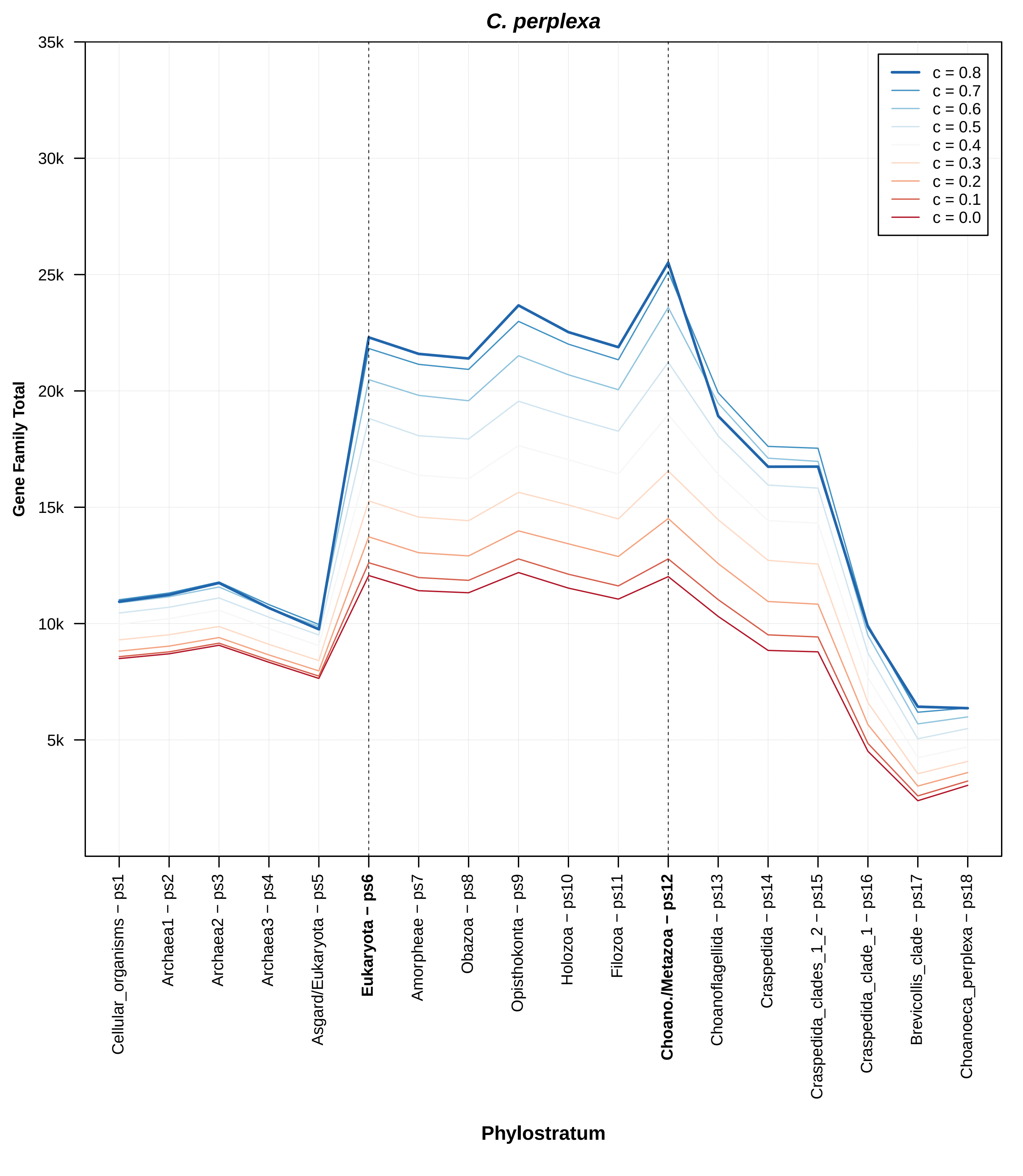
C. perplexa
Gene Family Total
35k
30k
25k
20k
15k
10k
5k
c = 0.8
c = 0.7
c = 0.6
c = 0.5
c = 0.4
c = 0.3
c = 0.2
c = 0.1
c = 0.0
Cellular_organisms – ps1
Archaea1 – ps2
Archaea2 – ps3
Archaea3 – ps4
Asgard/Eukaryota – ps5
Eukaryota – ps6
Amorpheae – ps7
Obazoa – ps8
Opisthokonta – ps9
Holozoa – ps10
Filozoa – ps11
Choano./Metazoa – ps12
Choanoflagellida – ps13
Craspedida – ps14
Craspedida_clades_1_2 – ps15
Craspedida_clade_1 – ps16
Brevicollis_clade – ps17
Choanoeca_perplexa – ps18
Phylostratum

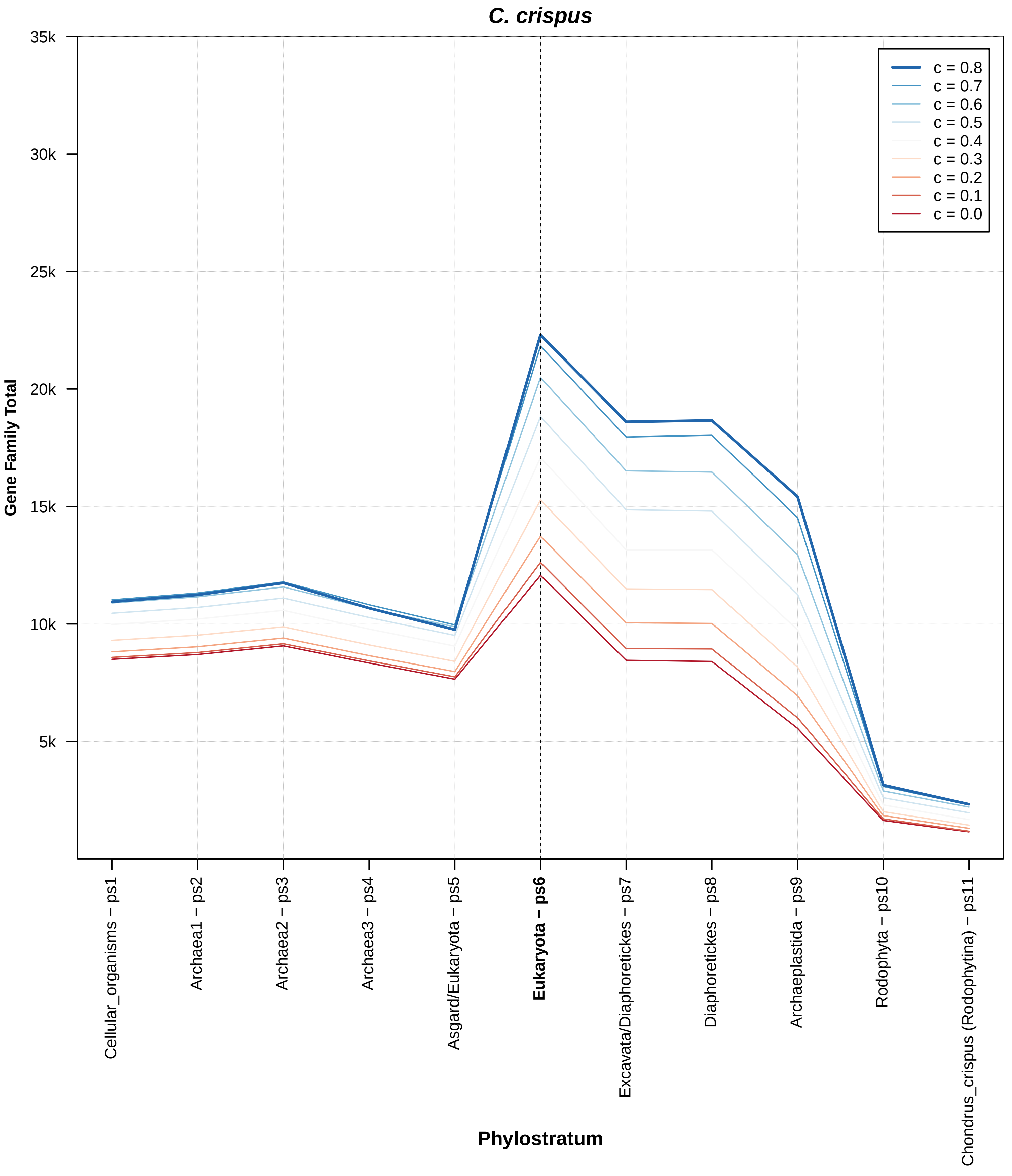
C. crispus
35k
30k
25k
20k
15k
10k
5k
Gene Family Total
c = 0.8
c = 0.7
c = 0.6
c = 0.5
c = 0.4
c = 0.3
c = 0.2
c = 0.1
c = 0.0
Cellular_organisms – ps1
Archaea1 – ps2
Archaea2 – ps3
Archaea3 – ps4
Asgard/Eukaryota – ps5
Eukaryota – ps6
Excavata/Diaphoretickes – ps7
Diaphoretickes – ps8
Archaeplastida – ps9
Rodophyta – ps10
Chondrus_crispus (Rodophytina) – ps11
Phylostratum

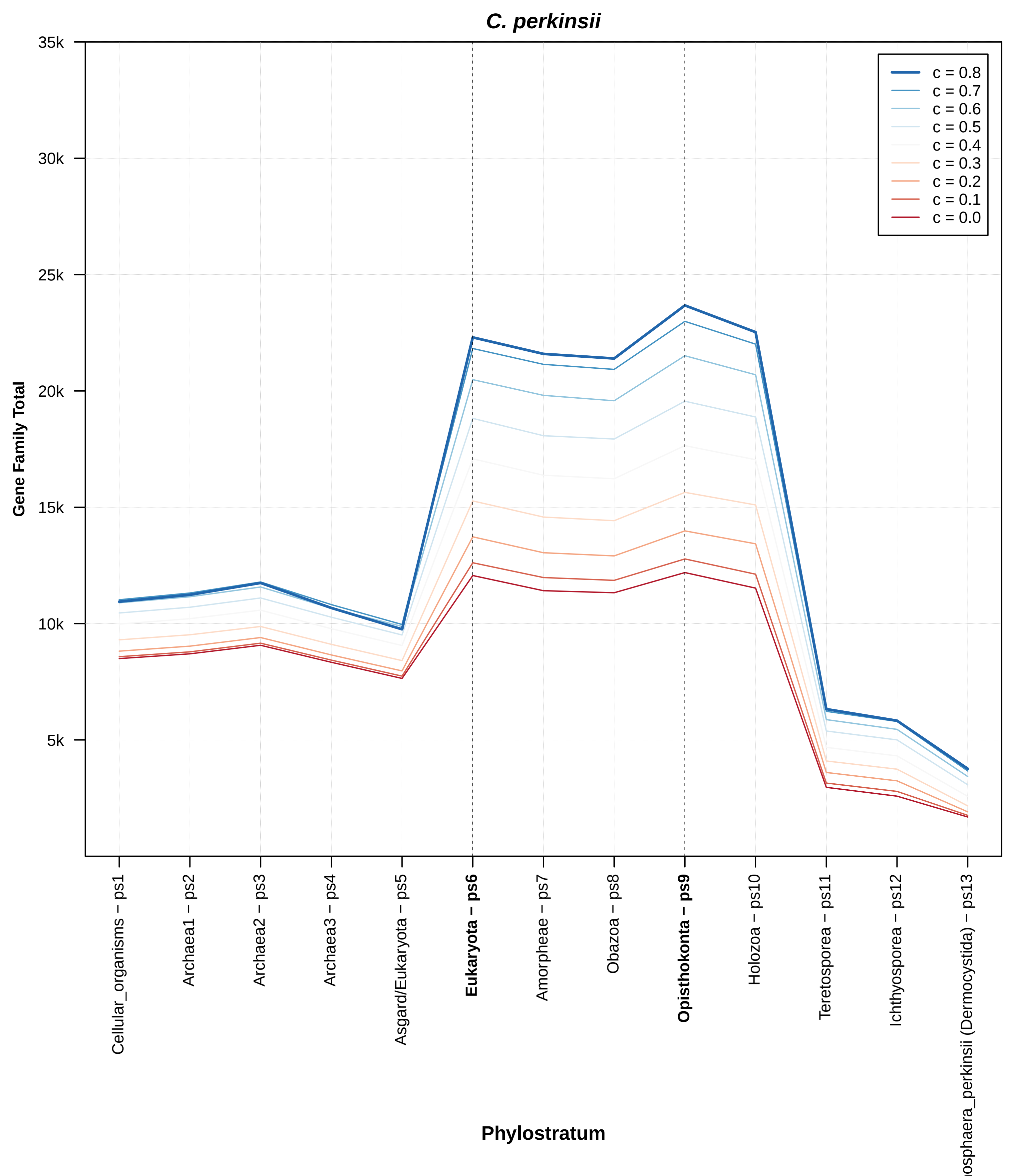

C. perkinsii
Gene Family Total
35k
30k
25k
20k
15k
10k
5k
c = 0.8
c = 0.7
c = 0.6
c = 0.5
c = 0.4
c = 0.3
c = 0.2
c = 0.1
c = 0.0
Cellular_organisms – ps1
Archaea1 – ps2
Archaea2 – ps3
Archaea3 – ps4
Asgard/Eukaryota – ps5
Eukaryota – ps6
Amorpheae – ps7
Obazoa – ps8
Opisthokonta – ps9
Holozoa – ps10
Teretosporea – ps11
Ichthyosporea – ps12
osphaera_perkinsii (Dermocystida) – ps13
Phylostratum

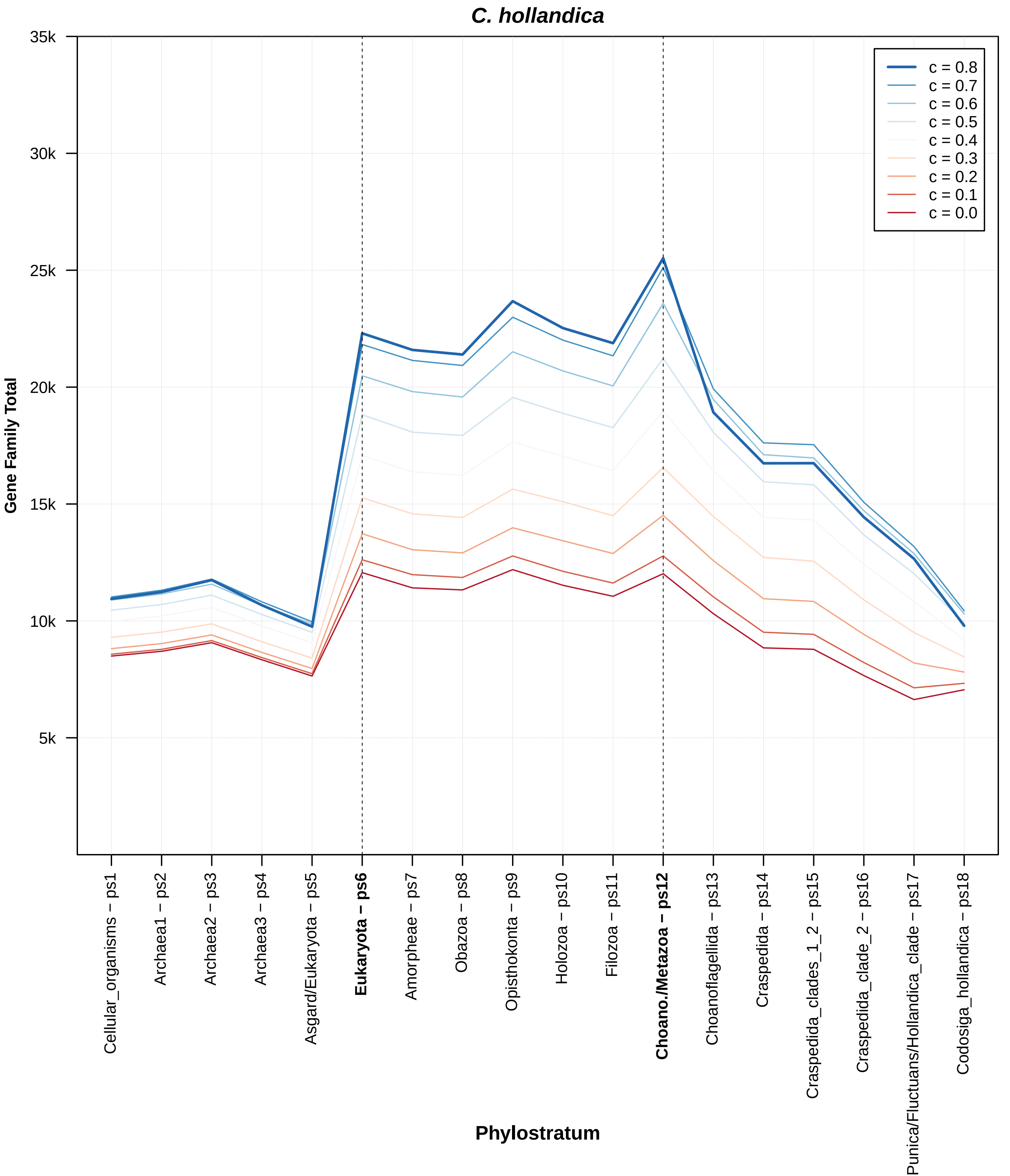
C. hollandica
35k
30k
25k
20k
15k
10k
5k
Gene Family Total
c = 0.8
c = 0.7
c = 0.6
c = 0.5
c = 0.4
c = 0.3
c = 0.2
c = 0.1
c = 0.0
Cellular_organisms – ps1
Archaea1 – ps2
Archaea2 – ps3
Archaea3 – ps4
Asgard/Eukaryota – ps5
Eukaryota – ps6
Amorpheae – ps7
Obazoa – ps8
Opisthokonta – ps9
Holozoa – ps10
Filozoa – ps11
Choano./Metazoa – ps12
Choanoflagellida – ps13
Craspedida – ps14
Craspedida_clades_1_2 – ps15
Craspedida_clade_2 – ps16
Punica/Fluctuans/Hollandica_clade – ps17
Codosiga_hollandica – ps18
Phylostratum

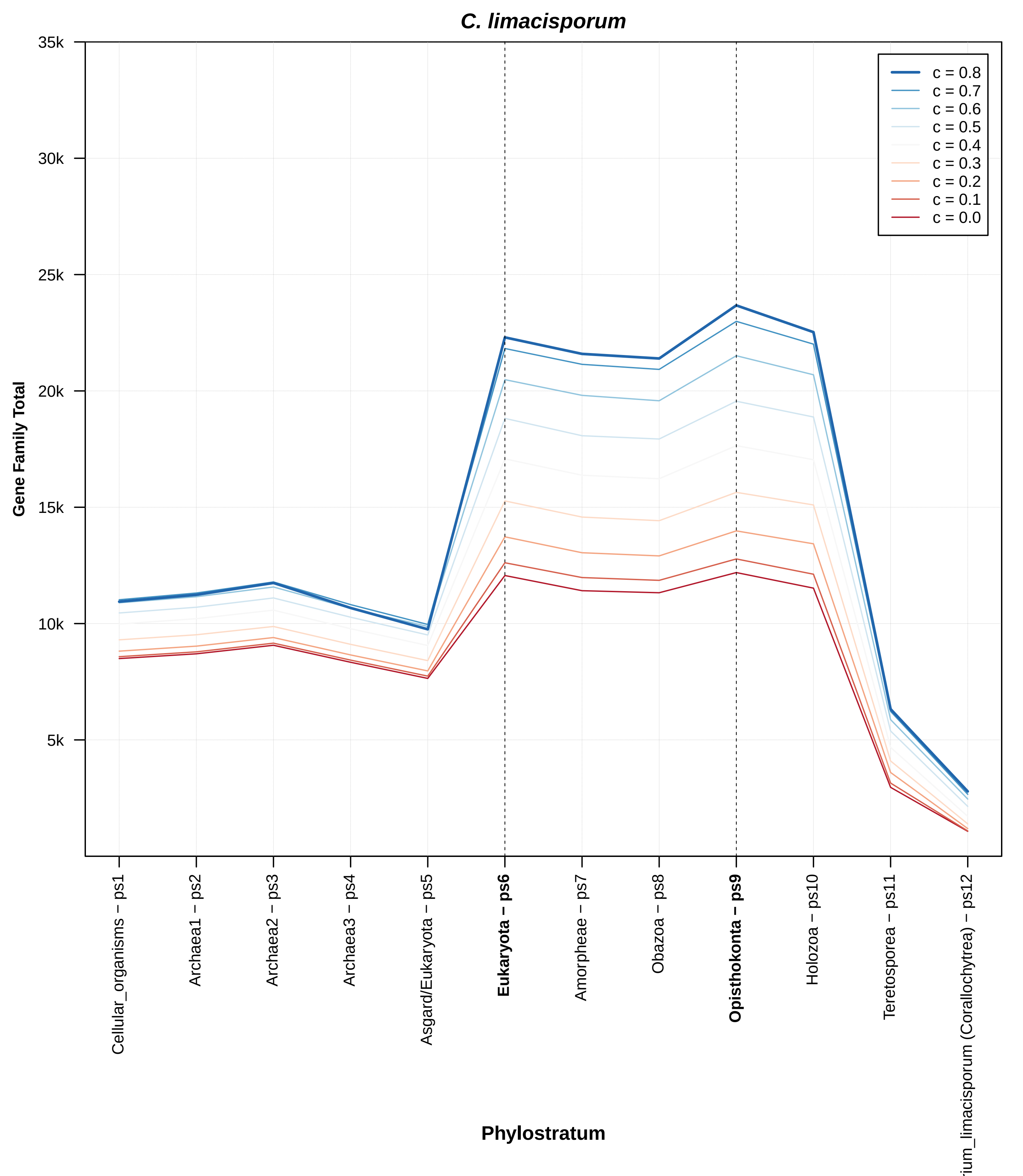

C. limacisporum
35k
30k
25k
20k
15k
10k
5k
Gene Family Total
c = 0.8
c = 0.7
c = 0.6
c = 0.5
c = 0.4
c = 0.3
c = 0.2
c = 0.1
c = 0.0
Cellular_organisms – ps1
Archaea1 – ps2
Archaea2 – ps3
Archaea3 – ps4
Asgard/Eukaryota – ps5
Eukaryota – ps6
Amorpheae – ps7
Obazoa – ps8
Opisthokonta – ps9
Holozoa – ps10
Teretosporea – ps11
rium_limacisporum (Corallochytrea) – ps12
Phylostratum

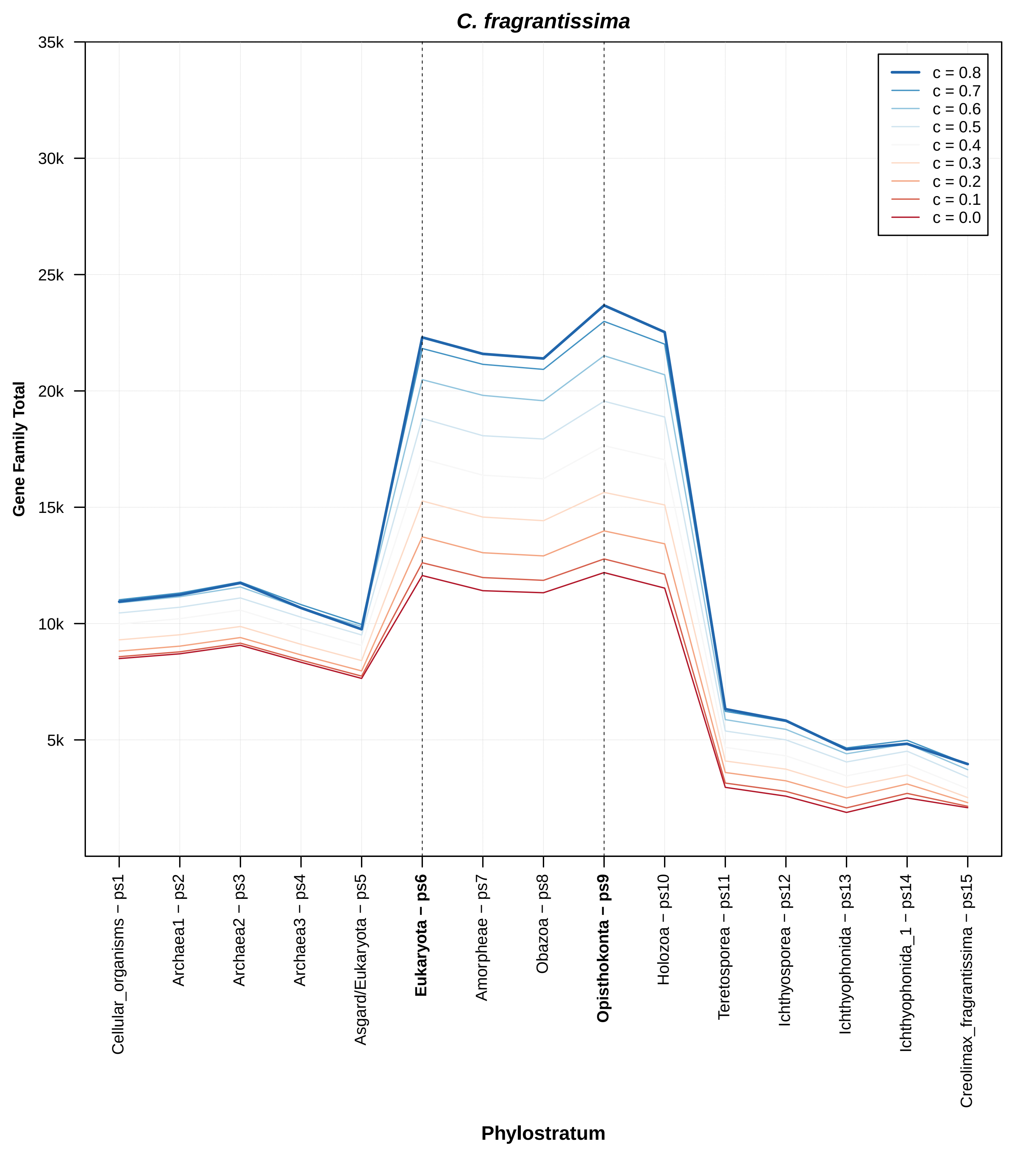
C. fragrantissima
35k
30k
25k
20k
15k
10k
5k
Gene Family Total
c = 0.8
c = 0.7
c = 0.6
c = 0.5
c = 0.4
c = 0.3
c = 0.2
c = 0.1
c = 0.0
Cellular_organisms – ps1
Archaea1 – ps2
Archaea2 – ps3
Archaea3 – ps4
Asgard/Eukaryota – ps5
Eukaryota – ps6
Amorpheae – ps7
Obazoa – ps8
Opisthokonta – ps9
Holozoa – ps10
Teretosporea – ps11
Ichthyosporea – ps12
Ichthyophonida – ps13
Ichthyophonida_1 – ps14
Creolimax_fragrantissima – ps15
Phylostratum

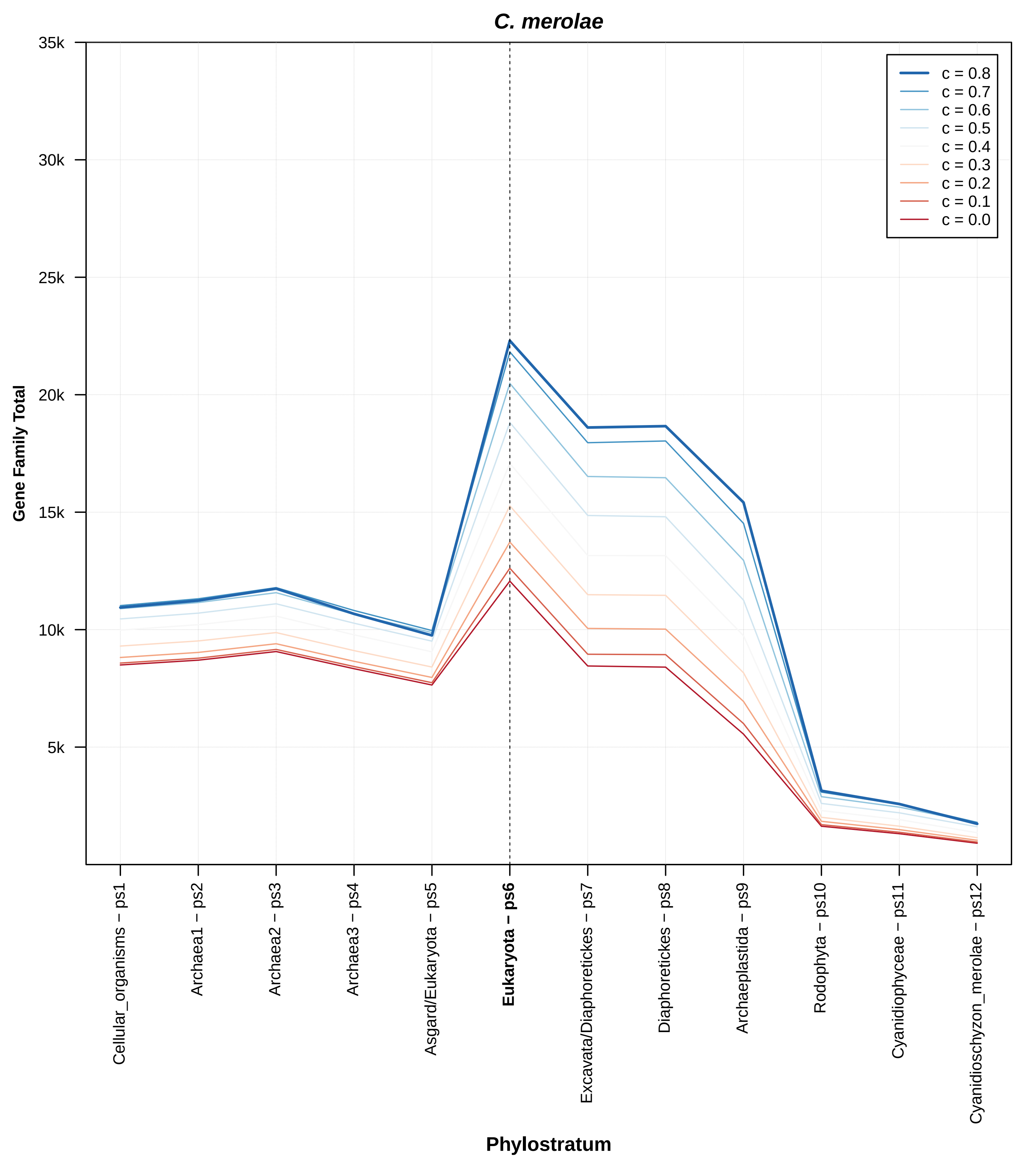
C. merolae
Gene Family Total
35k
30k
25k
20k
15k
10k
5k
c = 0.8
c = 0.7
c = 0.6
c = 0.5
c = 0.4
c = 0.3
c = 0.2
c = 0.1
c = 0.0
Cellular_organisms – ps1
Archaea1 – ps2
Archaea2 – ps3
Archaea3 – ps4
Asgard/Eukaryota – ps5
Eukaryota – ps6
Excavata/Diaphoretickes – ps7
Diaphoretickes – ps8
Archaeplastida – ps9
Rodophyta – ps10
Cyanidiophyceae – ps11
Cyanidioschyzon_merolae – ps12
Phylostratum

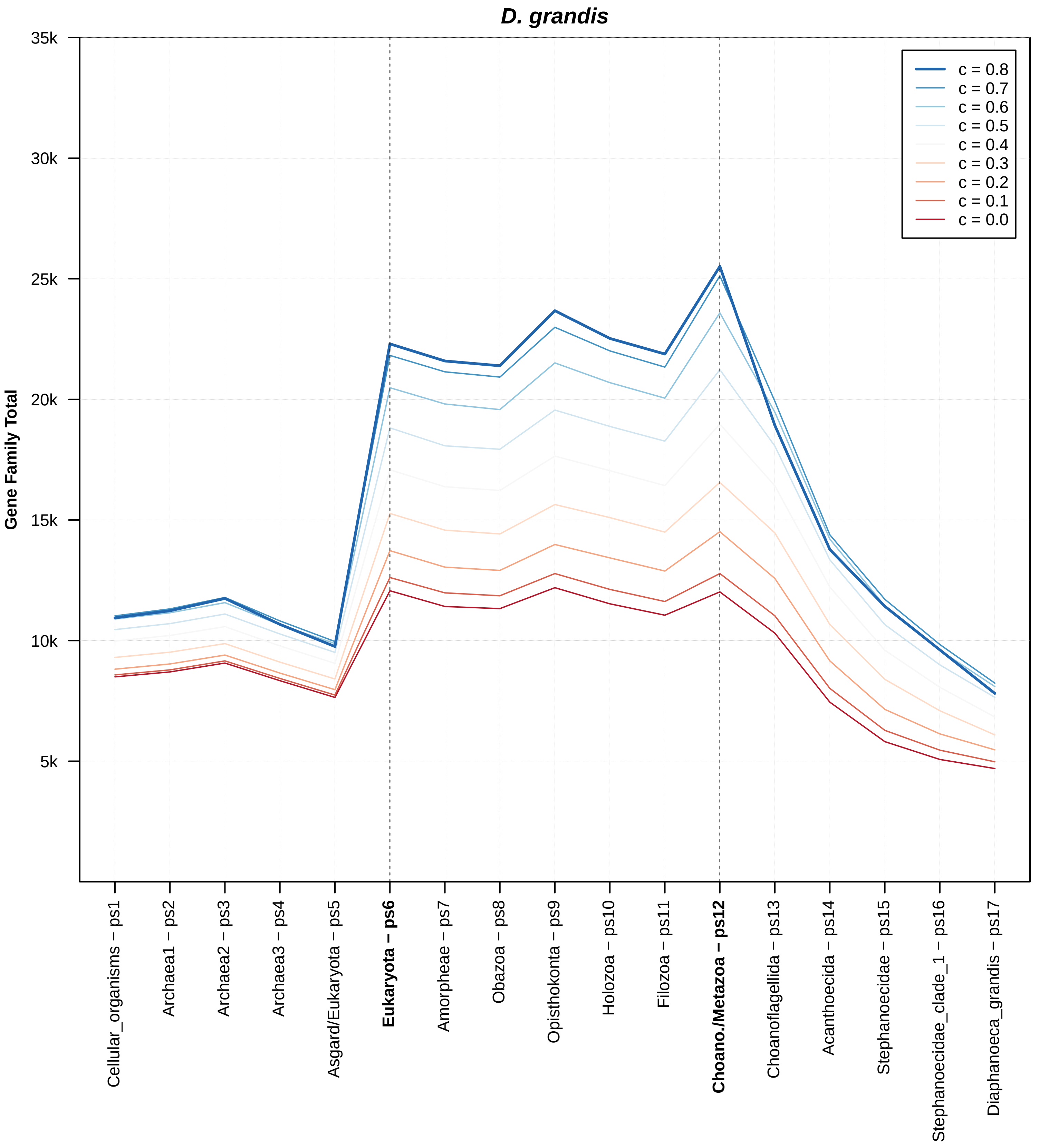

D. grandis
c = 0.8
c = 0.7
c = 0.6
c = 0.5
c = 0.4
c = 0.3
c = 0.2
c = 0.1
c = 0.0
Gene Family Total
35k
30k
25k
20k
15k
10k
5k
Cellular_organisms – ps1
Archaea1 – ps2
Archaea2 – ps3
Archaea3 – ps4
Asgard/Eukaryota – ps5
Eukaryota – ps6
Amorpheae – ps7
Obazoa – ps8
Opisthokonta – ps9
Holozoa – ps10
Filozoa – ps11
Choano./Metazoa – ps12
Choanoflagellida – ps13
Acanthoecida – ps14
Stephanoecidae – ps15
Stephanoecidae_clade_1 – ps16
Diaphanoeca_grandis – ps17
Phylostratum

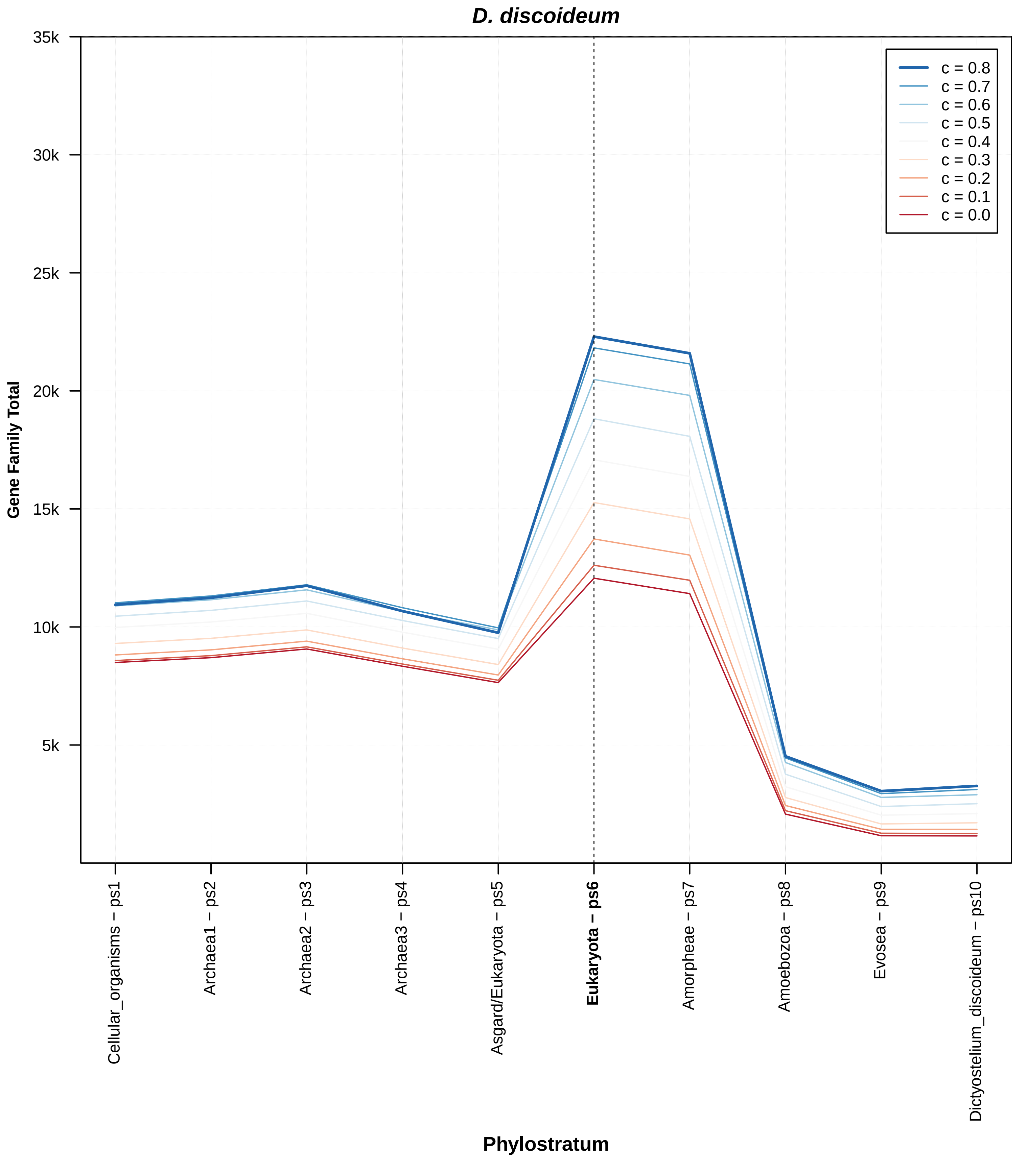
D. discoideum
35k
30k
25k
20k
15k
10k
5k
Gene Family Total
c = 0.8
c = 0.7
c = 0.6
c = 0.5
c = 0.4
c = 0.3
c = 0.2
c = 0.1
c = 0.0
Cellular_organisms – ps1
Archaea1 – ps2
Archaea2 – ps3
Archaea3 – ps4
Asgard/Eukaryota – ps5
Eukaryota – ps6
Amorpheae – ps7
Amoebozoa – ps8
Evosea – ps9
Dictyostelium_discoideum – ps10
Phylostratum

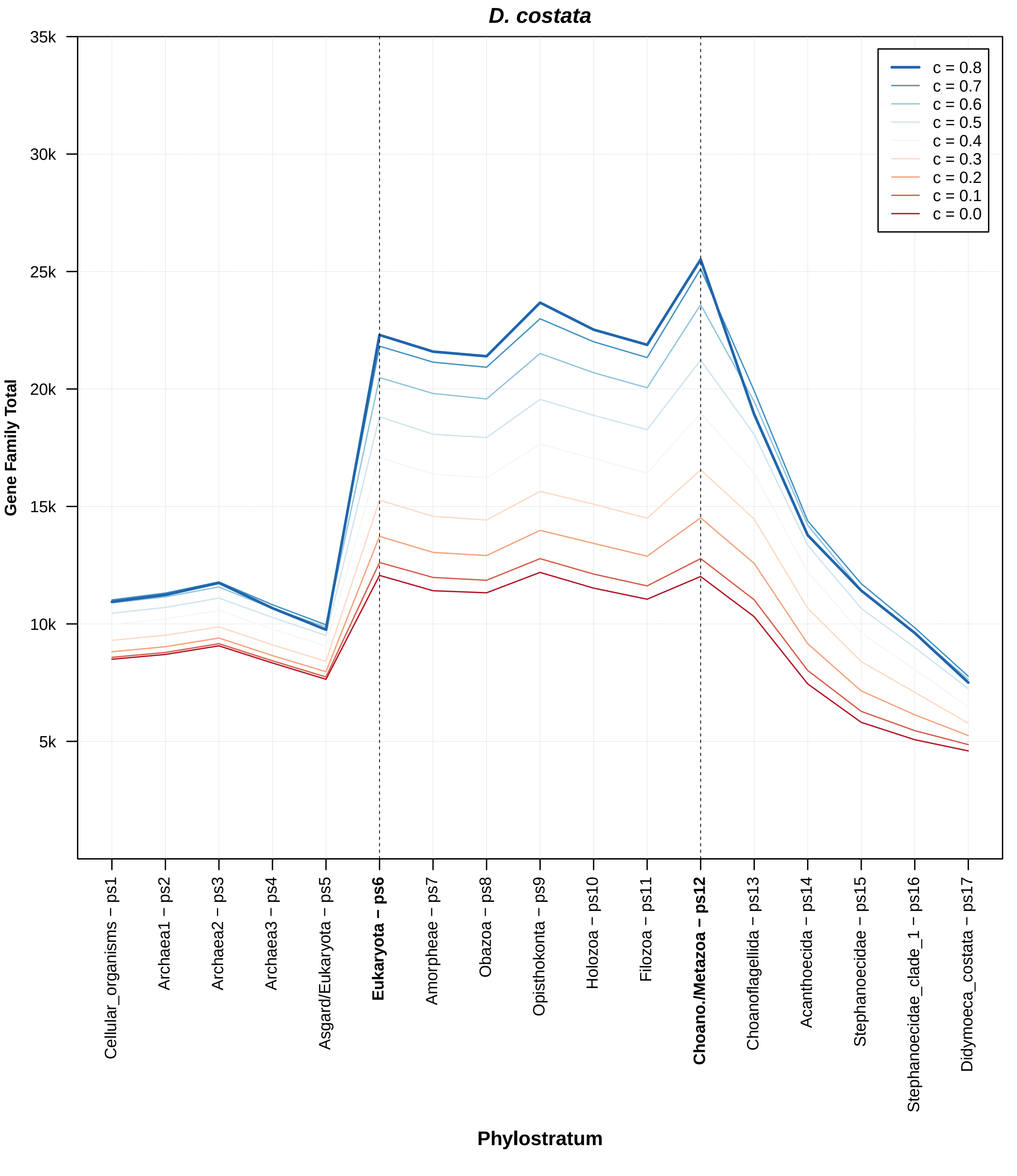

D. costata
Gene Family Total
35k
30k
25k
20k
15k
10k
5k
c = 0.8
c = 0.7
c = 0.6
c = 0.5
c = 0.4
c = 0.3
c = 0.2
c = 0.1
c = 0.0
Cellular_organisms – ps1
Archaea1 – ps2
Archaea2 – ps3
Archaea3 – ps4
Asgard/Eukaryota – ps5
Eukaryota – ps6
Amorpheae – ps7
Obazoa – ps8
Opisthokonta – ps9
Holozoa – ps10
Filozoa – ps11
Choano./Metazoa – ps12
Choanoflagellida – ps13
Acanthoecida – ps14
Stephanoecidae – ps15
Stephanoecidae_clade_1 – ps16
Didymoeca_costata – ps17
Phylostratum

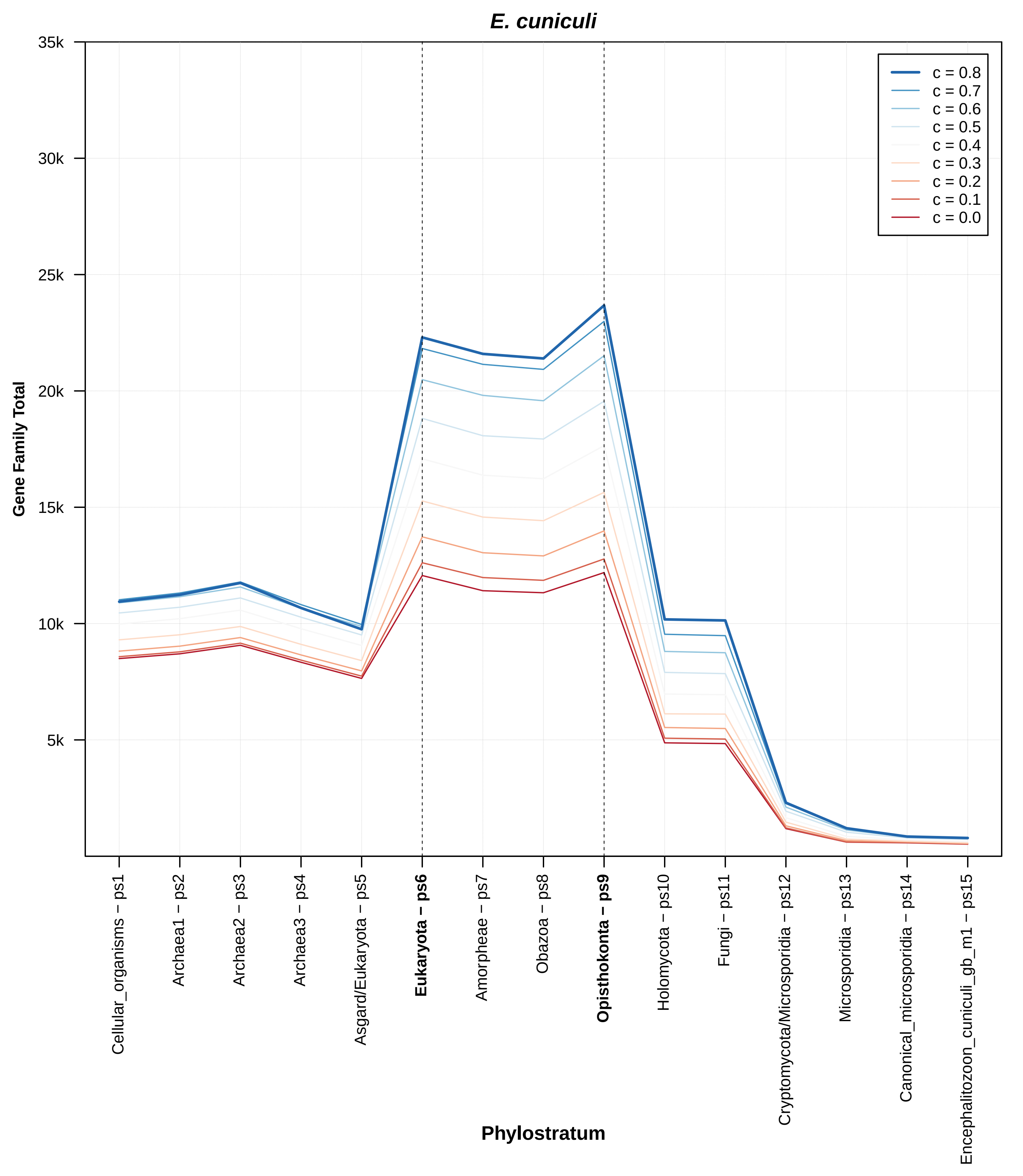

E. cuniculi
c = 0.8
c = 0.7
c = 0.6
c = 0.5
c = 0.4
c = 0.3
c = 0.2
c = 0.1
c = 0.0
35k
30k
25k
20k
15k
10k
5k
Gene Family Total
Cellular_organisms – ps1
Archaea1 – ps2
Archaea2 – ps3
Archaea3 – ps4
Asgard/Eukaryota – ps5
Eukaryota – ps6
Amorpheae – ps7
Obazoa – ps8
Opisthokonta – ps9
Holomycota – ps10
Fungi – ps11
Cryptomycota/Microsporidia – ps12
Microsporidia – ps13
Canonical_microsporidia – ps14
Encephalitozoon_cuniculi_gb_m1 – ps15
Phylostratum

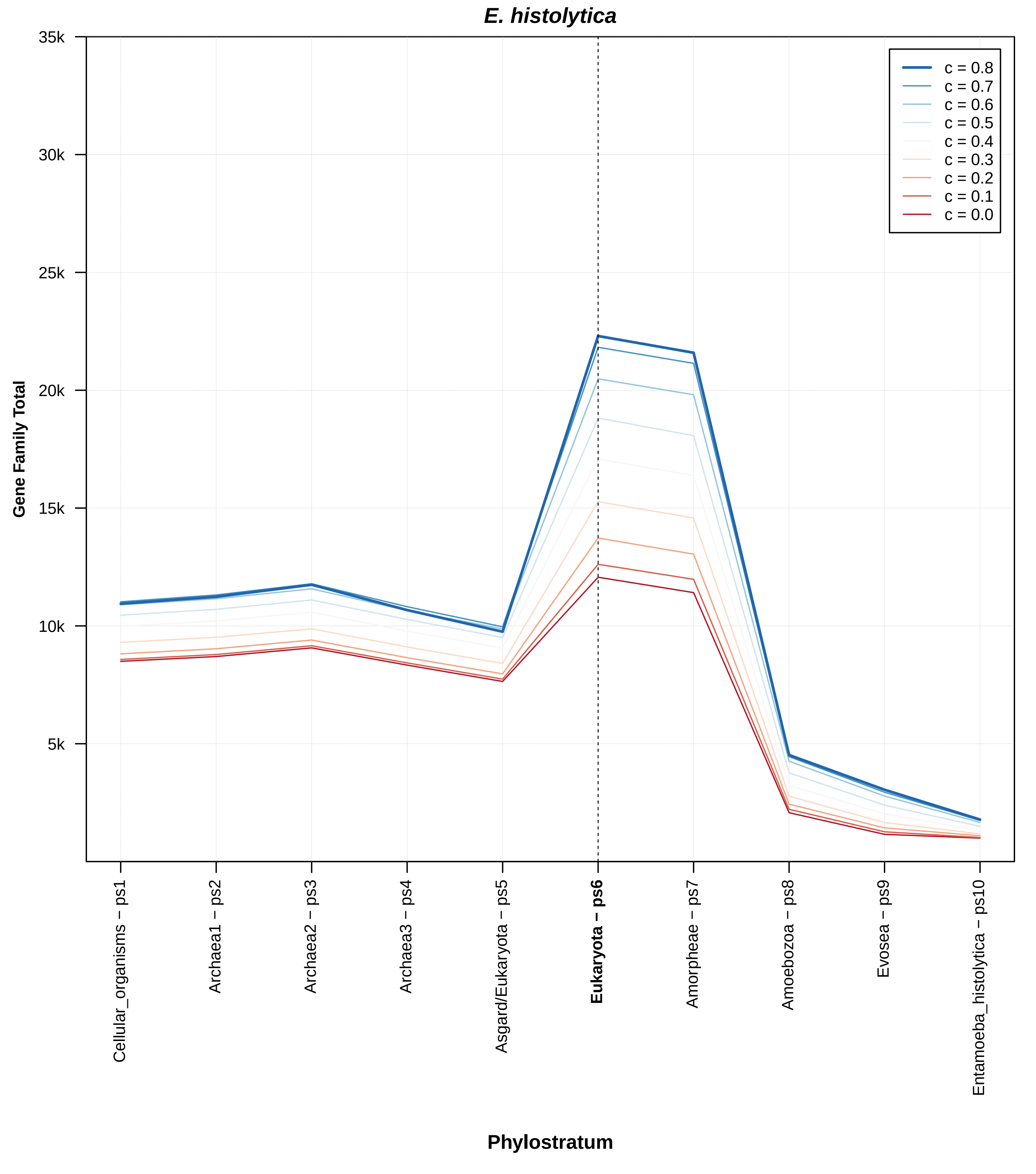
E. histolytica
Gene Family Total
35k
30k
25k
20k
15k
10k
5k
c = 0.8
c = 0.7
c = 0.6
c = 0.5
c = 0.4
c = 0.3
c = 0.2
c = 0.1
c = 0.0
Cellular_organisms – ps1
Archaea1 – ps2
Archaea2 – ps3
Archaea3 – ps4
Asgard/Eukaryota – ps5
Eukaryota – ps6
Amorpheae – ps7
Amoebozoa – ps8
Evosea – ps9
Entamoeba_histolytica – ps10
Phylostratum

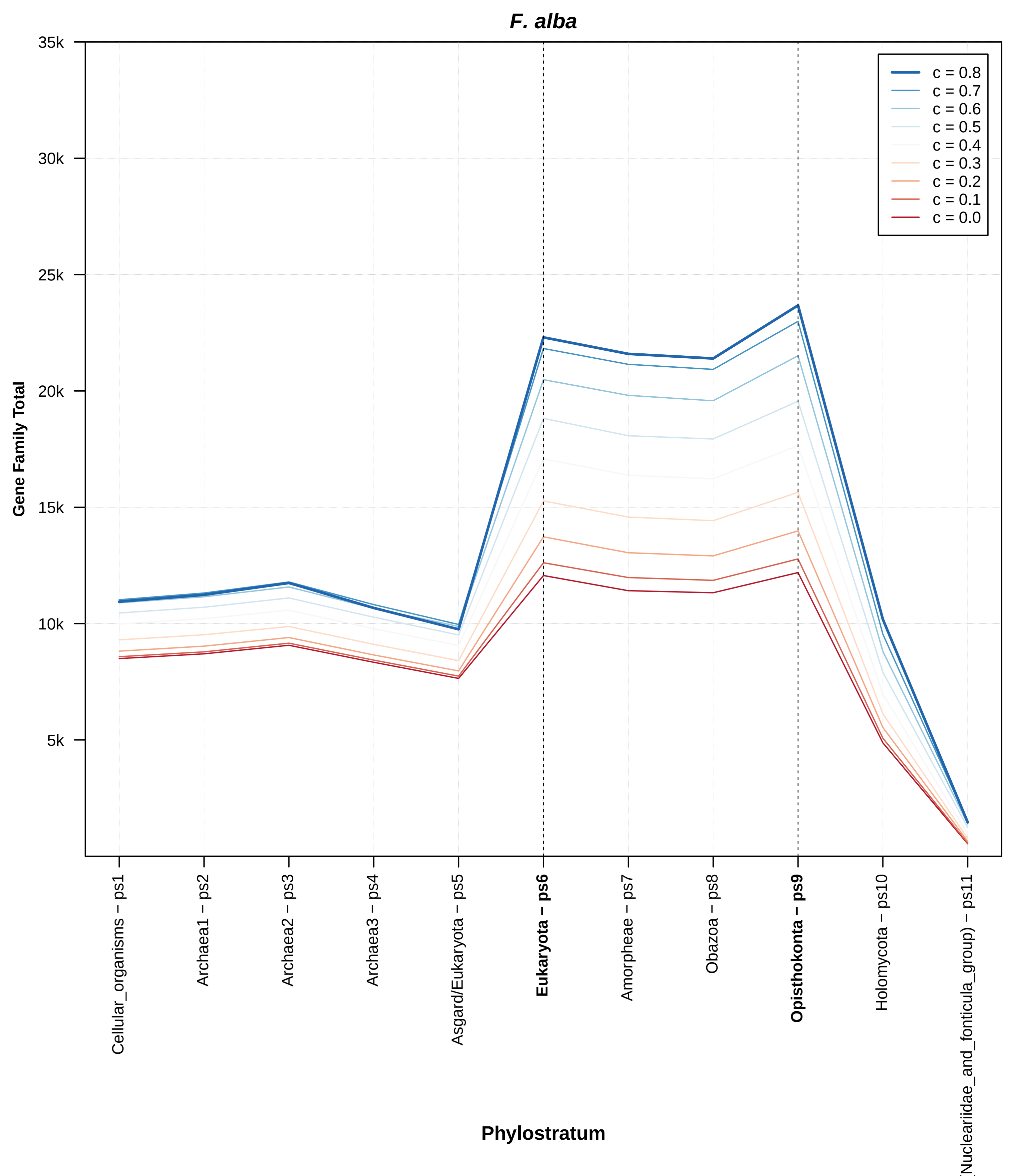

F. alba
35k
30k
25k
20k
15k
10k
5k
Gene Family Total
c = 0.8
c = 0.7
c = 0.6
c = 0.5
c = 0.4
c = 0.3
c = 0.2
c = 0.1
c = 0.0
Cellular_organisms – ps1
Archaea1 – ps2
Archaea2 – ps3
Archaea3 – ps4
Asgard/Eukaryota – ps5
Eukaryota – ps6
Amorpheae – ps7
Obazoa – ps8
Opisthokonta – ps9
Holomycota – ps10
(Nucleariidae_and_fonticula_group) – ps11
Phylostratum

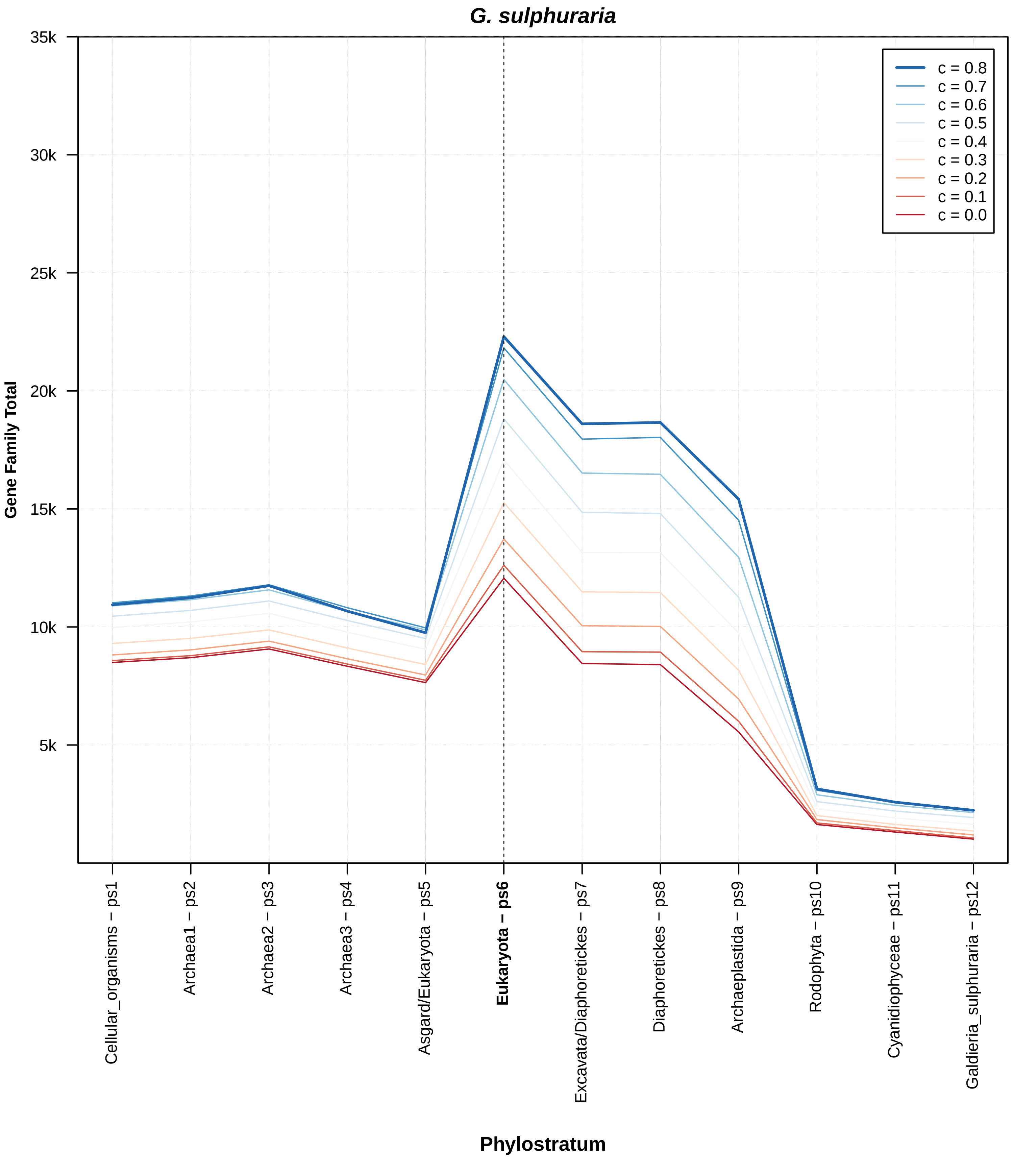
G. sulphuraria
Gene Family Total
35k
30k
25k
20k
15k
10k
5k
c = 0.8
c = 0.7
c = 0.6
c = 0.5
c = 0.4
c = 0.3
c = 0.2
c = 0.1
c = 0.0
Cellular_organisms – ps1
Archaea1 – ps2
Archaea2 – ps3
Archaea3 – ps4
Asgard/Eukaryota – ps5
Eukaryota – ps6
Excavata/Diaphoretickes – ps7
Diaphoretickes – ps8
Archaeplastida – ps9
Rodophyta – ps10
Cyanidiophyceae – ps11
Galdieria_sulphuraria – ps12
Phylostratum

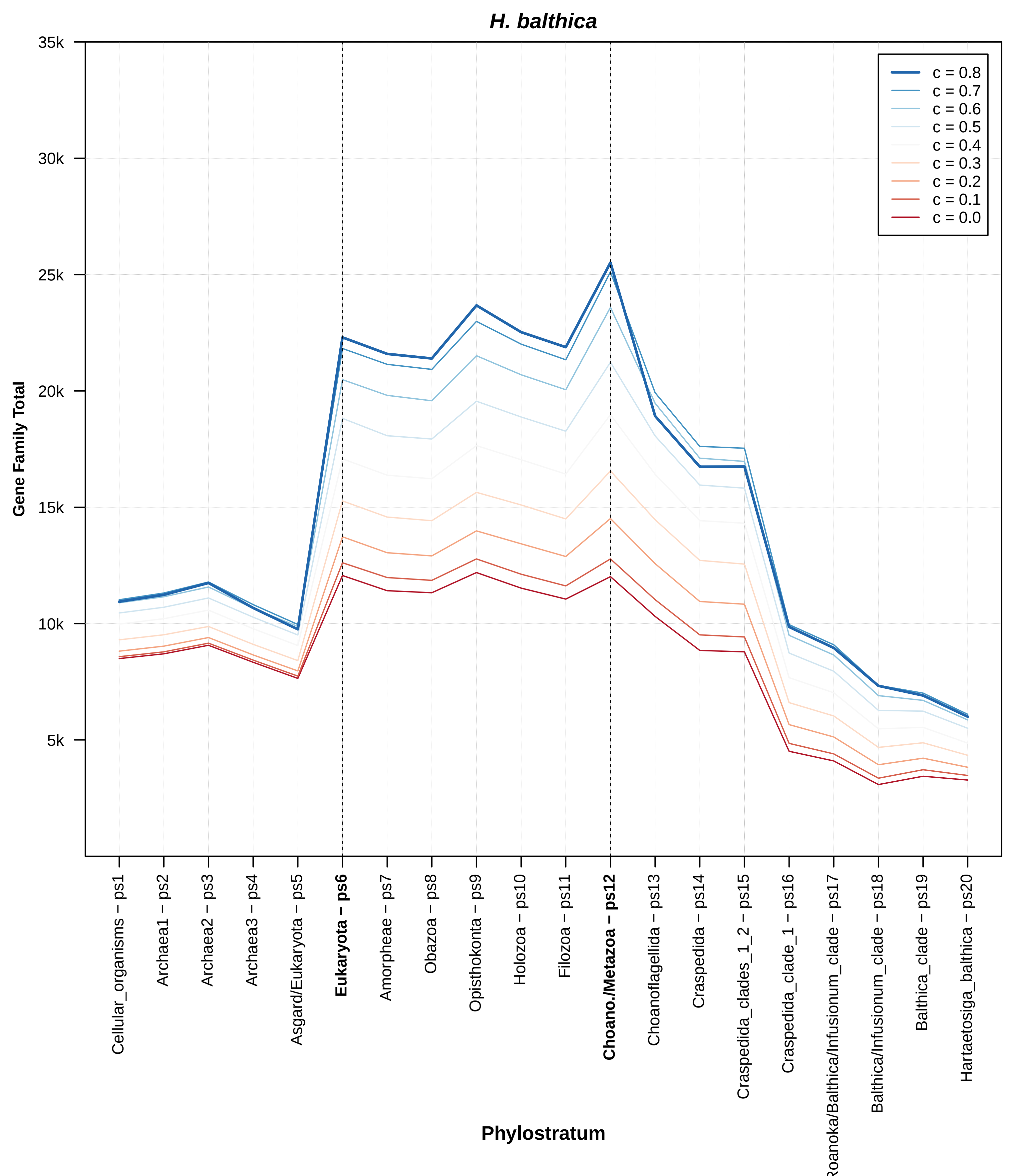
H. balthica
Gene Family Total
35k
30k
25k
20k
15k
10k
5k
c = 0.8
c = 0.7
c = 0.6
c = 0.5
c = 0.4
c = 0.3
c = 0.2
c = 0.1
c = 0.0
Cellular_organisms – ps1
Archaea1 – ps2
Archaea2 – ps3
Archaea3 – ps4
Asgard/Eukaryota – ps5
Eukaryota – ps6
Amorpheae – ps7
Obazoa – ps8
Opisthokonta – ps9
Holozoa – ps10
Filozoa – ps11
Choano./Metazoa – ps12
Choanoflagellida – ps13
Craspedida – ps14
Craspedida_clades_1_2 – ps15
Craspedida_clade_1 – ps16
Roanoka/Balthica/Infusionum_clade – ps17
Balthica/Infusionum_clade – ps18
Balthica_clade – ps19
Hartaetosiga_balthica – ps20
Phylostratum

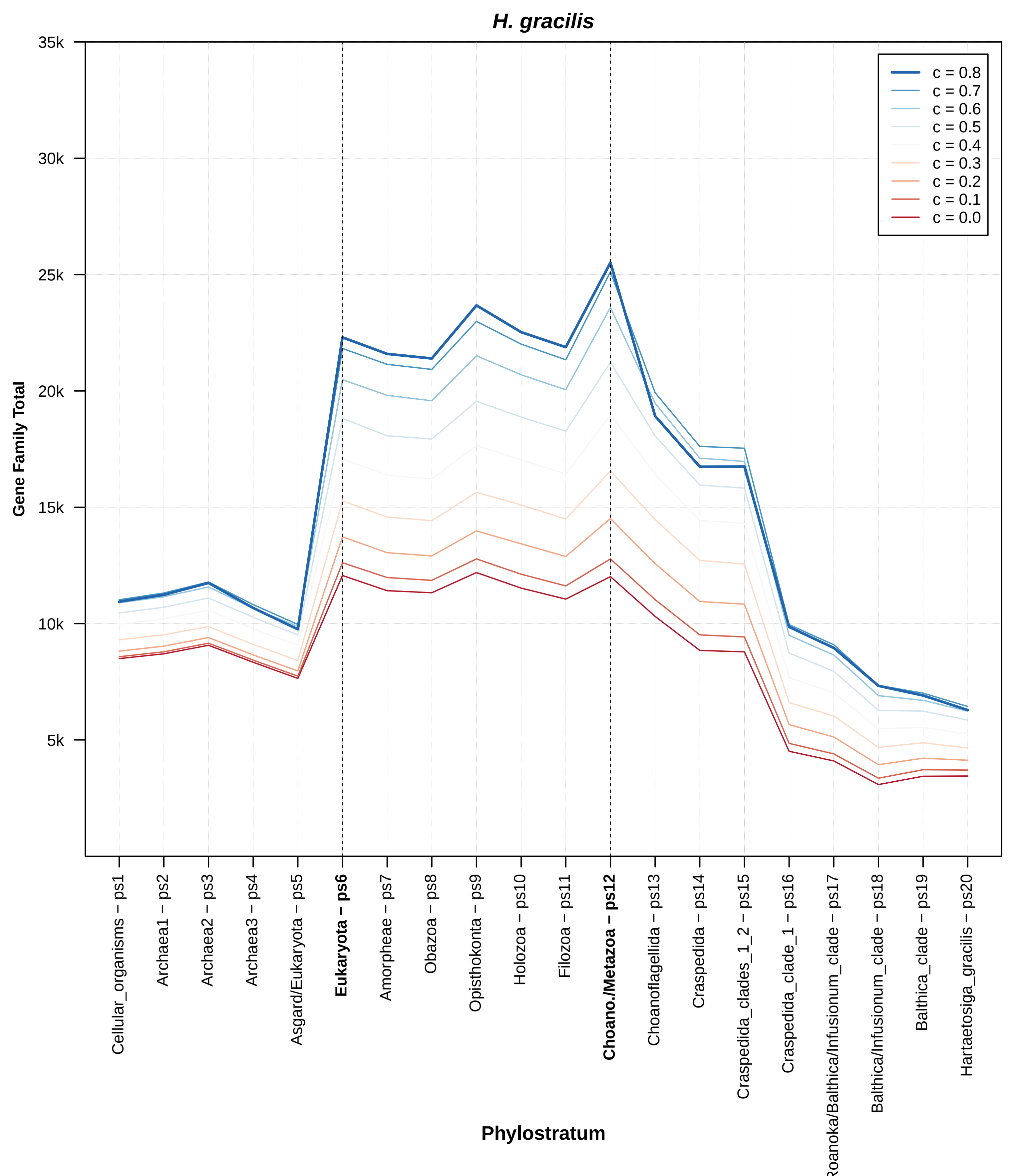

H. gracilis
Gene Family Total
35k
30k
25k
20k
15k
10k
5k
c = 0.8
c = 0.7
c = 0.6
c = 0.5
c = 0.4
c = 0.3
c = 0.2
c = 0.1
c = 0.0
Cellular_organisms – ps1
Archaea1 – ps2
Archaea2 – ps3
Archaea3 – ps4
Asgard/Eukaryota – ps5
Eukaryota – ps6
Amorpheae – ps7
Obazoa – ps8
Opisthokonta – ps9
Holozoa – ps10
Filozoa – ps11
Choano./Metazoa – ps12
Choanoflagellida – ps13
Craspedida – ps14
Craspedida_clades_1_2 – ps15
Craspedida_clade_1 – ps16
Roanoka/Balthica/Infusionum_clade – ps17
Balthica/Infusionum_clade – ps18
Balthica_clade – ps19
Hartaetosiga_gracilis – ps20
Phylostratum

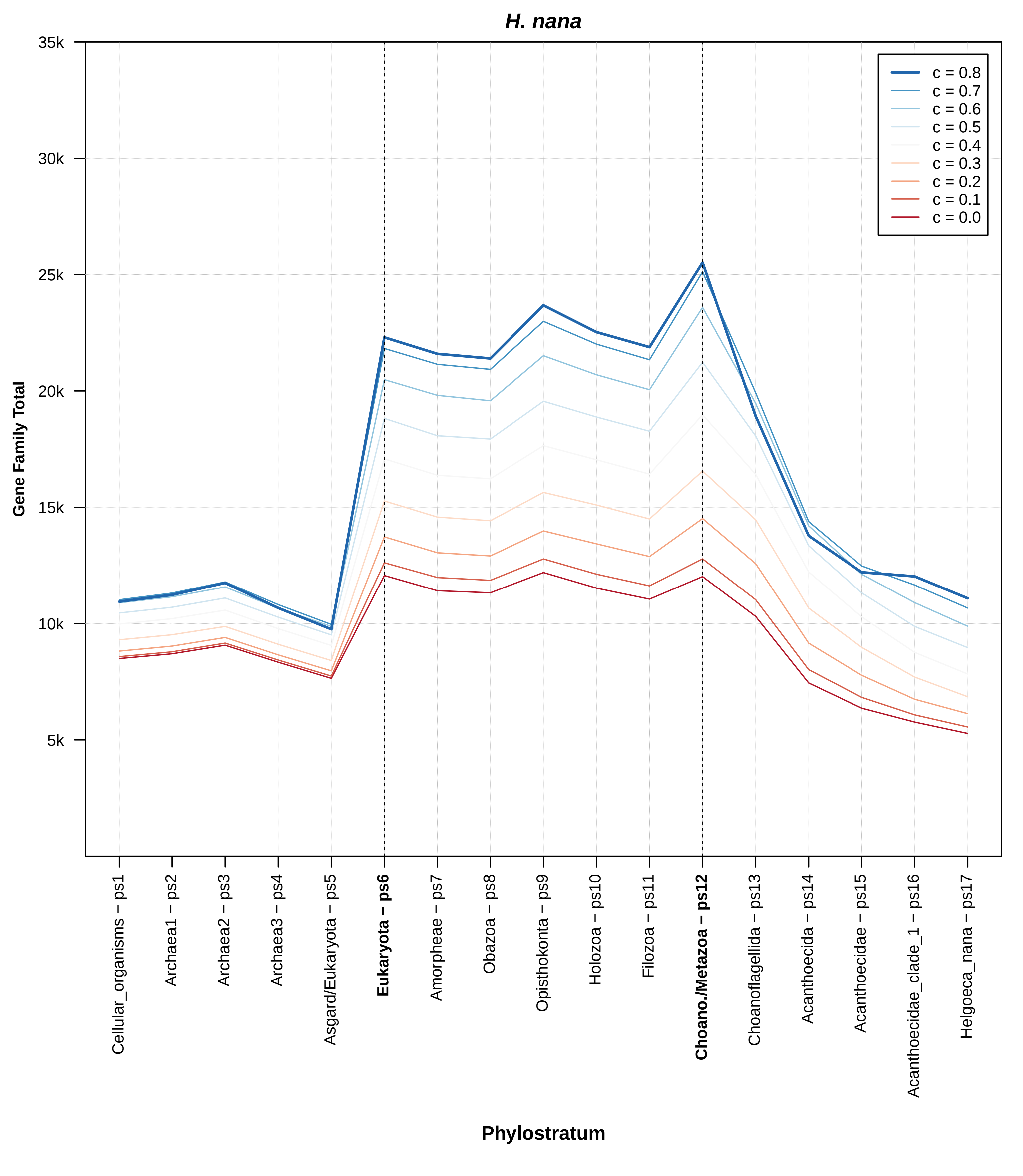

H. nana
Gene Family Total
35k
30k
25k
20k
15k
10k
5k
c = 0.8
c = 0.7
c = 0.6
c = 0.5
c = 0.4
c = 0.3
c = 0.2
c = 0.1
c = 0.0
Cellular_organisms – ps1
Archaea1 – ps2
Archaea2 – ps3
Archaea3 – ps4
Asgard/Eukaryota – ps5
Eukaryota – ps6
Amorpheae – ps7
Obazoa – ps8
Opisthokonta – ps9
Holozoa – ps10
Filozoa – ps11
Choano./Metazoa – ps12
Choanoflagellida – ps13
Acanthoecida – ps14
Acanthoecidae – ps15
Acanthoecidae_clade_1 – ps16
Helgoeca_nana – ps17
Phylostratum

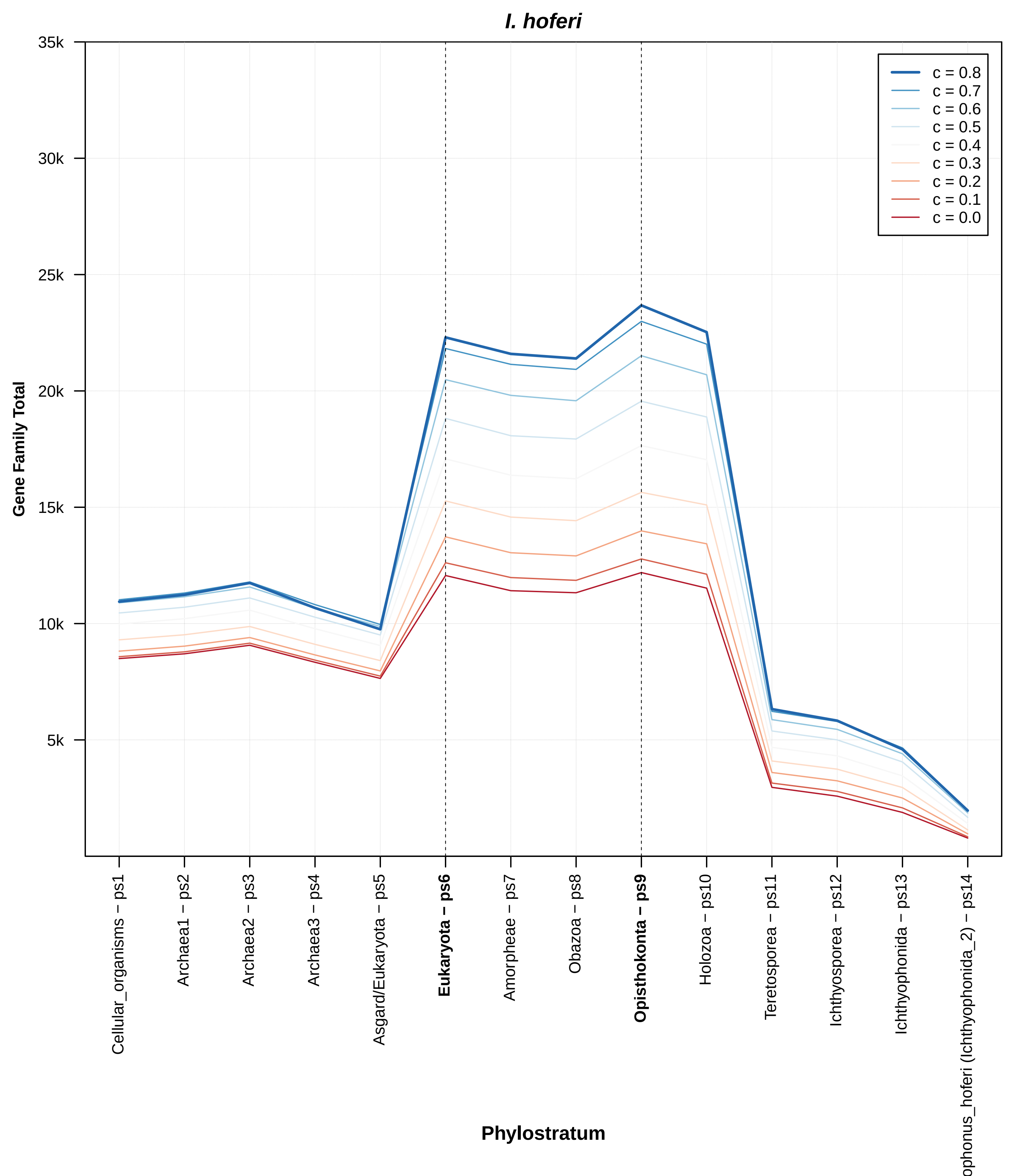

I. hoferi
Gene Family Total
35k
30k
25k
20k
15k
10k
5k
c = 0.8
c = 0.7
c = 0.6
c = 0.5
c = 0.4
c = 0.3
c = 0.2
c = 0.1
c = 0.0
Cellular_organisms – ps1
Archaea1 – ps2
Archaea2 – ps3
Archaea3 – ps4
Asgard/Eukaryota – ps5
Eukaryota – ps6
Amorpheae – ps7
Obazoa – ps8
Opisthokonta – ps9
Holozoa – ps10
Teretosporea – ps11
Ichthyosporea – ps12
Ichthyophonida – ps13
ophonus_hoferi (Ichthyophonida_2) – ps14
Phylostratum

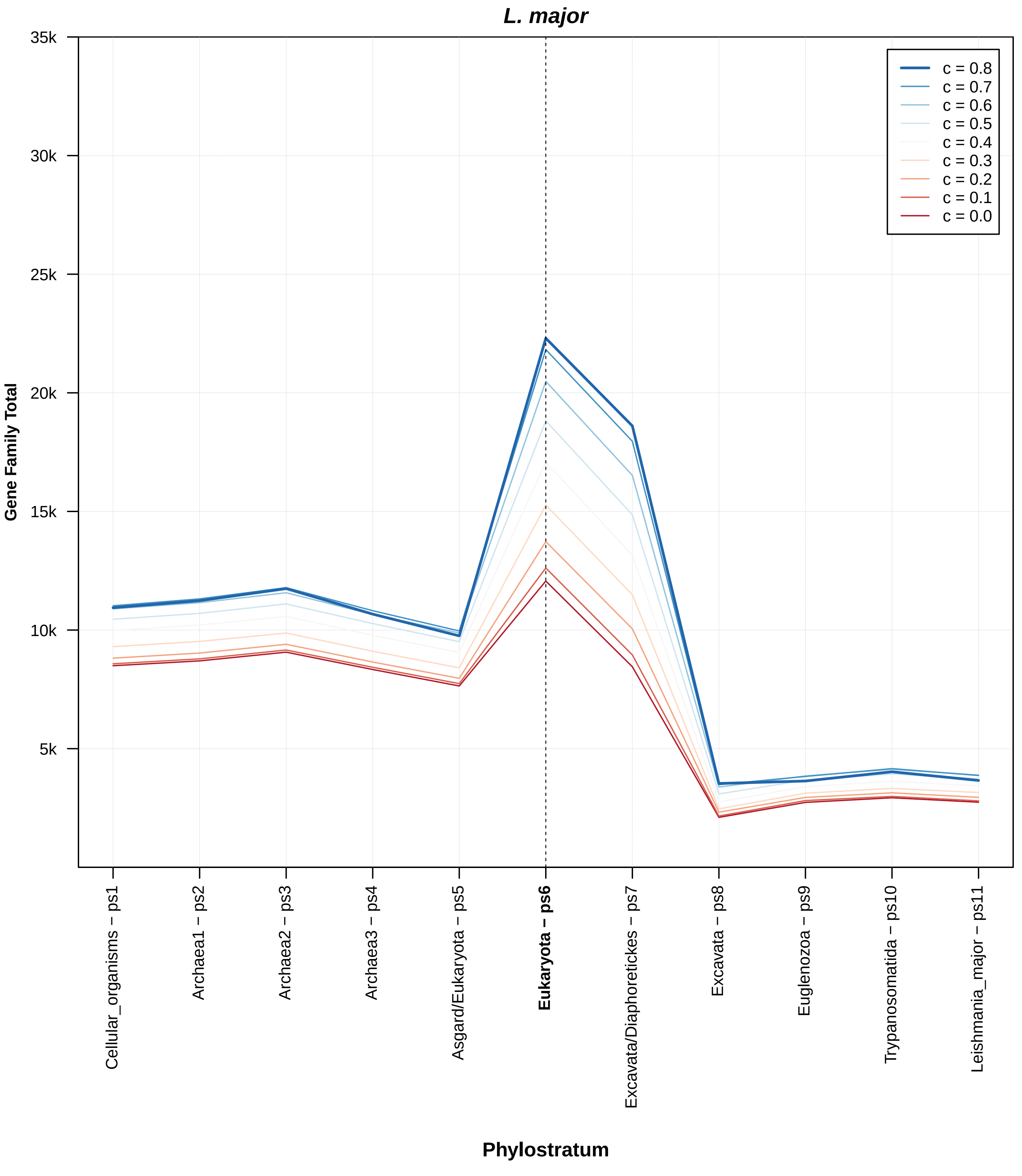
L. major
35k
30k
25k
20k
15k
10k
5k
Gene Family Total
c = 0.8
c = 0.7
c = 0.6
c = 0.5
c = 0.4
c = 0.3
c = 0.2
c = 0.1
c = 0.0
Cellular_organisms – ps1
Archaea1 – ps2
Archaea2 – ps3
Archaea3 – ps4
Asgard/Eukaryota – ps5
Eukaryota – ps6
Excavata/Diaphoretickes – ps7
Excavata – ps8
Euglenozoa – ps9
Trypanosomatida – ps10
Leishmania_major – ps11
Phylostratum

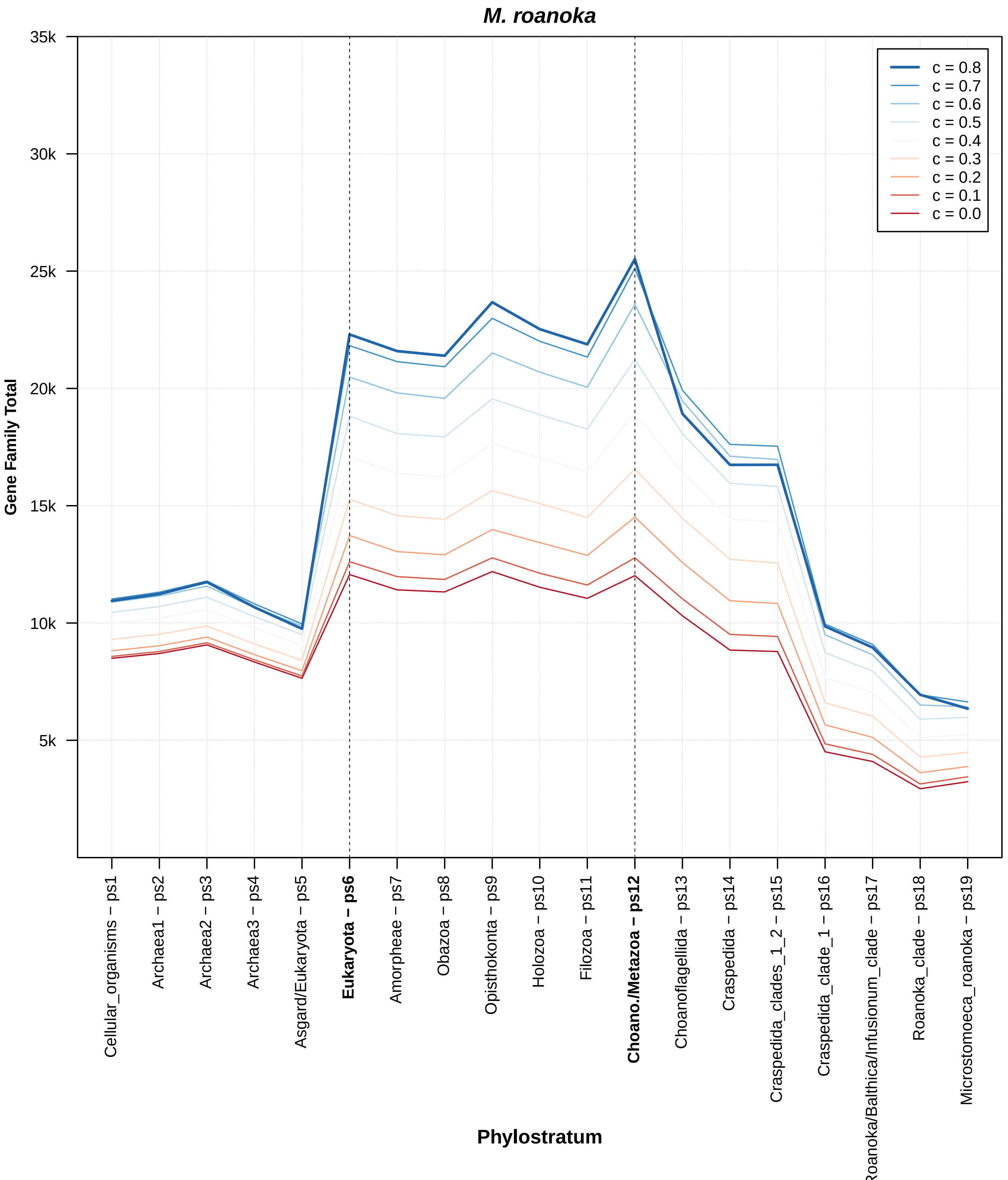

M. roanoka
Gene Family Total
35k
30k
25k
20k
15k
10k
5k
c = 0.8
c = 0.7
c = 0.6
c = 0.5
c = 0.4
c = 0.3
c = 0.2
c = 0.1
c = 0.0
Cellular_organisms – ps1
Archaea1 – ps2
Archaea2 – ps3
Archaea3 – ps4
Asgard/Eukaryota – ps5
Eukaryota – ps6
Amorpheae – ps7
Obazoa – ps8
Opisthokonta – ps9
Holozoa – ps10
Filozoa – ps11
Choano./Metazoa – ps12
Choanoflagellida – ps13
Craspedida – ps14
Craspedida_clades_1_2 – ps15
Craspedida_clade_1 – ps16
Roanoka/Balthica/Infusionum_clade – ps17
Roanoka_clade – ps18
Microstomoeca_roanoka – ps19
Phylostratum

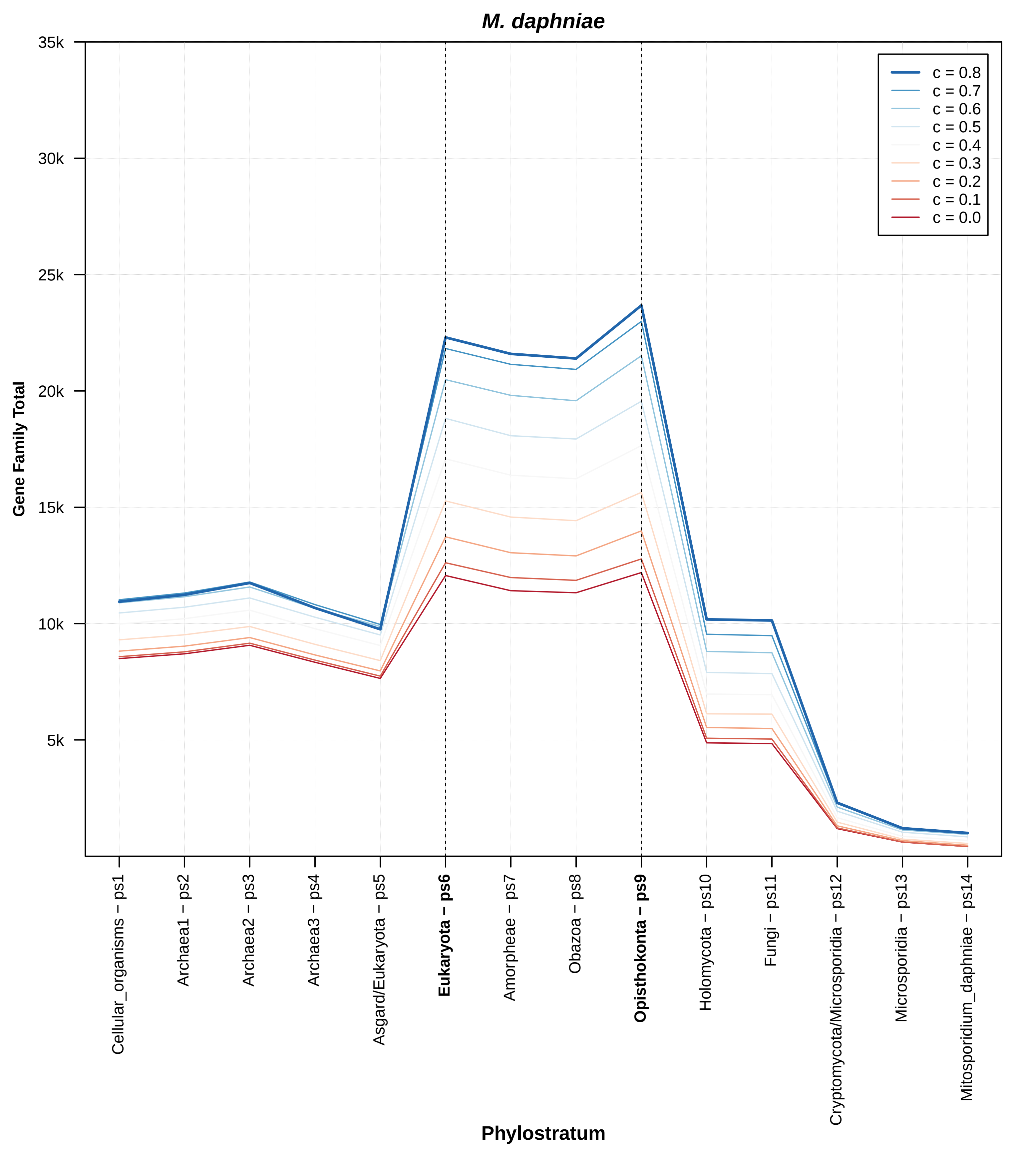
M. daphniae
c = 0.8
c = 0.7
c = 0.6
c = 0.5
c = 0.4
c = 0.3
c = 0.2
c = 0.1
c = 0.0
Gene Family Total
35k
30k
25k
20k
15k
10k
5k
Cellular_organisms – ps1
Archaea1 – ps2
Archaea2 – ps3
Archaea3 – ps4
Asgard/Eukaryota – ps5
Eukaryota – ps6
Amorpheae – ps7
Obazoa – ps8
Opisthokonta – ps9
Holomycota – ps10
Fungi – ps11
Cryptomycota/Microsporidia – ps12
Microsporidia – ps13
Mitosporidium_daphniae – ps14
Phylostratum

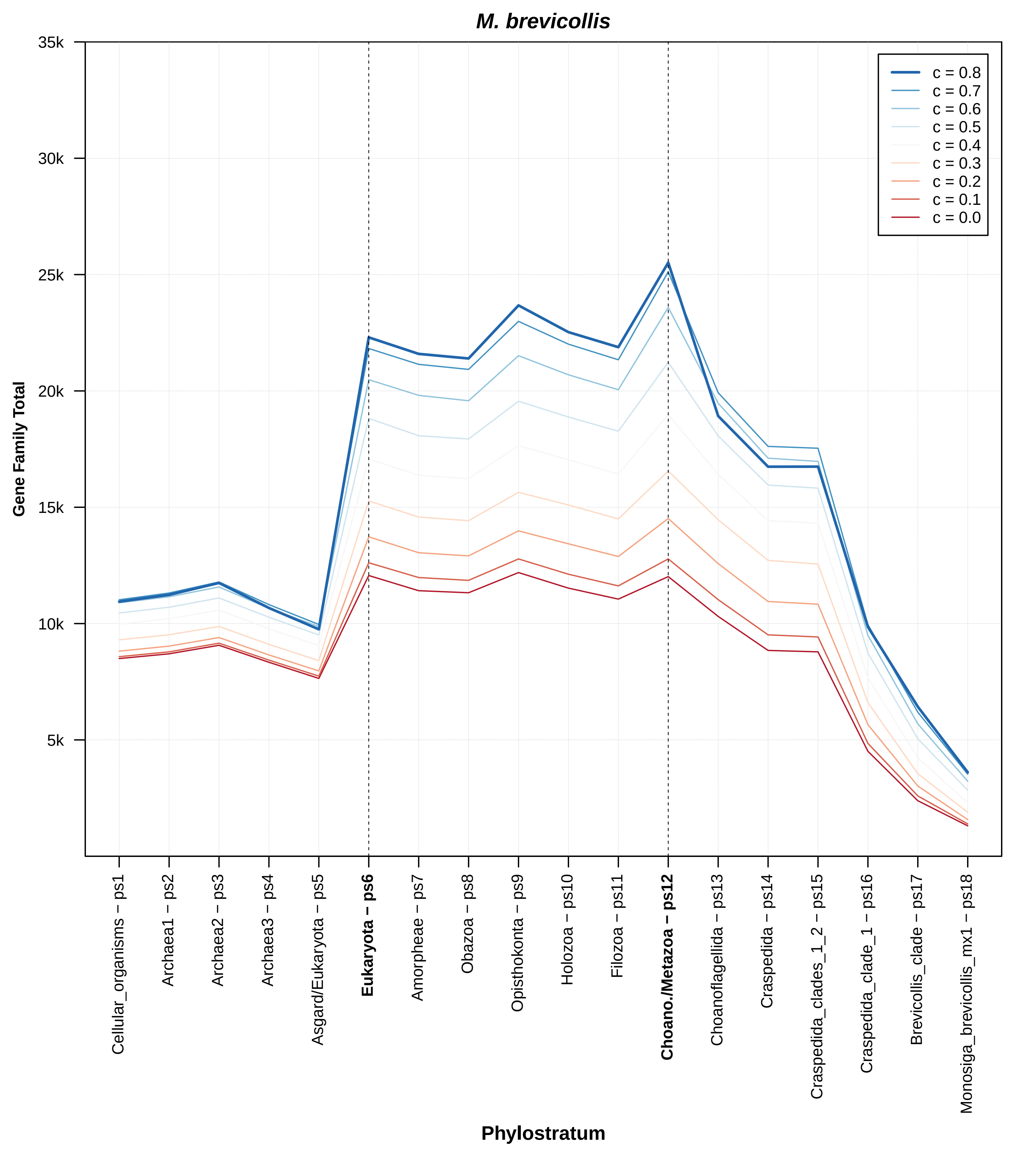

M. brevicollis
Gene Family Total
35k
30k
25k
20k
15k
10k
5k
c = 0.8
c = 0.7
c = 0.6
c = 0.5
c = 0.4
c = 0.3
c = 0.2
c = 0.1
c = 0.0
Cellular_organisms – ps1
Archaea1 – ps2
Archaea2 – ps3
Archaea3 – ps4
Asgard/Eukaryota – ps5
Eukaryota – ps6
Amorpheae – ps7
Obazoa – ps8
Opisthokonta – ps9
Holozoa – ps10
Filozoa – ps11
Choano./Metazoa – ps12
Choanoflagellida – ps13
Craspedida – ps14
Craspedida_clades_1_2 – ps15
Craspedida_clade_1 – ps16
Brevicollis_clade – ps17
Monosiga_brevicollis_mx1 – ps18
Phylostratum

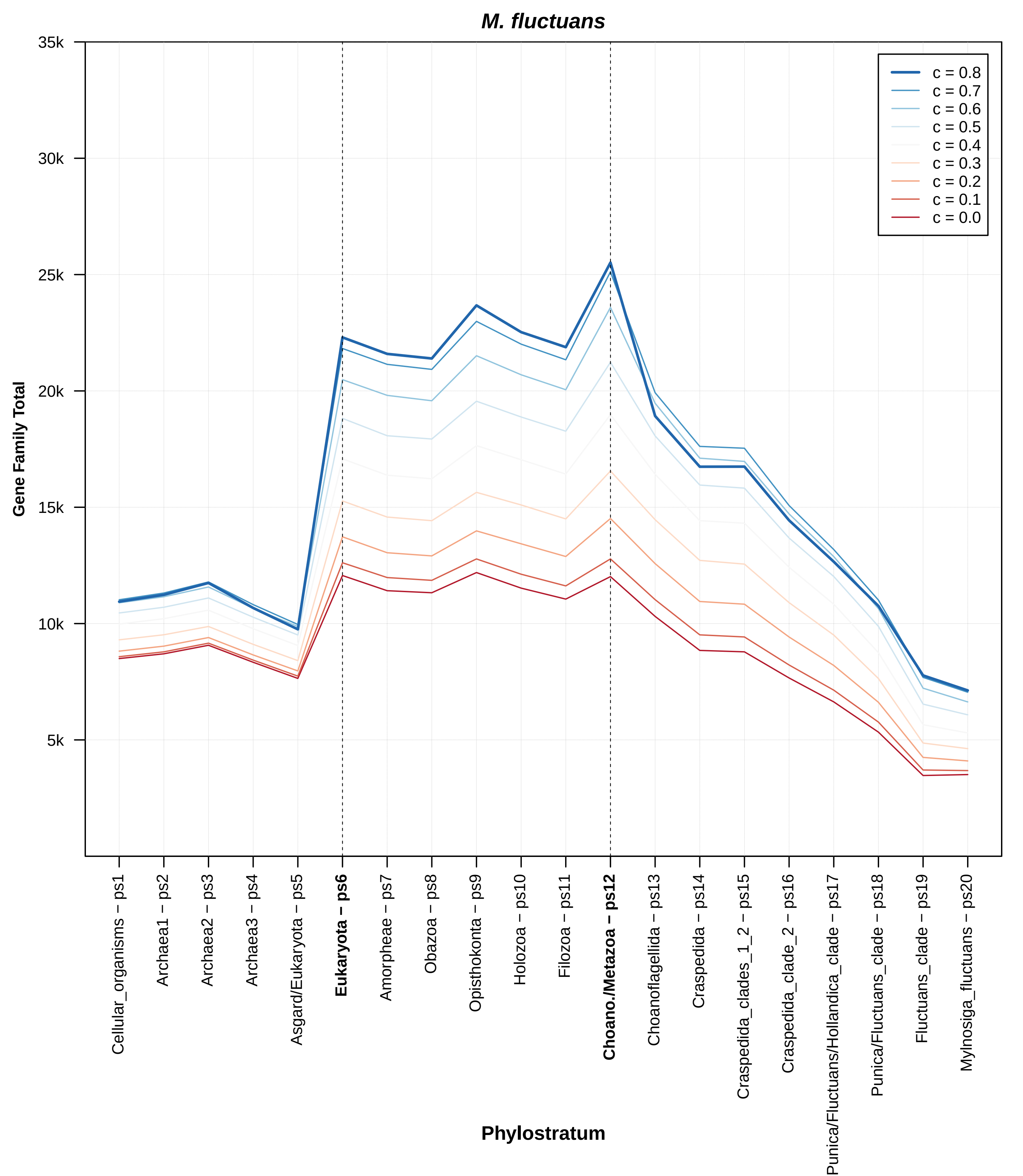

M. fluctuans
Gene Family Total
35k
30k
25k
20k
15k
10k
5k
c = 0.8
c = 0.7
c = 0.6
c = 0.5
c = 0.4
c = 0.3
c = 0.2
c = 0.1
c = 0.0
Cellular_organisms – ps1
Archaea1 – ps2
Archaea2 – ps3
Archaea3 – ps4
Asgard/Eukaryota – ps5
Eukaryota – ps6
Amorpheae – ps7
Obazoa – ps8
Opisthokonta – ps9
Holozoa – ps10
Filozoa – ps11
Choano./Metazoa – ps12
Choanoflagellida – ps13
Craspedida – ps14
Craspedida_clades_1_2 – ps15
Craspedida_clade_2 – ps16
Punica/Fluctuans/Hollandica_clade – ps17
Punica/Fluctuans_clade – ps18
Fluctuans_clade – ps19
Mylnosiga_fluctuans – ps20
Phylostratum

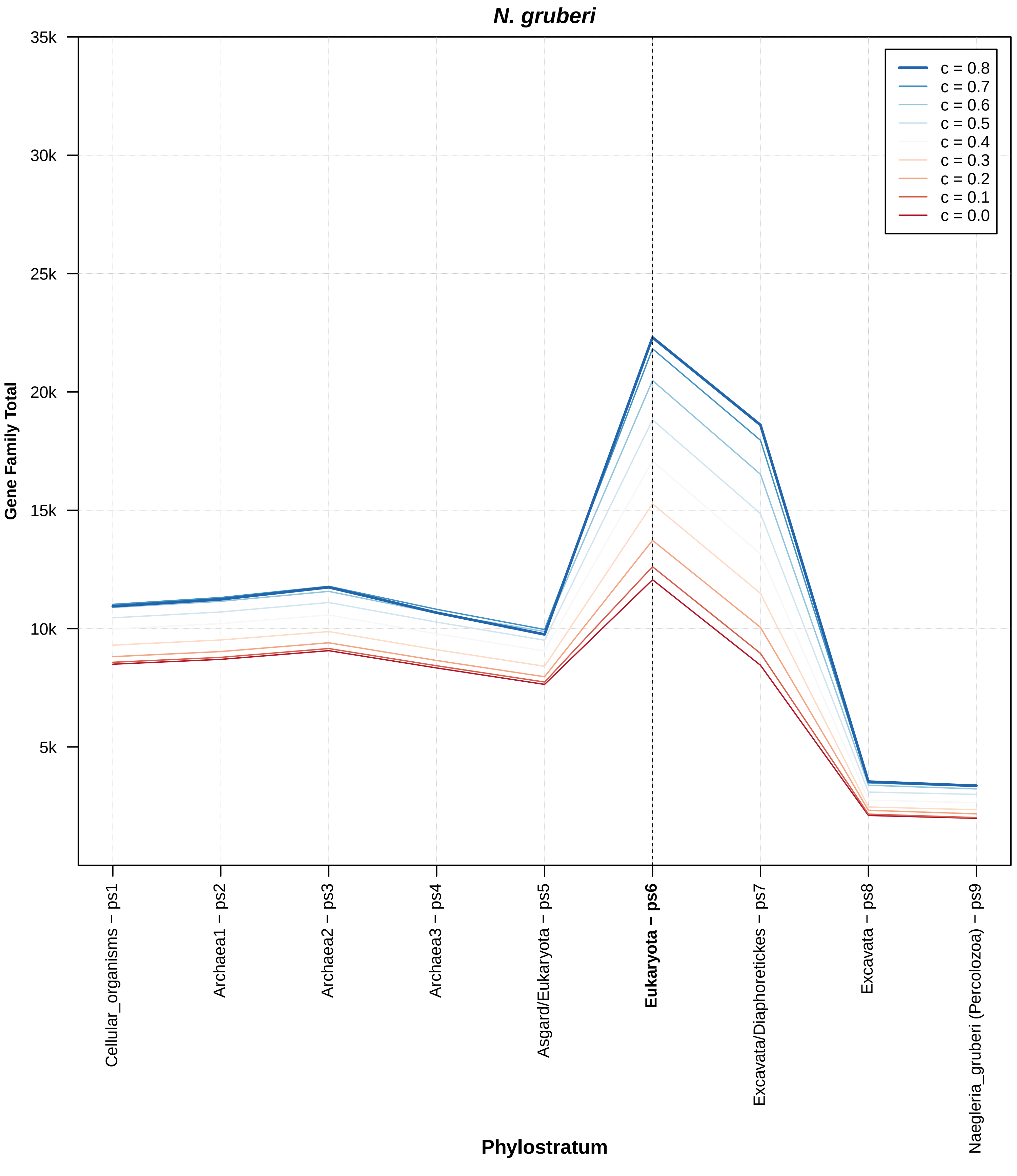
N. gruberi
35k
30k
25k
20k
15k
10k
5k
Gene Family Total
c = 0.8
c = 0.7
c = 0.6
c = 0.5
c = 0.4
c = 0.3
c = 0.2
c = 0.1
c = 0.0
Cellular_organisms – ps1
Archaea1 – ps2
Archaea2 – ps3
Archaea3 – ps4
Asgard/Eukaryota – ps5
Eukaryota – ps6
Excavata/Diaphoretickes – ps7
Excavata – ps8
Naegleria_gruberi (Percolozoa) – ps9
Phylostratum

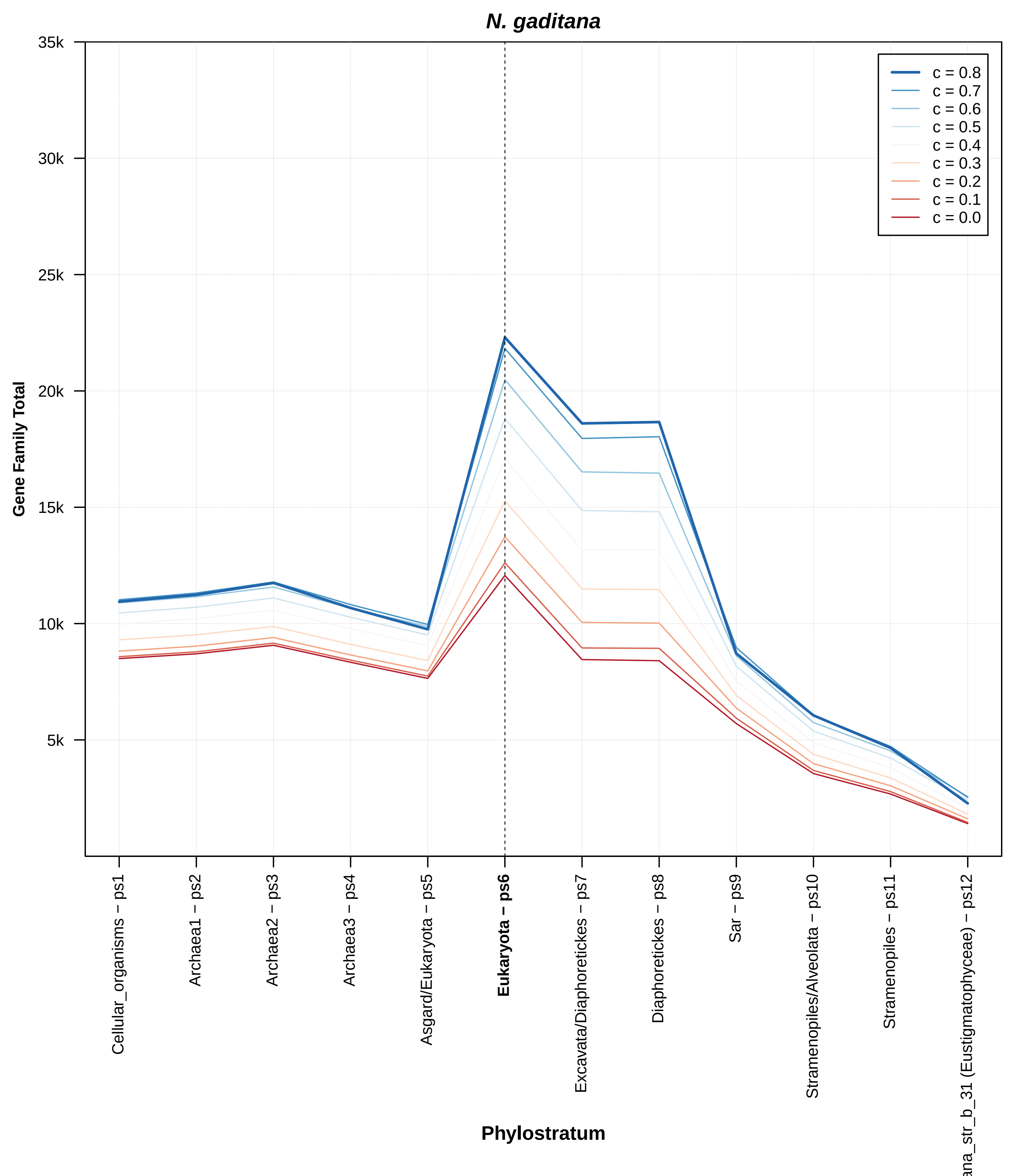
N. gaditana
35k
30k
25k
20k
15k
10k
5k
Gene Family Total
c = 0.8
c = 0.7
c = 0.6
c = 0.5
c = 0.4
c = 0.3
c = 0.2
c = 0.1
c = 0.0
Cellular_organisms – ps1
Archaea1 – ps2
Archaea2 – ps3
Archaea3 – ps4
Asgard/Eukaryota – ps5
Eukaryota – ps6
Excavata/Diaphoretickes – ps7
Diaphoretickes – ps8
Sar – ps9
Stramenopiles/Alveolata – ps10
Stramenopiles – ps11
ana_str_b_31 (Eustigmatophyceae) – ps12
Phylostratum

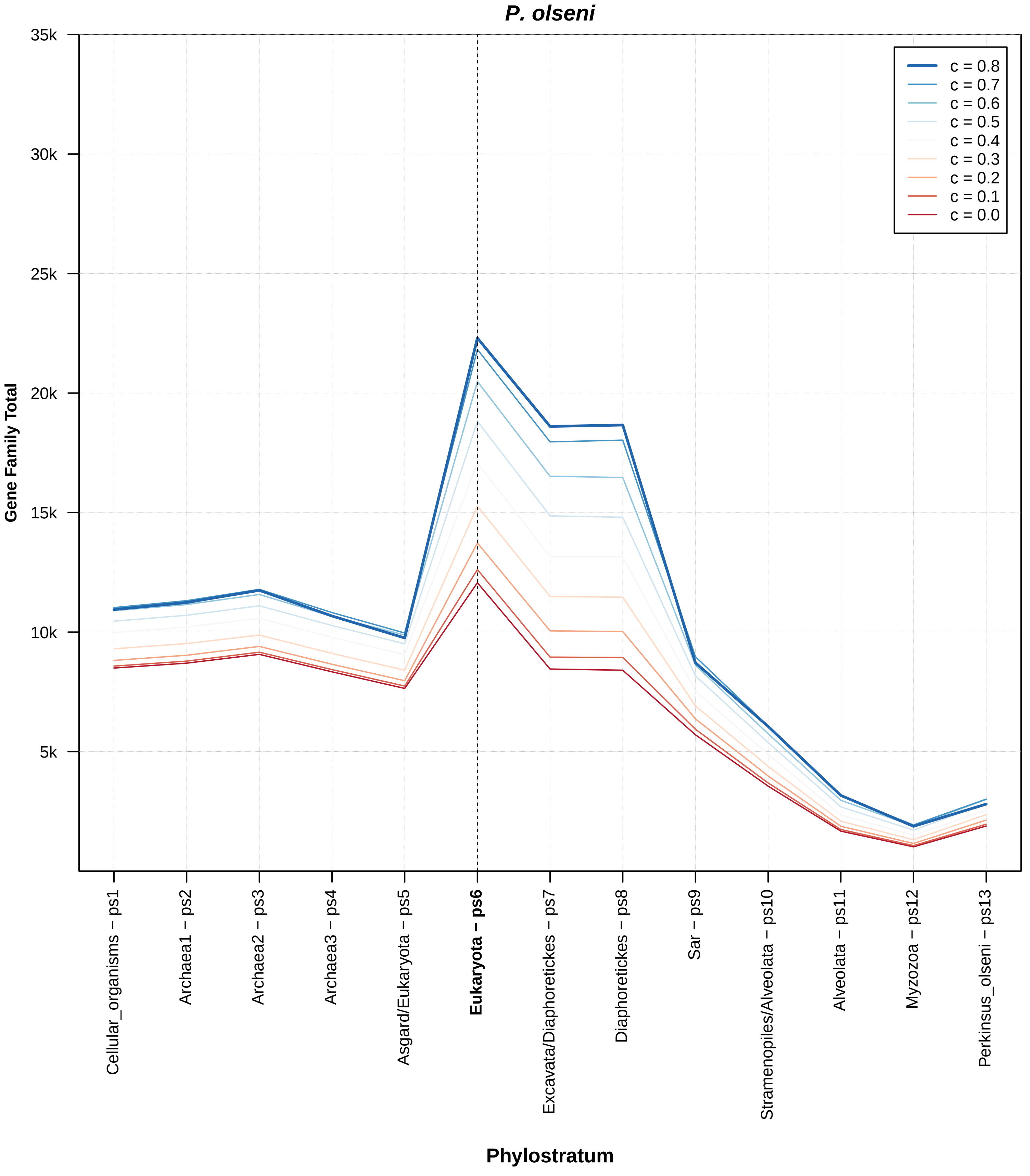
P. olseni
Gene Family Total
35k
30k
25k
20k
15k
10k
5k
c = 0.8
c = 0.7
c = 0.6
c = 0.5
c = 0.4
c = 0.3
c = 0.2
c = 0.1
c = 0.0
Cellular_organisms – ps1
Archaea1 – ps2
Archaea2 – ps3
Archaea3 – ps4
Asgard/Eukaryota – ps5
Eukaryota – ps6
Excavata/Diaphoretickes – ps7
Diaphoretickes – ps8
Sar – ps9
Stramenopiles/Alveolata – ps10
Alveolata – ps11
Myzozoa – ps12
Perkinsus_olseni – ps13
Phylostratum

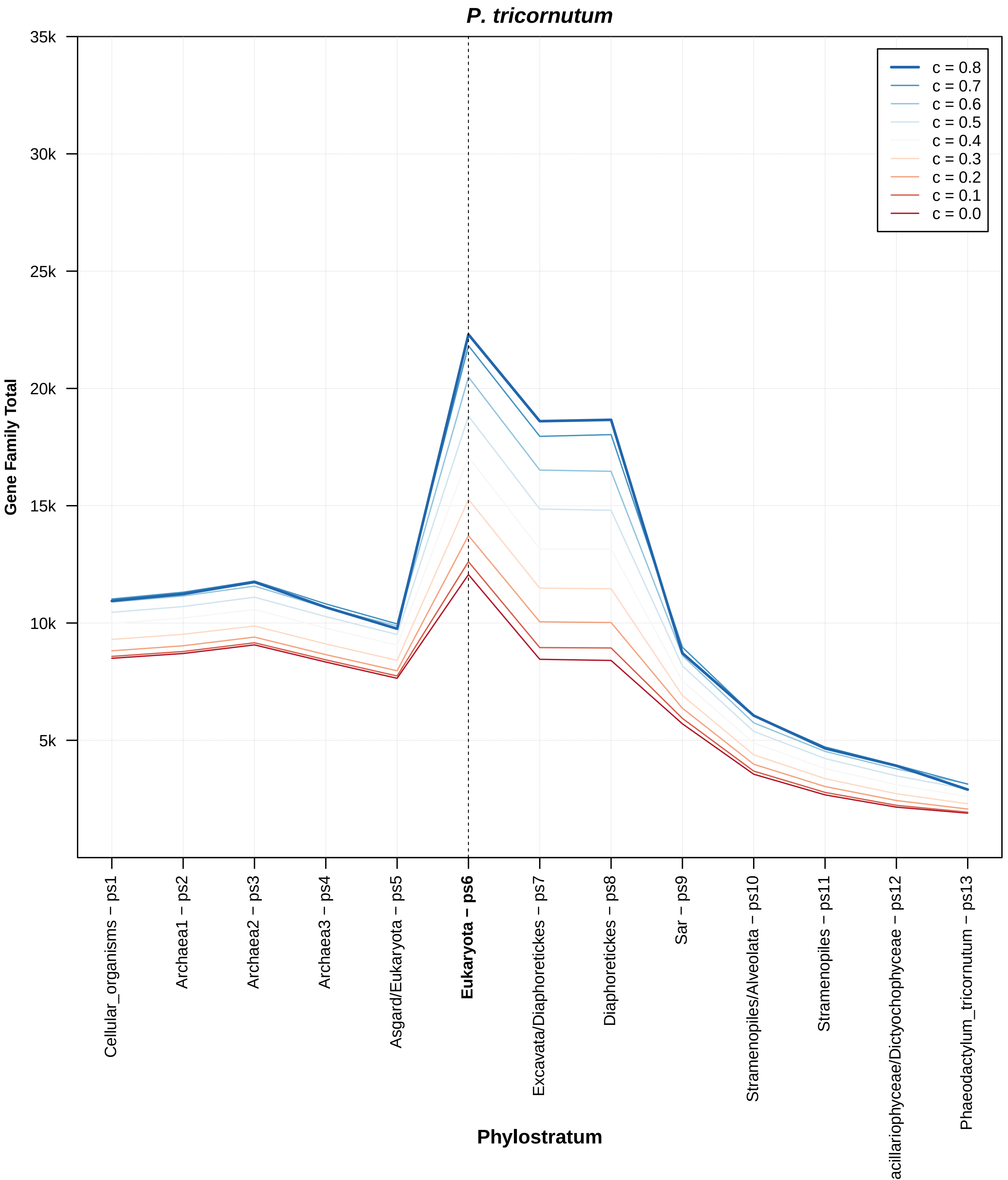

P. tricornutum
Gene Family Total
35k
30k
25k
20k
15k
10k
5k
c = 0.8
c = 0.7
c = 0.6
c = 0.5
c = 0.4
c = 0.3
c = 0.2
c = 0.1
c = 0.0
Cellular_organisms – ps1
Archaea1 – ps2
Archaea2 – ps3
Archaea3 – ps4
Asgard/Eukaryota – ps5
Eukaryota – ps6
Excavata/Diaphoretickes – ps7
Diaphoretickes – ps8
Sar – ps9
Stramenopiles/Alveolata – ps10
Stramenopiles – ps11
acillariophyceae/Dictyochophyceae – ps12
Phaeodactylum_tricornutum – ps13
Phylostratum

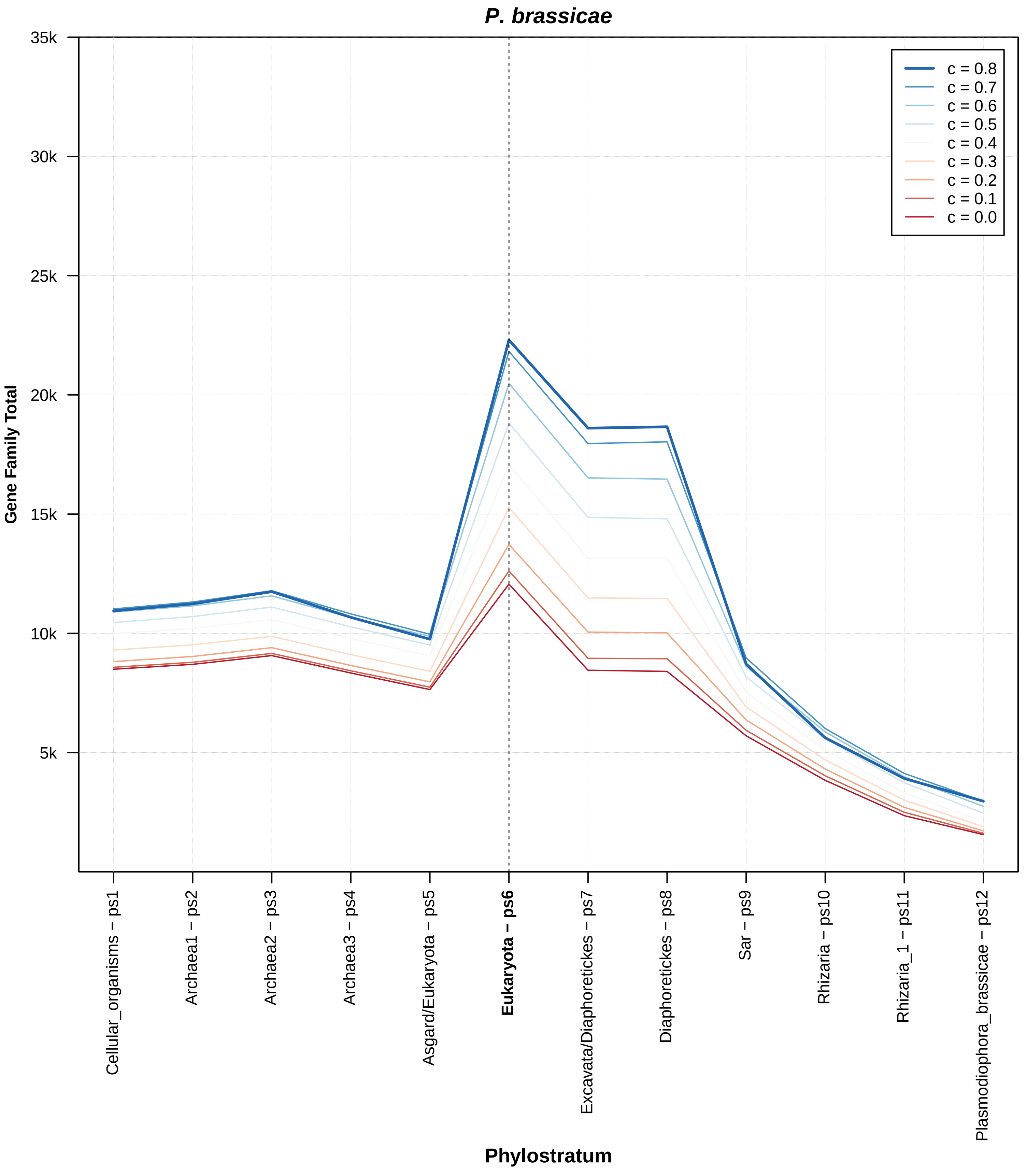
P. brassicae
Gene Family Total
35k
30k
25k
20k
15k
10k
5k
c = 0.8
c = 0.7
c = 0.6
c = 0.5
c = 0.4
c = 0.3
c = 0.2
c = 0.1
c = 0.0
Cellular_organisms – ps1
Archaea1 – ps2
Archaea2 – ps3
Archaea3 – ps4
Asgard/Eukaryota – ps5
Eukaryota – ps6
Excavata/Diaphoretickes – ps7
Diaphoretickes – ps8
Sar – ps9
Rhizaria – ps10
Rhizaria_1 – ps11
Plasmodiophora_brassicae – ps12
Phylostratum

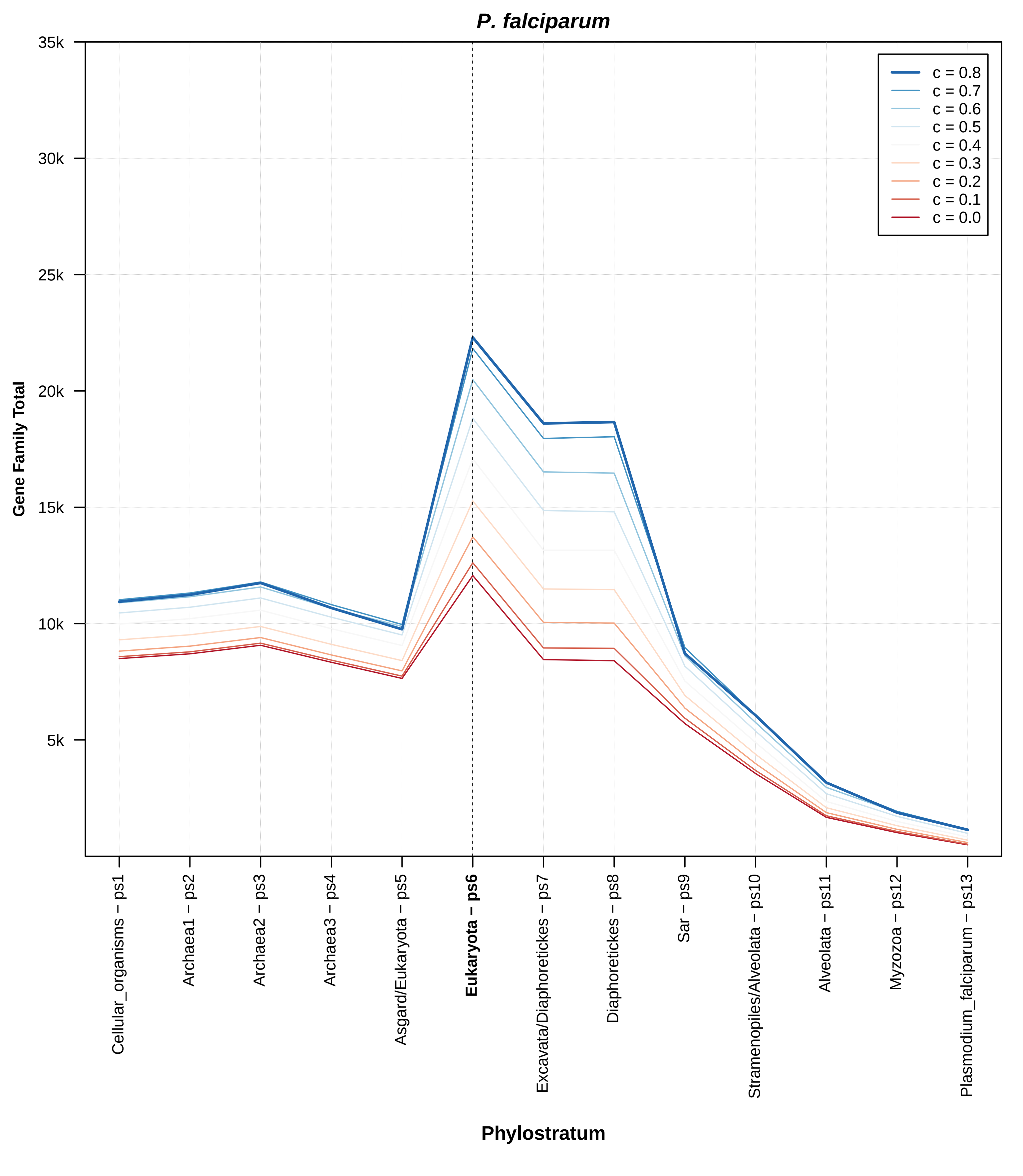
P. falciparum
Gene Family Total
35k
30k
25k
20k
15k
10k
5k
c = 0.8
c = 0.7
c = 0.6
c = 0.5
c = 0.4
c = 0.3
c = 0.2
c = 0.1
c = 0.0
Cellular_organisms – ps1
Archaea1 – ps2
Archaea2 – ps3
Archaea3 – ps4
Asgard/Eukaryota – ps5
Eukaryota – ps6
Excavata/Diaphoretickes – ps7
Diaphoretickes – ps8
Sar – ps9
Stramenopiles/Alveolata – ps10
Alveolata – ps11
Myzozoa – ps12
Plasmodium_falciparum – ps13
Phylostratum

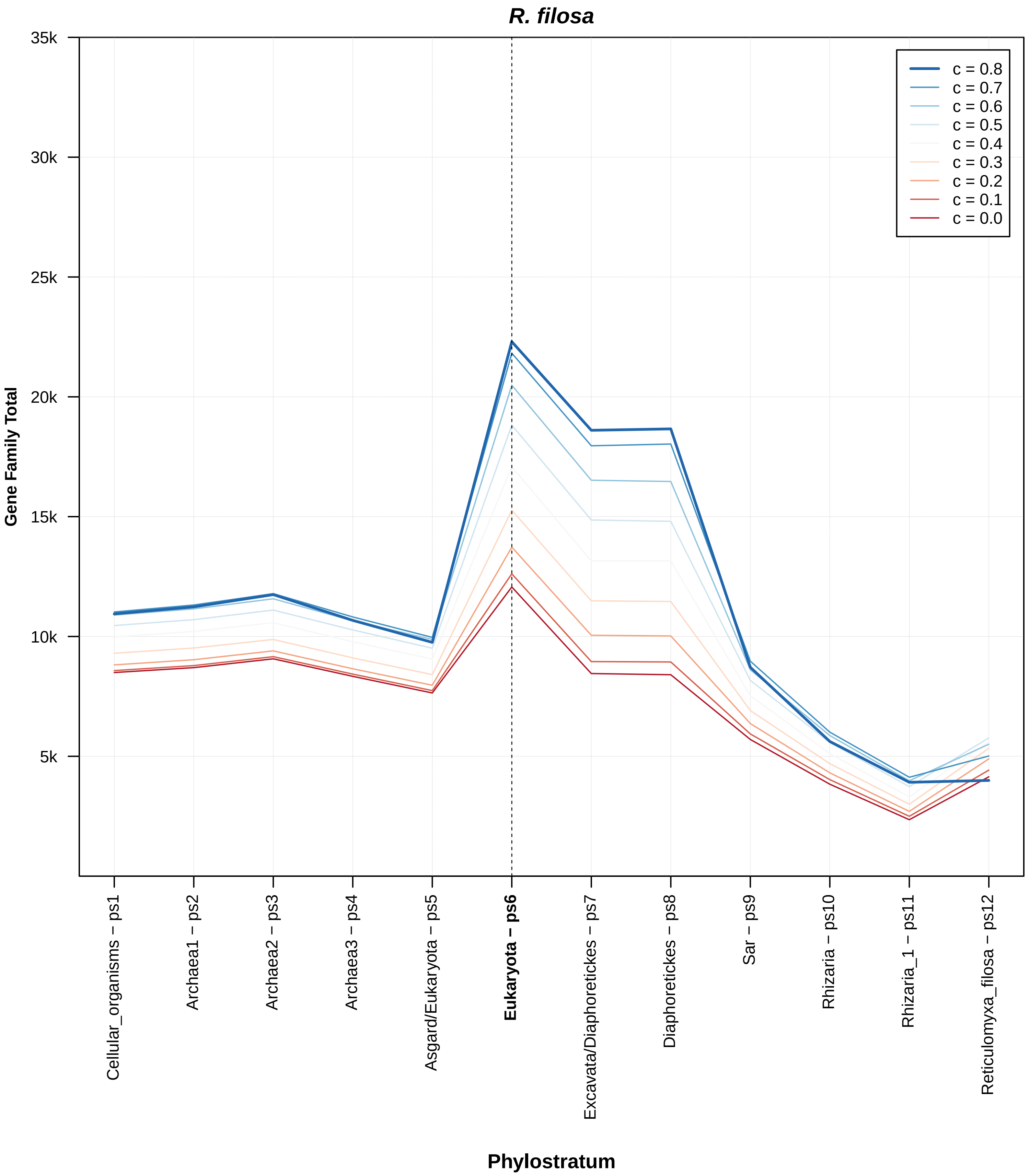
R. filosa
Gene Family Total
35k
30k
25k
20k
15k
10k
5k
c = 0.8
c = 0.7
c = 0.6
c = 0.5
c = 0.4
c = 0.3
c = 0.2
c = 0.1
c = 0.0
Cellular_organisms – ps1
Archaea1 – ps2
Archaea2 – ps3
Archaea3 – ps4
Asgard/Eukaryota – ps5
Eukaryota – ps6
Excavata/Diaphoretickes – ps7
Diaphoretickes – ps8
Sar – ps9
Rhizaria – ps10
Rhizaria_1 – ps11
Reticulomyxa_filosa – ps12
Phylostratum

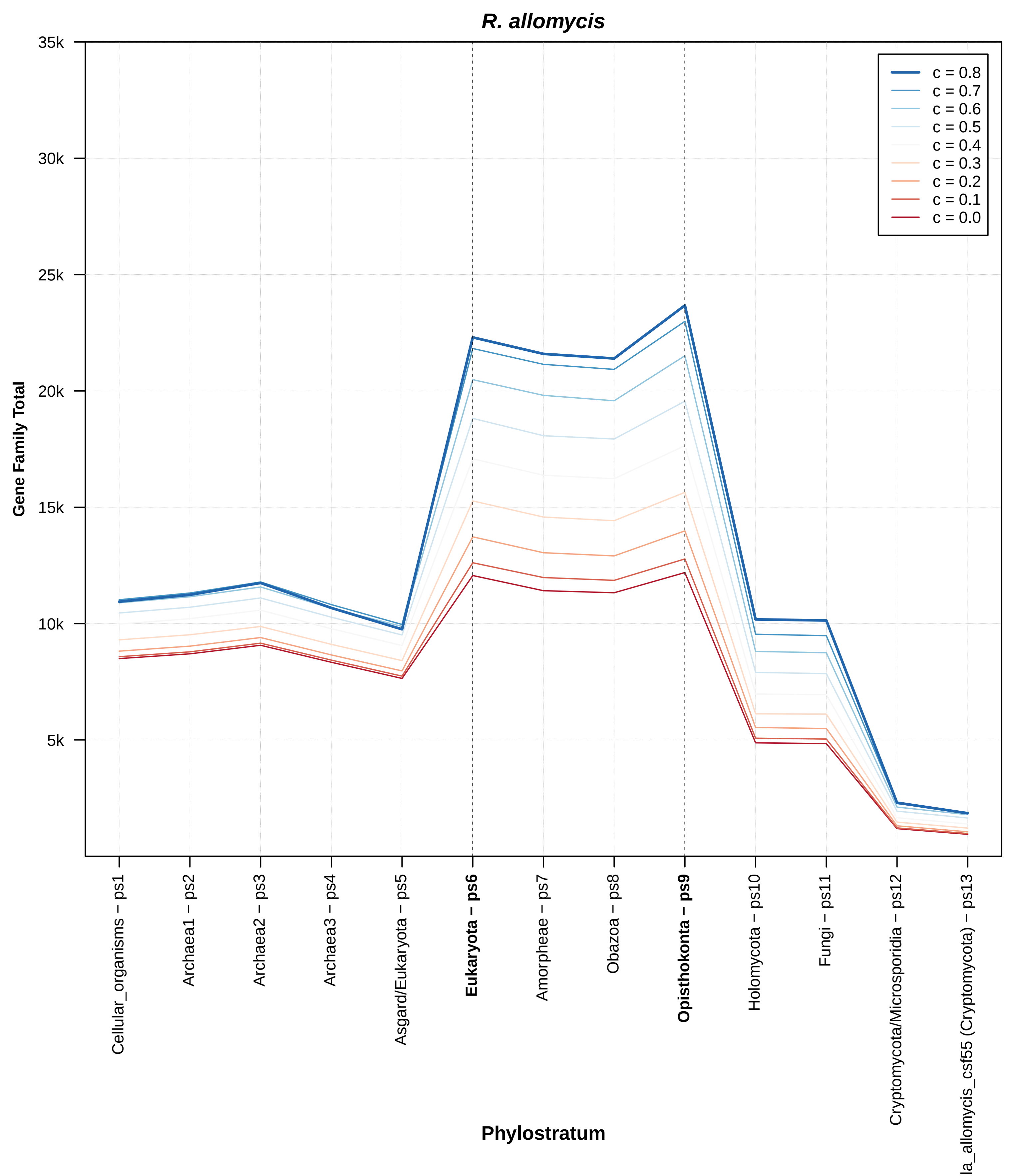

R. allomycis
35k
30k
25k
20k
15k
10k
5k
Gene Family Total
c = 0.8
c = 0.7
c = 0.6
c = 0.5
c = 0.4
c = 0.3
c = 0.2
c = 0.1
c = 0.0
Cellular_organisms – ps1
Archaea1 – ps2
Archaea2 – ps3
Archaea3 – ps4
Asgard/Eukaryota – ps5
Eukaryota – ps6
Amorpheae – ps7
Obazoa – ps8
Opisthokonta – ps9
Holomycota – ps10
Fungi – ps11
Cryptomycota/Microsporidia – ps12
lla_allomycis_csf55 (Cryptomycota) – ps13
Phylostratum

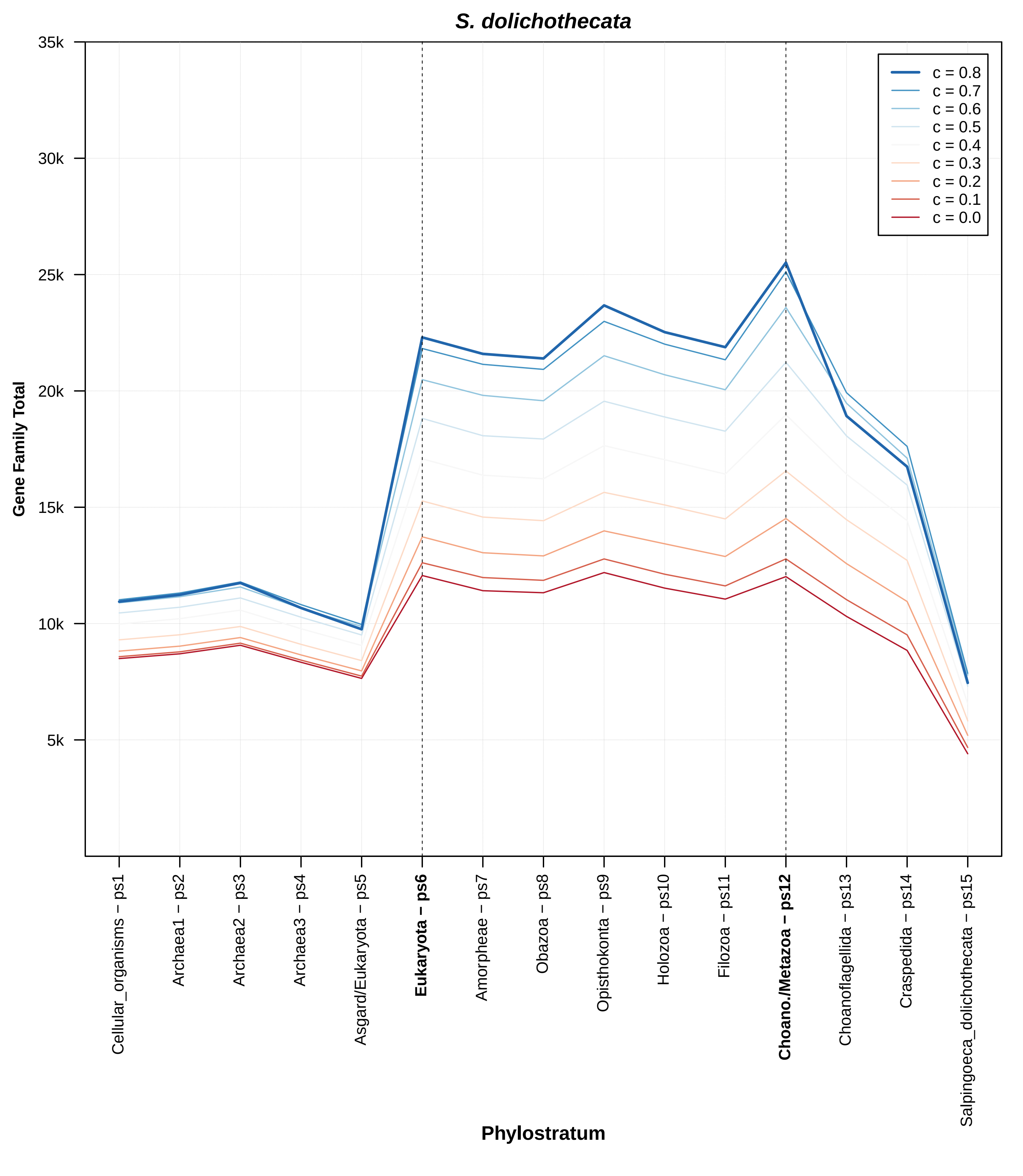
S. dolichothecata
Gene Family Total
35k
30k
25k
20k
15k
10k
5k
c = 0.8
c = 0.7
c = 0.6
c = 0.5
c = 0.4
c = 0.3
c = 0.2
c = 0.1
c = 0.0
Cellular_organisms – ps1
Archaea1 – ps2
Archaea2 – ps3
Archaea3 – ps4
Asgard/Eukaryota – ps5
Eukaryota – ps6
Amorpheae – ps7
Obazoa – ps8
Opisthokonta – ps9
Holozoa – ps10
Filozoa – ps11
Choano./Metazoa – ps12
Choanoflagellida – ps13
Craspedida – ps14
Salpingoeca_dolichothecata – ps15
Phylostratum

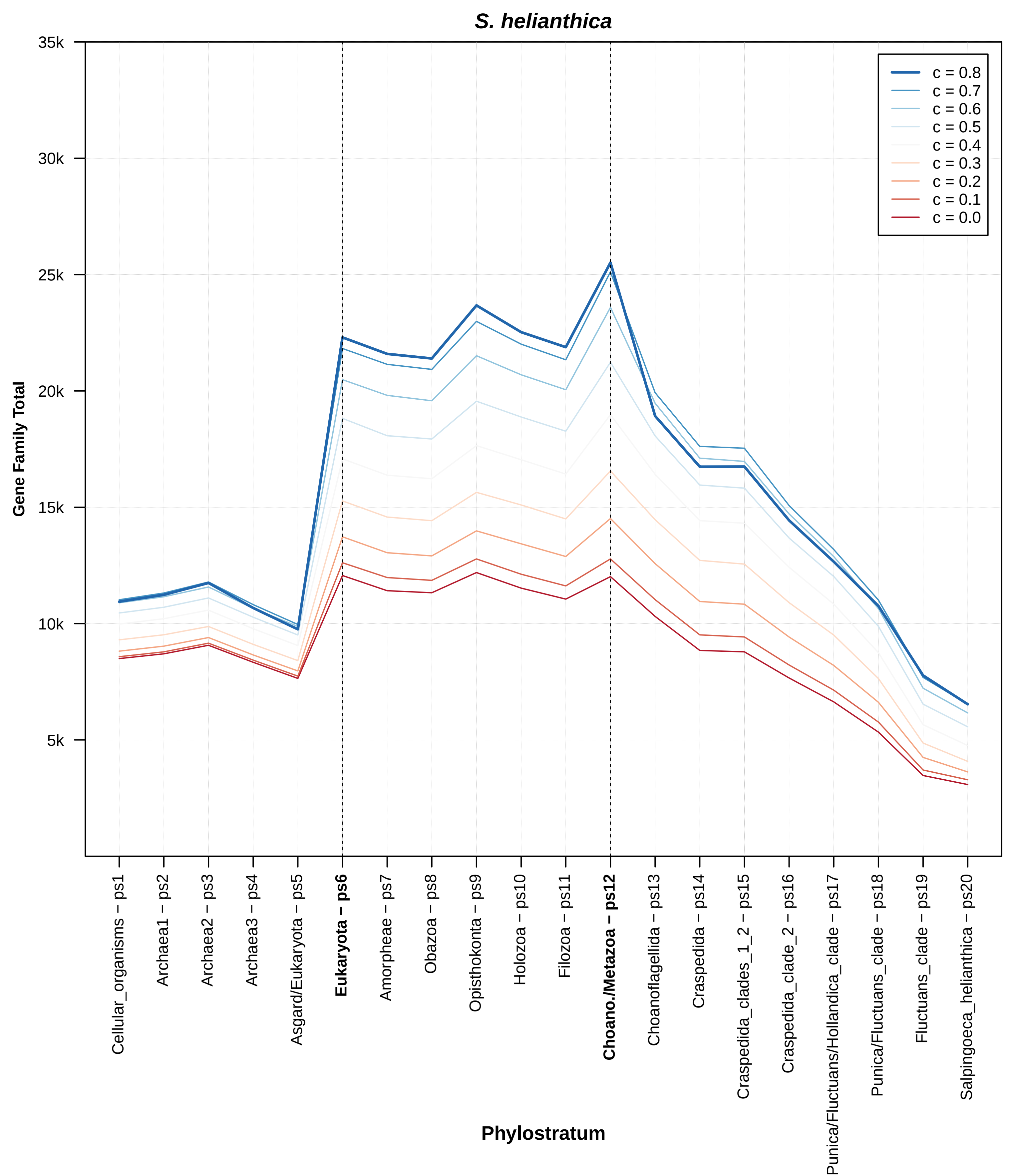
S. helianthica
Gene Family Total
35k
30k
25k
20k
15k
10k
5k
c = 0.8
c = 0.7
c = 0.6
c = 0.5
c = 0.4
c = 0.3
c = 0.2
c = 0.1
c = 0.0
Cellular_organisms – ps1
Archaea1 – ps2
Archaea2 – ps3
Archaea3 – ps4
Asgard/Eukaryota – ps5
Eukaryota – ps6
Amorpheae – ps7
Obazoa – ps8
Opisthokonta – ps9
Holozoa – ps10
Filozoa – ps11
Choano./Metazoa – ps12
Choanoflagellida – ps13
Craspedida – ps14
Craspedida_clades_1_2 – ps15
Craspedida_clade_2 – ps16
Punica/Fluctuans/Hollandica_clade – ps17
Punica/Fluctuans_clade – ps18
Fluctuans_clade – ps19
Salpingoeca_helianthica – ps20
Phylostratum

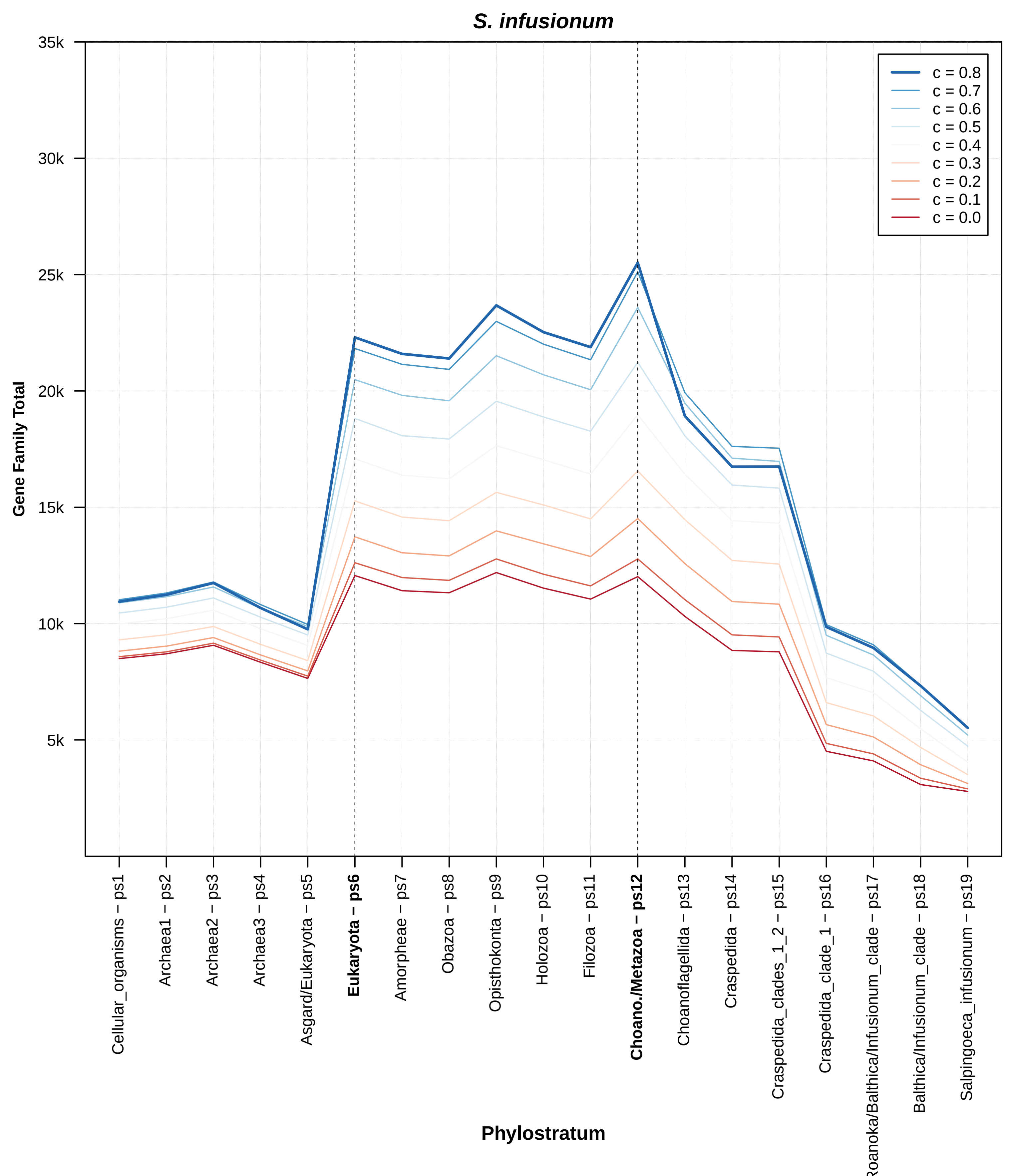

S. infusionum
Gene Family Total
35k
30k
25k
20k
15k
10k
5k
c = 0.8
c = 0.7
c = 0.6
c = 0.5
c = 0.4
c = 0.3
c = 0.2
c = 0.1
c = 0.0
Cellular_organisms – ps1
Archaea1 – ps2
Archaea2 – ps3
Archaea3 – ps4
Asgard/Eukaryota – ps5
Eukaryota – ps6
Amorpheae – ps7
Obazoa – ps8
Opisthokonta – ps9
Holozoa – ps10
Filozoa – ps11
Choano./Metazoa – ps12
Choanoflagellida – ps13
Craspedida – ps14
Craspedida_clades_1_2 – ps15
Craspedida_clade_1 – ps16
Roanoka/Balthica/Infusionum_clade – ps17
Balthica/Infusionum_clade – ps18
Salpingoeca_infusionum – ps19
Phylostratum

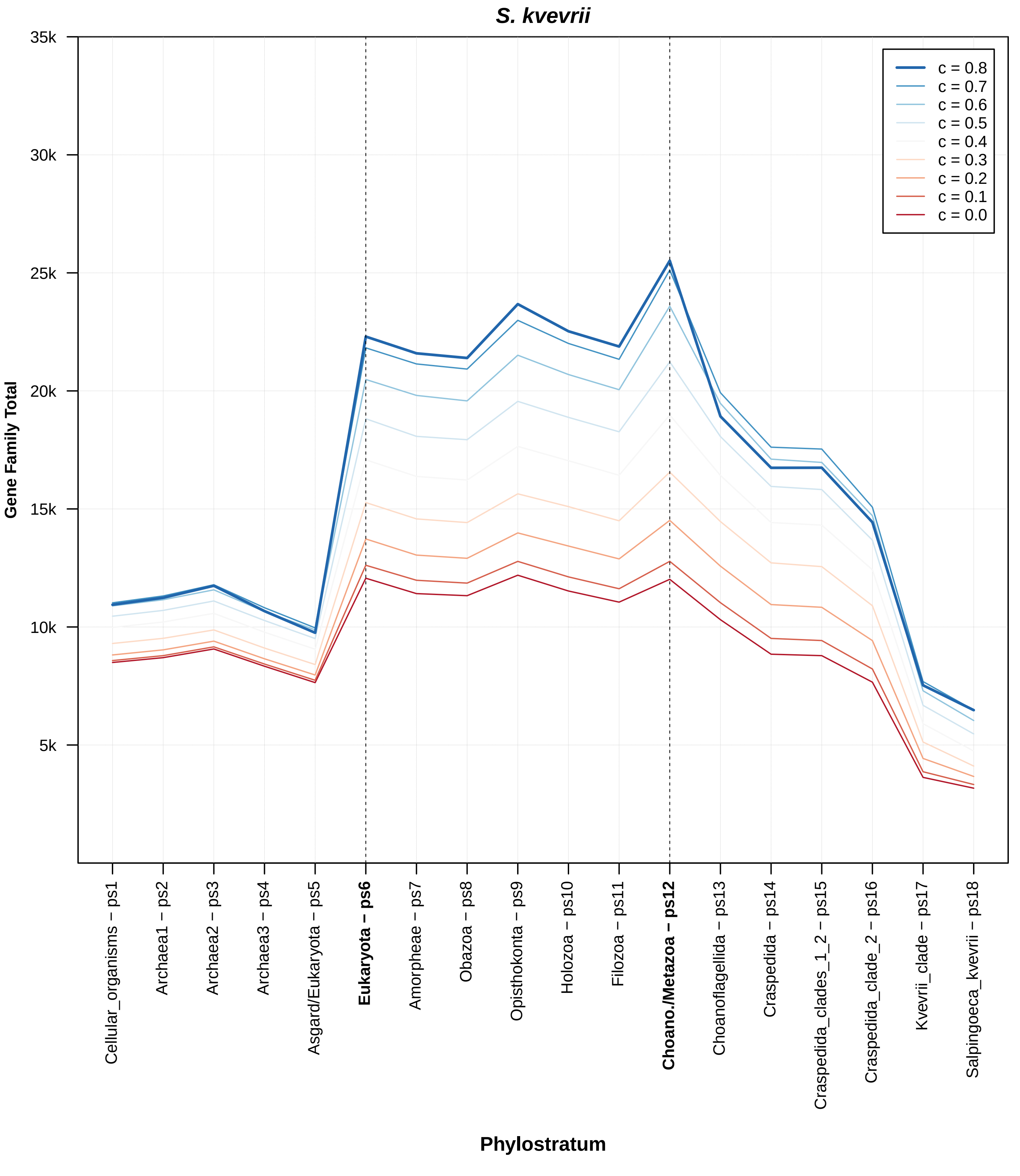

S. kvevrii
Gene Family Total
35k
30k
25k
20k
15k
10k
5k
c = 0.8
c = 0.7
c = 0.6
c = 0.5
c = 0.4
c = 0.3
c = 0.2
c = 0.1
c = 0.0
Cellular_organisms – ps1
Archaea1 – ps2
Archaea2 – ps3
Archaea3 – ps4
Asgard/Eukaryota – ps5
Eukaryota – ps6
Amorpheae – ps7
Obazoa – ps8
Opisthokonta – ps9
Holozoa – ps10
Filozoa – ps11
Choano./Metazoa – ps12
Choanoflagellida – ps13
Craspedida – ps14
Craspedida_clades_1_2 – ps15
Craspedida_clade_2 – ps16
Kvevrii_clade – ps17
Salpingoeca_kvevrii – ps18
Phylostratum

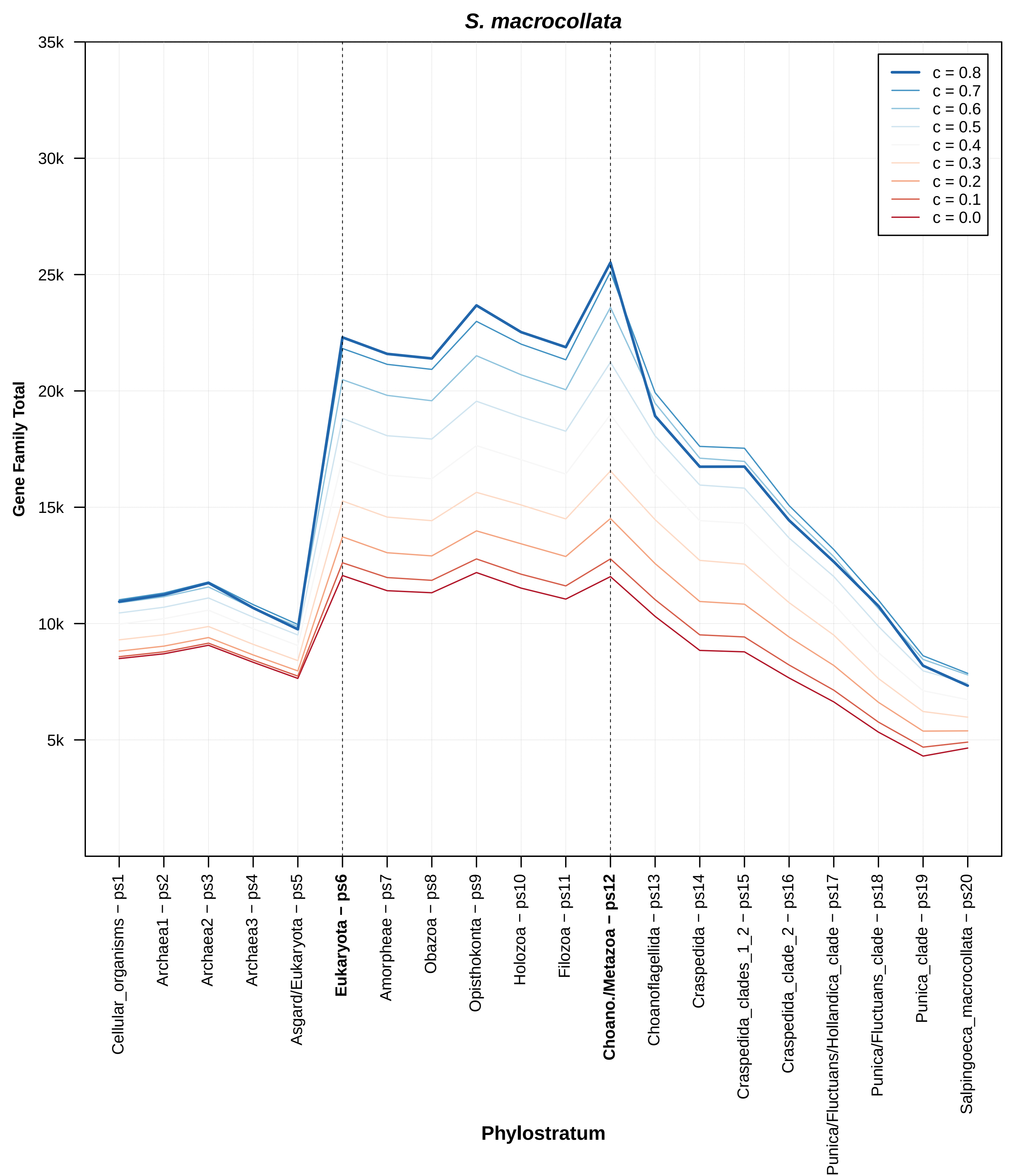

S. macrocollata
Gene Family Total
35k
30k
25k
20k
15k
10k
5k
c = 0.8
c = 0.7
c = 0.6
c = 0.5
c = 0.4
c = 0.3
c = 0.2
c = 0.1
c = 0.0
Cellular_organisms – ps1
Archaea1 – ps2
Archaea2 – ps3
Archaea3 – ps4
Asgard/Eukaryota – ps5
Eukaryota – ps6
Amorpheae – ps7
Obazoa – ps8
Opisthokonta – ps9
Holozoa – ps10
Filozoa – ps11
Choano./Metazoa – ps12
Choanoflagellida – ps13
Craspedida – ps14
Craspedida_clades_1_2 – ps15
Craspedida_clade_2 – ps16
Punica/Fluctuans/Hollandica_clade – ps17
Punica/Fluctuans_clade – ps18
Punica_clade – ps19
Salpingoeca_macrocollata – ps20
Phylostratum

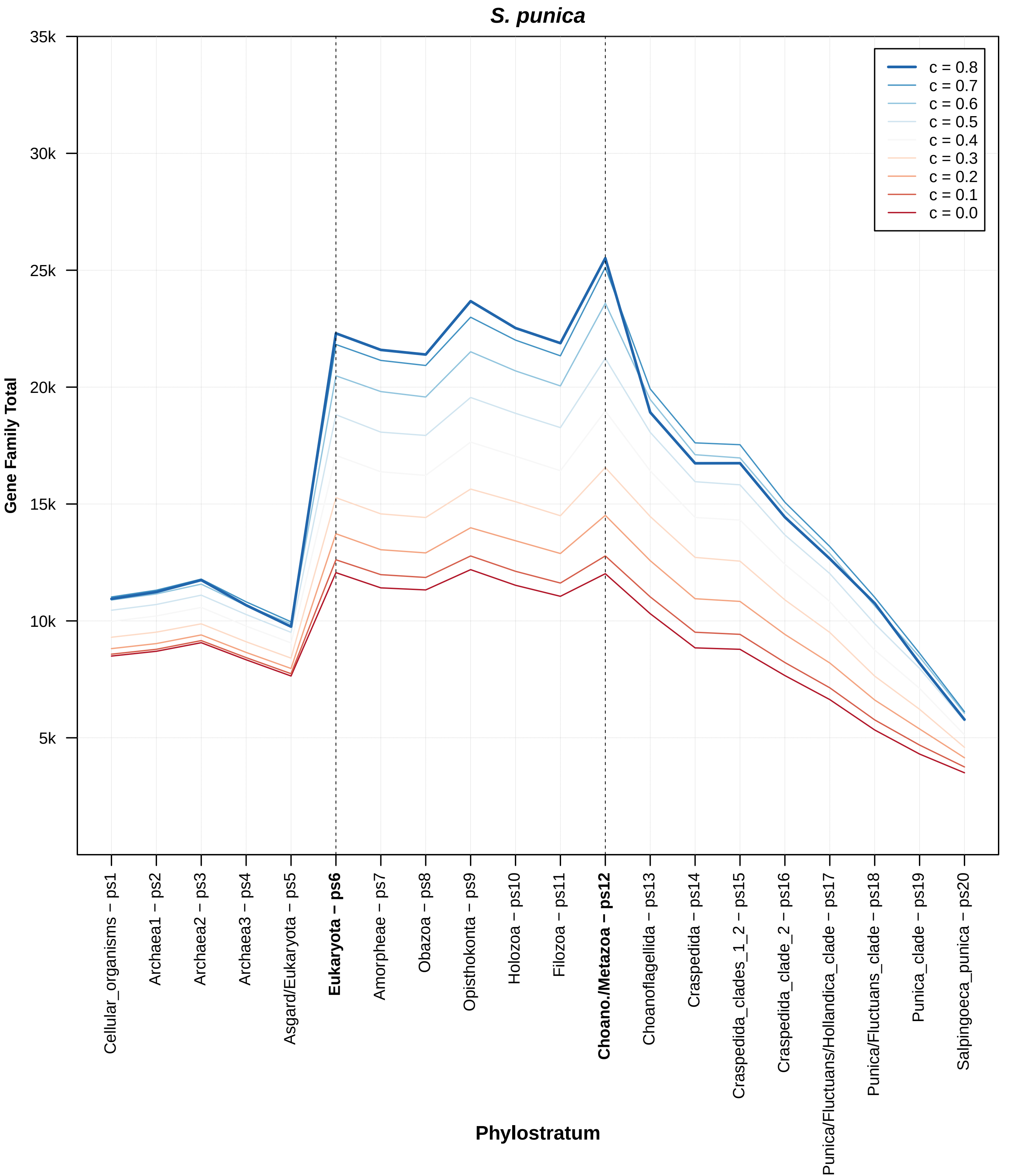

S. punica
Gene Family Total
35k
30k
25k
20k
15k
10k
5k
c = 0.8
c = 0.7
c = 0.6
c = 0.5
c = 0.4
c = 0.3
c = 0.2
c = 0.1
c = 0.0
Cellular_organisms – ps1
Archaea1 – ps2
Archaea2 – ps3
Archaea3 – ps4
Asgard/Eukaryota – ps5
Eukaryota – ps6
Amorpheae – ps7
Obazoa – ps8
Opisthokonta – ps9
Holozoa – ps10
Filozoa – ps11
Choano./Metazoa – ps12
Choanoflagellida – ps13
Craspedida – ps14
Craspedida_clades_1_2 – ps15
Craspedida_clade_2 – ps16
Punica/Fluctuans/Hollandica_clade – ps17
Punica/Fluctuans_clade – ps18
Punica_clade – ps19
Salpingoeca_punica – ps20
Phylostratum

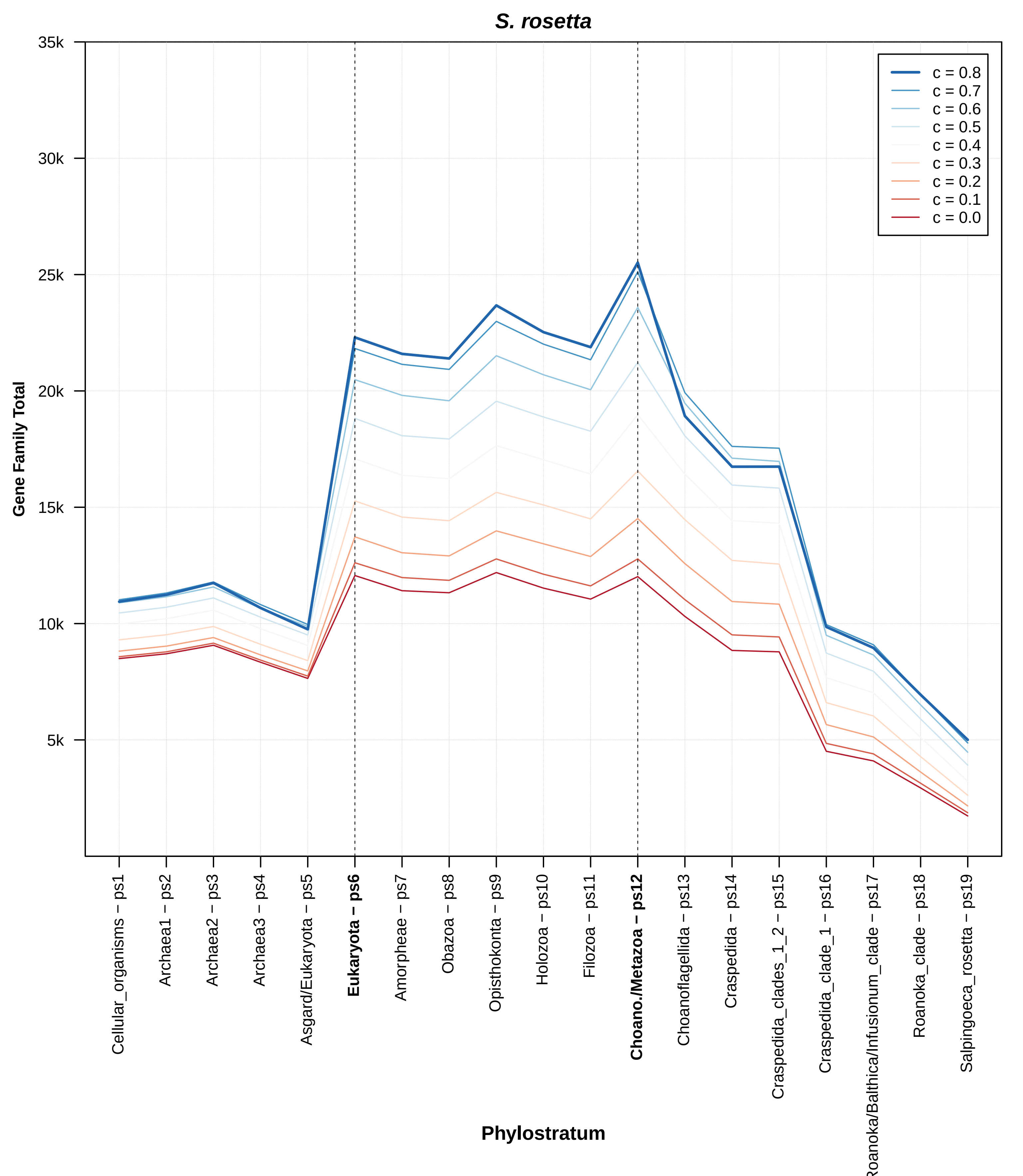
S. rosetta
35k
30k
25k
20k
15k
10k
5k
Gene Family Total
c = 0.8
c = 0.7
c = 0.6
c = 0.5
c = 0.4
c = 0.3
c = 0.2
c = 0.1
c = 0.0
Cellular_organisms – ps1
Archaea1 – ps2
Archaea2 – ps3
Archaea3 – ps4
Asgard/Eukaryota – ps5
Eukaryota – ps6
Amorpheae – ps7
Obazoa – ps8
Opisthokonta – ps9
Holozoa – ps10
Filozoa – ps11
Choano./Metazoa – ps12
Choanoflagellida – ps13
Craspedida – ps14
Craspedida_clades_1_2 – ps15
Craspedida_clade_1 – ps16
Roanoka/Balthica/Infusionum_clade – ps17
Roanoka_clade – ps18
Salpingoeca_rosetta – ps19
Phylostratum

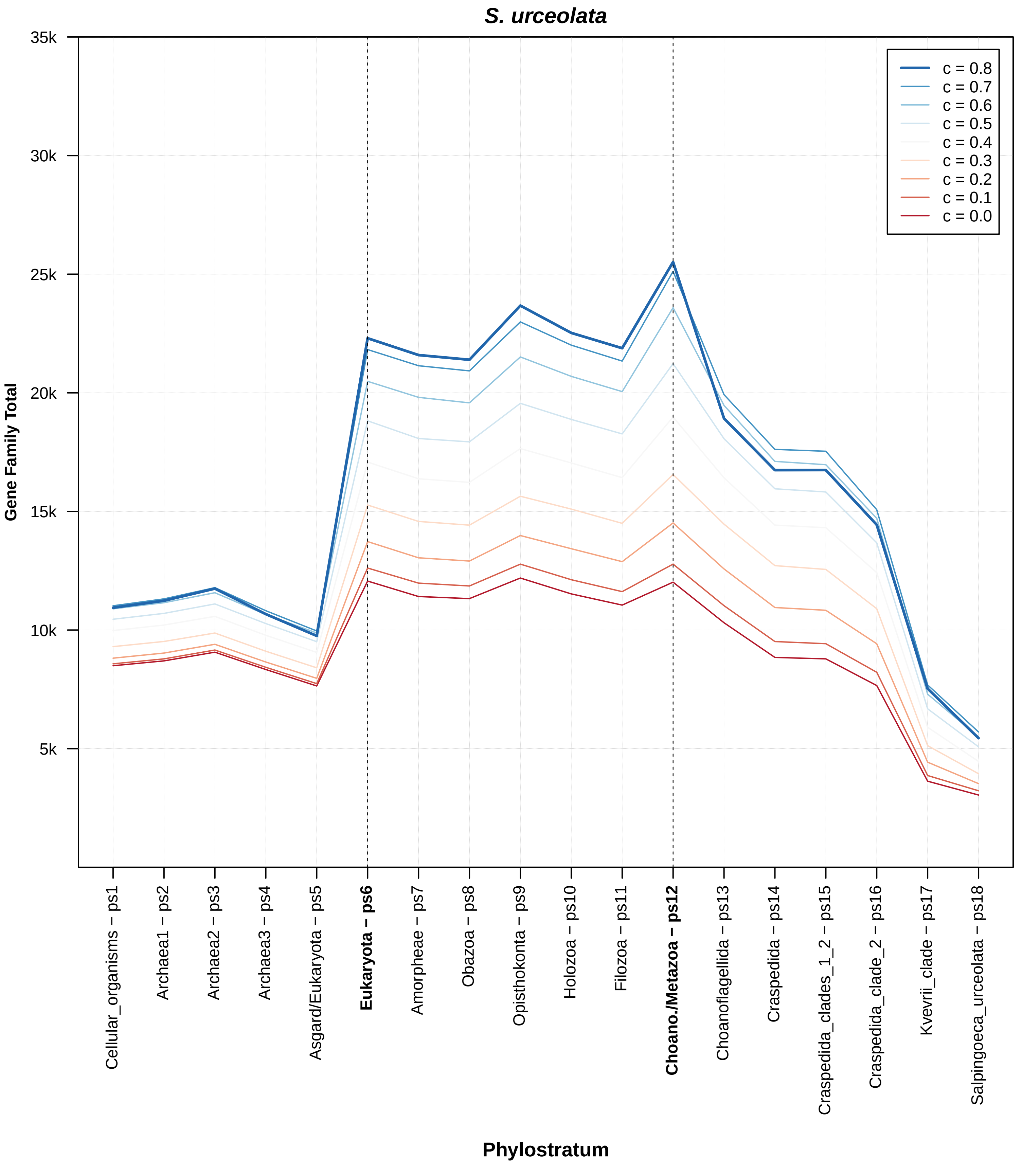

S. urceolata
Gene Family Total
35k
30k
25k
20k
15k
10k
5k
c = 0.8
c = 0.7
c = 0.6
c = 0.5
c = 0.4
c = 0.3
c = 0.2
c = 0.1
c = 0.0
Cellular_organisms – ps1
Archaea1 – ps2
Archaea2 – ps3
Archaea3 – ps4
Asgard/Eukaryota – ps5
Eukaryota – ps6
Amorpheae – ps7
Obazoa – ps8
Opisthokonta – ps9
Holozoa – ps10
Filozoa – ps11
Choano./Metazoa – ps12
Choanoflagellida – ps13
Craspedida – ps14
Craspedida_clades_1_2 – ps15
Craspedida_clade_2 – ps16
Kvevrii_clade – ps17
Salpingoeca_urceolata – ps18
Phylostratum

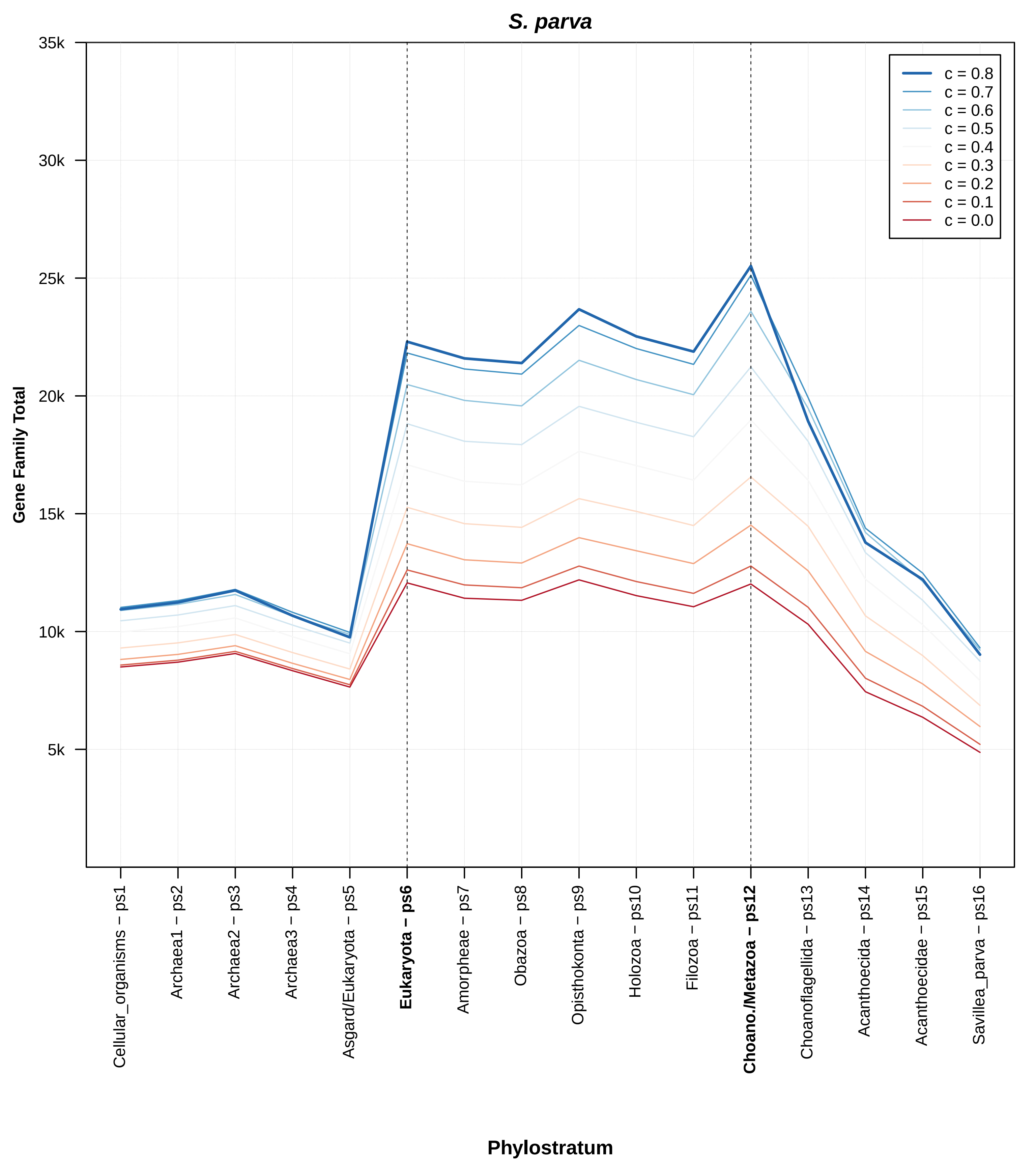
S. parva
Gene Family Total
35k
30k
25k
20k
15k
10k
5k
c = 0.8
c = 0.7
c = 0.6
c = 0.5
c = 0.4
c = 0.3
c = 0.2
c = 0.1
c = 0.0
Cellular_organisms – ps1
Archaea1 – ps2
Archaea2 – ps3
Archaea3 – ps4
Asgard/Eukaryota – ps5
Eukaryota – ps6
Amorpheae – ps7
Obazoa – ps8
Opisthokonta – ps9
Holozoa – ps10
Filozoa – ps11
Choano./Metazoa – ps12
Choanoflagellida – ps13
Acanthoecida – ps14
Acanthoecidae – ps15
Savillea_parva – ps16
Phylostratum

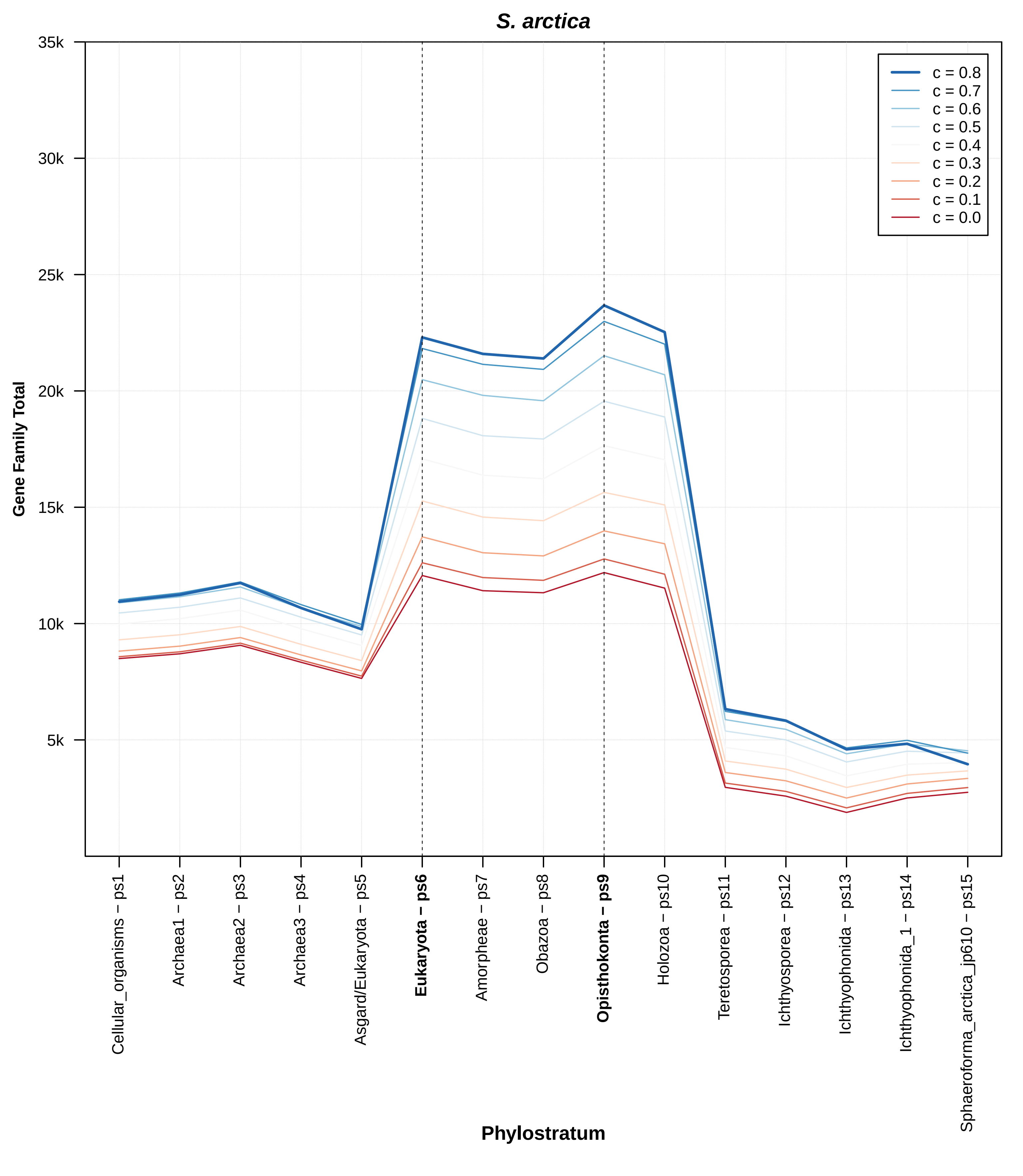
S. arctica
Gene Family Total
35k
30k
25k
20k
15k
10k
5k
c = 0.8
c = 0.7
c = 0.6
c = 0.5
c = 0.4
c = 0.3
c = 0.2
c = 0.1
c = 0.0
Cellular_organisms – ps1
Archaea1 – ps2
Archaea2 – ps3
Archaea3 – ps4
Asgard/Eukaryota – ps5
Eukaryota – ps6
Amorpheae – ps7
Obazoa – ps8
Opisthokonta – ps9
Holozoa – ps10
Teretosporea – ps11
Ichthyosporea – ps12
Ichthyophonida – ps13
Ichthyophonida_1 – ps14
Sphaeroforma_arctica_jp610 – ps15
Phylostratum

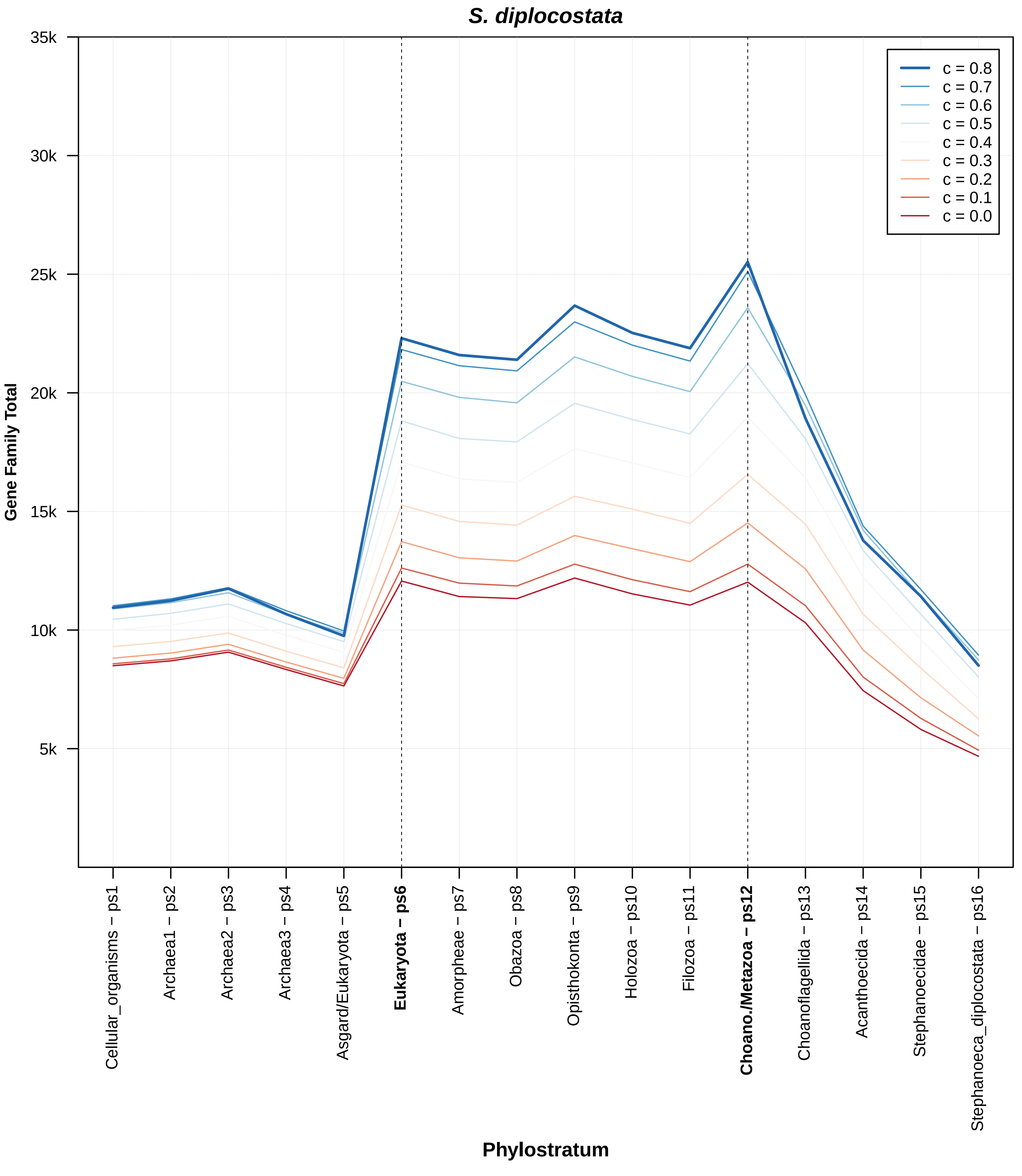
S. diplocostata
35k
30k
25k
20k
15k
10k
5k
Gene Family Total
c = 0.8
c = 0.7
c = 0.6
c = 0.5
c = 0.4
c = 0.3
c = 0.2
c = 0.1
c = 0.0
Cellular_organisms – ps1
Archaea1 – ps2
Archaea2 – ps3
Archaea3 – ps4
Asgard/Eukaryota – ps5
Eukaryota – ps6
Amorpheae – ps7
Obazoa – ps8
Opisthokonta – ps9
Holozoa – ps10
Filozoa – ps11
Choano./Metazoa – ps12
Choanoflagellida – ps13
Acanthoecida – ps14
Stephanoecidae – ps15
Stephanoeca_diplocostata – ps16
Phylostratum

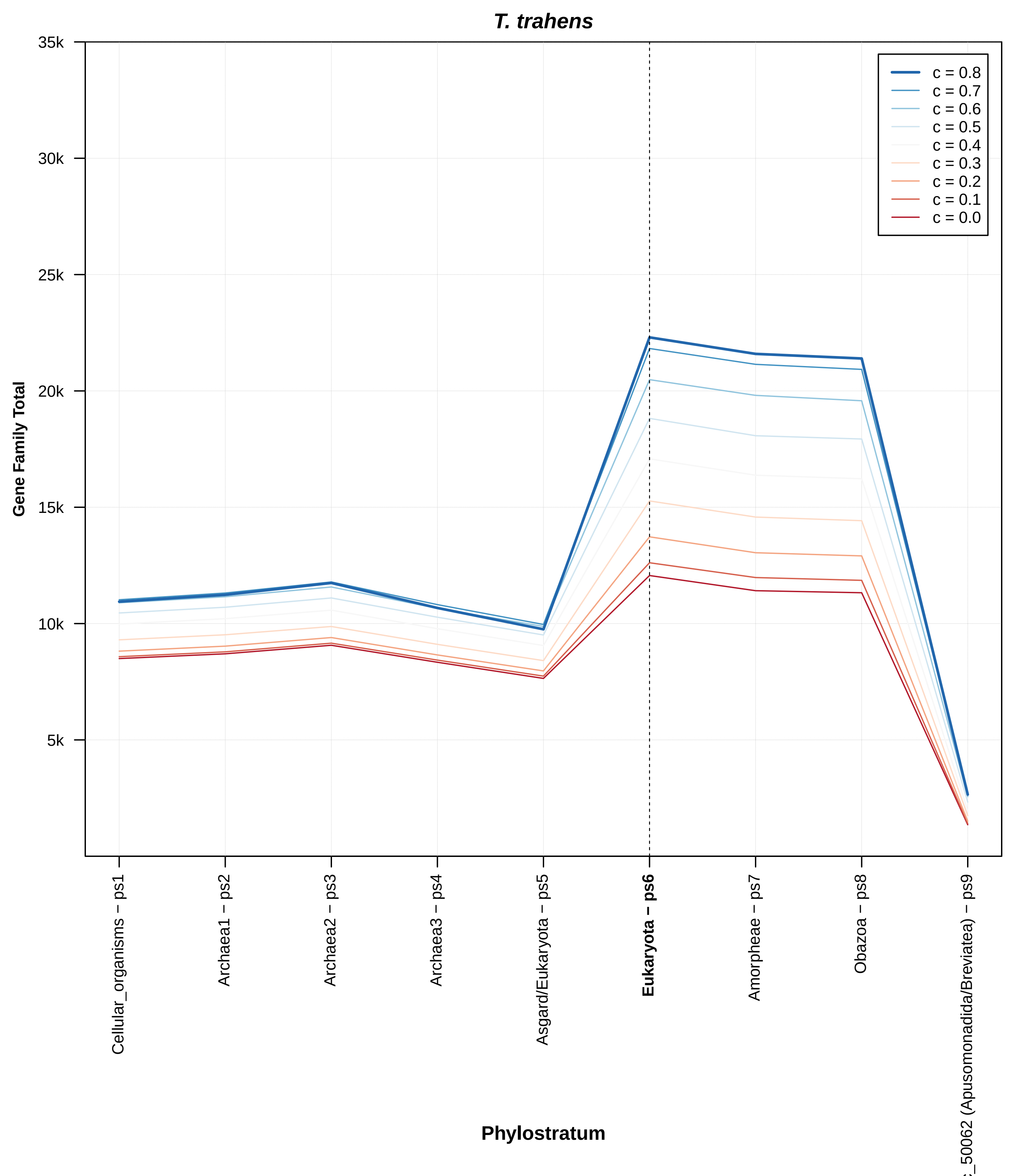

T. trahens
Gene Family Total
35k
30k
25k
20k
15k
10k
5k
c = 0.8
c = 0.7
c = 0.6
c = 0.5
c = 0.4
c = 0.3
c = 0.2
c = 0.1
c = 0.0
Cellular_organisms – ps1
Archaea1 – ps2
Archaea2 – ps3
Archaea3 – ps4
Asgard/Eukaryota – ps5
Eukaryota – ps6
Amorpheae – ps7
Obazoa – ps8
50062 (Apusomonadida/Breviatea) – ps9
Phylostratum

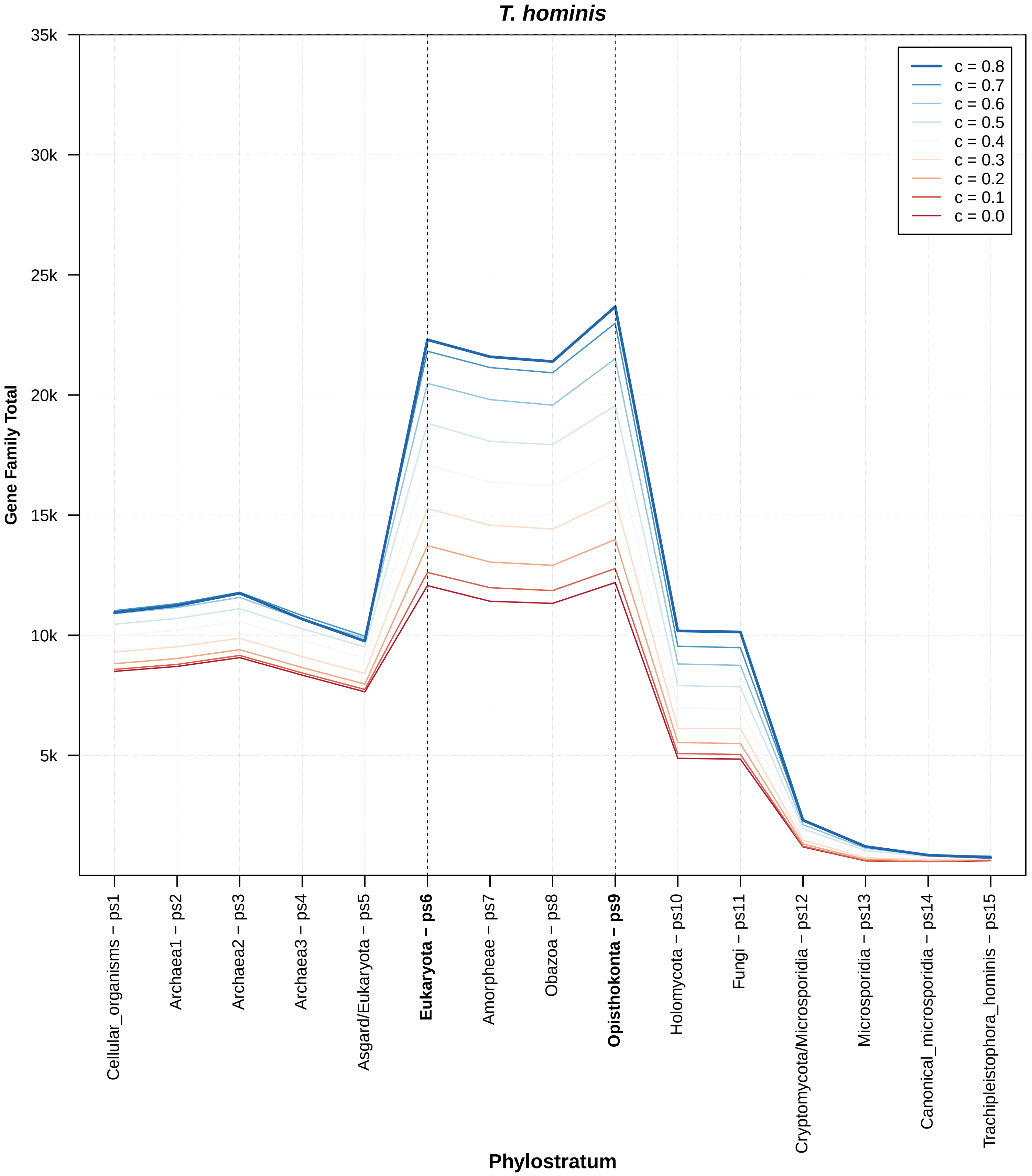

T. hominis
35k
30k
25k
20k
15k
10k
5k
Gene Family Total
c = 0.8
c = 0.7
c = 0.6
c = 0.5
c = 0.4
c = 0.3
c = 0.2
c = 0.1
c = 0.0
Cellular_organisms – ps1
Archaea1 – ps2
Archaea2 – ps3
Archaea3 – ps4
Asgard/Eukaryota – ps5
Eukaryota – ps6
Amorpheae – ps7
Obazoa – ps8
Opisthokonta – ps9
Holomycota – ps10
Fungi – ps11
Cryptomycota/Microsporidia – ps12
Microsporidia – ps13
Canonical_microsporidia – ps14
Trachipleistophora_hominis – ps15
Phylostratum

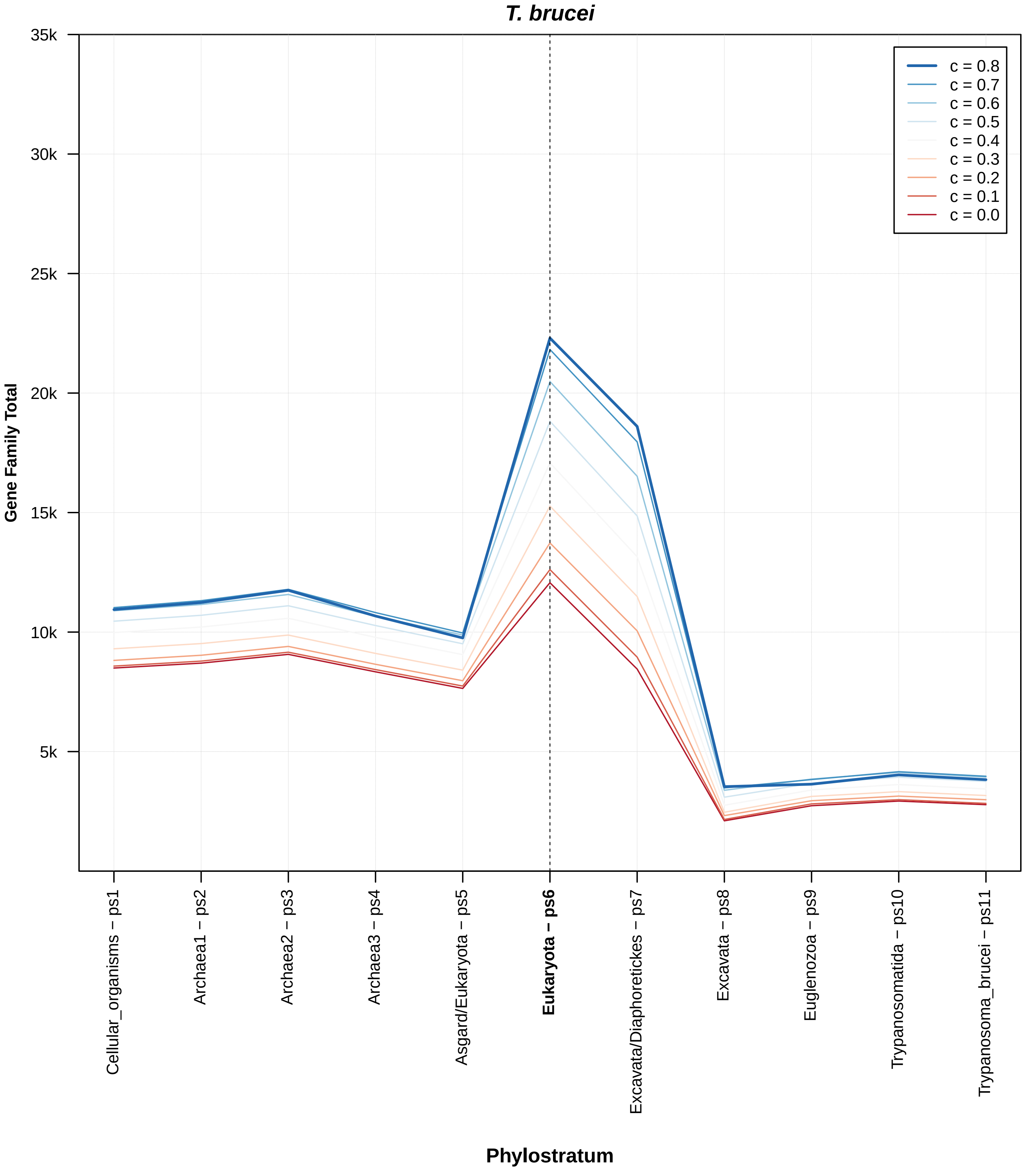
T. brucei
35k
30k
25k
20k
15k
10k
5k
Gene Family Total
c = 0.8
c = 0.7
c = 0.6
c = 0.5
c = 0.4
c = 0.3
c = 0.2
c = 0.1
c = 0.0
Cellular_organisms – ps1
Archaea1 – ps2
Archaea2 – ps3
Archaea3 – ps4
Asgard/Eukaryota – ps5
Eukaryota – ps6
Excavata/Diaphoretickes – ps7
Excavata – ps8
Euglenozoa – ps9
Trypanosomatida – ps10
Trypanosoma_brucei – ps11
Phylostratum
