# Supplementary material for: Macroevolutionary dynamics of gene family gain and loss along multicellular eukaryotic lineages
Source: Nat Commun. 2024 Mar 26;15:2663. doi: 10.1038/s41467-024-47017-w (PMC10966110; doi:10.1038/s41467-024-47017-w)
Supplement: Supplementary file 13 — Supplementary Dataset 8 [file 41467_2024_47017_MOESM13_ESM.pdf]

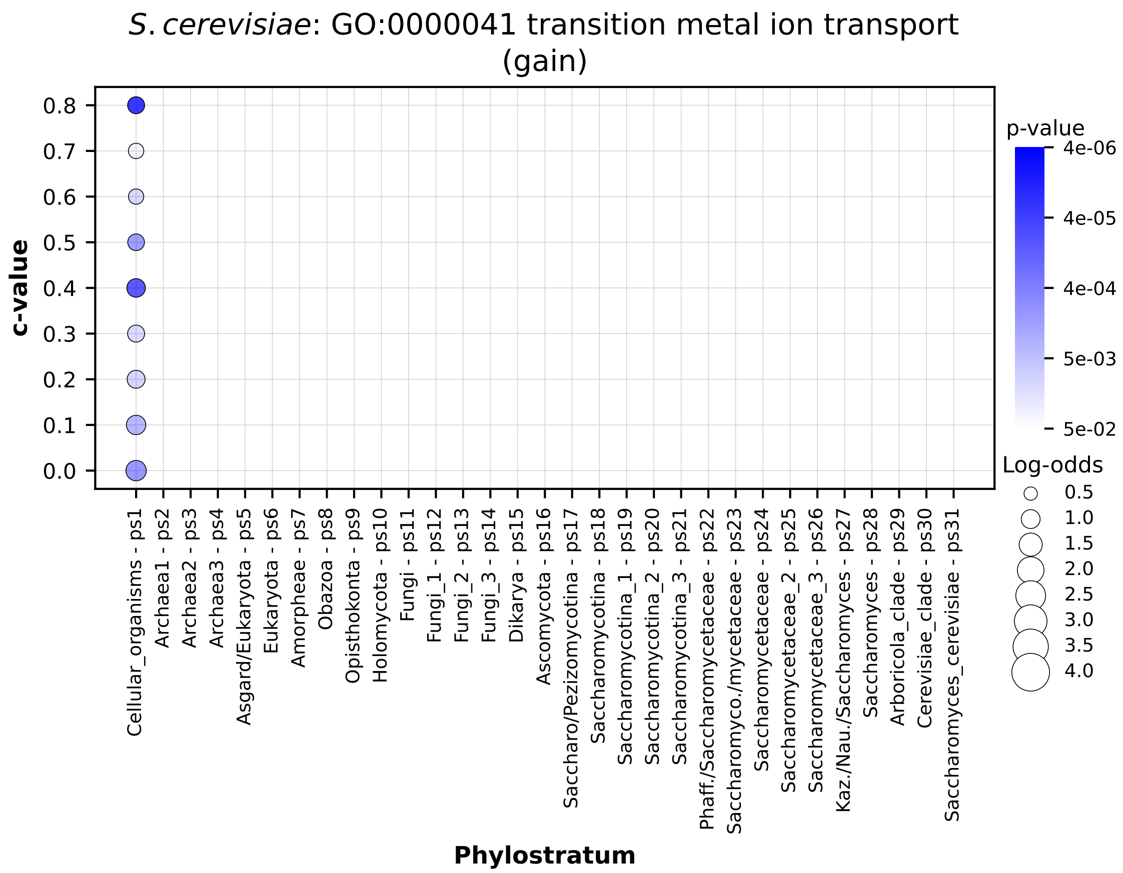
S. cerevisiae: GO:0000041 transition metal ion transport (gain)
c-value
0.8
0.7
0.6
0.5
0.4
0.3
0.2
0.1
0.0
p-value
4e-06
4e-05
4e-04
5e-03
5e-02
Log-odds
0.5
1.0
1.5
2.0
2.5
3.0
3.5
4.0
Cellular_organisms - ps1
Archaea1 - ps2
Archaea2 - ps3
Archaea3 - ps4
Asgard/Eukaryota - ps5
Eukaryota - ps6
Amorpheae - ps7
Obazoa - ps8
Opisthokonta - ps9
Holomycota - ps10
Fungi - ps11
Fungi_1 - ps12
Fungi_2 - ps13
Fungi_3 - ps14
Dikarya - ps15
Ascomycota - ps16
Saccharo/Pezizomycotina - ps17
Saccharomycotina - ps18
Saccharomycotina_1 - ps19
Saccharomycotina_2 - ps20
Saccharomycotina_3 - ps21
Phaff./Saccharomycetaceae - ps22
Saccharomyco./mycetaceae - ps23
Saccharomycetaceae - ps24
Saccharomycetaceae_2 - ps25
Saccharomycetaceae_3 - ps26
Kaz./Nau./Saccharomyces - ps27
Saccharomyces - ps28
Arboricola_clade - ps29
Cerevisiae_clade - ps30
Saccharomyces_cerevisiae - ps31
Phylostratum

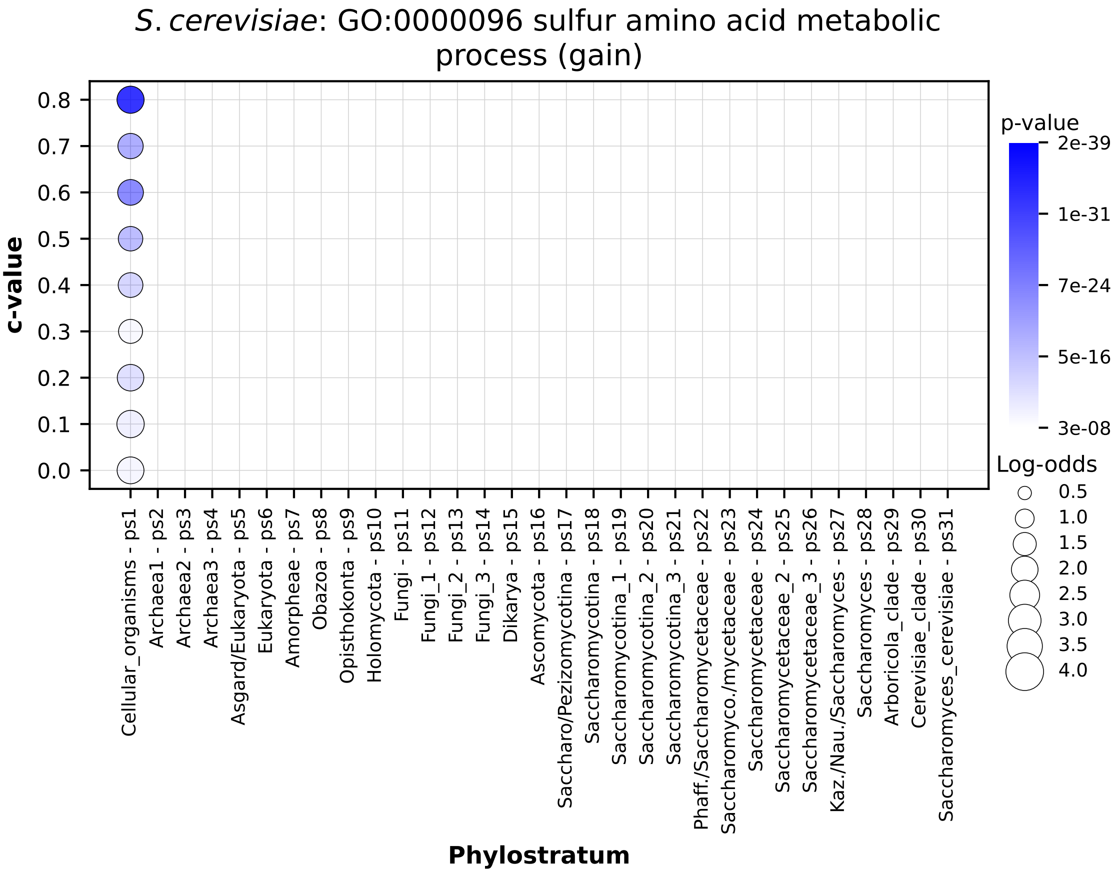
S. cerevisiae: GO:0000096 sulfur amino acid metabolic process (gain)
c-value
0.0
0.1
0.2
0.3
0.4
0.5
0.6
0.7
0.8
Phylostratum
Cellular_organisms - ps1
Archaea1 - ps2
Archaea2 - ps3
Archaea3 - ps4
Asgard/Eukaryota - ps5
Eukaryota - ps6
Amorpheae - ps7
Obazoa - ps8
Opisthokonta - ps9
Holomycota - ps10
Fungi - ps11
Fungi_1 - ps12
Fungi_2 - ps13
Fungi_3 - ps14
Dikarya - ps15
Ascomycota - ps16
Saccharo/Pezizomycotina - ps17
Saccharomycotina - ps18
Saccharomycotina_1 - ps19
Saccharomycotina_2 - ps20
Saccharomycotina_3 - ps21
Phaff./Saccharomycetaceae - ps22
Saccharomyco./mycetaceae - ps23
Saccharomycetaceae - ps24
Saccharomycetaceae_2 - ps25
Saccharomycetaceae_3 - ps26
Kaz./Nau./Saccharomyces - ps27
Saccharomyces - ps28
Arboricola_clade - ps29
Cerevisiae_clade - ps30
Saccharomyces_cerevisiae - ps31
p-value
2e-39
1e-31
7e-24
5e-16
3e-08
Log-odds
0.5
1.0
1.5
2.0
2.5
3.0
3.5
4.0

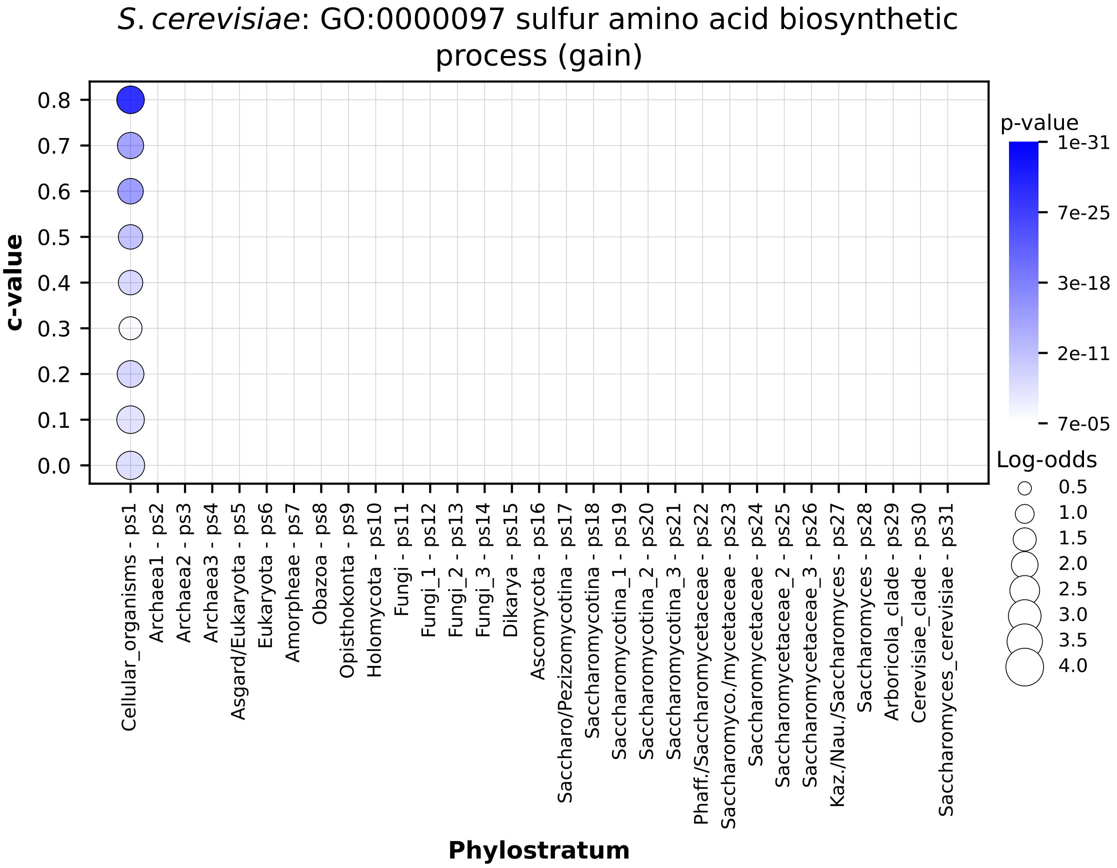

S. cerevisiae: GO:0000097 sulfur amino acid biosynthetic process (gain)
c-value
0.0
0.1
0.2
0.3
0.4
0.5
0.6
0.7
0.8
p-value
1e-31
7e-25
3e-18
2e-11
7e-05
Log-odds
0.5
1.0
1.5
2.0
2.5
3.0
3.5
4.0
Cellular_organisms - ps1
Archaea1 - ps2
Archaea2 - ps3
Archaea3 - ps4
Asgard/Eukaryota - ps5
Eukaryota - ps6
Amorpheae - ps7
Obazoa - ps8
Opisthokonta - ps9
Holomycota - ps10
Fungi - ps11
Fungi_1 - ps12
Fungi_2 - ps13
Fungi_3 - ps14
Dikarya - ps15
Ascomycota - ps16
Saccharo/Pezizomycotina - ps17
Saccharomycotina - ps18
Saccharomycotina_1 - ps19
Saccharomycotina_2 - ps20
Saccharomycotina_3 - ps21
Phaff./Saccharomycetaceae - ps22
Saccharomyco./mycetaceae - ps23
Saccharomycetaceae - ps24
Saccharomycetaceae_2 - ps25
Saccharomycetaceae_3 - ps26
Kaz./Nau./Saccharomyces - ps27
Saccharomyces - ps28
Arboricola_clade - ps29
Cerevisiae_clade - ps30
Saccharomyces_cerevisiae - ps31
Phylostratum

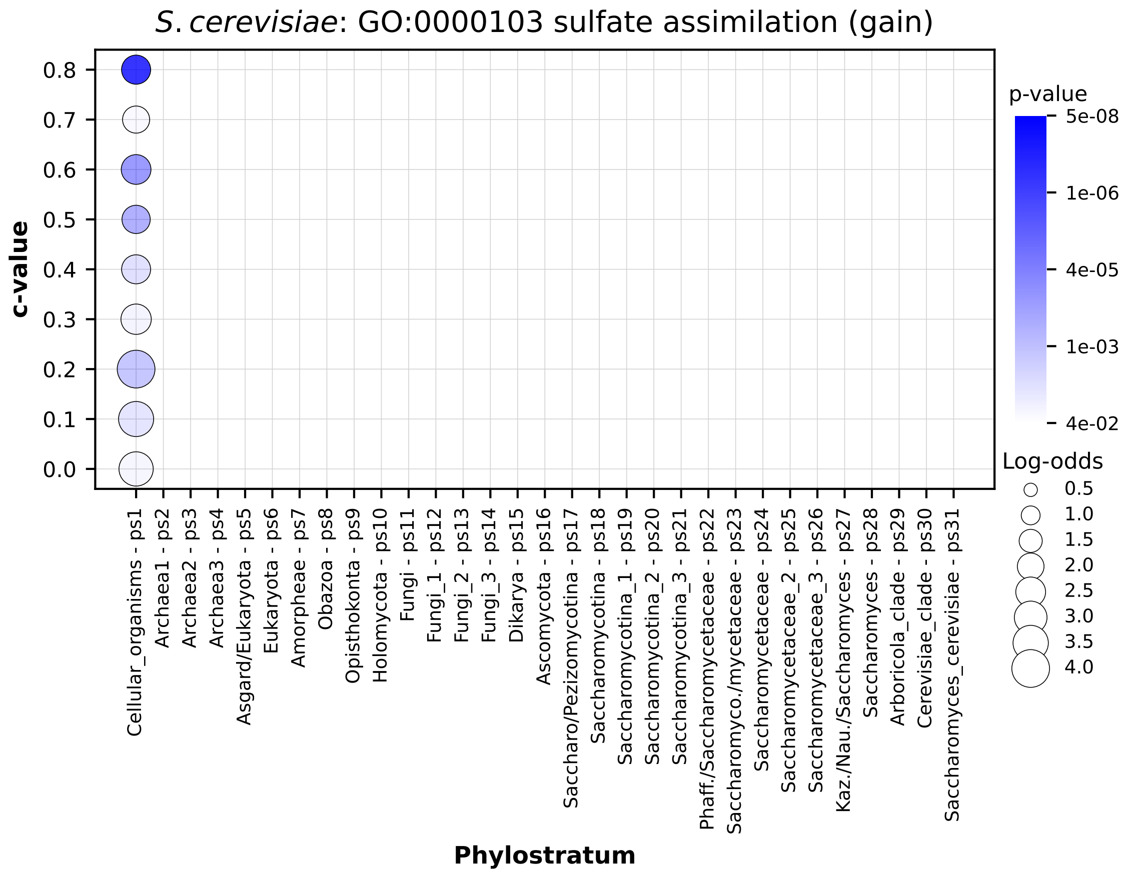

S. cerevisiae: GO:0000103 sulfate assimilation (gain)
c-value
0.0
0.1
0.2
0.3
0.4
0.5
0.6
0.7
0.8
Phylostratum
Cellular_organisms - ps1
Archaea1 - ps2
Archaea2 - ps3
Archaea3 - ps4
Asgard/Eukaryota - ps5
Eukaryota - ps6
Amorpheae - ps7
Obazoa - ps8
Opisthokonta - ps9
Holomycota - ps10
Fungi - ps11
Fungi_1 - ps12
Fungi_2 - ps13
Fungi_3 - ps14
Dikarya - ps15
Ascomycota - ps16
Saccharo/Pezizomycotina - ps17
Saccharomycotina - ps18
Saccharomycotina_1 - ps19
Saccharomycotina_2 - ps20
Saccharomycotina_3 - ps21
Phaff./Saccharomycetaceae - ps22
Saccharomyco./mycetaceae - ps23
Saccharomycetaceae - ps24
Saccharomycetaceae_2 - ps25
Saccharomycetaceae_3 - ps26
Kaz./Nau./Saccharomyces - ps27
Saccharomyces - ps28
Arboricola_clade - ps29
Cerevisiae_clade - ps30
Saccharomyces_cerevisiae - ps31
p-value
5e-08
1e-06
4e-05
1e-03
4e-02
Log-odds
0.5
1.0
1.5
2.0
2.5
3.0
3.5
4.0

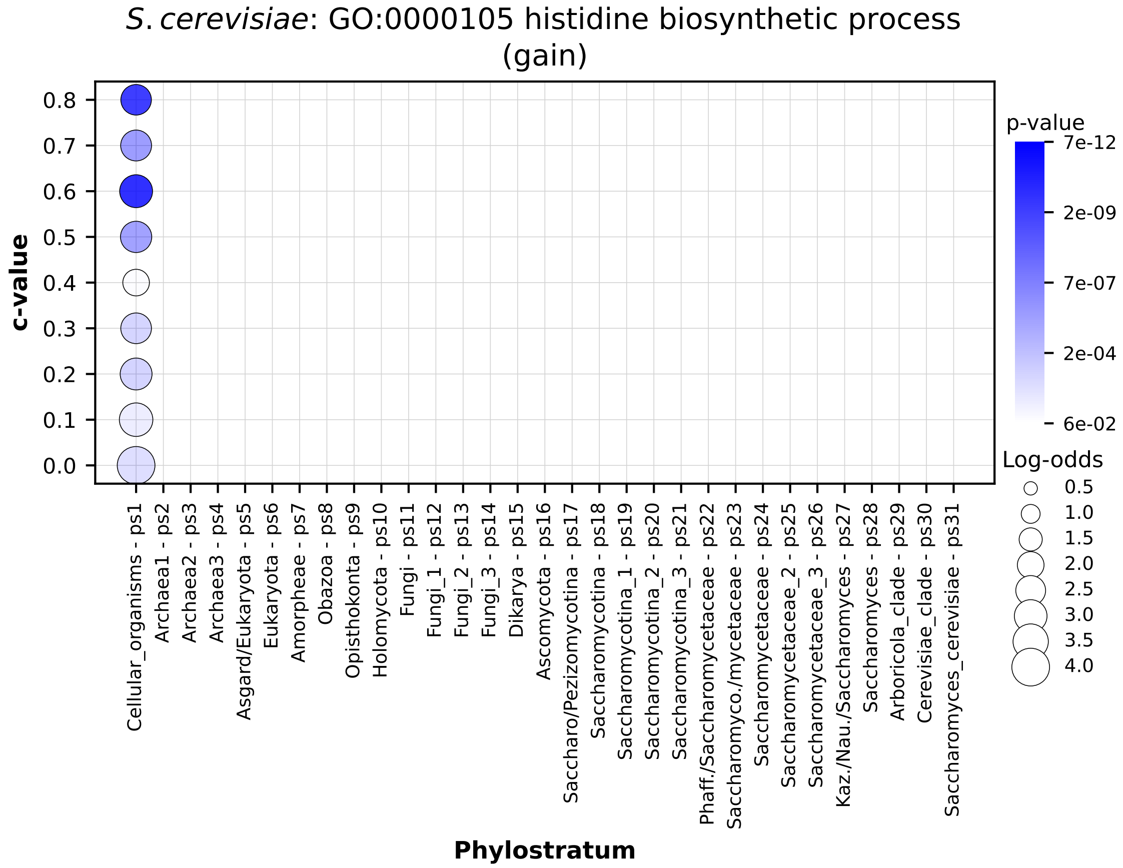
S. cerevisiae: GO:0000105 histidine biosynthetic process
(gain)
c-value
0.0
0.1
0.2
0.3
0.4
0.5
0.6
0.7
0.8
p-value
7e-12
2e-09
7e-07
2e-04
6e-02
Log-odds
0.5
1.0
1.5
2.0
2.5
3.0
3.5
4.0
Cellular_organisms - ps1
Archaea1 - ps2
Archaea2 - ps3
Archaea3 - ps4
Asgard/Eukaryota - ps5
Eukaryota - ps6
Amorpheae - ps7
Obazoa - ps8
Opisthokonta - ps9
Holomycota - ps10
Fungi - ps11
Fungi_1 - ps12
Fungi_2 - ps13
Fungi_3 - ps14
Dikarya - ps15
Ascomycota - ps16
Saccharo/Pezizomycotina - ps17
Saccharomycotina - ps18
Saccharomycotina_1 - ps19
Saccharomycotina_2 - ps20
Saccharomycotina_3 - ps21
Phaff./Saccharomycetaceae - ps22
Saccharomyco./mycetaceae - ps23
Saccharomycetaceae - ps24
Saccharomycetaceae_2 - ps25
Saccharomycetaceae_3 - ps26
Kaz./Nau./Saccharomyces - ps27
Saccharomyces - ps28
Arboricola_clade - ps29
Cerevisiae_clade - ps30
Saccharomyces_cerevisiae - ps31
Phylostratum

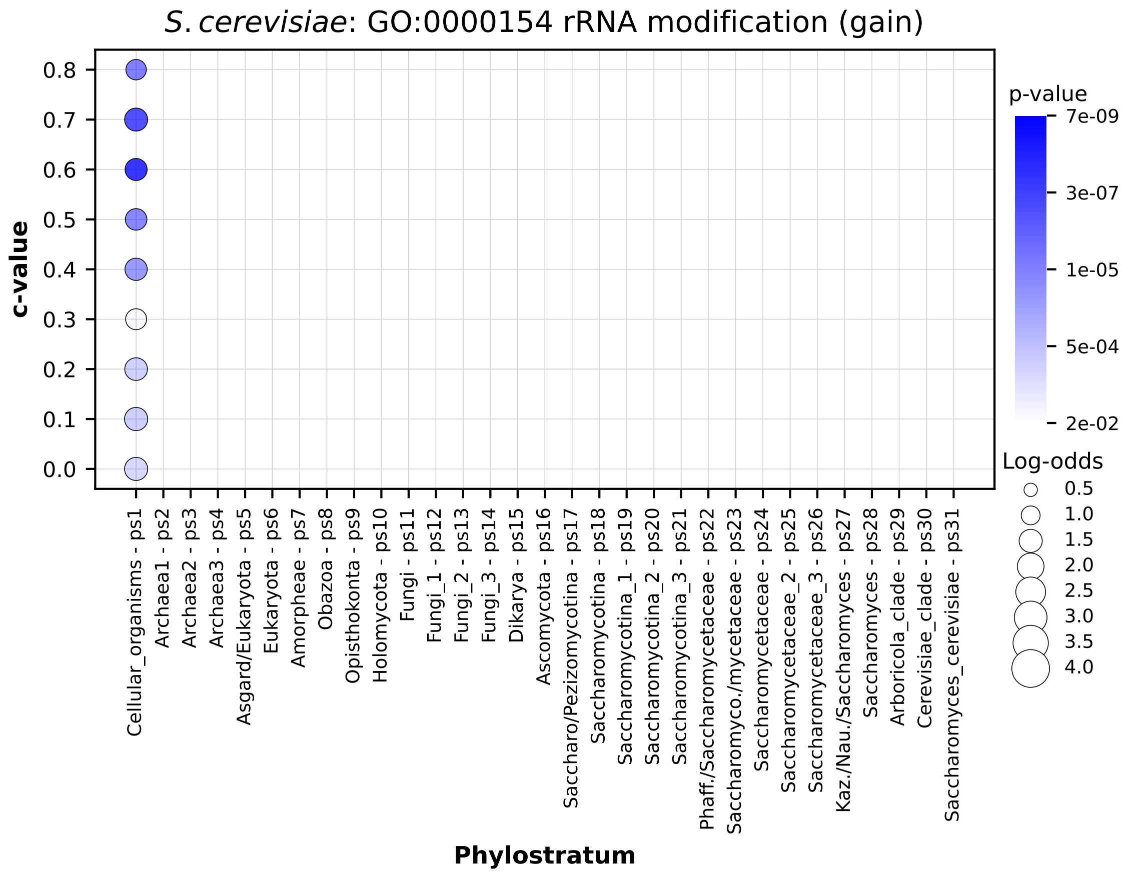

S. cerevisiae: GO:0000154 rRNA modification (gain)
c-value
0.8
0.7
0.6
0.5
0.4
0.3
0.2
0.1
0.0
p-value
7e-09
3e-07
1e-05
5e-04
2e-02
Log-odds
0.5
1.0
1.5
2.0
2.5
3.0
3.5
4.0
Cellular_organisms - ps1
Archaea1 - ps2
Archaea2 - ps3
Archaea3 - ps4
Asgard/Eukaryota - ps5
Eukaryota - ps6
Amorpheae - ps7
Obazoa - ps8
Opisthokonta - ps9
Holomycota - ps10
Fungi - ps11
Fungi_1 - ps12
Fungi_2 - ps13
Fungi_3 - ps14
Dikarya - ps15
Ascomycota - ps16
Saccharo/Pezizomycotina - ps17
Saccharomycotina - ps18
Saccharomycotina_1 - ps19
Saccharomycotina_2 - ps20
Saccharomycotina_3 - ps21
Phaff./Saccharomycetaceae - ps22
Saccharomyco./mycetaceae - ps23
Saccharomycetaceae - ps24
Saccharomycetaceae_2 - ps25
Saccharomycetaceae_3 - ps26
Kaz./Nau./Saccharomyces - ps27
Saccharomyces - ps28
Arboricola_clade - ps29
Cerevisiae_clade - ps30
Saccharomyces_cerevisiae - ps31
Phylostratum

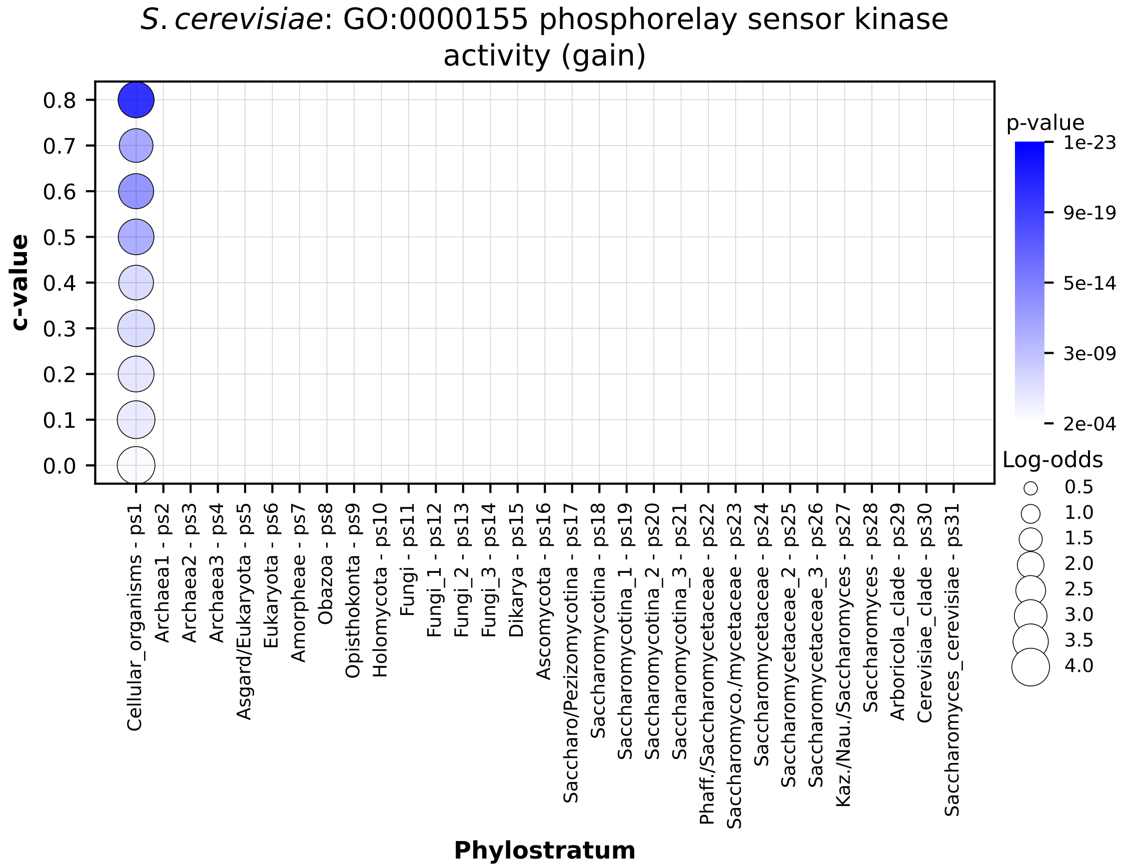
S. cerevisiae: GO:0000155 phosphorelay sensor kinase activity (gain)
c-value
0.8
0.7
0.6
0.5
0.4
0.3
0.2
0.1
0.0
p-value
1e-23
9e-19
5e-14
3e-09
2e-04
Log-odds
0.5
1.0
1.5
2.0
2.5
3.0
3.5
4.0
Cellular_organisms - ps1
Archaea1 - ps2
Archaea2 - ps3
Archaea3 - ps4
Asgard/Eukaryota - ps5
Eukaryota - ps6
Amorpheae - ps7
Obazoa - ps8
Opisthokonta - ps9
Holomycota - ps10
Fungi - ps11
Fungi_1 - ps12
Fungi_2 - ps13
Fungi_3 - ps14
Dikarya - ps15
Ascomycota - ps16
Saccharo/Pezizomycotina - ps17
Saccharomycotina - ps18
Saccharomycotina_1 - ps19
Saccharomycotina_2 - ps20
Saccharomycotina_3 - ps21
Phaff./Saccharomycetaceae - ps22
Saccharomyco./mycetaceae - ps23
Saccharomycetaceae - ps24
Saccharomycetaceae_2 - ps25
Saccharomycetaceae_3 - ps26
Kaz./Nau./Saccharomyces - ps27
Saccharomyces - ps28
Arboricola_clade - ps29
Cerevisiae_clade - ps30
Saccharomyces_cerevisiae - ps31
Phylostratum

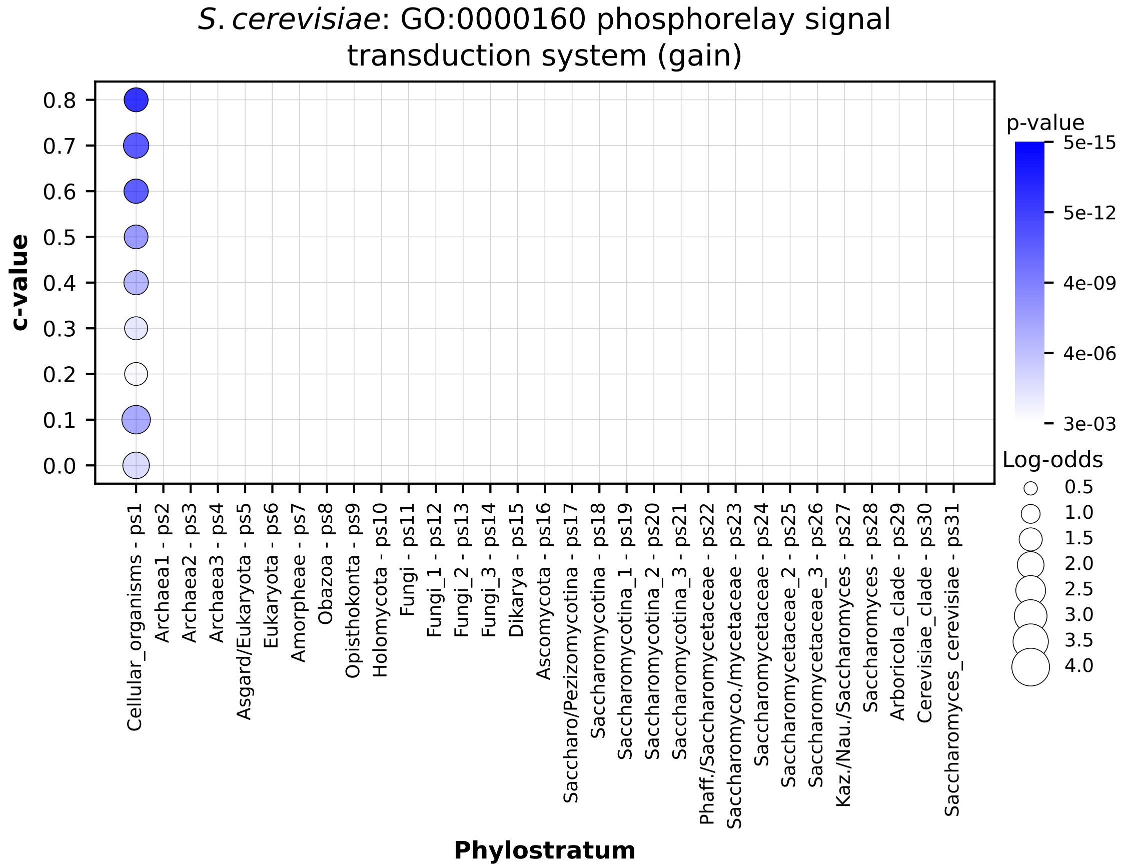
S. cerevisiae: GO:0000160 phosphorelay signal transduction system (gain)
c-value
0.0
0.1
0.2
0.3
0.4
0.5
0.6
0.7
0.8
p-value
5e-15
5e-12
4e-09
4e-06
3e-03
Log-odds
0.5
1.0
1.5
2.0
2.5
3.0
3.5
4.0
Cellular_organisms - ps1
Archaea1 - ps2
Archaea2 - ps3
Archaea3 - ps4
Asgard/Eukaryota - ps5
Eukaryota - ps6
Amorpheae - ps7
Obazoa - ps8
Opisthokonta - ps9
Holomycota - ps10
Fungi - ps11
Fungi_1 - ps12
Fungi_2 - ps13
Fungi_3 - ps14
Dikarya - ps15
Ascomycota - ps16
Saccharo/Pezizomycotina - ps17
Saccharomycotina - ps18
Saccharomycotina_1 - ps19
Saccharomycotina_2 - ps20
Saccharomycotina_3 - ps21
Phaff./Saccharomycetaceae - ps22
Saccharomyco./mycetaceae - ps23
Saccharomycetaceae - ps24
Saccharomycetaceae_2 - ps25
Saccharomycetaceae_3 - ps26
Kaz./Nau./Saccharomyces - ps27
Saccharomyces - ps28
Arboricola_clade - ps29
Cerevisiae_clade - ps30
Saccharomyces_cerevisiae - ps31
Phylostratum

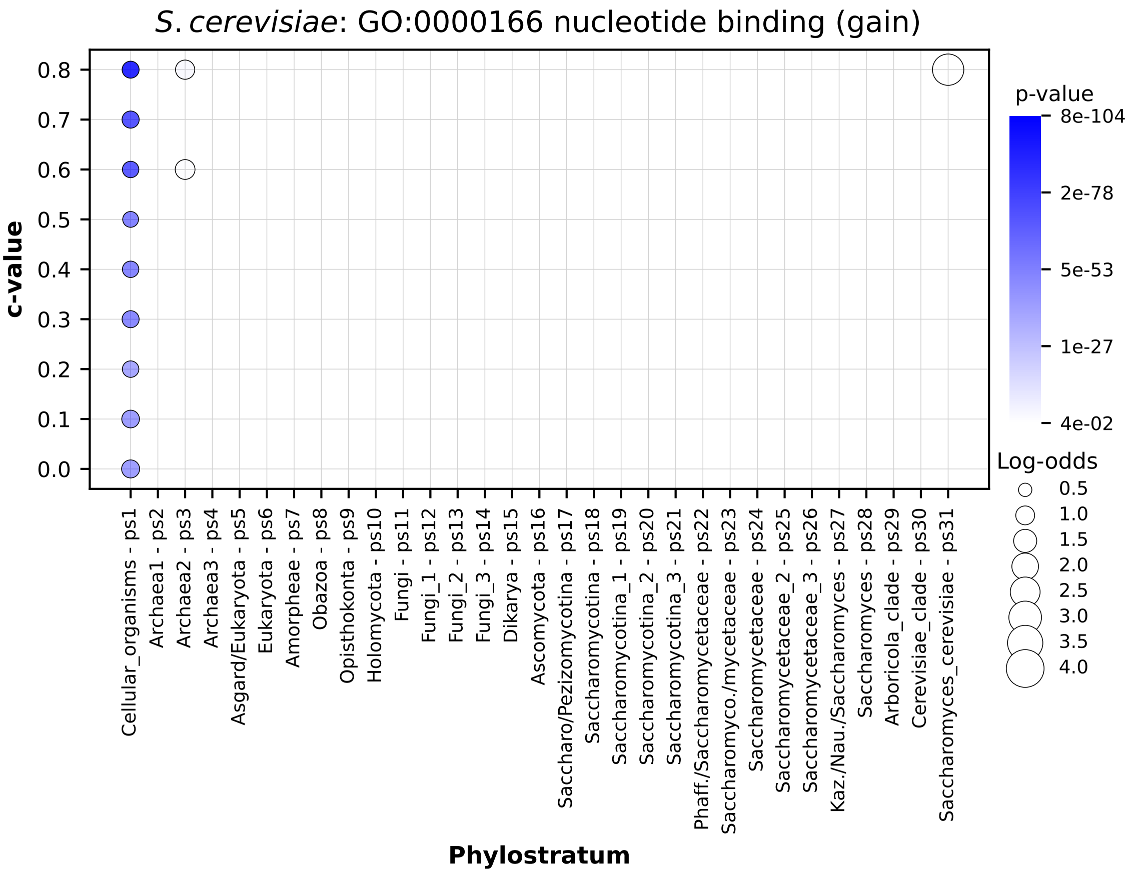
S. cerevisiae: GO:0000166 nucleotide binding (gain)
c-value
0.8
0.7
0.6
0.5
0.4
0.3
0.2
0.1
0.0
p-value
8e-104
2e-78
5e-53
1e-27
4e-02
Log-odds
0.5
1.0
1.5
2.0
2.5
3.0
3.5
4.0
Cellular_organisms - ps1
Archaea1 - ps2
Archaea2 - ps3
Archaea3 - ps4
Asgard/Eukaryota - ps5
Eukaryota - ps6
Amorpheae - ps7
Obazoa - ps8
Opisthokonta - ps9
Holomycota - ps10
Fungi - ps11
Fungi_1 - ps12
Fungi_2 - ps13
Fungi_3 - ps14
Dikarya - ps15
Ascomycota - ps16
Saccharo/Pezizomycotina - ps17
Saccharomycotina - ps18
Saccharomycotina_1 - ps19
Saccharomycotina_2 - ps20
Saccharomycotina_3 - ps21
Phaff./Saccharomycetaceae - ps22
Saccharomyco./mycetaceae - ps23
Saccharomycetaceae - ps24
Saccharomycetaceae_2 - ps25
Saccharomycetaceae_3 - ps26
Kaz./Nau./Saccharomyces - ps27
Saccharomyces - ps28
Arboricola_clade - ps29
Cerevisiae_clade - ps30
Saccharomyces_cerevisiae - ps31
Phylostratum

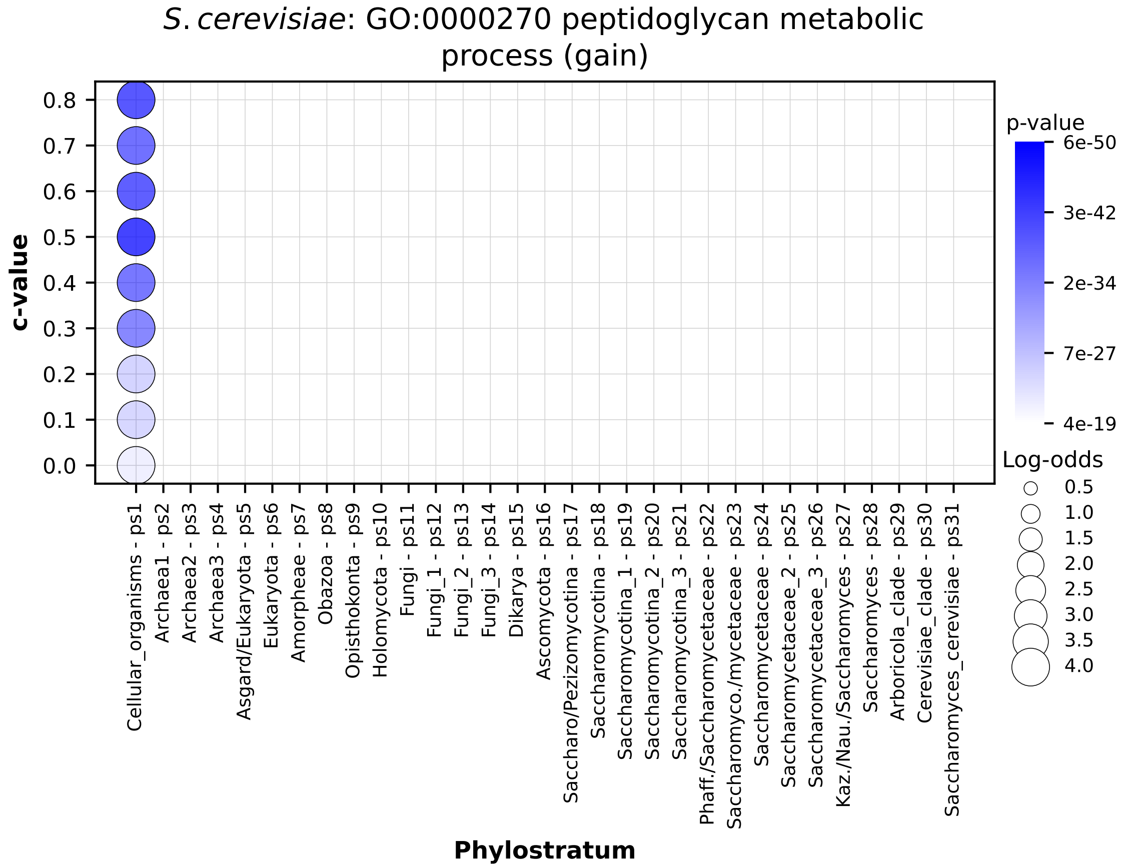
S. cerevisiae: GO:0000270 peptidoglycan metabolic process (gain)
c-value
0.8
0.7
0.6
0.5
0.4
0.3
0.2
0.1
0.0
p-value
6e-50
3e-42
2e-34
7e-27
4e-19
Log-odds
0.5
1.0
1.5
2.0
2.5
3.0
3.5
4.0
Cellular_organisms - ps1
Archaea1 - ps2
Archaea2 - ps3
Archaea3 - ps4
Asgard/Eukaryota - ps5
Eukaryota - ps6
Amorpheae - ps7
Obazoa - ps8
Opisthokonta - ps9
Holomycota - ps10
Fungi - ps11
Fungi_1 - ps12
Fungi_2 - ps13
Fungi_3 - ps14
Dikarya - ps15
Ascomycota - ps16
Saccharo/Pezizomycotina - ps17
Saccharomycotina - ps18
Saccharomycotina_1 - ps19
Saccharomycotina_2 - ps20
Saccharomycotina_3 - ps21
Phaff./Saccharomycetaceae - ps22
Saccharomyco./mycetaceae - ps23
Saccharomycetaceae - ps24
Saccharomycetaceae_2 - ps25
Saccharomycetaceae_3 - ps26
Kaz./Nau./Saccharomyces - ps27
Saccharomyces - ps28
Arboricola_clade - ps29
Cerevisiae_clade - ps30
Saccharomyces_cerevisiae - ps31
Phylostratum

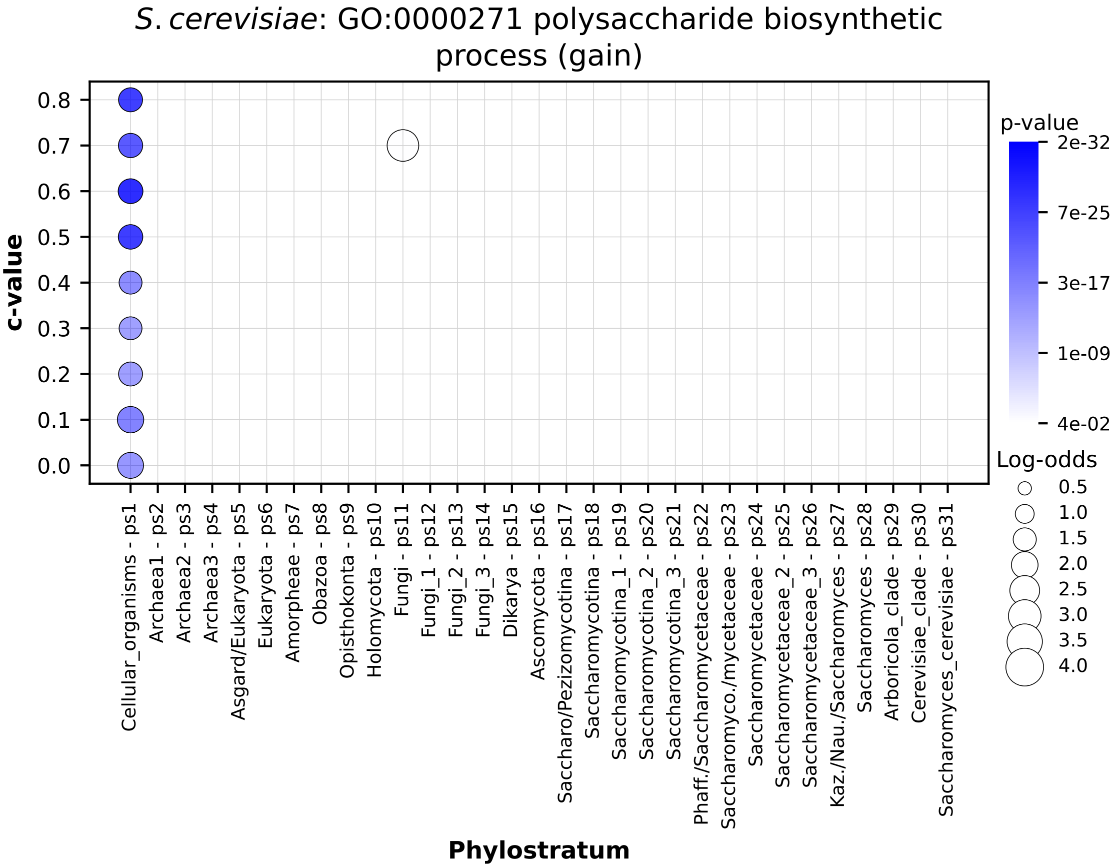

$S. cerevisiae$: GO:0000271 polysaccharide biosynthetic process (gain)
c-value
0.0
0.1
0.2
0.3
0.4
0.5
0.6
0.7
0.8
p-value
2e-32
7e-25
3e-17
1e-09
4e-02
Log-odds
0.5
1.0
1.5
2.0
2.5
3.0
3.5
4.0
Cellular_organisms - ps1
Archaea1 - ps2
Archaea2 - ps3
Archaea3 - ps4
Asgard/Eukaryota - ps5
Eukaryota - ps6
Amorpheae - ps7
Obazoa - ps8
Opisthokonta - ps9
Holomycota - ps10
Fungi - ps11
Fungi_1 - ps12
Fungi_2 - ps13
Fungi_3 - ps14
Dikarya - ps15
Ascomycota - ps16
Saccharo/Pezizomycotina - ps17
Saccharomycotina - ps18
Saccharomycotina_1 - ps19
Saccharomycotina_2 - ps20
Saccharomycotina_3 - ps21
Phaff./Saccharomycetaceae - ps22
Saccharomyco./mycetaceae - ps23
Saccharomycetaceae - ps24
Saccharomycetaceae_2 - ps25
Saccharomycetaceae_3 - ps26
Kaz./Nau./Saccharomyces - ps27
Saccharomyces - ps28
Arboricola_clade - ps29
Cerevisiae_clade - ps30
Saccharomyces_cerevisiae - ps31
Phylostratum

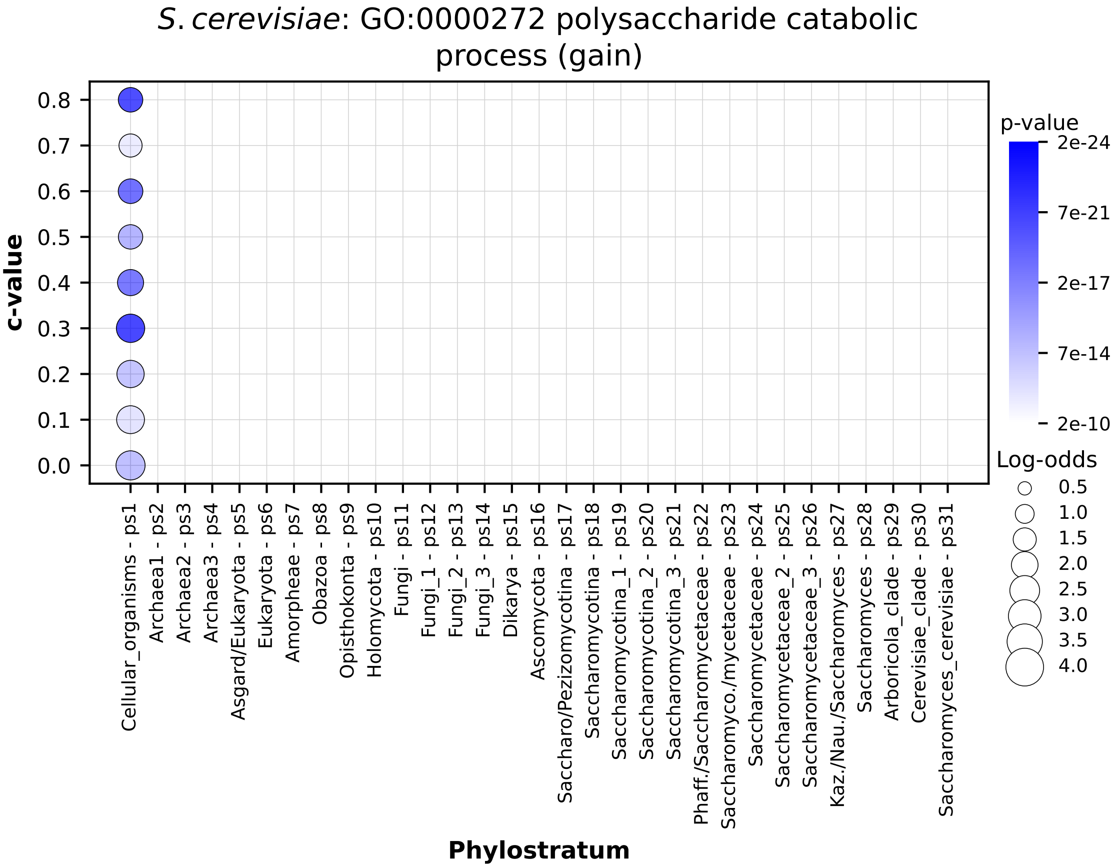

S. cerevisiae: GO:0000272 polysaccharide catabolic process (gain)
c-value
0.8
0.7
0.6
0.5
0.4
0.3
0.2
0.1
0.0
p-value
2e-24
7e-21
2e-17
7e-14
2e-10
Log-odds
0.5
1.0
1.5
2.0
2.5
3.0
3.5
4.0
Cellular_organisms - ps1
Archaea1 - ps2
Archaea2 - ps3
Archaea3 - ps4
Asgard/Eukaryota - ps5
Eukaryota - ps6
Amorpheae - ps7
Obazoa - ps8
Opisthokonta - ps9
Holomycota - ps10
Fungi - ps11
Fungi_1 - ps12
Fungi_2 - ps13
Fungi_3 - ps14
Dikarya - ps15
Ascomycota - ps16
Saccharo/Pezizomycotina - ps17
Saccharomycotina - ps18
Saccharomycotina_1 - ps19
Saccharomycotina_2 - ps20
Saccharomycotina_3 - ps21
Phaff./Saccharomycetaceae - ps22
Saccharomyco./mycetaceae - ps23
Saccharomycetaceae - ps24
Saccharomycetaceae_2 - ps25
Saccharomycetaceae_3 - ps26
Kaz./Nau./Saccharomyces - ps27
Saccharomyces - ps28
Arboricola_clade - ps29
Cerevisiae_clade - ps30
Saccharomyces_cerevisiae - ps31
Phylostratum

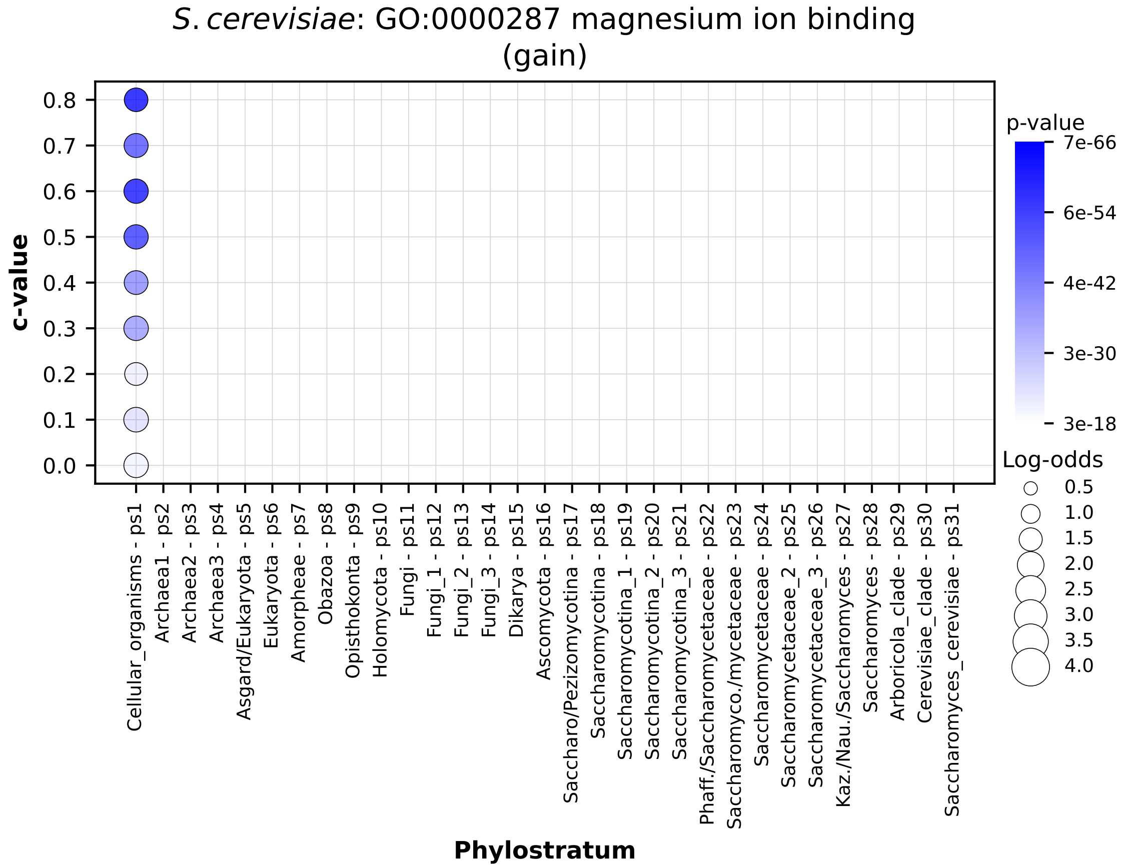

S. cerevisiae: GO:0000287 magnesium ion binding (gain)
c-value
0.8
0.7
0.6
0.5
0.4
0.3
0.2
0.1
0.0
p-value
7e-66
6e-54
4e-42
3e-30
3e-18
Log-odds
0.5
1.0
1.5
2.0
2.5
3.0
3.5
4.0
Cellular_organisms - ps1
Archaea1 - ps2
Archaea2 - ps3
Archaea3 - ps4
Asgard/Eukaryota - ps5
Eukaryota - ps6
Amorpheae - ps7
Obazoa - ps8
Opisthokonta - ps9
Holomycota - ps10
Fungi - ps11
Fungi_1 - ps12
Fungi_2 - ps13
Fungi_3 - ps14
Dikarya - ps15
Ascomycota - ps16
Saccharo/Pezizomycotina - ps17
Saccharomycotina - ps18
Saccharomycotina_1 - ps19
Saccharomycotina_2 - ps20
Saccharomycotina_3 - ps21
Phaff./Saccharomycetaceae - ps22
Saccharomyco./mycetaceae - ps23
Saccharomycetaceae - ps24
Saccharomycetaceae_2 - ps25
Saccharomycetaceae_3 - ps26
Kaz./Nau./Saccharomyces - ps27
Saccharomyces - ps28
Arboricola_clade - ps29
Cerevisiae_clade - ps30
Saccharomyces_cerevisiae - ps31
Phylostratum

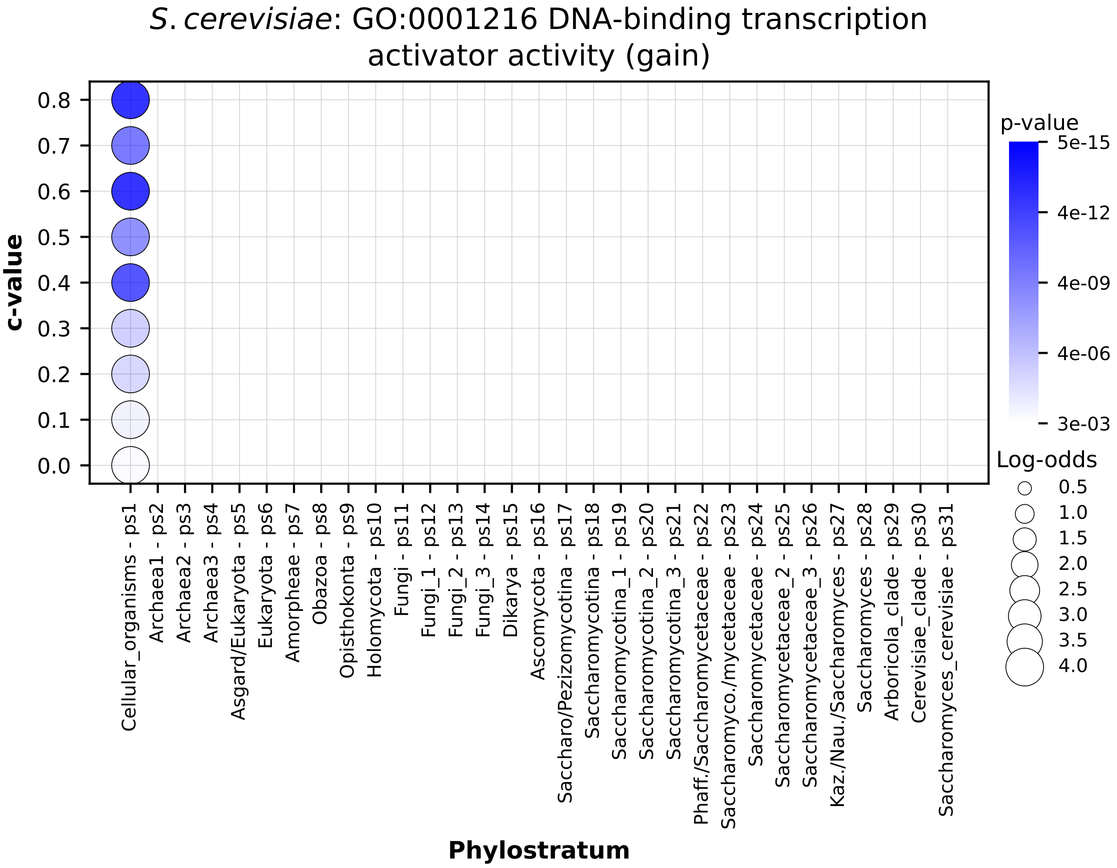
S. cerevisiae: GO:0001216 DNA-binding transcription activator activity (gain)
c-value
0.8
0.7
0.6
0.5
0.4
0.3
0.2
0.1
0.0
p-value
5e-15
4e-12
4e-09
4e-06
3e-03
Log-odds
0.5
1.0
1.5
2.0
2.5
3.0
3.5
4.0
Cellular_organisms - ps1
Archaea1 - ps2
Archaea2 - ps3
Archaea3 - ps4
Asgard/Eukaryota - ps5
Eukaryota - ps6
Amorpheae - ps7
Obazoa - ps8
Opisthokonta - ps9
Holomycota - ps10
Fungi - ps11
Fungi_1 - ps12
Fungi_2 - ps13
Fungi_3 - ps14
Dikarya - ps15
Ascomycota - ps16
Saccharo/Pezizomycotina - ps17
Saccharomycotina - ps18
Saccharomycotina_1 - ps19
Saccharomycotina_2 - ps20
Saccharomycotina_3 - ps21
Phaff./Saccharomycetaceae - ps22
Saccharomyco./mycetaceae - ps23
Saccharomycetaceae - ps24
Saccharomycetaceae_2 - ps25
Saccharomycetaceae_3 - ps26
Kaz./Nau./Saccharomyces - ps27
Saccharomyces - ps28
Arboricola_clade - ps29
Cerevisiae_clade - ps30
Saccharomyces_cerevisiae - ps31
Phylostratum

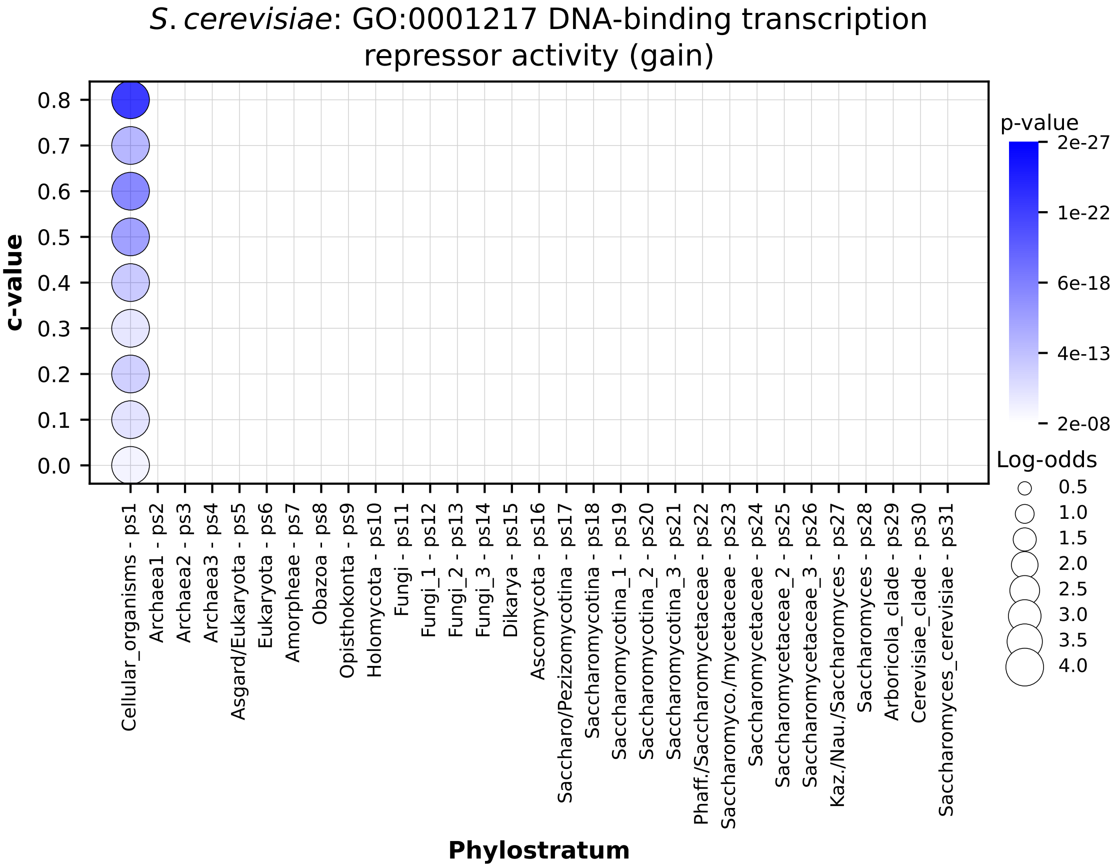
S. cerevisiae: GO:0001217 DNA-binding transcription repressor activity (gain)
c-value
0.0
0.1
0.2
0.3
0.4
0.5
0.6
0.7
0.8
Phylostratum
Cellular_organisms - ps1
Archaea1 - ps2
Archaea2 - ps3
Archaea3 - ps4
Asgard/Eukaryota - ps5
Eukaryota - ps6
Amorpheae - ps7
Obazoa - ps8
Opisthokonta - ps9
Holomycota - ps10
Fungi - ps11
Fungi_1 - ps12
Fungi_2 - ps13
Fungi_3 - ps14
Dikarya - ps15
Ascomycota - ps16
Saccharo/Pezizomycotina - ps17
Saccharomycotina - ps18
Saccharomycotina_1 - ps19
Saccharomycotina_2 - ps20
Saccharomycotina_3 - ps21
Phaff./Saccharomycetaceae - ps22
Saccharomyco./mycetaceae - ps23
Saccharomycetaceae - ps24
Saccharomycetaceae_2 - ps25
Saccharomycetaceae_3 - ps26
Kaz./Nau./Saccharomyces - ps27
Saccharomyces - ps28
Arboricola_clade - ps29
Cerevisiae_clade - ps30
Saccharomyces_cerevisiae - ps31
p-value
2e-27
1e-22
6e-18
4e-13
2e-08
Log-odds
0.5
1.0
1.5
2.0
2.5
3.0
3.5
4.0

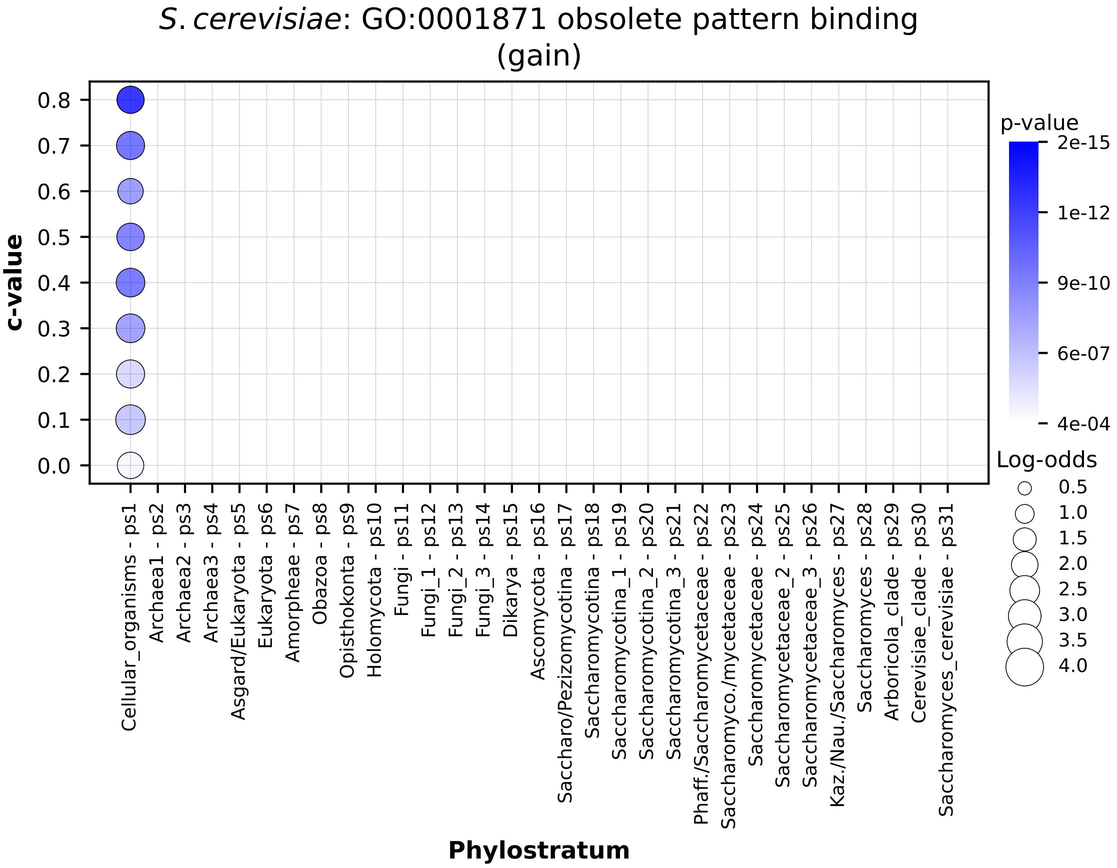
S. cerevisiae: GO:0001871 obsolete pattern binding
(gain)
c-value
0.0
0.1
0.2
0.3
0.4
0.5
0.6
0.7
0.8
p-value
2e-15
1e-12
9e-10
6e-07
4e-04
Log-odds
0.5
1.0
1.5
2.0
2.5
3.0
3.5
4.0
Cellular_organisms - ps1
Archaea1 - ps2
Archaea2 - ps3
Archaea3 - ps4
Asgard/Eukaryota - ps5
Eukaryota - ps6
Amorpheae - ps7
Obazoa - ps8
Opisthokonta - ps9
Holomycota - ps10
Fungi - ps11
Fungi_1 - ps12
Fungi_2 - ps13
Fungi_3 - ps14
Dikarya - ps15
Ascomycota - ps16
Saccharo/Pezizomycotina - ps17
Saccharomycotina - ps18
Saccharomycotina_1 - ps19
Saccharomycotina_2 - ps20
Saccharomycotina_3 - ps21
Phaff./Saccharomycetaceae - ps22
Saccharomyco./mycetaceae - ps23
Saccharomycetaceae - ps24
Saccharomycetaceae_2 - ps25
Saccharomycetaceae_3 - ps26
Kaz./Nau./Saccharomyces - ps27
Saccharomyces - ps28
Arboricola_clade - ps29
Cerevisiae_clade - ps30
Saccharomyces_cerevisiae - ps31
Phylostratum

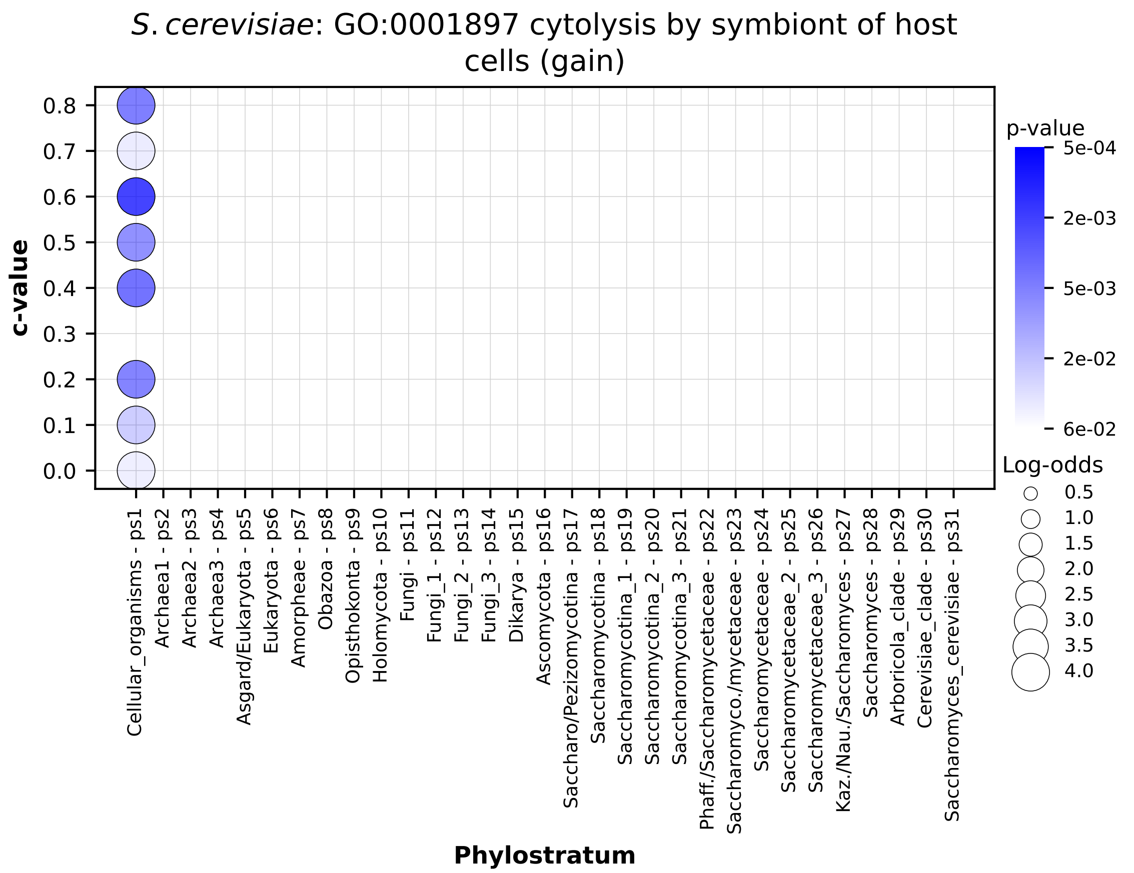

S. cerevisiae: GO:0001897 cytolysis by symbiont of host cells (gain)
c-value
0.0
0.1
0.2
0.3
0.4
0.5
0.6
0.7
0.8
Phylostratum
Cellular_organisms - ps1
Archaea1 - ps2
Archaea2 - ps3
Archaea3 - ps4
Asgard/Eukaryota - ps5
Eukaryota - ps6
Amorpheae - ps7
Obazoa - ps8
Opisthokonta - ps9
Holomycota - ps10
Fungi - ps11
Fungi_1 - ps12
Fungi_2 - ps13
Fungi_3 - ps14
Dikarya - ps15
Ascomycota - ps16
Saccharo/Pezizomycotina - ps17
Saccharomycotina - ps18
Saccharomycotina_1 - ps19
Saccharomycotina_2 - ps20
Saccharomycotina_3 - ps21
Phaff./Saccharomycetaceae - ps22
Saccharomyco./mycetaceae - ps23
Saccharomycetaceae - ps24
Saccharomycetaceae_2 - ps25
Saccharomycetaceae_3 - ps26
Kaz./Nau./Saccharomyces - ps27
Saccharomyces - ps28
Arboricola_clade - ps29
Cerevisiae_clade - ps30
Saccharomyces_cerevisiae - ps31
p-value
5e-04
2e-03
5e-03
2e-02
6e-02
Log-odds
0.5
1.0
1.5
2.0
2.5
3.0
3.5
4.0

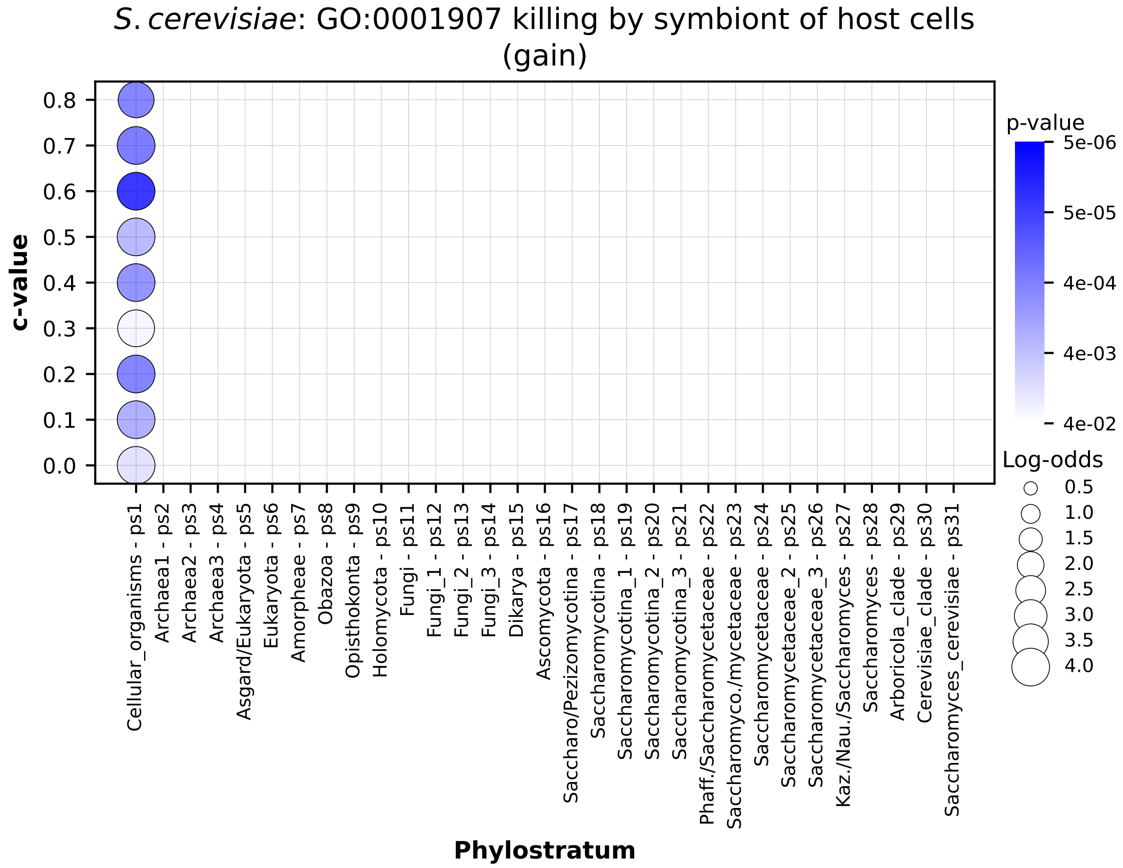

S. cerevisiae: GO:0001907 killing by symbiont of host cells (gain)
c-value
0.8
0.7
0.6
0.5
0.4
0.3
0.2
0.1
0.0
p-value
5e-06
5e-05
4e-04
4e-03
4e-02
Log-odds
0.5
1.0
1.5
2.0
2.5
3.0
3.5
4.0
Cellular_organisms - ps1
Archaea1 - ps2
Archaea2 - ps3
Archaea3 - ps4
Asgard/Eukaryota - ps5
Eukaryota - ps6
Amorpheae - ps7
Obazoa - ps8
Opisthokonta - ps9
Holomycota - ps10
Fungi - ps11
Fungi_1 - ps12
Fungi_2 - ps13
Fungi_3 - ps14
Dikarya - ps15
Ascomycota - ps16
Saccharo/Pezizomycotina - ps17
Saccharomycotina - ps18
Saccharomycotina_1 - ps19
Saccharomycotina_2 - ps20
Saccharomycotina_3 - ps21
Phaff./Saccharomycetaceae - ps22
Saccharomyco./mycetaceae - ps23
Saccharomycetaceae - ps24
Saccharomycetaceae_2 - ps25
Saccharomycetaceae_3 - ps26
Kaz./Nau./Saccharomyces - ps27
Saccharomyces - ps28
Arboricola_clade - ps29
Cerevisiae_clade - ps30
Saccharomyces_cerevisiae - ps31
Phylostratum

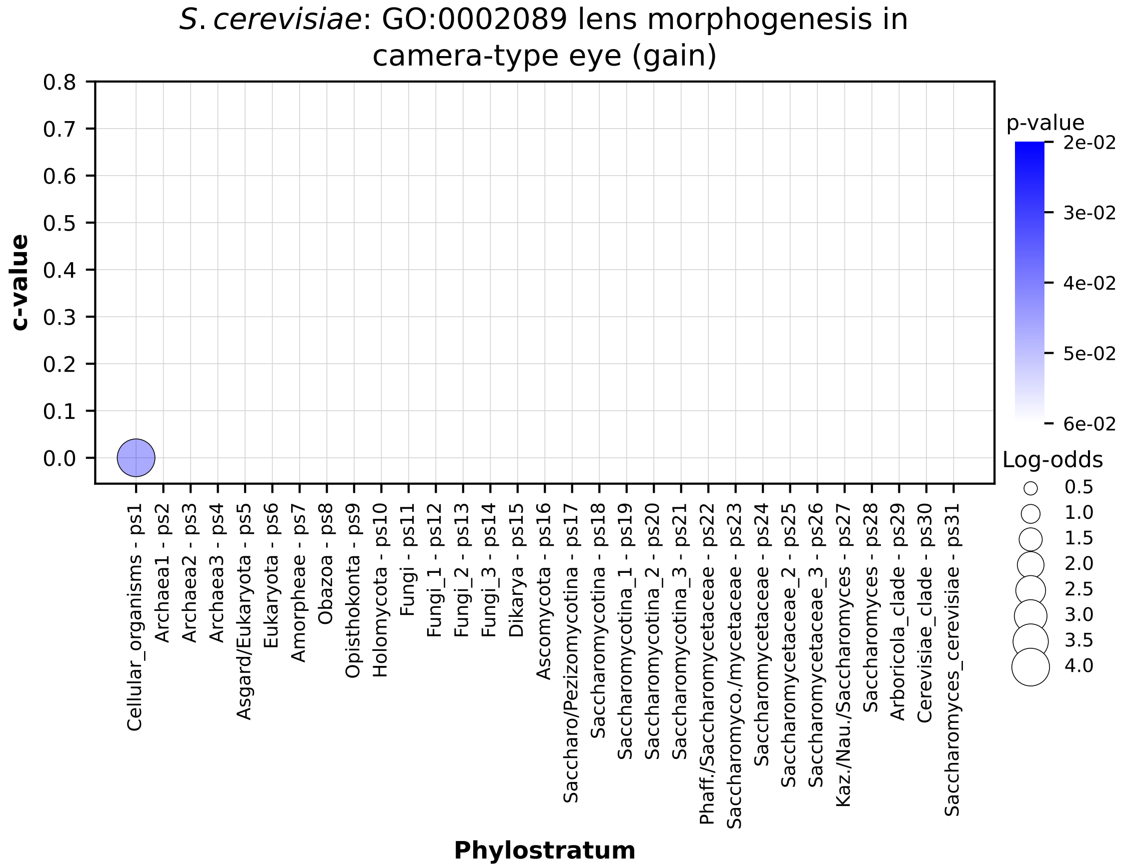
S. cerevisiae: GO:0002089 lens morphogenesis in camera-type eye (gain)
c-value
0.8
0.7
0.6
0.5
0.4
0.3
0.2
0.1
0.0
p-value
2e-02
3e-02
4e-02
5e-02
6e-02
Log-odds
0.5
1.0
1.5
2.0
2.5
3.0
3.5
4.0
Cellular_organisms - ps1
Archaea1 - ps2
Archaea2 - ps3
Archaea3 - ps4
Asgard/Eukaryota - ps5
Eukaryota - ps6
Amorpheae - ps7
Obazoa - ps8
Opisthokonta - ps9
Holomycota - ps10
Fungi - ps11
Fungi_1 - ps12
Fungi_2 - ps13
Fungi_3 - ps14
Dikarya - ps15
Ascomycota - ps16
Saccharo/Pezizomycotina - ps17
Saccharomycotina - ps18
Saccharomycotina_1 - ps19
Saccharomycotina_2 - ps20
Saccharomycotina_3 - ps21
Phaff./Saccharomycetaceae - ps22
Saccharomyco./mycetaceae - ps23
Saccharomycetaceae - ps24
Saccharomycetaceae_2 - ps25
Saccharomycetaceae_3 - ps26
Kaz./Nau./Saccharomyces - ps27
Saccharomyces - ps28
Arboricola_clade - ps29
Cerevisiae_clade - ps30
Saccharomyces_cerevisiae - ps31
Phylostratum

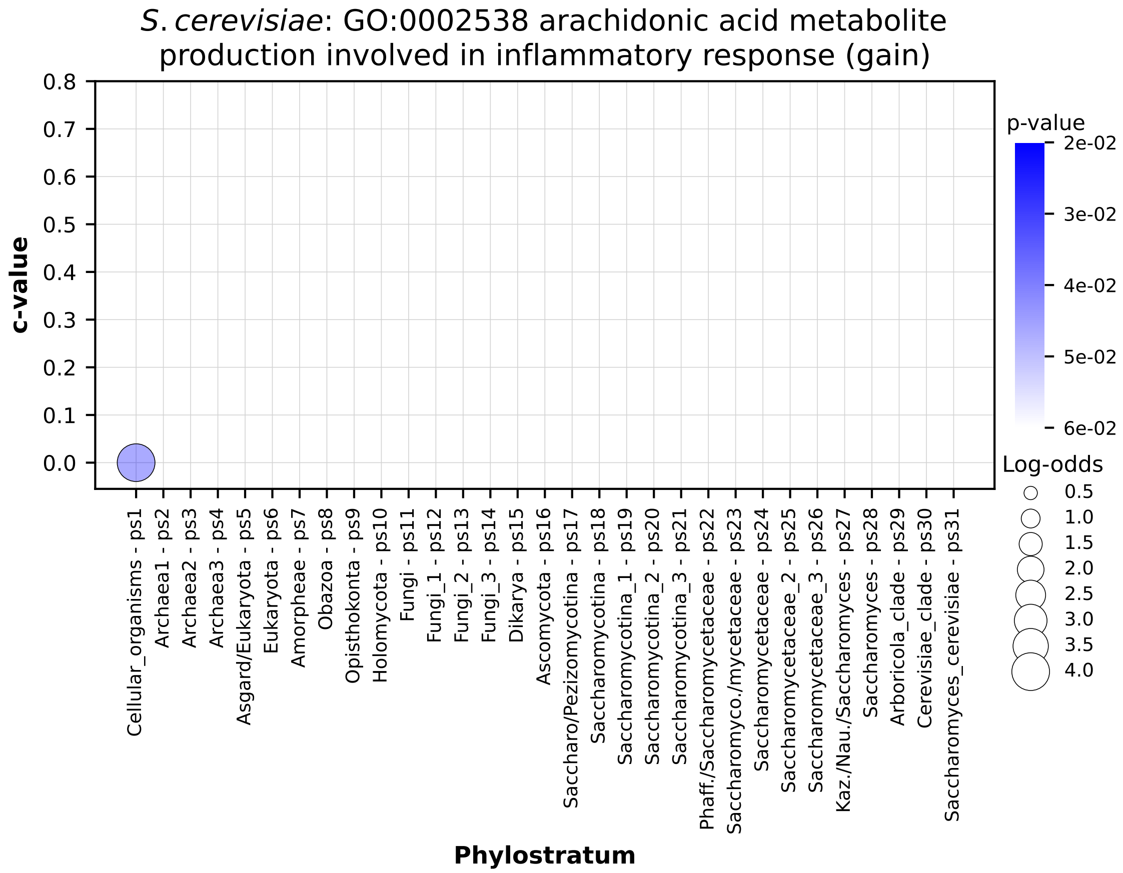

S. cerevisiae: GO:0002538 arachidonic acid metabolite production involved in inflammatory response (gain)
c-value
0.8
0.7
0.6
0.5
0.4
0.3
0.2
0.1
0.0
p-value
2e-02
3e-02
4e-02
5e-02
6e-02
Log-odds
0.5
1.0
1.5
2.0
2.5
3.0
3.5
4.0
Cellular_organisms - ps1
Archaea1 - ps2
Archaea2 - ps3
Archaea3 - ps4
Asgard/Eukaryota - ps5
Eukaryota - ps6
Amorpheae - ps7
Obazoa - ps8
Opisthokonta - ps9
Holomycota - ps10
Fungi - ps11
Fungi_1 - ps12
Fungi_2 - ps13
Fungi_3 - ps14
Dikarya - ps15
Ascomycota - ps16
Saccharo/Pezizomycotina - ps17
Saccharomycotina - ps18
Saccharomycotina_1 - ps19
Saccharomycotina_2 - ps20
Saccharomycotina_3 - ps21
Phaff./Saccharomycetaceae - ps22
Saccharomyco./mycetaceae - ps23
Saccharomycetaceae - ps24
Saccharomycetaceae_2 - ps25
Saccharomycetaceae_3 - ps26
Kaz./Nau./Saccharomyces - ps27
Saccharomyces - ps28
Arboricola_clade - ps29
Cerevisiae_clade - ps30
Saccharomyces_cerevisiae - ps31
Phylostratum

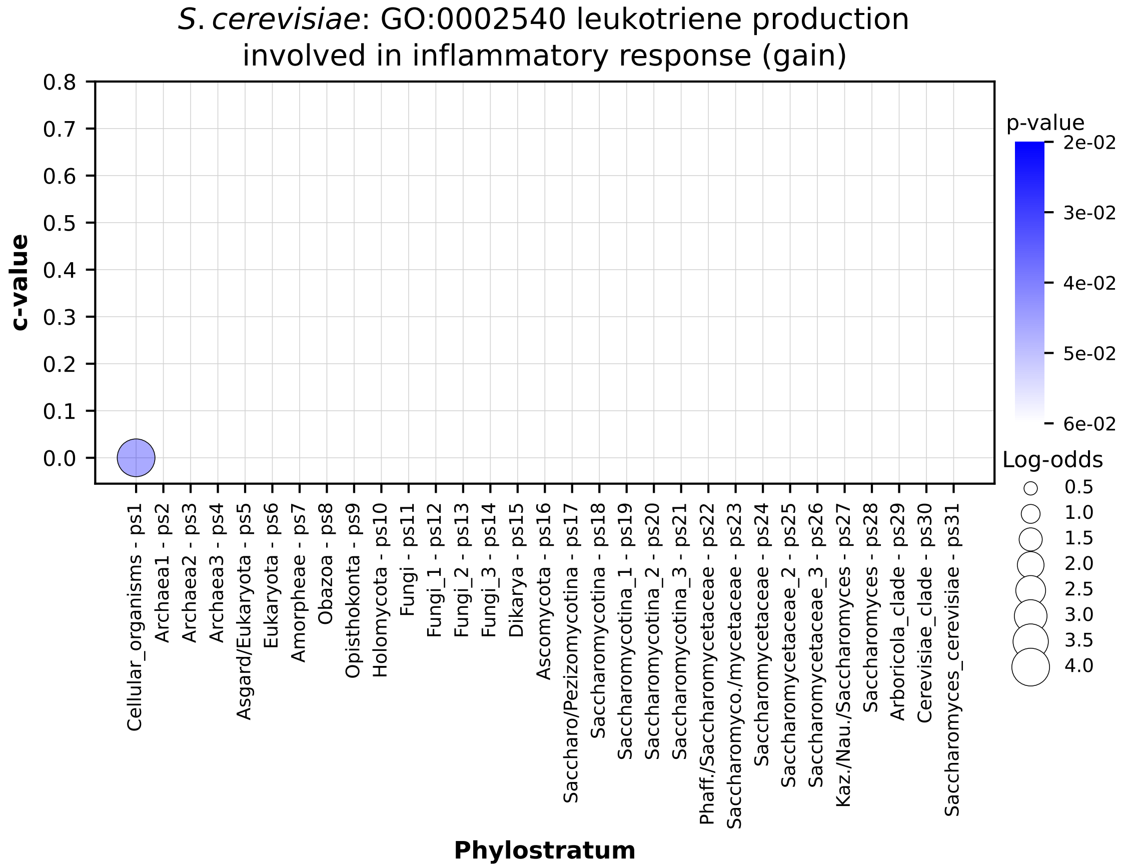

S. cerevisiae: GO:0002540 leukotriene production involved in inflammatory response (gain)
c-value
0.8
0.7
0.6
0.5
0.4
0.3
0.2
0.1
0.0
p-value
2e-02
3e-02
4e-02
5e-02
6e-02
Log-odds
0.5
1.0
1.5
2.0
2.5
3.0
3.5
4.0
Cellular_organisms - ps1
Archaea1 - ps2
Archaea2 - ps3
Archaea3 - ps4
Asgard/Eukaryota - ps5
Eukaryota - ps6
Amorpheae - ps7
Obazoa - ps8
Opisthokonta - ps9
Holomycota - ps10
Fungi - ps11
Fungi_1 - ps12
Fungi_2 - ps13
Fungi_3 - ps14
Dikarya - ps15
Ascomycota - ps16
Saccharo/Pezizomycotina - ps17
Saccharomycotina - ps18
Saccharomycotina_1 - ps19
Saccharomycotina_2 - ps20
Saccharomycotina_3 - ps21
Phaff./Saccharomycetaceae - ps22
Saccharomyco./mycetaceae - ps23
Saccharomycetaceae - ps24
Saccharomycetaceae_2 - ps25
Saccharomycetaceae_3 - ps26
Kaz./Nau./Saccharomyces - ps27
Saccharomyces - ps28
Arboricola_clade - ps29
Cerevisiae_clade - ps30
Saccharomyces_cerevisiae - ps31
Phylostratum

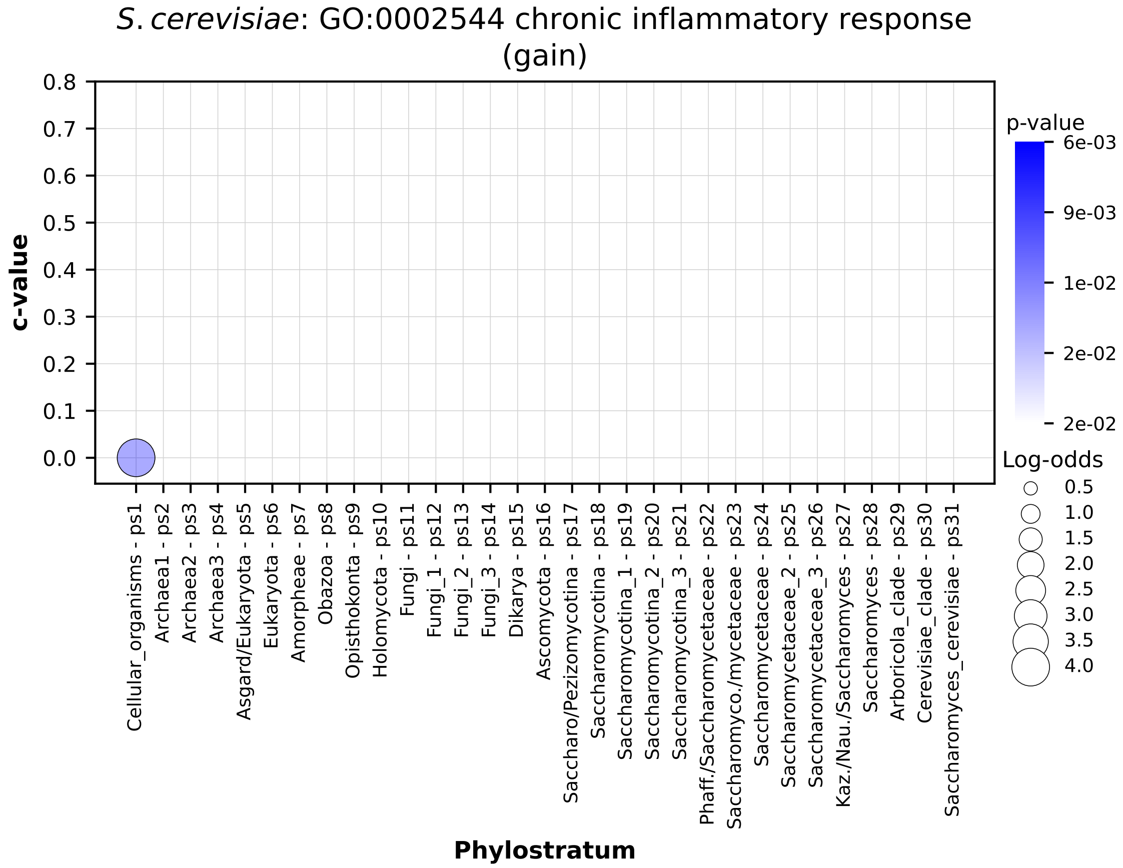

S. cerevisiae: GO:0002544 chronic inflammatory response (gain)
c-value
0.8
0.7
0.6
0.5
0.4
0.3
0.2
0.1
0.0
p-value
6e-03
9e-03
1e-02
2e-02
2e-02
Log-odds
0.5
1.0
1.5
2.0
2.5
3.0
3.5
4.0
Cellular_organisms - ps1
Archaea1 - ps2
Archaea2 - ps3
Archaea3 - ps4
Asgard/Eukaryota - ps5
Eukaryota - ps6
Amorpheae - ps7
Obazoa - ps8
Opisthokonta - ps9
Holomycota - ps10
Fungi - ps11
Fungi_1 - ps12
Fungi_2 - ps13
Fungi_3 - ps14
Dikarya - ps15
Ascomycota - ps16
Saccharo/Pezizomycotina - ps17
Saccharomycotina - ps18
Saccharomycotina_1 - ps19
Saccharomycotina_2 - ps20
Saccharomycotina_3 - ps21
Phaff./Saccharomycetaceae - ps22
Saccharomyco./mycetaceae - ps23
Saccharomycetaceae - ps24
Saccharomycetaceae_2 - ps25
Saccharomycetaceae_3 - ps26
Kaz./Nau./Saccharomyces - ps27
Saccharomyces - ps28
Arboricola_clade - ps29
Cerevisiae_clade - ps30
Saccharomyces_cerevisiae - ps31
Phylostratum

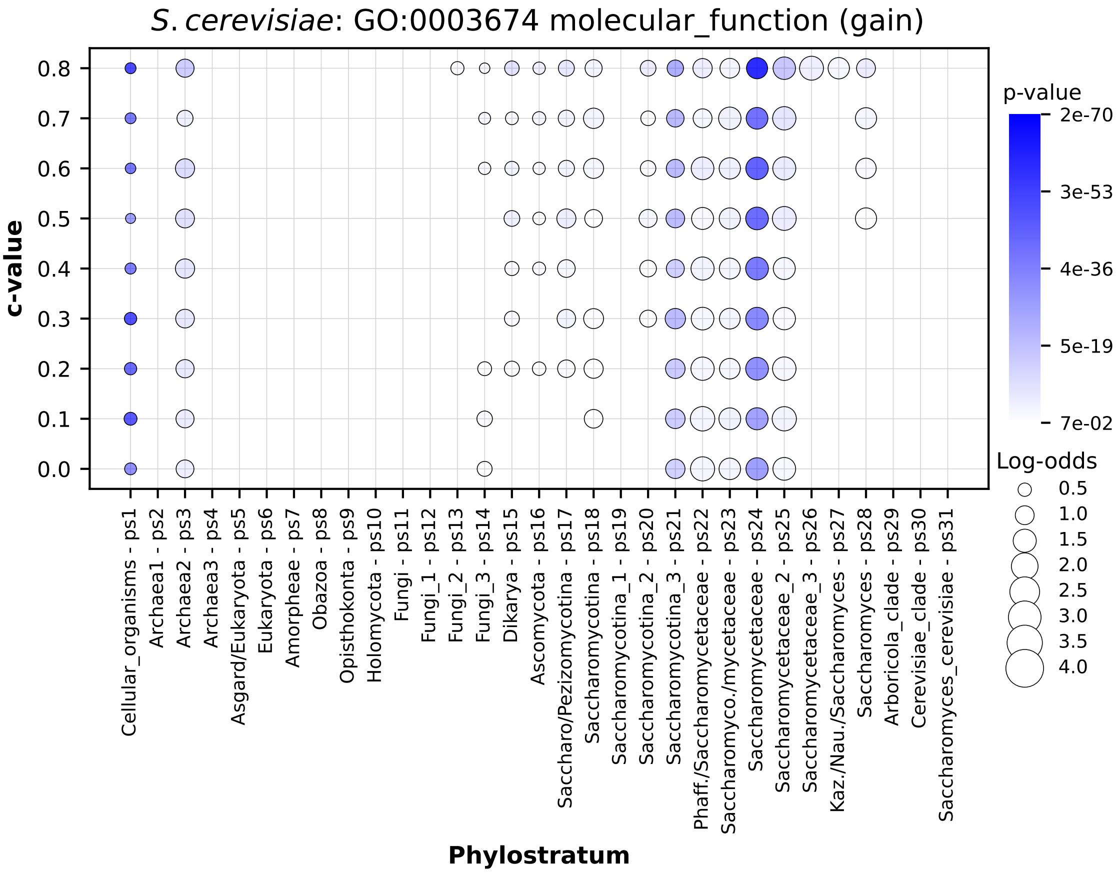
S. cerevisiae: GO:0003674 molecular_function (gain)
c-value
0.8
0.7
0.6
0.5
0.4
0.3
0.2
0.1
0.0
Phylostratum
Cellular_organisms - ps1
Archaea1 - ps2
Archaea2 - ps3
Archaea3 - ps4
Asgard/Eukaryota - ps5
Eukaryota - ps6
Amorpheae - ps7
Obazoa - ps8
Opisthokonta - ps9
Holomycota - ps10
Fungi - ps11
Fungi_1 - ps12
Fungi_2 - ps13
Fungi_3 - ps14
Dikarya - ps15
Ascomycota - ps16
Saccharo/Pezizomycotina - ps17
Saccharomycotina - ps18
Saccharomycotina_1 - ps19
Saccharomycotina_2 - ps20
Saccharomycotina_3 - ps21
Phaff./Saccharomycetaceae - ps22
Saccharomyco./mycetaceae - ps23
Saccharomycetaceae - ps24
Saccharomycetaceae_2 - ps25
Saccharomycetaceae_3 - ps26
Kaz./Nau./Saccharomyces - ps27
Saccharomyces - ps28
Arboricola_clade - ps29
Cerevisiae_clade - ps30
Saccharomyces_cerevisiae - ps31
p-value
2e-70
3e-53
4e-36
5e-19
7e-02
Log-odds
0.5
1.0
1.5
2.0
2.5
3.0
3.5
4.0

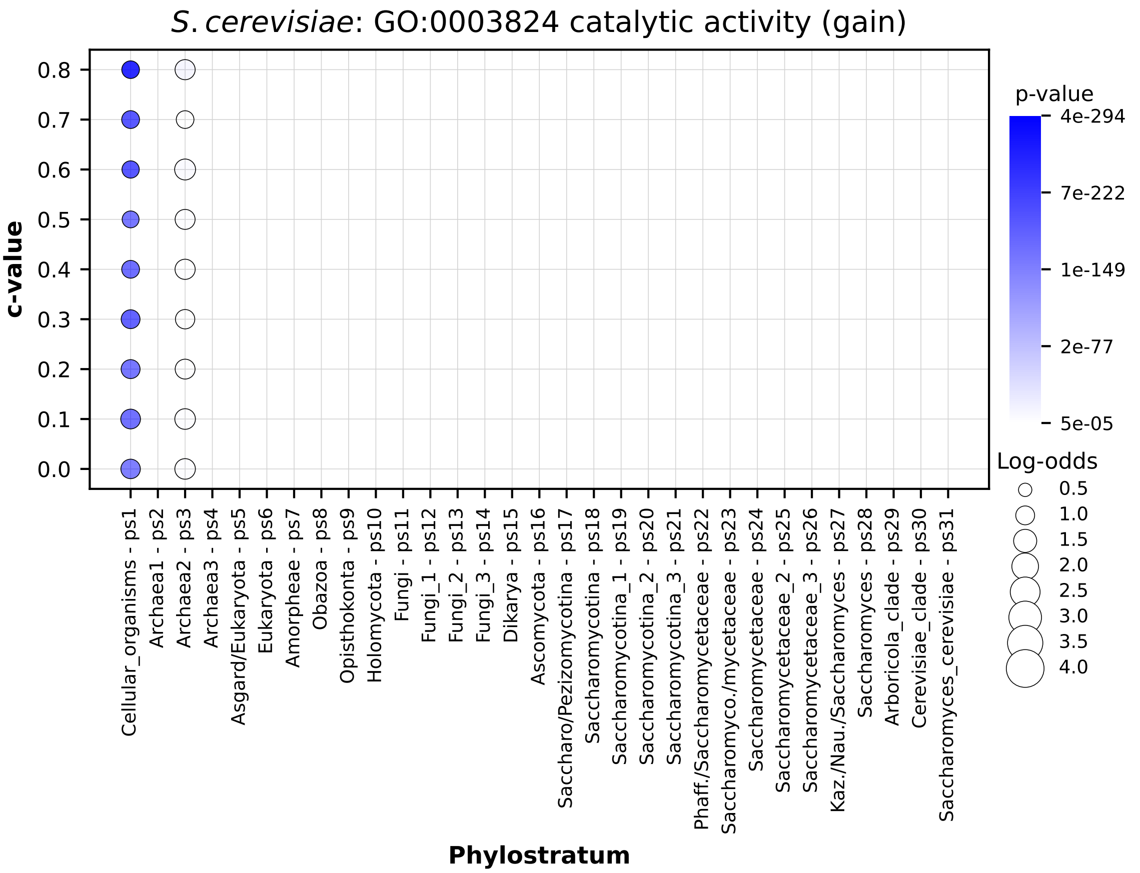
S. cerevisiae: GO:0003824 catalytic activity (gain)
c-value
0.8
0.7
0.6
0.5
0.4
0.3
0.2
0.1
0.0
p-value
4e-294
7e-222
1e-149
2e-77
5e-05
Log-odds
0.5
1.0
1.5
2.0
2.5
3.0
3.5
4.0
Cellular_organisms - ps1
Archaea1 - ps2
Archaea2 - ps3
Archaea3 - ps4
Asgard/Eukaryota - ps5
Eukaryota - ps6
Amorpheae - ps7
Obazoa - ps8
Opisthokonta - ps9
Holomycota - ps10
Fungi - ps11
Fungi_1 - ps12
Fungi_2 - ps13
Fungi_3 - ps14
Dikarya - ps15
Ascomycota - ps16
Saccharo/Pezizomycotina - ps17
Saccharomycotina - ps18
Saccharomycotina_1 - ps19
Saccharomycotina_2 - ps20
Saccharomycotina_3 - ps21
Phaff./Saccharomycetaceae - ps22
Saccharomyco./mycetaceae - ps23
Saccharomycetaceae - ps24
Saccharomycetaceae_2 - ps25
Saccharomycetaceae_3 - ps26
Kaz./Nau./Saccharomyces - ps27
Saccharomyces - ps28
Arboricola_clade - ps29
Cerevisiae_clade - ps30
Saccharomyces_cerevisiae - ps31
Phylostratum

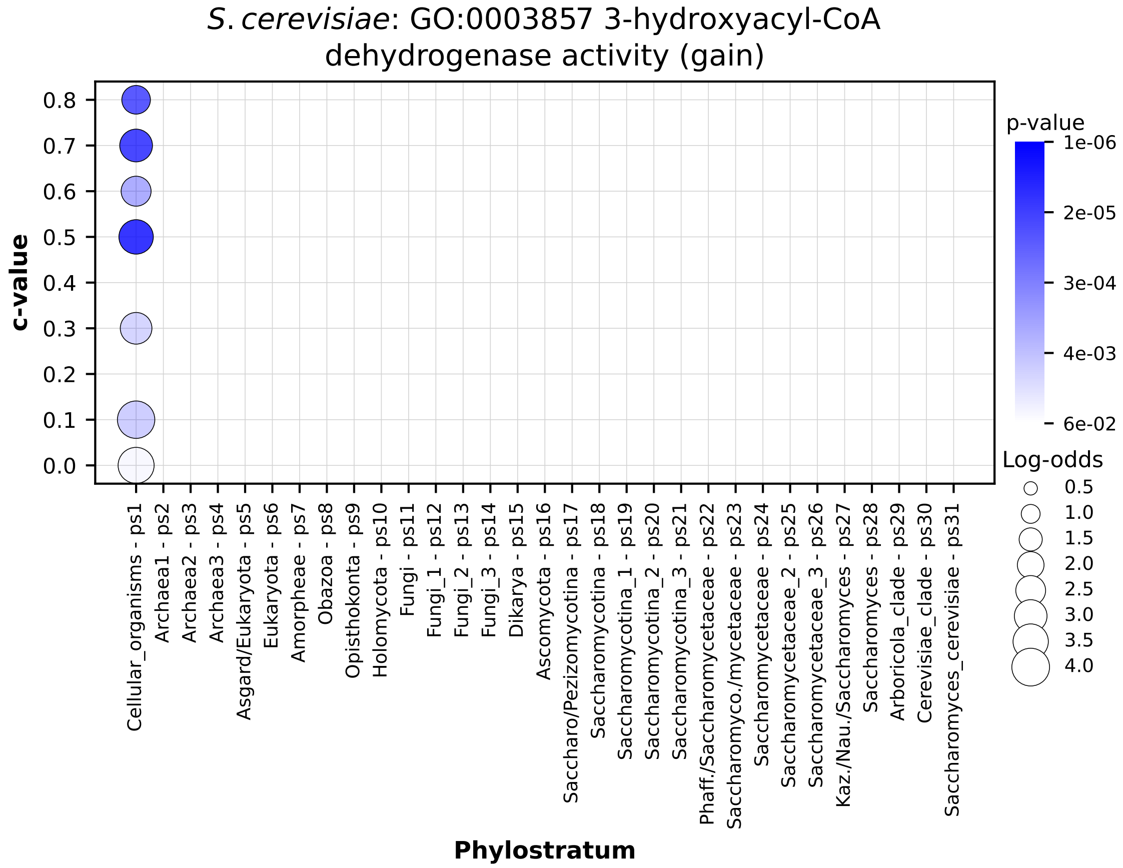
S. cerevisiae: GO:0003857 3-hydroxyacyl-CoA dehydrogenase activity (gain)
c-value
0.0
0.1
0.2
0.3
0.4
0.5
0.6
0.7
0.8
p-value
1e-06
2e-05
3e-04
4e-03
6e-02
Log-odds
0.5
1.0
1.5
2.0
2.5
3.0
3.5
4.0
Cellular_organisms - ps1
Archaea1 - ps2
Archaea2 - ps3
Archaea3 - ps4
Asgard/Eukaryota - ps5
Eukaryota - ps6
Amorpheae - ps7
Obazoa - ps8
Opisthokonta - ps9
Holomycota - ps10
Fungi - ps11
Fungi_1 - ps12
Fungi_2 - ps13
Fungi_3 - ps14
Dikarya - ps15
Ascomycota - ps16
Saccharo/Pezizomycotina - ps17
Saccharomycotina - ps18
Saccharomycotina_1 - ps19
Saccharomycotina_2 - ps20
Saccharomycotina_3 - ps21
Phaff./Saccharomycetaceae - ps22
Saccharomyco./mycetaceae - ps23
Saccharomycetaceae - ps24
Saccharomycetaceae_2 - ps25
Saccharomycetaceae_3 - ps26
Kaz./Nau./Saccharomyces - ps27
Saccharomyces - ps28
Arboricola_clade - ps29
Cerevisiae_clade - ps30
Saccharomyces_cerevisiae - ps31
Phylostratum

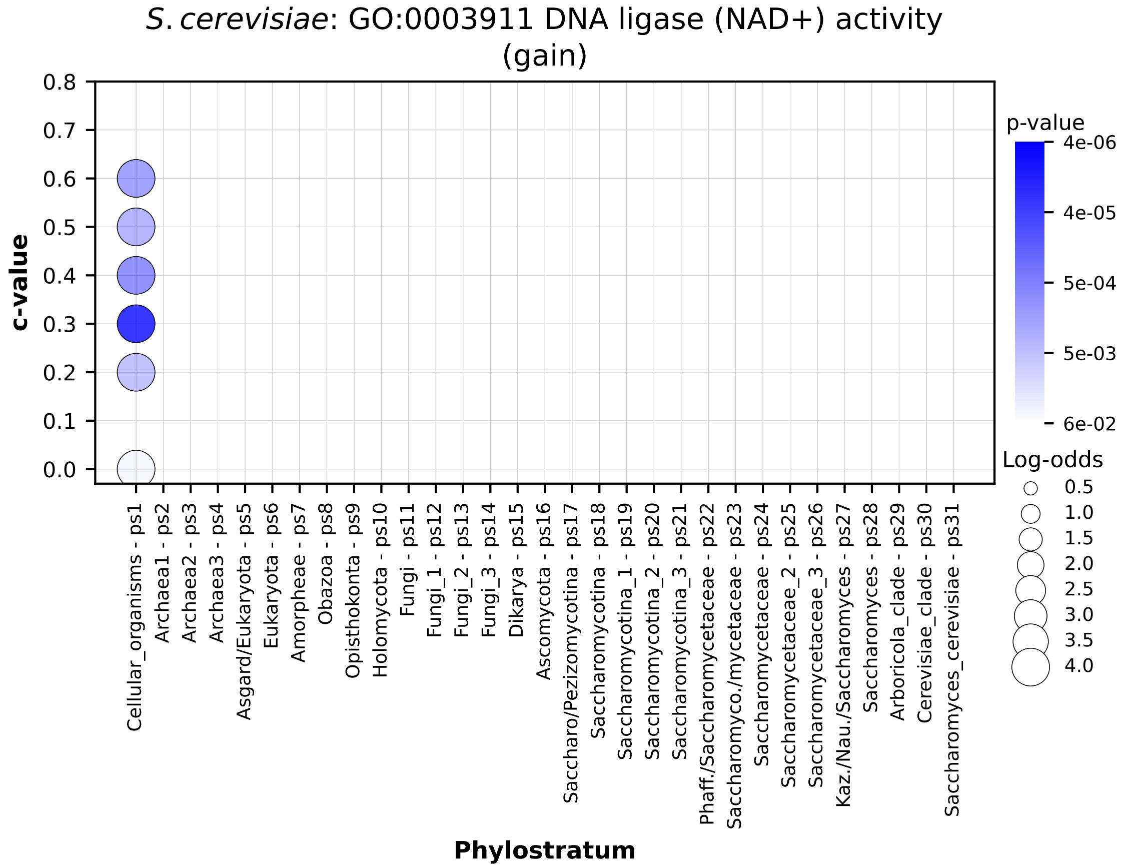

S. cerevisiae: GO:0003911 DNA ligase (NAD+) activity (gain)
c-value
0.8
0.7
0.6
0.5
0.4
0.3
0.2
0.1
0.0
p-value
4e-06
4e-05
5e-04
5e-03
6e-02
Log-odds
0.5
1.0
1.5
2.0
2.5
3.0
3.5
4.0
Cellular_organisms - ps1
Archaea1 - ps2
Archaea2 - ps3
Archaea3 - ps4
Asgard/Eukaryota - ps5
Eukaryota - ps6
Amorpheae - ps7
Obazoa - ps8
Opisthokonta - ps9
Holomycota - ps10
Fungi - ps11
Fungi_1 - ps12
Fungi_2 - ps13
Fungi_3 - ps14
Dikarya - ps15
Ascomycota - ps16
Saccharo/Pezizomycotina - ps17
Saccharomycotina - ps18
Saccharomycotina_1 - ps19
Saccharomycotina_2 - ps20
Saccharomycotina_3 - ps21
Phaff./Saccharomycetaceae - ps22
Saccharomyco./mycetaceae - ps23
Saccharomycetaceae - ps24
Saccharomycetaceae_2 - ps25
Saccharomycetaceae_3 - ps26
Kaz./Nau./Saccharomyces - ps27
Saccharomyces - ps28
Arboricola_clade - ps29
Cerevisiae_clade - ps30
Saccharomyces_cerevisiae - ps31
Phylostratum

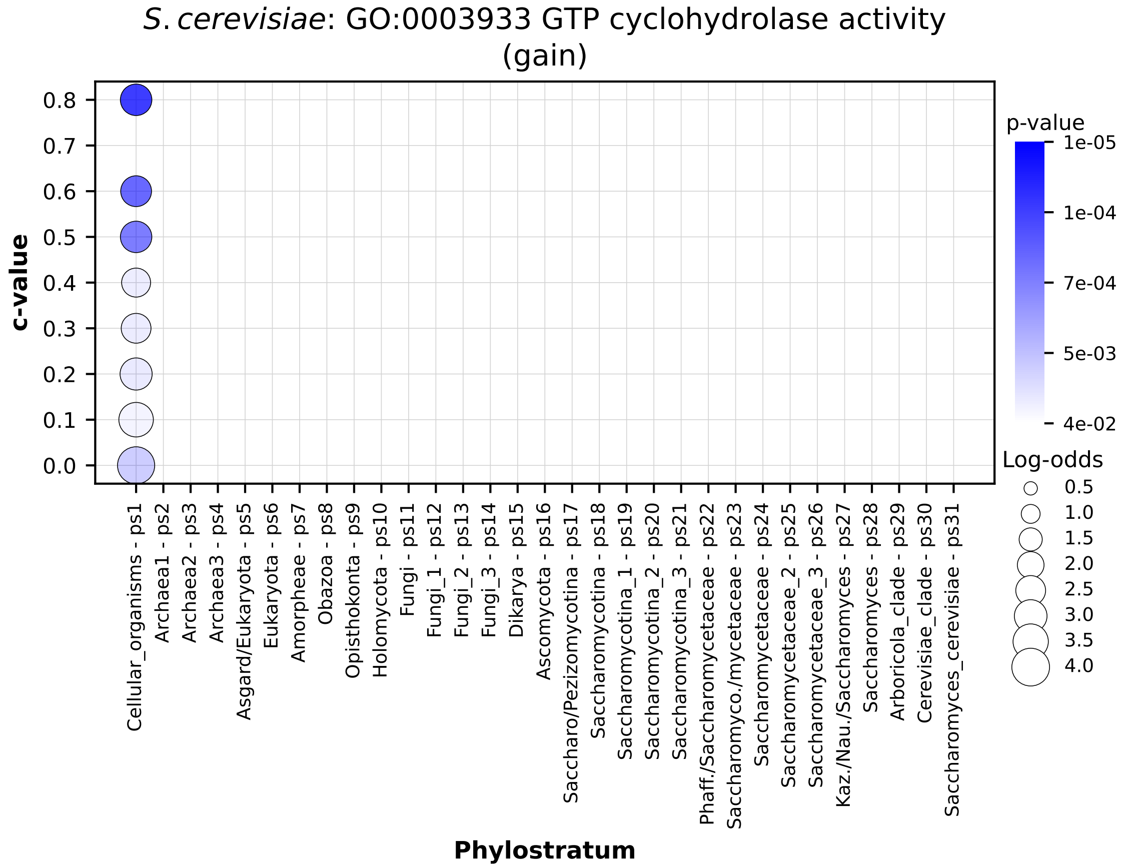
S. cerevisiae: GO:0003933 GTP cyclohydrolase activity
(gain)
c-value
0.8
0.7
0.6
0.5
0.4
0.3
0.2
0.1
0.0
p-value
1e-05
1e-04
7e-04
5e-03
4e-02
Log-odds
0.5
1.0
1.5
2.0
2.5
3.0
3.5
4.0
Cellular_organisms - ps1
Archaea1 - ps2
Archaea2 - ps3
Archaea3 - ps4
Asgard/Eukaryota - ps5
Eukaryota - ps6
Amorpheae - ps7
Obazoa - ps8
Opisthokonta - ps9
Holomycota - ps10
Fungi - ps11
Fungi_1 - ps12
Fungi_2 - ps13
Fungi_3 - ps14
Dikarya - ps15
Ascomycota - ps16
Saccharo/Pezizomycotina - ps17
Saccharomycotina - ps18
Saccharomycotina_1 - ps19
Saccharomycotina_2 - ps20
Saccharomycotina_3 - ps21
Phaff./Saccharomycetaceae - ps22
Saccharomyco./mycetaceae - ps23
Saccharomycetaceae - ps24
Saccharomycetaceae_2 - ps25
Saccharomycetaceae_3 - ps26
Kaz./Nau./Saccharomyces - ps27
Saccharomyces - ps28
Arboricola_clade - ps29
Cerevisiae_clade - ps30
Saccharomyces_cerevisiae - ps31
Phylostratum

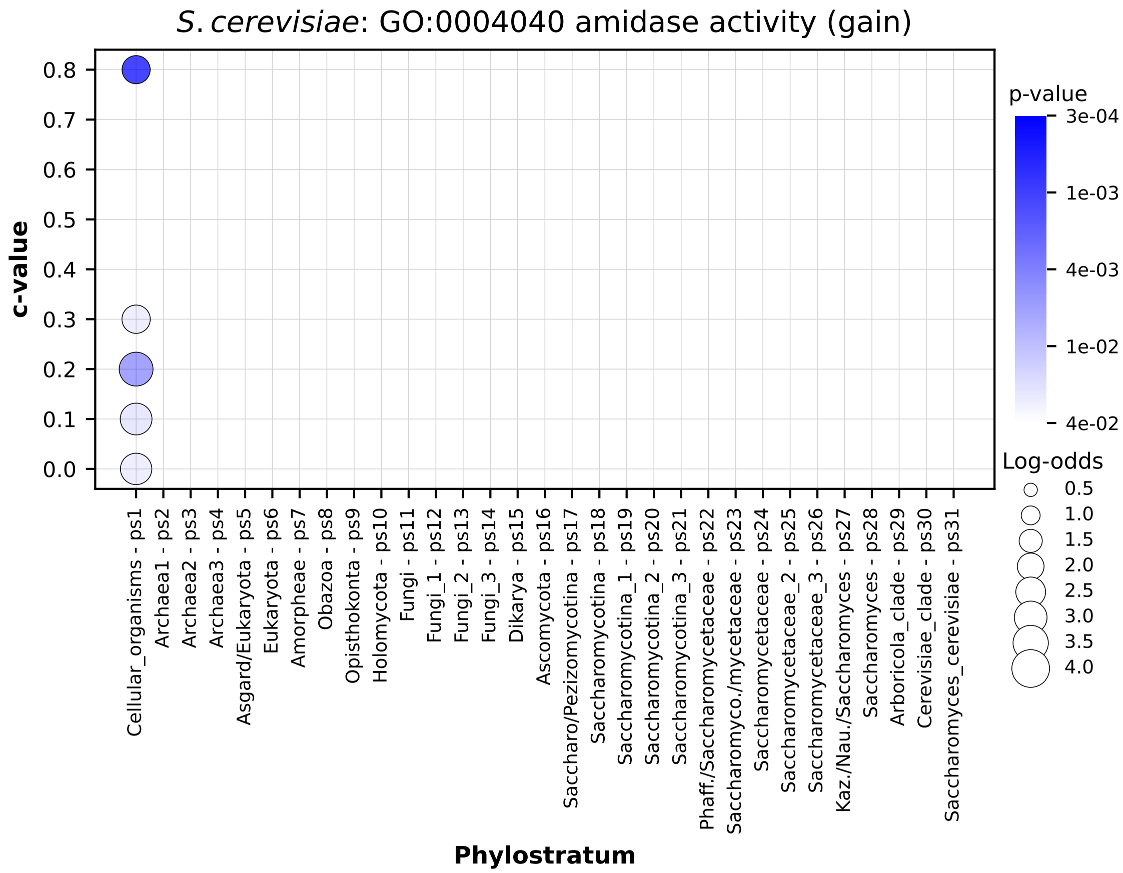
S. cerevisiae: GO:0004040 amidase activity (gain)
c-value
0.8
0.7
0.6
0.5
0.4
0.3
0.2
0.1
0.0
p-value
3e-04
1e-03
4e-03
1e-02
4e-02
Log-odds
0.5
1.0
1.5
2.0
2.5
3.0
3.5
4.0
Cellular_organisms - ps1
Archaea1 - ps2
Archaea2 - ps3
Archaea3 - ps4
Asgard/Eukaryota - ps5
Eukaryota - ps6
Amorpheae - ps7
Obazoa - ps8
Opisthokonta - ps9
Holomycota - ps10
Fungi - ps11
Fungi_1 - ps12
Fungi_2 - ps13
Fungi_3 - ps14
Dikarya - ps15
Ascomycota - ps16
Saccharo/Pezizomycotina - ps17
Saccharomycotina - ps18
Saccharomycotina_1 - ps19
Saccharomycotina_2 - ps20
Saccharomycotina_3 - ps21
Phaff./Saccharomycetaceae - ps22
Saccharomyco./mycetaceae - ps23
Saccharomycetaceae - ps24
Saccharomycetaceae_2 - ps25
Saccharomycetaceae_3 - ps26
Kaz./Nau./Saccharomyces - ps27
Saccharomyces - ps28
Arboricola_clade - ps29
Cerevisiae_clade - ps30
Saccharomyces_cerevisiae - ps31
Phylostratum

S. cerevisiae: GO:0004051 arachidonate 5-lipoxygenase activity (gain)

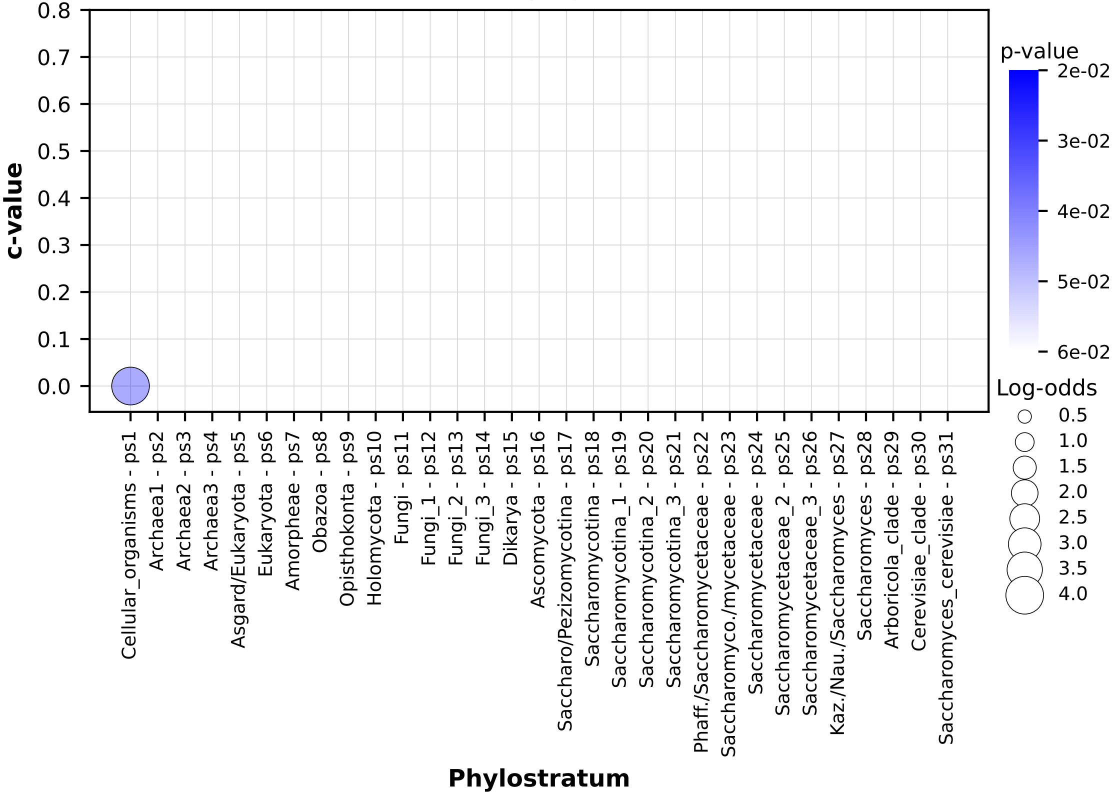

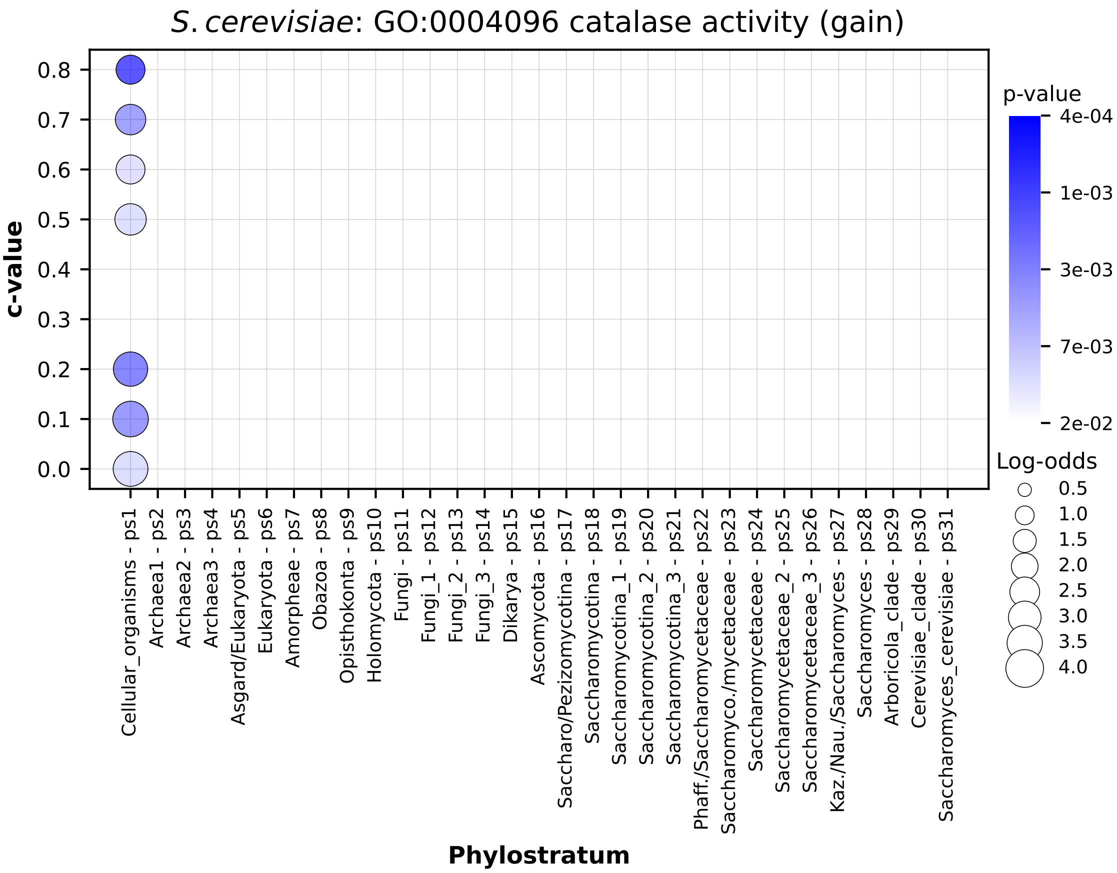
S. cerevisiae: GO:0004096 catalase activity (gain)
c-value
0.0
0.1
0.2
0.3
0.4
0.5
0.6
0.7
0.8
Phylostratum
Cellular_organisms - ps1
Archaea1 - ps2
Archaea2 - ps3
Archaea3 - ps4
Asgard/Eukaryota - ps5
Eukaryota - ps6
Amorpheae - ps7
Obazoa - ps8
Opisthokonta - ps9
Holomycota - ps10
Fungi - ps11
Fungi_1 - ps12
Fungi_2 - ps13
Fungi_3 - ps14
Dikarya - ps15
Ascomycota - ps16
Saccharo/Pezizomycotina - ps17
Saccharomycotina - ps18
Saccharomycotina_1 - ps19
Saccharomycotina_2 - ps20
Saccharomycotina_3 - ps21
Phaff./Saccharomycetaceae - ps22
Saccharomyco./mycetaceae - ps23
Saccharomycetaceae - ps24
Saccharomycetaceae_2 - ps25
Saccharomycetaceae_3 - ps26
Kaz./Nau./Saccharomyces - ps27
Saccharomyces - ps28
Arboricola_clade - ps29
Cerevisiae_clade - ps30
Saccharomyces_cerevisiae - ps31
p-value
4e-04
1e-03
3e-03
7e-03
2e-02
Log-odds
0.5
1.0
1.5
2.0
2.5
3.0
3.5
4.0

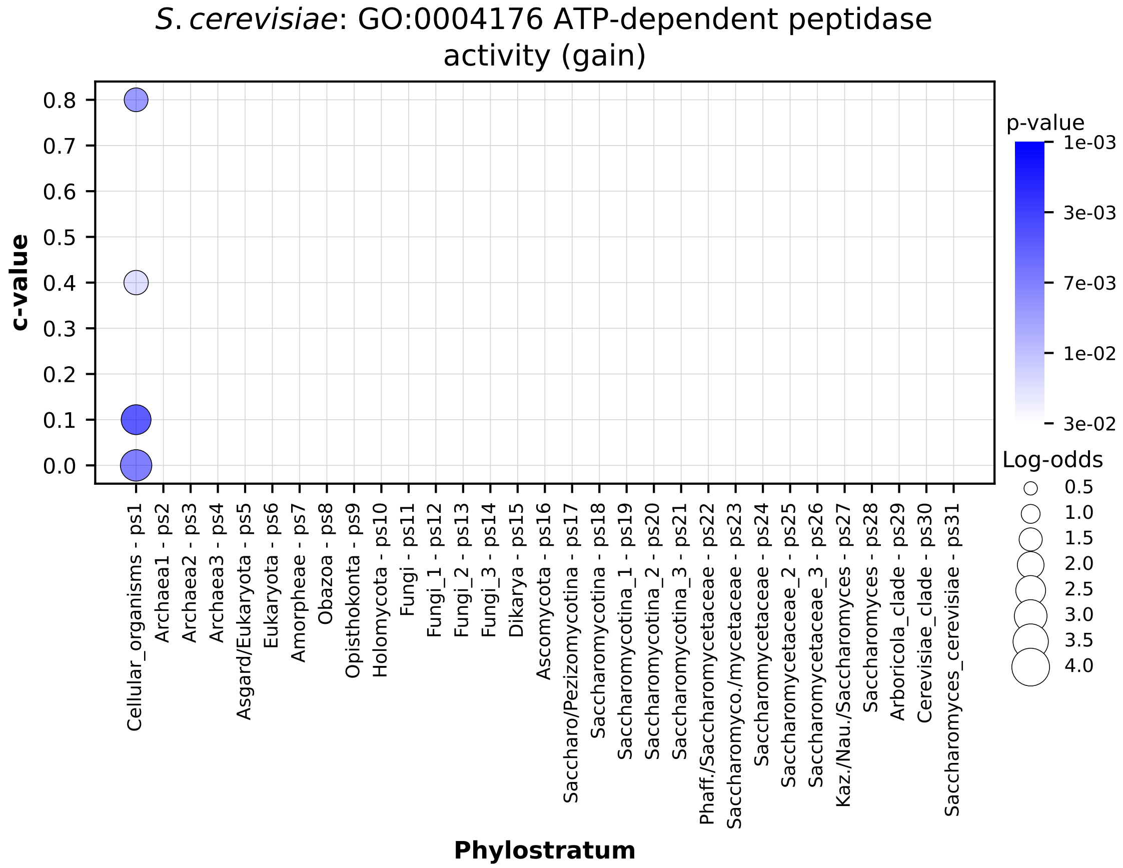
S. cerevisiae: GO:0004176 ATP-dependent peptidase activity (gain)
c-value
0.0
0.1
0.2
0.3
0.4
0.5
0.6
0.7
0.8
Phylostratum
Cellular_organisms - ps1
Archaea1 - ps2
Archaea2 - ps3
Archaea3 - ps4
Asgard/Eukaryota - ps5
Eukaryota - ps6
Amorpheae - ps7
Obazoa - ps8
Opisthokonta - ps9
Holomycota - ps10
Fungi - ps11
Fungi_1 - ps12
Fungi_2 - ps13
Fungi_3 - ps14
Dikarya - ps15
Ascomycota - ps16
Saccharo/Pezizomycotina - ps17
Saccharomycotina - ps18
Saccharomycotina_1 - ps19
Saccharomycotina_2 - ps20
Saccharomycotina_3 - ps21
Phaff./Saccharomycetaceae - ps22
Saccharomyco./mycetaceae - ps23
Saccharomycetaceae - ps24
Saccharomycetaceae_2 - ps25
Saccharomycetaceae_3 - ps26
Kaz./Nau./Saccharomyces - ps27
Saccharomyces - ps28
Arboricola_clade - ps29
Cerevisiae_clade - ps30
Saccharomyces_cerevisiae - ps31
p-value
1e-03
3e-03
7e-03
1e-02
3e-02
Log-odds
0.5
1.0
1.5
2.0
2.5
3.0
3.5
4.0

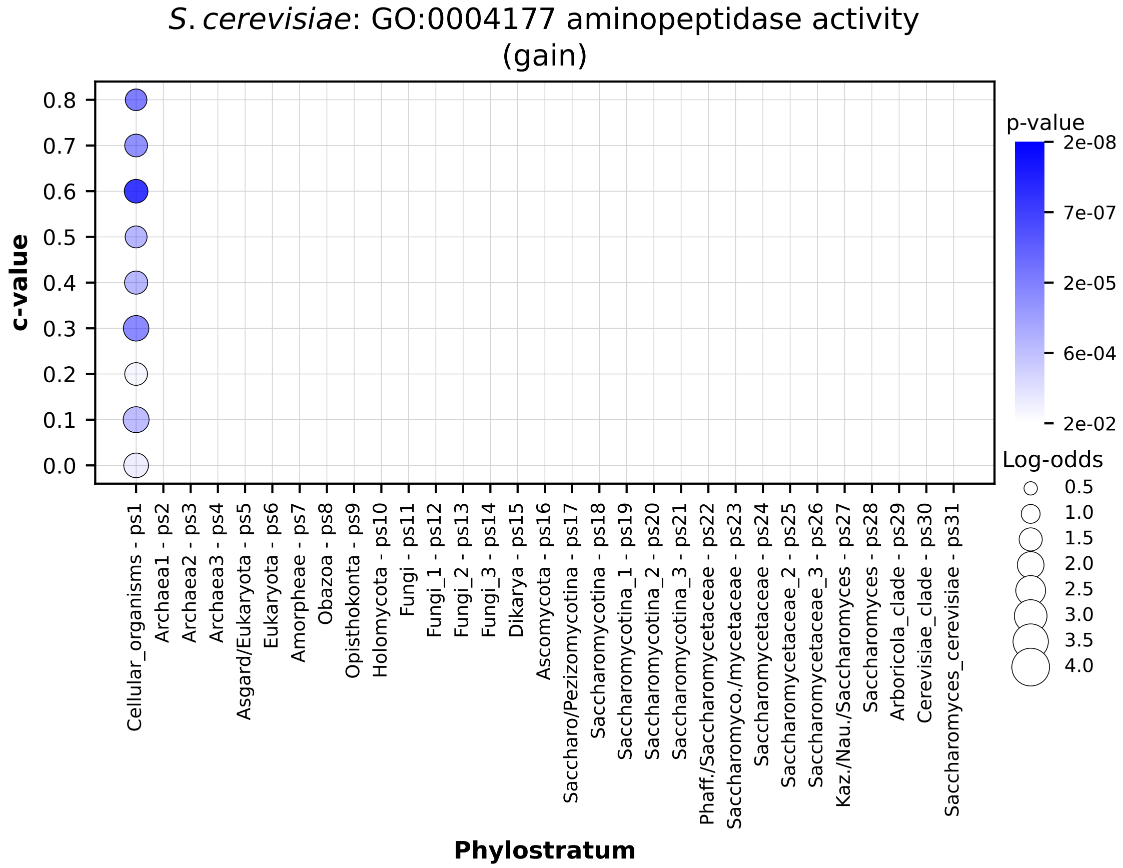
S. cerevisiae: GO:0004177 aminopeptidase activity
(gain)
c-value
0.8
0.7
0.6
0.5
0.4
0.3
0.2
0.1
0.0
p-value
2e-08
7e-07
2e-05
6e-04
2e-02
Log-odds
0.5
1.0
1.5
2.0
2.5
3.0
3.5
4.0
Cellular_organisms - ps1
Archaea1 - ps2
Archaea2 - ps3
Archaea3 - ps4
Asgard/Eukaryota - ps5
Eukaryota - ps6
Amorpheae - ps7
Obazoa - ps8
Opisthokonta - ps9
Holomycota - ps10
Fungi - ps11
Fungi_1 - ps12
Fungi_2 - ps13
Fungi_3 - ps14
Dikarya - ps15
Ascomycota - ps16
Saccharo/Pezizomycotina - ps17
Saccharomycotina - ps18
Saccharomycotina_1 - ps19
Saccharomycotina_2 - ps20
Saccharomycotina_3 - ps21
Phaff./Saccharomycetaceae - ps22
Saccharomyco./mycetaceae - ps23
Saccharomycetaceae - ps24
Saccharomycetaceae_2 - ps25
Saccharomycetaceae_3 - ps26
Kaz./Nau./Saccharomyces - ps27
Saccharomyces - ps28
Arboricola_clade - ps29
Cerevisiae_clade - ps30
Saccharomyces_cerevisiae - ps31
Phylostratum

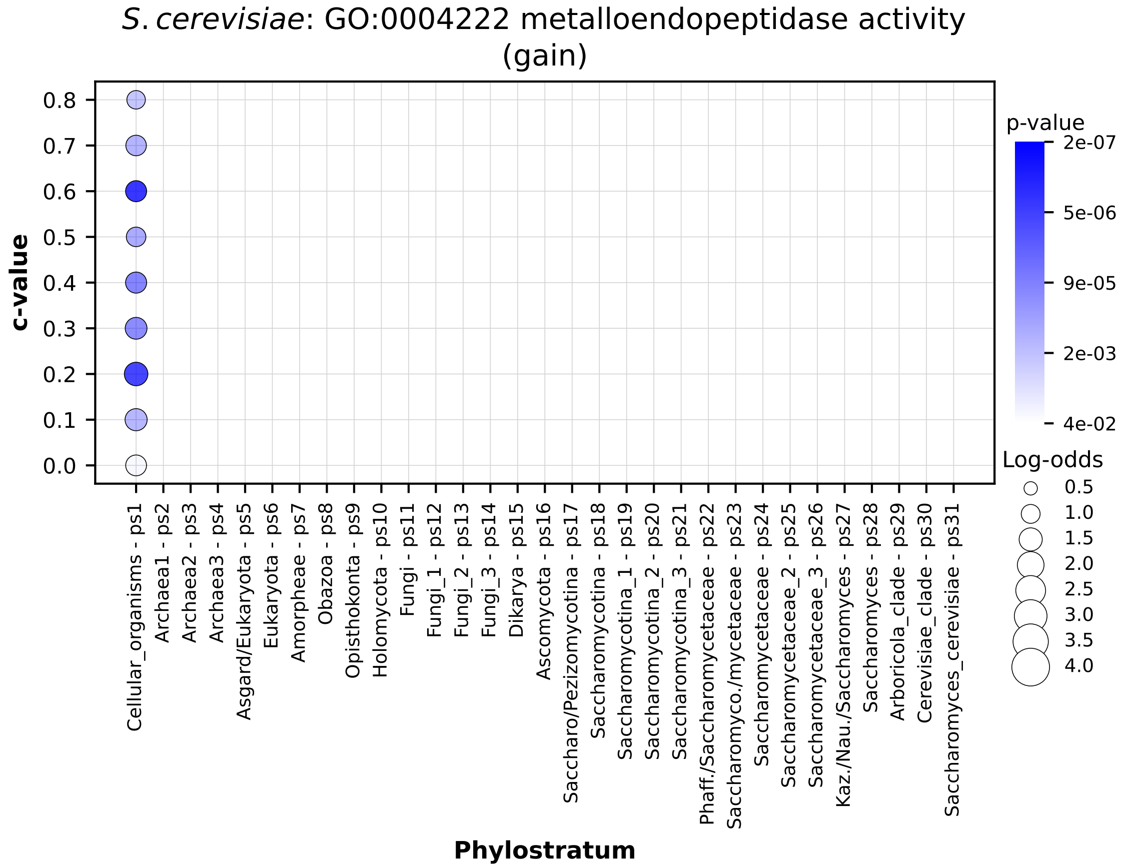

S. cerevisiae: GO:0004222 metalloendopeptidase activity
(gain)
c-value
0.8
0.7
0.6
0.5
0.4
0.3
0.2
0.1
0.0
p-value
2e-07
5e-06
9e-05
2e-03
4e-02
Log-odds
0.5
1.0
1.5
2.0
2.5
3.0
3.5
4.0
Cellular_organisms - ps1
Archaea1 - ps2
Archaea2 - ps3
Archaea3 - ps4
Asgard/Eukaryota - ps5
Eukaryota - ps6
Amorpheae - ps7
Obazoa - ps8
Opisthokonta - ps9
Holomycota - ps10
Fungi - ps11
Fungi_1 - ps12
Fungi_2 - ps13
Fungi_3 - ps14
Dikarya - ps15
Ascomycota - ps16
Saccharo/Pezizomycotina - ps17
Saccharomycotina - ps18
Saccharomycotina_1 - ps19
Saccharomycotina_2 - ps20
Saccharomycotina_3 - ps21
Phaff./Saccharomycetaceae - ps22
Saccharomyco./mycetaceae - ps23
Saccharomycetaceae - ps24
Saccharomycetaceae_2 - ps25
Saccharomycetaceae_3 - ps26
Kaz./Nau./Saccharomyces - ps27
Saccharomyces - ps28
Arboricola_clade - ps29
Cerevisiae_clade - ps30
Saccharomyces_cerevisiae - ps31
Phylostratum

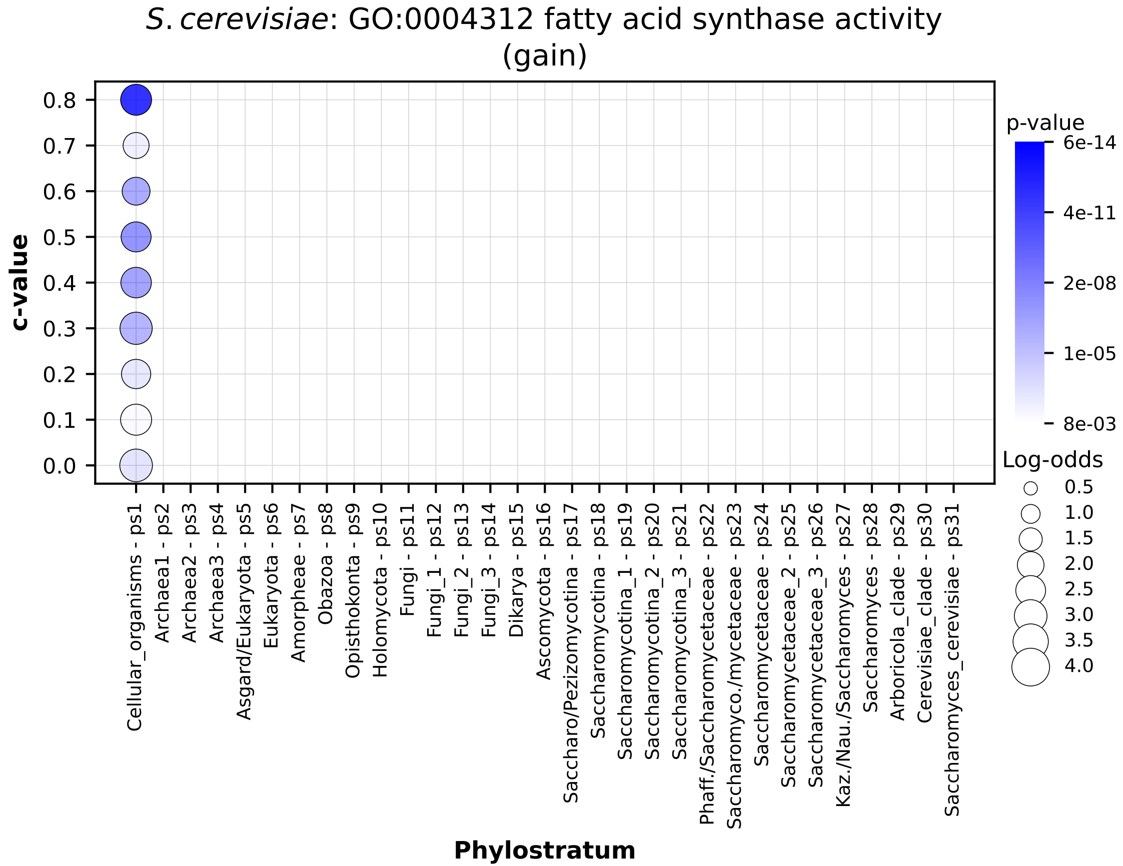
S. cerevisiae: GO:0004312 fatty acid synthase activity (gain)
c-value
0.8
0.7
0.6
0.5
0.4
0.3
0.2
0.1
0.0
p-value
6e-14
4e-11
2e-08
1e-05
8e-03
Log-odds
0.5
1.0
1.5
2.0
2.5
3.0
3.5
4.0
Cellular_organisms - ps1
Archaea1 - ps2
Archaea2 - ps3
Archaea3 - ps4
Asgard/Eukaryota - ps5
Eukaryota - ps6
Amorpheae - ps7
Obazoa - ps8
Opisthokonta - ps9
Holomycota - ps10
Fungi - ps11
Fungi_1 - ps12
Fungi_2 - ps13
Fungi_3 - ps14
Dikarya - ps15
Ascomycota - ps16
Saccharo/Pezizomycotina - ps17
Saccharomycotina - ps18
Saccharomycotina_1 - ps19
Saccharomycotina_2 - ps20
Saccharomycotina_3 - ps21
Phaff./Saccharomycetaceae - ps22
Saccharomyco./mycetaceae - ps23
Saccharomycetaceae - ps24
Saccharomycetaceae_2 - ps25
Saccharomycetaceae_3 - ps26
Kaz./Nau./Saccharomyces - ps27
Saccharomyces - ps28
Arboricola_clade - ps29
Cerevisiae_clade - ps30
Saccharomyces_cerevisiae - ps31
Phylostratum

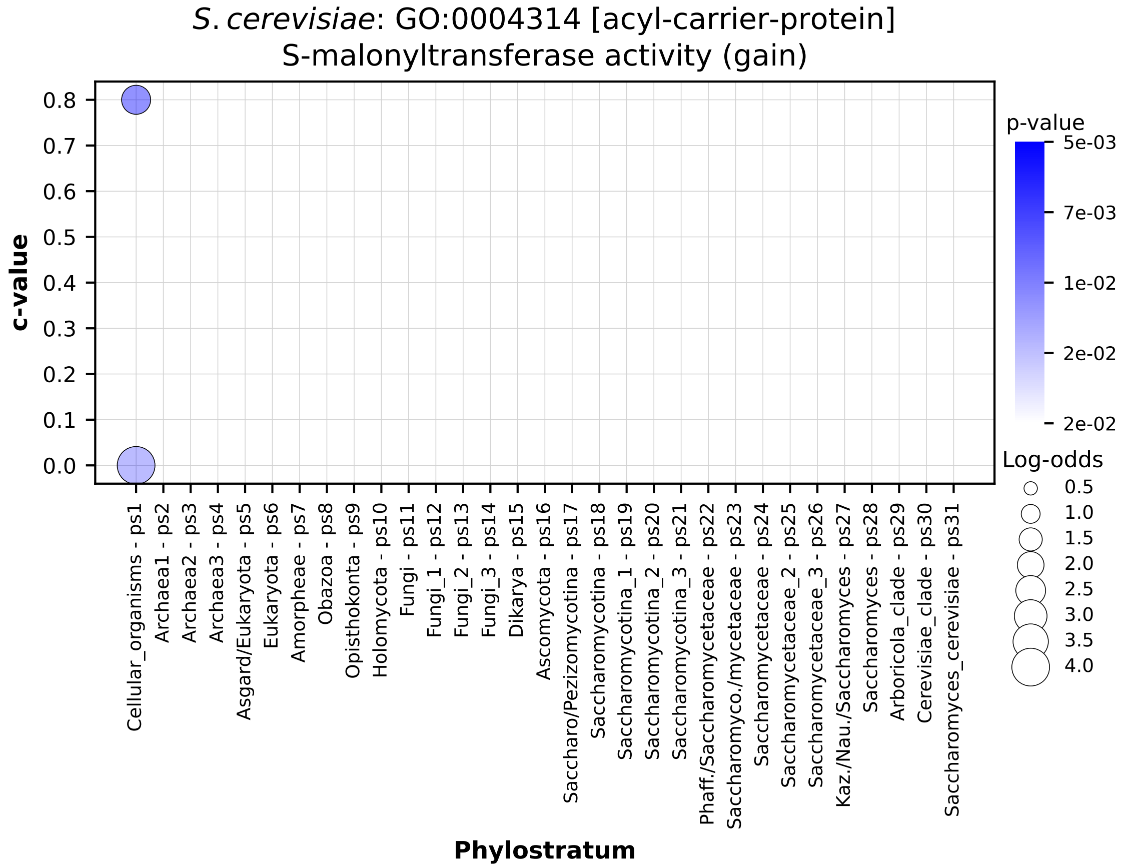

S. cerevisiae: GO:0004314 [acyl-carrier-protein] S-malonyltransferase activity (gain)
c-value
0.8
0.7
0.6
0.5
0.4
0.3
0.2
0.1
0.0
p-value
5e-03
7e-03
1e-02
2e-02
2e-02
Log-odds
0.5
1.0
1.5
2.0
2.5
3.0
3.5
4.0
Cellular_organisms - ps1
Archaea1 - ps2
Archaea2 - ps3
Archaea3 - ps4
Asgard/Eukaryota - ps5
Eukaryota - ps6
Amorpheae - ps7
Obazoa - ps8
Opisthokonta - ps9
Holomycota - ps10
Fungi - ps11
Fungi_1 - ps12
Fungi_2 - ps13
Fungi_3 - ps14
Dikarya - ps15
Ascomycota - ps16
Saccharo/Pezizomycotina - ps17
Saccharomycotina - ps18
Saccharomycotina_1 - ps19
Saccharomycotina_2 - ps20
Saccharomycotina_3 - ps21
Phaff./Saccharomycetaceae - ps22
Saccharomyco./mycetaceae - ps23
Saccharomycetaceae - ps24
Saccharomycetaceae_2 - ps25
Saccharomycetaceae_3 - ps26
Kaz./Nau./Saccharomyces - ps27
Saccharomyces - ps28
Arboricola_clade - ps29
Cerevisiae_clade - ps30
Saccharomyces_cerevisiae - ps31
Phylostratum

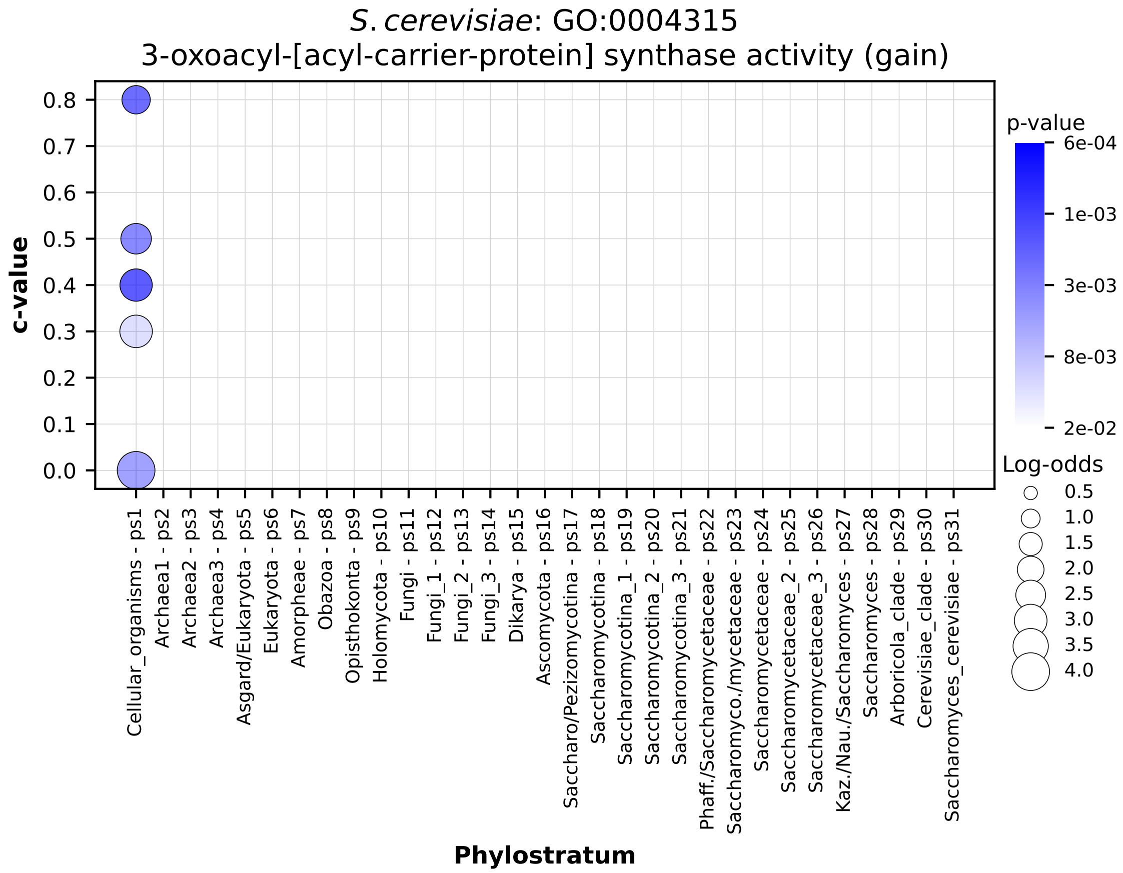

S. cerevisiae: GO:0004315
3-oxoacyl-[acyl-carrier-protein] synthase activity (gain)
c-value
0.0
0.1
0.2
0.3
0.4
0.5
0.6
0.7
0.8
Phylostratum
Cellular_organisms - ps1
Archaea1 - ps2
Archaea2 - ps3
Archaea3 - ps4
Asgard/Eukaryota - ps5
Eukaryota - ps6
Amorpheae - ps7
Obazoa - ps8
Opisthokonta - ps9
Holomycota - ps10
Fungi - ps11
Fungi_1 - ps12
Fungi_2 - ps13
Fungi_3 - ps14
Dikarya - ps15
Ascomycota - ps16
Saccharo/Pezizomycotina - ps17
Saccharomycotina - ps18
Saccharomycotina_1 - ps19
Saccharomycotina_2 - ps20
Saccharomycotina_3 - ps21
Phaff./Saccharomycetaceae - ps22
Saccharomyco./mycetaceae - ps23
Saccharomycetaceae - ps24
Saccharomycetaceae_2 - ps25
Saccharomycetaceae_3 - ps26
Kaz./Nau./Saccharomyces - ps27
Saccharomyces - ps28
Arboricola_clade - ps29
Cerevisiae_clade - ps30
Saccharomyces_cerevisiae - ps31
p-value
6e-04
1e-03
3e-03
8e-03
2e-02
Log-odds
0.5
1.0
1.5
2.0
2.5
3.0
3.5
4.0

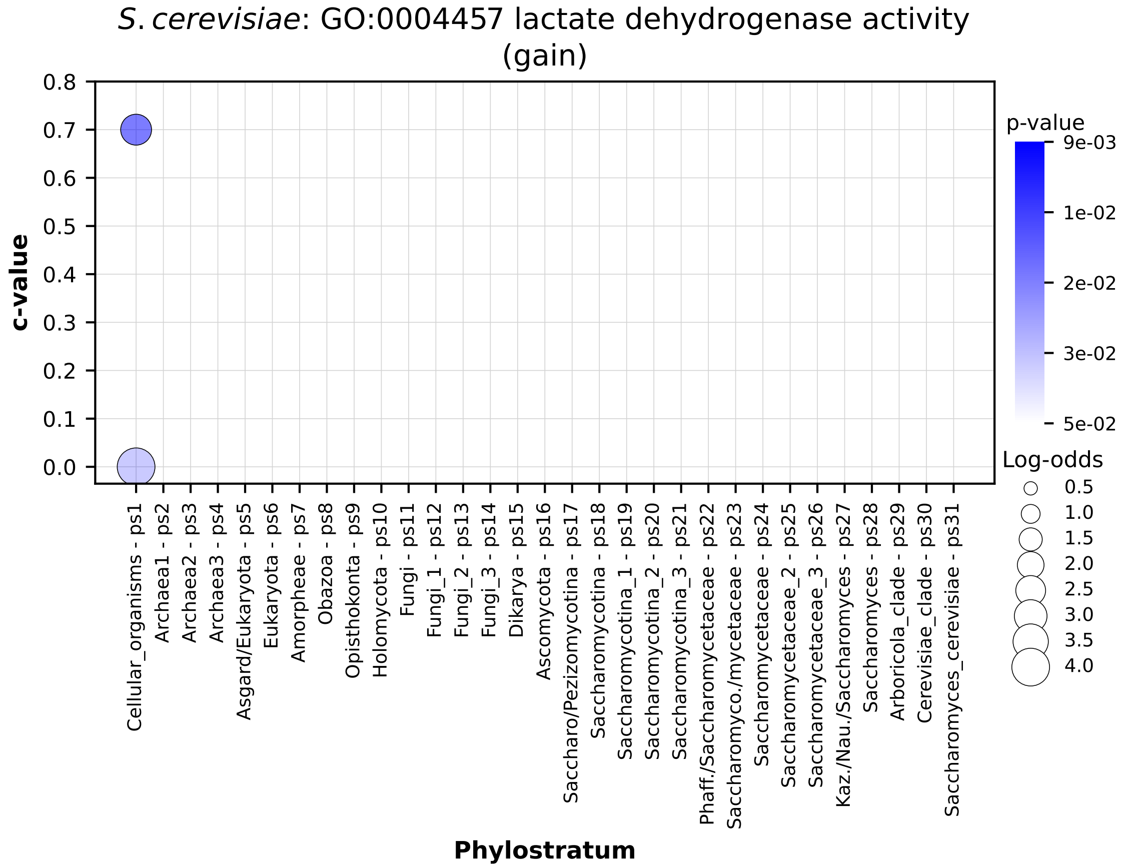
S. cerevisiae: GO:0004457 lactate dehydrogenase activity (gain)
c-value
0.8
0.7
0.6
0.5
0.4
0.3
0.2
0.1
0.0
p-value
9e-03
1e-02
2e-02
3e-02
5e-02
Log-odds
0.5
1.0
1.5
2.0
2.5
3.0
3.5
4.0
Cellular_organisms - ps1
Archaea1 - ps2
Archaea2 - ps3
Archaea3 - ps4
Asgard/Eukaryota - ps5
Eukaryota - ps6
Amorpheae - ps7
Obazoa - ps8
Opisthokonta - ps9
Holomycota - ps10
Fungi - ps11
Fungi_1 - ps12
Fungi_2 - ps13
Fungi_3 - ps14
Dikarya - ps15
Ascomycota - ps16
Saccharo/Pezizomycotina - ps17
Saccharomycotina - ps18
Saccharomycotina_1 - ps19
Saccharomycotina_2 - ps20
Saccharomycotina_3 - ps21
Phaff./Saccharomycetaceae - ps22
Saccharomyco./mycetaceae - ps23
Saccharomycetaceae - ps24
Saccharomycetaceae_2 - ps25
Saccharomycetaceae_3 - ps26
Kaz./Nau./Saccharomyces - ps27
Saccharomyces - ps28
Arboricola_clade - ps29
Cerevisiae_clade - ps30
Saccharomyces_cerevisiae - ps31
Phylostratum

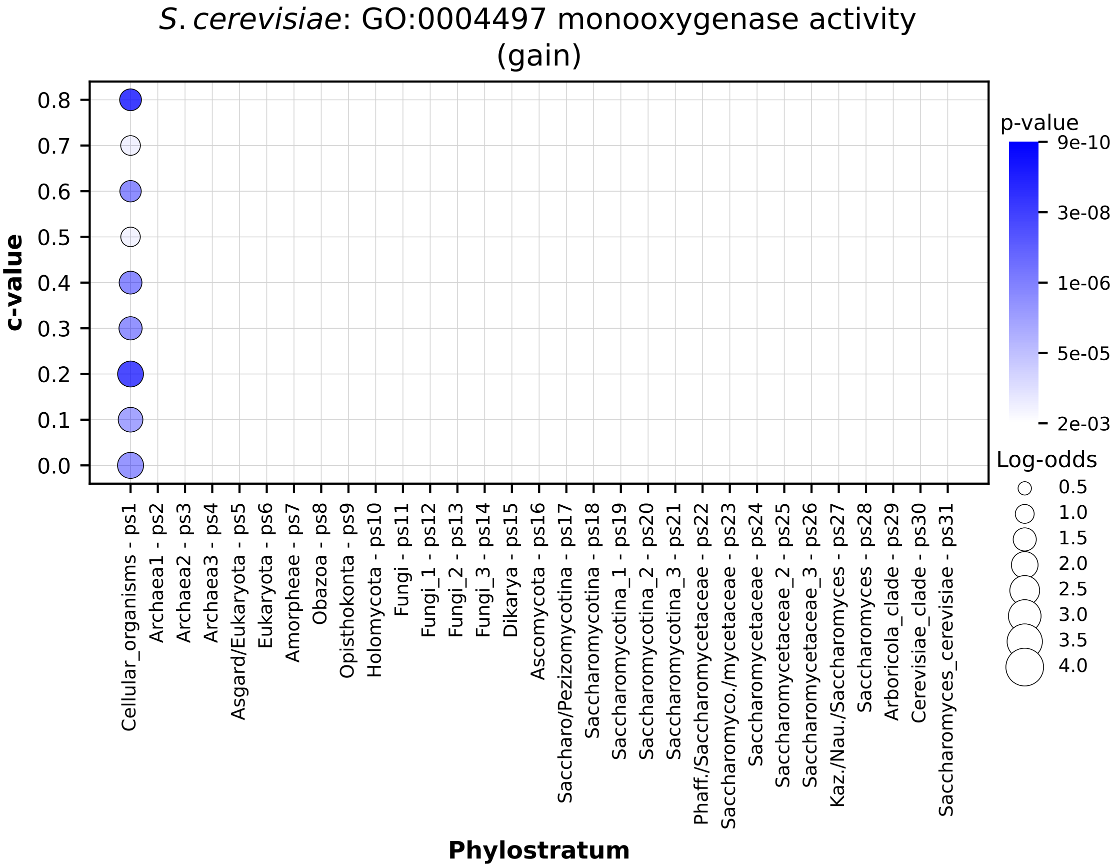
S. cerevisiae: GO:0004497 monooxygenase activity (gain)
c-value
0.8
0.7
0.6
0.5
0.4
0.3
0.2
0.1
0.0
p-value
9e-10
3e-08
1e-06
5e-05
2e-03
Log-odds
0.5
1.0
1.5
2.0
2.5
3.0
3.5
4.0
Cellular_organisms - ps1
Archaea1 - ps2
Archaea2 - ps3
Archaea3 - ps4
Asgard/Eukaryota - ps5
Eukaryota - ps6
Amorpheae - ps7
Obazoa - ps8
Opisthokonta - ps9
Holomycota - ps10
Fungi - ps11
Fungi_1 - ps12
Fungi_2 - ps13
Fungi_3 - ps14
Dikarya - ps15
Ascomycota - ps16
Saccharo/Pezizomycotina - ps17
Saccharomycotina - ps18
Saccharomycotina_1 - ps19
Saccharomycotina_2 - ps20
Saccharomycotina_3 - ps21
Phaff./Saccharomycetaceae - ps22
Saccharomyco./mycetaceae - ps23
Saccharomycetaceae - ps24
Saccharomycetaceae_2 - ps25
Saccharomycetaceae_3 - ps26
Kaz./Nau./Saccharomyces - ps27
Saccharomyces - ps28
Arboricola_clade - ps29
Cerevisiae_clade - ps30
Saccharomyces_cerevisiae - ps31
Phylostratum

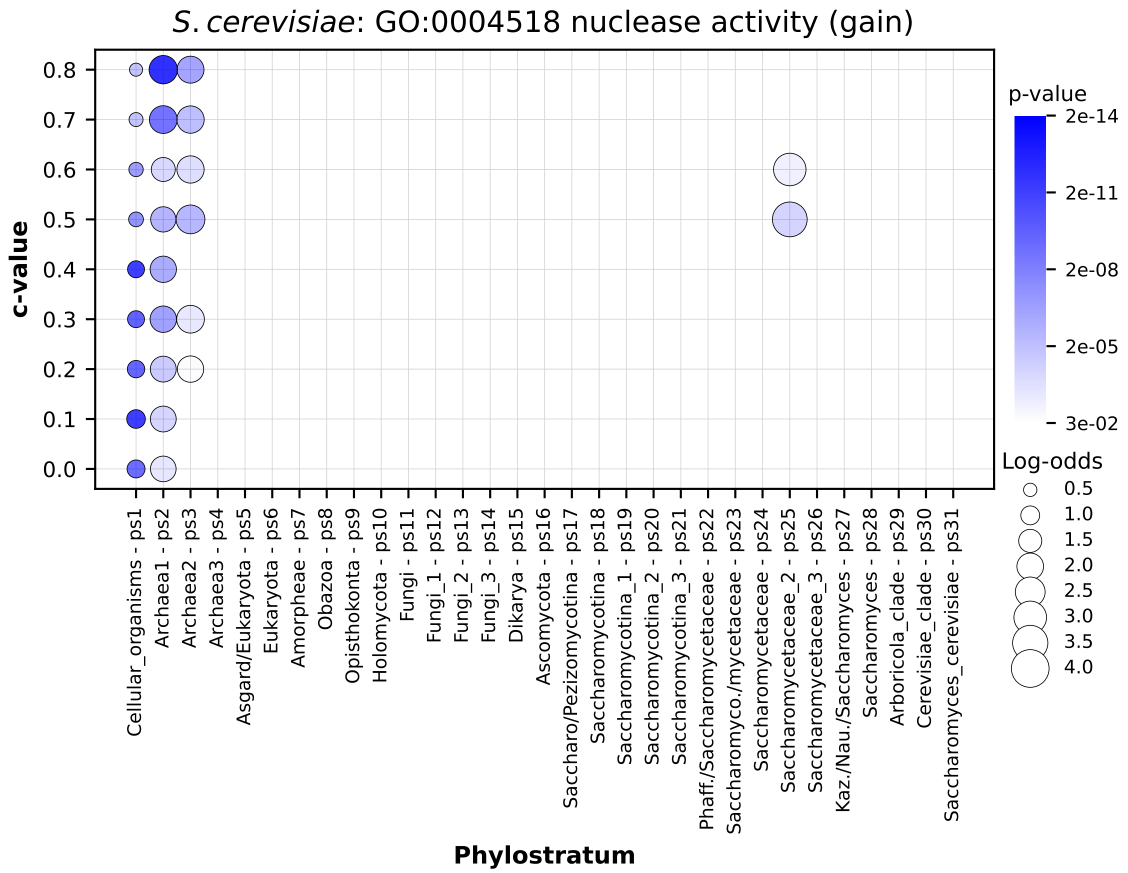

S. cerevisiae: GO:0004518 nuclease activity (gain)
c-value
0.8
0.7
0.6
0.5
0.4
0.3
0.2
0.1
0.0
p-value
2e-14
2e-11
2e-08
2e-05
3e-02
Log-odds
0.5
1.0
1.5
2.0
2.5
3.0
3.5
4.0
Cellular_organisms - ps1
Archaea1 - ps2
Archaea2 - ps3
Archaea3 - ps4
Asgard/Eukaryota - ps5
Eukaryota - ps6
Amorpheae - ps7
Obazoa - ps8
Opisthokonta - ps9
Holomycota - ps10
Fungi - ps11
Fungi_1 - ps12
Fungi_2 - ps13
Fungi_3 - ps14
Dikarya - ps15
Ascomycota - ps16
Saccharo/Pezizomycotina - ps17
Saccharomycotina - ps18
Saccharomycotina_1 - ps19
Saccharomycotina_2 - ps20
Saccharomycotina_3 - ps21
Phaff./Saccharomycetaceae - ps22
Saccharomyco./mycetaceae - ps23
Saccharomycetaceae - ps24
Saccharomycetaceae_2 - ps25
Saccharomycetaceae_3 - ps26
Kaz./Nau./Saccharomyces - ps27
Saccharomyces - ps28
Arboricola_clade - ps29
Cerevisiae_clade - ps30
Saccharomyces_cerevisiae - ps31
Phylostratum

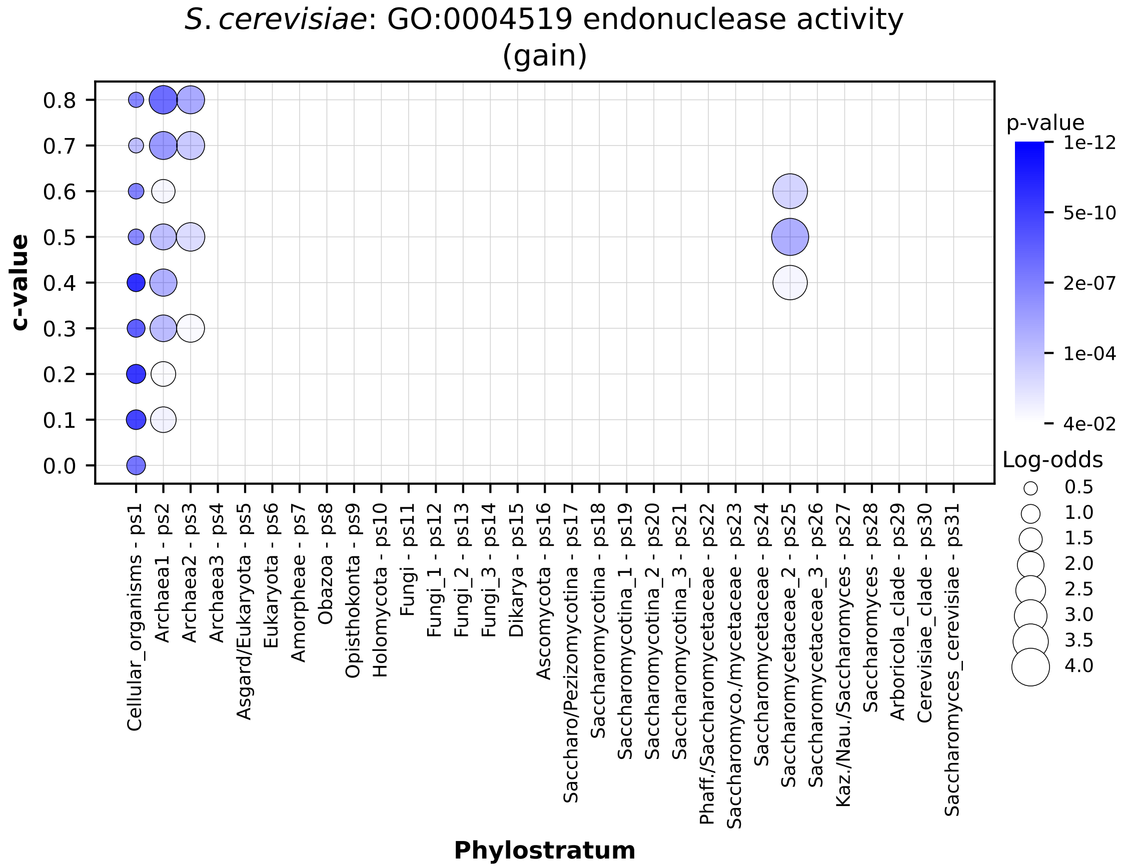

S. cerevisiae: GO:0004519 endonuclease activity
(gain)
c-value
0.8
0.7
0.6
0.5
0.4
0.3
0.2
0.1
0.0
p-value
1e-12
5e-10
2e-07
1e-04
4e-02
Log-odds
0.5
1.0
1.5
2.0
2.5
3.0
3.5
4.0
Cellular_organisms - ps1
Archaea1 - ps2
Archaea2 - ps3
Archaea3 - ps4
Asgard/Eukaryota - ps5
Eukaryota - ps6
Amorpheae - ps7
Obazoa - ps8
Opisthokonta - ps9
Holomycota - ps10
Fungi - ps11
Fungi_1 - ps12
Fungi_2 - ps13
Fungi_3 - ps14
Dikarya - ps15
Ascomycota - ps16
Saccharo/Pezizomycotina - ps17
Saccharomycotina - ps18
Saccharomycotina_1 - ps19
Saccharomycotina_2 - ps20
Saccharomycotina_3 - ps21
Phaff./Saccharomycetaceae - ps22
Saccharomyco./mycetaceae - ps23
Saccharomycetaceae - ps24
Saccharomycetaceae_2 - ps25
Saccharomycetaceae_3 - ps26
Kaz./Nau./Saccharomyces - ps27
Saccharomyces - ps28
Arboricola_clade - ps29
Cerevisiae_clade - ps30
Saccharomyces_cerevisiae - ps31
Phylostratum

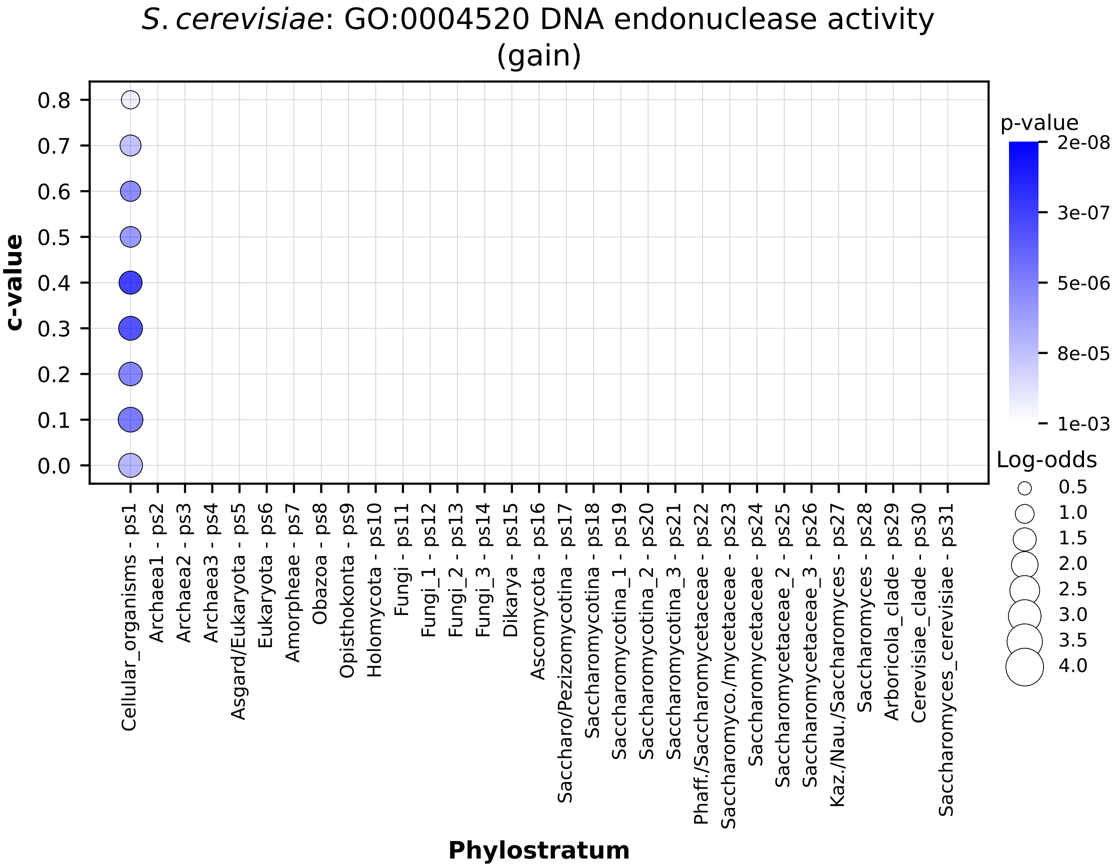

S. cerevisiae: GO:0004520 DNA endonuclease activity (gain)
c-value
0.8
0.7
0.6
0.5
0.4
0.3
0.2
0.1
0.0
p-value
2e-08
3e-07
5e-06
8e-05
1e-03
Log-odds
0.5
1.0
1.5
2.0
2.5
3.0
3.5
4.0
Cellular_organisms - ps1
Archaea1 - ps2
Archaea2 - ps3
Archaea3 - ps4
Asgard/Eukaryota - ps5
Eukaryota - ps6
Amorpheae - ps7
Obazoa - ps8
Opisthokonta - ps9
Holomycota - ps10
Fungi - ps11
Fungi_1 - ps12
Fungi_2 - ps13
Fungi_3 - ps14
Dikarya - ps15
Ascomycota - ps16
Saccharo/Pezizomycotina - ps17
Saccharomycotina - ps18
Saccharomycotina_1 - ps19
Saccharomycotina_2 - ps20
Saccharomycotina_3 - ps21
Phaff./Saccharomycetaceae - ps22
Saccharomyco./mycetaceae - ps23
Saccharomycetaceae - ps24
Saccharomycetaceae_2 - ps25
Saccharomycetaceae_3 - ps26
Kaz./Nau./Saccharomyces - ps27
Saccharomyces - ps28
Arboricola_clade - ps29
Cerevisiae_clade - ps30
Saccharomyces_cerevisiae - ps31
Phylostratum

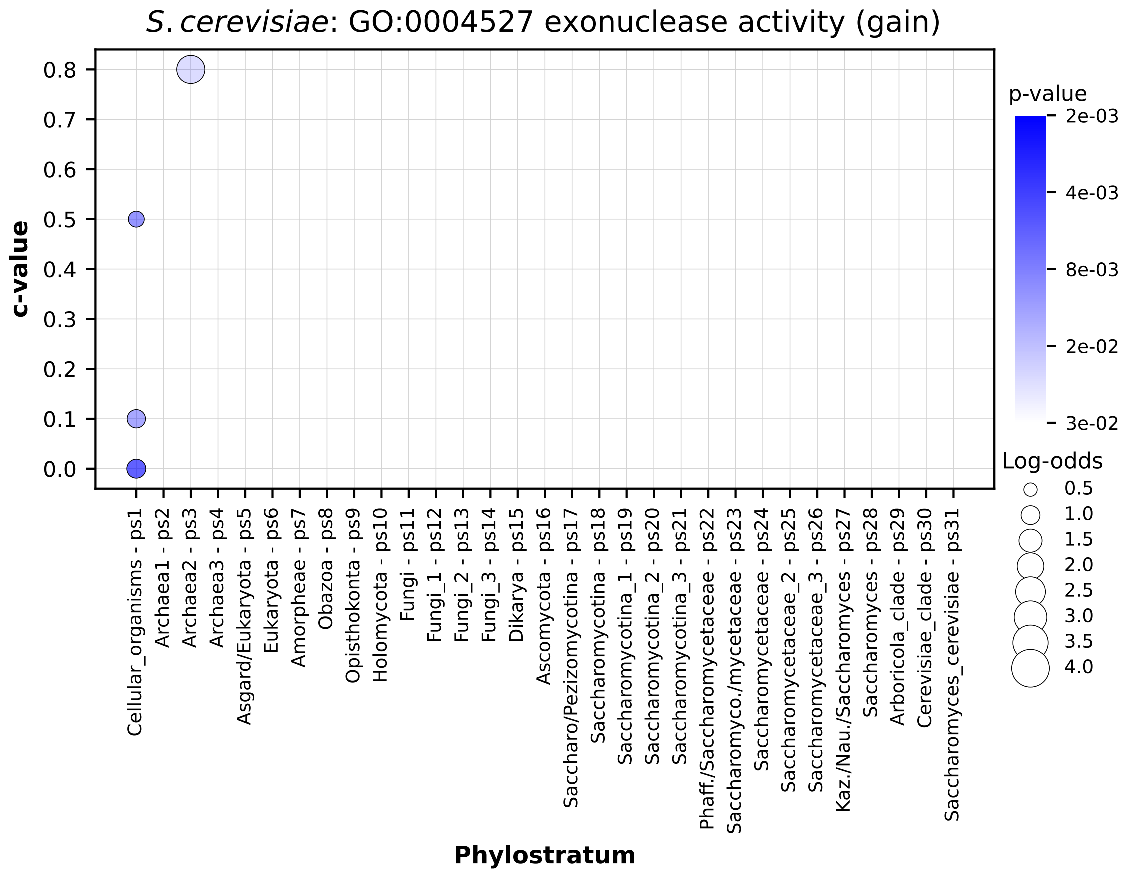
S. cerevisiae: GO:0004527 exonuclease activity (gain)
c-value
0.0
0.1
0.2
0.3
0.4
0.5
0.6
0.7
0.8
Phylostratum
Cellular_organisms - ps1
Archaea1 - ps2
Archaea2 - ps3
Archaea3 - ps4
Asgard/Eukaryota - ps5
Eukaryota - ps6
Amorpheae - ps7
Obazoa - ps8
Opisthokonta - ps9
Holomycota - ps10
Fungi - ps11
Fungi_1 - ps12
Fungi_2 - ps13
Fungi_3 - ps14
Dikarya - ps15
Ascomycota - ps16
Saccharo/Pezizomycotina - ps17
Saccharomycotina - ps18
Saccharomycotina_1 - ps19
Saccharomycotina_2 - ps20
Saccharomycotina_3 - ps21
Phaff./Saccharomycetaceae - ps22
Saccharomyco./mycetaceae - ps23
Saccharomycetaceae - ps24
Saccharomycetaceae_2 - ps25
Saccharomycetaceae_3 - ps26
Kaz./Nau./Saccharomyces - ps27
Saccharomyces - ps28
Arboricola_clade - ps29
Cerevisiae_clade - ps30
Saccharomyces_cerevisiae - ps31
p-value
2e-03
4e-03
8e-03
2e-02
3e-02
Log-odds
0.5
1.0
1.5
2.0
2.5
3.0
3.5
4.0

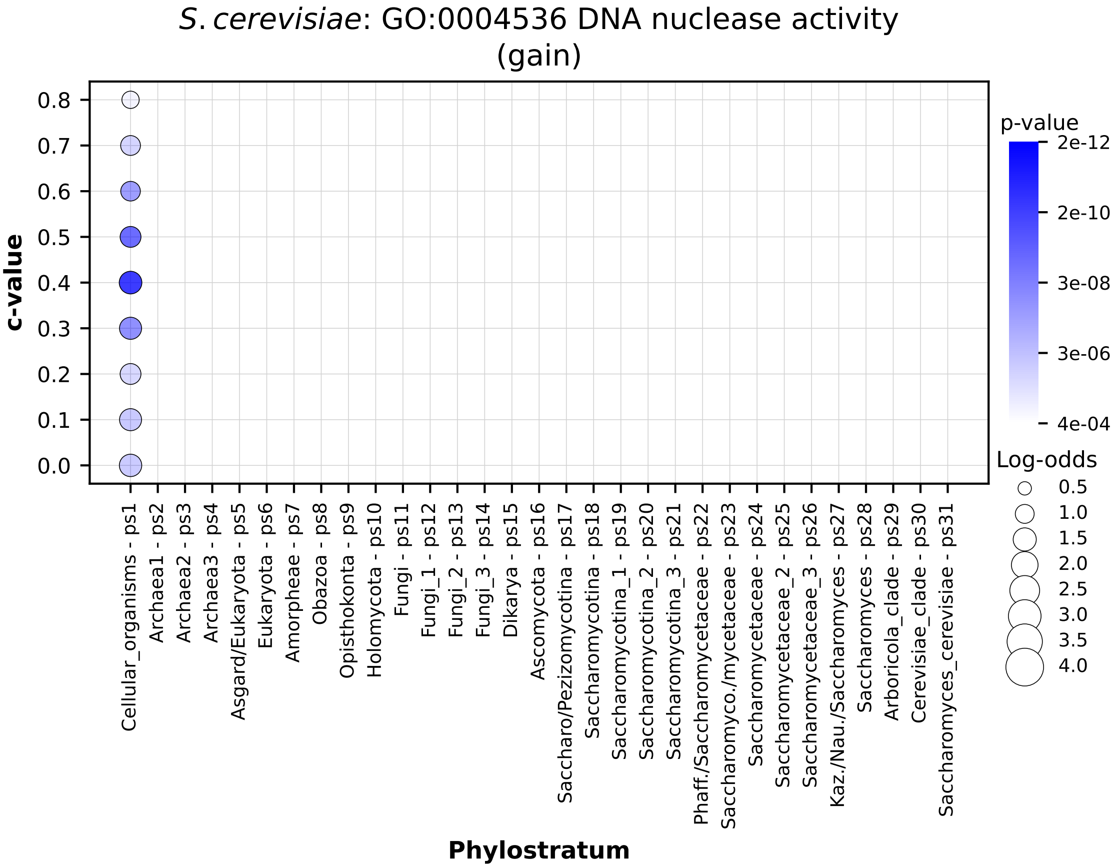

S. cerevisiae: GO:0004536 DNA nuclease activity
(gain)
c-value
0.0
0.1
0.2
0.3
0.4
0.5
0.6
0.7
0.8
p-value
2e-12
2e-10
3e-08
3e-06
4e-04
Log-odds
0.5
1.0
1.5
2.0
2.5
3.0
3.5
4.0
Cellular_organisms - ps1
Archaea1 - ps2
Archaea2 - ps3
Archaea3 - ps4
Asgard/Eukaryota - ps5
Eukaryota - ps6
Amorpheae - ps7
Obazoa - ps8
Opisthokonta - ps9
Holomycota - ps10
Fungi - ps11
Fungi_1 - ps12
Fungi_2 - ps13
Fungi_3 - ps14
Dikarya - ps15
Ascomycota - ps16
Saccharo/Pezizomycotina - ps17
Saccharomycotina - ps18
Saccharomycotina_1 - ps19
Saccharomycotina_2 - ps20
Saccharomycotina_3 - ps21
Phaff./Saccharomycetaceae - ps22
Saccharomyco./mycetaceae - ps23
Saccharomycetaceae - ps24
Saccharomycetaceae_2 - ps25
Saccharomycetaceae_3 - ps26
Kaz./Nau./Saccharomyces - ps27
Saccharomyces - ps28
Arboricola_clade - ps29
Cerevisiae_clade - ps30
Saccharomyces_cerevisiae - ps31
Phylostratum

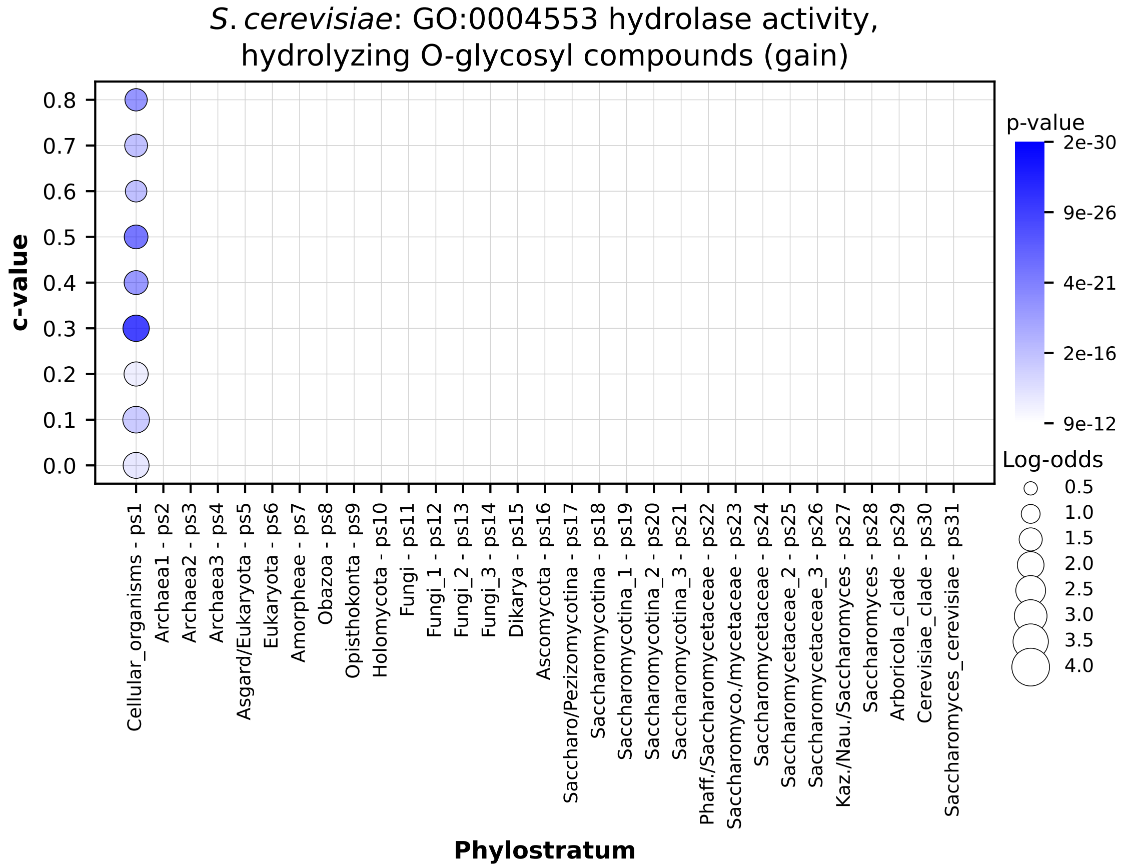
S. cerevisiae: GO:0004553 hydrolase activity, hydrolyzing O-glycosyl compounds (gain)
c-value
0.8
0.7
0.6
0.5
0.4
0.3
0.2
0.1
0.0
p-value
2e-30
9e-26
4e-21
2e-16
9e-12
Log-odds
0.5
1.0
1.5
2.0
2.5
3.0
3.5
4.0
Cellular_organisms - ps1
Archaea1 - ps2
Archaea2 - ps3
Archaea3 - ps4
Asgard/Eukaryota - ps5
Eukaryota - ps6
Amorpheae - ps7
Obazoa - ps8
Opisthokonta - ps9
Holomycota - ps10
Fungi - ps11
Fungi_1 - ps12
Fungi_2 - ps13
Fungi_3 - ps14
Dikarya - ps15
Ascomycota - ps16
Saccharo/Pezizomycotina - ps17
Saccharomycotina - ps18
Saccharomycotina_1 - ps19
Saccharomycotina_2 - ps20
Saccharomycotina_3 - ps21
Phaff./Saccharomycetaceae - ps22
Saccharomyco./mycetaceae - ps23
Saccharomycetaceae - ps24
Saccharomycetaceae_2 - ps25
Saccharomycetaceae_3 - ps26
Kaz./Nau./Saccharomyces - ps27
Saccharomyces - ps28
Arboricola_clade - ps29
Cerevisiae_clade - ps30
Saccharomyces_cerevisiae - ps31
Phylostratum

S. cerevisiae: GO:0004590 orotidine-5'-phosphate decarboxylase activity (gain)

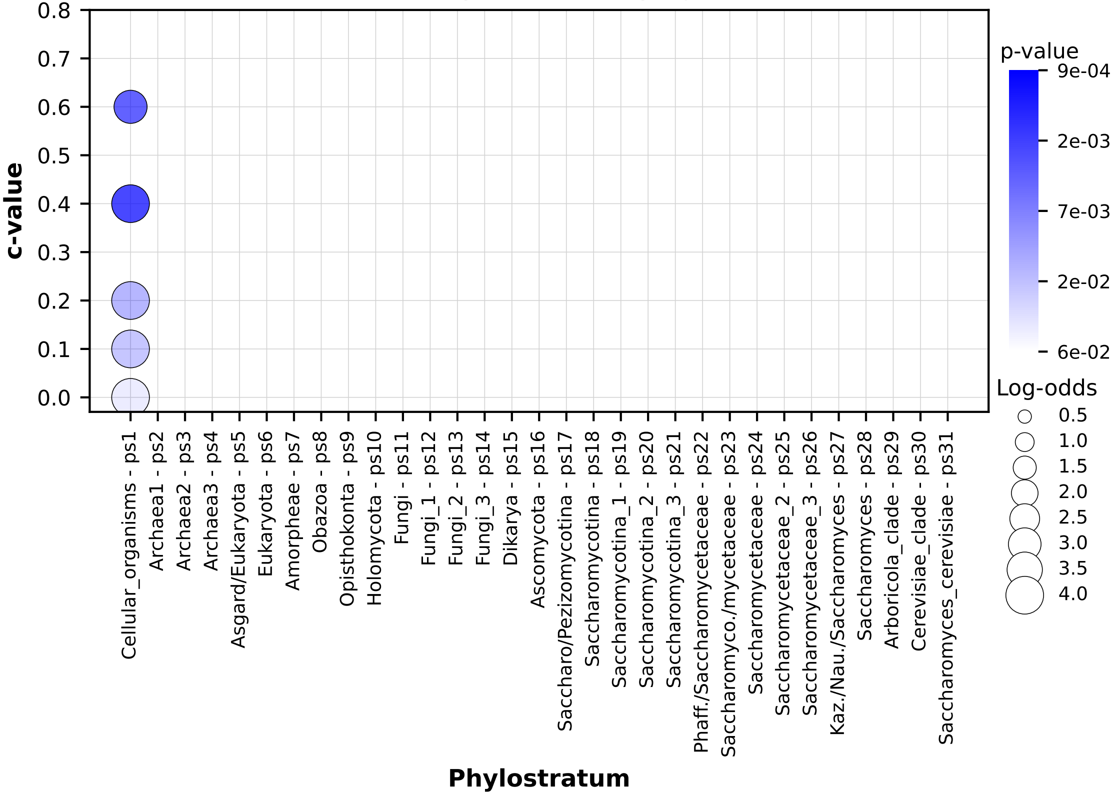

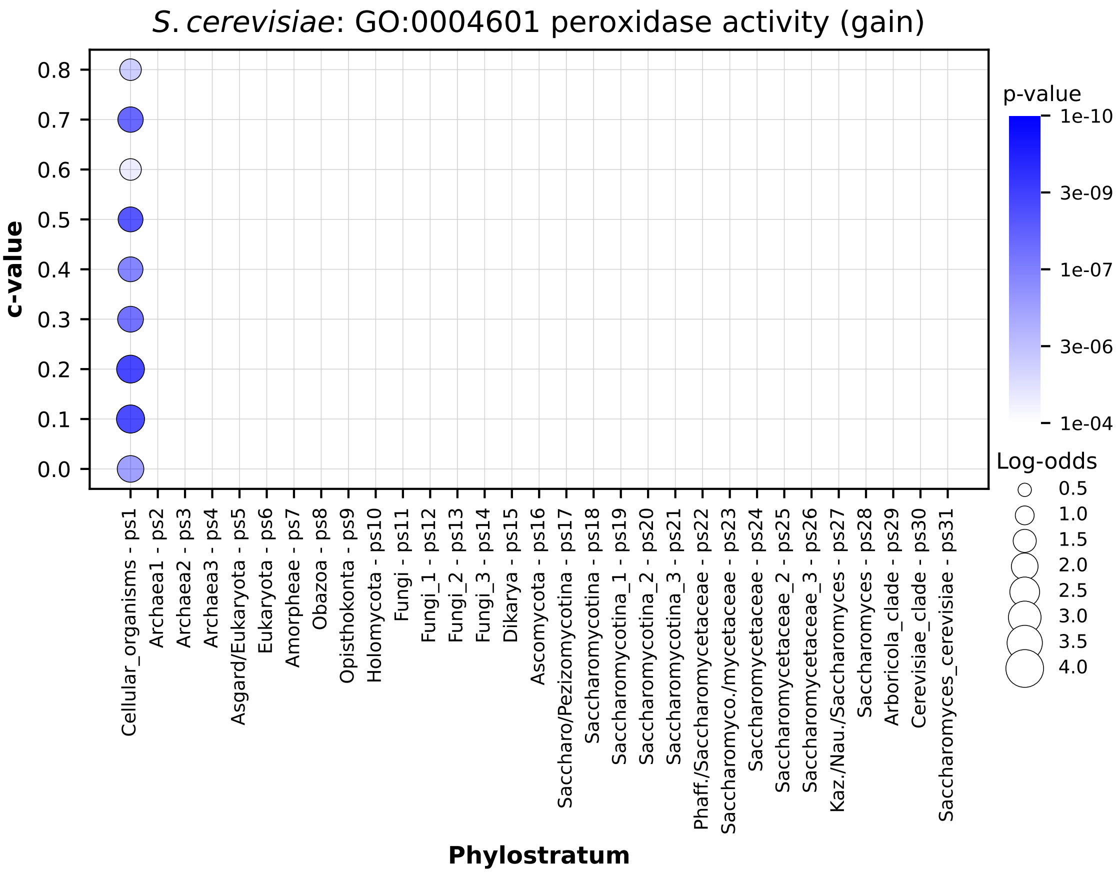

S. cerevisiae: GO:0004601 peroxidase activity (gain)
c-value
0.0
0.1
0.2
0.3
0.4
0.5
0.6
0.7
0.8
p-value
1e-10
3e-09
1e-07
3e-06
1e-04
Log-odds
0.5
1.0
1.5
2.0
2.5
3.0
3.5
4.0
Cellular_organisms - ps1
Archaea1 - ps2
Archaea2 - ps3
Archaea3 - ps4
Asgard/Eukaryota - ps5
Eukaryota - ps6
Amorpheae - ps7
Obazoa - ps8
Opisthokonta - ps9
Holomycota - ps10
Fungi - ps11
Fungi_1 - ps12
Fungi_2 - ps13
Fungi_3 - ps14
Dikarya - ps15
Ascomycota - ps16
Saccharo/Pezizomycotina - ps17
Saccharomycotina - ps18
Saccharomycotina_1 - ps19
Saccharomycotina_2 - ps20
Saccharomycotina_3 - ps21
Phaff./Saccharomycetaceae - ps22
Saccharomyco./mycetaceae - ps23
Saccharomycetaceae - ps24
Saccharomycetaceae_2 - ps25
Saccharomycetaceae_3 - ps26
Kaz./Nau./Saccharomyces - ps27
Saccharomyces - ps28
Arboricola_clade - ps29
Cerevisiae_clade - ps30
Saccharomyces_cerevisiae - ps31
Phylostratum

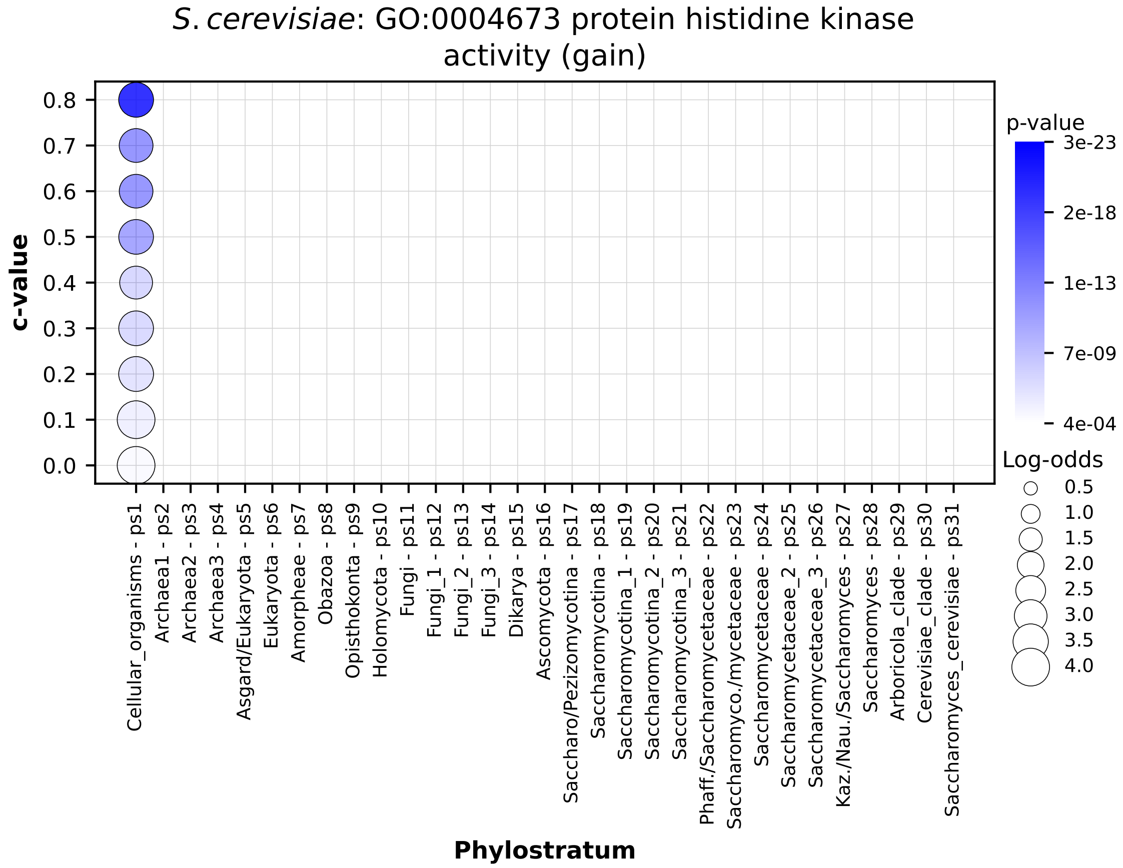

S. cerevisiae: GO:0004673 protein histidine kinase activity (gain)
c-value
0.8
0.7
0.6
0.5
0.4
0.3
0.2
0.1
0.0
p-value
3e-23
2e-18
1e-13
7e-09
4e-04
Log-odds
0.5
1.0
1.5
2.0
2.5
3.0
3.5
4.0
Cellular_organisms - ps1
Archaea1 - ps2
Archaea2 - ps3
Archaea3 - ps4
Asgard/Eukaryota - ps5
Eukaryota - ps6
Amorpheae - ps7
Obazoa - ps8
Opisthokonta - ps9
Holomycota - ps10
Fungi - ps11
Fungi_1 - ps12
Fungi_2 - ps13
Fungi_3 - ps14
Dikarya - ps15
Ascomycota - ps16
Saccharo/Pezizomycotina - ps17
Saccharomycotina - ps18
Saccharomycotina_1 - ps19
Saccharomycotina_2 - ps20
Saccharomycotina_3 - ps21
Phaff./Saccharomycetaceae - ps22
Saccharomyco./mycetaceae - ps23
Saccharomycetaceae - ps24
Saccharomycetaceae_2 - ps25
Saccharomycetaceae_3 - ps26
Kaz./Nau./Saccharomyces - ps27
Saccharomyces - ps28
Arboricola_clade - ps29
Cerevisiae_clade - ps30
Saccharomyces_cerevisiae - ps31
Phylostratum

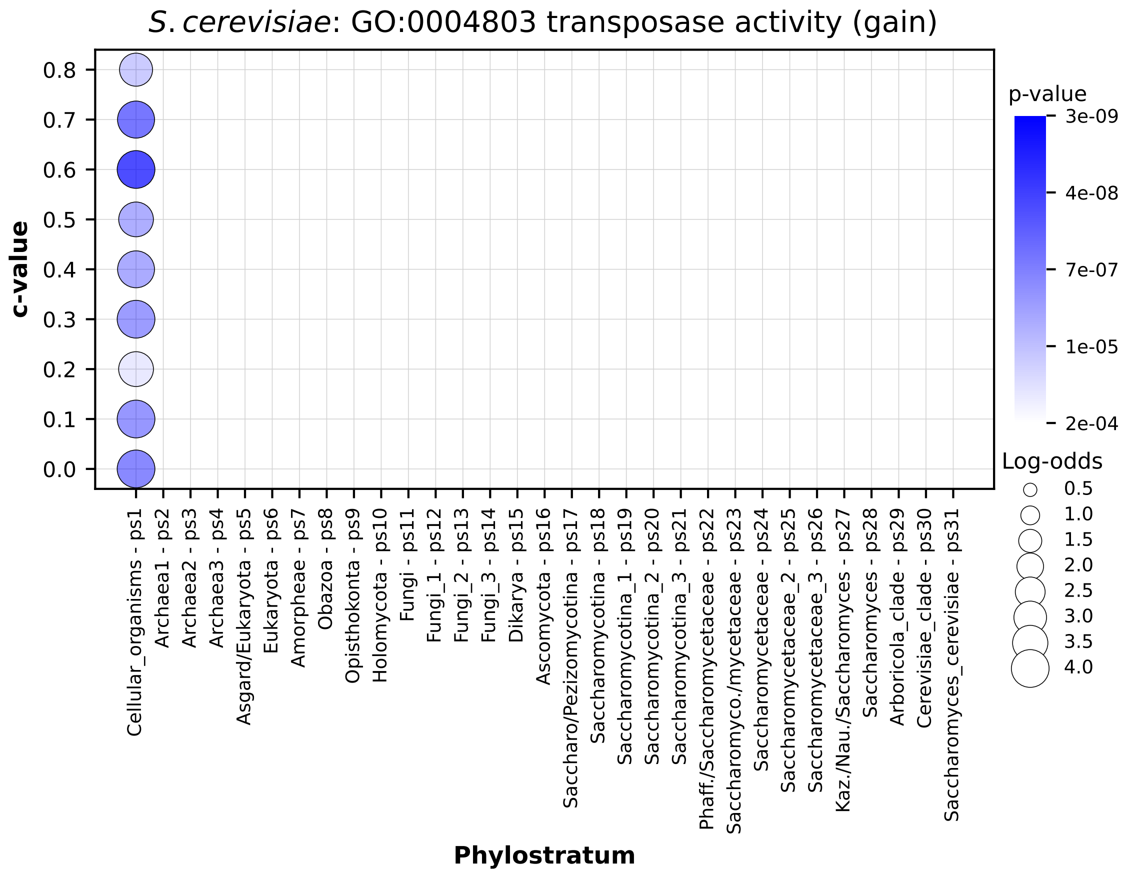

S. cerevisiae: GO:0004803 transposase activity (gain)
c-value
0.0
0.1
0.2
0.3
0.4
0.5
0.6
0.7
0.8
p-value
3e-09
4e-08
7e-07
1e-05
2e-04
Log-odds
0.5
1.0
1.5
2.0
2.5
3.0
3.5
4.0
Cellular_organisms - ps1
Archaea1 - ps2
Archaea2 - ps3
Archaea3 - ps4
Asgard/Eukaryota - ps5
Eukaryota - ps6
Amorpheae - ps7
Obazoa - ps8
Opisthokonta - ps9
Holomycota - ps10
Fungi - ps11
Fungi_1 - ps12
Fungi_2 - ps13
Fungi_3 - ps14
Dikarya - ps15
Ascomycota - ps16
Saccharo/Pezizomycotina - ps17
Saccharomycotina - ps18
Saccharomycotina_1 - ps19
Saccharomycotina_2 - ps20
Saccharomycotina_3 - ps21
Phaff./Saccharomycetaceae - ps22
Saccharomyco./mycetaceae - ps23
Saccharomycetaceae - ps24
Saccharomycetaceae_2 - ps25
Saccharomycetaceae_3 - ps26
Kaz./Nau./Saccharomyces - ps27
Saccharomyces - ps28
Arboricola_clade - ps29
Cerevisiae_clade - ps30
Saccharomyces_cerevisiae - ps31
Phylostratum

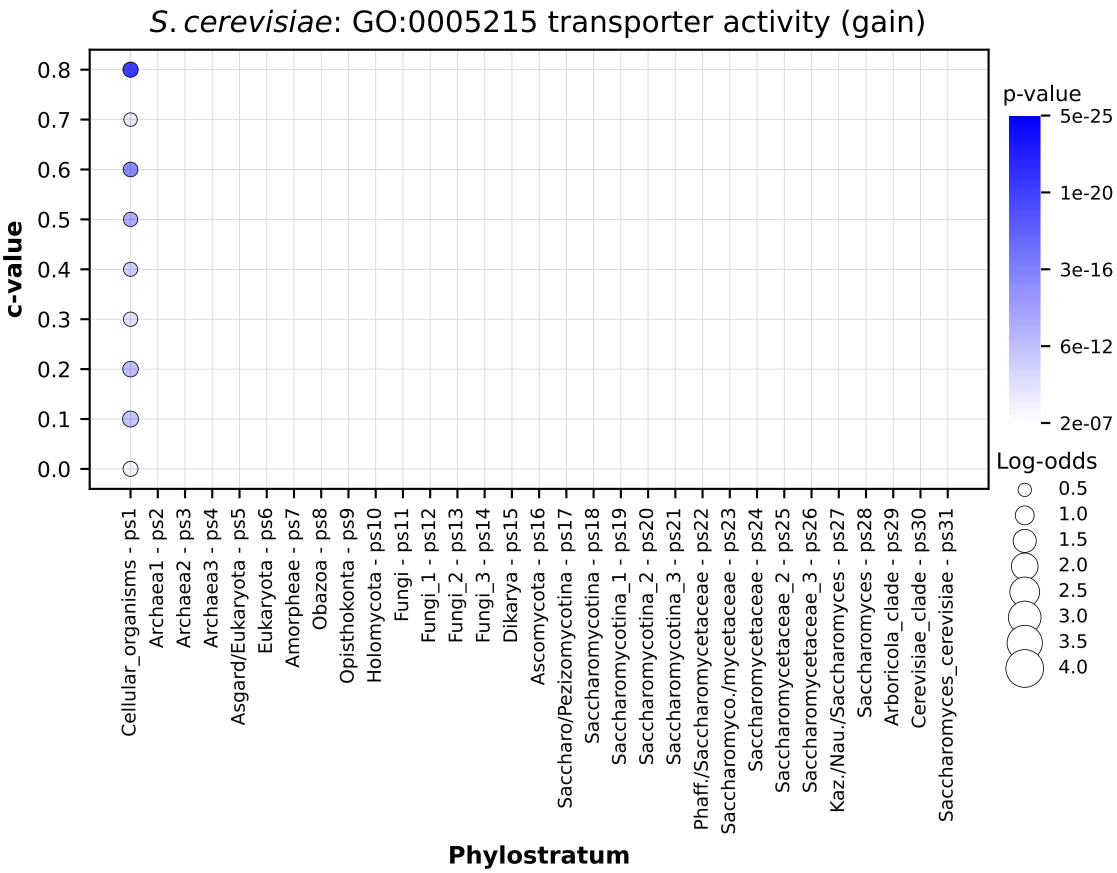
S. cerevisiae: GO:0005215 transporter activity (gain)
c-value
0.0
0.1
0.2
0.3
0.4
0.5
0.6
0.7
0.8
p-value
5e-25
1e-20
3e-16
6e-12
2e-07
Log-odds
0.5
1.0
1.5
2.0
2.5
3.0
3.5
4.0
Cellular_organisms - ps1
Archaea1 - ps2
Archaea2 - ps3
Archaea3 - ps4
Asgard/Eukaryota - ps5
Eukaryota - ps6
Amorpheae - ps7
Obazoa - ps8
Opisthokonta - ps9
Holomycota - ps10
Fungi - ps11
Fungi_1 - ps12
Fungi_2 - ps13
Fungi_3 - ps14
Dikarya - ps15
Ascomycota - ps16
Saccharo/Pezizomycotina - ps17
Saccharomycotina - ps18
Saccharomycotina_1 - ps19
Saccharomycotina_2 - ps20
Saccharomycotina_3 - ps21
Phaff./Saccharomycetaceae - ps22
Saccharomyco./mycetaceae - ps23
Saccharomycetaceae - ps24
Saccharomycetaceae_2 - ps25
Saccharomycetaceae_3 - ps26
Kaz./Nau./Saccharomyces - ps27
Saccharomyces - ps28
Arboricola_clade - ps29
Cerevisiae_clade - ps30
Saccharomyces_cerevisiae - ps31
Phylostratum

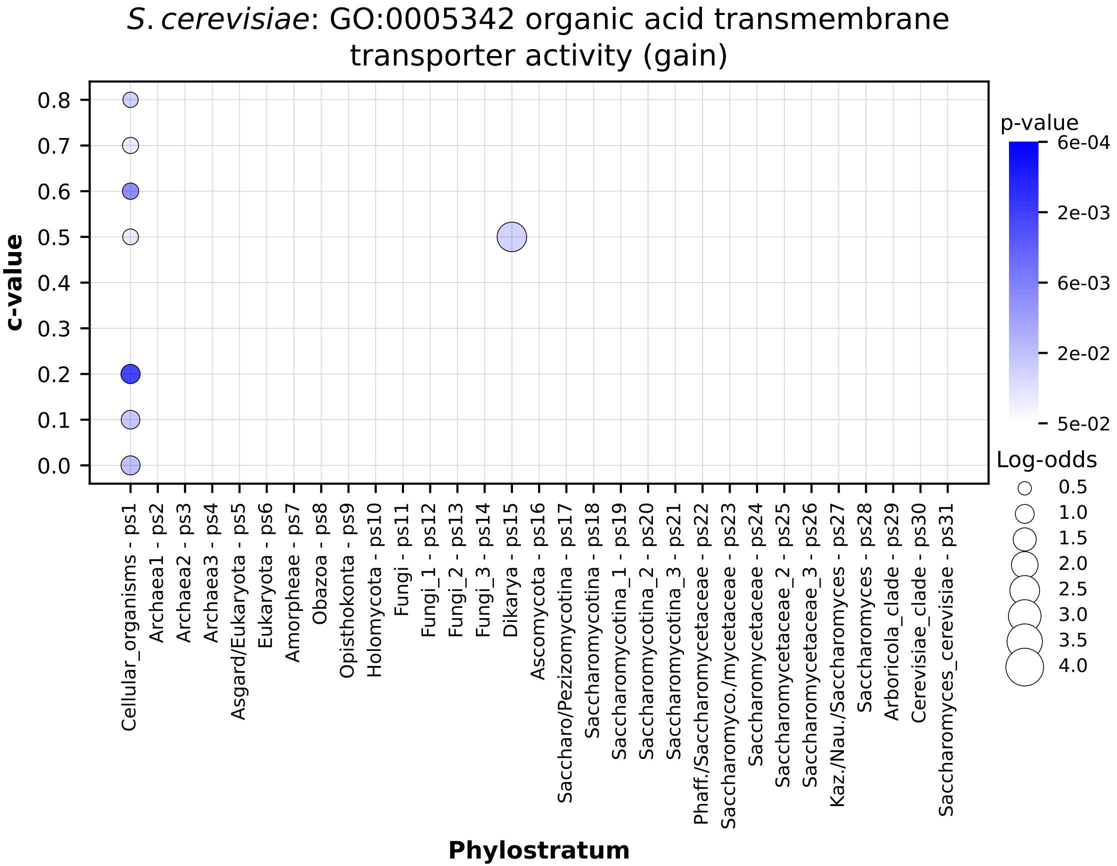
S. cerevisiae: GO:0005342 organic acid transmembrane transporter activity (gain)
c-value
0.0
0.1
0.2
0.3
0.4
0.5
0.6
0.7
0.8
p-value
6e-04
2e-03
6e-03
2e-02
5e-02
Log-odds
0.5
1.0
1.5
2.0
2.5
3.0
3.5
4.0
Cellular_organisms - ps1
Archaea1 - ps2
Archaea2 - ps3
Archaea3 - ps4
Asgard/Eukaryota - ps5
Eukaryota - ps6
Amorpheae - ps7
Obazoa - ps8
Opisthokonta - ps9
Holomycota - ps10
Fungi - ps11
Fungi_1 - ps12
Fungi_2 - ps13
Fungi_3 - ps14
Dikarya - ps15
Ascomycota - ps16
Saccharo/Pezizomycotina - ps17
Saccharomycotina - ps18
Saccharomycotina_1 - ps19
Saccharomycotina_2 - ps20
Saccharomycotina_3 - ps21
Phaff./Saccharomycetaceae - ps22
Saccharomyco./mycetaceae - ps23
Saccharomycetaceae - ps24
Saccharomycetaceae_2 - ps25
Saccharomycetaceae_3 - ps26
Kaz./Nau./Saccharomyces - ps27
Saccharomyces - ps28
Arboricola_clade - ps29
Cerevisiae_clade - ps30
Saccharomyces_cerevisiae - ps31
Phylostratum

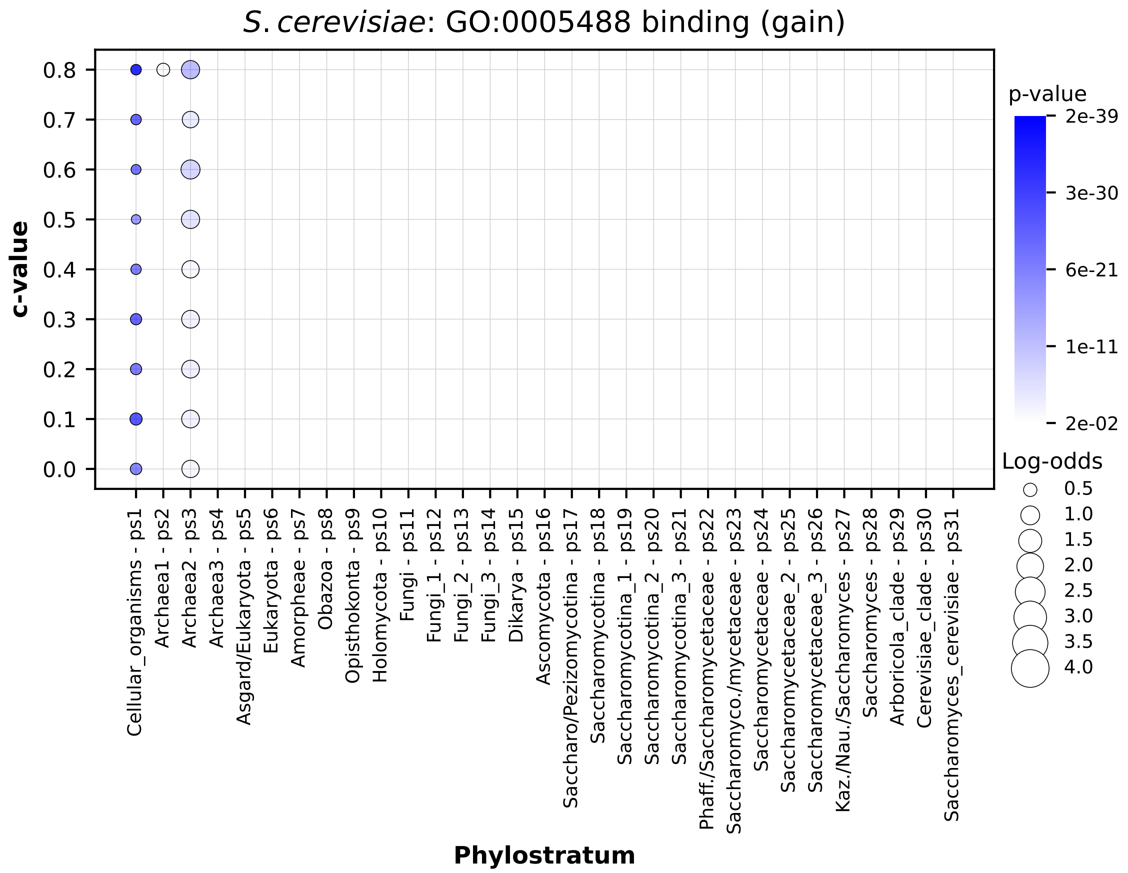
S. cerevisiae: GO:0005488 binding (gain)
c-value
0.8
0.7
0.6
0.5
0.4
0.3
0.2
0.1
0.0
p-value
2e-39
3e-30
6e-21
1e-11
2e-02
Log-odds
0.5
1.0
1.5
2.0
2.5
3.0
3.5
4.0
Cellular_organisms - ps1
Archaea1 - ps2
Archaea2 - ps3
Archaea3 - ps4
Asgard/Eukaryota - ps5
Eukaryota - ps6
Amorpheae - ps7
Obazoa - ps8
Opisthokonta - ps9
Holomycota - ps10
Fungi - ps11
Fungi_1 - ps12
Fungi_2 - ps13
Fungi_3 - ps14
Dikarya - ps15
Ascomycota - ps16
Saccharo/Pezizomycotina - ps17
Saccharomycotina - ps18
Saccharomycotina_1 - ps19
Saccharomycotina_2 - ps20
Saccharomycotina_3 - ps21
Phaff./Saccharomycetaceae - ps22
Saccharomyco./mycetaceae - ps23
Saccharomycetaceae - ps24
Saccharomycetaceae_2 - ps25
Saccharomycetaceae_3 - ps26
Kaz./Nau./Saccharomyces - ps27
Saccharomyces - ps28
Arboricola_clade - ps29
Cerevisiae_clade - ps30
Saccharomyces_cerevisiae - ps31
Phylostratum

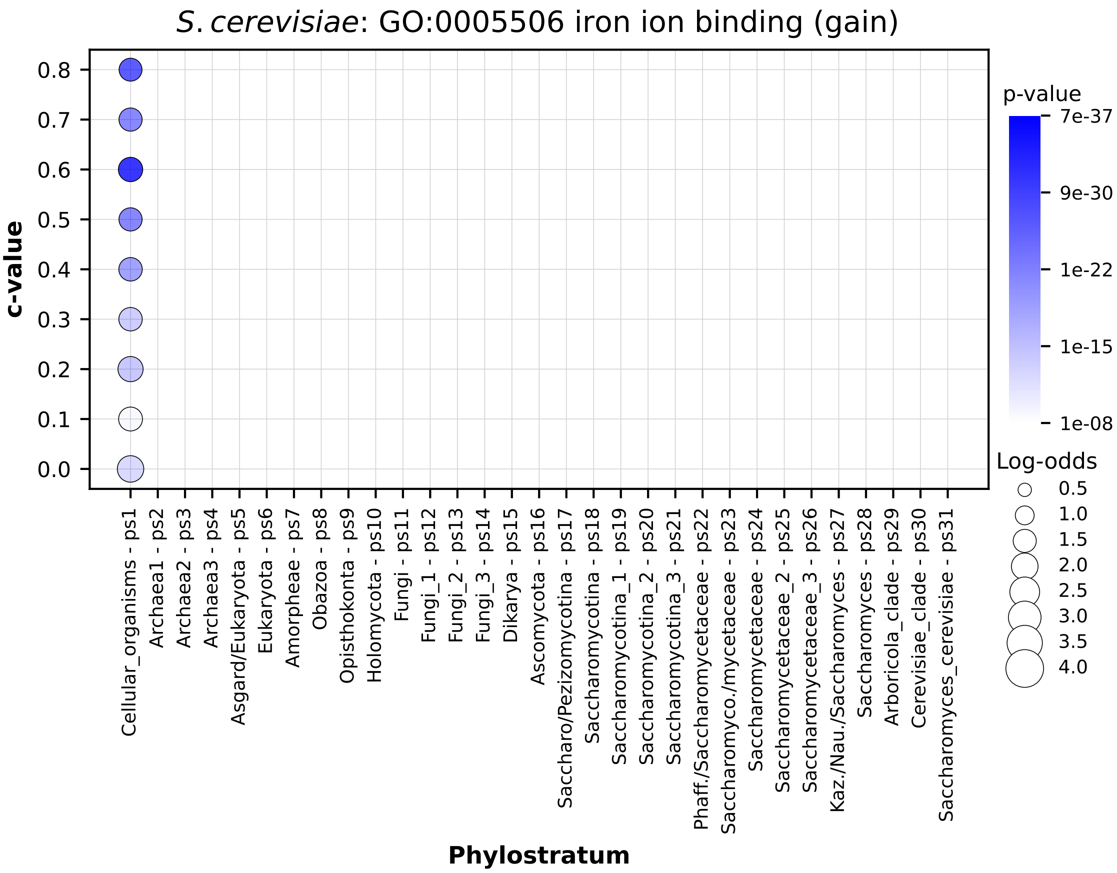
S. cerevisiae: GO:0005506 iron ion binding (gain)
c-value
0.0
0.1
0.2
0.3
0.4
0.5
0.6
0.7
0.8
p-value
7e-37
9e-30
1e-22
1e-15
1e-08
Log-odds
0.5
1.0
1.5
2.0
2.5
3.0
3.5
4.0
Cellular_organisms - ps1
Archaea1 - ps2
Archaea2 - ps3
Archaea3 - ps4
Asgard/Eukaryota - ps5
Eukaryota - ps6
Amorpheae - ps7
Obazoa - ps8
Opisthokonta - ps9
Holomycota - ps10
Fungi - ps11
Fungi_1 - ps12
Fungi_2 - ps13
Fungi_3 - ps14
Dikarya - ps15
Ascomycota - ps16
Saccharo/Pezizomycotina - ps17
Saccharomycotina - ps18
Saccharomycotina_1 - ps19
Saccharomycotina_2 - ps20
Saccharomycotina_3 - ps21
Phaff./Saccharomycetaceae - ps22
Saccharomyco./mycetaceae - ps23
Saccharomycetaceae - ps24
Saccharomycetaceae_2 - ps25
Saccharomycetaceae_3 - ps26
Kaz./Nau./Saccharomyces - ps27
Saccharomyces - ps28
Arboricola_clade - ps29
Cerevisiae_clade - ps30
Saccharomyces_cerevisiae - ps31
Phylostratum

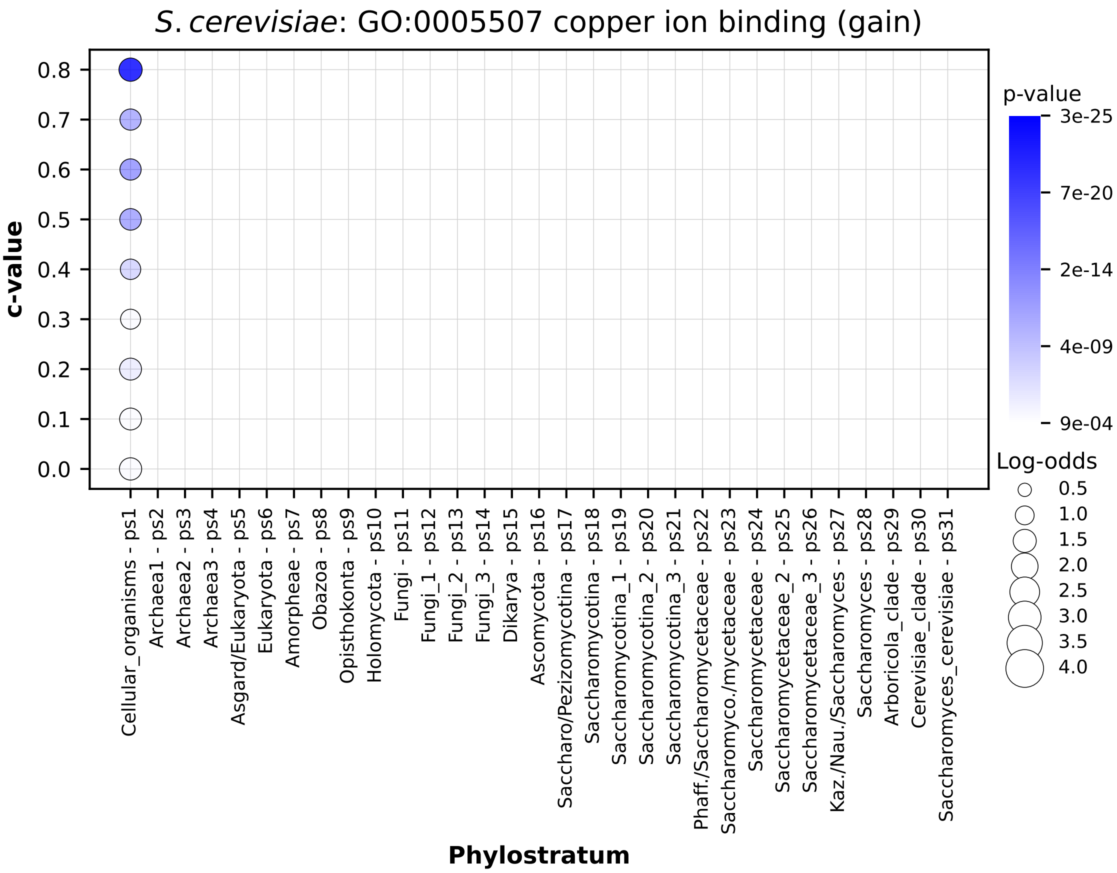
S. cerevisiae: GO:0005507 copper ion binding (gain)
c-value
0.0
0.1
0.2
0.3
0.4
0.5
0.6
0.7
0.8
Phylostratum
Cellular_organisms - ps1
Archaea1 - ps2
Archaea2 - ps3
Archaea3 - ps4
Asgard/Eukaryota - ps5
Eukaryota - ps6
Amorpheae - ps7
Obazoa - ps8
Opisthokonta - ps9
Holomycota - ps10
Fungi - ps11
Fungi_1 - ps12
Fungi_2 - ps13
Fungi_3 - ps14
Dikarya - ps15
Ascomycota - ps16
Saccharo/Pezizomycotina - ps17
Saccharomycotina - ps18
Saccharomycotina_1 - ps19
Saccharomycotina_2 - ps20
Saccharomycotina_3 - ps21
Phaff./Saccharomycetaceae - ps22
Saccharomyco./mycetaceae - ps23
Saccharomycetaceae - ps24
Saccharomycetaceae_2 - ps25
Saccharomycetaceae_3 - ps26
Kaz./Nau./Saccharomyces - ps27
Saccharomyces - ps28
Arboricola_clade - ps29
Cerevisiae_clade - ps30
Saccharomyces_cerevisiae - ps31
p-value
3e-25
7e-20
2e-14
4e-09
9e-04
Log-odds
0.5
1.0
1.5
2.0
2.5
3.0
3.5
4.0

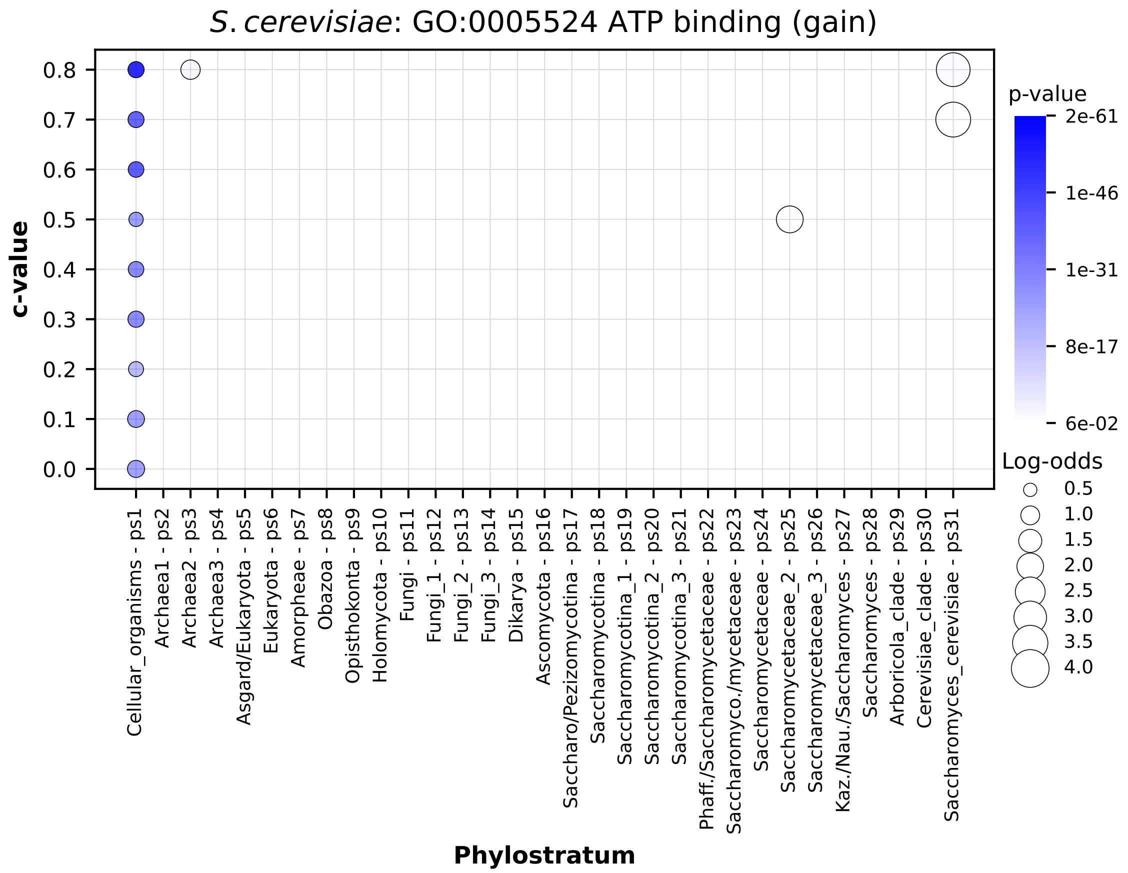
S. cerevisiae: GO:0005524 ATP binding (gain)
c-value
0.8
0.7
0.6
0.5
0.4
0.3
0.2
0.1
0.0
p-value
2e-61
1e-46
1e-31
8e-17
6e-02
Log-odds
0.5
1.0
1.5
2.0
2.5
3.0
3.5
4.0
Cellular_organisms - ps1
Archaea1 - ps2
Archaea2 - ps3
Archaea3 - ps4
Asgard/Eukaryota - ps5
Eukaryota - ps6
Amorpheae - ps7
Obazoa - ps8
Opisthokonta - ps9
Holomycota - ps10
Fungi - ps11
Fungi_1 - ps12
Fungi_2 - ps13
Fungi_3 - ps14
Dikarya - ps15
Ascomycota - ps16
Saccharo/Pezizomycotina - ps17
Saccharomycotina - ps18
Saccharomycotina_1 - ps19
Saccharomycotina_2 - ps20
Saccharomycotina_3 - ps21
Phaff./Saccharomycetaceae - ps22
Saccharomyco./mycetaceae - ps23
Saccharomycetaceae - ps24
Saccharomycetaceae_2 - ps25
Saccharomycetaceae_3 - ps26
Kaz./Nau./Saccharomyces - ps27
Saccharomyces - ps28
Arboricola_clade - ps29
Cerevisiae_clade - ps30
Saccharomyces_cerevisiae - ps31
Phylostratum

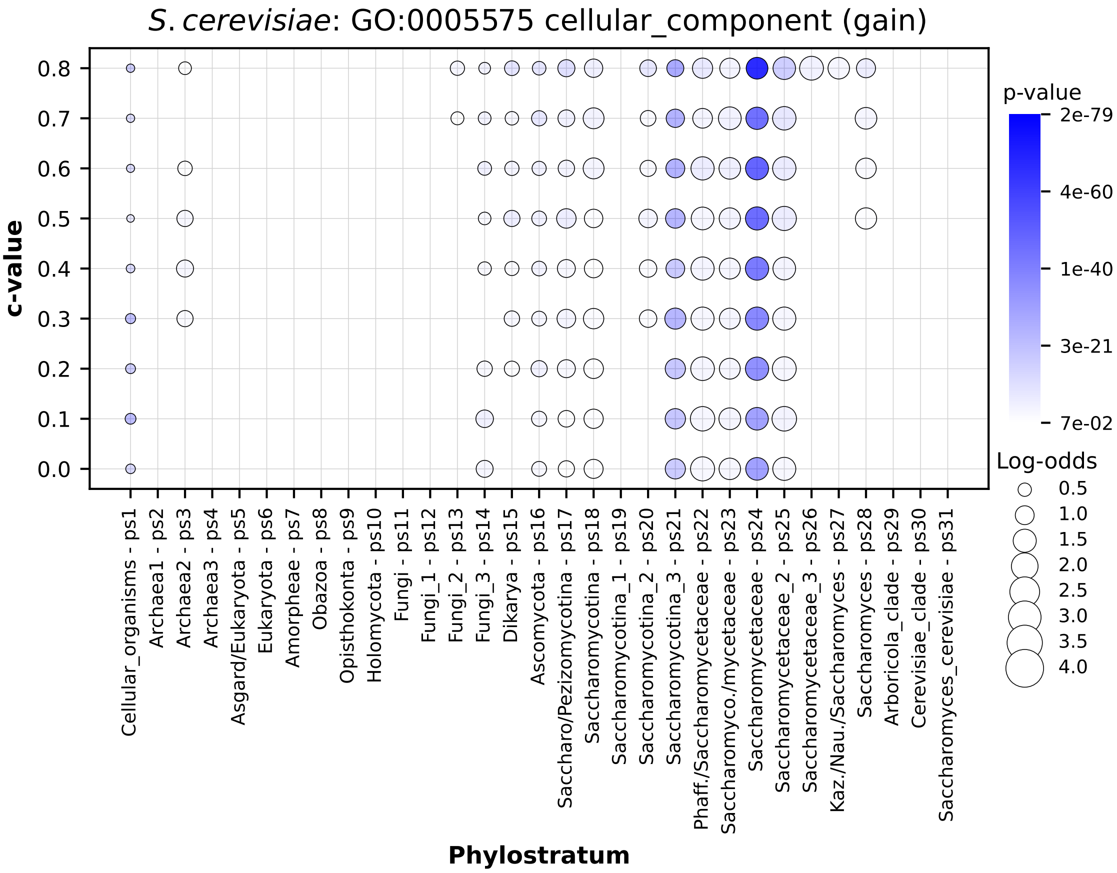
S. cerevisiae: GO:0005575 cellular_component (gain)
c-value
0.8
0.7
0.6
0.5
0.4
0.3
0.2
0.1
0.0
Phylostratum
Cellular_organisms - ps1
Archaea1 - ps2
Archaea2 - ps3
Archaea3 - ps4
Asgard/Eukaryota - ps5
Eukaryota - ps6
Amorpheae - ps7
Obazoa - ps8
Opisthokonta - ps9
Holomycota - ps10
Fungi - ps11
Fungi_1 - ps12
Fungi_2 - ps13
Fungi_3 - ps14
Dikarya - ps15
Ascomycota - ps16
Saccharo/Pezizomycotina - ps17
Saccharomycotina - ps18
Saccharomycotina_1 - ps19
Saccharomycotina_2 - ps20
Saccharomycotina_3 - ps21
Phaff./Saccharomycetaceae - ps22
Saccharomyco./mycetaceae - ps23
Saccharomycetaceae - ps24
Saccharomycetaceae_2 - ps25
Saccharomycetaceae_3 - ps26
Kaz./Nau./Saccharomyces - ps27
Saccharomyces - ps28
Arboricola_clade - ps29
Cerevisiae_clade - ps30
Saccharomyces_cerevisiae - ps31
p-value
2e-79
4e-60
1e-40
3e-21
7e-02
Log-odds
0.5
1.0
1.5
2.0
2.5
3.0
3.5
4.0

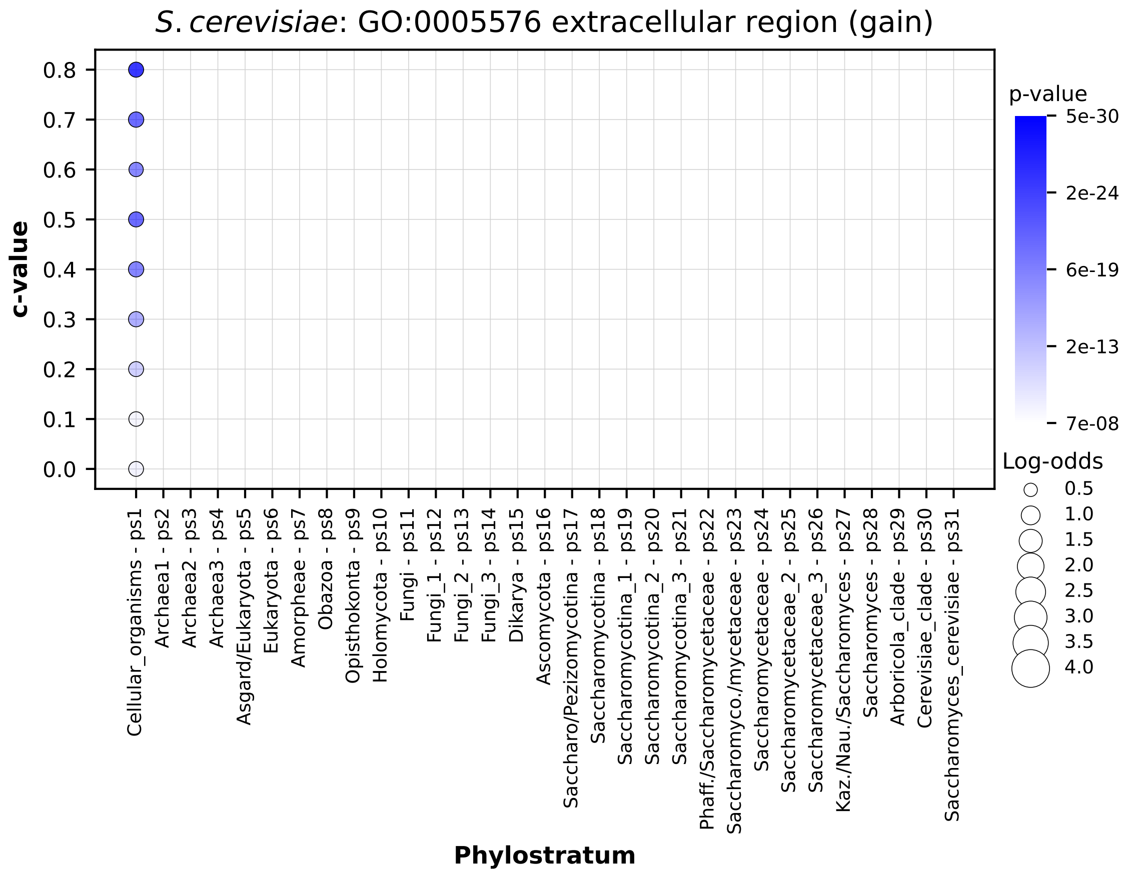
S. cerevisiae: GO:0005576 extracellular region (gain)
c-value
0.0
0.1
0.2
0.3
0.4
0.5
0.6
0.7
0.8
p-value
5e-30
2e-24
6e-19
2e-13
7e-08
Log-odds
0.5
1.0
1.5
2.0
2.5
3.0
3.5
4.0
Cellular_organisms - ps1
Archaea1 - ps2
Archaea2 - ps3
Archaea3 - ps4
Asgard/Eukaryota - ps5
Eukaryota - ps6
Amorpheae - ps7
Obazoa - ps8
Opisthokonta - ps9
Holomycota - ps10
Fungi - ps11
Fungi_1 - ps12
Fungi_2 - ps13
Fungi_3 - ps14
Dikarya - ps15
Ascomycota - ps16
Saccharo/Pezizomycotina - ps17
Saccharomycotina - ps18
Saccharomycotina_1 - ps19
Saccharomycotina_2 - ps20
Saccharomycotina_3 - ps21
Phaff./Saccharomycetaceae - ps22
Saccharomyco./mycetaceae - ps23
Saccharomycetaceae - ps24
Saccharomycetaceae_2 - ps25
Saccharomycetaceae_3 - ps26
Kaz./Nau./Saccharomyces - ps27
Saccharomyces - ps28
Arboricola_clade - ps29
Cerevisiae_clade - ps30
Saccharomyces_cerevisiae - ps31
Phylostratum

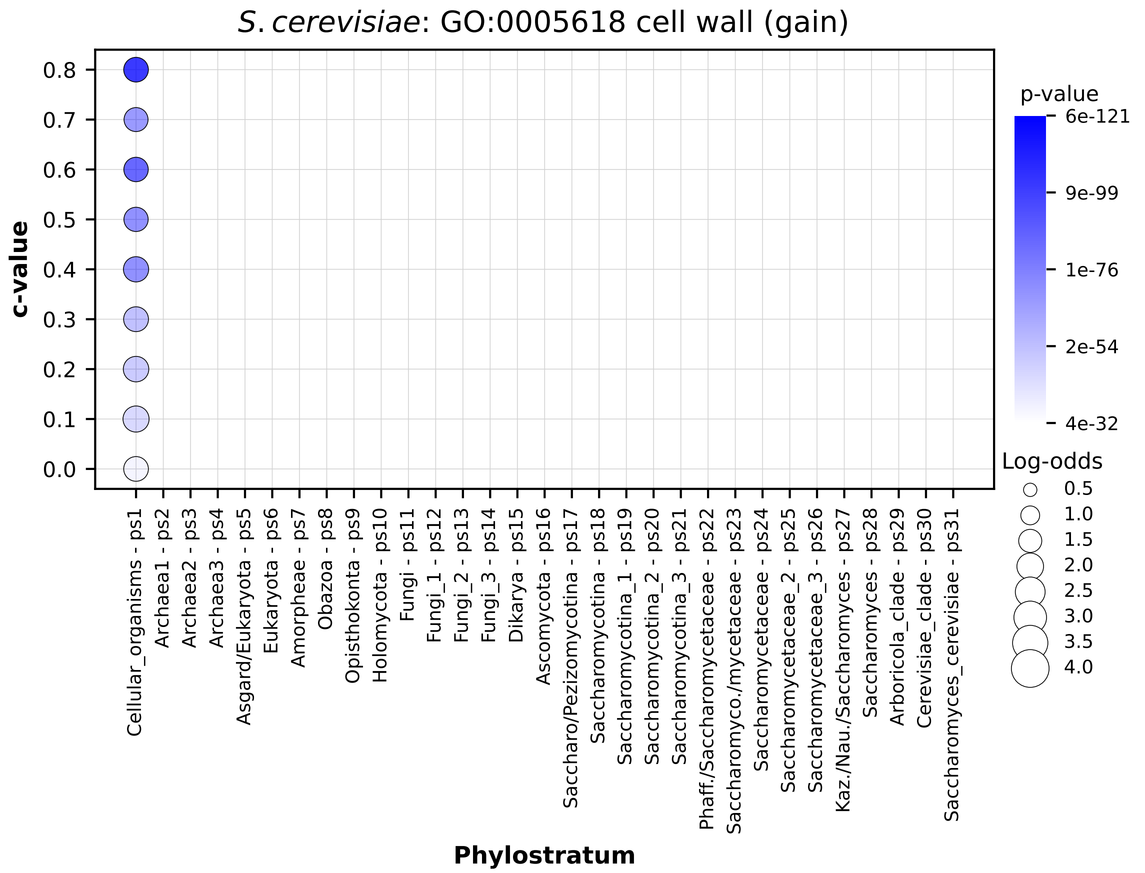

S. cerevisiae: GO:0005618 cell wall (gain)
c-value
0.8
0.7
0.6
0.5
0.4
0.3
0.2
0.1
0.0
p-value
6e-121
9e-99
1e-76
2e-54
4e-32
Log-odds
0.5
1.0
1.5
2.0
2.5
3.0
3.5
4.0
Cellular_organisms - ps1
Archaea1 - ps2
Archaea2 - ps3
Archaea3 - ps4
Asgard/Eukaryota - ps5
Eukaryota - ps6
Amorpheae - ps7
Obazoa - ps8
Opisthokonta - ps9
Holomycota - ps10
Fungi - ps11
Fungi_1 - ps12
Fungi_2 - ps13
Fungi_3 - ps14
Dikarya - ps15
Ascomycota - ps16
Saccharo/Pezizomycotina - ps17
Saccharomycotina - ps18
Saccharomycotina_1 - ps19
Saccharomycotina_2 - ps20
Saccharomycotina_3 - ps21
Phaff./Saccharomycetaceae - ps22
Saccharomyco./mycetaceae - ps23
Saccharomycetaceae - ps24
Saccharomycetaceae_2 - ps25
Saccharomycetaceae_3 - ps26
Kaz./Nau./Saccharomyces - ps27
Saccharomyces - ps28
Arboricola_clade - ps29
Cerevisiae_clade - ps30
Saccharomyces_cerevisiae - ps31
Phylostratum

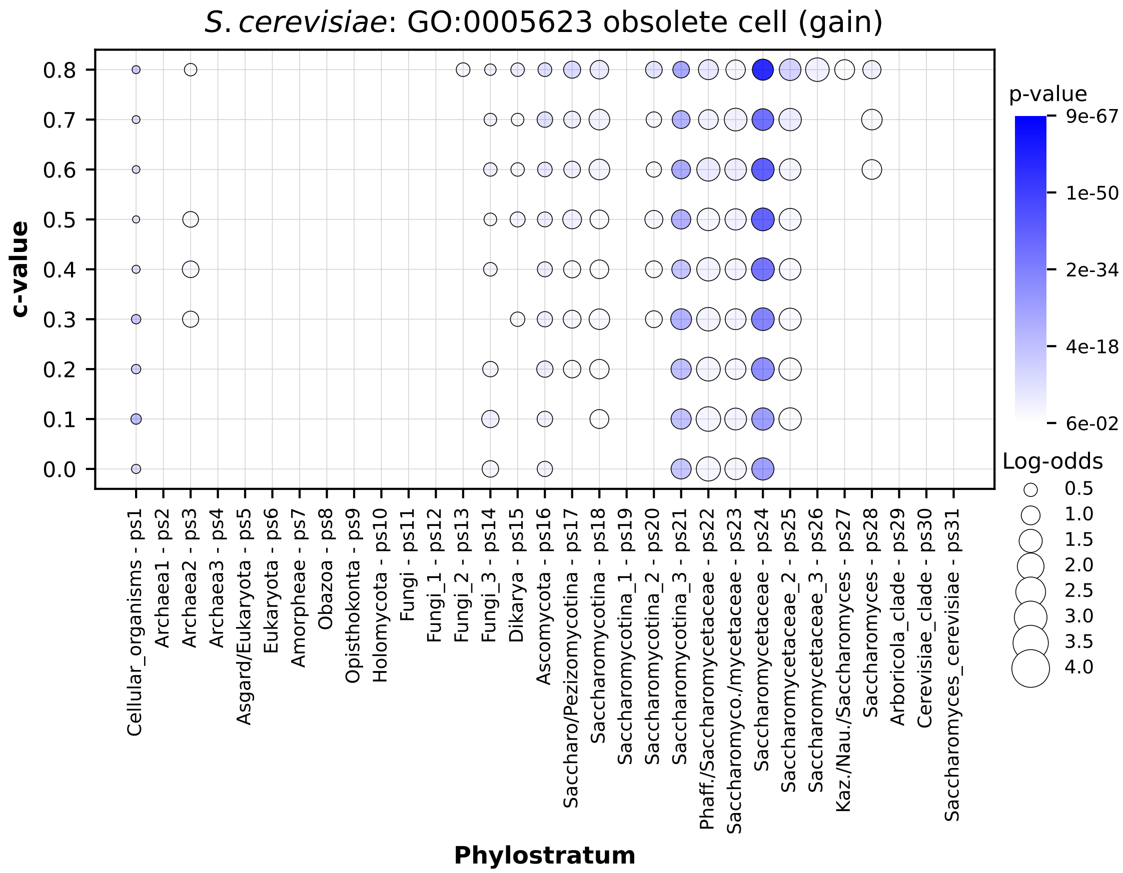
S. cerevisiae: GO:0005623 obsolete cell (gain)
c-value
0.8
0.7
0.6
0.5
0.4
0.3
0.2
0.1
0.0
p-value
9e-67
1e-50
2e-34
4e-18
6e-02
Log-odds
0.5
1.0
1.5
2.0
2.5
3.0
3.5
4.0
Cellular_organisms - ps1
Archaea1 - ps2
Archaea2 - ps3
Archaea3 - ps4
Asgard/Eukaryota - ps5
Eukaryota - ps6
Amorpheae - ps7
Obazoa - ps8
Opisthokonta - ps9
Holomycota - ps10
Fungi - ps11
Fungi_1 - ps12
Fungi_2 - ps13
Fungi_3 - ps14
Dikarya - ps15
Ascomycota - ps16
Saccharo/Pezizomycotina - ps17
Saccharomycotina - ps18
Saccharomycotina_1 - ps19
Saccharomycotina_2 - ps20
Saccharomycotina_3 - ps21
Phaff./Saccharomycetaceae - ps22
Saccharomyco./mycetaceae - ps23
Saccharomycetaceae - ps24
Saccharomycetaceae_2 - ps25
Saccharomycetaceae_3 - ps26
Kaz./Nau./Saccharomyces - ps27
Saccharomyces - ps28
Arboricola_clade - ps29
Cerevisiae_clade - ps30
Saccharomyces_cerevisiae - ps31
Phylostratum

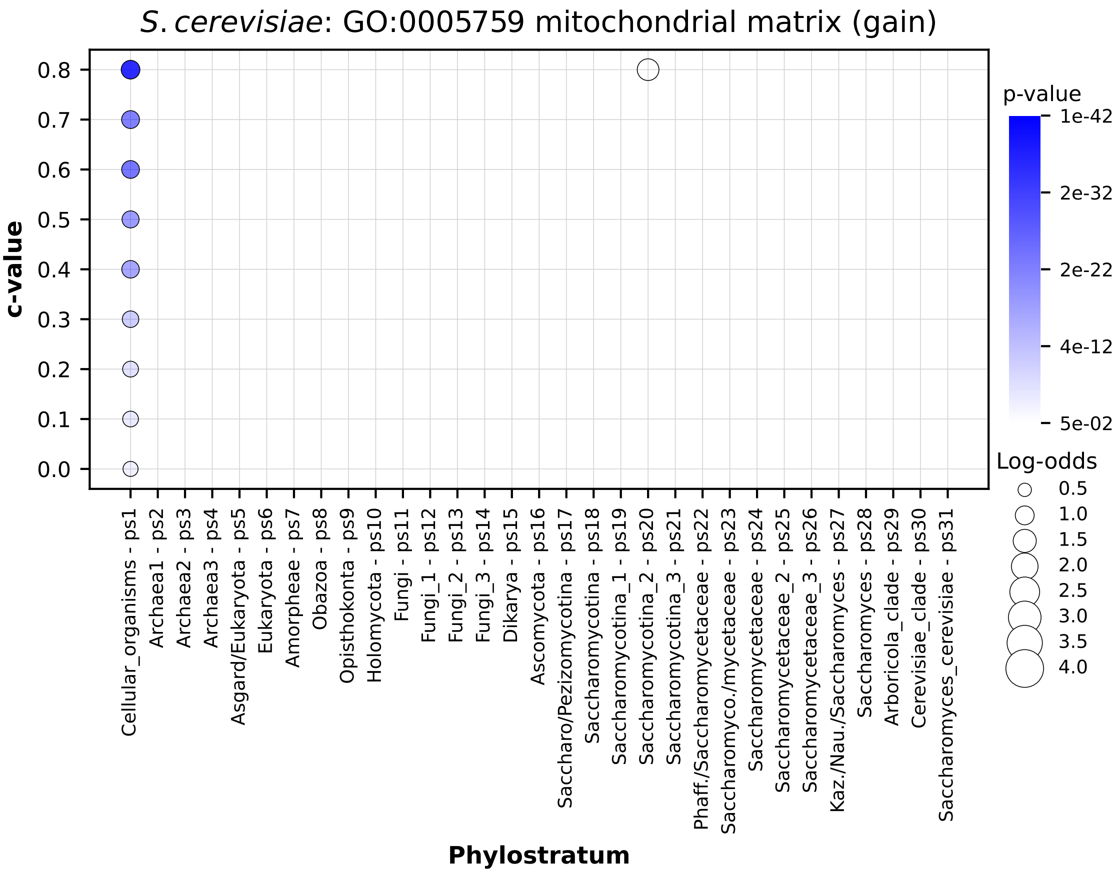
S. cerevisiae: GO:0005759 mitochondrial matrix (gain)
c-value
0.8
0.7
0.6
0.5
0.4
0.3
0.2
0.1
0.0
p-value
1e-42
2e-32
2e-22
4e-12
5e-02
Log-odds
0.5
1.0
1.5
2.0
2.5
3.0
3.5
4.0
Cellular_organisms - ps1
Archaea1 - ps2
Archaea2 - ps3
Archaea3 - ps4
Asgard/Eukaryota - ps5
Eukaryota - ps6
Amorpheae - ps7
Obazoa - ps8
Opisthokonta - ps9
Holomycota - ps10
Fungi - ps11
Fungi_1 - ps12
Fungi_2 - ps13
Fungi_3 - ps14
Dikarya - ps15
Ascomycota - ps16
Saccharo/Pezizomycotina - ps17
Saccharomycotina - ps18
Saccharomycotina_1 - ps19
Saccharomycotina_2 - ps20
Saccharomycotina_3 - ps21
Phaff./Saccharomycetaceae - ps22
Saccharomyco./mycetaceae - ps23
Saccharomycetaceae - ps24
Saccharomycetaceae_2 - ps25
Saccharomycetaceae_3 - ps26
Kaz./Nau./Saccharomyces - ps27
Saccharomyces - ps28
Arboricola_clade - ps29
Cerevisiae_clade - ps30
Saccharomyces_cerevisiae - ps31
Phylostratum

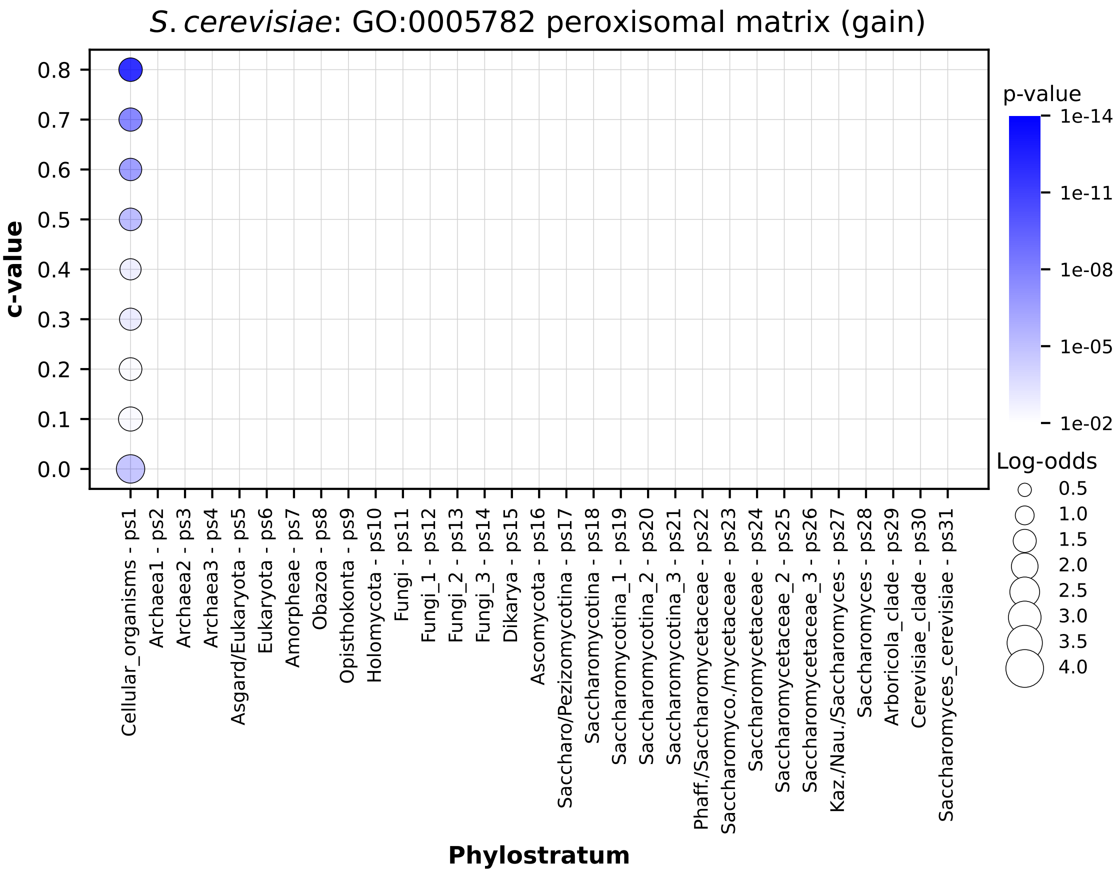

S. cerevisiae: GO:0005782 peroxisomal matrix (gain)
c-value
0.0
0.1
0.2
0.3
0.4
0.5
0.6
0.7
0.8
Phylostratum
Cellular_organisms - ps1
Archaea1 - ps2
Archaea2 - ps3
Archaea3 - ps4
Asgard/Eukaryota - ps5
Eukaryota - ps6
Amorpheae - ps7
Obazoa - ps8
Opisthokonta - ps9
Holomycota - ps10
Fungi - ps11
Fungi_1 - ps12
Fungi_2 - ps13
Fungi_3 - ps14
Dikarya - ps15
Ascomycota - ps16
Saccharo/Pezizomycotina - ps17
Saccharomycotina - ps18
Saccharomycotina_1 - ps19
Saccharomycotina_2 - ps20
Saccharomycotina_3 - ps21
Phaff./Saccharomycetaceae - ps22
Saccharomyco./mycetaceae - ps23
Saccharomycetaceae - ps24
Saccharomycetaceae_2 - ps25
Saccharomycetaceae_3 - ps26
Kaz./Nau./Saccharomyces - ps27
Saccharomyces - ps28
Arboricola_clade - ps29
Cerevisiae_clade - ps30
Saccharomyces_cerevisiae - ps31
p-value
1e-14
1e-11
1e-08
1e-05
1e-02
Log-odds
0.5
1.0
1.5
2.0
2.5
3.0
3.5
4.0

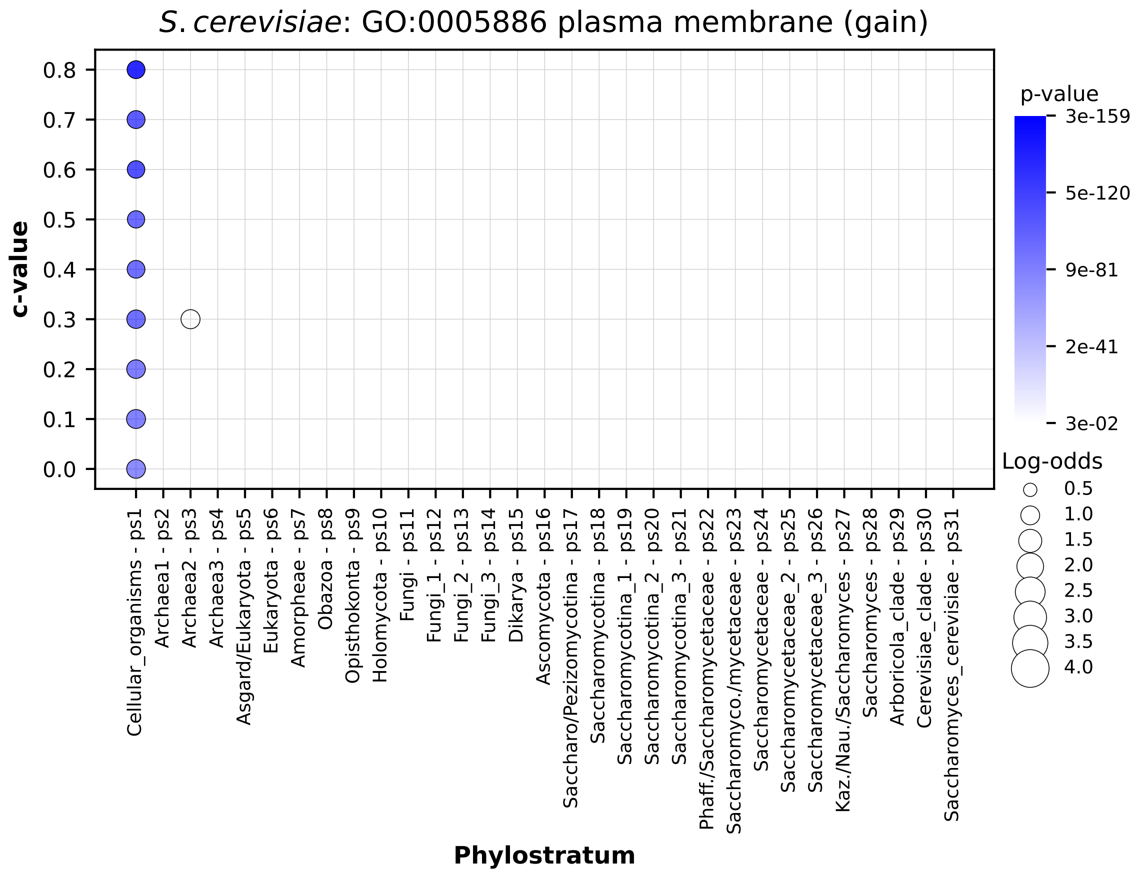
S. cerevisiae: GO:0005886 plasma membrane (gain)
c-value
0.8
0.7
0.6
0.5
0.4
0.3
0.2
0.1
0.0
Phylostratum
Cellular_organisms - ps1
Archaea1 - ps2
Archaea2 - ps3
Archaea3 - ps4
Asgard/Eukaryota - ps5
Eukaryota - ps6
Amorpheae - ps7
Obazoa - ps8
Opisthokonta - ps9
Holomycota - ps10
Fungi - ps11
Fungi_1 - ps12
Fungi_2 - ps13
Fungi_3 - ps14
Dikarya - ps15
Ascomycota - ps16
Saccharo/Pezizomycotina - ps17
Saccharomycotina - ps18
Saccharomycotina_1 - ps19
Saccharomycotina_2 - ps20
Saccharomycotina_3 - ps21
Phaff./Saccharomycetaceae - ps22
Saccharomyco./mycetaceae - ps23
Saccharomycetaceae - ps24
Saccharomycetaceae_2 - ps25
Saccharomycetaceae_3 - ps26
Kaz./Nau./Saccharomyces - ps27
Saccharomyces - ps28
Arboricola_clade - ps29
Cerevisiae_clade - ps30
Saccharomyces_cerevisiae - ps31
p-value
3e-159
5e-120
9e-81
2e-41
3e-02
Log-odds
0.5
1.0
1.5
2.0
2.5
3.0
3.5
4.0

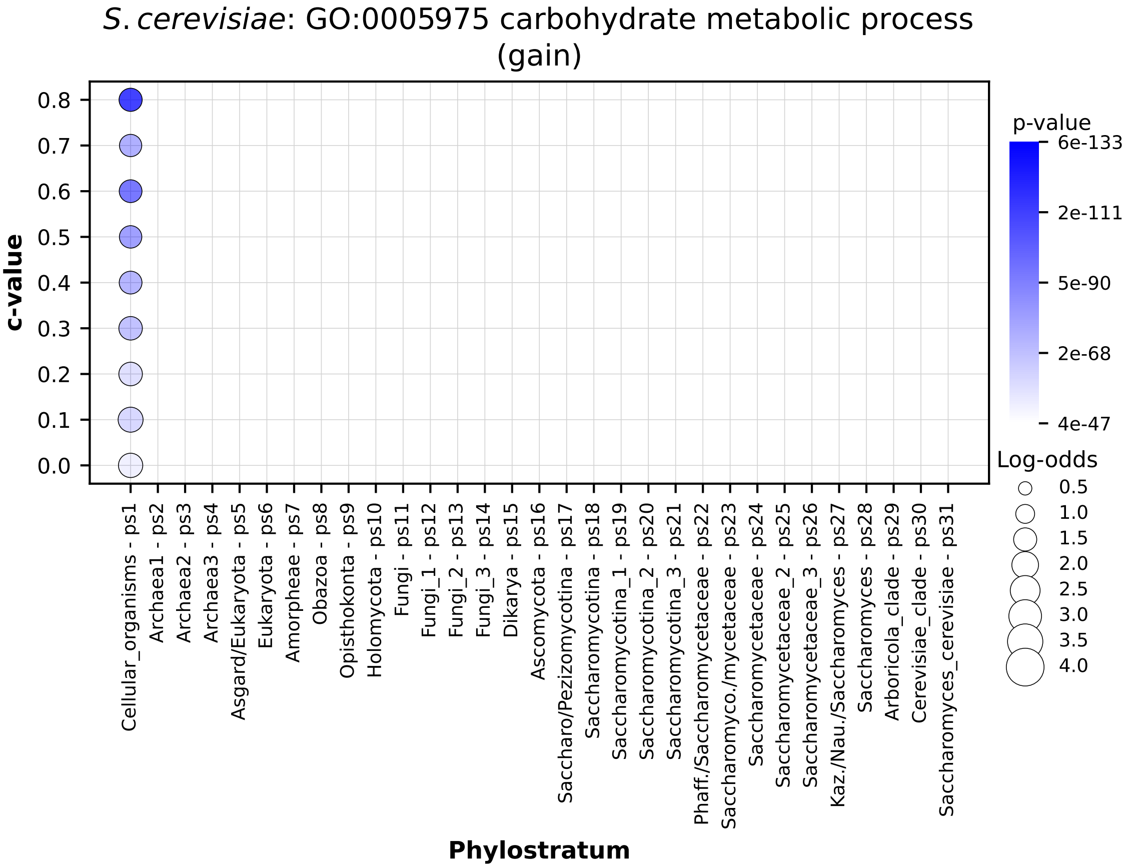

S. cerevisiae: GO:0005975 carbohydrate metabolic process (gain)
c-value
0.8
0.7
0.6
0.5
0.4
0.3
0.2
0.1
0.0
p-value
6e-133
2e-111
5e-90
2e-68
4e-47
Log-odds
0.5
1.0
1.5
2.0
2.5
3.0
3.5
4.0
Cellular_organisms - ps1
Archaea1 - ps2
Archaea2 - ps3
Archaea3 - ps4
Asgard/Eukaryota - ps5
Eukaryota - ps6
Amorpheae - ps7
Obazoa - ps8
Opisthokonta - ps9
Holomycota - ps10
Fungi - ps11
Fungi_1 - ps12
Fungi_2 - ps13
Fungi_3 - ps14
Dikarya - ps15
Ascomycota - ps16
Saccharo/Pezizomycotina - ps17
Saccharomycotina - ps18
Saccharomycotina_1 - ps19
Saccharomycotina_2 - ps20
Saccharomycotina_3 - ps21
Phaff./Saccharomycetaceae - ps22
Saccharomyco./mycetaceae - ps23
Saccharomycetaceae - ps24
Saccharomycetaceae_2 - ps25
Saccharomycetaceae_3 - ps26
Kaz./Nau./Saccharomyces - ps27
Saccharomyces - ps28
Arboricola_clade - ps29
Cerevisiae_clade - ps30
Saccharomyces_cerevisiae - ps31
Phylostratum

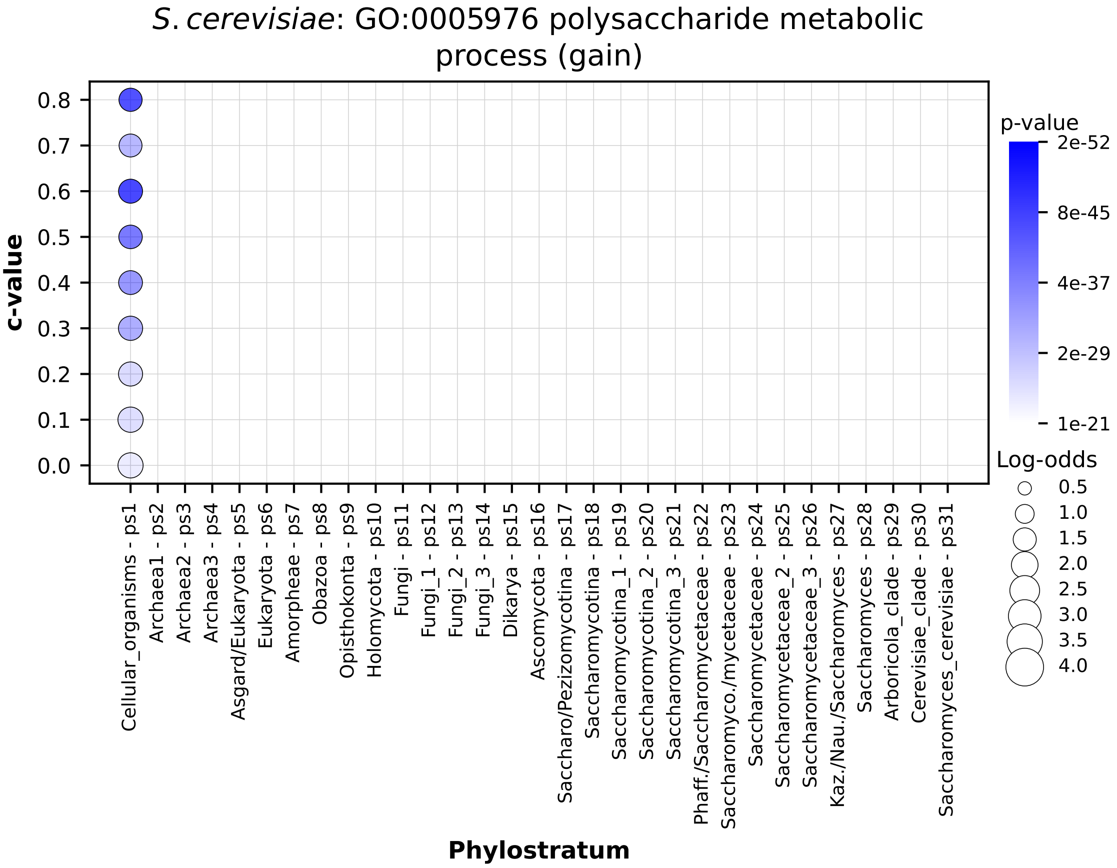
S. cerevisiae: GO:0005976 polysaccharide metabolic process (gain)
c-value
0.8
0.7
0.6
0.5
0.4
0.3
0.2
0.1
0.0
Phylostratum
Cellular_organisms - ps1
Archaea1 - ps2
Archaea2 - ps3
Archaea3 - ps4
Asgard/Eukaryota - ps5
Eukaryota - ps6
Amorpheae - ps7
Obazoa - ps8
Opisthokonta - ps9
Holomycota - ps10
Fungi - ps11
Fungi_1 - ps12
Fungi_2 - ps13
Fungi_3 - ps14
Dikarya - ps15
Ascomycota - ps16
Saccharo/Pezizomycotina - ps17
Saccharomycotina - ps18
Saccharomycotina_1 - ps19
Saccharomycotina_2 - ps20
Saccharomycotina_3 - ps21
Phaff./Saccharomycetaceae - ps22
Saccharomyco./mycetaceae - ps23
Saccharomycetaceae - ps24
Saccharomycetaceae_2 - ps25
Saccharomycetaceae_3 - ps26
Kaz./Nau./Saccharomyces - ps27
Saccharomyces - ps28
Arboricola_clade - ps29
Cerevisiae_clade - ps30
Saccharomyces_cerevisiae - ps31
p-value
2e-52
8e-45
4e-37
2e-29
1e-21
Log-odds
0.5
1.0
1.5
2.0
2.5
3.0
3.5
4.0

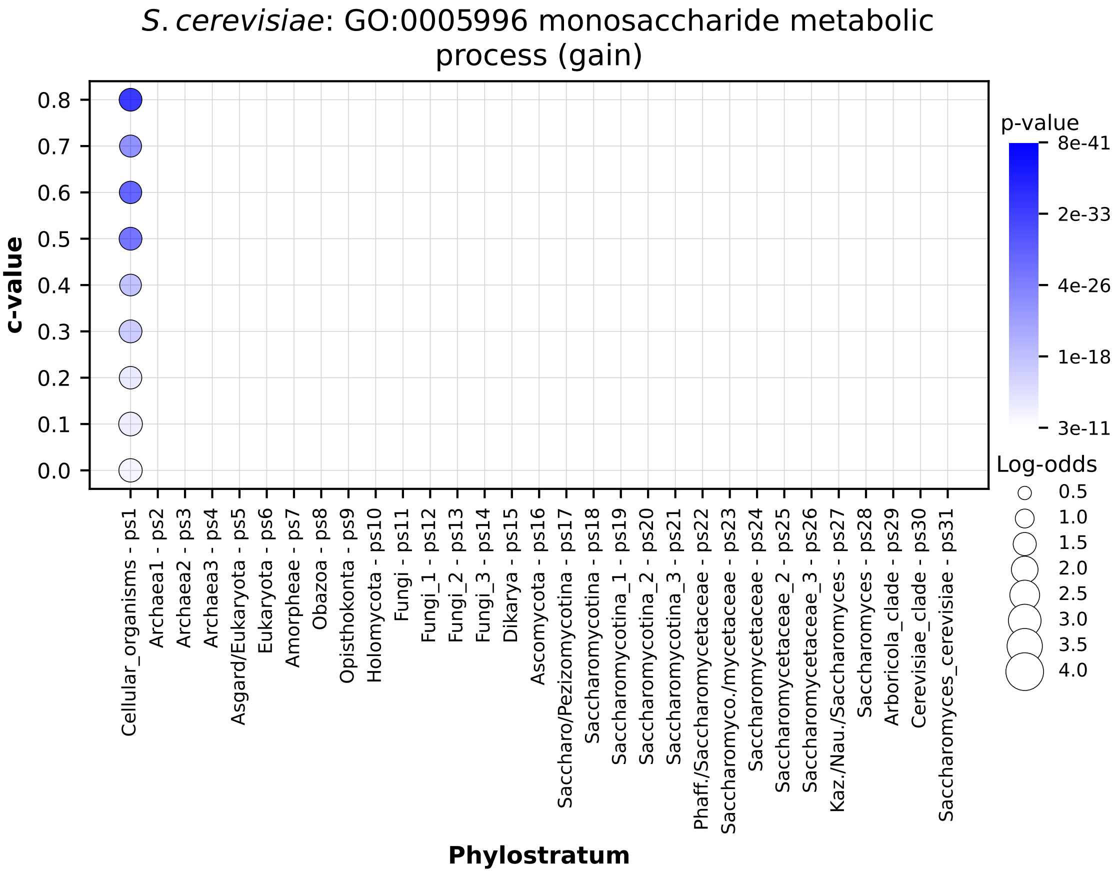

S. cerevisiae: GO:0005996 monosaccharide metabolic process (gain)
c-value
0.8
0.7
0.6
0.5
0.4
0.3
0.2
0.1
0.0
p-value
8e-41
2e-33
4e-26
1e-18
3e-11
Log-odds
0.5
1.0
1.5
2.0
2.5
3.0
3.5
4.0
Cellular_organisms - ps1
Archaea1 - ps2
Archaea2 - ps3
Archaea3 - ps4
Asgard/Eukaryota - ps5
Eukaryota - ps6
Amorpheae - ps7
Obazoa - ps8
Opisthokonta - ps9
Holomycota - ps10
Fungi - ps11
Fungi_1 - ps12
Fungi_2 - ps13
Fungi_3 - ps14
Dikarya - ps15
Ascomycota - ps16
Saccharo/Pezizomycotina - ps17
Saccharomycotina - ps18
Saccharomycotina_1 - ps19
Saccharomycotina_2 - ps20
Saccharomycotina_3 - ps21
Phaff./Saccharomycetaceae - ps22
Saccharomyco./mycetaceae - ps23
Saccharomycetaceae - ps24
Saccharomycetaceae_2 - ps25
Saccharomycetaceae_3 - ps26
Kaz./Nau./Saccharomyces - ps27
Saccharomyces - ps28
Arboricola_clade - ps29
Cerevisiae_clade - ps30
Saccharomyces_cerevisiae - ps31
Phylostratum

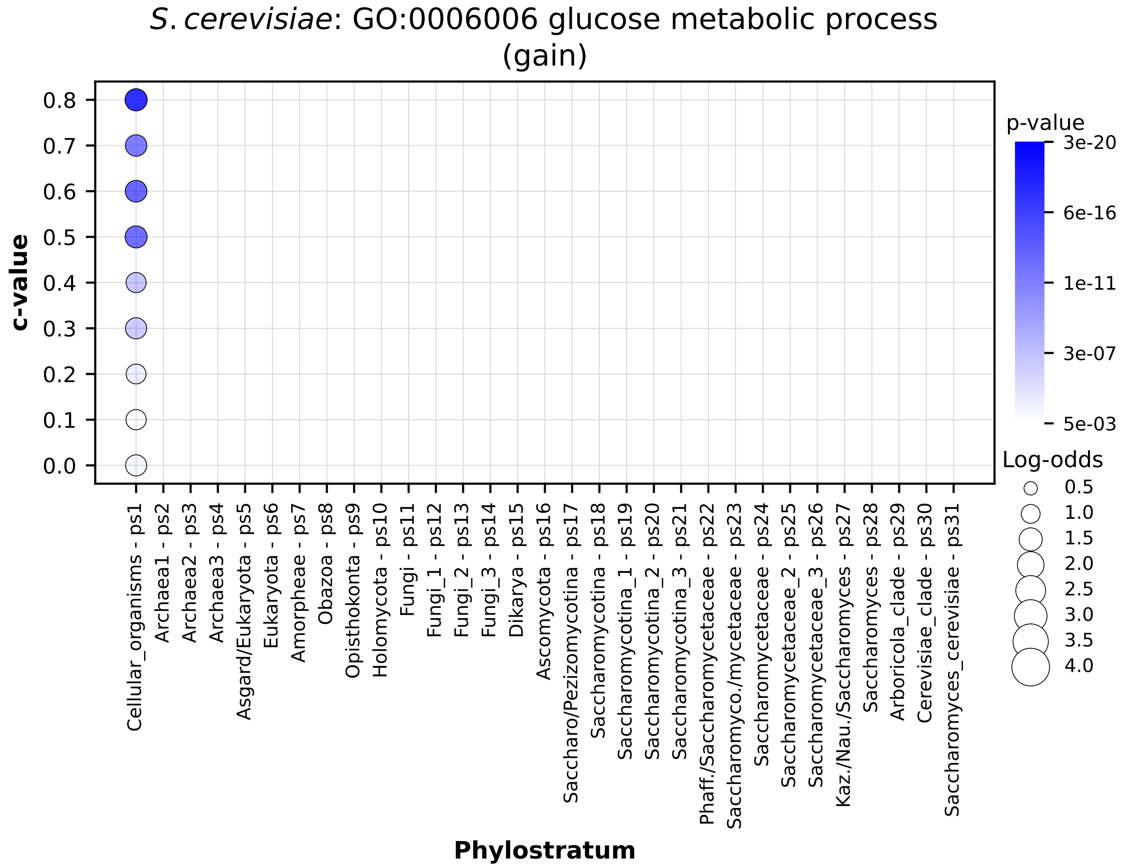

S. cerevisiae: GO:0006006 glucose metabolic process
(gain)
c-value
0.0
0.1
0.2
0.3
0.4
0.5
0.6
0.7
0.8
p-value
3e-20
6e-16
1e-11
3e-07
5e-03
Log-odds
0.5
1.0
1.5
2.0
2.5
3.0
3.5
4.0
Cellular_organisms - ps1
Archaea1 - ps2
Archaea2 - ps3
Archaea3 - ps4
Asgard/Eukaryota - ps5
Eukaryota - ps6
Amorpheae - ps7
Obazoa - ps8
Opisthokonta - ps9
Holomycota - ps10
Fungi - ps11
Fungi_1 - ps12
Fungi_2 - ps13
Fungi_3 - ps14
Dikarya - ps15
Ascomycota - ps16
Saccharo/Pezizomycotina - ps17
Saccharomycotina - ps18
Saccharomycotina_1 - ps19
Saccharomycotina_2 - ps20
Saccharomycotina_3 - ps21
Phaff./Saccharomycetaceae - ps22
Saccharomyco./mycetaceae - ps23
Saccharomycetaceae - ps24
Saccharomycetaceae_2 - ps25
Saccharomycetaceae_3 - ps26
Kaz./Nau./Saccharomyces - ps27
Saccharomyces - ps28
Arboricola_clade - ps29
Cerevisiae_clade - ps30
Saccharomyces_cerevisiae - ps31
Phylostratum

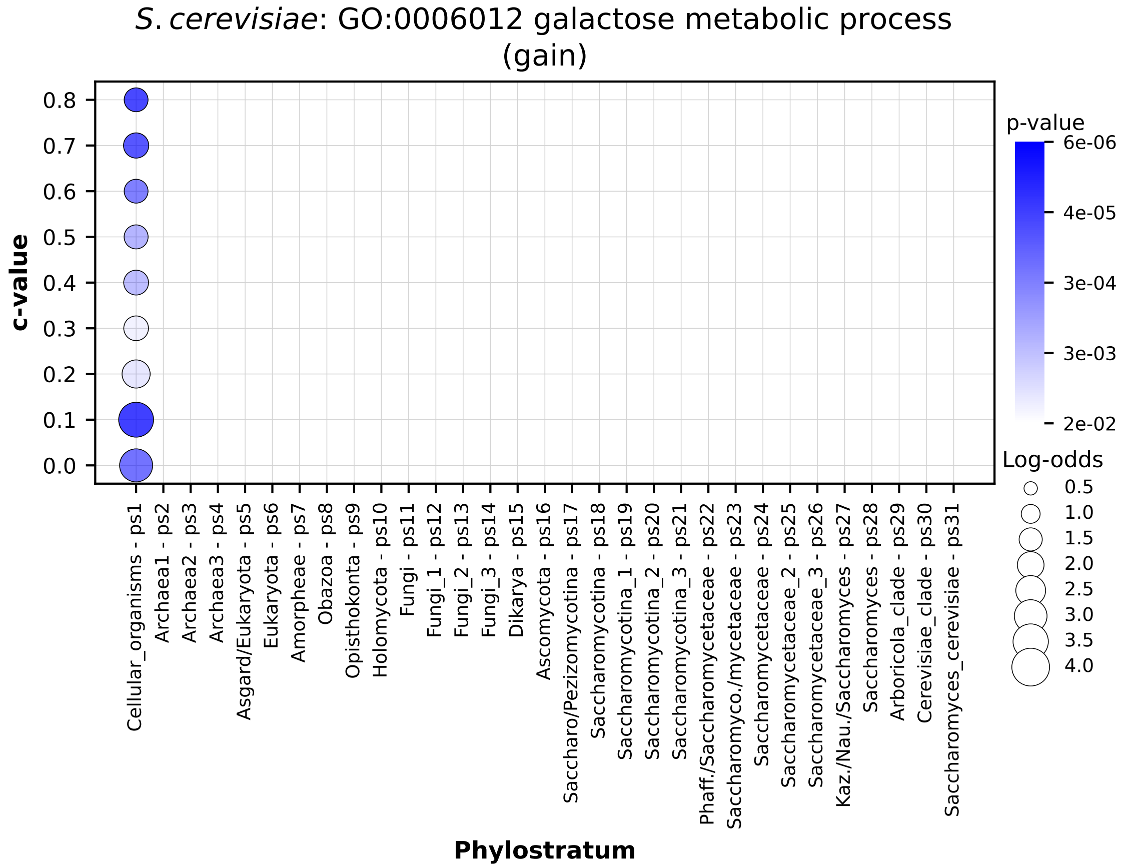

S. cerevisiae: GO:0006012 galactose metabolic process (gain)
c-value
0.8
0.7
0.6
0.5
0.4
0.3
0.2
0.1
0.0
p-value
6e-06
4e-05
3e-04
3e-03
2e-02
Log-odds
0.5
1.0
1.5
2.0
2.5
3.0
3.5
4.0
Cellular_organisms - ps1
Archaea1 - ps2
Archaea2 - ps3
Archaea3 - ps4
Asgard/Eukaryota - ps5
Eukaryota - ps6
Amorpheae - ps7
Obazoa - ps8
Opisthokonta - ps9
Holomycota - ps10
Fungi - ps11
Fungi_1 - ps12
Fungi_2 - ps13
Fungi_3 - ps14
Dikarya - ps15
Ascomycota - ps16
Saccharo/Pezizomycotina - ps17
Saccharomycotina - ps18
Saccharomycotina_1 - ps19
Saccharomycotina_2 - ps20
Saccharomycotina_3 - ps21
Phaff./Saccharomycetaceae - ps22
Saccharomyco./mycetaceae - ps23
Saccharomycetaceae - ps24
Saccharomycetaceae_2 - ps25
Saccharomycetaceae_3 - ps26
Kaz./Nau./Saccharomyces - ps27
Saccharomyces - ps28
Arboricola_clade - ps29
Cerevisiae_clade - ps30
Saccharomyces_cerevisiae - ps31
Phylostratum

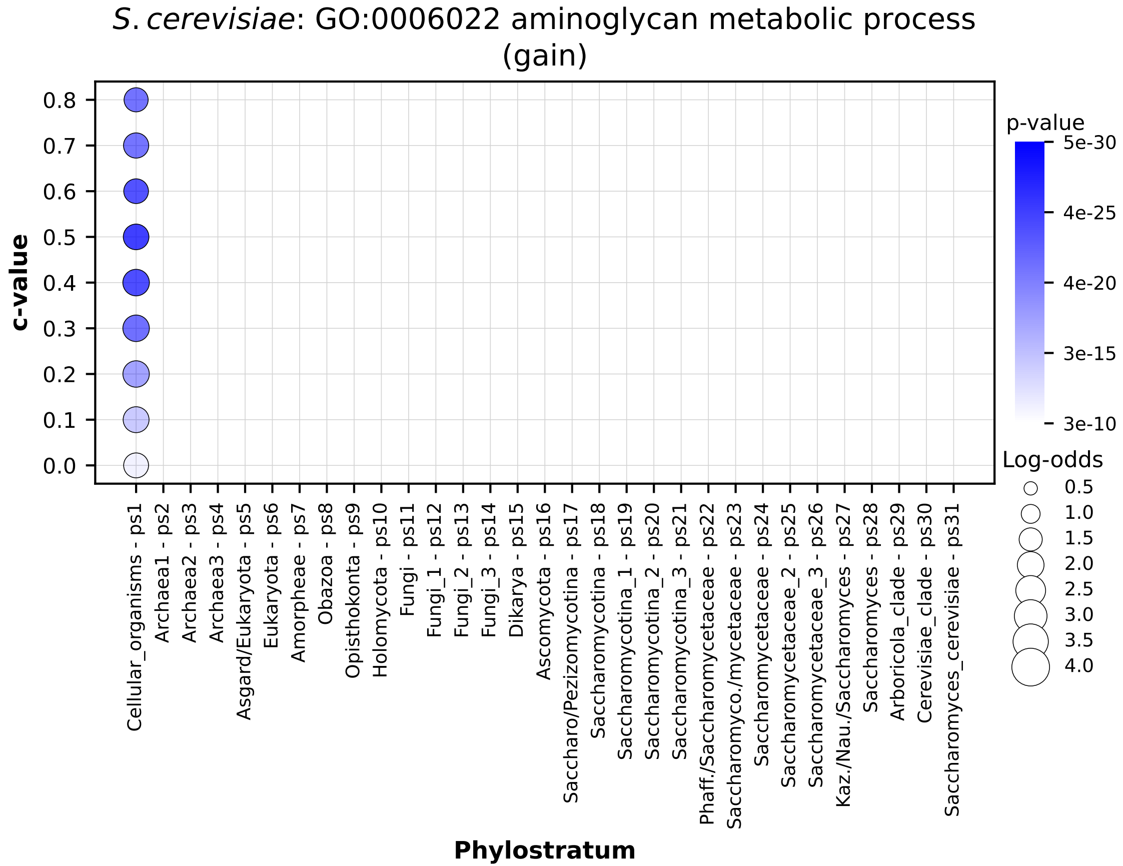
S. cerevisiae: GO:0006022 aminoglycan metabolic process
(gain)
c-value
0.8
0.7
0.6
0.5
0.4
0.3
0.2
0.1
0.0
p-value
5e-30
4e-25
4e-20
3e-15
3e-10
Log-odds
0.5
1.0
1.5
2.0
2.5
3.0
3.5
4.0
Cellular_organisms - ps1
Archaea1 - ps2
Archaea2 - ps3
Archaea3 - ps4
Asgard/Eukaryota - ps5
Eukaryota - ps6
Amorpheae - ps7
Obazoa - ps8
Opisthokonta - ps9
Holomycota - ps10
Fungi - ps11
Fungi_1 - ps12
Fungi_2 - ps13
Fungi_3 - ps14
Dikarya - ps15
Ascomycota - ps16
Saccharo/Pezizomycotina - ps17
Saccharomycotina - ps18
Saccharomycotina_1 - ps19
Saccharomycotina_2 - ps20
Saccharomycotina_3 - ps21
Phaff./Saccharomycetaceae - ps22
Saccharomyco./mycetaceae - ps23
Saccharomycetaceae - ps24
Saccharomycetaceae_2 - ps25
Saccharomycetaceae_3 - ps26
Kaz./Nau./Saccharomyces - ps27
Saccharomyces - ps28
Arboricola_clade - ps29
Cerevisiae_clade - ps30
Saccharomyces_cerevisiae - ps31
Phylostratum

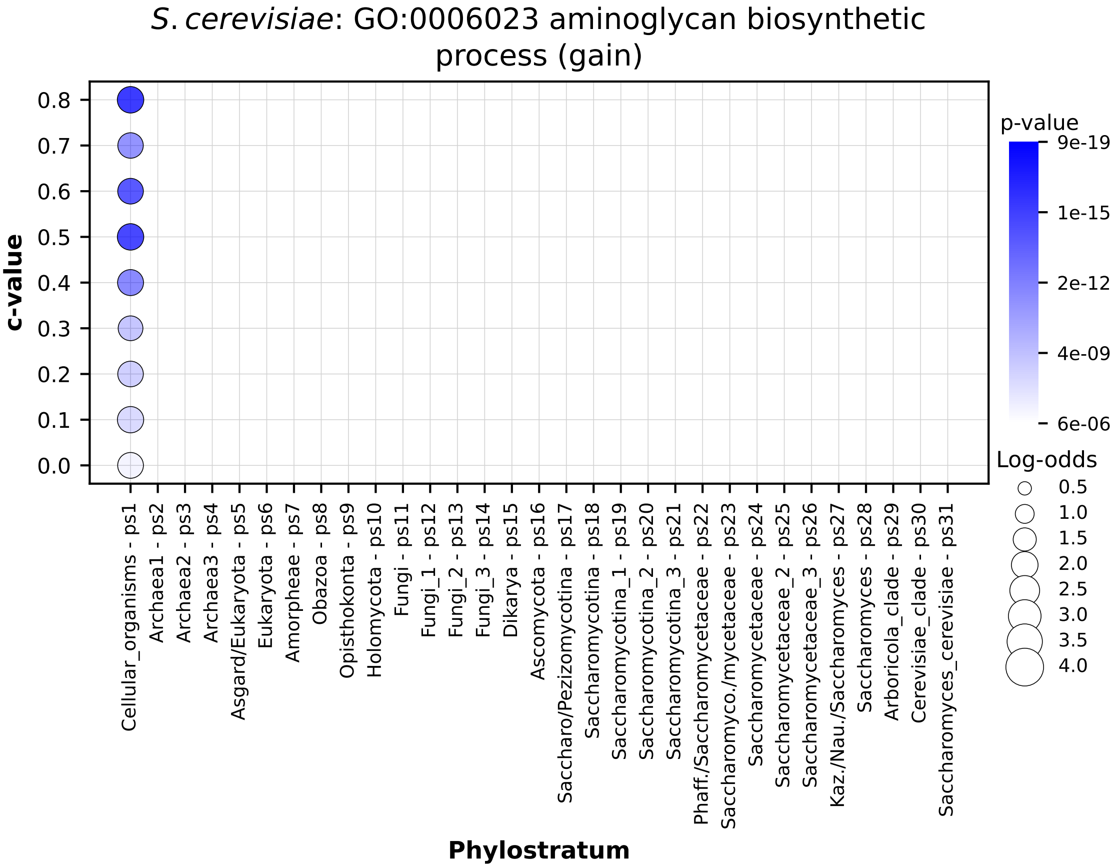

S. cerevisiae: GO:0006023 aminoglycan biosynthetic process (gain)
c-value
0.8
0.7
0.6
0.5
0.4
0.3
0.2
0.1
0.0
p-value
9e-19
1e-15
2e-12
4e-09
6e-06
Log-odds
0.5
1.0
1.5
2.0
2.5
3.0
3.5
4.0
Cellular_organisms - ps1
Archaea1 - ps2
Archaea2 - ps3
Archaea3 - ps4
Asgard/Eukaryota - ps5
Eukaryota - ps6
Amorpheae - ps7
Obazoa - ps8
Opisthokonta - ps9
Holomycota - ps10
Fungi - ps11
Fungi_1 - ps12
Fungi_2 - ps13
Fungi_3 - ps14
Dikarya - ps15
Ascomycota - ps16
Saccharo/Pezizomycotina - ps17
Saccharomycotina - ps18
Saccharomycotina_1 - ps19
Saccharomycotina_2 - ps20
Saccharomycotina_3 - ps21
Phaff./Saccharomycetaceae - ps22
Saccharomyco./mycetaceae - ps23
Saccharomycetaceae - ps24
Saccharomycetaceae_2 - ps25
Saccharomycetaceae_3 - ps26
Kaz./Nau./Saccharomyces - ps27
Saccharomyces - ps28
Arboricola_clade - ps29
Cerevisiae_clade - ps30
Saccharomyces_cerevisiae - ps31
Phylostratum

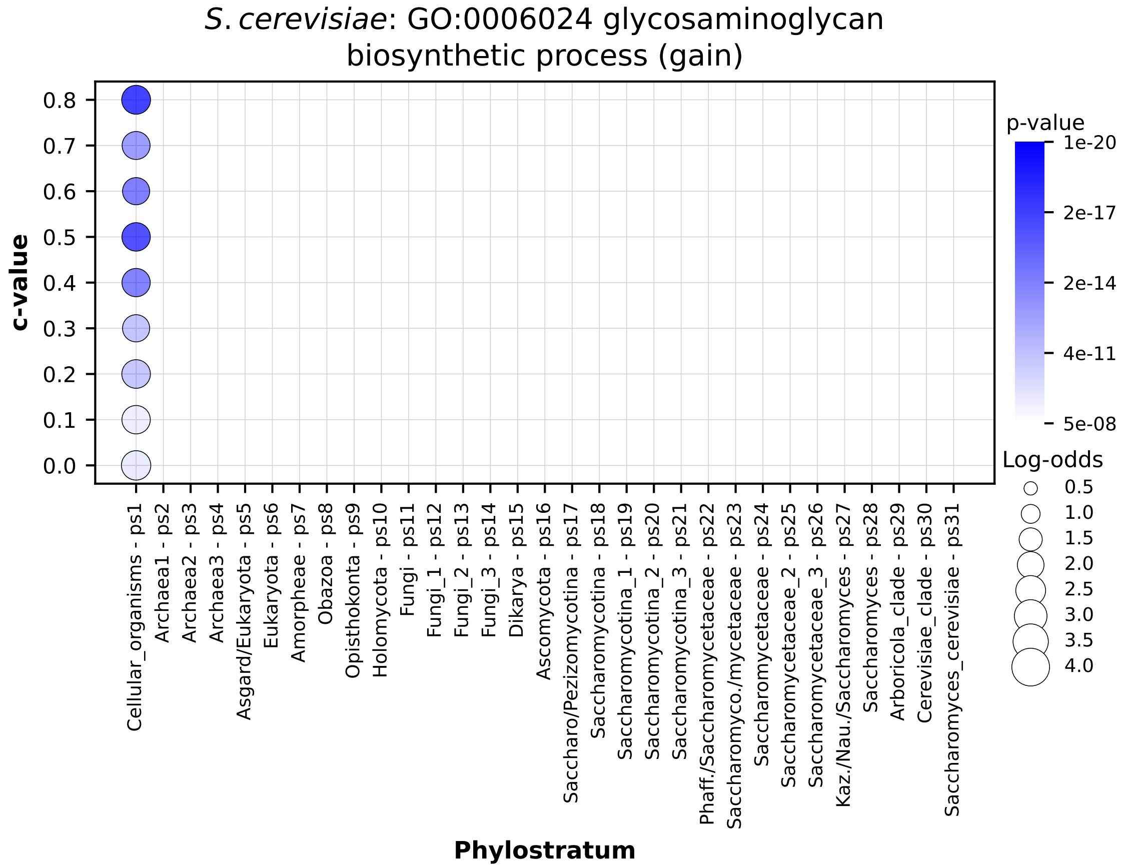

S. cerevisiae: GO:0006024 glycosaminoglycan biosynthetic process (gain)
c-value
0.8
0.7
0.6
0.5
0.4
0.3
0.2
0.1
0.0
Phylostratum
Cellular_organisms - ps1
Archaea1 - ps2
Archaea2 - ps3
Archaea3 - ps4
Asgard/Eukaryota - ps5
Eukaryota - ps6
Amorpheae - ps7
Obazoa - ps8
Opisthokonta - ps9
Holomycota - ps10
Fungi - ps11
Fungi_1 - ps12
Fungi_2 - ps13
Fungi_3 - ps14
Dikarya - ps15
Ascomycota - ps16
Saccharo/Pezizomycotina - ps17
Saccharomycotina - ps18
Saccharomycotina_1 - ps19
Saccharomycotina_2 - ps20
Saccharomycotina_3 - ps21
Phaff./Saccharomycetaceae - ps22
Saccharomyco./mycetaceae - ps23
Saccharomycetaceae - ps24
Saccharomycetaceae_2 - ps25
Saccharomycetaceae_3 - ps26
Kaz./Nau./Saccharomyces - ps27
Saccharomyces - ps28
Arboricola_clade - ps29
Cerevisiae_clade - ps30
Saccharomyces_cerevisiae - ps31
p-value
1e-20
2e-17
2e-14
4e-11
5e-08
Log-odds
0.5
1.0
1.5
2.0
2.5
3.0
3.5
4.0

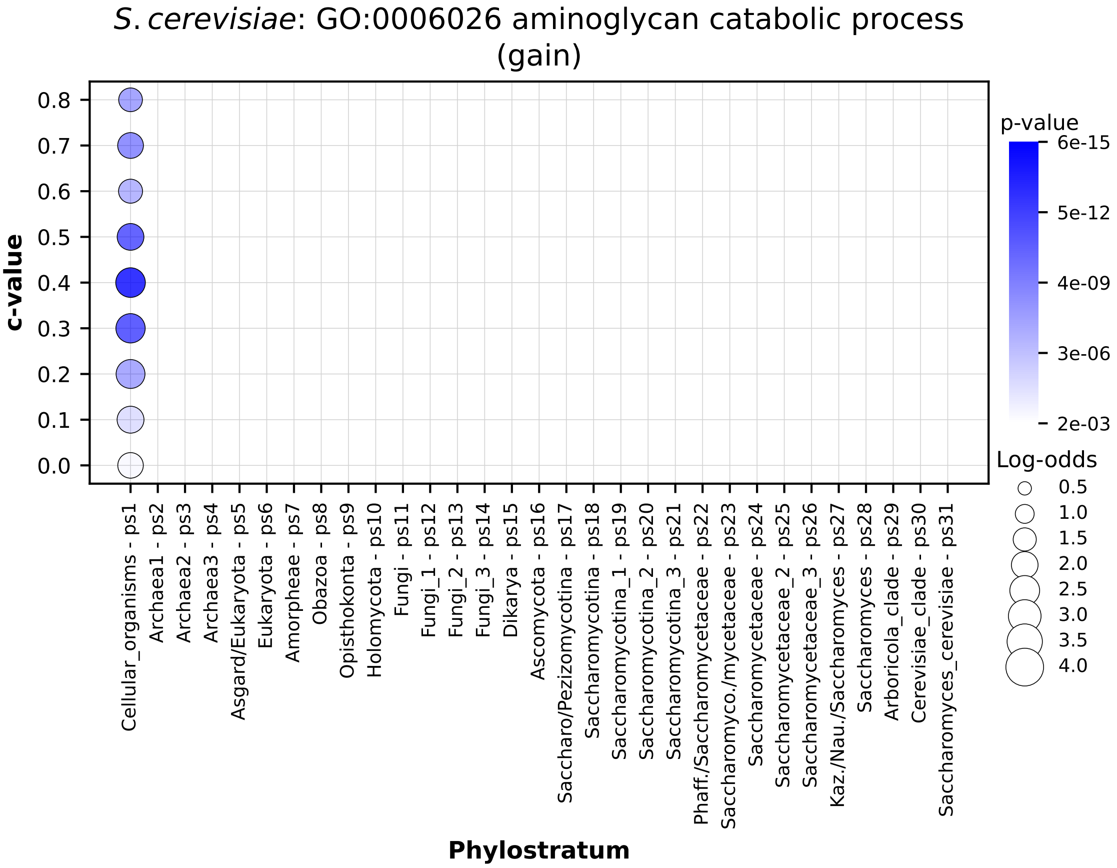
S. cerevisiae: GO:0006026 aminoglycan catabolic process
(gain)
c-value
0.0
0.1
0.2
0.3
0.4
0.5
0.6
0.7
0.8
p-value
6e-15
5e-12
4e-09
3e-06
2e-03
Log-odds
0.5
1.0
1.5
2.0
2.5
3.0
3.5
4.0
Cellular_organisms - ps1
Archaea1 - ps2
Archaea2 - ps3
Archaea3 - ps4
Asgard/Eukaryota - ps5
Eukaryota - ps6
Amorpheae - ps7
Obazoa - ps8
Opisthokonta - ps9
Holomycota - ps10
Fungi - ps11
Fungi_1 - ps12
Fungi_2 - ps13
Fungi_3 - ps14
Dikarya - ps15
Ascomycota - ps16
Saccharo/Pezizomycotina - ps17
Saccharomycotina - ps18
Saccharomycotina_1 - ps19
Saccharomycotina_2 - ps20
Saccharomycotina_3 - ps21
Phaff./Saccharomycetaceae - ps22
Saccharomyco./mycetaceae - ps23
Saccharomycetaceae - ps24
Saccharomycetaceae_2 - ps25
Saccharomycetaceae_3 - ps26
Kaz./Nau./Saccharomyces - ps27
Saccharomyces - ps28
Arboricola_clade - ps29
Cerevisiae_clade - ps30
Saccharomyces_cerevisiae - ps31
Phylostratum

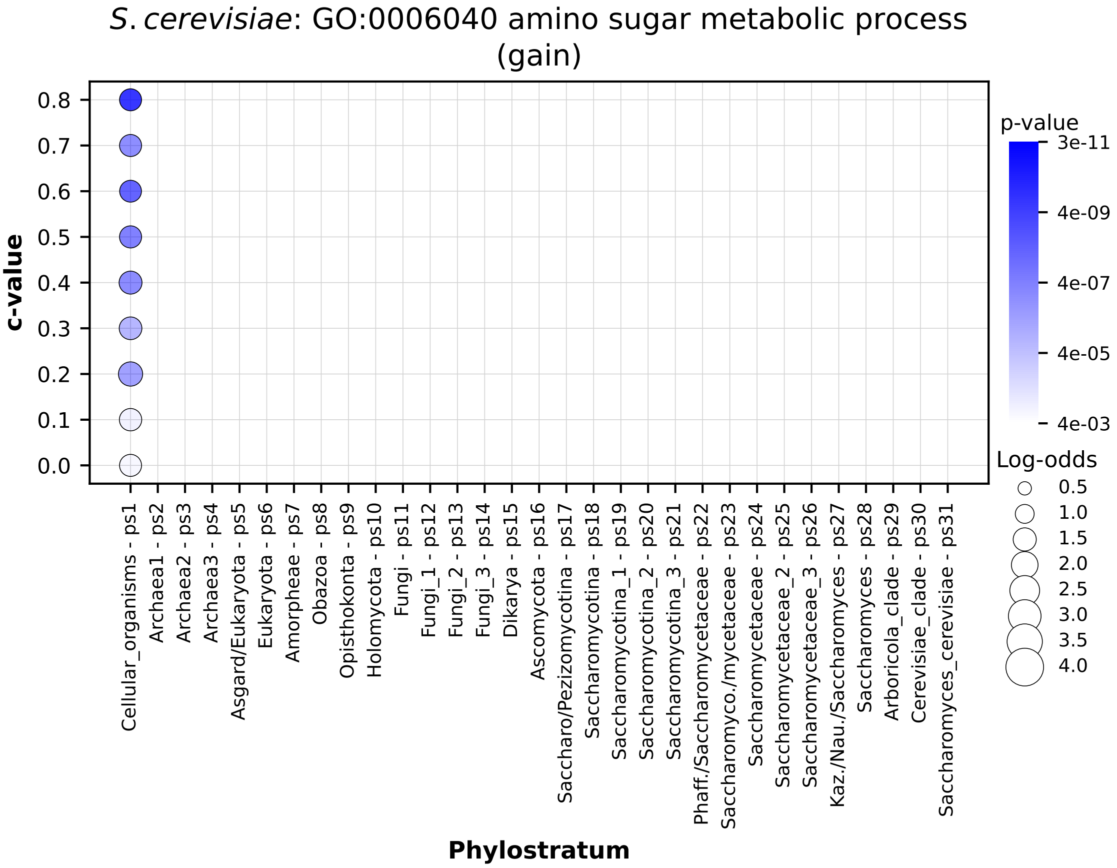
S. cerevisiae: GO:0006040 amino sugar metabolic process
(gain)
c-value
0.8
0.7
0.6
0.5
0.4
0.3
0.2
0.1
0.0
p-value
3e-11
4e-09
4e-07
4e-05
4e-03
Log-odds
0.5
1.0
1.5
2.0
2.5
3.0
3.5
4.0
Cellular_organisms - ps1
Archaea1 - ps2
Archaea2 - ps3
Archaea3 - ps4
Asgard/Eukaryota - ps5
Eukaryota - ps6
Amorpheae - ps7
Obazoa - ps8
Opisthokonta - ps9
Holomycota - ps10
Fungi - ps11
Fungi_1 - ps12
Fungi_2 - ps13
Fungi_3 - ps14
Dikarya - ps15
Ascomycota - ps16
Saccharo/Pezizomycotina - ps17
Saccharomycotina - ps18
Saccharomycotina_1 - ps19
Saccharomycotina_2 - ps20
Saccharomycotina_3 - ps21
Phaff./Saccharomycetaceae - ps22
Saccharomyco./mycetaceae - ps23
Saccharomycetaceae - ps24
Saccharomycetaceae_2 - ps25
Saccharomycetaceae_3 - ps26
Kaz./Nau./Saccharomyces - ps27
Saccharomyces - ps28
Arboricola_clade - ps29
Cerevisiae_clade - ps30
Saccharomyces_cerevisiae - ps31
Phylostratum

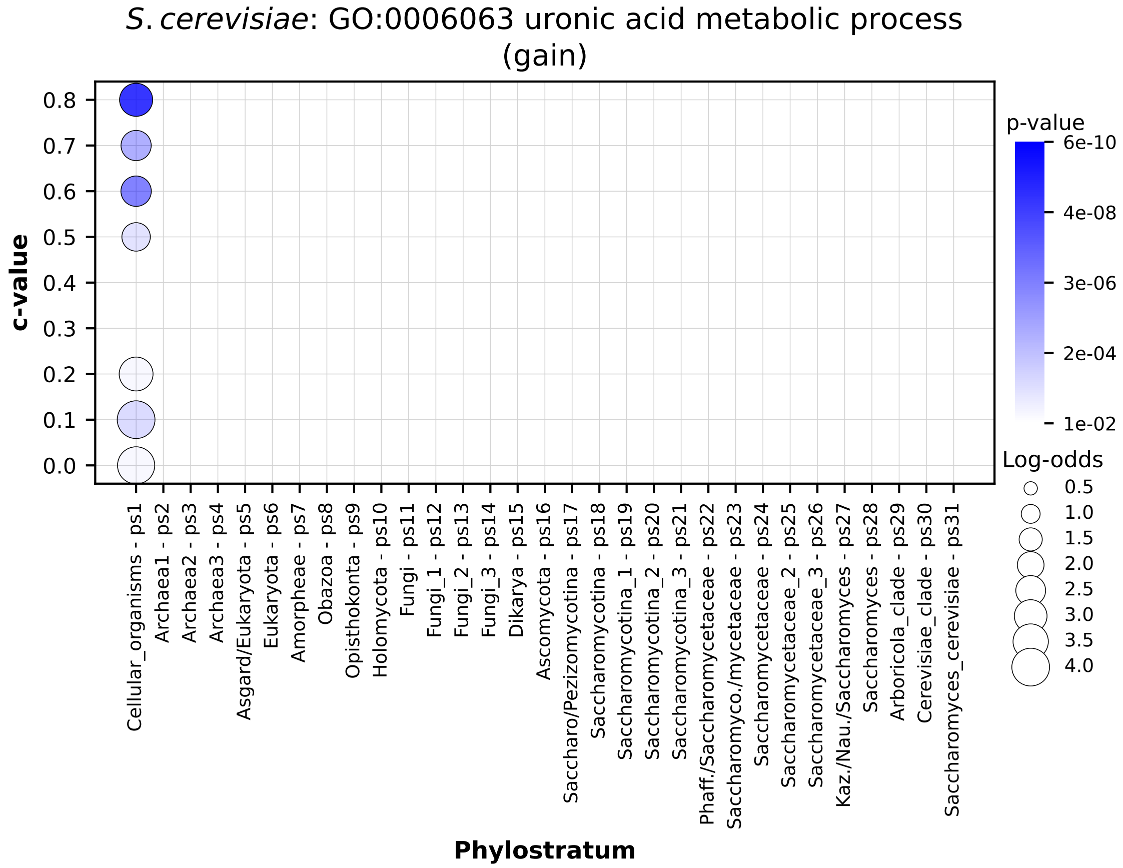

S. cerevisiae: GO:0006063 uronic acid metabolic process
(gain)
c-value
0.0
0.1
0.2
0.3
0.4
0.5
0.6
0.7
0.8
p-value
6e-10
4e-08
3e-06
2e-04
1e-02
Log-odds
0.5
1.0
1.5
2.0
2.5
3.0
3.5
4.0
Cellular_organisms - ps1
Archaea1 - ps2
Archaea2 - ps3
Archaea3 - ps4
Asgard/Eukaryota - ps5
Eukaryota - ps6
Amorpheae - ps7
Obazoa - ps8
Opisthokonta - ps9
Holomycota - ps10
Fungi - ps11
Fungi_1 - ps12
Fungi_2 - ps13
Fungi_3 - ps14
Dikarya - ps15
Ascomycota - ps16
Saccharo/Pezizomycotina - ps17
Saccharomycotina - ps18
Saccharomycotina_1 - ps19
Saccharomycotina_2 - ps20
Saccharomycotina_3 - ps21
Phaff./Saccharomycetaceae - ps22
Saccharomyco./mycetaceae - ps23
Saccharomycetaceae - ps24
Saccharomycetaceae_2 - ps25
Saccharomycetaceae_3 - ps26
Kaz./Nau./Saccharomyces - ps27
Saccharomyces - ps28
Arboricola_clade - ps29
Cerevisiae_clade - ps30
Saccharomyces_cerevisiae - ps31
Phylostratum

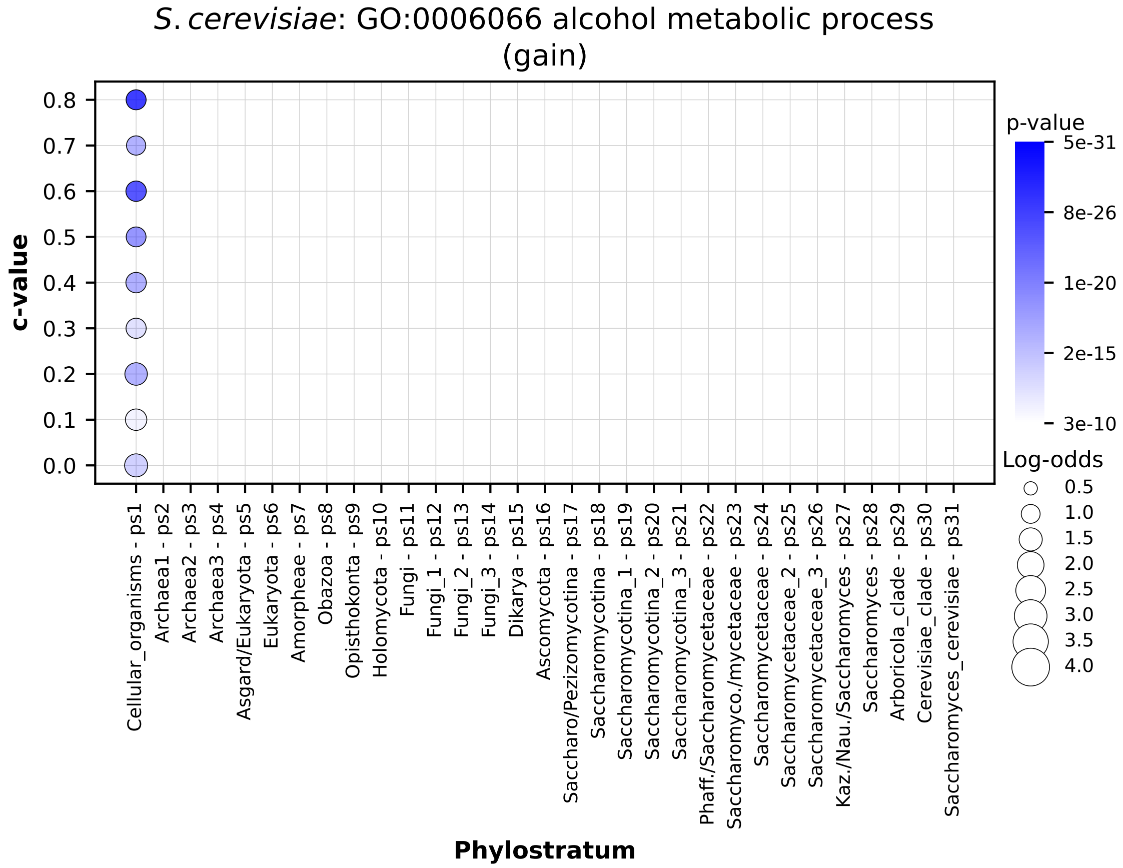

S. cerevisiae: GO:0006066 alcohol metabolic process
(gain)
c-value
0.8
0.7
0.6
0.5
0.4
0.3
0.2
0.1
0.0
Phylostratum
Cellular_organisms - ps1
Archaea1 - ps2
Archaea2 - ps3
Archaea3 - ps4
Asgard/Eukaryota - ps5
Eukaryota - ps6
Amorpheae - ps7
Obazoa - ps8
Opisthokonta - ps9
Holomycota - ps10
Fungi - ps11
Fungi_1 - ps12
Fungi_2 - ps13
Fungi_3 - ps14
Dikarya - ps15
Ascomycota - ps16
Saccharo/Pezizomycotina - ps17
Saccharomycotina - ps18
Saccharomycotina_1 - ps19
Saccharomycotina_2 - ps20
Saccharomycotina_3 - ps21
Phaff./Saccharomycetaceae - ps22
Saccharomyco./mycetaceae - ps23
Saccharomycetaceae - ps24
Saccharomycetaceae_2 - ps25
Saccharomycetaceae_3 - ps26
Kaz./Nau./Saccharomyces - ps27
Saccharomyces - ps28
Arboricola_clade - ps29
Cerevisiae_clade - ps30
Saccharomyces_cerevisiae - ps31
p-value
5e-31
8e-26
1e-20
2e-15
3e-10
Log-odds
0.5
1.0
1.5
2.0
2.5
3.0
3.5
4.0

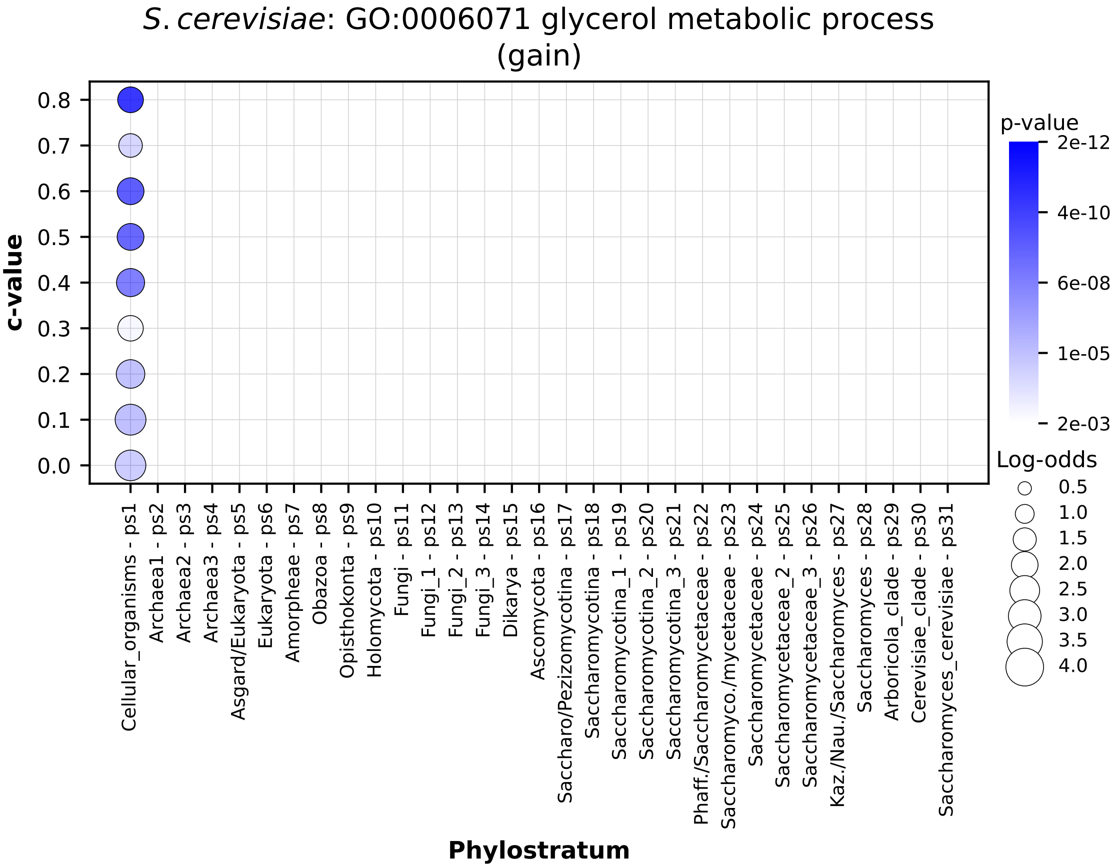

S. cerevisiae: GO:0006071 glycerol metabolic process (gain)
c-value
0.8
0.7
0.6
0.5
0.4
0.3
0.2
0.1
0.0
p-value
2e-12
4e-10
6e-08
1e-05
2e-03
Log-odds
0.5
1.0
1.5
2.0
2.5
3.0
3.5
4.0
Cellular_organisms - ps1
Archaea1 - ps2
Archaea2 - ps3
Archaea3 - ps4
Asgard/Eukaryota - ps5
Eukaryota - ps6
Amorpheae - ps7
Obazoa - ps8
Opisthokonta - ps9
Holomycota - ps10
Fungi - ps11
Fungi_1 - ps12
Fungi_2 - ps13
Fungi_3 - ps14
Dikarya - ps15
Ascomycota - ps16
Saccharo/Pezizomycotina - ps17
Saccharomycotina - ps18
Saccharomycotina_1 - ps19
Saccharomycotina_2 - ps20
Saccharomycotina_3 - ps21
Phaff./Saccharomycetaceae - ps22
Saccharomyco./mycetaceae - ps23
Saccharomycetaceae - ps24
Saccharomycetaceae_2 - ps25
Saccharomycetaceae_3 - ps26
Kaz./Nau./Saccharomyces - ps27
Saccharomyces - ps28
Arboricola_clade - ps29
Cerevisiae_clade - ps30
Saccharomyces_cerevisiae - ps31
Phylostratum

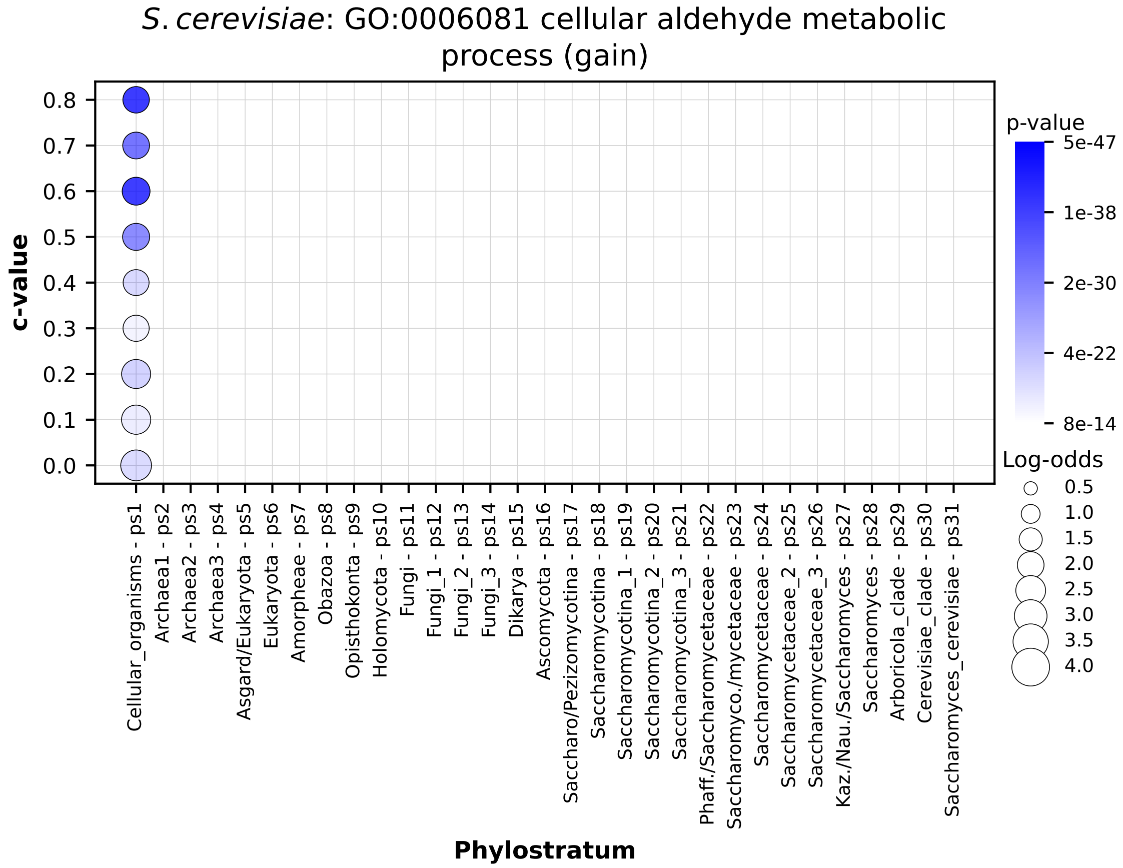

S. cerevisiae: GO:0006081 cellular aldehyde metabolic process (gain)
c-value
0.8
0.7
0.6
0.5
0.4
0.3
0.2
0.1
0.0
Phylostratum
Cellular_organisms - ps1
Archaea1 - ps2
Archaea2 - ps3
Archaea3 - ps4
Asgard/Eukaryota - ps5
Eukaryota - ps6
Amorpheae - ps7
Obazoa - ps8
Opisthokonta - ps9
Holomycota - ps10
Fungi - ps11
Fungi_1 - ps12
Fungi_2 - ps13
Fungi_3 - ps14
Dikarya - ps15
Ascomycota - ps16
Saccharo/Pezizomycotina - ps17
Saccharomycotina - ps18
Saccharomycotina_1 - ps19
Saccharomycotina_2 - ps20
Saccharomycotina_3 - ps21
Phaff./Saccharomycetaceae - ps22
Saccharomyco./mycetaceae - ps23
Saccharomycetaceae - ps24
Saccharomycetaceae_2 - ps25
Saccharomycetaceae_3 - ps26
Kaz./Nau./Saccharomyces - ps27
Saccharomyces - ps28
Arboricola_clade - ps29
Cerevisiae_clade - ps30
Saccharomyces_cerevisiae - ps31
p-value
5e-47
1e-38
2e-30
4e-22
8e-14
Log-odds
0.5
1.0
1.5
2.0
2.5
3.0
3.5
4.0

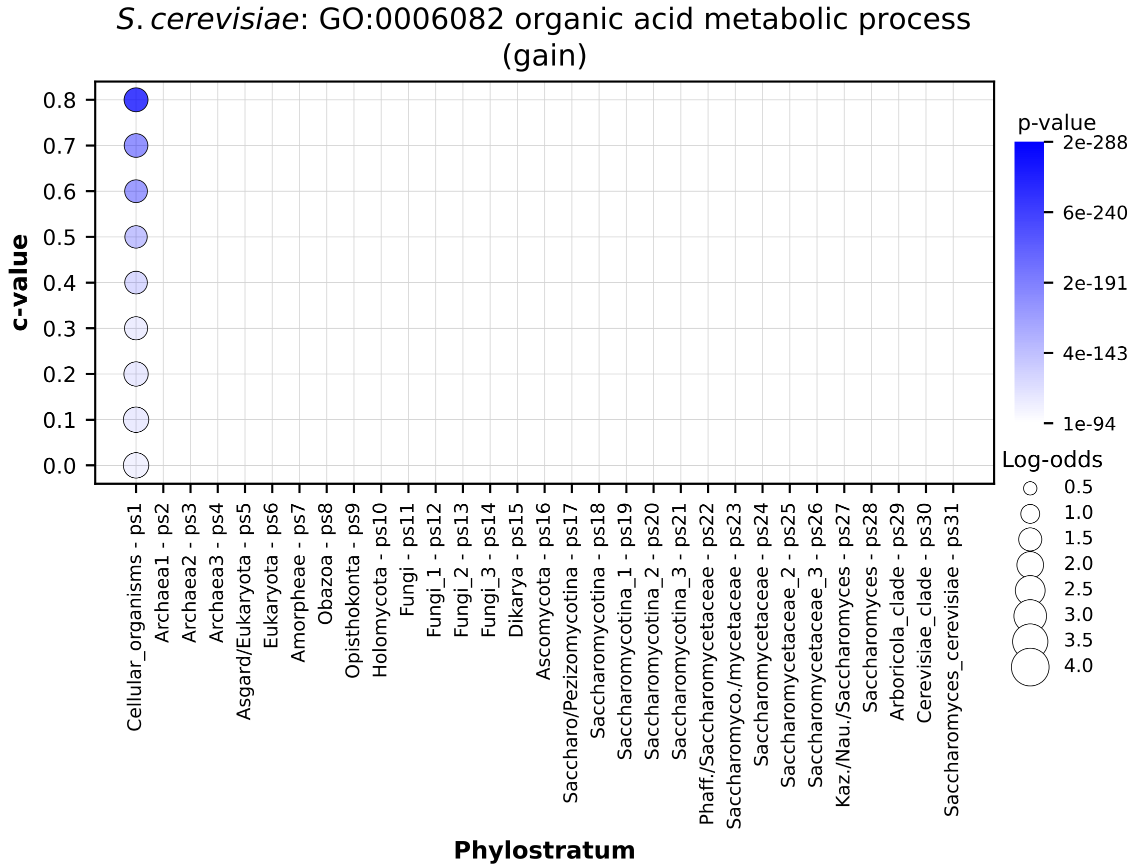

S. cerevisiae: GO:0006082 organic acid metabolic process (gain)
c-value
0.0
0.1
0.2
0.3
0.4
0.5
0.6
0.7
0.8
p-value
2e-288
6e-240
2e-191
4e-143
1e-94
Log-odds
0.5
1.0
1.5
2.0
2.5
3.0
3.5
4.0
Cellular_organisms - ps1
Archaea1 - ps2
Archaea2 - ps3
Archaea3 - ps4
Asgard/Eukaryota - ps5
Eukaryota - ps6
Amorpheae - ps7
Obazoa - ps8
Opisthokonta - ps9
Holomycota - ps10
Fungi - ps11
Fungi_1 - ps12
Fungi_2 - ps13
Fungi_3 - ps14
Dikarya - ps15
Ascomycota - ps16
Saccharo/Pezizomycotina - ps17
Saccharomycotina - ps18
Saccharomycotina_1 - ps19
Saccharomycotina_2 - ps20
Saccharomycotina_3 - ps21
Phaff./Saccharomycetaceae - ps22
Saccharomyco./mycetaceae - ps23
Saccharomycetaceae - ps24
Saccharomycetaceae_2 - ps25
Saccharomycetaceae_3 - ps26
Kaz./Nau./Saccharomyces - ps27
Saccharomyces - ps28
Arboricola_clade - ps29
Cerevisiae_clade - ps30
Saccharomyces_cerevisiae - ps31
Phylostratum

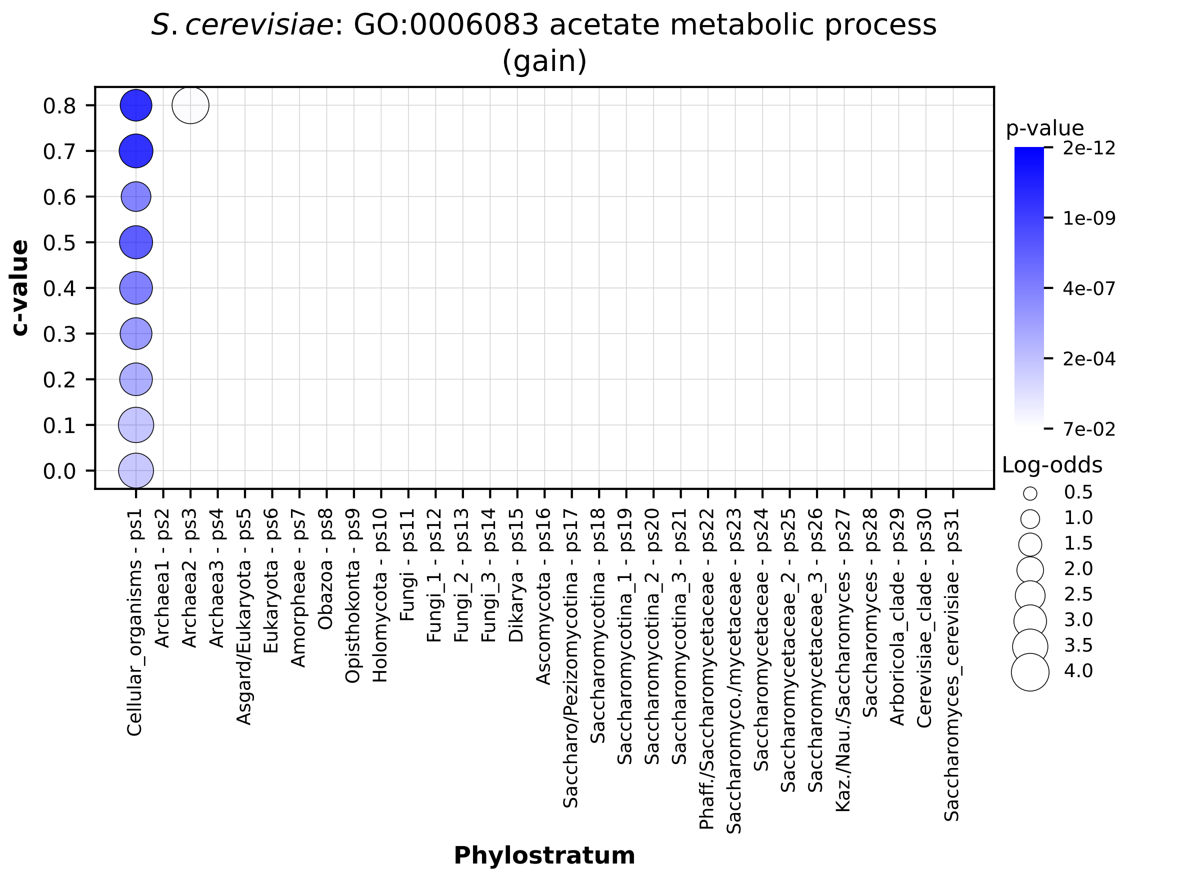

S. cerevisiae: GO:0006083 acetate metabolic process
(gain)
c-value
0.8
0.7
0.6
0.5
0.4
0.3
0.2
0.1
0.0
p-value
2e-12
1e-09
4e-07
2e-04
7e-02
Log-odds
0.5
1.0
1.5
2.0
2.5
3.0
3.5
4.0
Cellular_organisms - ps1
Archaea1 - ps2
Archaea2 - ps3
Archaea3 - ps4
Asgard/Eukaryota - ps5
Eukaryota - ps6
Amorpheae - ps7
Obazoa - ps8
Opisthokonta - ps9
Holomycota - ps10
Fungi - ps11
Fungi_1 - ps12
Fungi_2 - ps13
Fungi_3 - ps14
Dikarya - ps15
Ascomycota - ps16
Saccharo/Pezizomycotina - ps17
Saccharomycotina - ps18
Saccharomycotina_1 - ps19
Saccharomycotina_2 - ps20
Saccharomycotina_3 - ps21
Phaff./Saccharomycetaceae - ps22
Saccharomyco./mycetaceae - ps23
Saccharomycetaceae - ps24
Saccharomycetaceae_2 - ps25
Saccharomycetaceae_3 - ps26
Kaz./Nau./Saccharomyces - ps27
Saccharomyces - ps28
Arboricola_clade - ps29
Cerevisiae_clade - ps30
Saccharomyces_cerevisiae - ps31
Phylostratum

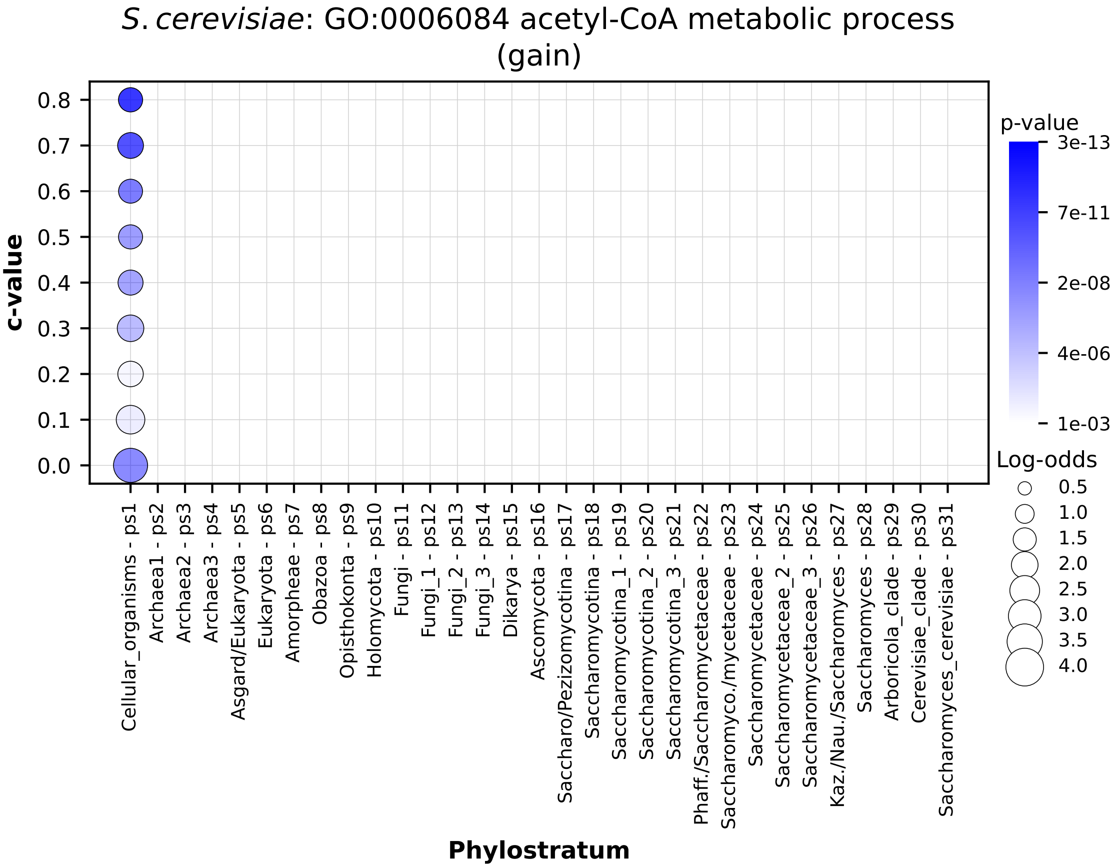
S. cerevisiae: GO:0006084 acetyl-CoA metabolic process
(gain)
c-value
0.8
0.7
0.6
0.5
0.4
0.3
0.2
0.1
0.0
p-value
3e-13
7e-11
2e-08
4e-06
1e-03
Log-odds
0.5
1.0
1.5
2.0
2.5
3.0
3.5
4.0
Cellular_organisms - ps1
Archaea1 - ps2
Archaea2 - ps3
Archaea3 - ps4
Asgard/Eukaryota - ps5
Eukaryota - ps6
Amorpheae - ps7
Obazoa - ps8
Opisthokonta - ps9
Holomycota - ps10
Fungi - ps11
Fungi_1 - ps12
Fungi_2 - ps13
Fungi_3 - ps14
Dikarya - ps15
Ascomycota - ps16
Saccharo/Pezizomycotina - ps17
Saccharomycotina - ps18
Saccharomycotina_1 - ps19
Saccharomycotina_2 - ps20
Saccharomycotina_3 - ps21
Phaff./Saccharomycetaceae - ps22
Saccharomyco./mycetaceae - ps23
Saccharomycetaceae - ps24
Saccharomycetaceae_2 - ps25
Saccharomycetaceae_3 - ps26
Kaz./Nau./Saccharomyces - ps27
Saccharomyces - ps28
Arboricola_clade - ps29
Cerevisiae_clade - ps30
Saccharomyces_cerevisiae - ps31
Phylostratum

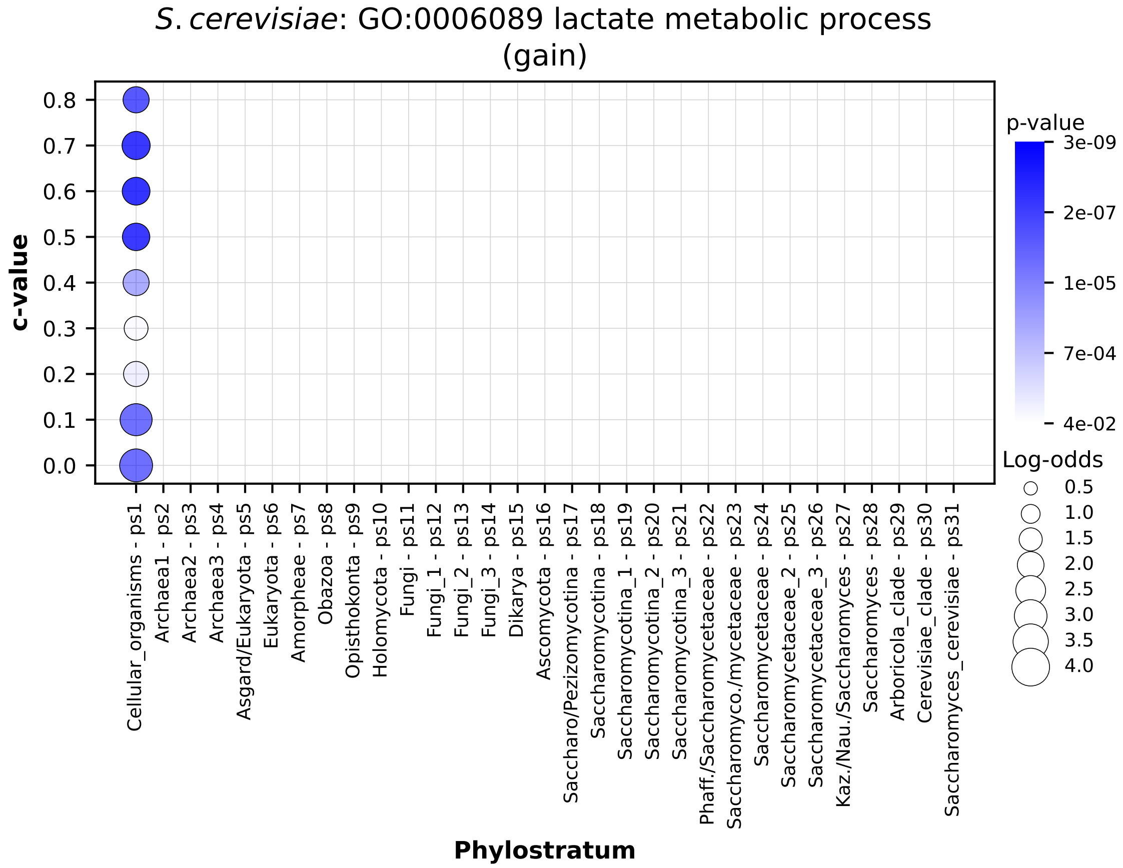
S. cerevisiae: GO:0006089 lactate metabolic process
(gain)
c-value
0.8
0.7
0.6
0.5
0.4
0.3
0.2
0.1
0.0
p-value
3e-09
2e-07
1e-05
7e-04
4e-02
Log-odds
0.5
1.0
1.5
2.0
2.5
3.0
3.5
4.0
Cellular_organisms - ps1
Archaea1 - ps2
Archaea2 - ps3
Archaea3 - ps4
Asgard/Eukaryota - ps5
Eukaryota - ps6
Amorpheae - ps7
Obazoa - ps8
Opisthokonta - ps9
Holomycota - ps10
Fungi - ps11
Fungi_1 - ps12
Fungi_2 - ps13
Fungi_3 - ps14
Dikarya - ps15
Ascomycota - ps16
Saccharo/Pezizomycotina - ps17
Saccharomycotina - ps18
Saccharomycotina_1 - ps19
Saccharomycotina_2 - ps20
Saccharomycotina_3 - ps21
Phaff./Saccharomycetaceae - ps22
Saccharomyco./mycetaceae - ps23
Saccharomycetaceae - ps24
Saccharomycetaceae_2 - ps25
Saccharomycetaceae_3 - ps26
Kaz./Nau./Saccharomyces - ps27
Saccharomyces - ps28
Arboricola_clade - ps29
Cerevisiae_clade - ps30
Saccharomyces_cerevisiae - ps31
Phylostratum

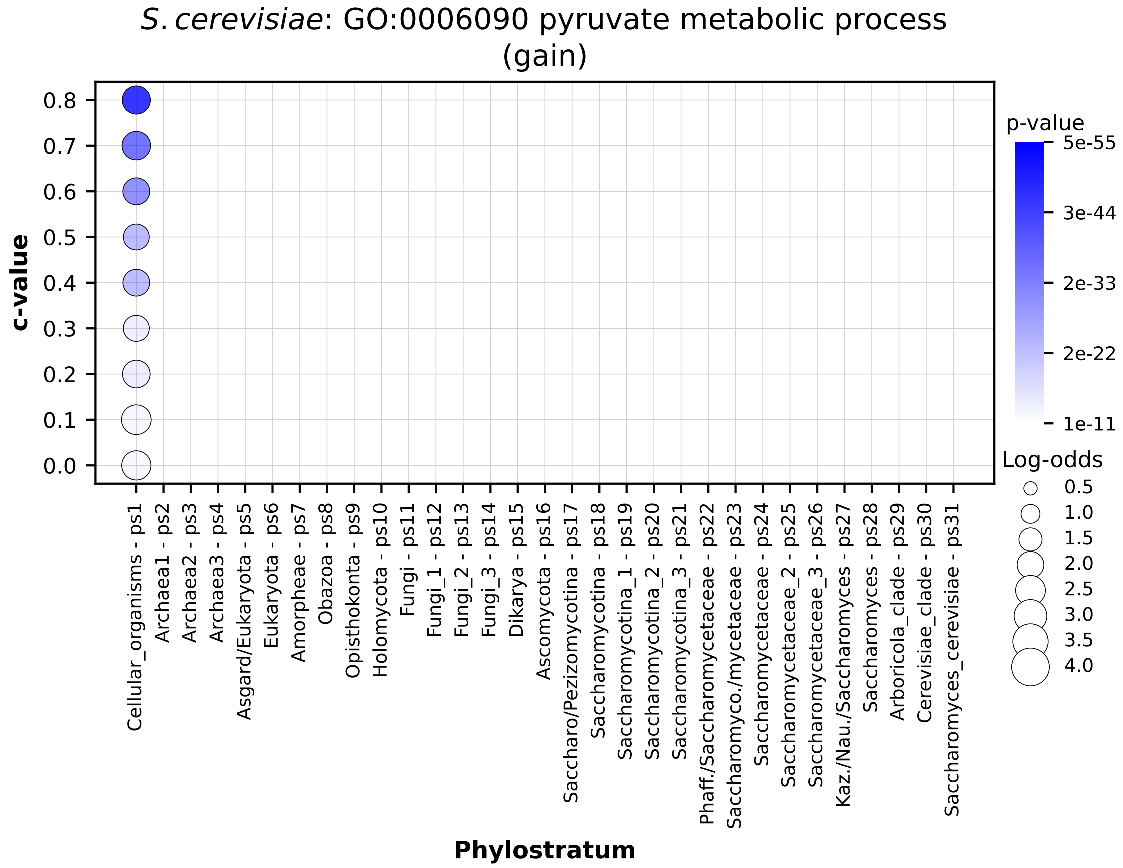

S. cerevisiae: GO:0006090 pyruvate metabolic process
(gain)
c-value
0.8
0.7
0.6
0.5
0.4
0.3
0.2
0.1
0.0
p-value
5e-55
3e-44
2e-33
2e-22
1e-11
Log-odds
0.5
1.0
1.5
2.0
2.5
3.0
3.5
4.0
Cellular_organisms - ps1
Archaea1 - ps2
Archaea2 - ps3
Archaea3 - ps4
Asgard/Eukaryota - ps5
Eukaryota - ps6
Amorpheae - ps7
Obazoa - ps8
Opisthokonta - ps9
Holomycota - ps10
Fungi - ps11
Fungi_1 - ps12
Fungi_2 - ps13
Fungi_3 - ps14
Dikarya - ps15
Ascomycota - ps16
Saccharo/Pezizomycotina - ps17
Saccharomycotina - ps18
Saccharomycotina_1 - ps19
Saccharomycotina_2 - ps20
Saccharomycotina_3 - ps21
Phaff./Saccharomycetaceae - ps22
Saccharomyco./mycetaceae - ps23
Saccharomycetaceae - ps24
Saccharomycetaceae_2 - ps25
Saccharomycetaceae_3 - ps26
Kaz./Nau./Saccharomyces - ps27
Saccharomyces - ps28
Arboricola_clade - ps29
Cerevisiae_clade - ps30
Saccharomyces_cerevisiae - ps31
Phylostratum

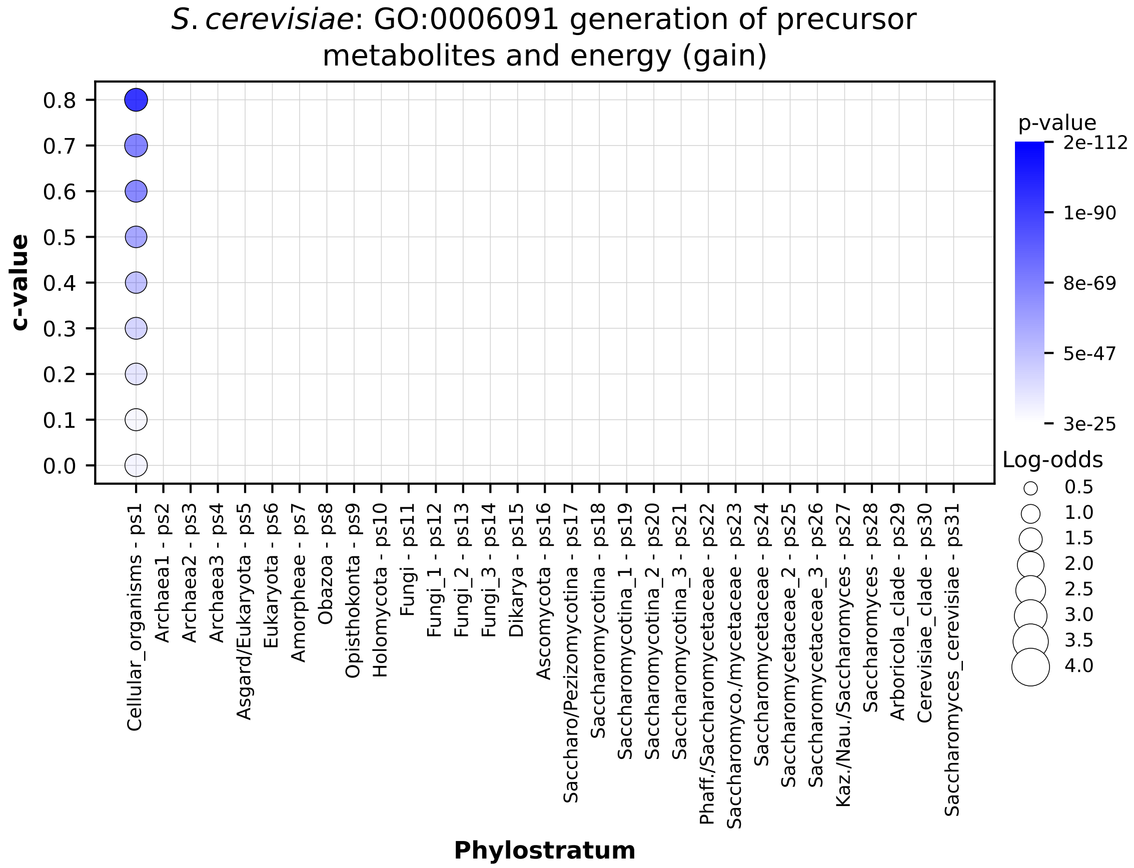

S. cerevisiae: GO:0006091 generation of precursor metabolites and energy (gain)
c-value
0.8
0.7
0.6
0.5
0.4
0.3
0.2
0.1
0.0
p-value
2e-112
1e-90
8e-69
5e-47
3e-25
Log-odds
0.5
1.0
1.5
2.0
2.5
3.0
3.5
4.0
Cellular_organisms - ps1
Archaea1 - ps2
Archaea2 - ps3
Archaea3 - ps4
Asgard/Eukaryota - ps5
Eukaryota - ps6
Amorpheae - ps7
Obazoa - ps8
Opisthokonta - ps9
Holomycota - ps10
Fungi - ps11
Fungi_1 - ps12
Fungi_2 - ps13
Fungi_3 - ps14
Dikarya - ps15
Ascomycota - ps16
Saccharo/Pezizomycotina - ps17
Saccharomycotina - ps18
Saccharomycotina_1 - ps19
Saccharomycotina_2 - ps20
Saccharomycotina_3 - ps21
Phaff./Saccharomycetaceae - ps22
Saccharomyco./mycetaceae - ps23
Saccharomycetaceae - ps24
Saccharomycetaceae_2 - ps25
Saccharomycetaceae_3 - ps26
Kaz./Nau./Saccharomyces - ps27
Saccharomyces - ps28
Arboricola_clade - ps29
Cerevisiae_clade - ps30
Saccharomyces_cerevisiae - ps31
Phylostratum

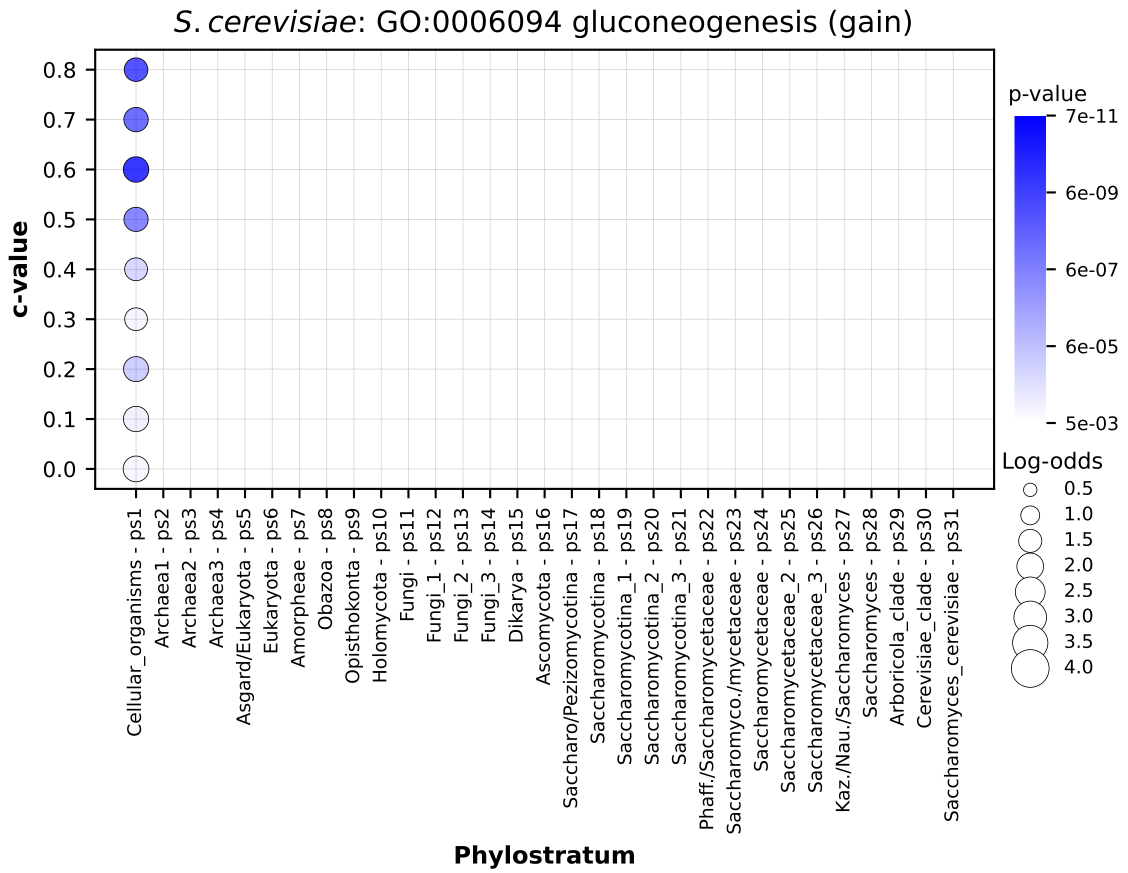

S. cerevisiae: GO:0006094 gluconeogenesis (gain)
c-value
0.8
0.7
0.6
0.5
0.4
0.3
0.2
0.1
0.0
p-value
7e-11
6e-09
6e-07
6e-05
5e-03
Log-odds
0.5
1.0
1.5
2.0
2.5
3.0
3.5
4.0
Cellular_organisms - ps1
Archaea1 - ps2
Archaea2 - ps3
Archaea3 - ps4
Asgard/Eukaryota - ps5
Eukaryota - ps6
Amorpheae - ps7
Obazoa - ps8
Opisthokonta - ps9
Holomycota - ps10
Fungi - ps11
Fungi_1 - ps12
Fungi_2 - ps13
Fungi_3 - ps14
Dikarya - ps15
Ascomycota - ps16
Saccharo/Pezizomycotina - ps17
Saccharomycotina - ps18
Saccharomycotina_1 - ps19
Saccharomycotina_2 - ps20
Saccharomycotina_3 - ps21
Phaff./Saccharomycetaceae - ps22
Saccharomyco./mycetaceae - ps23
Saccharomycetaceae - ps24
Saccharomycetaceae_2 - ps25
Saccharomycetaceae_3 - ps26
Kaz./Nau./Saccharomyces - ps27
Saccharomyces - ps28
Arboricola_clade - ps29
Cerevisiae_clade - ps30
Saccharomyces_cerevisiae - ps31
Phylostratum

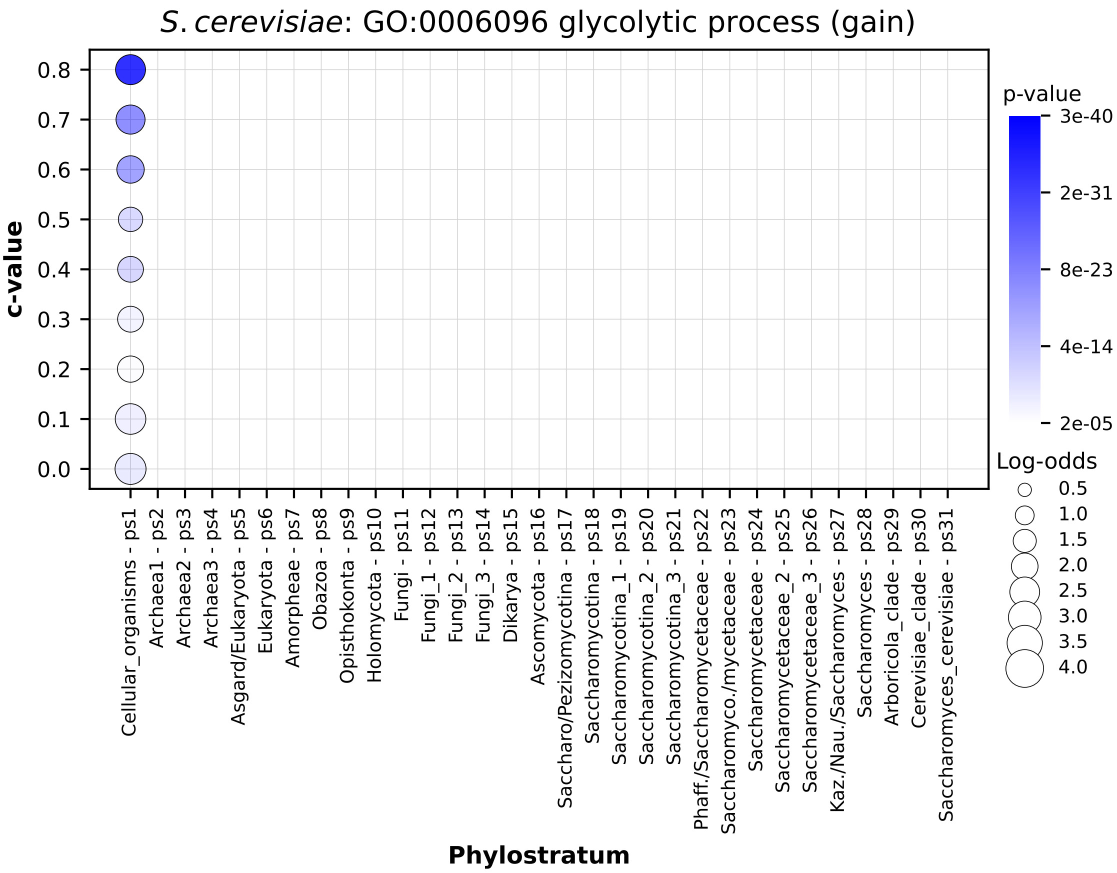
S. cerevisiae: GO:0006096 glycolytic process (gain)
c-value
0.8
0.7
0.6
0.5
0.4
0.3
0.2
0.1
0.0
p-value
3e-40
2e-31
8e-23
4e-14
2e-05
Log-odds
0.5
1.0
1.5
2.0
2.5
3.0
3.5
4.0
Cellular_organisms - ps1
Archaea1 - ps2
Archaea2 - ps3
Archaea3 - ps4
Asgard/Eukaryota - ps5
Eukaryota - ps6
Amorpheae - ps7
Obazoa - ps8
Opisthokonta - ps9
Holomycota - ps10
Fungi - ps11
Fungi_1 - ps12
Fungi_2 - ps13
Fungi_3 - ps14
Dikarya - ps15
Ascomycota - ps16
Saccharo/Pezizomycotina - ps17
Saccharomycotina - ps18
Saccharomycotina_1 - ps19
Saccharomycotina_2 - ps20
Saccharomycotina_3 - ps21
Phaff./Saccharomycetaceae - ps22
Saccharomyco./mycetaceae - ps23
Saccharomycetaceae - ps24
Saccharomycetaceae_2 - ps25
Saccharomycetaceae_3 - ps26
Kaz./Nau./Saccharomyces - ps27
Saccharomyces - ps28
Arboricola_clade - ps29
Cerevisiae_clade - ps30
Saccharomyces_cerevisiae - ps31
Phylostratum

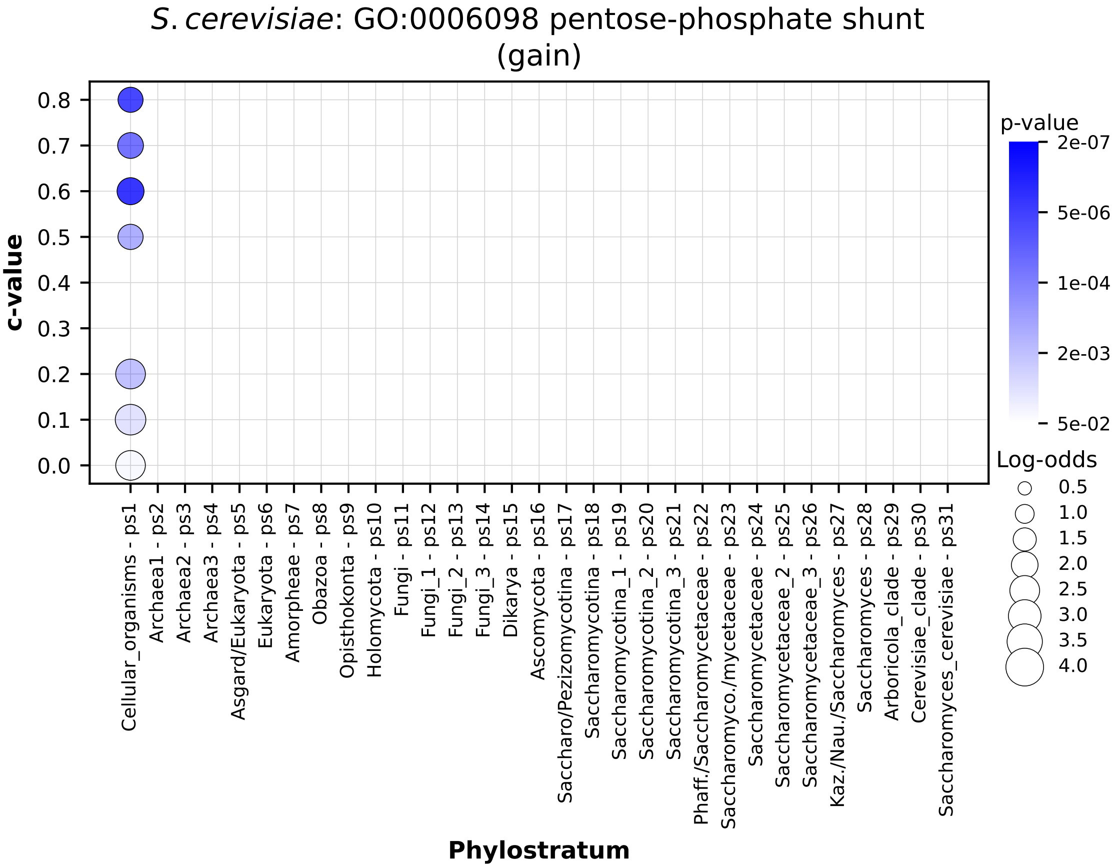
S. cerevisiae: GO:0006098 pentose-phosphate shunt
(gain)
c-value
0.8
0.7
0.6
0.5
0.4
0.3
0.2
0.1
0.0
Phylostratum
Cellular_organisms - ps1
Archaea1 - ps2
Archaea2 - ps3
Archaea3 - ps4
Asgard/Eukaryota - ps5
Eukaryota - ps6
Amorpheae - ps7
Obazoa - ps8
Opisthokonta - ps9
Holomycota - ps10
Fungi - ps11
Fungi_1 - ps12
Fungi_2 - ps13
Fungi_3 - ps14
Dikarya - ps15
Ascomycota - ps16
Saccharo/Pezizomycotina - ps17
Saccharomycotina - ps18
Saccharomycotina_1 - ps19
Saccharomycotina_2 - ps20
Saccharomycotina_3 - ps21
Phaff./Saccharomycetaceae - ps22
Saccharomyco./mycetaceae - ps23
Saccharomycetaceae - ps24
Saccharomycetaceae_2 - ps25
Saccharomycetaceae_3 - ps26
Kaz./Nau./Saccharomyces - ps27
Saccharomyces - ps28
Arboricola_clade - ps29
Cerevisiae_clade - ps30
Saccharomyces_cerevisiae - ps31
p-value
2e-07
5e-06
1e-04
2e-03
5e-02
Log-odds
0.5
1.0
1.5
2.0
2.5
3.0
3.5
4.0

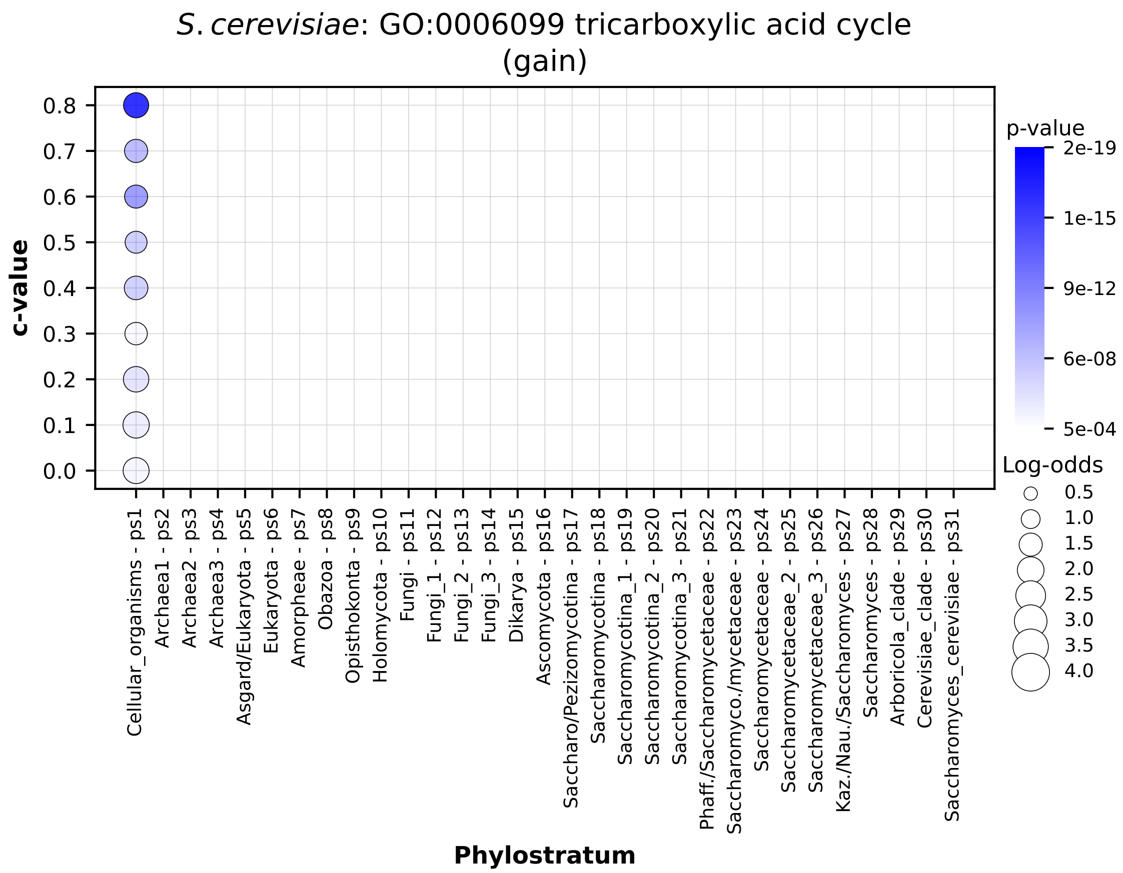

S. cerevisiae: GO:0006099 tricarboxylic acid cycle
(gain)
c-value
0.0
0.1
0.2
0.3
0.4
0.5
0.6
0.7
0.8
p-value
2e-19
1e-15
9e-12
6e-08
5e-04
Log-odds
0.5
1.0
1.5
2.0
2.5
3.0
3.5
4.0
Cellular_organisms - ps1
Archaea1 - ps2
Archaea2 - ps3
Archaea3 - ps4
Asgard/Eukaryota - ps5
Eukaryota - ps6
Amorpheae - ps7
Obazoa - ps8
Opisthokonta - ps9
Holomycota - ps10
Fungi - ps11
Fungi_1 - ps12
Fungi_2 - ps13
Fungi_3 - ps14
Dikarya - ps15
Ascomycota - ps16
Saccharo/Pezizomycotina - ps17
Saccharomycotina - ps18
Saccharomycotina_1 - ps19
Saccharomycotina_2 - ps20
Saccharomycotina_3 - ps21
Phaff./Saccharomycetaceae - ps22
Saccharomyco./mycetaceae - ps23
Saccharomycetaceae - ps24
Saccharomycetaceae_2 - ps25
Saccharomycetaceae_3 - ps26
Kaz./Nau./Saccharomyces - ps27
Saccharomyces - ps28
Arboricola_clade - ps29
Cerevisiae_clade - ps30
Saccharomyces_cerevisiae - ps31
Phylostratum

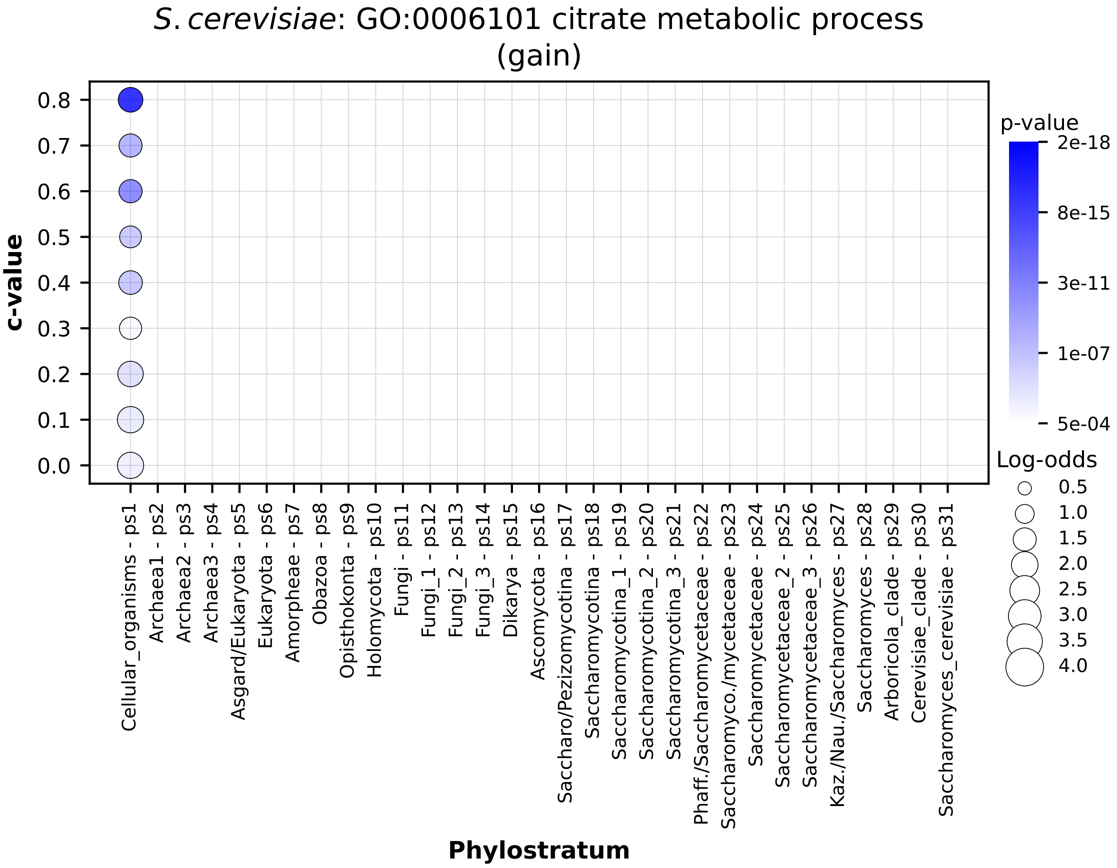
S. cerevisiae: GO:0006101 citrate metabolic process
(gain)
c-value
0.0
0.1
0.2
0.3
0.4
0.5
0.6
0.7
0.8
p-value
2e-18
8e-15
3e-11
1e-07
5e-04
Log-odds
0.5
1.0
1.5
2.0
2.5
3.0
3.5
4.0
Cellular_organisms - ps1
Archaea1 - ps2
Archaea2 - ps3
Archaea3 - ps4
Asgard/Eukaryota - ps5
Eukaryota - ps6
Amorpheae - ps7
Obazoa - ps8
Opisthokonta - ps9
Holomycota - ps10
Fungi - ps11
Fungi_1 - ps12
Fungi_2 - ps13
Fungi_3 - ps14
Dikarya - ps15
Ascomycota - ps16
Saccharo/Pezizomycotina - ps17
Saccharomycotina - ps18
Saccharomycotina_1 - ps19
Saccharomycotina_2 - ps20
Saccharomycotina_3 - ps21
Phaff./Saccharomycetaceae - ps22
Saccharomyco./mycetaceae - ps23
Saccharomycetaceae - ps24
Saccharomycetaceae_2 - ps25
Saccharomycetaceae_3 - ps26
Kaz./Nau./Saccharomyces - ps27
Saccharomyces - ps28
Arboricola_clade - ps29
Cerevisiae_clade - ps30
Saccharomyces_cerevisiae - ps31
Phylostratum

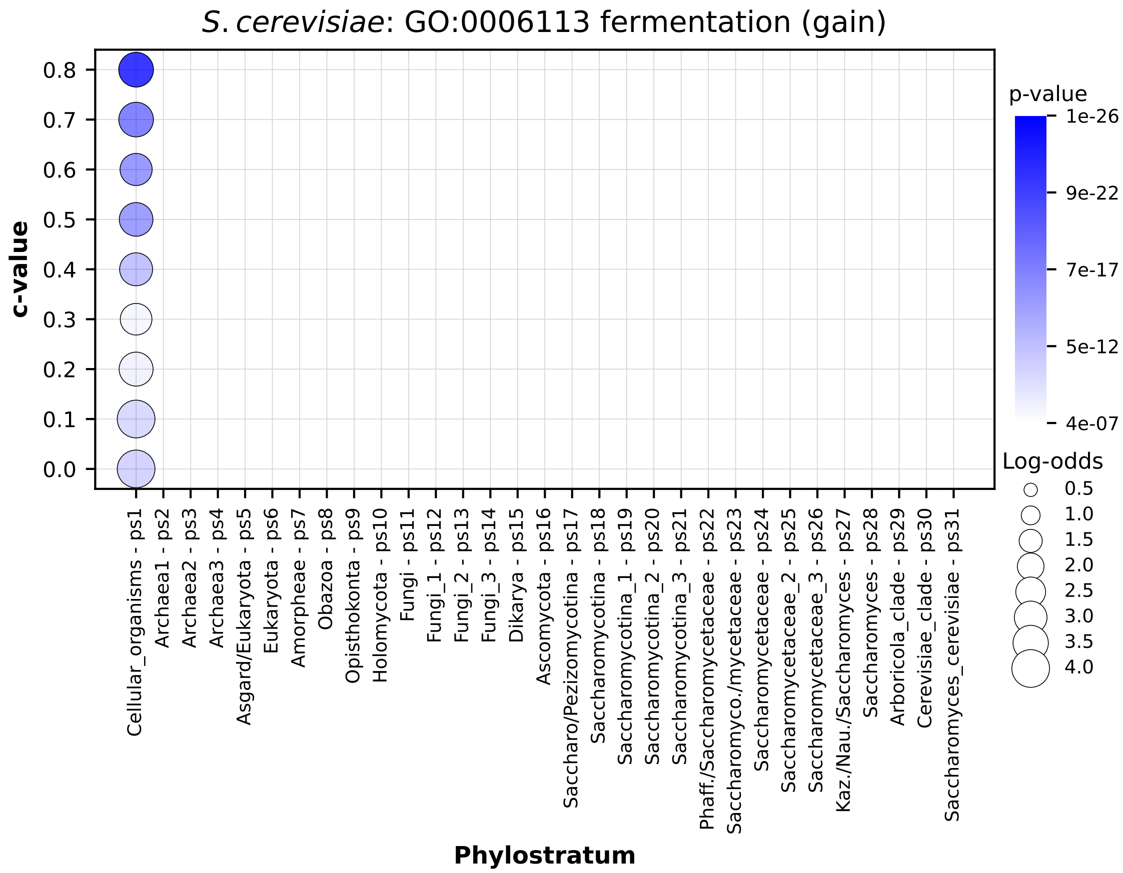
S. cerevisiae: GO:0006113 fermentation (gain)
c-value
0.8
0.7
0.6
0.5
0.4
0.3
0.2
0.1
0.0
p-value
1e-26
9e-22
7e-17
5e-12
4e-07
Log-odds
0.5
1.0
1.5
2.0
2.5
3.0
3.5
4.0
Cellular_organisms - ps1
Archaea1 - ps2
Archaea2 - ps3
Archaea3 - ps4
Asgard/Eukaryota - ps5
Eukaryota - ps6
Amorpheae - ps7
Obazoa - ps8
Opisthokonta - ps9
Holomycota - ps10
Fungi - ps11
Fungi_1 - ps12
Fungi_2 - ps13
Fungi_3 - ps14
Dikarya - ps15
Ascomycota - ps16
Saccharo/Pezizomycotina - ps17
Saccharomycotina - ps18
Saccharomycotina_1 - ps19
Saccharomycotina_2 - ps20
Saccharomycotina_3 - ps21
Phaff./Saccharomycetaceae - ps22
Saccharomyco./mycetaceae - ps23
Saccharomycetaceae - ps24
Saccharomycetaceae_2 - ps25
Saccharomycetaceae_3 - ps26
Kaz./Nau./Saccharomyces - ps27
Saccharomyces - ps28
Arboricola_clade - ps29
Cerevisiae_clade - ps30
Saccharomyces_cerevisiae - ps31
Phylostratum

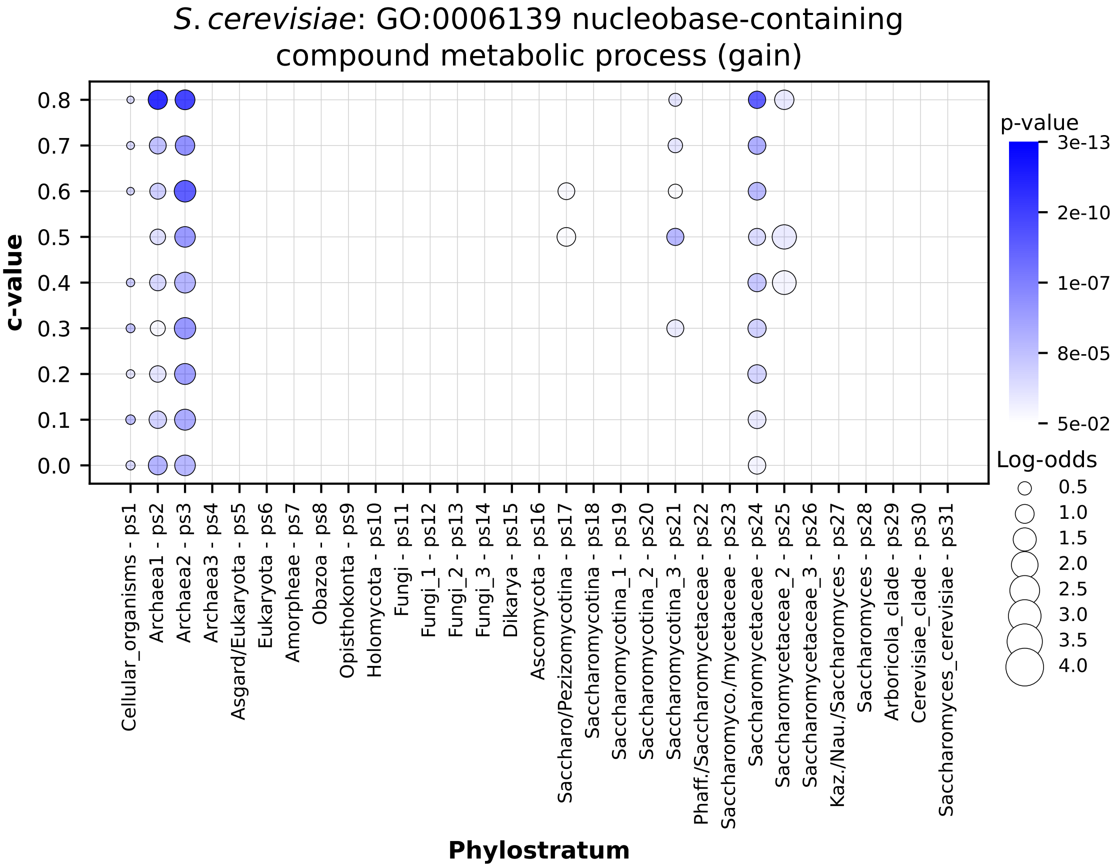
S. cerevisiae: GO:0006139 nucleobase-containing compound metabolic process (gain)
c-value
0.8
0.7
0.6
0.5
0.4
0.3
0.2
0.1
0.0
p-value
3e-13
2e-10
1e-07
8e-05
5e-02
Log-odds
0.5
1.0
1.5
2.0
2.5
3.0
3.5
4.0
Cellular_organisms - ps1
Archaea1 - ps2
Archaea2 - ps3
Archaea3 - ps4
Asgard/Eukaryota - ps5
Eukaryota - ps6
Amorpheae - ps7
Obazoa - ps8
Opisthokonta - ps9
Holomycota - ps10
Fungi - ps11
Fungi_1 - ps12
Fungi_2 - ps13
Fungi_3 - ps14
Dikarya - ps15
Ascomycota - ps16
Saccharo/Pezizomycotina - ps17
Saccharomycotina - ps18
Saccharomycotina_1 - ps19
Saccharomycotina_2 - ps20
Saccharomycotina_3 - ps21
Phaff./Saccharomycetaceae - ps22
Saccharomyco./mycetaceae - ps23
Saccharomycetaceae - ps24
Saccharomycetaceae_2 - ps25
Saccharomycetaceae_3 - ps26
Kaz./Nau./Saccharomyces - ps27
Saccharomyces - ps28
Arboricola_clade - ps29
Cerevisiae_clade - ps30
Saccharomyces_cerevisiae - ps31
Phylostratum

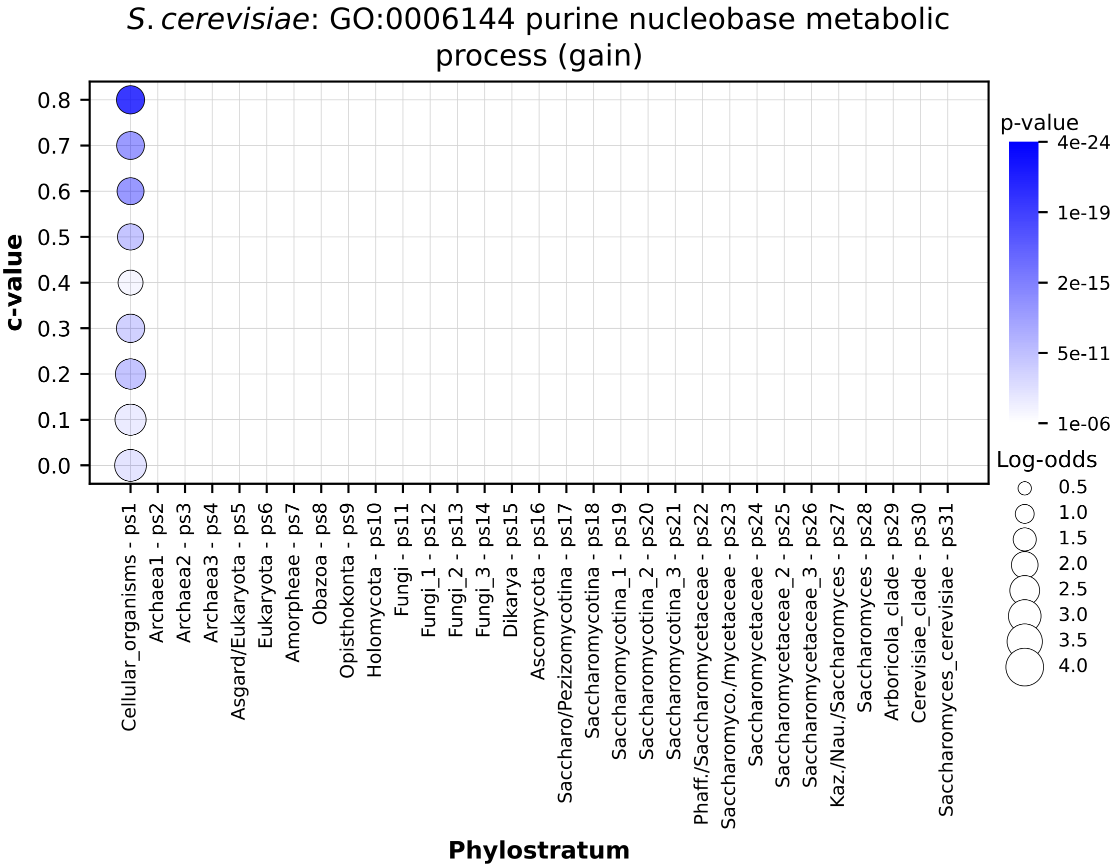

S. cerevisiae: GO:0006144 purine nucleobase metabolic process (gain)
c-value
0.8
0.7
0.6
0.5
0.4
0.3
0.2
0.1
0.0
p-value
4e-24
1e-19
2e-15
5e-11
1e-06
Log-odds
0.5
1.0
1.5
2.0
2.5
3.0
3.5
4.0
Cellular_organisms - ps1
Archaea1 - ps2
Archaea2 - ps3
Archaea3 - ps4
Asgard/Eukaryota - ps5
Eukaryota - ps6
Amorpheae - ps7
Obazoa - ps8
Opisthokonta - ps9
Holomycota - ps10
Fungi - ps11
Fungi_1 - ps12
Fungi_2 - ps13
Fungi_3 - ps14
Dikarya - ps15
Ascomycota - ps16
Saccharo/Pezizomycotina - ps17
Saccharomycotina - ps18
Saccharomycotina_1 - ps19
Saccharomycotina_2 - ps20
Saccharomycotina_3 - ps21
Phaff./Saccharomycetaceae - ps22
Saccharomyco./mycetaceae - ps23
Saccharomycetaceae - ps24
Saccharomycetaceae_2 - ps25
Saccharomycetaceae_3 - ps26
Kaz./Nau./Saccharomyces - ps27
Saccharomyces - ps28
Arboricola_clade - ps29
Cerevisiae_clade - ps30
Saccharomyces_cerevisiae - ps31
Phylostratum

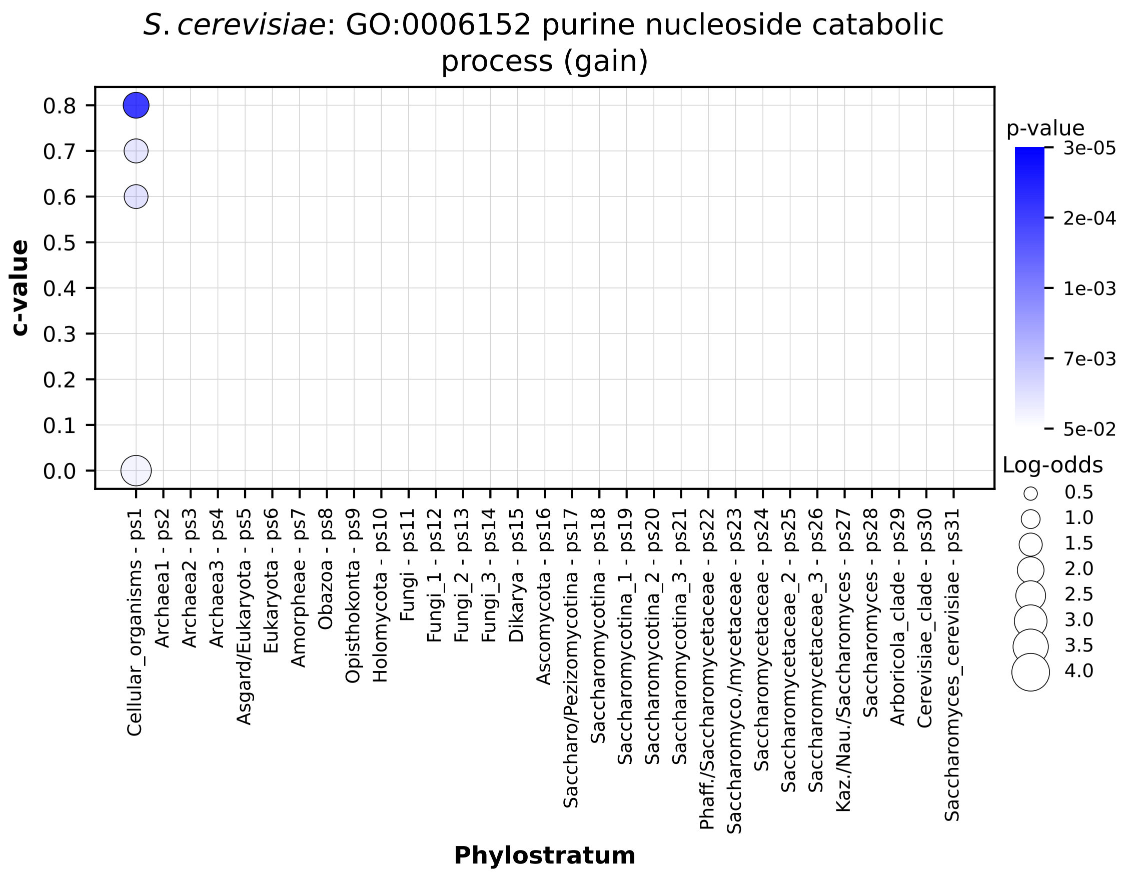

S. cerevisiae: GO:0006152 purine nucleoside catabolic process (gain)
c-value
0.8
0.7
0.6
0.5
0.4
0.3
0.2
0.1
0.0
p-value
3e-05
2e-04
1e-03
7e-03
5e-02
Log-odds
0.5
1.0
1.5
2.0
2.5
3.0
3.5
4.0
Cellular_organisms - ps1
Archaea1 - ps2
Archaea2 - ps3
Archaea3 - ps4
Asgard/Eukaryota - ps5
Eukaryota - ps6
Amorpheae - ps7
Obazoa - ps8
Opisthokonta - ps9
Holomycota - ps10
Fungi - ps11
Fungi_1 - ps12
Fungi_2 - ps13
Fungi_3 - ps14
Dikarya - ps15
Ascomycota - ps16
Saccharo/Pezizomycotina - ps17
Saccharomycotina - ps18
Saccharomycotina_1 - ps19
Saccharomycotina_2 - ps20
Saccharomycotina_3 - ps21
Phaff./Saccharomycetaceae - ps22
Saccharomyco./mycetaceae - ps23
Saccharomycetaceae - ps24
Saccharomycetaceae_2 - ps25
Saccharomycetaceae_3 - ps26
Kaz./Nau./Saccharomyces - ps27
Saccharomyces - ps28
Arboricola_clade - ps29
Cerevisiae_clade - ps30
Saccharomyces_cerevisiae - ps31
Phylostratum

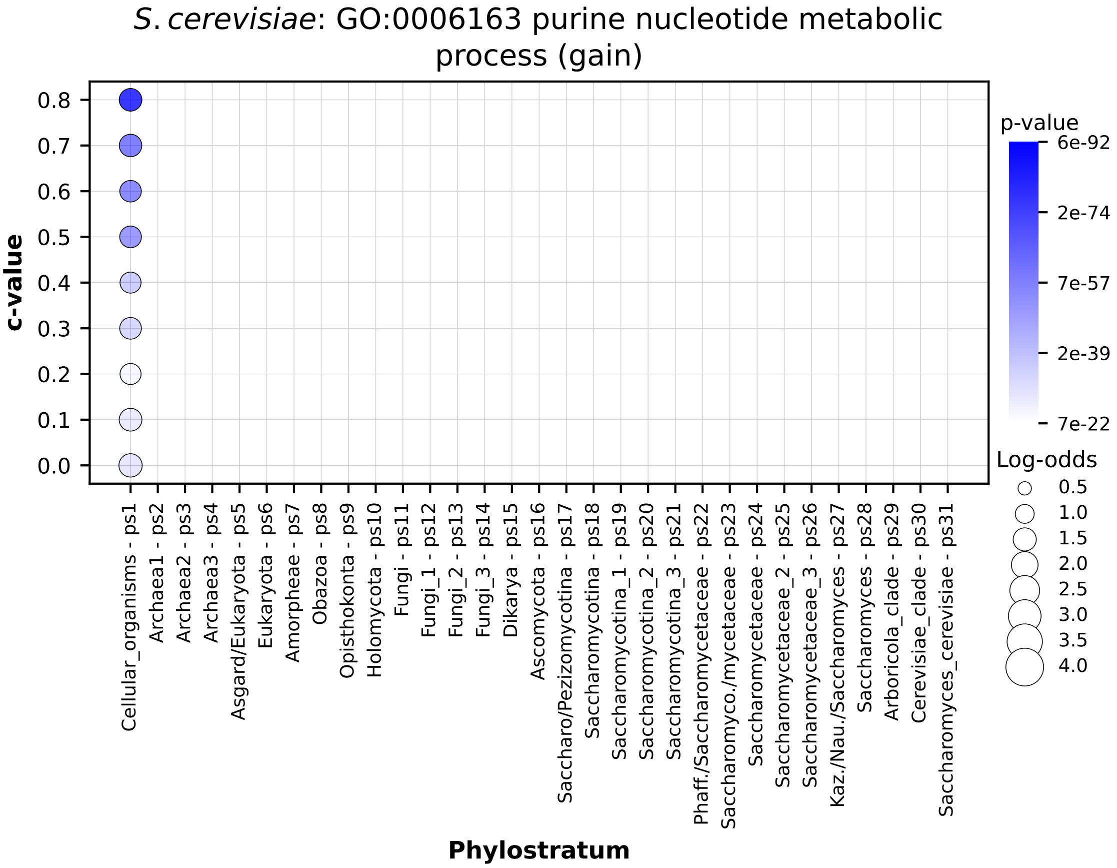
S. cerevisiae: GO:0006163 purine nucleotide metabolic process (gain)
c-value
0.8
0.7
0.6
0.5
0.4
0.3
0.2
0.1
0.0
p-value
6e-92
2e-74
7e-57
2e-39
7e-22
Log-odds
0.5
1.0
1.5
2.0
2.5
3.0
3.5
4.0
Cellular_organisms - ps1
Archaea1 - ps2
Archaea2 - ps3
Archaea3 - ps4
Asgard/Eukaryota - ps5
Eukaryota - ps6
Amorpheae - ps7
Obazoa - ps8
Opisthokonta - ps9
Holomycota - ps10
Fungi - ps11
Fungi_1 - ps12
Fungi_2 - ps13
Fungi_3 - ps14
Dikarya - ps15
Ascomycota - ps16
Saccharo/Pezizomycotina - ps17
Saccharomycotina - ps18
Saccharomycotina_1 - ps19
Saccharomycotina_2 - ps20
Saccharomycotina_3 - ps21
Phaff./Saccharomycetaceae - ps22
Saccharomyco./mycetaceae - ps23
Saccharomycetaceae - ps24
Saccharomycetaceae_2 - ps25
Saccharomycetaceae_3 - ps26
Kaz./Nau./Saccharomyces - ps27
Saccharomyces - ps28
Arboricola_clade - ps29
Cerevisiae_clade - ps30
Saccharomyces_cerevisiae - ps31
Phylostratum

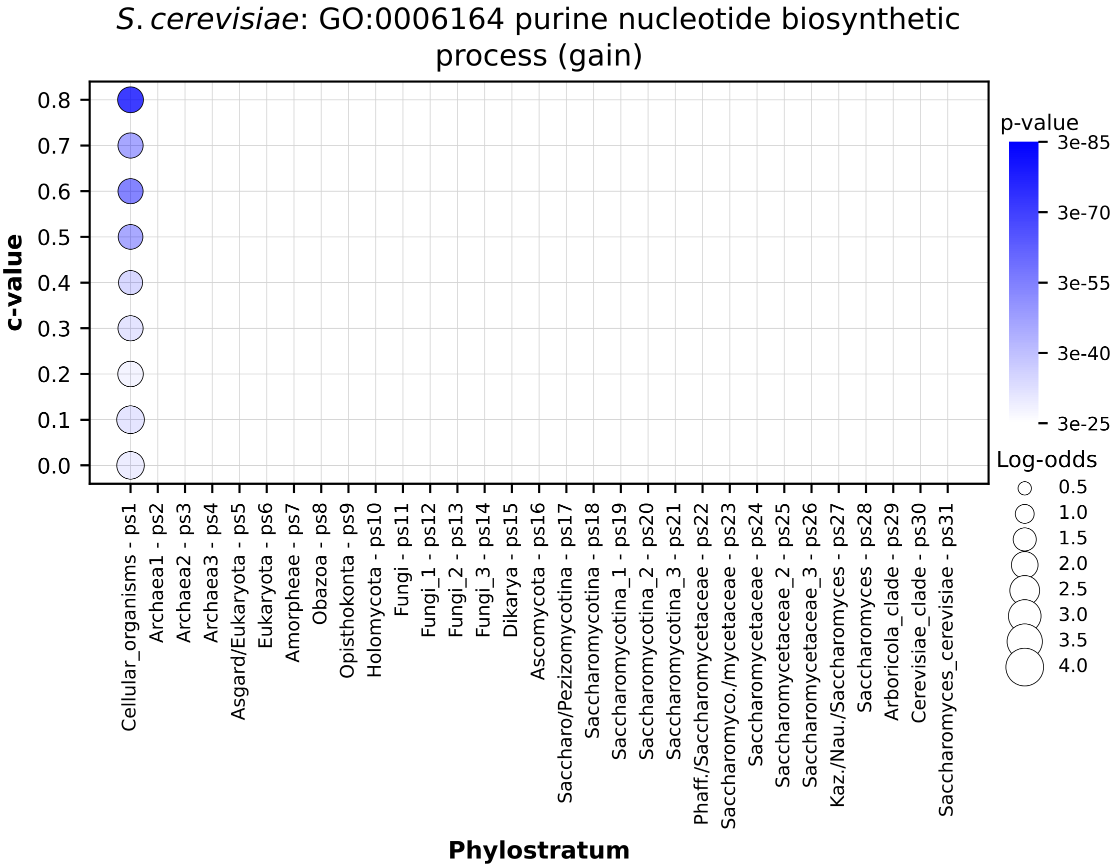

S. cerevisiae: GO:0006164 purine nucleotide biosynthetic process (gain)
c-value
0.8
0.7
0.6
0.5
0.4
0.3
0.2
0.1
0.0
p-value
3e-85
3e-70
3e-55
3e-40
3e-25
Log-odds
0.5
1.0
1.5
2.0
2.5
3.0
3.5
4.0
Cellular_organisms - ps1
Archaea1 - ps2
Archaea2 - ps3
Archaea3 - ps4
Asgard/Eukaryota - ps5
Eukaryota - ps6
Amorpheae - ps7
Obazoa - ps8
Opisthokonta - ps9
Holomycota - ps10
Fungi - ps11
Fungi_1 - ps12
Fungi_2 - ps13
Fungi_3 - ps14
Dikarya - ps15
Ascomycota - ps16
Saccharo/Pezizomycotina - ps17
Saccharomycotina - ps18
Saccharomycotina_1 - ps19
Saccharomycotina_2 - ps20
Saccharomycotina_3 - ps21
Phaff./Saccharomycetaceae - ps22
Saccharomyco./mycetaceae - ps23
Saccharomycetaceae - ps24
Saccharomycetaceae_2 - ps25
Saccharomycetaceae_3 - ps26
Kaz./Nau./Saccharomyces - ps27
Saccharomyces - ps28
Arboricola_clade - ps29
Cerevisiae_clade - ps30
Saccharomyces_cerevisiae - ps31
Phylostratum

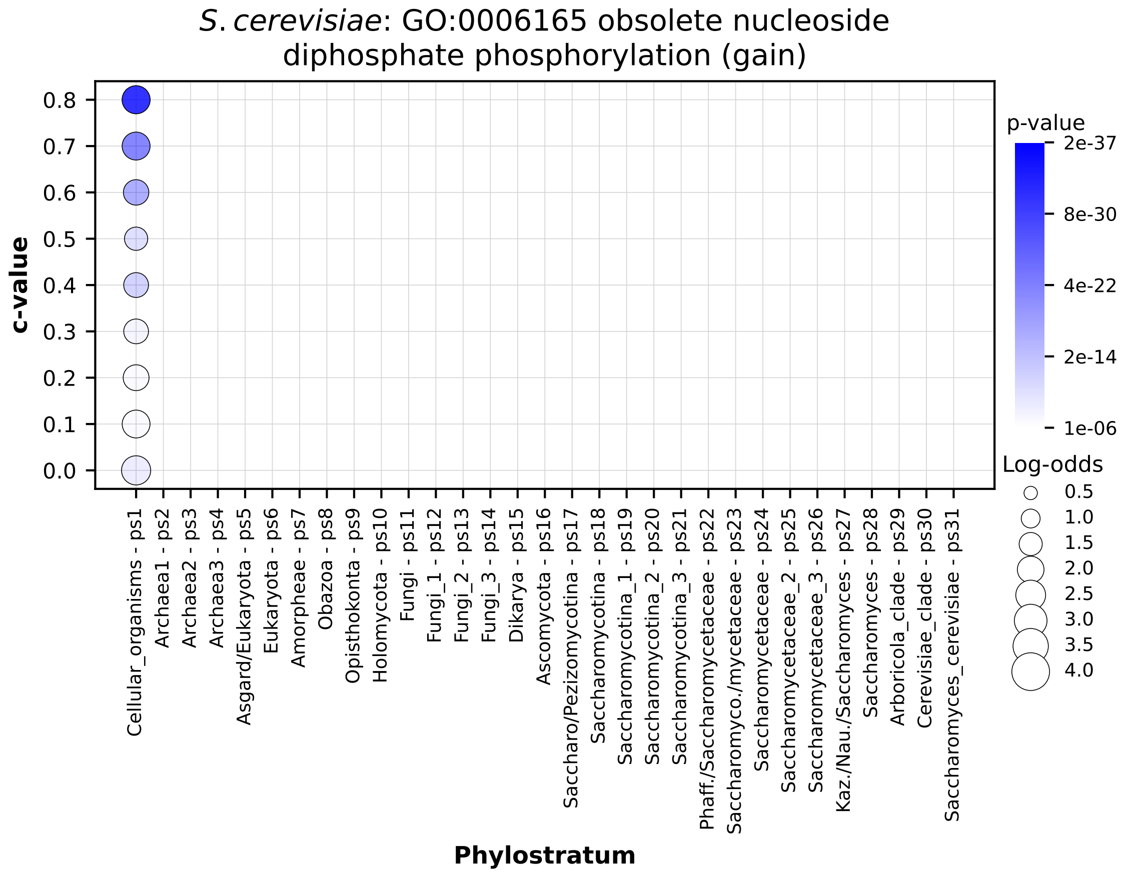

S. cerevisiae: GO:0006165 obsolete nucleoside diphosphate phosphorylation (gain)
c-value
0.8
0.7
0.6
0.5
0.4
0.3
0.2
0.1
0.0
p-value
2e-37
8e-30
4e-22
2e-14
1e-06
Log-odds
0.5
1.0
1.5
2.0
2.5
3.0
3.5
4.0
Cellular_organisms - ps1
Archaea1 - ps2
Archaea2 - ps3
Archaea3 - ps4
Asgard/Eukaryota - ps5
Eukaryota - ps6
Amorpheae - ps7
Obazoa - ps8
Opisthokonta - ps9
Holomycota - ps10
Fungi - ps11
Fungi_1 - ps12
Fungi_2 - ps13
Fungi_3 - ps14
Dikarya - ps15
Ascomycota - ps16
Saccharo/Pezizomycotina - ps17
Saccharomycotina - ps18
Saccharomycotina_1 - ps19
Saccharomycotina_2 - ps20
Saccharomycotina_3 - ps21
Phaff./Saccharomycetaceae - ps22
Saccharomyco./mycetaceae - ps23
Saccharomycetaceae - ps24
Saccharomycetaceae_2 - ps25
Saccharomycetaceae_3 - ps26
Kaz./Nau./Saccharomyces - ps27
Saccharomyces - ps28
Arboricola_clade - ps29
Cerevisiae_clade - ps30
Saccharomyces_cerevisiae - ps31
Phylostratum

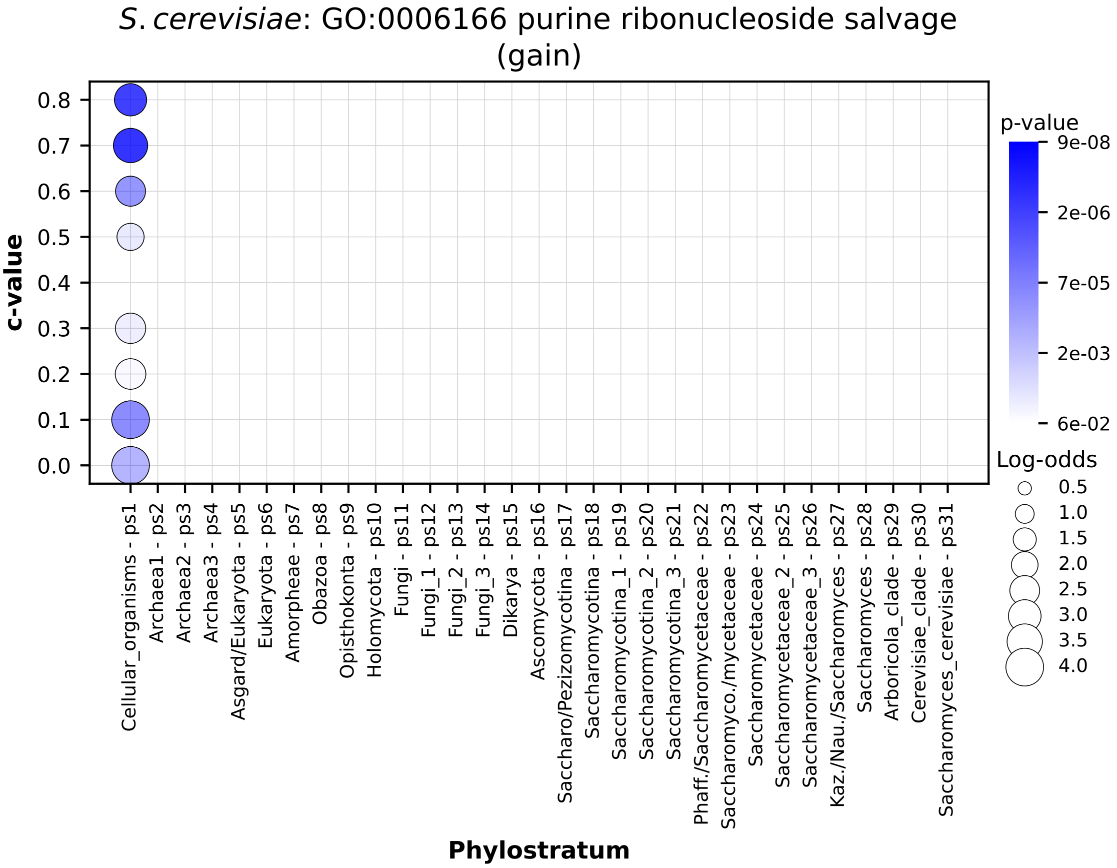

S. cerevisiae: GO:0006166 purine ribonucleoside salvage
(gain)
c-value
0.8
0.7
0.6
0.5
0.4
0.3
0.2
0.1
0.0
p-value
9e-08
2e-06
7e-05
2e-03
6e-02
Log-odds
0.5
1.0
1.5
2.0
2.5
3.0
3.5
4.0
Cellular_organisms - ps1
Archaea1 - ps2
Archaea2 - ps3
Archaea3 - ps4
Asgard/Eukaryota - ps5
Eukaryota - ps6
Amorpheae - ps7
Obazoa - ps8
Opisthokonta - ps9
Holomycota - ps10
Fungi - ps11
Fungi_1 - ps12
Fungi_2 - ps13
Fungi_3 - ps14
Dikarya - ps15
Ascomycota - ps16
Saccharo/Pezizomycotina - ps17
Saccharomycotina - ps18
Saccharomycotina_1 - ps19
Saccharomycotina_2 - ps20
Saccharomycotina_3 - ps21
Phaff./Saccharomycetaceae - ps22
Saccharomyco./mycetaceae - ps23
Saccharomycetaceae - ps24
Saccharomycetaceae_2 - ps25
Saccharomycetaceae_3 - ps26
Kaz./Nau./Saccharomyces - ps27
Saccharomyces - ps28
Arboricola_clade - ps29
Cerevisiae_clade - ps30
Saccharomyces_cerevisiae - ps31
Phylostratum

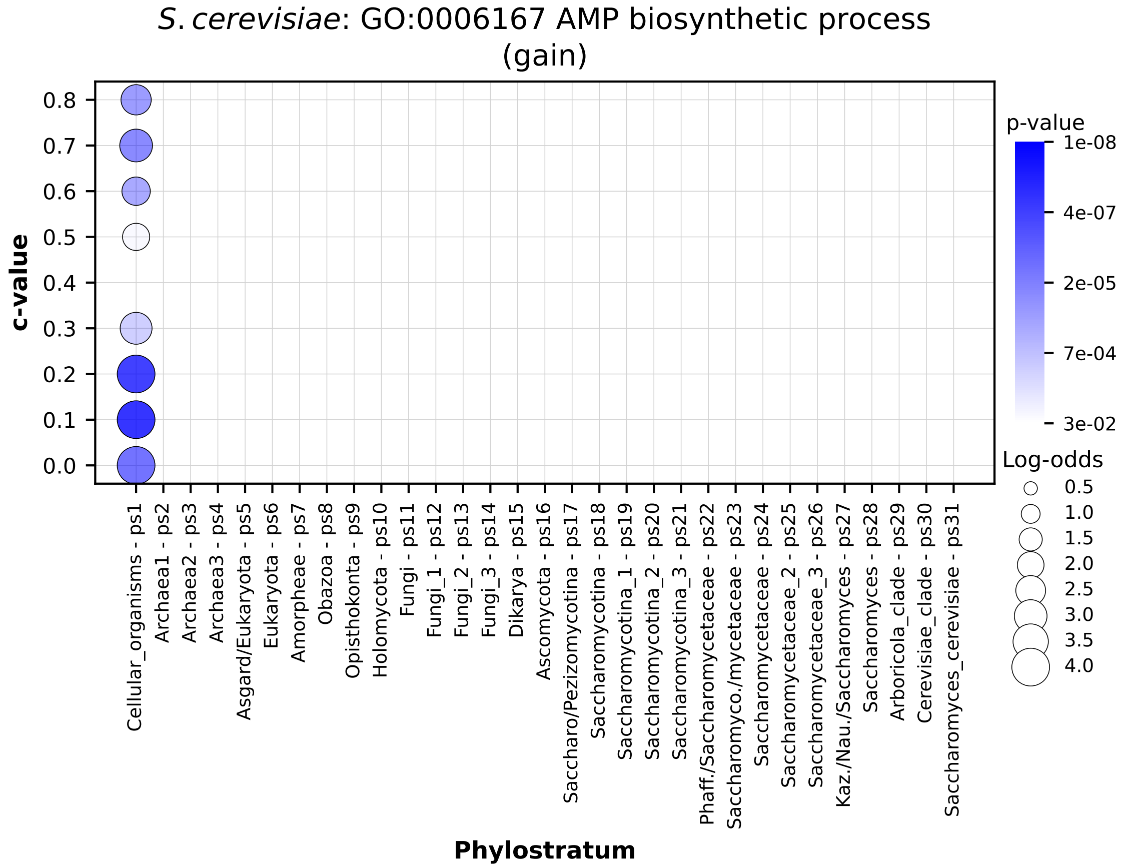

S. cerevisiae: GO:0006167 AMP biosynthetic process
(gain)
c-value
0.0
0.1
0.2
0.3
0.4
0.5
0.6
0.7
0.8
p-value
1e-08
4e-07
2e-05
7e-04
3e-02
Log-odds
0.5
1.0
1.5
2.0
2.5
3.0
3.5
4.0
Cellular_organisms - ps1
Archaea1 - ps2
Archaea2 - ps3
Archaea3 - ps4
Asgard/Eukaryota - ps5
Eukaryota - ps6
Amorpheae - ps7
Obazoa - ps8
Opisthokonta - ps9
Holomycota - ps10
Fungi - ps11
Fungi_1 - ps12
Fungi_2 - ps13
Fungi_3 - ps14
Dikarya - ps15
Ascomycota - ps16
Saccharo/Pezizomycotina - ps17
Saccharomycotina - ps18
Saccharomycotina_1 - ps19
Saccharomycotina_2 - ps20
Saccharomycotina_3 - ps21
Phaff./Saccharomycetaceae - ps22
Saccharomyco./mycetaceae - ps23
Saccharomycetaceae - ps24
Saccharomycetaceae_2 - ps25
Saccharomycetaceae_3 - ps26
Kaz./Nau./Saccharomyces - ps27
Saccharomyces - ps28
Arboricola_clade - ps29
Cerevisiae_clade - ps30
Saccharomyces_cerevisiae - ps31
Phylostratum

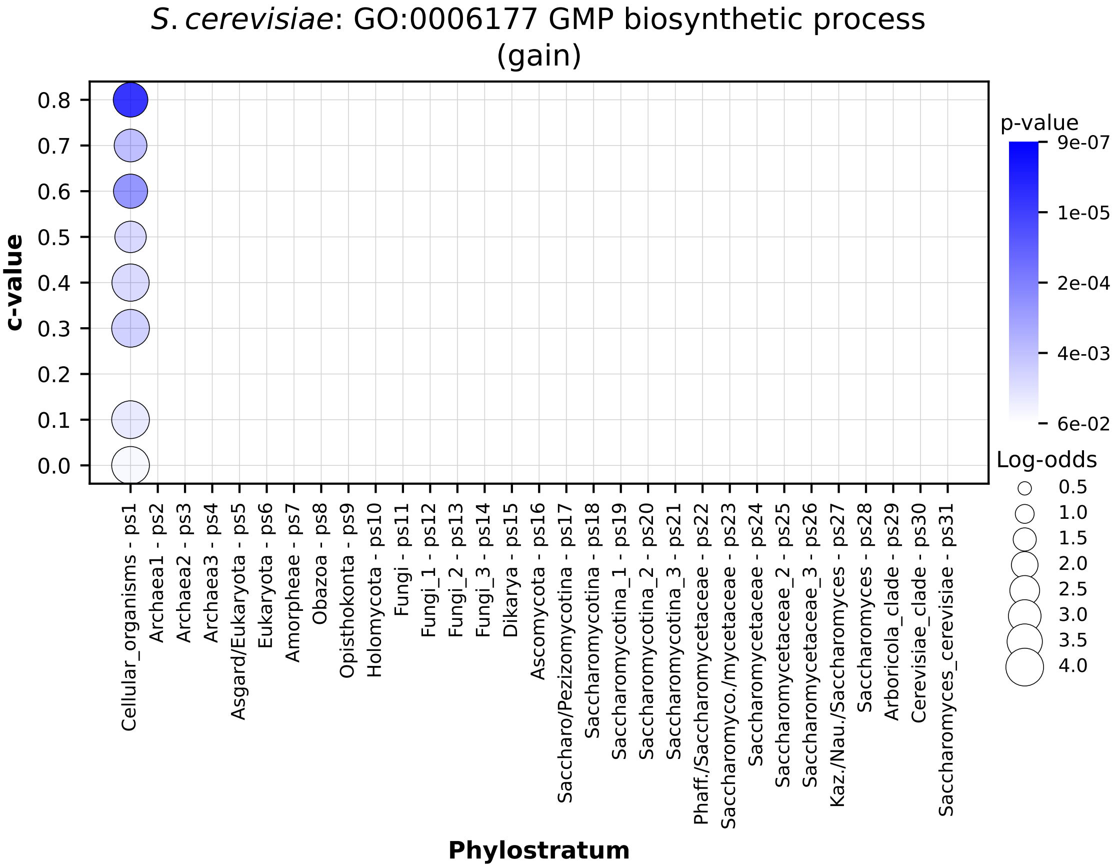
S. cerevisiae: GO:0006177 GMP biosynthetic process
(gain)
c-value
0.8
0.7
0.6
0.5
0.4
0.3
0.2
0.1
0.0
p-value
9e-07
1e-05
2e-04
4e-03
6e-02
Log-odds
0.5
1.0
1.5
2.0
2.5
3.0
3.5
4.0
Cellular_organisms - ps1
Archaea1 - ps2
Archaea2 - ps3
Archaea3 - ps4
Asgard/Eukaryota - ps5
Eukaryota - ps6
Amorpheae - ps7
Obazoa - ps8
Opisthokonta - ps9
Holomycota - ps10
Fungi - ps11
Fungi_1 - ps12
Fungi_2 - ps13
Fungi_3 - ps14
Dikarya - ps15
Ascomycota - ps16
Saccharo/Pezizomycotina - ps17
Saccharomycotina - ps18
Saccharomycotina_1 - ps19
Saccharomycotina_2 - ps20
Saccharomycotina_3 - ps21
Phaff./Saccharomycetaceae - ps22
Saccharomyco./mycetaceae - ps23
Saccharomycetaceae - ps24
Saccharomycetaceae_2 - ps25
Saccharomycetaceae_3 - ps26
Kaz./Nau./Saccharomyces - ps27
Saccharomyces - ps28
Arboricola_clade - ps29
Cerevisiae_clade - ps30
Saccharomyces_cerevisiae - ps31
Phylostratum

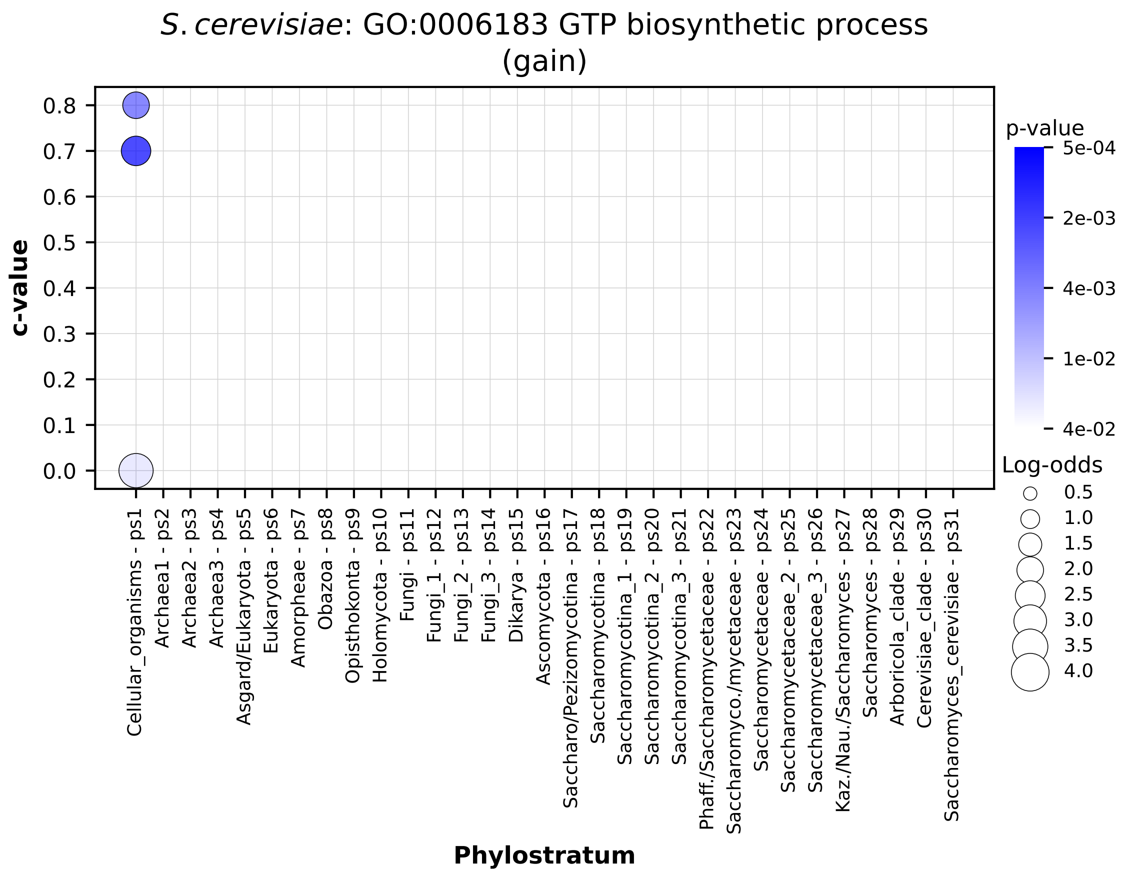
S. cerevisiae: GO:0006183 GTP biosynthetic process
(gain)
c-value
0.8
0.7
0.6
0.5
0.4
0.3
0.2
0.1
0.0
p-value
5e-04
2e-03
4e-03
1e-02
4e-02
Log-odds
0.5
1.0
1.5
2.0
2.5
3.0
3.5
4.0
Cellular_organisms - ps1
Archaea1 - ps2
Archaea2 - ps3
Archaea3 - ps4
Asgard/Eukaryota - ps5
Eukaryota - ps6
Amorpheae - ps7
Obazoa - ps8
Opisthokonta - ps9
Holomycota - ps10
Fungi - ps11
Fungi_1 - ps12
Fungi_2 - ps13
Fungi_3 - ps14
Dikarya - ps15
Ascomycota - ps16
Saccharo/Pezizomycotina - ps17
Saccharomycotina - ps18
Saccharomycotina_1 - ps19
Saccharomycotina_2 - ps20
Saccharomycotina_3 - ps21
Phaff./Saccharomycetaceae - ps22
Saccharomyco./mycetaceae - ps23
Saccharomycetaceae - ps24
Saccharomycetaceae_2 - ps25
Saccharomycetaceae_3 - ps26
Kaz./Nau./Saccharomyces - ps27
Saccharomyces - ps28
Arboricola_clade - ps29
Cerevisiae_clade - ps30
Saccharomyces_cerevisiae - ps31
Phylostratum

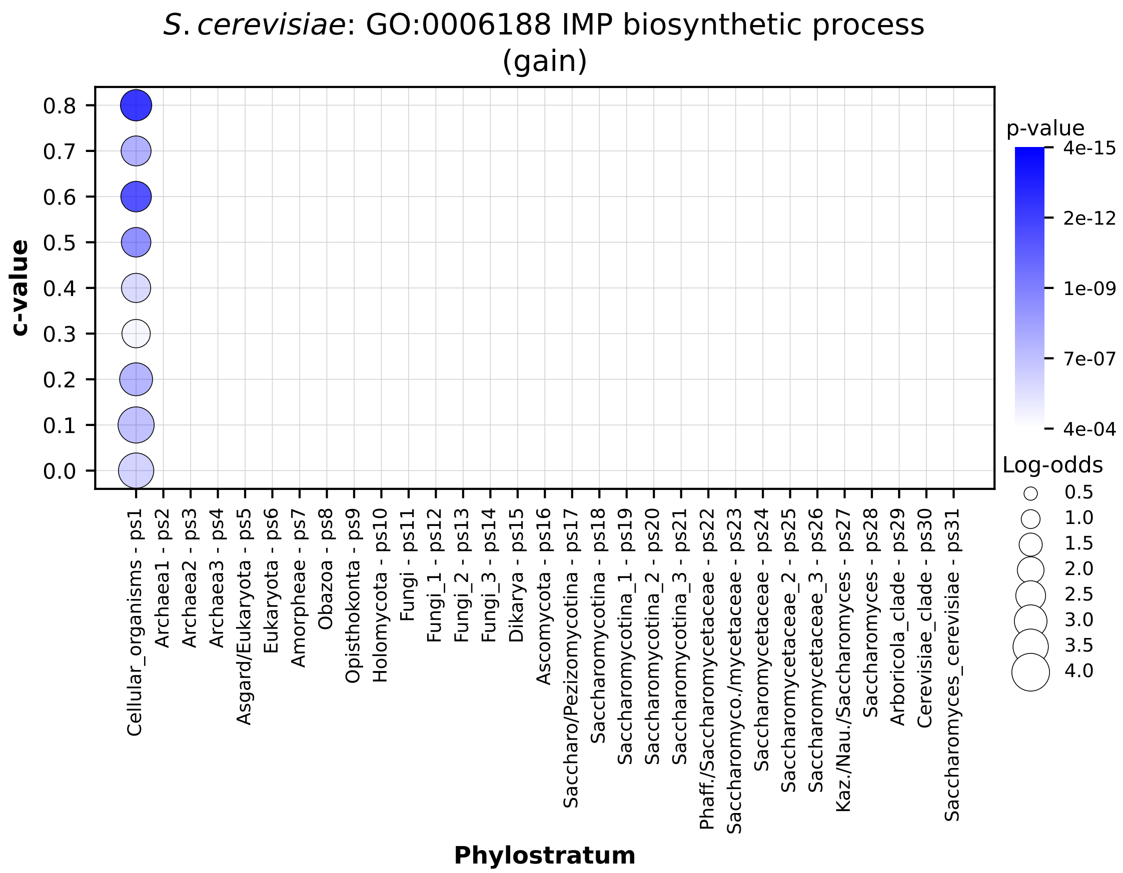
S. cerevisiae: GO:0006188 IMP biosynthetic process
(gain)
c-value
0.8
0.7
0.6
0.5
0.4
0.3
0.2
0.1
0.0
p-value
4e-15
2e-12
1e-09
7e-07
4e-04
Log-odds
0.5
1.0
1.5
2.0
2.5
3.0
3.5
4.0
Cellular_organisms - ps1
Archaea1 - ps2
Archaea2 - ps3
Archaea3 - ps4
Asgard/Eukaryota - ps5
Eukaryota - ps6
Amorpheae - ps7
Obazoa - ps8
Opisthokonta - ps9
Holomycota - ps10
Fungi - ps11
Fungi_1 - ps12
Fungi_2 - ps13
Fungi_3 - ps14
Dikarya - ps15
Ascomycota - ps16
Saccharo/Pezizomycotina - ps17
Saccharomycotina - ps18
Saccharomycotina_1 - ps19
Saccharomycotina_2 - ps20
Saccharomycotina_3 - ps21
Phaff./Saccharomycetaceae - ps22
Saccharomyco./mycetaceae - ps23
Saccharomycetaceae - ps24
Saccharomycetaceae_2 - ps25
Saccharomycetaceae_3 - ps26
Kaz./Nau./Saccharomyces - ps27
Saccharomyces - ps28
Arboricola_clade - ps29
Cerevisiae_clade - ps30
Saccharomyces_cerevisiae - ps31
Phylostratum

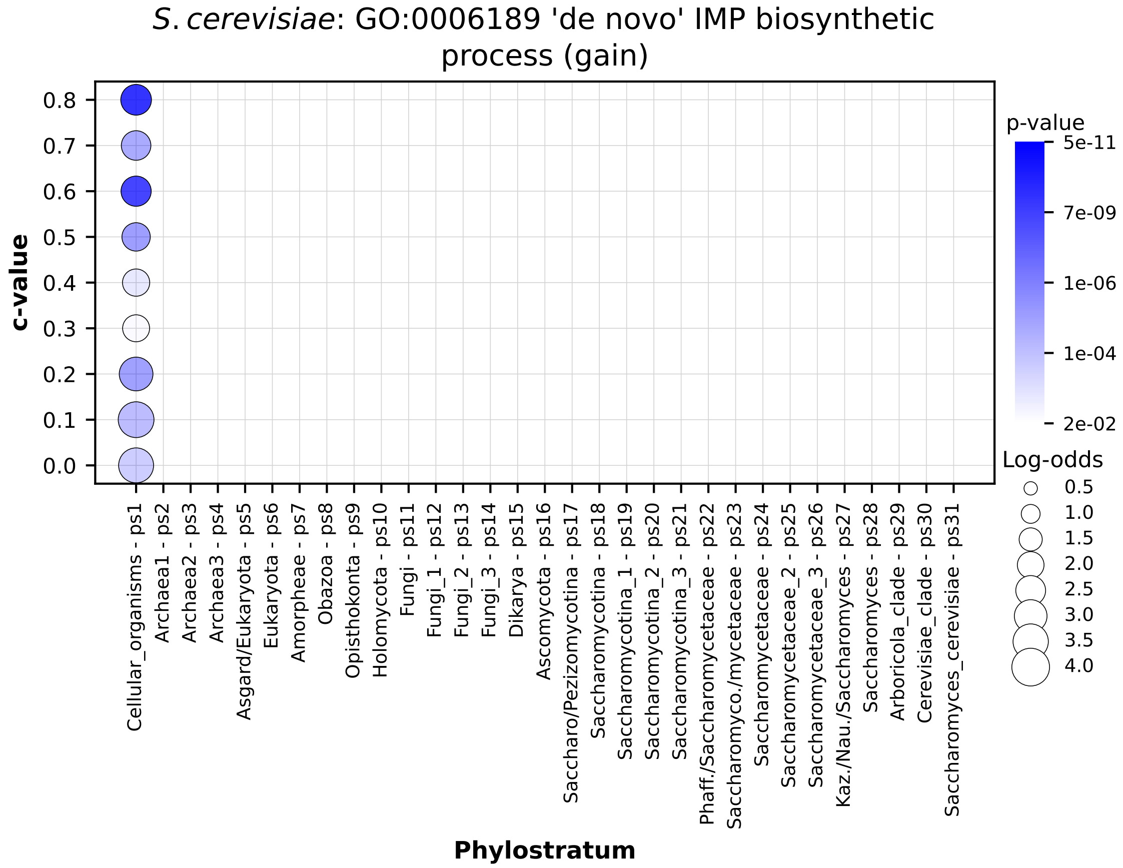
S. cerevisiae: GO:0006189 'de novo' IMP biosynthetic process (gain)
c-value
0.8
0.7
0.6
0.5
0.4
0.3
0.2
0.1
0.0
p-value
5e-11
7e-09
1e-06
1e-04
2e-02
Log-odds
0.5
1.0
1.5
2.0
2.5
3.0
3.5
4.0
Cellular_organisms - ps1
Archaea1 - ps2
Archaea2 - ps3
Archaea3 - ps4
Asgard/Eukaryota - ps5
Eukaryota - ps6
Amorpheae - ps7
Obazoa - ps8
Opisthokonta - ps9
Holomycota - ps10
Fungi - ps11
Fungi_1 - ps12
Fungi_2 - ps13
Fungi_3 - ps14
Dikarya - ps15
Ascomycota - ps16
Saccharo/Pezizomycotina - ps17
Saccharomycotina - ps18
Saccharomycotina_1 - ps19
Saccharomycotina_2 - ps20
Saccharomycotina_3 - ps21
Phaff./Saccharomycetaceae - ps22
Saccharomyco./mycetaceae - ps23
Saccharomycetaceae - ps24
Saccharomycetaceae_2 - ps25
Saccharomycetaceae_3 - ps26
Kaz./Nau./Saccharomyces - ps27
Saccharomyces - ps28
Arboricola_clade - ps29
Cerevisiae_clade - ps30
Saccharomyces_cerevisiae - ps31
Phylostratum

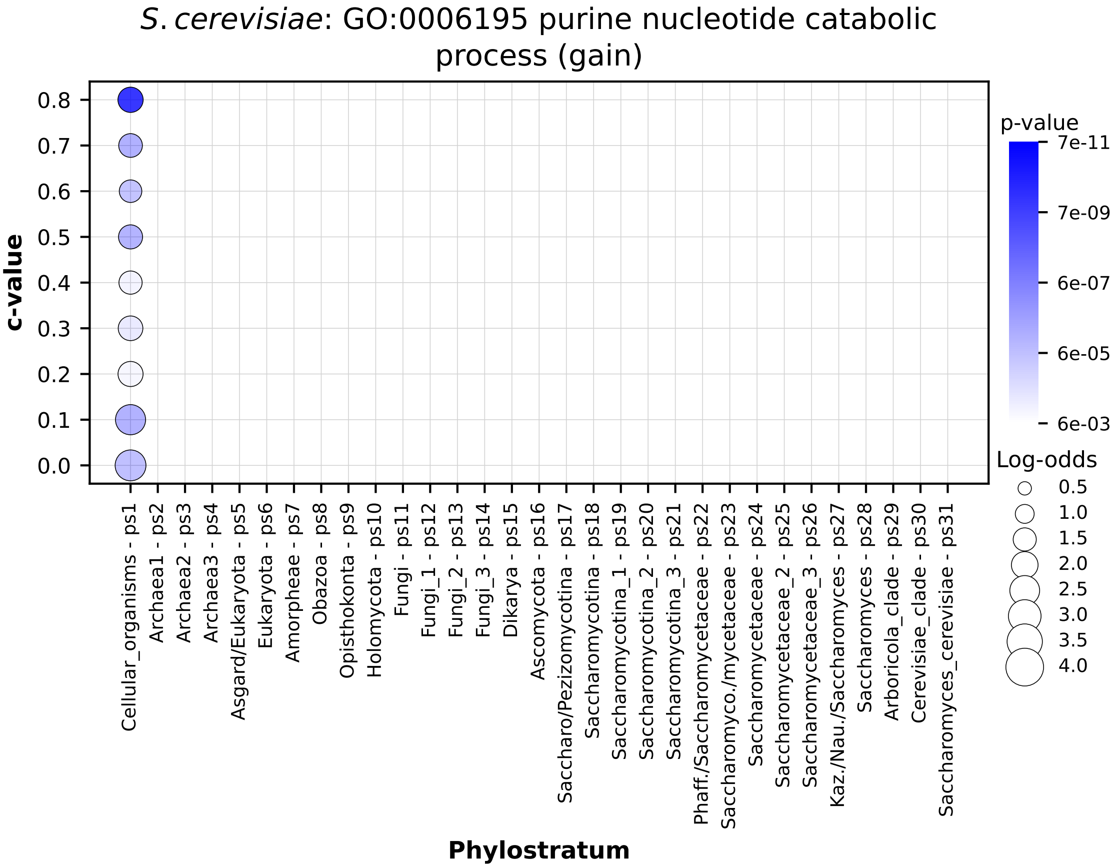

S. cerevisiae: GO:0006195 purine nucleotide catabolic process (gain)
c-value
0.0
0.1
0.2
0.3
0.4
0.5
0.6
0.7
0.8
p-value
7e-11
7e-09
6e-07
6e-05
6e-03
Log-odds
0.5
1.0
1.5
2.0
2.5
3.0
3.5
4.0
Cellular_organisms - ps1
Archaea1 - ps2
Archaea2 - ps3
Archaea3 - ps4
Asgard/Eukaryota - ps5
Eukaryota - ps6
Amorpheae - ps7
Obazoa - ps8
Opisthokonta - ps9
Holomycota - ps10
Fungi - ps11
Fungi_1 - ps12
Fungi_2 - ps13
Fungi_3 - ps14
Dikarya - ps15
Ascomycota - ps16
Saccharo/Pezizomycotina - ps17
Saccharomycotina - ps18
Saccharomycotina_1 - ps19
Saccharomycotina_2 - ps20
Saccharomycotina_3 - ps21
Phaff./Saccharomycetaceae - ps22
Saccharomyco./mycetaceae - ps23
Saccharomycetaceae - ps24
Saccharomycetaceae_2 - ps25
Saccharomycetaceae_3 - ps26
Kaz./Nau./Saccharomyces - ps27
Saccharomyces - ps28
Arboricola_clade - ps29
Cerevisiae_clade - ps30
Saccharomyces_cerevisiae - ps31
Phylostratum

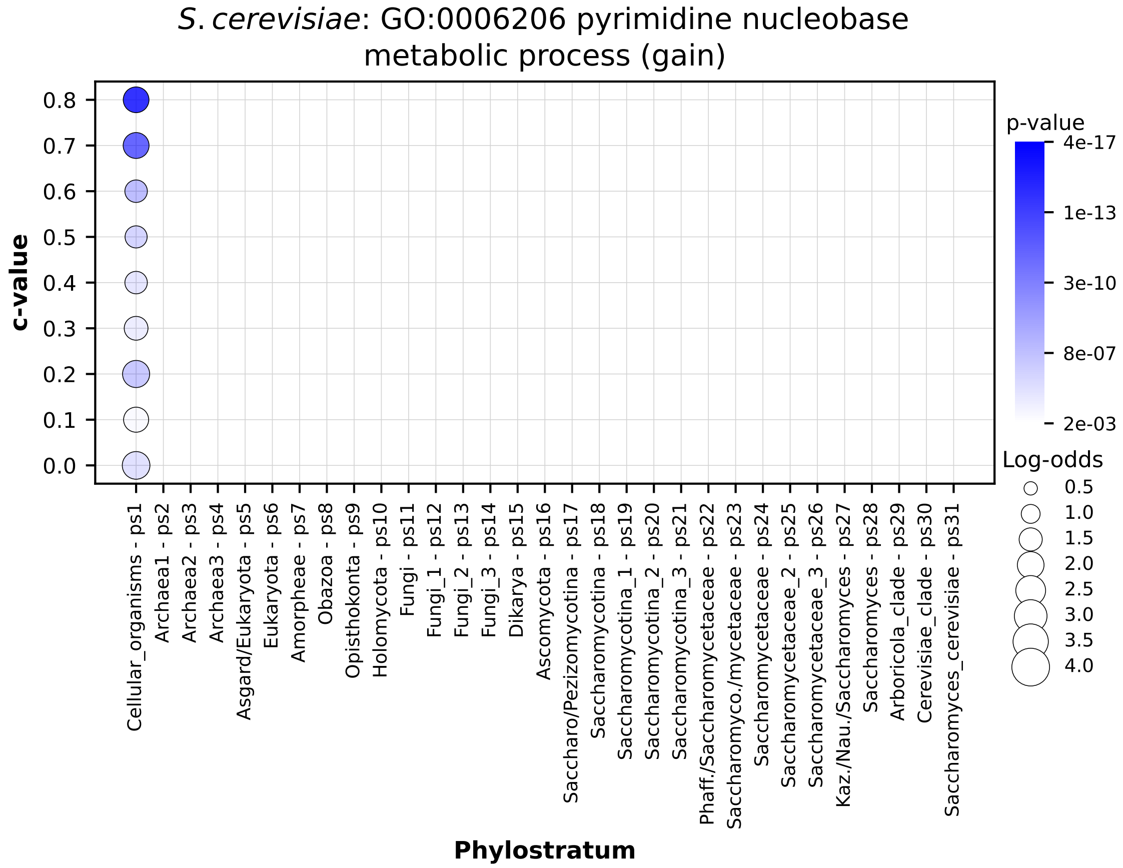

S. cerevisiae: GO:0006206 pyrimidine nucleobase metabolic process (gain)
c-value
0.8
0.7
0.6
0.5
0.4
0.3
0.2
0.1
0.0
p-value
4e-17
1e-13
3e-10
8e-07
2e-03
Log-odds
0.5
1.0
1.5
2.0
2.5
3.0
3.5
4.0
Cellular_organisms - ps1
Archaea1 - ps2
Archaea2 - ps3
Archaea3 - ps4
Asgard/Eukaryota - ps5
Eukaryota - ps6
Amorpheae - ps7
Obazoa - ps8
Opisthokonta - ps9
Holomycota - ps10
Fungi - ps11
Fungi_1 - ps12
Fungi_2 - ps13
Fungi_3 - ps14
Dikarya - ps15
Ascomycota - ps16
Saccharo/Pezizomycotina - ps17
Saccharomycotina - ps18
Saccharomycotina_1 - ps19
Saccharomycotina_2 - ps20
Saccharomycotina_3 - ps21
Phaff./Saccharomycetaceae - ps22
Saccharomyco./mycetaceae - ps23
Saccharomycetaceae - ps24
Saccharomycetaceae_2 - ps25
Saccharomycetaceae_3 - ps26
Kaz./Nau./Saccharomyces - ps27
Saccharomyces - ps28
Arboricola_clade - ps29
Cerevisiae_clade - ps30
Saccharomyces_cerevisiae - ps31
Phylostratum

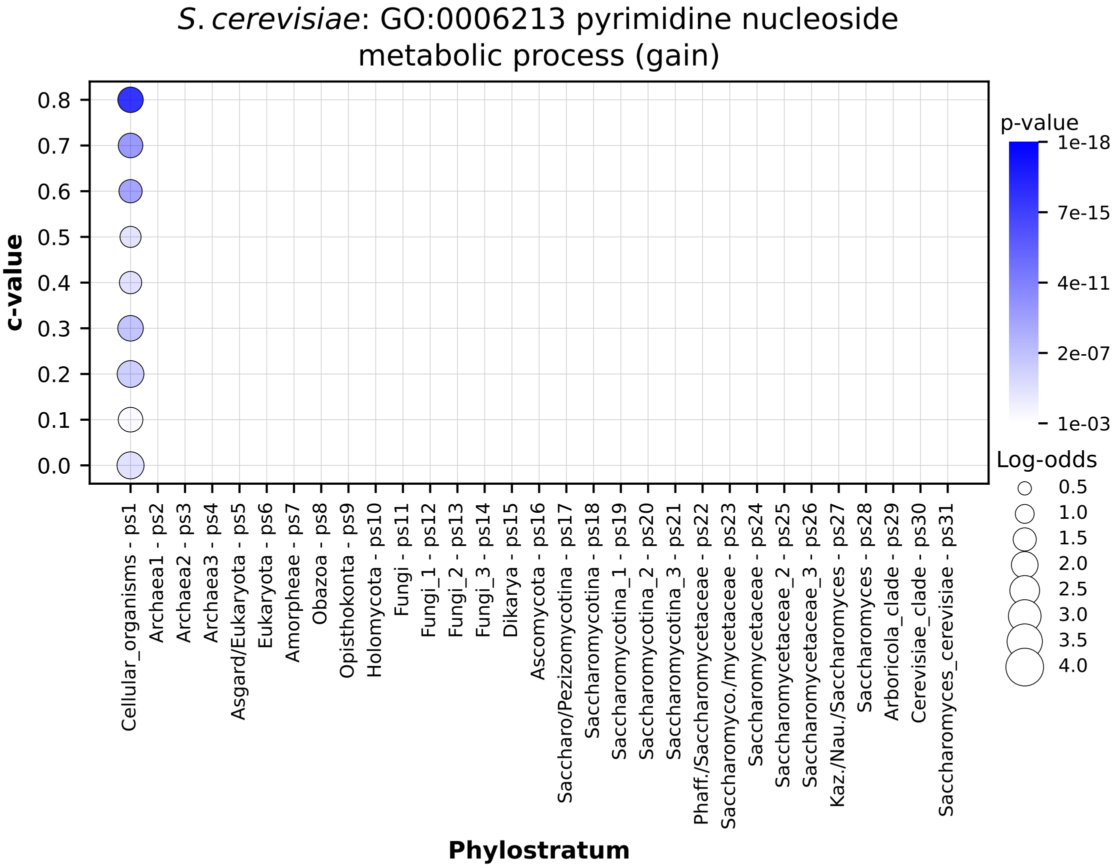

S. cerevisiae: GO:0006213 pyrimidine nucleoside metabolic process (gain)
c-value
0.8
0.7
0.6
0.5
0.4
0.3
0.2
0.1
0.0
p-value
1e-18
7e-15
4e-11
2e-07
1e-03
Log-odds
0.5
1.0
1.5
2.0
2.5
3.0
3.5
4.0
Cellular_organisms - ps1
Archaea1 - ps2
Archaea2 - ps3
Archaea3 - ps4
Asgard/Eukaryota - ps5
Eukaryota - ps6
Amorpheae - ps7
Obazoa - ps8
Opisthokonta - ps9
Holomycota - ps10
Fungi - ps11
Fungi_1 - ps12
Fungi_2 - ps13
Fungi_3 - ps14
Dikarya - ps15
Ascomycota - ps16
Saccharo/Pezizomycotina - ps17
Saccharomycotina - ps18
Saccharomycotina_1 - ps19
Saccharomycotina_2 - ps20
Saccharomycotina_3 - ps21
Phaff./Saccharomycetaceae - ps22
Saccharomyco./mycetaceae - ps23
Saccharomycetaceae - ps24
Saccharomycetaceae_2 - ps25
Saccharomycetaceae_3 - ps26
Kaz./Nau./Saccharomyces - ps27
Saccharomyces - ps28
Arboricola_clade - ps29
Cerevisiae_clade - ps30
Saccharomyces_cerevisiae - ps31
Phylostratum

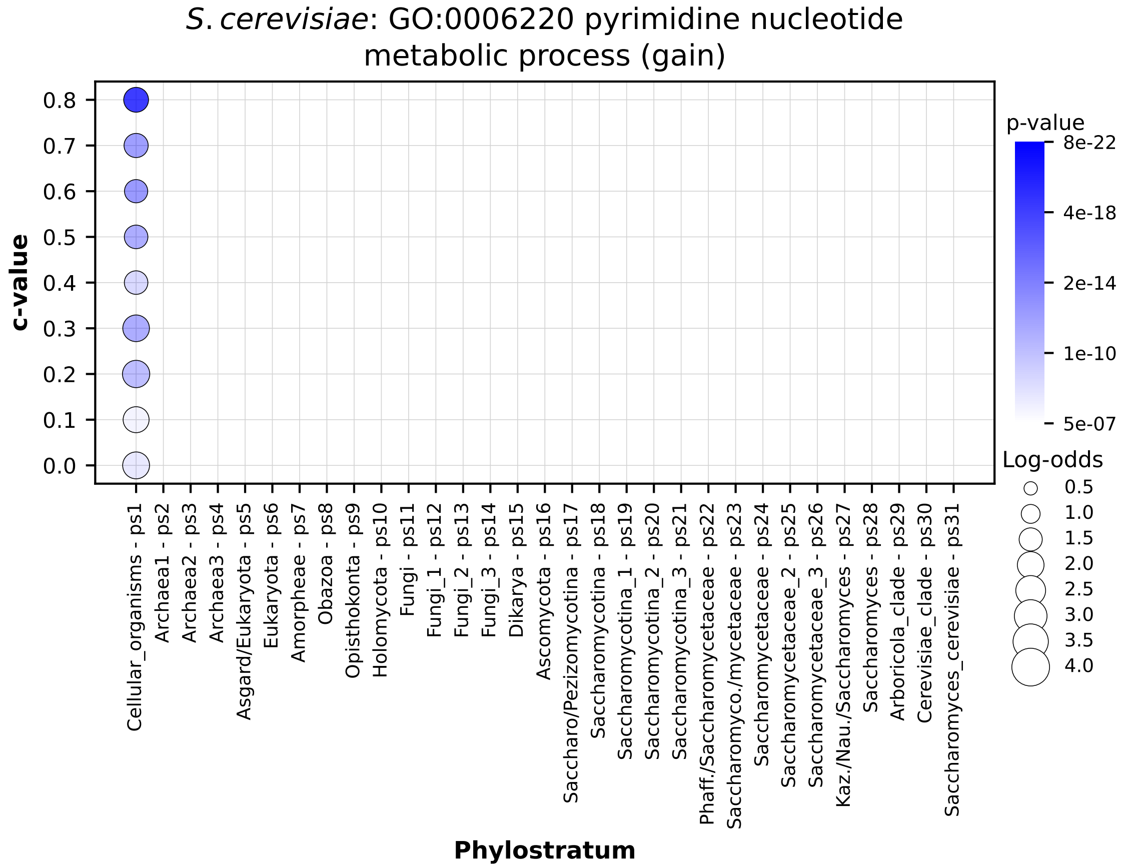

S. cerevisiae: GO:0006220 pyrimidine nucleotide metabolic process (gain)
c-value
0.0
0.1
0.2
0.3
0.4
0.5
0.6
0.7
0.8
p-value
8e-22
4e-18
2e-14
1e-10
5e-07
Log-odds
0.5
1.0
1.5
2.0
2.5
3.0
3.5
4.0
Cellular_organisms - ps1
Archaea1 - ps2
Archaea2 - ps3
Archaea3 - ps4
Asgard/Eukaryota - ps5
Eukaryota - ps6
Amorpheae - ps7
Obazoa - ps8
Opisthokonta - ps9
Holomycota - ps10
Fungi - ps11
Fungi_1 - ps12
Fungi_2 - ps13
Fungi_3 - ps14
Dikarya - ps15
Ascomycota - ps16
Saccharo/Pezizomycotina - ps17
Saccharomycotina - ps18
Saccharomycotina_1 - ps19
Saccharomycotina_2 - ps20
Saccharomycotina_3 - ps21
Phaff./Saccharomycetaceae - ps22
Saccharomyco./mycetaceae - ps23
Saccharomycetaceae - ps24
Saccharomycetaceae_2 - ps25
Saccharomycetaceae_3 - ps26
Kaz./Nau./Saccharomyces - ps27
Saccharomyces - ps28
Arboricola_clade - ps29
Cerevisiae_clade - ps30
Saccharomyces_cerevisiae - ps31
Phylostratum

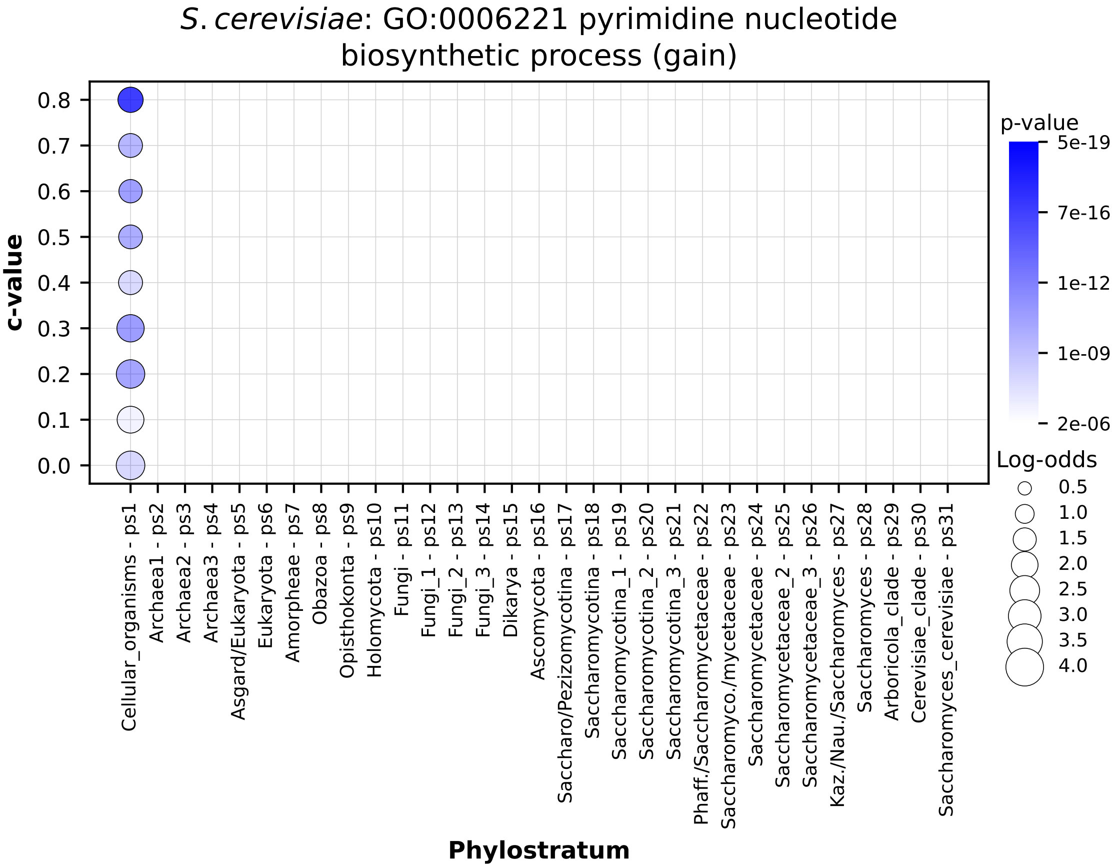

S. cerevisiae: GO:0006221 pyrimidine nucleotide biosynthetic process (gain)
c-value
0.8
0.7
0.6
0.5
0.4
0.3
0.2
0.1
0.0
Phylostratum
Cellular_organisms - ps1
Archaea1 - ps2
Archaea2 - ps3
Archaea3 - ps4
Asgard/Eukaryota - ps5
Eukaryota - ps6
Amorpheae - ps7
Obazoa - ps8
Opisthokonta - ps9
Holomycota - ps10
Fungi - ps11
Fungi_1 - ps12
Fungi_2 - ps13
Fungi_3 - ps14
Dikarya - ps15
Ascomycota - ps16
Saccharo/Pezizomycotina - ps17
Saccharomycotina - ps18
Saccharomycotina_1 - ps19
Saccharomycotina_2 - ps20
Saccharomycotina_3 - ps21
Phaff./Saccharomycetaceae - ps22
Saccharomyco./mycetaceae - ps23
Saccharomycetaceae - ps24
Saccharomycetaceae_2 - ps25
Saccharomycetaceae_3 - ps26
Kaz./Nau./Saccharomyces - ps27
Saccharomyces - ps28
Arboricola_clade - ps29
Cerevisiae_clade - ps30
Saccharomyces_cerevisiae - ps31
p-value
5e-19
7e-16
1e-12
1e-09
2e-06
Log-odds
0.5
1.0
1.5
2.0
2.5
3.0
3.5
4.0

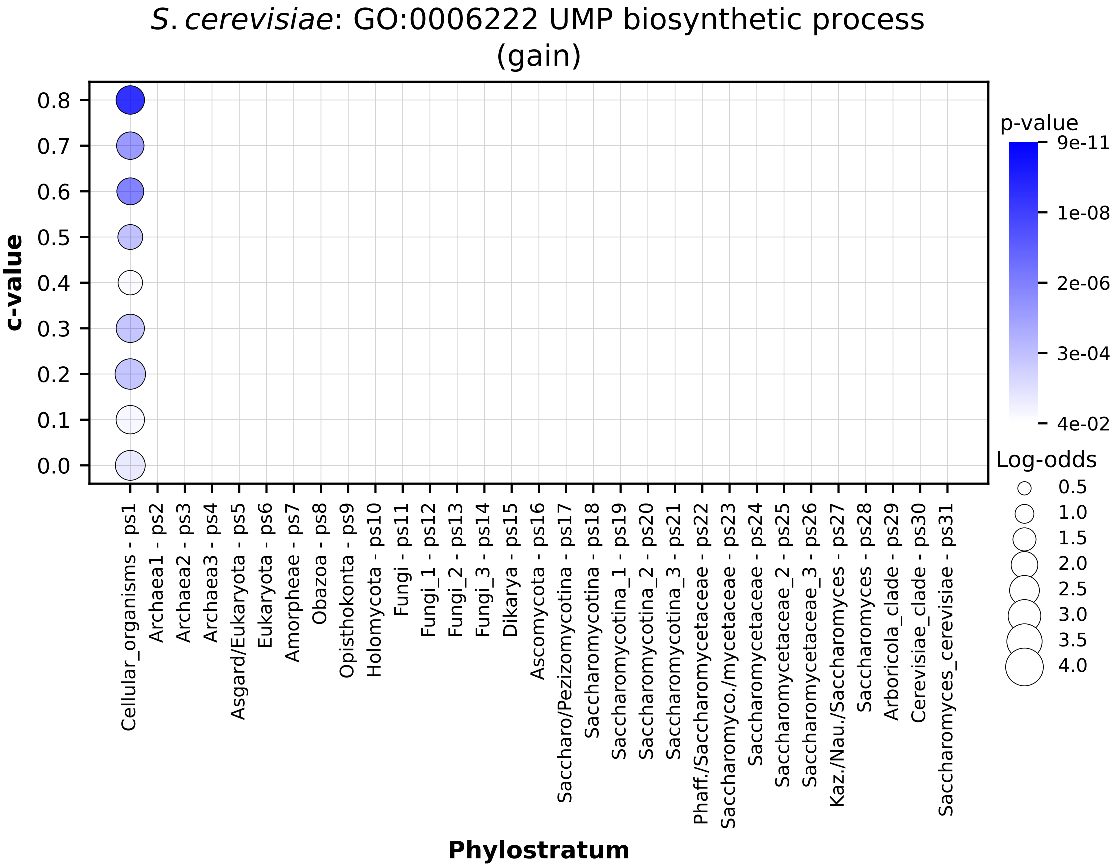

S. cerevisiae: GO:0006222 UMP biosynthetic process
(gain)
c-value
0.8
0.7
0.6
0.5
0.4
0.3
0.2
0.1
0.0
Phylostratum
Cellular_organisms - ps1
Archaea1 - ps2
Archaea2 - ps3
Archaea3 - ps4
Asgard/Eukaryota - ps5
Eukaryota - ps6
Amorpheae - ps7
Obazoa - ps8
Opisthokonta - ps9
Holomycota - ps10
Fungi - ps11
Fungi_1 - ps12
Fungi_2 - ps13
Fungi_3 - ps14
Dikarya - ps15
Ascomycota - ps16
Saccharo/Pezizomycotina - ps17
Saccharomycotina - ps18
Saccharomycotina_1 - ps19
Saccharomycotina_2 - ps20
Saccharomycotina_3 - ps21
Phaff./Saccharomycetaceae - ps22
Saccharomyco./mycetaceae - ps23
Saccharomycetaceae - ps24
Saccharomycetaceae_2 - ps25
Saccharomycetaceae_3 - ps26
Kaz./Nau./Saccharomyces - ps27
Saccharomyces - ps28
Arboricola_clade - ps29
Cerevisiae_clade - ps30
Saccharomyces_cerevisiae - ps31
p-value
9e-11
1e-08
2e-06
3e-04
4e-02
Log-odds
0.5
1.0
1.5
2.0
2.5
3.0
3.5
4.0

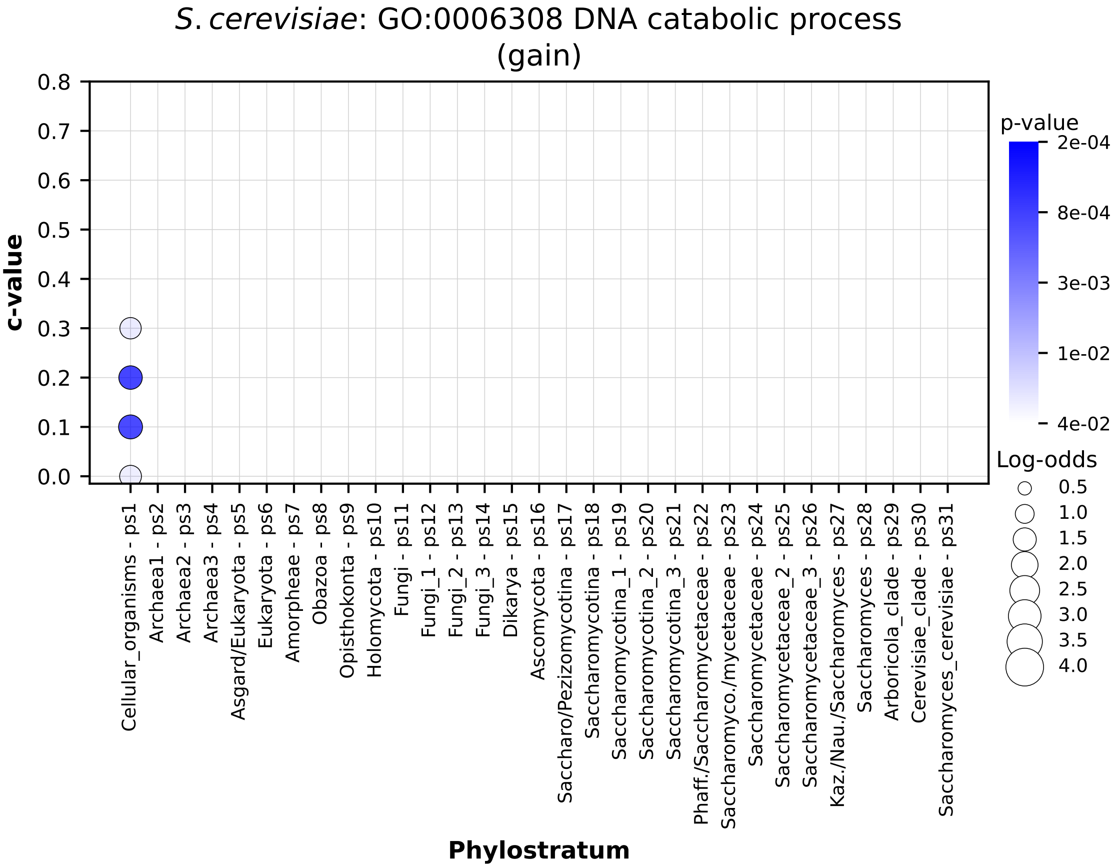
S. cerevisiae: GO:0006308 DNA catabolic process
(gain)
c-value
0.8
0.7
0.6
0.5
0.4
0.3
0.2
0.1
0.0
p-value
2e-04
8e-04
3e-03
1e-02
4e-02
Log-odds
0.5
1.0
1.5
2.0
2.5
3.0
3.5
4.0
Cellular_organisms - ps1
Archaea1 - ps2
Archaea2 - ps3
Archaea3 - ps4
Asgard/Eukaryota - ps5
Eukaryota - ps6
Amorpheae - ps7
Obazoa - ps8
Opisthokonta - ps9
Holomycota - ps10
Fungi - ps11
Fungi_1 - ps12
Fungi_2 - ps13
Fungi_3 - ps14
Dikarya - ps15
Ascomycota - ps16
Saccharo/Pezizomycotina - ps17
Saccharomycotina - ps18
Saccharomycotina_1 - ps19
Saccharomycotina_2 - ps20
Saccharomycotina_3 - ps21
Phaff./Saccharomycetaceae - ps22
Saccharomyco./mycetaceae - ps23
Saccharomycetaceae - ps24
Saccharomycetaceae_2 - ps25
Saccharomycetaceae_3 - ps26
Kaz./Nau./Saccharomyces - ps27
Saccharomyces - ps28
Arboricola_clade - ps29
Cerevisiae_clade - ps30
Saccharomyces_cerevisiae - ps31
Phylostratum

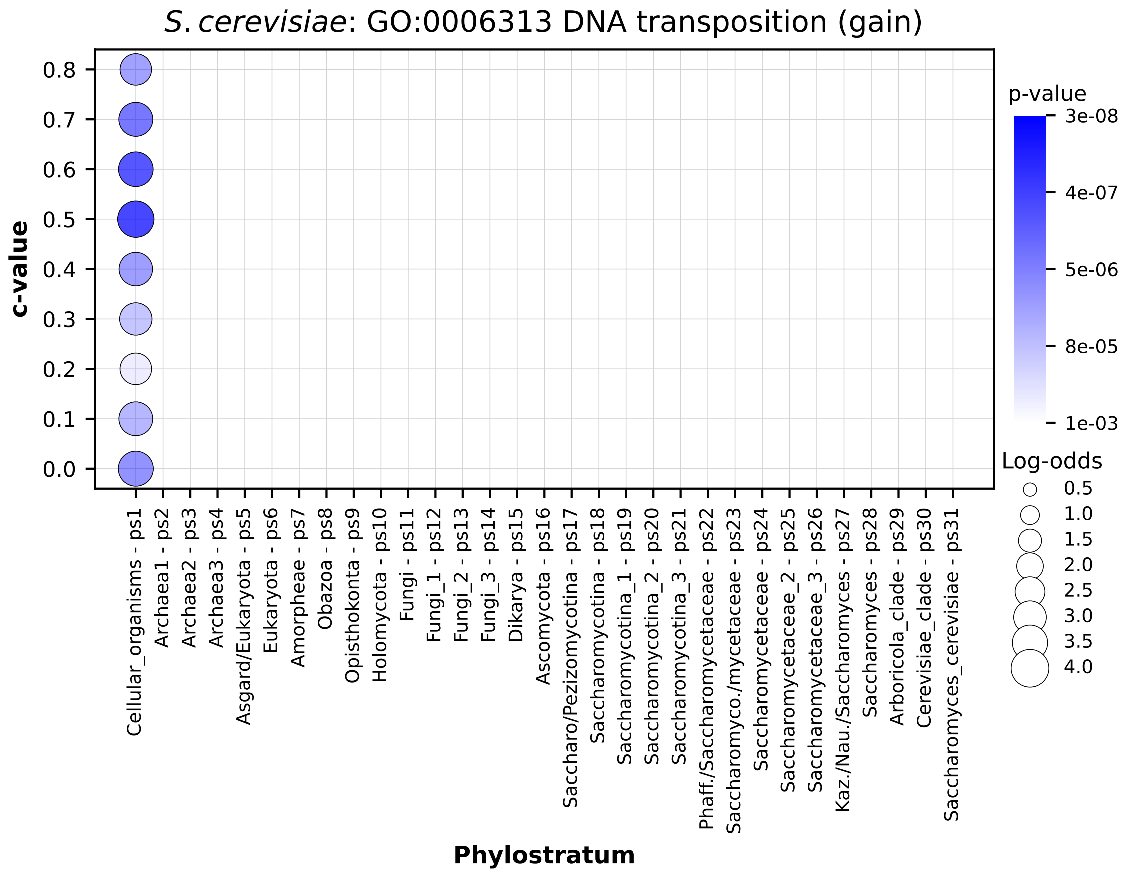

S. cerevisiae: GO:0006313 DNA transposition (gain)
c-value
0.0
0.1
0.2
0.3
0.4
0.5
0.6
0.7
0.8
Phylostratum
Cellular_organisms - ps1
Archaea1 - ps2
Archaea2 - ps3
Archaea3 - ps4
Asgard/Eukaryota - ps5
Eukaryota - ps6
Amorpheae - ps7
Obazoa - ps8
Opisthokonta - ps9
Holomycota - ps10
Fungi - ps11
Fungi_1 - ps12
Fungi_2 - ps13
Fungi_3 - ps14
Dikarya - ps15
Ascomycota - ps16
Saccharo/Pezizomycotina - ps17
Saccharomycotina - ps18
Saccharomycotina_1 - ps19
Saccharomycotina_2 - ps20
Saccharomycotina_3 - ps21
Phaff./Saccharomycetaceae - ps22
Saccharomyco./mycetaceae - ps23
Saccharomycetaceae - ps24
Saccharomycetaceae_2 - ps25
Saccharomycetaceae_3 - ps26
Kaz./Nau./Saccharomyces - ps27
Saccharomyces - ps28
Arboricola_clade - ps29
Cerevisiae_clade - ps30
Saccharomyces_cerevisiae - ps31
p-value
3e-08
4e-07
5e-06
8e-05
1e-03
Log-odds
0.5
1.0
1.5
2.0
2.5
3.0
3.5
4.0

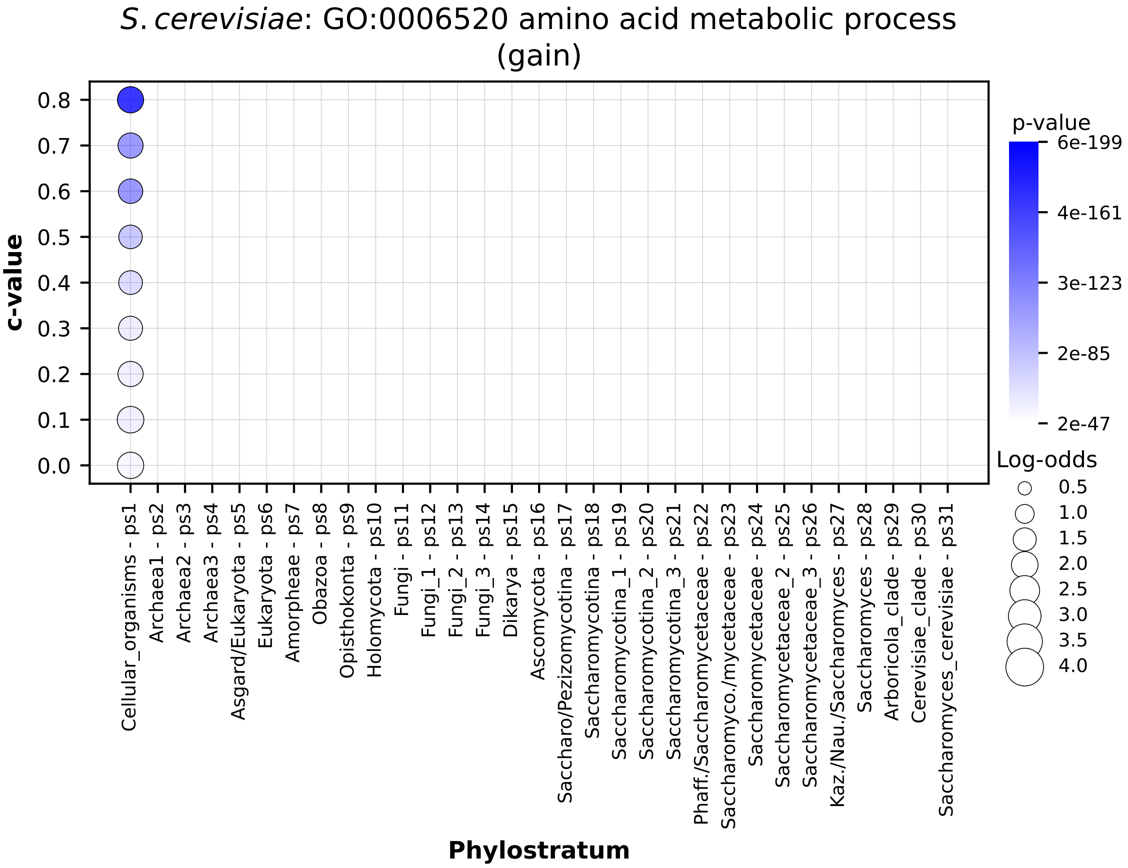
S. cerevisiae: GO:0006520 amino acid metabolic process
(gain)
c-value
0.8
0.7
0.6
0.5
0.4
0.3
0.2
0.1
0.0
p-value
6e-199
4e-161
3e-123
2e-85
2e-47
Log-odds
0.5
1.0
1.5
2.0
2.5
3.0
3.5
4.0
Cellular_organisms - ps1
Archaea1 - ps2
Archaea2 - ps3
Archaea3 - ps4
Asgard/Eukaryota - ps5
Eukaryota - ps6
Amorpheae - ps7
Obazoa - ps8
Opisthokonta - ps9
Holomycota - ps10
Fungi - ps11
Fungi_1 - ps12
Fungi_2 - ps13
Fungi_3 - ps14
Dikarya - ps15
Ascomycota - ps16
Saccharo/Pezizomycotina - ps17
Saccharomycotina - ps18
Saccharomycotina_1 - ps19
Saccharomycotina_2 - ps20
Saccharomycotina_3 - ps21
Phaff./Saccharomycetaceae - ps22
Saccharomyco./mycetaceae - ps23
Saccharomycetaceae - ps24
Saccharomycetaceae_2 - ps25
Saccharomycetaceae_3 - ps26
Kaz./Nau./Saccharomyces - ps27
Saccharomyces - ps28
Arboricola_clade - ps29
Cerevisiae_clade - ps30
Saccharomyces_cerevisiae - ps31
Phylostratum

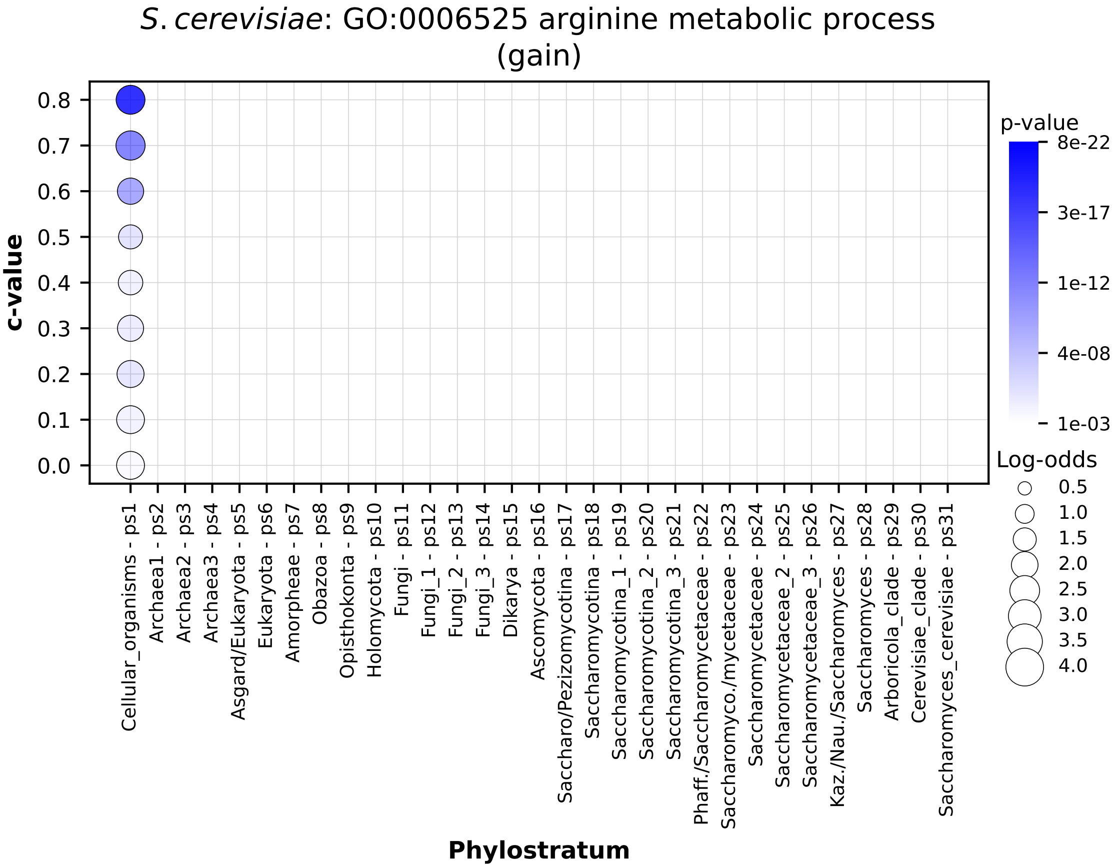

S. cerevisiae: GO:0006525 arginine metabolic process
(gain)
c-value
0.8
0.7
0.6
0.5
0.4
0.3
0.2
0.1
0.0
p-value
8e-22
3e-17
1e-12
4e-08
1e-03
Log-odds
0.5
1.0
1.5
2.0
2.5
3.0
3.5
4.0
Cellular_organisms - ps1
Archaea1 - ps2
Archaea2 - ps3
Archaea3 - ps4
Asgard/Eukaryota - ps5
Eukaryota - ps6
Amorpheae - ps7
Obazoa - ps8
Opisthokonta - ps9
Holomycota - ps10
Fungi - ps11
Fungi_1 - ps12
Fungi_2 - ps13
Fungi_3 - ps14
Dikarya - ps15
Ascomycota - ps16
Saccharo/Pezizomycotina - ps17
Saccharomycotina - ps18
Saccharomycotina_1 - ps19
Saccharomycotina_2 - ps20
Saccharomycotina_3 - ps21
Phaff./Saccharomycetaceae - ps22
Saccharomyco./mycetaceae - ps23
Saccharomycetaceae - ps24
Saccharomycetaceae_2 - ps25
Saccharomycetaceae_3 - ps26
Kaz./Nau./Saccharomyces - ps27
Saccharomyces - ps28
Arboricola_clade - ps29
Cerevisiae_clade - ps30
Saccharomyces_cerevisiae - ps31
Phylostratum

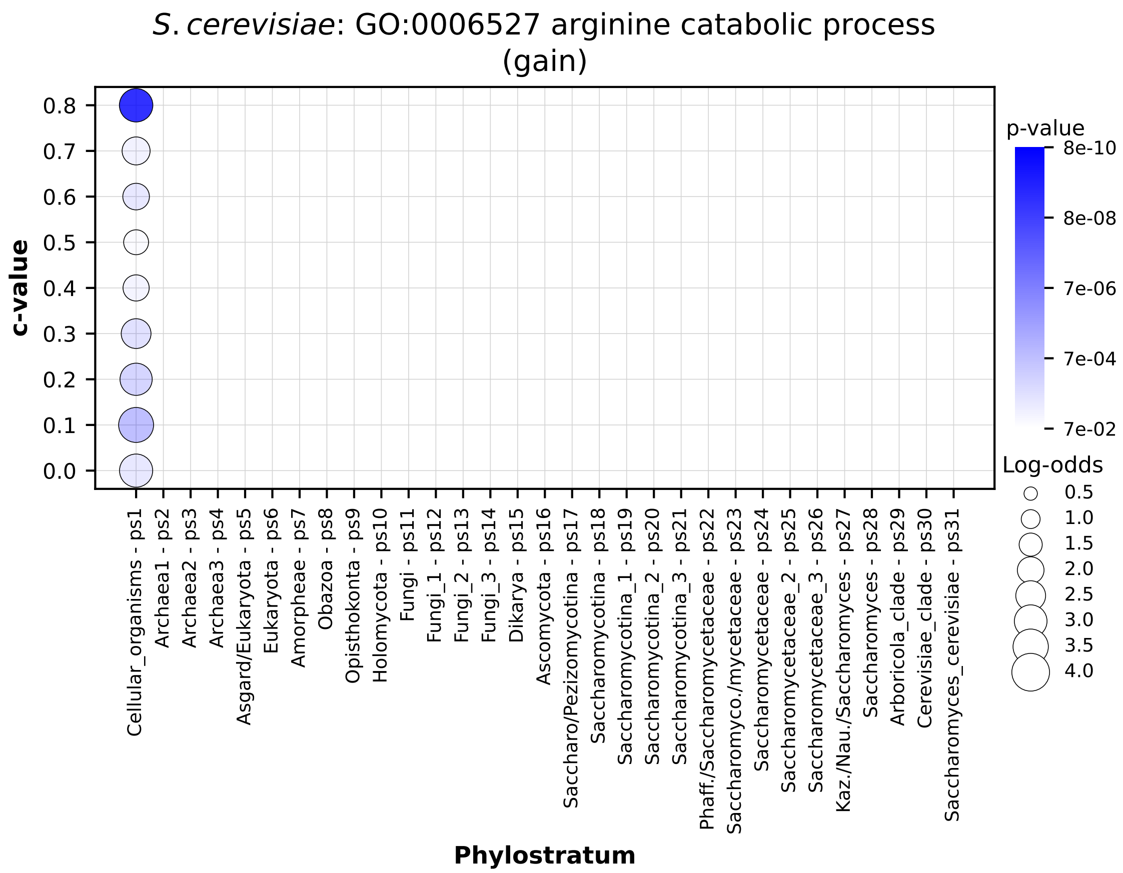

S. cerevisiae: GO:0006527 arginine catabolic process (gain)
c-value
0.0
0.1
0.2
0.3
0.4
0.5
0.6
0.7
0.8
p-value
8e-10
8e-08
7e-06
7e-04
7e-02
Log-odds
0.5
1.0
1.5
2.0
2.5
3.0
3.5
4.0
Cellular_organisms - ps1
Archaea1 - ps2
Archaea2 - ps3
Archaea3 - ps4
Asgard/Eukaryota - ps5
Eukaryota - ps6
Amorpheae - ps7
Obazoa - ps8
Opisthokonta - ps9
Holomycota - ps10
Fungi - ps11
Fungi_1 - ps12
Fungi_2 - ps13
Fungi_3 - ps14
Dikarya - ps15
Ascomycota - ps16
Saccharo/Pezizomycotina - ps17
Saccharomycotina - ps18
Saccharomycotina_1 - ps19
Saccharomycotina_2 - ps20
Saccharomycotina_3 - ps21
Phaff./Saccharomycetaceae - ps22
Saccharomyco./mycetaceae - ps23
Saccharomycetaceae - ps24
Saccharomycetaceae_2 - ps25
Saccharomycetaceae_3 - ps26
Kaz./Nau./Saccharomyces - ps27
Saccharomyces - ps28
Arboricola_clade - ps29
Cerevisiae_clade - ps30
Saccharomyces_cerevisiae - ps31
Phylostratum

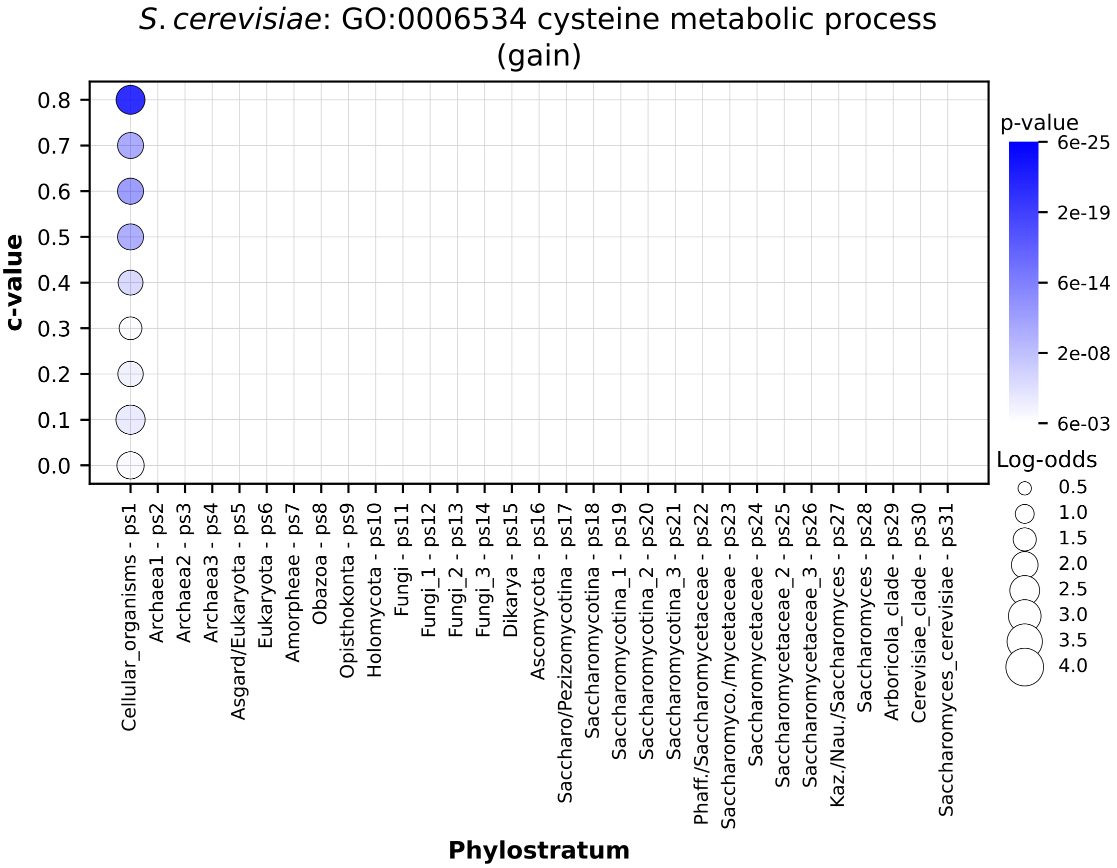

S. cerevisiae: GO:0006534 cysteine metabolic process
(gain)
c-value
0.8
0.7
0.6
0.5
0.4
0.3
0.2
0.1
0.0
p-value
6e-25
2e-19
6e-14
2e-08
6e-03
Log-odds
0.5
1.0
1.5
2.0
2.5
3.0
3.5
4.0
Cellular_organisms - ps1
Archaea1 - ps2
Archaea2 - ps3
Archaea3 - ps4
Asgard/Eukaryota - ps5
Eukaryota - ps6
Amorpheae - ps7
Obazoa - ps8
Opisthokonta - ps9
Holomycota - ps10
Fungi - ps11
Fungi_1 - ps12
Fungi_2 - ps13
Fungi_3 - ps14
Dikarya - ps15
Ascomycota - ps16
Saccharo/Pezizomycotina - ps17
Saccharomycotina - ps18
Saccharomycotina_1 - ps19
Saccharomycotina_2 - ps20
Saccharomycotina_3 - ps21
Phaff./Saccharomycetaceae - ps22
Saccharomyco./mycetaceae - ps23
Saccharomycetaceae - ps24
Saccharomycetaceae_2 - ps25
Saccharomycetaceae_3 - ps26
Kaz./Nau./Saccharomyces - ps27
Saccharomyces - ps28
Arboricola_clade - ps29
Cerevisiae_clade - ps30
Saccharomyces_cerevisiae - ps31
Phylostratum

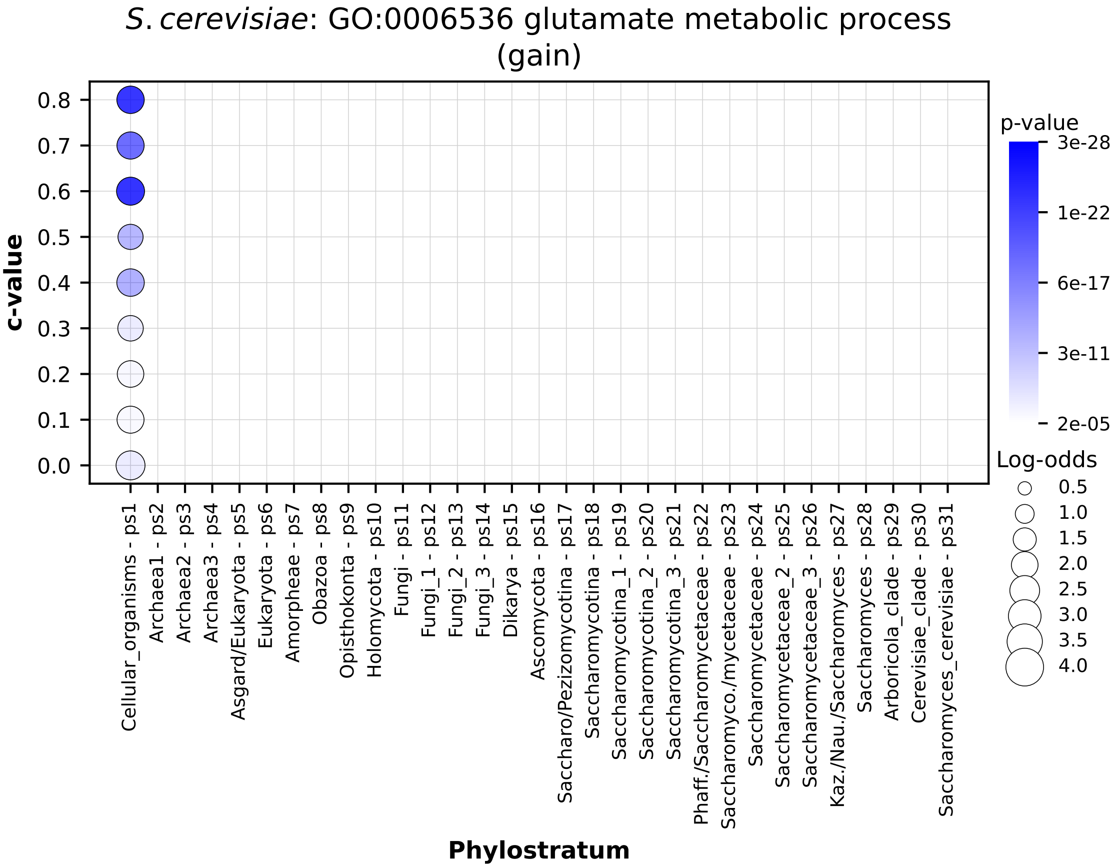

S. cerevisiae: GO:0006536 glutamate metabolic process
(gain)
c-value
0.0
0.1
0.2
0.3
0.4
0.5
0.6
0.7
0.8
p-value
3e-28
1e-22
6e-17
3e-11
2e-05
Log-odds
0.5
1.0
1.5
2.0
2.5
3.0
3.5
4.0
Cellular_organisms - ps1
Archaea1 - ps2
Archaea2 - ps3
Archaea3 - ps4
Asgard/Eukaryota - ps5
Eukaryota - ps6
Amorpheae - ps7
Obazoa - ps8
Opisthokonta - ps9
Holomycota - ps10
Fungi - ps11
Fungi_1 - ps12
Fungi_2 - ps13
Fungi_3 - ps14
Dikarya - ps15
Ascomycota - ps16
Saccharo/Pezizomycotina - ps17
Saccharomycotina - ps18
Saccharomycotina_1 - ps19
Saccharomycotina_2 - ps20
Saccharomycotina_3 - ps21
Phaff./Saccharomycetaceae - ps22
Saccharomyco./mycetaceae - ps23
Saccharomycetaceae - ps24
Saccharomycetaceae_2 - ps25
Saccharomycetaceae_3 - ps26
Kaz./Nau./Saccharomyces - ps27
Saccharomyces - ps28
Arboricola_clade - ps29
Cerevisiae_clade - ps30
Saccharomyces_cerevisiae - ps31
Phylostratum

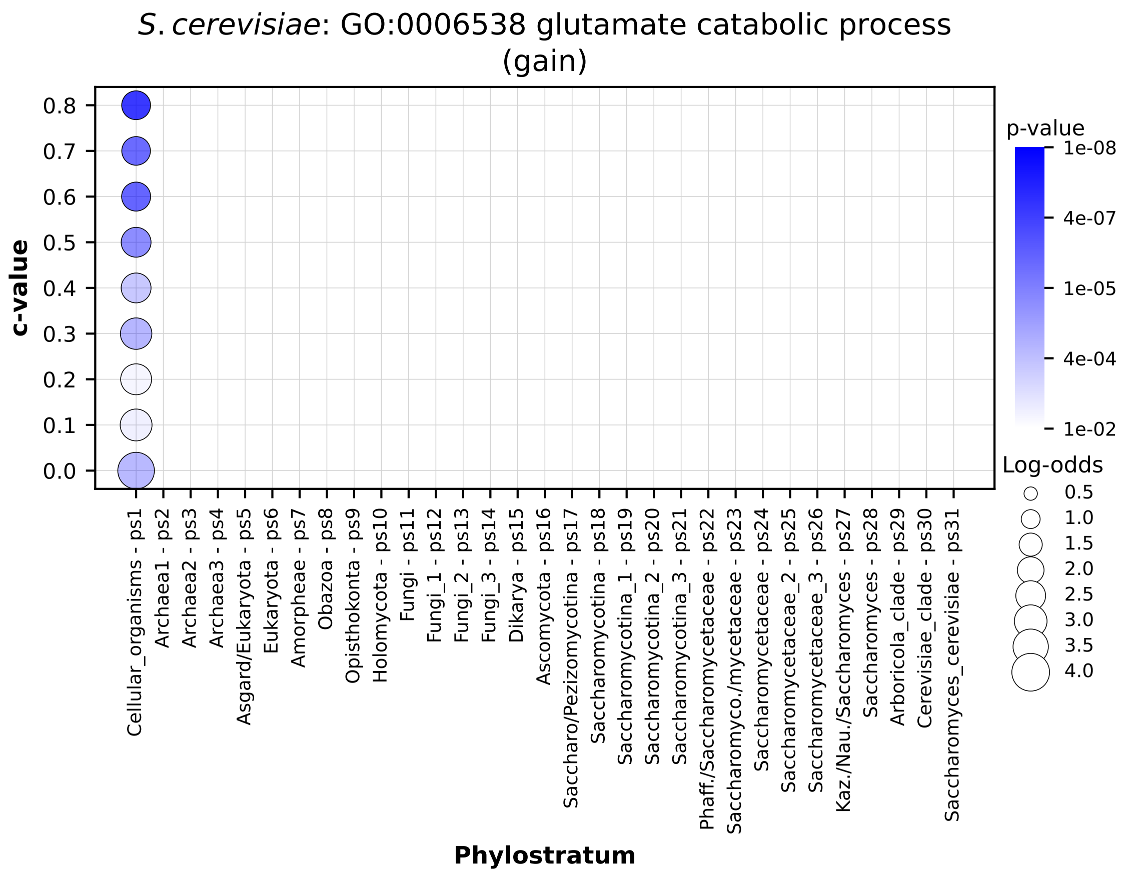

S. cerevisiae: GO:0006538 glutamate catabolic process
(gain)
c-value
0.8
0.7
0.6
0.5
0.4
0.3
0.2
0.1
0.0
p-value
1e-08
4e-07
1e-05
4e-04
1e-02
Log-odds
0.5
1.0
1.5
2.0
2.5
3.0
3.5
4.0
Cellular_organisms - ps1
Archaea1 - ps2
Archaea2 - ps3
Archaea3 - ps4
Asgard/Eukaryota - ps5
Eukaryota - ps6
Amorpheae - ps7
Obazoa - ps8
Opisthokonta - ps9
Holomycota - ps10
Fungi - ps11
Fungi_1 - ps12
Fungi_2 - ps13
Fungi_3 - ps14
Dikarya - ps15
Ascomycota - ps16
Saccharo/Pezizomycotina - ps17
Saccharomycotina - ps18
Saccharomycotina_1 - ps19
Saccharomycotina_2 - ps20
Saccharomycotina_3 - ps21
Phaff./Saccharomycetaceae - ps22
Saccharomyco./mycetaceae - ps23
Saccharomycetaceae - ps24
Saccharomycetaceae_2 - ps25
Saccharomycetaceae_3 - ps26
Kaz./Nau./Saccharomyces - ps27
Saccharomyces - ps28
Arboricola_clade - ps29
Cerevisiae_clade - ps30
Saccharomyces_cerevisiae - ps31
Phylostratum

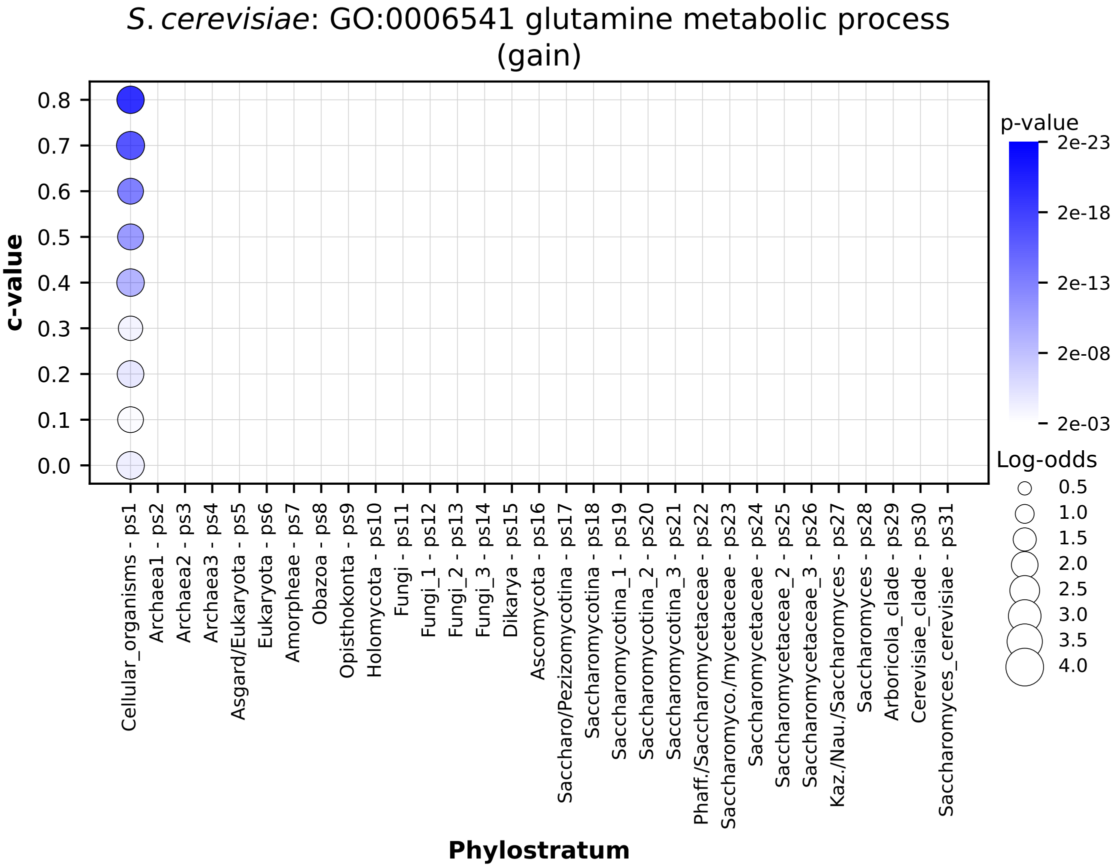

$S. cerevisiae$: GO:0006541 glutamine metabolic process (gain)
c-value
0.0
0.1
0.2
0.3
0.4
0.5
0.6
0.7
0.8
p-value
2e-23
2e-18
2e-13
2e-08
2e-03
Log-odds
0.5
1.0
1.5
2.0
2.5
3.0
3.5
4.0
Cellular_organisms - ps1
Archaea1 - ps2
Archaea2 - ps3
Archaea3 - ps4
Asgard/Eukaryota - ps5
Eukaryota - ps6
Amorpheae - ps7
Obazoa - ps8
Opisthokonta - ps9
Holomycota - ps10
Fungi - ps11
Fungi_1 - ps12
Fungi_2 - ps13
Fungi_3 - ps14
Dikarya - ps15
Ascomycota - ps16
Saccharo/Pezizomycotina - ps17
Saccharomycotina - ps18
Saccharomycotina_1 - ps19
Saccharomycotina_2 - ps20
Saccharomycotina_3 - ps21
Phaff./Saccharomycetaceae - ps22
Saccharomyco./mycetaceae - ps23
Saccharomycetaceae - ps24
Saccharomycetaceae_2 - ps25
Saccharomycetaceae_3 - ps26
Kaz./Nau./Saccharomyces - ps27
Saccharomyces - ps28
Arboricola_clade - ps29
Cerevisiae_clade - ps30
Saccharomyces_cerevisiae - ps31
Phylostratum

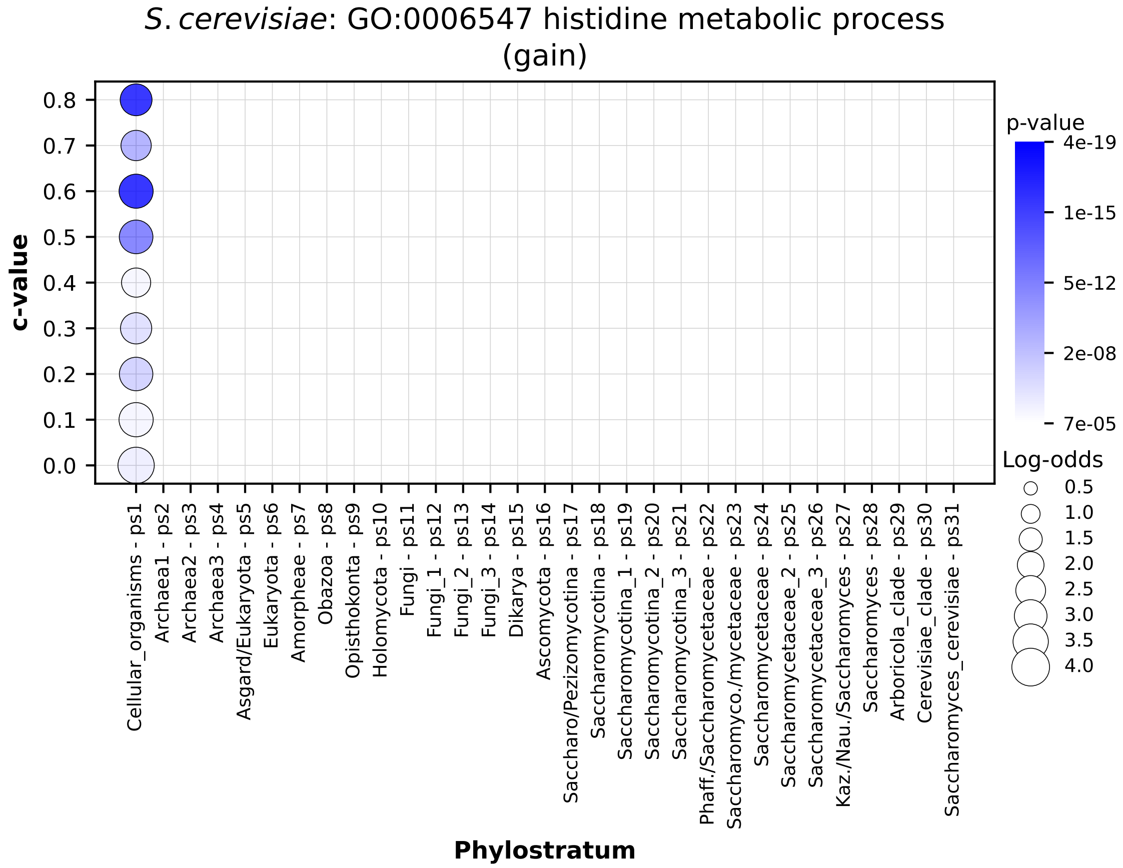

S. cerevisiae: GO:0006547 histidine metabolic process (gain)
c-value
0.8
0.7
0.6
0.5
0.4
0.3
0.2
0.1
0.0
p-value
4e-19
1e-15
5e-12
2e-08
7e-05
Log-odds
0.5
1.0
1.5
2.0
2.5
3.0
3.5
4.0
Cellular_organisms - ps1
Archaea1 - ps2
Archaea2 - ps3
Archaea3 - ps4
Asgard/Eukaryota - ps5
Eukaryota - ps6
Amorpheae - ps7
Obazoa - ps8
Opisthokonta - ps9
Holomycota - ps10
Fungi - ps11
Fungi_1 - ps12
Fungi_2 - ps13
Fungi_3 - ps14
Dikarya - ps15
Ascomycota - ps16
Saccharo/Pezizomycotina - ps17
Saccharomycotina - ps18
Saccharomycotina_1 - ps19
Saccharomycotina_2 - ps20
Saccharomycotina_3 - ps21
Phaff./Saccharomycetaceae - ps22
Saccharomyco./mycetaceae - ps23
Saccharomycetaceae - ps24
Saccharomycetaceae_2 - ps25
Saccharomycetaceae_3 - ps26
Kaz./Nau./Saccharomyces - ps27
Saccharomyces - ps28
Arboricola_clade - ps29
Cerevisiae_clade - ps30
Saccharomyces_cerevisiae - ps31
Phylostratum

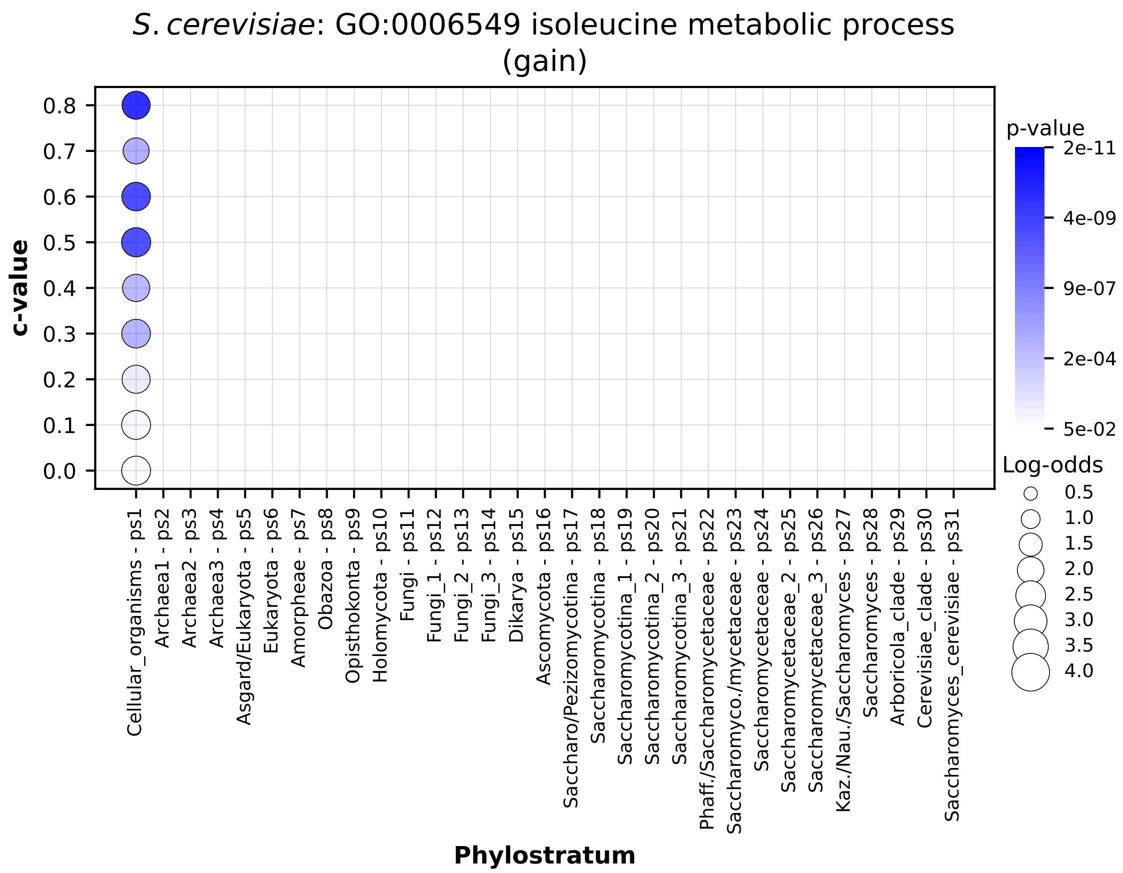

S. cerevisiae: GO:0006549 isoleucine metabolic process
(gain)
c-value
0.8
0.7
0.6
0.5
0.4
0.3
0.2
0.1
0.0
p-value
2e-11
4e-09
9e-07
2e-04
5e-02
Log-odds
0.5
1.0
1.5
2.0
2.5
3.0
3.5
4.0
Cellular_organisms - ps1
Archaea1 - ps2
Archaea2 - ps3
Archaea3 - ps4
Asgard/Eukaryota - ps5
Eukaryota - ps6
Amorpheae - ps7
Obazoa - ps8
Opisthokonta - ps9
Holomycota - ps10
Fungi - ps11
Fungi_1 - ps12
Fungi_2 - ps13
Fungi_3 - ps14
Dikarya - ps15
Ascomycota - ps16
Saccharo/Pezizomycotina - ps17
Saccharomycotina - ps18
Saccharomycotina_1 - ps19
Saccharomycotina_2 - ps20
Saccharomycotina_3 - ps21
Phaff./Saccharomycetaceae - ps22
Saccharomyco./mycetaceae - ps23
Saccharomycetaceae - ps24
Saccharomycetaceae_2 - ps25
Saccharomycetaceae_3 - ps26
Kaz./Nau./Saccharomyces - ps27
Saccharomyces - ps28
Arboricola_clade - ps29
Cerevisiae_clade - ps30
Saccharomyces_cerevisiae - ps31
Phylostratum

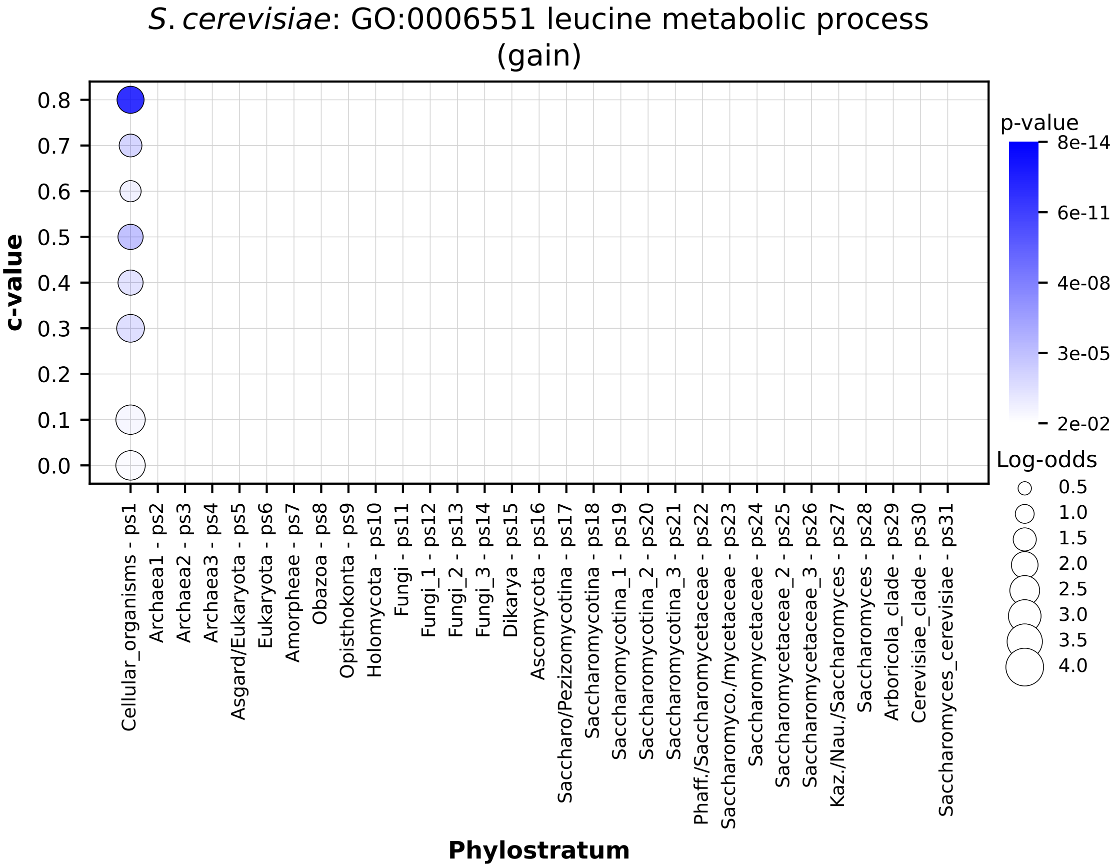

S. cerevisiae: GO:0006551 leucine metabolic process
(gain)
c-value
0.8
0.7
0.6
0.5
0.4
0.3
0.2
0.1
0.0
p-value
8e-14
6e-11
4e-08
3e-05
2e-02
Log-odds
0.5
1.0
1.5
2.0
2.5
3.0
3.5
4.0
Cellular_organisms - ps1
Archaea1 - ps2
Archaea2 - ps3
Archaea3 - ps4
Asgard/Eukaryota - ps5
Eukaryota - ps6
Amorpheae - ps7
Obazoa - ps8
Opisthokonta - ps9
Holomycota - ps10
Fungi - ps11
Fungi_1 - ps12
Fungi_2 - ps13
Fungi_3 - ps14
Dikarya - ps15
Ascomycota - ps16
Saccharo/Pezizomycotina - ps17
Saccharomycotina - ps18
Saccharomycotina_1 - ps19
Saccharomycotina_2 - ps20
Saccharomycotina_3 - ps21
Phaff./Saccharomycetaceae - ps22
Saccharomyco./mycetaceae - ps23
Saccharomycetaceae - ps24
Saccharomycetaceae_2 - ps25
Saccharomycetaceae_3 - ps26
Kaz./Nau./Saccharomyces - ps27
Saccharomyces - ps28
Arboricola_clade - ps29
Cerevisiae_clade - ps30
Saccharomyces_cerevisiae - ps31
Phylostratum

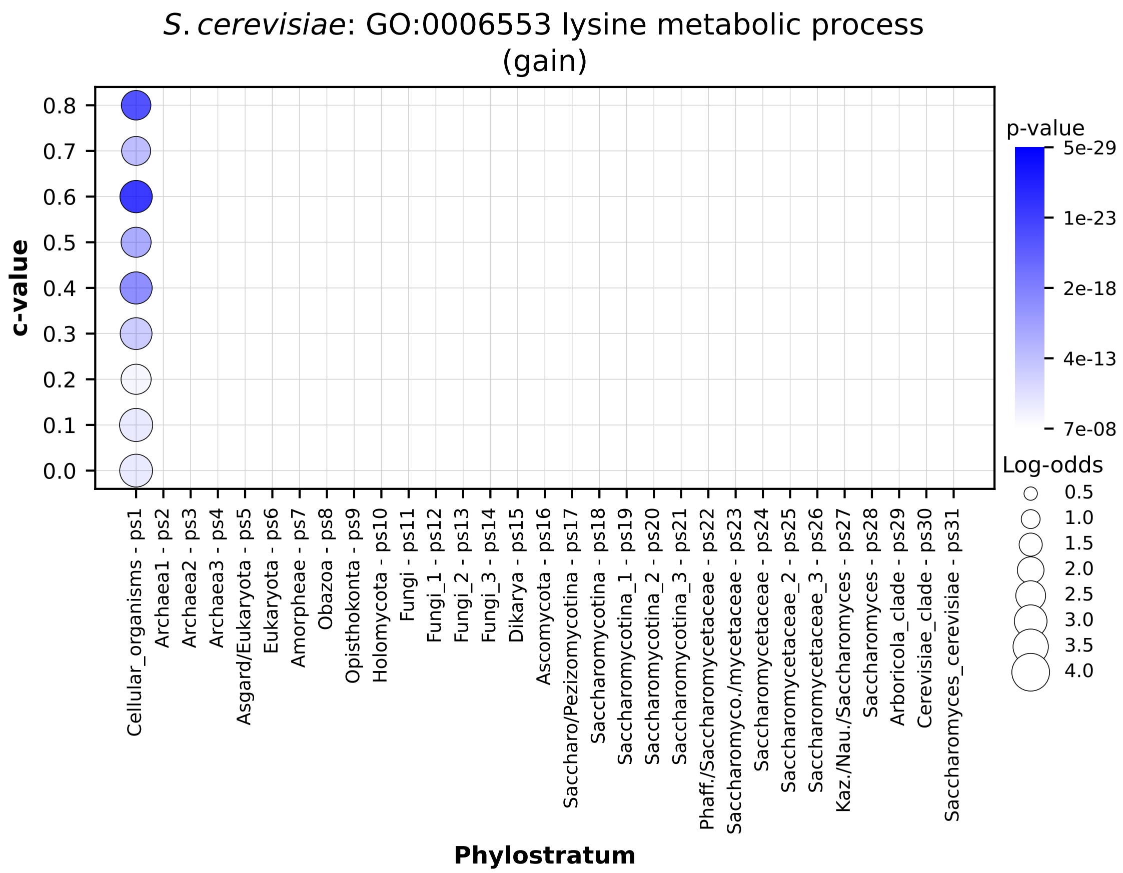

S. cerevisiae: GO:0006553 lysine metabolic process
(gain)
c-value
0.0
0.1
0.2
0.3
0.4
0.5
0.6
0.7
0.8
Phylostratum
Cellular_organisms - ps1
Archaea1 - ps2
Archaea2 - ps3
Archaea3 - ps4
Asgard/Eukaryota - ps5
Eukaryota - ps6
Amorpheae - ps7
Obazoa - ps8
Opisthokonta - ps9
Holomycota - ps10
Fungi - ps11
Fungi_1 - ps12
Fungi_2 - ps13
Fungi_3 - ps14
Dikarya - ps15
Ascomycota - ps16
Saccharo/Pezizomycotina - ps17
Saccharomycotina - ps18
Saccharomycotina_1 - ps19
Saccharomycotina_2 - ps20
Saccharomycotina_3 - ps21
Phaff./Saccharomycetaceae - ps22
Saccharomyco./mycetaceae - ps23
Saccharomycetaceae - ps24
Saccharomycetaceae_2 - ps25
Saccharomycetaceae_3 - ps26
Kaz./Nau./Saccharomyces - ps27
Saccharomyces - ps28
Arboricola_clade - ps29
Cerevisiae_clade - ps30
Saccharomyces_cerevisiae - ps31
p-value
5e-29
1e-23
2e-18
4e-13
7e-08
Log-odds
0.5
1.0
1.5
2.0
2.5
3.0
3.5
4.0

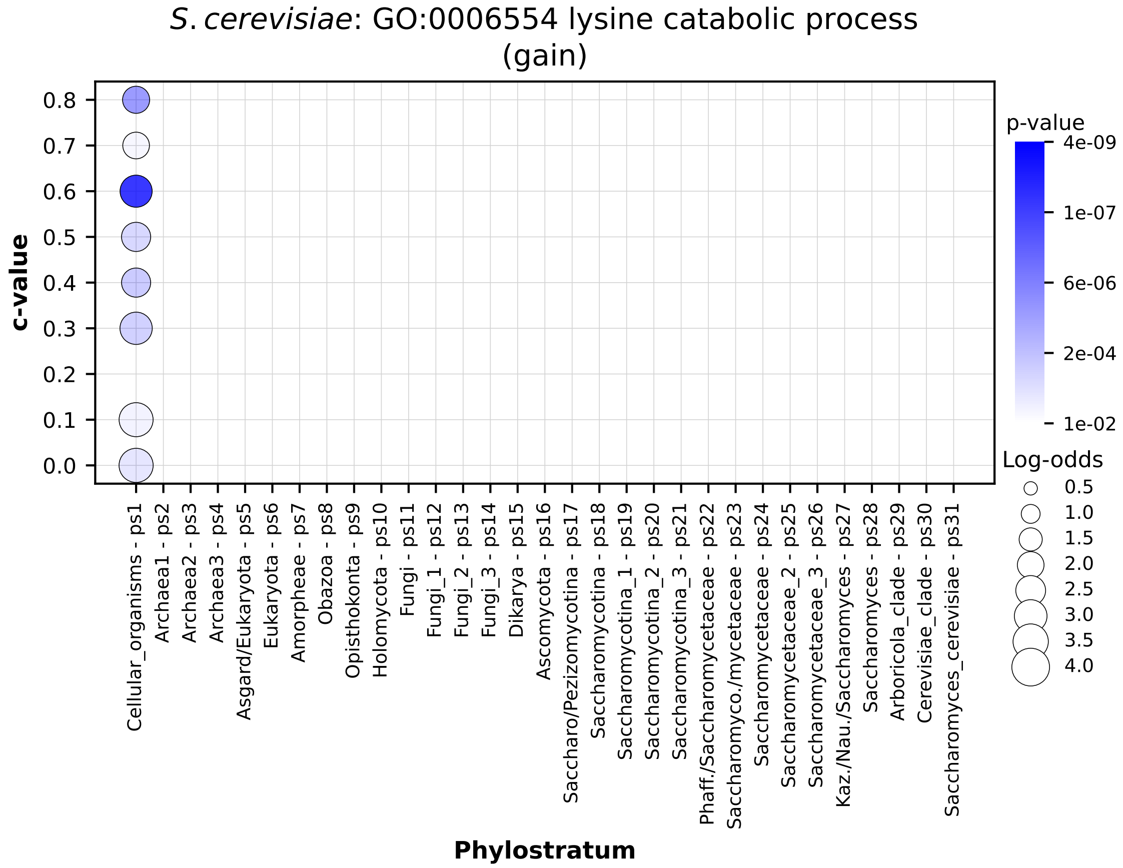
S. cerevisiae: GO:0006554 lysine catabolic process
(gain)
c-value
0.8
0.7
0.6
0.5
0.4
0.3
0.2
0.1
0.0
Phylostratum
Cellular_organisms - ps1
Archaea1 - ps2
Archaea2 - ps3
Archaea3 - ps4
Asgard/Eukaryota - ps5
Eukaryota - ps6
Amorpheae - ps7
Obazoa - ps8
Opisthokonta - ps9
Holomycota - ps10
Fungi - ps11
Fungi_1 - ps12
Fungi_2 - ps13
Fungi_3 - ps14
Dikarya - ps15
Ascomycota - ps16
Saccharo/Pezizomycotina - ps17
Saccharomycotina - ps18
Saccharomycotina_1 - ps19
Saccharomycotina_2 - ps20
Saccharomycotina_3 - ps21
Phaff./Saccharomycetaceae - ps22
Saccharomyco./mycetaceae - ps23
Saccharomycetaceae - ps24
Saccharomycetaceae_2 - ps25
Saccharomycetaceae_3 - ps26
Kaz./Nau./Saccharomyces - ps27
Saccharomyces - ps28
Arboricola_clade - ps29
Cerevisiae_clade - ps30
Saccharomyces_cerevisiae - ps31
p-value
4e-09
1e-07
6e-06
2e-04
1e-02
Log-odds
0.5
1.0
1.5
2.0
2.5
3.0
3.5
4.0

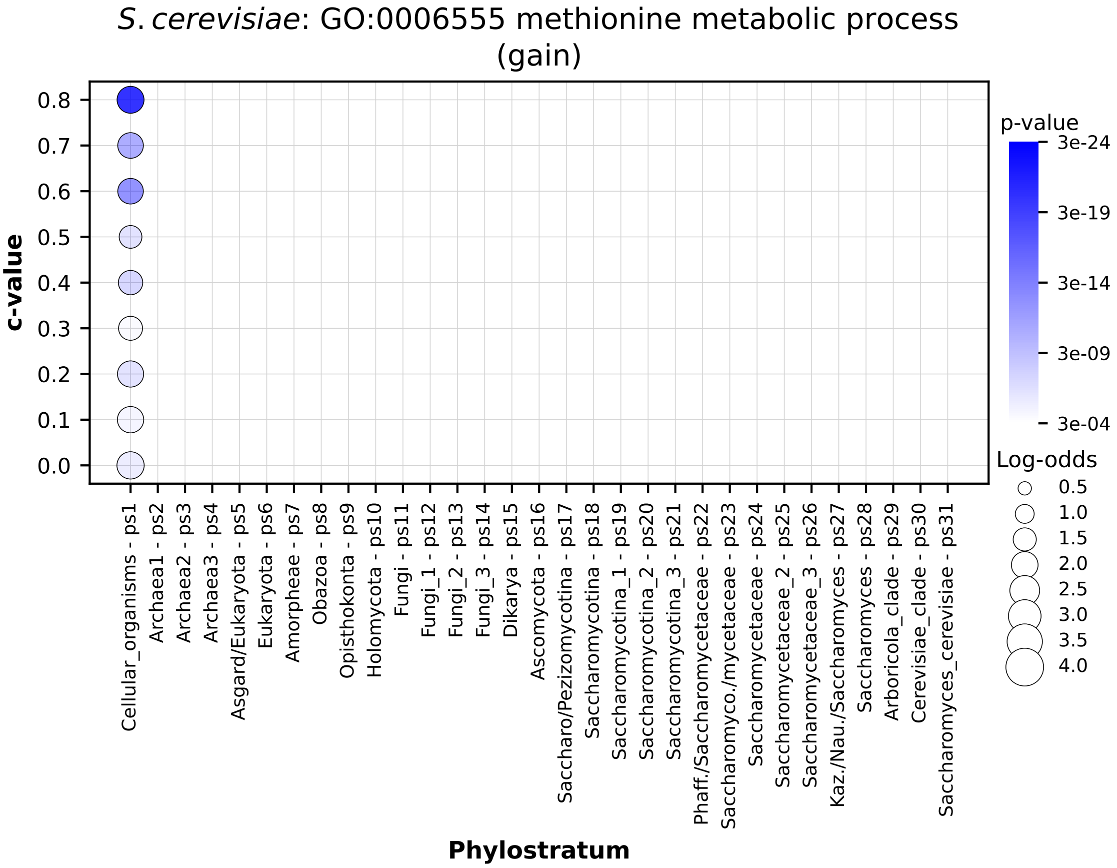
S. cerevisiae: GO:0006555 methionine metabolic process
(gain)
c-value
0.8
0.7
0.6
0.5
0.4
0.3
0.2
0.1
0.0
p-value
3e-24
3e-19
3e-14
3e-09
3e-04
Log-odds
0.5
1.0
1.5
2.0
2.5
3.0
3.5
4.0
Cellular_organisms - ps1
Archaea1 - ps2
Archaea2 - ps3
Archaea3 - ps4
Asgard/Eukaryota - ps5
Eukaryota - ps6
Amorpheae - ps7
Obazoa - ps8
Opisthokonta - ps9
Holomycota - ps10
Fungi - ps11
Fungi_1 - ps12
Fungi_2 - ps13
Fungi_3 - ps14
Dikarya - ps15
Ascomycota - ps16
Saccharo/Pezizomycotina - ps17
Saccharomycotina - ps18
Saccharomycotina_1 - ps19
Saccharomycotina_2 - ps20
Saccharomycotina_3 - ps21
Phaff./Saccharomycetaceae - ps22
Saccharomyco./mycetaceae - ps23
Saccharomycetaceae - ps24
Saccharomycetaceae_2 - ps25
Saccharomycetaceae_3 - ps26
Kaz./Nau./Saccharomyces - ps27
Saccharomyces - ps28
Arboricola_clade - ps29
Cerevisiae_clade - ps30
Saccharomyces_cerevisiae - ps31
Phylostratum

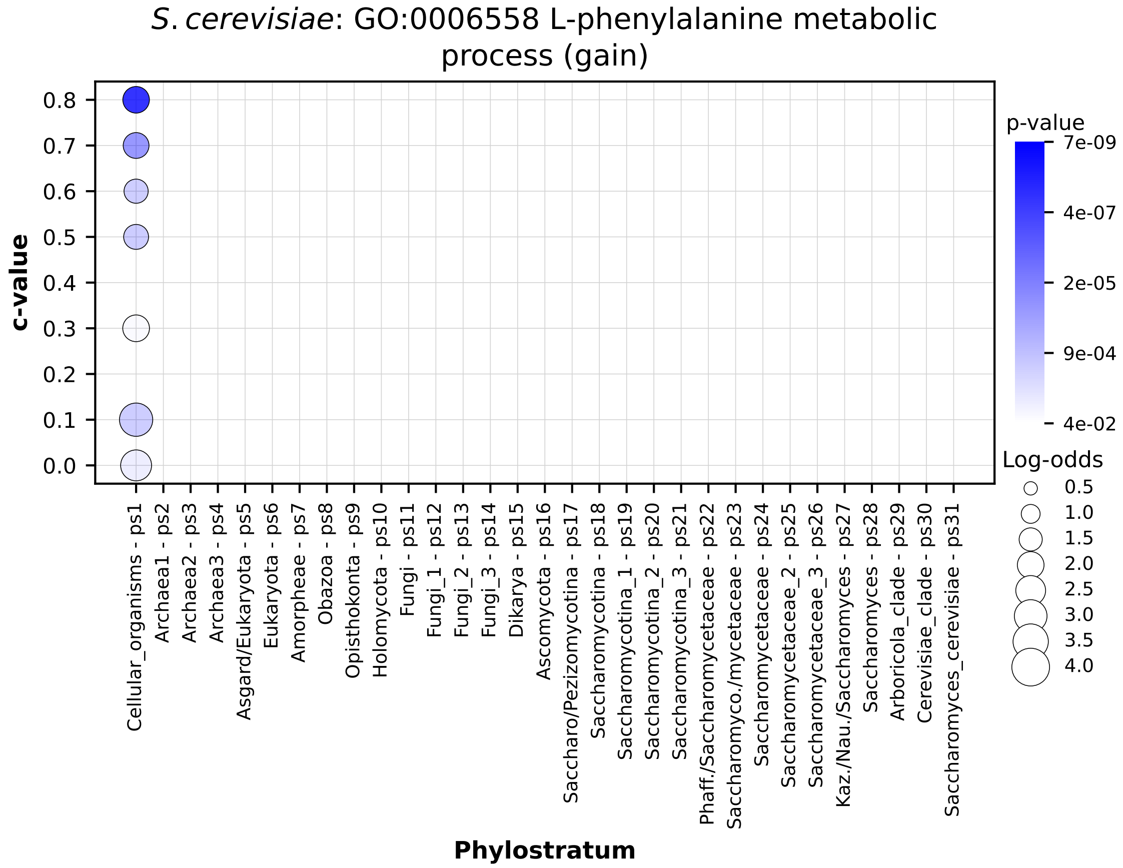
S. cerevisiae: GO:0006558 L-phenylalanine metabolic process (gain)
c-value
0.0
0.1
0.2
0.3
0.4
0.5
0.6
0.7
0.8
p-value
7e-09
4e-07
2e-05
9e-04
4e-02
Log-odds
0.5
1.0
1.5
2.0
2.5
3.0
3.5
4.0
Cellular_organisms - ps1
Archaea1 - ps2
Archaea2 - ps3
Archaea3 - ps4
Asgard/Eukaryota - ps5
Eukaryota - ps6
Amorpheae - ps7
Obazoa - ps8
Opisthokonta - ps9
Holomycota - ps10
Fungi - ps11
Fungi_1 - ps12
Fungi_2 - ps13
Fungi_3 - ps14
Dikarya - ps15
Ascomycota - ps16
Saccharo/Pezizomycotina - ps17
Saccharomycotina - ps18
Saccharomycotina_1 - ps19
Saccharomycotina_2 - ps20
Saccharomycotina_3 - ps21
Phaff./Saccharomycetaceae - ps22
Saccharomyco./mycetaceae - ps23
Saccharomycetaceae - ps24
Saccharomycetaceae_2 - ps25
Saccharomycetaceae_3 - ps26
Kaz./Nau./Saccharomyces - ps27
Saccharomyces - ps28
Arboricola_clade - ps29
Cerevisiae_clade - ps30
Saccharomyces_cerevisiae - ps31
Phylostratum

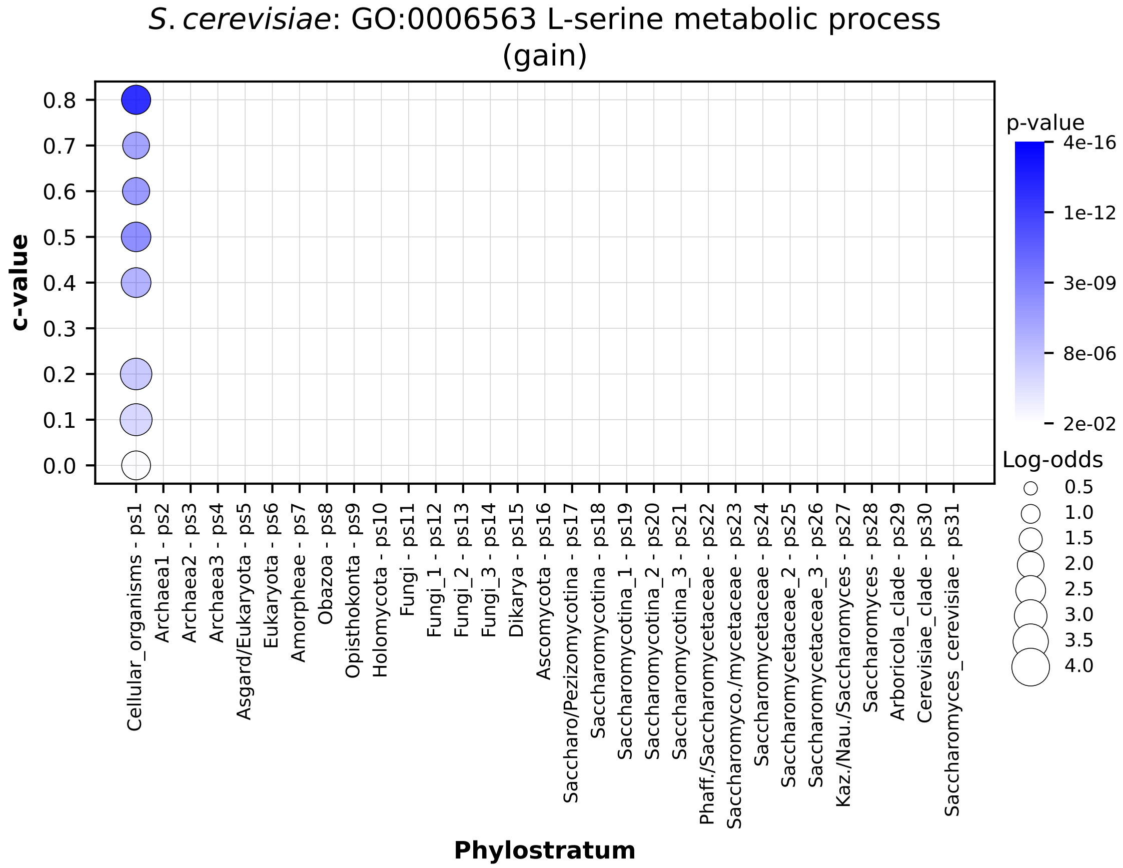

S. cerevisiae: GO:0006563 L-serine metabolic process
(gain)
c-value
0.8
0.7
0.6
0.5
0.4
0.3
0.2
0.1
0.0
p-value
4e-16
1e-12
3e-09
8e-06
2e-02
Log-odds
0.5
1.0
1.5
2.0
2.5
3.0
3.5
4.0
Cellular_organisms - ps1
Archaea1 - ps2
Archaea2 - ps3
Archaea3 - ps4
Asgard/Eukaryota - ps5
Eukaryota - ps6
Amorpheae - ps7
Obazoa - ps8
Opisthokonta - ps9
Holomycota - ps10
Fungi - ps11
Fungi_1 - ps12
Fungi_2 - ps13
Fungi_3 - ps14
Dikarya - ps15
Ascomycota - ps16
Saccharo/Pezizomycotina - ps17
Saccharomycotina - ps18
Saccharomycotina_1 - ps19
Saccharomycotina_2 - ps20
Saccharomycotina_3 - ps21
Phaff./Saccharomycetaceae - ps22
Saccharomyco./mycetaceae - ps23
Saccharomycetaceae - ps24
Saccharomycetaceae_2 - ps25
Saccharomycetaceae_3 - ps26
Kaz./Nau./Saccharomyces - ps27
Saccharomyces - ps28
Arboricola_clade - ps29
Cerevisiae_clade - ps30
Saccharomyces_cerevisiae - ps31
Phylostratum

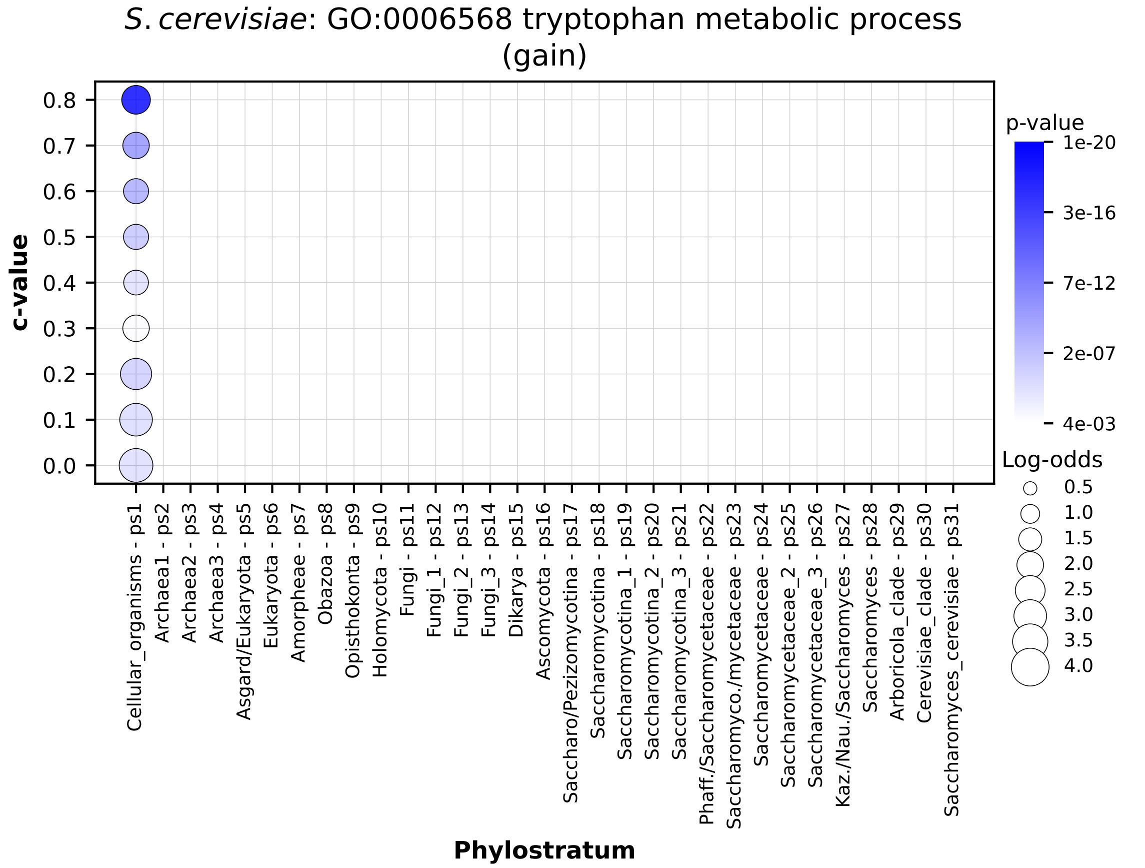

S. cerevisiae: GO:0006568 tryptophan metabolic process
(gain)
c-value
0.8
0.7
0.6
0.5
0.4
0.3
0.2
0.1
0.0
p-value
1e-20
3e-16
7e-12
2e-07
4e-03
Log-odds
0.5
1.0
1.5
2.0
2.5
3.0
3.5
4.0
Cellular_organisms - ps1
Archaea1 - ps2
Archaea2 - ps3
Archaea3 - ps4
Asgard/Eukaryota - ps5
Eukaryota - ps6
Amorpheae - ps7
Obazoa - ps8
Opisthokonta - ps9
Holomycota - ps10
Fungi - ps11
Fungi_1 - ps12
Fungi_2 - ps13
Fungi_3 - ps14
Dikarya - ps15
Ascomycota - ps16
Saccharo/Pezizomycotina - ps17
Saccharomycotina - ps18
Saccharomycotina_1 - ps19
Saccharomycotina_2 - ps20
Saccharomycotina_3 - ps21
Phaff./Saccharomycetaceae - ps22
Saccharomyco./mycetaceae - ps23
Saccharomycetaceae - ps24
Saccharomycetaceae_2 - ps25
Saccharomycetaceae_3 - ps26
Kaz./Nau./Saccharomyces - ps27
Saccharomyces - ps28
Arboricola_clade - ps29
Cerevisiae_clade - ps30
Saccharomyces_cerevisiae - ps31
Phylostratum

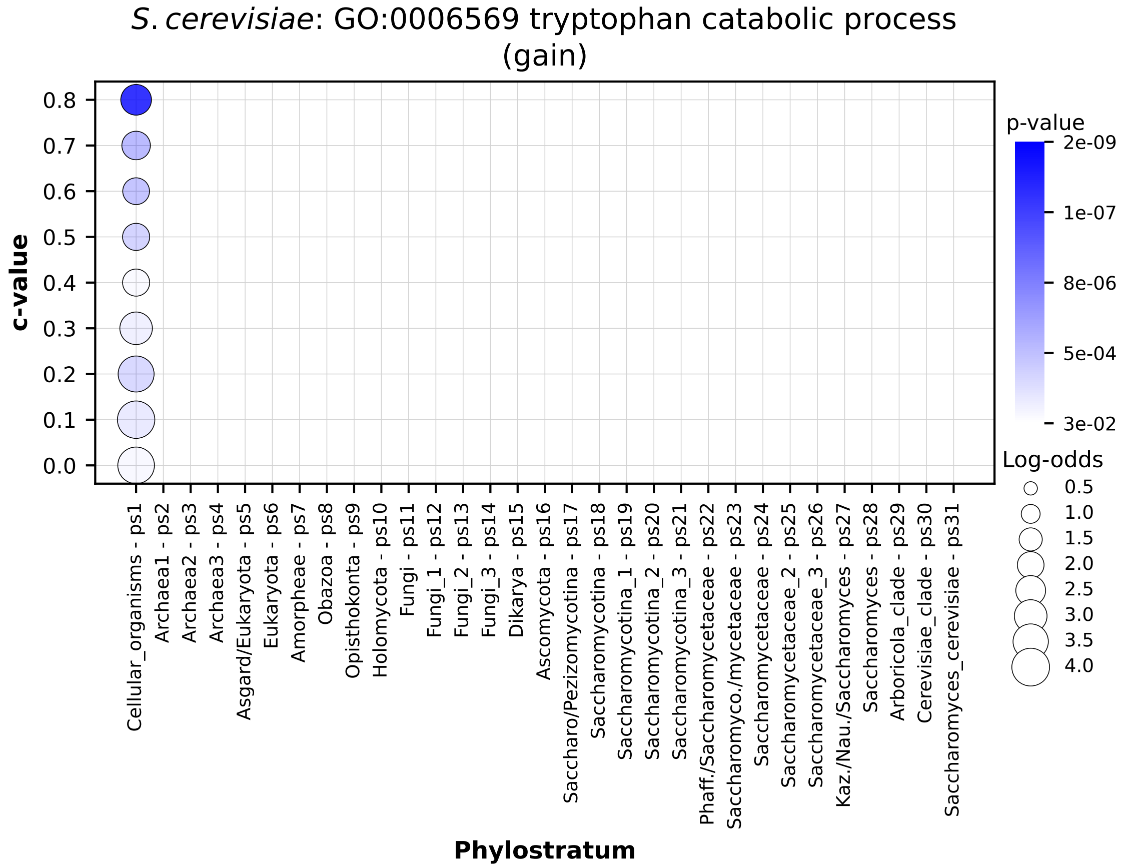
S. cerevisiae: GO:0006569 tryptophan catabolic process
(gain)
c-value
0.8
0.7
0.6
0.5
0.4
0.3
0.2
0.1
0.0
p-value
2e-09
1e-07
8e-06
5e-04
3e-02
Log-odds
0.5
1.0
1.5
2.0
2.5
3.0
3.5
4.0
Cellular_organisms - ps1
Archaea1 - ps2
Archaea2 - ps3
Archaea3 - ps4
Asgard/Eukaryota - ps5
Eukaryota - ps6
Amorpheae - ps7
Obazoa - ps8
Opisthokonta - ps9
Holomycota - ps10
Fungi - ps11
Fungi_1 - ps12
Fungi_2 - ps13
Fungi_3 - ps14
Dikarya - ps15
Ascomycota - ps16
Saccharo/Pezizomycotina - ps17
Saccharomycotina - ps18
Saccharomycotina_1 - ps19
Saccharomycotina_2 - ps20
Saccharomycotina_3 - ps21
Phaff./Saccharomycetaceae - ps22
Saccharomyco./mycetaceae - ps23
Saccharomycetaceae - ps24
Saccharomycetaceae_2 - ps25
Saccharomycetaceae_3 - ps26
Kaz./Nau./Saccharomyces - ps27
Saccharomyces - ps28
Arboricola_clade - ps29
Cerevisiae_clade - ps30
Saccharomyces_cerevisiae - ps31
Phylostratum

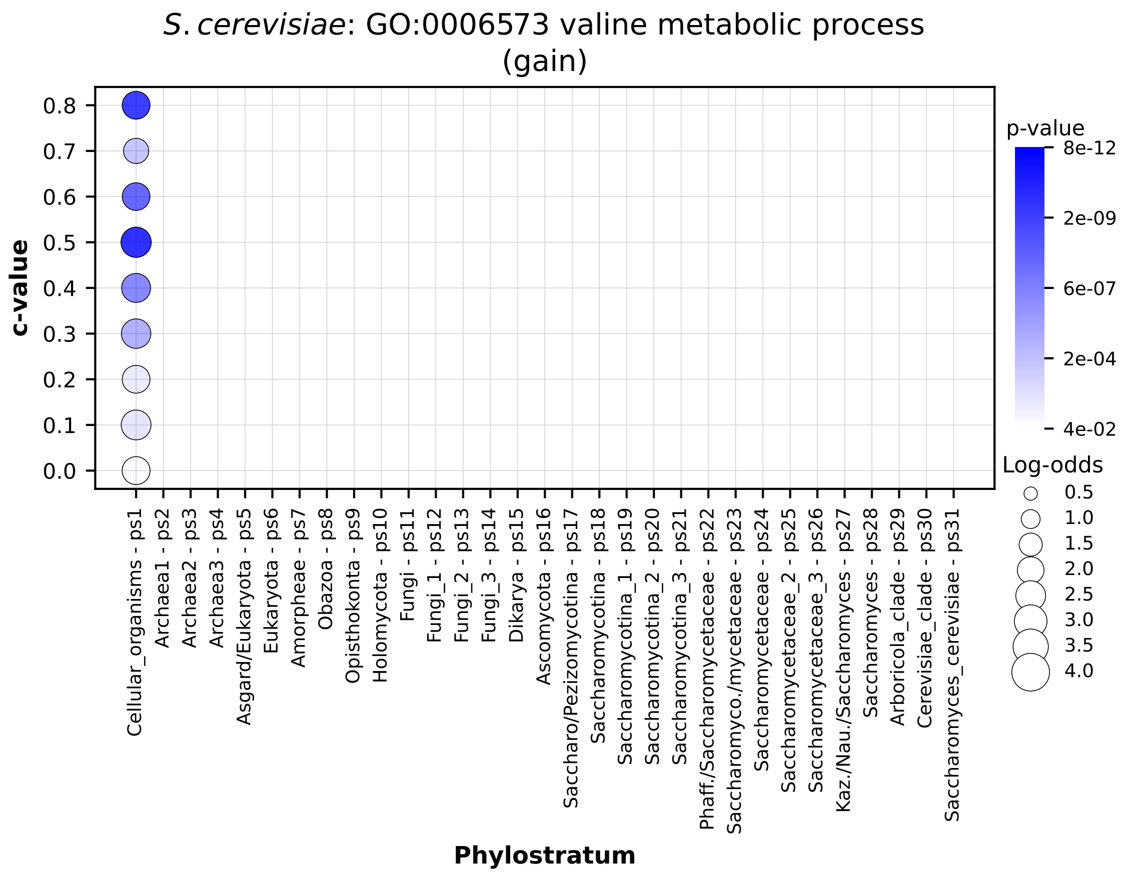

S. cerevisiae: GO:0006573 valine metabolic process
(gain)
c-value
0.8
0.7
0.6
0.5
0.4
0.3
0.2
0.1
0.0
p-value
8e-12
2e-09
6e-07
2e-04
4e-02
Log-odds
0.5
1.0
1.5
2.0
2.5
3.0
3.5
4.0
Cellular_organisms - ps1
Archaea1 - ps2
Archaea2 - ps3
Archaea3 - ps4
Asgard/Eukaryota - ps5
Eukaryota - ps6
Amorpheae - ps7
Obazoa - ps8
Opisthokonta - ps9
Holomycota - ps10
Fungi - ps11
Fungi_1 - ps12
Fungi_2 - ps13
Fungi_3 - ps14
Dikarya - ps15
Ascomycota - ps16
Saccharo/Pezizomycotina - ps17
Saccharomycotina - ps18
Saccharomycotina_1 - ps19
Saccharomycotina_2 - ps20
Saccharomycotina_3 - ps21
Phaff./Saccharomycetaceae - ps22
Saccharomyco./mycetaceae - ps23
Saccharomycetaceae - ps24
Saccharomycetaceae_2 - ps25
Saccharomycetaceae_3 - ps26
Kaz./Nau./Saccharomyces - ps27
Saccharomyces - ps28
Arboricola_clade - ps29
Cerevisiae_clade - ps30
Saccharomyces_cerevisiae - ps31
Phylostratum

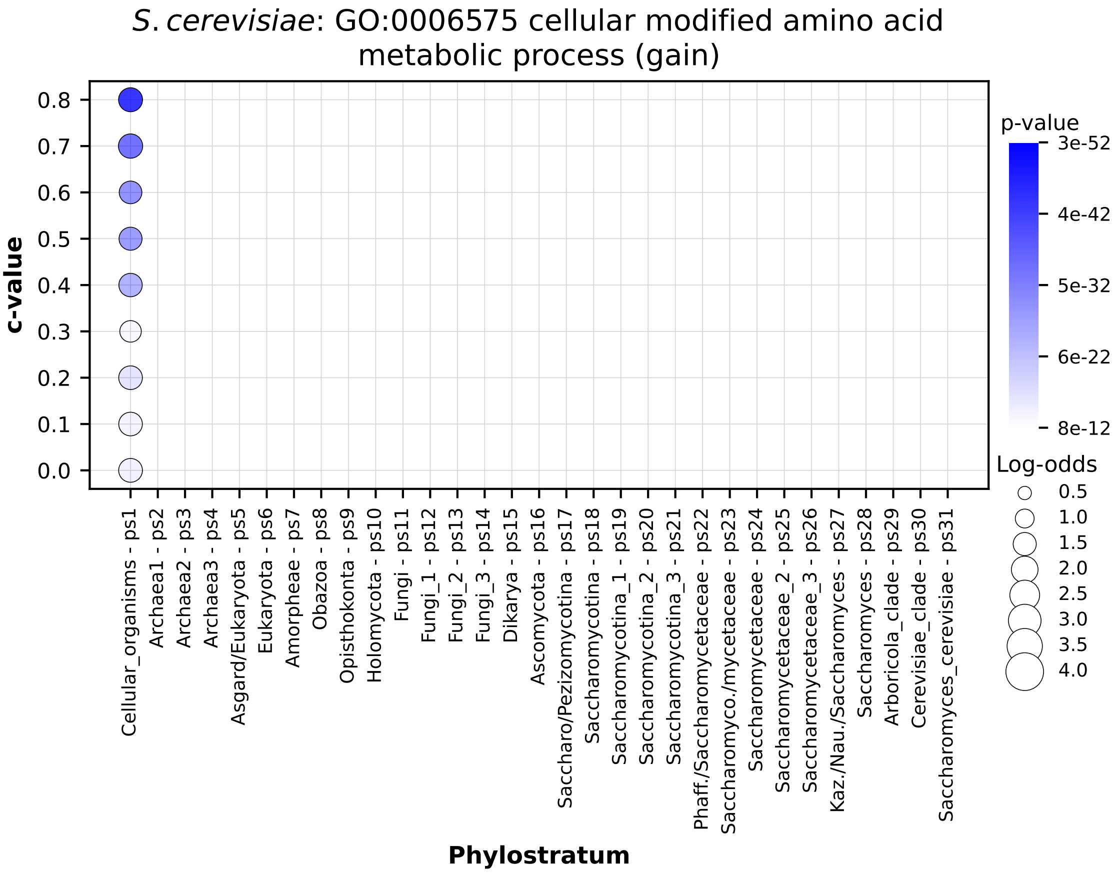

S. cerevisiae: GO:0006575 cellular modified amino acid metabolic process (gain)
c-value
0.8
0.7
0.6
0.5
0.4
0.3
0.2
0.1
0.0
Phylostratum
Cellular_organisms - ps1
Archaea1 - ps2
Archaea2 - ps3
Archaea3 - ps4
Asgard/Eukaryota - ps5
Eukaryota - ps6
Amorpheae - ps7
Obazoa - ps8
Opisthokonta - ps9
Holomycota - ps10
Fungi - ps11
Fungi_1 - ps12
Fungi_2 - ps13
Fungi_3 - ps14
Dikarya - ps15
Ascomycota - ps16
Saccharo/Pezizomycotina - ps17
Saccharomycotina - ps18
Saccharomycotina_1 - ps19
Saccharomycotina_2 - ps20
Saccharomycotina_3 - ps21
Phaff./Saccharomycetaceae - ps22
Saccharomyco./mycetaceae - ps23
Saccharomycetaceae - ps24
Saccharomycetaceae_2 - ps25
Saccharomycetaceae_3 - ps26
Kaz./Nau./Saccharomyces - ps27
Saccharomyces - ps28
Arboricola_clade - ps29
Cerevisiae_clade - ps30
Saccharomyces_cerevisiae - ps31
p-value
3e-52
4e-42
5e-32
6e-22
8e-12
Log-odds
0.5
1.0
1.5
2.0
2.5
3.0
3.5
4.0

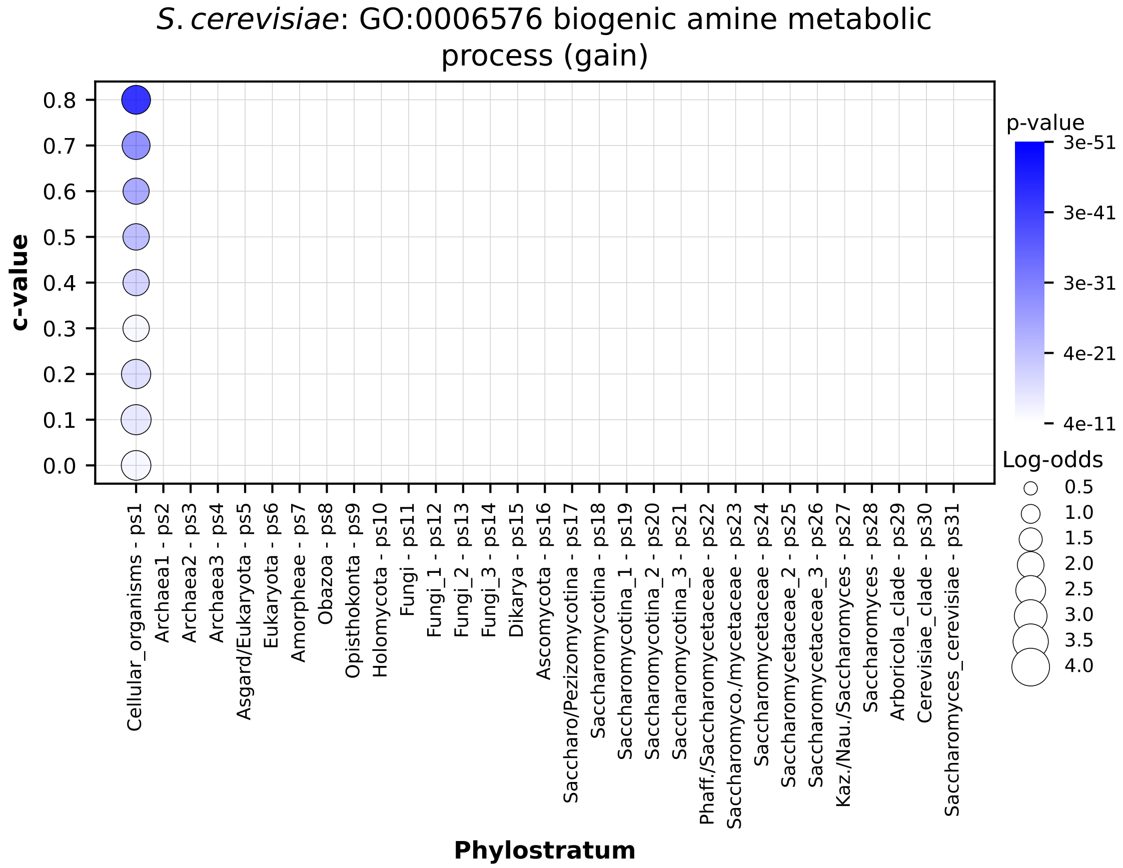
S. cerevisiae: GO:0006576 biogenic amine metabolic process (gain)
c-value
0.8
0.7
0.6
0.5
0.4
0.3
0.2
0.1
0.0
p-value
3e-51
3e-41
3e-31
4e-21
4e-11
Log-odds
0.5
1.0
1.5
2.0
2.5
3.0
3.5
4.0
Cellular_organisms - ps1
Archaea1 - ps2
Archaea2 - ps3
Archaea3 - ps4
Asgard/Eukaryota - ps5
Eukaryota - ps6
Amorpheae - ps7
Obazoa - ps8
Opisthokonta - ps9
Holomycota - ps10
Fungi - ps11
Fungi_1 - ps12
Fungi_2 - ps13
Fungi_3 - ps14
Dikarya - ps15
Ascomycota - ps16
Saccharo/Pezizomycotina - ps17
Saccharomycotina - ps18
Saccharomycotina_1 - ps19
Saccharomycotina_2 - ps20
Saccharomycotina_3 - ps21
Phaff./Saccharomycetaceae - ps22
Saccharomyco./mycetaceae - ps23
Saccharomycetaceae - ps24
Saccharomycetaceae_2 - ps25
Saccharomycetaceae_3 - ps26
Kaz./Nau./Saccharomyces - ps27
Saccharomyces - ps28
Arboricola_clade - ps29
Cerevisiae_clade - ps30
Saccharomyces_cerevisiae - ps31
Phylostratum

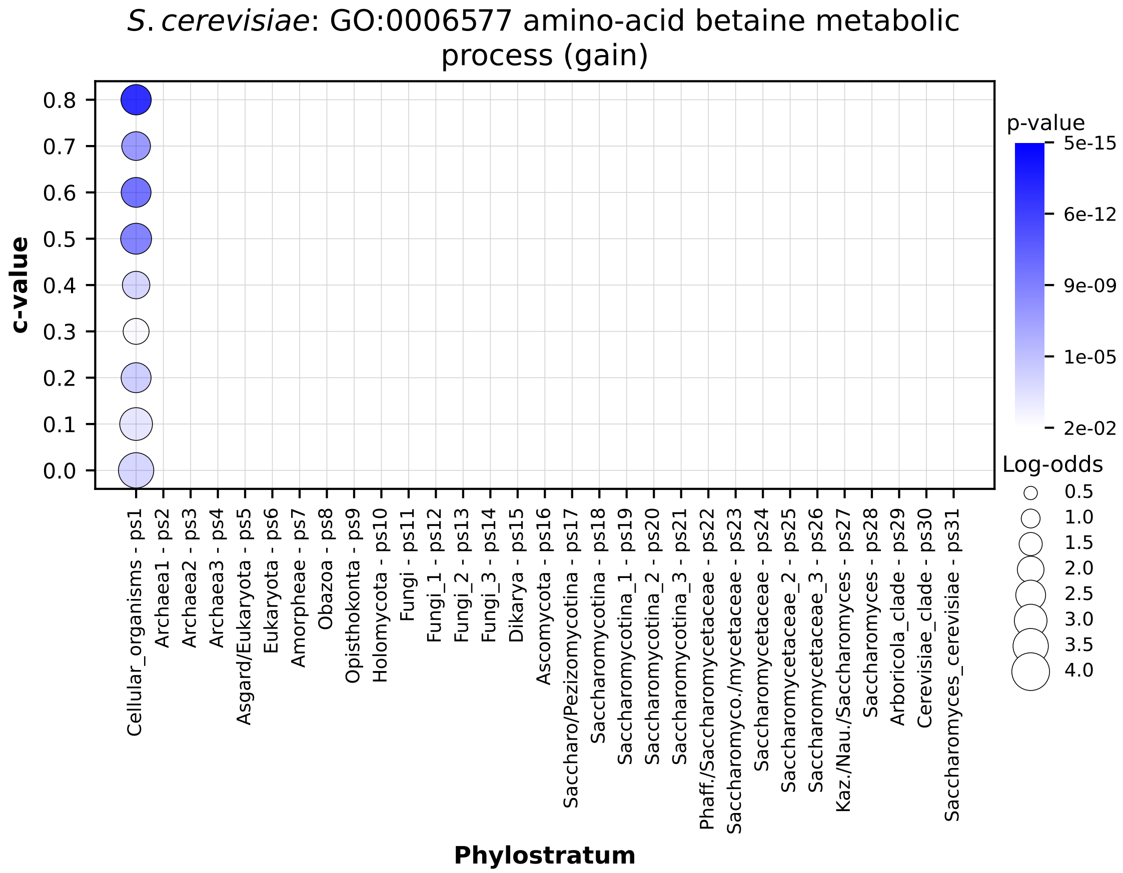

S. cerevisiae: GO:0006577 amino-acid betaine metabolic process (gain)
c-value
0.8
0.7
0.6
0.5
0.4
0.3
0.2
0.1
0.0
Phylostratum
Cellular_organisms - ps1
Archaea1 - ps2
Archaea2 - ps3
Archaea3 - ps4
Asgard/Eukaryota - ps5
Eukaryota - ps6
Amorpheae - ps7
Obazoa - ps8
Opisthokonta - ps9
Holomycota - ps10
Fungi - ps11
Fungi_1 - ps12
Fungi_2 - ps13
Fungi_3 - ps14
Dikarya - ps15
Ascomycota - ps16
Saccharo/Pezizomycotina - ps17
Saccharomycotina - ps18
Saccharomycotina_1 - ps19
Saccharomycotina_2 - ps20
Saccharomycotina_3 - ps21
Phaff./Saccharomycetaceae - ps22
Saccharomyco./mycetaceae - ps23
Saccharomycetaceae - ps24
Saccharomycetaceae_2 - ps25
Saccharomycetaceae_3 - ps26
Kaz./Nau./Saccharomyces - ps27
Saccharomyces - ps28
Arboricola_clade - ps29
Cerevisiae_clade - ps30
Saccharomyces_cerevisiae - ps31
p-value
5e-15
6e-12
9e-09
1e-05
2e-02
Log-odds
0.5
1.0
1.5
2.0
2.5
3.0
3.5
4.0

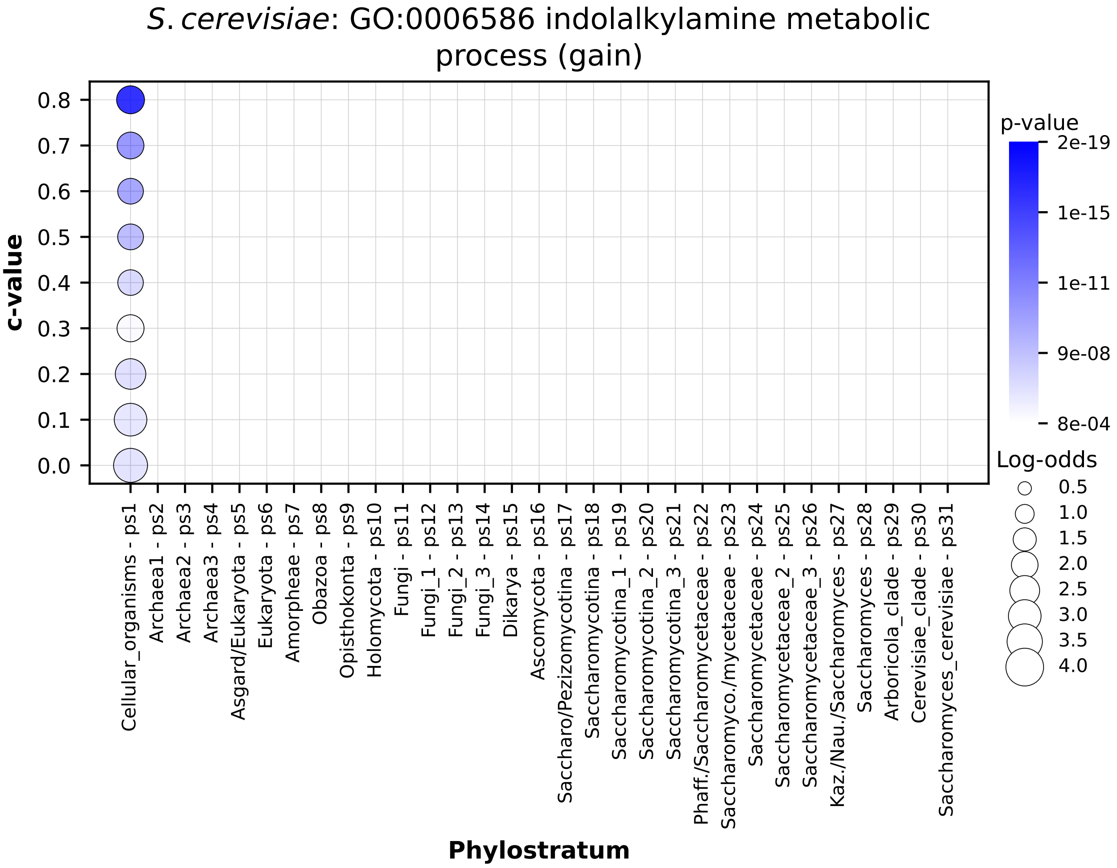

S. cerevisiae: GO:0006586 indolalkylamine metabolic process (gain)
c-value
0.8
0.7
0.6
0.5
0.4
0.3
0.2
0.1
0.0
p-value
2e-19
1e-15
1e-11
9e-08
8e-04
Log-odds
0.5
1.0
1.5
2.0
2.5
3.0
3.5
4.0
Cellular_organisms - ps1
Archaea1 - ps2
Archaea2 - ps3
Archaea3 - ps4
Asgard/Eukaryota - ps5
Eukaryota - ps6
Amorpheae - ps7
Obazoa - ps8
Opisthokonta - ps9
Holomycota - ps10
Fungi - ps11
Fungi_1 - ps12
Fungi_2 - ps13
Fungi_3 - ps14
Dikarya - ps15
Ascomycota - ps16
Saccharo/Pezizomycotina - ps17
Saccharomycotina - ps18
Saccharomycotina_1 - ps19
Saccharomycotina_2 - ps20
Saccharomycotina_3 - ps21
Phaff./Saccharomycetaceae - ps22
Saccharomyco./mycetaceae - ps23
Saccharomycetaceae - ps24
Saccharomycetaceae_2 - ps25
Saccharomycetaceae_3 - ps26
Kaz./Nau./Saccharomyces - ps27
Saccharomyces - ps28
Arboricola_clade - ps29
Cerevisiae_clade - ps30
Saccharomyces_cerevisiae - ps31
Phylostratum

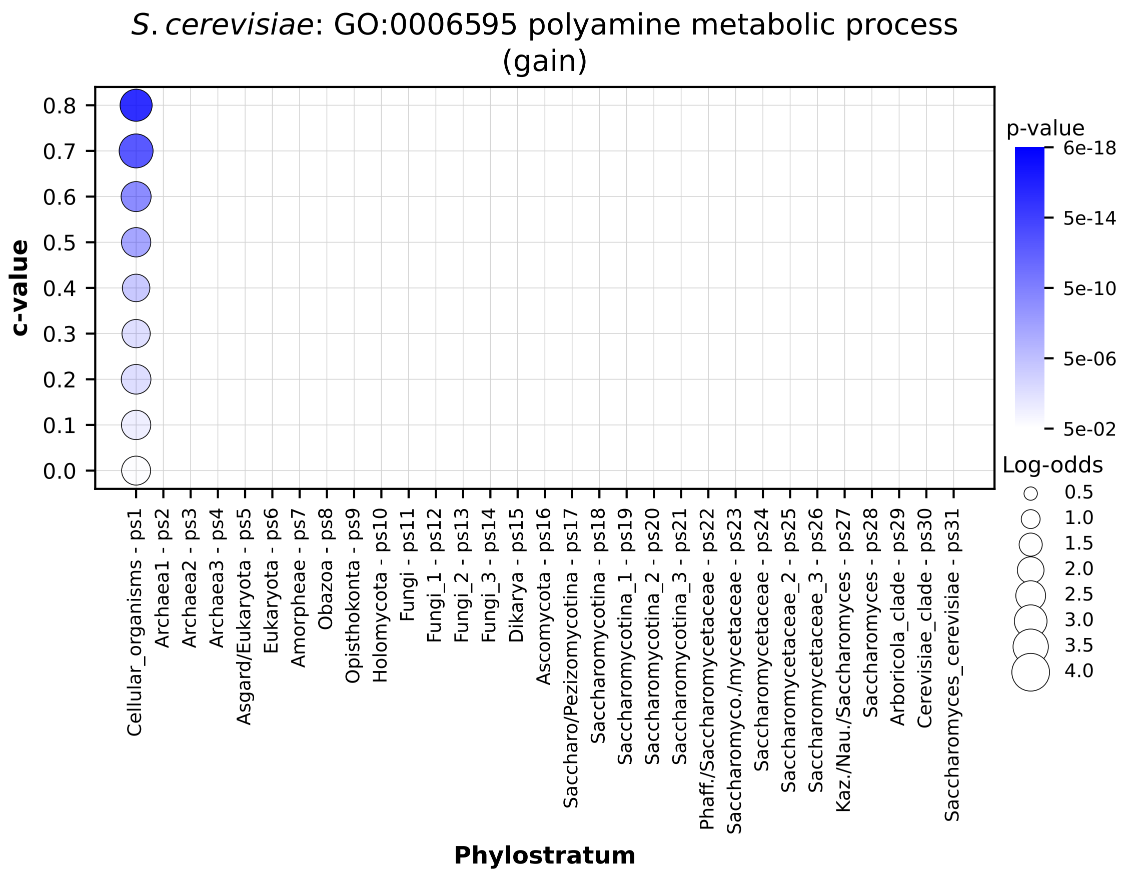

S. cerevisiae: GO:0006595 polyamine metabolic process
(gain)
c-value
0.8
0.7
0.6
0.5
0.4
0.3
0.2
0.1
0.0
p-value
6e-18
5e-14
5e-10
5e-06
5e-02
Log-odds
0.5
1.0
1.5
2.0
2.5
3.0
3.5
4.0
Cellular_organisms - ps1
Archaea1 - ps2
Archaea2 - ps3
Archaea3 - ps4
Asgard/Eukaryota - ps5
Eukaryota - ps6
Amorpheae - ps7
Obazoa - ps8
Opisthokonta - ps9
Holomycota - ps10
Fungi - ps11
Fungi_1 - ps12
Fungi_2 - ps13
Fungi_3 - ps14
Dikarya - ps15
Ascomycota - ps16
Saccharo/Pezizomycotina - ps17
Saccharomycotina - ps18
Saccharomycotina_1 - ps19
Saccharomycotina_2 - ps20
Saccharomycotina_3 - ps21
Phaff./Saccharomycetaceae - ps22
Saccharomyco./mycetaceae - ps23
Saccharomycetaceae - ps24
Saccharomycetaceae_2 - ps25
Saccharomycetaceae_3 - ps26
Kaz./Nau./Saccharomyces - ps27
Saccharomyces - ps28
Arboricola_clade - ps29
Cerevisiae_clade - ps30
Saccharomyces_cerevisiae - ps31
Phylostratum

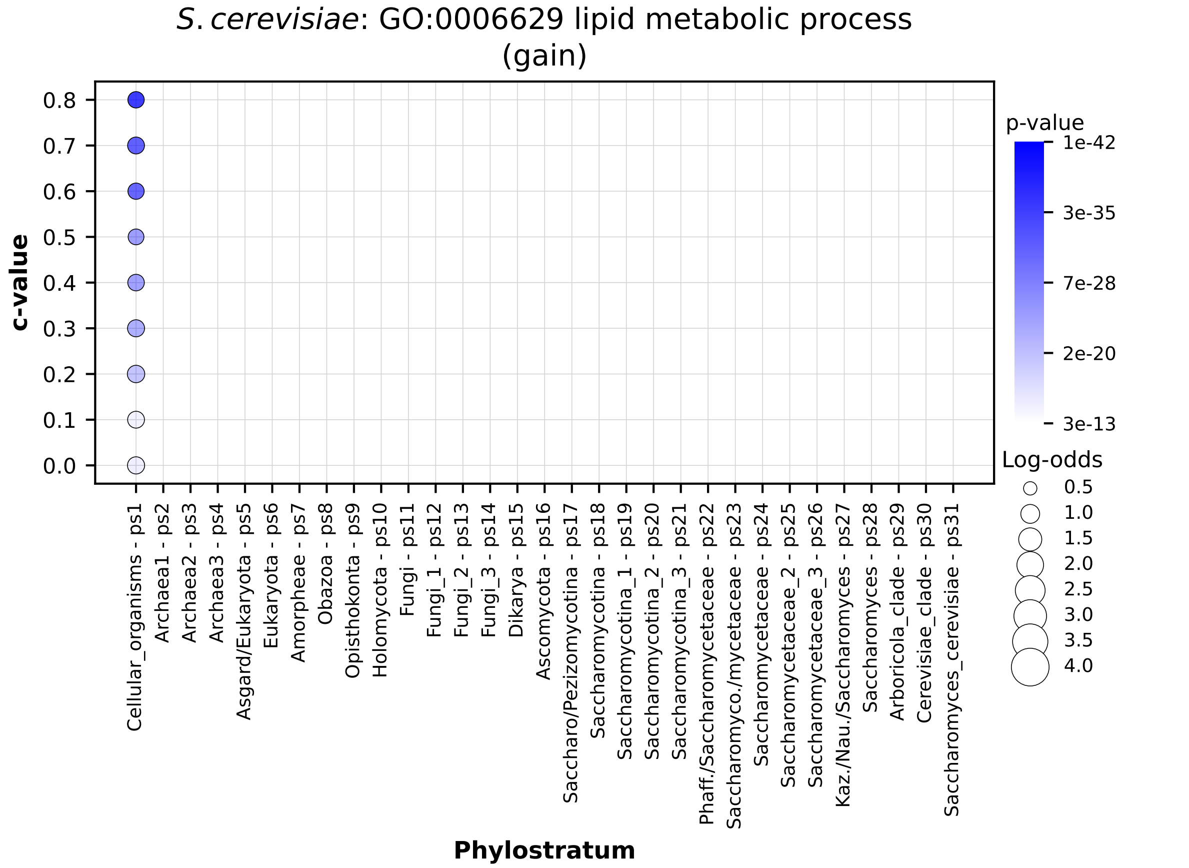

S. cerevisiae: GO:0006629 lipid metabolic process
(gain)
c-value
0.8
0.7
0.6
0.5
0.4
0.3
0.2
0.1
0.0
p-value
1e-42
3e-35
7e-28
2e-20
3e-13
Log-odds
0.5
1.0
1.5
2.0
2.5
3.0
3.5
4.0
Cellular_organisms - ps1
Archaea1 - ps2
Archaea2 - ps3
Archaea3 - ps4
Asgard/Eukaryota - ps5
Eukaryota - ps6
Amorpheae - ps7
Obazoa - ps8
Opisthokonta - ps9
Holomycota - ps10
Fungi - ps11
Fungi_1 - ps12
Fungi_2 - ps13
Fungi_3 - ps14
Dikarya - ps15
Ascomycota - ps16
Saccharo/Pezizomycotina - ps17
Saccharomycotina - ps18
Saccharomycotina_1 - ps19
Saccharomycotina_2 - ps20
Saccharomycotina_3 - ps21
Phaff./Saccharomycetaceae - ps22
Saccharomyco./mycetaceae - ps23
Saccharomycetaceae - ps24
Saccharomycetaceae_2 - ps25
Saccharomycetaceae_3 - ps26
Kaz./Nau./Saccharomyces - ps27
Saccharomyces - ps28
Arboricola_clade - ps29
Cerevisiae_clade - ps30
Saccharomyces_cerevisiae - ps31
Phylostratum

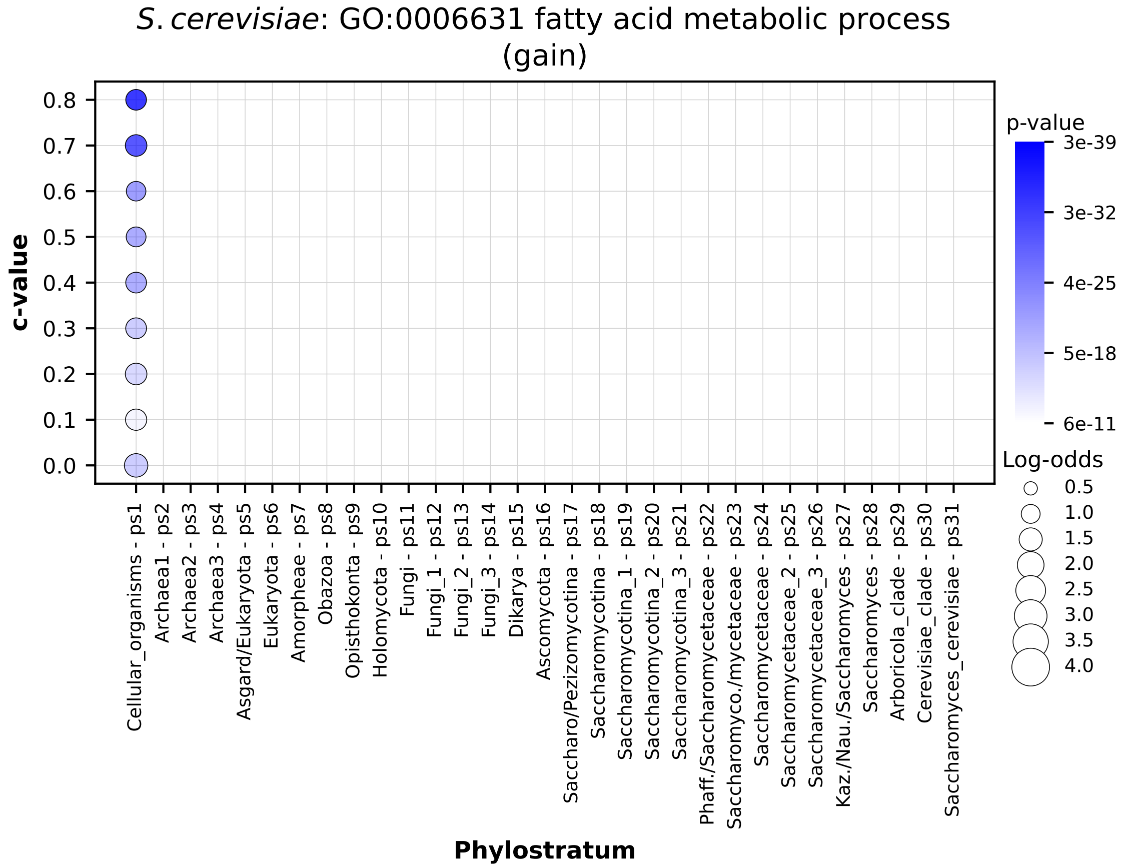
S. cerevisiae: GO:0006631 fatty acid metabolic process
(gain)
c-value
0.8
0.7
0.6
0.5
0.4
0.3
0.2
0.1
0.0
p-value
3e-39
3e-32
4e-25
5e-18
6e-11
Log-odds
0.5
1.0
1.5
2.0
2.5
3.0
3.5
4.0
Cellular_organisms - ps1
Archaea1 - ps2
Archaea2 - ps3
Archaea3 - ps4
Asgard/Eukaryota - ps5
Eukaryota - ps6
Amorpheae - ps7
Obazoa - ps8
Opisthokonta - ps9
Holomycota - ps10
Fungi - ps11
Fungi_1 - ps12
Fungi_2 - ps13
Fungi_3 - ps14
Dikarya - ps15
Ascomycota - ps16
Saccharo/Pezizomycotina - ps17
Saccharomycotina - ps18
Saccharomycotina_1 - ps19
Saccharomycotina_2 - ps20
Saccharomycotina_3 - ps21
Phaff./Saccharomycetaceae - ps22
Saccharomyco./mycetaceae - ps23
Saccharomycetaceae - ps24
Saccharomycetaceae_2 - ps25
Saccharomycetaceae_3 - ps26
Kaz./Nau./Saccharomyces - ps27
Saccharomyces - ps28
Arboricola_clade - ps29
Cerevisiae_clade - ps30
Saccharomyces_cerevisiae - ps31
Phylostratum

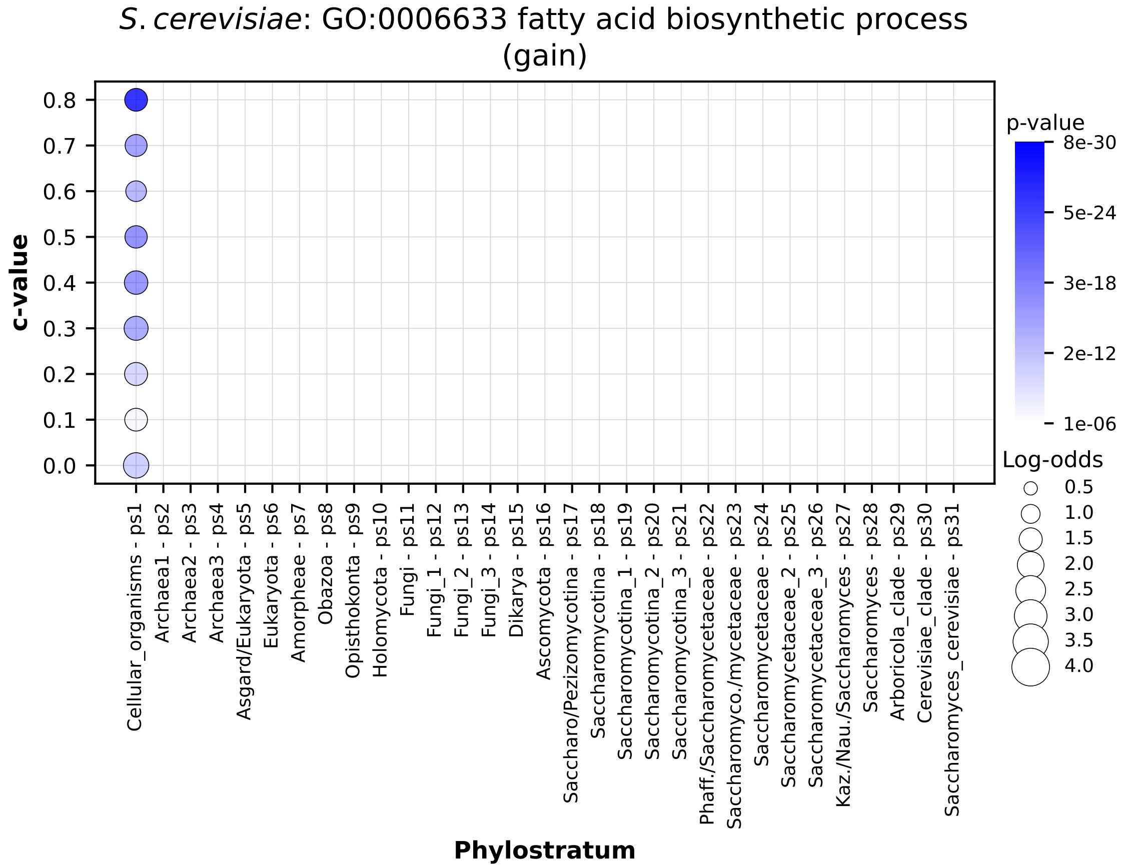

S. cerevisiae: GO:0006633 fatty acid biosynthetic process
(gain)
c-value
0.0
0.1
0.2
0.3
0.4
0.5
0.6
0.7
0.8
p-value
8e-30
5e-24
3e-18
2e-12
1e-06
Log-odds
0.5
1.0
1.5
2.0
2.5
3.0
3.5
4.0
Cellular_organisms - ps1
Archaea1 - ps2
Archaea2 - ps3
Archaea3 - ps4
Asgard/Eukaryota - ps5
Eukaryota - ps6
Amorpheae - ps7
Obazoa - ps8
Opisthokonta - ps9
Holomycota - ps10
Fungi - ps11
Fungi_1 - ps12
Fungi_2 - ps13
Fungi_3 - ps14
Dikarya - ps15
Ascomycota - ps16
Saccharo/Pezizomycotina - ps17
Saccharomycotina - ps18
Saccharomycotina_1 - ps19
Saccharomycotina_2 - ps20
Saccharomycotina_3 - ps21
Phaff./Saccharomycetaceae - ps22
Saccharomyco./mycetaceae - ps23
Saccharomycetaceae - ps24
Saccharomycetaceae_2 - ps25
Saccharomycetaceae_3 - ps26
Kaz./Nau./Saccharomyces - ps27
Saccharomyces - ps28
Arboricola_clade - ps29
Cerevisiae_clade - ps30
Saccharomyces_cerevisiae - ps31
Phylostratum

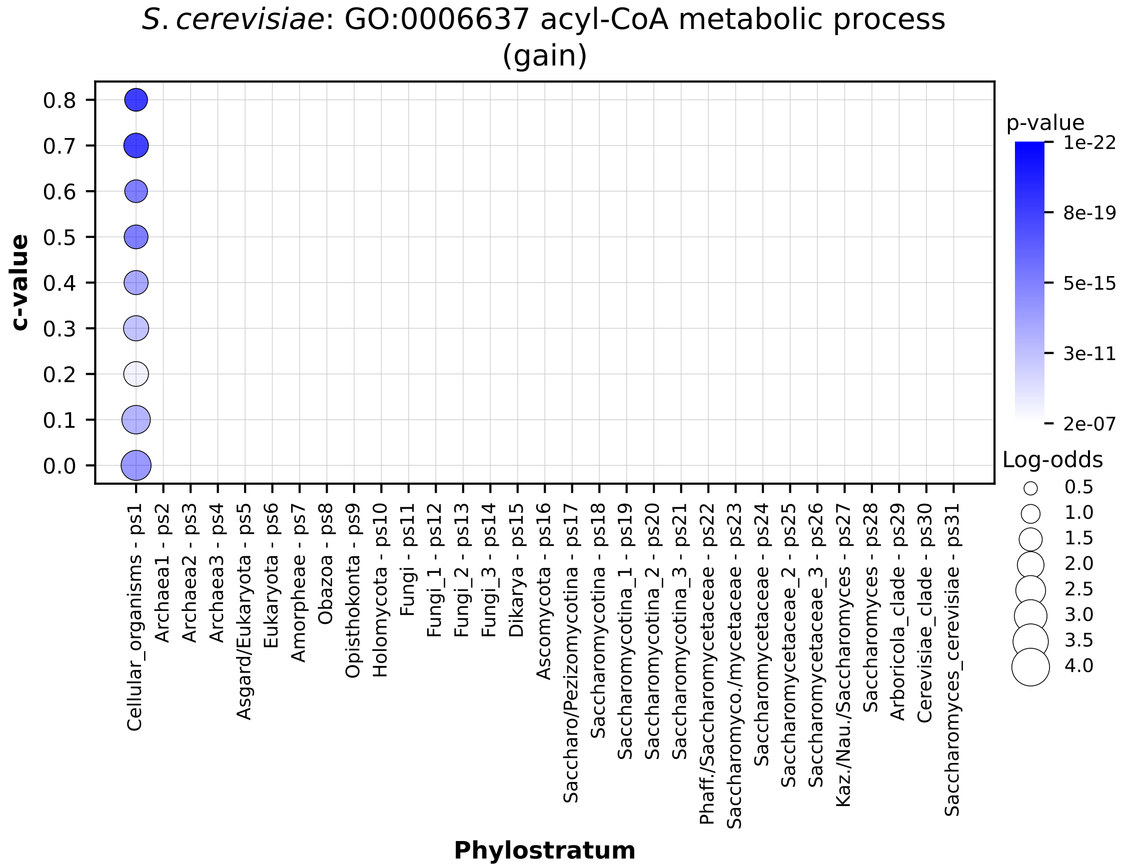

S. cerevisiae: GO:0006637 acyl-CoA metabolic process (gain)
c-value
0.8
0.7
0.6
0.5
0.4
0.3
0.2
0.1
0.0
Phylostratum
Cellular_organisms - ps1
Archaea1 - ps2
Archaea2 - ps3
Archaea3 - ps4
Asgard/Eukaryota - ps5
Eukaryota - ps6
Amorpheae - ps7
Obazoa - ps8
Opisthokonta - ps9
Holomycota - ps10
Fungi - ps11
Fungi_1 - ps12
Fungi_2 - ps13
Fungi_3 - ps14
Dikarya - ps15
Ascomycota - ps16
Saccharo/Pezizomycotina - ps17
Saccharomycotina - ps18
Saccharomycotina_1 - ps19
Saccharomycotina_2 - ps20
Saccharomycotina_3 - ps21
Phaff./Saccharomycetaceae - ps22
Saccharomyco./mycetaceae - ps23
Saccharomycetaceae - ps24
Saccharomycetaceae_2 - ps25
Saccharomycetaceae_3 - ps26
Kaz./Nau./Saccharomyces - ps27
Saccharomyces - ps28
Arboricola_clade - ps29
Cerevisiae_clade - ps30
Saccharomyces_cerevisiae - ps31
p-value
1e-22
8e-19
5e-15
3e-11
2e-07
Log-odds
0.5
1.0
1.5
2.0
2.5
3.0
3.5
4.0

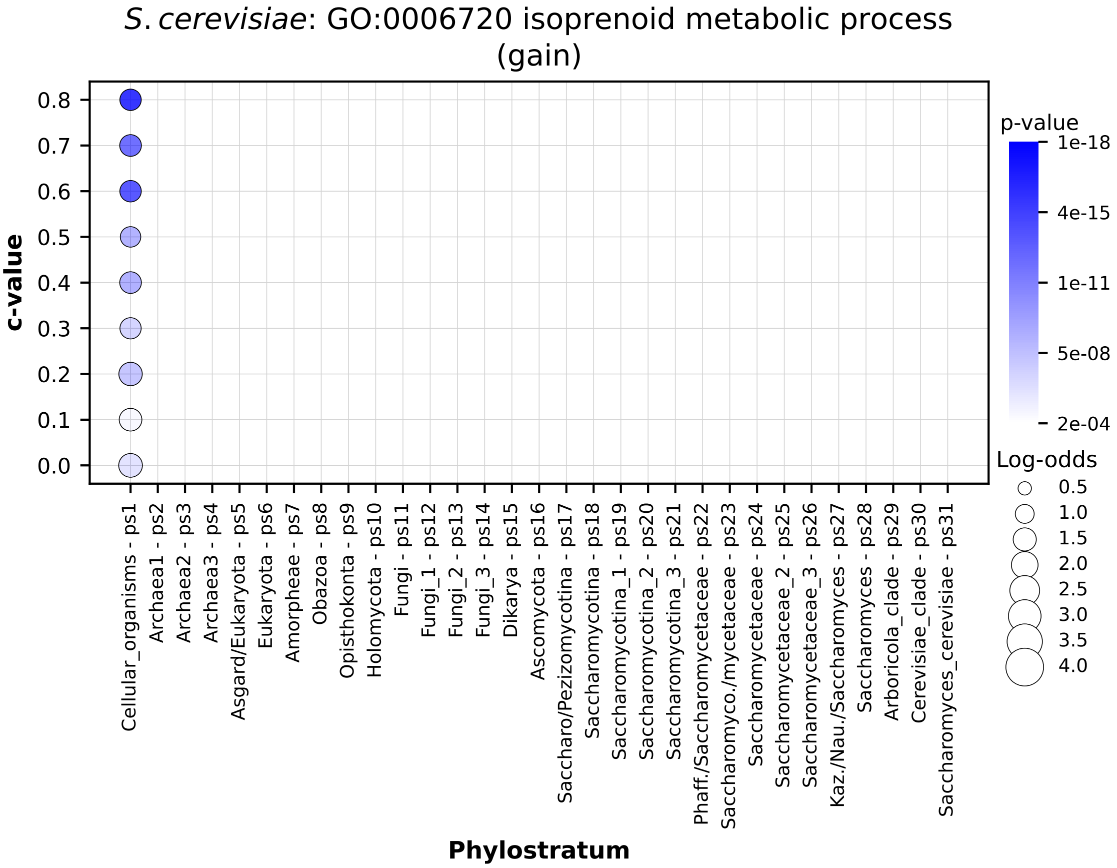

S. cerevisiae: GO:0006720 isoprenoid metabolic process
(gain)
c-value
0.8
0.7
0.6
0.5
0.4
0.3
0.2
0.1
0.0
p-value
1e-18
4e-15
1e-11
5e-08
2e-04
Log-odds
0.5
1.0
1.5
2.0
2.5
3.0
3.5
4.0
Cellular_organisms - ps1
Archaea1 - ps2
Archaea2 - ps3
Archaea3 - ps4
Asgard/Eukaryota - ps5
Eukaryota - ps6
Amorpheae - ps7
Obazoa - ps8
Opisthokonta - ps9
Holomycota - ps10
Fungi - ps11
Fungi_1 - ps12
Fungi_2 - ps13
Fungi_3 - ps14
Dikarya - ps15
Ascomycota - ps16
Saccharo/Pezizomycotina - ps17
Saccharomycotina - ps18
Saccharomycotina_1 - ps19
Saccharomycotina_2 - ps20
Saccharomycotina_3 - ps21
Phaff./Saccharomycetaceae - ps22
Saccharomyco./mycetaceae - ps23
Saccharomycetaceae - ps24
Saccharomycetaceae_2 - ps25
Saccharomycetaceae_3 - ps26
Kaz./Nau./Saccharomyces - ps27
Saccharomyces - ps28
Arboricola_clade - ps29
Cerevisiae_clade - ps30
Saccharomyces_cerevisiae - ps31
Phylostratum

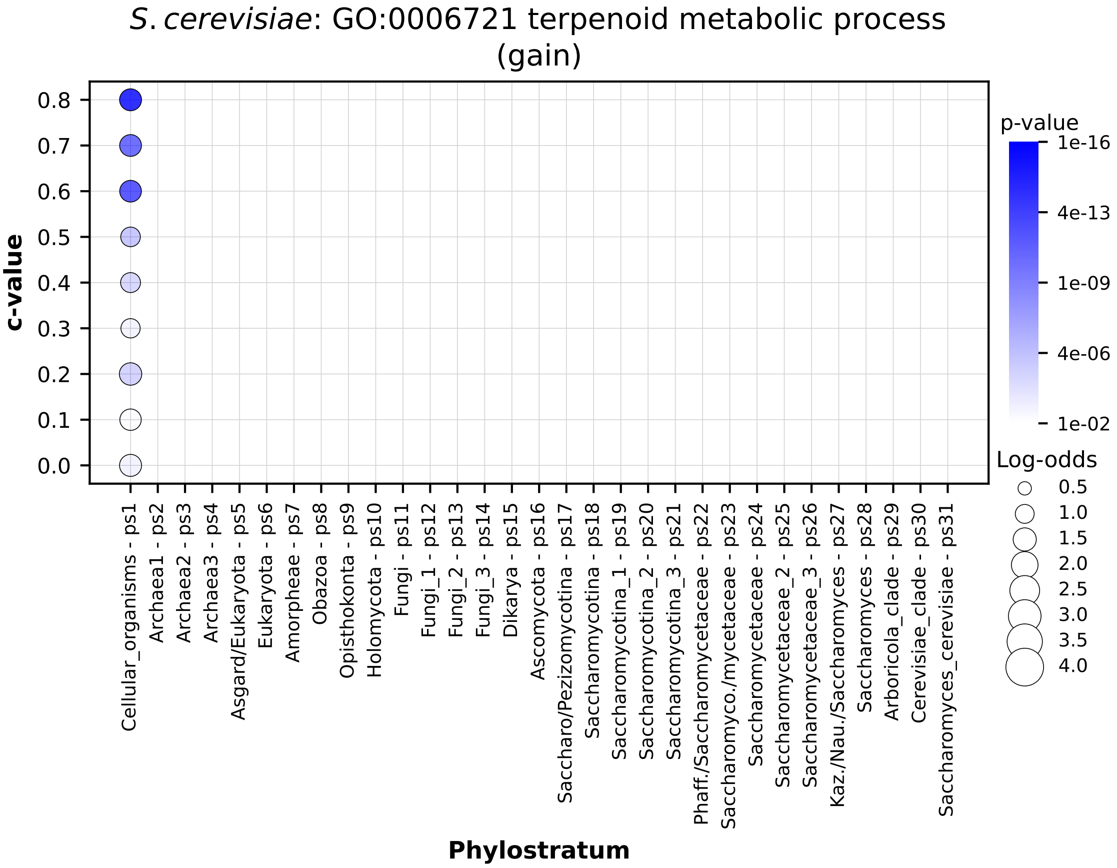
S. cerevisiae: GO:0006721 terpenoid metabolic process
(gain)
c-value
0.0
0.1
0.2
0.3
0.4
0.5
0.6
0.7
0.8
p-value
1e-16
4e-13
1e-09
4e-06
1e-02
Log-odds
0.5
1.0
1.5
2.0
2.5
3.0
3.5
4.0
Cellular_organisms - ps1
Archaea1 - ps2
Archaea2 - ps3
Archaea3 - ps4
Asgard/Eukaryota - ps5
Eukaryota - ps6
Amorpheae - ps7
Obazoa - ps8
Opisthokonta - ps9
Holomycota - ps10
Fungi - ps11
Fungi_1 - ps12
Fungi_2 - ps13
Fungi_3 - ps14
Dikarya - ps15
Ascomycota - ps16
Saccharo/Pezizomycotina - ps17
Saccharomycotina - ps18
Saccharomycotina_1 - ps19
Saccharomycotina_2 - ps20
Saccharomycotina_3 - ps21
Phaff./Saccharomycetaceae - ps22
Saccharomyco./mycetaceae - ps23
Saccharomycetaceae - ps24
Saccharomycetaceae_2 - ps25
Saccharomycetaceae_3 - ps26
Kaz./Nau./Saccharomyces - ps27
Saccharomyces - ps28
Arboricola_clade - ps29
Cerevisiae_clade - ps30
Saccharomyces_cerevisiae - ps31
Phylostratum

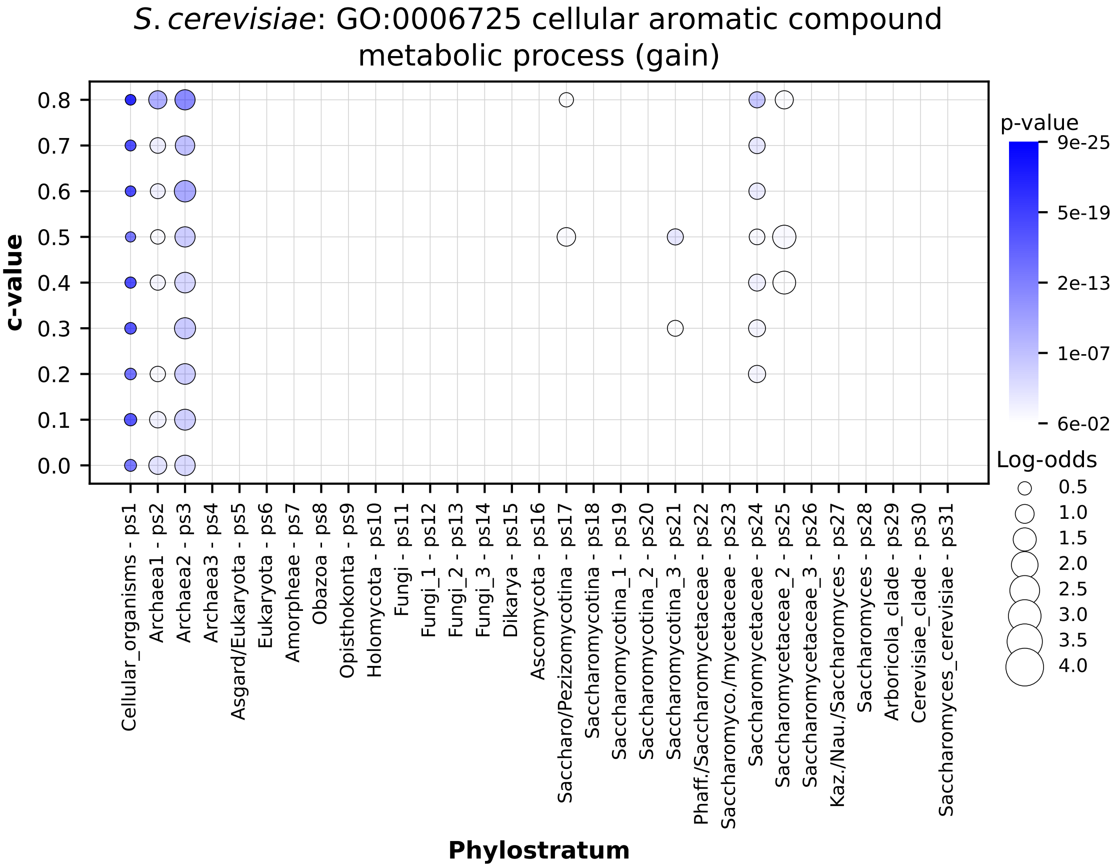
S. cerevisiae: GO:0006725 cellular aromatic compound metabolic process (gain)
c-value
Phylostratum
p-value
9e-25
5e-19
2e-13
1e-07
6e-02
Log-odds
0.5
1.0
1.5
2.0
2.5
3.0
3.5
4.0
0.8
0.7
0.6
0.5
0.4
0.3
0.2
0.1
0.0
Cellular_organisms - ps1
Archaea1 - ps2
Archaea2 - ps3
Archaea3 - ps4
Asgard/Eukaryota - ps5
Eukaryota - ps6
Amorpheae - ps7
Obazoa - ps8
Opisthokonta - ps9
Holomycota - ps10
Fungi - ps11
Fungi_1 - ps12
Fungi_2 - ps13
Fungi_3 - ps14
Dikarya - ps15
Ascomycota - ps16
Saccharo/Pezizomycotina - ps17
Saccharomycotina - ps18
Saccharomycotina_1 - ps19
Saccharomycotina_2 - ps20
Saccharomycotina_3 - ps21
Phaff./Saccharomycetaceae - ps22
Saccharomyco./mycetaceae - ps23
Saccharomycetaceae - ps24
Saccharomycetaceae_2 - ps25
Saccharomycetaceae_3 - ps26
Kaz./Nau./Saccharomyces - ps27
Saccharomyces - ps28
Arboricola_clade - ps29
Cerevisiae_clade - ps30
Saccharomyces_cerevisiae - ps31

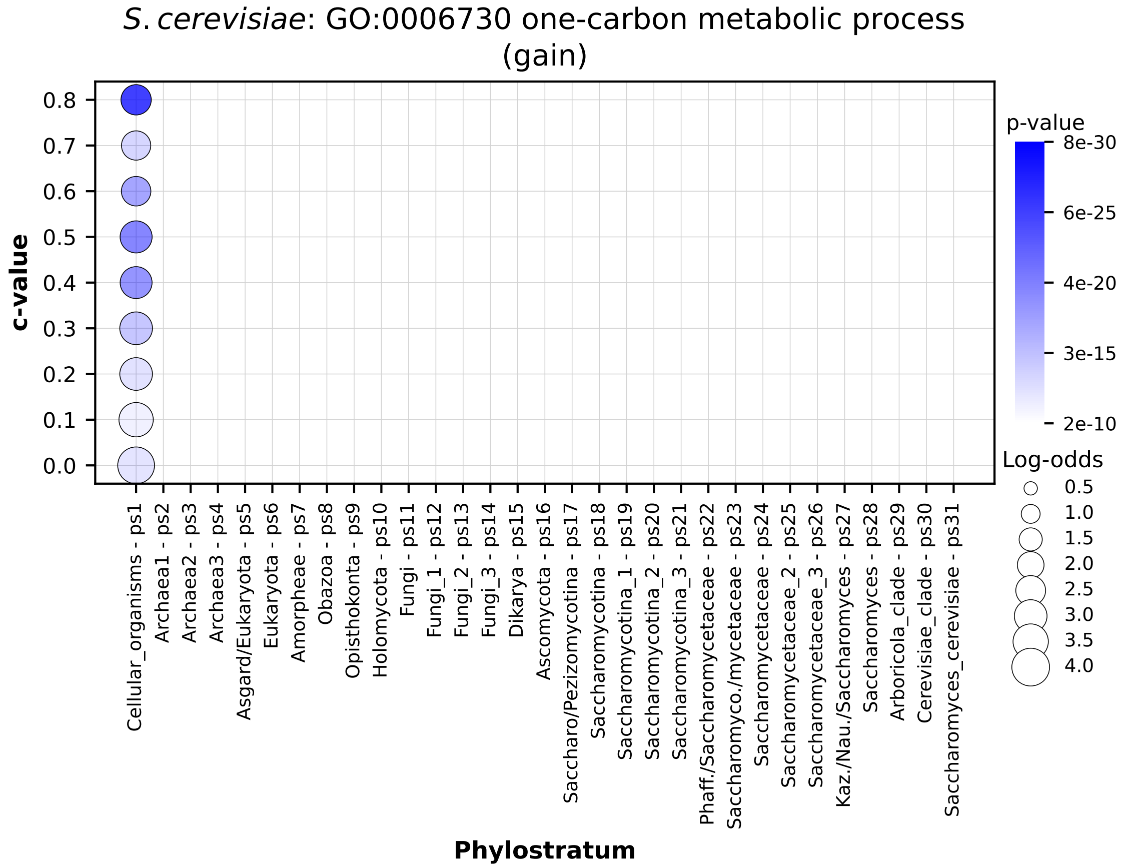

S. cerevisiae: GO:0006730 one-carbon metabolic process (gain)
c-value
0.0
0.1
0.2
0.3
0.4
0.5
0.6
0.7
0.8
Phylostratum
Cellular_organisms - ps1
Archaea1 - ps2
Archaea2 - ps3
Archaea3 - ps4
Asgard/Eukaryota - ps5
Eukaryota - ps6
Amorpheae - ps7
Obazoa - ps8
Opisthokonta - ps9
Holomycota - ps10
Fungi - ps11
Fungi_1 - ps12
Fungi_2 - ps13
Fungi_3 - ps14
Dikarya - ps15
Ascomycota - ps16
Saccharo/Pezizomycotina - ps17
Saccharomycotina - ps18
Saccharomycotina_1 - ps19
Saccharomycotina_2 - ps20
Saccharomycotina_3 - ps21
Phaff./Saccharomycetaceae - ps22
Saccharomyco./mycetaceae - ps23
Saccharomycetaceae - ps24
Saccharomycetaceae_2 - ps25
Saccharomycetaceae_3 - ps26
Kaz./Nau./Saccharomyces - ps27
Saccharomyces - ps28
Arboricola_clade - ps29
Cerevisiae_clade - ps30
Saccharomyces_cerevisiae - ps31
p-value
8e-30
6e-25
4e-20
3e-15
2e-10
Log-odds
0.5
1.0
1.5
2.0
2.5
3.0
3.5
4.0

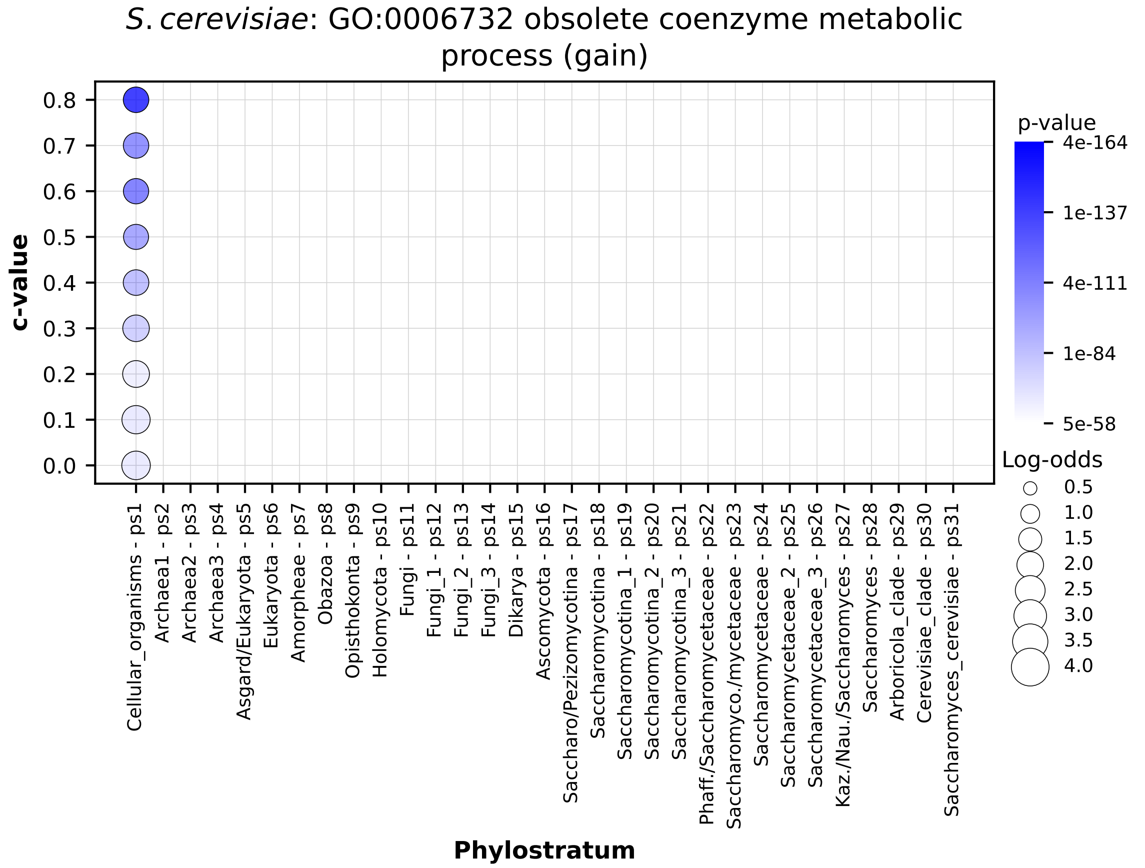
S. cerevisiae: GO:0006732 obsolete coenzyme metabolic process (gain)
c-value
0.8
0.7
0.6
0.5
0.4
0.3
0.2
0.1
0.0
p-value
4e-164
1e-137
4e-111
1e-84
5e-58
Log-odds
0.5
1.0
1.5
2.0
2.5
3.0
3.5
4.0
Cellular_organisms - ps1
Archaea1 - ps2
Archaea2 - ps3
Archaea3 - ps4
Asgard/Eukaryota - ps5
Eukaryota - ps6
Amorpheae - ps7
Obazoa - ps8
Opisthokonta - ps9
Holomycota - ps10
Fungi - ps11
Fungi_1 - ps12
Fungi_2 - ps13
Fungi_3 - ps14
Dikarya - ps15
Ascomycota - ps16
Saccharo/Pezizomycotina - ps17
Saccharomycotina - ps18
Saccharomycotina_1 - ps19
Saccharomycotina_2 - ps20
Saccharomycotina_3 - ps21
Phaff./Saccharomycetaceae - ps22
Saccharomyco./mycetaceae - ps23
Saccharomycetaceae - ps24
Saccharomycetaceae_2 - ps25
Saccharomycetaceae_3 - ps26
Kaz./Nau./Saccharomyces - ps27
Saccharomyces - ps28
Arboricola_clade - ps29
Cerevisiae_clade - ps30
Saccharomyces_cerevisiae - ps31
Phylostratum

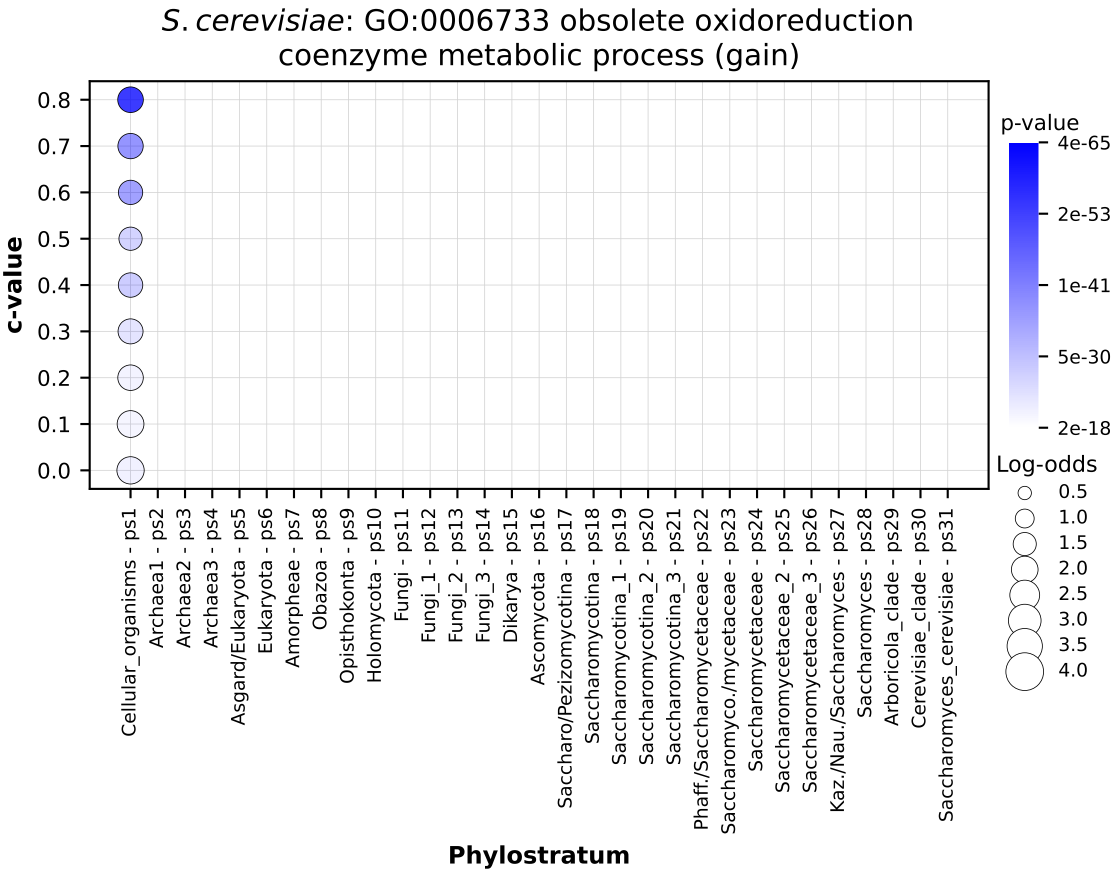
S. cerevisiae: GO:0006733 obsolete oxidoreduction coenzyme metabolic process (gain)
c-value
0.8
0.7
0.6
0.5
0.4
0.3
0.2
0.1
0.0
p-value
4e-65
2e-53
1e-41
5e-30
2e-18
Log-odds
0.5
1.0
1.5
2.0
2.5
3.0
3.5
4.0
Cellular_organisms - ps1
Archaea1 - ps2
Archaea2 - ps3
Archaea3 - ps4
Asgard/Eukaryota - ps5
Eukaryota - ps6
Amorpheae - ps7
Obazoa - ps8
Opisthokonta - ps9
Holomycota - ps10
Fungi - ps11
Fungi_1 - ps12
Fungi_2 - ps13
Fungi_3 - ps14
Dikarya - ps15
Ascomycota - ps16
Saccharo/Pezizomycotina - ps17
Saccharomycotina - ps18
Saccharomycotina_1 - ps19
Saccharomycotina_2 - ps20
Saccharomycotina_3 - ps21
Phaff./Saccharomycetaceae - ps22
Saccharomyco./mycetaceae - ps23
Saccharomycetaceae - ps24
Saccharomycetaceae_2 - ps25
Saccharomycetaceae_3 - ps26
Kaz./Nau./Saccharomyces - ps27
Saccharomyces - ps28
Arboricola_clade - ps29
Cerevisiae_clade - ps30
Saccharomyces_cerevisiae - ps31
Phylostratum

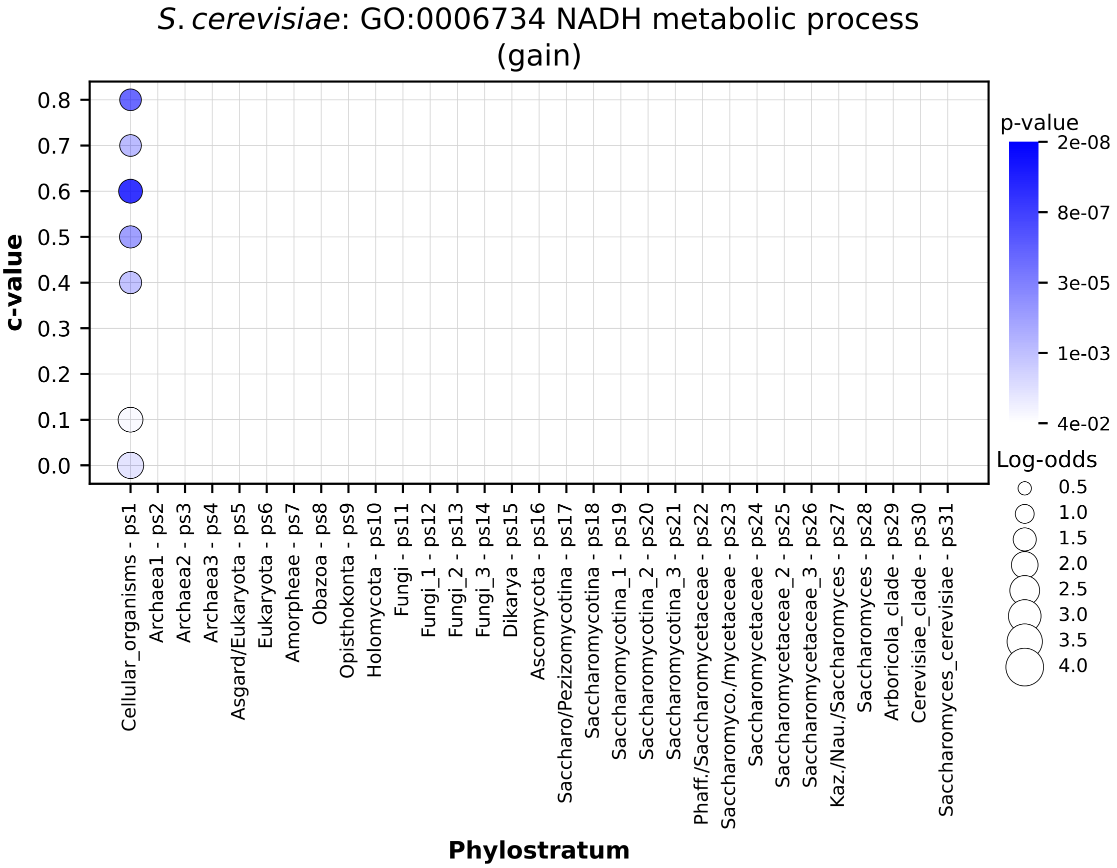

S. cerevisiae: GO:0006734 NADH metabolic process (gain)
c-value
0.8
0.7
0.6
0.5
0.4
0.3
0.2
0.1
0.0
Phylostratum
Cellular_organisms - ps1
Archaea1 - ps2
Archaea2 - ps3
Archaea3 - ps4
Asgard/Eukaryota - ps5
Eukaryota - ps6
Amorpheae - ps7
Obazoa - ps8
Opisthokonta - ps9
Holomycota - ps10
Fungi - ps11
Fungi_1 - ps12
Fungi_2 - ps13
Fungi_3 - ps14
Dikarya - ps15
Ascomycota - ps16
Saccharo/Pezizomycotina - ps17
Saccharomycotina - ps18
Saccharomycotina_1 - ps19
Saccharomycotina_2 - ps20
Saccharomycotina_3 - ps21
Phaff./Saccharomycetaceae - ps22
Saccharomyco./mycetaceae - ps23
Saccharomycetaceae - ps24
Saccharomycetaceae_2 - ps25
Saccharomycetaceae_3 - ps26
Kaz./Nau./Saccharomyces - ps27
Saccharomyces - ps28
Arboricola_clade - ps29
Cerevisiae_clade - ps30
Saccharomyces_cerevisiae - ps31
p-value
2e-08
8e-07
3e-05
1e-03
4e-02
Log-odds
0.5
1.0
1.5
2.0
2.5
3.0
3.5
4.0

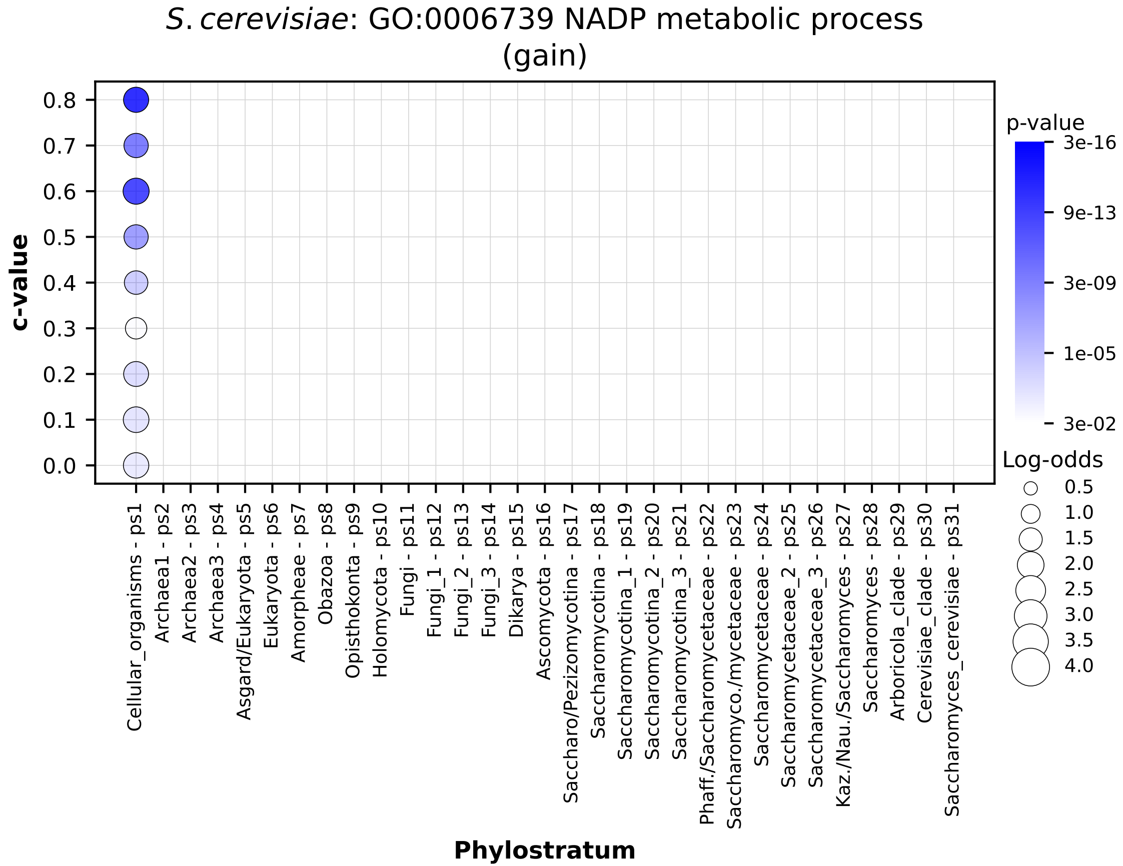

S. cerevisiae: GO:0006739 NADP metabolic process
(gain)
c-value
0.8
0.7
0.6
0.5
0.4
0.3
0.2
0.1
0.0
p-value
3e-16
9e-13
3e-09
1e-05
3e-02
Log-odds
0.5
1.0
1.5
2.0
2.5
3.0
3.5
4.0
Cellular_organisms - ps1
Archaea1 - ps2
Archaea2 - ps3
Archaea3 - ps4
Asgard/Eukaryota - ps5
Eukaryota - ps6
Amorpheae - ps7
Obazoa - ps8
Opisthokonta - ps9
Holomycota - ps10
Fungi - ps11
Fungi_1 - ps12
Fungi_2 - ps13
Fungi_3 - ps14
Dikarya - ps15
Ascomycota - ps16
Saccharo/Pezizomycotina - ps17
Saccharomycotina - ps18
Saccharomycotina_1 - ps19
Saccharomycotina_2 - ps20
Saccharomycotina_3 - ps21
Phaff./Saccharomycetaceae - ps22
Saccharomyco./mycetaceae - ps23
Saccharomycetaceae - ps24
Saccharomycetaceae_2 - ps25
Saccharomycetaceae_3 - ps26
Kaz./Nau./Saccharomyces - ps27
Saccharomyces - ps28
Arboricola_clade - ps29
Cerevisiae_clade - ps30
Saccharomyces_cerevisiae - ps31
Phylostratum

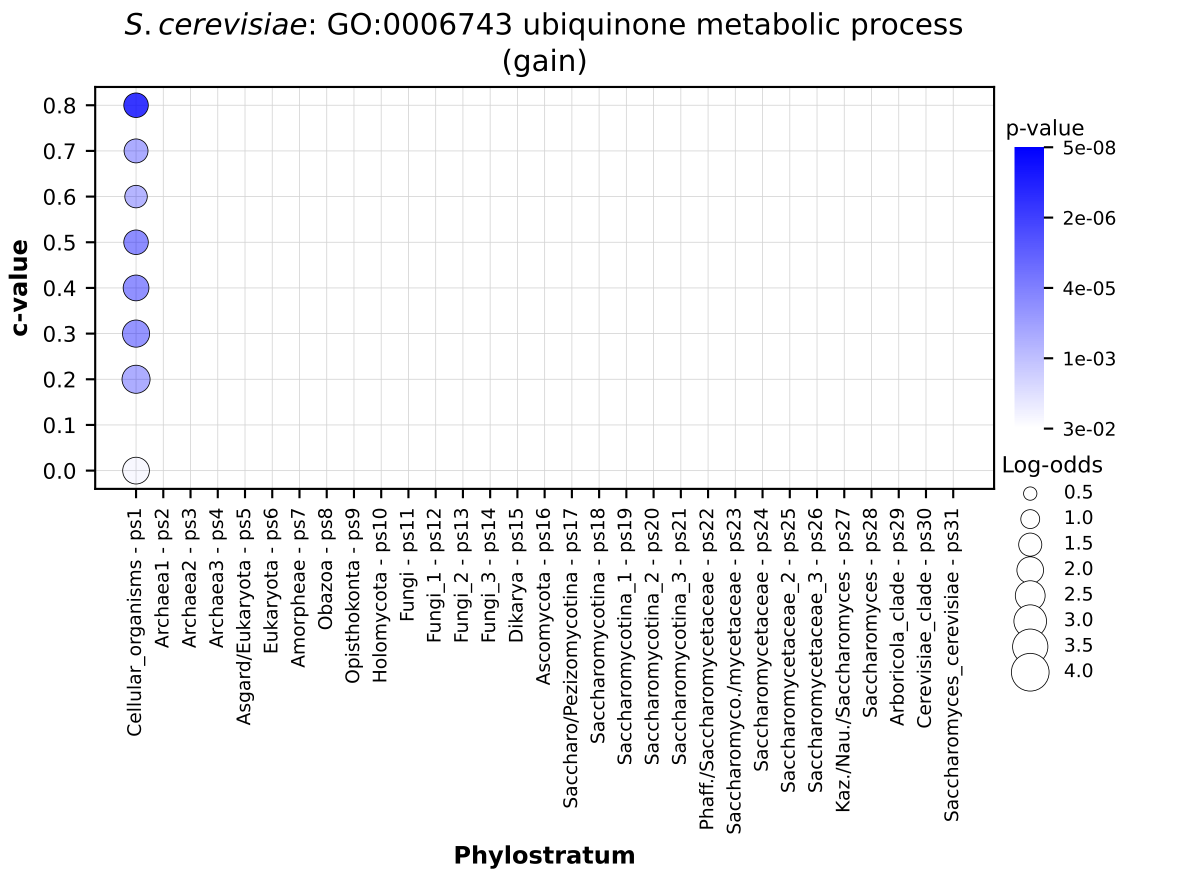
S. cerevisiae: GO:0006743 ubiquinone metabolic process
(gain)
c-value
0.8
0.7
0.6
0.5
0.4
0.3
0.2
0.1
0.0
p-value
5e-08
2e-06
4e-05
1e-03
3e-02
Log-odds
0.5
1.0
1.5
2.0
2.5
3.0
3.5
4.0
Cellular_organisms - ps1
Archaea1 - ps2
Archaea2 - ps3
Archaea3 - ps4
Asgard/Eukaryota - ps5
Eukaryota - ps6
Amorpheae - ps7
Obazoa - ps8
Opisthokonta - ps9
Holomycota - ps10
Fungi - ps11
Fungi_1 - ps12
Fungi_2 - ps13
Fungi_3 - ps14
Dikarya - ps15
Ascomycota - ps16
Saccharo/Pezizomycotina - ps17
Saccharomycotina - ps18
Saccharomycotina_1 - ps19
Saccharomycotina_2 - ps20
Saccharomycotina_3 - ps21
Phaff./Saccharomycetaceae - ps22
Saccharomyco./mycetaceae - ps23
Saccharomycetaceae - ps24
Saccharomycetaceae_2 - ps25
Saccharomycetaceae_3 - ps26
Kaz./Nau./Saccharomyces - ps27
Saccharomyces - ps28
Arboricola_clade - ps29
Cerevisiae_clade - ps30
Saccharomyces_cerevisiae - ps31
Phylostratum

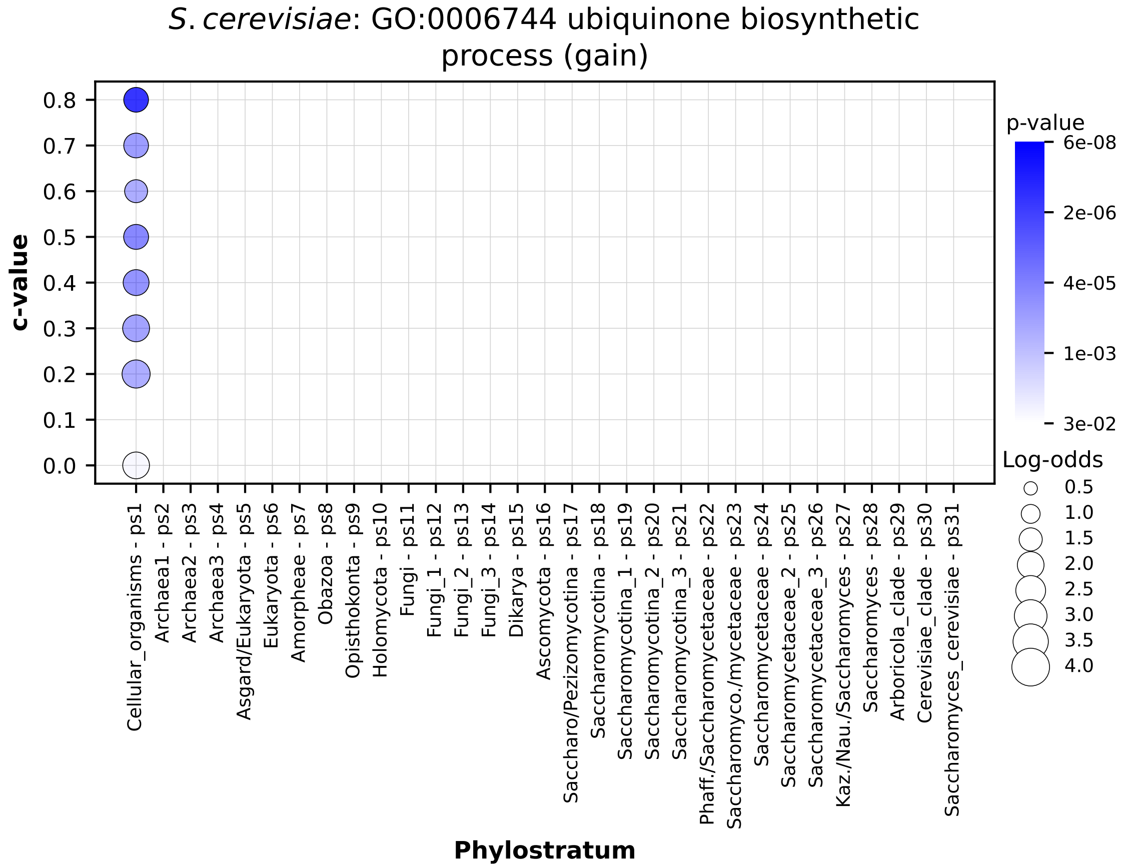
S. cerevisiae: GO:0006744 ubiquinone biosynthetic process (gain)
c-value
0.8
0.7
0.6
0.5
0.4
0.3
0.2
0.1
0.0
Phylostratum
Cellular_organisms - ps1
Archaea1 - ps2
Archaea2 - ps3
Archaea3 - ps4
Asgard/Eukaryota - ps5
Eukaryota - ps6
Amorpheae - ps7
Obazoa - ps8
Opisthokonta - ps9
Holomycota - ps10
Fungi - ps11
Fungi_1 - ps12
Fungi_2 - ps13
Fungi_3 - ps14
Dikarya - ps15
Ascomycota - ps16
Saccharo/Pezizomycotina - ps17
Saccharomycotina - ps18
Saccharomycotina_1 - ps19
Saccharomycotina_2 - ps20
Saccharomycotina_3 - ps21
Phaff./Saccharomycetaceae - ps22
Saccharomyco./mycetaceae - ps23
Saccharomycetaceae - ps24
Saccharomycetaceae_2 - ps25
Saccharomycetaceae_3 - ps26
Kaz./Nau./Saccharomyces - ps27
Saccharomyces - ps28
Arboricola_clade - ps29
Cerevisiae_clade - ps30
Saccharomyces_cerevisiae - ps31
p-value
6e-08
2e-06
4e-05
1e-03
3e-02
Log-odds
0.5
1.0
1.5
2.0
2.5
3.0
3.5
4.0

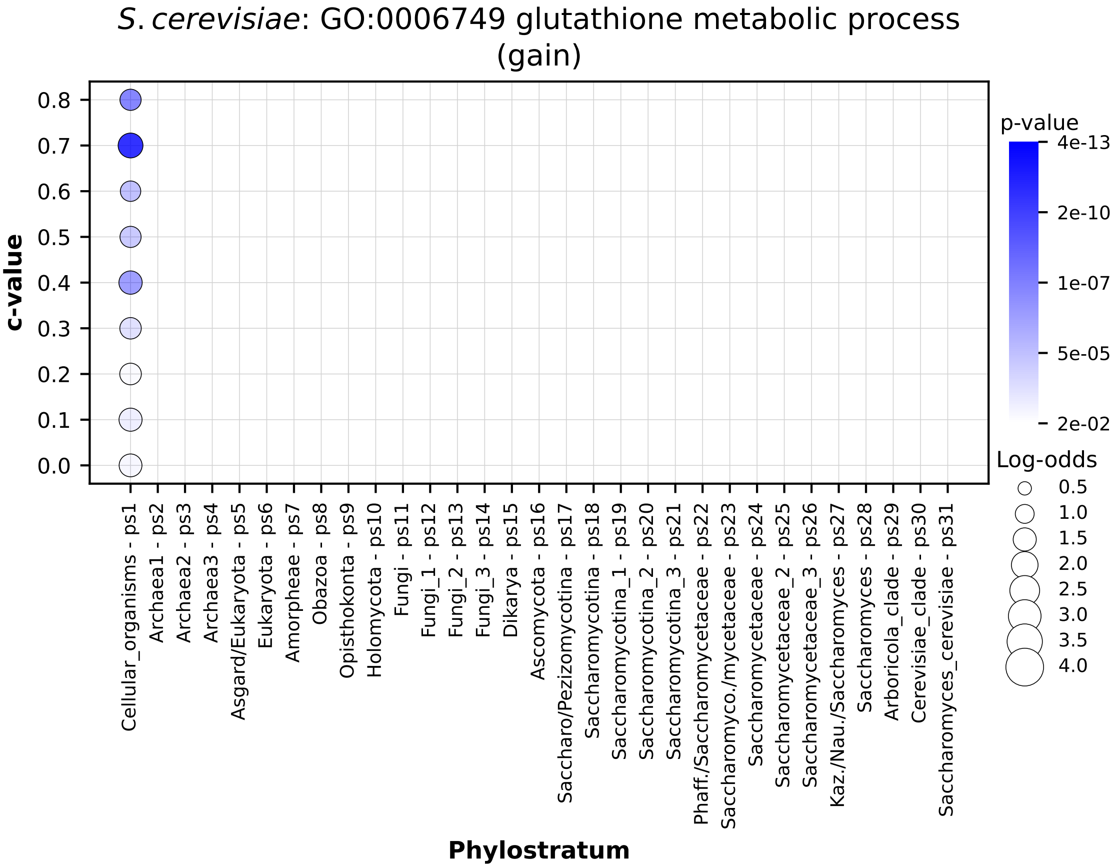

S. cerevisiae: GO:0006749 glutathione metabolic process
(gain)
c-value
0.8
0.7
0.6
0.5
0.4
0.3
0.2
0.1
0.0
p-value
4e-13
2e-10
1e-07
5e-05
2e-02
Log-odds
0.5
1.0
1.5
2.0
2.5
3.0
3.5
4.0
Cellular_organisms - ps1
Archaea1 - ps2
Archaea2 - ps3
Archaea3 - ps4
Asgard/Eukaryota - ps5
Eukaryota - ps6
Amorpheae - ps7
Obazoa - ps8
Opisthokonta - ps9
Holomycota - ps10
Fungi - ps11
Fungi_1 - ps12
Fungi_2 - ps13
Fungi_3 - ps14
Dikarya - ps15
Ascomycota - ps16
Saccharo/Pezizomycotina - ps17
Saccharomycotina - ps18
Saccharomycotina_1 - ps19
Saccharomycotina_2 - ps20
Saccharomycotina_3 - ps21
Phaff./Saccharomycetaceae - ps22
Saccharomyco./mycetaceae - ps23
Saccharomycetaceae - ps24
Saccharomycetaceae_2 - ps25
Saccharomycetaceae_3 - ps26
Kaz./Nau./Saccharomyces - ps27
Saccharomyces - ps28
Arboricola_clade - ps29
Cerevisiae_clade - ps30
Saccharomyces_cerevisiae - ps31
Phylostratum

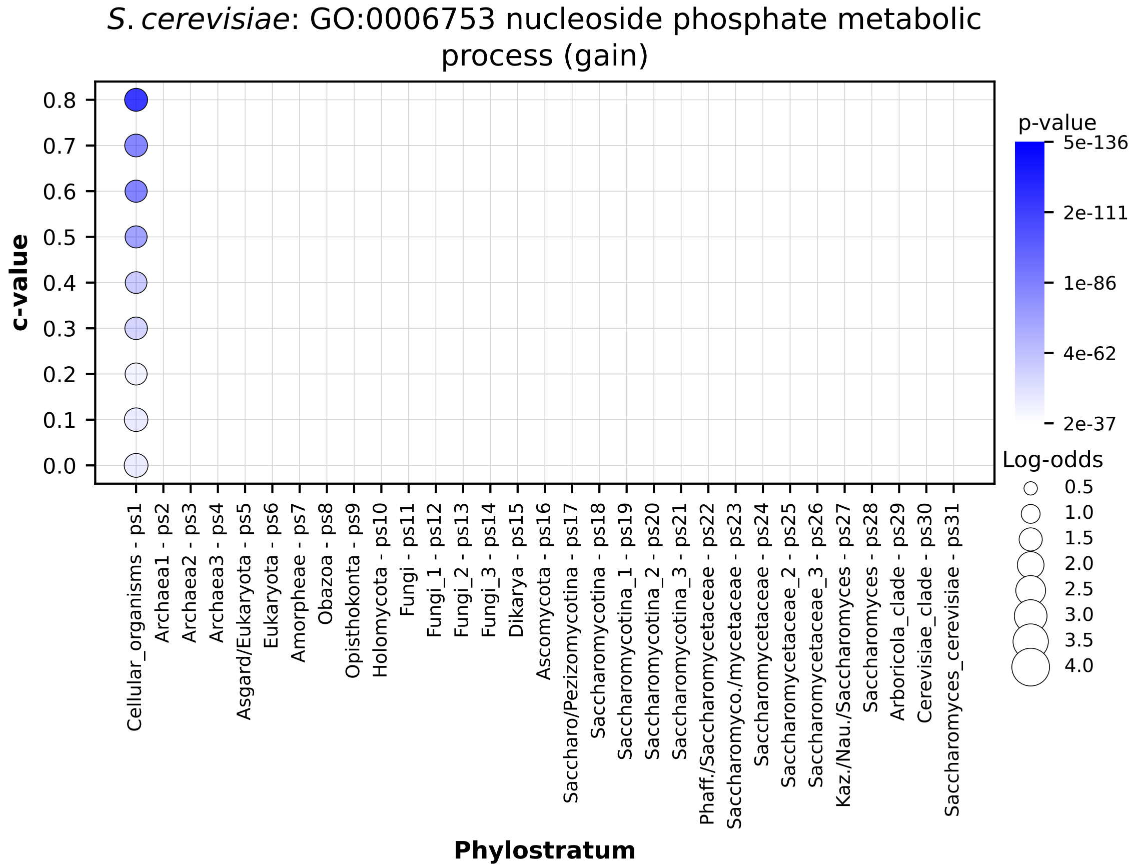
S. cerevisiae: GO:0006753 nucleoside phosphate metabolic process (gain)
c-value
0.8
0.7
0.6
0.5
0.4
0.3
0.2
0.1
0.0
p-value
5e-136
2e-111
1e-86
4e-62
2e-37
Log-odds
0.5
1.0
1.5
2.0
2.5
3.0
3.5
4.0
Cellular_organisms - ps1
Archaea1 - ps2
Archaea2 - ps3
Archaea3 - ps4
Asgard/Eukaryota - ps5
Eukaryota - ps6
Amorpheae - ps7
Obazoa - ps8
Opisthokonta - ps9
Holomycota - ps10
Fungi - ps11
Fungi_1 - ps12
Fungi_2 - ps13
Fungi_3 - ps14
Dikarya - ps15
Ascomycota - ps16
Saccharo/Pezizomycotina - ps17
Saccharomycotina - ps18
Saccharomycotina_1 - ps19
Saccharomycotina_2 - ps20
Saccharomycotina_3 - ps21
Phaff./Saccharomycetaceae - ps22
Saccharomyco./mycetaceae - ps23
Saccharomycetaceae - ps24
Saccharomycetaceae_2 - ps25
Saccharomycetaceae_3 - ps26
Kaz./Nau./Saccharomyces - ps27
Saccharomyces - ps28
Arboricola_clade - ps29
Cerevisiae_clade - ps30
Saccharomyces_cerevisiae - ps31
Phylostratum

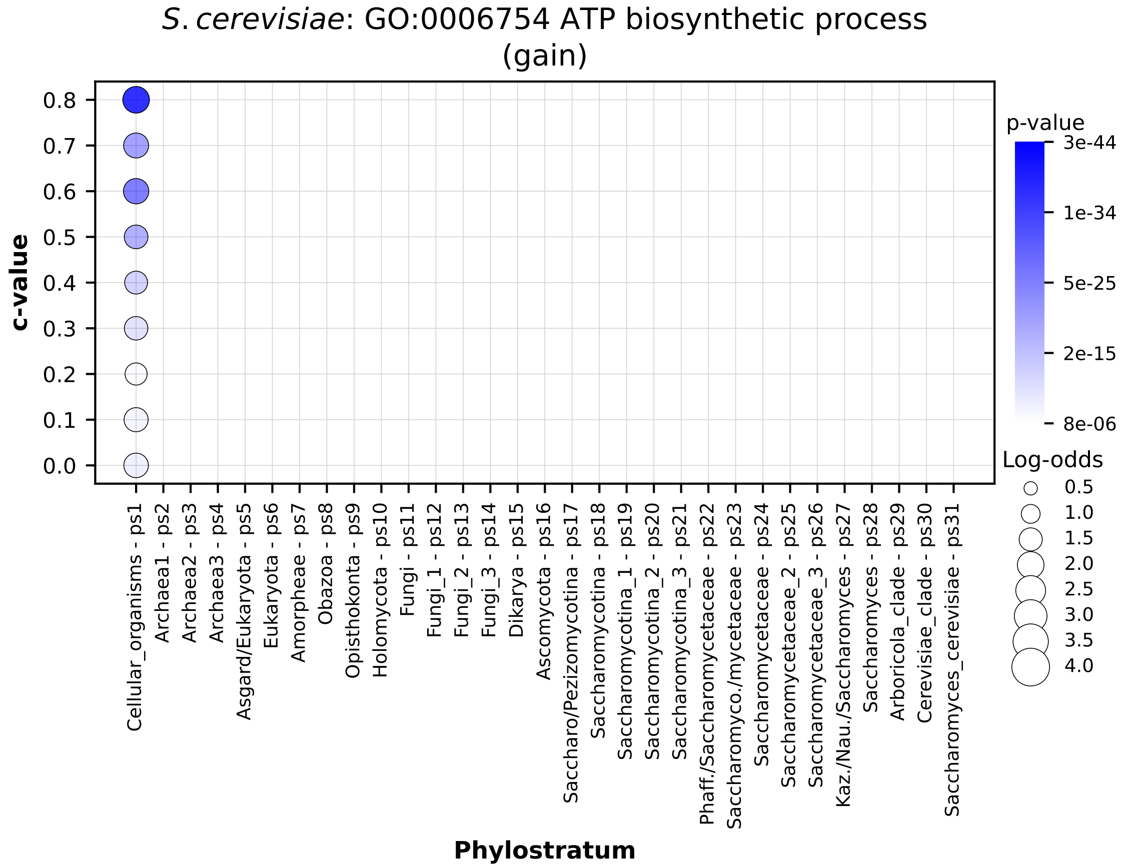

$S. cerevisiae$: GO:0006754 ATP biosynthetic process (gain)
c-value
0.8
0.7
0.6
0.5
0.4
0.3
0.2
0.1
0.0
Phylostratum
Cellular_organisms - ps1
Archaea1 - ps2
Archaea2 - ps3
Archaea3 - ps4
Asgard/Eukaryota - ps5
Eukaryota - ps6
Amorpheae - ps7
Obazoa - ps8
Opisthokonta - ps9
Holomycota - ps10
Fungi - ps11
Fungi_1 - ps12
Fungi_2 - ps13
Fungi_3 - ps14
Dikarya - ps15
Ascomycota - ps16
Saccharo/Pezizomycotina - ps17
Saccharomycotina - ps18
Saccharomycotina_1 - ps19
Saccharomycotina_2 - ps20
Saccharomycotina_3 - ps21
Phaff./Saccharomycetaceae - ps22
Saccharomyco./mycetaceae - ps23
Saccharomycetaceae - ps24
Saccharomycetaceae_2 - ps25
Saccharomycetaceae_3 - ps26
Kaz./Nau./Saccharomyces - ps27
Saccharomyces - ps28
Arboricola_clade - ps29
Cerevisiae_clade - ps30
Saccharomyces_cerevisiae - ps31
p-value
3e-44
1e-34
5e-25
2e-15
8e-06
Log-odds
0.5
1.0
1.5
2.0
2.5
3.0
3.5
4.0

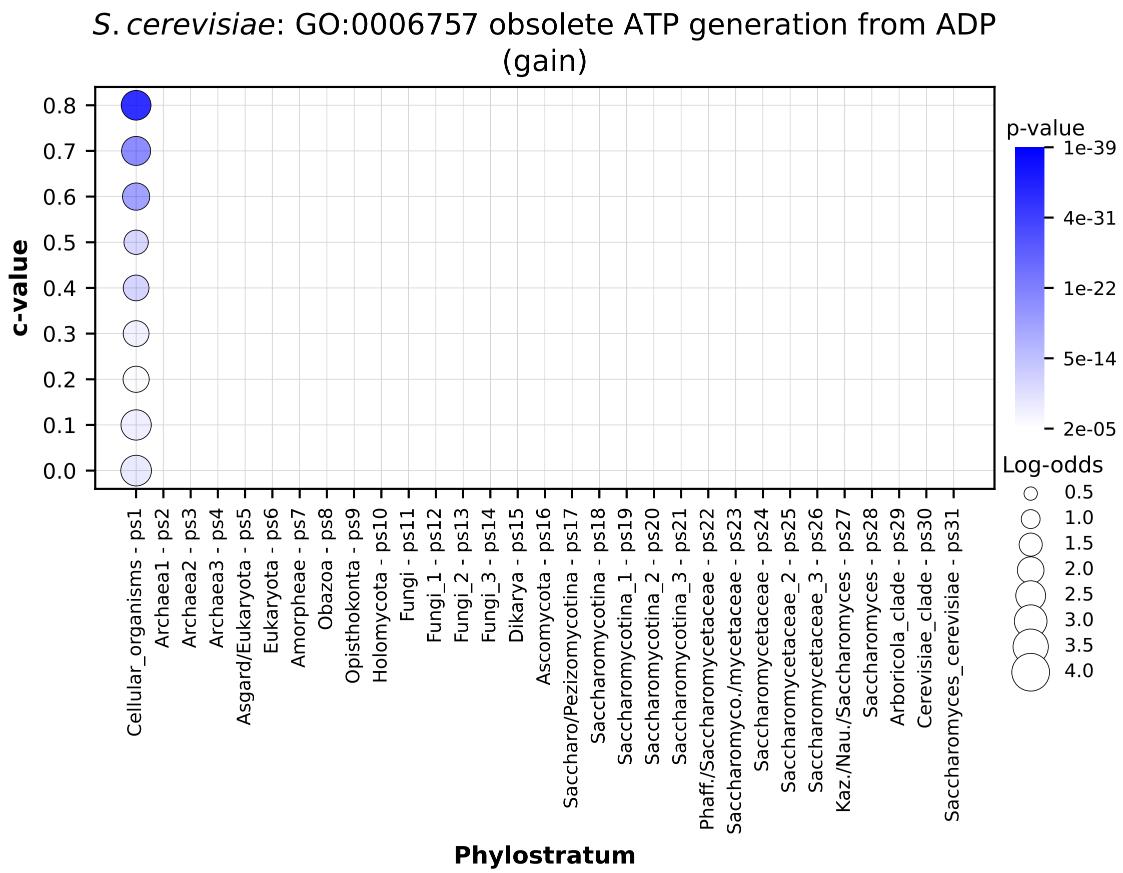

S. cerevisiae: GO:0006757 obsolete ATP generation from ADP (gain)
c-value
0.8
0.7
0.6
0.5
0.4
0.3
0.2
0.1
0.0
Phylostratum
Cellular_organisms - ps1
Archaea1 - ps2
Archaea2 - ps3
Archaea3 - ps4
Asgard/Eukaryota - ps5
Eukaryota - ps6
Amorpheae - ps7
Obazoa - ps8
Opisthokonta - ps9
Holomycota - ps10
Fungi - ps11
Fungi_1 - ps12
Fungi_2 - ps13
Fungi_3 - ps14
Dikarya - ps15
Ascomycota - ps16
Saccharo/Pezizomycotina - ps17
Saccharomycotina - ps18
Saccharomycotina_1 - ps19
Saccharomycotina_2 - ps20
Saccharomycotina_3 - ps21
Phaff./Saccharomycetaceae - ps22
Saccharomyco./mycetaceae - ps23
Saccharomycetaceae - ps24
Saccharomycetaceae_2 - ps25
Saccharomycetaceae_3 - ps26
Kaz./Nau./Saccharomyces - ps27
Saccharomyces - ps28
Arboricola_clade - ps29
Cerevisiae_clade - ps30
Saccharomyces_cerevisiae - ps31
p-value
1e-39
4e-31
1e-22
5e-14
2e-05
Log-odds
0.5
1.0
1.5
2.0
2.5
3.0
3.5
4.0

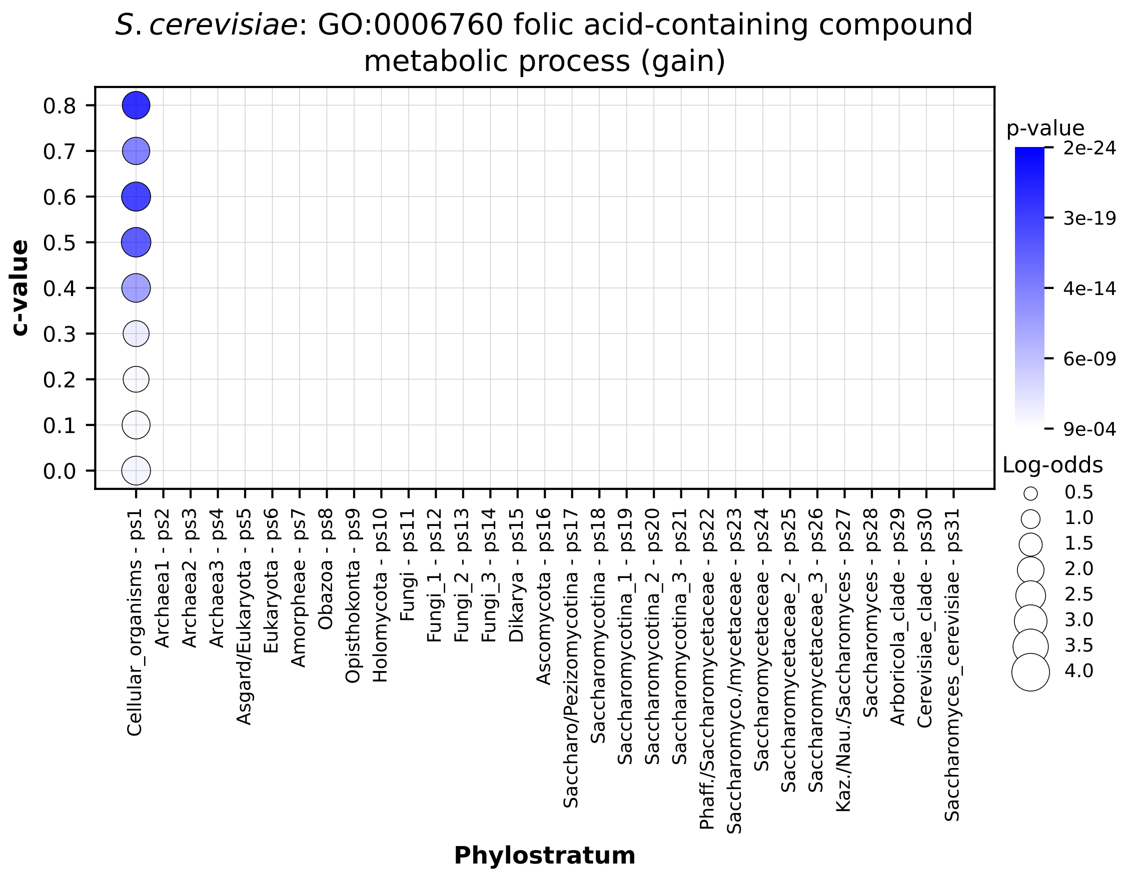

S. cerevisiae: GO:0006760 folic acid-containing compound metabolic process (gain)
c-value
0.8
0.7
0.6
0.5
0.4
0.3
0.2
0.1
0.0
p-value
2e-24
3e-19
4e-14
6e-09
9e-04
Log-odds
0.5
1.0
1.5
2.0
2.5
3.0
3.5
4.0
Cellular_organisms - ps1
Archaea1 - ps2
Archaea2 - ps3
Archaea3 - ps4
Asgard/Eukaryota - ps5
Eukaryota - ps6
Amorpheae - ps7
Obazoa - ps8
Opisthokonta - ps9
Holomycota - ps10
Fungi - ps11
Fungi_1 - ps12
Fungi_2 - ps13
Fungi_3 - ps14
Dikarya - ps15
Ascomycota - ps16
Saccharo/Pezizomycotina - ps17
Saccharomycotina - ps18
Saccharomycotina_1 - ps19
Saccharomycotina_2 - ps20
Saccharomycotina_3 - ps21
Phaff./Saccharomycetaceae - ps22
Saccharomyco./mycetaceae - ps23
Saccharomycetaceae - ps24
Saccharomycetaceae_2 - ps25
Saccharomycetaceae_3 - ps26
Kaz./Nau./Saccharomyces - ps27
Saccharomyces - ps28
Arboricola_clade - ps29
Cerevisiae_clade - ps30
Saccharomyces_cerevisiae - ps31
Phylostratum

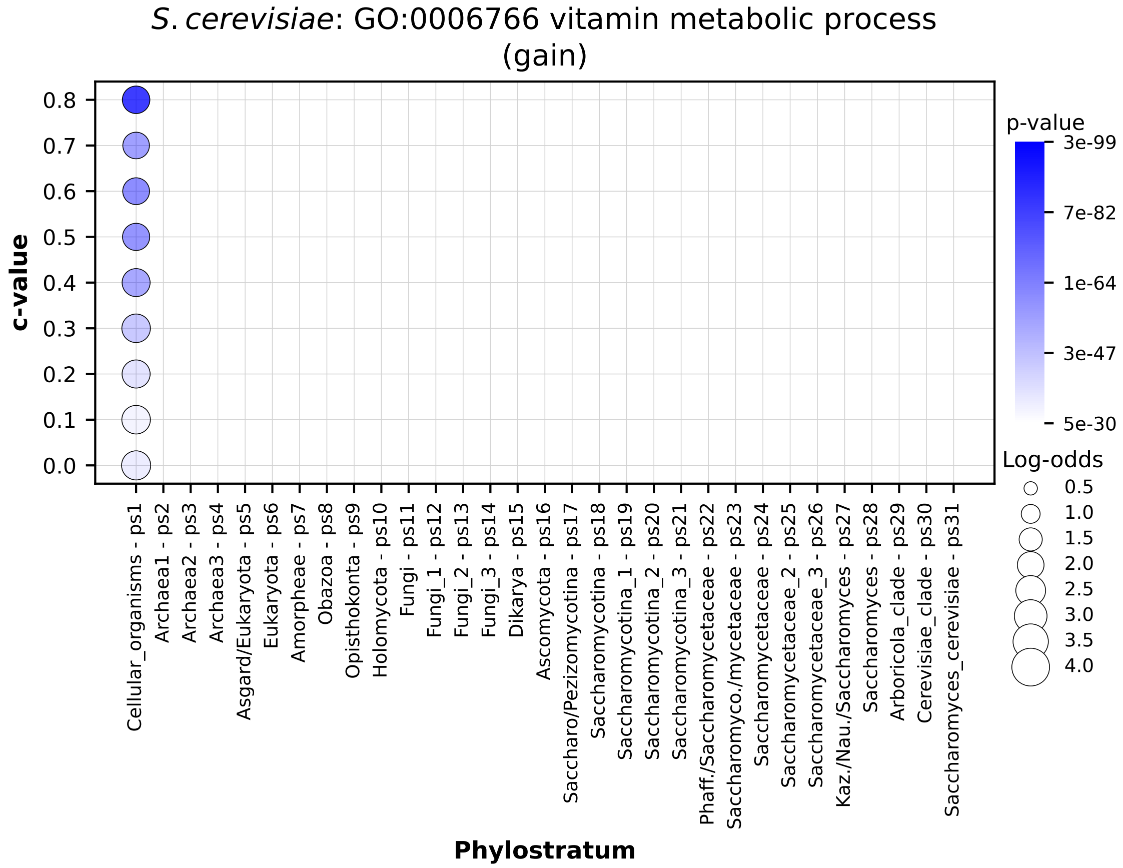

S. cerevisiae: GO:0006766 vitamin metabolic process
(gain)
c-value
0.8
0.7
0.6
0.5
0.4
0.3
0.2
0.1
0.0
p-value
3e-99
7e-82
1e-64
3e-47
5e-30
Log-odds
0.5
1.0
1.5
2.0
2.5
3.0
3.5
4.0
Cellular_organisms - ps1
Archaea1 - ps2
Archaea2 - ps3
Archaea3 - ps4
Asgard/Eukaryota - ps5
Eukaryota - ps6
Amorpheae - ps7
Obazoa - ps8
Opisthokonta - ps9
Holomycota - ps10
Fungi - ps11
Fungi_1 - ps12
Fungi_2 - ps13
Fungi_3 - ps14
Dikarya - ps15
Ascomycota - ps16
Saccharo/Pezizomycotina - ps17
Saccharomycotina - ps18
Saccharomycotina_1 - ps19
Saccharomycotina_2 - ps20
Saccharomycotina_3 - ps21
Phaff./Saccharomycetaceae - ps22
Saccharomyco./mycetaceae - ps23
Saccharomycetaceae - ps24
Saccharomycetaceae_2 - ps25
Saccharomycetaceae_3 - ps26
Kaz./Nau./Saccharomyces - ps27
Saccharomyces - ps28
Arboricola_clade - ps29
Cerevisiae_clade - ps30
Saccharomyces_cerevisiae - ps31
Phylostratum

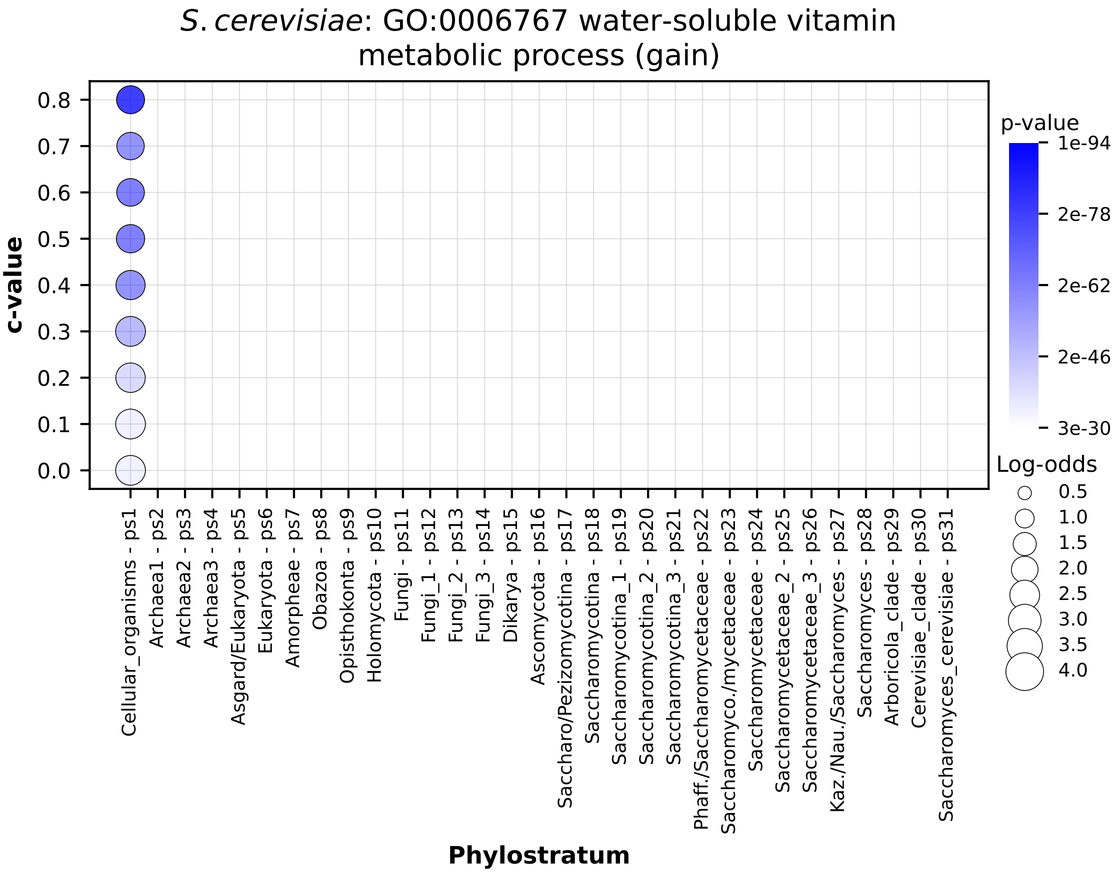

S. cerevisiae: GO:0006767 water-soluble vitamin metabolic process (gain)
c-value
0.8
0.7
0.6
0.5
0.4
0.3
0.2
0.1
0.0
p-value
1e-94
2e-78
2e-62
2e-46
3e-30
Log-odds
0.5
1.0
1.5
2.0
2.5
3.0
3.5
4.0
Cellular_organisms - ps1
Archaea1 - ps2
Archaea2 - ps3
Archaea3 - ps4
Asgard/Eukaryota - ps5
Eukaryota - ps6
Amorpheae - ps7
Obazoa - ps8
Opisthokonta - ps9
Holomycota - ps10
Fungi - ps11
Fungi_1 - ps12
Fungi_2 - ps13
Fungi_3 - ps14
Dikarya - ps15
Ascomycota - ps16
Saccharo/Pezizomycotina - ps17
Saccharomycotina - ps18
Saccharomycotina_1 - ps19
Saccharomycotina_2 - ps20
Saccharomycotina_3 - ps21
Phaff./Saccharomycetaceae - ps22
Saccharomyco./mycetaceae - ps23
Saccharomycetaceae - ps24
Saccharomycetaceae_2 - ps25
Saccharomycetaceae_3 - ps26
Kaz./Nau./Saccharomyces - ps27
Saccharomyces - ps28
Arboricola_clade - ps29
Cerevisiae_clade - ps30
Saccharomyces_cerevisiae - ps31
Phylostratum

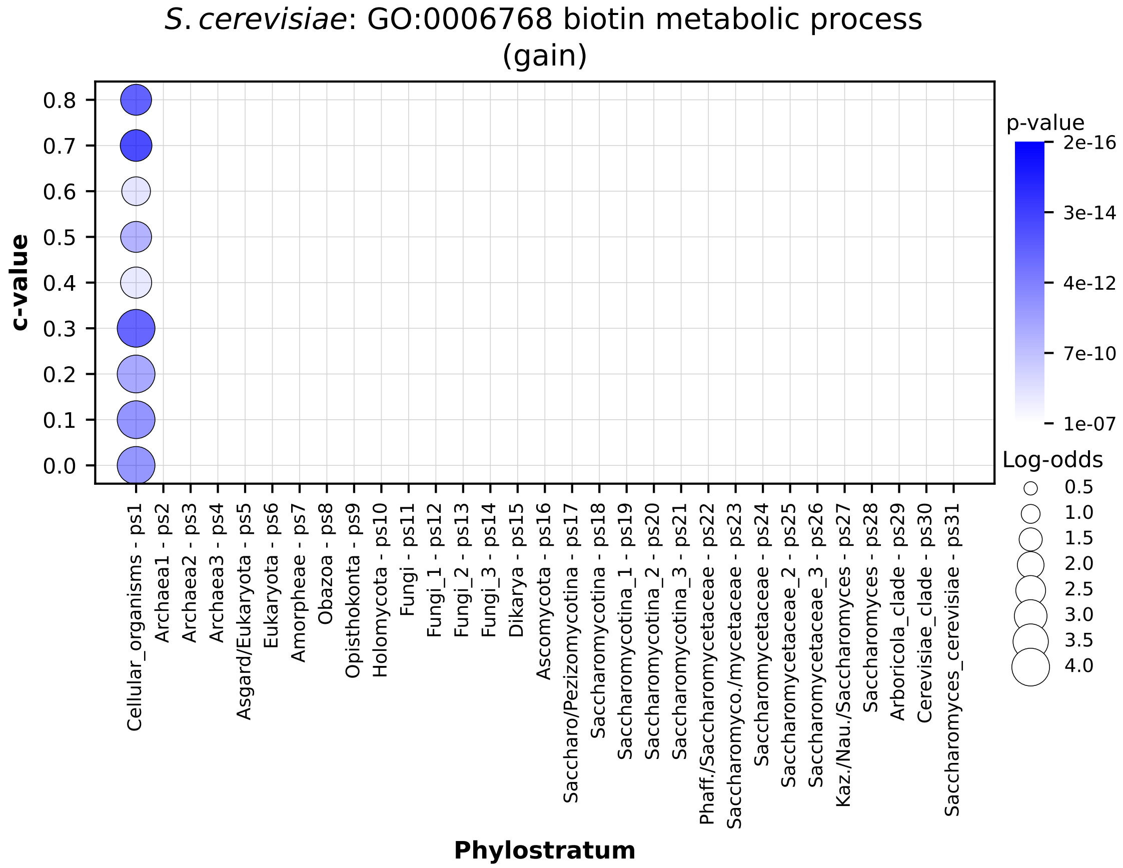

S. cerevisiae: GO:0006768 biotin metabolic process
(gain)
c-value
0.8
0.7
0.6
0.5
0.4
0.3
0.2
0.1
0.0
p-value
2e-16
3e-14
4e-12
7e-10
1e-07
Log-odds
0.5
1.0
1.5
2.0
2.5
3.0
3.5
4.0
Cellular_organisms - ps1
Archaea1 - ps2
Archaea2 - ps3
Archaea3 - ps4
Asgard/Eukaryota - ps5
Eukaryota - ps6
Amorpheae - ps7
Obazoa - ps8
Opisthokonta - ps9
Holomycota - ps10
Fungi - ps11
Fungi_1 - ps12
Fungi_2 - ps13
Fungi_3 - ps14
Dikarya - ps15
Ascomycota - ps16
Saccharo/Pezizomycotina - ps17
Saccharomycotina - ps18
Saccharomycotina_1 - ps19
Saccharomycotina_2 - ps20
Saccharomycotina_3 - ps21
Phaff./Saccharomycetaceae - ps22
Saccharomyco./mycetaceae - ps23
Saccharomycetaceae - ps24
Saccharomycetaceae_2 - ps25
Saccharomycetaceae_3 - ps26
Kaz./Nau./Saccharomyces - ps27
Saccharomyces - ps28
Arboricola_clade - ps29
Cerevisiae_clade - ps30
Saccharomyces_cerevisiae - ps31
Phylostratum

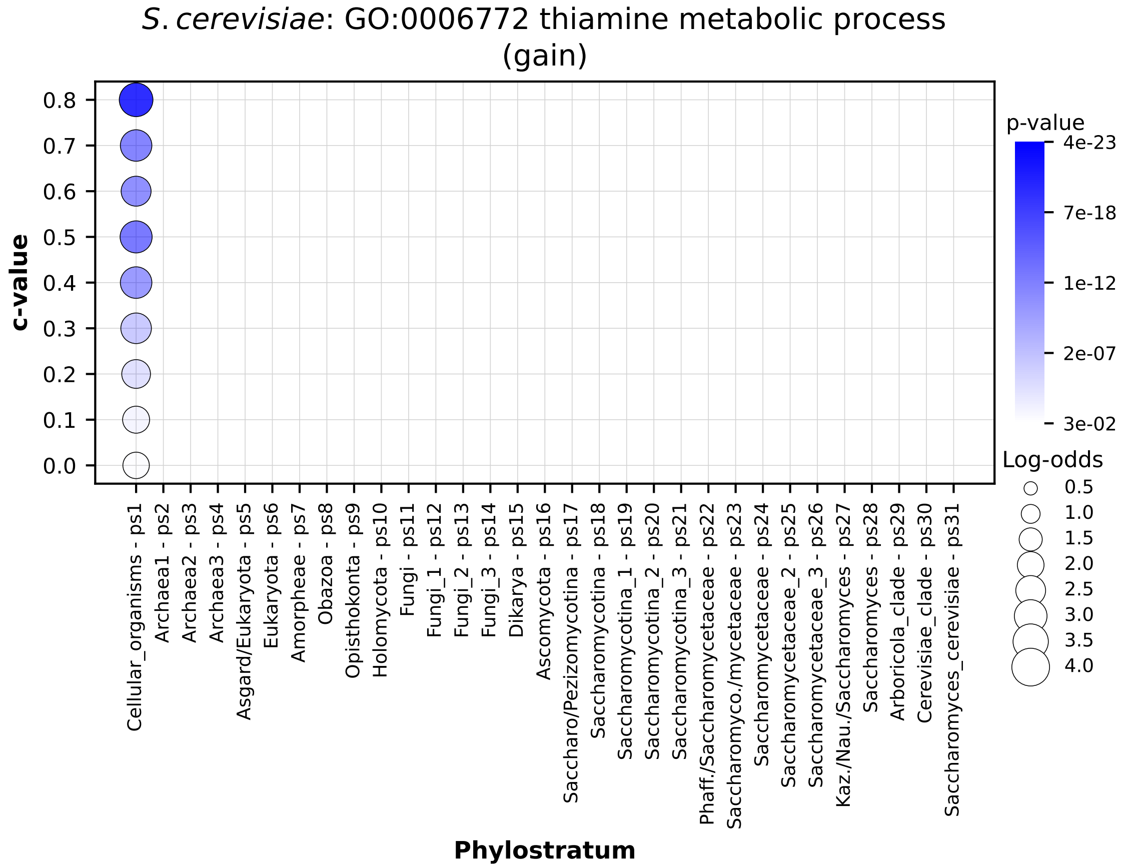
S. cerevisiae: GO:0006772 thiamine metabolic process
(gain)
c-value
0.0
0.1
0.2
0.3
0.4
0.5
0.6
0.7
0.8
p-value
4e-23
7e-18
1e-12
2e-07
3e-02
Log-odds
0.5
1.0
1.5
2.0
2.5
3.0
3.5
4.0
Cellular_organisms - ps1
Archaea1 - ps2
Archaea2 - ps3
Archaea3 - ps4
Asgard/Eukaryota - ps5
Eukaryota - ps6
Amorpheae - ps7
Obazoa - ps8
Opisthokonta - ps9
Holomycota - ps10
Fungi - ps11
Fungi_1 - ps12
Fungi_2 - ps13
Fungi_3 - ps14
Dikarya - ps15
Ascomycota - ps16
Saccharo/Pezizomycotina - ps17
Saccharomycotina - ps18
Saccharomycotina_1 - ps19
Saccharomycotina_2 - ps20
Saccharomycotina_3 - ps21
Phaff./Saccharomycetaceae - ps22
Saccharomyco./mycetaceae - ps23
Saccharomycetaceae - ps24
Saccharomycetaceae_2 - ps25
Saccharomycetaceae_3 - ps26
Kaz./Nau./Saccharomyces - ps27
Saccharomyces - ps28
Arboricola_clade - ps29
Cerevisiae_clade - ps30
Saccharomyces_cerevisiae - ps31
Phylostratum

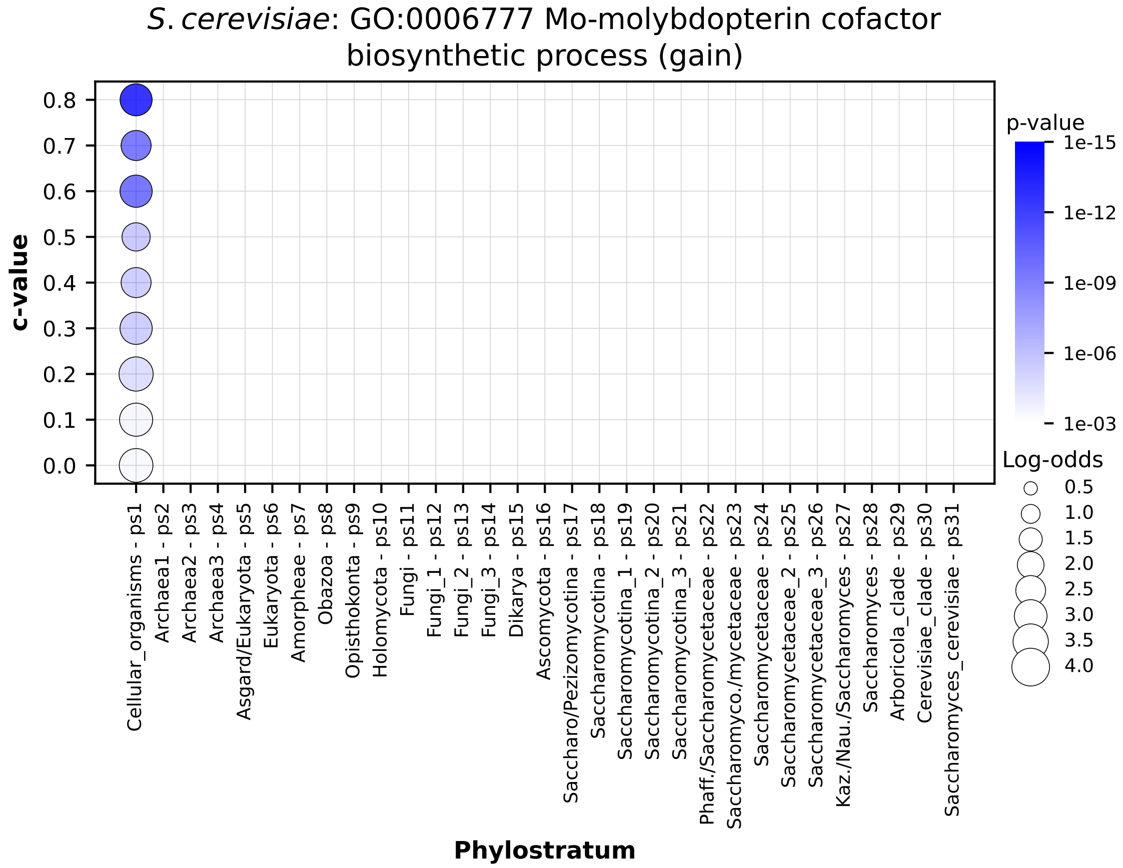
S. cerevisiae: GO:0006777 Mo-molybdopterin cofactor biosynthetic process (gain)
c-value
0.0
0.1
0.2
0.3
0.4
0.5
0.6
0.7
0.8
p-value
1e-15
1e-12
1e-09
1e-06
1e-03
Log-odds
0.5
1.0
1.5
2.0
2.5
3.0
3.5
4.0
Cellular_organisms - ps1
Archaea1 - ps2
Archaea2 - ps3
Archaea3 - ps4
Asgard/Eukaryota - ps5
Eukaryota - ps6
Amorpheae - ps7
Obazoa - ps8
Opisthokonta - ps9
Holomycota - ps10
Fungi - ps11
Fungi_1 - ps12
Fungi_2 - ps13
Fungi_3 - ps14
Dikarya - ps15
Ascomycota - ps16
Saccharo/Pezizomycotina - ps17
Saccharomycotina - ps18
Saccharomycotina_1 - ps19
Saccharomycotina_2 - ps20
Saccharomycotina_3 - ps21
Phaff./Saccharomycetaceae - ps22
Saccharomyco./mycetaceae - ps23
Saccharomycetaceae - ps24
Saccharomycetaceae_2 - ps25
Saccharomycetaceae_3 - ps26
Kaz./Nau./Saccharomyces - ps27
Saccharomyces - ps28
Arboricola_clade - ps29
Cerevisiae_clade - ps30
Saccharomyces_cerevisiae - ps31
Phylostratum

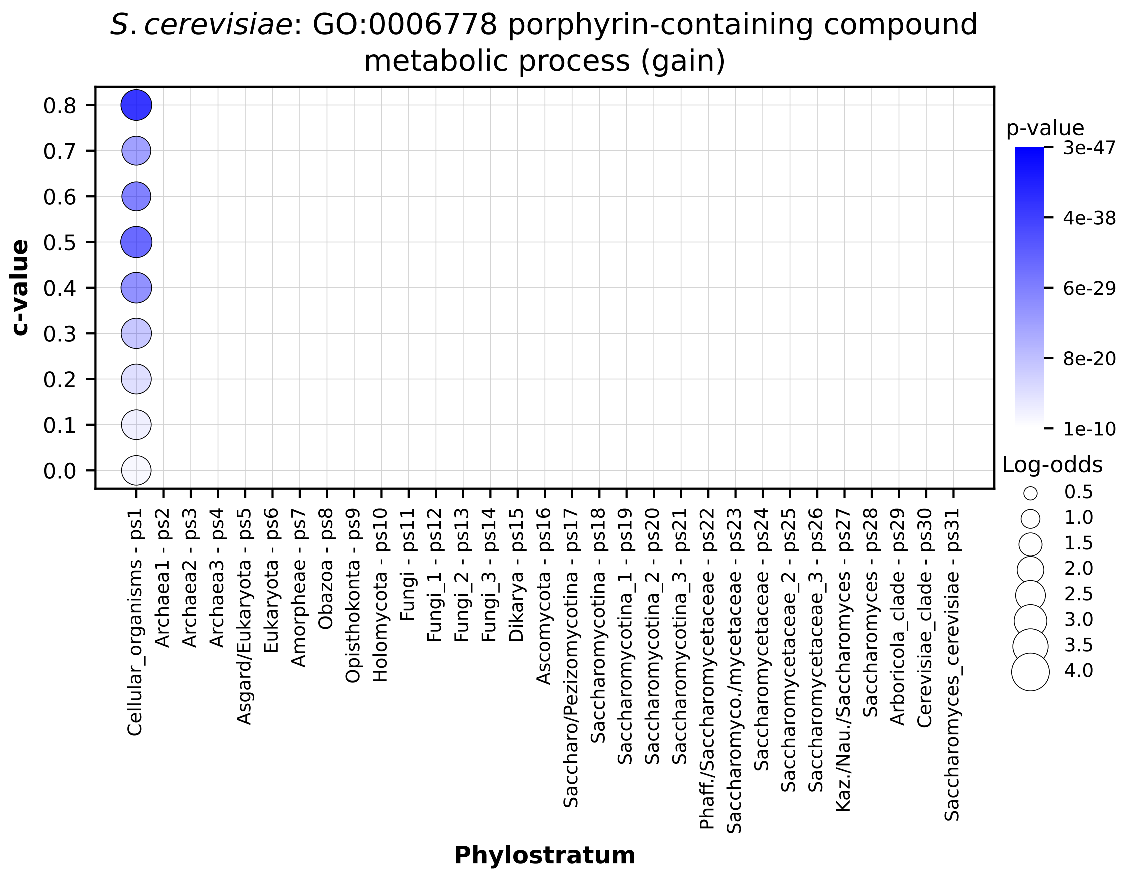
S. cerevisiae: GO:0006778 porphyrin-containing compound metabolic process (gain)
c-value
0.8
0.7
0.6
0.5
0.4
0.3
0.2
0.1
0.0
p-value
3e-47
4e-38
6e-29
8e-20
1e-10
Log-odds
0.5
1.0
1.5
2.0
2.5
3.0
3.5
4.0
Cellular_organisms - ps1
Archaea1 - ps2
Archaea2 - ps3
Archaea3 - ps4
Asgard/Eukaryota - ps5
Eukaryota - ps6
Amorpheae - ps7
Obazoa - ps8
Opisthokonta - ps9
Holomycota - ps10
Fungi - ps11
Fungi_1 - ps12
Fungi_2 - ps13
Fungi_3 - ps14
Dikarya - ps15
Ascomycota - ps16
Saccharo/Pezizomycotina - ps17
Saccharomycotina - ps18
Saccharomycotina_1 - ps19
Saccharomycotina_2 - ps20
Saccharomycotina_3 - ps21
Phaff./Saccharomycetaceae - ps22
Saccharomyco./mycetaceae - ps23
Saccharomycetaceae - ps24
Saccharomycetaceae_2 - ps25
Saccharomycetaceae_3 - ps26
Kaz./Nau./Saccharomyces - ps27
Saccharomyces - ps28
Arboricola_clade - ps29
Cerevisiae_clade - ps30
Saccharomyces_cerevisiae - ps31
Phylostratum

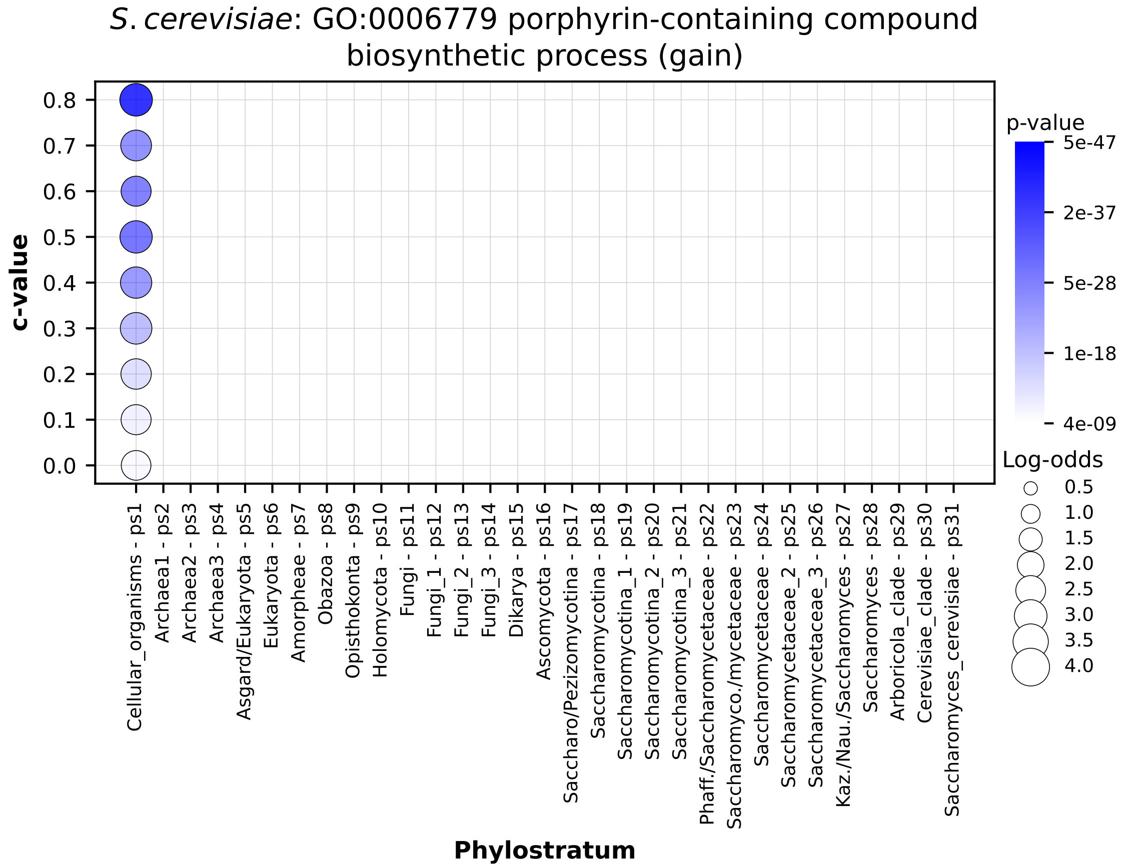
S. cerevisiae: GO:0006779 porphyrin-containing compound biosynthetic process (gain)
c-value
0.0
0.1
0.2
0.3
0.4
0.5
0.6
0.7
0.8
p-value
5e-47
2e-37
5e-28
1e-18
4e-09
Log-odds
0.5
1.0
1.5
2.0
2.5
3.0
3.5
4.0
Cellular_organisms - ps1
Archaea1 - ps2
Archaea2 - ps3
Archaea3 - ps4
Asgard/Eukaryota - ps5
Eukaryota - ps6
Amorpheae - ps7
Obazoa - ps8
Opisthokonta - ps9
Holomycota - ps10
Fungi - ps11
Fungi_1 - ps12
Fungi_2 - ps13
Fungi_3 - ps14
Dikarya - ps15
Ascomycota - ps16
Saccharo/Pezizomycotina - ps17
Saccharomycotina - ps18
Saccharomycotina_1 - ps19
Saccharomycotina_2 - ps20
Saccharomycotina_3 - ps21
Phaff./Saccharomycetaceae - ps22
Saccharomyco./mycetaceae - ps23
Saccharomycetaceae - ps24
Saccharomycetaceae_2 - ps25
Saccharomycetaceae_3 - ps26
Kaz./Nau./Saccharomyces - ps27
Saccharomyces - ps28
Arboricola_clade - ps29
Cerevisiae_clade - ps30
Saccharomyces_cerevisiae - ps31
Phylostratum

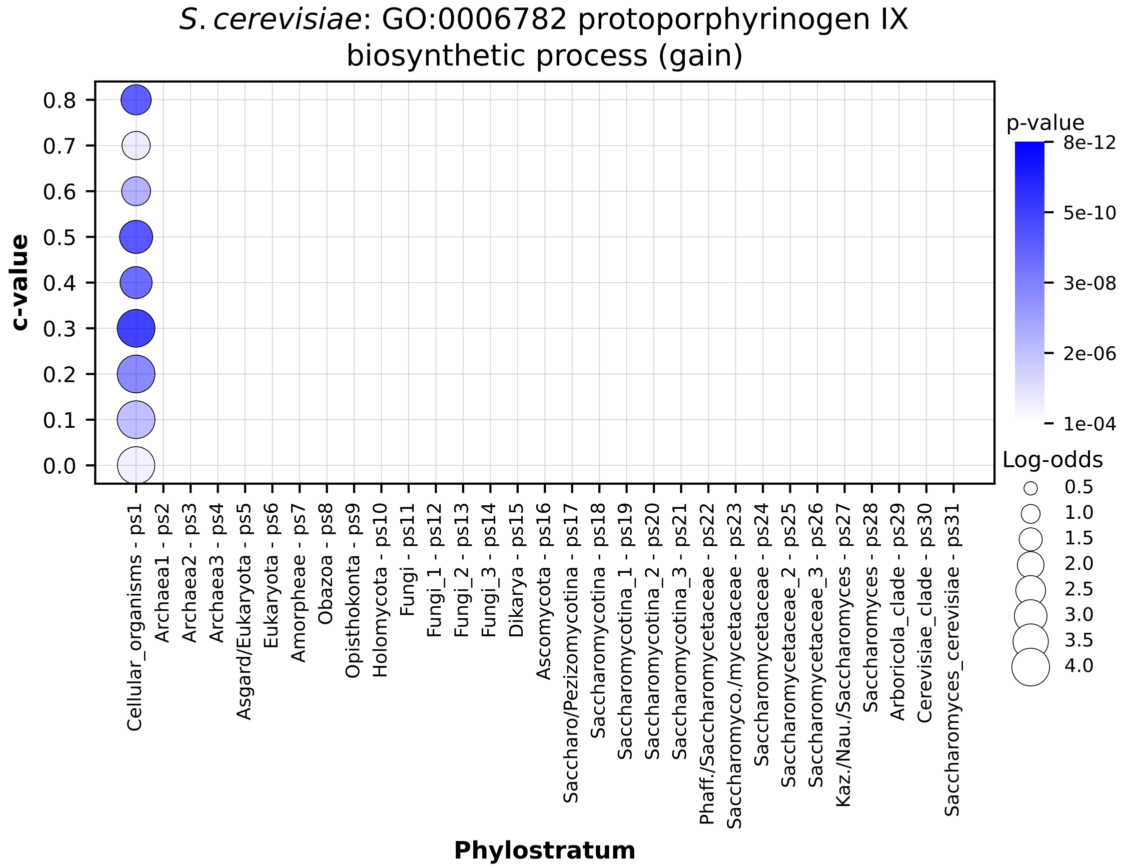

S. cerevisiae: GO:0006782 protoporphyrinogen IX biosynthetic process (gain)
c-value
0.8
0.7
0.6
0.5
0.4
0.3
0.2
0.1
0.0
p-value
8e-12
5e-10
3e-08
2e-06
1e-04
Log-odds
0.5
1.0
1.5
2.0
2.5
3.0
3.5
4.0
Cellular_organisms - ps1
Archaea1 - ps2
Archaea2 - ps3
Archaea3 - ps4
Asgard/Eukaryota - ps5
Eukaryota - ps6
Amorpheae - ps7
Obazoa - ps8
Opisthokonta - ps9
Holomycota - ps10
Fungi - ps11
Fungi_1 - ps12
Fungi_2 - ps13
Fungi_3 - ps14
Dikarya - ps15
Ascomycota - ps16
Saccharo/Pezizomycotina - ps17
Saccharomycotina - ps18
Saccharomycotina_1 - ps19
Saccharomycotina_2 - ps20
Saccharomycotina_3 - ps21
Phaff./Saccharomycetaceae - ps22
Saccharomyco./mycetaceae - ps23
Saccharomycetaceae - ps24
Saccharomycetaceae_2 - ps25
Saccharomycetaceae_3 - ps26
Kaz./Nau./Saccharomyces - ps27
Saccharomyces - ps28
Arboricola_clade - ps29
Cerevisiae_clade - ps30
Saccharomyces_cerevisiae - ps31
Phylostratum

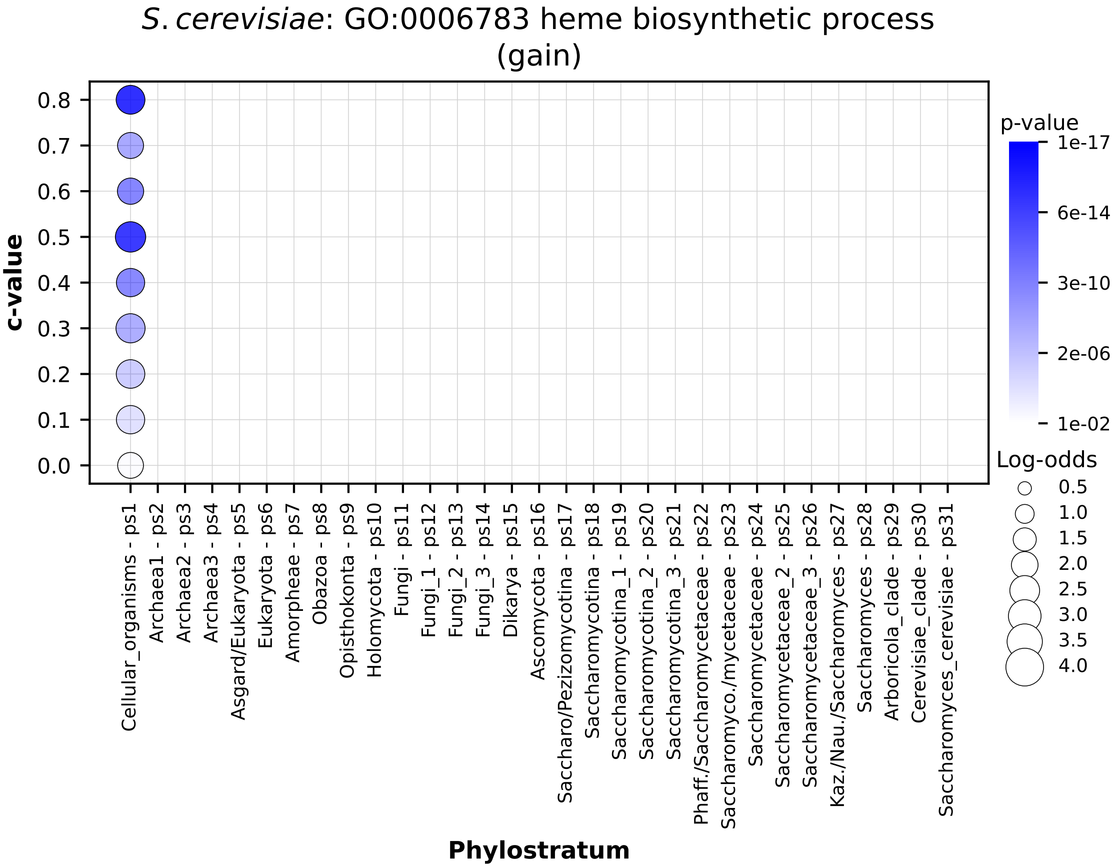

S. cerevisiae: GO:0006783 heme biosynthetic process (gain)
c-value
0.8
0.7
0.6
0.5
0.4
0.3
0.2
0.1
0.0
p-value
1e-17
6e-14
3e-10
2e-06
1e-02
Log-odds
0.5
1.0
1.5
2.0
2.5
3.0
3.5
4.0
Cellular_organisms - ps1
Archaea1 - ps2
Archaea2 - ps3
Archaea3 - ps4
Asgard/Eukaryota - ps5
Eukaryota - ps6
Amorpheae - ps7
Obazoa - ps8
Opisthokonta - ps9
Holomycota - ps10
Fungi - ps11
Fungi_1 - ps12
Fungi_2 - ps13
Fungi_3 - ps14
Dikarya - ps15
Ascomycota - ps16
Saccharo/Pezizomycotina - ps17
Saccharomycotina - ps18
Saccharomycotina_1 - ps19
Saccharomycotina_2 - ps20
Saccharomycotina_3 - ps21
Phaff./Saccharomycetaceae - ps22
Saccharomyco./mycetaceae - ps23
Saccharomycetaceae - ps24
Saccharomycetaceae_2 - ps25
Saccharomycetaceae_3 - ps26
Kaz./Nau./Saccharomyces - ps27
Saccharomyces - ps28
Arboricola_clade - ps29
Cerevisiae_clade - ps30
Saccharomyces_cerevisiae - ps31
Phylostratum

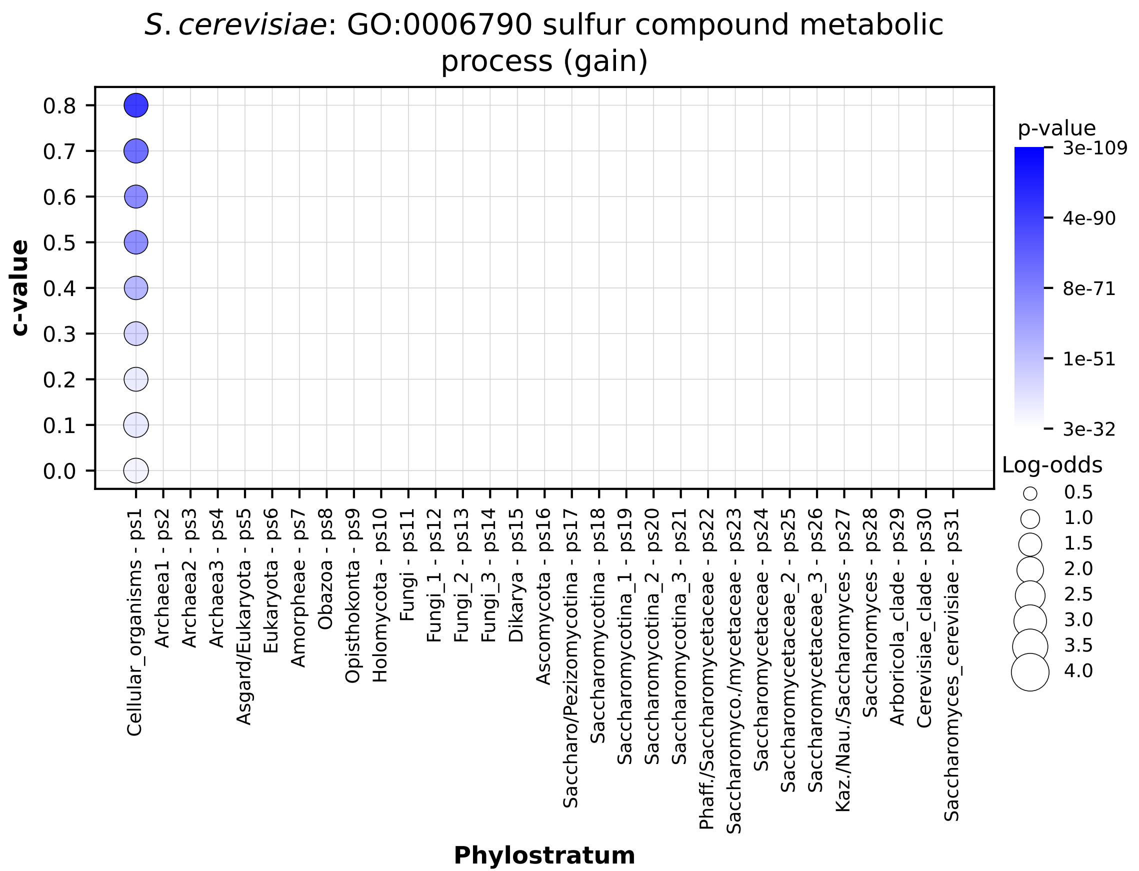
S. cerevisiae: GO:0006790 sulfur compound metabolic process (gain)
c-value
0.0
0.1
0.2
0.3
0.4
0.5
0.6
0.7
0.8
p-value
3e-109
4e-90
8e-71
1e-51
3e-32
Log-odds
0.5
1.0
1.5
2.0
2.5
3.0
3.5
4.0
Cellular_organisms - ps1
Archaea1 - ps2
Archaea2 - ps3
Archaea3 - ps4
Asgard/Eukaryota - ps5
Eukaryota - ps6
Amorpheae - ps7
Obazoa - ps8
Opisthokonta - ps9
Holomycota - ps10
Fungi - ps11
Fungi_1 - ps12
Fungi_2 - ps13
Fungi_3 - ps14
Dikarya - ps15
Ascomycota - ps16
Saccharo/Pezizomycotina - ps17
Saccharomycotina - ps18
Saccharomycotina_1 - ps19
Saccharomycotina_2 - ps20
Saccharomycotina_3 - ps21
Phaff./Saccharomycetaceae - ps22
Saccharomyco./mycetaceae - ps23
Saccharomycetaceae - ps24
Saccharomycetaceae_2 - ps25
Saccharomycetaceae_3 - ps26
Kaz./Nau./Saccharomyces - ps27
Saccharomyces - ps28
Arboricola_clade - ps29
Cerevisiae_clade - ps30
Saccharomyces_cerevisiae - ps31
Phylostratum

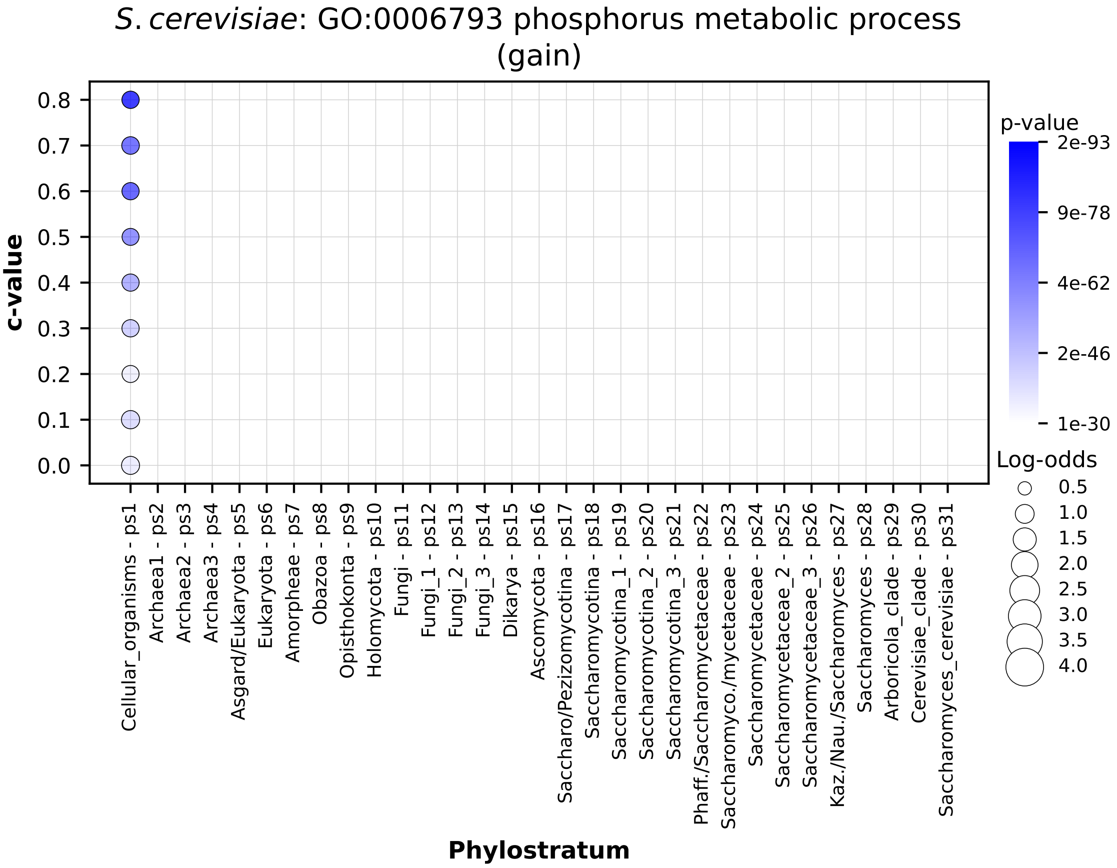
S. cerevisiae: GO:0006793 phosphorus metabolic process
(gain)
c-value
0.8
0.7
0.6
0.5
0.4
0.3
0.2
0.1
0.0
p-value
2e-93
9e-78
4e-62
2e-46
1e-30
Log-odds
0.5
1.0
1.5
2.0
2.5
3.0
3.5
4.0
Cellular_organisms - ps1
Archaea1 - ps2
Archaea2 - ps3
Archaea3 - ps4
Asgard/Eukaryota - ps5
Eukaryota - ps6
Amorpheae - ps7
Obazoa - ps8
Opisthokonta - ps9
Holomycota - ps10
Fungi - ps11
Fungi_1 - ps12
Fungi_2 - ps13
Fungi_3 - ps14
Dikarya - ps15
Ascomycota - ps16
Saccharo/Pezizomycotina - ps17
Saccharomycotina - ps18
Saccharomycotina_1 - ps19
Saccharomycotina_2 - ps20
Saccharomycotina_3 - ps21
Phaff./Saccharomycetaceae - ps22
Saccharomyco./mycetaceae - ps23
Saccharomycetaceae - ps24
Saccharomycetaceae_2 - ps25
Saccharomycetaceae_3 - ps26
Kaz./Nau./Saccharomyces - ps27
Saccharomyces - ps28
Arboricola_clade - ps29
Cerevisiae_clade - ps30
Saccharomyces_cerevisiae - ps31
Phylostratum

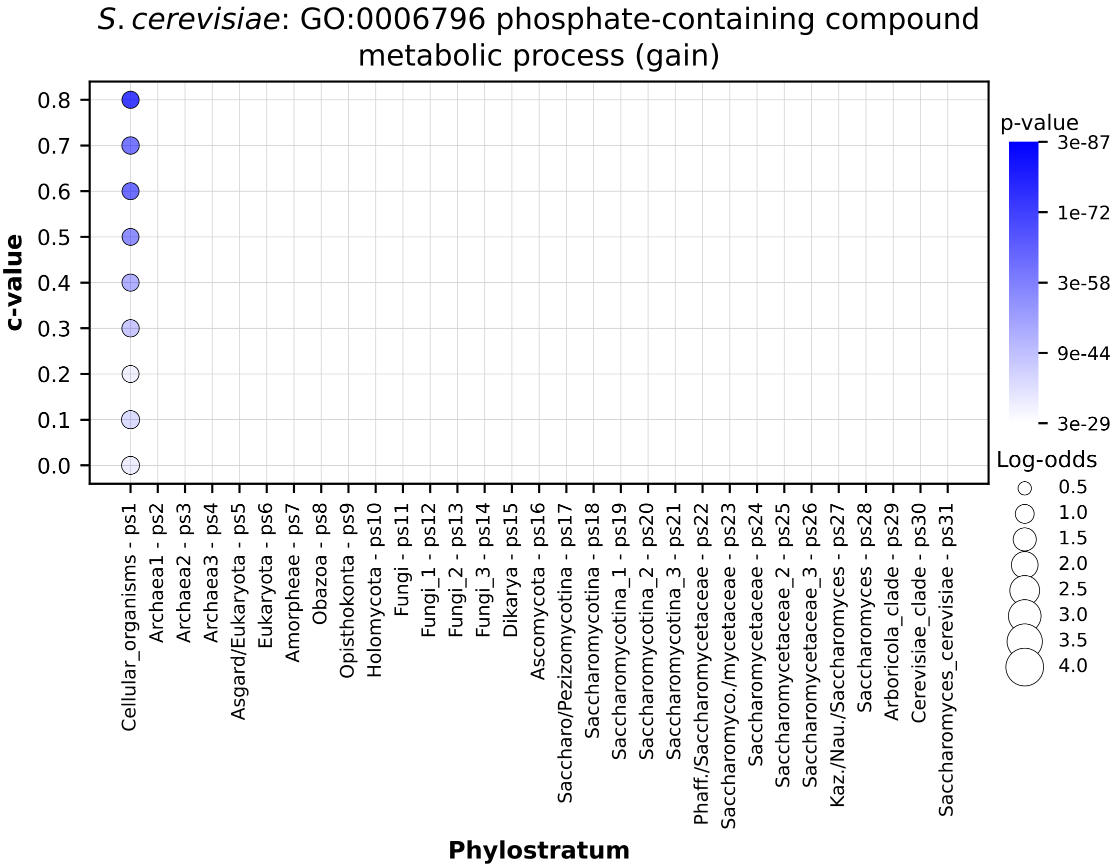
S. cerevisiae: GO:0006796 phosphate-containing compound metabolic process (gain)
c-value
0.0
0.1
0.2
0.3
0.4
0.5
0.6
0.7
0.8
Phylostratum
Cellular_organisms - ps1
Archaea1 - ps2
Archaea2 - ps3
Archaea3 - ps4
Asgard/Eukaryota - ps5
Eukaryota - ps6
Amorpheae - ps7
Obazoa - ps8
Opisthokonta - ps9
Holomycota - ps10
Fungi - ps11
Fungi_1 - ps12
Fungi_2 - ps13
Fungi_3 - ps14
Dikarya - ps15
Ascomycota - ps16
Saccharo/Pezizomycotina - ps17
Saccharomycotina - ps18
Saccharomycotina_1 - ps19
Saccharomycotina_2 - ps20
Saccharomycotina_3 - ps21
Phaff./Saccharomycetaceae - ps22
Saccharomyco./mycetaceae - ps23
Saccharomycetaceae - ps24
Saccharomycetaceae_2 - ps25
Saccharomycetaceae_3 - ps26
Kaz./Nau./Saccharomyces - ps27
Saccharomyces - ps28
Arboricola_clade - ps29
Cerevisiae_clade - ps30
Saccharomyces_cerevisiae - ps31
p-value
3e-87
1e-72
3e-58
9e-44
3e-29
Log-odds
0.5
1.0
1.5
2.0
2.5
3.0
3.5
4.0

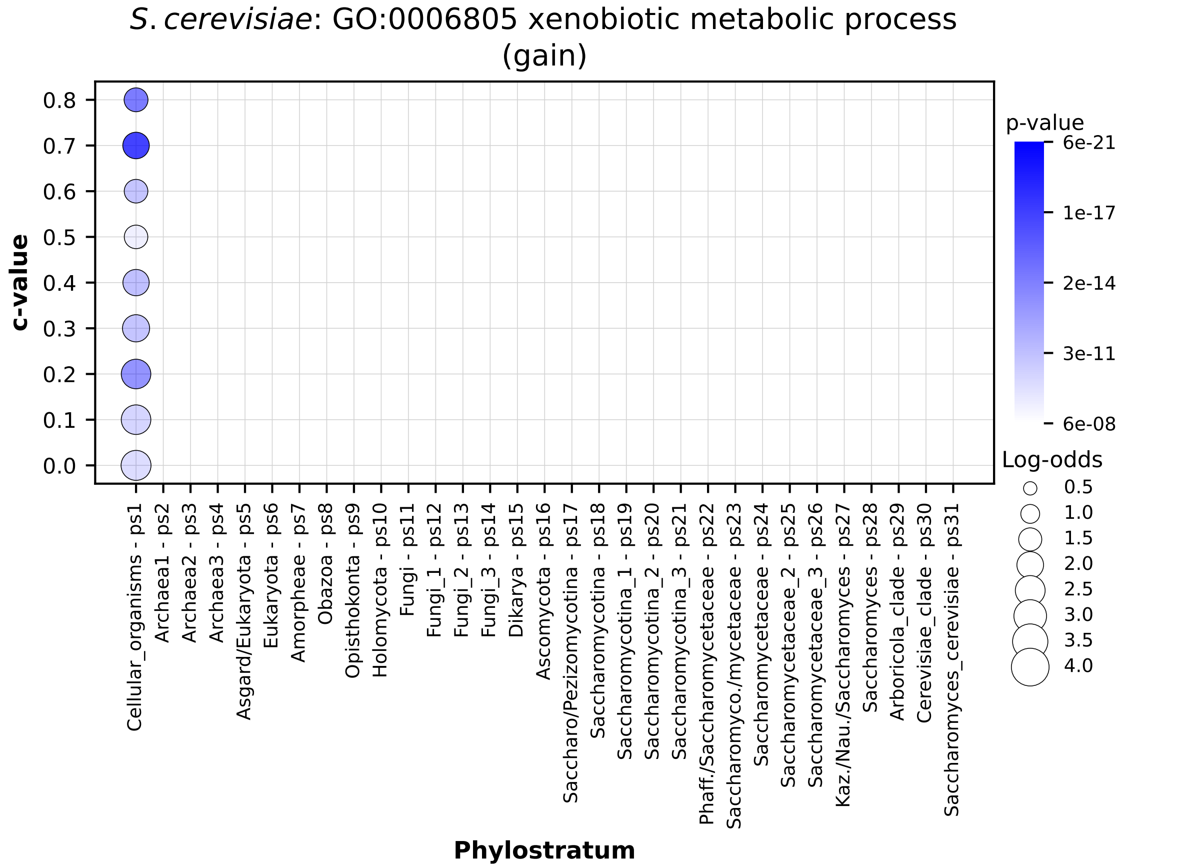
S. cerevisiae: GO:0006805 xenobiotic metabolic process (gain)
c-value
0.8
0.7
0.6
0.5
0.4
0.3
0.2
0.1
0.0
p-value
6e-21
1e-17
2e-14
3e-11
6e-08
Log-odds
0.5
1.0
1.5
2.0
2.5
3.0
3.5
4.0
Cellular_organisms - ps1
Archaea1 - ps2
Archaea2 - ps3
Archaea3 - ps4
Asgard/Eukaryota - ps5
Eukaryota - ps6
Amorpheae - ps7
Obazoa - ps8
Opisthokonta - ps9
Holomycota - ps10
Fungi - ps11
Fungi_1 - ps12
Fungi_2 - ps13
Fungi_3 - ps14
Dikarya - ps15
Ascomycota - ps16
Saccharo/Pezizomycotina - ps17
Saccharomycotina - ps18
Saccharomycotina_1 - ps19
Saccharomycotina_2 - ps20
Saccharomycotina_3 - ps21
Phaff./Saccharomycetaceae - ps22
Saccharomyco./mycetaceae - ps23
Saccharomycetaceae - ps24
Saccharomycetaceae_2 - ps25
Saccharomycetaceae_3 - ps26
Kaz./Nau./Saccharomyces - ps27
Saccharomyces - ps28
Arboricola_clade - ps29
Cerevisiae_clade - ps30
Saccharomyces_cerevisiae - ps31
Phylostratum

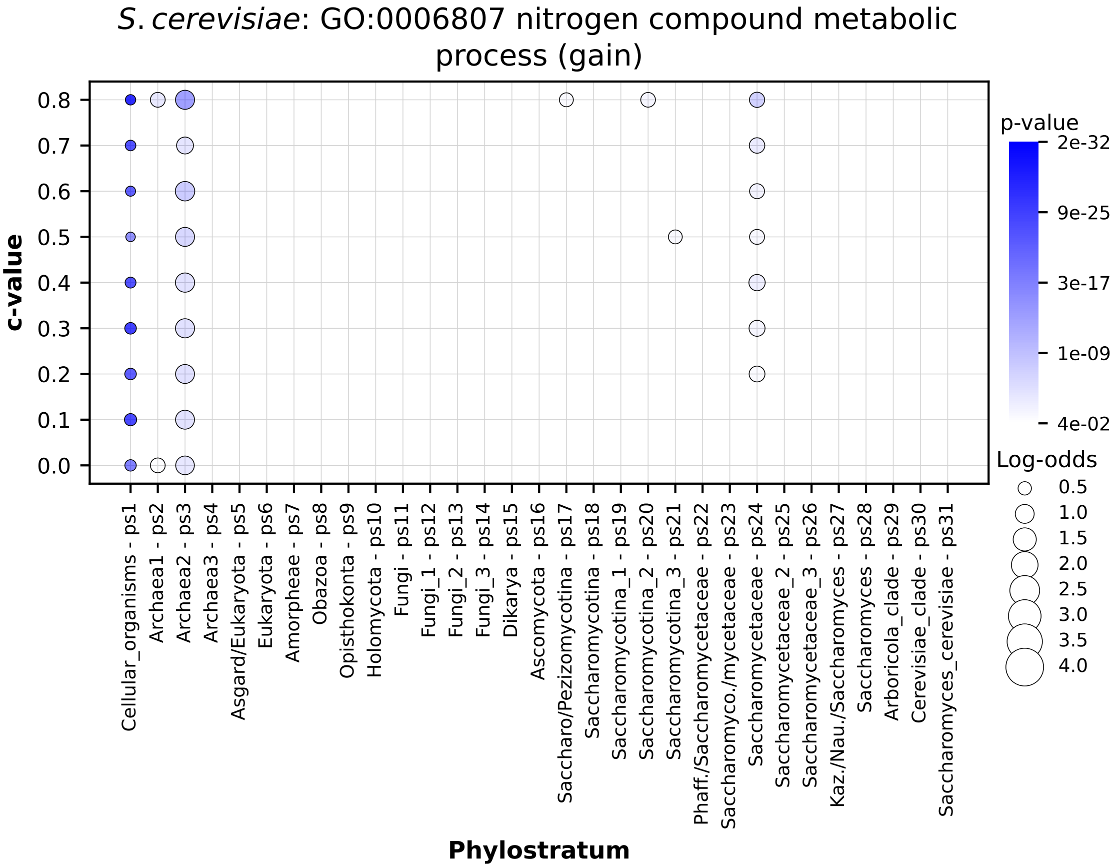

S. cerevisiae: GO:0006807 nitrogen compound metabolic process (gain)
c-value
0.8
0.7
0.6
0.5
0.4
0.3
0.2
0.1
0.0
p-value
2e-32
9e-25
3e-17
1e-09
4e-02
Log-odds
0.5
1.0
1.5
2.0
2.5
3.0
3.5
4.0
Cellular_organisms - ps1
Archaea1 - ps2
Archaea2 - ps3
Archaea3 - ps4
Asgard/Eukaryota - ps5
Eukaryota - ps6
Amorpheae - ps7
Obazoa - ps8
Opisthokonta - ps9
Holomycota - ps10
Fungi - ps11
Fungi_1 - ps12
Fungi_2 - ps13
Fungi_3 - ps14
Dikarya - ps15
Ascomycota - ps16
Saccharo/Pezizomycotina - ps17
Saccharomycotina - ps18
Saccharomycotina_1 - ps19
Saccharomycotina_2 - ps20
Saccharomycotina_3 - ps21
Phaff./Saccharomycetaceae - ps22
Saccharomyco./mycetaceae - ps23
Saccharomycetaceae - ps24
Saccharomycetaceae_2 - ps25
Saccharomycetaceae_3 - ps26
Kaz./Nau./Saccharomyces - ps27
Saccharomyces - ps28
Arboricola_clade - ps29
Cerevisiae_clade - ps30
Saccharomyces_cerevisiae - ps31
Phylostratum

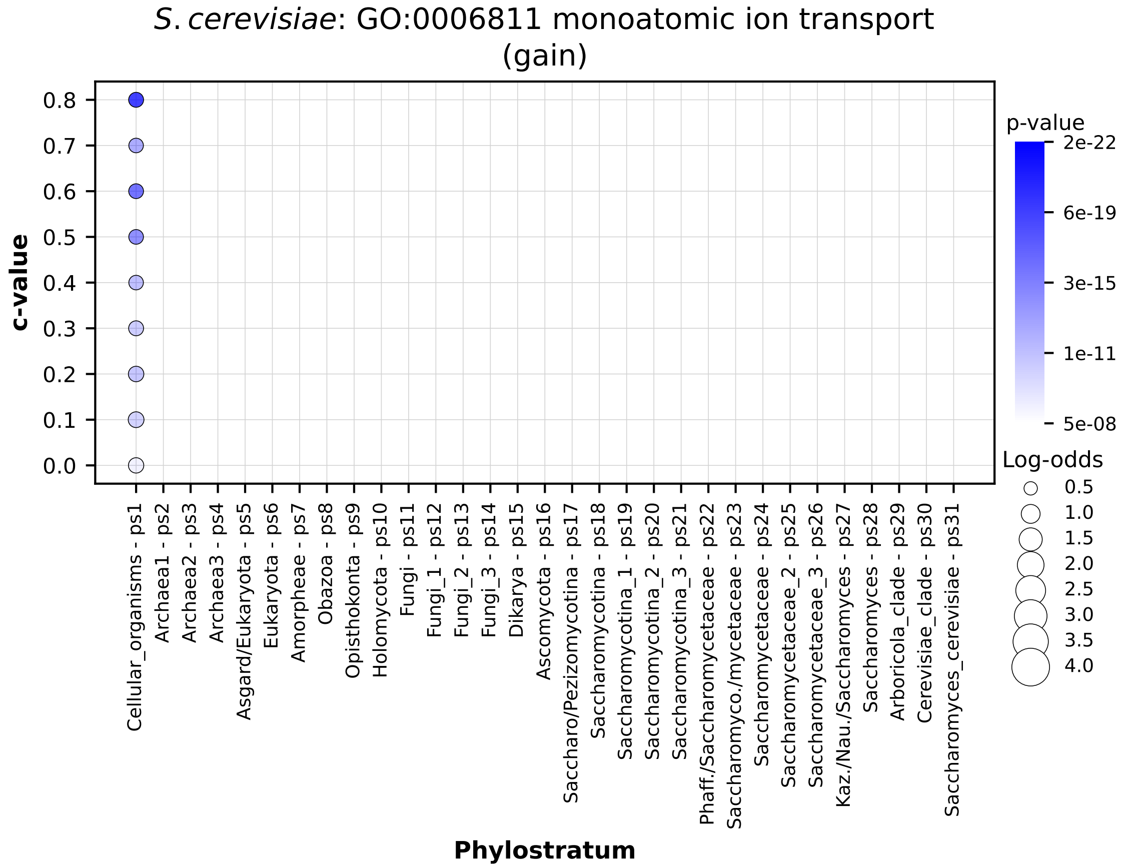

S. cerevisiae: GO:0006811 monoatomic ion transport
(gain)
c-value
0.8
0.7
0.6
0.5
0.4
0.3
0.2
0.1
0.0
p-value
2e-22
6e-19
3e-15
1e-11
5e-08
Log-odds
0.5
1.0
1.5
2.0
2.5
3.0
3.5
4.0
Cellular_organisms - ps1
Archaea1 - ps2
Archaea2 - ps3
Archaea3 - ps4
Asgard/Eukaryota - ps5
Eukaryota - ps6
Amorpheae - ps7
Obazoa - ps8
Opisthokonta - ps9
Holomycota - ps10
Fungi - ps11
Fungi_1 - ps12
Fungi_2 - ps13
Fungi_3 - ps14
Dikarya - ps15
Ascomycota - ps16
Saccharo/Pezizomycotina - ps17
Saccharomycotina - ps18
Saccharomycotina_1 - ps19
Saccharomycotina_2 - ps20
Saccharomycotina_3 - ps21
Phaff./Saccharomycetaceae - ps22
Saccharomyco./mycetaceae - ps23
Saccharomycetaceae - ps24
Saccharomycetaceae_2 - ps25
Saccharomycetaceae_3 - ps26
Kaz./Nau./Saccharomyces - ps27
Saccharomyces - ps28
Arboricola_clade - ps29
Cerevisiae_clade - ps30
Saccharomyces_cerevisiae - ps31
Phylostratum

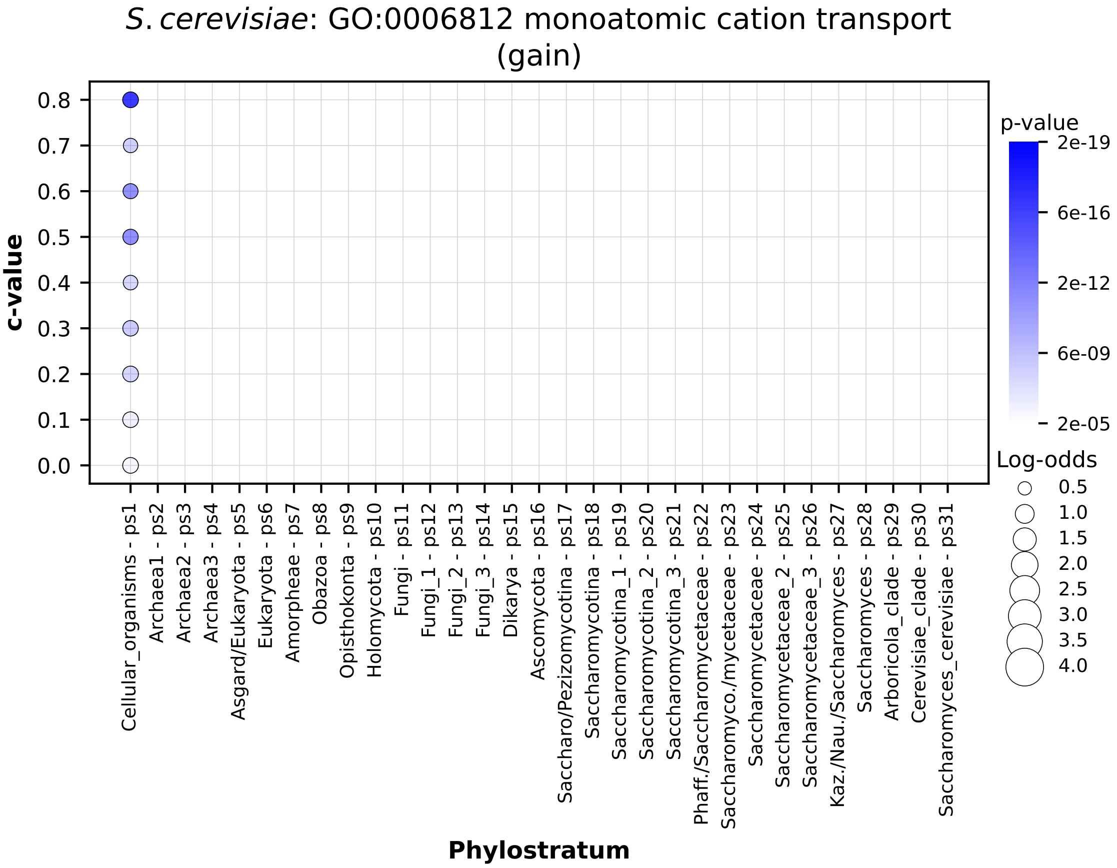
S. cerevisiae: GO:0006812 monoatomic cation transport
(gain)
c-value
0.8
0.7
0.6
0.5
0.4
0.3
0.2
0.1
0.0
p-value
2e-19
6e-16
2e-12
6e-09
2e-05
Log-odds
0.5
1.0
1.5
2.0
2.5
3.0
3.5
4.0
Cellular_organisms - ps1
Archaea1 - ps2
Archaea2 - ps3
Archaea3 - ps4
Asgard/Eukaryota - ps5
Eukaryota - ps6
Amorpheae - ps7
Obazoa - ps8
Opisthokonta - ps9
Holomycota - ps10
Fungi - ps11
Fungi_1 - ps12
Fungi_2 - ps13
Fungi_3 - ps14
Dikarya - ps15
Ascomycota - ps16
Saccharo/Pezizomycotina - ps17
Saccharomycotina - ps18
Saccharomycotina_1 - ps19
Saccharomycotina_2 - ps20
Saccharomycotina_3 - ps21
Phaff./Saccharomycetaceae - ps22
Saccharomyco./mycetaceae - ps23
Saccharomycetaceae - ps24
Saccharomycetaceae_2 - ps25
Saccharomycetaceae_3 - ps26
Kaz./Nau./Saccharomyces - ps27
Saccharomyces - ps28
Arboricola_clade - ps29
Cerevisiae_clade - ps30
Saccharomyces_cerevisiae - ps31
Phylostratum

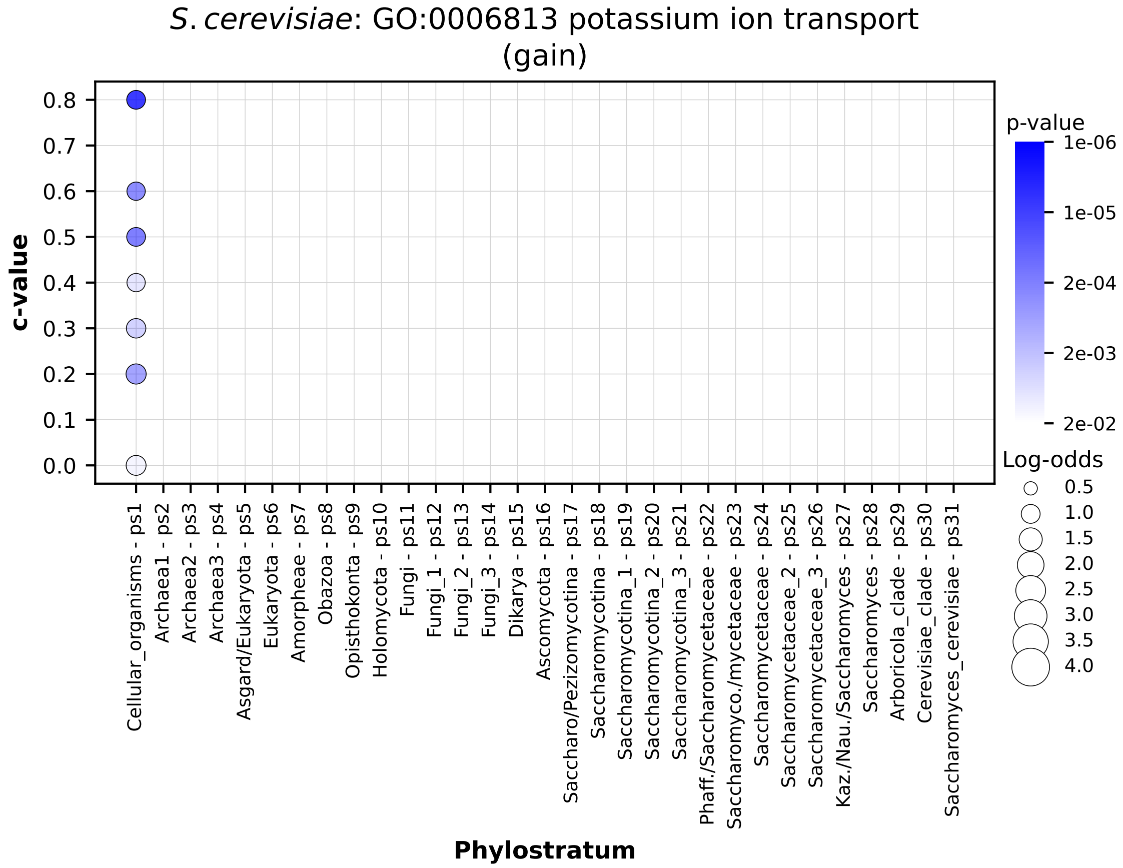
S. cerevisiae: GO:0006813 potassium ion transport
(gain)
c-value
0.8
0.7
0.6
0.5
0.4
0.3
0.2
0.1
0.0
p-value
1e-06
1e-05
2e-04
2e-03
2e-02
Log-odds
0.5
1.0
1.5
2.0
2.5
3.0
3.5
4.0
Cellular_organisms - ps1
Archaea1 - ps2
Archaea2 - ps3
Archaea3 - ps4
Asgard/Eukaryota - ps5
Eukaryota - ps6
Amorpheae - ps7
Obazoa - ps8
Opisthokonta - ps9
Holomycota - ps10
Fungi - ps11
Fungi_1 - ps12
Fungi_2 - ps13
Fungi_3 - ps14
Dikarya - ps15
Ascomycota - ps16
Saccharo/Pezizomycotina - ps17
Saccharomycotina - ps18
Saccharomycotina_1 - ps19
Saccharomycotina_2 - ps20
Saccharomycotina_3 - ps21
Phaff./Saccharomycetaceae - ps22
Saccharomyco./mycetaceae - ps23
Saccharomycetaceae - ps24
Saccharomycetaceae_2 - ps25
Saccharomycetaceae_3 - ps26
Kaz./Nau./Saccharomyces - ps27
Saccharomyces - ps28
Arboricola_clade - ps29
Cerevisiae_clade - ps30
Saccharomyces_cerevisiae - ps31
Phylostratum

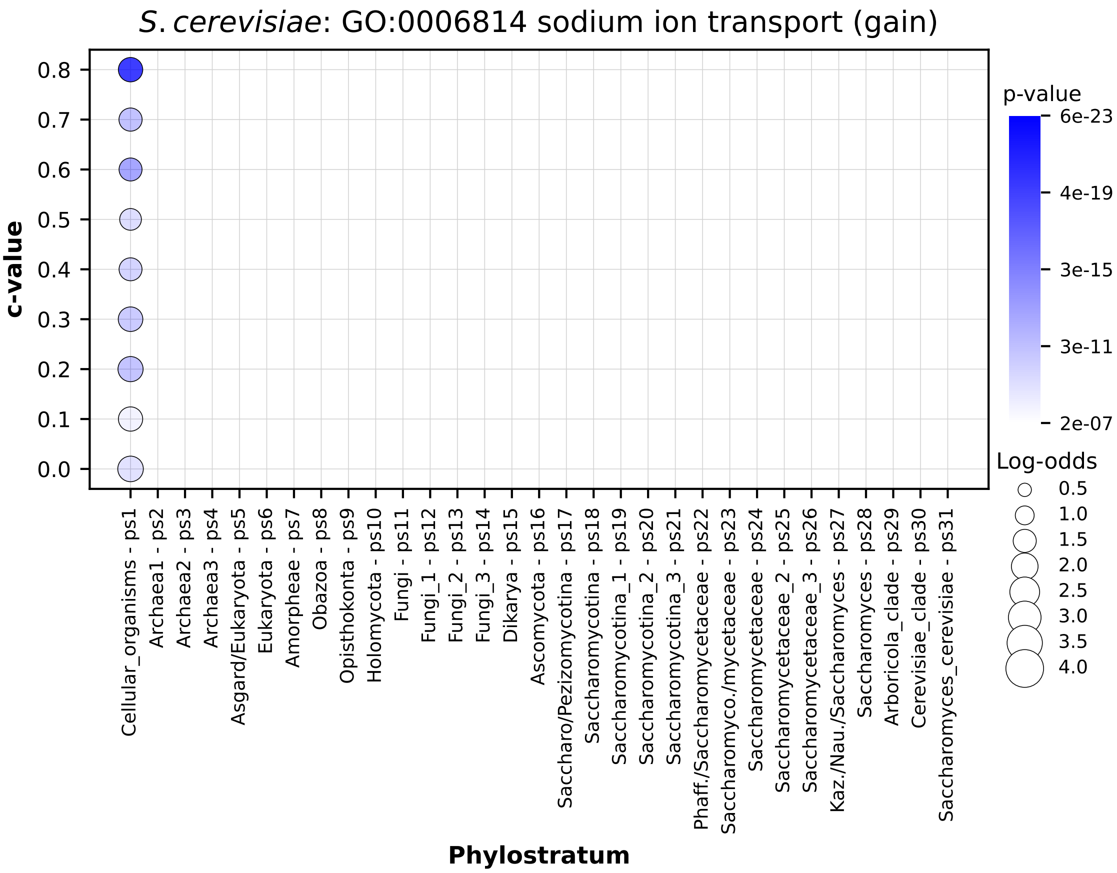

S. cerevisiae: GO:0006814 sodium ion transport (gain)
c-value
0.8
0.7
0.6
0.5
0.4
0.3
0.2
0.1
0.0
Phylostratum
Cellular_organisms - ps1
Archaea1 - ps2
Archaea2 - ps3
Archaea3 - ps4
Asgard/Eukaryota - ps5
Eukaryota - ps6
Amorpheae - ps7
Obazoa - ps8
Opisthokonta - ps9
Holomycota - ps10
Fungi - ps11
Fungi_1 - ps12
Fungi_2 - ps13
Fungi_3 - ps14
Dikarya - ps15
Ascomycota - ps16
Saccharo/Pezizomycotina - ps17
Saccharomycotina - ps18
Saccharomycotina_1 - ps19
Saccharomycotina_2 - ps20
Saccharomycotina_3 - ps21
Phaff./Saccharomycetaceae - ps22
Saccharomyco./mycetaceae - ps23
Saccharomycetaceae - ps24
Saccharomycetaceae_2 - ps25
Saccharomycetaceae_3 - ps26
Kaz./Nau./Saccharomyces - ps27
Saccharomyces - ps28
Arboricola_clade - ps29
Cerevisiae_clade - ps30
Saccharomyces_cerevisiae - ps31
p-value
6e-23
4e-19
3e-15
3e-11
2e-07
Log-odds
0.5
1.0
1.5
2.0
2.5
3.0
3.5
4.0

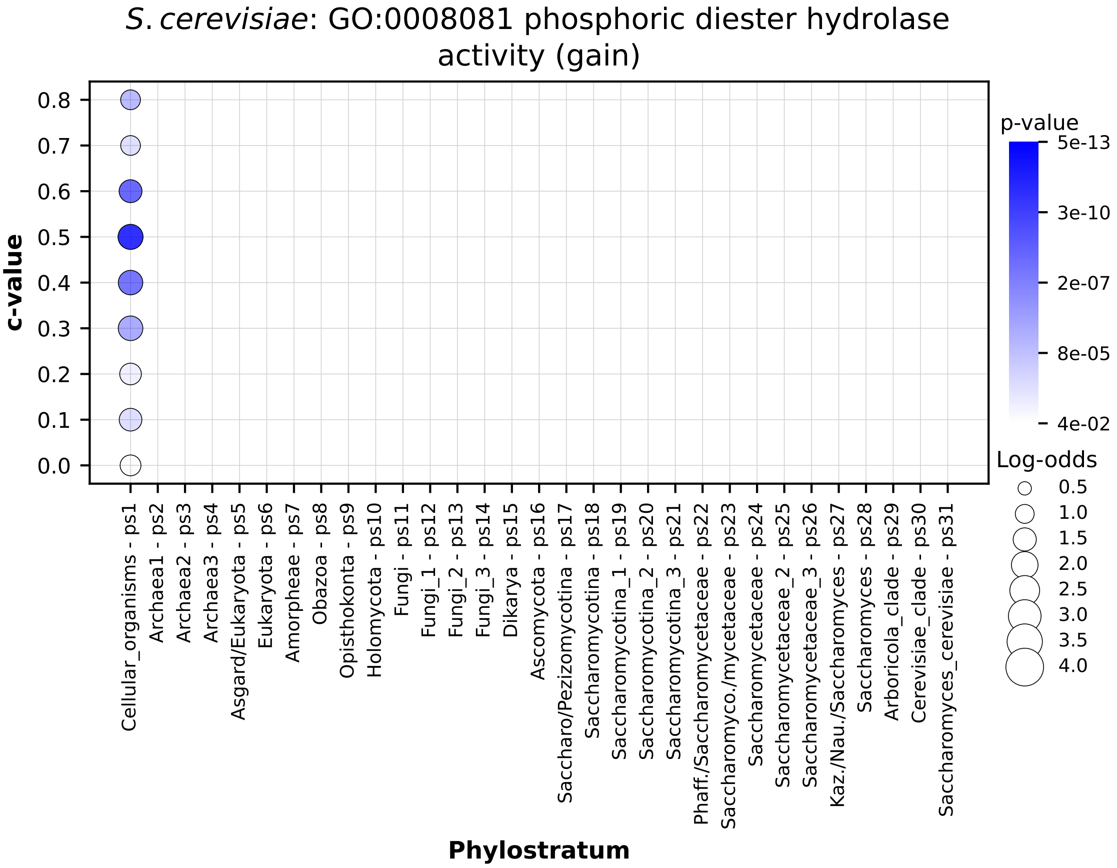

S. cerevisiae: GO:0008081 phosphoric diester hydrolase activity (gain)
c-value
0.8
0.7
0.6
0.5
0.4
0.3
0.2
0.1
0.0
p-value
5e-13
3e-10
2e-07
8e-05
4e-02
Log-odds
0.5
1.0
1.5
2.0
2.5
3.0
3.5
4.0
Cellular_organisms - ps1
Archaea1 - ps2
Archaea2 - ps3
Archaea3 - ps4
Asgard/Eukaryota - ps5
Eukaryota - ps6
Amorpheae - ps7
Obazoa - ps8
Opisthokonta - ps9
Holomycota - ps10
Fungi - ps11
Fungi_1 - ps12
Fungi_2 - ps13
Fungi_3 - ps14
Dikarya - ps15
Ascomycota - ps16
Saccharo/Pezizomycotina - ps17
Saccharomycotina - ps18
Saccharomycotina_1 - ps19
Saccharomycotina_2 - ps20
Saccharomycotina_3 - ps21
Phaff./Saccharomycetaceae - ps22
Saccharomyco./mycetaceae - ps23
Saccharomycetaceae - ps24
Saccharomycetaceae_2 - ps25
Saccharomycetaceae_3 - ps26
Kaz./Nau./Saccharomyces - ps27
Saccharomyces - ps28
Arboricola_clade - ps29
Cerevisiae_clade - ps30
Saccharomyces_cerevisiae - ps31
Phylostratum

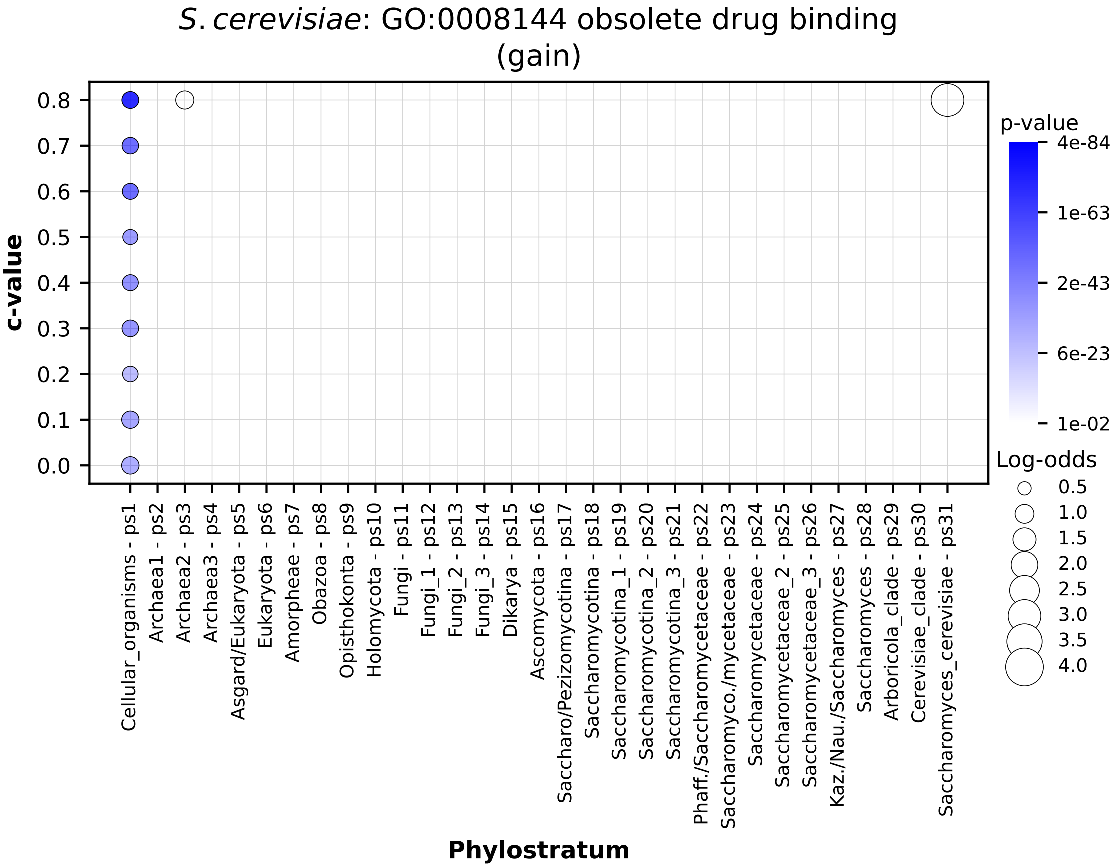
S. cerevisiae: GO:0008144 obsolete drug binding
(gain)
c-value
0.8
0.7
0.6
0.5
0.4
0.3
0.2
0.1
0.0
Phylostratum
Cellular_organisms - ps1
Archaea1 - ps2
Archaea2 - ps3
Archaea3 - ps4
Asgard/Eukaryota - ps5
Eukaryota - ps6
Amorpheae - ps7
Obazoa - ps8
Opisthokonta - ps9
Holomycota - ps10
Fungi - ps11
Fungi_1 - ps12
Fungi_2 - ps13
Fungi_3 - ps14
Dikarya - ps15
Ascomycota - ps16
Saccharo/Pezizomycotina - ps17
Saccharomycotina - ps18
Saccharomycotina_1 - ps19
Saccharomycotina_2 - ps20
Saccharomycotina_3 - ps21
Phaff./Saccharomycetaceae - ps22
Saccharomyco./mycetaceae - ps23
Saccharomycetaceae - ps24
Saccharomycetaceae_2 - ps25
Saccharomycetaceae_3 - ps26
Kaz./Nau./Saccharomyces - ps27
Saccharomyces - ps28
Arboricola_clade - ps29
Cerevisiae_clade - ps30
Saccharomyces_cerevisiae - ps31
p-value
4e-84
1e-63
2e-43
6e-23
1e-02
Log-odds
0.5
1.0
1.5
2.0
2.5
3.0
3.5
4.0

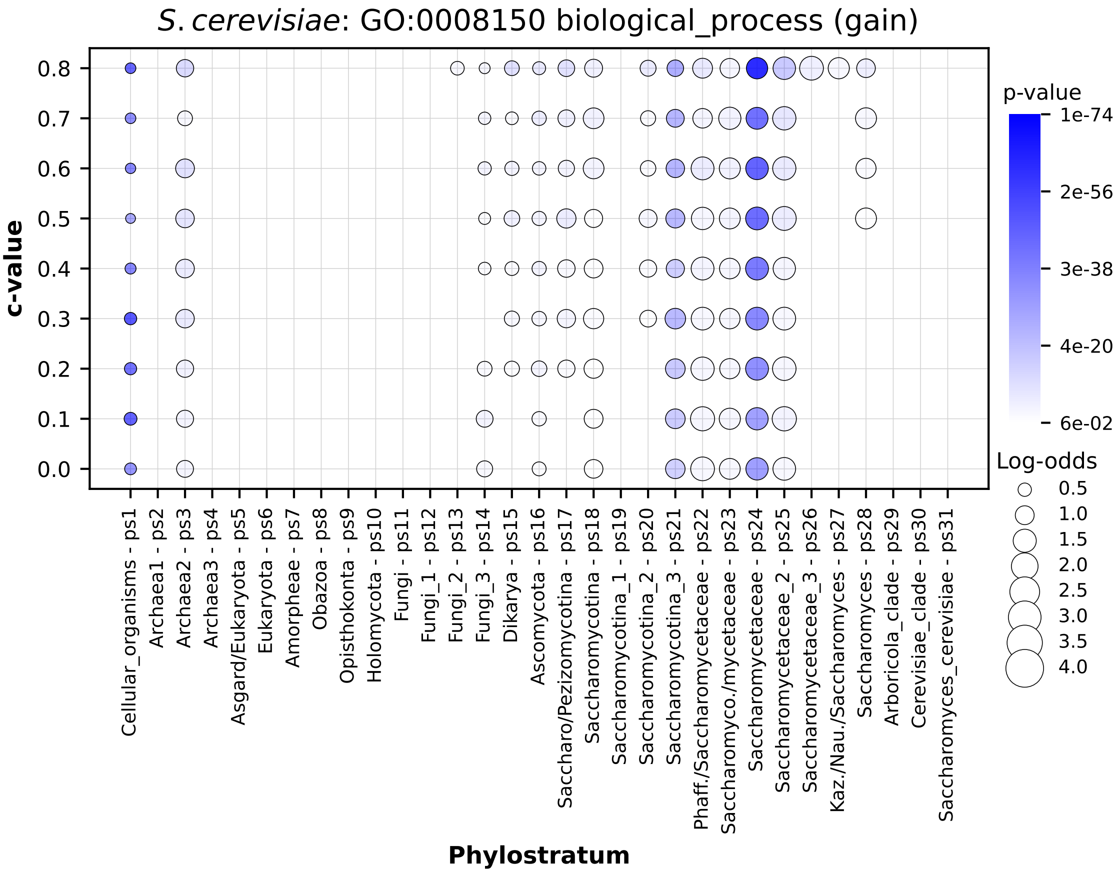

S. cerevisiae: GO:0008150 biological_process (gain)
c-value
0.8
0.7
0.6
0.5
0.4
0.3
0.2
0.1
0.0
p-value
1e-74
2e-56
3e-38
4e-20
6e-02
Log-odds
0.5
1.0
1.5
2.0
2.5
3.0
3.5
4.0
Cellular_organisms - ps1
Archaea1 - ps2
Archaea2 - ps3
Archaea3 - ps4
Asgard/Eukaryota - ps5
Eukaryota - ps6
Amorpheae - ps7
Obazoa - ps8
Opisthokonta - ps9
Holomycota - ps10
Fungi - ps11
Fungi_1 - ps12
Fungi_2 - ps13
Fungi_3 - ps14
Dikarya - ps15
Ascomycota - ps16
Saccharo/Pezizomycotina - ps17
Saccharomycotina - ps18
Saccharomycotina_1 - ps19
Saccharomycotina_2 - ps20
Saccharomycotina_3 - ps21
Phaff./Saccharomycetaceae - ps22
Saccharomyco./mycetaceae - ps23
Saccharomycetaceae - ps24
Saccharomycetaceae_2 - ps25
Saccharomycetaceae_3 - ps26
Kaz./Nau./Saccharomyces - ps27
Saccharomyces - ps28
Arboricola_clade - ps29
Cerevisiae_clade - ps30
Saccharomyces_cerevisiae - ps31
Phylostratum

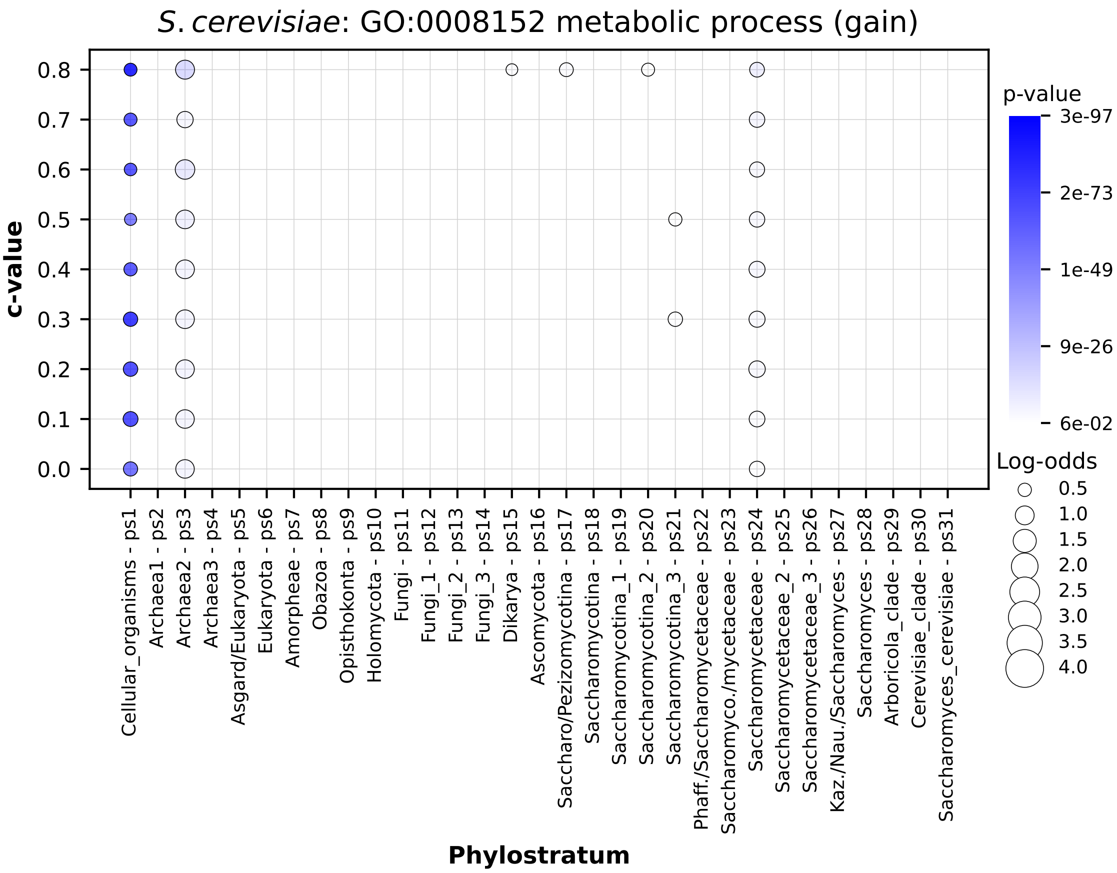

S. cerevisiae: GO:0008152 metabolic process (gain)
c-value
0.8
0.7
0.6
0.5
0.4
0.3
0.2
0.1
0.0
Phylostratum
Cellular_organisms - ps1
Archaea1 - ps2
Archaea2 - ps3
Archaea3 - ps4
Asgard/Eukaryota - ps5
Eukaryota - ps6
Amorpheae - ps7
Obazoa - ps8
Opisthokonta - ps9
Holomycota - ps10
Fungi - ps11
Fungi_1 - ps12
Fungi_2 - ps13
Fungi_3 - ps14
Dikarya - ps15
Ascomycota - ps16
Saccharo/Pezizomycotina - ps17
Saccharomycotina - ps18
Saccharomycotina_1 - ps19
Saccharomycotina_2 - ps20
Saccharomycotina_3 - ps21
Phaff./Saccharomycetaceae - ps22
Saccharomyco./mycetaceae - ps23
Saccharomycetaceae - ps24
Saccharomycetaceae_2 - ps25
Saccharomycetaceae_3 - ps26
Kaz./Nau./Saccharomyces - ps27
Saccharomyces - ps28
Arboricola_clade - ps29
Cerevisiae_clade - ps30
Saccharomyces_cerevisiae - ps31
p-value
3e-97
2e-73
1e-49
9e-26
6e-02
Log-odds
0.5
1.0
1.5
2.0
2.5
3.0
3.5
4.0

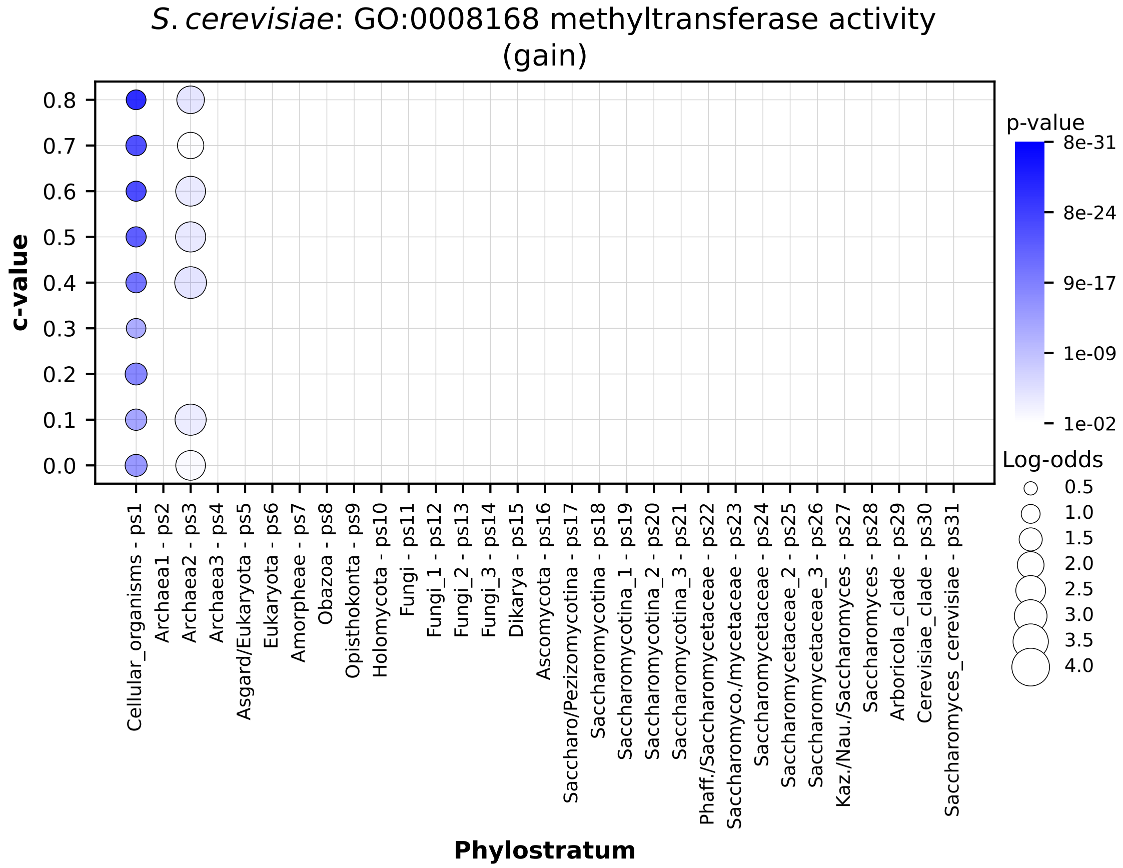
S. cerevisiae: GO:0008168 methyltransferase activity (gain)
c-value
0.8
0.7
0.6
0.5
0.4
0.3
0.2
0.1
0.0
Phylostratum
Cellular_organisms - ps1
Archaea1 - ps2
Archaea2 - ps3
Archaea3 - ps4
Asgard/Eukaryota - ps5
Eukaryota - ps6
Amorpheae - ps7
Obazoa - ps8
Opisthokonta - ps9
Holomycota - ps10
Fungi - ps11
Fungi_1 - ps12
Fungi_2 - ps13
Fungi_3 - ps14
Dikarya - ps15
Ascomycota - ps16
Saccharo/Pezizomycotina - ps17
Saccharomycotina - ps18
Saccharomycotina_1 - ps19
Saccharomycotina_2 - ps20
Saccharomycotina_3 - ps21
Phaff./Saccharomycetaceae - ps22
Saccharomyco./mycetaceae - ps23
Saccharomycetaceae - ps24
Saccharomycetaceae_2 - ps25
Saccharomycetaceae_3 - ps26
Kaz./Nau./Saccharomyces - ps27
Saccharomyces - ps28
Arboricola_clade - ps29
Cerevisiae_clade - ps30
Saccharomyces_cerevisiae - ps31
p-value
8e-31
8e-24
9e-17
1e-09
1e-02
Log-odds
0.5
1.0
1.5
2.0
2.5
3.0
3.5
4.0

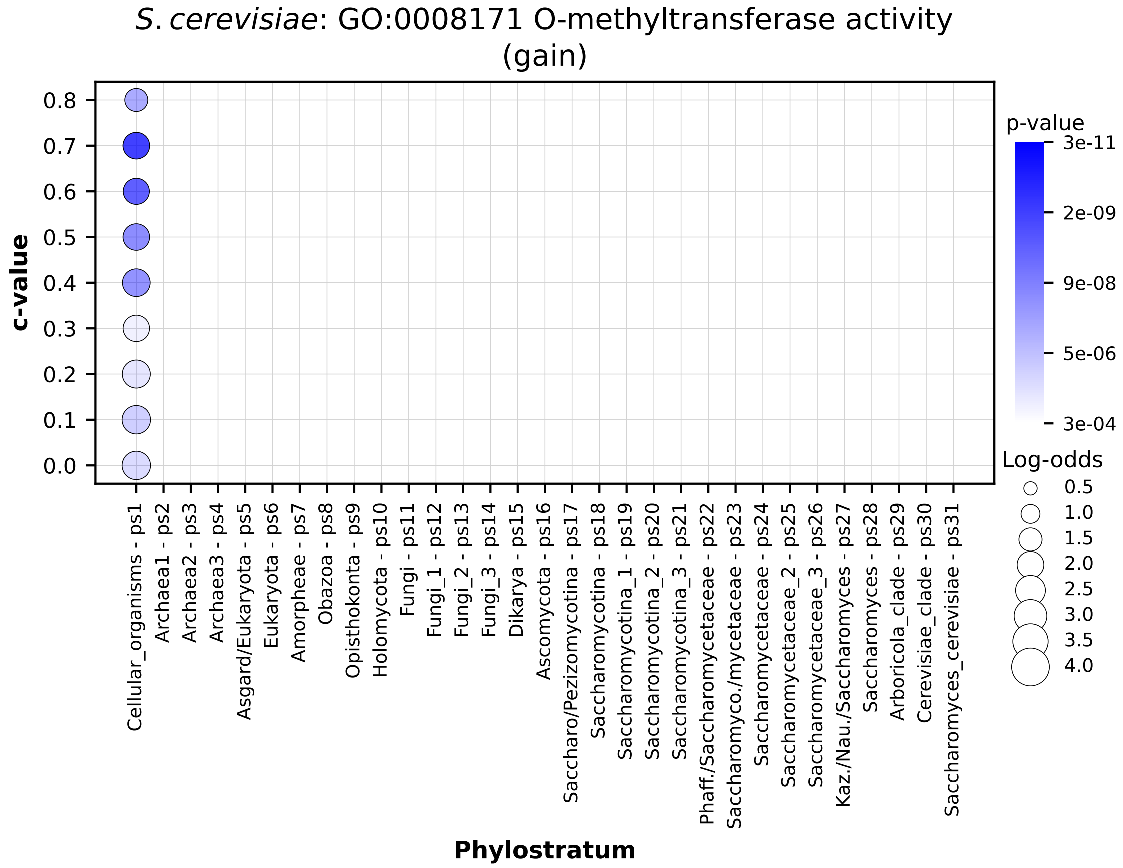

S. cerevisiae: GO:0008171 O-methyltransferase activity
(gain)
c-value
0.8
0.7
0.6
0.5
0.4
0.3
0.2
0.1
0.0
p-value
3e-11
2e-09
9e-08
5e-06
3e-04
Log-odds
0.5
1.0
1.5
2.0
2.5
3.0
3.5
4.0
Cellular_organisms - ps1
Archaea1 - ps2
Archaea2 - ps3
Archaea3 - ps4
Asgard/Eukaryota - ps5
Eukaryota - ps6
Amorpheae - ps7
Obazoa - ps8
Opisthokonta - ps9
Holomycota - ps10
Fungi - ps11
Fungi_1 - ps12
Fungi_2 - ps13
Fungi_3 - ps14
Dikarya - ps15
Ascomycota - ps16
Saccharo/Pezizomycotina - ps17
Saccharomycotina - ps18
Saccharomycotina_1 - ps19
Saccharomycotina_2 - ps20
Saccharomycotina_3 - ps21
Phaff./Saccharomycetaceae - ps22
Saccharomyco./mycetaceae - ps23
Saccharomycetaceae - ps24
Saccharomycetaceae_2 - ps25
Saccharomycetaceae_3 - ps26
Kaz./Nau./Saccharomyces - ps27
Saccharomyces - ps28
Arboricola_clade - ps29
Cerevisiae_clade - ps30
Saccharomyces_cerevisiae - ps31
Phylostratum

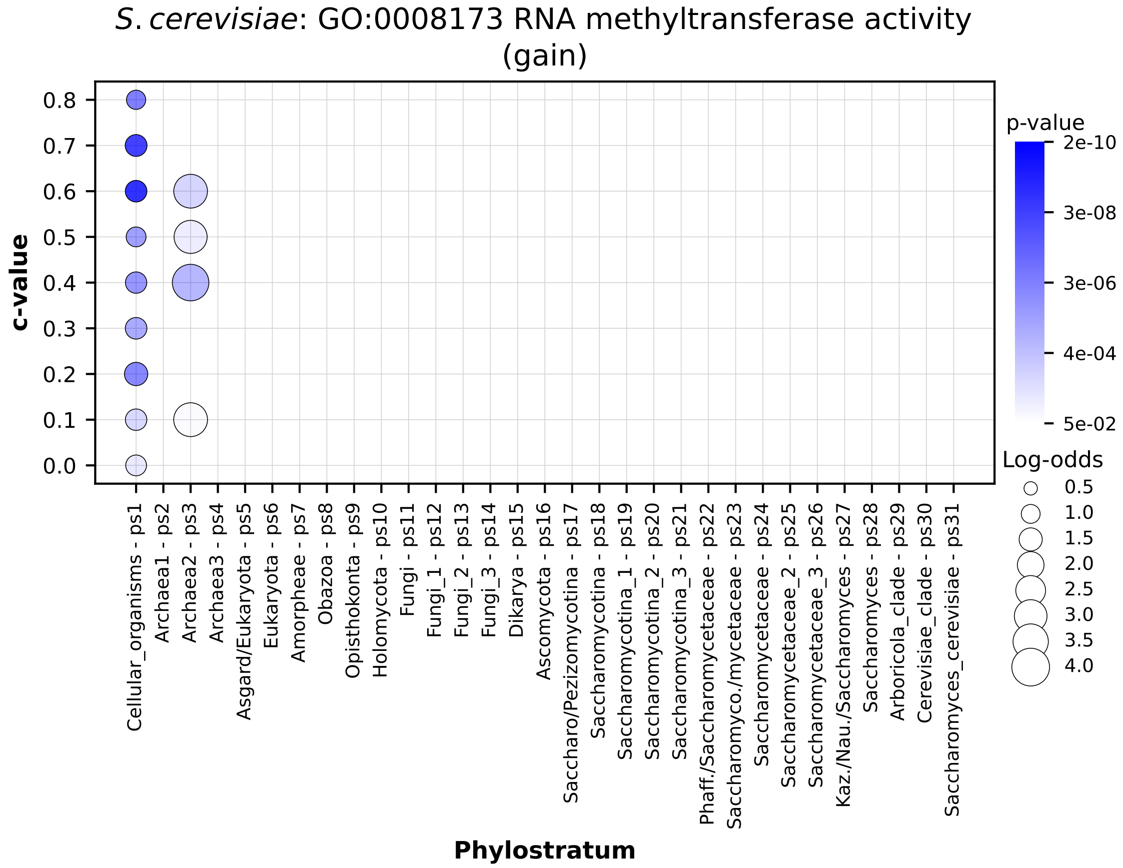

S. cerevisiae: GO:0008173 RNA methyltransferase activity
(gain)
c-value
0.8
0.7
0.6
0.5
0.4
0.3
0.2
0.1
0.0
p-value
2e-10
3e-08
3e-06
4e-04
5e-02
Log-odds
0.5
1.0
1.5
2.0
2.5
3.0
3.5
4.0
Cellular_organisms - ps1
Archaea1 - ps2
Archaea2 - ps3
Archaea3 - ps4
Asgard/Eukaryota - ps5
Eukaryota - ps6
Amorpheae - ps7
Obazoa - ps8
Opisthokonta - ps9
Holomycota - ps10
Fungi - ps11
Fungi_1 - ps12
Fungi_2 - ps13
Fungi_3 - ps14
Dikarya - ps15
Ascomycota - ps16
Saccharo/Pezizomycotina - ps17
Saccharomycotina - ps18
Saccharomycotina_1 - ps19
Saccharomycotina_2 - ps20
Saccharomycotina_3 - ps21
Phaff./Saccharomycetaceae - ps22
Saccharomyco./mycetaceae - ps23
Saccharomycetaceae - ps24
Saccharomycetaceae_2 - ps25
Saccharomycetaceae_3 - ps26
Kaz./Nau./Saccharomyces - ps27
Saccharomyces - ps28
Arboricola_clade - ps29
Cerevisiae_clade - ps30
Saccharomyces_cerevisiae - ps31
Phylostratum

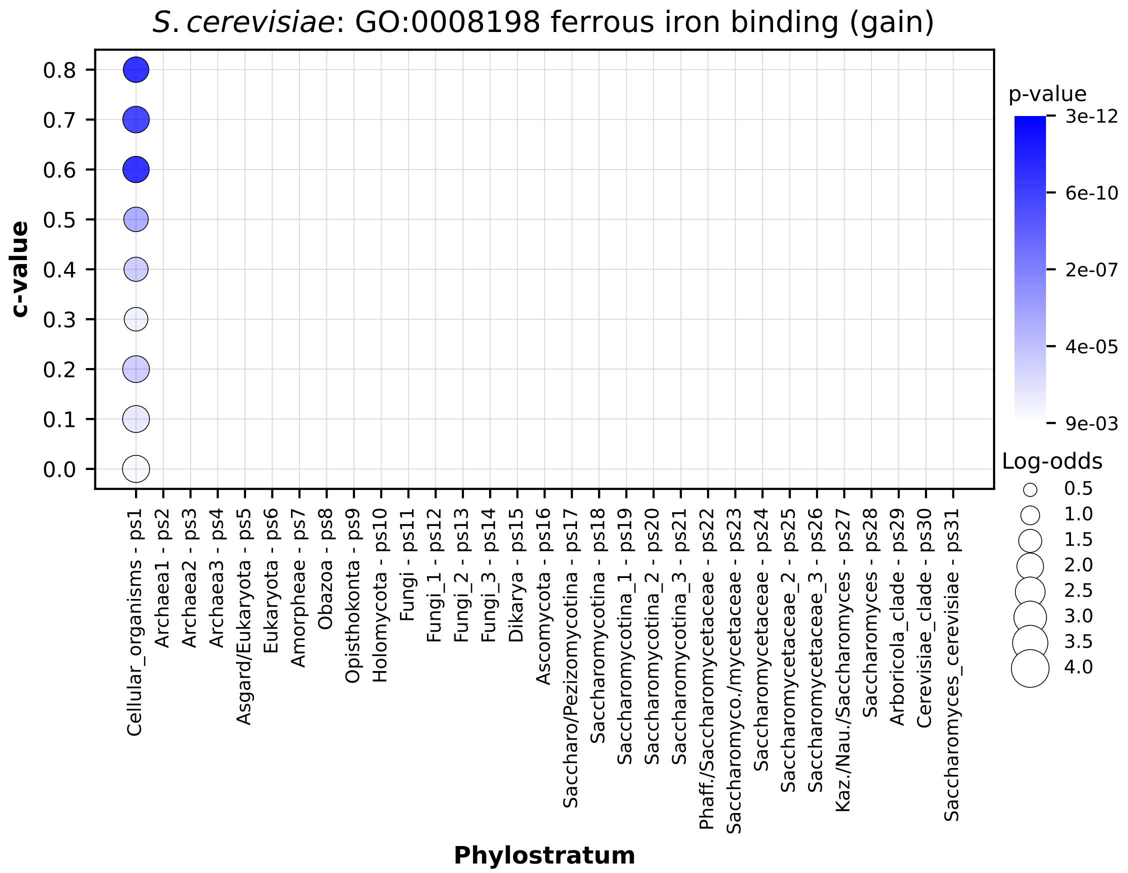
S. cerevisiae: GO:0008198 ferrous iron binding (gain)
c-value
0.8
0.7
0.6
0.5
0.4
0.3
0.2
0.1
0.0
p-value
3e-12
6e-10
2e-07
4e-05
9e-03
Log-odds
0.5
1.0
1.5
2.0
2.5
3.0
3.5
4.0
Cellular_organisms - ps1
Archaea1 - ps2
Archaea2 - ps3
Archaea3 - ps4
Asgard/Eukaryota - ps5
Eukaryota - ps6
Amorpheae - ps7
Obazoa - ps8
Opisthokonta - ps9
Holomycota - ps10
Fungi - ps11
Fungi_1 - ps12
Fungi_2 - ps13
Fungi_3 - ps14
Dikarya - ps15
Ascomycota - ps16
Saccharo/Pezizomycotina - ps17
Saccharomycotina - ps18
Saccharomycotina_1 - ps19
Saccharomycotina_2 - ps20
Saccharomycotina_3 - ps21
Phaff./Saccharomycetaceae - ps22
Saccharomyco./mycetaceae - ps23
Saccharomycetaceae - ps24
Saccharomycetaceae_2 - ps25
Saccharomycetaceae_3 - ps26
Kaz./Nau./Saccharomyces - ps27
Saccharomyces - ps28
Arboricola_clade - ps29
Cerevisiae_clade - ps30
Saccharomyces_cerevisiae - ps31
Phylostratum

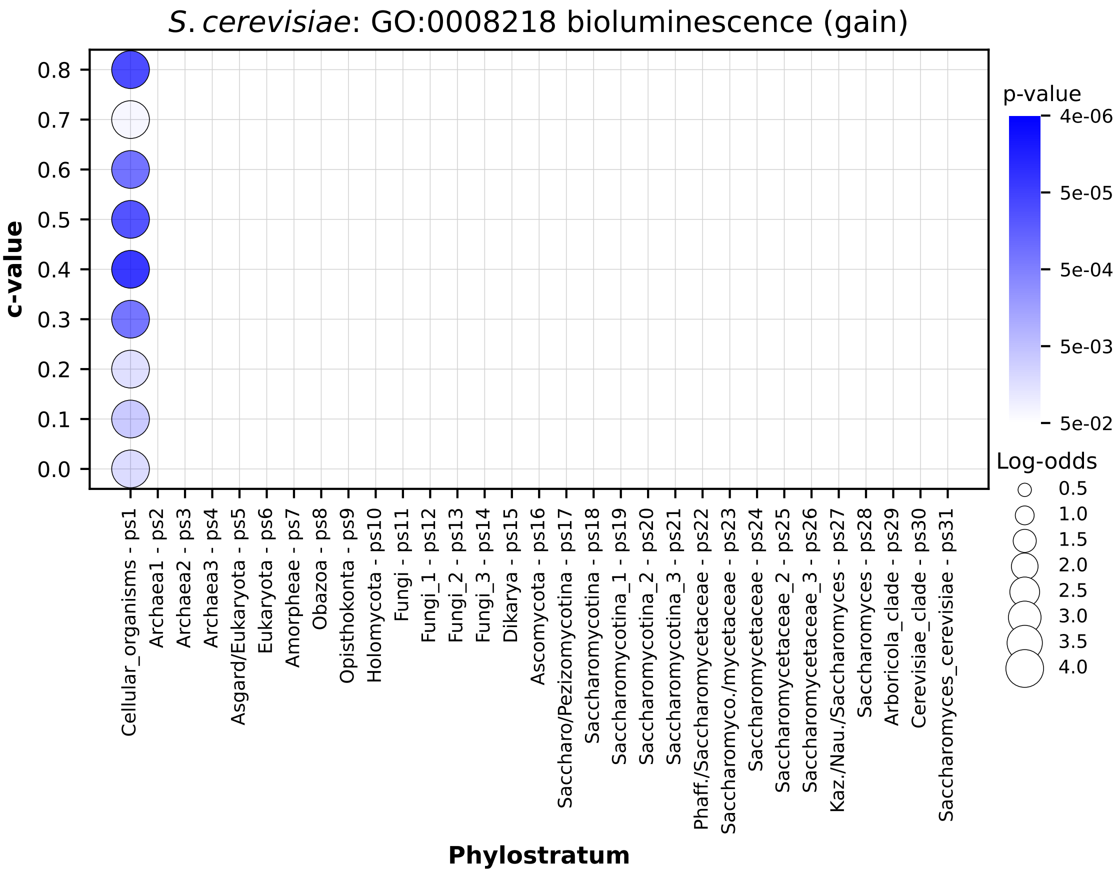
S. cerevisiae: GO:0008218 bioluminescence (gain)
c-value
0.8
0.7
0.6
0.5
0.4
0.3
0.2
0.1
0.0
p-value
4e-06
5e-05
5e-04
5e-03
5e-02
Log-odds
0.5
1.0
1.5
2.0
2.5
3.0
3.5
4.0
Cellular_organisms - ps1
Archaea1 - ps2
Archaea2 - ps3
Archaea3 - ps4
Asgard/Eukaryota - ps5
Eukaryota - ps6
Amorpheae - ps7
Obazoa - ps8
Opisthokonta - ps9
Holomycota - ps10
Fungi - ps11
Fungi_1 - ps12
Fungi_2 - ps13
Fungi_3 - ps14
Dikarya - ps15
Ascomycota - ps16
Saccharo/Pezizomycotina - ps17
Saccharomycotina - ps18
Saccharomycotina_1 - ps19
Saccharomycotina_2 - ps20
Saccharomycotina_3 - ps21
Phaff./Saccharomycetaceae - ps22
Saccharomyco./mycetaceae - ps23
Saccharomycetaceae - ps24
Saccharomycetaceae_2 - ps25
Saccharomycetaceae_3 - ps26
Kaz./Nau./Saccharomyces - ps27
Saccharomyces - ps28
Arboricola_clade - ps29
Cerevisiae_clade - ps30
Saccharomyces_cerevisiae - ps31
Phylostratum

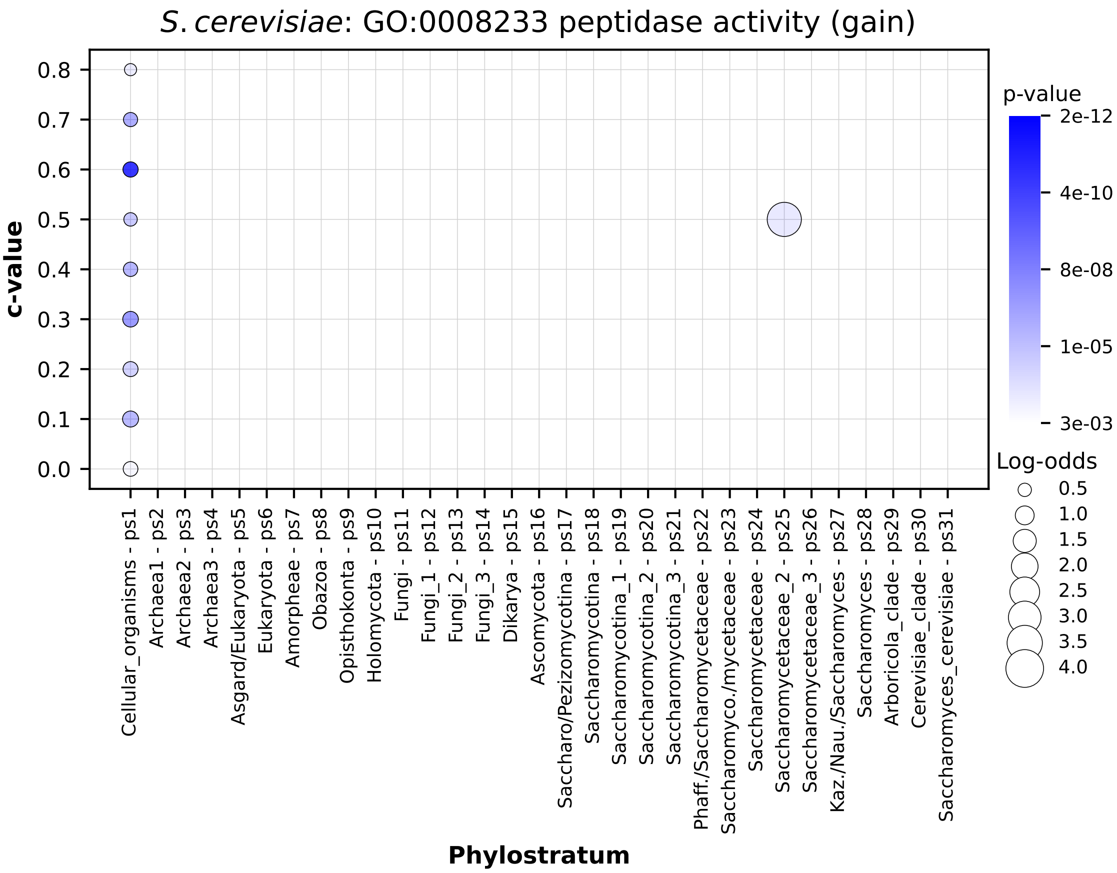

S. cerevisiae: GO:0008233 peptidase activity (gain)
c-value
0.8
0.7
0.6
0.5
0.4
0.3
0.2
0.1
0.0
p-value
2e-12
4e-10
8e-08
1e-05
3e-03
Log-odds
0.5
1.0
1.5
2.0
2.5
3.0
3.5
4.0
Cellular_organisms - ps1
Archaea1 - ps2
Archaea2 - ps3
Archaea3 - ps4
Asgard/Eukaryota - ps5
Eukaryota - ps6
Amorpheae - ps7
Obazoa - ps8
Opisthokonta - ps9
Holomycota - ps10
Fungi - ps11
Fungi_1 - ps12
Fungi_2 - ps13
Fungi_3 - ps14
Dikarya - ps15
Ascomycota - ps16
Saccharo/Pezizomycotina - ps17
Saccharomycotina - ps18
Saccharomycotina_1 - ps19
Saccharomycotina_2 - ps20
Saccharomycotina_3 - ps21
Phaff./Saccharomycetaceae - ps22
Saccharomyco./mycetaceae - ps23
Saccharomycetaceae - ps24
Saccharomycetaceae_2 - ps25
Saccharomycetaceae_3 - ps26
Kaz./Nau./Saccharomyces - ps27
Saccharomyces - ps28
Arboricola_clade - ps29
Cerevisiae_clade - ps30
Saccharomyces_cerevisiae - ps31
Phylostratum

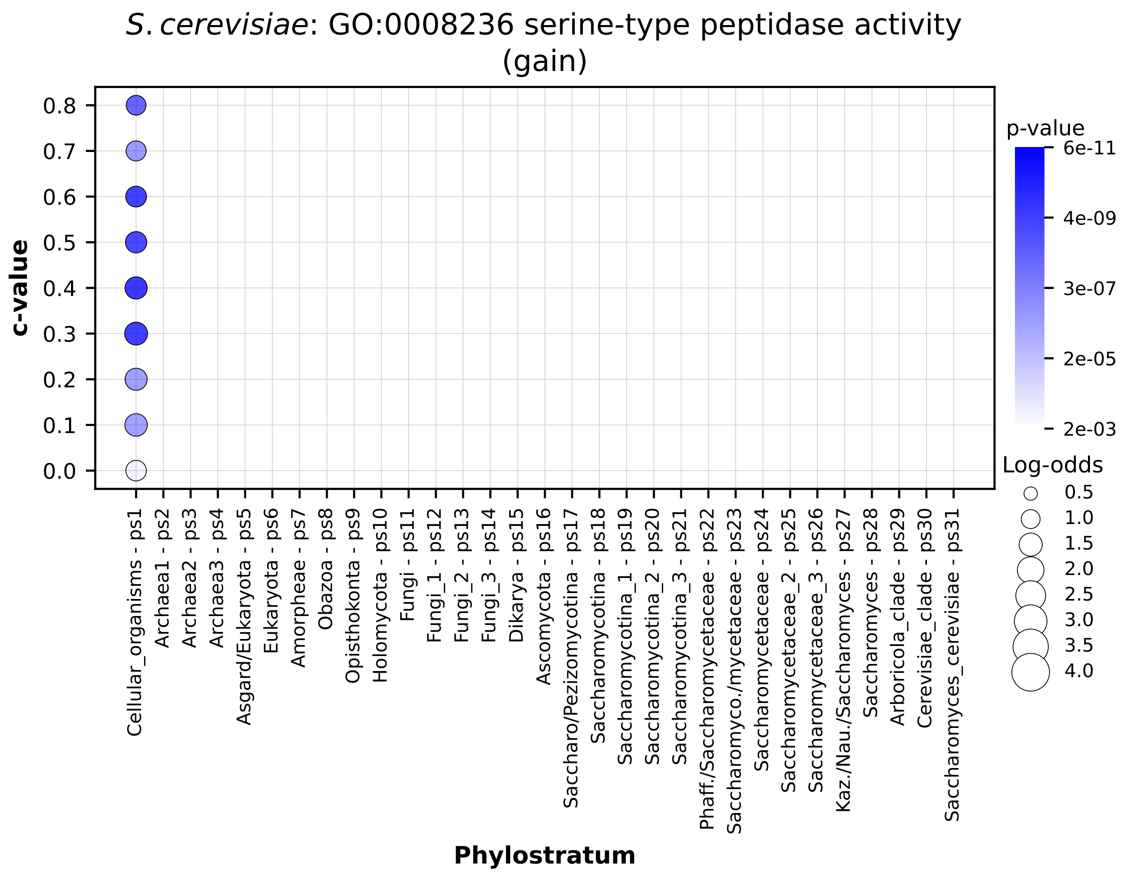
S. cerevisiae: GO:0008236 serine-type peptidase activity
(gain)
c-value
0.8
0.7
0.6
0.5
0.4
0.3
0.2
0.1
0.0
p-value
6e-11
4e-09
3e-07
2e-05
2e-03
Log-odds
0.5
1.0
1.5
2.0
2.5
3.0
3.5
4.0
Cellular_organisms - ps1
Archaea1 - ps2
Archaea2 - ps3
Archaea3 - ps4
Asgard/Eukaryota - ps5
Eukaryota - ps6
Amorpheae - ps7
Obazoa - ps8
Opisthokonta - ps9
Holomycota - ps10
Fungi - ps11
Fungi_1 - ps12
Fungi_2 - ps13
Fungi_3 - ps14
Dikarya - ps15
Ascomycota - ps16
Saccharo/Pezizomycotina - ps17
Saccharomycotina - ps18
Saccharomycotina_1 - ps19
Saccharomycotina_2 - ps20
Saccharomycotina_3 - ps21
Phaff./Saccharomycetaceae - ps22
Saccharomyco./mycetaceae - ps23
Saccharomycetaceae - ps24
Saccharomycetaceae_2 - ps25
Saccharomycetaceae_3 - ps26
Kaz./Nau./Saccharomyces - ps27
Saccharomyces - ps28
Arboricola_clade - ps29
Cerevisiae_clade - ps30
Saccharomyces_cerevisiae - ps31
Phylostratum

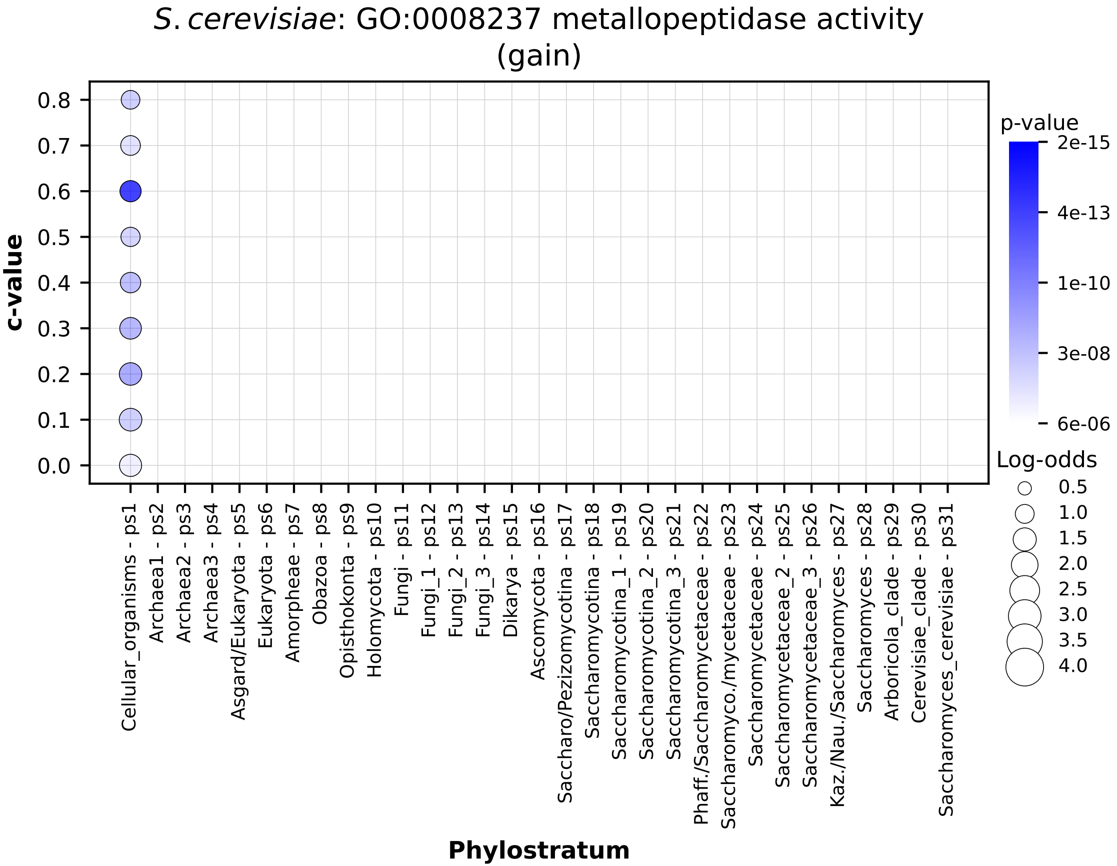

S. cerevisiae: GO:0008237 metallopeptidase activity
(gain)
c-value
0.8
0.7
0.6
0.5
0.4
0.3
0.2
0.1
0.0
p-value
2e-15
4e-13
1e-10
3e-08
6e-06
Log-odds
0.5
1.0
1.5
2.0
2.5
3.0
3.5
4.0
Cellular_organisms - ps1
Archaea1 - ps2
Archaea2 - ps3
Archaea3 - ps4
Asgard/Eukaryota - ps5
Eukaryota - ps6
Amorpheae - ps7
Obazoa - ps8
Opisthokonta - ps9
Holomycota - ps10
Fungi - ps11
Fungi_1 - ps12
Fungi_2 - ps13
Fungi_3 - ps14
Dikarya - ps15
Ascomycota - ps16
Saccharo/Pezizomycotina - ps17
Saccharomycotina - ps18
Saccharomycotina_1 - ps19
Saccharomycotina_2 - ps20
Saccharomycotina_3 - ps21
Phaff./Saccharomycetaceae - ps22
Saccharomyco./mycetaceae - ps23
Saccharomycetaceae - ps24
Saccharomycetaceae_2 - ps25
Saccharomycetaceae_3 - ps26
Kaz./Nau./Saccharomyces - ps27
Saccharomyces - ps28
Arboricola_clade - ps29
Cerevisiae_clade - ps30
Saccharomyces_cerevisiae - ps31
Phylostratum

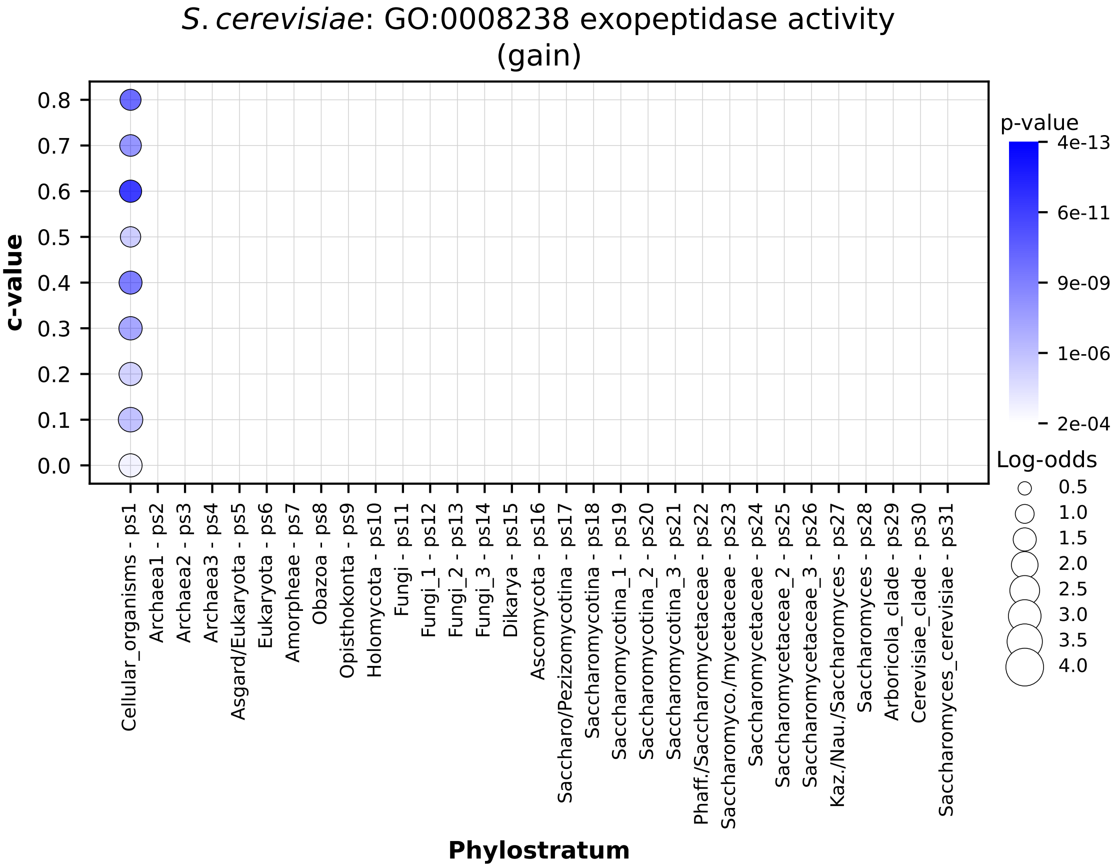

S. cerevisiae: GO:0008238 exopeptidase activity
(gain)
c-value
0.0
0.1
0.2
0.3
0.4
0.5
0.6
0.7
0.8
p-value
4e-13
6e-11
9e-09
1e-06
2e-04
Log-odds
0.5
1.0
1.5
2.0
2.5
3.0
3.5
4.0
Cellular_organisms - ps1
Archaea1 - ps2
Archaea2 - ps3
Archaea3 - ps4
Asgard/Eukaryota - ps5
Eukaryota - ps6
Amorpheae - ps7
Obazoa - ps8
Opisthokonta - ps9
Holomycota - ps10
Fungi - ps11
Fungi_1 - ps12
Fungi_2 - ps13
Fungi_3 - ps14
Dikarya - ps15
Ascomycota - ps16
Saccharo/Pezizomycotina - ps17
Saccharomycotina - ps18
Saccharomycotina_1 - ps19
Saccharomycotina_2 - ps20
Saccharomycotina_3 - ps21
Phaff./Saccharomycetaceae - ps22
Saccharomyco./mycetaceae - ps23
Saccharomycetaceae - ps24
Saccharomycetaceae_2 - ps25
Saccharomycetaceae_3 - ps26
Kaz./Nau./Saccharomyces - ps27
Saccharomyces - ps28
Arboricola_clade - ps29
Cerevisiae_clade - ps30
Saccharomyces_cerevisiae - ps31
Phylostratum

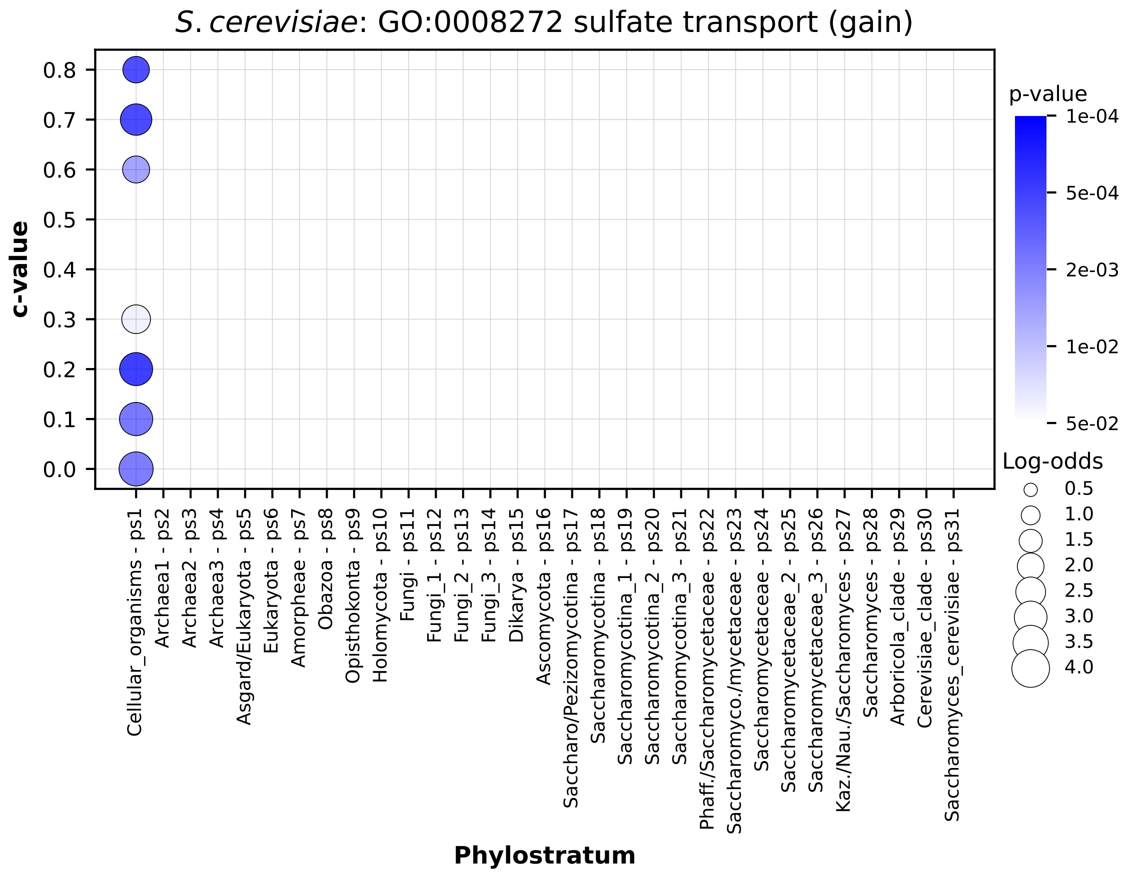
S. cerevisiae: GO:0008272 sulfate transport (gain)
c-value
0.0
0.1
0.2
0.3
0.4
0.5
0.6
0.7
0.8
p-value
1e-04
5e-04
2e-03
1e-02
5e-02
Log-odds
0.5
1.0
1.5
2.0
2.5
3.0
3.5
4.0
Cellular_organisms - ps1
Archaea1 - ps2
Archaea2 - ps3
Archaea3 - ps4
Asgard/Eukaryota - ps5
Eukaryota - ps6
Amorpheae - ps7
Obazoa - ps8
Opisthokonta - ps9
Holomycota - ps10
Fungi - ps11
Fungi_1 - ps12
Fungi_2 - ps13
Fungi_3 - ps14
Dikarya - ps15
Ascomycota - ps16
Saccharo/Pezizomycotina - ps17
Saccharomycotina - ps18
Saccharomycotina_1 - ps19
Saccharomycotina_2 - ps20
Saccharomycotina_3 - ps21
Phaff./Saccharomycetaceae - ps22
Saccharomyco./mycetaceae - ps23
Saccharomycetaceae - ps24
Saccharomycetaceae_2 - ps25
Saccharomycetaceae_3 - ps26
Kaz./Nau./Saccharomyces - ps27
Saccharomyces - ps28
Arboricola_clade - ps29
Cerevisiae_clade - ps30
Saccharomyces_cerevisiae - ps31
Phylostratum

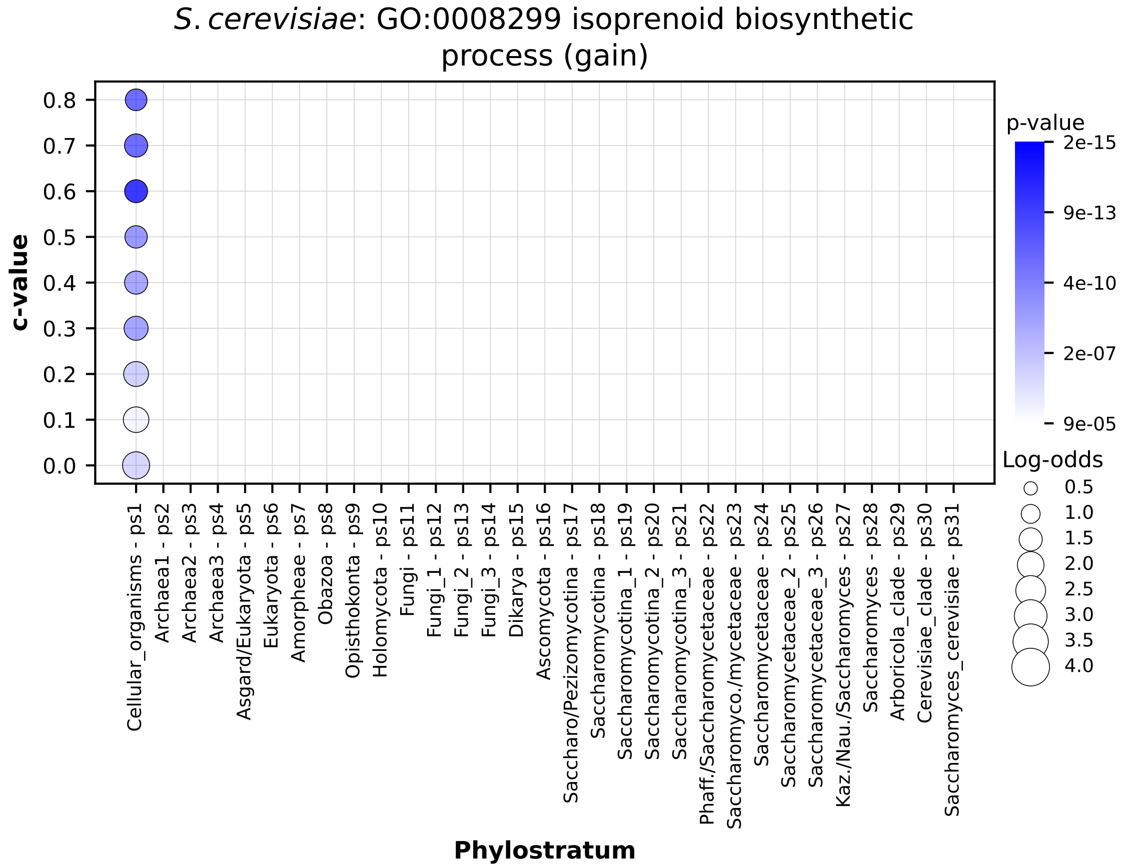

S. cerevisiae: GO:0008299 isoprenoid biosynthetic process (gain)
c-value
0.8
0.7
0.6
0.5
0.4
0.3
0.2
0.1
0.0
p-value
2e-15
9e-13
4e-10
2e-07
9e-05
Log-odds
0.5
1.0
1.5
2.0
2.5
3.0
3.5
4.0
Cellular_organisms - ps1
Archaea1 - ps2
Archaea2 - ps3
Archaea3 - ps4
Asgard/Eukaryota - ps5
Eukaryota - ps6
Amorpheae - ps7
Obazoa - ps8
Opisthokonta - ps9
Holomycota - ps10
Fungi - ps11
Fungi_1 - ps12
Fungi_2 - ps13
Fungi_3 - ps14
Dikarya - ps15
Ascomycota - ps16
Saccharo/Pezizomycotina - ps17
Saccharomycotina - ps18
Saccharomycotina_1 - ps19
Saccharomycotina_2 - ps20
Saccharomycotina_3 - ps21
Phaff./Saccharomycetaceae - ps22
Saccharomyco./mycetaceae - ps23
Saccharomycetaceae - ps24
Saccharomycetaceae_2 - ps25
Saccharomycetaceae_3 - ps26
Kaz./Nau./Saccharomyces - ps27
Saccharomyces - ps28
Arboricola_clade - ps29
Cerevisiae_clade - ps30
Saccharomyces_cerevisiae - ps31
Phylostratum

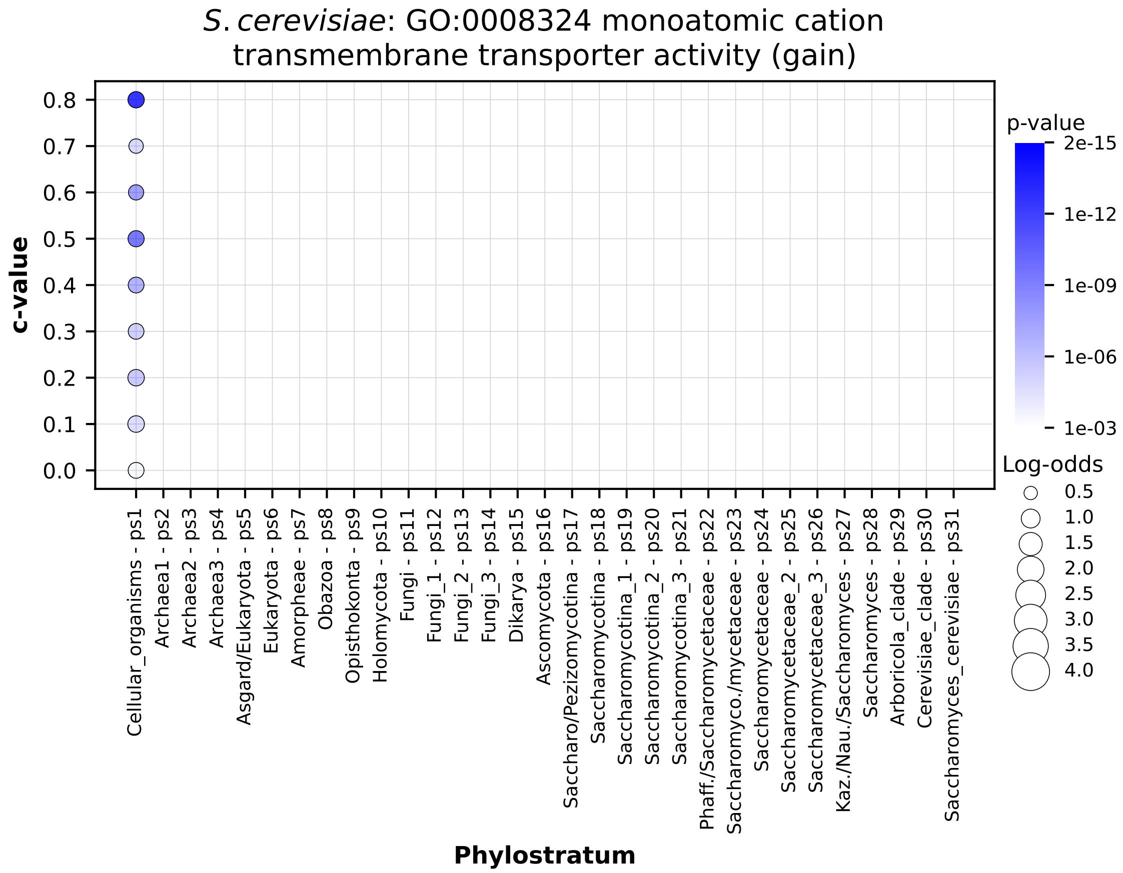
S. cerevisiae: GO:0008324 monoatomic cation transmembrane transporter activity (gain)
c-value
0.8
0.7
0.6
0.5
0.4
0.3
0.2
0.1
0.0
p-value
2e-15
1e-12
1e-09
1e-06
1e-03
Log-odds
0.5
1.0
1.5
2.0
2.5
3.0
3.5
4.0
Cellular_organisms - ps1
Archaea1 - ps2
Archaea2 - ps3
Archaea3 - ps4
Asgard/Eukaryota - ps5
Eukaryota - ps6
Amorpheae - ps7
Obazoa - ps8
Opisthokonta - ps9
Holomycota - ps10
Fungi - ps11
Fungi_1 - ps12
Fungi_2 - ps13
Fungi_3 - ps14
Dikarya - ps15
Ascomycota - ps16
Saccharo/Pezizomycotina - ps17
Saccharomycotina - ps18
Saccharomycotina_1 - ps19
Saccharomycotina_2 - ps20
Saccharomycotina_3 - ps21
Phaff./Saccharomycetaceae - ps22
Saccharomyco./mycetaceae - ps23
Saccharomycetaceae - ps24
Saccharomycetaceae_2 - ps25
Saccharomycetaceae_3 - ps26
Kaz./Nau./Saccharomyces - ps27
Saccharomyces - ps28
Arboricola_clade - ps29
Cerevisiae_clade - ps30
Saccharomyces_cerevisiae - ps31
Phylostratum

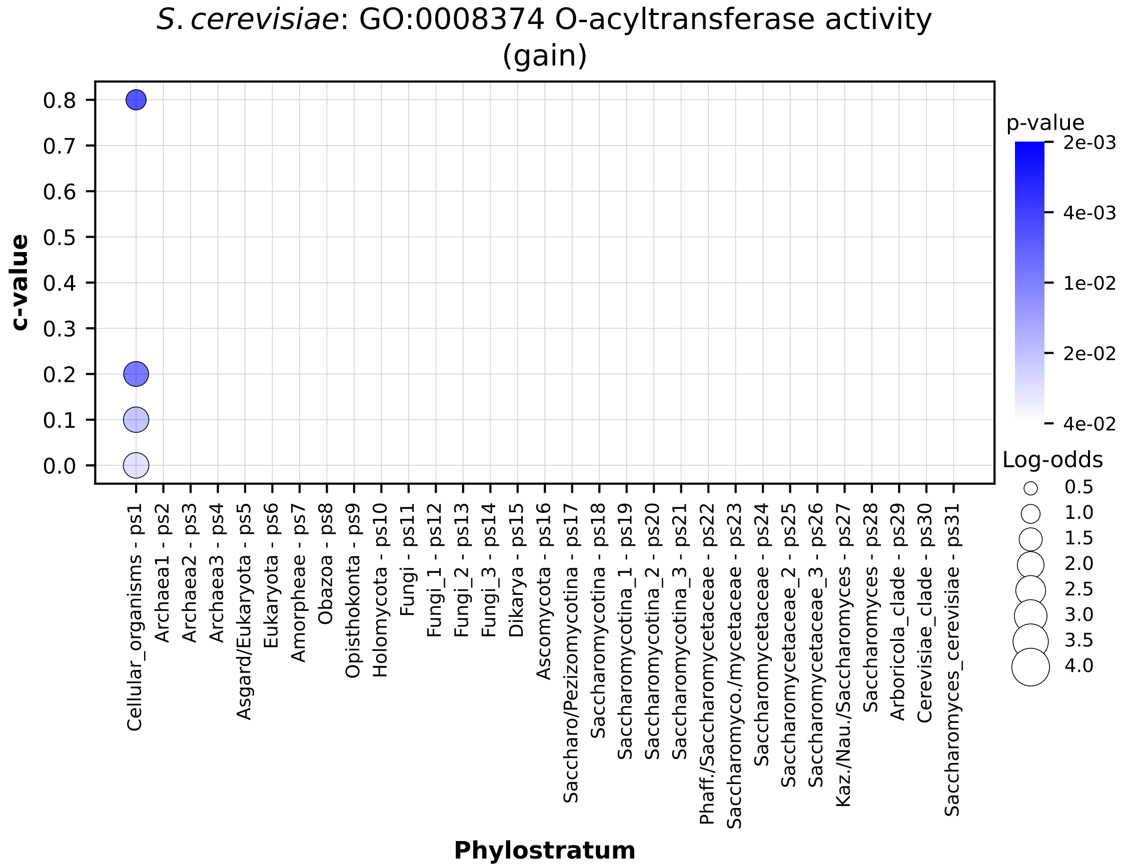
S. cerevisiae: GO:0008374 O-acyltransferase activity (gain)
c-value
0.0
0.1
0.2
0.3
0.4
0.5
0.6
0.7
0.8
p-value
2e-03
4e-03
1e-02
2e-02
4e-02
Log-odds
0.5
1.0
1.5
2.0
2.5
3.0
3.5
4.0
Cellular_organisms - ps1
Archaea1 - ps2
Archaea2 - ps3
Archaea3 - ps4
Asgard/Eukaryota - ps5
Eukaryota - ps6
Amorpheae - ps7
Obazoa - ps8
Opisthokonta - ps9
Holomycota - ps10
Fungi - ps11
Fungi_1 - ps12
Fungi_2 - ps13
Fungi_3 - ps14
Dikarya - ps15
Ascomycota - ps16
Saccharo/Pezizomycotina - ps17
Saccharomycotina - ps18
Saccharomycotina_1 - ps19
Saccharomycotina_2 - ps20
Saccharomycotina_3 - ps21
Phaff./Saccharomycetaceae - ps22
Saccharomyco./mycetaceae - ps23
Saccharomycetaceae - ps24
Saccharomycetaceae_2 - ps25
Saccharomycetaceae_3 - ps26
Kaz./Nau./Saccharomyces - ps27
Saccharomyces - ps28
Arboricola_clade - ps29
Cerevisiae_clade - ps30
Saccharomyces_cerevisiae - ps31
Phylostratum

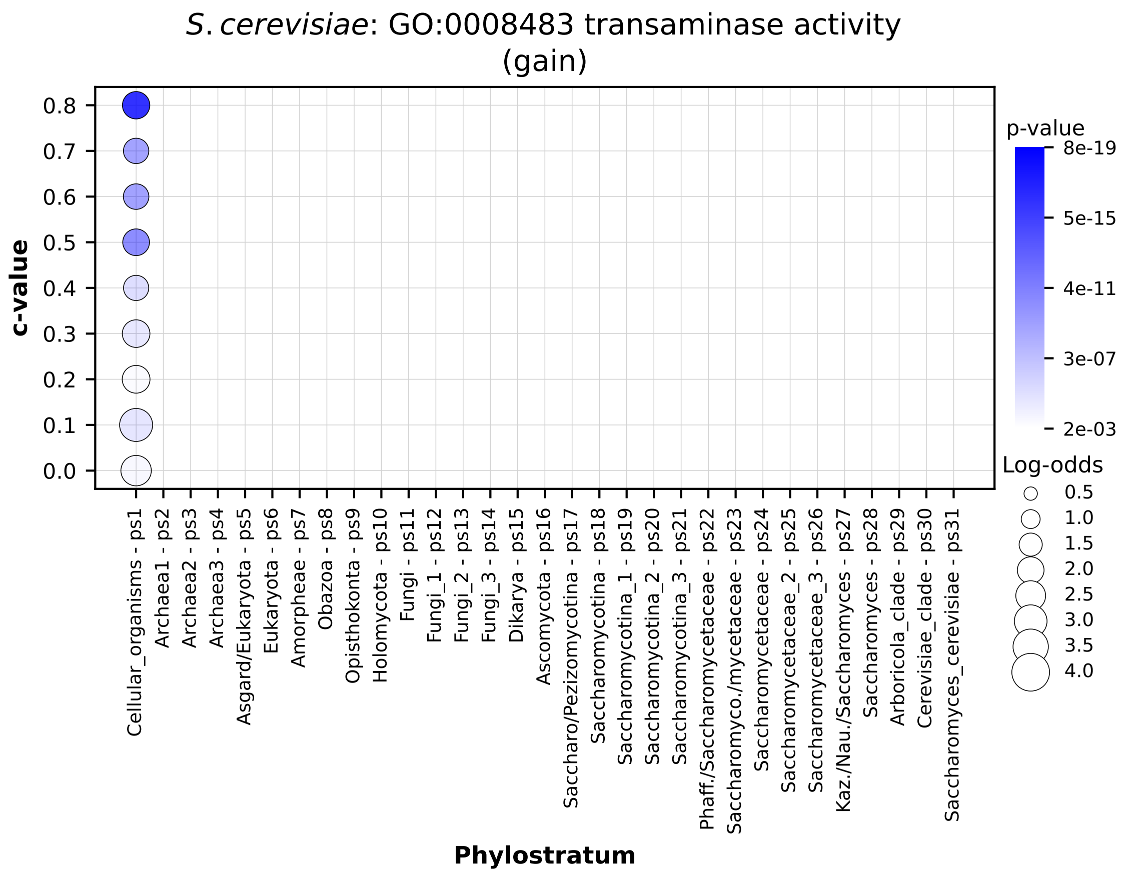

S. cerevisiae: GO:0008483 transaminase activity
(gain)
c-value
0.8
0.7
0.6
0.5
0.4
0.3
0.2
0.1
0.0
p-value
8e-19
5e-15
4e-11
3e-07
2e-03
Log-odds
0.5
1.0
1.5
2.0
2.5
3.0
3.5
4.0
Cellular_organisms - ps1
Archaea1 - ps2
Archaea2 - ps3
Archaea3 - ps4
Asgard/Eukaryota - ps5
Eukaryota - ps6
Amorpheae - ps7
Obazoa - ps8
Opisthokonta - ps9
Holomycota - ps10
Fungi - ps11
Fungi_1 - ps12
Fungi_2 - ps13
Fungi_3 - ps14
Dikarya - ps15
Ascomycota - ps16
Saccharo/Pezizomycotina - ps17
Saccharomycotina - ps18
Saccharomycotina_1 - ps19
Saccharomycotina_2 - ps20
Saccharomycotina_3 - ps21
Phaff./Saccharomycetaceae - ps22
Saccharomyco./mycetaceae - ps23
Saccharomycetaceae - ps24
Saccharomycetaceae_2 - ps25
Saccharomycetaceae_3 - ps26
Kaz./Nau./Saccharomyces - ps27
Saccharomyces - ps28
Arboricola_clade - ps29
Cerevisiae_clade - ps30
Saccharomyces_cerevisiae - ps31
Phylostratum

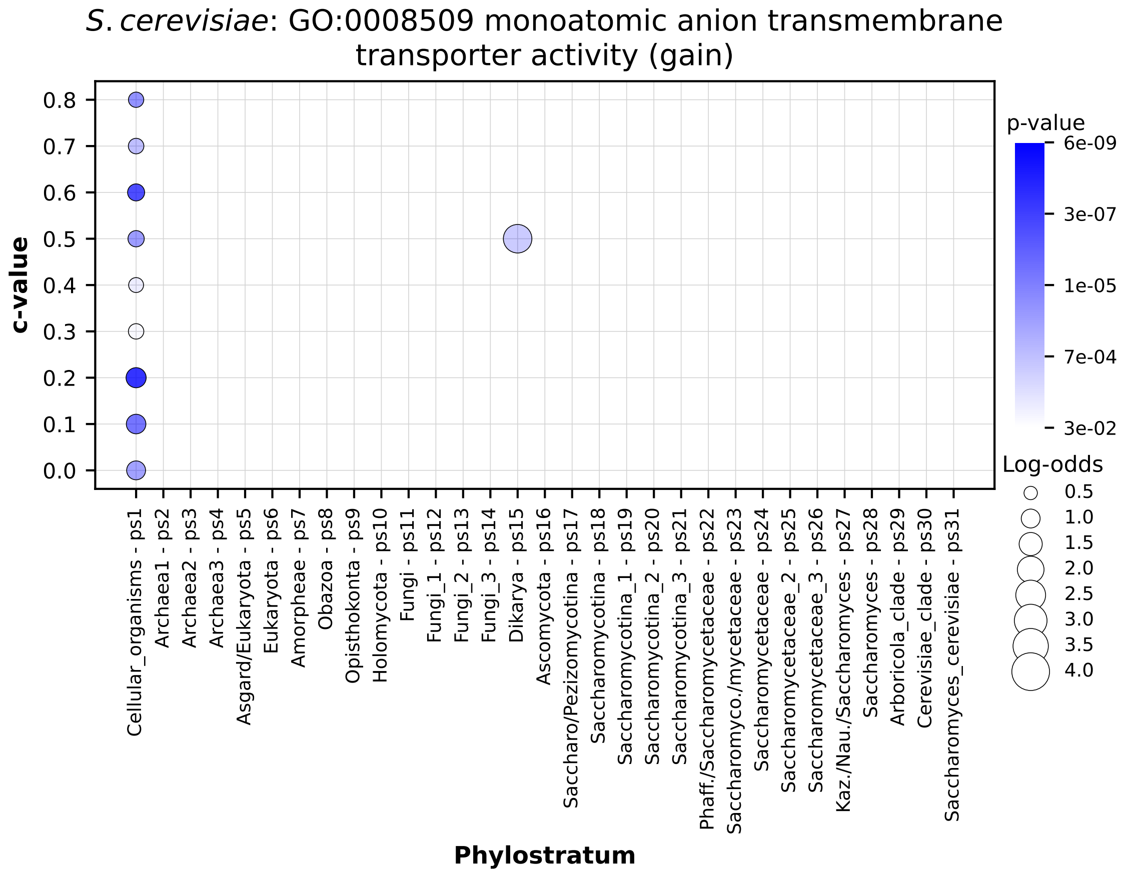
S. cerevisiae: GO:0008509 monoatomic anion transmembrane transporter activity (gain)
c-value
0.8
0.7
0.6
0.5
0.4
0.3
0.2
0.1
0.0
Phylostratum
Cellular_organisms - ps1
Archaea1 - ps2
Archaea2 - ps3
Archaea3 - ps4
Asgard/Eukaryota - ps5
Eukaryota - ps6
Amorpheae - ps7
Obazoa - ps8
Opisthokonta - ps9
Holomycota - ps10
Fungi - ps11
Fungi_1 - ps12
Fungi_2 - ps13
Fungi_3 - ps14
Dikarya - ps15
Ascomycota - ps16
Saccharo/Pezizomycotina - ps17
Saccharomycotina - ps18
Saccharomycotina_1 - ps19
Saccharomycotina_2 - ps20
Saccharomycotina_3 - ps21
Phaff./Saccharomycetaceae - ps22
Saccharomyco./mycetaceae - ps23
Saccharomycetaceae - ps24
Saccharomycetaceae_2 - ps25
Saccharomycetaceae_3 - ps26
Kaz./Nau./Saccharomyces - ps27
Saccharomyces - ps28
Arboricola_clade - ps29
Cerevisiae_clade - ps30
Saccharomyces_cerevisiae - ps31
p-value
6e-09
3e-07
1e-05
7e-04
3e-02
Log-odds
0.5
1.0
1.5
2.0
2.5
3.0
3.5
4.0

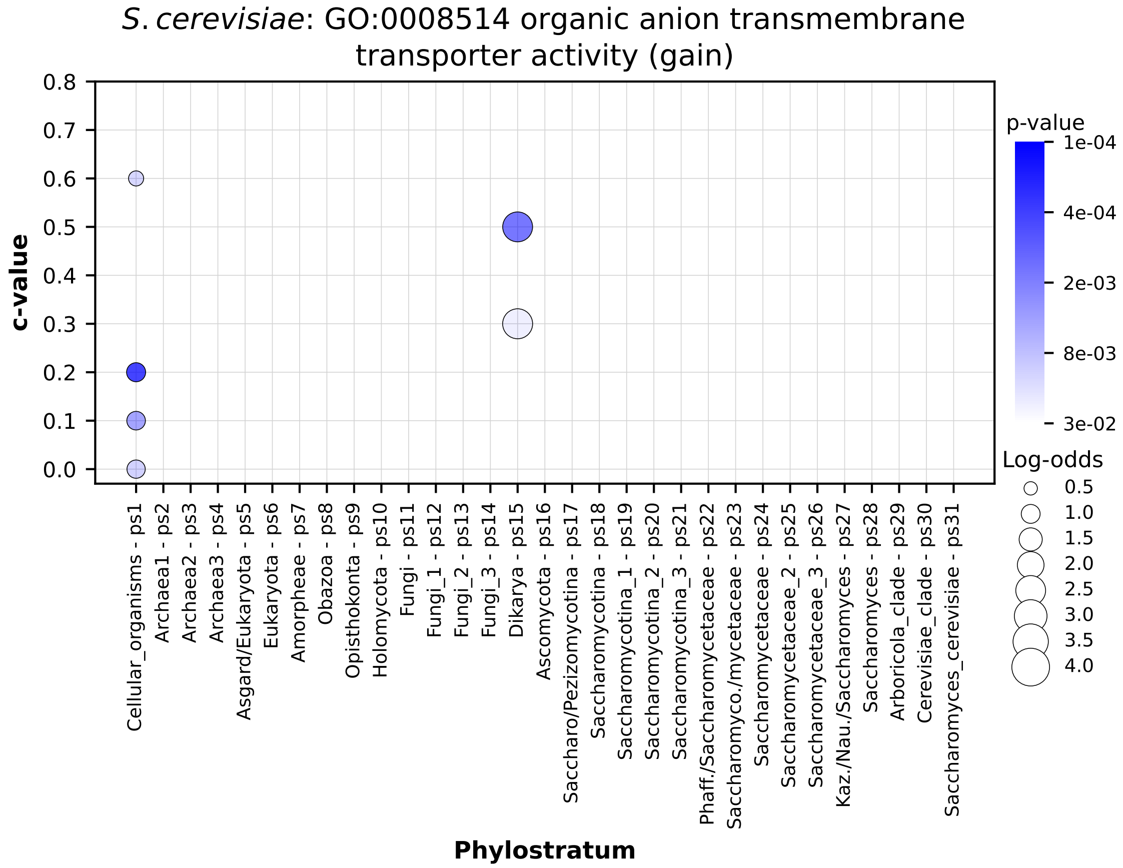
S. cerevisiae: GO:0008514 organic anion transmembrane transporter activity (gain)
c-value
0.8
0.7
0.6
0.5
0.4
0.3
0.2
0.1
0.0
p-value
1e-04
4e-04
2e-03
8e-03
3e-02
Log-odds
0.5
1.0
1.5
2.0
2.5
3.0
3.5
4.0
Cellular_organisms - ps1
Archaea1 - ps2
Archaea2 - ps3
Archaea3 - ps4
Asgard/Eukaryota - ps5
Eukaryota - ps6
Amorpheae - ps7
Obazoa - ps8
Opisthokonta - ps9
Holomycota - ps10
Fungi - ps11
Fungi_1 - ps12
Fungi_2 - ps13
Fungi_3 - ps14
Dikarya - ps15
Ascomycota - ps16
Saccharo/Pezizomycotina - ps17
Saccharomycotina - ps18
Saccharomycotina_1 - ps19
Saccharomycotina_2 - ps20
Saccharomycotina_3 - ps21
Phaff./Saccharomycetaceae - ps22
Saccharomyco./mycetaceae - ps23
Saccharomycetaceae - ps24
Saccharomycetaceae_2 - ps25
Saccharomycetaceae_3 - ps26
Kaz./Nau./Saccharomyces - ps27
Saccharomyces - ps28
Arboricola_clade - ps29
Cerevisiae_clade - ps30
Saccharomyces_cerevisiae - ps31
Phylostratum

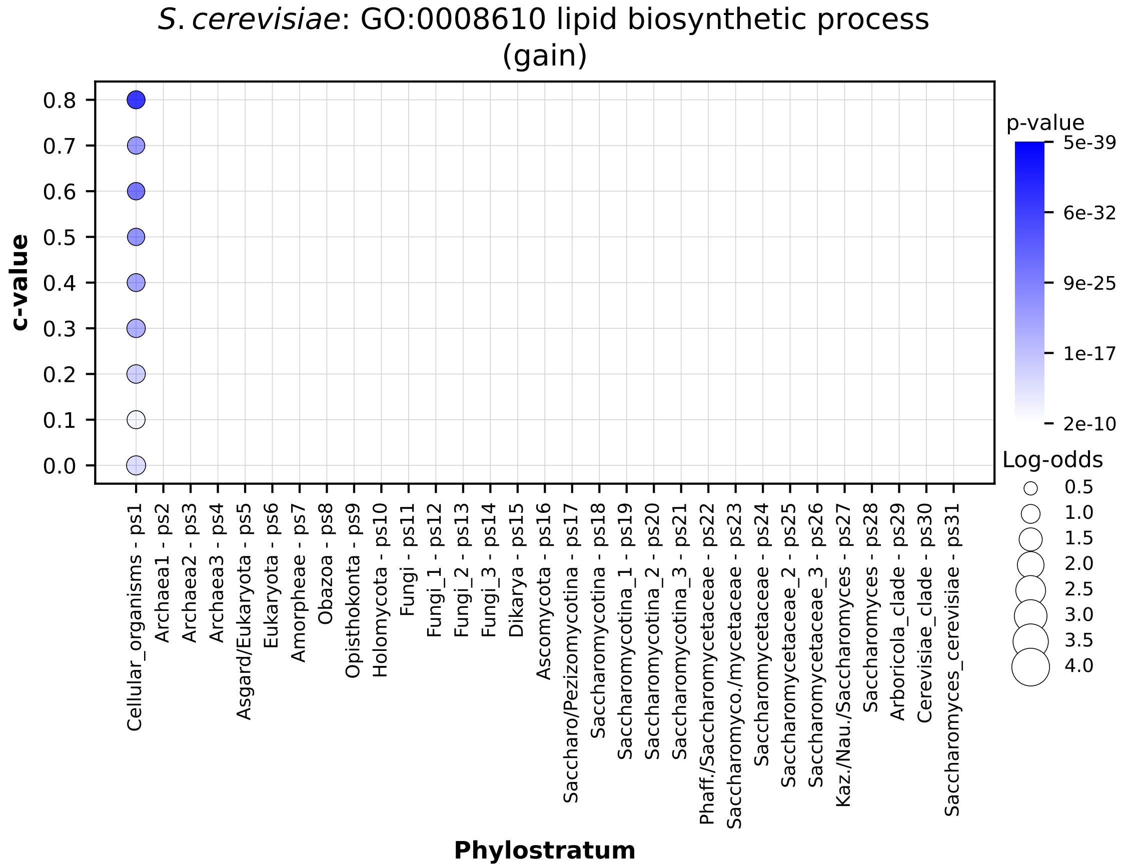
S. cerevisiae: GO:0008610 lipid biosynthetic process
(gain)
c-value
0.8
0.7
0.6
0.5
0.4
0.3
0.2
0.1
0.0
p-value
5e-39
6e-32
9e-25
1e-17
2e-10
Log-odds
0.5
1.0
1.5
2.0
2.5
3.0
3.5
4.0
Cellular_organisms - ps1
Archaea1 - ps2
Archaea2 - ps3
Archaea3 - ps4
Asgard/Eukaryota - ps5
Eukaryota - ps6
Amorpheae - ps7
Obazoa - ps8
Opisthokonta - ps9
Holomycota - ps10
Fungi - ps11
Fungi_1 - ps12
Fungi_2 - ps13
Fungi_3 - ps14
Dikarya - ps15
Ascomycota - ps16
Saccharo/Pezizomycotina - ps17
Saccharomycotina - ps18
Saccharomycotina_1 - ps19
Saccharomycotina_2 - ps20
Saccharomycotina_3 - ps21
Phaff./Saccharomycetaceae - ps22
Saccharomyco./mycetaceae - ps23
Saccharomycetaceae - ps24
Saccharomycetaceae_2 - ps25
Saccharomycetaceae_3 - ps26
Kaz./Nau./Saccharomyces - ps27
Saccharomyces - ps28
Arboricola_clade - ps29
Cerevisiae_clade - ps30
Saccharomyces_cerevisiae - ps31
Phylostratum

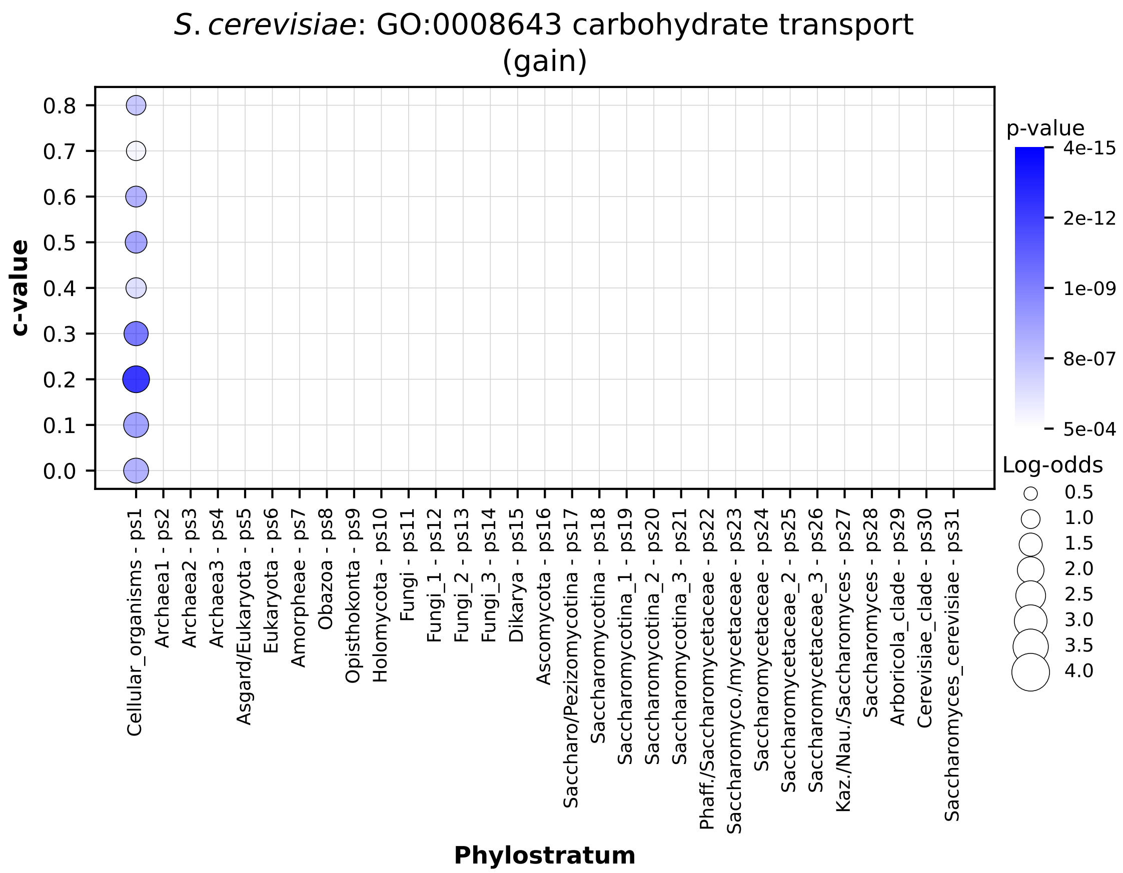

S. cerevisiae: GO:0008643 carbohydrate transport
(gain)
c-value
0.8
0.7
0.6
0.5
0.4
0.3
0.2
0.1
0.0
p-value
4e-15
2e-12
1e-09
8e-07
5e-04
Log-odds
0.5
1.0
1.5
2.0
2.5
3.0
3.5
4.0
Cellular_organisms - ps1
Archaea1 - ps2
Archaea2 - ps3
Archaea3 - ps4
Asgard/Eukaryota - ps5
Eukaryota - ps6
Amorpheae - ps7
Obazoa - ps8
Opisthokonta - ps9
Holomycota - ps10
Fungi - ps11
Fungi_1 - ps12
Fungi_2 - ps13
Fungi_3 - ps14
Dikarya - ps15
Ascomycota - ps16
Saccharo/Pezizomycotina - ps17
Saccharomycotina - ps18
Saccharomycotina_1 - ps19
Saccharomycotina_2 - ps20
Saccharomycotina_3 - ps21
Phaff./Saccharomycetaceae - ps22
Saccharomyco./mycetaceae - ps23
Saccharomycetaceae - ps24
Saccharomycetaceae_2 - ps25
Saccharomycetaceae_3 - ps26
Kaz./Nau./Saccharomyces - ps27
Saccharomyces - ps28
Arboricola_clade - ps29
Cerevisiae_clade - ps30
Saccharomyces_cerevisiae - ps31
Phylostratum

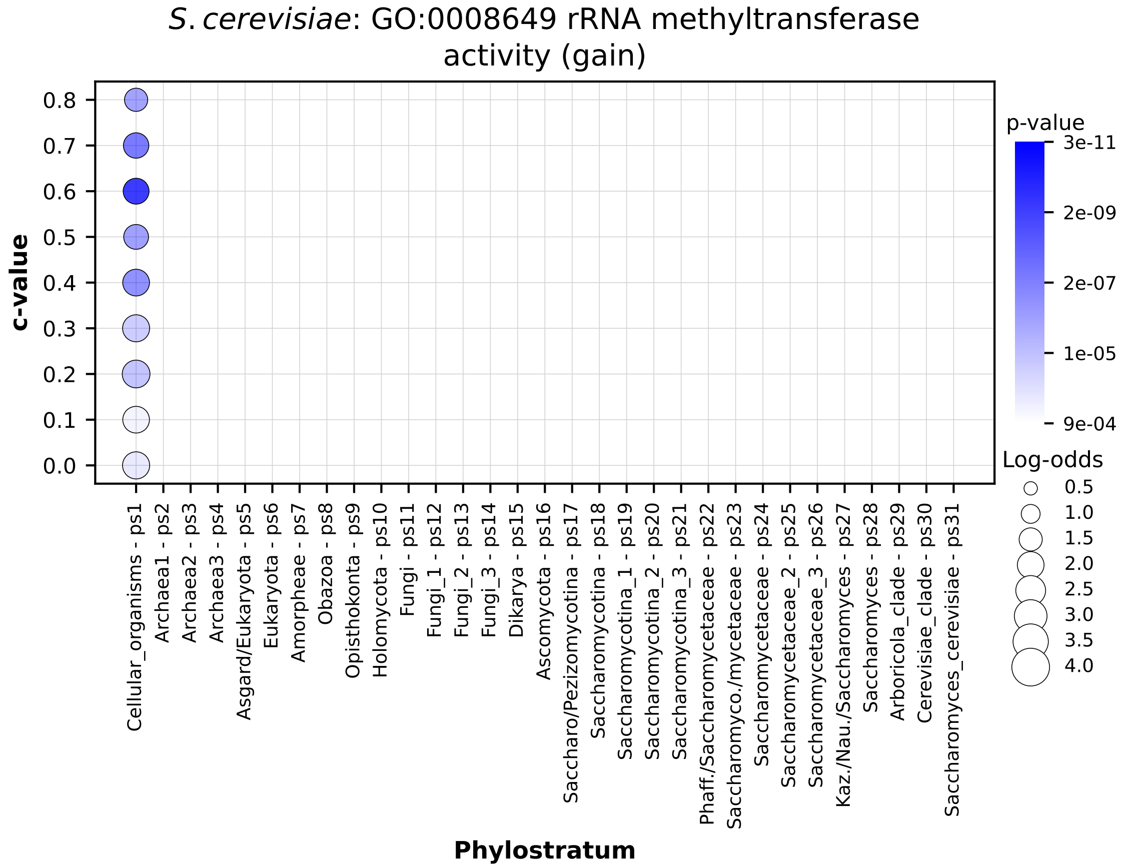

S. cerevisiae: GO:0008649 rRNA methyltransferase activity (gain)
c-value
0.8
0.7
0.6
0.5
0.4
0.3
0.2
0.1
0.0
p-value
3e-11
2e-09
2e-07
1e-05
9e-04
Log-odds
0.5
1.0
1.5
2.0
2.5
3.0
3.5
4.0
Cellular_organisms - ps1
Archaea1 - ps2
Archaea2 - ps3
Archaea3 - ps4
Asgard/Eukaryota - ps5
Eukaryota - ps6
Amorpheae - ps7
Obazoa - ps8
Opisthokonta - ps9
Holomycota - ps10
Fungi - ps11
Fungi_1 - ps12
Fungi_2 - ps13
Fungi_3 - ps14
Dikarya - ps15
Ascomycota - ps16
Saccharo/Pezizomycotina - ps17
Saccharomycotina - ps18
Saccharomycotina_1 - ps19
Saccharomycotina_2 - ps20
Saccharomycotina_3 - ps21
Phaff./Saccharomycetaceae - ps22
Saccharomyco./mycetaceae - ps23
Saccharomycetaceae - ps24
Saccharomycetaceae_2 - ps25
Saccharomycetaceae_3 - ps26
Kaz./Nau./Saccharomyces - ps27
Saccharomyces - ps28
Arboricola_clade - ps29
Cerevisiae_clade - ps30
Saccharomyces_cerevisiae - ps31
Phylostratum

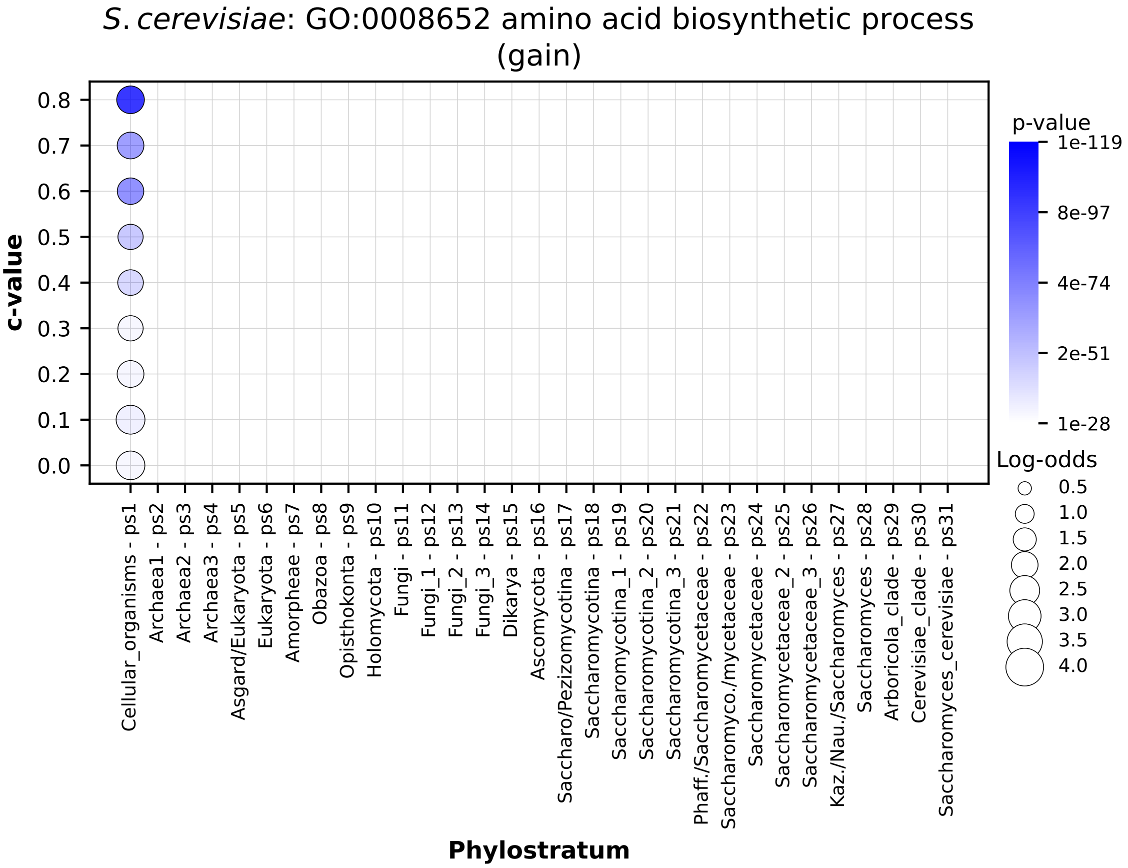
S. cerevisiae: GO:0008652 amino acid biosynthetic process
(gain)
c-value
0.8
0.7
0.6
0.5
0.4
0.3
0.2
0.1
0.0
p-value
1e-119
8e-97
4e-74
2e-51
1e-28
Log-odds
0.5
1.0
1.5
2.0
2.5
3.0
3.5
4.0
Cellular_organisms - ps1
Archaea1 - ps2
Archaea2 - ps3
Archaea3 - ps4
Asgard/Eukaryota - ps5
Eukaryota - ps6
Amorpheae - ps7
Obazoa - ps8
Opisthokonta - ps9
Holomycota - ps10
Fungi - ps11
Fungi_1 - ps12
Fungi_2 - ps13
Fungi_3 - ps14
Dikarya - ps15
Ascomycota - ps16
Saccharo/Pezizomycotina - ps17
Saccharomycotina - ps18
Saccharomycotina_1 - ps19
Saccharomycotina_2 - ps20
Saccharomycotina_3 - ps21
Phaff./Saccharomycetaceae - ps22
Saccharomyco./mycetaceae - ps23
Saccharomycetaceae - ps24
Saccharomycetaceae_2 - ps25
Saccharomycetaceae_3 - ps26
Kaz./Nau./Saccharomyces - ps27
Saccharomyces - ps28
Arboricola_clade - ps29
Cerevisiae_clade - ps30
Saccharomyces_cerevisiae - ps31
Phylostratum

S. cerevisiae: GO:0008653 lipopolysaccharide metabolic process (gain)

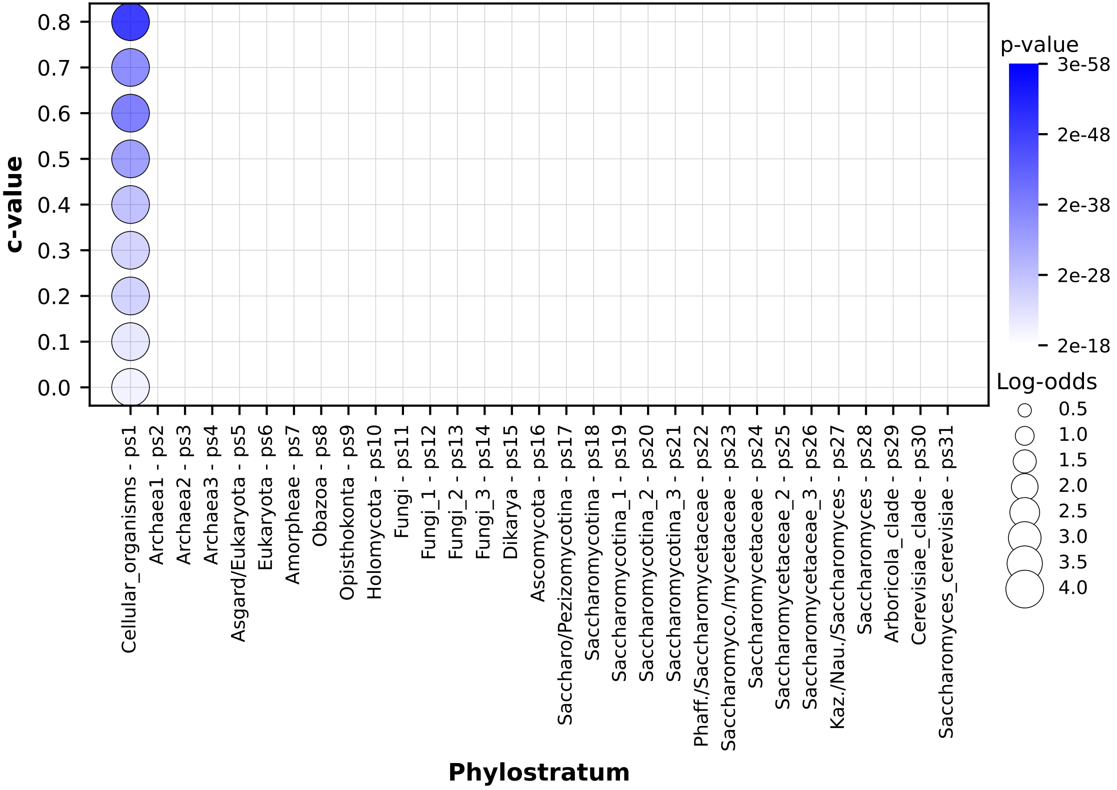

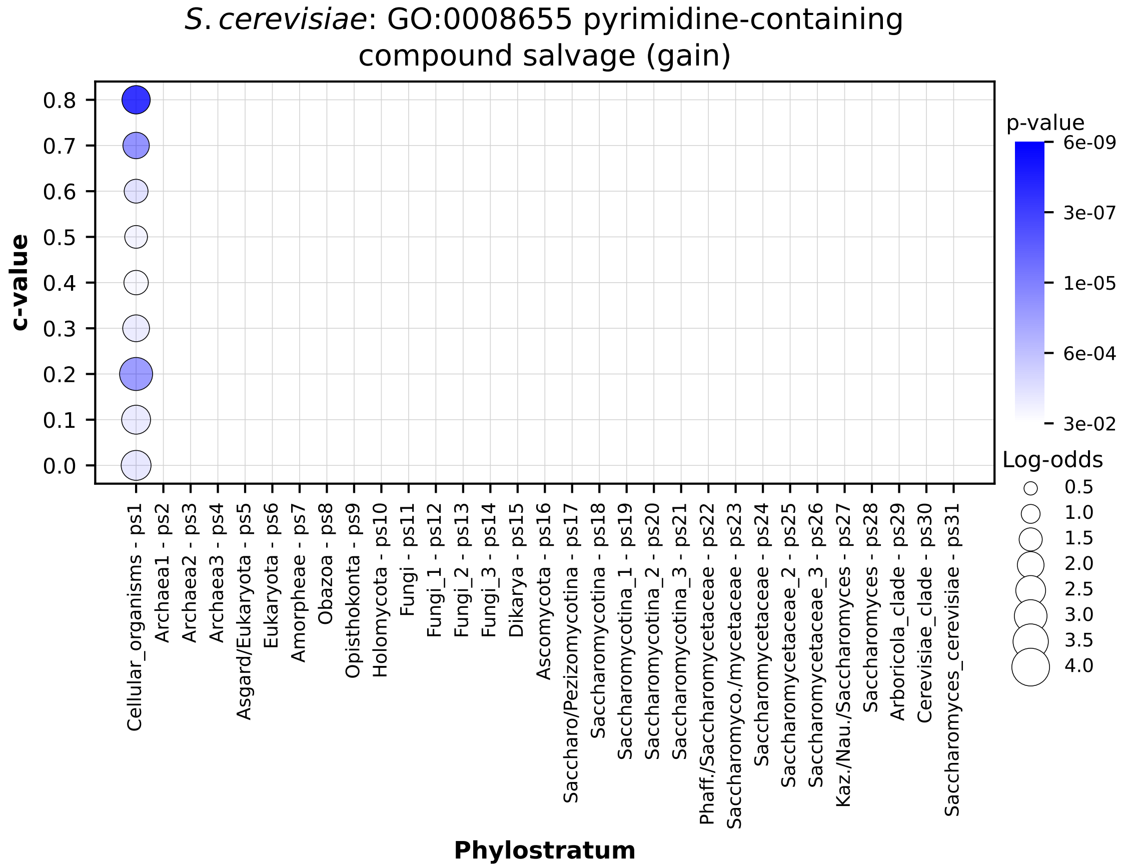

S. cerevisiae: GO:0008655 pyrimidine-containing compound salvage (gain)
c-value
0.8
0.7
0.6
0.5
0.4
0.3
0.2
0.1
0.0
p-value
6e-09
3e-07
1e-05
6e-04
3e-02
Log-odds
0.5
1.0
1.5
2.0
2.5
3.0
3.5
4.0
Cellular_organisms - ps1
Archaea1 - ps2
Archaea2 - ps3
Archaea3 - ps4
Asgard/Eukaryota - ps5
Eukaryota - ps6
Amorpheae - ps7
Obazoa - ps8
Opisthokonta - ps9
Holomycota - ps10
Fungi - ps11
Fungi_1 - ps12
Fungi_2 - ps13
Fungi_3 - ps14
Dikarya - ps15
Ascomycota - ps16
Saccharo/Pezizomycotina - ps17
Saccharomycotina - ps18
Saccharomycotina_1 - ps19
Saccharomycotina_2 - ps20
Saccharomycotina_3 - ps21
Phaff./Saccharomycetaceae - ps22
Saccharomyco./mycetaceae - ps23
Saccharomycetaceae - ps24
Saccharomycetaceae_2 - ps25
Saccharomycetaceae_3 - ps26
Kaz./Nau./Saccharomyces - ps27
Saccharomyces - ps28
Arboricola_clade - ps29
Cerevisiae_clade - ps30
Saccharomyces_cerevisiae - ps31
Phylostratum

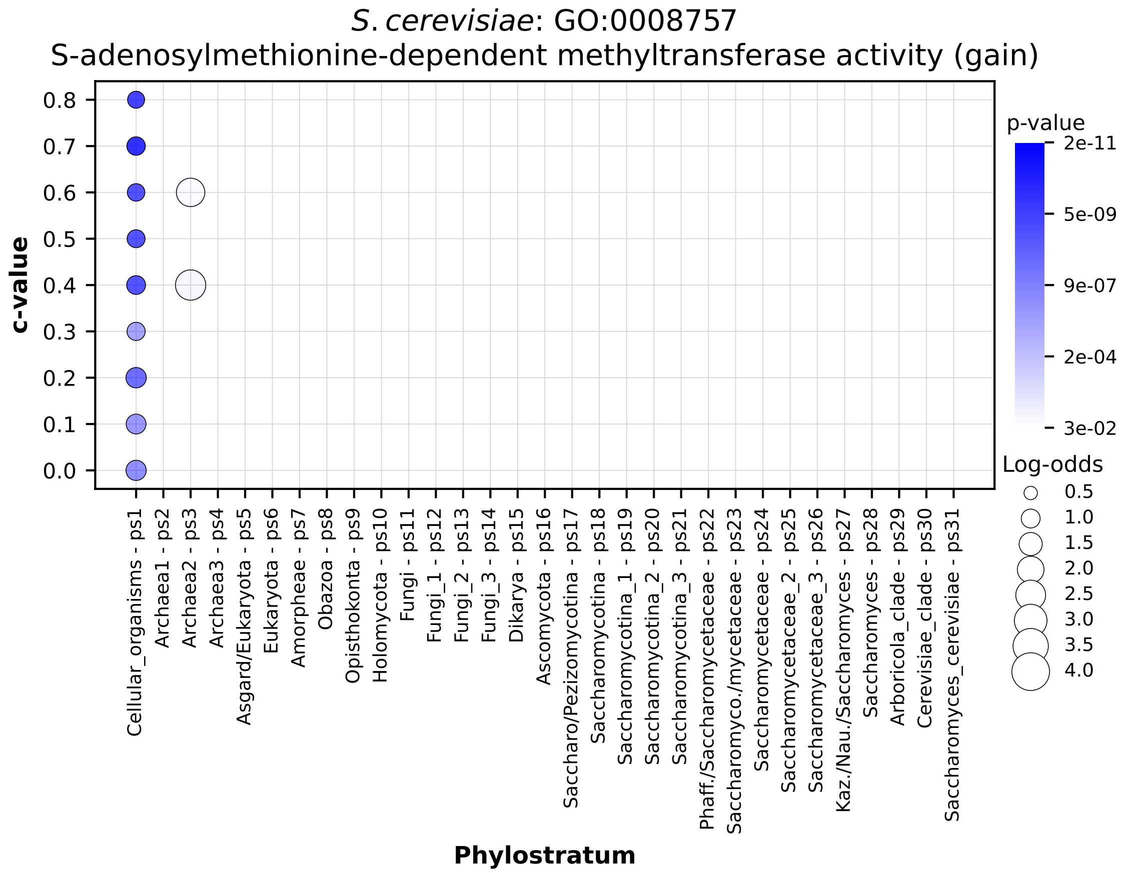

S. cerevisiae: GO:0008757
S-adenosylmethionine-dependent methyltransferase activity (gain)
c-value
0.8
0.7
0.6
0.5
0.4
0.3
0.2
0.1
0.0
p-value
2e-11
5e-09
9e-07
2e-04
3e-02
Log-odds
0.5
1.0
1.5
2.0
2.5
3.0
3.5
4.0
Cellular_organisms - ps1
Archaea1 - ps2
Archaea2 - ps3
Archaea3 - ps4
Asgard/Eukaryota - ps5
Eukaryota - ps6
Amorpheae - ps7
Obazoa - ps8
Opisthokonta - ps9
Holomycota - ps10
Fungi - ps11
Fungi_1 - ps12
Fungi_2 - ps13
Fungi_3 - ps14
Dikarya - ps15
Ascomycota - ps16
Saccharo/Pezizomycotina - ps17
Saccharomycotina - ps18
Saccharomycotina_1 - ps19
Saccharomycotina_2 - ps20
Saccharomycotina_3 - ps21
Phaff./Saccharomycetaceae - ps22
Saccharomyco./mycetaceae - ps23
Saccharomycetaceae - ps24
Saccharomycetaceae_2 - ps25
Saccharomycetaceae_3 - ps26
Kaz./Nau./Saccharomyces - ps27
Saccharomyces - ps28
Arboricola_clade - ps29
Cerevisiae_clade - ps30
Saccharomyces_cerevisiae - ps31
Phylostratum

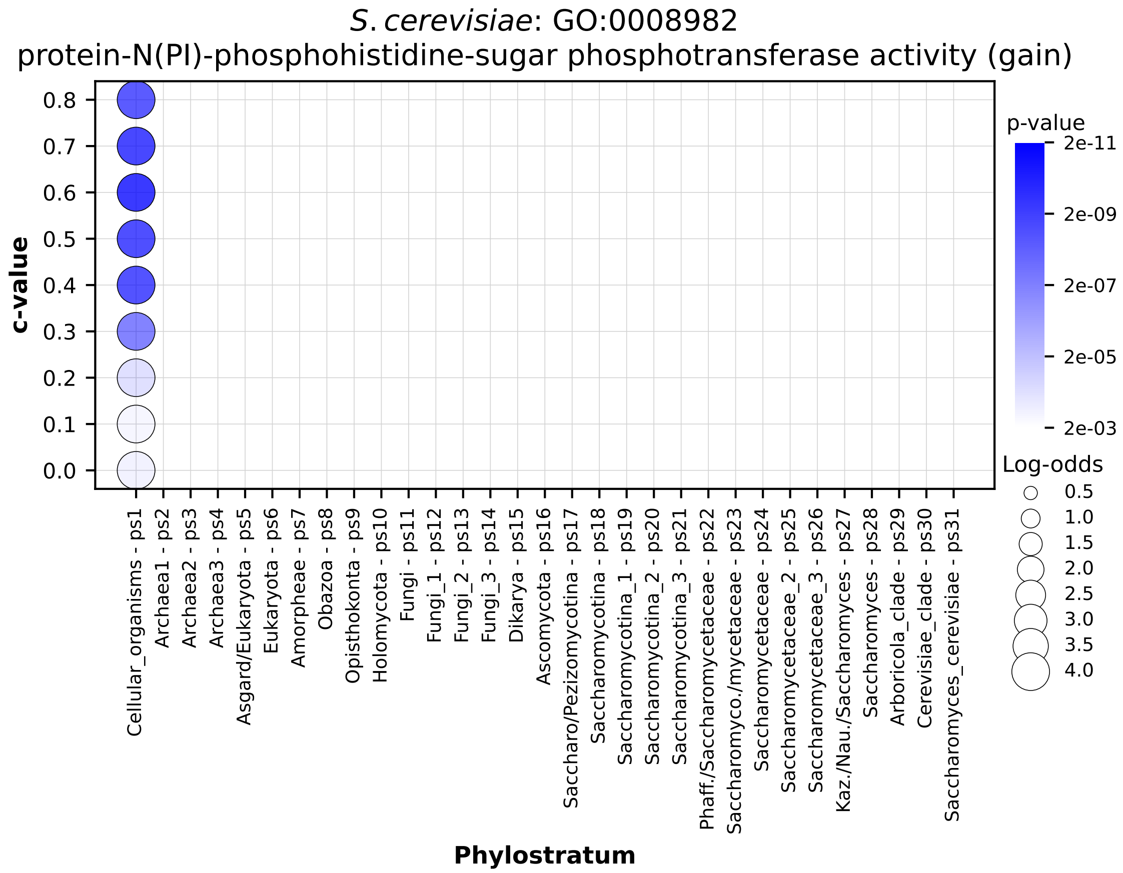

S. cerevisiae: GO:0008982
protein-N(PI)-phosphohistidine-sugar phosphotransferase activity (gain)
c-value
0.8
0.7
0.6
0.5
0.4
0.3
0.2
0.1
0.0
p-value
2e-11
2e-09
2e-07
2e-05
2e-03
Log-odds
0.5
1.0
1.5
2.0
2.5
3.0
3.5
4.0
Cellular_organisms - ps1
Archaea1 - ps2
Archaea2 - ps3
Archaea3 - ps4
Asgard/Eukaryota - ps5
Eukaryota - ps6
Amorpheae - ps7
Obazoa - ps8
Opisthokonta - ps9
Holomycota - ps10
Fungi - ps11
Fungi_1 - ps12
Fungi_2 - ps13
Fungi_3 - ps14
Dikarya - ps15
Ascomycota - ps16
Saccharo/Pezizomycotina - ps17
Saccharomycotina - ps18
Saccharomycotina_1 - ps19
Saccharomycotina_2 - ps20
Saccharomycotina_3 - ps21
Phaff./Saccharomycetaceae - ps22
Saccharomyco./mycetaceae - ps23
Saccharomycetaceae - ps24
Saccharomycetaceae_2 - ps25
Saccharomycetaceae_3 - ps26
Kaz./Nau./Saccharomyces - ps27
Saccharomyces - ps28
Arboricola_clade - ps29
Cerevisiae_clade - ps30
Saccharomyces_cerevisiae - ps31
Phylostratum

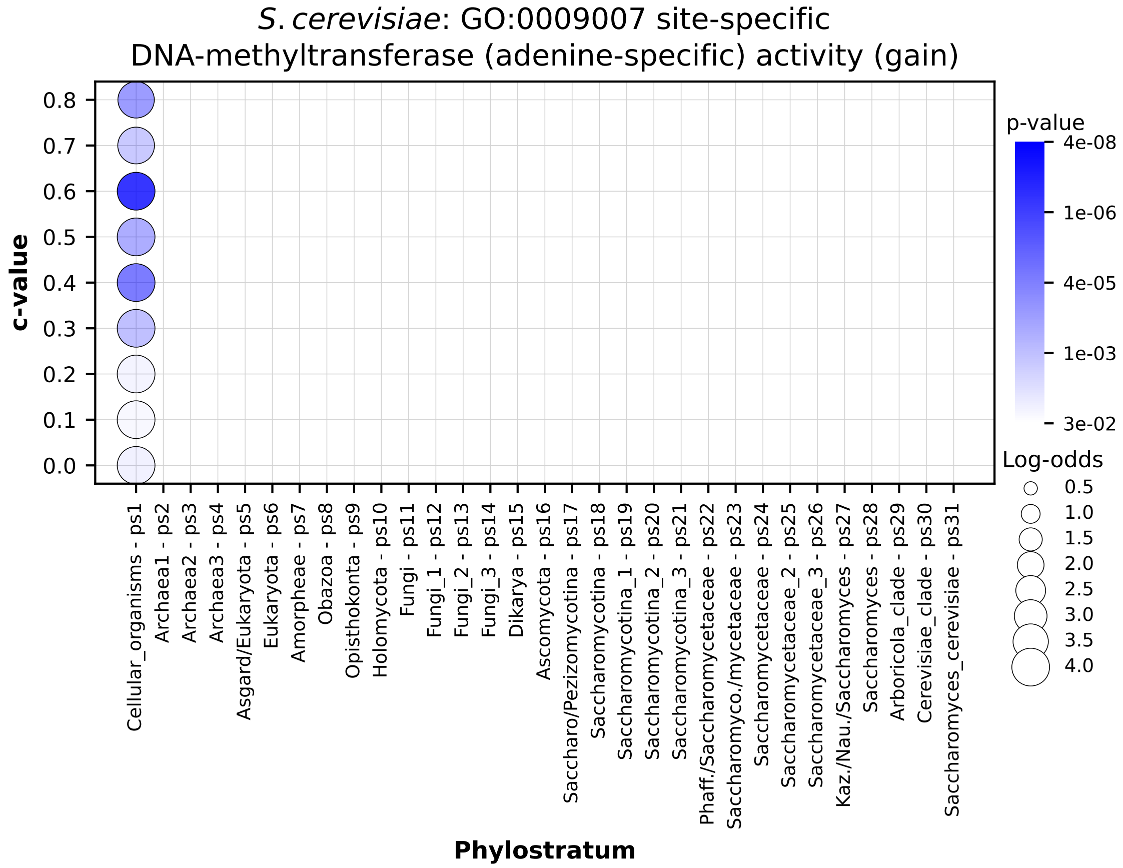
S. cerevisiae: GO:0009007 site-specific
DNA-methyltransferase (adenine-specific) activity (gain)
c-value
0.8
0.7
0.6
0.5
0.4
0.3
0.2
0.1
0.0
p-value
4e-08
1e-06
4e-05
1e-03
3e-02
Log-odds
0.5
1.0
1.5
2.0
2.5
3.0
3.5
4.0
Cellular_organisms - ps1
Archaea1 - ps2
Archaea2 - ps3
Archaea3 - ps4
Asgard/Eukaryota - ps5
Eukaryota - ps6
Amorpheae - ps7
Obazoa - ps8
Opisthokonta - ps9
Holomycota - ps10
Fungi - ps11
Fungi_1 - ps12
Fungi_2 - ps13
Fungi_3 - ps14
Dikarya - ps15
Ascomycota - ps16
Saccharo/Pezizomycotina - ps17
Saccharomycotina - ps18
Saccharomycotina_1 - ps19
Saccharomycotina_2 - ps20
Saccharomycotina_3 - ps21
Phaff./Saccharomycetaceae - ps22
Saccharomyco./mycetaceae - ps23
Saccharomycetaceae - ps24
Saccharomycetaceae_2 - ps25
Saccharomycetaceae_3 - ps26
Kaz./Nau./Saccharomyces - ps27
Saccharomyces - ps28
Arboricola_clade - ps29
Cerevisiae_clade - ps30
Saccharomyces_cerevisiae - ps31
Phylostratum

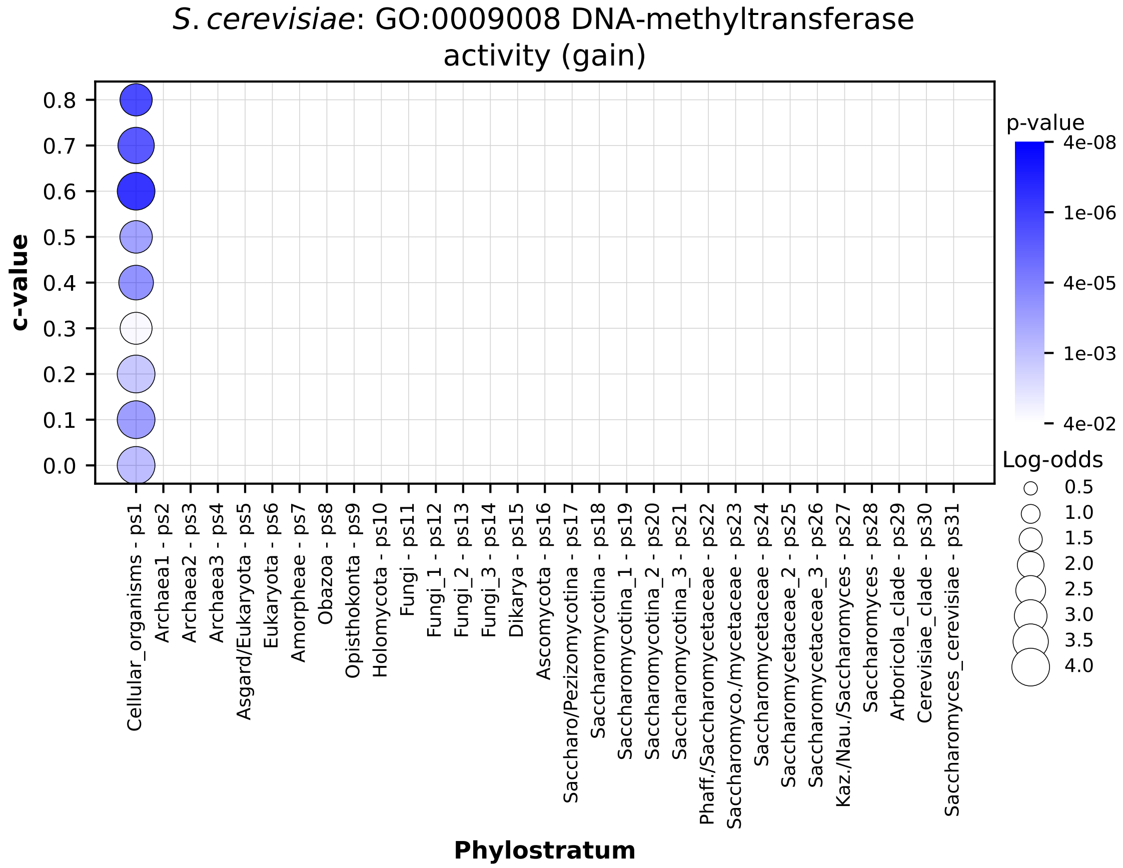

S. cerevisiae: GO:0009008 DNA-methyltransferase activity (gain)
c-value
0.0
0.1
0.2
0.3
0.4
0.5
0.6
0.7
0.8
p-value
4e-08
1e-06
4e-05
1e-03
4e-02
Log-odds
0.5
1.0
1.5
2.0
2.5
3.0
3.5
4.0
Cellular_organisms - ps1
Archaea1 - ps2
Archaea2 - ps3
Archaea3 - ps4
Asgard/Eukaryota - ps5
Eukaryota - ps6
Amorpheae - ps7
Obazoa - ps8
Opisthokonta - ps9
Holomycota - ps10
Fungi - ps11
Fungi_1 - ps12
Fungi_2 - ps13
Fungi_3 - ps14
Dikarya - ps15
Ascomycota - ps16
Saccharo/Pezizomycotina - ps17
Saccharomycotina - ps18
Saccharomycotina_1 - ps19
Saccharomycotina_2 - ps20
Saccharomycotina_3 - ps21
Phaff./Saccharomycetaceae - ps22
Saccharomyco./mycetaceae - ps23
Saccharomycetaceae - ps24
Saccharomycetaceae_2 - ps25
Saccharomycetaceae_3 - ps26
Kaz./Nau./Saccharomyces - ps27
Saccharomyces - ps28
Arboricola_clade - ps29
Cerevisiae_clade - ps30
Saccharomyces_cerevisiae - ps31
Phylostratum

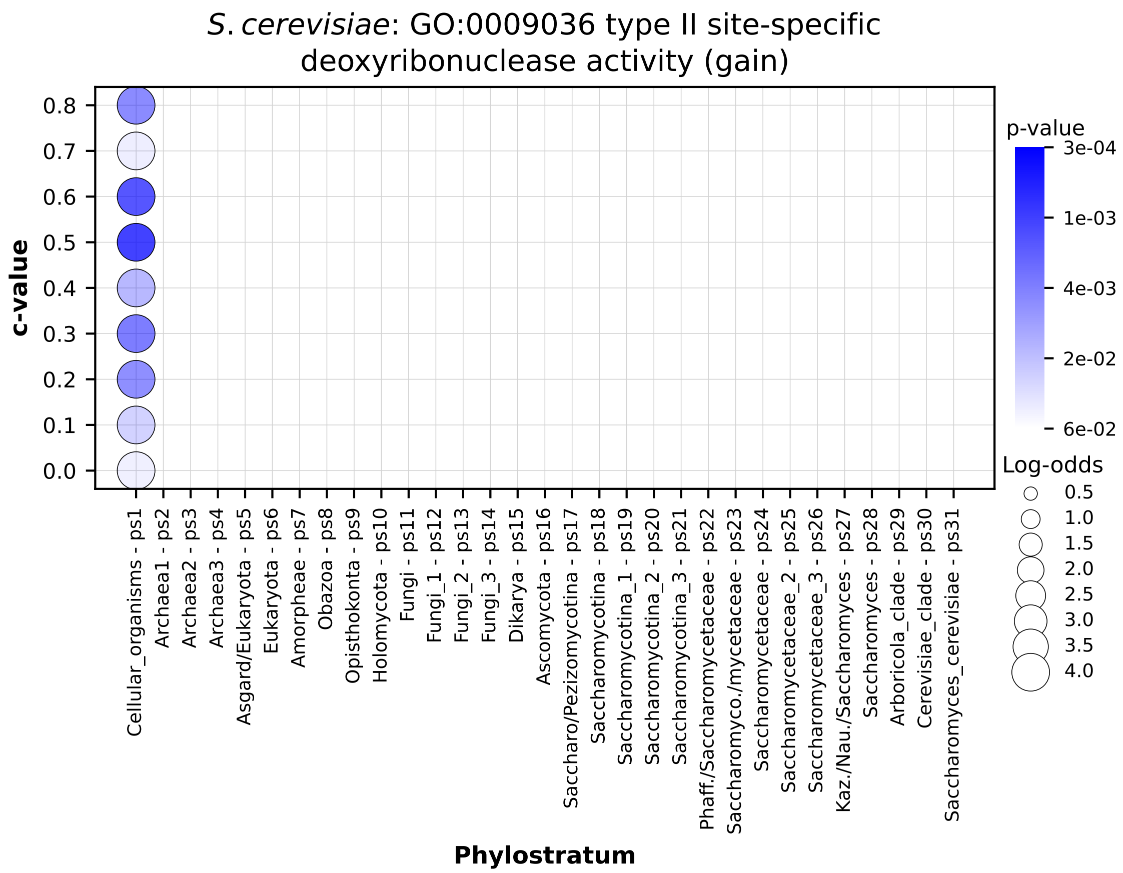

S. cerevisiae: GO:0009036 type II site-specific deoxyribonuclease activity (gain)
c-value
0.8
0.7
0.6
0.5
0.4
0.3
0.2
0.1
0.0
p-value
3e-04
1e-03
4e-03
2e-02
6e-02
Log-odds
0.5
1.0
1.5
2.0
2.5
3.0
3.5
4.0
Cellular_organisms - ps1
Archaea1 - ps2
Archaea2 - ps3
Archaea3 - ps4
Asgard/Eukaryota - ps5
Eukaryota - ps6
Amorpheae - ps7
Obazoa - ps8
Opisthokonta - ps9
Holomycota - ps10
Fungi - ps11
Fungi_1 - ps12
Fungi_2 - ps13
Fungi_3 - ps14
Dikarya - ps15
Ascomycota - ps16
Saccharo/Pezizomycotina - ps17
Saccharomycotina - ps18
Saccharomycotina_1 - ps19
Saccharomycotina_2 - ps20
Saccharomycotina_3 - ps21
Phaff./Saccharomycetaceae - ps22
Saccharomyco./mycetaceae - ps23
Saccharomycetaceae - ps24
Saccharomycetaceae_2 - ps25
Saccharomycetaceae_3 - ps26
Kaz./Nau./Saccharomyces - ps27
Saccharomyces - ps28
Arboricola_clade - ps29
Cerevisiae_clade - ps30
Saccharomyces_cerevisiae - ps31
Phylostratum

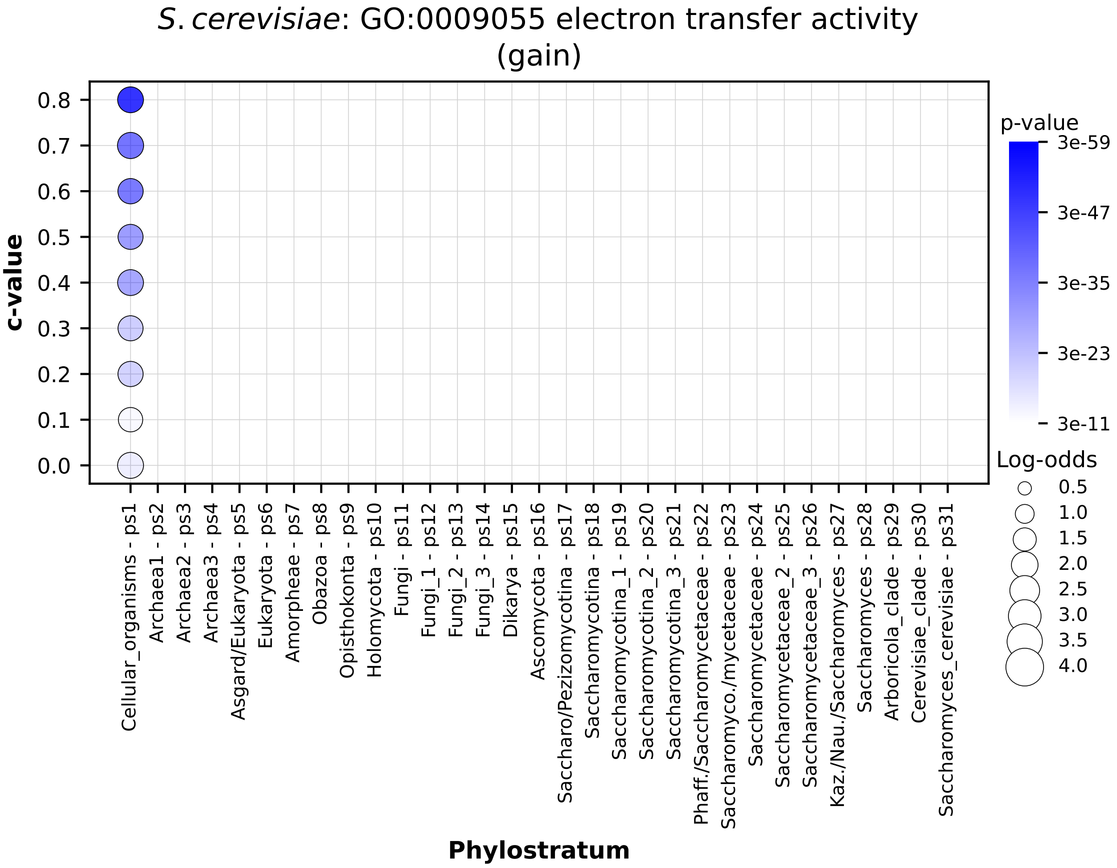

S. cerevisiae: GO:0009055 electron transfer activity (gain)
c-value
0.0
0.1
0.2
0.3
0.4
0.5
0.6
0.7
0.8
p-value
3e-59
3e-47
3e-35
3e-23
3e-11
Log-odds
0.5
1.0
1.5
2.0
2.5
3.0
3.5
4.0
Cellular_organisms - ps1
Archaea1 - ps2
Archaea2 - ps3
Archaea3 - ps4
Asgard/Eukaryota - ps5
Eukaryota - ps6
Amorpheae - ps7
Obazoa - ps8
Opisthokonta - ps9
Holomycota - ps10
Fungi - ps11
Fungi_1 - ps12
Fungi_2 - ps13
Fungi_3 - ps14
Dikarya - ps15
Ascomycota - ps16
Saccharo/Pezizomycotina - ps17
Saccharomycotina - ps18
Saccharomycotina_1 - ps19
Saccharomycotina_2 - ps20
Saccharomycotina_3 - ps21
Phaff./Saccharomycetaceae - ps22
Saccharomyco./mycetaceae - ps23
Saccharomycetaceae - ps24
Saccharomycetaceae_2 - ps25
Saccharomycetaceae_3 - ps26
Kaz./Nau./Saccharomyces - ps27
Saccharomyces - ps28
Arboricola_clade - ps29
Cerevisiae_clade - ps30
Saccharomyces_cerevisiae - ps31
Phylostratum

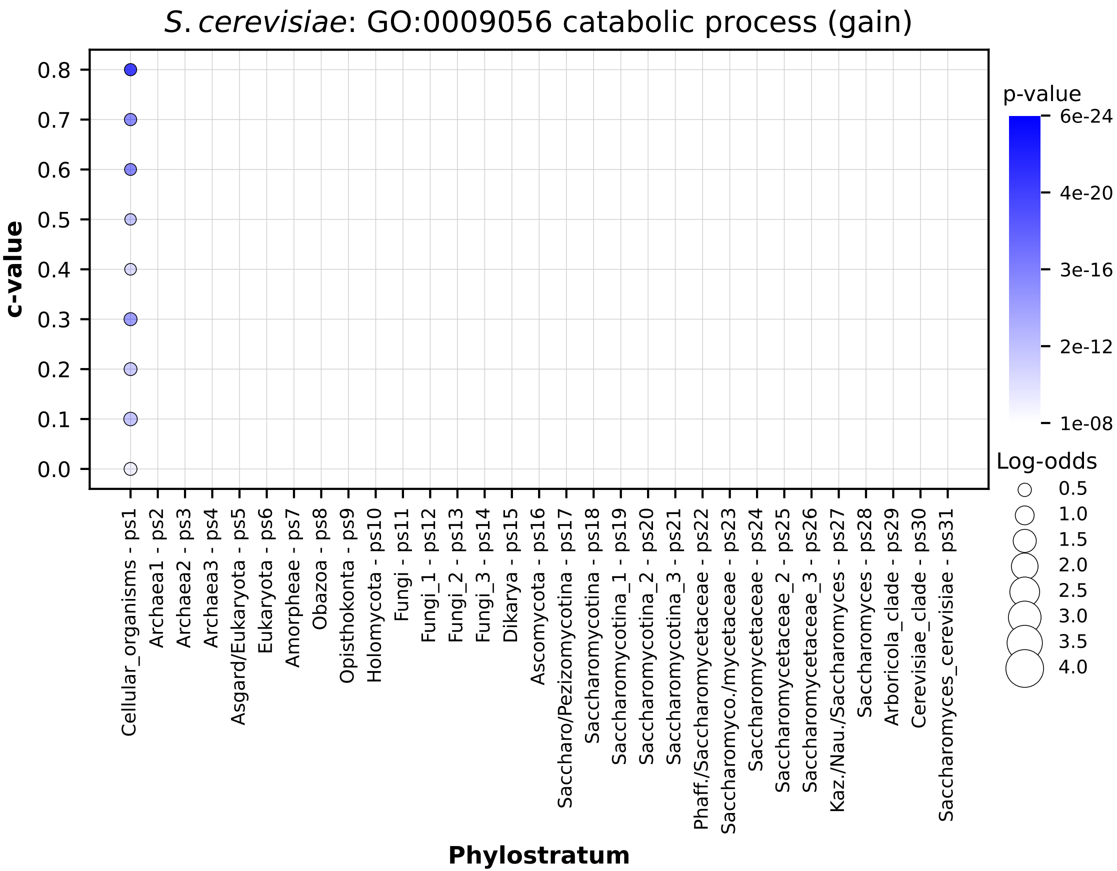

S. cerevisiae: GO:0009056 catabolic process (gain)
c-value
0.0
0.1
0.2
0.3
0.4
0.5
0.6
0.7
0.8
Phylostratum
Cellular_organisms - ps1
Archaea1 - ps2
Archaea2 - ps3
Archaea3 - ps4
Asgard/Eukaryota - ps5
Eukaryota - ps6
Amorpheae - ps7
Obazoa - ps8
Opisthokonta - ps9
Holomycota - ps10
Fungi - ps11
Fungi_1 - ps12
Fungi_2 - ps13
Fungi_3 - ps14
Dikarya - ps15
Ascomycota - ps16
Saccharo/Pezizomycotina - ps17
Saccharomycotina - ps18
Saccharomycotina_1 - ps19
Saccharomycotina_2 - ps20
Saccharomycotina_3 - ps21
Phaff./Saccharomycetaceae - ps22
Saccharomyco./mycetaceae - ps23
Saccharomycetaceae - ps24
Saccharomycetaceae_2 - ps25
Saccharomycetaceae_3 - ps26
Kaz./Nau./Saccharomyces - ps27
Saccharomyces - ps28
Arboricola_clade - ps29
Cerevisiae_clade - ps30
Saccharomyces_cerevisiae - ps31
p-value
6e-24
4e-20
3e-16
2e-12
1e-08
Log-odds
0.5
1.0
1.5
2.0
2.5
3.0
3.5
4.0

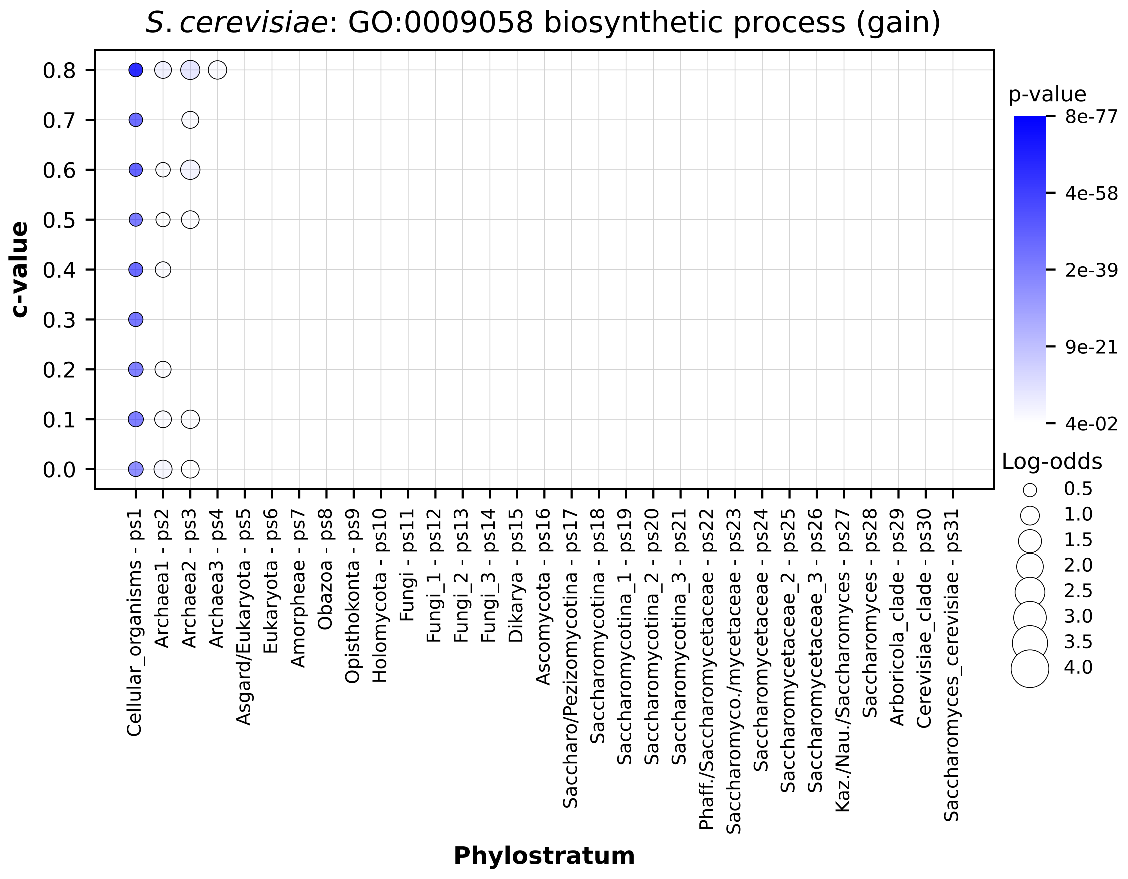

S. cerevisiae: GO:0009058 biosynthetic process (gain)
c-value
0.8
0.7
0.6
0.5
0.4
0.3
0.2
0.1
0.0
p-value
8e-77
4e-58
2e-39
9e-21
4e-02
Log-odds
0.5
1.0
1.5
2.0
2.5
3.0
3.5
4.0
Cellular_organisms - ps1
Archaea1 - ps2
Archaea2 - ps3
Archaea3 - ps4
Asgard/Eukaryota - ps5
Eukaryota - ps6
Amorpheae - ps7
Obazoa - ps8
Opisthokonta - ps9
Holomycota - ps10
Fungi - ps11
Fungi_1 - ps12
Fungi_2 - ps13
Fungi_3 - ps14
Dikarya - ps15
Ascomycota - ps16
Saccharo/Pezizomycotina - ps17
Saccharomycotina - ps18
Saccharomycotina_1 - ps19
Saccharomycotina_2 - ps20
Saccharomycotina_3 - ps21
Phaff./Saccharomycetaceae - ps22
Saccharomyco./mycetaceae - ps23
Saccharomycetaceae - ps24
Saccharomycetaceae_2 - ps25
Saccharomycetaceae_3 - ps26
Kaz./Nau./Saccharomyces - ps27
Saccharomyces - ps28
Arboricola_clade - ps29
Cerevisiae_clade - ps30
Saccharomyces_cerevisiae - ps31
Phylostratum

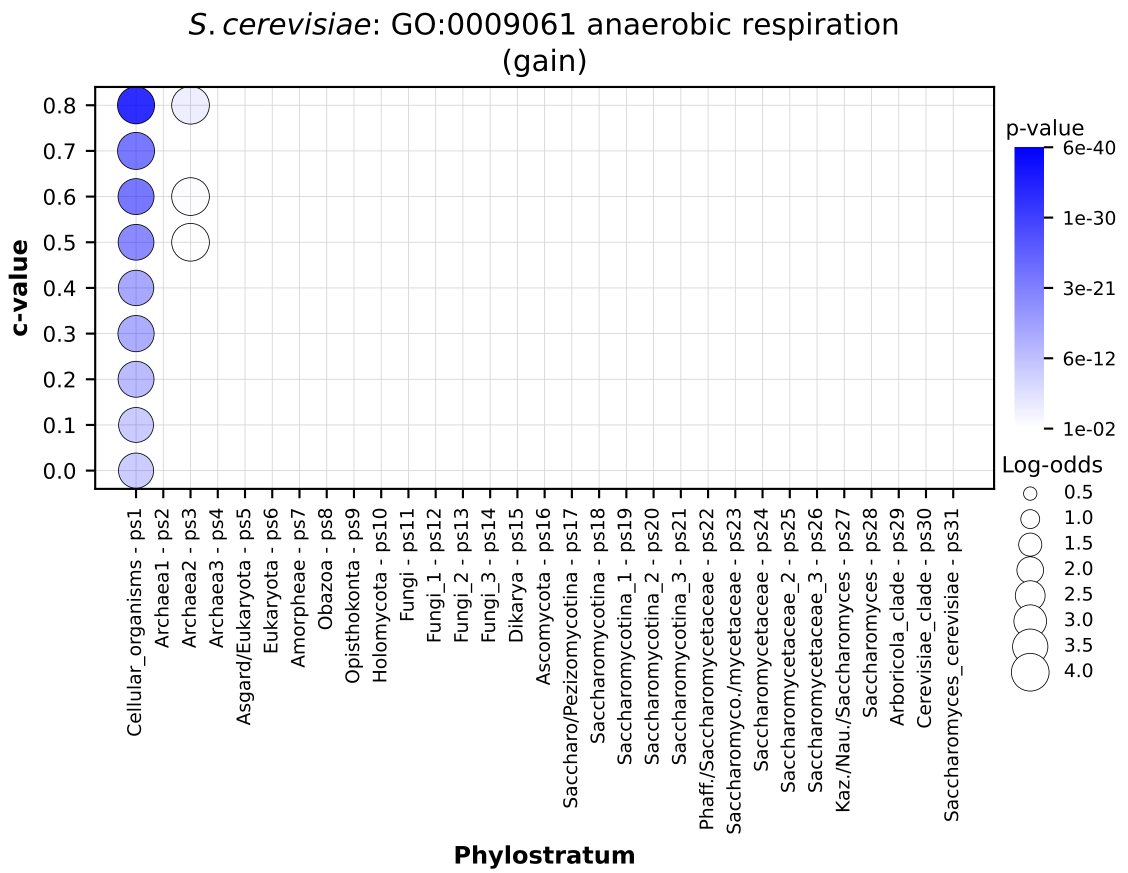
S. cerevisiae: GO:0009061 anaerobic respiration
(gain)
c-value
0.0
0.1
0.2
0.3
0.4
0.5
0.6
0.7
0.8
p-value
6e-40
1e-30
3e-21
6e-12
1e-02
Log-odds
0.5
1.0
1.5
2.0
2.5
3.0
3.5
4.0
Cellular_organisms - ps1
Archaea1 - ps2
Archaea2 - ps3
Archaea3 - ps4
Asgard/Eukaryota - ps5
Eukaryota - ps6
Amorpheae - ps7
Obazoa - ps8
Opisthokonta - ps9
Holomycota - ps10
Fungi - ps11
Fungi_1 - ps12
Fungi_2 - ps13
Fungi_3 - ps14
Dikarya - ps15
Ascomycota - ps16
Saccharo/Pezizomycotina - ps17
Saccharomycotina - ps18
Saccharomycotina_1 - ps19
Saccharomycotina_2 - ps20
Saccharomycotina_3 - ps21
Phaff./Saccharomycetaceae - ps22
Saccharomyco./mycetaceae - ps23
Saccharomycetaceae - ps24
Saccharomycetaceae_2 - ps25
Saccharomycetaceae_3 - ps26
Kaz./Nau./Saccharomyces - ps27
Saccharomyces - ps28
Arboricola_clade - ps29
Cerevisiae_clade - ps30
Saccharomyces_cerevisiae - ps31
Phylostratum

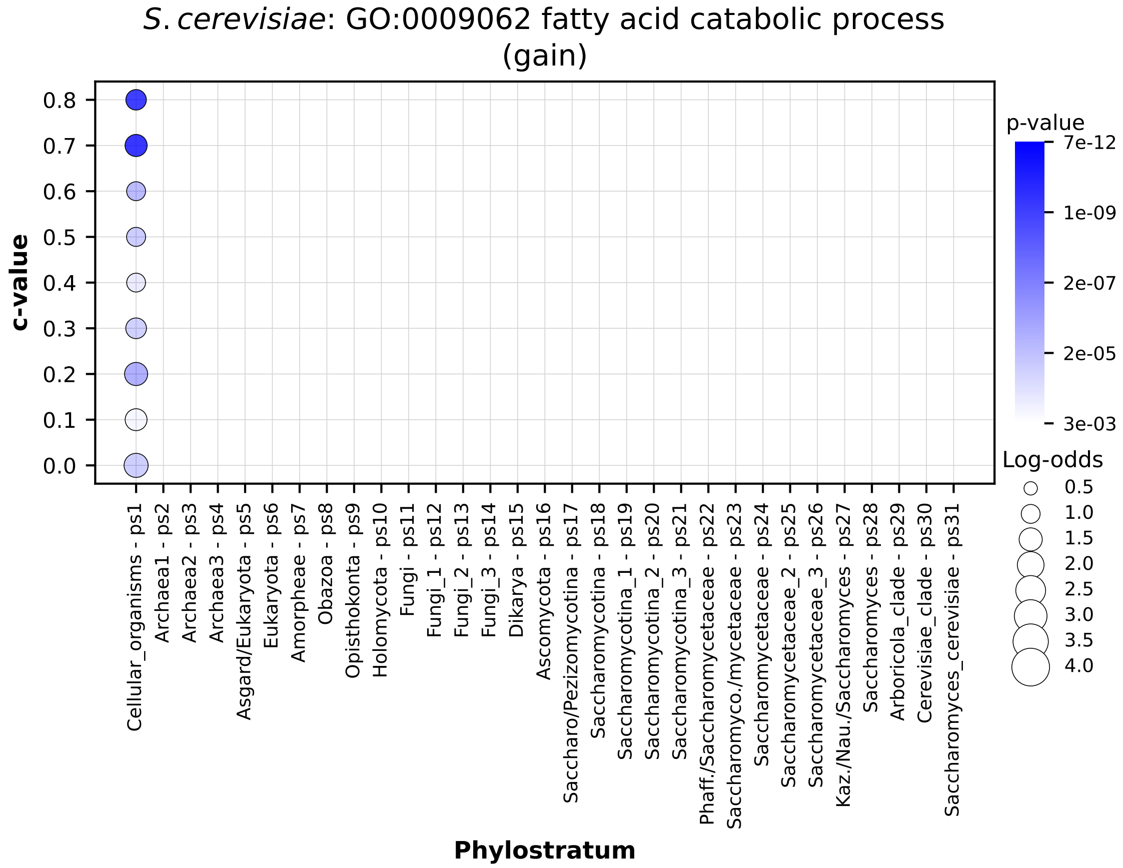
S. cerevisiae: GO:0009062 fatty acid catabolic process
(gain)
c-value
0.8
0.7
0.6
0.5
0.4
0.3
0.2
0.1
0.0
p-value
7e-12
1e-09
2e-07
2e-05
3e-03
Log-odds
0.5
1.0
1.5
2.0
2.5
3.0
3.5
4.0
Cellular_organisms - ps1
Archaea1 - ps2
Archaea2 - ps3
Archaea3 - ps4
Asgard/Eukaryota - ps5
Eukaryota - ps6
Amorpheae - ps7
Obazoa - ps8
Opisthokonta - ps9
Holomycota - ps10
Fungi - ps11
Fungi_1 - ps12
Fungi_2 - ps13
Fungi_3 - ps14
Dikarya - ps15
Ascomycota - ps16
Saccharo/Pezizomycotina - ps17
Saccharomycotina - ps18
Saccharomycotina_1 - ps19
Saccharomycotina_2 - ps20
Saccharomycotina_3 - ps21
Phaff./Saccharomycetaceae - ps22
Saccharomyco./mycetaceae - ps23
Saccharomycetaceae - ps24
Saccharomycetaceae_2 - ps25
Saccharomycetaceae_3 - ps26
Kaz./Nau./Saccharomyces - ps27
Saccharomyces - ps28
Arboricola_clade - ps29
Cerevisiae_clade - ps30
Saccharomyces_cerevisiae - ps31
Phylostratum

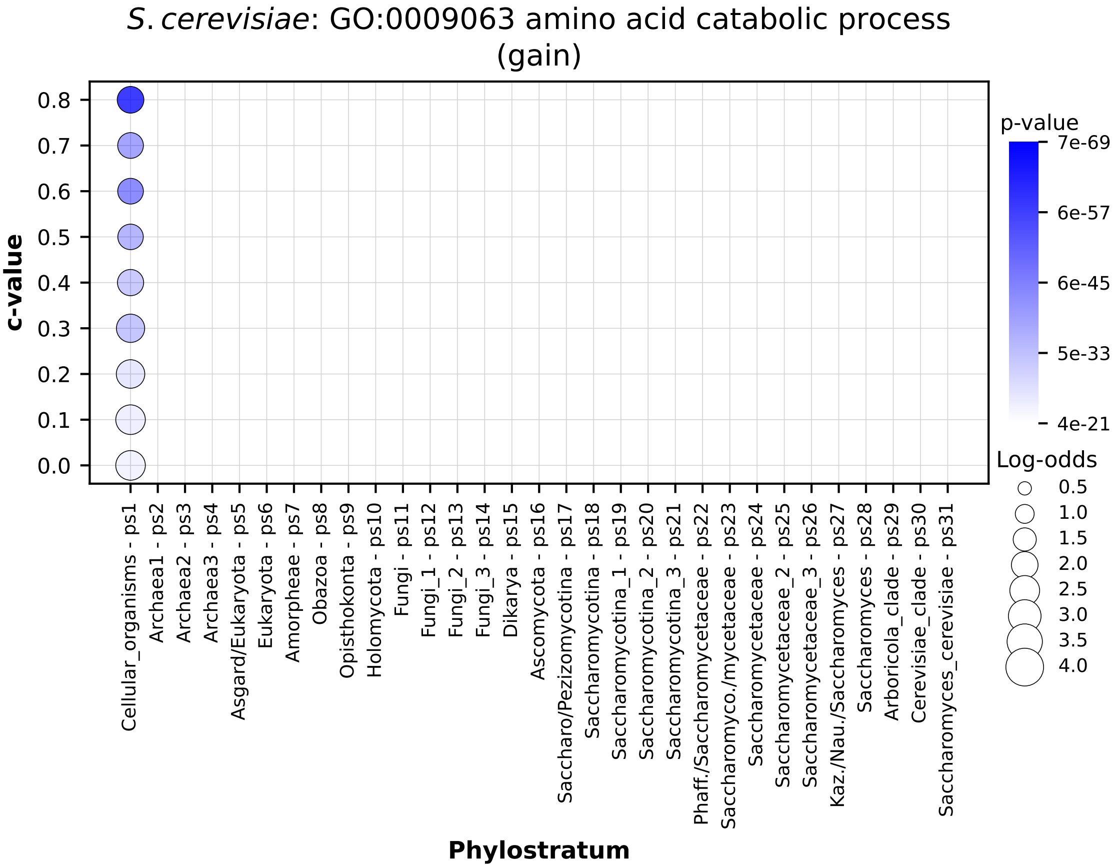

S. cerevisiae: GO:0009063 amino acid catabolic process
(gain)
c-value
0.8
0.7
0.6
0.5
0.4
0.3
0.2
0.1
0.0
p-value
7e-69
6e-57
6e-45
5e-33
4e-21
Log-odds
0.5
1.0
1.5
2.0
2.5
3.0
3.5
4.0
Cellular_organisms - ps1
Archaea1 - ps2
Archaea2 - ps3
Archaea3 - ps4
Asgard/Eukaryota - ps5
Eukaryota - ps6
Amorpheae - ps7
Obazoa - ps8
Opisthokonta - ps9
Holomycota - ps10
Fungi - ps11
Fungi_1 - ps12
Fungi_2 - ps13
Fungi_3 - ps14
Dikarya - ps15
Ascomycota - ps16
Saccharo/Pezizomycotina - ps17
Saccharomycotina - ps18
Saccharomycotina_1 - ps19
Saccharomycotina_2 - ps20
Saccharomycotina_3 - ps21
Phaff./Saccharomycetaceae - ps22
Saccharomyco./mycetaceae - ps23
Saccharomycetaceae - ps24
Saccharomycetaceae_2 - ps25
Saccharomycetaceae_3 - ps26
Kaz./Nau./Saccharomyces - ps27
Saccharomyces - ps28
Arboricola_clade - ps29
Cerevisiae_clade - ps30
Saccharomyces_cerevisiae - ps31
Phylostratum

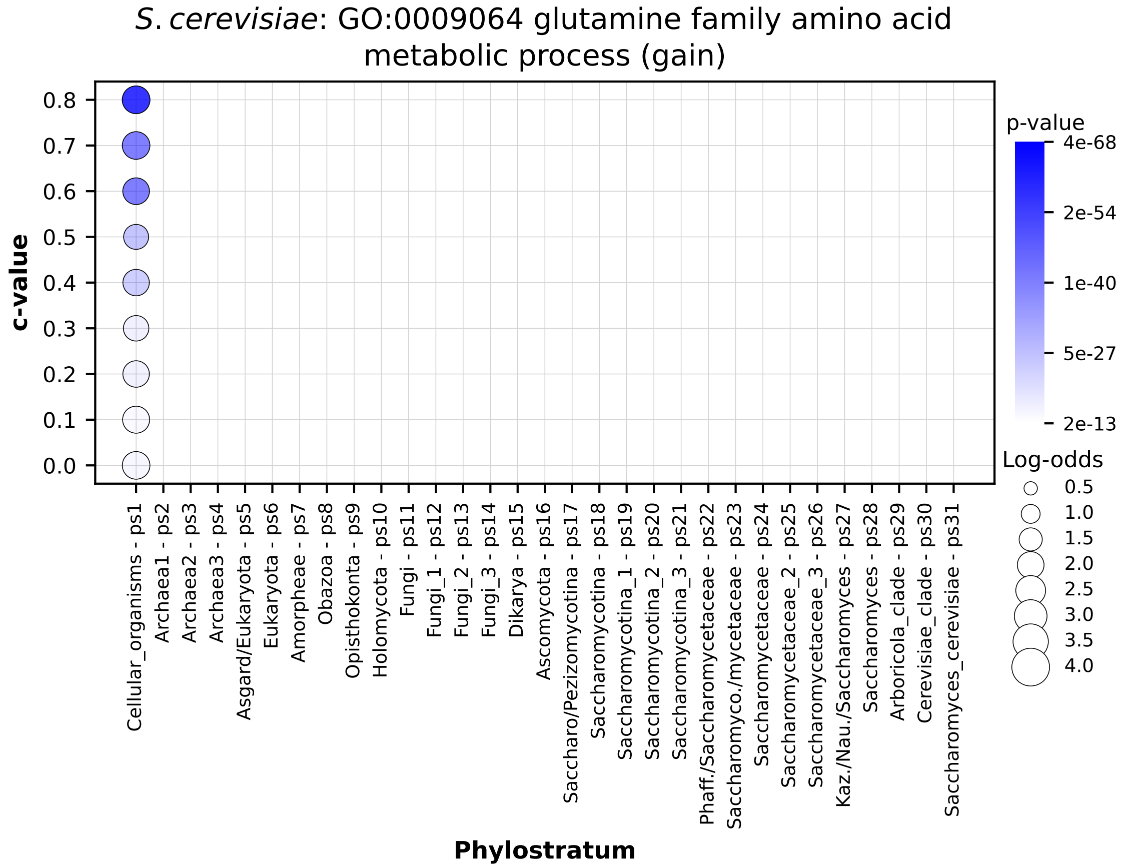

S. cerevisiae: GO:0009064 glutamine family amino acid metabolic process (gain)
c-value
0.0
0.1
0.2
0.3
0.4
0.5
0.6
0.7
0.8
p-value
4e-68
2e-54
1e-40
5e-27
2e-13
Log-odds
0.5
1.0
1.5
2.0
2.5
3.0
3.5
4.0
Cellular_organisms - ps1
Archaea1 - ps2
Archaea2 - ps3
Archaea3 - ps4
Asgard/Eukaryota - ps5
Eukaryota - ps6
Amorpheae - ps7
Obazoa - ps8
Opisthokonta - ps9
Holomycota - ps10
Fungi - ps11
Fungi_1 - ps12
Fungi_2 - ps13
Fungi_3 - ps14
Dikarya - ps15
Ascomycota - ps16
Saccharo/Pezizomycotina - ps17
Saccharomycotina - ps18
Saccharomycotina_1 - ps19
Saccharomycotina_2 - ps20
Saccharomycotina_3 - ps21
Phaff./Saccharomycetaceae - ps22
Saccharomyco./mycetaceae - ps23
Saccharomycetaceae - ps24
Saccharomycetaceae_2 - ps25
Saccharomycetaceae_3 - ps26
Kaz./Nau./Saccharomyces - ps27
Saccharomyces - ps28
Arboricola_clade - ps29
Cerevisiae_clade - ps30
Saccharomyces_cerevisiae - ps31
Phylostratum

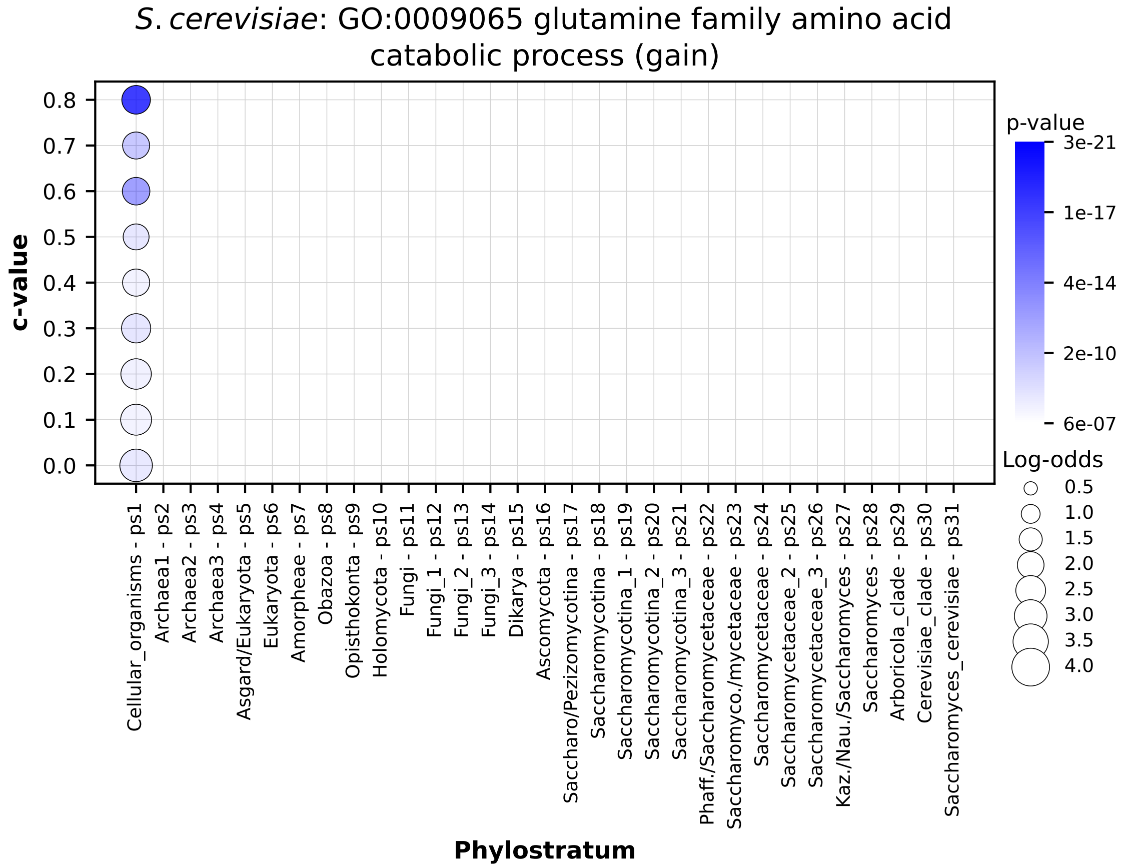

S. cerevisiae: GO:0009065 glutamine family amino acid catabolic process (gain)
c-value
0.8
0.7
0.6
0.5
0.4
0.3
0.2
0.1
0.0
p-value
3e-21
1e-17
4e-14
2e-10
6e-07
Log-odds
0.5
1.0
1.5
2.0
2.5
3.0
3.5
4.0
Cellular_organisms - ps1
Archaea1 - ps2
Archaea2 - ps3
Archaea3 - ps4
Asgard/Eukaryota - ps5
Eukaryota - ps6
Amorpheae - ps7
Obazoa - ps8
Opisthokonta - ps9
Holomycota - ps10
Fungi - ps11
Fungi_1 - ps12
Fungi_2 - ps13
Fungi_3 - ps14
Dikarya - ps15
Ascomycota - ps16
Saccharo/Pezizomycotina - ps17
Saccharomycotina - ps18
Saccharomycotina_1 - ps19
Saccharomycotina_2 - ps20
Saccharomycotina_3 - ps21
Phaff./Saccharomycetaceae - ps22
Saccharomyco./mycetaceae - ps23
Saccharomycetaceae - ps24
Saccharomycetaceae_2 - ps25
Saccharomycetaceae_3 - ps26
Kaz./Nau./Saccharomyces - ps27
Saccharomyces - ps28
Arboricola_clade - ps29
Cerevisiae_clade - ps30
Saccharomyces_cerevisiae - ps31
Phylostratum

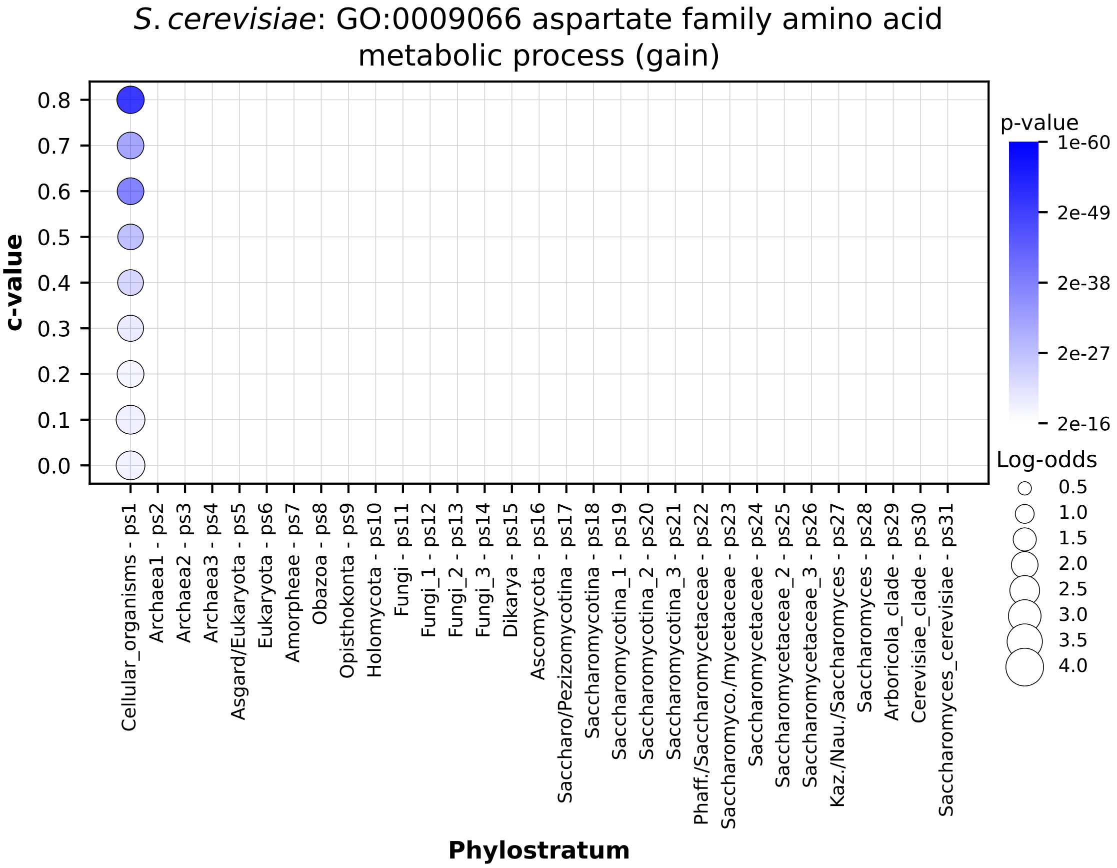
S. cerevisiae: GO:0009066 aspartate family amino acid metabolic process (gain)
c-value
0.8
0.7
0.6
0.5
0.4
0.3
0.2
0.1
0.0
p-value
1e-60
2e-49
2e-38
2e-27
2e-16
Log-odds
0.5
1.0
1.5
2.0
2.5
3.0
3.5
4.0
Cellular_organisms - ps1
Archaea1 - ps2
Archaea2 - ps3
Archaea3 - ps4
Asgard/Eukaryota - ps5
Eukaryota - ps6
Amorpheae - ps7
Obazoa - ps8
Opisthokonta - ps9
Holomycota - ps10
Fungi - ps11
Fungi_1 - ps12
Fungi_2 - ps13
Fungi_3 - ps14
Dikarya - ps15
Ascomycota - ps16
Saccharo/Pezizomycotina - ps17
Saccharomycotina - ps18
Saccharomycotina_1 - ps19
Saccharomycotina_2 - ps20
Saccharomycotina_3 - ps21
Phaff./Saccharomycetaceae - ps22
Saccharomyco./mycetaceae - ps23
Saccharomycetaceae - ps24
Saccharomycetaceae_2 - ps25
Saccharomycetaceae_3 - ps26
Kaz./Nau./Saccharomyces - ps27
Saccharomyces - ps28
Arboricola_clade - ps29
Cerevisiae_clade - ps30
Saccharomyces_cerevisiae - ps31
Phylostratum

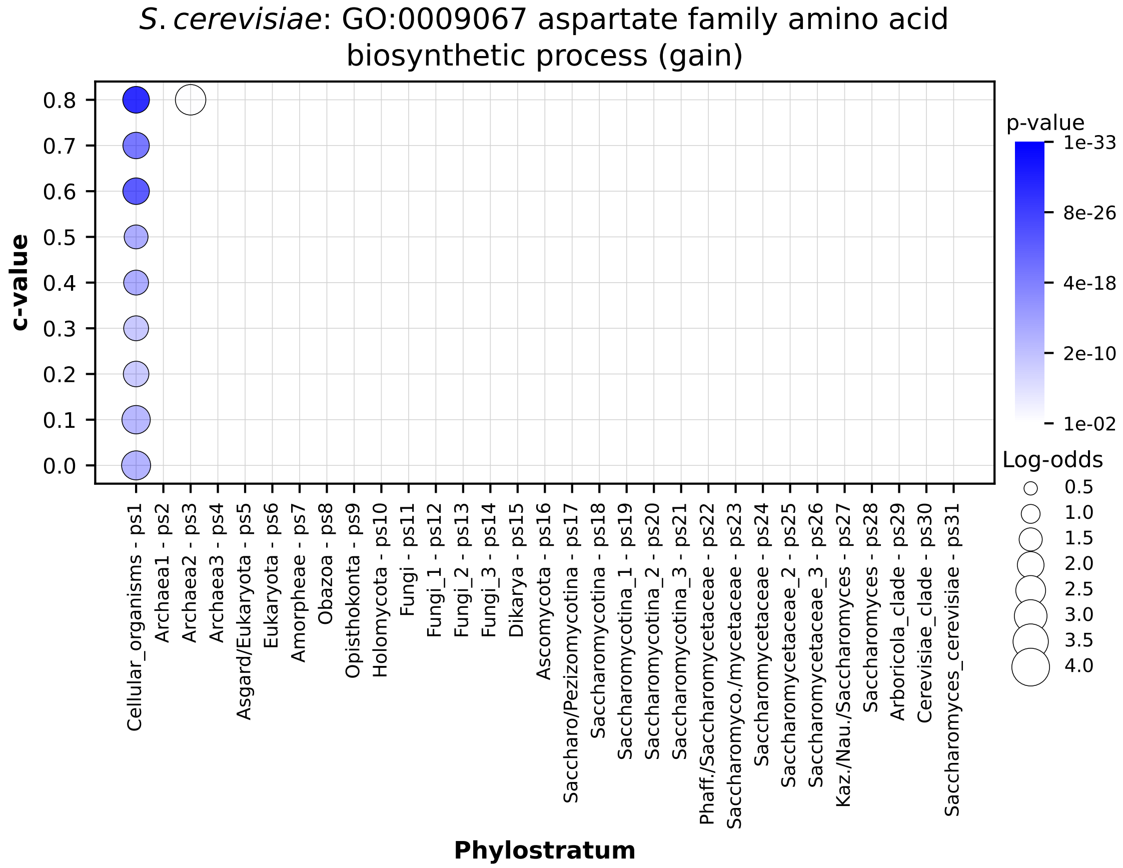

S. cerevisiae: GO:0009067 aspartate family amino acid biosynthetic process (gain)
c-value
0.8
0.7
0.6
0.5
0.4
0.3
0.2
0.1
0.0
p-value
1e-33
8e-26
4e-18
2e-10
1e-02
Log-odds
0.5
1.0
1.5
2.0
2.5
3.0
3.5
4.0
Cellular_organisms - ps1
Archaea1 - ps2
Archaea2 - ps3
Archaea3 - ps4
Asgard/Eukaryota - ps5
Eukaryota - ps6
Amorpheae - ps7
Obazoa - ps8
Opisthokonta - ps9
Holomycota - ps10
Fungi - ps11
Fungi_1 - ps12
Fungi_2 - ps13
Fungi_3 - ps14
Dikarya - ps15
Ascomycota - ps16
Saccharo/Pezizomycotina - ps17
Saccharomycotina - ps18
Saccharomycotina_1 - ps19
Saccharomycotina_2 - ps20
Saccharomycotina_3 - ps21
Phaff./Saccharomycetaceae - ps22
Saccharomyco./mycetaceae - ps23
Saccharomycetaceae - ps24
Saccharomycetaceae_2 - ps25
Saccharomycetaceae_3 - ps26
Kaz./Nau./Saccharomyces - ps27
Saccharomyces - ps28
Arboricola_clade - ps29
Cerevisiae_clade - ps30
Saccharomyces_cerevisiae - ps31
Phylostratum

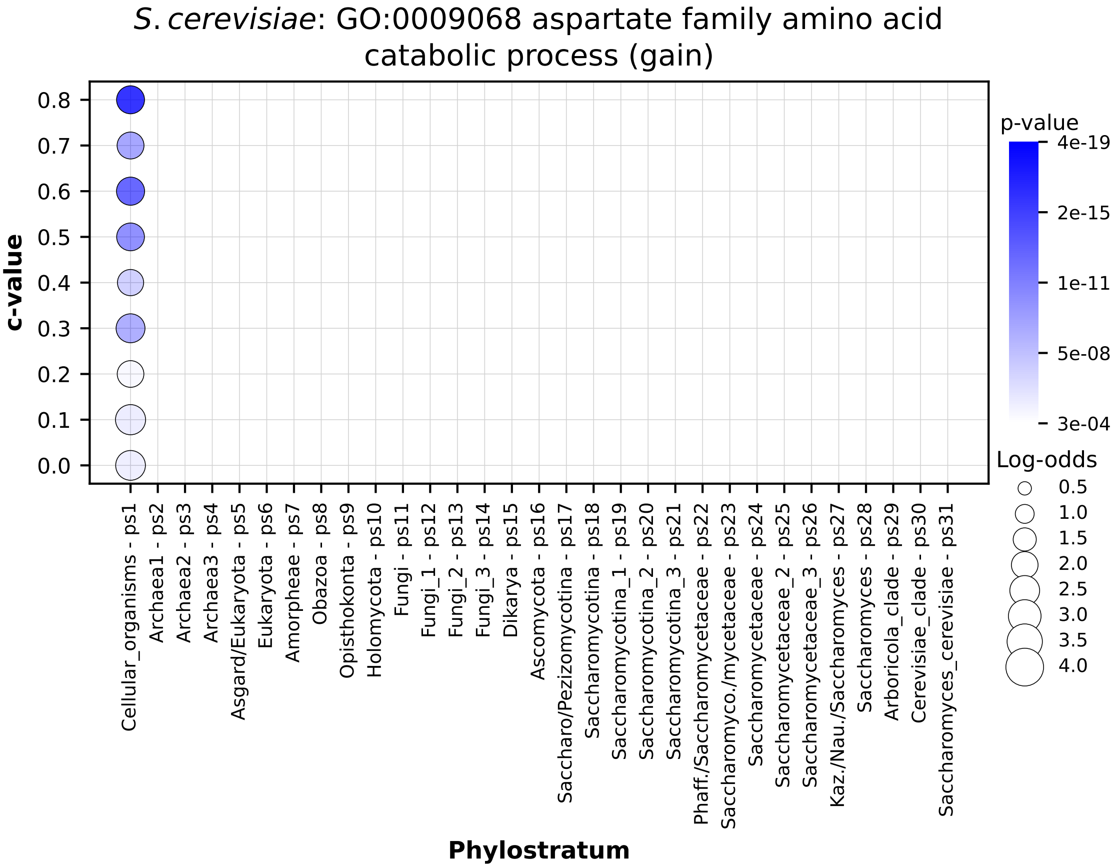

S. cerevisiae: GO:0009068 aspartate family amino acid catabolic process (gain)
c-value
0.8
0.7
0.6
0.5
0.4
0.3
0.2
0.1
0.0
p-value
4e-19
2e-15
1e-11
5e-08
3e-04
Log-odds
0.5
1.0
1.5
2.0
2.5
3.0
3.5
4.0
Cellular_organisms - ps1
Archaea1 - ps2
Archaea2 - ps3
Archaea3 - ps4
Asgard/Eukaryota - ps5
Eukaryota - ps6
Amorpheae - ps7
Obazoa - ps8
Opisthokonta - ps9
Holomycota - ps10
Fungi - ps11
Fungi_1 - ps12
Fungi_2 - ps13
Fungi_3 - ps14
Dikarya - ps15
Ascomycota - ps16
Saccharo/Pezizomycotina - ps17
Saccharomycotina - ps18
Saccharomycotina_1 - ps19
Saccharomycotina_2 - ps20
Saccharomycotina_3 - ps21
Phaff./Saccharomycetaceae - ps22
Saccharomyco./mycetaceae - ps23
Saccharomycetaceae - ps24
Saccharomycetaceae_2 - ps25
Saccharomycetaceae_3 - ps26
Kaz./Nau./Saccharomyces - ps27
Saccharomyces - ps28
Arboricola_clade - ps29
Cerevisiae_clade - ps30
Saccharomyces_cerevisiae - ps31
Phylostratum

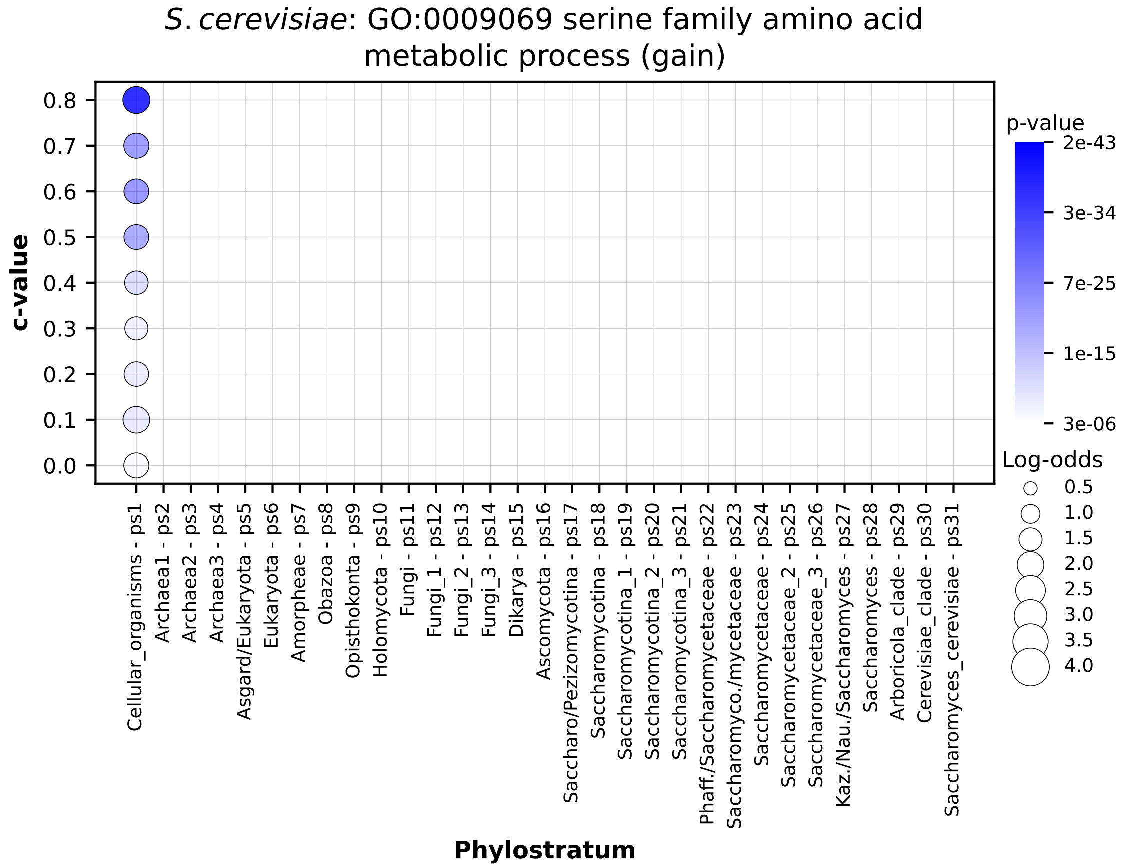

S. cerevisiae: GO:0009069 serine family amino acid metabolic process (gain)
c-value
0.8
0.7
0.6
0.5
0.4
0.3
0.2
0.1
0.0
p-value
2e-43
3e-34
7e-25
1e-15
3e-06
Log-odds
0.5
1.0
1.5
2.0
2.5
3.0
3.5
4.0
Cellular_organisms - ps1
Archaea1 - ps2
Archaea2 - ps3
Archaea3 - ps4
Asgard/Eukaryota - ps5
Eukaryota - ps6
Amorpheae - ps7
Obazoa - ps8
Opisthokonta - ps9
Holomycota - ps10
Fungi - ps11
Fungi_1 - ps12
Fungi_2 - ps13
Fungi_3 - ps14
Dikarya - ps15
Ascomycota - ps16
Saccharo/Pezizomycotina - ps17
Saccharomycotina - ps18
Saccharomycotina_1 - ps19
Saccharomycotina_2 - ps20
Saccharomycotina_3 - ps21
Phaff./Saccharomycetaceae - ps22
Saccharomyco./mycetaceae - ps23
Saccharomycetaceae - ps24
Saccharomycetaceae_2 - ps25
Saccharomycetaceae_3 - ps26
Kaz./Nau./Saccharomyces - ps27
Saccharomyces - ps28
Arboricola_clade - ps29
Cerevisiae_clade - ps30
Saccharomyces_cerevisiae - ps31
Phylostratum

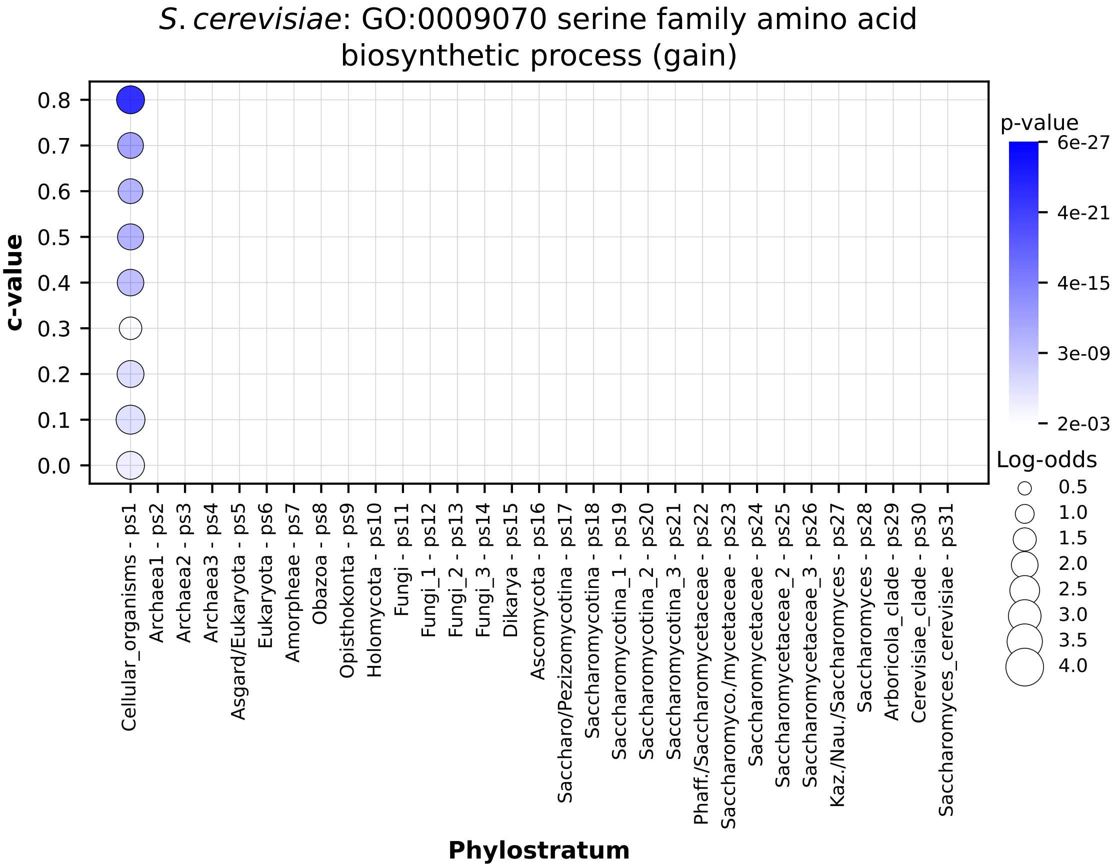
S. cerevisiae: GO:0009070 serine family amino acid biosynthetic process (gain)
c-value
0.8
0.7
0.6
0.5
0.4
0.3
0.2
0.1
0.0
p-value
6e-27
4e-21
4e-15
3e-09
2e-03
Log-odds
0.5
1.0
1.5
2.0
2.5
3.0
3.5
4.0
Cellular_organisms - ps1
Archaea1 - ps2
Archaea2 - ps3
Archaea3 - ps4
Asgard/Eukaryota - ps5
Eukaryota - ps6
Amorpheae - ps7
Obazoa - ps8
Opisthokonta - ps9
Holomycota - ps10
Fungi - ps11
Fungi_1 - ps12
Fungi_2 - ps13
Fungi_3 - ps14
Dikarya - ps15
Ascomycota - ps16
Saccharo/Pezizomycotina - ps17
Saccharomycotina - ps18
Saccharomycotina_1 - ps19
Saccharomycotina_2 - ps20
Saccharomycotina_3 - ps21
Phaff./Saccharomycetaceae - ps22
Saccharomyco./mycetaceae - ps23
Saccharomycetaceae - ps24
Saccharomycetaceae_2 - ps25
Saccharomycetaceae_3 - ps26
Kaz./Nau./Saccharomyces - ps27
Saccharomyces - ps28
Arboricola_clade - ps29
Cerevisiae_clade - ps30
Saccharomyces_cerevisiae - ps31
Phylostratum

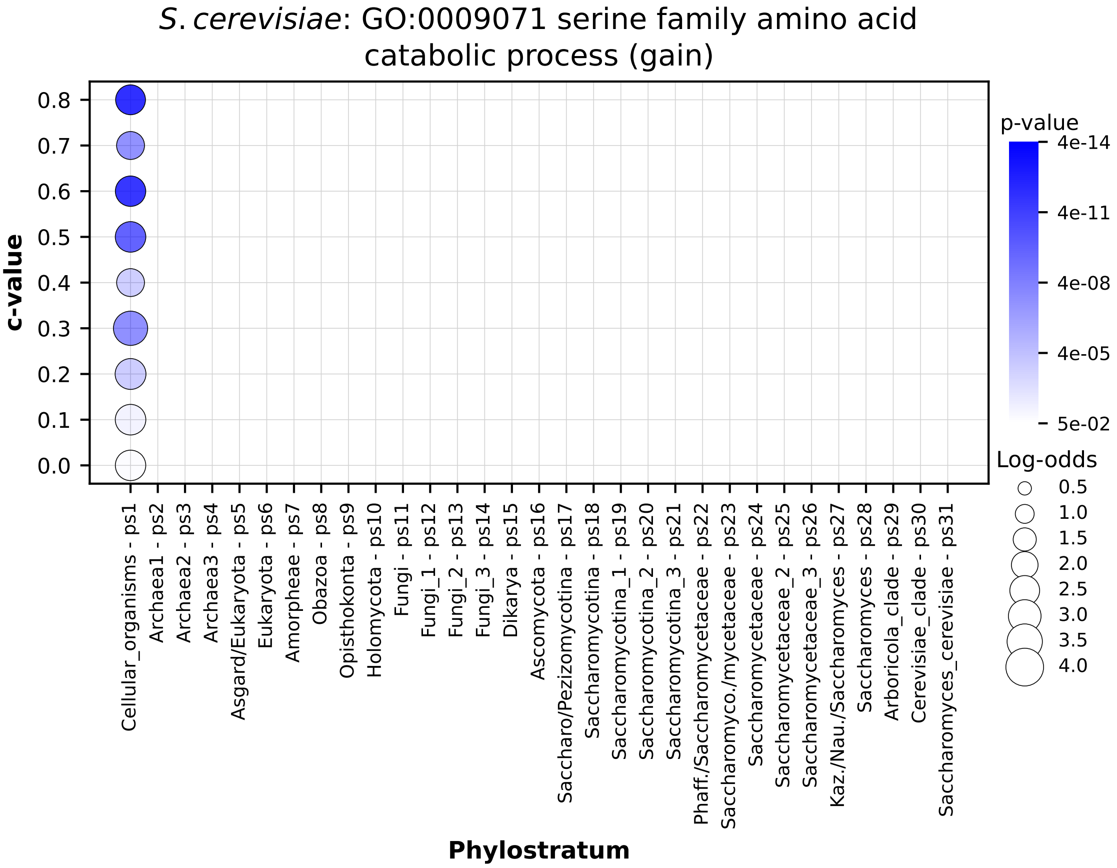
S. cerevisiae: GO:0009071 serine family amino acid catabolic process (gain)
c-value
0.8
0.7
0.6
0.5
0.4
0.3
0.2
0.1
0.0
p-value
4e-14
4e-11
4e-08
4e-05
5e-02
Log-odds
0.5
1.0
1.5
2.0
2.5
3.0
3.5
4.0
Cellular_organisms - ps1
Archaea1 - ps2
Archaea2 - ps3
Archaea3 - ps4
Asgard/Eukaryota - ps5
Eukaryota - ps6
Amorpheae - ps7
Obazoa - ps8
Opisthokonta - ps9
Holomycota - ps10
Fungi - ps11
Fungi_1 - ps12
Fungi_2 - ps13
Fungi_3 - ps14
Dikarya - ps15
Ascomycota - ps16
Saccharo/Pezizomycotina - ps17
Saccharomycotina - ps18
Saccharomycotina_1 - ps19
Saccharomycotina_2 - ps20
Saccharomycotina_3 - ps21
Phaff./Saccharomycetaceae - ps22
Saccharomyco./mycetaceae - ps23
Saccharomycetaceae - ps24
Saccharomycetaceae_2 - ps25
Saccharomycetaceae_3 - ps26
Kaz./Nau./Saccharomyces - ps27
Saccharomyces - ps28
Arboricola_clade - ps29
Cerevisiae_clade - ps30
Saccharomyces_cerevisiae - ps31
Phylostratum

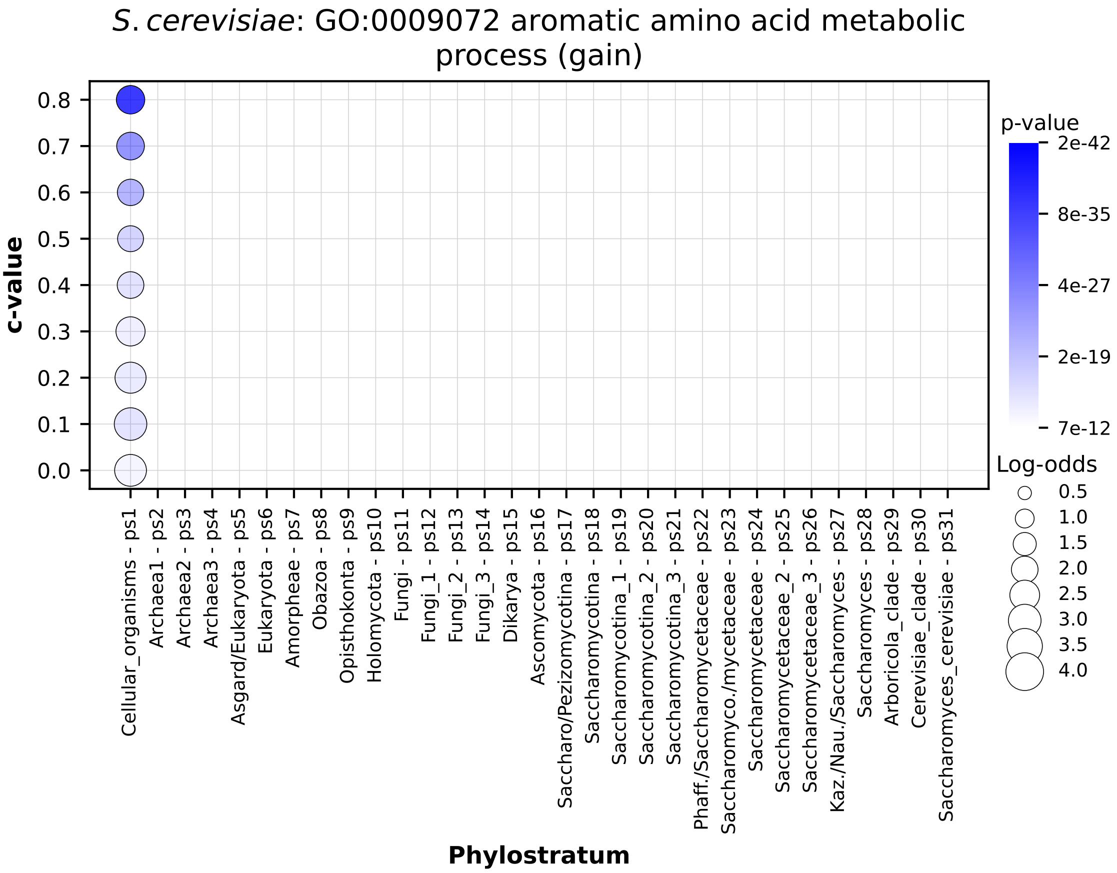

S. cerevisiae: GO:0009072 aromatic amino acid metabolic process (gain)
c-value
0.8
0.7
0.6
0.5
0.4
0.3
0.2
0.1
0.0
p-value
2e-42
8e-35
4e-27
2e-19
7e-12
Log-odds
0.5
1.0
1.5
2.0
2.5
3.0
3.5
4.0
Cellular_organisms - ps1
Archaea1 - ps2
Archaea2 - ps3
Archaea3 - ps4
Asgard/Eukaryota - ps5
Eukaryota - ps6
Amorpheae - ps7
Obazoa - ps8
Opisthokonta - ps9
Holomycota - ps10
Fungi - ps11
Fungi_1 - ps12
Fungi_2 - ps13
Fungi_3 - ps14
Dikarya - ps15
Ascomycota - ps16
Saccharo/Pezizomycotina - ps17
Saccharomycotina - ps18
Saccharomycotina_1 - ps19
Saccharomycotina_2 - ps20
Saccharomycotina_3 - ps21
Phaff./Saccharomycetaceae - ps22
Saccharomyco./mycetaceae - ps23
Saccharomycetaceae - ps24
Saccharomycetaceae_2 - ps25
Saccharomycetaceae_3 - ps26
Kaz./Nau./Saccharomyces - ps27
Saccharomyces - ps28
Arboricola_clade - ps29
Cerevisiae_clade - ps30
Saccharomyces_cerevisiae - ps31
Phylostratum

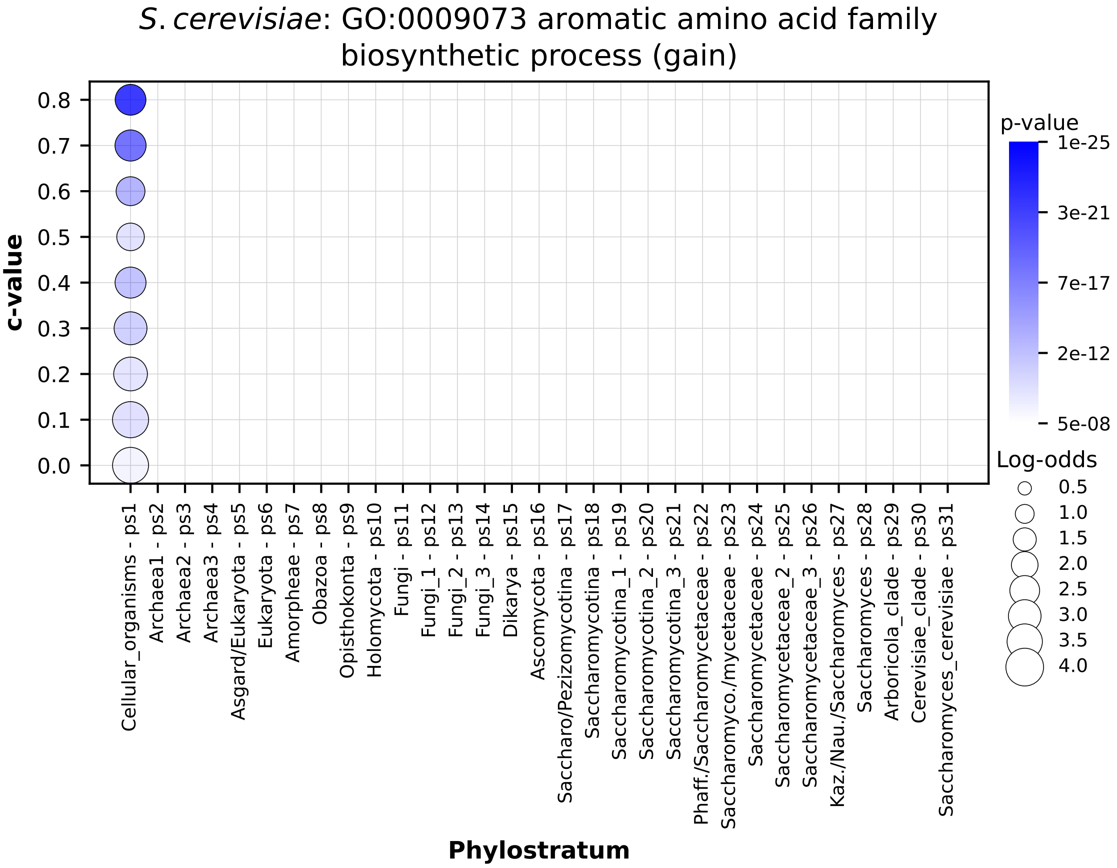

S. cerevisiae: GO:0009073 aromatic amino acid family biosynthetic process (gain)
c-value
0.0
0.1
0.2
0.3
0.4
0.5
0.6
0.7
0.8
p-value
1e-25
3e-21
7e-17
2e-12
5e-08
Log-odds
0.5
1.0
1.5
2.0
2.5
3.0
3.5
4.0
Cellular_organisms - ps1
Archaea1 - ps2
Archaea2 - ps3
Archaea3 - ps4
Asgard/Eukaryota - ps5
Eukaryota - ps6
Amorpheae - ps7
Obazoa - ps8
Opisthokonta - ps9
Holomycota - ps10
Fungi - ps11
Fungi_1 - ps12
Fungi_2 - ps13
Fungi_3 - ps14
Dikarya - ps15
Ascomycota - ps16
Saccharo/Pezizomycotina - ps17
Saccharomycotina - ps18
Saccharomycotina_1 - ps19
Saccharomycotina_2 - ps20
Saccharomycotina_3 - ps21
Phaff./Saccharomycetaceae - ps22
Saccharomyco./mycetaceae - ps23
Saccharomycetaceae - ps24
Saccharomycetaceae_2 - ps25
Saccharomycetaceae_3 - ps26
Kaz./Nau./Saccharomyces - ps27
Saccharomyces - ps28
Arboricola_clade - ps29
Cerevisiae_clade - ps30
Saccharomyces_cerevisiae - ps31
Phylostratum

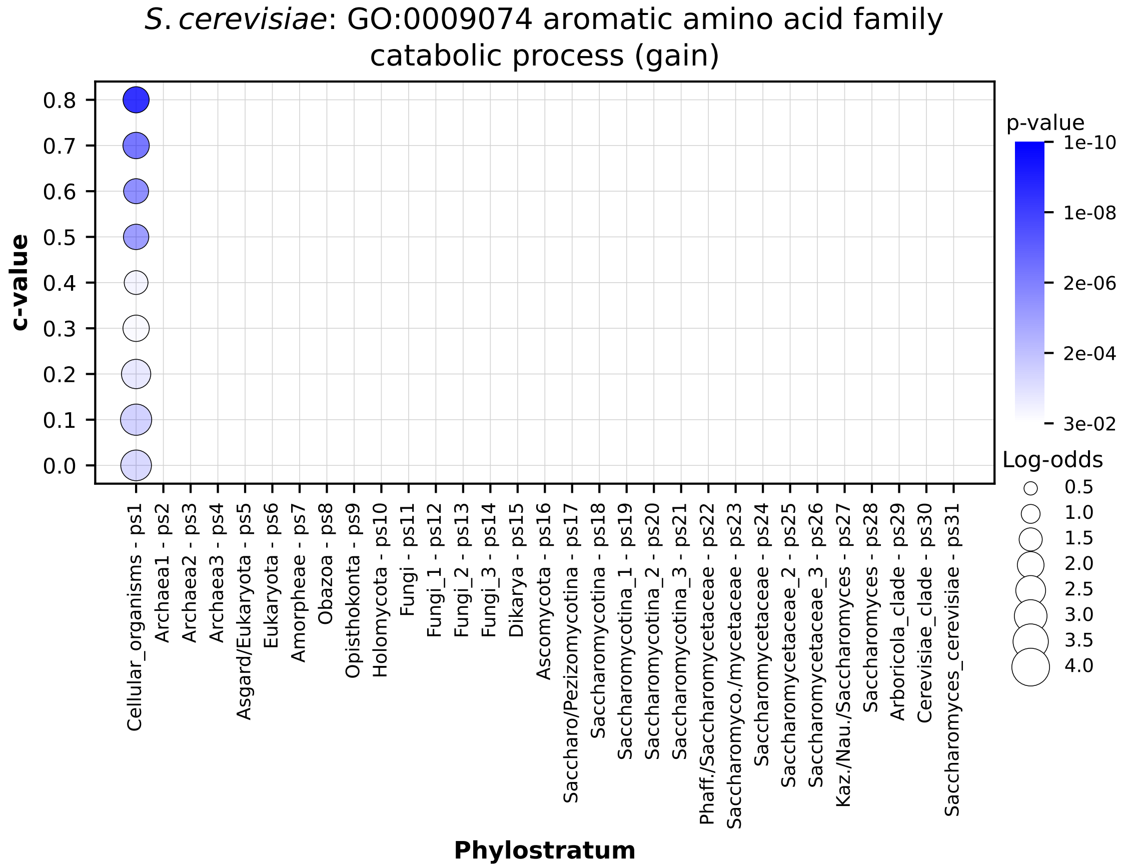

S. cerevisiae: GO:0009074 aromatic amino acid family catabolic process (gain)
c-value
0.8
0.7
0.6
0.5
0.4
0.3
0.2
0.1
0.0
p-value
1e-10
1e-08
2e-06
2e-04
3e-02
Log-odds
0.5
1.0
1.5
2.0
2.5
3.0
3.5
4.0
Cellular_organisms - ps1
Archaea1 - ps2
Archaea2 - ps3
Archaea3 - ps4
Asgard/Eukaryota - ps5
Eukaryota - ps6
Amorpheae - ps7
Obazoa - ps8
Opisthokonta - ps9
Holomycota - ps10
Fungi - ps11
Fungi_1 - ps12
Fungi_2 - ps13
Fungi_3 - ps14
Dikarya - ps15
Ascomycota - ps16
Saccharo/Pezizomycotina - ps17
Saccharomycotina - ps18
Saccharomycotina_1 - ps19
Saccharomycotina_2 - ps20
Saccharomycotina_3 - ps21
Phaff./Saccharomycetaceae - ps22
Saccharomyco./mycetaceae - ps23
Saccharomycetaceae - ps24
Saccharomycetaceae_2 - ps25
Saccharomycetaceae_3 - ps26
Kaz./Nau./Saccharomyces - ps27
Saccharomyces - ps28
Arboricola_clade - ps29
Cerevisiae_clade - ps30
Saccharomyces_cerevisiae - ps31
Phylostratum

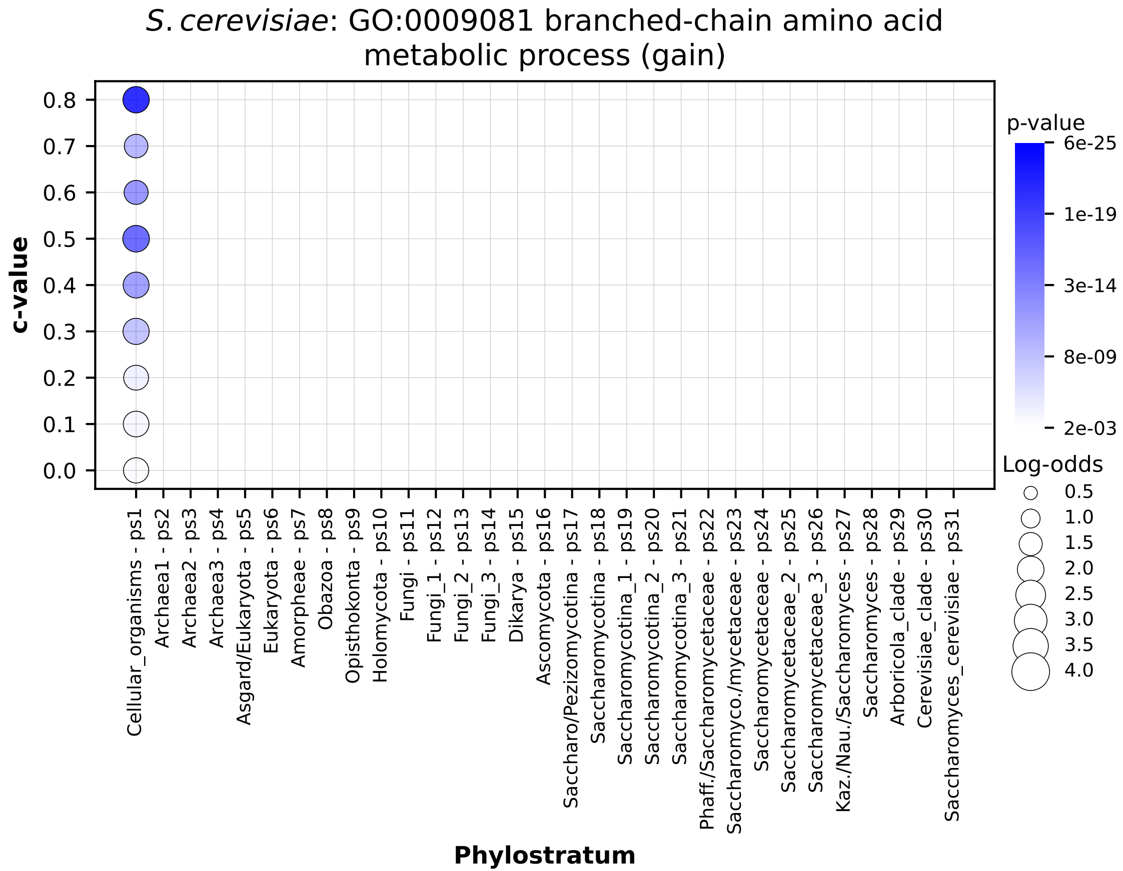

S. cerevisiae: GO:0009081 branched-chain amino acid metabolic process (gain)
c-value
0.8
0.7
0.6
0.5
0.4
0.3
0.2
0.1
0.0
p-value
6e-25
1e-19
3e-14
8e-09
2e-03
Log-odds
0.5
1.0
1.5
2.0
2.5
3.0
3.5
4.0
Cellular_organisms - ps1
Archaea1 - ps2
Archaea2 - ps3
Archaea3 - ps4
Asgard/Eukaryota - ps5
Eukaryota - ps6
Amorpheae - ps7
Obazoa - ps8
Opisthokonta - ps9
Holomycota - ps10
Fungi - ps11
Fungi_1 - ps12
Fungi_2 - ps13
Fungi_3 - ps14
Dikarya - ps15
Ascomycota - ps16
Saccharo/Pezizomycotina - ps17
Saccharomycotina - ps18
Saccharomycotina_1 - ps19
Saccharomycotina_2 - ps20
Saccharomycotina_3 - ps21
Phaff./Saccharomycetaceae - ps22
Saccharomyco./mycetaceae - ps23
Saccharomycetaceae - ps24
Saccharomycetaceae_2 - ps25
Saccharomycetaceae_3 - ps26
Kaz./Nau./Saccharomyces - ps27
Saccharomyces - ps28
Arboricola_clade - ps29
Cerevisiae_clade - ps30
Saccharomyces_cerevisiae - ps31
Phylostratum

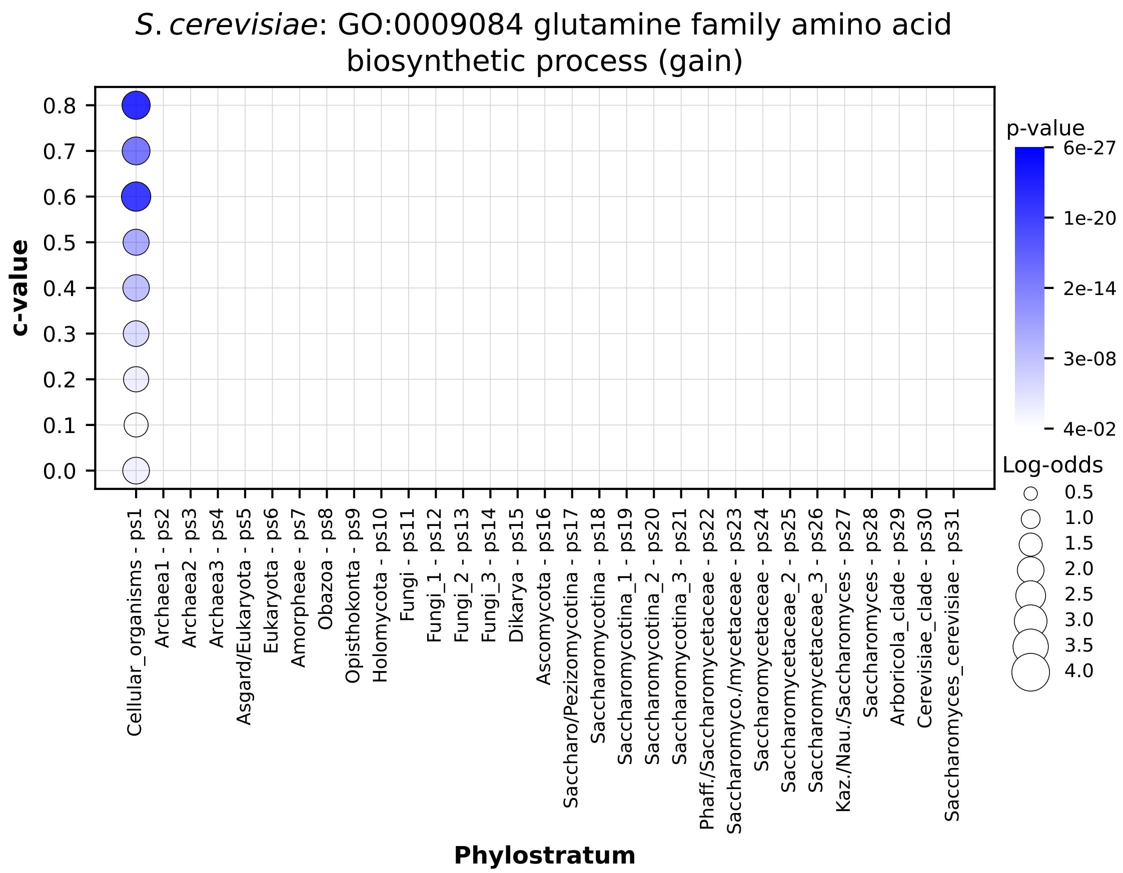

S. cerevisiae: GO:0009084 glutamine family amino acid biosynthetic process (gain)
c-value
0.8
0.7
0.6
0.5
0.4
0.3
0.2
0.1
0.0
p-value
6e-27
1e-20
2e-14
3e-08
4e-02
Log-odds
0.5
1.0
1.5
2.0
2.5
3.0
3.5
4.0
Cellular_organisms - ps1
Archaea1 - ps2
Archaea2 - ps3
Archaea3 - ps4
Asgard/Eukaryota - ps5
Eukaryota - ps6
Amorpheae - ps7
Obazoa - ps8
Opisthokonta - ps9
Holomycota - ps10
Fungi - ps11
Fungi_1 - ps12
Fungi_2 - ps13
Fungi_3 - ps14
Dikarya - ps15
Ascomycota - ps16
Saccharo/Pezizomycotina - ps17
Saccharomycotina - ps18
Saccharomycotina_1 - ps19
Saccharomycotina_2 - ps20
Saccharomycotina_3 - ps21
Phaff./Saccharomycetaceae - ps22
Saccharomyco./mycetaceae - ps23
Saccharomycetaceae - ps24
Saccharomycetaceae_2 - ps25
Saccharomycetaceae_3 - ps26
Kaz./Nau./Saccharomyces - ps27
Saccharomyces - ps28
Arboricola_clade - ps29
Cerevisiae_clade - ps30
Saccharomyces_cerevisiae - ps31
Phylostratum

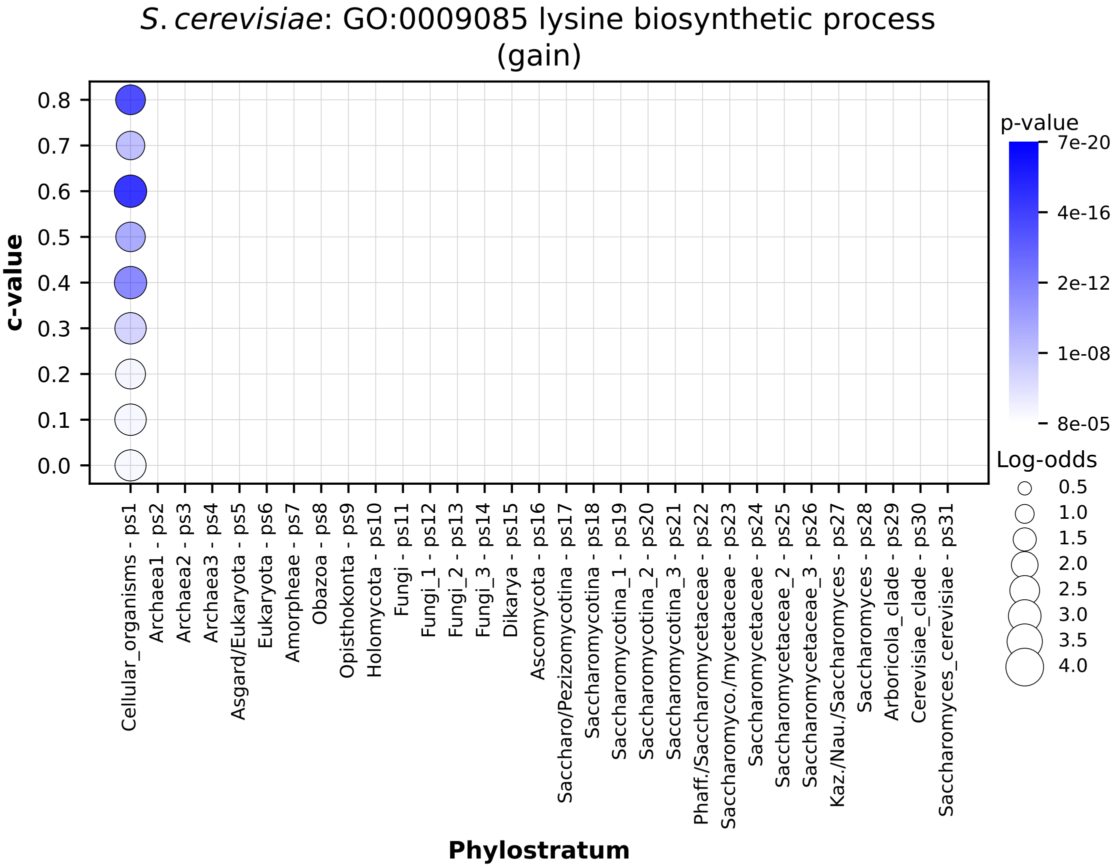

S. cerevisiae: GO:0009085 lysine biosynthetic process
(gain)
c-value
0.8
0.7
0.6
0.5
0.4
0.3
0.2
0.1
0.0
p-value
7e-20
4e-16
2e-12
1e-08
8e-05
Log-odds
0.5
1.0
1.5
2.0
2.5
3.0
3.5
4.0
Cellular_organisms - ps1
Archaea1 - ps2
Archaea2 - ps3
Archaea3 - ps4
Asgard/Eukaryota - ps5
Eukaryota - ps6
Amorpheae - ps7
Obazoa - ps8
Opisthokonta - ps9
Holomycota - ps10
Fungi - ps11
Fungi_1 - ps12
Fungi_2 - ps13
Fungi_3 - ps14
Dikarya - ps15
Ascomycota - ps16
Saccharo/Pezizomycotina - ps17
Saccharomycotina - ps18
Saccharomycotina_1 - ps19
Saccharomycotina_2 - ps20
Saccharomycotina_3 - ps21
Phaff./Saccharomycetaceae - ps22
Saccharomyco./mycetaceae - ps23
Saccharomycetaceae - ps24
Saccharomycetaceae_2 - ps25
Saccharomycetaceae_3 - ps26
Kaz./Nau./Saccharomyces - ps27
Saccharomyces - ps28
Arboricola_clade - ps29
Cerevisiae_clade - ps30
Saccharomyces_cerevisiae - ps31
Phylostratum

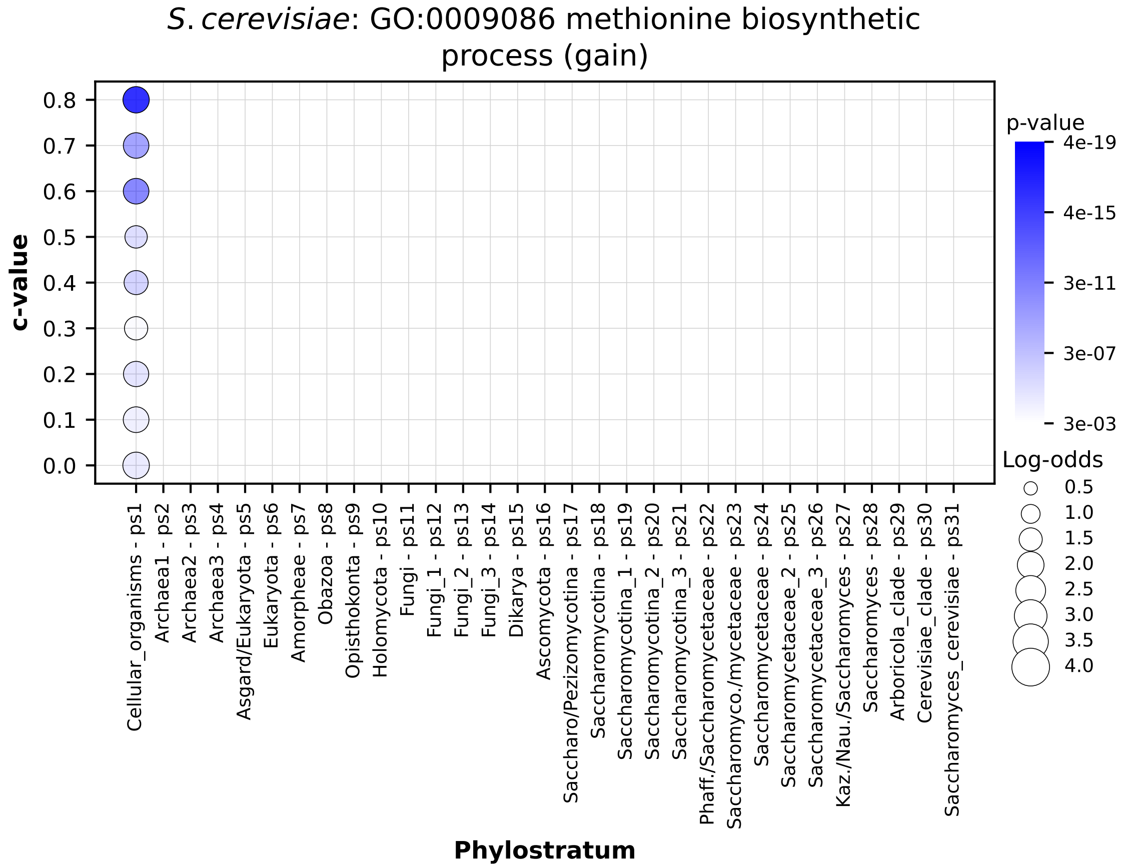
S. cerevisiae: GO:0009086 methionine biosynthetic process (gain)
c-value
0.8
0.7
0.6
0.5
0.4
0.3
0.2
0.1
0.0
p-value
4e-19
4e-15
3e-11
3e-07
3e-03
Log-odds
0.5
1.0
1.5
2.0
2.5
3.0
3.5
4.0
Cellular_organisms - ps1
Archaea1 - ps2
Archaea2 - ps3
Archaea3 - ps4
Asgard/Eukaryota - ps5
Eukaryota - ps6
Amorpheae - ps7
Obazoa - ps8
Opisthokonta - ps9
Holomycota - ps10
Fungi - ps11
Fungi_1 - ps12
Fungi_2 - ps13
Fungi_3 - ps14
Dikarya - ps15
Ascomycota - ps16
Saccharo/Pezizomycotina - ps17
Saccharomycotina - ps18
Saccharomycotina_1 - ps19
Saccharomycotina_2 - ps20
Saccharomycotina_3 - ps21
Phaff./Saccharomycetaceae - ps22
Saccharomyco./mycetaceae - ps23
Saccharomycetaceae - ps24
Saccharomycetaceae_2 - ps25
Saccharomycetaceae_3 - ps26
Kaz./Nau./Saccharomyces - ps27
Saccharomyces - ps28
Arboricola_clade - ps29
Cerevisiae_clade - ps30
Saccharomyces_cerevisiae - ps31
Phylostratum

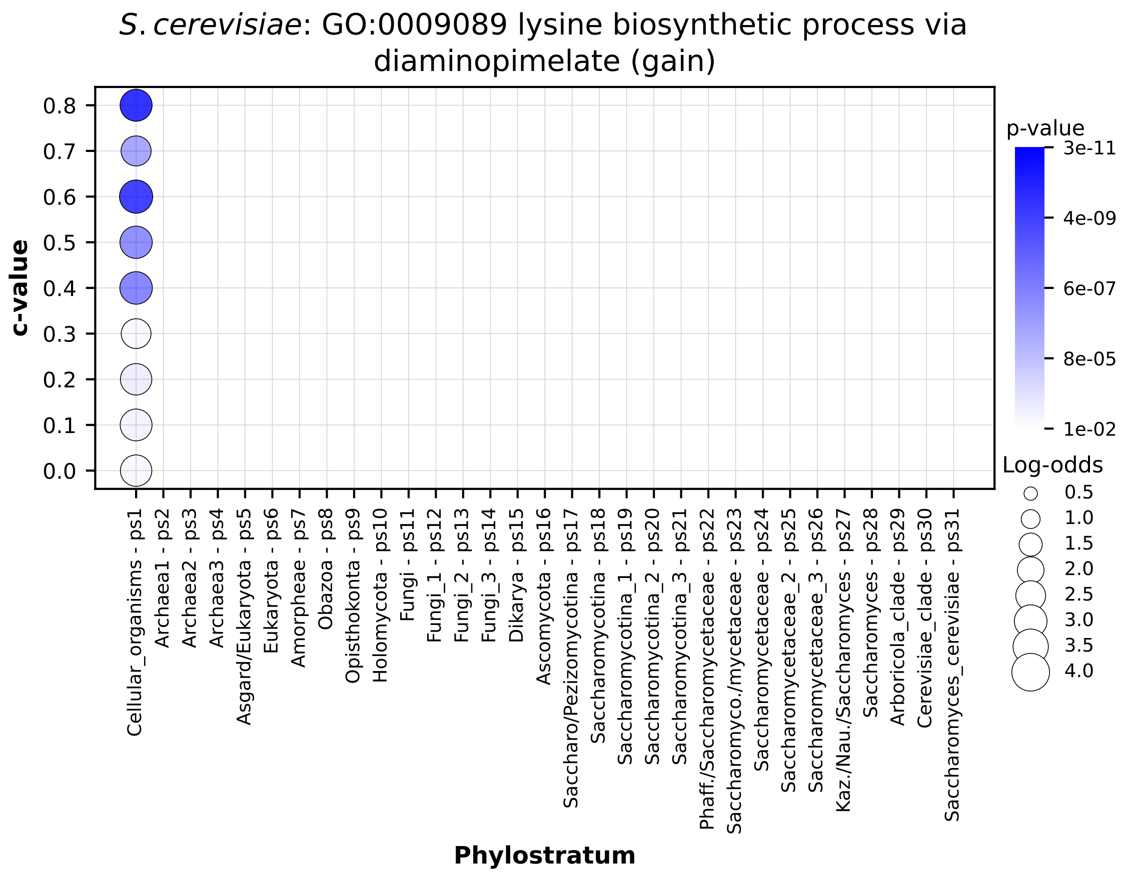

S. cerevisiae: GO:0009089 lysine biosynthetic process via diaminopimelate (gain)
c-value
0.8
0.7
0.6
0.5
0.4
0.3
0.2
0.1
0.0
p-value
3e-11
4e-09
6e-07
8e-05
1e-02
Log-odds
0.5
1.0
1.5
2.0
2.5
3.0
3.5
4.0
Cellular_organisms - ps1
Archaea1 - ps2
Archaea2 - ps3
Archaea3 - ps4
Asgard/Eukaryota - ps5
Eukaryota - ps6
Amorpheae - ps7
Obazoa - ps8
Opisthokonta - ps9
Holomycota - ps10
Fungi - ps11
Fungi_1 - ps12
Fungi_2 - ps13
Fungi_3 - ps14
Dikarya - ps15
Ascomycota - ps16
Saccharo/Pezizomycotina - ps17
Saccharomycotina - ps18
Saccharomycotina_1 - ps19
Saccharomycotina_2 - ps20
Saccharomycotina_3 - ps21
Phaff./Saccharomycetaceae - ps22
Saccharomyco./mycetaceae - ps23
Saccharomycetaceae - ps24
Saccharomycetaceae_2 - ps25
Saccharomycetaceae_3 - ps26
Kaz./Nau./Saccharomyces - ps27
Saccharomyces - ps28
Arboricola_clade - ps29
Cerevisiae_clade - ps30
Saccharomyces_cerevisiae - ps31
Phylostratum

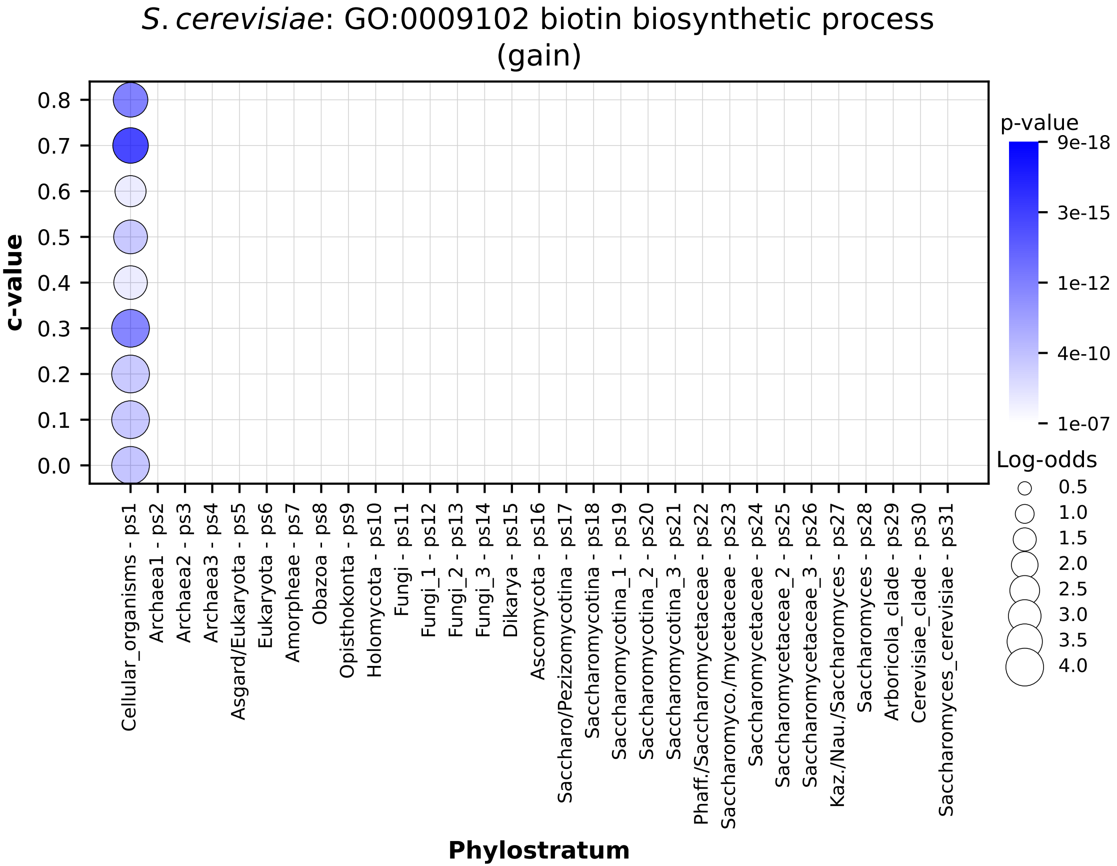

S. cerevisiae: GO:0009102 biotin biosynthetic process (gain)
c-value
0.0
0.1
0.2
0.3
0.4
0.5
0.6
0.7
0.8
Phylostratum
Cellular_organisms - ps1
Archaea1 - ps2
Archaea2 - ps3
Archaea3 - ps4
Asgard/Eukaryota - ps5
Eukaryota - ps6
Amorpheae - ps7
Obazoa - ps8
Opisthokonta - ps9
Holomycota - ps10
Fungi - ps11
Fungi_1 - ps12
Fungi_2 - ps13
Fungi_3 - ps14
Dikarya - ps15
Ascomycota - ps16
Saccharo/Pezizomycotina - ps17
Saccharomycotina - ps18
Saccharomycotina_1 - ps19
Saccharomycotina_2 - ps20
Saccharomycotina_3 - ps21
Phaff./Saccharomycetaceae - ps22
Saccharomyco./mycetaceae - ps23
Saccharomycetaceae - ps24
Saccharomycetaceae_2 - ps25
Saccharomycetaceae_3 - ps26
Kaz./Nau./Saccharomyces - ps27
Saccharomyces - ps28
Arboricola_clade - ps29
Cerevisiae_clade - ps30
Saccharomyces_cerevisiae - ps31
p-value
9e-18
3e-15
1e-12
4e-10
1e-07
Log-odds
0.5
1.0
1.5
2.0
2.5
3.0
3.5
4.0

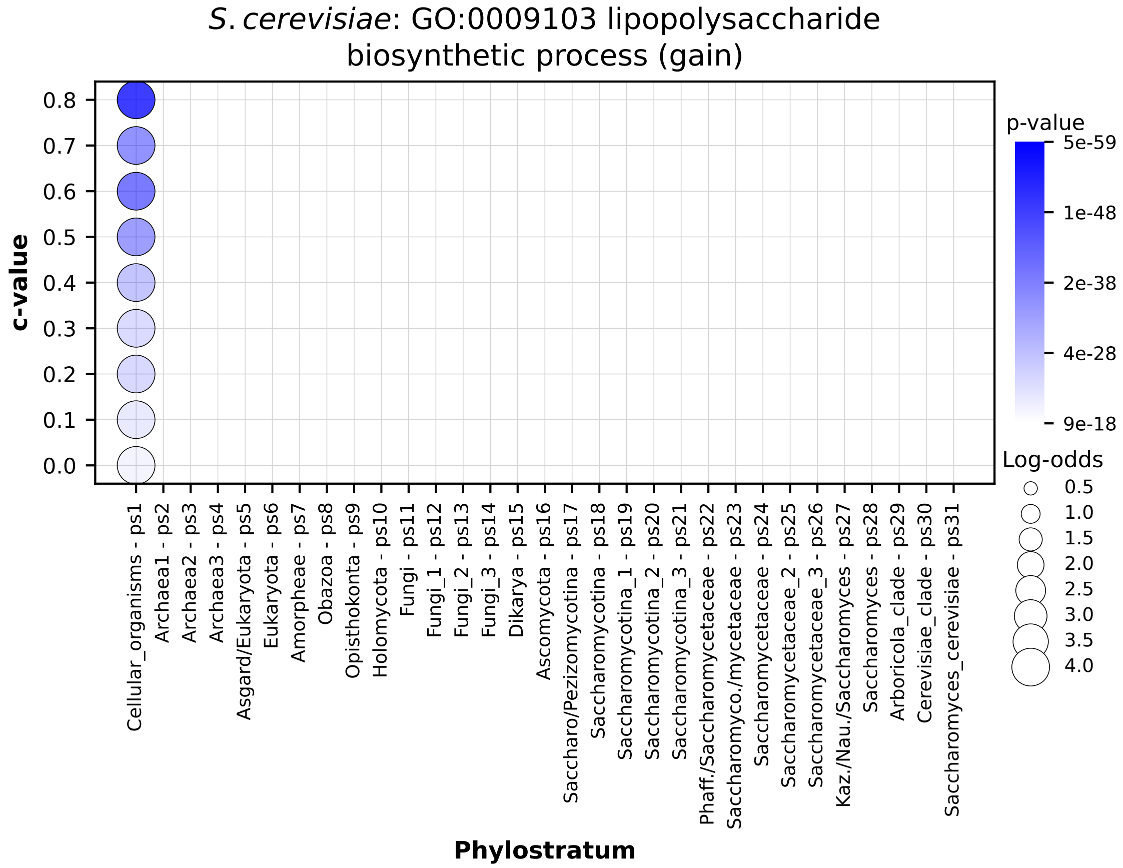

S. cerevisiae: GO:0009103 lipopolysaccharide biosynthetic process (gain)
c-value
0.8
0.7
0.6
0.5
0.4
0.3
0.2
0.1
0.0
p-value
5e-59
1e-48
2e-38
4e-28
9e-18
Log-odds
0.5
1.0
1.5
2.0
2.5
3.0
3.5
4.0
Cellular_organisms - ps1
Archaea1 - ps2
Archaea2 - ps3
Archaea3 - ps4
Asgard/Eukaryota - ps5
Eukaryota - ps6
Amorpheae - ps7
Obazoa - ps8
Opisthokonta - ps9
Holomycota - ps10
Fungi - ps11
Fungi_1 - ps12
Fungi_2 - ps13
Fungi_3 - ps14
Dikarya - ps15
Ascomycota - ps16
Saccharo/Pezizomycotina - ps17
Saccharomycotina - ps18
Saccharomycotina_1 - ps19
Saccharomycotina_2 - ps20
Saccharomycotina_3 - ps21
Phaff./Saccharomycetaceae - ps22
Saccharomyco./mycetaceae - ps23
Saccharomycetaceae - ps24
Saccharomycetaceae_2 - ps25
Saccharomycetaceae_3 - ps26
Kaz./Nau./Saccharomyces - ps27
Saccharomyces - ps28
Arboricola_clade - ps29
Cerevisiae_clade - ps30
Saccharomyces_cerevisiae - ps31
Phylostratum

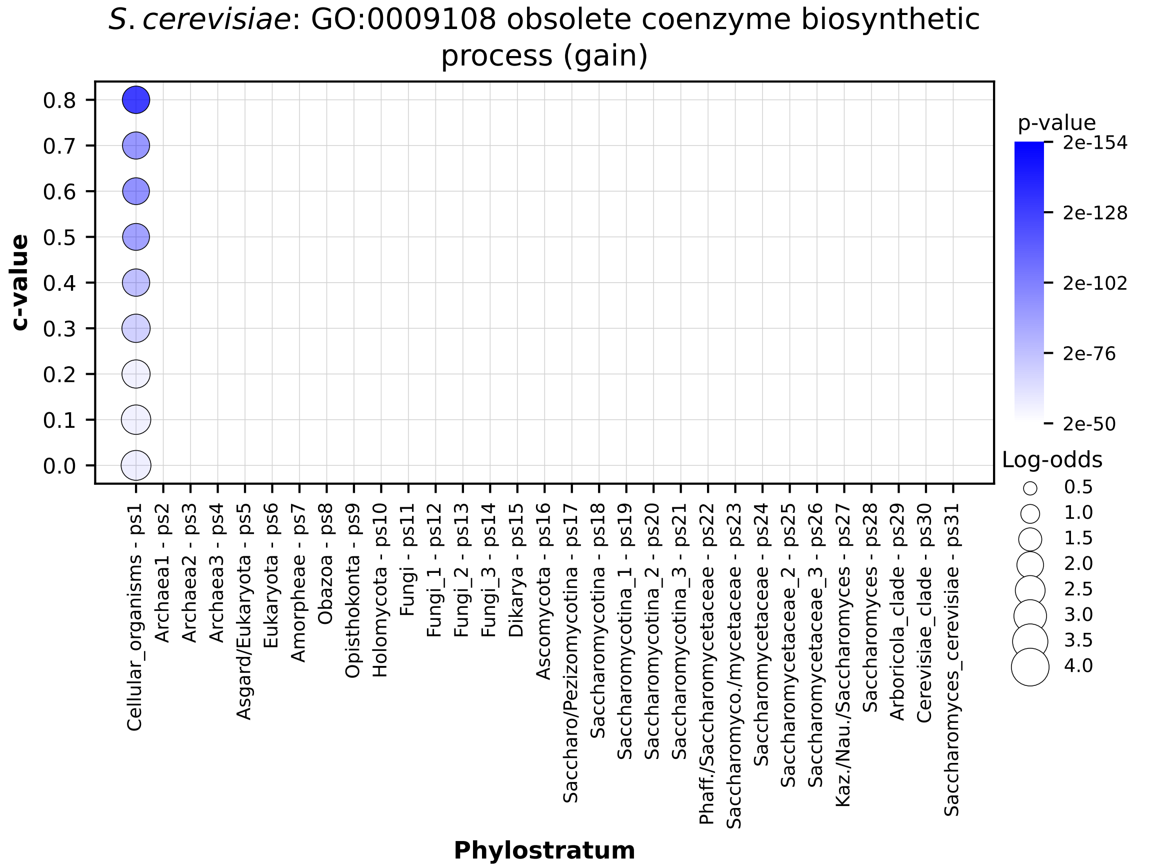
S. cerevisiae: GO:0009108 obsolete coenzyme biosynthetic process (gain)
c-value
0.8
0.7
0.6
0.5
0.4
0.3
0.2
0.1
0.0
Phylostratum
Cellular_organisms - ps1
Archaea1 - ps2
Archaea2 - ps3
Archaea3 - ps4
Asgard/Eukaryota - ps5
Eukaryota - ps6
Amorpheae - ps7
Obazoa - ps8
Opisthokonta - ps9
Holomycota - ps10
Fungi - ps11
Fungi_1 - ps12
Fungi_2 - ps13
Fungi_3 - ps14
Dikarya - ps15
Ascomycota - ps16
Saccharo/Pezizomycotina - ps17
Saccharomycotina - ps18
Saccharomycotina_1 - ps19
Saccharomycotina_2 - ps20
Saccharomycotina_3 - ps21
Phaff./Saccharomycetaceae - ps22
Saccharomyco./mycetaceae - ps23
Saccharomycetaceae - ps24
Saccharomycetaceae_2 - ps25
Saccharomycetaceae_3 - ps26
Kaz./Nau./Saccharomyces - ps27
Saccharomyces - ps28
Arboricola_clade - ps29
Cerevisiae_clade - ps30
Saccharomyces_cerevisiae - ps31
p-value
2e-154
2e-128
2e-102
2e-76
2e-50
Log-odds
0.5
1.0
1.5
2.0
2.5
3.0
3.5
4.0

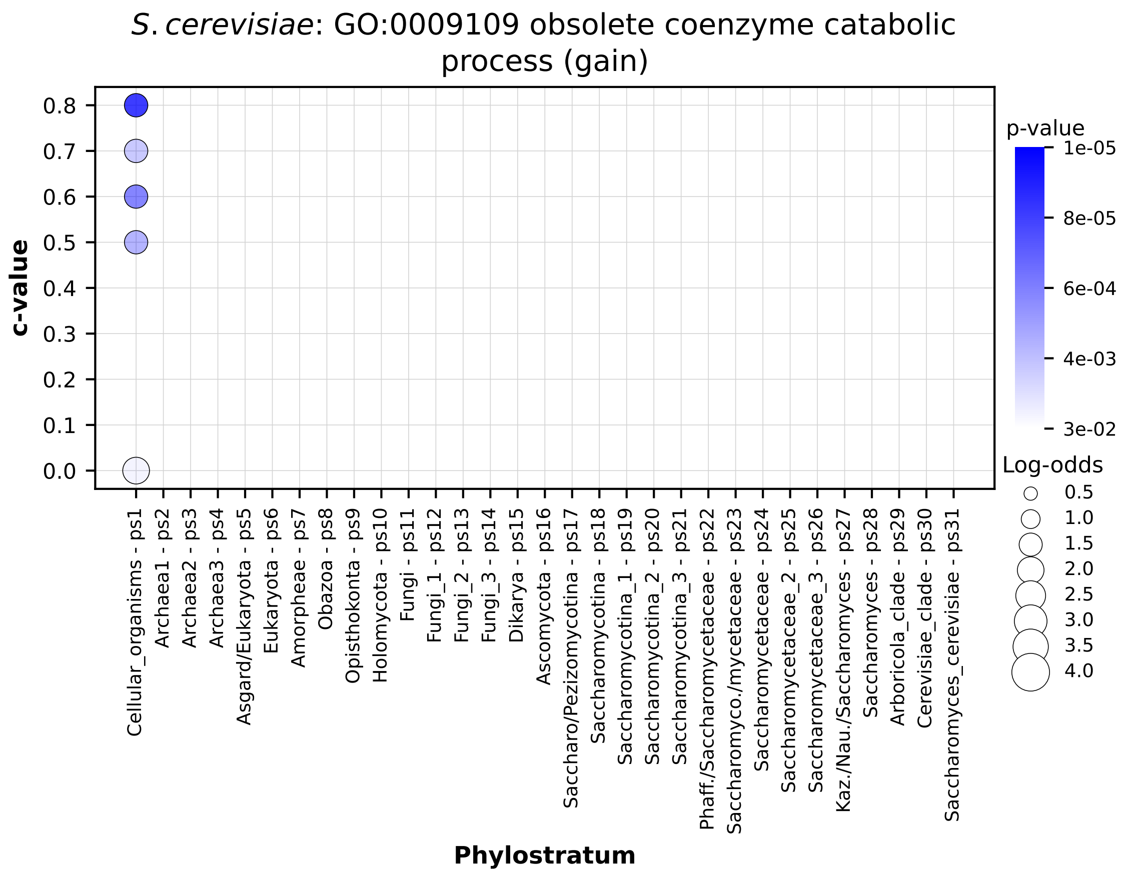

S. cerevisiae: GO:0009109 obsolete coenzyme catabolic process (gain)
c-value
0.0
0.1
0.2
0.3
0.4
0.5
0.6
0.7
0.8
p-value
1e-05
8e-05
6e-04
4e-03
3e-02
Log-odds
0.5
1.0
1.5
2.0
2.5
3.0
3.5
4.0
Cellular_organisms - ps1
Archaea1 - ps2
Archaea2 - ps3
Archaea3 - ps4
Asgard/Eukaryota - ps5
Eukaryota - ps6
Amorpheae - ps7
Obazoa - ps8
Opisthokonta - ps9
Holomycota - ps10
Fungi - ps11
Fungi_1 - ps12
Fungi_2 - ps13
Fungi_3 - ps14
Dikarya - ps15
Ascomycota - ps16
Saccharo/Pezizomycotina - ps17
Saccharomycotina - ps18
Saccharomycotina_1 - ps19
Saccharomycotina_2 - ps20
Saccharomycotina_3 - ps21
Phaff./Saccharomycetaceae - ps22
Saccharomyco./mycetaceae - ps23
Saccharomycetaceae - ps24
Saccharomycetaceae_2 - ps25
Saccharomycetaceae_3 - ps26
Kaz./Nau./Saccharomyces - ps27
Saccharomyces - ps28
Arboricola_clade - ps29
Cerevisiae_clade - ps30
Saccharomyces_cerevisiae - ps31
Phylostratum

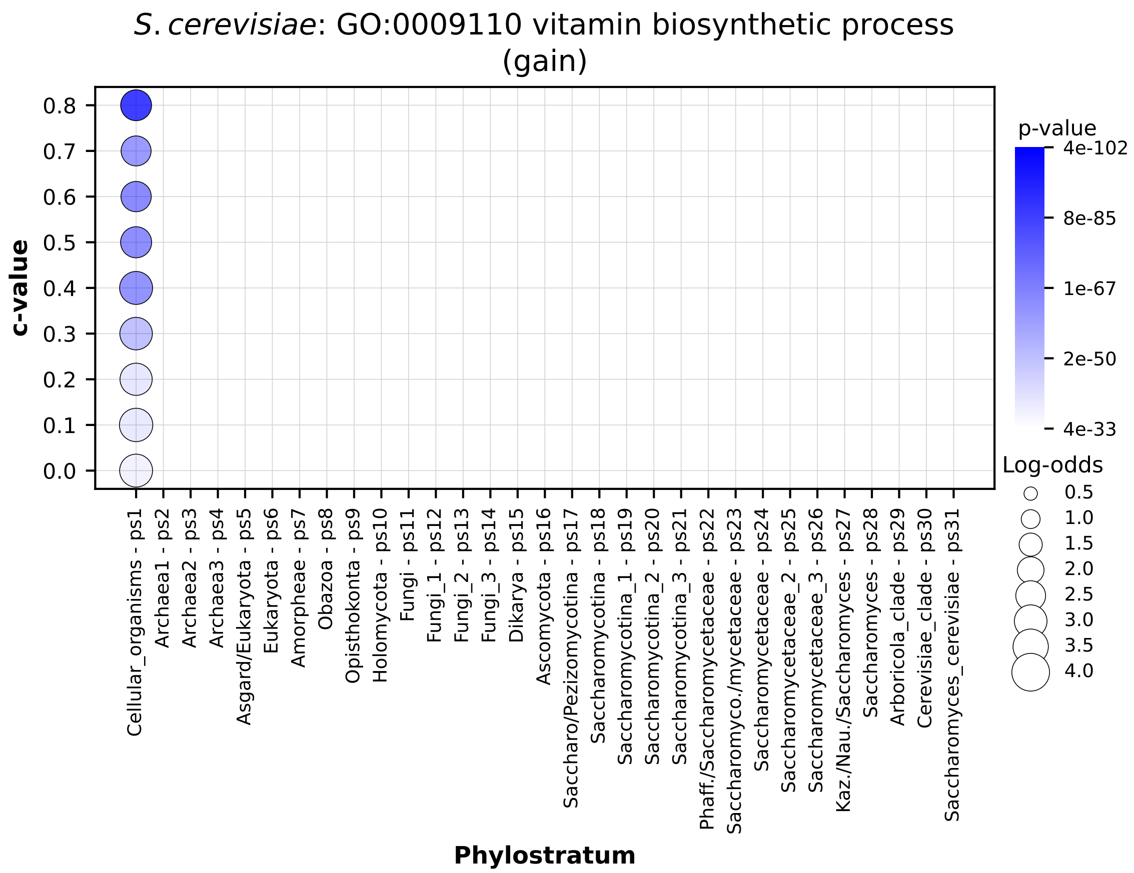
S. cerevisiae: GO:0009110 vitamin biosynthetic process (gain)
c-value
0.8
0.7
0.6
0.5
0.4
0.3
0.2
0.1
0.0
Phylostratum
Cellular_organisms - ps1
Archaea1 - ps2
Archaea2 - ps3
Archaea3 - ps4
Asgard/Eukaryota - ps5
Eukaryota - ps6
Amorpheae - ps7
Obazoa - ps8
Opisthokonta - ps9
Holomycota - ps10
Fungi - ps11
Fungi_1 - ps12
Fungi_2 - ps13
Fungi_3 - ps14
Dikarya - ps15
Ascomycota - ps16
Saccharo/Pezizomycotina - ps17
Saccharomycotina - ps18
Saccharomycotina_1 - ps19
Saccharomycotina_2 - ps20
Saccharomycotina_3 - ps21
Phaff./Saccharomycetaceae - ps22
Saccharomyco./mycetaceae - ps23
Saccharomycetaceae - ps24
Saccharomycetaceae_2 - ps25
Saccharomycetaceae_3 - ps26
Kaz./Nau./Saccharomyces - ps27
Saccharomyces - ps28
Arboricola_clade - ps29
Cerevisiae_clade - ps30
Saccharomyces_cerevisiae - ps31
p-value
4e-102
8e-85
1e-67
2e-50
4e-33
Log-odds
0.5
1.0
1.5
2.0
2.5
3.0
3.5
4.0

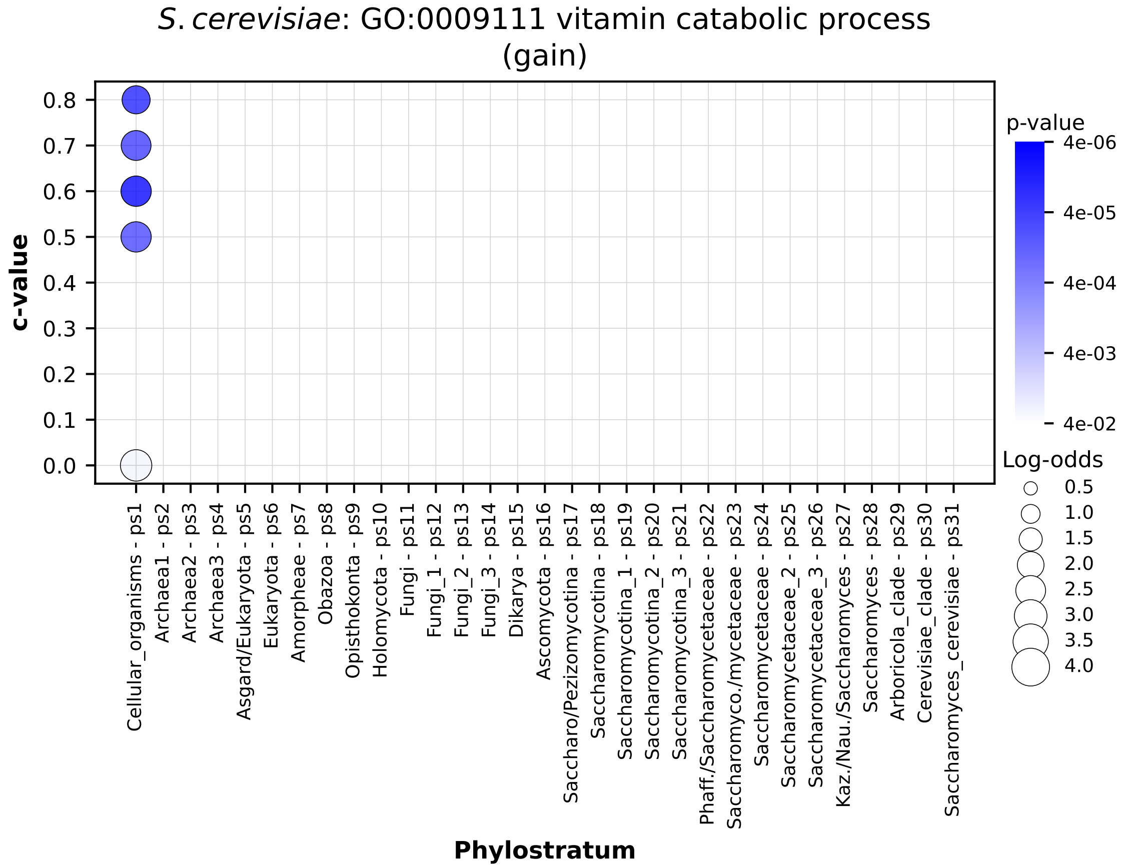

S. cerevisiae: GO:0009111 vitamin catabolic process
(gain)
c-value
0.8
0.7
0.6
0.5
0.4
0.3
0.2
0.1
0.0
p-value
4e-06
4e-05
4e-04
4e-03
4e-02
Log-odds
0.5
1.0
1.5
2.0
2.5
3.0
3.5
4.0
Cellular_organisms - ps1
Archaea1 - ps2
Archaea2 - ps3
Archaea3 - ps4
Asgard/Eukaryota - ps5
Eukaryota - ps6
Amorpheae - ps7
Obazoa - ps8
Opisthokonta - ps9
Holomycota - ps10
Fungi - ps11
Fungi_1 - ps12
Fungi_2 - ps13
Fungi_3 - ps14
Dikarya - ps15
Ascomycota - ps16
Saccharo/Pezizomycotina - ps17
Saccharomycotina - ps18
Saccharomycotina_1 - ps19
Saccharomycotina_2 - ps20
Saccharomycotina_3 - ps21
Phaff./Saccharomycetaceae - ps22
Saccharomyco./mycetaceae - ps23
Saccharomycetaceae - ps24
Saccharomycetaceae_2 - ps25
Saccharomycetaceae_3 - ps26
Kaz./Nau./Saccharomyces - ps27
Saccharomyces - ps28
Arboricola_clade - ps29
Cerevisiae_clade - ps30
Saccharomyces_cerevisiae - ps31
Phylostratum

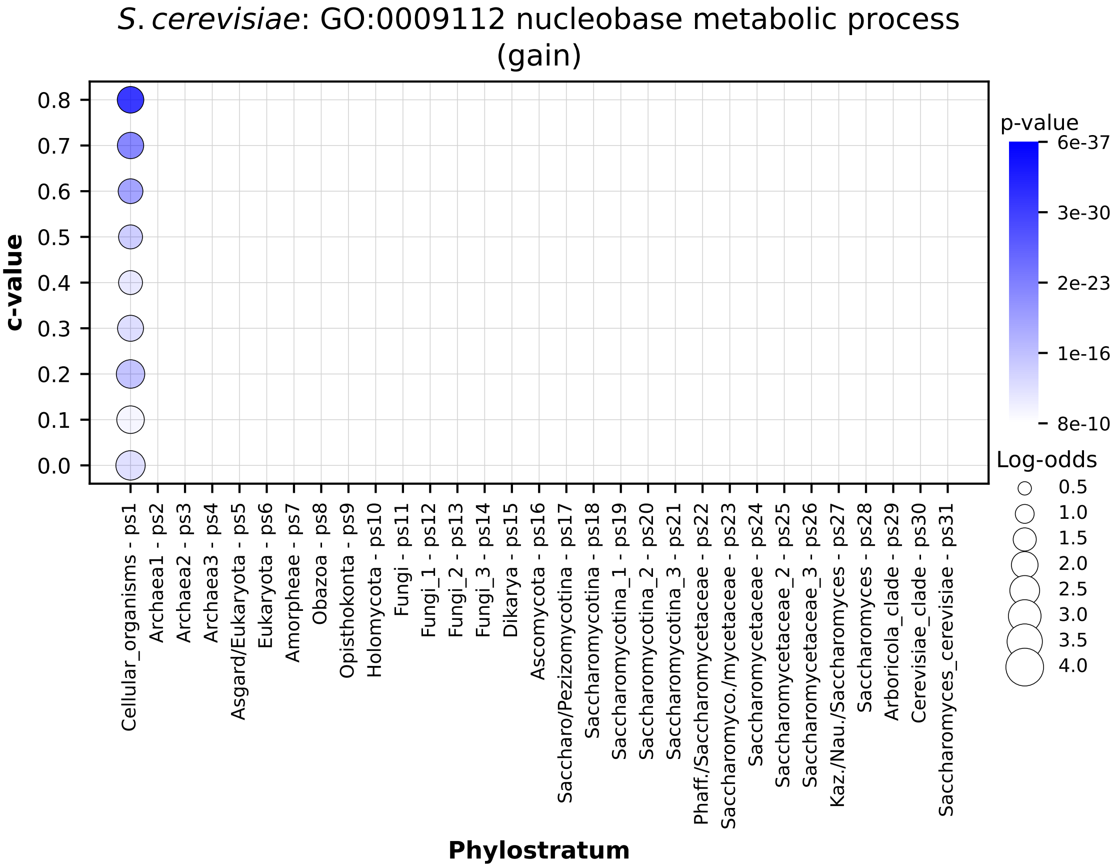

S. cerevisiae: GO:0009112 nucleobase metabolic process (gain)
c-value
0.8
0.7
0.6
0.5
0.4
0.3
0.2
0.1
0.0
p-value
6e-37
3e-30
2e-23
1e-16
8e-10
Log-odds
0.5
1.0
1.5
2.0
2.5
3.0
3.5
4.0
Cellular_organisms - ps1
Archaea1 - ps2
Archaea2 - ps3
Archaea3 - ps4
Asgard/Eukaryota - ps5
Eukaryota - ps6
Amorpheae - ps7
Obazoa - ps8
Opisthokonta - ps9
Holomycota - ps10
Fungi - ps11
Fungi_1 - ps12
Fungi_2 - ps13
Fungi_3 - ps14
Dikarya - ps15
Ascomycota - ps16
Saccharo/Pezizomycotina - ps17
Saccharomycotina - ps18
Saccharomycotina_1 - ps19
Saccharomycotina_2 - ps20
Saccharomycotina_3 - ps21
Phaff./Saccharomycetaceae - ps22
Saccharomyco./mycetaceae - ps23
Saccharomycetaceae - ps24
Saccharomycetaceae_2 - ps25
Saccharomycetaceae_3 - ps26
Kaz./Nau./Saccharomyces - ps27
Saccharomyces - ps28
Arboricola_clade - ps29
Cerevisiae_clade - ps30
Saccharomyces_cerevisiae - ps31
Phylostratum

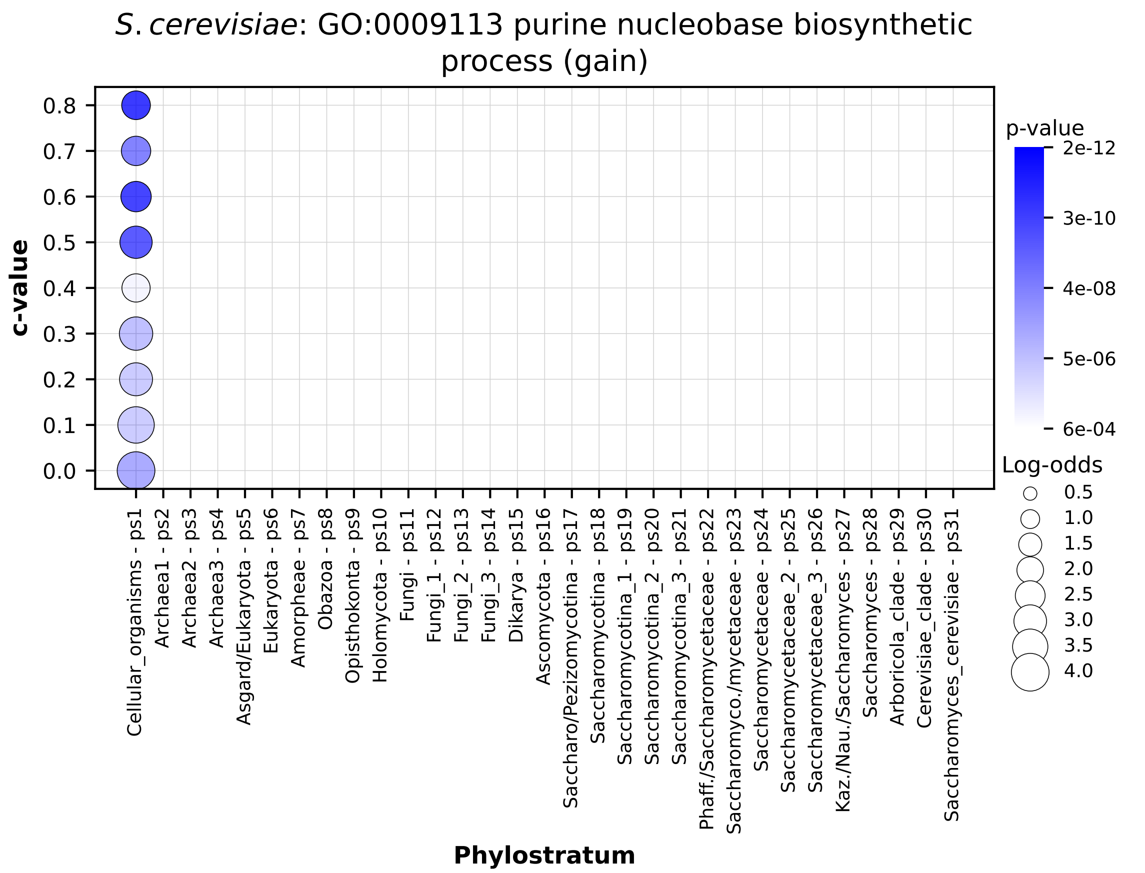
S. cerevisiae: GO:0009113 purine nucleobase biosynthetic process (gain)
c-value
0.8
0.7
0.6
0.5
0.4
0.3
0.2
0.1
0.0
p-value
2e-12
3e-10
4e-08
5e-06
6e-04
Log-odds
0.5
1.0
1.5
2.0
2.5
3.0
3.5
4.0
Cellular_organisms - ps1
Archaea1 - ps2
Archaea2 - ps3
Archaea3 - ps4
Asgard/Eukaryota - ps5
Eukaryota - ps6
Amorpheae - ps7
Obazoa - ps8
Opisthokonta - ps9
Holomycota - ps10
Fungi - ps11
Fungi_1 - ps12
Fungi_2 - ps13
Fungi_3 - ps14
Dikarya - ps15
Ascomycota - ps16
Saccharo/Pezizomycotina - ps17
Saccharomycotina - ps18
Saccharomycotina_1 - ps19
Saccharomycotina_2 - ps20
Saccharomycotina_3 - ps21
Phaff./Saccharomycetaceae - ps22
Saccharomyco./mycetaceae - ps23
Saccharomycetaceae - ps24
Saccharomycetaceae_2 - ps25
Saccharomycetaceae_3 - ps26
Kaz./Nau./Saccharomyces - ps27
Saccharomyces - ps28
Arboricola_clade - ps29
Cerevisiae_clade - ps30
Saccharomyces_cerevisiae - ps31
Phylostratum

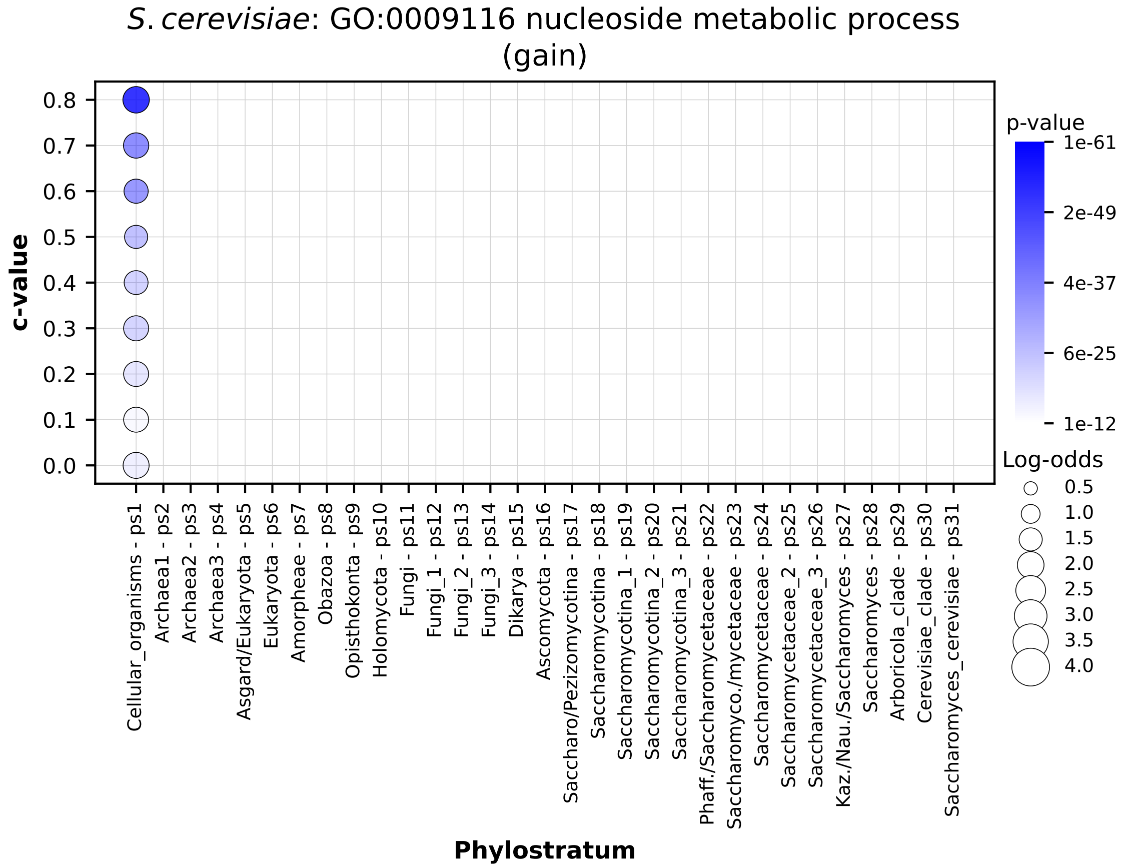

S. cerevisiae: GO:0009116 nucleoside metabolic process (gain)
c-value
0.0
0.1
0.2
0.3
0.4
0.5
0.6
0.7
0.8
p-value
1e-61
2e-49
4e-37
6e-25
1e-12
Log-odds
0.5
1.0
1.5
2.0
2.5
3.0
3.5
4.0
Cellular_organisms - ps1
Archaea1 - ps2
Archaea2 - ps3
Archaea3 - ps4
Asgard/Eukaryota - ps5
Eukaryota - ps6
Amorpheae - ps7
Obazoa - ps8
Opisthokonta - ps9
Holomycota - ps10
Fungi - ps11
Fungi_1 - ps12
Fungi_2 - ps13
Fungi_3 - ps14
Dikarya - ps15
Ascomycota - ps16
Saccharo/Pezizomycotina - ps17
Saccharomycotina - ps18
Saccharomycotina_1 - ps19
Saccharomycotina_2 - ps20
Saccharomycotina_3 - ps21
Phaff./Saccharomycetaceae - ps22
Saccharomyco./mycetaceae - ps23
Saccharomycetaceae - ps24
Saccharomycetaceae_2 - ps25
Saccharomycetaceae_3 - ps26
Kaz./Nau./Saccharomyces - ps27
Saccharomyces - ps28
Arboricola_clade - ps29
Cerevisiae_clade - ps30
Saccharomyces_cerevisiae - ps31
Phylostratum

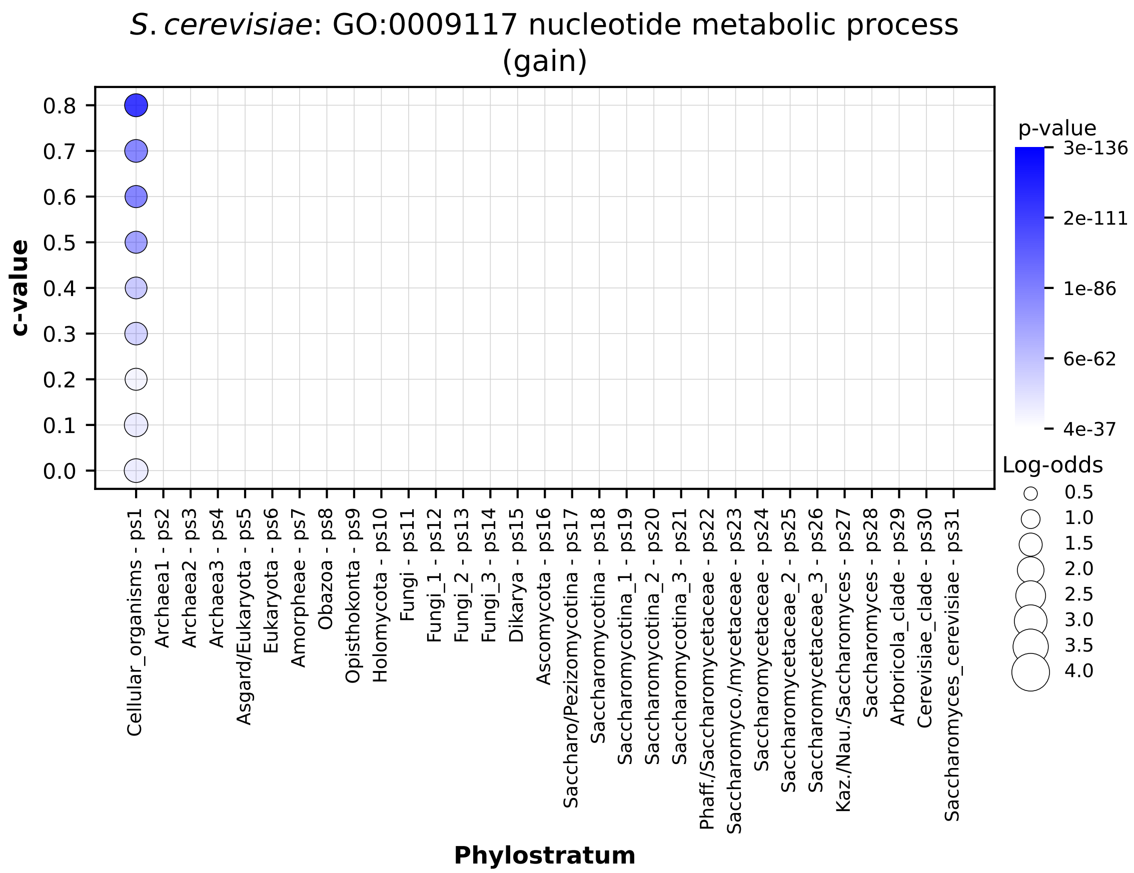

S. cerevisiae: GO:0009117 nucleotide metabolic process
(gain)
c-value
0.0
0.1
0.2
0.3
0.4
0.5
0.6
0.7
0.8
p-value
3e-136
2e-111
1e-86
6e-62
4e-37
Log-odds
0.5
1.0
1.5
2.0
2.5
3.0
3.5
4.0
Cellular_organisms - ps1
Archaea1 - ps2
Archaea2 - ps3
Archaea3 - ps4
Asgard/Eukaryota - ps5
Eukaryota - ps6
Amorpheae - ps7
Obazoa - ps8
Opisthokonta - ps9
Holomycota - ps10
Fungi - ps11
Fungi_1 - ps12
Fungi_2 - ps13
Fungi_3 - ps14
Dikarya - ps15
Ascomycota - ps16
Saccharo/Pezizomycotina - ps17
Saccharomycotina - ps18
Saccharomycotina_1 - ps19
Saccharomycotina_2 - ps20
Saccharomycotina_3 - ps21
Phaff./Saccharomycetaceae - ps22
Saccharomyco./mycetaceae - ps23
Saccharomycetaceae - ps24
Saccharomycetaceae_2 - ps25
Saccharomycetaceae_3 - ps26
Kaz./Nau./Saccharomyces - ps27
Saccharomyces - ps28
Arboricola_clade - ps29
Cerevisiae_clade - ps30
Saccharomyces_cerevisiae - ps31
Phylostratum

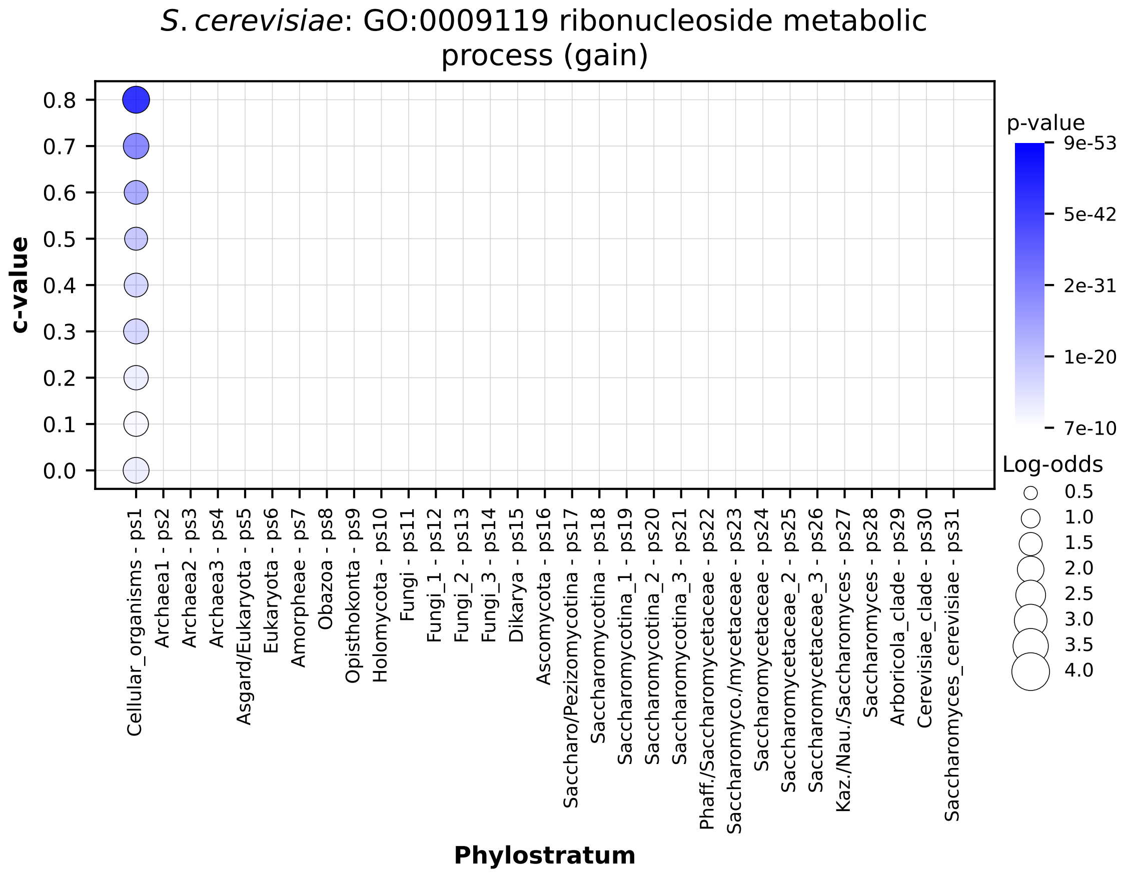

S. cerevisiae: GO:0009119 ribonucleoside metabolic process (gain)
c-value
0.8
0.7
0.6
0.5
0.4
0.3
0.2
0.1
0.0
p-value
9e-53
5e-42
2e-31
1e-20
7e-10
Log-odds
0.5
1.0
1.5
2.0
2.5
3.0
3.5
4.0
Cellular_organisms - ps1
Archaea1 - ps2
Archaea2 - ps3
Archaea3 - ps4
Asgard/Eukaryota - ps5
Eukaryota - ps6
Amorpheae - ps7
Obazoa - ps8
Opisthokonta - ps9
Holomycota - ps10
Fungi - ps11
Fungi_1 - ps12
Fungi_2 - ps13
Fungi_3 - ps14
Dikarya - ps15
Ascomycota - ps16
Saccharo/Pezizomycotina - ps17
Saccharomycotina - ps18
Saccharomycotina_1 - ps19
Saccharomycotina_2 - ps20
Saccharomycotina_3 - ps21
Phaff./Saccharomycetaceae - ps22
Saccharomyco./mycetaceae - ps23
Saccharomycetaceae - ps24
Saccharomycetaceae_2 - ps25
Saccharomycetaceae_3 - ps26
Kaz./Nau./Saccharomyces - ps27
Saccharomyces - ps28
Arboricola_clade - ps29
Cerevisiae_clade - ps30
Saccharomyces_cerevisiae - ps31
Phylostratum

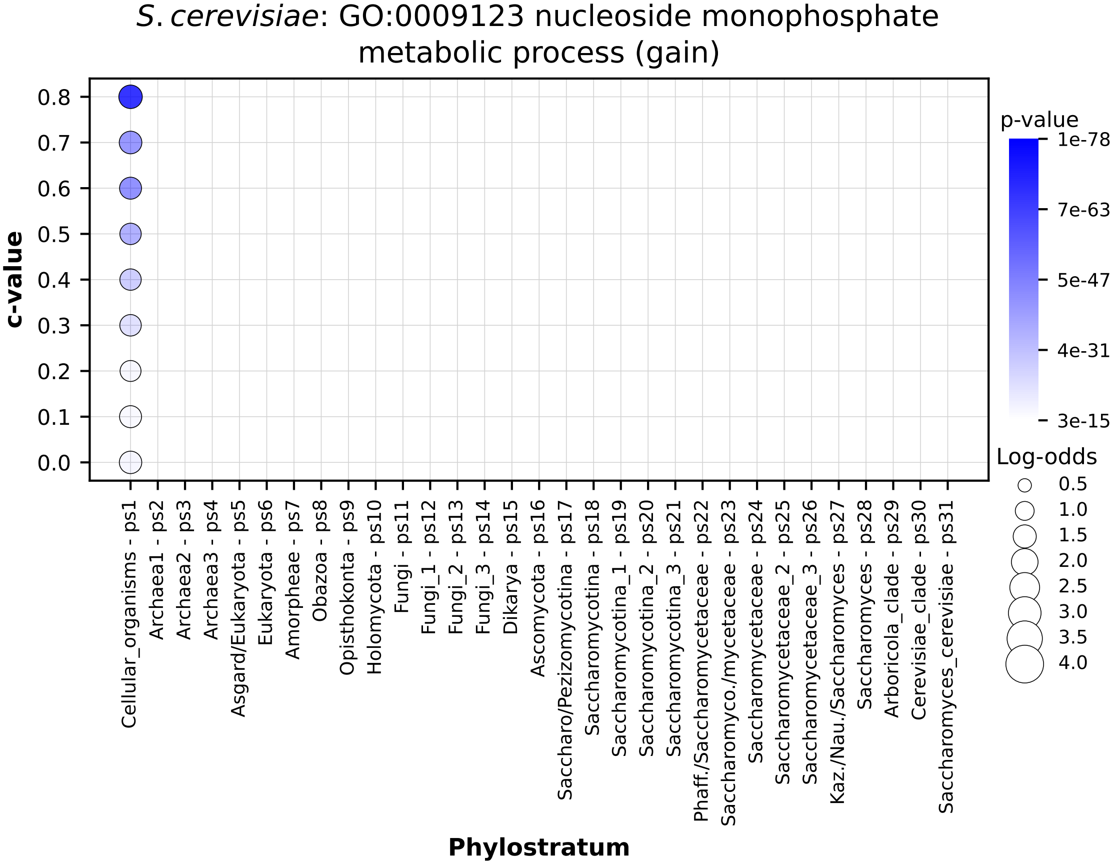

S. cerevisiae: GO:0009123 nucleoside monophosphate metabolic process (gain)
c-value
0.8
0.7
0.6
0.5
0.4
0.3
0.2
0.1
0.0
p-value
1e-78
7e-63
5e-47
4e-31
3e-15
Log-odds
0.5
1.0
1.5
2.0
2.5
3.0
3.5
4.0
Cellular_organisms - ps1
Archaea1 - ps2
Archaea2 - ps3
Archaea3 - ps4
Asgard/Eukaryota - ps5
Eukaryota - ps6
Amorpheae - ps7
Obazoa - ps8
Opisthokonta - ps9
Holomycota - ps10
Fungi - ps11
Fungi_1 - ps12
Fungi_2 - ps13
Fungi_3 - ps14
Dikarya - ps15
Ascomycota - ps16
Saccharo/Pezizomycotina - ps17
Saccharomycotina - ps18
Saccharomycotina_1 - ps19
Saccharomycotina_2 - ps20
Saccharomycotina_3 - ps21
Phaff./Saccharomycetaceae - ps22
Saccharomyco./mycetaceae - ps23
Saccharomycetaceae - ps24
Saccharomycetaceae_2 - ps25
Saccharomycetaceae_3 - ps26
Kaz./Nau./Saccharomyces - ps27
Saccharomyces - ps28
Arboricola_clade - ps29
Cerevisiae_clade - ps30
Saccharomyces_cerevisiae - ps31
Phylostratum

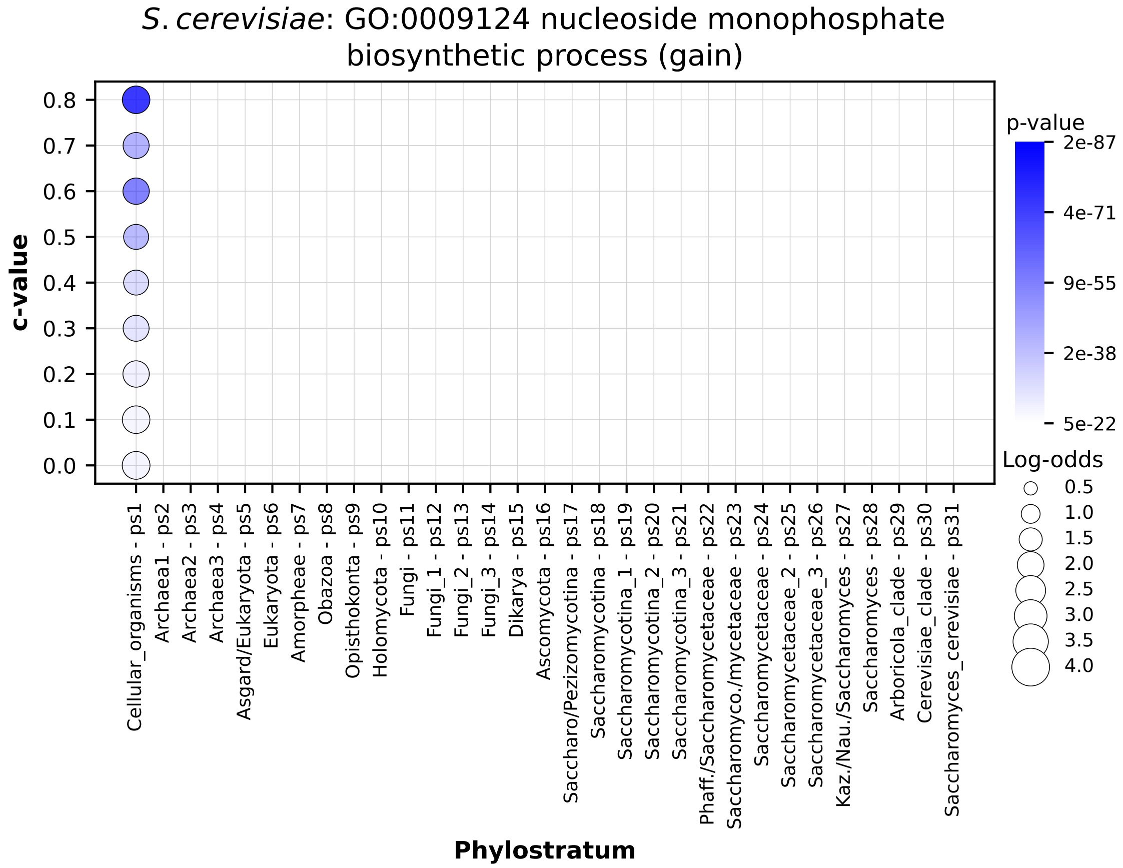
S. cerevisiae: GO:0009124 nucleoside monophosphate biosynthetic process (gain)
c-value
0.8
0.7
0.6
0.5
0.4
0.3
0.2
0.1
0.0
p-value
2e-87
4e-71
9e-55
2e-38
5e-22
Log-odds
0.5
1.0
1.5
2.0
2.5
3.0
3.5
4.0
Cellular_organisms - ps1
Archaea1 - ps2
Archaea2 - ps3
Archaea3 - ps4
Asgard/Eukaryota - ps5
Eukaryota - ps6
Amorpheae - ps7
Obazoa - ps8
Opisthokonta - ps9
Holomycota - ps10
Fungi - ps11
Fungi_1 - ps12
Fungi_2 - ps13
Fungi_3 - ps14
Dikarya - ps15
Ascomycota - ps16
Saccharo/Pezizomycotina - ps17
Saccharomycotina - ps18
Saccharomycotina_1 - ps19
Saccharomycotina_2 - ps20
Saccharomycotina_3 - ps21
Phaff./Saccharomycetaceae - ps22
Saccharomyco./mycetaceae - ps23
Saccharomycetaceae - ps24
Saccharomycetaceae_2 - ps25
Saccharomycetaceae_3 - ps26
Kaz./Nau./Saccharomyces - ps27
Saccharomyces - ps28
Arboricola_clade - ps29
Cerevisiae_clade - ps30
Saccharomyces_cerevisiae - ps31
Phylostratum

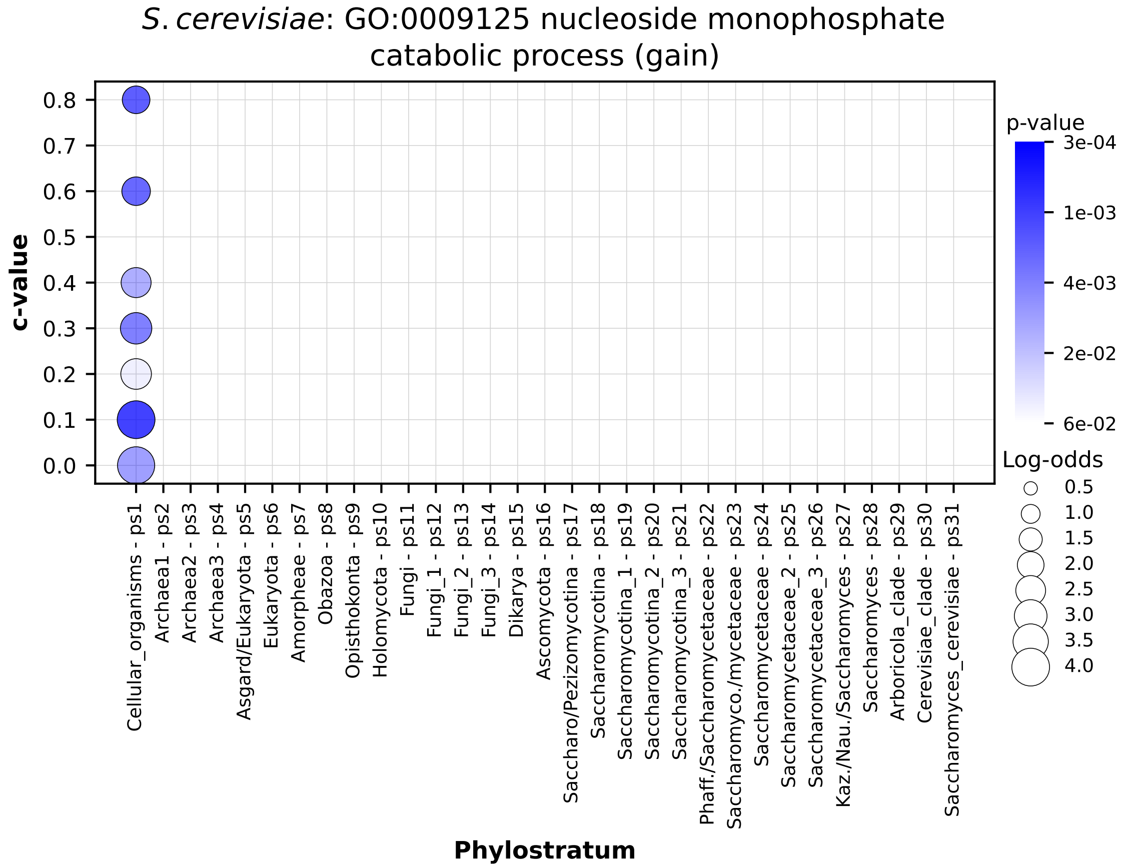

S. cerevisiae: GO:0009125 nucleoside monophosphate catabolic process (gain)
c-value
0.0
0.1
0.2
0.3
0.4
0.5
0.6
0.7
0.8
Phylostratum
Cellular_organisms - ps1
Archaea1 - ps2
Archaea2 - ps3
Archaea3 - ps4
Asgard/Eukaryota - ps5
Eukaryota - ps6
Amorpheae - ps7
Obazoa - ps8
Opisthokonta - ps9
Holomycota - ps10
Fungi - ps11
Fungi_1 - ps12
Fungi_2 - ps13
Fungi_3 - ps14
Dikarya - ps15
Ascomycota - ps16
Saccharo/Pezizomycotina - ps17
Saccharomycotina - ps18
Saccharomycotina_1 - ps19
Saccharomycotina_2 - ps20
Saccharomycotina_3 - ps21
Phaff./Saccharomycetaceae - ps22
Saccharomyco./mycetaceae - ps23
Saccharomycetaceae - ps24
Saccharomycetaceae_2 - ps25
Saccharomycetaceae_3 - ps26
Kaz./Nau./Saccharomyces - ps27
Saccharomyces - ps28
Arboricola_clade - ps29
Cerevisiae_clade - ps30
Saccharomyces_cerevisiae - ps31
p-value
3e-04
1e-03
4e-03
2e-02
6e-02
Log-odds
0.5
1.0
1.5
2.0
2.5
3.0
3.5
4.0

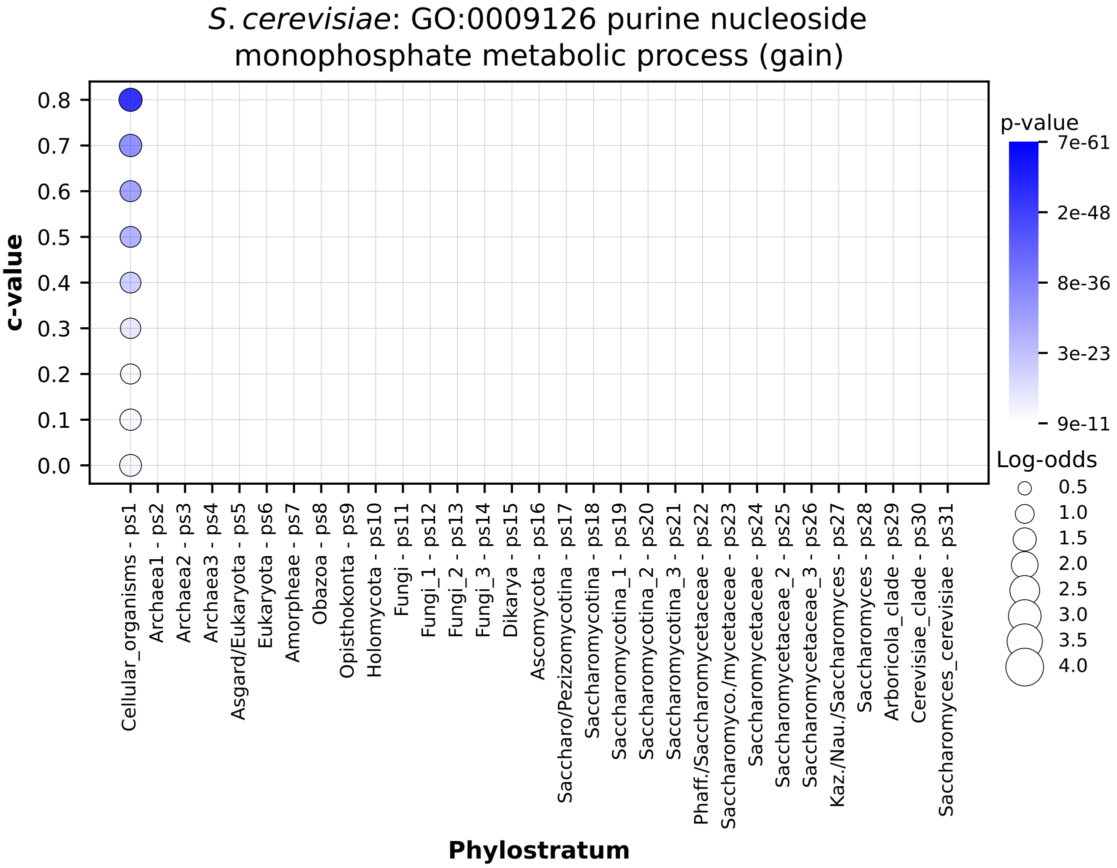
S. cerevisiae: GO:0009126 purine nucleoside monophosphate metabolic process (gain)
c-value
0.8
0.7
0.6
0.5
0.4
0.3
0.2
0.1
0.0
p-value
7e-61
2e-48
8e-36
3e-23
9e-11
Log-odds
0.5
1.0
1.5
2.0
2.5
3.0
3.5
4.0
Cellular_organisms - ps1
Archaea1 - ps2
Archaea2 - ps3
Archaea3 - ps4
Asgard/Eukaryota - ps5
Eukaryota - ps6
Amorpheae - ps7
Obazoa - ps8
Opisthokonta - ps9
Holomycota - ps10
Fungi - ps11
Fungi_1 - ps12
Fungi_2 - ps13
Fungi_3 - ps14
Dikarya - ps15
Ascomycota - ps16
Saccharo/Pezizomycotina - ps17
Saccharomycotina - ps18
Saccharomycotina_1 - ps19
Saccharomycotina_2 - ps20
Saccharomycotina_3 - ps21
Phaff./Saccharomycetaceae - ps22
Saccharomyco./mycetaceae - ps23
Saccharomycetaceae - ps24
Saccharomycetaceae_2 - ps25
Saccharomycetaceae_3 - ps26
Kaz./Nau./Saccharomyces - ps27
Saccharomyces - ps28
Arboricola_clade - ps29
Cerevisiae_clade - ps30
Saccharomyces_cerevisiae - ps31
Phylostratum

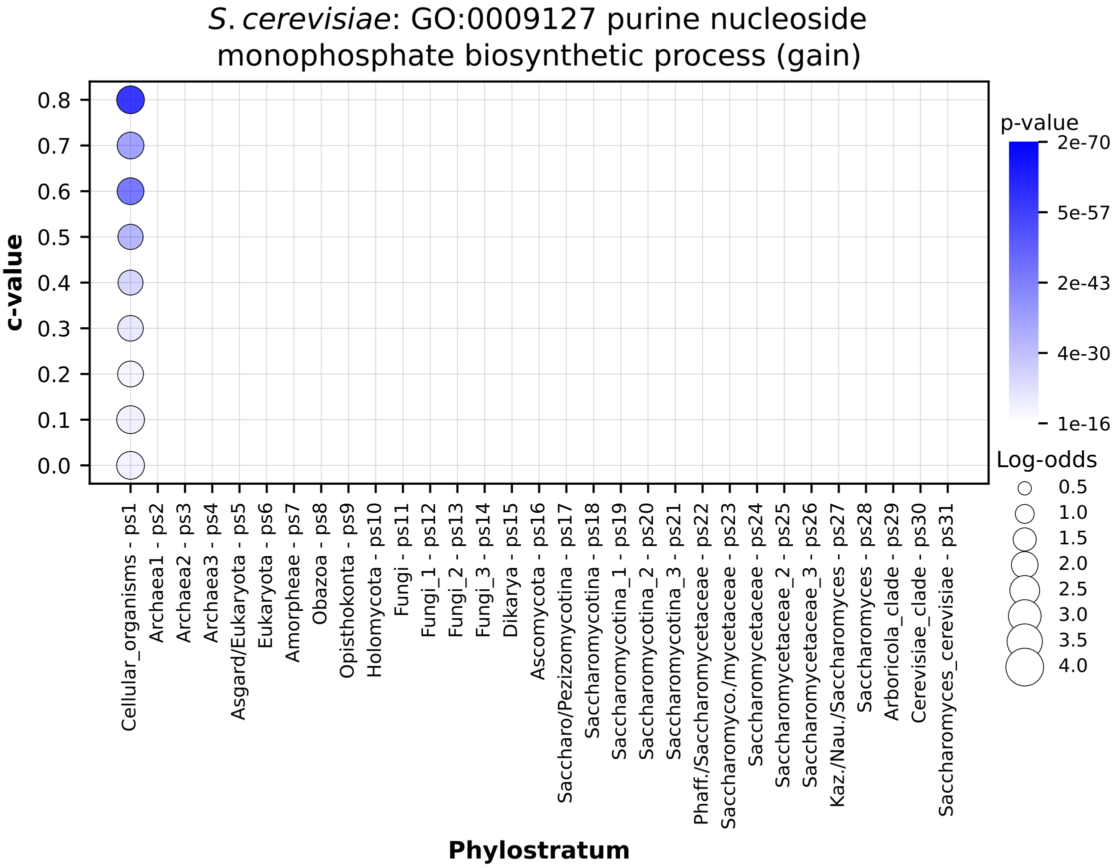

S. cerevisiae: GO:0009127 purine nucleoside monophosphate biosynthetic process (gain)
c-value
0.8
0.7
0.6
0.5
0.4
0.3
0.2
0.1
0.0
p-value
2e-70
5e-57
2e-43
4e-30
1e-16
Log-odds
0.5
1.0
1.5
2.0
2.5
3.0
3.5
4.0
Cellular_organisms - ps1
Archaea1 - ps2
Archaea2 - ps3
Archaea3 - ps4
Asgard/Eukaryota - ps5
Eukaryota - ps6
Amorpheae - ps7
Obazoa - ps8
Opisthokonta - ps9
Holomycota - ps10
Fungi - ps11
Fungi_1 - ps12
Fungi_2 - ps13
Fungi_3 - ps14
Dikarya - ps15
Ascomycota - ps16
Saccharo/Pezizomycotina - ps17
Saccharomycotina - ps18
Saccharomycotina_1 - ps19
Saccharomycotina_2 - ps20
Saccharomycotina_3 - ps21
Phaff./Saccharomycetaceae - ps22
Saccharomyco./mycetaceae - ps23
Saccharomycetaceae - ps24
Saccharomycetaceae_2 - ps25
Saccharomycetaceae_3 - ps26
Kaz./Nau./Saccharomyces - ps27
Saccharomyces - ps28
Arboricola_clade - ps29
Cerevisiae_clade - ps30
Saccharomyces_cerevisiae - ps31
Phylostratum

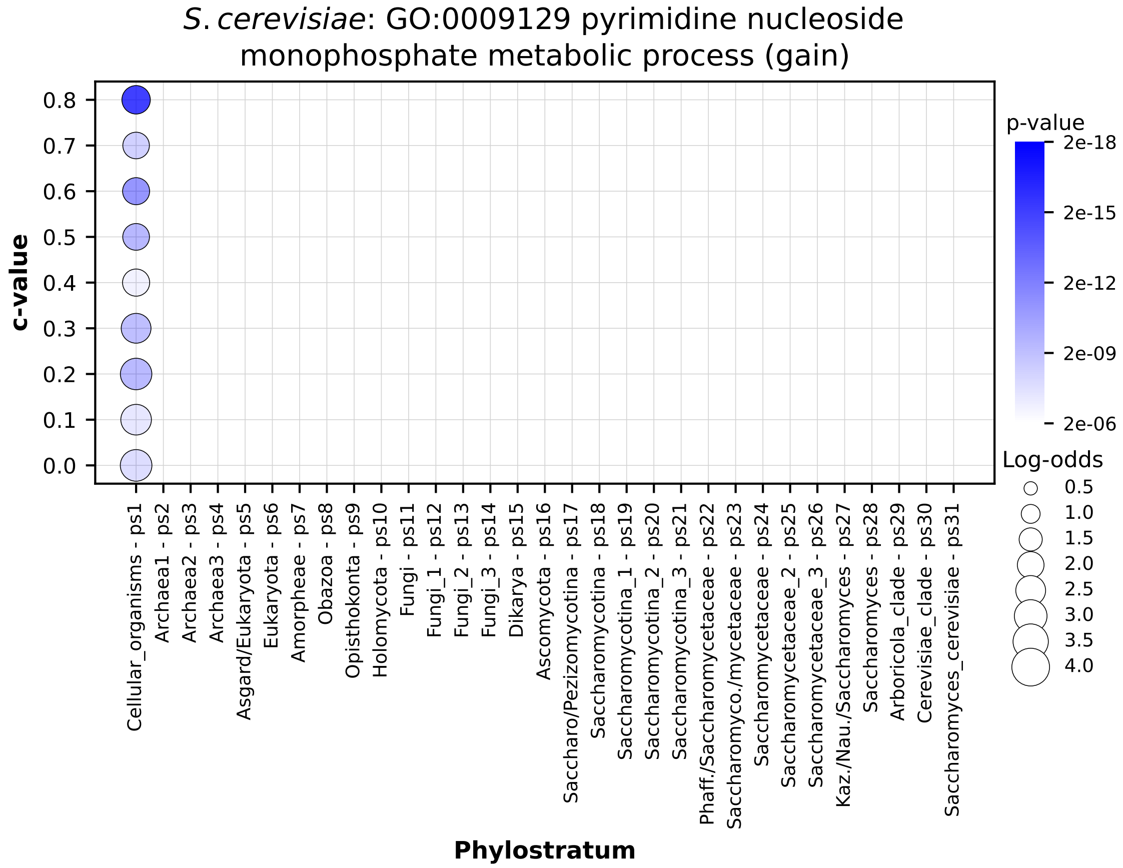

S. cerevisiae: GO:0009129 pyrimidine nucleoside monophosphate metabolic process (gain)
c-value
0.8
0.7
0.6
0.5
0.4
0.3
0.2
0.1
0.0
p-value
2e-18
2e-15
2e-12
2e-09
2e-06
Log-odds
0.5
1.0
1.5
2.0
2.5
3.0
3.5
4.0
Cellular_organisms - ps1
Archaea1 - ps2
Archaea2 - ps3
Archaea3 - ps4
Asgard/Eukaryota - ps5
Eukaryota - ps6
Amorpheae - ps7
Obazoa - ps8
Opisthokonta - ps9
Holomycota - ps10
Fungi - ps11
Fungi_1 - ps12
Fungi_2 - ps13
Fungi_3 - ps14
Dikarya - ps15
Ascomycota - ps16
Saccharo/Pezizomycotina - ps17
Saccharomycotina - ps18
Saccharomycotina_1 - ps19
Saccharomycotina_2 - ps20
Saccharomycotina_3 - ps21
Phaff./Saccharomycetaceae - ps22
Saccharomyco./mycetaceae - ps23
Saccharomycetaceae - ps24
Saccharomycetaceae_2 - ps25
Saccharomycetaceae_3 - ps26
Kaz./Nau./Saccharomyces - ps27
Saccharomyces - ps28
Arboricola_clade - ps29
Cerevisiae_clade - ps30
Saccharomyces_cerevisiae - ps31
Phylostratum

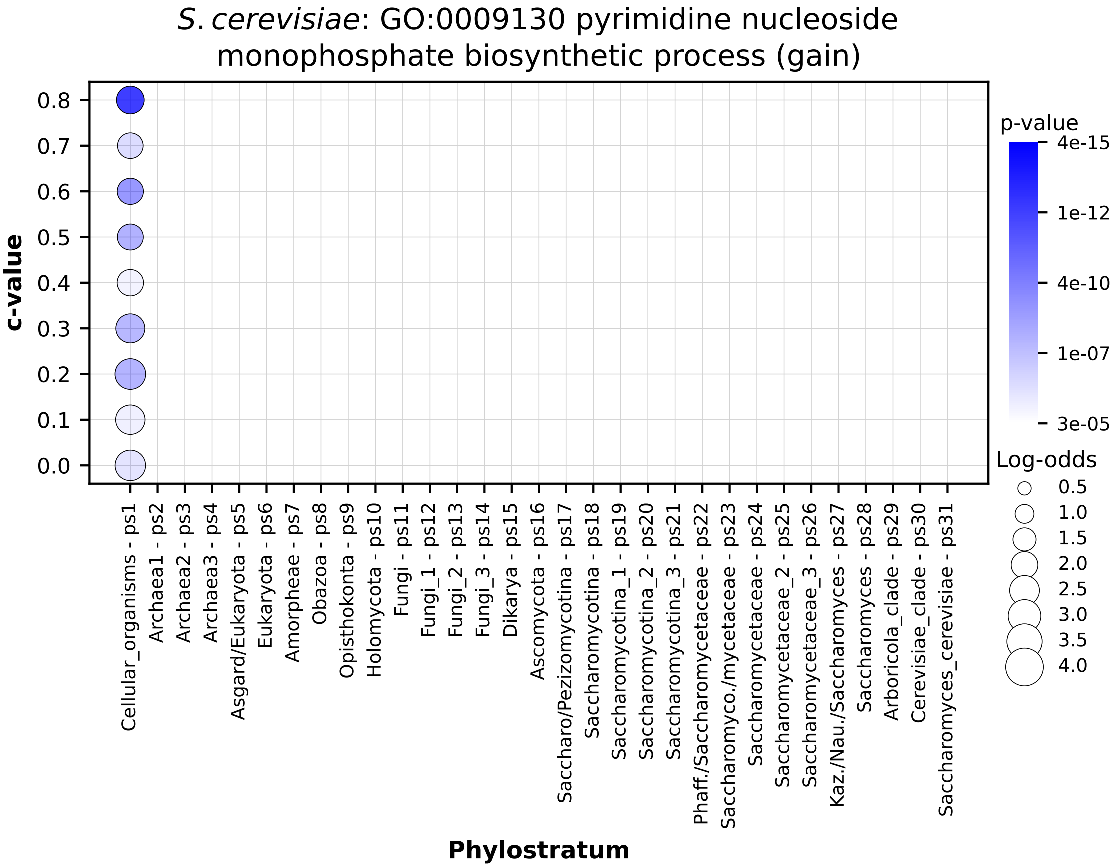

S. cerevisiae: GO:0009130 pyrimidine nucleoside monophosphate biosynthetic process (gain)
c-value
0.8
0.7
0.6
0.5
0.4
0.3
0.2
0.1
0.0
p-value
4e-15
1e-12
4e-10
1e-07
3e-05
Log-odds
0.5
1.0
1.5
2.0
2.5
3.0
3.5
4.0
Cellular_organisms - ps1
Archaea1 - ps2
Archaea2 - ps3
Archaea3 - ps4
Asgard/Eukaryota - ps5
Eukaryota - ps6
Amorpheae - ps7
Obazoa - ps8
Opisthokonta - ps9
Holomycota - ps10
Fungi - ps11
Fungi_1 - ps12
Fungi_2 - ps13
Fungi_3 - ps14
Dikarya - ps15
Ascomycota - ps16
Saccharo/Pezizomycotina - ps17
Saccharomycotina - ps18
Saccharomycotina_1 - ps19
Saccharomycotina_2 - ps20
Saccharomycotina_3 - ps21
Phaff./Saccharomycetaceae - ps22
Saccharomyco./mycetaceae - ps23
Saccharomycetaceae - ps24
Saccharomycetaceae_2 - ps25
Saccharomycetaceae_3 - ps26
Kaz./Nau./Saccharomyces - ps27
Saccharomyces - ps28
Arboricola_clade - ps29
Cerevisiae_clade - ps30
Saccharomyces_cerevisiae - ps31
Phylostratum

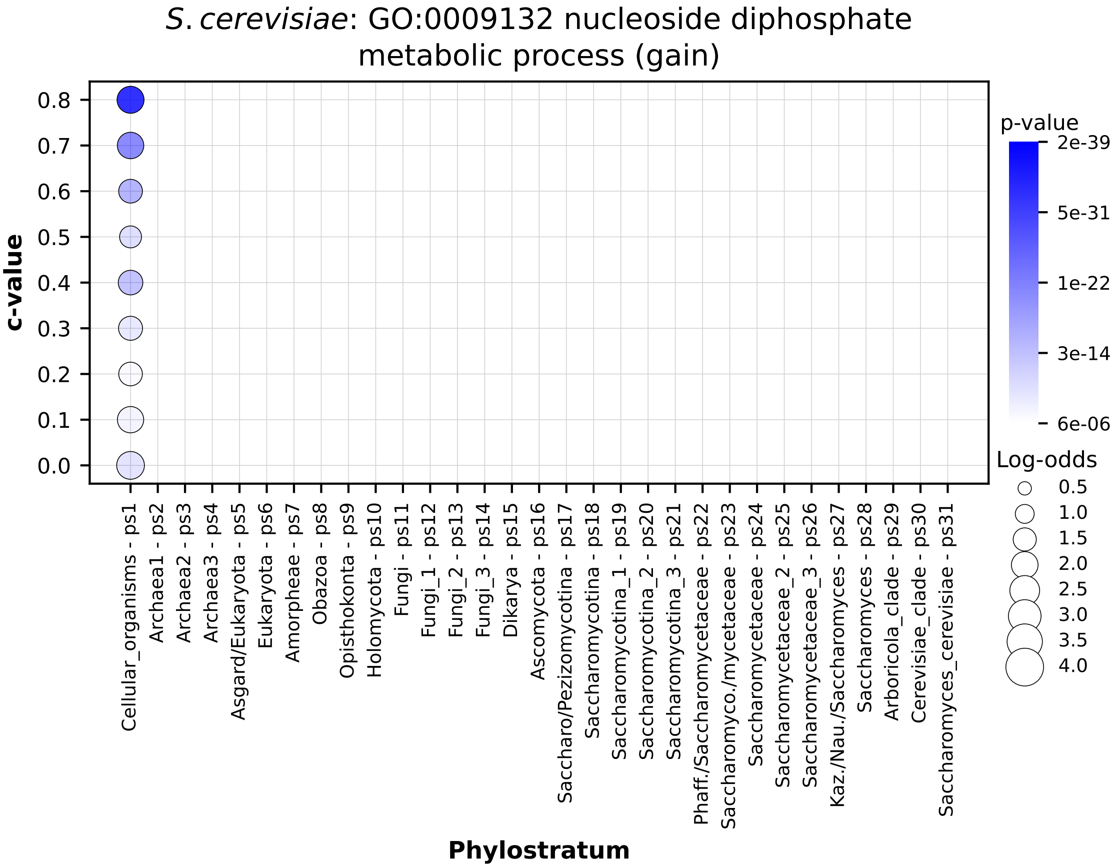

S. cerevisiae: GO:0009132 nucleoside diphosphate metabolic process (gain)
c-value
0.8
0.7
0.6
0.5
0.4
0.3
0.2
0.1
0.0
p-value
2e-39
5e-31
1e-22
3e-14
6e-06
Log-odds
0.5
1.0
1.5
2.0
2.5
3.0
3.5
4.0
Cellular_organisms - ps1
Archaea1 - ps2
Archaea2 - ps3
Archaea3 - ps4
Asgard/Eukaryota - ps5
Eukaryota - ps6
Amorpheae - ps7
Obazoa - ps8
Opisthokonta - ps9
Holomycota - ps10
Fungi - ps11
Fungi_1 - ps12
Fungi_2 - ps13
Fungi_3 - ps14
Dikarya - ps15
Ascomycota - ps16
Saccharo/Pezizomycotina - ps17
Saccharomycotina - ps18
Saccharomycotina_1 - ps19
Saccharomycotina_2 - ps20
Saccharomycotina_3 - ps21
Phaff./Saccharomycetaceae - ps22
Saccharomyco./mycetaceae - ps23
Saccharomycetaceae - ps24
Saccharomycetaceae_2 - ps25
Saccharomycetaceae_3 - ps26
Kaz./Nau./Saccharomyces - ps27
Saccharomyces - ps28
Arboricola_clade - ps29
Cerevisiae_clade - ps30
Saccharomyces_cerevisiae - ps31
Phylostratum

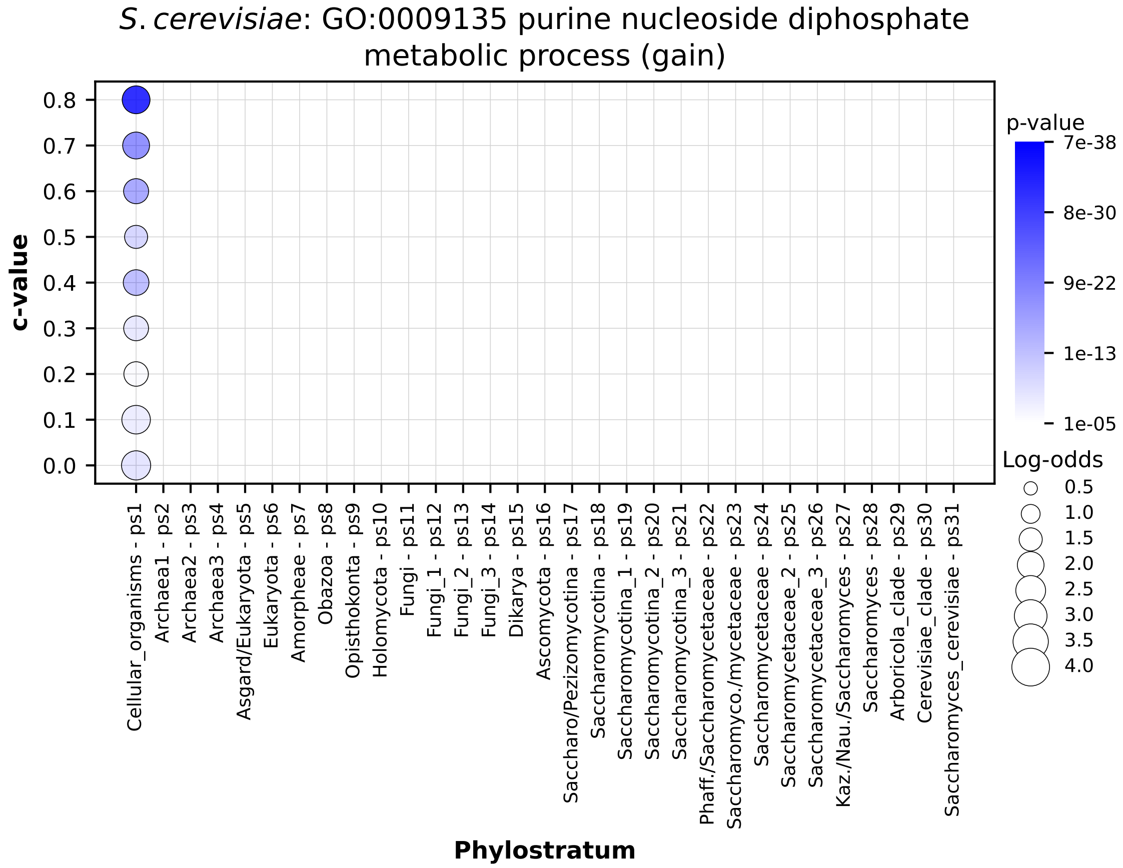

S. cerevisiae: GO:0009135 purine nucleoside diphosphate metabolic process (gain)
c-value
0.0
0.1
0.2
0.3
0.4
0.5
0.6
0.7
0.8
p-value
7e-38
8e-30
9e-22
1e-13
1e-05
Log-odds
0.5
1.0
1.5
2.0
2.5
3.0
3.5
4.0
Cellular_organisms - ps1
Archaea1 - ps2
Archaea2 - ps3
Archaea3 - ps4
Asgard/Eukaryota - ps5
Eukaryota - ps6
Amorpheae - ps7
Obazoa - ps8
Opisthokonta - ps9
Holomycota - ps10
Fungi - ps11
Fungi_1 - ps12
Fungi_2 - ps13
Fungi_3 - ps14
Dikarya - ps15
Ascomycota - ps16
Saccharo/Pezizomycotina - ps17
Saccharomycotina - ps18
Saccharomycotina_1 - ps19
Saccharomycotina_2 - ps20
Saccharomycotina_3 - ps21
Phaff./Saccharomycetaceae - ps22
Saccharomyco./mycetaceae - ps23
Saccharomycetaceae - ps24
Saccharomycetaceae_2 - ps25
Saccharomycetaceae_3 - ps26
Kaz./Nau./Saccharomyces - ps27
Saccharomyces - ps28
Arboricola_clade - ps29
Cerevisiae_clade - ps30
Saccharomyces_cerevisiae - ps31
Phylostratum

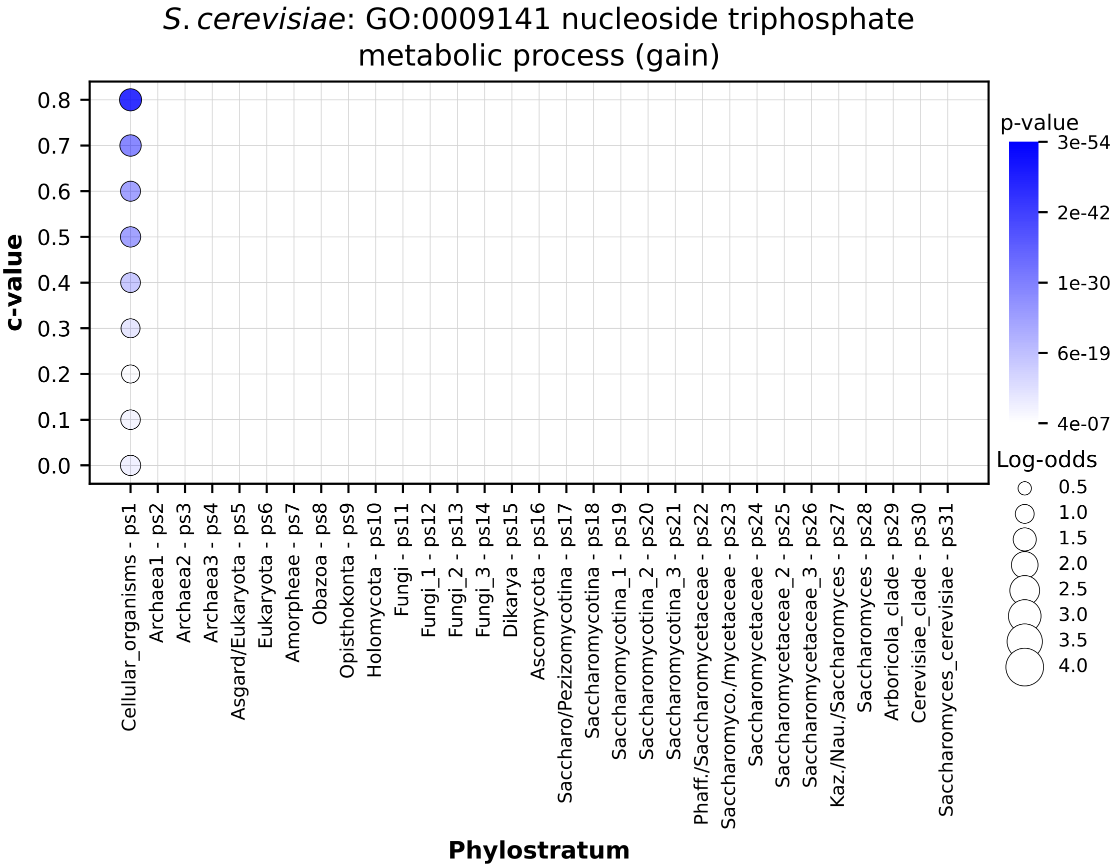
S. cerevisiae: GO:0009141 nucleoside triphosphate metabolic process (gain)
c-value
0.8
0.7
0.6
0.5
0.4
0.3
0.2
0.1
0.0
p-value
3e-54
2e-42
1e-30
6e-19
4e-07
Log-odds
0.5
1.0
1.5
2.0
2.5
3.0
3.5
4.0
Cellular_organisms - ps1
Archaea1 - ps2
Archaea2 - ps3
Archaea3 - ps4
Asgard/Eukaryota - ps5
Eukaryota - ps6
Amorpheae - ps7
Obazoa - ps8
Opisthokonta - ps9
Holomycota - ps10
Fungi - ps11
Fungi_1 - ps12
Fungi_2 - ps13
Fungi_3 - ps14
Dikarya - ps15
Ascomycota - ps16
Saccharo/Pezizomycotina - ps17
Saccharomycotina - ps18
Saccharomycotina_1 - ps19
Saccharomycotina_2 - ps20
Saccharomycotina_3 - ps21
Phaff./Saccharomycetaceae - ps22
Saccharomyco./mycetaceae - ps23
Saccharomycetaceae - ps24
Saccharomycetaceae_2 - ps25
Saccharomycetaceae_3 - ps26
Kaz./Nau./Saccharomyces - ps27
Saccharomyces - ps28
Arboricola_clade - ps29
Cerevisiae_clade - ps30
Saccharomyces_cerevisiae - ps31
Phylostratum

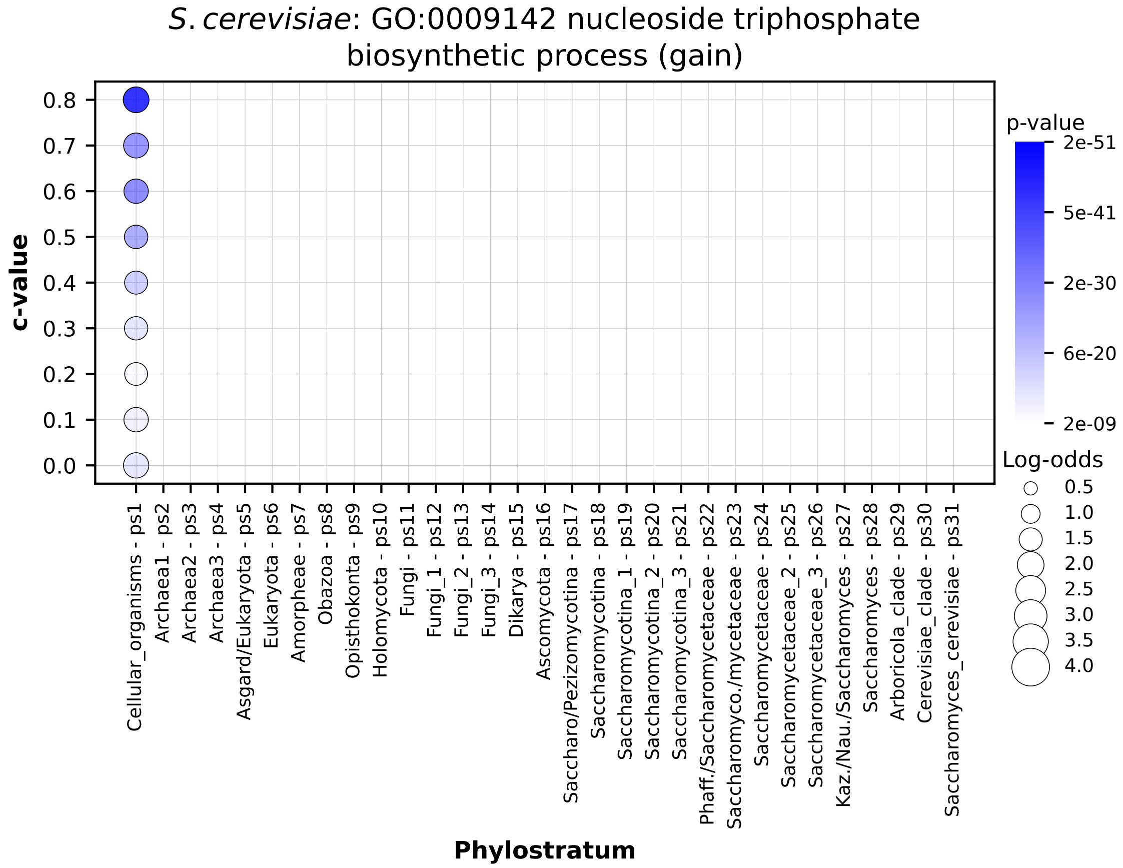

S. cerevisiae: GO:0009142 nucleoside triphosphate biosynthetic process (gain)
c-value
0.8
0.7
0.6
0.5
0.4
0.3
0.2
0.1
0.0
Phylostratum
Cellular_organisms - ps1
Archaea1 - ps2
Archaea2 - ps3
Archaea3 - ps4
Asgard/Eukaryota - ps5
Eukaryota - ps6
Amorpheae - ps7
Obazoa - ps8
Opisthokonta - ps9
Holomycota - ps10
Fungi - ps11
Fungi_1 - ps12
Fungi_2 - ps13
Fungi_3 - ps14
Dikarya - ps15
Ascomycota - ps16
Saccharo/Pezizomycotina - ps17
Saccharomycotina - ps18
Saccharomycotina_1 - ps19
Saccharomycotina_2 - ps20
Saccharomycotina_3 - ps21
Phaff./Saccharomycetaceae - ps22
Saccharomyco./mycetaceae - ps23
Saccharomycetaceae - ps24
Saccharomycetaceae_2 - ps25
Saccharomycetaceae_3 - ps26
Kaz./Nau./Saccharomyces - ps27
Saccharomyces - ps28
Arboricola_clade - ps29
Cerevisiae_clade - ps30
Saccharomyces_cerevisiae - ps31
p-value
2e-51
5e-41
2e-30
6e-20
2e-09
Log-odds
0.5
1.0
1.5
2.0
2.5
3.0
3.5
4.0

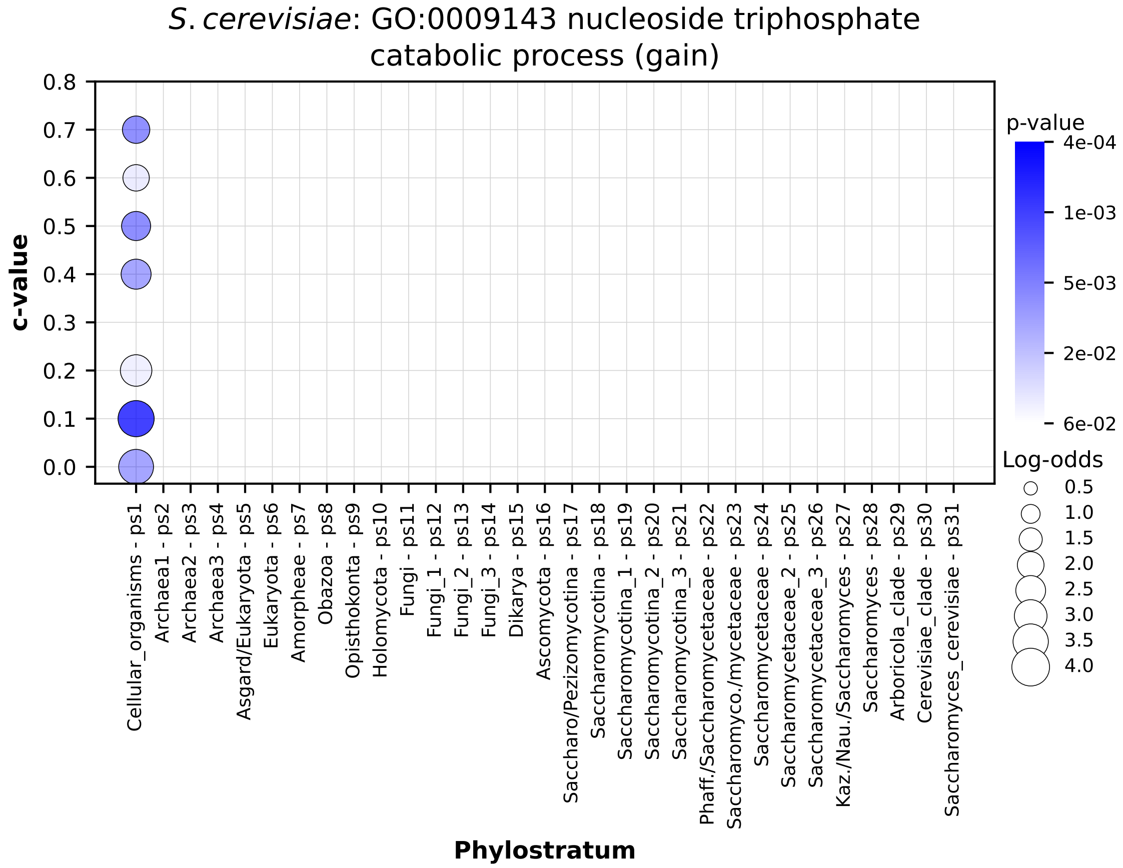

S. cerevisiae: GO:0009143 nucleoside triphosphate catabolic process (gain)
c-value
0.8
0.7
0.6
0.5
0.4
0.3
0.2
0.1
0.0
p-value
4e-04
1e-03
5e-03
2e-02
6e-02
Log-odds
0.5
1.0
1.5
2.0
2.5
3.0
3.5
4.0
Cellular_organisms - ps1
Archaea1 - ps2
Archaea2 - ps3
Archaea3 - ps4
Asgard/Eukaryota - ps5
Eukaryota - ps6
Amorpheae - ps7
Obazoa - ps8
Opisthokonta - ps9
Holomycota - ps10
Fungi - ps11
Fungi_1 - ps12
Fungi_2 - ps13
Fungi_3 - ps14
Dikarya - ps15
Ascomycota - ps16
Saccharo/Pezizomycotina - ps17
Saccharomycotina - ps18
Saccharomycotina_1 - ps19
Saccharomycotina_2 - ps20
Saccharomycotina_3 - ps21
Phaff./Saccharomycetaceae - ps22
Saccharomyco./mycetaceae - ps23
Saccharomycetaceae - ps24
Saccharomycetaceae_2 - ps25
Saccharomycetaceae_3 - ps26
Kaz./Nau./Saccharomyces - ps27
Saccharomyces - ps28
Arboricola_clade - ps29
Cerevisiae_clade - ps30
Saccharomyces_cerevisiae - ps31
Phylostratum

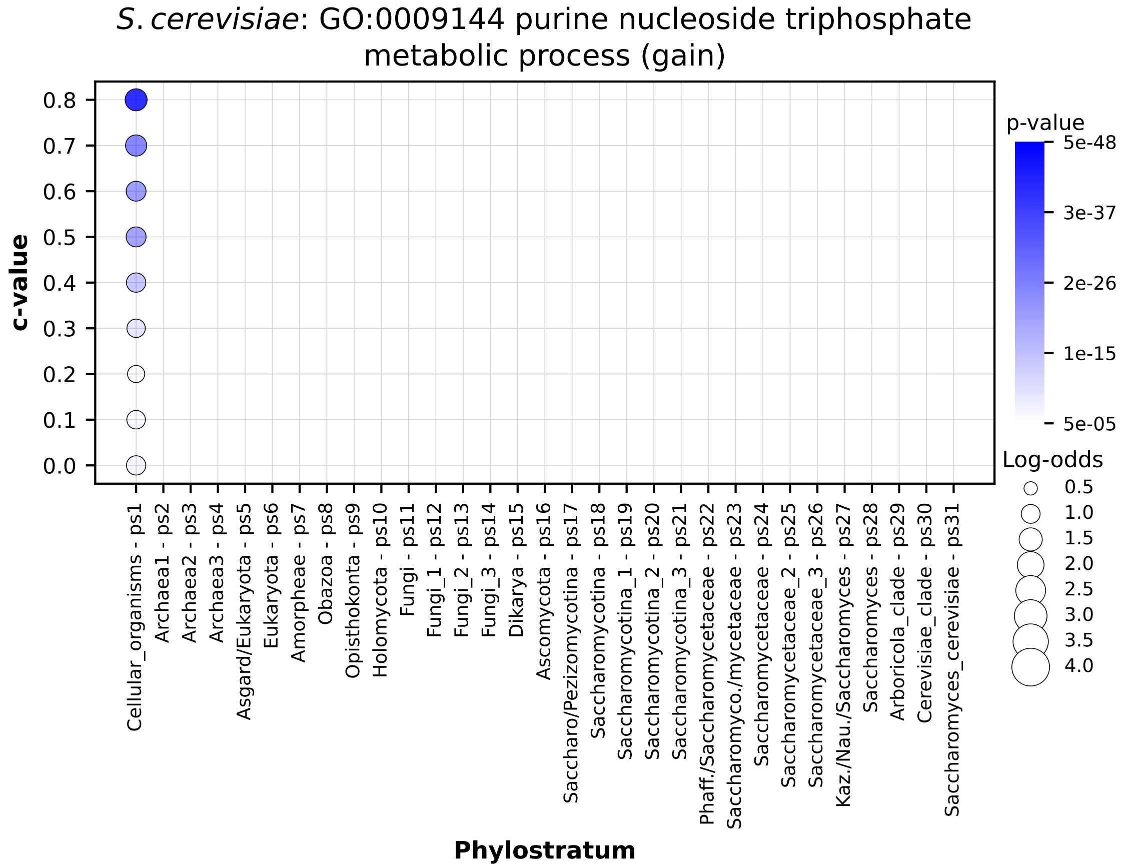
S. cerevisiae: GO:0009144 purine nucleoside triphosphate metabolic process (gain)
c-value
0.8
0.7
0.6
0.5
0.4
0.3
0.2
0.1
0.0
p-value
5e-48
3e-37
2e-26
1e-15
5e-05
Log-odds
0.5
1.0
1.5
2.0
2.5
3.0
3.5
4.0
Cellular_organisms - ps1
Archaea1 - ps2
Archaea2 - ps3
Archaea3 - ps4
Asgard/Eukaryota - ps5
Eukaryota - ps6
Amorpheae - ps7
Obazoa - ps8
Opisthokonta - ps9
Holomycota - ps10
Fungi - ps11
Fungi_1 - ps12
Fungi_2 - ps13
Fungi_3 - ps14
Dikarya - ps15
Ascomycota - ps16
Saccharo/Pezizomycotina - ps17
Saccharomycotina - ps18
Saccharomycotina_1 - ps19
Saccharomycotina_2 - ps20
Saccharomycotina_3 - ps21
Phaff./Saccharomycetaceae - ps22
Saccharomyco./mycetaceae - ps23
Saccharomycetaceae - ps24
Saccharomycetaceae_2 - ps25
Saccharomycetaceae_3 - ps26
Kaz./Nau./Saccharomyces - ps27
Saccharomyces - ps28
Arboricola_clade - ps29
Cerevisiae_clade - ps30
Saccharomyces_cerevisiae - ps31
Phylostratum

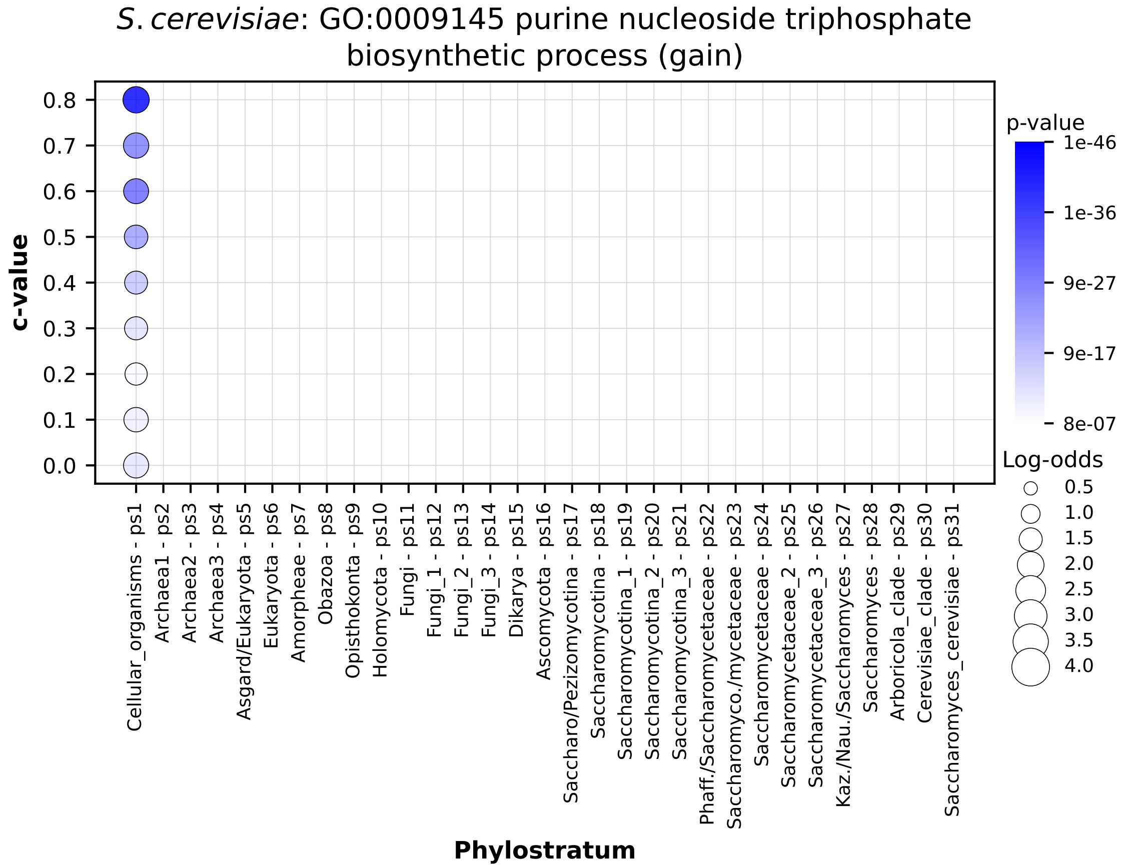

S. cerevisiae: GO:0009145 purine nucleoside triphosphate biosynthetic process (gain)
c-value
0.8
0.7
0.6
0.5
0.4
0.3
0.2
0.1
0.0
p-value
1e-46
1e-36
9e-27
9e-17
8e-07
Log-odds
0.5
1.0
1.5
2.0
2.5
3.0
3.5
4.0
Cellular_organisms - ps1
Archaea1 - ps2
Archaea2 - ps3
Archaea3 - ps4
Asgard/Eukaryota - ps5
Eukaryota - ps6
Amorpheae - ps7
Obazoa - ps8
Opisthokonta - ps9
Holomycota - ps10
Fungi - ps11
Fungi_1 - ps12
Fungi_2 - ps13
Fungi_3 - ps14
Dikarya - ps15
Ascomycota - ps16
Saccharo/Pezizomycotina - ps17
Saccharomycotina - ps18
Saccharomycotina_1 - ps19
Saccharomycotina_2 - ps20
Saccharomycotina_3 - ps21
Phaff./Saccharomycetaceae - ps22
Saccharomyco./mycetaceae - ps23
Saccharomycetaceae - ps24
Saccharomycetaceae_2 - ps25
Saccharomycetaceae_3 - ps26
Kaz./Nau./Saccharomyces - ps27
Saccharomyces - ps28
Arboricola_clade - ps29
Cerevisiae_clade - ps30
Saccharomyces_cerevisiae - ps31
Phylostratum

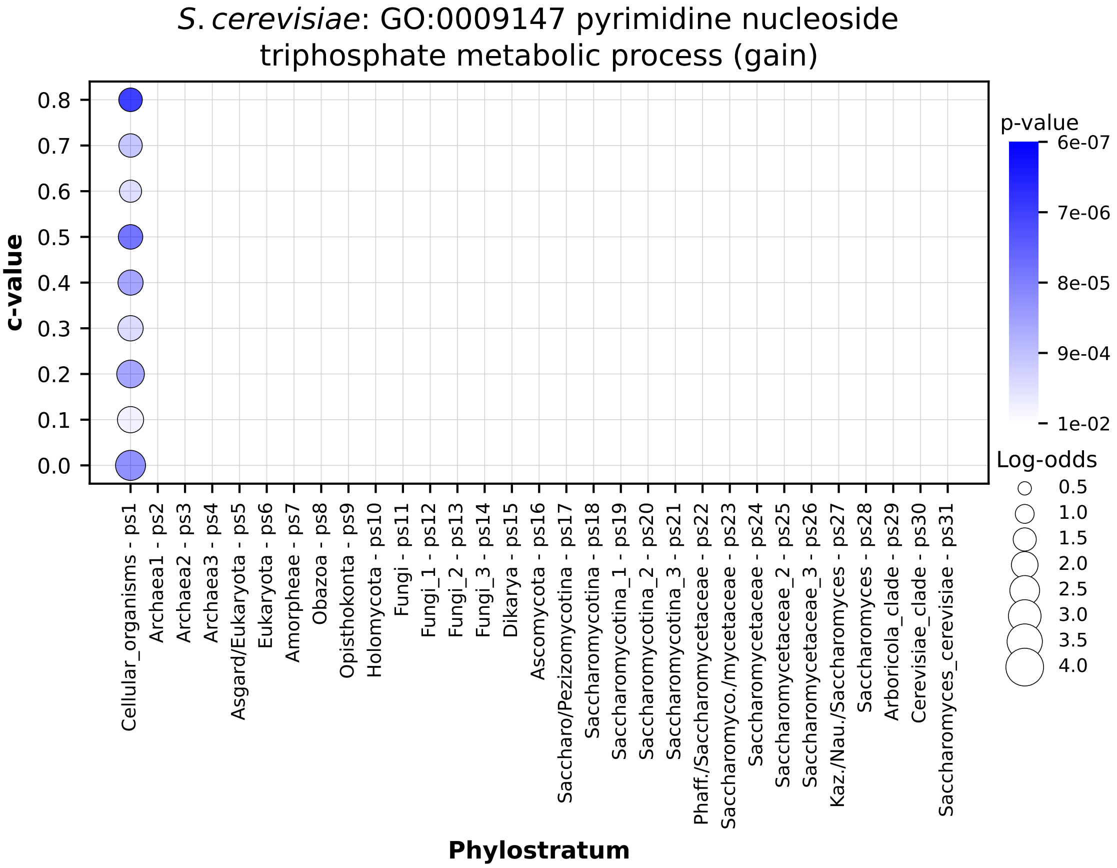

S. cerevisiae: GO:0009147 pyrimidine nucleoside triphosphate metabolic process (gain)
c-value
0.8
0.7
0.6
0.5
0.4
0.3
0.2
0.1
0.0
p-value
6e-07
7e-06
8e-05
9e-04
1e-02
Log-odds
0.5
1.0
1.5
2.0
2.5
3.0
3.5
4.0
Cellular_organisms - ps1
Archaea1 - ps2
Archaea2 - ps3
Archaea3 - ps4
Asgard/Eukaryota - ps5
Eukaryota - ps6
Amorpheae - ps7
Obazoa - ps8
Opisthokonta - ps9
Holomycota - ps10
Fungi - ps11
Fungi_1 - ps12
Fungi_2 - ps13
Fungi_3 - ps14
Dikarya - ps15
Ascomycota - ps16
Saccharo/Pezizomycotina - ps17
Saccharomycotina - ps18
Saccharomycotina_1 - ps19
Saccharomycotina_2 - ps20
Saccharomycotina_3 - ps21
Phaff./Saccharomycetaceae - ps22
Saccharomyco./mycetaceae - ps23
Saccharomycetaceae - ps24
Saccharomycetaceae_2 - ps25
Saccharomycetaceae_3 - ps26
Kaz./Nau./Saccharomyces - ps27
Saccharomyces - ps28
Arboricola_clade - ps29
Cerevisiae_clade - ps30
Saccharomyces_cerevisiae - ps31
Phylostratum

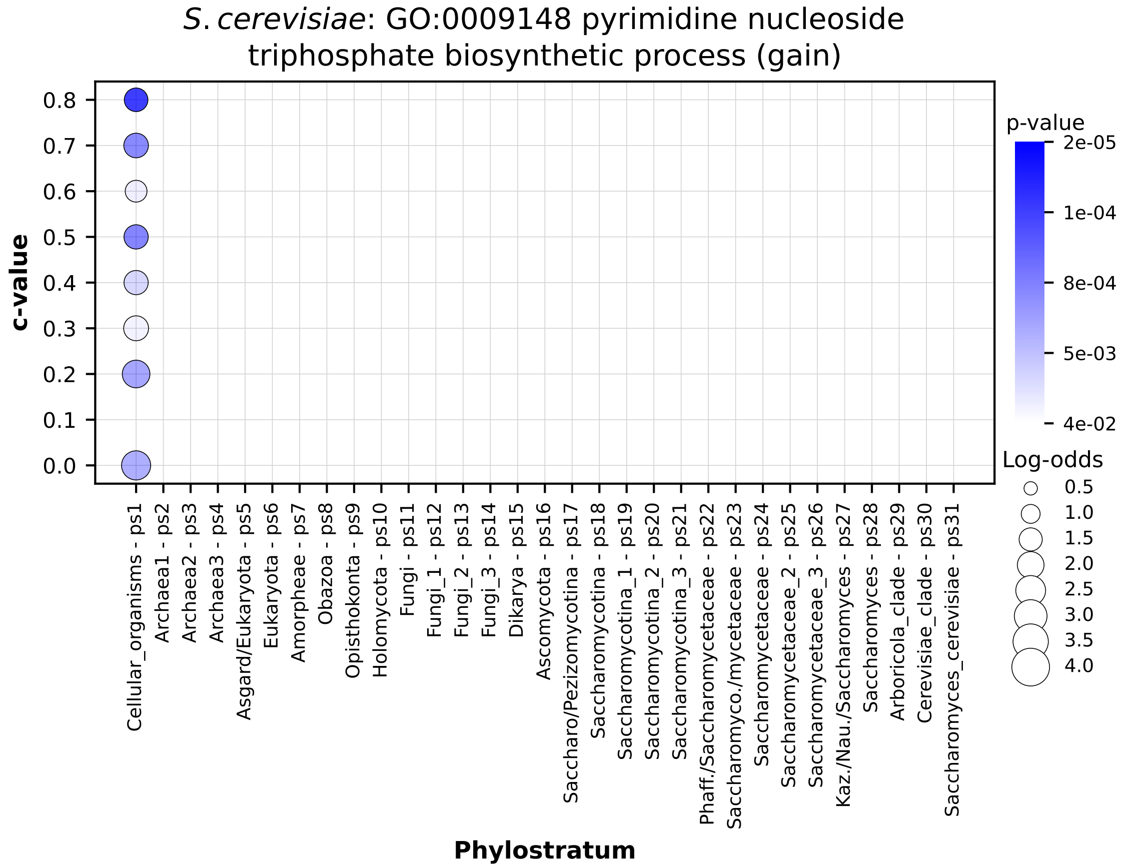
S. cerevisiae: GO:0009148 pyrimidine nucleoside triphosphate biosynthetic process (gain)
c-value
0.8
0.7
0.6
0.5
0.4
0.3
0.2
0.1
0.0
p-value
2e-05
1e-04
8e-04
5e-03
4e-02
Log-odds
0.5
1.0
1.5
2.0
2.5
3.0
3.5
4.0
Cellular_organisms - ps1
Archaea1 - ps2
Archaea2 - ps3
Archaea3 - ps4
Asgard/Eukaryota - ps5
Eukaryota - ps6
Amorpheae - ps7
Obazoa - ps8
Opisthokonta - ps9
Holomycota - ps10
Fungi - ps11
Fungi_1 - ps12
Fungi_2 - ps13
Fungi_3 - ps14
Dikarya - ps15
Ascomycota - ps16
Saccharo/Pezizomycotina - ps17
Saccharomycotina - ps18
Saccharomycotina_1 - ps19
Saccharomycotina_2 - ps20
Saccharomycotina_3 - ps21
Phaff./Saccharomycetaceae - ps22
Saccharomyco./mycetaceae - ps23
Saccharomycetaceae - ps24
Saccharomycetaceae_2 - ps25
Saccharomycetaceae_3 - ps26
Kaz./Nau./Saccharomyces - ps27
Saccharomyces - ps28
Arboricola_clade - ps29
Cerevisiae_clade - ps30
Saccharomyces_cerevisiae - ps31
Phylostratum

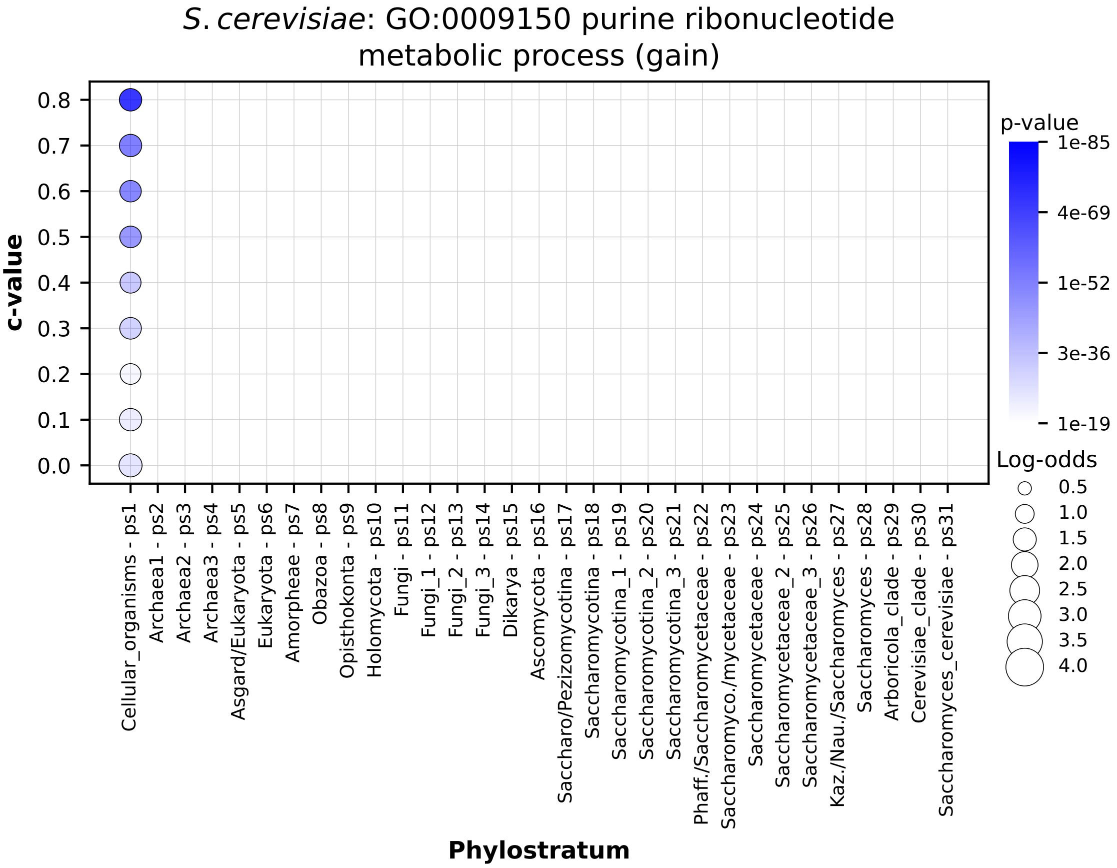

S. cerevisiae: GO:0009150 purine ribonucleotide metabolic process (gain)
c-value
0.8
0.7
0.6
0.5
0.4
0.3
0.2
0.1
0.0
p-value
1e-85
4e-69
1e-52
3e-36
1e-19
Log-odds
0.5
1.0
1.5
2.0
2.5
3.0
3.5
4.0
Cellular_organisms - ps1
Archaea1 - ps2
Archaea2 - ps3
Archaea3 - ps4
Asgard/Eukaryota - ps5
Eukaryota - ps6
Amorpheae - ps7
Obazoa - ps8
Opisthokonta - ps9
Holomycota - ps10
Fungi - ps11
Fungi_1 - ps12
Fungi_2 - ps13
Fungi_3 - ps14
Dikarya - ps15
Ascomycota - ps16
Saccharo/Pezizomycotina - ps17
Saccharomycotina - ps18
Saccharomycotina_1 - ps19
Saccharomycotina_2 - ps20
Saccharomycotina_3 - ps21
Phaff./Saccharomycetaceae - ps22
Saccharomyco./mycetaceae - ps23
Saccharomycetaceae - ps24
Saccharomycetaceae_2 - ps25
Saccharomycetaceae_3 - ps26
Kaz./Nau./Saccharomyces - ps27
Saccharomyces - ps28
Arboricola_clade - ps29
Cerevisiae_clade - ps30
Saccharomyces_cerevisiae - ps31
Phylostratum

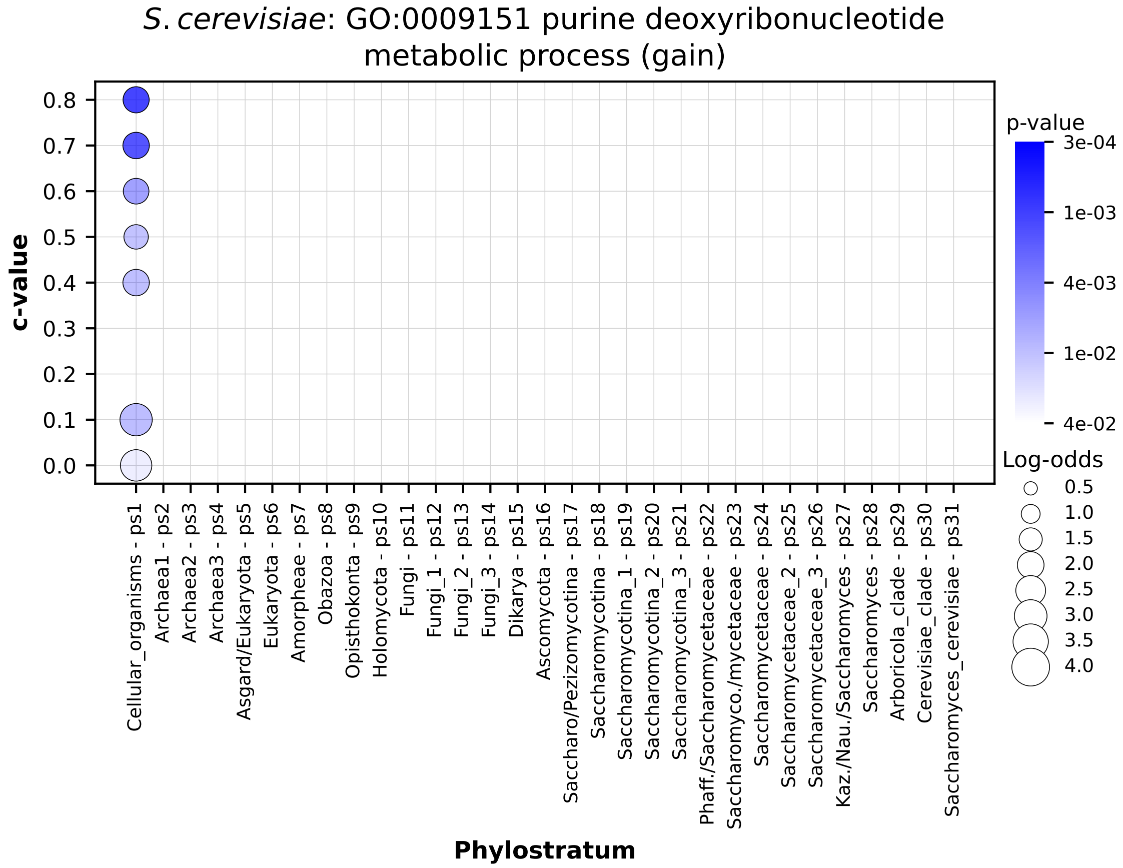
S. cerevisiae: GO:0009151 purine deoxyribonucleotide metabolic process (gain)
c-value
0.0
0.1
0.2
0.3
0.4
0.5
0.6
0.7
0.8
p-value
3e-04
1e-03
4e-03
1e-02
4e-02
Log-odds
0.5
1.0
1.5
2.0
2.5
3.0
3.5
4.0
Cellular_organisms - ps1
Archaea1 - ps2
Archaea2 - ps3
Archaea3 - ps4
Asgard/Eukaryota - ps5
Eukaryota - ps6
Amorpheae - ps7
Obazoa - ps8
Opisthokonta - ps9
Holomycota - ps10
Fungi - ps11
Fungi_1 - ps12
Fungi_2 - ps13
Fungi_3 - ps14
Dikarya - ps15
Ascomycota - ps16
Saccharo/Pezizomycotina - ps17
Saccharomycotina - ps18
Saccharomycotina_1 - ps19
Saccharomycotina_2 - ps20
Saccharomycotina_3 - ps21
Phaff./Saccharomycetaceae - ps22
Saccharomyco./mycetaceae - ps23
Saccharomycetaceae - ps24
Saccharomycetaceae_2 - ps25
Saccharomycetaceae_3 - ps26
Kaz./Nau./Saccharomyces - ps27
Saccharomyces - ps28
Arboricola_clade - ps29
Cerevisiae_clade - ps30
Saccharomyces_cerevisiae - ps31
Phylostratum

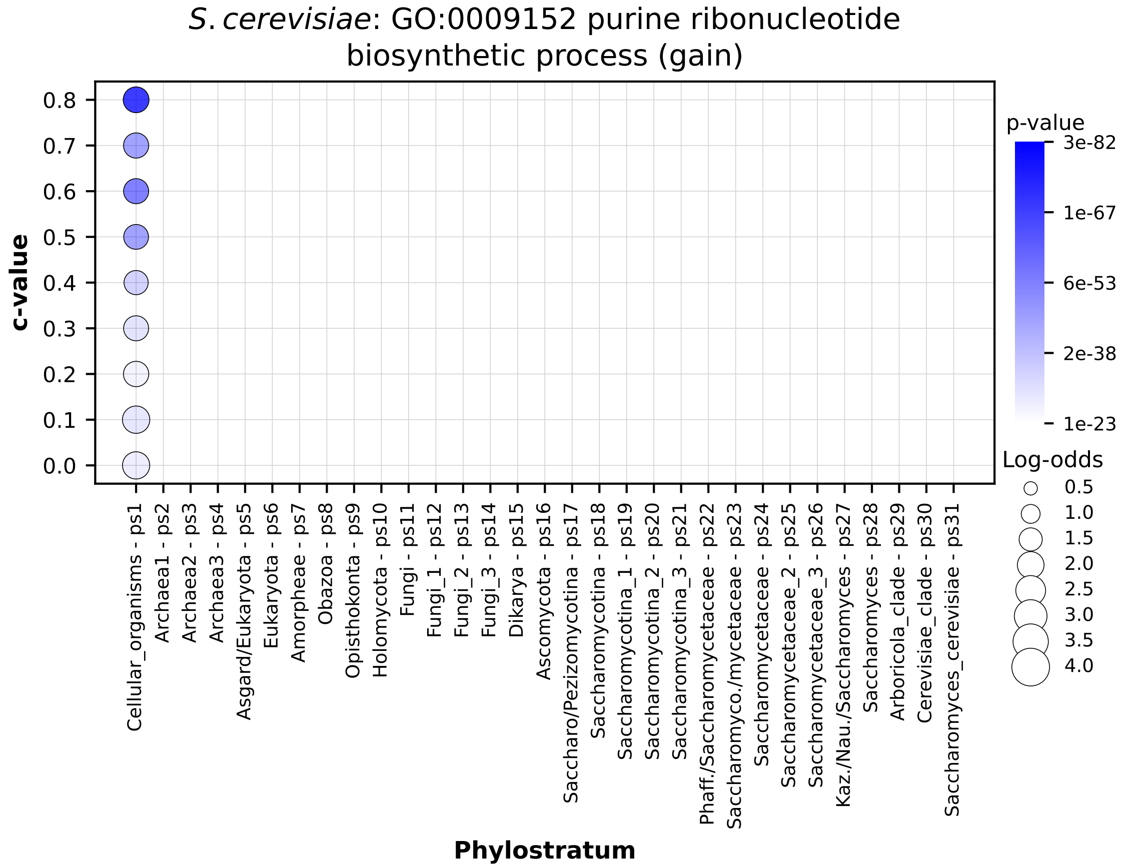

S. cerevisiae: GO:0009152 purine ribonucleotide biosynthetic process (gain)
c-value
0.8
0.7
0.6
0.5
0.4
0.3
0.2
0.1
0.0
p-value
3e-82
1e-67
6e-53
2e-38
1e-23
Log-odds
0.5
1.0
1.5
2.0
2.5
3.0
3.5
4.0
Cellular_organisms - ps1
Archaea1 - ps2
Archaea2 - ps3
Archaea3 - ps4
Asgard/Eukaryota - ps5
Eukaryota - ps6
Amorpheae - ps7
Obazoa - ps8
Opisthokonta - ps9
Holomycota - ps10
Fungi - ps11
Fungi_1 - ps12
Fungi_2 - ps13
Fungi_3 - ps14
Dikarya - ps15
Ascomycota - ps16
Saccharo/Pezizomycotina - ps17
Saccharomycotina - ps18
Saccharomycotina_1 - ps19
Saccharomycotina_2 - ps20
Saccharomycotina_3 - ps21
Phaff./Saccharomycetaceae - ps22
Saccharomyco./mycetaceae - ps23
Saccharomycetaceae - ps24
Saccharomycetaceae_2 - ps25
Saccharomycetaceae_3 - ps26
Kaz./Nau./Saccharomyces - ps27
Saccharomyces - ps28
Arboricola_clade - ps29
Cerevisiae_clade - ps30
Saccharomyces_cerevisiae - ps31
Phylostratum

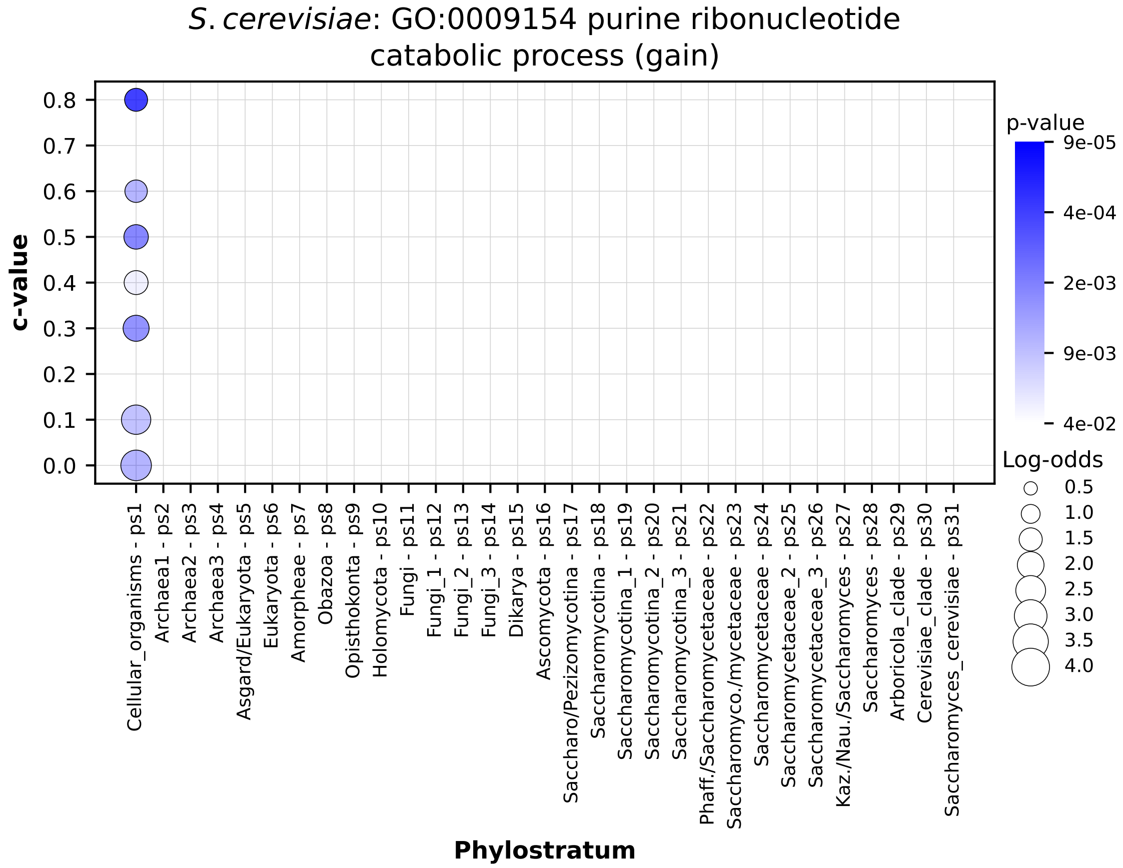

S. cerevisiae: GO:0009154 purine ribonucleotide catabolic process (gain)
c-value
0.8
0.7
0.6
0.5
0.4
0.3
0.2
0.1
0.0
p-value
9e-05
4e-04
2e-03
9e-03
4e-02
Log-odds
0.5
1.0
1.5
2.0
2.5
3.0
3.5
4.0
Cellular_organisms - ps1
Archaea1 - ps2
Archaea2 - ps3
Archaea3 - ps4
Asgard/Eukaryota - ps5
Eukaryota - ps6
Amorpheae - ps7
Obazoa - ps8
Opisthokonta - ps9
Holomycota - ps10
Fungi - ps11
Fungi_1 - ps12
Fungi_2 - ps13
Fungi_3 - ps14
Dikarya - ps15
Ascomycota - ps16
Saccharo/Pezizomycotina - ps17
Saccharomycotina - ps18
Saccharomycotina_1 - ps19
Saccharomycotina_2 - ps20
Saccharomycotina_3 - ps21
Phaff./Saccharomycetaceae - ps22
Saccharomyco./mycetaceae - ps23
Saccharomycetaceae - ps24
Saccharomycetaceae_2 - ps25
Saccharomycetaceae_3 - ps26
Kaz./Nau./Saccharomyces - ps27
Saccharomyces - ps28
Arboricola_clade - ps29
Cerevisiae_clade - ps30
Saccharomyces_cerevisiae - ps31
Phylostratum

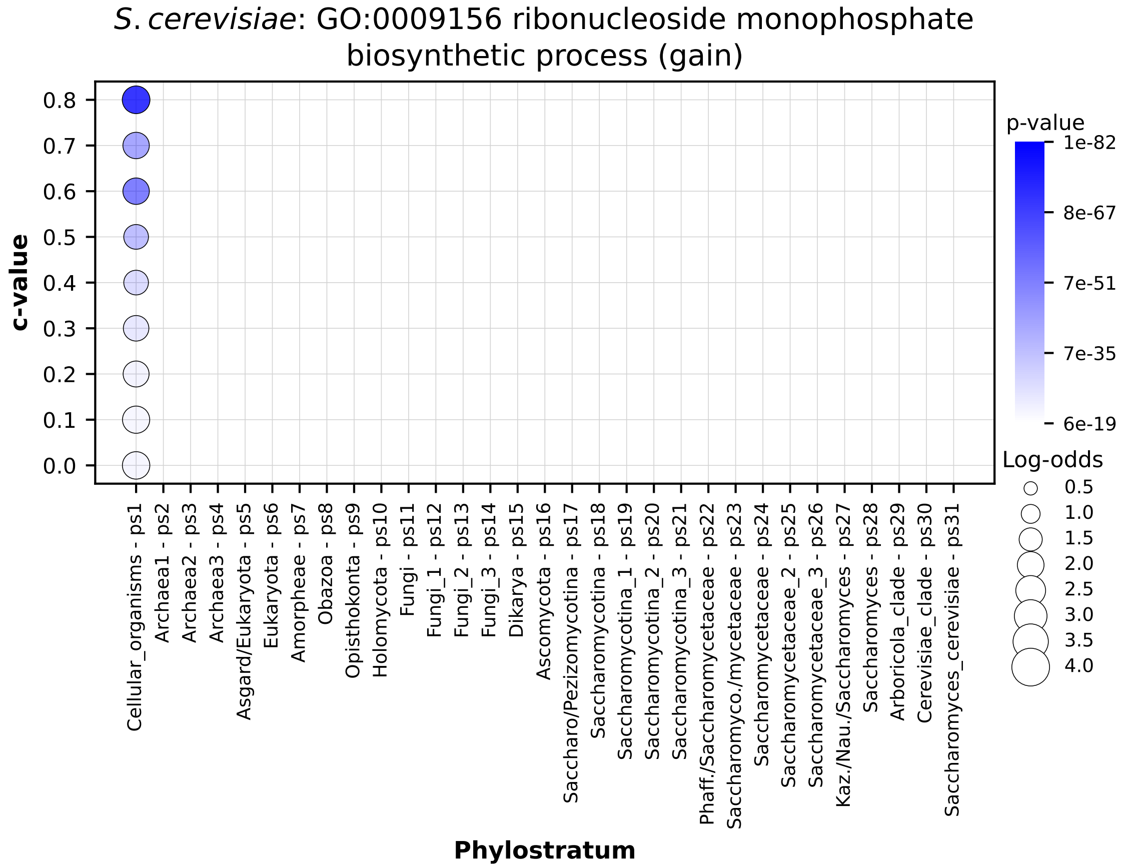

S. cerevisiae: GO:0009156 ribonucleoside monophosphate biosynthetic process (gain)
c-value
0.8
0.7
0.6
0.5
0.4
0.3
0.2
0.1
0.0
p-value
1e-82
8e-67
7e-51
7e-35
6e-19
Log-odds
0.5
1.0
1.5
2.0
2.5
3.0
3.5
4.0
Cellular_organisms - ps1
Archaea1 - ps2
Archaea2 - ps3
Archaea3 - ps4
Asgard/Eukaryota - ps5
Eukaryota - ps6
Amorpheae - ps7
Obazoa - ps8
Opisthokonta - ps9
Holomycota - ps10
Fungi - ps11
Fungi_1 - ps12
Fungi_2 - ps13
Fungi_3 - ps14
Dikarya - ps15
Ascomycota - ps16
Saccharo/Pezizomycotina - ps17
Saccharomycotina - ps18
Saccharomycotina_1 - ps19
Saccharomycotina_2 - ps20
Saccharomycotina_3 - ps21
Phaff./Saccharomycetaceae - ps22
Saccharomyco./mycetaceae - ps23
Saccharomycetaceae - ps24
Saccharomycetaceae_2 - ps25
Saccharomycetaceae_3 - ps26
Kaz./Nau./Saccharomyces - ps27
Saccharomyces - ps28
Arboricola_clade - ps29
Cerevisiae_clade - ps30
Saccharomyces_cerevisiae - ps31
Phylostratum

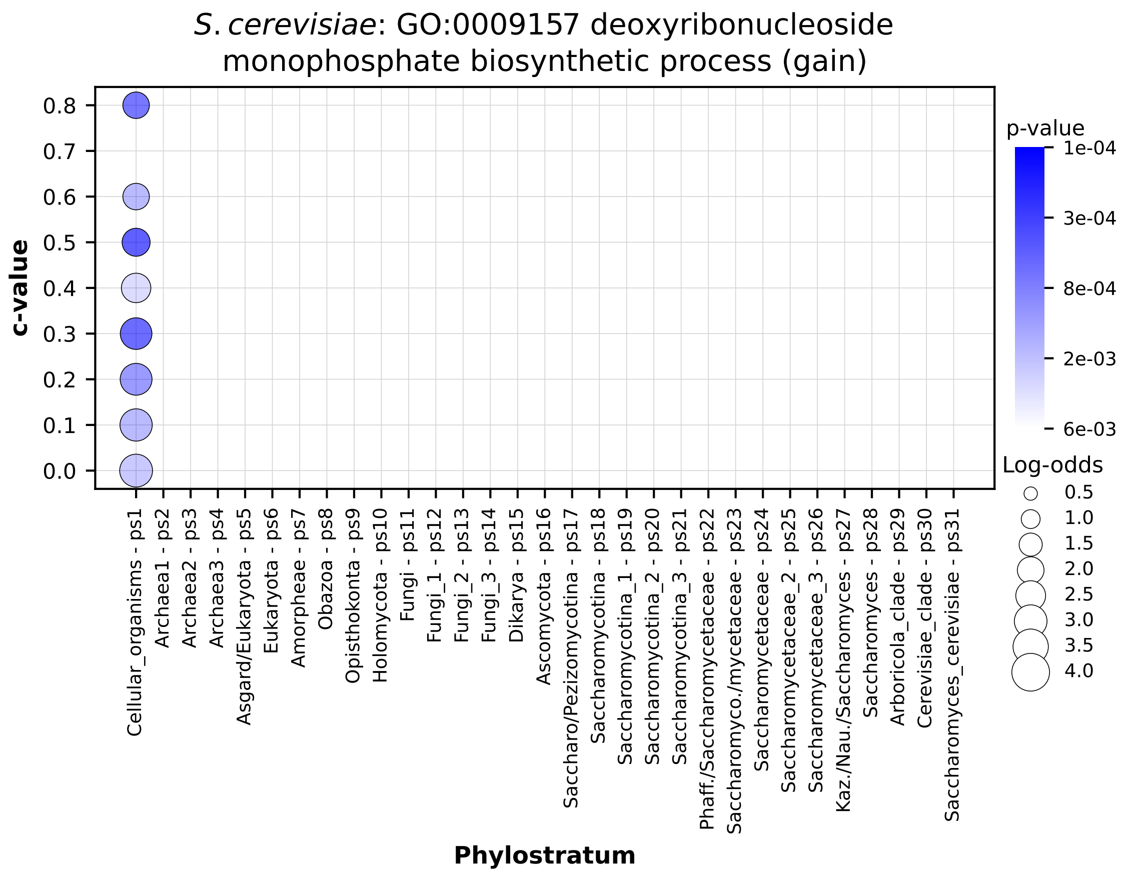

S. cerevisiae: GO:0009157 deoxyribonucleoside monophosphate biosynthetic process (gain)
c-value
0.8
0.7
0.6
0.5
0.4
0.3
0.2
0.1
0.0
p-value
1e-04
3e-04
8e-04
2e-03
6e-03
Log-odds
0.5
1.0
1.5
2.0
2.5
3.0
3.5
4.0
Cellular_organisms - ps1
Archaea1 - ps2
Archaea2 - ps3
Archaea3 - ps4
Asgard/Eukaryota - ps5
Eukaryota - ps6
Amorpheae - ps7
Obazoa - ps8
Opisthokonta - ps9
Holomycota - ps10
Fungi - ps11
Fungi_1 - ps12
Fungi_2 - ps13
Fungi_3 - ps14
Dikarya - ps15
Ascomycota - ps16
Saccharo/Pezizomycotina - ps17
Saccharomycotina - ps18
Saccharomycotina_1 - ps19
Saccharomycotina_2 - ps20
Saccharomycotina_3 - ps21
Phaff./Saccharomycetaceae - ps22
Saccharomyco./mycetaceae - ps23
Saccharomycetaceae - ps24
Saccharomycetaceae_2 - ps25
Saccharomycetaceae_3 - ps26
Kaz./Nau./Saccharomyces - ps27
Saccharomyces - ps28
Arboricola_clade - ps29
Cerevisiae_clade - ps30
Saccharomyces_cerevisiae - ps31
Phylostratum

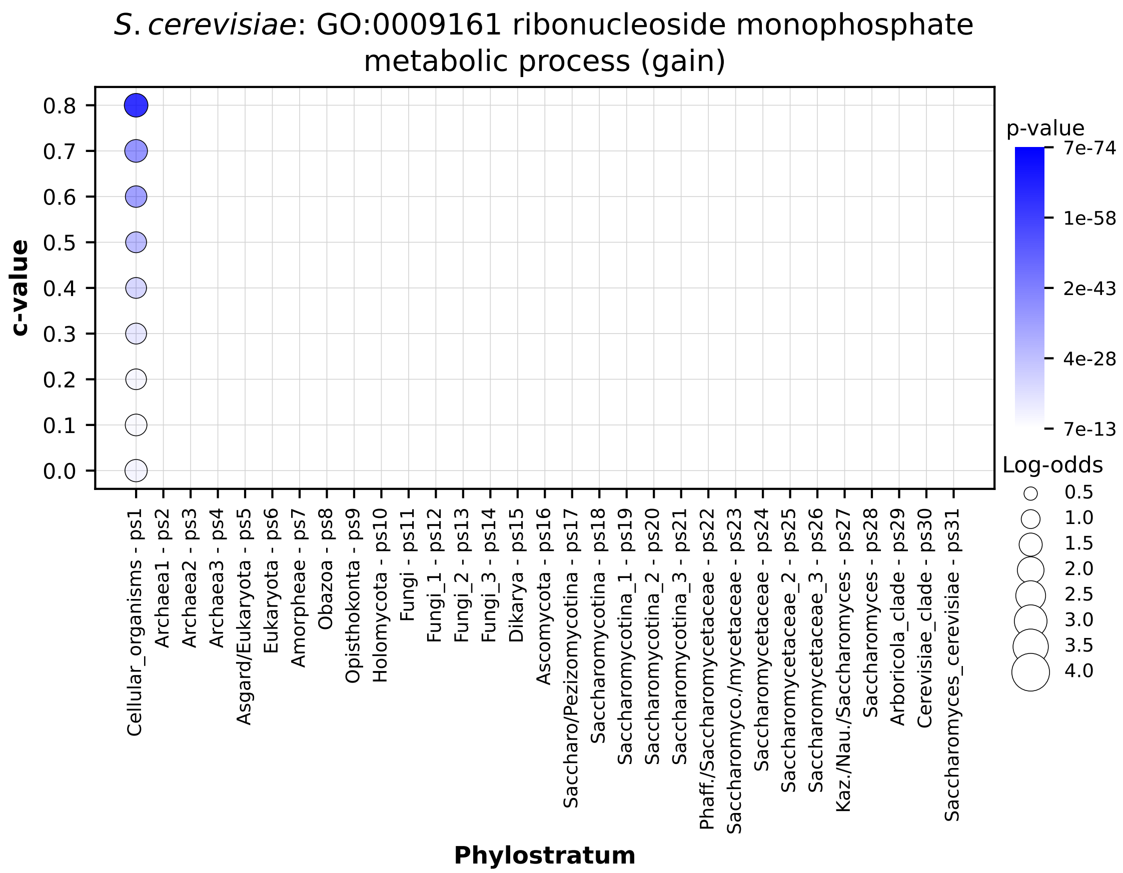

S. cerevisiae: GO:0009161 ribonucleoside monophosphate metabolic process (gain)
c-value
0.8
0.7
0.6
0.5
0.4
0.3
0.2
0.1
0.0
p-value
7e-74
1e-58
2e-43
4e-28
7e-13
Log-odds
0.5
1.0
1.5
2.0
2.5
3.0
3.5
4.0
Cellular_organisms - ps1
Archaea1 - ps2
Archaea2 - ps3
Archaea3 - ps4
Asgard/Eukaryota - ps5
Eukaryota - ps6
Amorpheae - ps7
Obazoa - ps8
Opisthokonta - ps9
Holomycota - ps10
Fungi - ps11
Fungi_1 - ps12
Fungi_2 - ps13
Fungi_3 - ps14
Dikarya - ps15
Ascomycota - ps16
Saccharo/Pezizomycotina - ps17
Saccharomycotina - ps18
Saccharomycotina_1 - ps19
Saccharomycotina_2 - ps20
Saccharomycotina_3 - ps21
Phaff./Saccharomycetaceae - ps22
Saccharomyco./mycetaceae - ps23
Saccharomycetaceae - ps24
Saccharomycetaceae_2 - ps25
Saccharomycetaceae_3 - ps26
Kaz./Nau./Saccharomyces - ps27
Saccharomyces - ps28
Arboricola_clade - ps29
Cerevisiae_clade - ps30
Saccharomyces_cerevisiae - ps31
Phylostratum

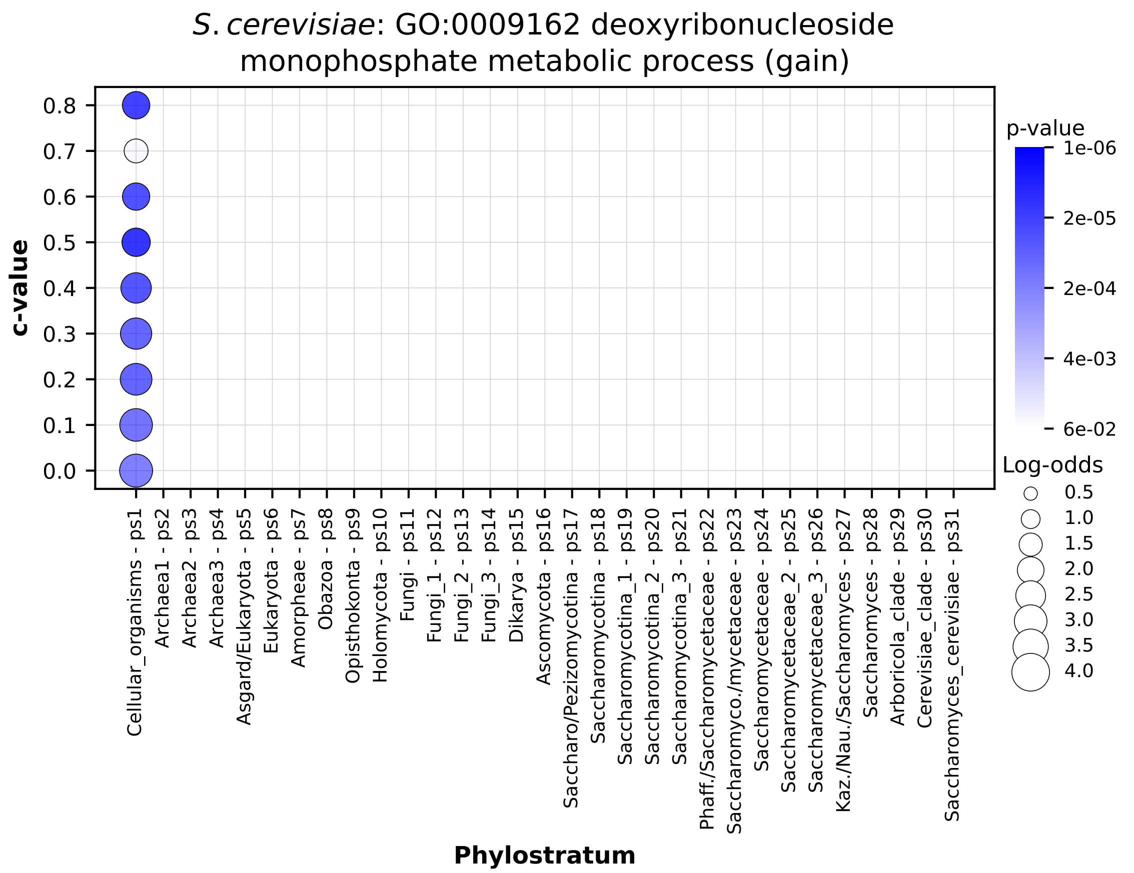

S. cerevisiae: GO:0009162 deoxyribonucleoside monophosphate metabolic process (gain)
c-value
0.8
0.7
0.6
0.5
0.4
0.3
0.2
0.1
0.0
p-value
1e-06
2e-05
2e-04
4e-03
6e-02
Log-odds
0.5
1.0
1.5
2.0
2.5
3.0
3.5
4.0
Cellular_organisms - ps1
Archaea1 - ps2
Archaea2 - ps3
Archaea3 - ps4
Asgard/Eukaryota - ps5
Eukaryota - ps6
Amorpheae - ps7
Obazoa - ps8
Opisthokonta - ps9
Holomycota - ps10
Fungi - ps11
Fungi_1 - ps12
Fungi_2 - ps13
Fungi_3 - ps14
Dikarya - ps15
Ascomycota - ps16
Saccharo/Pezizomycotina - ps17
Saccharomycotina - ps18
Saccharomycotina_1 - ps19
Saccharomycotina_2 - ps20
Saccharomycotina_3 - ps21
Phaff./Saccharomycetaceae - ps22
Saccharomyco./mycetaceae - ps23
Saccharomycetaceae - ps24
Saccharomycetaceae_2 - ps25
Saccharomycetaceae_3 - ps26
Kaz./Nau./Saccharomyces - ps27
Saccharomyces - ps28
Arboricola_clade - ps29
Cerevisiae_clade - ps30
Saccharomyces_cerevisiae - ps31
Phylostratum

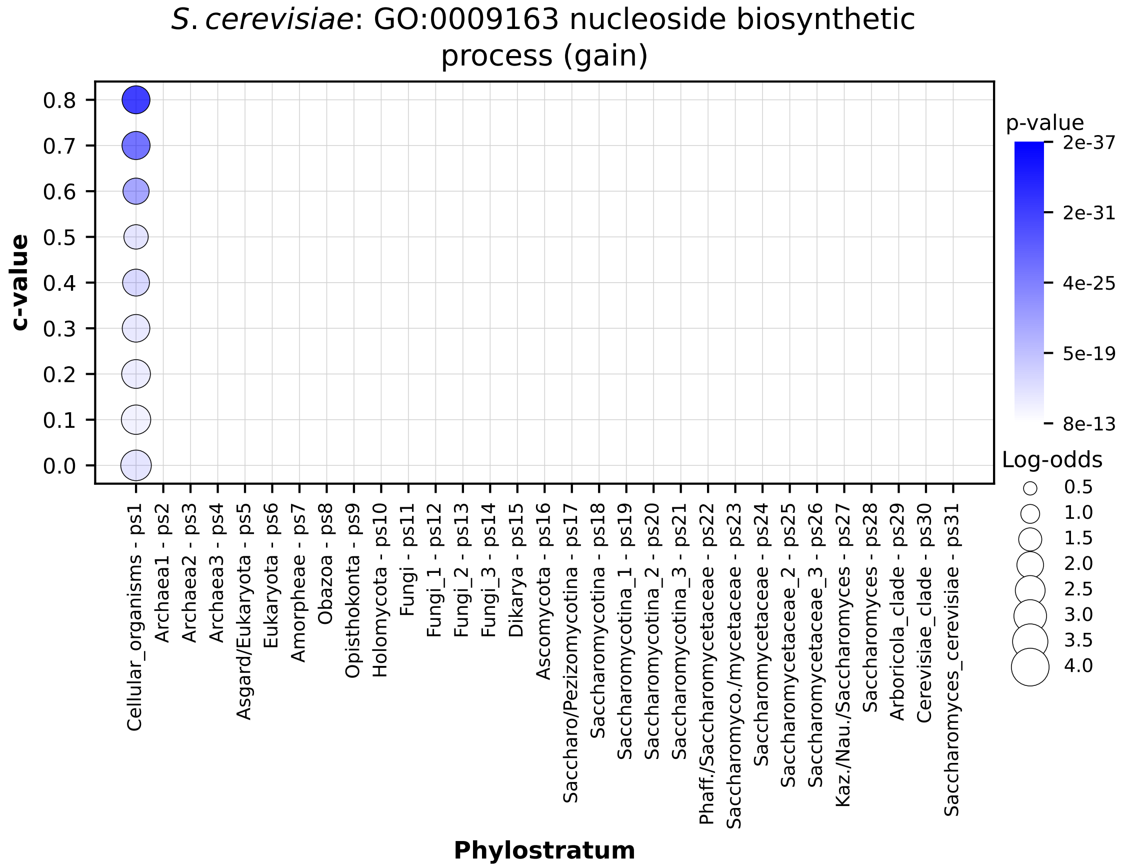

S. cerevisiae: GO:0009163 nucleoside biosynthetic process (gain)
c-value
0.8
0.7
0.6
0.5
0.4
0.3
0.2
0.1
0.0
p-value
2e-37
2e-31
4e-25
5e-19
8e-13
Log-odds
0.5
1.0
1.5
2.0
2.5
3.0
3.5
4.0
Cellular_organisms - ps1
Archaea1 - ps2
Archaea2 - ps3
Archaea3 - ps4
Asgard/Eukaryota - ps5
Eukaryota - ps6
Amorpheae - ps7
Obazoa - ps8
Opisthokonta - ps9
Holomycota - ps10
Fungi - ps11
Fungi_1 - ps12
Fungi_2 - ps13
Fungi_3 - ps14
Dikarya - ps15
Ascomycota - ps16
Saccharo/Pezizomycotina - ps17
Saccharomycotina - ps18
Saccharomycotina_1 - ps19
Saccharomycotina_2 - ps20
Saccharomycotina_3 - ps21
Phaff./Saccharomycetaceae - ps22
Saccharomyco./mycetaceae - ps23
Saccharomycetaceae - ps24
Saccharomycetaceae_2 - ps25
Saccharomycetaceae_3 - ps26
Kaz./Nau./Saccharomyces - ps27
Saccharomyces - ps28
Arboricola_clade - ps29
Cerevisiae_clade - ps30
Saccharomyces_cerevisiae - ps31
Phylostratum

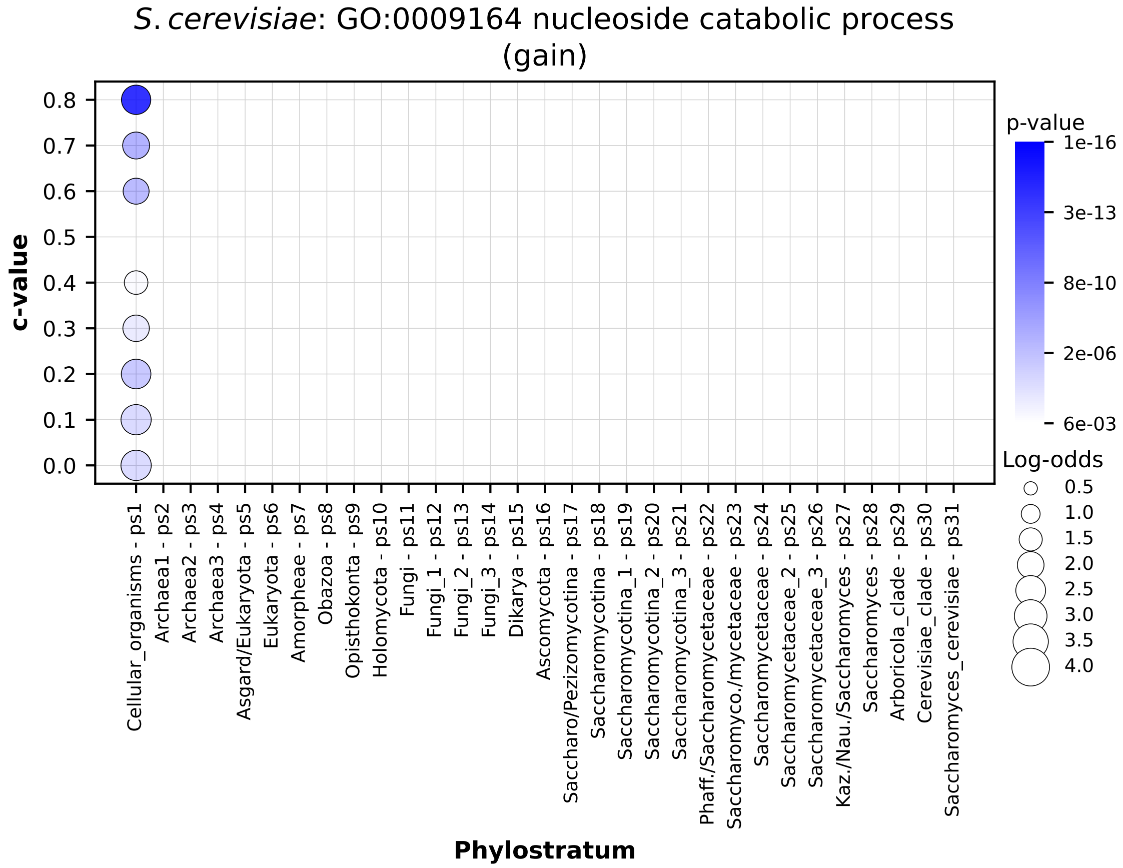

S. cerevisiae: GO:0009164 nucleoside catabolic process (gain)
c-value
0.0
0.1
0.2
0.3
0.4
0.5
0.6
0.7
0.8
p-value
1e-16
3e-13
8e-10
2e-06
6e-03
Log-odds
0.5
1.0
1.5
2.0
2.5
3.0
3.5
4.0
Cellular_organisms - ps1
Archaea1 - ps2
Archaea2 - ps3
Archaea3 - ps4
Asgard/Eukaryota - ps5
Eukaryota - ps6
Amorpheae - ps7
Obazoa - ps8
Opisthokonta - ps9
Holomycota - ps10
Fungi - ps11
Fungi_1 - ps12
Fungi_2 - ps13
Fungi_3 - ps14
Dikarya - ps15
Ascomycota - ps16
Saccharo/Pezizomycotina - ps17
Saccharomycotina - ps18
Saccharomycotina_1 - ps19
Saccharomycotina_2 - ps20
Saccharomycotina_3 - ps21
Phaff./Saccharomycetaceae - ps22
Saccharomyco./mycetaceae - ps23
Saccharomycetaceae - ps24
Saccharomycetaceae_2 - ps25
Saccharomycetaceae_3 - ps26
Kaz./Nau./Saccharomyces - ps27
Saccharomyces - ps28
Arboricola_clade - ps29
Cerevisiae_clade - ps30
Saccharomyces_cerevisiae - ps31
Phylostratum

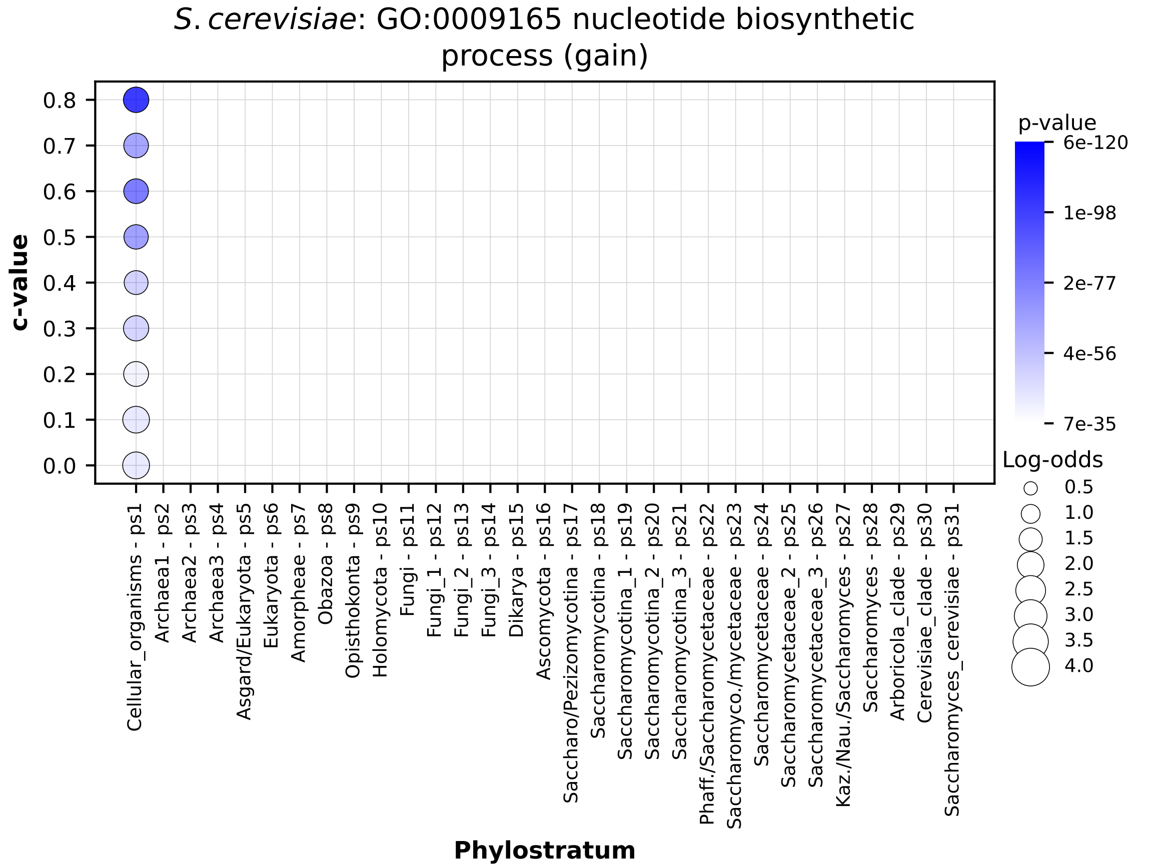

S. cerevisiae: GO:0009165 nucleotide biosynthetic process (gain)
c-value
0.8
0.7
0.6
0.5
0.4
0.3
0.2
0.1
0.0
p-value
6e-120
1e-98
2e-77
4e-56
7e-35
Log-odds
0.5
1.0
1.5
2.0
2.5
3.0
3.5
4.0
Cellular_organisms - ps1
Archaea1 - ps2
Archaea2 - ps3
Archaea3 - ps4
Asgard/Eukaryota - ps5
Eukaryota - ps6
Amorpheae - ps7
Obazoa - ps8
Opisthokonta - ps9
Holomycota - ps10
Fungi - ps11
Fungi_1 - ps12
Fungi_2 - ps13
Fungi_3 - ps14
Dikarya - ps15
Ascomycota - ps16
Saccharo/Pezizomycotina - ps17
Saccharomycotina - ps18
Saccharomycotina_1 - ps19
Saccharomycotina_2 - ps20
Saccharomycotina_3 - ps21
Phaff./Saccharomycetaceae - ps22
Saccharomyco./mycetaceae - ps23
Saccharomycetaceae - ps24
Saccharomycetaceae_2 - ps25
Saccharomycetaceae_3 - ps26
Kaz./Nau./Saccharomyces - ps27
Saccharomyces - ps28
Arboricola_clade - ps29
Cerevisiae_clade - ps30
Saccharomyces_cerevisiae - ps31
Phylostratum

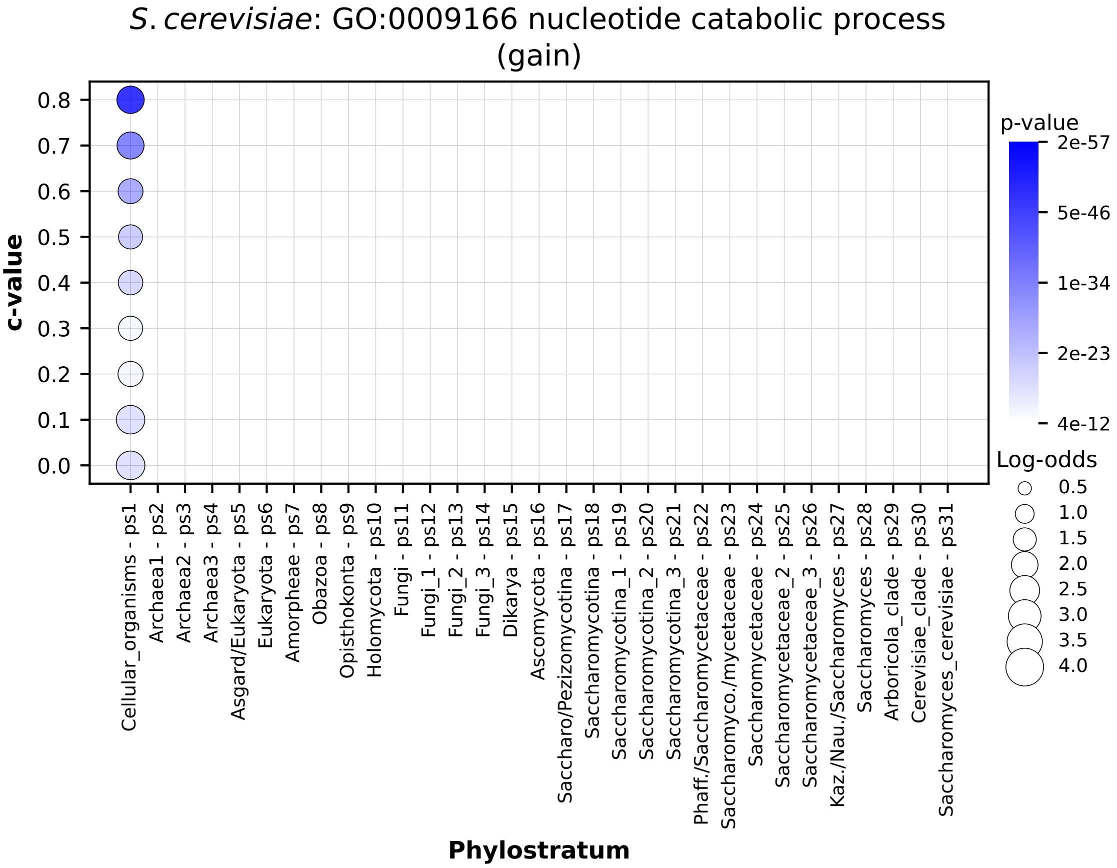

S. cerevisiae: GO:0009166 nucleotide catabolic process
(gain)
c-value
0.8
0.7
0.6
0.5
0.4
0.3
0.2
0.1
0.0
p-value
2e-57
5e-46
1e-34
2e-23
4e-12
Log-odds
0.5
1.0
1.5
2.0
2.5
3.0
3.5
4.0
Cellular_organisms - ps1
Archaea1 - ps2
Archaea2 - ps3
Archaea3 - ps4
Asgard/Eukaryota - ps5
Eukaryota - ps6
Amorpheae - ps7
Obazoa - ps8
Opisthokonta - ps9
Holomycota - ps10
Fungi - ps11
Fungi_1 - ps12
Fungi_2 - ps13
Fungi_3 - ps14
Dikarya - ps15
Ascomycota - ps16
Saccharo/Pezizomycotina - ps17
Saccharomycotina - ps18
Saccharomycotina_1 - ps19
Saccharomycotina_2 - ps20
Saccharomycotina_3 - ps21
Phaff./Saccharomycetaceae - ps22
Saccharomyco./mycetaceae - ps23
Saccharomycetaceae - ps24
Saccharomycetaceae_2 - ps25
Saccharomycetaceae_3 - ps26
Kaz./Nau./Saccharomyces - ps27
Saccharomyces - ps28
Arboricola_clade - ps29
Cerevisiae_clade - ps30
Saccharomyces_cerevisiae - ps31
Phylostratum

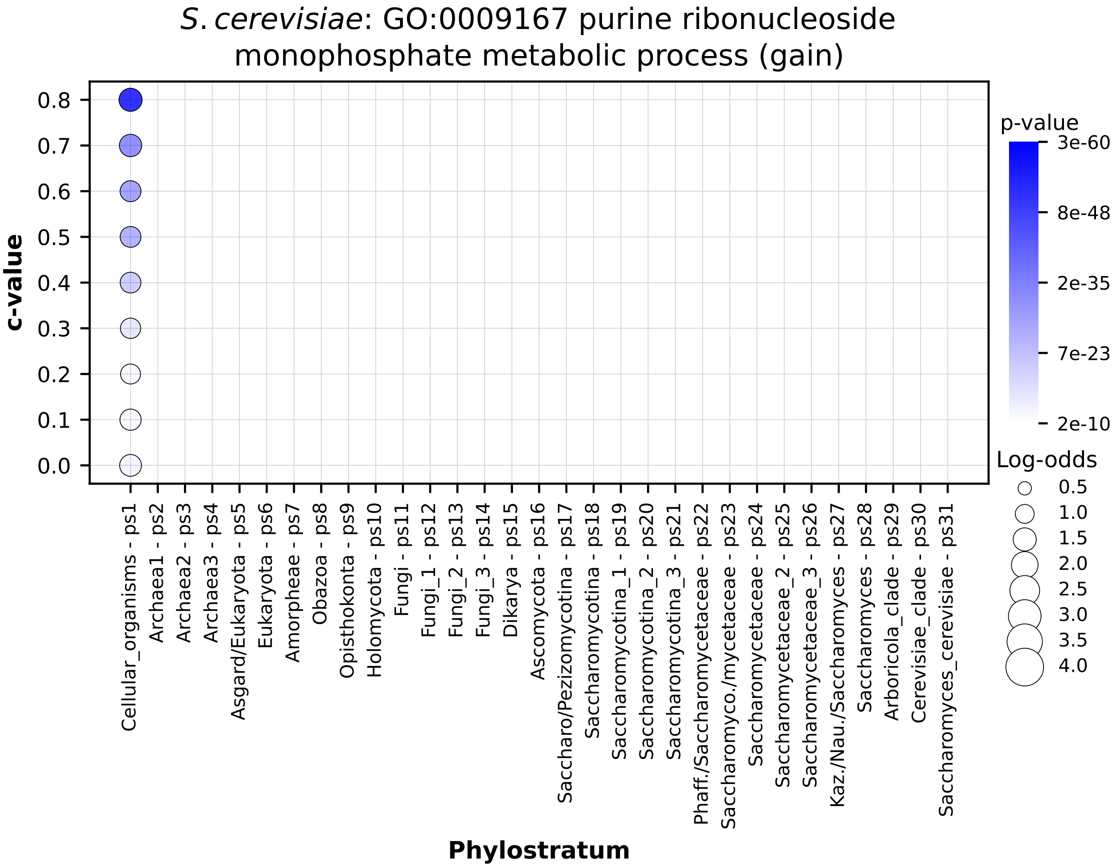
S. cerevisiae: GO:0009167 purine ribonucleoside monophosphate metabolic process (gain)
c-value
0.8
0.7
0.6
0.5
0.4
0.3
0.2
0.1
0.0
p-value
3e-60
8e-48
2e-35
7e-23
2e-10
Log-odds
0.5
1.0
1.5
2.0
2.5
3.0
3.5
4.0
Cellular_organisms - ps1
Archaea1 - ps2
Archaea2 - ps3
Archaea3 - ps4
Asgard/Eukaryota - ps5
Eukaryota - ps6
Amorpheae - ps7
Obazoa - ps8
Opisthokonta - ps9
Holomycota - ps10
Fungi - ps11
Fungi_1 - ps12
Fungi_2 - ps13
Fungi_3 - ps14
Dikarya - ps15
Ascomycota - ps16
Saccharo/Pezizomycotina - ps17
Saccharomycotina - ps18
Saccharomycotina_1 - ps19
Saccharomycotina_2 - ps20
Saccharomycotina_3 - ps21
Phaff./Saccharomycetaceae - ps22
Saccharomyco./mycetaceae - ps23
Saccharomycetaceae - ps24
Saccharomycetaceae_2 - ps25
Saccharomycetaceae_3 - ps26
Kaz./Nau./Saccharomyces - ps27
Saccharomyces - ps28
Arboricola_clade - ps29
Cerevisiae_clade - ps30
Saccharomyces_cerevisiae - ps31
Phylostratum

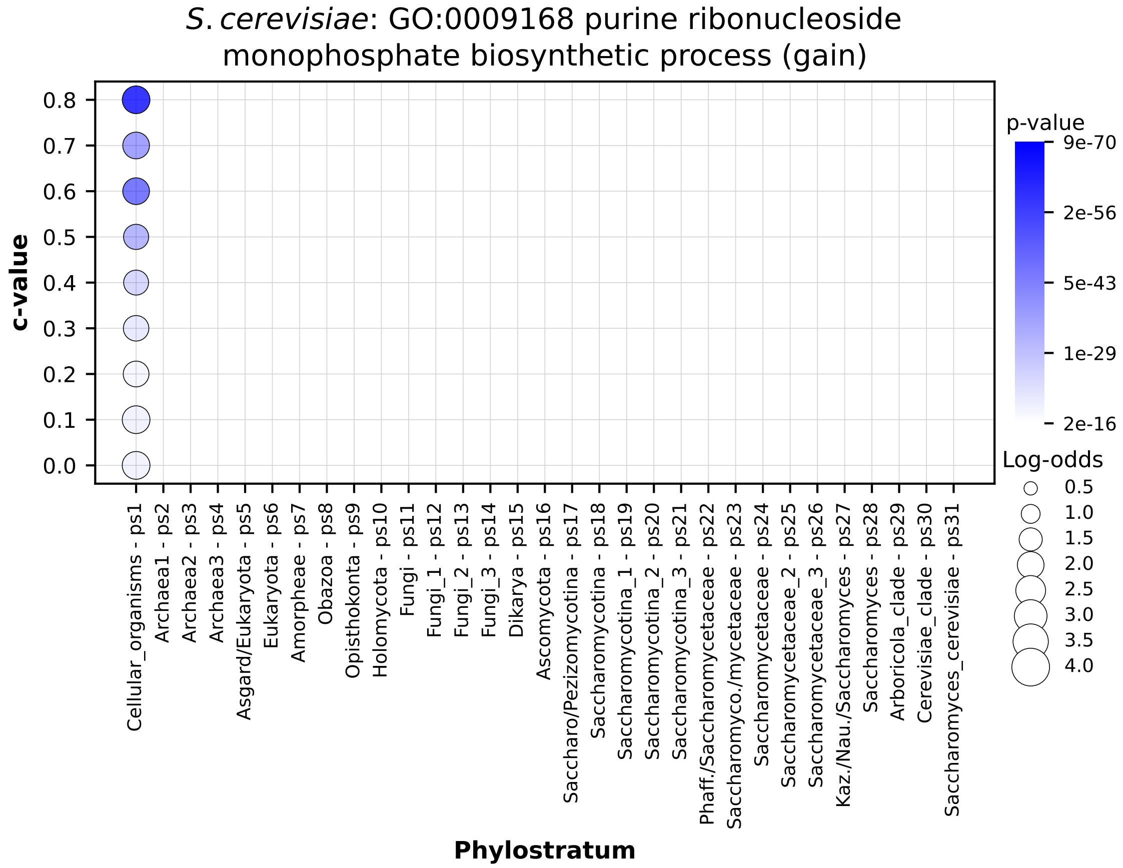

S. cerevisiae: GO:0009168 purine ribonucleoside monophosphate biosynthetic process (gain)
c-value
0.8
0.7
0.6
0.5
0.4
0.3
0.2
0.1
0.0
p-value
9e-70
2e-56
5e-43
1e-29
2e-16
Log-odds
0.5
1.0
1.5
2.0
2.5
3.0
3.5
4.0
Cellular_organisms - ps1
Archaea1 - ps2
Archaea2 - ps3
Archaea3 - ps4
Asgard/Eukaryota - ps5
Eukaryota - ps6
Amorpheae - ps7
Obazoa - ps8
Opisthokonta - ps9
Holomycota - ps10
Fungi - ps11
Fungi_1 - ps12
Fungi_2 - ps13
Fungi_3 - ps14
Dikarya - ps15
Ascomycota - ps16
Saccharo/Pezizomycotina - ps17
Saccharomycotina - ps18
Saccharomycotina_1 - ps19
Saccharomycotina_2 - ps20
Saccharomycotina_3 - ps21
Phaff./Saccharomycetaceae - ps22
Saccharomyco./mycetaceae - ps23
Saccharomycetaceae - ps24
Saccharomycetaceae_2 - ps25
Saccharomycetaceae_3 - ps26
Kaz./Nau./Saccharomyces - ps27
Saccharomyces - ps28
Arboricola_clade - ps29
Cerevisiae_clade - ps30
Saccharomyces_cerevisiae - ps31
Phylostratum

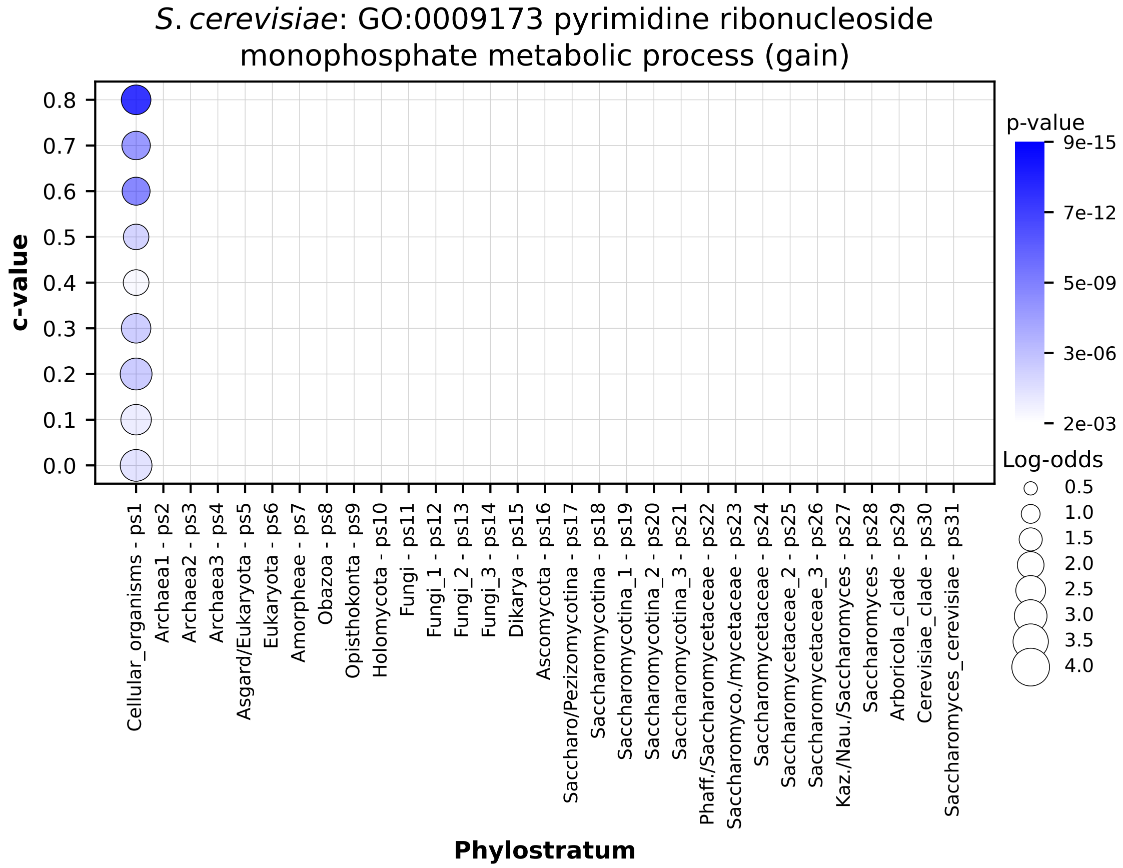

S. cerevisiae: GO:0009173 pyrimidine ribonucleoside monophosphate metabolic process (gain)
c-value
0.0
0.1
0.2
0.3
0.4
0.5
0.6
0.7
0.8
p-value
9e-15
7e-12
5e-09
3e-06
2e-03
Log-odds
0.5
1.0
1.5
2.0
2.5
3.0
3.5
4.0
Cellular_organisms - ps1
Archaea1 - ps2
Archaea2 - ps3
Archaea3 - ps4
Asgard/Eukaryota - ps5
Eukaryota - ps6
Amorpheae - ps7
Obazoa - ps8
Opisthokonta - ps9
Holomycota - ps10
Fungi - ps11
Fungi_1 - ps12
Fungi_2 - ps13
Fungi_3 - ps14
Dikarya - ps15
Ascomycota - ps16
Saccharo/Pezizomycotina - ps17
Saccharomycotina - ps18
Saccharomycotina_1 - ps19
Saccharomycotina_2 - ps20
Saccharomycotina_3 - ps21
Phaff./Saccharomycetaceae - ps22
Saccharomyco./mycetaceae - ps23
Saccharomycetaceae - ps24
Saccharomycetaceae_2 - ps25
Saccharomycetaceae_3 - ps26
Kaz./Nau./Saccharomyces - ps27
Saccharomyces - ps28
Arboricola_clade - ps29
Cerevisiae_clade - ps30
Saccharomyces_cerevisiae - ps31
Phylostratum

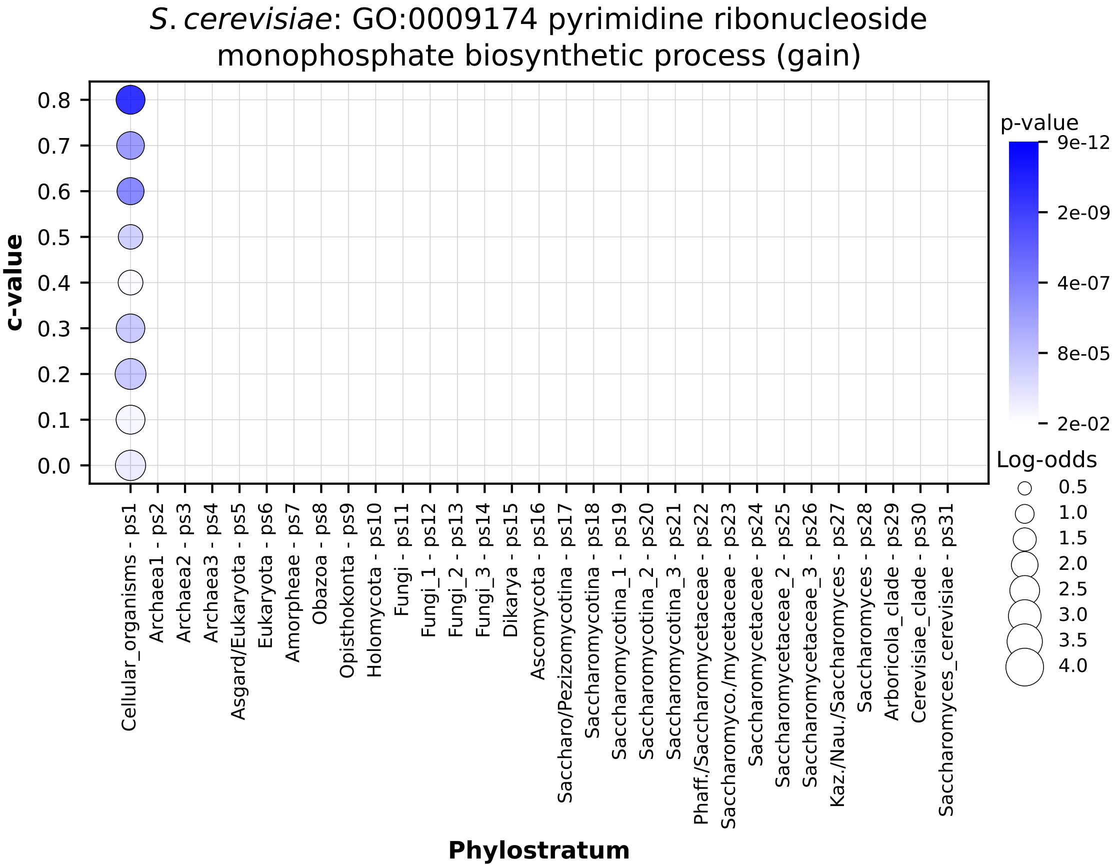

S. cerevisiae: GO:0009174 pyrimidine ribonucleoside monophosphate biosynthetic process (gain)
c-value
0.8
0.7
0.6
0.5
0.4
0.3
0.2
0.1
0.0
p-value
9e-12
2e-09
4e-07
8e-05
2e-02
Log-odds
0.5
1.0
1.5
2.0
2.5
3.0
3.5
4.0
Cellular_organisms - ps1
Archaea1 - ps2
Archaea2 - ps3
Archaea3 - ps4
Asgard/Eukaryota - ps5
Eukaryota - ps6
Amorpheae - ps7
Obazoa - ps8
Opisthokonta - ps9
Holomycota - ps10
Fungi - ps11
Fungi_1 - ps12
Fungi_2 - ps13
Fungi_3 - ps14
Dikarya - ps15
Ascomycota - ps16
Saccharo/Pezizomycotina - ps17
Saccharomycotina - ps18
Saccharomycotina_1 - ps19
Saccharomycotina_2 - ps20
Saccharomycotina_3 - ps21
Phaff./Saccharomycetaceae - ps22
Saccharomyco./mycetaceae - ps23
Saccharomycetaceae - ps24
Saccharomycetaceae_2 - ps25
Saccharomycetaceae_3 - ps26
Kaz./Nau./Saccharomyces - ps27
Saccharomyces - ps28
Arboricola_clade - ps29
Cerevisiae_clade - ps30
Saccharomyces_cerevisiae - ps31
Phylostratum

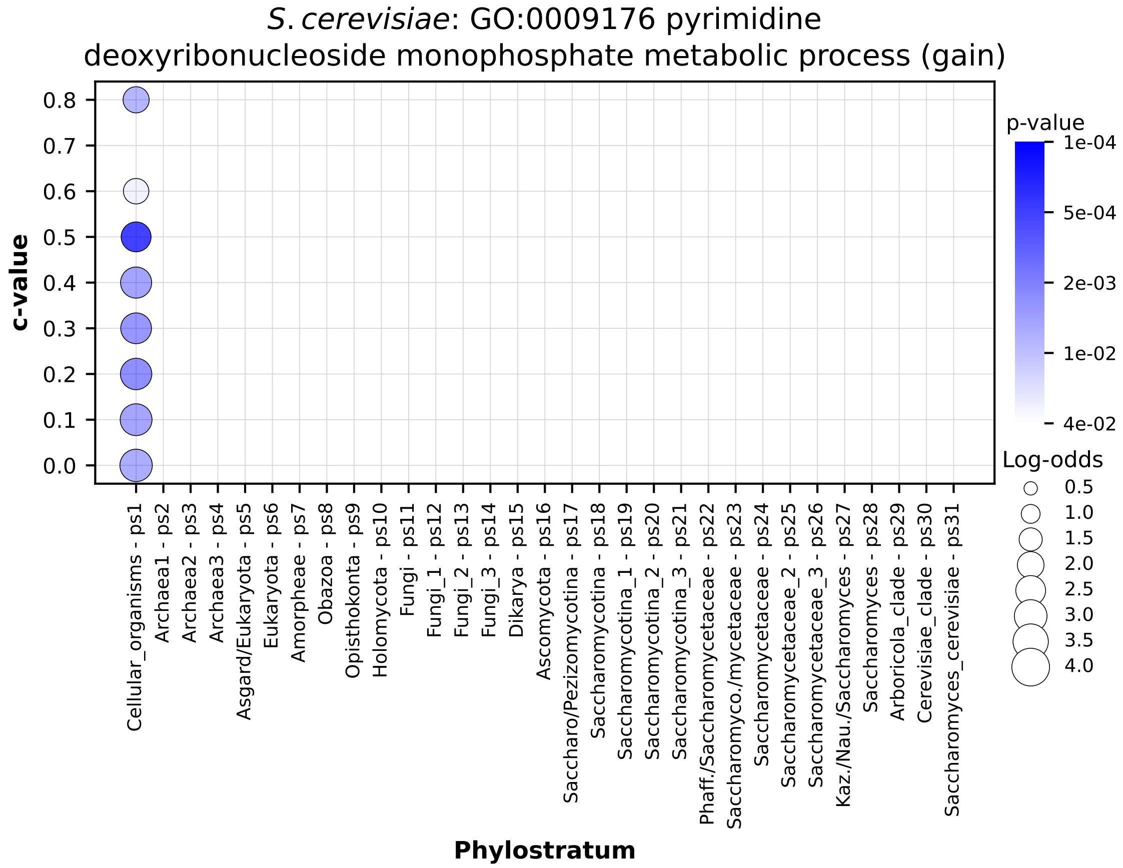
S. cerevisiae: GO:0009176 pyrimidine deoxyribonucleoside monophosphate metabolic process (gain)
c-value
0.8
0.7
0.6
0.5
0.4
0.3
0.2
0.1
0.0
p-value
1e-04
5e-04
2e-03
1e-02
4e-02
Log-odds
0.5
1.0
1.5
2.0
2.5
3.0
3.5
4.0
Cellular_organisms - ps1
Archaea1 - ps2
Archaea2 - ps3
Archaea3 - ps4
Asgard/Eukaryota - ps5
Eukaryota - ps6
Amorpheae - ps7
Obazoa - ps8
Opisthokonta - ps9
Holomycota - ps10
Fungi - ps11
Fungi_1 - ps12
Fungi_2 - ps13
Fungi_3 - ps14
Dikarya - ps15
Ascomycota - ps16
Saccharo/Pezizomycotina - ps17
Saccharomycotina - ps18
Saccharomycotina_1 - ps19
Saccharomycotina_2 - ps20
Saccharomycotina_3 - ps21
Phaff./Saccharomycetaceae - ps22
Saccharomyco./mycetaceae - ps23
Saccharomycetaceae - ps24
Saccharomycetaceae_2 - ps25
Saccharomycetaceae_3 - ps26
Kaz./Nau./Saccharomyces - ps27
Saccharomyces - ps28
Arboricola_clade - ps29
Cerevisiae_clade - ps30
Saccharomyces_cerevisiae - ps31
Phylostratum

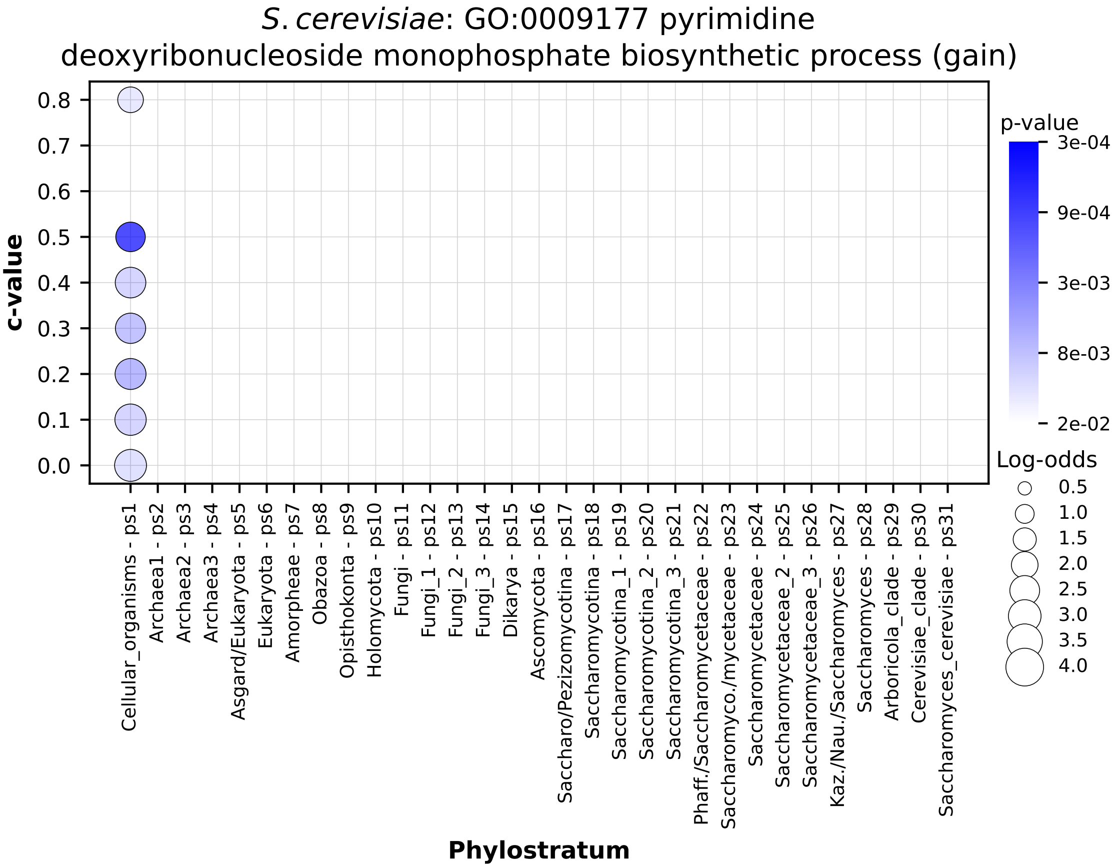
S. cerevisiae: GO:0009177 pyrimidine deoxyribonucleoside monophosphate biosynthetic process (gain)
c-value
0.0
0.1
0.2
0.3
0.4
0.5
0.6
0.7
0.8
p-value
3e-04
9e-04
3e-03
8e-03
2e-02
Log-odds
0.5
1.0
1.5
2.0
2.5
3.0
3.5
4.0
Cellular_organisms - ps1
Archaea1 - ps2
Archaea2 - ps3
Archaea3 - ps4
Asgard/Eukaryota - ps5
Eukaryota - ps6
Amorpheae - ps7
Obazoa - ps8
Opisthokonta - ps9
Holomycota - ps10
Fungi - ps11
Fungi_1 - ps12
Fungi_2 - ps13
Fungi_3 - ps14
Dikarya - ps15
Ascomycota - ps16
Saccharo/Pezizomycotina - ps17
Saccharomycotina - ps18
Saccharomycotina_1 - ps19
Saccharomycotina_2 - ps20
Saccharomycotina_3 - ps21
Phaff./Saccharomycetaceae - ps22
Saccharomyco./mycetaceae - ps23
Saccharomycetaceae - ps24
Saccharomycetaceae_2 - ps25
Saccharomycetaceae_3 - ps26
Kaz./Nau./Saccharomyces - ps27
Saccharomyces - ps28
Arboricola_clade - ps29
Cerevisiae_clade - ps30
Saccharomyces_cerevisiae - ps31
Phylostratum

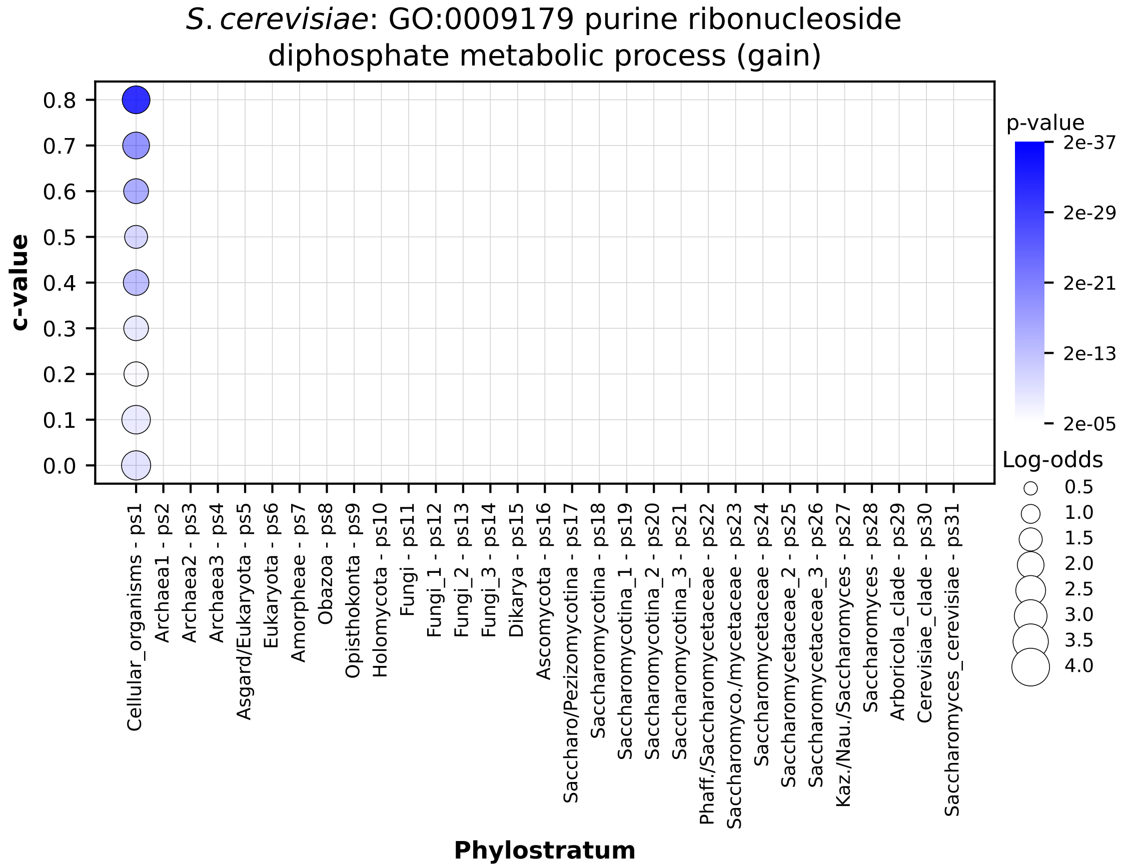
S. cerevisiae: GO:0009179 purine ribonucleoside diphosphate metabolic process (gain)
c-value
0.8
0.7
0.6
0.5
0.4
0.3
0.2
0.1
0.0
p-value
2e-37
2e-29
2e-21
2e-13
2e-05
Log-odds
0.5
1.0
1.5
2.0
2.5
3.0
3.5
4.0
Cellular_organisms - ps1
Archaea1 - ps2
Archaea2 - ps3
Archaea3 - ps4
Asgard/Eukaryota - ps5
Eukaryota - ps6
Amorpheae - ps7
Obazoa - ps8
Opisthokonta - ps9
Holomycota - ps10
Fungi - ps11
Fungi_1 - ps12
Fungi_2 - ps13
Fungi_3 - ps14
Dikarya - ps15
Ascomycota - ps16
Saccharo/Pezizomycotina - ps17
Saccharomycotina - ps18
Saccharomycotina_1 - ps19
Saccharomycotina_2 - ps20
Saccharomycotina_3 - ps21
Phaff./Saccharomycetaceae - ps22
Saccharomyco./mycetaceae - ps23
Saccharomycetaceae - ps24
Saccharomycetaceae_2 - ps25
Saccharomycetaceae_3 - ps26
Kaz./Nau./Saccharomyces - ps27
Saccharomyces - ps28
Arboricola_clade - ps29
Cerevisiae_clade - ps30
Saccharomyces_cerevisiae - ps31
Phylostratum

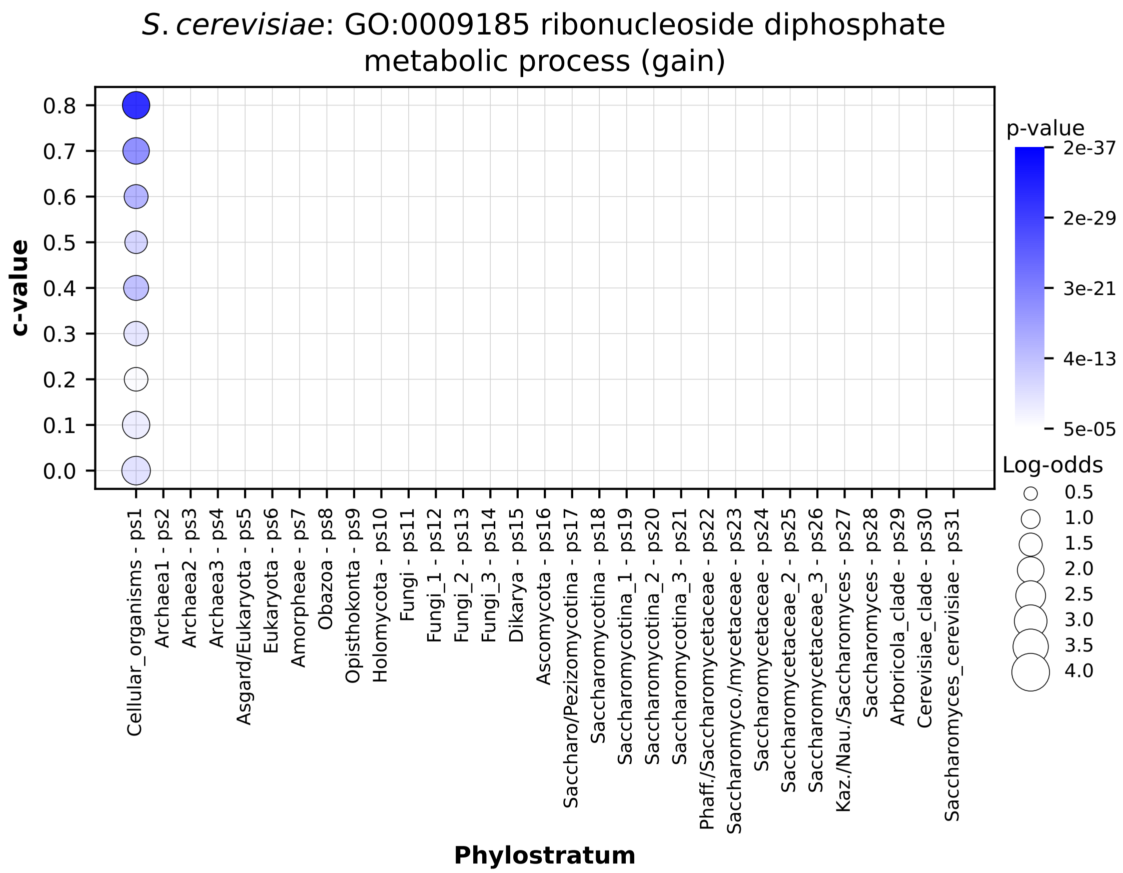
S. cerevisiae: GO:0009185 ribonucleoside diphosphate metabolic process (gain)
c-value
0.8
0.7
0.6
0.5
0.4
0.3
0.2
0.1
0.0
p-value
2e-37
2e-29
3e-21
4e-13
5e-05
Log-odds
0.5
1.0
1.5
2.0
2.5
3.0
3.5
4.0
Cellular_organisms - ps1
Archaea1 - ps2
Archaea2 - ps3
Archaea3 - ps4
Asgard/Eukaryota - ps5
Eukaryota - ps6
Amorpheae - ps7
Obazoa - ps8
Opisthokonta - ps9
Holomycota - ps10
Fungi - ps11
Fungi_1 - ps12
Fungi_2 - ps13
Fungi_3 - ps14
Dikarya - ps15
Ascomycota - ps16
Saccharo/Pezizomycotina - ps17
Saccharomycotina - ps18
Saccharomycotina_1 - ps19
Saccharomycotina_2 - ps20
Saccharomycotina_3 - ps21
Phaff./Saccharomycetaceae - ps22
Saccharomyco./mycetaceae - ps23
Saccharomycetaceae - ps24
Saccharomycetaceae_2 - ps25
Saccharomycetaceae_3 - ps26
Kaz./Nau./Saccharomyces - ps27
Saccharomyces - ps28
Arboricola_clade - ps29
Cerevisiae_clade - ps30
Saccharomyces_cerevisiae - ps31
Phylostratum

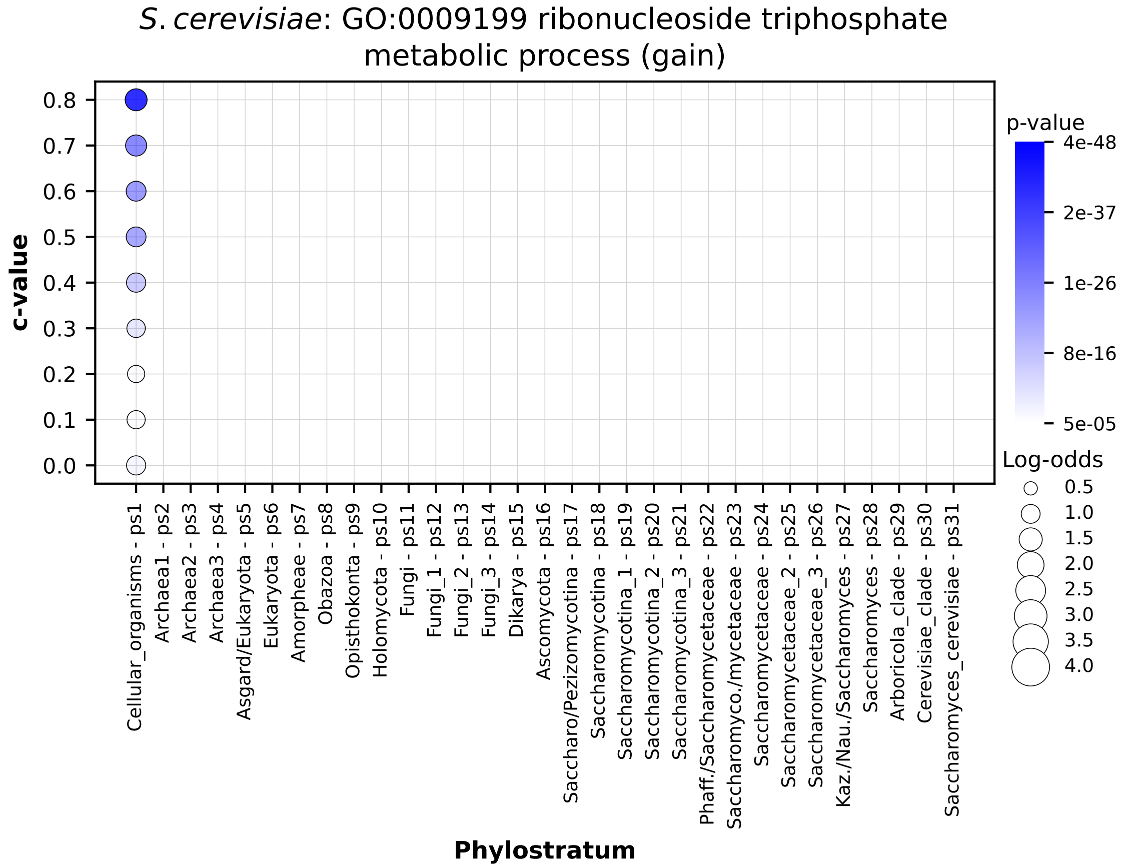

S. cerevisiae: GO:0009199 ribonucleoside triphosphate metabolic process (gain)
c-value
0.8
0.7
0.6
0.5
0.4
0.3
0.2
0.1
0.0
p-value
4e-48
2e-37
1e-26
8e-16
5e-05
Log-odds
0.5
1.0
1.5
2.0
2.5
3.0
3.5
4.0
Cellular_organisms - ps1
Archaea1 - ps2
Archaea2 - ps3
Archaea3 - ps4
Asgard/Eukaryota - ps5
Eukaryota - ps6
Amorpheae - ps7
Obazoa - ps8
Opisthokonta - ps9
Holomycota - ps10
Fungi - ps11
Fungi_1 - ps12
Fungi_2 - ps13
Fungi_3 - ps14
Dikarya - ps15
Ascomycota - ps16
Saccharo/Pezizomycotina - ps17
Saccharomycotina - ps18
Saccharomycotina_1 - ps19
Saccharomycotina_2 - ps20
Saccharomycotina_3 - ps21
Phaff./Saccharomycetaceae - ps22
Saccharomyco./mycetaceae - ps23
Saccharomycetaceae - ps24
Saccharomycetaceae_2 - ps25
Saccharomycetaceae_3 - ps26
Kaz./Nau./Saccharomyces - ps27
Saccharomyces - ps28
Arboricola_clade - ps29
Cerevisiae_clade - ps30
Saccharomyces_cerevisiae - ps31
Phylostratum

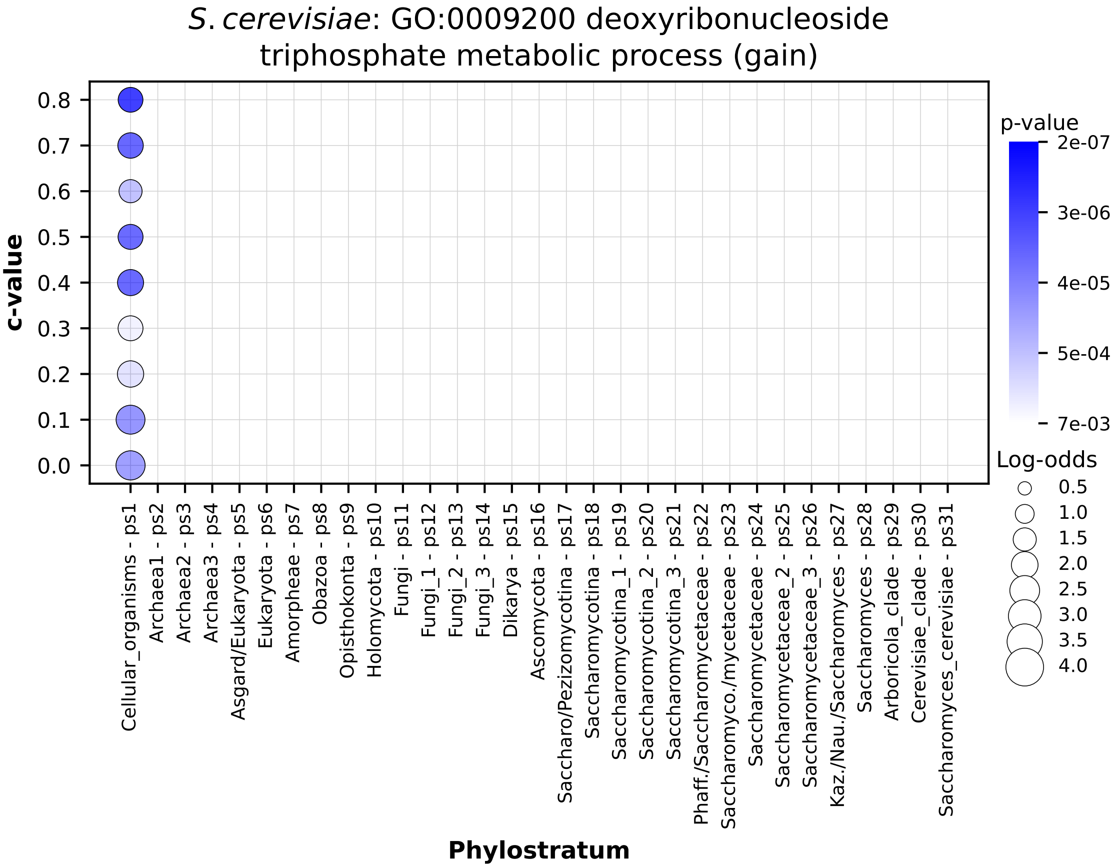

S. cerevisiae: GO:0009200 deoxyribonucleoside triphosphate metabolic process (gain)
c-value
0.0
0.1
0.2
0.3
0.4
0.5
0.6
0.7
0.8
p-value
2e-07
3e-06
4e-05
5e-04
7e-03
Log-odds
0.5
1.0
1.5
2.0
2.5
3.0
3.5
4.0
Cellular_organisms - ps1
Archaea1 - ps2
Archaea2 - ps3
Archaea3 - ps4
Asgard/Eukaryota - ps5
Eukaryota - ps6
Amorpheae - ps7
Obazoa - ps8
Opisthokonta - ps9
Holomycota - ps10
Fungi - ps11
Fungi_1 - ps12
Fungi_2 - ps13
Fungi_3 - ps14
Dikarya - ps15
Ascomycota - ps16
Saccharo/Pezizomycotina - ps17
Saccharomycotina - ps18
Saccharomycotina_1 - ps19
Saccharomycotina_2 - ps20
Saccharomycotina_3 - ps21
Phaff./Saccharomycetaceae - ps22
Saccharomyco./mycetaceae - ps23
Saccharomycetaceae - ps24
Saccharomycetaceae_2 - ps25
Saccharomycetaceae_3 - ps26
Kaz./Nau./Saccharomyces - ps27
Saccharomyces - ps28
Arboricola_clade - ps29
Cerevisiae_clade - ps30
Saccharomyces_cerevisiae - ps31
Phylostratum

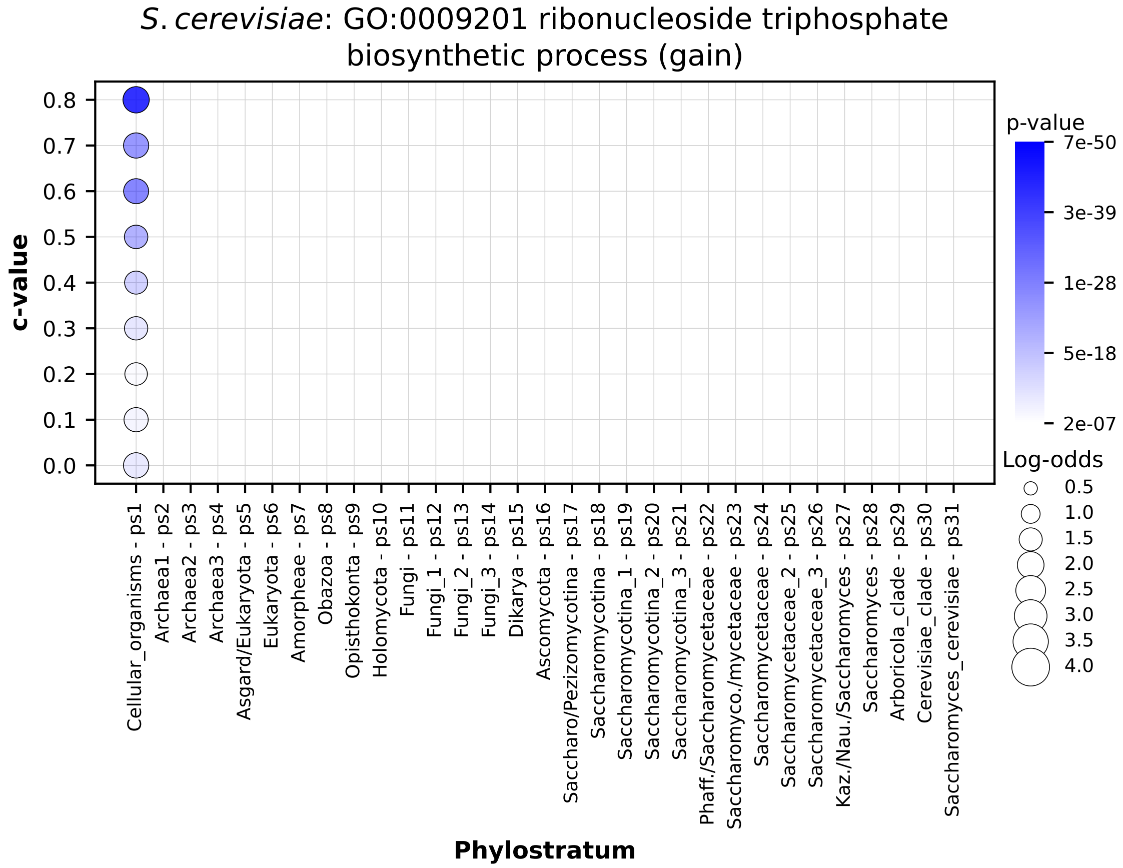

S. cerevisiae: GO:0009201 ribonucleoside triphosphate biosynthetic process (gain)
c-value
0.0
0.1
0.2
0.3
0.4
0.5
0.6
0.7
0.8
p-value
7e-50
3e-39
1e-28
5e-18
2e-07
Log-odds
0.5
1.0
1.5
2.0
2.5
3.0
3.5
4.0
Cellular_organisms - ps1
Archaea1 - ps2
Archaea2 - ps3
Archaea3 - ps4
Asgard/Eukaryota - ps5
Eukaryota - ps6
Amorpheae - ps7
Obazoa - ps8
Opisthokonta - ps9
Holomycota - ps10
Fungi - ps11
Fungi_1 - ps12
Fungi_2 - ps13
Fungi_3 - ps14
Dikarya - ps15
Ascomycota - ps16
Saccharo/Pezizomycotina - ps17
Saccharomycotina - ps18
Saccharomycotina_1 - ps19
Saccharomycotina_2 - ps20
Saccharomycotina_3 - ps21
Phaff./Saccharomycetaceae - ps22
Saccharomyco./mycetaceae - ps23
Saccharomycetaceae - ps24
Saccharomycetaceae_2 - ps25
Saccharomycetaceae_3 - ps26
Kaz./Nau./Saccharomyces - ps27
Saccharomyces - ps28
Arboricola_clade - ps29
Cerevisiae_clade - ps30
Saccharomyces_cerevisiae - ps31
Phylostratum

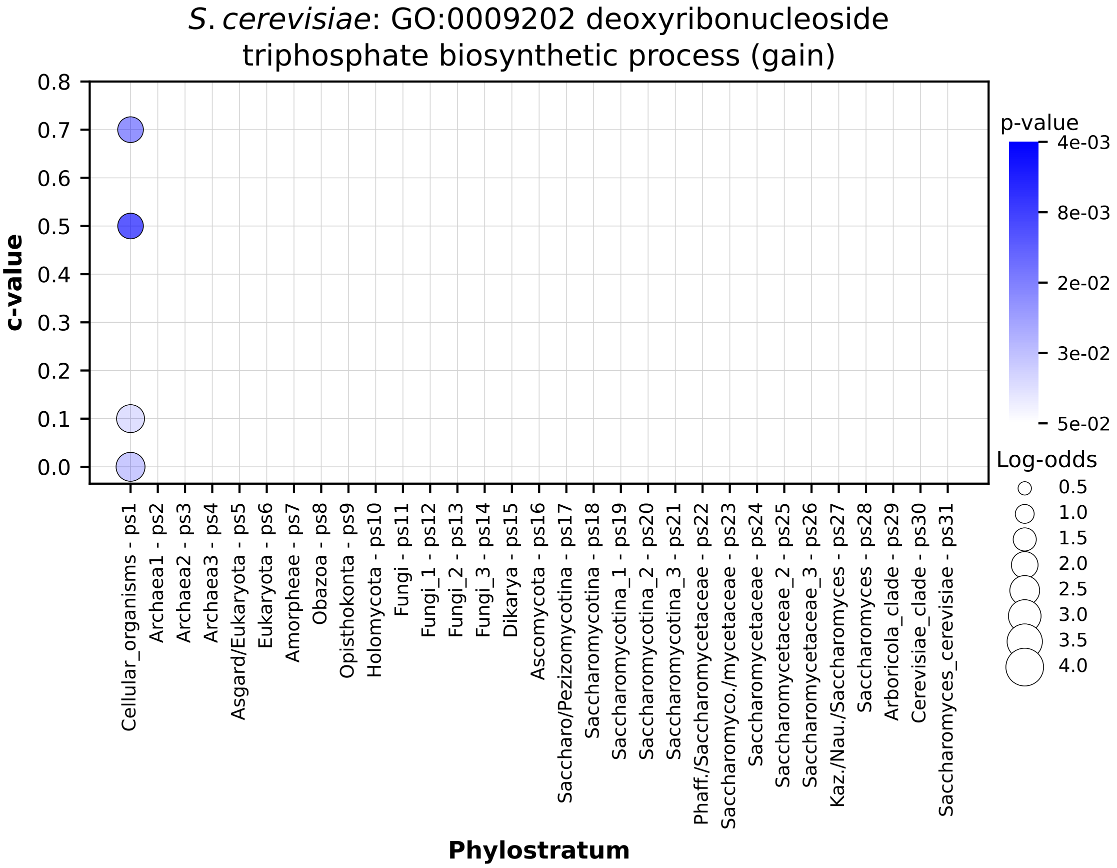
S. cerevisiae: GO:0009202 deoxyribonucleoside triphosphate biosynthetic process (gain)
c-value
0.0
0.1
0.2
0.3
0.4
0.5
0.6
0.7
0.8
p-value
4e-03
8e-03
2e-02
3e-02
5e-02
Log-odds
0.5
1.0
1.5
2.0
2.5
3.0
3.5
4.0
Cellular_organisms - ps1
Archaea1 - ps2
Archaea2 - ps3
Archaea3 - ps4
Asgard/Eukaryota - ps5
Eukaryota - ps6
Amorpheae - ps7
Obazoa - ps8
Opisthokonta - ps9
Holomycota - ps10
Fungi - ps11
Fungi_1 - ps12
Fungi_2 - ps13
Fungi_3 - ps14
Dikarya - ps15
Ascomycota - ps16
Saccharo/Pezizomycotina - ps17
Saccharomycotina - ps18
Saccharomycotina_1 - ps19
Saccharomycotina_2 - ps20
Saccharomycotina_3 - ps21
Phaff./Saccharomycetaceae - ps22
Saccharomyco./mycetaceae - ps23
Saccharomycetaceae - ps24
Saccharomycetaceae_2 - ps25
Saccharomycetaceae_3 - ps26
Kaz./Nau./Saccharomyces - ps27
Saccharomyces - ps28
Arboricola_clade - ps29
Cerevisiae_clade - ps30
Saccharomyces_cerevisiae - ps31
Phylostratum

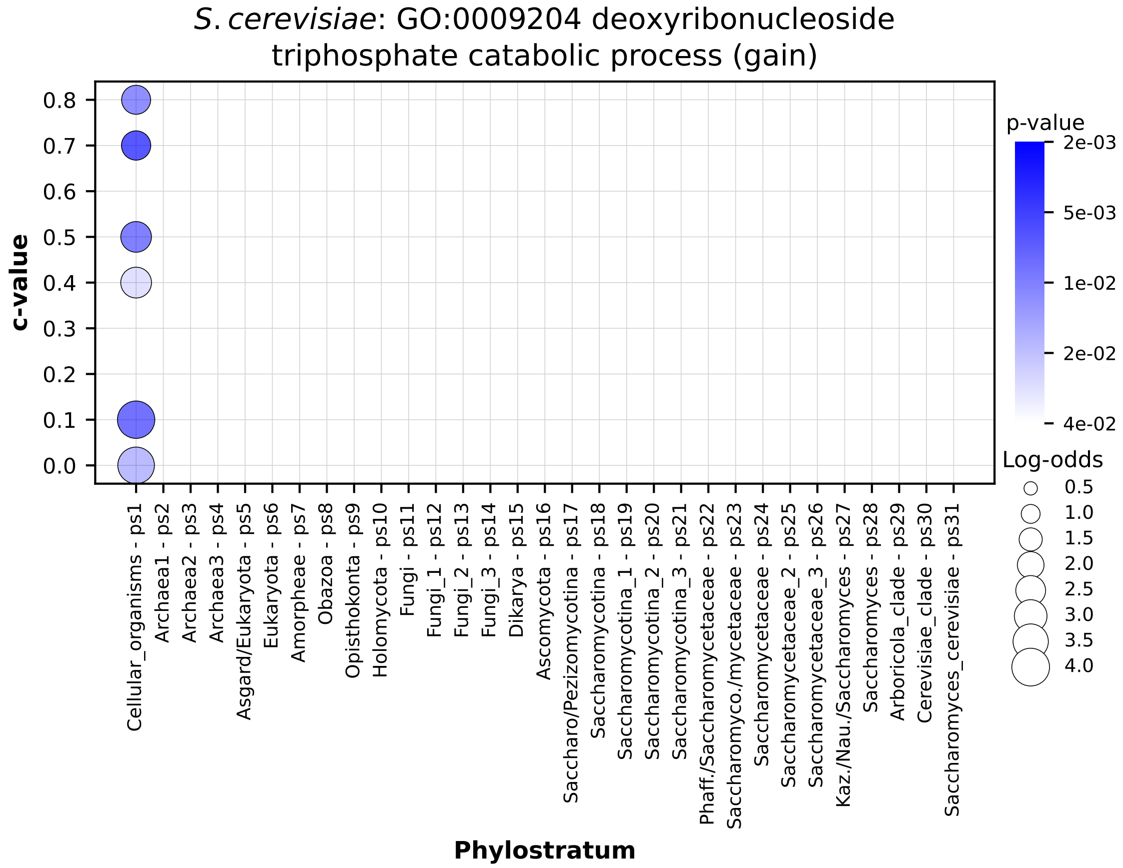

S. cerevisiae: GO:0009204 deoxyribonucleoside triphosphate catabolic process (gain)
c-value
0.8
0.7
0.6
0.5
0.4
0.3
0.2
0.1
0.0
p-value
2e-03
5e-03
1e-02
2e-02
4e-02
Log-odds
0.5
1.0
1.5
2.0
2.5
3.0
3.5
4.0
Cellular_organisms - ps1
Archaea1 - ps2
Archaea2 - ps3
Archaea3 - ps4
Asgard/Eukaryota - ps5
Eukaryota - ps6
Amorpheae - ps7
Obazoa - ps8
Opisthokonta - ps9
Holomycota - ps10
Fungi - ps11
Fungi_1 - ps12
Fungi_2 - ps13
Fungi_3 - ps14
Dikarya - ps15
Ascomycota - ps16
Saccharo/Pezizomycotina - ps17
Saccharomycotina - ps18
Saccharomycotina_1 - ps19
Saccharomycotina_2 - ps20
Saccharomycotina_3 - ps21
Phaff./Saccharomycetaceae - ps22
Saccharomyco./mycetaceae - ps23
Saccharomycetaceae - ps24
Saccharomycetaceae_2 - ps25
Saccharomycetaceae_3 - ps26
Kaz./Nau./Saccharomyces - ps27
Saccharomyces - ps28
Arboricola_clade - ps29
Cerevisiae_clade - ps30
Saccharomyces_cerevisiae - ps31
Phylostratum

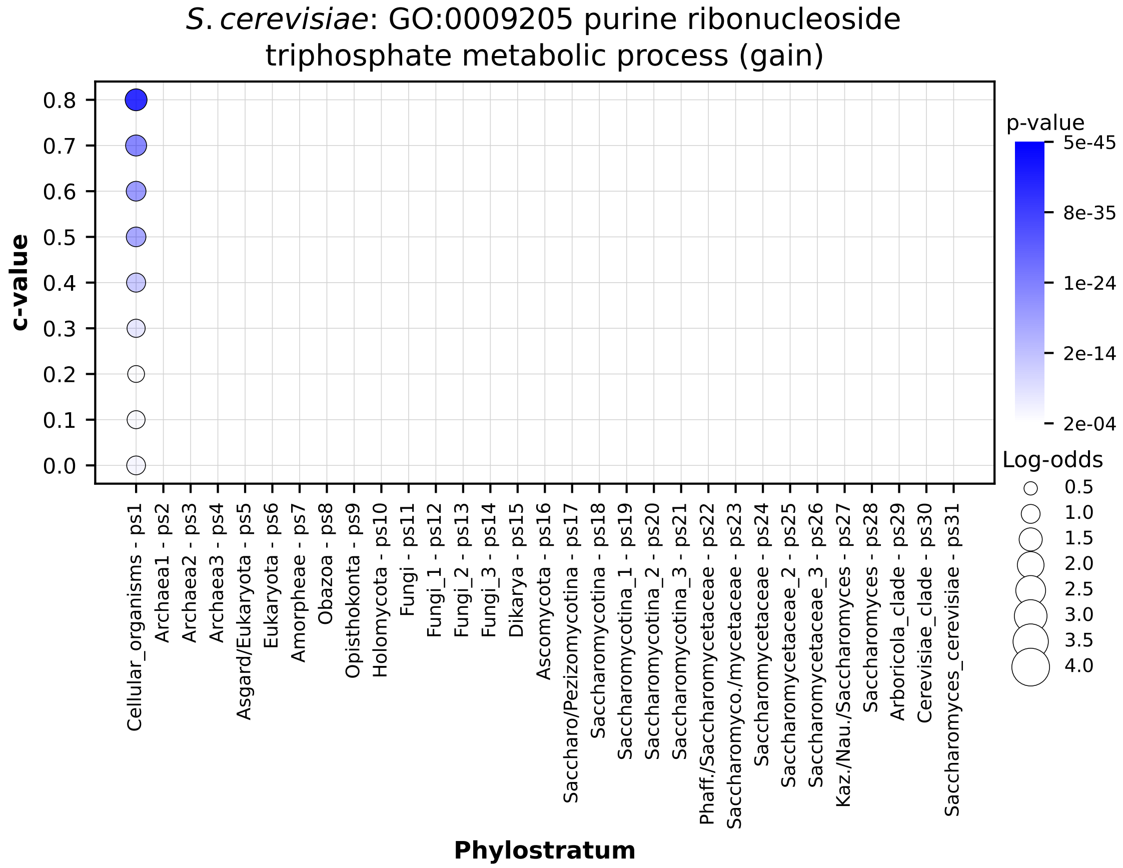
S. cerevisiae: GO:0009205 purine ribonucleoside triphosphate metabolic process (gain)
c-value
0.8
0.7
0.6
0.5
0.4
0.3
0.2
0.1
0.0
p-value
5e-45
8e-35
1e-24
2e-14
2e-04
Log-odds
0.5
1.0
1.5
2.0
2.5
3.0
3.5
4.0
Cellular_organisms - ps1
Archaea1 - ps2
Archaea2 - ps3
Archaea3 - ps4
Asgard/Eukaryota - ps5
Eukaryota - ps6
Amorpheae - ps7
Obazoa - ps8
Opisthokonta - ps9
Holomycota - ps10
Fungi - ps11
Fungi_1 - ps12
Fungi_2 - ps13
Fungi_3 - ps14
Dikarya - ps15
Ascomycota - ps16
Saccharo/Pezizomycotina - ps17
Saccharomycotina - ps18
Saccharomycotina_1 - ps19
Saccharomycotina_2 - ps20
Saccharomycotina_3 - ps21
Phaff./Saccharomycetaceae - ps22
Saccharomyco./mycetaceae - ps23
Saccharomycetaceae - ps24
Saccharomycetaceae_2 - ps25
Saccharomycetaceae_3 - ps26
Kaz./Nau./Saccharomyces - ps27
Saccharomyces - ps28
Arboricola_clade - ps29
Cerevisiae_clade - ps30
Saccharomyces_cerevisiae - ps31
Phylostratum

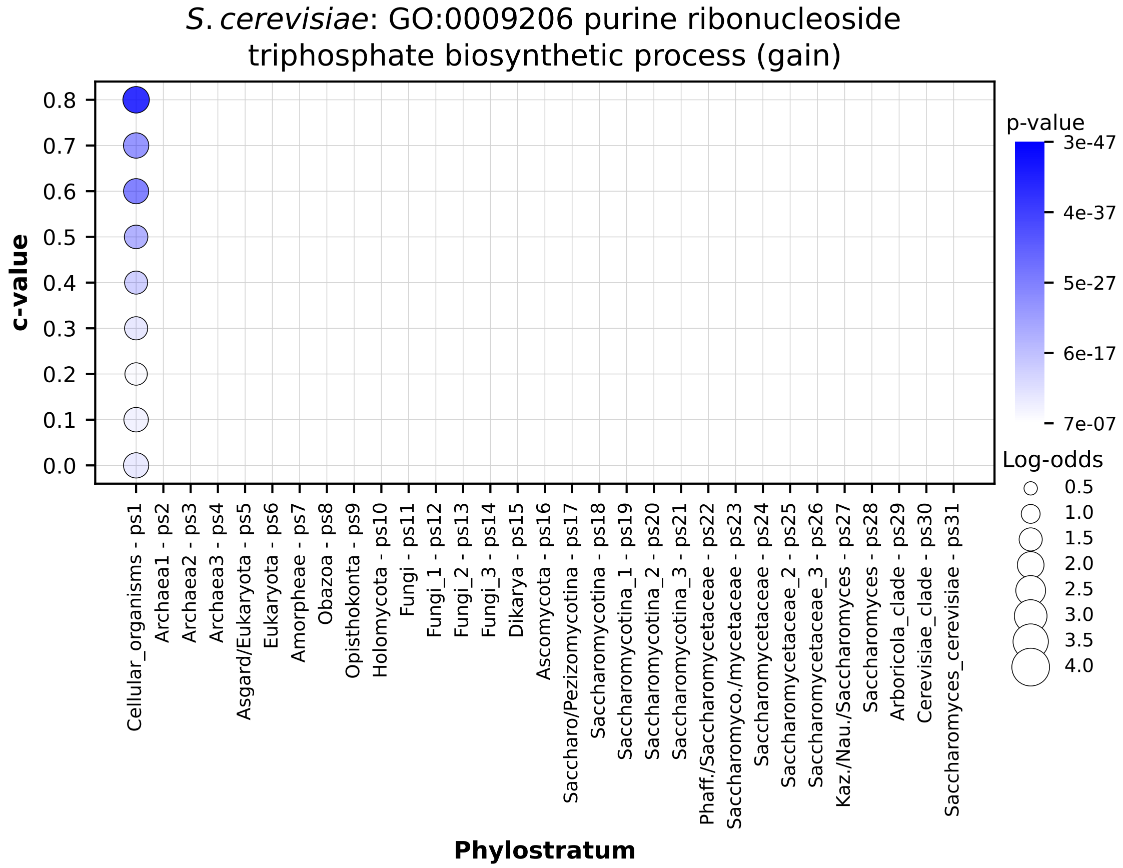

S. cerevisiae: GO:0009206 purine ribonucleoside triphosphate biosynthetic process (gain)
c-value
0.8
0.7
0.6
0.5
0.4
0.3
0.2
0.1
0.0
p-value
3e-47
4e-37
5e-27
6e-17
7e-07
Log-odds
0.5
1.0
1.5
2.0
2.5
3.0
3.5
4.0
Cellular_organisms - ps1
Archaea1 - ps2
Archaea2 - ps3
Archaea3 - ps4
Asgard/Eukaryota - ps5
Eukaryota - ps6
Amorpheae - ps7
Obazoa - ps8
Opisthokonta - ps9
Holomycota - ps10
Fungi - ps11
Fungi_1 - ps12
Fungi_2 - ps13
Fungi_3 - ps14
Dikarya - ps15
Ascomycota - ps16
Saccharo/Pezizomycotina - ps17
Saccharomycotina - ps18
Saccharomycotina_1 - ps19
Saccharomycotina_2 - ps20
Saccharomycotina_3 - ps21
Phaff./Saccharomycetaceae - ps22
Saccharomyco./mycetaceae - ps23
Saccharomycetaceae - ps24
Saccharomycetaceae_2 - ps25
Saccharomycetaceae_3 - ps26
Kaz./Nau./Saccharomyces - ps27
Saccharomyces - ps28
Arboricola_clade - ps29
Cerevisiae_clade - ps30
Saccharomyces_cerevisiae - ps31
Phylostratum

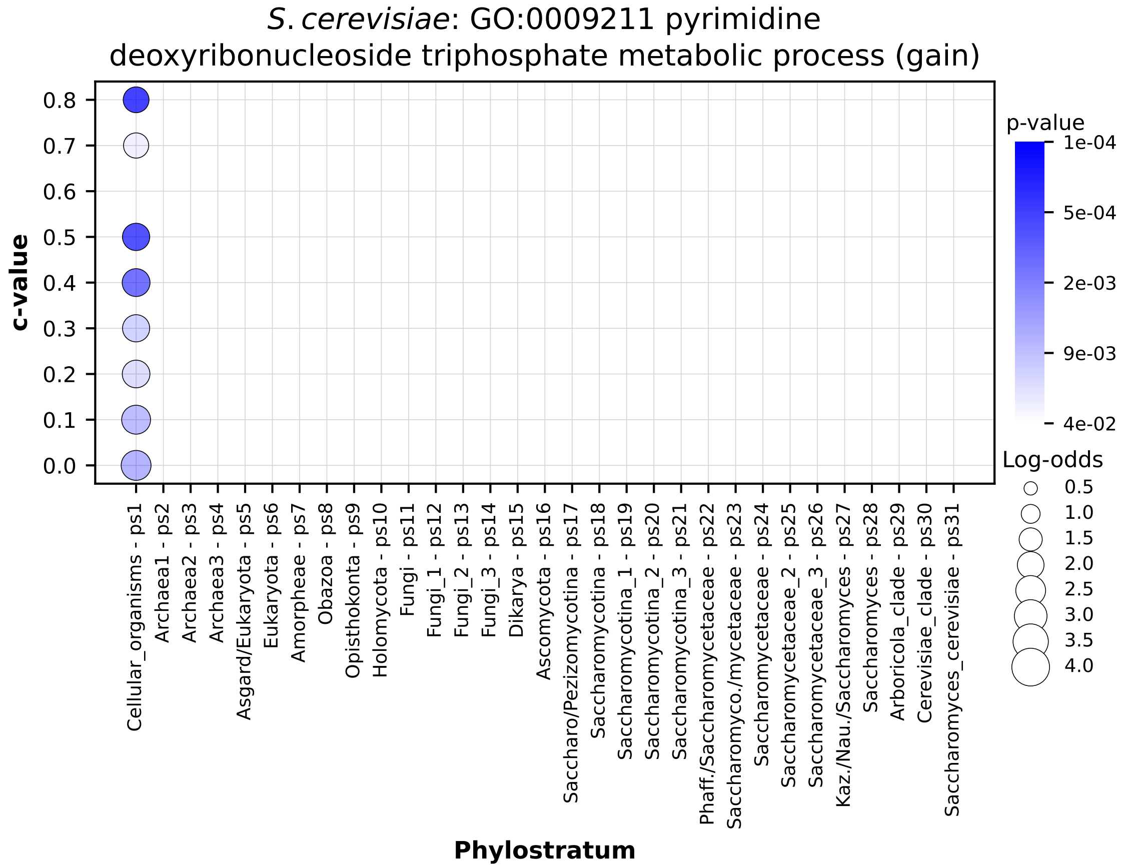

S. cerevisiae: GO:0009211 pyrimidine deoxyribonucleoside triphosphate metabolic process (gain)
c-value
0.8
0.7
0.6
0.5
0.4
0.3
0.2
0.1
0.0
p-value
1e-04
5e-04
2e-03
9e-03
4e-02
Log-odds
0.5
1.0
1.5
2.0
2.5
3.0
3.5
4.0
Cellular_organisms - ps1
Archaea1 - ps2
Archaea2 - ps3
Archaea3 - ps4
Asgard/Eukaryota - ps5
Eukaryota - ps6
Amorpheae - ps7
Obazoa - ps8
Opisthokonta - ps9
Holomycota - ps10
Fungi - ps11
Fungi_1 - ps12
Fungi_2 - ps13
Fungi_3 - ps14
Dikarya - ps15
Ascomycota - ps16
Saccharo/Pezizomycotina - ps17
Saccharomycotina - ps18
Saccharomycotina_1 - ps19
Saccharomycotina_2 - ps20
Saccharomycotina_3 - ps21
Phaff./Saccharomycetaceae - ps22
Saccharomyco./mycetaceae - ps23
Saccharomycetaceae - ps24
Saccharomycetaceae_2 - ps25
Saccharomycetaceae_3 - ps26
Kaz./Nau./Saccharomyces - ps27
Saccharomyces - ps28
Arboricola_clade - ps29
Cerevisiae_clade - ps30
Saccharomyces_cerevisiae - ps31
Phylostratum

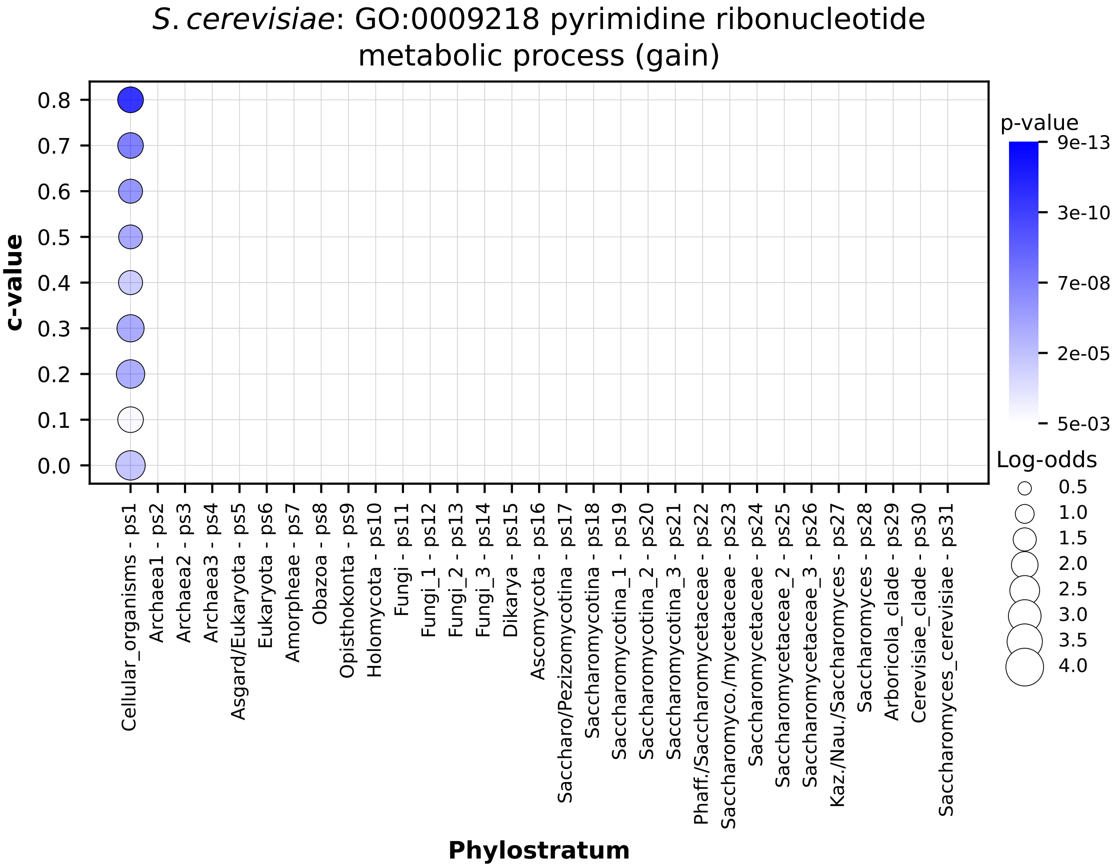

S. cerevisiae: GO:0009218 pyrimidine ribonucleotide metabolic process (gain)
c-value
0.8
0.7
0.6
0.5
0.4
0.3
0.2
0.1
0.0
p-value
9e-13
3e-10
7e-08
2e-05
5e-03
Log-odds
0.5
1.0
1.5
2.0
2.5
3.0
3.5
4.0
Cellular_organisms - ps1
Archaea1 - ps2
Archaea2 - ps3
Archaea3 - ps4
Asgard/Eukaryota - ps5
Eukaryota - ps6
Amorpheae - ps7
Obazoa - ps8
Opisthokonta - ps9
Holomycota - ps10
Fungi - ps11
Fungi_1 - ps12
Fungi_2 - ps13
Fungi_3 - ps14
Dikarya - ps15
Ascomycota - ps16
Saccharo/Pezizomycotina - ps17
Saccharomycotina - ps18
Saccharomycotina_1 - ps19
Saccharomycotina_2 - ps20
Saccharomycotina_3 - ps21
Phaff./Saccharomycetaceae - ps22
Saccharomyco./mycetaceae - ps23
Saccharomycetaceae - ps24
Saccharomycetaceae_2 - ps25
Saccharomycetaceae_3 - ps26
Kaz./Nau./Saccharomyces - ps27
Saccharomyces - ps28
Arboricola_clade - ps29
Cerevisiae_clade - ps30
Saccharomyces_cerevisiae - ps31
Phylostratum

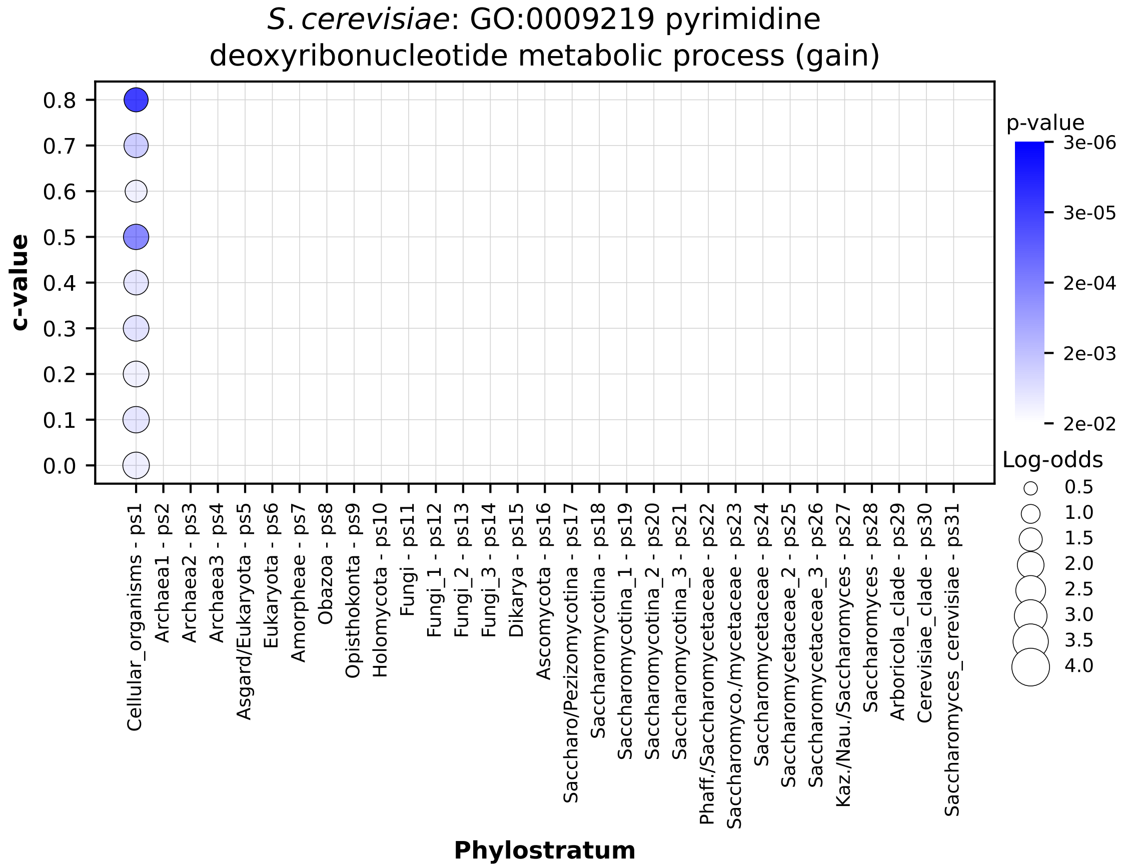

S. cerevisiae: GO:0009219 pyrimidine deoxyribonucleotide metabolic process (gain)
c-value
0.8
0.7
0.6
0.5
0.4
0.3
0.2
0.1
0.0
Phylostratum
Cellular_organisms - ps1
Archaea1 - ps2
Archaea2 - ps3
Archaea3 - ps4
Asgard/Eukaryota - ps5
Eukaryota - ps6
Amorpheae - ps7
Obazoa - ps8
Opisthokonta - ps9
Holomycota - ps10
Fungi - ps11
Fungi_1 - ps12
Fungi_2 - ps13
Fungi_3 - ps14
Dikarya - ps15
Ascomycota - ps16
Saccharo/Pezizomycotina - ps17
Saccharomycotina - ps18
Saccharomycotina_1 - ps19
Saccharomycotina_2 - ps20
Saccharomycotina_3 - ps21
Phaff./Saccharomycetaceae - ps22
Saccharomyco./mycetaceae - ps23
Saccharomycetaceae - ps24
Saccharomycetaceae_2 - ps25
Saccharomycetaceae_3 - ps26
Kaz./Nau./Saccharomyces - ps27
Saccharomyces - ps28
Arboricola_clade - ps29
Cerevisiae_clade - ps30
Saccharomyces_cerevisiae - ps31
p-value
3e-06
3e-05
2e-04
2e-03
2e-02
Log-odds
0.5
1.0
1.5
2.0
2.5
3.0
3.5
4.0

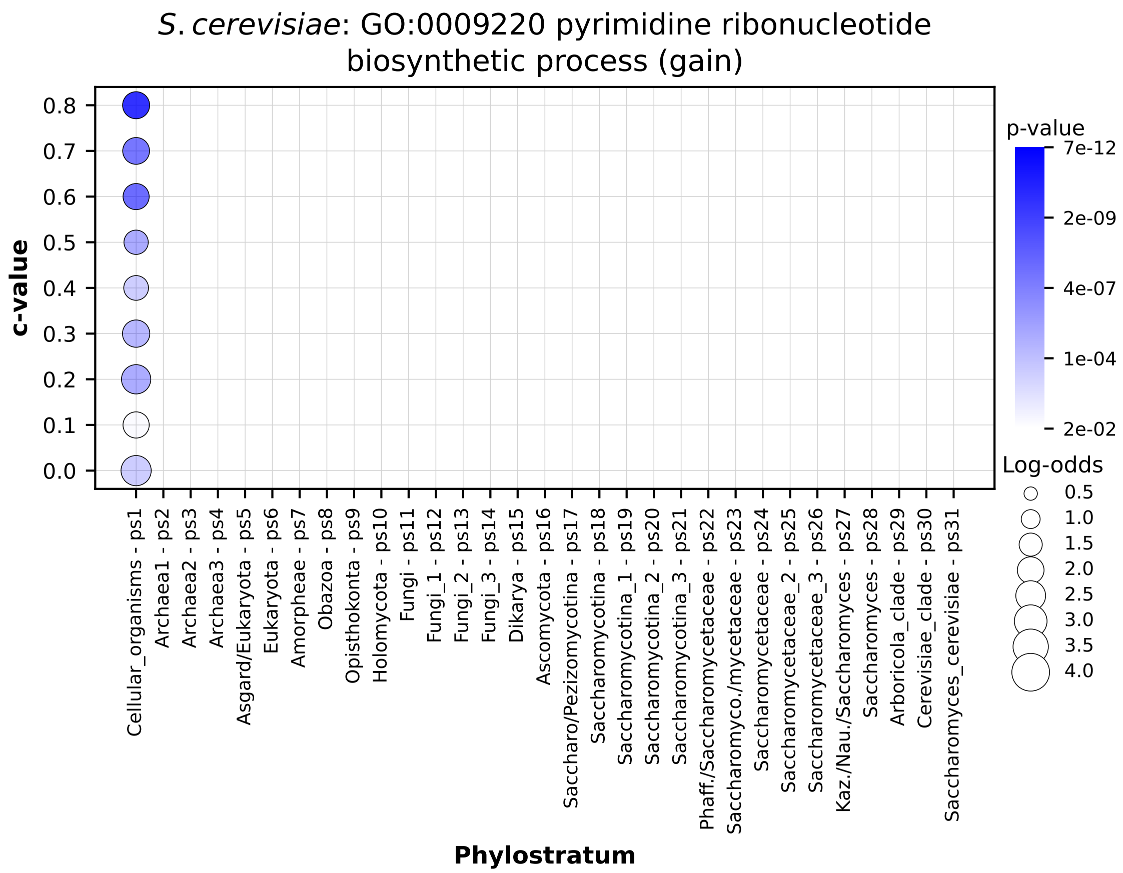

S. cerevisiae: GO:0009220 pyrimidine ribonucleotide biosynthetic process (gain)
c-value
0.8
0.7
0.6
0.5
0.4
0.3
0.2
0.1
0.0
p-value
7e-12
2e-09
4e-07
1e-04
2e-02
Log-odds
0.5
1.0
1.5
2.0
2.5
3.0
3.5
4.0
Cellular_organisms - ps1
Archaea1 - ps2
Archaea2 - ps3
Archaea3 - ps4
Asgard/Eukaryota - ps5
Eukaryota - ps6
Amorpheae - ps7
Obazoa - ps8
Opisthokonta - ps9
Holomycota - ps10
Fungi - ps11
Fungi_1 - ps12
Fungi_2 - ps13
Fungi_3 - ps14
Dikarya - ps15
Ascomycota - ps16
Saccharo/Pezizomycotina - ps17
Saccharomycotina - ps18
Saccharomycotina_1 - ps19
Saccharomycotina_2 - ps20
Saccharomycotina_3 - ps21
Phaff./Saccharomycetaceae - ps22
Saccharomyco./mycetaceae - ps23
Saccharomycetaceae - ps24
Saccharomycetaceae_2 - ps25
Saccharomycetaceae_3 - ps26
Kaz./Nau./Saccharomyces - ps27
Saccharomyces - ps28
Arboricola_clade - ps29
Cerevisiae_clade - ps30
Saccharomyces_cerevisiae - ps31
Phylostratum

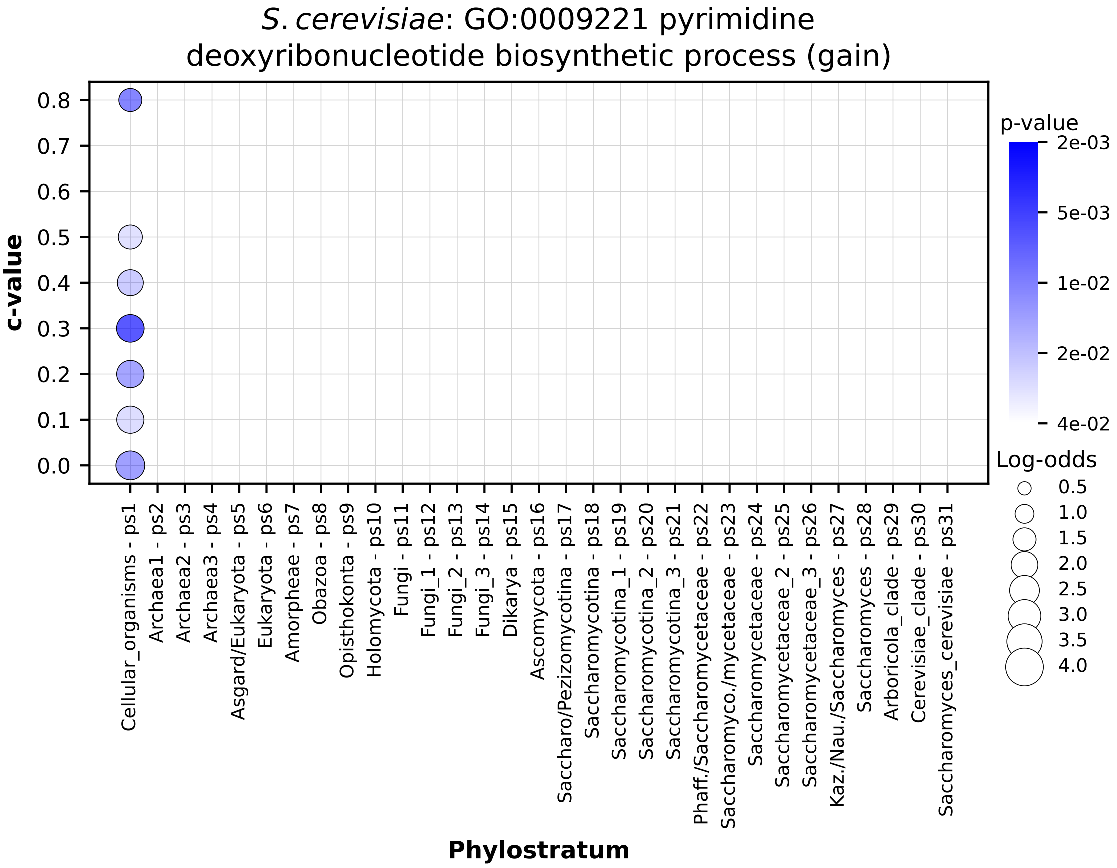
S. cerevisiae: GO:0009221 pyrimidine deoxyribonucleotide biosynthetic process (gain)
c-value
0.8
0.7
0.6
0.5
0.4
0.3
0.2
0.1
0.0
p-value
2e-03
5e-03
1e-02
2e-02
4e-02
Log-odds
0.5
1.0
1.5
2.0
2.5
3.0
3.5
4.0
Cellular_organisms - ps1
Archaea1 - ps2
Archaea2 - ps3
Archaea3 - ps4
Asgard/Eukaryota - ps5
Eukaryota - ps6
Amorpheae - ps7
Obazoa - ps8
Opisthokonta - ps9
Holomycota - ps10
Fungi - ps11
Fungi_1 - ps12
Fungi_2 - ps13
Fungi_3 - ps14
Dikarya - ps15
Ascomycota - ps16
Saccharo/Pezizomycotina - ps17
Saccharomycotina - ps18
Saccharomycotina_1 - ps19
Saccharomycotina_2 - ps20
Saccharomycotina_3 - ps21
Phaff./Saccharomycetaceae - ps22
Saccharomyco./mycetaceae - ps23
Saccharomycetaceae - ps24
Saccharomycetaceae_2 - ps25
Saccharomycetaceae_3 - ps26
Kaz./Nau./Saccharomyces - ps27
Saccharomyces - ps28
Arboricola_clade - ps29
Cerevisiae_clade - ps30
Saccharomyces_cerevisiae - ps31
Phylostratum

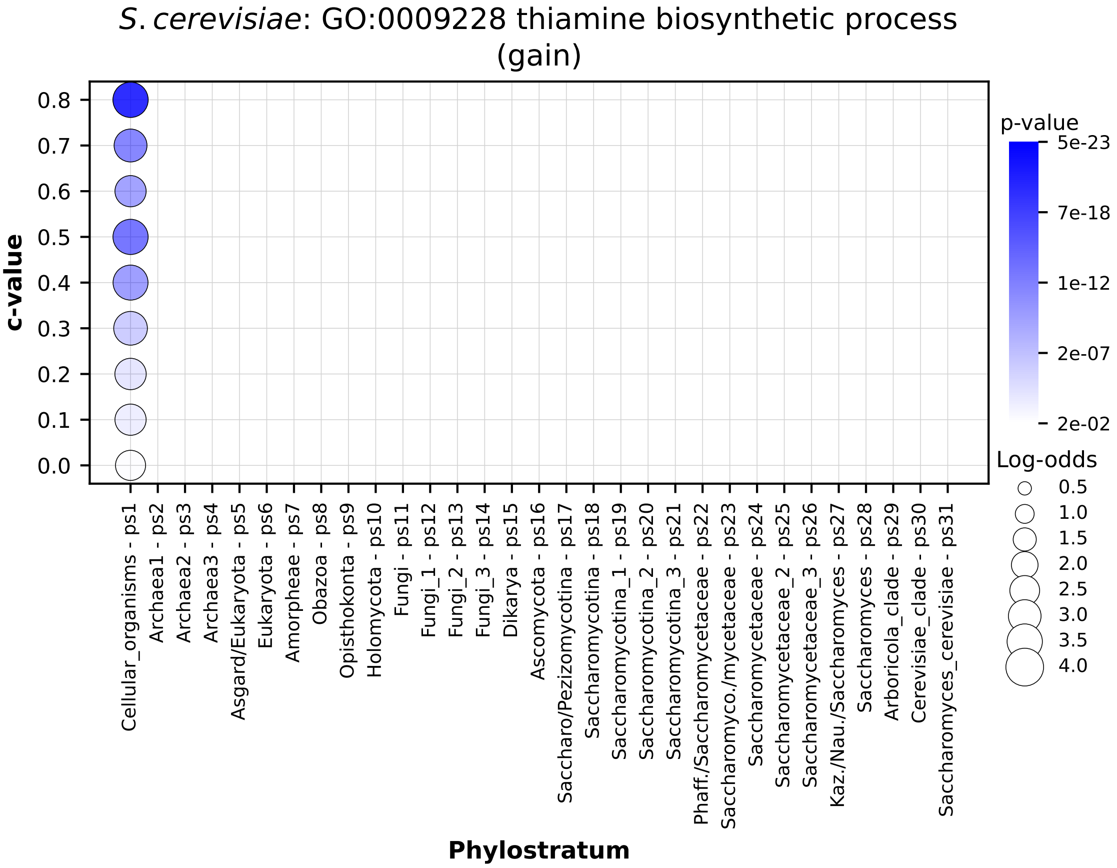
S. cerevisiae: GO:0009228 thiamine biosynthetic process
(gain)
c-value
0.8
0.7
0.6
0.5
0.4
0.3
0.2
0.1
0.0
p-value
5e-23
7e-18
1e-12
2e-07
2e-02
Log-odds
0.5
1.0
1.5
2.0
2.5
3.0
3.5
4.0
Cellular_organisms - ps1
Archaea1 - ps2
Archaea2 - ps3
Archaea3 - ps4
Asgard/Eukaryota - ps5
Eukaryota - ps6
Amorpheae - ps7
Obazoa - ps8
Opisthokonta - ps9
Holomycota - ps10
Fungi - ps11
Fungi_1 - ps12
Fungi_2 - ps13
Fungi_3 - ps14
Dikarya - ps15
Ascomycota - ps16
Saccharo/Pezizomycotina - ps17
Saccharomycotina - ps18
Saccharomycotina_1 - ps19
Saccharomycotina_2 - ps20
Saccharomycotina_3 - ps21
Phaff./Saccharomycetaceae - ps22
Saccharomyco./mycetaceae - ps23
Saccharomycetaceae - ps24
Saccharomycetaceae_2 - ps25
Saccharomycetaceae_3 - ps26
Kaz./Nau./Saccharomyces - ps27
Saccharomyces - ps28
Arboricola_clade - ps29
Cerevisiae_clade - ps30
Saccharomyces_cerevisiae - ps31
Phylostratum

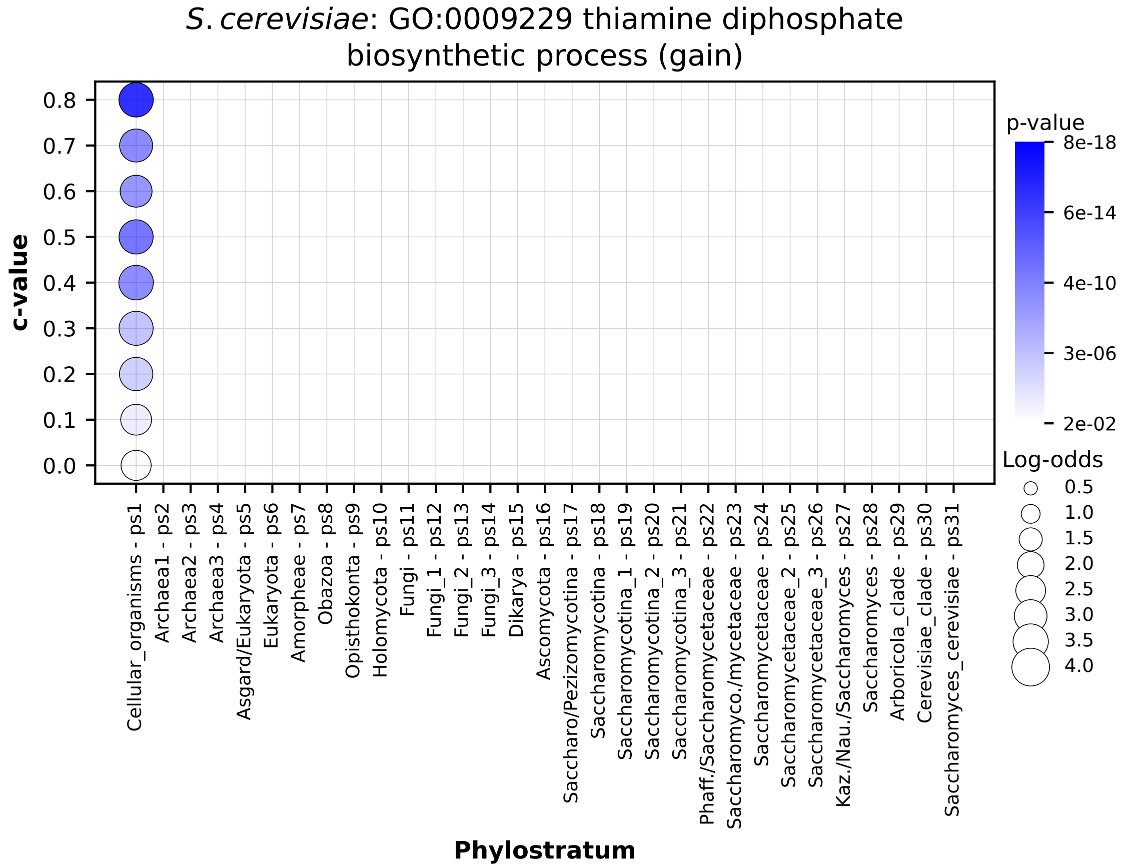

S. cerevisiae: GO:0009229 thiamine diphosphate biosynthetic process (gain)
c-value
0.8
0.7
0.6
0.5
0.4
0.3
0.2
0.1
0.0
p-value
8e-18
6e-14
4e-10
3e-06
2e-02
Log-odds
0.5
1.0
1.5
2.0
2.5
3.0
3.5
4.0
Cellular_organisms - ps1
Archaea1 - ps2
Archaea2 - ps3
Archaea3 - ps4
Asgard/Eukaryota - ps5
Eukaryota - ps6
Amorpheae - ps7
Obazoa - ps8
Opisthokonta - ps9
Holomycota - ps10
Fungi - ps11
Fungi_1 - ps12
Fungi_2 - ps13
Fungi_3 - ps14
Dikarya - ps15
Ascomycota - ps16
Saccharo/Pezizomycotina - ps17
Saccharomycotina - ps18
Saccharomycotina_1 - ps19
Saccharomycotina_2 - ps20
Saccharomycotina_3 - ps21
Phaff./Saccharomycetaceae - ps22
Saccharomyco./mycetaceae - ps23
Saccharomycetaceae - ps24
Saccharomycetaceae_2 - ps25
Saccharomycetaceae_3 - ps26
Kaz./Nau./Saccharomyces - ps27
Saccharomyces - ps28
Arboricola_clade - ps29
Cerevisiae_clade - ps30
Saccharomyces_cerevisiae - ps31
Phylostratum

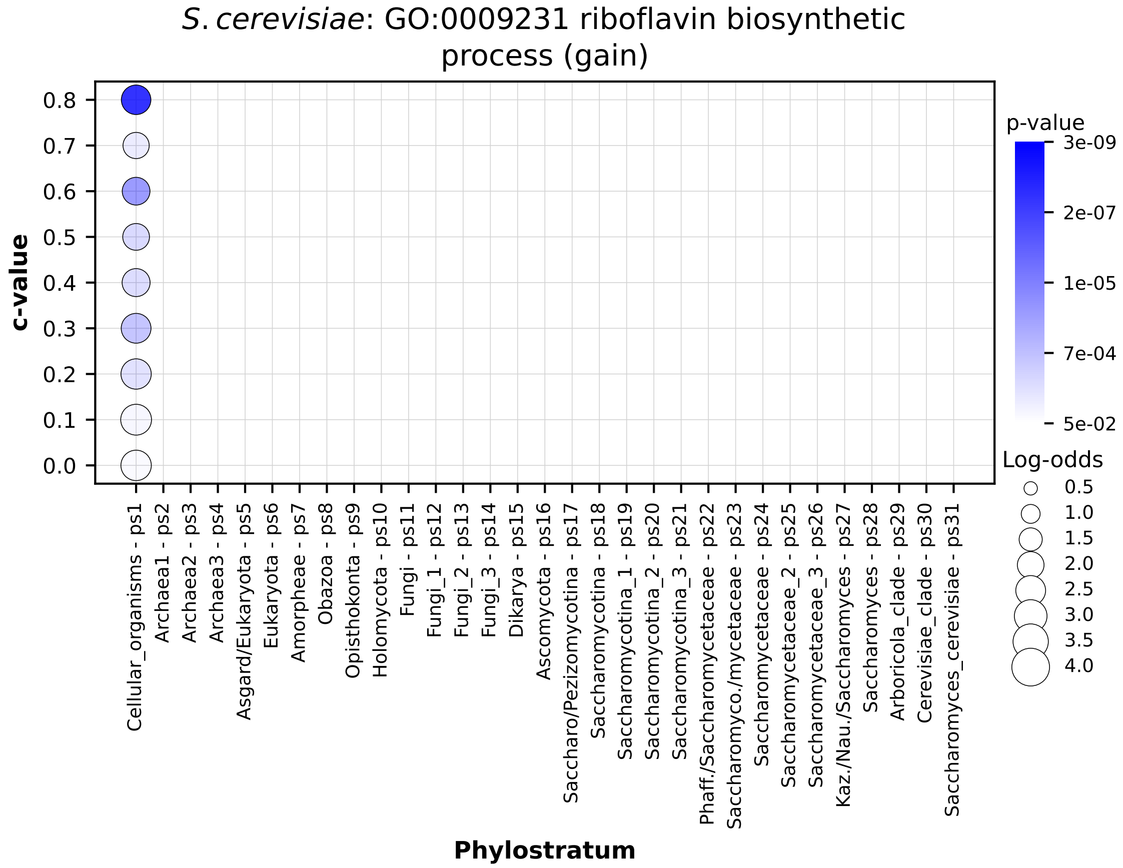
S. cerevisiae: GO:0009231 riboflavin biosynthetic process (gain)
c-value
0.8
0.7
0.6
0.5
0.4
0.3
0.2
0.1
0.0
p-value
3e-09
2e-07
1e-05
7e-04
5e-02
Log-odds
0.5
1.0
1.5
2.0
2.5
3.0
3.5
4.0
Cellular_organisms - ps1
Archaea1 - ps2
Archaea2 - ps3
Archaea3 - ps4
Asgard/Eukaryota - ps5
Eukaryota - ps6
Amorpheae - ps7
Obazoa - ps8
Opisthokonta - ps9
Holomycota - ps10
Fungi - ps11
Fungi_1 - ps12
Fungi_2 - ps13
Fungi_3 - ps14
Dikarya - ps15
Ascomycota - ps16
Saccharo/Pezizomycotina - ps17
Saccharomycotina - ps18
Saccharomycotina_1 - ps19
Saccharomycotina_2 - ps20
Saccharomycotina_3 - ps21
Phaff./Saccharomycetaceae - ps22
Saccharomyco./mycetaceae - ps23
Saccharomycetaceae - ps24
Saccharomycetaceae_2 - ps25
Saccharomycetaceae_3 - ps26
Kaz./Nau./Saccharomyces - ps27
Saccharomyces - ps28
Arboricola_clade - ps29
Cerevisiae_clade - ps30
Saccharomyces_cerevisiae - ps31
Phylostratum

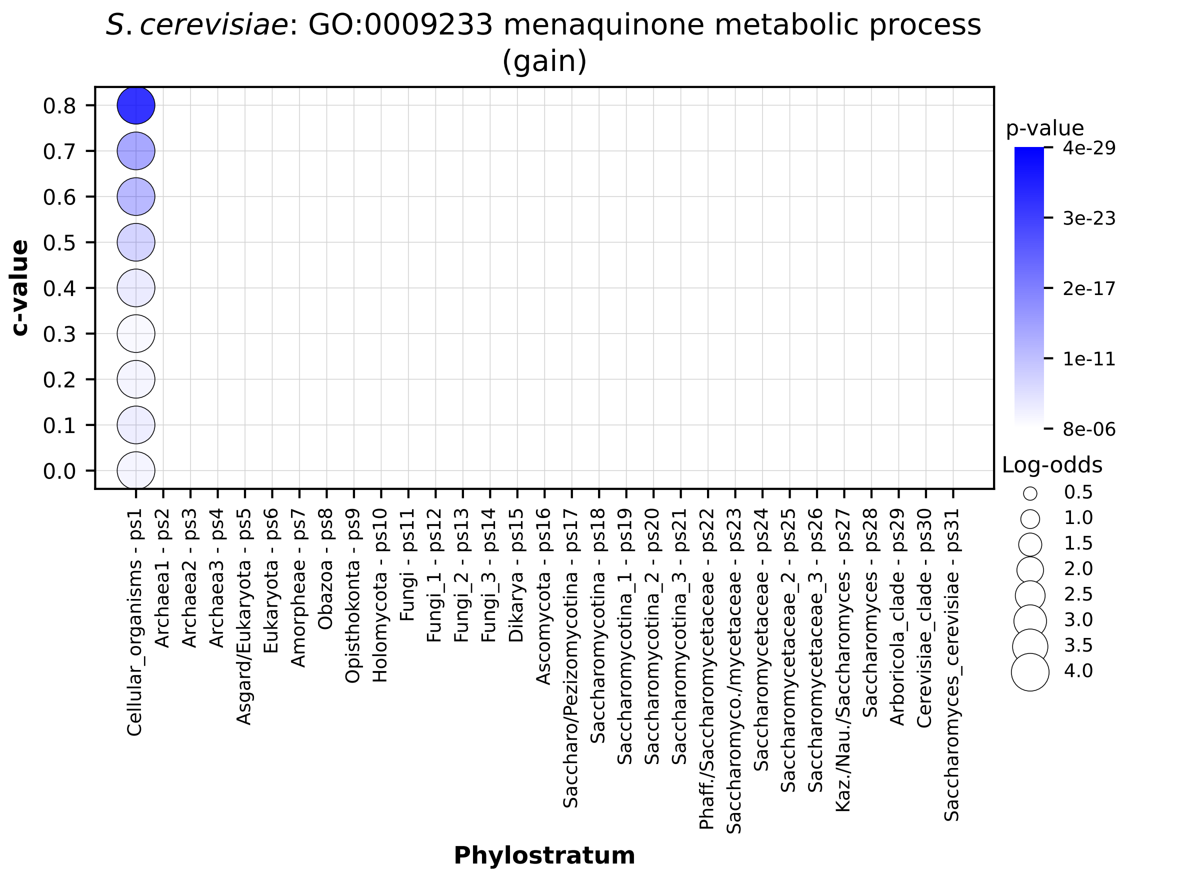

S. cerevisiae: GO:0009233 menaquinone metabolic process (gain)
c-value
0.8
0.7
0.6
0.5
0.4
0.3
0.2
0.1
0.0
p-value
4e-29
3e-23
2e-17
1e-11
8e-06
Log-odds
0.5
1.0
1.5
2.0
2.5
3.0
3.5
4.0
Cellular_organisms - ps1
Archaea1 - ps2
Archaea2 - ps3
Archaea3 - ps4
Asgard/Eukaryota - ps5
Eukaryota - ps6
Amorpheae - ps7
Obazoa - ps8
Opisthokonta - ps9
Holomycota - ps10
Fungi - ps11
Fungi_1 - ps12
Fungi_2 - ps13
Fungi_3 - ps14
Dikarya - ps15
Ascomycota - ps16
Saccharo/Pezizomycotina - ps17
Saccharomycotina - ps18
Saccharomycotina_1 - ps19
Saccharomycotina_2 - ps20
Saccharomycotina_3 - ps21
Phaff./Saccharomycetaceae - ps22
Saccharomyco./mycetaceae - ps23
Saccharomycetaceae - ps24
Saccharomycetaceae_2 - ps25
Saccharomycetaceae_3 - ps26
Kaz./Nau./Saccharomyces - ps27
Saccharomyces - ps28
Arboricola_clade - ps29
Cerevisiae_clade - ps30
Saccharomyces_cerevisiae - ps31
Phylostratum

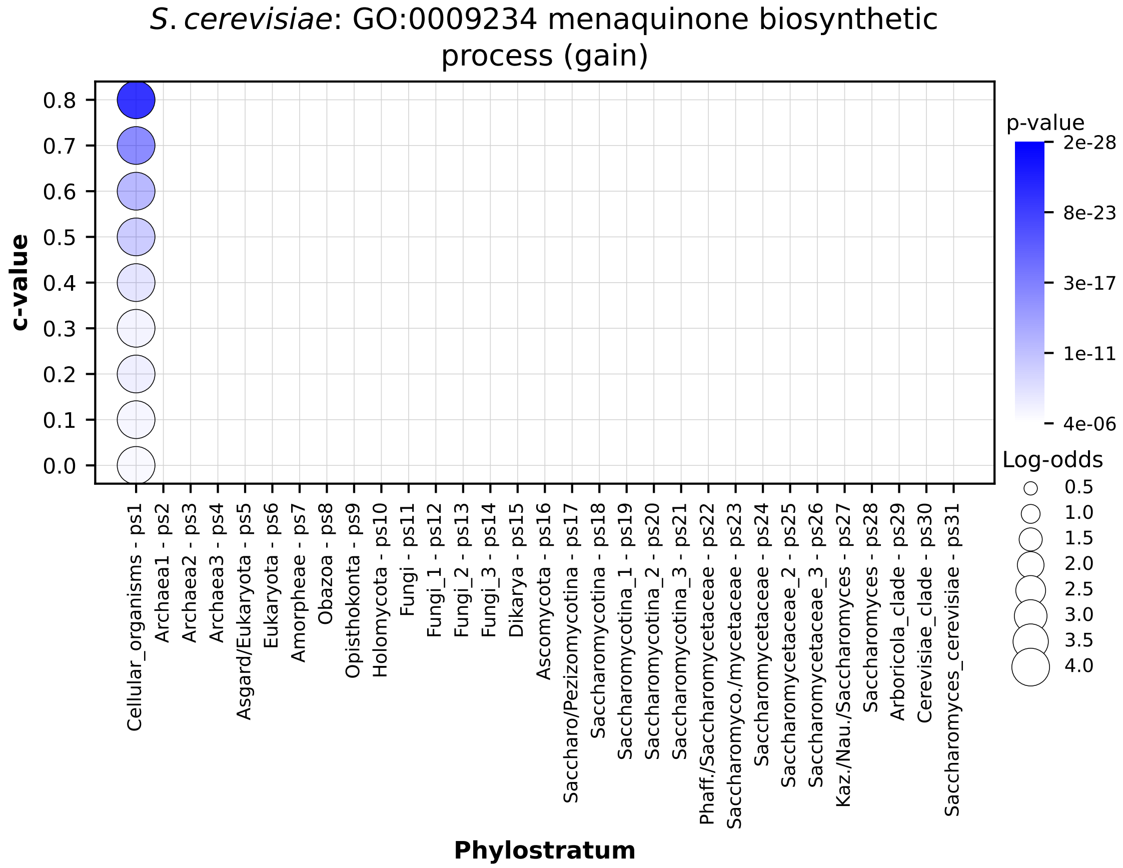
S. cerevisiae: GO:0009234 menaquinone biosynthetic process (gain)
c-value
0.8
0.7
0.6
0.5
0.4
0.3
0.2
0.1
0.0
p-value
2e-28
8e-23
3e-17
1e-11
4e-06
Log-odds
0.5
1.0
1.5
2.0
2.5
3.0
3.5
4.0
Cellular_organisms - ps1
Archaea1 - ps2
Archaea2 - ps3
Archaea3 - ps4
Asgard/Eukaryota - ps5
Eukaryota - ps6
Amorpheae - ps7
Obazoa - ps8
Opisthokonta - ps9
Holomycota - ps10
Fungi - ps11
Fungi_1 - ps12
Fungi_2 - ps13
Fungi_3 - ps14
Dikarya - ps15
Ascomycota - ps16
Saccharo/Pezizomycotina - ps17
Saccharomycotina - ps18
Saccharomycotina_1 - ps19
Saccharomycotina_2 - ps20
Saccharomycotina_3 - ps21
Phaff./Saccharomycetaceae - ps22
Saccharomyco./mycetaceae - ps23
Saccharomycetaceae - ps24
Saccharomycetaceae_2 - ps25
Saccharomycetaceae_3 - ps26
Kaz./Nau./Saccharomyces - ps27
Saccharomyces - ps28
Arboricola_clade - ps29
Cerevisiae_clade - ps30
Saccharomyces_cerevisiae - ps31
Phylostratum

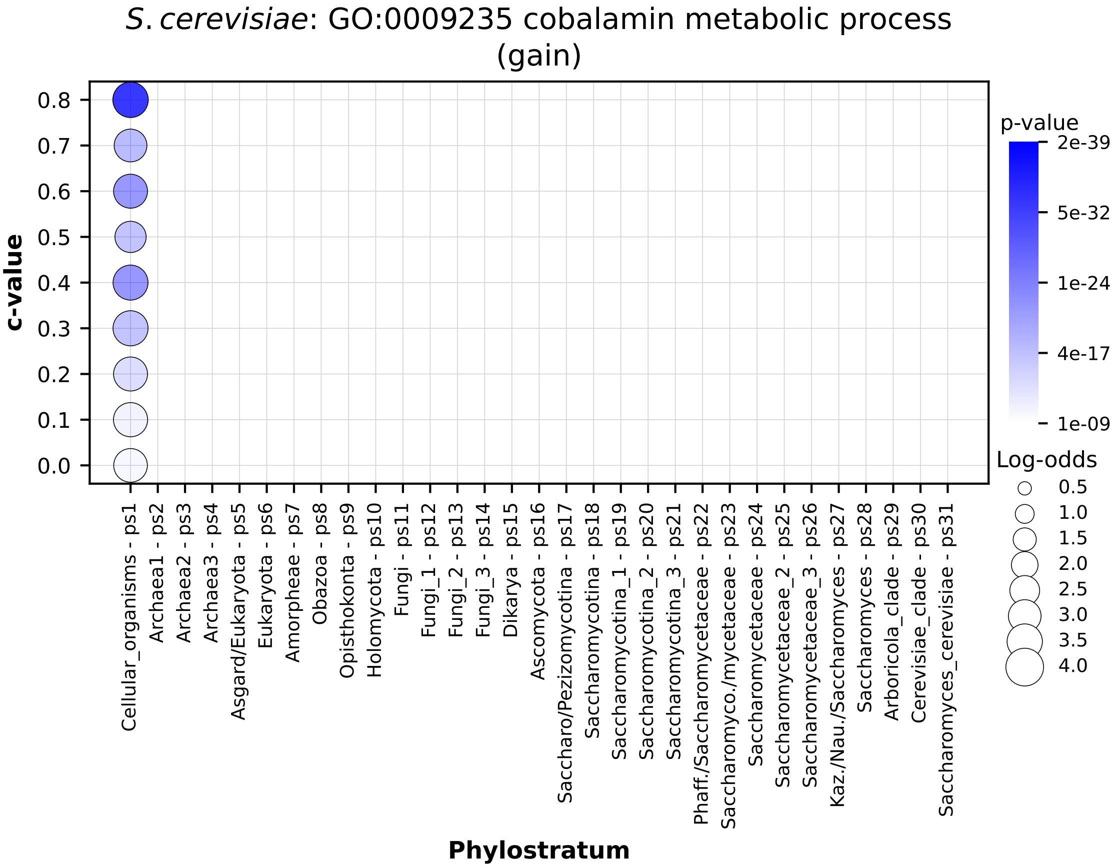

S. cerevisiae: GO:0009235 cobalamin metabolic process (gain)
c-value
0.8
0.7
0.6
0.5
0.4
0.3
0.2
0.1
0.0
Phylostratum
Cellular_organisms - ps1
Archaea1 - ps2
Archaea2 - ps3
Archaea3 - ps4
Asgard/Eukaryota - ps5
Eukaryota - ps6
Amorpheae - ps7
Obazoa - ps8
Opisthokonta - ps9
Holomycota - ps10
Fungi - ps11
Fungi_1 - ps12
Fungi_2 - ps13
Fungi_3 - ps14
Dikarya - ps15
Ascomycota - ps16
Saccharo/Pezizomycotina - ps17
Saccharomycotina - ps18
Saccharomycotina_1 - ps19
Saccharomycotina_2 - ps20
Saccharomycotina_3 - ps21
Phaff./Saccharomycetaceae - ps22
Saccharomyco./mycetaceae - ps23
Saccharomycetaceae - ps24
Saccharomycetaceae_2 - ps25
Saccharomycetaceae_3 - ps26
Kaz./Nau./Saccharomyces - ps27
Saccharomyces - ps28
Arboricola_clade - ps29
Cerevisiae_clade - ps30
Saccharomyces_cerevisiae - ps31
p-value
2e-39
5e-32
1e-24
4e-17
1e-09
Log-odds
0.5
1.0
1.5
2.0
2.5
3.0
3.5
4.0

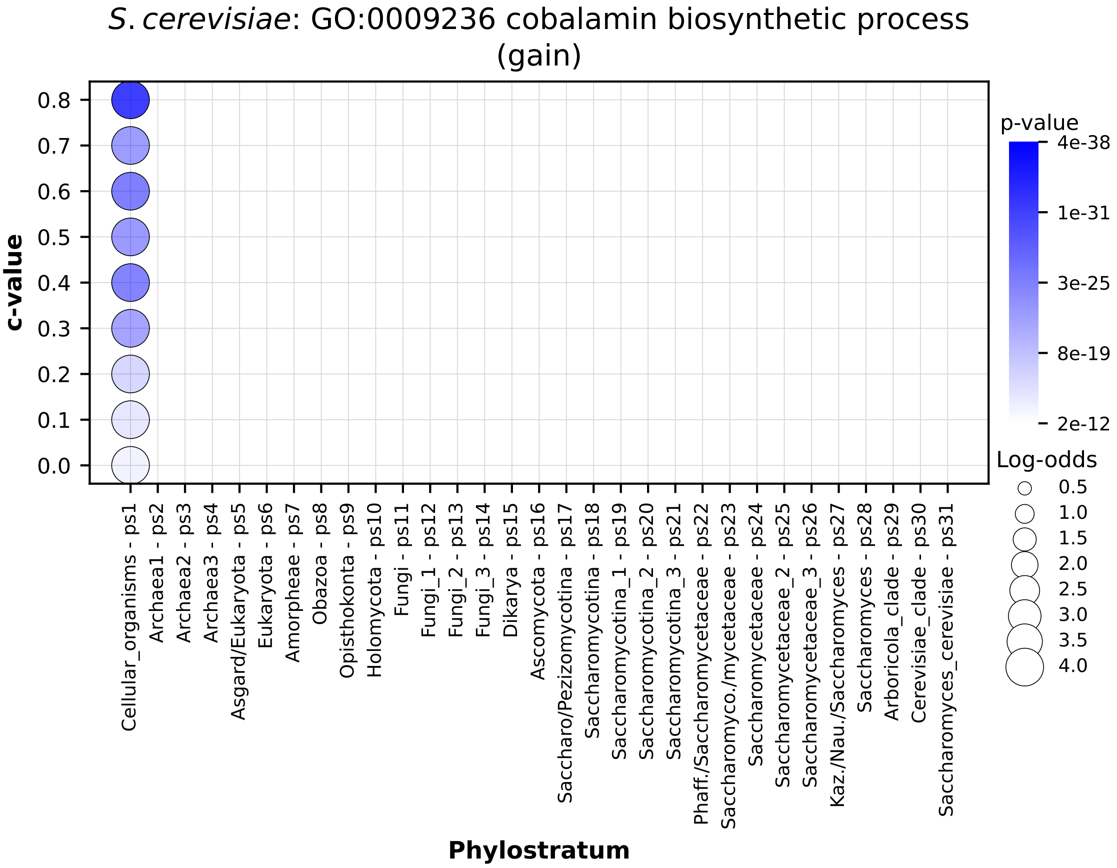

S. cerevisiae: GO:0009236 cobalamin biosynthetic process
(gain)
c-value
0.8
0.7
0.6
0.5
0.4
0.3
0.2
0.1
0.0
p-value
4e-38
1e-31
3e-25
8e-19
2e-12
Log-odds
0.5
1.0
1.5
2.0
2.5
3.0
3.5
4.0
Cellular_organisms - ps1
Archaea1 - ps2
Archaea2 - ps3
Archaea3 - ps4
Asgard/Eukaryota - ps5
Eukaryota - ps6
Amorpheae - ps7
Obazoa - ps8
Opisthokonta - ps9
Holomycota - ps10
Fungi - ps11
Fungi_1 - ps12
Fungi_2 - ps13
Fungi_3 - ps14
Dikarya - ps15
Ascomycota - ps16
Saccharo/Pezizomycotina - ps17
Saccharomycotina - ps18
Saccharomycotina_1 - ps19
Saccharomycotina_2 - ps20
Saccharomycotina_3 - ps21
Phaff./Saccharomycetaceae - ps22
Saccharomyco./mycetaceae - ps23
Saccharomycetaceae - ps24
Saccharomycetaceae_2 - ps25
Saccharomycetaceae_3 - ps26
Kaz./Nau./Saccharomyces - ps27
Saccharomyces - ps28
Arboricola_clade - ps29
Cerevisiae_clade - ps30
Saccharomyces_cerevisiae - ps31
Phylostratum

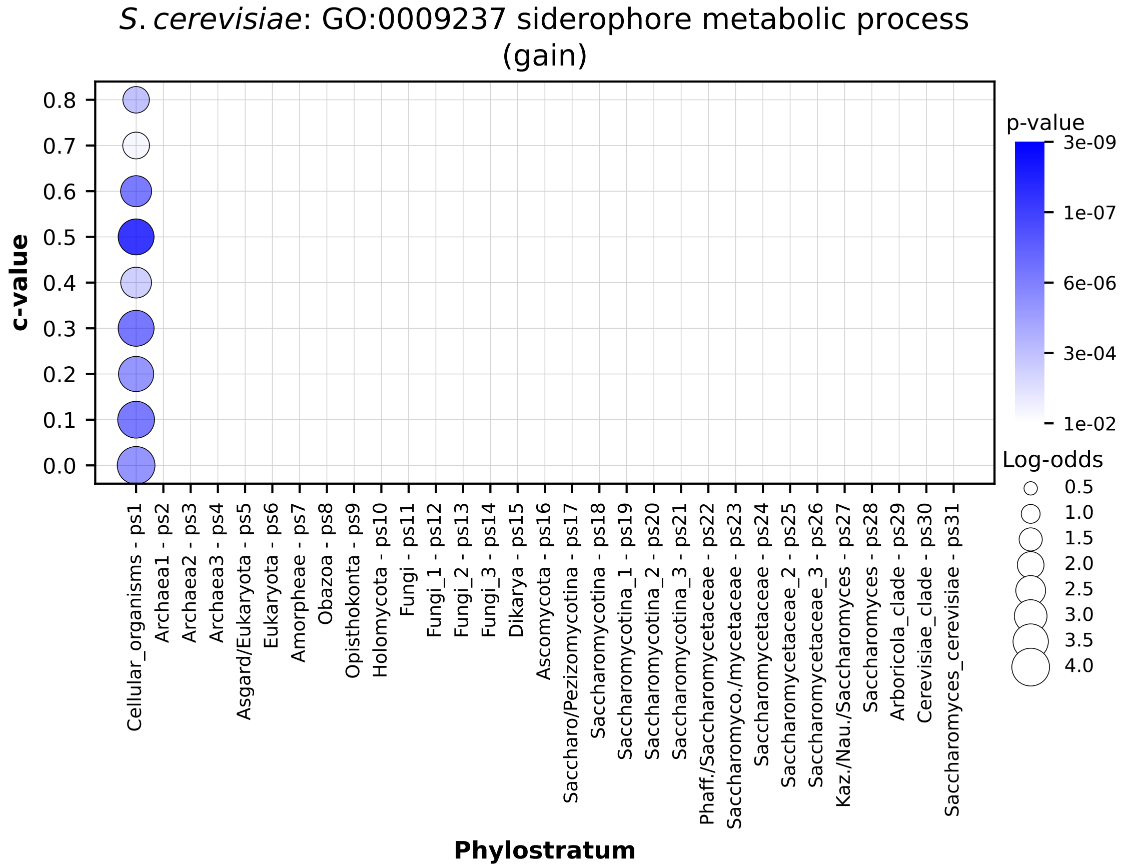
S. cerevisiae: GO:0009237 siderophore metabolic process (gain)
c-value
0.8
0.7
0.6
0.5
0.4
0.3
0.2
0.1
0.0
p-value
3e-09
1e-07
6e-06
3e-04
1e-02
Log-odds
0.5
1.0
1.5
2.0
2.5
3.0
3.5
4.0
Cellular_organisms - ps1
Archaea1 - ps2
Archaea2 - ps3
Archaea3 - ps4
Asgard/Eukaryota - ps5
Eukaryota - ps6
Amorpheae - ps7
Obazoa - ps8
Opisthokonta - ps9
Holomycota - ps10
Fungi - ps11
Fungi_1 - ps12
Fungi_2 - ps13
Fungi_3 - ps14
Dikarya - ps15
Ascomycota - ps16
Saccharo/Pezizomycotina - ps17
Saccharomycotina - ps18
Saccharomycotina_1 - ps19
Saccharomycotina_2 - ps20
Saccharomycotina_3 - ps21
Phaff./Saccharomycetaceae - ps22
Saccharomyco./mycetaceae - ps23
Saccharomycetaceae - ps24
Saccharomycetaceae_2 - ps25
Saccharomycetaceae_3 - ps26
Kaz./Nau./Saccharomyces - ps27
Saccharomyces - ps28
Arboricola_clade - ps29
Cerevisiae_clade - ps30
Saccharomyces_cerevisiae - ps31
Phylostratum

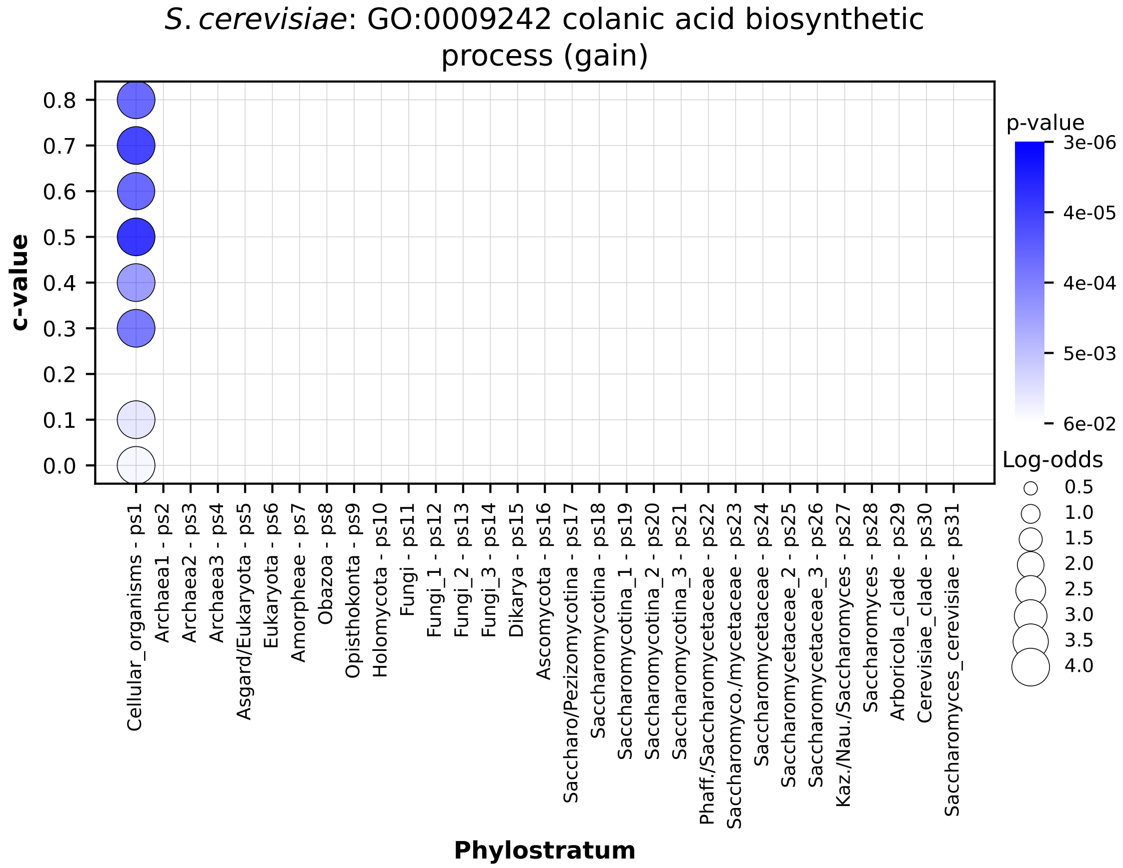

S. cerevisiae: GO:0009242 colanic acid biosynthetic process (gain)
c-value
0.8
0.7
0.6
0.5
0.4
0.3
0.2
0.1
0.0
p-value
3e-06
4e-05
4e-04
5e-03
6e-02
Log-odds
0.5
1.0
1.5
2.0
2.5
3.0
3.5
4.0
Cellular_organisms - ps1
Archaea1 - ps2
Archaea2 - ps3
Archaea3 - ps4
Asgard/Eukaryota - ps5
Eukaryota - ps6
Amorpheae - ps7
Obazoa - ps8
Opisthokonta - ps9
Holomycota - ps10
Fungi - ps11
Fungi_1 - ps12
Fungi_2 - ps13
Fungi_3 - ps14
Dikarya - ps15
Ascomycota - ps16
Saccharo/Pezizomycotina - ps17
Saccharomycotina - ps18
Saccharomycotina_1 - ps19
Saccharomycotina_2 - ps20
Saccharomycotina_3 - ps21
Phaff./Saccharomycetaceae - ps22
Saccharomyco./mycetaceae - ps23
Saccharomycetaceae - ps24
Saccharomycetaceae_2 - ps25
Saccharomycetaceae_3 - ps26
Kaz./Nau./Saccharomyces - ps27
Saccharomyces - ps28
Arboricola_clade - ps29
Cerevisiae_clade - ps30
Saccharomyces_cerevisiae - ps31
Phylostratum

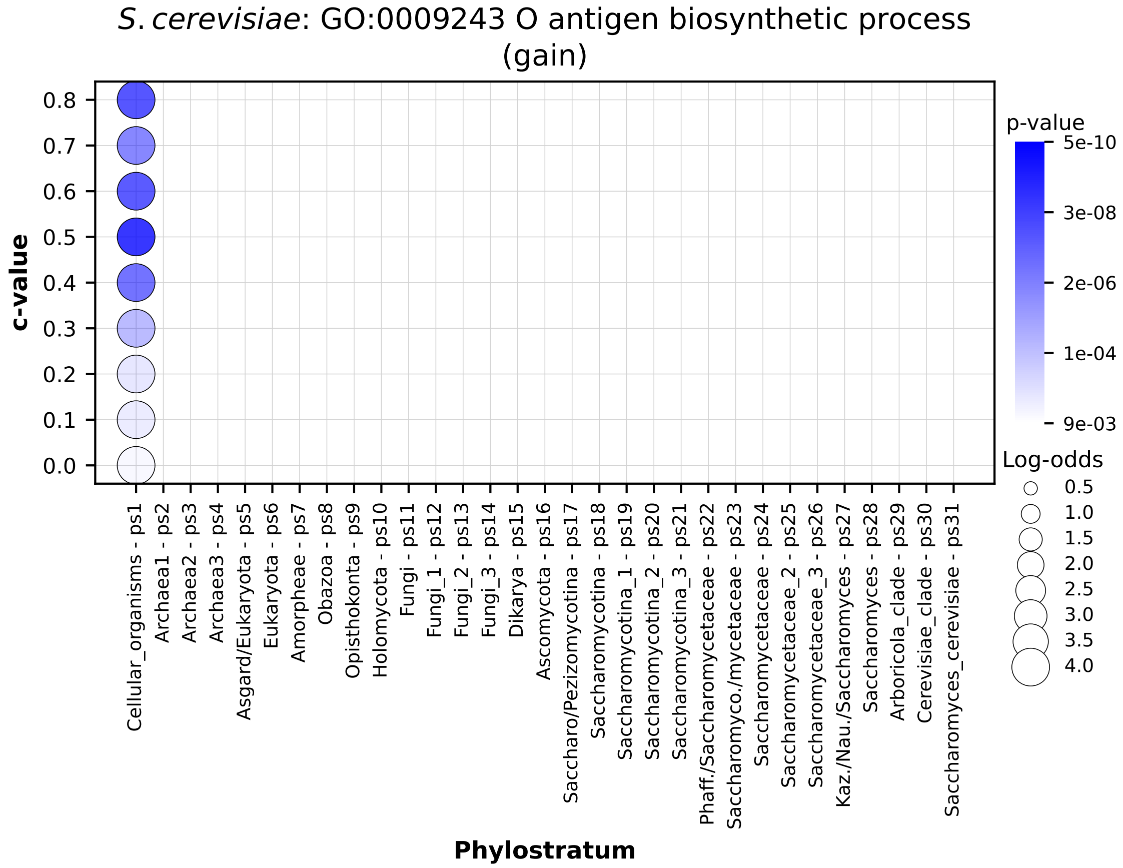

S. cerevisiae: GO:0009243 O antigen biosynthetic process
(gain)
c-value
0.8
0.7
0.6
0.5
0.4
0.3
0.2
0.1
0.0
p-value
5e-10
3e-08
2e-06
1e-04
9e-03
Log-odds
0.5
1.0
1.5
2.0
2.5
3.0
3.5
4.0
Cellular_organisms - ps1
Archaea1 - ps2
Archaea2 - ps3
Archaea3 - ps4
Asgard/Eukaryota - ps5
Eukaryota - ps6
Amorpheae - ps7
Obazoa - ps8
Opisthokonta - ps9
Holomycota - ps10
Fungi - ps11
Fungi_1 - ps12
Fungi_2 - ps13
Fungi_3 - ps14
Dikarya - ps15
Ascomycota - ps16
Saccharo/Pezizomycotina - ps17
Saccharomycotina - ps18
Saccharomycotina_1 - ps19
Saccharomycotina_2 - ps20
Saccharomycotina_3 - ps21
Phaff./Saccharomycetaceae - ps22
Saccharomyco./mycetaceae - ps23
Saccharomycetaceae - ps24
Saccharomycetaceae_2 - ps25
Saccharomycetaceae_3 - ps26
Kaz./Nau./Saccharomyces - ps27
Saccharomyces - ps28
Arboricola_clade - ps29
Cerevisiae_clade - ps30
Saccharomyces_cerevisiae - ps31
Phylostratum

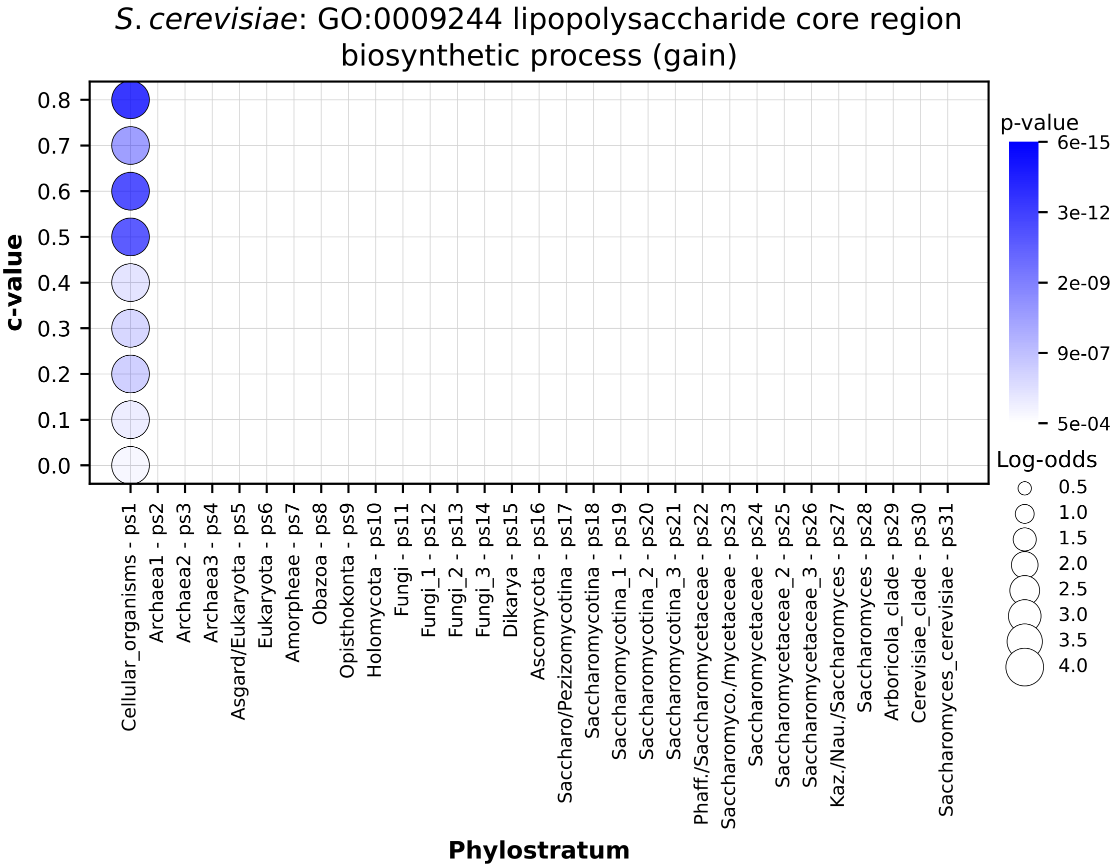

S. cerevisiae: GO:0009244 lipopolysaccharide core region biosynthetic process (gain)
c-value
0.8
0.7
0.6
0.5
0.4
0.3
0.2
0.1
0.0
p-value
6e-15
3e-12
2e-09
9e-07
5e-04
Log-odds
0.5
1.0
1.5
2.0
2.5
3.0
3.5
4.0
Cellular_organisms - ps1
Archaea1 - ps2
Archaea2 - ps3
Archaea3 - ps4
Asgard/Eukaryota - ps5
Eukaryota - ps6
Amorpheae - ps7
Obazoa - ps8
Opisthokonta - ps9
Holomycota - ps10
Fungi - ps11
Fungi_1 - ps12
Fungi_2 - ps13
Fungi_3 - ps14
Dikarya - ps15
Ascomycota - ps16
Saccharo/Pezizomycotina - ps17
Saccharomycotina - ps18
Saccharomycotina_1 - ps19
Saccharomycotina_2 - ps20
Saccharomycotina_3 - ps21
Phaff./Saccharomycetaceae - ps22
Saccharomyco./mycetaceae - ps23
Saccharomycetaceae - ps24
Saccharomycetaceae_2 - ps25
Saccharomycetaceae_3 - ps26
Kaz./Nau./Saccharomyces - ps27
Saccharomyces - ps28
Arboricola_clade - ps29
Cerevisiae_clade - ps30
Saccharomyces_cerevisiae - ps31
Phylostratum

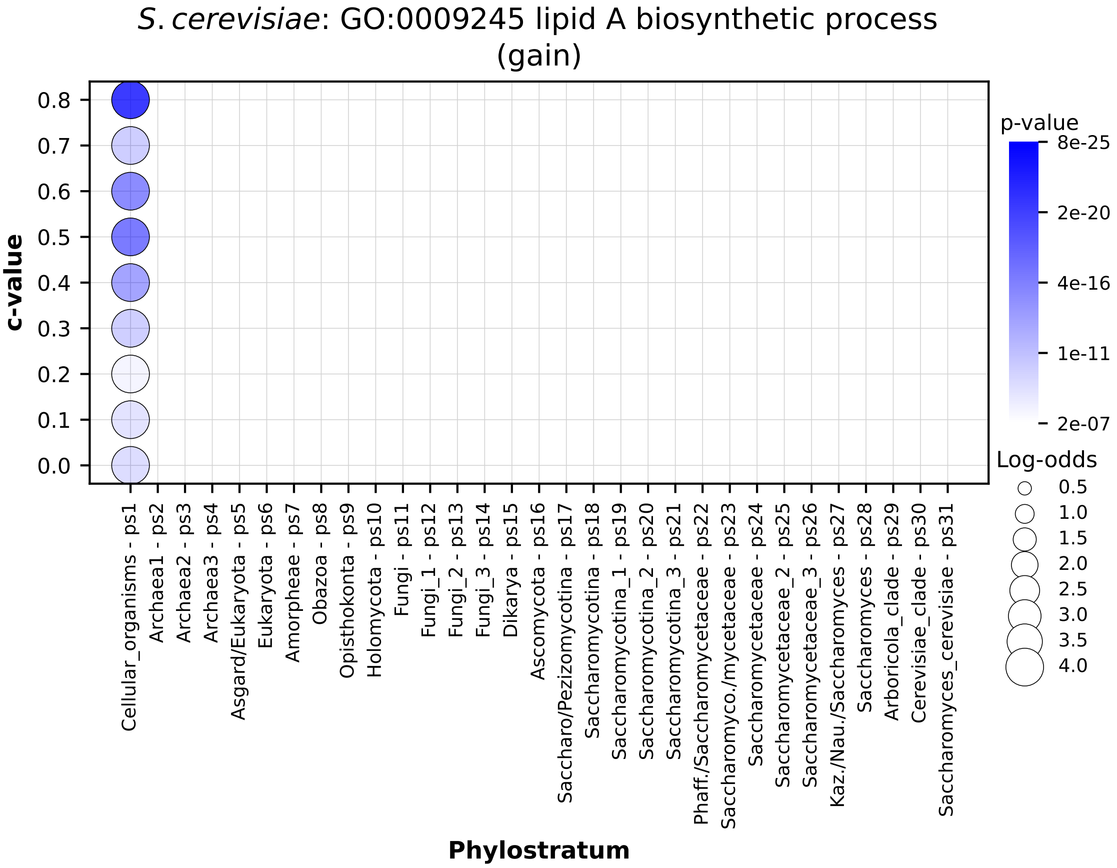

S. cerevisiae: GO:0009245 lipid A biosynthetic process
(gain)
c-value
0.0
0.1
0.2
0.3
0.4
0.5
0.6
0.7
0.8
p-value
8e-25
2e-20
4e-16
1e-11
2e-07
Log-odds
0.5
1.0
1.5
2.0
2.5
3.0
3.5
4.0
Cellular_organisms - ps1
Archaea1 - ps2
Archaea2 - ps3
Archaea3 - ps4
Asgard/Eukaryota - ps5
Eukaryota - ps6
Amorpheae - ps7
Obazoa - ps8
Opisthokonta - ps9
Holomycota - ps10
Fungi - ps11
Fungi_1 - ps12
Fungi_2 - ps13
Fungi_3 - ps14
Dikarya - ps15
Ascomycota - ps16
Saccharo/Pezizomycotina - ps17
Saccharomycotina - ps18
Saccharomycotina_1 - ps19
Saccharomycotina_2 - ps20
Saccharomycotina_3 - ps21
Phaff./Saccharomycetaceae - ps22
Saccharomyco./mycetaceae - ps23
Saccharomycetaceae - ps24
Saccharomycetaceae_2 - ps25
Saccharomycetaceae_3 - ps26
Kaz./Nau./Saccharomyces - ps27
Saccharomyces - ps28
Arboricola_clade - ps29
Cerevisiae_clade - ps30
Saccharomyces_cerevisiae - ps31
Phylostratum

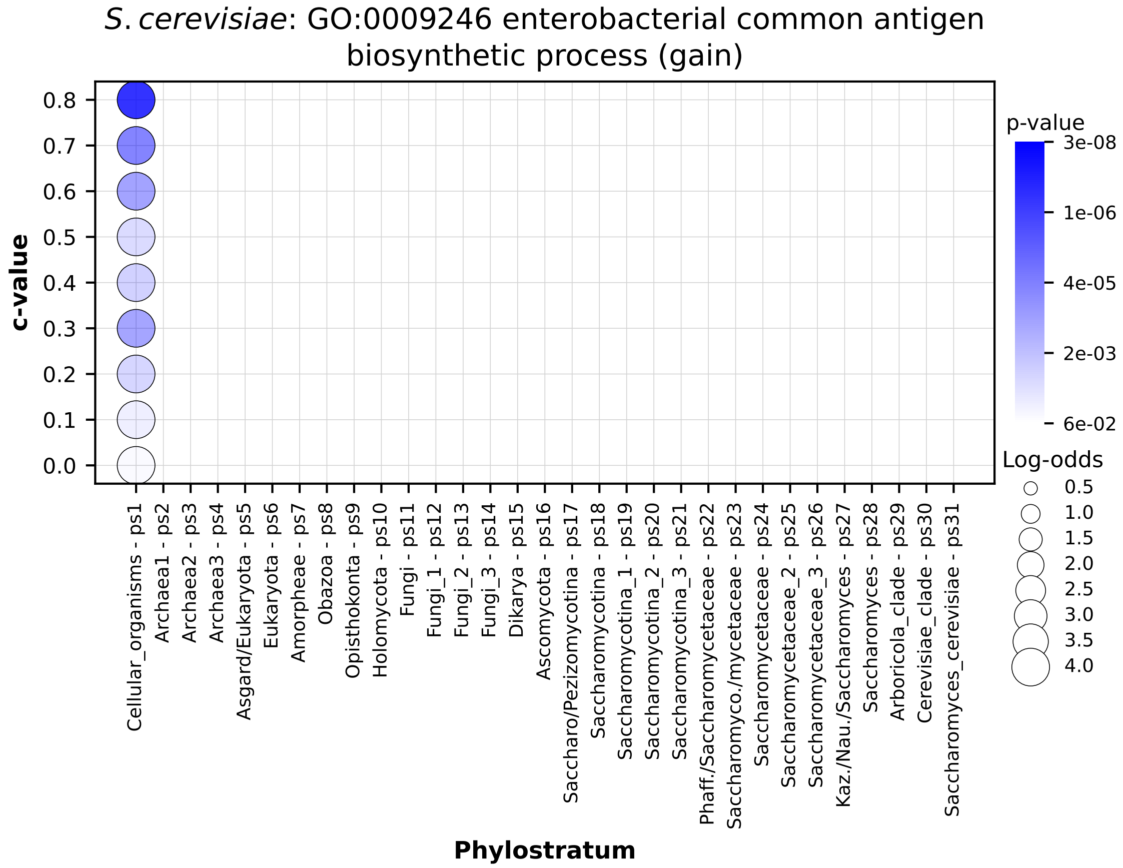
S. cerevisiae: GO:0009246 enterobacterial common antigen biosynthetic process (gain)
c-value
0.8
0.7
0.6
0.5
0.4
0.3
0.2
0.1
0.0
p-value
3e-08
1e-06
4e-05
2e-03
6e-02
Log-odds
0.5
1.0
1.5
2.0
2.5
3.0
3.5
4.0
Cellular_organisms - ps1
Archaea1 - ps2
Archaea2 - ps3
Archaea3 - ps4
Asgard/Eukaryota - ps5
Eukaryota - ps6
Amorpheae - ps7
Obazoa - ps8
Opisthokonta - ps9
Holomycota - ps10
Fungi - ps11
Fungi_1 - ps12
Fungi_2 - ps13
Fungi_3 - ps14
Dikarya - ps15
Ascomycota - ps16
Saccharo/Pezizomycotina - ps17
Saccharomycotina - ps18
Saccharomycotina_1 - ps19
Saccharomycotina_2 - ps20
Saccharomycotina_3 - ps21
Phaff./Saccharomycetaceae - ps22
Saccharomyco./mycetaceae - ps23
Saccharomycetaceae - ps24
Saccharomycetaceae_2 - ps25
Saccharomycetaceae_3 - ps26
Kaz./Nau./Saccharomyces - ps27
Saccharomyces - ps28
Arboricola_clade - ps29
Cerevisiae_clade - ps30
Saccharomyces_cerevisiae - ps31
Phylostratum

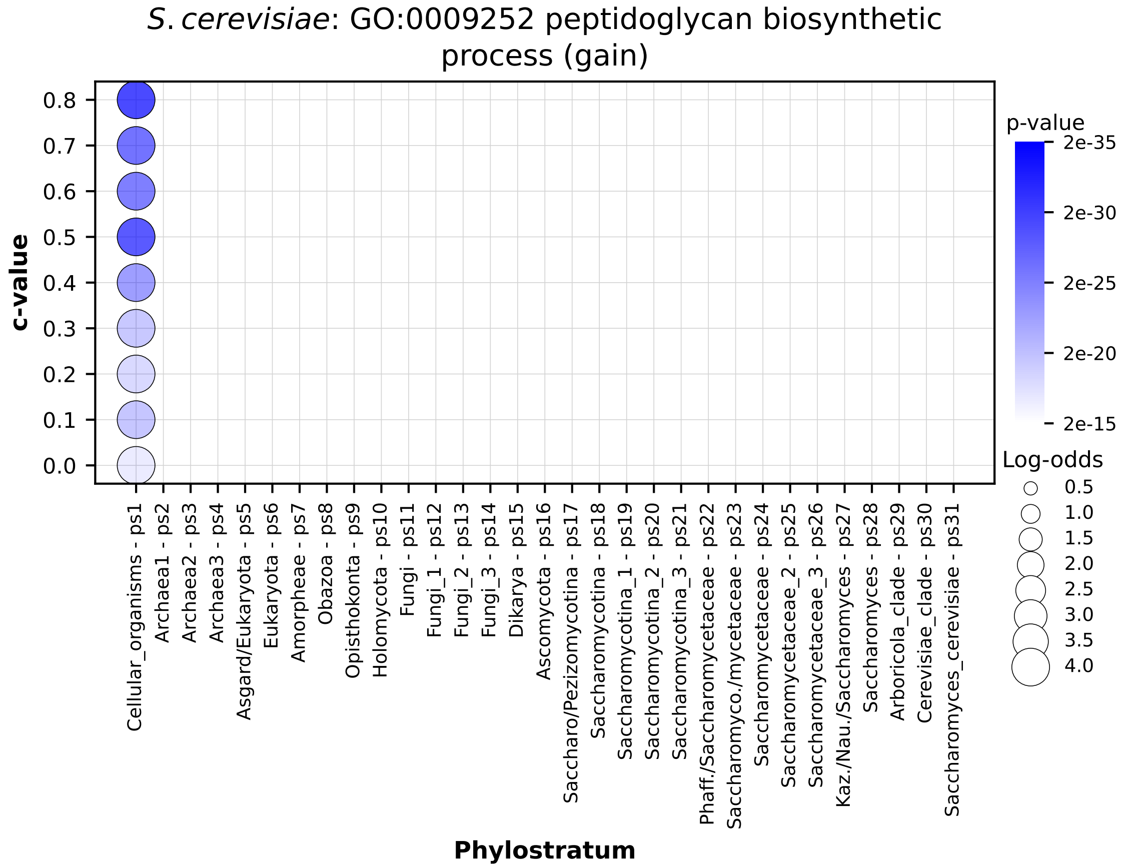

S. cerevisiae: GO:0009252 peptidoglycan biosynthetic process (gain)
c-value
0.8
0.7
0.6
0.5
0.4
0.3
0.2
0.1
0.0
Phylostratum
Cellular_organisms - ps1
Archaea1 - ps2
Archaea2 - ps3
Archaea3 - ps4
Asgard/Eukaryota - ps5
Eukaryota - ps6
Amorpheae - ps7
Obazoa - ps8
Opisthokonta - ps9
Holomycota - ps10
Fungi - ps11
Fungi_1 - ps12
Fungi_2 - ps13
Fungi_3 - ps14
Dikarya - ps15
Ascomycota - ps16
Saccharo/Pezizomycotina - ps17
Saccharomycotina - ps18
Saccharomycotina_1 - ps19
Saccharomycotina_2 - ps20
Saccharomycotina_3 - ps21
Phaff./Saccharomycetaceae - ps22
Saccharomyco./mycetaceae - ps23
Saccharomycetaceae - ps24
Saccharomycetaceae_2 - ps25
Saccharomycetaceae_3 - ps26
Kaz./Nau./Saccharomyces - ps27
Saccharomyces - ps28
Arboricola_clade - ps29
Cerevisiae_clade - ps30
Saccharomyces_cerevisiae - ps31
p-value
2e-35
2e-30
2e-25
2e-20
2e-15
Log-odds
0.5
1.0
1.5
2.0
2.5
3.0
3.5
4.0

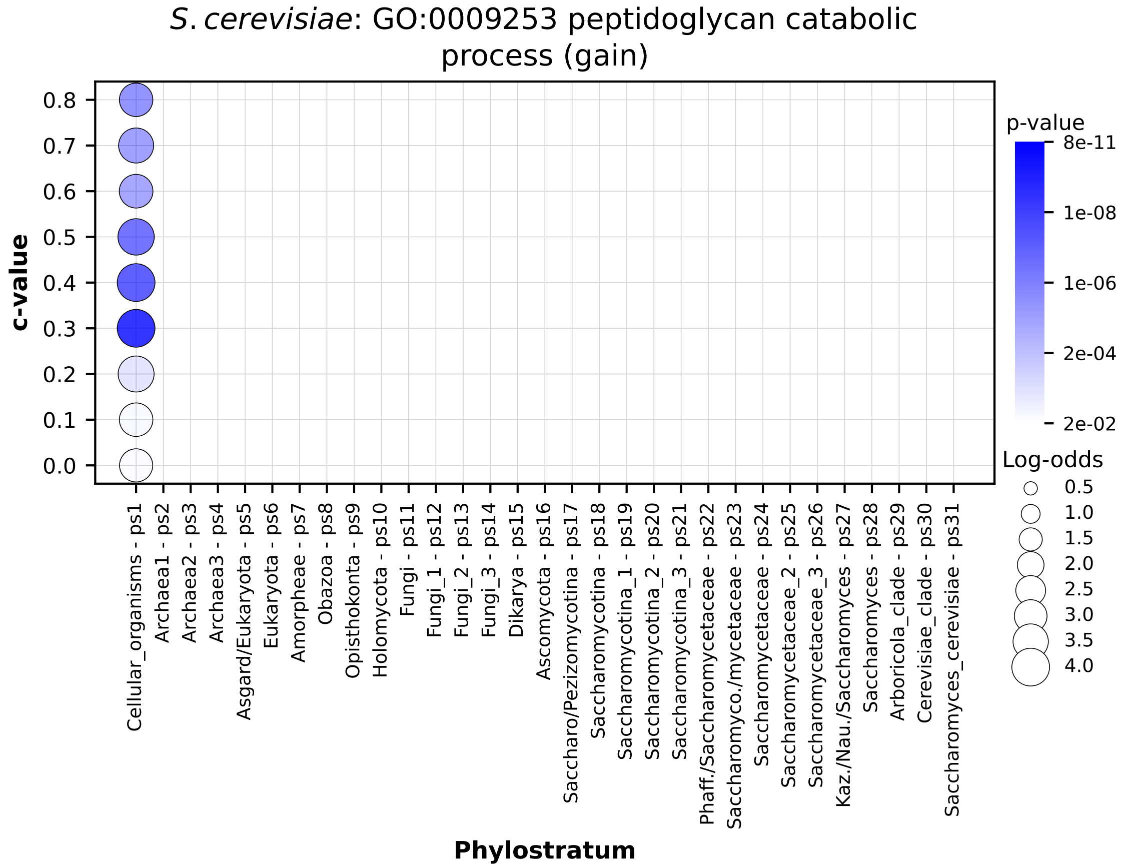

S. cerevisiae: GO:0009253 peptidoglycan catabolic process (gain)
c-value
0.8
0.7
0.6
0.5
0.4
0.3
0.2
0.1
0.0
p-value
8e-11
1e-08
1e-06
2e-04
2e-02
Log-odds
0.5
1.0
1.5
2.0
2.5
3.0
3.5
4.0
Cellular_organisms - ps1
Archaea1 - ps2
Archaea2 - ps3
Archaea3 - ps4
Asgard/Eukaryota - ps5
Eukaryota - ps6
Amorpheae - ps7
Obazoa - ps8
Opisthokonta - ps9
Holomycota - ps10
Fungi - ps11
Fungi_1 - ps12
Fungi_2 - ps13
Fungi_3 - ps14
Dikarya - ps15
Ascomycota - ps16
Saccharo/Pezizomycotina - ps17
Saccharomycotina - ps18
Saccharomycotina_1 - ps19
Saccharomycotina_2 - ps20
Saccharomycotina_3 - ps21
Phaff./Saccharomycetaceae - ps22
Saccharomyco./mycetaceae - ps23
Saccharomycetaceae - ps24
Saccharomycetaceae_2 - ps25
Saccharomycetaceae_3 - ps26
Kaz./Nau./Saccharomyces - ps27
Saccharomyces - ps28
Arboricola_clade - ps29
Cerevisiae_clade - ps30
Saccharomyces_cerevisiae - ps31
Phylostratum

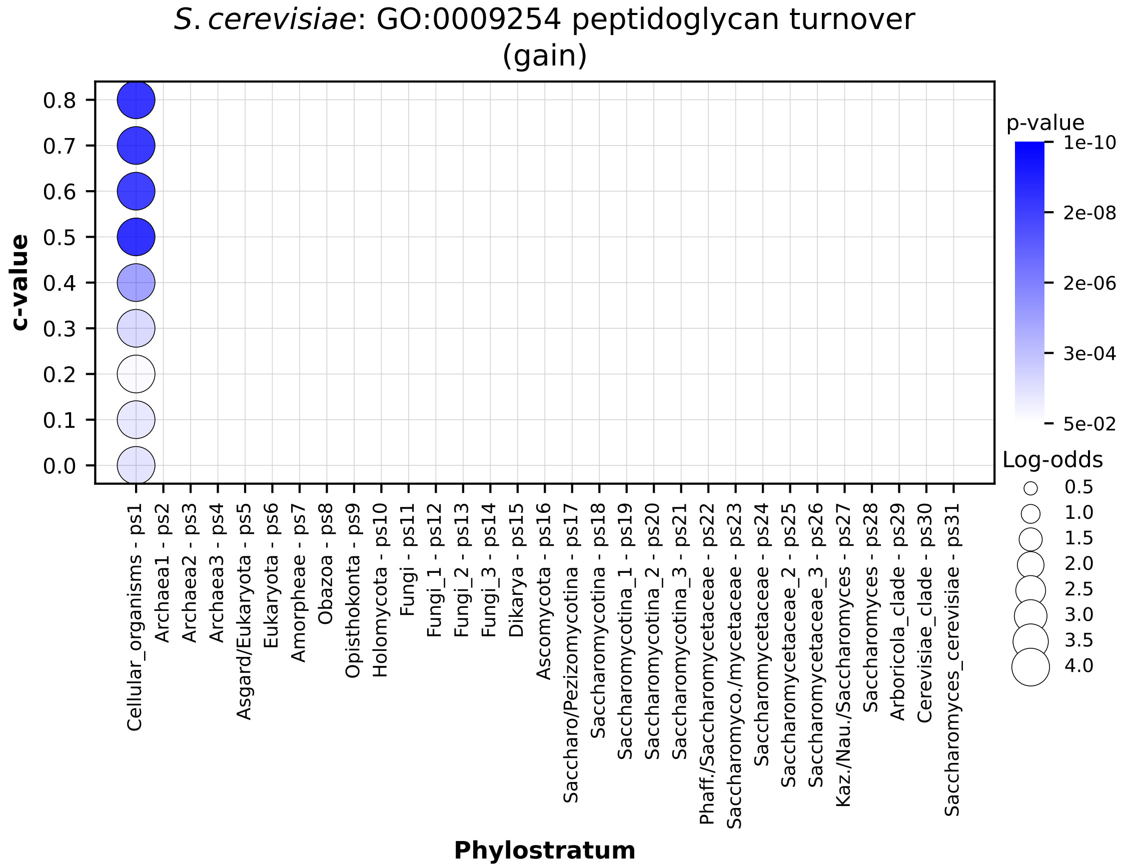

S. cerevisiae: GO:0009254 peptidoglycan turnover
(gain)
c-value
0.0
0.1
0.2
0.3
0.4
0.5
0.6
0.7
0.8
p-value
1e-10
2e-08
2e-06
3e-04
5e-02
Log-odds
0.5
1.0
1.5
2.0
2.5
3.0
3.5
4.0
Cellular_organisms - ps1
Archaea1 - ps2
Archaea2 - ps3
Archaea3 - ps4
Asgard/Eukaryota - ps5
Eukaryota - ps6
Amorpheae - ps7
Obazoa - ps8
Opisthokonta - ps9
Holomycota - ps10
Fungi - ps11
Fungi_1 - ps12
Fungi_2 - ps13
Fungi_3 - ps14
Dikarya - ps15
Ascomycota - ps16
Saccharo/Pezizomycotina - ps17
Saccharomycotina - ps18
Saccharomycotina_1 - ps19
Saccharomycotina_2 - ps20
Saccharomycotina_3 - ps21
Phaff./Saccharomycetaceae - ps22
Saccharomyco./mycetaceae - ps23
Saccharomycetaceae - ps24
Saccharomycetaceae_2 - ps25
Saccharomycetaceae_3 - ps26
Kaz./Nau./Saccharomyces - ps27
Saccharomyces - ps28
Arboricola_clade - ps29
Cerevisiae_clade - ps30
Saccharomyces_cerevisiae - ps31
Phylostratum

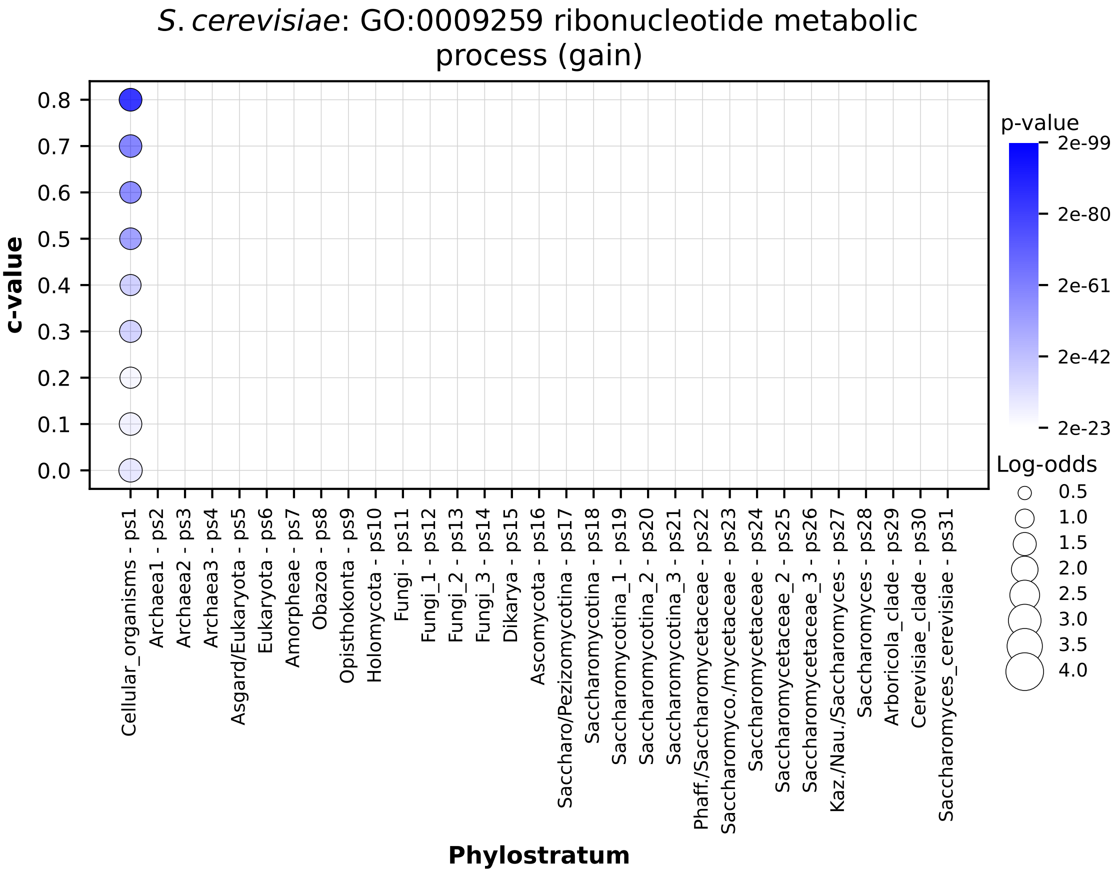
S. cerevisiae: GO:0009259 ribonucleotide metabolic process (gain)
c-value
0.8
0.7
0.6
0.5
0.4
0.3
0.2
0.1
0.0
p-value
2e-99
2e-80
2e-61
2e-42
2e-23
Log-odds
0.5
1.0
1.5
2.0
2.5
3.0
3.5
4.0
Cellular_organisms - ps1
Archaea1 - ps2
Archaea2 - ps3
Archaea3 - ps4
Asgard/Eukaryota - ps5
Eukaryota - ps6
Amorpheae - ps7
Obazoa - ps8
Opisthokonta - ps9
Holomycota - ps10
Fungi - ps11
Fungi_1 - ps12
Fungi_2 - ps13
Fungi_3 - ps14
Dikarya - ps15
Ascomycota - ps16
Saccharo/Pezizomycotina - ps17
Saccharomycotina - ps18
Saccharomycotina_1 - ps19
Saccharomycotina_2 - ps20
Saccharomycotina_3 - ps21
Phaff./Saccharomycetaceae - ps22
Saccharomyco./mycetaceae - ps23
Saccharomycetaceae - ps24
Saccharomycetaceae_2 - ps25
Saccharomycetaceae_3 - ps26
Kaz./Nau./Saccharomyces - ps27
Saccharomyces - ps28
Arboricola_clade - ps29
Cerevisiae_clade - ps30
Saccharomyces_cerevisiae - ps31
Phylostratum

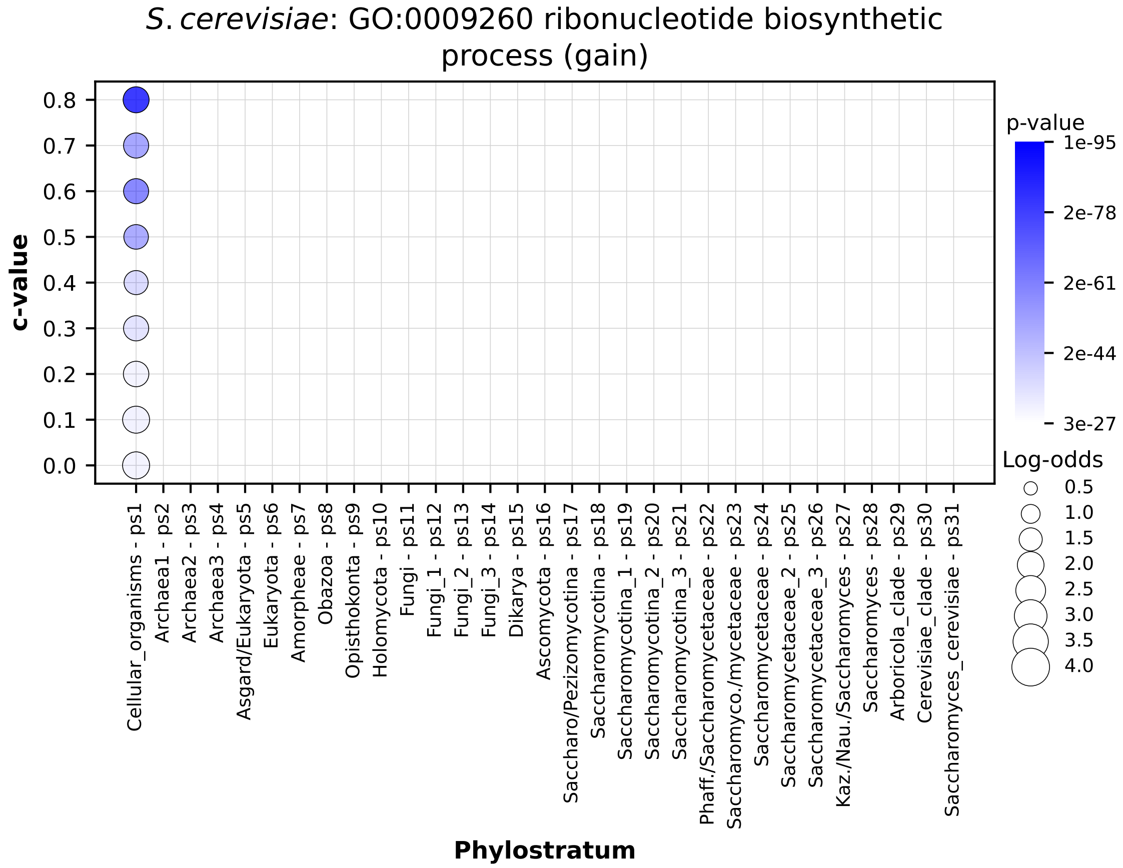
S. cerevisiae: GO:0009260 ribonucleotide biosynthetic process (gain)
c-value
0.8
0.7
0.6
0.5
0.4
0.3
0.2
0.1
0.0
p-value
1e-95
2e-78
2e-61
2e-44
3e-27
Log-odds
0.5
1.0
1.5
2.0
2.5
3.0
3.5
4.0
Cellular_organisms - ps1
Archaea1 - ps2
Archaea2 - ps3
Archaea3 - ps4
Asgard/Eukaryota - ps5
Eukaryota - ps6
Amorpheae - ps7
Obazoa - ps8
Opisthokonta - ps9
Holomycota - ps10
Fungi - ps11
Fungi_1 - ps12
Fungi_2 - ps13
Fungi_3 - ps14
Dikarya - ps15
Ascomycota - ps16
Saccharo/Pezizomycotina - ps17
Saccharomycotina - ps18
Saccharomycotina_1 - ps19
Saccharomycotina_2 - ps20
Saccharomycotina_3 - ps21
Phaff./Saccharomycetaceae - ps22
Saccharomyco./mycetaceae - ps23
Saccharomycetaceae - ps24
Saccharomycetaceae_2 - ps25
Saccharomycetaceae_3 - ps26
Kaz./Nau./Saccharomyces - ps27
Saccharomyces - ps28
Arboricola_clade - ps29
Cerevisiae_clade - ps30
Saccharomyces_cerevisiae - ps31
Phylostratum

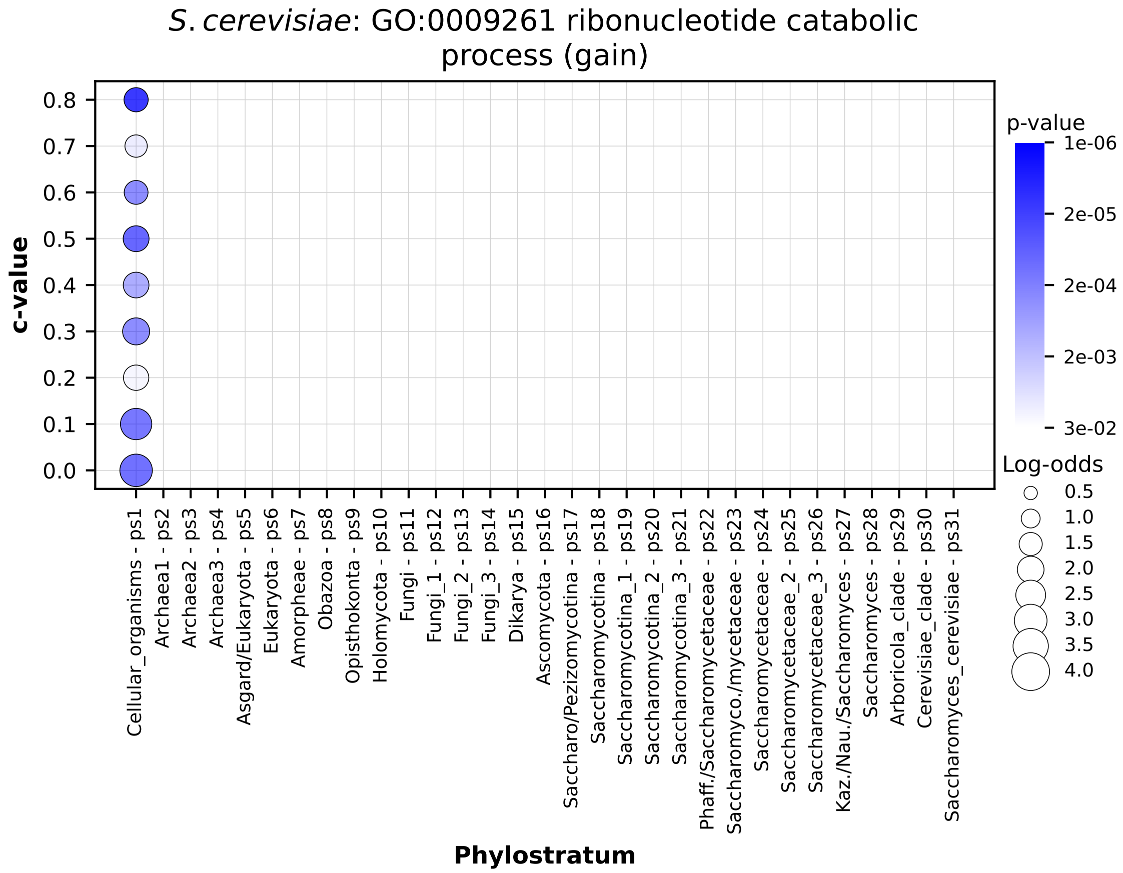

S. cerevisiae: GO:0009261 ribonucleotide catabolic process (gain)
c-value
0.8
0.7
0.6
0.5
0.4
0.3
0.2
0.1
0.0
p-value
1e-06
2e-05
2e-04
2e-03
3e-02
Log-odds
0.5
1.0
1.5
2.0
2.5
3.0
3.5
4.0
Cellular_organisms - ps1
Archaea1 - ps2
Archaea2 - ps3
Archaea3 - ps4
Asgard/Eukaryota - ps5
Eukaryota - ps6
Amorpheae - ps7
Obazoa - ps8
Opisthokonta - ps9
Holomycota - ps10
Fungi - ps11
Fungi_1 - ps12
Fungi_2 - ps13
Fungi_3 - ps14
Dikarya - ps15
Ascomycota - ps16
Saccharo/Pezizomycotina - ps17
Saccharomycotina - ps18
Saccharomycotina_1 - ps19
Saccharomycotina_2 - ps20
Saccharomycotina_3 - ps21
Phaff./Saccharomycetaceae - ps22
Saccharomyco./mycetaceae - ps23
Saccharomycetaceae - ps24
Saccharomycetaceae_2 - ps25
Saccharomycetaceae_3 - ps26
Kaz./Nau./Saccharomyces - ps27
Saccharomyces - ps28
Arboricola_clade - ps29
Cerevisiae_clade - ps30
Saccharomyces_cerevisiae - ps31
Phylostratum

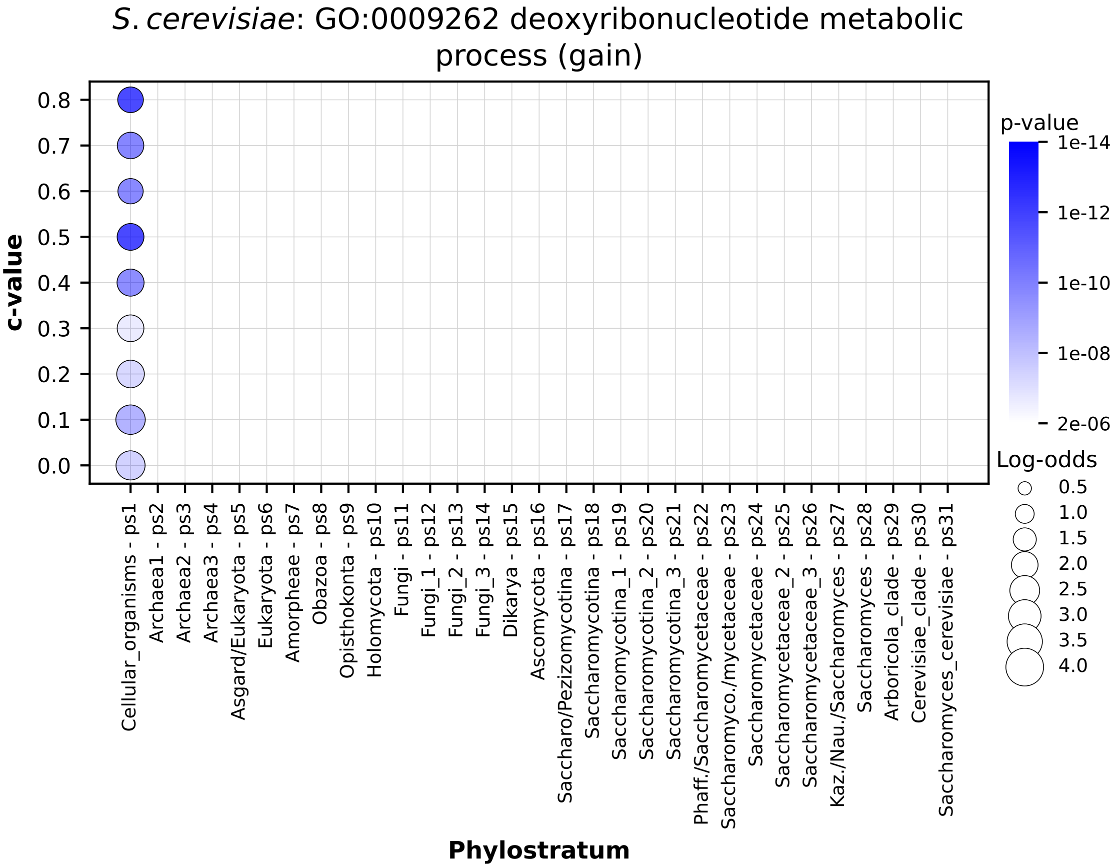

S. cerevisiae: GO:0009262 deoxyribonucleotide metabolic process (gain)
c-value
0.0
0.1
0.2
0.3
0.4
0.5
0.6
0.7
0.8
p-value
1e-14
1e-12
1e-10
1e-08
2e-06
Log-odds
0.5
1.0
1.5
2.0
2.5
3.0
3.5
4.0
Cellular_organisms - ps1
Archaea1 - ps2
Archaea2 - ps3
Archaea3 - ps4
Asgard/Eukaryota - ps5
Eukaryota - ps6
Amorpheae - ps7
Obazoa - ps8
Opisthokonta - ps9
Holomycota - ps10
Fungi - ps11
Fungi_1 - ps12
Fungi_2 - ps13
Fungi_3 - ps14
Dikarya - ps15
Ascomycota - ps16
Saccharo/Pezizomycotina - ps17
Saccharomycotina - ps18
Saccharomycotina_1 - ps19
Saccharomycotina_2 - ps20
Saccharomycotina_3 - ps21
Phaff./Saccharomycetaceae - ps22
Saccharomyco./mycetaceae - ps23
Saccharomycetaceae - ps24
Saccharomycetaceae_2 - ps25
Saccharomycetaceae_3 - ps26
Kaz./Nau./Saccharomyces - ps27
Saccharomyces - ps28
Arboricola_clade - ps29
Cerevisiae_clade - ps30
Saccharomyces_cerevisiae - ps31
Phylostratum

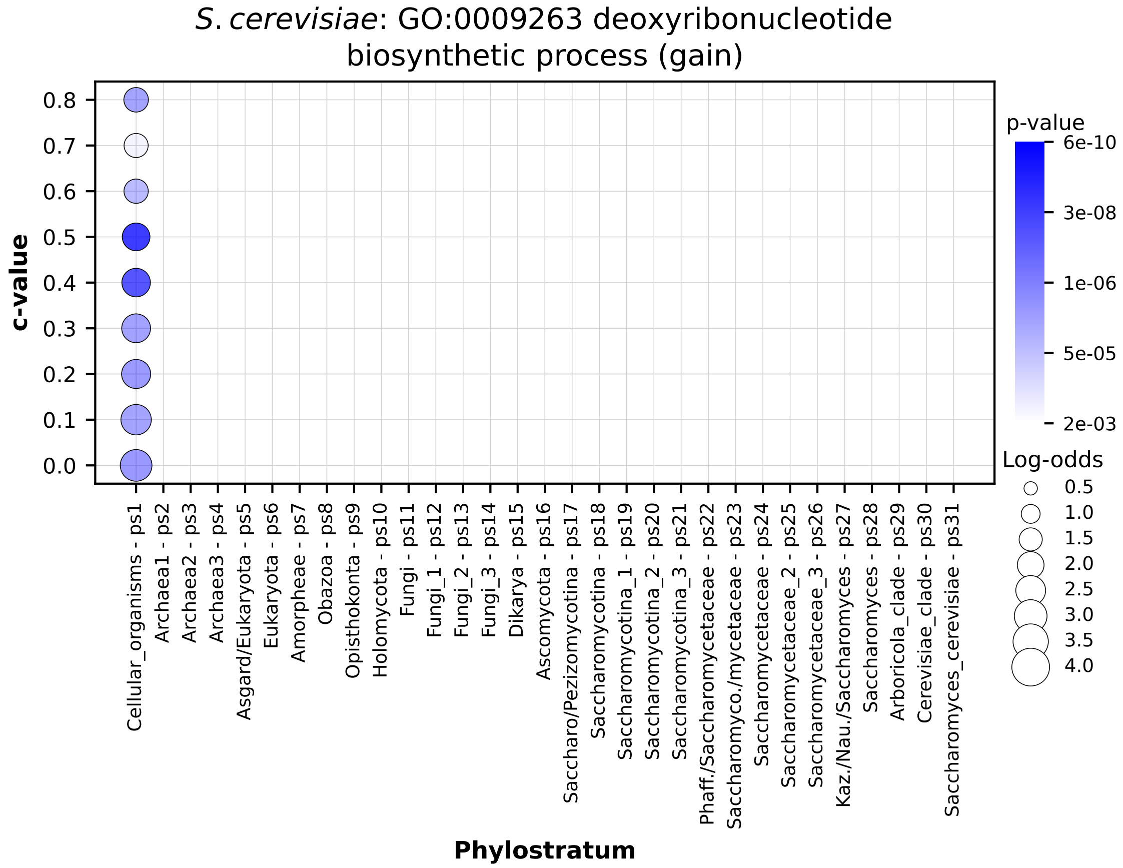
S. cerevisiae: GO:0009263 deoxyribonucleotide biosynthetic process (gain)
c-value
0.0
0.1
0.2
0.3
0.4
0.5
0.6
0.7
0.8
p-value
6e-10
3e-08
1e-06
5e-05
2e-03
Log-odds
0.5
1.0
1.5
2.0
2.5
3.0
3.5
4.0
Cellular_organisms - ps1
Archaea1 - ps2
Archaea2 - ps3
Archaea3 - ps4
Asgard/Eukaryota - ps5
Eukaryota - ps6
Amorpheae - ps7
Obazoa - ps8
Opisthokonta - ps9
Holomycota - ps10
Fungi - ps11
Fungi_1 - ps12
Fungi_2 - ps13
Fungi_3 - ps14
Dikarya - ps15
Ascomycota - ps16
Saccharo/Pezizomycotina - ps17
Saccharomycotina - ps18
Saccharomycotina_1 - ps19
Saccharomycotina_2 - ps20
Saccharomycotina_3 - ps21
Phaff./Saccharomycetaceae - ps22
Saccharomyco./mycetaceae - ps23
Saccharomycetaceae - ps24
Saccharomycetaceae_2 - ps25
Saccharomycetaceae_3 - ps26
Kaz./Nau./Saccharomyces - ps27
Saccharomyces - ps28
Arboricola_clade - ps29
Cerevisiae_clade - ps30
Saccharomyces_cerevisiae - ps31
Phylostratum

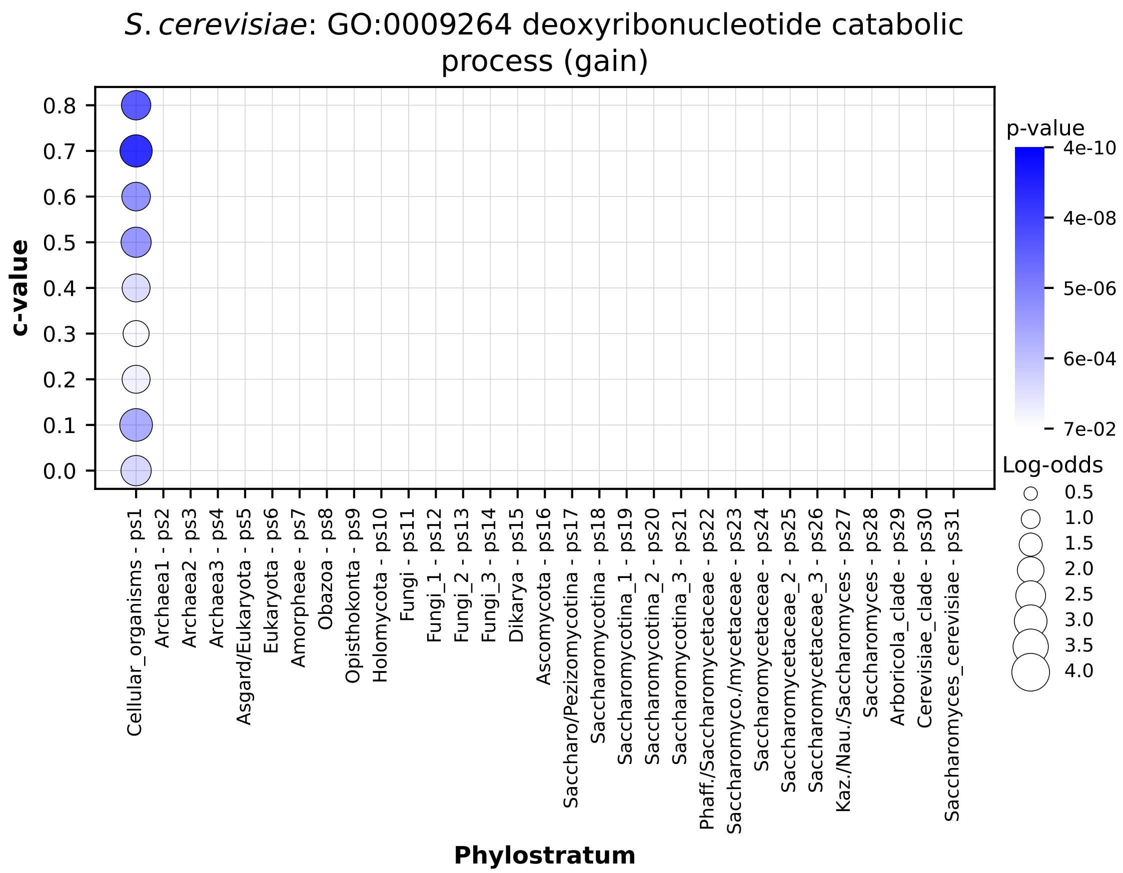
S. cerevisiae: GO:0009264 deoxyribonucleotide catabolic process (gain)
c-value
0.0
0.1
0.2
0.3
0.4
0.5
0.6
0.7
0.8
p-value
4e-10
4e-08
5e-06
6e-04
7e-02
Log-odds
0.5
1.0
1.5
2.0
2.5
3.0
3.5
4.0
Cellular_organisms - ps1
Archaea1 - ps2
Archaea2 - ps3
Archaea3 - ps4
Asgard/Eukaryota - ps5
Eukaryota - ps6
Amorpheae - ps7
Obazoa - ps8
Opisthokonta - ps9
Holomycota - ps10
Fungi - ps11
Fungi_1 - ps12
Fungi_2 - ps13
Fungi_3 - ps14
Dikarya - ps15
Ascomycota - ps16
Saccharo/Pezizomycotina - ps17
Saccharomycotina - ps18
Saccharomycotina_1 - ps19
Saccharomycotina_2 - ps20
Saccharomycotina_3 - ps21
Phaff./Saccharomycetaceae - ps22
Saccharomyco./mycetaceae - ps23
Saccharomycetaceae - ps24
Saccharomycetaceae_2 - ps25
Saccharomycetaceae_3 - ps26
Kaz./Nau./Saccharomyces - ps27
Saccharomyces - ps28
Arboricola_clade - ps29
Cerevisiae_clade - ps30
Saccharomyces_cerevisiae - ps31
Phylostratum

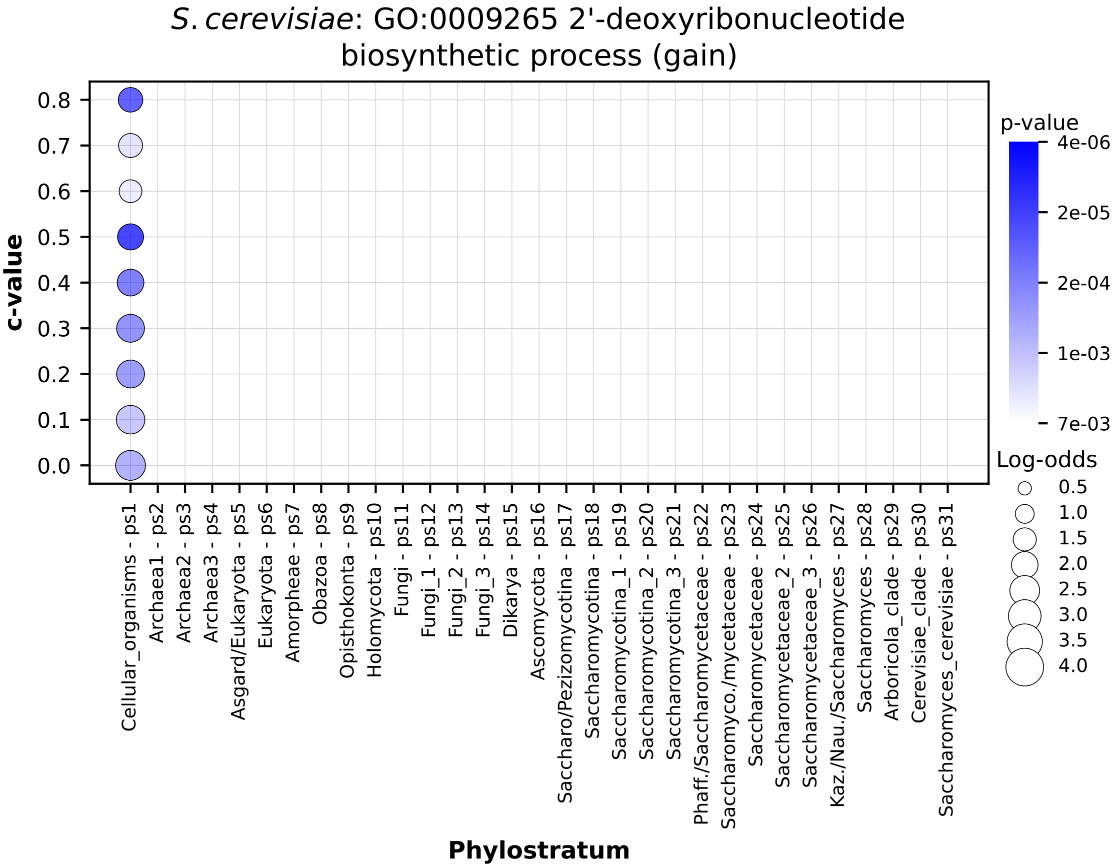

S. cerevisiae: GO:0009265 2'-deoxyribonucleotide biosynthetic process (gain)
c-value
0.8
0.7
0.6
0.5
0.4
0.3
0.2
0.1
0.0
p-value
4e-06
2e-05
2e-04
1e-03
7e-03
Log-odds
0.5
1.0
1.5
2.0
2.5
3.0
3.5
4.0
Cellular_organisms - ps1
Archaea1 - ps2
Archaea2 - ps3
Archaea3 - ps4
Asgard/Eukaryota - ps5
Eukaryota - ps6
Amorpheae - ps7
Obazoa - ps8
Opisthokonta - ps9
Holomycota - ps10
Fungi - ps11
Fungi_1 - ps12
Fungi_2 - ps13
Fungi_3 - ps14
Dikarya - ps15
Ascomycota - ps16
Saccharo/Pezizomycotina - ps17
Saccharomycotina - ps18
Saccharomycotina_1 - ps19
Saccharomycotina_2 - ps20
Saccharomycotina_3 - ps21
Phaff./Saccharomycetaceae - ps22
Saccharomyco./mycetaceae - ps23
Saccharomycetaceae - ps24
Saccharomycetaceae_2 - ps25
Saccharomycetaceae_3 - ps26
Kaz./Nau./Saccharomyces - ps27
Saccharomyces - ps28
Arboricola_clade - ps29
Cerevisiae_clade - ps30
Saccharomyces_cerevisiae - ps31
Phylostratum

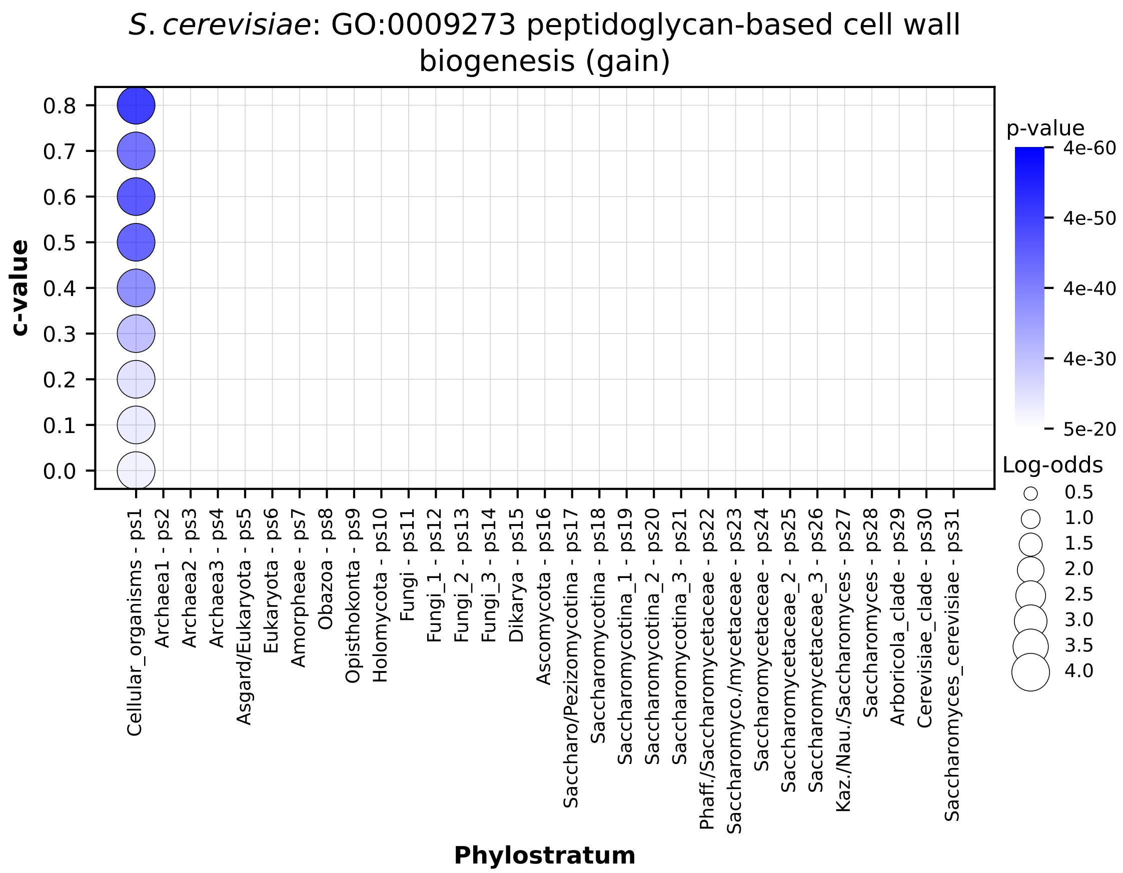
S. cerevisiae: GO:0009273 peptidoglycan-based cell wall biogenesis (gain)
c-value
0.8
0.7
0.6
0.5
0.4
0.3
0.2
0.1
0.0
p-value
4e-60
4e-50
4e-40
4e-30
5e-20
Log-odds
0.5
1.0
1.5
2.0
2.5
3.0
3.5
4.0
Cellular_organisms - ps1
Archaea1 - ps2
Archaea2 - ps3
Archaea3 - ps4
Asgard/Eukaryota - ps5
Eukaryota - ps6
Amorpheae - ps7
Obazoa - ps8
Opisthokonta - ps9
Holomycota - ps10
Fungi - ps11
Fungi_1 - ps12
Fungi_2 - ps13
Fungi_3 - ps14
Dikarya - ps15
Ascomycota - ps16
Saccharo/Pezizomycotina - ps17
Saccharomycotina - ps18
Saccharomycotina_1 - ps19
Saccharomycotina_2 - ps20
Saccharomycotina_3 - ps21
Phaff./Saccharomycetaceae - ps22
Saccharomyco./mycetaceae - ps23
Saccharomycetaceae - ps24
Saccharomycetaceae_2 - ps25
Saccharomycetaceae_3 - ps26
Kaz./Nau./Saccharomyces - ps27
Saccharomyces - ps28
Arboricola_clade - ps29
Cerevisiae_clade - ps30
Saccharomyces_cerevisiae - ps31
Phylostratum

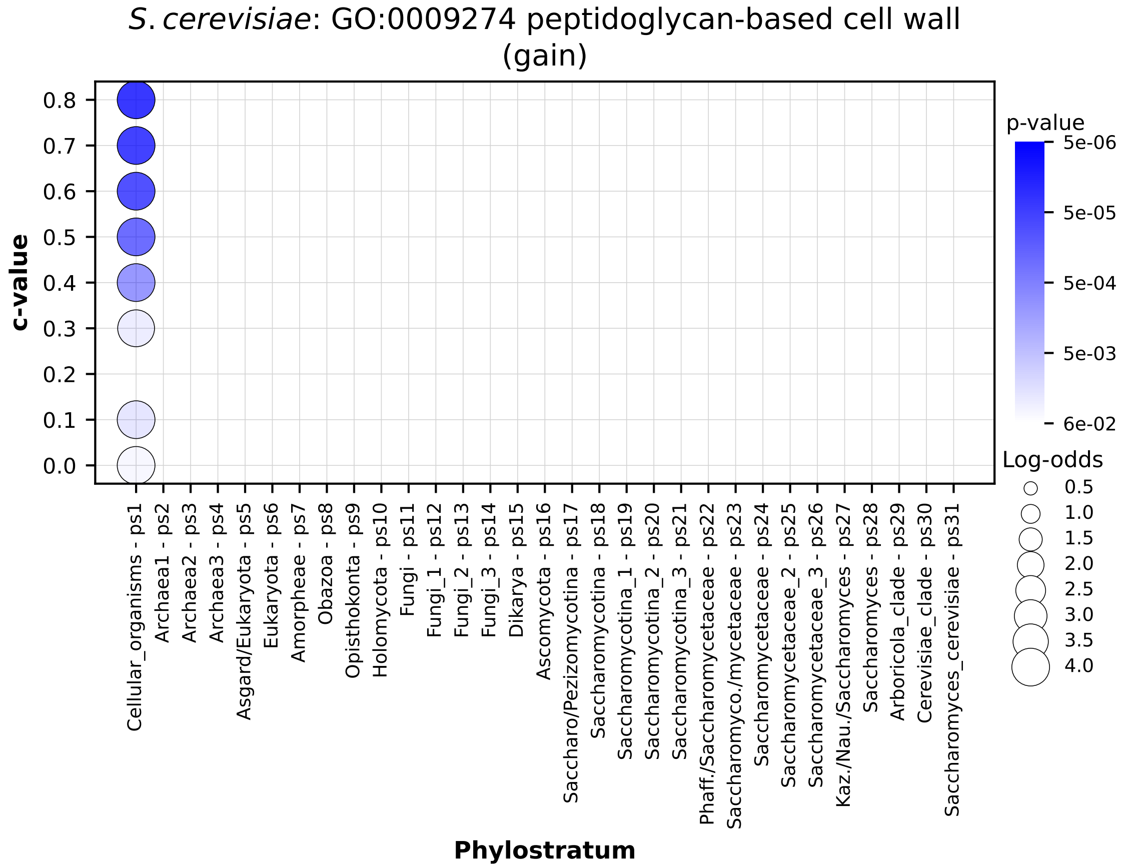
S. cerevisiae: GO:0009274 peptidoglycan-based cell wall
(gain)
c-value
0.8
0.7
0.6
0.5
0.4
0.3
0.2
0.1
0.0
p-value
5e-06
5e-05
5e-04
5e-03
6e-02
Log-odds
0.5
1.0
1.5
2.0
2.5
3.0
3.5
4.0
Cellular_organisms - ps1
Archaea1 - ps2
Archaea2 - ps3
Archaea3 - ps4
Asgard/Eukaryota - ps5
Eukaryota - ps6
Amorpheae - ps7
Obazoa - ps8
Opisthokonta - ps9
Holomycota - ps10
Fungi - ps11
Fungi_1 - ps12
Fungi_2 - ps13
Fungi_3 - ps14
Dikarya - ps15
Ascomycota - ps16
Saccharo/Pezizomycotina - ps17
Saccharomycotina - ps18
Saccharomycotina_1 - ps19
Saccharomycotina_2 - ps20
Saccharomycotina_3 - ps21
Phaff./Saccharomycetaceae - ps22
Saccharomyco./mycetaceae - ps23
Saccharomycetaceae - ps24
Saccharomycetaceae_2 - ps25
Saccharomycetaceae_3 - ps26
Kaz./Nau./Saccharomyces - ps27
Saccharomyces - ps28
Arboricola_clade - ps29
Cerevisiae_clade - ps30
Saccharomyces_cerevisiae - ps31
Phylostratum

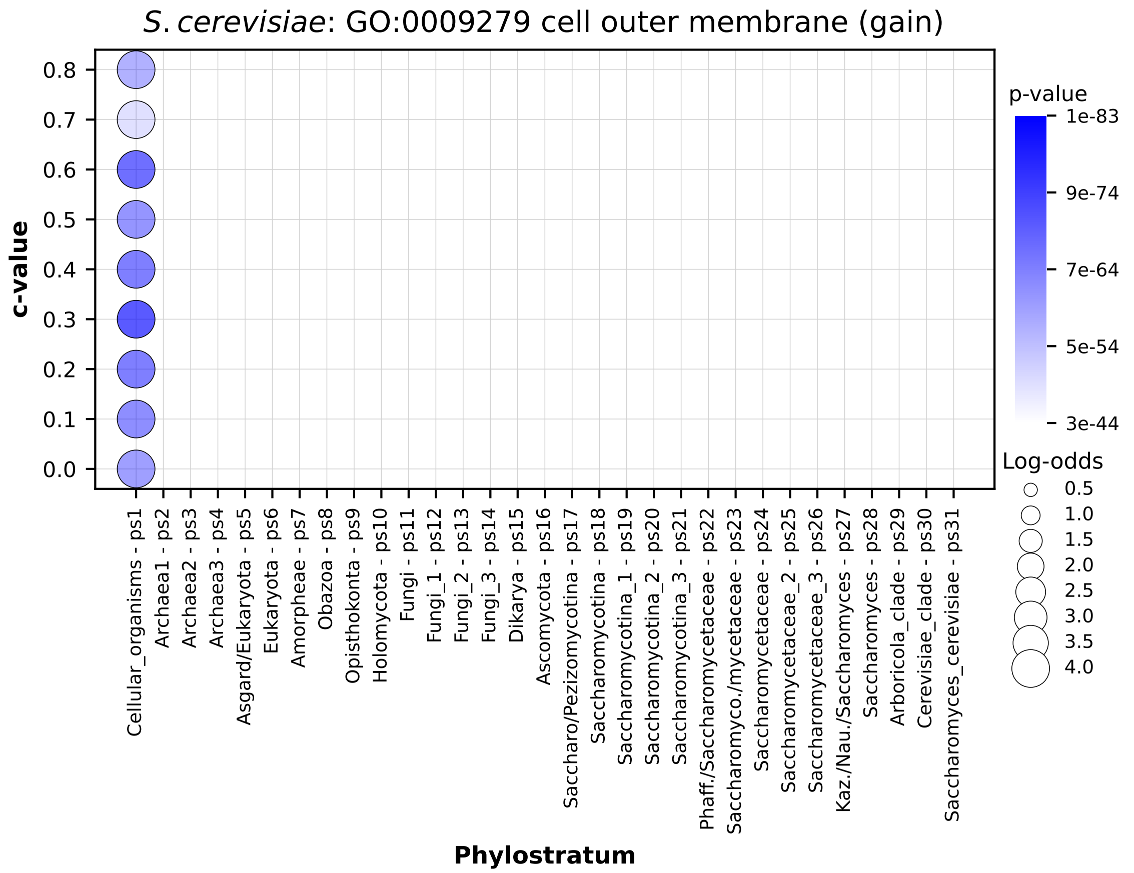

S. cerevisiae: GO:0009279 cell outer membrane (gain)
c-value
0.0
0.1
0.2
0.3
0.4
0.5
0.6
0.7
0.8
Phylostratum
Cellular_organisms - ps1
Archaea1 - ps2
Archaea2 - ps3
Archaea3 - ps4
Asgard/Eukaryota - ps5
Eukaryota - ps6
Amorpheae - ps7
Obazoa - ps8
Opisthokonta - ps9
Holomycota - ps10
Fungi - ps11
Fungi_1 - ps12
Fungi_2 - ps13
Fungi_3 - ps14
Dikarya - ps15
Ascomycota - ps16
Saccharo/Pezizomycotina - ps17
Saccharomycotina - ps18
Saccharomycotina_1 - ps19
Saccharomycotina_2 - ps20
Saccharomycotina_3 - ps21
Phaff./Saccharomycetaceae - ps22
Saccharomyco./mycetaceae - ps23
Saccharomycetaceae - ps24
Saccharomycetaceae_2 - ps25
Saccharomycetaceae_3 - ps26
Kaz./Nau./Saccharomyces - ps27
Saccharomyces - ps28
Arboricola_clade - ps29
Cerevisiae_clade - ps30
Saccharomyces_cerevisiae - ps31
p-value
1e-83
9e-74
7e-64
5e-54
3e-44
Log-odds
0.5
1.0
1.5
2.0
2.5
3.0
3.5
4.0

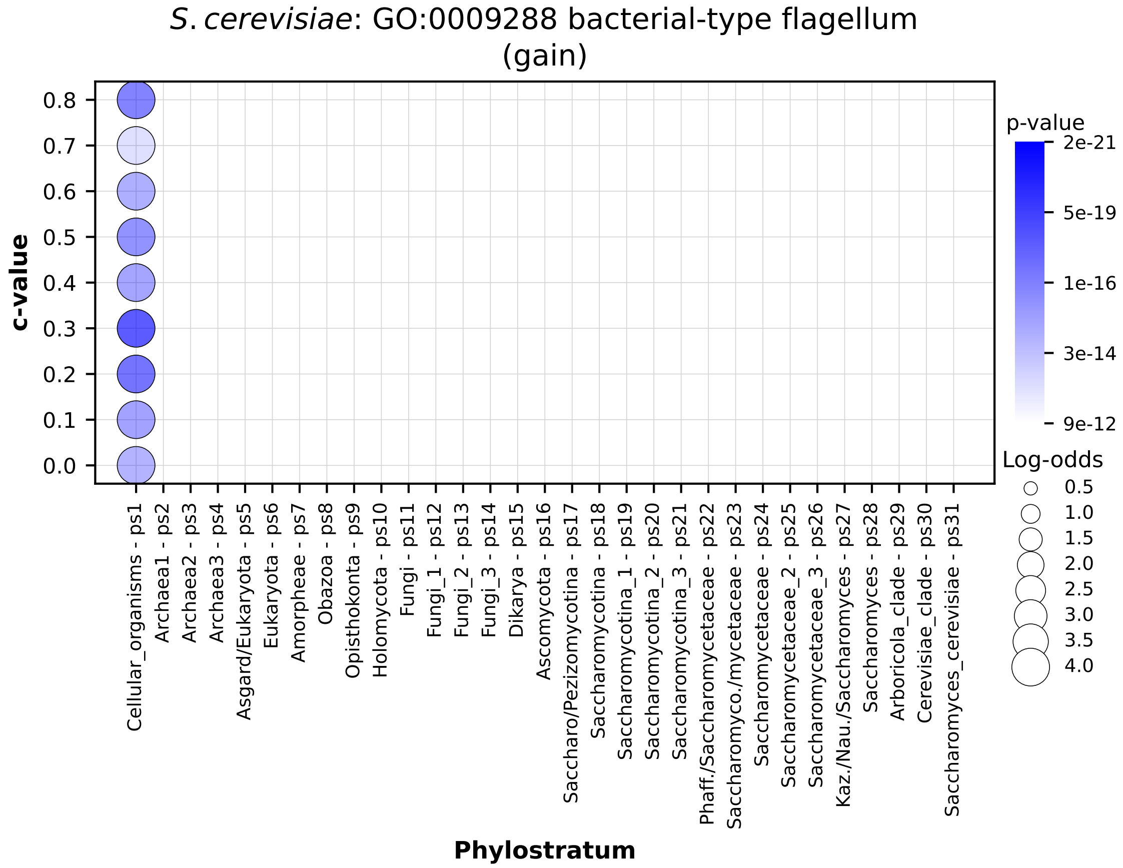

S. cerevisiae: GO:0009288 bacterial-type flagellum
(gain)
c-value
0.8
0.7
0.6
0.5
0.4
0.3
0.2
0.1
0.0
p-value
2e-21
5e-19
1e-16
3e-14
9e-12
Log-odds
0.5
1.0
1.5
2.0
2.5
3.0
3.5
4.0
Cellular_organisms - ps1
Archaea1 - ps2
Archaea2 - ps3
Archaea3 - ps4
Asgard/Eukaryota - ps5
Eukaryota - ps6
Amorpheae - ps7
Obazoa - ps8
Opisthokonta - ps9
Holomycota - ps10
Fungi - ps11
Fungi_1 - ps12
Fungi_2 - ps13
Fungi_3 - ps14
Dikarya - ps15
Ascomycota - ps16
Saccharo/Pezizomycotina - ps17
Saccharomycotina - ps18
Saccharomycotina_1 - ps19
Saccharomycotina_2 - ps20
Saccharomycotina_3 - ps21
Phaff./Saccharomycetaceae - ps22
Saccharomyco./mycetaceae - ps23
Saccharomycetaceae - ps24
Saccharomycetaceae_2 - ps25
Saccharomycetaceae_3 - ps26
Kaz./Nau./Saccharomyces - ps27
Saccharomyces - ps28
Arboricola_clade - ps29
Cerevisiae_clade - ps30
Saccharomyces_cerevisiae - ps31
Phylostratum

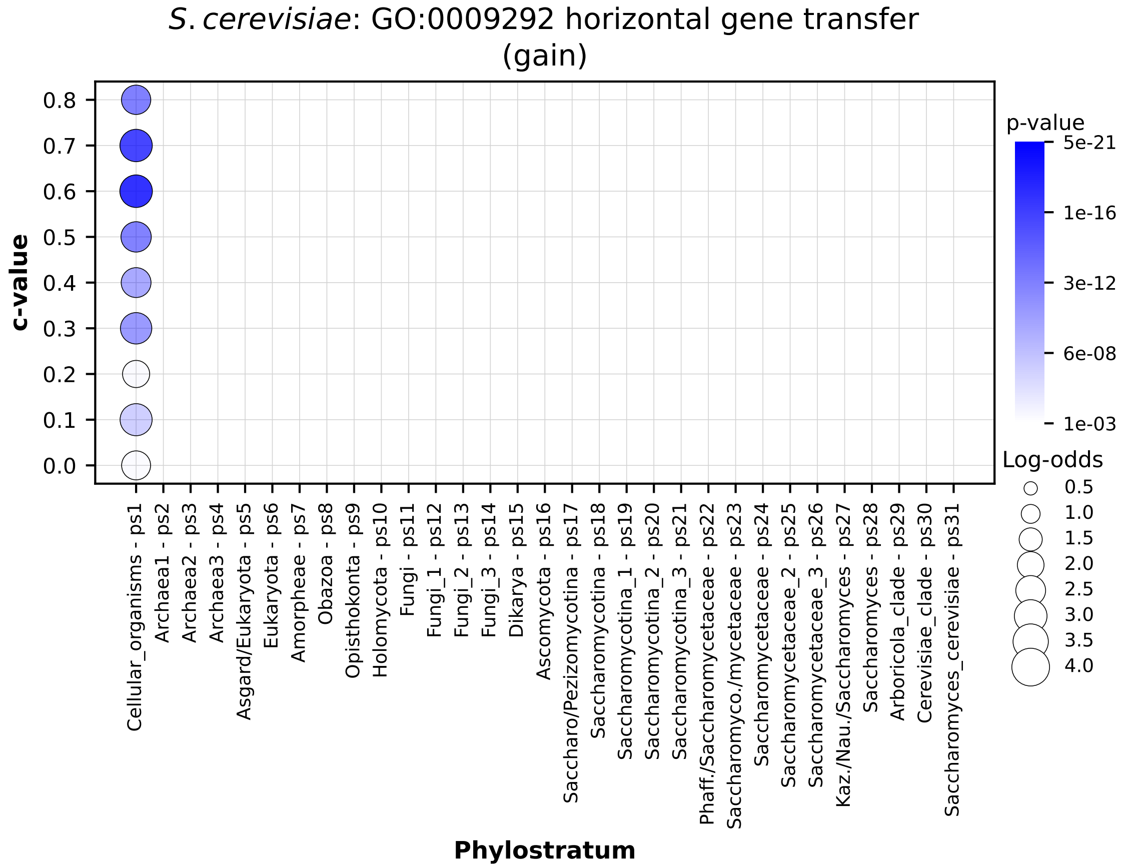
S. cerevisiae: GO:0009292 horizontal gene transfer (gain)
c-value
0.8
0.7
0.6
0.5
0.4
0.3
0.2
0.1
0.0
p-value
5e-21
1e-16
3e-12
6e-08
1e-03
Log-odds
0.5
1.0
1.5
2.0
2.5
3.0
3.5
4.0
Cellular_organisms - ps1
Archaea1 - ps2
Archaea2 - ps3
Archaea3 - ps4
Asgard/Eukaryota - ps5
Eukaryota - ps6
Amorpheae - ps7
Obazoa - ps8
Opisthokonta - ps9
Holomycota - ps10
Fungi - ps11
Fungi_1 - ps12
Fungi_2 - ps13
Fungi_3 - ps14
Dikarya - ps15
Ascomycota - ps16
Saccharo/Pezizomycotina - ps17
Saccharomycotina - ps18
Saccharomycotina_1 - ps19
Saccharomycotina_2 - ps20
Saccharomycotina_3 - ps21
Phaff./Saccharomycetaceae - ps22
Saccharomyco./mycetaceae - ps23
Saccharomycetaceae - ps24
Saccharomycetaceae_2 - ps25
Saccharomycetaceae_3 - ps26
Kaz./Nau./Saccharomyces - ps27
Saccharomyces - ps28
Arboricola_clade - ps29
Cerevisiae_clade - ps30
Saccharomyces_cerevisiae - ps31
Phylostratum

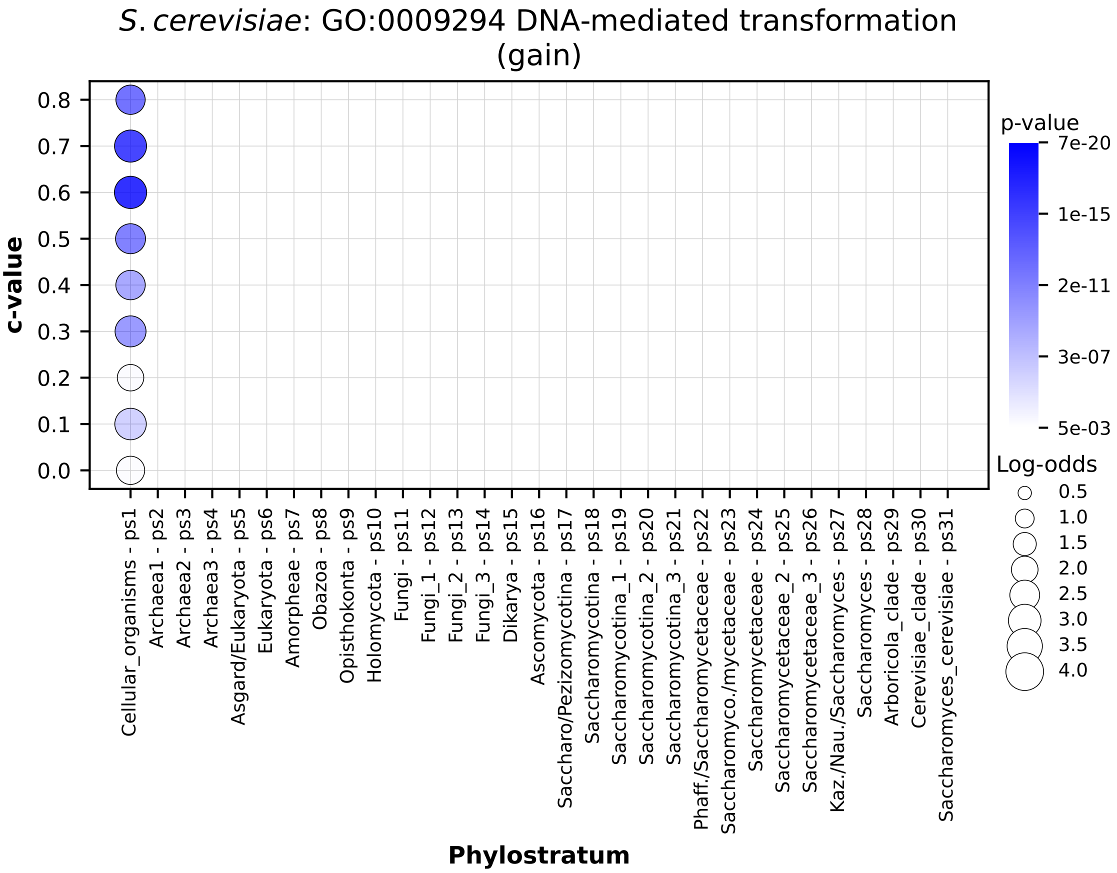

S. cerevisiae: GO:0009294 DNA-mediated transformation
(gain)
c-value
0.0
0.1
0.2
0.3
0.4
0.5
0.6
0.7
0.8
p-value
7e-20
1e-15
2e-11
3e-07
5e-03
Log-odds
0.5
1.0
1.5
2.0
2.5
3.0
3.5
4.0
Cellular_organisms - ps1
Archaea1 - ps2
Archaea2 - ps3
Archaea3 - ps4
Asgard/Eukaryota - ps5
Eukaryota - ps6
Amorpheae - ps7
Obazoa - ps8
Opisthokonta - ps9
Holomycota - ps10
Fungi - ps11
Fungi_1 - ps12
Fungi_2 - ps13
Fungi_3 - ps14
Dikarya - ps15
Ascomycota - ps16
Saccharo/Pezizomycotina - ps17
Saccharomycotina - ps18
Saccharomycotina_1 - ps19
Saccharomycotina_2 - ps20
Saccharomycotina_3 - ps21
Phaff./Saccharomycetaceae - ps22
Saccharomyco./mycetaceae - ps23
Saccharomycetaceae - ps24
Saccharomycetaceae_2 - ps25
Saccharomycetaceae_3 - ps26
Kaz./Nau./Saccharomyces - ps27
Saccharomyces - ps28
Arboricola_clade - ps29
Cerevisiae_clade - ps30
Saccharomyces_cerevisiae - ps31
Phylostratum

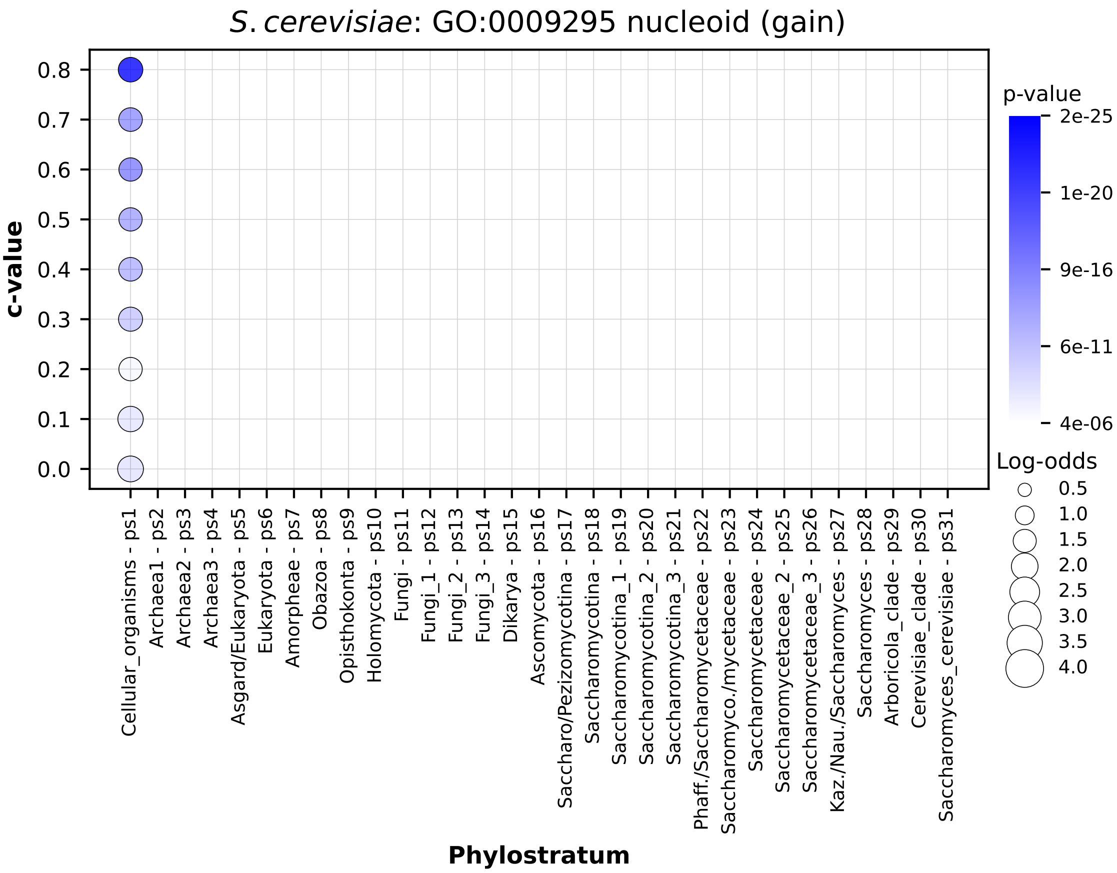

S. cerevisiae: GO:0009295 nucleoid (gain)
c-value
0.8
0.7
0.6
0.5
0.4
0.3
0.2
0.1
0.0
p-value
2e-25
1e-20
9e-16
6e-11
4e-06
Log-odds
0.5
1.0
1.5
2.0
2.5
3.0
3.5
4.0
Cellular_organisms - ps1
Archaea1 - ps2
Archaea2 - ps3
Archaea3 - ps4
Asgard/Eukaryota - ps5
Eukaryota - ps6
Amorpheae - ps7
Obazoa - ps8
Opisthokonta - ps9
Holomycota - ps10
Fungi - ps11
Fungi_1 - ps12
Fungi_2 - ps13
Fungi_3 - ps14
Dikarya - ps15
Ascomycota - ps16
Saccharo/Pezizomycotina - ps17
Saccharomycotina - ps18
Saccharomycotina_1 - ps19
Saccharomycotina_2 - ps20
Saccharomycotina_3 - ps21
Phaff./Saccharomycetaceae - ps22
Saccharomyco./mycetaceae - ps23
Saccharomycetaceae - ps24
Saccharomycetaceae_2 - ps25
Saccharomycetaceae_3 - ps26
Kaz./Nau./Saccharomyces - ps27
Saccharomyces - ps28
Arboricola_clade - ps29
Cerevisiae_clade - ps30
Saccharomyces_cerevisiae - ps31
Phylostratum

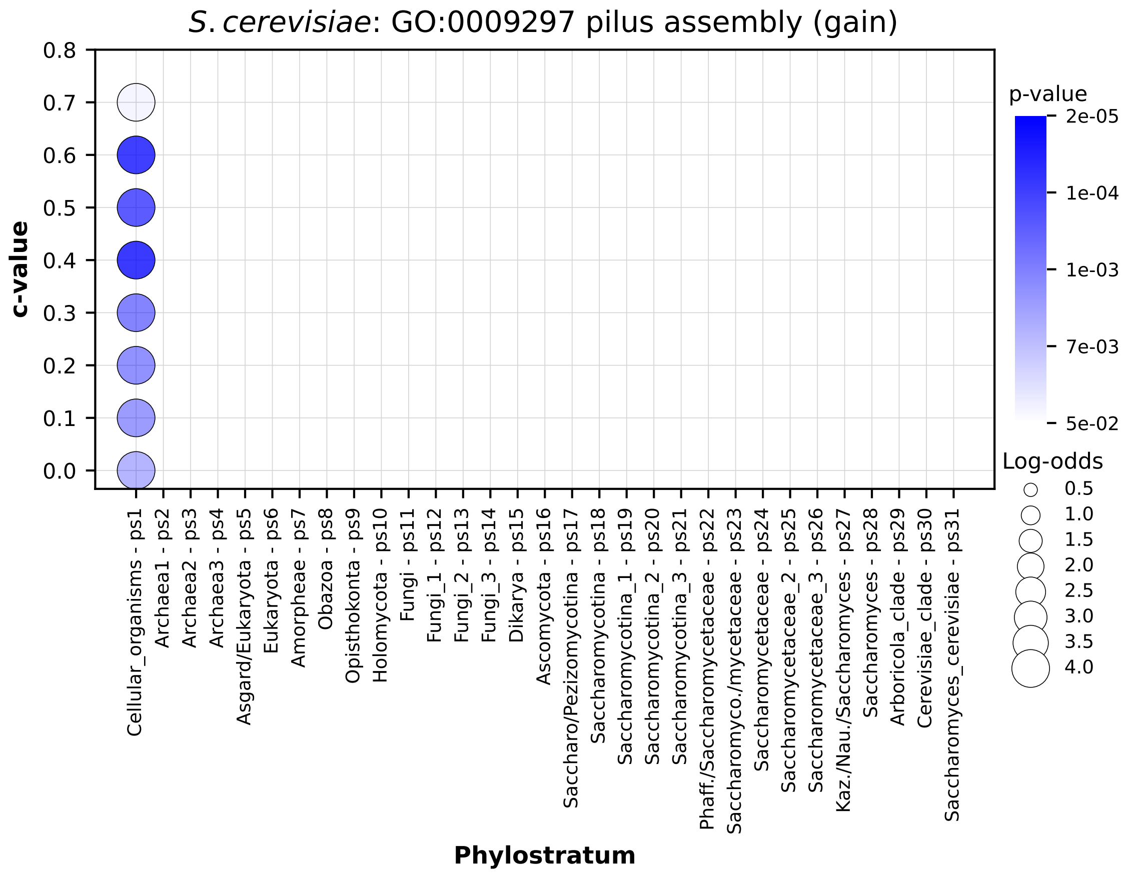

S. cerevisiae: GO:0009297 pilus assembly (gain)
c-value
0.8
0.7
0.6
0.5
0.4
0.3
0.2
0.1
0.0
p-value
2e-05
1e-04
1e-03
7e-03
5e-02
Log-odds
0.5
1.0
1.5
2.0
2.5
3.0
3.5
4.0
Cellular_organisms - ps1
Archaea1 - ps2
Archaea2 - ps3
Archaea3 - ps4
Asgard/Eukaryota - ps5
Eukaryota - ps6
Amorpheae - ps7
Obazoa - ps8
Opisthokonta - ps9
Holomycota - ps10
Fungi - ps11
Fungi_1 - ps12
Fungi_2 - ps13
Fungi_3 - ps14
Dikarya - ps15
Ascomycota - ps16
Saccharo/Pezizomycotina - ps17
Saccharomycotina - ps18
Saccharomycotina_1 - ps19
Saccharomycotina_2 - ps20
Saccharomycotina_3 - ps21
Phaff./Saccharomycetaceae - ps22
Saccharomyco./mycetaceae - ps23
Saccharomycetaceae - ps24
Saccharomycetaceae_2 - ps25
Saccharomycetaceae_3 - ps26
Kaz./Nau./Saccharomyces - ps27
Saccharomyces - ps28
Arboricola_clade - ps29
Cerevisiae_clade - ps30
Saccharomyces_cerevisiae - ps31
Phylostratum

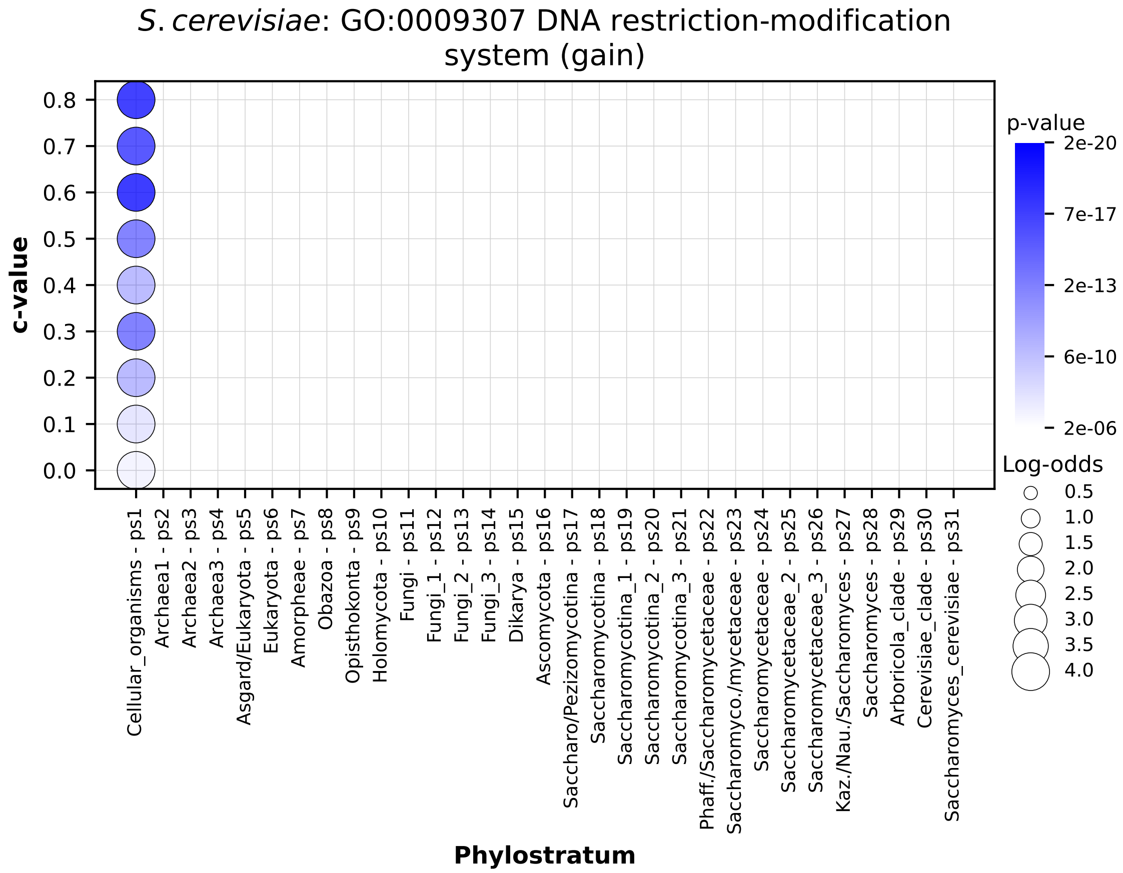

S. cerevisiae: GO:0009307 DNA restriction-modification system (gain)
c-value
0.8
0.7
0.6
0.5
0.4
0.3
0.2
0.1
0.0
p-value
2e-20
7e-17
2e-13
6e-10
2e-06
Log-odds
0.5
1.0
1.5
2.0
2.5
3.0
3.5
4.0
Cellular_organisms - ps1
Archaea1 - ps2
Archaea2 - ps3
Archaea3 - ps4
Asgard/Eukaryota - ps5
Eukaryota - ps6
Amorpheae - ps7
Obazoa - ps8
Opisthokonta - ps9
Holomycota - ps10
Fungi - ps11
Fungi_1 - ps12
Fungi_2 - ps13
Fungi_3 - ps14
Dikarya - ps15
Ascomycota - ps16
Saccharo/Pezizomycotina - ps17
Saccharomycotina - ps18
Saccharomycotina_1 - ps19
Saccharomycotina_2 - ps20
Saccharomycotina_3 - ps21
Phaff./Saccharomycetaceae - ps22
Saccharomyco./mycetaceae - ps23
Saccharomycetaceae - ps24
Saccharomycetaceae_2 - ps25
Saccharomycetaceae_3 - ps26
Kaz./Nau./Saccharomyces - ps27
Saccharomyces - ps28
Arboricola_clade - ps29
Cerevisiae_clade - ps30
Saccharomyces_cerevisiae - ps31
Phylostratum

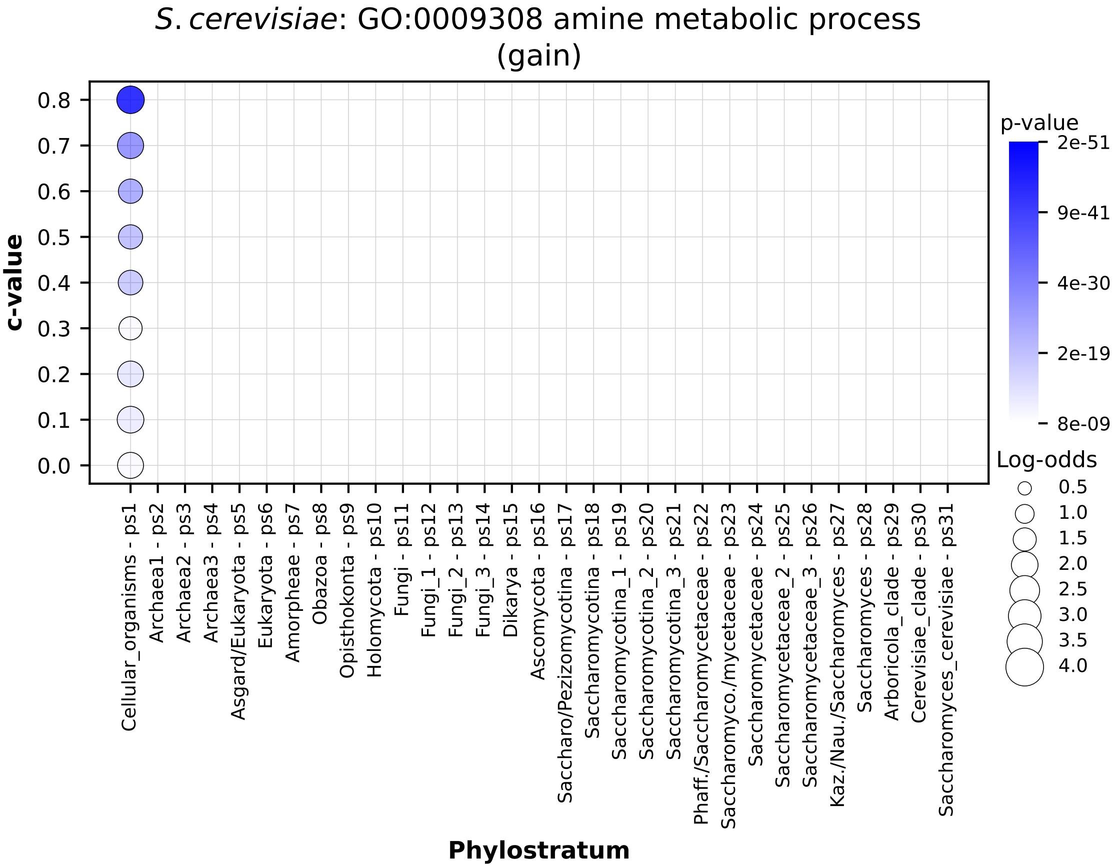

S. cerevisiae: GO:0009308 amine metabolic process
(gain)
c-value
0.8
0.7
0.6
0.5
0.4
0.3
0.2
0.1
0.0
p-value
2e-51
9e-41
4e-30
2e-19
8e-09
Log-odds
0.5
1.0
1.5
2.0
2.5
3.0
3.5
4.0
Cellular_organisms - ps1
Archaea1 - ps2
Archaea2 - ps3
Archaea3 - ps4
Asgard/Eukaryota - ps5
Eukaryota - ps6
Amorpheae - ps7
Obazoa - ps8
Opisthokonta - ps9
Holomycota - ps10
Fungi - ps11
Fungi_1 - ps12
Fungi_2 - ps13
Fungi_3 - ps14
Dikarya - ps15
Ascomycota - ps16
Saccharo/Pezizomycotina - ps17
Saccharomycotina - ps18
Saccharomycotina_1 - ps19
Saccharomycotina_2 - ps20
Saccharomycotina_3 - ps21
Phaff./Saccharomycetaceae - ps22
Saccharomyco./mycetaceae - ps23
Saccharomycetaceae - ps24
Saccharomycetaceae_2 - ps25
Saccharomycetaceae_3 - ps26
Kaz./Nau./Saccharomyces - ps27
Saccharomyces - ps28
Arboricola_clade - ps29
Cerevisiae_clade - ps30
Saccharomyces_cerevisiae - ps31
Phylostratum

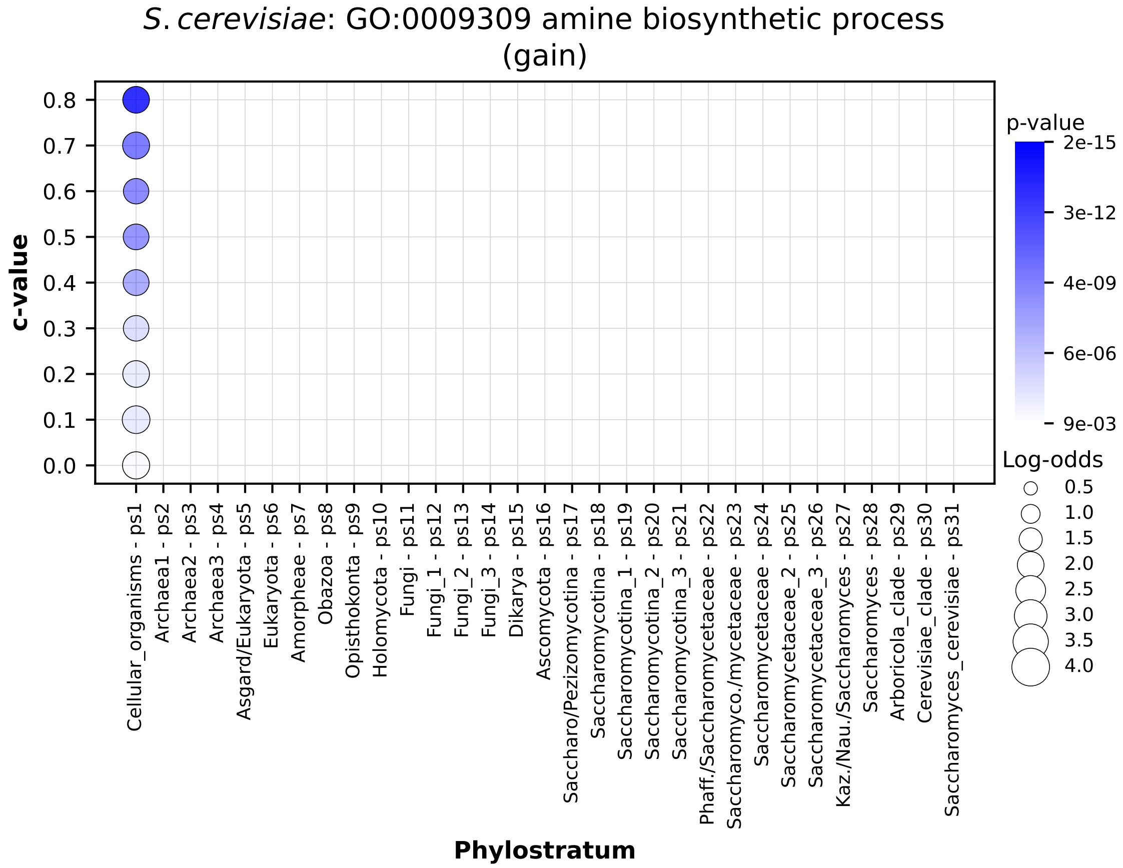

S. cerevisiae: GO:0009309 amine biosynthetic process
(gain)
c-value
0.8
0.7
0.6
0.5
0.4
0.3
0.2
0.1
0.0
p-value
2e-15
3e-12
4e-09
6e-06
9e-03
Log-odds
0.5
1.0
1.5
2.0
2.5
3.0
3.5
4.0
Cellular_organisms - ps1
Archaea1 - ps2
Archaea2 - ps3
Archaea3 - ps4
Asgard/Eukaryota - ps5
Eukaryota - ps6
Amorpheae - ps7
Obazoa - ps8
Opisthokonta - ps9
Holomycota - ps10
Fungi - ps11
Fungi_1 - ps12
Fungi_2 - ps13
Fungi_3 - ps14
Dikarya - ps15
Ascomycota - ps16
Saccharo/Pezizomycotina - ps17
Saccharomycotina - ps18
Saccharomycotina_1 - ps19
Saccharomycotina_2 - ps20
Saccharomycotina_3 - ps21
Phaff./Saccharomycetaceae - ps22
Saccharomyco./mycetaceae - ps23
Saccharomycetaceae - ps24
Saccharomycetaceae_2 - ps25
Saccharomycetaceae_3 - ps26
Kaz./Nau./Saccharomyces - ps27
Saccharomyces - ps28
Arboricola_clade - ps29
Cerevisiae_clade - ps30
Saccharomyces_cerevisiae - ps31
Phylostratum

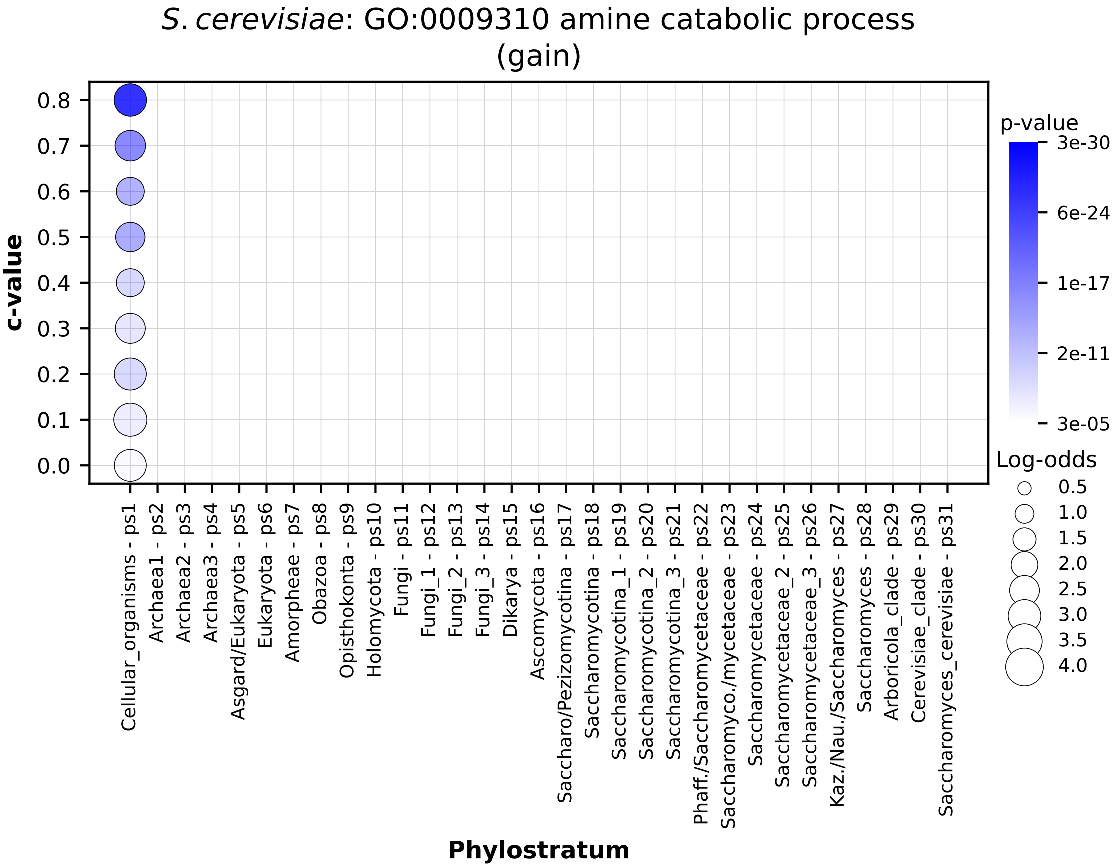
S. cerevisiae: GO:0009310 amine catabolic process
(gain)
c-value
0.8
0.7
0.6
0.5
0.4
0.3
0.2
0.1
0.0
p-value
3e-30
6e-24
1e-17
2e-11
3e-05
Log-odds
0.5
1.0
1.5
2.0
2.5
3.0
3.5
4.0
Cellular_organisms - ps1
Archaea1 - ps2
Archaea2 - ps3
Archaea3 - ps4
Asgard/Eukaryota - ps5
Eukaryota - ps6
Amorpheae - ps7
Obazoa - ps8
Opisthokonta - ps9
Holomycota - ps10
Fungi - ps11
Fungi_1 - ps12
Fungi_2 - ps13
Fungi_3 - ps14
Dikarya - ps15
Ascomycota - ps16
Saccharo/Pezizomycotina - ps17
Saccharomycotina - ps18
Saccharomycotina_1 - ps19
Saccharomycotina_2 - ps20
Saccharomycotina_3 - ps21
Phaff./Saccharomycetaceae - ps22
Saccharomyco./mycetaceae - ps23
Saccharomycetaceae - ps24
Saccharomycetaceae_2 - ps25
Saccharomycetaceae_3 - ps26
Kaz./Nau./Saccharomyces - ps27
Saccharomyces - ps28
Arboricola_clade - ps29
Cerevisiae_clade - ps30
Saccharomyces_cerevisiae - ps31
Phylostratum

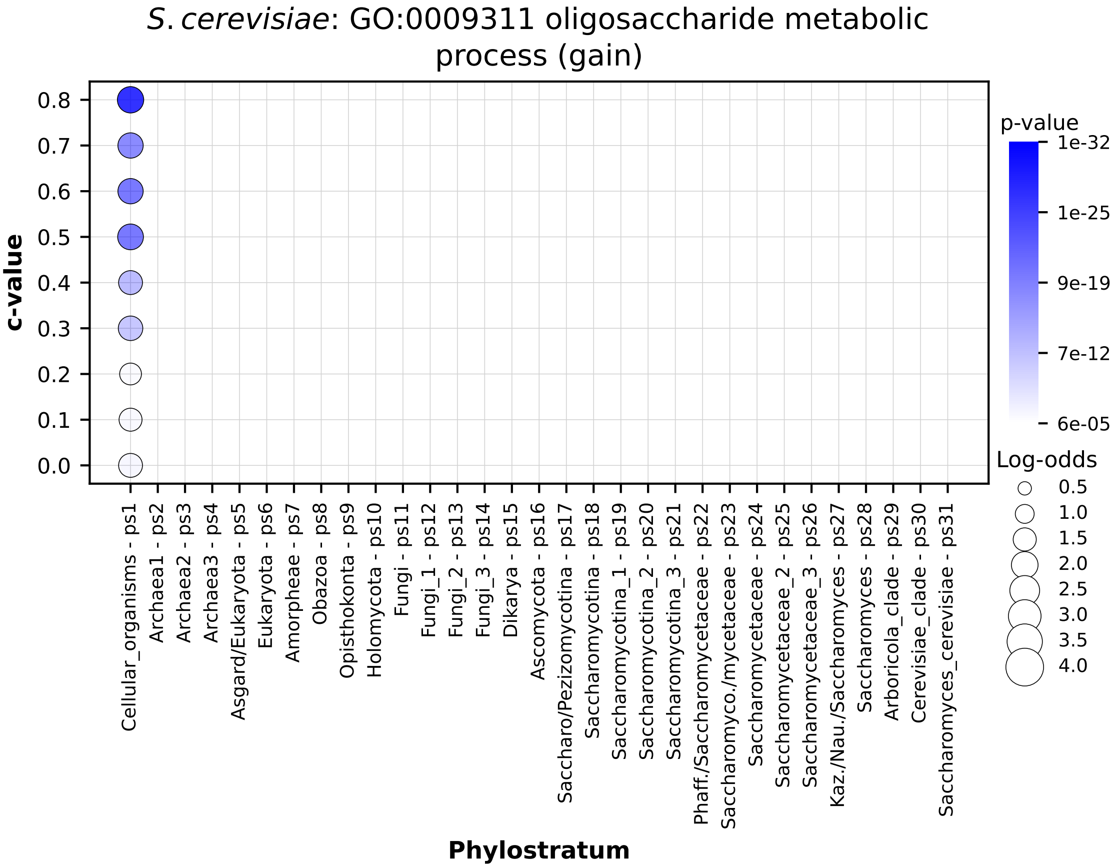
S. cerevisiae: GO:0009311 oligosaccharide metabolic process (gain)
c-value
0.0
0.1
0.2
0.3
0.4
0.5
0.6
0.7
0.8
p-value
1e-32
1e-25
9e-19
7e-12
6e-05
Log-odds
0.5
1.0
1.5
2.0
2.5
3.0
3.5
4.0
Cellular_organisms - ps1
Archaea1 - ps2
Archaea2 - ps3
Archaea3 - ps4
Asgard/Eukaryota - ps5
Eukaryota - ps6
Amorpheae - ps7
Obazoa - ps8
Opisthokonta - ps9
Holomycota - ps10
Fungi - ps11
Fungi_1 - ps12
Fungi_2 - ps13
Fungi_3 - ps14
Dikarya - ps15
Ascomycota - ps16
Saccharo/Pezizomycotina - ps17
Saccharomycotina - ps18
Saccharomycotina_1 - ps19
Saccharomycotina_2 - ps20
Saccharomycotina_3 - ps21
Phaff./Saccharomycetaceae - ps22
Saccharomyco./mycetaceae - ps23
Saccharomycetaceae - ps24
Saccharomycetaceae_2 - ps25
Saccharomycetaceae_3 - ps26
Kaz./Nau./Saccharomyces - ps27
Saccharomyces - ps28
Arboricola_clade - ps29
Cerevisiae_clade - ps30
Saccharomyces_cerevisiae - ps31
Phylostratum

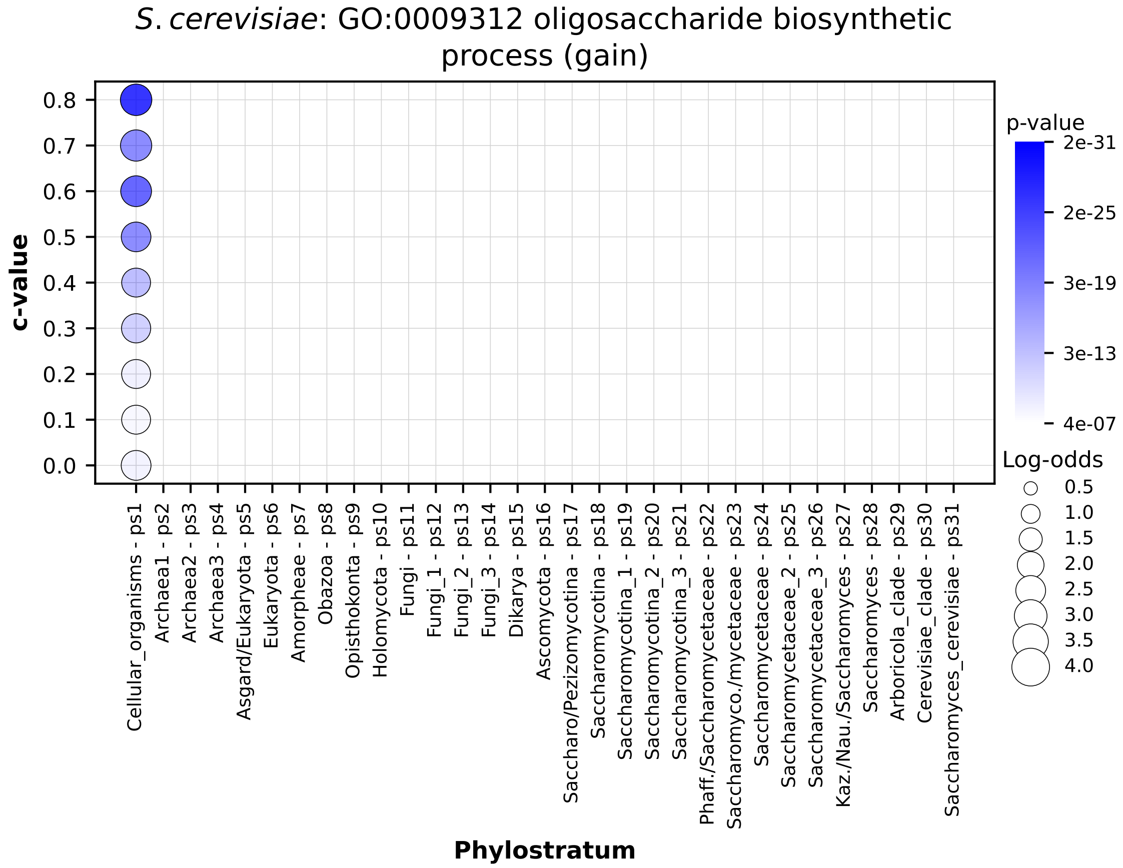
S. cerevisiae: GO:0009312 oligosaccharide biosynthetic process (gain)
c-value
0.8
0.7
0.6
0.5
0.4
0.3
0.2
0.1
0.0
Phylostratum
Cellular_organisms - ps1
Archaea1 - ps2
Archaea2 - ps3
Archaea3 - ps4
Asgard/Eukaryota - ps5
Eukaryota - ps6
Amorpheae - ps7
Obazoa - ps8
Opisthokonta - ps9
Holomycota - ps10
Fungi - ps11
Fungi_1 - ps12
Fungi_2 - ps13
Fungi_3 - ps14
Dikarya - ps15
Ascomycota - ps16
Saccharo/Pezizomycotina - ps17
Saccharomycotina - ps18
Saccharomycotina_1 - ps19
Saccharomycotina_2 - ps20
Saccharomycotina_3 - ps21
Phaff./Saccharomycetaceae - ps22
Saccharomyco./mycetaceae - ps23
Saccharomycetaceae - ps24
Saccharomycetaceae_2 - ps25
Saccharomycetaceae_3 - ps26
Kaz./Nau./Saccharomyces - ps27
Saccharomyces - ps28
Arboricola_clade - ps29
Cerevisiae_clade - ps30
Saccharomyces_cerevisiae - ps31
p-value
2e-31
2e-25
3e-19
3e-13
4e-07
Log-odds
0.5
1.0
1.5
2.0
2.5
3.0
3.5
4.0

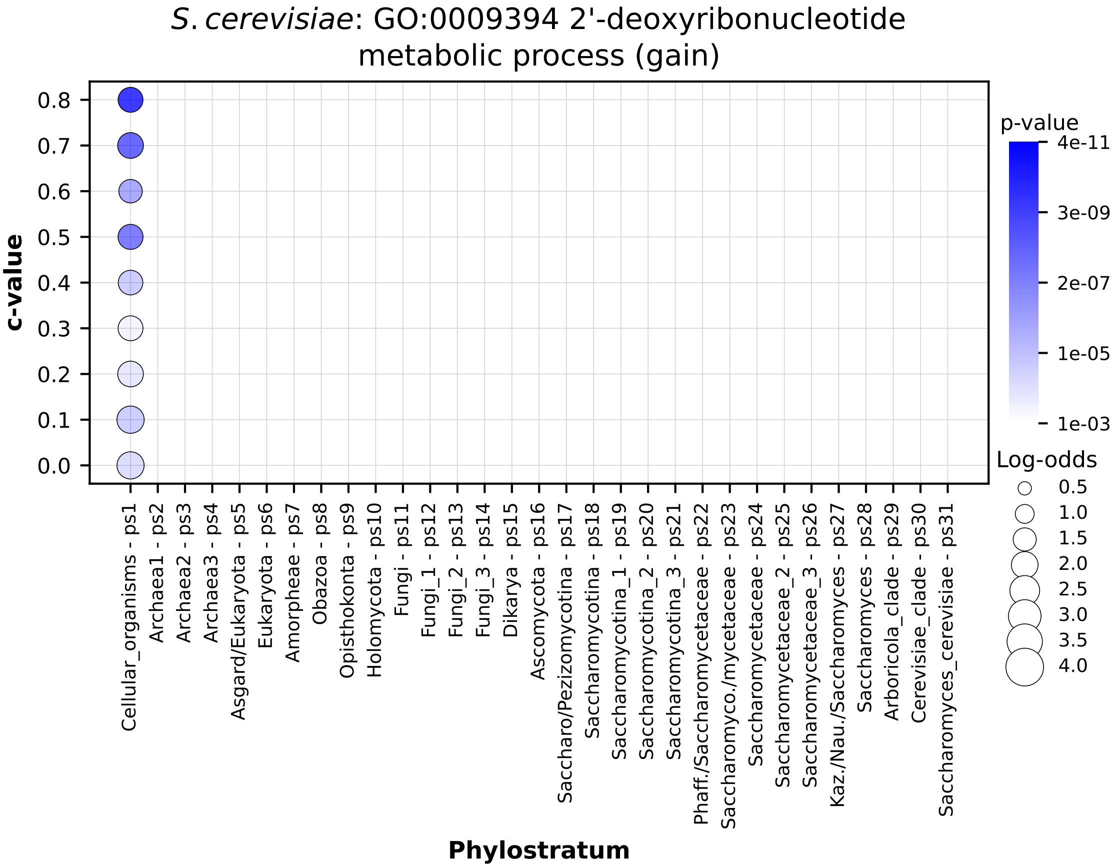
S. cerevisiae: GO:0009394 2'-deoxyribonucleotide metabolic process (gain)
c-value
0.8
0.7
0.6
0.5
0.4
0.3
0.2
0.1
0.0
p-value
4e-11
3e-09
2e-07
1e-05
1e-03
Log-odds
0.5
1.0
1.5
2.0
2.5
3.0
3.5
4.0
Cellular_organisms - ps1
Archaea1 - ps2
Archaea2 - ps3
Archaea3 - ps4
Asgard/Eukaryota - ps5
Eukaryota - ps6
Amorpheae - ps7
Obazoa - ps8
Opisthokonta - ps9
Holomycota - ps10
Fungi - ps11
Fungi_1 - ps12
Fungi_2 - ps13
Fungi_3 - ps14
Dikarya - ps15
Ascomycota - ps16
Saccharo/Pezizomycotina - ps17
Saccharomycotina - ps18
Saccharomycotina_1 - ps19
Saccharomycotina_2 - ps20
Saccharomycotina_3 - ps21
Phaff./Saccharomycetaceae - ps22
Saccharomyco./mycetaceae - ps23
Saccharomycetaceae - ps24
Saccharomycetaceae_2 - ps25
Saccharomycetaceae_3 - ps26
Kaz./Nau./Saccharomyces - ps27
Saccharomyces - ps28
Arboricola_clade - ps29
Cerevisiae_clade - ps30
Saccharomyces_cerevisiae - ps31
Phylostratum

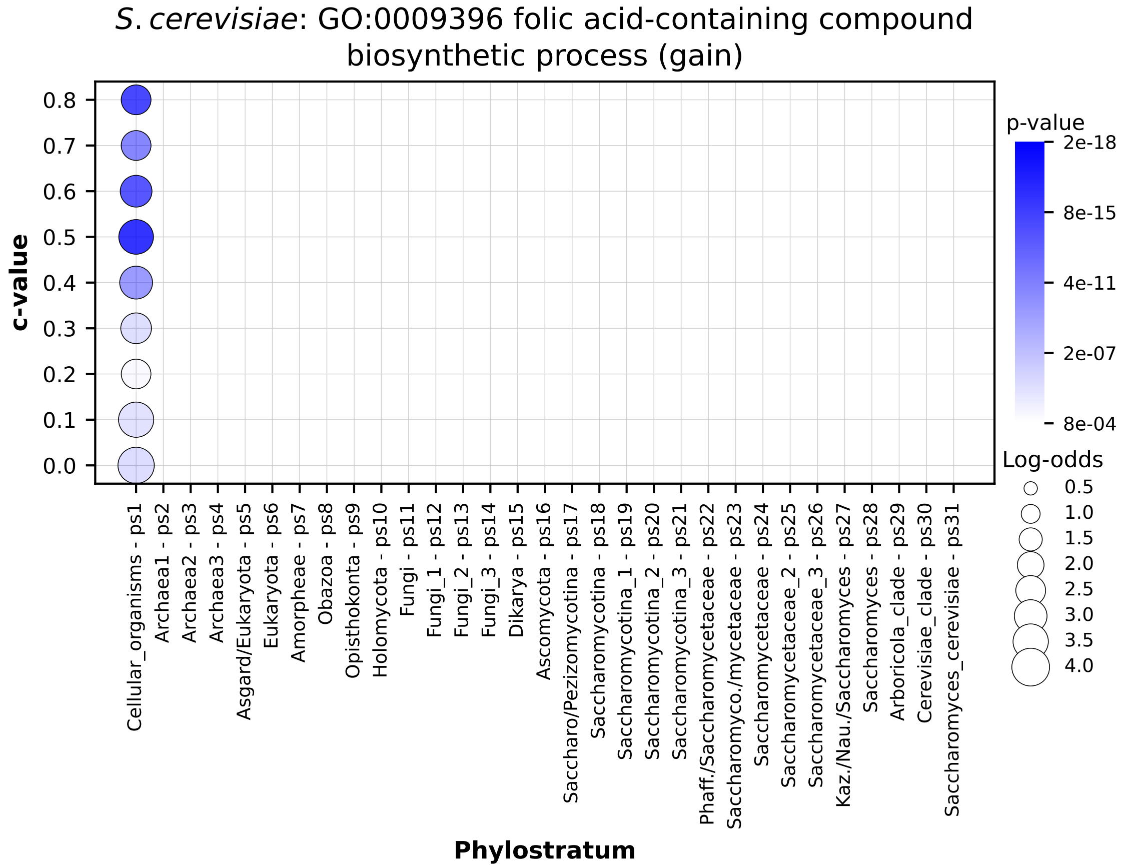

S. cerevisiae: GO:0009396 folic acid-containing compound biosynthetic process (gain)
c-value
0.8
0.7
0.6
0.5
0.4
0.3
0.2
0.1
0.0
p-value
2e-18
8e-15
4e-11
2e-07
8e-04
Log-odds
0.5
1.0
1.5
2.0
2.5
3.0
3.5
4.0
Cellular_organisms - ps1
Archaea1 - ps2
Archaea2 - ps3
Archaea3 - ps4
Asgard/Eukaryota - ps5
Eukaryota - ps6
Amorpheae - ps7
Obazoa - ps8
Opisthokonta - ps9
Holomycota - ps10
Fungi - ps11
Fungi_1 - ps12
Fungi_2 - ps13
Fungi_3 - ps14
Dikarya - ps15
Ascomycota - ps16
Saccharo/Pezizomycotina - ps17
Saccharomycotina - ps18
Saccharomycotina_1 - ps19
Saccharomycotina_2 - ps20
Saccharomycotina_3 - ps21
Phaff./Saccharomycetaceae - ps22
Saccharomyco./mycetaceae - ps23
Saccharomycetaceae - ps24
Saccharomycetaceae_2 - ps25
Saccharomycetaceae_3 - ps26
Kaz./Nau./Saccharomyces - ps27
Saccharomyces - ps28
Arboricola_clade - ps29
Cerevisiae_clade - ps30
Saccharomyces_cerevisiae - ps31
Phylostratum

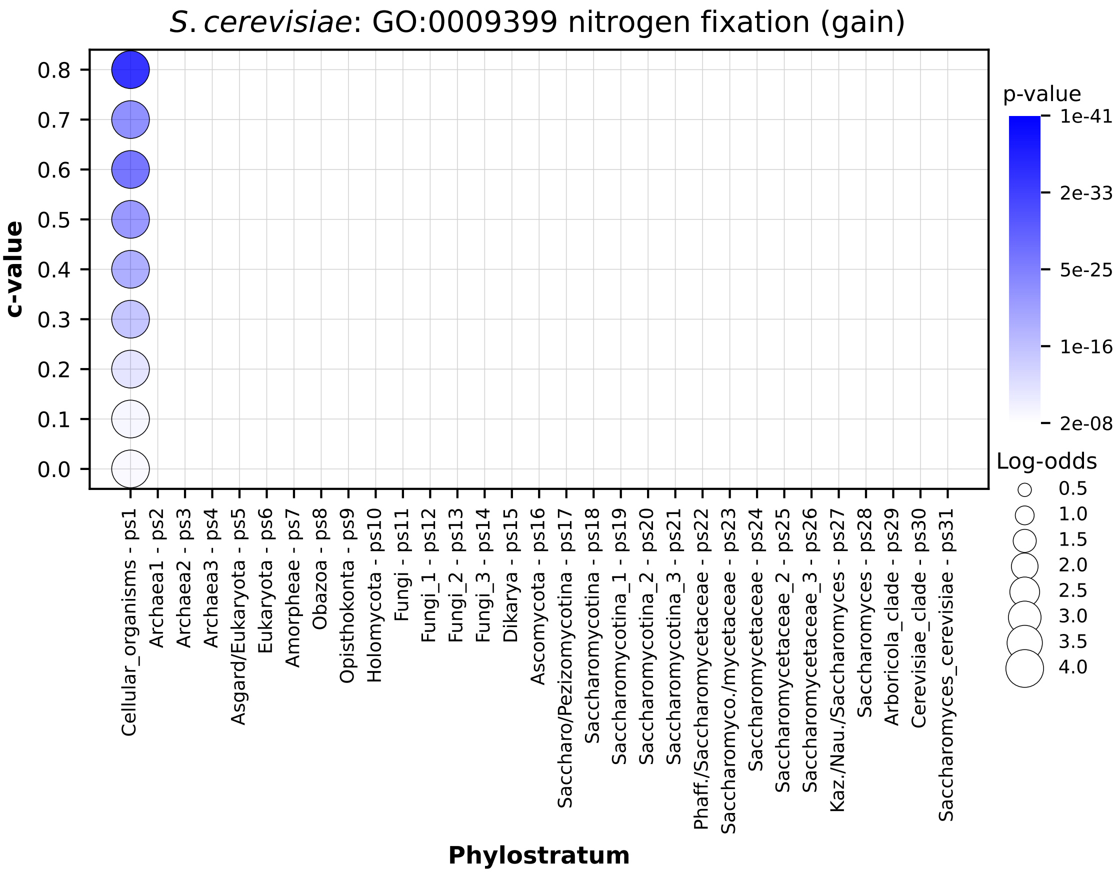

S. cerevisiae: GO:0009399 nitrogen fixation (gain)
c-value
0.0
0.1
0.2
0.3
0.4
0.5
0.6
0.7
0.8
p-value
1e-41
2e-33
5e-25
1e-16
2e-08
Log-odds
0.5
1.0
1.5
2.0
2.5
3.0
3.5
4.0
Cellular_organisms - ps1
Archaea1 - ps2
Archaea2 - ps3
Archaea3 - ps4
Asgard/Eukaryota - ps5
Eukaryota - ps6
Amorpheae - ps7
Obazoa - ps8
Opisthokonta - ps9
Holomycota - ps10
Fungi - ps11
Fungi_1 - ps12
Fungi_2 - ps13
Fungi_3 - ps14
Dikarya - ps15
Ascomycota - ps16
Saccharo/Pezizomycotina - ps17
Saccharomycotina - ps18
Saccharomycotina_1 - ps19
Saccharomycotina_2 - ps20
Saccharomycotina_3 - ps21
Phaff./Saccharomycetaceae - ps22
Saccharomyco./mycetaceae - ps23
Saccharomycetaceae - ps24
Saccharomycetaceae_2 - ps25
Saccharomycetaceae_3 - ps26
Kaz./Nau./Saccharomyces - ps27
Saccharomyces - ps28
Arboricola_clade - ps29
Cerevisiae_clade - ps30
Saccharomyces_cerevisiae - ps31
Phylostratum

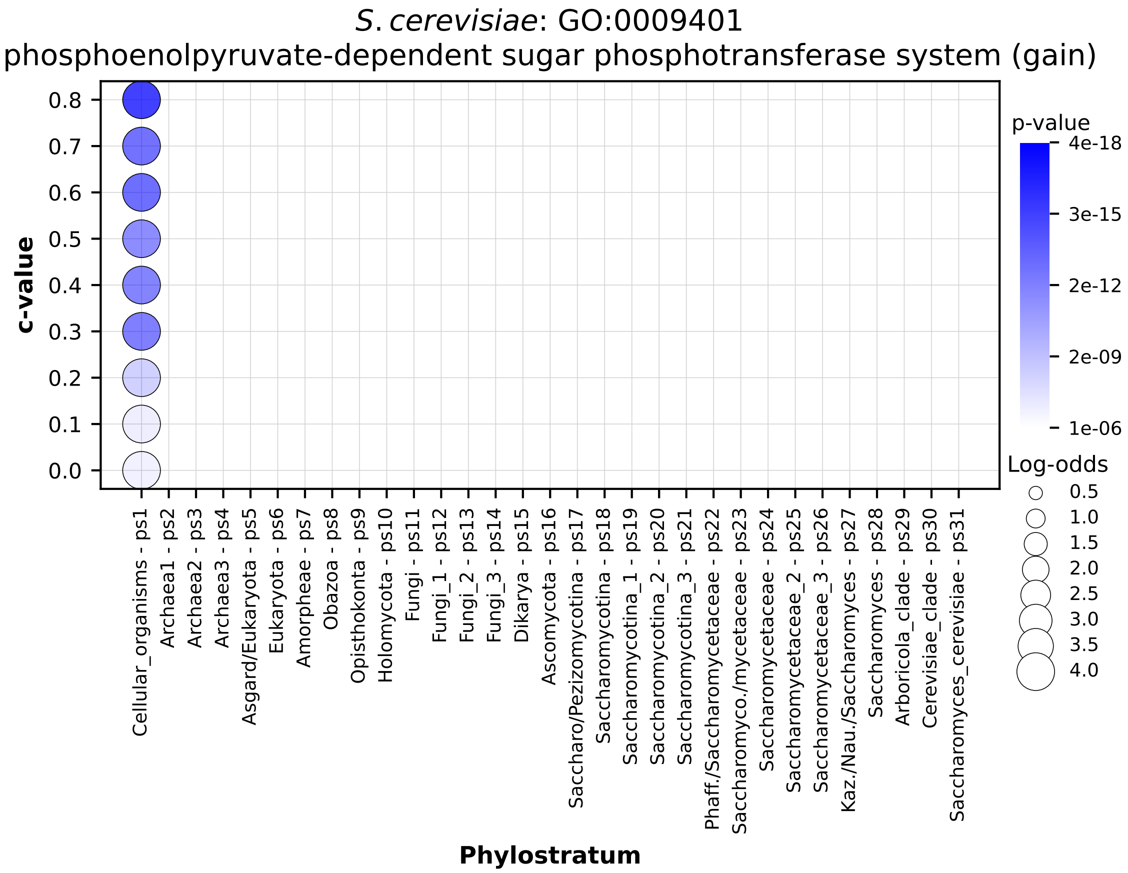

S. cerevisiae: GO:0009401
phosphoenolpyruvate-dependent sugar phosphotransferase system (gain)
c-value
0.8
0.7
0.6
0.5
0.4
0.3
0.2
0.1
0.0
p-value
4e-18
3e-15
2e-12
2e-09
1e-06
Log-odds
0.5
1.0
1.5
2.0
2.5
3.0
3.5
4.0
Cellular_organisms - ps1
Archaea1 - ps2
Archaea2 - ps3
Archaea3 - ps4
Asgard/Eukaryota - ps5
Eukaryota - ps6
Amorpheae - ps7
Obazoa - ps8
Opisthokonta - ps9
Holomycota - ps10
Fungi - ps11
Fungi_1 - ps12
Fungi_2 - ps13
Fungi_3 - ps14
Dikarya - ps15
Ascomycota - ps16
Saccharo/Pezizomycotina - ps17
Saccharomycotina - ps18
Saccharomycotina_1 - ps19
Saccharomycotina_2 - ps20
Saccharomycotina_3 - ps21
Phaff./Saccharomycetaceae - ps22
Saccharomyco./mycetaceae - ps23
Saccharomycetaceae - ps24
Saccharomycetaceae_2 - ps25
Saccharomycetaceae_3 - ps26
Kaz./Nau./Saccharomyces - ps27
Saccharomyces - ps28
Arboricola_clade - ps29
Cerevisiae_clade - ps30
Saccharomyces_cerevisiae - ps31
Phylostratum

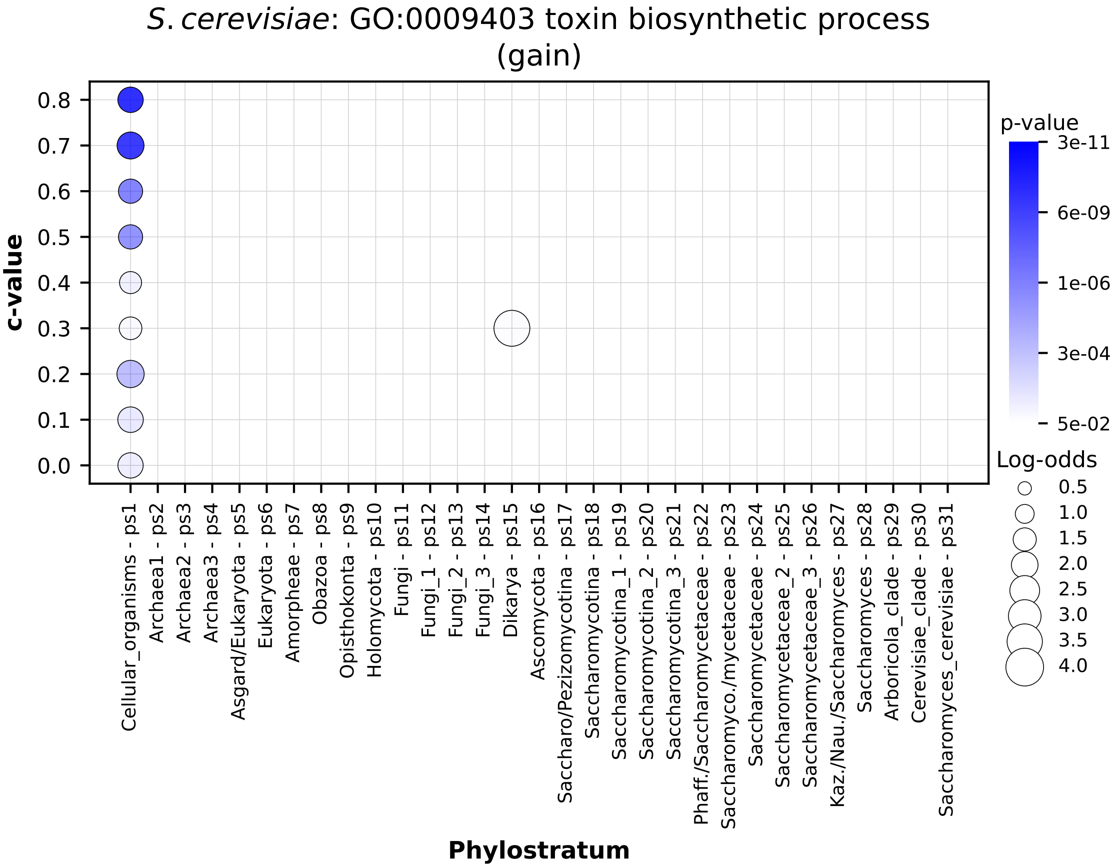

S. cerevisiae: GO:0009403 toxin biosynthetic process (gain)
c-value
0.8
0.7
0.6
0.5
0.4
0.3
0.2
0.1
0.0
p-value
3e-11
6e-09
1e-06
3e-04
5e-02
Log-odds
0.5
1.0
1.5
2.0
2.5
3.0
3.5
4.0
Cellular_organisms - ps1
Archaea1 - ps2
Archaea2 - ps3
Archaea3 - ps4
Asgard/Eukaryota - ps5
Eukaryota - ps6
Amorpheae - ps7
Obazoa - ps8
Opisthokonta - ps9
Holomycota - ps10
Fungi - ps11
Fungi_1 - ps12
Fungi_2 - ps13
Fungi_3 - ps14
Dikarya - ps15
Ascomycota - ps16
Saccharo/Pezizomycotina - ps17
Saccharomycotina - ps18
Saccharomycotina_1 - ps19
Saccharomycotina_2 - ps20
Saccharomycotina_3 - ps21
Phaff./Saccharomycetaceae - ps22
Saccharomyco./mycetaceae - ps23
Saccharomycetaceae - ps24
Saccharomycetaceae_2 - ps25
Saccharomycetaceae_3 - ps26
Kaz./Nau./Saccharomyces - ps27
Saccharomyces - ps28
Arboricola_clade - ps29
Cerevisiae_clade - ps30
Saccharomyces_cerevisiae - ps31
Phylostratum

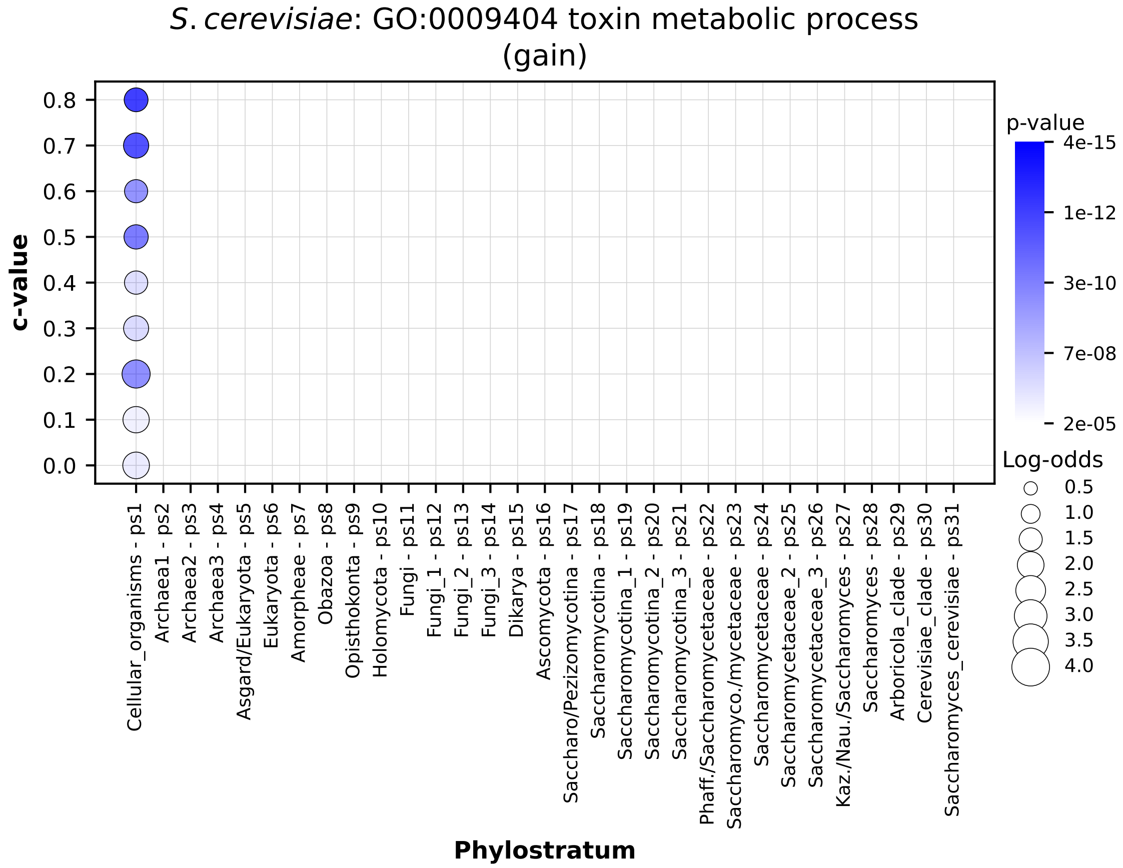

S. cerevisiae: GO:0009404 toxin metabolic process
(gain)
c-value
0.8
0.7
0.6
0.5
0.4
0.3
0.2
0.1
0.0
p-value
4e-15
1e-12
3e-10
7e-08
2e-05
Log-odds
0.5
1.0
1.5
2.0
2.5
3.0
3.5
4.0
Cellular_organisms - ps1
Archaea1 - ps2
Archaea2 - ps3
Archaea3 - ps4
Asgard/Eukaryota - ps5
Eukaryota - ps6
Amorpheae - ps7
Obazoa - ps8
Opisthokonta - ps9
Holomycota - ps10
Fungi - ps11
Fungi_1 - ps12
Fungi_2 - ps13
Fungi_3 - ps14
Dikarya - ps15
Ascomycota - ps16
Saccharo/Pezizomycotina - ps17
Saccharomycotina - ps18
Saccharomycotina_1 - ps19
Saccharomycotina_2 - ps20
Saccharomycotina_3 - ps21
Phaff./Saccharomycetaceae - ps22
Saccharomyco./mycetaceae - ps23
Saccharomycetaceae - ps24
Saccharomycetaceae_2 - ps25
Saccharomycetaceae_3 - ps26
Kaz./Nau./Saccharomyces - ps27
Saccharomyces - ps28
Arboricola_clade - ps29
Cerevisiae_clade - ps30
Saccharomyces_cerevisiae - ps31
Phylostratum

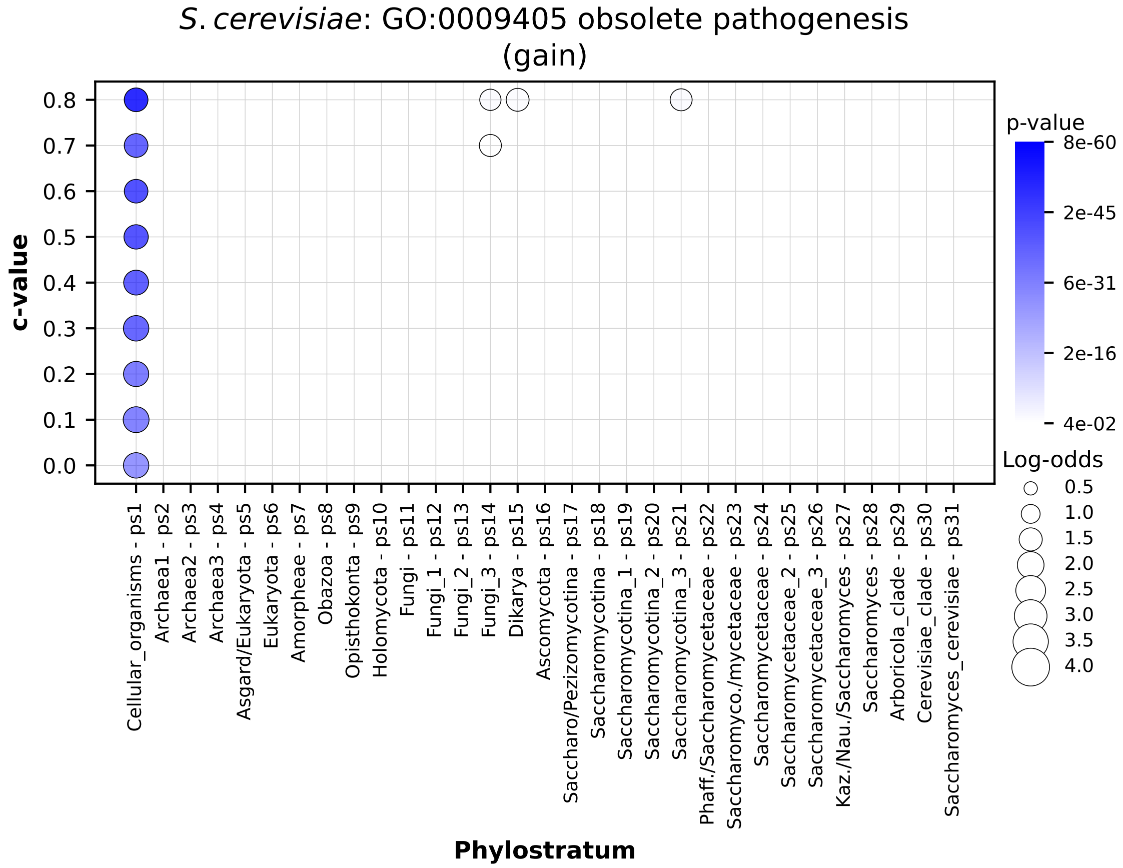

S. cerevisiae: GO:0009405 obsolete pathogenesis
(gain)
c-value
0.8
0.7
0.6
0.5
0.4
0.3
0.2
0.1
0.0
p-value
8e-60
2e-45
6e-31
2e-16
4e-02
Log-odds
0.5
1.0
1.5
2.0
2.5
3.0
3.5
4.0
Cellular_organisms - ps1
Archaea1 - ps2
Archaea2 - ps3
Archaea3 - ps4
Asgard/Eukaryota - ps5
Eukaryota - ps6
Amorpheae - ps7
Obazoa - ps8
Opisthokonta - ps9
Holomycota - ps10
Fungi - ps11
Fungi_1 - ps12
Fungi_2 - ps13
Fungi_3 - ps14
Dikarya - ps15
Ascomycota - ps16
Saccharo/Pezizomycotina - ps17
Saccharomycotina - ps18
Saccharomycotina_1 - ps19
Saccharomycotina_2 - ps20
Saccharomycotina_3 - ps21
Phaff./Saccharomycetaceae - ps22
Saccharomyco./mycetaceae - ps23
Saccharomycetaceae - ps24
Saccharomycetaceae_2 - ps25
Saccharomycetaceae_3 - ps26
Kaz./Nau./Saccharomyces - ps27
Saccharomyces - ps28
Arboricola_clade - ps29
Cerevisiae_clade - ps30
Saccharomyces_cerevisiae - ps31
Phylostratum

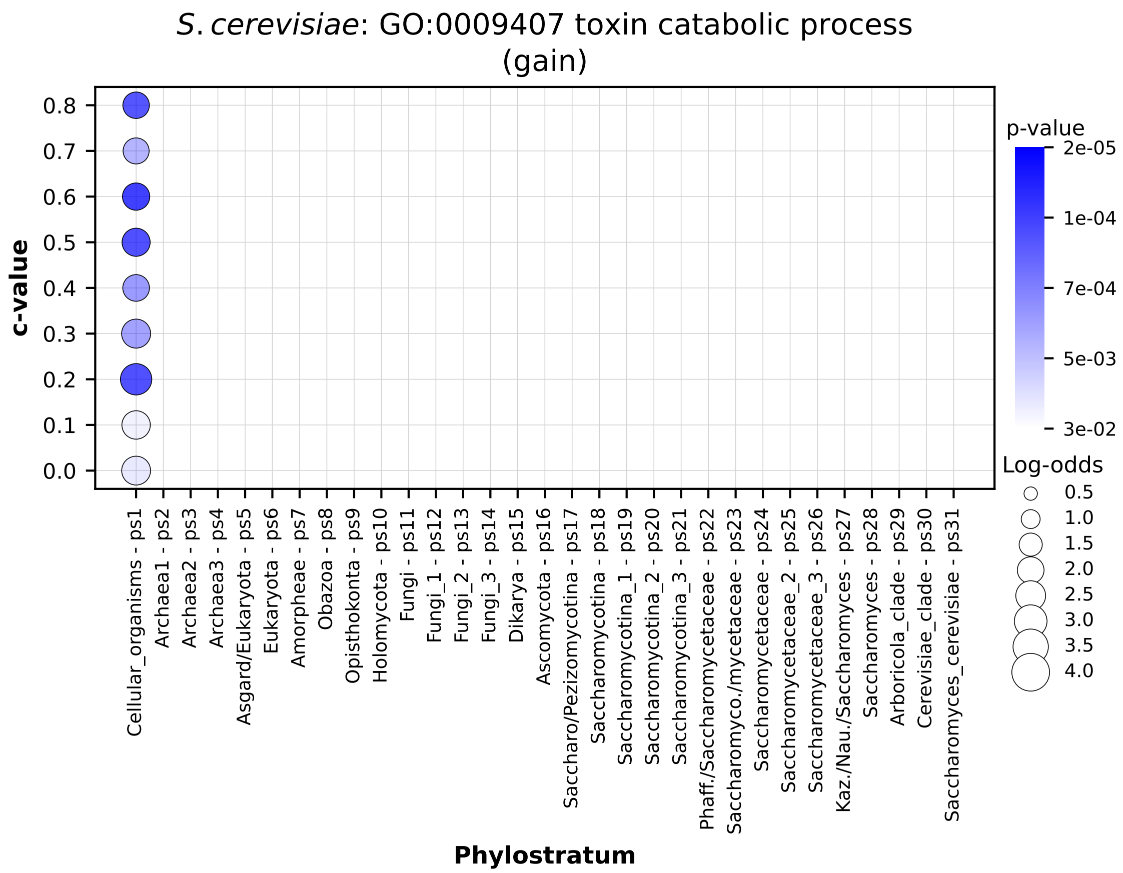

S. cerevisiae: GO:0009407 toxin catabolic process
(gain)
c-value
0.8
0.7
0.6
0.5
0.4
0.3
0.2
0.1
0.0
p-value
2e-05
1e-04
7e-04
5e-03
3e-02
Log-odds
0.5
1.0
1.5
2.0
2.5
3.0
3.5
4.0
Cellular_organisms - ps1
Archaea1 - ps2
Archaea2 - ps3
Archaea3 - ps4
Asgard/Eukaryota - ps5
Eukaryota - ps6
Amorpheae - ps7
Obazoa - ps8
Opisthokonta - ps9
Holomycota - ps10
Fungi - ps11
Fungi_1 - ps12
Fungi_2 - ps13
Fungi_3 - ps14
Dikarya - ps15
Ascomycota - ps16
Saccharo/Pezizomycotina - ps17
Saccharomycotina - ps18
Saccharomycotina_1 - ps19
Saccharomycotina_2 - ps20
Saccharomycotina_3 - ps21
Phaff./Saccharomycetaceae - ps22
Saccharomyco./mycetaceae - ps23
Saccharomycetaceae - ps24
Saccharomycetaceae_2 - ps25
Saccharomycetaceae_3 - ps26
Kaz./Nau./Saccharomyces - ps27
Saccharomyces - ps28
Arboricola_clade - ps29
Cerevisiae_clade - ps30
Saccharomyces_cerevisiae - ps31
Phylostratum

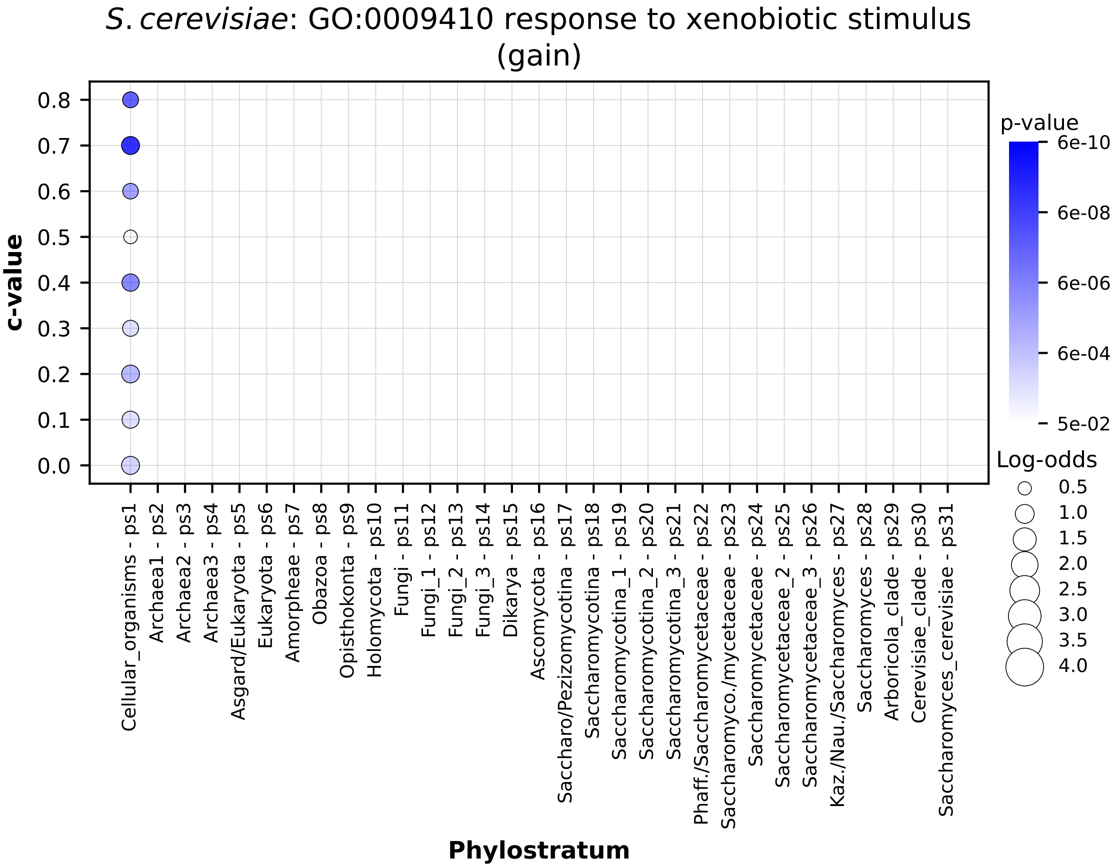

S. cerevisiae: GO:0009410 response to xenobiotic stimulus (gain)
c-value
0.8
0.7
0.6
0.5
0.4
0.3
0.2
0.1
0.0
Phylostratum
Cellular_organisms - ps1
Archaea1 - ps2
Archaea2 - ps3
Archaea3 - ps4
Asgard/Eukaryota - ps5
Eukaryota - ps6
Amorpheae - ps7
Obazoa - ps8
Opisthokonta - ps9
Holomycota - ps10
Fungi - ps11
Fungi_1 - ps12
Fungi_2 - ps13
Fungi_3 - ps14
Dikarya - ps15
Ascomycota - ps16
Saccharo/Pezizomycotina - ps17
Saccharomycotina - ps18
Saccharomycotina_1 - ps19
Saccharomycotina_2 - ps20
Saccharomycotina_3 - ps21
Phaff./Saccharomycetaceae - ps22
Saccharomyco./mycetaceae - ps23
Saccharomycetaceae - ps24
Saccharomycetaceae_2 - ps25
Saccharomycetaceae_3 - ps26
Kaz./Nau./Saccharomyces - ps27
Saccharomyces - ps28
Arboricola_clade - ps29
Cerevisiae_clade - ps30
Saccharomyces_cerevisiae - ps31
p-value
6e-10
6e-08
6e-06
6e-04
5e-02
Log-odds
0.5
1.0
1.5
2.0
2.5
3.0
3.5
4.0

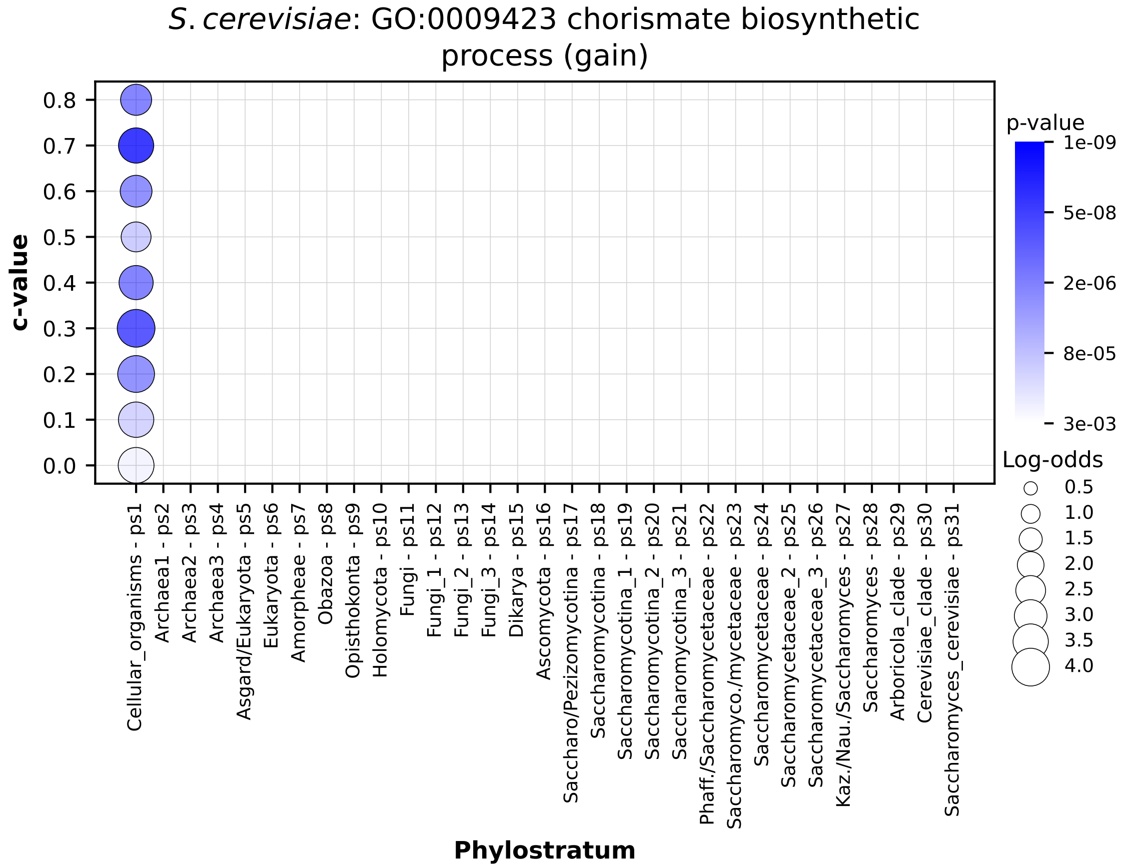
S. cerevisiae: GO:0009423 chorismate biosynthetic process (gain)
c-value
0.8
0.7
0.6
0.5
0.4
0.3
0.2
0.1
0.0
p-value
1e-09
5e-08
2e-06
8e-05
3e-03
Log-odds
0.5
1.0
1.5
2.0
2.5
3.0
3.5
4.0
Cellular_organisms - ps1
Archaea1 - ps2
Archaea2 - ps3
Archaea3 - ps4
Asgard/Eukaryota - ps5
Eukaryota - ps6
Amorpheae - ps7
Obazoa - ps8
Opisthokonta - ps9
Holomycota - ps10
Fungi - ps11
Fungi_1 - ps12
Fungi_2 - ps13
Fungi_3 - ps14
Dikarya - ps15
Ascomycota - ps16
Saccharo/Pezizomycotina - ps17
Saccharomycotina - ps18
Saccharomycotina_1 - ps19
Saccharomycotina_2 - ps20
Saccharomycotina_3 - ps21
Phaff./Saccharomycetaceae - ps22
Saccharomyco./mycetaceae - ps23
Saccharomycetaceae - ps24
Saccharomycetaceae_2 - ps25
Saccharomycetaceae_3 - ps26
Kaz./Nau./Saccharomyces - ps27
Saccharomyces - ps28
Arboricola_clade - ps29
Cerevisiae_clade - ps30
Saccharomyces_cerevisiae - ps31
Phylostratum

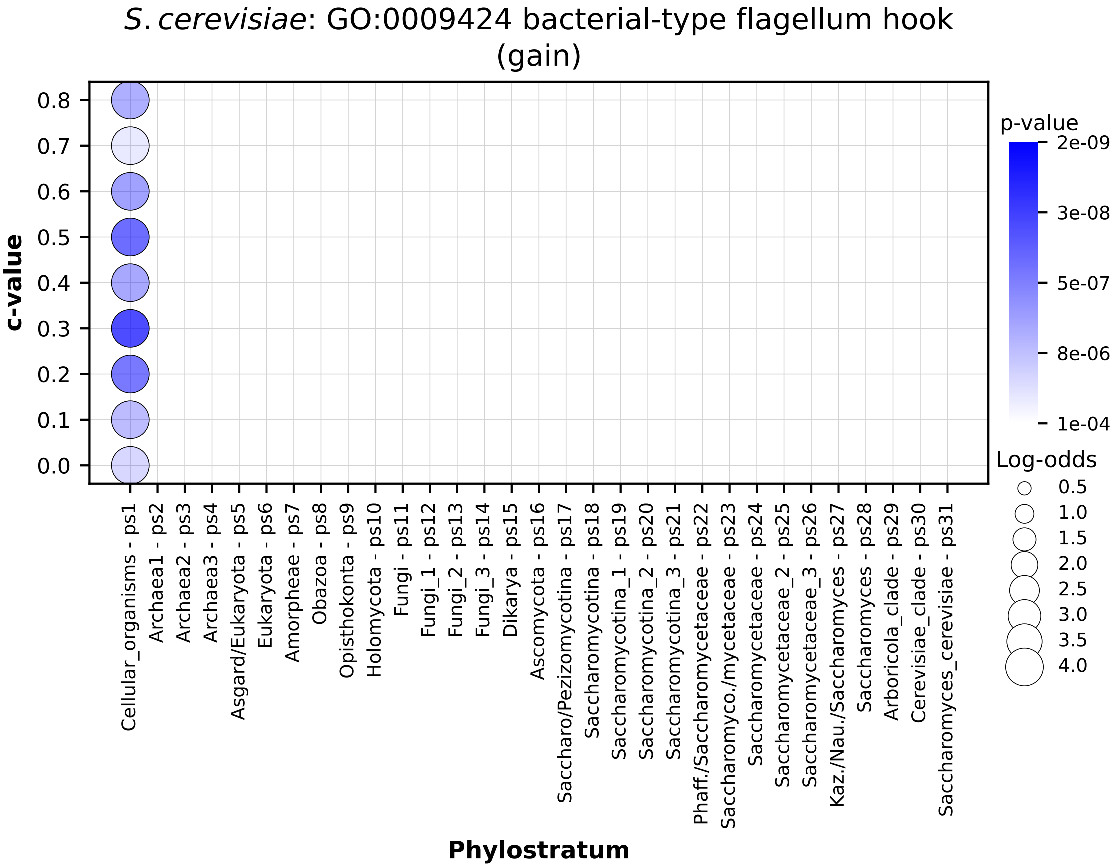
S. cerevisiae: GO:0009424 bacterial-type flagellum hook (gain)
c-value
0.8
0.7
0.6
0.5
0.4
0.3
0.2
0.1
0.0
p-value
2e-09
3e-08
5e-07
8e-06
1e-04
Log-odds
0.5
1.0
1.5
2.0
2.5
3.0
3.5
4.0
Cellular_organisms - ps1
Archaea1 - ps2
Archaea2 - ps3
Archaea3 - ps4
Asgard/Eukaryota - ps5
Eukaryota - ps6
Amorpheae - ps7
Obazoa - ps8
Opisthokonta - ps9
Holomycota - ps10
Fungi - ps11
Fungi_1 - ps12
Fungi_2 - ps13
Fungi_3 - ps14
Dikarya - ps15
Ascomycota - ps16
Saccharo/Pezizomycotina - ps17
Saccharomycotina - ps18
Saccharomycotina_1 - ps19
Saccharomycotina_2 - ps20
Saccharomycotina_3 - ps21
Phaff./Saccharomycetaceae - ps22
Saccharomyco./mycetaceae - ps23
Saccharomycetaceae - ps24
Saccharomycetaceae_2 - ps25
Saccharomycetaceae_3 - ps26
Kaz./Nau./Saccharomyces - ps27
Saccharomyces - ps28
Arboricola_clade - ps29
Cerevisiae_clade - ps30
Saccharomyces_cerevisiae - ps31
Phylostratum

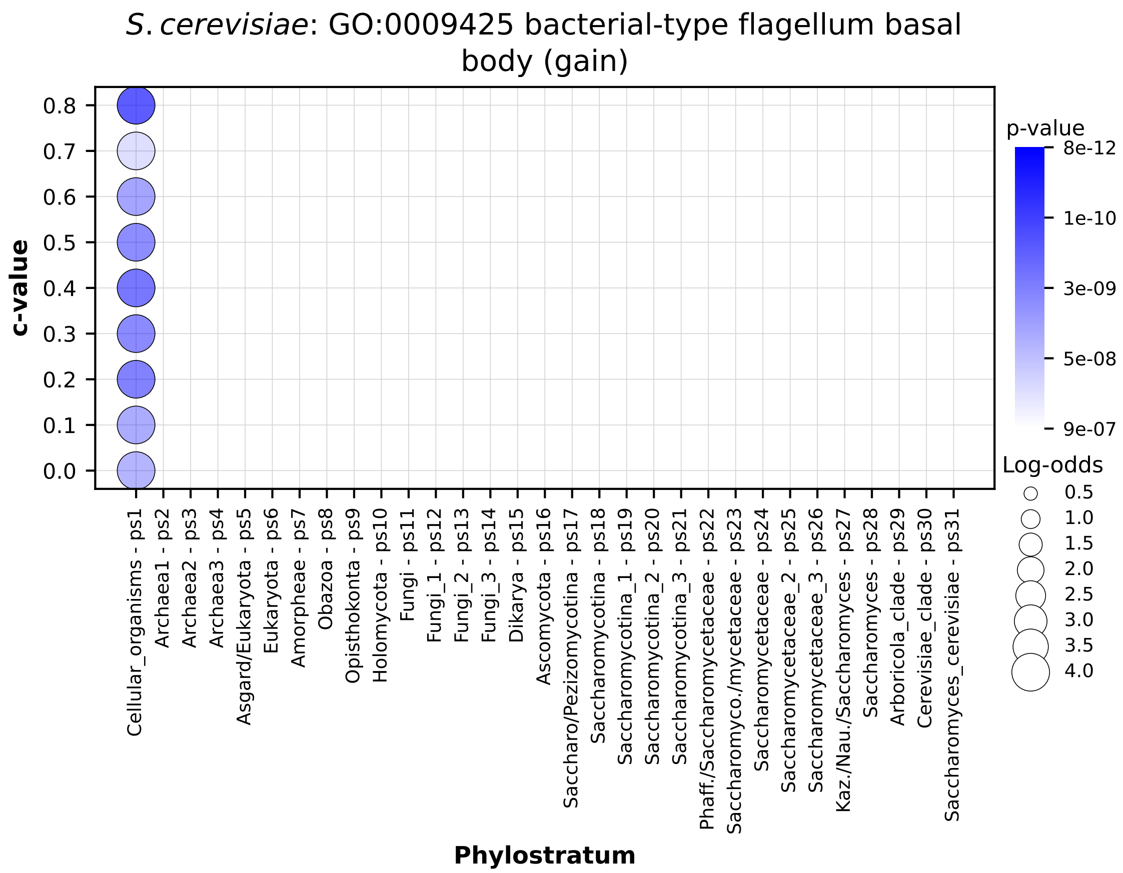
S. cerevisiae: GO:0009425 bacterial-type flagellum basal body (gain)
c-value
0.8
0.7
0.6
0.5
0.4
0.3
0.2
0.1
0.0
Phylostratum
Cellular_organisms - ps1
Archaea1 - ps2
Archaea2 - ps3
Archaea3 - ps4
Asgard/Eukaryota - ps5
Eukaryota - ps6
Amorpheae - ps7
Obazoa - ps8
Opisthokonta - ps9
Holomycota - ps10
Fungi - ps11
Fungi_1 - ps12
Fungi_2 - ps13
Fungi_3 - ps14
Dikarya - ps15
Ascomycota - ps16
Saccharo/Pezizomycotina - ps17
Saccharomycotina - ps18
Saccharomycotina_1 - ps19
Saccharomycotina_2 - ps20
Saccharomycotina_3 - ps21
Phaff./Saccharomycetaceae - ps22
Saccharomyco./mycetaceae - ps23
Saccharomycetaceae - ps24
Saccharomycetaceae_2 - ps25
Saccharomycetaceae_3 - ps26
Kaz./Nau./Saccharomyces - ps27
Saccharomyces - ps28
Arboricola_clade - ps29
Cerevisiae_clade - ps30
Saccharomyces_cerevisiae - ps31
p-value
8e-12
1e-10
3e-09
5e-08
9e-07
Log-odds
0.5
1.0
1.5
2.0
2.5
3.0
3.5
4.0

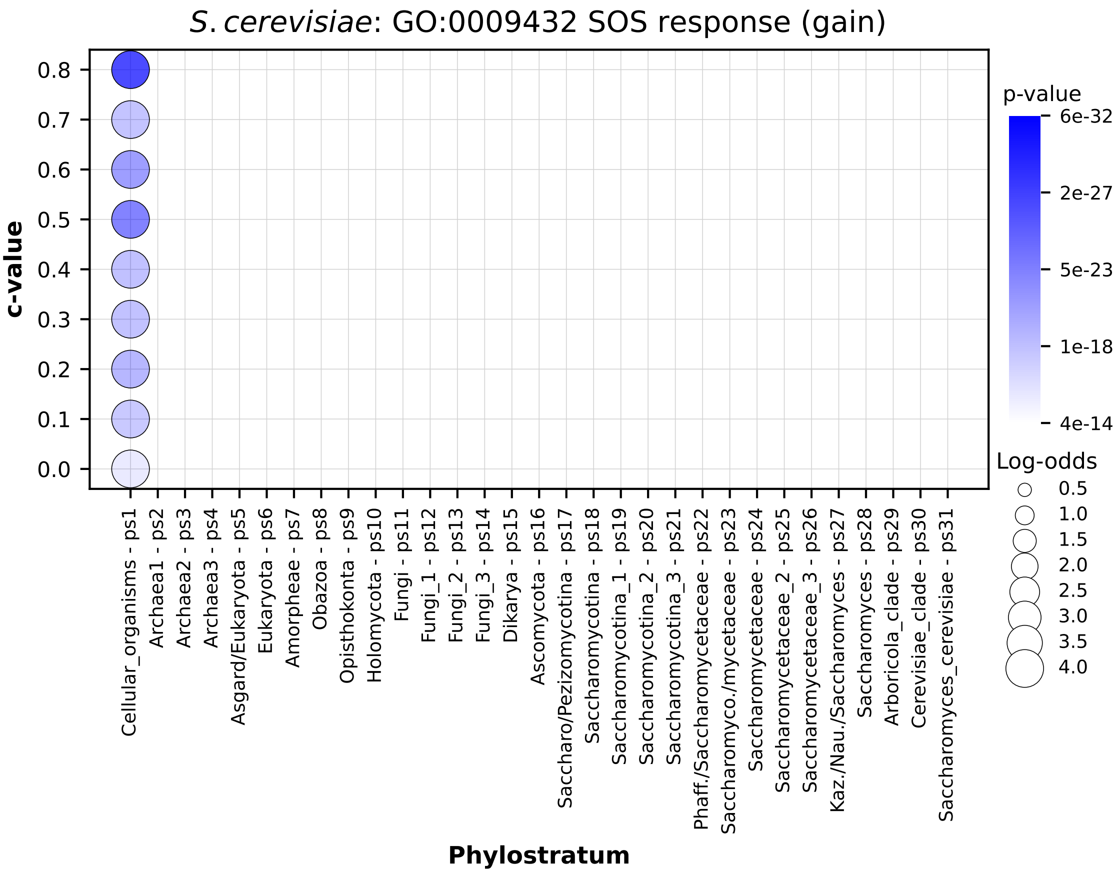

S. cerevisiae: GO:0009432 SOS response (gain)
c-value
0.0
0.1
0.2
0.3
0.4
0.5
0.6
0.7
0.8
p-value
6e-32
2e-27
5e-23
1e-18
4e-14
Log-odds
0.5
1.0
1.5
2.0
2.5
3.0
3.5
4.0
Cellular_organisms - ps1
Archaea1 - ps2
Archaea2 - ps3
Archaea3 - ps4
Asgard/Eukaryota - ps5
Eukaryota - ps6
Amorpheae - ps7
Obazoa - ps8
Opisthokonta - ps9
Holomycota - ps10
Fungi - ps11
Fungi_1 - ps12
Fungi_2 - ps13
Fungi_3 - ps14
Dikarya - ps15
Ascomycota - ps16
Saccharo/Pezizomycotina - ps17
Saccharomycotina - ps18
Saccharomycotina_1 - ps19
Saccharomycotina_2 - ps20
Saccharomycotina_3 - ps21
Phaff./Saccharomycetaceae - ps22
Saccharomyco./mycetaceae - ps23
Saccharomycetaceae - ps24
Saccharomycetaceae_2 - ps25
Saccharomycetaceae_3 - ps26
Kaz./Nau./Saccharomyces - ps27
Saccharomyces - ps28
Arboricola_clade - ps29
Cerevisiae_clade - ps30
Saccharomyces_cerevisiae - ps31
Phylostratum

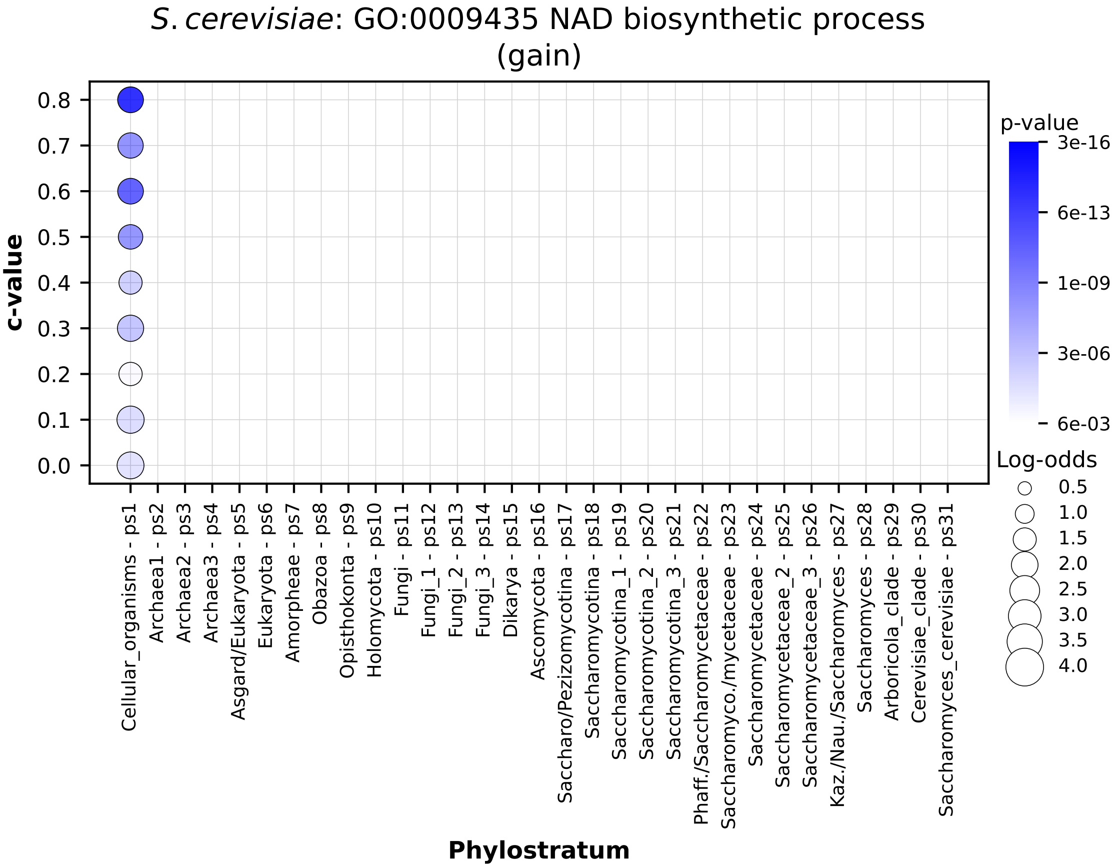

S. cerevisiae: GO:0009435 NAD biosynthetic process
(gain)
c-value
0.8
0.7
0.6
0.5
0.4
0.3
0.2
0.1
0.0
p-value
3e-16
6e-13
1e-09
3e-06
6e-03
Log-odds
0.5
1.0
1.5
2.0
2.5
3.0
3.5
4.0
Cellular_organisms - ps1
Archaea1 - ps2
Archaea2 - ps3
Archaea3 - ps4
Asgard/Eukaryota - ps5
Eukaryota - ps6
Amorpheae - ps7
Obazoa - ps8
Opisthokonta - ps9
Holomycota - ps10
Fungi - ps11
Fungi_1 - ps12
Fungi_2 - ps13
Fungi_3 - ps14
Dikarya - ps15
Ascomycota - ps16
Saccharo/Pezizomycotina - ps17
Saccharomycotina - ps18
Saccharomycotina_1 - ps19
Saccharomycotina_2 - ps20
Saccharomycotina_3 - ps21
Phaff./Saccharomycetaceae - ps22
Saccharomyco./mycetaceae - ps23
Saccharomycetaceae - ps24
Saccharomycetaceae_2 - ps25
Saccharomycetaceae_3 - ps26
Kaz./Nau./Saccharomyces - ps27
Saccharomyces - ps28
Arboricola_clade - ps29
Cerevisiae_clade - ps30
Saccharomyces_cerevisiae - ps31
Phylostratum

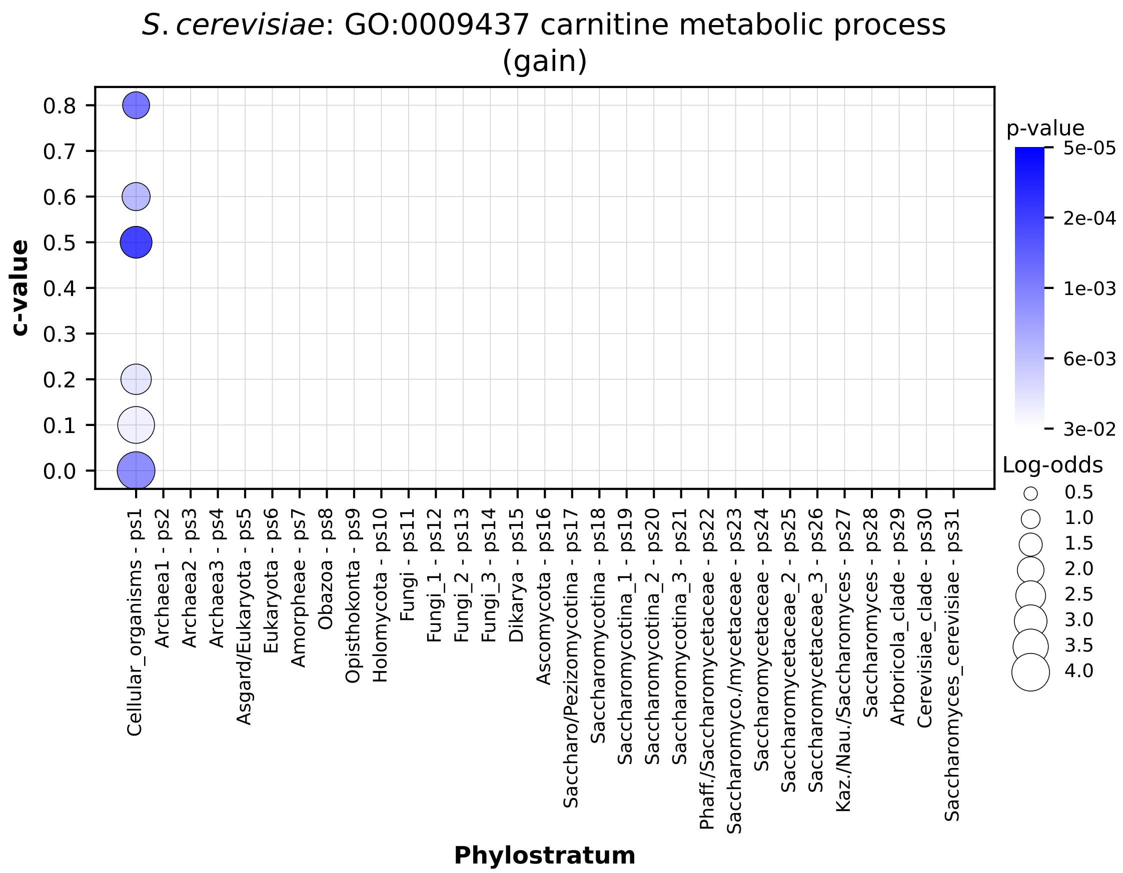

S. cerevisiae: GO:0009437 carnitine metabolic process (gain)
c-value
0.8
0.7
0.6
0.5
0.4
0.3
0.2
0.1
0.0
p-value
5e-05
2e-04
1e-03
6e-03
3e-02
Log-odds
0.5
1.0
1.5
2.0
2.5
3.0
3.5
4.0
Cellular_organisms - ps1
Archaea1 - ps2
Archaea2 - ps3
Archaea3 - ps4
Asgard/Eukaryota - ps5
Eukaryota - ps6
Amorpheae - ps7
Obazoa - ps8
Opisthokonta - ps9
Holomycota - ps10
Fungi - ps11
Fungi_1 - ps12
Fungi_2 - ps13
Fungi_3 - ps14
Dikarya - ps15
Ascomycota - ps16
Saccharo/Pezizomycotina - ps17
Saccharomycotina - ps18
Saccharomycotina_1 - ps19
Saccharomycotina_2 - ps20
Saccharomycotina_3 - ps21
Phaff./Saccharomycetaceae - ps22
Saccharomyco./mycetaceae - ps23
Saccharomycetaceae - ps24
Saccharomycetaceae_2 - ps25
Saccharomycetaceae_3 - ps26
Kaz./Nau./Saccharomyces - ps27
Saccharomyces - ps28
Arboricola_clade - ps29
Cerevisiae_clade - ps30
Saccharomyces_cerevisiae - ps31
Phylostratum

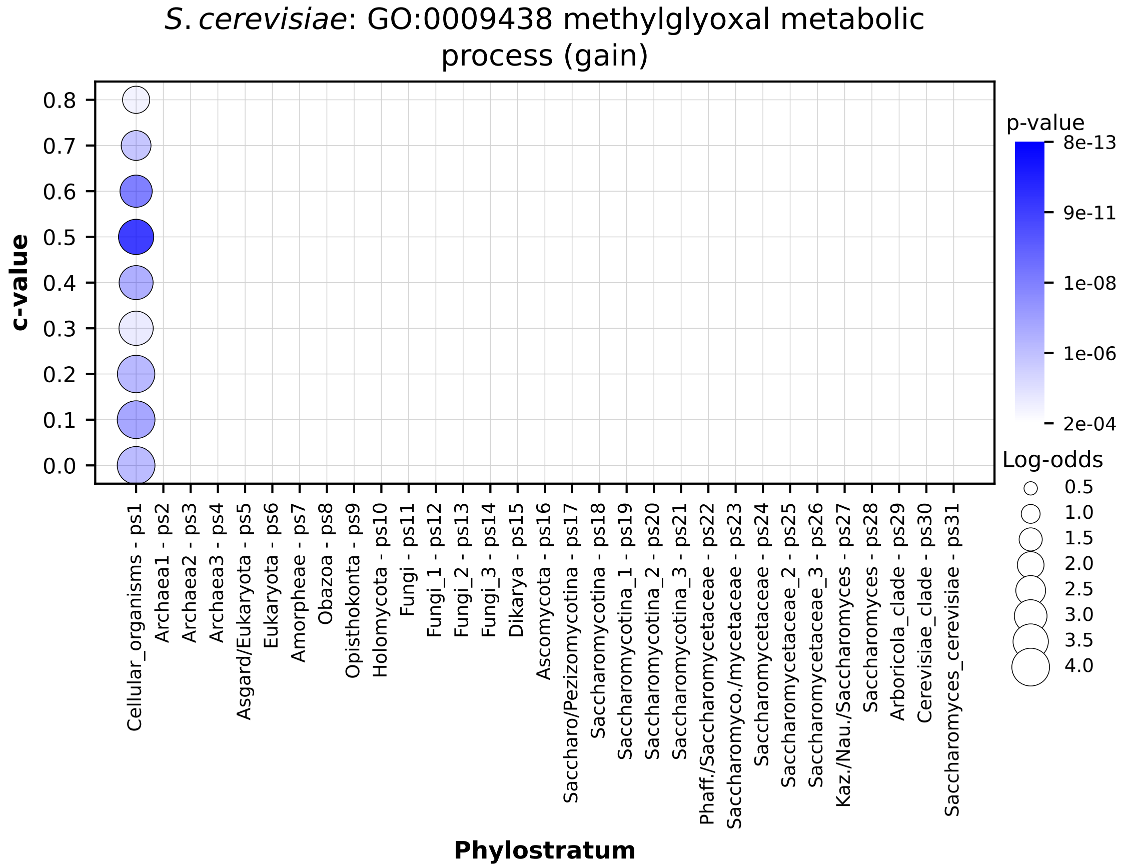
S. cerevisiae: GO:0009438 methylglyoxal metabolic process (gain)
c-value
0.8
0.7
0.6
0.5
0.4
0.3
0.2
0.1
0.0
p-value
8e-13
9e-11
1e-08
1e-06
2e-04
Log-odds
0.5
1.0
1.5
2.0
2.5
3.0
3.5
4.0
Cellular_organisms - ps1
Archaea1 - ps2
Archaea2 - ps3
Archaea3 - ps4
Asgard/Eukaryota - ps5
Eukaryota - ps6
Amorpheae - ps7
Obazoa - ps8
Opisthokonta - ps9
Holomycota - ps10
Fungi - ps11
Fungi_1 - ps12
Fungi_2 - ps13
Fungi_3 - ps14
Dikarya - ps15
Ascomycota - ps16
Saccharo/Pezizomycotina - ps17
Saccharomycotina - ps18
Saccharomycotina_1 - ps19
Saccharomycotina_2 - ps20
Saccharomycotina_3 - ps21
Phaff./Saccharomycetaceae - ps22
Saccharomyco./mycetaceae - ps23
Saccharomycetaceae - ps24
Saccharomycetaceae_2 - ps25
Saccharomycetaceae_3 - ps26
Kaz./Nau./Saccharomyces - ps27
Saccharomyces - ps28
Arboricola_clade - ps29
Cerevisiae_clade - ps30
Saccharomyces_cerevisiae - ps31
Phylostratum

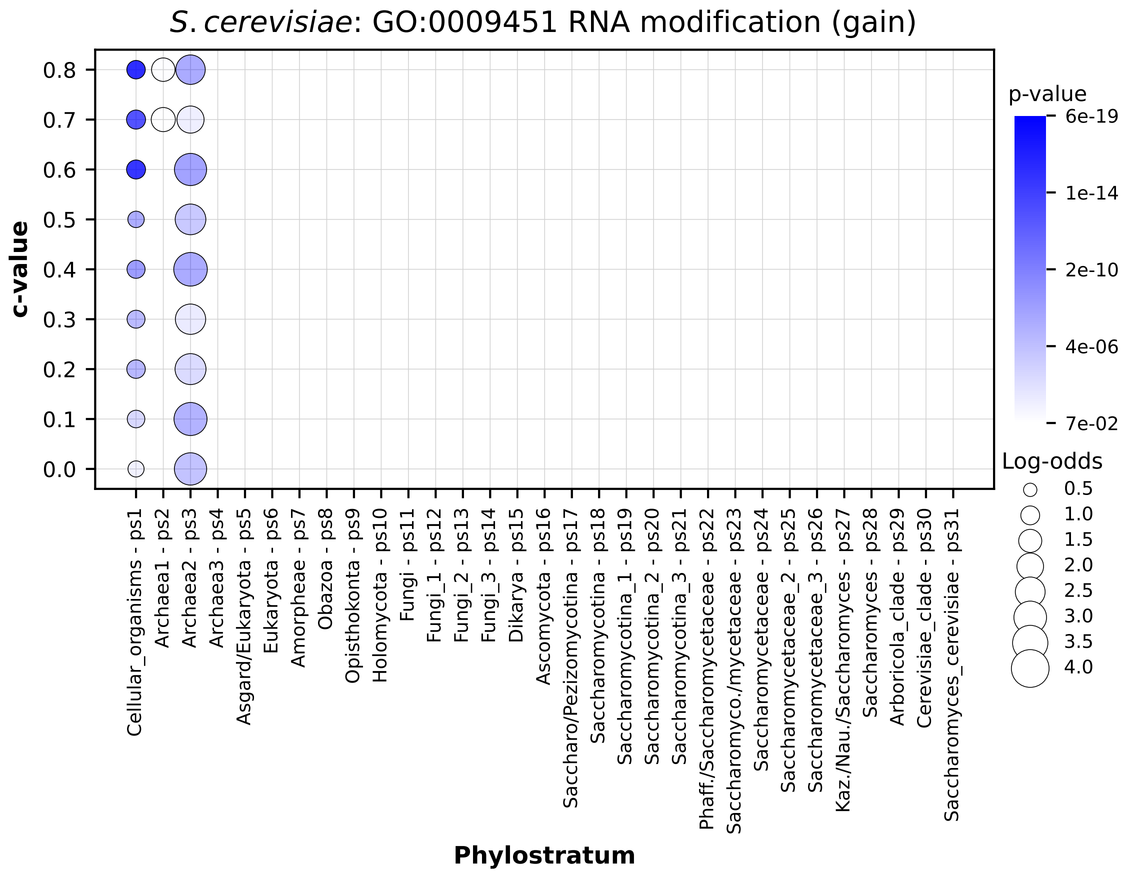

S. cerevisiae: GO:0009451 RNA modification (gain)
c-value
0.0
0.1
0.2
0.3
0.4
0.5
0.6
0.7
0.8
Phylostratum
Cellular_organisms - ps1
Archaea1 - ps2
Archaea2 - ps3
Archaea3 - ps4
Asgard/Eukaryota - ps5
Eukaryota - ps6
Amorpheae - ps7
Obazoa - ps8
Opisthokonta - ps9
Holomycota - ps10
Fungi - ps11
Fungi_1 - ps12
Fungi_2 - ps13
Fungi_3 - ps14
Dikarya - ps15
Ascomycota - ps16
Saccharo/Pezizomycotina - ps17
Saccharomycotina - ps18
Saccharomycotina_1 - ps19
Saccharomycotina_2 - ps20
Saccharomycotina_3 - ps21
Phaff./Saccharomycetaceae - ps22
Saccharomyco./mycetaceae - ps23
Saccharomycetaceae - ps24
Saccharomycetaceae_2 - ps25
Saccharomycetaceae_3 - ps26
Kaz./Nau./Saccharomyces - ps27
Saccharomyces - ps28
Arboricola_clade - ps29
Cerevisiae_clade - ps30
Saccharomyces_cerevisiae - ps31
p-value
6e-19
1e-14
2e-10
4e-06
7e-02
Log-odds
0.5
1.0
1.5
2.0
2.5
3.0
3.5
4.0

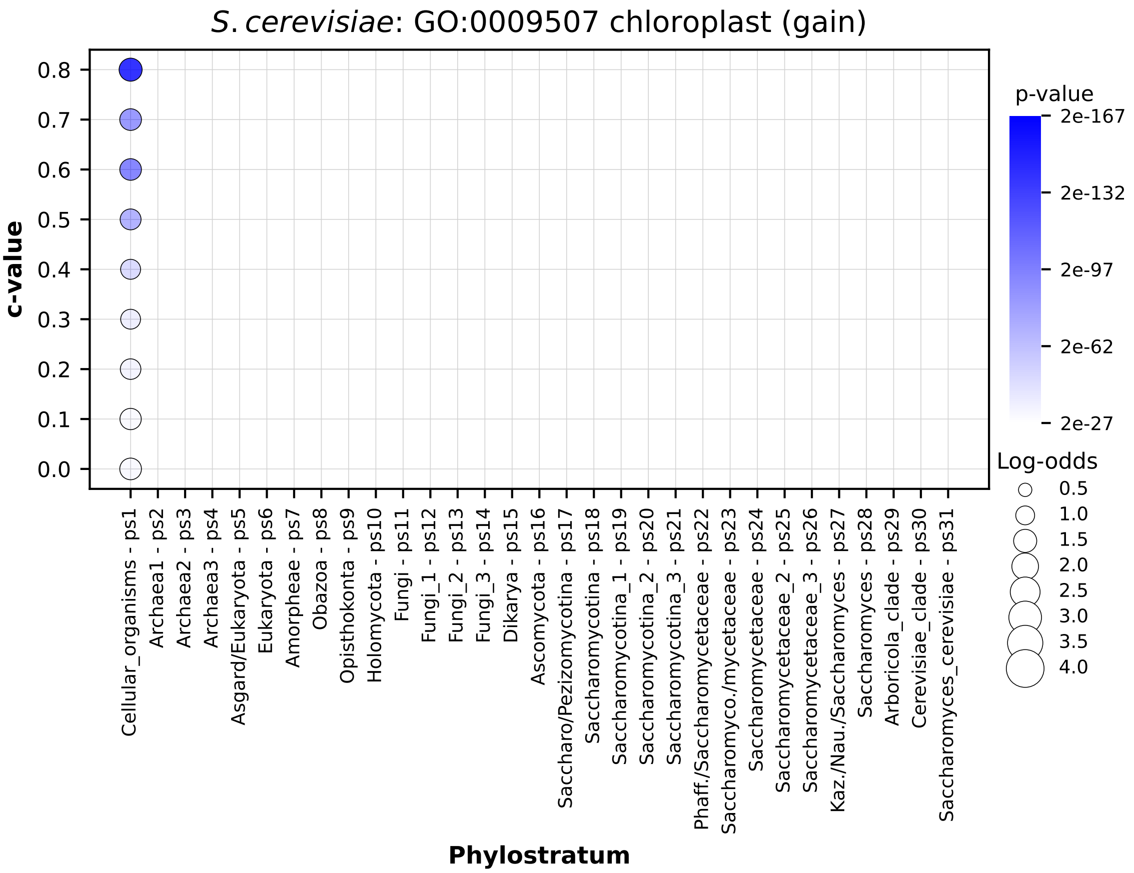

S. cerevisiae: GO:0009507 chloroplast (gain)
c-value
0.8
0.7
0.6
0.5
0.4
0.3
0.2
0.1
0.0
p-value
2e-167
2e-132
2e-97
2e-62
2e-27
Log-odds
0.5
1.0
1.5
2.0
2.5
3.0
3.5
4.0
Cellular_organisms - ps1
Archaea1 - ps2
Archaea2 - ps3
Archaea3 - ps4
Asgard/Eukaryota - ps5
Eukaryota - ps6
Amorpheae - ps7
Obazoa - ps8
Opisthokonta - ps9
Holomycota - ps10
Fungi - ps11
Fungi_1 - ps12
Fungi_2 - ps13
Fungi_3 - ps14
Dikarya - ps15
Ascomycota - ps16
Saccharo/Pezizomycotina - ps17
Saccharomycotina - ps18
Saccharomycotina_1 - ps19
Saccharomycotina_2 - ps20
Saccharomycotina_3 - ps21
Phaff./Saccharomycetaceae - ps22
Saccharomyco./mycetaceae - ps23
Saccharomycetaceae - ps24
Saccharomycetaceae_2 - ps25
Saccharomycetaceae_3 - ps26
Kaz./Nau./Saccharomyces - ps27
Saccharomyces - ps28
Arboricola_clade - ps29
Cerevisiae_clade - ps30
Saccharomyces_cerevisiae - ps31
Phylostratum

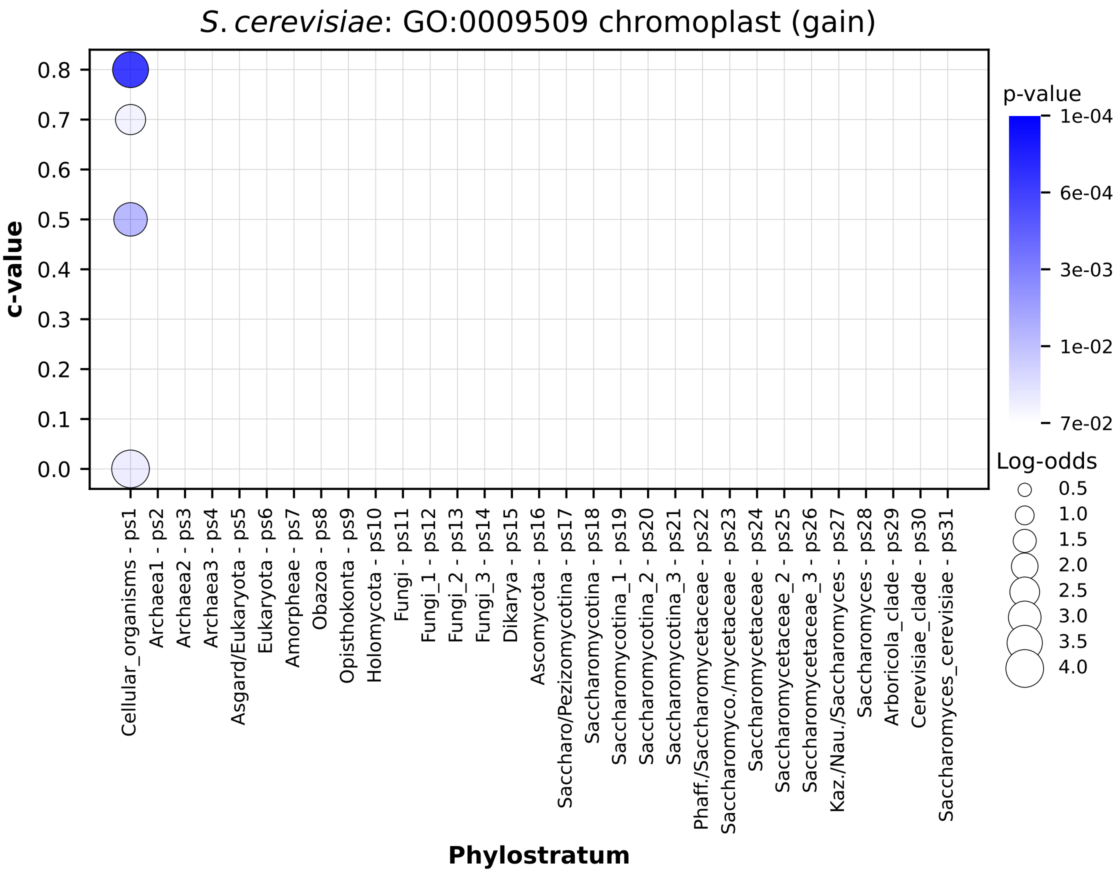

S. cerevisiae: GO:0009509 chromoplast (gain)
c-value
0.0
0.1
0.2
0.3
0.4
0.5
0.6
0.7
0.8
p-value
1e-04
6e-04
3e-03
1e-02
7e-02
Log-odds
0.5
1.0
1.5
2.0
2.5
3.0
3.5
4.0
Cellular_organisms - ps1
Archaea1 - ps2
Archaea2 - ps3
Archaea3 - ps4
Asgard/Eukaryota - ps5
Eukaryota - ps6
Amorpheae - ps7
Obazoa - ps8
Opisthokonta - ps9
Holomycota - ps10
Fungi - ps11
Fungi_1 - ps12
Fungi_2 - ps13
Fungi_3 - ps14
Dikarya - ps15
Ascomycota - ps16
Saccharo/Pezizomycotina - ps17
Saccharomycotina - ps18
Saccharomycotina_1 - ps19
Saccharomycotina_2 - ps20
Saccharomycotina_3 - ps21
Phaff./Saccharomycetaceae - ps22
Saccharomyco./mycetaceae - ps23
Saccharomycetaceae - ps24
Saccharomycetaceae_2 - ps25
Saccharomycetaceae_3 - ps26
Kaz./Nau./Saccharomyces - ps27
Saccharomyces - ps28
Arboricola_clade - ps29
Cerevisiae_clade - ps30
Saccharomyces_cerevisiae - ps31
Phylostratum

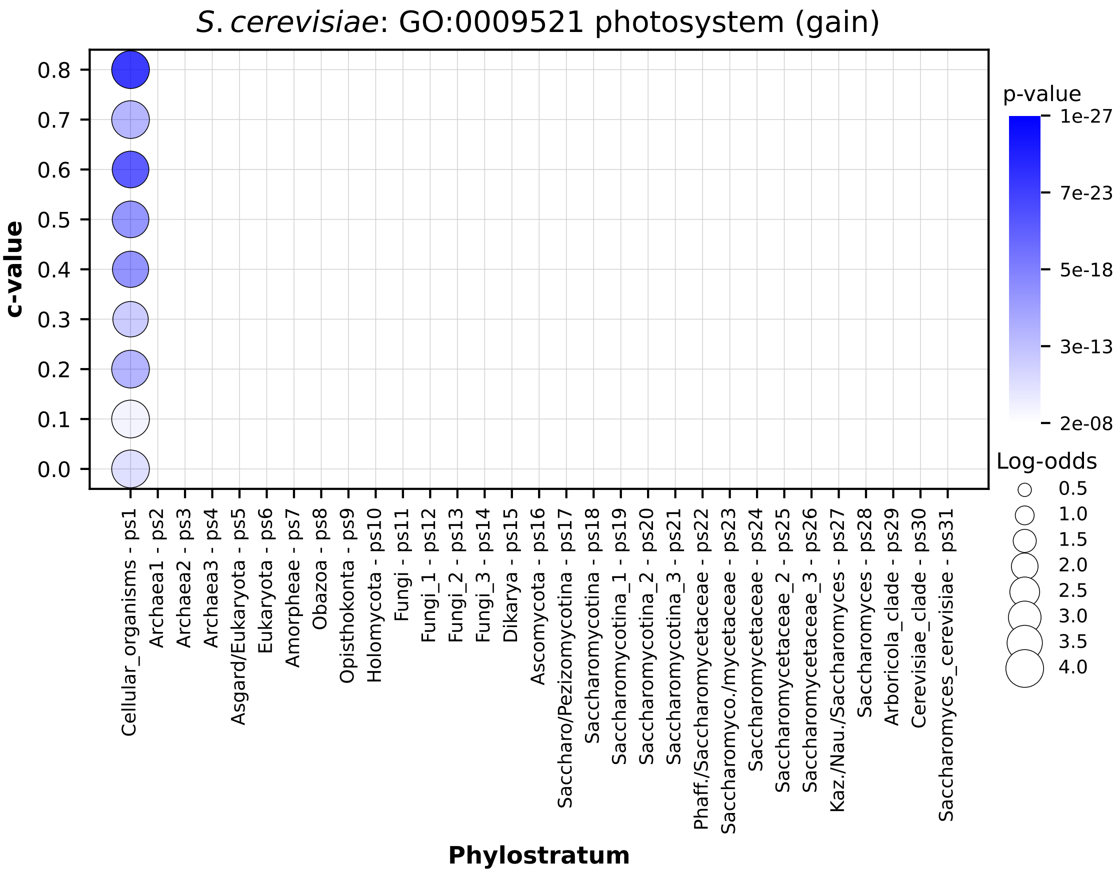

S. cerevisiae: GO:0009521 photosystem (gain)
c-value
0.8
0.7
0.6
0.5
0.4
0.3
0.2
0.1
0.0
Phylostratum
Cellular_organisms - ps1
Archaea1 - ps2
Archaea2 - ps3
Archaea3 - ps4
Asgard/Eukaryota - ps5
Eukaryota - ps6
Amorpheae - ps7
Obazoa - ps8
Opisthokonta - ps9
Holomycota - ps10
Fungi - ps11
Fungi_1 - ps12
Fungi_2 - ps13
Fungi_3 - ps14
Dikarya - ps15
Ascomycota - ps16
Saccharo/Pezizomycotina - ps17
Saccharomycotina - ps18
Saccharomycotina_1 - ps19
Saccharomycotina_2 - ps20
Saccharomycotina_3 - ps21
Phaff./Saccharomycetaceae - ps22
Saccharomyco./mycetaceae - ps23
Saccharomycetaceae - ps24
Saccharomycetaceae_2 - ps25
Saccharomycetaceae_3 - ps26
Kaz./Nau./Saccharomyces - ps27
Saccharomyces - ps28
Arboricola_clade - ps29
Cerevisiae_clade - ps30
Saccharomyces_cerevisiae - ps31
p-value
1e-27
7e-23
5e-18
3e-13
2e-08
Log-odds
0.5
1.0
1.5
2.0
2.5
3.0
3.5
4.0

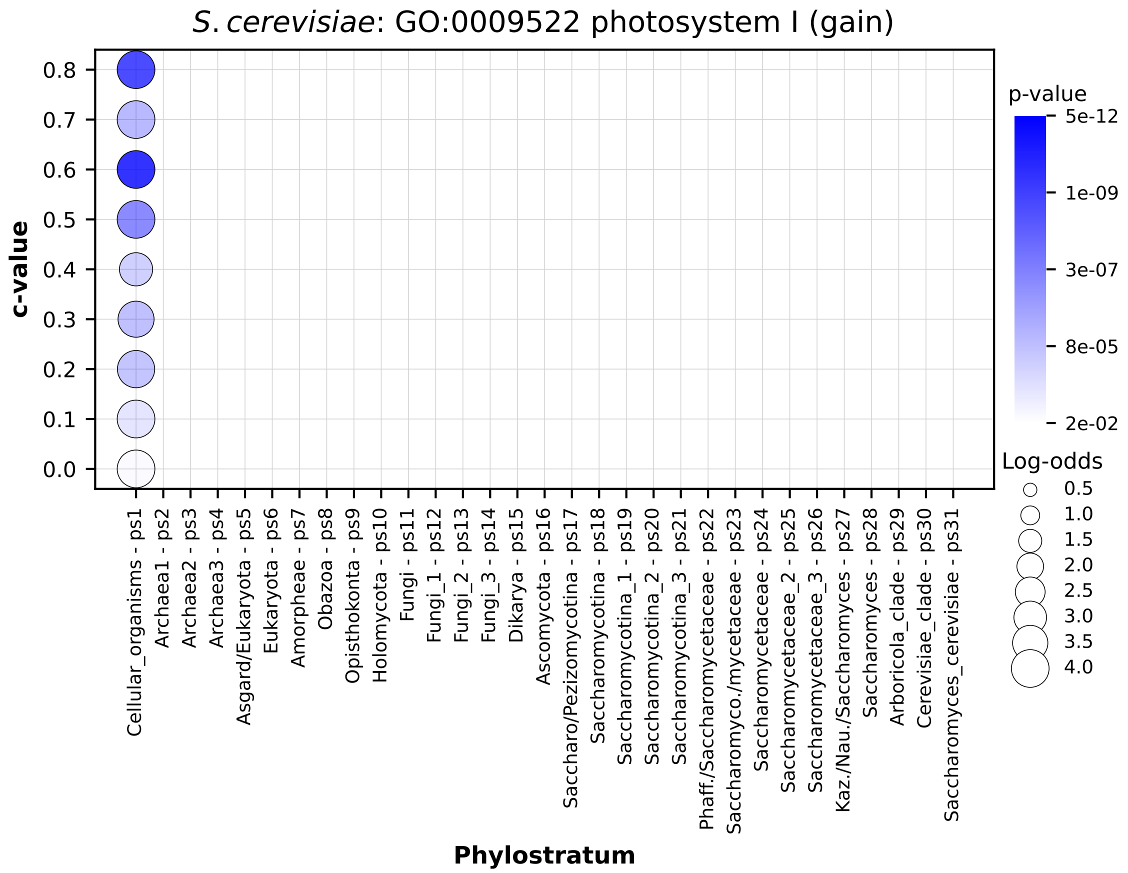
S. cerevisiae: GO:0009522 photosystem I (gain)
c-value
0.8
0.7
0.6
0.5
0.4
0.3
0.2
0.1
0.0
p-value
5e-12
1e-09
3e-07
8e-05
2e-02
Log-odds
0.5
1.0
1.5
2.0
2.5
3.0
3.5
4.0
Cellular_organisms - ps1
Archaea1 - ps2
Archaea2 - ps3
Archaea3 - ps4
Asgard/Eukaryota - ps5
Eukaryota - ps6
Amorpheae - ps7
Obazoa - ps8
Opisthokonta - ps9
Holomycota - ps10
Fungi - ps11
Fungi_1 - ps12
Fungi_2 - ps13
Fungi_3 - ps14
Dikarya - ps15
Ascomycota - ps16
Saccharo/Pezizomycotina - ps17
Saccharomycotina - ps18
Saccharomycotina_1 - ps19
Saccharomycotina_2 - ps20
Saccharomycotina_3 - ps21
Phaff./Saccharomycetaceae - ps22
Saccharomyco./mycetaceae - ps23
Saccharomycetaceae - ps24
Saccharomycetaceae_2 - ps25
Saccharomycetaceae_3 - ps26
Kaz./Nau./Saccharomyces - ps27
Saccharomyces - ps28
Arboricola_clade - ps29
Cerevisiae_clade - ps30
Saccharomyces_cerevisiae - ps31
Phylostratum

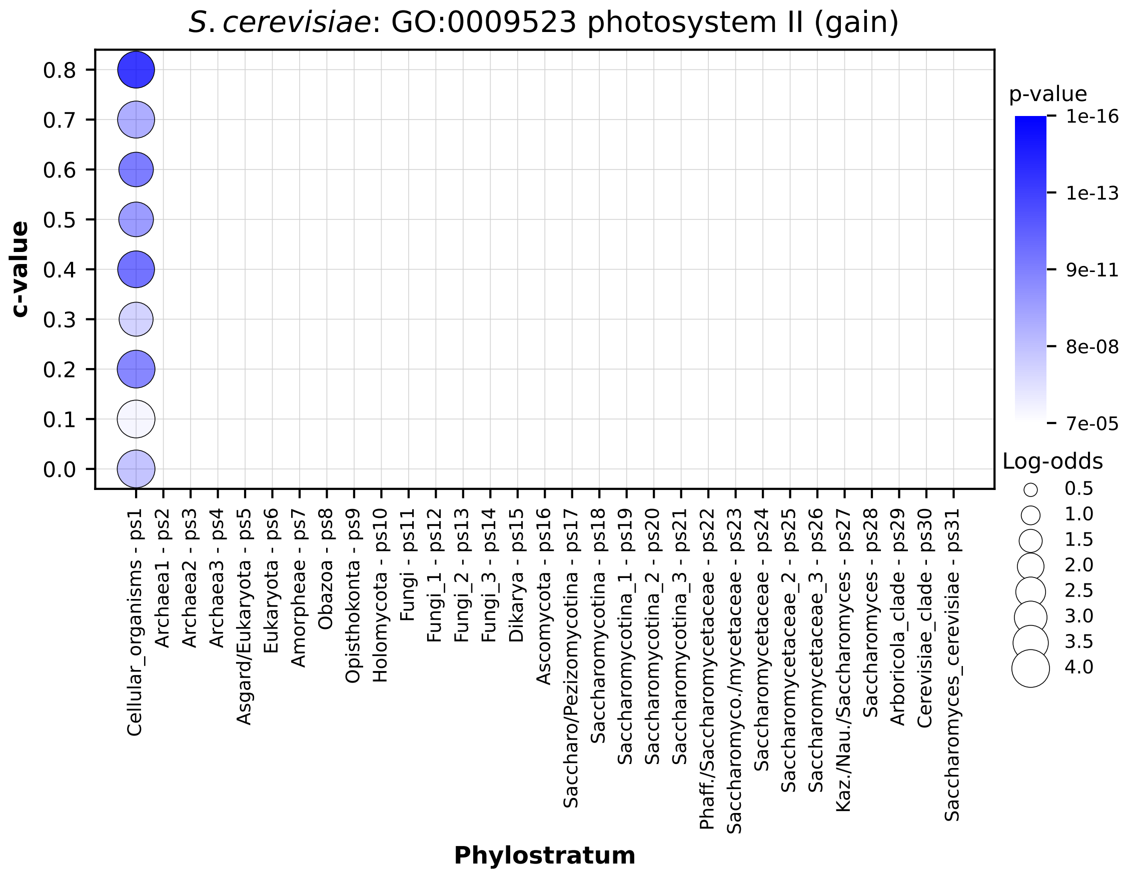
S. cerevisiae: GO:0009523 photosystem II (gain)
c-value
0.0
0.1
0.2
0.3
0.4
0.5
0.6
0.7
0.8
Phylostratum
Cellular_organisms - ps1
Archaea1 - ps2
Archaea2 - ps3
Archaea3 - ps4
Asgard/Eukaryota - ps5
Eukaryota - ps6
Amorpheae - ps7
Obazoa - ps8
Opisthokonta - ps9
Holomycota - ps10
Fungi - ps11
Fungi_1 - ps12
Fungi_2 - ps13
Fungi_3 - ps14
Dikarya - ps15
Ascomycota - ps16
Saccharo/Pezizomycotina - ps17
Saccharomycotina - ps18
Saccharomycotina_1 - ps19
Saccharomycotina_2 - ps20
Saccharomycotina_3 - ps21
Phaff./Saccharomycetaceae - ps22
Saccharomyco./mycetaceae - ps23
Saccharomycetaceae - ps24
Saccharomycetaceae_2 - ps25
Saccharomycetaceae_3 - ps26
Kaz./Nau./Saccharomyces - ps27
Saccharomyces - ps28
Arboricola_clade - ps29
Cerevisiae_clade - ps30
Saccharomyces_cerevisiae - ps31
p-value
1e-16
1e-13
9e-11
8e-08
7e-05
Log-odds
0.5
1.0
1.5
2.0
2.5
3.0
3.5
4.0

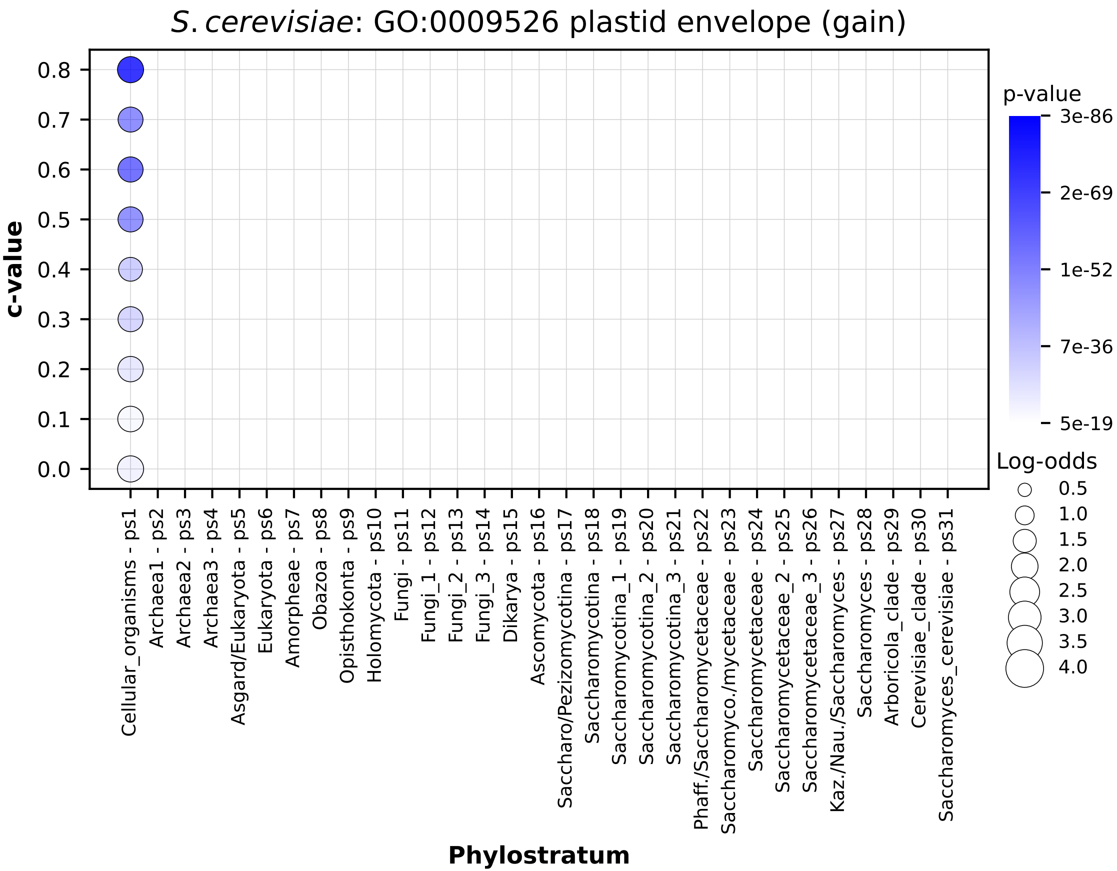

S. cerevisiae: GO:0009526 plastid envelope (gain)
c-value
0.0
0.1
0.2
0.3
0.4
0.5
0.6
0.7
0.8
Phylostratum
Cellular_organisms - ps1
Archaea1 - ps2
Archaea2 - ps3
Archaea3 - ps4
Asgard/Eukaryota - ps5
Eukaryota - ps6
Amorpheae - ps7
Obazoa - ps8
Opisthokonta - ps9
Holomycota - ps10
Fungi - ps11
Fungi_1 - ps12
Fungi_2 - ps13
Fungi_3 - ps14
Dikarya - ps15
Ascomycota - ps16
Saccharo/Pezizomycotina - ps17
Saccharomycotina - ps18
Saccharomycotina_1 - ps19
Saccharomycotina_2 - ps20
Saccharomycotina_3 - ps21
Phaff./Saccharomycetaceae - ps22
Saccharomyco./mycetaceae - ps23
Saccharomycetaceae - ps24
Saccharomycetaceae_2 - ps25
Saccharomycetaceae_3 - ps26
Kaz./Nau./Saccharomyces - ps27
Saccharomyces - ps28
Arboricola_clade - ps29
Cerevisiae_clade - ps30
Saccharomyces_cerevisiae - ps31
p-value
3e-86
2e-69
1e-52
7e-36
5e-19
Log-odds
0.5
1.0
1.5
2.0
2.5
3.0
3.5
4.0

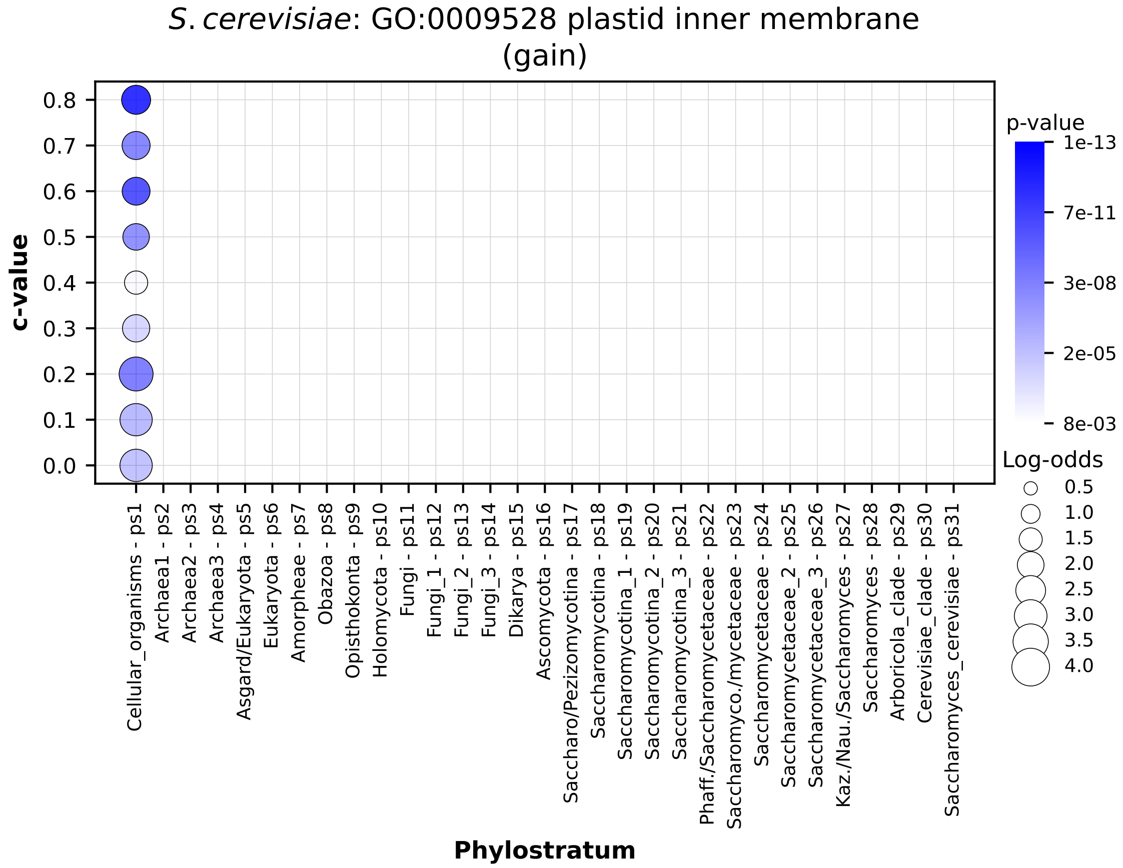
S. cerevisiae: GO:0009528 plastid inner membrane
(gain)
c-value
0.8
0.7
0.6
0.5
0.4
0.3
0.2
0.1
0.0
p-value
1e-13
7e-11
3e-08
2e-05
8e-03
Log-odds
0.5
1.0
1.5
2.0
2.5
3.0
3.5
4.0
Cellular_organisms - ps1
Archaea1 - ps2
Archaea2 - ps3
Archaea3 - ps4
Asgard/Eukaryota - ps5
Eukaryota - ps6
Amorpheae - ps7
Obazoa - ps8
Opisthokonta - ps9
Holomycota - ps10
Fungi - ps11
Fungi_1 - ps12
Fungi_2 - ps13
Fungi_3 - ps14
Dikarya - ps15
Ascomycota - ps16
Saccharo/Pezizomycotina - ps17
Saccharomycotina - ps18
Saccharomycotina_1 - ps19
Saccharomycotina_2 - ps20
Saccharomycotina_3 - ps21
Phaff./Saccharomycetaceae - ps22
Saccharomyco./mycetaceae - ps23
Saccharomycetaceae - ps24
Saccharomycetaceae_2 - ps25
Saccharomycetaceae_3 - ps26
Kaz./Nau./Saccharomyces - ps27
Saccharomyces - ps28
Arboricola_clade - ps29
Cerevisiae_clade - ps30
Saccharomyces_cerevisiae - ps31
Phylostratum

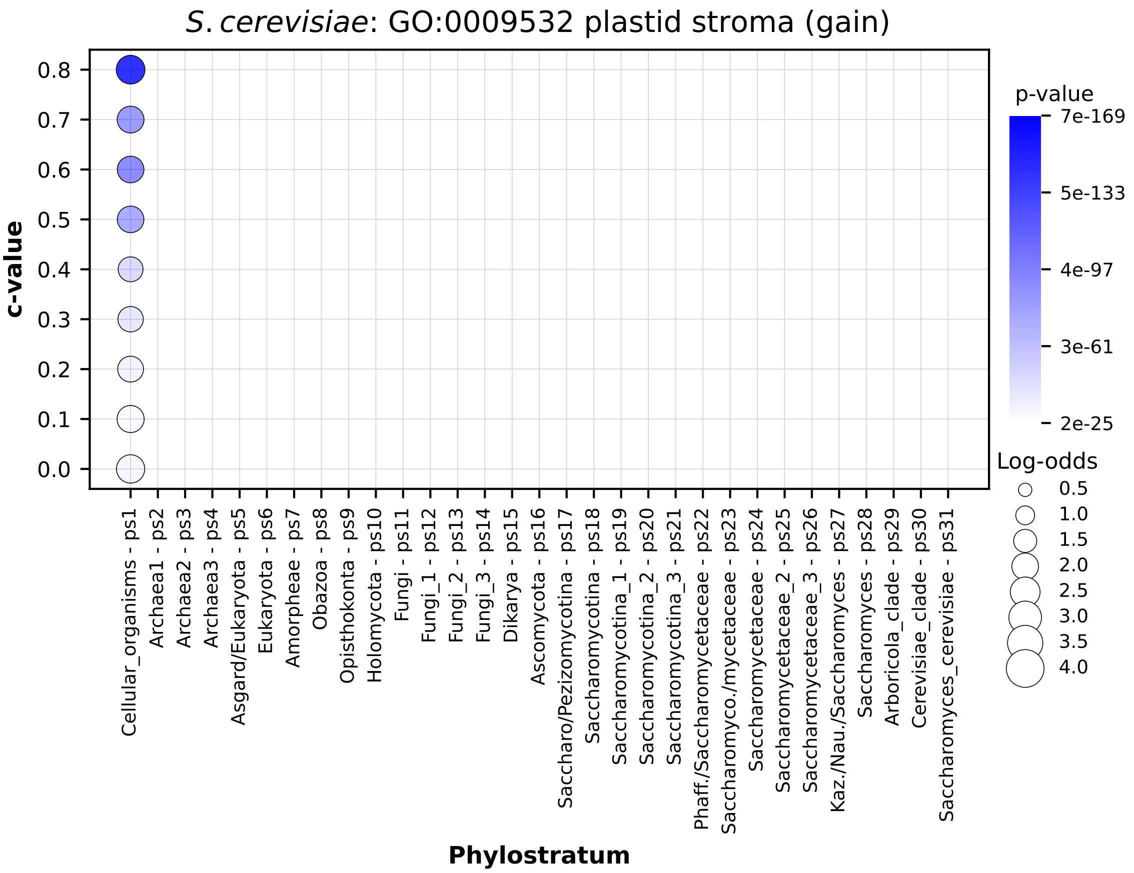
S. cerevisiae: GO:0009532 plastid stroma (gain)
c-value
0.8
0.7
0.6
0.5
0.4
0.3
0.2
0.1
0.0
Phylostratum
Cellular_organisms - ps1
Archaea1 - ps2
Archaea2 - ps3
Archaea3 - ps4
Asgard/Eukaryota - ps5
Eukaryota - ps6
Amorpheae - ps7
Obazoa - ps8
Opisthokonta - ps9
Holomycota - ps10
Fungi - ps11
Fungi_1 - ps12
Fungi_2 - ps13
Fungi_3 - ps14
Dikarya - ps15
Ascomycota - ps16
Saccharo/Pezizomycotina - ps17
Saccharomycotina - ps18
Saccharomycotina_1 - ps19
Saccharomycotina_2 - ps20
Saccharomycotina_3 - ps21
Phaff./Saccharomycetaceae - ps22
Saccharomyco./mycetaceae - ps23
Saccharomycetaceae - ps24
Saccharomycetaceae_2 - ps25
Saccharomycetaceae_3 - ps26
Kaz./Nau./Saccharomyces - ps27
Saccharomyces - ps28
Arboricola_clade - ps29
Cerevisiae_clade - ps30
Saccharomyces_cerevisiae - ps31
p-value
7e-169
5e-133
4e-97
3e-61
2e-25
Log-odds
0.5
1.0
1.5
2.0
2.5
3.0
3.5
4.0

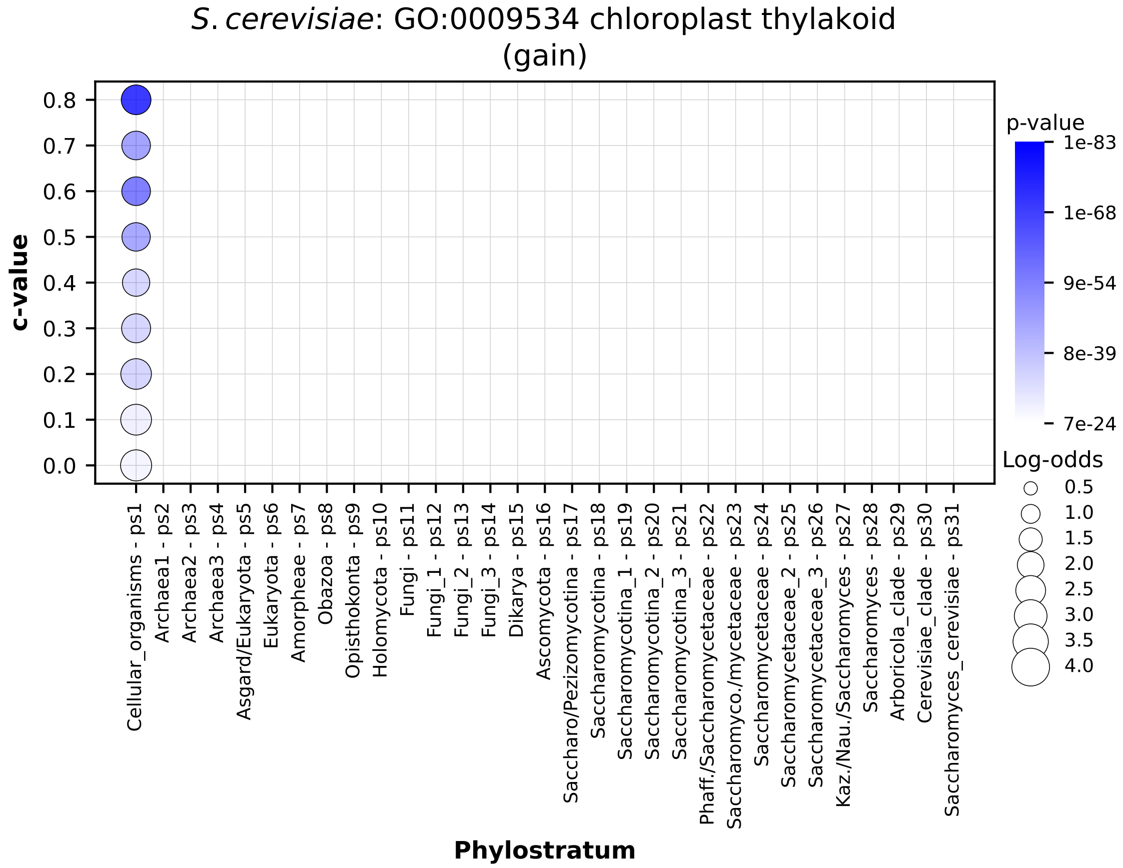

S. cerevisiae: GO:0009534 chloroplast thylakoid
(gain)
c-value
0.8
0.7
0.6
0.5
0.4
0.3
0.2
0.1
0.0
p-value
1e-83
1e-68
9e-54
8e-39
7e-24
Log-odds
0.5
1.0
1.5
2.0
2.5
3.0
3.5
4.0
Cellular_organisms - ps1
Archaea1 - ps2
Archaea2 - ps3
Archaea3 - ps4
Asgard/Eukaryota - ps5
Eukaryota - ps6
Amorpheae - ps7
Obazoa - ps8
Opisthokonta - ps9
Holomycota - ps10
Fungi - ps11
Fungi_1 - ps12
Fungi_2 - ps13
Fungi_3 - ps14
Dikarya - ps15
Ascomycota - ps16
Saccharo/Pezizomycotina - ps17
Saccharomycotina - ps18
Saccharomycotina_1 - ps19
Saccharomycotina_2 - ps20
Saccharomycotina_3 - ps21
Phaff./Saccharomycetaceae - ps22
Saccharomyco./mycetaceae - ps23
Saccharomycetaceae - ps24
Saccharomycetaceae_2 - ps25
Saccharomycetaceae_3 - ps26
Kaz./Nau./Saccharomyces - ps27
Saccharomyces - ps28
Arboricola_clade - ps29
Cerevisiae_clade - ps30
Saccharomyces_cerevisiae - ps31
Phylostratum

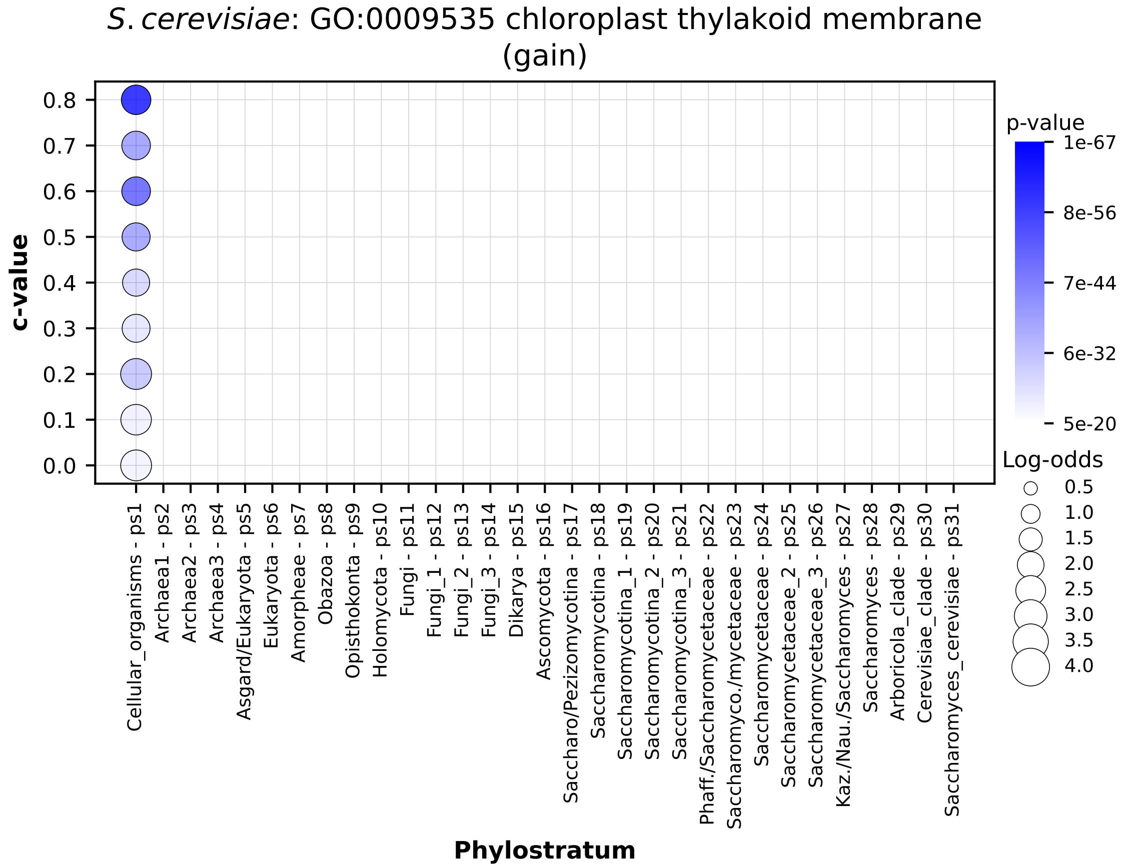
S. cerevisiae: GO:0009535 chloroplast thylakoid membrane (gain)
c-value
0.0
0.1
0.2
0.3
0.4
0.5
0.6
0.7
0.8
p-value
1e-67
8e-56
7e-44
6e-32
5e-20
Log-odds
0.5
1.0
1.5
2.0
2.5
3.0
3.5
4.0
Cellular_organisms - ps1
Archaea1 - ps2
Archaea2 - ps3
Archaea3 - ps4
Asgard/Eukaryota - ps5
Eukaryota - ps6
Amorpheae - ps7
Obazoa - ps8
Opisthokonta - ps9
Holomycota - ps10
Fungi - ps11
Fungi_1 - ps12
Fungi_2 - ps13
Fungi_3 - ps14
Dikarya - ps15
Ascomycota - ps16
Saccharo/Pezizomycotina - ps17
Saccharomycotina - ps18
Saccharomycotina_1 - ps19
Saccharomycotina_2 - ps20
Saccharomycotina_3 - ps21
Phaff./Saccharomycetaceae - ps22
Saccharomyco./mycetaceae - ps23
Saccharomycetaceae - ps24
Saccharomycetaceae_2 - ps25
Saccharomycetaceae_3 - ps26
Kaz./Nau./Saccharomyces - ps27
Saccharomyces - ps28
Arboricola_clade - ps29
Cerevisiae_clade - ps30
Saccharomyces_cerevisiae - ps31
Phylostratum

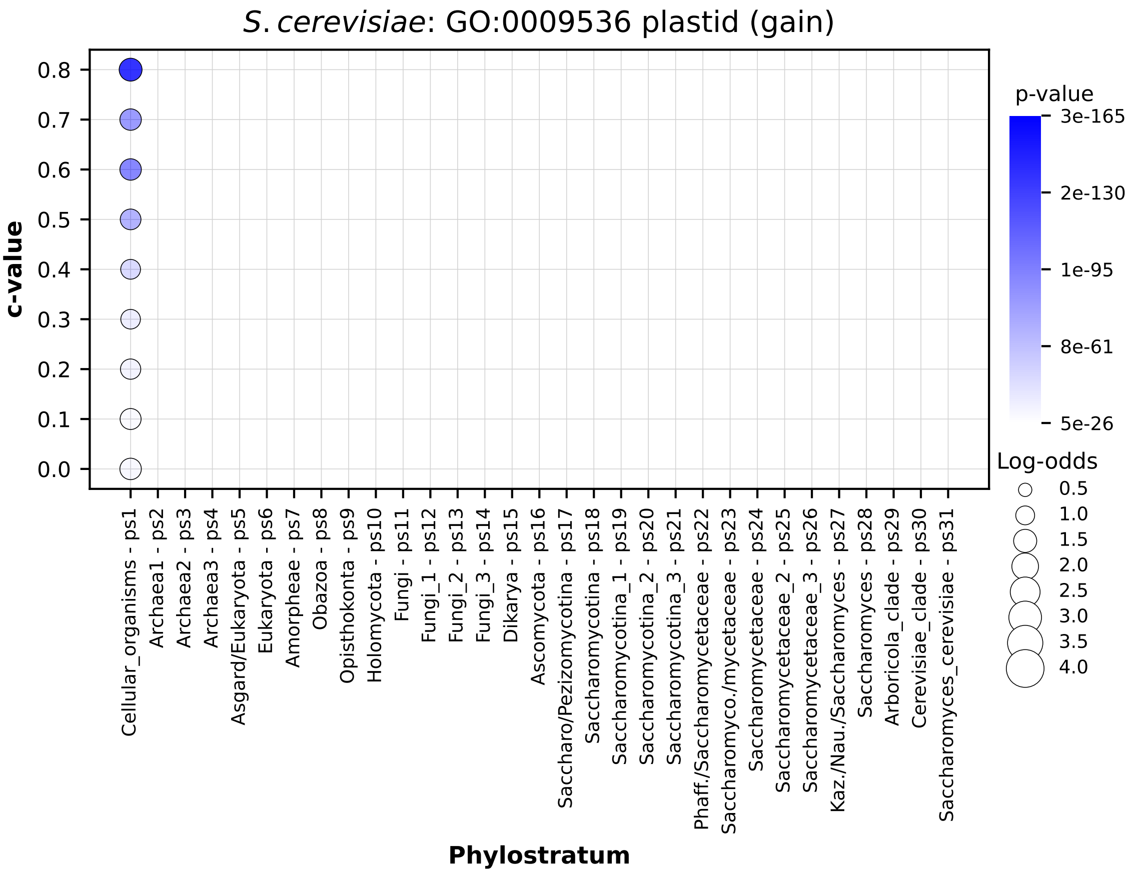

S. cerevisiae: GO:0009536 plastid (gain)
c-value
0.8
0.7
0.6
0.5
0.4
0.3
0.2
0.1
0.0
Phylostratum
Cellular_organisms - ps1
Archaea1 - ps2
Archaea2 - ps3
Archaea3 - ps4
Asgard/Eukaryota - ps5
Eukaryota - ps6
Amorpheae - ps7
Obazoa - ps8
Opisthokonta - ps9
Holomycota - ps10
Fungi - ps11
Fungi_1 - ps12
Fungi_2 - ps13
Fungi_3 - ps14
Dikarya - ps15
Ascomycota - ps16
Saccharo/Pezizomycotina - ps17
Saccharomycotina - ps18
Saccharomycotina_1 - ps19
Saccharomycotina_2 - ps20
Saccharomycotina_3 - ps21
Phaff./Saccharomycetaceae - ps22
Saccharomyco./mycetaceae - ps23
Saccharomycetaceae - ps24
Saccharomycetaceae_2 - ps25
Saccharomycetaceae_3 - ps26
Kaz./Nau./Saccharomyces - ps27
Saccharomyces - ps28
Arboricola_clade - ps29
Cerevisiae_clade - ps30
Saccharomyces_cerevisiae - ps31
p-value
3e-165
2e-130
1e-95
8e-61
5e-26
Log-odds
0.5
1.0
1.5
2.0
2.5
3.0
3.5
4.0

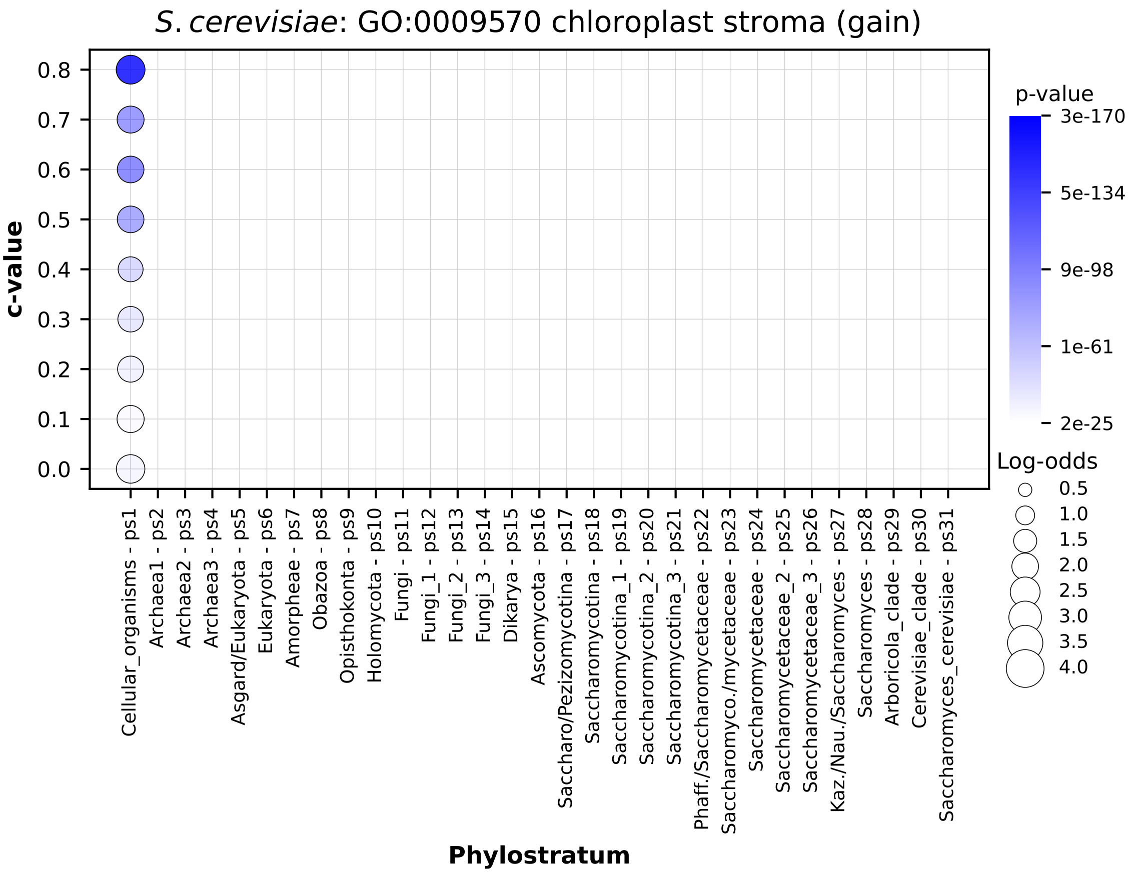

S. cerevisiae: GO:0009570 chloroplast stroma (gain)
c-value
0.8
0.7
0.6
0.5
0.4
0.3
0.2
0.1
0.0
p-value
3e-170
5e-134
9e-98
1e-61
2e-25
Log-odds
0.5
1.0
1.5
2.0
2.5
3.0
3.5
4.0
Cellular_organisms - ps1
Archaea1 - ps2
Archaea2 - ps3
Archaea3 - ps4
Asgard/Eukaryota - ps5
Eukaryota - ps6
Amorpheae - ps7
Obazoa - ps8
Opisthokonta - ps9
Holomycota - ps10
Fungi - ps11
Fungi_1 - ps12
Fungi_2 - ps13
Fungi_3 - ps14
Dikarya - ps15
Ascomycota - ps16
Saccharo/Pezizomycotina - ps17
Saccharomycotina - ps18
Saccharomycotina_1 - ps19
Saccharomycotina_2 - ps20
Saccharomycotina_3 - ps21
Phaff./Saccharomycetaceae - ps22
Saccharomyco./mycetaceae - ps23
Saccharomycetaceae - ps24
Saccharomycetaceae_2 - ps25
Saccharomycetaceae_3 - ps26
Kaz./Nau./Saccharomyces - ps27
Saccharomyces - ps28
Arboricola_clade - ps29
Cerevisiae_clade - ps30
Saccharomyces_cerevisiae - ps31
Phylostratum

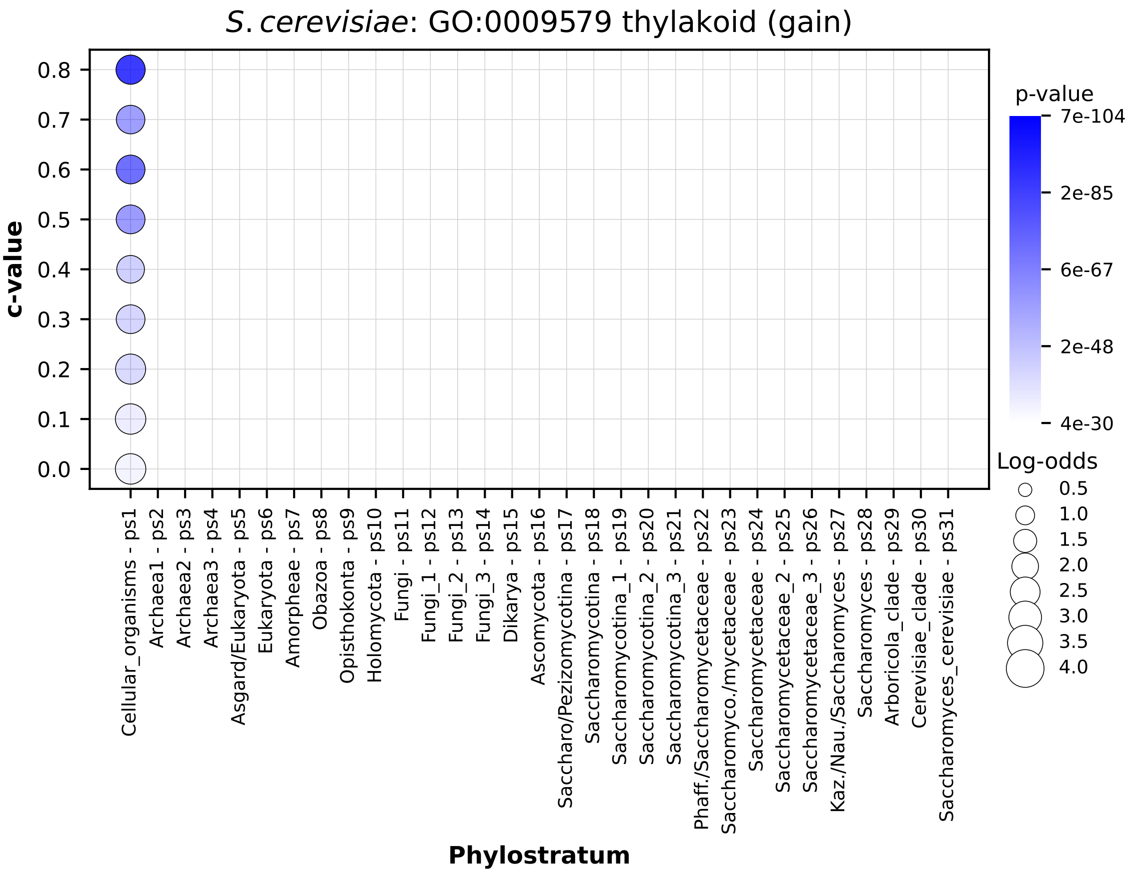

S. cerevisiae: GO:0009579 thylakoid (gain)
c-value
0.8
0.7
0.6
0.5
0.4
0.3
0.2
0.1
0.0
Phylostratum
Cellular_organisms - ps1
Archaea1 - ps2
Archaea2 - ps3
Archaea3 - ps4
Asgard/Eukaryota - ps5
Eukaryota - ps6
Amorpheae - ps7
Obazoa - ps8
Opisthokonta - ps9
Holomycota - ps10
Fungi - ps11
Fungi_1 - ps12
Fungi_2 - ps13
Fungi_3 - ps14
Dikarya - ps15
Ascomycota - ps16
Saccharo/Pezizomycotina - ps17
Saccharomycotina - ps18
Saccharomycotina_1 - ps19
Saccharomycotina_2 - ps20
Saccharomycotina_3 - ps21
Phaff./Saccharomycetaceae - ps22
Saccharomyco./mycetaceae - ps23
Saccharomycetaceae - ps24
Saccharomycetaceae_2 - ps25
Saccharomycetaceae_3 - ps26
Kaz./Nau./Saccharomyces - ps27
Saccharomyces - ps28
Arboricola_clade - ps29
Cerevisiae_clade - ps30
Saccharomyces_cerevisiae - ps31
p-value
7e-104
2e-85
6e-67
2e-48
4e-30
Log-odds
0.5
1.0
1.5
2.0
2.5
3.0
3.5
4.0

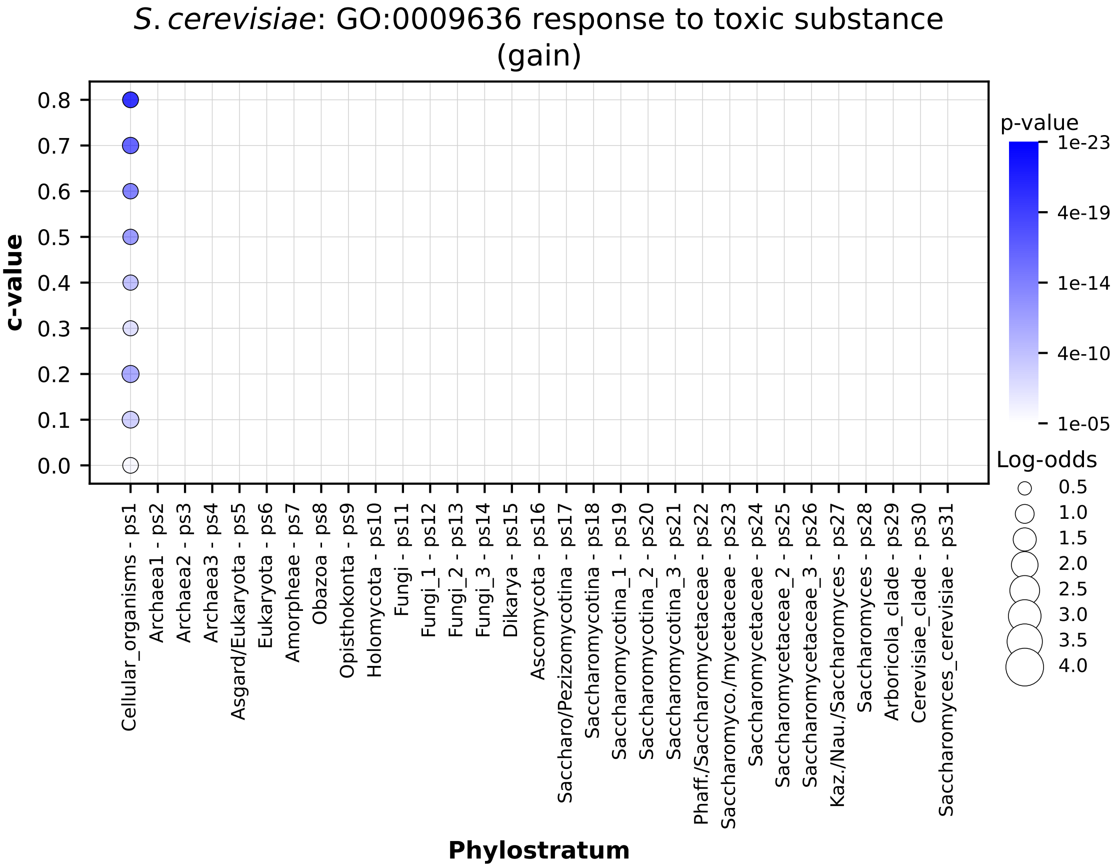

S. cerevisiae: GO:0009636 response to toxic substance
(gain)
c-value
0.8
0.7
0.6
0.5
0.4
0.3
0.2
0.1
0.0
p-value
1e-23
4e-19
1e-14
4e-10
1e-05
Log-odds
0.5
1.0
1.5
2.0
2.5
3.0
3.5
4.0
Cellular_organisms - ps1
Archaea1 - ps2
Archaea2 - ps3
Archaea3 - ps4
Asgard/Eukaryota - ps5
Eukaryota - ps6
Amorpheae - ps7
Obazoa - ps8
Opisthokonta - ps9
Holomycota - ps10
Fungi - ps11
Fungi_1 - ps12
Fungi_2 - ps13
Fungi_3 - ps14
Dikarya - ps15
Ascomycota - ps16
Saccharo/Pezizomycotina - ps17
Saccharomycotina - ps18
Saccharomycotina_1 - ps19
Saccharomycotina_2 - ps20
Saccharomycotina_3 - ps21
Phaff./Saccharomycetaceae - ps22
Saccharomyco./mycetaceae - ps23
Saccharomycetaceae - ps24
Saccharomycetaceae_2 - ps25
Saccharomycetaceae_3 - ps26
Kaz./Nau./Saccharomyces - ps27
Saccharomyces - ps28
Arboricola_clade - ps29
Cerevisiae_clade - ps30
Saccharomyces_cerevisiae - ps31
Phylostratum

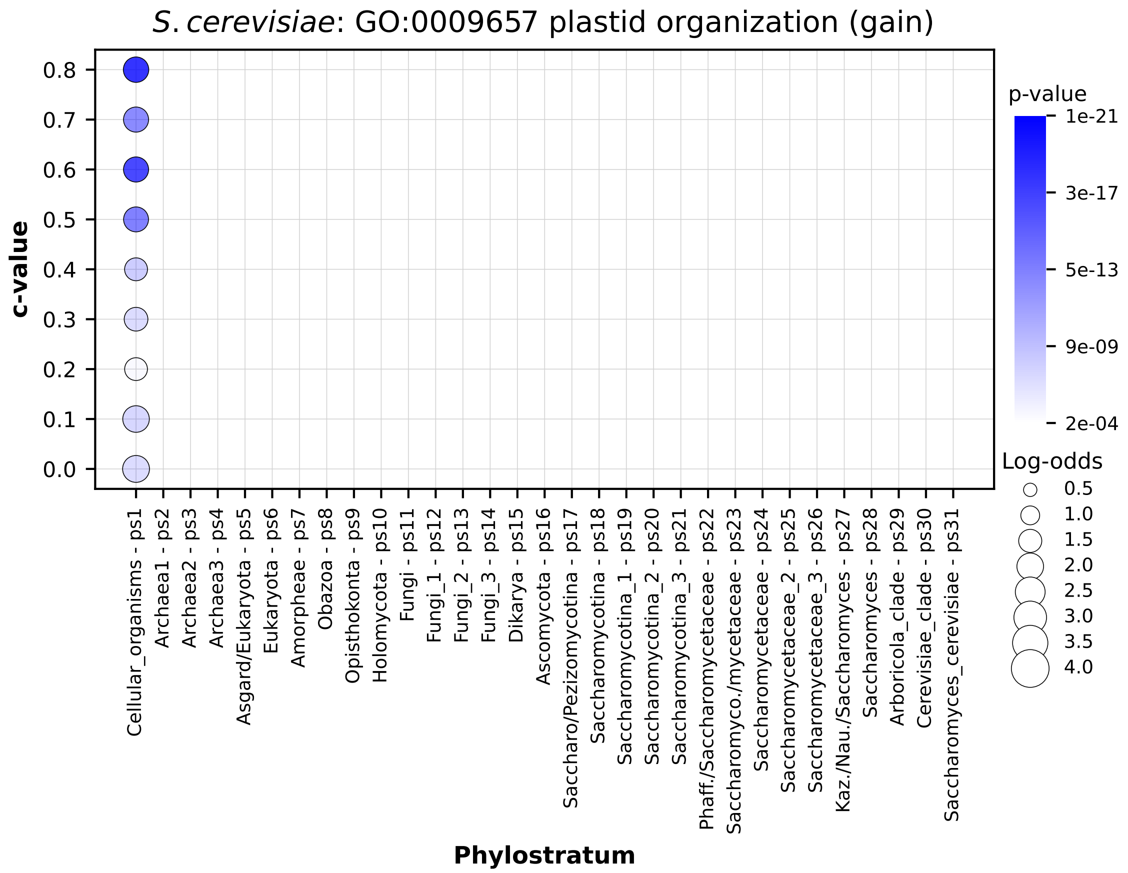

S. cerevisiae: GO:0009657 plastid organization (gain)
c-value
0.0
0.1
0.2
0.3
0.4
0.5
0.6
0.7
0.8
p-value
1e-21
3e-17
5e-13
9e-09
2e-04
Log-odds
0.5
1.0
1.5
2.0
2.5
3.0
3.5
4.0
Cellular_organisms - ps1
Archaea1 - ps2
Archaea2 - ps3
Archaea3 - ps4
Asgard/Eukaryota - ps5
Eukaryota - ps6
Amorpheae - ps7
Obazoa - ps8
Opisthokonta - ps9
Holomycota - ps10
Fungi - ps11
Fungi_1 - ps12
Fungi_2 - ps13
Fungi_3 - ps14
Dikarya - ps15
Ascomycota - ps16
Saccharo/Pezizomycotina - ps17
Saccharomycotina - ps18
Saccharomycotina_1 - ps19
Saccharomycotina_2 - ps20
Saccharomycotina_3 - ps21
Phaff./Saccharomycetaceae - ps22
Saccharomyco./mycetaceae - ps23
Saccharomycetaceae - ps24
Saccharomycetaceae_2 - ps25
Saccharomycetaceae_3 - ps26
Kaz./Nau./Saccharomyces - ps27
Saccharomyces - ps28
Arboricola_clade - ps29
Cerevisiae_clade - ps30
Saccharomyces_cerevisiae - ps31
Phylostratum

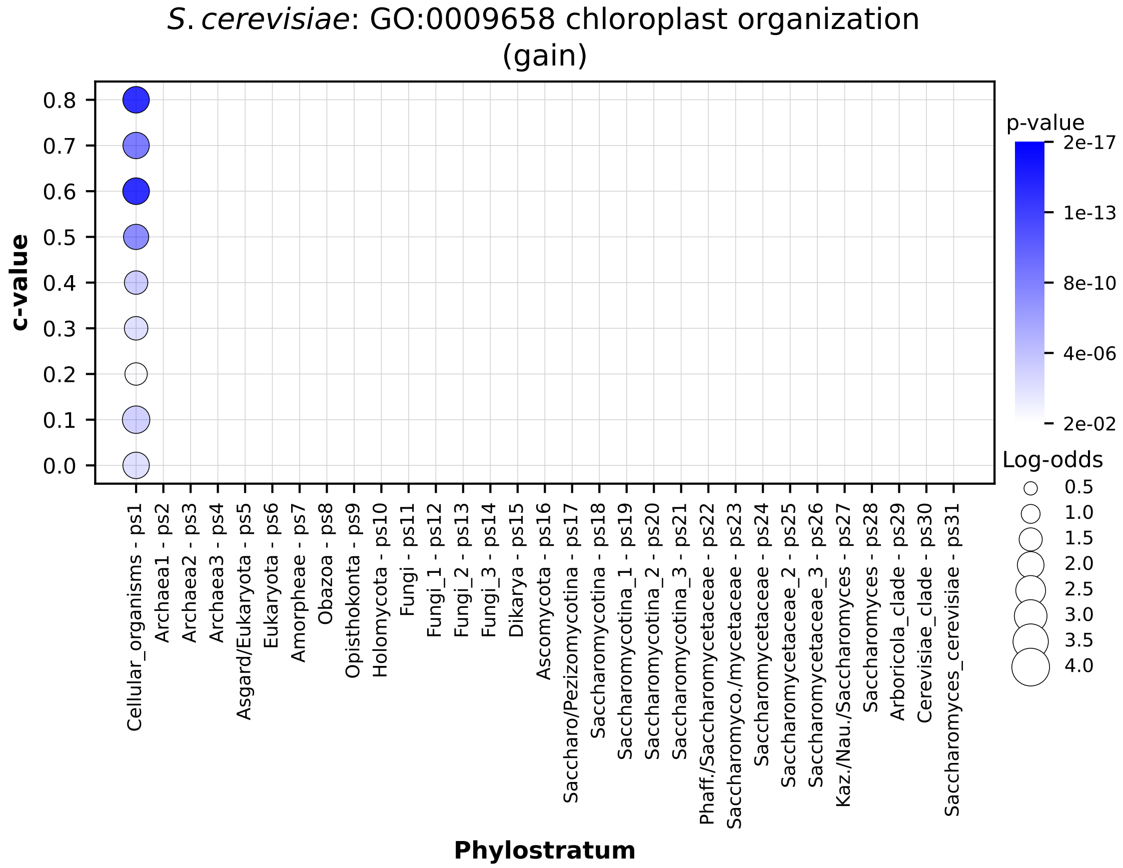

S. cerevisiae: GO:0009658 chloroplast organization
(gain)
c-value
0.0
0.1
0.2
0.3
0.4
0.5
0.6
0.7
0.8
p-value
2e-17
1e-13
8e-10
4e-06
2e-02
Log-odds
0.5
1.0
1.5
2.0
2.5
3.0
3.5
4.0
Cellular_organisms - ps1
Archaea1 - ps2
Archaea2 - ps3
Archaea3 - ps4
Asgard/Eukaryota - ps5
Eukaryota - ps6
Amorpheae - ps7
Obazoa - ps8
Opisthokonta - ps9
Holomycota - ps10
Fungi - ps11
Fungi_1 - ps12
Fungi_2 - ps13
Fungi_3 - ps14
Dikarya - ps15
Ascomycota - ps16
Saccharo/Pezizomycotina - ps17
Saccharomycotina - ps18
Saccharomycotina_1 - ps19
Saccharomycotina_2 - ps20
Saccharomycotina_3 - ps21
Phaff./Saccharomycetaceae - ps22
Saccharomyco./mycetaceae - ps23
Saccharomycetaceae - ps24
Saccharomycetaceae_2 - ps25
Saccharomycetaceae_3 - ps26
Kaz./Nau./Saccharomyces - ps27
Saccharomyces - ps28
Arboricola_clade - ps29
Cerevisiae_clade - ps30
Saccharomyces_cerevisiae - ps31
Phylostratum

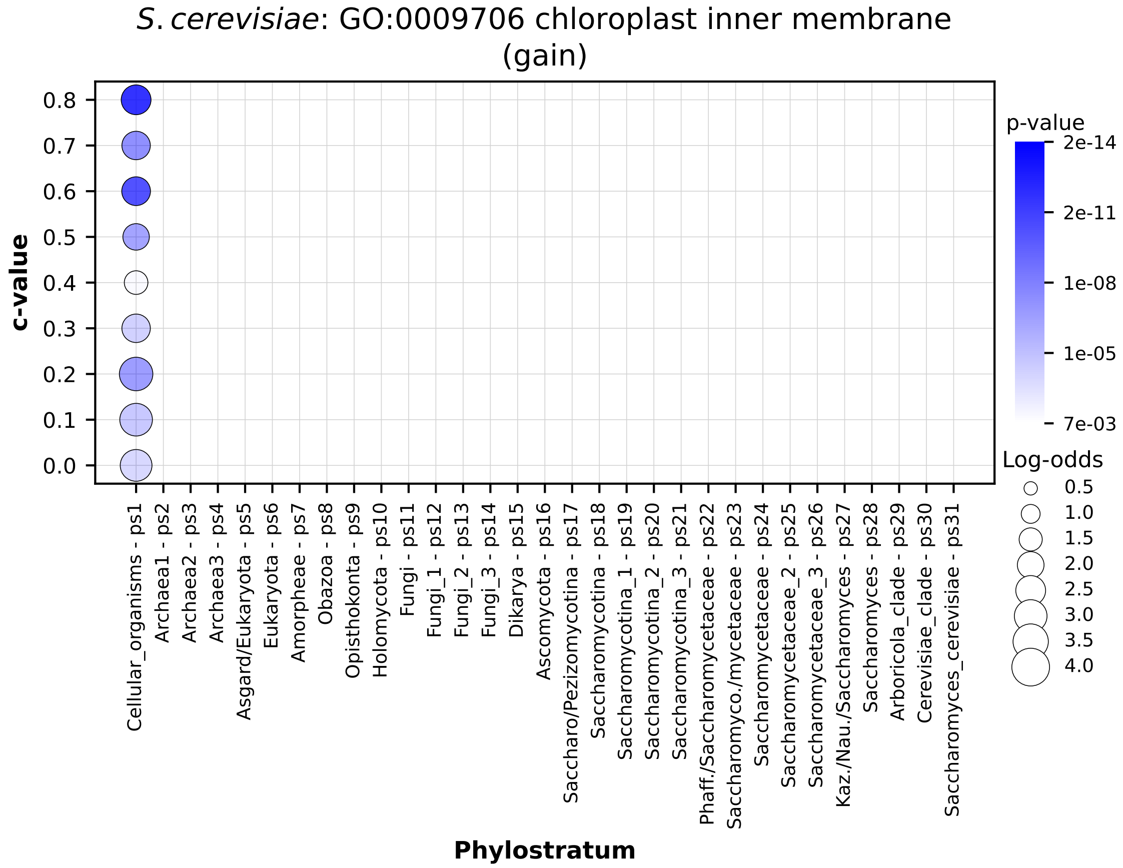
S. cerevisiae: GO:0009706 chloroplast inner membrane (gain)
c-value
0.8
0.7
0.6
0.5
0.4
0.3
0.2
0.1
0.0
Phylostratum
Cellular_organisms - ps1
Archaea1 - ps2
Archaea2 - ps3
Archaea3 - ps4
Asgard/Eukaryota - ps5
Eukaryota - ps6
Amorpheae - ps7
Obazoa - ps8
Opisthokonta - ps9
Holomycota - ps10
Fungi - ps11
Fungi_1 - ps12
Fungi_2 - ps13
Fungi_3 - ps14
Dikarya - ps15
Ascomycota - ps16
Saccharo/Pezizomycotina - ps17
Saccharomycotina - ps18
Saccharomycotina_1 - ps19
Saccharomycotina_2 - ps20
Saccharomycotina_3 - ps21
Phaff./Saccharomycetaceae - ps22
Saccharomyco./mycetaceae - ps23
Saccharomycetaceae - ps24
Saccharomycetaceae_2 - ps25
Saccharomycetaceae_3 - ps26
Kaz./Nau./Saccharomyces - ps27
Saccharomyces - ps28
Arboricola_clade - ps29
Cerevisiae_clade - ps30
Saccharomyces_cerevisiae - ps31
p-value
2e-14
2e-11
1e-08
1e-05
7e-03
Log-odds
0.5
1.0
1.5
2.0
2.5
3.0
3.5
4.0

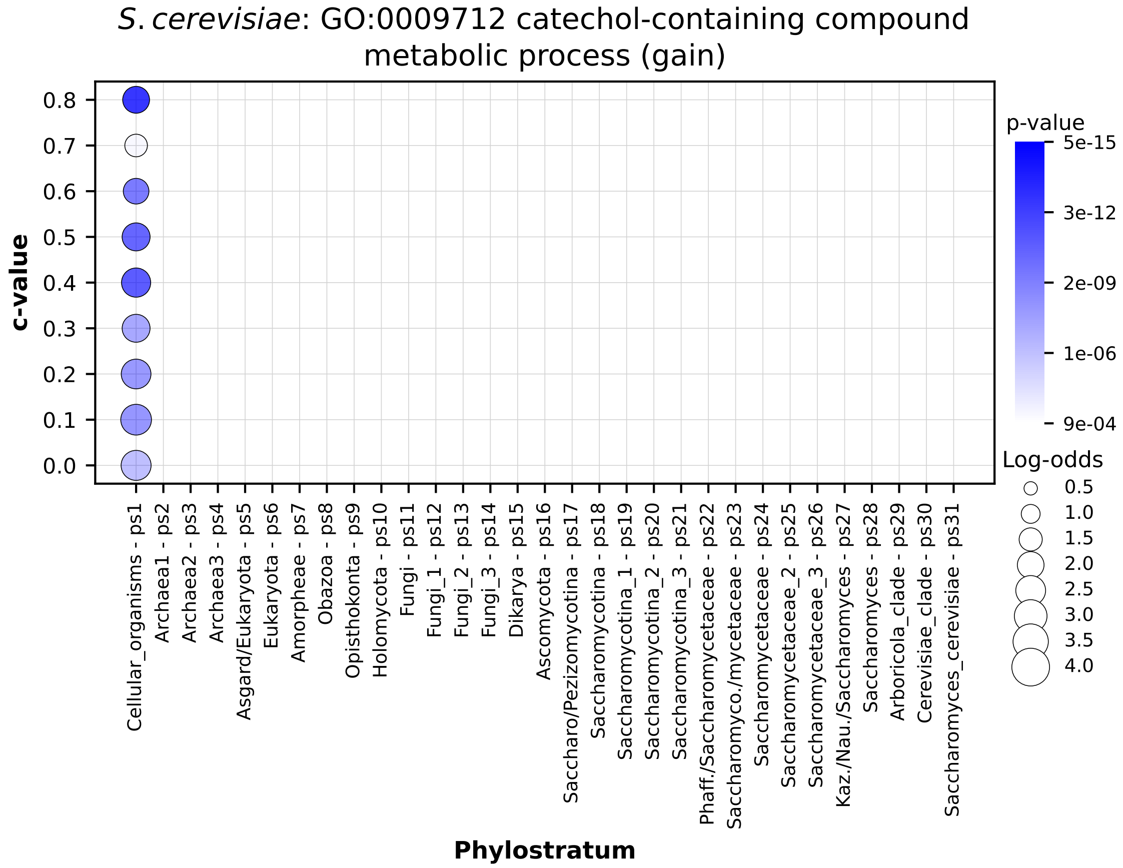
S. cerevisiae: GO:0009712 catechol-containing compound metabolic process (gain)
c-value
0.8
0.7
0.6
0.5
0.4
0.3
0.2
0.1
0.0
Phylostratum
Cellular_organisms - ps1
Archaea1 - ps2
Archaea2 - ps3
Archaea3 - ps4
Asgard/Eukaryota - ps5
Eukaryota - ps6
Amorpheae - ps7
Obazoa - ps8
Opisthokonta - ps9
Holomycota - ps10
Fungi - ps11
Fungi_1 - ps12
Fungi_2 - ps13
Fungi_3 - ps14
Dikarya - ps15
Ascomycota - ps16
Saccharo/Pezizomycotina - ps17
Saccharomycotina - ps18
Saccharomycotina_1 - ps19
Saccharomycotina_2 - ps20
Saccharomycotina_3 - ps21
Phaff./Saccharomycetaceae - ps22
Saccharomyco./mycetaceae - ps23
Saccharomycetaceae - ps24
Saccharomycetaceae_2 - ps25
Saccharomycetaceae_3 - ps26
Kaz./Nau./Saccharomyces - ps27
Saccharomyces - ps28
Arboricola_clade - ps29
Cerevisiae_clade - ps30
Saccharomyces_cerevisiae - ps31
p-value
5e-15
3e-12
2e-09
1e-06
9e-04
Log-odds
0.5
1.0
1.5
2.0
2.5
3.0
3.5
4.0

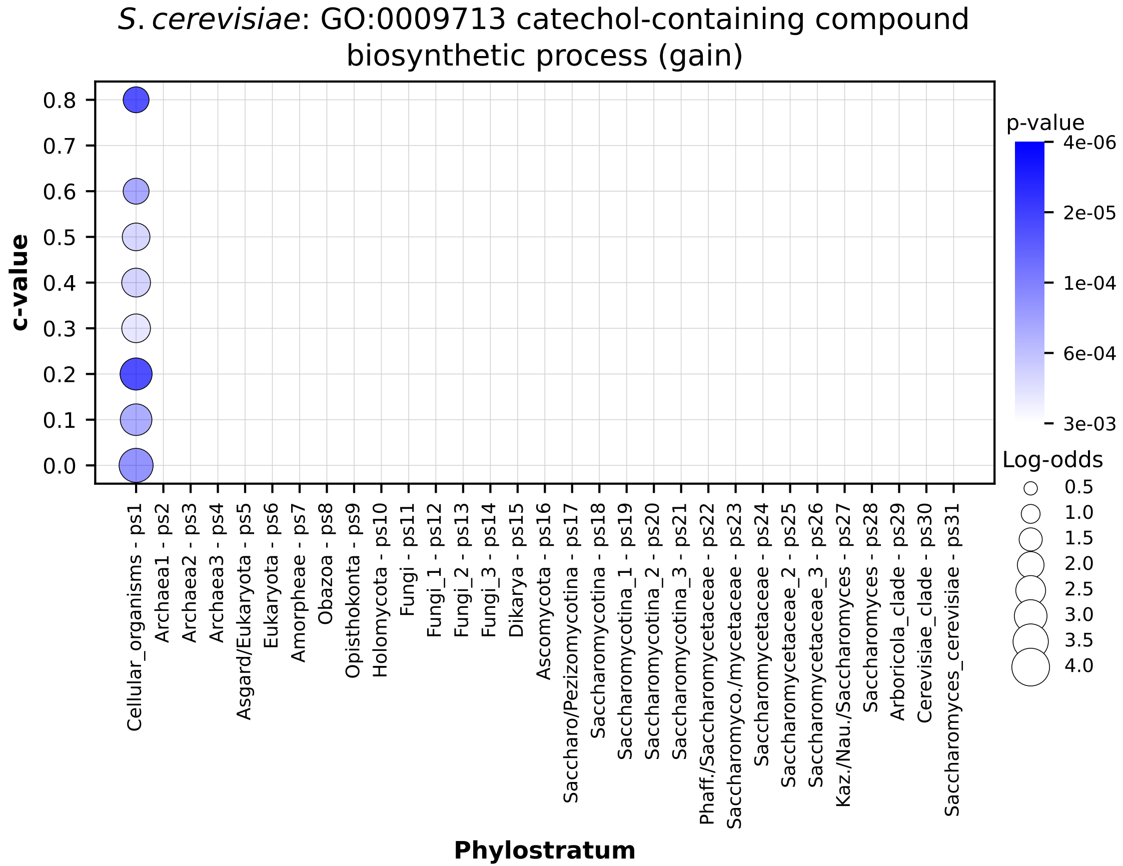

S. cerevisiae: GO:0009713 catechol-containing compound biosynthetic process (gain)
c-value
0.8
0.7
0.6
0.5
0.4
0.3
0.2
0.1
0.0
p-value
4e-06
2e-05
1e-04
6e-04
3e-03
Log-odds
0.5
1.0
1.5
2.0
2.5
3.0
3.5
4.0
Cellular_organisms - ps1
Archaea1 - ps2
Archaea2 - ps3
Archaea3 - ps4
Asgard/Eukaryota - ps5
Eukaryota - ps6
Amorpheae - ps7
Obazoa - ps8
Opisthokonta - ps9
Holomycota - ps10
Fungi - ps11
Fungi_1 - ps12
Fungi_2 - ps13
Fungi_3 - ps14
Dikarya - ps15
Ascomycota - ps16
Saccharo/Pezizomycotina - ps17
Saccharomycotina - ps18
Saccharomycotina_1 - ps19
Saccharomycotina_2 - ps20
Saccharomycotina_3 - ps21
Phaff./Saccharomycetaceae - ps22
Saccharomyco./mycetaceae - ps23
Saccharomycetaceae - ps24
Saccharomycetaceae_2 - ps25
Saccharomycetaceae_3 - ps26
Kaz./Nau./Saccharomyces - ps27
Saccharomyces - ps28
Arboricola_clade - ps29
Cerevisiae_clade - ps30
Saccharomyces_cerevisiae - ps31
Phylostratum

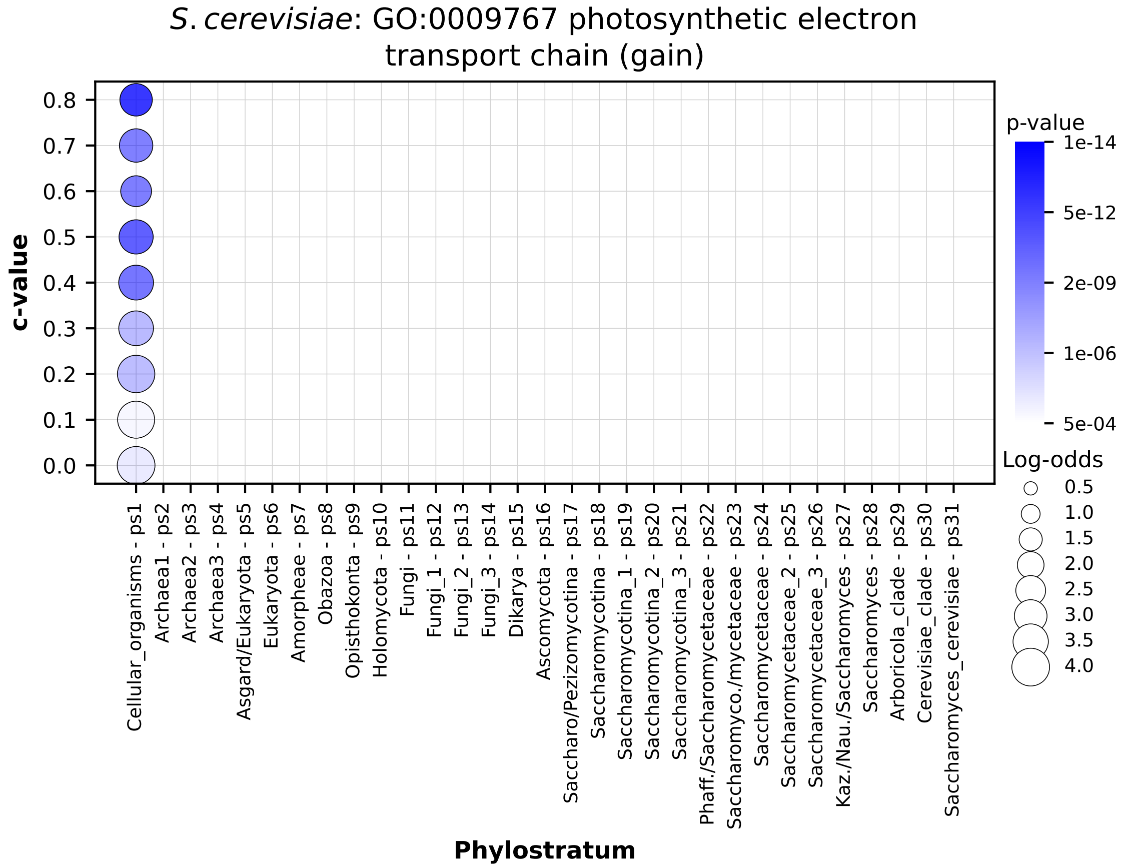

S. cerevisiae: GO:0009767 photosynthetic electron transport chain (gain)
c-value
0.0
0.1
0.2
0.3
0.4
0.5
0.6
0.7
0.8
Phylostratum
Cellular_organisms - ps1
Archaea1 - ps2
Archaea2 - ps3
Archaea3 - ps4
Asgard/Eukaryota - ps5
Eukaryota - ps6
Amorpheae - ps7
Obazoa - ps8
Opisthokonta - ps9
Holomycota - ps10
Fungi - ps11
Fungi_1 - ps12
Fungi_2 - ps13
Fungi_3 - ps14
Dikarya - ps15
Ascomycota - ps16
Saccharo/Pezizomycotina - ps17
Saccharomycotina - ps18
Saccharomycotina_1 - ps19
Saccharomycotina_2 - ps20
Saccharomycotina_3 - ps21
Phaff./Saccharomycetaceae - ps22
Saccharomyco./mycetaceae - ps23
Saccharomycetaceae - ps24
Saccharomycetaceae_2 - ps25
Saccharomycetaceae_3 - ps26
Kaz./Nau./Saccharomyces - ps27
Saccharomyces - ps28
Arboricola_clade - ps29
Cerevisiae_clade - ps30
Saccharomyces_cerevisiae - ps31
p-value
1e-14
5e-12
2e-09
1e-06
5e-04
Log-odds
0.5
1.0
1.5
2.0
2.5
3.0
3.5
4.0

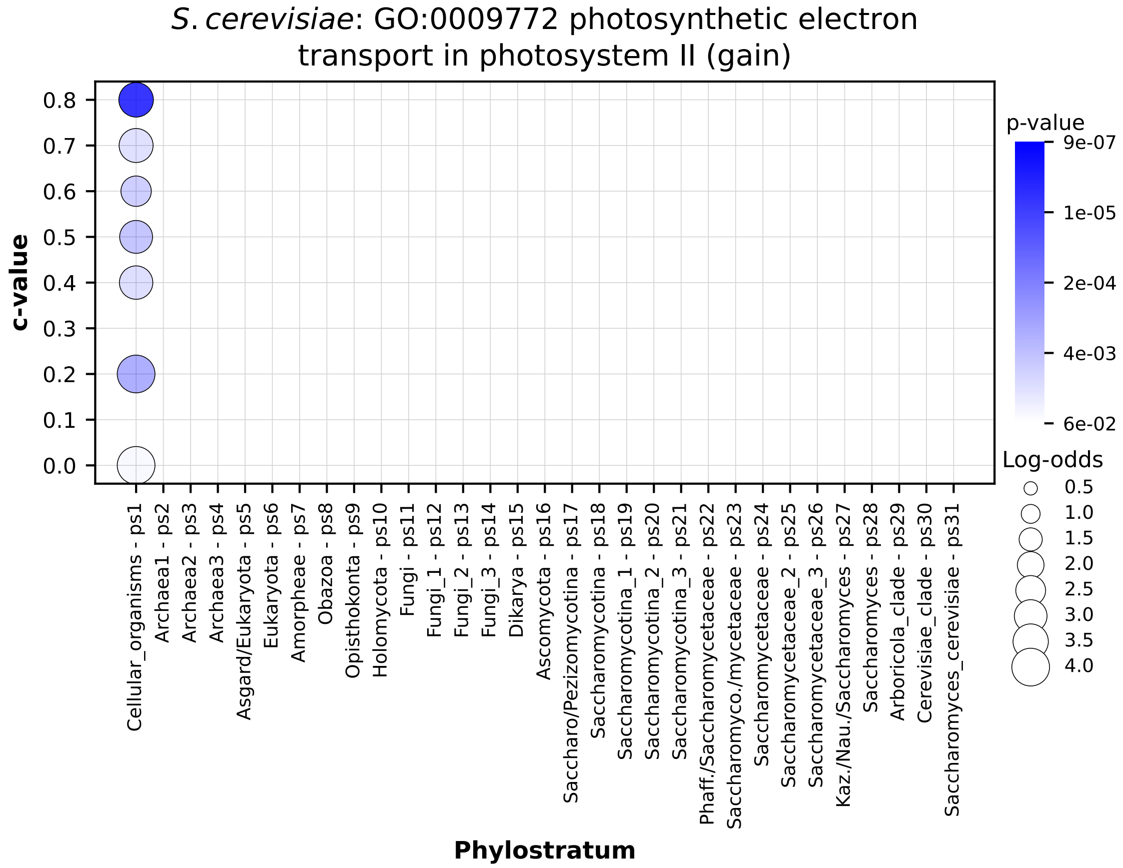

S. cerevisiae: GO:0009772 photosynthetic electron transport in photosystem II (gain)
c-value
0.8
0.7
0.6
0.5
0.4
0.3
0.2
0.1
0.0
p-value
9e-07
1e-05
2e-04
4e-03
6e-02
Log-odds
0.5
1.0
1.5
2.0
2.5
3.0
3.5
4.0
Cellular_organisms - ps1
Archaea1 - ps2
Archaea2 - ps3
Archaea3 - ps4
Asgard/Eukaryota - ps5
Eukaryota - ps6
Amorpheae - ps7
Obazoa - ps8
Opisthokonta - ps9
Holomycota - ps10
Fungi - ps11
Fungi_1 - ps12
Fungi_2 - ps13
Fungi_3 - ps14
Dikarya - ps15
Ascomycota - ps16
Saccharo/Pezizomycotina - ps17
Saccharomycotina - ps18
Saccharomycotina_1 - ps19
Saccharomycotina_2 - ps20
Saccharomycotina_3 - ps21
Phaff./Saccharomycetaceae - ps22
Saccharomyco./mycetaceae - ps23
Saccharomycetaceae - ps24
Saccharomycetaceae_2 - ps25
Saccharomycetaceae_3 - ps26
Kaz./Nau./Saccharomyces - ps27
Saccharomyces - ps28
Arboricola_clade - ps29
Cerevisiae_clade - ps30
Saccharomyces_cerevisiae - ps31
Phylostratum

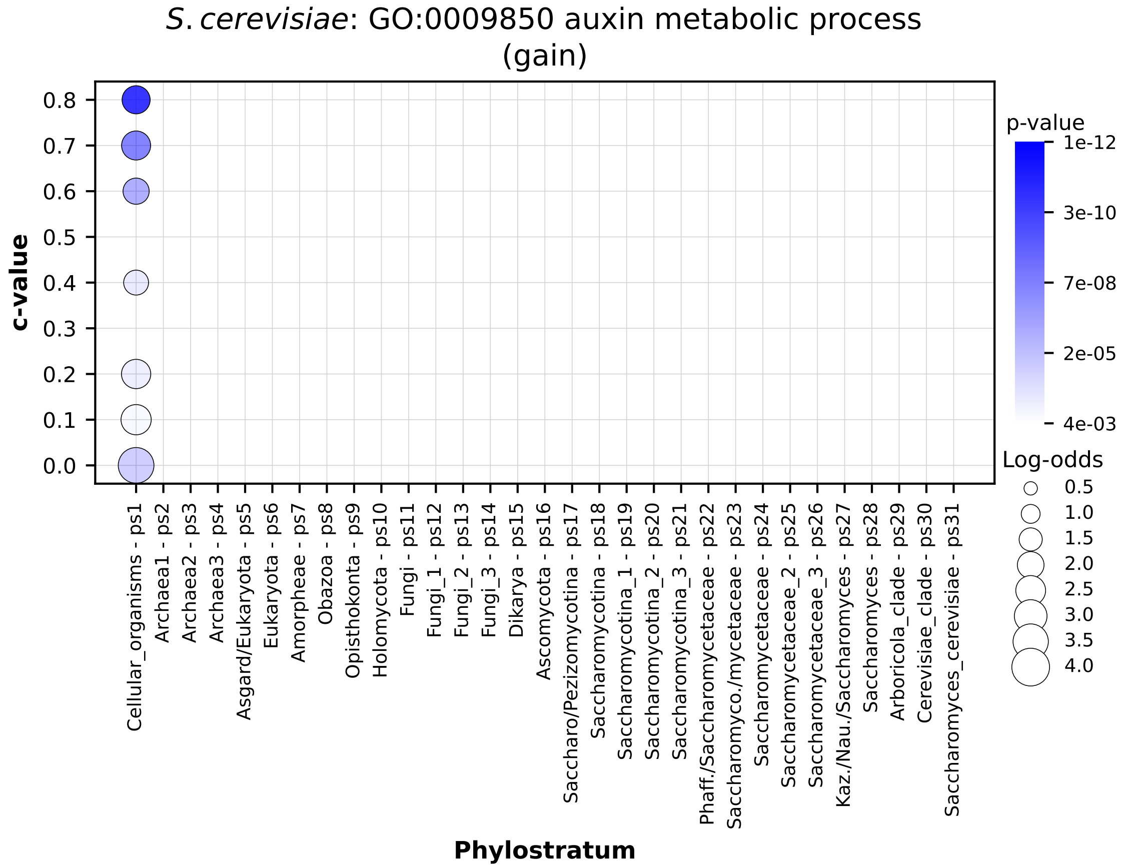

S. cerevisiae: GO:0009850 auxin metabolic process
(gain)
c-value
0.0
0.1
0.2
0.3
0.4
0.5
0.6
0.7
0.8
p-value
1e-12
3e-10
7e-08
2e-05
4e-03
Log-odds
0.5
1.0
1.5
2.0
2.5
3.0
3.5
4.0
Cellular_organisms - ps1
Archaea1 - ps2
Archaea2 - ps3
Archaea3 - ps4
Asgard/Eukaryota - ps5
Eukaryota - ps6
Amorpheae - ps7
Obazoa - ps8
Opisthokonta - ps9
Holomycota - ps10
Fungi - ps11
Fungi_1 - ps12
Fungi_2 - ps13
Fungi_3 - ps14
Dikarya - ps15
Ascomycota - ps16
Saccharo/Pezizomycotina - ps17
Saccharomycotina - ps18
Saccharomycotina_1 - ps19
Saccharomycotina_2 - ps20
Saccharomycotina_3 - ps21
Phaff./Saccharomycetaceae - ps22
Saccharomyco./mycetaceae - ps23
Saccharomycetaceae - ps24
Saccharomycetaceae_2 - ps25
Saccharomycetaceae_3 - ps26
Kaz./Nau./Saccharomyces - ps27
Saccharomyces - ps28
Arboricola_clade - ps29
Cerevisiae_clade - ps30
Saccharomyces_cerevisiae - ps31
Phylostratum

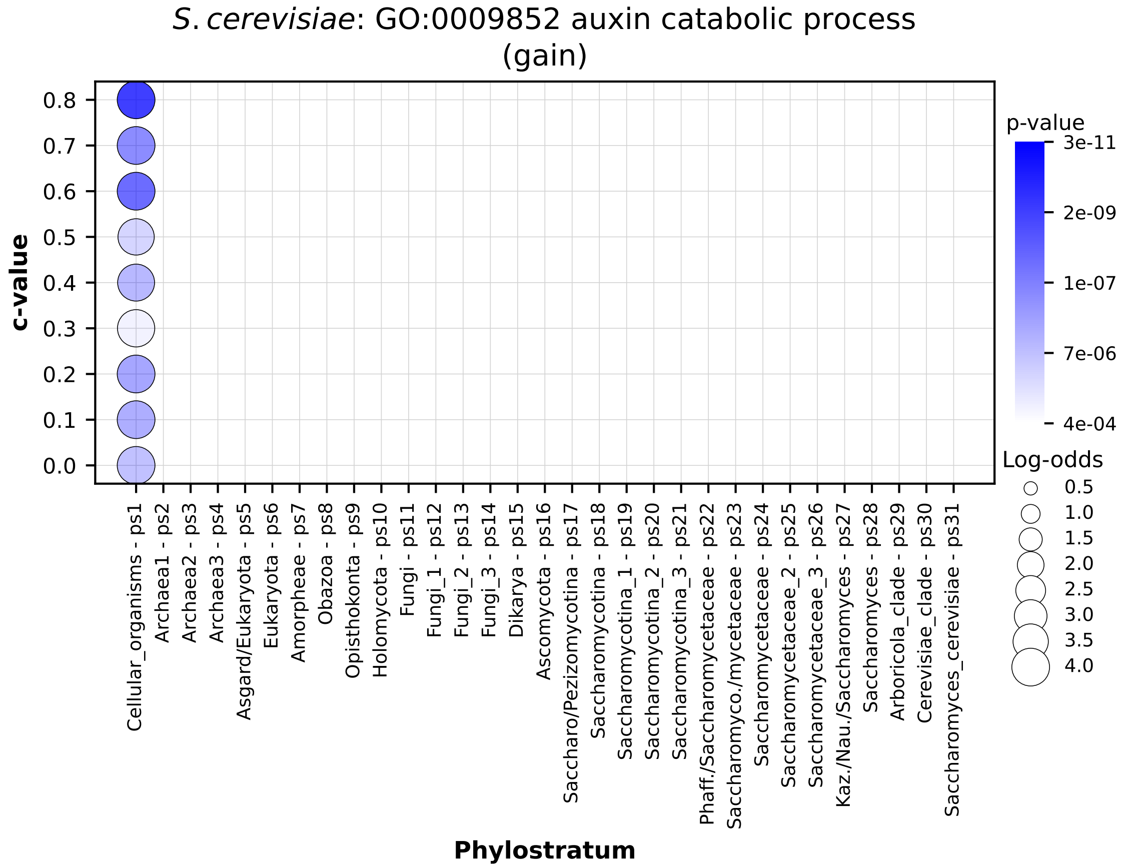

S. cerevisiae: GO:0009852 auxin catabolic process
(gain)
c-value
0.8
0.7
0.6
0.5
0.4
0.3
0.2
0.1
0.0
Phylostratum
Cellular_organisms - ps1
Archaea1 - ps2
Archaea2 - ps3
Archaea3 - ps4
Asgard/Eukaryota - ps5
Eukaryota - ps6
Amorpheae - ps7
Obazoa - ps8
Opisthokonta - ps9
Holomycota - ps10
Fungi - ps11
Fungi_1 - ps12
Fungi_2 - ps13
Fungi_3 - ps14
Dikarya - ps15
Ascomycota - ps16
Saccharo/Pezizomycotina - ps17
Saccharomycotina - ps18
Saccharomycotina_1 - ps19
Saccharomycotina_2 - ps20
Saccharomycotina_3 - ps21
Phaff./Saccharomycetaceae - ps22
Saccharomyco./mycetaceae - ps23
Saccharomycetaceae - ps24
Saccharomycetaceae_2 - ps25
Saccharomycetaceae_3 - ps26
Kaz./Nau./Saccharomyces - ps27
Saccharomyces - ps28
Arboricola_clade - ps29
Cerevisiae_clade - ps30
Saccharomyces_cerevisiae - ps31
p-value
3e-11
2e-09
1e-07
7e-06
4e-04
Log-odds
0.5
1.0
1.5
2.0
2.5
3.0
3.5
4.0

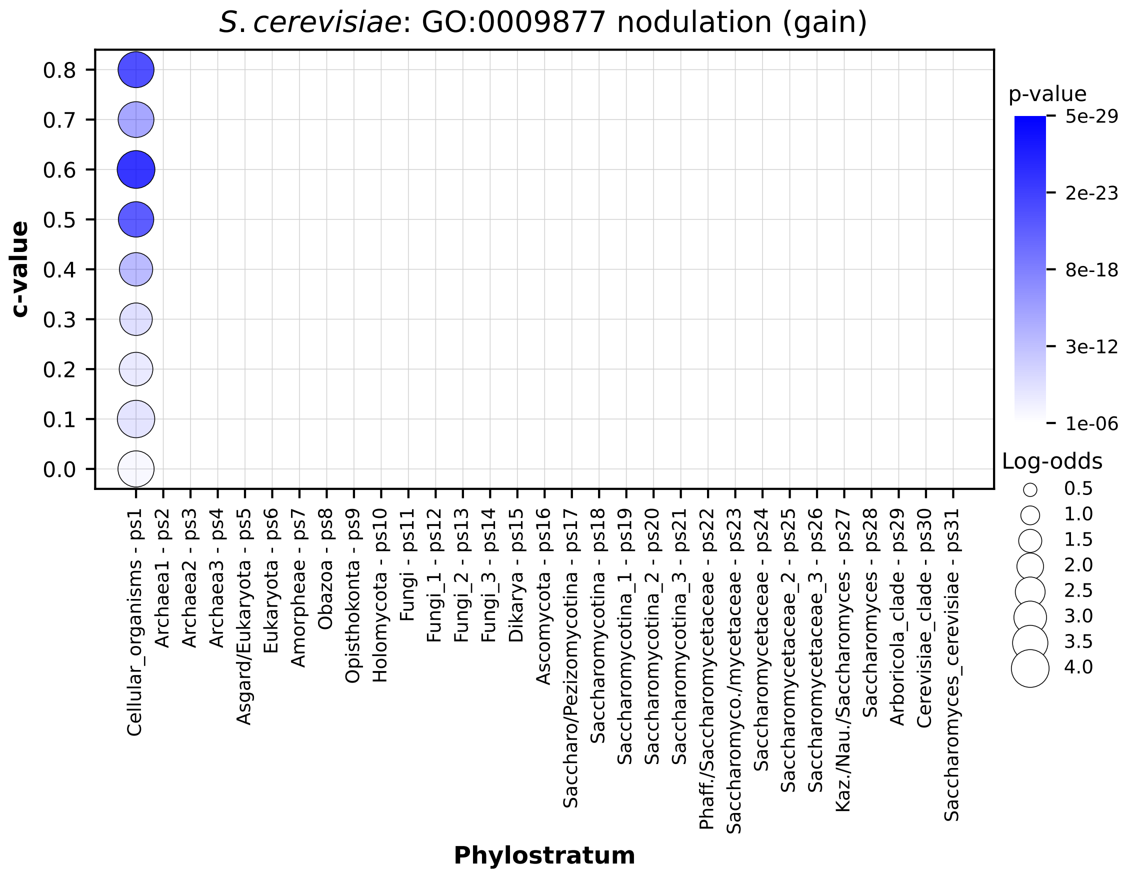
S. cerevisiae: GO:0009877 nodulation (gain)
c-value
0.8
0.7
0.6
0.5
0.4
0.3
0.2
0.1
0.0
p-value
5e-29
2e-23
8e-18
3e-12
1e-06
Log-odds
0.5
1.0
1.5
2.0
2.5
3.0
3.5
4.0
Cellular_organisms - ps1
Archaea1 - ps2
Archaea2 - ps3
Archaea3 - ps4
Asgard/Eukaryota - ps5
Eukaryota - ps6
Amorpheae - ps7
Obazoa - ps8
Opisthokonta - ps9
Holomycota - ps10
Fungi - ps11
Fungi_1 - ps12
Fungi_2 - ps13
Fungi_3 - ps14
Dikarya - ps15
Ascomycota - ps16
Saccharo/Pezizomycotina - ps17
Saccharomycotina - ps18
Saccharomycotina_1 - ps19
Saccharomycotina_2 - ps20
Saccharomycotina_3 - ps21
Phaff./Saccharomycetaceae - ps22
Saccharomyco./mycetaceae - ps23
Saccharomycetaceae - ps24
Saccharomycetaceae_2 - ps25
Saccharomycetaceae_3 - ps26
Kaz./Nau./Saccharomyces - ps27
Saccharomyces - ps28
Arboricola_clade - ps29
Cerevisiae_clade - ps30
Saccharomyces_cerevisiae - ps31
Phylostratum

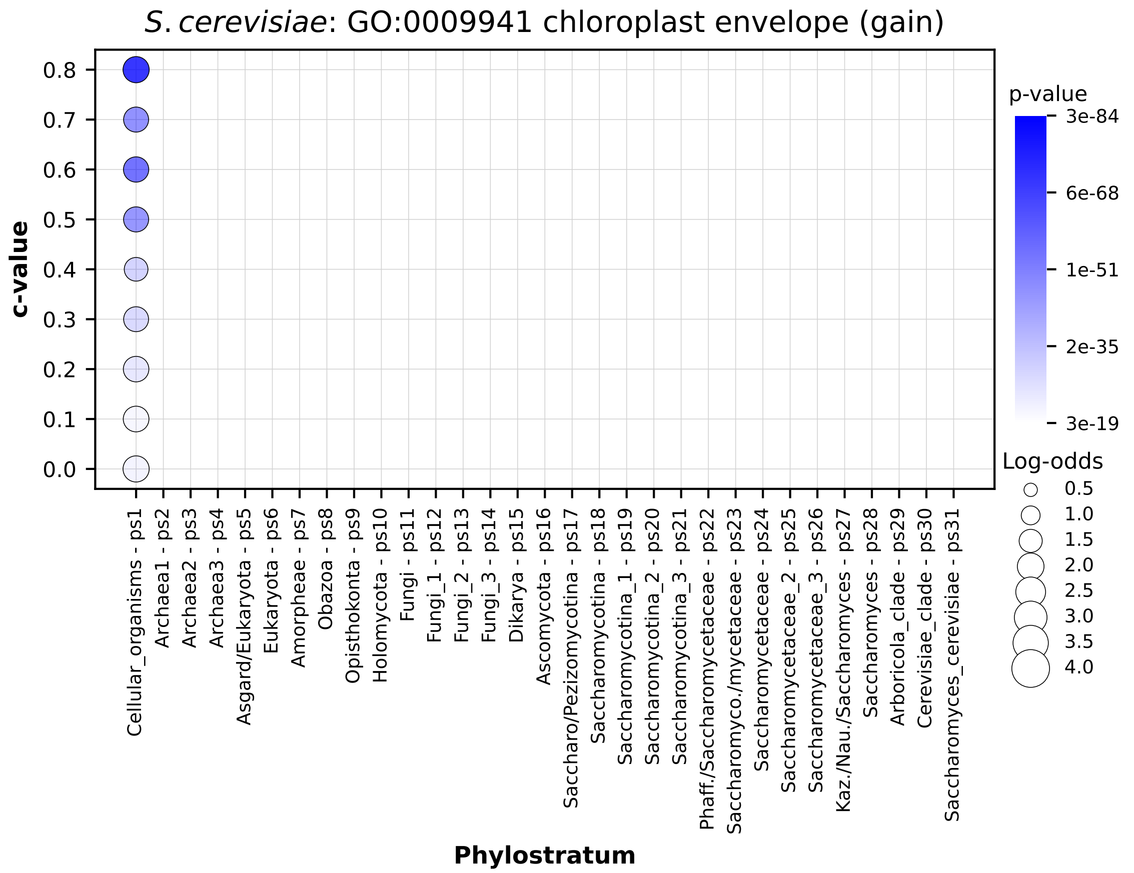

S. cerevisiae: GO:0009941 chloroplast envelope (gain)
c-value
0.0
0.1
0.2
0.3
0.4
0.5
0.6
0.7
0.8
Phylostratum
Cellular_organisms - ps1
Archaea1 - ps2
Archaea2 - ps3
Archaea3 - ps4
Asgard/Eukaryota - ps5
Eukaryota - ps6
Amorpheae - ps7
Obazoa - ps8
Opisthokonta - ps9
Holomycota - ps10
Fungi - ps11
Fungi_1 - ps12
Fungi_2 - ps13
Fungi_3 - ps14
Dikarya - ps15
Ascomycota - ps16
Saccharo/Pezizomycotina - ps17
Saccharomycotina - ps18
Saccharomycotina_1 - ps19
Saccharomycotina_2 - ps20
Saccharomycotina_3 - ps21
Phaff./Saccharomycetaceae - ps22
Saccharomyco./mycetaceae - ps23
Saccharomycetaceae - ps24
Saccharomycetaceae_2 - ps25
Saccharomycetaceae_3 - ps26
Kaz./Nau./Saccharomyces - ps27
Saccharomyces - ps28
Arboricola_clade - ps29
Cerevisiae_clade - ps30
Saccharomyces_cerevisiae - ps31
p-value
3e-84
6e-68
1e-51
2e-35
3e-19
Log-odds
0.5
1.0
1.5
2.0
2.5
3.0
3.5
4.0

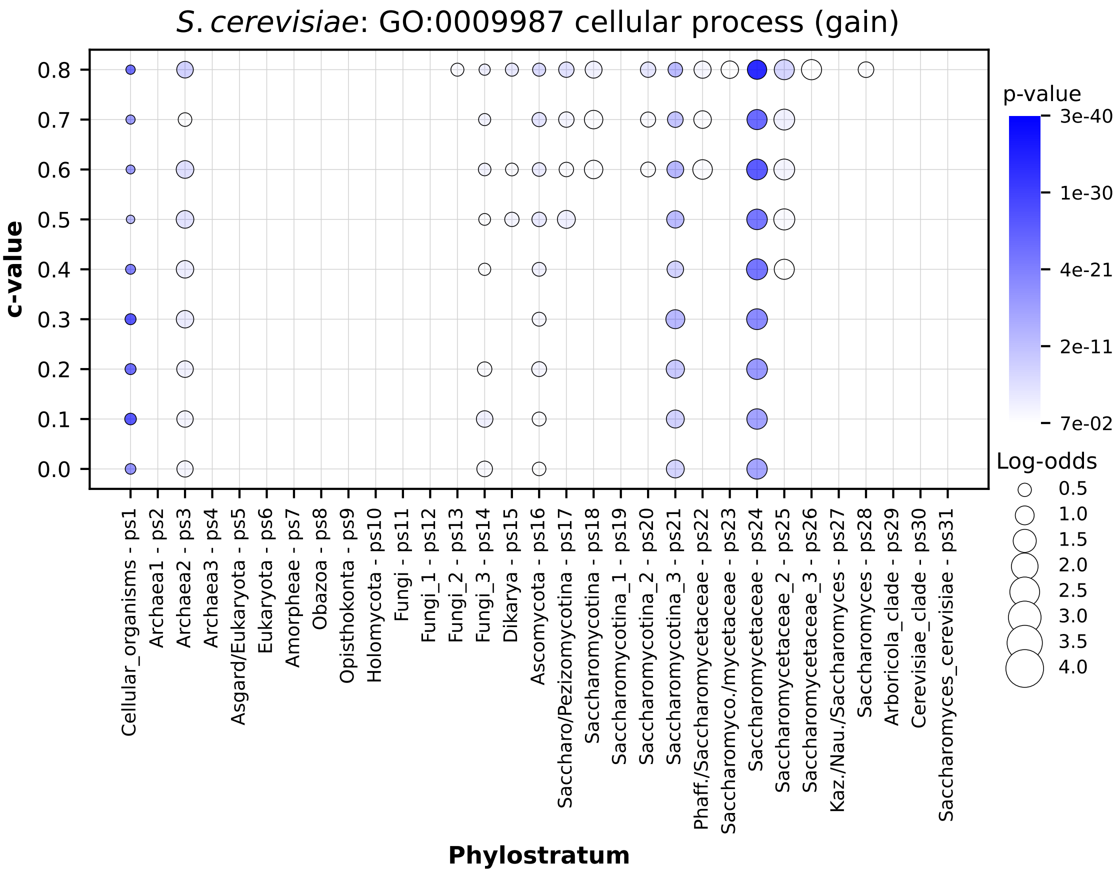
S. cerevisiae: GO:0009987 cellular process (gain)
c-value
0.8
0.7
0.6
0.5
0.4
0.3
0.2
0.1
0.0
p-value
3e-40
1e-30
4e-21
2e-11
7e-02
Log-odds
0.5
1.0
1.5
2.0
2.5
3.0
3.5
4.0
Cellular_organisms - ps1
Archaea1 - ps2
Archaea2 - ps3
Archaea3 - ps4
Asgard/Eukaryota - ps5
Eukaryota - ps6
Amorpheae - ps7
Obazoa - ps8
Opisthokonta - ps9
Holomycota - ps10
Fungi - ps11
Fungi_1 - ps12
Fungi_2 - ps13
Fungi_3 - ps14
Dikarya - ps15
Ascomycota - ps16
Saccharo/Pezizomycotina - ps17
Saccharomycotina - ps18
Saccharomycotina_1 - ps19
Saccharomycotina_2 - ps20
Saccharomycotina_3 - ps21
Phaff./Saccharomycetaceae - ps22
Saccharomyco./mycetaceae - ps23
Saccharomycetaceae - ps24
Saccharomycetaceae_2 - ps25
Saccharomycetaceae_3 - ps26
Kaz./Nau./Saccharomyces - ps27
Saccharomyces - ps28
Arboricola_clade - ps29
Cerevisiae_clade - ps30
Saccharomyces_cerevisiae - ps31
Phylostratum

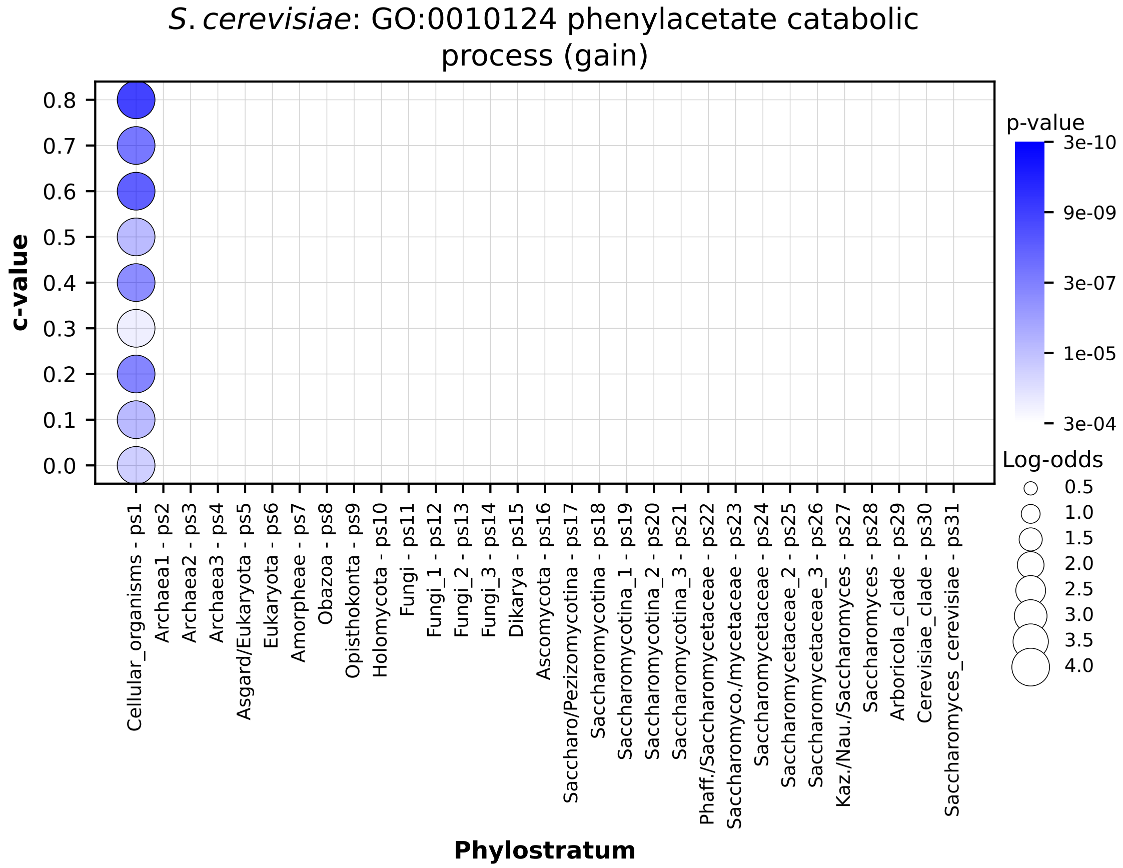

S. cerevisiae: GO:0010124 phenylacetate catabolic process (gain)
c-value
0.8
0.7
0.6
0.5
0.4
0.3
0.2
0.1
0.0
p-value
3e-10
9e-09
3e-07
1e-05
3e-04
Log-odds
0.5
1.0
1.5
2.0
2.5
3.0
3.5
4.0
Cellular_organisms - ps1
Archaea1 - ps2
Archaea2 - ps3
Archaea3 - ps4
Asgard/Eukaryota - ps5
Eukaryota - ps6
Amorpheae - ps7
Obazoa - ps8
Opisthokonta - ps9
Holomycota - ps10
Fungi - ps11
Fungi_1 - ps12
Fungi_2 - ps13
Fungi_3 - ps14
Dikarya - ps15
Ascomycota - ps16
Saccharo/Pezizomycotina - ps17
Saccharomycotina - ps18
Saccharomycotina_1 - ps19
Saccharomycotina_2 - ps20
Saccharomycotina_3 - ps21
Phaff./Saccharomycetaceae - ps22
Saccharomyco./mycetaceae - ps23
Saccharomycetaceae - ps24
Saccharomycetaceae_2 - ps25
Saccharomycetaceae_3 - ps26
Kaz./Nau./Saccharomyces - ps27
Saccharomyces - ps28
Arboricola_clade - ps29
Cerevisiae_clade - ps30
Saccharomyces_cerevisiae - ps31
Phylostratum

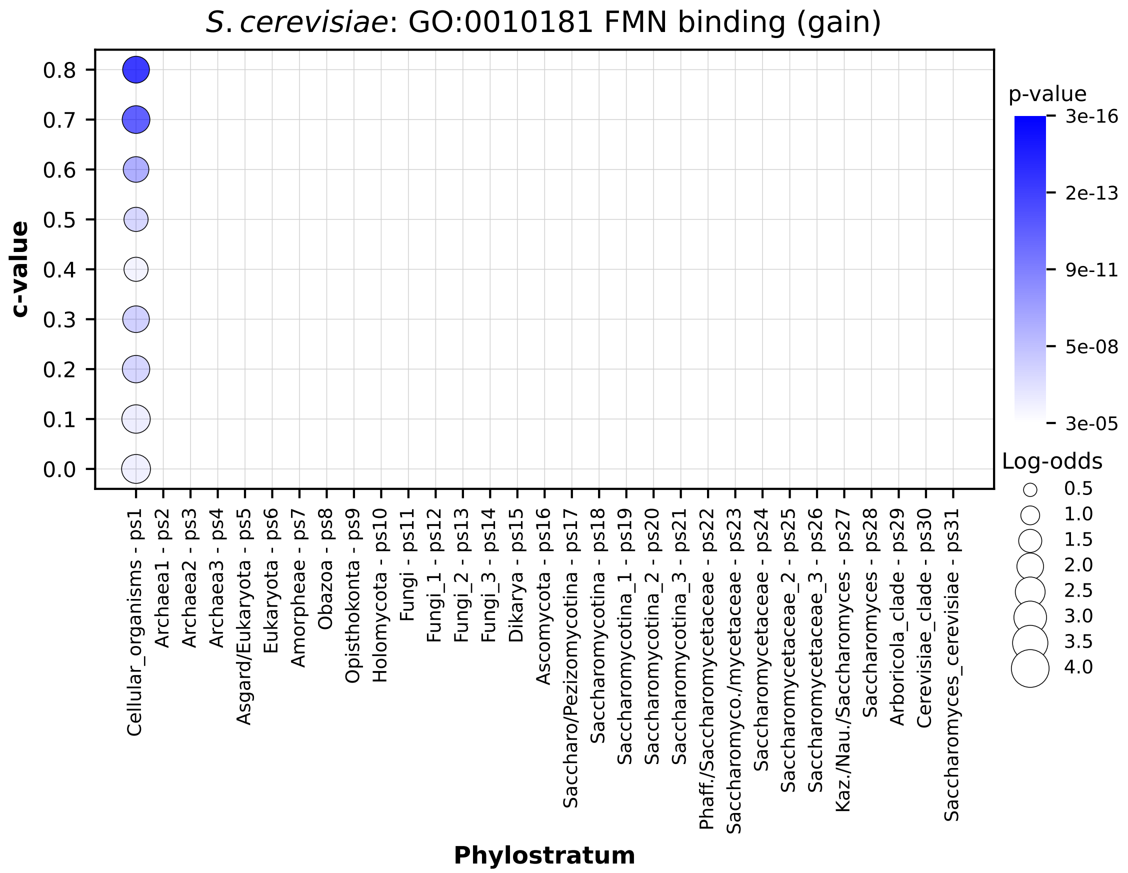

S. cerevisiae: GO:0010181 FMN binding (gain)
c-value
0.8
0.7
0.6
0.5
0.4
0.3
0.2
0.1
0.0
p-value
3e-16
2e-13
9e-11
5e-08
3e-05
Log-odds
0.5
1.0
1.5
2.0
2.5
3.0
3.5
4.0
Cellular_organisms - ps1
Archaea1 - ps2
Archaea2 - ps3
Archaea3 - ps4
Asgard/Eukaryota - ps5
Eukaryota - ps6
Amorpheae - ps7
Obazoa - ps8
Opisthokonta - ps9
Holomycota - ps10
Fungi - ps11
Fungi_1 - ps12
Fungi_2 - ps13
Fungi_3 - ps14
Dikarya - ps15
Ascomycota - ps16
Saccharo/Pezizomycotina - ps17
Saccharomycotina - ps18
Saccharomycotina_1 - ps19
Saccharomycotina_2 - ps20
Saccharomycotina_3 - ps21
Phaff./Saccharomycetaceae - ps22
Saccharomyco./mycetaceae - ps23
Saccharomycetaceae - ps24
Saccharomycetaceae_2 - ps25
Saccharomycetaceae_3 - ps26
Kaz./Nau./Saccharomyces - ps27
Saccharomyces - ps28
Arboricola_clade - ps29
Cerevisiae_clade - ps30
Saccharomyces_cerevisiae - ps31
Phylostratum

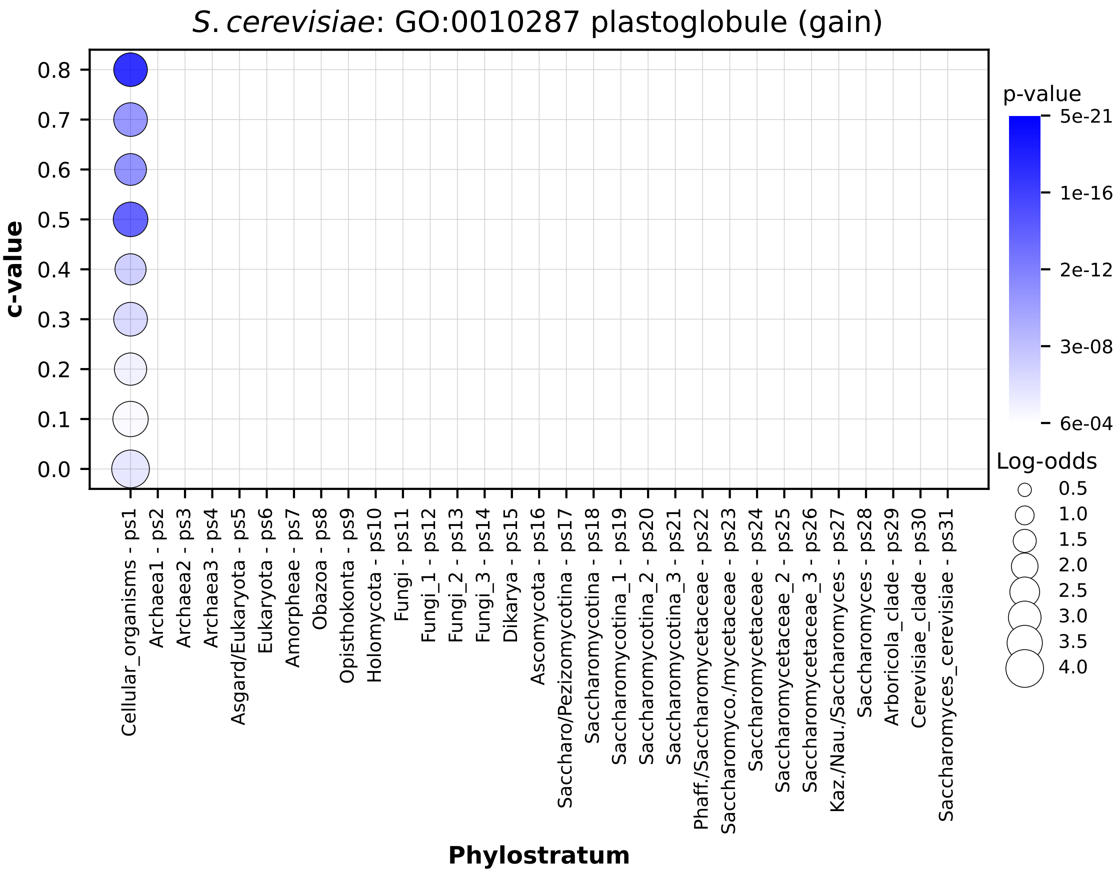
S. cerevisiae: GO:0010287 plastoglobule (gain)
c-value
0.8
0.7
0.6
0.5
0.4
0.3
0.2
0.1
0.0
p-value
5e-21
1e-16
2e-12
3e-08
6e-04
Log-odds
0.5
1.0
1.5
2.0
2.5
3.0
3.5
4.0
Cellular_organisms - ps1
Archaea1 - ps2
Archaea2 - ps3
Archaea3 - ps4
Asgard/Eukaryota - ps5
Eukaryota - ps6
Amorpheae - ps7
Obazoa - ps8
Opisthokonta - ps9
Holomycota - ps10
Fungi - ps11
Fungi_1 - ps12
Fungi_2 - ps13
Fungi_3 - ps14
Dikarya - ps15
Ascomycota - ps16
Saccharo/Pezizomycotina - ps17
Saccharomycotina - ps18
Saccharomycotina_1 - ps19
Saccharomycotina_2 - ps20
Saccharomycotina_3 - ps21
Phaff./Saccharomycetaceae - ps22
Saccharomyco./mycetaceae - ps23
Saccharomycetaceae - ps24
Saccharomycetaceae_2 - ps25
Saccharomycetaceae_3 - ps26
Kaz./Nau./Saccharomyces - ps27
Saccharomyces - ps28
Arboricola_clade - ps29
Cerevisiae_clade - ps30
Saccharomyces_cerevisiae - ps31
Phylostratum

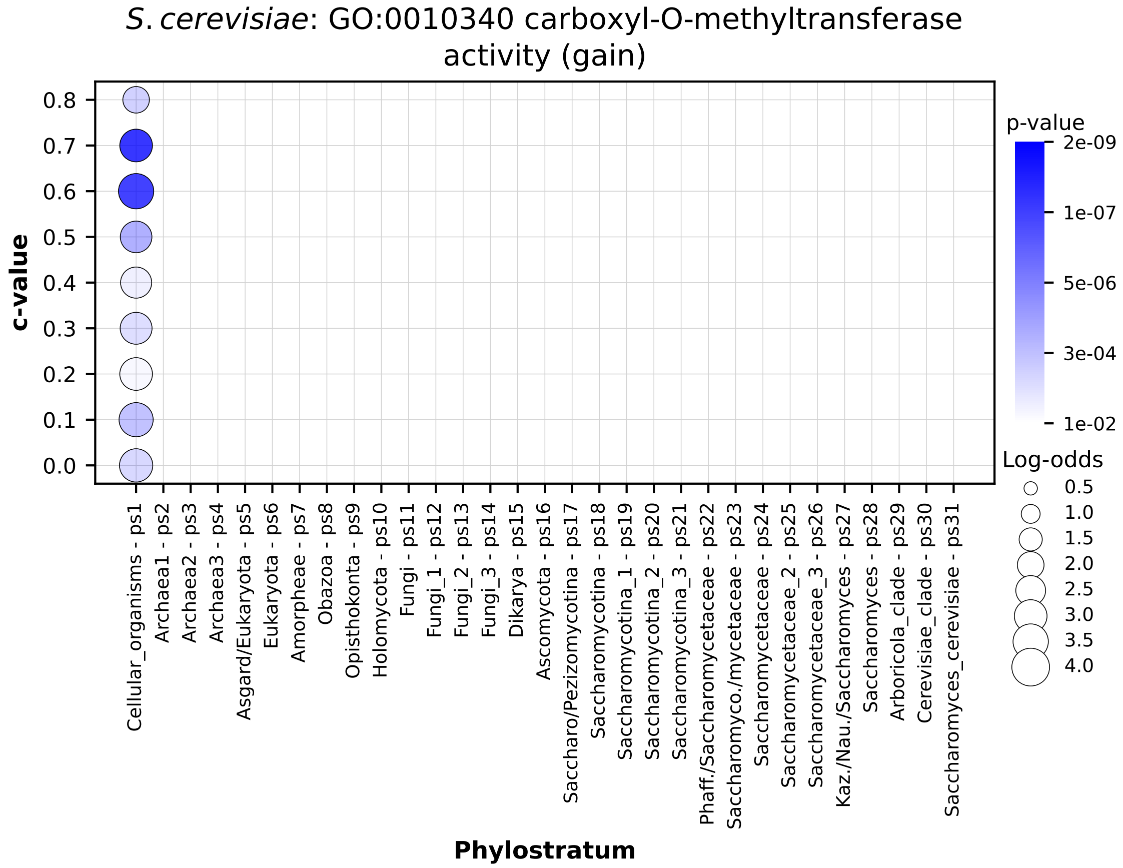
S. cerevisiae: GO:0010340 carboxyl-O-methyltransferase activity (gain)
c-value
0.0
0.1
0.2
0.3
0.4
0.5
0.6
0.7
0.8
Phylostratum
Cellular_organisms - ps1
Archaea1 - ps2
Archaea2 - ps3
Archaea3 - ps4
Asgard/Eukaryota - ps5
Eukaryota - ps6
Amorpheae - ps7
Obazoa - ps8
Opisthokonta - ps9
Holomycota - ps10
Fungi - ps11
Fungi_1 - ps12
Fungi_2 - ps13
Fungi_3 - ps14
Dikarya - ps15
Ascomycota - ps16
Saccharo/Pezizomycotina - ps17
Saccharomycotina - ps18
Saccharomycotina_1 - ps19
Saccharomycotina_2 - ps20
Saccharomycotina_3 - ps21
Phaff./Saccharomycetaceae - ps22
Saccharomyco./mycetaceae - ps23
Saccharomycetaceae - ps24
Saccharomycetaceae_2 - ps25
Saccharomycetaceae_3 - ps26
Kaz./Nau./Saccharomyces - ps27
Saccharomyces - ps28
Arboricola_clade - ps29
Cerevisiae_clade - ps30
Saccharomyces_cerevisiae - ps31
p-value
2e-09
1e-07
5e-06
3e-04
1e-02
Log-odds
0.5
1.0
1.5
2.0
2.5
3.0
3.5
4.0

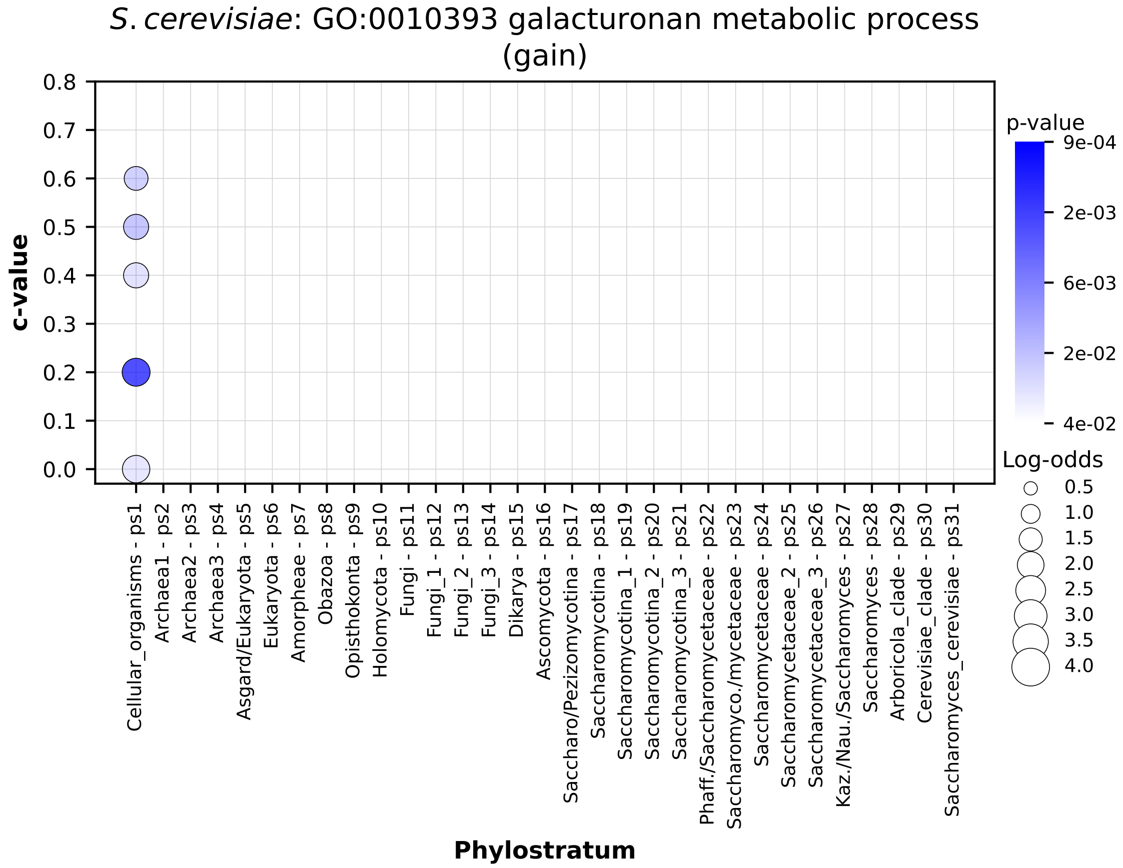
S. cerevisiae: GO:0010393 galacturonan metabolic process
(gain)
c-value
0.8
0.7
0.6
0.5
0.4
0.3
0.2
0.1
0.0
p-value
9e-04
2e-03
6e-03
2e-02
4e-02
Log-odds
0.5
1.0
1.5
2.0
2.5
3.0
3.5
4.0
Cellular_organisms - ps1
Archaea1 - ps2
Archaea2 - ps3
Archaea3 - ps4
Asgard/Eukaryota - ps5
Eukaryota - ps6
Amorpheae - ps7
Obazoa - ps8
Opisthokonta - ps9
Holomycota - ps10
Fungi - ps11
Fungi_1 - ps12
Fungi_2 - ps13
Fungi_3 - ps14
Dikarya - ps15
Ascomycota - ps16
Saccharo/Pezizomycotina - ps17
Saccharomycotina - ps18
Saccharomycotina_1 - ps19
Saccharomycotina_2 - ps20
Saccharomycotina_3 - ps21
Phaff./Saccharomycetaceae - ps22
Saccharomyco./mycetaceae - ps23
Saccharomycetaceae - ps24
Saccharomycetaceae_2 - ps25
Saccharomycetaceae_3 - ps26
Kaz./Nau./Saccharomyces - ps27
Saccharomyces - ps28
Arboricola_clade - ps29
Cerevisiae_clade - ps30
Saccharomyces_cerevisiae - ps31
Phylostratum

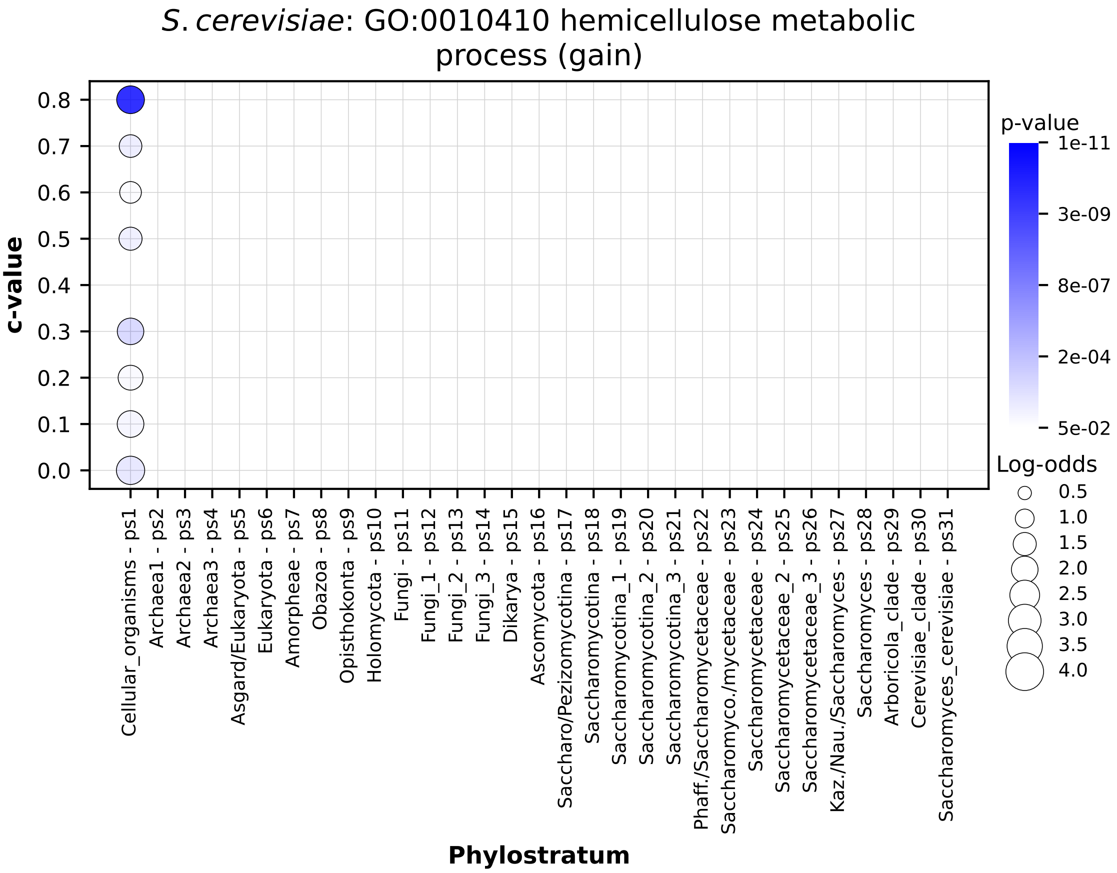
S. cerevisiae: GO:0010410 hemicellulose metabolic process (gain)
c-value
Phylostratum
p-value
1e-11
3e-09
8e-07
2e-04
5e-02
Log-odds
0.5
1.0
1.5
2.0
2.5
3.0
3.5
4.0
Cellular_organisms - ps1
Archaea1 - ps2
Archaea2 - ps3
Archaea3 - ps4
Asgard/Eukaryota - ps5
Eukaryota - ps6
Amorpheae - ps7
Obazoa - ps8
Opisthokonta - ps9
Holomycota - ps10
Fungi - ps11
Fungi_1 - ps12
Fungi_2 - ps13
Fungi_3 - ps14
Dikarya - ps15
Ascomycota - ps16
Saccharo/Pezizomycotina - ps17
Saccharomycotina - ps18
Saccharomycotina_1 - ps19
Saccharomycotina_2 - ps20
Saccharomycotina_3 - ps21
Phaff./Saccharomycetaceae - ps22
Saccharomyco./mycetaceae - ps23
Saccharomycetaceae - ps24
Saccharomycetaceae_2 - ps25
Saccharomycetaceae_3 - ps26
Kaz./Nau./Saccharomyces - ps27
Saccharomyces - ps28
Arboricola_clade - ps29
Cerevisiae_clade - ps30
Saccharomyces_cerevisiae - ps31

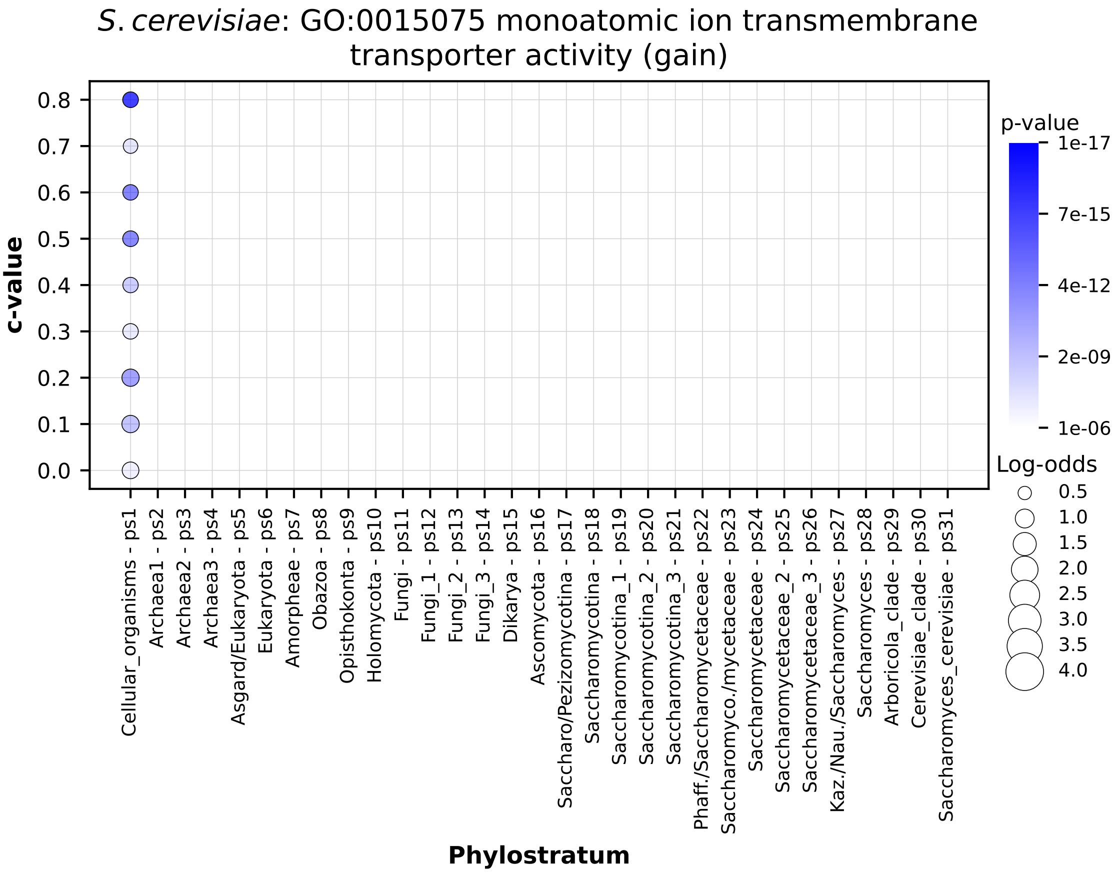

S. cerevisiae: GO:0015075 monoatomic ion transmembrane transporter activity (gain)
c-value
0.8
0.7
0.6
0.5
0.4
0.3
0.2
0.1
0.0
p-value
1e-17
7e-15
4e-12
2e-09
1e-06
Log-odds
0.5
1.0
1.5
2.0
2.5
3.0
3.5
4.0
Cellular_organisms - ps1
Archaea1 - ps2
Archaea2 - ps3
Archaea3 - ps4
Asgard/Eukaryota - ps5
Eukaryota - ps6
Amorpheae - ps7
Obazoa - ps8
Opisthokonta - ps9
Holomycota - ps10
Fungi - ps11
Fungi_1 - ps12
Fungi_2 - ps13
Fungi_3 - ps14
Dikarya - ps15
Ascomycota - ps16
Saccharo/Pezizomycotina - ps17
Saccharomycotina - ps18
Saccharomycotina_1 - ps19
Saccharomycotina_2 - ps20
Saccharomycotina_3 - ps21
Phaff./Saccharomycetaceae - ps22
Saccharomyco./mycetaceae - ps23
Saccharomycetaceae - ps24
Saccharomycetaceae_2 - ps25
Saccharomycetaceae_3 - ps26
Kaz./Nau./Saccharomyces - ps27
Saccharomyces - ps28
Arboricola_clade - ps29
Cerevisiae_clade - ps30
Saccharomyces_cerevisiae - ps31
Phylostratum

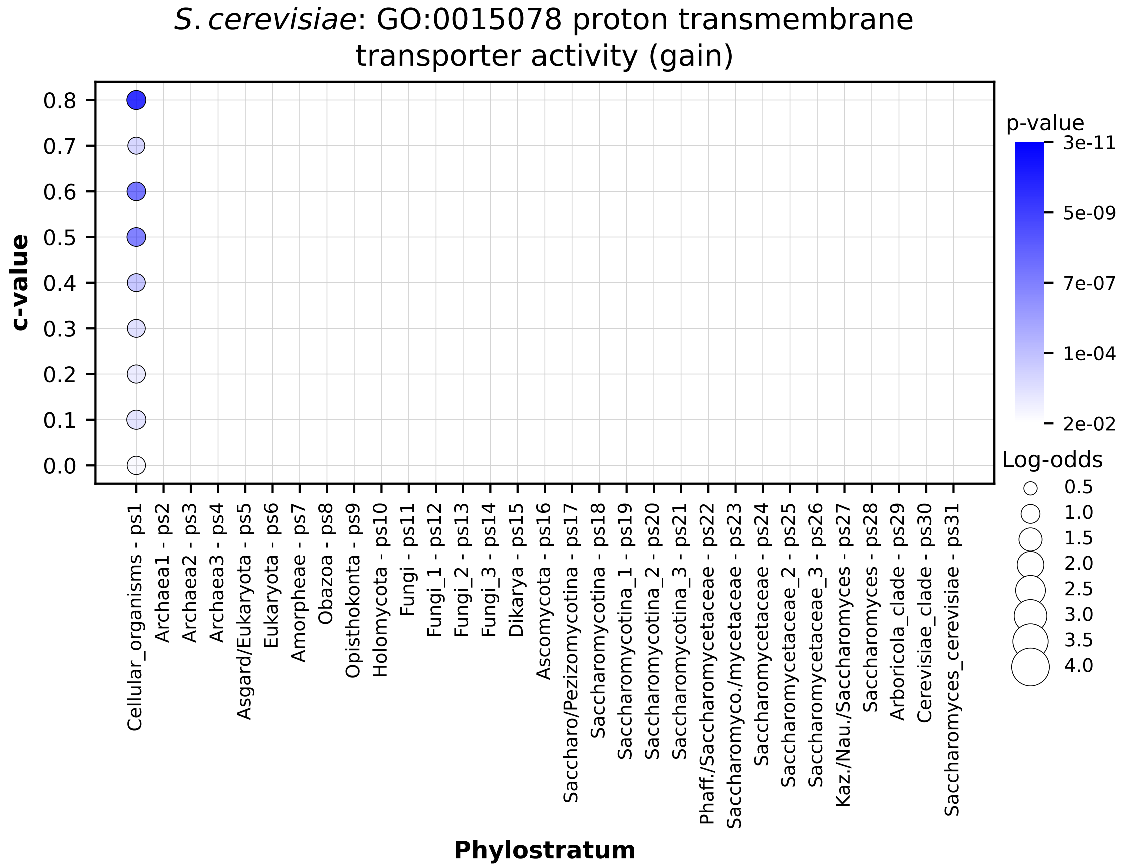

S. cerevisiae: GO:0015078 proton transmembrane transporter activity (gain)
c-value
0.0
0.1
0.2
0.3
0.4
0.5
0.6
0.7
0.8
Phylostratum
Cellular_organisms - ps1
Archaea1 - ps2
Archaea2 - ps3
Archaea3 - ps4
Asgard/Eukaryota - ps5
Eukaryota - ps6
Amorpheae - ps7
Obazoa - ps8
Opisthokonta - ps9
Holomycota - ps10
Fungi - ps11
Fungi_1 - ps12
Fungi_2 - ps13
Fungi_3 - ps14
Dikarya - ps15
Ascomycota - ps16
Saccharo/Pezizomycotina - ps17
Saccharomycotina - ps18
Saccharomycotina_1 - ps19
Saccharomycotina_2 - ps20
Saccharomycotina_3 - ps21
Phaff./Saccharomycetaceae - ps22
Saccharomyco./mycetaceae - ps23
Saccharomycetaceae - ps24
Saccharomycetaceae_2 - ps25
Saccharomycetaceae_3 - ps26
Kaz./Nau./Saccharomyces - ps27
Saccharomyces - ps28
Arboricola_clade - ps29
Cerevisiae_clade - ps30
Saccharomyces_cerevisiae - ps31
p-value
3e-11
5e-09
7e-07
1e-04
2e-02
Log-odds
0.5
1.0
1.5
2.0
2.5
3.0
3.5
4.0

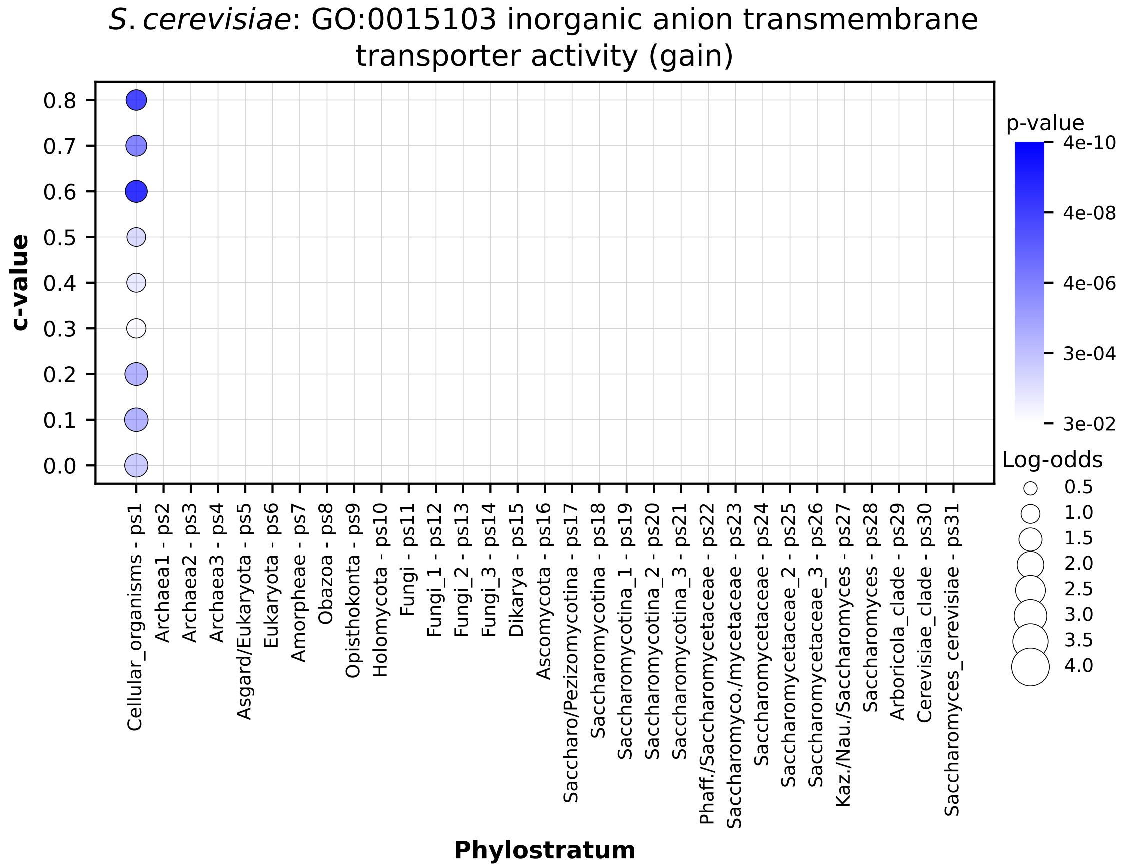

S. cerevisiae: GO:0015103 inorganic anion transmembrane transporter activity (gain)
c-value
0.8
0.7
0.6
0.5
0.4
0.3
0.2
0.1
0.0
p-value
4e-10
4e-08
4e-06
3e-04
3e-02
Log-odds
0.5
1.0
1.5
2.0
2.5
3.0
3.5
4.0
Cellular_organisms - ps1
Archaea1 - ps2
Archaea2 - ps3
Archaea3 - ps4
Asgard/Eukaryota - ps5
Eukaryota - ps6
Amorpheae - ps7
Obazoa - ps8
Opisthokonta - ps9
Holomycota - ps10
Fungi - ps11
Fungi_1 - ps12
Fungi_2 - ps13
Fungi_3 - ps14
Dikarya - ps15
Ascomycota - ps16
Saccharo/Pezizomycotina - ps17
Saccharomycotina - ps18
Saccharomycotina_1 - ps19
Saccharomycotina_2 - ps20
Saccharomycotina_3 - ps21
Phaff./Saccharomycetaceae - ps22
Saccharomyco./mycetaceae - ps23
Saccharomycetaceae - ps24
Saccharomycetaceae_2 - ps25
Saccharomycetaceae_3 - ps26
Kaz./Nau./Saccharomyces - ps27
Saccharomyces - ps28
Arboricola_clade - ps29
Cerevisiae_clade - ps30
Saccharomyces_cerevisiae - ps31
Phylostratum

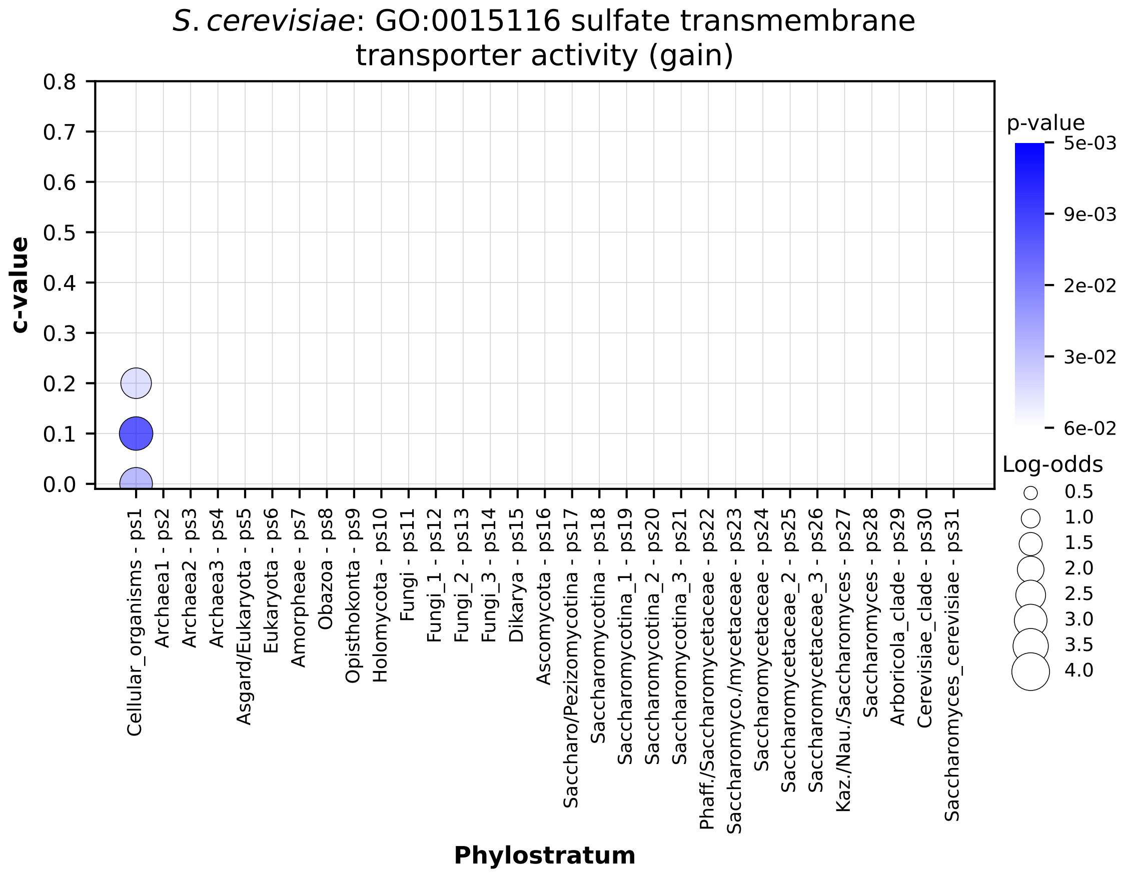

S. cerevisiae: GO:0015116 sulfate transmembrane transporter activity (gain)
c-value
0.0
0.1
0.2
0.3
0.4
0.5
0.6
0.7
0.8
Phylostratum
Cellular_organisms - ps1
Archaea1 - ps2
Archaea2 - ps3
Archaea3 - ps4
Asgard/Eukaryota - ps5
Eukaryota - ps6
Amorpheae - ps7
Obazoa - ps8
Opisthokonta - ps9
Holomycota - ps10
Fungi - ps11
Fungi_1 - ps12
Fungi_2 - ps13
Fungi_3 - ps14
Dikarya - ps15
Ascomycota - ps16
Saccharo/Pezizomycotina - ps17
Saccharomycotina - ps18
Saccharomycotina_1 - ps19
Saccharomycotina_2 - ps20
Saccharomycotina_3 - ps21
Phaff./Saccharomycetaceae - ps22
Saccharomyco./mycetaceae - ps23
Saccharomycetaceae - ps24
Saccharomycetaceae_2 - ps25
Saccharomycetaceae_3 - ps26
Kaz./Nau./Saccharomyces - ps27
Saccharomyces - ps28
Arboricola_clade - ps29
Cerevisiae_clade - ps30
Saccharomyces_cerevisiae - ps31
p-value
5e-03
9e-03
2e-02
3e-02
6e-02
Log-odds
0.5
1.0
1.5
2.0
2.5
3.0
3.5
4.0

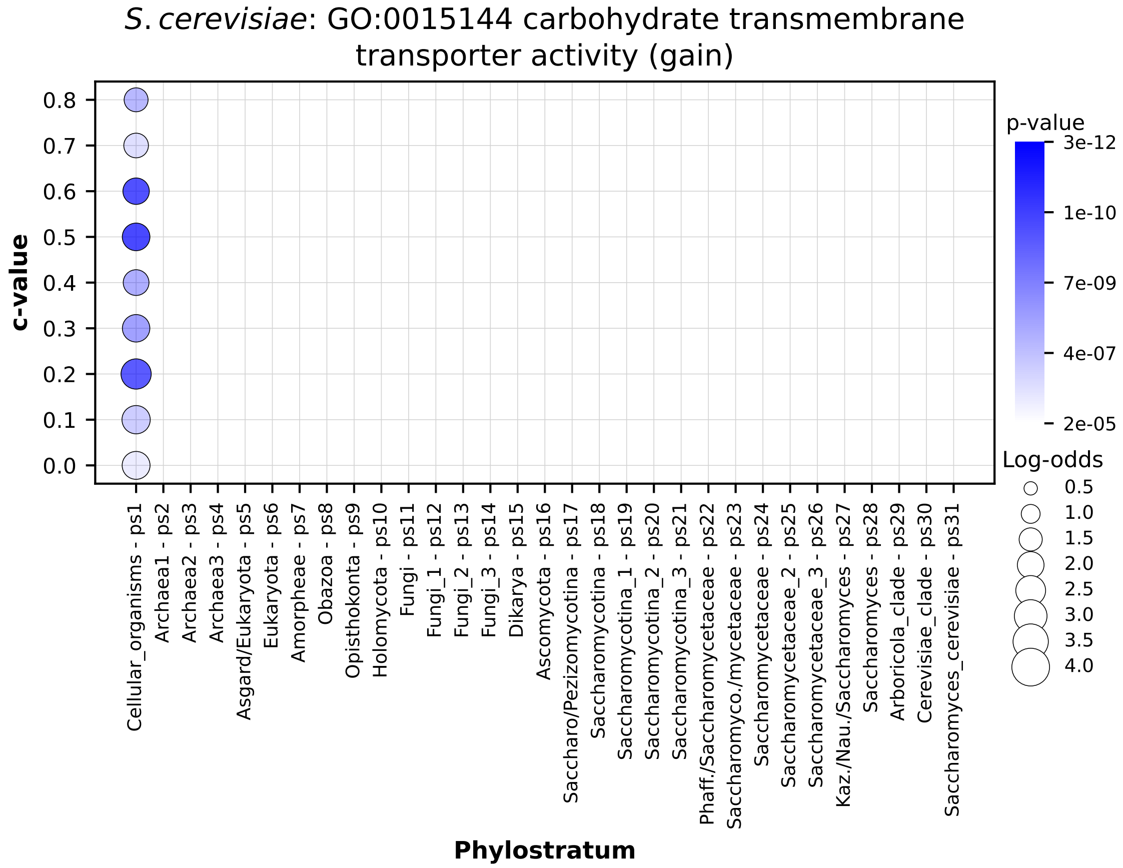
S. cerevisiae: GO:0015144 carbohydrate transmembrane transporter activity (gain)
c-value
0.8
0.7
0.6
0.5
0.4
0.3
0.2
0.1
0.0
p-value
3e-12
1e-10
7e-09
4e-07
2e-05
Log-odds
0.5
1.0
1.5
2.0
2.5
3.0
3.5
4.0
Cellular_organisms - ps1
Archaea1 - ps2
Archaea2 - ps3
Archaea3 - ps4
Asgard/Eukaryota - ps5
Eukaryota - ps6
Amorpheae - ps7
Obazoa - ps8
Opisthokonta - ps9
Holomycota - ps10
Fungi - ps11
Fungi_1 - ps12
Fungi_2 - ps13
Fungi_3 - ps14
Dikarya - ps15
Ascomycota - ps16
Saccharo/Pezizomycotina - ps17
Saccharomycotina - ps18
Saccharomycotina_1 - ps19
Saccharomycotina_2 - ps20
Saccharomycotina_3 - ps21
Phaff./Saccharomycetaceae - ps22
Saccharomyco./mycetaceae - ps23
Saccharomycetaceae - ps24
Saccharomycetaceae_2 - ps25
Saccharomycetaceae_3 - ps26
Kaz./Nau./Saccharomyces - ps27
Saccharomyces - ps28
Arboricola_clade - ps29
Cerevisiae_clade - ps30
Saccharomyces_cerevisiae - ps31
Phylostratum

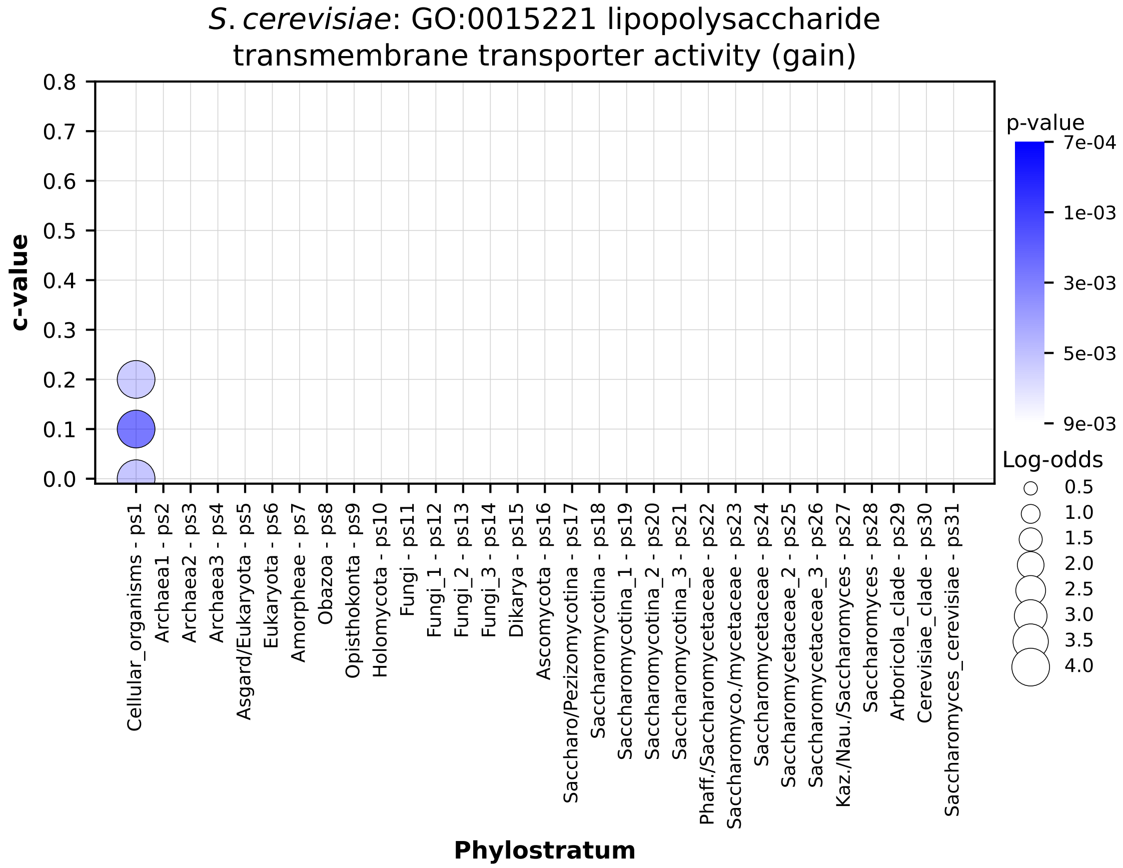
S. cerevisiae: GO:0015221 lipopolysaccharide transmembrane transporter activity (gain)
c-value
0.8
0.7
0.6
0.5
0.4
0.3
0.2
0.1
0.0
Phylostratum
Cellular_organisms - ps1
Archaea1 - ps2
Archaea2 - ps3
Archaea3 - ps4
Asgard/Eukaryota - ps5
Eukaryota - ps6
Amorpheae - ps7
Obazoa - ps8
Opisthokonta - ps9
Holomycota - ps10
Fungi - ps11
Fungi_1 - ps12
Fungi_2 - ps13
Fungi_3 - ps14
Dikarya - ps15
Ascomycota - ps16
Saccharo/Pezizomycotina - ps17
Saccharomycotina - ps18
Saccharomycotina_1 - ps19
Saccharomycotina_2 - ps20
Saccharomycotina_3 - ps21
Phaff./Saccharomycetaceae - ps22
Saccharomyco./mycetaceae - ps23
Saccharomycetaceae - ps24
Saccharomycetaceae_2 - ps25
Saccharomycetaceae_3 - ps26
Kaz./Nau./Saccharomyces - ps27
Saccharomyces - ps28
Arboricola_clade - ps29
Cerevisiae_clade - ps30
Saccharomyces_cerevisiae - ps31
p-value
7e-04
1e-03
3e-03
5e-03
9e-03
Log-odds
0.5
1.0
1.5
2.0
2.5
3.0
3.5
4.0

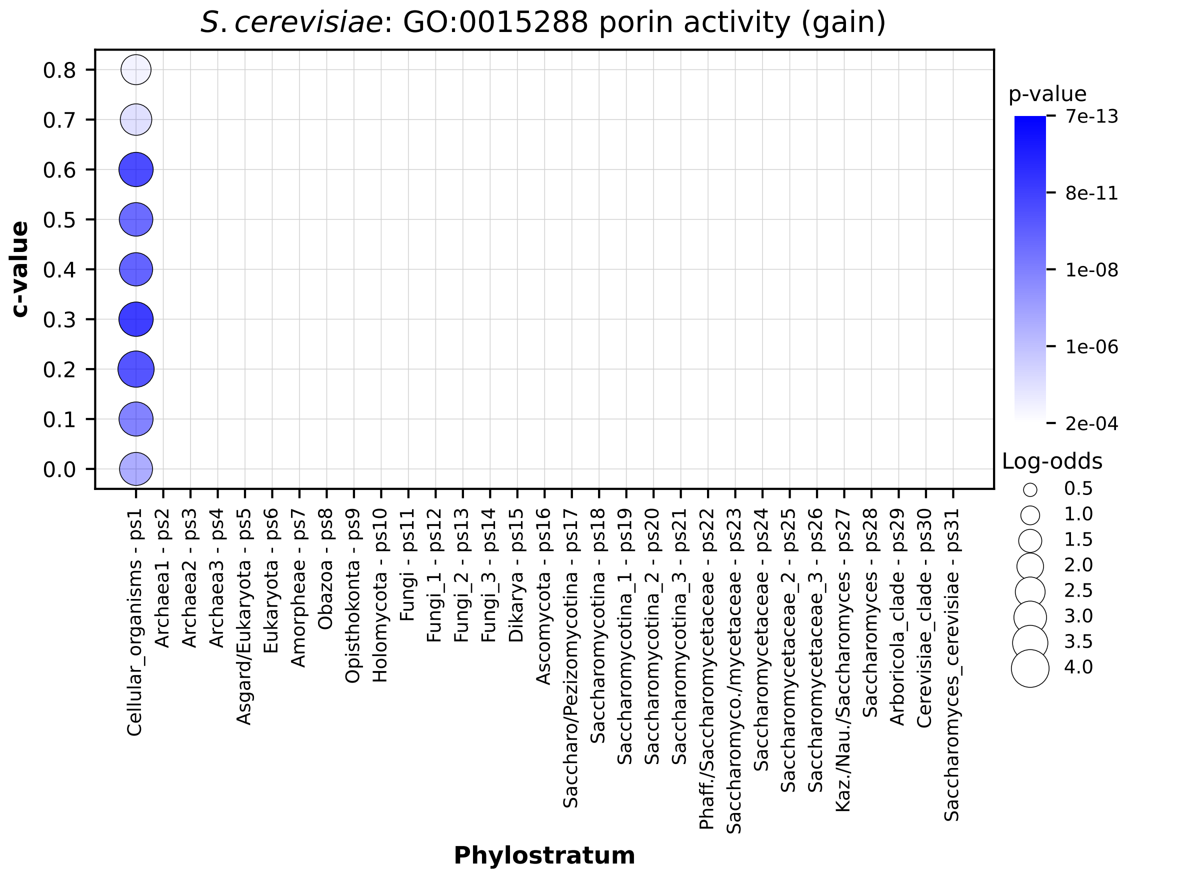
S. cerevisiae: GO:0015288 porin activity (gain)
c-value
0.8
0.7
0.6
0.5
0.4
0.3
0.2
0.1
0.0
p-value
7e-13
8e-11
1e-08
1e-06
2e-04
Log-odds
0.5
1.0
1.5
2.0
2.5
3.0
3.5
4.0
Cellular_organisms - ps1
Archaea1 - ps2
Archaea2 - ps3
Archaea3 - ps4
Asgard/Eukaryota - ps5
Eukaryota - ps6
Amorpheae - ps7
Obazoa - ps8
Opisthokonta - ps9
Holomycota - ps10
Fungi - ps11
Fungi_1 - ps12
Fungi_2 - ps13
Fungi_3 - ps14
Dikarya - ps15
Ascomycota - ps16
Saccharo/Pezizomycotina - ps17
Saccharomycotina - ps18
Saccharomycotina_1 - ps19
Saccharomycotina_2 - ps20
Saccharomycotina_3 - ps21
Phaff./Saccharomycetaceae - ps22
Saccharomyco./mycetaceae - ps23
Saccharomycetaceae - ps24
Saccharomycetaceae_2 - ps25
Saccharomycetaceae_3 - ps26
Kaz./Nau./Saccharomyces - ps27
Saccharomyces - ps28
Arboricola_clade - ps29
Cerevisiae_clade - ps30
Saccharomyces_cerevisiae - ps31
Phylostratum

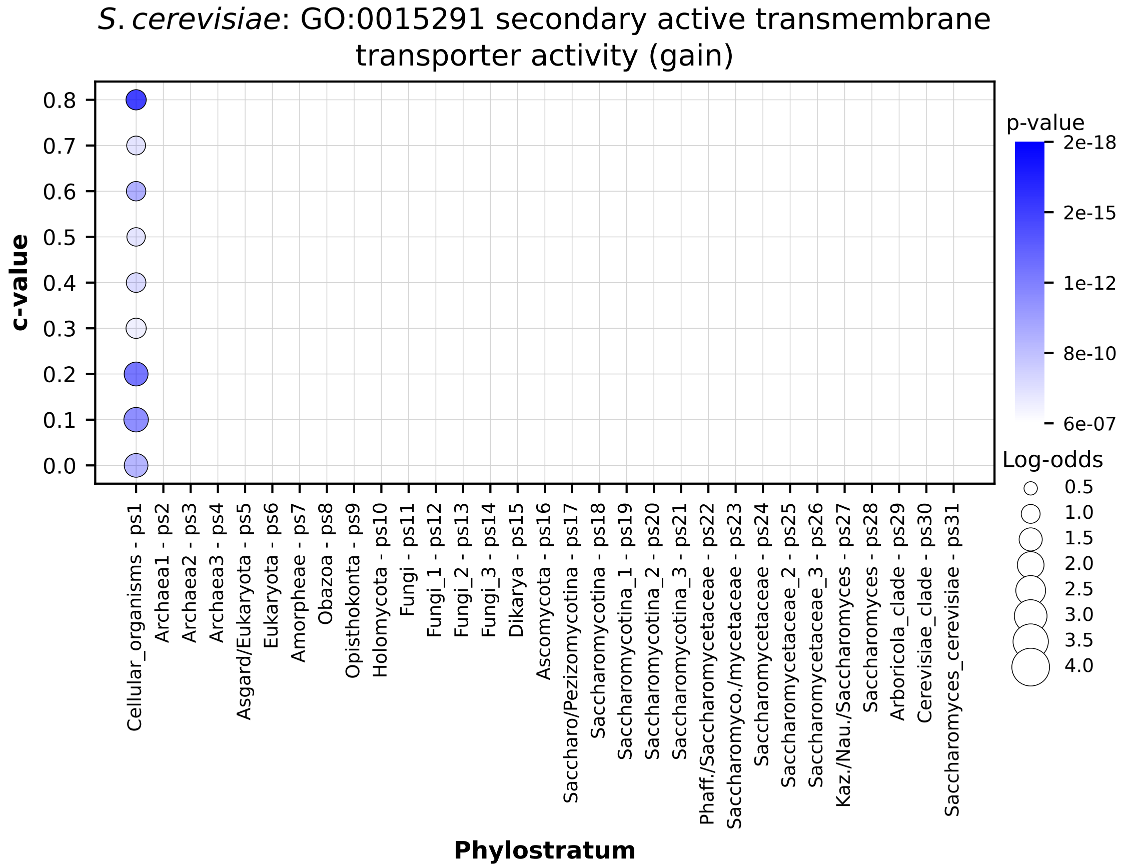

S. cerevisiae: GO:0015291 secondary active transmembrane transporter activity (gain)
c-value
0.8
0.7
0.6
0.5
0.4
0.3
0.2
0.1
0.0
p-value
2e-18
2e-15
1e-12
8e-10
6e-07
Log-odds
0.5
1.0
1.5
2.0
2.5
3.0
3.5
4.0
Cellular_organisms - ps1
Archaea1 - ps2
Archaea2 - ps3
Archaea3 - ps4
Asgard/Eukaryota - ps5
Eukaryota - ps6
Amorpheae - ps7
Obazoa - ps8
Opisthokonta - ps9
Holomycota - ps10
Fungi - ps11
Fungi_1 - ps12
Fungi_2 - ps13
Fungi_3 - ps14
Dikarya - ps15
Ascomycota - ps16
Saccharo/Pezizomycotina - ps17
Saccharomycotina - ps18
Saccharomycotina_1 - ps19
Saccharomycotina_2 - ps20
Saccharomycotina_3 - ps21
Phaff./Saccharomycetaceae - ps22
Saccharomyco./mycetaceae - ps23
Saccharomycetaceae - ps24
Saccharomycetaceae_2 - ps25
Saccharomycetaceae_3 - ps26
Kaz./Nau./Saccharomyces - ps27
Saccharomyces - ps28
Arboricola_clade - ps29
Cerevisiae_clade - ps30
Saccharomyces_cerevisiae - ps31
Phylostratum

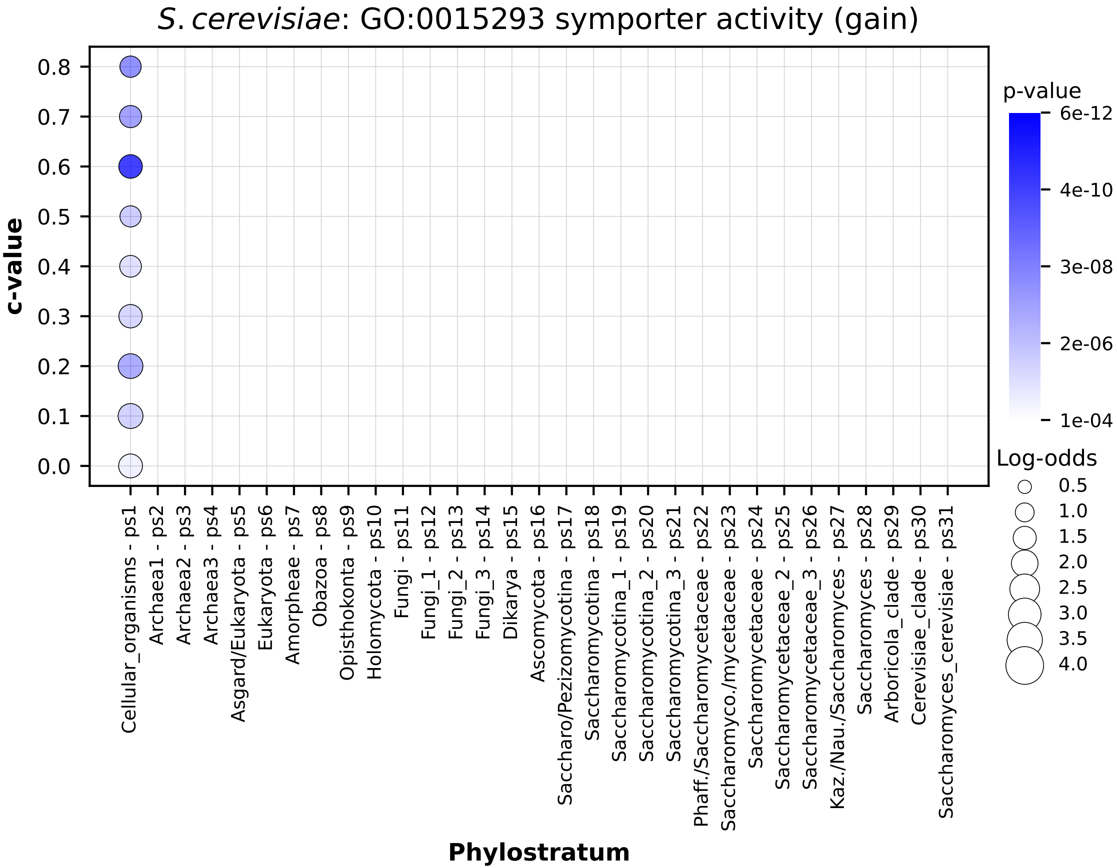

S. cerevisiae: GO:0015293 symporter activity (gain)
c-value
0.8
0.7
0.6
0.5
0.4
0.3
0.2
0.1
0.0
p-value
6e-12
4e-10
3e-08
2e-06
1e-04
Log-odds
0.5
1.0
1.5
2.0
2.5
3.0
3.5
4.0
Cellular_organisms - ps1
Archaea1 - ps2
Archaea2 - ps3
Archaea3 - ps4
Asgard/Eukaryota - ps5
Eukaryota - ps6
Amorpheae - ps7
Obazoa - ps8
Opisthokonta - ps9
Holomycota - ps10
Fungi - ps11
Fungi_1 - ps12
Fungi_2 - ps13
Fungi_3 - ps14
Dikarya - ps15
Ascomycota - ps16
Saccharo/Pezizomycotina - ps17
Saccharomycotina - ps18
Saccharomycotina_1 - ps19
Saccharomycotina_2 - ps20
Saccharomycotina_3 - ps21
Phaff./Saccharomycetaceae - ps22
Saccharomyco./mycetaceae - ps23
Saccharomycetaceae - ps24
Saccharomycetaceae_2 - ps25
Saccharomycetaceae_3 - ps26
Kaz./Nau./Saccharomyces - ps27
Saccharomyces - ps28
Arboricola_clade - ps29
Cerevisiae_clade - ps30
Saccharomyces_cerevisiae - ps31
Phylostratum

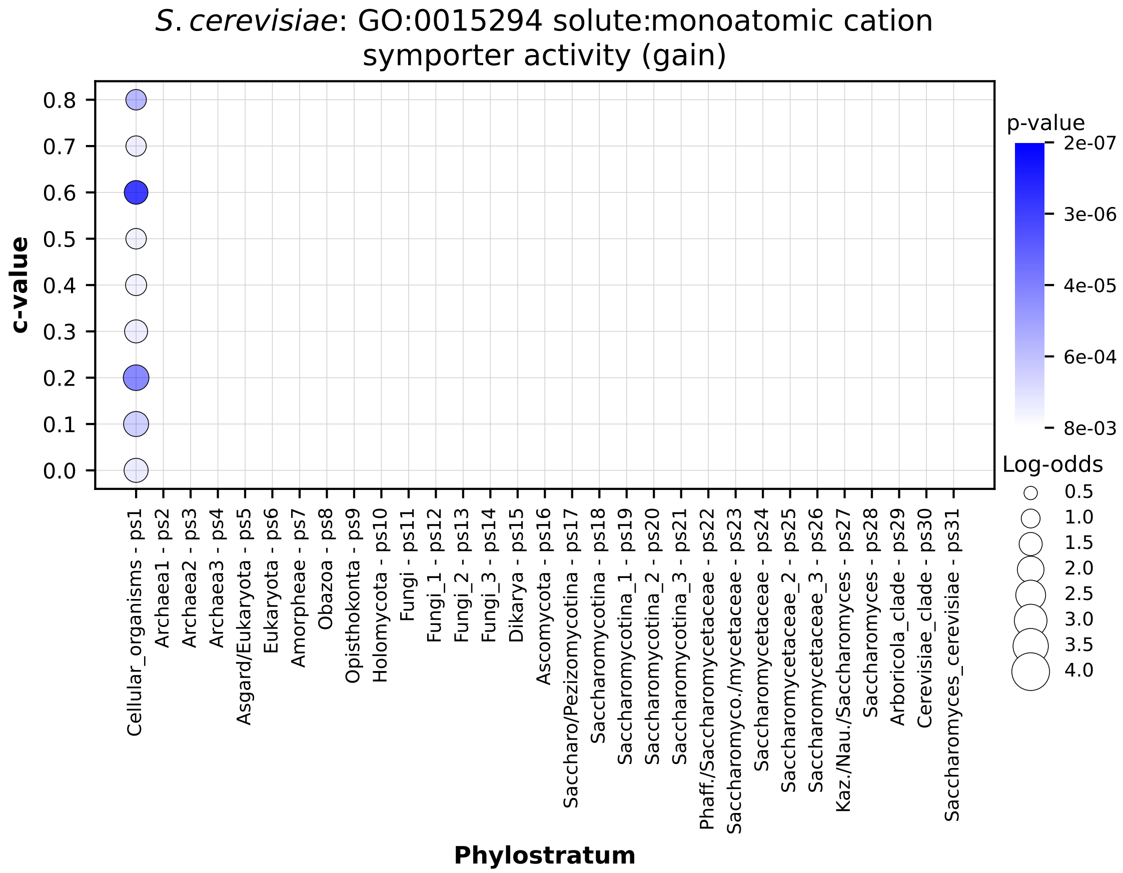
S. cerevisiae: GO:0015294 solute:monoatomic cation symporter activity (gain)
c-value
0.8
0.7
0.6
0.5
0.4
0.3
0.2
0.1
0.0
Phylostratum
Cellular_organisms - ps1
Archaea1 - ps2
Archaea2 - ps3
Archaea3 - ps4
Asgard/Eukaryota - ps5
Eukaryota - ps6
Amorpheae - ps7
Obazoa - ps8
Opisthokonta - ps9
Holomycota - ps10
Fungi - ps11
Fungi_1 - ps12
Fungi_2 - ps13
Fungi_3 - ps14
Dikarya - ps15
Ascomycota - ps16
Saccharo/Pezizomycotina - ps17
Saccharomycotina - ps18
Saccharomycotina_1 - ps19
Saccharomycotina_2 - ps20
Saccharomycotina_3 - ps21
Phaff./Saccharomycetaceae - ps22
Saccharomyco./mycetaceae - ps23
Saccharomycetaceae - ps24
Saccharomycetaceae_2 - ps25
Saccharomycetaceae_3 - ps26
Kaz./Nau./Saccharomyces - ps27
Saccharomyces - ps28
Arboricola_clade - ps29
Cerevisiae_clade - ps30
Saccharomyces_cerevisiae - ps31
p-value
2e-07
3e-06
4e-05
6e-04
8e-03
Log-odds
0.5
1.0
1.5
2.0
2.5
3.0
3.5
4.0

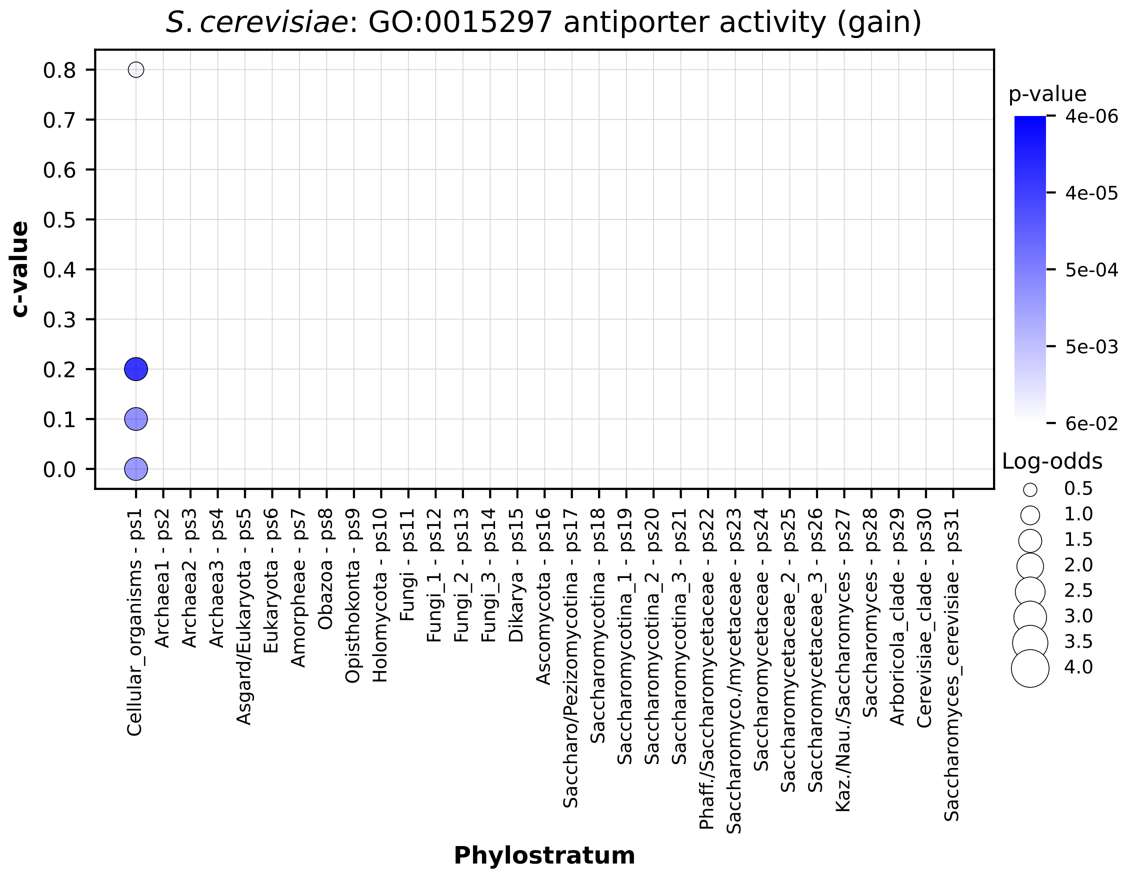

S. cerevisiae: GO:0015297 antiporter activity (gain)
c-value
0.8
0.7
0.6
0.5
0.4
0.3
0.2
0.1
0.0
p-value
4e-06
4e-05
5e-04
5e-03
6e-02
Log-odds
0.5
1.0
1.5
2.0
2.5
3.0
3.5
4.0
Cellular_organisms - ps1
Archaea1 - ps2
Archaea2 - ps3
Archaea3 - ps4
Asgard/Eukaryota - ps5
Eukaryota - ps6
Amorpheae - ps7
Obazoa - ps8
Opisthokonta - ps9
Holomycota - ps10
Fungi - ps11
Fungi_1 - ps12
Fungi_2 - ps13
Fungi_3 - ps14
Dikarya - ps15
Ascomycota - ps16
Saccharo/Pezizomycotina - ps17
Saccharomycotina - ps18
Saccharomycotina_1 - ps19
Saccharomycotina_2 - ps20
Saccharomycotina_3 - ps21
Phaff./Saccharomycetaceae - ps22
Saccharomyco./mycetaceae - ps23
Saccharomycetaceae - ps24
Saccharomycetaceae_2 - ps25
Saccharomycetaceae_3 - ps26
Kaz./Nau./Saccharomyces - ps27
Saccharomyces - ps28
Arboricola_clade - ps29
Cerevisiae_clade - ps30
Saccharomyces_cerevisiae - ps31
Phylostratum

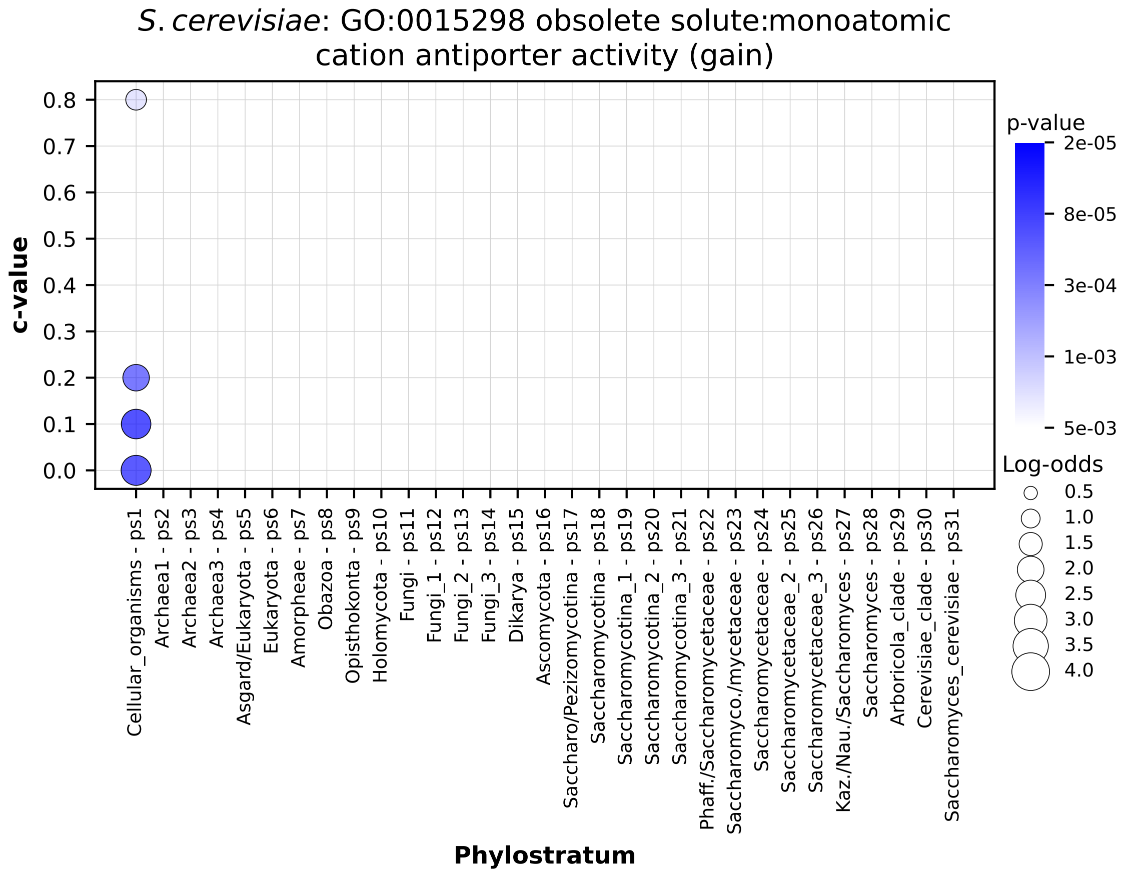

S. cerevisiae: GO:0015298 obsolete solute:monoatomic cation antiporter activity (gain)
c-value
0.8
0.7
0.6
0.5
0.4
0.3
0.2
0.1
0.0
Phylostratum
Cellular_organisms - ps1
Archaea1 - ps2
Archaea2 - ps3
Archaea3 - ps4
Asgard/Eukaryota - ps5
Eukaryota - ps6
Amorpheae - ps7
Obazoa - ps8
Opisthokonta - ps9
Holomycota - ps10
Fungi - ps11
Fungi_1 - ps12
Fungi_2 - ps13
Fungi_3 - ps14
Dikarya - ps15
Ascomycota - ps16
Saccharo/Pezizomycotina - ps17
Saccharomycotina - ps18
Saccharomycotina_1 - ps19
Saccharomycotina_2 - ps20
Saccharomycotina_3 - ps21
Phaff./Saccharomycetaceae - ps22
Saccharomyco./mycetaceae - ps23
Saccharomycetaceae - ps24
Saccharomycetaceae_2 - ps25
Saccharomycetaceae_3 - ps26
Kaz./Nau./Saccharomyces - ps27
Saccharomyces - ps28
Arboricola_clade - ps29
Cerevisiae_clade - ps30
Saccharomyces_cerevisiae - ps31
p-value
2e-05
8e-05
3e-04
1e-03
5e-03
Log-odds
0.5
1.0
1.5
2.0
2.5
3.0
3.5
4.0

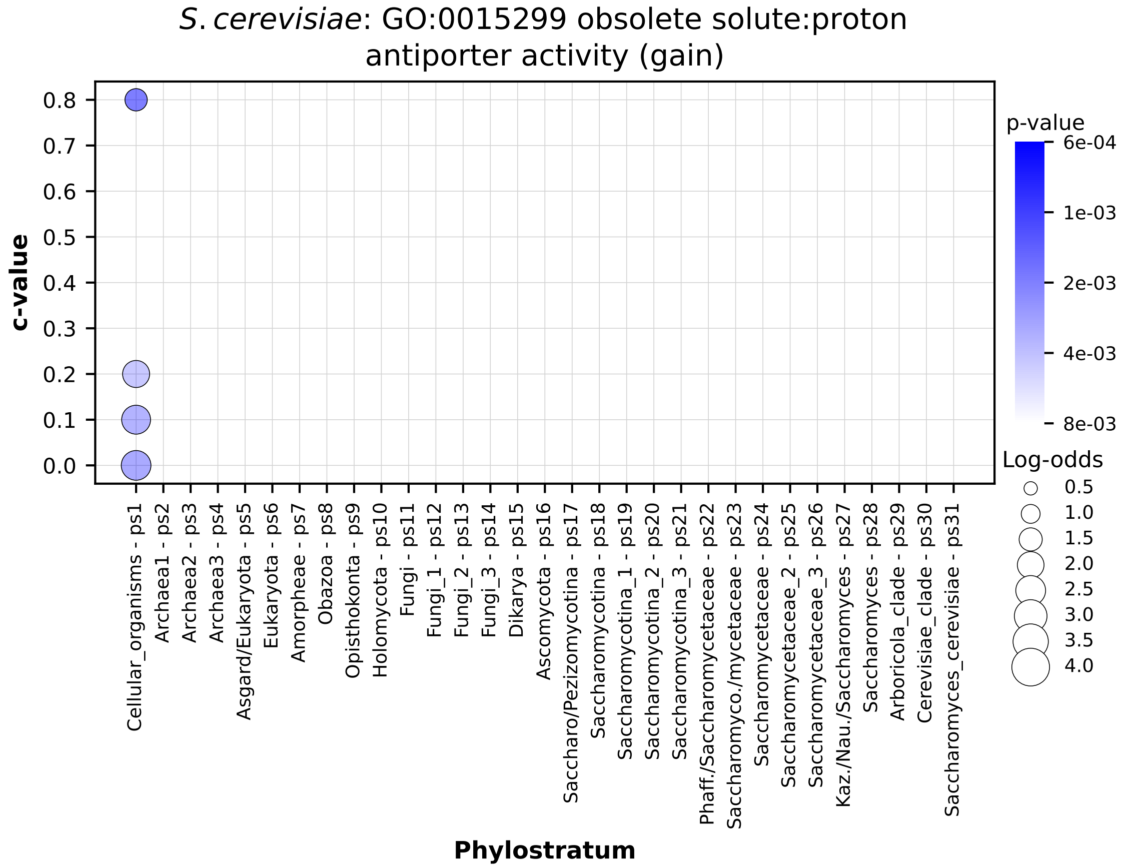

S. cerevisiae: GO:0015299 obsolete solute:proton antiporter activity (gain)
c-value
0.0
0.1
0.2
0.3
0.4
0.5
0.6
0.7
0.8
p-value
6e-04
1e-03
2e-03
4e-03
8e-03
Log-odds
0.5
1.0
1.5
2.0
2.5
3.0
3.5
4.0
Cellular_organisms - ps1
Archaea1 - ps2
Archaea2 - ps3
Archaea3 - ps4
Asgard/Eukaryota - ps5
Eukaryota - ps6
Amorpheae - ps7
Obazoa - ps8
Opisthokonta - ps9
Holomycota - ps10
Fungi - ps11
Fungi_1 - ps12
Fungi_2 - ps13
Fungi_3 - ps14
Dikarya - ps15
Ascomycota - ps16
Saccharo/Pezizomycotina - ps17
Saccharomycotina - ps18
Saccharomycotina_1 - ps19
Saccharomycotina_2 - ps20
Saccharomycotina_3 - ps21
Phaff./Saccharomycetaceae - ps22
Saccharomyco./mycetaceae - ps23
Saccharomycetaceae - ps24
Saccharomycetaceae_2 - ps25
Saccharomycetaceae_3 - ps26
Kaz./Nau./Saccharomyces - ps27
Saccharomyces - ps28
Arboricola_clade - ps29
Cerevisiae_clade - ps30
Saccharomyces_cerevisiae - ps31
Phylostratum

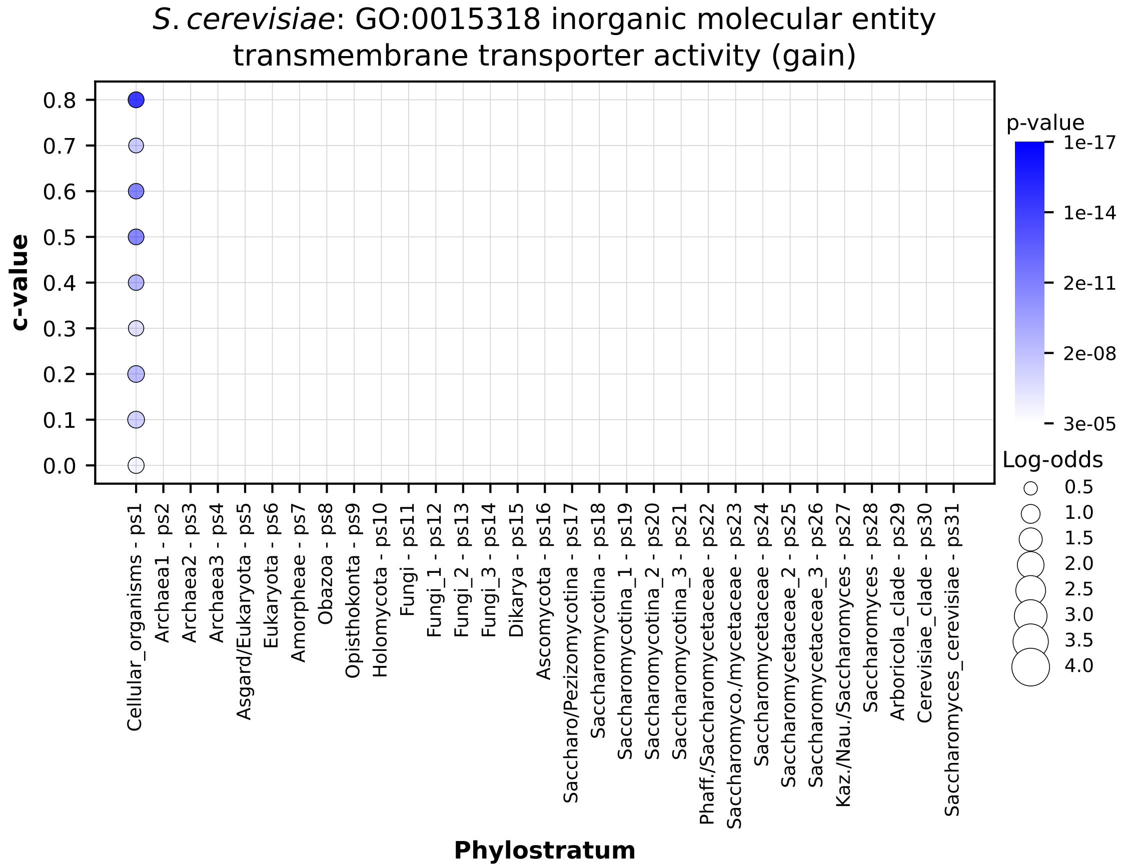

S. cerevisiae: GO:0015318 inorganic molecular entity transmembrane transporter activity (gain)
c-value
0.8
0.7
0.6
0.5
0.4
0.3
0.2
0.1
0.0
p-value
1e-17
1e-14
2e-11
2e-08
3e-05
Log-odds
0.5
1.0
1.5
2.0
2.5
3.0
3.5
4.0
Cellular_organisms - ps1
Archaea1 - ps2
Archaea2 - ps3
Archaea3 - ps4
Asgard/Eukaryota - ps5
Eukaryota - ps6
Amorpheae - ps7
Obazoa - ps8
Opisthokonta - ps9
Holomycota - ps10
Fungi - ps11
Fungi_1 - ps12
Fungi_2 - ps13
Fungi_3 - ps14
Dikarya - ps15
Ascomycota - ps16
Saccharo/Pezizomycotina - ps17
Saccharomycotina - ps18
Saccharomycotina_1 - ps19
Saccharomycotina_2 - ps20
Saccharomycotina_3 - ps21
Phaff./Saccharomycetaceae - ps22
Saccharomyco./mycetaceae - ps23
Saccharomycetaceae - ps24
Saccharomycetaceae_2 - ps25
Saccharomycetaceae_3 - ps26
Kaz./Nau./Saccharomyces - ps27
Saccharomyces - ps28
Arboricola_clade - ps29
Cerevisiae_clade - ps30
Saccharomyces_cerevisiae - ps31
Phylostratum

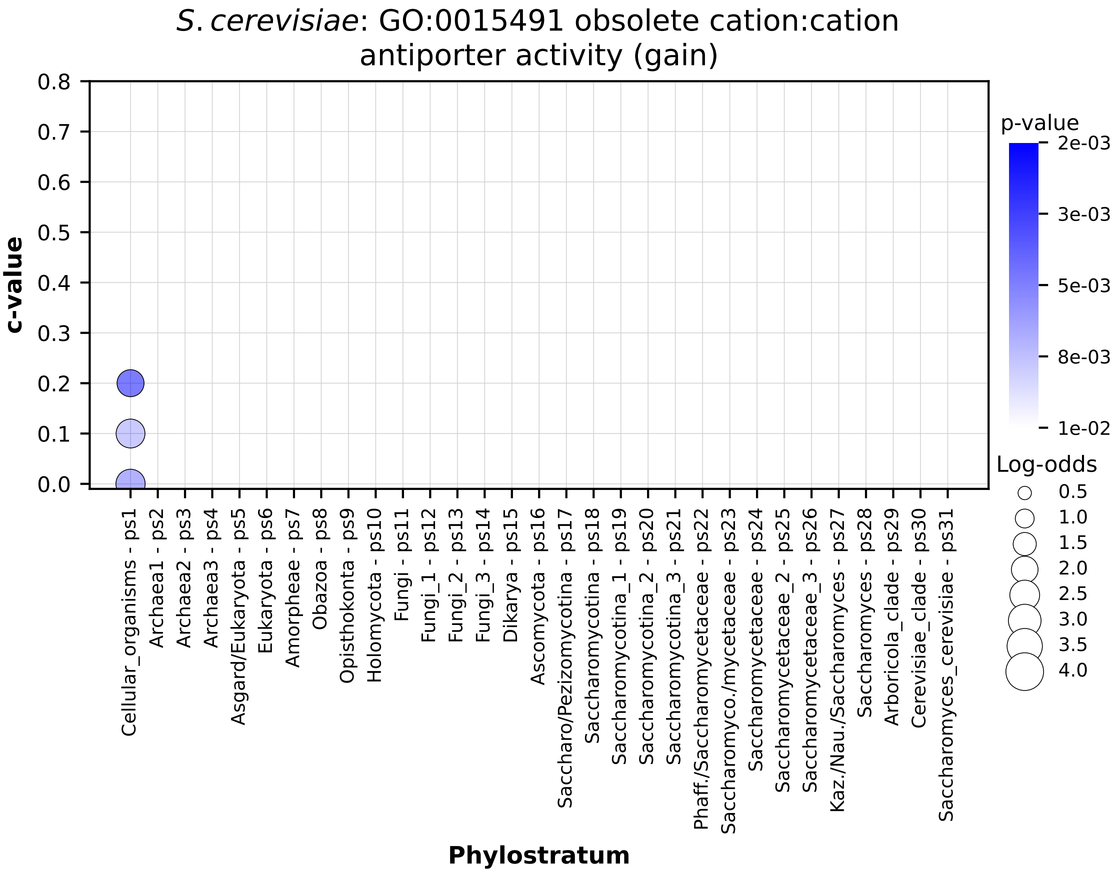

S. cerevisiae: GO:0015491 obsolete cation:cation antiporter activity (gain)
c-value
0.8
0.7
0.6
0.5
0.4
0.3
0.2
0.1
0.0
p-value
2e-03
3e-03
5e-03
8e-03
1e-02
Log-odds
0.5
1.0
1.5
2.0
2.5
3.0
3.5
4.0
Cellular_organisms - ps1
Archaea1 - ps2
Archaea2 - ps3
Archaea3 - ps4
Asgard/Eukaryota - ps5
Eukaryota - ps6
Amorpheae - ps7
Obazoa - ps8
Opisthokonta - ps9
Holomycota - ps10
Fungi - ps11
Fungi_1 - ps12
Fungi_2 - ps13
Fungi_3 - ps14
Dikarya - ps15
Ascomycota - ps16
Saccharo/Pezizomycotina - ps17
Saccharomycotina - ps18
Saccharomycotina_1 - ps19
Saccharomycotina_2 - ps20
Saccharomycotina_3 - ps21
Phaff./Saccharomycetaceae - ps22
Saccharomyco./mycetaceae - ps23
Saccharomycetaceae - ps24
Saccharomycetaceae_2 - ps25
Saccharomycetaceae_3 - ps26
Kaz./Nau./Saccharomyces - ps27
Saccharomyces - ps28
Arboricola_clade - ps29
Cerevisiae_clade - ps30
Saccharomyces_cerevisiae - ps31
Phylostratum

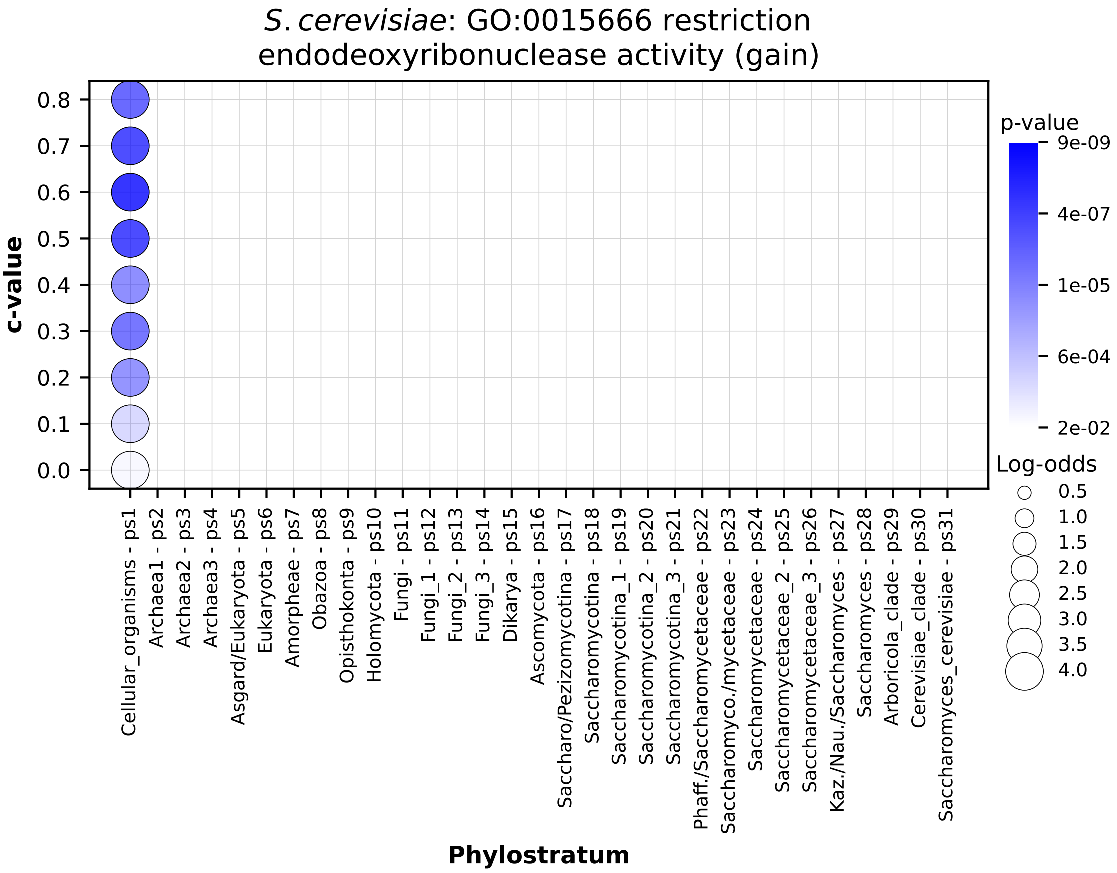
S. cerevisiae: GO:0015666 restriction endodeoxyribonuclease activity (gain)
c-value
0.8
0.7
0.6
0.5
0.4
0.3
0.2
0.1
0.0
p-value
9e-09
4e-07
1e-05
6e-04
2e-02
Log-odds
0.5
1.0
1.5
2.0
2.5
3.0
3.5
4.0
Cellular_organisms - ps1
Archaea1 - ps2
Archaea2 - ps3
Archaea3 - ps4
Asgard/Eukaryota - ps5
Eukaryota - ps6
Amorpheae - ps7
Obazoa - ps8
Opisthokonta - ps9
Holomycota - ps10
Fungi - ps11
Fungi_1 - ps12
Fungi_2 - ps13
Fungi_3 - ps14
Dikarya - ps15
Ascomycota - ps16
Saccharo/Pezizomycotina - ps17
Saccharomycotina - ps18
Saccharomycotina_1 - ps19
Saccharomycotina_2 - ps20
Saccharomycotina_3 - ps21
Phaff./Saccharomycetaceae - ps22
Saccharomyco./mycetaceae - ps23
Saccharomycetaceae - ps24
Saccharomycetaceae_2 - ps25
Saccharomycetaceae_3 - ps26
Kaz./Nau./Saccharomyces - ps27
Saccharomyces - ps28
Arboricola_clade - ps29
Cerevisiae_clade - ps30
Saccharomyces_cerevisiae - ps31
Phylostratum

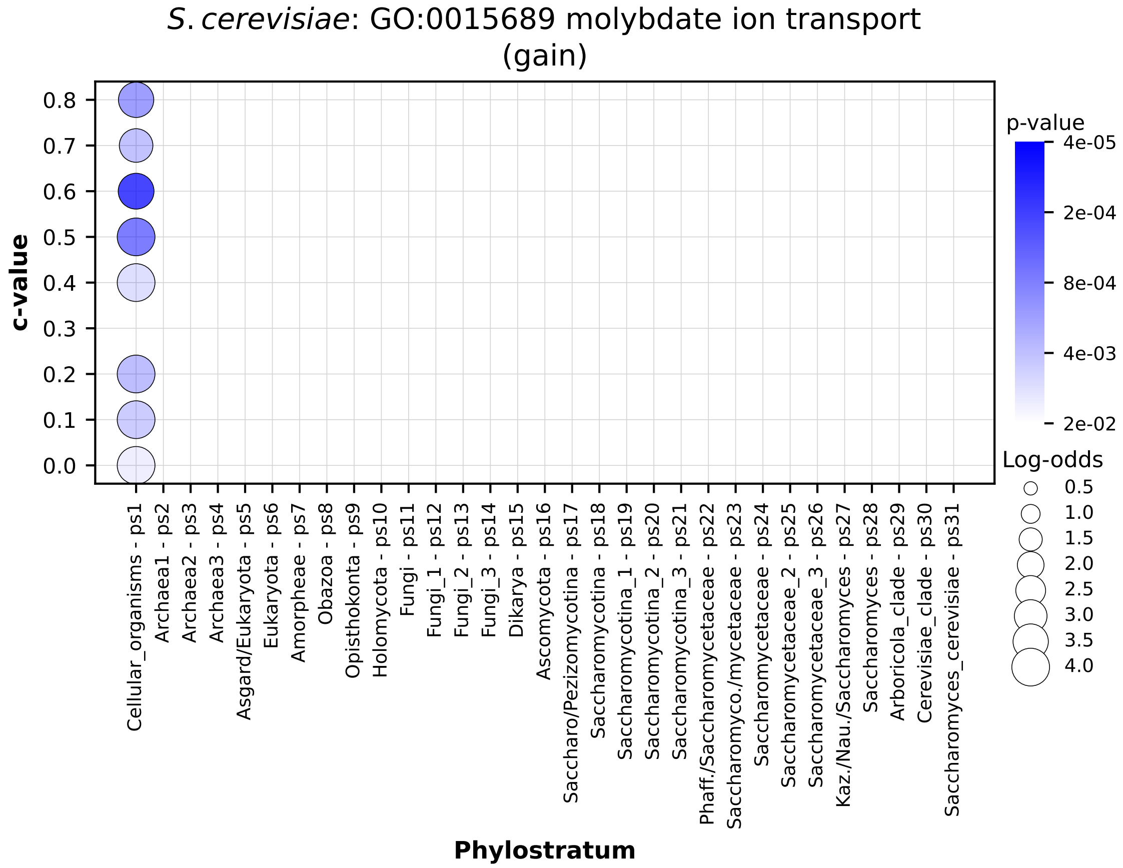
S. cerevisiae: GO:0015689 molybdate ion transport
(gain)
c-value
0.8
0.7
0.6
0.5
0.4
0.3
0.2
0.1
0.0
p-value
4e-05
2e-04
8e-04
4e-03
2e-02
Log-odds
0.5
1.0
1.5
2.0
2.5
3.0
3.5
4.0
Cellular_organisms - ps1
Archaea1 - ps2
Archaea2 - ps3
Archaea3 - ps4
Asgard/Eukaryota - ps5
Eukaryota - ps6
Amorpheae - ps7
Obazoa - ps8
Opisthokonta - ps9
Holomycota - ps10
Fungi - ps11
Fungi_1 - ps12
Fungi_2 - ps13
Fungi_3 - ps14
Dikarya - ps15
Ascomycota - ps16
Saccharo/Pezizomycotina - ps17
Saccharomycotina - ps18
Saccharomycotina_1 - ps19
Saccharomycotina_2 - ps20
Saccharomycotina_3 - ps21
Phaff./Saccharomycetaceae - ps22
Saccharomyco./mycetaceae - ps23
Saccharomycetaceae - ps24
Saccharomycetaceae_2 - ps25
Saccharomycetaceae_3 - ps26
Kaz./Nau./Saccharomyces - ps27
Saccharomyces - ps28
Arboricola_clade - ps29
Cerevisiae_clade - ps30
Saccharomyces_cerevisiae - ps31
Phylostratum

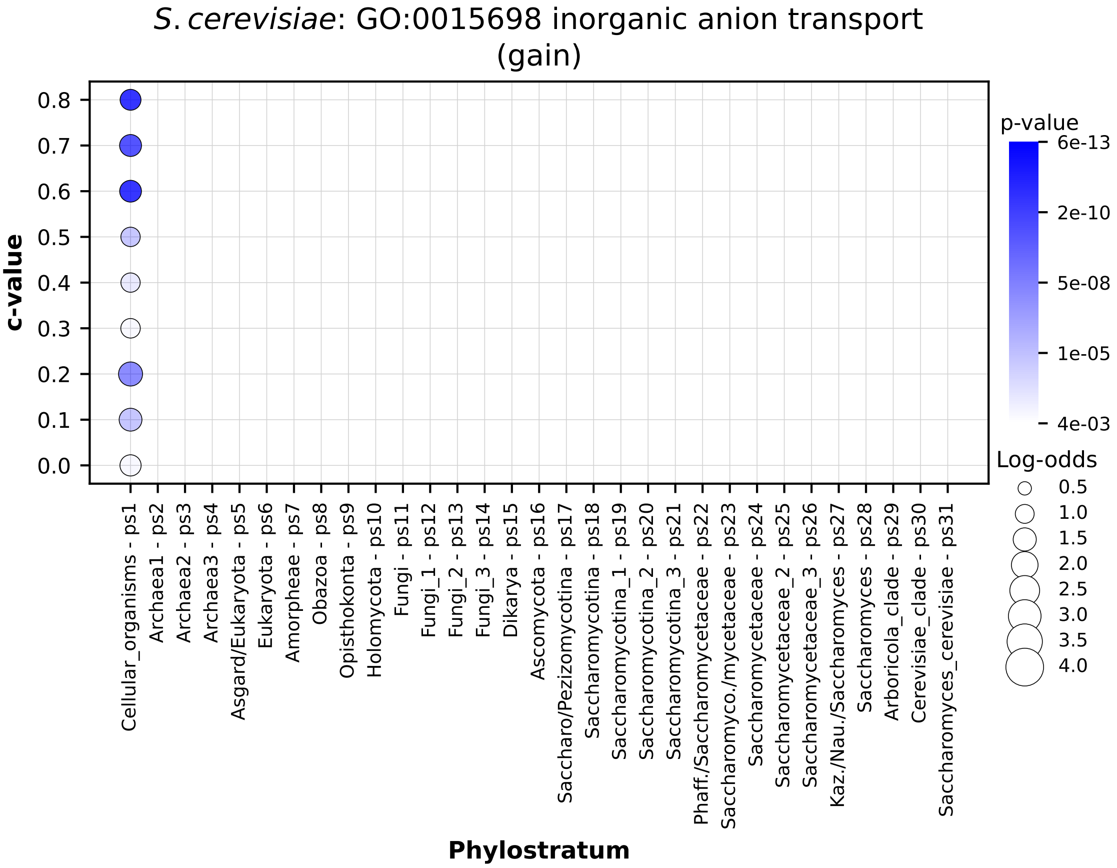
S. cerevisiae: GO:0015698 inorganic anion transport
(gain)
c-value
0.0
0.1
0.2
0.3
0.4
0.5
0.6
0.7
0.8
p-value
6e-13
2e-10
5e-08
1e-05
4e-03
Log-odds
0.5
1.0
1.5
2.0
2.5
3.0
3.5
4.0
Cellular_organisms - ps1
Archaea1 - ps2
Archaea2 - ps3
Archaea3 - ps4
Asgard/Eukaryota - ps5
Eukaryota - ps6
Amorpheae - ps7
Obazoa - ps8
Opisthokonta - ps9
Holomycota - ps10
Fungi - ps11
Fungi_1 - ps12
Fungi_2 - ps13
Fungi_3 - ps14
Dikarya - ps15
Ascomycota - ps16
Saccharo/Pezizomycotina - ps17
Saccharomycotina - ps18
Saccharomycotina_1 - ps19
Saccharomycotina_2 - ps20
Saccharomycotina_3 - ps21
Phaff./Saccharomycetaceae - ps22
Saccharomyco./mycetaceae - ps23
Saccharomycetaceae - ps24
Saccharomycetaceae_2 - ps25
Saccharomycetaceae_3 - ps26
Kaz./Nau./Saccharomyces - ps27
Saccharomyces - ps28
Arboricola_clade - ps29
Cerevisiae_clade - ps30
Saccharomyces_cerevisiae - ps31
Phylostratum

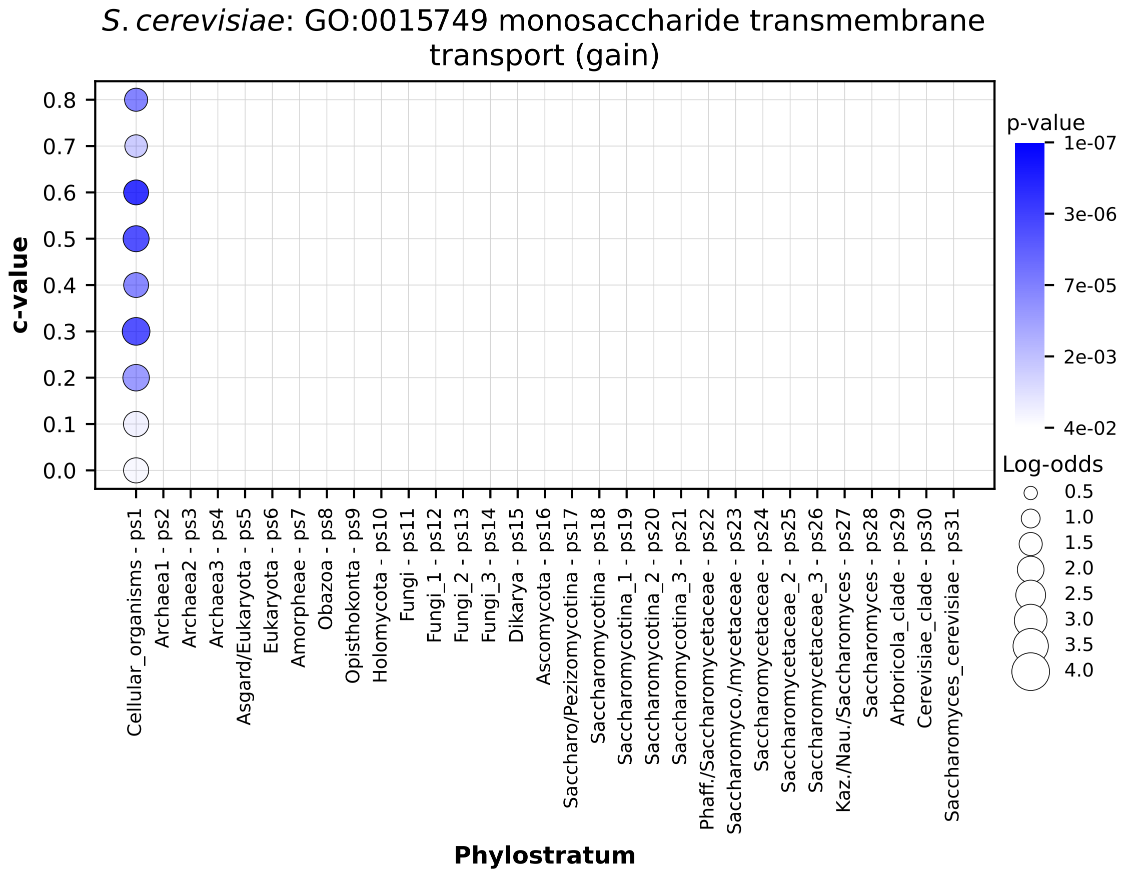

S. cerevisiae: GO:0015749 monosaccharide transmembrane transport (gain)
c-value
0.8
0.7
0.6
0.5
0.4
0.3
0.2
0.1
0.0
p-value
1e-07
3e-06
7e-05
2e-03
4e-02
Log-odds
0.5
1.0
1.5
2.0
2.5
3.0
3.5
4.0
Cellular_organisms - ps1
Archaea1 - ps2
Archaea2 - ps3
Archaea3 - ps4
Asgard/Eukaryota - ps5
Eukaryota - ps6
Amorpheae - ps7
Obazoa - ps8
Opisthokonta - ps9
Holomycota - ps10
Fungi - ps11
Fungi_1 - ps12
Fungi_2 - ps13
Fungi_3 - ps14
Dikarya - ps15
Ascomycota - ps16
Saccharo/Pezizomycotina - ps17
Saccharomycotina - ps18
Saccharomycotina_1 - ps19
Saccharomycotina_2 - ps20
Saccharomycotina_3 - ps21
Phaff./Saccharomycetaceae - ps22
Saccharomyco./mycetaceae - ps23
Saccharomycetaceae - ps24
Saccharomycetaceae_2 - ps25
Saccharomycetaceae_3 - ps26
Kaz./Nau./Saccharomyces - ps27
Saccharomyces - ps28
Arboricola_clade - ps29
Cerevisiae_clade - ps30
Saccharomyces_cerevisiae - ps31
Phylostratum

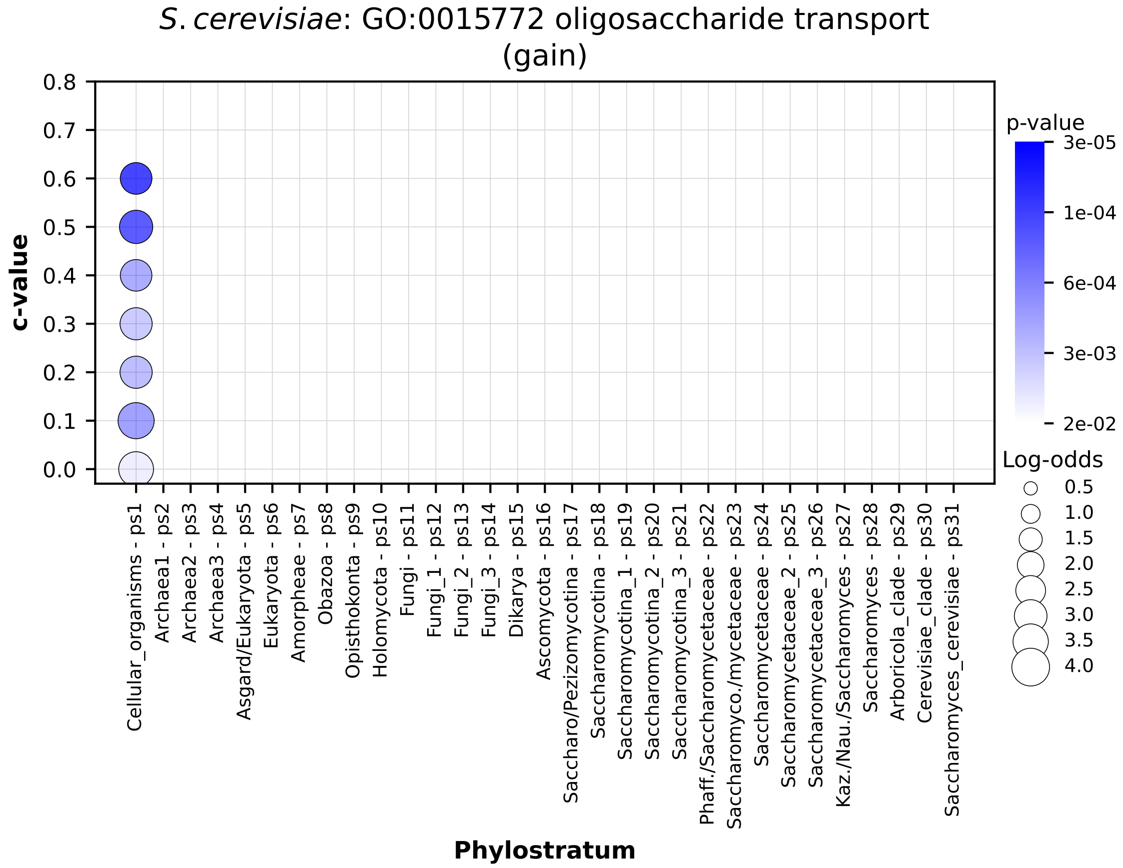
S. cerevisiae: GO:0015772 oligosaccharide transport
(gain)
c-value
0.8
0.7
0.6
0.5
0.4
0.3
0.2
0.1
0.0
p-value
3e-05
1e-04
6e-04
3e-03
2e-02
Log-odds
0.5
1.0
1.5
2.0
2.5
3.0
3.5
4.0
Cellular_organisms - ps1
Archaea1 - ps2
Archaea2 - ps3
Archaea3 - ps4
Asgard/Eukaryota - ps5
Eukaryota - ps6
Amorpheae - ps7
Obazoa - ps8
Opisthokonta - ps9
Holomycota - ps10
Fungi - ps11
Fungi_1 - ps12
Fungi_2 - ps13
Fungi_3 - ps14
Dikarya - ps15
Ascomycota - ps16
Saccharo/Pezizomycotina - ps17
Saccharomycotina - ps18
Saccharomycotina_1 - ps19
Saccharomycotina_2 - ps20
Saccharomycotina_3 - ps21
Phaff./Saccharomycetaceae - ps22
Saccharomyco./mycetaceae - ps23
Saccharomycetaceae - ps24
Saccharomycetaceae_2 - ps25
Saccharomycetaceae_3 - ps26
Kaz./Nau./Saccharomyces - ps27
Saccharomyces - ps28
Arboricola_clade - ps29
Cerevisiae_clade - ps30
Saccharomyces_cerevisiae - ps31
Phylostratum

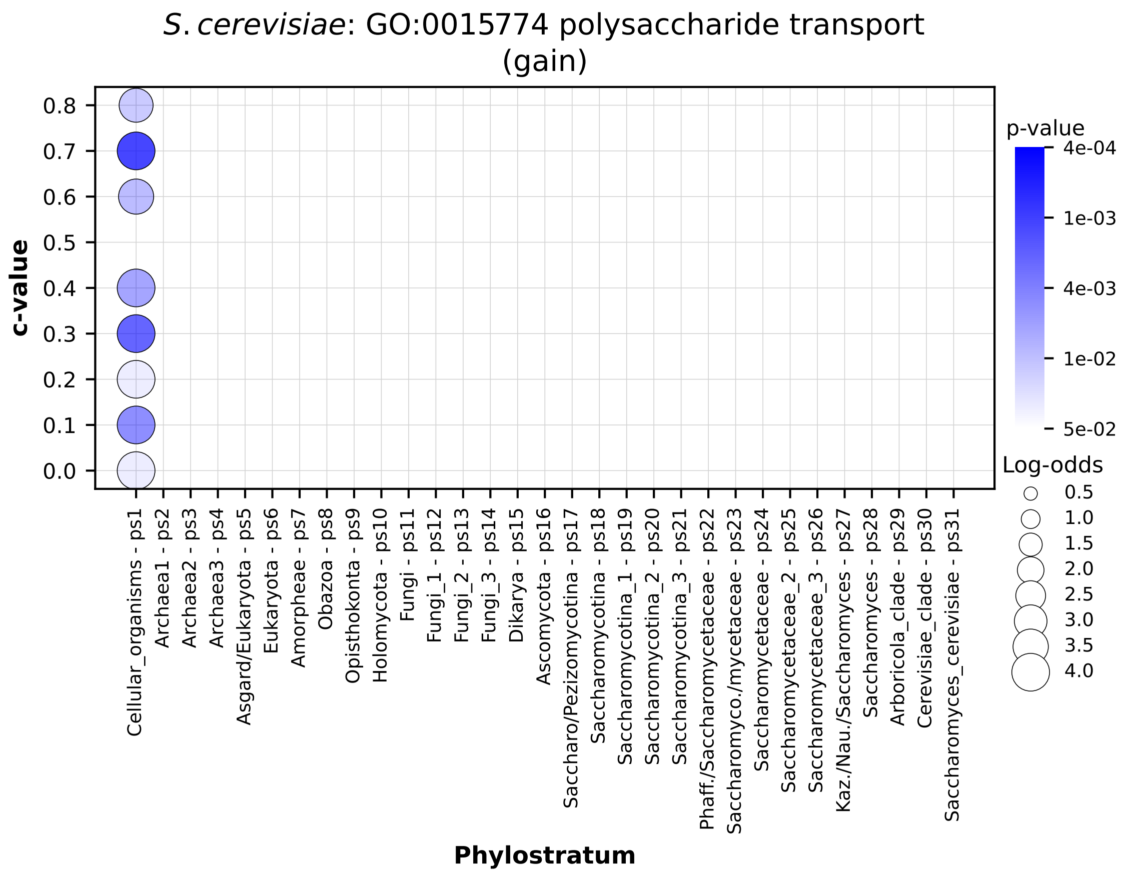

S. cerevisiae: GO:0015774 polysaccharide transport
(gain)
c-value
0.8
0.7
0.6
0.5
0.4
0.3
0.2
0.1
0.0
p-value
4e-04
1e-03
4e-03
1e-02
5e-02
Log-odds
0.5
1.0
1.5
2.0
2.5
3.0
3.5
4.0
Cellular_organisms - ps1
Archaea1 - ps2
Archaea2 - ps3
Archaea3 - ps4
Asgard/Eukaryota - ps5
Eukaryota - ps6
Amorpheae - ps7
Obazoa - ps8
Opisthokonta - ps9
Holomycota - ps10
Fungi - ps11
Fungi_1 - ps12
Fungi_2 - ps13
Fungi_3 - ps14
Dikarya - ps15
Ascomycota - ps16
Saccharo/Pezizomycotina - ps17
Saccharomycotina - ps18
Saccharomycotina_1 - ps19
Saccharomycotina_2 - ps20
Saccharomycotina_3 - ps21
Phaff./Saccharomycetaceae - ps22
Saccharomyco./mycetaceae - ps23
Saccharomycetaceae - ps24
Saccharomycetaceae_2 - ps25
Saccharomycetaceae_3 - ps26
Kaz./Nau./Saccharomyces - ps27
Saccharomyces - ps28
Arboricola_clade - ps29
Cerevisiae_clade - ps30
Saccharomyces_cerevisiae - ps31
Phylostratum

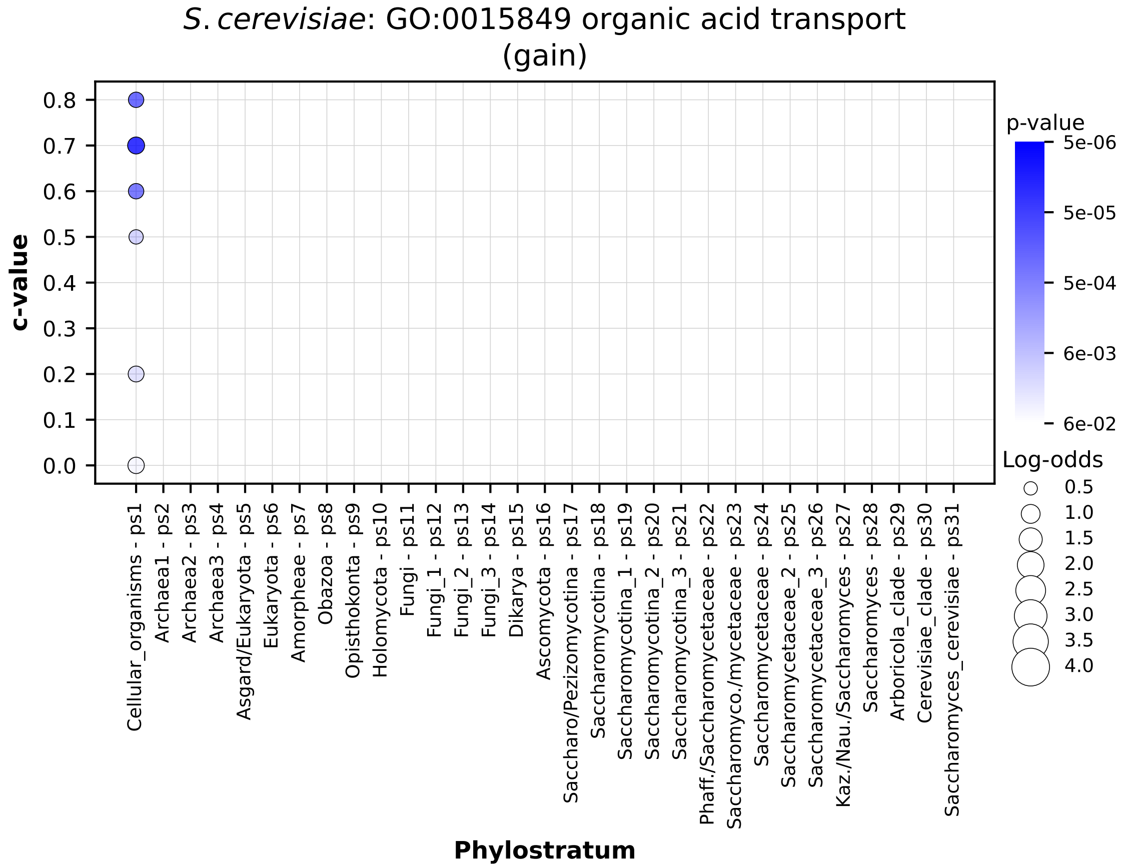
S. cerevisiae: GO:0015849 organic acid transport
(gain)
c-value
0.0
0.1
0.2
0.3
0.4
0.5
0.6
0.7
0.8
p-value
5e-06
5e-05
5e-04
6e-03
6e-02
Log-odds
0.5
1.0
1.5
2.0
2.5
3.0
3.5
4.0
Cellular_organisms - ps1
Archaea1 - ps2
Archaea2 - ps3
Archaea3 - ps4
Asgard/Eukaryota - ps5
Eukaryota - ps6
Amorpheae - ps7
Obazoa - ps8
Opisthokonta - ps9
Holomycota - ps10
Fungi - ps11
Fungi_1 - ps12
Fungi_2 - ps13
Fungi_3 - ps14
Dikarya - ps15
Ascomycota - ps16
Saccharo/Pezizomycotina - ps17
Saccharomycotina - ps18
Saccharomycotina_1 - ps19
Saccharomycotina_2 - ps20
Saccharomycotina_3 - ps21
Phaff./Saccharomycetaceae - ps22
Saccharomyco./mycetaceae - ps23
Saccharomycetaceae - ps24
Saccharomycetaceae_2 - ps25
Saccharomycetaceae_3 - ps26
Kaz./Nau./Saccharomyces - ps27
Saccharomyces - ps28
Arboricola_clade - ps29
Cerevisiae_clade - ps30
Saccharomyces_cerevisiae - ps31
Phylostratum

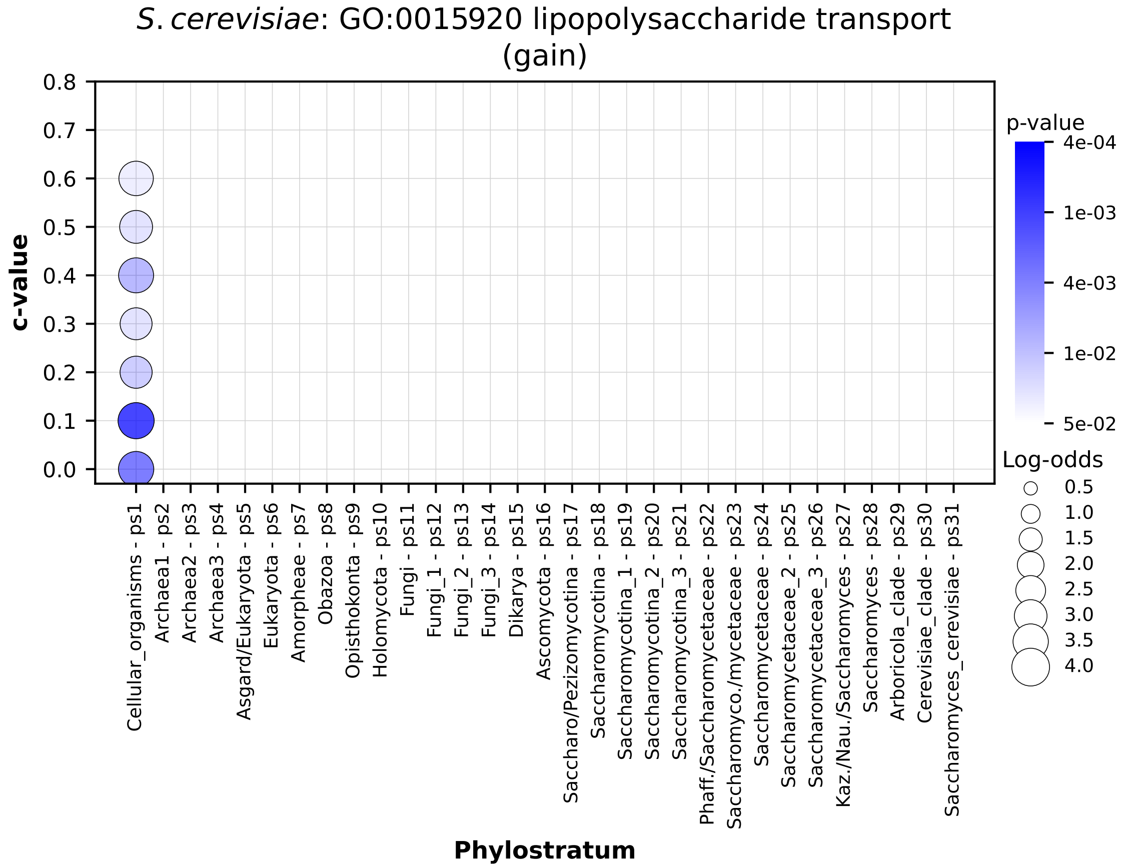
S. cerevisiae: GO:0015920 lipopolysaccharide transport
(gain)
c-value
0.8
0.7
0.6
0.5
0.4
0.3
0.2
0.1
0.0
p-value
4e-04
1e-03
4e-03
1e-02
5e-02
Log-odds
0.5
1.0
1.5
2.0
2.5
3.0
3.5
4.0
Cellular_organisms - ps1
Archaea1 - ps2
Archaea2 - ps3
Archaea3 - ps4
Asgard/Eukaryota - ps5
Eukaryota - ps6
Amorpheae - ps7
Obazoa - ps8
Opisthokonta - ps9
Holomycota - ps10
Fungi - ps11
Fungi_1 - ps12
Fungi_2 - ps13
Fungi_3 - ps14
Dikarya - ps15
Ascomycota - ps16
Saccharo/Pezizomycotina - ps17
Saccharomycotina - ps18
Saccharomycotina_1 - ps19
Saccharomycotina_2 - ps20
Saccharomycotina_3 - ps21
Phaff./Saccharomycetaceae - ps22
Saccharomyco./mycetaceae - ps23
Saccharomycetaceae - ps24
Saccharomycetaceae_2 - ps25
Saccharomycetaceae_3 - ps26
Kaz./Nau./Saccharomyces - ps27
Saccharomyces - ps28
Arboricola_clade - ps29
Cerevisiae_clade - ps30
Saccharomyces_cerevisiae - ps31
Phylostratum

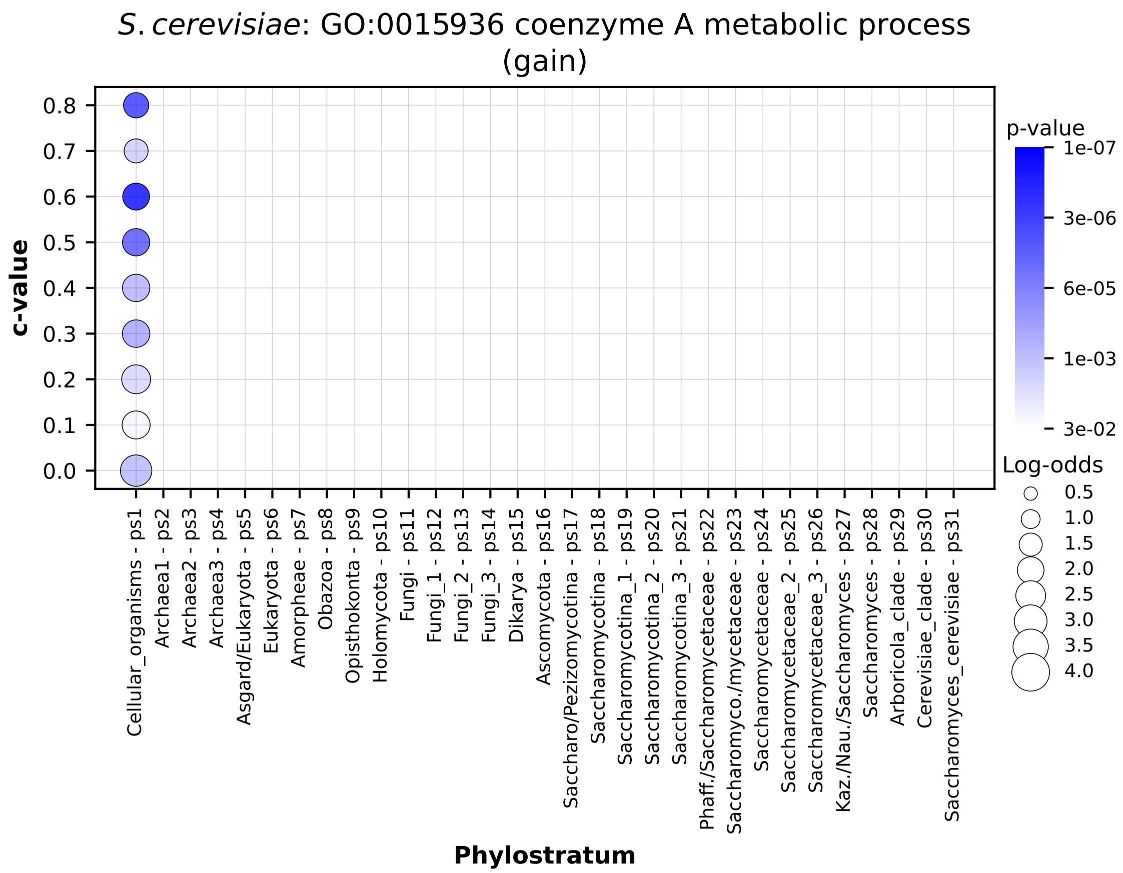

S. cerevisiae: GO:0015936 coenzyme A metabolic process (gain)
c-value
0.0
0.1
0.2
0.3
0.4
0.5
0.6
0.7
0.8
p-value
1e-07
3e-06
6e-05
1e-03
3e-02
Log-odds
0.5
1.0
1.5
2.0
2.5
3.0
3.5
4.0
Cellular_organisms - ps1
Archaea1 - ps2
Archaea2 - ps3
Archaea3 - ps4
Asgard/Eukaryota - ps5
Eukaryota - ps6
Amorpheae - ps7
Obazoa - ps8
Opisthokonta - ps9
Holomycota - ps10
Fungi - ps11
Fungi_1 - ps12
Fungi_2 - ps13
Fungi_3 - ps14
Dikarya - ps15
Ascomycota - ps16
Saccharo/Pezizomycotina - ps17
Saccharomycotina - ps18
Saccharomycotina_1 - ps19
Saccharomycotina_2 - ps20
Saccharomycotina_3 - ps21
Phaff./Saccharomycetaceae - ps22
Saccharomyco./mycetaceae - ps23
Saccharomycetaceae - ps24
Saccharomycetaceae_2 - ps25
Saccharomycetaceae_3 - ps26
Kaz./Nau./Saccharomyces - ps27
Saccharomyces - ps28
Arboricola_clade - ps29
Cerevisiae_clade - ps30
Saccharomyces_cerevisiae - ps31
Phylostratum

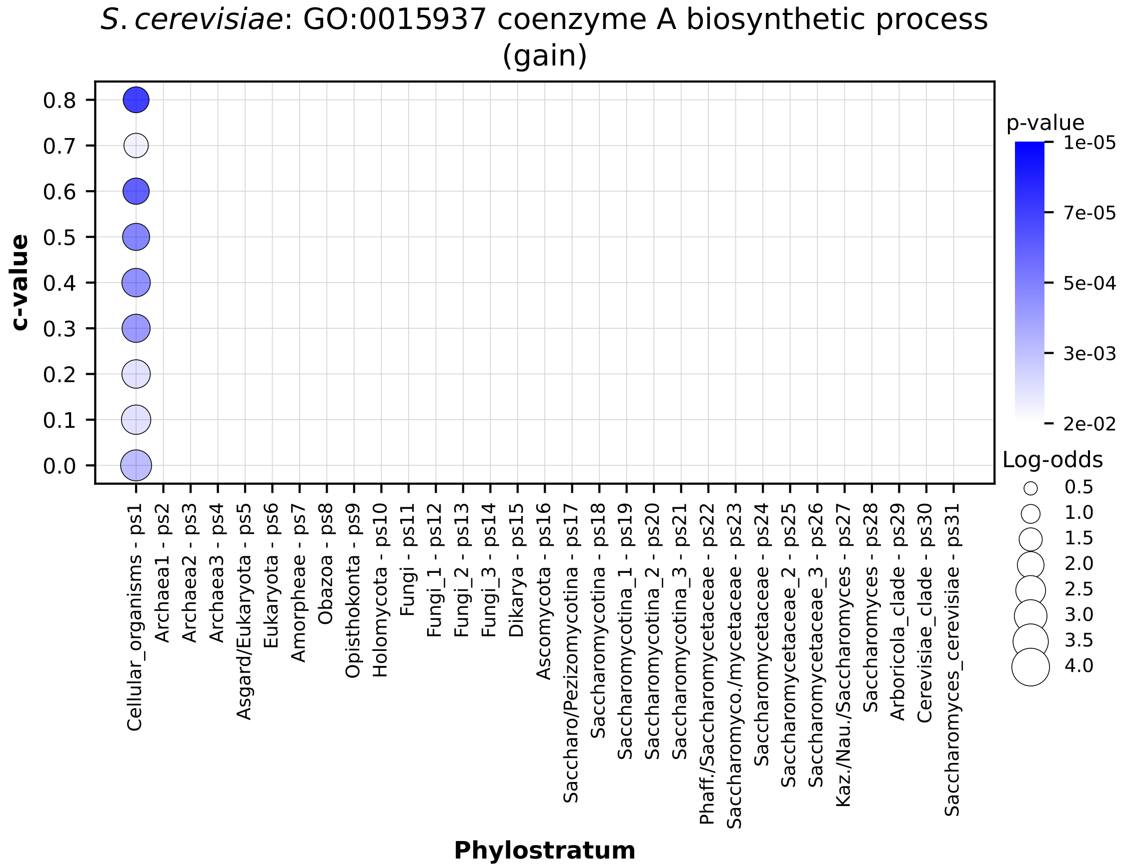

S. cerevisiae: GO:0015937 coenzyme A biosynthetic process (gain)
c-value
0.8
0.7
0.6
0.5
0.4
0.3
0.2
0.1
0.0
Phylostratum
Cellular_organisms - ps1
Archaea1 - ps2
Archaea2 - ps3
Archaea3 - ps4
Asgard/Eukaryota - ps5
Eukaryota - ps6
Amorpheae - ps7
Obazoa - ps8
Opisthokonta - ps9
Holomycota - ps10
Fungi - ps11
Fungi_1 - ps12
Fungi_2 - ps13
Fungi_3 - ps14
Dikarya - ps15
Ascomycota - ps16
Saccharo/Pezizomycotina - ps17
Saccharomycotina - ps18
Saccharomycotina_1 - ps19
Saccharomycotina_2 - ps20
Saccharomycotina_3 - ps21
Phaff./Saccharomycetaceae - ps22
Saccharomyco./mycetaceae - ps23
Saccharomycetaceae - ps24
Saccharomycetaceae_2 - ps25
Saccharomycetaceae_3 - ps26
Kaz./Nau./Saccharomyces - ps27
Saccharomyces - ps28
Arboricola_clade - ps29
Cerevisiae_clade - ps30
Saccharomyces_cerevisiae - ps31
p-value
1e-05
7e-05
5e-04
3e-03
2e-02
Log-odds
0.5
1.0
1.5
2.0
2.5
3.0
3.5
4.0

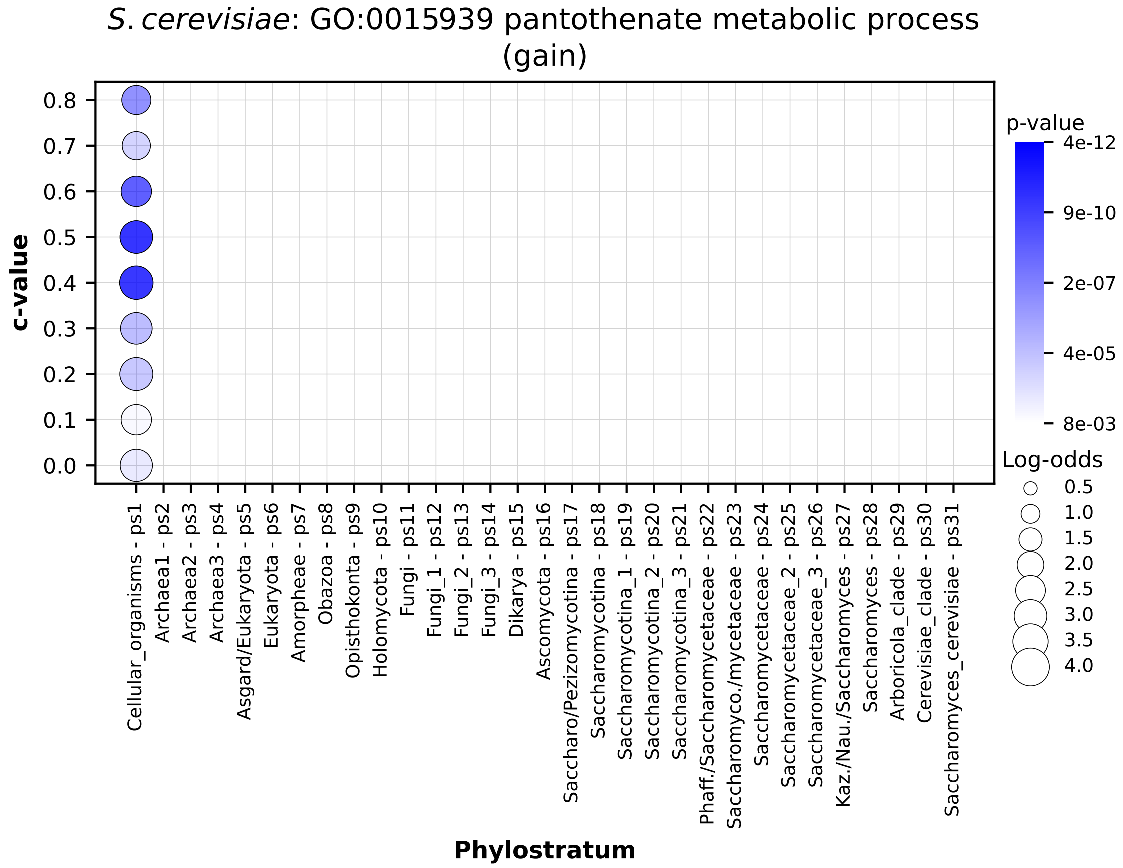

S. cerevisiae: GO:0015939 pantothenate metabolic process (gain)
c-value
0.8
0.7
0.6
0.5
0.4
0.3
0.2
0.1
0.0
p-value
4e-12
9e-10
2e-07
4e-05
8e-03
Log-odds
0.5
1.0
1.5
2.0
2.5
3.0
3.5
4.0
Cellular_organisms - ps1
Archaea1 - ps2
Archaea2 - ps3
Archaea3 - ps4
Asgard/Eukaryota - ps5
Eukaryota - ps6
Amorpheae - ps7
Obazoa - ps8
Opisthokonta - ps9
Holomycota - ps10
Fungi - ps11
Fungi_1 - ps12
Fungi_2 - ps13
Fungi_3 - ps14
Dikarya - ps15
Ascomycota - ps16
Saccharo/Pezizomycotina - ps17
Saccharomycotina - ps18
Saccharomycotina_1 - ps19
Saccharomycotina_2 - ps20
Saccharomycotina_3 - ps21
Phaff./Saccharomycetaceae - ps22
Saccharomyco./mycetaceae - ps23
Saccharomycetaceae - ps24
Saccharomycetaceae_2 - ps25
Saccharomycetaceae_3 - ps26
Kaz./Nau./Saccharomyces - ps27
Saccharomyces - ps28
Arboricola_clade - ps29
Cerevisiae_clade - ps30
Saccharomyces_cerevisiae - ps31
Phylostratum

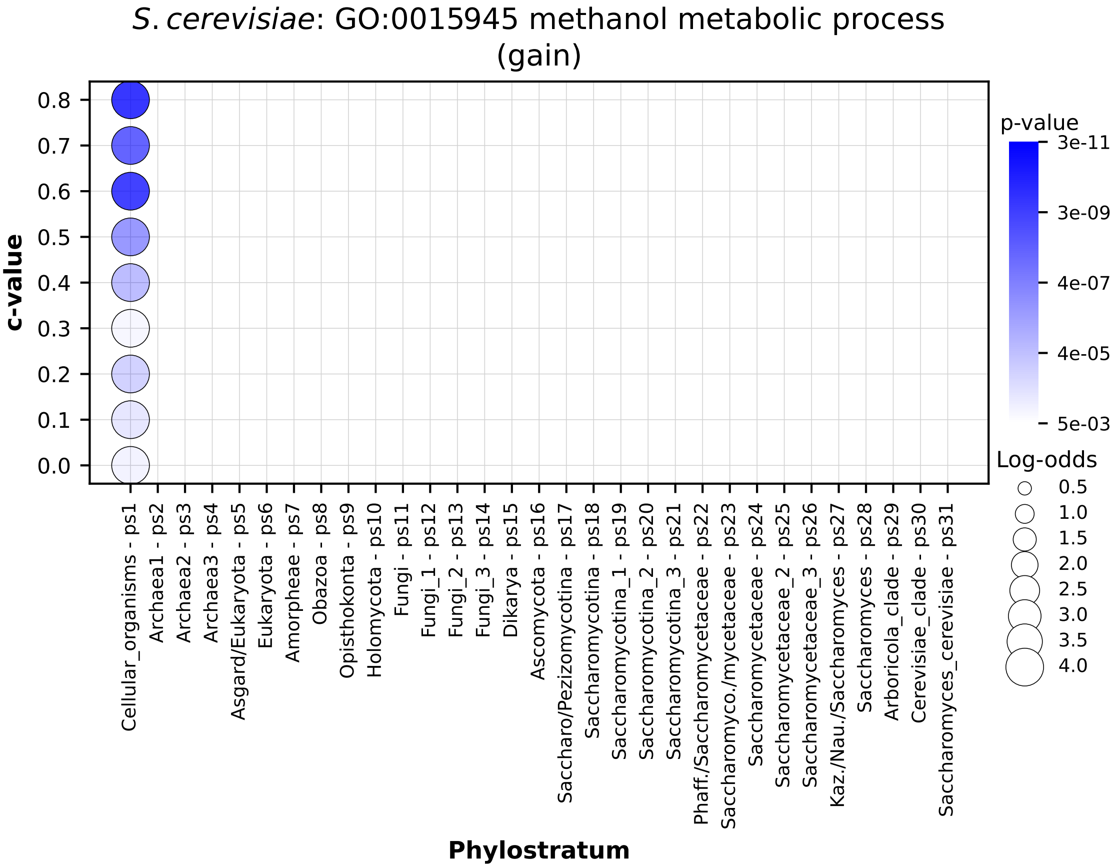

S. cerevisiae: GO:0015945 methanol metabolic process (gain)
c-value
0.0
0.1
0.2
0.3
0.4
0.5
0.6
0.7
0.8
p-value
3e-11
3e-09
4e-07
4e-05
5e-03
Log-odds
0.5
1.0
1.5
2.0
2.5
3.0
3.5
4.0
Cellular_organisms - ps1
Archaea1 - ps2
Archaea2 - ps3
Archaea3 - ps4
Asgard/Eukaryota - ps5
Eukaryota - ps6
Amorpheae - ps7
Obazoa - ps8
Opisthokonta - ps9
Holomycota - ps10
Fungi - ps11
Fungi_1 - ps12
Fungi_2 - ps13
Fungi_3 - ps14
Dikarya - ps15
Ascomycota - ps16
Saccharo/Pezizomycotina - ps17
Saccharomycotina - ps18
Saccharomycotina_1 - ps19
Saccharomycotina_2 - ps20
Saccharomycotina_3 - ps21
Phaff./Saccharomycetaceae - ps22
Saccharomyco./mycetaceae - ps23
Saccharomycetaceae - ps24
Saccharomycetaceae_2 - ps25
Saccharomycetaceae_3 - ps26
Kaz./Nau./Saccharomyces - ps27
Saccharomyces - ps28
Arboricola_clade - ps29
Cerevisiae_clade - ps30
Saccharomyces_cerevisiae - ps31
Phylostratum

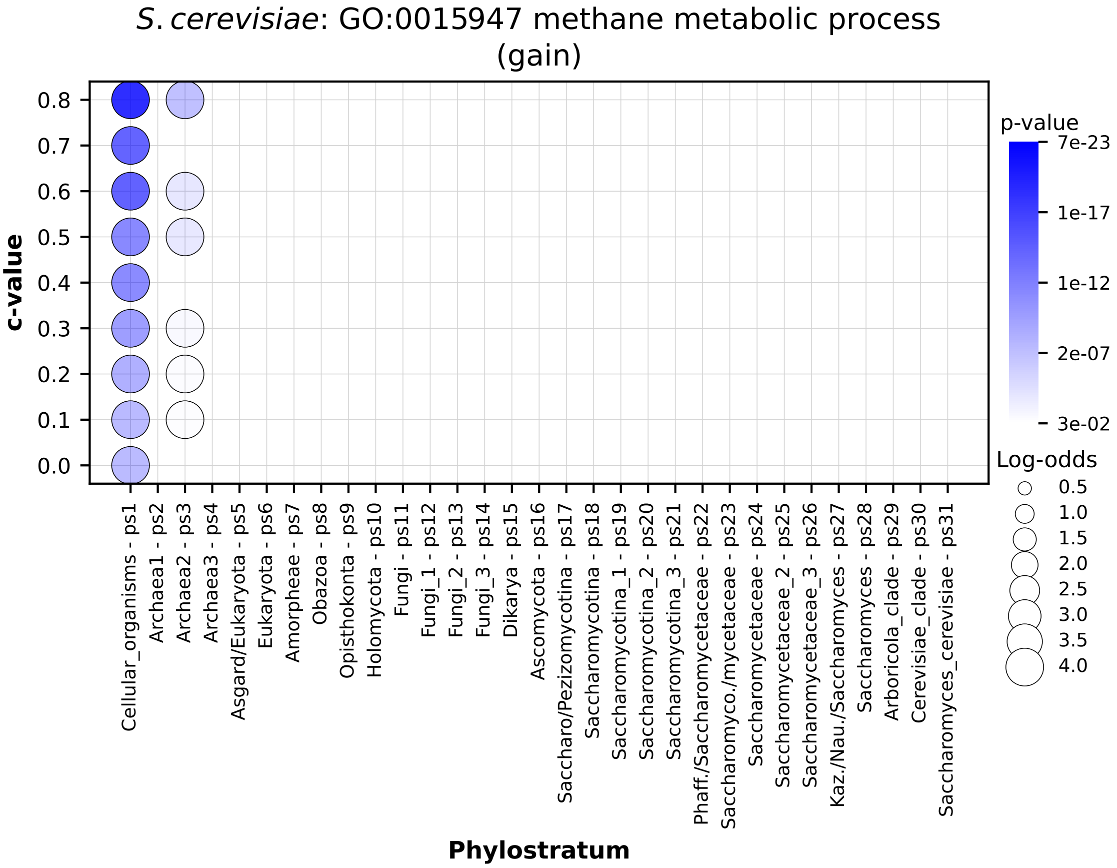
S. cerevisiae: GO:0015947 methane metabolic process
(gain)
c-value
0.8
0.7
0.6
0.5
0.4
0.3
0.2
0.1
0.0
p-value
7e-23
1e-17
1e-12
2e-07
3e-02
Log-odds
0.5
1.0
1.5
2.0
2.5
3.0
3.5
4.0
Cellular_organisms - ps1
Archaea1 - ps2
Archaea2 - ps3
Archaea3 - ps4
Asgard/Eukaryota - ps5
Eukaryota - ps6
Amorpheae - ps7
Obazoa - ps8
Opisthokonta - ps9
Holomycota - ps10
Fungi - ps11
Fungi_1 - ps12
Fungi_2 - ps13
Fungi_3 - ps14
Dikarya - ps15
Ascomycota - ps16
Saccharo/Pezizomycotina - ps17
Saccharomycotina - ps18
Saccharomycotina_1 - ps19
Saccharomycotina_2 - ps20
Saccharomycotina_3 - ps21
Phaff./Saccharomycetaceae - ps22
Saccharomyco./mycetaceae - ps23
Saccharomycetaceae - ps24
Saccharomycetaceae_2 - ps25
Saccharomycetaceae_3 - ps26
Kaz./Nau./Saccharomyces - ps27
Saccharomyces - ps28
Arboricola_clade - ps29
Cerevisiae_clade - ps30
Saccharomyces_cerevisiae - ps31
Phylostratum

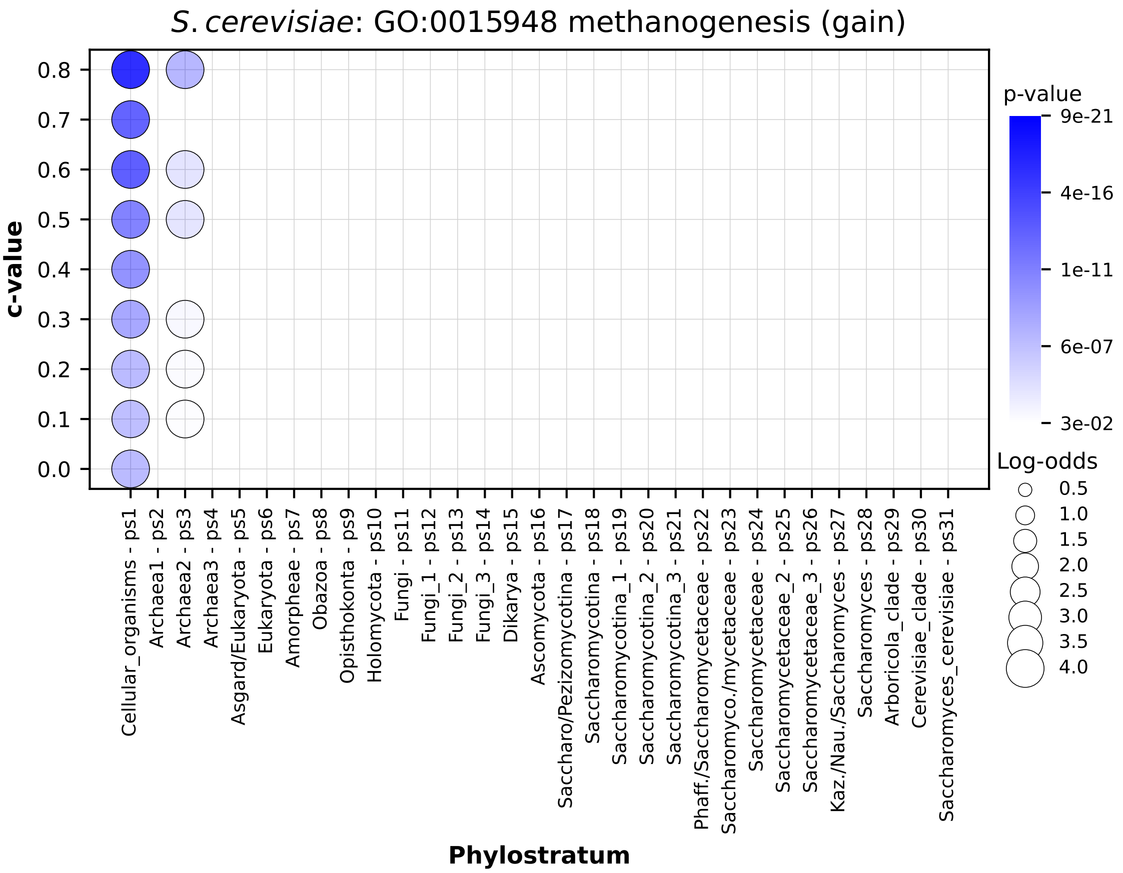

S. cerevisiae: GO:0015948 methanogenesis (gain)
c-value
0.8
0.7
0.6
0.5
0.4
0.3
0.2
0.1
0.0
p-value
9e-21
4e-16
1e-11
6e-07
3e-02
Log-odds
0.5
1.0
1.5
2.0
2.5
3.0
3.5
4.0
Cellular_organisms - ps1
Archaea1 - ps2
Archaea2 - ps3
Archaea3 - ps4
Asgard/Eukaryota - ps5
Eukaryota - ps6
Amorpheae - ps7
Obazoa - ps8
Opisthokonta - ps9
Holomycota - ps10
Fungi - ps11
Fungi_1 - ps12
Fungi_2 - ps13
Fungi_3 - ps14
Dikarya - ps15
Ascomycota - ps16
Saccharo/Pezizomycotina - ps17
Saccharomycotina - ps18
Saccharomycotina_1 - ps19
Saccharomycotina_2 - ps20
Saccharomycotina_3 - ps21
Phaff./Saccharomycetaceae - ps22
Saccharomyco./mycetaceae - ps23
Saccharomycetaceae - ps24
Saccharomycetaceae_2 - ps25
Saccharomycetaceae_3 - ps26
Kaz./Nau./Saccharomyces - ps27
Saccharomyces - ps28
Arboricola_clade - ps29
Cerevisiae_clade - ps30
Saccharomyces_cerevisiae - ps31
Phylostratum

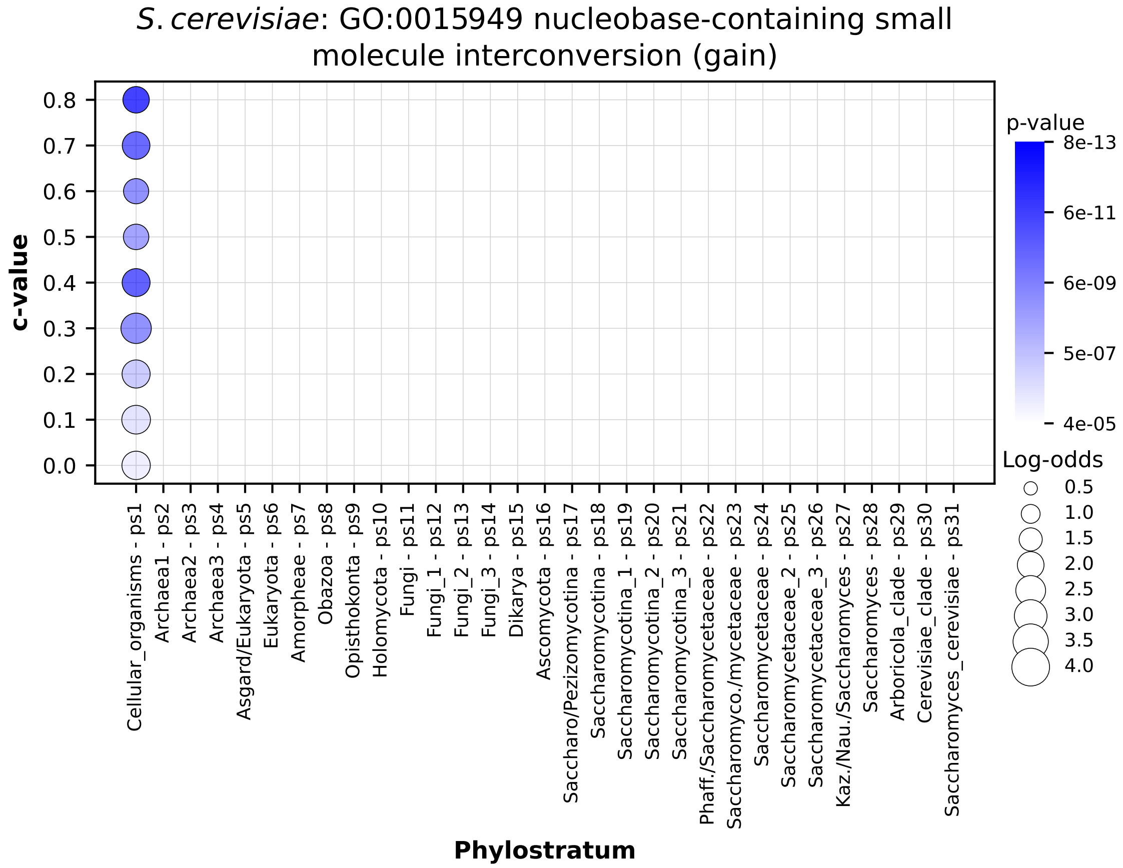
S. cerevisiae: GO:0015949 nucleobase-containing small molecule interconversion (gain)
c-value
0.8
0.7
0.6
0.5
0.4
0.3
0.2
0.1
0.0
p-value
8e-13
6e-11
6e-09
5e-07
4e-05
Log-odds
0.5
1.0
1.5
2.0
2.5
3.0
3.5
4.0
Cellular_organisms - ps1
Archaea1 - ps2
Archaea2 - ps3
Archaea3 - ps4
Asgard/Eukaryota - ps5
Eukaryota - ps6
Amorpheae - ps7
Obazoa - ps8
Opisthokonta - ps9
Holomycota - ps10
Fungi - ps11
Fungi_1 - ps12
Fungi_2 - ps13
Fungi_3 - ps14
Dikarya - ps15
Ascomycota - ps16
Saccharo/Pezizomycotina - ps17
Saccharomycotina - ps18
Saccharomycotina_1 - ps19
Saccharomycotina_2 - ps20
Saccharomycotina_3 - ps21
Phaff./Saccharomycetaceae - ps22
Saccharomyco./mycetaceae - ps23
Saccharomycetaceae - ps24
Saccharomycetaceae_2 - ps25
Saccharomycetaceae_3 - ps26
Kaz./Nau./Saccharomyces - ps27
Saccharomyces - ps28
Arboricola_clade - ps29
Cerevisiae_clade - ps30
Saccharomyces_cerevisiae - ps31
Phylostratum

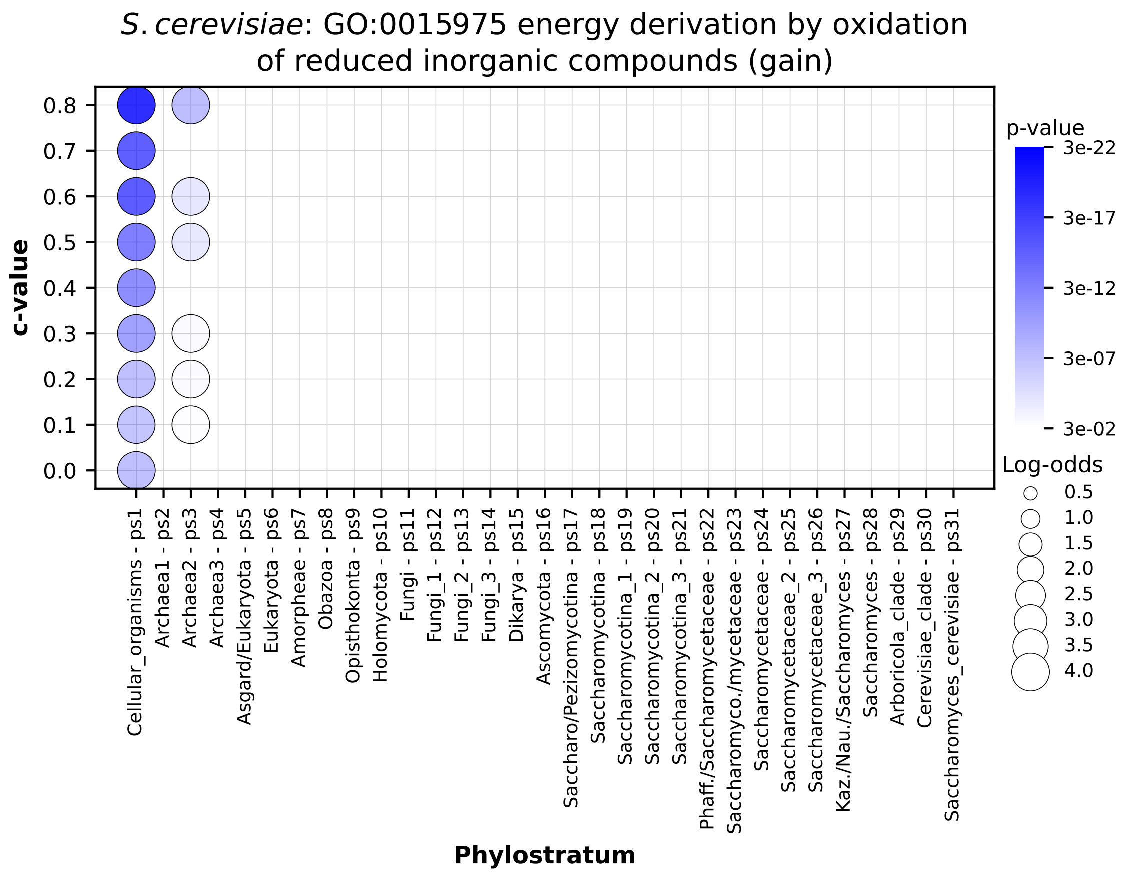

S. cerevisiae: GO:0015975 energy derivation by oxidation of reduced inorganic compounds (gain)
c-value
0.8
0.7
0.6
0.5
0.4
0.3
0.2
0.1
0.0
p-value
3e-22
3e-17
3e-12
3e-07
3e-02
Log-odds
0.5
1.0
1.5
2.0
2.5
3.0
3.5
4.0
Cellular_organisms - ps1
Archaea1 - ps2
Archaea2 - ps3
Archaea3 - ps4
Asgard/Eukaryota - ps5
Eukaryota - ps6
Amorpheae - ps7
Obazoa - ps8
Opisthokonta - ps9
Holomycota - ps10
Fungi - ps11
Fungi_1 - ps12
Fungi_2 - ps13
Fungi_3 - ps14
Dikarya - ps15
Ascomycota - ps16
Saccharo/Pezizomycotina - ps17
Saccharomycotina - ps18
Saccharomycotina_1 - ps19
Saccharomycotina_2 - ps20
Saccharomycotina_3 - ps21
Phaff./Saccharomycetaceae - ps22
Saccharomyco./mycetaceae - ps23
Saccharomycetaceae - ps24
Saccharomycetaceae_2 - ps25
Saccharomycetaceae_3 - ps26
Kaz./Nau./Saccharomyces - ps27
Saccharomyces - ps28
Arboricola_clade - ps29
Cerevisiae_clade - ps30
Saccharomyces_cerevisiae - ps31
Phylostratum

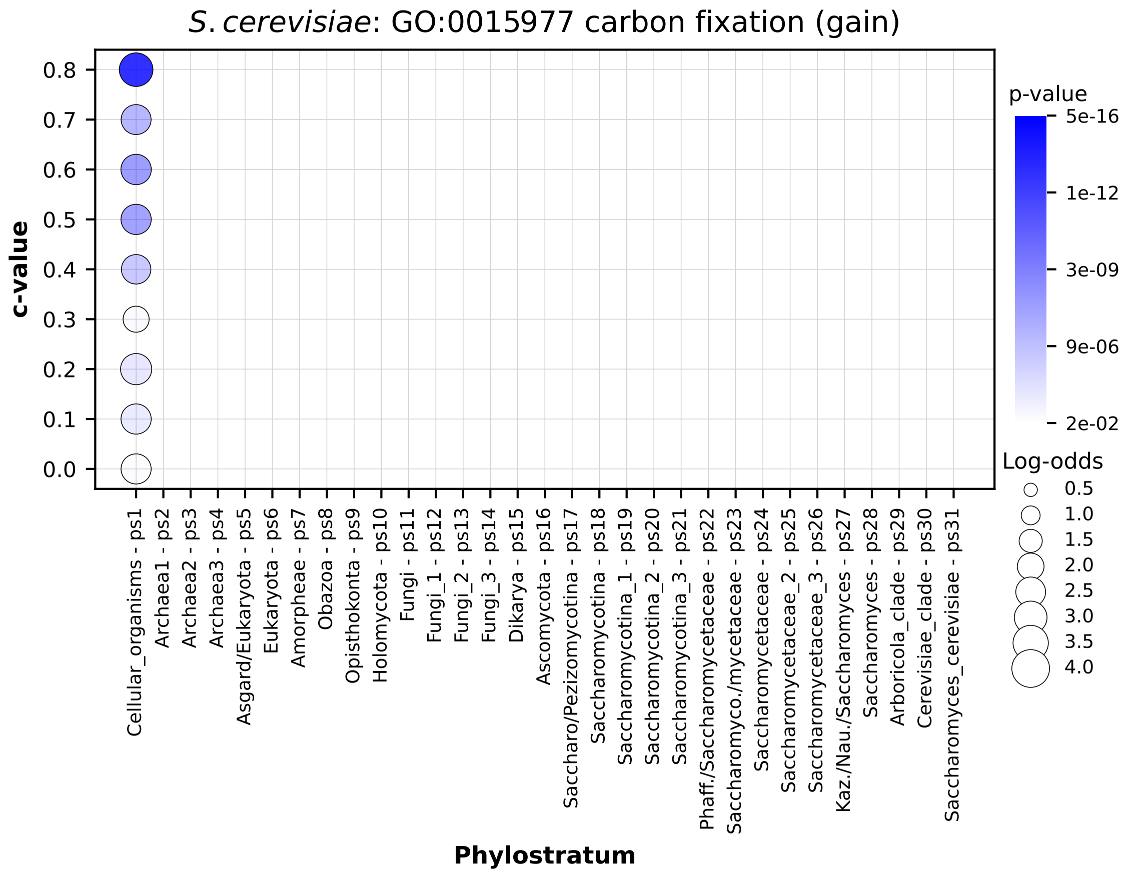

S. cerevisiae: GO:0015977 carbon fixation (gain)
c-value
0.8
0.7
0.6
0.5
0.4
0.3
0.2
0.1
0.0
p-value
5e-16
1e-12
3e-09
9e-06
2e-02
Log-odds
0.5
1.0
1.5
2.0
2.5
3.0
3.5
4.0
Cellular_organisms - ps1
Archaea1 - ps2
Archaea2 - ps3
Archaea3 - ps4
Asgard/Eukaryota - ps5
Eukaryota - ps6
Amorpheae - ps7
Obazoa - ps8
Opisthokonta - ps9
Holomycota - ps10
Fungi - ps11
Fungi_1 - ps12
Fungi_2 - ps13
Fungi_3 - ps14
Dikarya - ps15
Ascomycota - ps16
Saccharo/Pezizomycotina - ps17
Saccharomycotina - ps18
Saccharomycotina_1 - ps19
Saccharomycotina_2 - ps20
Saccharomycotina_3 - ps21
Phaff./Saccharomycetaceae - ps22
Saccharomyco./mycetaceae - ps23
Saccharomycetaceae - ps24
Saccharomycetaceae_2 - ps25
Saccharomycetaceae_3 - ps26
Kaz./Nau./Saccharomyces - ps27
Saccharomyces - ps28
Arboricola_clade - ps29
Cerevisiae_clade - ps30
Saccharomyces_cerevisiae - ps31
Phylostratum

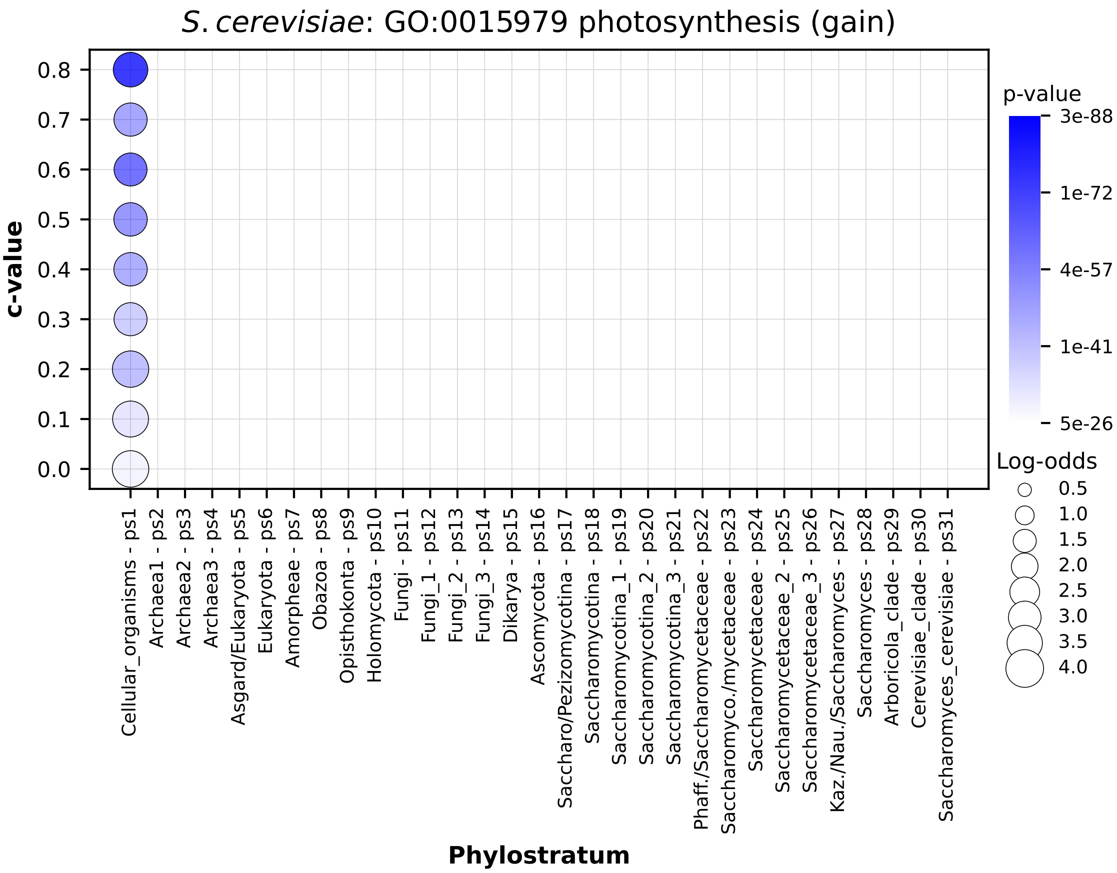
S. cerevisiae: GO:0015979 photosynthesis (gain)
c-value
0.0
0.1
0.2
0.3
0.4
0.5
0.6
0.7
0.8
p-value
3e-88
1e-72
4e-57
1e-41
5e-26
Log-odds
0.5
1.0
1.5
2.0
2.5
3.0
3.5
4.0
Cellular_organisms - ps1
Archaea1 - ps2
Archaea2 - ps3
Archaea3 - ps4
Asgard/Eukaryota - ps5
Eukaryota - ps6
Amorpheae - ps7
Obazoa - ps8
Opisthokonta - ps9
Holomycota - ps10
Fungi - ps11
Fungi_1 - ps12
Fungi_2 - ps13
Fungi_3 - ps14
Dikarya - ps15
Ascomycota - ps16
Saccharo/Pezizomycotina - ps17
Saccharomycotina - ps18
Saccharomycotina_1 - ps19
Saccharomycotina_2 - ps20
Saccharomycotina_3 - ps21
Phaff./Saccharomycetaceae - ps22
Saccharomyco./mycetaceae - ps23
Saccharomycetaceae - ps24
Saccharomycetaceae_2 - ps25
Saccharomycetaceae_3 - ps26
Kaz./Nau./Saccharomyces - ps27
Saccharomyces - ps28
Arboricola_clade - ps29
Cerevisiae_clade - ps30
Saccharomyces_cerevisiae - ps31
Phylostratum

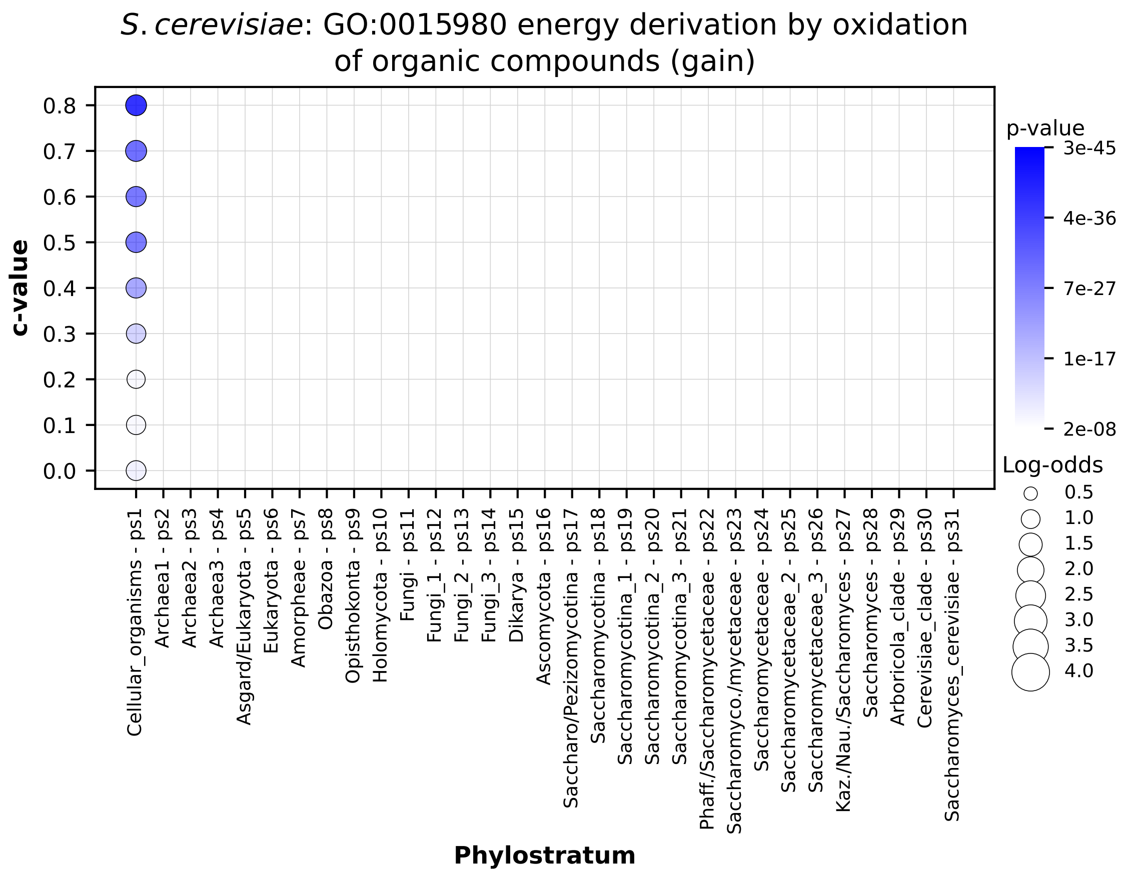
S. cerevisiae: GO:0015980 energy derivation by oxidation of organic compounds (gain)
c-value
0.8
0.7
0.6
0.5
0.4
0.3
0.2
0.1
0.0
p-value
3e-45
4e-36
7e-27
1e-17
2e-08
Log-odds
0.5
1.0
1.5
2.0
2.5
3.0
3.5
4.0
Cellular_organisms - ps1
Archaea1 - ps2
Archaea2 - ps3
Archaea3 - ps4
Asgard/Eukaryota - ps5
Eukaryota - ps6
Amorpheae - ps7
Obazoa - ps8
Opisthokonta - ps9
Holomycota - ps10
Fungi - ps11
Fungi_1 - ps12
Fungi_2 - ps13
Fungi_3 - ps14
Dikarya - ps15
Ascomycota - ps16
Saccharo/Pezizomycotina - ps17
Saccharomycotina - ps18
Saccharomycotina_1 - ps19
Saccharomycotina_2 - ps20
Saccharomycotina_3 - ps21
Phaff./Saccharomycetaceae - ps22
Saccharomyco./mycetaceae - ps23
Saccharomycetaceae - ps24
Saccharomycetaceae_2 - ps25
Saccharomycetaceae_3 - ps26
Kaz./Nau./Saccharomyces - ps27
Saccharomyces - ps28
Arboricola_clade - ps29
Cerevisiae_clade - ps30
Saccharomyces_cerevisiae - ps31
Phylostratum

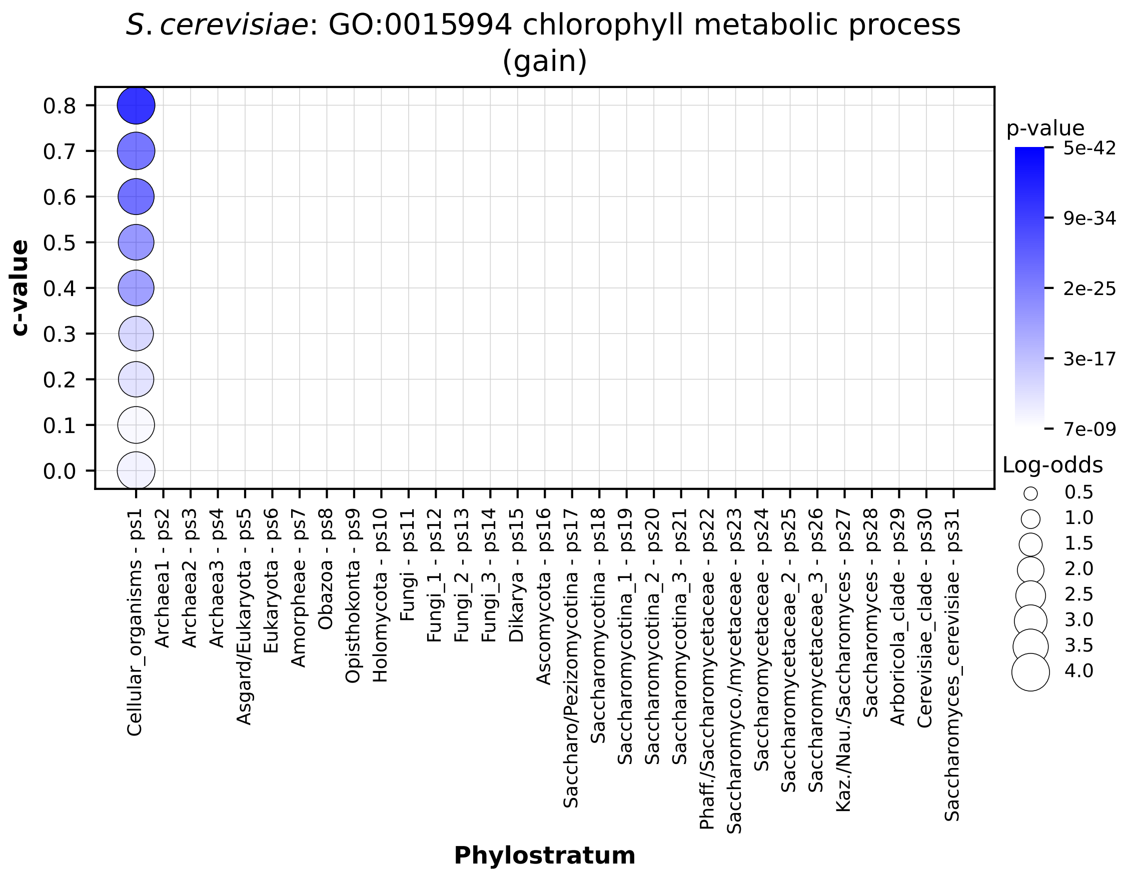
S. cerevisiae: GO:0015994 chlorophyll metabolic process
(gain)
c-value
0.8
0.7
0.6
0.5
0.4
0.3
0.2
0.1
0.0
p-value
5e-42
9e-34
2e-25
3e-17
7e-09
Log-odds
0.5
1.0
1.5
2.0
2.5
3.0
3.5
4.0
Cellular_organisms - ps1
Archaea1 - ps2
Archaea2 - ps3
Archaea3 - ps4
Asgard/Eukaryota - ps5
Eukaryota - ps6
Amorpheae - ps7
Obazoa - ps8
Opisthokonta - ps9
Holomycota - ps10
Fungi - ps11
Fungi_1 - ps12
Fungi_2 - ps13
Fungi_3 - ps14
Dikarya - ps15
Ascomycota - ps16
Saccharo/Pezizomycotina - ps17
Saccharomycotina - ps18
Saccharomycotina_1 - ps19
Saccharomycotina_2 - ps20
Saccharomycotina_3 - ps21
Phaff./Saccharomycetaceae - ps22
Saccharomyco./mycetaceae - ps23
Saccharomycetaceae - ps24
Saccharomycetaceae_2 - ps25
Saccharomycetaceae_3 - ps26
Kaz./Nau./Saccharomyces - ps27
Saccharomyces - ps28
Arboricola_clade - ps29
Cerevisiae_clade - ps30
Saccharomyces_cerevisiae - ps31
Phylostratum

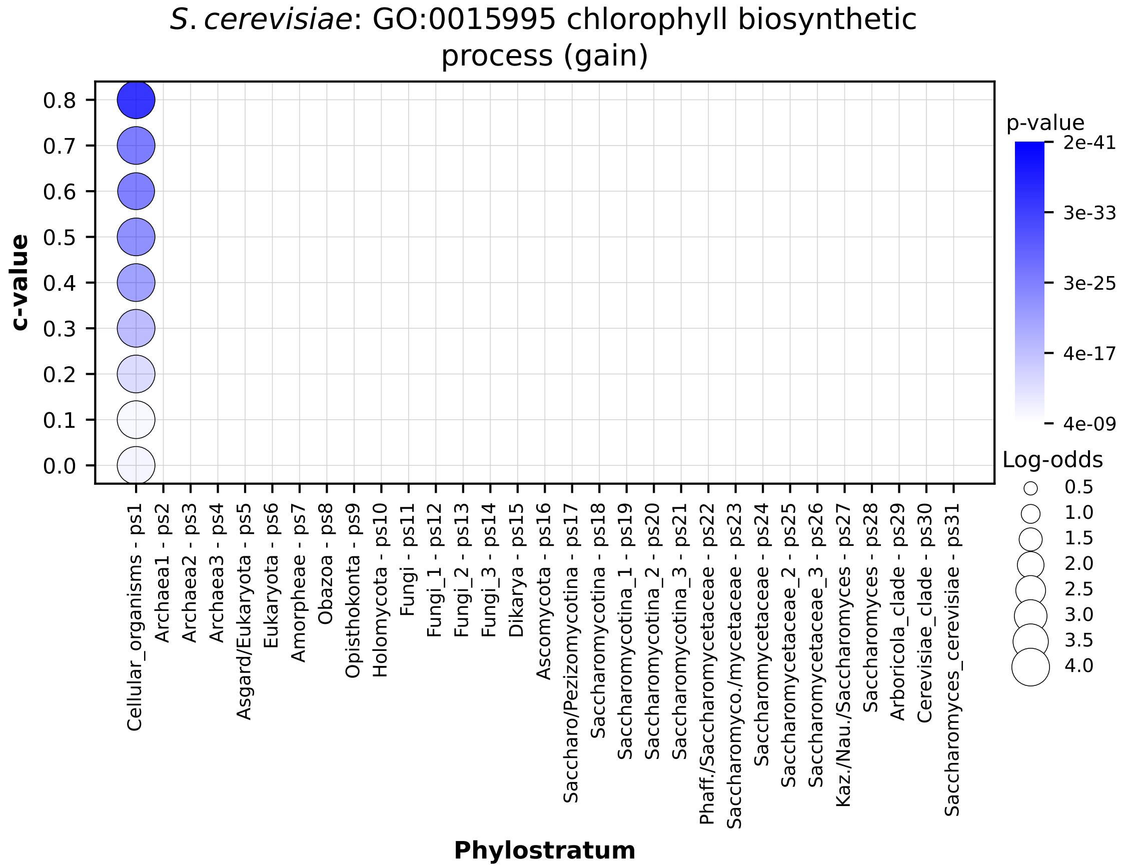

S. cerevisiae: GO:0015995 chlorophyll biosynthetic process (gain)
c-value
0.8
0.7
0.6
0.5
0.4
0.3
0.2
0.1
0.0
p-value
2e-41
3e-33
3e-25
4e-17
4e-09
Log-odds
0.5
1.0
1.5
2.0
2.5
3.0
3.5
4.0
Cellular_organisms - ps1
Archaea1 - ps2
Archaea2 - ps3
Archaea3 - ps4
Asgard/Eukaryota - ps5
Eukaryota - ps6
Amorpheae - ps7
Obazoa - ps8
Opisthokonta - ps9
Holomycota - ps10
Fungi - ps11
Fungi_1 - ps12
Fungi_2 - ps13
Fungi_3 - ps14
Dikarya - ps15
Ascomycota - ps16
Saccharo/Pezizomycotina - ps17
Saccharomycotina - ps18
Saccharomycotina_1 - ps19
Saccharomycotina_2 - ps20
Saccharomycotina_3 - ps21
Phaff./Saccharomycetaceae - ps22
Saccharomyco./mycetaceae - ps23
Saccharomycetaceae - ps24
Saccharomycetaceae_2 - ps25
Saccharomycetaceae_3 - ps26
Kaz./Nau./Saccharomyces - ps27
Saccharomyces - ps28
Arboricola_clade - ps29
Cerevisiae_clade - ps30
Saccharomyces_cerevisiae - ps31
Phylostratum

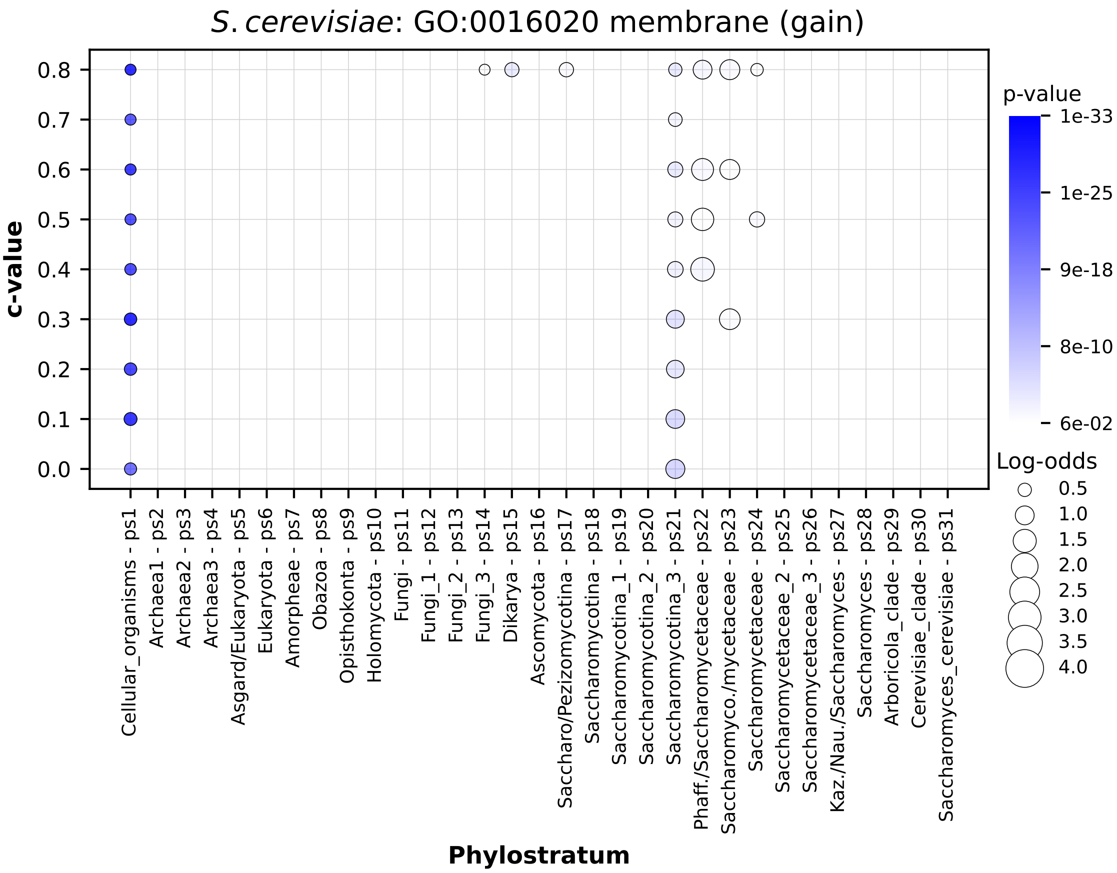

S. cerevisiae: GO:0016020 membrane (gain)
c-value
0.0
0.1
0.2
0.3
0.4
0.5
0.6
0.7
0.8
p-value
1e-33
1e-25
9e-18
8e-10
6e-02
Log-odds
0.5
1.0
1.5
2.0
2.5
3.0
3.5
4.0
Cellular_organisms - ps1
Archaea1 - ps2
Archaea2 - ps3
Archaea3 - ps4
Asgard/Eukaryota - ps5
Eukaryota - ps6
Amorpheae - ps7
Obazoa - ps8
Opisthokonta - ps9
Holomycota - ps10
Fungi - ps11
Fungi_1 - ps12
Fungi_2 - ps13
Fungi_3 - ps14
Dikarya - ps15
Ascomycota - ps16
Saccharo/Pezizomycotina - ps17
Saccharomycotina - ps18
Saccharomycotina_1 - ps19
Saccharomycotina_2 - ps20
Saccharomycotina_3 - ps21
Phaff./Saccharomycetaceae - ps22
Saccharomyco./mycetaceae - ps23
Saccharomycetaceae - ps24
Saccharomycetaceae_2 - ps25
Saccharomycetaceae_3 - ps26
Kaz./Nau./Saccharomyces - ps27
Saccharomyces - ps28
Arboricola_clade - ps29
Cerevisiae_clade - ps30
Saccharomyces_cerevisiae - ps31
Phylostratum

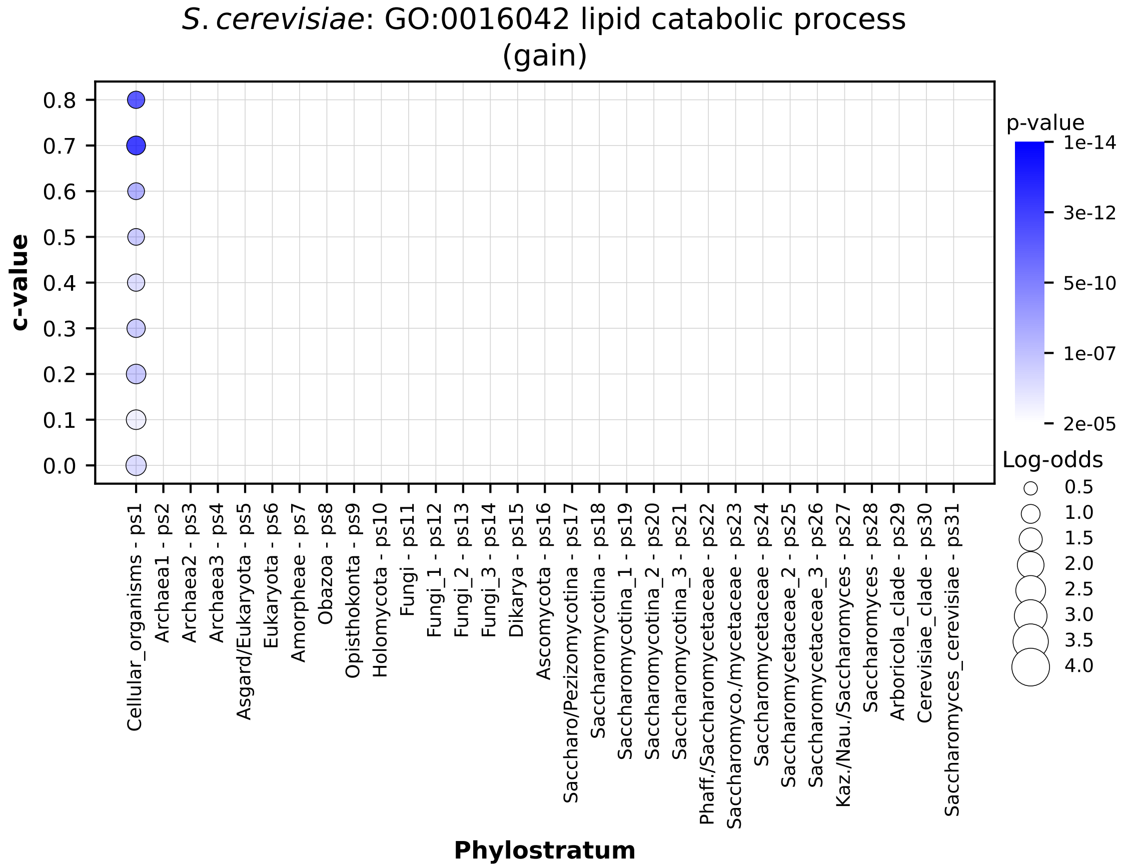
S. cerevisiae: GO:0016042 lipid catabolic process
(gain)
c-value
0.8
0.7
0.6
0.5
0.4
0.3
0.2
0.1
0.0
p-value
1e-14
3e-12
5e-10
1e-07
2e-05
Log-odds
0.5
1.0
1.5
2.0
2.5
3.0
3.5
4.0
Cellular_organisms - ps1
Archaea1 - ps2
Archaea2 - ps3
Archaea3 - ps4
Asgard/Eukaryota - ps5
Eukaryota - ps6
Amorpheae - ps7
Obazoa - ps8
Opisthokonta - ps9
Holomycota - ps10
Fungi - ps11
Fungi_1 - ps12
Fungi_2 - ps13
Fungi_3 - ps14
Dikarya - ps15
Ascomycota - ps16
Saccharo/Pezizomycotina - ps17
Saccharomycotina - ps18
Saccharomycotina_1 - ps19
Saccharomycotina_2 - ps20
Saccharomycotina_3 - ps21
Phaff./Saccharomycetaceae - ps22
Saccharomyco./mycetaceae - ps23
Saccharomycetaceae - ps24
Saccharomycetaceae_2 - ps25
Saccharomycetaceae_3 - ps26
Kaz./Nau./Saccharomyces - ps27
Saccharomyces - ps28
Arboricola_clade - ps29
Cerevisiae_clade - ps30
Saccharomyces_cerevisiae - ps31
Phylostratum

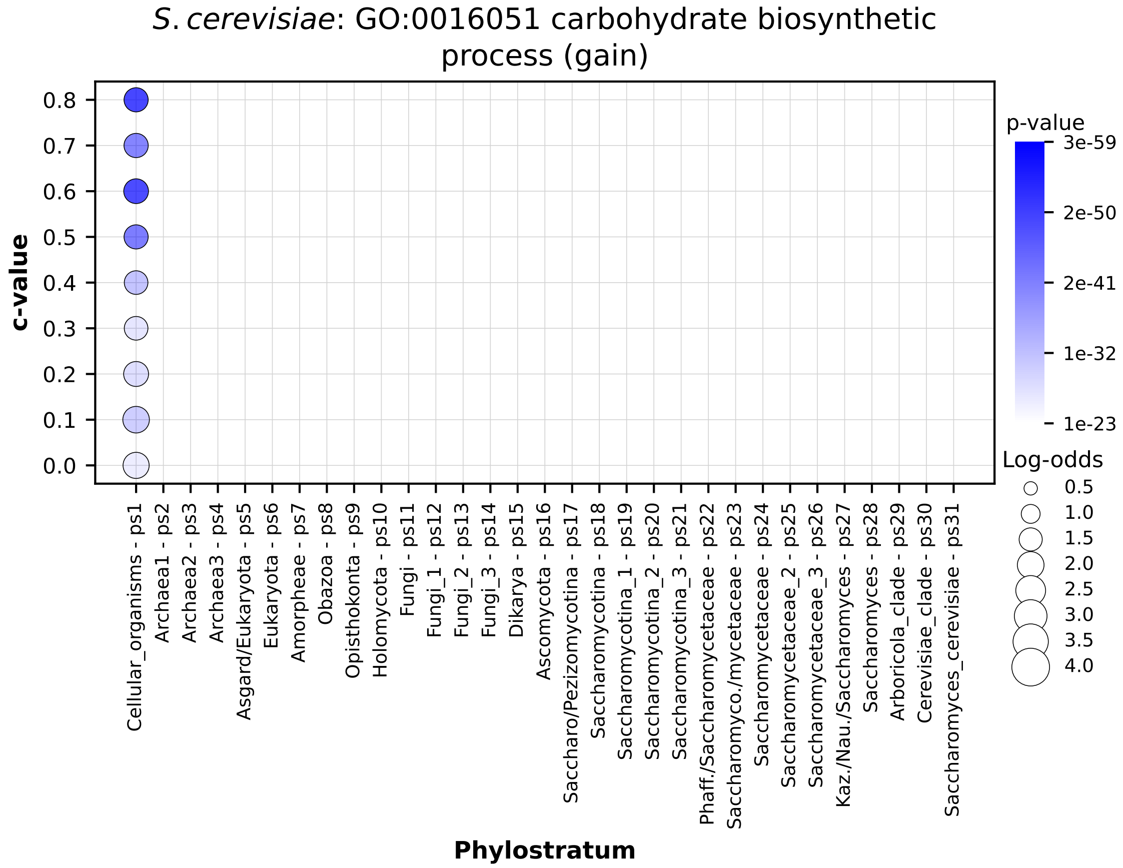

S. cerevisiae: GO:0016051 carbohydrate biosynthetic process (gain)
c-value
0.8
0.7
0.6
0.5
0.4
0.3
0.2
0.1
0.0
p-value
3e-59
2e-50
2e-41
1e-32
1e-23
Log-odds
0.5
1.0
1.5
2.0
2.5
3.0
3.5
4.0
Cellular_organisms - ps1
Archaea1 - ps2
Archaea2 - ps3
Archaea3 - ps4
Asgard/Eukaryota - ps5
Eukaryota - ps6
Amorpheae - ps7
Obazoa - ps8
Opisthokonta - ps9
Holomycota - ps10
Fungi - ps11
Fungi_1 - ps12
Fungi_2 - ps13
Fungi_3 - ps14
Dikarya - ps15
Ascomycota - ps16
Saccharo/Pezizomycotina - ps17
Saccharomycotina - ps18
Saccharomycotina_1 - ps19
Saccharomycotina_2 - ps20
Saccharomycotina_3 - ps21
Phaff./Saccharomycetaceae - ps22
Saccharomyco./mycetaceae - ps23
Saccharomycetaceae - ps24
Saccharomycetaceae_2 - ps25
Saccharomycetaceae_3 - ps26
Kaz./Nau./Saccharomyces - ps27
Saccharomyces - ps28
Arboricola_clade - ps29
Cerevisiae_clade - ps30
Saccharomyces_cerevisiae - ps31
Phylostratum

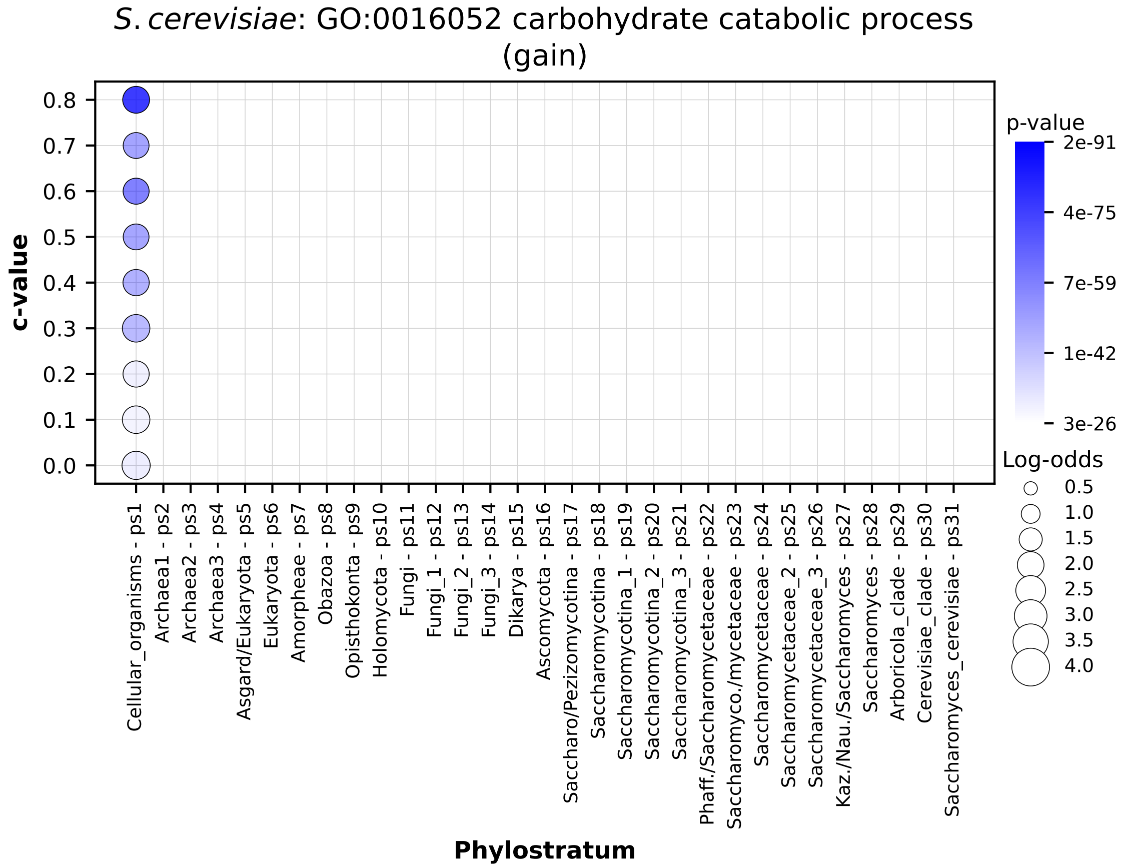
S. cerevisiae: GO:0016052 carbohydrate catabolic process
(gain)
c-value
0.8
0.7
0.6
0.5
0.4
0.3
0.2
0.1
0.0
p-value
2e-91
4e-75
7e-59
1e-42
3e-26
Log-odds
0.5
1.0
1.5
2.0
2.5
3.0
3.5
4.0
Cellular_organisms - ps1
Archaea1 - ps2
Archaea2 - ps3
Archaea3 - ps4
Asgard/Eukaryota - ps5
Eukaryota - ps6
Amorpheae - ps7
Obazoa - ps8
Opisthokonta - ps9
Holomycota - ps10
Fungi - ps11
Fungi_1 - ps12
Fungi_2 - ps13
Fungi_3 - ps14
Dikarya - ps15
Ascomycota - ps16
Saccharo/Pezizomycotina - ps17
Saccharomycotina - ps18
Saccharomycotina_1 - ps19
Saccharomycotina_2 - ps20
Saccharomycotina_3 - ps21
Phaff./Saccharomycetaceae - ps22
Saccharomyco./mycetaceae - ps23
Saccharomycetaceae - ps24
Saccharomycetaceae_2 - ps25
Saccharomycetaceae_3 - ps26
Kaz./Nau./Saccharomyces - ps27
Saccharomyces - ps28
Arboricola_clade - ps29
Cerevisiae_clade - ps30
Saccharomyces_cerevisiae - ps31
Phylostratum

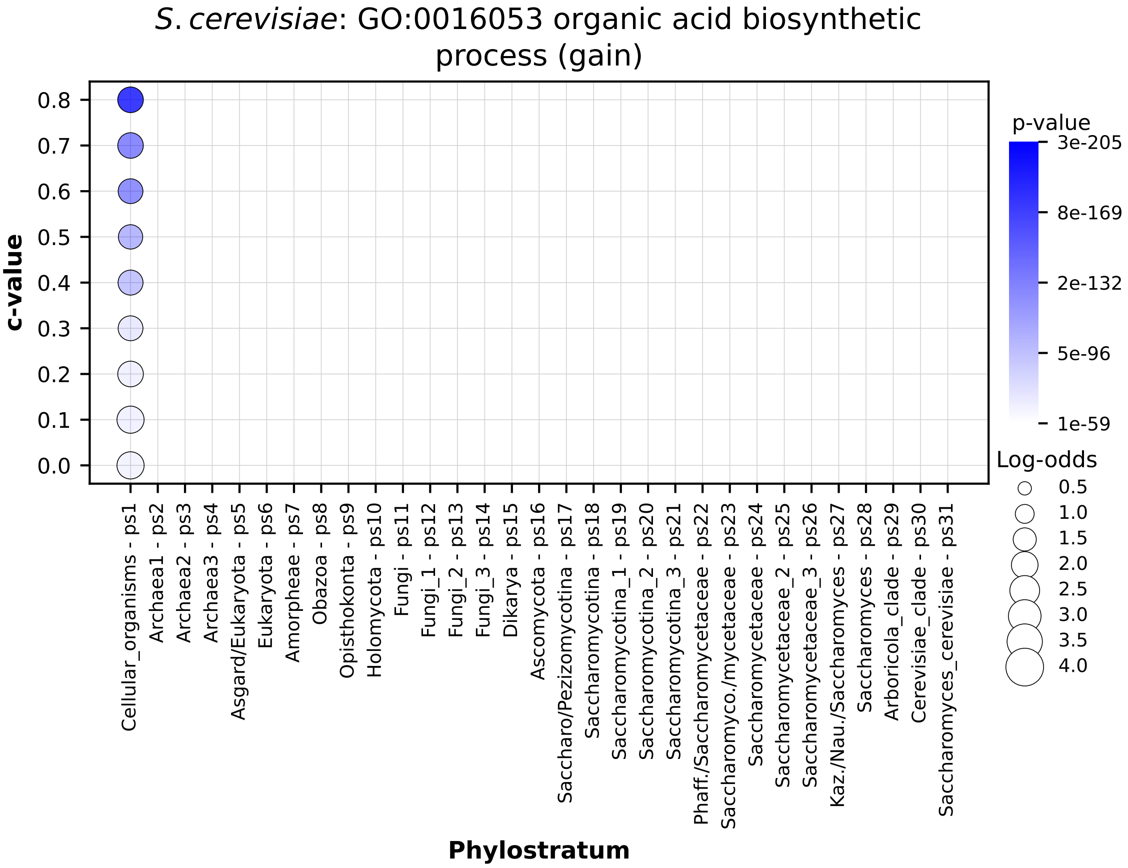

S. cerevisiae: GO:0016053 organic acid biosynthetic process (gain)
c-value
0.0
0.1
0.2
0.3
0.4
0.5
0.6
0.7
0.8
Phylostratum
Cellular_organisms - ps1
Archaea1 - ps2
Archaea2 - ps3
Archaea3 - ps4
Asgard/Eukaryota - ps5
Eukaryota - ps6
Amorpheae - ps7
Obazoa - ps8
Opisthokonta - ps9
Holomycota - ps10
Fungi - ps11
Fungi_1 - ps12
Fungi_2 - ps13
Fungi_3 - ps14
Dikarya - ps15
Ascomycota - ps16
Saccharo/Pezizomycotina - ps17
Saccharomycotina - ps18
Saccharomycotina_1 - ps19
Saccharomycotina_2 - ps20
Saccharomycotina_3 - ps21
Phaff./Saccharomycetaceae - ps22
Saccharomyco./mycetaceae - ps23
Saccharomycetaceae - ps24
Saccharomycetaceae_2 - ps25
Saccharomycetaceae_3 - ps26
Kaz./Nau./Saccharomyces - ps27
Saccharomyces - ps28
Arboricola_clade - ps29
Cerevisiae_clade - ps30
Saccharomyces_cerevisiae - ps31
p-value
3e-205
8e-169
2e-132
5e-96
1e-59
Log-odds
0.5
1.0
1.5
2.0
2.5
3.0
3.5
4.0

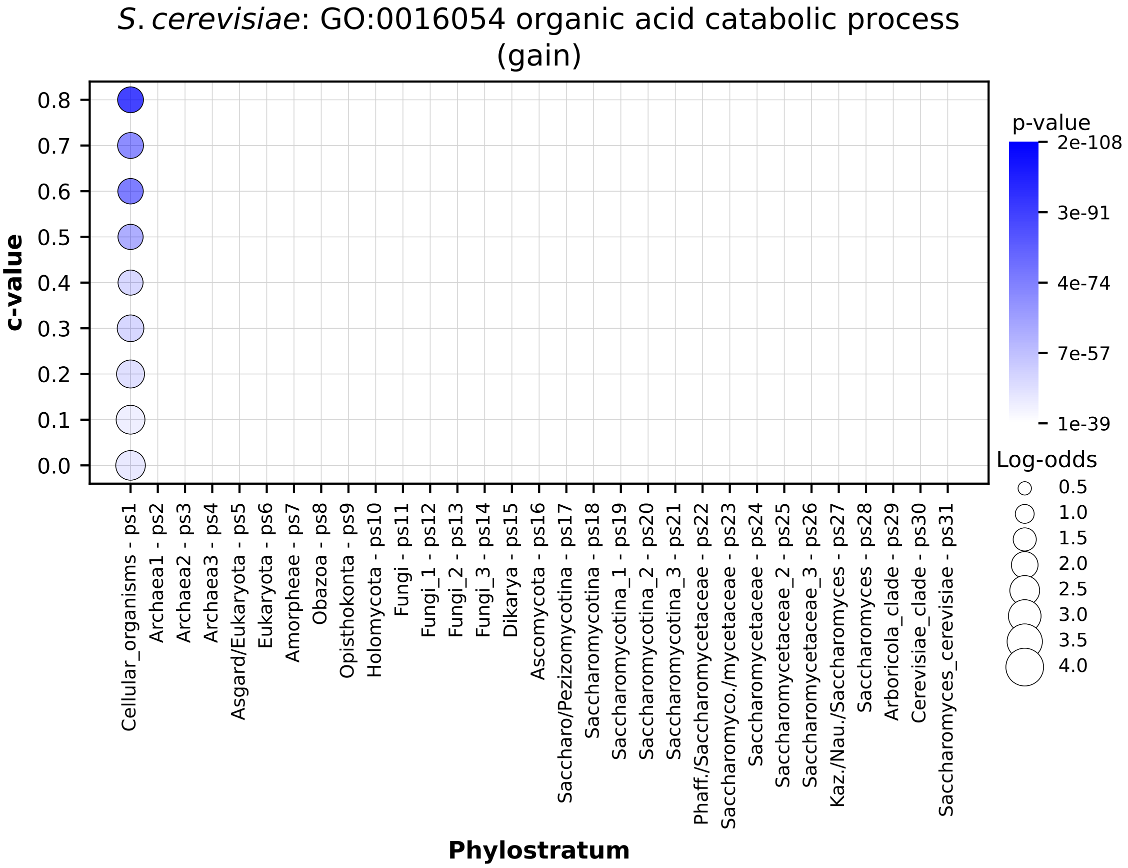
S. cerevisiae: GO:0016054 organic acid catabolic process
(gain)
c-value
0.8
0.7
0.6
0.5
0.4
0.3
0.2
0.1
0.0
Phylostratum
Cellular_organisms - ps1
Archaea1 - ps2
Archaea2 - ps3
Archaea3 - ps4
Asgard/Eukaryota - ps5
Eukaryota - ps6
Amorpheae - ps7
Obazoa - ps8
Opisthokonta - ps9
Holomycota - ps10
Fungi - ps11
Fungi_1 - ps12
Fungi_2 - ps13
Fungi_3 - ps14
Dikarya - ps15
Ascomycota - ps16
Saccharo/Pezizomycotina - ps17
Saccharomycotina - ps18
Saccharomycotina_1 - ps19
Saccharomycotina_2 - ps20
Saccharomycotina_3 - ps21
Phaff./Saccharomycetaceae - ps22
Saccharomyco./mycetaceae - ps23
Saccharomycetaceae - ps24
Saccharomycetaceae_2 - ps25
Saccharomycetaceae_3 - ps26
Kaz./Nau./Saccharomyces - ps27
Saccharomyces - ps28
Arboricola_clade - ps29
Cerevisiae_clade - ps30
Saccharomyces_cerevisiae - ps31
p-value
2e-108
3e-91
4e-74
7e-57
1e-39
Log-odds
0.5
1.0
1.5
2.0
2.5
3.0
3.5
4.0

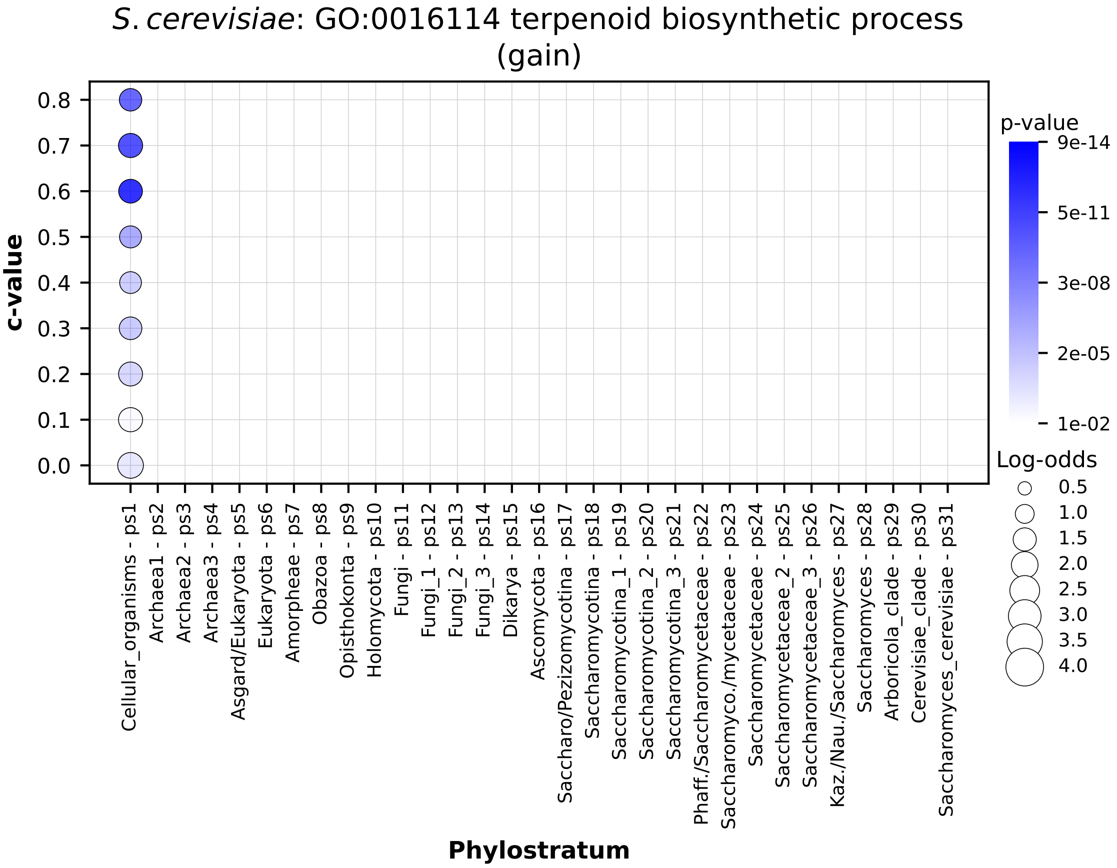

S. cerevisiae: GO:0016114 terpenoid biosynthetic process
(gain)
c-value
0.8
0.7
0.6
0.5
0.4
0.3
0.2
0.1
0.0
p-value
9e-14
5e-11
3e-08
2e-05
1e-02
Log-odds
0.5
1.0
1.5
2.0
2.5
3.0
3.5
4.0
Cellular_organisms - ps1
Archaea1 - ps2
Archaea2 - ps3
Archaea3 - ps4
Asgard/Eukaryota - ps5
Eukaryota - ps6
Amorpheae - ps7
Obazoa - ps8
Opisthokonta - ps9
Holomycota - ps10
Fungi - ps11
Fungi_1 - ps12
Fungi_2 - ps13
Fungi_3 - ps14
Dikarya - ps15
Ascomycota - ps16
Saccharo/Pezizomycotina - ps17
Saccharomycotina - ps18
Saccharomycotina_1 - ps19
Saccharomycotina_2 - ps20
Saccharomycotina_3 - ps21
Phaff./Saccharomycetaceae - ps22
Saccharomyco./mycetaceae - ps23
Saccharomycetaceae - ps24
Saccharomycetaceae_2 - ps25
Saccharomycetaceae_3 - ps26
Kaz./Nau./Saccharomyces - ps27
Saccharomyces - ps28
Arboricola_clade - ps29
Cerevisiae_clade - ps30
Saccharomyces_cerevisiae - ps31
Phylostratum

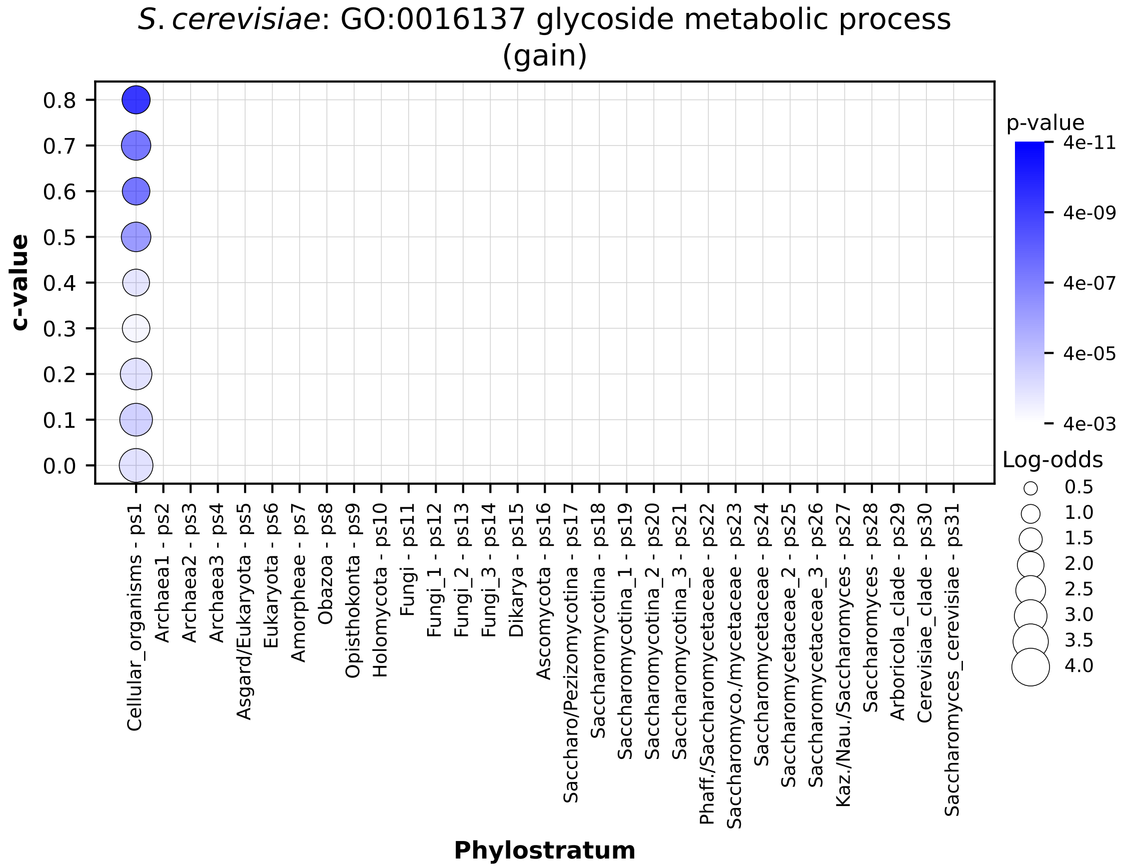
S. cerevisiae: GO:0016137 glycoside metabolic process
(gain)
c-value
0.8
0.7
0.6
0.5
0.4
0.3
0.2
0.1
0.0
p-value
4e-11
4e-09
4e-07
4e-05
4e-03
Log-odds
0.5
1.0
1.5
2.0
2.5
3.0
3.5
4.0
Cellular_organisms - ps1
Archaea1 - ps2
Archaea2 - ps3
Archaea3 - ps4
Asgard/Eukaryota - ps5
Eukaryota - ps6
Amorpheae - ps7
Obazoa - ps8
Opisthokonta - ps9
Holomycota - ps10
Fungi - ps11
Fungi_1 - ps12
Fungi_2 - ps13
Fungi_3 - ps14
Dikarya - ps15
Ascomycota - ps16
Saccharo/Pezizomycotina - ps17
Saccharomycotina - ps18
Saccharomycotina_1 - ps19
Saccharomycotina_2 - ps20
Saccharomycotina_3 - ps21
Phaff./Saccharomycetaceae - ps22
Saccharomyco./mycetaceae - ps23
Saccharomycetaceae - ps24
Saccharomycetaceae_2 - ps25
Saccharomycetaceae_3 - ps26
Kaz./Nau./Saccharomyces - ps27
Saccharomyces - ps28
Arboricola_clade - ps29
Cerevisiae_clade - ps30
Saccharomyces_cerevisiae - ps31
Phylostratum

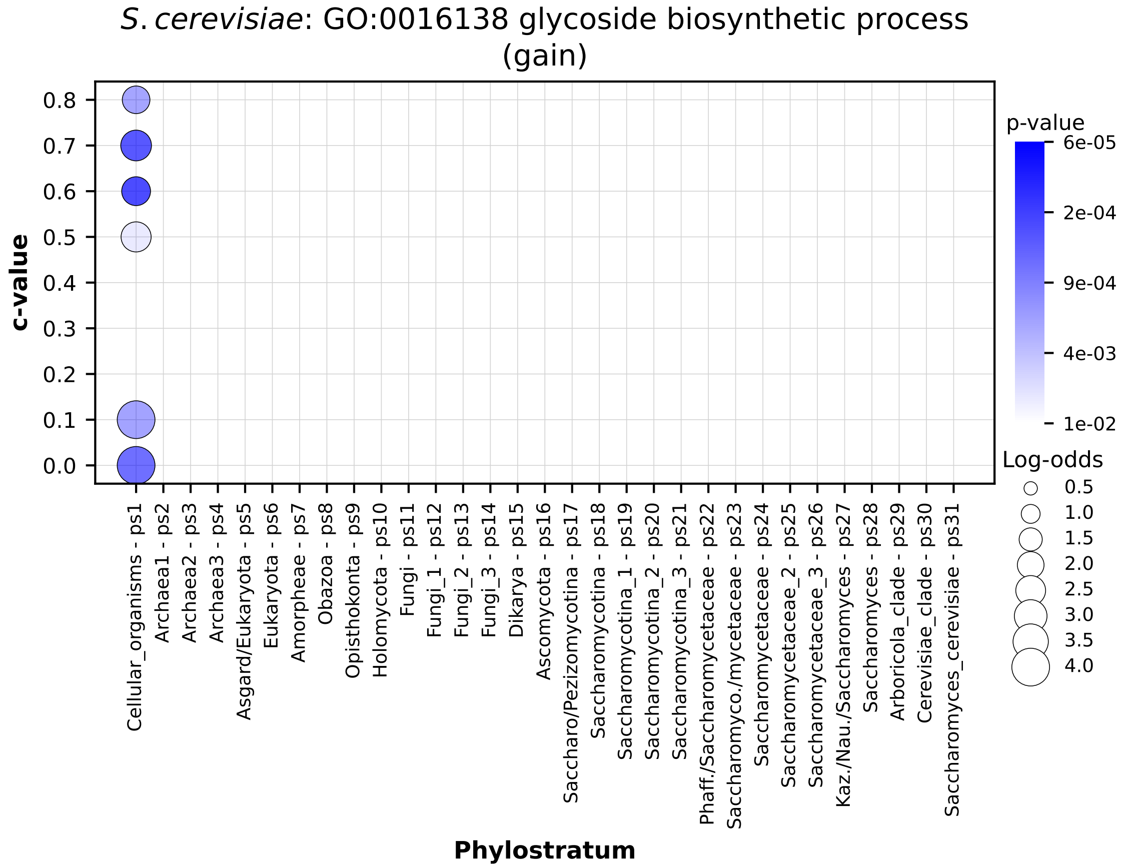
S. cerevisiae: GO:0016138 glycoside biosynthetic process (gain)
c-value
0.8
0.7
0.6
0.5
0.4
0.3
0.2
0.1
0.0
p-value
6e-05
2e-04
9e-04
4e-03
1e-02
Log-odds
0.5
1.0
1.5
2.0
2.5
3.0
3.5
4.0
Cellular_organisms - ps1
Archaea1 - ps2
Archaea2 - ps3
Archaea3 - ps4
Asgard/Eukaryota - ps5
Eukaryota - ps6
Amorpheae - ps7
Obazoa - ps8
Opisthokonta - ps9
Holomycota - ps10
Fungi - ps11
Fungi_1 - ps12
Fungi_2 - ps13
Fungi_3 - ps14
Dikarya - ps15
Ascomycota - ps16
Saccharo/Pezizomycotina - ps17
Saccharomycotina - ps18
Saccharomycotina_1 - ps19
Saccharomycotina_2 - ps20
Saccharomycotina_3 - ps21
Phaff./Saccharomycetaceae - ps22
Saccharomyco./mycetaceae - ps23
Saccharomycetaceae - ps24
Saccharomycetaceae_2 - ps25
Saccharomycetaceae_3 - ps26
Kaz./Nau./Saccharomyces - ps27
Saccharomyces - ps28
Arboricola_clade - ps29
Cerevisiae_clade - ps30
Saccharomyces_cerevisiae - ps31
Phylostratum

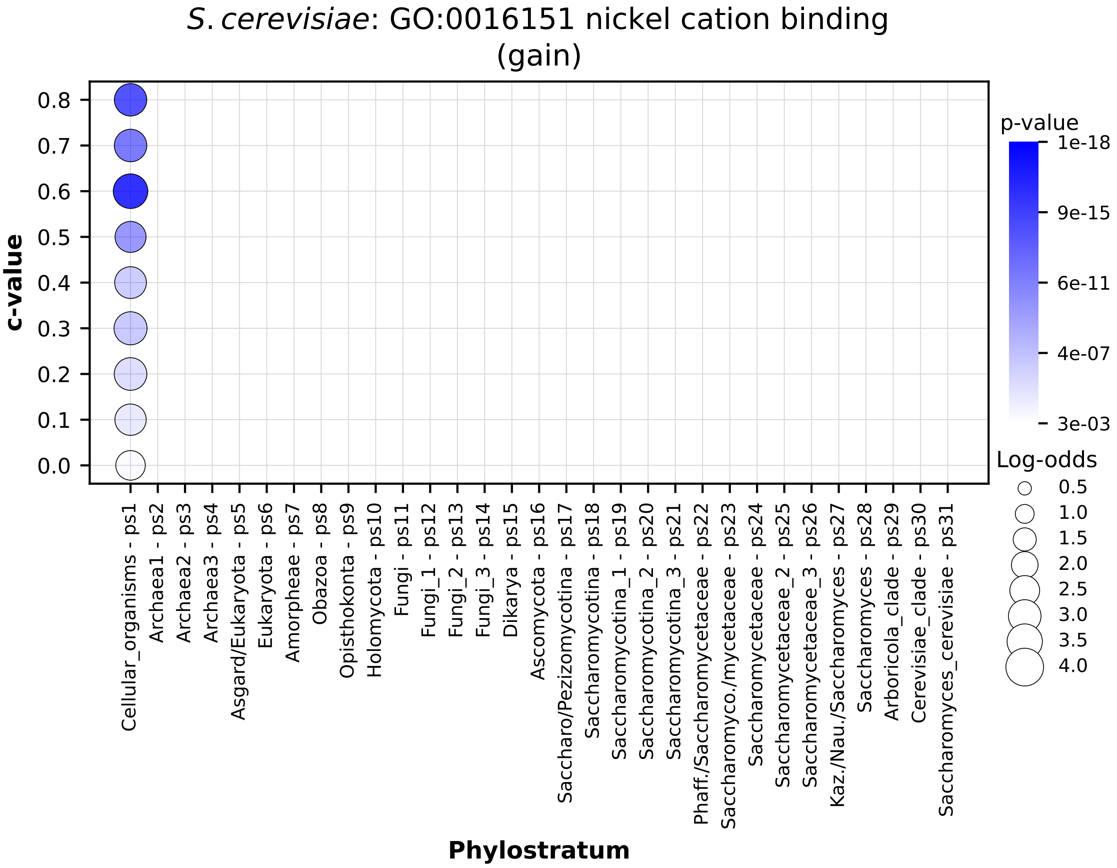

S. cerevisiae: GO:0016151 nickel cation binding (gain)
c-value
0.8
0.7
0.6
0.5
0.4
0.3
0.2
0.1
0.0
p-value
1e-18
9e-15
6e-11
4e-07
3e-03
Log-odds
0.5
1.0
1.5
2.0
2.5
3.0
3.5
4.0
Cellular_organisms - ps1
Archaea1 - ps2
Archaea2 - ps3
Archaea3 - ps4
Asgard/Eukaryota - ps5
Eukaryota - ps6
Amorpheae - ps7
Obazoa - ps8
Opisthokonta - ps9
Holomycota - ps10
Fungi - ps11
Fungi_1 - ps12
Fungi_2 - ps13
Fungi_3 - ps14
Dikarya - ps15
Ascomycota - ps16
Saccharo/Pezizomycotina - ps17
Saccharomycotina - ps18
Saccharomycotina_1 - ps19
Saccharomycotina_2 - ps20
Saccharomycotina_3 - ps21
Phaff./Saccharomycetaceae - ps22
Saccharomyco./mycetaceae - ps23
Saccharomycetaceae - ps24
Saccharomycetaceae_2 - ps25
Saccharomycetaceae_3 - ps26
Kaz./Nau./Saccharomyces - ps27
Saccharomyces - ps28
Arboricola_clade - ps29
Cerevisiae_clade - ps30
Saccharomyces_cerevisiae - ps31
Phylostratum

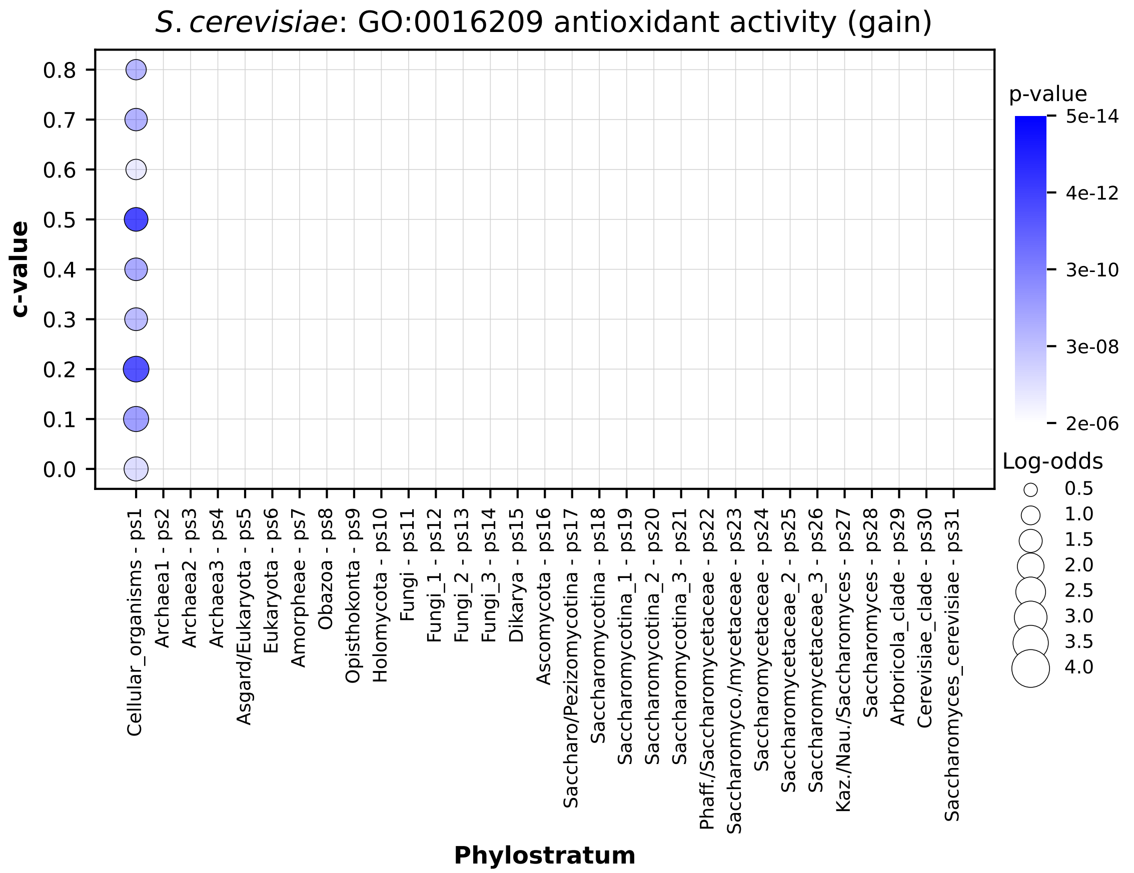

S. cerevisiae: GO:0016209 antioxidant activity (gain)
c-value
0.8
0.7
0.6
0.5
0.4
0.3
0.2
0.1
0.0
p-value
5e-14
4e-12
3e-10
3e-08
2e-06
Log-odds
0.5
1.0
1.5
2.0
2.5
3.0
3.5
4.0
Cellular_organisms - ps1
Archaea1 - ps2
Archaea2 - ps3
Archaea3 - ps4
Asgard/Eukaryota - ps5
Eukaryota - ps6
Amorpheae - ps7
Obazoa - ps8
Opisthokonta - ps9
Holomycota - ps10
Fungi - ps11
Fungi_1 - ps12
Fungi_2 - ps13
Fungi_3 - ps14
Dikarya - ps15
Ascomycota - ps16
Saccharo/Pezizomycotina - ps17
Saccharomycotina - ps18
Saccharomycotina_1 - ps19
Saccharomycotina_2 - ps20
Saccharomycotina_3 - ps21
Phaff./Saccharomycetaceae - ps22
Saccharomyco./mycetaceae - ps23
Saccharomycetaceae - ps24
Saccharomycetaceae_2 - ps25
Saccharomycetaceae_3 - ps26
Kaz./Nau./Saccharomyces - ps27
Saccharomyces - ps28
Arboricola_clade - ps29
Cerevisiae_clade - ps30
Saccharomyces_cerevisiae - ps31
Phylostratum

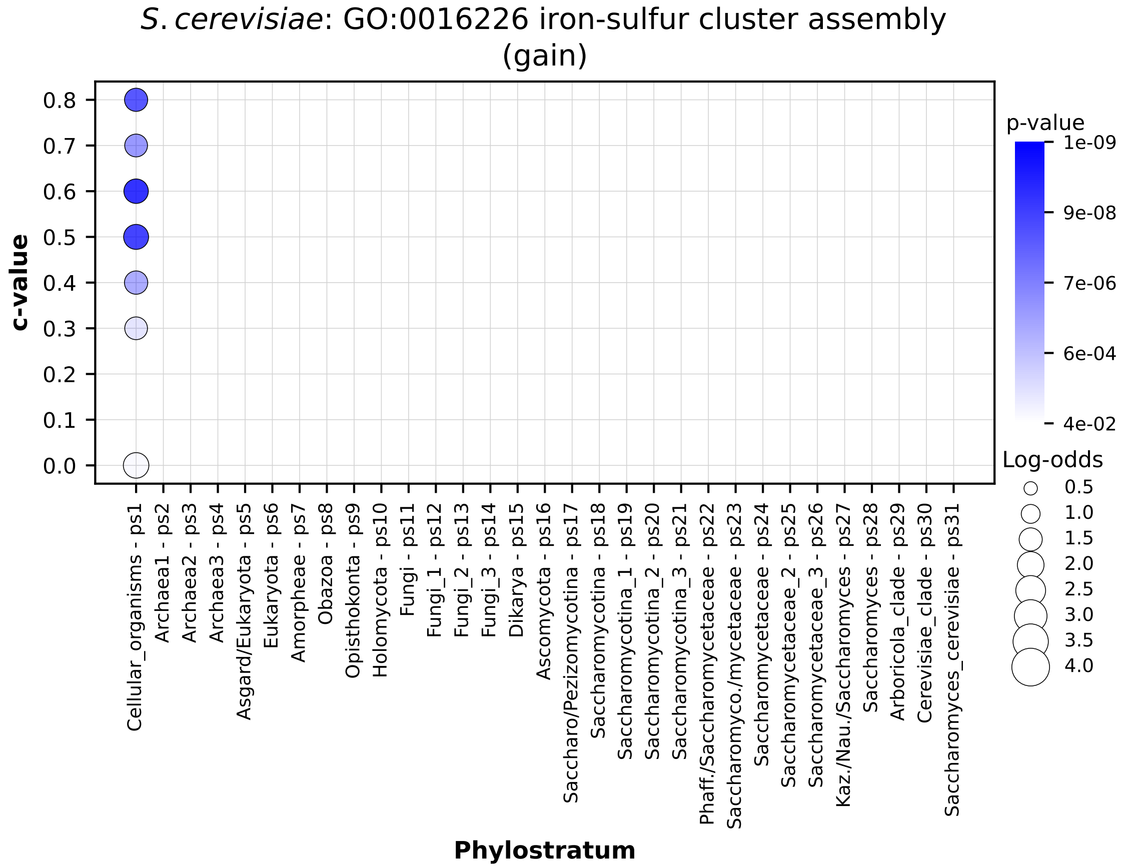

S. cerevisiae: GO:0016226 iron-sulfur cluster assembly (gain)
c-value
0.8
0.7
0.6
0.5
0.4
0.3
0.2
0.1
0.0
p-value
1e-09
9e-08
7e-06
6e-04
4e-02
Log-odds
0.5
1.0
1.5
2.0
2.5
3.0
3.5
4.0
Cellular_organisms - ps1
Archaea1 - ps2
Archaea2 - ps3
Archaea3 - ps4
Asgard/Eukaryota - ps5
Eukaryota - ps6
Amorpheae - ps7
Obazoa - ps8
Opisthokonta - ps9
Holomycota - ps10
Fungi - ps11
Fungi_1 - ps12
Fungi_2 - ps13
Fungi_3 - ps14
Dikarya - ps15
Ascomycota - ps16
Saccharo/Pezizomycotina - ps17
Saccharomycotina - ps18
Saccharomycotina_1 - ps19
Saccharomycotina_2 - ps20
Saccharomycotina_3 - ps21
Phaff./Saccharomycetaceae - ps22
Saccharomyco./mycetaceae - ps23
Saccharomycetaceae - ps24
Saccharomycetaceae_2 - ps25
Saccharomycetaceae_3 - ps26
Kaz./Nau./Saccharomyces - ps27
Saccharomyces - ps28
Arboricola_clade - ps29
Cerevisiae_clade - ps30
Saccharomyces_cerevisiae - ps31
Phylostratum

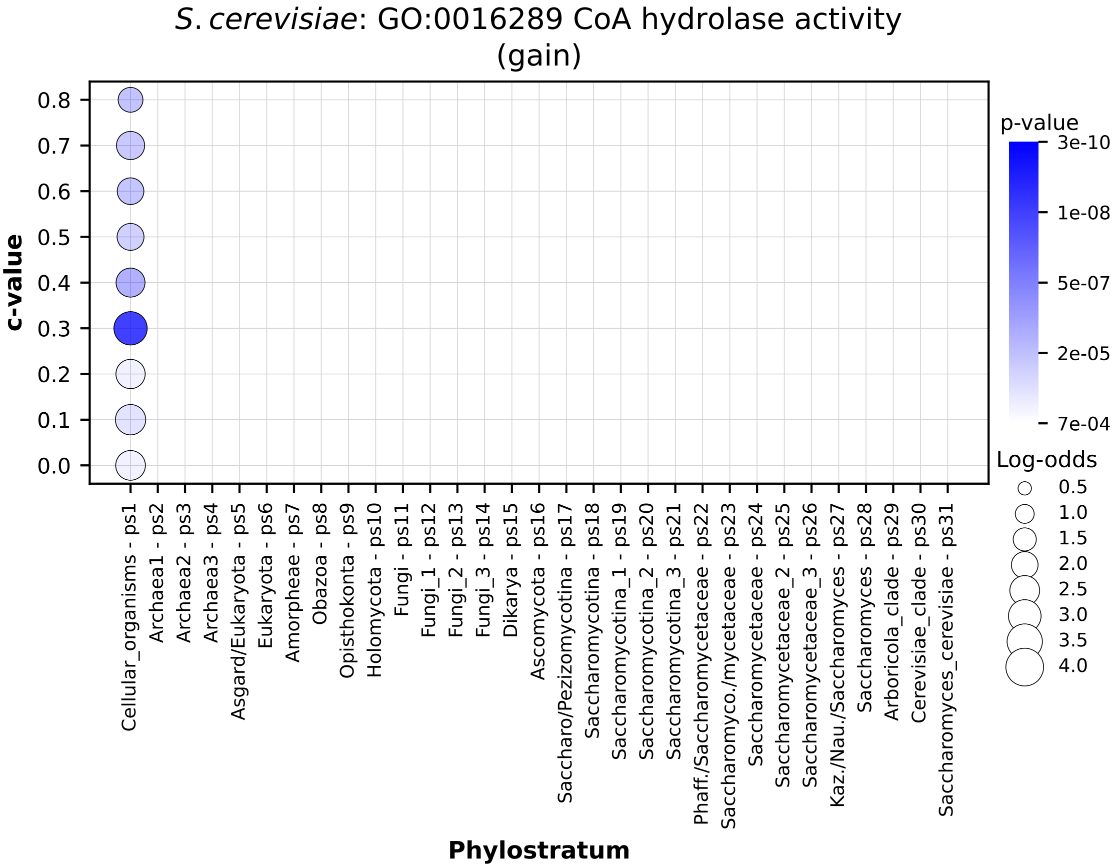

S. cerevisiae: GO:0016289 CoA hydrolase activity
(gain)
c-value
0.8
0.7
0.6
0.5
0.4
0.3
0.2
0.1
0.0
p-value
3e-10
1e-08
5e-07
2e-05
7e-04
Log-odds
0.5
1.0
1.5
2.0
2.5
3.0
3.5
4.0
Cellular_organisms - ps1
Archaea1 - ps2
Archaea2 - ps3
Archaea3 - ps4
Asgard/Eukaryota - ps5
Eukaryota - ps6
Amorpheae - ps7
Obazoa - ps8
Opisthokonta - ps9
Holomycota - ps10
Fungi - ps11
Fungi_1 - ps12
Fungi_2 - ps13
Fungi_3 - ps14
Dikarya - ps15
Ascomycota - ps16
Saccharo/Pezizomycotina - ps17
Saccharomycotina - ps18
Saccharomycotina_1 - ps19
Saccharomycotina_2 - ps20
Saccharomycotina_3 - ps21
Phaff./Saccharomycetaceae - ps22
Saccharomyco./mycetaceae - ps23
Saccharomycetaceae - ps24
Saccharomycetaceae_2 - ps25
Saccharomycetaceae_3 - ps26
Kaz./Nau./Saccharomyces - ps27
Saccharomyces - ps28
Arboricola_clade - ps29
Cerevisiae_clade - ps30
Saccharomyces_cerevisiae - ps31
Phylostratum

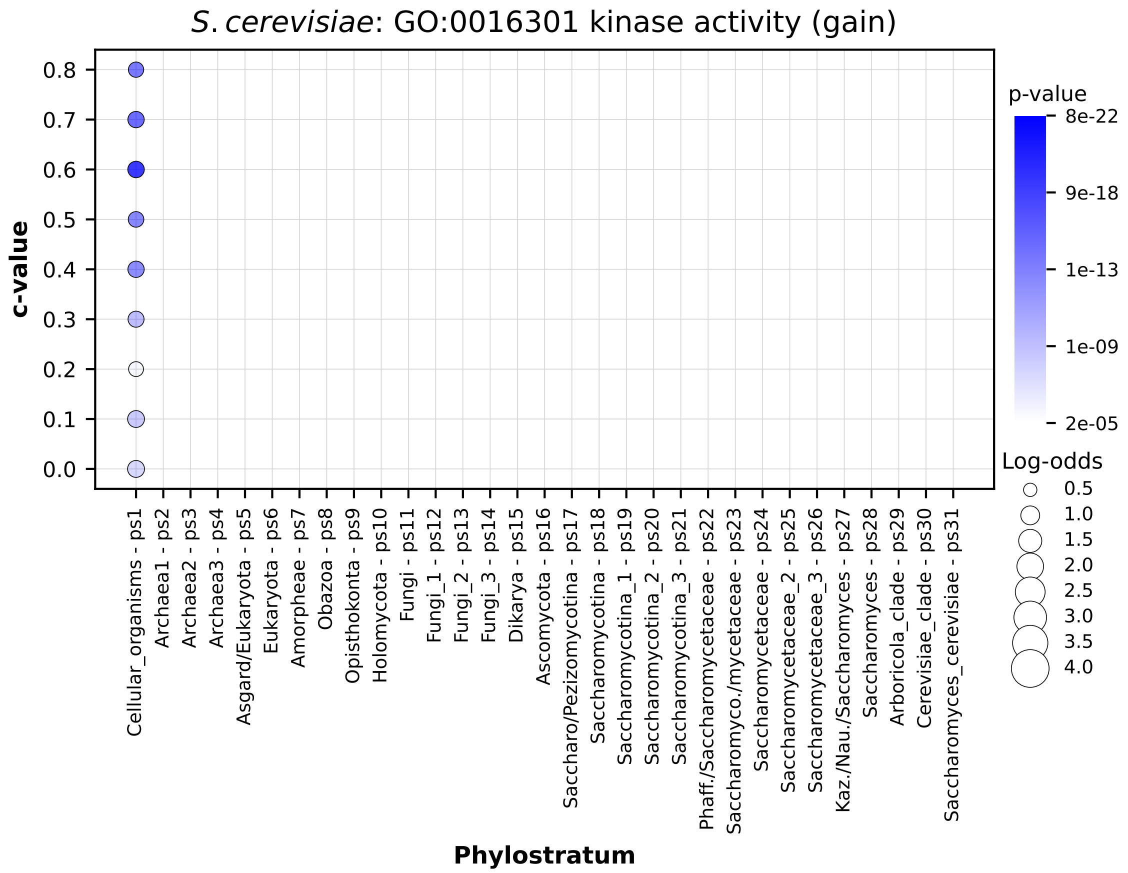
S. cerevisiae: GO:0016301 kinase activity (gain)
c-value
0.8
0.7
0.6
0.5
0.4
0.3
0.2
0.1
0.0
p-value
8e-22
9e-18
1e-13
1e-09
2e-05
Log-odds
0.5
1.0
1.5
2.0
2.5
3.0
3.5
4.0
Cellular_organisms - ps1
Archaea1 - ps2
Archaea2 - ps3
Archaea3 - ps4
Asgard/Eukaryota - ps5
Eukaryota - ps6
Amorpheae - ps7
Obazoa - ps8
Opisthokonta - ps9
Holomycota - ps10
Fungi - ps11
Fungi_1 - ps12
Fungi_2 - ps13
Fungi_3 - ps14
Dikarya - ps15
Ascomycota - ps16
Saccharo/Pezizomycotina - ps17
Saccharomycotina - ps18
Saccharomycotina_1 - ps19
Saccharomycotina_2 - ps20
Saccharomycotina_3 - ps21
Phaff./Saccharomycetaceae - ps22
Saccharomyco./mycetaceae - ps23
Saccharomycetaceae - ps24
Saccharomycetaceae_2 - ps25
Saccharomycetaceae_3 - ps26
Kaz./Nau./Saccharomyces - ps27
Saccharomyces - ps28
Arboricola_clade - ps29
Cerevisiae_clade - ps30
Saccharomyces_cerevisiae - ps31
Phylostratum

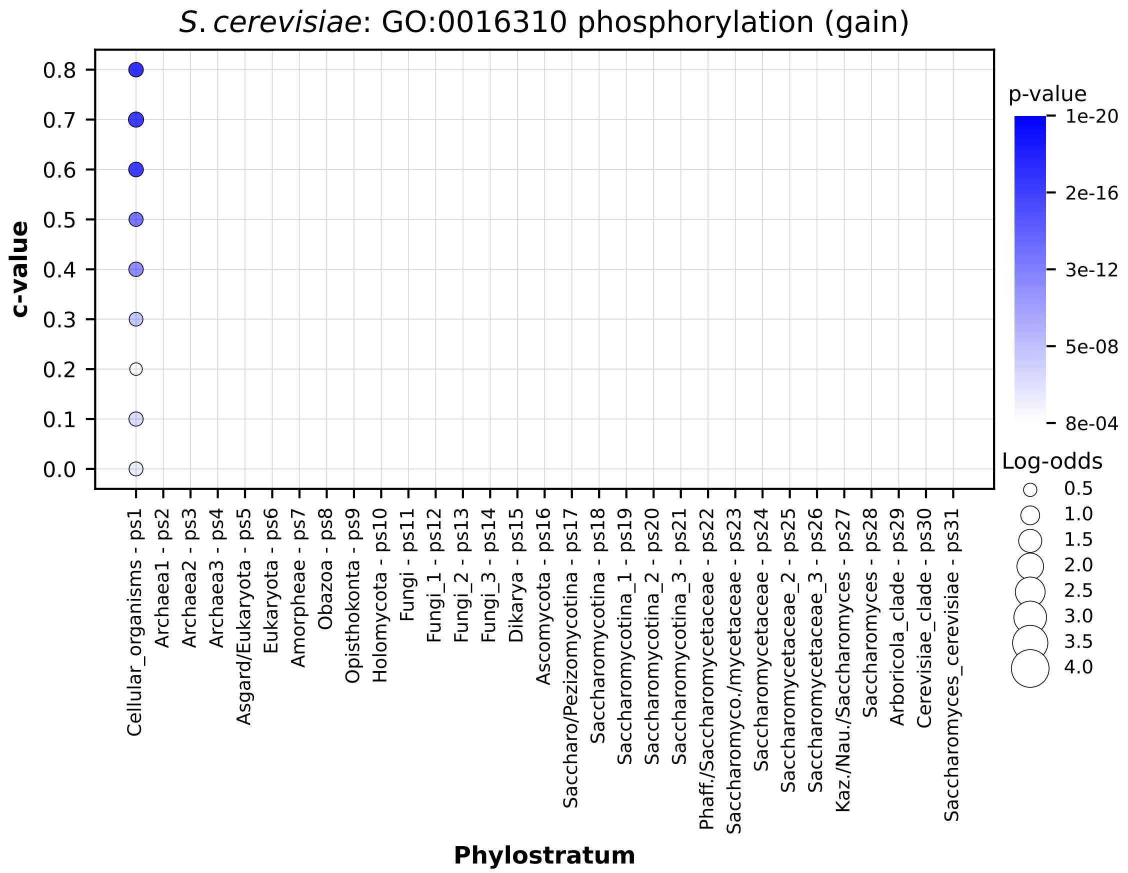
S. cerevisiae: GO:0016310 phosphorylation (gain)
c-value
0.8
0.7
0.6
0.5
0.4
0.3
0.2
0.1
0.0
p-value
1e-20
2e-16
3e-12
5e-08
8e-04
Log-odds
0.5
1.0
1.5
2.0
2.5
3.0
3.5
4.0
Cellular_organisms - ps1
Archaea1 - ps2
Archaea2 - ps3
Archaea3 - ps4
Asgard/Eukaryota - ps5
Eukaryota - ps6
Amorpheae - ps7
Obazoa - ps8
Opisthokonta - ps9
Holomycota - ps10
Fungi - ps11
Fungi_1 - ps12
Fungi_2 - ps13
Fungi_3 - ps14
Dikarya - ps15
Ascomycota - ps16
Saccharo/Pezizomycotina - ps17
Saccharomycotina - ps18
Saccharomycotina_1 - ps19
Saccharomycotina_2 - ps20
Saccharomycotina_3 - ps21
Phaff./Saccharomycetaceae - ps22
Saccharomyco./mycetaceae - ps23
Saccharomycetaceae - ps24
Saccharomycetaceae_2 - ps25
Saccharomycetaceae_3 - ps26
Kaz./Nau./Saccharomyces - ps27
Saccharomyces - ps28
Arboricola_clade - ps29
Cerevisiae_clade - ps30
Saccharomyces_cerevisiae - ps31
Phylostratum

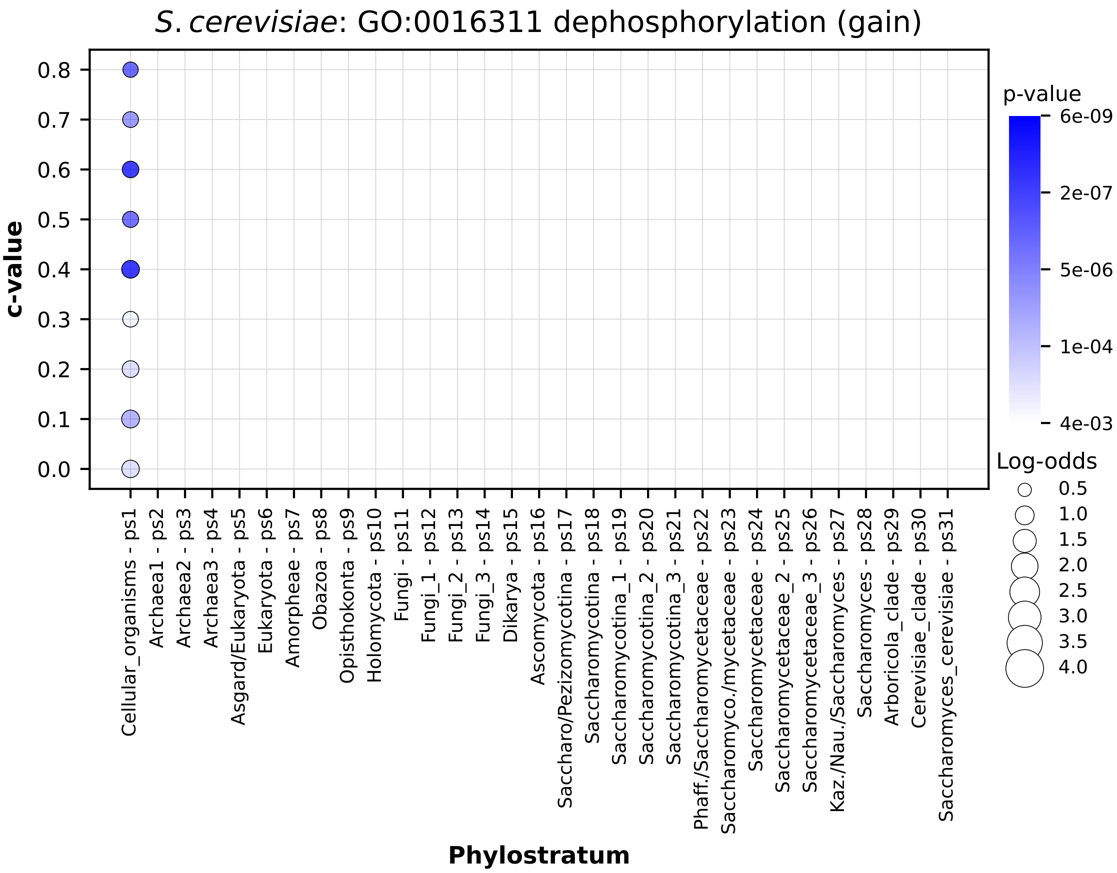
S. cerevisiae: GO:0016311 dephosphorylation (gain)
c-value
0.8
0.7
0.6
0.5
0.4
0.3
0.2
0.1
0.0
p-value
6e-09
2e-07
5e-06
1e-04
4e-03
Log-odds
0.5
1.0
1.5
2.0
2.5
3.0
3.5
4.0
Cellular_organisms - ps1
Archaea1 - ps2
Archaea2 - ps3
Archaea3 - ps4
Asgard/Eukaryota - ps5
Eukaryota - ps6
Amorpheae - ps7
Obazoa - ps8
Opisthokonta - ps9
Holomycota - ps10
Fungi - ps11
Fungi_1 - ps12
Fungi_2 - ps13
Fungi_3 - ps14
Dikarya - ps15
Ascomycota - ps16
Saccharo/Pezizomycotina - ps17
Saccharomycotina - ps18
Saccharomycotina_1 - ps19
Saccharomycotina_2 - ps20
Saccharomycotina_3 - ps21
Phaff./Saccharomycetaceae - ps22
Saccharomyco./mycetaceae - ps23
Saccharomycetaceae - ps24
Saccharomycetaceae_2 - ps25
Saccharomycetaceae_3 - ps26
Kaz./Nau./Saccharomyces - ps27
Saccharomyces - ps28
Arboricola_clade - ps29
Cerevisiae_clade - ps30
Saccharomyces_cerevisiae - ps31
Phylostratum

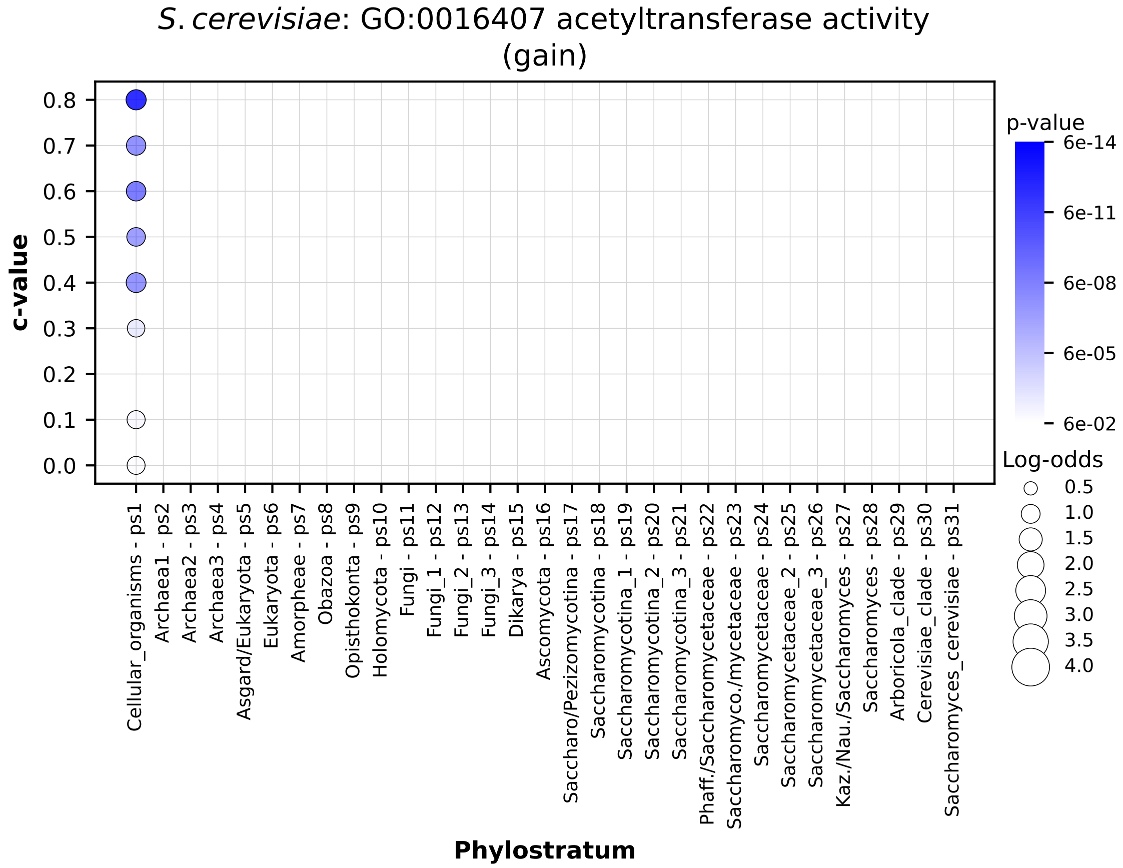
S. cerevisiae: GO:0016407 acetyltransferase activity (gain)
c-value
0.8
0.7
0.6
0.5
0.4
0.3
0.2
0.1
0.0
p-value
6e-14
6e-11
6e-08
6e-05
6e-02
Log-odds
0.5
1.0
1.5
2.0
2.5
3.0
3.5
4.0
Cellular_organisms - ps1
Archaea1 - ps2
Archaea2 - ps3
Archaea3 - ps4
Asgard/Eukaryota - ps5
Eukaryota - ps6
Amorpheae - ps7
Obazoa - ps8
Opisthokonta - ps9
Holomycota - ps10
Fungi - ps11
Fungi_1 - ps12
Fungi_2 - ps13
Fungi_3 - ps14
Dikarya - ps15
Ascomycota - ps16
Saccharo/Pezizomycotina - ps17
Saccharomycotina - ps18
Saccharomycotina_1 - ps19
Saccharomycotina_2 - ps20
Saccharomycotina_3 - ps21
Phaff./Saccharomycetaceae - ps22
Saccharomyco./mycetaceae - ps23
Saccharomycetaceae - ps24
Saccharomycetaceae_2 - ps25
Saccharomycetaceae_3 - ps26
Kaz./Nau./Saccharomyces - ps27
Saccharomyces - ps28
Arboricola_clade - ps29
Cerevisiae_clade - ps30
Saccharomyces_cerevisiae - ps31
Phylostratum

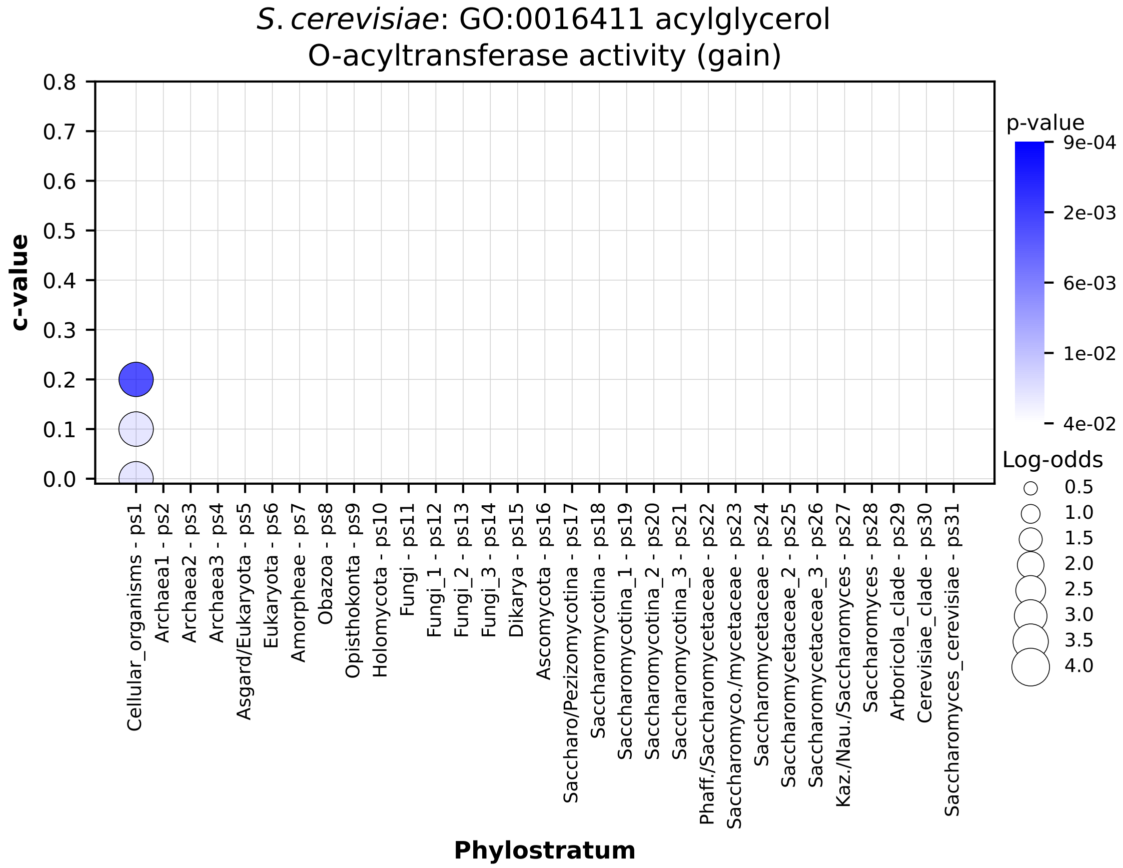

S. cerevisiae: GO:0016411 acylglycerol O-acyltransferase activity (gain)
c-value
0.8
0.7
0.6
0.5
0.4
0.3
0.2
0.1
0.0
p-value
9e-04
2e-03
6e-03
1e-02
4e-02
Log-odds
0.5
1.0
1.5
2.0
2.5
3.0
3.5
4.0
Cellular_organisms - ps1
Archaea1 - ps2
Archaea2 - ps3
Archaea3 - ps4
Asgard/Eukaryota - ps5
Eukaryota - ps6
Amorpheae - ps7
Obazoa - ps8
Opisthokonta - ps9
Holomycota - ps10
Fungi - ps11
Fungi_1 - ps12
Fungi_2 - ps13
Fungi_3 - ps14
Dikarya - ps15
Ascomycota - ps16
Saccharo/Pezizomycotina - ps17
Saccharomycotina - ps18
Saccharomycotina_1 - ps19
Saccharomycotina_2 - ps20
Saccharomycotina_3 - ps21
Phaff./Saccharomycetaceae - ps22
Saccharomyco./mycetaceae - ps23
Saccharomycetaceae - ps24
Saccharomycetaceae_2 - ps25
Saccharomycetaceae_3 - ps26
Kaz./Nau./Saccharomyces - ps27
Saccharomyces - ps28
Arboricola_clade - ps29
Cerevisiae_clade - ps30
Saccharomyces_cerevisiae - ps31
Phylostratum

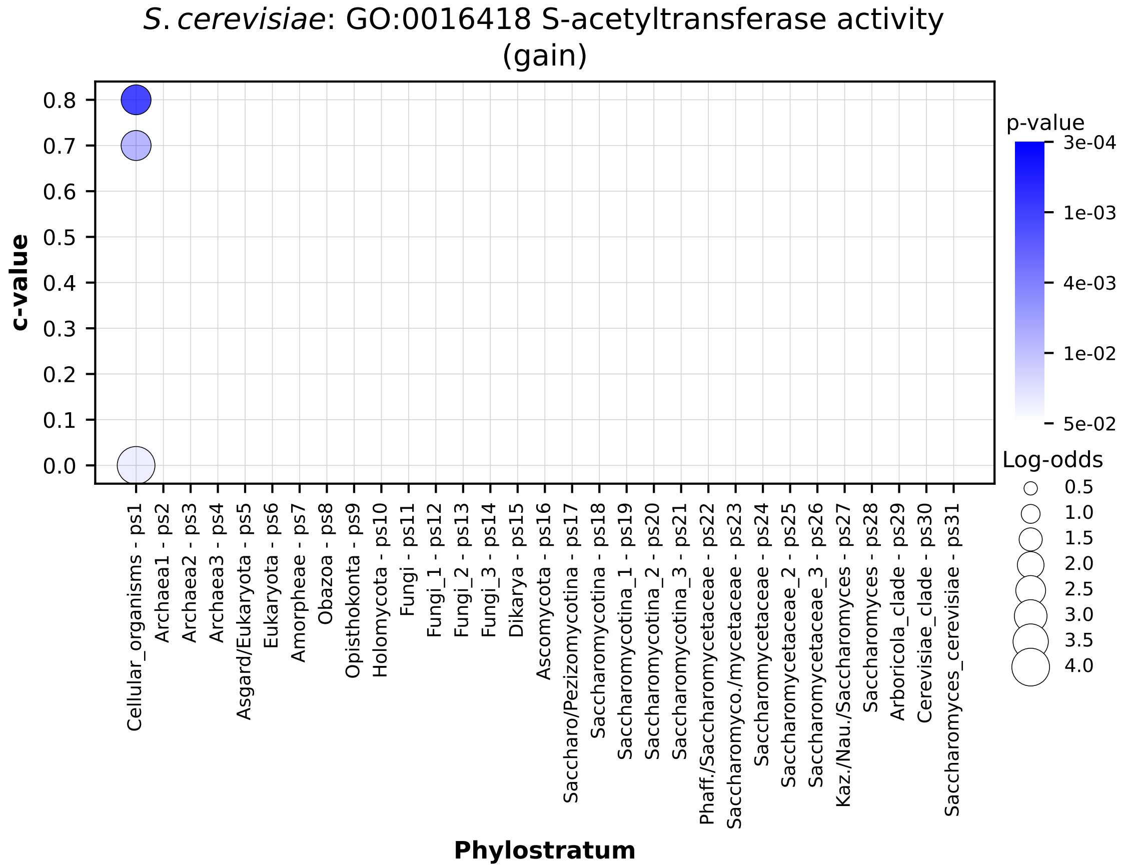
S. cerevisiae: GO:0016418 S-acetyltransferase activity
(gain)
c-value
0.8
0.7
0.6
0.5
0.4
0.3
0.2
0.1
0.0
p-value
3e-04
1e-03
4e-03
1e-02
5e-02
Log-odds
0.5
1.0
1.5
2.0
2.5
3.0
3.5
4.0
Cellular_organisms - ps1
Archaea1 - ps2
Archaea2 - ps3
Archaea3 - ps4
Asgard/Eukaryota - ps5
Eukaryota - ps6
Amorpheae - ps7
Obazoa - ps8
Opisthokonta - ps9
Holomycota - ps10
Fungi - ps11
Fungi_1 - ps12
Fungi_2 - ps13
Fungi_3 - ps14
Dikarya - ps15
Ascomycota - ps16
Saccharo/Pezizomycotina - ps17
Saccharomycotina - ps18
Saccharomycotina_1 - ps19
Saccharomycotina_2 - ps20
Saccharomycotina_3 - ps21
Phaff./Saccharomycetaceae - ps22
Saccharomyco./mycetaceae - ps23
Saccharomycetaceae - ps24
Saccharomycetaceae_2 - ps25
Saccharomycetaceae_3 - ps26
Kaz./Nau./Saccharomyces - ps27
Saccharomyces - ps28
Arboricola_clade - ps29
Cerevisiae_clade - ps30
Saccharomyces_cerevisiae - ps31
Phylostratum

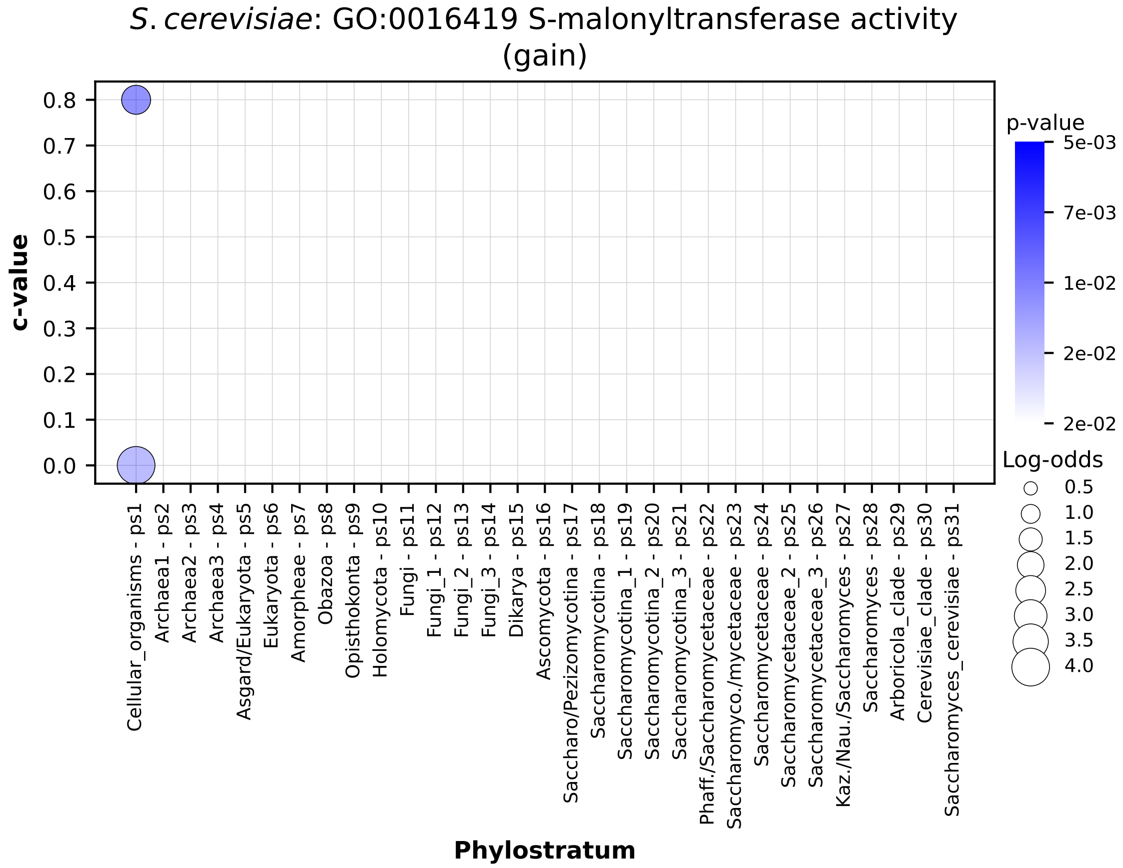

S. cerevisiae: GO:0016419 S-malonyltransferase activity (gain)
c-value
0.0
0.1
0.2
0.3
0.4
0.5
0.6
0.7
0.8
p-value
5e-03
7e-03
1e-02
2e-02
2e-02
Log-odds
0.5
1.0
1.5
2.0
2.5
3.0
3.5
4.0
Cellular_organisms - ps1
Archaea1 - ps2
Archaea2 - ps3
Archaea3 - ps4
Asgard/Eukaryota - ps5
Eukaryota - ps6
Amorpheae - ps7
Obazoa - ps8
Opisthokonta - ps9
Holomycota - ps10
Fungi - ps11
Fungi_1 - ps12
Fungi_2 - ps13
Fungi_3 - ps14
Dikarya - ps15
Ascomycota - ps16
Saccharo/Pezizomycotina - ps17
Saccharomycotina - ps18
Saccharomycotina_1 - ps19
Saccharomycotina_2 - ps20
Saccharomycotina_3 - ps21
Phaff./Saccharomycetaceae - ps22
Saccharomyco./mycetaceae - ps23
Saccharomycetaceae - ps24
Saccharomycetaceae_2 - ps25
Saccharomycetaceae_3 - ps26
Kaz./Nau./Saccharomyces - ps27
Saccharomyces - ps28
Arboricola_clade - ps29
Cerevisiae_clade - ps30
Saccharomyces_cerevisiae - ps31
Phylostratum

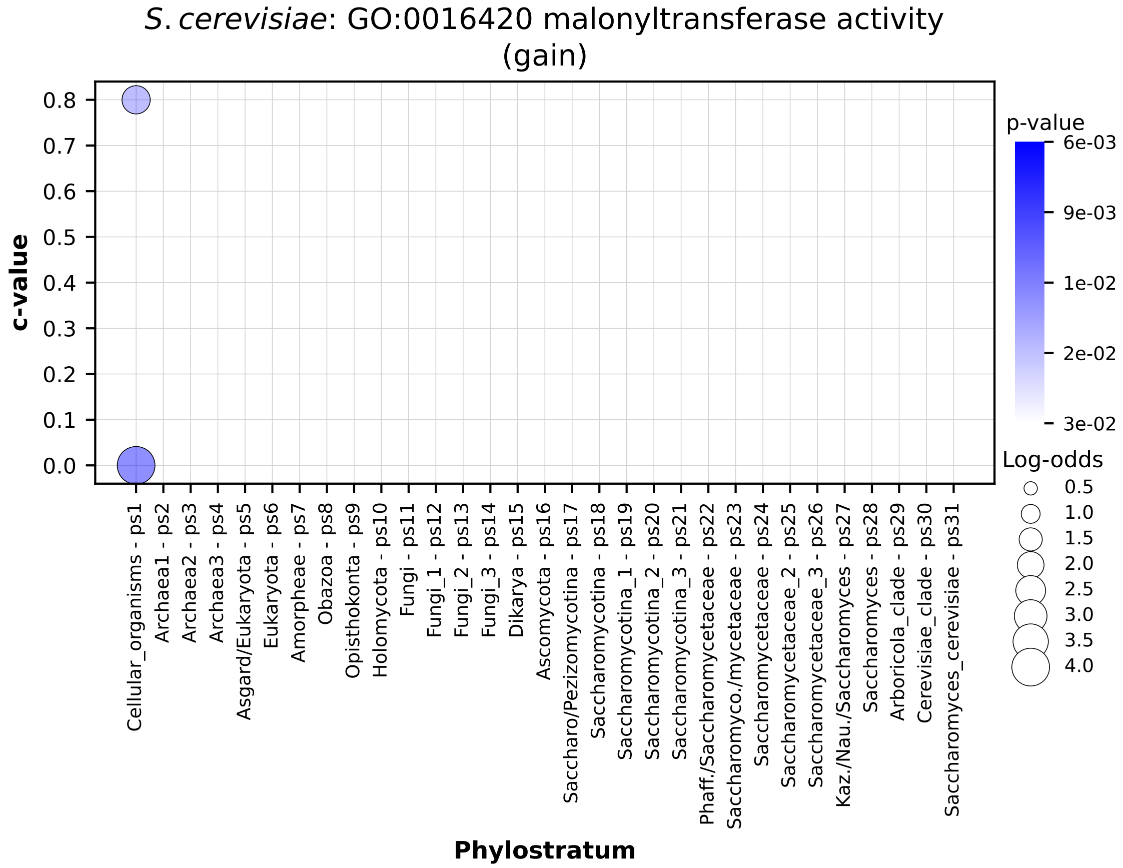
S. cerevisiae: GO:0016420 malonyltransferase activity
(gain)
c-value
0.8
0.7
0.6
0.5
0.4
0.3
0.2
0.1
0.0
p-value
6e-03
9e-03
1e-02
2e-02
3e-02
Log-odds
0.5
1.0
1.5
2.0
2.5
3.0
3.5
4.0
Cellular_organisms - ps1
Archaea1 - ps2
Archaea2 - ps3
Archaea3 - ps4
Asgard/Eukaryota - ps5
Eukaryota - ps6
Amorpheae - ps7
Obazoa - ps8
Opisthokonta - ps9
Holomycota - ps10
Fungi - ps11
Fungi_1 - ps12
Fungi_2 - ps13
Fungi_3 - ps14
Dikarya - ps15
Ascomycota - ps16
Saccharo/Pezizomycotina - ps17
Saccharomycotina - ps18
Saccharomycotina_1 - ps19
Saccharomycotina_2 - ps20
Saccharomycotina_3 - ps21
Phaff./Saccharomycetaceae - ps22
Saccharomyco./mycetaceae - ps23
Saccharomycetaceae - ps24
Saccharomycetaceae_2 - ps25
Saccharomycetaceae_3 - ps26
Kaz./Nau./Saccharomyces - ps27
Saccharomyces - ps28
Arboricola_clade - ps29
Cerevisiae_clade - ps30
Saccharomyces_cerevisiae - ps31
Phylostratum

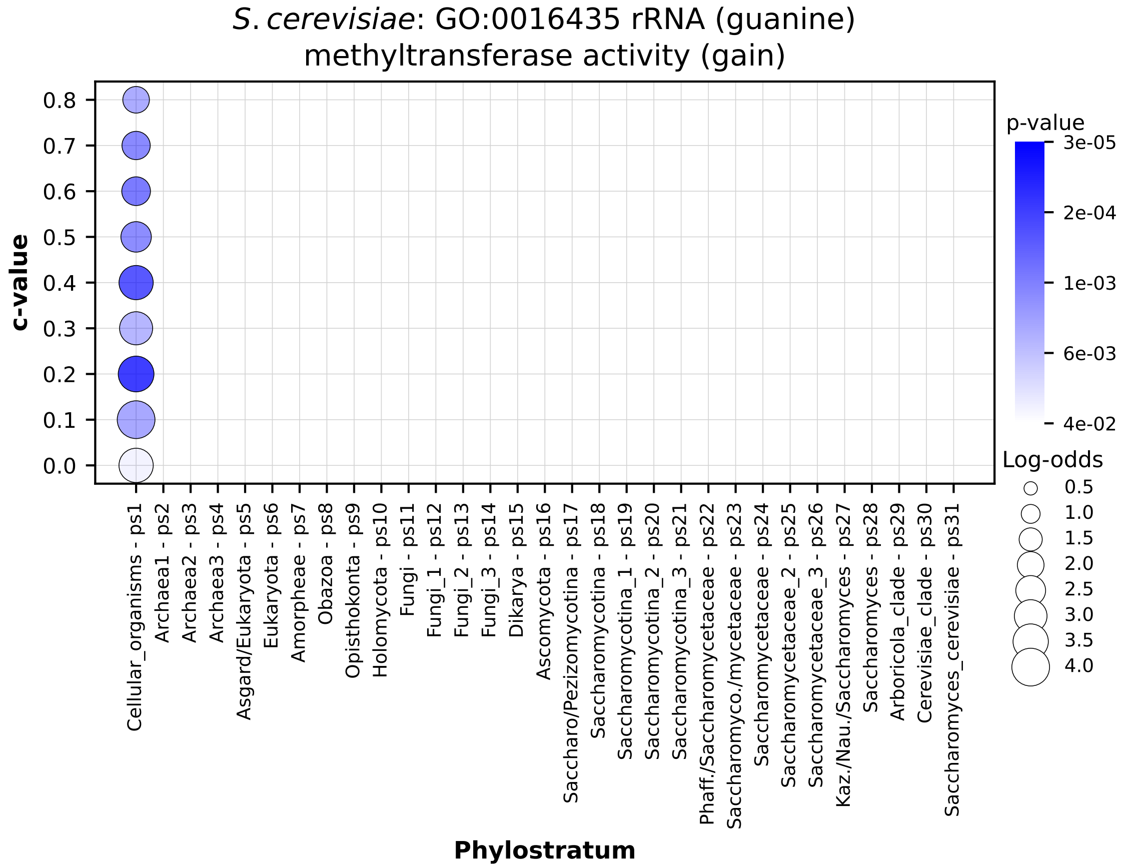
S. cerevisiae: GO:0016435 rRNA (guanine) methyltransferase activity (gain)
c-value
0.8
0.7
0.6
0.5
0.4
0.3
0.2
0.1
0.0
p-value
3e-05
2e-04
1e-03
6e-03
4e-02
Log-odds
0.5
1.0
1.5
2.0
2.5
3.0
3.5
4.0
Cellular_organisms - ps1
Archaea1 - ps2
Archaea2 - ps3
Archaea3 - ps4
Asgard/Eukaryota - ps5
Eukaryota - ps6
Amorpheae - ps7
Obazoa - ps8
Opisthokonta - ps9
Holomycota - ps10
Fungi - ps11
Fungi_1 - ps12
Fungi_2 - ps13
Fungi_3 - ps14
Dikarya - ps15
Ascomycota - ps16
Saccharo/Pezizomycotina - ps17
Saccharomycotina - ps18
Saccharomycotina_1 - ps19
Saccharomycotina_2 - ps20
Saccharomycotina_3 - ps21
Phaff./Saccharomycetaceae - ps22
Saccharomyco./mycetaceae - ps23
Saccharomycetaceae - ps24
Saccharomycetaceae_2 - ps25
Saccharomycetaceae_3 - ps26
Kaz./Nau./Saccharomyces - ps27
Saccharomyces - ps28
Arboricola_clade - ps29
Cerevisiae_clade - ps30
Saccharomyces_cerevisiae - ps31
Phylostratum

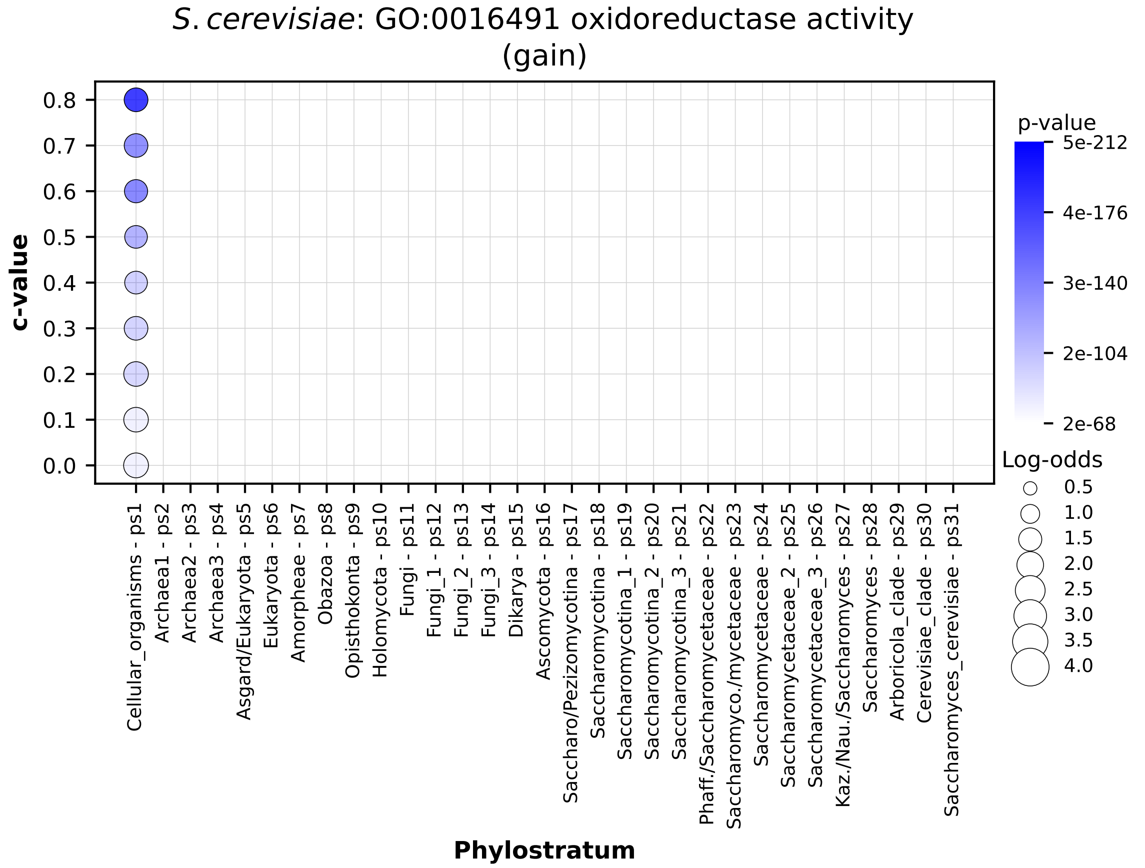

S. cerevisiae: GO:0016491 oxidoreductase activity
(gain)
c-value
0.8
0.7
0.6
0.5
0.4
0.3
0.2
0.1
0.0
p-value
5e-212
4e-176
3e-140
2e-104
2e-68
Log-odds
0.5
1.0
1.5
2.0
2.5
3.0
3.5
4.0
Cellular_organisms - ps1
Archaea1 - ps2
Archaea2 - ps3
Archaea3 - ps4
Asgard/Eukaryota - ps5
Eukaryota - ps6
Amorpheae - ps7
Obazoa - ps8
Opisthokonta - ps9
Holomycota - ps10
Fungi - ps11
Fungi_1 - ps12
Fungi_2 - ps13
Fungi_3 - ps14
Dikarya - ps15
Ascomycota - ps16
Saccharo/Pezizomycotina - ps17
Saccharomycotina - ps18
Saccharomycotina_1 - ps19
Saccharomycotina_2 - ps20
Saccharomycotina_3 - ps21
Phaff./Saccharomycetaceae - ps22
Saccharomyco./mycetaceae - ps23
Saccharomycetaceae - ps24
Saccharomycetaceae_2 - ps25
Saccharomycetaceae_3 - ps26
Kaz./Nau./Saccharomyces - ps27
Saccharomyces - ps28
Arboricola_clade - ps29
Cerevisiae_clade - ps30
Saccharomyces_cerevisiae - ps31
Phylostratum

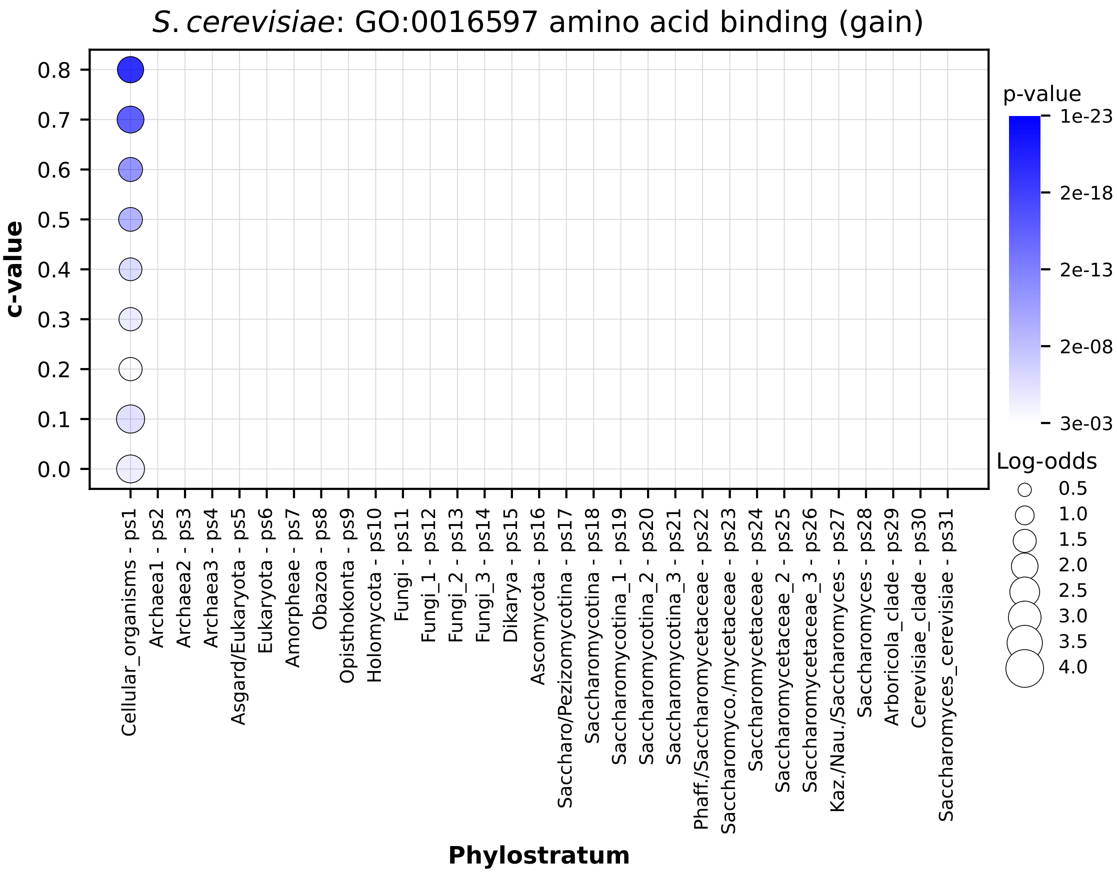

S. cerevisiae: GO:0016597 amino acid binding (gain)
c-value
0.8
0.7
0.6
0.5
0.4
0.3
0.2
0.1
0.0
p-value
1e-23
2e-18
2e-13
2e-08
3e-03
Log-odds
0.5
1.0
1.5
2.0
2.5
3.0
3.5
4.0
Cellular_organisms - ps1
Archaea1 - ps2
Archaea2 - ps3
Archaea3 - ps4
Asgard/Eukaryota - ps5
Eukaryota - ps6
Amorpheae - ps7
Obazoa - ps8
Opisthokonta - ps9
Holomycota - ps10
Fungi - ps11
Fungi_1 - ps12
Fungi_2 - ps13
Fungi_3 - ps14
Dikarya - ps15
Ascomycota - ps16
Saccharo/Pezizomycotina - ps17
Saccharomycotina - ps18
Saccharomycotina_1 - ps19
Saccharomycotina_2 - ps20
Saccharomycotina_3 - ps21
Phaff./Saccharomycetaceae - ps22
Saccharomyco./mycetaceae - ps23
Saccharomycetaceae - ps24
Saccharomycetaceae_2 - ps25
Saccharomycetaceae_3 - ps26
Kaz./Nau./Saccharomyces - ps27
Saccharomyces - ps28
Arboricola_clade - ps29
Cerevisiae_clade - ps30
Saccharomyces_cerevisiae - ps31
Phylostratum

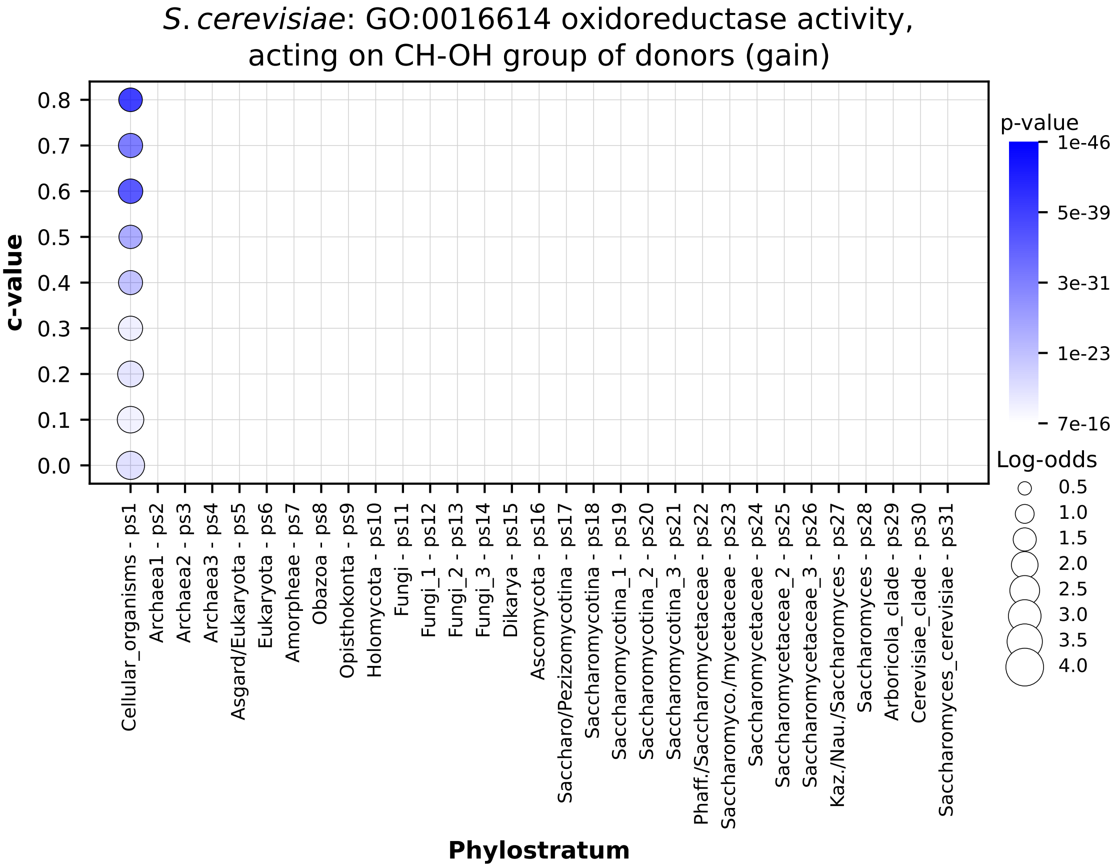

S. cerevisiae: GO:0016614 oxidoreductase activity, acting on CH-OH group of donors (gain)
c-value
0.8
0.7
0.6
0.5
0.4
0.3
0.2
0.1
0.0
p-value
1e-46
5e-39
3e-31
1e-23
7e-16
Log-odds
0.5
1.0
1.5
2.0
2.5
3.0
3.5
4.0
Cellular_organisms - ps1
Archaea1 - ps2
Archaea2 - ps3
Archaea3 - ps4
Asgard/Eukaryota - ps5
Eukaryota - ps6
Amorpheae - ps7
Obazoa - ps8
Opisthokonta - ps9
Holomycota - ps10
Fungi - ps11
Fungi_1 - ps12
Fungi_2 - ps13
Fungi_3 - ps14
Dikarya - ps15
Ascomycota - ps16
Saccharo/Pezizomycotina - ps17
Saccharomycotina - ps18
Saccharomycotina_1 - ps19
Saccharomycotina_2 - ps20
Saccharomycotina_3 - ps21
Phaff./Saccharomycetaceae - ps22
Saccharomyco./mycetaceae - ps23
Saccharomycetaceae - ps24
Saccharomycetaceae_2 - ps25
Saccharomycetaceae_3 - ps26
Kaz./Nau./Saccharomyces - ps27
Saccharomyces - ps28
Arboricola_clade - ps29
Cerevisiae_clade - ps30
Saccharomyces_cerevisiae - ps31
Phylostratum

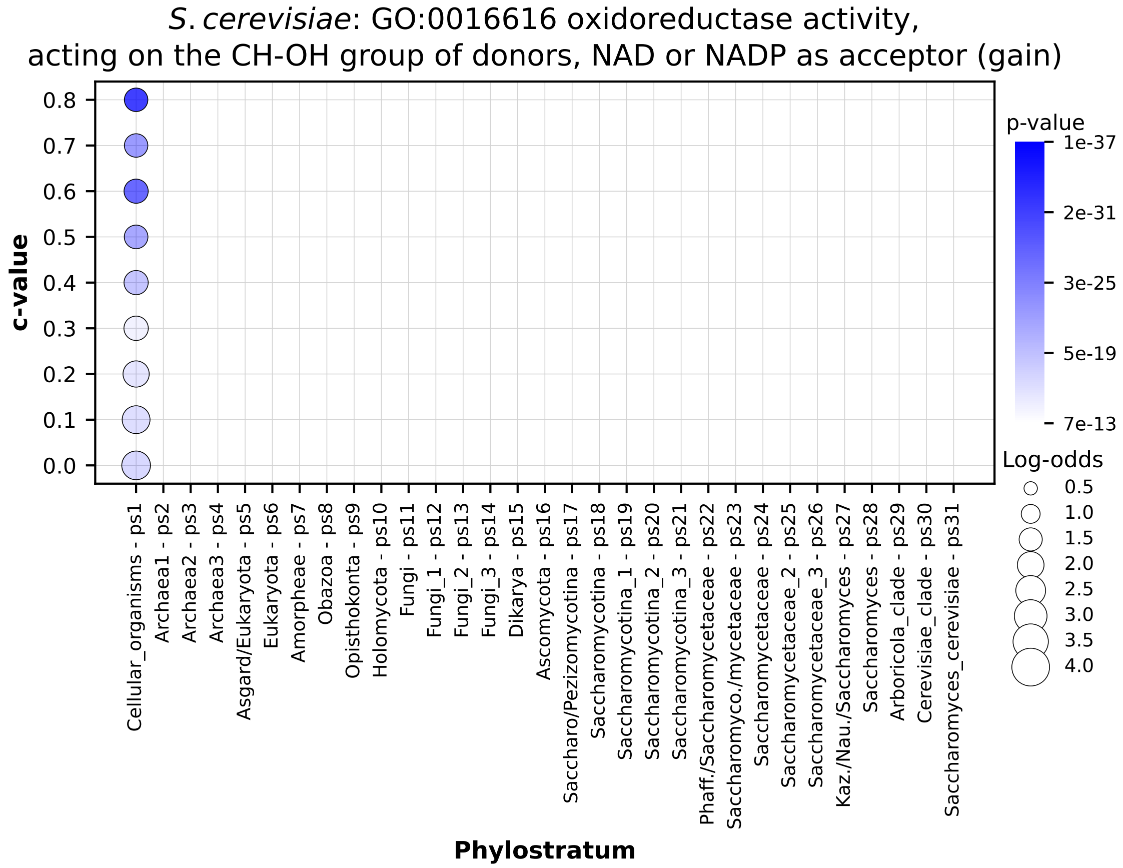

S. cerevisiae: GO:0016616 oxidoreductase activity,
acting on the CH-OH group of donors, NAD or NADP as acceptor (gain)
c-value
0.8
0.7
0.6
0.5
0.4
0.3
0.2
0.1
0.0
p-value
1e-37
2e-31
3e-25
5e-19
7e-13
Log-odds
0.5
1.0
1.5
2.0
2.5
3.0
3.5
4.0
Cellular_organisms - ps1
Archaea1 - ps2
Archaea2 - ps3
Archaea3 - ps4
Asgard/Eukaryota - ps5
Eukaryota - ps6
Amorpheae - ps7
Obazoa - ps8
Opisthokonta - ps9
Holomycota - ps10
Fungi - ps11
Fungi_1 - ps12
Fungi_2 - ps13
Fungi_3 - ps14
Dikarya - ps15
Ascomycota - ps16
Saccharo/Pezizomycotina - ps17
Saccharomycotina - ps18
Saccharomycotina_1 - ps19
Saccharomycotina_2 - ps20
Saccharomycotina_3 - ps21
Phaff./Saccharomycetaceae - ps22
Saccharomyco./mycetaceae - ps23
Saccharomycetaceae - ps24
Saccharomycetaceae_2 - ps25
Saccharomycetaceae_3 - ps26
Kaz./Nau./Saccharomyces - ps27
Saccharomyces - ps28
Arboricola_clade - ps29
Cerevisiae_clade - ps30
Saccharomyces_cerevisiae - ps31
Phylostratum

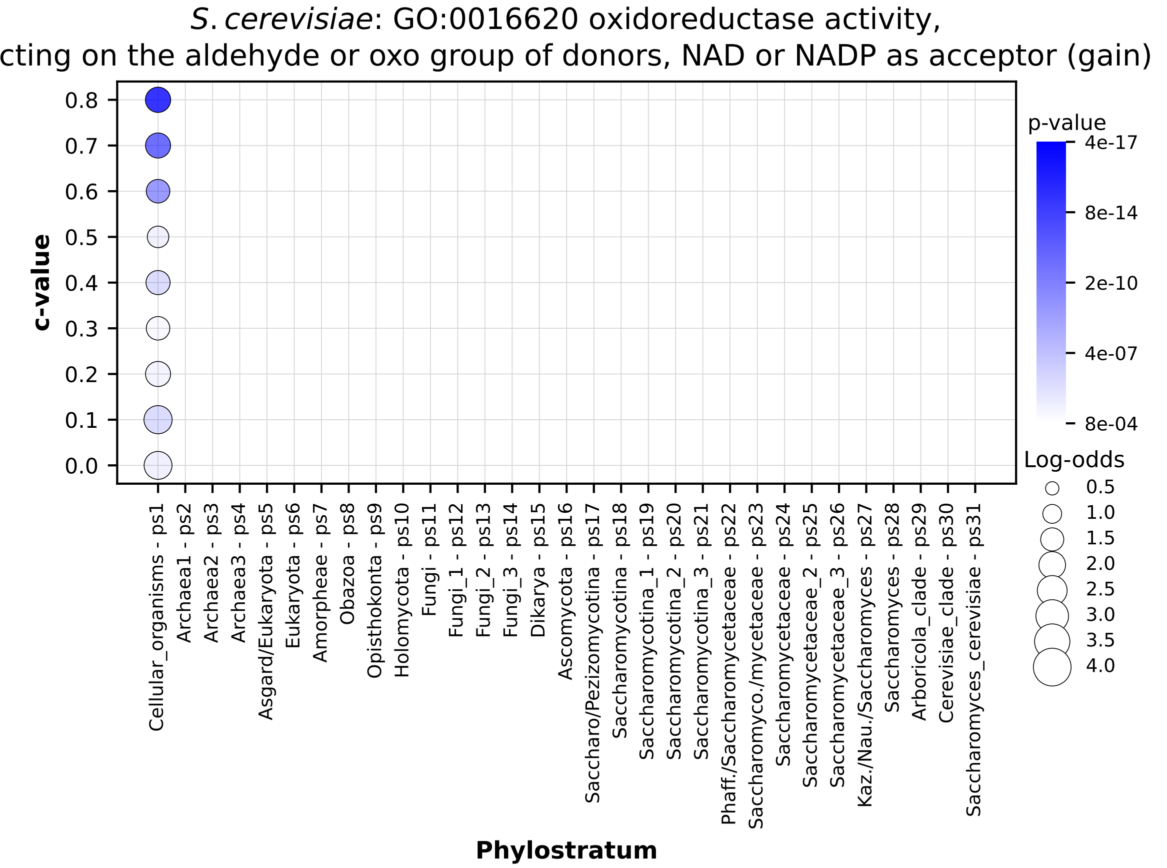

S. cerevisiae: GO:0016620 oxidoreductase activity,
cting on the aldehyde or oxo group of donors, NAD or NADP as acceptor (gain)
c-value
0.8
0.7
0.6
0.5
0.4
0.3
0.2
0.1
0.0
p-value
4e-17
8e-14
2e-10
4e-07
8e-04
Log-odds
0.5
1.0
1.5
2.0
2.5
3.0
3.5
4.0
Cellular_organisms - ps1
Archaea1 - ps2
Archaea2 - ps3
Archaea3 - ps4
Asgard/Eukaryota - ps5
Eukaryota - ps6
Amorpheae - ps7
Obazoa - ps8
Opisthokonta - ps9
Holomycota - ps10
Fungi - ps11
Fungi_1 - ps12
Fungi_2 - ps13
Fungi_3 - ps14
Dikarya - ps15
Ascomycota - ps16
Saccharo/Pezizomycotina - ps17
Saccharomycotina - ps18
Saccharomycotina_1 - ps19
Saccharomycotina_2 - ps20
Saccharomycotina_3 - ps21
Phaff./Saccharomycetaceae - ps22
Saccharomyco./mycetaceae - ps23
Saccharomycetaceae - ps24
Saccharomycetaceae_2 - ps25
Saccharomycetaceae_3 - ps26
Kaz./Nau./Saccharomyces - ps27
Saccharomyces - ps28
Arboricola_clade - ps29
Cerevisiae_clade - ps30
Saccharomyces_cerevisiae - ps31
Phylostratum

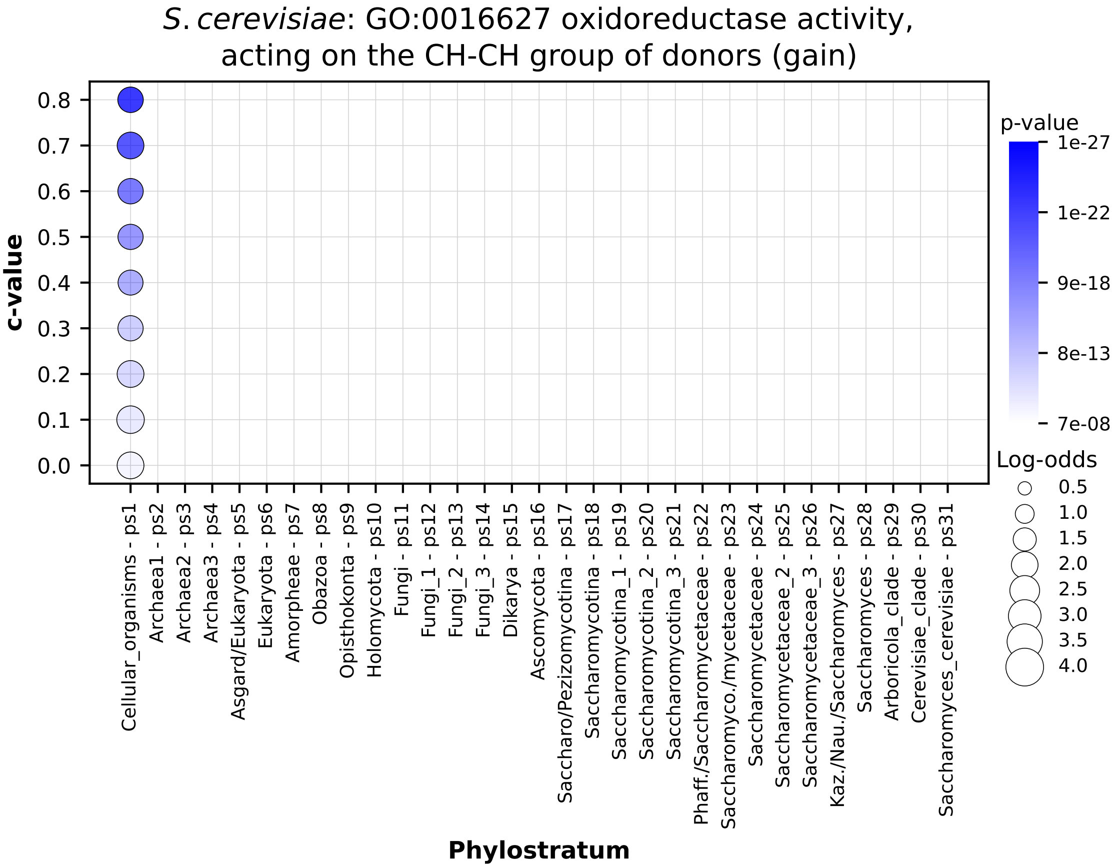
S. cerevisiae: GO:0016627 oxidoreductase activity, acting on the CH-CH group of donors (gain)
c-value
0.0
0.1
0.2
0.3
0.4
0.5
0.6
0.7
0.8
p-value
1e-27
1e-22
9e-18
8e-13
7e-08
Log-odds
0.5
1.0
1.5
2.0
2.5
3.0
3.5
4.0
Cellular_organisms - ps1
Archaea1 - ps2
Archaea2 - ps3
Archaea3 - ps4
Asgard/Eukaryota - ps5
Eukaryota - ps6
Amorpheae - ps7
Obazoa - ps8
Opisthokonta - ps9
Holomycota - ps10
Fungi - ps11
Fungi_1 - ps12
Fungi_2 - ps13
Fungi_3 - ps14
Dikarya - ps15
Ascomycota - ps16
Saccharo/Pezizomycotina - ps17
Saccharomycotina - ps18
Saccharomycotina_1 - ps19
Saccharomycotina_2 - ps20
Saccharomycotina_3 - ps21
Phaff./Saccharomycetaceae - ps22
Saccharomyco./mycetaceae - ps23
Saccharomycetaceae - ps24
Saccharomycetaceae_2 - ps25
Saccharomycetaceae_3 - ps26
Kaz./Nau./Saccharomyces - ps27
Saccharomyces - ps28
Arboricola_clade - ps29
Cerevisiae_clade - ps30
Saccharomyces_cerevisiae - ps31
Phylostratum

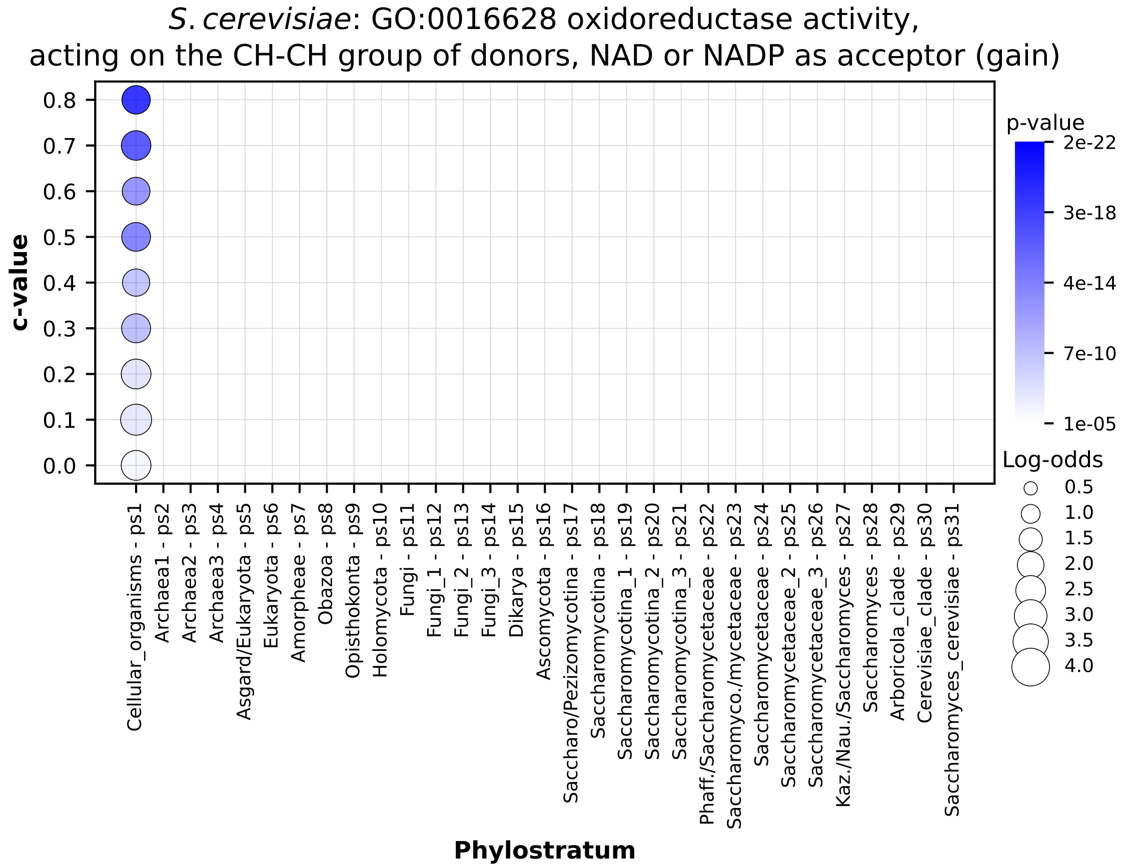

S. cerevisiae: GO:0016628 oxidoreductase activity,
acting on the CH-CH group of donors, NAD or NADP as acceptor (gain)
c-value
0.8
0.7
0.6
0.5
0.4
0.3
0.2
0.1
0.0
p-value
2e-22
3e-18
4e-14
7e-10
1e-05
Log-odds
0.5
1.0
1.5
2.0
2.5
3.0
3.5
4.0
Cellular_organisms - ps1
Archaea1 - ps2
Archaea2 - ps3
Archaea3 - ps4
Asgard/Eukaryota - ps5
Eukaryota - ps6
Amorpheae - ps7
Obazoa - ps8
Opisthokonta - ps9
Holomycota - ps10
Fungi - ps11
Fungi_1 - ps12
Fungi_2 - ps13
Fungi_3 - ps14
Dikarya - ps15
Ascomycota - ps16
Saccharo/Pezizomycotina - ps17
Saccharomycotina - ps18
Saccharomycotina_1 - ps19
Saccharomycotina_2 - ps20
Saccharomycotina_3 - ps21
Phaff./Saccharomycetaceae - ps22
Saccharomyco./mycetaceae - ps23
Saccharomycetaceae - ps24
Saccharomycetaceae_2 - ps25
Saccharomycetaceae_3 - ps26
Kaz./Nau./Saccharomyces - ps27
Saccharomyces - ps28
Arboricola_clade - ps29
Cerevisiae_clade - ps30
Saccharomyces_cerevisiae - ps31
Phylostratum

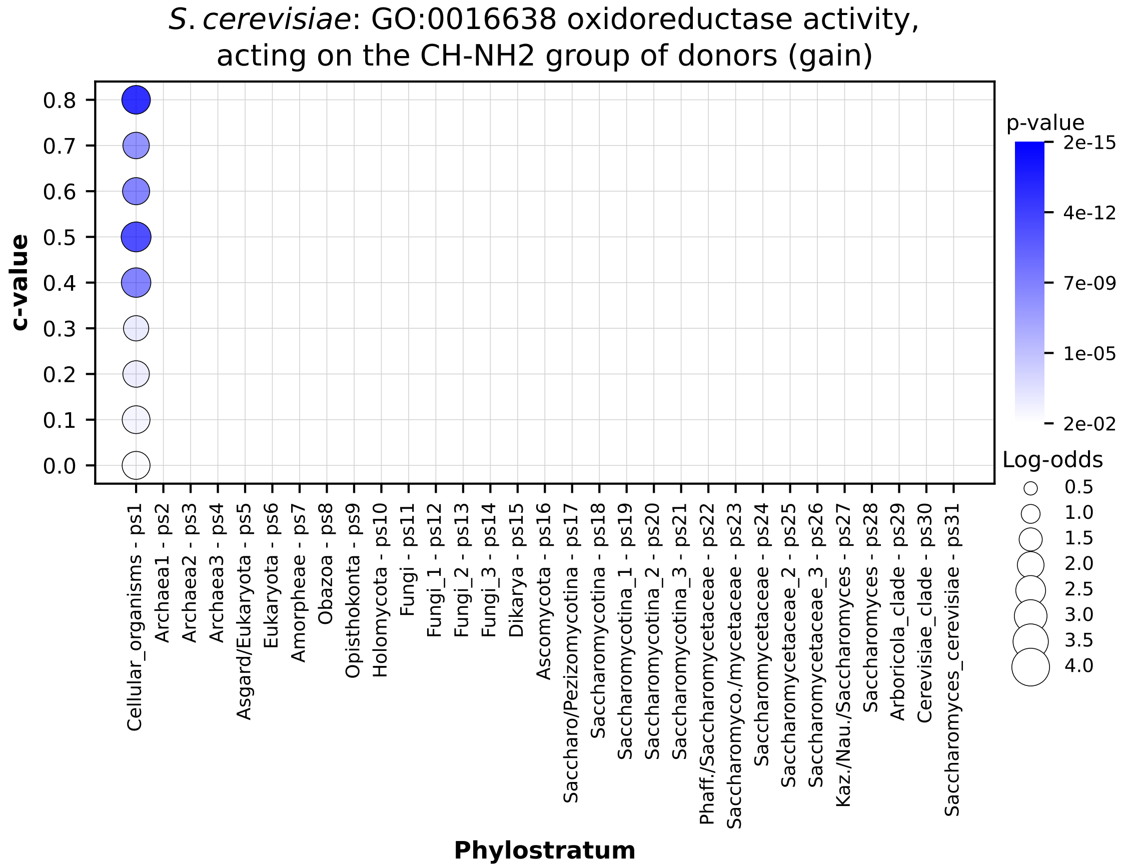

S. cerevisiae: GO:0016638 oxidoreductase activity, acting on the CH-NH2 group of donors (gain)
c-value
0.8
0.7
0.6
0.5
0.4
0.3
0.2
0.1
0.0
p-value
2e-15
4e-12
7e-09
1e-05
2e-02
Log-odds
0.5
1.0
1.5
2.0
2.5
3.0
3.5
4.0
Cellular_organisms - ps1
Archaea1 - ps2
Archaea2 - ps3
Archaea3 - ps4
Asgard/Eukaryota - ps5
Eukaryota - ps6
Amorpheae - ps7
Obazoa - ps8
Opisthokonta - ps9
Holomycota - ps10
Fungi - ps11
Fungi_1 - ps12
Fungi_2 - ps13
Fungi_3 - ps14
Dikarya - ps15
Ascomycota - ps16
Saccharo/Pezizomycotina - ps17
Saccharomycotina - ps18
Saccharomycotina_1 - ps19
Saccharomycotina_2 - ps20
Saccharomycotina_3 - ps21
Phaff./Saccharomycetaceae - ps22
Saccharomyco./mycetaceae - ps23
Saccharomycetaceae - ps24
Saccharomycetaceae_2 - ps25
Saccharomycetaceae_3 - ps26
Kaz./Nau./Saccharomyces - ps27
Saccharomyces - ps28
Arboricola_clade - ps29
Cerevisiae_clade - ps30
Saccharomyces_cerevisiae - ps31
Phylostratum

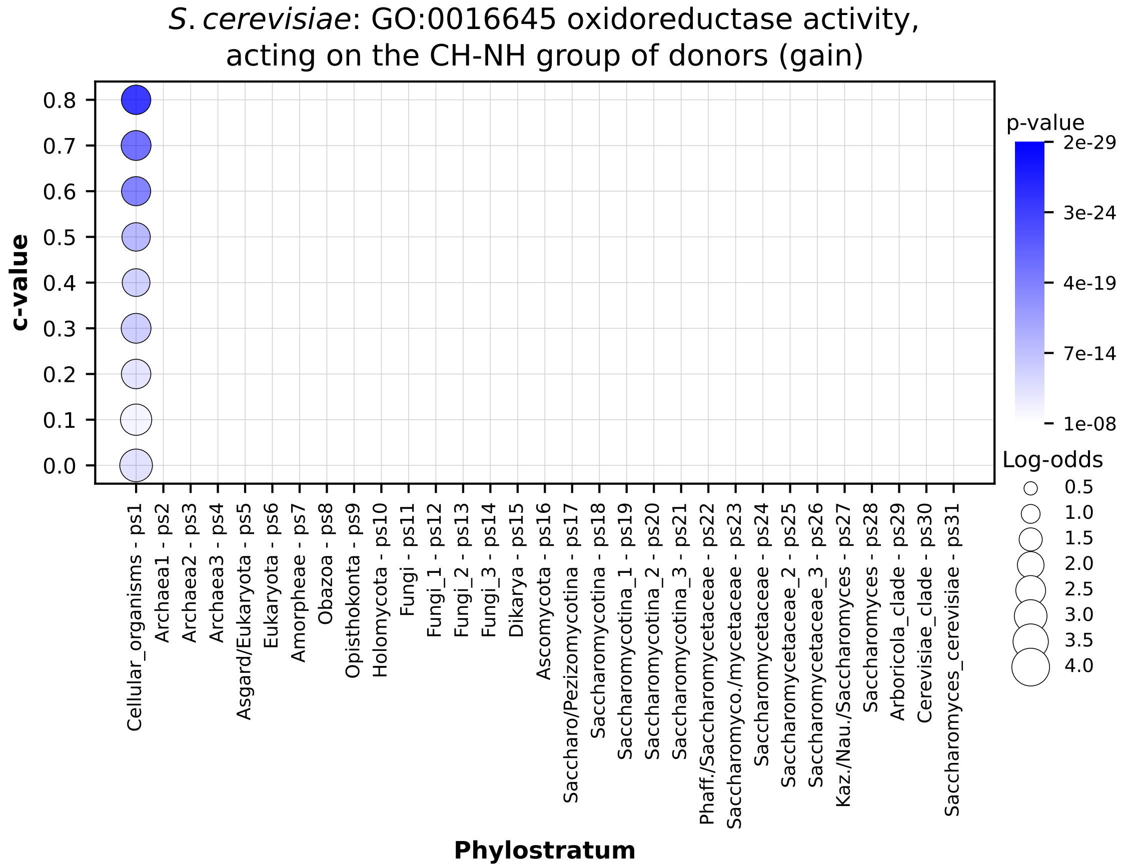
S. cerevisiae: GO:0016645 oxidoreductase activity, acting on the CH-NH group of donors (gain)
c-value
0.8
0.7
0.6
0.5
0.4
0.3
0.2
0.1
0.0
p-value
2e-29
3e-24
4e-19
7e-14
1e-08
Log-odds
0.5
1.0
1.5
2.0
2.5
3.0
3.5
4.0
Cellular_organisms - ps1
Archaea1 - ps2
Archaea2 - ps3
Archaea3 - ps4
Asgard/Eukaryota - ps5
Eukaryota - ps6
Amorpheae - ps7
Obazoa - ps8
Opisthokonta - ps9
Holomycota - ps10
Fungi - ps11
Fungi_1 - ps12
Fungi_2 - ps13
Fungi_3 - ps14
Dikarya - ps15
Ascomycota - ps16
Saccharo/Pezizomycotina - ps17
Saccharomycotina - ps18
Saccharomycotina_1 - ps19
Saccharomycotina_2 - ps20
Saccharomycotina_3 - ps21
Phaff./Saccharomycetaceae - ps22
Saccharomyco./mycetaceae - ps23
Saccharomycetaceae - ps24
Saccharomycetaceae_2 - ps25
Saccharomycetaceae_3 - ps26
Kaz./Nau./Saccharomyces - ps27
Saccharomyces - ps28
Arboricola_clade - ps29
Cerevisiae_clade - ps30
Saccharomyces_cerevisiae - ps31
Phylostratum

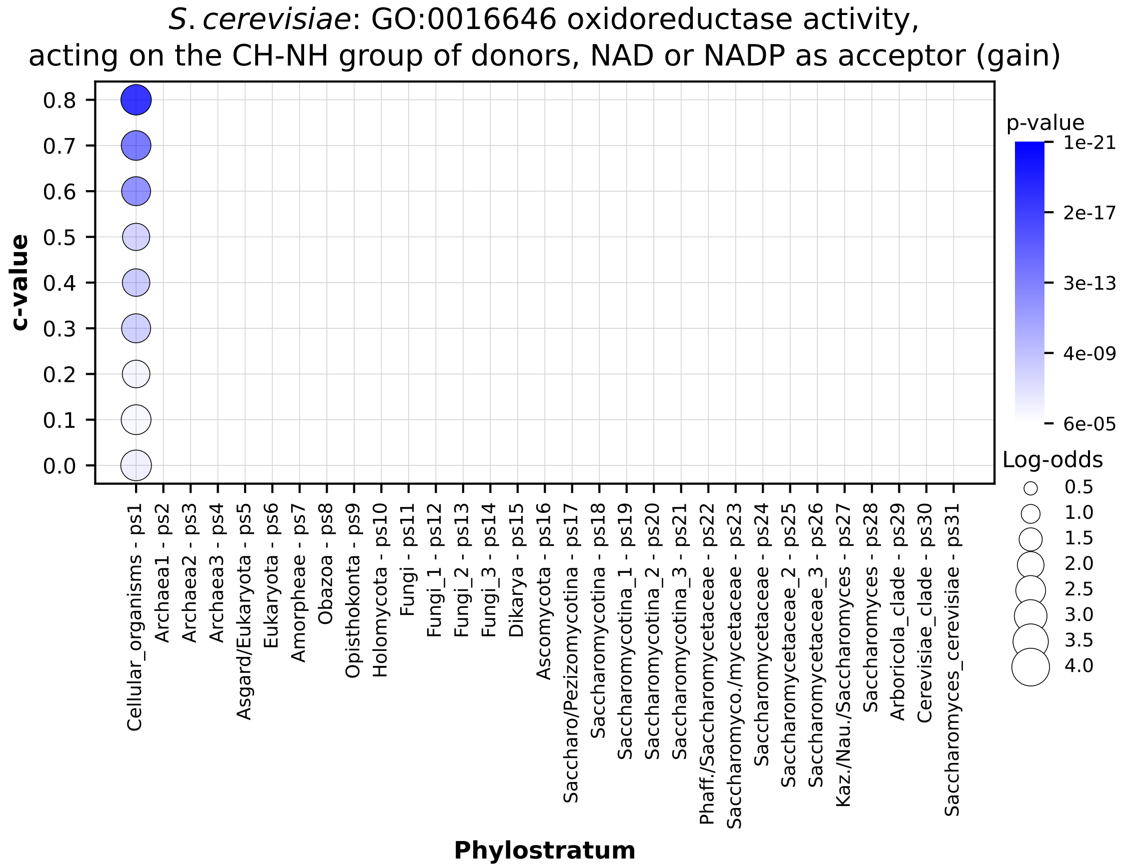

S. cerevisiae: GO:0016646 oxidoreductase activity,
acting on the CH-NH group of donors, NAD or NADP as acceptor (gain)
c-value
0.8
0.7
0.6
0.5
0.4
0.3
0.2
0.1
0.0
p-value
1e-21
2e-17
3e-13
4e-09
6e-05
Log-odds
0.5
1.0
1.5
2.0
2.5
3.0
3.5
4.0
Cellular_organisms - ps1
Archaea1 - ps2
Archaea2 - ps3
Archaea3 - ps4
Asgard/Eukaryota - ps5
Eukaryota - ps6
Amorpheae - ps7
Obazoa - ps8
Opisthokonta - ps9
Holomycota - ps10
Fungi - ps11
Fungi_1 - ps12
Fungi_2 - ps13
Fungi_3 - ps14
Dikarya - ps15
Ascomycota - ps16
Saccharo/Pezizomycotina - ps17
Saccharomycotina - ps18
Saccharomycotina_1 - ps19
Saccharomycotina_2 - ps20
Saccharomycotina_3 - ps21
Phaff./Saccharomycetaceae - ps22
Saccharomyco./mycetaceae - ps23
Saccharomycetaceae - ps24
Saccharomycetaceae_2 - ps25
Saccharomycetaceae_3 - ps26
Kaz./Nau./Saccharomyces - ps27
Saccharomyces - ps28
Arboricola_clade - ps29
Cerevisiae_clade - ps30
Saccharomyces_cerevisiae - ps31
Phylostratum

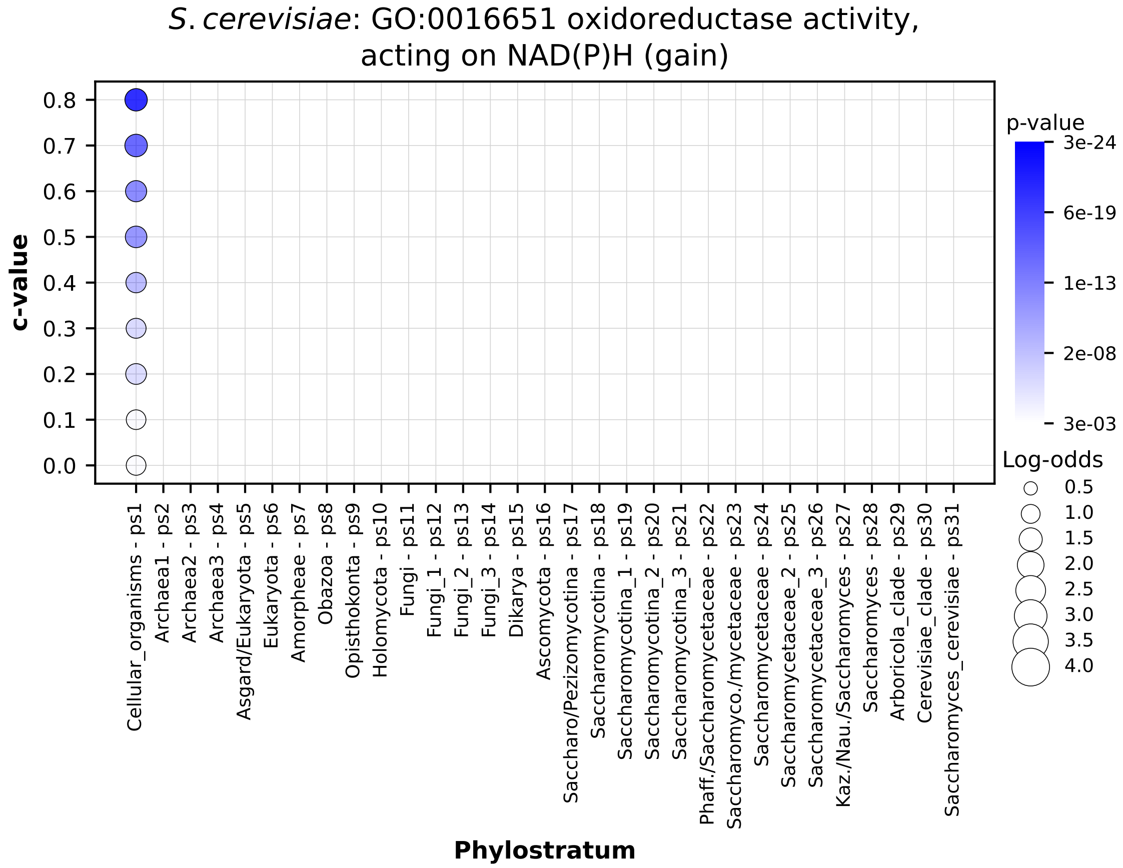
S. cerevisiae: GO:0016651 oxidoreductase activity, acting on NAD(P)H (gain)
c-value
0.8
0.7
0.6
0.5
0.4
0.3
0.2
0.1
0.0
p-value
3e-24
6e-19
1e-13
2e-08
3e-03
Log-odds
0.5
1.0
1.5
2.0
2.5
3.0
3.5
4.0
Cellular_organisms - ps1
Archaea1 - ps2
Archaea2 - ps3
Archaea3 - ps4
Asgard/Eukaryota - ps5
Eukaryota - ps6
Amorpheae - ps7
Obazoa - ps8
Opisthokonta - ps9
Holomycota - ps10
Fungi - ps11
Fungi_1 - ps12
Fungi_2 - ps13
Fungi_3 - ps14
Dikarya - ps15
Ascomycota - ps16
Saccharo/Pezizomycotina - ps17
Saccharomycotina - ps18
Saccharomycotina_1 - ps19
Saccharomycotina_2 - ps20
Saccharomycotina_3 - ps21
Phaff./Saccharomycetaceae - ps22
Saccharomyco./mycetaceae - ps23
Saccharomycetaceae - ps24
Saccharomycetaceae_2 - ps25
Saccharomycetaceae_3 - ps26
Kaz./Nau./Saccharomyces - ps27
Saccharomyces - ps28
Arboricola_clade - ps29
Cerevisiae_clade - ps30
Saccharomyces_cerevisiae - ps31
Phylostratum

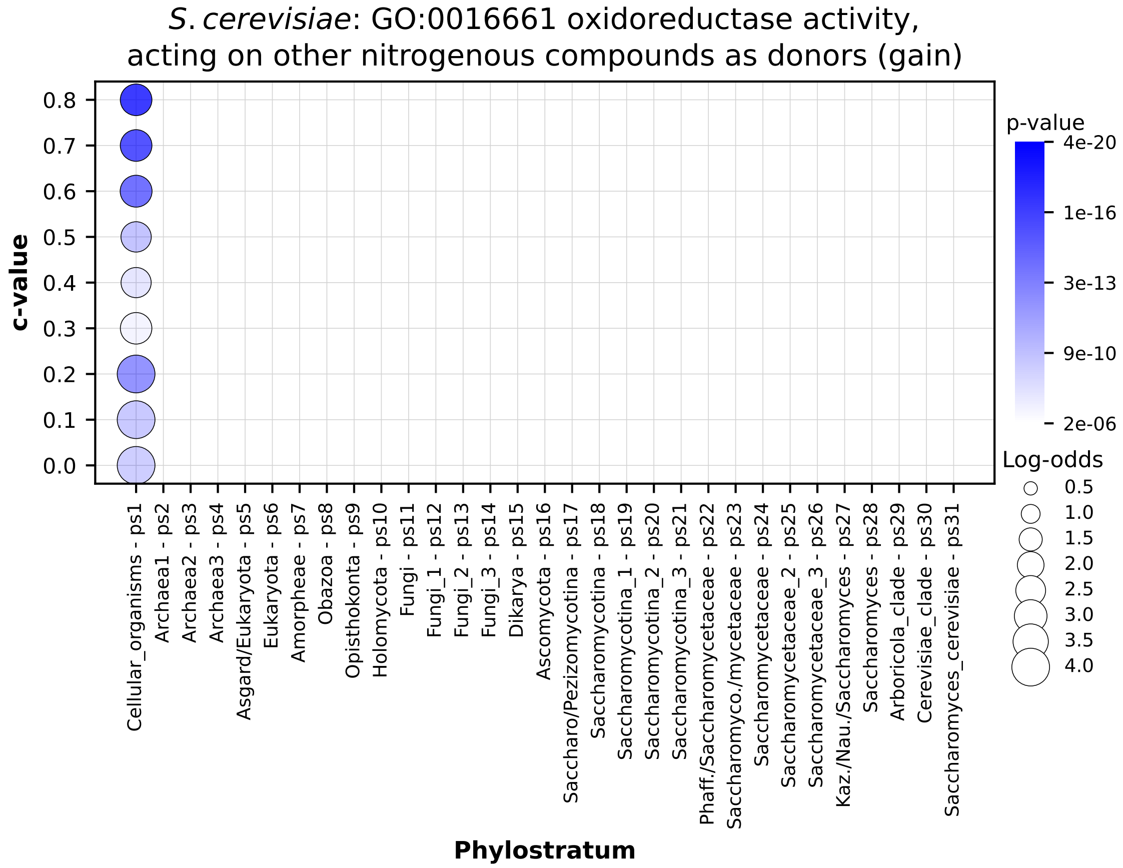
S. cerevisiae: GO:0016661 oxidoreductase activity,
acting on other nitrogenous compounds as donors (gain)
c-value
0.8
0.7
0.6
0.5
0.4
0.3
0.2
0.1
0.0
p-value
4e-20
1e-16
3e-13
9e-10
2e-06
Log-odds
0.5
1.0
1.5
2.0
2.5
3.0
3.5
4.0
Cellular_organisms - ps1
Archaea1 - ps2
Archaea2 - ps3
Archaea3 - ps4
Asgard/Eukaryota - ps5
Eukaryota - ps6
Amorpheae - ps7
Obazoa - ps8
Opisthokonta - ps9
Holomycota - ps10
Fungi - ps11
Fungi_1 - ps12
Fungi_2 - ps13
Fungi_3 - ps14
Dikarya - ps15
Ascomycota - ps16
Saccharo/Pezizomycotina - ps17
Saccharomycotina - ps18
Saccharomycotina_1 - ps19
Saccharomycotina_2 - ps20
Saccharomycotina_3 - ps21
Phaff./Saccharomycetaceae - ps22
Saccharomyco./mycetaceae - ps23
Saccharomycetaceae - ps24
Saccharomycetaceae_2 - ps25
Saccharomycetaceae_3 - ps26
Kaz./Nau./Saccharomyces - ps27
Saccharomyces - ps28
Arboricola_clade - ps29
Cerevisiae_clade - ps30
Saccharomyces_cerevisiae - ps31
Phylostratum

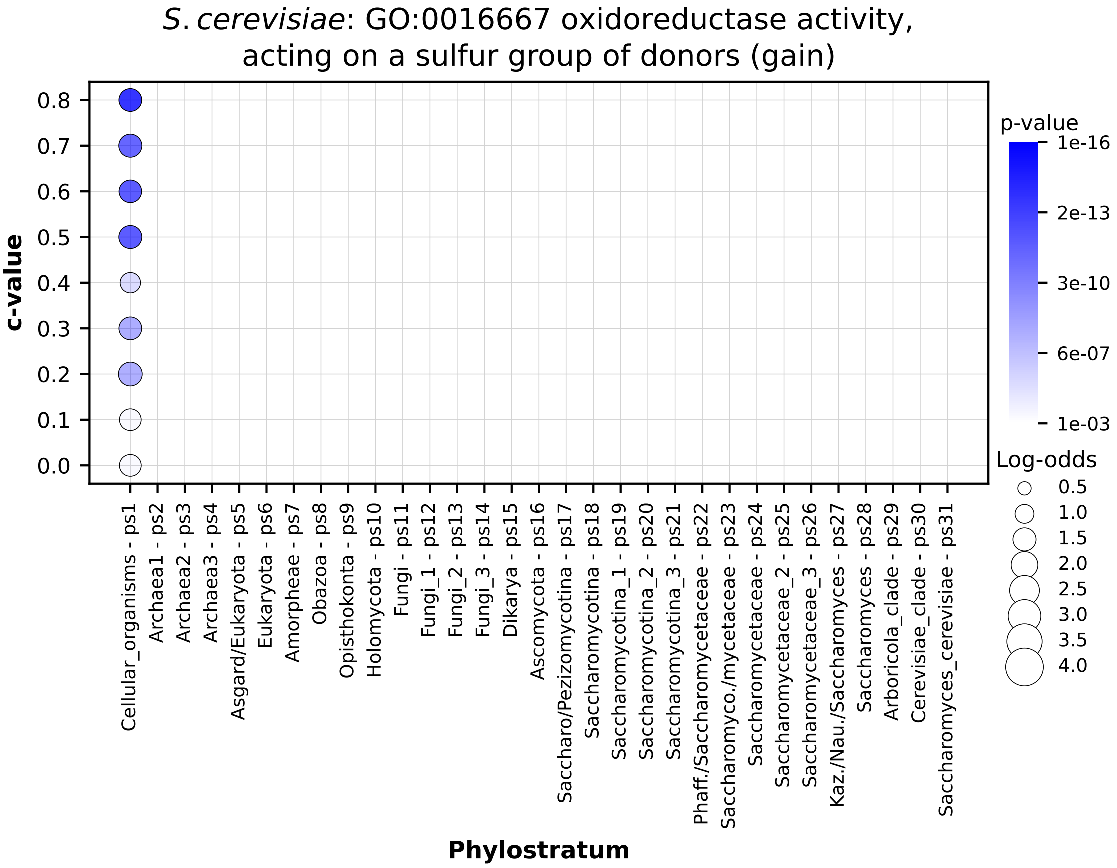

S. cerevisiae: GO:0016667 oxidoreductase activity, acting on a sulfur group of donors (gain)
c-value
0.8
0.7
0.6
0.5
0.4
0.3
0.2
0.1
0.0
p-value
1e-16
2e-13
3e-10
6e-07
1e-03
Log-odds
0.5
1.0
1.5
2.0
2.5
3.0
3.5
4.0
Cellular_organisms - ps1
Archaea1 - ps2
Archaea2 - ps3
Archaea3 - ps4
Asgard/Eukaryota - ps5
Eukaryota - ps6
Amorpheae - ps7
Obazoa - ps8
Opisthokonta - ps9
Holomycota - ps10
Fungi - ps11
Fungi_1 - ps12
Fungi_2 - ps13
Fungi_3 - ps14
Dikarya - ps15
Ascomycota - ps16
Saccharo/Pezizomycotina - ps17
Saccharomycotina - ps18
Saccharomycotina_1 - ps19
Saccharomycotina_2 - ps20
Saccharomycotina_3 - ps21
Phaff./Saccharomycetaceae - ps22
Saccharomyco./mycetaceae - ps23
Saccharomycetaceae - ps24
Saccharomycetaceae_2 - ps25
Saccharomycetaceae_3 - ps26
Kaz./Nau./Saccharomyces - ps27
Saccharomyces - ps28
Arboricola_clade - ps29
Cerevisiae_clade - ps30
Saccharomyces_cerevisiae - ps31
Phylostratum

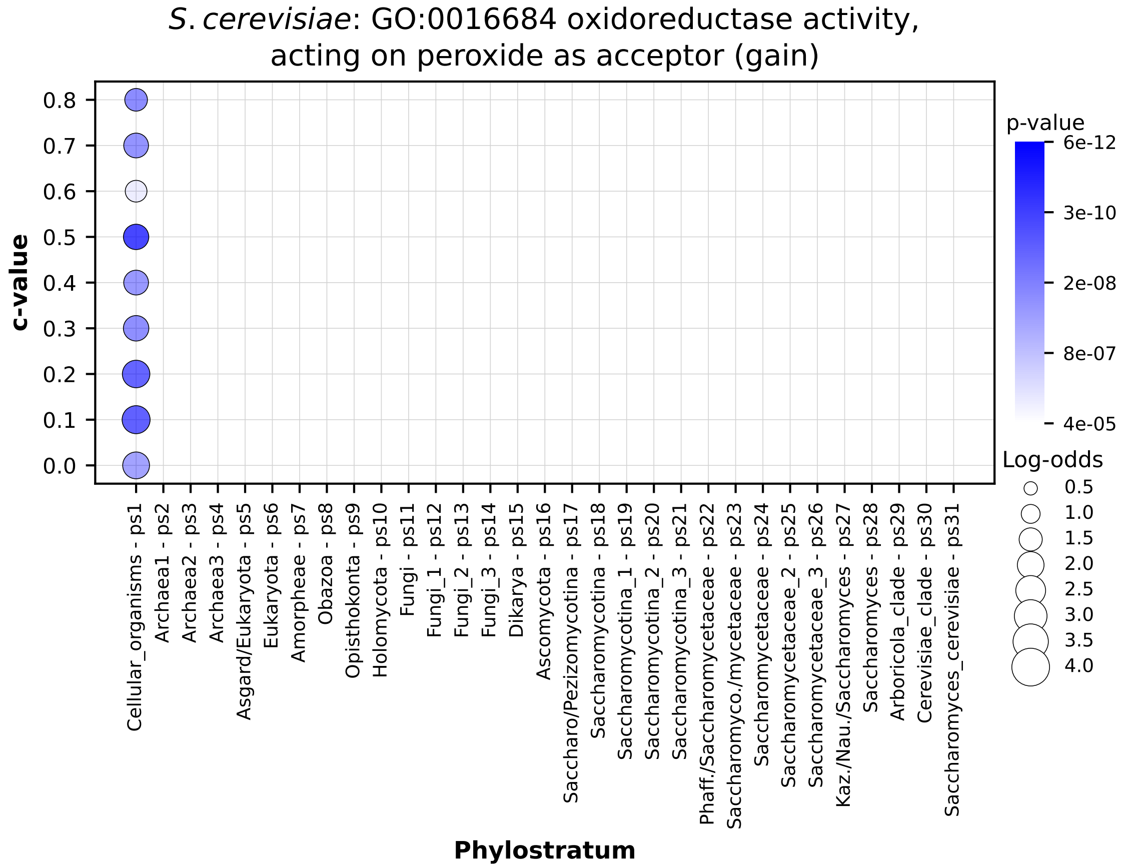
S. cerevisiae: GO:0016684 oxidoreductase activity, acting on peroxide as acceptor (gain)
c-value
0.8
0.7
0.6
0.5
0.4
0.3
0.2
0.1
0.0
Phylostratum
Cellular_organisms - ps1
Archaea1 - ps2
Archaea2 - ps3
Archaea3 - ps4
Asgard/Eukaryota - ps5
Eukaryota - ps6
Amorpheae - ps7
Obazoa - ps8
Opisthokonta - ps9
Holomycota - ps10
Fungi - ps11
Fungi_1 - ps12
Fungi_2 - ps13
Fungi_3 - ps14
Dikarya - ps15
Ascomycota - ps16
Saccharo/Pezizomycotina - ps17
Saccharomycotina - ps18
Saccharomycotina_1 - ps19
Saccharomycotina_2 - ps20
Saccharomycotina_3 - ps21
Phaff./Saccharomycetaceae - ps22
Saccharomyco./mycetaceae - ps23
Saccharomycetaceae - ps24
Saccharomycetaceae_2 - ps25
Saccharomycetaceae_3 - ps26
Kaz./Nau./Saccharomyces - ps27
Saccharomyces - ps28
Arboricola_clade - ps29
Cerevisiae_clade - ps30
Saccharomyces_cerevisiae - ps31
p-value
6e-12
3e-10
2e-08
8e-07
4e-05
Log-odds
0.5
1.0
1.5
2.0
2.5
3.0
3.5
4.0

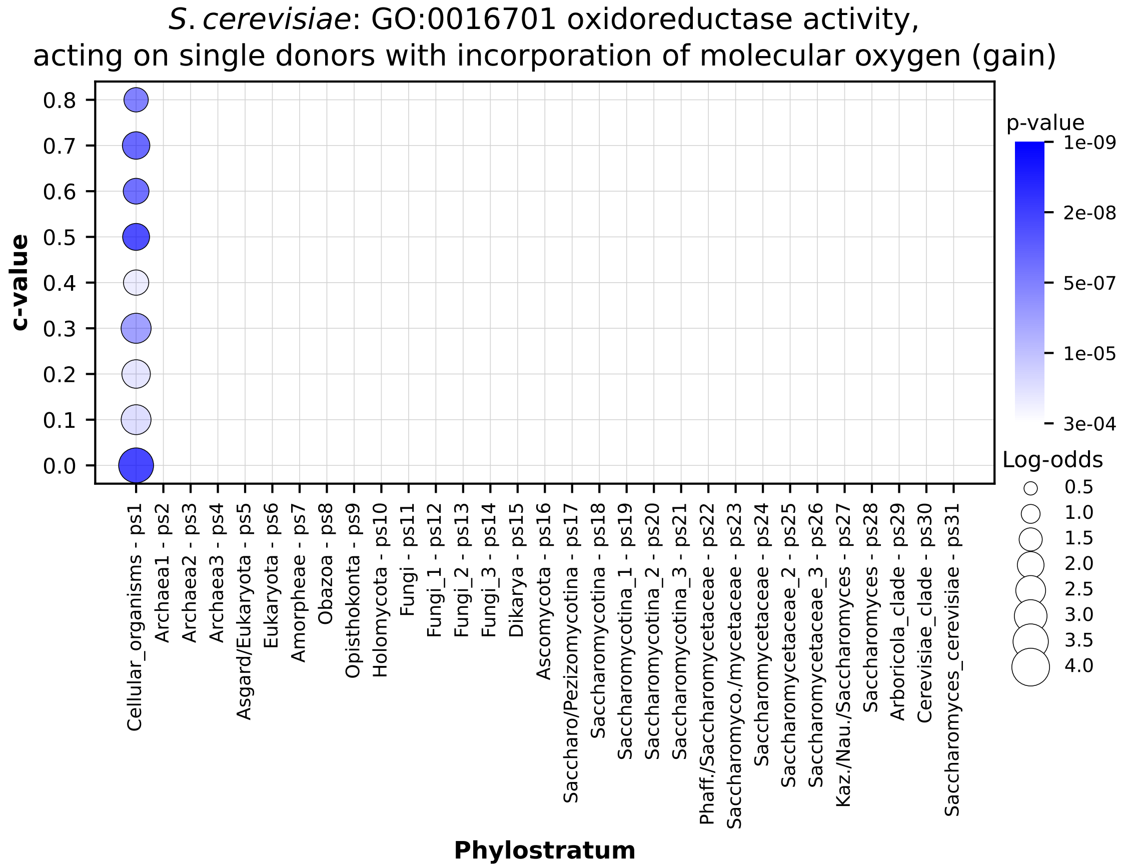
S. cerevisiae: GO:0016701 oxidoreductase activity,
acting on single donors with incorporation of molecular oxygen (gain)
c-value
0.0
0.1
0.2
0.3
0.4
0.5
0.6
0.7
0.8
Phylostratum
Cellular_organisms - ps1
Archaea1 - ps2
Archaea2 - ps3
Archaea3 - ps4
Asgard/Eukaryota - ps5
Eukaryota - ps6
Amorpheae - ps7
Obazoa - ps8
Opisthokonta - ps9
Holomycota - ps10
Fungi - ps11
Fungi_1 - ps12
Fungi_2 - ps13
Fungi_3 - ps14
Dikarya - ps15
Ascomycota - ps16
Saccharo/Pezizomycotina - ps17
Saccharomycotina - ps18
Saccharomycotina_1 - ps19
Saccharomycotina_2 - ps20
Saccharomycotina_3 - ps21
Phaff./Saccharomycetaceae - ps22
Saccharomyco./mycetaceae - ps23
Saccharomycetaceae - ps24
Saccharomycetaceae_2 - ps25
Saccharomycetaceae_3 - ps26
Kaz./Nau./Saccharomyces - ps27
Saccharomyces - ps28
Arboricola_clade - ps29
Cerevisiae_clade - ps30
Saccharomyces_cerevisiae - ps31
p-value
1e-09
2e-08
5e-07
1e-05
3e-04
Log-odds
0.5
1.0
1.5
2.0
2.5
3.0
3.5
4.0

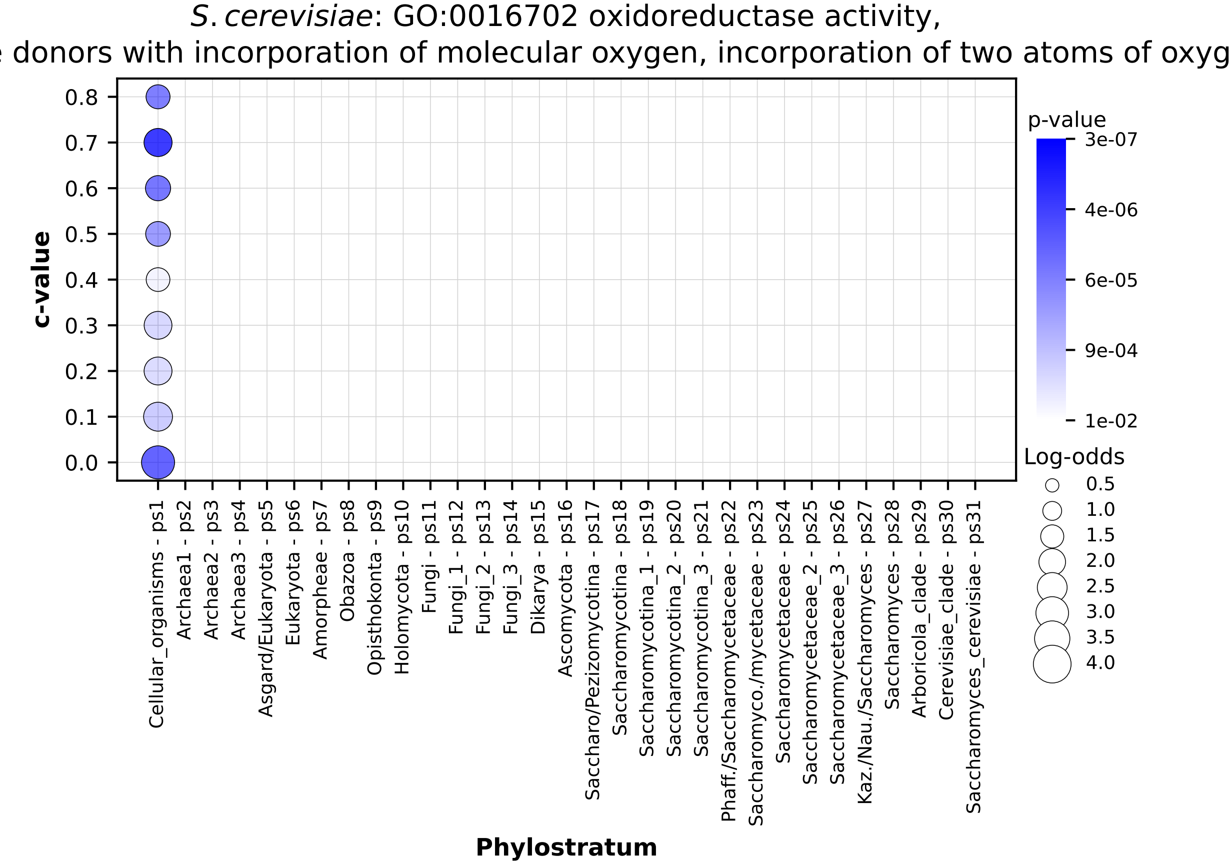

S. cerevisiae: GO:0016702 oxidoreductase activity,
e donors with incorporation of molecular oxygen, incorporation of two atoms of oxyg
c-value
0.8
0.7
0.6
0.5
0.4
0.3
0.2
0.1
0.0
p-value
3e-07
4e-06
6e-05
9e-04
1e-02
Log-odds
0.5
1.0
1.5
2.0
2.5
3.0
3.5
4.0
Cellular_organisms - ps1
Archaea1 - ps2
Archaea2 - ps3
Archaea3 - ps4
Asgard/Eukaryota - ps5
Eukaryota - ps6
Amorpheae - ps7
Obazoa - ps8
Opisthokonta - ps9
Holomycota - ps10
Fungi - ps11
Fungi_1 - ps12
Fungi_2 - ps13
Fungi_3 - ps14
Dikarya - ps15
Ascomycota - ps16
Saccharo/Pezizomycotina - ps17
Saccharomycotina - ps18
Saccharomycotina_1 - ps19
Saccharomycotina_2 - ps20
Saccharomycotina_3 - ps21
Phaff./Saccharomycetaceae - ps22
Saccharomyco./mycetaceae - ps23
Saccharomycetaceae - ps24
Saccharomycetaceae_2 - ps25
Saccharomycetaceae_3 - ps26
Kaz./Nau./Saccharomyces - ps27
Saccharomyces - ps28
Arboricola_clade - ps29
Cerevisiae_clade - ps30
Saccharomyces_cerevisiae - ps31
Phylostratum

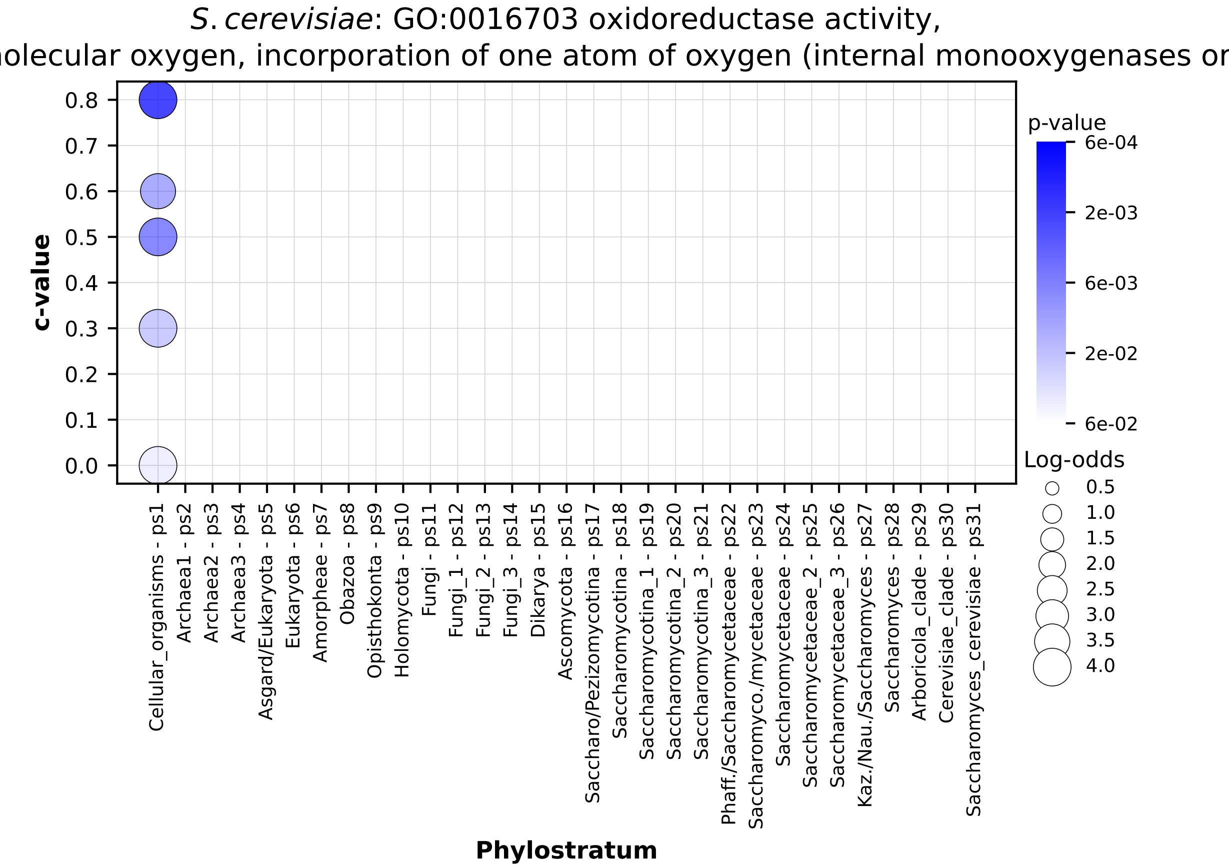
S. cerevisiae: GO:0016703 oxidoreductase activity,
olecular oxygen, incorporation of one atom of oxygen (internal monooxygenases or
c-value
0.8
0.7
0.6
0.5
0.4
0.3
0.2
0.1
0.0
p-value
6e-04
2e-03
6e-03
2e-02
6e-02
Log-odds
0.5
1.0
1.5
2.0
2.5
3.0
3.5
4.0
Cellular_organisms - ps1
Archaea1 - ps2
Archaea2 - ps3
Archaea3 - ps4
Asgard/Eukaryota - ps5
Eukaryota - ps6
Amorpheae - ps7
Obazoa - ps8
Opisthokonta - ps9
Holomycota - ps10
Fungi - ps11
Fungi_1 - ps12
Fungi_2 - ps13
Fungi_3 - ps14
Dikarya - ps15
Ascomycota - ps16
Saccharo/Pezizomycotina - ps17
Saccharomycotina - ps18
Saccharomycotina_1 - ps19
Saccharomycotina_2 - ps20
Saccharomycotina_3 - ps21
Phaff./Saccharomycetaceae - ps22
Saccharomyco./mycetaceae - ps23
Saccharomycetaceae - ps24
Saccharomycetaceae_2 - ps25
Saccharomycetaceae_3 - ps26
Kaz./Nau./Saccharomyces - ps27
Saccharomyces - ps28
Arboricola_clade - ps29
Cerevisiae_clade - ps30
Saccharomyces_cerevisiae - ps31
Phylostratum

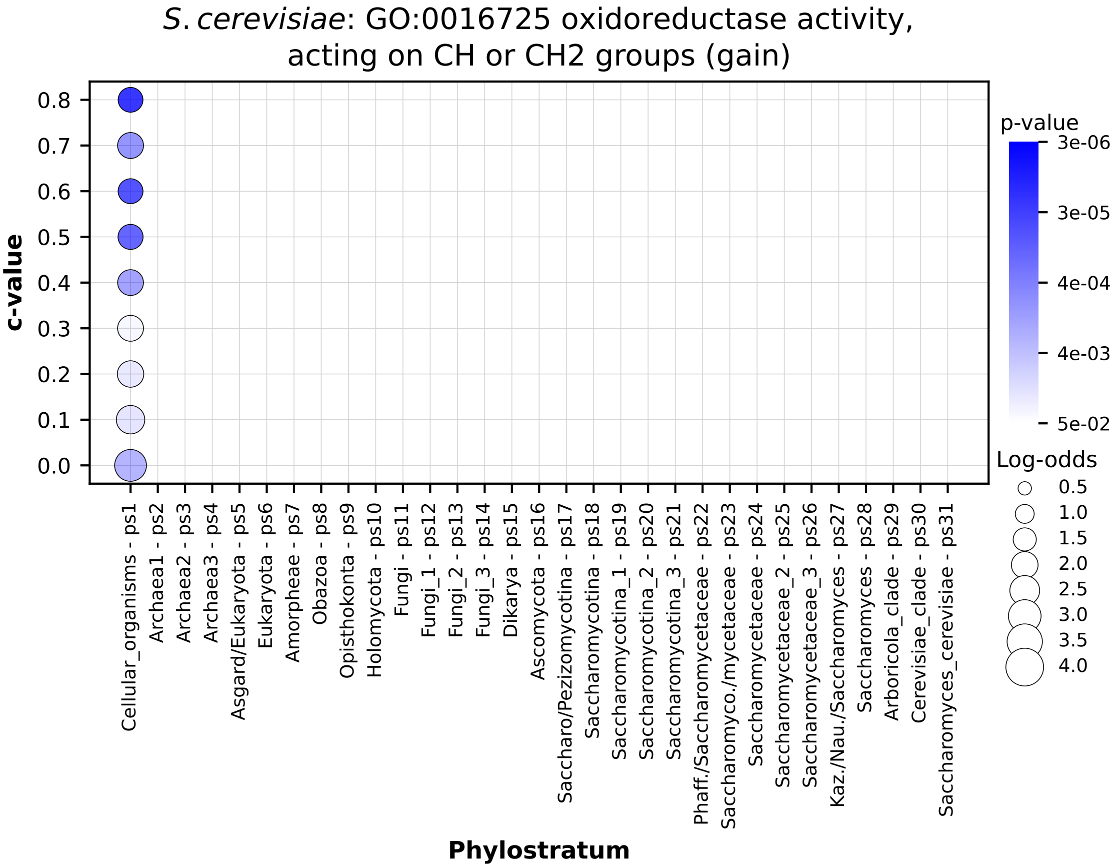

S. cerevisiae: GO:0016725 oxidoreductase activity, acting on CH or CH2 groups (gain)
c-value
0.8
0.7
0.6
0.5
0.4
0.3
0.2
0.1
0.0
p-value
3e-06
3e-05
4e-04
4e-03
5e-02
Log-odds
0.5
1.0
1.5
2.0
2.5
3.0
3.5
4.0
Cellular_organisms - ps1
Archaea1 - ps2
Archaea2 - ps3
Archaea3 - ps4
Asgard/Eukaryota - ps5
Eukaryota - ps6
Amorpheae - ps7
Obazoa - ps8
Opisthokonta - ps9
Holomycota - ps10
Fungi - ps11
Fungi_1 - ps12
Fungi_2 - ps13
Fungi_3 - ps14
Dikarya - ps15
Ascomycota - ps16
Saccharo/Pezizomycotina - ps17
Saccharomycotina - ps18
Saccharomycotina_1 - ps19
Saccharomycotina_2 - ps20
Saccharomycotina_3 - ps21
Phaff./Saccharomycetaceae - ps22
Saccharomyco./mycetaceae - ps23
Saccharomycetaceae - ps24
Saccharomycetaceae_2 - ps25
Saccharomycetaceae_3 - ps26
Kaz./Nau./Saccharomyces - ps27
Saccharomyces - ps28
Arboricola_clade - ps29
Cerevisiae_clade - ps30
Saccharomyces_cerevisiae - ps31
Phylostratum

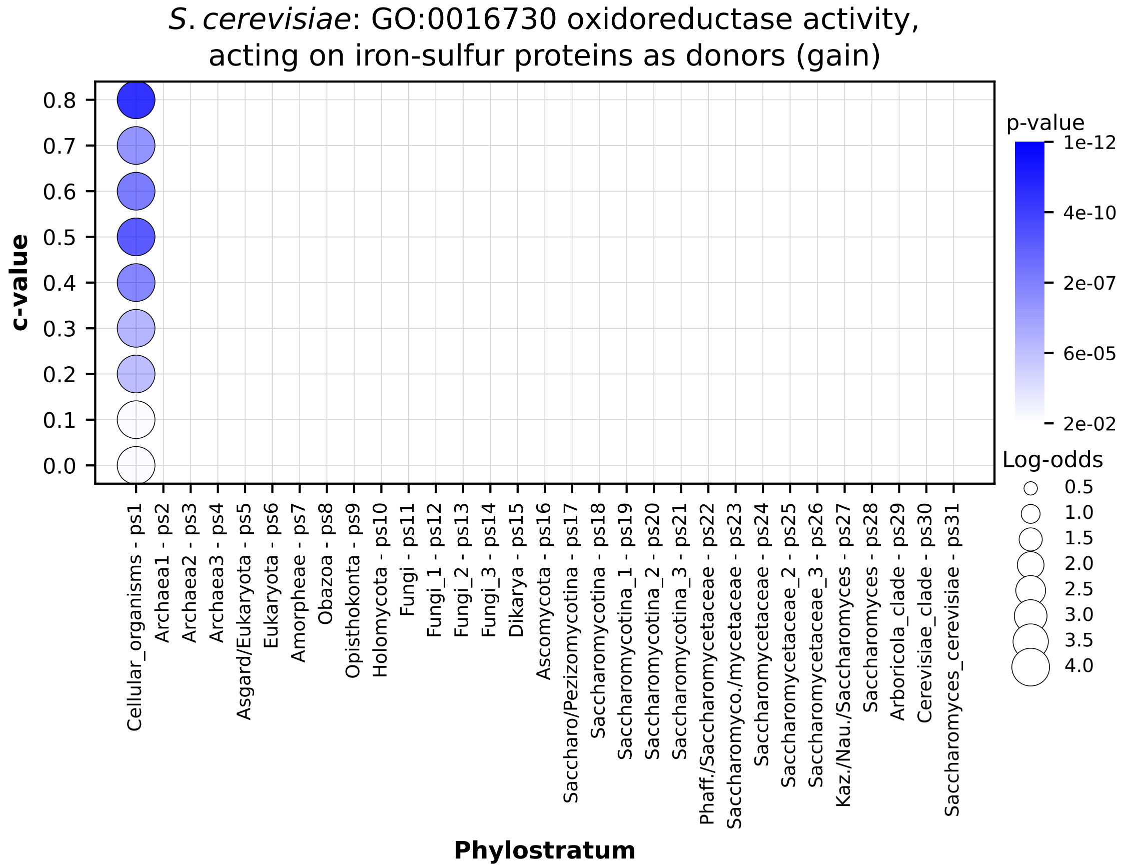

S. cerevisiae: GO:0016730 oxidoreductase activity, acting on iron-sulfur proteins as donors (gain)
c-value
0.8
0.7
0.6
0.5
0.4
0.3
0.2
0.1
0.0
p-value
1e-12
4e-10
2e-07
6e-05
2e-02
Log-odds
0.5
1.0
1.5
2.0
2.5
3.0
3.5
4.0
Cellular_organisms - ps1
Archaea1 - ps2
Archaea2 - ps3
Archaea3 - ps4
Asgard/Eukaryota - ps5
Eukaryota - ps6
Amorpheae - ps7
Obazoa - ps8
Opisthokonta - ps9
Holomycota - ps10
Fungi - ps11
Fungi_1 - ps12
Fungi_2 - ps13
Fungi_3 - ps14
Dikarya - ps15
Ascomycota - ps16
Saccharo/Pezizomycotina - ps17
Saccharomycotina - ps18
Saccharomycotina_1 - ps19
Saccharomycotina_2 - ps20
Saccharomycotina_3 - ps21
Phaff./Saccharomycetaceae - ps22
Saccharomyco./mycetaceae - ps23
Saccharomycetaceae - ps24
Saccharomycetaceae_2 - ps25
Saccharomycetaceae_3 - ps26
Kaz./Nau./Saccharomyces - ps27
Saccharomyces - ps28
Arboricola_clade - ps29
Cerevisiae_clade - ps30
Saccharomyces_cerevisiae - ps31
Phylostratum

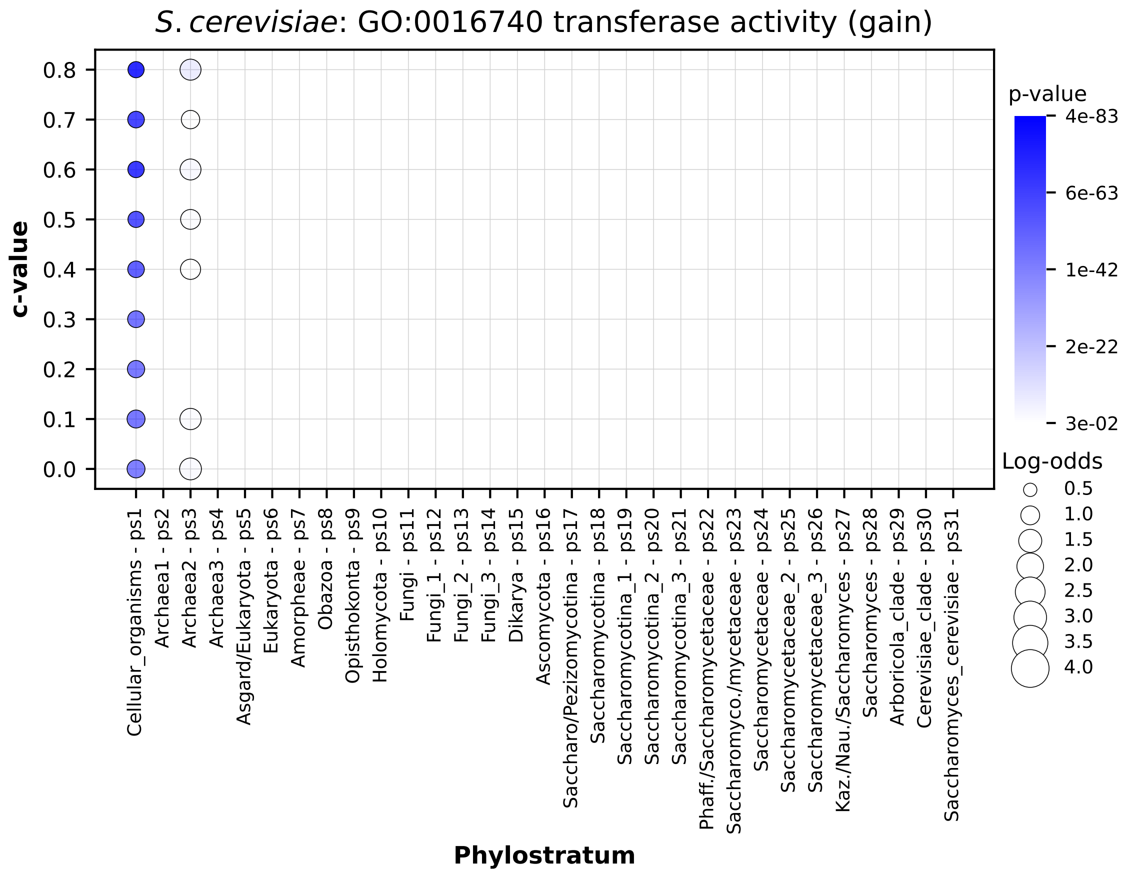
S. cerevisiae: GO:0016740 transferase activity (gain)
c-value
0.8
0.7
0.6
0.5
0.4
0.3
0.2
0.1
0.0
p-value
4e-83
6e-63
1e-42
2e-22
3e-02
Log-odds
0.5
1.0
1.5
2.0
2.5
3.0
3.5
4.0
Cellular_organisms - ps1
Archaea1 - ps2
Archaea2 - ps3
Archaea3 - ps4
Asgard/Eukaryota - ps5
Eukaryota - ps6
Amorpheae - ps7
Obazoa - ps8
Opisthokonta - ps9
Holomycota - ps10
Fungi - ps11
Fungi_1 - ps12
Fungi_2 - ps13
Fungi_3 - ps14
Dikarya - ps15
Ascomycota - ps16
Saccharo/Pezizomycotina - ps17
Saccharomycotina - ps18
Saccharomycotina_1 - ps19
Saccharomycotina_2 - ps20
Saccharomycotina_3 - ps21
Phaff./Saccharomycetaceae - ps22
Saccharomyco./mycetaceae - ps23
Saccharomycetaceae - ps24
Saccharomycetaceae_2 - ps25
Saccharomycetaceae_3 - ps26
Kaz./Nau./Saccharomyces - ps27
Saccharomyces - ps28
Arboricola_clade - ps29
Cerevisiae_clade - ps30
Saccharomyces_cerevisiae - ps31
Phylostratum

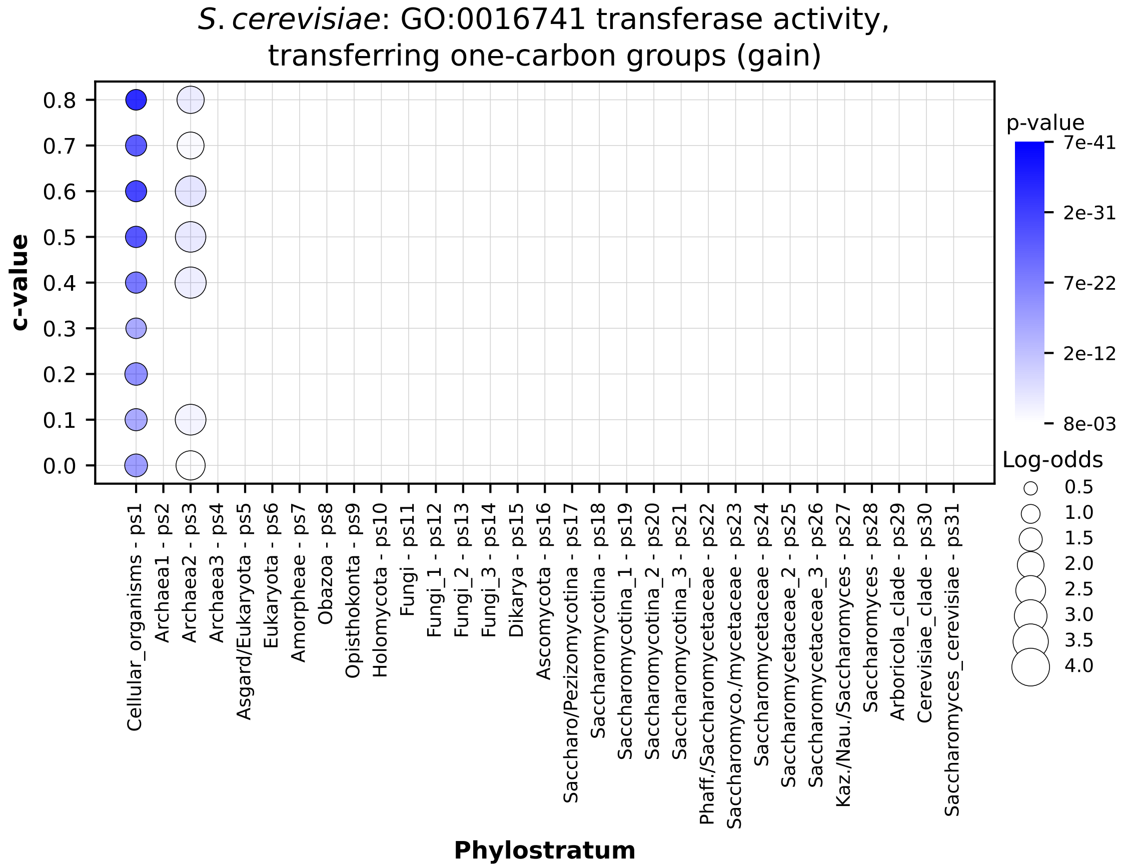
S. cerevisiae: GO:0016741 transferase activity, transferring one-carbon groups (gain)
c-value
0.8
0.7
0.6
0.5
0.4
0.3
0.2
0.1
0.0
p-value
7e-41
2e-31
7e-22
2e-12
8e-03
Log-odds
0.5
1.0
1.5
2.0
2.5
3.0
3.5
4.0
Cellular_organisms - ps1
Archaea1 - ps2
Archaea2 - ps3
Archaea3 - ps4
Asgard/Eukaryota - ps5
Eukaryota - ps6
Amorpheae - ps7
Obazoa - ps8
Opisthokonta - ps9
Holomycota - ps10
Fungi - ps11
Fungi_1 - ps12
Fungi_2 - ps13
Fungi_3 - ps14
Dikarya - ps15
Ascomycota - ps16
Saccharo/Pezizomycotina - ps17
Saccharomycotina - ps18
Saccharomycotina_1 - ps19
Saccharomycotina_2 - ps20
Saccharomycotina_3 - ps21
Phaff./Saccharomycetaceae - ps22
Saccharomyco./mycetaceae - ps23
Saccharomycetaceae - ps24
Saccharomycetaceae_2 - ps25
Saccharomycetaceae_3 - ps26
Kaz./Nau./Saccharomyces - ps27
Saccharomyces - ps28
Arboricola_clade - ps29
Cerevisiae_clade - ps30
Saccharomyces_cerevisiae - ps31
Phylostratum

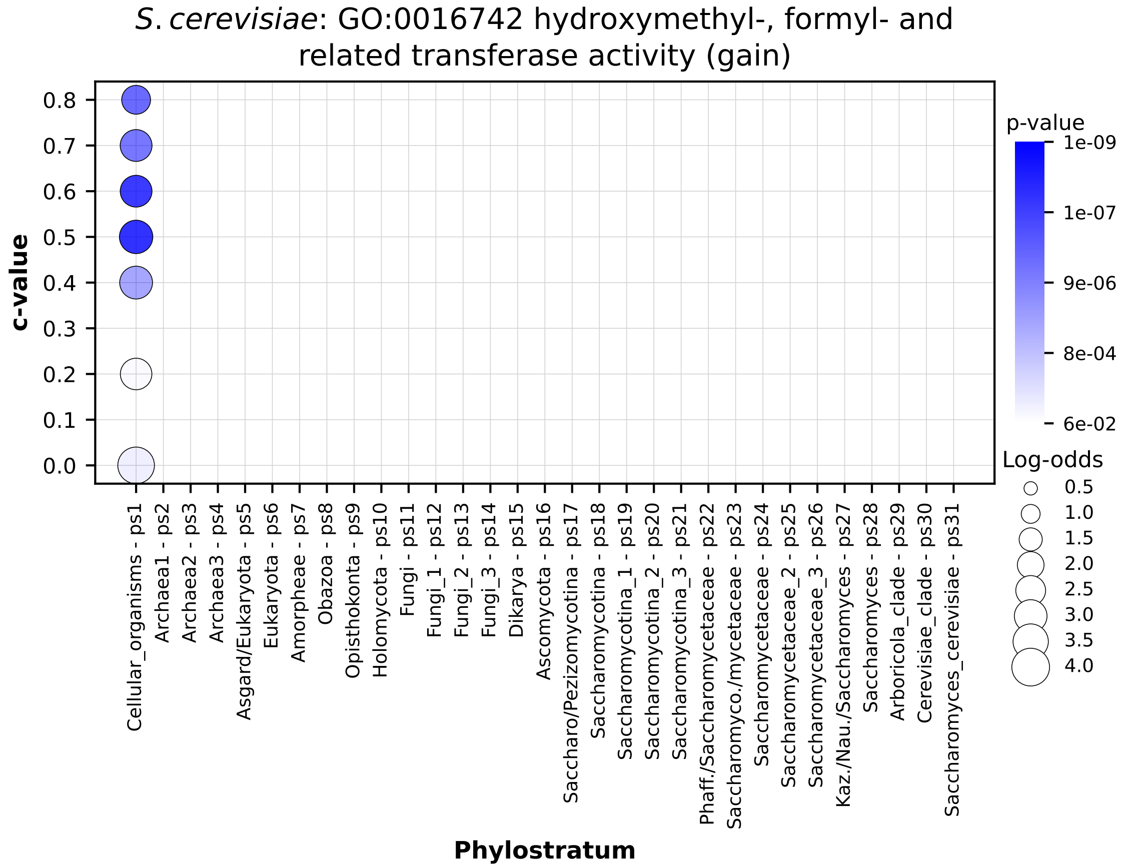

S. cerevisiae: GO:0016742 hydroxymethyl-, formyl- and related transferase activity (gain)
c-value
0.8
0.7
0.6
0.5
0.4
0.3
0.2
0.1
0.0
p-value
1e-09
1e-07
9e-06
8e-04
6e-02
Log-odds
0.5
1.0
1.5
2.0
2.5
3.0
3.5
4.0
Cellular_organisms - ps1
Archaea1 - ps2
Archaea2 - ps3
Archaea3 - ps4
Asgard/Eukaryota - ps5
Eukaryota - ps6
Amorpheae - ps7
Obazoa - ps8
Opisthokonta - ps9
Holomycota - ps10
Fungi - ps11
Fungi_1 - ps12
Fungi_2 - ps13
Fungi_3 - ps14
Dikarya - ps15
Ascomycota - ps16
Saccharo/Pezizomycotina - ps17
Saccharomycotina - ps18
Saccharomycotina_1 - ps19
Saccharomycotina_2 - ps20
Saccharomycotina_3 - ps21
Phaff./Saccharomycetaceae - ps22
Saccharomyco./mycetaceae - ps23
Saccharomycetaceae - ps24
Saccharomycetaceae_2 - ps25
Saccharomycetaceae_3 - ps26
Kaz./Nau./Saccharomyces - ps27
Saccharomyces - ps28
Arboricola_clade - ps29
Cerevisiae_clade - ps30
Saccharomyces_cerevisiae - ps31
Phylostratum

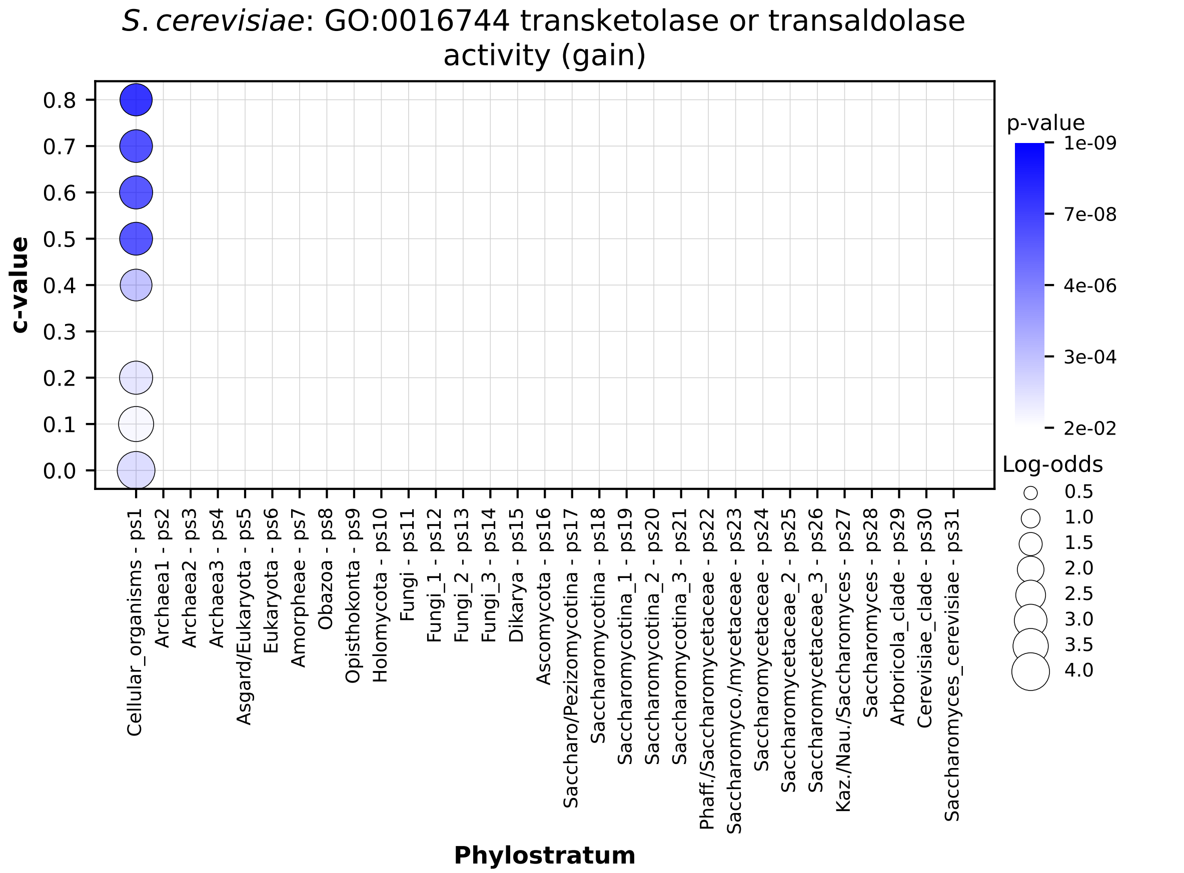
S. cerevisiae: GO:0016744 transketolase or transaldolase activity (gain)
c-value
0.8
0.7
0.6
0.5
0.4
0.3
0.2
0.1
0.0
p-value
1e-09
7e-08
4e-06
3e-04
2e-02
Log-odds
0.5
1.0
1.5
2.0
2.5
3.0
3.5
4.0
Cellular_organisms - ps1
Archaea1 - ps2
Archaea2 - ps3
Archaea3 - ps4
Asgard/Eukaryota - ps5
Eukaryota - ps6
Amorpheae - ps7
Obazoa - ps8
Opisthokonta - ps9
Holomycota - ps10
Fungi - ps11
Fungi_1 - ps12
Fungi_2 - ps13
Fungi_3 - ps14
Dikarya - ps15
Ascomycota - ps16
Saccharo/Pezizomycotina - ps17
Saccharomycotina - ps18
Saccharomycotina_1 - ps19
Saccharomycotina_2 - ps20
Saccharomycotina_3 - ps21
Phaff./Saccharomycetaceae - ps22
Saccharomyco./mycetaceae - ps23
Saccharomycetaceae - ps24
Saccharomycetaceae_2 - ps25
Saccharomycetaceae_3 - ps26
Kaz./Nau./Saccharomyces - ps27
Saccharomyces - ps28
Arboricola_clade - ps29
Cerevisiae_clade - ps30
Saccharomyces_cerevisiae - ps31
Phylostratum

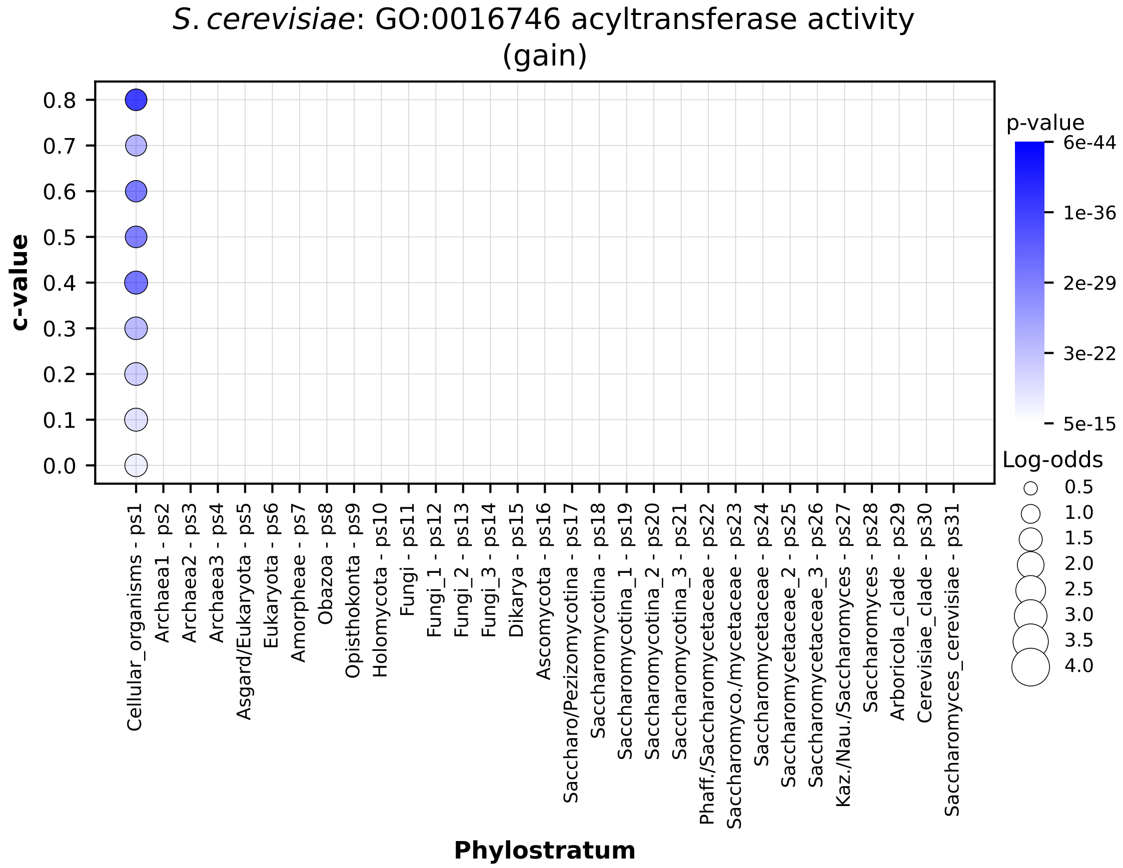
S. cerevisiae: GO:0016746 acyltransferase activity
(gain)
c-value
0.8
0.7
0.6
0.5
0.4
0.3
0.2
0.1
0.0
p-value
6e-44
1e-36
2e-29
3e-22
5e-15
Log-odds
0.5
1.0
1.5
2.0
2.5
3.0
3.5
4.0
Cellular_organisms - ps1
Archaea1 - ps2
Archaea2 - ps3
Archaea3 - ps4
Asgard/Eukaryota - ps5
Eukaryota - ps6
Amorpheae - ps7
Obazoa - ps8
Opisthokonta - ps9
Holomycota - ps10
Fungi - ps11
Fungi_1 - ps12
Fungi_2 - ps13
Fungi_3 - ps14
Dikarya - ps15
Ascomycota - ps16
Saccharo/Pezizomycotina - ps17
Saccharomycotina - ps18
Saccharomycotina_1 - ps19
Saccharomycotina_2 - ps20
Saccharomycotina_3 - ps21
Phaff./Saccharomycetaceae - ps22
Saccharomyco./mycetaceae - ps23
Saccharomycetaceae - ps24
Saccharomycetaceae_2 - ps25
Saccharomycetaceae_3 - ps26
Kaz./Nau./Saccharomyces - ps27
Saccharomyces - ps28
Arboricola_clade - ps29
Cerevisiae_clade - ps30
Saccharomyces_cerevisiae - ps31
Phylostratum

S. cerevisiae: GO:0016747 acyltransferase activity, transferring groups other than amino-acyl groups (gain)

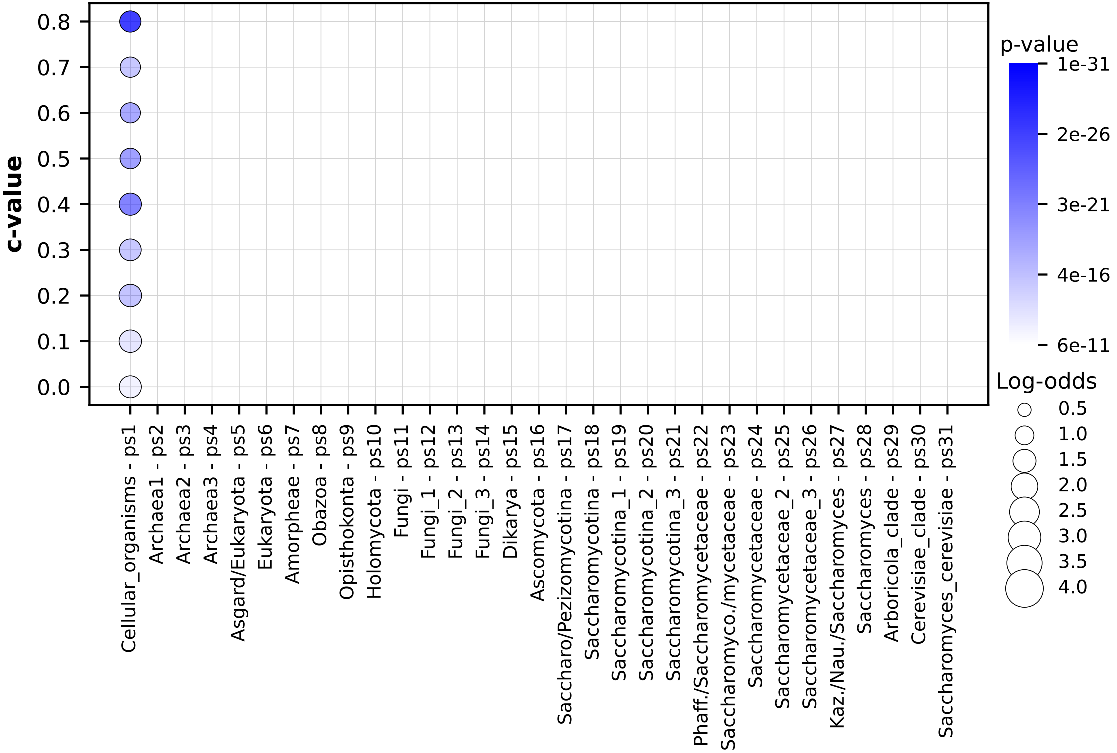

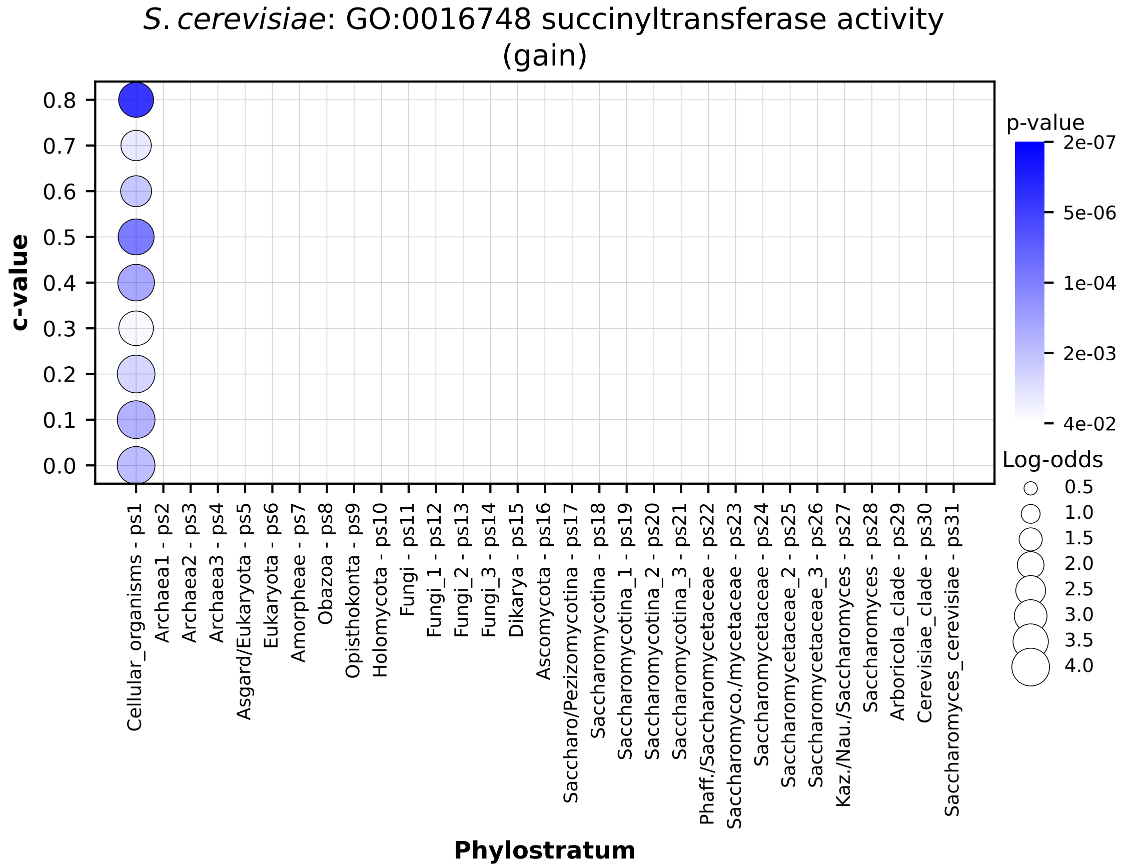

S. cerevisiae: GO:0016748 succinyltransferase activity (gain)
c-value
0.8
0.7
0.6
0.5
0.4
0.3
0.2
0.1
0.0
p-value
2e-07
5e-06
1e-04
2e-03
4e-02
Log-odds
0.5
1.0
1.5
2.0
2.5
3.0
3.5
4.0
Cellular_organisms - ps1
Archaea1 - ps2
Archaea2 - ps3
Archaea3 - ps4
Asgard/Eukaryota - ps5
Eukaryota - ps6
Amorpheae - ps7
Obazoa - ps8
Opisthokonta - ps9
Holomycota - ps10
Fungi - ps11
Fungi_1 - ps12
Fungi_2 - ps13
Fungi_3 - ps14
Dikarya - ps15
Ascomycota - ps16
Saccharo/Pezizomycotina - ps17
Saccharomycotina - ps18
Saccharomycotina_1 - ps19
Saccharomycotina_2 - ps20
Saccharomycotina_3 - ps21
Phaff./Saccharomycetaceae - ps22
Saccharomyco./mycetaceae - ps23
Saccharomycetaceae - ps24
Saccharomycetaceae_2 - ps25
Saccharomycetaceae_3 - ps26
Kaz./Nau./Saccharomyces - ps27
Saccharomyces - ps28
Arboricola_clade - ps29
Cerevisiae_clade - ps30
Saccharomyces_cerevisiae - ps31
Phylostratum

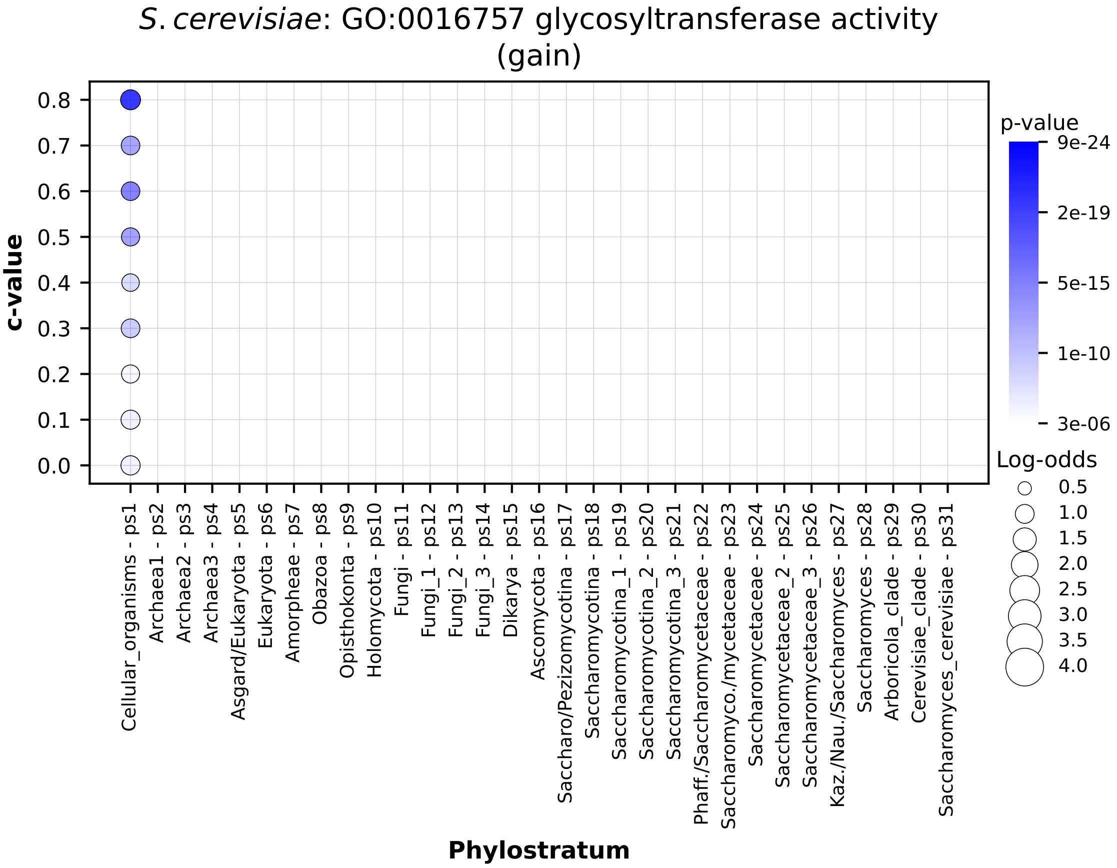

S. cerevisiae: GO:0016757 glycosyltransferase activity
(gain)
c-value
0.8
0.7
0.6
0.5
0.4
0.3
0.2
0.1
0.0
p-value
9e-24
2e-19
5e-15
1e-10
3e-06
Log-odds
0.5
1.0
1.5
2.0
2.5
3.0
3.5
4.0
Cellular_organisms - ps1
Archaea1 - ps2
Archaea2 - ps3
Archaea3 - ps4
Asgard/Eukaryota - ps5
Eukaryota - ps6
Amorpheae - ps7
Obazoa - ps8
Opisthokonta - ps9
Holomycota - ps10
Fungi - ps11
Fungi_1 - ps12
Fungi_2 - ps13
Fungi_3 - ps14
Dikarya - ps15
Ascomycota - ps16
Saccharo/Pezizomycotina - ps17
Saccharomycotina - ps18
Saccharomycotina_1 - ps19
Saccharomycotina_2 - ps20
Saccharomycotina_3 - ps21
Phaff./Saccharomycetaceae - ps22
Saccharomyco./mycetaceae - ps23
Saccharomycetaceae - ps24
Saccharomycetaceae_2 - ps25
Saccharomycetaceae_3 - ps26
Kaz./Nau./Saccharomyces - ps27
Saccharomyces - ps28
Arboricola_clade - ps29
Cerevisiae_clade - ps30
Saccharomyces_cerevisiae - ps31
Phylostratum

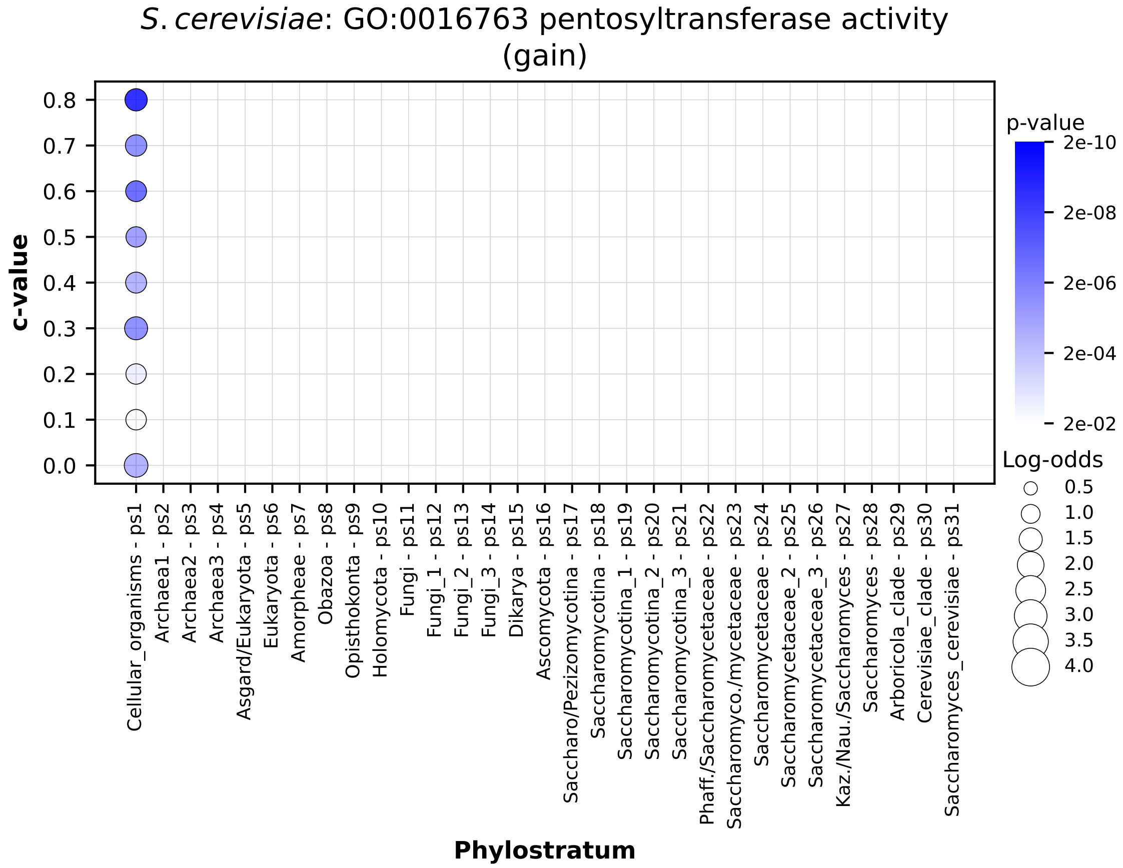

S. cerevisiae: GO:0016763 pentosyltransferase activity
(gain)
c-value
0.8
0.7
0.6
0.5
0.4
0.3
0.2
0.1
0.0
p-value
2e-10
2e-08
2e-06
2e-04
2e-02
Log-odds
0.5
1.0
1.5
2.0
2.5
3.0
3.5
4.0
Cellular_organisms - ps1
Archaea1 - ps2
Archaea2 - ps3
Archaea3 - ps4
Asgard/Eukaryota - ps5
Eukaryota - ps6
Amorpheae - ps7
Obazoa - ps8
Opisthokonta - ps9
Holomycota - ps10
Fungi - ps11
Fungi_1 - ps12
Fungi_2 - ps13
Fungi_3 - ps14
Dikarya - ps15
Ascomycota - ps16
Saccharo/Pezizomycotina - ps17
Saccharomycotina - ps18
Saccharomycotina_1 - ps19
Saccharomycotina_2 - ps20
Saccharomycotina_3 - ps21
Phaff./Saccharomycetaceae - ps22
Saccharomyco./mycetaceae - ps23
Saccharomycetaceae - ps24
Saccharomycetaceae_2 - ps25
Saccharomycetaceae_3 - ps26
Kaz./Nau./Saccharomyces - ps27
Saccharomyces - ps28
Arboricola_clade - ps29
Cerevisiae_clade - ps30
Saccharomyces_cerevisiae - ps31
Phylostratum

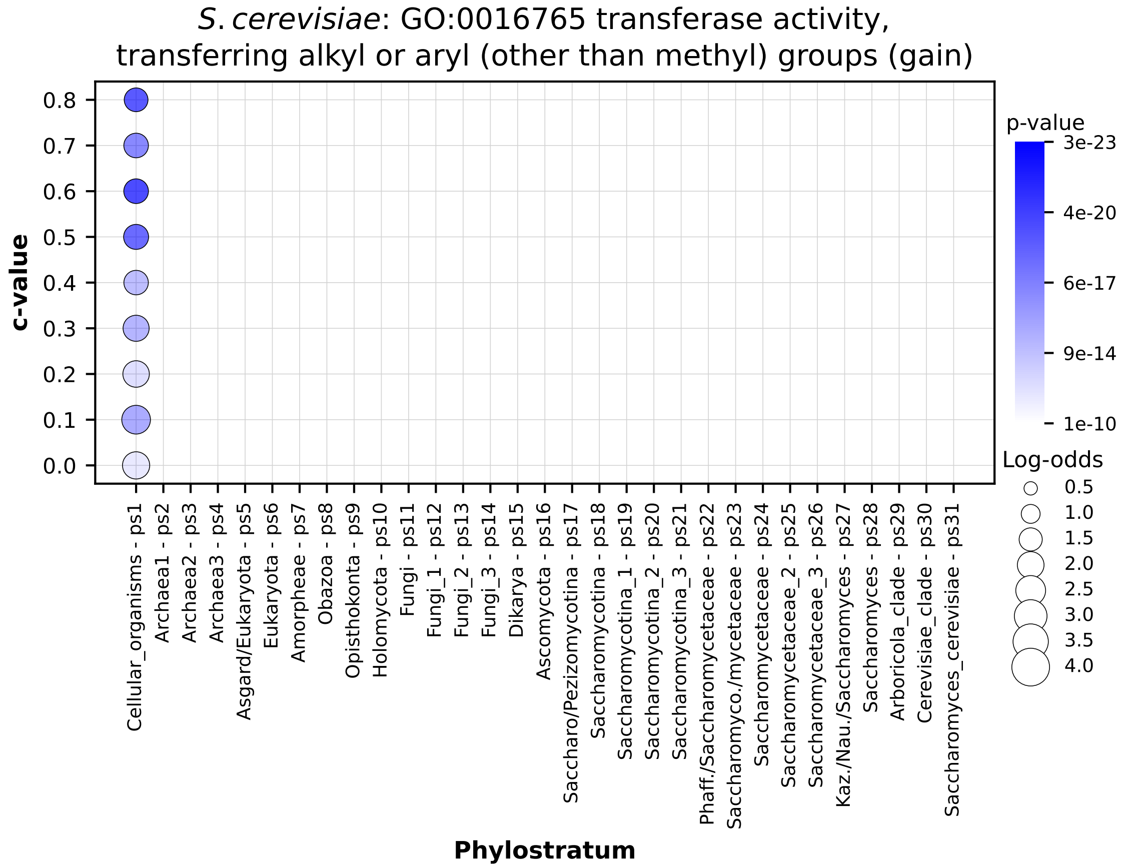

S. cerevisiae: GO:0016765 transferase activity, transferring alkyl or aryl (other than methyl) groups (gain)
c-value
0.8
0.7
0.6
0.5
0.4
0.3
0.2
0.1
0.0
p-value
3e-23
4e-20
6e-17
9e-14
1e-10
Log-odds
0.5
1.0
1.5
2.0
2.5
3.0
3.5
4.0
Cellular_organisms - ps1
Archaea1 - ps2
Archaea2 - ps3
Archaea3 - ps4
Asgard/Eukaryota - ps5
Eukaryota - ps6
Amorpheae - ps7
Obazoa - ps8
Opisthokonta - ps9
Holomycota - ps10
Fungi - ps11
Fungi_1 - ps12
Fungi_2 - ps13
Fungi_3 - ps14
Dikarya - ps15
Ascomycota - ps16
Saccharo/Pezizomycotina - ps17
Saccharomycotina - ps18
Saccharomycotina_1 - ps19
Saccharomycotina_2 - ps20
Saccharomycotina_3 - ps21
Phaff./Saccharomycetaceae - ps22
Saccharomyco./mycetaceae - ps23
Saccharomycetaceae - ps24
Saccharomycetaceae_2 - ps25
Saccharomycetaceae_3 - ps26
Kaz./Nau./Saccharomyces - ps27
Saccharomyces - ps28
Arboricola_clade - ps29
Cerevisiae_clade - ps30
Saccharomyces_cerevisiae - ps31
Phylostratum

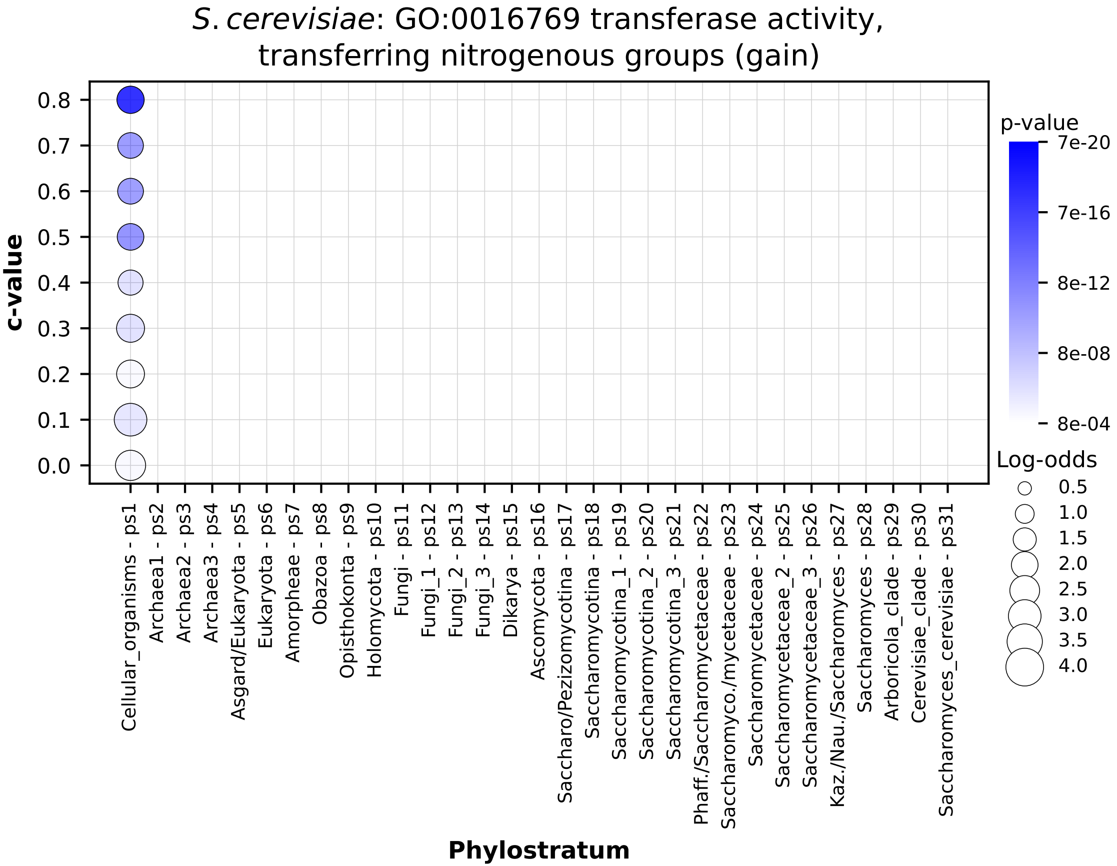

S. cerevisiae: GO:0016769 transferase activity, transferring nitrogenous groups (gain)
c-value
0.8
0.7
0.6
0.5
0.4
0.3
0.2
0.1
0.0
p-value
7e-20
7e-16
8e-12
8e-08
8e-04
Log-odds
0.5
1.0
1.5
2.0
2.5
3.0
3.5
4.0
Cellular_organisms - ps1
Archaea1 - ps2
Archaea2 - ps3
Archaea3 - ps4
Asgard/Eukaryota - ps5
Eukaryota - ps6
Amorpheae - ps7
Obazoa - ps8
Opisthokonta - ps9
Holomycota - ps10
Fungi - ps11
Fungi_1 - ps12
Fungi_2 - ps13
Fungi_3 - ps14
Dikarya - ps15
Ascomycota - ps16
Saccharo/Pezizomycotina - ps17
Saccharomycotina - ps18
Saccharomycotina_1 - ps19
Saccharomycotina_2 - ps20
Saccharomycotina_3 - ps21
Phaff./Saccharomycetaceae - ps22
Saccharomyco./mycetaceae - ps23
Saccharomycetaceae - ps24
Saccharomycetaceae_2 - ps25
Saccharomycetaceae_3 - ps26
Kaz./Nau./Saccharomyces - ps27
Saccharomyces - ps28
Arboricola_clade - ps29
Cerevisiae_clade - ps30
Saccharomyces_cerevisiae - ps31
Phylostratum

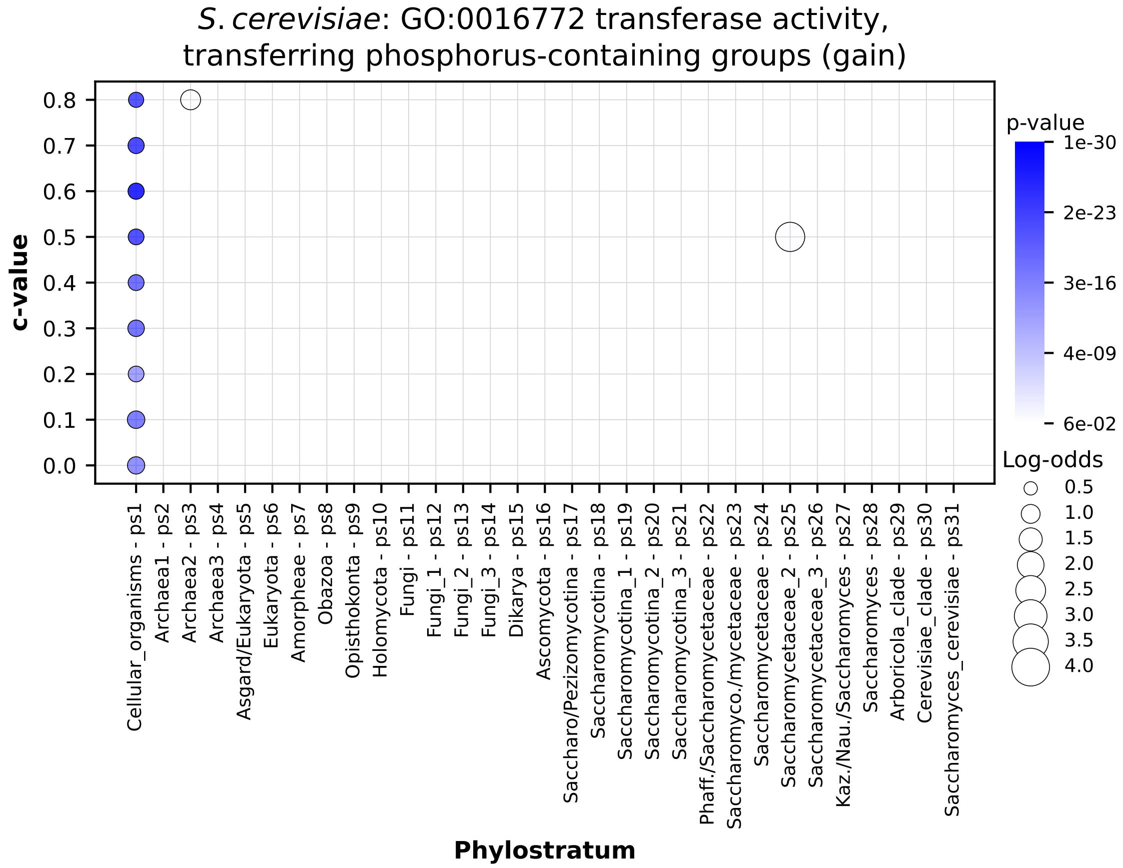

S. cerevisiae: GO:0016772 transferase activity, transferring phosphorus-containing groups (gain)
c-value
0.8
0.7
0.6
0.5
0.4
0.3
0.2
0.1
0.0
Phylostratum
Cellular_organisms - ps1
Archaea1 - ps2
Archaea2 - ps3
Archaea3 - ps4
Asgard/Eukaryota - ps5
Eukaryota - ps6
Amorpheae - ps7
Obazoa - ps8
Opisthokonta - ps9
Holomycota - ps10
Fungi - ps11
Fungi_1 - ps12
Fungi_2 - ps13
Fungi_3 - ps14
Dikarya - ps15
Ascomycota - ps16
Saccharo/Pezizomycotina - ps17
Saccharomycotina - ps18
Saccharomycotina_1 - ps19
Saccharomycotina_2 - ps20
Saccharomycotina_3 - ps21
Phaff./Saccharomycetaceae - ps22
Saccharomyco./mycetaceae - ps23
Saccharomycetaceae - ps24
Saccharomycetaceae_2 - ps25
Saccharomycetaceae_3 - ps26
Kaz./Nau./Saccharomyces - ps27
Saccharomyces - ps28
Arboricola_clade - ps29
Cerevisiae_clade - ps30
Saccharomyces_cerevisiae - ps31
p-value
1e-30
2e-23
3e-16
4e-09
6e-02
Log-odds
0.5
1.0
1.5
2.0
2.5
3.0
3.5
4.0

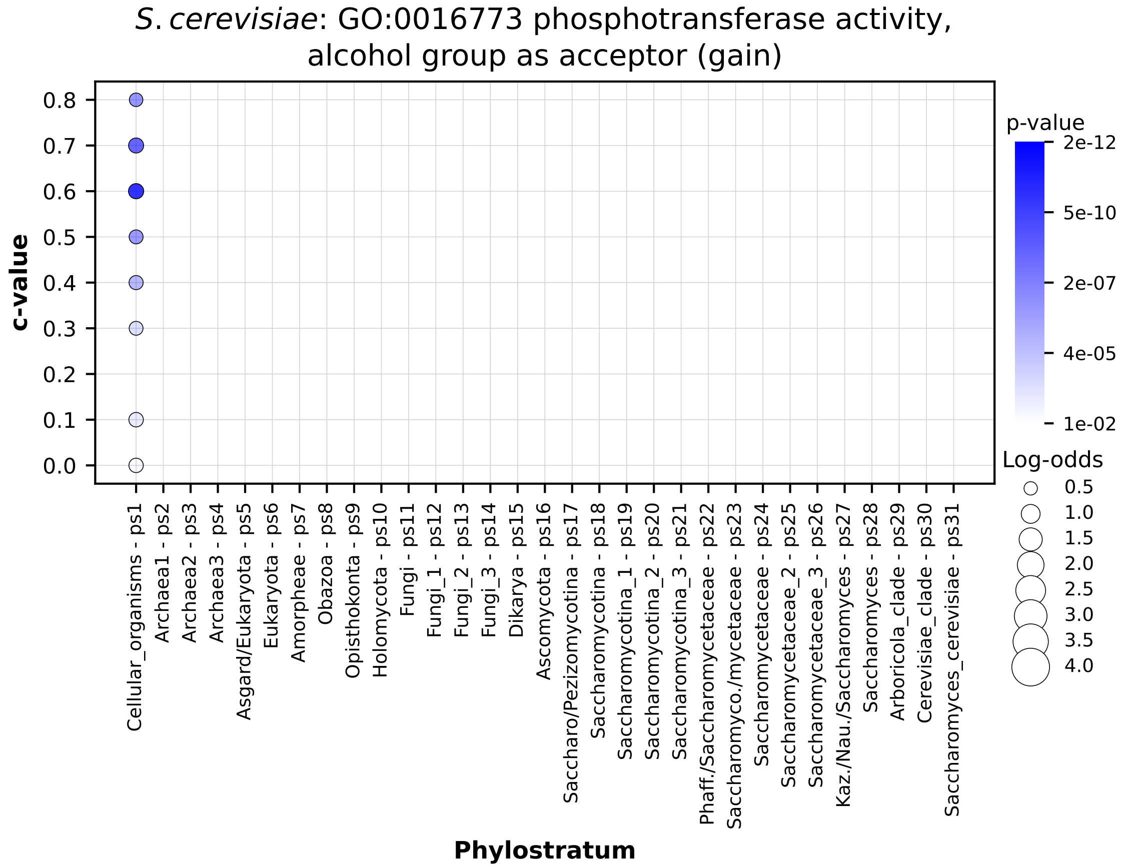

S. cerevisiae: GO:0016773 phosphotransferase activity, alcohol group as acceptor (gain)
c-value
0.0
0.1
0.2
0.3
0.4
0.5
0.6
0.7
0.8
Phylostratum
Cellular_organisms - ps1
Archaea1 - ps2
Archaea2 - ps3
Archaea3 - ps4
Asgard/Eukaryota - ps5
Eukaryota - ps6
Amorpheae - ps7
Obazoa - ps8
Opisthokonta - ps9
Holomycota - ps10
Fungi - ps11
Fungi_1 - ps12
Fungi_2 - ps13
Fungi_3 - ps14
Dikarya - ps15
Ascomycota - ps16
Saccharo/Pezizomycotina - ps17
Saccharomycotina - ps18
Saccharomycotina_1 - ps19
Saccharomycotina_2 - ps20
Saccharomycotina_3 - ps21
Phaff./Saccharomycetaceae - ps22
Saccharomyco./mycetaceae - ps23
Saccharomycetaceae - ps24
Saccharomycetaceae_2 - ps25
Saccharomycetaceae_3 - ps26
Kaz./Nau./Saccharomyces - ps27
Saccharomyces - ps28
Arboricola_clade - ps29
Cerevisiae_clade - ps30
Saccharomyces_cerevisiae - ps31
p-value
2e-12
5e-10
2e-07
4e-05
1e-02
Log-odds
0.5
1.0
1.5
2.0
2.5
3.0
3.5
4.0

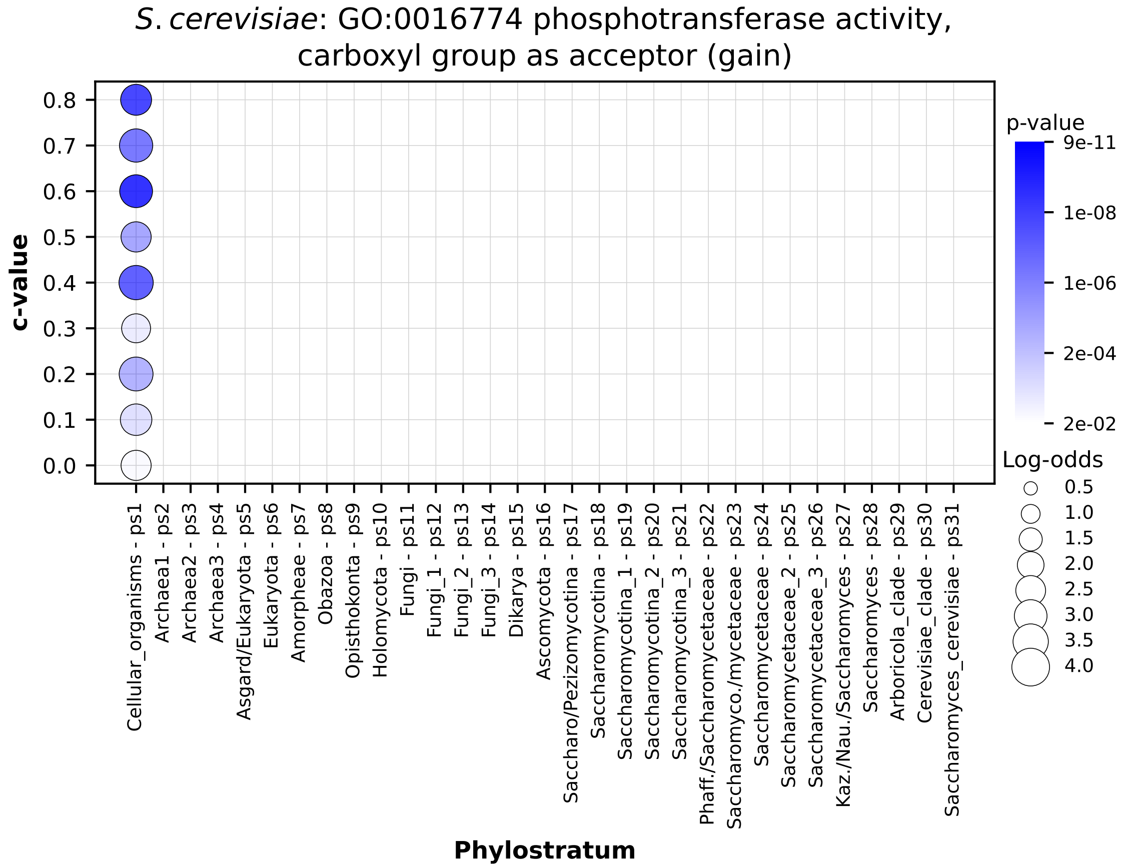

S. cerevisiae: GO:0016774 phosphotransferase activity, carboxyl group as acceptor (gain)
c-value
0.0
0.1
0.2
0.3
0.4
0.5
0.6
0.7
0.8
Phylostratum
Cellular_organisms - ps1
Archaea1 - ps2
Archaea2 - ps3
Archaea3 - ps4
Asgard/Eukaryota - ps5
Eukaryota - ps6
Amorpheae - ps7
Obazoa - ps8
Opisthokonta - ps9
Holomycota - ps10
Fungi - ps11
Fungi_1 - ps12
Fungi_2 - ps13
Fungi_3 - ps14
Dikarya - ps15
Ascomycota - ps16
Saccharo/Pezizomycotina - ps17
Saccharomycotina - ps18
Saccharomycotina_1 - ps19
Saccharomycotina_2 - ps20
Saccharomycotina_3 - ps21
Phaff./Saccharomycetaceae - ps22
Saccharomyco./mycetaceae - ps23
Saccharomycetaceae - ps24
Saccharomycetaceae_2 - ps25
Saccharomycetaceae_3 - ps26
Kaz./Nau./Saccharomyces - ps27
Saccharomyces - ps28
Arboricola_clade - ps29
Cerevisiae_clade - ps30
Saccharomyces_cerevisiae - ps31
p-value
9e-11
1e-08
1e-06
2e-04
2e-02
Log-odds
0.5
1.0
1.5
2.0
2.5
3.0
3.5
4.0

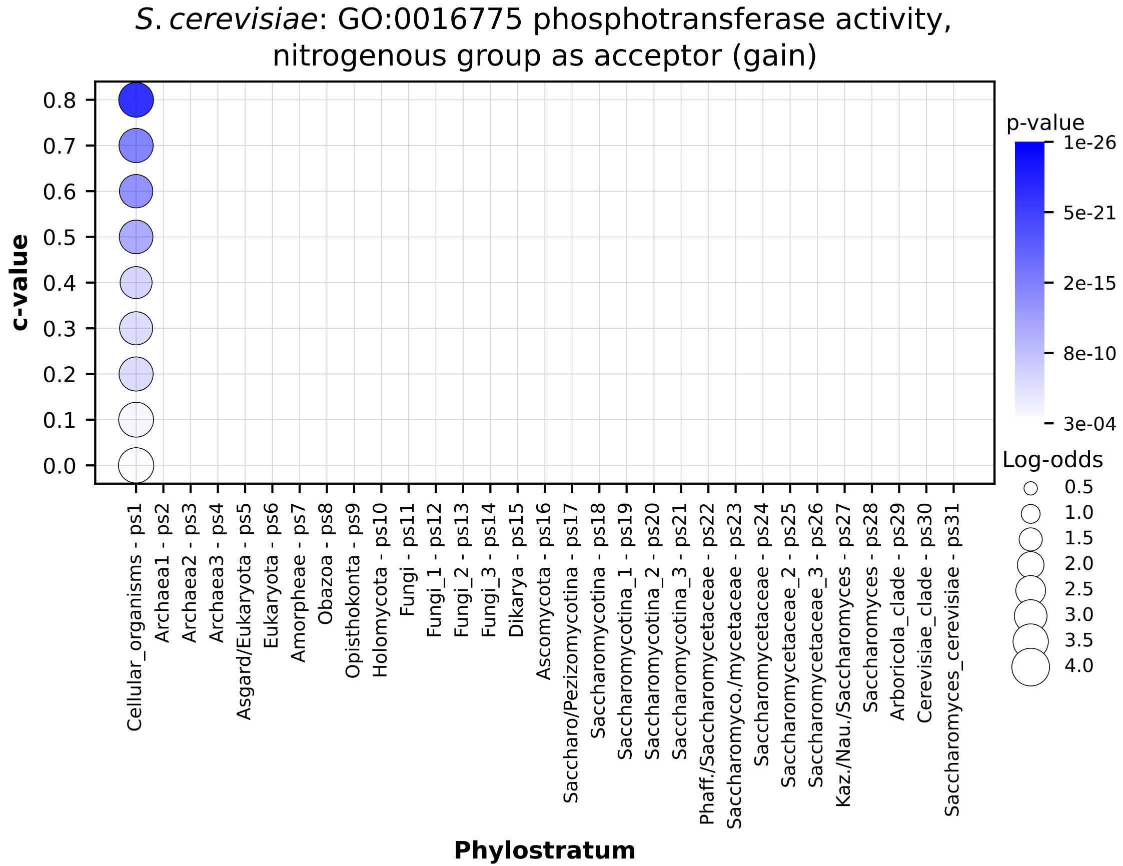
S. cerevisiae: GO:0016775 phosphotransferase activity, nitrogenous group as acceptor (gain)
c-value
0.8
0.7
0.6
0.5
0.4
0.3
0.2
0.1
0.0
Phylostratum
Cellular_organisms - ps1
Archaea1 - ps2
Archaea2 - ps3
Archaea3 - ps4
Asgard/Eukaryota - ps5
Eukaryota - ps6
Amorpheae - ps7
Obazoa - ps8
Opisthokonta - ps9
Holomycota - ps10
Fungi - ps11
Fungi_1 - ps12
Fungi_2 - ps13
Fungi_3 - ps14
Dikarya - ps15
Ascomycota - ps16
Saccharo/Pezizomycotina - ps17
Saccharomycotina - ps18
Saccharomycotina_1 - ps19
Saccharomycotina_2 - ps20
Saccharomycotina_3 - ps21
Phaff./Saccharomycetaceae - ps22
Saccharomyco./mycetaceae - ps23
Saccharomycetaceae - ps24
Saccharomycetaceae_2 - ps25
Saccharomycetaceae_3 - ps26
Kaz./Nau./Saccharomyces - ps27
Saccharomyces - ps28
Arboricola_clade - ps29
Cerevisiae_clade - ps30
Saccharomyces_cerevisiae - ps31
p-value
1e-26
5e-21
2e-15
8e-10
3e-04
Log-odds
0.5
1.0
1.5
2.0
2.5
3.0
3.5
4.0

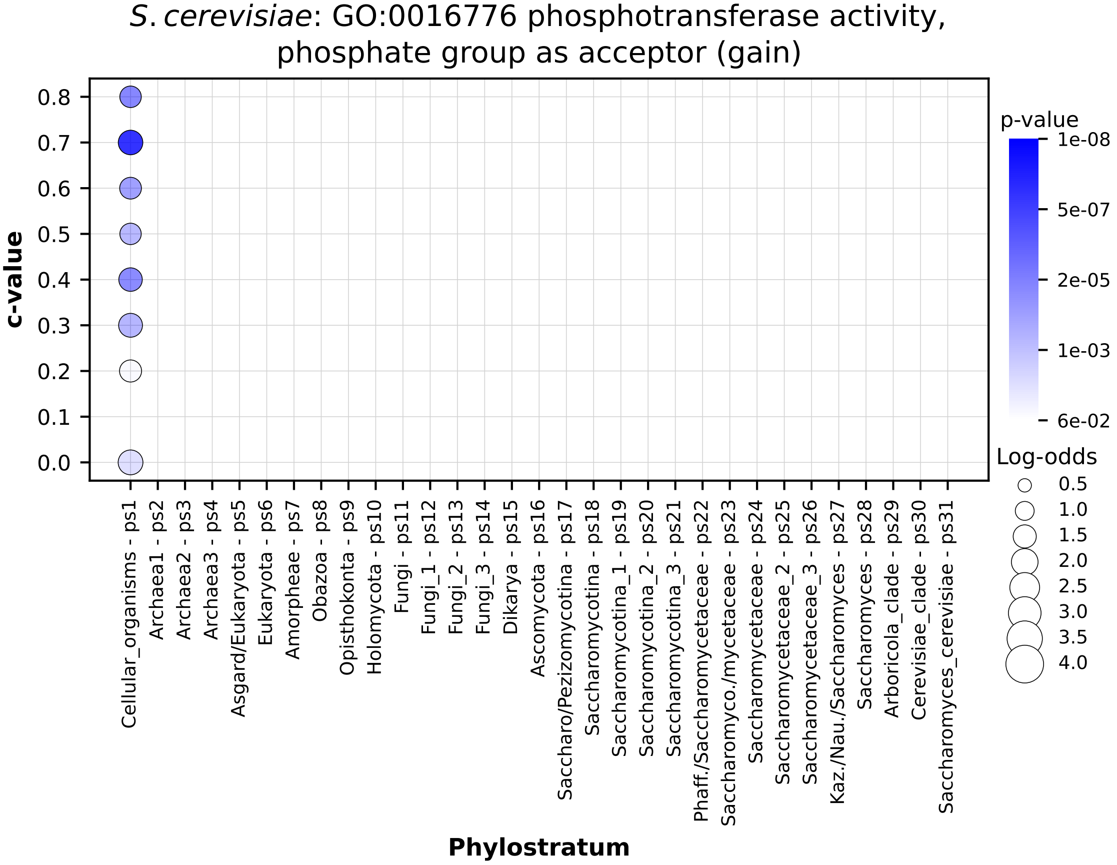
S. cerevisiae: GO:0016776 phosphotransferase activity, phosphate group as acceptor (gain)
c-value
0.8
0.7
0.6
0.5
0.4
0.3
0.2
0.1
0.0
p-value
1e-08
5e-07
2e-05
1e-03
6e-02
Log-odds
0.5
1.0
1.5
2.0
2.5
3.0
3.5
4.0
Cellular_organisms - ps1
Archaea1 - ps2
Archaea2 - ps3
Archaea3 - ps4
Asgard/Eukaryota - ps5
Eukaryota - ps6
Amorpheae - ps7
Obazoa - ps8
Opisthokonta - ps9
Holomycota - ps10
Fungi - ps11
Fungi_1 - ps12
Fungi_2 - ps13
Fungi_3 - ps14
Dikarya - ps15
Ascomycota - ps16
Saccharo/Pezizomycotina - ps17
Saccharomycotina - ps18
Saccharomycotina_1 - ps19
Saccharomycotina_2 - ps20
Saccharomycotina_3 - ps21
Phaff./Saccharomycetaceae - ps22
Saccharomyco./mycetaceae - ps23
Saccharomycetaceae - ps24
Saccharomycetaceae_2 - ps25
Saccharomycetaceae_3 - ps26
Kaz./Nau./Saccharomyces - ps27
Saccharomyces - ps28
Arboricola_clade - ps29
Cerevisiae_clade - ps30
Saccharomyces_cerevisiae - ps31
Phylostratum

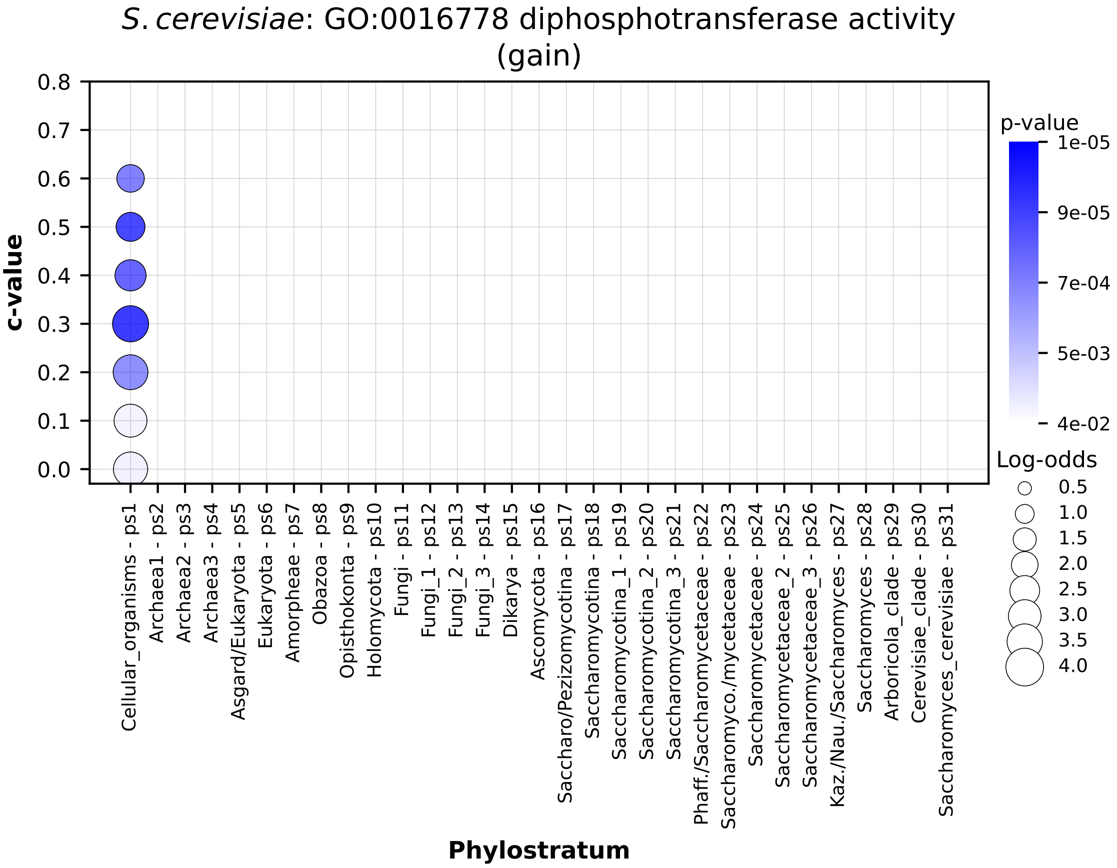

S. cerevisiae: GO:0016778 diphosphotransferase activity (gain)
c-value
0.8
0.7
0.6
0.5
0.4
0.3
0.2
0.1
0.0
p-value
1e-05
9e-05
7e-04
5e-03
4e-02
Log-odds
0.5
1.0
1.5
2.0
2.5
3.0
3.5
4.0
Cellular_organisms - ps1
Archaea1 - ps2
Archaea2 - ps3
Archaea3 - ps4
Asgard/Eukaryota - ps5
Eukaryota - ps6
Amorpheae - ps7
Obazoa - ps8
Opisthokonta - ps9
Holomycota - ps10
Fungi - ps11
Fungi_1 - ps12
Fungi_2 - ps13
Fungi_3 - ps14
Dikarya - ps15
Ascomycota - ps16
Saccharo/Pezizomycotina - ps17
Saccharomycotina - ps18
Saccharomycotina_1 - ps19
Saccharomycotina_2 - ps20
Saccharomycotina_3 - ps21
Phaff./Saccharomycetaceae - ps22
Saccharomyco./mycetaceae - ps23
Saccharomycetaceae - ps24
Saccharomycetaceae_2 - ps25
Saccharomycetaceae_3 - ps26
Kaz./Nau./Saccharomyces - ps27
Saccharomyces - ps28
Arboricola_clade - ps29
Cerevisiae_clade - ps30
Saccharomyces_cerevisiae - ps31
Phylostratum

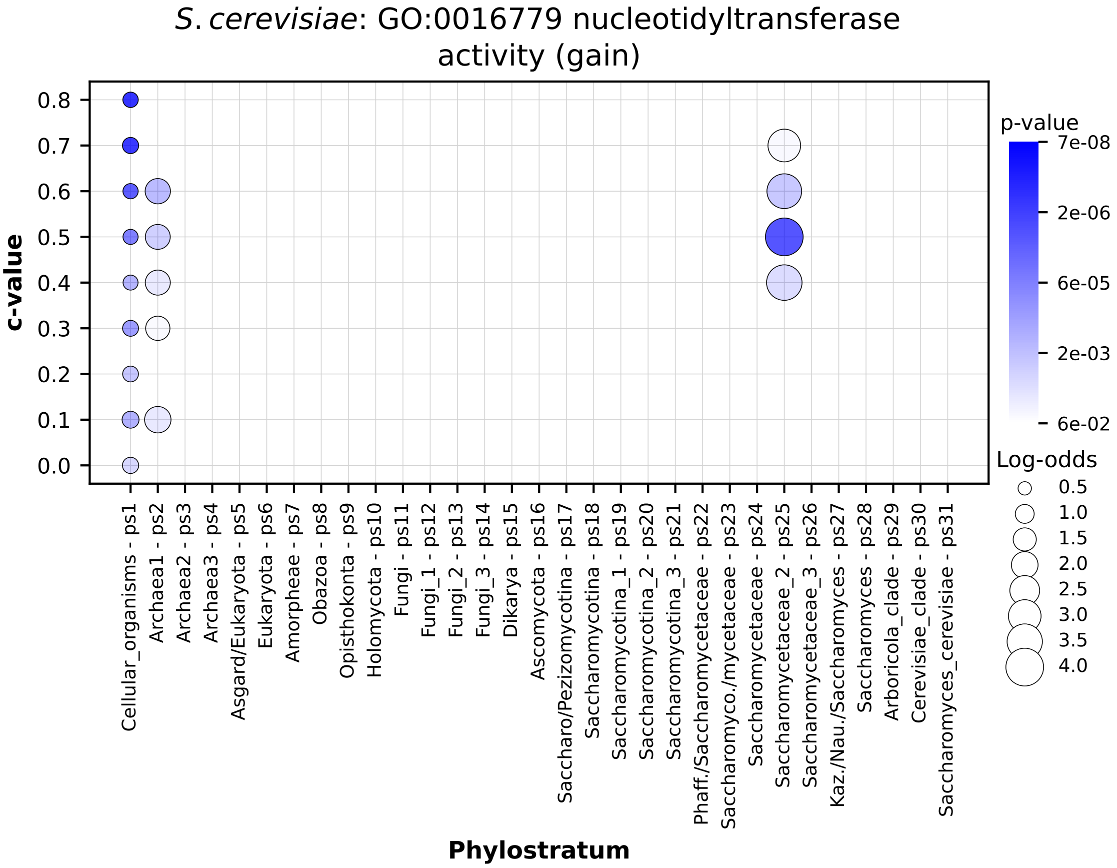
S. cerevisiae: GO:0016779 nucleotidyltransferase activity (gain)
c-value
Phylostratum
p-value
7e-08
2e-06
6e-05
2e-03
6e-02
Log-odds
0.5
1.0
1.5
2.0
2.5
3.0
3.5
4.0
Cellular_organisms - ps1
Archaea1 - ps2
Archaea2 - ps3
Archaea3 - ps4
Asgard/Eukaryota - ps5
Eukaryota - ps6
Amorpheae - ps7
Obazoa - ps8
Opisthokonta - ps9
Holomycota - ps10
Fungi - ps11
Fungi_1 - ps12
Fungi_2 - ps13
Fungi_3 - ps14
Dikarya - ps15
Ascomycota - ps16
Saccharo/Pezizomycotina - ps17
Saccharomycotina - ps18
Saccharomycotina_1 - ps19
Saccharomycotina_2 - ps20
Saccharomycotina_3 - ps21
Phaff./Saccharomycetaceae - ps22
Saccharomyco./mycetaceae - ps23
Saccharomycetaceae - ps24
Saccharomycetaceae_2 - ps25
Saccharomycetaceae_3 - ps26
Kaz./Nau./Saccharomyces - ps27
Saccharomyces - ps28
Arboricola_clade - ps29
Cerevisiae_clade - ps30
Saccharomyces_cerevisiae - ps31

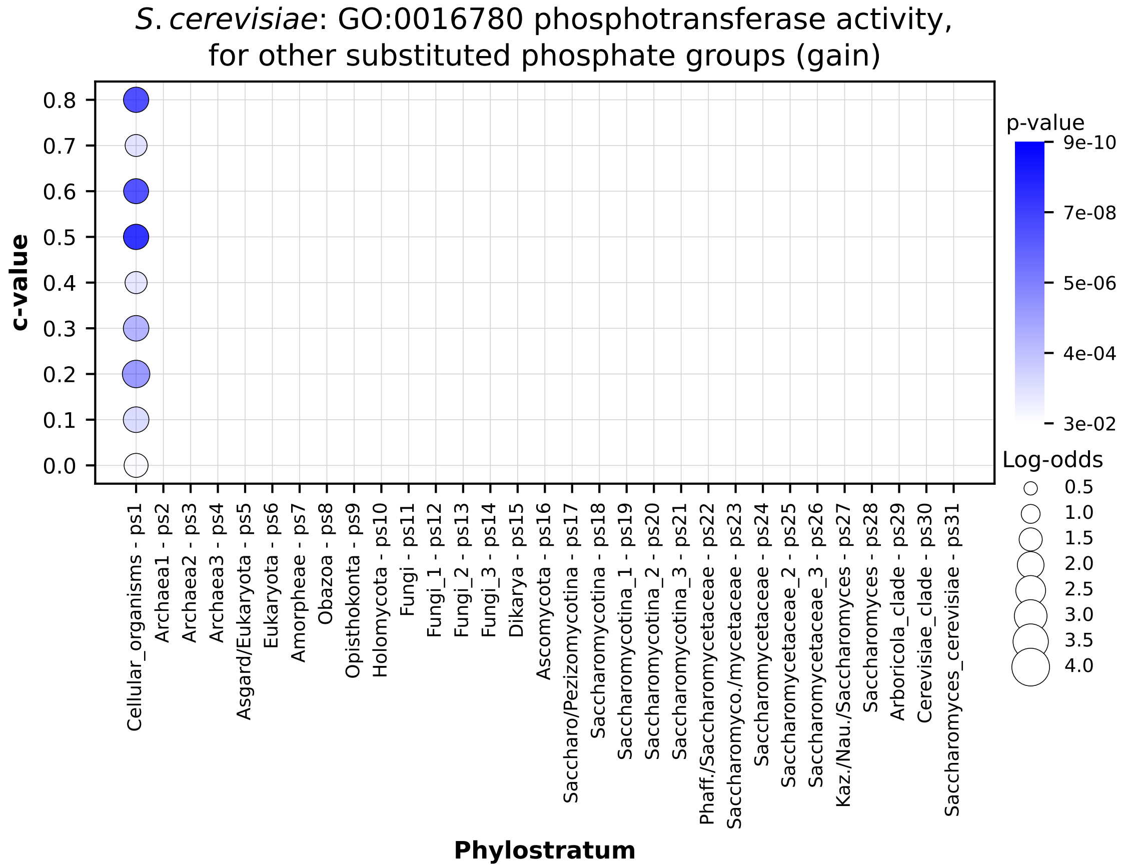
S. cerevisiae: GO:0016780 phosphotransferase activity, for other substituted phosphate groups (gain)
c-value
0.8
0.7
0.6
0.5
0.4
0.3
0.2
0.1
0.0
p-value
9e-10
7e-08
5e-06
4e-04
3e-02
Log-odds
0.5
1.0
1.5
2.0
2.5
3.0
3.5
4.0
Cellular_organisms - ps1
Archaea1 - ps2
Archaea2 - ps3
Archaea3 - ps4
Asgard/Eukaryota - ps5
Eukaryota - ps6
Amorpheae - ps7
Obazoa - ps8
Opisthokonta - ps9
Holomycota - ps10
Fungi - ps11
Fungi_1 - ps12
Fungi_2 - ps13
Fungi_3 - ps14
Dikarya - ps15
Ascomycota - ps16
Saccharo/Pezizomycotina - ps17
Saccharomycotina - ps18
Saccharomycotina_1 - ps19
Saccharomycotina_2 - ps20
Saccharomycotina_3 - ps21
Phaff./Saccharomycetaceae - ps22
Saccharomyco./mycetaceae - ps23
Saccharomycetaceae - ps24
Saccharomycetaceae_2 - ps25
Saccharomycetaceae_3 - ps26
Kaz./Nau./Saccharomyces - ps27
Saccharomyces - ps28
Arboricola_clade - ps29
Cerevisiae_clade - ps30
Saccharomyces_cerevisiae - ps31
Phylostratum

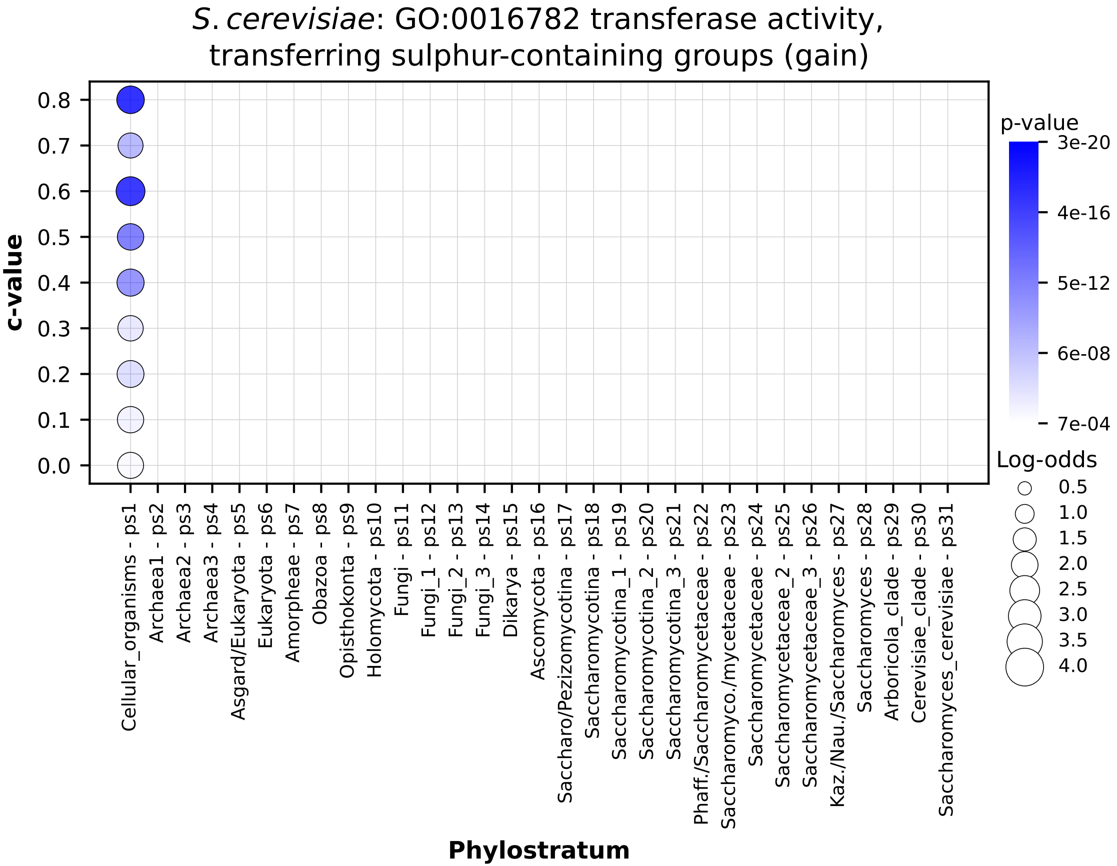

S. cerevisiae: GO:0016782 transferase activity, transferring sulphur-containing groups (gain)
c-value
0.0
0.1
0.2
0.3
0.4
0.5
0.6
0.7
0.8
Phylostratum
Cellular_organisms - ps1
Archaea1 - ps2
Archaea2 - ps3
Archaea3 - ps4
Asgard/Eukaryota - ps5
Eukaryota - ps6
Amorpheae - ps7
Obazoa - ps8
Opisthokonta - ps9
Holomycota - ps10
Fungi - ps11
Fungi_1 - ps12
Fungi_2 - ps13
Fungi_3 - ps14
Dikarya - ps15
Ascomycota - ps16
Saccharo/Pezizomycotina - ps17
Saccharomycotina - ps18
Saccharomycotina_1 - ps19
Saccharomycotina_2 - ps20
Saccharomycotina_3 - ps21
Phaff./Saccharomycetaceae - ps22
Saccharomyco./mycetaceae - ps23
Saccharomycetaceae - ps24
Saccharomycetaceae_2 - ps25
Saccharomycetaceae_3 - ps26
Kaz./Nau./Saccharomyces - ps27
Saccharomyces - ps28
Arboricola_clade - ps29
Cerevisiae_clade - ps30
Saccharomyces_cerevisiae - ps31
p-value
3e-20
4e-16
5e-12
6e-08
7e-04
Log-odds
0.5
1.0
1.5
2.0
2.5
3.0
3.5
4.0

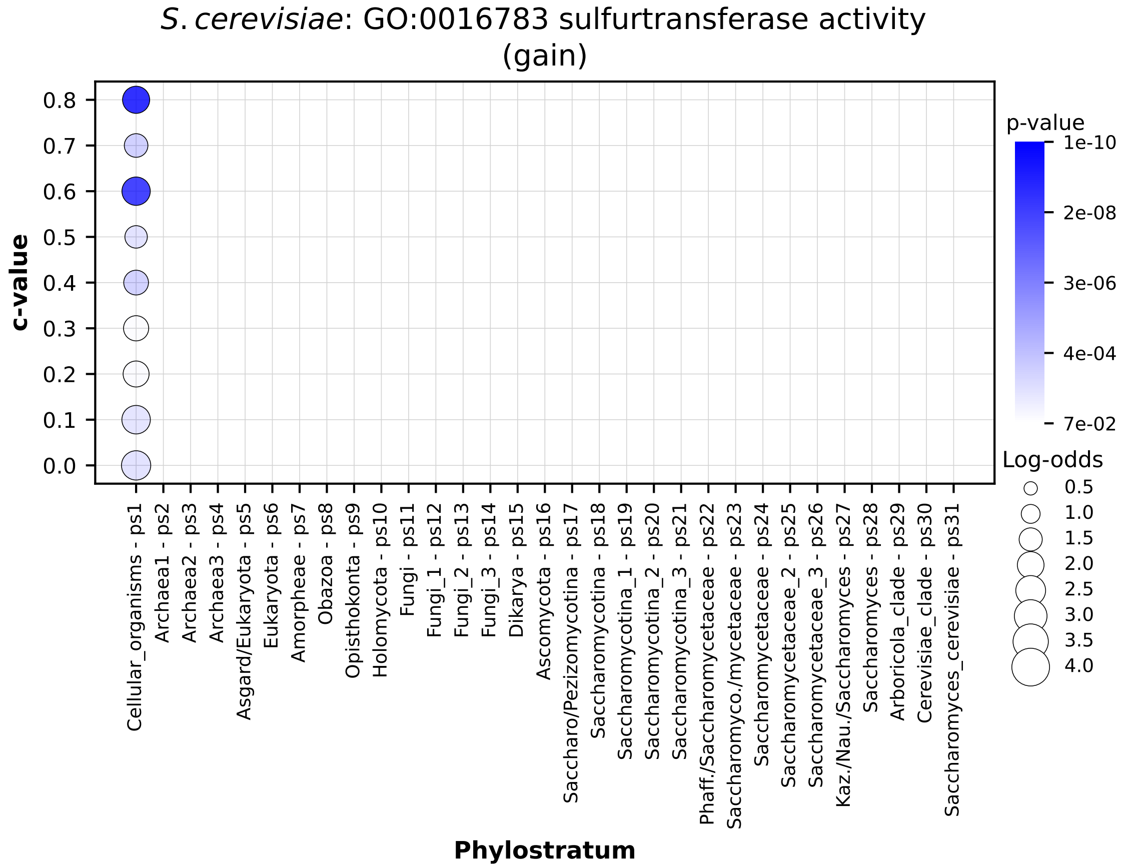
S. cerevisiae: GO:0016783 sulfurtransferase activity (gain)
c-value
0.8
0.7
0.6
0.5
0.4
0.3
0.2
0.1
0.0
p-value
1e-10
2e-08
3e-06
4e-04
7e-02
Log-odds
0.5
1.0
1.5
2.0
2.5
3.0
3.5
4.0
Cellular_organisms - ps1
Archaea1 - ps2
Archaea2 - ps3
Archaea3 - ps4
Asgard/Eukaryota - ps5
Eukaryota - ps6
Amorpheae - ps7
Obazoa - ps8
Opisthokonta - ps9
Holomycota - ps10
Fungi - ps11
Fungi_1 - ps12
Fungi_2 - ps13
Fungi_3 - ps14
Dikarya - ps15
Ascomycota - ps16
Saccharo/Pezizomycotina - ps17
Saccharomycotina - ps18
Saccharomycotina_1 - ps19
Saccharomycotina_2 - ps20
Saccharomycotina_3 - ps21
Phaff./Saccharomycetaceae - ps22
Saccharomyco./mycetaceae - ps23
Saccharomycetaceae - ps24
Saccharomycetaceae_2 - ps25
Saccharomycetaceae_3 - ps26
Kaz./Nau./Saccharomyces - ps27
Saccharomyces - ps28
Arboricola_clade - ps29
Cerevisiae_clade - ps30
Saccharomyces_cerevisiae - ps31
Phylostratum

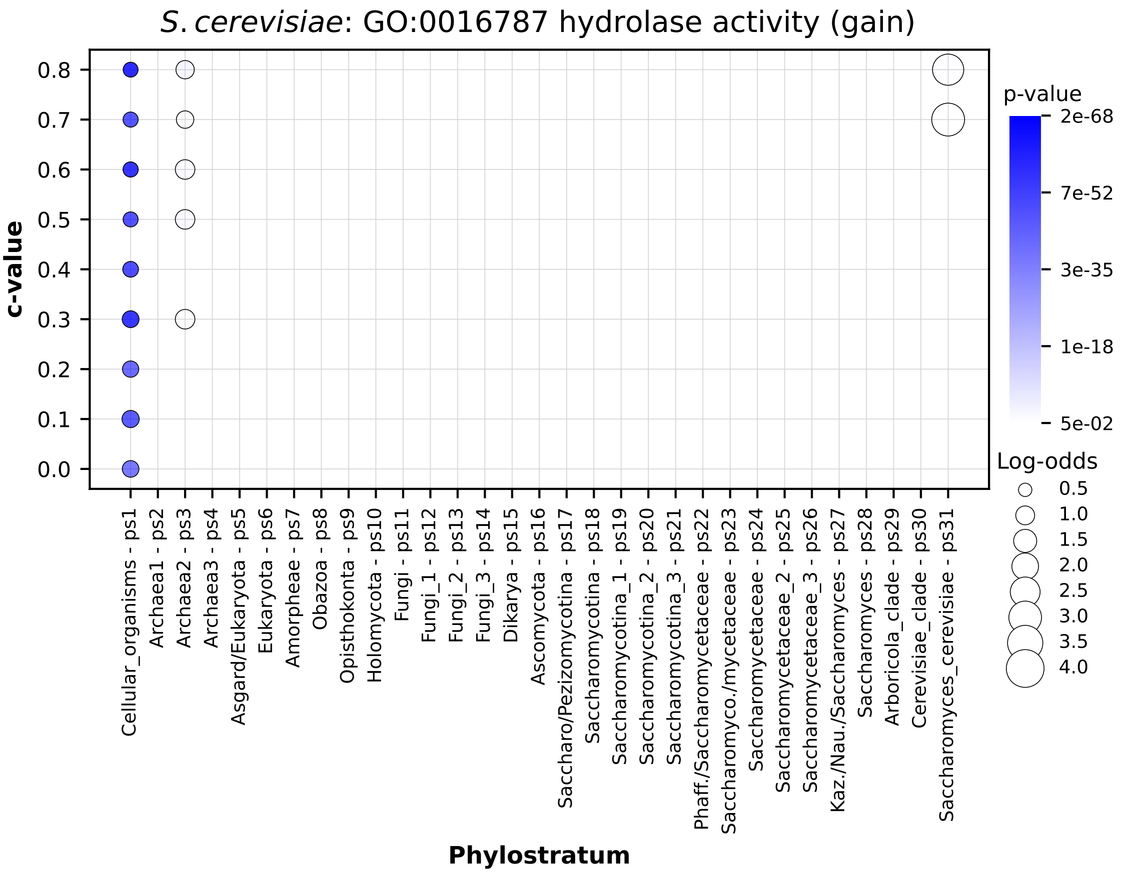

S. cerevisiae: GO:0016787 hydrolase activity (gain)
c-value
0.8
0.7
0.6
0.5
0.4
0.3
0.2
0.1
0.0
p-value
2e-68
7e-52
3e-35
1e-18
5e-02
Log-odds
0.5
1.0
1.5
2.0
2.5
3.0
3.5
4.0
Cellular_organisms - ps1
Archaea1 - ps2
Archaea2 - ps3
Archaea3 - ps4
Asgard/Eukaryota - ps5
Eukaryota - ps6
Amorpheae - ps7
Obazoa - ps8
Opisthokonta - ps9
Holomycota - ps10
Fungi - ps11
Fungi_1 - ps12
Fungi_2 - ps13
Fungi_3 - ps14
Dikarya - ps15
Ascomycota - ps16
Saccharo/Pezizomycotina - ps17
Saccharomycotina - ps18
Saccharomycotina_1 - ps19
Saccharomycotina_2 - ps20
Saccharomycotina_3 - ps21
Phaff./Saccharomycetaceae - ps22
Saccharomyco./mycetaceae - ps23
Saccharomycetaceae - ps24
Saccharomycetaceae_2 - ps25
Saccharomycetaceae_3 - ps26
Kaz./Nau./Saccharomyces - ps27
Saccharomyces - ps28
Arboricola_clade - ps29
Cerevisiae_clade - ps30
Saccharomyces_cerevisiae - ps31
Phylostratum

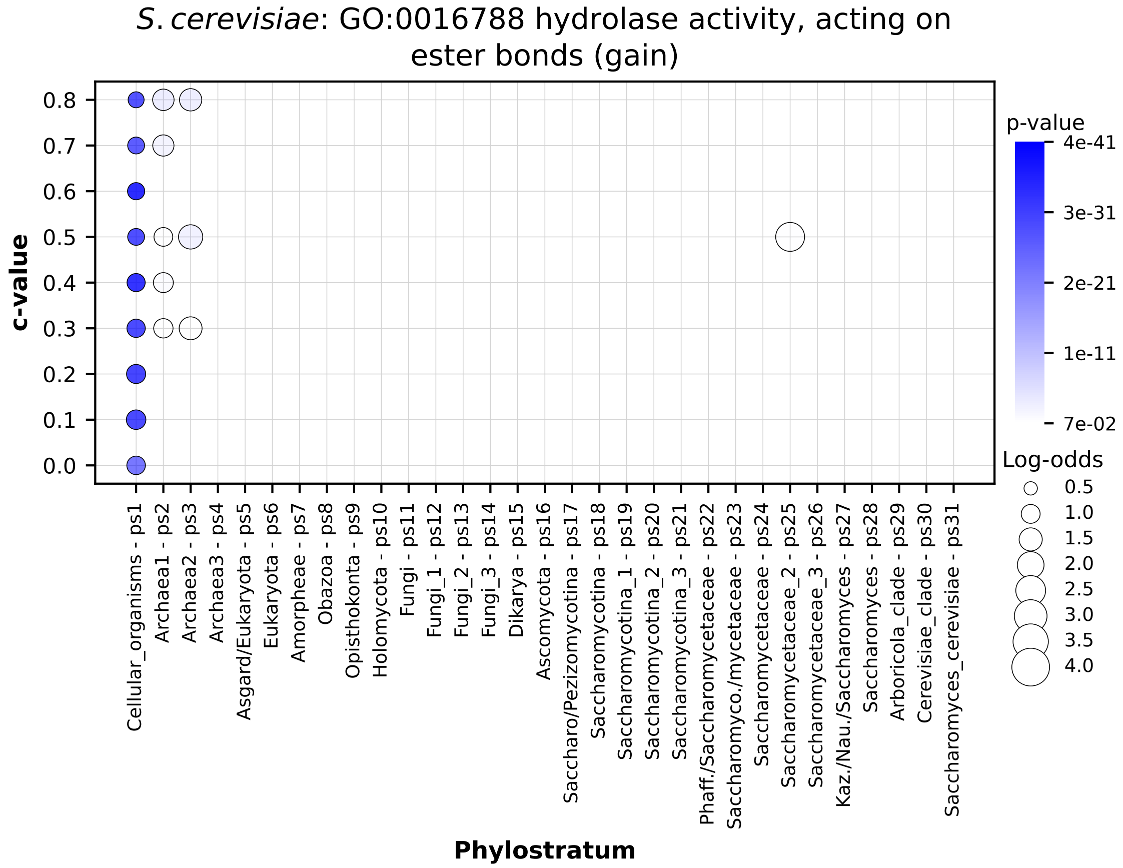

S. cerevisiae: GO:0016788 hydrolase activity, acting on ester bonds (gain)
c-value
0.8
0.7
0.6
0.5
0.4
0.3
0.2
0.1
0.0
p-value
4e-41
3e-31
2e-21
1e-11
7e-02
Log-odds
0.5
1.0
1.5
2.0
2.5
3.0
3.5
4.0
Cellular_organisms - ps1
Archaea1 - ps2
Archaea2 - ps3
Archaea3 - ps4
Asgard/Eukaryota - ps5
Eukaryota - ps6
Amorpheae - ps7
Obazoa - ps8
Opisthokonta - ps9
Holomycota - ps10
Fungi - ps11
Fungi_1 - ps12
Fungi_2 - ps13
Fungi_3 - ps14
Dikarya - ps15
Ascomycota - ps16
Saccharo/Pezizomycotina - ps17
Saccharomycotina - ps18
Saccharomycotina_1 - ps19
Saccharomycotina_2 - ps20
Saccharomycotina_3 - ps21
Phaff./Saccharomycetaceae - ps22
Saccharomyco./mycetaceae - ps23
Saccharomycetaceae - ps24
Saccharomycetaceae_2 - ps25
Saccharomycetaceae_3 - ps26
Kaz./Nau./Saccharomyces - ps27
Saccharomyces - ps28
Arboricola_clade - ps29
Cerevisiae_clade - ps30
Saccharomyces_cerevisiae - ps31
Phylostratum

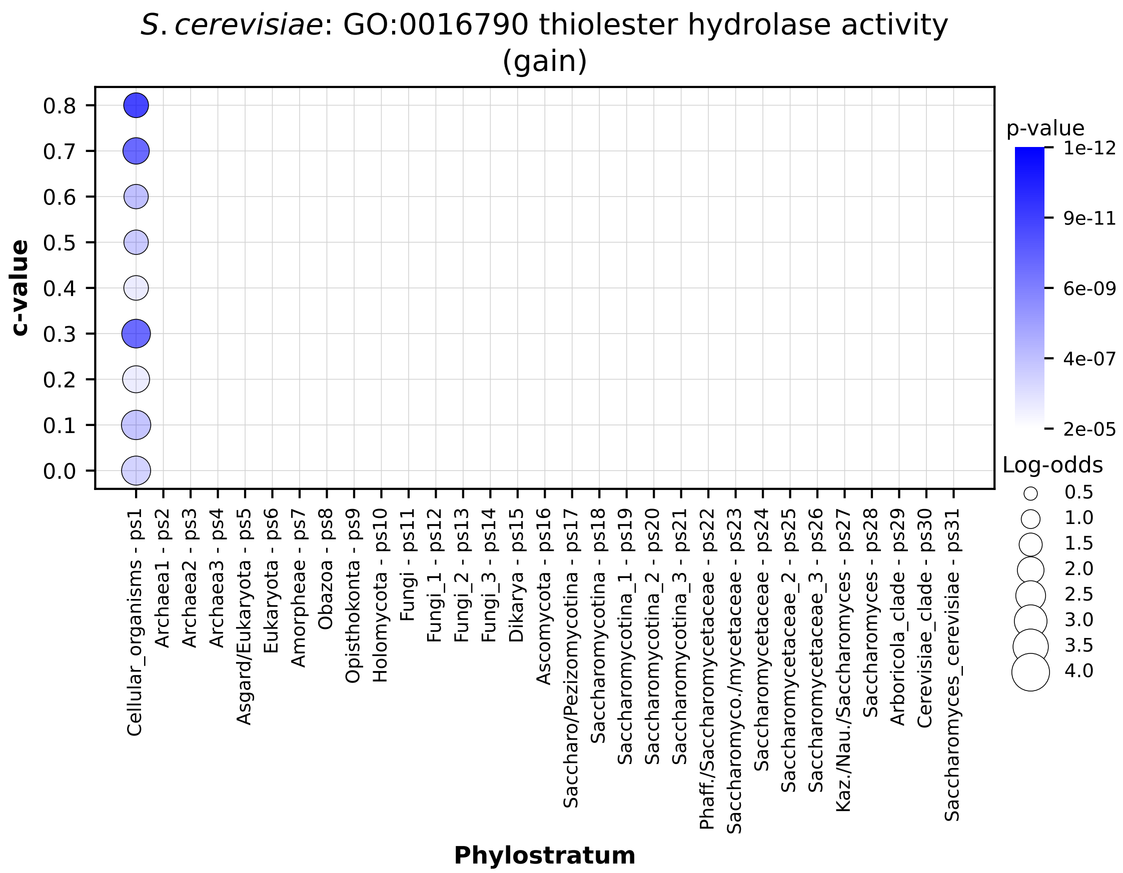
S. cerevisiae: GO:0016790 thiolester hydrolase activity (gain)
c-value
0.0
0.1
0.2
0.3
0.4
0.5
0.6
0.7
0.8
p-value
1e-12
9e-11
6e-09
4e-07
2e-05
Log-odds
0.5
1.0
1.5
2.0
2.5
3.0
3.5
4.0
Cellular_organisms - ps1
Archaea1 - ps2
Archaea2 - ps3
Archaea3 - ps4
Asgard/Eukaryota - ps5
Eukaryota - ps6
Amorpheae - ps7
Obazoa - ps8
Opisthokonta - ps9
Holomycota - ps10
Fungi - ps11
Fungi_1 - ps12
Fungi_2 - ps13
Fungi_3 - ps14
Dikarya - ps15
Ascomycota - ps16
Saccharo/Pezizomycotina - ps17
Saccharomycotina - ps18
Saccharomycotina_1 - ps19
Saccharomycotina_2 - ps20
Saccharomycotina_3 - ps21
Phaff./Saccharomycetaceae - ps22
Saccharomyco./mycetaceae - ps23
Saccharomycetaceae - ps24
Saccharomycetaceae_2 - ps25
Saccharomycetaceae_3 - ps26
Kaz./Nau./Saccharomyces - ps27
Saccharomyces - ps28
Arboricola_clade - ps29
Cerevisiae_clade - ps30
Saccharomyces_cerevisiae - ps31
Phylostratum

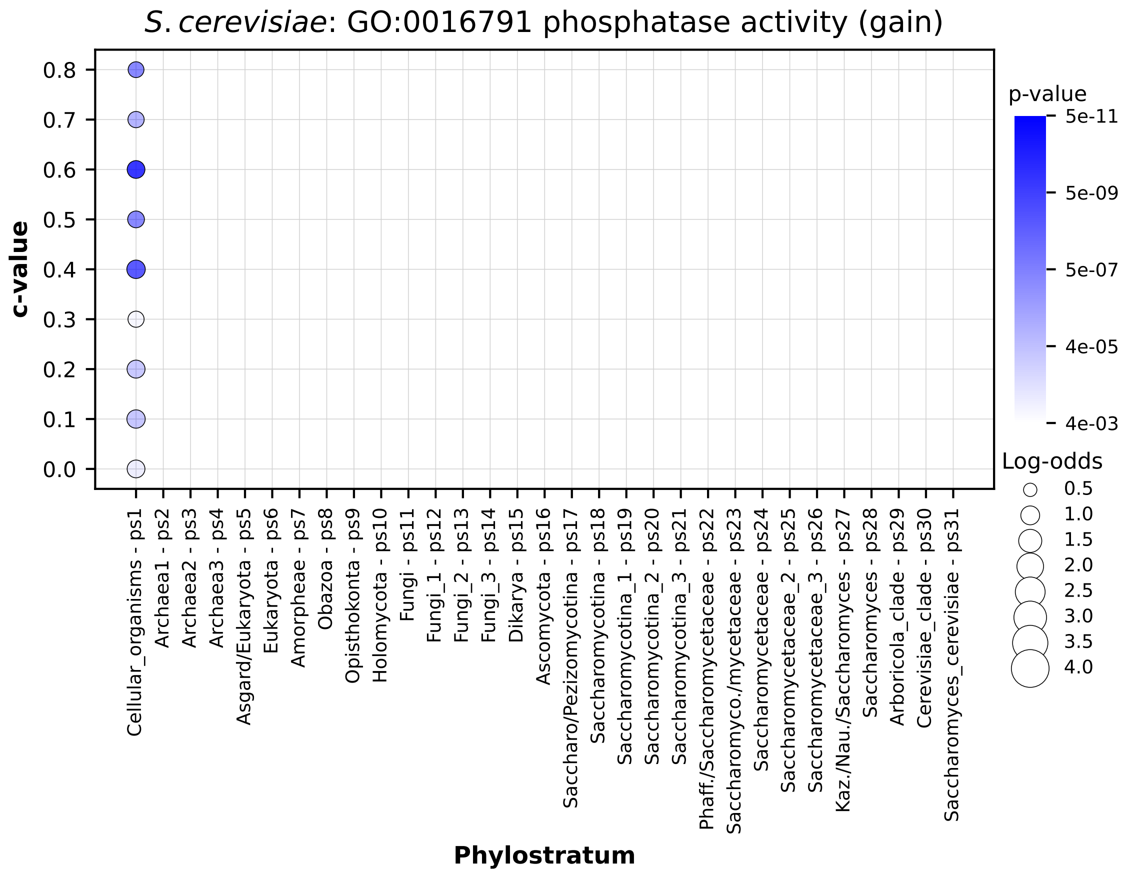
S. cerevisiae: GO:0016791 phosphatase activity (gain)
c-value
0.8
0.7
0.6
0.5
0.4
0.3
0.2
0.1
0.0
p-value
5e-11
5e-09
5e-07
4e-05
4e-03
Log-odds
0.5
1.0
1.5
2.0
2.5
3.0
3.5
4.0
Cellular_organisms - ps1
Archaea1 - ps2
Archaea2 - ps3
Archaea3 - ps4
Asgard/Eukaryota - ps5
Eukaryota - ps6
Amorpheae - ps7
Obazoa - ps8
Opisthokonta - ps9
Holomycota - ps10
Fungi - ps11
Fungi_1 - ps12
Fungi_2 - ps13
Fungi_3 - ps14
Dikarya - ps15
Ascomycota - ps16
Saccharo/Pezizomycotina - ps17
Saccharomycotina - ps18
Saccharomycotina_1 - ps19
Saccharomycotina_2 - ps20
Saccharomycotina_3 - ps21
Phaff./Saccharomycetaceae - ps22
Saccharomyco./mycetaceae - ps23
Saccharomycetaceae - ps24
Saccharomycetaceae_2 - ps25
Saccharomycetaceae_3 - ps26
Kaz./Nau./Saccharomyces - ps27
Saccharomyces - ps28
Arboricola_clade - ps29
Cerevisiae_clade - ps30
Saccharomyces_cerevisiae - ps31
Phylostratum

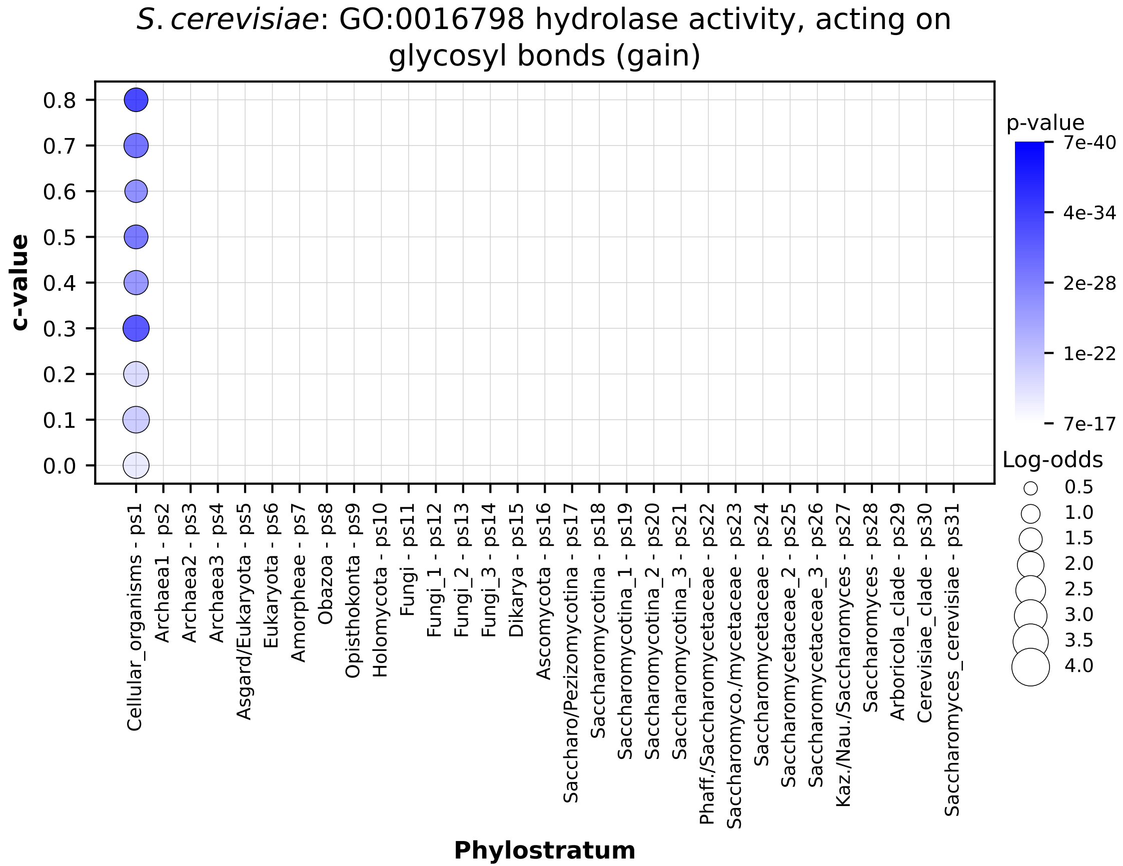
S. cerevisiae: GO:0016798 hydrolase activity, acting on glycosyl bonds (gain)
c-value
0.8
0.7
0.6
0.5
0.4
0.3
0.2
0.1
0.0
Phylostratum
Cellular_organisms - ps1
Archaea1 - ps2
Archaea2 - ps3
Archaea3 - ps4
Asgard/Eukaryota - ps5
Eukaryota - ps6
Amorpheae - ps7
Obazoa - ps8
Opisthokonta - ps9
Holomycota - ps10
Fungi - ps11
Fungi_1 - ps12
Fungi_2 - ps13
Fungi_3 - ps14
Dikarya - ps15
Ascomycota - ps16
Saccharo/Pezizomycotina - ps17
Saccharomycotina - ps18
Saccharomycotina_1 - ps19
Saccharomycotina_2 - ps20
Saccharomycotina_3 - ps21
Phaff./Saccharomycetaceae - ps22
Saccharomyco./mycetaceae - ps23
Saccharomycetaceae - ps24
Saccharomycetaceae_2 - ps25
Saccharomycetaceae_3 - ps26
Kaz./Nau./Saccharomyces - ps27
Saccharomyces - ps28
Arboricola_clade - ps29
Cerevisiae_clade - ps30
Saccharomyces_cerevisiae - ps31
p-value
7e-40
4e-34
2e-28
1e-22
7e-17
Log-odds
0.5
1.0
1.5
2.0
2.5
3.0
3.5
4.0

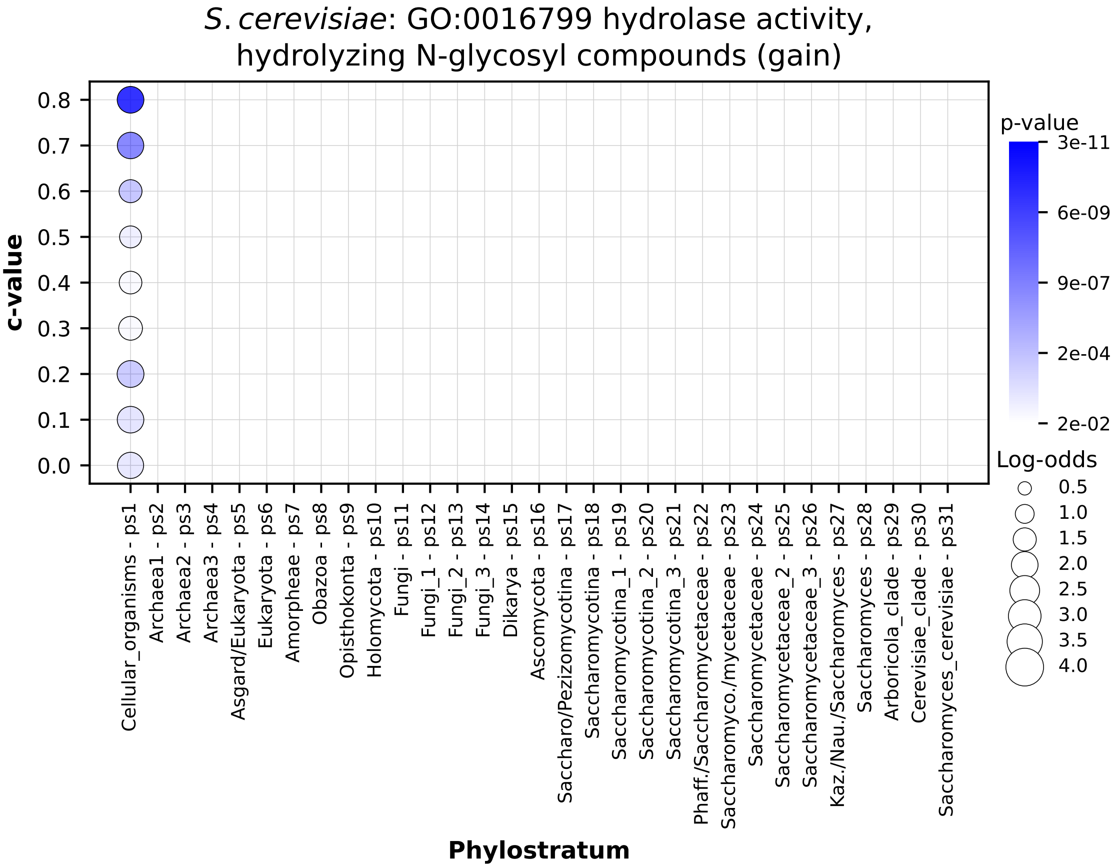
S. cerevisiae: GO:0016799 hydrolase activity, hydrolyzing N-glycosyl compounds (gain)
c-value
0.8
0.7
0.6
0.5
0.4
0.3
0.2
0.1
0.0
p-value
3e-11
6e-09
9e-07
2e-04
2e-02
Log-odds
0.5
1.0
1.5
2.0
2.5
3.0
3.5
4.0
Cellular_organisms - ps1
Archaea1 - ps2
Archaea2 - ps3
Archaea3 - ps4
Asgard/Eukaryota - ps5
Eukaryota - ps6
Amorpheae - ps7
Obazoa - ps8
Opisthokonta - ps9
Holomycota - ps10
Fungi - ps11
Fungi_1 - ps12
Fungi_2 - ps13
Fungi_3 - ps14
Dikarya - ps15
Ascomycota - ps16
Saccharo/Pezizomycotina - ps17
Saccharomycotina - ps18
Saccharomycotina_1 - ps19
Saccharomycotina_2 - ps20
Saccharomycotina_3 - ps21
Phaff./Saccharomycetaceae - ps22
Saccharomyco./mycetaceae - ps23
Saccharomycetaceae - ps24
Saccharomycetaceae_2 - ps25
Saccharomycetaceae_3 - ps26
Kaz./Nau./Saccharomyces - ps27
Saccharomyces - ps28
Arboricola_clade - ps29
Cerevisiae_clade - ps30
Saccharomyces_cerevisiae - ps31
Phylostratum

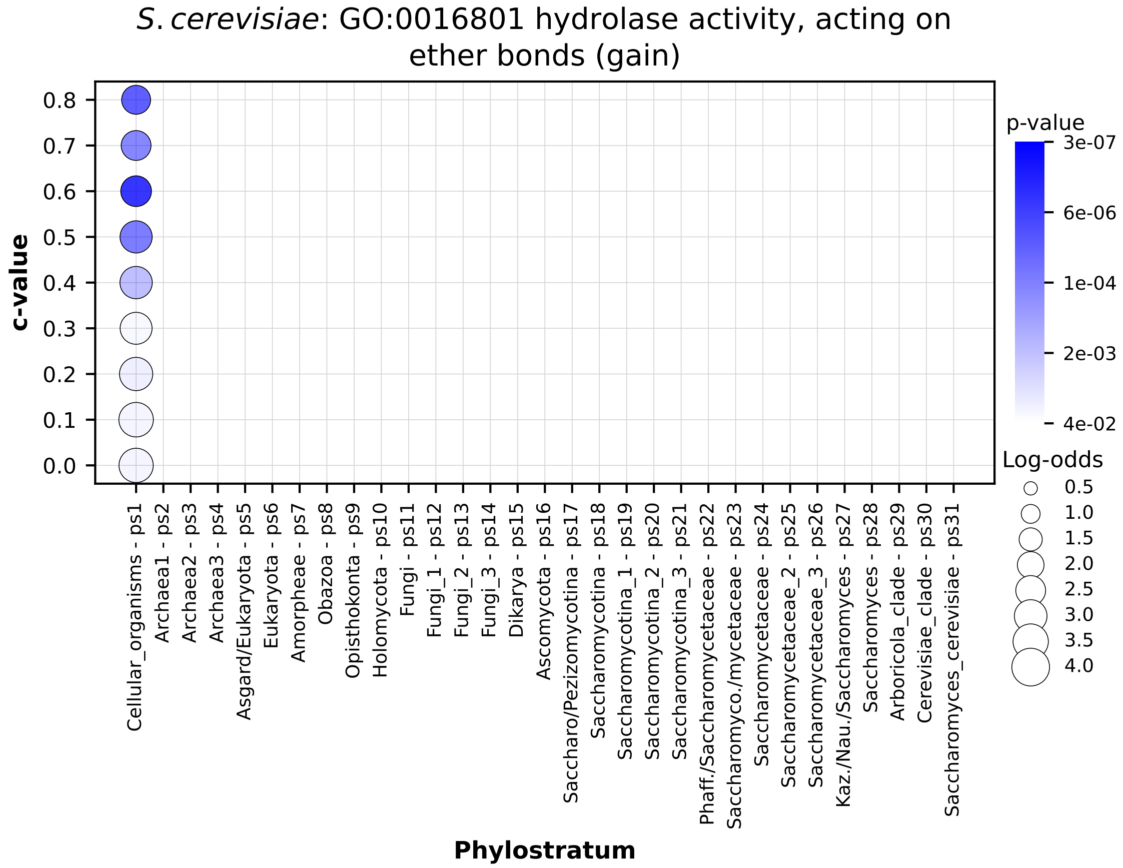
S. cerevisiae: GO:0016801 hydrolase activity, acting on ether bonds (gain)
c-value
0.8
0.7
0.6
0.5
0.4
0.3
0.2
0.1
0.0
p-value
3e-07
6e-06
1e-04
2e-03
4e-02
Log-odds
0.5
1.0
1.5
2.0
2.5
3.0
3.5
4.0
Cellular_organisms - ps1
Archaea1 - ps2
Archaea2 - ps3
Archaea3 - ps4
Asgard/Eukaryota - ps5
Eukaryota - ps6
Amorpheae - ps7
Obazoa - ps8
Opisthokonta - ps9
Holomycota - ps10
Fungi - ps11
Fungi_1 - ps12
Fungi_2 - ps13
Fungi_3 - ps14
Dikarya - ps15
Ascomycota - ps16
Saccharo/Pezizomycotina - ps17
Saccharomycotina - ps18
Saccharomycotina_1 - ps19
Saccharomycotina_2 - ps20
Saccharomycotina_3 - ps21
Phaff./Saccharomycetaceae - ps22
Saccharomyco./mycetaceae - ps23
Saccharomycetaceae - ps24
Saccharomycetaceae_2 - ps25
Saccharomycetaceae_3 - ps26
Kaz./Nau./Saccharomyces - ps27
Saccharomyces - ps28
Arboricola_clade - ps29
Cerevisiae_clade - ps30
Saccharomyces_cerevisiae - ps31
Phylostratum

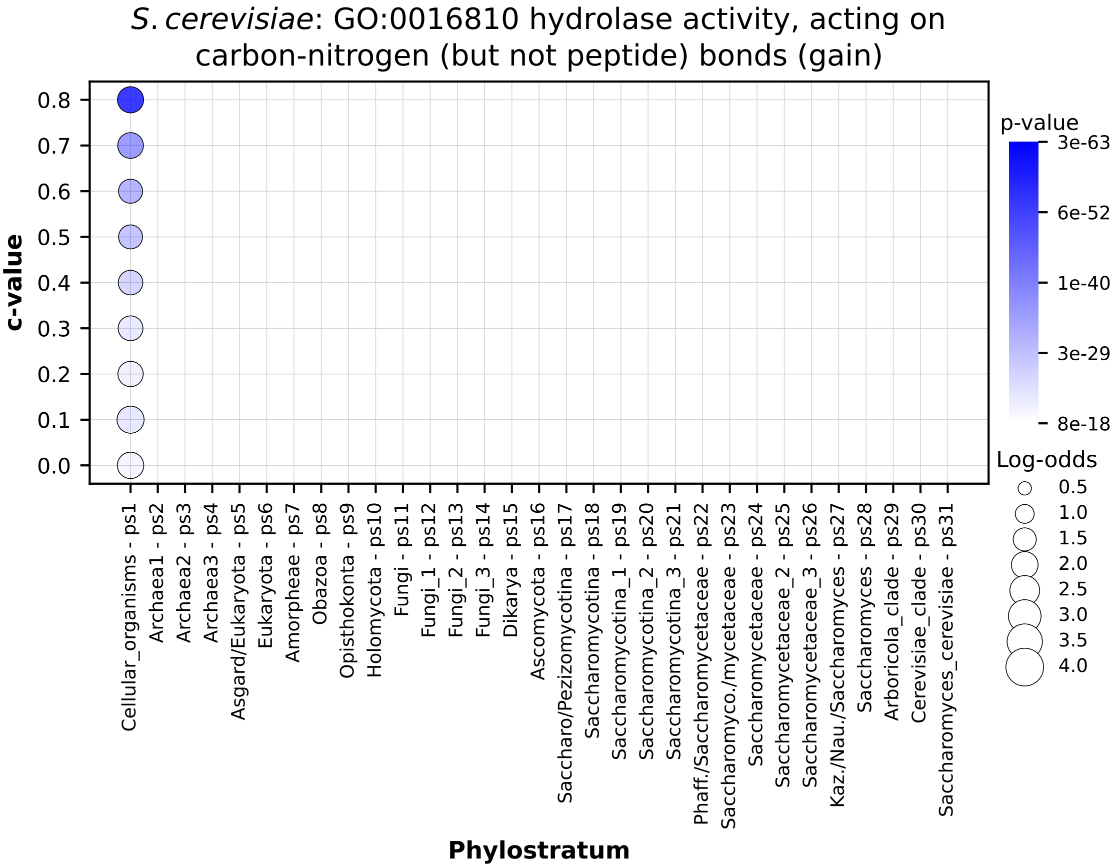
S. cerevisiae: GO:0016810 hydrolase activity, acting on carbon-nitrogen (but not peptide) bonds (gain)
c-value
0.8
0.7
0.6
0.5
0.4
0.3
0.2
0.1
0.0
p-value
3e-63
6e-52
1e-40
3e-29
8e-18
Log-odds
0.5
1.0
1.5
2.0
2.5
3.0
3.5
4.0
Cellular_organisms - ps1
Archaea1 - ps2
Archaea2 - ps3
Archaea3 - ps4
Asgard/Eukaryota - ps5
Eukaryota - ps6
Amorpheae - ps7
Obazoa - ps8
Opisthokonta - ps9
Holomycota - ps10
Fungi - ps11
Fungi_1 - ps12
Fungi_2 - ps13
Fungi_3 - ps14
Dikarya - ps15
Ascomycota - ps16
Saccharo/Pezizomycotina - ps17
Saccharomycotina - ps18
Saccharomycotina_1 - ps19
Saccharomycotina_2 - ps20
Saccharomycotina_3 - ps21
Phaff./Saccharomycetaceae - ps22
Saccharomyco./mycetaceae - ps23
Saccharomycetaceae - ps24
Saccharomycetaceae_2 - ps25
Saccharomycetaceae_3 - ps26
Kaz./Nau./Saccharomyces - ps27
Saccharomyces - ps28
Arboricola_clade - ps29
Cerevisiae_clade - ps30
Saccharomyces_cerevisiae - ps31
Phylostratum

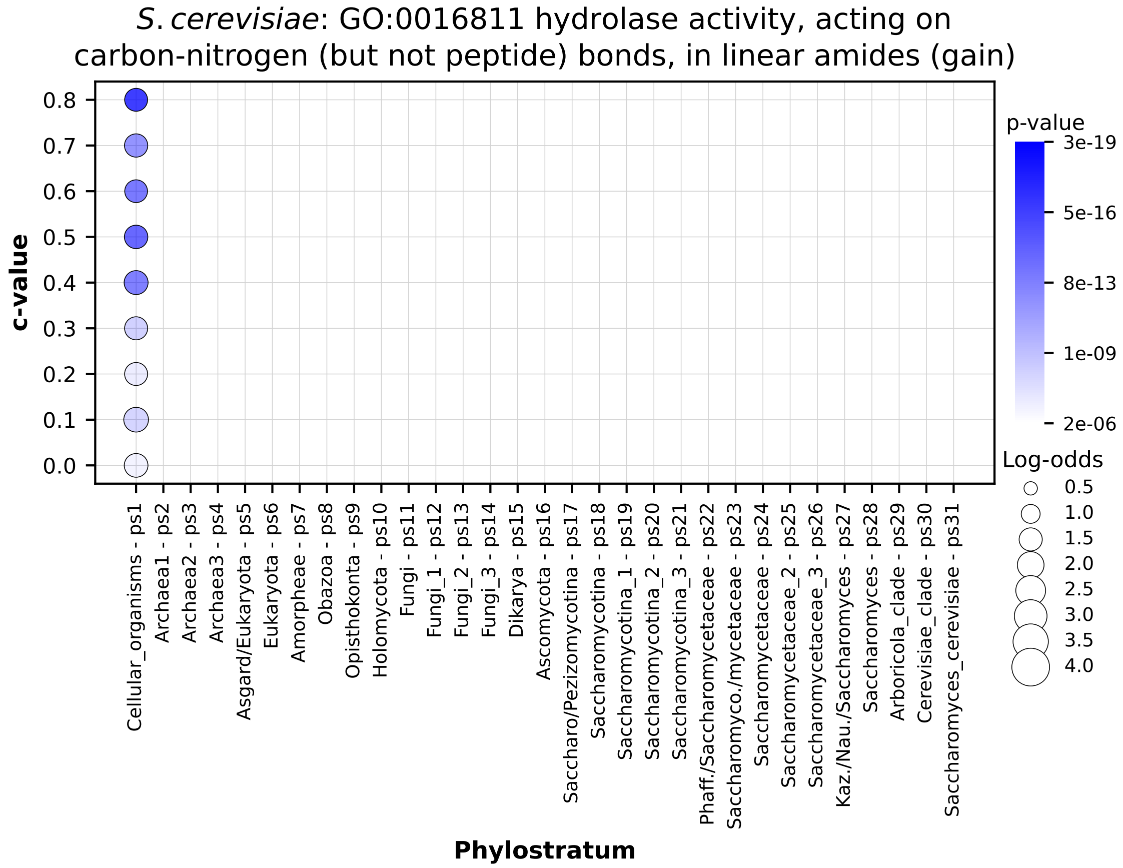
S. cerevisiae: GO:0016811 hydrolase activity, acting on carbon-nitrogen (but not peptide) bonds, in linear amides (gain)
c-value
0.0
0.1
0.2
0.3
0.4
0.5
0.6
0.7
0.8
p-value
3e-19
5e-16
8e-13
1e-09
2e-06
Log-odds
0.5
1.0
1.5
2.0
2.5
3.0
3.5
4.0
Cellular_organisms - ps1
Archaea1 - ps2
Archaea2 - ps3
Archaea3 - ps4
Asgard/Eukaryota - ps5
Eukaryota - ps6
Amorpheae - ps7
Obazoa - ps8
Opisthokonta - ps9
Holomycota - ps10
Fungi - ps11
Fungi_1 - ps12
Fungi_2 - ps13
Fungi_3 - ps14
Dikarya - ps15
Ascomycota - ps16
Saccharo/Pezizomycotina - ps17
Saccharomycotina - ps18
Saccharomycotina_1 - ps19
Saccharomycotina_2 - ps20
Saccharomycotina_3 - ps21
Phaff./Saccharomycetaceae - ps22
Saccharomyco./mycetaceae - ps23
Saccharomycetaceae - ps24
Saccharomycetaceae_2 - ps25
Saccharomycetaceae_3 - ps26
Kaz./Nau./Saccharomyces - ps27
Saccharomyces - ps28
Arboricola_clade - ps29
Cerevisiae_clade - ps30
Saccharomyces_cerevisiae - ps31
Phylostratum

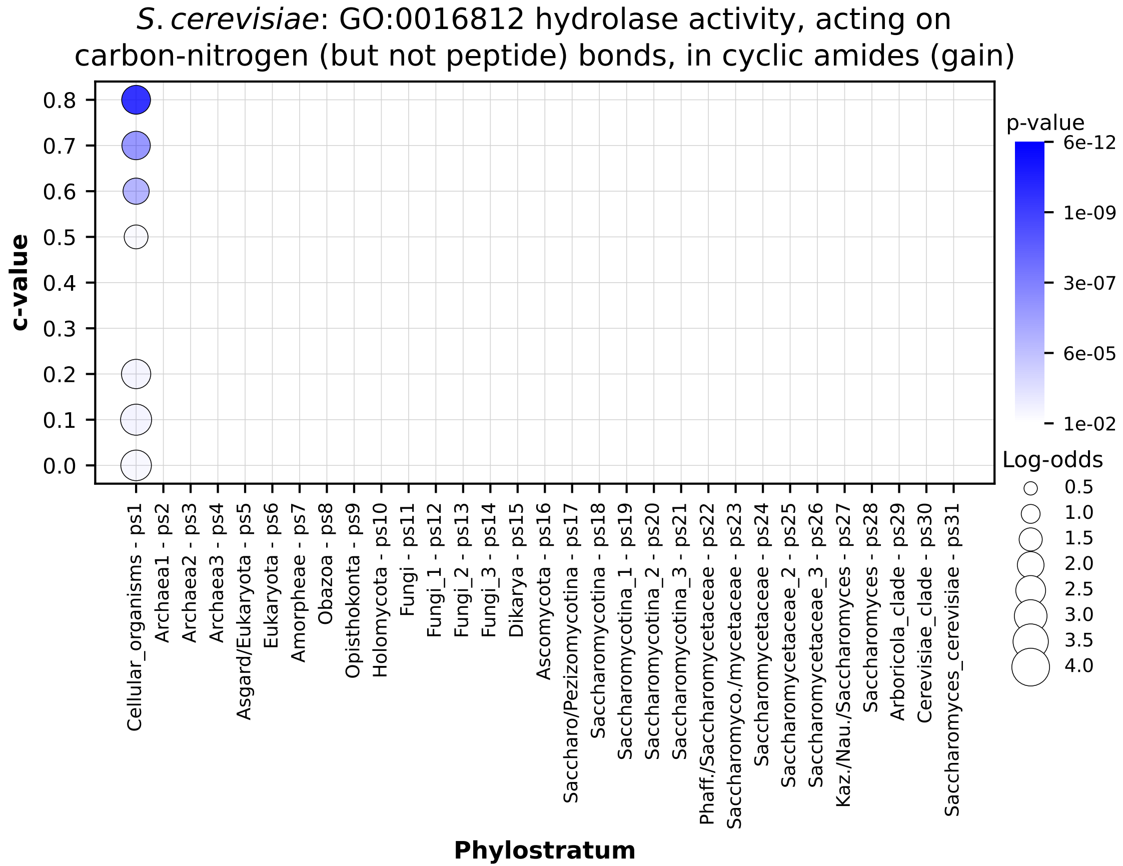

S. cerevisiae: GO:0016812 hydrolase activity, acting on carbon-nitrogen (but not peptide) bonds, in cyclic amides (gain)
c-value
0.8
0.7
0.6
0.5
0.4
0.3
0.2
0.1
0.0
p-value
6e-12
1e-09
3e-07
6e-05
1e-02
Log-odds
0.5
1.0
1.5
2.0
2.5
3.0
3.5
4.0
Cellular_organisms - ps1
Archaea1 - ps2
Archaea2 - ps3
Archaea3 - ps4
Asgard/Eukaryota - ps5
Eukaryota - ps6
Amorpheae - ps7
Obazoa - ps8
Opisthokonta - ps9
Holomycota - ps10
Fungi - ps11
Fungi_1 - ps12
Fungi_2 - ps13
Fungi_3 - ps14
Dikarya - ps15
Ascomycota - ps16
Saccharo/Pezizomycotina - ps17
Saccharomycotina - ps18
Saccharomycotina_1 - ps19
Saccharomycotina_2 - ps20
Saccharomycotina_3 - ps21
Phaff./Saccharomycetaceae - ps22
Saccharomyco./mycetaceae - ps23
Saccharomycetaceae - ps24
Saccharomycetaceae_2 - ps25
Saccharomycetaceae_3 - ps26
Kaz./Nau./Saccharomyces - ps27
Saccharomyces - ps28
Arboricola_clade - ps29
Cerevisiae_clade - ps30
Saccharomyces_cerevisiae - ps31
Phylostratum

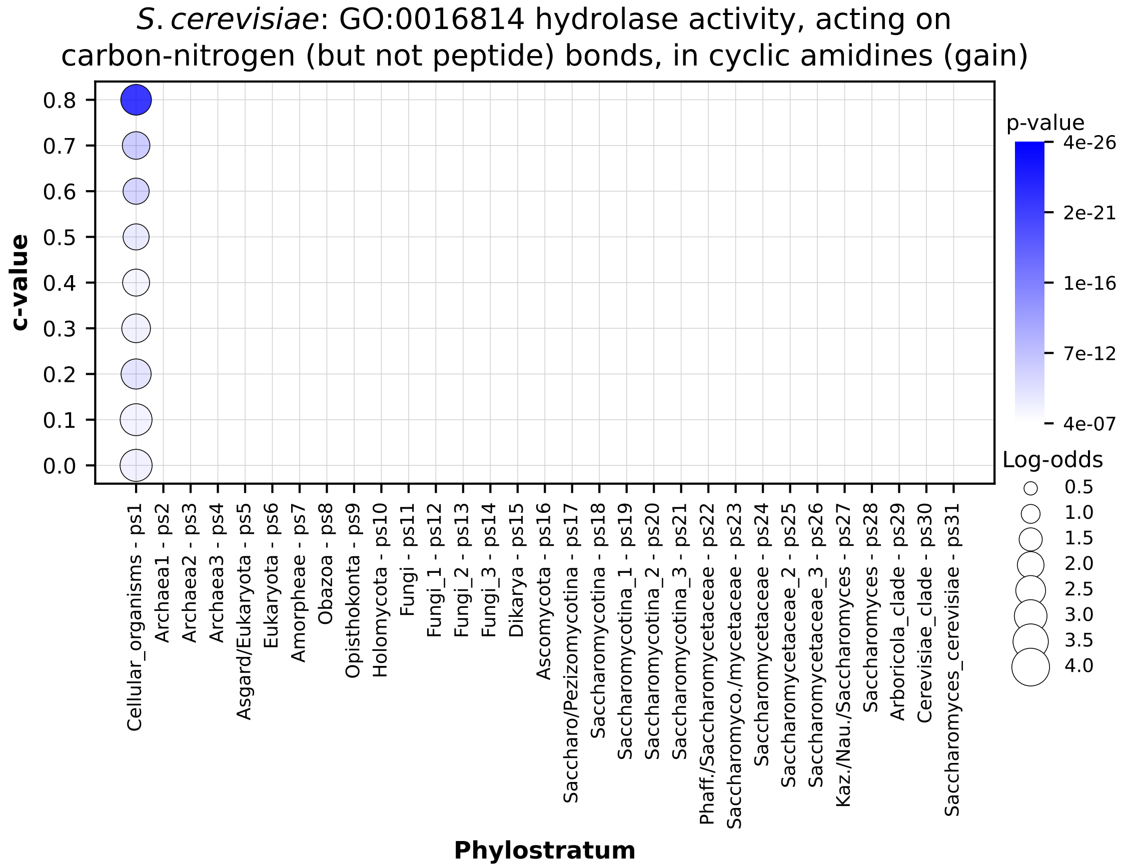

S. cerevisiae: GO:0016814 hydrolase activity, acting on carbon-nitrogen (but not peptide) bonds, in cyclic amidines (gain)
c-value
0.8
0.7
0.6
0.5
0.4
0.3
0.2
0.1
0.0
p-value
4e-26
2e-21
1e-16
7e-12
4e-07
Log-odds
0.5
1.0
1.5
2.0
2.5
3.0
3.5
4.0
Cellular_organisms - ps1
Archaea1 - ps2
Archaea2 - ps3
Archaea3 - ps4
Asgard/Eukaryota - ps5
Eukaryota - ps6
Amorpheae - ps7
Obazoa - ps8
Opisthokonta - ps9
Holomycota - ps10
Fungi - ps11
Fungi_1 - ps12
Fungi_2 - ps13
Fungi_3 - ps14
Dikarya - ps15
Ascomycota - ps16
Saccharo/Pezizomycotina - ps17
Saccharomycotina - ps18
Saccharomycotina_1 - ps19
Saccharomycotina_2 - ps20
Saccharomycotina_3 - ps21
Phaff./Saccharomycetaceae - ps22
Saccharomyco./mycetaceae - ps23
Saccharomycetaceae - ps24
Saccharomycetaceae_2 - ps25
Saccharomycetaceae_3 - ps26
Kaz./Nau./Saccharomyces - ps27
Saccharomyces - ps28
Arboricola_clade - ps29
Cerevisiae_clade - ps30
Saccharomyces_cerevisiae - ps31
Phylostratum

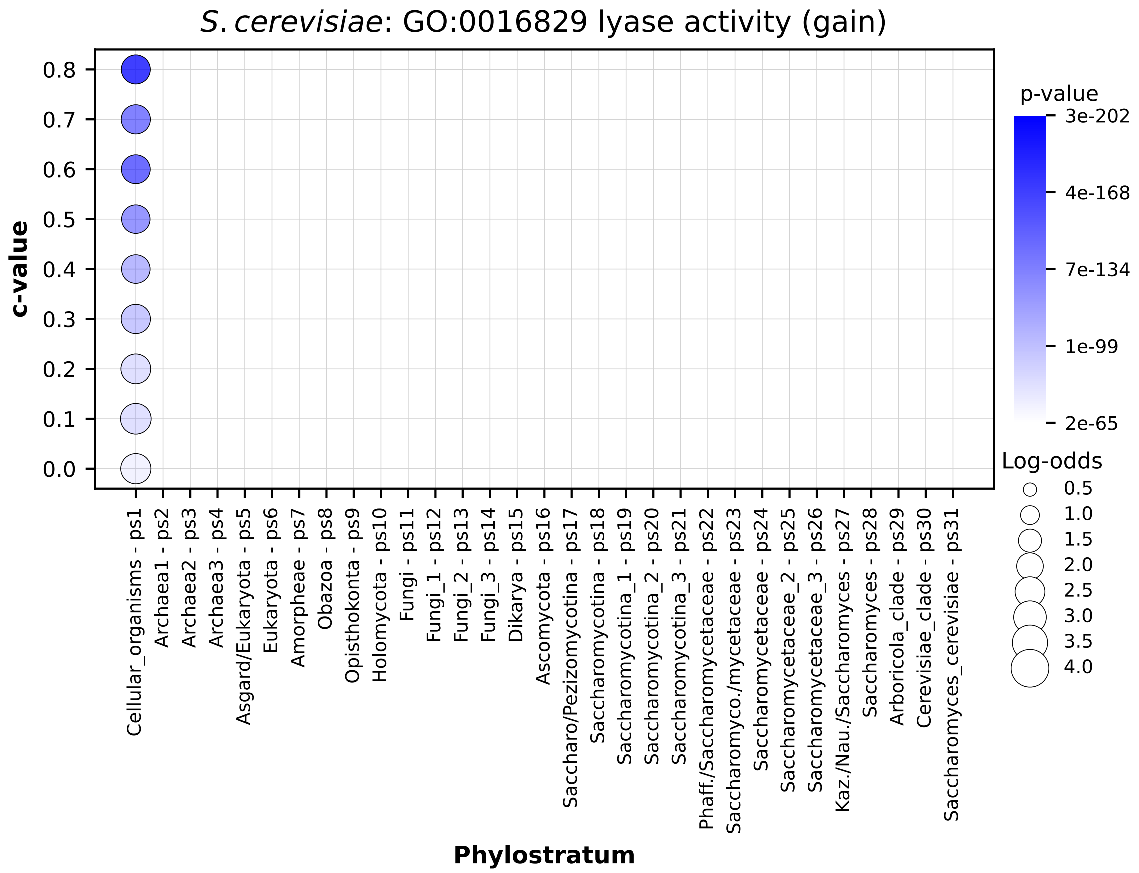
S. cerevisiae: GO:0016829 lyase activity (gain)
c-value
0.8
0.7
0.6
0.5
0.4
0.3
0.2
0.1
0.0
p-value
3e-202
4e-168
7e-134
1e-99
2e-65
Log-odds
0.5
1.0
1.5
2.0
2.5
3.0
3.5
4.0
Cellular_organisms - ps1
Archaea1 - ps2
Archaea2 - ps3
Archaea3 - ps4
Asgard/Eukaryota - ps5
Eukaryota - ps6
Amorpheae - ps7
Obazoa - ps8
Opisthokonta - ps9
Holomycota - ps10
Fungi - ps11
Fungi_1 - ps12
Fungi_2 - ps13
Fungi_3 - ps14
Dikarya - ps15
Ascomycota - ps16
Saccharo/Pezizomycotina - ps17
Saccharomycotina - ps18
Saccharomycotina_1 - ps19
Saccharomycotina_2 - ps20
Saccharomycotina_3 - ps21
Phaff./Saccharomycetaceae - ps22
Saccharomyco./mycetaceae - ps23
Saccharomycetaceae - ps24
Saccharomycetaceae_2 - ps25
Saccharomycetaceae_3 - ps26
Kaz./Nau./Saccharomyces - ps27
Saccharomyces - ps28
Arboricola_clade - ps29
Cerevisiae_clade - ps30
Saccharomyces_cerevisiae - ps31
Phylostratum

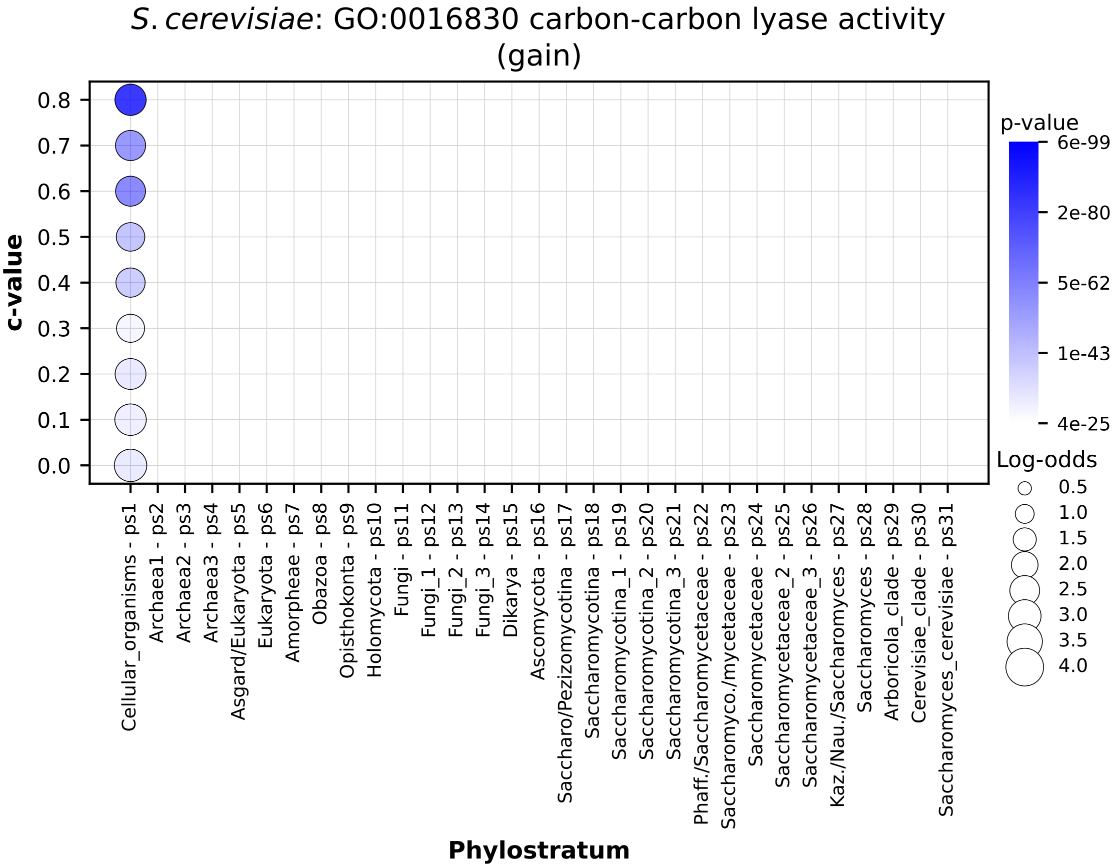

S. cerevisiae: GO:0016830 carbon-carbon lyase activity (gain)
c-value
0.0
0.1
0.2
0.3
0.4
0.5
0.6
0.7
0.8
Phylostratum
Cellular_organisms - ps1
Archaea1 - ps2
Archaea2 - ps3
Archaea3 - ps4
Asgard/Eukaryota - ps5
Eukaryota - ps6
Amorpheae - ps7
Obazoa - ps8
Opisthokonta - ps9
Holomycota - ps10
Fungi - ps11
Fungi_1 - ps12
Fungi_2 - ps13
Fungi_3 - ps14
Dikarya - ps15
Ascomycota - ps16
Saccharo/Pezizomycotina - ps17
Saccharomycotina - ps18
Saccharomycotina_1 - ps19
Saccharomycotina_2 - ps20
Saccharomycotina_3 - ps21
Phaff./Saccharomycetaceae - ps22
Saccharomyco./mycetaceae - ps23
Saccharomycetaceae - ps24
Saccharomycetaceae_2 - ps25
Saccharomycetaceae_3 - ps26
Kaz./Nau./Saccharomyces - ps27
Saccharomyces - ps28
Arboricola_clade - ps29
Cerevisiae_clade - ps30
Saccharomyces_cerevisiae - ps31
p-value
6e-99
2e-80
5e-62
1e-43
4e-25
Log-odds
0.5
1.0
1.5
2.0
2.5
3.0
3.5
4.0

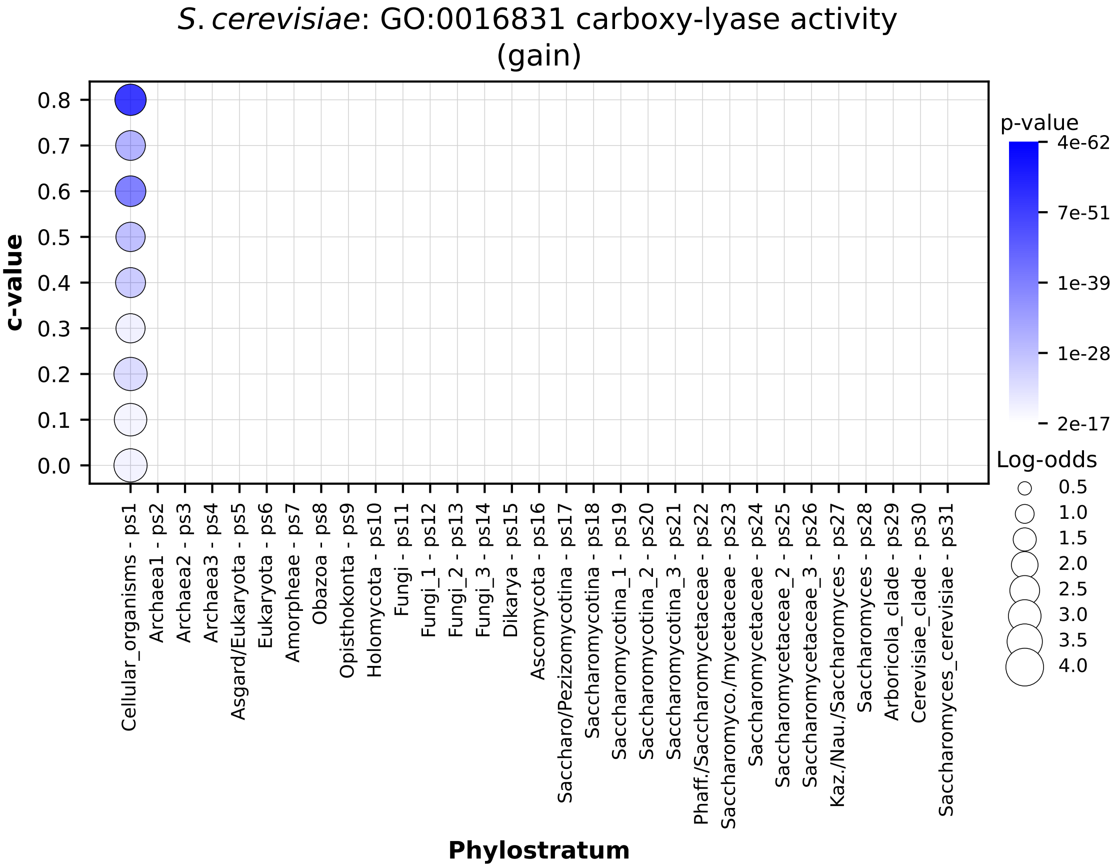

S. cerevisiae: GO:0016831 carboxy-lyase activity
(gain)
c-value
0.0
0.1
0.2
0.3
0.4
0.5
0.6
0.7
0.8
p-value
4e-62
7e-51
1e-39
1e-28
2e-17
Log-odds
0.5
1.0
1.5
2.0
2.5
3.0
3.5
4.0
Cellular_organisms - ps1
Archaea1 - ps2
Archaea2 - ps3
Archaea3 - ps4
Asgard/Eukaryota - ps5
Eukaryota - ps6
Amorpheae - ps7
Obazoa - ps8
Opisthokonta - ps9
Holomycota - ps10
Fungi - ps11
Fungi_1 - ps12
Fungi_2 - ps13
Fungi_3 - ps14
Dikarya - ps15
Ascomycota - ps16
Saccharo/Pezizomycotina - ps17
Saccharomycotina - ps18
Saccharomycotina_1 - ps19
Saccharomycotina_2 - ps20
Saccharomycotina_3 - ps21
Phaff./Saccharomycetaceae - ps22
Saccharomyco./mycetaceae - ps23
Saccharomycetaceae - ps24
Saccharomycetaceae_2 - ps25
Saccharomycetaceae_3 - ps26
Kaz./Nau./Saccharomyces - ps27
Saccharomyces - ps28
Arboricola_clade - ps29
Cerevisiae_clade - ps30
Saccharomyces_cerevisiae - ps31
Phylostratum

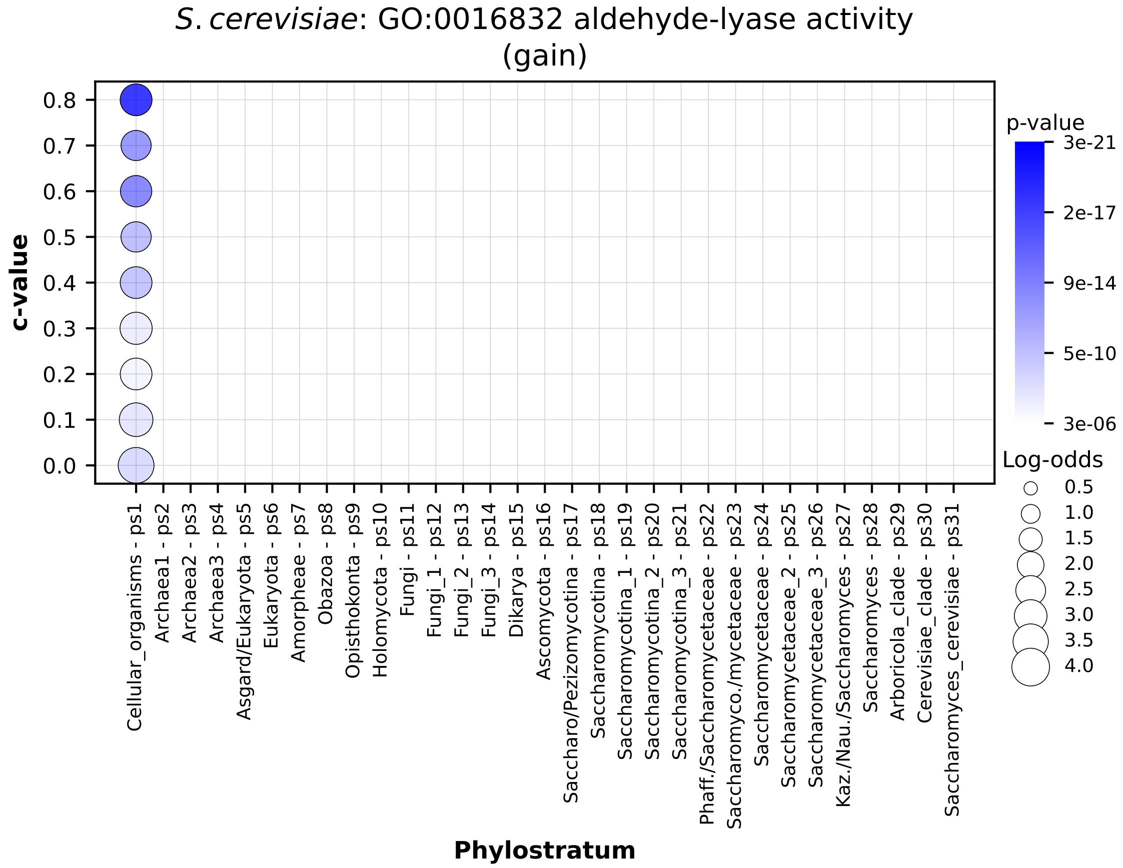

S. cerevisiae: GO:0016832 aldehyde-lyase activity
(gain)
c-value
0.0
0.1
0.2
0.3
0.4
0.5
0.6
0.7
0.8
p-value
3e-21
2e-17
9e-14
5e-10
3e-06
Log-odds
0.5
1.0
1.5
2.0
2.5
3.0
3.5
4.0
Cellular_organisms - ps1
Archaea1 - ps2
Archaea2 - ps3
Archaea3 - ps4
Asgard/Eukaryota - ps5
Eukaryota - ps6
Amorpheae - ps7
Obazoa - ps8
Opisthokonta - ps9
Holomycota - ps10
Fungi - ps11
Fungi_1 - ps12
Fungi_2 - ps13
Fungi_3 - ps14
Dikarya - ps15
Ascomycota - ps16
Saccharo/Pezizomycotina - ps17
Saccharomycotina - ps18
Saccharomycotina_1 - ps19
Saccharomycotina_2 - ps20
Saccharomycotina_3 - ps21
Phaff./Saccharomycetaceae - ps22
Saccharomyco./mycetaceae - ps23
Saccharomycetaceae - ps24
Saccharomycetaceae_2 - ps25
Saccharomycetaceae_3 - ps26
Kaz./Nau./Saccharomyces - ps27
Saccharomyces - ps28
Arboricola_clade - ps29
Cerevisiae_clade - ps30
Saccharomyces_cerevisiae - ps31
Phylostratum

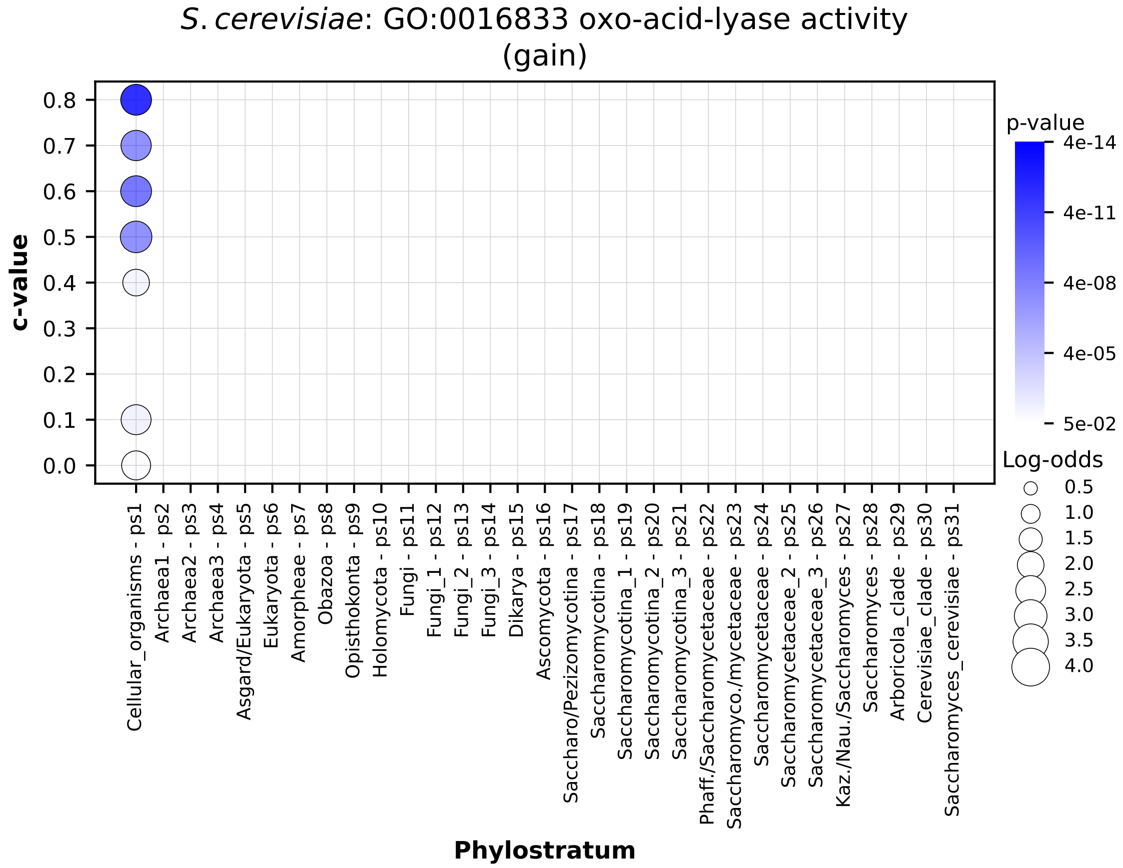

S. cerevisiae: GO:0016833 oxo-acid-lyase activity (gain)
c-value
0.8
0.7
0.6
0.5
0.4
0.3
0.2
0.1
0.0
p-value
4e-14
4e-11
4e-08
4e-05
5e-02
Log-odds
0.5
1.0
1.5
2.0
2.5
3.0
3.5
4.0
Cellular_organisms - ps1
Archaea1 - ps2
Archaea2 - ps3
Archaea3 - ps4
Asgard/Eukaryota - ps5
Eukaryota - ps6
Amorpheae - ps7
Obazoa - ps8
Opisthokonta - ps9
Holomycota - ps10
Fungi - ps11
Fungi_1 - ps12
Fungi_2 - ps13
Fungi_3 - ps14
Dikarya - ps15
Ascomycota - ps16
Saccharo/Pezizomycotina - ps17
Saccharomycotina - ps18
Saccharomycotina_1 - ps19
Saccharomycotina_2 - ps20
Saccharomycotina_3 - ps21
Phaff./Saccharomycetaceae - ps22
Saccharomyco./mycetaceae - ps23
Saccharomycetaceae - ps24
Saccharomycetaceae_2 - ps25
Saccharomycetaceae_3 - ps26
Kaz./Nau./Saccharomyces - ps27
Saccharomyces - ps28
Arboricola_clade - ps29
Cerevisiae_clade - ps30
Saccharomyces_cerevisiae - ps31
Phylostratum

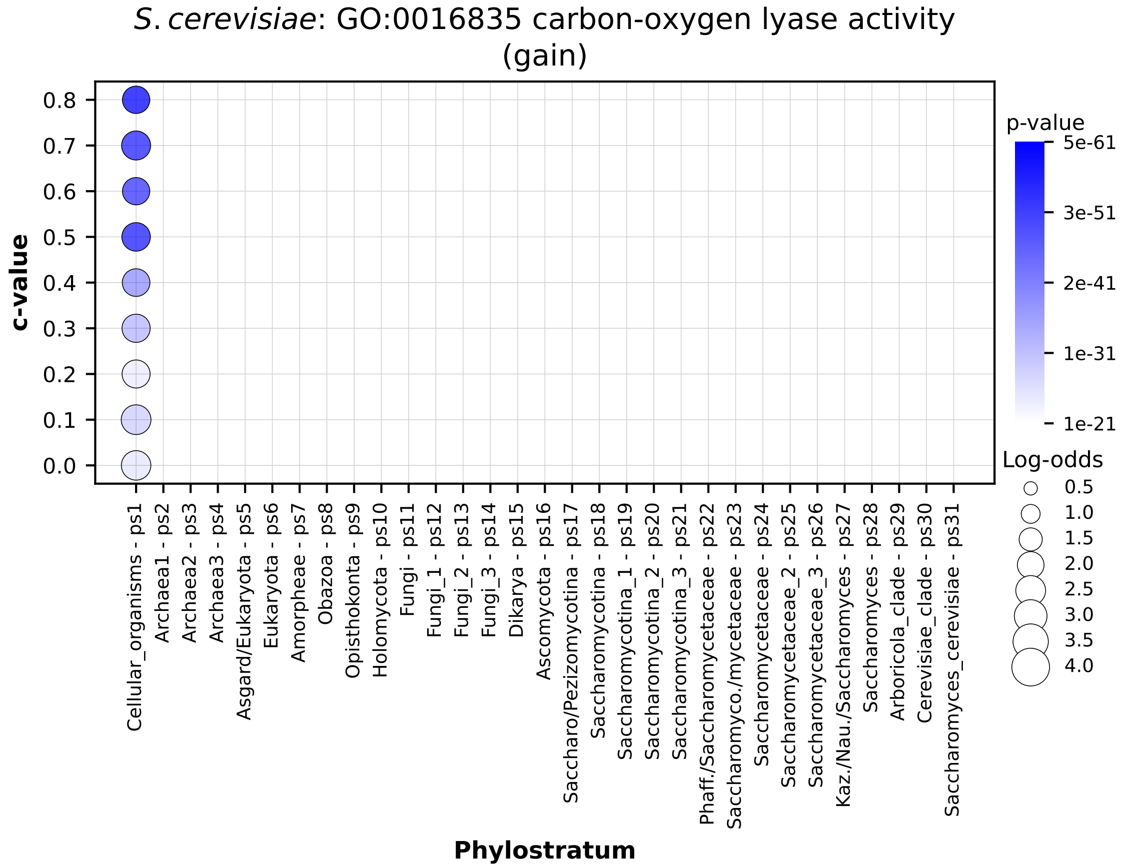
S. cerevisiae: GO:0016835 carbon-oxygen lyase activity
(gain)
c-value
0.8
0.7
0.6
0.5
0.4
0.3
0.2
0.1
0.0
p-value
5e-61
3e-51
2e-41
1e-31
1e-21
Log-odds
0.5
1.0
1.5
2.0
2.5
3.0
3.5
4.0
Cellular_organisms - ps1
Archaea1 - ps2
Archaea2 - ps3
Archaea3 - ps4
Asgard/Eukaryota - ps5
Eukaryota - ps6
Amorpheae - ps7
Obazoa - ps8
Opisthokonta - ps9
Holomycota - ps10
Fungi - ps11
Fungi_1 - ps12
Fungi_2 - ps13
Fungi_3 - ps14
Dikarya - ps15
Ascomycota - ps16
Saccharo/Pezizomycotina - ps17
Saccharomycotina - ps18
Saccharomycotina_1 - ps19
Saccharomycotina_2 - ps20
Saccharomycotina_3 - ps21
Phaff./Saccharomycetaceae - ps22
Saccharomyco./mycetaceae - ps23
Saccharomycetaceae - ps24
Saccharomycetaceae_2 - ps25
Saccharomycetaceae_3 - ps26
Kaz./Nau./Saccharomyces - ps27
Saccharomyces - ps28
Arboricola_clade - ps29
Cerevisiae_clade - ps30
Saccharomyces_cerevisiae - ps31
Phylostratum

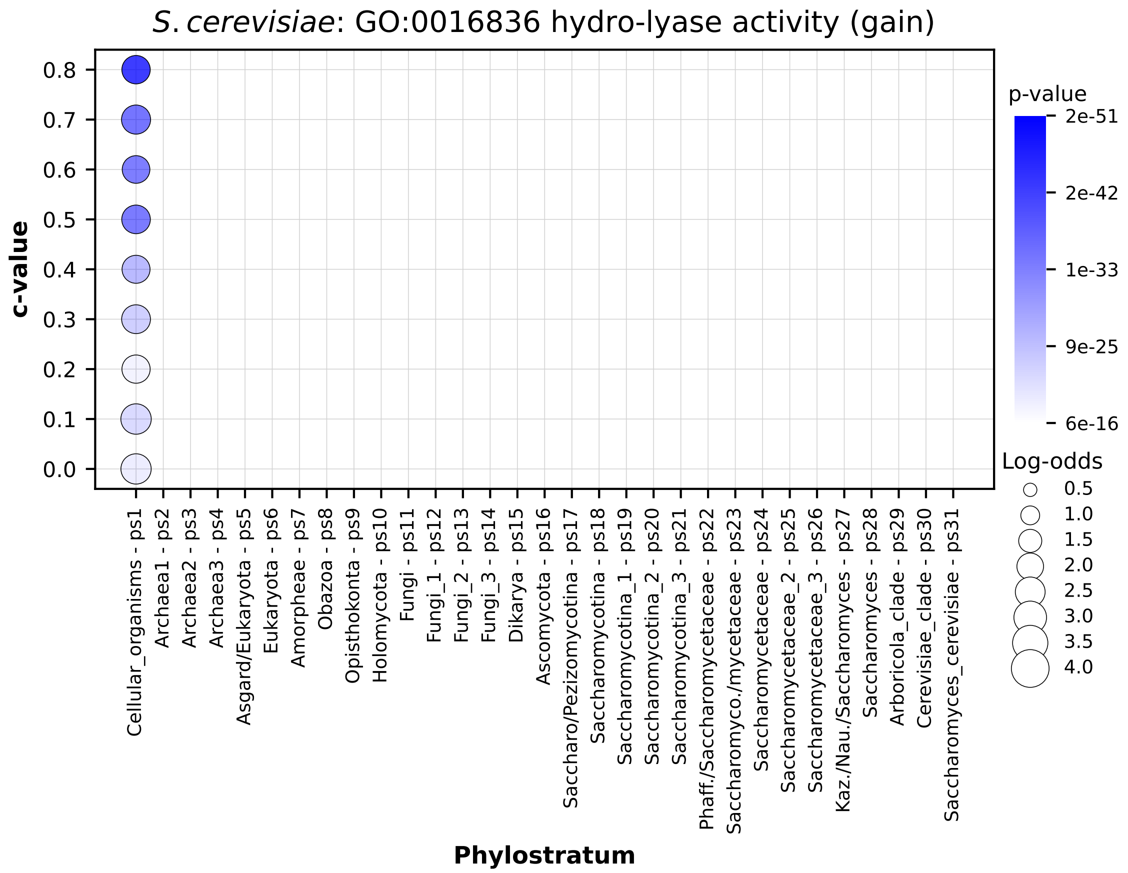

S. cerevisiae: GO:0016836 hydro-lyase activity (gain)
c-value
0.8
0.7
0.6
0.5
0.4
0.3
0.2
0.1
0.0
p-value
2e-51
2e-42
1e-33
9e-25
6e-16
Log-odds
0.5
1.0
1.5
2.0
2.5
3.0
3.5
4.0
Cellular_organisms - ps1
Archaea1 - ps2
Archaea2 - ps3
Archaea3 - ps4
Asgard/Eukaryota - ps5
Eukaryota - ps6
Amorpheae - ps7
Obazoa - ps8
Opisthokonta - ps9
Holomycota - ps10
Fungi - ps11
Fungi_1 - ps12
Fungi_2 - ps13
Fungi_3 - ps14
Dikarya - ps15
Ascomycota - ps16
Saccharo/Pezizomycotina - ps17
Saccharomycotina - ps18
Saccharomycotina_1 - ps19
Saccharomycotina_2 - ps20
Saccharomycotina_3 - ps21
Phaff./Saccharomycetaceae - ps22
Saccharomyco./mycetaceae - ps23
Saccharomycetaceae - ps24
Saccharomycetaceae_2 - ps25
Saccharomycetaceae_3 - ps26
Kaz./Nau./Saccharomyces - ps27
Saccharomyces - ps28
Arboricola_clade - ps29
Cerevisiae_clade - ps30
Saccharomyces_cerevisiae - ps31
Phylostratum

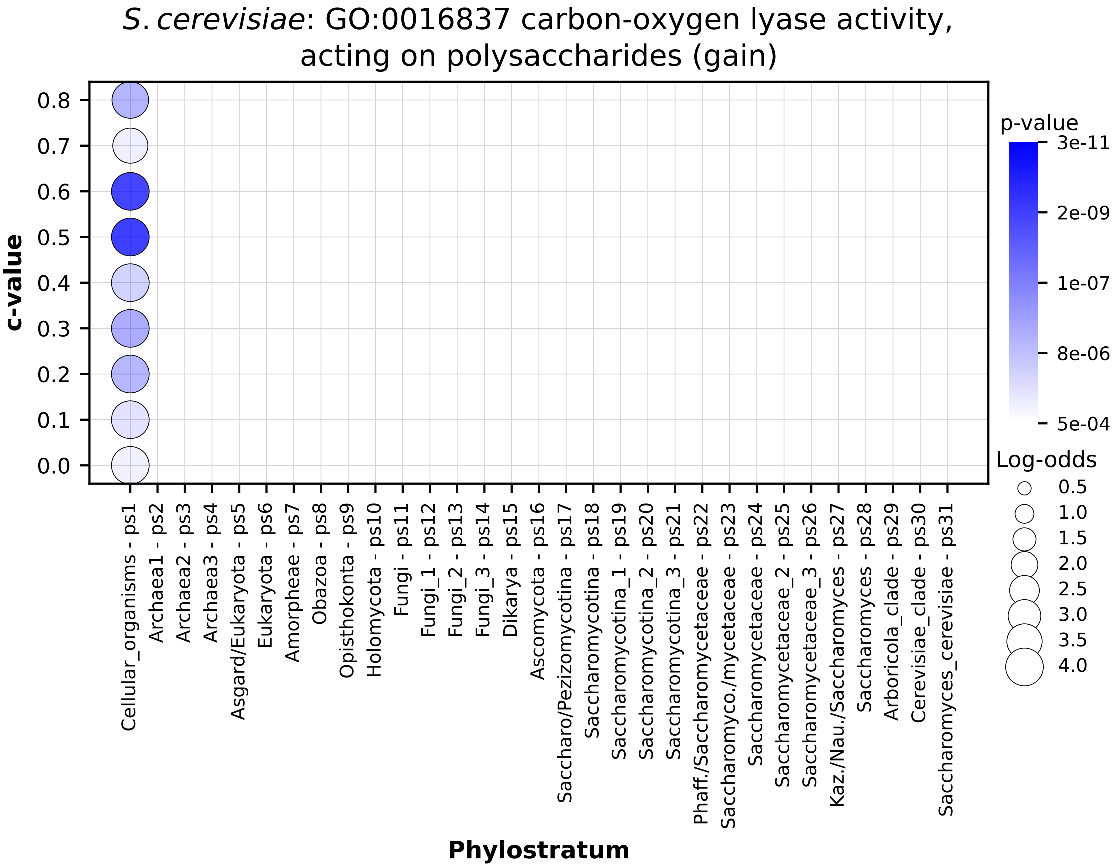
S. cerevisiae: GO:0016837 carbon-oxygen lyase activity, acting on polysaccharides (gain)
c-value
0.0
0.1
0.2
0.3
0.4
0.5
0.6
0.7
0.8
p-value
3e-11
2e-09
1e-07
8e-06
5e-04
Log-odds
0.5
1.0
1.5
2.0
2.5
3.0
3.5
4.0
Cellular_organisms - ps1
Archaea1 - ps2
Archaea2 - ps3
Archaea3 - ps4
Asgard/Eukaryota - ps5
Eukaryota - ps6
Amorpheae - ps7
Obazoa - ps8
Opisthokonta - ps9
Holomycota - ps10
Fungi - ps11
Fungi_1 - ps12
Fungi_2 - ps13
Fungi_3 - ps14
Dikarya - ps15
Ascomycota - ps16
Saccharo/Pezizomycotina - ps17
Saccharomycotina - ps18
Saccharomycotina_1 - ps19
Saccharomycotina_2 - ps20
Saccharomycotina_3 - ps21
Phaff./Saccharomycetaceae - ps22
Saccharomyco./mycetaceae - ps23
Saccharomycetaceae - ps24
Saccharomycetaceae_2 - ps25
Saccharomycetaceae_3 - ps26
Kaz./Nau./Saccharomyces - ps27
Saccharomyces - ps28
Arboricola_clade - ps29
Cerevisiae_clade - ps30
Saccharomyces_cerevisiae - ps31
Phylostratum

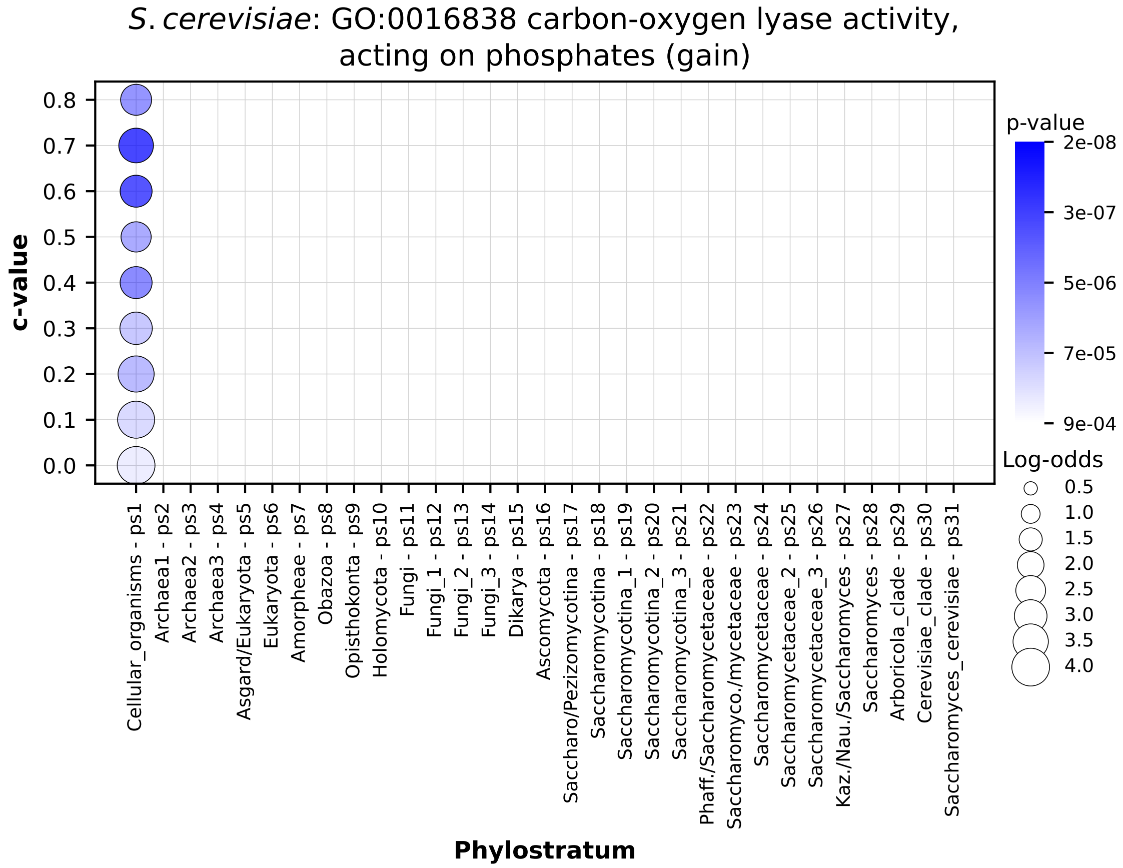

S. cerevisiae: GO:0016838 carbon-oxygen lyase activity, acting on phosphates (gain)
c-value
0.8
0.7
0.6
0.5
0.4
0.3
0.2
0.1
0.0
p-value
2e-08
3e-07
5e-06
7e-05
9e-04
Log-odds
0.5
1.0
1.5
2.0
2.5
3.0
3.5
4.0
Cellular_organisms - ps1
Archaea1 - ps2
Archaea2 - ps3
Archaea3 - ps4
Asgard/Eukaryota - ps5
Eukaryota - ps6
Amorpheae - ps7
Obazoa - ps8
Opisthokonta - ps9
Holomycota - ps10
Fungi - ps11
Fungi_1 - ps12
Fungi_2 - ps13
Fungi_3 - ps14
Dikarya - ps15
Ascomycota - ps16
Saccharo/Pezizomycotina - ps17
Saccharomycotina - ps18
Saccharomycotina_1 - ps19
Saccharomycotina_2 - ps20
Saccharomycotina_3 - ps21
Phaff./Saccharomycetaceae - ps22
Saccharomyco./mycetaceae - ps23
Saccharomycetaceae - ps24
Saccharomycetaceae_2 - ps25
Saccharomycetaceae_3 - ps26
Kaz./Nau./Saccharomyces - ps27
Saccharomyces - ps28
Arboricola_clade - ps29
Cerevisiae_clade - ps30
Saccharomyces_cerevisiae - ps31
Phylostratum

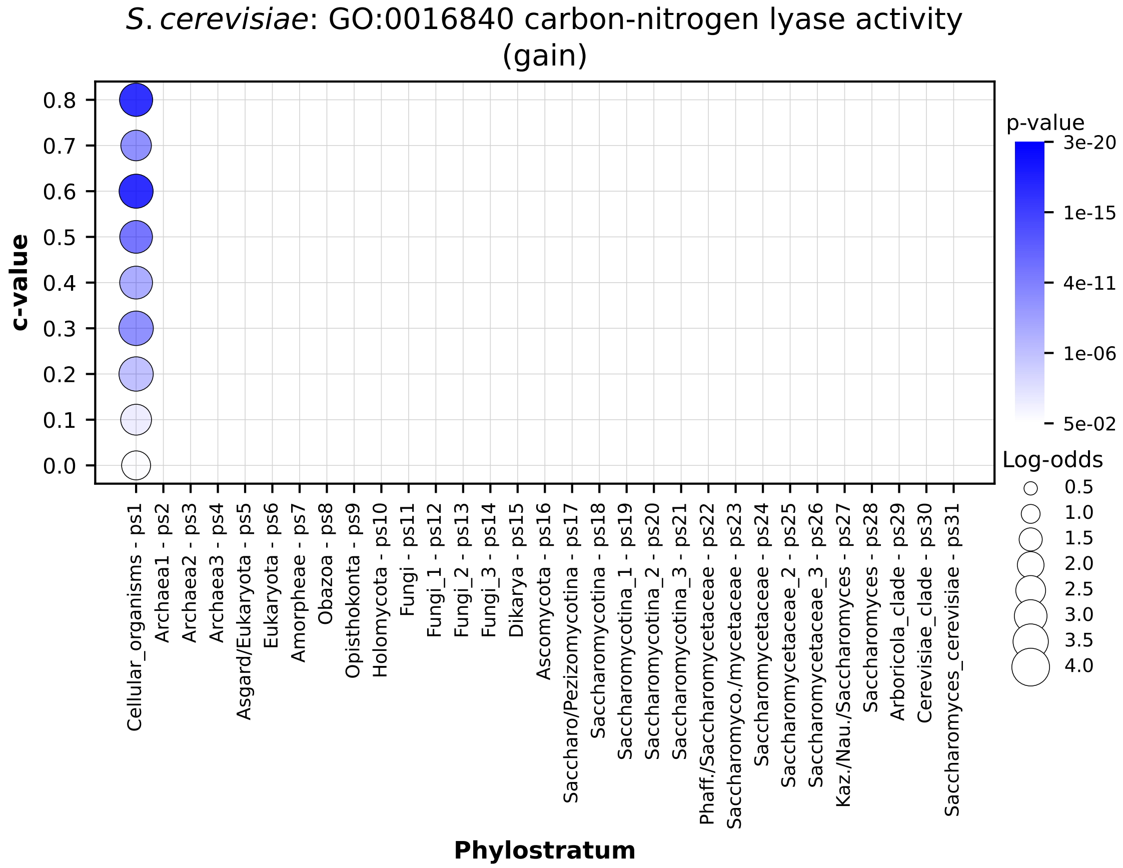

S. cerevisiae: GO:0016840 carbon-nitrogen lyase activity
(gain)
c-value
0.8
0.7
0.6
0.5
0.4
0.3
0.2
0.1
0.0
p-value
3e-20
1e-15
4e-11
1e-06
5e-02
Log-odds
0.5
1.0
1.5
2.0
2.5
3.0
3.5
4.0
Cellular_organisms - ps1
Archaea1 - ps2
Archaea2 - ps3
Archaea3 - ps4
Asgard/Eukaryota - ps5
Eukaryota - ps6
Amorpheae - ps7
Obazoa - ps8
Opisthokonta - ps9
Holomycota - ps10
Fungi - ps11
Fungi_1 - ps12
Fungi_2 - ps13
Fungi_3 - ps14
Dikarya - ps15
Ascomycota - ps16
Saccharo/Pezizomycotina - ps17
Saccharomycotina - ps18
Saccharomycotina_1 - ps19
Saccharomycotina_2 - ps20
Saccharomycotina_3 - ps21
Phaff./Saccharomycetaceae - ps22
Saccharomyco./mycetaceae - ps23
Saccharomycetaceae - ps24
Saccharomycetaceae_2 - ps25
Saccharomycetaceae_3 - ps26
Kaz./Nau./Saccharomyces - ps27
Saccharomyces - ps28
Arboricola_clade - ps29
Cerevisiae_clade - ps30
Saccharomyces_cerevisiae - ps31
Phylostratum

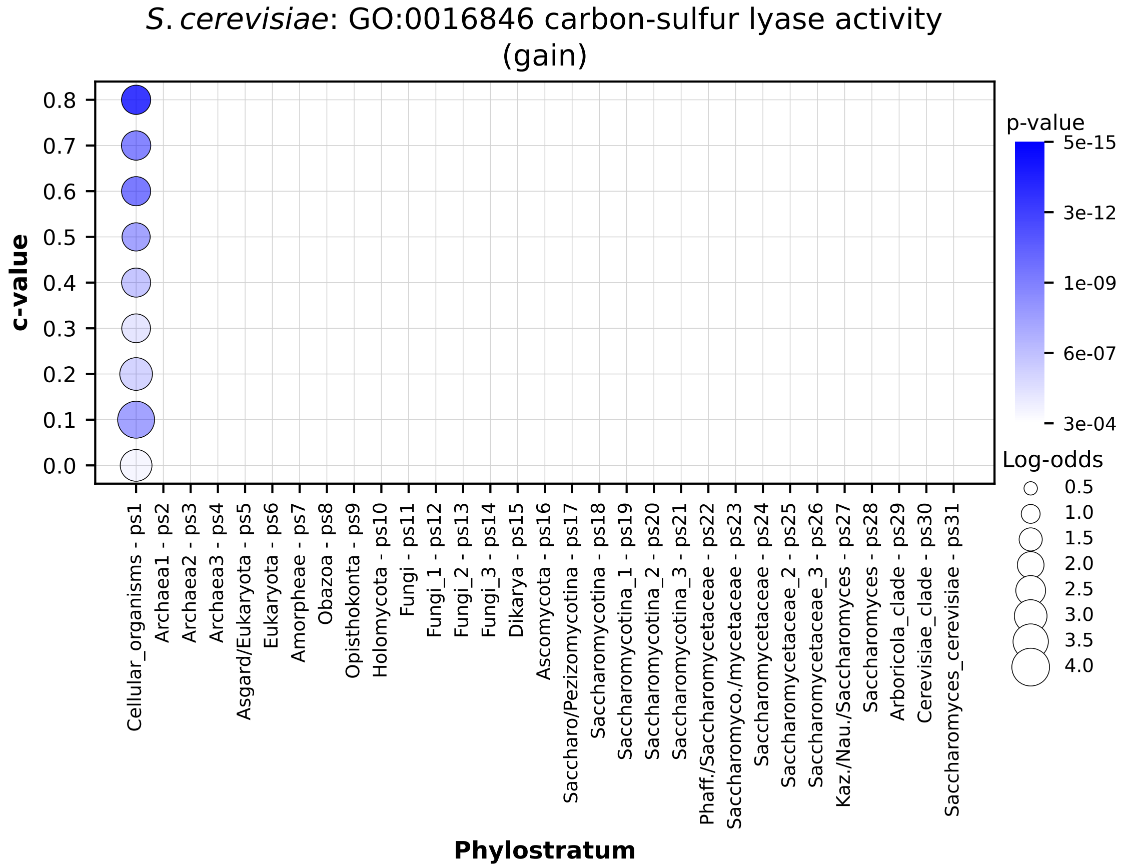
S. cerevisiae: GO:0016846 carbon-sulfur lyase activity
(gain)
c-value
0.8
0.7
0.6
0.5
0.4
0.3
0.2
0.1
0.0
p-value
5e-15
3e-12
1e-09
6e-07
3e-04
Log-odds
0.5
1.0
1.5
2.0
2.5
3.0
3.5
4.0
Cellular_organisms - ps1
Archaea1 - ps2
Archaea2 - ps3
Archaea3 - ps4
Asgard/Eukaryota - ps5
Eukaryota - ps6
Amorpheae - ps7
Obazoa - ps8
Opisthokonta - ps9
Holomycota - ps10
Fungi - ps11
Fungi_1 - ps12
Fungi_2 - ps13
Fungi_3 - ps14
Dikarya - ps15
Ascomycota - ps16
Saccharo/Pezizomycotina - ps17
Saccharomycotina - ps18
Saccharomycotina_1 - ps19
Saccharomycotina_2 - ps20
Saccharomycotina_3 - ps21
Phaff./Saccharomycetaceae - ps22
Saccharomyco./mycetaceae - ps23
Saccharomycetaceae - ps24
Saccharomycetaceae_2 - ps25
Saccharomycetaceae_3 - ps26
Kaz./Nau./Saccharomyces - ps27
Saccharomyces - ps28
Arboricola_clade - ps29
Cerevisiae_clade - ps30
Saccharomyces_cerevisiae - ps31
Phylostratum

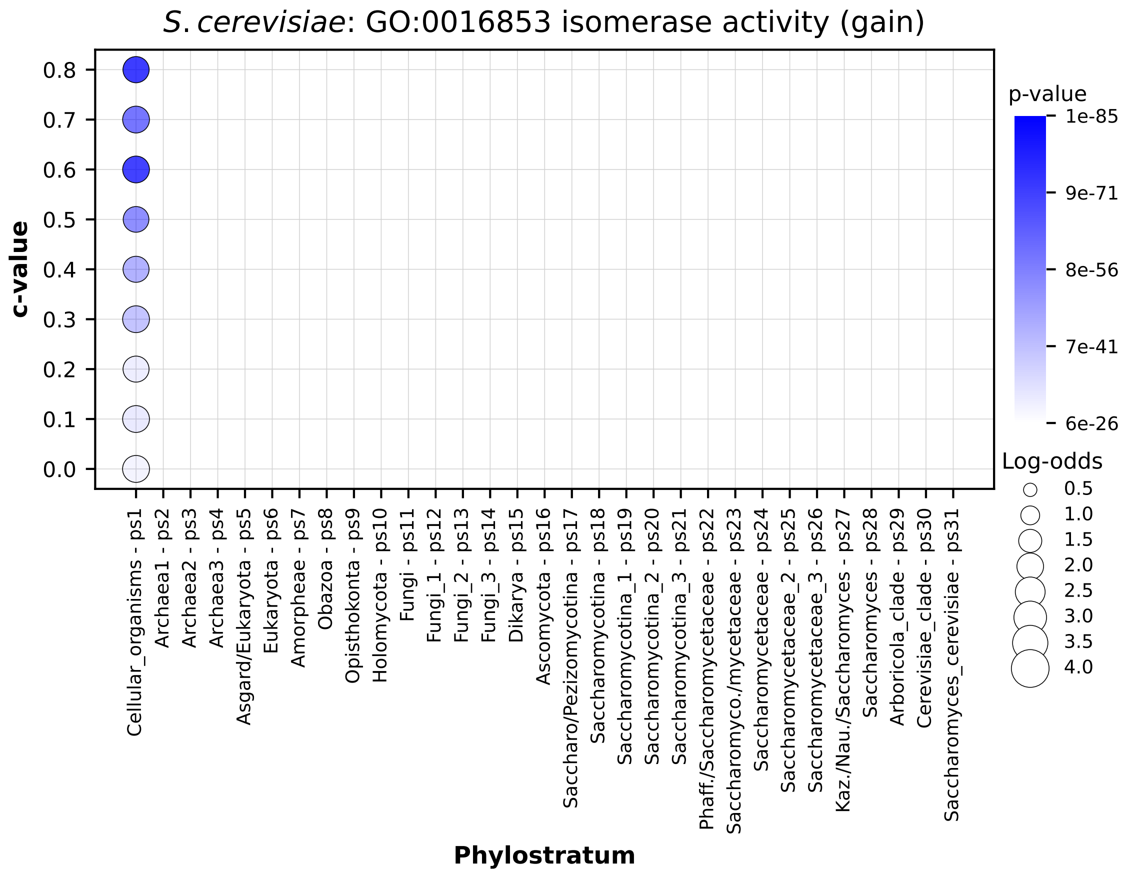
S. cerevisiae: GO:0016853 isomerase activity (gain)
c-value
0.0
0.1
0.2
0.3
0.4
0.5
0.6
0.7
0.8
p-value
1e-85
9e-71
8e-56
7e-41
6e-26
Log-odds
0.5
1.0
1.5
2.0
2.5
3.0
3.5
4.0
Cellular_organisms - ps1
Archaea1 - ps2
Archaea2 - ps3
Archaea3 - ps4
Asgard/Eukaryota - ps5
Eukaryota - ps6
Amorpheae - ps7
Obazoa - ps8
Opisthokonta - ps9
Holomycota - ps10
Fungi - ps11
Fungi_1 - ps12
Fungi_2 - ps13
Fungi_3 - ps14
Dikarya - ps15
Ascomycota - ps16
Saccharo/Pezizomycotina - ps17
Saccharomycotina - ps18
Saccharomycotina_1 - ps19
Saccharomycotina_2 - ps20
Saccharomycotina_3 - ps21
Phaff./Saccharomycetaceae - ps22
Saccharomyco./mycetaceae - ps23
Saccharomycetaceae - ps24
Saccharomycetaceae_2 - ps25
Saccharomycetaceae_3 - ps26
Kaz./Nau./Saccharomyces - ps27
Saccharomyces - ps28
Arboricola_clade - ps29
Cerevisiae_clade - ps30
Saccharomyces_cerevisiae - ps31
Phylostratum

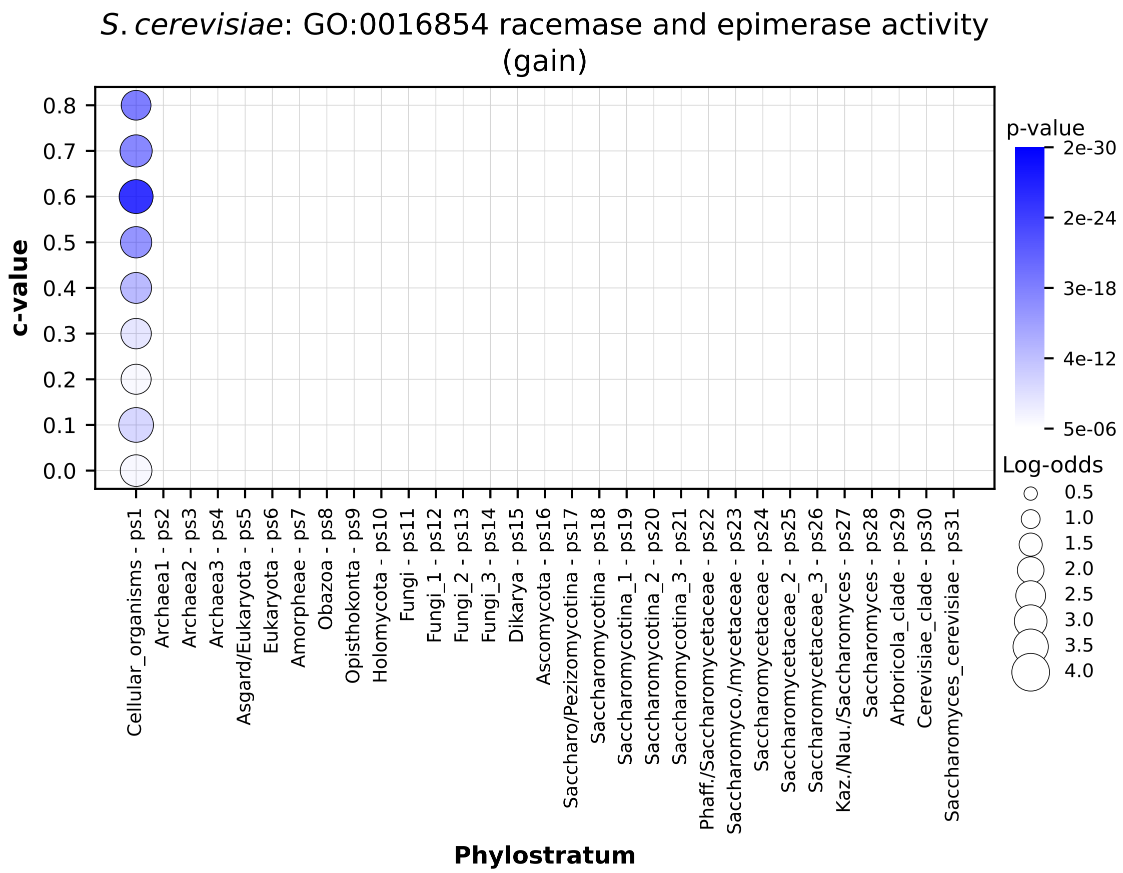

S. cerevisiae: GO:0016854 racemase and epimerase activity (gain)
c-value
0.0
0.1
0.2
0.3
0.4
0.5
0.6
0.7
0.8
p-value
2e-30
2e-24
3e-18
4e-12
5e-06
Log-odds
0.5
1.0
1.5
2.0
2.5
3.0
3.5
4.0
Cellular_organisms - ps1
Archaea1 - ps2
Archaea2 - ps3
Archaea3 - ps4
Asgard/Eukaryota - ps5
Eukaryota - ps6
Amorpheae - ps7
Obazoa - ps8
Opisthokonta - ps9
Holomycota - ps10
Fungi - ps11
Fungi_1 - ps12
Fungi_2 - ps13
Fungi_3 - ps14
Dikarya - ps15
Ascomycota - ps16
Saccharo/Pezizomycotina - ps17
Saccharomycotina - ps18
Saccharomycotina_1 - ps19
Saccharomycotina_2 - ps20
Saccharomycotina_3 - ps21
Phaff./Saccharomycetaceae - ps22
Saccharomyco./mycetaceae - ps23
Saccharomycetaceae - ps24
Saccharomycetaceae_2 - ps25
Saccharomycetaceae_3 - ps26
Kaz./Nau./Saccharomyces - ps27
Saccharomyces - ps28
Arboricola_clade - ps29
Cerevisiae_clade - ps30
Saccharomyces_cerevisiae - ps31
Phylostratum

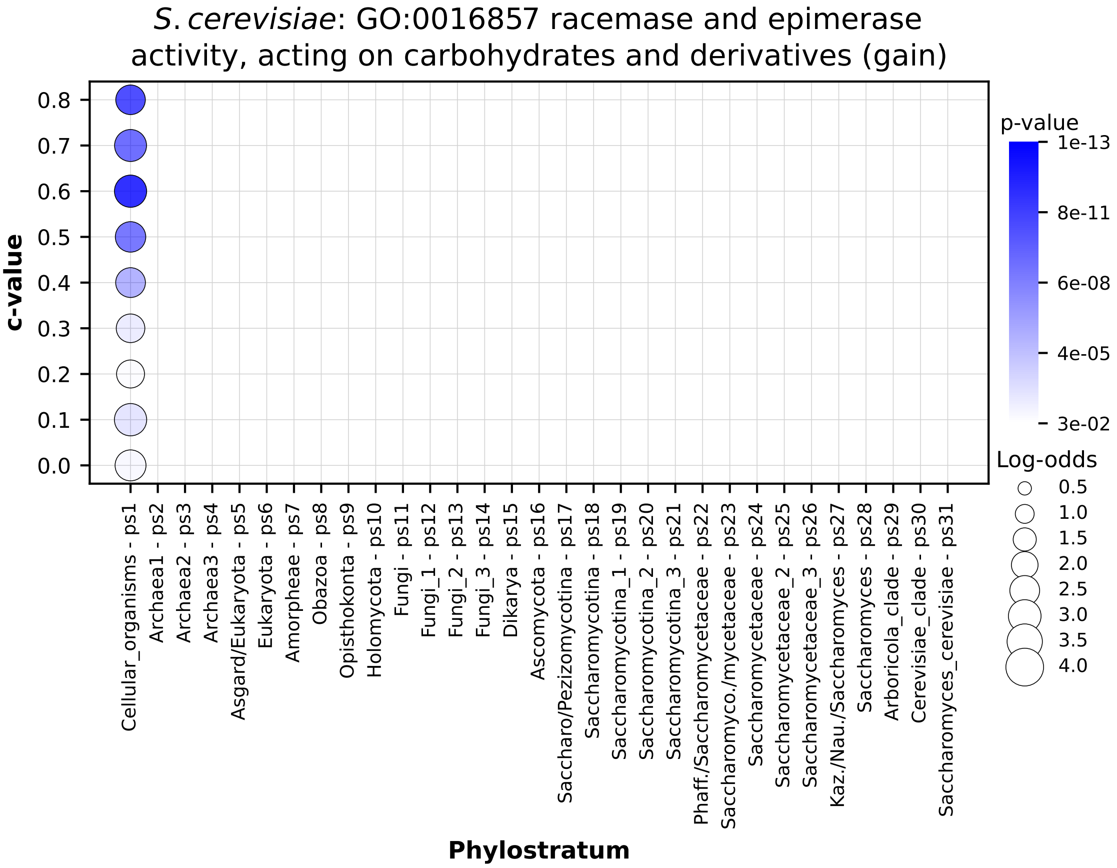
S. cerevisiae: GO:0016857 racemase and epimerase activity, acting on carbohydrates and derivatives (gain)
c-value
0.8
0.7
0.6
0.5
0.4
0.3
0.2
0.1
0.0
p-value
1e-13
8e-11
6e-08
4e-05
3e-02
Log-odds
0.5
1.0
1.5
2.0
2.5
3.0
3.5
4.0
Cellular_organisms - ps1
Archaea1 - ps2
Archaea2 - ps3
Archaea3 - ps4
Asgard/Eukaryota - ps5
Eukaryota - ps6
Amorpheae - ps7
Obazoa - ps8
Opisthokonta - ps9
Holomycota - ps10
Fungi - ps11
Fungi_1 - ps12
Fungi_2 - ps13
Fungi_3 - ps14
Dikarya - ps15
Ascomycota - ps16
Saccharo/Pezizomycotina - ps17
Saccharomycotina - ps18
Saccharomycotina_1 - ps19
Saccharomycotina_2 - ps20
Saccharomycotina_3 - ps21
Phaff./Saccharomycetaceae - ps22
Saccharomyco./mycetaceae - ps23
Saccharomycetaceae - ps24
Saccharomycetaceae_2 - ps25
Saccharomycetaceae_3 - ps26
Kaz./Nau./Saccharomyces - ps27
Saccharomyces - ps28
Arboricola_clade - ps29
Cerevisiae_clade - ps30
Saccharomyces_cerevisiae - ps31
Phylostratum

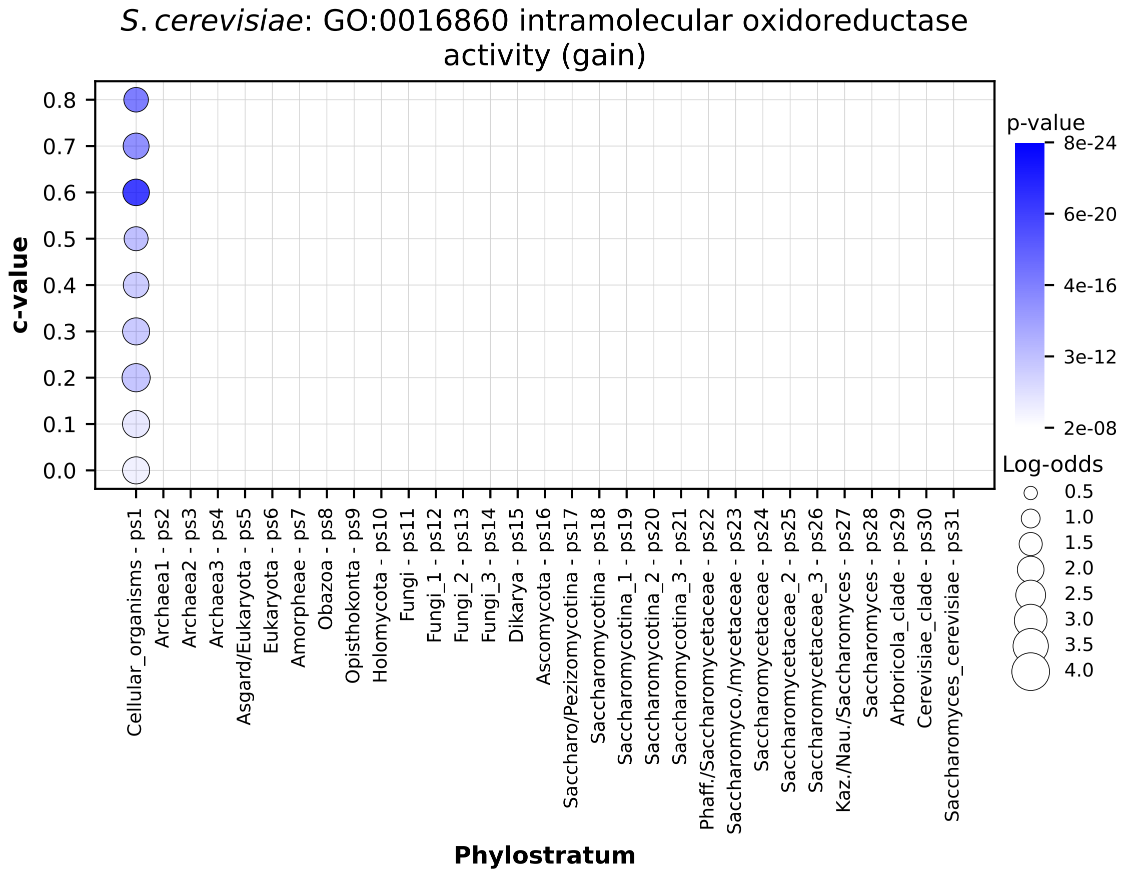

S. cerevisiae: GO:0016860 intramolecular oxidoreductase activity (gain)
c-value
0.8
0.7
0.6
0.5
0.4
0.3
0.2
0.1
0.0
p-value
8e-24
6e-20
4e-16
3e-12
2e-08
Log-odds
0.5
1.0
1.5
2.0
2.5
3.0
3.5
4.0
Cellular_organisms - ps1
Archaea1 - ps2
Archaea2 - ps3
Archaea3 - ps4
Asgard/Eukaryota - ps5
Eukaryota - ps6
Amorpheae - ps7
Obazoa - ps8
Opisthokonta - ps9
Holomycota - ps10
Fungi - ps11
Fungi_1 - ps12
Fungi_2 - ps13
Fungi_3 - ps14
Dikarya - ps15
Ascomycota - ps16
Saccharo/Pezizomycotina - ps17
Saccharomycotina - ps18
Saccharomycotina_1 - ps19
Saccharomycotina_2 - ps20
Saccharomycotina_3 - ps21
Phaff./Saccharomycetaceae - ps22
Saccharomyco./mycetaceae - ps23
Saccharomycetaceae - ps24
Saccharomycetaceae_2 - ps25
Saccharomycetaceae_3 - ps26
Kaz./Nau./Saccharomyces - ps27
Saccharomyces - ps28
Arboricola_clade - ps29
Cerevisiae_clade - ps30
Saccharomyces_cerevisiae - ps31
Phylostratum

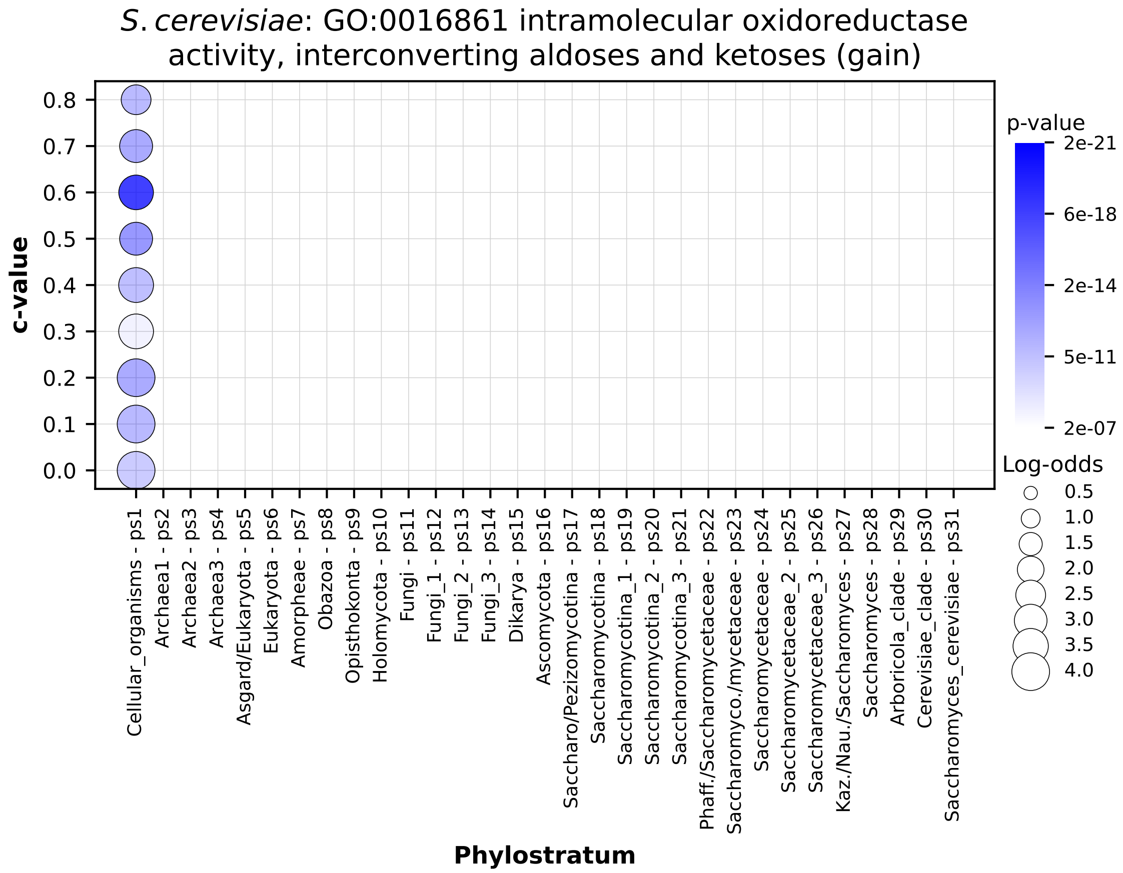

S. cerevisiae: GO:0016861 intramolecular oxidoreductase activity, interconverting aldoses and ketoses (gain)
c-value
0.8
0.7
0.6
0.5
0.4
0.3
0.2
0.1
0.0
p-value
2e-21
6e-18
2e-14
5e-11
2e-07
Log-odds
0.5
1.0
1.5
2.0
2.5
3.0
3.5
4.0
Cellular_organisms - ps1
Archaea1 - ps2
Archaea2 - ps3
Archaea3 - ps4
Asgard/Eukaryota - ps5
Eukaryota - ps6
Amorpheae - ps7
Obazoa - ps8
Opisthokonta - ps9
Holomycota - ps10
Fungi - ps11
Fungi_1 - ps12
Fungi_2 - ps13
Fungi_3 - ps14
Dikarya - ps15
Ascomycota - ps16
Saccharo/Pezizomycotina - ps17
Saccharomycotina - ps18
Saccharomycotina_1 - ps19
Saccharomycotina_2 - ps20
Saccharomycotina_3 - ps21
Phaff./Saccharomycetaceae - ps22
Saccharomyco./mycetaceae - ps23
Saccharomycetaceae - ps24
Saccharomycetaceae_2 - ps25
Saccharomycetaceae_3 - ps26
Kaz./Nau./Saccharomyces - ps27
Saccharomyces - ps28
Arboricola_clade - ps29
Cerevisiae_clade - ps30
Saccharomyces_cerevisiae - ps31
Phylostratum

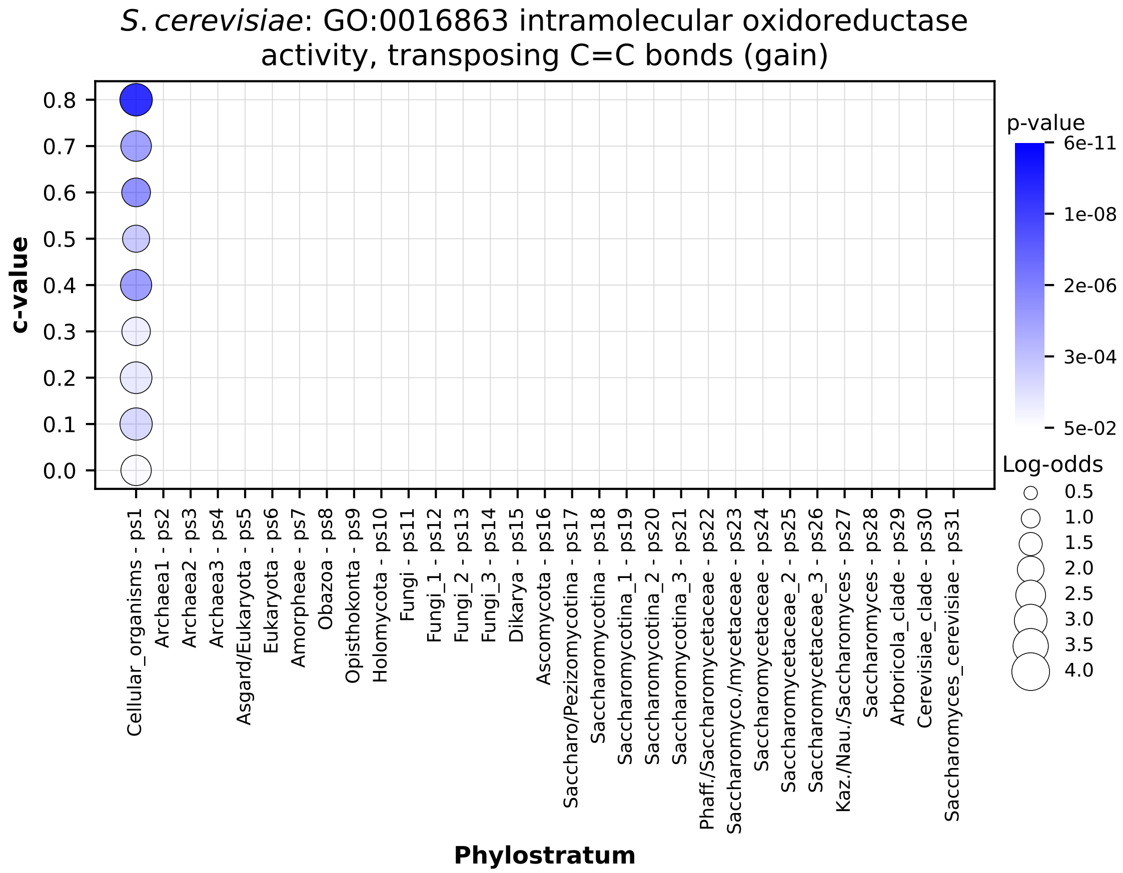

S. cerevisiae: GO:0016863 intramolecular oxidoreductase activity, transposing C=C bonds (gain)
c-value
0.0
0.1
0.2
0.3
0.4
0.5
0.6
0.7
0.8
p-value
6e-11
1e-08
2e-06
3e-04
5e-02
Log-odds
0.5
1.0
1.5
2.0
2.5
3.0
3.5
4.0
Cellular_organisms - ps1
Archaea1 - ps2
Archaea2 - ps3
Archaea3 - ps4
Asgard/Eukaryota - ps5
Eukaryota - ps6
Amorpheae - ps7
Obazoa - ps8
Opisthokonta - ps9
Holomycota - ps10
Fungi - ps11
Fungi_1 - ps12
Fungi_2 - ps13
Fungi_3 - ps14
Dikarya - ps15
Ascomycota - ps16
Saccharo/Pezizomycotina - ps17
Saccharomycotina - ps18
Saccharomycotina_1 - ps19
Saccharomycotina_2 - ps20
Saccharomycotina_3 - ps21
Phaff./Saccharomycetaceae - ps22
Saccharomyco./mycetaceae - ps23
Saccharomycetaceae - ps24
Saccharomycetaceae_2 - ps25
Saccharomycetaceae_3 - ps26
Kaz./Nau./Saccharomyces - ps27
Saccharomyces - ps28
Arboricola_clade - ps29
Cerevisiae_clade - ps30
Saccharomyces_cerevisiae - ps31
Phylostratum

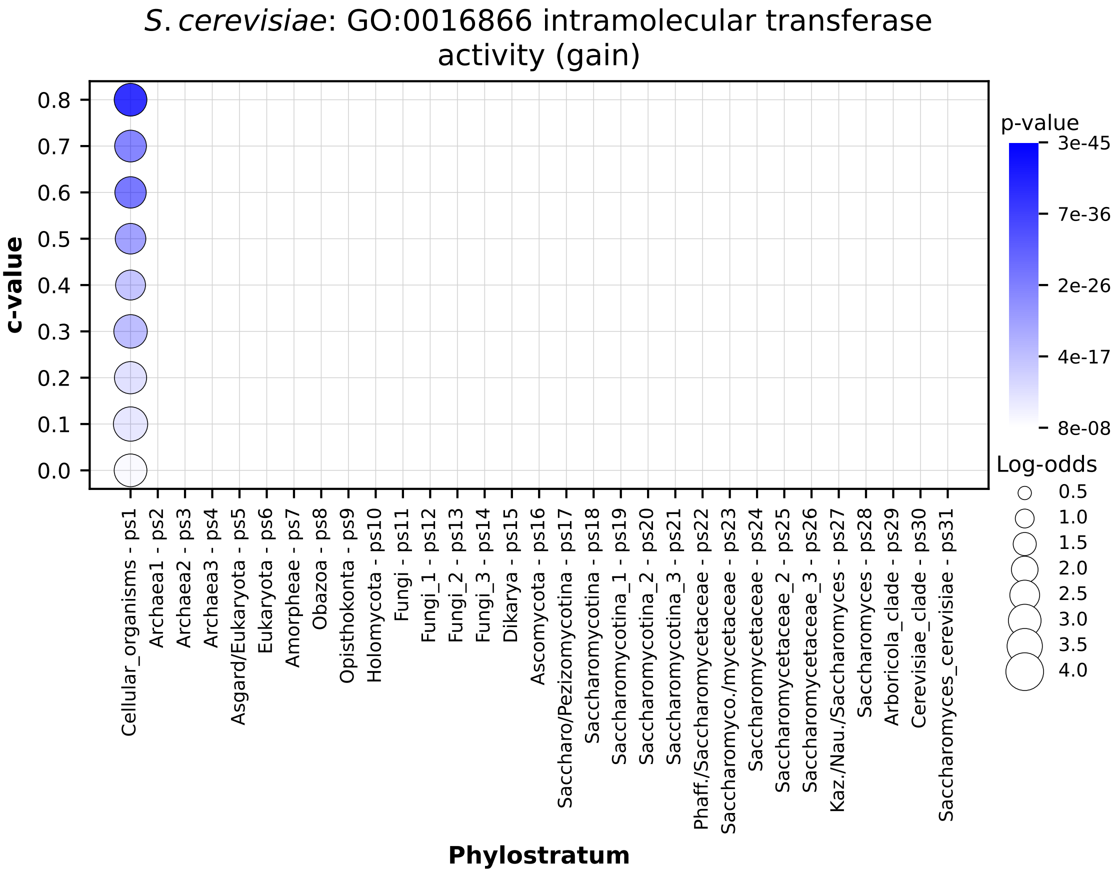
S. cerevisiae: GO:0016866 intramolecular transferase activity (gain)
c-value
0.8
0.7
0.6
0.5
0.4
0.3
0.2
0.1
0.0
p-value
3e-45
7e-36
2e-26
4e-17
8e-08
Log-odds
0.5
1.0
1.5
2.0
2.5
3.0
3.5
4.0
Cellular_organisms - ps1
Archaea1 - ps2
Archaea2 - ps3
Archaea3 - ps4
Asgard/Eukaryota - ps5
Eukaryota - ps6
Amorpheae - ps7
Obazoa - ps8
Opisthokonta - ps9
Holomycota - ps10
Fungi - ps11
Fungi_1 - ps12
Fungi_2 - ps13
Fungi_3 - ps14
Dikarya - ps15
Ascomycota - ps16
Saccharo/Pezizomycotina - ps17
Saccharomycotina - ps18
Saccharomycotina_1 - ps19
Saccharomycotina_2 - ps20
Saccharomycotina_3 - ps21
Phaff./Saccharomycetaceae - ps22
Saccharomyco./mycetaceae - ps23
Saccharomycetaceae - ps24
Saccharomycetaceae_2 - ps25
Saccharomycetaceae_3 - ps26
Kaz./Nau./Saccharomyces - ps27
Saccharomyces - ps28
Arboricola_clade - ps29
Cerevisiae_clade - ps30
Saccharomyces_cerevisiae - ps31
Phylostratum

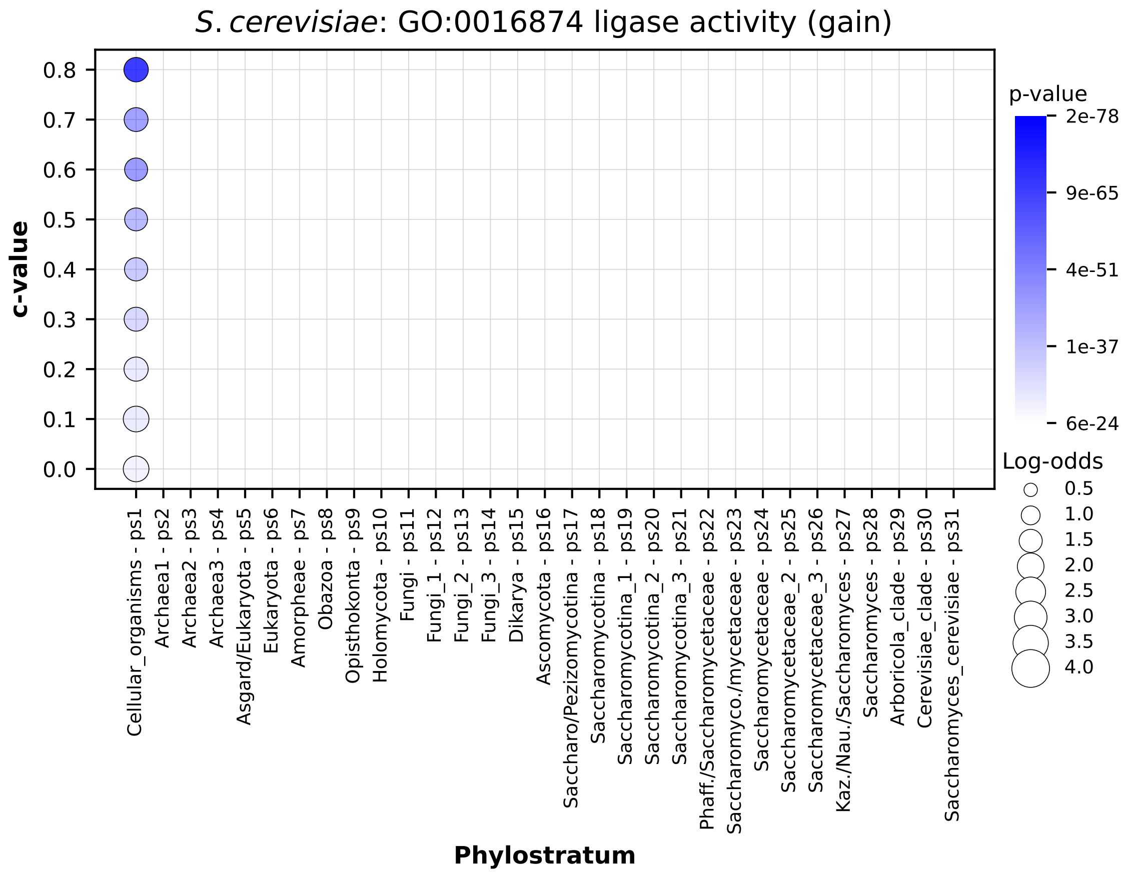
S. cerevisiae: GO:0016874 ligase activity (gain)
c-value
0.0
0.1
0.2
0.3
0.4
0.5
0.6
0.7
0.8
Phylostratum
Cellular_organisms - ps1
Archaea1 - ps2
Archaea2 - ps3
Archaea3 - ps4
Asgard/Eukaryota - ps5
Eukaryota - ps6
Amorpheae - ps7
Obazoa - ps8
Opisthokonta - ps9
Holomycota - ps10
Fungi - ps11
Fungi_1 - ps12
Fungi_2 - ps13
Fungi_3 - ps14
Dikarya - ps15
Ascomycota - ps16
Saccharo/Pezizomycotina - ps17
Saccharomycotina - ps18
Saccharomycotina_1 - ps19
Saccharomycotina_2 - ps20
Saccharomycotina_3 - ps21
Phaff./Saccharomycetaceae - ps22
Saccharomyco./mycetaceae - ps23
Saccharomycetaceae - ps24
Saccharomycetaceae_2 - ps25
Saccharomycetaceae_3 - ps26
Kaz./Nau./Saccharomyces - ps27
Saccharomyces - ps28
Arboricola_clade - ps29
Cerevisiae_clade - ps30
Saccharomyces_cerevisiae - ps31
p-value
2e-78
9e-65
4e-51
1e-37
6e-24
Log-odds
0.5
1.0
1.5
2.0
2.5
3.0
3.5
4.0

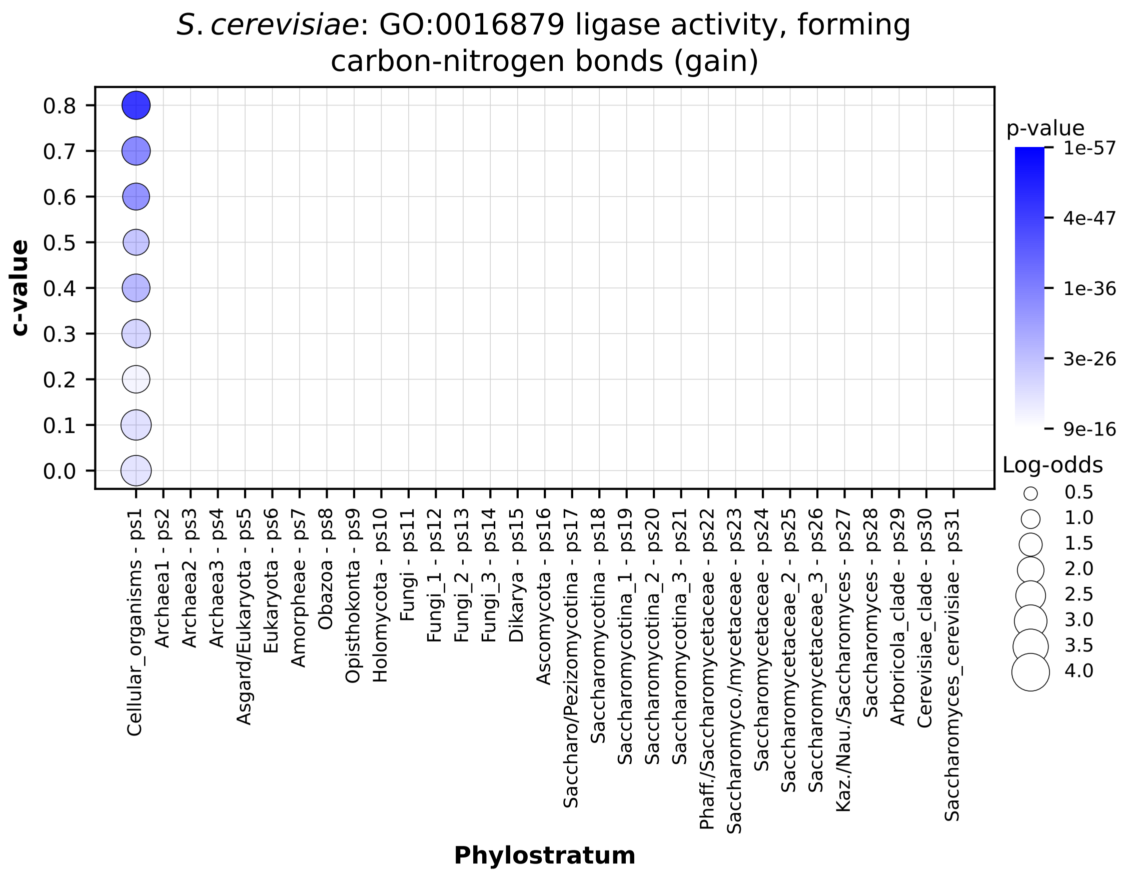

S. cerevisiae: GO:0016879 ligase activity, forming carbon-nitrogen bonds (gain)
c-value
0.8
0.7
0.6
0.5
0.4
0.3
0.2
0.1
0.0
p-value
1e-57
4e-47
1e-36
3e-26
9e-16
Log-odds
0.5
1.0
1.5
2.0
2.5
3.0
3.5
4.0
Cellular_organisms - ps1
Archaea1 - ps2
Archaea2 - ps3
Archaea3 - ps4
Asgard/Eukaryota - ps5
Eukaryota - ps6
Amorpheae - ps7
Obazoa - ps8
Opisthokonta - ps9
Holomycota - ps10
Fungi - ps11
Fungi_1 - ps12
Fungi_2 - ps13
Fungi_3 - ps14
Dikarya - ps15
Ascomycota - ps16
Saccharo/Pezizomycotina - ps17
Saccharomycotina - ps18
Saccharomycotina_1 - ps19
Saccharomycotina_2 - ps20
Saccharomycotina_3 - ps21
Phaff./Saccharomycetaceae - ps22
Saccharomyco./mycetaceae - ps23
Saccharomycetaceae - ps24
Saccharomycetaceae_2 - ps25
Saccharomycetaceae_3 - ps26
Kaz./Nau./Saccharomyces - ps27
Saccharomyces - ps28
Arboricola_clade - ps29
Cerevisiae_clade - ps30
Saccharomyces_cerevisiae - ps31
Phylostratum

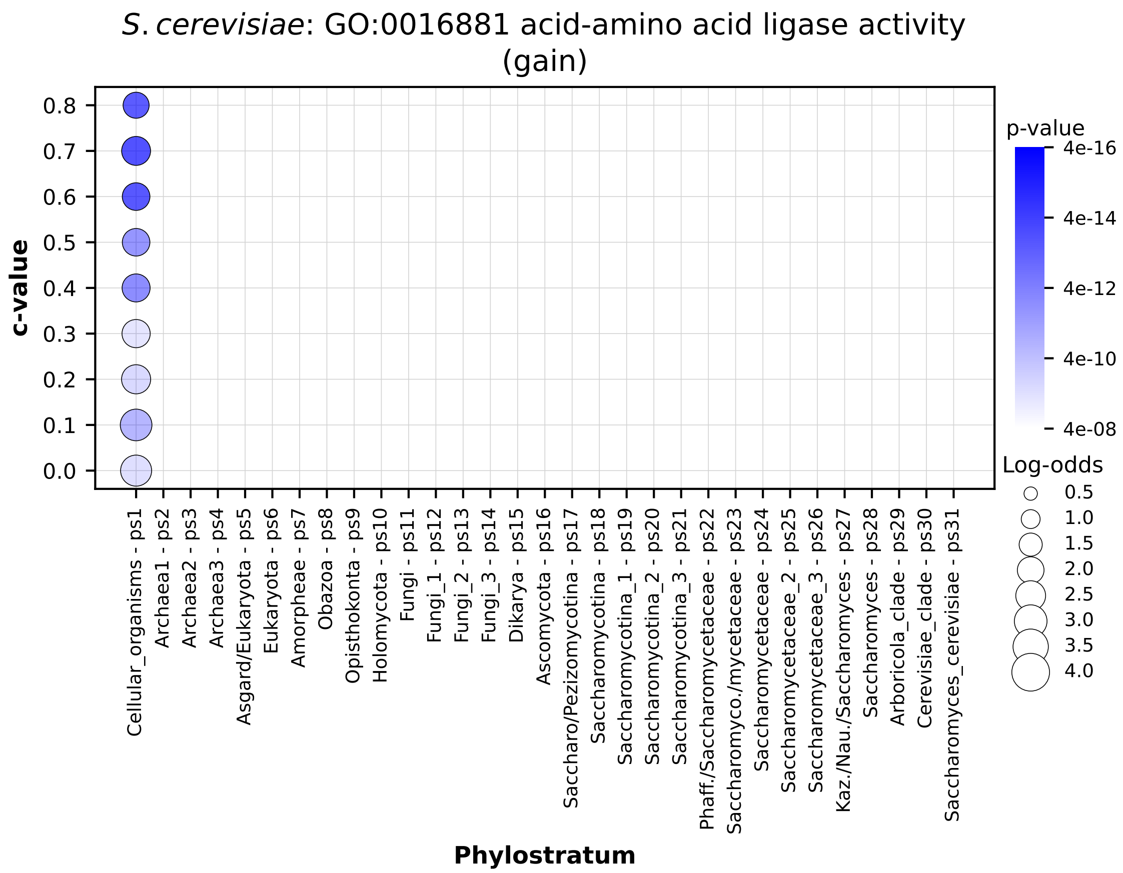
S. cerevisiae: GO:0016881 acid-amino acid ligase activity (gain)
c-value
0.8
0.7
0.6
0.5
0.4
0.3
0.2
0.1
0.0
p-value
4e-16
4e-14
4e-12
4e-10
4e-08
Log-odds
0.5
1.0
1.5
2.0
2.5
3.0
3.5
4.0
Cellular_organisms - ps1
Archaea1 - ps2
Archaea2 - ps3
Archaea3 - ps4
Asgard/Eukaryota - ps5
Eukaryota - ps6
Amorpheae - ps7
Obazoa - ps8
Opisthokonta - ps9
Holomycota - ps10
Fungi - ps11
Fungi_1 - ps12
Fungi_2 - ps13
Fungi_3 - ps14
Dikarya - ps15
Ascomycota - ps16
Saccharo/Pezizomycotina - ps17
Saccharomycotina - ps18
Saccharomycotina_1 - ps19
Saccharomycotina_2 - ps20
Saccharomycotina_3 - ps21
Phaff./Saccharomycetaceae - ps22
Saccharomyco./mycetaceae - ps23
Saccharomycetaceae - ps24
Saccharomycetaceae_2 - ps25
Saccharomycetaceae_3 - ps26
Kaz./Nau./Saccharomyces - ps27
Saccharomyces - ps28
Arboricola_clade - ps29
Cerevisiae_clade - ps30
Saccharomyces_cerevisiae - ps31
Phylostratum

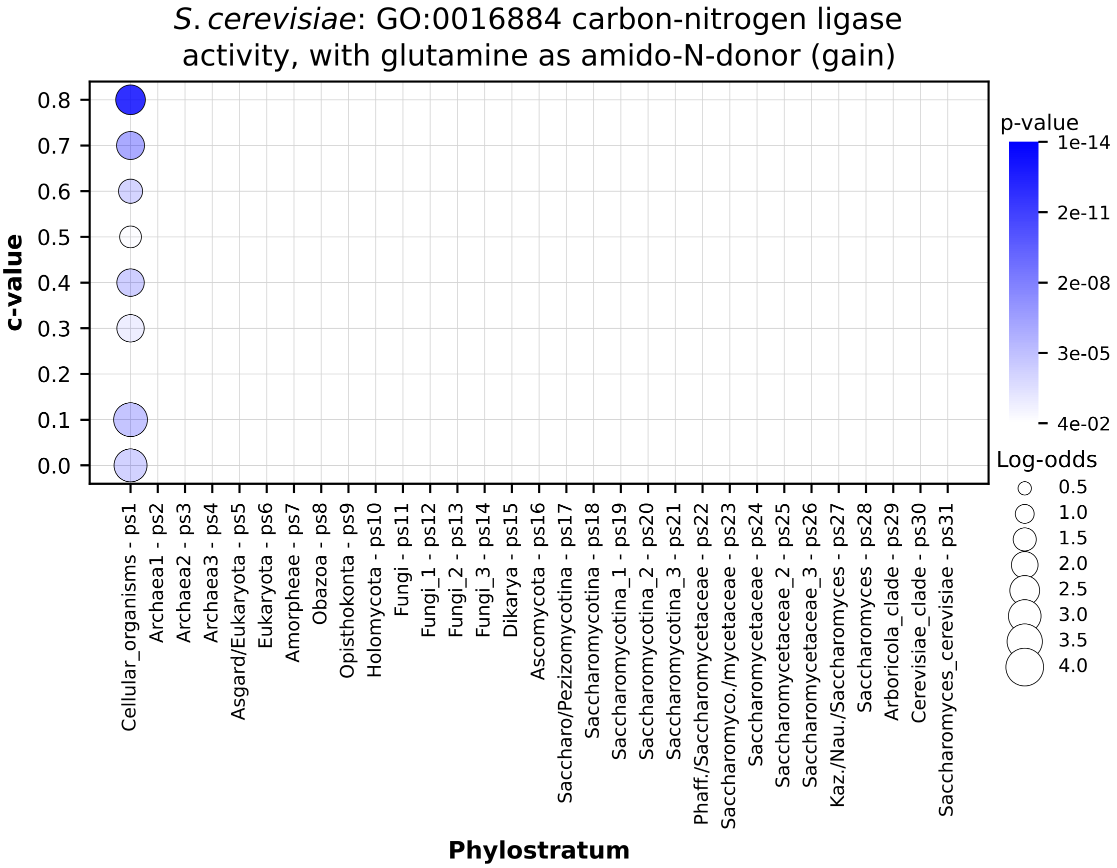
S. cerevisiae: GO:0016884 carbon-nitrogen ligase activity, with glutamine as amido-N-donor (gain)
c-value
0.0
0.1
0.2
0.3
0.4
0.5
0.6
0.7
0.8
Phylostratum
Cellular_organisms - ps1
Archaea1 - ps2
Archaea2 - ps3
Archaea3 - ps4
Asgard/Eukaryota - ps5
Eukaryota - ps6
Amorpheae - ps7
Obazoa - ps8
Opisthokonta - ps9
Holomycota - ps10
Fungi - ps11
Fungi_1 - ps12
Fungi_2 - ps13
Fungi_3 - ps14
Dikarya - ps15
Ascomycota - ps16
Saccharo/Pezizomycotina - ps17
Saccharomycotina - ps18
Saccharomycotina_1 - ps19
Saccharomycotina_2 - ps20
Saccharomycotina_3 - ps21
Phaff./Saccharomycetaceae - ps22
Saccharomyco./mycetaceae - ps23
Saccharomycetaceae - ps24
Saccharomycetaceae_2 - ps25
Saccharomycetaceae_3 - ps26
Kaz./Nau./Saccharomyces - ps27
Saccharomyces - ps28
Arboricola_clade - ps29
Cerevisiae_clade - ps30
Saccharomyces_cerevisiae - ps31
p-value
1e-14
2e-11
2e-08
3e-05
4e-02
Log-odds
0.5
1.0
1.5
2.0
2.5
3.0
3.5
4.0

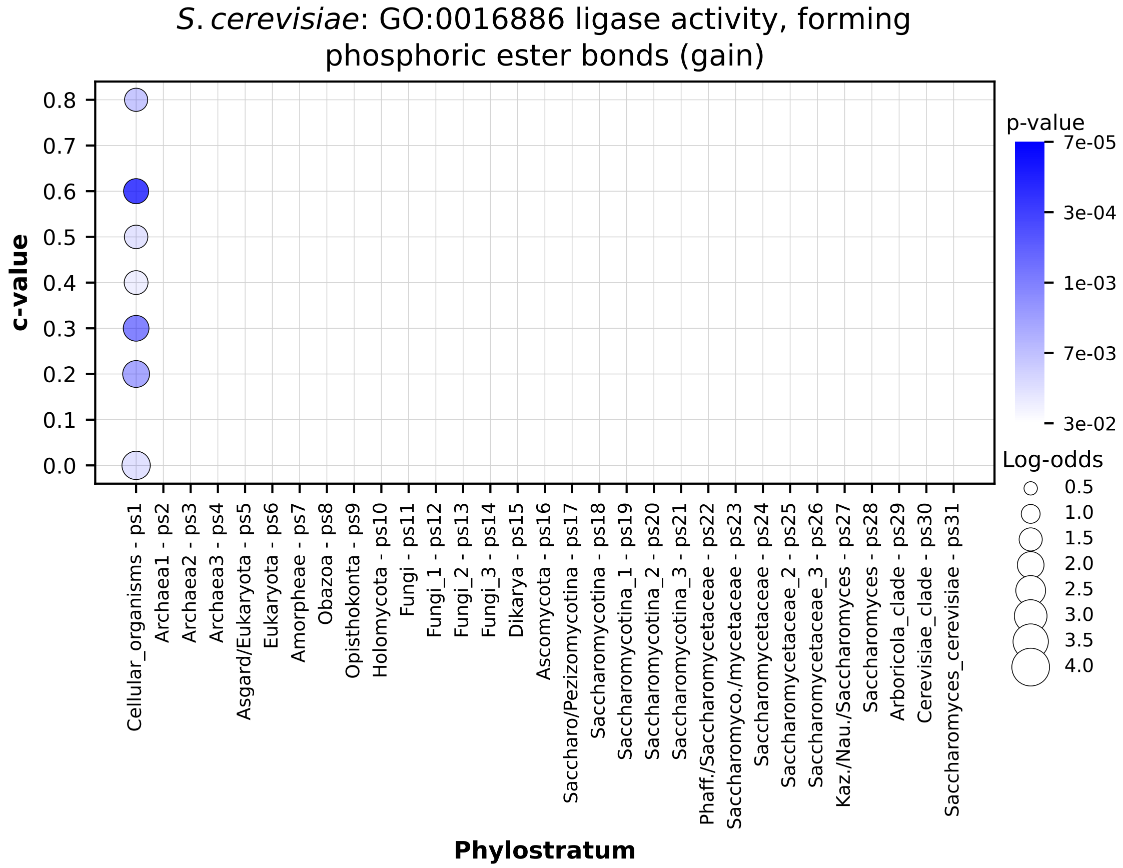
S. cerevisiae: GO:0016886 ligase activity, forming phosphoric ester bonds (gain)
c-value
0.8
0.7
0.6
0.5
0.4
0.3
0.2
0.1
0.0
p-value
7e-05
3e-04
1e-03
7e-03
3e-02
Log-odds
0.5
1.0
1.5
2.0
2.5
3.0
3.5
4.0
Cellular_organisms - ps1
Archaea1 - ps2
Archaea2 - ps3
Archaea3 - ps4
Asgard/Eukaryota - ps5
Eukaryota - ps6
Amorpheae - ps7
Obazoa - ps8
Opisthokonta - ps9
Holomycota - ps10
Fungi - ps11
Fungi_1 - ps12
Fungi_2 - ps13
Fungi_3 - ps14
Dikarya - ps15
Ascomycota - ps16
Saccharo/Pezizomycotina - ps17
Saccharomycotina - ps18
Saccharomycotina_1 - ps19
Saccharomycotina_2 - ps20
Saccharomycotina_3 - ps21
Phaff./Saccharomycetaceae - ps22
Saccharomyco./mycetaceae - ps23
Saccharomycetaceae - ps24
Saccharomycetaceae_2 - ps25
Saccharomycetaceae_3 - ps26
Kaz./Nau./Saccharomyces - ps27
Saccharomyces - ps28
Arboricola_clade - ps29
Cerevisiae_clade - ps30
Saccharomyces_cerevisiae - ps31
Phylostratum

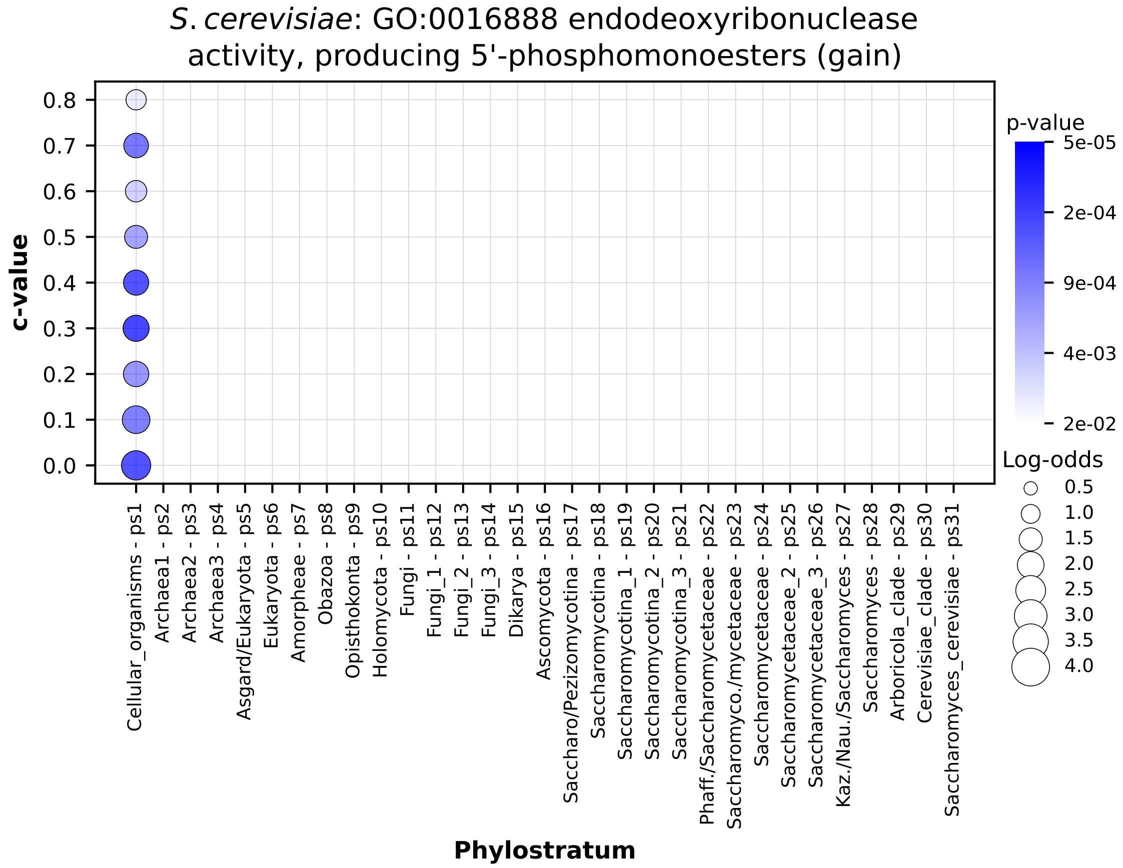

S. cerevisiae: GO:0016888 endodeoxyribonuclease activity, producing 5'-phosphomonoesters (gain)
c-value
0.8
0.7
0.6
0.5
0.4
0.3
0.2
0.1
0.0
p-value
5e-05
2e-04
9e-04
4e-03
2e-02
Log-odds
0.5
1.0
1.5
2.0
2.5
3.0
3.5
4.0
Cellular_organisms - ps1
Archaea1 - ps2
Archaea2 - ps3
Archaea3 - ps4
Asgard/Eukaryota - ps5
Eukaryota - ps6
Amorpheae - ps7
Obazoa - ps8
Opisthokonta - ps9
Holomycota - ps10
Fungi - ps11
Fungi_1 - ps12
Fungi_2 - ps13
Fungi_3 - ps14
Dikarya - ps15
Ascomycota - ps16
Saccharo/Pezizomycotina - ps17
Saccharomycotina - ps18
Saccharomycotina_1 - ps19
Saccharomycotina_2 - ps20
Saccharomycotina_3 - ps21
Phaff./Saccharomycetaceae - ps22
Saccharomyco./mycetaceae - ps23
Saccharomycetaceae - ps24
Saccharomycetaceae_2 - ps25
Saccharomycetaceae_3 - ps26
Kaz./Nau./Saccharomyces - ps27
Saccharomyces - ps28
Arboricola_clade - ps29
Cerevisiae_clade - ps30
Saccharomyces_cerevisiae - ps31
Phylostratum

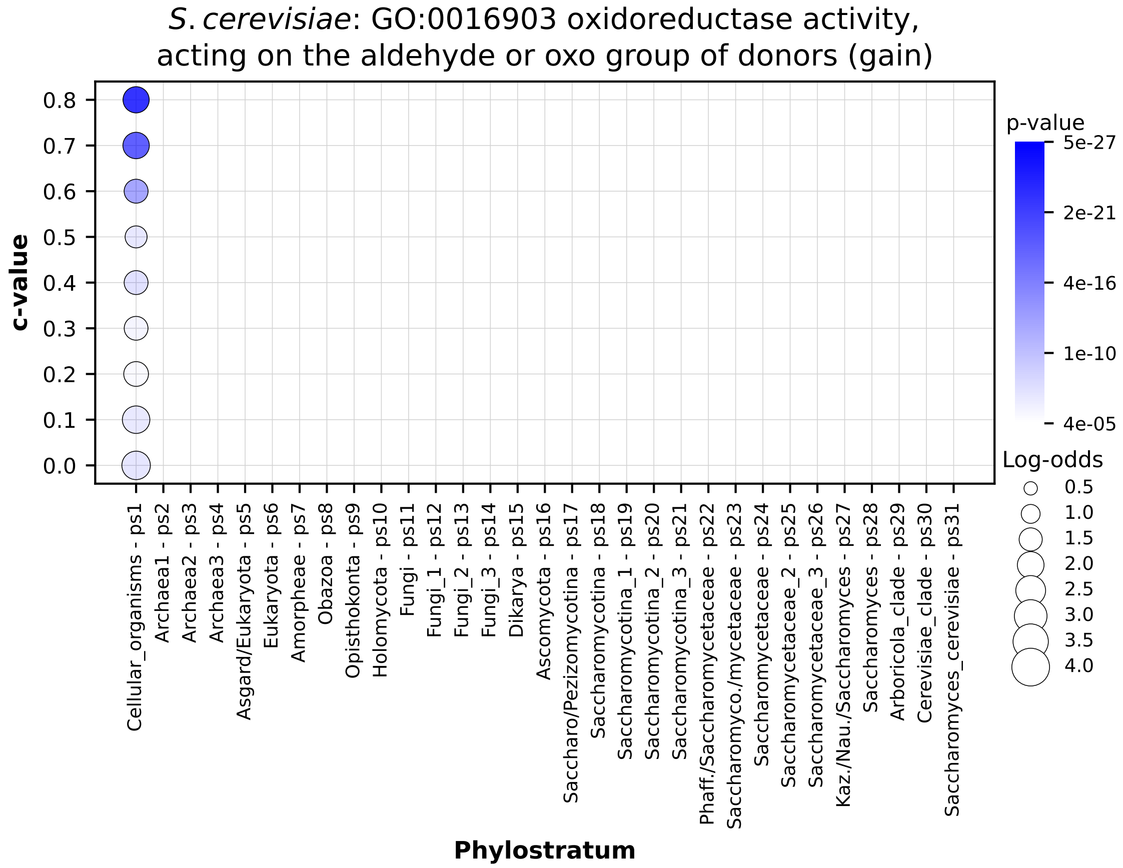
S. cerevisiae: GO:0016903 oxidoreductase activity, acting on the aldehyde or oxo group of donors (gain)
c-value
0.8
0.7
0.6
0.5
0.4
0.3
0.2
0.1
0.0
p-value
5e-27
2e-21
4e-16
1e-10
4e-05
Log-odds
0.5
1.0
1.5
2.0
2.5
3.0
3.5
4.0
Cellular_organisms - ps1
Archaea1 - ps2
Archaea2 - ps3
Archaea3 - ps4
Asgard/Eukaryota - ps5
Eukaryota - ps6
Amorpheae - ps7
Obazoa - ps8
Opisthokonta - ps9
Holomycota - ps10
Fungi - ps11
Fungi_1 - ps12
Fungi_2 - ps13
Fungi_3 - ps14
Dikarya - ps15
Ascomycota - ps16
Saccharo/Pezizomycotina - ps17
Saccharomycotina - ps18
Saccharomycotina_1 - ps19
Saccharomycotina_2 - ps20
Saccharomycotina_3 - ps21
Phaff./Saccharomycetaceae - ps22
Saccharomyco./mycetaceae - ps23
Saccharomycetaceae - ps24
Saccharomycetaceae_2 - ps25
Saccharomycetaceae_3 - ps26
Kaz./Nau./Saccharomyces - ps27
Saccharomyces - ps28
Arboricola_clade - ps29
Cerevisiae_clade - ps30
Saccharomyces_cerevisiae - ps31
Phylostratum

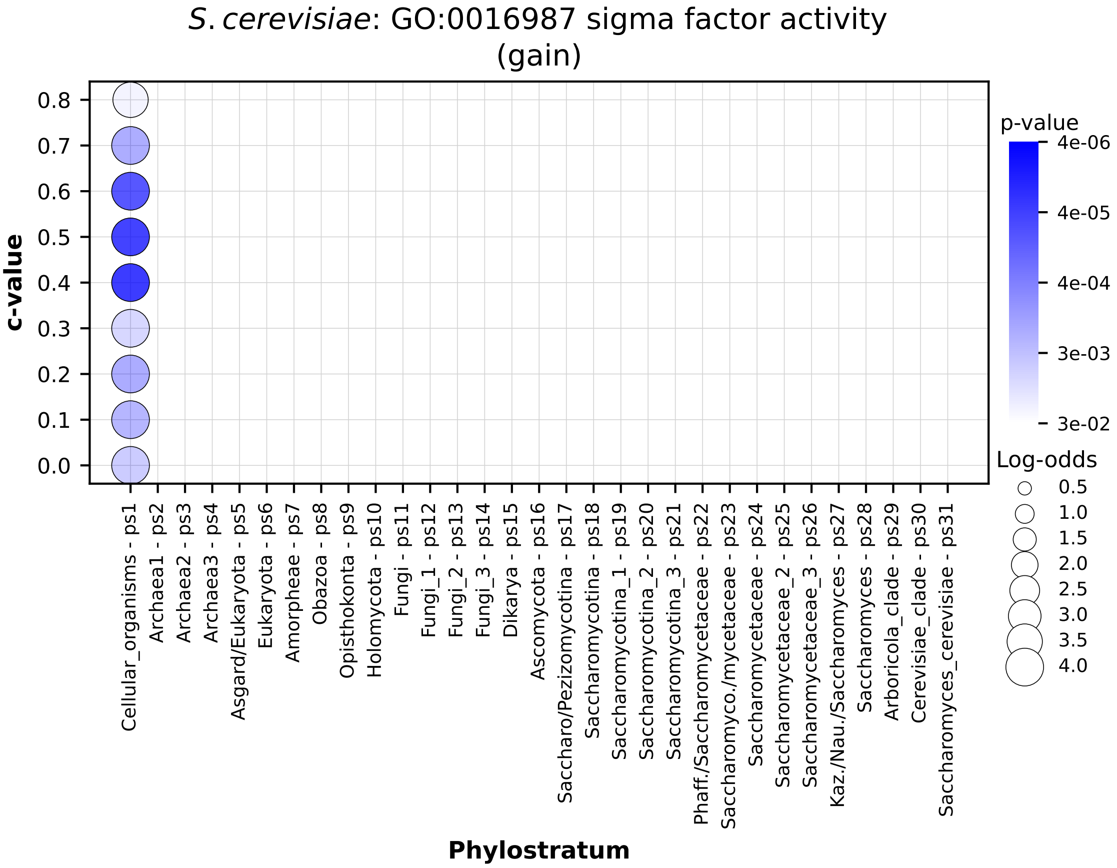

S. cerevisiae: GO:0016987 sigma factor activity
(gain)
c-value
0.8
0.7
0.6
0.5
0.4
0.3
0.2
0.1
0.0
p-value
4e-06
4e-05
4e-04
3e-03
3e-02
Log-odds
0.5
1.0
1.5
2.0
2.5
3.0
3.5
4.0
Cellular_organisms - ps1
Archaea1 - ps2
Archaea2 - ps3
Archaea3 - ps4
Asgard/Eukaryota - ps5
Eukaryota - ps6
Amorpheae - ps7
Obazoa - ps8
Opisthokonta - ps9
Holomycota - ps10
Fungi - ps11
Fungi_1 - ps12
Fungi_2 - ps13
Fungi_3 - ps14
Dikarya - ps15
Ascomycota - ps16
Saccharo/Pezizomycotina - ps17
Saccharomycotina - ps18
Saccharomycotina_1 - ps19
Saccharomycotina_2 - ps20
Saccharomycotina_3 - ps21
Phaff./Saccharomycetaceae - ps22
Saccharomyco./mycetaceae - ps23
Saccharomycetaceae - ps24
Saccharomycetaceae_2 - ps25
Saccharomycetaceae_3 - ps26
Kaz./Nau./Saccharomyces - ps27
Saccharomyces - ps28
Arboricola_clade - ps29
Cerevisiae_clade - ps30
Saccharomyces_cerevisiae - ps31
Phylostratum

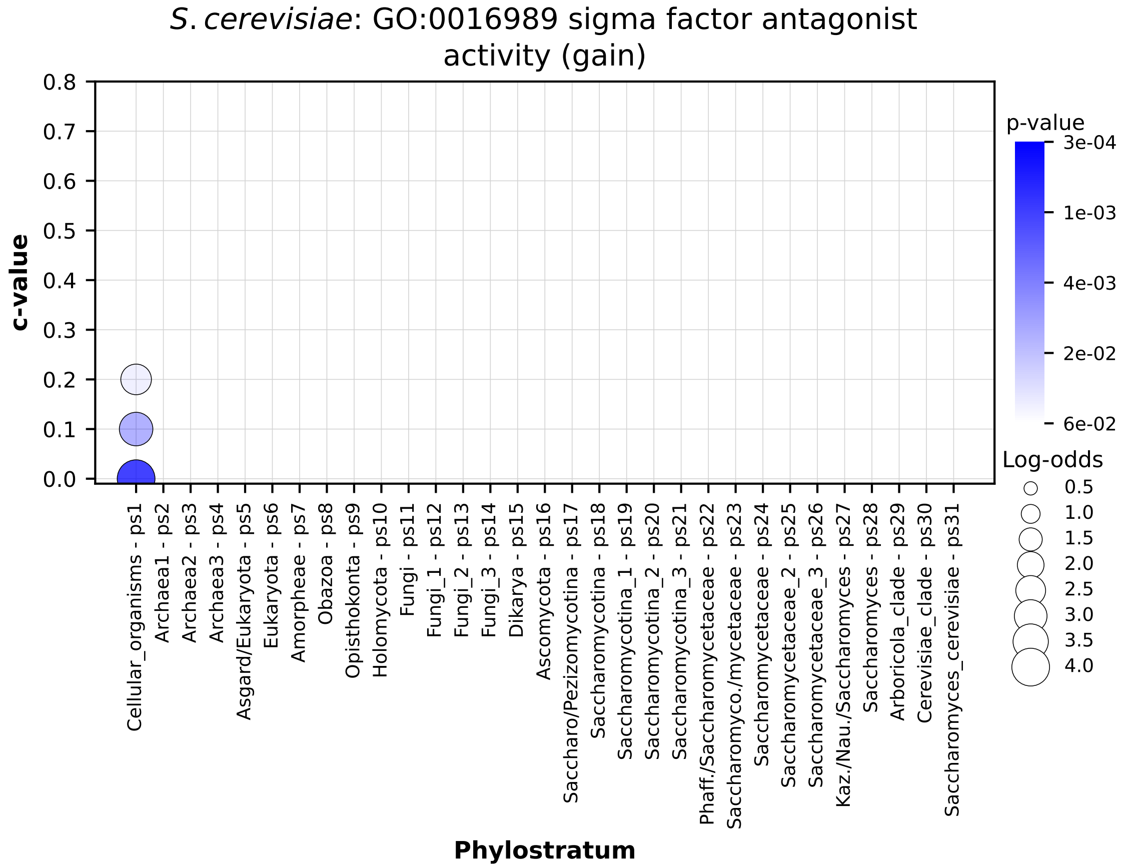
S. cerevisiae: GO:0016989 sigma factor antagonist activity (gain)
c-value
0.8
0.7
0.6
0.5
0.4
0.3
0.2
0.1
0.0
p-value
3e-04
1e-03
4e-03
2e-02
6e-02
Log-odds
0.5
1.0
1.5
2.0
2.5
3.0
3.5
4.0
Cellular_organisms - ps1
Archaea1 - ps2
Archaea2 - ps3
Archaea3 - ps4
Asgard/Eukaryota - ps5
Eukaryota - ps6
Amorpheae - ps7
Obazoa - ps8
Opisthokonta - ps9
Holomycota - ps10
Fungi - ps11
Fungi_1 - ps12
Fungi_2 - ps13
Fungi_3 - ps14
Dikarya - ps15
Ascomycota - ps16
Saccharo/Pezizomycotina - ps17
Saccharomycotina - ps18
Saccharomycotina_1 - ps19
Saccharomycotina_2 - ps20
Saccharomycotina_3 - ps21
Phaff./Saccharomycetaceae - ps22
Saccharomyco./mycetaceae - ps23
Saccharomycetaceae - ps24
Saccharomycetaceae_2 - ps25
Saccharomycetaceae_3 - ps26
Kaz./Nau./Saccharomyces - ps27
Saccharomyces - ps28
Arboricola_clade - ps29
Cerevisiae_clade - ps30
Saccharomyces_cerevisiae - ps31
Phylostratum

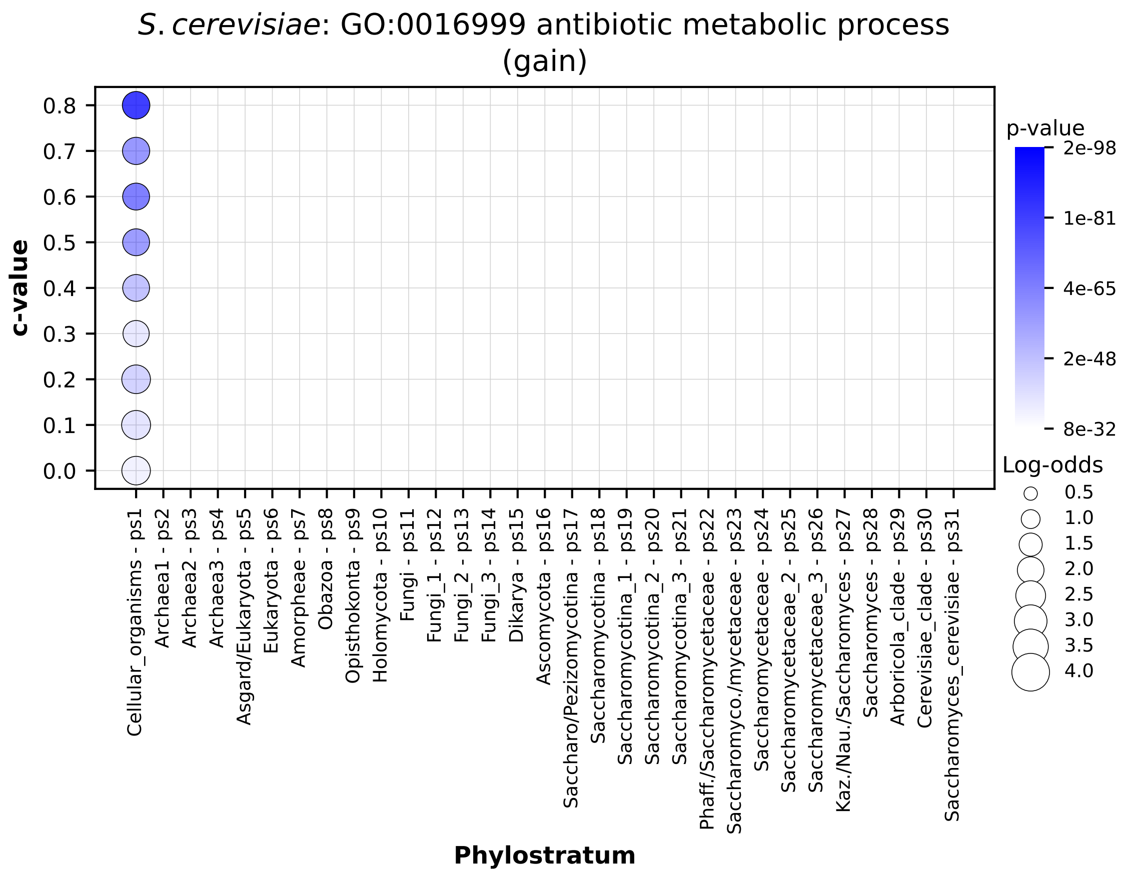

S. cerevisiae: GO:0016999 antibiotic metabolic process
(gain)
c-value
0.0
0.1
0.2
0.3
0.4
0.5
0.6
0.7
0.8
p-value
2e-98
1e-81
4e-65
2e-48
8e-32
Log-odds
0.5
1.0
1.5
2.0
2.5
3.0
3.5
4.0
Cellular_organisms - ps1
Archaea1 - ps2
Archaea2 - ps3
Archaea3 - ps4
Asgard/Eukaryota - ps5
Eukaryota - ps6
Amorpheae - ps7
Obazoa - ps8
Opisthokonta - ps9
Holomycota - ps10
Fungi - ps11
Fungi_1 - ps12
Fungi_2 - ps13
Fungi_3 - ps14
Dikarya - ps15
Ascomycota - ps16
Saccharo/Pezizomycotina - ps17
Saccharomycotina - ps18
Saccharomycotina_1 - ps19
Saccharomycotina_2 - ps20
Saccharomycotina_3 - ps21
Phaff./Saccharomycetaceae - ps22
Saccharomyco./mycetaceae - ps23
Saccharomycetaceae - ps24
Saccharomycetaceae_2 - ps25
Saccharomycetaceae_3 - ps26
Kaz./Nau./Saccharomyces - ps27
Saccharomyces - ps28
Arboricola_clade - ps29
Cerevisiae_clade - ps30
Saccharomyces_cerevisiae - ps31
Phylostratum

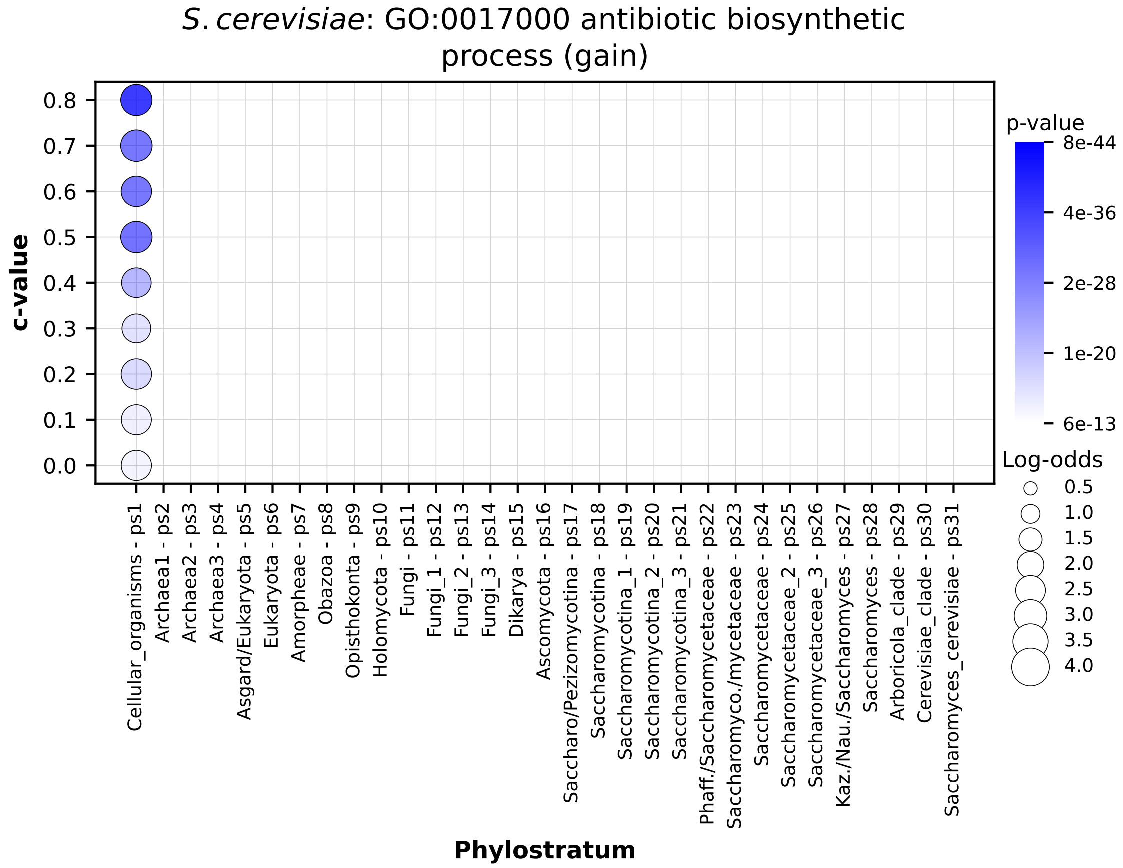
S. cerevisiae: GO:0017000 antibiotic biosynthetic process (gain)
c-value
0.0
0.1
0.2
0.3
0.4
0.5
0.6
0.7
0.8
p-value
8e-44
4e-36
2e-28
1e-20
6e-13
Log-odds
0.5
1.0
1.5
2.0
2.5
3.0
3.5
4.0
Cellular_organisms - ps1
Archaea1 - ps2
Archaea2 - ps3
Archaea3 - ps4
Asgard/Eukaryota - ps5
Eukaryota - ps6
Amorpheae - ps7
Obazoa - ps8
Opisthokonta - ps9
Holomycota - ps10
Fungi - ps11
Fungi_1 - ps12
Fungi_2 - ps13
Fungi_3 - ps14
Dikarya - ps15
Ascomycota - ps16
Saccharo/Pezizomycotina - ps17
Saccharomycotina - ps18
Saccharomycotina_1 - ps19
Saccharomycotina_2 - ps20
Saccharomycotina_3 - ps21
Phaff./Saccharomycetaceae - ps22
Saccharomyco./mycetaceae - ps23
Saccharomycetaceae - ps24
Saccharomycetaceae_2 - ps25
Saccharomycetaceae_3 - ps26
Kaz./Nau./Saccharomyces - ps27
Saccharomyces - ps28
Arboricola_clade - ps29
Cerevisiae_clade - ps30
Saccharomyces_cerevisiae - ps31
Phylostratum

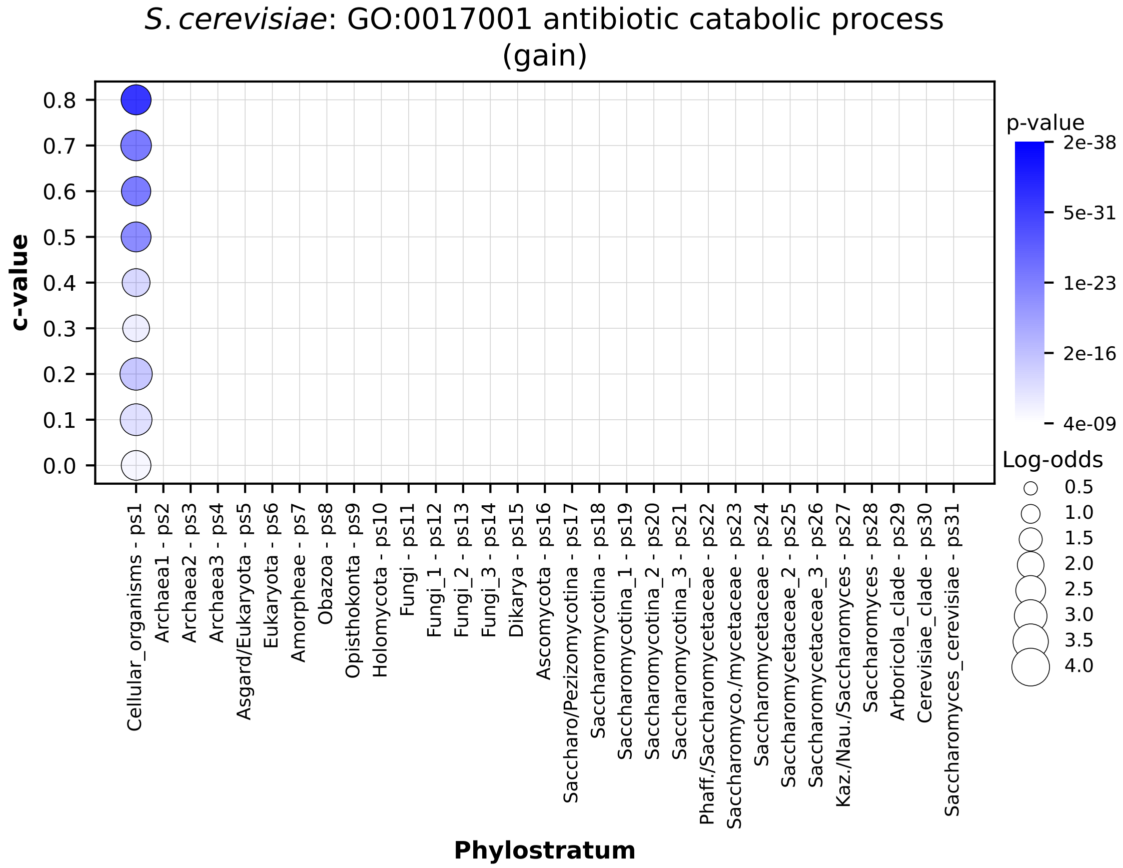

S. cerevisiae: GO:0017001 antibiotic catabolic process (gain)
c-value
0.8
0.7
0.6
0.5
0.4
0.3
0.2
0.1
0.0
p-value
2e-38
5e-31
1e-23
2e-16
4e-09
Log-odds
0.5
1.0
1.5
2.0
2.5
3.0
3.5
4.0
Cellular_organisms - ps1
Archaea1 - ps2
Archaea2 - ps3
Archaea3 - ps4
Asgard/Eukaryota - ps5
Eukaryota - ps6
Amorpheae - ps7
Obazoa - ps8
Opisthokonta - ps9
Holomycota - ps10
Fungi - ps11
Fungi_1 - ps12
Fungi_2 - ps13
Fungi_3 - ps14
Dikarya - ps15
Ascomycota - ps16
Saccharo/Pezizomycotina - ps17
Saccharomycotina - ps18
Saccharomycotina_1 - ps19
Saccharomycotina_2 - ps20
Saccharomycotina_3 - ps21
Phaff./Saccharomycetaceae - ps22
Saccharomyco./mycetaceae - ps23
Saccharomycetaceae - ps24
Saccharomycetaceae_2 - ps25
Saccharomycetaceae_3 - ps26
Kaz./Nau./Saccharomyces - ps27
Saccharomyces - ps28
Arboricola_clade - ps29
Cerevisiae_clade - ps30
Saccharomyces_cerevisiae - ps31
Phylostratum

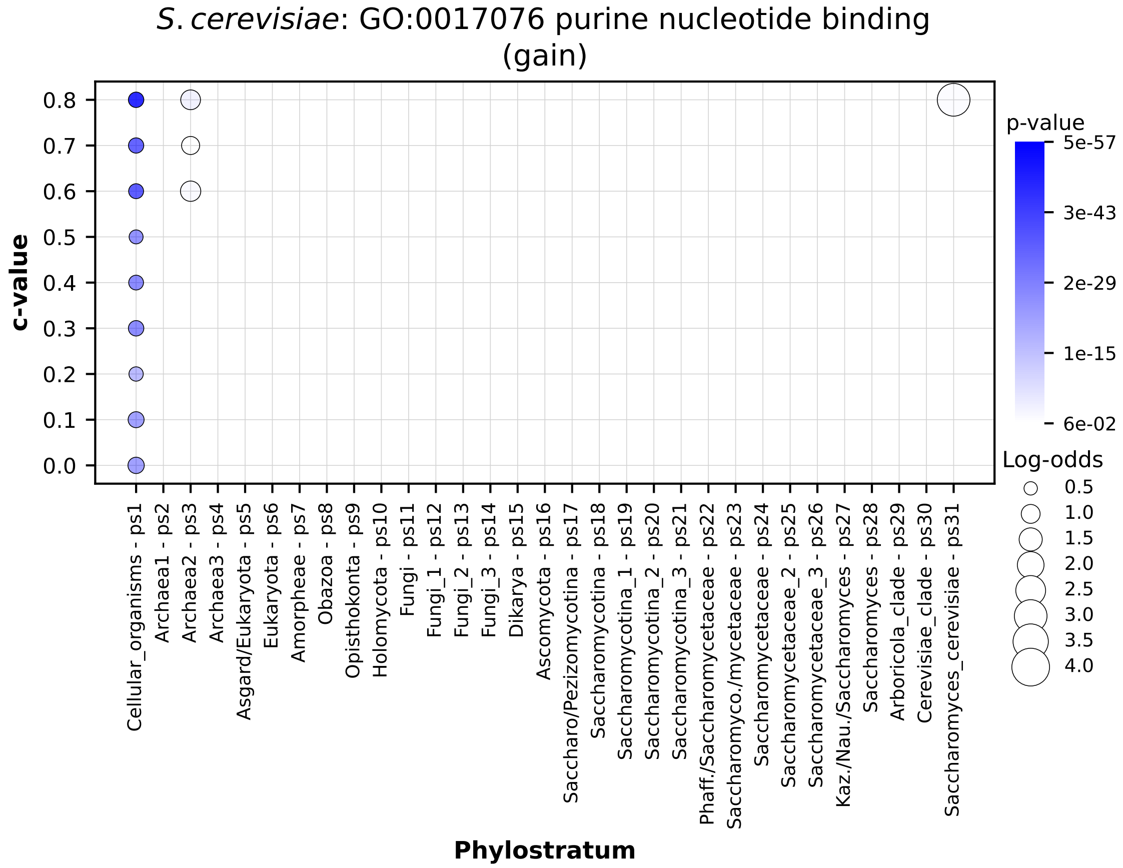
S. cerevisiae: GO:0017076 purine nucleotide binding (gain)
c-value
0.0
0.1
0.2
0.3
0.4
0.5
0.6
0.7
0.8
Phylostratum
Cellular_organisms - ps1
Archaea1 - ps2
Archaea2 - ps3
Archaea3 - ps4
Asgard/Eukaryota - ps5
Eukaryota - ps6
Amorpheae - ps7
Obazoa - ps8
Opisthokonta - ps9
Holomycota - ps10
Fungi - ps11
Fungi_1 - ps12
Fungi_2 - ps13
Fungi_3 - ps14
Dikarya - ps15
Ascomycota - ps16
Saccharo/Pezizomycotina - ps17
Saccharomycotina - ps18
Saccharomycotina_1 - ps19
Saccharomycotina_2 - ps20
Saccharomycotina_3 - ps21
Phaff./Saccharomycetaceae - ps22
Saccharomyco./mycetaceae - ps23
Saccharomycetaceae - ps24
Saccharomycetaceae_2 - ps25
Saccharomycetaceae_3 - ps26
Kaz./Nau./Saccharomyces - ps27
Saccharomyces - ps28
Arboricola_clade - ps29
Cerevisiae_clade - ps30
Saccharomyces_cerevisiae - ps31
p-value
5e-57
3e-43
2e-29
1e-15
6e-02
Log-odds
0.5
1.0
1.5
2.0
2.5
3.0
3.5
4.0

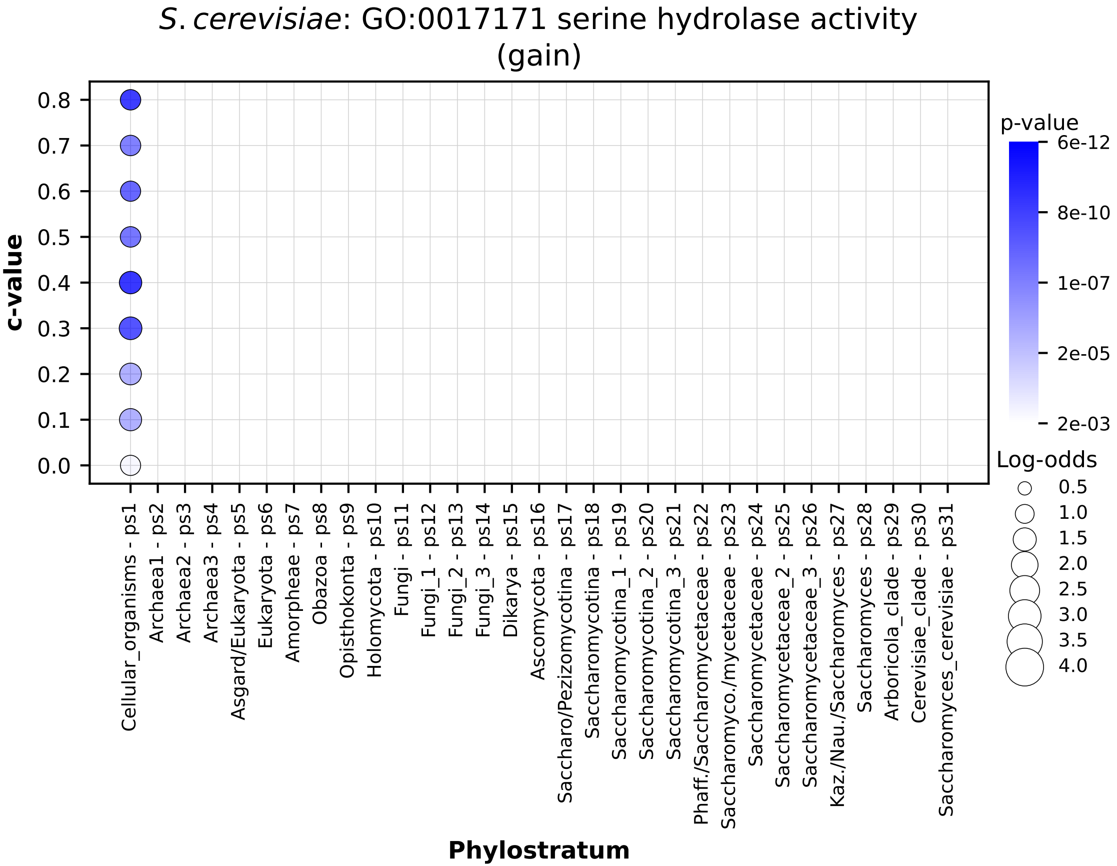
S. cerevisiae: GO:0017171 serine hydrolase activity
(gain)
c-value
0.8
0.7
0.6
0.5
0.4
0.3
0.2
0.1
0.0
p-value
6e-12
8e-10
1e-07
2e-05
2e-03
Log-odds
0.5
1.0
1.5
2.0
2.5
3.0
3.5
4.0
Cellular_organisms - ps1
Archaea1 - ps2
Archaea2 - ps3
Archaea3 - ps4
Asgard/Eukaryota - ps5
Eukaryota - ps6
Amorpheae - ps7
Obazoa - ps8
Opisthokonta - ps9
Holomycota - ps10
Fungi - ps11
Fungi_1 - ps12
Fungi_2 - ps13
Fungi_3 - ps14
Dikarya - ps15
Ascomycota - ps16
Saccharo/Pezizomycotina - ps17
Saccharomycotina - ps18
Saccharomycotina_1 - ps19
Saccharomycotina_2 - ps20
Saccharomycotina_3 - ps21
Phaff./Saccharomycetaceae - ps22
Saccharomyco./mycetaceae - ps23
Saccharomycetaceae - ps24
Saccharomycetaceae_2 - ps25
Saccharomycetaceae_3 - ps26
Kaz./Nau./Saccharomyces - ps27
Saccharomyces - ps28
Arboricola_clade - ps29
Cerevisiae_clade - ps30
Saccharomyces_cerevisiae - ps31
Phylostratum

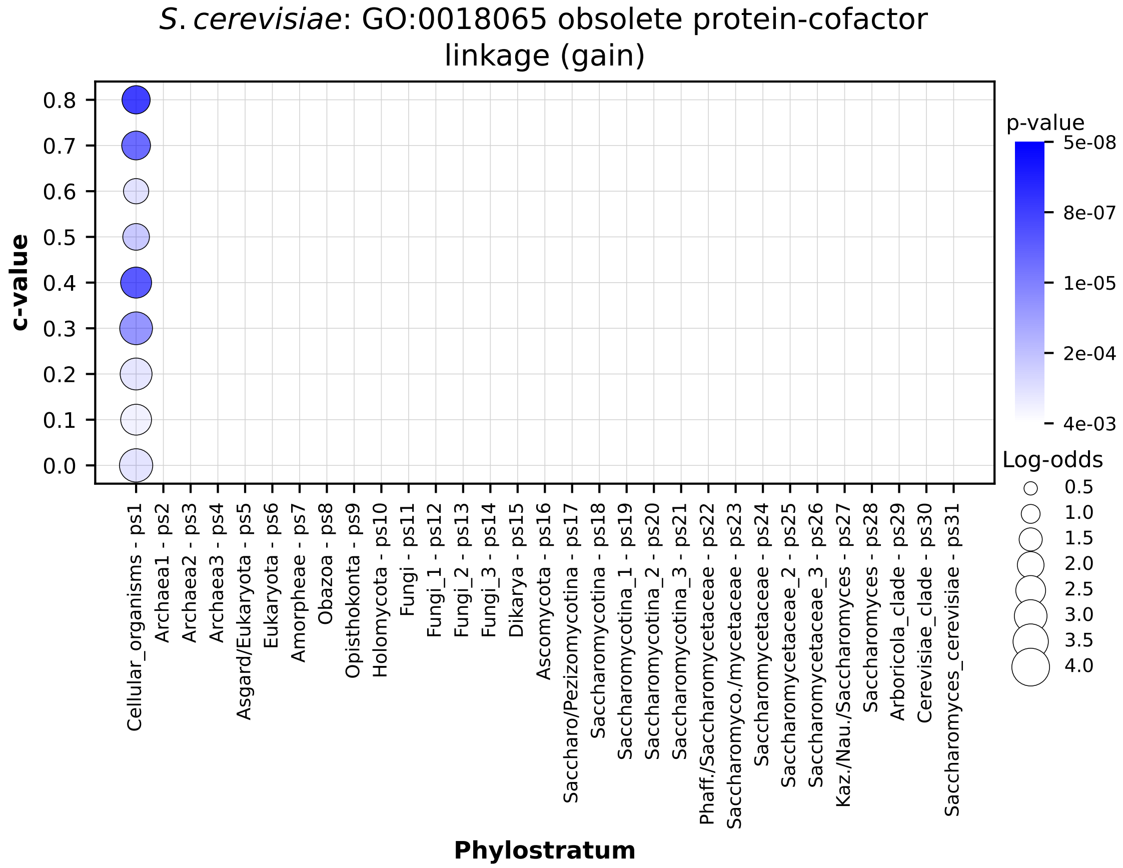

S. cerevisiae: GO:0018065 obsolete protein-cofactor linkage (gain)
c-value
0.8
0.7
0.6
0.5
0.4
0.3
0.2
0.1
0.0
p-value
5e-08
8e-07
1e-05
2e-04
4e-03
Log-odds
0.5
1.0
1.5
2.0
2.5
3.0
3.5
4.0
Cellular_organisms - ps1
Archaea1 - ps2
Archaea2 - ps3
Archaea3 - ps4
Asgard/Eukaryota - ps5
Eukaryota - ps6
Amorpheae - ps7
Obazoa - ps8
Opisthokonta - ps9
Holomycota - ps10
Fungi - ps11
Fungi_1 - ps12
Fungi_2 - ps13
Fungi_3 - ps14
Dikarya - ps15
Ascomycota - ps16
Saccharo/Pezizomycotina - ps17
Saccharomycotina - ps18
Saccharomycotina_1 - ps19
Saccharomycotina_2 - ps20
Saccharomycotina_3 - ps21
Phaff./Saccharomycetaceae - ps22
Saccharomyco./mycetaceae - ps23
Saccharomycetaceae - ps24
Saccharomycetaceae_2 - ps25
Saccharomycetaceae_3 - ps26
Kaz./Nau./Saccharomyces - ps27
Saccharomyces - ps28
Arboricola_clade - ps29
Cerevisiae_clade - ps30
Saccharomyces_cerevisiae - ps31
Phylostratum

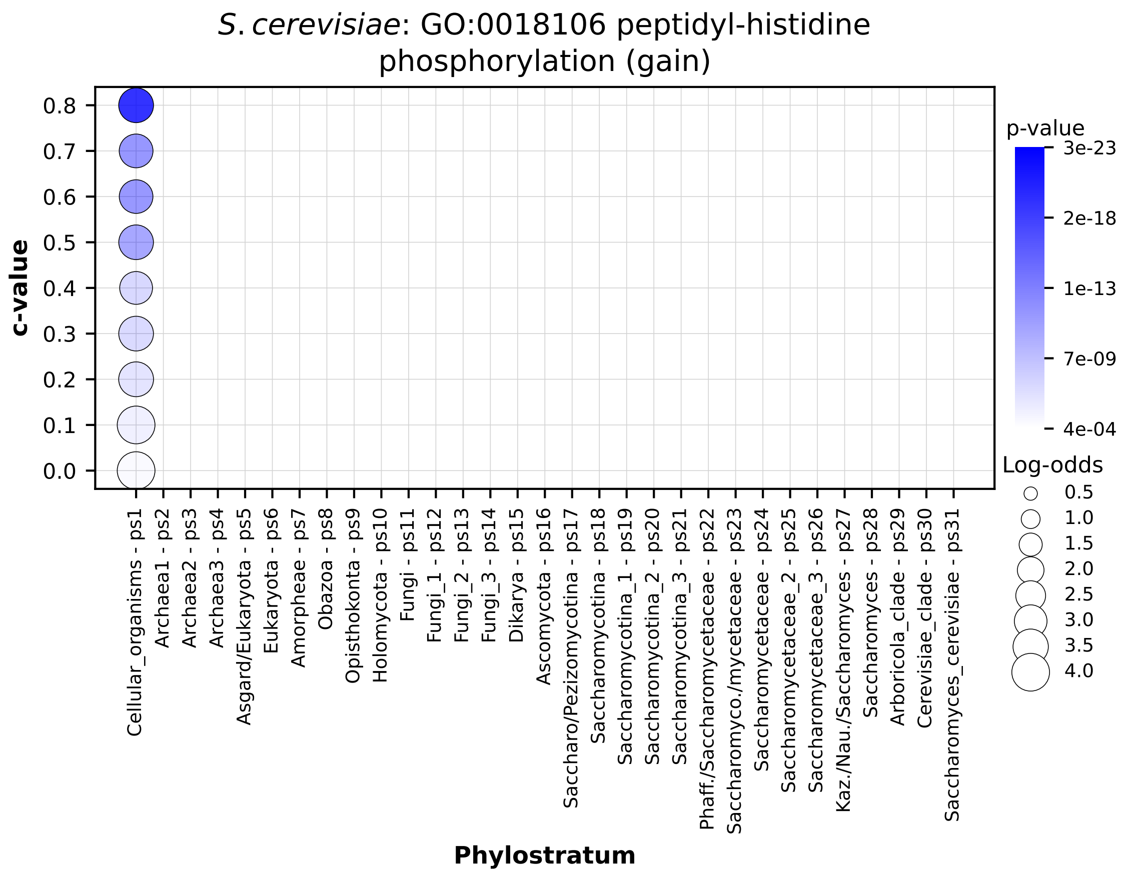
S. cerevisiae: GO:0018106 peptidyl-histidine phosphorylation (gain)
c-value
0.8
0.7
0.6
0.5
0.4
0.3
0.2
0.1
0.0
p-value
3e-23
2e-18
1e-13
7e-09
4e-04
Log-odds
0.5
1.0
1.5
2.0
2.5
3.0
3.5
4.0
Cellular_organisms - ps1
Archaea1 - ps2
Archaea2 - ps3
Archaea3 - ps4
Asgard/Eukaryota - ps5
Eukaryota - ps6
Amorpheae - ps7
Obazoa - ps8
Opisthokonta - ps9
Holomycota - ps10
Fungi - ps11
Fungi_1 - ps12
Fungi_2 - ps13
Fungi_3 - ps14
Dikarya - ps15
Ascomycota - ps16
Saccharo/Pezizomycotina - ps17
Saccharomycotina - ps18
Saccharomycotina_1 - ps19
Saccharomycotina_2 - ps20
Saccharomycotina_3 - ps21
Phaff./Saccharomycetaceae - ps22
Saccharomyco./mycetaceae - ps23
Saccharomycetaceae - ps24
Saccharomycetaceae_2 - ps25
Saccharomycetaceae_3 - ps26
Kaz./Nau./Saccharomyces - ps27
Saccharomyces - ps28
Arboricola_clade - ps29
Cerevisiae_clade - ps30
Saccharomyces_cerevisiae - ps31
Phylostratum

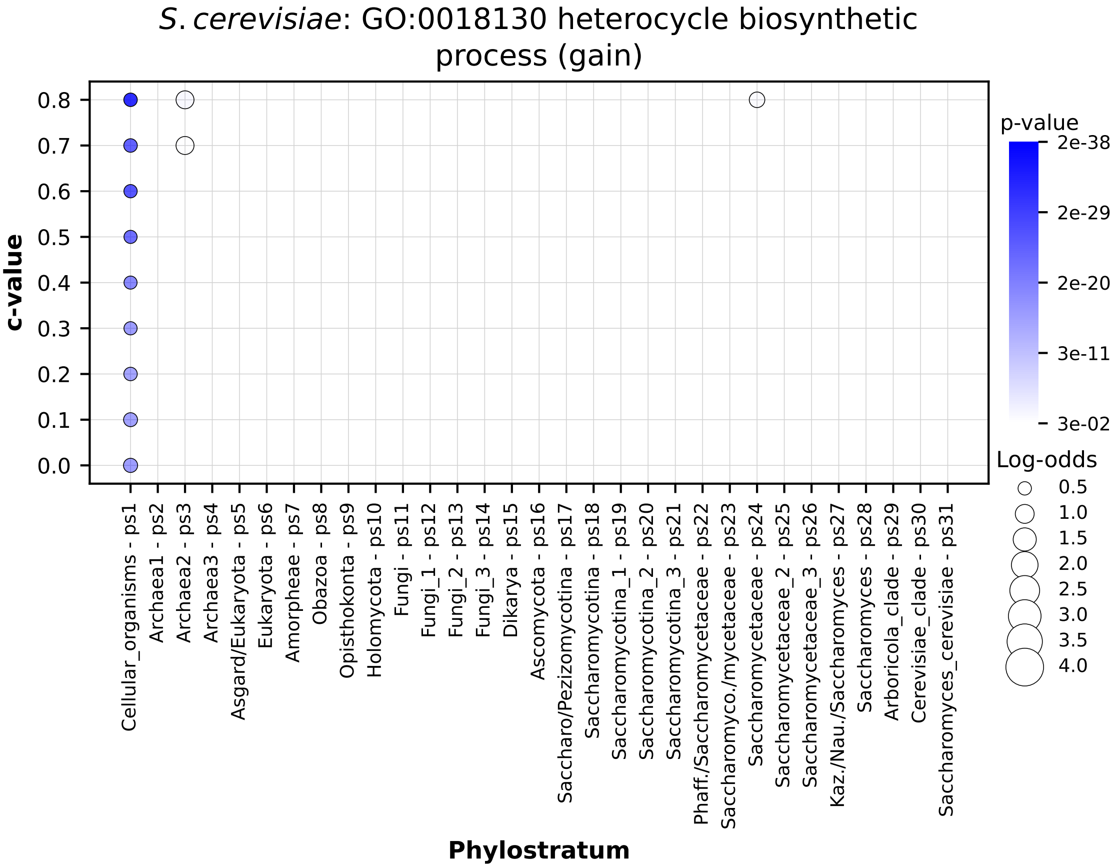
S. cerevisiae: GO:0018130 heterocycle biosynthetic process (gain)
c-value
0.8
0.7
0.6
0.5
0.4
0.3
0.2
0.1
0.0
p-value
2e-38
2e-29
2e-20
3e-11
3e-02
Log-odds
0.5
1.0
1.5
2.0
2.5
3.0
3.5
4.0
Cellular_organisms - ps1
Archaea1 - ps2
Archaea2 - ps3
Archaea3 - ps4
Asgard/Eukaryota - ps5
Eukaryota - ps6
Amorpheae - ps7
Obazoa - ps8
Opisthokonta - ps9
Holomycota - ps10
Fungi - ps11
Fungi_1 - ps12
Fungi_2 - ps13
Fungi_3 - ps14
Dikarya - ps15
Ascomycota - ps16
Saccharo/Pezizomycotina - ps17
Saccharomycotina - ps18
Saccharomycotina_1 - ps19
Saccharomycotina_2 - ps20
Saccharomycotina_3 - ps21
Phaff./Saccharomycetaceae - ps22
Saccharomyco./mycetaceae - ps23
Saccharomycetaceae - ps24
Saccharomycetaceae_2 - ps25
Saccharomycetaceae_3 - ps26
Kaz./Nau./Saccharomyces - ps27
Saccharomyces - ps28
Arboricola_clade - ps29
Cerevisiae_clade - ps30
Saccharomyces_cerevisiae - ps31
Phylostratum

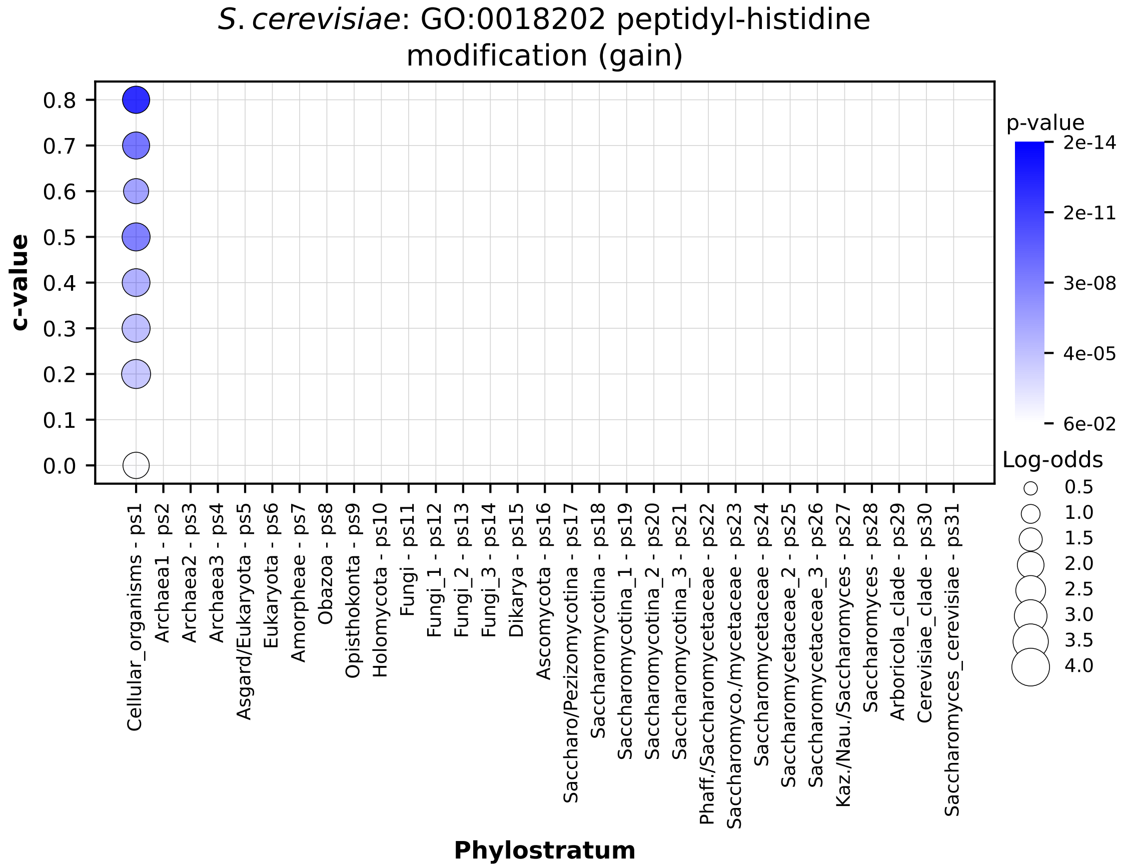

S. cerevisiae: GO:0018202 peptidyl-histidine modification (gain)
c-value
0.0
0.1
0.2
0.3
0.4
0.5
0.6
0.7
0.8
p-value
2e-14
2e-11
3e-08
4e-05
6e-02
Log-odds
0.5
1.0
1.5
2.0
2.5
3.0
3.5
4.0
Cellular_organisms - ps1
Archaea1 - ps2
Archaea2 - ps3
Archaea3 - ps4
Asgard/Eukaryota - ps5
Eukaryota - ps6
Amorpheae - ps7
Obazoa - ps8
Opisthokonta - ps9
Holomycota - ps10
Fungi - ps11
Fungi_1 - ps12
Fungi_2 - ps13
Fungi_3 - ps14
Dikarya - ps15
Ascomycota - ps16
Saccharo/Pezizomycotina - ps17
Saccharomycotina - ps18
Saccharomycotina_1 - ps19
Saccharomycotina_2 - ps20
Saccharomycotina_3 - ps21
Phaff./Saccharomycetaceae - ps22
Saccharomyco./mycetaceae - ps23
Saccharomycetaceae - ps24
Saccharomycetaceae_2 - ps25
Saccharomycetaceae_3 - ps26
Kaz./Nau./Saccharomyces - ps27
Saccharomyces - ps28
Arboricola_clade - ps29
Cerevisiae_clade - ps30
Saccharomyces_cerevisiae - ps31
Phylostratum

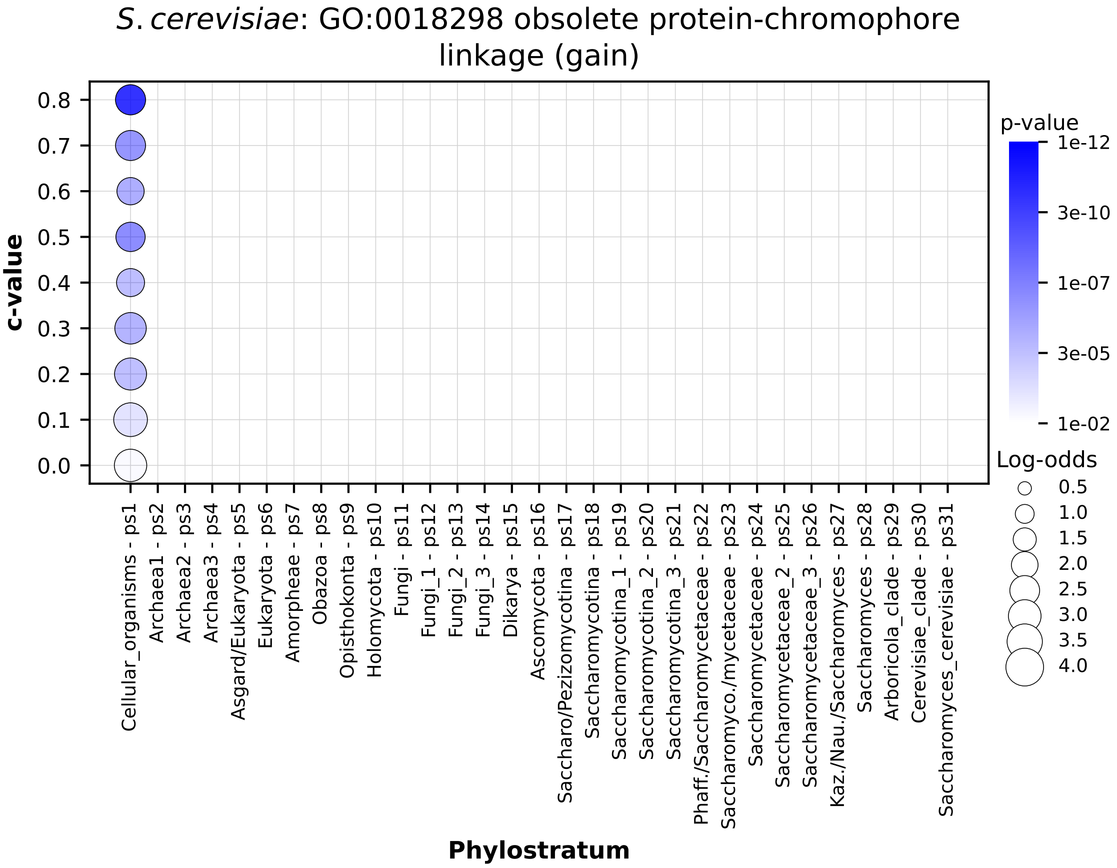

S. cerevisiae: GO:0018298 obsolete protein-chromophore linkage (gain)
c-value
0.0
0.1
0.2
0.3
0.4
0.5
0.6
0.7
0.8
p-value
1e-12
3e-10
1e-07
3e-05
1e-02
Log-odds
0.5
1.0
1.5
2.0
2.5
3.0
3.5
4.0
Cellular_organisms - ps1
Archaea1 - ps2
Archaea2 - ps3
Archaea3 - ps4
Asgard/Eukaryota - ps5
Eukaryota - ps6
Amorpheae - ps7
Obazoa - ps8
Opisthokonta - ps9
Holomycota - ps10
Fungi - ps11
Fungi_1 - ps12
Fungi_2 - ps13
Fungi_3 - ps14
Dikarya - ps15
Ascomycota - ps16
Saccharo/Pezizomycotina - ps17
Saccharomycotina - ps18
Saccharomycotina_1 - ps19
Saccharomycotina_2 - ps20
Saccharomycotina_3 - ps21
Phaff./Saccharomycetaceae - ps22
Saccharomyco./mycetaceae - ps23
Saccharomycetaceae - ps24
Saccharomycetaceae_2 - ps25
Saccharomycetaceae_3 - ps26
Kaz./Nau./Saccharomyces - ps27
Saccharomyces - ps28
Arboricola_clade - ps29
Cerevisiae_clade - ps30
Saccharomyces_cerevisiae - ps31
Phylostratum

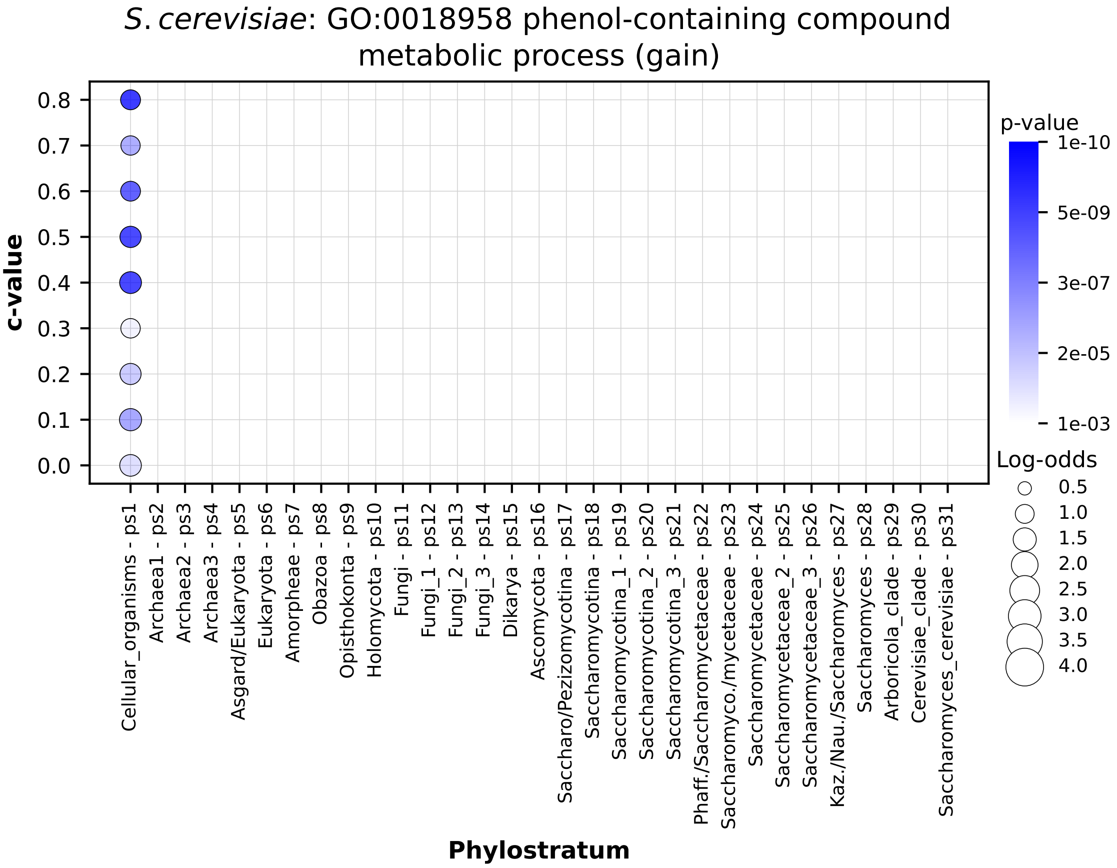

S. cerevisiae: GO:0018958 phenol-containing compound metabolic process (gain)
c-value
0.0
0.1
0.2
0.3
0.4
0.5
0.6
0.7
0.8
Phylostratum
Cellular_organisms - ps1
Archaea1 - ps2
Archaea2 - ps3
Archaea3 - ps4
Asgard/Eukaryota - ps5
Eukaryota - ps6
Amorpheae - ps7
Obazoa - ps8
Opisthokonta - ps9
Holomycota - ps10
Fungi - ps11
Fungi_1 - ps12
Fungi_2 - ps13
Fungi_3 - ps14
Dikarya - ps15
Ascomycota - ps16
Saccharo/Pezizomycotina - ps17
Saccharomycotina - ps18
Saccharomycotina_1 - ps19
Saccharomycotina_2 - ps20
Saccharomycotina_3 - ps21
Phaff./Saccharomycetaceae - ps22
Saccharomyco./mycetaceae - ps23
Saccharomycetaceae - ps24
Saccharomycetaceae_2 - ps25
Saccharomycetaceae_3 - ps26
Kaz./Nau./Saccharomyces - ps27
Saccharomyces - ps28
Arboricola_clade - ps29
Cerevisiae_clade - ps30
Saccharomyces_cerevisiae - ps31
p-value
1e-10
5e-09
3e-07
2e-05
1e-03
Log-odds
0.5
1.0
1.5
2.0
2.5
3.0
3.5
4.0

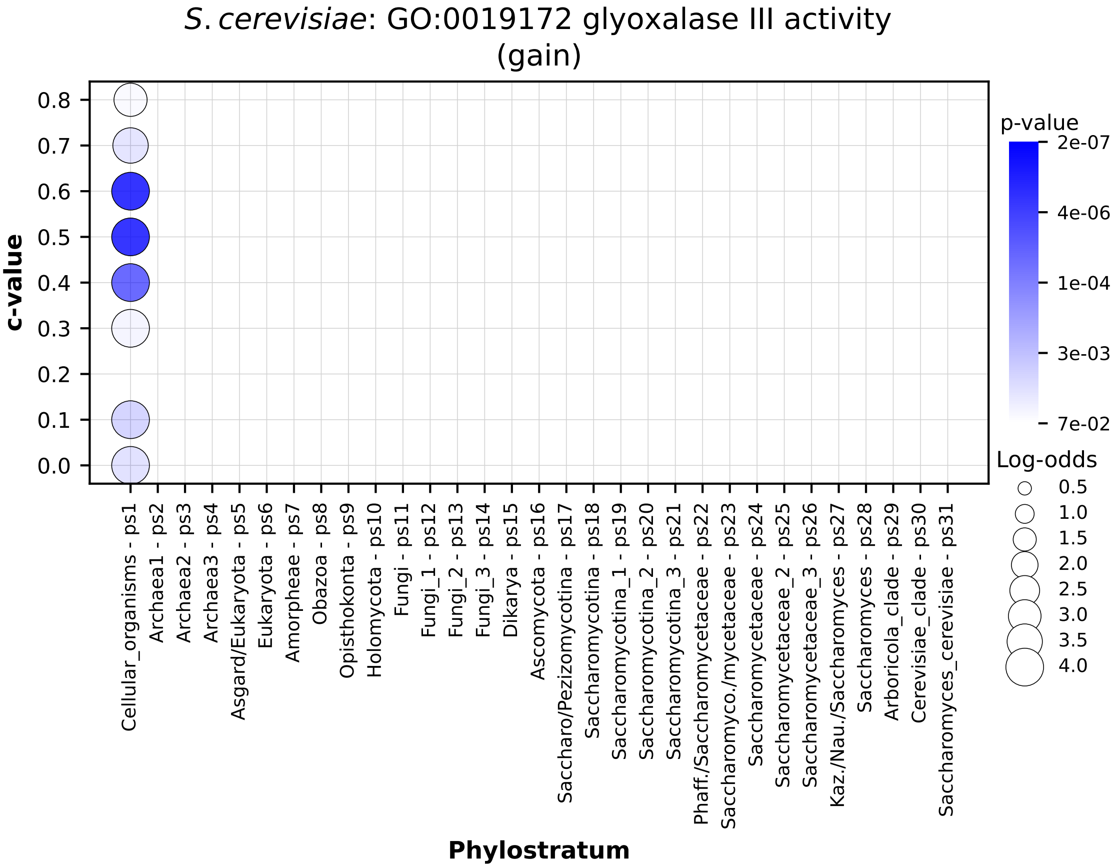

S. cerevisiae: GO:0019172 glyoxalase III activity
(gain)
c-value
0.8
0.7
0.6
0.5
0.4
0.3
0.2
0.1
0.0
Phylostratum
Cellular_organisms - ps1
Archaea1 - ps2
Archaea2 - ps3
Archaea3 - ps4
Asgard/Eukaryota - ps5
Eukaryota - ps6
Amorpheae - ps7
Obazoa - ps8
Opisthokonta - ps9
Holomycota - ps10
Fungi - ps11
Fungi_1 - ps12
Fungi_2 - ps13
Fungi_3 - ps14
Dikarya - ps15
Ascomycota - ps16
Saccharo/Pezizomycotina - ps17
Saccharomycotina - ps18
Saccharomycotina_1 - ps19
Saccharomycotina_2 - ps20
Saccharomycotina_3 - ps21
Phaff./Saccharomycetaceae - ps22
Saccharomyco./mycetaceae - ps23
Saccharomycetaceae - ps24
Saccharomycetaceae_2 - ps25
Saccharomycetaceae_3 - ps26
Kaz./Nau./Saccharomyces - ps27
Saccharomyces - ps28
Arboricola_clade - ps29
Cerevisiae_clade - ps30
Saccharomyces_cerevisiae - ps31
p-value
2e-07
4e-06
1e-04
3e-03
7e-02
Log-odds
0.5
1.0
1.5
2.0
2.5
3.0
3.5
4.0

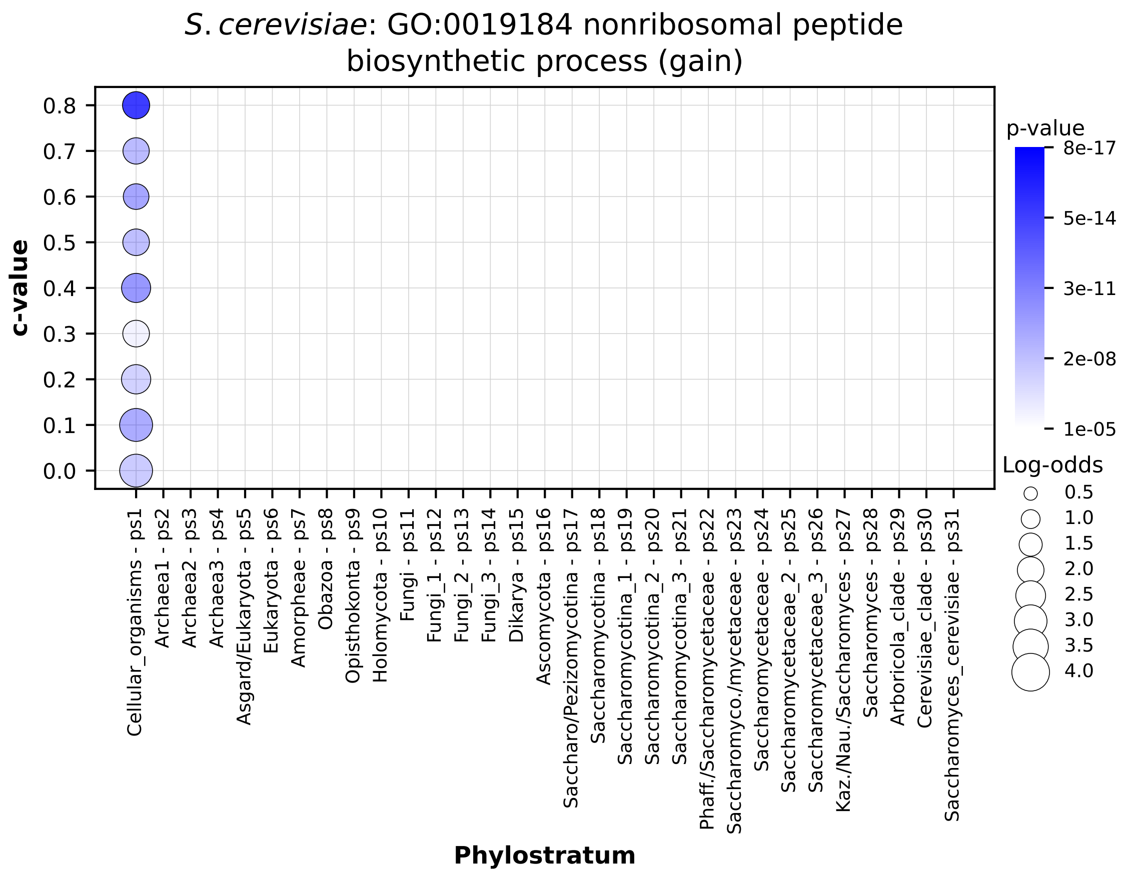

S. cerevisiae: GO:0019184 nonribosomal peptide biosynthetic process (gain)
c-value
0.8
0.7
0.6
0.5
0.4
0.3
0.2
0.1
0.0
p-value
8e-17
5e-14
3e-11
2e-08
1e-05
Log-odds
0.5
1.0
1.5
2.0
2.5
3.0
3.5
4.0
Cellular_organisms - ps1
Archaea1 - ps2
Archaea2 - ps3
Archaea3 - ps4
Asgard/Eukaryota - ps5
Eukaryota - ps6
Amorpheae - ps7
Obazoa - ps8
Opisthokonta - ps9
Holomycota - ps10
Fungi - ps11
Fungi_1 - ps12
Fungi_2 - ps13
Fungi_3 - ps14
Dikarya - ps15
Ascomycota - ps16
Saccharo/Pezizomycotina - ps17
Saccharomycotina - ps18
Saccharomycotina_1 - ps19
Saccharomycotina_2 - ps20
Saccharomycotina_3 - ps21
Phaff./Saccharomycetaceae - ps22
Saccharomyco./mycetaceae - ps23
Saccharomycetaceae - ps24
Saccharomycetaceae_2 - ps25
Saccharomycetaceae_3 - ps26
Kaz./Nau./Saccharomyces - ps27
Saccharomyces - ps28
Arboricola_clade - ps29
Cerevisiae_clade - ps30
Saccharomyces_cerevisiae - ps31
Phylostratum

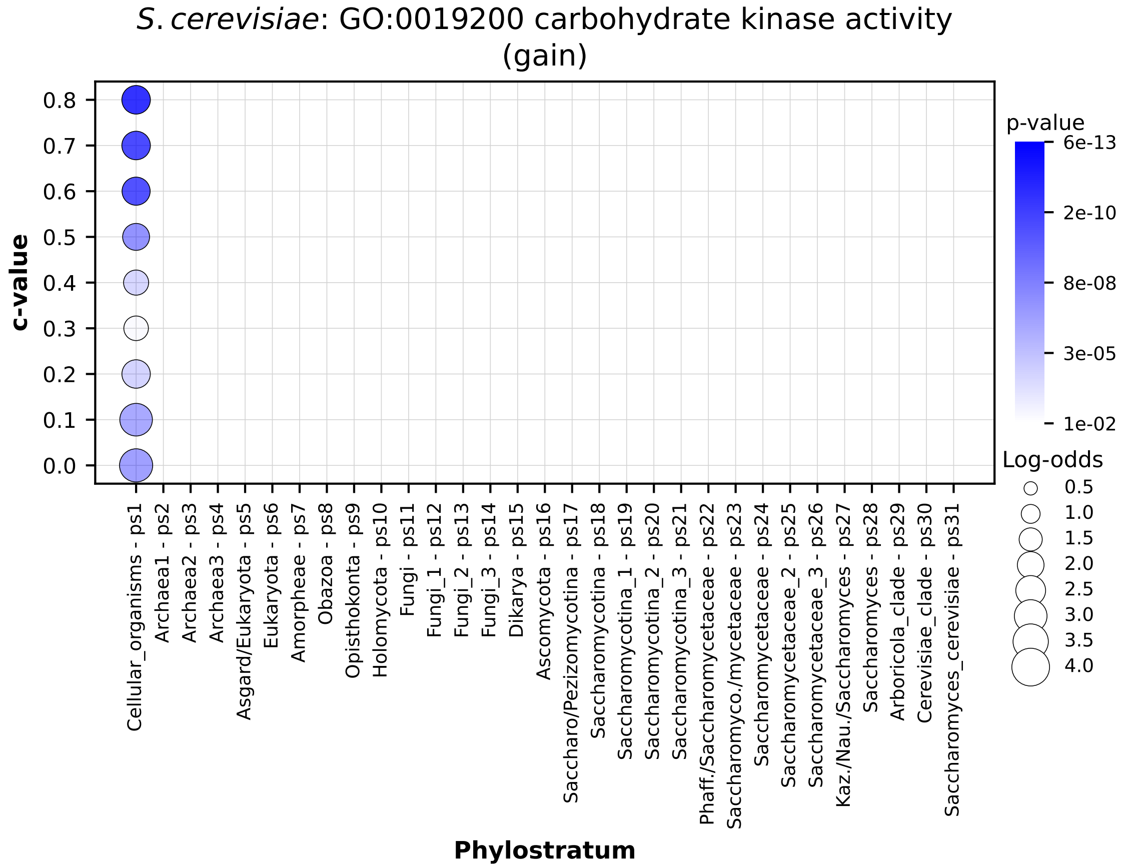
S. cerevisiae: GO:0019200 carbohydrate kinase activity (gain)
c-value
0.8
0.7
0.6
0.5
0.4
0.3
0.2
0.1
0.0
p-value
6e-13
2e-10
8e-08
3e-05
1e-02
Log-odds
0.5
1.0
1.5
2.0
2.5
3.0
3.5
4.0
Cellular_organisms - ps1
Archaea1 - ps2
Archaea2 - ps3
Archaea3 - ps4
Asgard/Eukaryota - ps5
Eukaryota - ps6
Amorpheae - ps7
Obazoa - ps8
Opisthokonta - ps9
Holomycota - ps10
Fungi - ps11
Fungi_1 - ps12
Fungi_2 - ps13
Fungi_3 - ps14
Dikarya - ps15
Ascomycota - ps16
Saccharo/Pezizomycotina - ps17
Saccharomycotina - ps18
Saccharomycotina_1 - ps19
Saccharomycotina_2 - ps20
Saccharomycotina_3 - ps21
Phaff./Saccharomycetaceae - ps22
Saccharomyco./mycetaceae - ps23
Saccharomycetaceae - ps24
Saccharomycetaceae_2 - ps25
Saccharomycetaceae_3 - ps26
Kaz./Nau./Saccharomyces - ps27
Saccharomyces - ps28
Arboricola_clade - ps29
Cerevisiae_clade - ps30
Saccharomyces_cerevisiae - ps31
Phylostratum

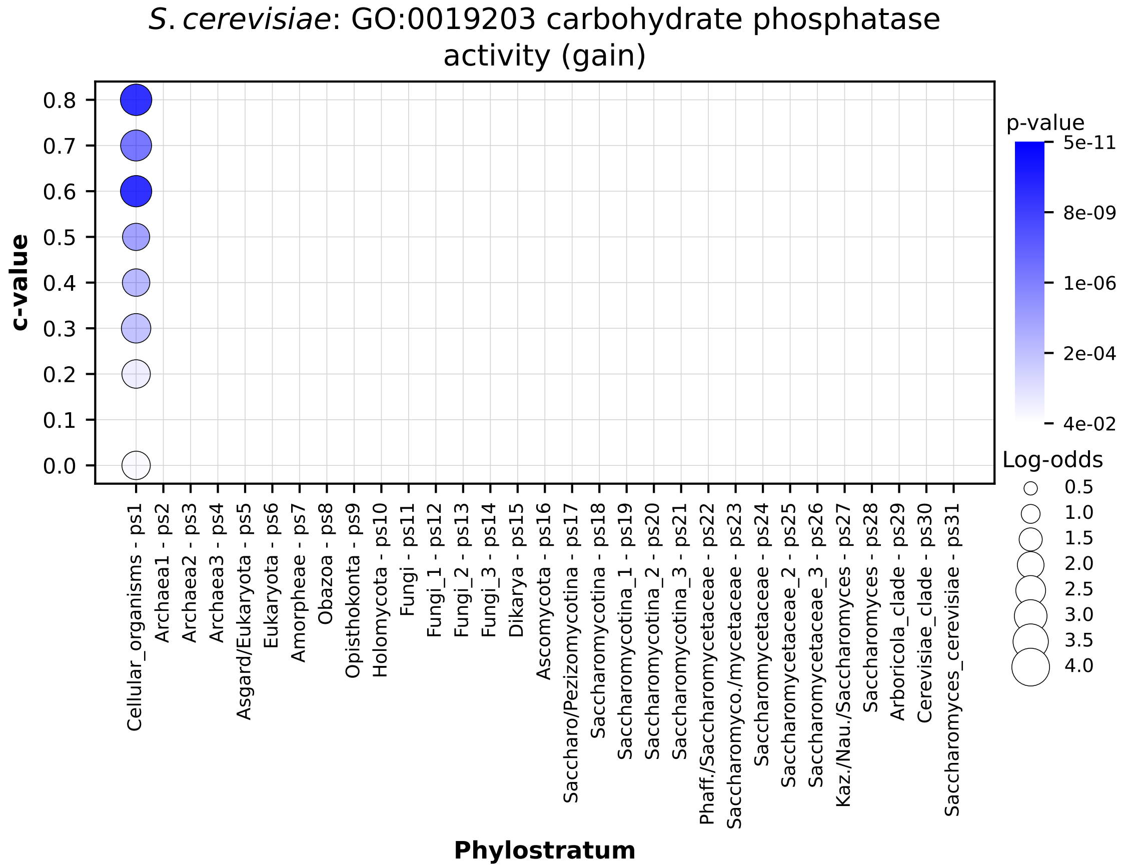

S. cerevisiae: GO:0019203 carbohydrate phosphatase activity (gain)
c-value
0.8
0.7
0.6
0.5
0.4
0.3
0.2
0.1
0.0
p-value
5e-11
8e-09
1e-06
2e-04
4e-02
Log-odds
0.5
1.0
1.5
2.0
2.5
3.0
3.5
4.0
Cellular_organisms - ps1
Archaea1 - ps2
Archaea2 - ps3
Archaea3 - ps4
Asgard/Eukaryota - ps5
Eukaryota - ps6
Amorpheae - ps7
Obazoa - ps8
Opisthokonta - ps9
Holomycota - ps10
Fungi - ps11
Fungi_1 - ps12
Fungi_2 - ps13
Fungi_3 - ps14
Dikarya - ps15
Ascomycota - ps16
Saccharo/Pezizomycotina - ps17
Saccharomycotina - ps18
Saccharomycotina_1 - ps19
Saccharomycotina_2 - ps20
Saccharomycotina_3 - ps21
Phaff./Saccharomycetaceae - ps22
Saccharomyco./mycetaceae - ps23
Saccharomycetaceae - ps24
Saccharomycetaceae_2 - ps25
Saccharomycetaceae_3 - ps26
Kaz./Nau./Saccharomyces - ps27
Saccharomyces - ps28
Arboricola_clade - ps29
Cerevisiae_clade - ps30
Saccharomyces_cerevisiae - ps31
Phylostratum

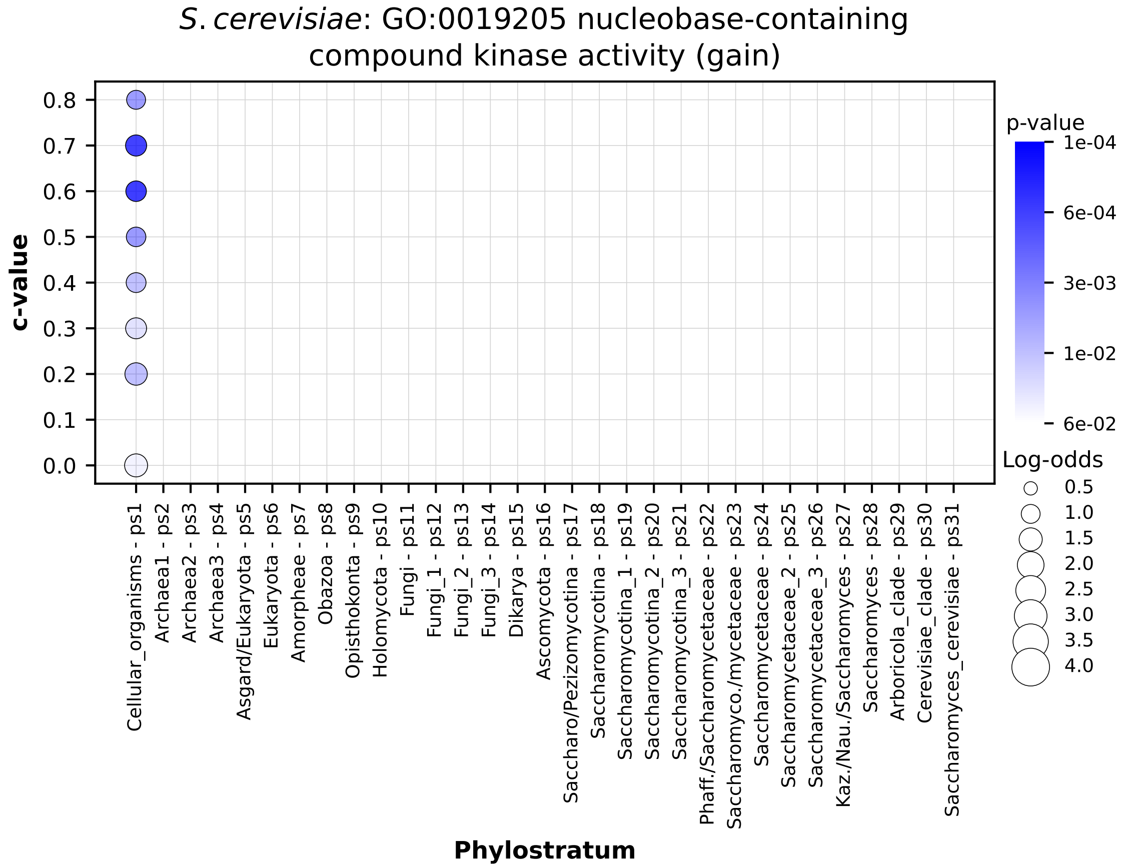
S. cerevisiae: GO:0019205 nucleobase-containing compound kinase activity (gain)
c-value
0.8
0.7
0.6
0.5
0.4
0.3
0.2
0.1
0.0
p-value
1e-04
6e-04
3e-03
1e-02
6e-02
Log-odds
0.5
1.0
1.5
2.0
2.5
3.0
3.5
4.0
Cellular_organisms - ps1
Archaea1 - ps2
Archaea2 - ps3
Archaea3 - ps4
Asgard/Eukaryota - ps5
Eukaryota - ps6
Amorpheae - ps7
Obazoa - ps8
Opisthokonta - ps9
Holomycota - ps10
Fungi - ps11
Fungi_1 - ps12
Fungi_2 - ps13
Fungi_3 - ps14
Dikarya - ps15
Ascomycota - ps16
Saccharo/Pezizomycotina - ps17
Saccharomycotina - ps18
Saccharomycotina_1 - ps19
Saccharomycotina_2 - ps20
Saccharomycotina_3 - ps21
Phaff./Saccharomycetaceae - ps22
Saccharomyco./mycetaceae - ps23
Saccharomycetaceae - ps24
Saccharomycetaceae_2 - ps25
Saccharomycetaceae_3 - ps26
Kaz./Nau./Saccharomyces - ps27
Saccharomyces - ps28
Arboricola_clade - ps29
Cerevisiae_clade - ps30
Saccharomyces_cerevisiae - ps31
Phylostratum

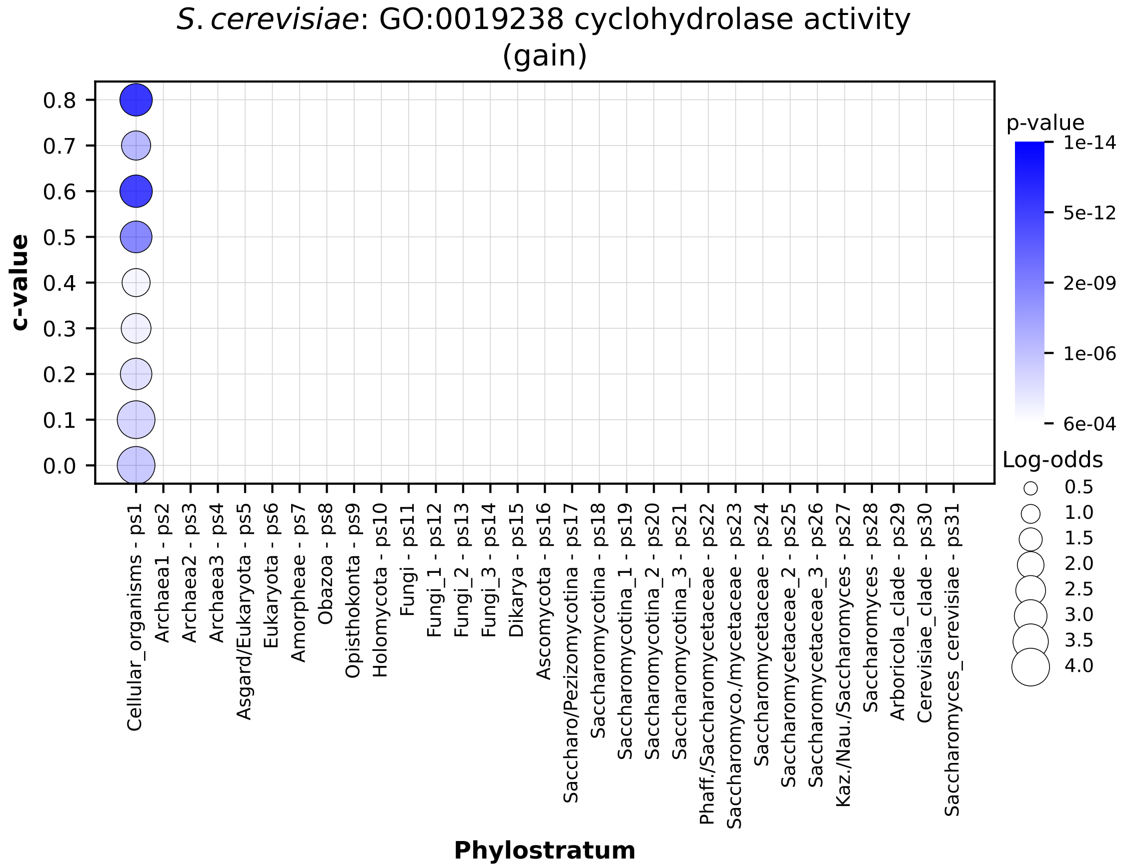

S. cerevisiae: GO:0019238 cyclohydrolase activity
(gain)
c-value
0.8
0.7
0.6
0.5
0.4
0.3
0.2
0.1
0.0
p-value
1e-14
5e-12
2e-09
1e-06
6e-04
Log-odds
0.5
1.0
1.5
2.0
2.5
3.0
3.5
4.0
Cellular_organisms - ps1
Archaea1 - ps2
Archaea2 - ps3
Archaea3 - ps4
Asgard/Eukaryota - ps5
Eukaryota - ps6
Amorpheae - ps7
Obazoa - ps8
Opisthokonta - ps9
Holomycota - ps10
Fungi - ps11
Fungi_1 - ps12
Fungi_2 - ps13
Fungi_3 - ps14
Dikarya - ps15
Ascomycota - ps16
Saccharo/Pezizomycotina - ps17
Saccharomycotina - ps18
Saccharomycotina_1 - ps19
Saccharomycotina_2 - ps20
Saccharomycotina_3 - ps21
Phaff./Saccharomycetaceae - ps22
Saccharomyco./mycetaceae - ps23
Saccharomycetaceae - ps24
Saccharomycetaceae_2 - ps25
Saccharomycetaceae_3 - ps26
Kaz./Nau./Saccharomyces - ps27
Saccharomyces - ps28
Arboricola_clade - ps29
Cerevisiae_clade - ps30
Saccharomyces_cerevisiae - ps31
Phylostratum

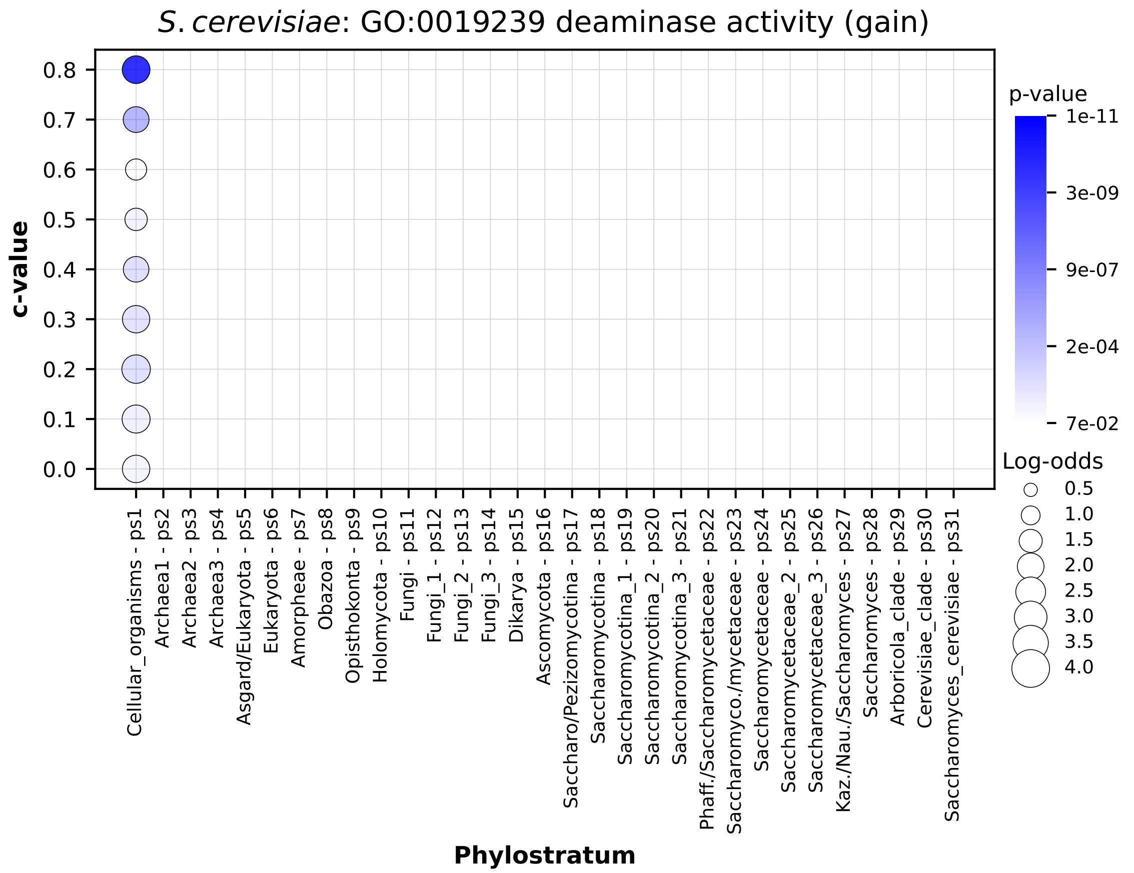

S. cerevisiae: GO:0019239 deaminase activity (gain)
c-value
0.8
0.7
0.6
0.5
0.4
0.3
0.2
0.1
0.0
p-value
1e-11
3e-09
9e-07
2e-04
7e-02
Log-odds
0.5
1.0
1.5
2.0
2.5
3.0
3.5
4.0
Cellular_organisms - ps1
Archaea1 - ps2
Archaea2 - ps3
Archaea3 - ps4
Asgard/Eukaryota - ps5
Eukaryota - ps6
Amorpheae - ps7
Obazoa - ps8
Opisthokonta - ps9
Holomycota - ps10
Fungi - ps11
Fungi_1 - ps12
Fungi_2 - ps13
Fungi_3 - ps14
Dikarya - ps15
Ascomycota - ps16
Saccharo/Pezizomycotina - ps17
Saccharomycotina - ps18
Saccharomycotina_1 - ps19
Saccharomycotina_2 - ps20
Saccharomycotina_3 - ps21
Phaff./Saccharomycetaceae - ps22
Saccharomyco./mycetaceae - ps23
Saccharomycetaceae - ps24
Saccharomycetaceae_2 - ps25
Saccharomycetaceae_3 - ps26
Kaz./Nau./Saccharomyces - ps27
Saccharomyces - ps28
Arboricola_clade - ps29
Cerevisiae_clade - ps30
Saccharomyces_cerevisiae - ps31
Phylostratum

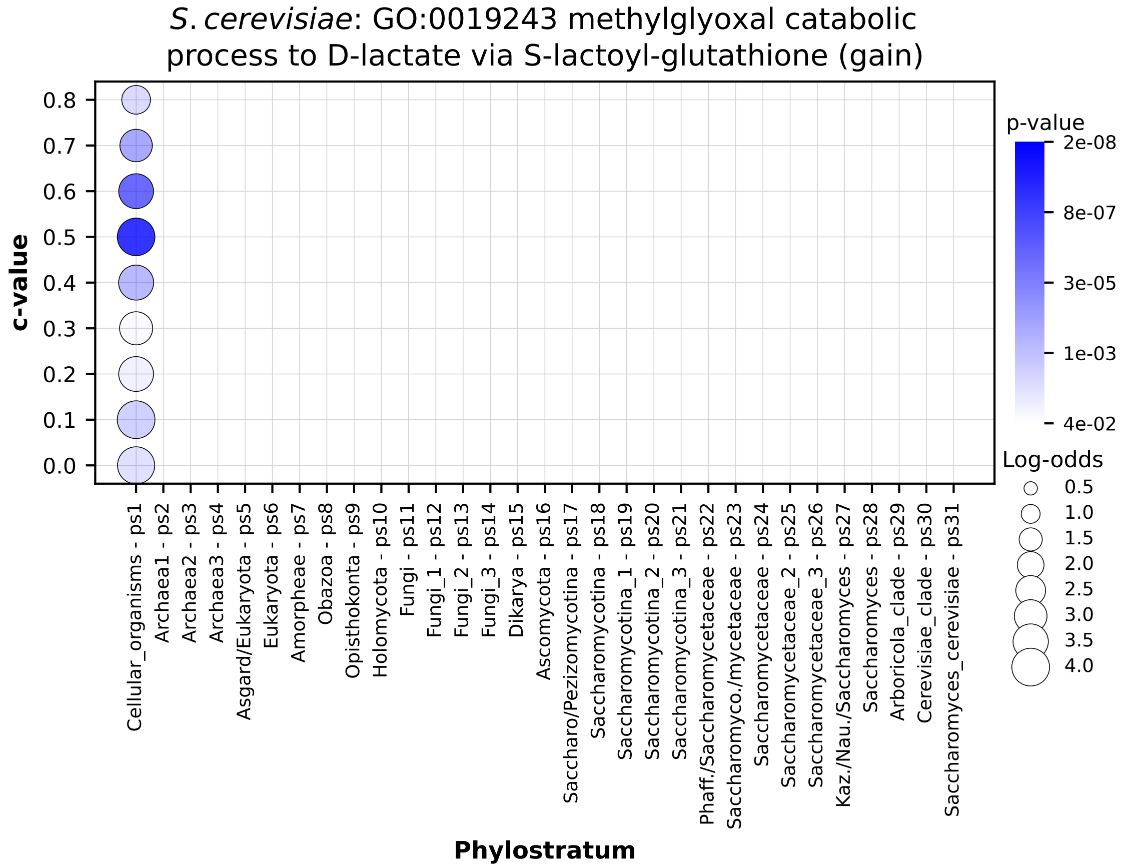

S. cerevisiae: GO:0019243 methylglyoxal catabolic process to D-lactate via S-lactoyl-glutathione (gain)
c-value
0.0
0.1
0.2
0.3
0.4
0.5
0.6
0.7
0.8
Phylostratum
Cellular_organisms - ps1
Archaea1 - ps2
Archaea2 - ps3
Archaea3 - ps4
Asgard/Eukaryota - ps5
Eukaryota - ps6
Amorpheae - ps7
Obazoa - ps8
Opisthokonta - ps9
Holomycota - ps10
Fungi - ps11
Fungi_1 - ps12
Fungi_2 - ps13
Fungi_3 - ps14
Dikarya - ps15
Ascomycota - ps16
Saccharo/Pezizomycotina - ps17
Saccharomycotina - ps18
Saccharomycotina_1 - ps19
Saccharomycotina_2 - ps20
Saccharomycotina_3 - ps21
Phaff./Saccharomycetaceae - ps22
Saccharomyco./mycetaceae - ps23
Saccharomycetaceae - ps24
Saccharomycetaceae_2 - ps25
Saccharomycetaceae_3 - ps26
Kaz./Nau./Saccharomyces - ps27
Saccharomyces - ps28
Arboricola_clade - ps29
Cerevisiae_clade - ps30
Saccharomyces_cerevisiae - ps31
p-value
2e-08
8e-07
3e-05
1e-03
4e-02
Log-odds
0.5
1.0
1.5
2.0
2.5
3.0
3.5
4.0

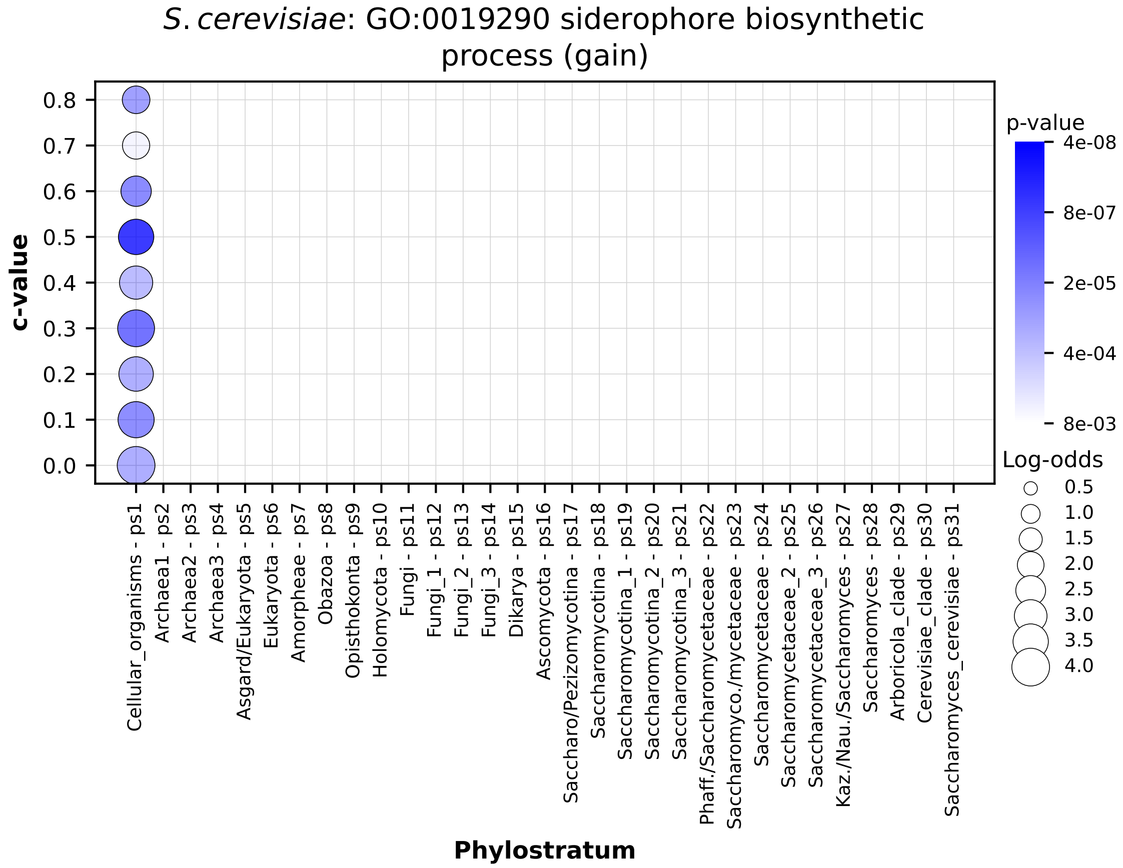
S. cerevisiae: GO:0019290 siderophore biosynthetic process (gain)
c-value
0.8
0.7
0.6
0.5
0.4
0.3
0.2
0.1
0.0
p-value
4e-08
8e-07
2e-05
4e-04
8e-03
Log-odds
0.5
1.0
1.5
2.0
2.5
3.0
3.5
4.0
Cellular_organisms - ps1
Archaea1 - ps2
Archaea2 - ps3
Archaea3 - ps4
Asgard/Eukaryota - ps5
Eukaryota - ps6
Amorpheae - ps7
Obazoa - ps8
Opisthokonta - ps9
Holomycota - ps10
Fungi - ps11
Fungi_1 - ps12
Fungi_2 - ps13
Fungi_3 - ps14
Dikarya - ps15
Ascomycota - ps16
Saccharo/Pezizomycotina - ps17
Saccharomycotina - ps18
Saccharomycotina_1 - ps19
Saccharomycotina_2 - ps20
Saccharomycotina_3 - ps21
Phaff./Saccharomycetaceae - ps22
Saccharomyco./mycetaceae - ps23
Saccharomycetaceae - ps24
Saccharomycetaceae_2 - ps25
Saccharomycetaceae_3 - ps26
Kaz./Nau./Saccharomyces - ps27
Saccharomyces - ps28
Arboricola_clade - ps29
Cerevisiae_clade - ps30
Saccharomyces_cerevisiae - ps31
Phylostratum

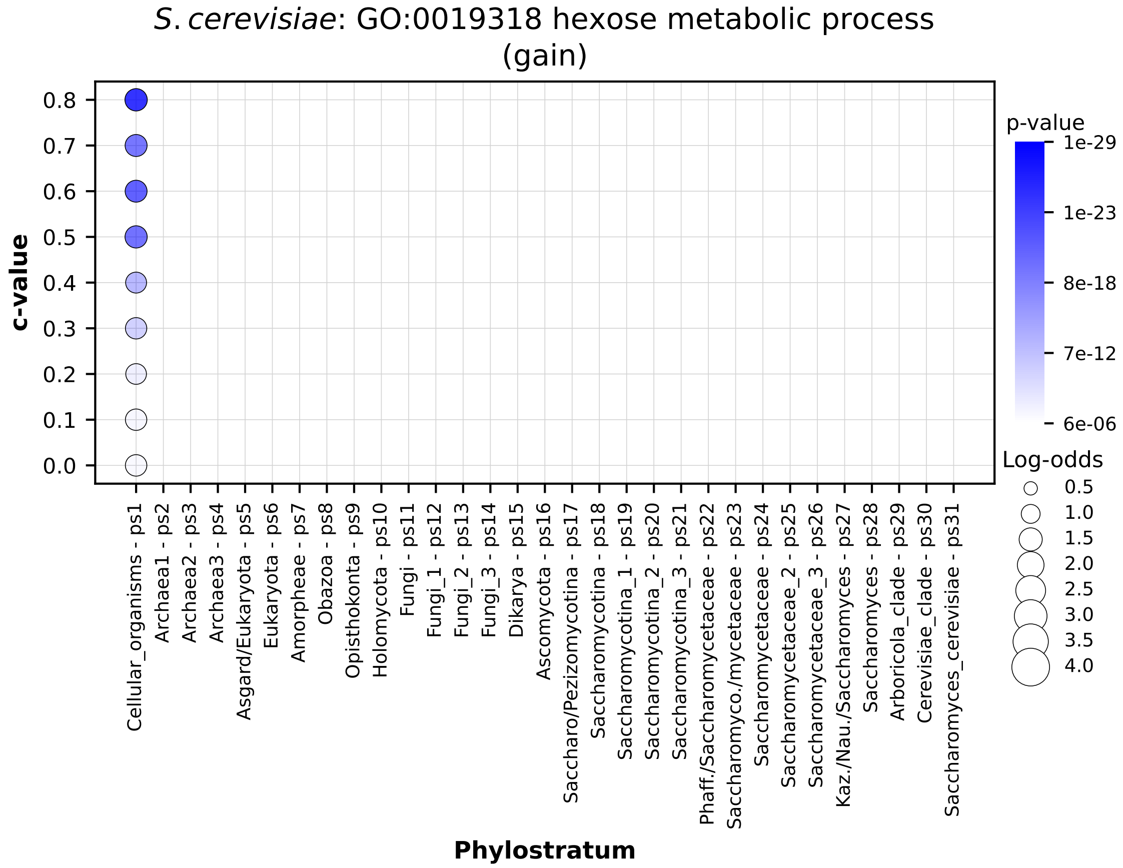

S. cerevisiae: GO:0019318 hexose metabolic process
(gain)
c-value
0.0
0.1
0.2
0.3
0.4
0.5
0.6
0.7
0.8
p-value
1e-29
1e-23
8e-18
7e-12
6e-06
Log-odds
0.5
1.0
1.5
2.0
2.5
3.0
3.5
4.0
Cellular_organisms - ps1
Archaea1 - ps2
Archaea2 - ps3
Archaea3 - ps4
Asgard/Eukaryota - ps5
Eukaryota - ps6
Amorpheae - ps7
Obazoa - ps8
Opisthokonta - ps9
Holomycota - ps10
Fungi - ps11
Fungi_1 - ps12
Fungi_2 - ps13
Fungi_3 - ps14
Dikarya - ps15
Ascomycota - ps16
Saccharo/Pezizomycotina - ps17
Saccharomycotina - ps18
Saccharomycotina_1 - ps19
Saccharomycotina_2 - ps20
Saccharomycotina_3 - ps21
Phaff./Saccharomycetaceae - ps22
Saccharomyco./mycetaceae - ps23
Saccharomycetaceae - ps24
Saccharomycetaceae_2 - ps25
Saccharomycetaceae_3 - ps26
Kaz./Nau./Saccharomyces - ps27
Saccharomyces - ps28
Arboricola_clade - ps29
Cerevisiae_clade - ps30
Saccharomyces_cerevisiae - ps31
Phylostratum

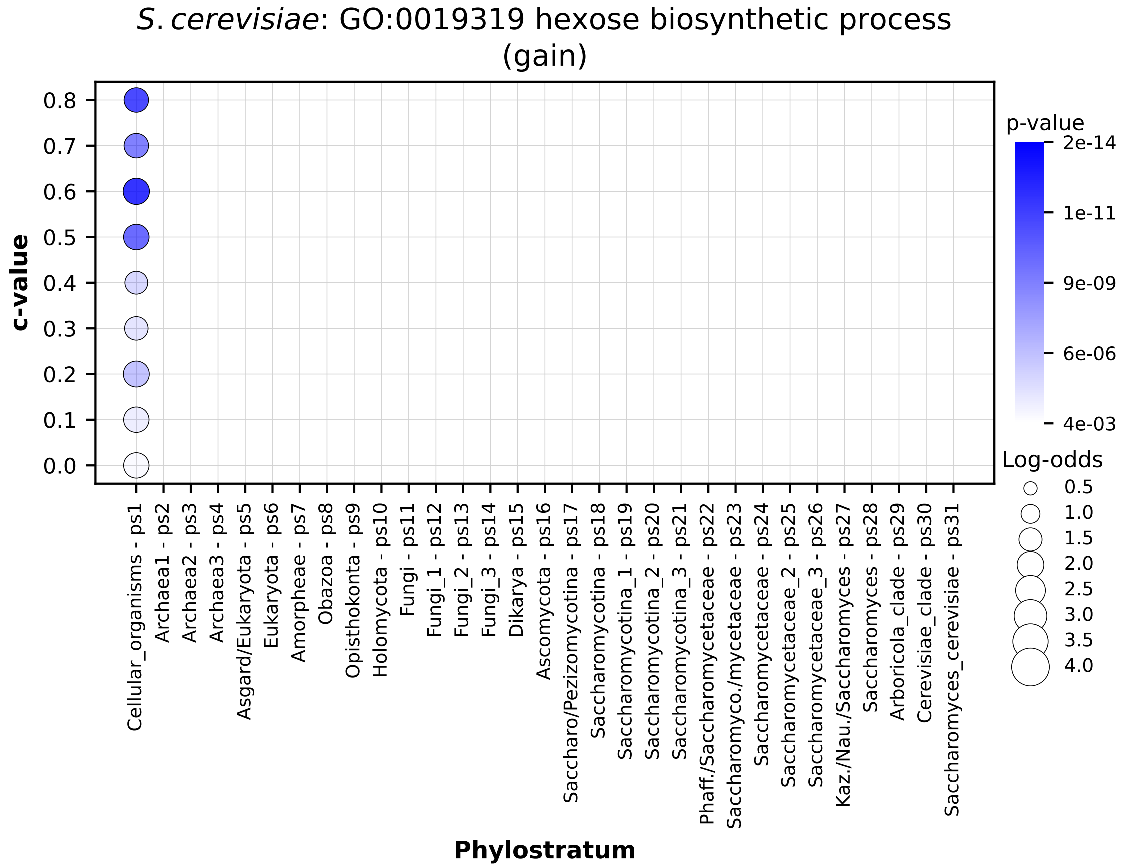

S. cerevisiae: GO:0019319 hexose biosynthetic process
(gain)
c-value
0.8
0.7
0.6
0.5
0.4
0.3
0.2
0.1
0.0
p-value
2e-14
1e-11
9e-09
6e-06
4e-03
Log-odds
0.5
1.0
1.5
2.0
2.5
3.0
3.5
4.0
Cellular_organisms - ps1
Archaea1 - ps2
Archaea2 - ps3
Archaea3 - ps4
Asgard/Eukaryota - ps5
Eukaryota - ps6
Amorpheae - ps7
Obazoa - ps8
Opisthokonta - ps9
Holomycota - ps10
Fungi - ps11
Fungi_1 - ps12
Fungi_2 - ps13
Fungi_3 - ps14
Dikarya - ps15
Ascomycota - ps16
Saccharo/Pezizomycotina - ps17
Saccharomycotina - ps18
Saccharomycotina_1 - ps19
Saccharomycotina_2 - ps20
Saccharomycotina_3 - ps21
Phaff./Saccharomycetaceae - ps22
Saccharomyco./mycetaceae - ps23
Saccharomycetaceae - ps24
Saccharomycetaceae_2 - ps25
Saccharomycetaceae_3 - ps26
Kaz./Nau./Saccharomyces - ps27
Saccharomyces - ps28
Arboricola_clade - ps29
Cerevisiae_clade - ps30
Saccharomyces_cerevisiae - ps31
Phylostratum

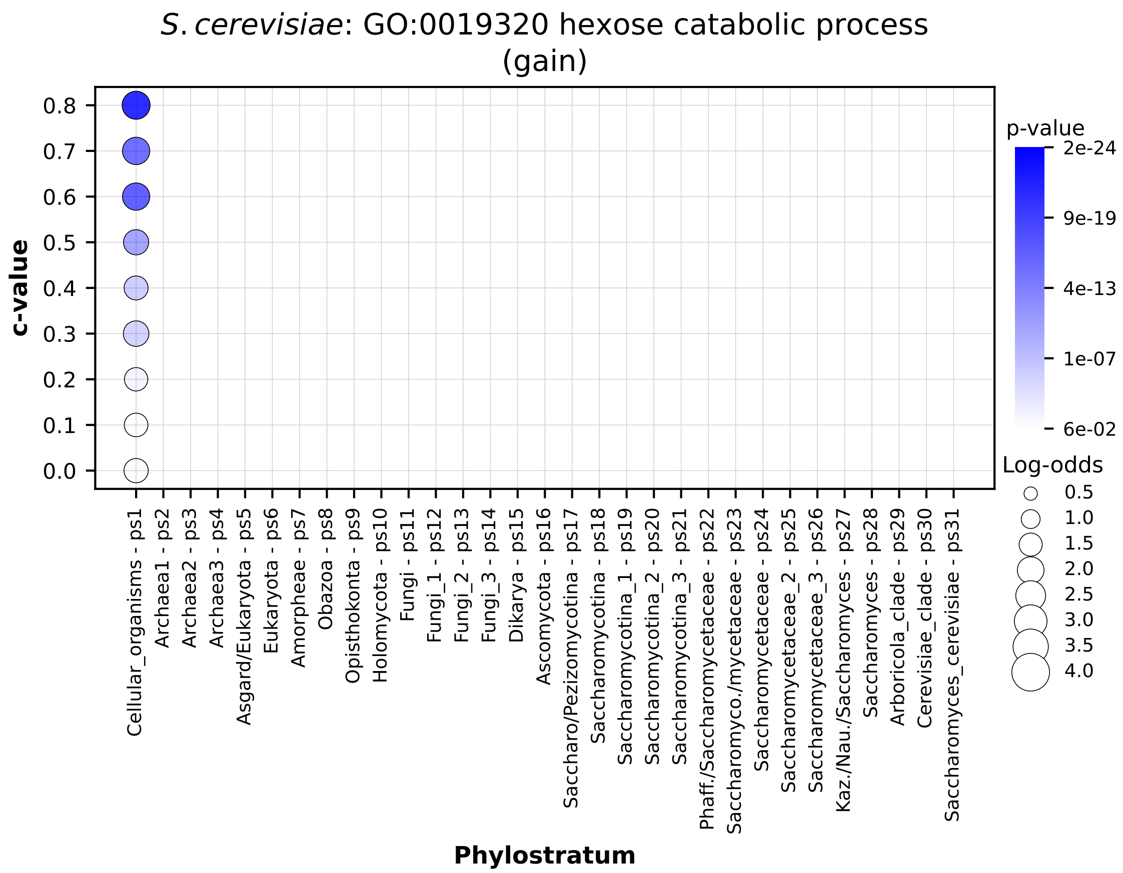
S. cerevisiae: GO:0019320 hexose catabolic process
(gain)
c-value
0.8
0.7
0.6
0.5
0.4
0.3
0.2
0.1
0.0
p-value
2e-24
9e-19
4e-13
1e-07
6e-02
Log-odds
0.5
1.0
1.5
2.0
2.5
3.0
3.5
4.0
Cellular_organisms - ps1
Archaea1 - ps2
Archaea2 - ps3
Archaea3 - ps4
Asgard/Eukaryota - ps5
Eukaryota - ps6
Amorpheae - ps7
Obazoa - ps8
Opisthokonta - ps9
Holomycota - ps10
Fungi - ps11
Fungi_1 - ps12
Fungi_2 - ps13
Fungi_3 - ps14
Dikarya - ps15
Ascomycota - ps16
Saccharo/Pezizomycotina - ps17
Saccharomycotina - ps18
Saccharomycotina_1 - ps19
Saccharomycotina_2 - ps20
Saccharomycotina_3 - ps21
Phaff./Saccharomycetaceae - ps22
Saccharomyco./mycetaceae - ps23
Saccharomycetaceae - ps24
Saccharomycetaceae_2 - ps25
Saccharomycetaceae_3 - ps26
Kaz./Nau./Saccharomyces - ps27
Saccharomyces - ps28
Arboricola_clade - ps29
Cerevisiae_clade - ps30
Saccharomyces_cerevisiae - ps31
Phylostratum

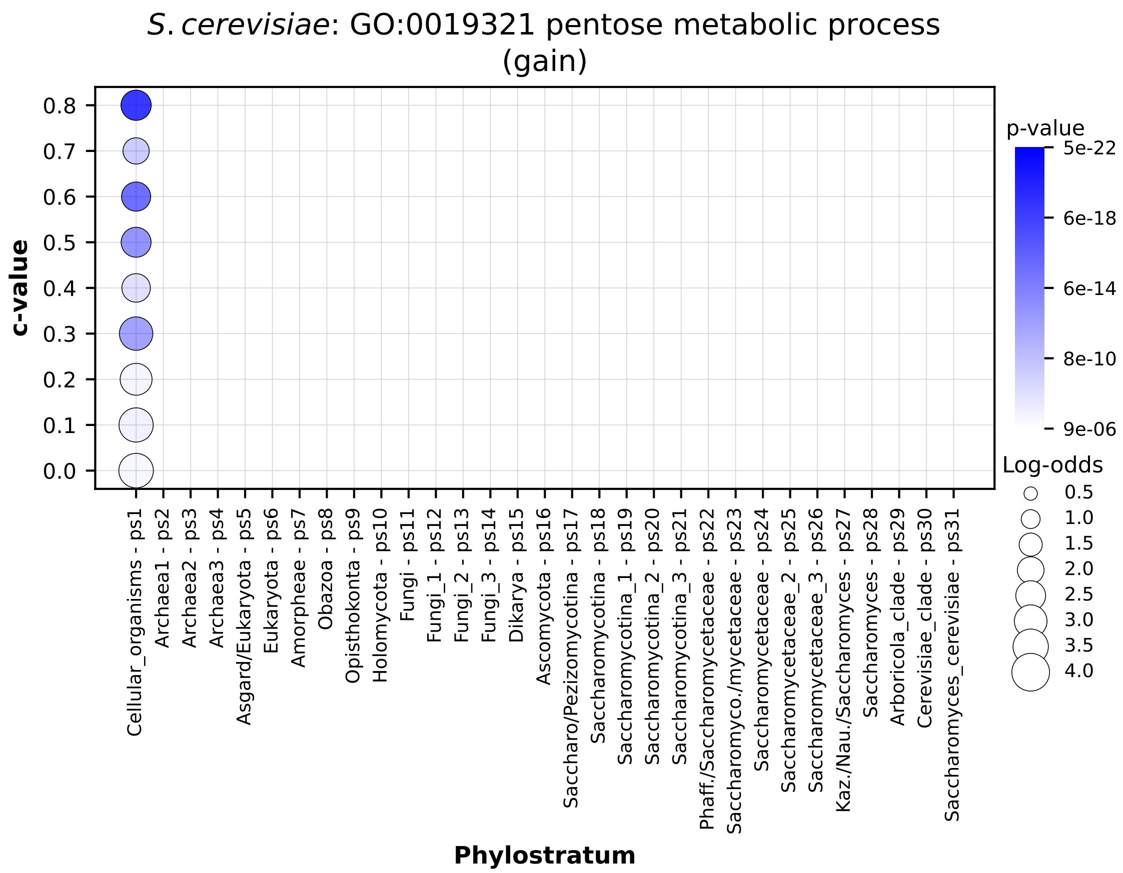
S. cerevisiae: GO:0019321 pentose metabolic process (gain)
c-value
0.8
0.7
0.6
0.5
0.4
0.3
0.2
0.1
0.0
p-value
5e-22
6e-18
6e-14
8e-10
9e-06
Log-odds
0.5
1.0
1.5
2.0
2.5
3.0
3.5
4.0
Cellular_organisms - ps1
Archaea1 - ps2
Archaea2 - ps3
Archaea3 - ps4
Asgard/Eukaryota - ps5
Eukaryota - ps6
Amorpheae - ps7
Obazoa - ps8
Opisthokonta - ps9
Holomycota - ps10
Fungi - ps11
Fungi_1 - ps12
Fungi_2 - ps13
Fungi_3 - ps14
Dikarya - ps15
Ascomycota - ps16
Saccharo/Pezizomycotina - ps17
Saccharomycotina - ps18
Saccharomycotina_1 - ps19
Saccharomycotina_2 - ps20
Saccharomycotina_3 - ps21
Phaff./Saccharomycetaceae - ps22
Saccharomyco./mycetaceae - ps23
Saccharomycetaceae - ps24
Saccharomycetaceae_2 - ps25
Saccharomycetaceae_3 - ps26
Kaz./Nau./Saccharomyces - ps27
Saccharomyces - ps28
Arboricola_clade - ps29
Cerevisiae_clade - ps30
Saccharomyces_cerevisiae - ps31
Phylostratum

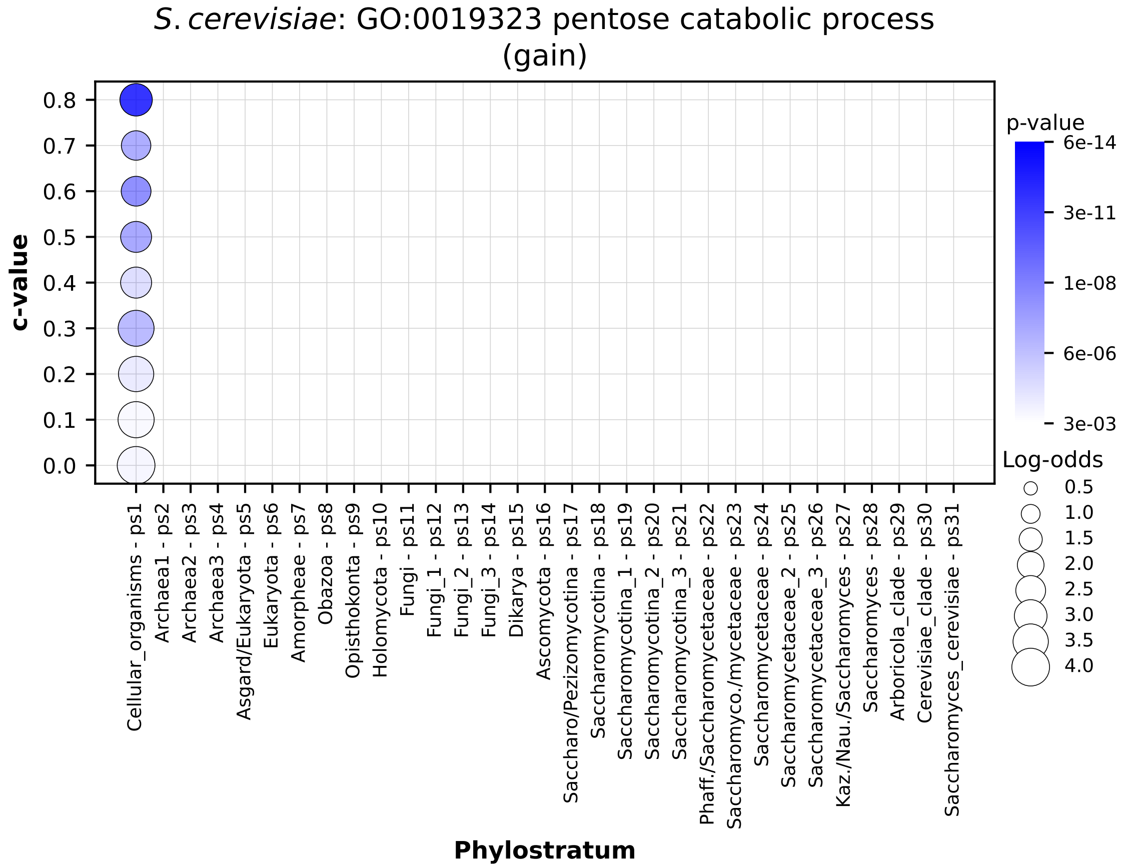

S. cerevisiae: GO:0019323 pentose catabolic process (gain)
c-value
0.0
0.1
0.2
0.3
0.4
0.5
0.6
0.7
0.8
p-value
6e-14
3e-11
1e-08
6e-06
3e-03
Log-odds
0.5
1.0
1.5
2.0
2.5
3.0
3.5
4.0
Cellular_organisms - ps1
Archaea1 - ps2
Archaea2 - ps3
Archaea3 - ps4
Asgard/Eukaryota - ps5
Eukaryota - ps6
Amorpheae - ps7
Obazoa - ps8
Opisthokonta - ps9
Holomycota - ps10
Fungi - ps11
Fungi_1 - ps12
Fungi_2 - ps13
Fungi_3 - ps14
Dikarya - ps15
Ascomycota - ps16
Saccharo/Pezizomycotina - ps17
Saccharomycotina - ps18
Saccharomycotina_1 - ps19
Saccharomycotina_2 - ps20
Saccharomycotina_3 - ps21
Phaff./Saccharomycetaceae - ps22
Saccharomyco./mycetaceae - ps23
Saccharomycetaceae - ps24
Saccharomycetaceae_2 - ps25
Saccharomycetaceae_3 - ps26
Kaz./Nau./Saccharomyces - ps27
Saccharomyces - ps28
Arboricola_clade - ps29
Cerevisiae_clade - ps30
Saccharomyces_cerevisiae - ps31
Phylostratum

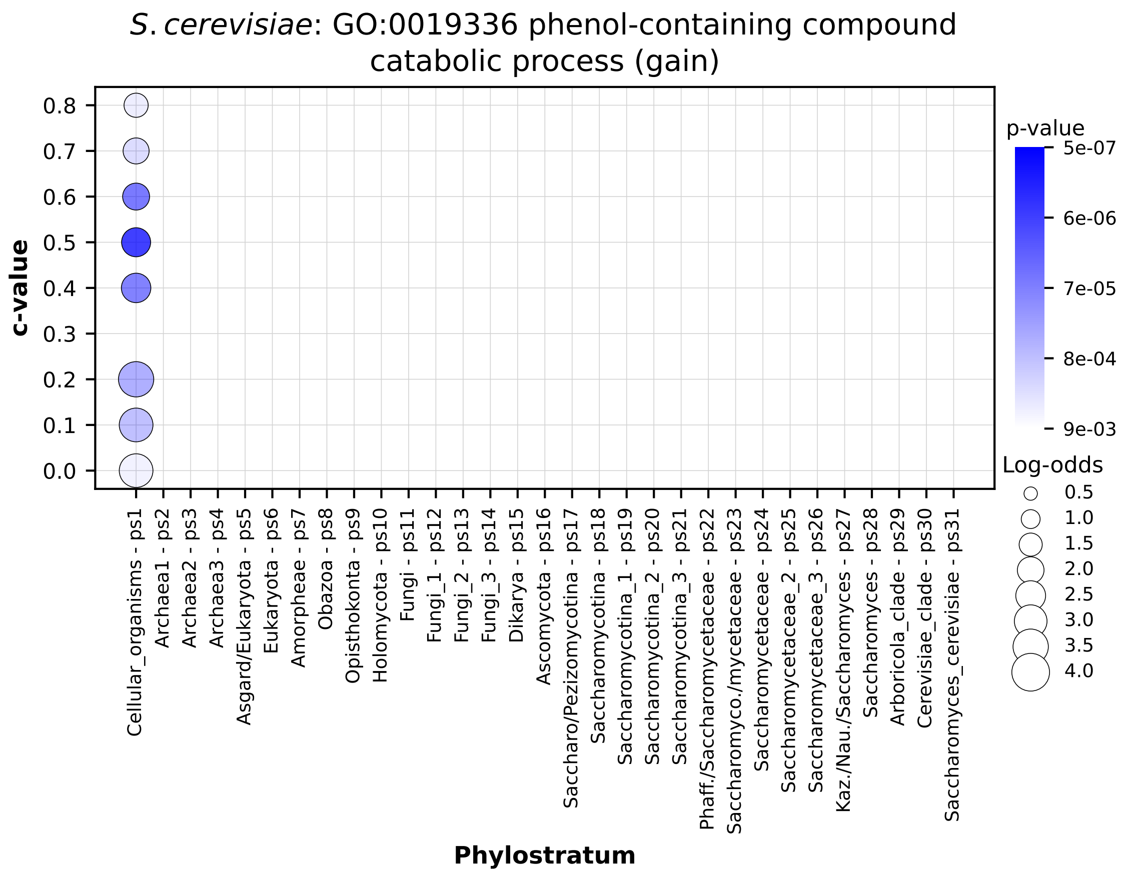

S. cerevisiae: GO:0019336 phenol-containing compound catabolic process (gain)
c-value
0.8
0.7
0.6
0.5
0.4
0.3
0.2
0.1
0.0
p-value
5e-07
6e-06
7e-05
8e-04
9e-03
Log-odds
0.5
1.0
1.5
2.0
2.5
3.0
3.5
4.0
Cellular_organisms - ps1
Archaea1 - ps2
Archaea2 - ps3
Archaea3 - ps4
Asgard/Eukaryota - ps5
Eukaryota - ps6
Amorpheae - ps7
Obazoa - ps8
Opisthokonta - ps9
Holomycota - ps10
Fungi - ps11
Fungi_1 - ps12
Fungi_2 - ps13
Fungi_3 - ps14
Dikarya - ps15
Ascomycota - ps16
Saccharo/Pezizomycotina - ps17
Saccharomycotina - ps18
Saccharomycotina_1 - ps19
Saccharomycotina_2 - ps20
Saccharomycotina_3 - ps21
Phaff./Saccharomycetaceae - ps22
Saccharomyco./mycetaceae - ps23
Saccharomycetaceae - ps24
Saccharomycetaceae_2 - ps25
Saccharomycetaceae_3 - ps26
Kaz./Nau./Saccharomyces - ps27
Saccharomyces - ps28
Arboricola_clade - ps29
Cerevisiae_clade - ps30
Saccharomyces_cerevisiae - ps31
Phylostratum

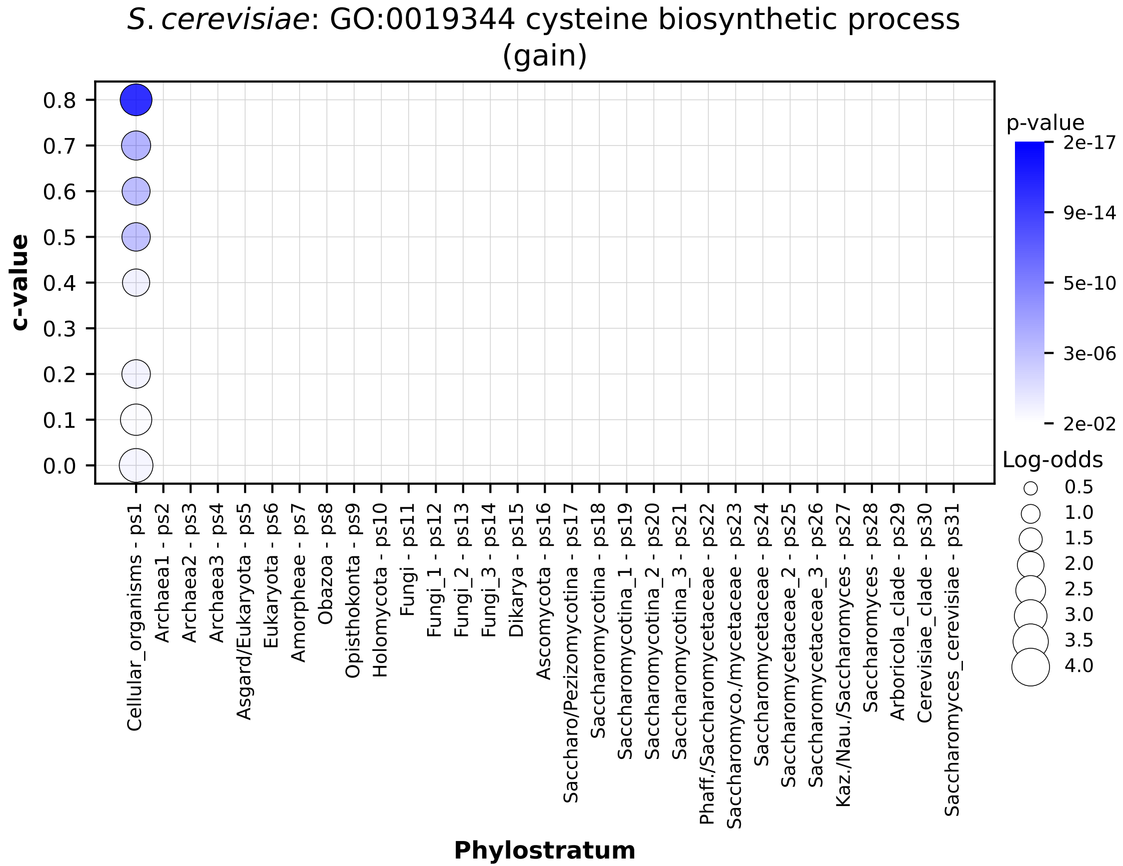

S. cerevisiae: GO:0019344 cysteine biosynthetic process (gain)
c-value
0.8
0.7
0.6
0.5
0.4
0.3
0.2
0.1
0.0
p-value
2e-17
9e-14
5e-10
3e-06
2e-02
Log-odds
0.5
1.0
1.5
2.0
2.5
3.0
3.5
4.0
Cellular_organisms - ps1
Archaea1 - ps2
Archaea2 - ps3
Archaea3 - ps4
Asgard/Eukaryota - ps5
Eukaryota - ps6
Amorpheae - ps7
Obazoa - ps8
Opisthokonta - ps9
Holomycota - ps10
Fungi - ps11
Fungi_1 - ps12
Fungi_2 - ps13
Fungi_3 - ps14
Dikarya - ps15
Ascomycota - ps16
Saccharo/Pezizomycotina - ps17
Saccharomycotina - ps18
Saccharomycotina_1 - ps19
Saccharomycotina_2 - ps20
Saccharomycotina_3 - ps21
Phaff./Saccharomycetaceae - ps22
Saccharomyco./mycetaceae - ps23
Saccharomycetaceae - ps24
Saccharomycetaceae_2 - ps25
Saccharomycetaceae_3 - ps26
Kaz./Nau./Saccharomyces - ps27
Saccharomyces - ps28
Arboricola_clade - ps29
Cerevisiae_clade - ps30
Saccharomyces_cerevisiae - ps31
Phylostratum

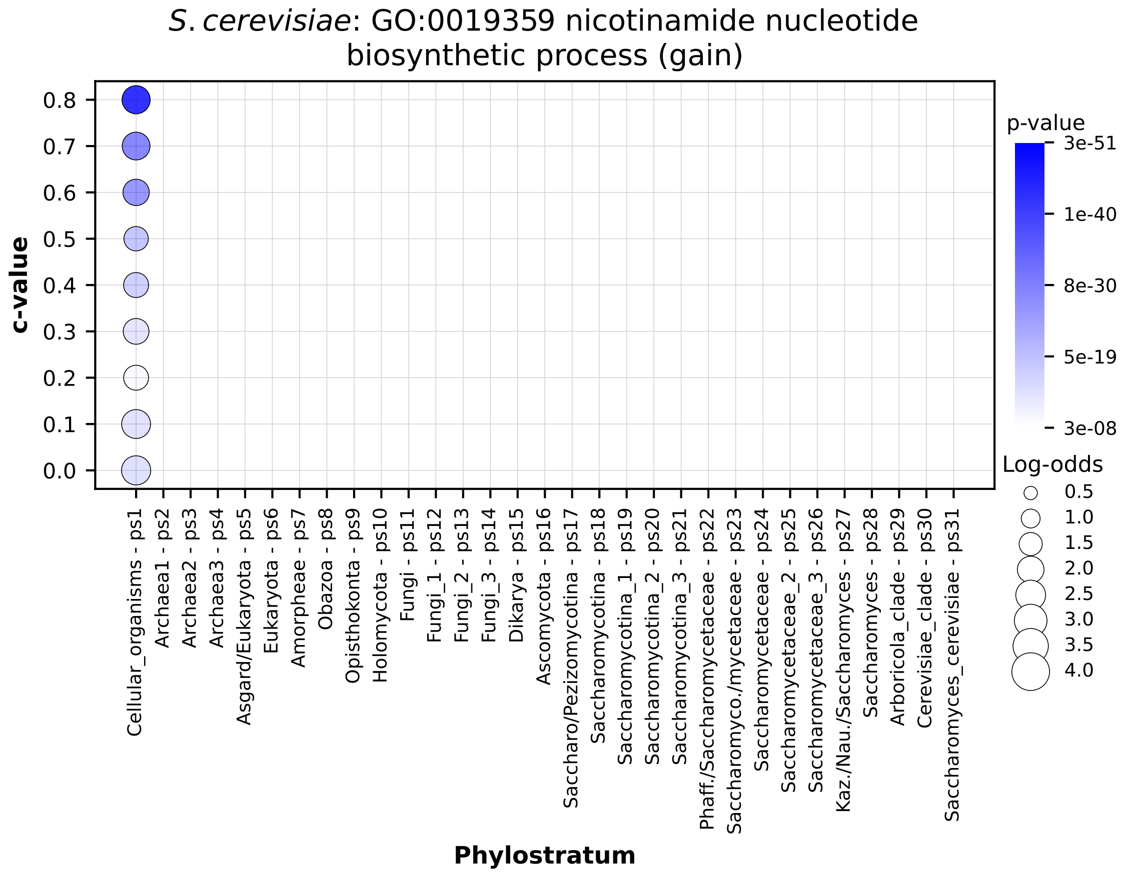

S. cerevisiae: GO:0019359 nicotinamide nucleotide biosynthetic process (gain)
c-value
0.8
0.7
0.6
0.5
0.4
0.3
0.2
0.1
0.0
p-value
3e-51
1e-40
8e-30
5e-19
3e-08
Log-odds
0.5
1.0
1.5
2.0
2.5
3.0
3.5
4.0
Cellular_organisms - ps1
Archaea1 - ps2
Archaea2 - ps3
Archaea3 - ps4
Asgard/Eukaryota - ps5
Eukaryota - ps6
Amorpheae - ps7
Obazoa - ps8
Opisthokonta - ps9
Holomycota - ps10
Fungi - ps11
Fungi_1 - ps12
Fungi_2 - ps13
Fungi_3 - ps14
Dikarya - ps15
Ascomycota - ps16
Saccharo/Pezizomycotina - ps17
Saccharomycotina - ps18
Saccharomycotina_1 - ps19
Saccharomycotina_2 - ps20
Saccharomycotina_3 - ps21
Phaff./Saccharomycetaceae - ps22
Saccharomyco./mycetaceae - ps23
Saccharomycetaceae - ps24
Saccharomycetaceae_2 - ps25
Saccharomycetaceae_3 - ps26
Kaz./Nau./Saccharomyces - ps27
Saccharomyces - ps28
Arboricola_clade - ps29
Cerevisiae_clade - ps30
Saccharomyces_cerevisiae - ps31
Phylostratum

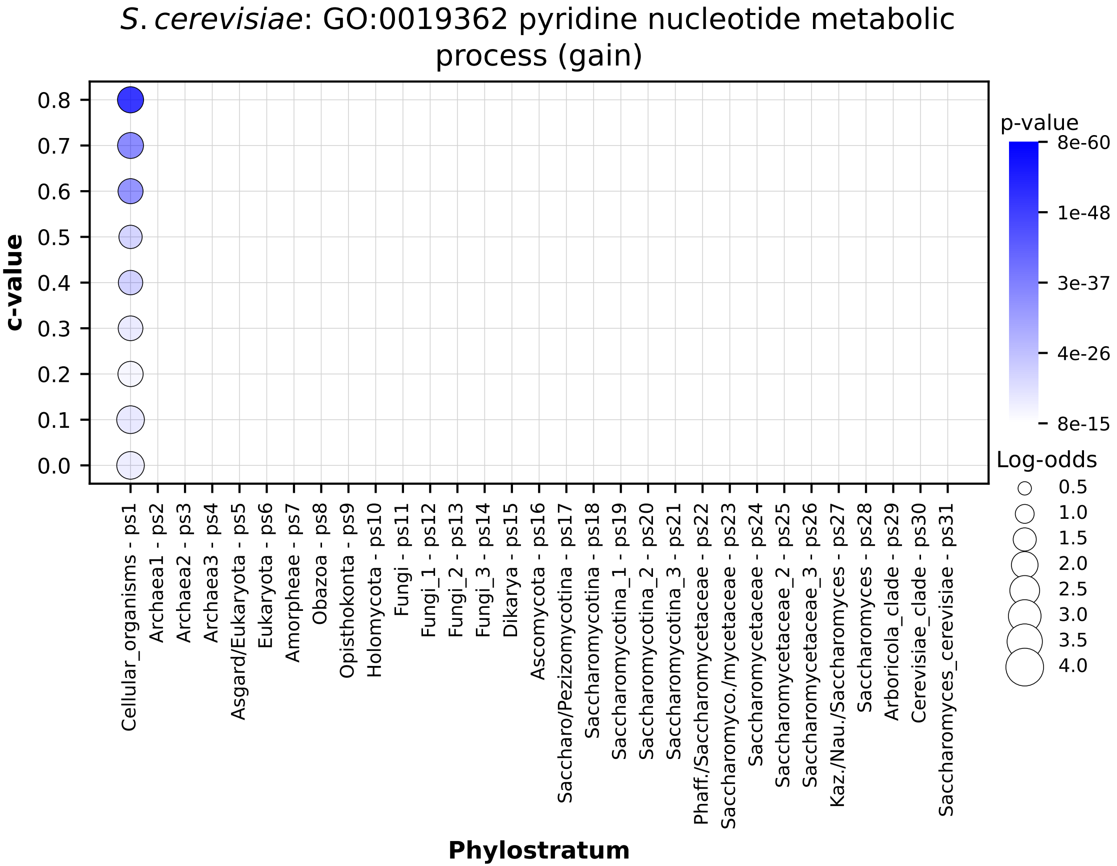
S. cerevisiae: GO:0019362 pyridine nucleotide metabolic process (gain)
c-value
0.8
0.7
0.6
0.5
0.4
0.3
0.2
0.1
0.0
Phylostratum
Cellular_organisms - ps1
Archaea1 - ps2
Archaea2 - ps3
Archaea3 - ps4
Asgard/Eukaryota - ps5
Eukaryota - ps6
Amorpheae - ps7
Obazoa - ps8
Opisthokonta - ps9
Holomycota - ps10
Fungi - ps11
Fungi_1 - ps12
Fungi_2 - ps13
Fungi_3 - ps14
Dikarya - ps15
Ascomycota - ps16
Saccharo/Pezizomycotina - ps17
Saccharomycotina - ps18
Saccharomycotina_1 - ps19
Saccharomycotina_2 - ps20
Saccharomycotina_3 - ps21
Phaff./Saccharomycetaceae - ps22
Saccharomyco./mycetaceae - ps23
Saccharomycetaceae - ps24
Saccharomycetaceae_2 - ps25
Saccharomycetaceae_3 - ps26
Kaz./Nau./Saccharomyces - ps27
Saccharomyces - ps28
Arboricola_clade - ps29
Cerevisiae_clade - ps30
Saccharomyces_cerevisiae - ps31
p-value
8e-60
1e-48
3e-37
4e-26
8e-15
Log-odds
0.5
1.0
1.5
2.0
2.5
3.0
3.5
4.0

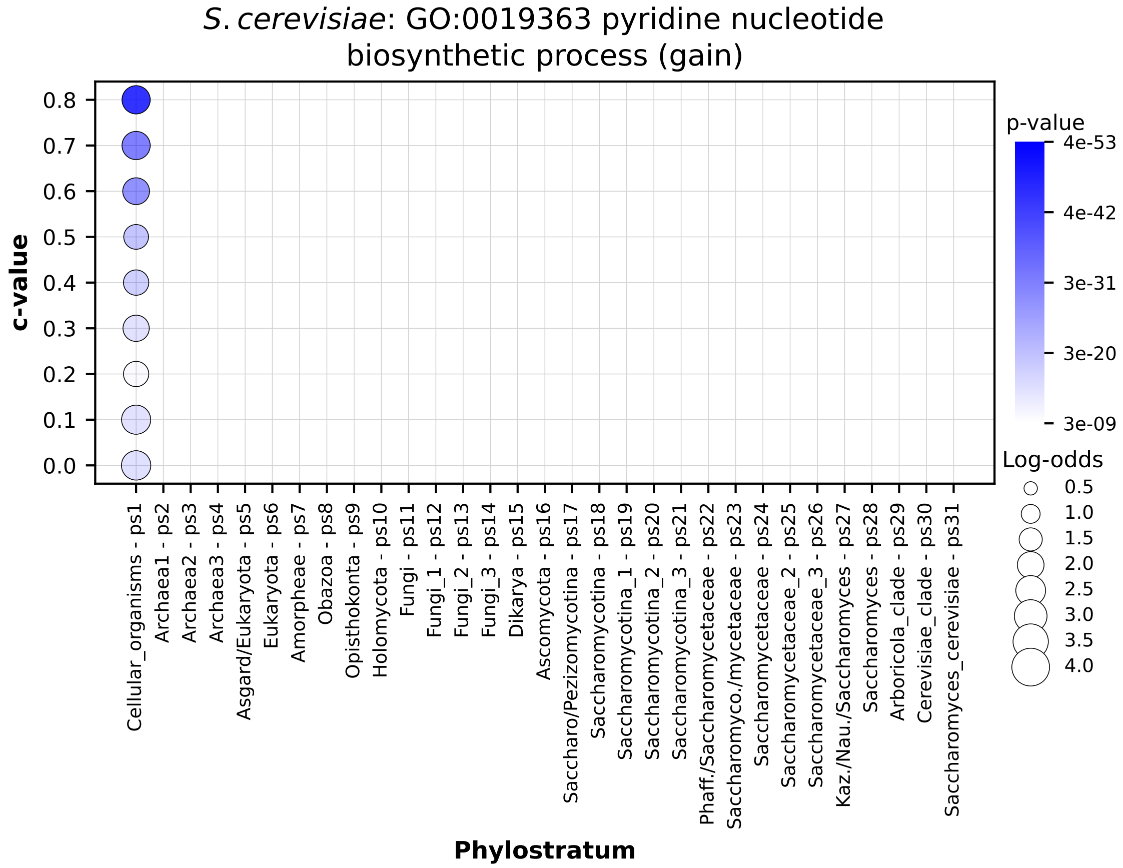

S. cerevisiae: GO:0019363 pyridine nucleotide biosynthetic process (gain)
c-value
0.8
0.7
0.6
0.5
0.4
0.3
0.2
0.1
0.0
p-value
4e-53
4e-42
3e-31
3e-20
3e-09
Log-odds
0.5
1.0
1.5
2.0
2.5
3.0
3.5
4.0
Cellular_organisms - ps1
Archaea1 - ps2
Archaea2 - ps3
Archaea3 - ps4
Asgard/Eukaryota - ps5
Eukaryota - ps6
Amorpheae - ps7
Obazoa - ps8
Opisthokonta - ps9
Holomycota - ps10
Fungi - ps11
Fungi_1 - ps12
Fungi_2 - ps13
Fungi_3 - ps14
Dikarya - ps15
Ascomycota - ps16
Saccharo/Pezizomycotina - ps17
Saccharomycotina - ps18
Saccharomycotina_1 - ps19
Saccharomycotina_2 - ps20
Saccharomycotina_3 - ps21
Phaff./Saccharomycetaceae - ps22
Saccharomyco./mycetaceae - ps23
Saccharomycetaceae - ps24
Saccharomycetaceae_2 - ps25
Saccharomycetaceae_3 - ps26
Kaz./Nau./Saccharomyces - ps27
Saccharomyces - ps28
Arboricola_clade - ps29
Cerevisiae_clade - ps30
Saccharomyces_cerevisiae - ps31
Phylostratum

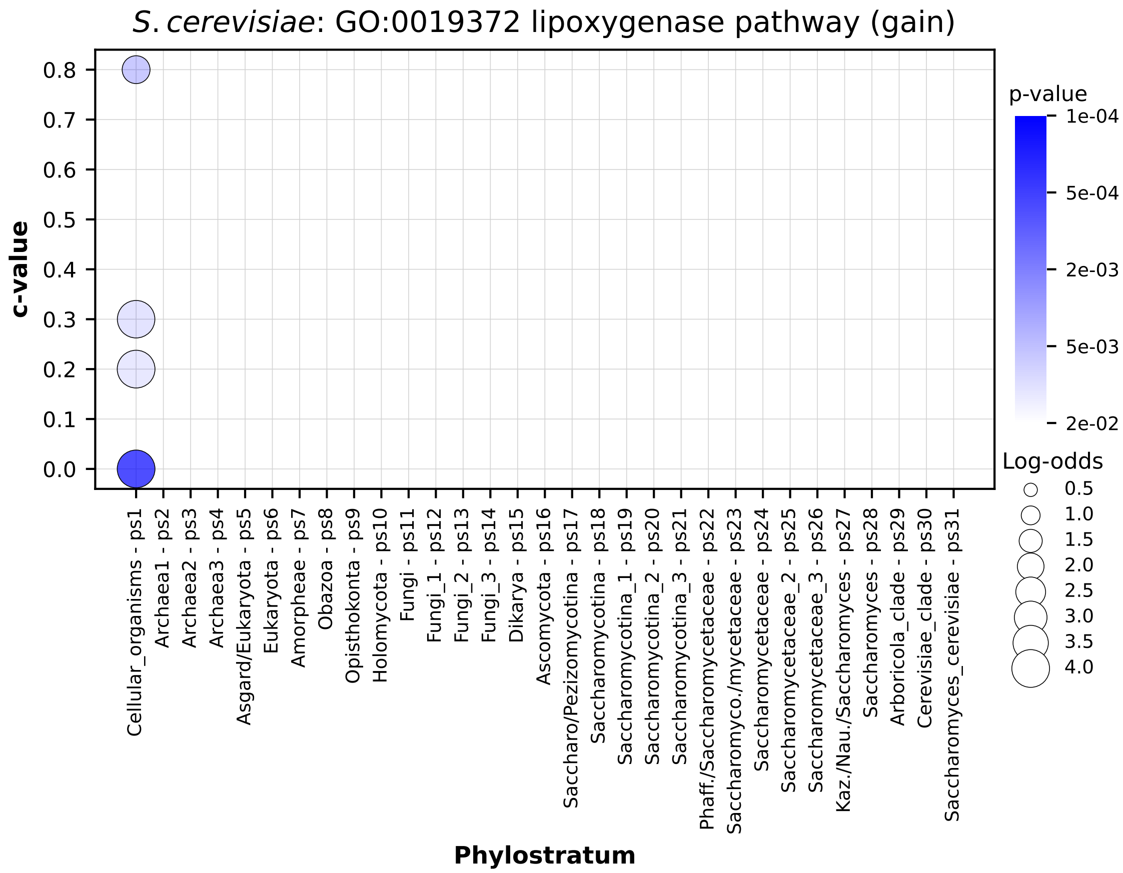

S. cerevisiae: GO:0019372 lipoxygenase pathway (gain)
c-value
0.8
0.7
0.6
0.5
0.4
0.3
0.2
0.1
0.0
p-value
1e-04
5e-04
2e-03
5e-03
2e-02
Log-odds
0.5
1.0
1.5
2.0
2.5
3.0
3.5
4.0
Cellular_organisms - ps1
Archaea1 - ps2
Archaea2 - ps3
Archaea3 - ps4
Asgard/Eukaryota - ps5
Eukaryota - ps6
Amorpheae - ps7
Obazoa - ps8
Opisthokonta - ps9
Holomycota - ps10
Fungi - ps11
Fungi_1 - ps12
Fungi_2 - ps13
Fungi_3 - ps14
Dikarya - ps15
Ascomycota - ps16
Saccharo/Pezizomycotina - ps17
Saccharomycotina - ps18
Saccharomycotina_1 - ps19
Saccharomycotina_2 - ps20
Saccharomycotina_3 - ps21
Phaff./Saccharomycetaceae - ps22
Saccharomyco./mycetaceae - ps23
Saccharomycetaceae - ps24
Saccharomycetaceae_2 - ps25
Saccharomycetaceae_3 - ps26
Kaz./Nau./Saccharomyces - ps27
Saccharomyces - ps28
Arboricola_clade - ps29
Cerevisiae_clade - ps30
Saccharomyces_cerevisiae - ps31
Phylostratum

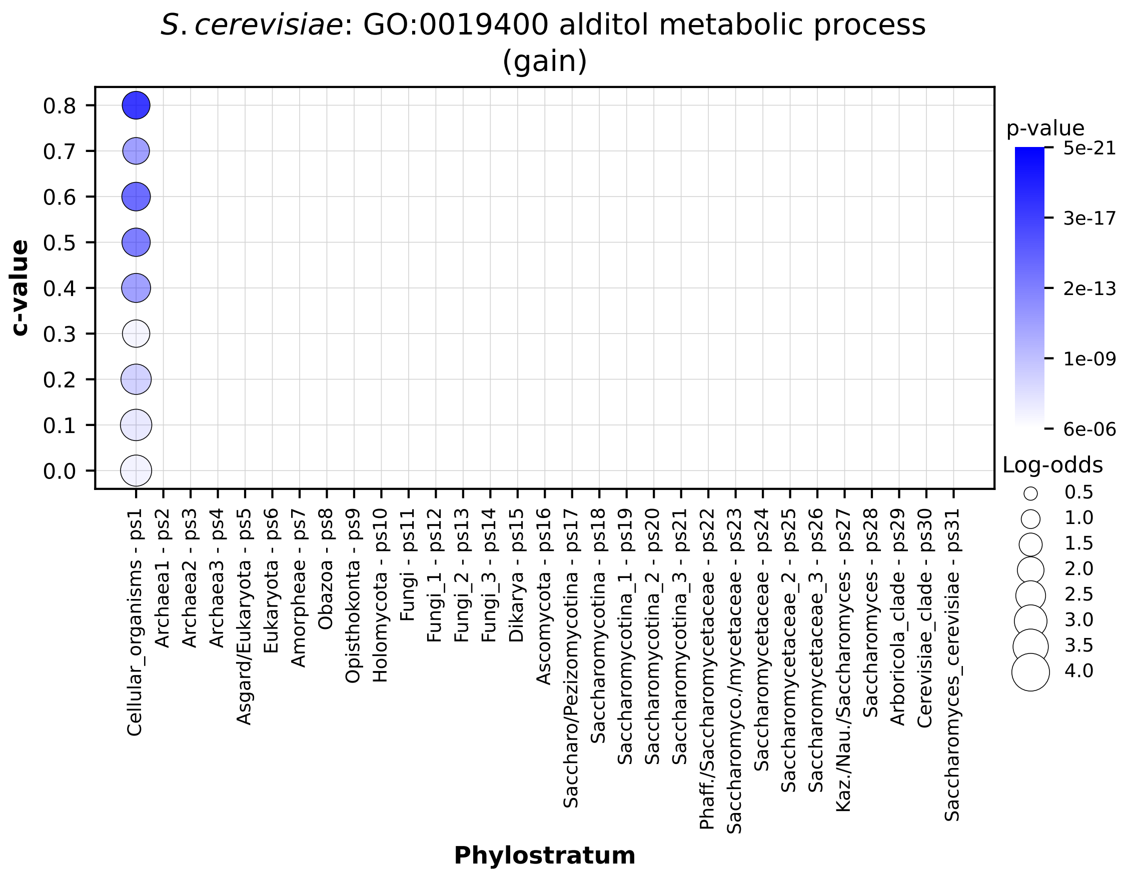

S. cerevisiae: GO:0019400 alditol metabolic process
(gain)
c-value
0.8
0.7
0.6
0.5
0.4
0.3
0.2
0.1
0.0
p-value
5e-21
3e-17
2e-13
1e-09
6e-06
Log-odds
0.5
1.0
1.5
2.0
2.5
3.0
3.5
4.0
Cellular_organisms - ps1
Archaea1 - ps2
Archaea2 - ps3
Archaea3 - ps4
Asgard/Eukaryota - ps5
Eukaryota - ps6
Amorpheae - ps7
Obazoa - ps8
Opisthokonta - ps9
Holomycota - ps10
Fungi - ps11
Fungi_1 - ps12
Fungi_2 - ps13
Fungi_3 - ps14
Dikarya - ps15
Ascomycota - ps16
Saccharo/Pezizomycotina - ps17
Saccharomycotina - ps18
Saccharomycotina_1 - ps19
Saccharomycotina_2 - ps20
Saccharomycotina_3 - ps21
Phaff./Saccharomycetaceae - ps22
Saccharomyco./mycetaceae - ps23
Saccharomycetaceae - ps24
Saccharomycetaceae_2 - ps25
Saccharomycetaceae_3 - ps26
Kaz./Nau./Saccharomyces - ps27
Saccharomyces - ps28
Arboricola_clade - ps29
Cerevisiae_clade - ps30
Saccharomyces_cerevisiae - ps31
Phylostratum

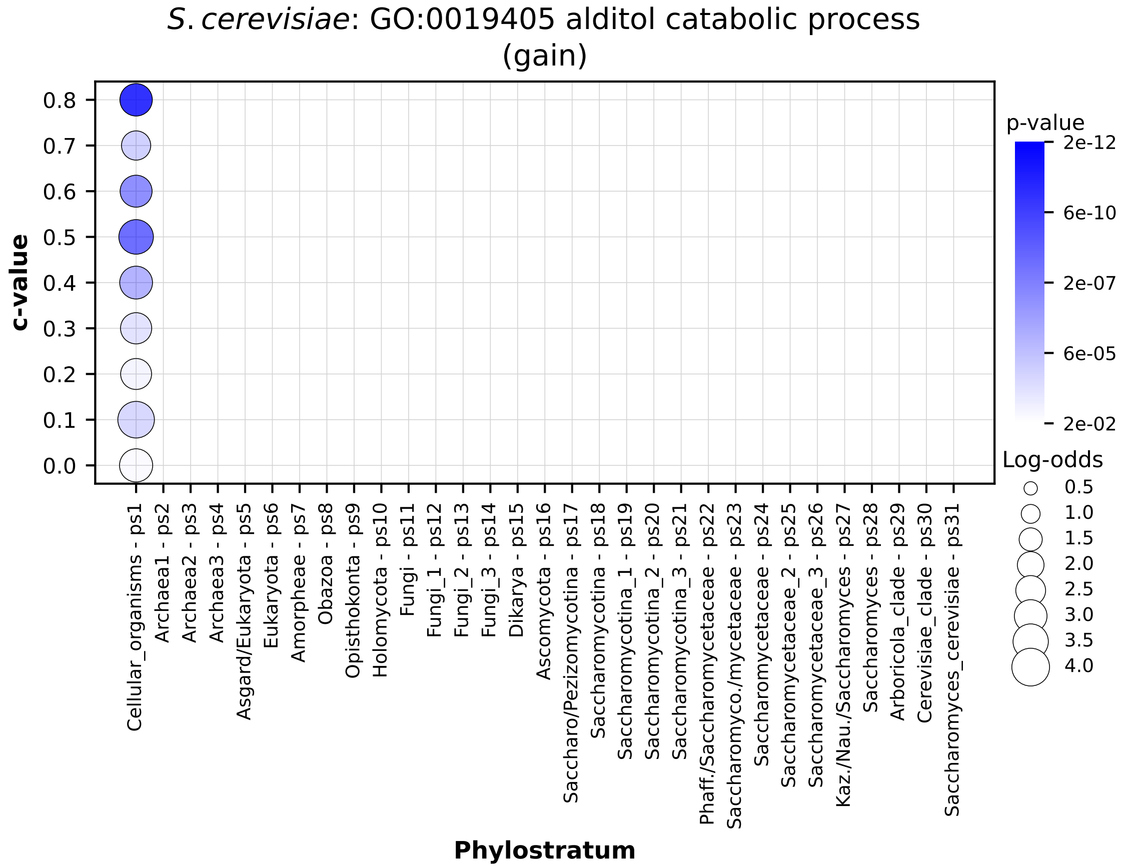

S. cerevisiae: GO:0019405 alditol catabolic process
(gain)
c-value
0.8
0.7
0.6
0.5
0.4
0.3
0.2
0.1
0.0
p-value
2e-12
6e-10
2e-07
6e-05
2e-02
Log-odds
0.5
1.0
1.5
2.0
2.5
3.0
3.5
4.0
Cellular_organisms - ps1
Archaea1 - ps2
Archaea2 - ps3
Archaea3 - ps4
Asgard/Eukaryota - ps5
Eukaryota - ps6
Amorpheae - ps7
Obazoa - ps8
Opisthokonta - ps9
Holomycota - ps10
Fungi - ps11
Fungi_1 - ps12
Fungi_2 - ps13
Fungi_3 - ps14
Dikarya - ps15
Ascomycota - ps16
Saccharo/Pezizomycotina - ps17
Saccharomycotina - ps18
Saccharomycotina_1 - ps19
Saccharomycotina_2 - ps20
Saccharomycotina_3 - ps21
Phaff./Saccharomycetaceae - ps22
Saccharomyco./mycetaceae - ps23
Saccharomycetaceae - ps24
Saccharomycetaceae_2 - ps25
Saccharomycetaceae_3 - ps26
Kaz./Nau./Saccharomyces - ps27
Saccharomyces - ps28
Arboricola_clade - ps29
Cerevisiae_clade - ps30
Saccharomyces_cerevisiae - ps31
Phylostratum

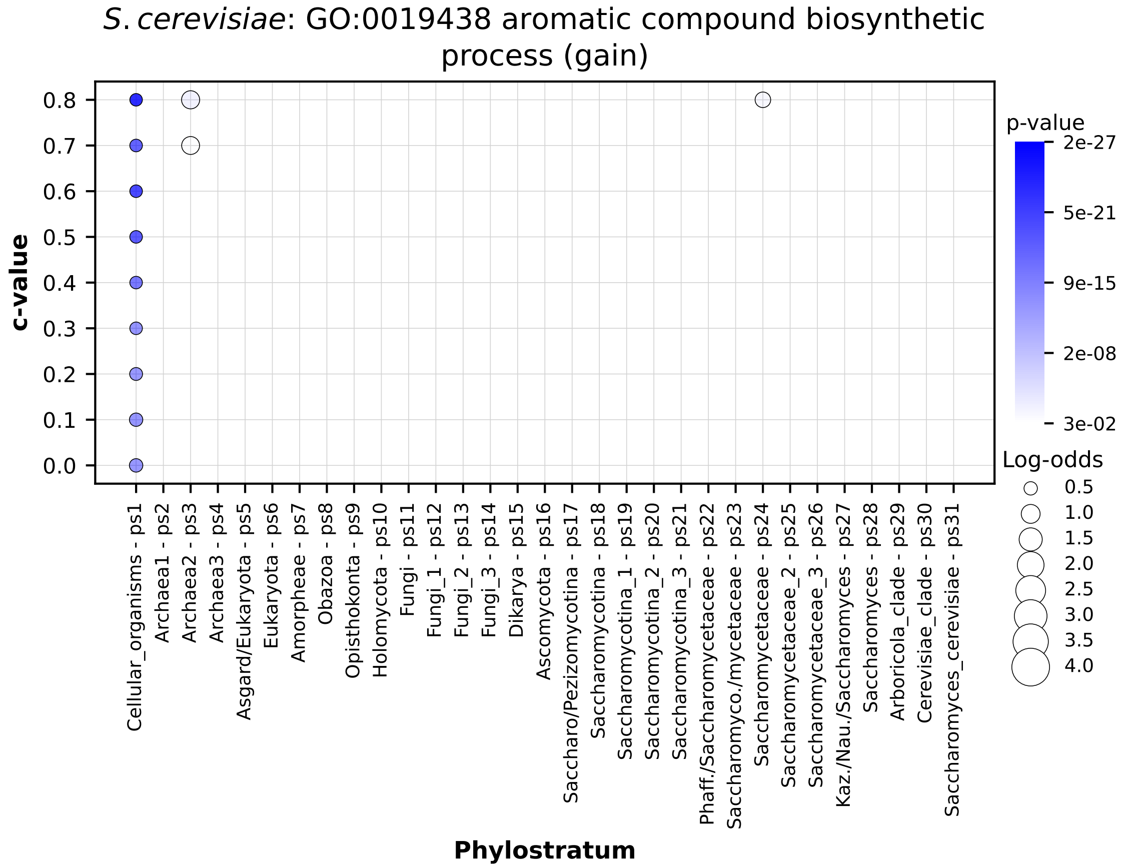

S. cerevisiae: GO:0019438 aromatic compound biosynthetic process (gain)
c-value
0.8
0.7
0.6
0.5
0.4
0.3
0.2
0.1
0.0
Phylostratum
Cellular_organisms - ps1
Archaea1 - ps2
Archaea2 - ps3
Archaea3 - ps4
Asgard/Eukaryota - ps5
Eukaryota - ps6
Amorpheae - ps7
Obazoa - ps8
Opisthokonta - ps9
Holomycota - ps10
Fungi - ps11
Fungi_1 - ps12
Fungi_2 - ps13
Fungi_3 - ps14
Dikarya - ps15
Ascomycota - ps16
Saccharo/Pezizomycotina - ps17
Saccharomycotina - ps18
Saccharomycotina_1 - ps19
Saccharomycotina_2 - ps20
Saccharomycotina_3 - ps21
Phaff./Saccharomycetaceae - ps22
Saccharomyco./mycetaceae - ps23
Saccharomycetaceae - ps24
Saccharomycetaceae_2 - ps25
Saccharomycetaceae_3 - ps26
Kaz./Nau./Saccharomyces - ps27
Saccharomyces - ps28
Arboricola_clade - ps29
Cerevisiae_clade - ps30
Saccharomyces_cerevisiae - ps31
p-value
2e-27
5e-21
9e-15
2e-08
3e-02
Log-odds
0.5
1.0
1.5
2.0
2.5
3.0
3.5
4.0

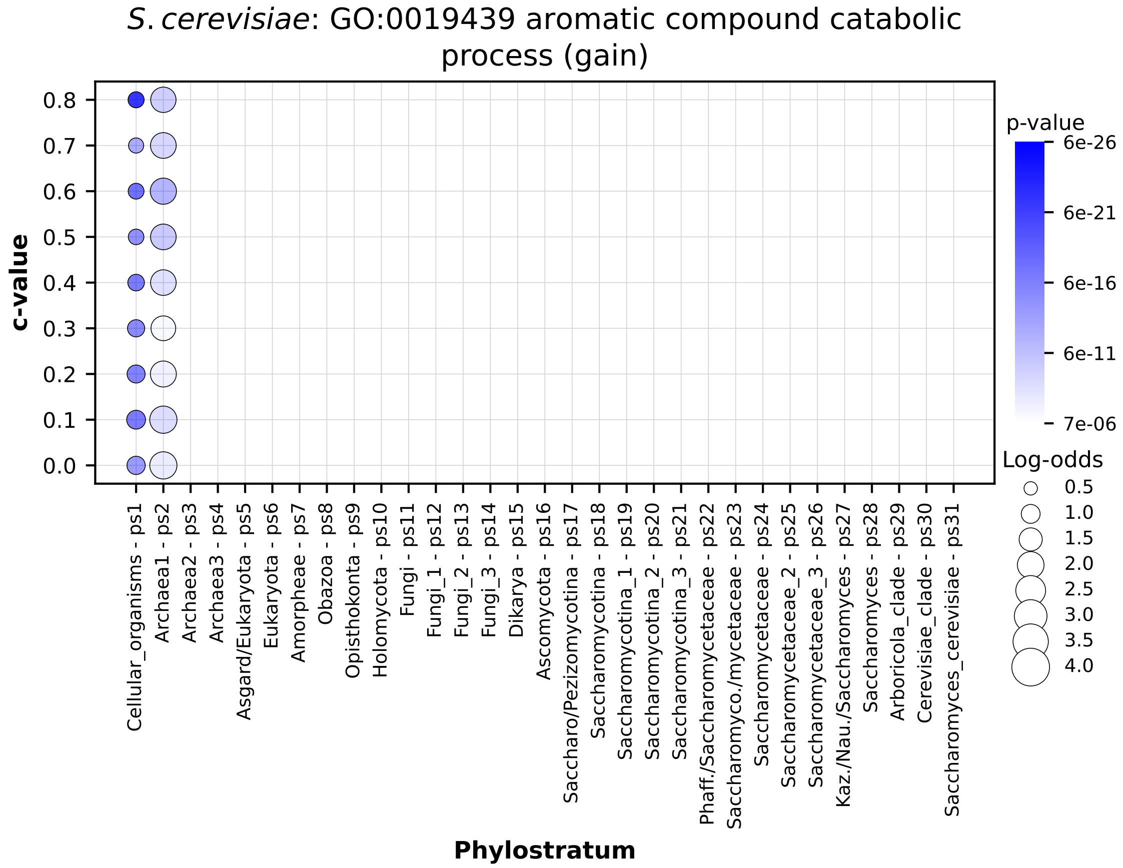
S. cerevisiae: GO:0019439 aromatic compound catabolic process (gain)
c-value
0.8
0.7
0.6
0.5
0.4
0.3
0.2
0.1
0.0
p-value
6e-26
6e-21
6e-16
6e-11
7e-06
Log-odds
0.5
1.0
1.5
2.0
2.5
3.0
3.5
4.0
Cellular_organisms - ps1
Archaea1 - ps2
Archaea2 - ps3
Archaea3 - ps4
Asgard/Eukaryota - ps5
Eukaryota - ps6
Amorpheae - ps7
Obazoa - ps8
Opisthokonta - ps9
Holomycota - ps10
Fungi - ps11
Fungi_1 - ps12
Fungi_2 - ps13
Fungi_3 - ps14
Dikarya - ps15
Ascomycota - ps16
Saccharo/Pezizomycotina - ps17
Saccharomycotina - ps18
Saccharomycotina_1 - ps19
Saccharomycotina_2 - ps20
Saccharomycotina_3 - ps21
Phaff./Saccharomycetaceae - ps22
Saccharomyco./mycetaceae - ps23
Saccharomycetaceae - ps24
Saccharomycetaceae_2 - ps25
Saccharomycetaceae_3 - ps26
Kaz./Nau./Saccharomyces - ps27
Saccharomyces - ps28
Arboricola_clade - ps29
Cerevisiae_clade - ps30
Saccharomyces_cerevisiae - ps31
Phylostratum

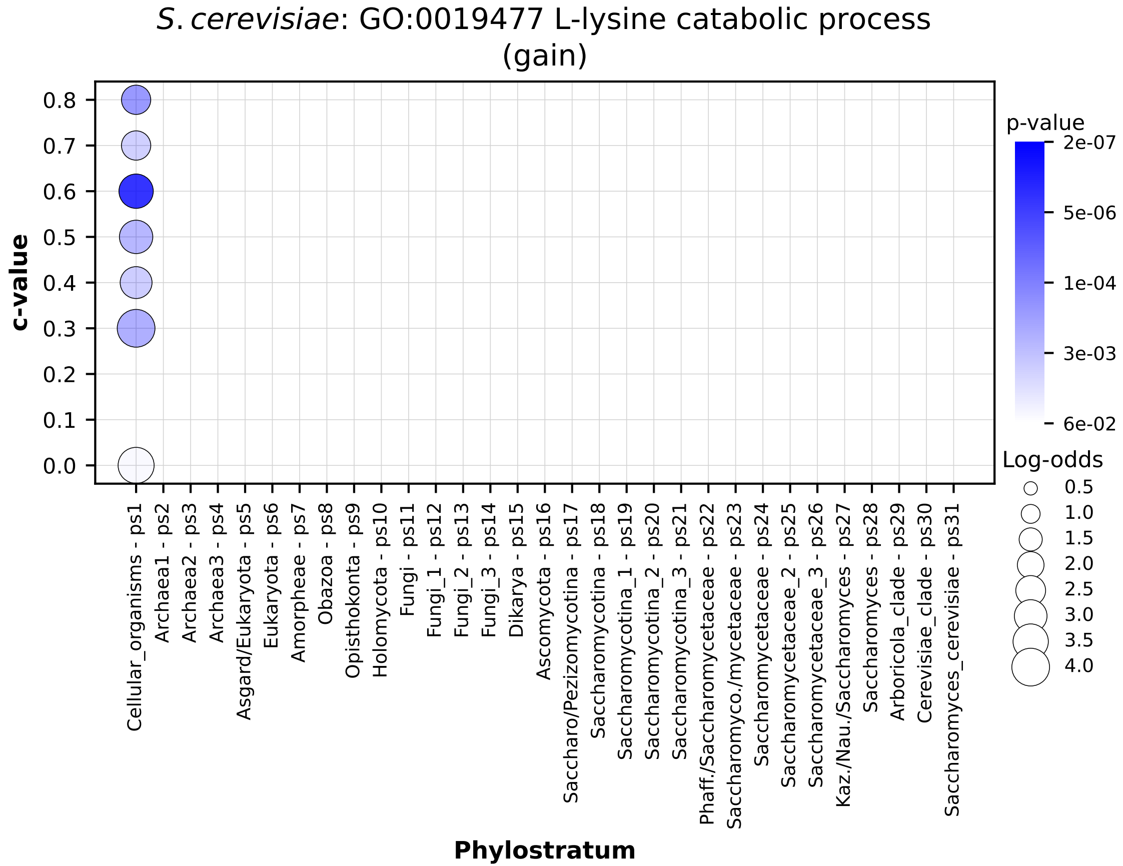
S. cerevisiae: GO:0019477 L-lysine catabolic process
(gain)
c-value
0.8
0.7
0.6
0.5
0.4
0.3
0.2
0.1
0.0
p-value
2e-07
5e-06
1e-04
3e-03
6e-02
Log-odds
0.5
1.0
1.5
2.0
2.5
3.0
3.5
4.0
Cellular_organisms - ps1
Archaea1 - ps2
Archaea2 - ps3
Archaea3 - ps4
Asgard/Eukaryota - ps5
Eukaryota - ps6
Amorpheae - ps7
Obazoa - ps8
Opisthokonta - ps9
Holomycota - ps10
Fungi - ps11
Fungi_1 - ps12
Fungi_2 - ps13
Fungi_3 - ps14
Dikarya - ps15
Ascomycota - ps16
Saccharo/Pezizomycotina - ps17
Saccharomycotina - ps18
Saccharomycotina_1 - ps19
Saccharomycotina_2 - ps20
Saccharomycotina_3 - ps21
Phaff./Saccharomycetaceae - ps22
Saccharomyco./mycetaceae - ps23
Saccharomycetaceae - ps24
Saccharomycetaceae_2 - ps25
Saccharomycetaceae_3 - ps26
Kaz./Nau./Saccharomyces - ps27
Saccharomyces - ps28
Arboricola_clade - ps29
Cerevisiae_clade - ps30
Saccharomyces_cerevisiae - ps31
Phylostratum

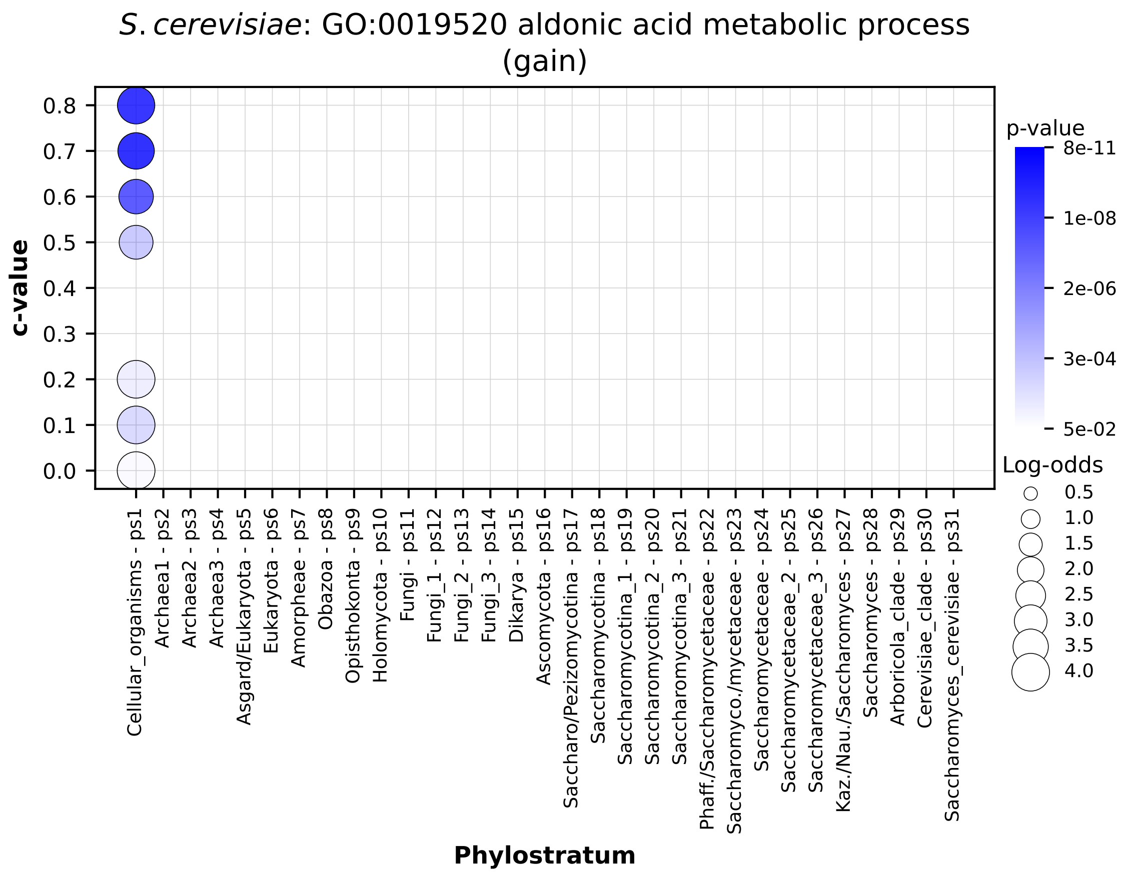
S. cerevisiae: GO:0019520 aldonic acid metabolic process (gain)
c-value
0.8
0.7
0.6
0.5
0.4
0.3
0.2
0.1
0.0
p-value
8e-11
1e-08
2e-06
3e-04
5e-02
Log-odds
0.5
1.0
1.5
2.0
2.5
3.0
3.5
4.0
Cellular_organisms - ps1
Archaea1 - ps2
Archaea2 - ps3
Archaea3 - ps4
Asgard/Eukaryota - ps5
Eukaryota - ps6
Amorpheae - ps7
Obazoa - ps8
Opisthokonta - ps9
Holomycota - ps10
Fungi - ps11
Fungi_1 - ps12
Fungi_2 - ps13
Fungi_3 - ps14
Dikarya - ps15
Ascomycota - ps16
Saccharo/Pezizomycotina - ps17
Saccharomycotina - ps18
Saccharomycotina_1 - ps19
Saccharomycotina_2 - ps20
Saccharomycotina_3 - ps21
Phaff./Saccharomycetaceae - ps22
Saccharomyco./mycetaceae - ps23
Saccharomycetaceae - ps24
Saccharomycetaceae_2 - ps25
Saccharomycetaceae_3 - ps26
Kaz./Nau./Saccharomyces - ps27
Saccharomyces - ps28
Arboricola_clade - ps29
Cerevisiae_clade - ps30
Saccharomyces_cerevisiae - ps31
Phylostratum

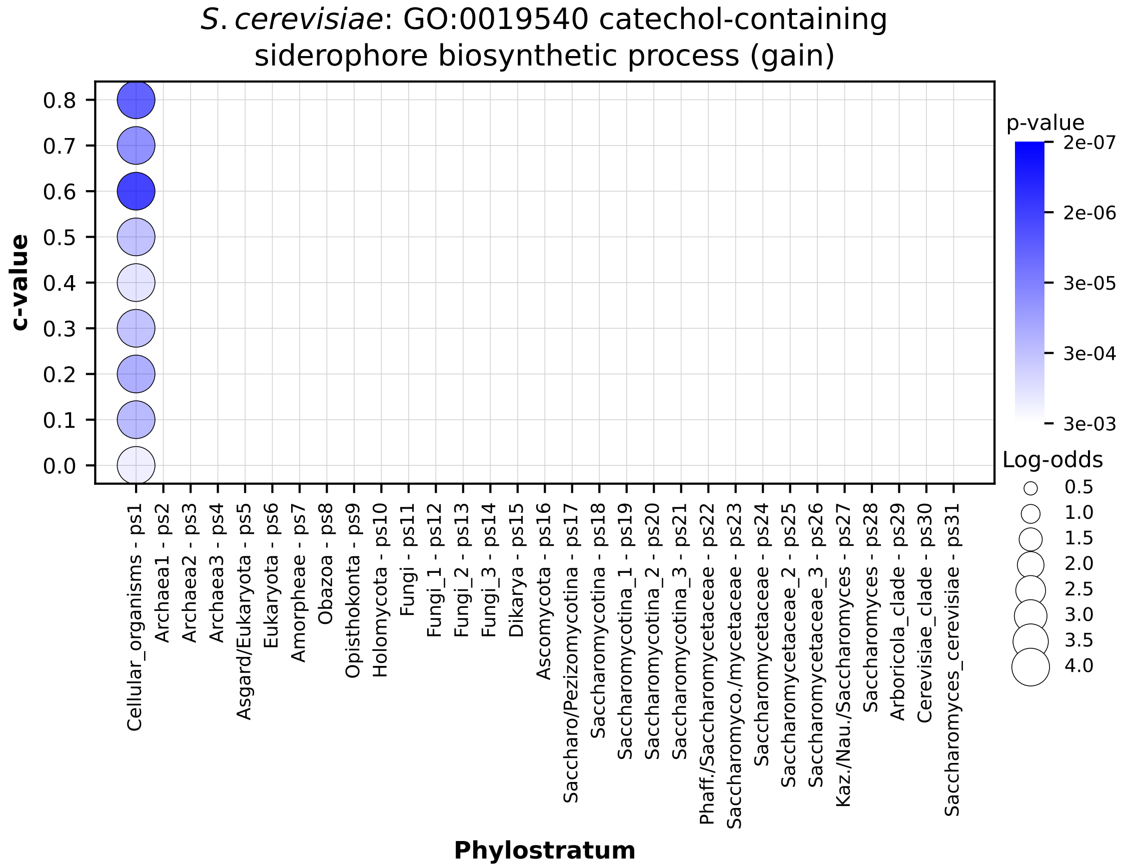
S. cerevisiae: GO:0019540 catechol-containing siderophore biosynthetic process (gain)
c-value
0.8
0.7
0.6
0.5
0.4
0.3
0.2
0.1
0.0
p-value
2e-07
2e-06
3e-05
3e-04
3e-03
Log-odds
0.5
1.0
1.5
2.0
2.5
3.0
3.5
4.0
Cellular_organisms - ps1
Archaea1 - ps2
Archaea2 - ps3
Archaea3 - ps4
Asgard/Eukaryota - ps5
Eukaryota - ps6
Amorpheae - ps7
Obazoa - ps8
Opisthokonta - ps9
Holomycota - ps10
Fungi - ps11
Fungi_1 - ps12
Fungi_2 - ps13
Fungi_3 - ps14
Dikarya - ps15
Ascomycota - ps16
Saccharo/Pezizomycotina - ps17
Saccharomycotina - ps18
Saccharomycotina_1 - ps19
Saccharomycotina_2 - ps20
Saccharomycotina_3 - ps21
Phaff./Saccharomycetaceae - ps22
Saccharomyco./mycetaceae - ps23
Saccharomycetaceae - ps24
Saccharomycetaceae_2 - ps25
Saccharomycetaceae_3 - ps26
Kaz./Nau./Saccharomyces - ps27
Saccharomyces - ps28
Arboricola_clade - ps29
Cerevisiae_clade - ps30
Saccharomyces_cerevisiae - ps31
Phylostratum

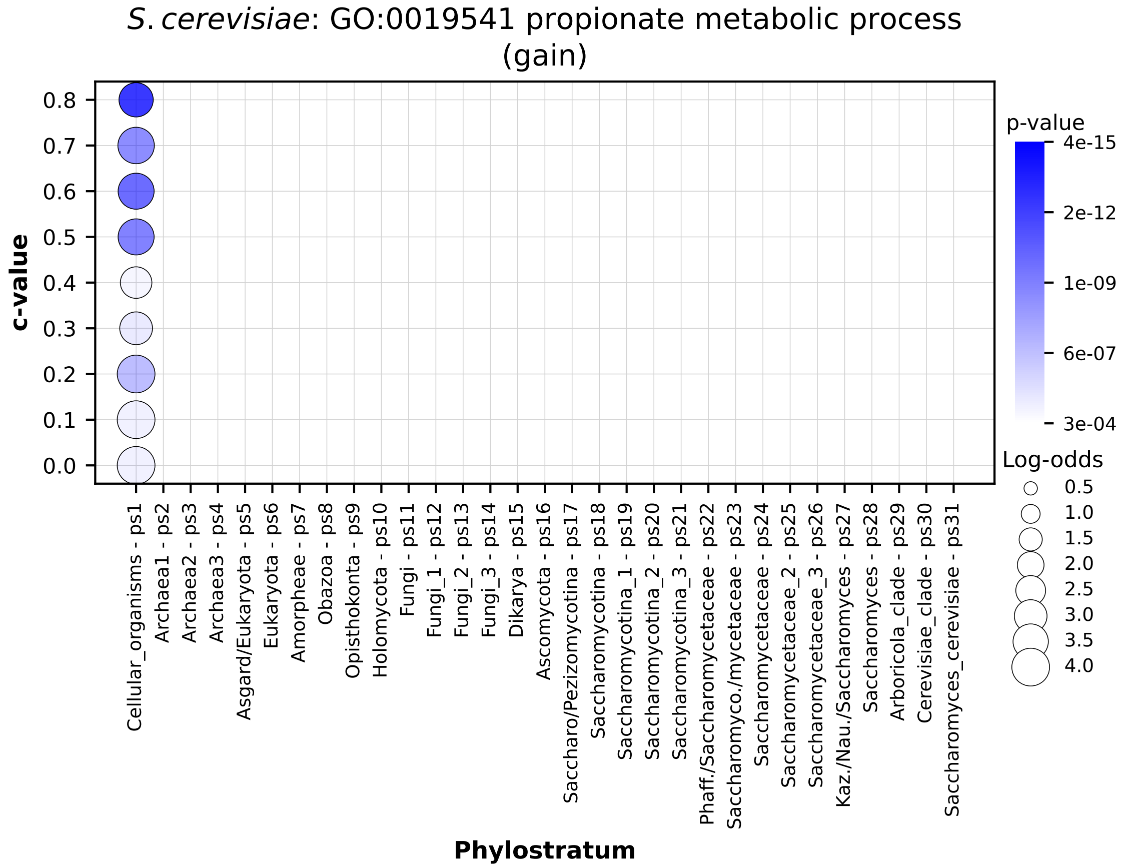

S. cerevisiae: GO:0019541 propionate metabolic process
(gain)
c-value
0.8
0.7
0.6
0.5
0.4
0.3
0.2
0.1
0.0
p-value
4e-15
2e-12
1e-09
6e-07
3e-04
Log-odds
0.5
1.0
1.5
2.0
2.5
3.0
3.5
4.0
Cellular_organisms - ps1
Archaea1 - ps2
Archaea2 - ps3
Archaea3 - ps4
Asgard/Eukaryota - ps5
Eukaryota - ps6
Amorpheae - ps7
Obazoa - ps8
Opisthokonta - ps9
Holomycota - ps10
Fungi - ps11
Fungi_1 - ps12
Fungi_2 - ps13
Fungi_3 - ps14
Dikarya - ps15
Ascomycota - ps16
Saccharo/Pezizomycotina - ps17
Saccharomycotina - ps18
Saccharomycotina_1 - ps19
Saccharomycotina_2 - ps20
Saccharomycotina_3 - ps21
Phaff./Saccharomycetaceae - ps22
Saccharomyco./mycetaceae - ps23
Saccharomycetaceae - ps24
Saccharomycetaceae_2 - ps25
Saccharomycetaceae_3 - ps26
Kaz./Nau./Saccharomyces - ps27
Saccharomyces - ps28
Arboricola_clade - ps29
Cerevisiae_clade - ps30
Saccharomyces_cerevisiae - ps31
Phylostratum

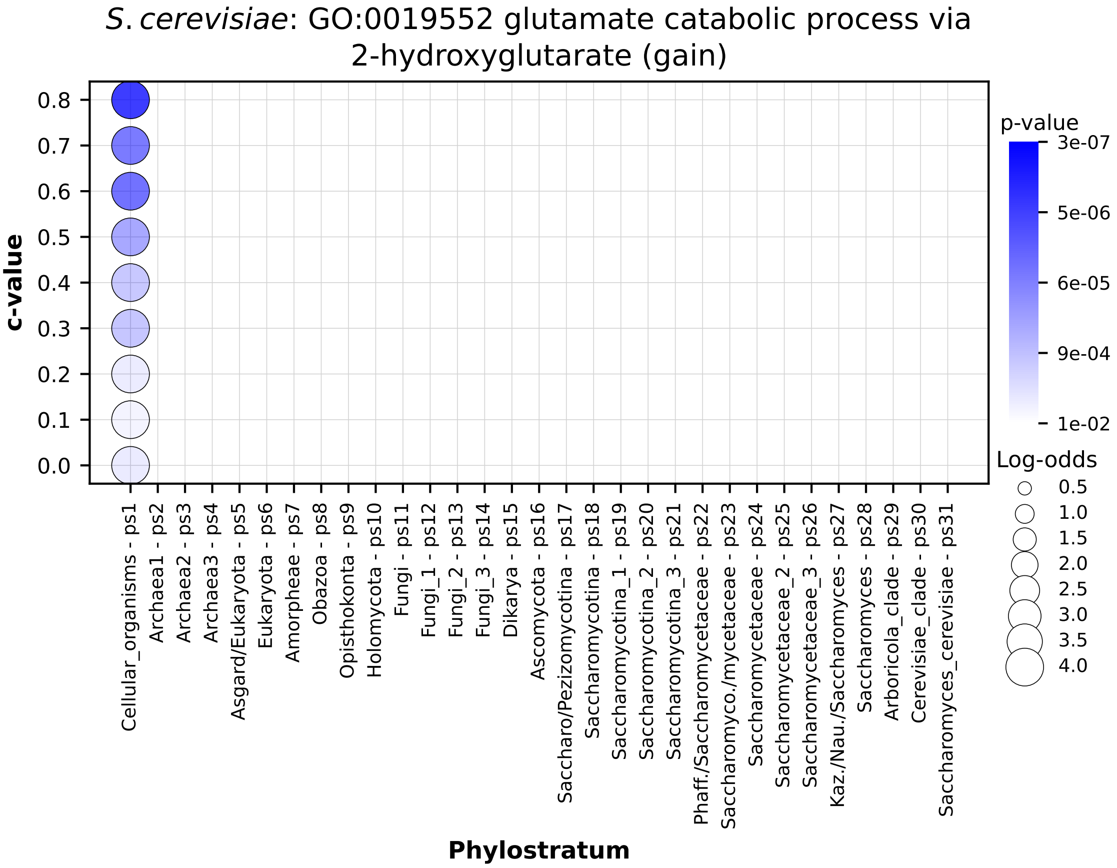
S. cerevisiae: GO:0019552 glutamate catabolic process via 2-hydroxyglutarate (gain)
c-value
0.0
0.1
0.2
0.3
0.4
0.5
0.6
0.7
0.8
p-value
3e-07
5e-06
6e-05
9e-04
1e-02
Log-odds
0.5
1.0
1.5
2.0
2.5
3.0
3.5
4.0
Cellular_organisms - ps1
Archaea1 - ps2
Archaea2 - ps3
Archaea3 - ps4
Asgard/Eukaryota - ps5
Eukaryota - ps6
Amorpheae - ps7
Obazoa - ps8
Opisthokonta - ps9
Holomycota - ps10
Fungi - ps11
Fungi_1 - ps12
Fungi_2 - ps13
Fungi_3 - ps14
Dikarya - ps15
Ascomycota - ps16
Saccharo/Pezizomycotina - ps17
Saccharomycotina - ps18
Saccharomycotina_1 - ps19
Saccharomycotina_2 - ps20
Saccharomycotina_3 - ps21
Phaff./Saccharomycetaceae - ps22
Saccharomyco./mycetaceae - ps23
Saccharomycetaceae - ps24
Saccharomycetaceae_2 - ps25
Saccharomycetaceae_3 - ps26
Kaz./Nau./Saccharomyces - ps27
Saccharomyces - ps28
Arboricola_clade - ps29
Cerevisiae_clade - ps30
Saccharomyces_cerevisiae - ps31
Phylostratum

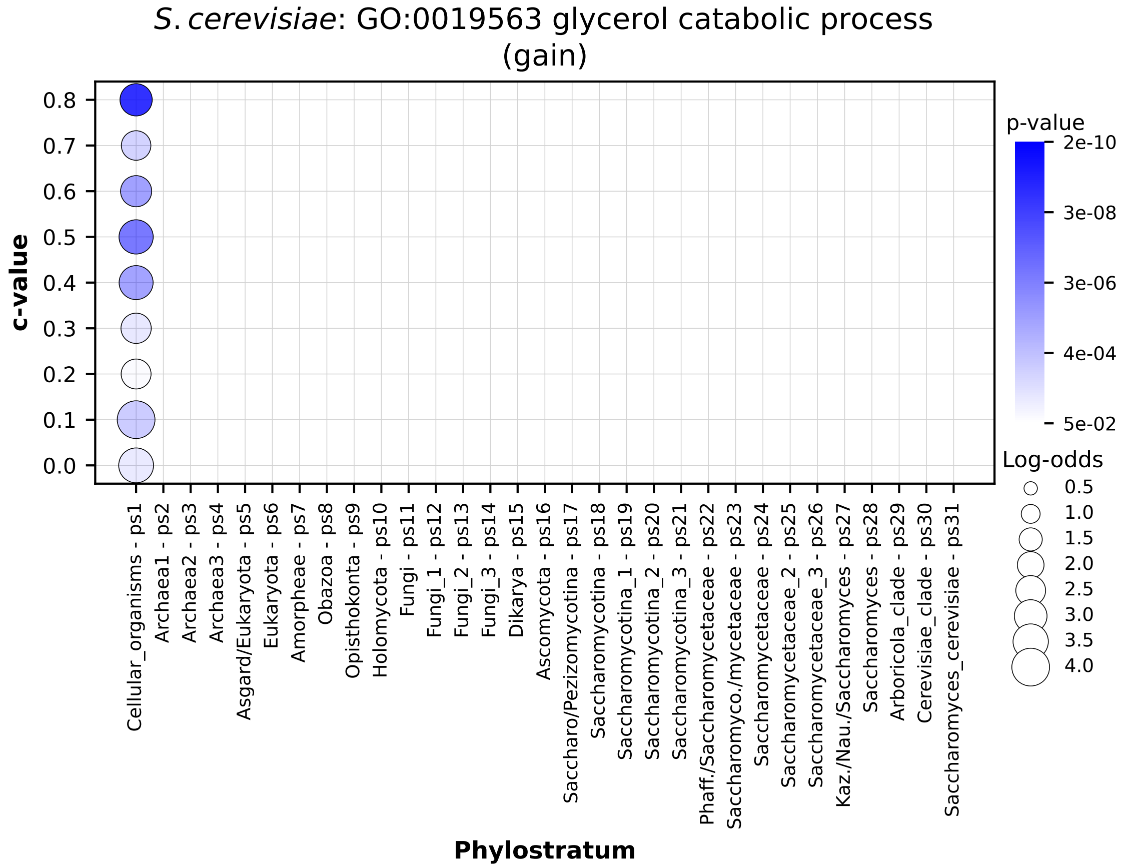

S. cerevisiae: GO:0019563 glycerol catabolic process (gain)
c-value
0.8
0.7
0.6
0.5
0.4
0.3
0.2
0.1
0.0
p-value
2e-10
3e-08
3e-06
4e-04
5e-02
Log-odds
0.5
1.0
1.5
2.0
2.5
3.0
3.5
4.0
Cellular_organisms - ps1
Archaea1 - ps2
Archaea2 - ps3
Archaea3 - ps4
Asgard/Eukaryota - ps5
Eukaryota - ps6
Amorpheae - ps7
Obazoa - ps8
Opisthokonta - ps9
Holomycota - ps10
Fungi - ps11
Fungi_1 - ps12
Fungi_2 - ps13
Fungi_3 - ps14
Dikarya - ps15
Ascomycota - ps16
Saccharo/Pezizomycotina - ps17
Saccharomycotina - ps18
Saccharomycotina_1 - ps19
Saccharomycotina_2 - ps20
Saccharomycotina_3 - ps21
Phaff./Saccharomycetaceae - ps22
Saccharomyco./mycetaceae - ps23
Saccharomycetaceae - ps24
Saccharomycetaceae_2 - ps25
Saccharomycetaceae_3 - ps26
Kaz./Nau./Saccharomyces - ps27
Saccharomyces - ps28
Arboricola_clade - ps29
Cerevisiae_clade - ps30
Saccharomyces_cerevisiae - ps31
Phylostratum

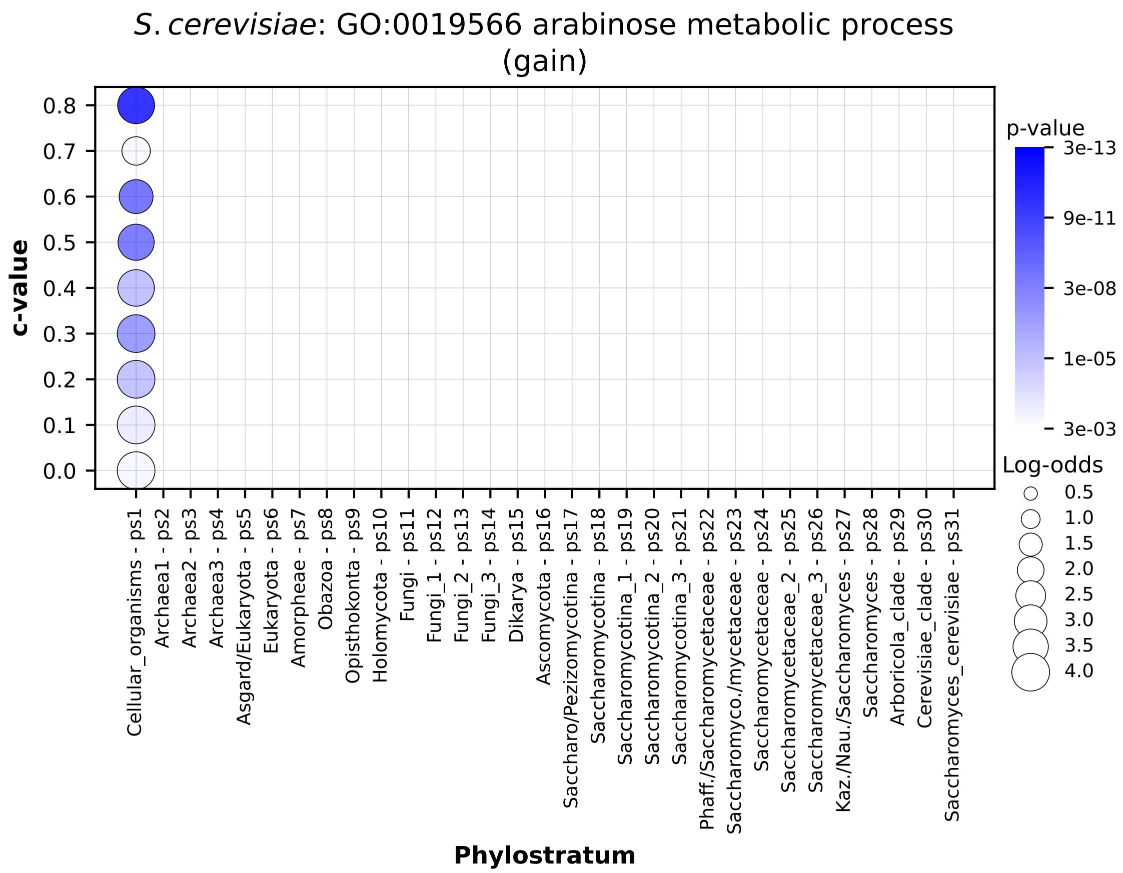
S. cerevisiae: GO:0019566 arabinose metabolic process (gain)
c-value
0.8
0.7
0.6
0.5
0.4
0.3
0.2
0.1
0.0
p-value
3e-13
9e-11
3e-08
1e-05
3e-03
Log-odds
0.5
1.0
1.5
2.0
2.5
3.0
3.5
4.0
Cellular_organisms - ps1
Archaea1 - ps2
Archaea2 - ps3
Archaea3 - ps4
Asgard/Eukaryota - ps5
Eukaryota - ps6
Amorpheae - ps7
Obazoa - ps8
Opisthokonta - ps9
Holomycota - ps10
Fungi - ps11
Fungi_1 - ps12
Fungi_2 - ps13
Fungi_3 - ps14
Dikarya - ps15
Ascomycota - ps16
Saccharo/Pezizomycotina - ps17
Saccharomycotina - ps18
Saccharomycotina_1 - ps19
Saccharomycotina_2 - ps20
Saccharomycotina_3 - ps21
Phaff./Saccharomycetaceae - ps22
Saccharomyco./mycetaceae - ps23
Saccharomycetaceae - ps24
Saccharomycetaceae_2 - ps25
Saccharomycetaceae_3 - ps26
Kaz./Nau./Saccharomyces - ps27
Saccharomyces - ps28
Arboricola_clade - ps29
Cerevisiae_clade - ps30
Saccharomyces_cerevisiae - ps31
Phylostratum

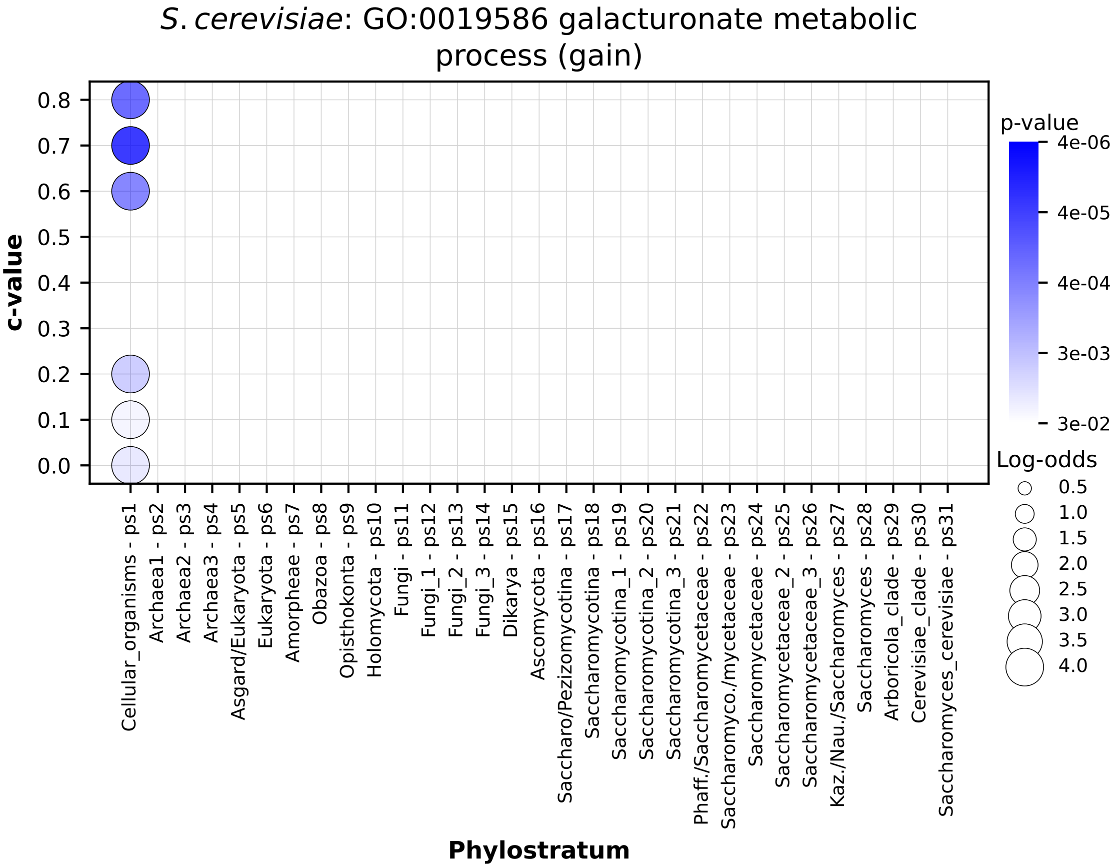

S. cerevisiae: GO:0019586 galacturonate metabolic process (gain)
c-value
0.8
0.7
0.6
0.5
0.4
0.3
0.2
0.1
0.0
p-value
4e-06
4e-05
4e-04
3e-03
3e-02
Log-odds
0.5
1.0
1.5
2.0
2.5
3.0
3.5
4.0
Cellular_organisms - ps1
Archaea1 - ps2
Archaea2 - ps3
Archaea3 - ps4
Asgard/Eukaryota - ps5
Eukaryota - ps6
Amorpheae - ps7
Obazoa - ps8
Opisthokonta - ps9
Holomycota - ps10
Fungi - ps11
Fungi_1 - ps12
Fungi_2 - ps13
Fungi_3 - ps14
Dikarya - ps15
Ascomycota - ps16
Saccharo/Pezizomycotina - ps17
Saccharomycotina - ps18
Saccharomycotina_1 - ps19
Saccharomycotina_2 - ps20
Saccharomycotina_3 - ps21
Phaff./Saccharomycetaceae - ps22
Saccharomyco./mycetaceae - ps23
Saccharomycetaceae - ps24
Saccharomycetaceae_2 - ps25
Saccharomycetaceae_3 - ps26
Kaz./Nau./Saccharomyces - ps27
Saccharomyces - ps28
Arboricola_clade - ps29
Cerevisiae_clade - ps30
Saccharomyces_cerevisiae - ps31
Phylostratum

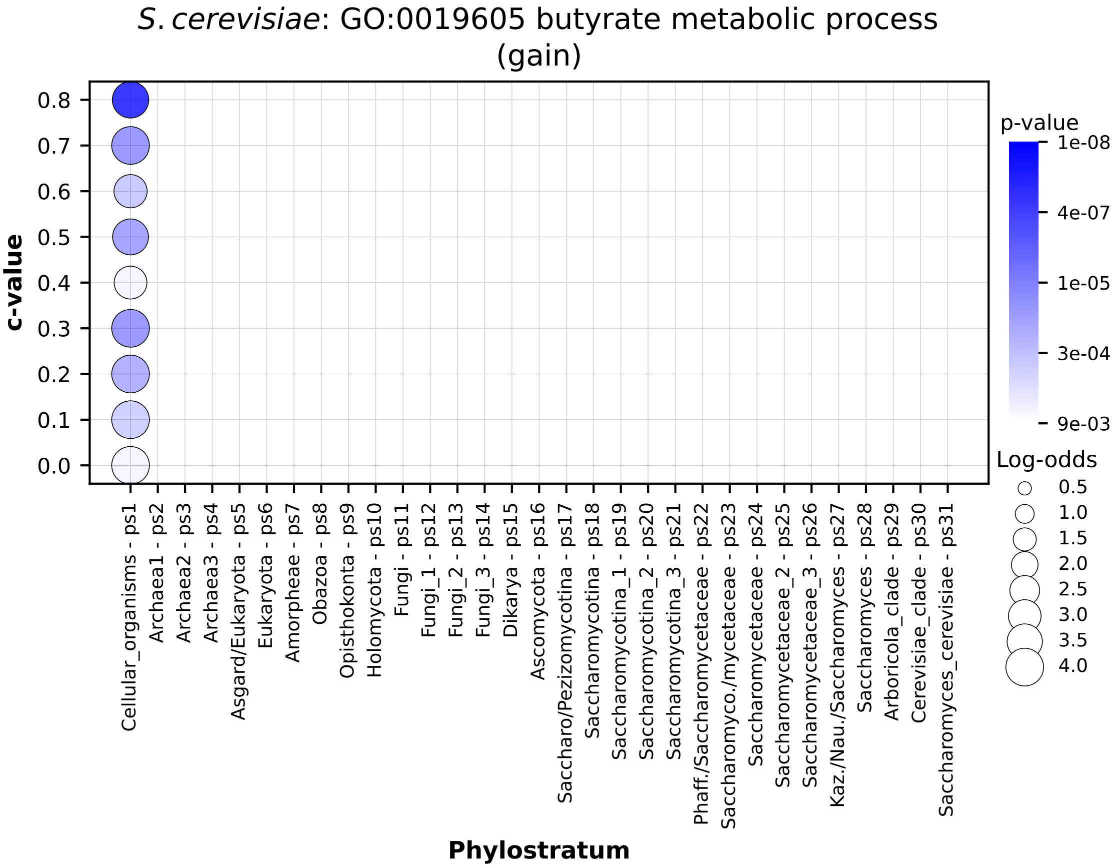

S. cerevisiae: GO:0019605 butyrate metabolic process
(gain)
c-value
0.8
0.7
0.6
0.5
0.4
0.3
0.2
0.1
0.0
p-value
1e-08
4e-07
1e-05
3e-04
9e-03
Log-odds
0.5
1.0
1.5
2.0
2.5
3.0
3.5
4.0
Cellular_organisms - ps1
Archaea1 - ps2
Archaea2 - ps3
Archaea3 - ps4
Asgard/Eukaryota - ps5
Eukaryota - ps6
Amorpheae - ps7
Obazoa - ps8
Opisthokonta - ps9
Holomycota - ps10
Fungi - ps11
Fungi_1 - ps12
Fungi_2 - ps13
Fungi_3 - ps14
Dikarya - ps15
Ascomycota - ps16
Saccharo/Pezizomycotina - ps17
Saccharomycotina - ps18
Saccharomycotina_1 - ps19
Saccharomycotina_2 - ps20
Saccharomycotina_3 - ps21
Phaff./Saccharomycetaceae - ps22
Saccharomyco./mycetaceae - ps23
Saccharomycetaceae - ps24
Saccharomycetaceae_2 - ps25
Saccharomycetaceae_3 - ps26
Kaz./Nau./Saccharomyces - ps27
Saccharomyces - ps28
Arboricola_clade - ps29
Cerevisiae_clade - ps30
Saccharomyces_cerevisiae - ps31
Phylostratum

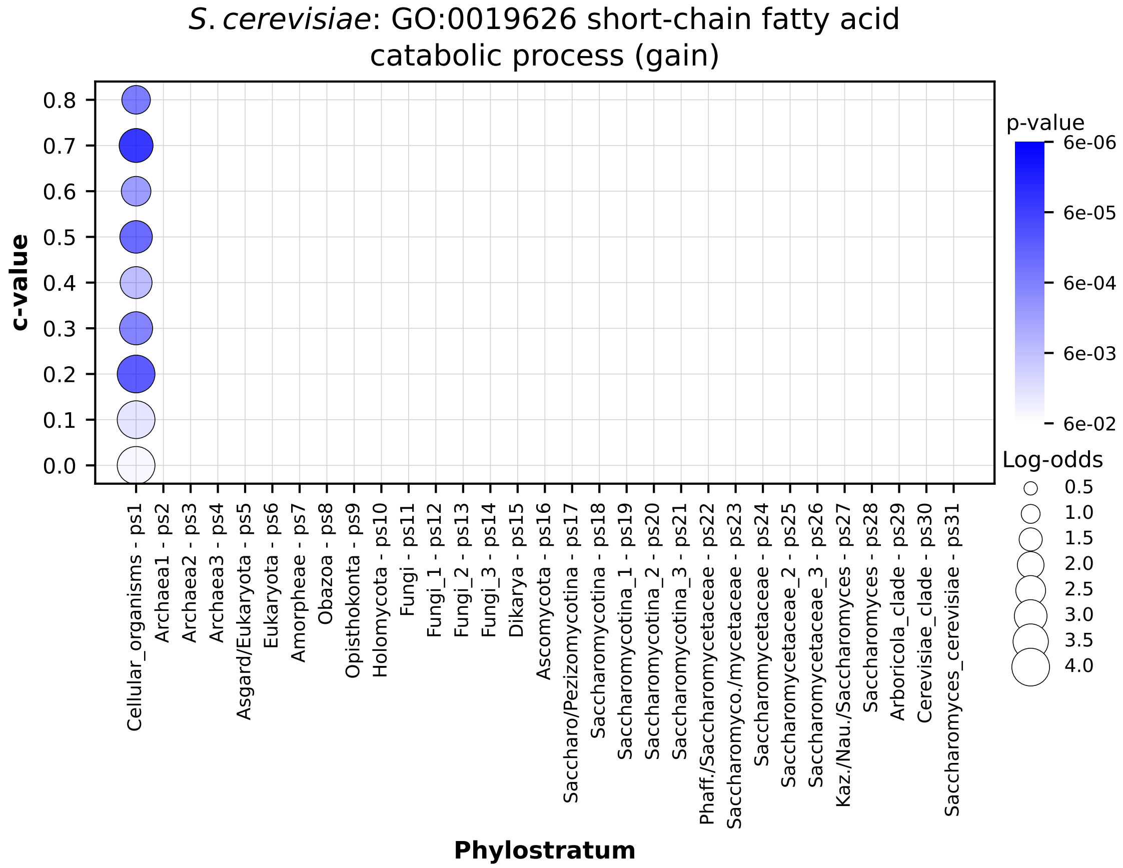
S. cerevisiae: GO:0019626 short-chain fatty acid catabolic process (gain)
c-value
0.0
0.1
0.2
0.3
0.4
0.5
0.6
0.7
0.8
p-value
6e-06
6e-05
6e-04
6e-03
6e-02
Log-odds
0.5
1.0
1.5
2.0
2.5
3.0
3.5
4.0
Cellular_organisms - ps1
Archaea1 - ps2
Archaea2 - ps3
Archaea3 - ps4
Asgard/Eukaryota - ps5
Eukaryota - ps6
Amorpheae - ps7
Obazoa - ps8
Opisthokonta - ps9
Holomycota - ps10
Fungi - ps11
Fungi_1 - ps12
Fungi_2 - ps13
Fungi_3 - ps14
Dikarya - ps15
Ascomycota - ps16
Saccharo/Pezizomycotina - ps17
Saccharomycotina - ps18
Saccharomycotina_1 - ps19
Saccharomycotina_2 - ps20
Saccharomycotina_3 - ps21
Phaff./Saccharomycetaceae - ps22
Saccharomyco./mycetaceae - ps23
Saccharomycetaceae - ps24
Saccharomycetaceae_2 - ps25
Saccharomycetaceae_3 - ps26
Kaz./Nau./Saccharomyces - ps27
Saccharomyces - ps28
Arboricola_clade - ps29
Cerevisiae_clade - ps30
Saccharomyces_cerevisiae - ps31
Phylostratum

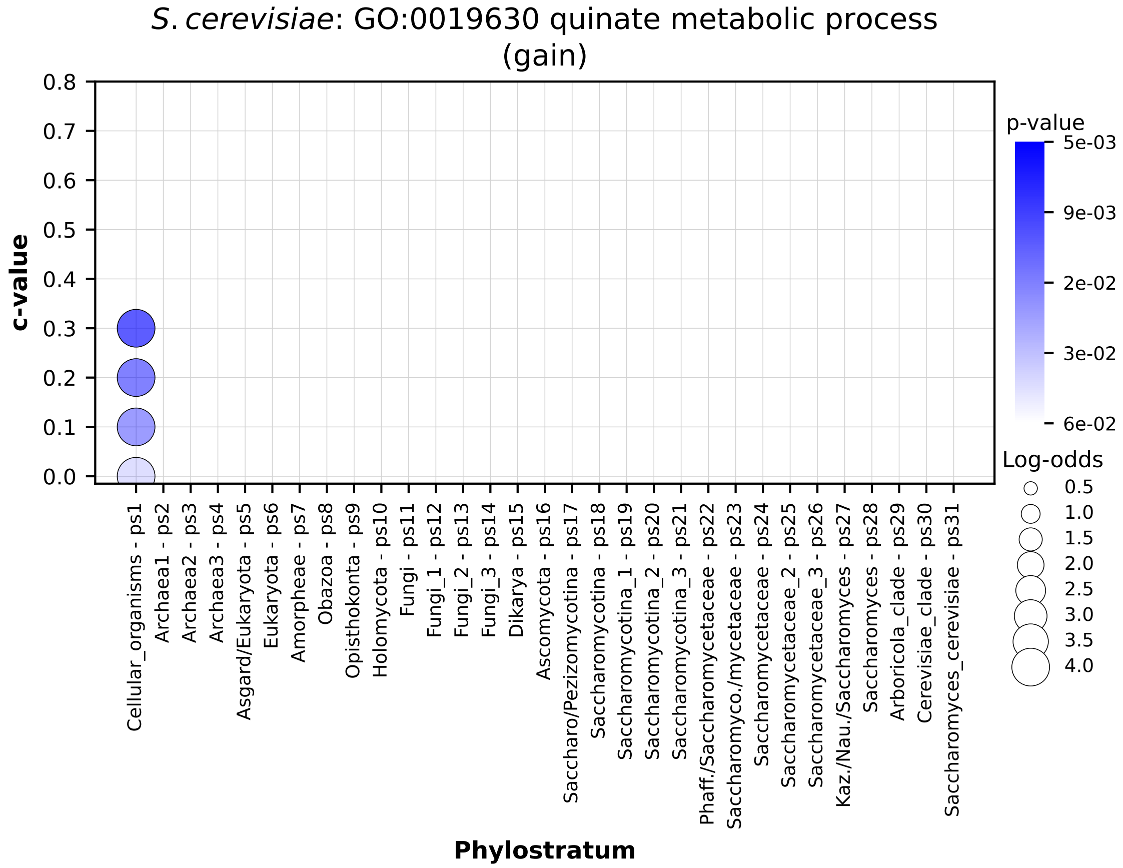
S. cerevisiae: GO:0019630 quinate metabolic process (gain)
c-value
0.8
0.7
0.6
0.5
0.4
0.3
0.2
0.1
0.0
Phylostratum
Cellular_organisms - ps1
Archaea1 - ps2
Archaea2 - ps3
Archaea3 - ps4
Asgard/Eukaryota - ps5
Eukaryota - ps6
Amorpheae - ps7
Obazoa - ps8
Opisthokonta - ps9
Holomycota - ps10
Fungi - ps11
Fungi_1 - ps12
Fungi_2 - ps13
Fungi_3 - ps14
Dikarya - ps15
Ascomycota - ps16
Saccharo/Pezizomycotina - ps17
Saccharomycotina - ps18
Saccharomycotina_1 - ps19
Saccharomycotina_2 - ps20
Saccharomycotina_3 - ps21
Phaff./Saccharomycetaceae - ps22
Saccharomyco./mycetaceae - ps23
Saccharomycetaceae - ps24
Saccharomycetaceae_2 - ps25
Saccharomycetaceae_3 - ps26
Kaz./Nau./Saccharomyces - ps27
Saccharomyces - ps28
Arboricola_clade - ps29
Cerevisiae_clade - ps30
Saccharomyces_cerevisiae - ps31
p-value
5e-03
9e-03
2e-02
3e-02
6e-02
Log-odds
0.5
1.0
1.5
2.0
2.5
3.0
3.5
4.0

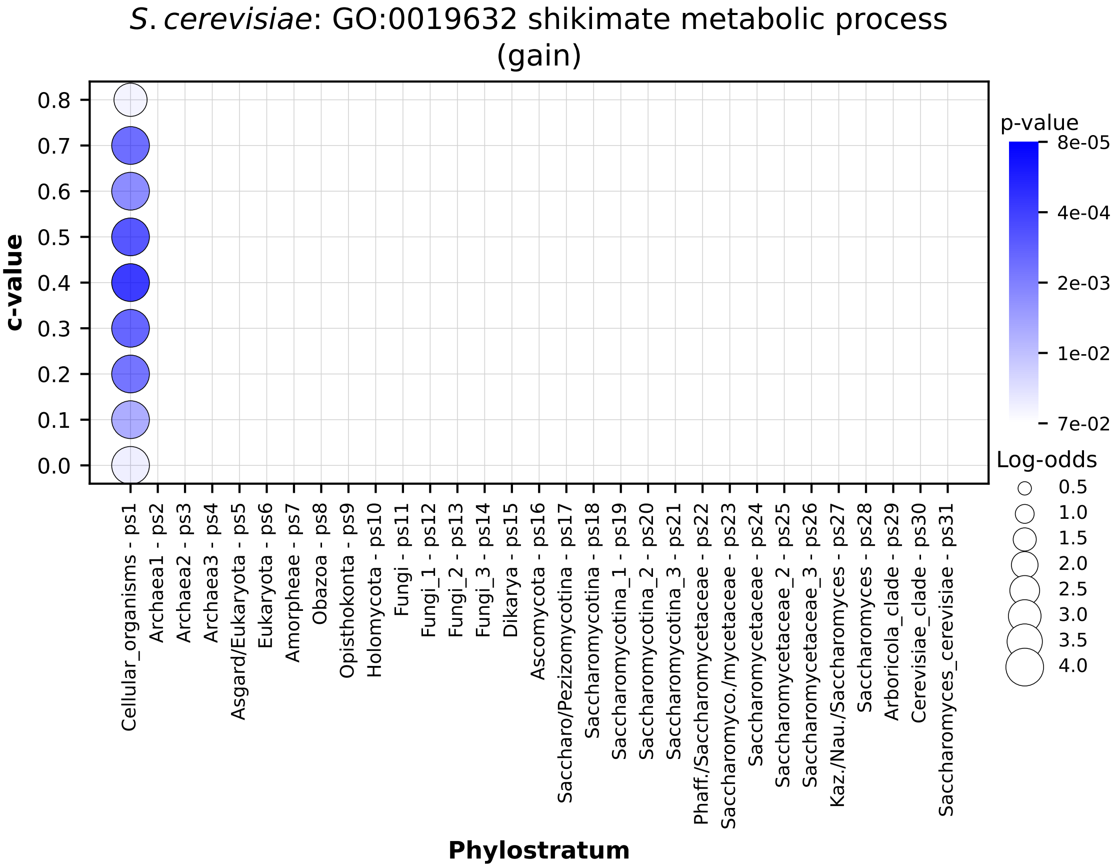

S. cerevisiae: GO:0019632 shikimate metabolic process (gain)
c-value
0.8
0.7
0.6
0.5
0.4
0.3
0.2
0.1
0.0
Phylostratum
Cellular_organisms - ps1
Archaea1 - ps2
Archaea2 - ps3
Archaea3 - ps4
Asgard/Eukaryota - ps5
Eukaryota - ps6
Amorpheae - ps7
Obazoa - ps8
Opisthokonta - ps9
Holomycota - ps10
Fungi - ps11
Fungi_1 - ps12
Fungi_2 - ps13
Fungi_3 - ps14
Dikarya - ps15
Ascomycota - ps16
Saccharo/Pezizomycotina - ps17
Saccharomycotina - ps18
Saccharomycotina_1 - ps19
Saccharomycotina_2 - ps20
Saccharomycotina_3 - ps21
Phaff./Saccharomycetaceae - ps22
Saccharomyco./mycetaceae - ps23
Saccharomycetaceae - ps24
Saccharomycetaceae_2 - ps25
Saccharomycetaceae_3 - ps26
Kaz./Nau./Saccharomyces - ps27
Saccharomyces - ps28
Arboricola_clade - ps29
Cerevisiae_clade - ps30
Saccharomyces_cerevisiae - ps31
p-value
8e-05
4e-04
2e-03
1e-02
7e-02
Log-odds
0.5
1.0
1.5
2.0
2.5
3.0
3.5
4.0

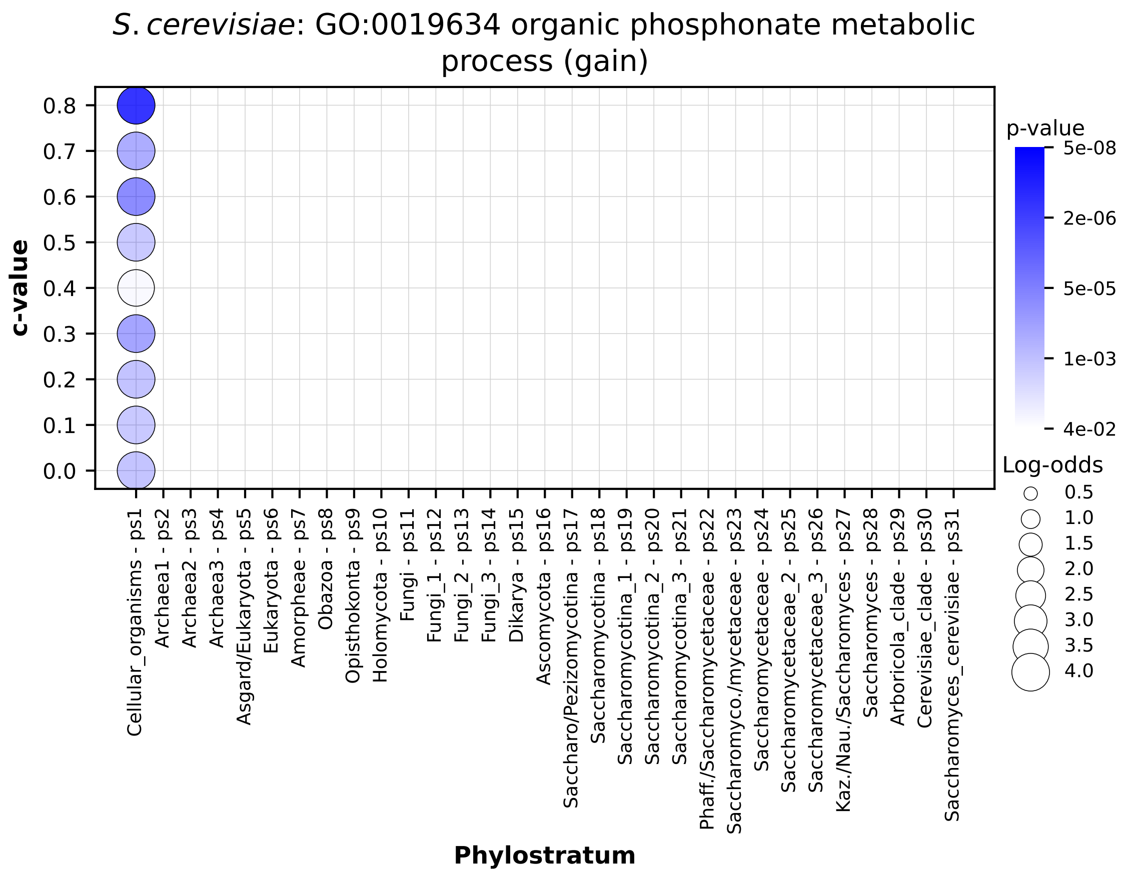
S. cerevisiae: GO:0019634 organic phosphonate metabolic process (gain)
c-value
0.8
0.7
0.6
0.5
0.4
0.3
0.2
0.1
0.0
p-value
5e-08
2e-06
5e-05
1e-03
4e-02
Log-odds
0.5
1.0
1.5
2.0
2.5
3.0
3.5
4.0
Cellular_organisms - ps1
Archaea1 - ps2
Archaea2 - ps3
Archaea3 - ps4
Asgard/Eukaryota - ps5
Eukaryota - ps6
Amorpheae - ps7
Obazoa - ps8
Opisthokonta - ps9
Holomycota - ps10
Fungi - ps11
Fungi_1 - ps12
Fungi_2 - ps13
Fungi_3 - ps14
Dikarya - ps15
Ascomycota - ps16
Saccharo/Pezizomycotina - ps17
Saccharomycotina - ps18
Saccharomycotina_1 - ps19
Saccharomycotina_2 - ps20
Saccharomycotina_3 - ps21
Phaff./Saccharomycetaceae - ps22
Saccharomyco./mycetaceae - ps23
Saccharomycetaceae - ps24
Saccharomycetaceae_2 - ps25
Saccharomycetaceae_3 - ps26
Kaz./Nau./Saccharomyces - ps27
Saccharomyces - ps28
Arboricola_clade - ps29
Cerevisiae_clade - ps30
Saccharomyces_cerevisiae - ps31
Phylostratum

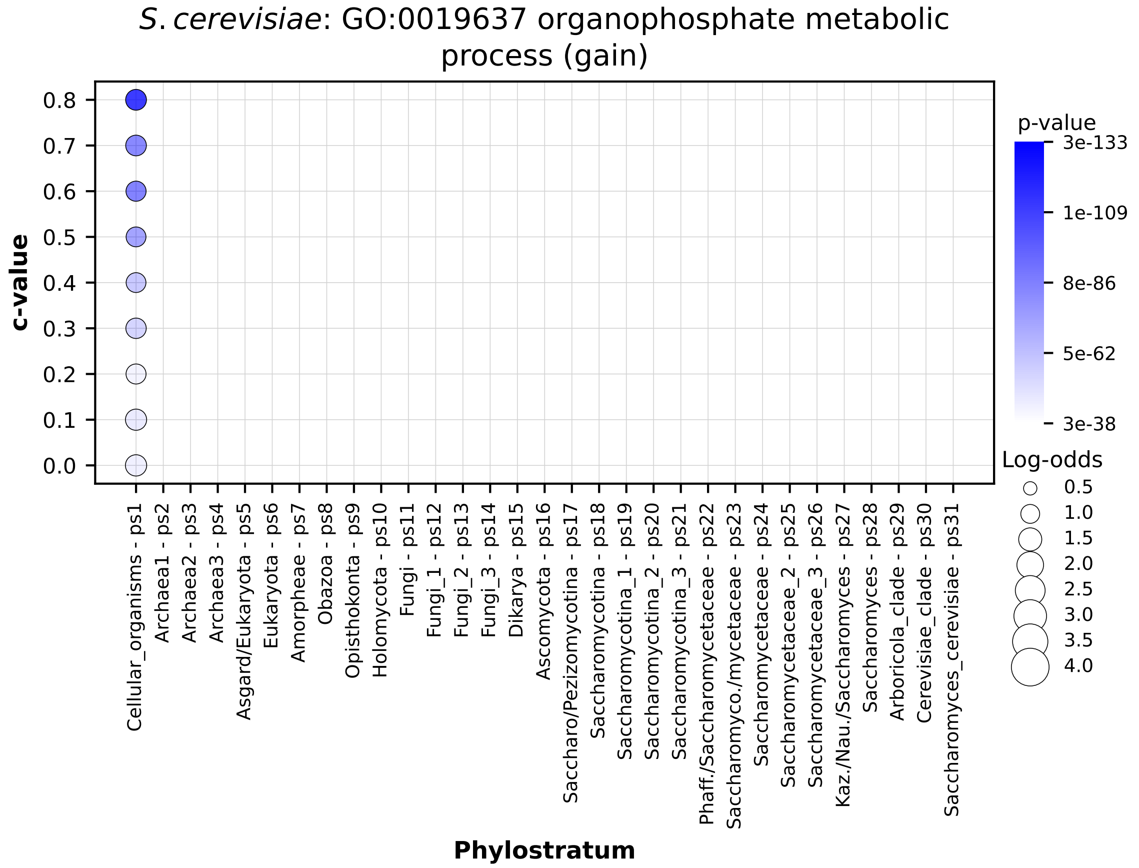

S. cerevisiae: GO:0019637 organophosphate metabolic process (gain)
c-value
0.8
0.7
0.6
0.5
0.4
0.3
0.2
0.1
0.0
p-value
3e-133
1e-109
8e-86
5e-62
3e-38
Log-odds
0.5
1.0
1.5
2.0
2.5
3.0
3.5
4.0
Cellular_organisms - ps1
Archaea1 - ps2
Archaea2 - ps3
Archaea3 - ps4
Asgard/Eukaryota - ps5
Eukaryota - ps6
Amorpheae - ps7
Obazoa - ps8
Opisthokonta - ps9
Holomycota - ps10
Fungi - ps11
Fungi_1 - ps12
Fungi_2 - ps13
Fungi_3 - ps14
Dikarya - ps15
Ascomycota - ps16
Saccharo/Pezizomycotina - ps17
Saccharomycotina - ps18
Saccharomycotina_1 - ps19
Saccharomycotina_2 - ps20
Saccharomycotina_3 - ps21
Phaff./Saccharomycetaceae - ps22
Saccharomyco./mycetaceae - ps23
Saccharomycetaceae - ps24
Saccharomycetaceae_2 - ps25
Saccharomycetaceae_3 - ps26
Kaz./Nau./Saccharomyces - ps27
Saccharomyces - ps28
Arboricola_clade - ps29
Cerevisiae_clade - ps30
Saccharomyces_cerevisiae - ps31
Phylostratum

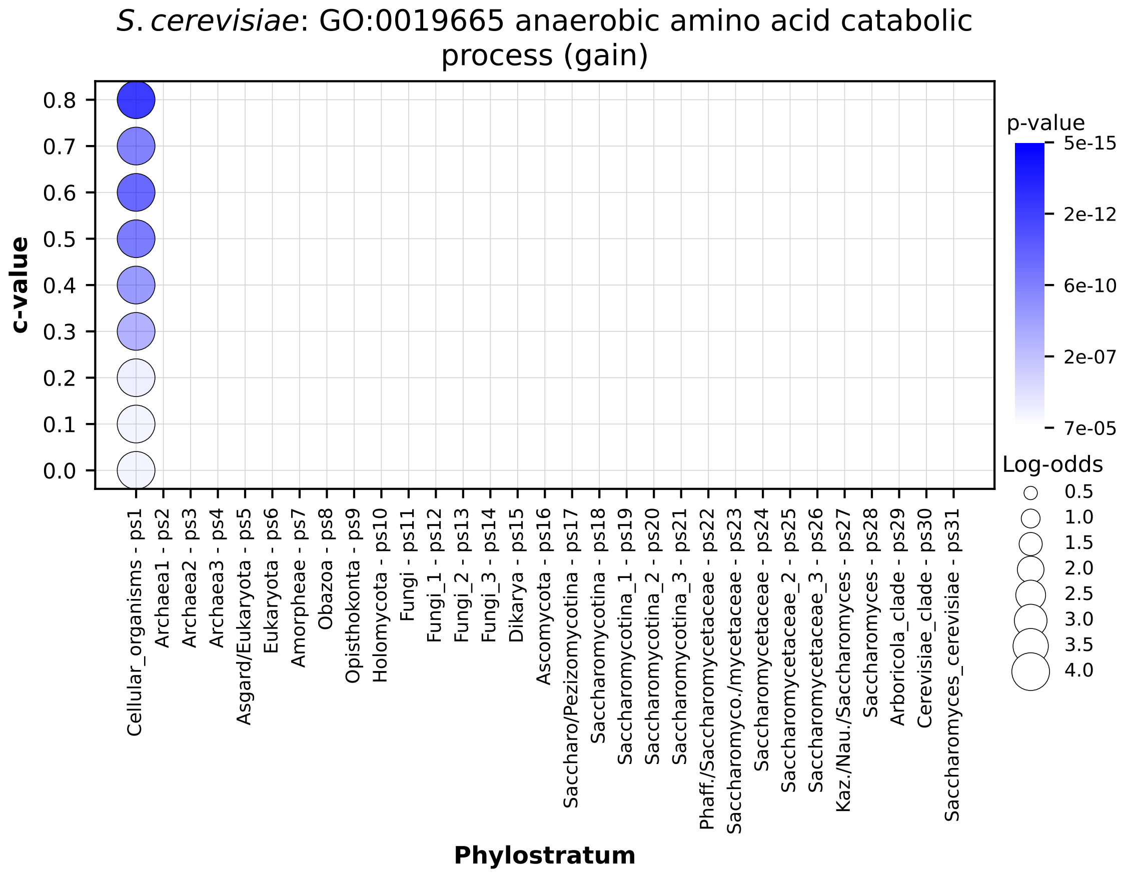

S. cerevisiae: GO:0019665 anaerobic amino acid catabolic process (gain)
c-value
0.8
0.7
0.6
0.5
0.4
0.3
0.2
0.1
0.0
p-value
5e-15
2e-12
6e-10
2e-07
7e-05
Log-odds
0.5
1.0
1.5
2.0
2.5
3.0
3.5
4.0
Cellular_organisms - ps1
Archaea1 - ps2
Archaea2 - ps3
Archaea3 - ps4
Asgard/Eukaryota - ps5
Eukaryota - ps6
Amorpheae - ps7
Obazoa - ps8
Opisthokonta - ps9
Holomycota - ps10
Fungi - ps11
Fungi_1 - ps12
Fungi_2 - ps13
Fungi_3 - ps14
Dikarya - ps15
Ascomycota - ps16
Saccharo/Pezizomycotina - ps17
Saccharomycotina - ps18
Saccharomycotina_1 - ps19
Saccharomycotina_2 - ps20
Saccharomycotina_3 - ps21
Phaff./Saccharomycetaceae - ps22
Saccharomyco./mycetaceae - ps23
Saccharomycetaceae - ps24
Saccharomycetaceae_2 - ps25
Saccharomycetaceae_3 - ps26
Kaz./Nau./Saccharomyces - ps27
Saccharomyces - ps28
Arboricola_clade - ps29
Cerevisiae_clade - ps30
Saccharomyces_cerevisiae - ps31
Phylostratum

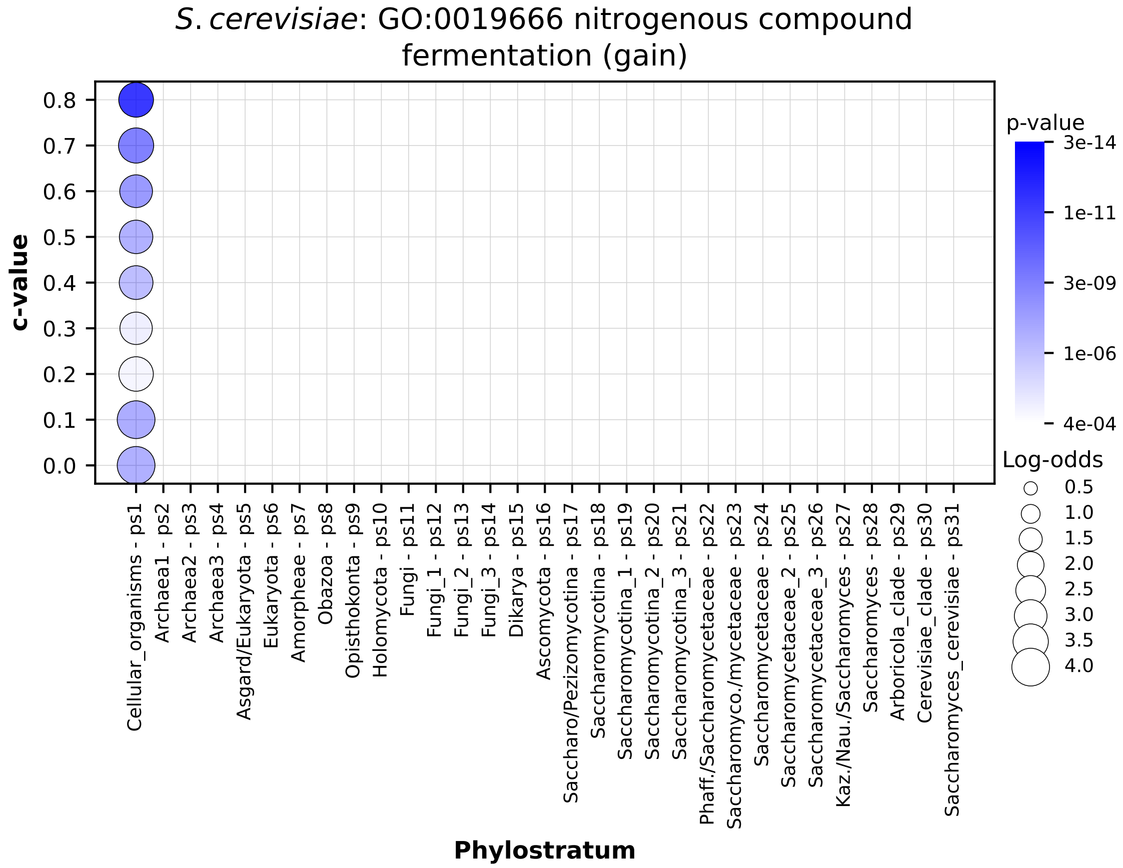
S. cerevisiae: GO:0019666 nitrogenous compound fermentation (gain)
c-value
0.8
0.7
0.6
0.5
0.4
0.3
0.2
0.1
0.0
p-value
3e-14
1e-11
3e-09
1e-06
4e-04
Log-odds
0.5
1.0
1.5
2.0
2.5
3.0
3.5
4.0
Cellular_organisms - ps1
Archaea1 - ps2
Archaea2 - ps3
Archaea3 - ps4
Asgard/Eukaryota - ps5
Eukaryota - ps6
Amorpheae - ps7
Obazoa - ps8
Opisthokonta - ps9
Holomycota - ps10
Fungi - ps11
Fungi_1 - ps12
Fungi_2 - ps13
Fungi_3 - ps14
Dikarya - ps15
Ascomycota - ps16
Saccharo/Pezizomycotina - ps17
Saccharomycotina - ps18
Saccharomycotina_1 - ps19
Saccharomycotina_2 - ps20
Saccharomycotina_3 - ps21
Phaff./Saccharomycetaceae - ps22
Saccharomyco./mycetaceae - ps23
Saccharomycetaceae - ps24
Saccharomycetaceae_2 - ps25
Saccharomycetaceae_3 - ps26
Kaz./Nau./Saccharomyces - ps27
Saccharomyces - ps28
Arboricola_clade - ps29
Cerevisiae_clade - ps30
Saccharomyces_cerevisiae - ps31
Phylostratum

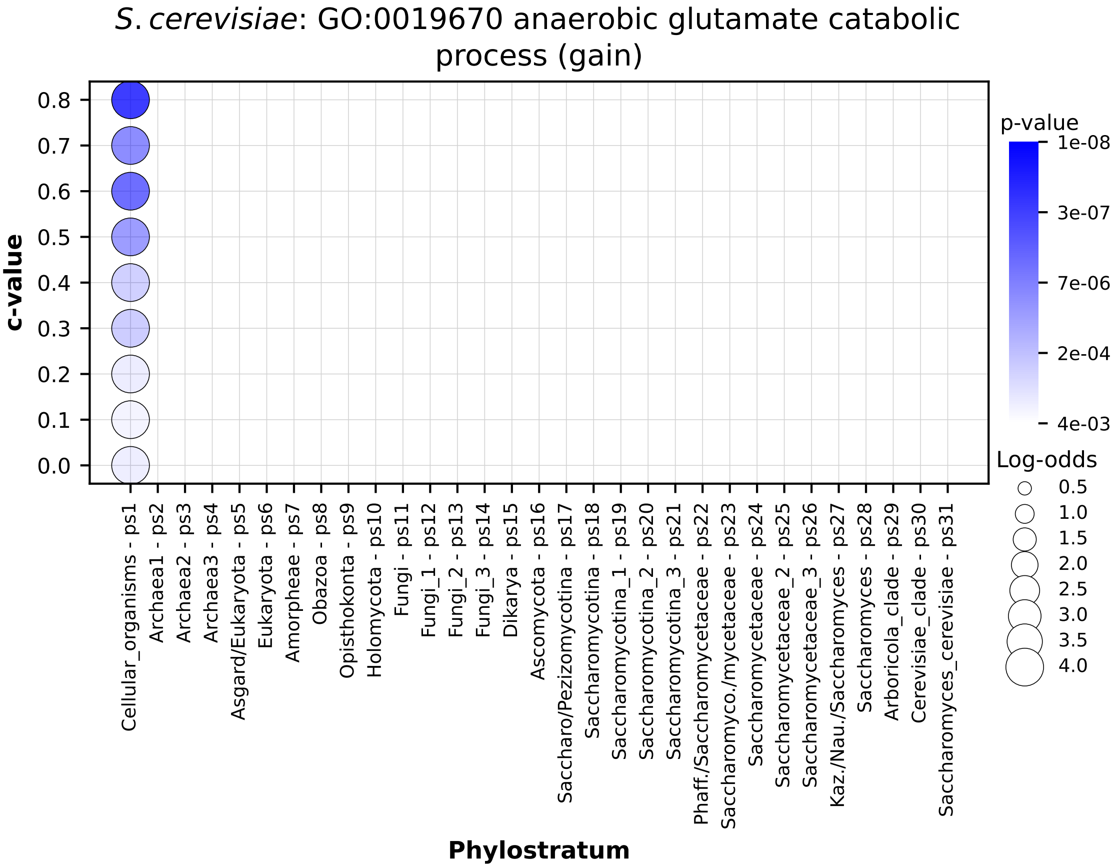
S. cerevisiae: GO:0019670 anaerobic glutamate catabolic process (gain)
c-value
0.8
0.7
0.6
0.5
0.4
0.3
0.2
0.1
0.0
p-value
1e-08
3e-07
7e-06
2e-04
4e-03
Log-odds
0.5
1.0
1.5
2.0
2.5
3.0
3.5
4.0
Cellular_organisms - ps1
Archaea1 - ps2
Archaea2 - ps3
Archaea3 - ps4
Asgard/Eukaryota - ps5
Eukaryota - ps6
Amorpheae - ps7
Obazoa - ps8
Opisthokonta - ps9
Holomycota - ps10
Fungi - ps11
Fungi_1 - ps12
Fungi_2 - ps13
Fungi_3 - ps14
Dikarya - ps15
Ascomycota - ps16
Saccharo/Pezizomycotina - ps17
Saccharomycotina - ps18
Saccharomycotina_1 - ps19
Saccharomycotina_2 - ps20
Saccharomycotina_3 - ps21
Phaff./Saccharomycetaceae - ps22
Saccharomyco./mycetaceae - ps23
Saccharomycetaceae - ps24
Saccharomycetaceae_2 - ps25
Saccharomycetaceae_3 - ps26
Kaz./Nau./Saccharomyces - ps27
Saccharomyces - ps28
Arboricola_clade - ps29
Cerevisiae_clade - ps30
Saccharomyces_cerevisiae - ps31
Phylostratum

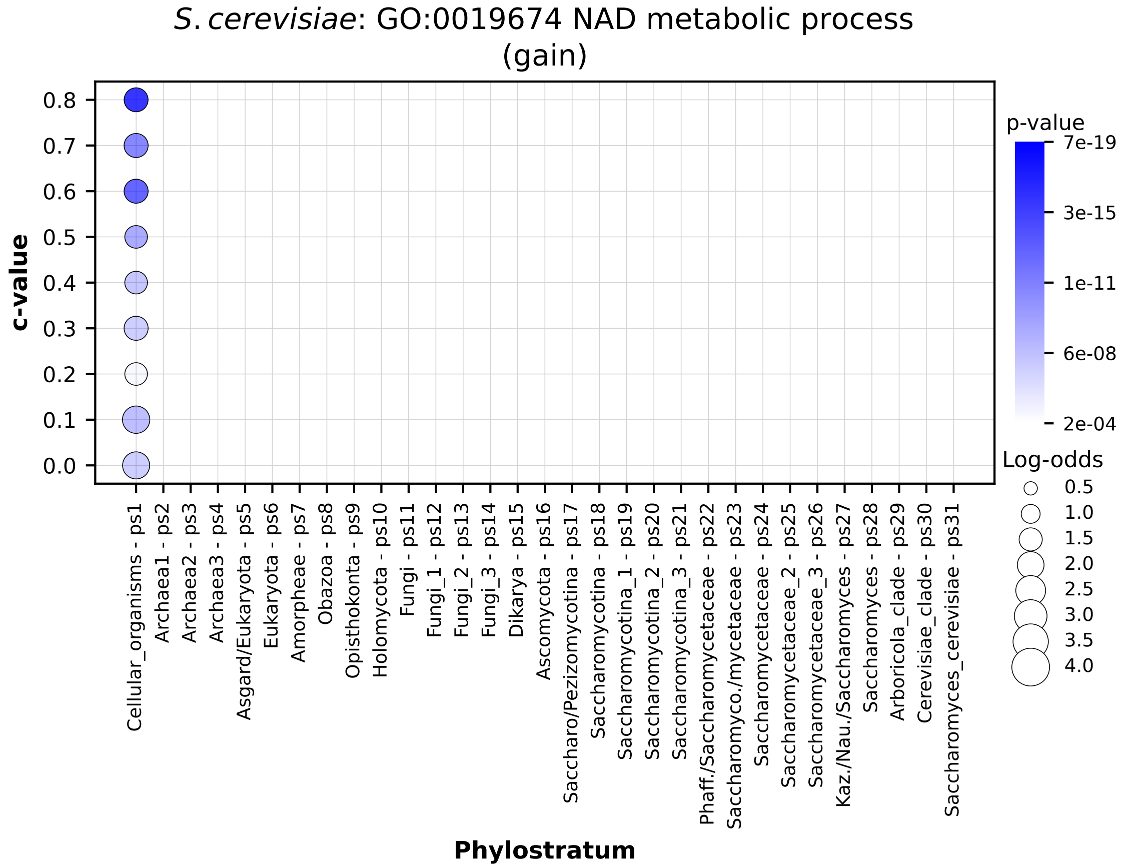
S. cerevisiae: GO:0019674 NAD metabolic process
(gain)
c-value
0.8
0.7
0.6
0.5
0.4
0.3
0.2
0.1
0.0
p-value
7e-19
3e-15
1e-11
6e-08
2e-04
Log-odds
0.5
1.0
1.5
2.0
2.5
3.0
3.5
4.0
Cellular_organisms - ps1
Archaea1 - ps2
Archaea2 - ps3
Archaea3 - ps4
Asgard/Eukaryota - ps5
Eukaryota - ps6
Amorpheae - ps7
Obazoa - ps8
Opisthokonta - ps9
Holomycota - ps10
Fungi - ps11
Fungi_1 - ps12
Fungi_2 - ps13
Fungi_3 - ps14
Dikarya - ps15
Ascomycota - ps16
Saccharo/Pezizomycotina - ps17
Saccharomycotina - ps18
Saccharomycotina_1 - ps19
Saccharomycotina_2 - ps20
Saccharomycotina_3 - ps21
Phaff./Saccharomycetaceae - ps22
Saccharomyco./mycetaceae - ps23
Saccharomycetaceae - ps24
Saccharomycetaceae_2 - ps25
Saccharomycetaceae_3 - ps26
Kaz./Nau./Saccharomyces - ps27
Saccharomyces - ps28
Arboricola_clade - ps29
Cerevisiae_clade - ps30
Saccharomyces_cerevisiae - ps31
Phylostratum

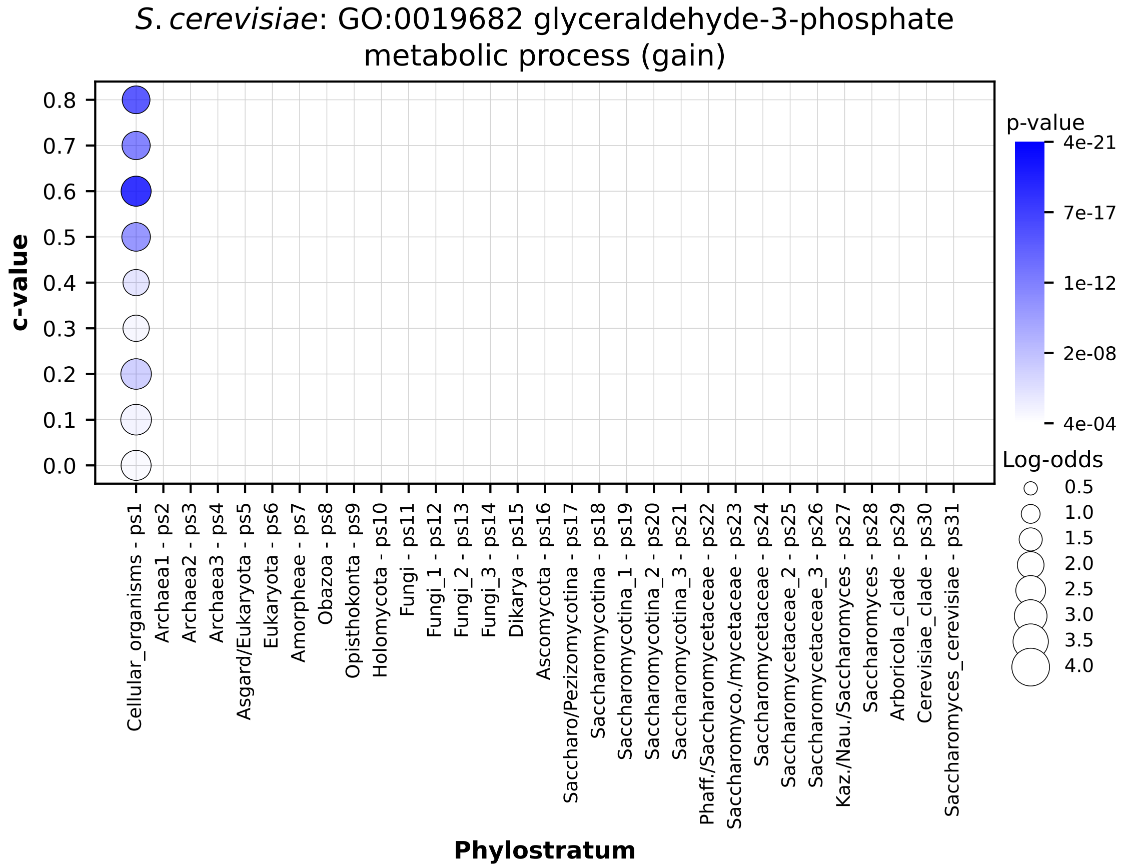
S. cerevisiae: GO:0019682 glyceraldehyde-3-phosphate metabolic process (gain)
c-value
0.8
0.7
0.6
0.5
0.4
0.3
0.2
0.1
0.0
p-value
4e-21
7e-17
1e-12
2e-08
4e-04
Log-odds
0.5
1.0
1.5
2.0
2.5
3.0
3.5
4.0
Cellular_organisms - ps1
Archaea1 - ps2
Archaea2 - ps3
Archaea3 - ps4
Asgard/Eukaryota - ps5
Eukaryota - ps6
Amorpheae - ps7
Obazoa - ps8
Opisthokonta - ps9
Holomycota - ps10
Fungi - ps11
Fungi_1 - ps12
Fungi_2 - ps13
Fungi_3 - ps14
Dikarya - ps15
Ascomycota - ps16
Saccharo/Pezizomycotina - ps17
Saccharomycotina - ps18
Saccharomycotina_1 - ps19
Saccharomycotina_2 - ps20
Saccharomycotina_3 - ps21
Phaff./Saccharomycetaceae - ps22
Saccharomyco./mycetaceae - ps23
Saccharomycetaceae - ps24
Saccharomycetaceae_2 - ps25
Saccharomycetaceae_3 - ps26
Kaz./Nau./Saccharomyces - ps27
Saccharomyces - ps28
Arboricola_clade - ps29
Cerevisiae_clade - ps30
Saccharomyces_cerevisiae - ps31
Phylostratum

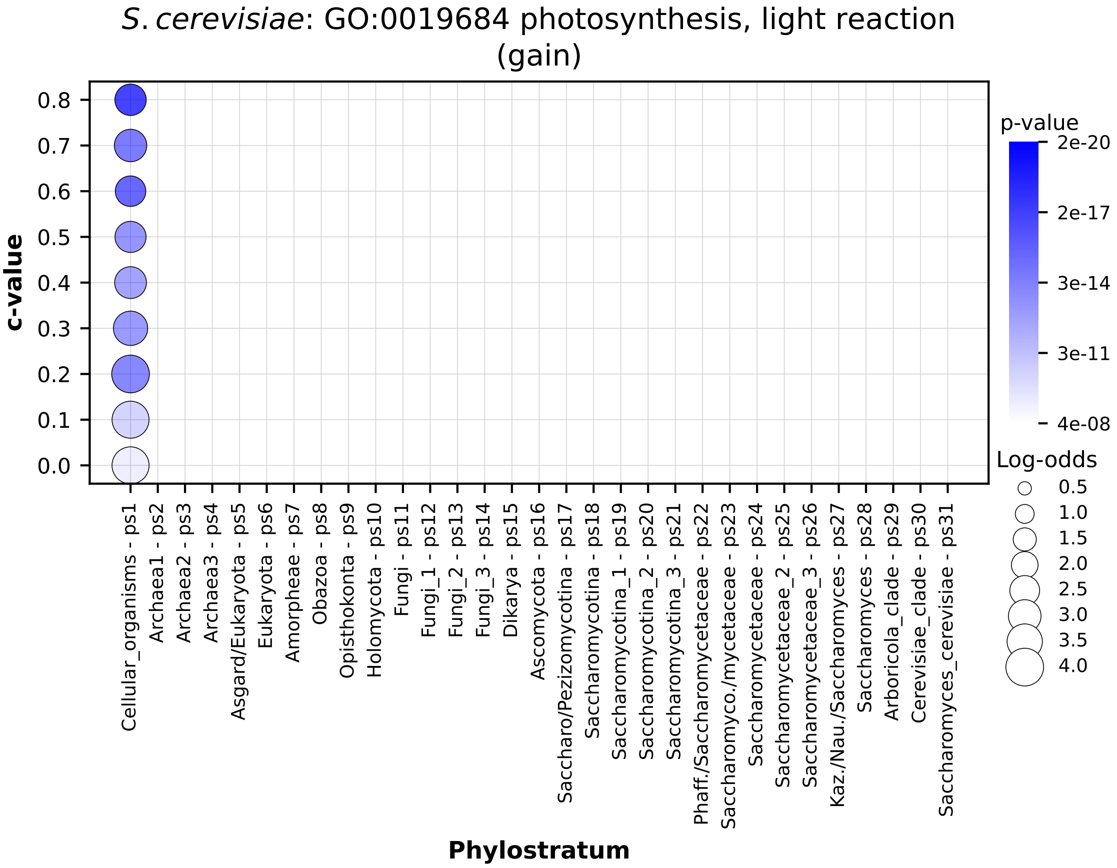

S. cerevisiae: GO:0019684 photosynthesis, light reaction (gain)
c-value
0.8
0.7
0.6
0.5
0.4
0.3
0.2
0.1
0.0
Phylostratum
Cellular_organisms - ps1
Archaea1 - ps2
Archaea2 - ps3
Archaea3 - ps4
Asgard/Eukaryota - ps5
Eukaryota - ps6
Amorpheae - ps7
Obazoa - ps8
Opisthokonta - ps9
Holomycota - ps10
Fungi - ps11
Fungi_1 - ps12
Fungi_2 - ps13
Fungi_3 - ps14
Dikarya - ps15
Ascomycota - ps16
Saccharo/Pezizomycotina - ps17
Saccharomycotina - ps18
Saccharomycotina_1 - ps19
Saccharomycotina_2 - ps20
Saccharomycotina_3 - ps21
Phaff./Saccharomycetaceae - ps22
Saccharomyco./mycetaceae - ps23
Saccharomycetaceae - ps24
Saccharomycetaceae_2 - ps25
Saccharomycetaceae_3 - ps26
Kaz./Nau./Saccharomyces - ps27
Saccharomyces - ps28
Arboricola_clade - ps29
Cerevisiae_clade - ps30
Saccharomyces_cerevisiae - ps31
p-value
2e-20
2e-17
3e-14
3e-11
4e-08
Log-odds
0.5
1.0
1.5
2.0
2.5
3.0
3.5
4.0

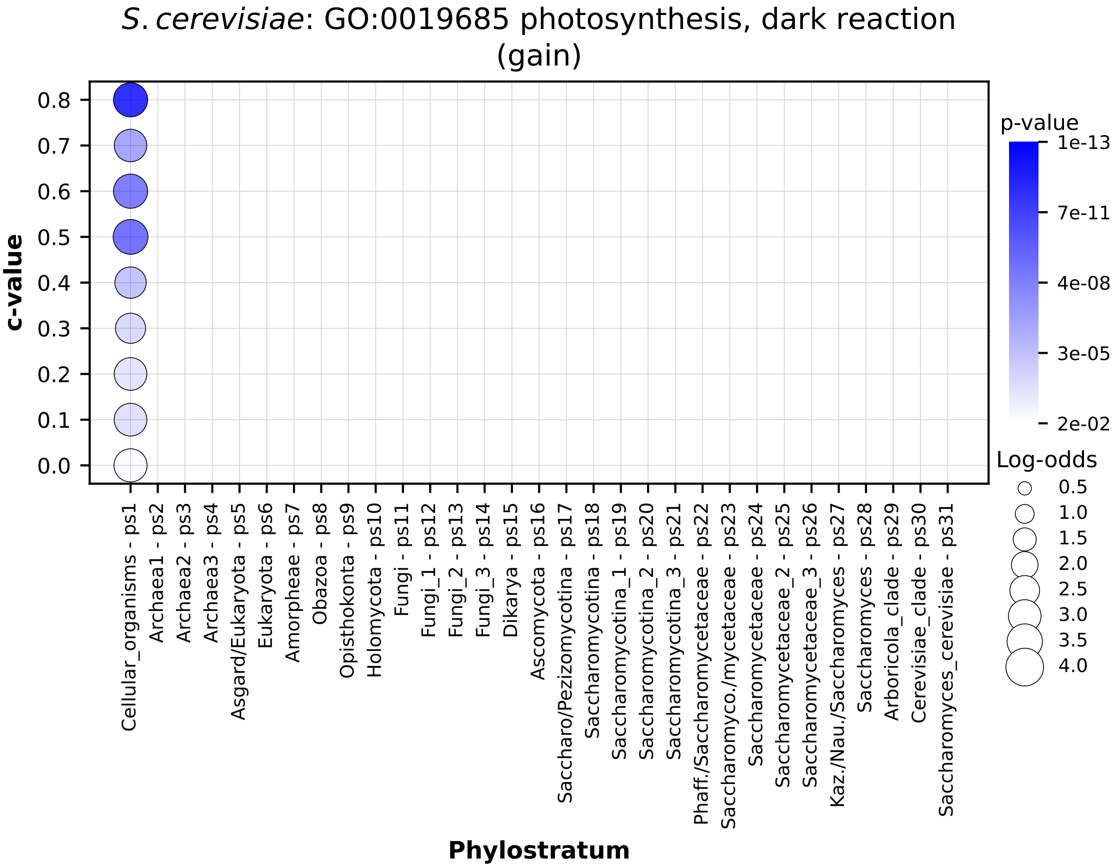

S. cerevisiae: GO:0019685 photosynthesis, dark reaction
(gain)
c-value
0.0
0.1
0.2
0.3
0.4
0.5
0.6
0.7
0.8
p-value
1e-13
7e-11
4e-08
3e-05
2e-02
Log-odds
0.5
1.0
1.5
2.0
2.5
3.0
3.5
4.0
Cellular_organisms - ps1
Archaea1 - ps2
Archaea2 - ps3
Archaea3 - ps4
Asgard/Eukaryota - ps5
Eukaryota - ps6
Amorpheae - ps7
Obazoa - ps8
Opisthokonta - ps9
Holomycota - ps10
Fungi - ps11
Fungi_1 - ps12
Fungi_2 - ps13
Fungi_3 - ps14
Dikarya - ps15
Ascomycota - ps16
Saccharo/Pezizomycotina - ps17
Saccharomycotina - ps18
Saccharomycotina_1 - ps19
Saccharomycotina_2 - ps20
Saccharomycotina_3 - ps21
Phaff./Saccharomycetaceae - ps22
Saccharomyco./mycetaceae - ps23
Saccharomycetaceae - ps24
Saccharomycetaceae_2 - ps25
Saccharomycetaceae_3 - ps26
Kaz./Nau./Saccharomyces - ps27
Saccharomyces - ps28
Arboricola_clade - ps29
Cerevisiae_clade - ps30
Saccharomyces_cerevisiae - ps31
Phylostratum

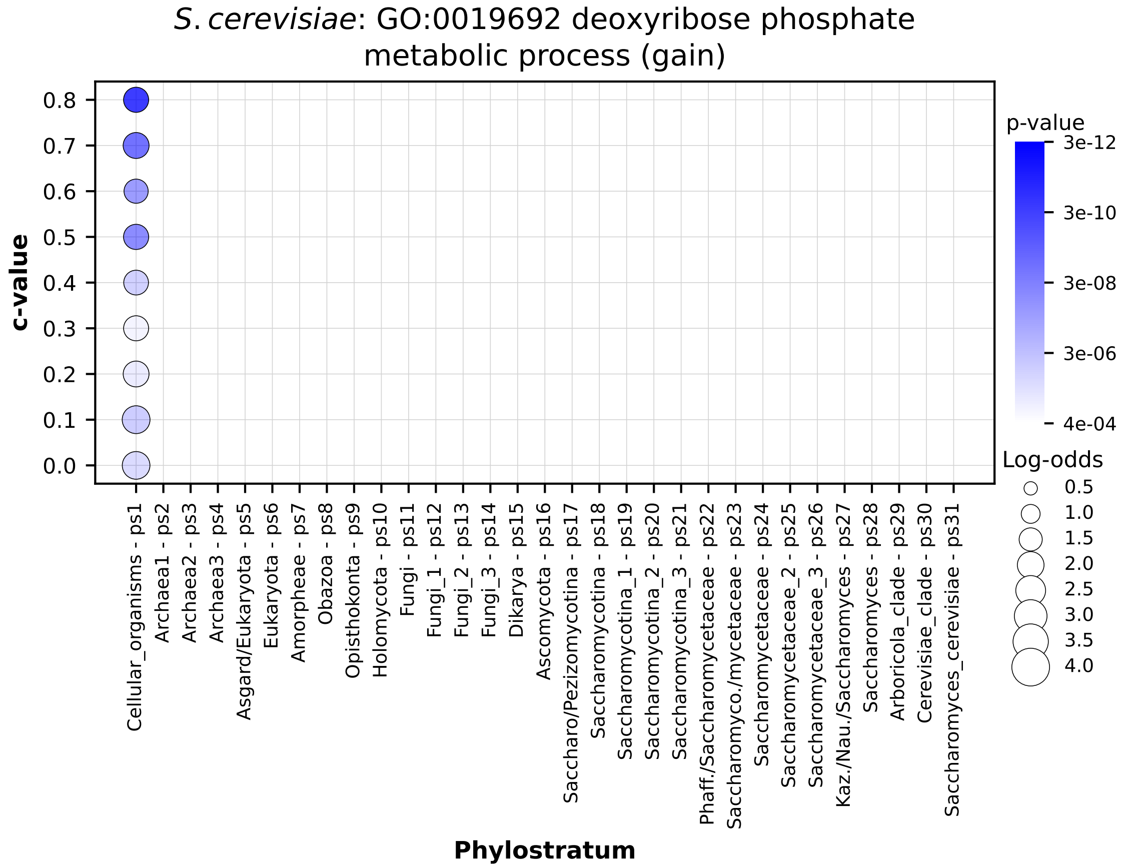
S. cerevisiae: GO:0019692 deoxyribose phosphate metabolic process (gain)
c-value
0.8
0.7
0.6
0.5
0.4
0.3
0.2
0.1
0.0
p-value
3e-12
3e-10
3e-08
3e-06
4e-04
Log-odds
0.5
1.0
1.5
2.0
2.5
3.0
3.5
4.0
Cellular_organisms - ps1
Archaea1 - ps2
Archaea2 - ps3
Archaea3 - ps4
Asgard/Eukaryota - ps5
Eukaryota - ps6
Amorpheae - ps7
Obazoa - ps8
Opisthokonta - ps9
Holomycota - ps10
Fungi - ps11
Fungi_1 - ps12
Fungi_2 - ps13
Fungi_3 - ps14
Dikarya - ps15
Ascomycota - ps16
Saccharo/Pezizomycotina - ps17
Saccharomycotina - ps18
Saccharomycotina_1 - ps19
Saccharomycotina_2 - ps20
Saccharomycotina_3 - ps21
Phaff./Saccharomycetaceae - ps22
Saccharomyco./mycetaceae - ps23
Saccharomycetaceae - ps24
Saccharomycetaceae_2 - ps25
Saccharomycetaceae_3 - ps26
Kaz./Nau./Saccharomyces - ps27
Saccharomyces - ps28
Arboricola_clade - ps29
Cerevisiae_clade - ps30
Saccharomyces_cerevisiae - ps31
Phylostratum

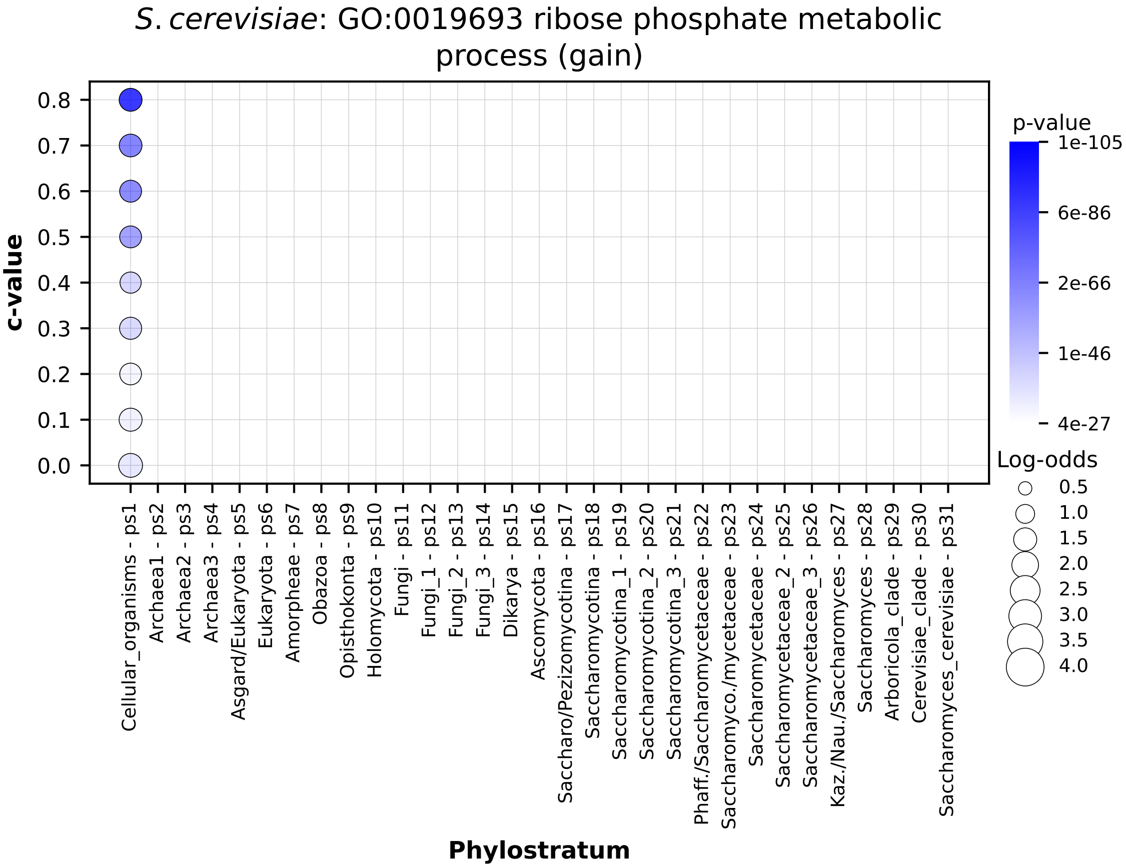
S. cerevisiae: GO:0019693 ribose phosphate metabolic process (gain)
c-value
0.8
0.7
0.6
0.5
0.4
0.3
0.2
0.1
0.0
p-value
1e-105
6e-86
2e-66
1e-46
4e-27
Log-odds
0.5
1.0
1.5
2.0
2.5
3.0
3.5
4.0
Cellular_organisms - ps1
Archaea1 - ps2
Archaea2 - ps3
Archaea3 - ps4
Asgard/Eukaryota - ps5
Eukaryota - ps6
Amorpheae - ps7
Obazoa - ps8
Opisthokonta - ps9
Holomycota - ps10
Fungi - ps11
Fungi_1 - ps12
Fungi_2 - ps13
Fungi_3 - ps14
Dikarya - ps15
Ascomycota - ps16
Saccharo/Pezizomycotina - ps17
Saccharomycotina - ps18
Saccharomycotina_1 - ps19
Saccharomycotina_2 - ps20
Saccharomycotina_3 - ps21
Phaff./Saccharomycetaceae - ps22
Saccharomyco./mycetaceae - ps23
Saccharomycetaceae - ps24
Saccharomycetaceae_2 - ps25
Saccharomycetaceae_3 - ps26
Kaz./Nau./Saccharomyces - ps27
Saccharomyces - ps28
Arboricola_clade - ps29
Cerevisiae_clade - ps30
Saccharomyces_cerevisiae - ps31
Phylostratum

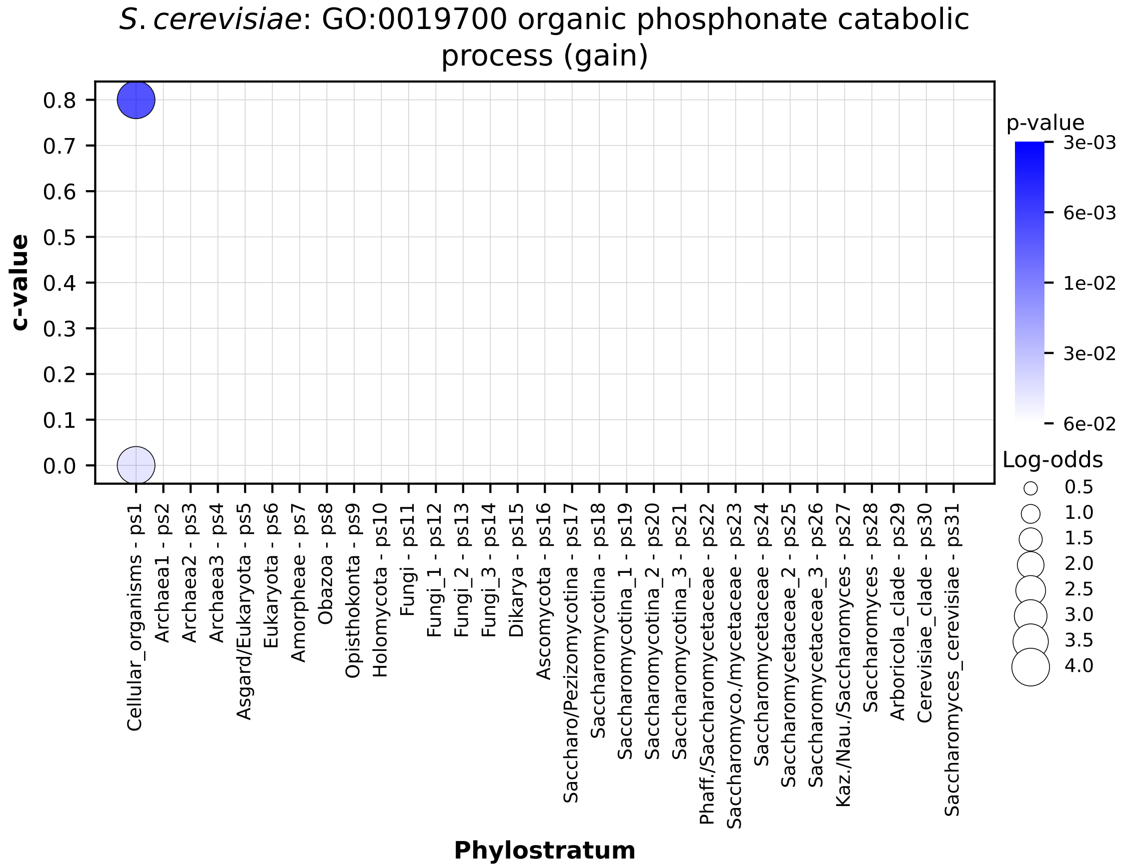

S. cerevisiae: GO:0019700 organic phosphonate catabolic process (gain)
c-value
0.8
0.7
0.6
0.5
0.4
0.3
0.2
0.1
0.0
p-value
3e-03
6e-03
1e-02
3e-02
6e-02
Log-odds
0.5
1.0
1.5
2.0
2.5
3.0
3.5
4.0
Cellular_organisms - ps1
Archaea1 - ps2
Archaea2 - ps3
Archaea3 - ps4
Asgard/Eukaryota - ps5
Eukaryota - ps6
Amorpheae - ps7
Obazoa - ps8
Opisthokonta - ps9
Holomycota - ps10
Fungi - ps11
Fungi_1 - ps12
Fungi_2 - ps13
Fungi_3 - ps14
Dikarya - ps15
Ascomycota - ps16
Saccharo/Pezizomycotina - ps17
Saccharomycotina - ps18
Saccharomycotina_1 - ps19
Saccharomycotina_2 - ps20
Saccharomycotina_3 - ps21
Phaff./Saccharomycetaceae - ps22
Saccharomyco./mycetaceae - ps23
Saccharomycetaceae - ps24
Saccharomycetaceae_2 - ps25
Saccharomycetaceae_3 - ps26
Kaz./Nau./Saccharomyces - ps27
Saccharomyces - ps28
Arboricola_clade - ps29
Cerevisiae_clade - ps30
Saccharomyces_cerevisiae - ps31
Phylostratum

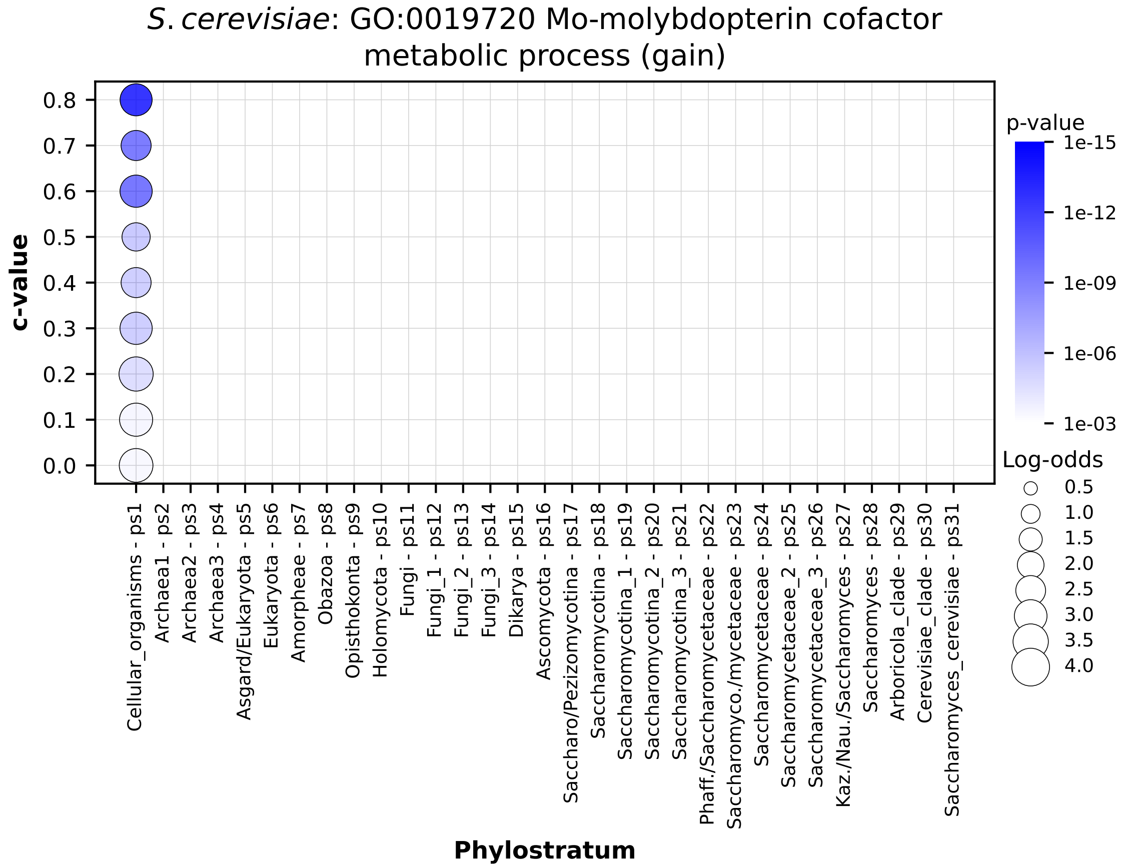
S. cerevisiae: GO:0019720 Mo-molybdopterin cofactor metabolic process (gain)
c-value
Phylostratum
p-value
1e-15
1e-12
1e-09
1e-06
1e-03
Log-odds
0.5
1.0
1.5
2.0
2.5
3.0
3.5
4.0
Cellular_organisms - ps1
Archaea1 - ps2
Archaea2 - ps3
Archaea3 - ps4
Asgard/Eukaryota - ps5
Eukaryota - ps6
Amorpheae - ps7
Obazoa - ps8
Opisthokonta - ps9
Holomycota - ps10
Fungi - ps11
Fungi_1 - ps12
Fungi_2 - ps13
Fungi_3 - ps14
Dikarya - ps15
Ascomycota - ps16
Saccharo/Pezizomycotina - ps17
Saccharomycotina - ps18
Saccharomycotina_1 - ps19
Saccharomycotina_2 - ps20
Saccharomycotina_3 - ps21
Phaff./Saccharomycetaceae - ps22
Saccharomyco./mycetaceae - ps23
Saccharomycetaceae - ps24
Saccharomycetaceae_2 - ps25
Saccharomycetaceae_3 - ps26
Kaz./Nau./Saccharomyces - ps27
Saccharomyces - ps28
Arboricola_clade - ps29
Cerevisiae_clade - ps30
Saccharomyces_cerevisiae - ps31

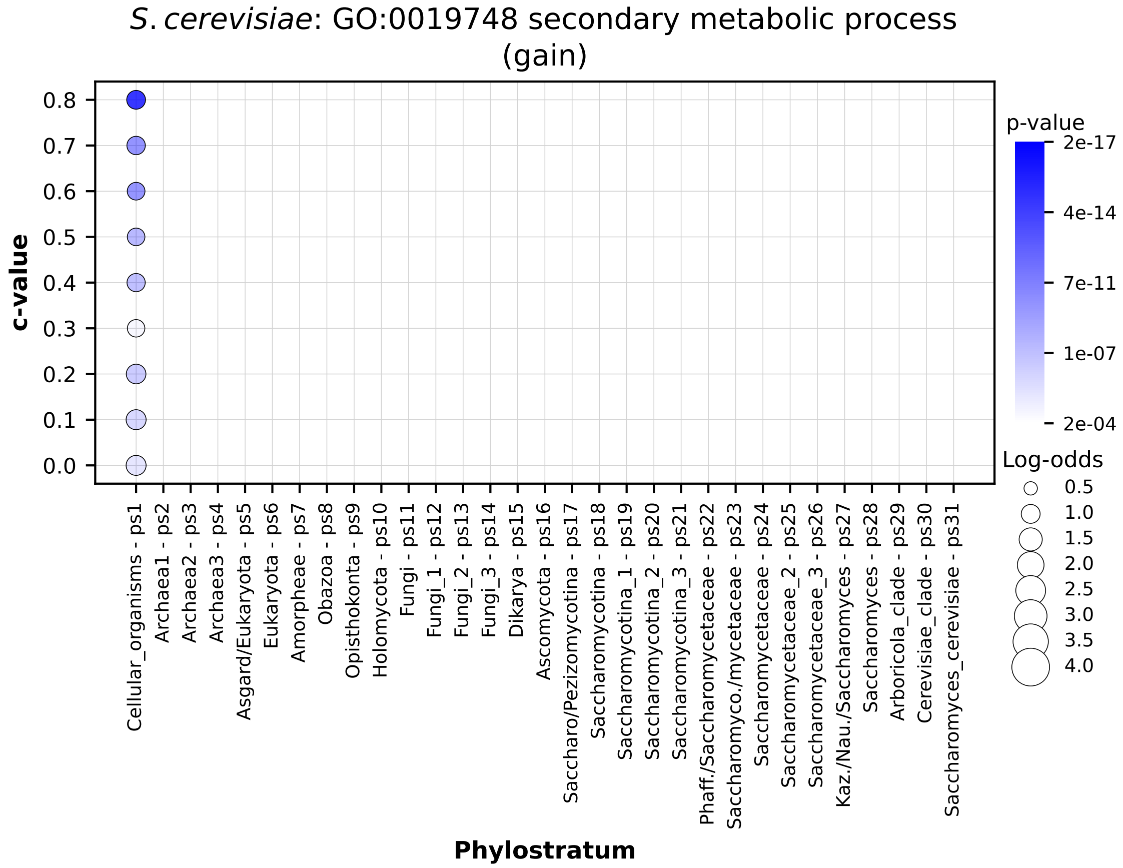
S. cerevisiae: GO:0019748 secondary metabolic process (gain)
c-value
0.8
0.7
0.6
0.5
0.4
0.3
0.2
0.1
0.0
p-value
2e-17
4e-14
7e-11
1e-07
2e-04
Log-odds
0.5
1.0
1.5
2.0
2.5
3.0
3.5
4.0
Cellular_organisms - ps1
Archaea1 - ps2
Archaea2 - ps3
Archaea3 - ps4
Asgard/Eukaryota - ps5
Eukaryota - ps6
Amorpheae - ps7
Obazoa - ps8
Opisthokonta - ps9
Holomycota - ps10
Fungi - ps11
Fungi_1 - ps12
Fungi_2 - ps13
Fungi_3 - ps14
Dikarya - ps15
Ascomycota - ps16
Saccharo/Pezizomycotina - ps17
Saccharomycotina - ps18
Saccharomycotina_1 - ps19
Saccharomycotina_2 - ps20
Saccharomycotina_3 - ps21
Phaff./Saccharomycetaceae - ps22
Saccharomyco./mycetaceae - ps23
Saccharomycetaceae - ps24
Saccharomycetaceae_2 - ps25
Saccharomycetaceae_3 - ps26
Kaz./Nau./Saccharomyces - ps27
Saccharomyces - ps28
Arboricola_clade - ps29
Cerevisiae_clade - ps30
Saccharomyces_cerevisiae - ps31
Phylostratum

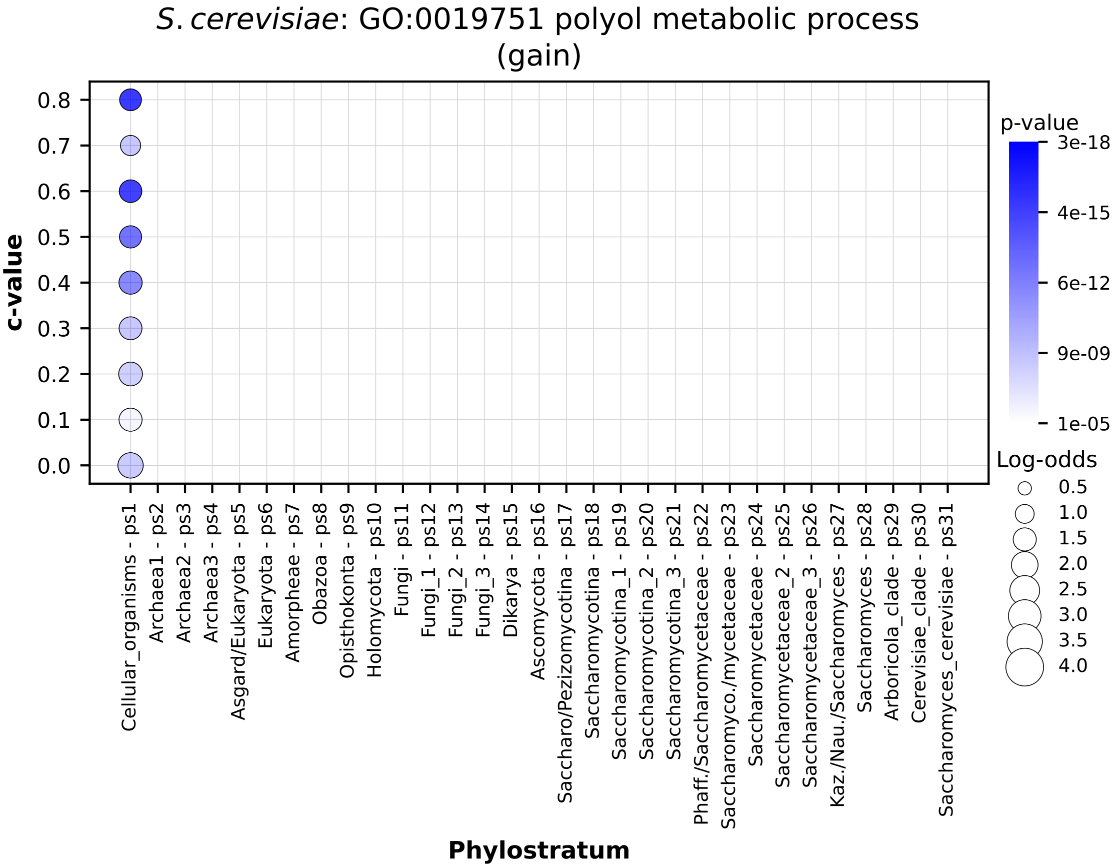
S. cerevisiae: GO:0019751 polyol metabolic process
(gain)
c-value
0.8
0.7
0.6
0.5
0.4
0.3
0.2
0.1
0.0
p-value
3e-18
4e-15
6e-12
9e-09
1e-05
Log-odds
0.5
1.0
1.5
2.0
2.5
3.0
3.5
4.0
Cellular_organisms - ps1
Archaea1 - ps2
Archaea2 - ps3
Archaea3 - ps4
Asgard/Eukaryota - ps5
Eukaryota - ps6
Amorpheae - ps7
Obazoa - ps8
Opisthokonta - ps9
Holomycota - ps10
Fungi - ps11
Fungi_1 - ps12
Fungi_2 - ps13
Fungi_3 - ps14
Dikarya - ps15
Ascomycota - ps16
Saccharo/Pezizomycotina - ps17
Saccharomycotina - ps18
Saccharomycotina_1 - ps19
Saccharomycotina_2 - ps20
Saccharomycotina_3 - ps21
Phaff./Saccharomycetaceae - ps22
Saccharomyco./mycetaceae - ps23
Saccharomycetaceae - ps24
Saccharomycetaceae_2 - ps25
Saccharomycetaceae_3 - ps26
Kaz./Nau./Saccharomyces - ps27
Saccharomyces - ps28
Arboricola_clade - ps29
Cerevisiae_clade - ps30
Saccharomyces_cerevisiae - ps31
Phylostratum

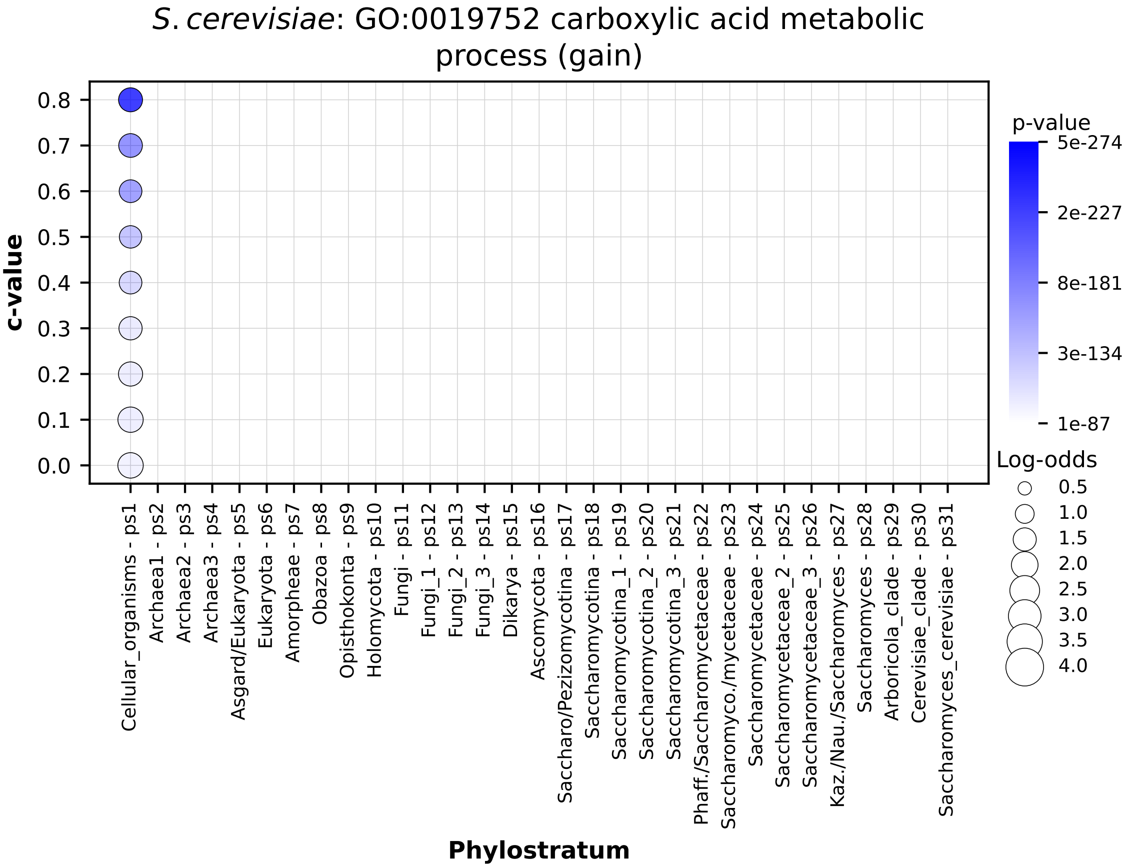
S. cerevisiae: GO:0019752 carboxylic acid metabolic process (gain)
c-value
0.0
0.1
0.2
0.3
0.4
0.5
0.6
0.7
0.8
p-value
5e-274
2e-227
8e-181
3e-134
1e-87
Log-odds
0.5
1.0
1.5
2.0
2.5
3.0
3.5
4.0
Cellular_organisms - ps1
Archaea1 - ps2
Archaea2 - ps3
Archaea3 - ps4
Asgard/Eukaryota - ps5
Eukaryota - ps6
Amorpheae - ps7
Obazoa - ps8
Opisthokonta - ps9
Holomycota - ps10
Fungi - ps11
Fungi_1 - ps12
Fungi_2 - ps13
Fungi_3 - ps14
Dikarya - ps15
Ascomycota - ps16
Saccharo/Pezizomycotina - ps17
Saccharomycotina - ps18
Saccharomycotina_1 - ps19
Saccharomycotina_2 - ps20
Saccharomycotina_3 - ps21
Phaff./Saccharomycetaceae - ps22
Saccharomyco./mycetaceae - ps23
Saccharomycetaceae - ps24
Saccharomycetaceae_2 - ps25
Saccharomycetaceae_3 - ps26
Kaz./Nau./Saccharomyces - ps27
Saccharomyces - ps28
Arboricola_clade - ps29
Cerevisiae_clade - ps30
Saccharomyces_cerevisiae - ps31
Phylostratum

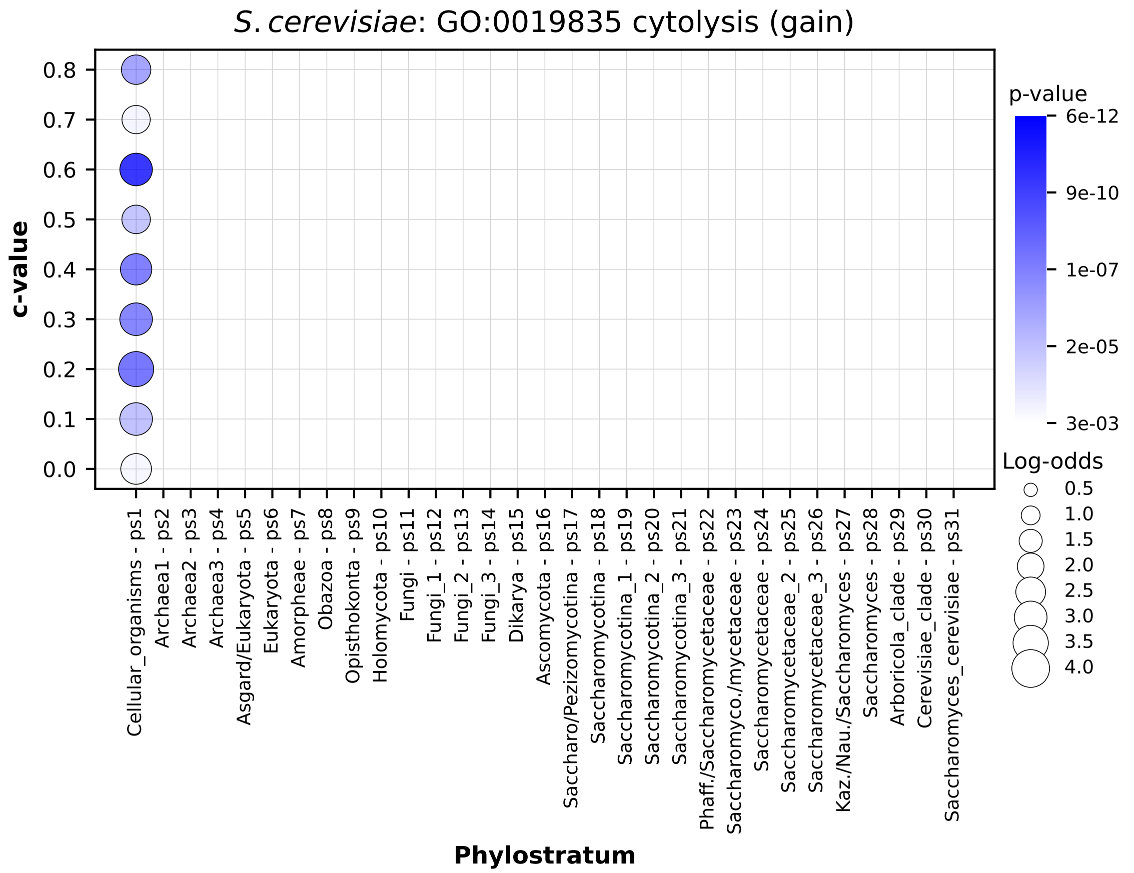

S. cerevisiae: GO:0019835 cytolysis (gain)
c-value
0.0
0.1
0.2
0.3
0.4
0.5
0.6
0.7
0.8
Phylostratum
Cellular_organisms - ps1
Archaea1 - ps2
Archaea2 - ps3
Archaea3 - ps4
Asgard/Eukaryota - ps5
Eukaryota - ps6
Amorpheae - ps7
Obazoa - ps8
Opisthokonta - ps9
Holomycota - ps10
Fungi - ps11
Fungi_1 - ps12
Fungi_2 - ps13
Fungi_3 - ps14
Dikarya - ps15
Ascomycota - ps16
Saccharo/Pezizomycotina - ps17
Saccharomycotina - ps18
Saccharomycotina_1 - ps19
Saccharomycotina_2 - ps20
Saccharomycotina_3 - ps21
Phaff./Saccharomycetaceae - ps22
Saccharomyco./mycetaceae - ps23
Saccharomycetaceae - ps24
Saccharomycetaceae_2 - ps25
Saccharomycetaceae_3 - ps26
Kaz./Nau./Saccharomyces - ps27
Saccharomyces - ps28
Arboricola_clade - ps29
Cerevisiae_clade - ps30
Saccharomyces_cerevisiae - ps31
p-value
6e-12
9e-10
1e-07
2e-05
3e-03
Log-odds
0.5
1.0
1.5
2.0
2.5
3.0
3.5
4.0

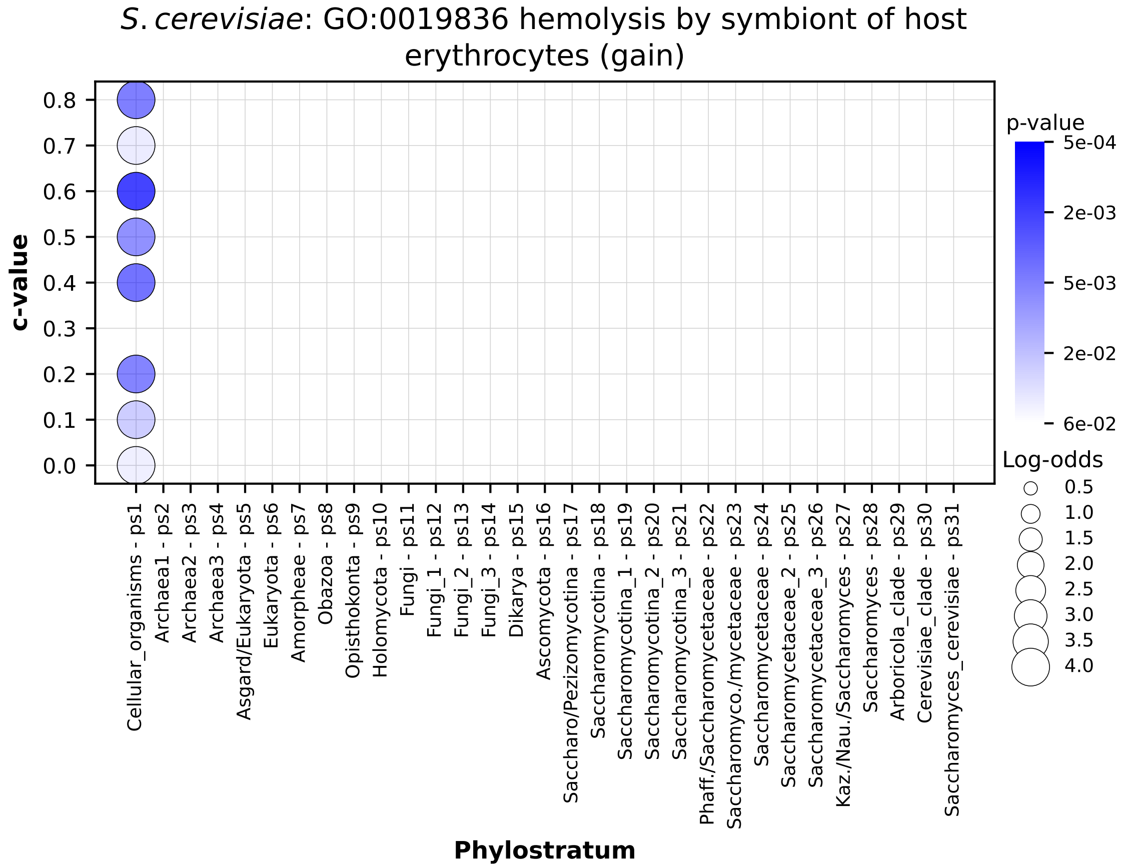
S. cerevisiae: GO:0019836 hemolysis by symbiont of host erythrocytes (gain)
c-value
0.8
0.7
0.6
0.5
0.4
0.3
0.2
0.1
0.0
p-value
5e-04
2e-03
5e-03
2e-02
6e-02
Log-odds
0.5
1.0
1.5
2.0
2.5
3.0
3.5
4.0
Cellular_organisms - ps1
Archaea1 - ps2
Archaea2 - ps3
Archaea3 - ps4
Asgard/Eukaryota - ps5
Eukaryota - ps6
Amorpheae - ps7
Obazoa - ps8
Opisthokonta - ps9
Holomycota - ps10
Fungi - ps11
Fungi_1 - ps12
Fungi_2 - ps13
Fungi_3 - ps14
Dikarya - ps15
Ascomycota - ps16
Saccharo/Pezizomycotina - ps17
Saccharomycotina - ps18
Saccharomycotina_1 - ps19
Saccharomycotina_2 - ps20
Saccharomycotina_3 - ps21
Phaff./Saccharomycetaceae - ps22
Saccharomyco./mycetaceae - ps23
Saccharomycetaceae - ps24
Saccharomycetaceae_2 - ps25
Saccharomycetaceae_3 - ps26
Kaz./Nau./Saccharomyces - ps27
Saccharomyces - ps28
Arboricola_clade - ps29
Cerevisiae_clade - ps30
Saccharomyces_cerevisiae - ps31
Phylostratum

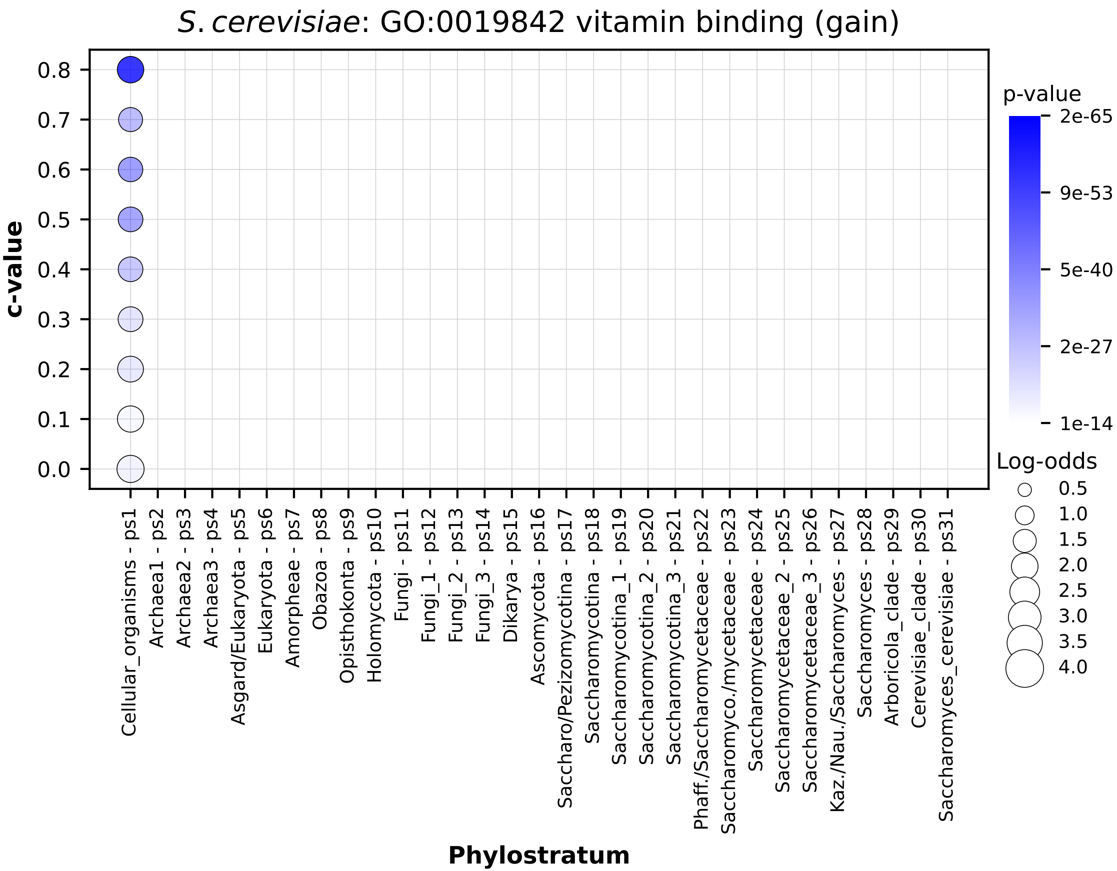

S. cerevisiae: GO:0019842 vitamin binding (gain)
c-value
0.0
0.1
0.2
0.3
0.4
0.5
0.6
0.7
0.8
p-value
2e-65
9e-53
5e-40
2e-27
1e-14
Log-odds
0.5
1.0
1.5
2.0
2.5
3.0
3.5
4.0
Cellular_organisms - ps1
Archaea1 - ps2
Archaea2 - ps3
Archaea3 - ps4
Asgard/Eukaryota - ps5
Eukaryota - ps6
Amorpheae - ps7
Obazoa - ps8
Opisthokonta - ps9
Holomycota - ps10
Fungi - ps11
Fungi_1 - ps12
Fungi_2 - ps13
Fungi_3 - ps14
Dikarya - ps15
Ascomycota - ps16
Saccharo/Pezizomycotina - ps17
Saccharomycotina - ps18
Saccharomycotina_1 - ps19
Saccharomycotina_2 - ps20
Saccharomycotina_3 - ps21
Phaff./Saccharomycetaceae - ps22
Saccharomyco./mycetaceae - ps23
Saccharomycetaceae - ps24
Saccharomycetaceae_2 - ps25
Saccharomycetaceae_3 - ps26
Kaz./Nau./Saccharomyces - ps27
Saccharomyces - ps28
Arboricola_clade - ps29
Cerevisiae_clade - ps30
Saccharomyces_cerevisiae - ps31
Phylostratum

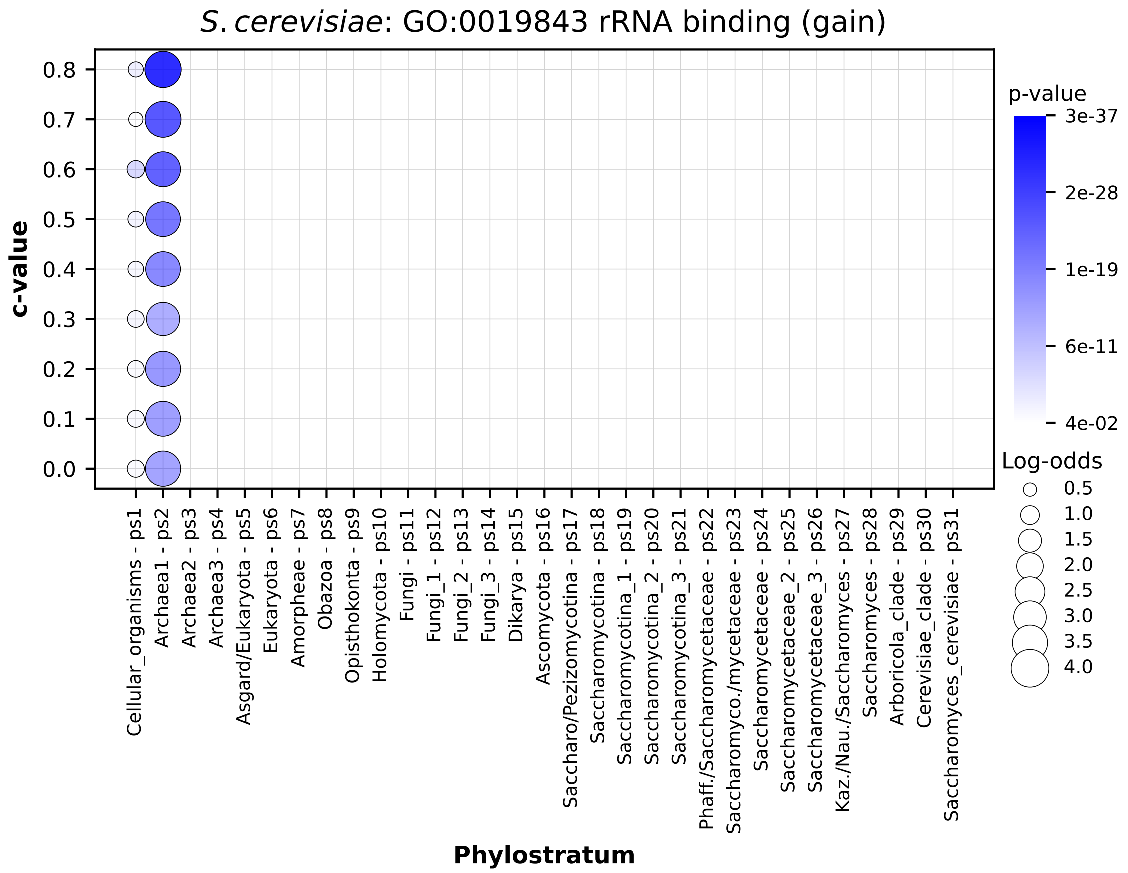
S. cerevisiae: GO:0019843 rRNA binding (gain)
c-value
0.8
0.7
0.6
0.5
0.4
0.3
0.2
0.1
0.0
p-value
3e-37
2e-28
1e-19
6e-11
4e-02
Log-odds
0.5
1.0
1.5
2.0
2.5
3.0
3.5
4.0
Cellular_organisms - ps1
Archaea1 - ps2
Archaea2 - ps3
Archaea3 - ps4
Asgard/Eukaryota - ps5
Eukaryota - ps6
Amorpheae - ps7
Obazoa - ps8
Opisthokonta - ps9
Holomycota - ps10
Fungi - ps11
Fungi_1 - ps12
Fungi_2 - ps13
Fungi_3 - ps14
Dikarya - ps15
Ascomycota - ps16
Saccharo/Pezizomycotina - ps17
Saccharomycotina - ps18
Saccharomycotina_1 - ps19
Saccharomycotina_2 - ps20
Saccharomycotina_3 - ps21
Phaff./Saccharomycetaceae - ps22
Saccharomyco./mycetaceae - ps23
Saccharomycetaceae - ps24
Saccharomycetaceae_2 - ps25
Saccharomycetaceae_3 - ps26
Kaz./Nau./Saccharomyces - ps27
Saccharomyces - ps28
Arboricola_clade - ps29
Cerevisiae_clade - ps30
Saccharomyces_cerevisiae - ps31
Phylostratum

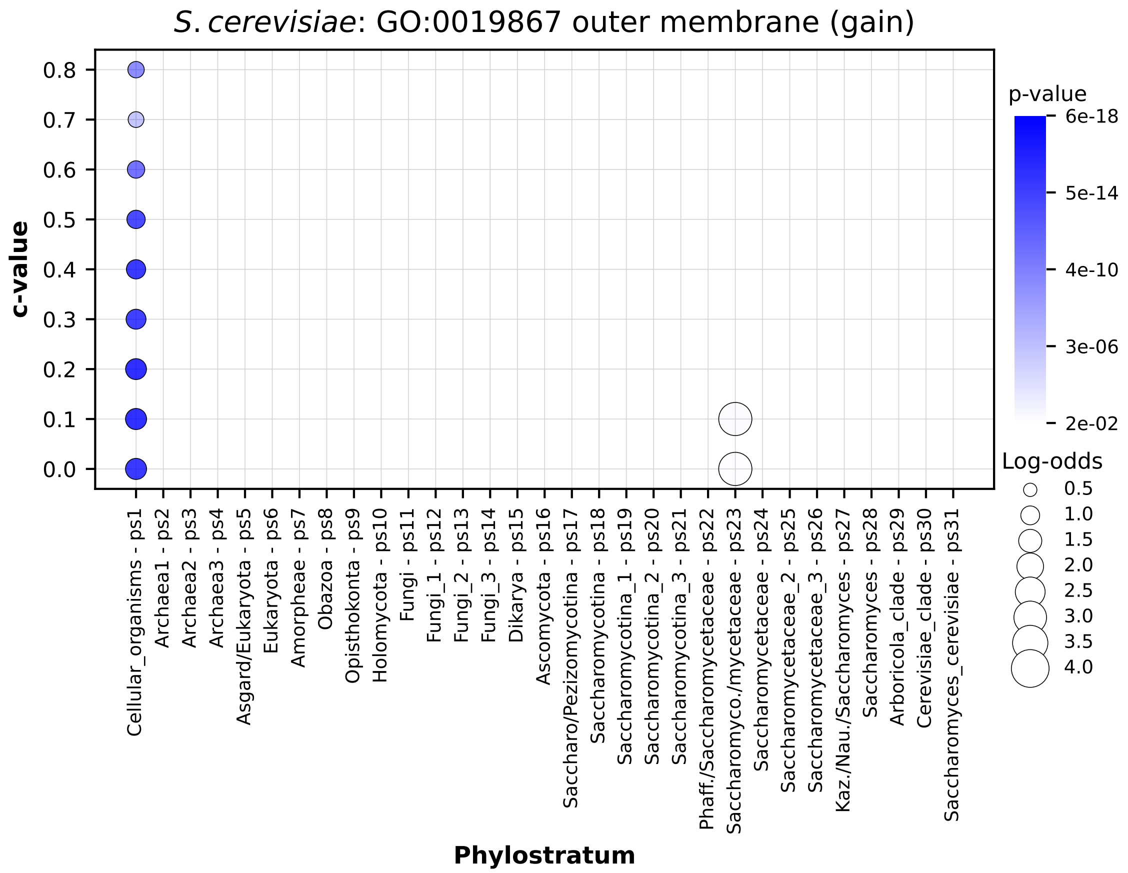

S. cerevisiae: GO:0019867 outer membrane (gain)
c-value
0.0
0.1
0.2
0.3
0.4
0.5
0.6
0.7
0.8
Phylostratum
Cellular_organisms - ps1
Archaea1 - ps2
Archaea2 - ps3
Archaea3 - ps4
Asgard/Eukaryota - ps5
Eukaryota - ps6
Amorpheae - ps7
Obazoa - ps8
Opisthokonta - ps9
Holomycota - ps10
Fungi - ps11
Fungi_1 - ps12
Fungi_2 - ps13
Fungi_3 - ps14
Dikarya - ps15
Ascomycota - ps16
Saccharo/Pezizomycotina - ps17
Saccharomycotina - ps18
Saccharomycotina_1 - ps19
Saccharomycotina_2 - ps20
Saccharomycotina_3 - ps21
Phaff./Saccharomycetaceae - ps22
Saccharomyco./mycetaceae - ps23
Saccharomycetaceae - ps24
Saccharomycetaceae_2 - ps25
Saccharomycetaceae_3 - ps26
Kaz./Nau./Saccharomyces - ps27
Saccharomyces - ps28
Arboricola_clade - ps29
Cerevisiae_clade - ps30
Saccharomyces_cerevisiae - ps31
p-value
6e-18
5e-14
4e-10
3e-06
2e-02
Log-odds
0.5
1.0
1.5
2.0
2.5
3.0
3.5
4.0

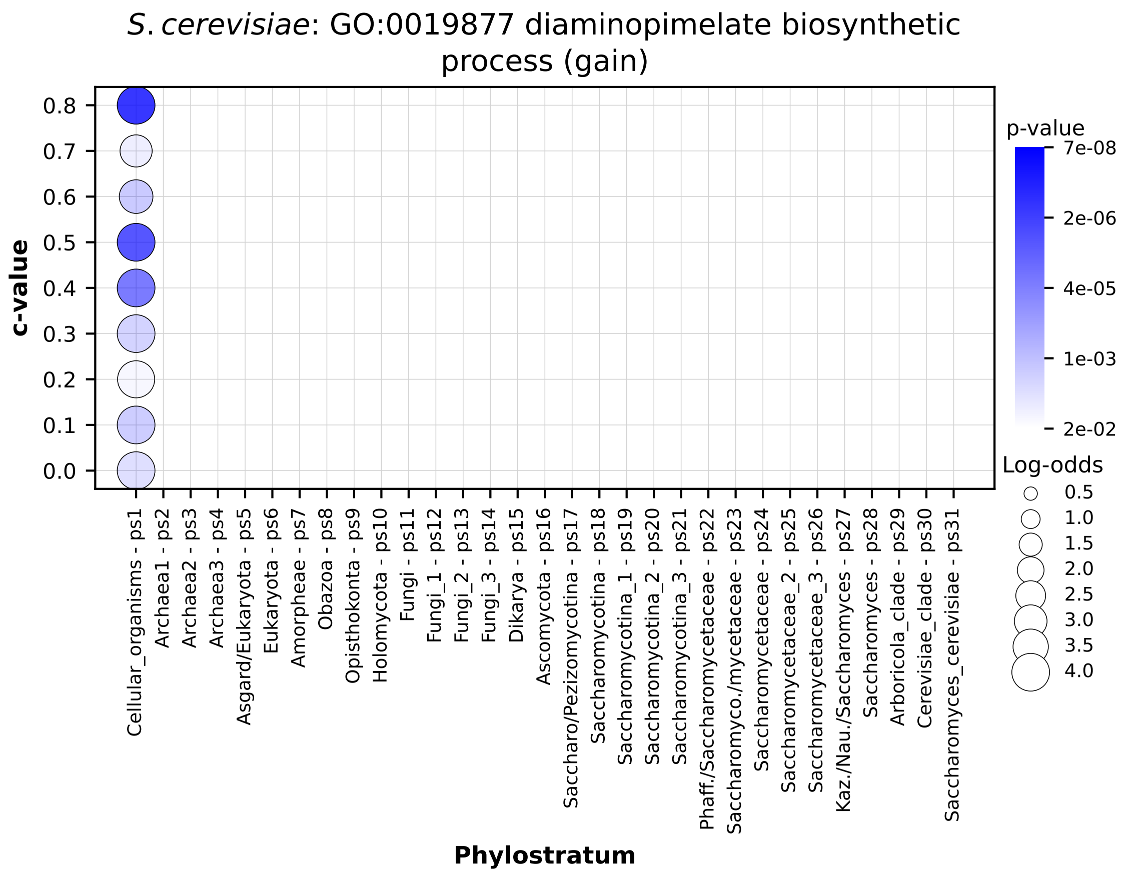
S. cerevisiae: GO:0019877 diaminopimelate biosynthetic process (gain)
c-value
0.8
0.7
0.6
0.5
0.4
0.3
0.2
0.1
0.0
p-value
7e-08
2e-06
4e-05
1e-03
2e-02
Log-odds
0.5
1.0
1.5
2.0
2.5
3.0
3.5
4.0
Cellular_organisms - ps1
Archaea1 - ps2
Archaea2 - ps3
Archaea3 - ps4
Asgard/Eukaryota - ps5
Eukaryota - ps6
Amorpheae - ps7
Obazoa - ps8
Opisthokonta - ps9
Holomycota - ps10
Fungi - ps11
Fungi_1 - ps12
Fungi_2 - ps13
Fungi_3 - ps14
Dikarya - ps15
Ascomycota - ps16
Saccharo/Pezizomycotina - ps17
Saccharomycotina - ps18
Saccharomycotina_1 - ps19
Saccharomycotina_2 - ps20
Saccharomycotina_3 - ps21
Phaff./Saccharomycetaceae - ps22
Saccharomyco./mycetaceae - ps23
Saccharomycetaceae - ps24
Saccharomycetaceae_2 - ps25
Saccharomycetaceae_3 - ps26
Kaz./Nau./Saccharomyces - ps27
Saccharomyces - ps28
Arboricola_clade - ps29
Cerevisiae_clade - ps30
Saccharomyces_cerevisiae - ps31
Phylostratum

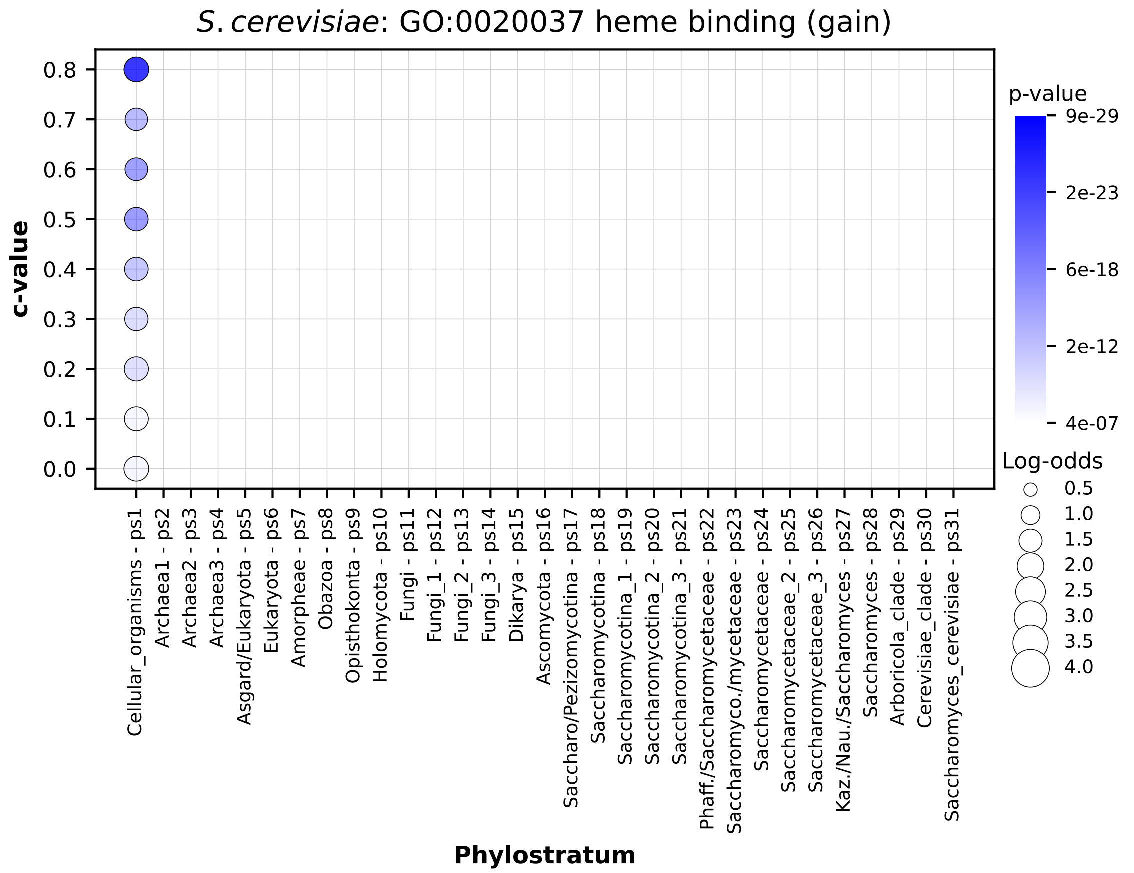

S. cerevisiae: GO:0020037 heme binding (gain)
c-value
0.0
0.1
0.2
0.3
0.4
0.5
0.6
0.7
0.8
p-value
9e-29
2e-23
6e-18
2e-12
4e-07
Log-odds
0.5
1.0
1.5
2.0
2.5
3.0
3.5
4.0
Cellular_organisms - ps1
Archaea1 - ps2
Archaea2 - ps3
Archaea3 - ps4
Asgard/Eukaryota - ps5
Eukaryota - ps6
Amorpheae - ps7
Obazoa - ps8
Opisthokonta - ps9
Holomycota - ps10
Fungi - ps11
Fungi_1 - ps12
Fungi_2 - ps13
Fungi_3 - ps14
Dikarya - ps15
Ascomycota - ps16
Saccharo/Pezizomycotina - ps17
Saccharomycotina - ps18
Saccharomycotina_1 - ps19
Saccharomycotina_2 - ps20
Saccharomycotina_3 - ps21
Phaff./Saccharomycetaceae - ps22
Saccharomyco./mycetaceae - ps23
Saccharomycetaceae - ps24
Saccharomycetaceae_2 - ps25
Saccharomycetaceae_3 - ps26
Kaz./Nau./Saccharomyces - ps27
Saccharomyces - ps28
Arboricola_clade - ps29
Cerevisiae_clade - ps30
Saccharomyces_cerevisiae - ps31
Phylostratum

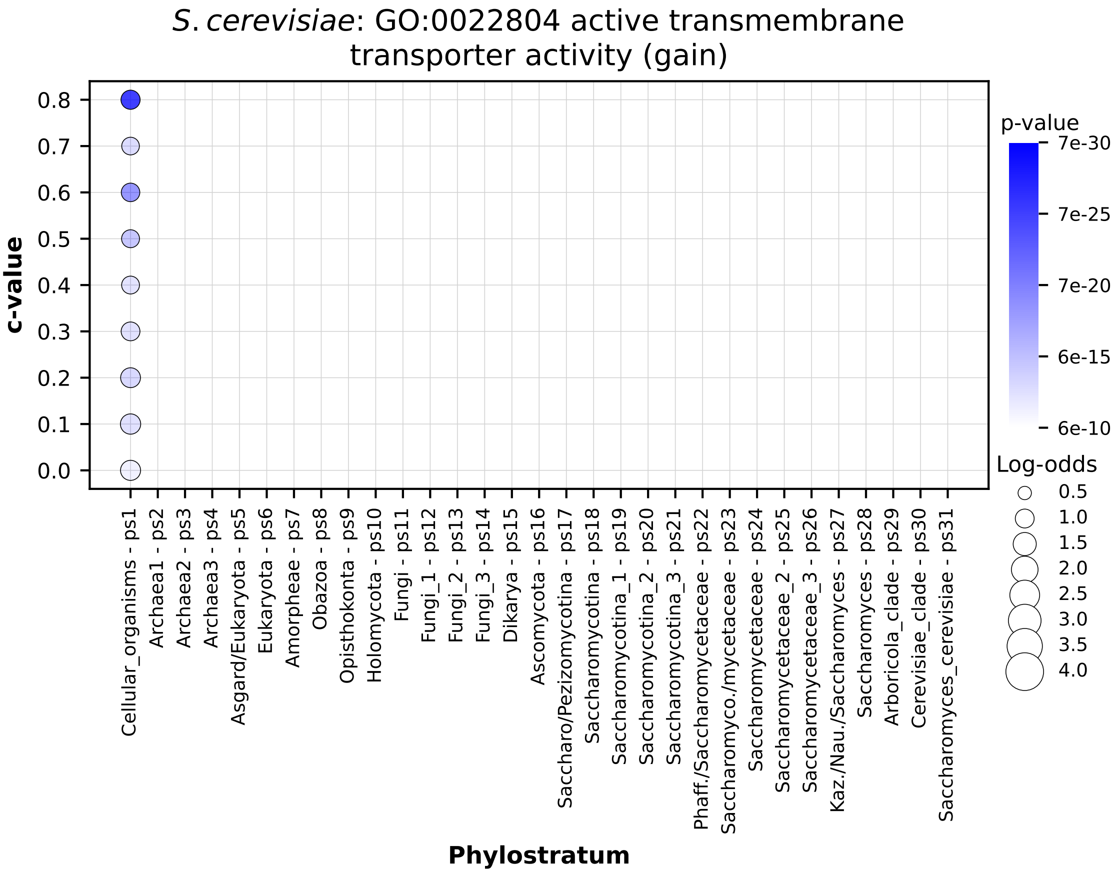

S. cerevisiae: GO:0022804 active transmembrane transporter activity (gain)
c-value
0.8
0.7
0.6
0.5
0.4
0.3
0.2
0.1
0.0
p-value
7e-30
7e-25
7e-20
6e-15
6e-10
Log-odds
0.5
1.0
1.5
2.0
2.5
3.0
3.5
4.0
Cellular_organisms - ps1
Archaea1 - ps2
Archaea2 - ps3
Archaea3 - ps4
Asgard/Eukaryota - ps5
Eukaryota - ps6
Amorpheae - ps7
Obazoa - ps8
Opisthokonta - ps9
Holomycota - ps10
Fungi - ps11
Fungi_1 - ps12
Fungi_2 - ps13
Fungi_3 - ps14
Dikarya - ps15
Ascomycota - ps16
Saccharo/Pezizomycotina - ps17
Saccharomycotina - ps18
Saccharomycotina_1 - ps19
Saccharomycotina_2 - ps20
Saccharomycotina_3 - ps21
Phaff./Saccharomycetaceae - ps22
Saccharomyco./mycetaceae - ps23
Saccharomycetaceae - ps24
Saccharomycetaceae_2 - ps25
Saccharomycetaceae_3 - ps26
Kaz./Nau./Saccharomyces - ps27
Saccharomyces - ps28
Arboricola_clade - ps29
Cerevisiae_clade - ps30
Saccharomyces_cerevisiae - ps31
Phylostratum

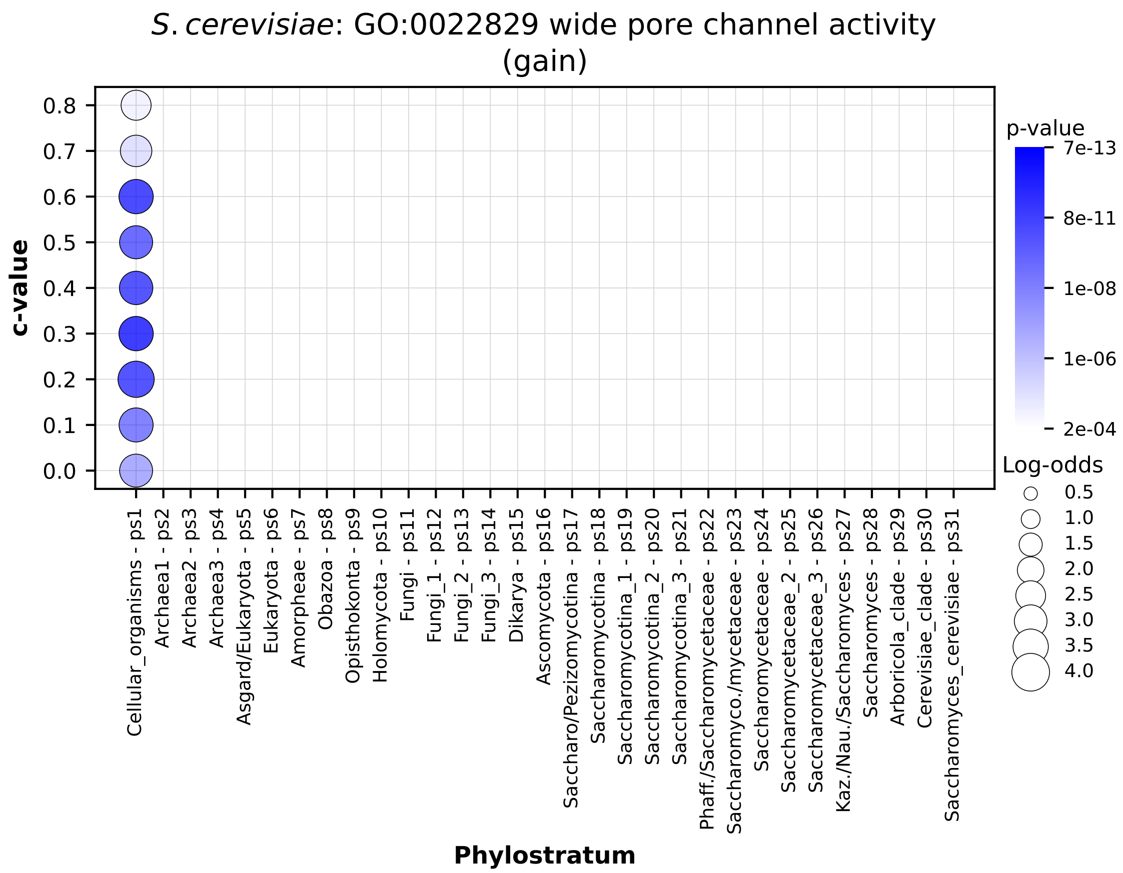

S. cerevisiae: GO:0022829 wide pore channel activity
(gain)
c-value
0.8
0.7
0.6
0.5
0.4
0.3
0.2
0.1
0.0
p-value
7e-13
8e-11
1e-08
1e-06
2e-04
Log-odds
0.5
1.0
1.5
2.0
2.5
3.0
3.5
4.0
Cellular_organisms - ps1
Archaea1 - ps2
Archaea2 - ps3
Archaea3 - ps4
Asgard/Eukaryota - ps5
Eukaryota - ps6
Amorpheae - ps7
Obazoa - ps8
Opisthokonta - ps9
Holomycota - ps10
Fungi - ps11
Fungi_1 - ps12
Fungi_2 - ps13
Fungi_3 - ps14
Dikarya - ps15
Ascomycota - ps16
Saccharo/Pezizomycotina - ps17
Saccharomycotina - ps18
Saccharomycotina_1 - ps19
Saccharomycotina_2 - ps20
Saccharomycotina_3 - ps21
Phaff./Saccharomycetaceae - ps22
Saccharomyco./mycetaceae - ps23
Saccharomycetaceae - ps24
Saccharomycetaceae_2 - ps25
Saccharomycetaceae_3 - ps26
Kaz./Nau./Saccharomyces - ps27
Saccharomyces - ps28
Arboricola_clade - ps29
Cerevisiae_clade - ps30
Saccharomyces_cerevisiae - ps31
Phylostratum

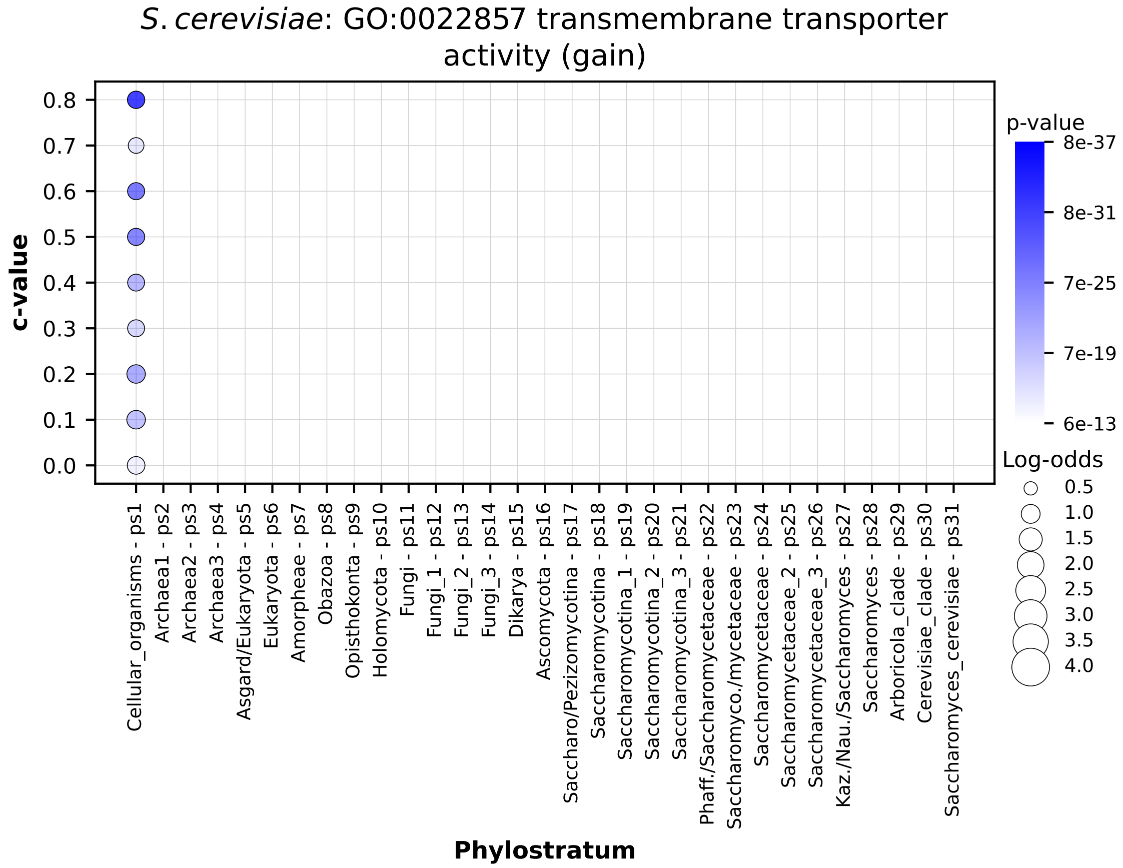
S. cerevisiae: GO:0022857 transmembrane transporter activity (gain)
c-value
0.0
0.1
0.2
0.3
0.4
0.5
0.6
0.7
0.8
p-value
8e-37
8e-31
7e-25
7e-19
6e-13
Log-odds
0.5
1.0
1.5
2.0
2.5
3.0
3.5
4.0
Cellular_organisms - ps1
Archaea1 - ps2
Archaea2 - ps3
Archaea3 - ps4
Asgard/Eukaryota - ps5
Eukaryota - ps6
Amorpheae - ps7
Obazoa - ps8
Opisthokonta - ps9
Holomycota - ps10
Fungi - ps11
Fungi_1 - ps12
Fungi_2 - ps13
Fungi_3 - ps14
Dikarya - ps15
Ascomycota - ps16
Saccharo/Pezizomycotina - ps17
Saccharomycotina - ps18
Saccharomycotina_1 - ps19
Saccharomycotina_2 - ps20
Saccharomycotina_3 - ps21
Phaff./Saccharomycetaceae - ps22
Saccharomyco./mycetaceae - ps23
Saccharomycetaceae - ps24
Saccharomycetaceae_2 - ps25
Saccharomycetaceae_3 - ps26
Kaz./Nau./Saccharomyces - ps27
Saccharomyces - ps28
Arboricola_clade - ps29
Cerevisiae_clade - ps30
Saccharomyces_cerevisiae - ps31
Phylostratum

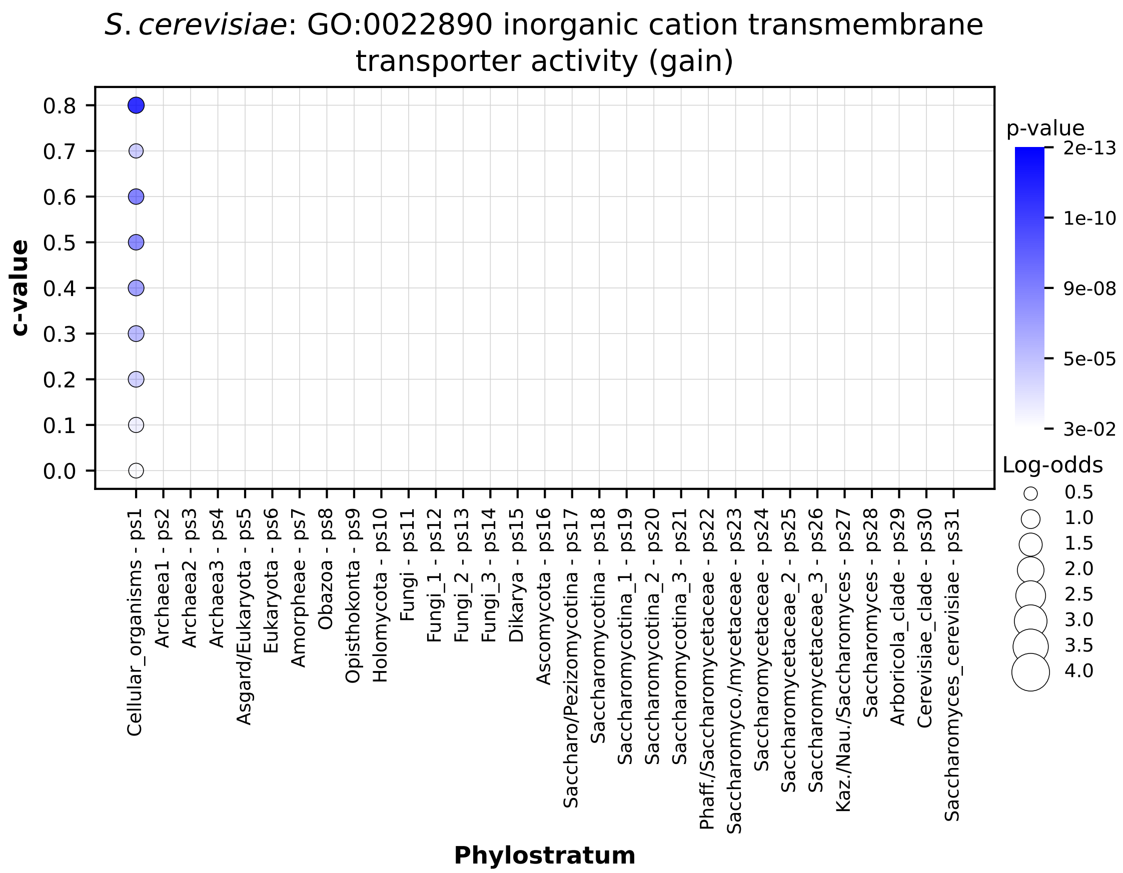

S. cerevisiae: GO:0022890 inorganic cation transmembrane transporter activity (gain)
c-value
0.8
0.7
0.6
0.5
0.4
0.3
0.2
0.1
0.0
Phylostratum
Cellular_organisms - ps1
Archaea1 - ps2
Archaea2 - ps3
Archaea3 - ps4
Asgard/Eukaryota - ps5
Eukaryota - ps6
Amorpheae - ps7
Obazoa - ps8
Opisthokonta - ps9
Holomycota - ps10
Fungi - ps11
Fungi_1 - ps12
Fungi_2 - ps13
Fungi_3 - ps14
Dikarya - ps15
Ascomycota - ps16
Saccharo/Pezizomycotina - ps17
Saccharomycotina - ps18
Saccharomycotina_1 - ps19
Saccharomycotina_2 - ps20
Saccharomycotina_3 - ps21
Phaff./Saccharomycetaceae - ps22
Saccharomyco./mycetaceae - ps23
Saccharomycetaceae - ps24
Saccharomycetaceae_2 - ps25
Saccharomycetaceae_3 - ps26
Kaz./Nau./Saccharomyces - ps27
Saccharomyces - ps28
Arboricola_clade - ps29
Cerevisiae_clade - ps30
Saccharomyces_cerevisiae - ps31
p-value
2e-13
1e-10
9e-08
5e-05
3e-02
Log-odds
0.5
1.0
1.5
2.0
2.5
3.0
3.5
4.0

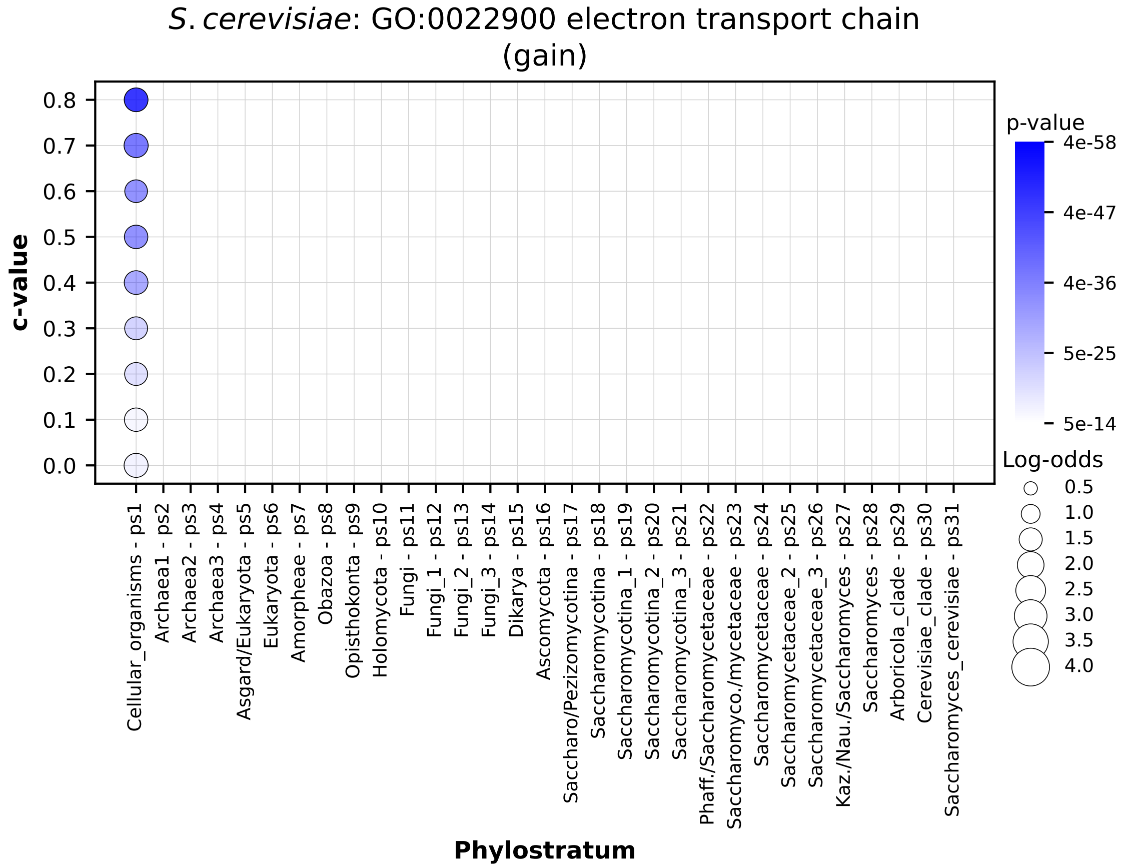

S. cerevisiae: GO:0022900 electron transport chain (gain)
c-value
0.8
0.7
0.6
0.5
0.4
0.3
0.2
0.1
0.0
p-value
4e-58
4e-47
4e-36
5e-25
5e-14
Log-odds
0.5
1.0
1.5
2.0
2.5
3.0
3.5
4.0
Cellular_organisms - ps1
Archaea1 - ps2
Archaea2 - ps3
Archaea3 - ps4
Asgard/Eukaryota - ps5
Eukaryota - ps6
Amorpheae - ps7
Obazoa - ps8
Opisthokonta - ps9
Holomycota - ps10
Fungi - ps11
Fungi_1 - ps12
Fungi_2 - ps13
Fungi_3 - ps14
Dikarya - ps15
Ascomycota - ps16
Saccharo/Pezizomycotina - ps17
Saccharomycotina - ps18
Saccharomycotina_1 - ps19
Saccharomycotina_2 - ps20
Saccharomycotina_3 - ps21
Phaff./Saccharomycetaceae - ps22
Saccharomyco./mycetaceae - ps23
Saccharomycetaceae - ps24
Saccharomycetaceae_2 - ps25
Saccharomycetaceae_3 - ps26
Kaz./Nau./Saccharomyces - ps27
Saccharomyces - ps28
Arboricola_clade - ps29
Cerevisiae_clade - ps30
Saccharomyces_cerevisiae - ps31
Phylostratum

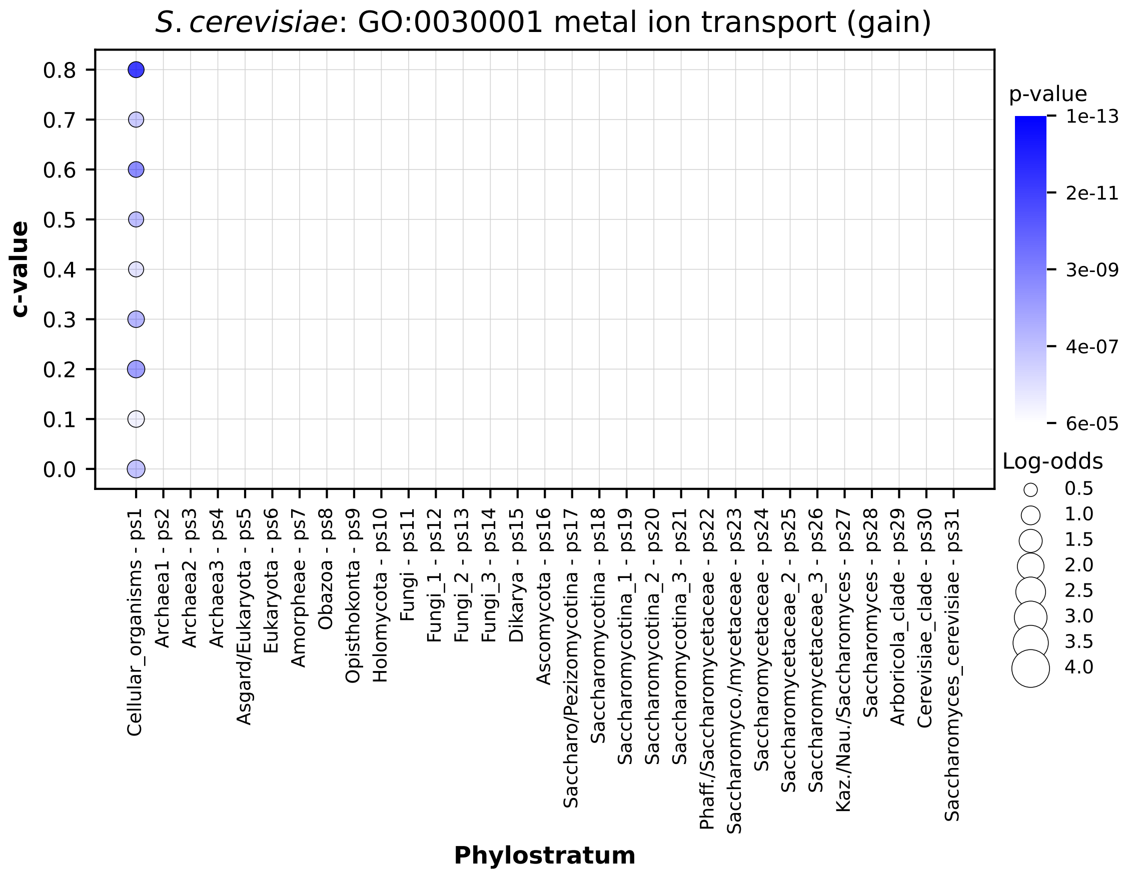
S. cerevisiae: GO:0030001 metal ion transport (gain)
c-value
0.8
0.7
0.6
0.5
0.4
0.3
0.2
0.1
0.0
p-value
1e-13
2e-11
3e-09
4e-07
6e-05
Log-odds
0.5
1.0
1.5
2.0
2.5
3.0
3.5
4.0
Cellular_organisms - ps1
Archaea1 - ps2
Archaea2 - ps3
Archaea3 - ps4
Asgard/Eukaryota - ps5
Eukaryota - ps6
Amorpheae - ps7
Obazoa - ps8
Opisthokonta - ps9
Holomycota - ps10
Fungi - ps11
Fungi_1 - ps12
Fungi_2 - ps13
Fungi_3 - ps14
Dikarya - ps15
Ascomycota - ps16
Saccharo/Pezizomycotina - ps17
Saccharomycotina - ps18
Saccharomycotina_1 - ps19
Saccharomycotina_2 - ps20
Saccharomycotina_3 - ps21
Phaff./Saccharomycetaceae - ps22
Saccharomyco./mycetaceae - ps23
Saccharomycetaceae - ps24
Saccharomycetaceae_2 - ps25
Saccharomycetaceae_3 - ps26
Kaz./Nau./Saccharomyces - ps27
Saccharomyces - ps28
Arboricola_clade - ps29
Cerevisiae_clade - ps30
Saccharomyces_cerevisiae - ps31
Phylostratum

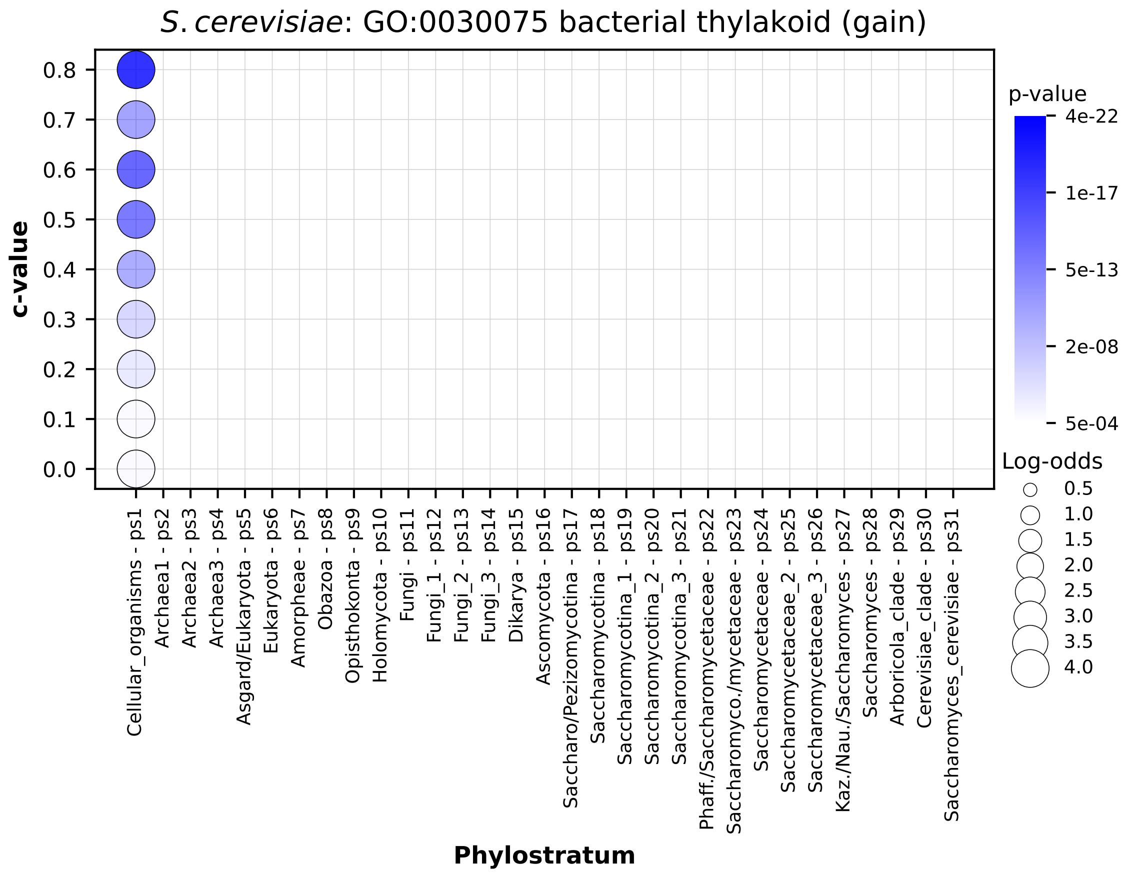

S. cerevisiae: GO:0030075 bacterial thylakoid (gain)
c-value
0.8
0.7
0.6
0.5
0.4
0.3
0.2
0.1
0.0
p-value
4e-22
1e-17
5e-13
2e-08
5e-04
Log-odds
0.5
1.0
1.5
2.0
2.5
3.0
3.5
4.0
Cellular_organisms - ps1
Archaea1 - ps2
Archaea2 - ps3
Archaea3 - ps4
Asgard/Eukaryota - ps5
Eukaryota - ps6
Amorpheae - ps7
Obazoa - ps8
Opisthokonta - ps9
Holomycota - ps10
Fungi - ps11
Fungi_1 - ps12
Fungi_2 - ps13
Fungi_3 - ps14
Dikarya - ps15
Ascomycota - ps16
Saccharo/Pezizomycotina - ps17
Saccharomycotina - ps18
Saccharomycotina_1 - ps19
Saccharomycotina_2 - ps20
Saccharomycotina_3 - ps21
Phaff./Saccharomycetaceae - ps22
Saccharomyco./mycetaceae - ps23
Saccharomycetaceae - ps24
Saccharomycetaceae_2 - ps25
Saccharomycetaceae_3 - ps26
Kaz./Nau./Saccharomyces - ps27
Saccharomyces - ps28
Arboricola_clade - ps29
Cerevisiae_clade - ps30
Saccharomyces_cerevisiae - ps31
Phylostratum

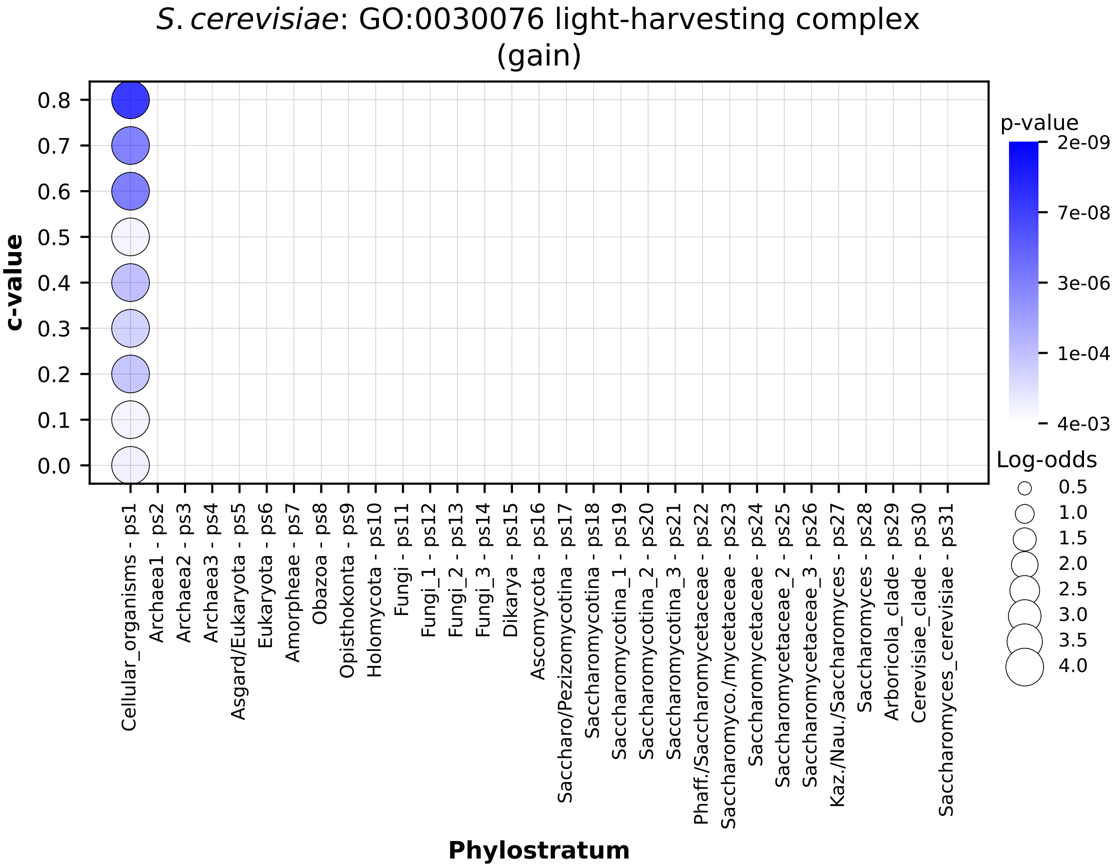

S. cerevisiae: GO:0030076 light-harvesting complex
(gain)
c-value
0.8
0.7
0.6
0.5
0.4
0.3
0.2
0.1
0.0
p-value
2e-09
7e-08
3e-06
1e-04
4e-03
Log-odds
0.5
1.0
1.5
2.0
2.5
3.0
3.5
4.0
Cellular_organisms - ps1
Archaea1 - ps2
Archaea2 - ps3
Archaea3 - ps4
Asgard/Eukaryota - ps5
Eukaryota - ps6
Amorpheae - ps7
Obazoa - ps8
Opisthokonta - ps9
Holomycota - ps10
Fungi - ps11
Fungi_1 - ps12
Fungi_2 - ps13
Fungi_3 - ps14
Dikarya - ps15
Ascomycota - ps16
Saccharo/Pezizomycotina - ps17
Saccharomycotina - ps18
Saccharomycotina_1 - ps19
Saccharomycotina_2 - ps20
Saccharomycotina_3 - ps21
Phaff./Saccharomycetaceae - ps22
Saccharomyco./mycetaceae - ps23
Saccharomycetaceae - ps24
Saccharomycetaceae_2 - ps25
Saccharomycetaceae_3 - ps26
Kaz./Nau./Saccharomyces - ps27
Saccharomyces - ps28
Arboricola_clade - ps29
Cerevisiae_clade - ps30
Saccharomyces_cerevisiae - ps31
Phylostratum

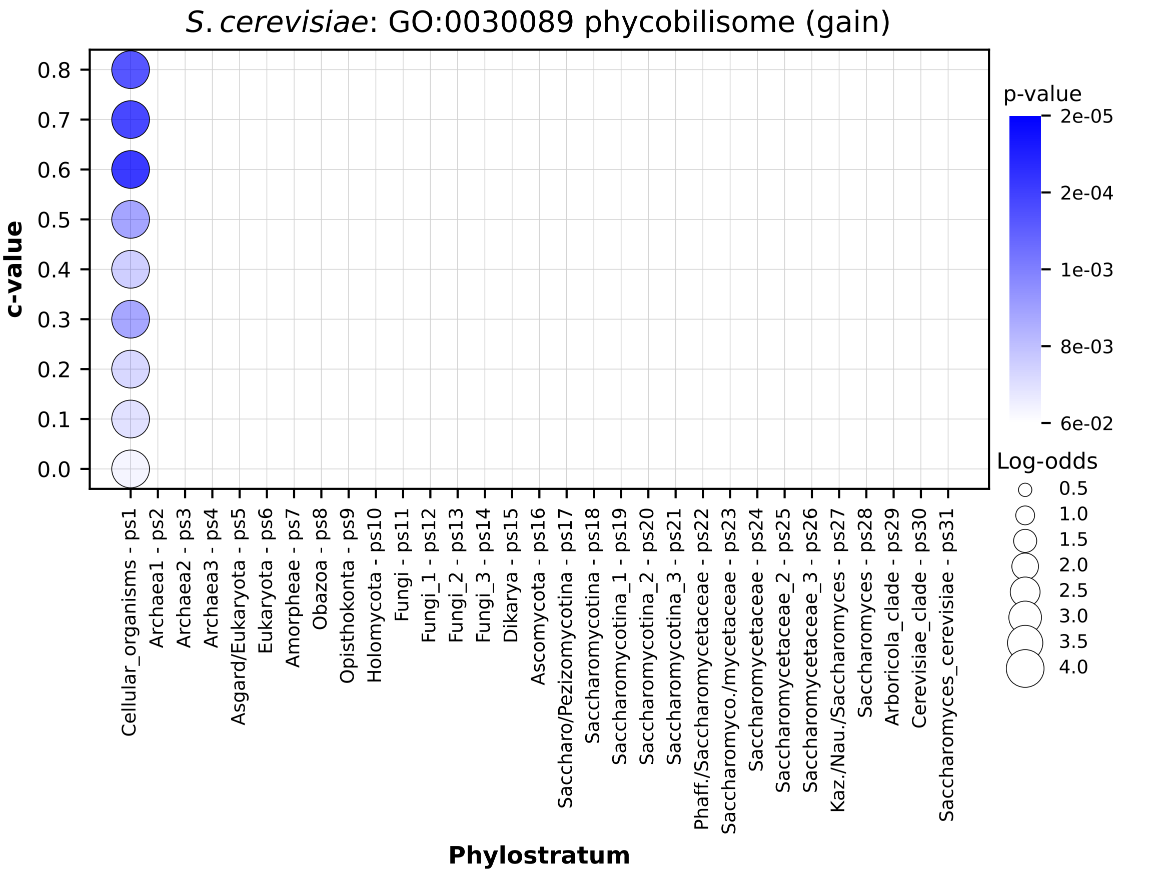
S. cerevisiae: GO:0030089 phycobilisome (gain)
c-value
0.0
0.1
0.2
0.3
0.4
0.5
0.6
0.7
0.8
Phylostratum
Cellular_organisms - ps1
Archaea1 - ps2
Archaea2 - ps3
Archaea3 - ps4
Asgard/Eukaryota - ps5
Eukaryota - ps6
Amorpheae - ps7
Obazoa - ps8
Opisthokonta - ps9
Holomycota - ps10
Fungi - ps11
Fungi_1 - ps12
Fungi_2 - ps13
Fungi_3 - ps14
Dikarya - ps15
Ascomycota - ps16
Saccharo/Pezizomycotina - ps17
Saccharomycotina - ps18
Saccharomycotina_1 - ps19
Saccharomycotina_2 - ps20
Saccharomycotina_3 - ps21
Phaff./Saccharomycetaceae - ps22
Saccharomyco./mycetaceae - ps23
Saccharomycetaceae - ps24
Saccharomycetaceae_2 - ps25
Saccharomycetaceae_3 - ps26
Kaz./Nau./Saccharomyces - ps27
Saccharomyces - ps28
Arboricola_clade - ps29
Cerevisiae_clade - ps30
Saccharomyces_cerevisiae - ps31
p-value
2e-05
2e-04
1e-03
8e-03
6e-02
Log-odds
0.5
1.0
1.5
2.0
2.5
3.0
3.5
4.0

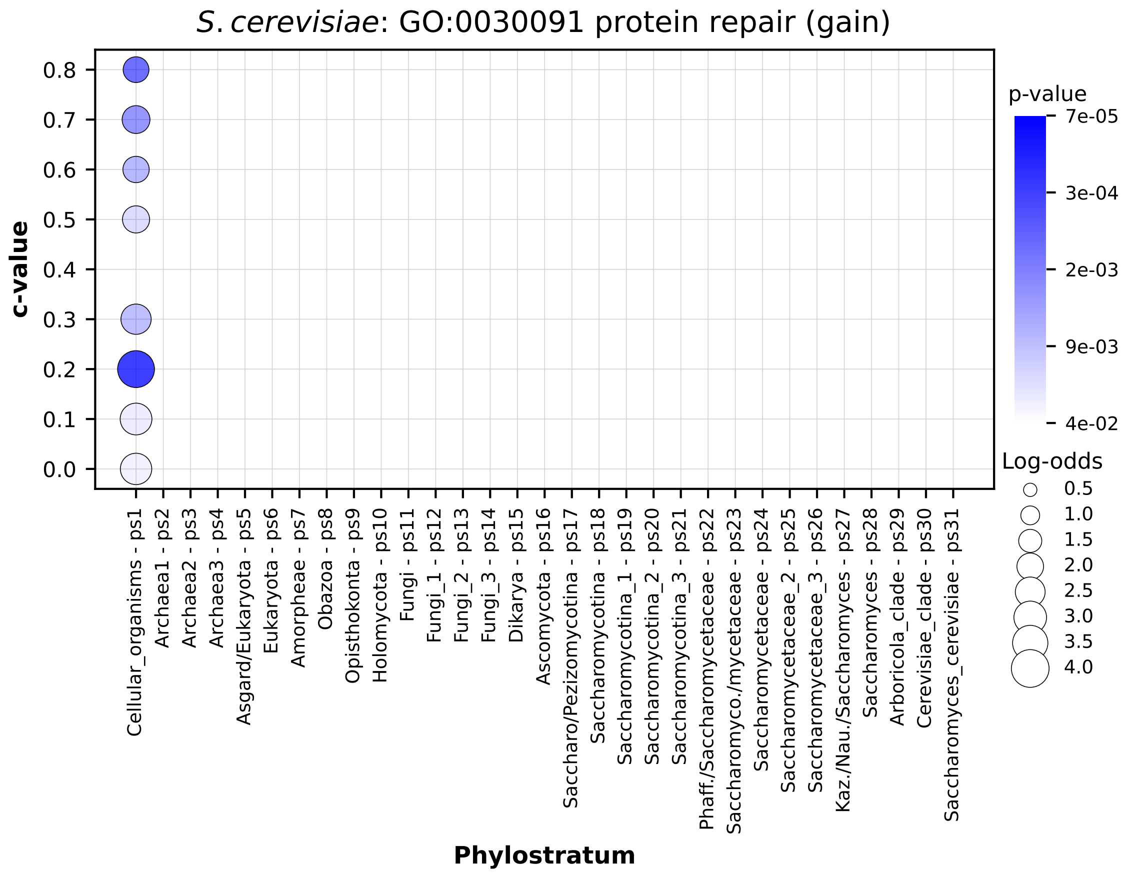

S. cerevisiae: GO:0030091 protein repair (gain)
c-value
0.0
0.1
0.2
0.3
0.4
0.5
0.6
0.7
0.8
Phylostratum
Cellular_organisms - ps1
Archaea1 - ps2
Archaea2 - ps3
Archaea3 - ps4
Asgard/Eukaryota - ps5
Eukaryota - ps6
Amorpheae - ps7
Obazoa - ps8
Opisthokonta - ps9
Holomycota - ps10
Fungi - ps11
Fungi_1 - ps12
Fungi_2 - ps13
Fungi_3 - ps14
Dikarya - ps15
Ascomycota - ps16
Saccharo/Pezizomycotina - ps17
Saccharomycotina - ps18
Saccharomycotina_1 - ps19
Saccharomycotina_2 - ps20
Saccharomycotina_3 - ps21
Phaff./Saccharomycetaceae - ps22
Saccharomyco./mycetaceae - ps23
Saccharomycetaceae - ps24
Saccharomycetaceae_2 - ps25
Saccharomycetaceae_3 - ps26
Kaz./Nau./Saccharomyces - ps27
Saccharomyces - ps28
Arboricola_clade - ps29
Cerevisiae_clade - ps30
Saccharomyces_cerevisiae - ps31
p-value
7e-05
3e-04
2e-03
9e-03
4e-02
Log-odds
0.5
1.0
1.5
2.0
2.5
3.0
3.5
4.0

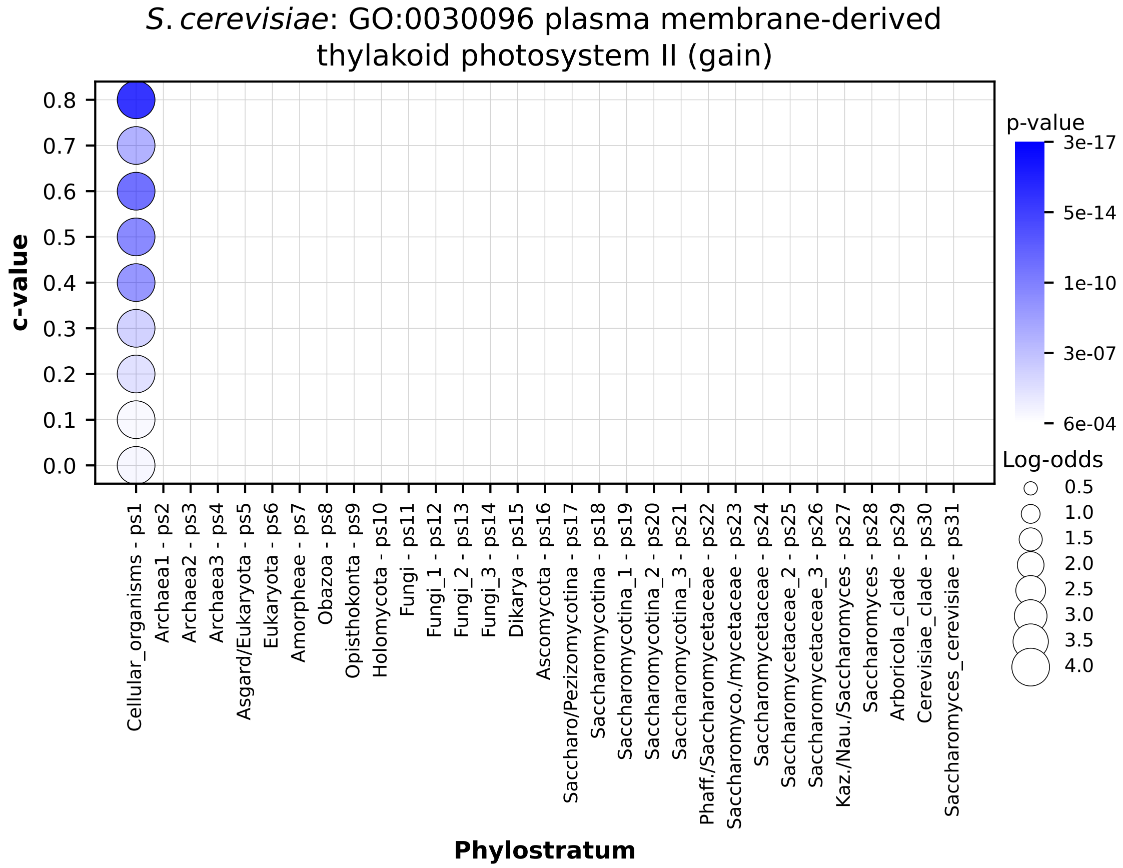

S. cerevisiae: GO:0030096 plasma membrane-derived thylakoid photosystem II (gain)
c-value
0.8
0.7
0.6
0.5
0.4
0.3
0.2
0.1
0.0
p-value
3e-17
5e-14
1e-10
3e-07
6e-04
Log-odds
0.5
1.0
1.5
2.0
2.5
3.0
3.5
4.0
Cellular_organisms - ps1
Archaea1 - ps2
Archaea2 - ps3
Archaea3 - ps4
Asgard/Eukaryota - ps5
Eukaryota - ps6
Amorpheae - ps7
Obazoa - ps8
Opisthokonta - ps9
Holomycota - ps10
Fungi - ps11
Fungi_1 - ps12
Fungi_2 - ps13
Fungi_3 - ps14
Dikarya - ps15
Ascomycota - ps16
Saccharo/Pezizomycotina - ps17
Saccharomycotina - ps18
Saccharomycotina_1 - ps19
Saccharomycotina_2 - ps20
Saccharomycotina_3 - ps21
Phaff./Saccharomycetaceae - ps22
Saccharomyco./mycetaceae - ps23
Saccharomycetaceae - ps24
Saccharomycetaceae_2 - ps25
Saccharomycetaceae_3 - ps26
Kaz./Nau./Saccharomyces - ps27
Saccharomyces - ps28
Arboricola_clade - ps29
Cerevisiae_clade - ps30
Saccharomyces_cerevisiae - ps31
Phylostratum

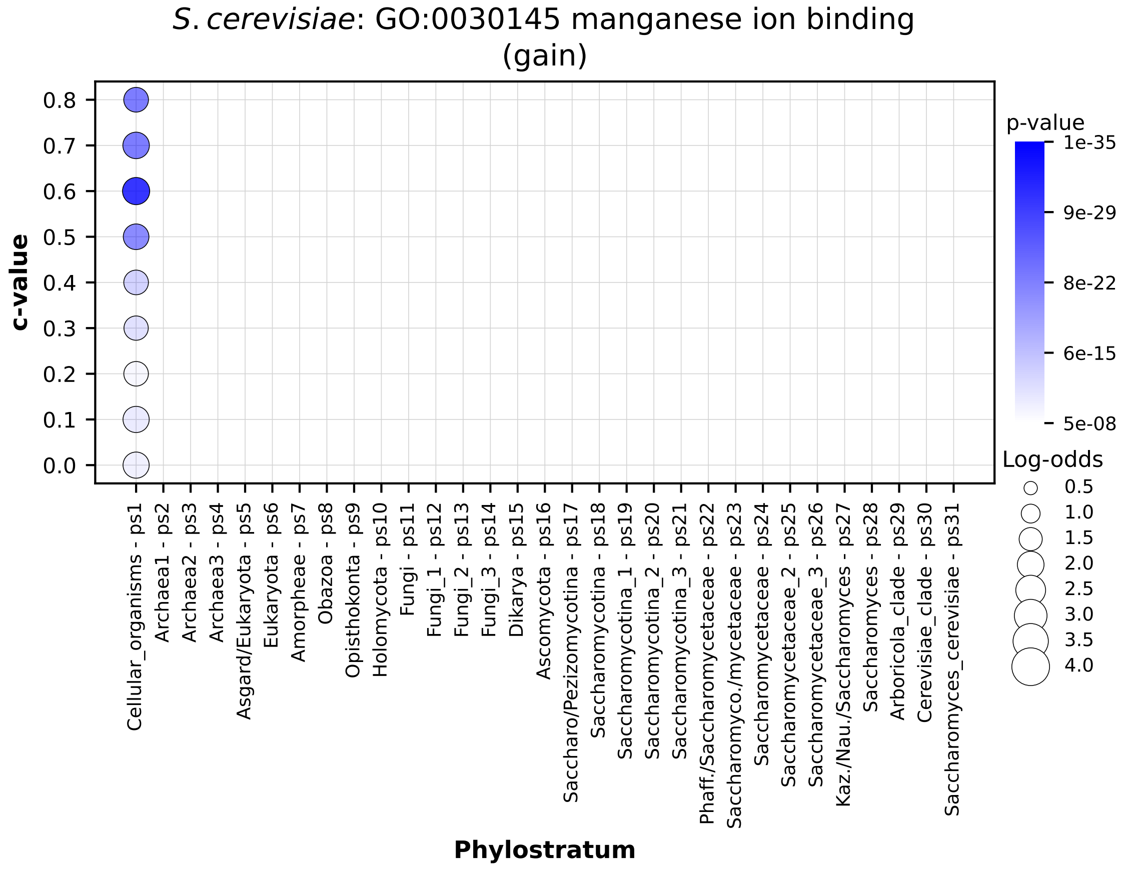

S. cerevisiae: GO:0030145 manganese ion binding (gain)
c-value
0.8
0.7
0.6
0.5
0.4
0.3
0.2
0.1
0.0
p-value
1e-35
9e-29
8e-22
6e-15
5e-08
Log-odds
0.5
1.0
1.5
2.0
2.5
3.0
3.5
4.0
Cellular_organisms - ps1
Archaea1 - ps2
Archaea2 - ps3
Archaea3 - ps4
Asgard/Eukaryota - ps5
Eukaryota - ps6
Amorpheae - ps7
Obazoa - ps8
Opisthokonta - ps9
Holomycota - ps10
Fungi - ps11
Fungi_1 - ps12
Fungi_2 - ps13
Fungi_3 - ps14
Dikarya - ps15
Ascomycota - ps16
Saccharo/Pezizomycotina - ps17
Saccharomycotina - ps18
Saccharomycotina_1 - ps19
Saccharomycotina_2 - ps20
Saccharomycotina_3 - ps21
Phaff./Saccharomycetaceae - ps22
Saccharomyco./mycetaceae - ps23
Saccharomycetaceae - ps24
Saccharomycetaceae_2 - ps25
Saccharomycetaceae_3 - ps26
Kaz./Nau./Saccharomyces - ps27
Saccharomyces - ps28
Arboricola_clade - ps29
Cerevisiae_clade - ps30
Saccharomyces_cerevisiae - ps31
Phylostratum

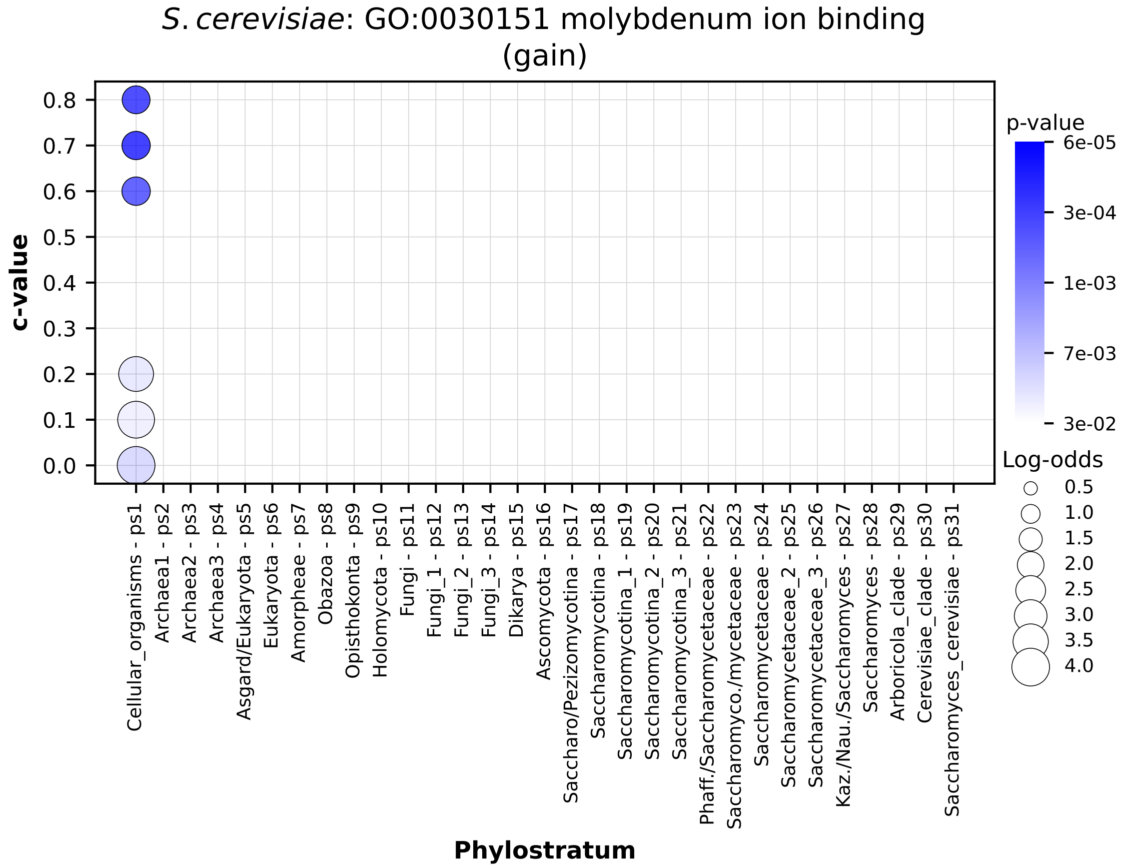

S. cerevisiae: GO:0030151 molybdenum ion binding (gain)
c-value
0.8
0.7
0.6
0.5
0.4
0.3
0.2
0.1
0.0
p-value
6e-05
3e-04
1e-03
7e-03
3e-02
Log-odds
0.5
1.0
1.5
2.0
2.5
3.0
3.5
4.0
Cellular_organisms - ps1
Archaea1 - ps2
Archaea2 - ps3
Archaea3 - ps4
Asgard/Eukaryota - ps5
Eukaryota - ps6
Amorpheae - ps7
Obazoa - ps8
Opisthokonta - ps9
Holomycota - ps10
Fungi - ps11
Fungi_1 - ps12
Fungi_2 - ps13
Fungi_3 - ps14
Dikarya - ps15
Ascomycota - ps16
Saccharo/Pezizomycotina - ps17
Saccharomycotina - ps18
Saccharomycotina_1 - ps19
Saccharomycotina_2 - ps20
Saccharomycotina_3 - ps21
Phaff./Saccharomycetaceae - ps22
Saccharomyco./mycetaceae - ps23
Saccharomycetaceae - ps24
Saccharomycetaceae_2 - ps25
Saccharomycetaceae_3 - ps26
Kaz./Nau./Saccharomyces - ps27
Saccharomyces - ps28
Arboricola_clade - ps29
Cerevisiae_clade - ps30
Saccharomyces_cerevisiae - ps31
Phylostratum

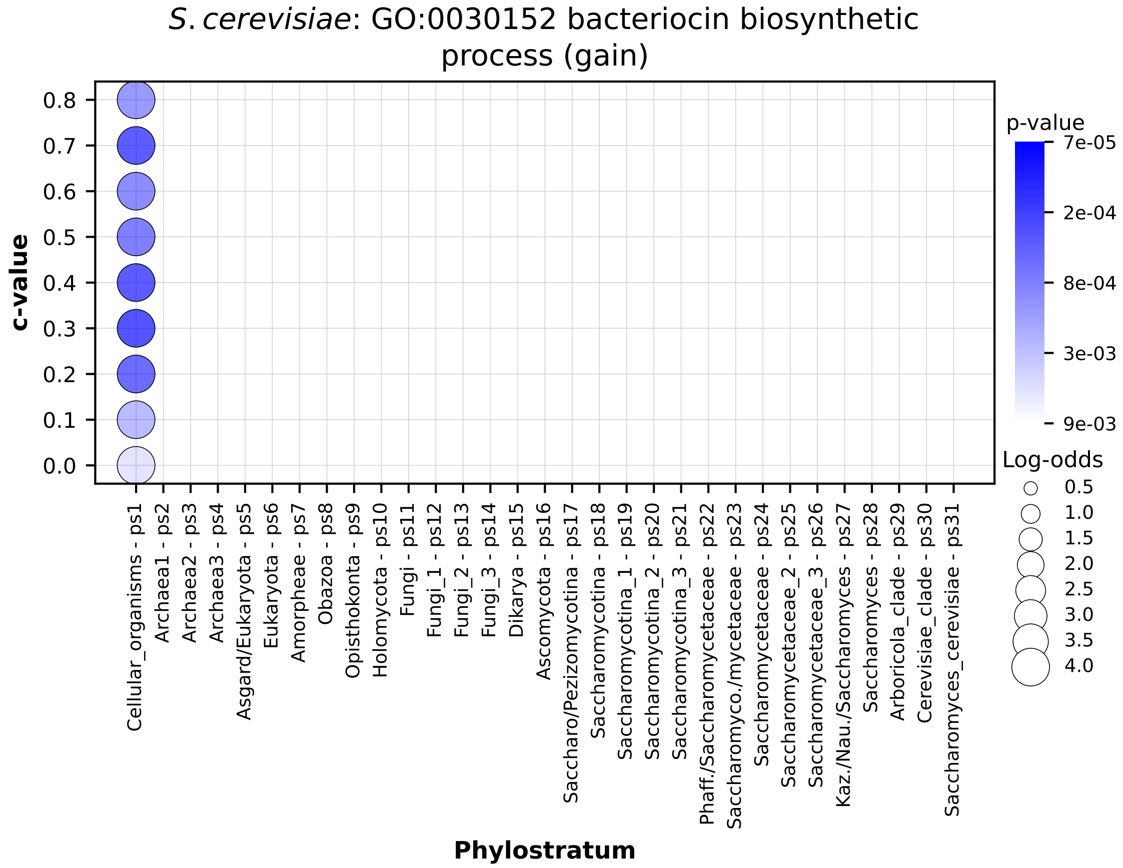

S. cerevisiae: GO:0030152 bacteriocin biosynthetic process (gain)
c-value
0.8
0.7
0.6
0.5
0.4
0.3
0.2
0.1
0.0
Phylostratum
Cellular_organisms - ps1
Archaea1 - ps2
Archaea2 - ps3
Archaea3 - ps4
Asgard/Eukaryota - ps5
Eukaryota - ps6
Amorpheae - ps7
Obazoa - ps8
Opisthokonta - ps9
Holomycota - ps10
Fungi - ps11
Fungi_1 - ps12
Fungi_2 - ps13
Fungi_3 - ps14
Dikarya - ps15
Ascomycota - ps16
Saccharo/Pezizomycotina - ps17
Saccharomycotina - ps18
Saccharomycotina_1 - ps19
Saccharomycotina_2 - ps20
Saccharomycotina_3 - ps21
Phaff./Saccharomycetaceae - ps22
Saccharomyco./mycetaceae - ps23
Saccharomycetaceae - ps24
Saccharomycetaceae_2 - ps25
Saccharomycetaceae_3 - ps26
Kaz./Nau./Saccharomyces - ps27
Saccharomyces - ps28
Arboricola_clade - ps29
Cerevisiae_clade - ps30
Saccharomyces_cerevisiae - ps31
p-value
7e-05
2e-04
8e-04
3e-03
9e-03
Log-odds
0.5
1.0
1.5
2.0
2.5
3.0
3.5
4.0

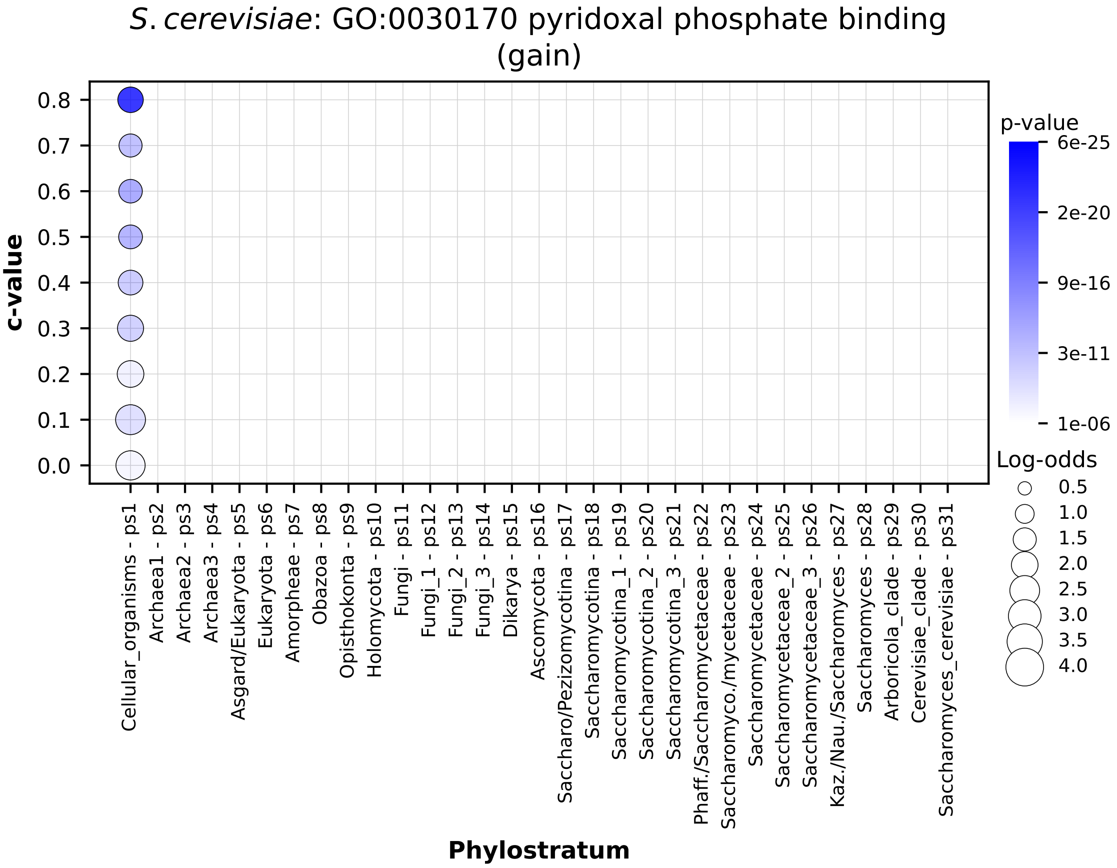
S. cerevisiae: GO:0030170 pyridoxal phosphate binding
(gain)
c-value
0.8
0.7
0.6
0.5
0.4
0.3
0.2
0.1
0.0
p-value
6e-25
2e-20
9e-16
3e-11
1e-06
Log-odds
0.5
1.0
1.5
2.0
2.5
3.0
3.5
4.0
Cellular_organisms - ps1
Archaea1 - ps2
Archaea2 - ps3
Archaea3 - ps4
Asgard/Eukaryota - ps5
Eukaryota - ps6
Amorpheae - ps7
Obazoa - ps8
Opisthokonta - ps9
Holomycota - ps10
Fungi - ps11
Fungi_1 - ps12
Fungi_2 - ps13
Fungi_3 - ps14
Dikarya - ps15
Ascomycota - ps16
Saccharo/Pezizomycotina - ps17
Saccharomycotina - ps18
Saccharomycotina_1 - ps19
Saccharomycotina_2 - ps20
Saccharomycotina_3 - ps21
Phaff./Saccharomycetaceae - ps22
Saccharomyco./mycetaceae - ps23
Saccharomycetaceae - ps24
Saccharomycetaceae_2 - ps25
Saccharomycetaceae_3 - ps26
Kaz./Nau./Saccharomyces - ps27
Saccharomyces - ps28
Arboricola_clade - ps29
Cerevisiae_clade - ps30
Saccharomyces_cerevisiae - ps31
Phylostratum

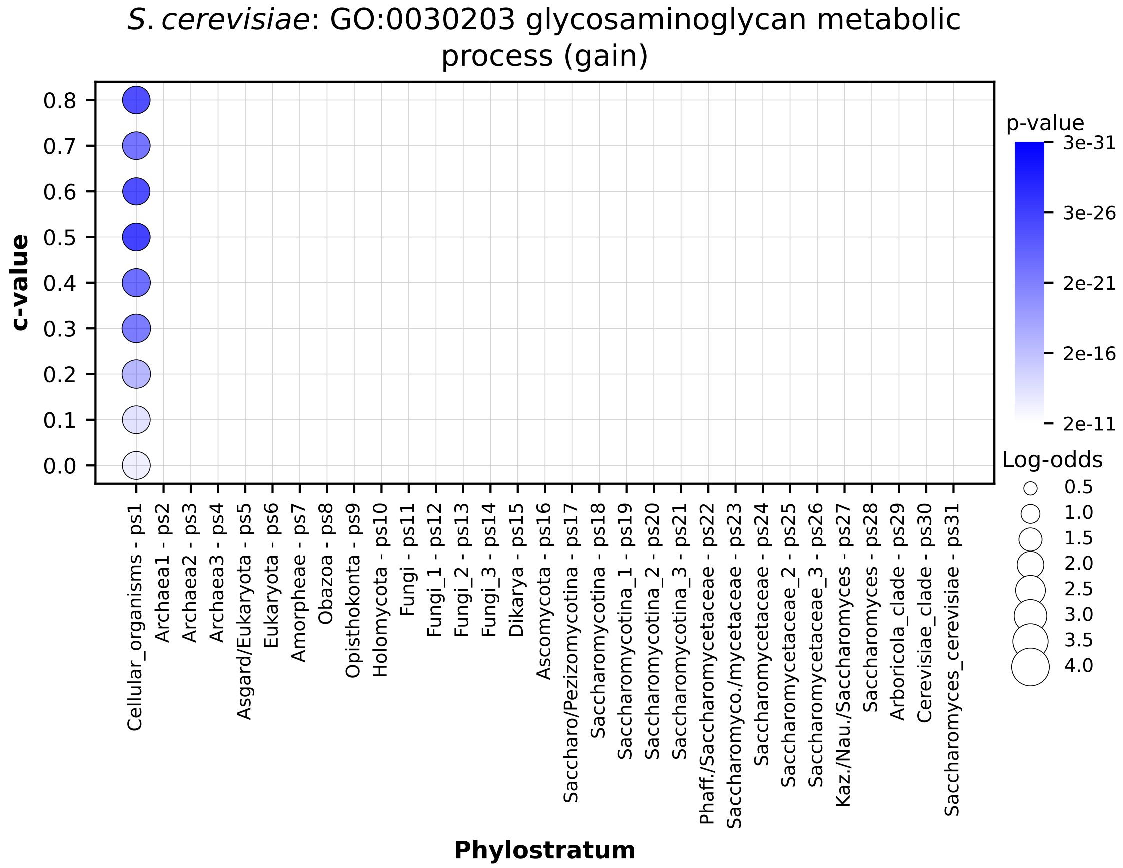
S. cerevisiae: GO:0030203 glycosaminoglycan metabolic process (gain)
c-value
0.8
0.7
0.6
0.5
0.4
0.3
0.2
0.1
0.0
p-value
3e-31
3e-26
2e-21
2e-16
2e-11
Log-odds
0.5
1.0
1.5
2.0
2.5
3.0
3.5
4.0
Cellular_organisms - ps1
Archaea1 - ps2
Archaea2 - ps3
Archaea3 - ps4
Asgard/Eukaryota - ps5
Eukaryota - ps6
Amorpheae - ps7
Obazoa - ps8
Opisthokonta - ps9
Holomycota - ps10
Fungi - ps11
Fungi_1 - ps12
Fungi_2 - ps13
Fungi_3 - ps14
Dikarya - ps15
Ascomycota - ps16
Saccharo/Pezizomycotina - ps17
Saccharomycotina - ps18
Saccharomycotina_1 - ps19
Saccharomycotina_2 - ps20
Saccharomycotina_3 - ps21
Phaff./Saccharomycetaceae - ps22
Saccharomyco./mycetaceae - ps23
Saccharomycetaceae - ps24
Saccharomycetaceae_2 - ps25
Saccharomycetaceae_3 - ps26
Kaz./Nau./Saccharomyces - ps27
Saccharomyces - ps28
Arboricola_clade - ps29
Cerevisiae_clade - ps30
Saccharomyces_cerevisiae - ps31
Phylostratum

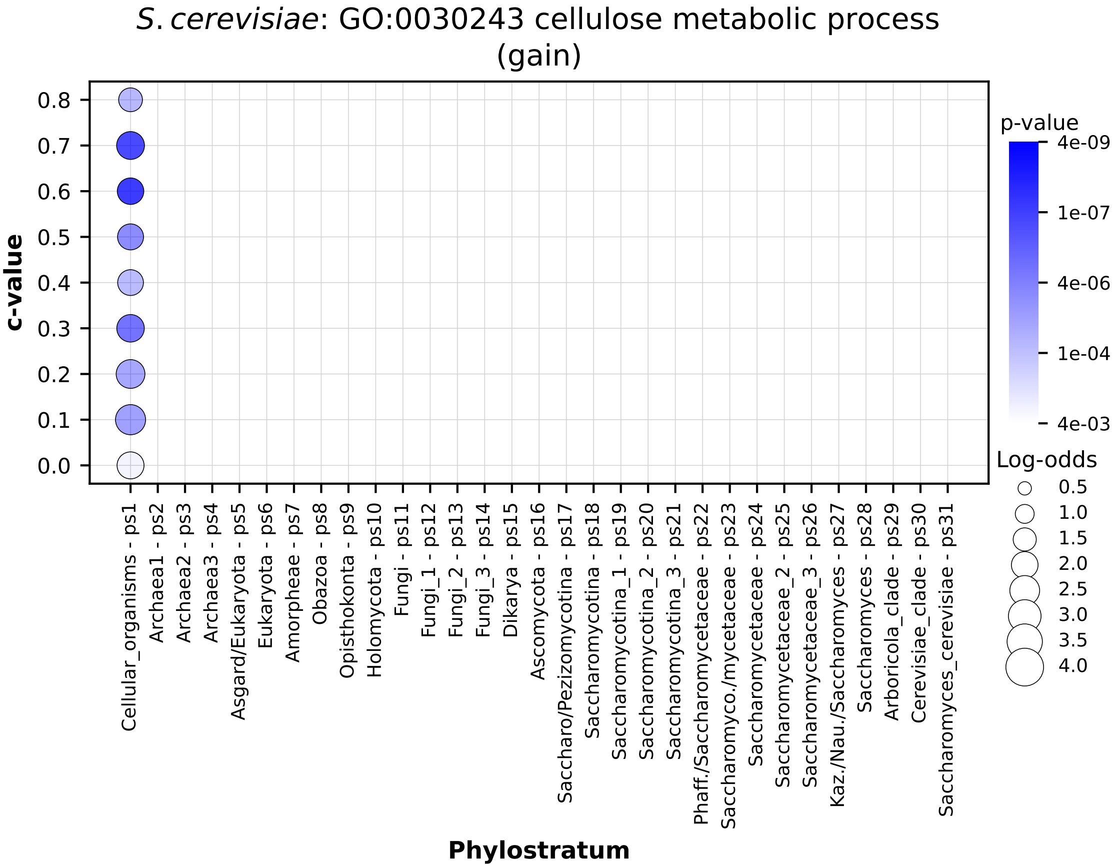

S. cerevisiae: GO:0030243 cellulose metabolic process (gain)
c-value
Phylostratum
p-value
4e-09
1e-07
4e-06
1e-04
4e-03
Log-odds
0.5
1.0
1.5
2.0
2.5
3.0
3.5
4.0
Cellular_organisms - ps1
Archaea1 - ps2
Archaea2 - ps3
Archaea3 - ps4
Asgard/Eukaryota - ps5
Eukaryota - ps6
Amorpheae - ps7
Obazoa - ps8
Opisthokonta - ps9
Holomycota - ps10
Fungi - ps11
Fungi_1 - ps12
Fungi_2 - ps13
Fungi_3 - ps14
Dikarya - ps15
Ascomycota - ps16
Saccharo/Pezizomycotina - ps17
Saccharomycotina - ps18
Saccharomycotina_1 - ps19
Saccharomycotina_2 - ps20
Saccharomycotina_3 - ps21
Phaff./Saccharomycetaceae - ps22
Saccharomyco./mycetaceae - ps23
Saccharomycetaceae - ps24
Saccharomycetaceae_2 - ps25
Saccharomycetaceae_3 - ps26
Kaz./Nau./Saccharomyces - ps27
Saccharomyces - ps28
Arboricola_clade - ps29
Cerevisiae_clade - ps30
Saccharomyces_cerevisiae - ps31

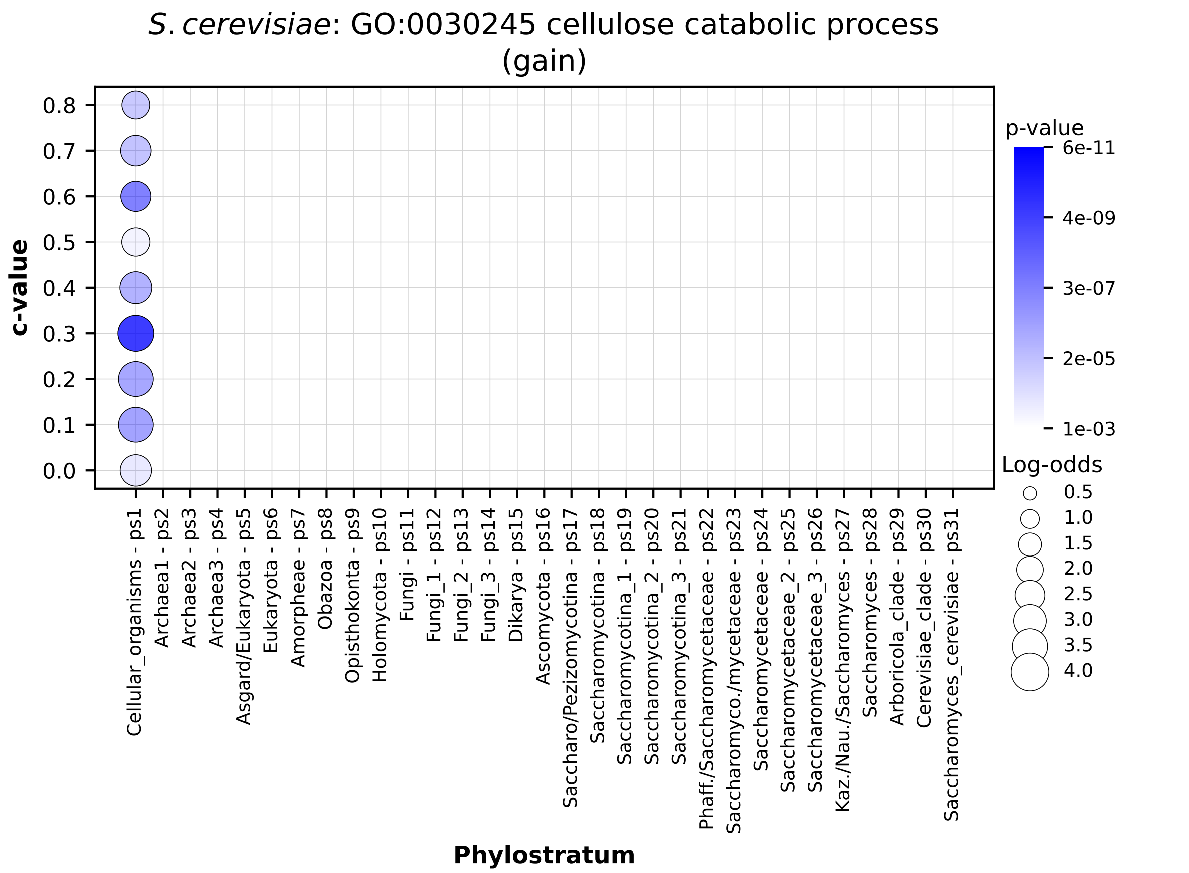

S. cerevisiae: GO:0030245 cellulose catabolic process
(gain)
c-value
0.8
0.7
0.6
0.5
0.4
0.3
0.2
0.1
0.0
p-value
6e-11
4e-09
3e-07
2e-05
1e-03
Log-odds
0.5
1.0
1.5
2.0
2.5
3.0
3.5
4.0
Cellular_organisms - ps1
Archaea1 - ps2
Archaea2 - ps3
Archaea3 - ps4
Asgard/Eukaryota - ps5
Eukaryota - ps6
Amorpheae - ps7
Obazoa - ps8
Opisthokonta - ps9
Holomycota - ps10
Fungi - ps11
Fungi_1 - ps12
Fungi_2 - ps13
Fungi_3 - ps14
Dikarya - ps15
Ascomycota - ps16
Saccharo/Pezizomycotina - ps17
Saccharomycotina - ps18
Saccharomycotina_1 - ps19
Saccharomycotina_2 - ps20
Saccharomycotina_3 - ps21
Phaff./Saccharomycetaceae - ps22
Saccharomyco./mycetaceae - ps23
Saccharomycetaceae - ps24
Saccharomycetaceae_2 - ps25
Saccharomycetaceae_3 - ps26
Kaz./Nau./Saccharomyces - ps27
Saccharomyces - ps28
Arboricola_clade - ps29
Cerevisiae_clade - ps30
Saccharomyces_cerevisiae - ps31
Phylostratum

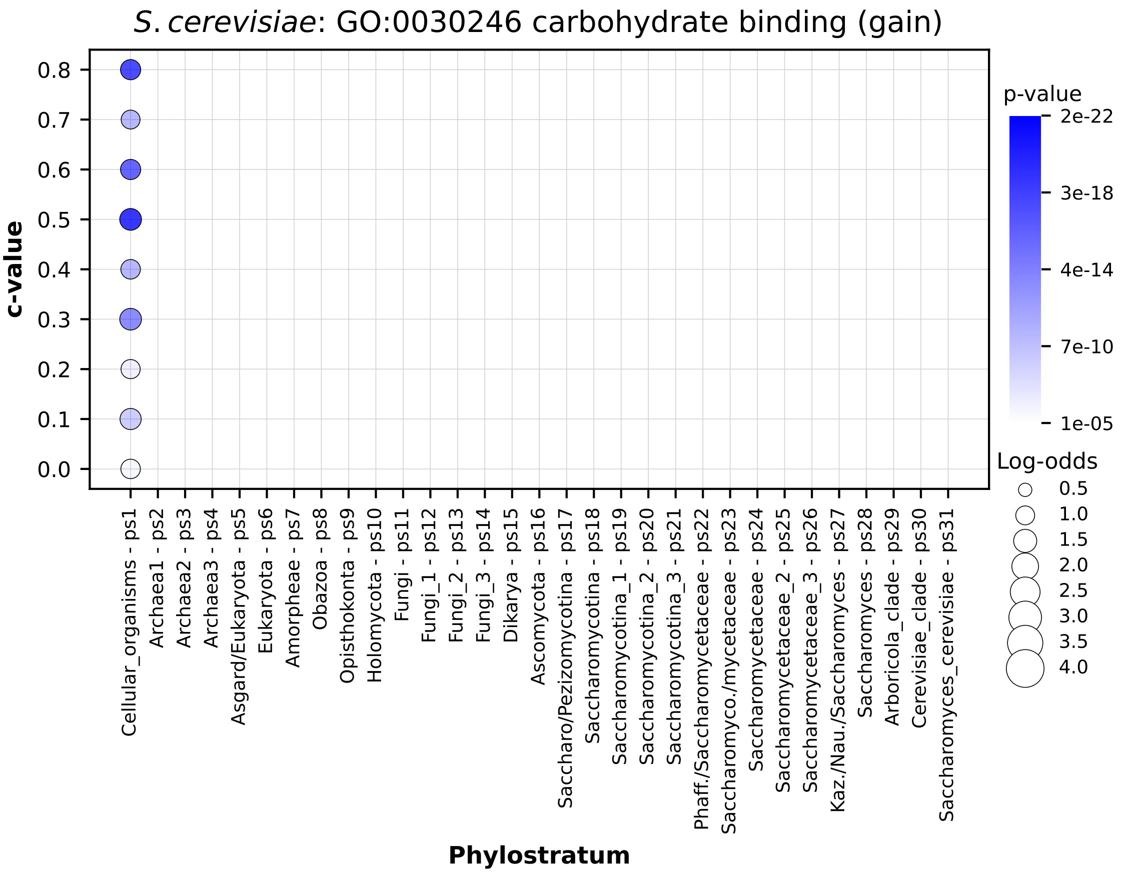
S. cerevisiae: GO:0030246 carbohydrate binding (gain)
c-value
0.8
0.7
0.6
0.5
0.4
0.3
0.2
0.1
0.0
p-value
2e-22
3e-18
4e-14
7e-10
1e-05
Log-odds
0.5
1.0
1.5
2.0
2.5
3.0
3.5
4.0
Cellular_organisms - ps1
Archaea1 - ps2
Archaea2 - ps3
Archaea3 - ps4
Asgard/Eukaryota - ps5
Eukaryota - ps6
Amorpheae - ps7
Obazoa - ps8
Opisthokonta - ps9
Holomycota - ps10
Fungi - ps11
Fungi_1 - ps12
Fungi_2 - ps13
Fungi_3 - ps14
Dikarya - ps15
Ascomycota - ps16
Saccharo/Pezizomycotina - ps17
Saccharomycotina - ps18
Saccharomycotina_1 - ps19
Saccharomycotina_2 - ps20
Saccharomycotina_3 - ps21
Phaff./Saccharomycetaceae - ps22
Saccharomyco./mycetaceae - ps23
Saccharomycetaceae - ps24
Saccharomycetaceae_2 - ps25
Saccharomycetaceae_3 - ps26
Kaz./Nau./Saccharomyces - ps27
Saccharomyces - ps28
Arboricola_clade - ps29
Cerevisiae_clade - ps30
Saccharomyces_cerevisiae - ps31
Phylostratum

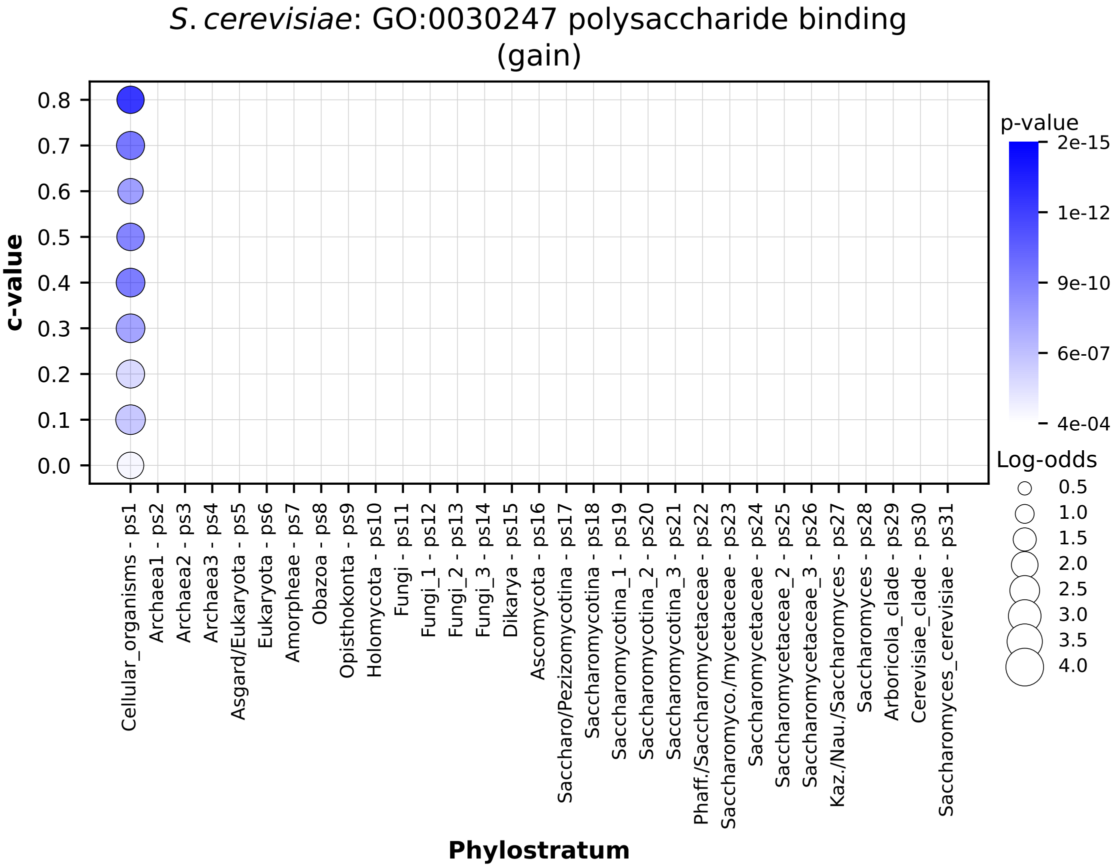
S. cerevisiae: GO:0030247 polysaccharide binding
(gain)
c-value
0.8
0.7
0.6
0.5
0.4
0.3
0.2
0.1
0.0
p-value
2e-15
1e-12
9e-10
6e-07
4e-04
Log-odds
0.5
1.0
1.5
2.0
2.5
3.0
3.5
4.0
Cellular_organisms - ps1
Archaea1 - ps2
Archaea2 - ps3
Archaea3 - ps4
Asgard/Eukaryota - ps5
Eukaryota - ps6
Amorpheae - ps7
Obazoa - ps8
Opisthokonta - ps9
Holomycota - ps10
Fungi - ps11
Fungi_1 - ps12
Fungi_2 - ps13
Fungi_3 - ps14
Dikarya - ps15
Ascomycota - ps16
Saccharo/Pezizomycotina - ps17
Saccharomycotina - ps18
Saccharomycotina_1 - ps19
Saccharomycotina_2 - ps20
Saccharomycotina_3 - ps21
Phaff./Saccharomycetaceae - ps22
Saccharomyco./mycetaceae - ps23
Saccharomycetaceae - ps24
Saccharomycetaceae_2 - ps25
Saccharomycetaceae_3 - ps26
Kaz./Nau./Saccharomyces - ps27
Saccharomyces - ps28
Arboricola_clade - ps29
Cerevisiae_clade - ps30
Saccharomyces_cerevisiae - ps31
Phylostratum

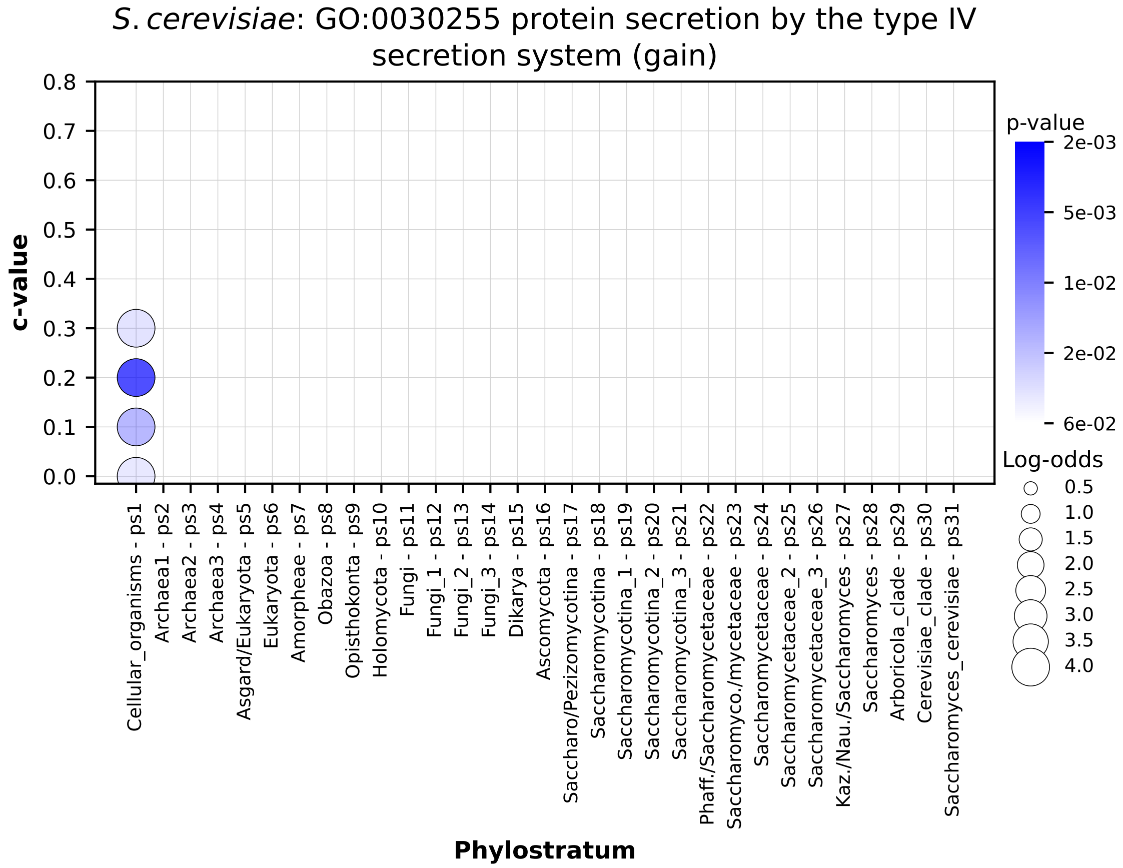
S. cerevisiae: GO:0030255 protein secretion by the type IV secretion system (gain)
c-value
0.8
0.7
0.6
0.5
0.4
0.3
0.2
0.1
0.0
p-value
2e-03
5e-03
1e-02
2e-02
6e-02
Log-odds
0.5
1.0
1.5
2.0
2.5
3.0
3.5
4.0
Cellular_organisms - ps1
Archaea1 - ps2
Archaea2 - ps3
Archaea3 - ps4
Asgard/Eukaryota - ps5
Eukaryota - ps6
Amorpheae - ps7
Obazoa - ps8
Opisthokonta - ps9
Holomycota - ps10
Fungi - ps11
Fungi_1 - ps12
Fungi_2 - ps13
Fungi_3 - ps14
Dikarya - ps15
Ascomycota - ps16
Saccharo/Pezizomycotina - ps17
Saccharomycotina - ps18
Saccharomycotina_1 - ps19
Saccharomycotina_2 - ps20
Saccharomycotina_3 - ps21
Phaff./Saccharomycetaceae - ps22
Saccharomyco./mycetaceae - ps23
Saccharomycetaceae - ps24
Saccharomycetaceae_2 - ps25
Saccharomycetaceae_3 - ps26
Kaz./Nau./Saccharomyces - ps27
Saccharomyces - ps28
Arboricola_clade - ps29
Cerevisiae_clade - ps30
Saccharomyces_cerevisiae - ps31
Phylostratum

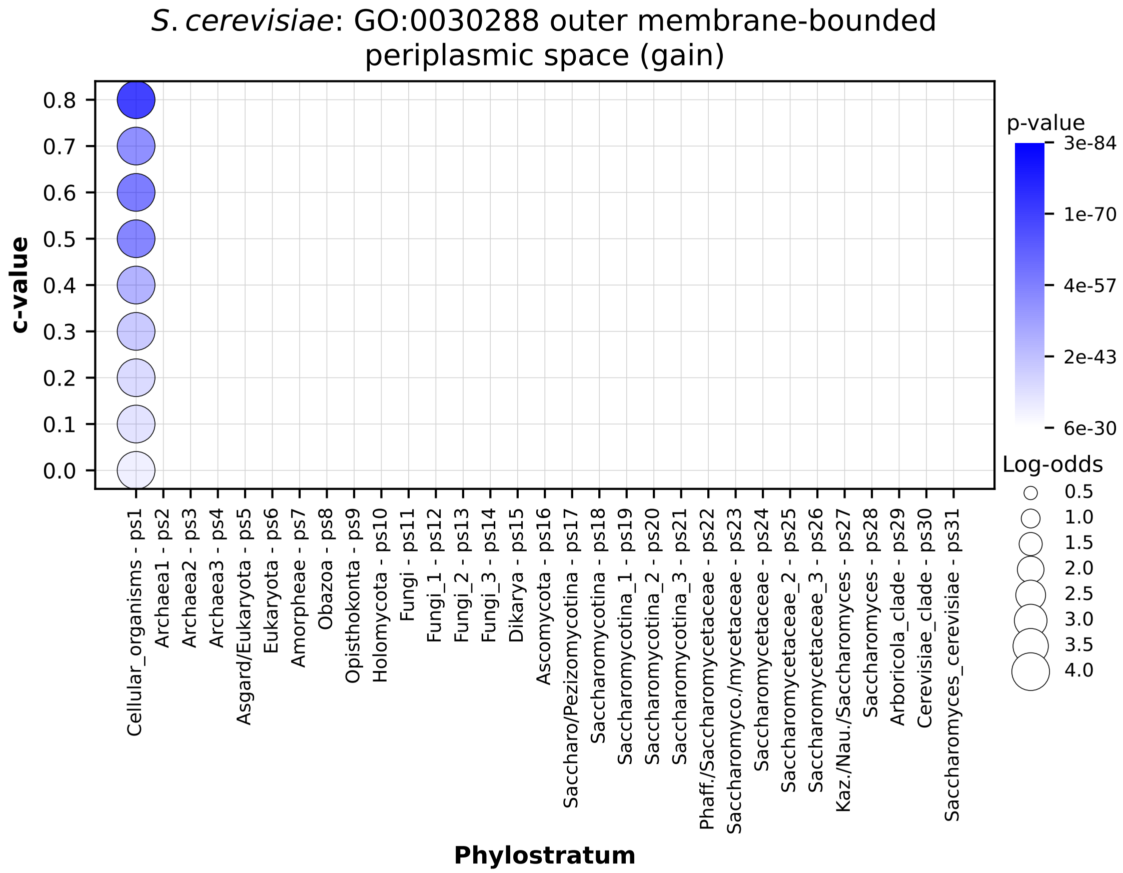

S. cerevisiae: GO:0030288 outer membrane-bounded periplasmic space (gain)
c-value
0.0
0.1
0.2
0.3
0.4
0.5
0.6
0.7
0.8
Phylostratum
Cellular_organisms - ps1
Archaea1 - ps2
Archaea2 - ps3
Archaea3 - ps4
Asgard/Eukaryota - ps5
Eukaryota - ps6
Amorpheae - ps7
Obazoa - ps8
Opisthokonta - ps9
Holomycota - ps10
Fungi - ps11
Fungi_1 - ps12
Fungi_2 - ps13
Fungi_3 - ps14
Dikarya - ps15
Ascomycota - ps16
Saccharo/Pezizomycotina - ps17
Saccharomycotina - ps18
Saccharomycotina_1 - ps19
Saccharomycotina_2 - ps20
Saccharomycotina_3 - ps21
Phaff./Saccharomycetaceae - ps22
Saccharomyco./mycetaceae - ps23
Saccharomycetaceae - ps24
Saccharomycetaceae_2 - ps25
Saccharomycetaceae_3 - ps26
Kaz./Nau./Saccharomyces - ps27
Saccharomyces - ps28
Arboricola_clade - ps29
Cerevisiae_clade - ps30
Saccharomyces_cerevisiae - ps31
p-value
3e-84
1e-70
4e-57
2e-43
6e-30
Log-odds
0.5
1.0
1.5
2.0
2.5
3.0
3.5
4.0

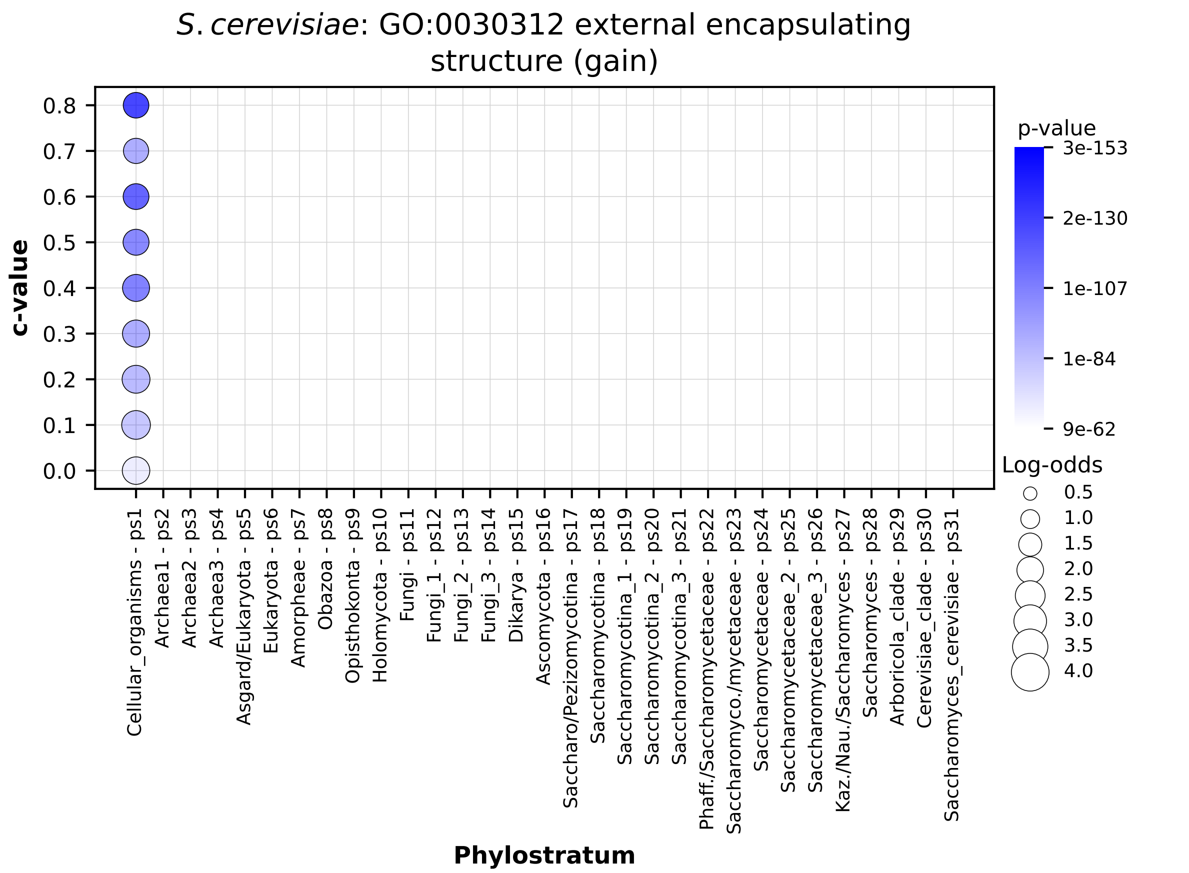

S. cerevisiae: GO:0030312 external encapsulating structure (gain)
c-value
0.8
0.7
0.6
0.5
0.4
0.3
0.2
0.1
0.0
p-value
3e-153
2e-130
1e-107
1e-84
9e-62
Log-odds
0.5
1.0
1.5
2.0
2.5
3.0
3.5
4.0
Cellular_organisms - ps1
Archaea1 - ps2
Archaea2 - ps3
Archaea3 - ps4
Asgard/Eukaryota - ps5
Eukaryota - ps6
Amorpheae - ps7
Obazoa - ps8
Opisthokonta - ps9
Holomycota - ps10
Fungi - ps11
Fungi_1 - ps12
Fungi_2 - ps13
Fungi_3 - ps14
Dikarya - ps15
Ascomycota - ps16
Saccharo/Pezizomycotina - ps17
Saccharomycotina - ps18
Saccharomycotina_1 - ps19
Saccharomycotina_2 - ps20
Saccharomycotina_3 - ps21
Phaff./Saccharomycetaceae - ps22
Saccharomyco./mycetaceae - ps23
Saccharomycetaceae - ps24
Saccharomycetaceae_2 - ps25
Saccharomycetaceae_3 - ps26
Kaz./Nau./Saccharomyces - ps27
Saccharomyces - ps28
Arboricola_clade - ps29
Cerevisiae_clade - ps30
Saccharomyces_cerevisiae - ps31
Phylostratum

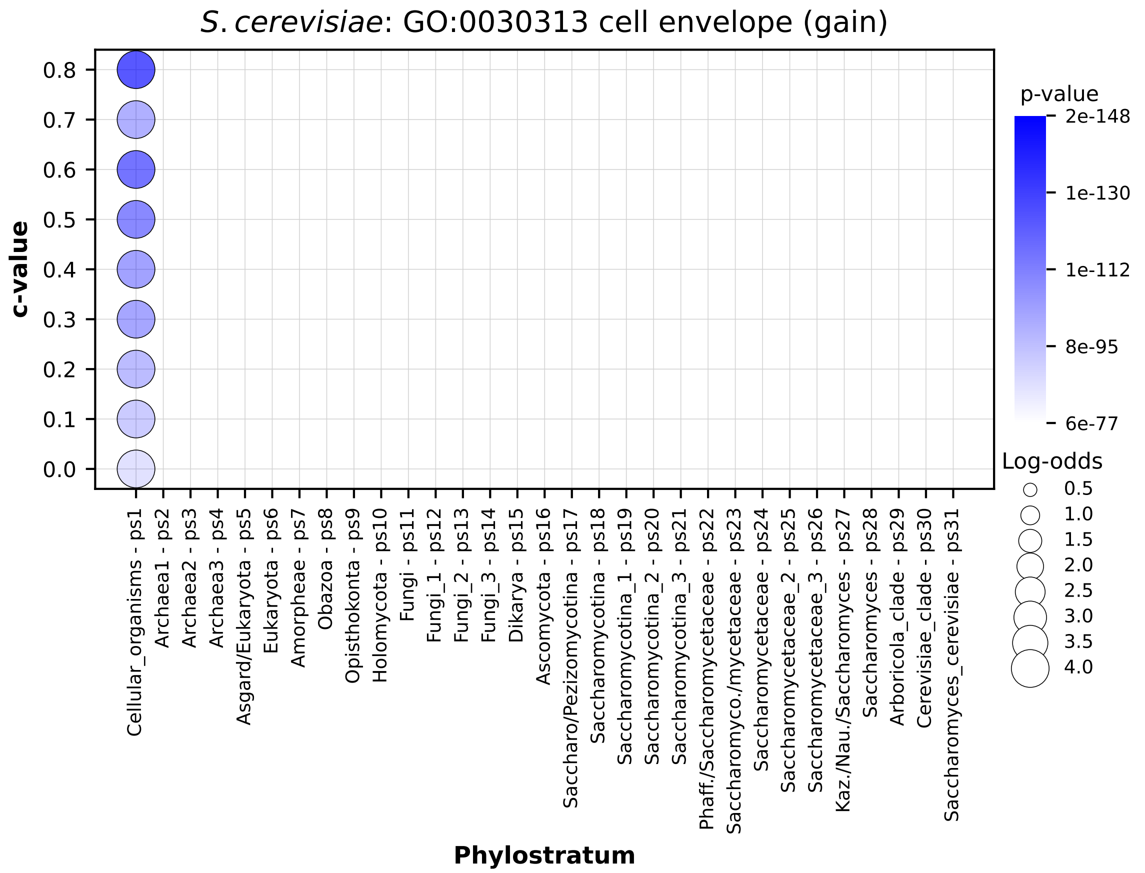
S. cerevisiae: GO:0030313 cell envelope (gain)
c-value
0.8
0.7
0.6
0.5
0.4
0.3
0.2
0.1
0.0
Phylostratum
Cellular_organisms - ps1
Archaea1 - ps2
Archaea2 - ps3
Archaea3 - ps4
Asgard/Eukaryota - ps5
Eukaryota - ps6
Amorpheae - ps7
Obazoa - ps8
Opisthokonta - ps9
Holomycota - ps10
Fungi - ps11
Fungi_1 - ps12
Fungi_2 - ps13
Fungi_3 - ps14
Dikarya - ps15
Ascomycota - ps16
Saccharo/Pezizomycotina - ps17
Saccharomycotina - ps18
Saccharomycotina_1 - ps19
Saccharomycotina_2 - ps20
Saccharomycotina_3 - ps21
Phaff./Saccharomycetaceae - ps22
Saccharomyco./mycetaceae - ps23
Saccharomycetaceae - ps24
Saccharomycetaceae_2 - ps25
Saccharomycetaceae_3 - ps26
Kaz./Nau./Saccharomyces - ps27
Saccharomyces - ps28
Arboricola_clade - ps29
Cerevisiae_clade - ps30
Saccharomyces_cerevisiae - ps31
p-value
2e-148
1e-130
1e-112
8e-95
6e-77
Log-odds
0.5
1.0
1.5
2.0
2.5
3.0
3.5
4.0

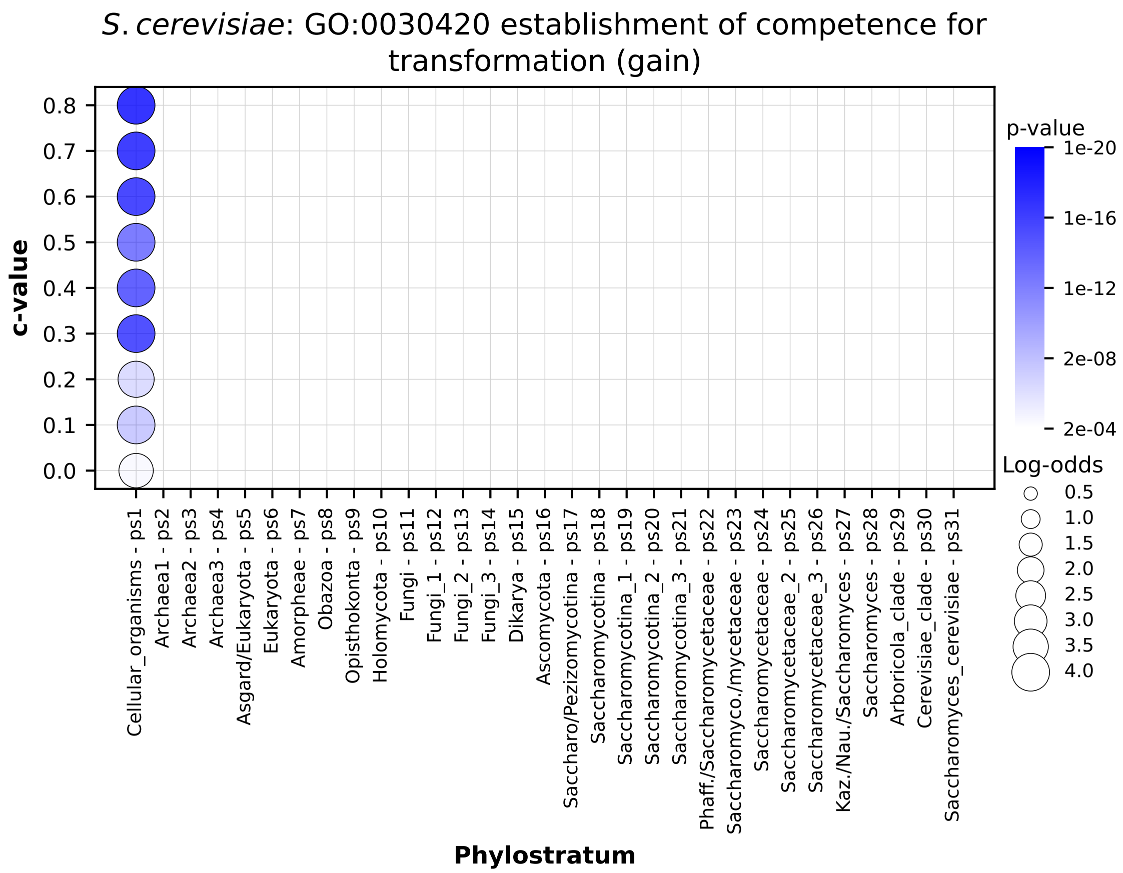
S. cerevisiae: GO:0030420 establishment of competence for transformation (gain)
c-value
0.8
0.7
0.6
0.5
0.4
0.3
0.2
0.1
0.0
p-value
1e-20
1e-16
1e-12
2e-08
2e-04
Log-odds
0.5
1.0
1.5
2.0
2.5
3.0
3.5
4.0
Cellular_organisms - ps1
Archaea1 - ps2
Archaea2 - ps3
Archaea3 - ps4
Asgard/Eukaryota - ps5
Eukaryota - ps6
Amorpheae - ps7
Obazoa - ps8
Opisthokonta - ps9
Holomycota - ps10
Fungi - ps11
Fungi_1 - ps12
Fungi_2 - ps13
Fungi_3 - ps14
Dikarya - ps15
Ascomycota - ps16
Saccharo/Pezizomycotina - ps17
Saccharomycotina - ps18
Saccharomycotina_1 - ps19
Saccharomycotina_2 - ps20
Saccharomycotina_3 - ps21
Phaff./Saccharomycetaceae - ps22
Saccharomyco./mycetaceae - ps23
Saccharomycetaceae - ps24
Saccharomycetaceae_2 - ps25
Saccharomycetaceae_3 - ps26
Kaz./Nau./Saccharomyces - ps27
Saccharomyces - ps28
Arboricola_clade - ps29
Cerevisiae_clade - ps30
Saccharomyces_cerevisiae - ps31
Phylostratum

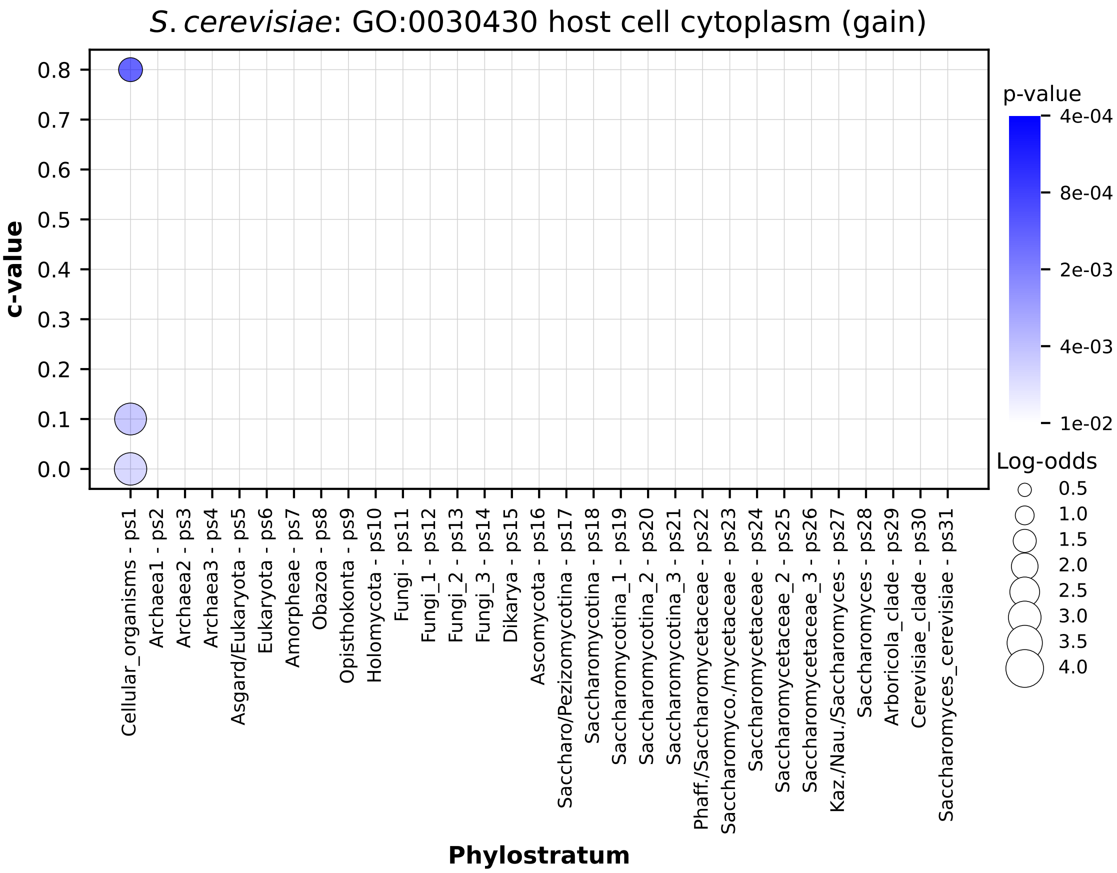

S. cerevisiae: GO:0030430 host cell cytoplasm (gain)
c-value
0.8
0.7
0.6
0.5
0.4
0.3
0.2
0.1
0.0
p-value
4e-04
8e-04
2e-03
4e-03
1e-02
Log-odds
0.5
1.0
1.5
2.0
2.5
3.0
3.5
4.0
Cellular_organisms - ps1
Archaea1 - ps2
Archaea2 - ps3
Archaea3 - ps4
Asgard/Eukaryota - ps5
Eukaryota - ps6
Amorpheae - ps7
Obazoa - ps8
Opisthokonta - ps9
Holomycota - ps10
Fungi - ps11
Fungi_1 - ps12
Fungi_2 - ps13
Fungi_3 - ps14
Dikarya - ps15
Ascomycota - ps16
Saccharo/Pezizomycotina - ps17
Saccharomycotina - ps18
Saccharomycotina_1 - ps19
Saccharomycotina_2 - ps20
Saccharomycotina_3 - ps21
Phaff./Saccharomycetaceae - ps22
Saccharomyco./mycetaceae - ps23
Saccharomycetaceae - ps24
Saccharomycetaceae_2 - ps25
Saccharomycetaceae_3 - ps26
Kaz./Nau./Saccharomyces - ps27
Saccharomyces - ps28
Arboricola_clade - ps29
Cerevisiae_clade - ps30
Saccharomyces_cerevisiae - ps31
Phylostratum

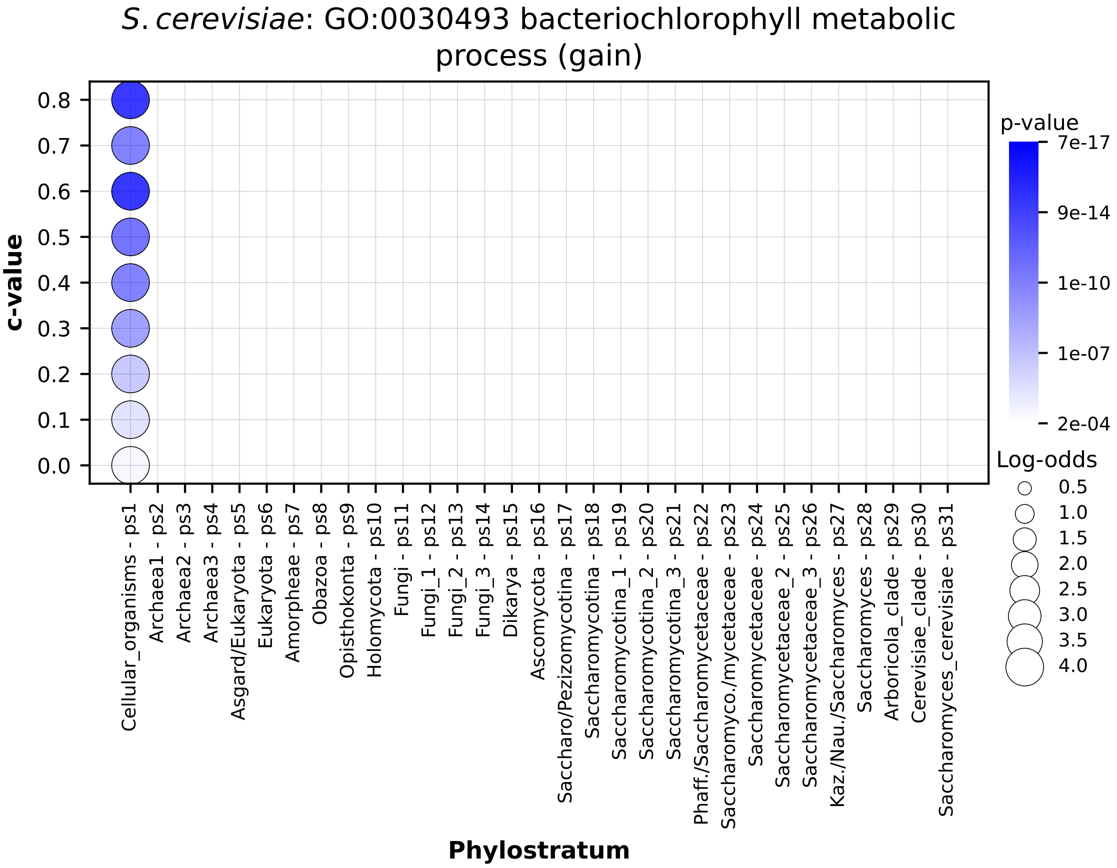

S. cerevisiae: GO:0030493 bacteriochlorophyll metabolic process (gain)
c-value
0.8
0.7
0.6
0.5
0.4
0.3
0.2
0.1
0.0
p-value
7e-17
9e-14
1e-10
1e-07
2e-04
Log-odds
0.5
1.0
1.5
2.0
2.5
3.0
3.5
4.0
Cellular_organisms - ps1
Archaea1 - ps2
Archaea2 - ps3
Archaea3 - ps4
Asgard/Eukaryota - ps5
Eukaryota - ps6
Amorpheae - ps7
Obazoa - ps8
Opisthokonta - ps9
Holomycota - ps10
Fungi - ps11
Fungi_1 - ps12
Fungi_2 - ps13
Fungi_3 - ps14
Dikarya - ps15
Ascomycota - ps16
Saccharo/Pezizomycotina - ps17
Saccharomycotina - ps18
Saccharomycotina_1 - ps19
Saccharomycotina_2 - ps20
Saccharomycotina_3 - ps21
Phaff./Saccharomycetaceae - ps22
Saccharomyco./mycetaceae - ps23
Saccharomycetaceae - ps24
Saccharomycetaceae_2 - ps25
Saccharomycetaceae_3 - ps26
Kaz./Nau./Saccharomyces - ps27
Saccharomyces - ps28
Arboricola_clade - ps29
Cerevisiae_clade - ps30
Saccharomyces_cerevisiae - ps31
Phylostratum

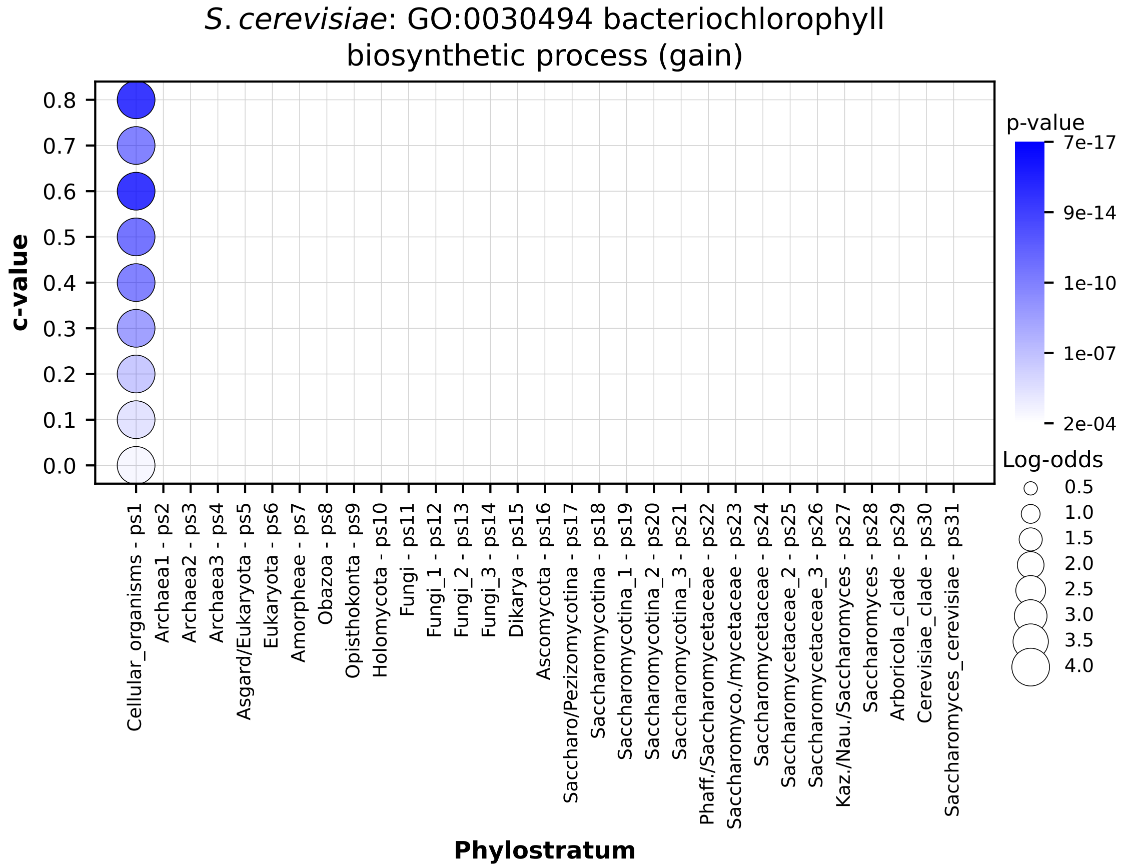
S. cerevisiae: GO:0030494 bacteriochlorophyll biosynthetic process (gain)
c-value
0.8
0.7
0.6
0.5
0.4
0.3
0.2
0.1
0.0
p-value
7e-17
9e-14
1e-10
1e-07
2e-04
Log-odds
0.5
1.0
1.5
2.0
2.5
3.0
3.5
4.0
Cellular_organisms - ps1
Archaea1 - ps2
Archaea2 - ps3
Archaea3 - ps4
Asgard/Eukaryota - ps5
Eukaryota - ps6
Amorpheae - ps7
Obazoa - ps8
Opisthokonta - ps9
Holomycota - ps10
Fungi - ps11
Fungi_1 - ps12
Fungi_2 - ps13
Fungi_3 - ps14
Dikarya - ps15
Ascomycota - ps16
Saccharo/Pezizomycotina - ps17
Saccharomycotina - ps18
Saccharomycotina_1 - ps19
Saccharomycotina_2 - ps20
Saccharomycotina_3 - ps21
Phaff./Saccharomycetaceae - ps22
Saccharomyco./mycetaceae - ps23
Saccharomycetaceae - ps24
Saccharomycetaceae_2 - ps25
Saccharomycetaceae_3 - ps26
Kaz./Nau./Saccharomyces - ps27
Saccharomyces - ps28
Arboricola_clade - ps29
Cerevisiae_clade - ps30
Saccharomyces_cerevisiae - ps31
Phylostratum

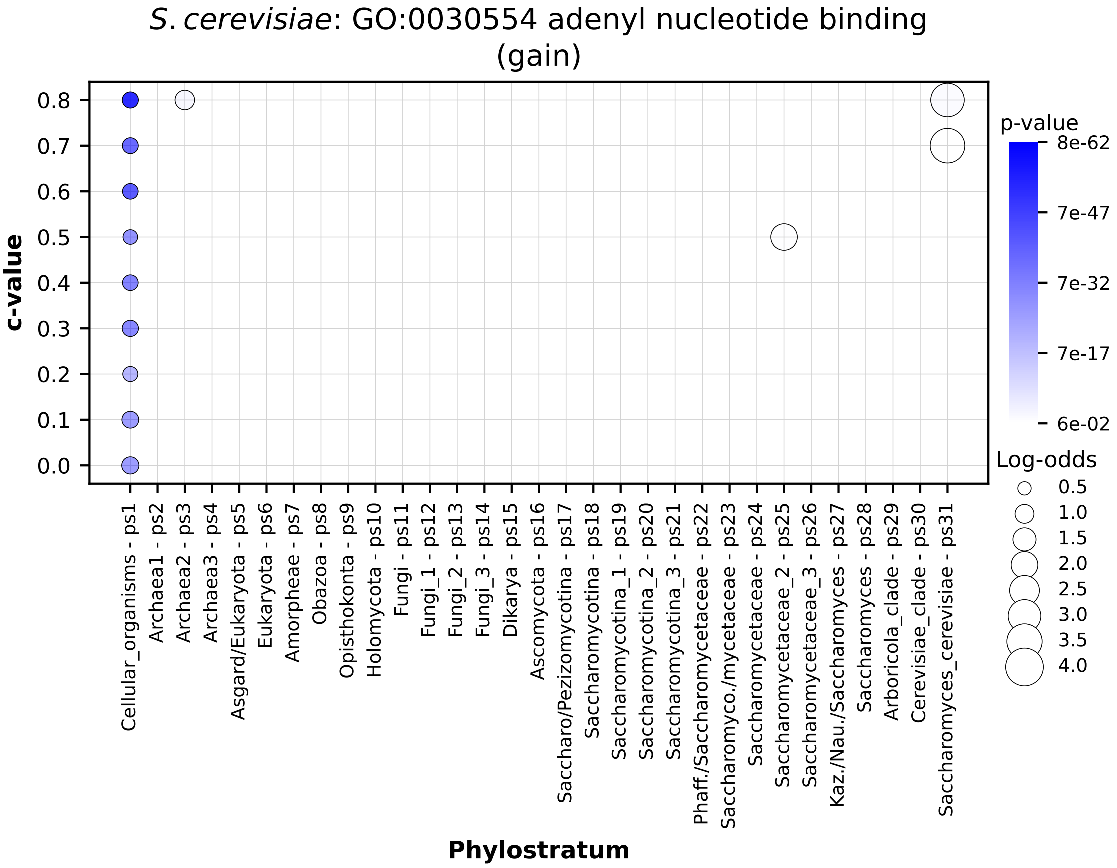
S. cerevisiae: GO:0030554 adenyl nucleotide binding
(gain)
c-value
0.8
0.7
0.6
0.5
0.4
0.3
0.2
0.1
0.0
p-value
8e-62
7e-47
7e-32
7e-17
6e-02
Log-odds
0.5
1.0
1.5
2.0
2.5
3.0
3.5
4.0
Cellular_organisms - ps1
Archaea1 - ps2
Archaea2 - ps3
Archaea3 - ps4
Asgard/Eukaryota - ps5
Eukaryota - ps6
Amorpheae - ps7
Obazoa - ps8
Opisthokonta - ps9
Holomycota - ps10
Fungi - ps11
Fungi_1 - ps12
Fungi_2 - ps13
Fungi_3 - ps14
Dikarya - ps15
Ascomycota - ps16
Saccharo/Pezizomycotina - ps17
Saccharomycotina - ps18
Saccharomycotina_1 - ps19
Saccharomycotina_2 - ps20
Saccharomycotina_3 - ps21
Phaff./Saccharomycetaceae - ps22
Saccharomyco./mycetaceae - ps23
Saccharomycetaceae - ps24
Saccharomycetaceae_2 - ps25
Saccharomycetaceae_3 - ps26
Kaz./Nau./Saccharomyces - ps27
Saccharomyces - ps28
Arboricola_clade - ps29
Cerevisiae_clade - ps30
Saccharomyces_cerevisiae - ps31
Phylostratum

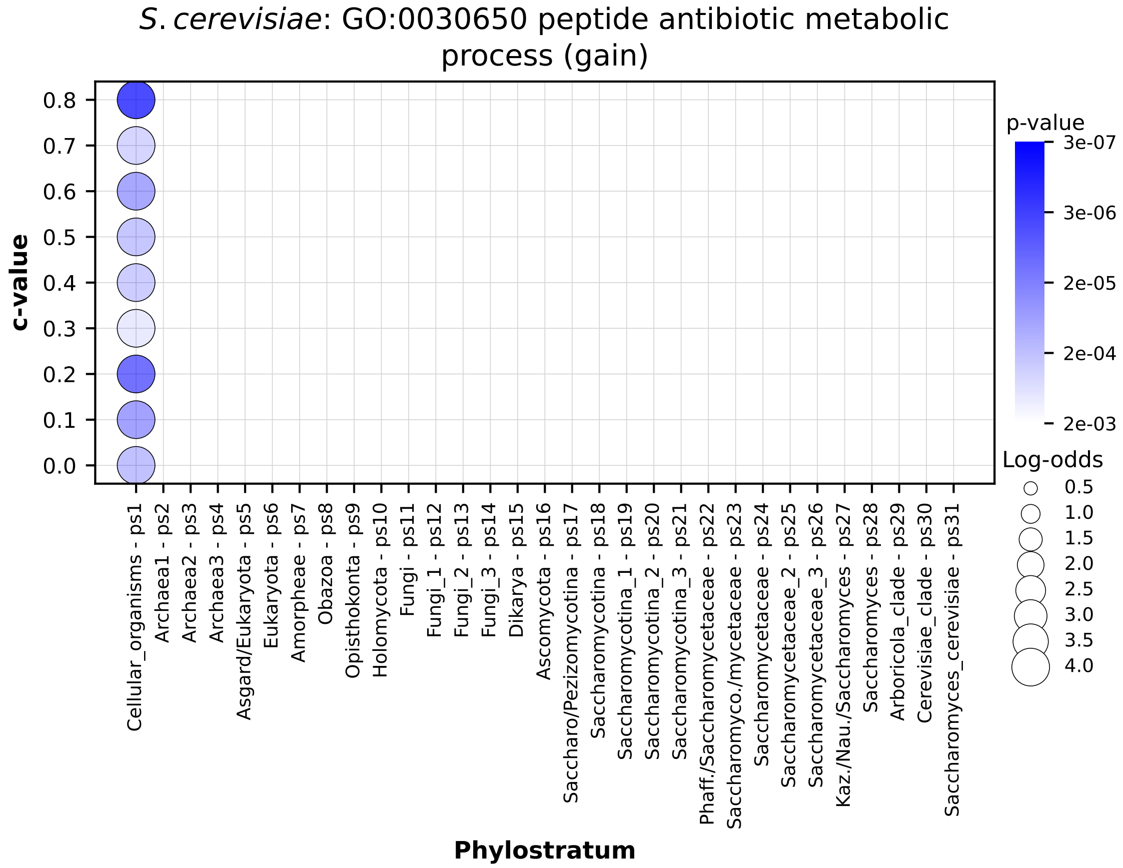
S. cerevisiae: GO:0030650 peptide antibiotic metabolic process (gain)
c-value
0.8
0.7
0.6
0.5
0.4
0.3
0.2
0.1
0.0
Phylostratum
Cellular_organisms - ps1
Archaea1 - ps2
Archaea2 - ps3
Archaea3 - ps4
Asgard/Eukaryota - ps5
Eukaryota - ps6
Amorpheae - ps7
Obazoa - ps8
Opisthokonta - ps9
Holomycota - ps10
Fungi - ps11
Fungi_1 - ps12
Fungi_2 - ps13
Fungi_3 - ps14
Dikarya - ps15
Ascomycota - ps16
Saccharo/Pezizomycotina - ps17
Saccharomycotina - ps18
Saccharomycotina_1 - ps19
Saccharomycotina_2 - ps20
Saccharomycotina_3 - ps21
Phaff./Saccharomycetaceae - ps22
Saccharomyco./mycetaceae - ps23
Saccharomycetaceae - ps24
Saccharomycetaceae_2 - ps25
Saccharomycetaceae_3 - ps26
Kaz./Nau./Saccharomyces - ps27
Saccharomyces - ps28
Arboricola_clade - ps29
Cerevisiae_clade - ps30
Saccharomyces_cerevisiae - ps31
p-value
3e-07
3e-06
2e-05
2e-04
2e-03
Log-odds
0.5
1.0
1.5
2.0
2.5
3.0
3.5
4.0

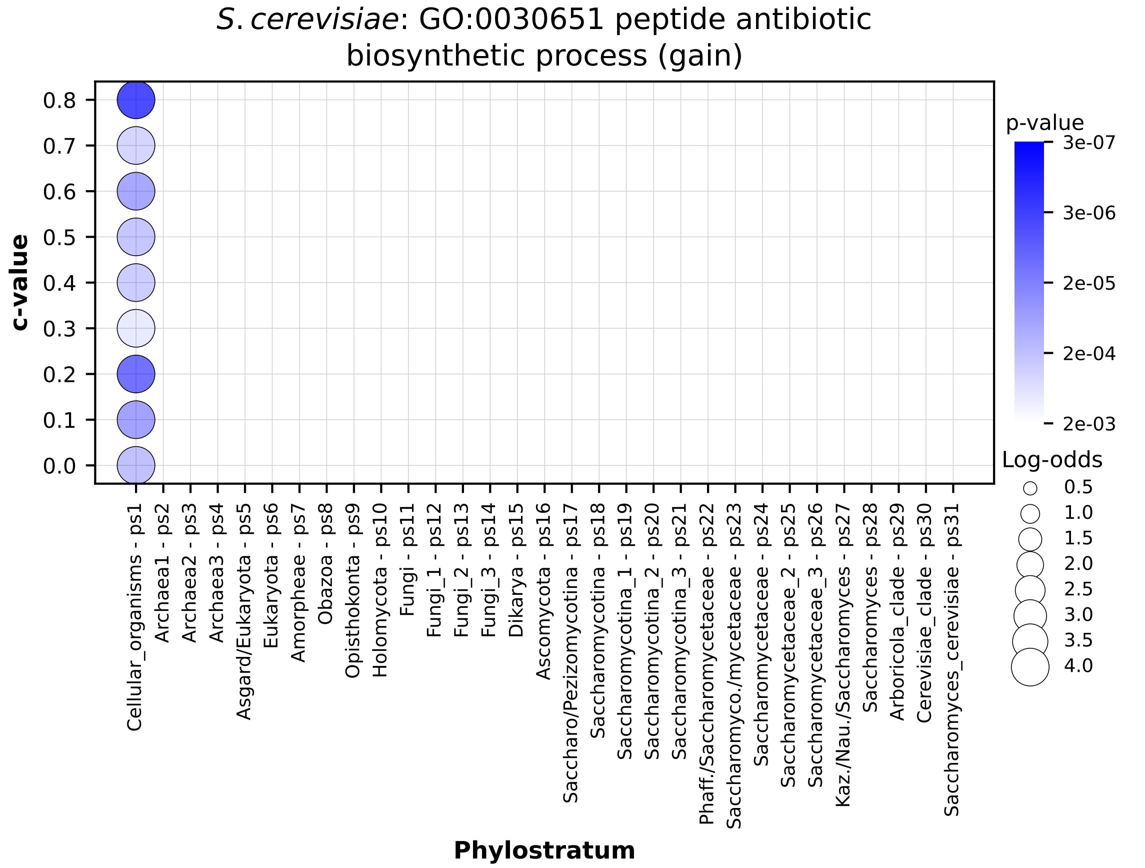

S. cerevisiae: GO:0030651 peptide antibiotic biosynthetic process (gain)
c-value
0.0
0.1
0.2
0.3
0.4
0.5
0.6
0.7
0.8
p-value
3e-07
3e-06
2e-05
2e-04
2e-03
Log-odds
0.5
1.0
1.5
2.0
2.5
3.0
3.5
4.0
Cellular_organisms - ps1
Archaea1 - ps2
Archaea2 - ps3
Archaea3 - ps4
Asgard/Eukaryota - ps5
Eukaryota - ps6
Amorpheae - ps7
Obazoa - ps8
Opisthokonta - ps9
Holomycota - ps10
Fungi - ps11
Fungi_1 - ps12
Fungi_2 - ps13
Fungi_3 - ps14
Dikarya - ps15
Ascomycota - ps16
Saccharo/Pezizomycotina - ps17
Saccharomycotina - ps18
Saccharomycotina_1 - ps19
Saccharomycotina_2 - ps20
Saccharomycotina_3 - ps21
Phaff./Saccharomycetaceae - ps22
Saccharomyco./mycetaceae - ps23
Saccharomycetaceae - ps24
Saccharomycetaceae_2 - ps25
Saccharomycetaceae_3 - ps26
Kaz./Nau./Saccharomyces - ps27
Saccharomyces - ps28
Arboricola_clade - ps29
Cerevisiae_clade - ps30
Saccharomyces_cerevisiae - ps31
Phylostratum

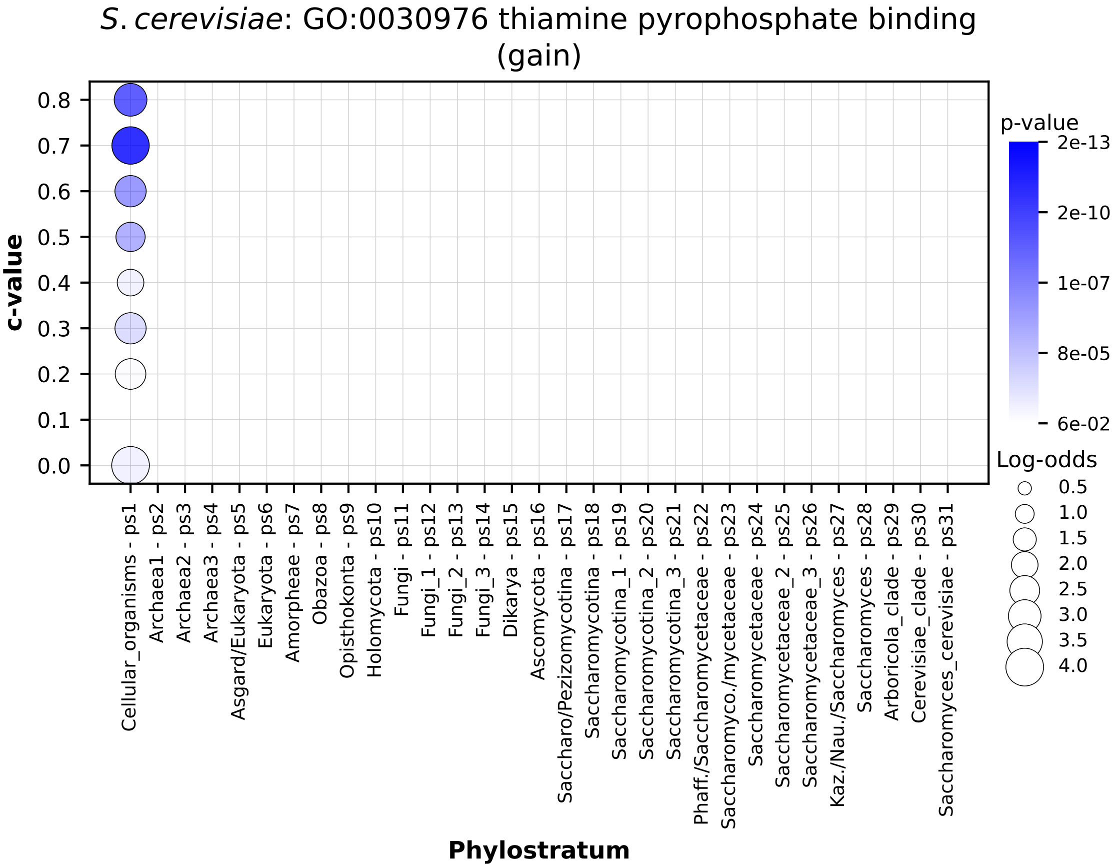
S. cerevisiae: GO:0030976 thiamine pyrophosphate binding (gain)
c-value
0.8
0.7
0.6
0.5
0.4
0.3
0.2
0.1
0.0
Phylostratum
Cellular_organisms - ps1
Archaea1 - ps2
Archaea2 - ps3
Archaea3 - ps4
Asgard/Eukaryota - ps5
Eukaryota - ps6
Amorpheae - ps7
Obazoa - ps8
Opisthokonta - ps9
Holomycota - ps10
Fungi - ps11
Fungi_1 - ps12
Fungi_2 - ps13
Fungi_3 - ps14
Dikarya - ps15
Ascomycota - ps16
Saccharo/Pezizomycotina - ps17
Saccharomycotina - ps18
Saccharomycotina_1 - ps19
Saccharomycotina_2 - ps20
Saccharomycotina_3 - ps21
Phaff./Saccharomycetaceae - ps22
Saccharomyco./mycetaceae - ps23
Saccharomycetaceae - ps24
Saccharomycetaceae_2 - ps25
Saccharomycetaceae_3 - ps26
Kaz./Nau./Saccharomyces - ps27
Saccharomyces - ps28
Arboricola_clade - ps29
Cerevisiae_clade - ps30
Saccharomyces_cerevisiae - ps31
p-value
2e-13
2e-10
1e-07
8e-05
6e-02
Log-odds
0.5
1.0
1.5
2.0
2.5
3.0
3.5
4.0

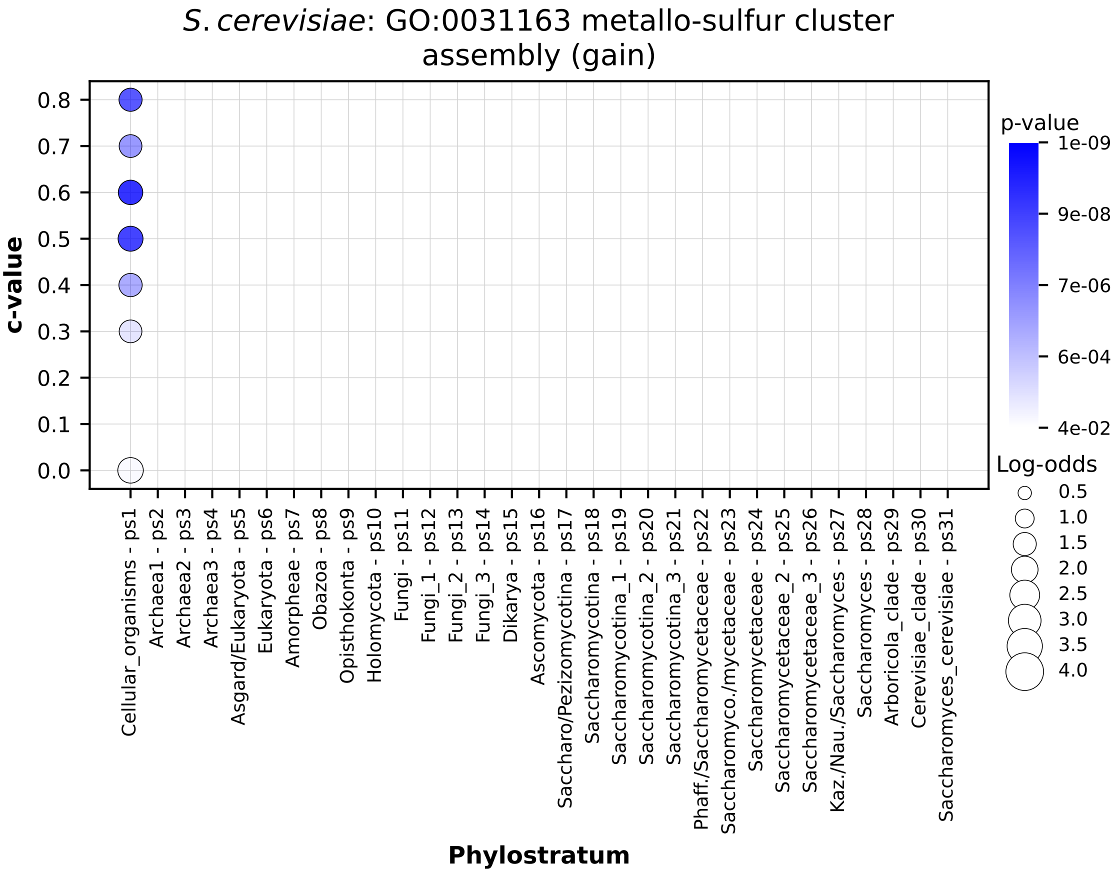

S. cerevisiae: GO:0031163 metallo-sulfur cluster assembly (gain)
c-value
0.8
0.7
0.6
0.5
0.4
0.3
0.2
0.1
0.0
Phylostratum
Cellular_organisms - ps1
Archaea1 - ps2
Archaea2 - ps3
Archaea3 - ps4
Asgard/Eukaryota - ps5
Eukaryota - ps6
Amorpheae - ps7
Obazoa - ps8
Opisthokonta - ps9
Holomycota - ps10
Fungi - ps11
Fungi_1 - ps12
Fungi_2 - ps13
Fungi_3 - ps14
Dikarya - ps15
Ascomycota - ps16
Saccharo/Pezizomycotina - ps17
Saccharomycotina - ps18
Saccharomycotina_1 - ps19
Saccharomycotina_2 - ps20
Saccharomycotina_3 - ps21
Phaff./Saccharomycetaceae - ps22
Saccharomyco./mycetaceae - ps23
Saccharomycetaceae - ps24
Saccharomycetaceae_2 - ps25
Saccharomycetaceae_3 - ps26
Kaz./Nau./Saccharomyces - ps27
Saccharomyces - ps28
Arboricola_clade - ps29
Cerevisiae_clade - ps30
Saccharomyces_cerevisiae - ps31
p-value
1e-09
9e-08
7e-06
6e-04
4e-02
Log-odds
0.5
1.0
1.5
2.0
2.5
3.0
3.5
4.0

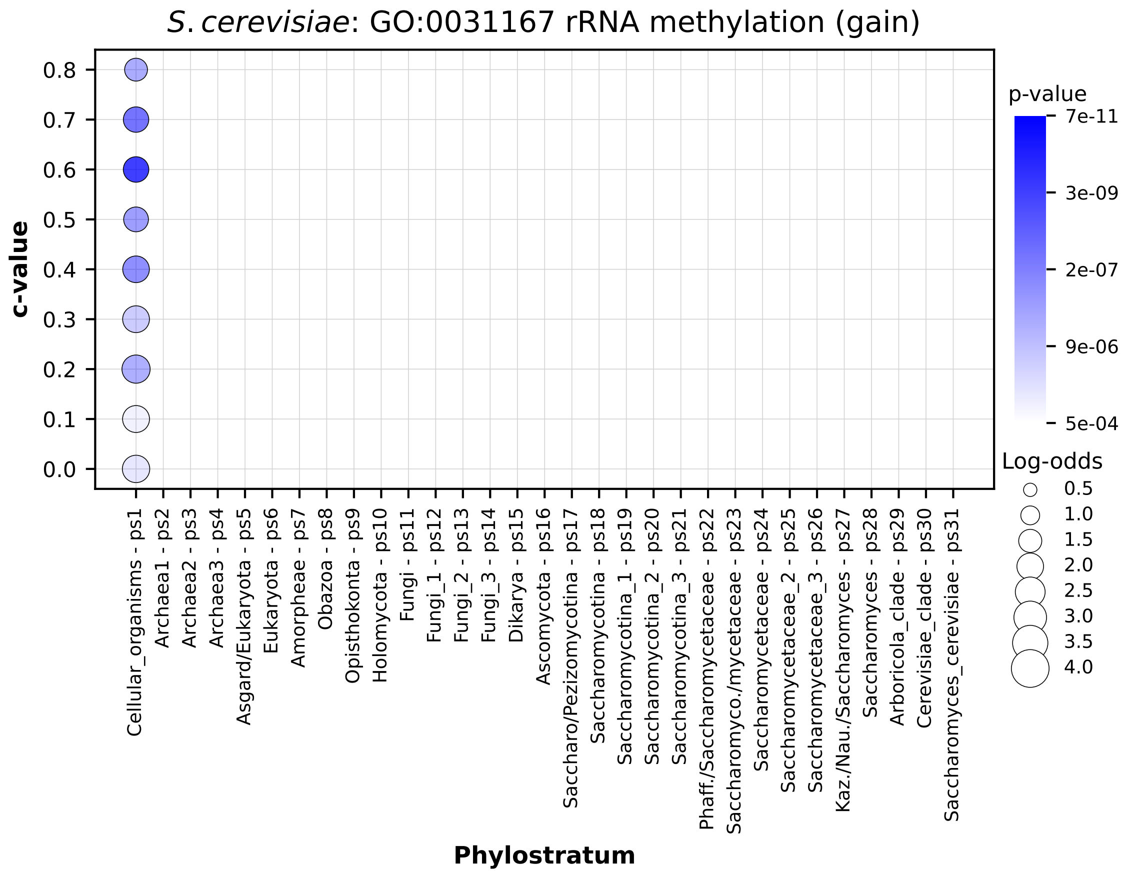

S. cerevisiae: GO:0031167 rRNA methylation (gain)
c-value
0.8
0.7
0.6
0.5
0.4
0.3
0.2
0.1
0.0
p-value
7e-11
3e-09
2e-07
9e-06
5e-04
Log-odds
0.5
1.0
1.5
2.0
2.5
3.0
3.5
4.0
Cellular_organisms - ps1
Archaea1 - ps2
Archaea2 - ps3
Archaea3 - ps4
Asgard/Eukaryota - ps5
Eukaryota - ps6
Amorpheae - ps7
Obazoa - ps8
Opisthokonta - ps9
Holomycota - ps10
Fungi - ps11
Fungi_1 - ps12
Fungi_2 - ps13
Fungi_3 - ps14
Dikarya - ps15
Ascomycota - ps16
Saccharo/Pezizomycotina - ps17
Saccharomycotina - ps18
Saccharomycotina_1 - ps19
Saccharomycotina_2 - ps20
Saccharomycotina_3 - ps21
Phaff./Saccharomycetaceae - ps22
Saccharomyco./mycetaceae - ps23
Saccharomycetaceae - ps24
Saccharomycetaceae_2 - ps25
Saccharomycetaceae_3 - ps26
Kaz./Nau./Saccharomyces - ps27
Saccharomyces - ps28
Arboricola_clade - ps29
Cerevisiae_clade - ps30
Saccharomyces_cerevisiae - ps31
Phylostratum

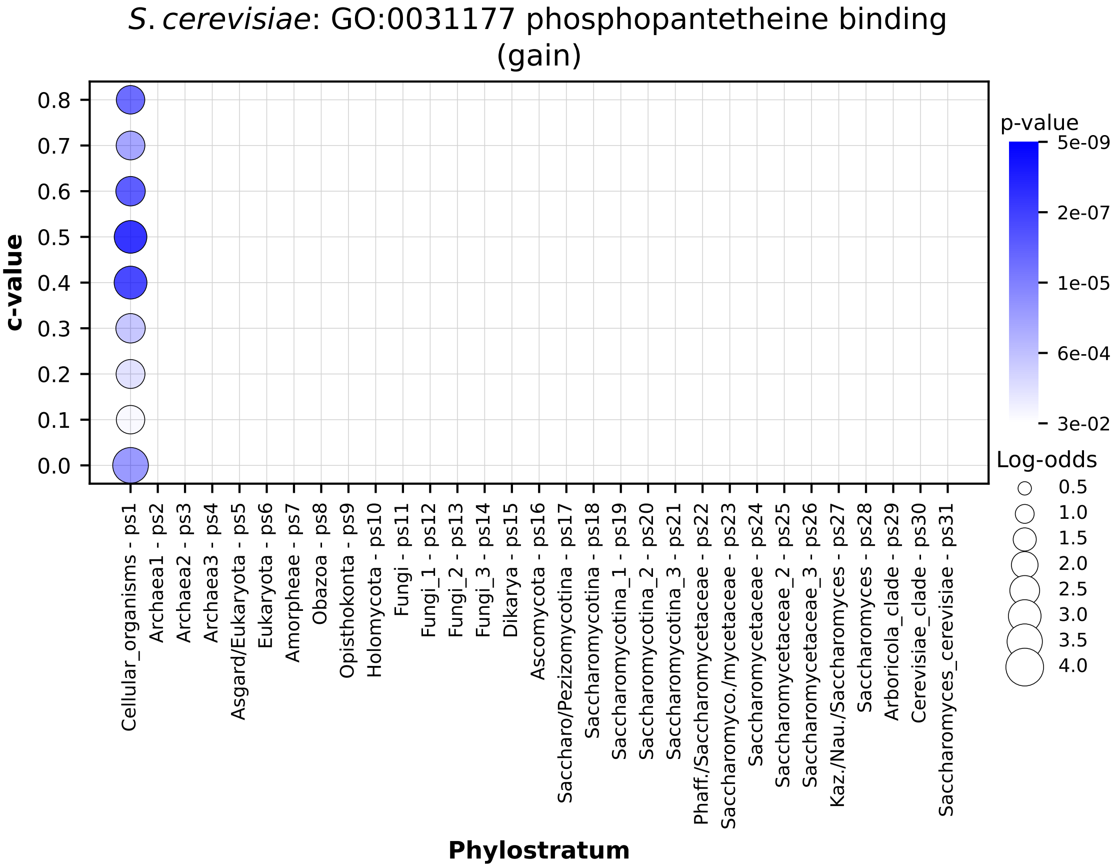
S. cerevisiae: GO:0031177 phosphopantetheine binding
(gain)
c-value
0.8
0.7
0.6
0.5
0.4
0.3
0.2
0.1
0.0
p-value
5e-09
2e-07
1e-05
6e-04
3e-02
Log-odds
0.5
1.0
1.5
2.0
2.5
3.0
3.5
4.0
Cellular_organisms - ps1
Archaea1 - ps2
Archaea2 - ps3
Archaea3 - ps4
Asgard/Eukaryota - ps5
Eukaryota - ps6
Amorpheae - ps7
Obazoa - ps8
Opisthokonta - ps9
Holomycota - ps10
Fungi - ps11
Fungi_1 - ps12
Fungi_2 - ps13
Fungi_3 - ps14
Dikarya - ps15
Ascomycota - ps16
Saccharo/Pezizomycotina - ps17
Saccharomycotina - ps18
Saccharomycotina_1 - ps19
Saccharomycotina_2 - ps20
Saccharomycotina_3 - ps21
Phaff./Saccharomycetaceae - ps22
Saccharomyco./mycetaceae - ps23
Saccharomycetaceae - ps24
Saccharomycetaceae_2 - ps25
Saccharomycetaceae_3 - ps26
Kaz./Nau./Saccharomyces - ps27
Saccharomyces - ps28
Arboricola_clade - ps29
Cerevisiae_clade - ps30
Saccharomyces_cerevisiae - ps31
Phylostratum

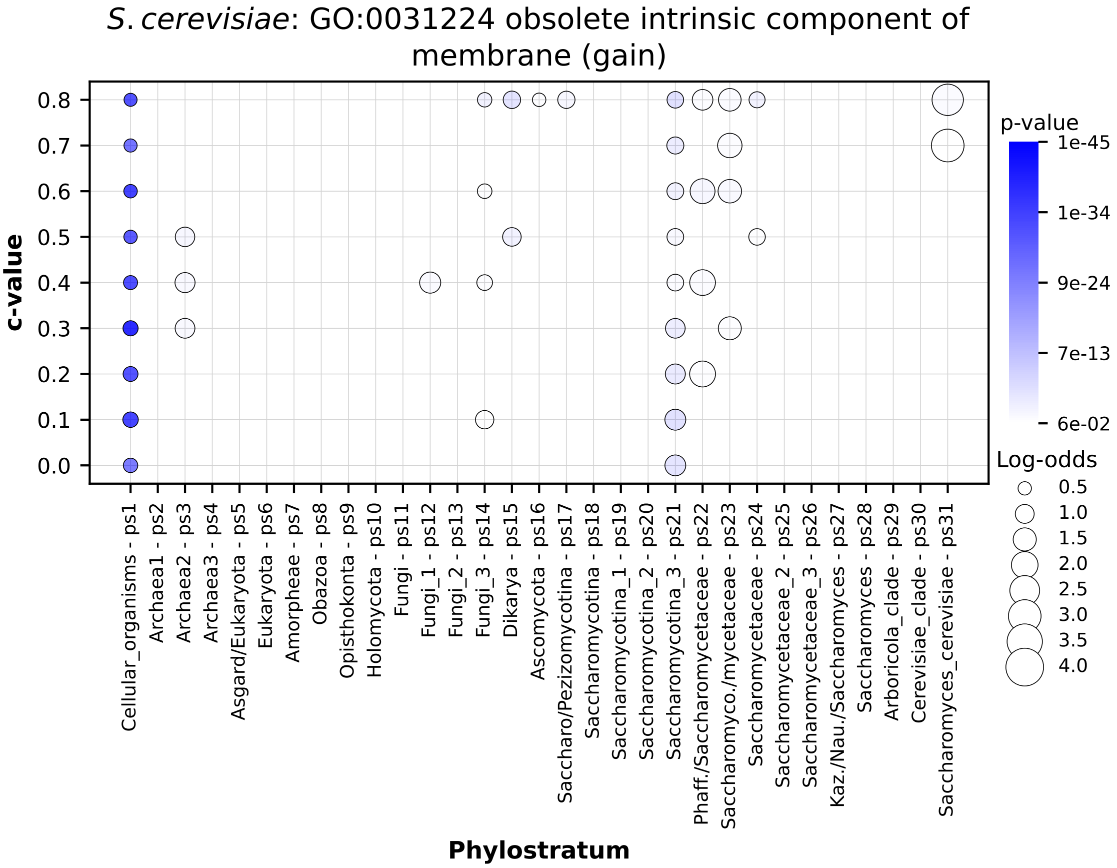
S. cerevisiae: GO:0031224 obsolete intrinsic component of membrane (gain)
c-value
0.8
0.7
0.6
0.5
0.4
0.3
0.2
0.1
0.0
p-value
1e-45
1e-34
9e-24
7e-13
6e-02
Log-odds
0.5
1.0
1.5
2.0
2.5
3.0
3.5
4.0
Cellular_organisms - ps1
Archaea1 - ps2
Archaea2 - ps3
Archaea3 - ps4
Asgard/Eukaryota - ps5
Eukaryota - ps6
Amorpheae - ps7
Obazoa - ps8
Opisthokonta - ps9
Holomycota - ps10
Fungi - ps11
Fungi_1 - ps12
Fungi_2 - ps13
Fungi_3 - ps14
Dikarya - ps15
Ascomycota - ps16
Saccharo/Pezizomycotina - ps17
Saccharomycotina - ps18
Saccharomycotina_1 - ps19
Saccharomycotina_2 - ps20
Saccharomycotina_3 - ps21
Phaff./Saccharomycetaceae - ps22
Saccharomyco./mycetaceae - ps23
Saccharomycetaceae - ps24
Saccharomycetaceae_2 - ps25
Saccharomycetaceae_3 - ps26
Kaz./Nau./Saccharomyces - ps27
Saccharomyces - ps28
Arboricola_clade - ps29
Cerevisiae_clade - ps30
Saccharomyces_cerevisiae - ps31
Phylostratum

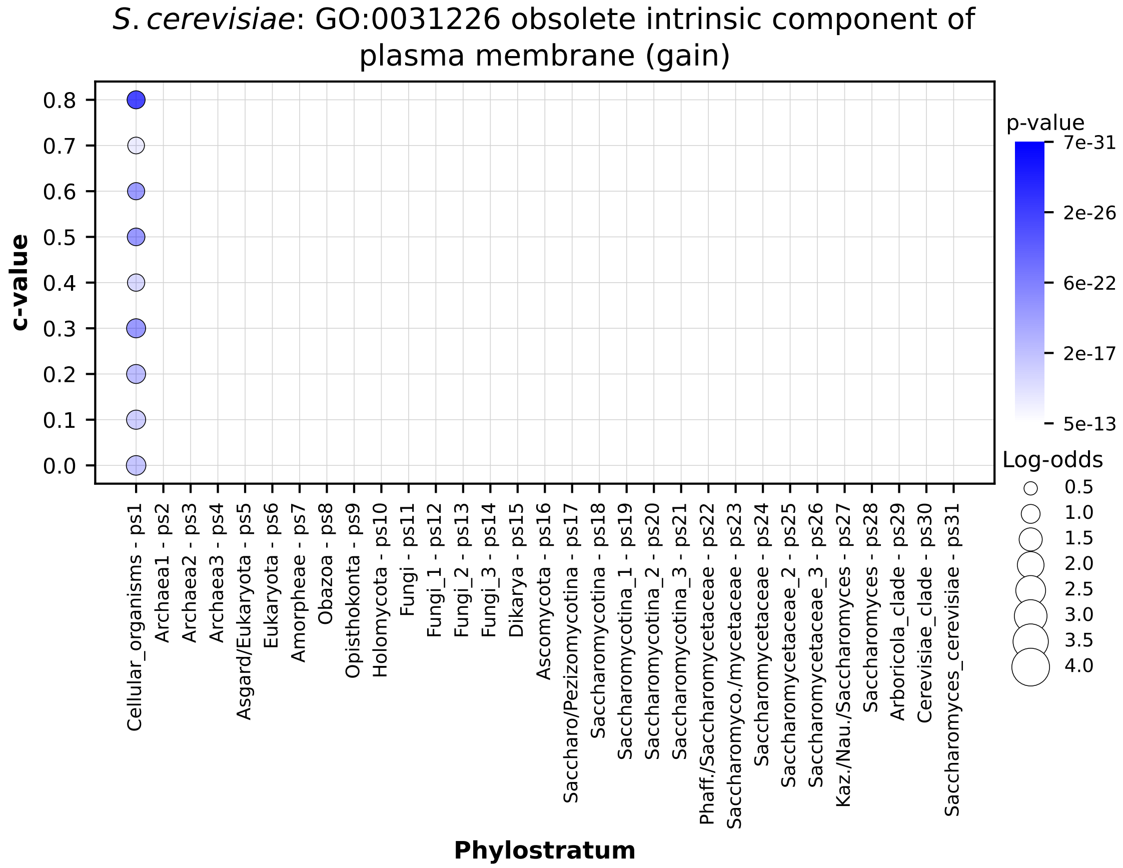
S. cerevisiae: GO:0031226 obsolete intrinsic component of plasma membrane (gain)
c-value
0.0
0.1
0.2
0.3
0.4
0.5
0.6
0.7
0.8
p-value
7e-31
2e-26
6e-22
2e-17
5e-13
Log-odds
0.5
1.0
1.5
2.0
2.5
3.0
3.5
4.0
Cellular_organisms - ps1
Archaea1 - ps2
Archaea2 - ps3
Archaea3 - ps4
Asgard/Eukaryota - ps5
Eukaryota - ps6
Amorpheae - ps7
Obazoa - ps8
Opisthokonta - ps9
Holomycota - ps10
Fungi - ps11
Fungi_1 - ps12
Fungi_2 - ps13
Fungi_3 - ps14
Dikarya - ps15
Ascomycota - ps16
Saccharo/Pezizomycotina - ps17
Saccharomycotina - ps18
Saccharomycotina_1 - ps19
Saccharomycotina_2 - ps20
Saccharomycotina_3 - ps21
Phaff./Saccharomycetaceae - ps22
Saccharomyco./mycetaceae - ps23
Saccharomycetaceae - ps24
Saccharomycetaceae_2 - ps25
Saccharomycetaceae_3 - ps26
Kaz./Nau./Saccharomyces - ps27
Saccharomyces - ps28
Arboricola_clade - ps29
Cerevisiae_clade - ps30
Saccharomyces_cerevisiae - ps31
Phylostratum

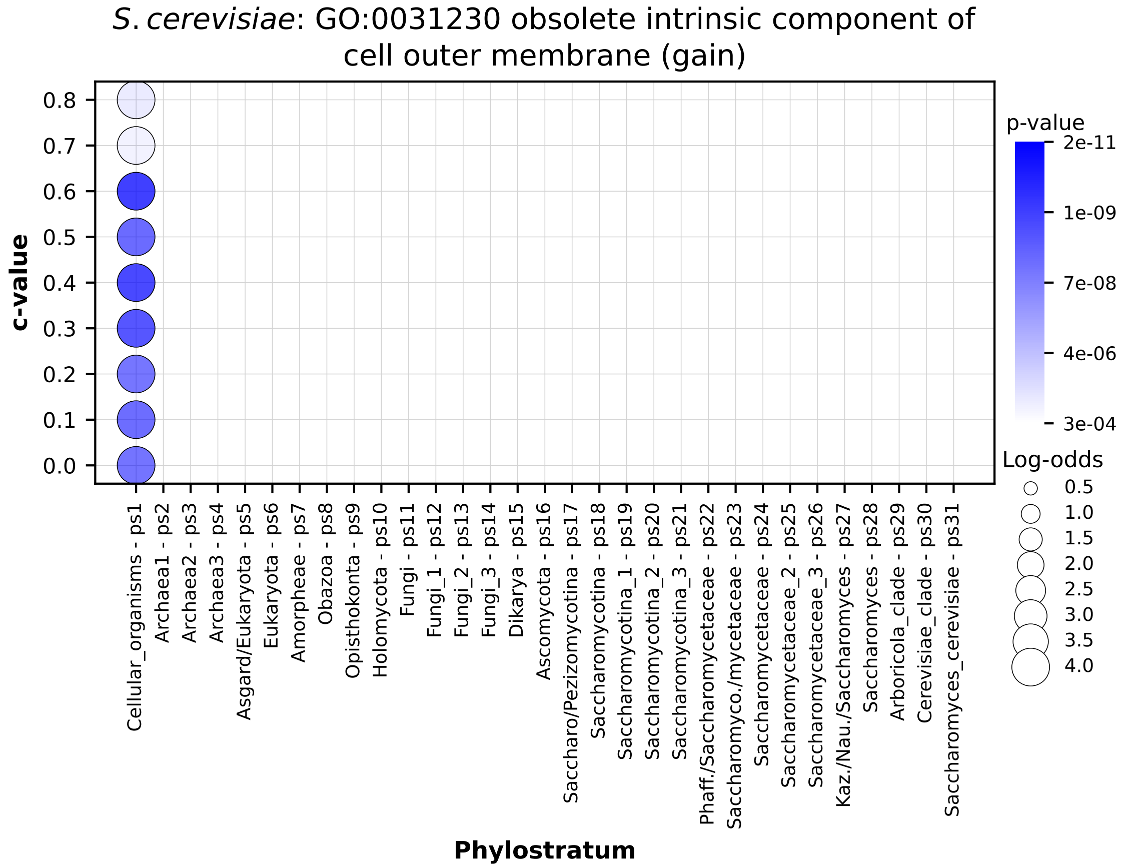

S. cerevisiae: GO:0031230 obsolete intrinsic component of cell outer membrane (gain)
c-value
0.8
0.7
0.6
0.5
0.4
0.3
0.2
0.1
0.0
p-value
2e-11
1e-09
7e-08
4e-06
3e-04
Log-odds
0.5
1.0
1.5
2.0
2.5
3.0
3.5
4.0
Cellular_organisms - ps1
Archaea1 - ps2
Archaea2 - ps3
Archaea3 - ps4
Asgard/Eukaryota - ps5
Eukaryota - ps6
Amorpheae - ps7
Obazoa - ps8
Opisthokonta - ps9
Holomycota - ps10
Fungi - ps11
Fungi_1 - ps12
Fungi_2 - ps13
Fungi_3 - ps14
Dikarya - ps15
Ascomycota - ps16
Saccharo/Pezizomycotina - ps17
Saccharomycotina - ps18
Saccharomycotina_1 - ps19
Saccharomycotina_2 - ps20
Saccharomycotina_3 - ps21
Phaff./Saccharomycetaceae - ps22
Saccharomyco./mycetaceae - ps23
Saccharomycetaceae - ps24
Saccharomycetaceae_2 - ps25
Saccharomycetaceae_3 - ps26
Kaz./Nau./Saccharomyces - ps27
Saccharomyces - ps28
Arboricola_clade - ps29
Cerevisiae_clade - ps30
Saccharomyces_cerevisiae - ps31
Phylostratum

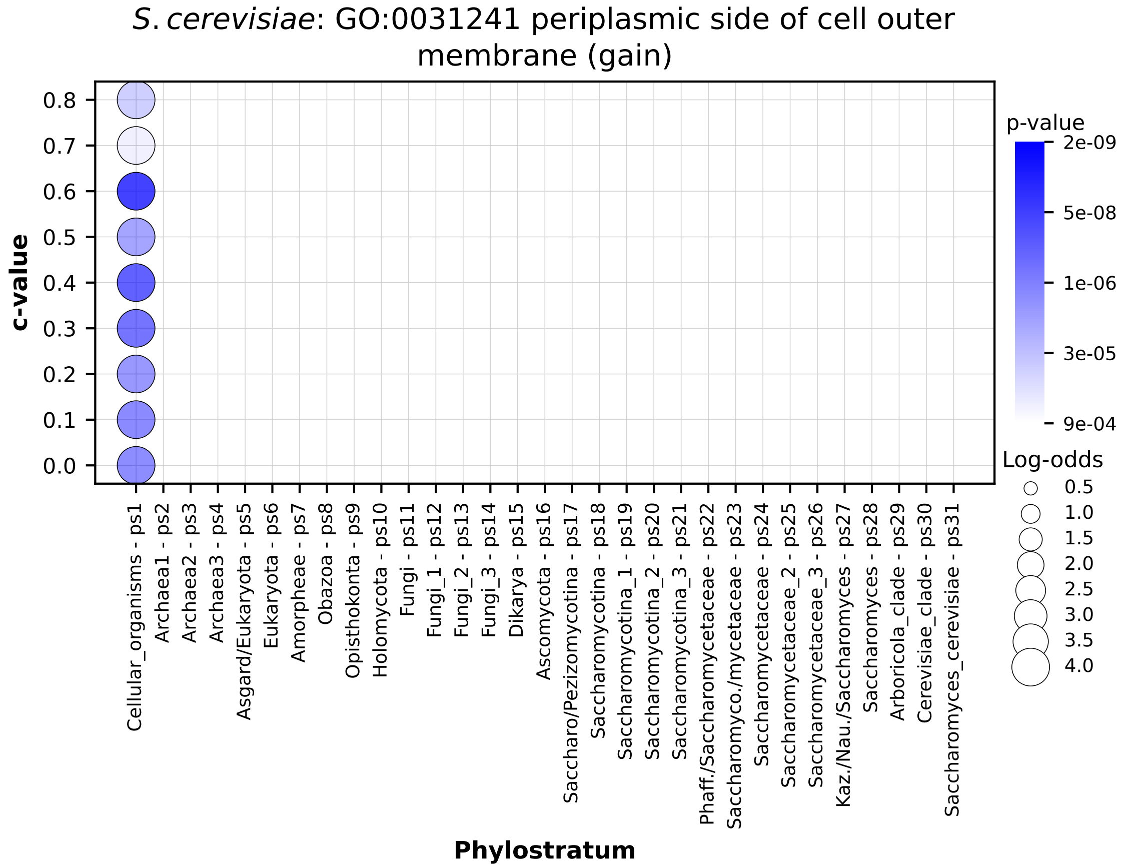
S. cerevisiae: GO:0031241 periplasmic side of cell outer membrane (gain)
c-value
0.8
0.7
0.6
0.5
0.4
0.3
0.2
0.1
0.0
p-value
2e-09
5e-08
1e-06
3e-05
9e-04
Log-odds
0.5
1.0
1.5
2.0
2.5
3.0
3.5
4.0
Cellular_organisms - ps1
Archaea1 - ps2
Archaea2 - ps3
Archaea3 - ps4
Asgard/Eukaryota - ps5
Eukaryota - ps6
Amorpheae - ps7
Obazoa - ps8
Opisthokonta - ps9
Holomycota - ps10
Fungi - ps11
Fungi_1 - ps12
Fungi_2 - ps13
Fungi_3 - ps14
Dikarya - ps15
Ascomycota - ps16
Saccharo/Pezizomycotina - ps17
Saccharomycotina - ps18
Saccharomycotina_1 - ps19
Saccharomycotina_2 - ps20
Saccharomycotina_3 - ps21
Phaff./Saccharomycetaceae - ps22
Saccharomyco./mycetaceae - ps23
Saccharomycetaceae - ps24
Saccharomycetaceae_2 - ps25
Saccharomycetaceae_3 - ps26
Kaz./Nau./Saccharomyces - ps27
Saccharomyces - ps28
Arboricola_clade - ps29
Cerevisiae_clade - ps30
Saccharomyces_cerevisiae - ps31
Phylostratum

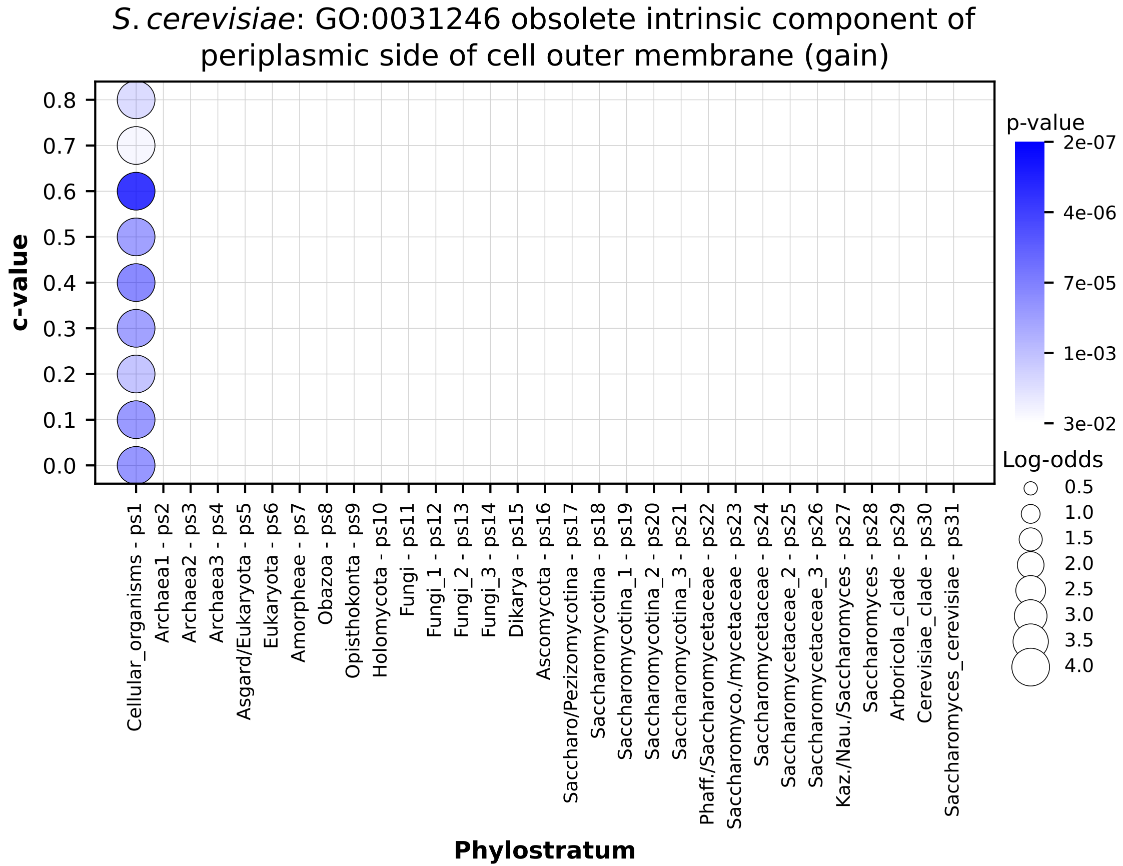
S. cerevisiae: GO:0031246 obsolete intrinsic component of periplasmic side of cell outer membrane (gain)
c-value
0.8
0.7
0.6
0.5
0.4
0.3
0.2
0.1
0.0
Phylostratum
Cellular_organisms - ps1
Archaea1 - ps2
Archaea2 - ps3
Archaea3 - ps4
Asgard/Eukaryota - ps5
Eukaryota - ps6
Amorpheae - ps7
Obazoa - ps8
Opisthokonta - ps9
Holomycota - ps10
Fungi - ps11
Fungi_1 - ps12
Fungi_2 - ps13
Fungi_3 - ps14
Dikarya - ps15
Ascomycota - ps16
Saccharo/Pezizomycotina - ps17
Saccharomycotina - ps18
Saccharomycotina_1 - ps19
Saccharomycotina_2 - ps20
Saccharomycotina_3 - ps21
Phaff./Saccharomycetaceae - ps22
Saccharomyco./mycetaceae - ps23
Saccharomycetaceae - ps24
Saccharomycetaceae_2 - ps25
Saccharomycetaceae_3 - ps26
Kaz./Nau./Saccharomyces - ps27
Saccharomyces - ps28
Arboricola_clade - ps29
Cerevisiae_clade - ps30
Saccharomyces_cerevisiae - ps31
p-value
2e-07
4e-06
7e-05
1e-03
3e-02
Log-odds
0.5
1.0
1.5
2.0
2.5
3.0
3.5
4.0

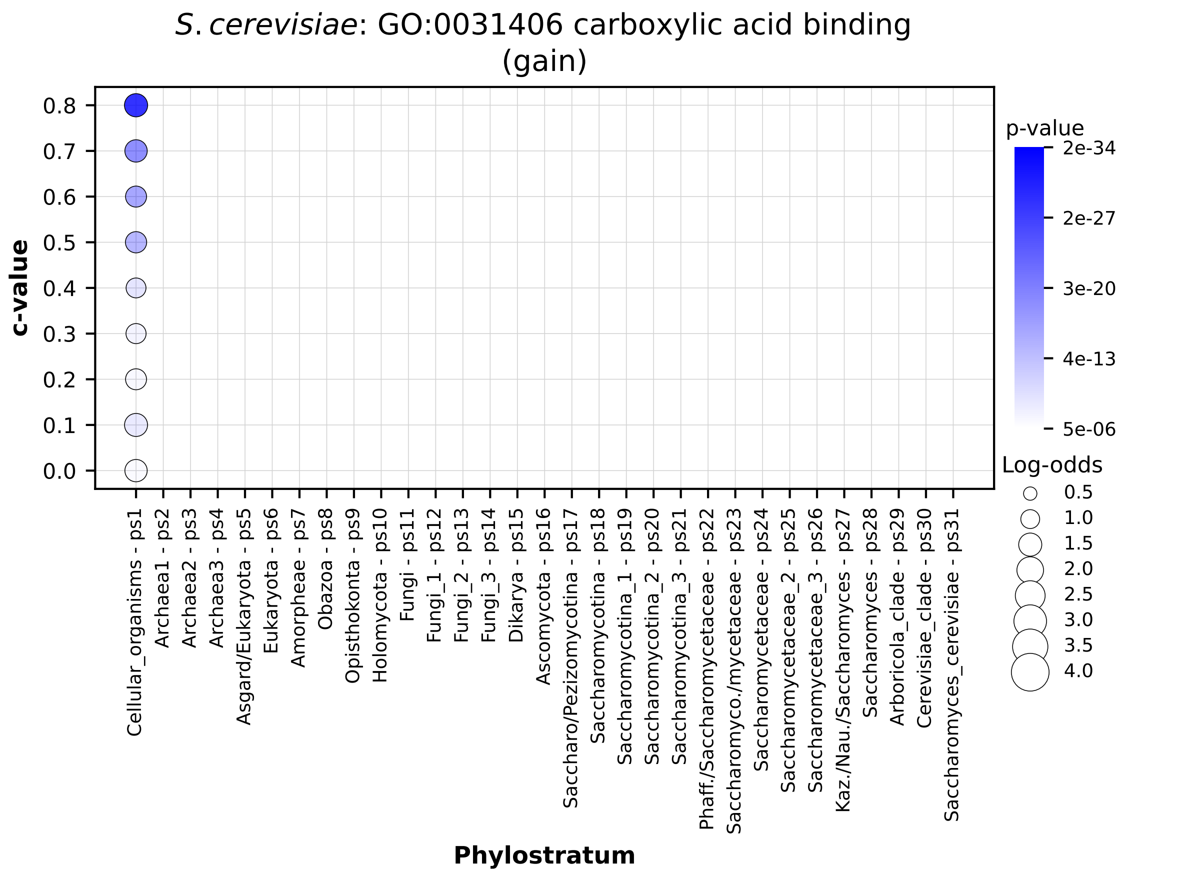
S. cerevisiae: GO:0031406 carboxylic acid binding
(gain)
c-value
0.0
0.1
0.2
0.3
0.4
0.5
0.6
0.7
0.8
p-value
2e-34
2e-27
3e-20
4e-13
5e-06
Log-odds
0.5
1.0
1.5
2.0
2.5
3.0
3.5
4.0
Cellular_organisms - ps1
Archaea1 - ps2
Archaea2 - ps3
Archaea3 - ps4
Asgard/Eukaryota - ps5
Eukaryota - ps6
Amorpheae - ps7
Obazoa - ps8
Opisthokonta - ps9
Holomycota - ps10
Fungi - ps11
Fungi_1 - ps12
Fungi_2 - ps13
Fungi_3 - ps14
Dikarya - ps15
Ascomycota - ps16
Saccharo/Pezizomycotina - ps17
Saccharomycotina - ps18
Saccharomycotina_1 - ps19
Saccharomycotina_2 - ps20
Saccharomycotina_3 - ps21
Phaff./Saccharomycetaceae - ps22
Saccharomyco./mycetaceae - ps23
Saccharomycetaceae - ps24
Saccharomycetaceae_2 - ps25
Saccharomycetaceae_3 - ps26
Kaz./Nau./Saccharomyces - ps27
Saccharomyces - ps28
Arboricola_clade - ps29
Cerevisiae_clade - ps30
Saccharomyces_cerevisiae - ps31
Phylostratum

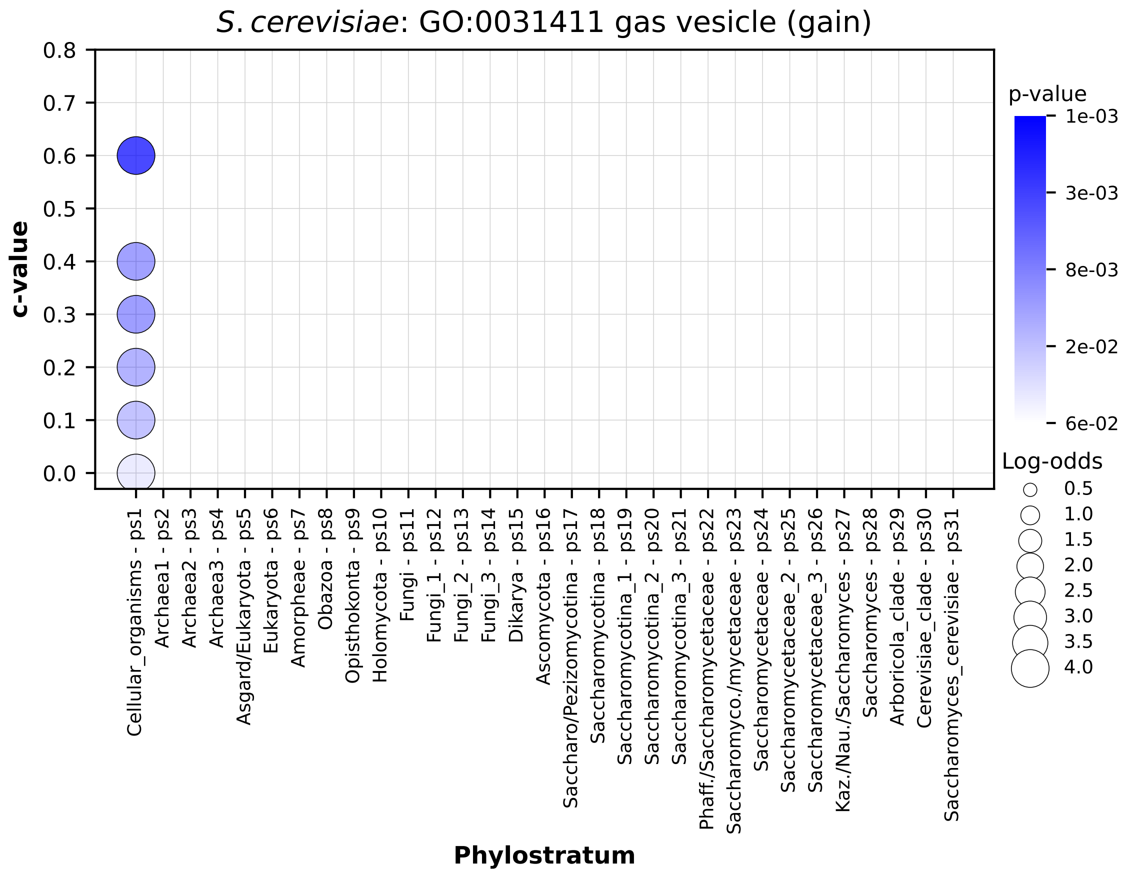

$S. cerevisiae$: GO:0031411 gas vesicle (gain)
c-value
0.8
0.7
0.6
0.5
0.4
0.3
0.2
0.1
0.0
p-value
1e-03
3e-03
8e-03
2e-02
6e-02
Log-odds
0.5
1.0
1.5
2.0
2.5
3.0
3.5
4.0
Cellular_organisms - ps1
Archaea1 - ps2
Archaea2 - ps3
Archaea3 - ps4
Asgard/Eukaryota - ps5
Eukaryota - ps6
Amorpheae - ps7
Obazoa - ps8
Opisthokonta - ps9
Holomycota - ps10
Fungi - ps11
Fungi_1 - ps12
Fungi_2 - ps13
Fungi_3 - ps14
Dikarya - ps15
Ascomycota - ps16
Saccharo/Pezizomycotina - ps17
Saccharomycotina - ps18
Saccharomycotina_1 - ps19
Saccharomycotina_2 - ps20
Saccharomycotina_3 - ps21
Phaff./Saccharomycetaceae - ps22
Saccharomyco./mycetaceae - ps23
Saccharomycetaceae - ps24
Saccharomycetaceae_2 - ps25
Saccharomycetaceae_3 - ps26
Kaz./Nau./Saccharomyces - ps27
Saccharomyces - ps28
Arboricola_clade - ps29
Cerevisiae_clade - ps30
Saccharomyces_cerevisiae - ps31
Phylostratum

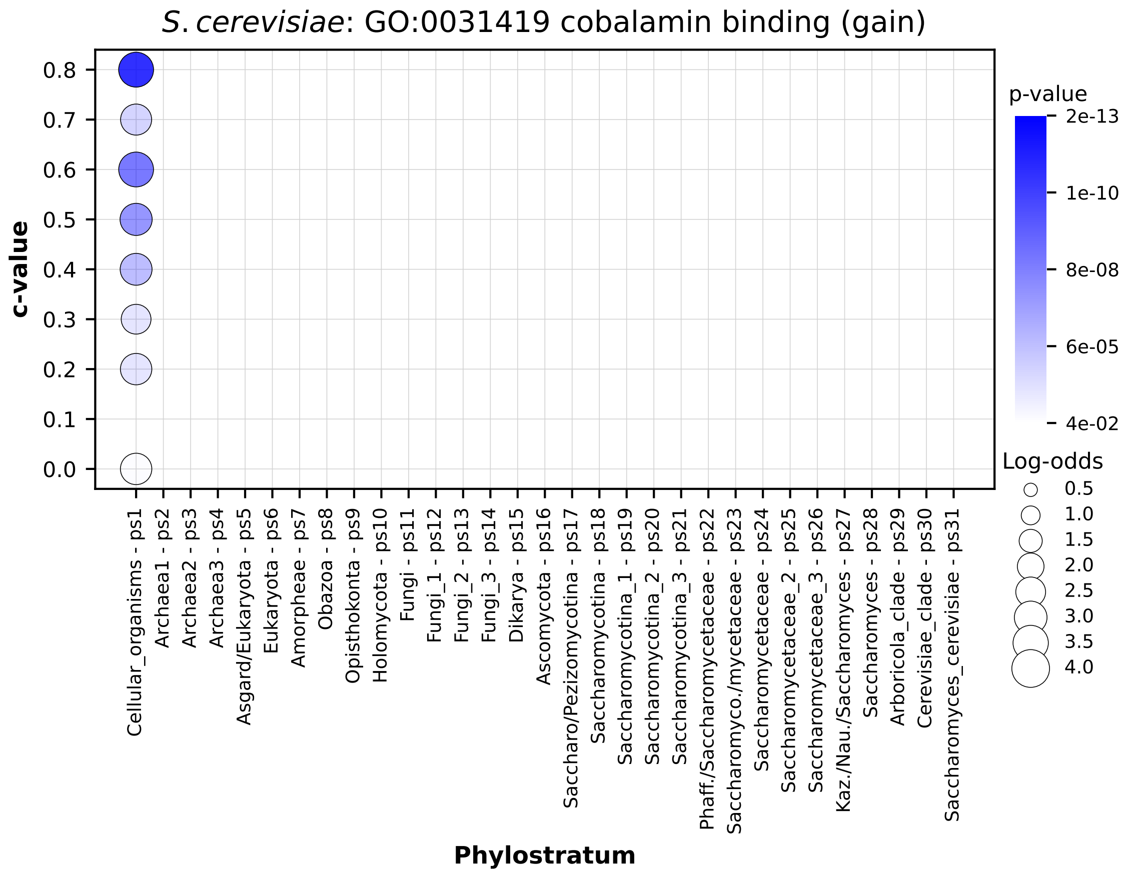

S. cerevisiae: GO:0031419 cobalamin binding (gain)
c-value
0.8
0.7
0.6
0.5
0.4
0.3
0.2
0.1
0.0
p-value
2e-13
1e-10
8e-08
6e-05
4e-02
Log-odds
0.5
1.0
1.5
2.0
2.5
3.0
3.5
4.0
Cellular_organisms - ps1
Archaea1 - ps2
Archaea2 - ps3
Archaea3 - ps4
Asgard/Eukaryota - ps5
Eukaryota - ps6
Amorpheae - ps7
Obazoa - ps8
Opisthokonta - ps9
Holomycota - ps10
Fungi - ps11
Fungi_1 - ps12
Fungi_2 - ps13
Fungi_3 - ps14
Dikarya - ps15
Ascomycota - ps16
Saccharo/Pezizomycotina - ps17
Saccharomycotina - ps18
Saccharomycotina_1 - ps19
Saccharomycotina_2 - ps20
Saccharomycotina_3 - ps21
Phaff./Saccharomycetaceae - ps22
Saccharomyco./mycetaceae - ps23
Saccharomycetaceae - ps24
Saccharomycetaceae_2 - ps25
Saccharomycetaceae_3 - ps26
Kaz./Nau./Saccharomyces - ps27
Saccharomyces - ps28
Arboricola_clade - ps29
Cerevisiae_clade - ps30
Saccharomyces_cerevisiae - ps31
Phylostratum

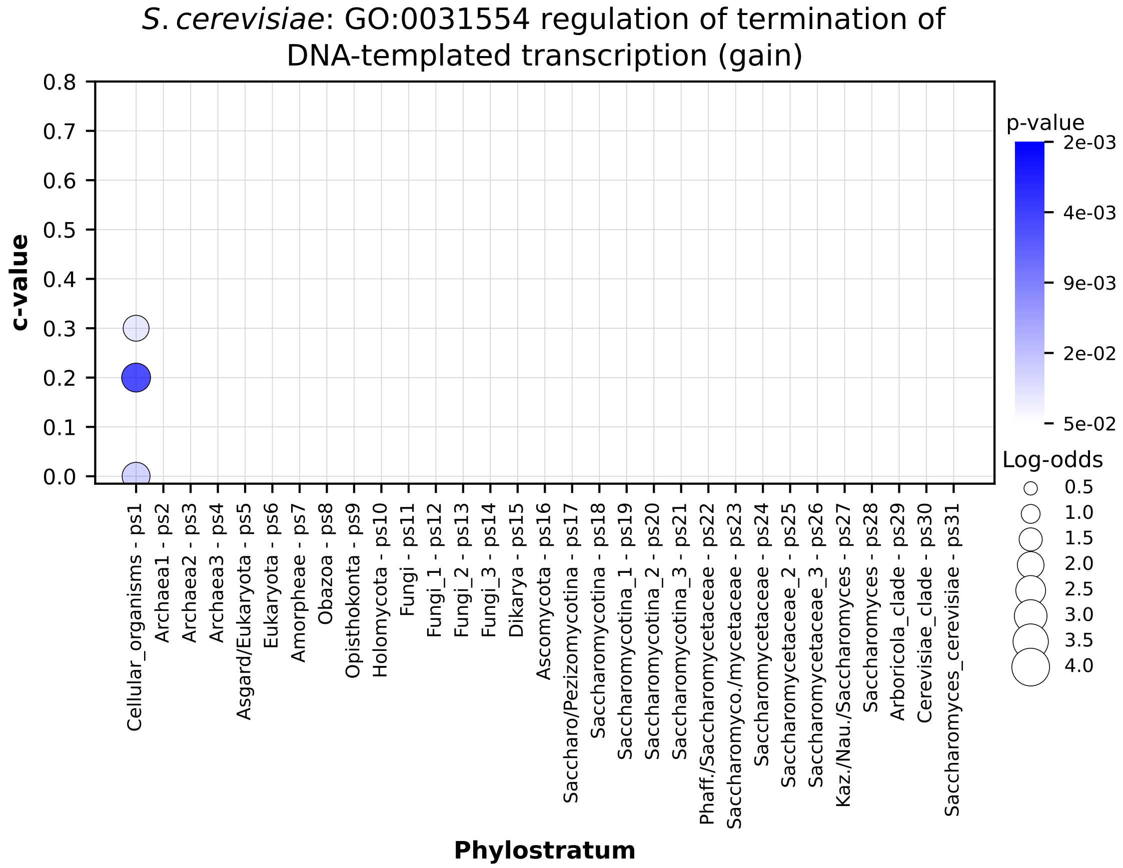

S. cerevisiae: GO:0031554 regulation of termination of DNA-templated transcription (gain)
c-value
0.8
0.7
0.6
0.5
0.4
0.3
0.2
0.1
0.0
p-value
2e-03
4e-03
9e-03
2e-02
5e-02
Log-odds
0.5
1.0
1.5
2.0
2.5
3.0
3.5
4.0
Cellular_organisms - ps1
Archaea1 - ps2
Archaea2 - ps3
Archaea3 - ps4
Asgard/Eukaryota - ps5
Eukaryota - ps6
Amorpheae - ps7
Obazoa - ps8
Opisthokonta - ps9
Holomycota - ps10
Fungi - ps11
Fungi_1 - ps12
Fungi_2 - ps13
Fungi_3 - ps14
Dikarya - ps15
Ascomycota - ps16
Saccharo/Pezizomycotina - ps17
Saccharomycotina - ps18
Saccharomycotina_1 - ps19
Saccharomycotina_2 - ps20
Saccharomycotina_3 - ps21
Phaff./Saccharomycetaceae - ps22
Saccharomyco./mycetaceae - ps23
Saccharomycetaceae - ps24
Saccharomycetaceae_2 - ps25
Saccharomycetaceae_3 - ps26
Kaz./Nau./Saccharomyces - ps27
Saccharomyces - ps28
Arboricola_clade - ps29
Cerevisiae_clade - ps30
Saccharomyces_cerevisiae - ps31
Phylostratum

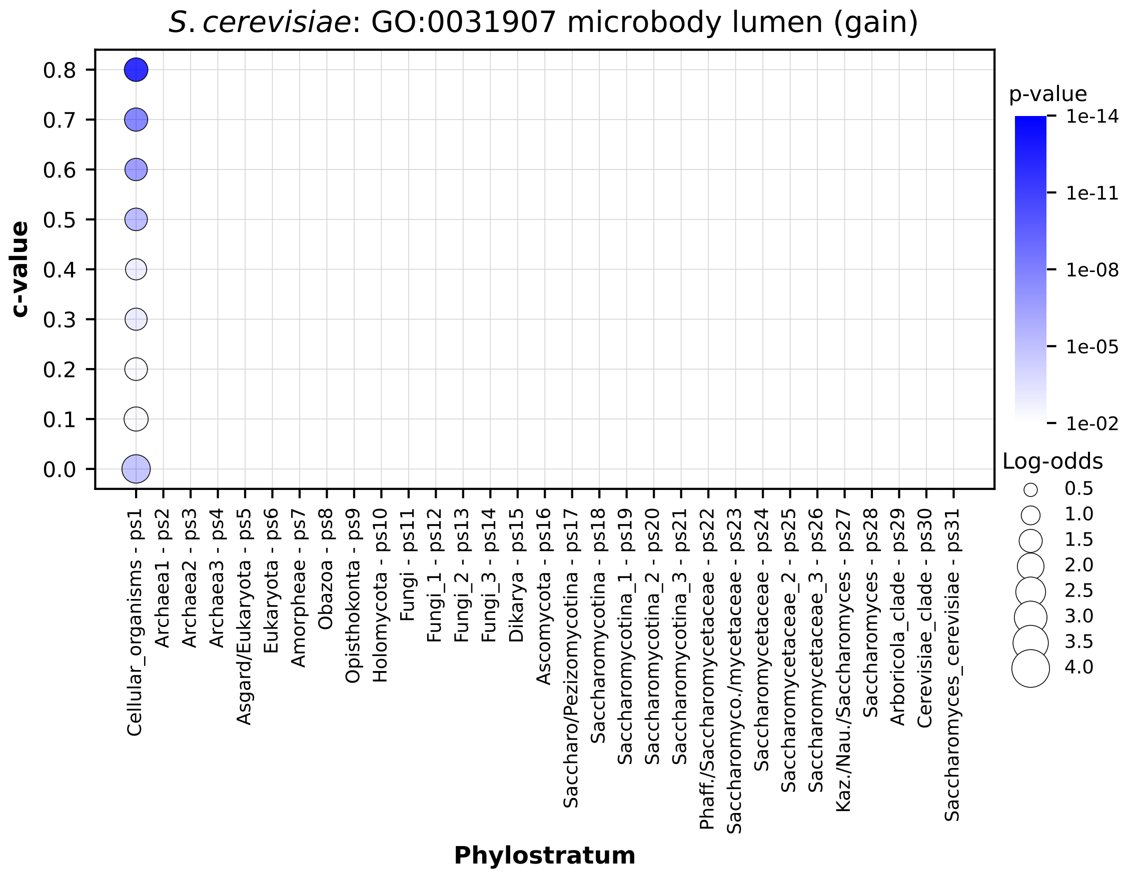

S. cerevisiae: GO:0031907 microbody lumen (gain)
c-value
0.8
0.7
0.6
0.5
0.4
0.3
0.2
0.1
0.0
p-value
1e-14
1e-11
1e-08
1e-05
1e-02
Log-odds
0.5
1.0
1.5
2.0
2.5
3.0
3.5
4.0
Cellular_organisms - ps1
Archaea1 - ps2
Archaea2 - ps3
Archaea3 - ps4
Asgard/Eukaryota - ps5
Eukaryota - ps6
Amorpheae - ps7
Obazoa - ps8
Opisthokonta - ps9
Holomycota - ps10
Fungi - ps11
Fungi_1 - ps12
Fungi_2 - ps13
Fungi_3 - ps14
Dikarya - ps15
Ascomycota - ps16
Saccharo/Pezizomycotina - ps17
Saccharomycotina - ps18
Saccharomycotina_1 - ps19
Saccharomycotina_2 - ps20
Saccharomycotina_3 - ps21
Phaff./Saccharomycetaceae - ps22
Saccharomyco./mycetaceae - ps23
Saccharomycetaceae - ps24
Saccharomycetaceae_2 - ps25
Saccharomycetaceae_3 - ps26
Kaz./Nau./Saccharomyces - ps27
Saccharomyces - ps28
Arboricola_clade - ps29
Cerevisiae_clade - ps30
Saccharomyces_cerevisiae - ps31
Phylostratum

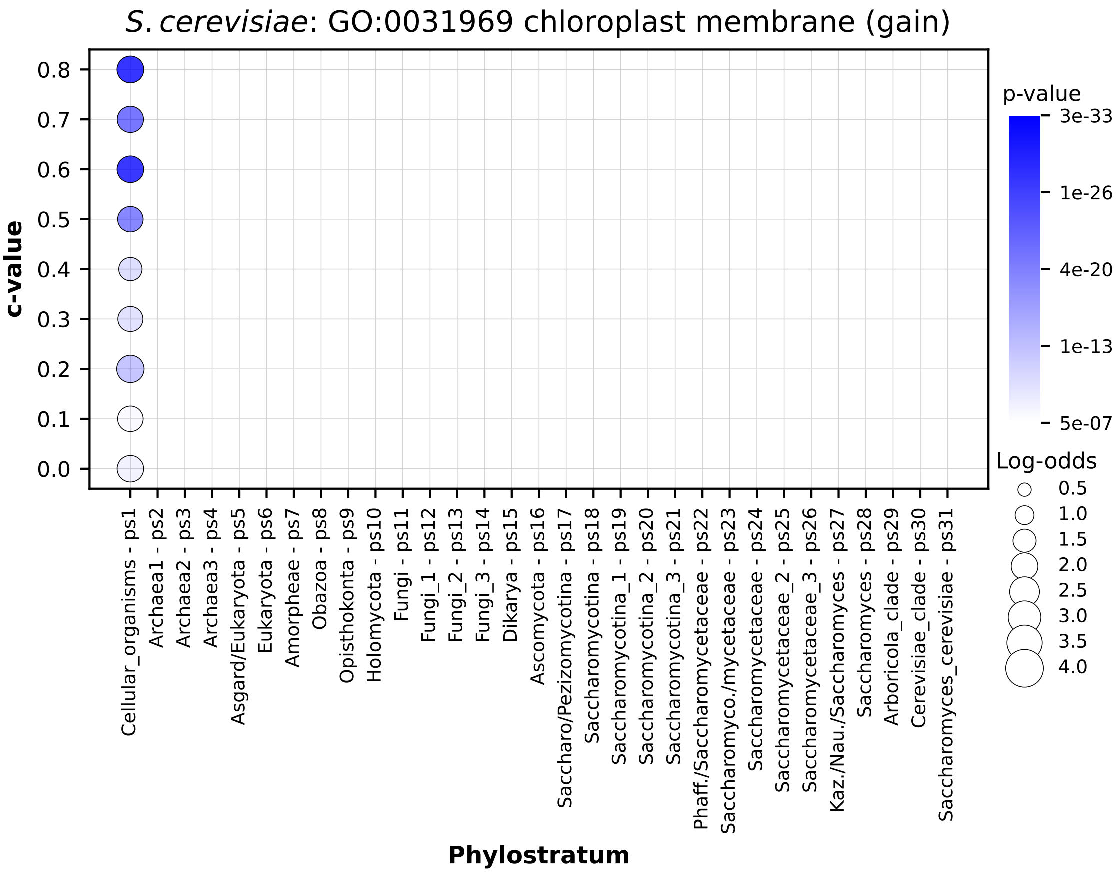
S. cerevisiae: GO:0031969 chloroplast membrane (gain)
c-value
0.0
0.1
0.2
0.3
0.4
0.5
0.6
0.7
0.8
Phylostratum
Cellular_organisms - ps1
Archaea1 - ps2
Archaea2 - ps3
Archaea3 - ps4
Asgard/Eukaryota - ps5
Eukaryota - ps6
Amorpheae - ps7
Obazoa - ps8
Opisthokonta - ps9
Holomycota - ps10
Fungi - ps11
Fungi_1 - ps12
Fungi_2 - ps13
Fungi_3 - ps14
Dikarya - ps15
Ascomycota - ps16
Saccharo/Pezizomycotina - ps17
Saccharomycotina - ps18
Saccharomycotina_1 - ps19
Saccharomycotina_2 - ps20
Saccharomycotina_3 - ps21
Phaff./Saccharomycetaceae - ps22
Saccharomyco./mycetaceae - ps23
Saccharomycetaceae - ps24
Saccharomycetaceae_2 - ps25
Saccharomycetaceae_3 - ps26
Kaz./Nau./Saccharomyces - ps27
Saccharomyces - ps28
Arboricola_clade - ps29
Cerevisiae_clade - ps30
Saccharomyces_cerevisiae - ps31
p-value
3e-33
1e-26
4e-20
1e-13
5e-07
Log-odds
0.5
1.0
1.5
2.0
2.5
3.0
3.5
4.0

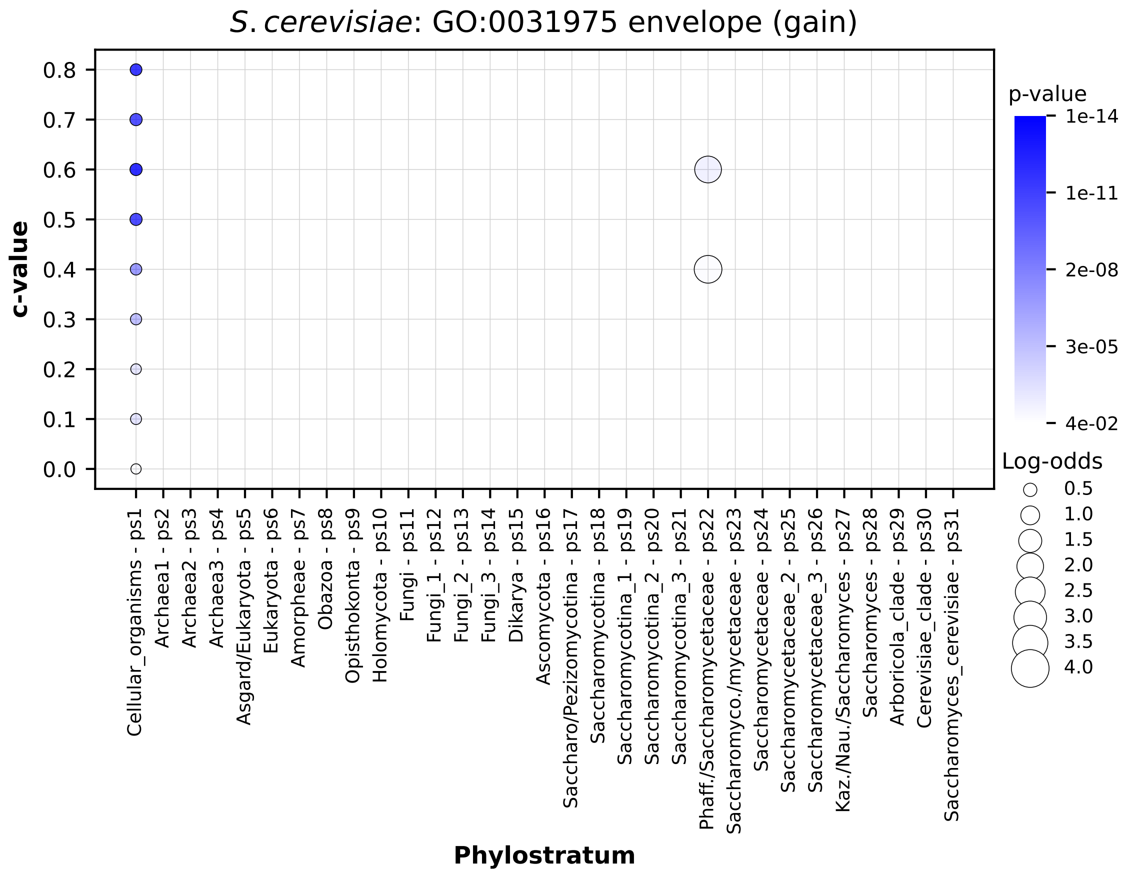

S. cerevisiae: GO:0031975 envelope (gain)
c-value
0.8
0.7
0.6
0.5
0.4
0.3
0.2
0.1
0.0
Phylostratum
Cellular_organisms - ps1
Archaea1 - ps2
Archaea2 - ps3
Archaea3 - ps4
Asgard/Eukaryota - ps5
Eukaryota - ps6
Amorpheae - ps7
Obazoa - ps8
Opisthokonta - ps9
Holomycota - ps10
Fungi - ps11
Fungi_1 - ps12
Fungi_2 - ps13
Fungi_3 - ps14
Dikarya - ps15
Ascomycota - ps16
Saccharo/Pezizomycotina - ps17
Saccharomycotina - ps18
Saccharomycotina_1 - ps19
Saccharomycotina_2 - ps20
Saccharomycotina_3 - ps21
Phaff./Saccharomycetaceae - ps22
Saccharomyco./mycetaceae - ps23
Saccharomycetaceae - ps24
Saccharomycetaceae_2 - ps25
Saccharomycetaceae_3 - ps26
Kaz./Nau./Saccharomyces - ps27
Saccharomyces - ps28
Arboricola_clade - ps29
Cerevisiae_clade - ps30
Saccharomyces_cerevisiae - ps31
p-value
1e-14
1e-11
2e-08
3e-05
4e-02
Log-odds
0.5
1.0
1.5
2.0
2.5
3.0
3.5
4.0

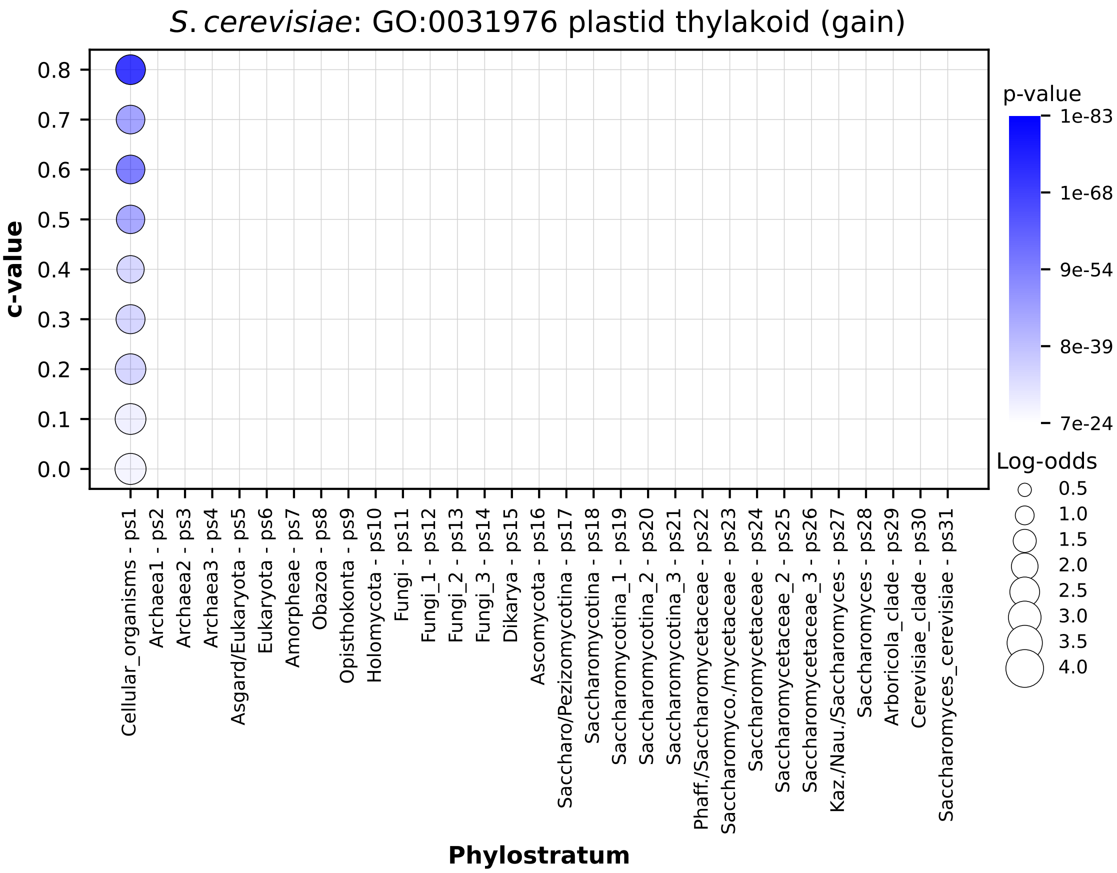

S. cerevisiae: GO:0031976 plastid thylakoid (gain)
c-value
0.0
0.1
0.2
0.3
0.4
0.5
0.6
0.7
0.8
Phylostratum
Cellular_organisms - ps1
Archaea1 - ps2
Archaea2 - ps3
Archaea3 - ps4
Asgard/Eukaryota - ps5
Eukaryota - ps6
Amorpheae - ps7
Obazoa - ps8
Opisthokonta - ps9
Holomycota - ps10
Fungi - ps11
Fungi_1 - ps12
Fungi_2 - ps13
Fungi_3 - ps14
Dikarya - ps15
Ascomycota - ps16
Saccharo/Pezizomycotina - ps17
Saccharomycotina - ps18
Saccharomycotina_1 - ps19
Saccharomycotina_2 - ps20
Saccharomycotina_3 - ps21
Phaff./Saccharomycetaceae - ps22
Saccharomyco./mycetaceae - ps23
Saccharomycetaceae - ps24
Saccharomycetaceae_2 - ps25
Saccharomycetaceae_3 - ps26
Kaz./Nau./Saccharomyces - ps27
Saccharomyces - ps28
Arboricola_clade - ps29
Cerevisiae_clade - ps30
Saccharomyces_cerevisiae - ps31
p-value
1e-83
1e-68
9e-54
8e-39
7e-24
Log-odds
0.5
1.0
1.5
2.0
2.5
3.0
3.5
4.0

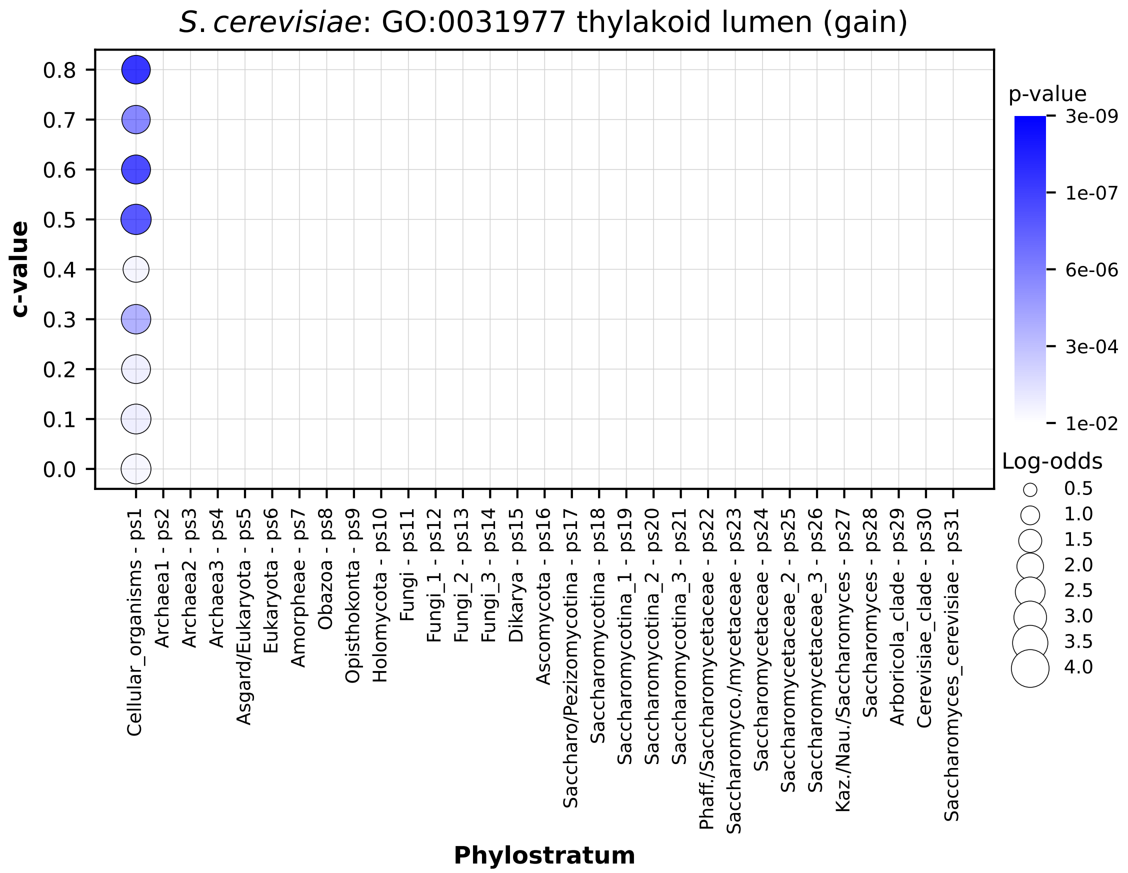

$S. cerevisiae$: GO:0031977 thylakoid lumen (gain)
c-value
0.8
0.7
0.6
0.5
0.4
0.3
0.2
0.1
0.0
p-value
3e-09
1e-07
6e-06
3e-04
1e-02
Log-odds
0.5
1.0
1.5
2.0
2.5
3.0
3.5
4.0
Cellular_organisms - ps1
Archaea1 - ps2
Archaea2 - ps3
Archaea3 - ps4
Asgard/Eukaryota - ps5
Eukaryota - ps6
Amorpheae - ps7
Obazoa - ps8
Opisthokonta - ps9
Holomycota - ps10
Fungi - ps11
Fungi_1 - ps12
Fungi_2 - ps13
Fungi_3 - ps14
Dikarya - ps15
Ascomycota - ps16
Saccharo/Pezizomycotina - ps17
Saccharomycotina - ps18
Saccharomycotina_1 - ps19
Saccharomycotina_2 - ps20
Saccharomycotina_3 - ps21
Phaff./Saccharomycetaceae - ps22
Saccharomyco./mycetaceae - ps23
Saccharomycetaceae - ps24
Saccharomycetaceae_2 - ps25
Saccharomycetaceae_3 - ps26
Kaz./Nau./Saccharomyces - ps27
Saccharomyces - ps28
Arboricola_clade - ps29
Cerevisiae_clade - ps30
Saccharomyces_cerevisiae - ps31
Phylostratum

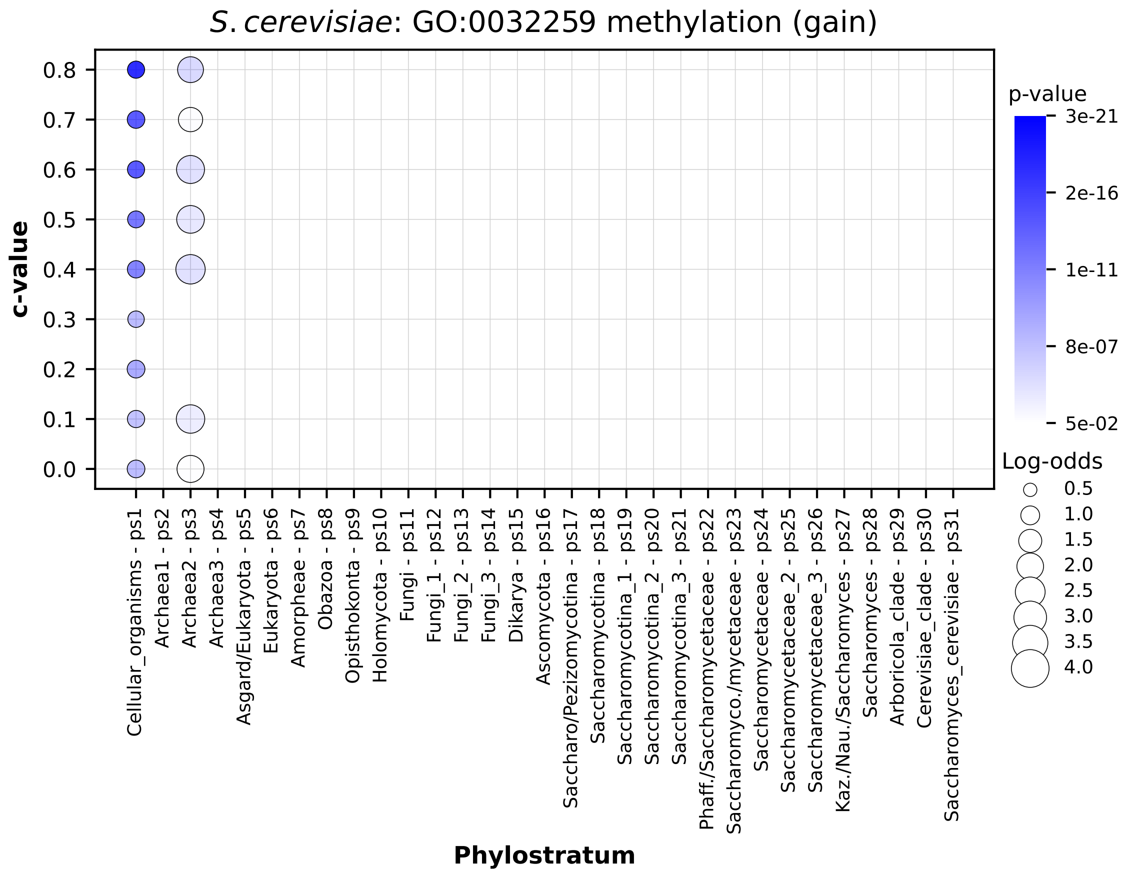

S. cerevisiae: GO:0032259 methylation (gain)
c-value
0.8
0.7
0.6
0.5
0.4
0.3
0.2
0.1
0.0
p-value
3e-21
2e-16
1e-11
8e-07
5e-02
Log-odds
0.5
1.0
1.5
2.0
2.5
3.0
3.5
4.0
Cellular_organisms - ps1
Archaea1 - ps2
Archaea2 - ps3
Archaea3 - ps4
Asgard/Eukaryota - ps5
Eukaryota - ps6
Amorpheae - ps7
Obazoa - ps8
Opisthokonta - ps9
Holomycota - ps10
Fungi - ps11
Fungi_1 - ps12
Fungi_2 - ps13
Fungi_3 - ps14
Dikarya - ps15
Ascomycota - ps16
Saccharo/Pezizomycotina - ps17
Saccharomycotina - ps18
Saccharomycotina_1 - ps19
Saccharomycotina_2 - ps20
Saccharomycotina_3 - ps21
Phaff./Saccharomycetaceae - ps22
Saccharomyco./mycetaceae - ps23
Saccharomycetaceae - ps24
Saccharomycetaceae_2 - ps25
Saccharomycetaceae_3 - ps26
Kaz./Nau./Saccharomyces - ps27
Saccharomyces - ps28
Arboricola_clade - ps29
Cerevisiae_clade - ps30
Saccharomyces_cerevisiae - ps31
Phylostratum

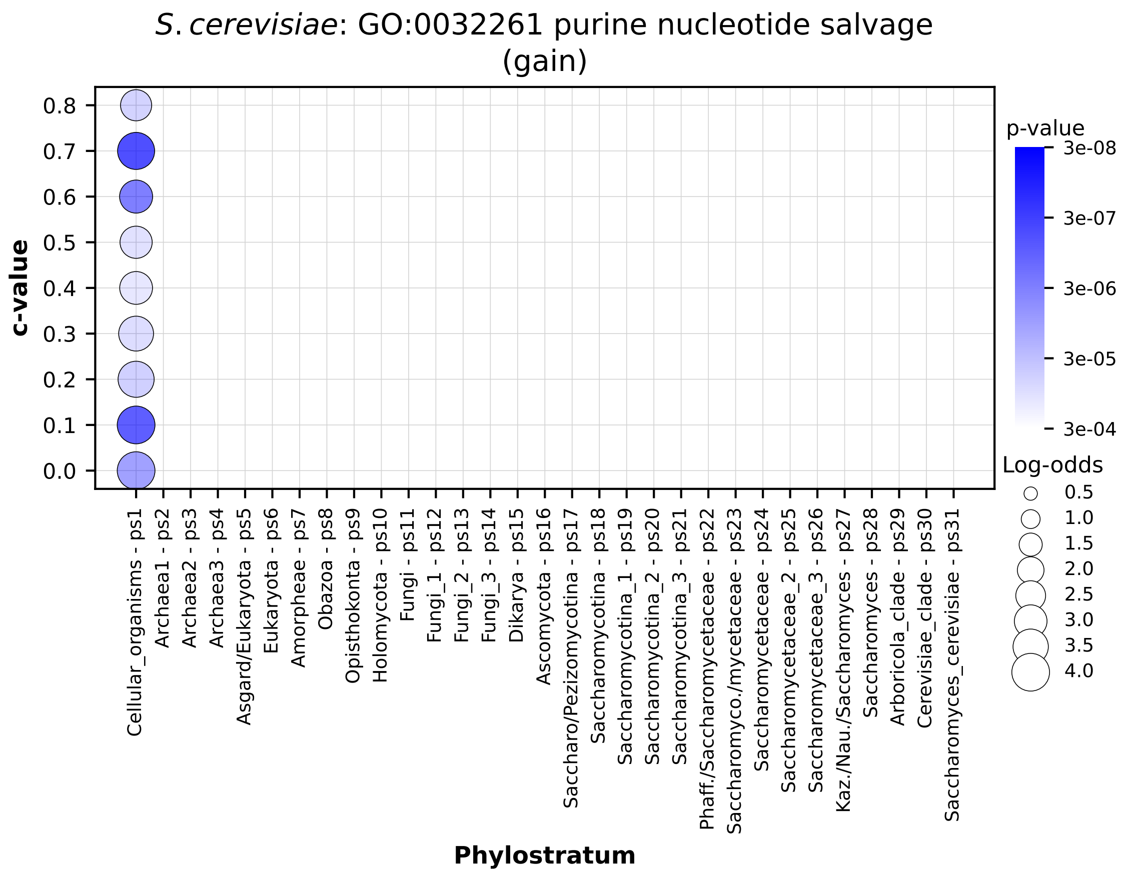

S. cerevisiae: GO:0032261 purine nucleotide salvage
(gain)
c-value
0.0
0.1
0.2
0.3
0.4
0.5
0.6
0.7
0.8
p-value
3e-08
3e-07
3e-06
3e-05
3e-04
Log-odds
0.5
1.0
1.5
2.0
2.5
3.0
3.5
4.0
Cellular_organisms - ps1
Archaea1 - ps2
Archaea2 - ps3
Archaea3 - ps4
Asgard/Eukaryota - ps5
Eukaryota - ps6
Amorpheae - ps7
Obazoa - ps8
Opisthokonta - ps9
Holomycota - ps10
Fungi - ps11
Fungi_1 - ps12
Fungi_2 - ps13
Fungi_3 - ps14
Dikarya - ps15
Ascomycota - ps16
Saccharo/Pezizomycotina - ps17
Saccharomycotina - ps18
Saccharomycotina_1 - ps19
Saccharomycotina_2 - ps20
Saccharomycotina_3 - ps21
Phaff./Saccharomycetaceae - ps22
Saccharomyco./mycetaceae - ps23
Saccharomycetaceae - ps24
Saccharomycetaceae_2 - ps25
Saccharomycetaceae_3 - ps26
Kaz./Nau./Saccharomyces - ps27
Saccharomyces - ps28
Arboricola_clade - ps29
Cerevisiae_clade - ps30
Saccharomyces_cerevisiae - ps31
Phylostratum

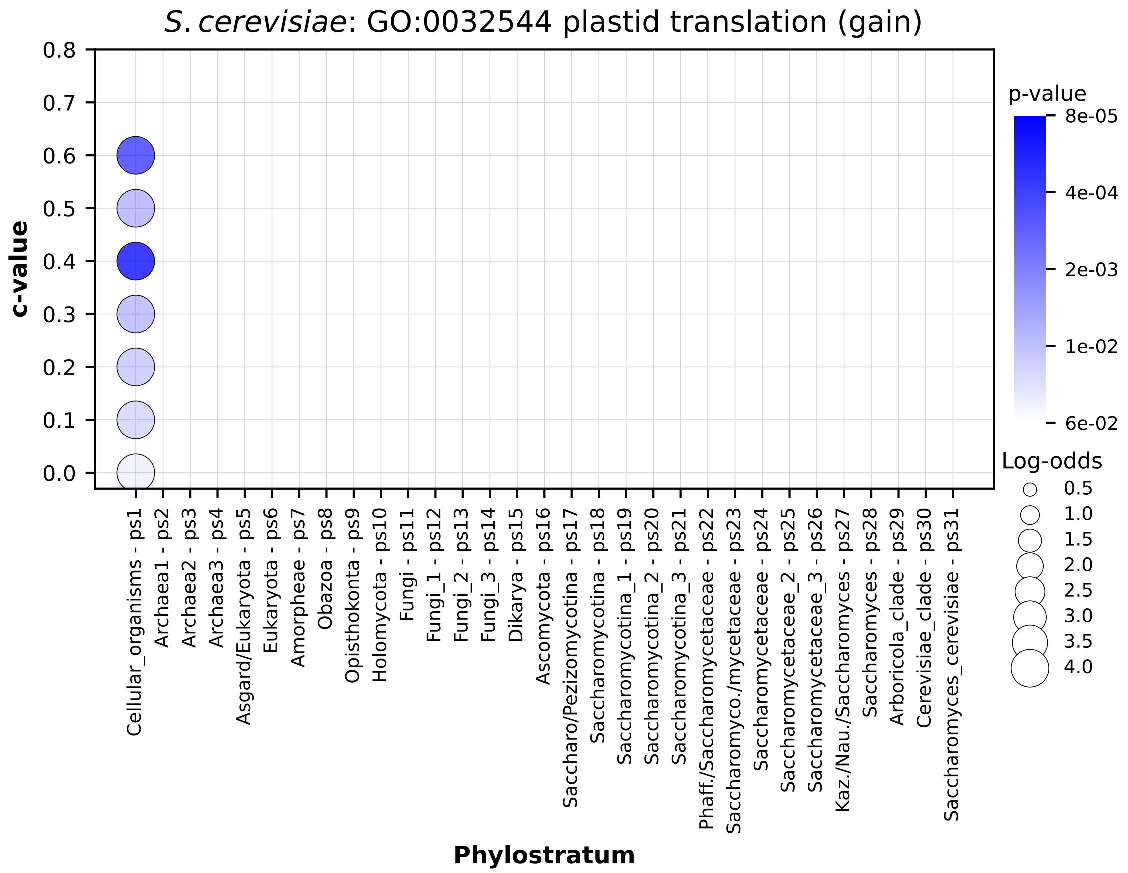
S. cerevisiae: GO:0032544 plastid translation (gain)
c-value
0.8
0.7
0.6
0.5
0.4
0.3
0.2
0.1
0.0
p-value
8e-05
4e-04
2e-03
1e-02
6e-02
Log-odds
0.5
1.0
1.5
2.0
2.5
3.0
3.5
4.0
Cellular_organisms - ps1
Archaea1 - ps2
Archaea2 - ps3
Archaea3 - ps4
Asgard/Eukaryota - ps5
Eukaryota - ps6
Amorpheae - ps7
Obazoa - ps8
Opisthokonta - ps9
Holomycota - ps10
Fungi - ps11
Fungi_1 - ps12
Fungi_2 - ps13
Fungi_3 - ps14
Dikarya - ps15
Ascomycota - ps16
Saccharo/Pezizomycotina - ps17
Saccharomycotina - ps18
Saccharomycotina_1 - ps19
Saccharomycotina_2 - ps20
Saccharomycotina_3 - ps21
Phaff./Saccharomycetaceae - ps22
Saccharomyco./mycetaceae - ps23
Saccharomycetaceae - ps24
Saccharomycetaceae_2 - ps25
Saccharomycetaceae_3 - ps26
Kaz./Nau./Saccharomyces - ps27
Saccharomyces - ps28
Arboricola_clade - ps29
Cerevisiae_clade - ps30
Saccharomyces_cerevisiae - ps31
Phylostratum

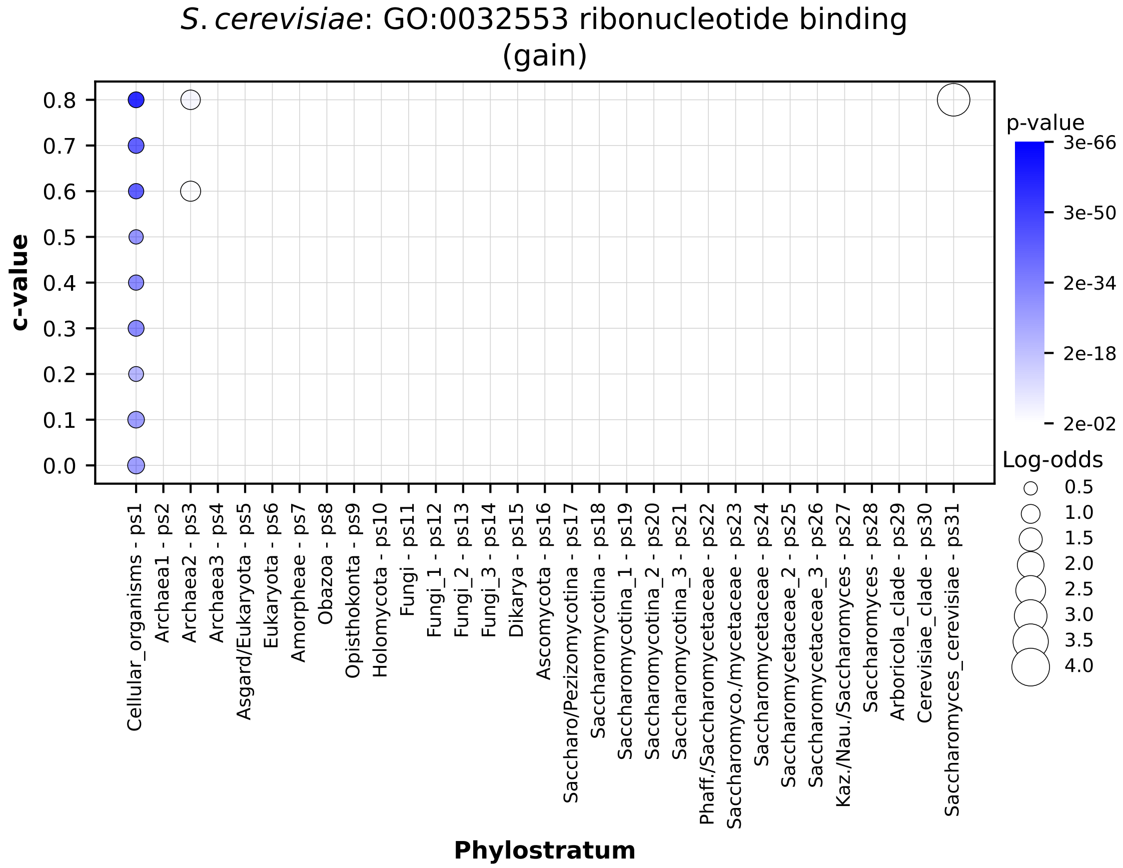
S. cerevisiae: GO:0032553 ribonucleotide binding
(gain)
c-value
0.8
0.7
0.6
0.5
0.4
0.3
0.2
0.1
0.0
p-value
3e-66
3e-50
2e-34
2e-18
2e-02
Log-odds
0.5
1.0
1.5
2.0
2.5
3.0
3.5
4.0
Cellular_organisms - ps1
Archaea1 - ps2
Archaea2 - ps3
Archaea3 - ps4
Asgard/Eukaryota - ps5
Eukaryota - ps6
Amorpheae - ps7
Obazoa - ps8
Opisthokonta - ps9
Holomycota - ps10
Fungi - ps11
Fungi_1 - ps12
Fungi_2 - ps13
Fungi_3 - ps14
Dikarya - ps15
Ascomycota - ps16
Saccharo/Pezizomycotina - ps17
Saccharomycotina - ps18
Saccharomycotina_1 - ps19
Saccharomycotina_2 - ps20
Saccharomycotina_3 - ps21
Phaff./Saccharomycetaceae - ps22
Saccharomyco./mycetaceae - ps23
Saccharomycetaceae - ps24
Saccharomycetaceae_2 - ps25
Saccharomycetaceae_3 - ps26
Kaz./Nau./Saccharomyces - ps27
Saccharomyces - ps28
Arboricola_clade - ps29
Cerevisiae_clade - ps30
Saccharomyces_cerevisiae - ps31
Phylostratum

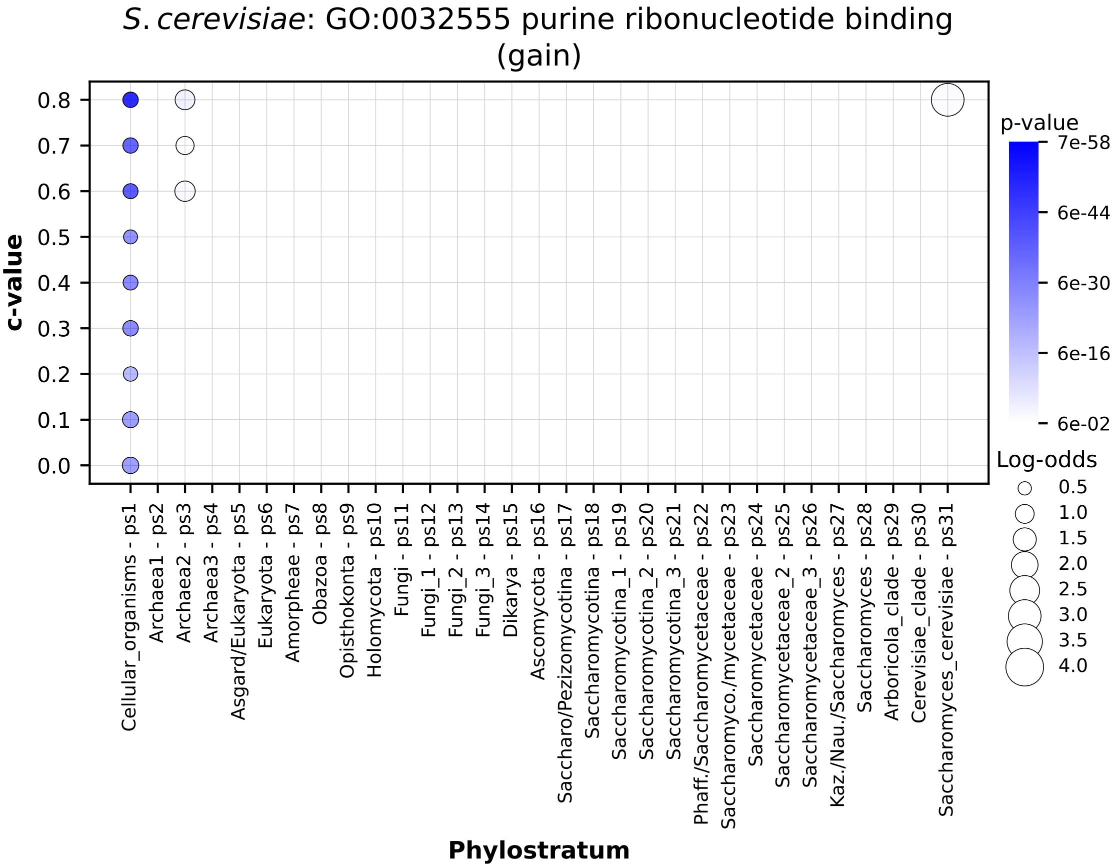

S. cerevisiae: GO:0032555 purine ribonucleotide binding (gain)
c-value
0.0
0.1
0.2
0.3
0.4
0.5
0.6
0.7
0.8
Phylostratum
Cellular_organisms - ps1
Archaea1 - ps2
Archaea2 - ps3
Archaea3 - ps4
Asgard/Eukaryota - ps5
Eukaryota - ps6
Amorpheae - ps7
Obazoa - ps8
Opisthokonta - ps9
Holomycota - ps10
Fungi - ps11
Fungi_1 - ps12
Fungi_2 - ps13
Fungi_3 - ps14
Dikarya - ps15
Ascomycota - ps16
Saccharo/Pezizomycotina - ps17
Saccharomycotina - ps18
Saccharomycotina_1 - ps19
Saccharomycotina_2 - ps20
Saccharomycotina_3 - ps21
Phaff./Saccharomycetaceae - ps22
Saccharomyco./mycetaceae - ps23
Saccharomycetaceae - ps24
Saccharomycetaceae_2 - ps25
Saccharomycetaceae_3 - ps26
Kaz./Nau./Saccharomyces - ps27
Saccharomyces - ps28
Arboricola_clade - ps29
Cerevisiae_clade - ps30
Saccharomyces_cerevisiae - ps31
p-value
7e-58
6e-44
6e-30
6e-16
6e-02
Log-odds
0.5
1.0
1.5
2.0
2.5
3.0
3.5
4.0

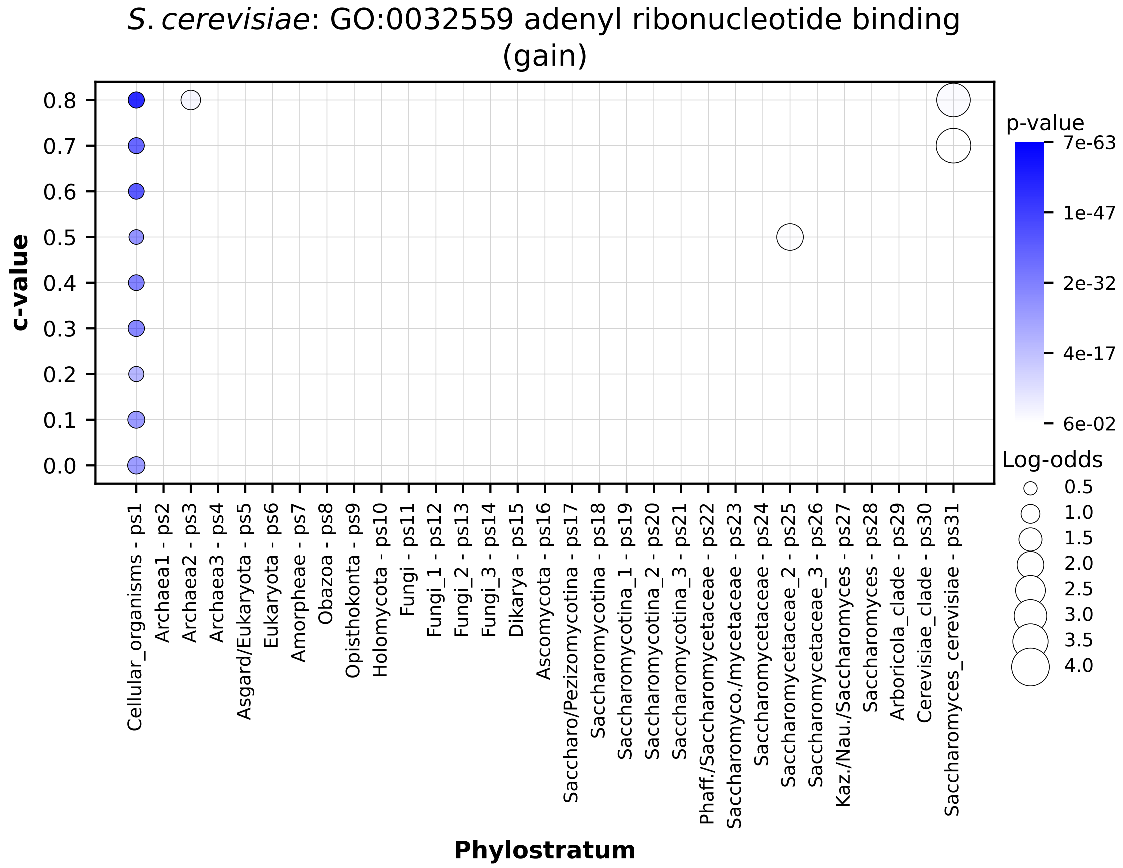

S. cerevisiae: GO:0032559 adenyl ribonucleotide binding (gain)
c-value
0.8
0.7
0.6
0.5
0.4
0.3
0.2
0.1
0.0
p-value
7e-63
1e-47
2e-32
4e-17
6e-02
Log-odds
0.5
1.0
1.5
2.0
2.5
3.0
3.5
4.0
Cellular_organisms - ps1
Archaea1 - ps2
Archaea2 - ps3
Archaea3 - ps4
Asgard/Eukaryota - ps5
Eukaryota - ps6
Amorpheae - ps7
Obazoa - ps8
Opisthokonta - ps9
Holomycota - ps10
Fungi - ps11
Fungi_1 - ps12
Fungi_2 - ps13
Fungi_3 - ps14
Dikarya - ps15
Ascomycota - ps16
Saccharo/Pezizomycotina - ps17
Saccharomycotina - ps18
Saccharomycotina_1 - ps19
Saccharomycotina_2 - ps20
Saccharomycotina_3 - ps21
Phaff./Saccharomycetaceae - ps22
Saccharomyco./mycetaceae - ps23
Saccharomycetaceae - ps24
Saccharomycetaceae_2 - ps25
Saccharomycetaceae_3 - ps26
Kaz./Nau./Saccharomyces - ps27
Saccharomyces - ps28
Arboricola_clade - ps29
Cerevisiae_clade - ps30
Saccharomyces_cerevisiae - ps31
Phylostratum

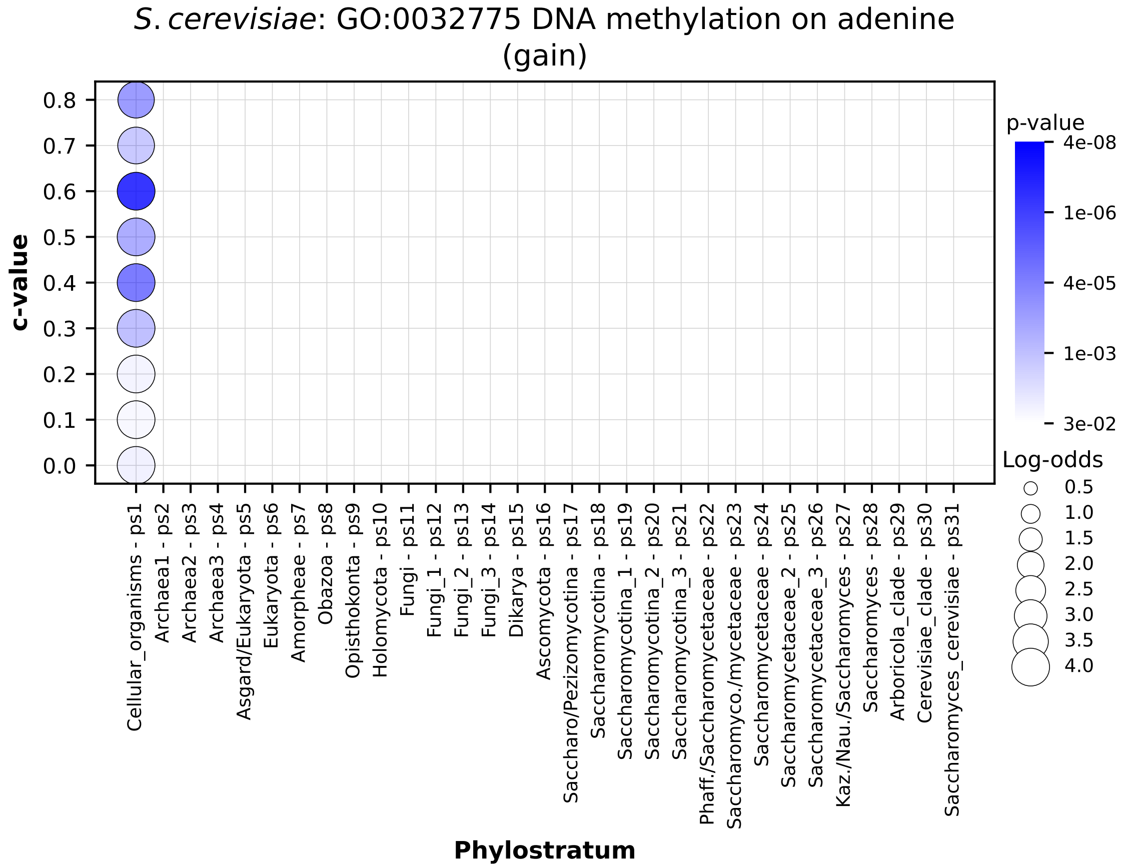

S. cerevisiae: GO:0032775 DNA methylation on adenine
(gain)
c-value
0.0
0.1
0.2
0.3
0.4
0.5
0.6
0.7
0.8
Phylostratum
Cellular_organisms - ps1
Archaea1 - ps2
Archaea2 - ps3
Archaea3 - ps4
Asgard/Eukaryota - ps5
Eukaryota - ps6
Amorpheae - ps7
Obazoa - ps8
Opisthokonta - ps9
Holomycota - ps10
Fungi - ps11
Fungi_1 - ps12
Fungi_2 - ps13
Fungi_3 - ps14
Dikarya - ps15
Ascomycota - ps16
Saccharo/Pezizomycotina - ps17
Saccharomycotina - ps18
Saccharomycotina_1 - ps19
Saccharomycotina_2 - ps20
Saccharomycotina_3 - ps21
Phaff./Saccharomycetaceae - ps22
Saccharomyco./mycetaceae - ps23
Saccharomycetaceae - ps24
Saccharomycetaceae_2 - ps25
Saccharomycetaceae_3 - ps26
Kaz./Nau./Saccharomyces - ps27
Saccharomyces - ps28
Arboricola_clade - ps29
Cerevisiae_clade - ps30
Saccharomyces_cerevisiae - ps31
p-value
4e-08
1e-06
4e-05
1e-03
3e-02
Log-odds
0.5
1.0
1.5
2.0
2.5
3.0
3.5
4.0

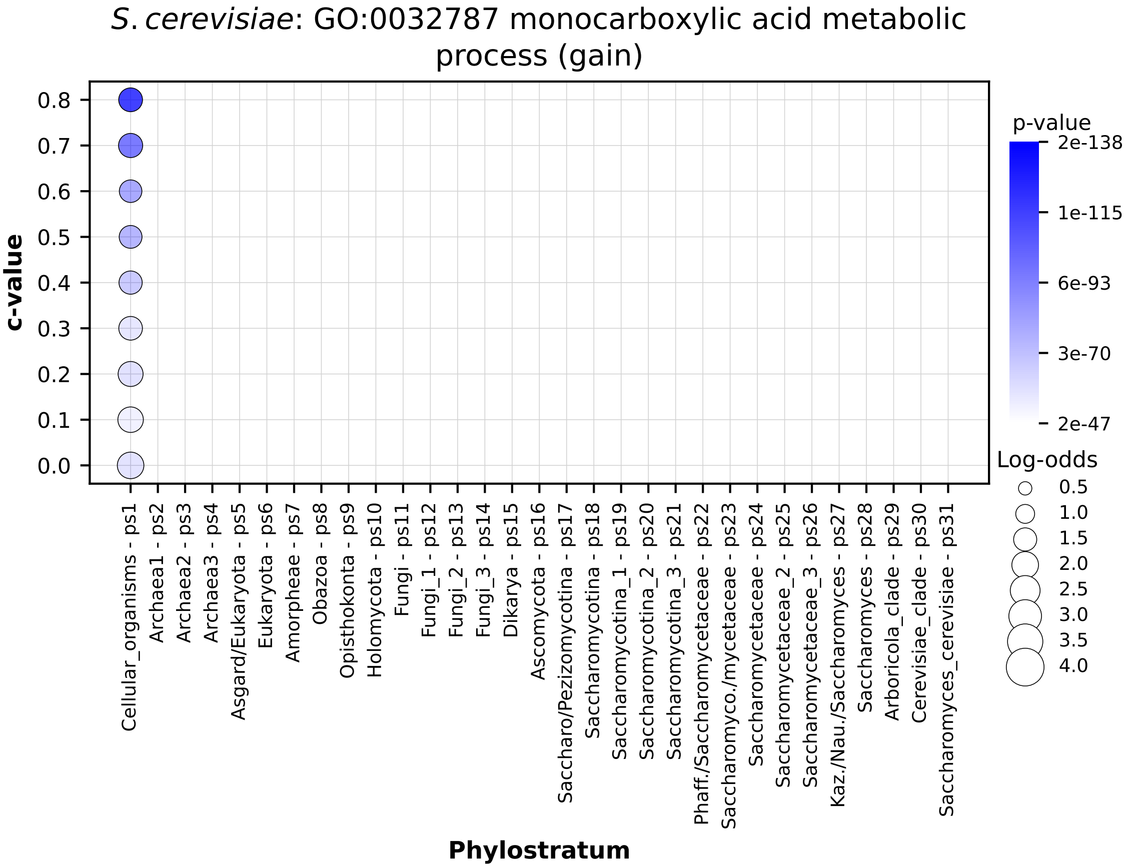
S. cerevisiae: GO:0032787 monocarboxylic acid metabolic process (gain)
c-value
0.8
0.7
0.6
0.5
0.4
0.3
0.2
0.1
0.0
Phylostratum
Cellular_organisms - ps1
Archaea1 - ps2
Archaea2 - ps3
Archaea3 - ps4
Asgard/Eukaryota - ps5
Eukaryota - ps6
Amorpheae - ps7
Obazoa - ps8
Opisthokonta - ps9
Holomycota - ps10
Fungi - ps11
Fungi_1 - ps12
Fungi_2 - ps13
Fungi_3 - ps14
Dikarya - ps15
Ascomycota - ps16
Saccharo/Pezizomycotina - ps17
Saccharomycotina - ps18
Saccharomycotina_1 - ps19
Saccharomycotina_2 - ps20
Saccharomycotina_3 - ps21
Phaff./Saccharomycetaceae - ps22
Saccharomyco./mycetaceae - ps23
Saccharomycetaceae - ps24
Saccharomycetaceae_2 - ps25
Saccharomycetaceae_3 - ps26
Kaz./Nau./Saccharomyces - ps27
Saccharomyces - ps28
Arboricola_clade - ps29
Cerevisiae_clade - ps30
Saccharomyces_cerevisiae - ps31
p-value
2e-138
1e-115
6e-93
3e-70
2e-47
Log-odds
0.5
1.0
1.5
2.0
2.5
3.0
3.5
4.0

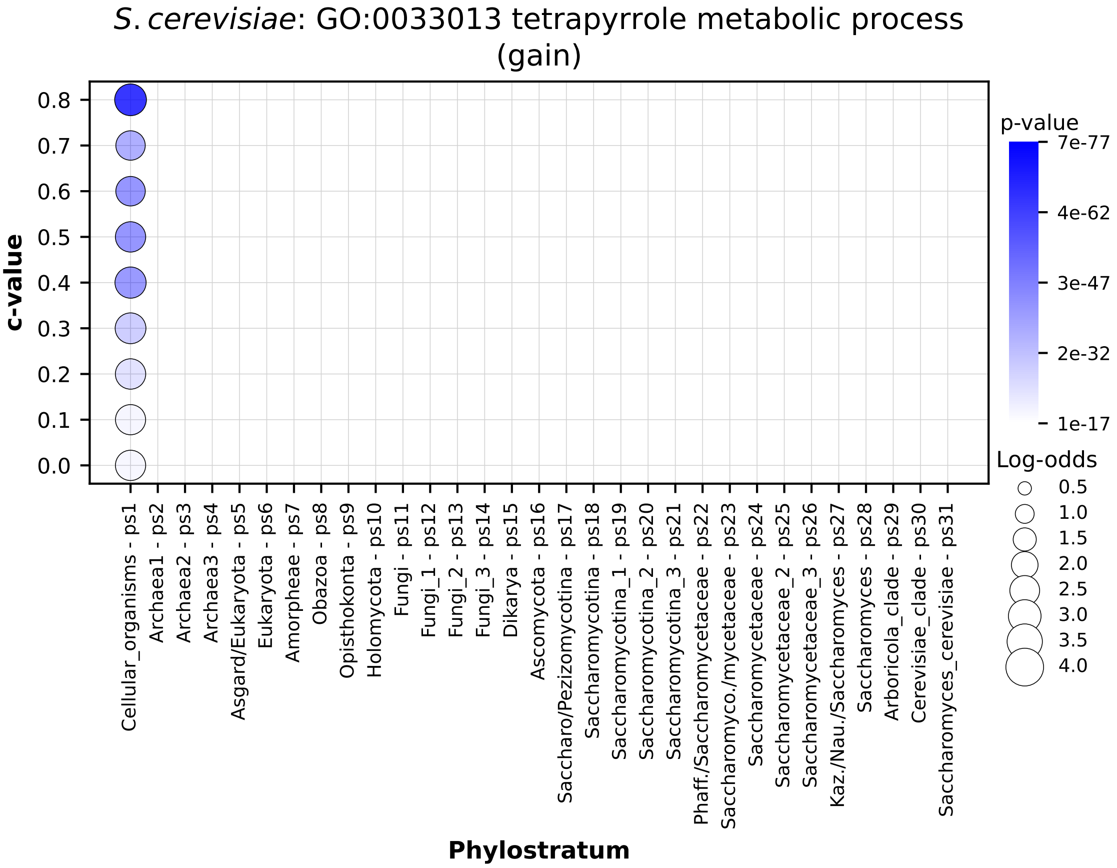
S. cerevisiae: GO:0033013 tetrapyrrole metabolic process
(gain)
c-value
0.8
0.7
0.6
0.5
0.4
0.3
0.2
0.1
0.0
p-value
7e-77
4e-62
3e-47
2e-32
1e-17
Log-odds
0.5
1.0
1.5
2.0
2.5
3.0
3.5
4.0
Cellular_organisms - ps1
Archaea1 - ps2
Archaea2 - ps3
Archaea3 - ps4
Asgard/Eukaryota - ps5
Eukaryota - ps6
Amorpheae - ps7
Obazoa - ps8
Opisthokonta - ps9
Holomycota - ps10
Fungi - ps11
Fungi_1 - ps12
Fungi_2 - ps13
Fungi_3 - ps14
Dikarya - ps15
Ascomycota - ps16
Saccharo/Pezizomycotina - ps17
Saccharomycotina - ps18
Saccharomycotina_1 - ps19
Saccharomycotina_2 - ps20
Saccharomycotina_3 - ps21
Phaff./Saccharomycetaceae - ps22
Saccharomyco./mycetaceae - ps23
Saccharomycetaceae - ps24
Saccharomycetaceae_2 - ps25
Saccharomycetaceae_3 - ps26
Kaz./Nau./Saccharomyces - ps27
Saccharomyces - ps28
Arboricola_clade - ps29
Cerevisiae_clade - ps30
Saccharomyces_cerevisiae - ps31
Phylostratum

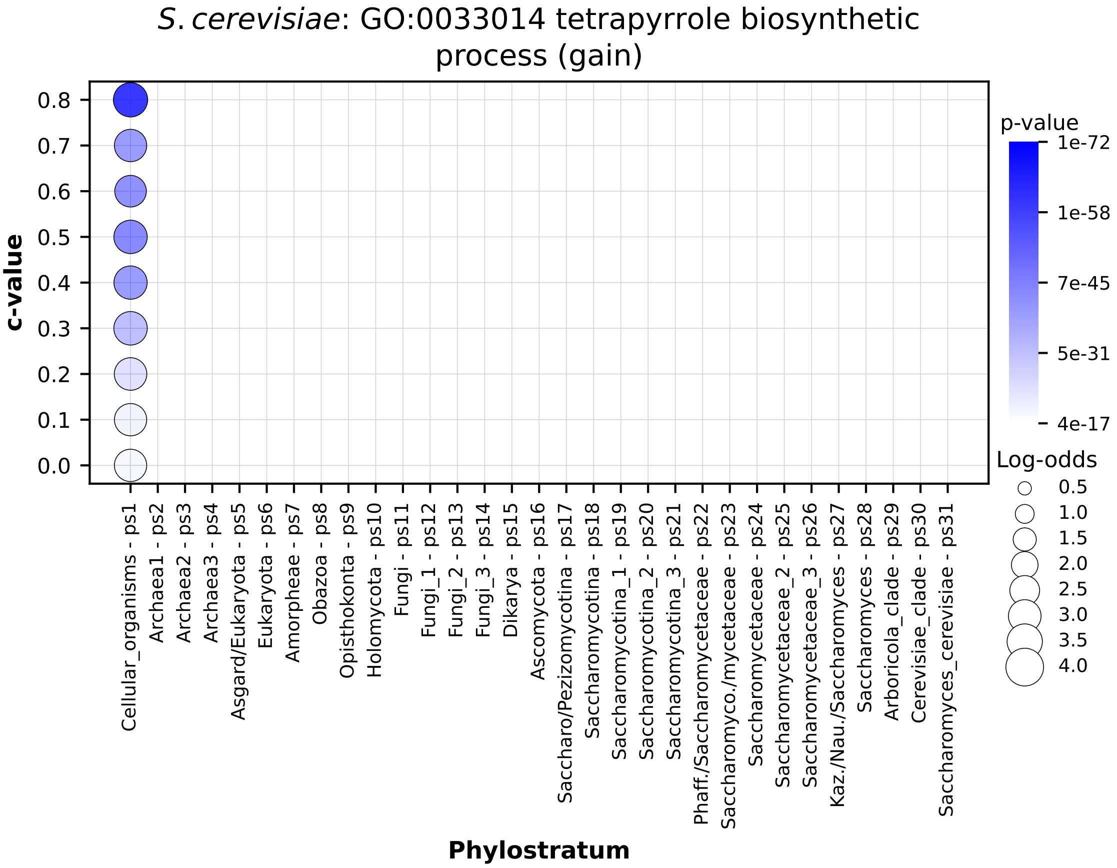

S. cerevisiae: GO:0033014 tetrapyrrole biosynthetic process (gain)
c-value
0.8
0.7
0.6
0.5
0.4
0.3
0.2
0.1
0.0
p-value
1e-72
1e-58
7e-45
5e-31
4e-17
Log-odds
0.5
1.0
1.5
2.0
2.5
3.0
3.5
4.0
Cellular_organisms - ps1
Archaea1 - ps2
Archaea2 - ps3
Archaea3 - ps4
Asgard/Eukaryota - ps5
Eukaryota - ps6
Amorpheae - ps7
Obazoa - ps8
Opisthokonta - ps9
Holomycota - ps10
Fungi - ps11
Fungi_1 - ps12
Fungi_2 - ps13
Fungi_3 - ps14
Dikarya - ps15
Ascomycota - ps16
Saccharo/Pezizomycotina - ps17
Saccharomycotina - ps18
Saccharomycotina_1 - ps19
Saccharomycotina_2 - ps20
Saccharomycotina_3 - ps21
Phaff./Saccharomycetaceae - ps22
Saccharomyco./mycetaceae - ps23
Saccharomycetaceae - ps24
Saccharomycetaceae_2 - ps25
Saccharomycetaceae_3 - ps26
Kaz./Nau./Saccharomyces - ps27
Saccharomyces - ps28
Arboricola_clade - ps29
Cerevisiae_clade - ps30
Saccharomyces_cerevisiae - ps31
Phylostratum

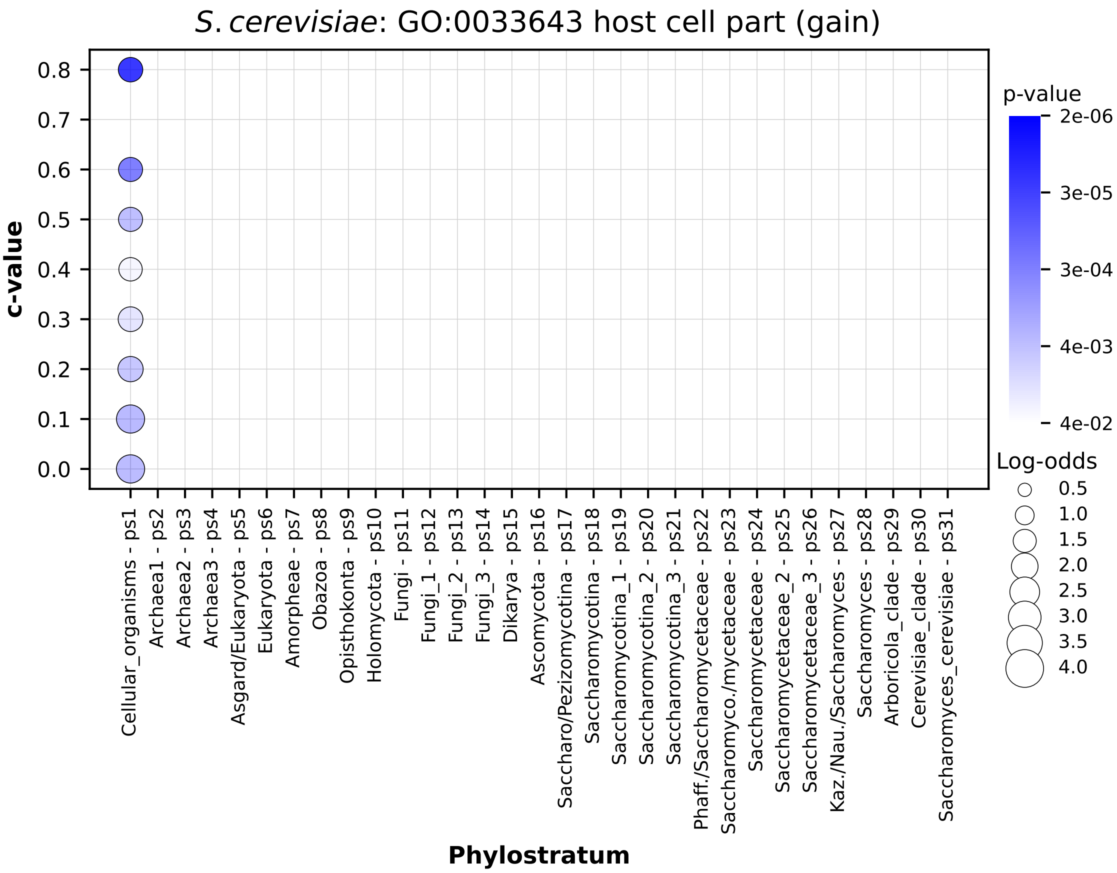

S. cerevisiae: GO:0033643 host cell part (gain)
c-value
0.0
0.1
0.2
0.3
0.4
0.5
0.6
0.7
0.8
p-value
2e-06
3e-05
3e-04
4e-03
4e-02
Log-odds
0.5
1.0
1.5
2.0
2.5
3.0
3.5
4.0
Cellular_organisms - ps1
Archaea1 - ps2
Archaea2 - ps3
Archaea3 - ps4
Asgard/Eukaryota - ps5
Eukaryota - ps6
Amorpheae - ps7
Obazoa - ps8
Opisthokonta - ps9
Holomycota - ps10
Fungi - ps11
Fungi_1 - ps12
Fungi_2 - ps13
Fungi_3 - ps14
Dikarya - ps15
Ascomycota - ps16
Saccharo/Pezizomycotina - ps17
Saccharomycotina - ps18
Saccharomycotina_1 - ps19
Saccharomycotina_2 - ps20
Saccharomycotina_3 - ps21
Phaff./Saccharomycetaceae - ps22
Saccharomyco./mycetaceae - ps23
Saccharomycetaceae - ps24
Saccharomycetaceae_2 - ps25
Saccharomycetaceae_3 - ps26
Kaz./Nau./Saccharomyces - ps27
Saccharomyces - ps28
Arboricola_clade - ps29
Cerevisiae_clade - ps30
Saccharomyces_cerevisiae - ps31
Phylostratum

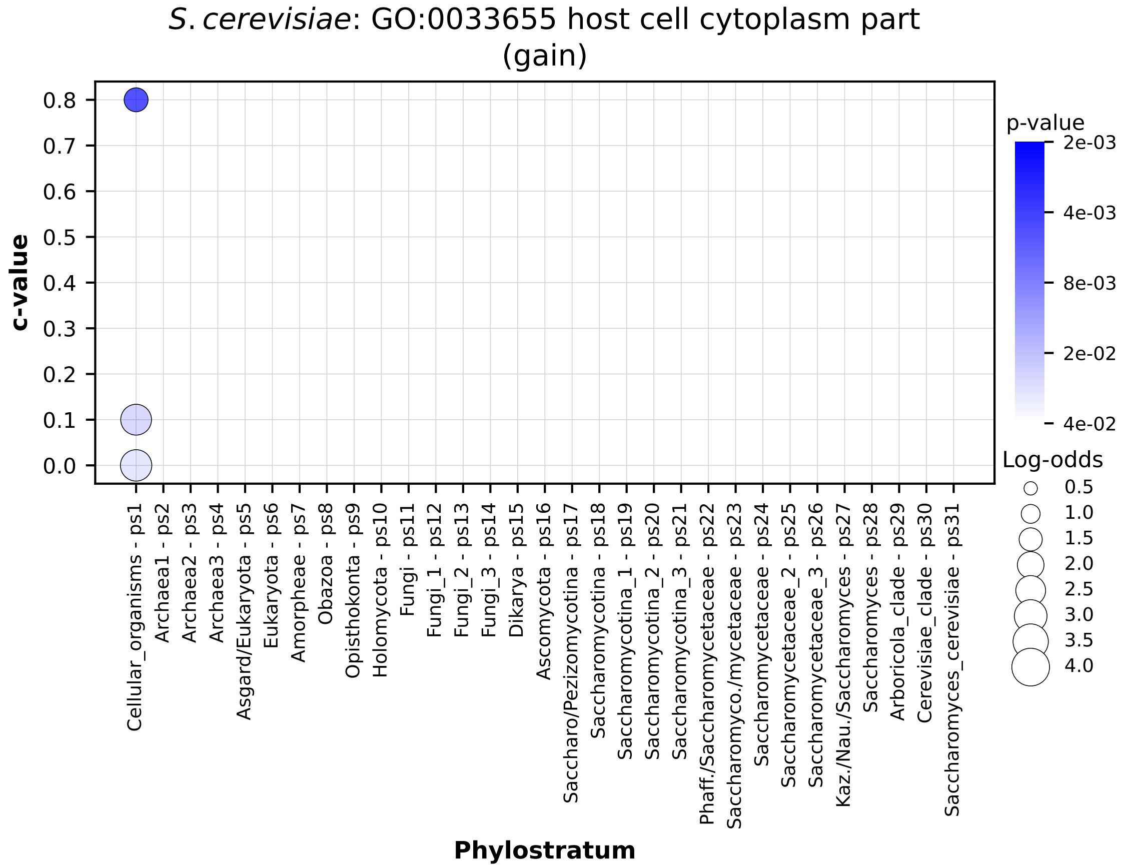
S. cerevisiae: GO:0033655 host cell cytoplasm part
(gain)
c-value
0.8
0.7
0.6
0.5
0.4
0.3
0.2
0.1
0.0
p-value
2e-03
4e-03
8e-03
2e-02
4e-02
Log-odds
0.5
1.0
1.5
2.0
2.5
3.0
3.5
4.0
Cellular_organisms - ps1
Archaea1 - ps2
Archaea2 - ps3
Archaea3 - ps4
Asgard/Eukaryota - ps5
Eukaryota - ps6
Amorpheae - ps7
Obazoa - ps8
Opisthokonta - ps9
Holomycota - ps10
Fungi - ps11
Fungi_1 - ps12
Fungi_2 - ps13
Fungi_3 - ps14
Dikarya - ps15
Ascomycota - ps16
Saccharo/Pezizomycotina - ps17
Saccharomycotina - ps18
Saccharomycotina_1 - ps19
Saccharomycotina_2 - ps20
Saccharomycotina_3 - ps21
Phaff./Saccharomycetaceae - ps22
Saccharomyco./mycetaceae - ps23
Saccharomycetaceae - ps24
Saccharomycetaceae_2 - ps25
Saccharomycetaceae_3 - ps26
Kaz./Nau./Saccharomyces - ps27
Saccharomyces - ps28
Arboricola_clade - ps29
Cerevisiae_clade - ps30
Saccharomyces_cerevisiae - ps31
Phylostratum

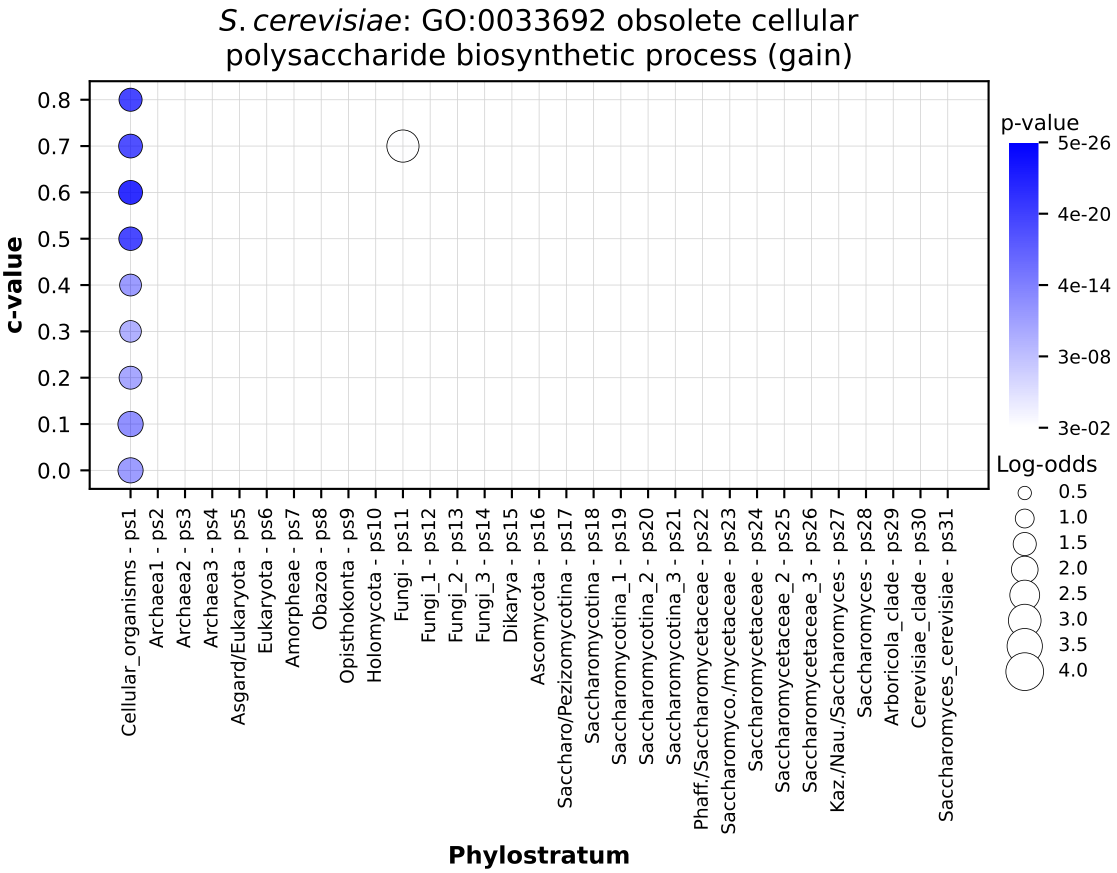
S. cerevisiae: GO:0033692 obsolete cellular polysaccharide biosynthetic process (gain)
c-value
0.8
0.7
0.6
0.5
0.4
0.3
0.2
0.1
0.0
p-value
5e-26
4e-20
4e-14
3e-08
3e-02
Log-odds
0.5
1.0
1.5
2.0
2.5
3.0
3.5
4.0
Cellular_organisms - ps1
Archaea1 - ps2
Archaea2 - ps3
Archaea3 - ps4
Asgard/Eukaryota - ps5
Eukaryota - ps6
Amorpheae - ps7
Obazoa - ps8
Opisthokonta - ps9
Holomycota - ps10
Fungi - ps11
Fungi_1 - ps12
Fungi_2 - ps13
Fungi_3 - ps14
Dikarya - ps15
Ascomycota - ps16
Saccharo/Pezizomycotina - ps17
Saccharomycotina - ps18
Saccharomycotina_1 - ps19
Saccharomycotina_2 - ps20
Saccharomycotina_3 - ps21
Phaff./Saccharomycetaceae - ps22
Saccharomyco./mycetaceae - ps23
Saccharomycetaceae - ps24
Saccharomycetaceae_2 - ps25
Saccharomycetaceae_3 - ps26
Kaz./Nau./Saccharomyces - ps27
Saccharomyces - ps28
Arboricola_clade - ps29
Cerevisiae_clade - ps30
Saccharomyces_cerevisiae - ps31
Phylostratum

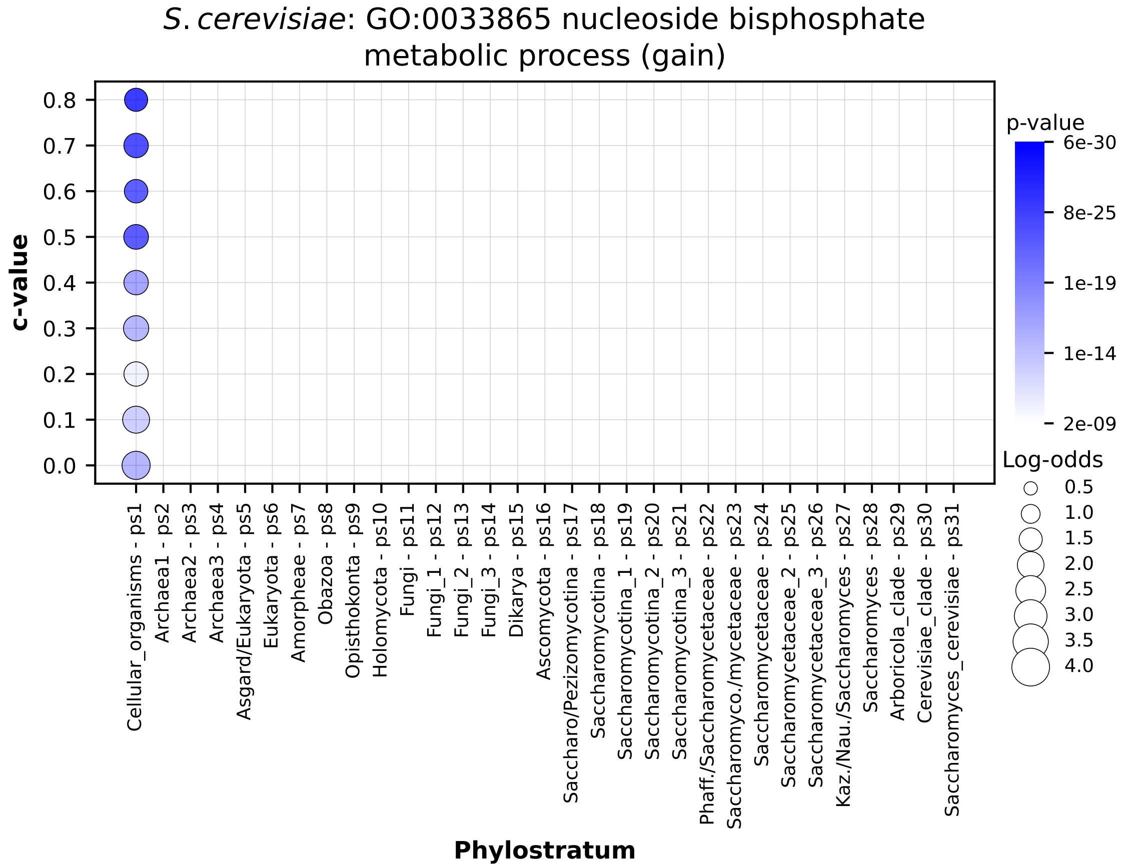

S. cerevisiae: GO:0033865 nucleoside bisphosphate metabolic process (gain)
c-value
0.8
0.7
0.6
0.5
0.4
0.3
0.2
0.1
0.0
p-value
6e-30
8e-25
1e-19
1e-14
2e-09
Log-odds
0.5
1.0
1.5
2.0
2.5
3.0
3.5
4.0
Cellular_organisms - ps1
Archaea1 - ps2
Archaea2 - ps3
Archaea3 - ps4
Asgard/Eukaryota - ps5
Eukaryota - ps6
Amorpheae - ps7
Obazoa - ps8
Opisthokonta - ps9
Holomycota - ps10
Fungi - ps11
Fungi_1 - ps12
Fungi_2 - ps13
Fungi_3 - ps14
Dikarya - ps15
Ascomycota - ps16
Saccharo/Pezizomycotina - ps17
Saccharomycotina - ps18
Saccharomycotina_1 - ps19
Saccharomycotina_2 - ps20
Saccharomycotina_3 - ps21
Phaff./Saccharomycetaceae - ps22
Saccharomyco./mycetaceae - ps23
Saccharomycetaceae - ps24
Saccharomycetaceae_2 - ps25
Saccharomycetaceae_3 - ps26
Kaz./Nau./Saccharomyces - ps27
Saccharomyces - ps28
Arboricola_clade - ps29
Cerevisiae_clade - ps30
Saccharomyces_cerevisiae - ps31
Phylostratum

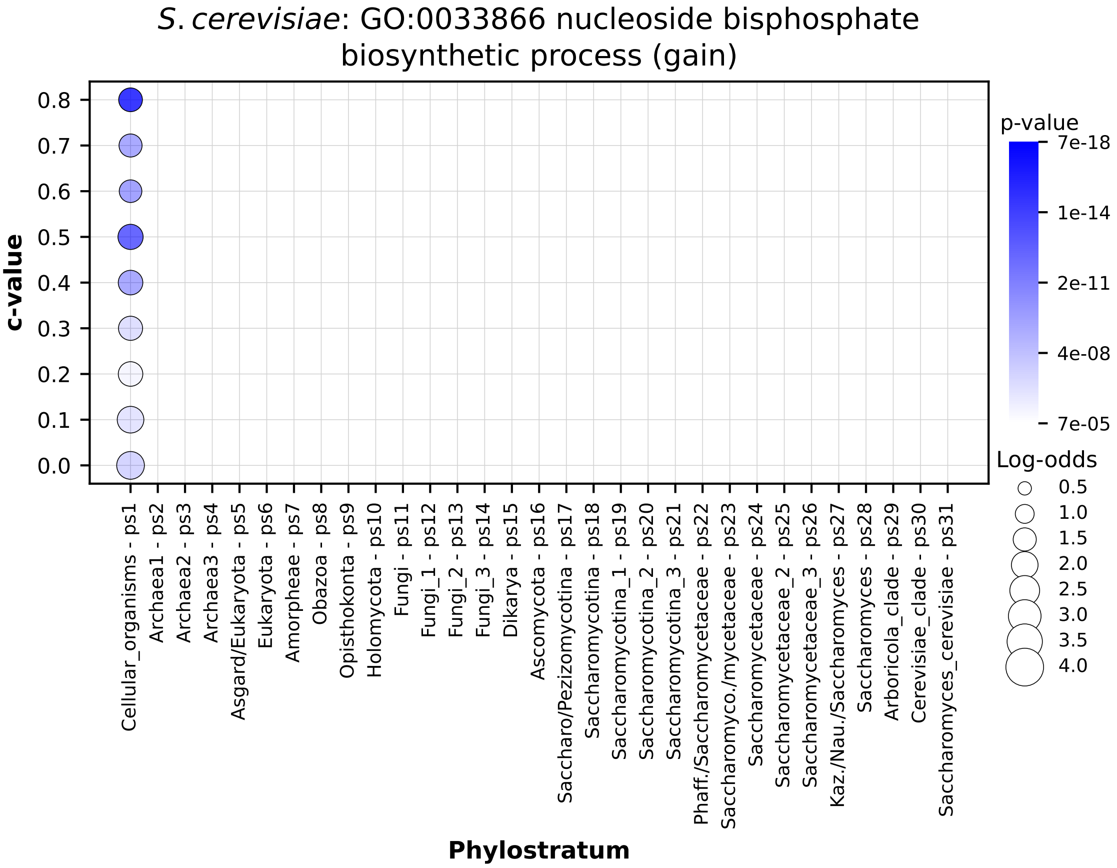

S. cerevisiae: GO:0033866 nucleoside bisphosphate biosynthetic process (gain)
c-value
0.8
0.7
0.6
0.5
0.4
0.3
0.2
0.1
0.0
Phylostratum
Cellular_organisms - ps1
Archaea1 - ps2
Archaea2 - ps3
Archaea3 - ps4
Asgard/Eukaryota - ps5
Eukaryota - ps6
Amorpheae - ps7
Obazoa - ps8
Opisthokonta - ps9
Holomycota - ps10
Fungi - ps11
Fungi_1 - ps12
Fungi_2 - ps13
Fungi_3 - ps14
Dikarya - ps15
Ascomycota - ps16
Saccharo/Pezizomycotina - ps17
Saccharomycotina - ps18
Saccharomycotina_1 - ps19
Saccharomycotina_2 - ps20
Saccharomycotina_3 - ps21
Phaff./Saccharomycetaceae - ps22
Saccharomyco./mycetaceae - ps23
Saccharomycetaceae - ps24
Saccharomycetaceae_2 - ps25
Saccharomycetaceae_3 - ps26
Kaz./Nau./Saccharomyces - ps27
Saccharomyces - ps28
Arboricola_clade - ps29
Cerevisiae_clade - ps30
Saccharomyces_cerevisiae - ps31
p-value
7e-18
1e-14
2e-11
4e-08
7e-05
Log-odds
0.5
1.0
1.5
2.0
2.5
3.0
3.5
4.0

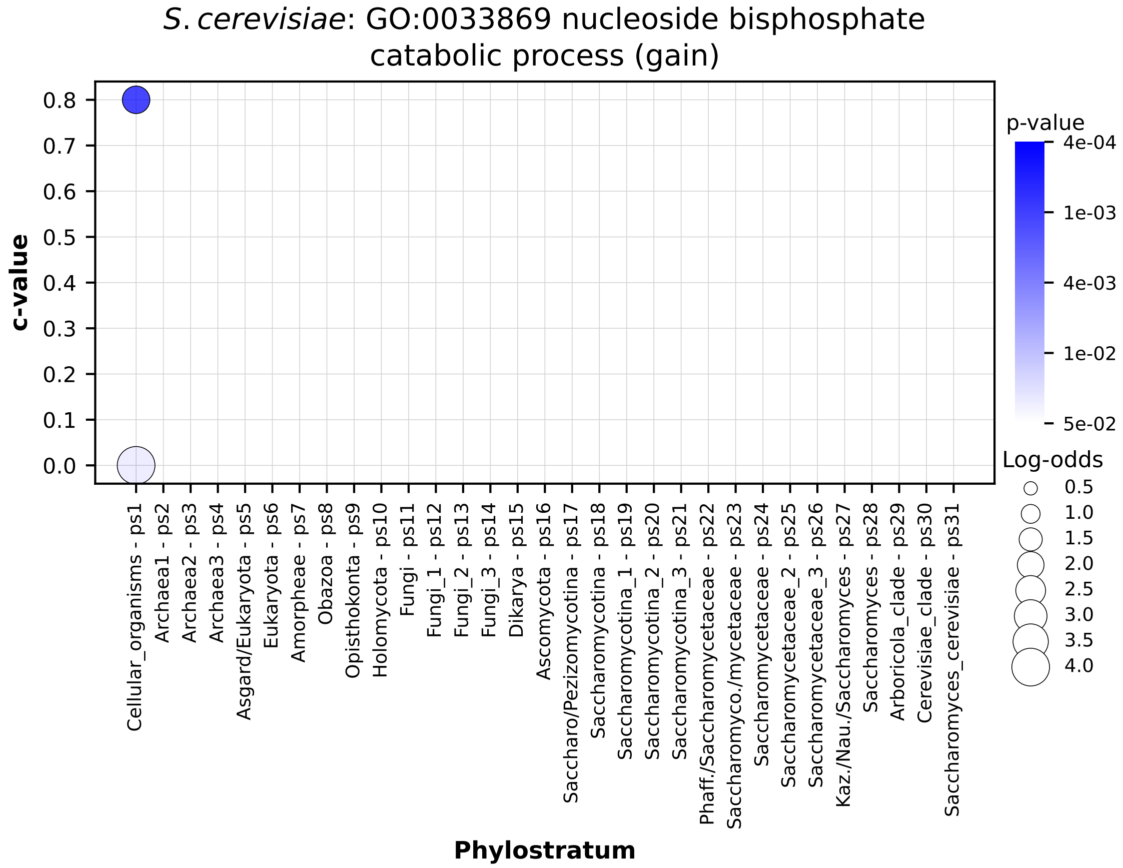
S. cerevisiae: GO:0033869 nucleoside bisphosphate catabolic process (gain)
c-value
0.8
0.7
0.6
0.5
0.4
0.3
0.2
0.1
0.0
p-value
4e-04
1e-03
4e-03
1e-02
5e-02
Log-odds
0.5
1.0
1.5
2.0
2.5
3.0
3.5
4.0
Cellular_organisms - ps1
Archaea1 - ps2
Archaea2 - ps3
Archaea3 - ps4
Asgard/Eukaryota - ps5
Eukaryota - ps6
Amorpheae - ps7
Obazoa - ps8
Opisthokonta - ps9
Holomycota - ps10
Fungi - ps11
Fungi_1 - ps12
Fungi_2 - ps13
Fungi_3 - ps14
Dikarya - ps15
Ascomycota - ps16
Saccharo/Pezizomycotina - ps17
Saccharomycotina - ps18
Saccharomycotina_1 - ps19
Saccharomycotina_2 - ps20
Saccharomycotina_3 - ps21
Phaff./Saccharomycetaceae - ps22
Saccharomyco./mycetaceae - ps23
Saccharomycetaceae - ps24
Saccharomycetaceae_2 - ps25
Saccharomycetaceae_3 - ps26
Kaz./Nau./Saccharomyces - ps27
Saccharomyces - ps28
Arboricola_clade - ps29
Cerevisiae_clade - ps30
Saccharomyces_cerevisiae - ps31
Phylostratum

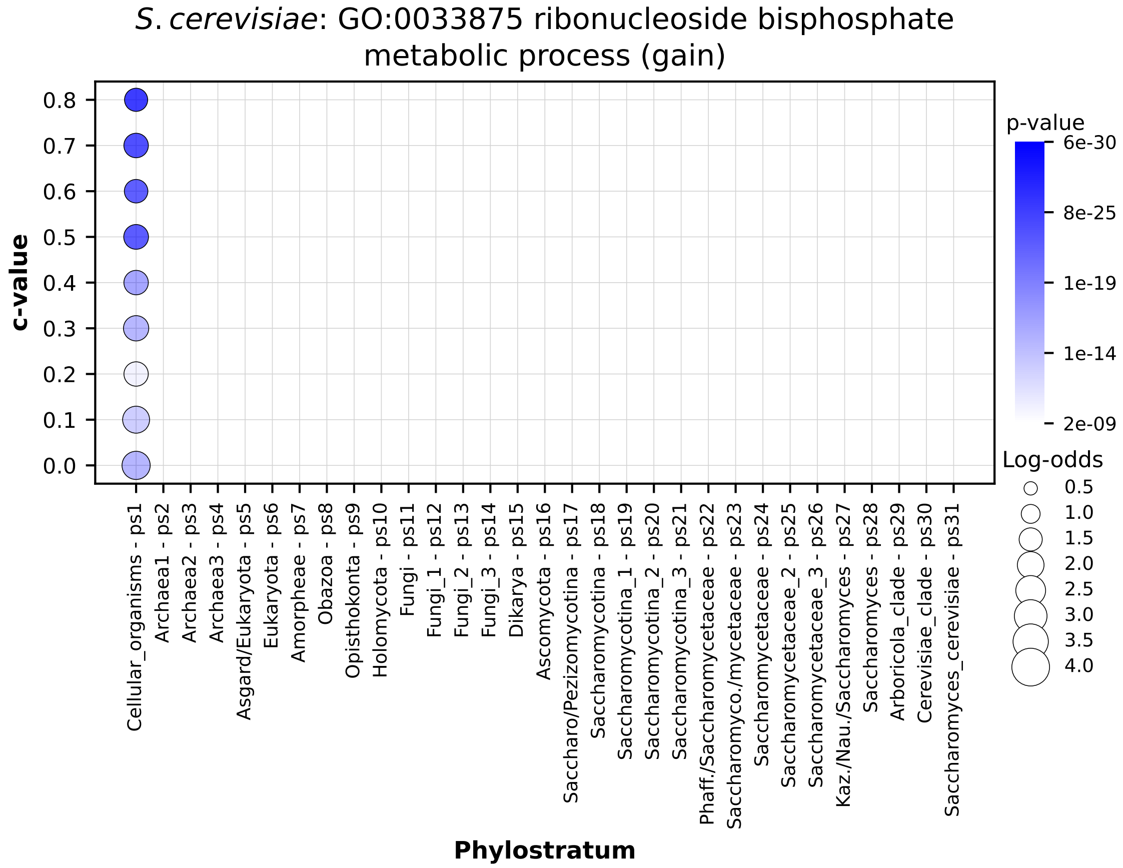

S. cerevisiae: GO:0033875 ribonucleoside bisphosphate metabolic process (gain)
c-value
0.0
0.1
0.2
0.3
0.4
0.5
0.6
0.7
0.8
p-value
6e-30
8e-25
1e-19
1e-14
2e-09
Log-odds
0.5
1.0
1.5
2.0
2.5
3.0
3.5
4.0
Cellular_organisms - ps1
Archaea1 - ps2
Archaea2 - ps3
Archaea3 - ps4
Asgard/Eukaryota - ps5
Eukaryota - ps6
Amorpheae - ps7
Obazoa - ps8
Opisthokonta - ps9
Holomycota - ps10
Fungi - ps11
Fungi_1 - ps12
Fungi_2 - ps13
Fungi_3 - ps14
Dikarya - ps15
Ascomycota - ps16
Saccharo/Pezizomycotina - ps17
Saccharomycotina - ps18
Saccharomycotina_1 - ps19
Saccharomycotina_2 - ps20
Saccharomycotina_3 - ps21
Phaff./Saccharomycetaceae - ps22
Saccharomyco./mycetaceae - ps23
Saccharomycetaceae - ps24
Saccharomycetaceae_2 - ps25
Saccharomycetaceae_3 - ps26
Kaz./Nau./Saccharomyces - ps27
Saccharomyces - ps28
Arboricola_clade - ps29
Cerevisiae_clade - ps30
Saccharomyces_cerevisiae - ps31
Phylostratum

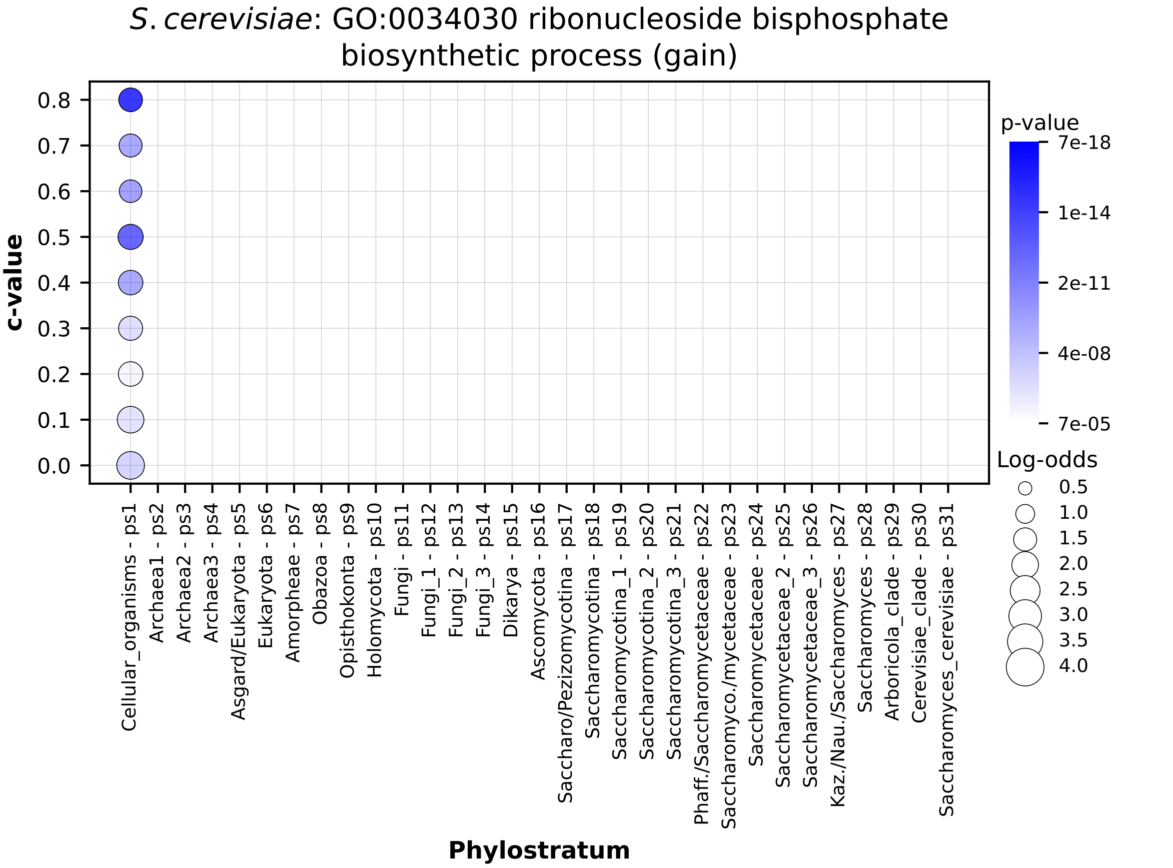
S. cerevisiae: GO:0034030 ribonucleoside bisphosphate biosynthetic process (gain)
c-value
0.8
0.7
0.6
0.5
0.4
0.3
0.2
0.1
0.0
p-value
7e-18
1e-14
2e-11
4e-08
7e-05
Log-odds
0.5
1.0
1.5
2.0
2.5
3.0
3.5
4.0
Cellular_organisms - ps1
Archaea1 - ps2
Archaea2 - ps3
Archaea3 - ps4
Asgard/Eukaryota - ps5
Eukaryota - ps6
Amorpheae - ps7
Obazoa - ps8
Opisthokonta - ps9
Holomycota - ps10
Fungi - ps11
Fungi_1 - ps12
Fungi_2 - ps13
Fungi_3 - ps14
Dikarya - ps15
Ascomycota - ps16
Saccharo/Pezizomycotina - ps17
Saccharomycotina - ps18
Saccharomycotina_1 - ps19
Saccharomycotina_2 - ps20
Saccharomycotina_3 - ps21
Phaff./Saccharomycetaceae - ps22
Saccharomyco./mycetaceae - ps23
Saccharomycetaceae - ps24
Saccharomycetaceae_2 - ps25
Saccharomycetaceae_3 - ps26
Kaz./Nau./Saccharomyces - ps27
Saccharomyces - ps28
Arboricola_clade - ps29
Cerevisiae_clade - ps30
Saccharomyces_cerevisiae - ps31
Phylostratum

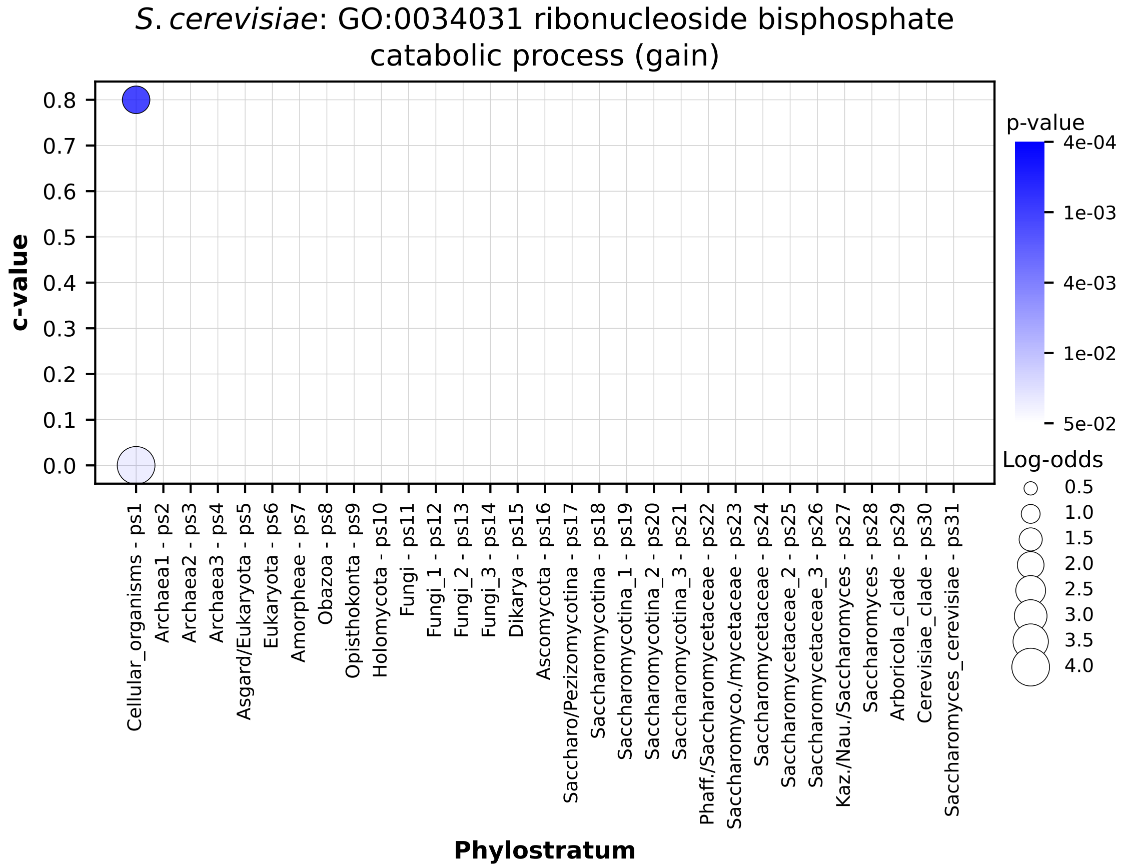
S. cerevisiae: GO:0034031 ribonucleoside bisphosphate catabolic process (gain)
c-value
0.8
0.7
0.6
0.5
0.4
0.3
0.2
0.1
0.0
p-value
4e-04
1e-03
4e-03
1e-02
5e-02
Log-odds
0.5
1.0
1.5
2.0
2.5
3.0
3.5
4.0
Cellular_organisms - ps1
Archaea1 - ps2
Archaea2 - ps3
Archaea3 - ps4
Asgard/Eukaryota - ps5
Eukaryota - ps6
Amorpheae - ps7
Obazoa - ps8
Opisthokonta - ps9
Holomycota - ps10
Fungi - ps11
Fungi_1 - ps12
Fungi_2 - ps13
Fungi_3 - ps14
Dikarya - ps15
Ascomycota - ps16
Saccharo/Pezizomycotina - ps17
Saccharomycotina - ps18
Saccharomycotina_1 - ps19
Saccharomycotina_2 - ps20
Saccharomycotina_3 - ps21
Phaff./Saccharomycetaceae - ps22
Saccharomyco./mycetaceae - ps23
Saccharomycetaceae - ps24
Saccharomycetaceae_2 - ps25
Saccharomycetaceae_3 - ps26
Kaz./Nau./Saccharomyces - ps27
Saccharomyces - ps28
Arboricola_clade - ps29
Cerevisiae_clade - ps30
Saccharomyces_cerevisiae - ps31
Phylostratum

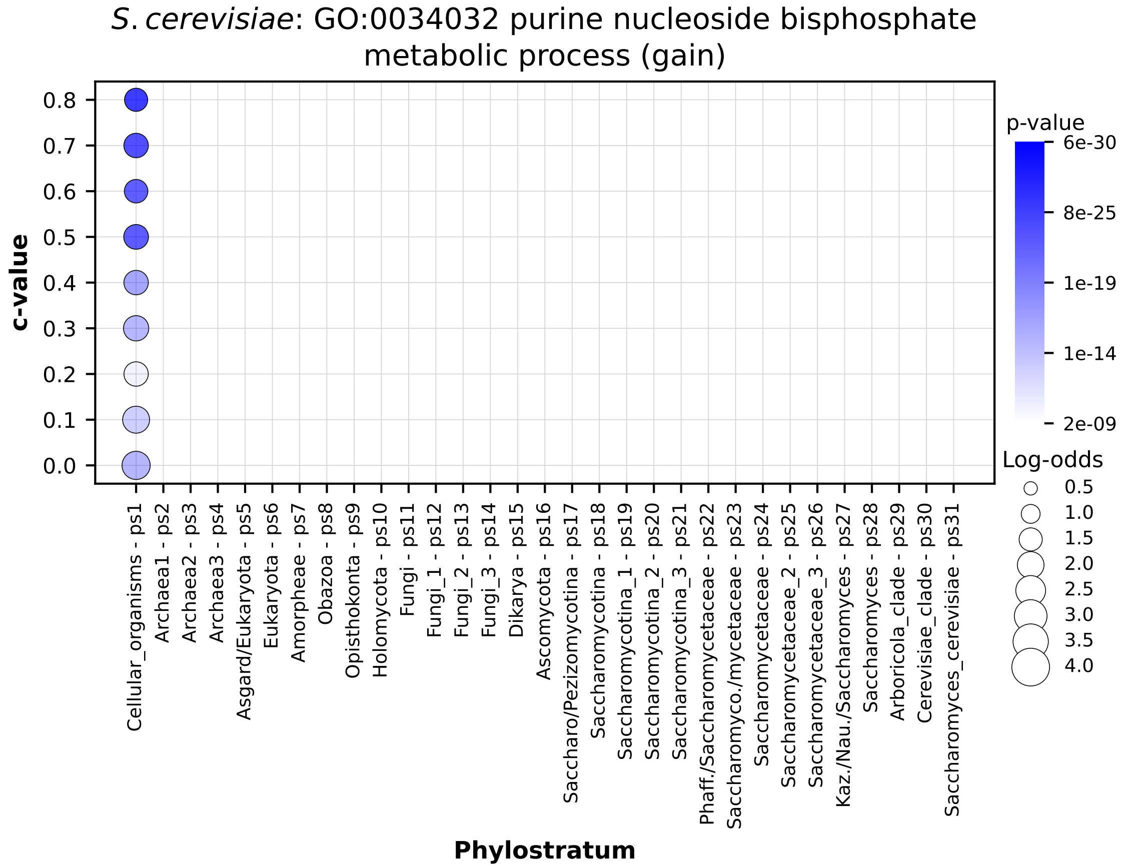

S. cerevisiae: GO:0034032 purine nucleoside bisphosphate metabolic process (gain)
c-value
0.0
0.1
0.2
0.3
0.4
0.5
0.6
0.7
0.8
Phylostratum
Cellular_organisms - ps1
Archaea1 - ps2
Archaea2 - ps3
Archaea3 - ps4
Asgard/Eukaryota - ps5
Eukaryota - ps6
Amorpheae - ps7
Obazoa - ps8
Opisthokonta - ps9
Holomycota - ps10
Fungi - ps11
Fungi_1 - ps12
Fungi_2 - ps13
Fungi_3 - ps14
Dikarya - ps15
Ascomycota - ps16
Saccharo/Pezizomycotina - ps17
Saccharomycotina - ps18
Saccharomycotina_1 - ps19
Saccharomycotina_2 - ps20
Saccharomycotina_3 - ps21
Phaff./Saccharomycetaceae - ps22
Saccharomyco./mycetaceae - ps23
Saccharomycetaceae - ps24
Saccharomycetaceae_2 - ps25
Saccharomycetaceae_3 - ps26
Kaz./Nau./Saccharomyces - ps27
Saccharomyces - ps28
Arboricola_clade - ps29
Cerevisiae_clade - ps30
Saccharomyces_cerevisiae - ps31
p-value
6e-30
8e-25
1e-19
1e-14
2e-09
Log-odds
0.5
1.0
1.5
2.0
2.5
3.0
3.5
4.0

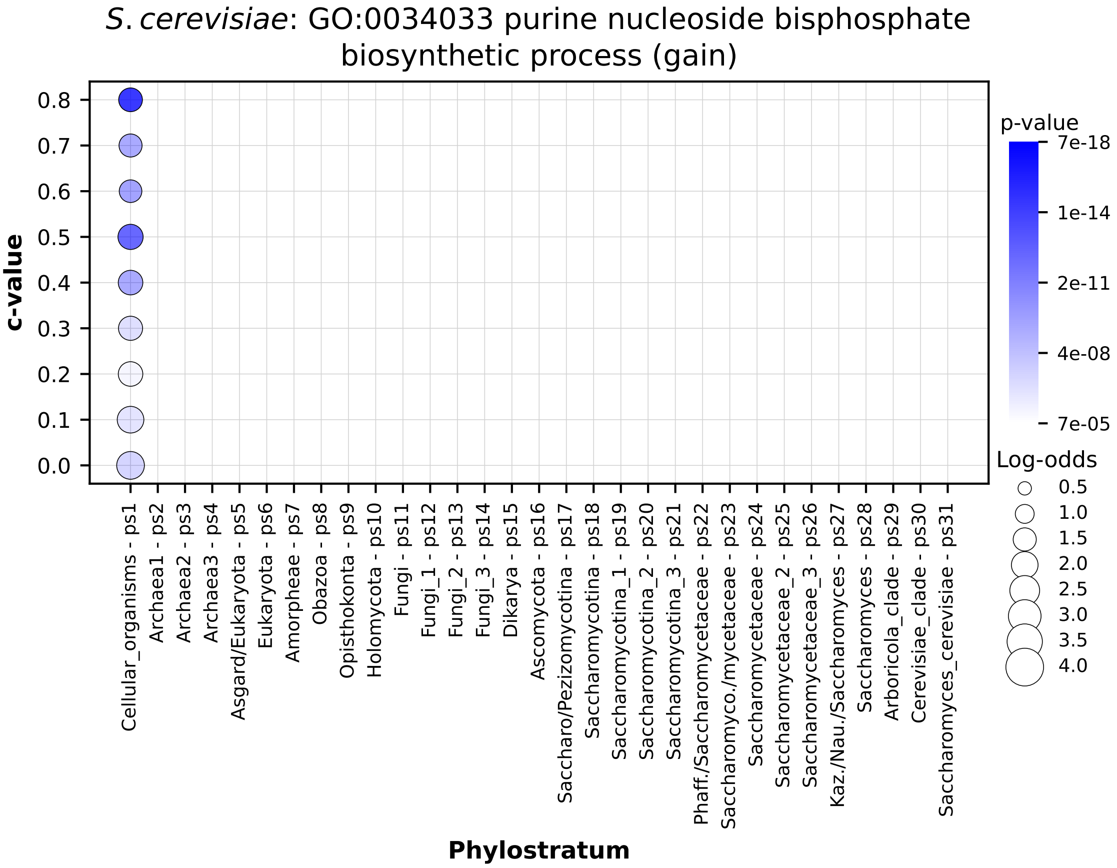
S. cerevisiae: GO:0034033 purine nucleoside bisphosphate biosynthetic process (gain)
c-value
0.0
0.1
0.2
0.3
0.4
0.5
0.6
0.7
0.8
p-value
7e-18
1e-14
2e-11
4e-08
7e-05
Log-odds
0.5
1.0
1.5
2.0
2.5
3.0
3.5
4.0
Cellular_organisms - ps1
Archaea1 - ps2
Archaea2 - ps3
Archaea3 - ps4
Asgard/Eukaryota - ps5
Eukaryota - ps6
Amorpheae - ps7
Obazoa - ps8
Opisthokonta - ps9
Holomycota - ps10
Fungi - ps11
Fungi_1 - ps12
Fungi_2 - ps13
Fungi_3 - ps14
Dikarya - ps15
Ascomycota - ps16
Saccharo/Pezizomycotina - ps17
Saccharomycotina - ps18
Saccharomycotina_1 - ps19
Saccharomycotina_2 - ps20
Saccharomycotina_3 - ps21
Phaff./Saccharomycetaceae - ps22
Saccharomyco./mycetaceae - ps23
Saccharomycetaceae - ps24
Saccharomycetaceae_2 - ps25
Saccharomycetaceae_3 - ps26
Kaz./Nau./Saccharomyces - ps27
Saccharomyces - ps28
Arboricola_clade - ps29
Cerevisiae_clade - ps30
Saccharomyces_cerevisiae - ps31
Phylostratum

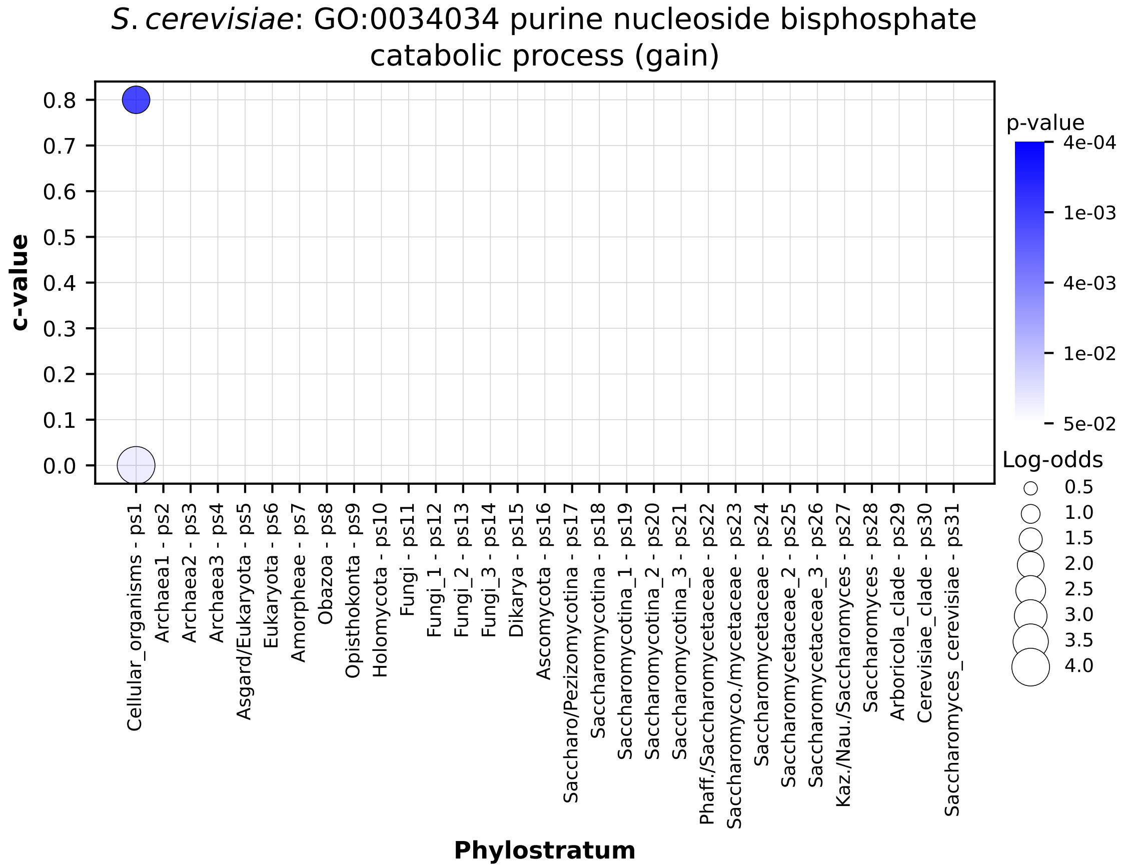
S. cerevisiae: GO:0034034 purine nucleoside bisphosphate catabolic process (gain)
c-value
0.0
0.1
0.2
0.3
0.4
0.5
0.6
0.7
0.8
p-value
4e-04
1e-03
4e-03
1e-02
5e-02
Log-odds
0.5
1.0
1.5
2.0
2.5
3.0
3.5
4.0
Cellular_organisms - ps1
Archaea1 - ps2
Archaea2 - ps3
Archaea3 - ps4
Asgard/Eukaryota - ps5
Eukaryota - ps6
Amorpheae - ps7
Obazoa - ps8
Opisthokonta - ps9
Holomycota - ps10
Fungi - ps11
Fungi_1 - ps12
Fungi_2 - ps13
Fungi_3 - ps14
Dikarya - ps15
Ascomycota - ps16
Saccharo/Pezizomycotina - ps17
Saccharomycotina - ps18
Saccharomycotina_1 - ps19
Saccharomycotina_2 - ps20
Saccharomycotina_3 - ps21
Phaff./Saccharomycetaceae - ps22
Saccharomyco./mycetaceae - ps23
Saccharomycetaceae - ps24
Saccharomycetaceae_2 - ps25
Saccharomycetaceae_3 - ps26
Kaz./Nau./Saccharomyces - ps27
Saccharomyces - ps28
Arboricola_clade - ps29
Cerevisiae_clade - ps30
Saccharomyces_cerevisiae - ps31
Phylostratum

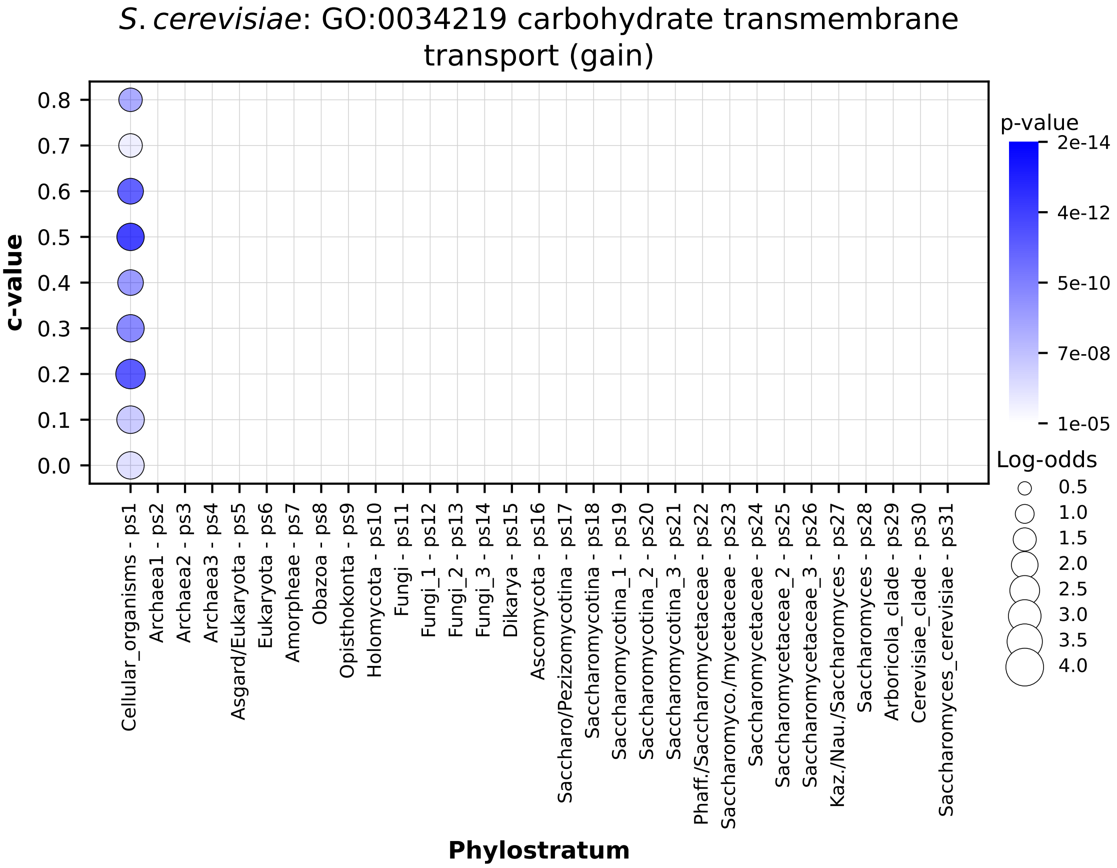
S. cerevisiae: GO:0034219 carbohydrate transmembrane transport (gain)
c-value
0.0
0.1
0.2
0.3
0.4
0.5
0.6
0.7
0.8
Phylostratum
Cellular_organisms - ps1
Archaea1 - ps2
Archaea2 - ps3
Archaea3 - ps4
Asgard/Eukaryota - ps5
Eukaryota - ps6
Amorpheae - ps7
Obazoa - ps8
Opisthokonta - ps9
Holomycota - ps10
Fungi - ps11
Fungi_1 - ps12
Fungi_2 - ps13
Fungi_3 - ps14
Dikarya - ps15
Ascomycota - ps16
Saccharo/Pezizomycotina - ps17
Saccharomycotina - ps18
Saccharomycotina_1 - ps19
Saccharomycotina_2 - ps20
Saccharomycotina_3 - ps21
Phaff./Saccharomycetaceae - ps22
Saccharomyco./mycetaceae - ps23
Saccharomycetaceae - ps24
Saccharomycetaceae_2 - ps25
Saccharomycetaceae_3 - ps26
Kaz./Nau./Saccharomyces - ps27
Saccharomyces - ps28
Arboricola_clade - ps29
Cerevisiae_clade - ps30
Saccharomyces_cerevisiae - ps31
p-value
2e-14
4e-12
5e-10
7e-08
1e-05
Log-odds
0.5
1.0
1.5
2.0
2.5
3.0
3.5
4.0

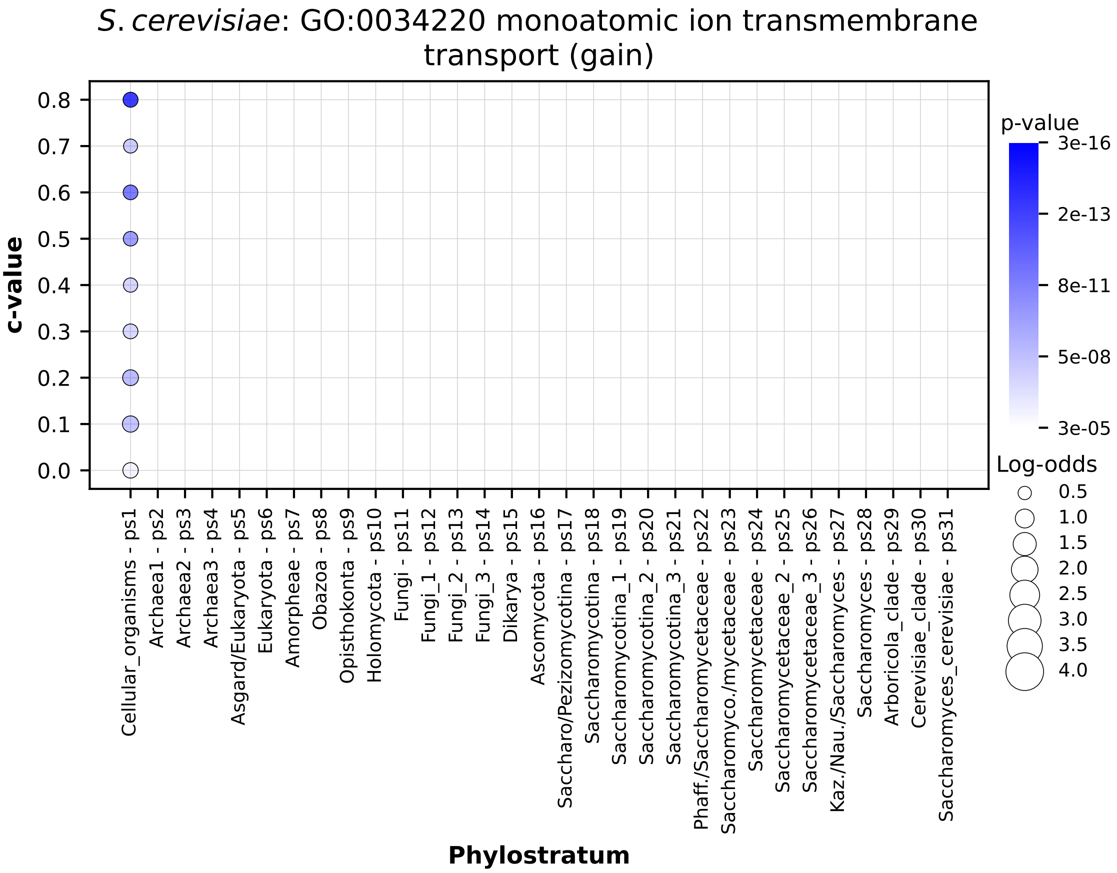

S. cerevisiae: GO:0034220 monoatomic ion transmembrane transport (gain)
c-value
0.8
0.7
0.6
0.5
0.4
0.3
0.2
0.1
0.0
p-value
3e-16
2e-13
8e-11
5e-08
3e-05
Log-odds
0.5
1.0
1.5
2.0
2.5
3.0
3.5
4.0
Cellular_organisms - ps1
Archaea1 - ps2
Archaea2 - ps3
Archaea3 - ps4
Asgard/Eukaryota - ps5
Eukaryota - ps6
Amorpheae - ps7
Obazoa - ps8
Opisthokonta - ps9
Holomycota - ps10
Fungi - ps11
Fungi_1 - ps12
Fungi_2 - ps13
Fungi_3 - ps14
Dikarya - ps15
Ascomycota - ps16
Saccharo/Pezizomycotina - ps17
Saccharomycotina - ps18
Saccharomycotina_1 - ps19
Saccharomycotina_2 - ps20
Saccharomycotina_3 - ps21
Phaff./Saccharomycetaceae - ps22
Saccharomyco./mycetaceae - ps23
Saccharomycetaceae - ps24
Saccharomycetaceae_2 - ps25
Saccharomycetaceae_3 - ps26
Kaz./Nau./Saccharomyces - ps27
Saccharomyces - ps28
Arboricola_clade - ps29
Cerevisiae_clade - ps30
Saccharomyces_cerevisiae - ps31
Phylostratum

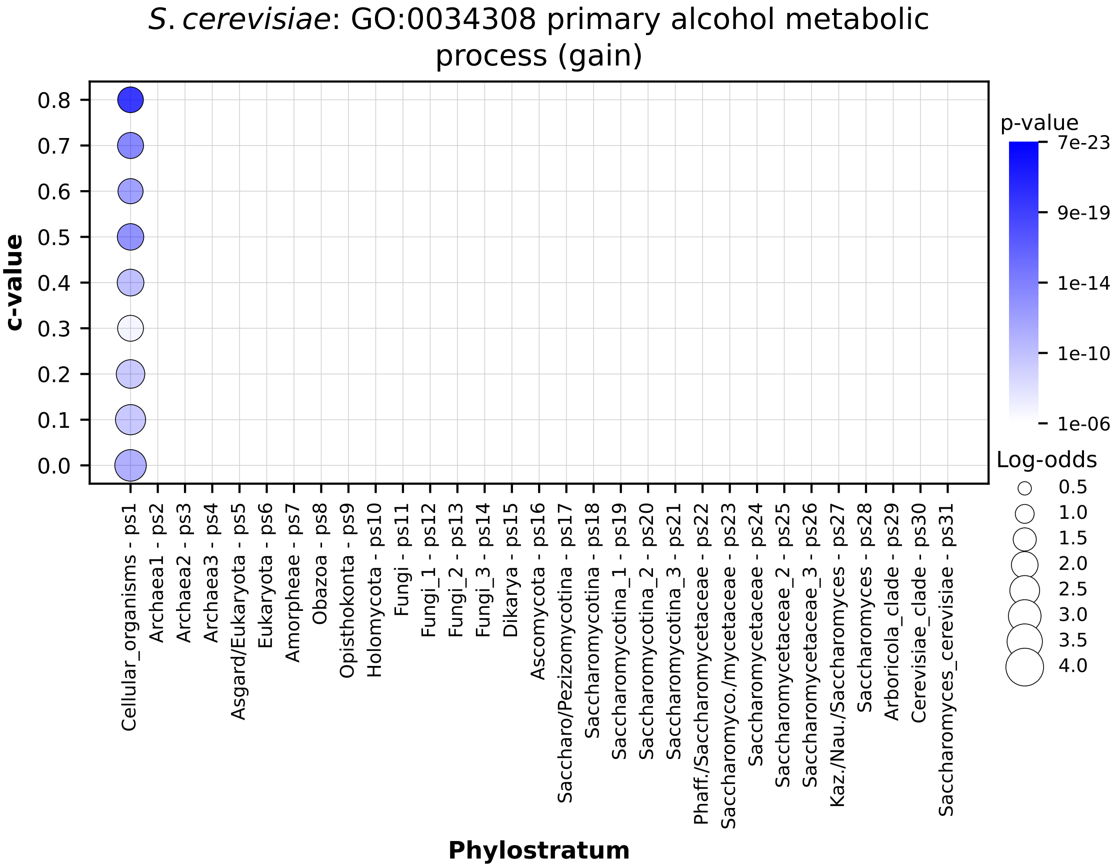
S. cerevisiae: GO:0034308 primary alcohol metabolic process (gain)
c-value
0.8
0.7
0.6
0.5
0.4
0.3
0.2
0.1
0.0
p-value
7e-23
9e-19
1e-14
1e-10
1e-06
Log-odds
0.5
1.0
1.5
2.0
2.5
3.0
3.5
4.0
Cellular_organisms - ps1
Archaea1 - ps2
Archaea2 - ps3
Archaea3 - ps4
Asgard/Eukaryota - ps5
Eukaryota - ps6
Amorpheae - ps7
Obazoa - ps8
Opisthokonta - ps9
Holomycota - ps10
Fungi - ps11
Fungi_1 - ps12
Fungi_2 - ps13
Fungi_3 - ps14
Dikarya - ps15
Ascomycota - ps16
Saccharo/Pezizomycotina - ps17
Saccharomycotina - ps18
Saccharomycotina_1 - ps19
Saccharomycotina_2 - ps20
Saccharomycotina_3 - ps21
Phaff./Saccharomycetaceae - ps22
Saccharomyco./mycetaceae - ps23
Saccharomycetaceae - ps24
Saccharomycetaceae_2 - ps25
Saccharomycetaceae_3 - ps26
Kaz./Nau./Saccharomyces - ps27
Saccharomyces - ps28
Arboricola_clade - ps29
Cerevisiae_clade - ps30
Saccharomyces_cerevisiae - ps31
Phylostratum

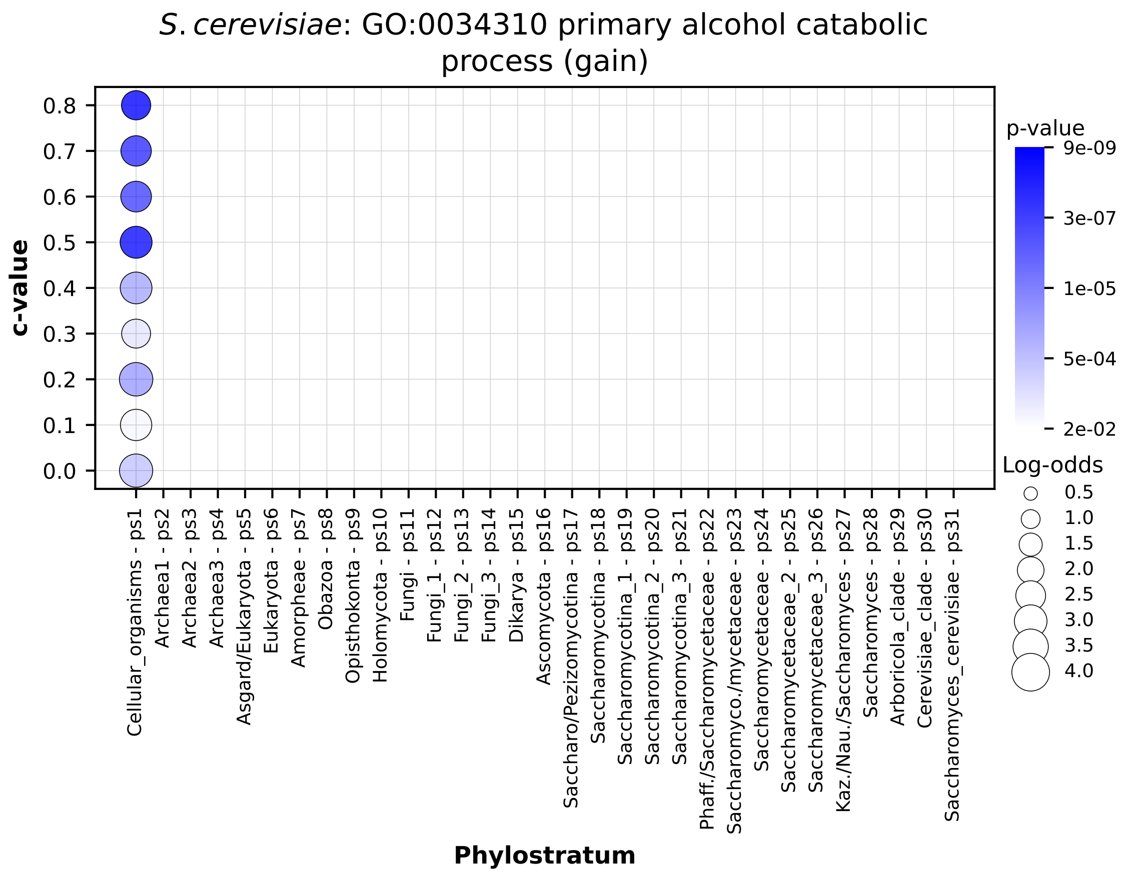
S. cerevisiae: GO:0034310 primary alcohol catabolic process (gain)
c-value
0.8
0.7
0.6
0.5
0.4
0.3
0.2
0.1
0.0
p-value
9e-09
3e-07
1e-05
5e-04
2e-02
Log-odds
0.5
1.0
1.5
2.0
2.5
3.0
3.5
4.0
Cellular_organisms - ps1
Archaea1 - ps2
Archaea2 - ps3
Archaea3 - ps4
Asgard/Eukaryota - ps5
Eukaryota - ps6
Amorpheae - ps7
Obazoa - ps8
Opisthokonta - ps9
Holomycota - ps10
Fungi - ps11
Fungi_1 - ps12
Fungi_2 - ps13
Fungi_3 - ps14
Dikarya - ps15
Ascomycota - ps16
Saccharo/Pezizomycotina - ps17
Saccharomycotina - ps18
Saccharomycotina_1 - ps19
Saccharomycotina_2 - ps20
Saccharomycotina_3 - ps21
Phaff./Saccharomycetaceae - ps22
Saccharomyco./mycetaceae - ps23
Saccharomycetaceae - ps24
Saccharomycetaceae_2 - ps25
Saccharomycetaceae_3 - ps26
Kaz./Nau./Saccharomyces - ps27
Saccharomyces - ps28
Arboricola_clade - ps29
Cerevisiae_clade - ps30
Saccharomyces_cerevisiae - ps31
Phylostratum

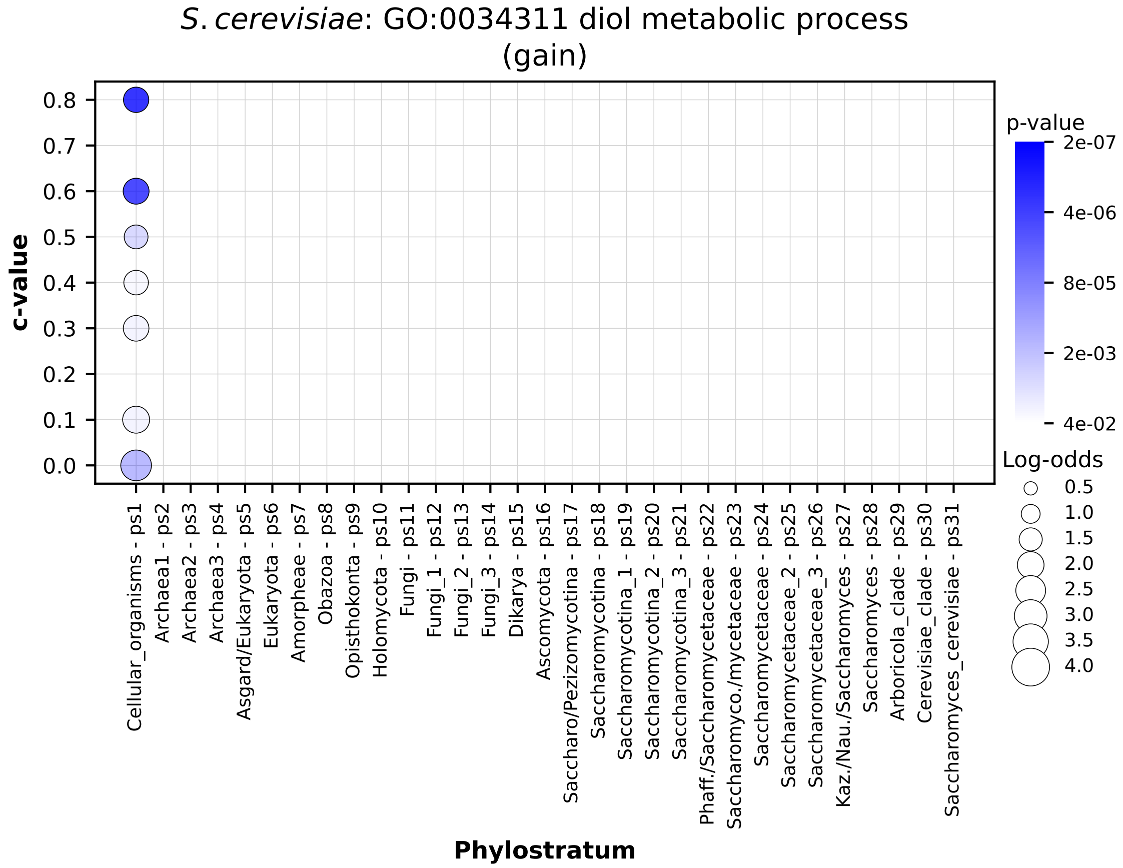

S. cerevisiae: GO:0034311 diol metabolic process
(gain)
c-value
0.0
0.1
0.2
0.3
0.4
0.5
0.6
0.7
0.8
Phylostratum
Cellular_organisms - ps1
Archaea1 - ps2
Archaea2 - ps3
Archaea3 - ps4
Asgard/Eukaryota - ps5
Eukaryota - ps6
Amorpheae - ps7
Obazoa - ps8
Opisthokonta - ps9
Holomycota - ps10
Fungi - ps11
Fungi_1 - ps12
Fungi_2 - ps13
Fungi_3 - ps14
Dikarya - ps15
Ascomycota - ps16
Saccharo/Pezizomycotina - ps17
Saccharomycotina - ps18
Saccharomycotina_1 - ps19
Saccharomycotina_2 - ps20
Saccharomycotina_3 - ps21
Phaff./Saccharomycetaceae - ps22
Saccharomyco./mycetaceae - ps23
Saccharomycetaceae - ps24
Saccharomycetaceae_2 - ps25
Saccharomycetaceae_3 - ps26
Kaz./Nau./Saccharomyces - ps27
Saccharomyces - ps28
Arboricola_clade - ps29
Cerevisiae_clade - ps30
Saccharomyces_cerevisiae - ps31
p-value
2e-07
4e-06
8e-05
2e-03
4e-02
Log-odds
0.5
1.0
1.5
2.0
2.5
3.0
3.5
4.0

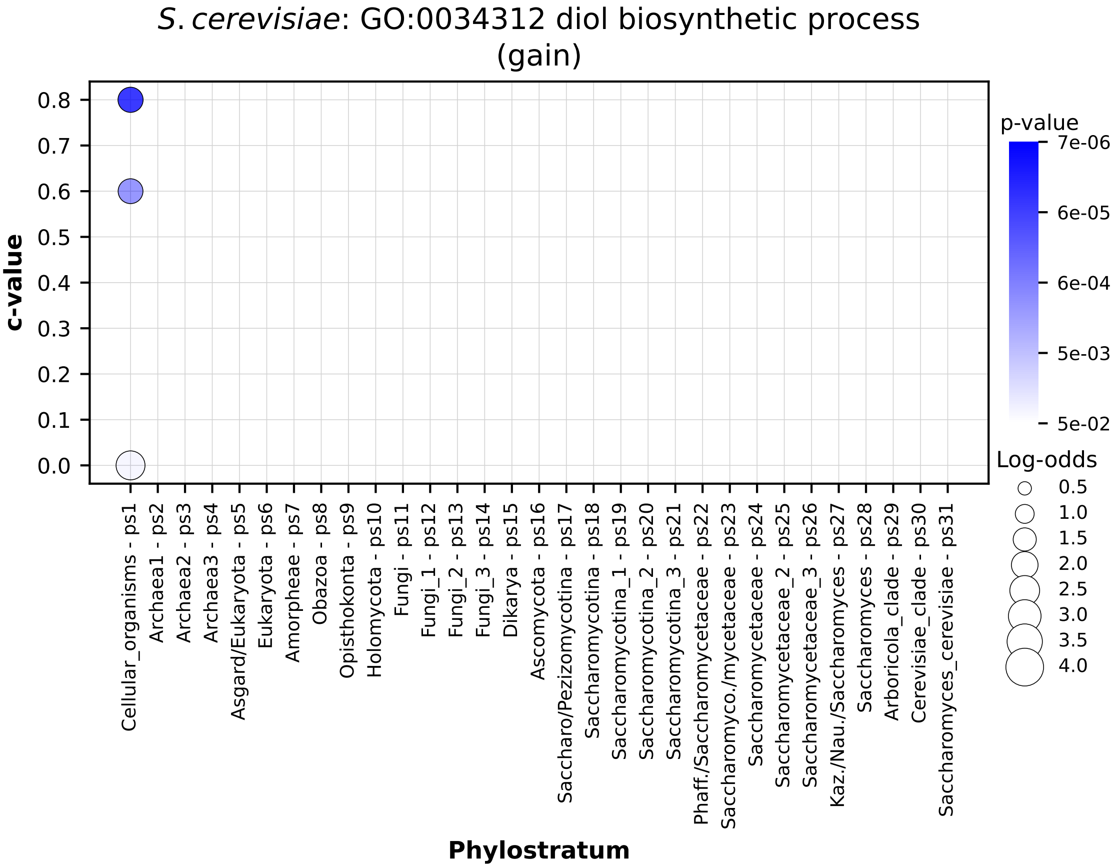

S. cerevisiae: GO:0034312 diol biosynthetic process
(gain)
c-value
0.8
0.7
0.6
0.5
0.4
0.3
0.2
0.1
0.0
p-value
7e-06
6e-05
6e-04
5e-03
5e-02
Log-odds
0.5
1.0
1.5
2.0
2.5
3.0
3.5
4.0
Cellular_organisms - ps1
Archaea1 - ps2
Archaea2 - ps3
Archaea3 - ps4
Asgard/Eukaryota - ps5
Eukaryota - ps6
Amorpheae - ps7
Obazoa - ps8
Opisthokonta - ps9
Holomycota - ps10
Fungi - ps11
Fungi_1 - ps12
Fungi_2 - ps13
Fungi_3 - ps14
Dikarya - ps15
Ascomycota - ps16
Saccharo/Pezizomycotina - ps17
Saccharomycotina - ps18
Saccharomycotina_1 - ps19
Saccharomycotina_2 - ps20
Saccharomycotina_3 - ps21
Phaff./Saccharomycetaceae - ps22
Saccharomyco./mycetaceae - ps23
Saccharomycetaceae - ps24
Saccharomycetaceae_2 - ps25
Saccharomycetaceae_3 - ps26
Kaz./Nau./Saccharomyces - ps27
Saccharomyces - ps28
Arboricola_clade - ps29
Cerevisiae_clade - ps30
Saccharomyces_cerevisiae - ps31
Phylostratum

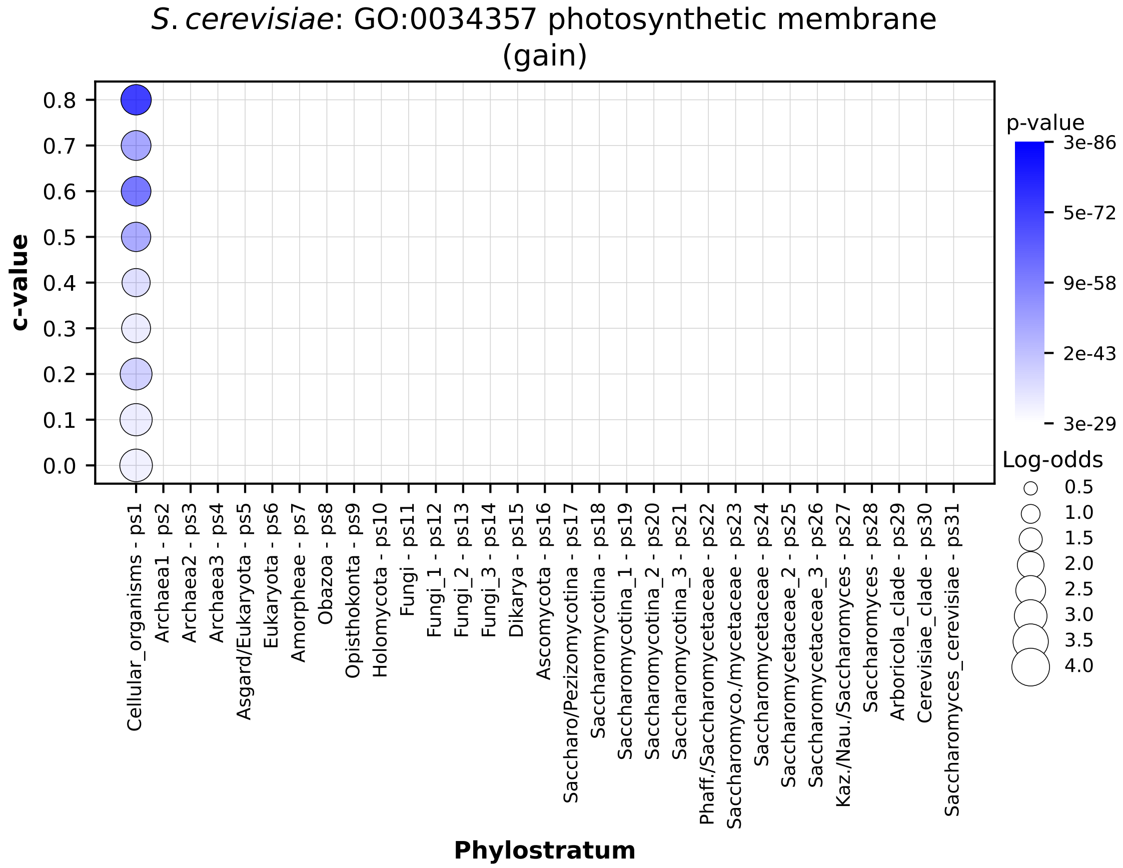
S. cerevisiae: GO:0034357 photosynthetic membrane
(gain)
c-value
0.8
0.7
0.6
0.5
0.4
0.3
0.2
0.1
0.0
p-value
3e-86
5e-72
9e-58
2e-43
3e-29
Log-odds
0.5
1.0
1.5
2.0
2.5
3.0
3.5
4.0
Cellular_organisms - ps1
Archaea1 - ps2
Archaea2 - ps3
Archaea3 - ps4
Asgard/Eukaryota - ps5
Eukaryota - ps6
Amorpheae - ps7
Obazoa - ps8
Opisthokonta - ps9
Holomycota - ps10
Fungi - ps11
Fungi_1 - ps12
Fungi_2 - ps13
Fungi_3 - ps14
Dikarya - ps15
Ascomycota - ps16
Saccharo/Pezizomycotina - ps17
Saccharomycotina - ps18
Saccharomycotina_1 - ps19
Saccharomycotina_2 - ps20
Saccharomycotina_3 - ps21
Phaff./Saccharomycetaceae - ps22
Saccharomyco./mycetaceae - ps23
Saccharomycetaceae - ps24
Saccharomycetaceae_2 - ps25
Saccharomycetaceae_3 - ps26
Kaz./Nau./Saccharomyces - ps27
Saccharomyces - ps28
Arboricola_clade - ps29
Cerevisiae_clade - ps30
Saccharomyces_cerevisiae - ps31
Phylostratum

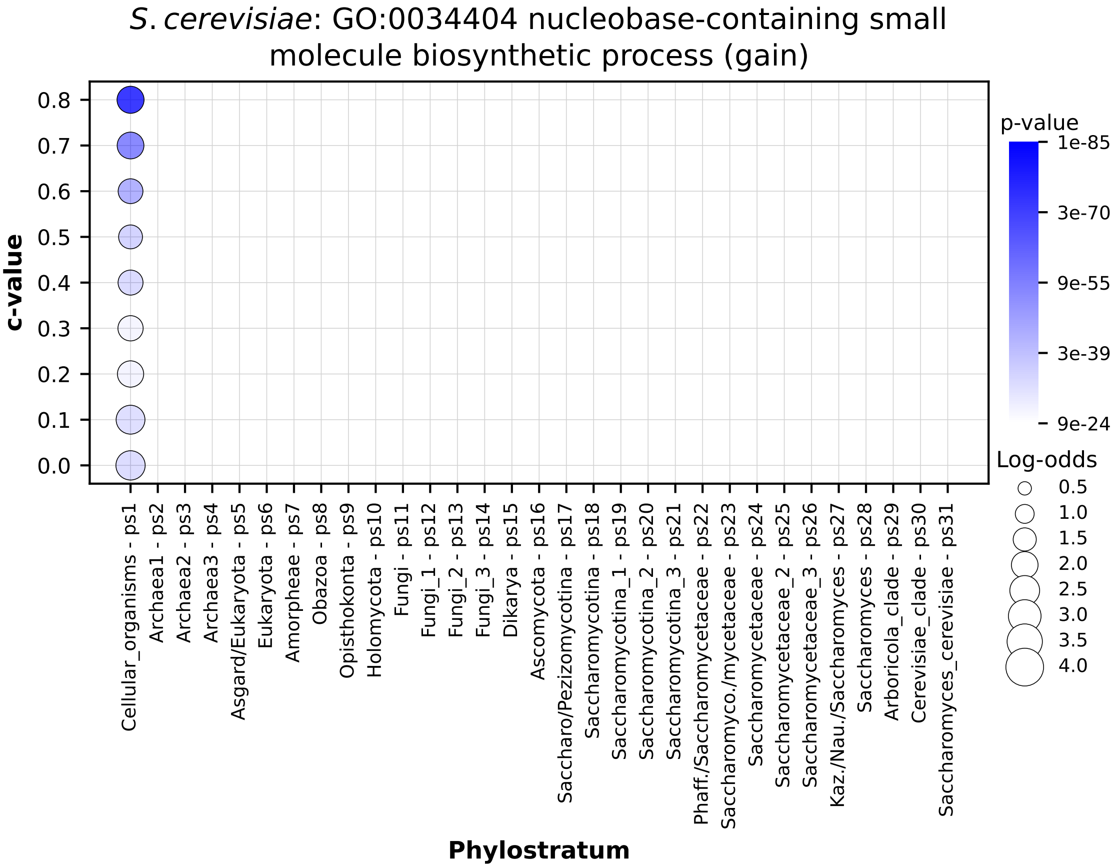
S. cerevisiae: GO:0034404 nucleobase-containing small molecule biosynthetic process (gain)
c-value
0.8
0.7
0.6
0.5
0.4
0.3
0.2
0.1
0.0
p-value
1e-85
3e-70
9e-55
3e-39
9e-24
Log-odds
0.5
1.0
1.5
2.0
2.5
3.0
3.5
4.0
Cellular_organisms - ps1
Archaea1 - ps2
Archaea2 - ps3
Archaea3 - ps4
Asgard/Eukaryota - ps5
Eukaryota - ps6
Amorpheae - ps7
Obazoa - ps8
Opisthokonta - ps9
Holomycota - ps10
Fungi - ps11
Fungi_1 - ps12
Fungi_2 - ps13
Fungi_3 - ps14
Dikarya - ps15
Ascomycota - ps16
Saccharo/Pezizomycotina - ps17
Saccharomycotina - ps18
Saccharomycotina_1 - ps19
Saccharomycotina_2 - ps20
Saccharomycotina_3 - ps21
Phaff./Saccharomycetaceae - ps22
Saccharomyco./mycetaceae - ps23
Saccharomycetaceae - ps24
Saccharomycetaceae_2 - ps25
Saccharomycetaceae_3 - ps26
Kaz./Nau./Saccharomyces - ps27
Saccharomyces - ps28
Arboricola_clade - ps29
Cerevisiae_clade - ps30
Saccharomyces_cerevisiae - ps31
Phylostratum

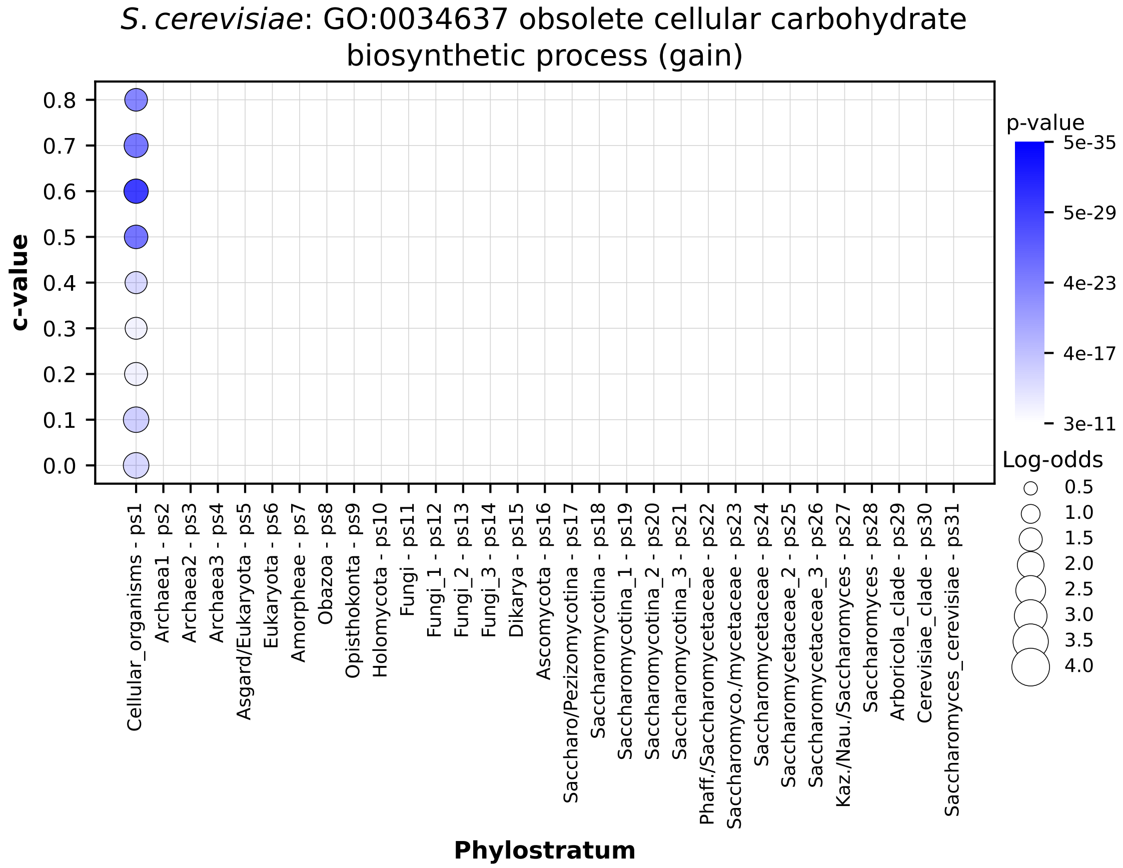

S. cerevisiae: GO:0034637 obsolete cellular carbohydrate biosynthetic process (gain)
c-value
0.8
0.7
0.6
0.5
0.4
0.3
0.2
0.1
0.0
p-value
5e-35
5e-29
4e-23
4e-17
3e-11
Log-odds
0.5
1.0
1.5
2.0
2.5
3.0
3.5
4.0
Cellular_organisms - ps1
Archaea1 - ps2
Archaea2 - ps3
Archaea3 - ps4
Asgard/Eukaryota - ps5
Eukaryota - ps6
Amorpheae - ps7
Obazoa - ps8
Opisthokonta - ps9
Holomycota - ps10
Fungi - ps11
Fungi_1 - ps12
Fungi_2 - ps13
Fungi_3 - ps14
Dikarya - ps15
Ascomycota - ps16
Saccharo/Pezizomycotina - ps17
Saccharomycotina - ps18
Saccharomycotina_1 - ps19
Saccharomycotina_2 - ps20
Saccharomycotina_3 - ps21
Phaff./Saccharomycetaceae - ps22
Saccharomyco./mycetaceae - ps23
Saccharomycetaceae - ps24
Saccharomycetaceae_2 - ps25
Saccharomycetaceae_3 - ps26
Kaz./Nau./Saccharomyces - ps27
Saccharomyces - ps28
Arboricola_clade - ps29
Cerevisiae_clade - ps30
Saccharomyces_cerevisiae - ps31
Phylostratum

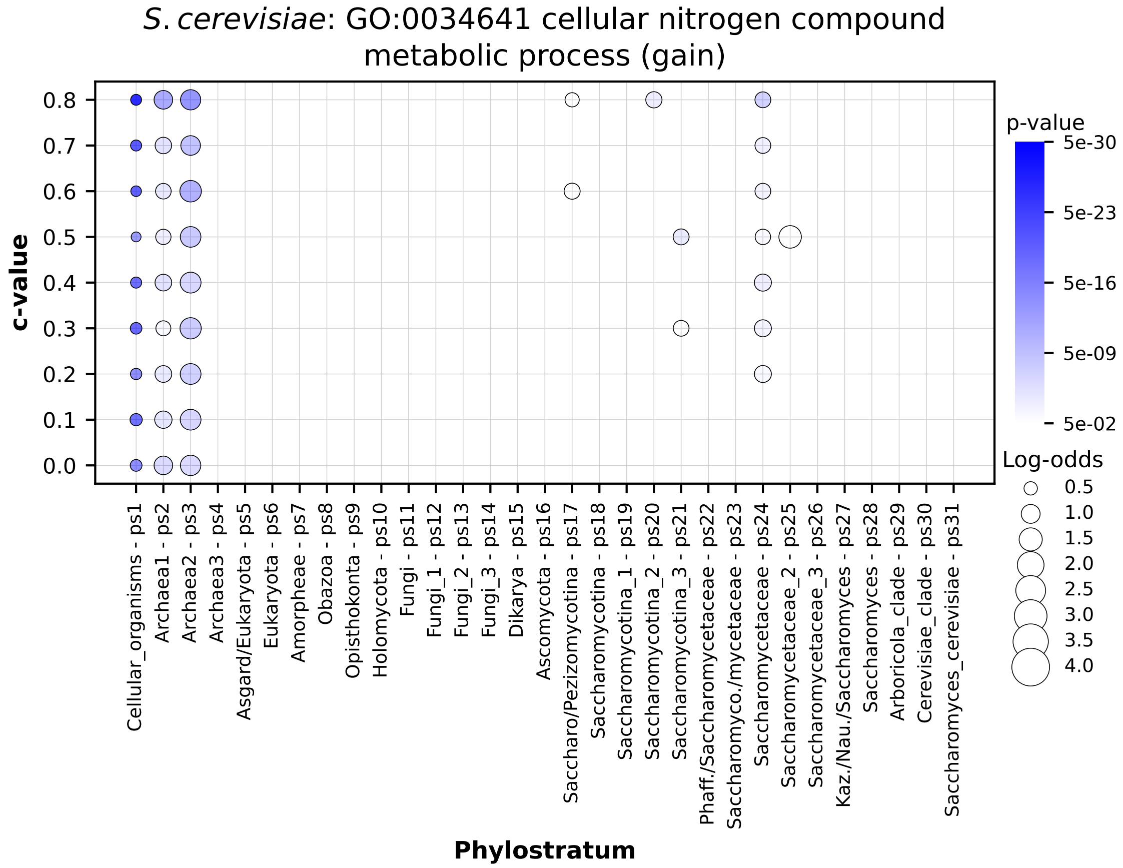
S. cerevisiae: GO:0034641 cellular nitrogen compound metabolic process (gain)
c-value
0.0
0.1
0.2
0.3
0.4
0.5
0.6
0.7
0.8
p-value
5e-30
5e-23
5e-16
5e-09
5e-02
Log-odds
0.5
1.0
1.5
2.0
2.5
3.0
3.5
4.0
Cellular_organisms - ps1
Archaea1 - ps2
Archaea2 - ps3
Archaea3 - ps4
Asgard/Eukaryota - ps5
Eukaryota - ps6
Amorpheae - ps7
Obazoa - ps8
Opisthokonta - ps9
Holomycota - ps10
Fungi - ps11
Fungi_1 - ps12
Fungi_2 - ps13
Fungi_3 - ps14
Dikarya - ps15
Ascomycota - ps16
Saccharo/Pezizomycotina - ps17
Saccharomycotina - ps18
Saccharomycotina_1 - ps19
Saccharomycotina_2 - ps20
Saccharomycotina_3 - ps21
Phaff./Saccharomycetaceae - ps22
Saccharomyco./mycetaceae - ps23
Saccharomycetaceae - ps24
Saccharomycetaceae_2 - ps25
Saccharomycetaceae_3 - ps26
Kaz./Nau./Saccharomyces - ps27
Saccharomyces - ps28
Arboricola_clade - ps29
Cerevisiae_clade - ps30
Saccharomyces_cerevisiae - ps31
Phylostratum

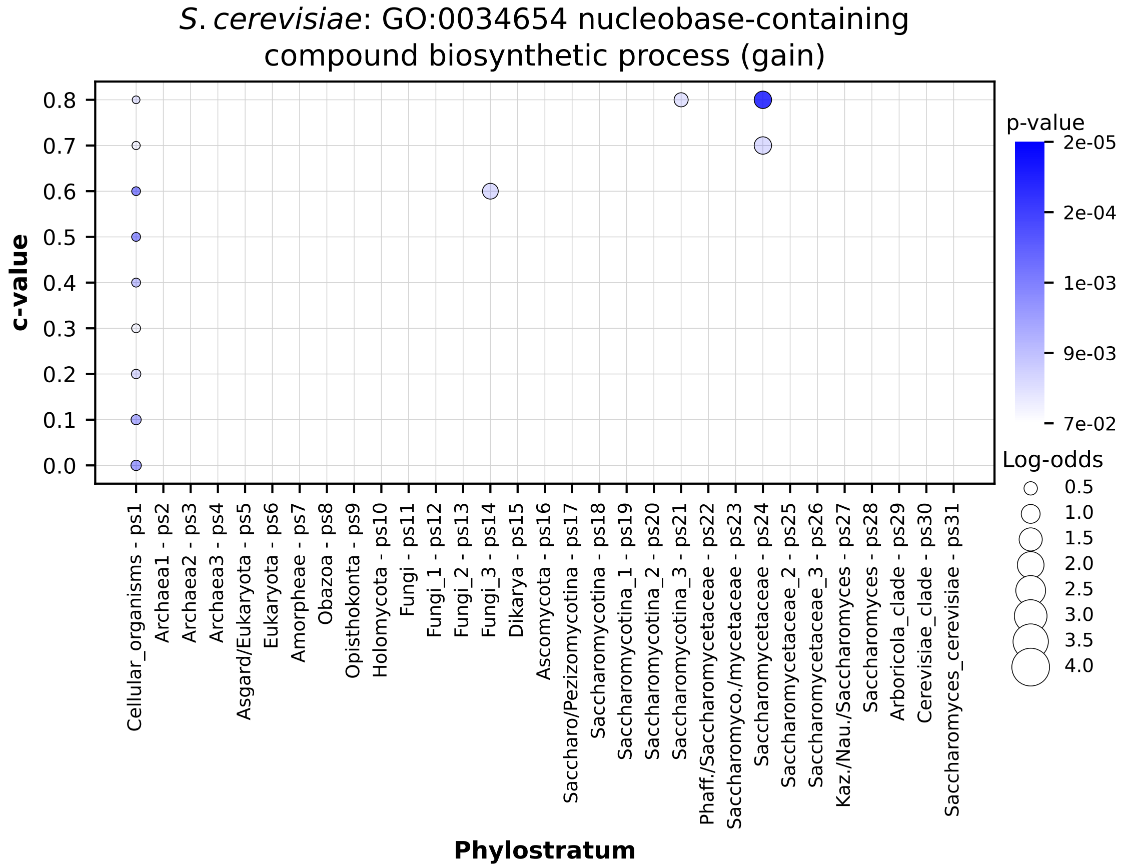
S. cerevisiae: GO:0034654 nucleobase-containing compound biosynthetic process (gain)
c-value
0.8
0.7
0.6
0.5
0.4
0.3
0.2
0.1
0.0
p-value
2e-05
2e-04
1e-03
9e-03
7e-02
Log-odds
0.5
1.0
1.5
2.0
2.5
3.0
3.5
4.0
Cellular_organisms - ps1
Archaea1 - ps2
Archaea2 - ps3
Archaea3 - ps4
Asgard/Eukaryota - ps5
Eukaryota - ps6
Amorpheae - ps7
Obazoa - ps8
Opisthokonta - ps9
Holomycota - ps10
Fungi - ps11
Fungi_1 - ps12
Fungi_2 - ps13
Fungi_3 - ps14
Dikarya - ps15
Ascomycota - ps16
Saccharo/Pezizomycotina - ps17
Saccharomycotina - ps18
Saccharomycotina_1 - ps19
Saccharomycotina_2 - ps20
Saccharomycotina_3 - ps21
Phaff./Saccharomycetaceae - ps22
Saccharomyco./mycetaceae - ps23
Saccharomycetaceae - ps24
Saccharomycetaceae_2 - ps25
Saccharomycetaceae_3 - ps26
Kaz./Nau./Saccharomyces - ps27
Saccharomyces - ps28
Arboricola_clade - ps29
Cerevisiae_clade - ps30
Saccharomyces_cerevisiae - ps31
Phylostratum

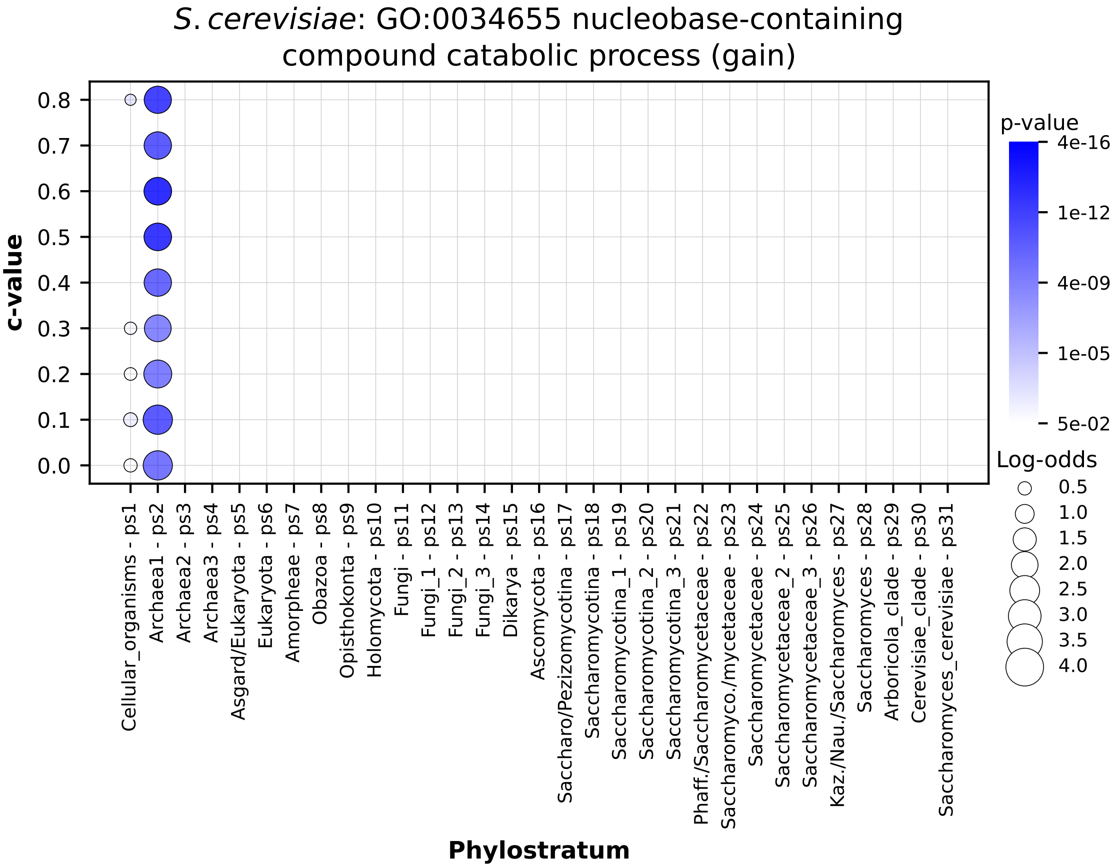

S. cerevisiae: GO:0034655 nucleobase-containing compound catabolic process (gain)
c-value
0.8
0.7
0.6
0.5
0.4
0.3
0.2
0.1
0.0
p-value
4e-16
1e-12
4e-09
1e-05
5e-02
Log-odds
0.5
1.0
1.5
2.0
2.5
3.0
3.5
4.0
Cellular_organisms - ps1
Archaea1 - ps2
Archaea2 - ps3
Archaea3 - ps4
Asgard/Eukaryota - ps5
Eukaryota - ps6
Amorpheae - ps7
Obazoa - ps8
Opisthokonta - ps9
Holomycota - ps10
Fungi - ps11
Fungi_1 - ps12
Fungi_2 - ps13
Fungi_3 - ps14
Dikarya - ps15
Ascomycota - ps16
Saccharo/Pezizomycotina - ps17
Saccharomycotina - ps18
Saccharomycotina_1 - ps19
Saccharomycotina_2 - ps20
Saccharomycotina_3 - ps21
Phaff./Saccharomycetaceae - ps22
Saccharomyco./mycetaceae - ps23
Saccharomycetaceae - ps24
Saccharomycetaceae_2 - ps25
Saccharomycetaceae_3 - ps26
Kaz./Nau./Saccharomyces - ps27
Saccharomyces - ps28
Arboricola_clade - ps29
Cerevisiae_clade - ps30
Saccharomyces_cerevisiae - ps31
Phylostratum

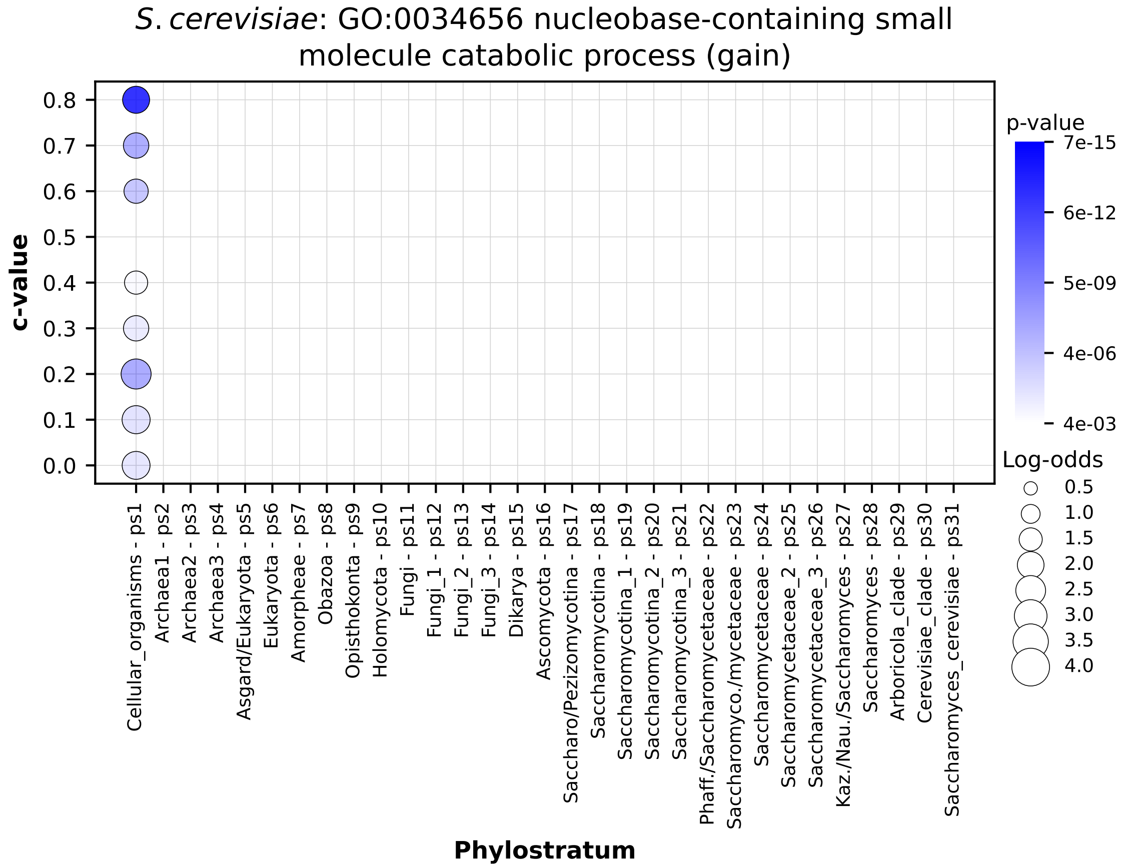
S. cerevisiae: GO:0034656 nucleobase-containing small molecule catabolic process (gain)
c-value
0.0
0.1
0.2
0.3
0.4
0.5
0.6
0.7
0.8
Phylostratum
Cellular_organisms - ps1
Archaea1 - ps2
Archaea2 - ps3
Archaea3 - ps4
Asgard/Eukaryota - ps5
Eukaryota - ps6
Amorpheae - ps7
Obazoa - ps8
Opisthokonta - ps9
Holomycota - ps10
Fungi - ps11
Fungi_1 - ps12
Fungi_2 - ps13
Fungi_3 - ps14
Dikarya - ps15
Ascomycota - ps16
Saccharo/Pezizomycotina - ps17
Saccharomycotina - ps18
Saccharomycotina_1 - ps19
Saccharomycotina_2 - ps20
Saccharomycotina_3 - ps21
Phaff./Saccharomycetaceae - ps22
Saccharomyco./mycetaceae - ps23
Saccharomycetaceae - ps24
Saccharomycetaceae_2 - ps25
Saccharomycetaceae_3 - ps26
Kaz./Nau./Saccharomyces - ps27
Saccharomyces - ps28
Arboricola_clade - ps29
Cerevisiae_clade - ps30
Saccharomyces_cerevisiae - ps31
p-value
7e-15
6e-12
5e-09
4e-06
4e-03
Log-odds
0.5
1.0
1.5
2.0
2.5
3.0
3.5
4.0

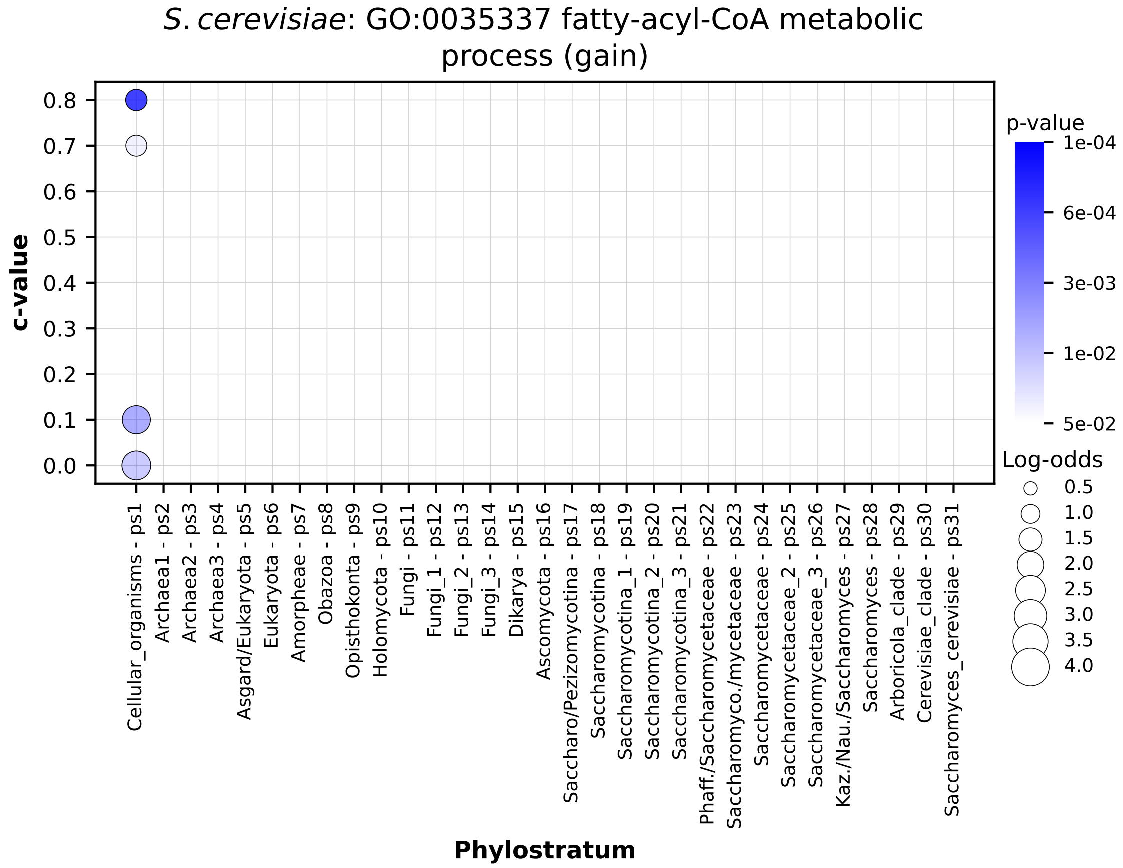

S. cerevisiae: GO:0035337 fatty-acyl-CoA metabolic process (gain)
c-value
0.0
0.1
0.2
0.3
0.4
0.5
0.6
0.7
0.8
Phylostratum
Cellular_organisms - ps1
Archaea1 - ps2
Archaea2 - ps3
Archaea3 - ps4
Asgard/Eukaryota - ps5
Eukaryota - ps6
Amorpheae - ps7
Obazoa - ps8
Opisthokonta - ps9
Holomycota - ps10
Fungi - ps11
Fungi_1 - ps12
Fungi_2 - ps13
Fungi_3 - ps14
Dikarya - ps15
Ascomycota - ps16
Saccharo/Pezizomycotina - ps17
Saccharomycotina - ps18
Saccharomycotina_1 - ps19
Saccharomycotina_2 - ps20
Saccharomycotina_3 - ps21
Phaff./Saccharomycetaceae - ps22
Saccharomyco./mycetaceae - ps23
Saccharomycetaceae - ps24
Saccharomycetaceae_2 - ps25
Saccharomycetaceae_3 - ps26
Kaz./Nau./Saccharomyces - ps27
Saccharomyces - ps28
Arboricola_clade - ps29
Cerevisiae_clade - ps30
Saccharomyces_cerevisiae - ps31
p-value
1e-04
6e-04
3e-03
1e-02
5e-02
Log-odds
0.5
1.0
1.5
2.0
2.5
3.0
3.5
4.0

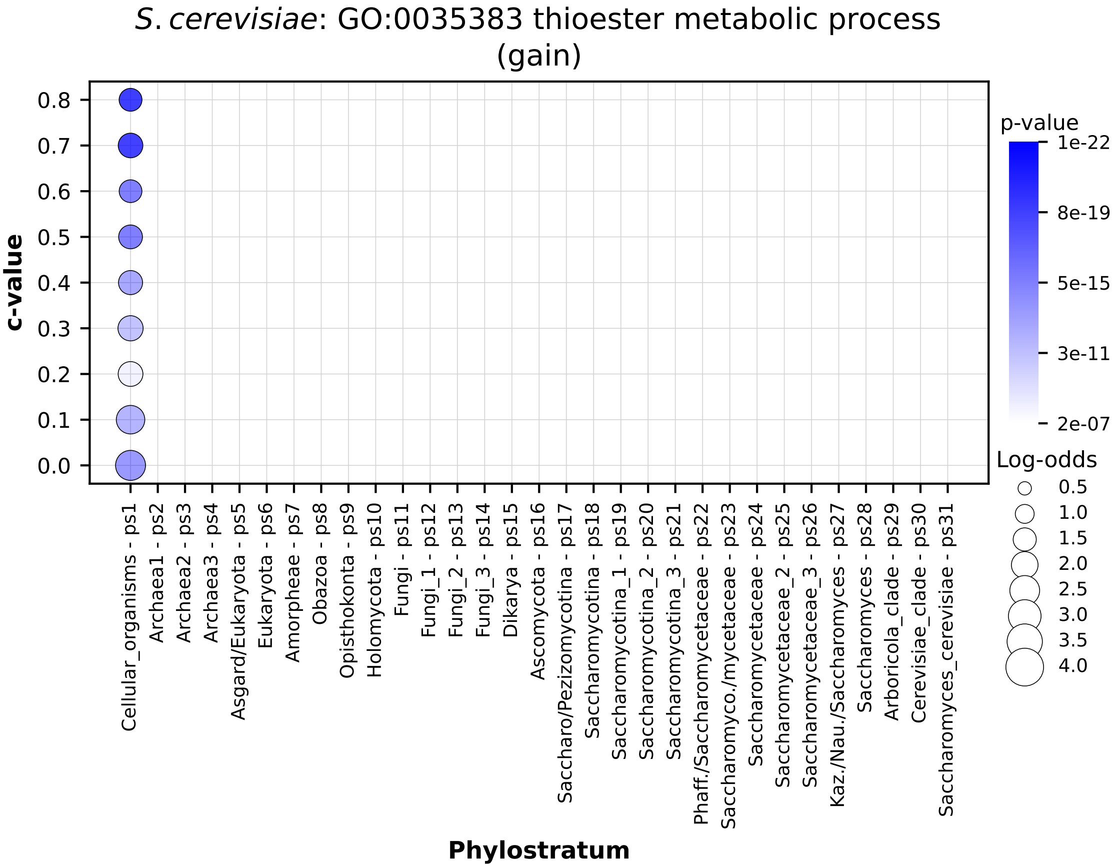

S. cerevisiae: GO:0035383 thioester metabolic process (gain)
c-value
0.8
0.7
0.6
0.5
0.4
0.3
0.2
0.1
0.0
p-value
1e-22
8e-19
5e-15
3e-11
2e-07
Log-odds
0.5
1.0
1.5
2.0
2.5
3.0
3.5
4.0
Cellular_organisms - ps1
Archaea1 - ps2
Archaea2 - ps3
Archaea3 - ps4
Asgard/Eukaryota - ps5
Eukaryota - ps6
Amorpheae - ps7
Obazoa - ps8
Opisthokonta - ps9
Holomycota - ps10
Fungi - ps11
Fungi_1 - ps12
Fungi_2 - ps13
Fungi_3 - ps14
Dikarya - ps15
Ascomycota - ps16
Saccharo/Pezizomycotina - ps17
Saccharomycotina - ps18
Saccharomycotina_1 - ps19
Saccharomycotina_2 - ps20
Saccharomycotina_3 - ps21
Phaff./Saccharomycetaceae - ps22
Saccharomyco./mycetaceae - ps23
Saccharomycetaceae - ps24
Saccharomycetaceae_2 - ps25
Saccharomycetaceae_3 - ps26
Kaz./Nau./Saccharomyces - ps27
Saccharomyces - ps28
Arboricola_clade - ps29
Cerevisiae_clade - ps30
Saccharomyces_cerevisiae - ps31
Phylostratum

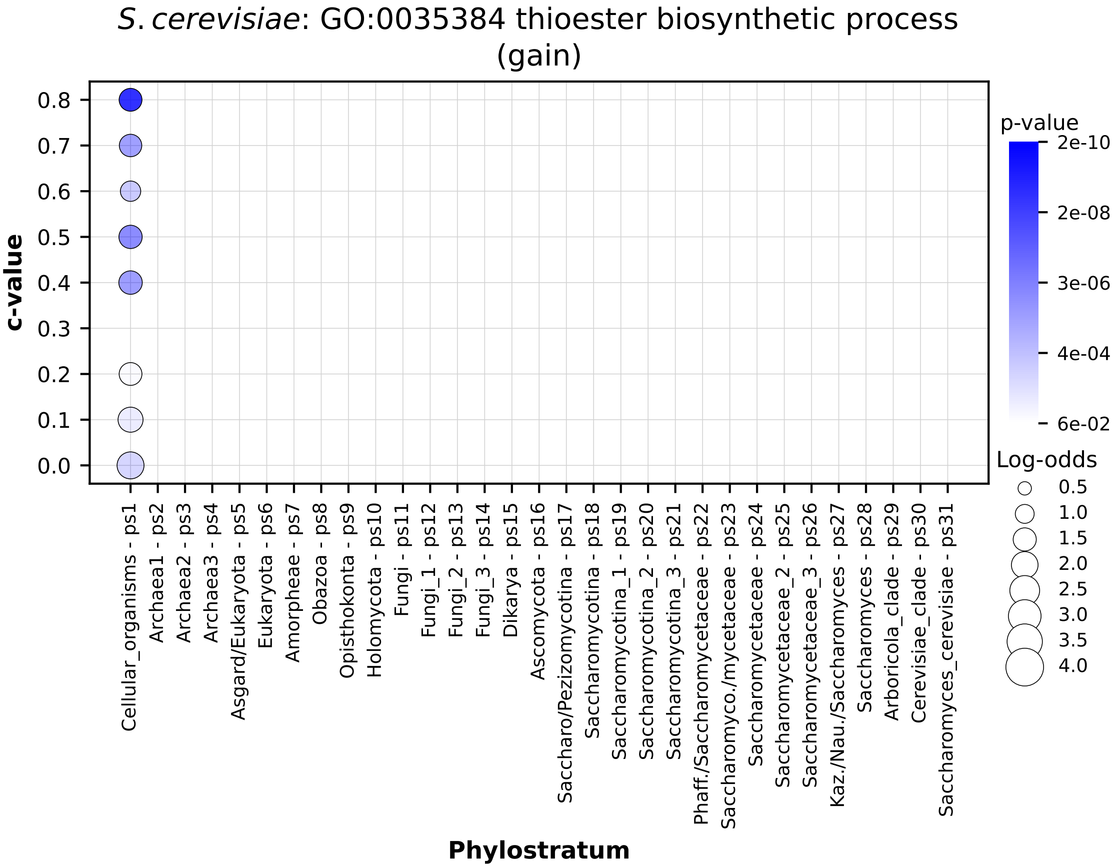

S. cerevisiae: GO:0035384 thioester biosynthetic process (gain)
c-value
0.8
0.7
0.6
0.5
0.4
0.3
0.2
0.1
0.0
p-value
2e-10
2e-08
3e-06
4e-04
6e-02
Log-odds
0.5
1.0
1.5
2.0
2.5
3.0
3.5
4.0
Cellular_organisms - ps1
Archaea1 - ps2
Archaea2 - ps3
Archaea3 - ps4
Asgard/Eukaryota - ps5
Eukaryota - ps6
Amorpheae - ps7
Obazoa - ps8
Opisthokonta - ps9
Holomycota - ps10
Fungi - ps11
Fungi_1 - ps12
Fungi_2 - ps13
Fungi_3 - ps14
Dikarya - ps15
Ascomycota - ps16
Saccharo/Pezizomycotina - ps17
Saccharomycotina - ps18
Saccharomycotina_1 - ps19
Saccharomycotina_2 - ps20
Saccharomycotina_3 - ps21
Phaff./Saccharomycetaceae - ps22
Saccharomyco./mycetaceae - ps23
Saccharomycetaceae - ps24
Saccharomycetaceae_2 - ps25
Saccharomycetaceae_3 - ps26
Kaz./Nau./Saccharomyces - ps27
Saccharomyces - ps28
Arboricola_clade - ps29
Cerevisiae_clade - ps30
Saccharomyces_cerevisiae - ps31
Phylostratum

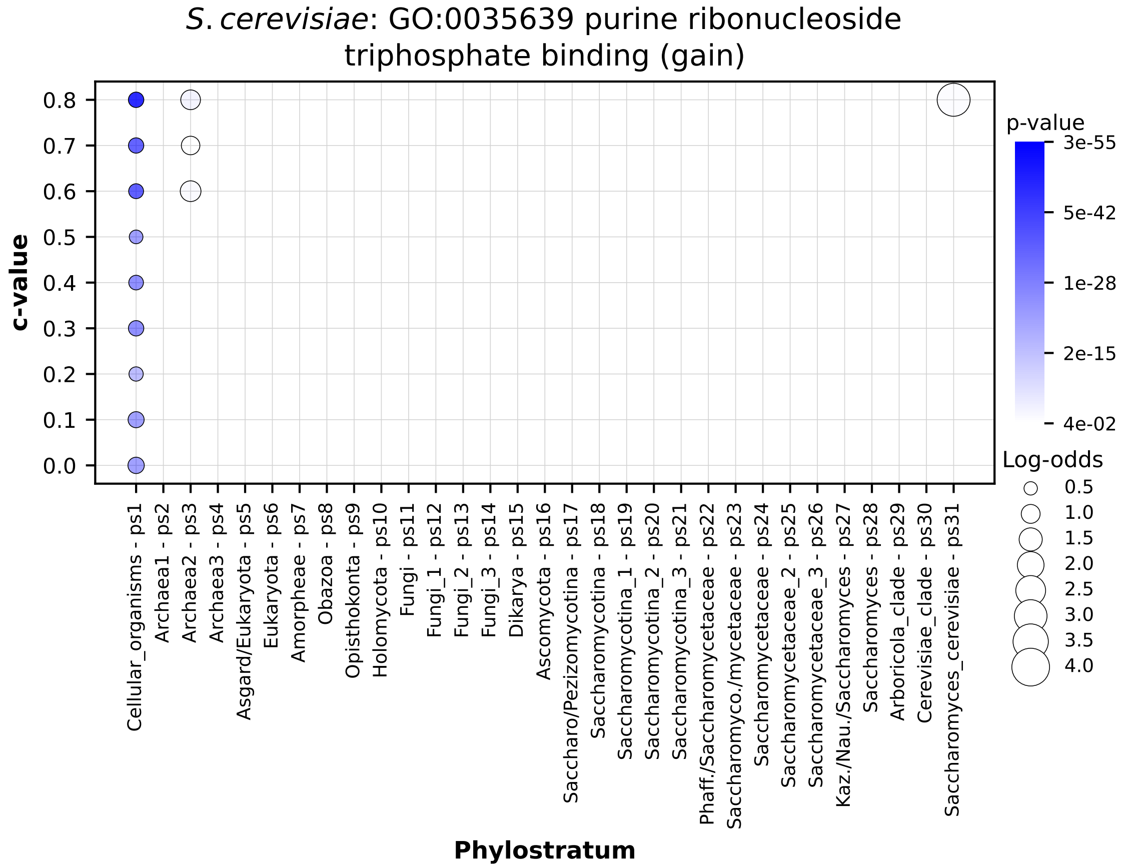

S. cerevisiae: GO:0035639 purine ribonucleoside triphosphate binding (gain)
c-value
0.8
0.7
0.6
0.5
0.4
0.3
0.2
0.1
0.0
p-value
3e-55
5e-42
1e-28
2e-15
4e-02
Log-odds
0.5
1.0
1.5
2.0
2.5
3.0
3.5
4.0
Cellular_organisms - ps1
Archaea1 - ps2
Archaea2 - ps3
Archaea3 - ps4
Asgard/Eukaryota - ps5
Eukaryota - ps6
Amorpheae - ps7
Obazoa - ps8
Opisthokonta - ps9
Holomycota - ps10
Fungi - ps11
Fungi_1 - ps12
Fungi_2 - ps13
Fungi_3 - ps14
Dikarya - ps15
Ascomycota - ps16
Saccharo/Pezizomycotina - ps17
Saccharomycotina - ps18
Saccharomycotina_1 - ps19
Saccharomycotina_2 - ps20
Saccharomycotina_3 - ps21
Phaff./Saccharomycetaceae - ps22
Saccharomyco./mycetaceae - ps23
Saccharomycetaceae - ps24
Saccharomycetaceae_2 - ps25
Saccharomycetaceae_3 - ps26
Kaz./Nau./Saccharomyces - ps27
Saccharomyces - ps28
Arboricola_clade - ps29
Cerevisiae_clade - ps30
Saccharomyces_cerevisiae - ps31
Phylostratum

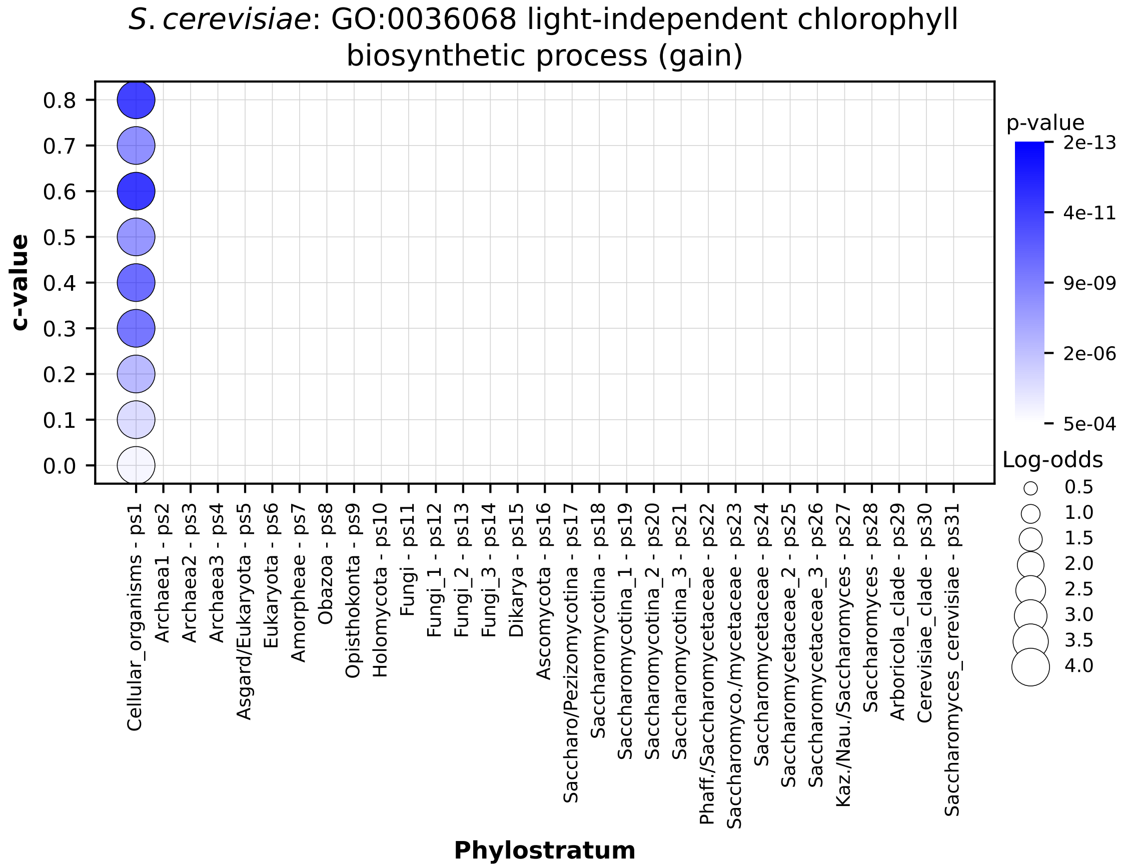
S. cerevisiae: GO:0036068 light-independent chlorophyll biosynthetic process (gain)
c-value
0.8
0.7
0.6
0.5
0.4
0.3
0.2
0.1
0.0
p-value
2e-13
4e-11
9e-09
2e-06
5e-04
Log-odds
0.5
1.0
1.5
2.0
2.5
3.0
3.5
4.0
Cellular_organisms - ps1
Archaea1 - ps2
Archaea2 - ps3
Archaea3 - ps4
Asgard/Eukaryota - ps5
Eukaryota - ps6
Amorpheae - ps7
Obazoa - ps8
Opisthokonta - ps9
Holomycota - ps10
Fungi - ps11
Fungi_1 - ps12
Fungi_2 - ps13
Fungi_3 - ps14
Dikarya - ps15
Ascomycota - ps16
Saccharo/Pezizomycotina - ps17
Saccharomycotina - ps18
Saccharomycotina_1 - ps19
Saccharomycotina_2 - ps20
Saccharomycotina_3 - ps21
Phaff./Saccharomycetaceae - ps22
Saccharomyco./mycetaceae - ps23
Saccharomycetaceae - ps24
Saccharomycetaceae_2 - ps25
Saccharomycetaceae_3 - ps26
Kaz./Nau./Saccharomyces - ps27
Saccharomyces - ps28
Arboricola_clade - ps29
Cerevisiae_clade - ps30
Saccharomyces_cerevisiae - ps31
Phylostratum

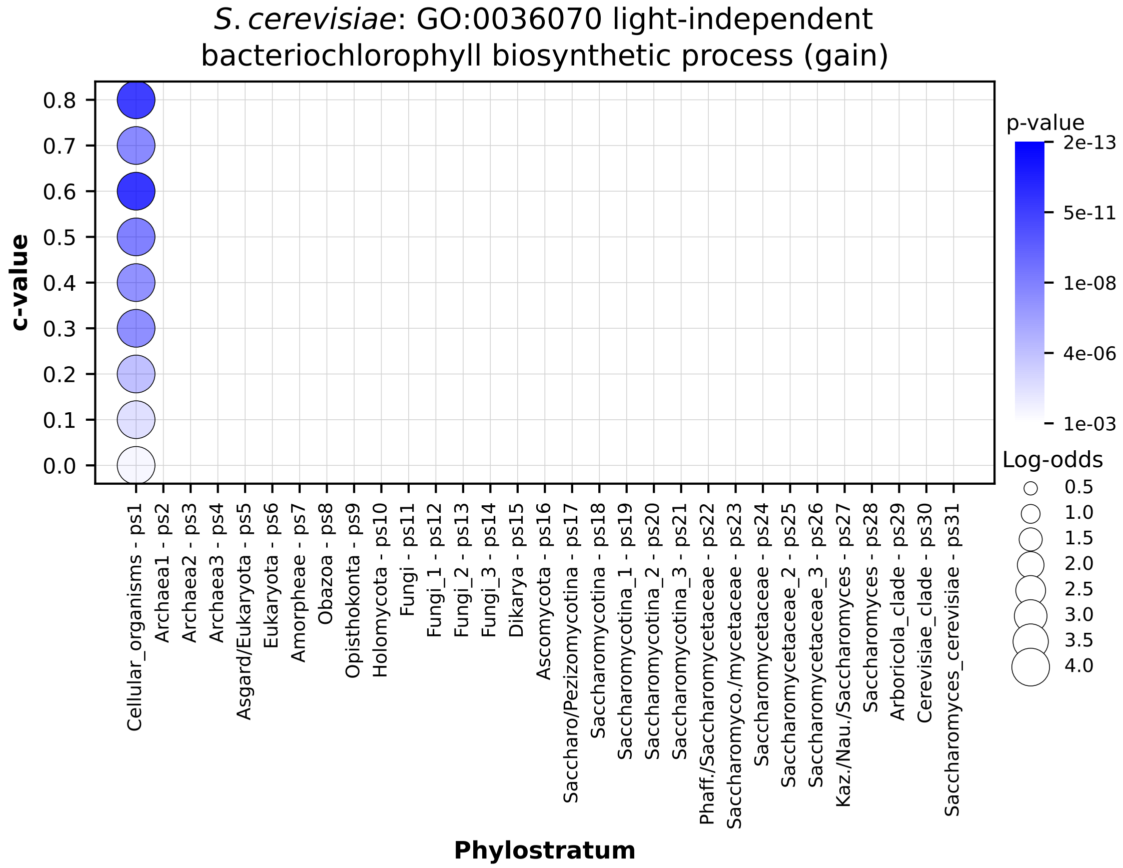

S. cerevisiae: GO:0036070 light-independent bacteriochlorophyll biosynthetic process (gain)
c-value
0.8
0.7
0.6
0.5
0.4
0.3
0.2
0.1
0.0
p-value
2e-13
5e-11
1e-08
4e-06
1e-03
Log-odds
0.5
1.0
1.5
2.0
2.5
3.0
3.5
4.0
Cellular_organisms - ps1
Archaea1 - ps2
Archaea2 - ps3
Archaea3 - ps4
Asgard/Eukaryota - ps5
Eukaryota - ps6
Amorpheae - ps7
Obazoa - ps8
Opisthokonta - ps9
Holomycota - ps10
Fungi - ps11
Fungi_1 - ps12
Fungi_2 - ps13
Fungi_3 - ps14
Dikarya - ps15
Ascomycota - ps16
Saccharo/Pezizomycotina - ps17
Saccharomycotina - ps18
Saccharomycotina_1 - ps19
Saccharomycotina_2 - ps20
Saccharomycotina_3 - ps21
Phaff./Saccharomycetaceae - ps22
Saccharomyco./mycetaceae - ps23
Saccharomycetaceae - ps24
Saccharomycetaceae_2 - ps25
Saccharomycetaceae_3 - ps26
Kaz./Nau./Saccharomyces - ps27
Saccharomyces - ps28
Arboricola_clade - ps29
Cerevisiae_clade - ps30
Saccharomyces_cerevisiae - ps31
Phylostratum

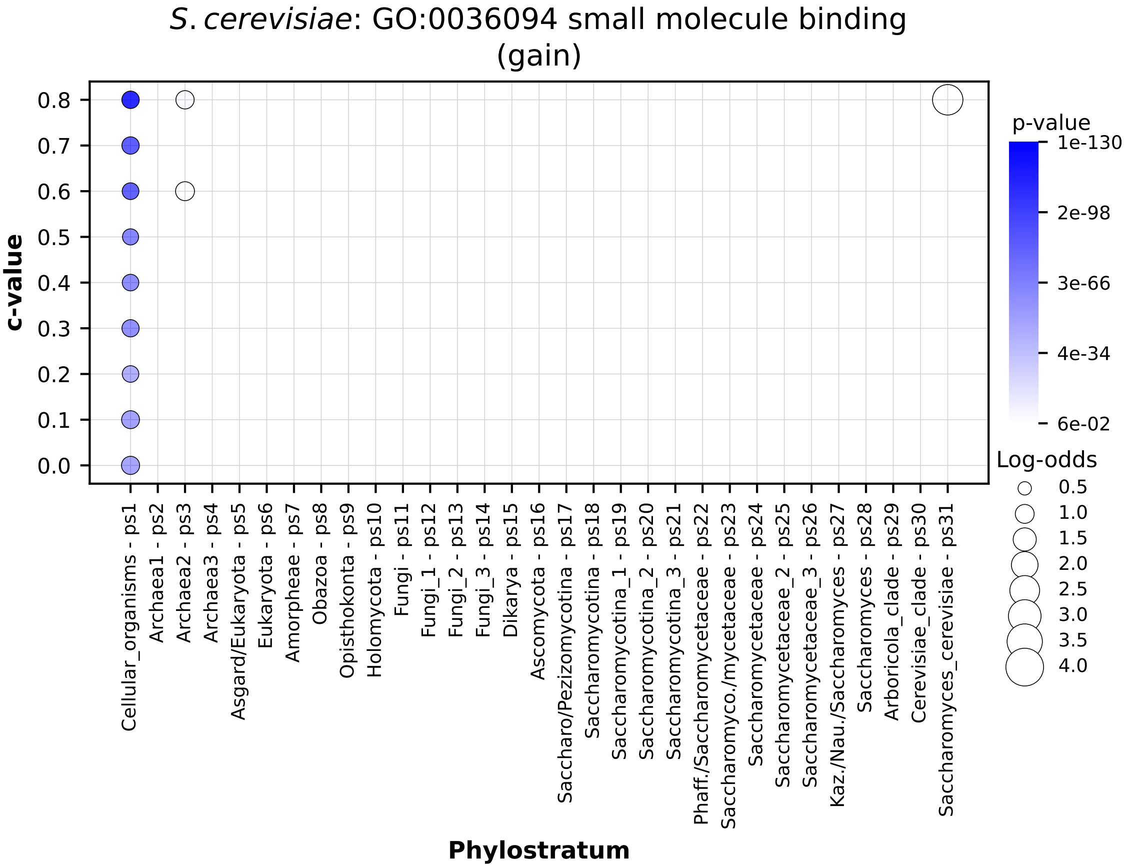
S. cerevisiae: GO:0036094 small molecule binding (gain)
c-value
0.8
0.7
0.6
0.5
0.4
0.3
0.2
0.1
0.0
p-value
1e-130
2e-98
3e-66
4e-34
6e-02
Log-odds
0.5
1.0
1.5
2.0
2.5
3.0
3.5
4.0
Cellular_organisms - ps1
Archaea1 - ps2
Archaea2 - ps3
Archaea3 - ps4
Asgard/Eukaryota - ps5
Eukaryota - ps6
Amorpheae - ps7
Obazoa - ps8
Opisthokonta - ps9
Holomycota - ps10
Fungi - ps11
Fungi_1 - ps12
Fungi_2 - ps13
Fungi_3 - ps14
Dikarya - ps15
Ascomycota - ps16
Saccharo/Pezizomycotina - ps17
Saccharomycotina - ps18
Saccharomycotina_1 - ps19
Saccharomycotina_2 - ps20
Saccharomycotina_3 - ps21
Phaff./Saccharomycetaceae - ps22
Saccharomyco./mycetaceae - ps23
Saccharomycetaceae - ps24
Saccharomycetaceae_2 - ps25
Saccharomycetaceae_3 - ps26
Kaz./Nau./Saccharomyces - ps27
Saccharomyces - ps28
Arboricola_clade - ps29
Cerevisiae_clade - ps30
Saccharomyces_cerevisiae - ps31
Phylostratum

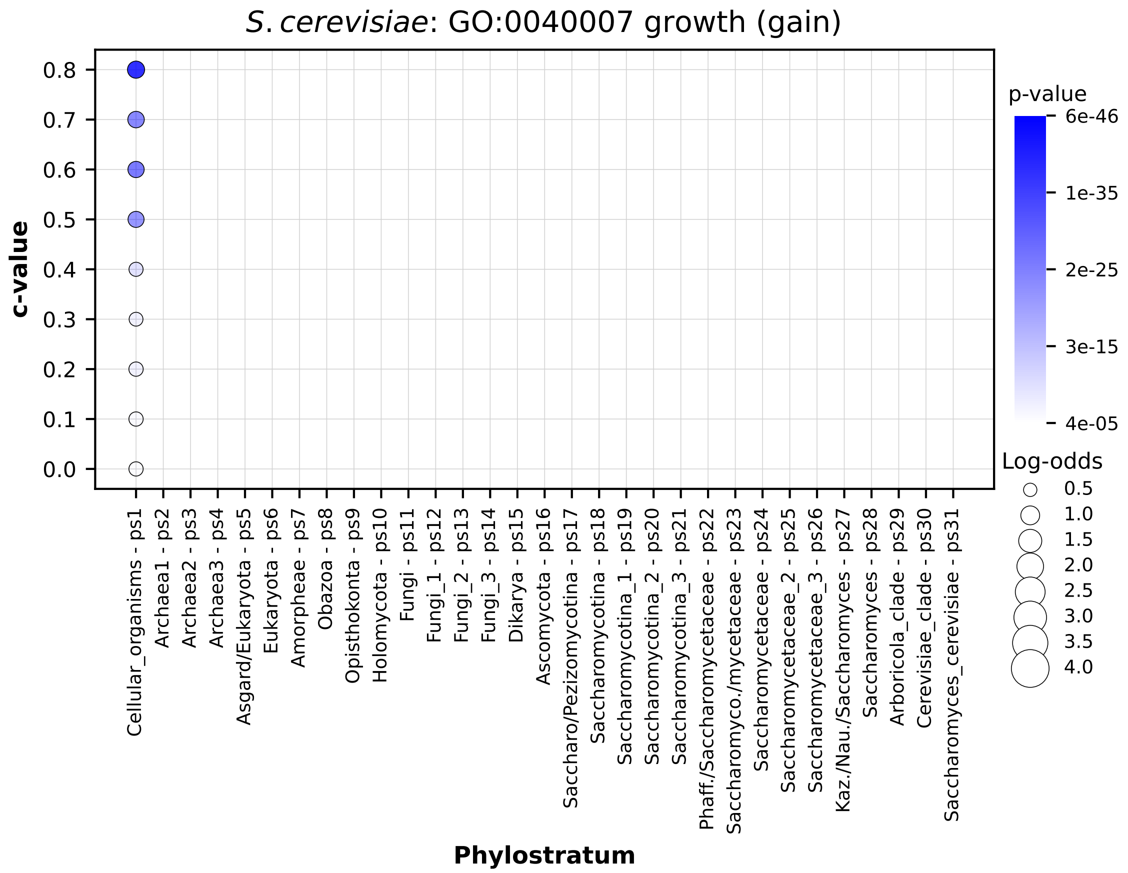
S. cerevisiae: GO:0040007 growth (gain)
c-value
0.8
0.7
0.6
0.5
0.4
0.3
0.2
0.1
0.0
p-value
6e-46
1e-35
2e-25
3e-15
4e-05
Log-odds
0.5
1.0
1.5
2.0
2.5
3.0
3.5
4.0
Cellular_organisms - ps1
Archaea1 - ps2
Archaea2 - ps3
Archaea3 - ps4
Asgard/Eukaryota - ps5
Eukaryota - ps6
Amorpheae - ps7
Obazoa - ps8
Opisthokonta - ps9
Holomycota - ps10
Fungi - ps11
Fungi_1 - ps12
Fungi_2 - ps13
Fungi_3 - ps14
Dikarya - ps15
Ascomycota - ps16
Saccharo/Pezizomycotina - ps17
Saccharomycotina - ps18
Saccharomycotina_1 - ps19
Saccharomycotina_2 - ps20
Saccharomycotina_3 - ps21
Phaff./Saccharomycetaceae - ps22
Saccharomyco./mycetaceae - ps23
Saccharomycetaceae - ps24
Saccharomycetaceae_2 - ps25
Saccharomycetaceae_3 - ps26
Kaz./Nau./Saccharomyces - ps27
Saccharomyces - ps28
Arboricola_clade - ps29
Cerevisiae_clade - ps30
Saccharomyces_cerevisiae - ps31
Phylostratum

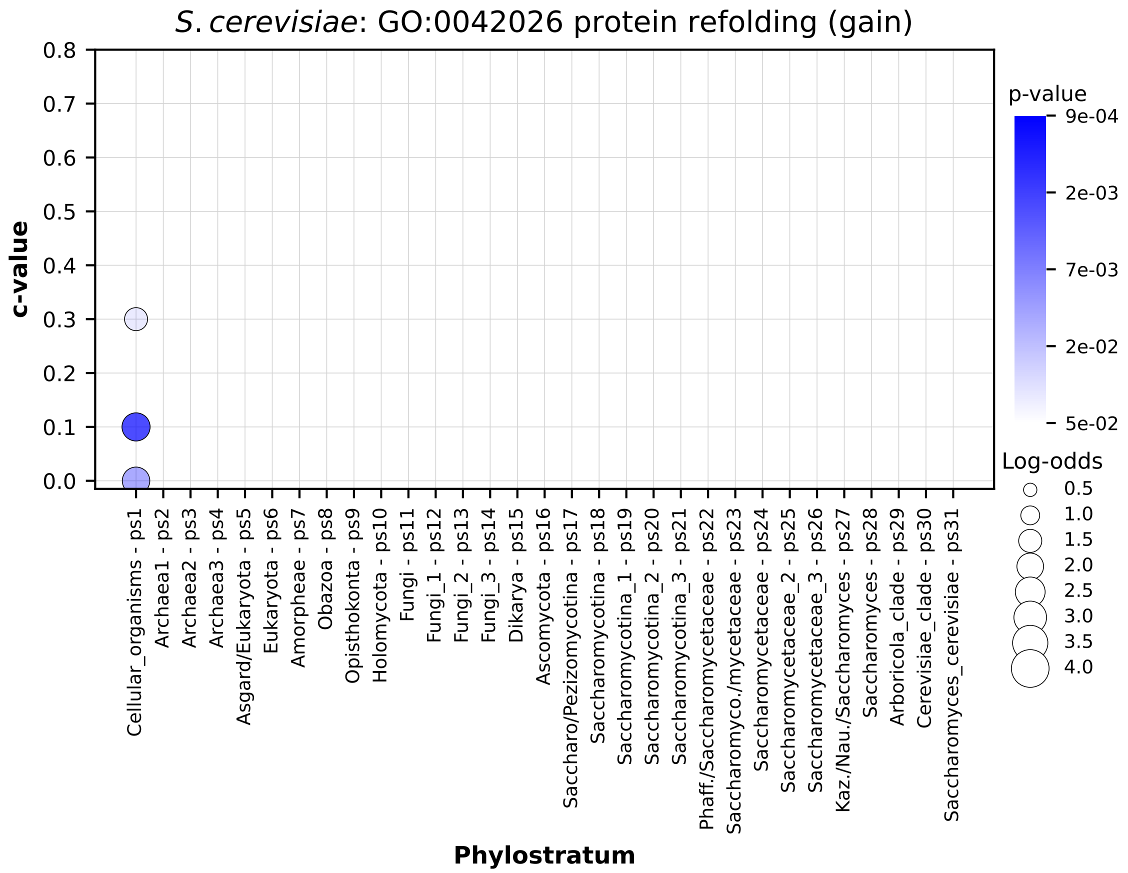
S. cerevisiae: GO:0042026 protein refolding (gain)
c-value
0.8
0.7
0.6
0.5
0.4
0.3
0.2
0.1
0.0
p-value
9e-04
2e-03
7e-03
2e-02
5e-02
Log-odds
0.5
1.0
1.5
2.0
2.5
3.0
3.5
4.0
Cellular_organisms - ps1
Archaea1 - ps2
Archaea2 - ps3
Archaea3 - ps4
Asgard/Eukaryota - ps5
Eukaryota - ps6
Amorpheae - ps7
Obazoa - ps8
Opisthokonta - ps9
Holomycota - ps10
Fungi - ps11
Fungi_1 - ps12
Fungi_2 - ps13
Fungi_3 - ps14
Dikarya - ps15
Ascomycota - ps16
Saccharo/Pezizomycotina - ps17
Saccharomycotina - ps18
Saccharomycotina_1 - ps19
Saccharomycotina_2 - ps20
Saccharomycotina_3 - ps21
Phaff./Saccharomycetaceae - ps22
Saccharomyco./mycetaceae - ps23
Saccharomycetaceae - ps24
Saccharomycetaceae_2 - ps25
Saccharomycetaceae_3 - ps26
Kaz./Nau./Saccharomyces - ps27
Saccharomyces - ps28
Arboricola_clade - ps29
Cerevisiae_clade - ps30
Saccharomyces_cerevisiae - ps31
Phylostratum

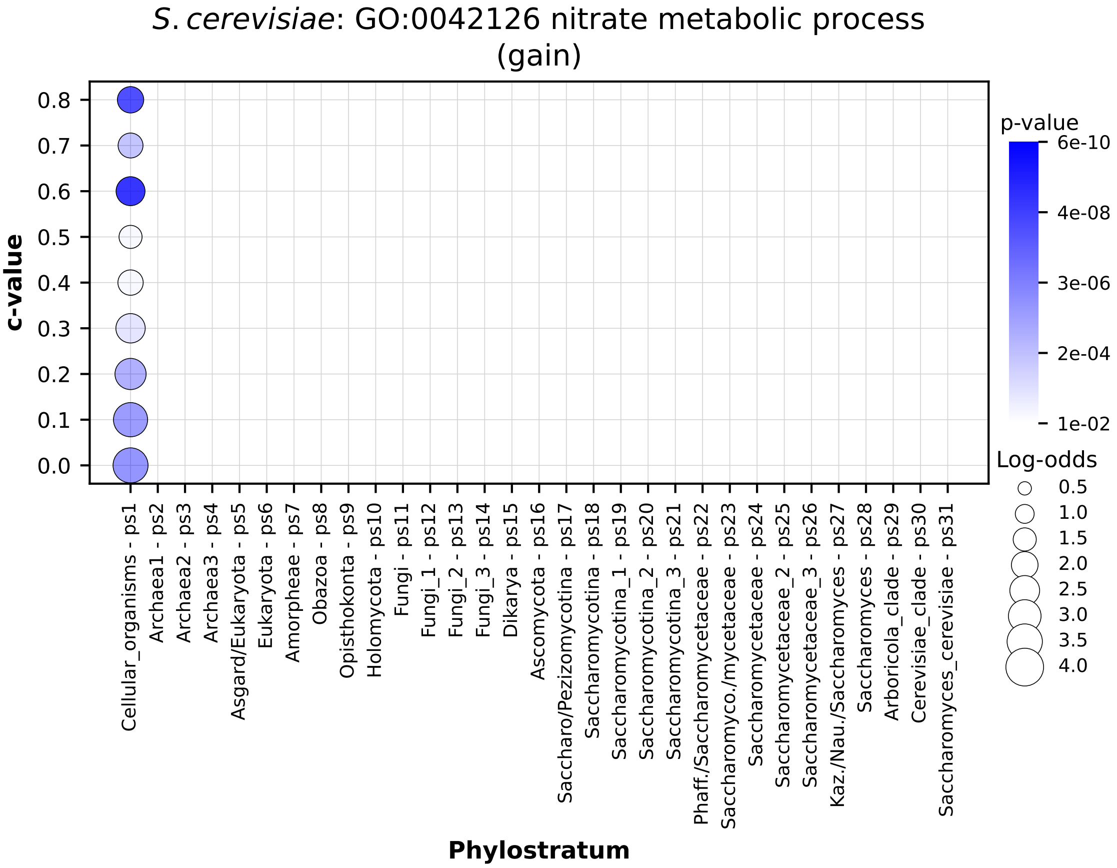

S. cerevisiae: GO:0042126 nitrate metabolic process
(gain)
c-value
0.8
0.7
0.6
0.5
0.4
0.3
0.2
0.1
0.0
p-value
6e-10
4e-08
3e-06
2e-04
1e-02
Log-odds
0.5
1.0
1.5
2.0
2.5
3.0
3.5
4.0
Cellular_organisms - ps1
Archaea1 - ps2
Archaea2 - ps3
Archaea3 - ps4
Asgard/Eukaryota - ps5
Eukaryota - ps6
Amorpheae - ps7
Obazoa - ps8
Opisthokonta - ps9
Holomycota - ps10
Fungi - ps11
Fungi_1 - ps12
Fungi_2 - ps13
Fungi_3 - ps14
Dikarya - ps15
Ascomycota - ps16
Saccharo/Pezizomycotina - ps17
Saccharomycotina - ps18
Saccharomycotina_1 - ps19
Saccharomycotina_2 - ps20
Saccharomycotina_3 - ps21
Phaff./Saccharomycetaceae - ps22
Saccharomyco./mycetaceae - ps23
Saccharomycetaceae - ps24
Saccharomycetaceae_2 - ps25
Saccharomycetaceae_3 - ps26
Kaz./Nau./Saccharomyces - ps27
Saccharomyces - ps28
Arboricola_clade - ps29
Cerevisiae_clade - ps30
Saccharomyces_cerevisiae - ps31
Phylostratum

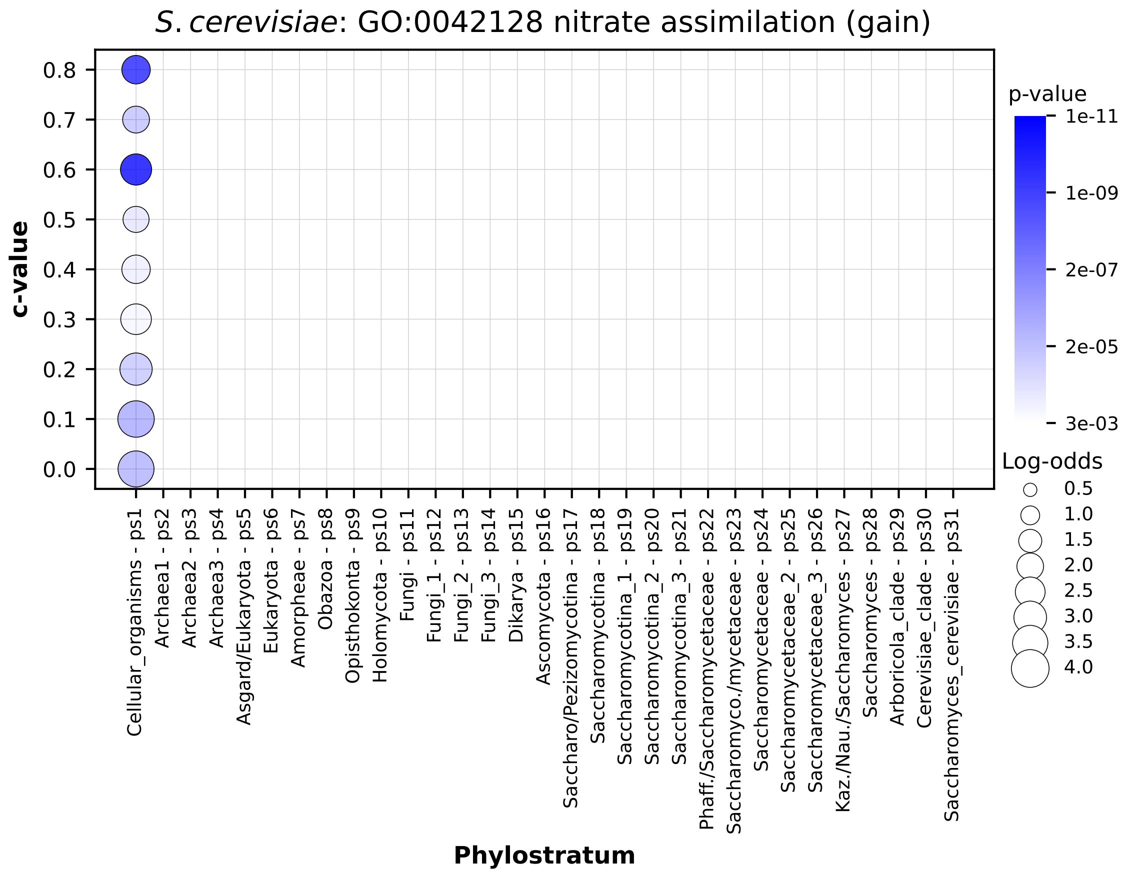

S. cerevisiae: GO:0042128 nitrate assimilation (gain)
c-value
0.8
0.7
0.6
0.5
0.4
0.3
0.2
0.1
0.0
p-value
1e-11
1e-09
2e-07
2e-05
3e-03
Log-odds
0.5
1.0
1.5
2.0
2.5
3.0
3.5
4.0
Cellular_organisms - ps1
Archaea1 - ps2
Archaea2 - ps3
Archaea3 - ps4
Asgard/Eukaryota - ps5
Eukaryota - ps6
Amorpheae - ps7
Obazoa - ps8
Opisthokonta - ps9
Holomycota - ps10
Fungi - ps11
Fungi_1 - ps12
Fungi_2 - ps13
Fungi_3 - ps14
Dikarya - ps15
Ascomycota - ps16
Saccharo/Pezizomycotina - ps17
Saccharomycotina - ps18
Saccharomycotina_1 - ps19
Saccharomycotina_2 - ps20
Saccharomycotina_3 - ps21
Phaff./Saccharomycetaceae - ps22
Saccharomyco./mycetaceae - ps23
Saccharomycetaceae - ps24
Saccharomycetaceae_2 - ps25
Saccharomycetaceae_3 - ps26
Kaz./Nau./Saccharomyces - ps27
Saccharomyces - ps28
Arboricola_clade - ps29
Cerevisiae_clade - ps30
Saccharomyces_cerevisiae - ps31
Phylostratum

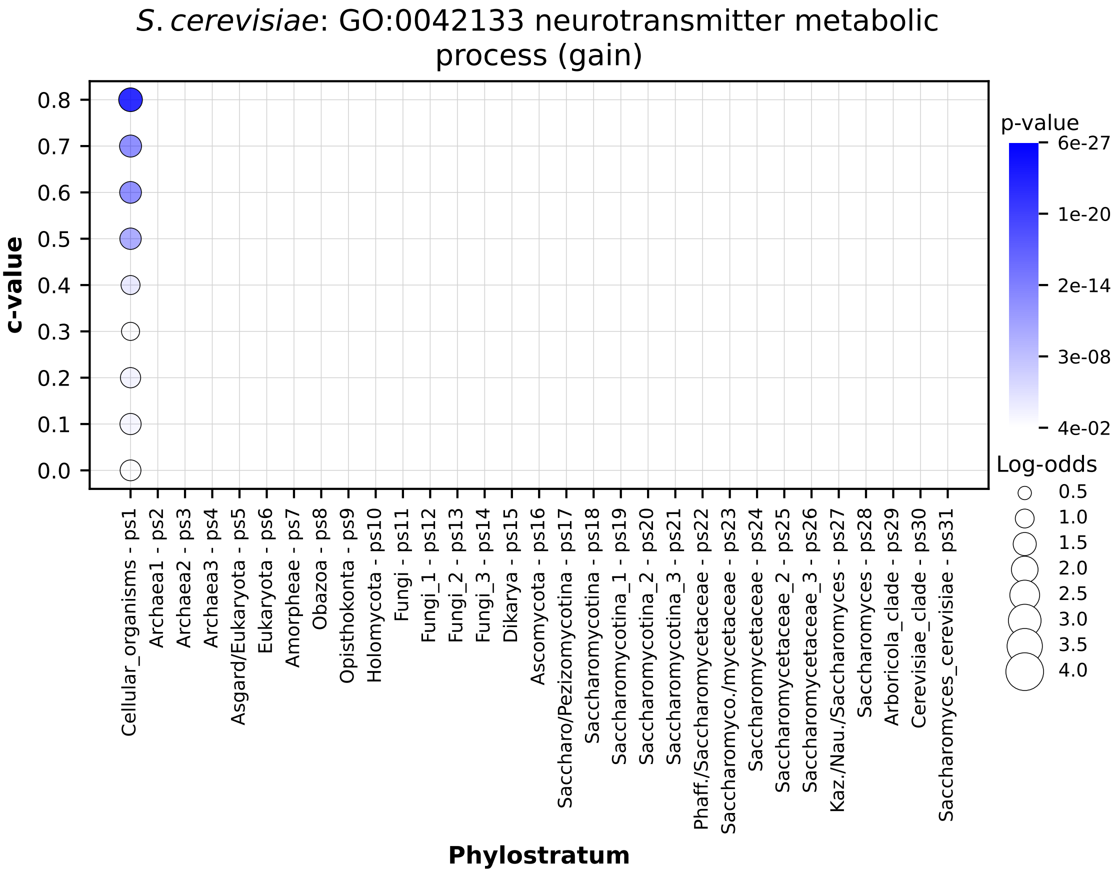

S. cerevisiae: GO:0042133 neurotransmitter metabolic process (gain)
c-value
0.0
0.1
0.2
0.3
0.4
0.5
0.6
0.7
0.8
Phylostratum
Cellular_organisms - ps1
Archaea1 - ps2
Archaea2 - ps3
Archaea3 - ps4
Asgard/Eukaryota - ps5
Eukaryota - ps6
Amorpheae - ps7
Obazoa - ps8
Opisthokonta - ps9
Holomycota - ps10
Fungi - ps11
Fungi_1 - ps12
Fungi_2 - ps13
Fungi_3 - ps14
Dikarya - ps15
Ascomycota - ps16
Saccharo/Pezizomycotina - ps17
Saccharomycotina - ps18
Saccharomycotina_1 - ps19
Saccharomycotina_2 - ps20
Saccharomycotina_3 - ps21
Phaff./Saccharomycetaceae - ps22
Saccharomyco./mycetaceae - ps23
Saccharomycetaceae - ps24
Saccharomycetaceae_2 - ps25
Saccharomycetaceae_3 - ps26
Kaz./Nau./Saccharomyces - ps27
Saccharomyces - ps28
Arboricola_clade - ps29
Cerevisiae_clade - ps30
Saccharomyces_cerevisiae - ps31
p-value
6e-27
1e-20
2e-14
3e-08
4e-02
Log-odds
0.5
1.0
1.5
2.0
2.5
3.0
3.5
4.0

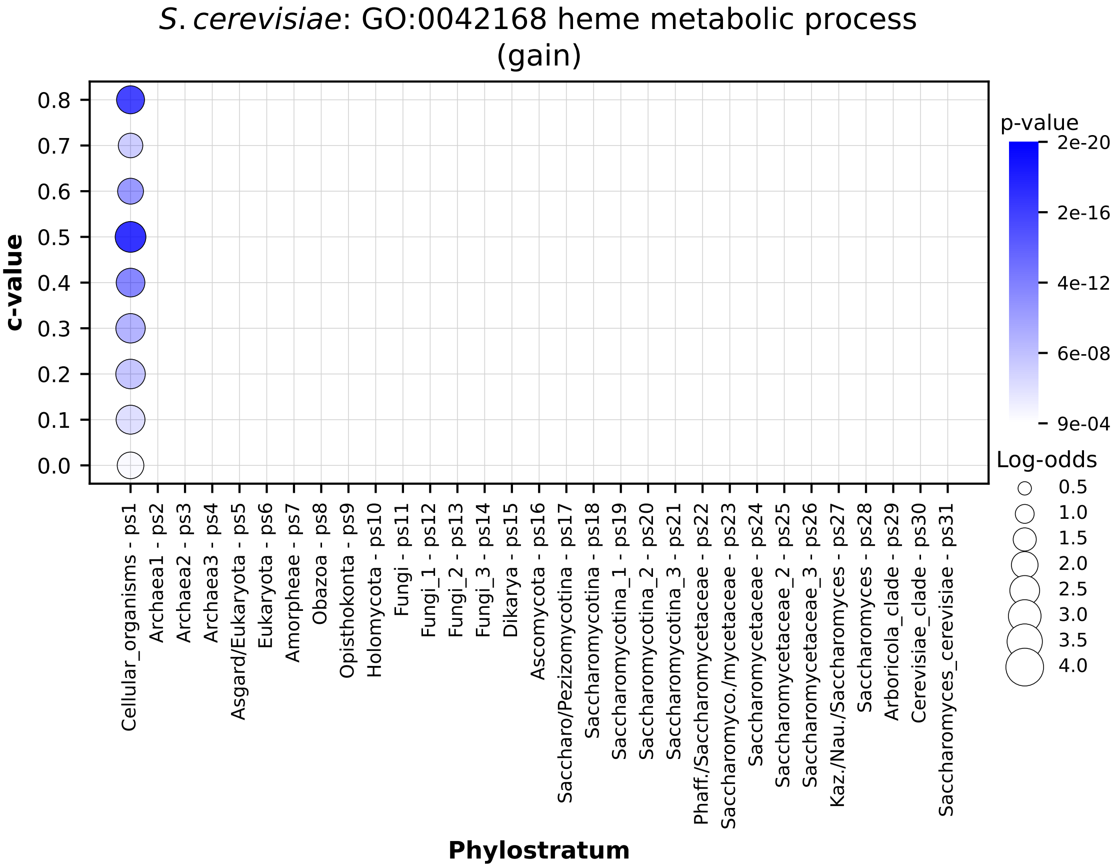
S. cerevisiae: GO:0042168 heme metabolic process
(gain)
c-value
0.8
0.7
0.6
0.5
0.4
0.3
0.2
0.1
0.0
p-value
2e-20
2e-16
4e-12
6e-08
9e-04
Log-odds
0.5
1.0
1.5
2.0
2.5
3.0
3.5
4.0
Cellular_organisms - ps1
Archaea1 - ps2
Archaea2 - ps3
Archaea3 - ps4
Asgard/Eukaryota - ps5
Eukaryota - ps6
Amorpheae - ps7
Obazoa - ps8
Opisthokonta - ps9
Holomycota - ps10
Fungi - ps11
Fungi_1 - ps12
Fungi_2 - ps13
Fungi_3 - ps14
Dikarya - ps15
Ascomycota - ps16
Saccharo/Pezizomycotina - ps17
Saccharomycotina - ps18
Saccharomycotina_1 - ps19
Saccharomycotina_2 - ps20
Saccharomycotina_3 - ps21
Phaff./Saccharomycetaceae - ps22
Saccharomyco./mycetaceae - ps23
Saccharomycetaceae - ps24
Saccharomycetaceae_2 - ps25
Saccharomycetaceae_3 - ps26
Kaz./Nau./Saccharomyces - ps27
Saccharomyces - ps28
Arboricola_clade - ps29
Cerevisiae_clade - ps30
Saccharomyces_cerevisiae - ps31
Phylostratum

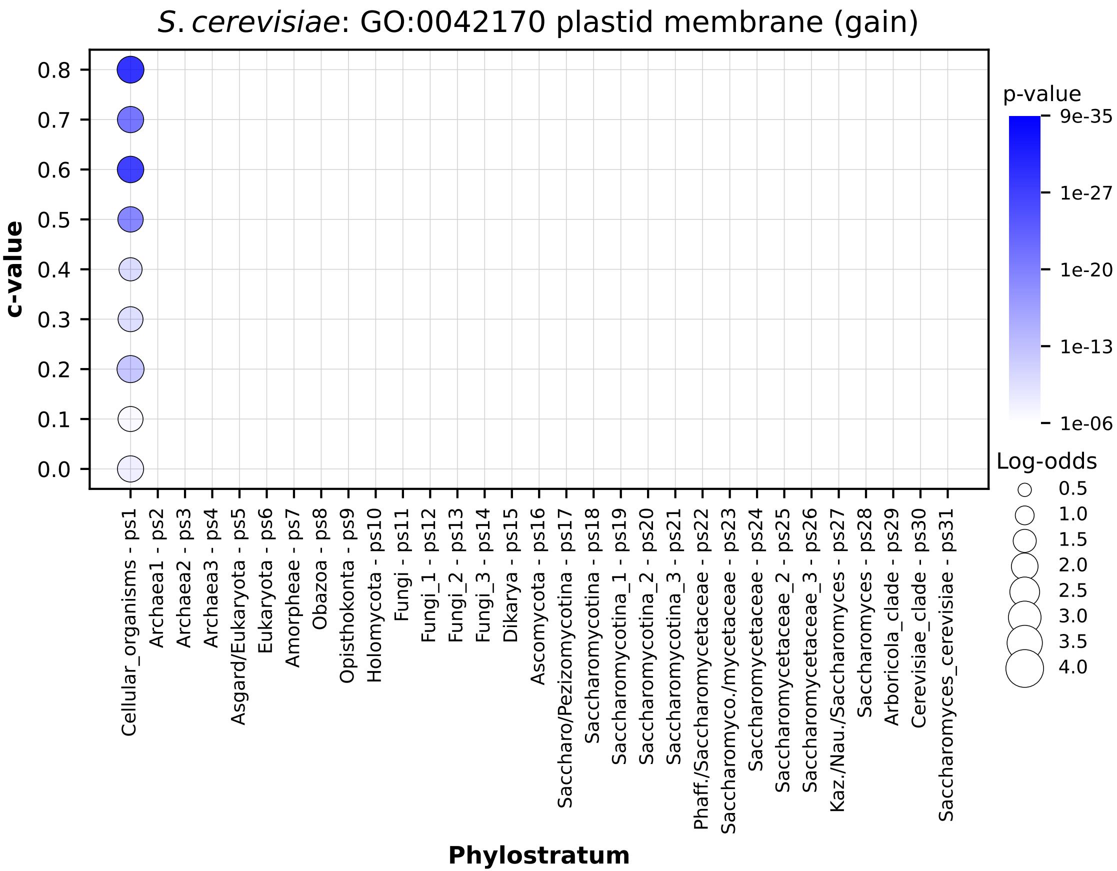

S. cerevisiae: GO:0042170 plastid membrane (gain)
c-value
0.8
0.7
0.6
0.5
0.4
0.3
0.2
0.1
0.0
Phylostratum
Cellular_organisms - ps1
Archaea1 - ps2
Archaea2 - ps3
Archaea3 - ps4
Asgard/Eukaryota - ps5
Eukaryota - ps6
Amorpheae - ps7
Obazoa - ps8
Opisthokonta - ps9
Holomycota - ps10
Fungi - ps11
Fungi_1 - ps12
Fungi_2 - ps13
Fungi_3 - ps14
Dikarya - ps15
Ascomycota - ps16
Saccharo/Pezizomycotina - ps17
Saccharomycotina - ps18
Saccharomycotina_1 - ps19
Saccharomycotina_2 - ps20
Saccharomycotina_3 - ps21
Phaff./Saccharomycetaceae - ps22
Saccharomyco./mycetaceae - ps23
Saccharomycetaceae - ps24
Saccharomycetaceae_2 - ps25
Saccharomycetaceae_3 - ps26
Kaz./Nau./Saccharomyces - ps27
Saccharomyces - ps28
Arboricola_clade - ps29
Cerevisiae_clade - ps30
Saccharomyces_cerevisiae - ps31
p-value
9e-35
1e-27
1e-20
1e-13
1e-06
Log-odds
0.5
1.0
1.5
2.0
2.5
3.0
3.5
4.0

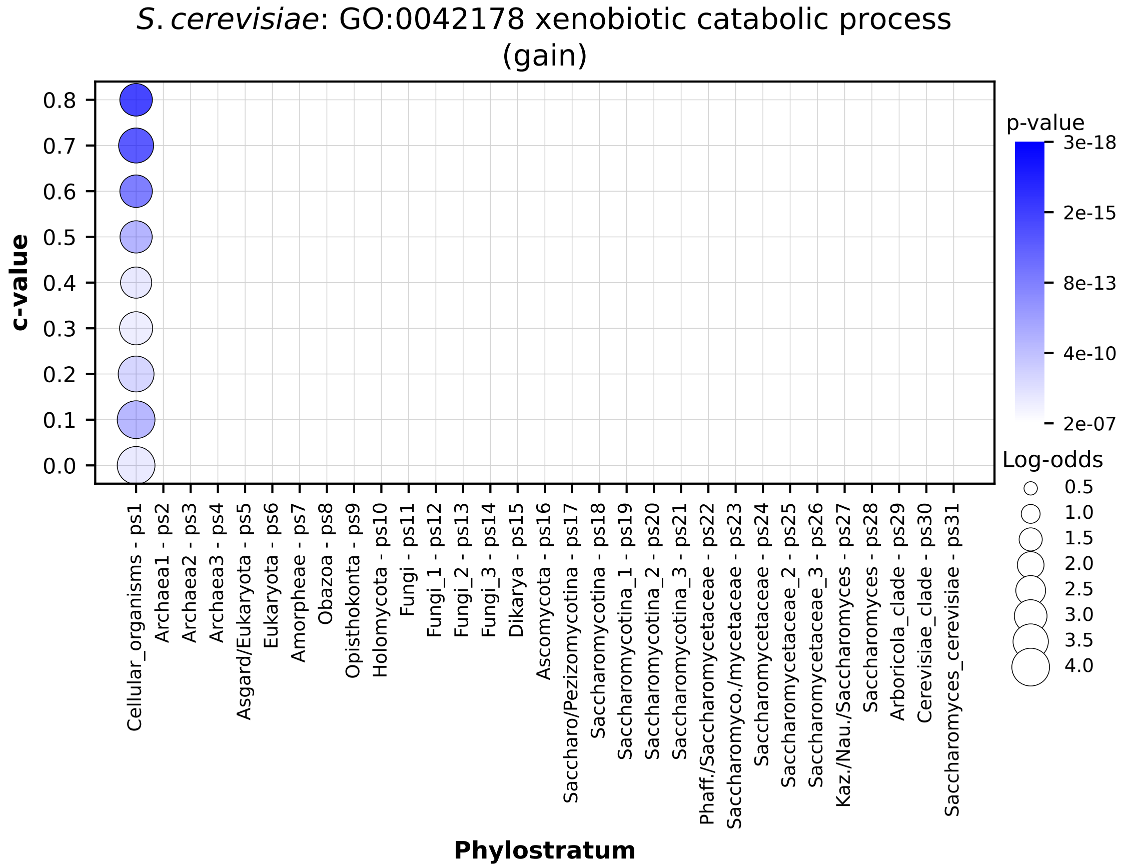

S. cerevisiae: GO:0042178 xenobiotic catabolic process
(gain)
c-value
0.8
0.7
0.6
0.5
0.4
0.3
0.2
0.1
0.0
p-value
3e-18
2e-15
8e-13
4e-10
2e-07
Log-odds
0.5
1.0
1.5
2.0
2.5
3.0
3.5
4.0
Cellular_organisms - ps1
Archaea1 - ps2
Archaea2 - ps3
Archaea3 - ps4
Asgard/Eukaryota - ps5
Eukaryota - ps6
Amorpheae - ps7
Obazoa - ps8
Opisthokonta - ps9
Holomycota - ps10
Fungi - ps11
Fungi_1 - ps12
Fungi_2 - ps13
Fungi_3 - ps14
Dikarya - ps15
Ascomycota - ps16
Saccharo/Pezizomycotina - ps17
Saccharomycotina - ps18
Saccharomycotina_1 - ps19
Saccharomycotina_2 - ps20
Saccharomycotina_3 - ps21
Phaff./Saccharomycetaceae - ps22
Saccharomyco./mycetaceae - ps23
Saccharomycetaceae - ps24
Saccharomycetaceae_2 - ps25
Saccharomycetaceae_3 - ps26
Kaz./Nau./Saccharomyces - ps27
Saccharomyces - ps28
Arboricola_clade - ps29
Cerevisiae_clade - ps30
Saccharomyces_cerevisiae - ps31
Phylostratum

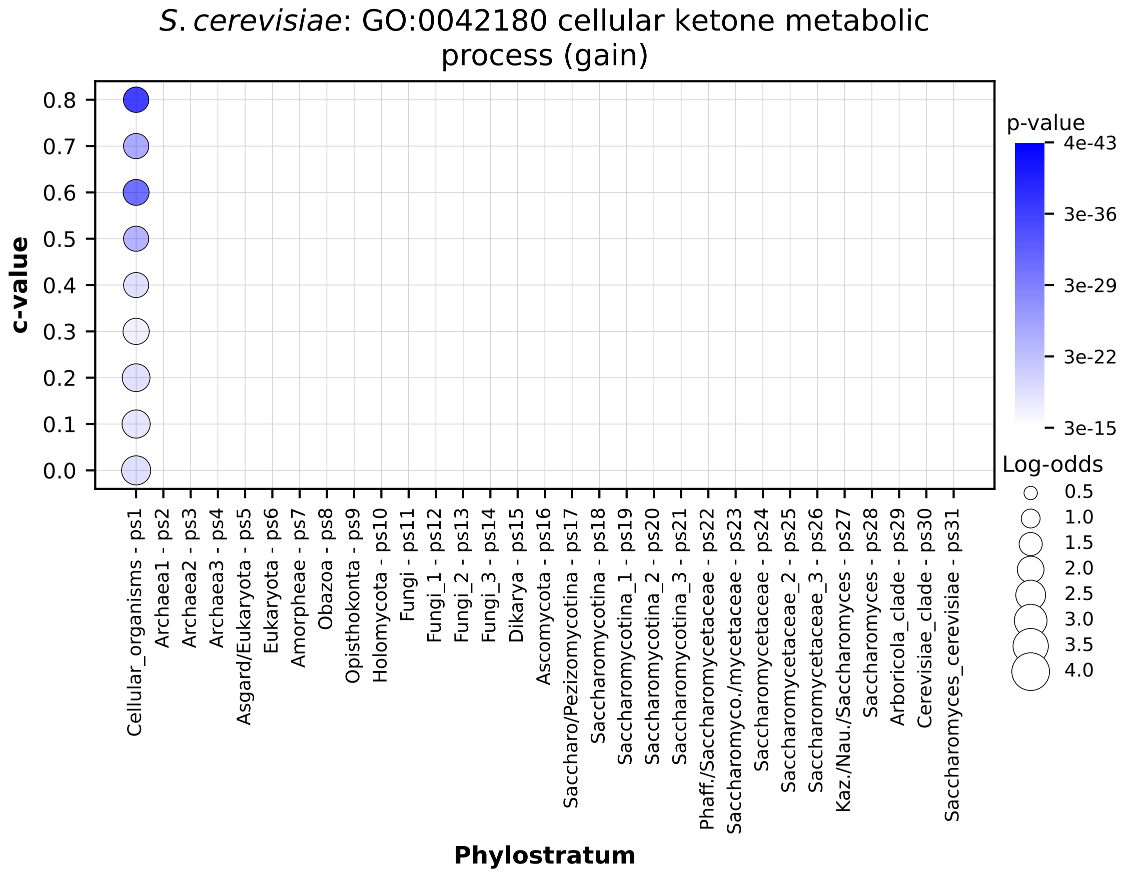
S. cerevisiae: GO:0042180 cellular ketone metabolic process (gain)
c-value
0.0
0.1
0.2
0.3
0.4
0.5
0.6
0.7
0.8
p-value
4e-43
3e-36
3e-29
3e-22
3e-15
Log-odds
0.5
1.0
1.5
2.0
2.5
3.0
3.5
4.0
Cellular_organisms - ps1
Archaea1 - ps2
Archaea2 - ps3
Archaea3 - ps4
Asgard/Eukaryota - ps5
Eukaryota - ps6
Amorpheae - ps7
Obazoa - ps8
Opisthokonta - ps9
Holomycota - ps10
Fungi - ps11
Fungi_1 - ps12
Fungi_2 - ps13
Fungi_3 - ps14
Dikarya - ps15
Ascomycota - ps16
Saccharo/Pezizomycotina - ps17
Saccharomycotina - ps18
Saccharomycotina_1 - ps19
Saccharomycotina_2 - ps20
Saccharomycotina_3 - ps21
Phaff./Saccharomycetaceae - ps22
Saccharomyco./mycetaceae - ps23
Saccharomycetaceae - ps24
Saccharomycetaceae_2 - ps25
Saccharomycetaceae_3 - ps26
Kaz./Nau./Saccharomyces - ps27
Saccharomyces - ps28
Arboricola_clade - ps29
Cerevisiae_clade - ps30
Saccharomyces_cerevisiae - ps31
Phylostratum

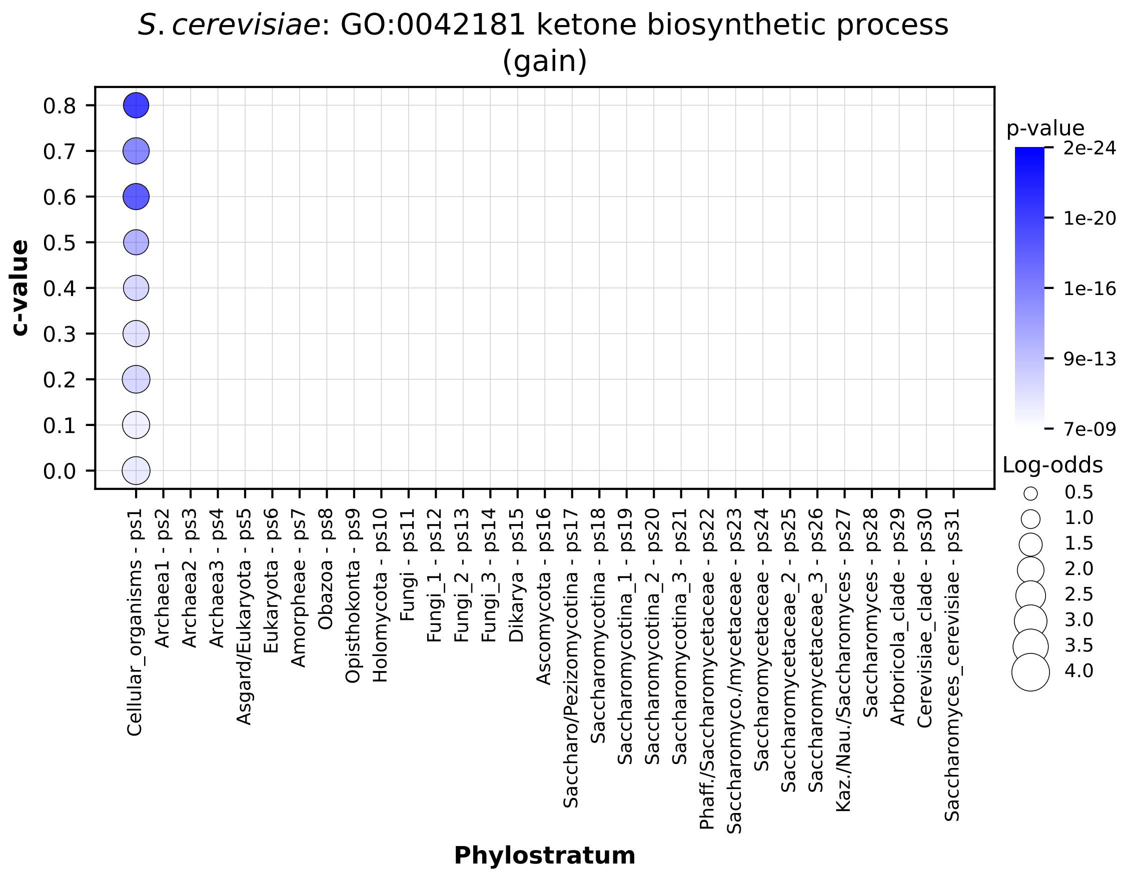
S. cerevisiae: GO:0042181 ketone biosynthetic process
(gain)
c-value
0.8
0.7
0.6
0.5
0.4
0.3
0.2
0.1
0.0
p-value
2e-24
1e-20
1e-16
9e-13
7e-09
Log-odds
0.5
1.0
1.5
2.0
2.5
3.0
3.5
4.0
Cellular_organisms - ps1
Archaea1 - ps2
Archaea2 - ps3
Archaea3 - ps4
Asgard/Eukaryota - ps5
Eukaryota - ps6
Amorpheae - ps7
Obazoa - ps8
Opisthokonta - ps9
Holomycota - ps10
Fungi - ps11
Fungi_1 - ps12
Fungi_2 - ps13
Fungi_3 - ps14
Dikarya - ps15
Ascomycota - ps16
Saccharo/Pezizomycotina - ps17
Saccharomycotina - ps18
Saccharomycotina_1 - ps19
Saccharomycotina_2 - ps20
Saccharomycotina_3 - ps21
Phaff./Saccharomycetaceae - ps22
Saccharomyco./mycetaceae - ps23
Saccharomycetaceae - ps24
Saccharomycetaceae_2 - ps25
Saccharomycetaceae_3 - ps26
Kaz./Nau./Saccharomyces - ps27
Saccharomyces - ps28
Arboricola_clade - ps29
Cerevisiae_clade - ps30
Saccharomyces_cerevisiae - ps31
Phylostratum

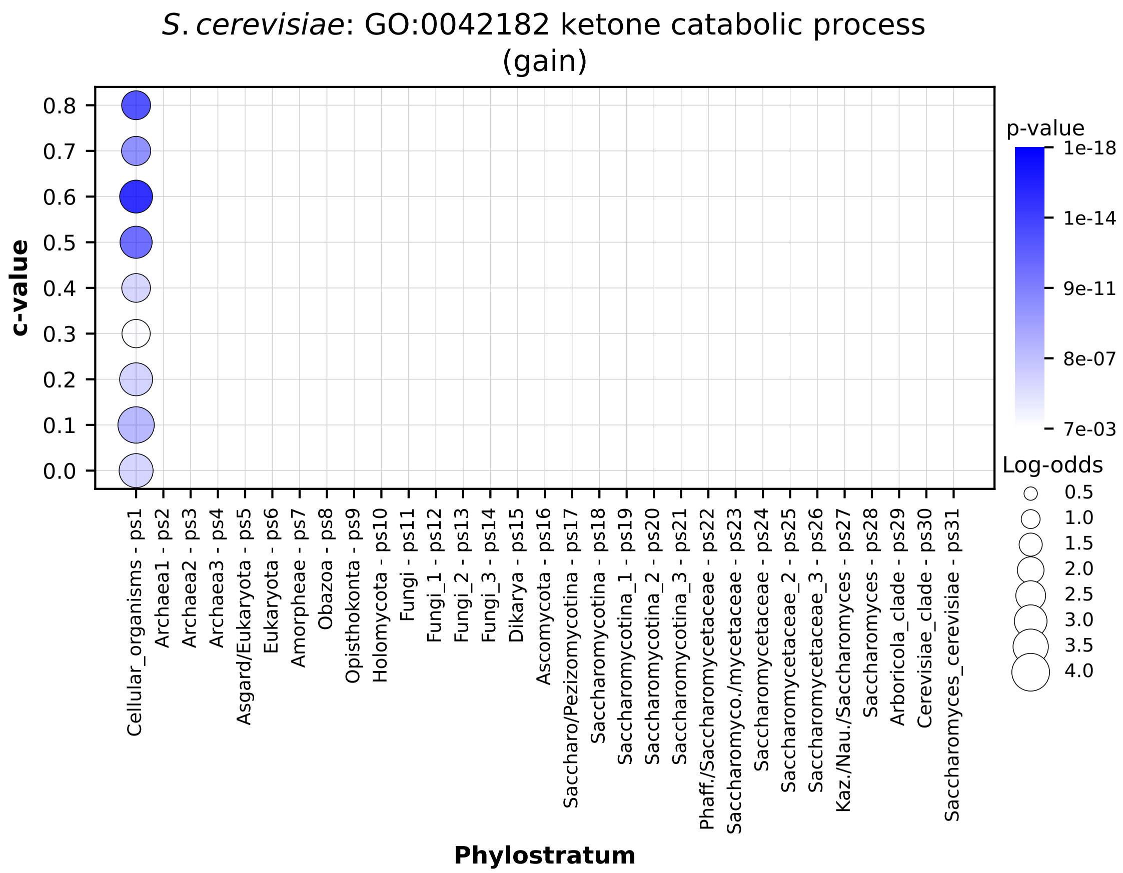
S. cerevisiae: GO:0042182 ketone catabolic process
(gain)
c-value
0.8
0.7
0.6
0.5
0.4
0.3
0.2
0.1
0.0
p-value
1e-18
1e-14
9e-11
8e-07
7e-03
Log-odds
0.5
1.0
1.5
2.0
2.5
3.0
3.5
4.0
Cellular_organisms - ps1
Archaea1 - ps2
Archaea2 - ps3
Archaea3 - ps4
Asgard/Eukaryota - ps5
Eukaryota - ps6
Amorpheae - ps7
Obazoa - ps8
Opisthokonta - ps9
Holomycota - ps10
Fungi - ps11
Fungi_1 - ps12
Fungi_2 - ps13
Fungi_3 - ps14
Dikarya - ps15
Ascomycota - ps16
Saccharo/Pezizomycotina - ps17
Saccharomycotina - ps18
Saccharomycotina_1 - ps19
Saccharomycotina_2 - ps20
Saccharomycotina_3 - ps21
Phaff./Saccharomycetaceae - ps22
Saccharomyco./mycetaceae - ps23
Saccharomycetaceae - ps24
Saccharomycetaceae_2 - ps25
Saccharomycetaceae_3 - ps26
Kaz./Nau./Saccharomyces - ps27
Saccharomyces - ps28
Arboricola_clade - ps29
Cerevisiae_clade - ps30
Saccharomyces_cerevisiae - ps31
Phylostratum

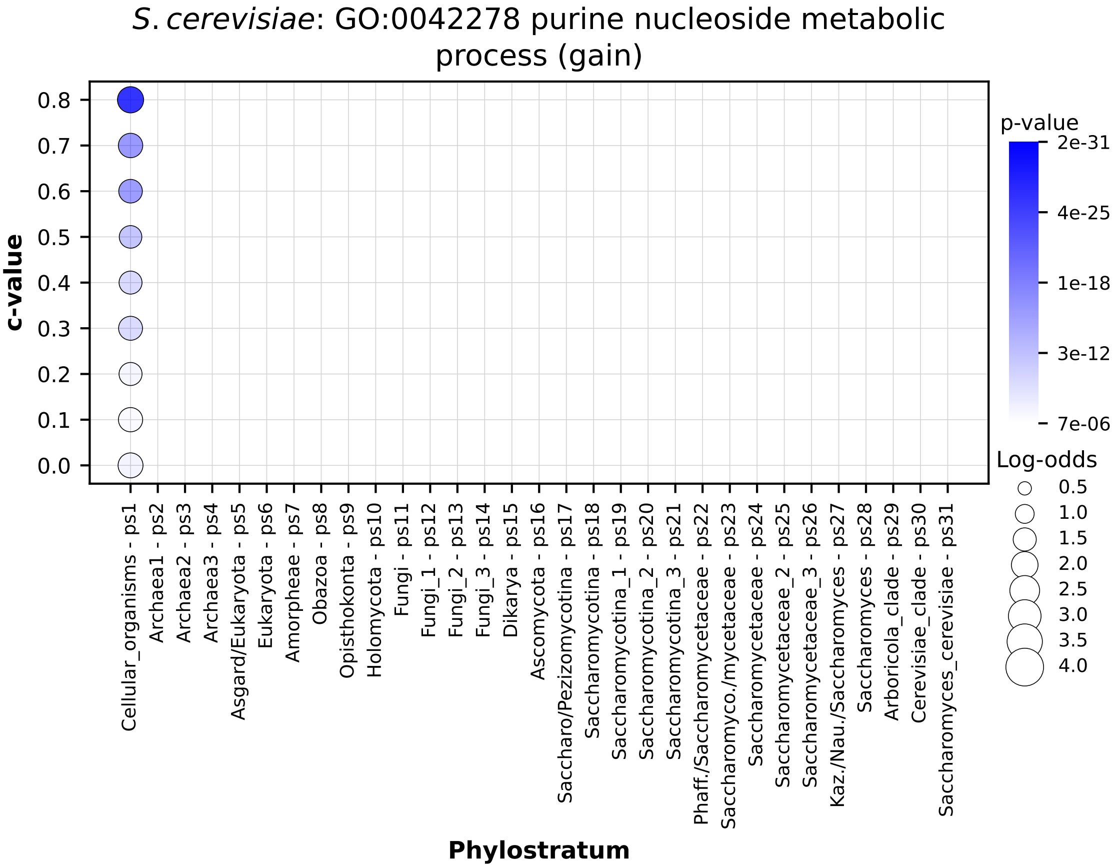
S. cerevisiae: GO:0042278 purine nucleoside metabolic process (gain)
c-value
0.8
0.7
0.6
0.5
0.4
0.3
0.2
0.1
0.0
p-value
2e-31
4e-25
1e-18
3e-12
7e-06
Log-odds
0.5
1.0
1.5
2.0
2.5
3.0
3.5
4.0
Cellular_organisms - ps1
Archaea1 - ps2
Archaea2 - ps3
Archaea3 - ps4
Asgard/Eukaryota - ps5
Eukaryota - ps6
Amorpheae - ps7
Obazoa - ps8
Opisthokonta - ps9
Holomycota - ps10
Fungi - ps11
Fungi_1 - ps12
Fungi_2 - ps13
Fungi_3 - ps14
Dikarya - ps15
Ascomycota - ps16
Saccharo/Pezizomycotina - ps17
Saccharomycotina - ps18
Saccharomycotina_1 - ps19
Saccharomycotina_2 - ps20
Saccharomycotina_3 - ps21
Phaff./Saccharomycetaceae - ps22
Saccharomyco./mycetaceae - ps23
Saccharomycetaceae - ps24
Saccharomycetaceae_2 - ps25
Saccharomycetaceae_3 - ps26
Kaz./Nau./Saccharomyces - ps27
Saccharomyces - ps28
Arboricola_clade - ps29
Cerevisiae_clade - ps30
Saccharomyces_cerevisiae - ps31
Phylostratum

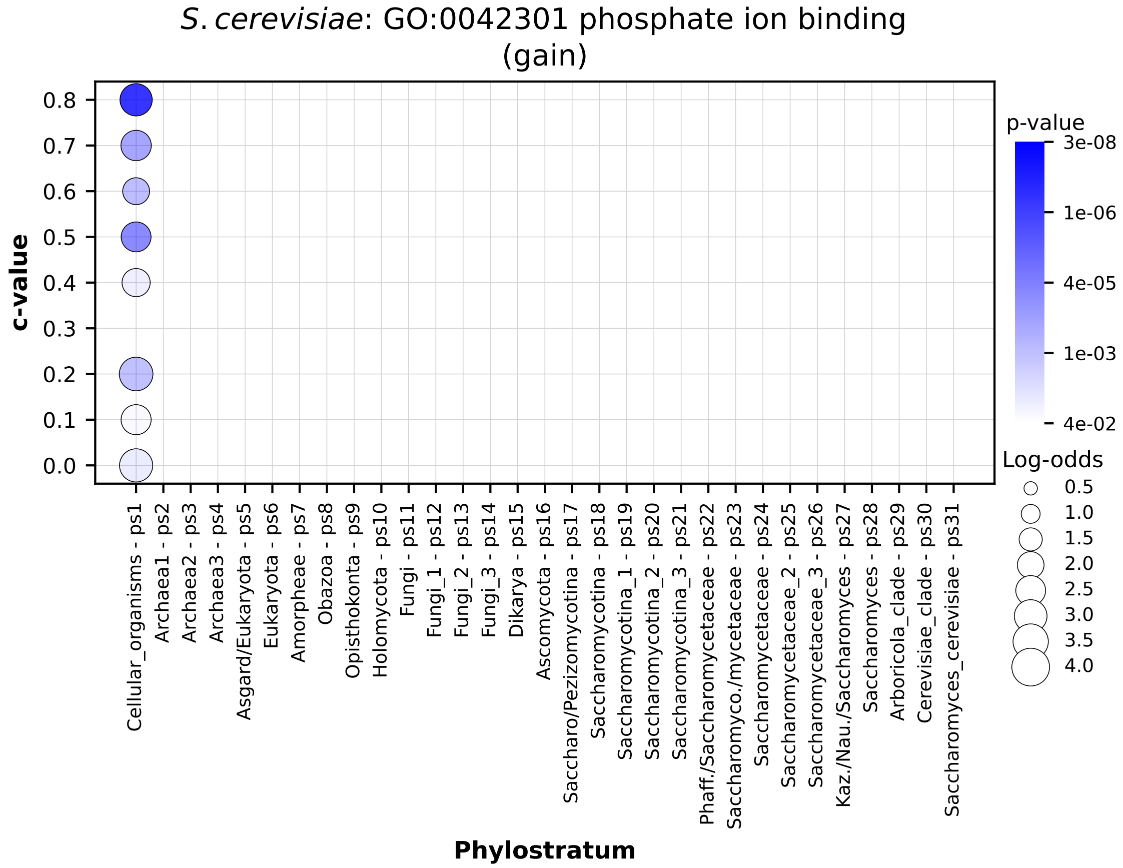

S. cerevisiae: GO:0042301 phosphate ion binding
(gain)
c-value
0.0
0.1
0.2
0.3
0.4
0.5
0.6
0.7
0.8
p-value
3e-08
1e-06
4e-05
1e-03
4e-02
Log-odds
0.5
1.0
1.5
2.0
2.5
3.0
3.5
4.0
Cellular_organisms - ps1
Archaea1 - ps2
Archaea2 - ps3
Archaea3 - ps4
Asgard/Eukaryota - ps5
Eukaryota - ps6
Amorpheae - ps7
Obazoa - ps8
Opisthokonta - ps9
Holomycota - ps10
Fungi - ps11
Fungi_1 - ps12
Fungi_2 - ps13
Fungi_3 - ps14
Dikarya - ps15
Ascomycota - ps16
Saccharo/Pezizomycotina - ps17
Saccharomycotina - ps18
Saccharomycotina_1 - ps19
Saccharomycotina_2 - ps20
Saccharomycotina_3 - ps21
Phaff./Saccharomycetaceae - ps22
Saccharomyco./mycetaceae - ps23
Saccharomycetaceae - ps24
Saccharomycetaceae_2 - ps25
Saccharomycetaceae_3 - ps26
Kaz./Nau./Saccharomyces - ps27
Saccharomyces - ps28
Arboricola_clade - ps29
Cerevisiae_clade - ps30
Saccharomyces_cerevisiae - ps31
Phylostratum

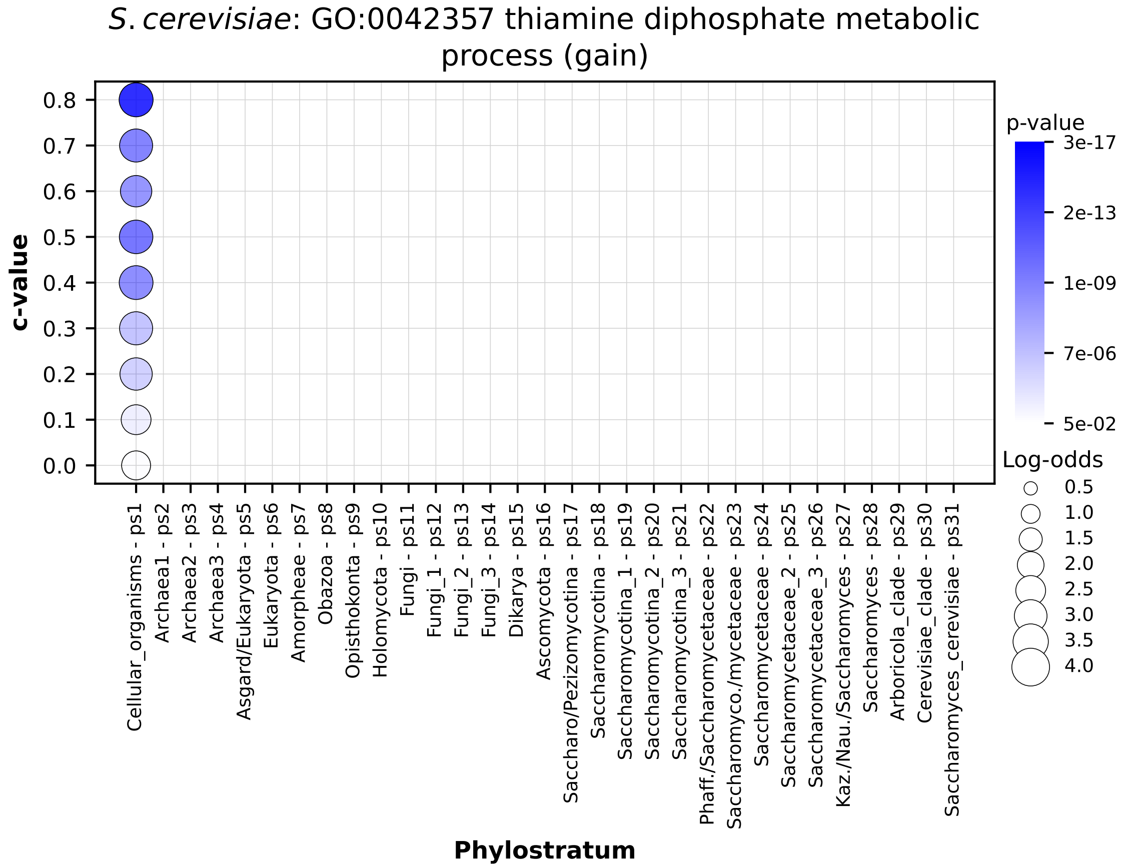
S. cerevisiae: GO:0042357 thiamine diphosphate metabolic process (gain)
c-value
0.8
0.7
0.6
0.5
0.4
0.3
0.2
0.1
0.0
p-value
3e-17
2e-13
1e-09
7e-06
5e-02
Log-odds
0.5
1.0
1.5
2.0
2.5
3.0
3.5
4.0
Cellular_organisms - ps1
Archaea1 - ps2
Archaea2 - ps3
Archaea3 - ps4
Asgard/Eukaryota - ps5
Eukaryota - ps6
Amorpheae - ps7
Obazoa - ps8
Opisthokonta - ps9
Holomycota - ps10
Fungi - ps11
Fungi_1 - ps12
Fungi_2 - ps13
Fungi_3 - ps14
Dikarya - ps15
Ascomycota - ps16
Saccharo/Pezizomycotina - ps17
Saccharomycotina - ps18
Saccharomycotina_1 - ps19
Saccharomycotina_2 - ps20
Saccharomycotina_3 - ps21
Phaff./Saccharomycetaceae - ps22
Saccharomyco./mycetaceae - ps23
Saccharomycetaceae - ps24
Saccharomycetaceae_2 - ps25
Saccharomycetaceae_3 - ps26
Kaz./Nau./Saccharomyces - ps27
Saccharomyces - ps28
Arboricola_clade - ps29
Cerevisiae_clade - ps30
Saccharomyces_cerevisiae - ps31
Phylostratum

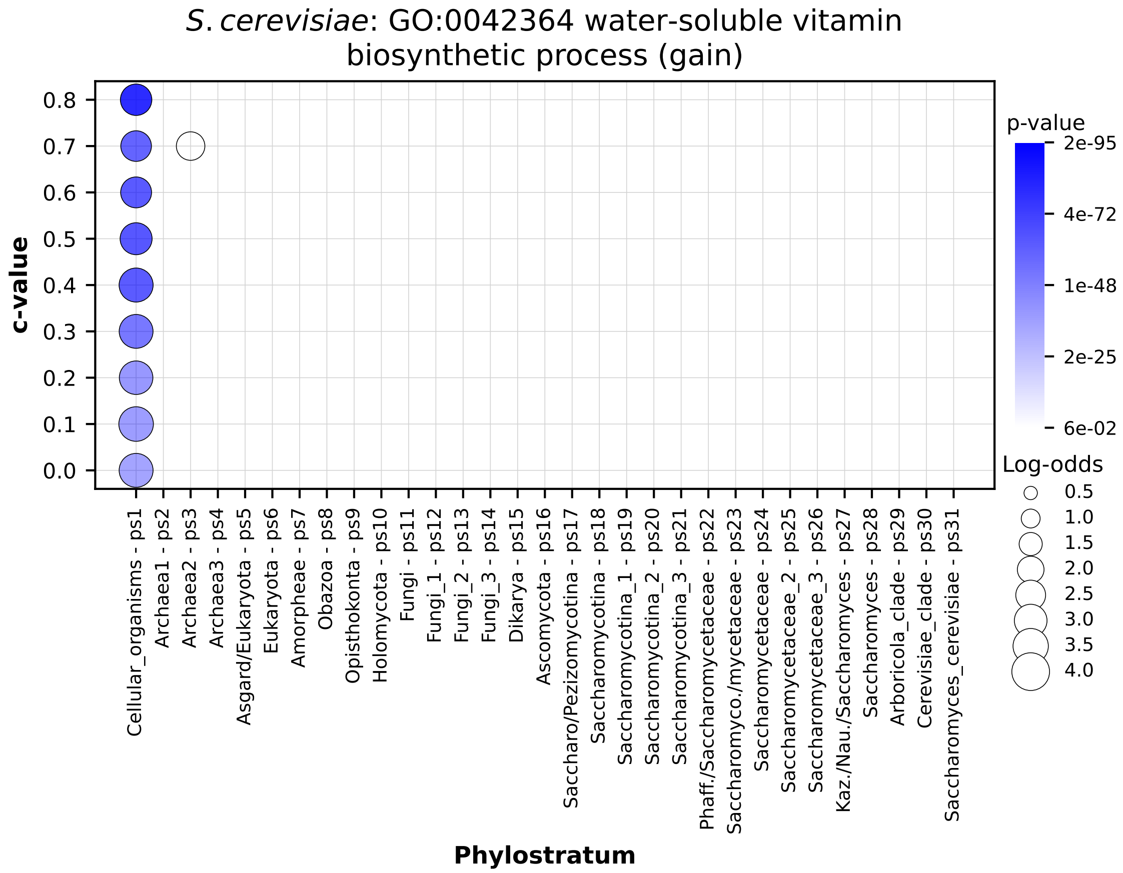
S. cerevisiae: GO:0042364 water-soluble vitamin biosynthetic process (gain)
c-value
0.8
0.7
0.6
0.5
0.4
0.3
0.2
0.1
0.0
p-value
2e-95
4e-72
1e-48
2e-25
6e-02
Log-odds
0.5
1.0
1.5
2.0
2.5
3.0
3.5
4.0
Cellular_organisms - ps1
Archaea1 - ps2
Archaea2 - ps3
Archaea3 - ps4
Asgard/Eukaryota - ps5
Eukaryota - ps6
Amorpheae - ps7
Obazoa - ps8
Opisthokonta - ps9
Holomycota - ps10
Fungi - ps11
Fungi_1 - ps12
Fungi_2 - ps13
Fungi_3 - ps14
Dikarya - ps15
Ascomycota - ps16
Saccharo/Pezizomycotina - ps17
Saccharomycotina - ps18
Saccharomycotina_1 - ps19
Saccharomycotina_2 - ps20
Saccharomycotina_3 - ps21
Phaff./Saccharomycetaceae - ps22
Saccharomyco./mycetaceae - ps23
Saccharomycetaceae - ps24
Saccharomycetaceae_2 - ps25
Saccharomycetaceae_3 - ps26
Kaz./Nau./Saccharomyces - ps27
Saccharomyces - ps28
Arboricola_clade - ps29
Cerevisiae_clade - ps30
Saccharomyces_cerevisiae - ps31
Phylostratum

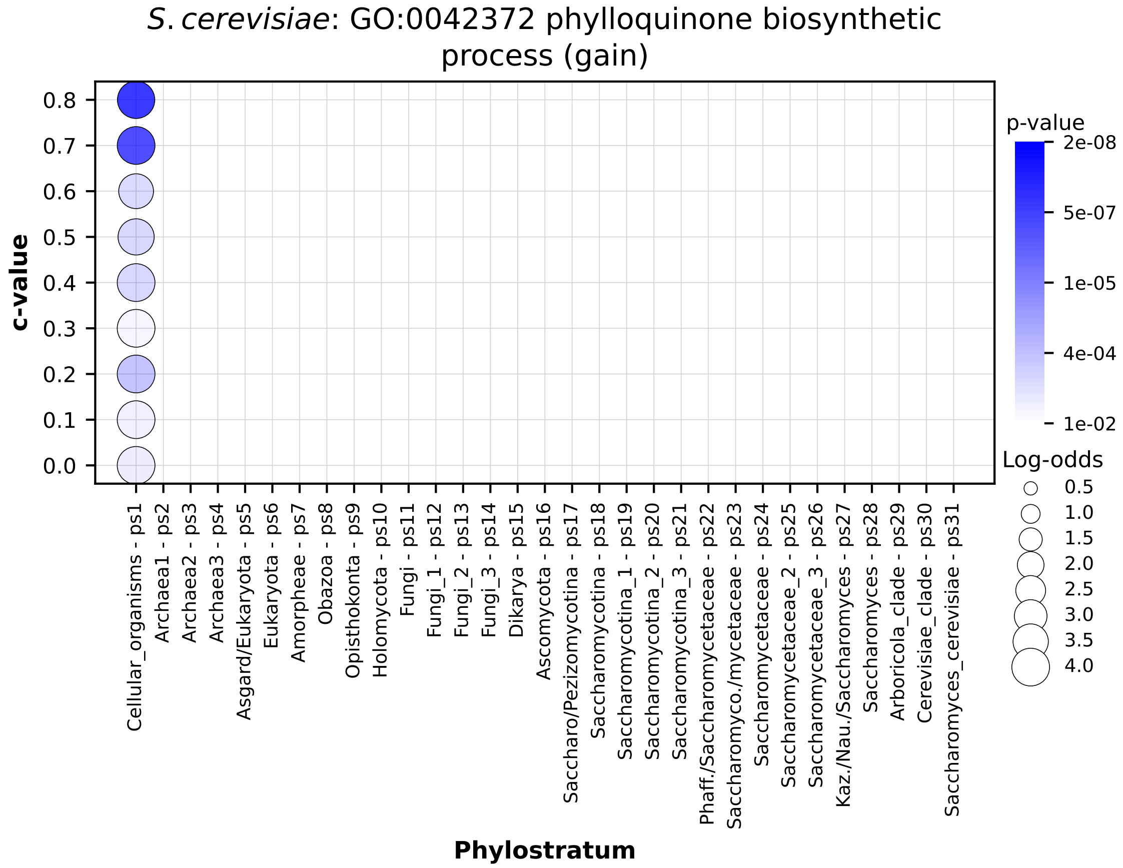

S. cerevisiae: GO:0042372 phylloquinone biosynthetic process (gain)
c-value
0.8
0.7
0.6
0.5
0.4
0.3
0.2
0.1
0.0
p-value
2e-08
5e-07
1e-05
4e-04
1e-02
Log-odds
0.5
1.0
1.5
2.0
2.5
3.0
3.5
4.0
Cellular_organisms - ps1
Archaea1 - ps2
Archaea2 - ps3
Archaea3 - ps4
Asgard/Eukaryota - ps5
Eukaryota - ps6
Amorpheae - ps7
Obazoa - ps8
Opisthokonta - ps9
Holomycota - ps10
Fungi - ps11
Fungi_1 - ps12
Fungi_2 - ps13
Fungi_3 - ps14
Dikarya - ps15
Ascomycota - ps16
Saccharo/Pezizomycotina - ps17
Saccharomycotina - ps18
Saccharomycotina_1 - ps19
Saccharomycotina_2 - ps20
Saccharomycotina_3 - ps21
Phaff./Saccharomycetaceae - ps22
Saccharomyco./mycetaceae - ps23
Saccharomycetaceae - ps24
Saccharomycetaceae_2 - ps25
Saccharomycetaceae_3 - ps26
Kaz./Nau./Saccharomyces - ps27
Saccharomyces - ps28
Arboricola_clade - ps29
Cerevisiae_clade - ps30
Saccharomyces_cerevisiae - ps31
Phylostratum

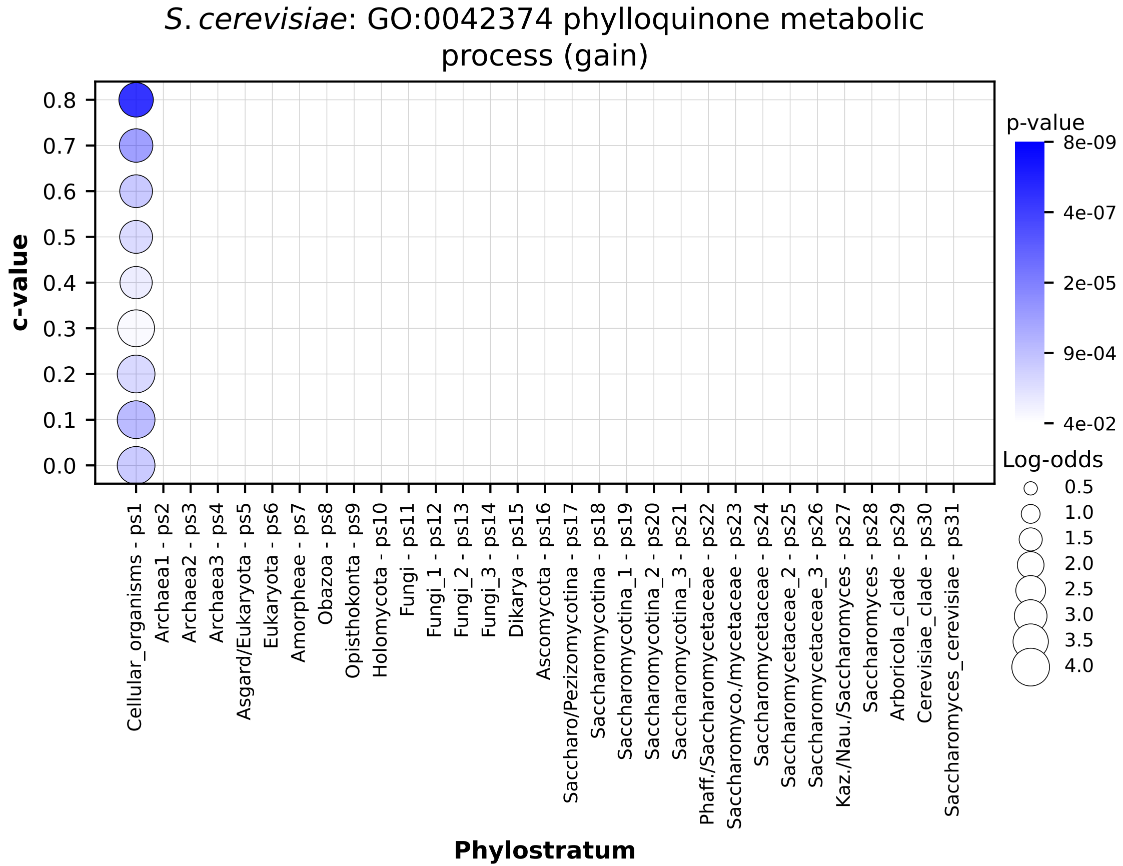

S. cerevisiae: GO:0042374 phylloquinone metabolic process (gain)
c-value
0.8
0.7
0.6
0.5
0.4
0.3
0.2
0.1
0.0
Phylostratum
Cellular_organisms - ps1
Archaea1 - ps2
Archaea2 - ps3
Archaea3 - ps4
Asgard/Eukaryota - ps5
Eukaryota - ps6
Amorpheae - ps7
Obazoa - ps8
Opisthokonta - ps9
Holomycota - ps10
Fungi - ps11
Fungi_1 - ps12
Fungi_2 - ps13
Fungi_3 - ps14
Dikarya - ps15
Ascomycota - ps16
Saccharo/Pezizomycotina - ps17
Saccharomycotina - ps18
Saccharomycotina_1 - ps19
Saccharomycotina_2 - ps20
Saccharomycotina_3 - ps21
Phaff./Saccharomycetaceae - ps22
Saccharomyco./mycetaceae - ps23
Saccharomycetaceae - ps24
Saccharomycetaceae_2 - ps25
Saccharomycetaceae_3 - ps26
Kaz./Nau./Saccharomyces - ps27
Saccharomyces - ps28
Arboricola_clade - ps29
Cerevisiae_clade - ps30
Saccharomyces_cerevisiae - ps31
p-value
8e-09
4e-07
2e-05
9e-04
4e-02
Log-odds
0.5
1.0
1.5
2.0
2.5
3.0
3.5
4.0

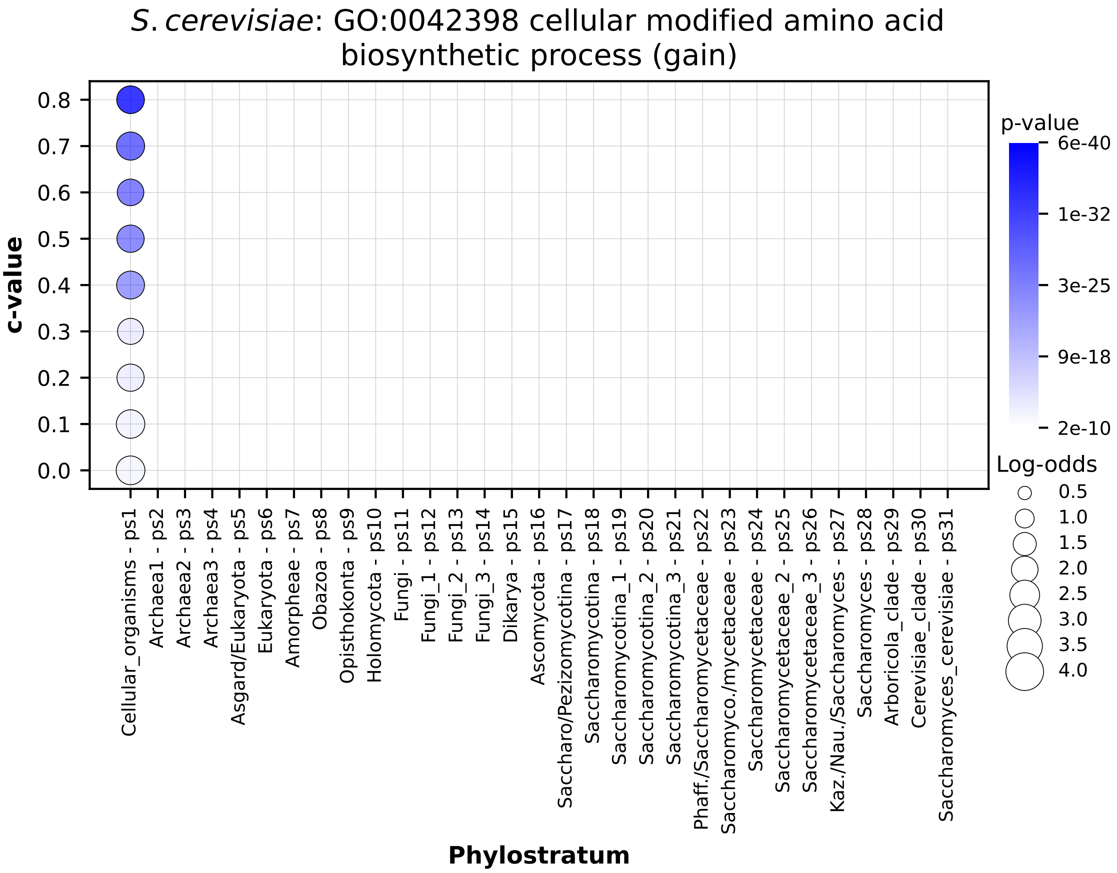

S. cerevisiae: GO:0042398 cellular modified amino acid biosynthetic process (gain)
c-value
0.8
0.7
0.6
0.5
0.4
0.3
0.2
0.1
0.0
p-value
6e-40
1e-32
3e-25
9e-18
2e-10
Log-odds
0.5
1.0
1.5
2.0
2.5
3.0
3.5
4.0
Cellular_organisms - ps1
Archaea1 - ps2
Archaea2 - ps3
Archaea3 - ps4
Asgard/Eukaryota - ps5
Eukaryota - ps6
Amorpheae - ps7
Obazoa - ps8
Opisthokonta - ps9
Holomycota - ps10
Fungi - ps11
Fungi_1 - ps12
Fungi_2 - ps13
Fungi_3 - ps14
Dikarya - ps15
Ascomycota - ps16
Saccharo/Pezizomycotina - ps17
Saccharomycotina - ps18
Saccharomycotina_1 - ps19
Saccharomycotina_2 - ps20
Saccharomycotina_3 - ps21
Phaff./Saccharomycetaceae - ps22
Saccharomyco./mycetaceae - ps23
Saccharomycetaceae - ps24
Saccharomycetaceae_2 - ps25
Saccharomycetaceae_3 - ps26
Kaz./Nau./Saccharomyces - ps27
Saccharomyces - ps28
Arboricola_clade - ps29
Cerevisiae_clade - ps30
Saccharomyces_cerevisiae - ps31
Phylostratum

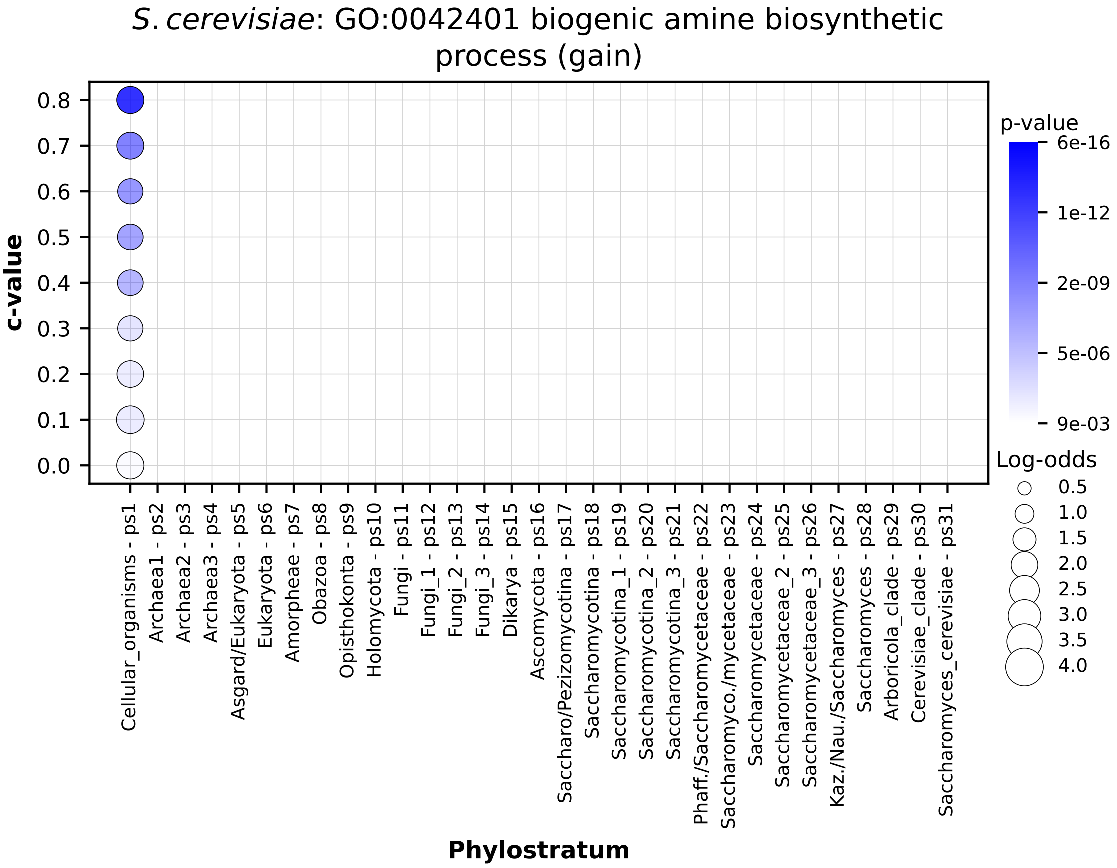
S. cerevisiae: GO:0042401 biogenic amine biosynthetic process (gain)
c-value
0.8
0.7
0.6
0.5
0.4
0.3
0.2
0.1
0.0
p-value
6e-16
1e-12
2e-09
5e-06
9e-03
Log-odds
0.5
1.0
1.5
2.0
2.5
3.0
3.5
4.0
Cellular_organisms - ps1
Archaea1 - ps2
Archaea2 - ps3
Archaea3 - ps4
Asgard/Eukaryota - ps5
Eukaryota - ps6
Amorpheae - ps7
Obazoa - ps8
Opisthokonta - ps9
Holomycota - ps10
Fungi - ps11
Fungi_1 - ps12
Fungi_2 - ps13
Fungi_3 - ps14
Dikarya - ps15
Ascomycota - ps16
Saccharo/Pezizomycotina - ps17
Saccharomycotina - ps18
Saccharomycotina_1 - ps19
Saccharomycotina_2 - ps20
Saccharomycotina_3 - ps21
Phaff./Saccharomycetaceae - ps22
Saccharomyco./mycetaceae - ps23
Saccharomycetaceae - ps24
Saccharomycetaceae_2 - ps25
Saccharomycetaceae_3 - ps26
Kaz./Nau./Saccharomyces - ps27
Saccharomyces - ps28
Arboricola_clade - ps29
Cerevisiae_clade - ps30
Saccharomyces_cerevisiae - ps31
Phylostratum

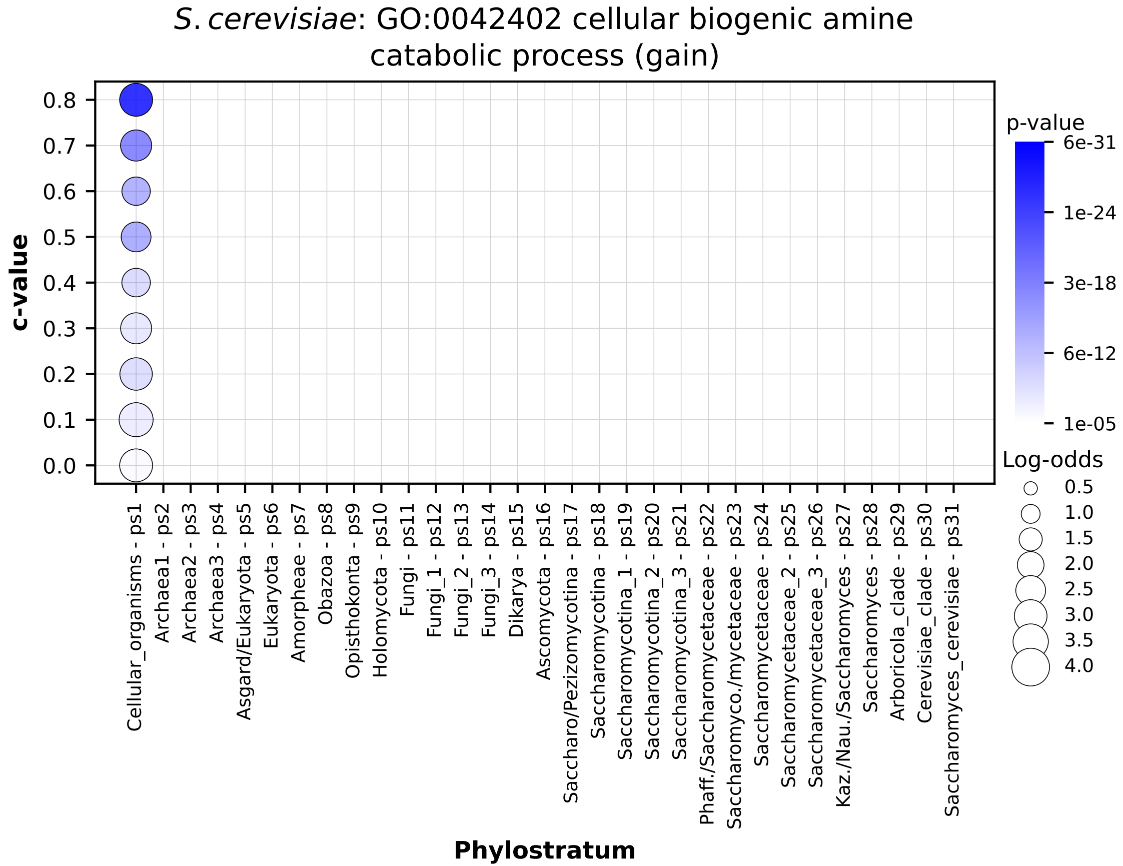

S. cerevisiae: GO:0042402 cellular biogenic amine catabolic process (gain)
c-value
0.8
0.7
0.6
0.5
0.4
0.3
0.2
0.1
0.0
p-value
6e-31
1e-24
3e-18
6e-12
1e-05
Log-odds
0.5
1.0
1.5
2.0
2.5
3.0
3.5
4.0
Cellular_organisms - ps1
Archaea1 - ps2
Archaea2 - ps3
Archaea3 - ps4
Asgard/Eukaryota - ps5
Eukaryota - ps6
Amorpheae - ps7
Obazoa - ps8
Opisthokonta - ps9
Holomycota - ps10
Fungi - ps11
Fungi_1 - ps12
Fungi_2 - ps13
Fungi_3 - ps14
Dikarya - ps15
Ascomycota - ps16
Saccharo/Pezizomycotina - ps17
Saccharomycotina - ps18
Saccharomycotina_1 - ps19
Saccharomycotina_2 - ps20
Saccharomycotina_3 - ps21
Phaff./Saccharomycetaceae - ps22
Saccharomyco./mycetaceae - ps23
Saccharomycetaceae - ps24
Saccharomycetaceae_2 - ps25
Saccharomycetaceae_3 - ps26
Kaz./Nau./Saccharomyces - ps27
Saccharomyces - ps28
Arboricola_clade - ps29
Cerevisiae_clade - ps30
Saccharomyces_cerevisiae - ps31
Phylostratum

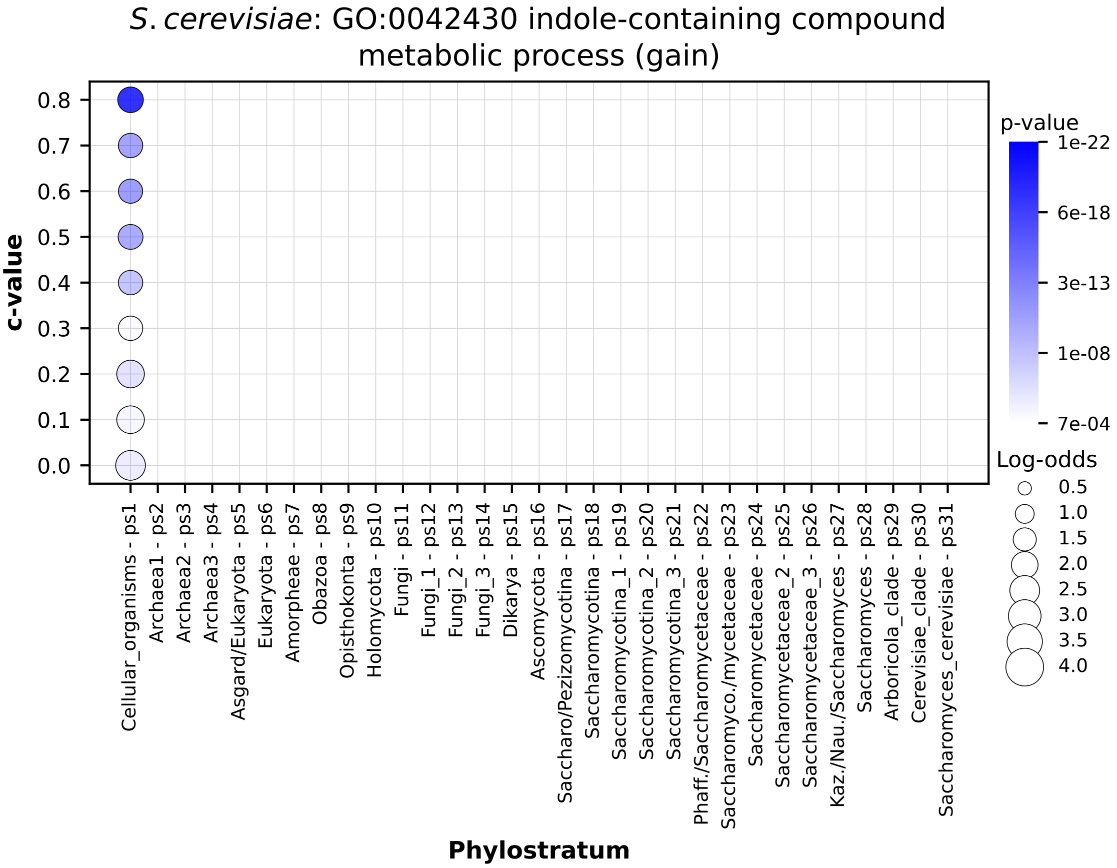

S. cerevisiae: GO:0042430 indole-containing compound metabolic process (gain)
c-value
0.8
0.7
0.6
0.5
0.4
0.3
0.2
0.1
0.0
p-value
1e-22
6e-18
3e-13
1e-08
7e-04
Log-odds
0.5
1.0
1.5
2.0
2.5
3.0
3.5
4.0
Cellular_organisms - ps1
Archaea1 - ps2
Archaea2 - ps3
Archaea3 - ps4
Asgard/Eukaryota - ps5
Eukaryota - ps6
Amorpheae - ps7
Obazoa - ps8
Opisthokonta - ps9
Holomycota - ps10
Fungi - ps11
Fungi_1 - ps12
Fungi_2 - ps13
Fungi_3 - ps14
Dikarya - ps15
Ascomycota - ps16
Saccharo/Pezizomycotina - ps17
Saccharomycotina - ps18
Saccharomycotina_1 - ps19
Saccharomycotina_2 - ps20
Saccharomycotina_3 - ps21
Phaff./Saccharomycetaceae - ps22
Saccharomyco./mycetaceae - ps23
Saccharomycetaceae - ps24
Saccharomycetaceae_2 - ps25
Saccharomycetaceae_3 - ps26
Kaz./Nau./Saccharomyces - ps27
Saccharomyces - ps28
Arboricola_clade - ps29
Cerevisiae_clade - ps30
Saccharomyces_cerevisiae - ps31
Phylostratum

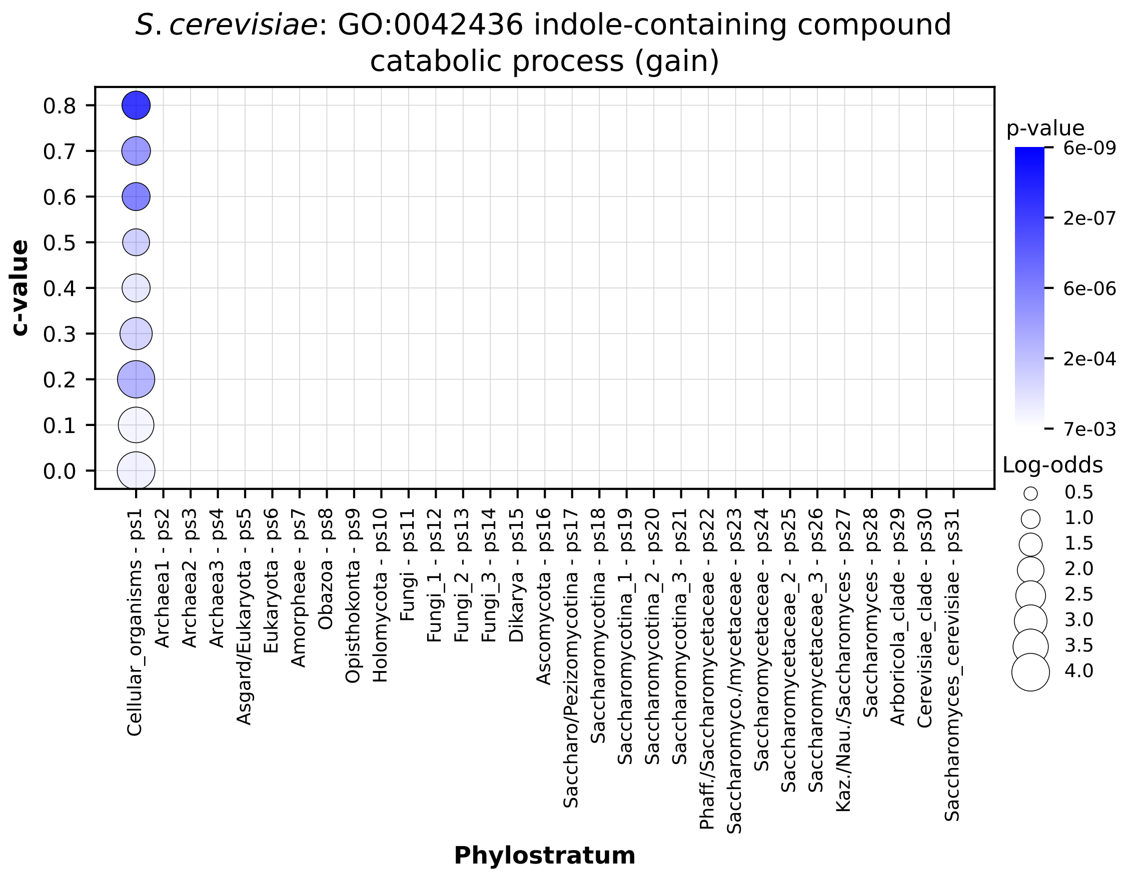

S. cerevisiae: GO:0042436 indole-containing compound catabolic process (gain)
c-value
0.0
0.1
0.2
0.3
0.4
0.5
0.6
0.7
0.8
p-value
6e-09
2e-07
6e-06
2e-04
7e-03
Log-odds
0.5
1.0
1.5
2.0
2.5
3.0
3.5
4.0
Cellular_organisms - ps1
Archaea1 - ps2
Archaea2 - ps3
Archaea3 - ps4
Asgard/Eukaryota - ps5
Eukaryota - ps6
Amorpheae - ps7
Obazoa - ps8
Opisthokonta - ps9
Holomycota - ps10
Fungi - ps11
Fungi_1 - ps12
Fungi_2 - ps13
Fungi_3 - ps14
Dikarya - ps15
Ascomycota - ps16
Saccharo/Pezizomycotina - ps17
Saccharomycotina - ps18
Saccharomycotina_1 - ps19
Saccharomycotina_2 - ps20
Saccharomycotina_3 - ps21
Phaff./Saccharomycetaceae - ps22
Saccharomyco./mycetaceae - ps23
Saccharomycetaceae - ps24
Saccharomycetaceae_2 - ps25
Saccharomycetaceae_3 - ps26
Kaz./Nau./Saccharomyces - ps27
Saccharomyces - ps28
Arboricola_clade - ps29
Cerevisiae_clade - ps30
Saccharomyces_cerevisiae - ps31
Phylostratum

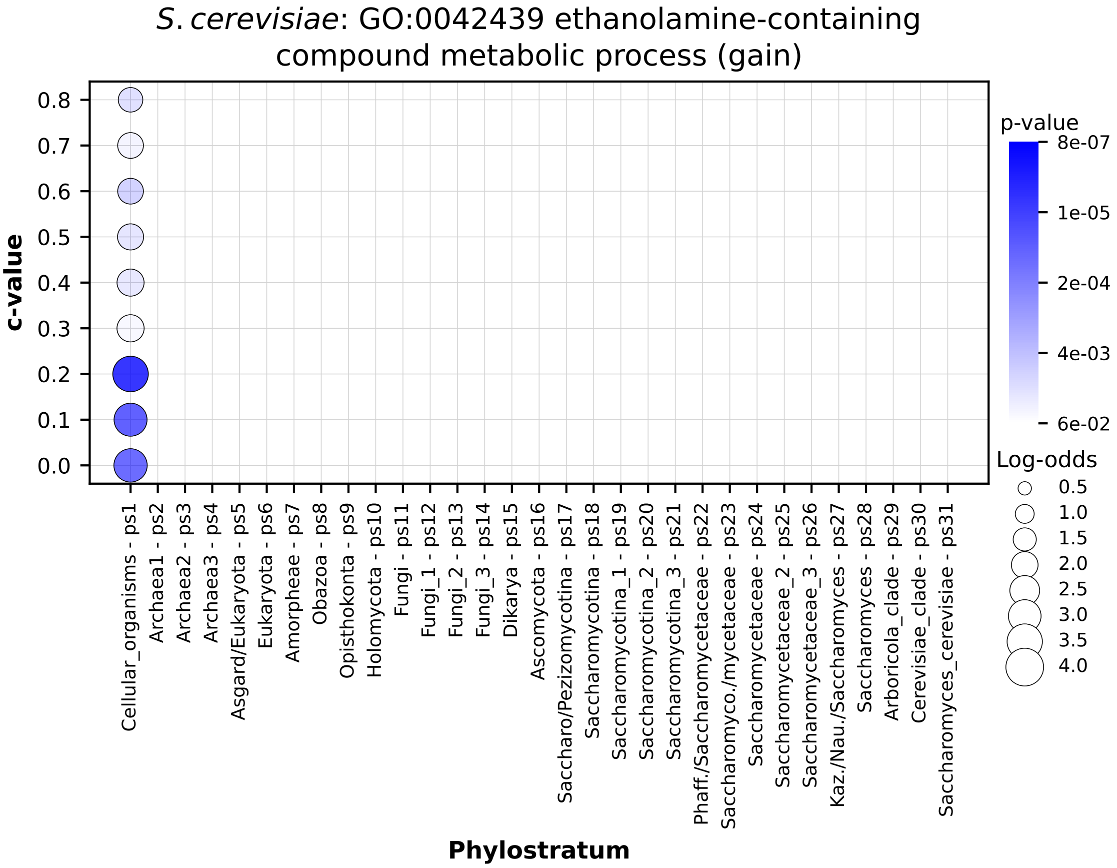

S. cerevisiae: GO:0042439 ethanolamine-containing compound metabolic process (gain)
c-value
0.8
0.7
0.6
0.5
0.4
0.3
0.2
0.1
0.0
p-value
8e-07
1e-05
2e-04
4e-03
6e-02
Log-odds
0.5
1.0
1.5
2.0
2.5
3.0
3.5
4.0
Cellular_organisms - ps1
Archaea1 - ps2
Archaea2 - ps3
Archaea3 - ps4
Asgard/Eukaryota - ps5
Eukaryota - ps6
Amorpheae - ps7
Obazoa - ps8
Opisthokonta - ps9
Holomycota - ps10
Fungi - ps11
Fungi_1 - ps12
Fungi_2 - ps13
Fungi_3 - ps14
Dikarya - ps15
Ascomycota - ps16
Saccharo/Pezizomycotina - ps17
Saccharomycotina - ps18
Saccharomycotina_1 - ps19
Saccharomycotina_2 - ps20
Saccharomycotina_3 - ps21
Phaff./Saccharomycetaceae - ps22
Saccharomyco./mycetaceae - ps23
Saccharomycetaceae - ps24
Saccharomycetaceae_2 - ps25
Saccharomycetaceae_3 - ps26
Kaz./Nau./Saccharomyces - ps27
Saccharomyces - ps28
Arboricola_clade - ps29
Cerevisiae_clade - ps30
Saccharomyces_cerevisiae - ps31
Phylostratum

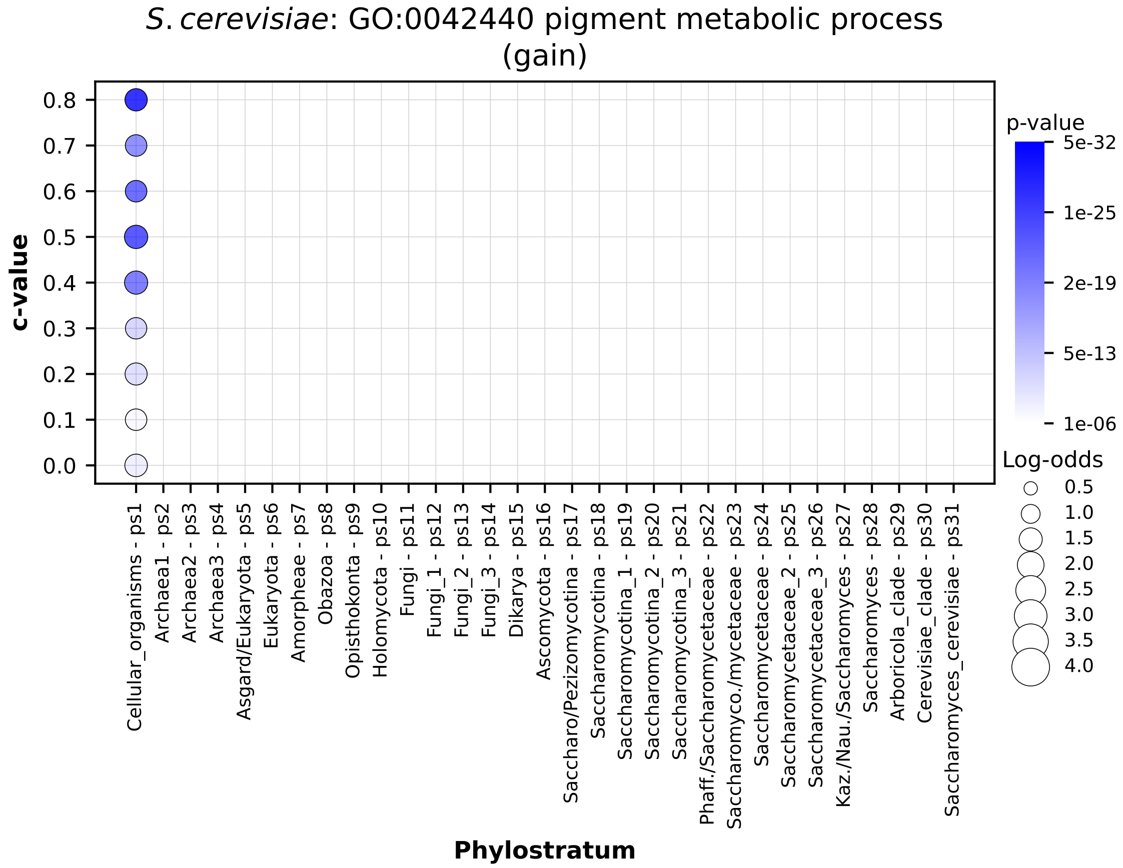
S. cerevisiae: GO:0042440 pigment metabolic process (gain)
c-value
0.8
0.7
0.6
0.5
0.4
0.3
0.2
0.1
0.0
p-value
5e-32
1e-25
2e-19
5e-13
1e-06
Log-odds
0.5
1.0
1.5
2.0
2.5
3.0
3.5
4.0
Cellular_organisms - ps1
Archaea1 - ps2
Archaea2 - ps3
Archaea3 - ps4
Asgard/Eukaryota - ps5
Eukaryota - ps6
Amorpheae - ps7
Obazoa - ps8
Opisthokonta - ps9
Holomycota - ps10
Fungi - ps11
Fungi_1 - ps12
Fungi_2 - ps13
Fungi_3 - ps14
Dikarya - ps15
Ascomycota - ps16
Saccharo/Pezizomycotina - ps17
Saccharomycotina - ps18
Saccharomycotina_1 - ps19
Saccharomycotina_2 - ps20
Saccharomycotina_3 - ps21
Phaff./Saccharomycetaceae - ps22
Saccharomyco./mycetaceae - ps23
Saccharomycetaceae - ps24
Saccharomycetaceae_2 - ps25
Saccharomycetaceae_3 - ps26
Kaz./Nau./Saccharomyces - ps27
Saccharomyces - ps28
Arboricola_clade - ps29
Cerevisiae_clade - ps30
Saccharomyces_cerevisiae - ps31
Phylostratum

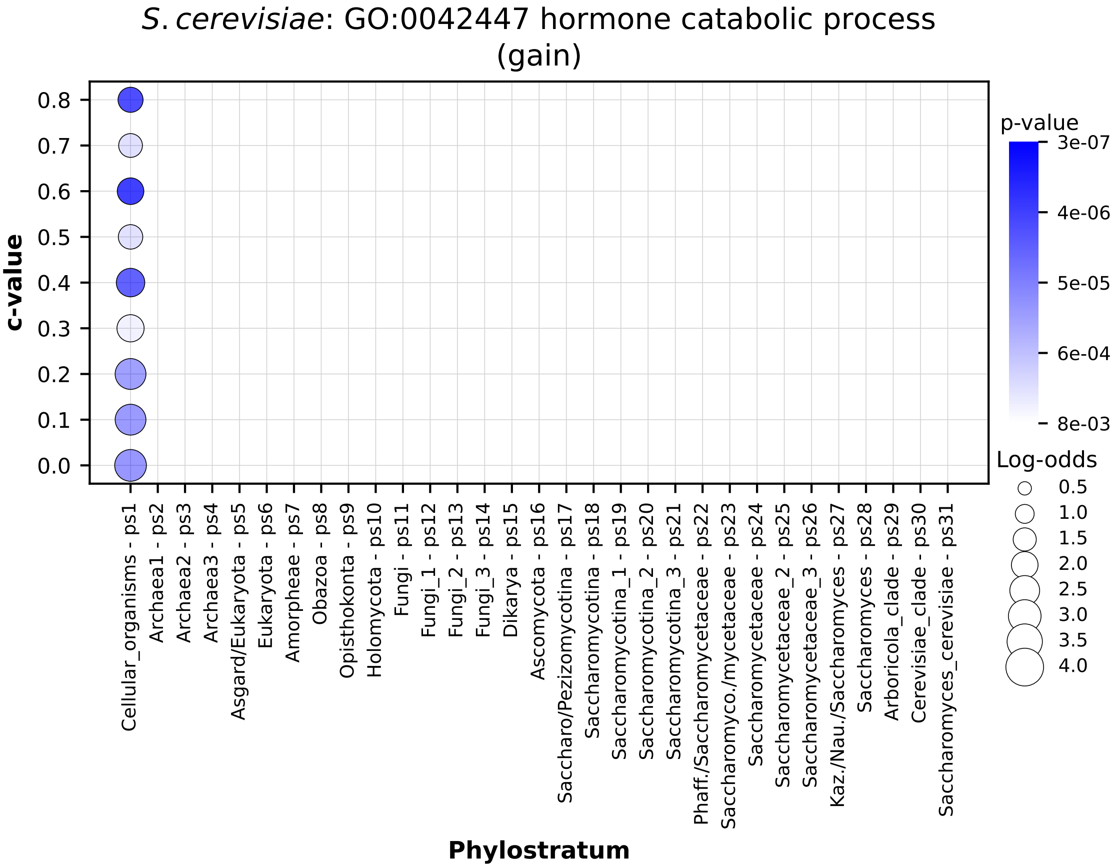
S. cerevisiae: GO:0042447 hormone catabolic process
(gain)
c-value
0.8
0.7
0.6
0.5
0.4
0.3
0.2
0.1
0.0
p-value
3e-07
4e-06
5e-05
6e-04
8e-03
Log-odds
0.5
1.0
1.5
2.0
2.5
3.0
3.5
4.0
Cellular_organisms - ps1
Archaea1 - ps2
Archaea2 - ps3
Archaea3 - ps4
Asgard/Eukaryota - ps5
Eukaryota - ps6
Amorpheae - ps7
Obazoa - ps8
Opisthokonta - ps9
Holomycota - ps10
Fungi - ps11
Fungi_1 - ps12
Fungi_2 - ps13
Fungi_3 - ps14
Dikarya - ps15
Ascomycota - ps16
Saccharo/Pezizomycotina - ps17
Saccharomycotina - ps18
Saccharomycotina_1 - ps19
Saccharomycotina_2 - ps20
Saccharomycotina_3 - ps21
Phaff./Saccharomycetaceae - ps22
Saccharomyco./mycetaceae - ps23
Saccharomycetaceae - ps24
Saccharomycetaceae_2 - ps25
Saccharomycetaceae_3 - ps26
Kaz./Nau./Saccharomyces - ps27
Saccharomyces - ps28
Arboricola_clade - ps29
Cerevisiae_clade - ps30
Saccharomyces_cerevisiae - ps31
Phylostratum

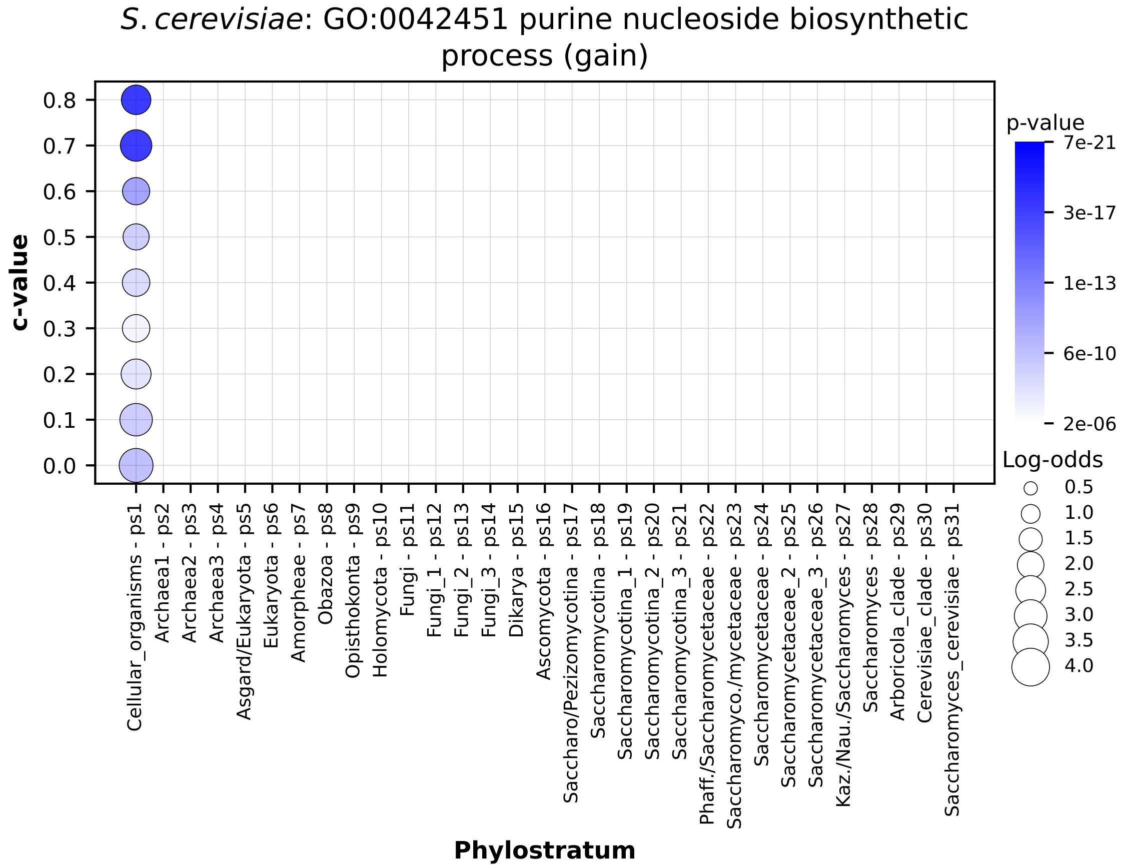

S. cerevisiae: GO:0042451 purine nucleoside biosynthetic process (gain)
c-value
0.0
0.1
0.2
0.3
0.4
0.5
0.6
0.7
0.8
Phylostratum
Cellular_organisms - ps1
Archaea1 - ps2
Archaea2 - ps3
Archaea3 - ps4
Asgard/Eukaryota - ps5
Eukaryota - ps6
Amorpheae - ps7
Obazoa - ps8
Opisthokonta - ps9
Holomycota - ps10
Fungi - ps11
Fungi_1 - ps12
Fungi_2 - ps13
Fungi_3 - ps14
Dikarya - ps15
Ascomycota - ps16
Saccharo/Pezizomycotina - ps17
Saccharomycotina - ps18
Saccharomycotina_1 - ps19
Saccharomycotina_2 - ps20
Saccharomycotina_3 - ps21
Phaff./Saccharomycetaceae - ps22
Saccharomyco./mycetaceae - ps23
Saccharomycetaceae - ps24
Saccharomycetaceae_2 - ps25
Saccharomycetaceae_3 - ps26
Kaz./Nau./Saccharomyces - ps27
Saccharomyces - ps28
Arboricola_clade - ps29
Cerevisiae_clade - ps30
Saccharomyces_cerevisiae - ps31
p-value
7e-21
3e-17
1e-13
6e-10
2e-06
Log-odds
0.5
1.0
1.5
2.0
2.5
3.0
3.5
4.0

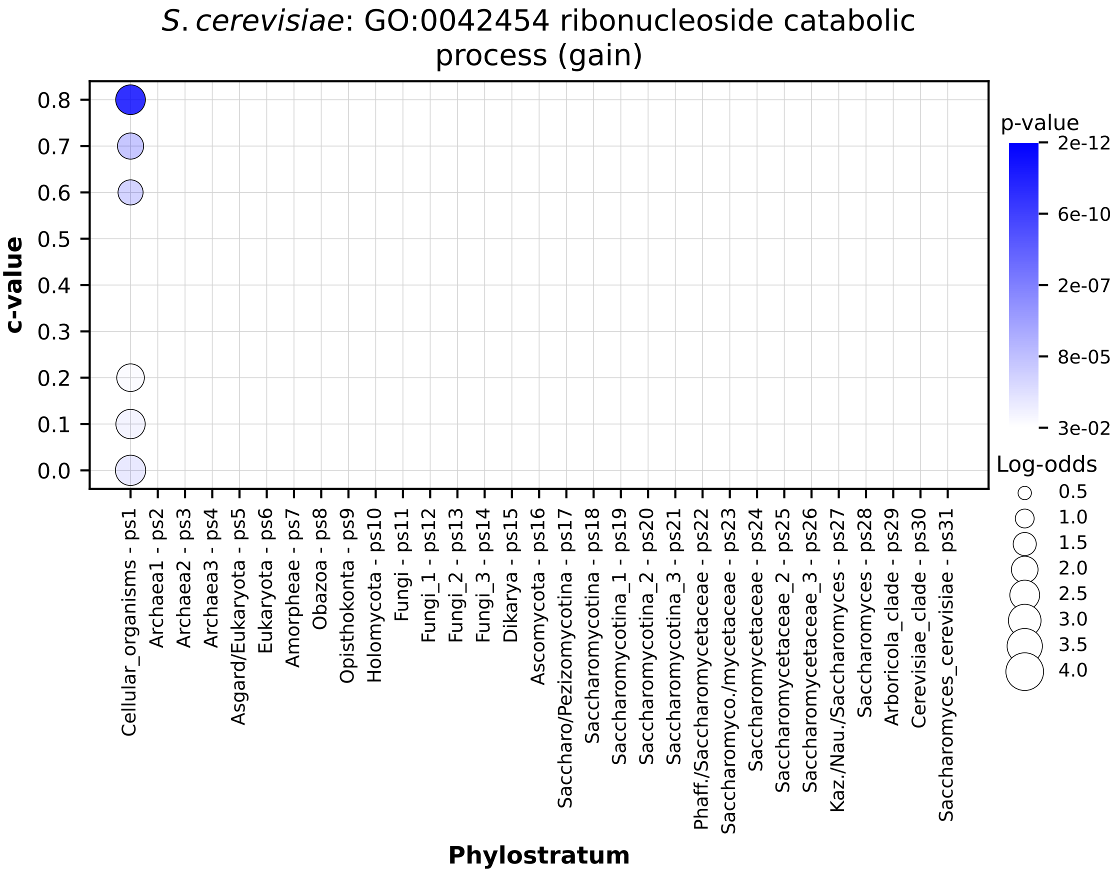

S. cerevisiae: GO:0042454 ribonucleoside catabolic process (gain)
c-value
0.8
0.7
0.6
0.5
0.4
0.3
0.2
0.1
0.0
p-value
2e-12
6e-10
2e-07
8e-05
3e-02
Log-odds
0.5
1.0
1.5
2.0
2.5
3.0
3.5
4.0
Cellular_organisms - ps1
Archaea1 - ps2
Archaea2 - ps3
Archaea3 - ps4
Asgard/Eukaryota - ps5
Eukaryota - ps6
Amorpheae - ps7
Obazoa - ps8
Opisthokonta - ps9
Holomycota - ps10
Fungi - ps11
Fungi_1 - ps12
Fungi_2 - ps13
Fungi_3 - ps14
Dikarya - ps15
Ascomycota - ps16
Saccharo/Pezizomycotina - ps17
Saccharomycotina - ps18
Saccharomycotina_1 - ps19
Saccharomycotina_2 - ps20
Saccharomycotina_3 - ps21
Phaff./Saccharomycetaceae - ps22
Saccharomyco./mycetaceae - ps23
Saccharomycetaceae - ps24
Saccharomycetaceae_2 - ps25
Saccharomycetaceae_3 - ps26
Kaz./Nau./Saccharomyces - ps27
Saccharomyces - ps28
Arboricola_clade - ps29
Cerevisiae_clade - ps30
Saccharomyces_cerevisiae - ps31
Phylostratum

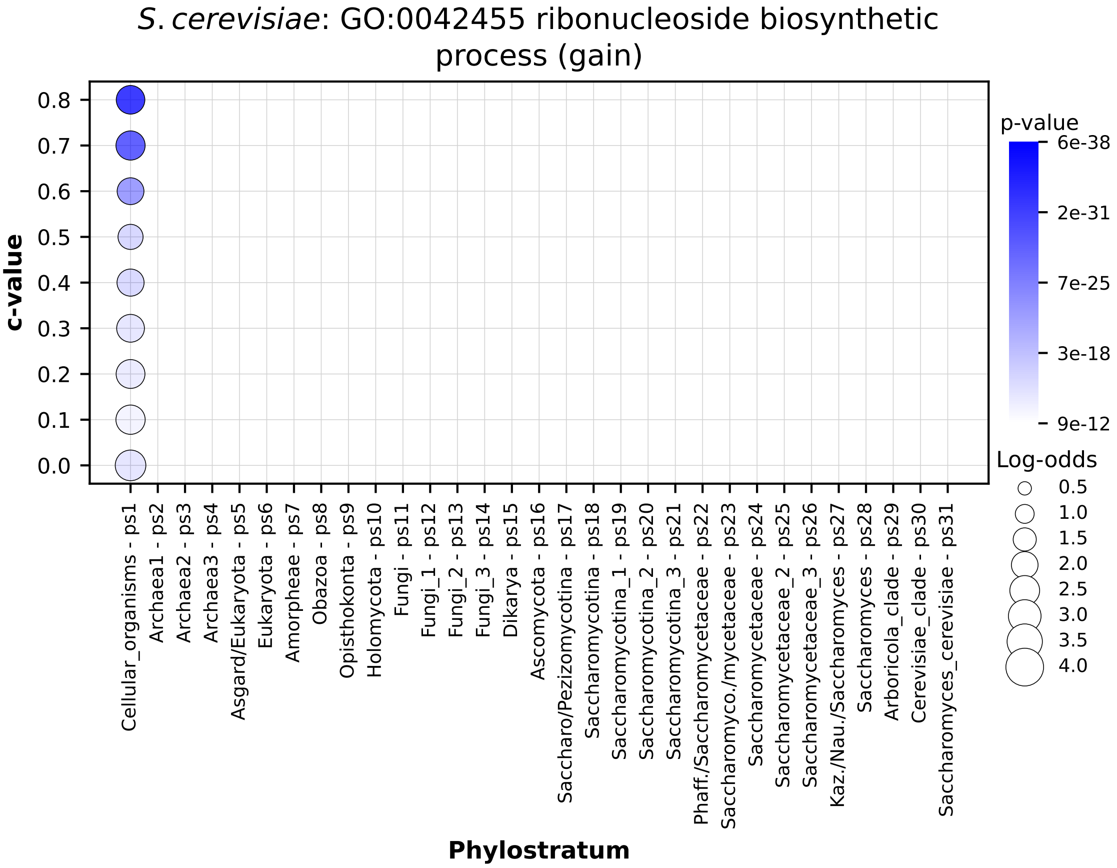

S. cerevisiae: GO:0042455 ribonucleoside biosynthetic process (gain)
c-value
0.8
0.7
0.6
0.5
0.4
0.3
0.2
0.1
0.0
p-value
6e-38
2e-31
7e-25
3e-18
9e-12
Log-odds
0.5
1.0
1.5
2.0
2.5
3.0
3.5
4.0
Cellular_organisms - ps1
Archaea1 - ps2
Archaea2 - ps3
Archaea3 - ps4
Asgard/Eukaryota - ps5
Eukaryota - ps6
Amorpheae - ps7
Obazoa - ps8
Opisthokonta - ps9
Holomycota - ps10
Fungi - ps11
Fungi_1 - ps12
Fungi_2 - ps13
Fungi_3 - ps14
Dikarya - ps15
Ascomycota - ps16
Saccharo/Pezizomycotina - ps17
Saccharomycotina - ps18
Saccharomycotina_1 - ps19
Saccharomycotina_2 - ps20
Saccharomycotina_3 - ps21
Phaff./Saccharomycetaceae - ps22
Saccharomyco./mycetaceae - ps23
Saccharomycetaceae - ps24
Saccharomycetaceae_2 - ps25
Saccharomycetaceae_3 - ps26
Kaz./Nau./Saccharomyces - ps27
Saccharomyces - ps28
Arboricola_clade - ps29
Cerevisiae_clade - ps30
Saccharomyces_cerevisiae - ps31
Phylostratum

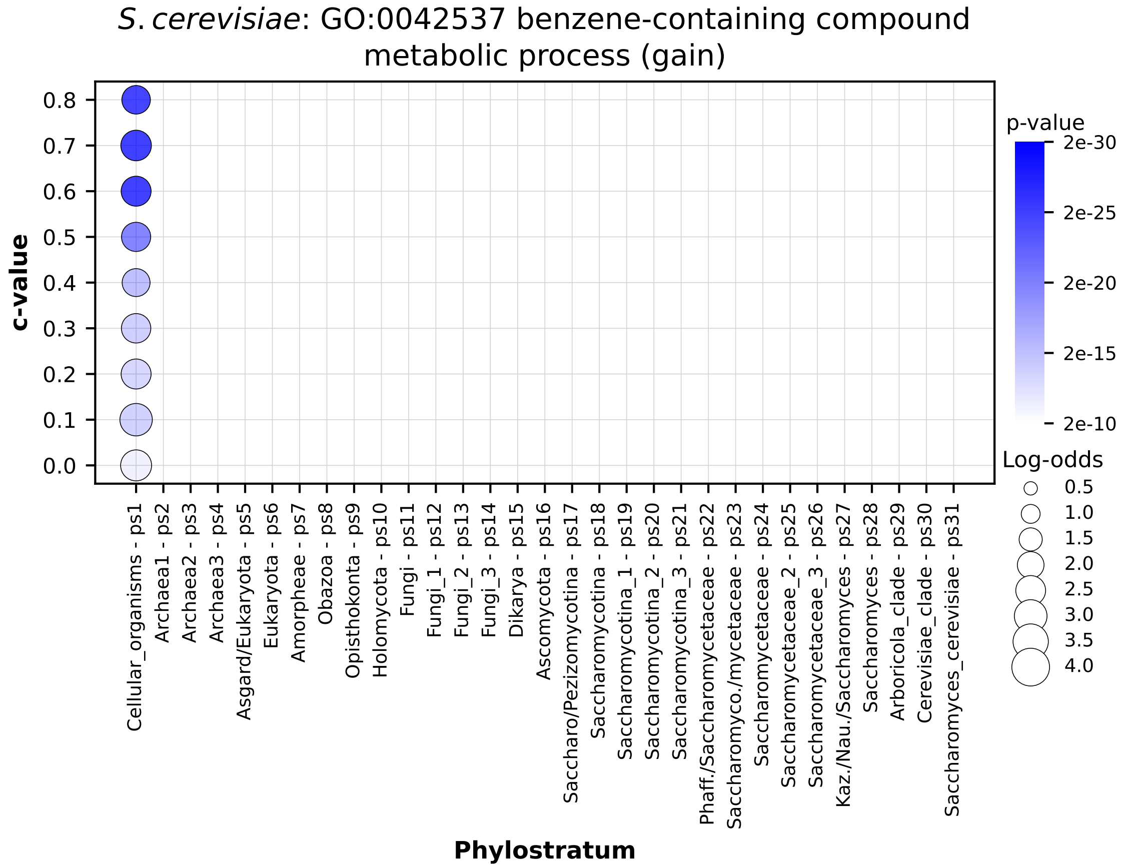
S. cerevisiae: GO:0042537 benzene-containing compound metabolic process (gain)
c-value
0.0
0.1
0.2
0.3
0.4
0.5
0.6
0.7
0.8
p-value
2e-30
2e-25
2e-20
2e-15
2e-10
Log-odds
0.5
1.0
1.5
2.0
2.5
3.0
3.5
4.0
Cellular_organisms - ps1
Archaea1 - ps2
Archaea2 - ps3
Archaea3 - ps4
Asgard/Eukaryota - ps5
Eukaryota - ps6
Amorpheae - ps7
Obazoa - ps8
Opisthokonta - ps9
Holomycota - ps10
Fungi - ps11
Fungi_1 - ps12
Fungi_2 - ps13
Fungi_3 - ps14
Dikarya - ps15
Ascomycota - ps16
Saccharo/Pezizomycotina - ps17
Saccharomycotina - ps18
Saccharomycotina_1 - ps19
Saccharomycotina_2 - ps20
Saccharomycotina_3 - ps21
Phaff./Saccharomycetaceae - ps22
Saccharomyco./mycetaceae - ps23
Saccharomycetaceae - ps24
Saccharomycetaceae_2 - ps25
Saccharomycetaceae_3 - ps26
Kaz./Nau./Saccharomyces - ps27
Saccharomyces - ps28
Arboricola_clade - ps29
Cerevisiae_clade - ps30
Saccharomyces_cerevisiae - ps31
Phylostratum

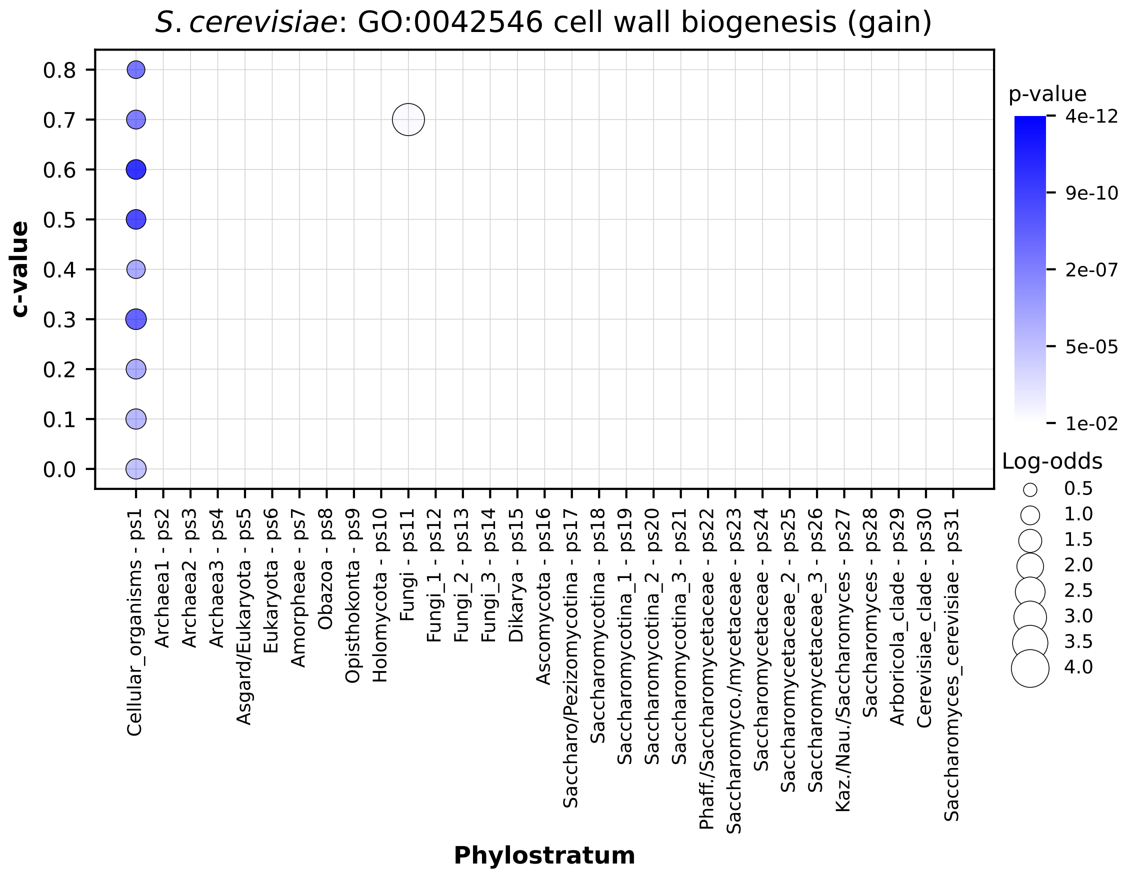
S. cerevisiae: GO:0042546 cell wall biogenesis (gain)
c-value
0.8
0.7
0.6
0.5
0.4
0.3
0.2
0.1
0.0
p-value
4e-12
9e-10
2e-07
5e-05
1e-02
Log-odds
0.5
1.0
1.5
2.0
2.5
3.0
3.5
4.0
Cellular_organisms - ps1
Archaea1 - ps2
Archaea2 - ps3
Archaea3 - ps4
Asgard/Eukaryota - ps5
Eukaryota - ps6
Amorpheae - ps7
Obazoa - ps8
Opisthokonta - ps9
Holomycota - ps10
Fungi - ps11
Fungi_1 - ps12
Fungi_2 - ps13
Fungi_3 - ps14
Dikarya - ps15
Ascomycota - ps16
Saccharo/Pezizomycotina - ps17
Saccharomycotina - ps18
Saccharomycotina_1 - ps19
Saccharomycotina_2 - ps20
Saccharomycotina_3 - ps21
Phaff./Saccharomycetaceae - ps22
Saccharomyco./mycetaceae - ps23
Saccharomycetaceae - ps24
Saccharomycetaceae_2 - ps25
Saccharomycetaceae_3 - ps26
Kaz./Nau./Saccharomyces - ps27
Saccharomyces - ps28
Arboricola_clade - ps29
Cerevisiae_clade - ps30
Saccharomyces_cerevisiae - ps31
Phylostratum

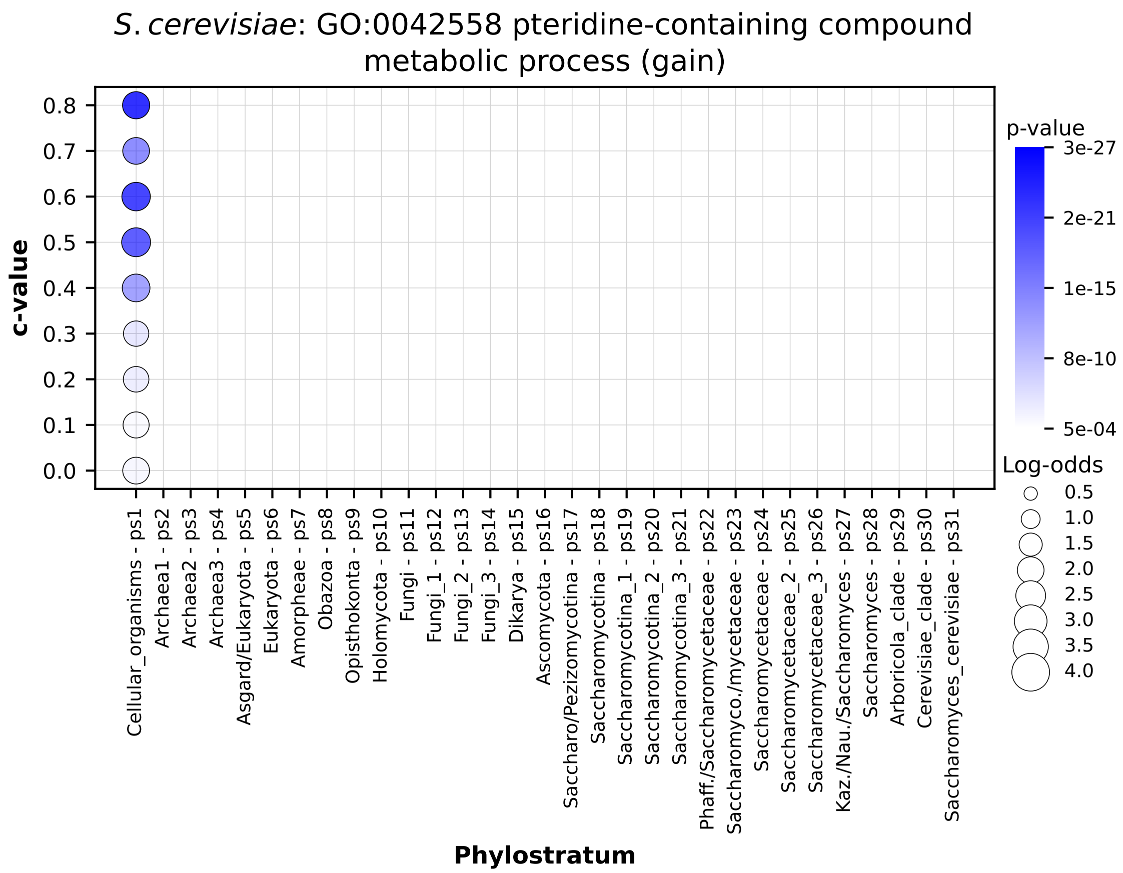
S. cerevisiae: GO:0042558 pteridine-containing compound metabolic process (gain)
c-value
0.8
0.7
0.6
0.5
0.4
0.3
0.2
0.1
0.0
p-value
3e-27
2e-21
1e-15
8e-10
5e-04
Log-odds
0.5
1.0
1.5
2.0
2.5
3.0
3.5
4.0
Cellular_organisms - ps1
Archaea1 - ps2
Archaea2 - ps3
Archaea3 - ps4
Asgard/Eukaryota - ps5
Eukaryota - ps6
Amorpheae - ps7
Obazoa - ps8
Opisthokonta - ps9
Holomycota - ps10
Fungi - ps11
Fungi_1 - ps12
Fungi_2 - ps13
Fungi_3 - ps14
Dikarya - ps15
Ascomycota - ps16
Saccharo/Pezizomycotina - ps17
Saccharomycotina - ps18
Saccharomycotina_1 - ps19
Saccharomycotina_2 - ps20
Saccharomycotina_3 - ps21
Phaff./Saccharomycetaceae - ps22
Saccharomyco./mycetaceae - ps23
Saccharomycetaceae - ps24
Saccharomycetaceae_2 - ps25
Saccharomycetaceae_3 - ps26
Kaz./Nau./Saccharomyces - ps27
Saccharomyces - ps28
Arboricola_clade - ps29
Cerevisiae_clade - ps30
Saccharomyces_cerevisiae - ps31
Phylostratum

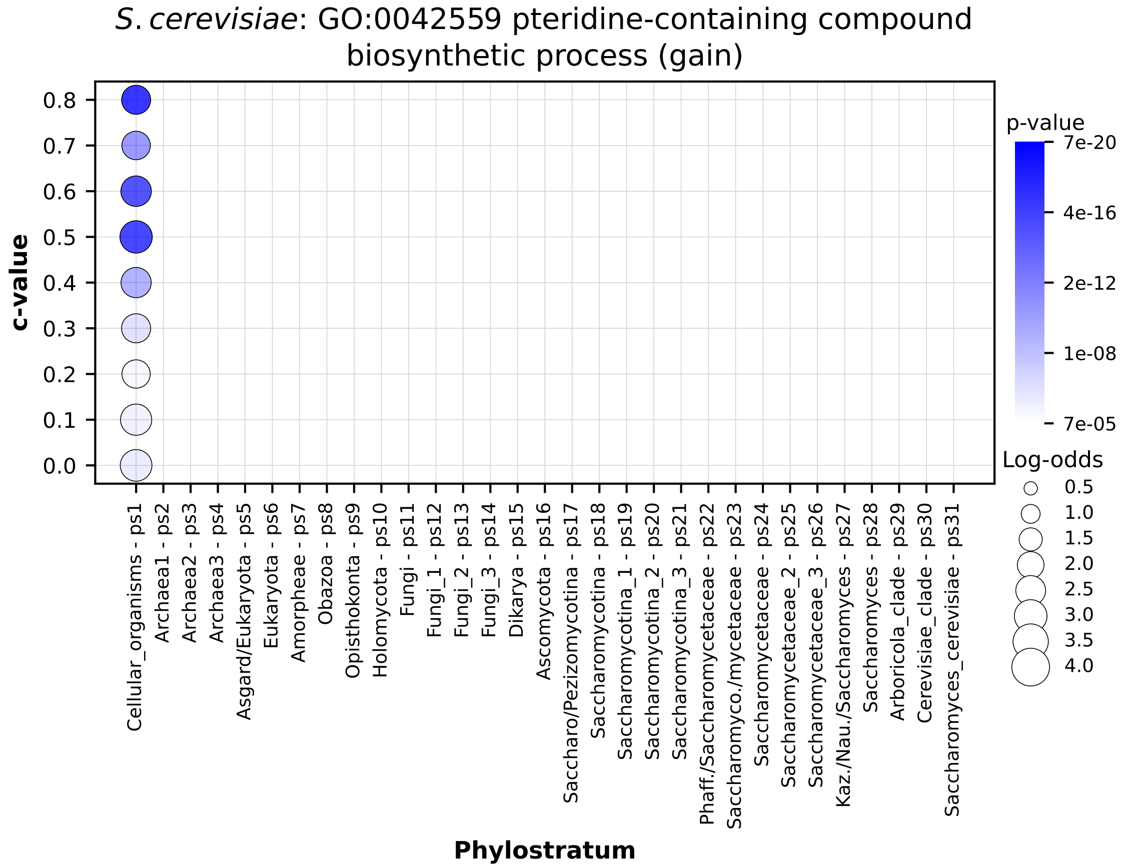
S. cerevisiae: GO:0042559 pteridine-containing compound biosynthetic process (gain)
c-value
0.0
0.1
0.2
0.3
0.4
0.5
0.6
0.7
0.8
Phylostratum
Cellular_organisms - ps1
Archaea1 - ps2
Archaea2 - ps3
Archaea3 - ps4
Asgard/Eukaryota - ps5
Eukaryota - ps6
Amorpheae - ps7
Obazoa - ps8
Opisthokonta - ps9
Holomycota - ps10
Fungi - ps11
Fungi_1 - ps12
Fungi_2 - ps13
Fungi_3 - ps14
Dikarya - ps15
Ascomycota - ps16
Saccharo/Pezizomycotina - ps17
Saccharomycotina - ps18
Saccharomycotina_1 - ps19
Saccharomycotina_2 - ps20
Saccharomycotina_3 - ps21
Phaff./Saccharomycetaceae - ps22
Saccharomyco./mycetaceae - ps23
Saccharomycetaceae - ps24
Saccharomycetaceae_2 - ps25
Saccharomycetaceae_3 - ps26
Kaz./Nau./Saccharomyces - ps27
Saccharomyces - ps28
Arboricola_clade - ps29
Cerevisiae_clade - ps30
Saccharomyces_cerevisiae - ps31
p-value
7e-20
4e-16
2e-12
1e-08
7e-05
Log-odds
0.5
1.0
1.5
2.0
2.5
3.0
3.5
4.0

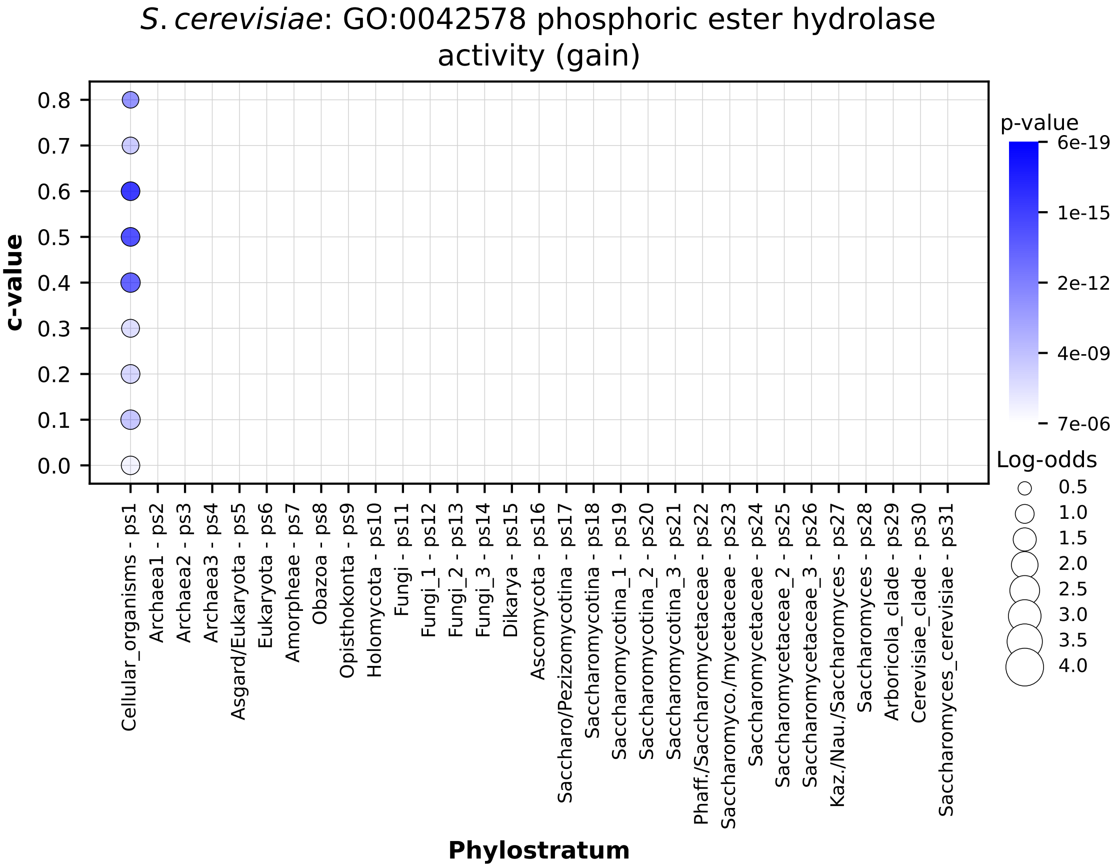
S. cerevisiae: GO:0042578 phosphoric ester hydrolase activity (gain)
c-value
0.0
0.1
0.2
0.3
0.4
0.5
0.6
0.7
0.8
Phylostratum
Cellular_organisms - ps1
Archaea1 - ps2
Archaea2 - ps3
Archaea3 - ps4
Asgard/Eukaryota - ps5
Eukaryota - ps6
Amorpheae - ps7
Obazoa - ps8
Opisthokonta - ps9
Holomycota - ps10
Fungi - ps11
Fungi_1 - ps12
Fungi_2 - ps13
Fungi_3 - ps14
Dikarya - ps15
Ascomycota - ps16
Saccharo/Pezizomycotina - ps17
Saccharomycotina - ps18
Saccharomycotina_1 - ps19
Saccharomycotina_2 - ps20
Saccharomycotina_3 - ps21
Phaff./Saccharomycetaceae - ps22
Saccharomyco./mycetaceae - ps23
Saccharomycetaceae - ps24
Saccharomycetaceae_2 - ps25
Saccharomycetaceae_3 - ps26
Kaz./Nau./Saccharomyces - ps27
Saccharomyces - ps28
Arboricola_clade - ps29
Cerevisiae_clade - ps30
Saccharomyces_cerevisiae - ps31
p-value
6e-19
1e-15
2e-12
4e-09
7e-06
Log-odds
0.5
1.0
1.5
2.0
2.5
3.0
3.5
4.0

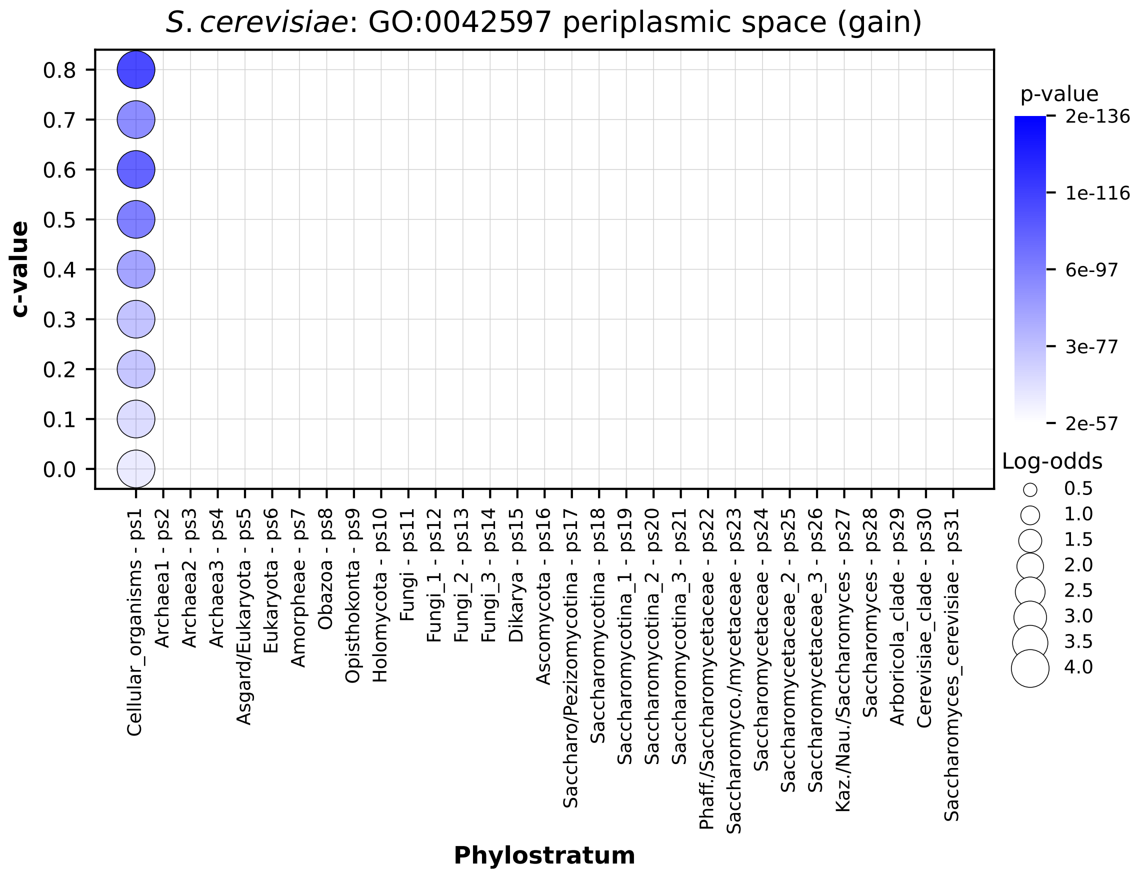

S. cerevisiae: GO:0042597 periplasmic space (gain)
c-value
0.0
0.1
0.2
0.3
0.4
0.5
0.6
0.7
0.8
p-value
2e-136
1e-116
6e-97
3e-77
2e-57
Log-odds
0.5
1.0
1.5
2.0
2.5
3.0
3.5
4.0
Cellular_organisms - ps1
Archaea1 - ps2
Archaea2 - ps3
Archaea3 - ps4
Asgard/Eukaryota - ps5
Eukaryota - ps6
Amorpheae - ps7
Obazoa - ps8
Opisthokonta - ps9
Holomycota - ps10
Fungi - ps11
Fungi_1 - ps12
Fungi_2 - ps13
Fungi_3 - ps14
Dikarya - ps15
Ascomycota - ps16
Saccharo/Pezizomycotina - ps17
Saccharomycotina - ps18
Saccharomycotina_1 - ps19
Saccharomycotina_2 - ps20
Saccharomycotina_3 - ps21
Phaff./Saccharomycetaceae - ps22
Saccharomyco./mycetaceae - ps23
Saccharomycetaceae - ps24
Saccharomycetaceae_2 - ps25
Saccharomycetaceae_3 - ps26
Kaz./Nau./Saccharomyces - ps27
Saccharomyces - ps28
Arboricola_clade - ps29
Cerevisiae_clade - ps30
Saccharomyces_cerevisiae - ps31
Phylostratum

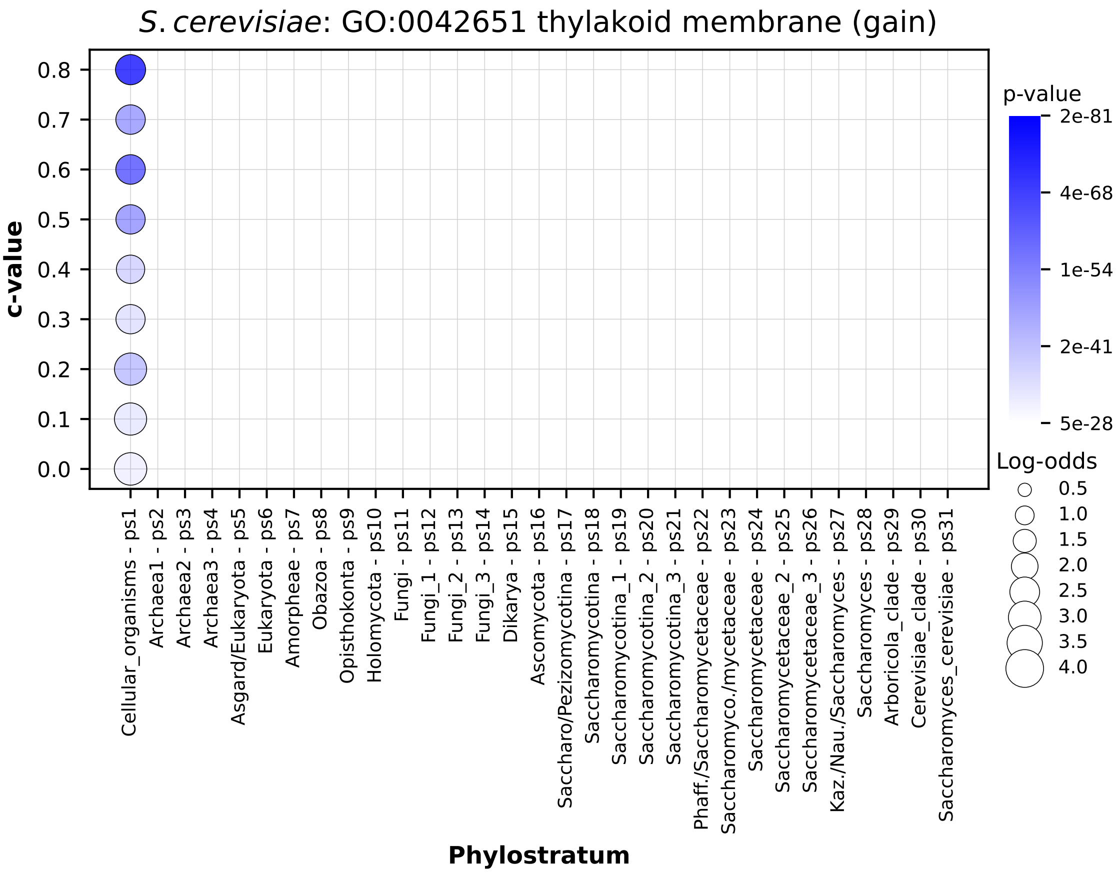

S. cerevisiae: GO:0042651 thylakoid membrane (gain)
c-value
0.8
0.7
0.6
0.5
0.4
0.3
0.2
0.1
0.0
p-value
2e-81
4e-68
1e-54
2e-41
5e-28
Log-odds
0.5
1.0
1.5
2.0
2.5
3.0
3.5
4.0
Cellular_organisms - ps1
Archaea1 - ps2
Archaea2 - ps3
Archaea3 - ps4
Asgard/Eukaryota - ps5
Eukaryota - ps6
Amorpheae - ps7
Obazoa - ps8
Opisthokonta - ps9
Holomycota - ps10
Fungi - ps11
Fungi_1 - ps12
Fungi_2 - ps13
Fungi_3 - ps14
Dikarya - ps15
Ascomycota - ps16
Saccharo/Pezizomycotina - ps17
Saccharomycotina - ps18
Saccharomycotina_1 - ps19
Saccharomycotina_2 - ps20
Saccharomycotina_3 - ps21
Phaff./Saccharomycetaceae - ps22
Saccharomyco./mycetaceae - ps23
Saccharomycetaceae - ps24
Saccharomycetaceae_2 - ps25
Saccharomycetaceae_3 - ps26
Kaz./Nau./Saccharomyces - ps27
Saccharomyces - ps28
Arboricola_clade - ps29
Cerevisiae_clade - ps30
Saccharomyces_cerevisiae - ps31
Phylostratum

S. cerevisiae: GO:0042716 plasma membrane-derived chromatophore (gain)

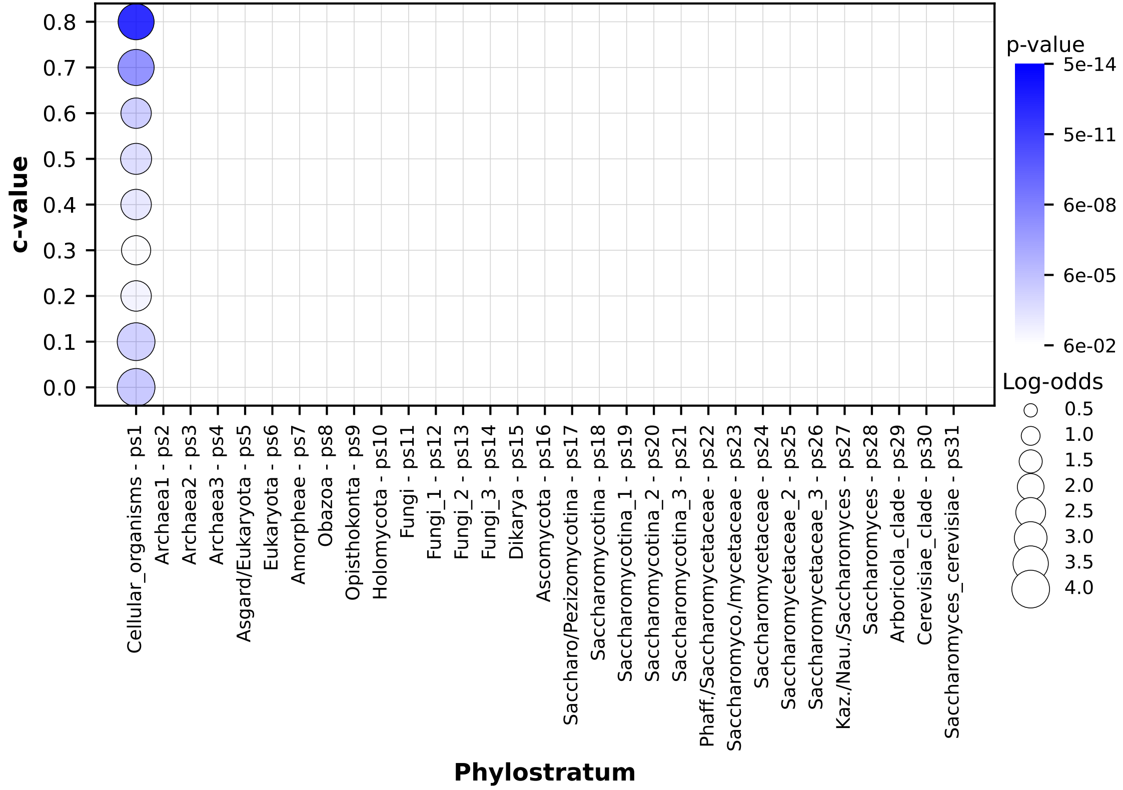

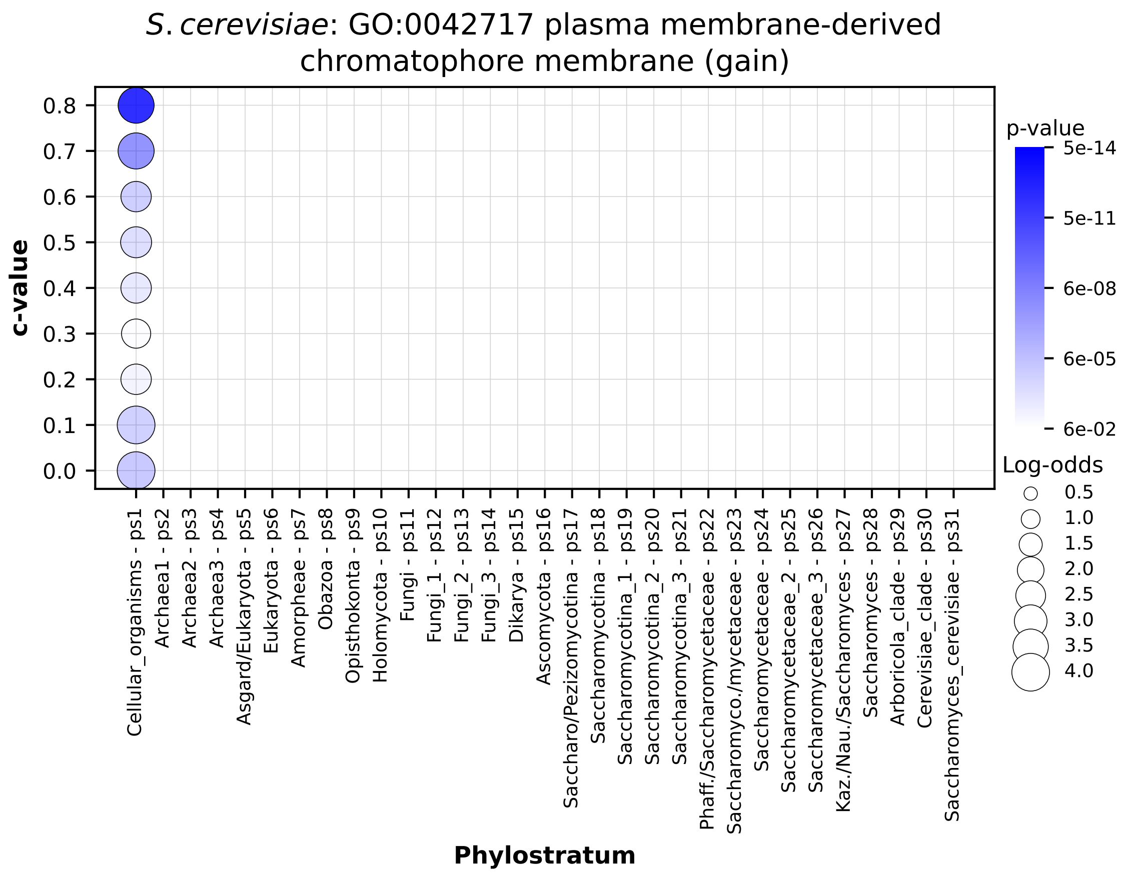

S. cerevisiae: GO:0042717 plasma membrane-derived chromatophore membrane (gain)
c-value
0.8
0.7
0.6
0.5
0.4
0.3
0.2
0.1
0.0
p-value
5e-14
5e-11
6e-08
6e-05
6e-02
Log-odds
0.5
1.0
1.5
2.0
2.5
3.0
3.5
4.0
Cellular_organisms - ps1
Archaea1 - ps2
Archaea2 - ps3
Archaea3 - ps4
Asgard/Eukaryota - ps5
Eukaryota - ps6
Amorpheae - ps7
Obazoa - ps8
Opisthokonta - ps9
Holomycota - ps10
Fungi - ps11
Fungi_1 - ps12
Fungi_2 - ps13
Fungi_3 - ps14
Dikarya - ps15
Ascomycota - ps16
Saccharo/Pezizomycotina - ps17
Saccharomycotina - ps18
Saccharomycotina_1 - ps19
Saccharomycotina_2 - ps20
Saccharomycotina_3 - ps21
Phaff./Saccharomycetaceae - ps22
Saccharomyco./mycetaceae - ps23
Saccharomycetaceae - ps24
Saccharomycetaceae_2 - ps25
Saccharomycetaceae_3 - ps26
Kaz./Nau./Saccharomyces - ps27
Saccharomyces - ps28
Arboricola_clade - ps29
Cerevisiae_clade - ps30
Saccharomyces_cerevisiae - ps31
Phylostratum

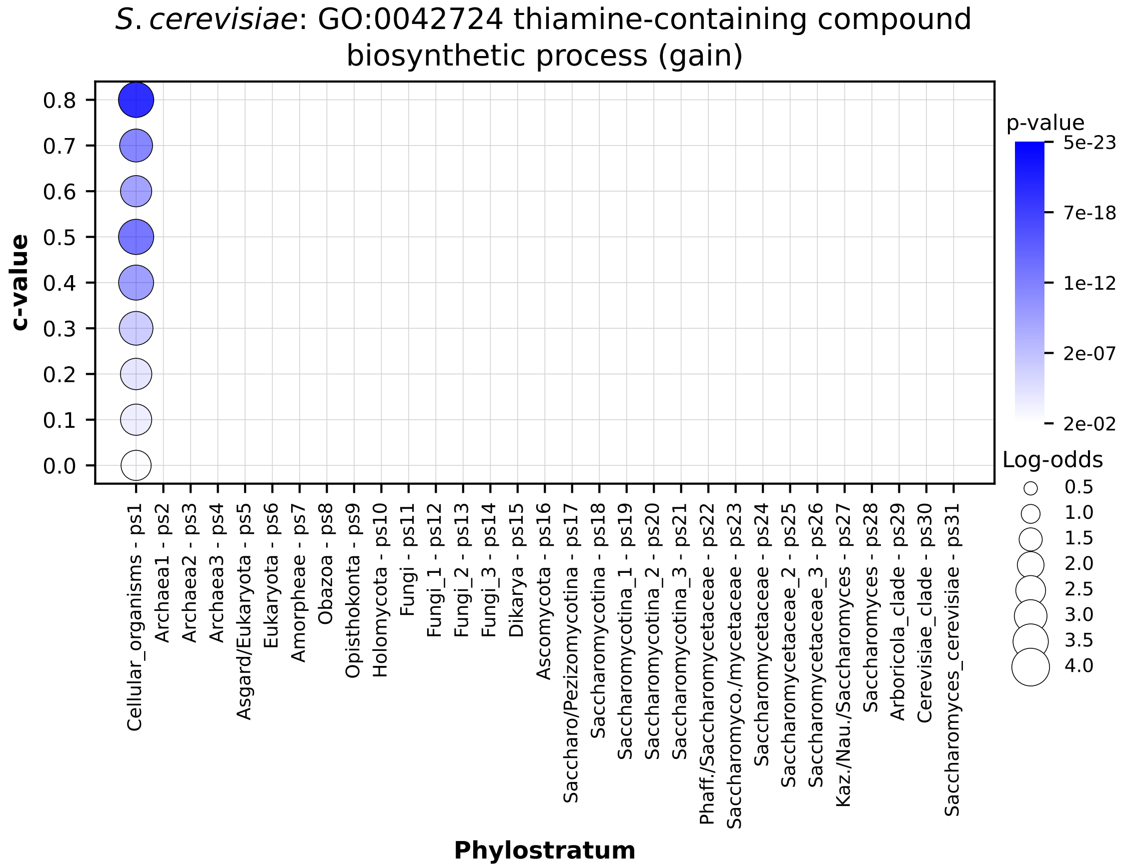
S. cerevisiae: GO:0042724 thiamine-containing compound biosynthetic process (gain)
c-value
0.0
0.1
0.2
0.3
0.4
0.5
0.6
0.7
0.8
p-value
5e-23
7e-18
1e-12
2e-07
2e-02
Log-odds
0.5
1.0
1.5
2.0
2.5
3.0
3.5
4.0
Cellular_organisms - ps1
Archaea1 - ps2
Archaea2 - ps3
Archaea3 - ps4
Asgard/Eukaryota - ps5
Eukaryota - ps6
Amorpheae - ps7
Obazoa - ps8
Opisthokonta - ps9
Holomycota - ps10
Fungi - ps11
Fungi_1 - ps12
Fungi_2 - ps13
Fungi_3 - ps14
Dikarya - ps15
Ascomycota - ps16
Saccharo/Pezizomycotina - ps17
Saccharomycotina - ps18
Saccharomycotina_1 - ps19
Saccharomycotina_2 - ps20
Saccharomycotina_3 - ps21
Phaff./Saccharomycetaceae - ps22
Saccharomyco./mycetaceae - ps23
Saccharomycetaceae - ps24
Saccharomycetaceae_2 - ps25
Saccharomycetaceae_3 - ps26
Kaz./Nau./Saccharomyces - ps27
Saccharomyces - ps28
Arboricola_clade - ps29
Cerevisiae_clade - ps30
Saccharomyces_cerevisiae - ps31
Phylostratum

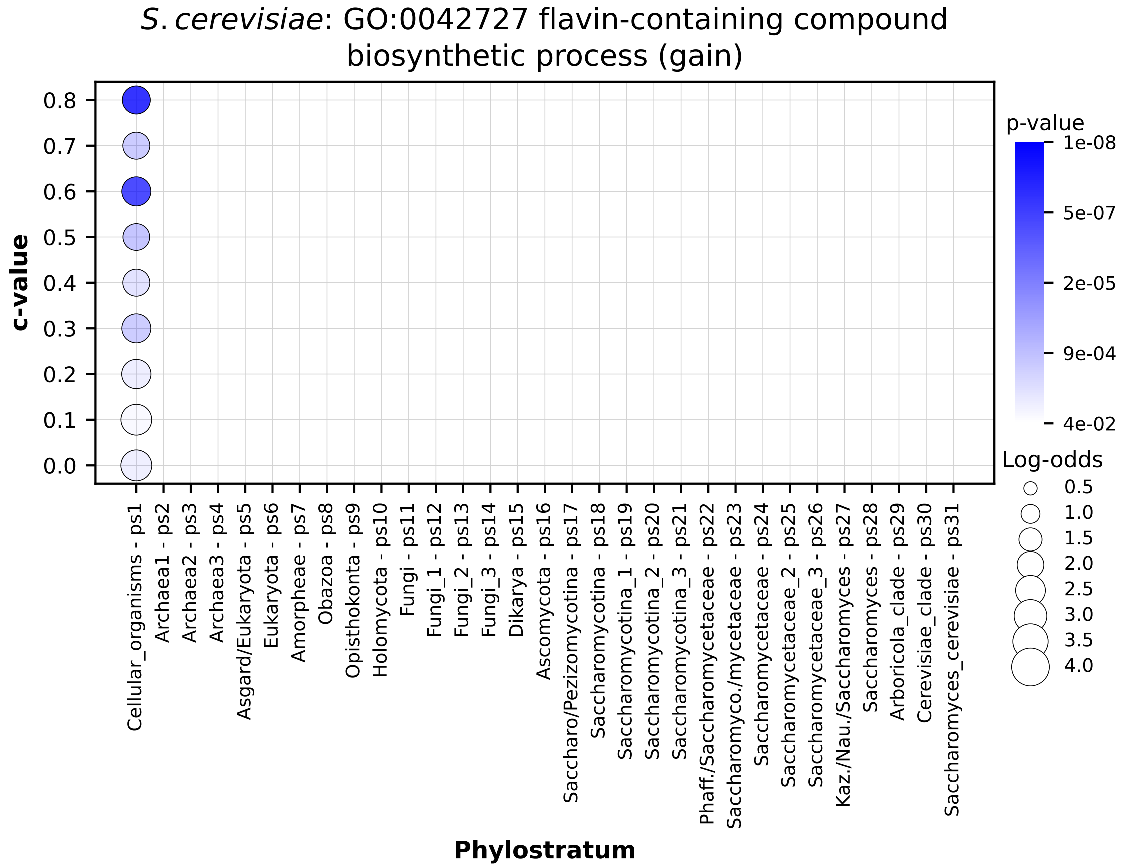

S. cerevisiae: GO:0042727 flavin-containing compound biosynthetic process (gain)
c-value
0.0
0.1
0.2
0.3
0.4
0.5
0.6
0.7
0.8
p-value
1e-08
5e-07
2e-05
9e-04
4e-02
Log-odds
0.5
1.0
1.5
2.0
2.5
3.0
3.5
4.0
Cellular_organisms - ps1
Archaea1 - ps2
Archaea2 - ps3
Archaea3 - ps4
Asgard/Eukaryota - ps5
Eukaryota - ps6
Amorpheae - ps7
Obazoa - ps8
Opisthokonta - ps9
Holomycota - ps10
Fungi - ps11
Fungi_1 - ps12
Fungi_2 - ps13
Fungi_3 - ps14
Dikarya - ps15
Ascomycota - ps16
Saccharo/Pezizomycotina - ps17
Saccharomycotina - ps18
Saccharomycotina_1 - ps19
Saccharomycotina_2 - ps20
Saccharomycotina_3 - ps21
Phaff./Saccharomycetaceae - ps22
Saccharomyco./mycetaceae - ps23
Saccharomycetaceae - ps24
Saccharomycetaceae_2 - ps25
Saccharomycetaceae_3 - ps26
Kaz./Nau./Saccharomyces - ps27
Saccharomyces - ps28
Arboricola_clade - ps29
Cerevisiae_clade - ps30
Saccharomyces_cerevisiae - ps31
Phylostratum

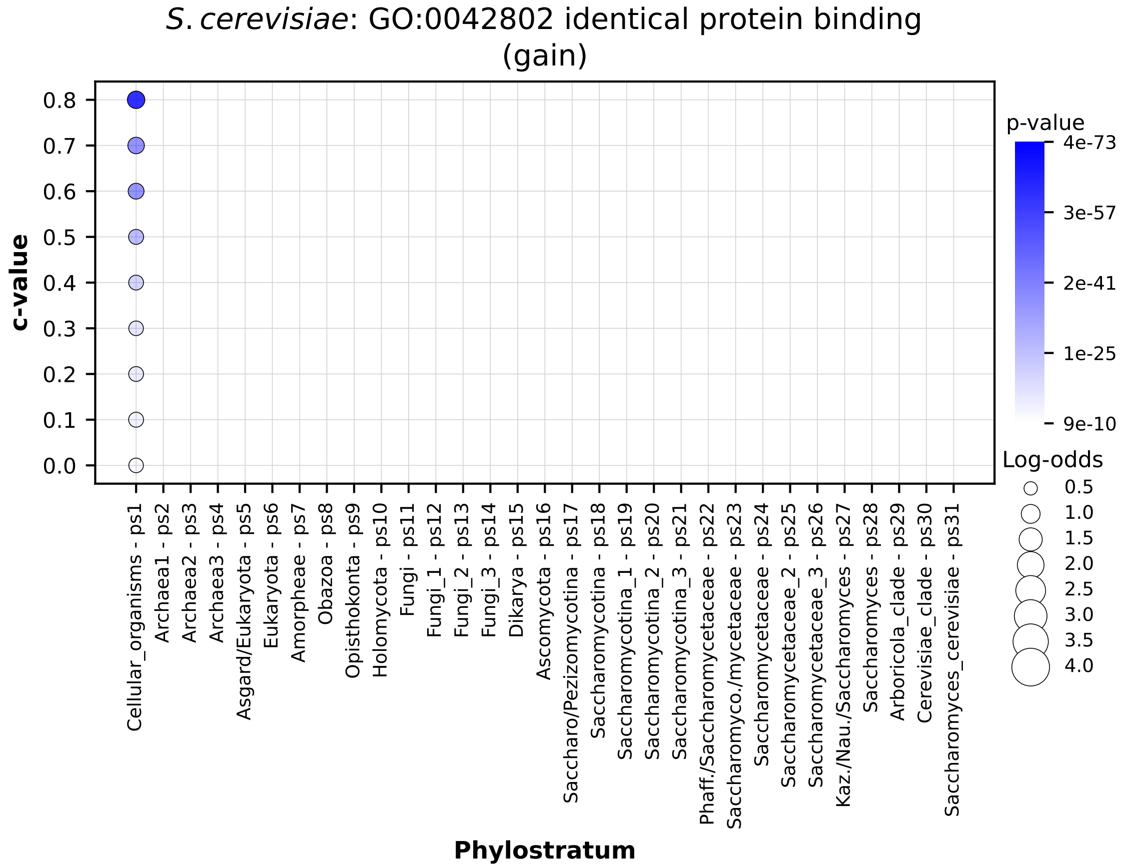

S. cerevisiae: GO:0042802 identical protein binding
(gain)
c-value
0.8
0.7
0.6
0.5
0.4
0.3
0.2
0.1
0.0
p-value
4e-73
3e-57
2e-41
1e-25
9e-10
Log-odds
0.5
1.0
1.5
2.0
2.5
3.0
3.5
4.0
Cellular_organisms - ps1
Archaea1 - ps2
Archaea2 - ps3
Archaea3 - ps4
Asgard/Eukaryota - ps5
Eukaryota - ps6
Amorpheae - ps7
Obazoa - ps8
Opisthokonta - ps9
Holomycota - ps10
Fungi - ps11
Fungi_1 - ps12
Fungi_2 - ps13
Fungi_3 - ps14
Dikarya - ps15
Ascomycota - ps16
Saccharo/Pezizomycotina - ps17
Saccharomycotina - ps18
Saccharomycotina_1 - ps19
Saccharomycotina_2 - ps20
Saccharomycotina_3 - ps21
Phaff./Saccharomycetaceae - ps22
Saccharomyco./mycetaceae - ps23
Saccharomycetaceae - ps24
Saccharomycetaceae_2 - ps25
Saccharomycetaceae_3 - ps26
Kaz./Nau./Saccharomyces - ps27
Saccharomyces - ps28
Arboricola_clade - ps29
Cerevisiae_clade - ps30
Saccharomyces_cerevisiae - ps31
Phylostratum

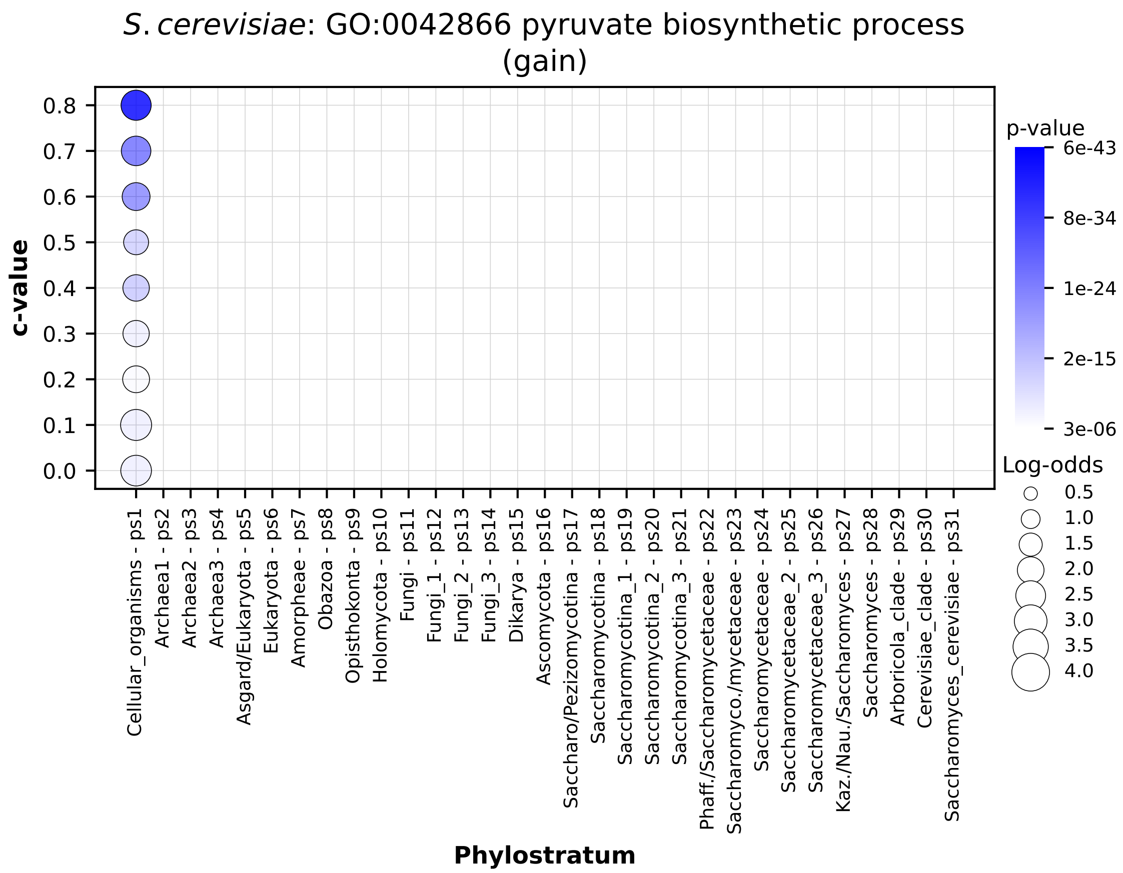

S. cerevisiae: GO:0042866 pyruvate biosynthetic process
(gain)
c-value
0.0
0.1
0.2
0.3
0.4
0.5
0.6
0.7
0.8
p-value
6e-43
8e-34
1e-24
2e-15
3e-06
Log-odds
0.5
1.0
1.5
2.0
2.5
3.0
3.5
4.0
Cellular_organisms - ps1
Archaea1 - ps2
Archaea2 - ps3
Archaea3 - ps4
Asgard/Eukaryota - ps5
Eukaryota - ps6
Amorpheae - ps7
Obazoa - ps8
Opisthokonta - ps9
Holomycota - ps10
Fungi - ps11
Fungi_1 - ps12
Fungi_2 - ps13
Fungi_3 - ps14
Dikarya - ps15
Ascomycota - ps16
Saccharo/Pezizomycotina - ps17
Saccharomycotina - ps18
Saccharomycotina_1 - ps19
Saccharomycotina_2 - ps20
Saccharomycotina_3 - ps21
Phaff./Saccharomycetaceae - ps22
Saccharomyco./mycetaceae - ps23
Saccharomycetaceae - ps24
Saccharomycetaceae_2 - ps25
Saccharomycetaceae_3 - ps26
Kaz./Nau./Saccharomyces - ps27
Saccharomyces - ps28
Arboricola_clade - ps29
Cerevisiae_clade - ps30
Saccharomyces_cerevisiae - ps31
Phylostratum

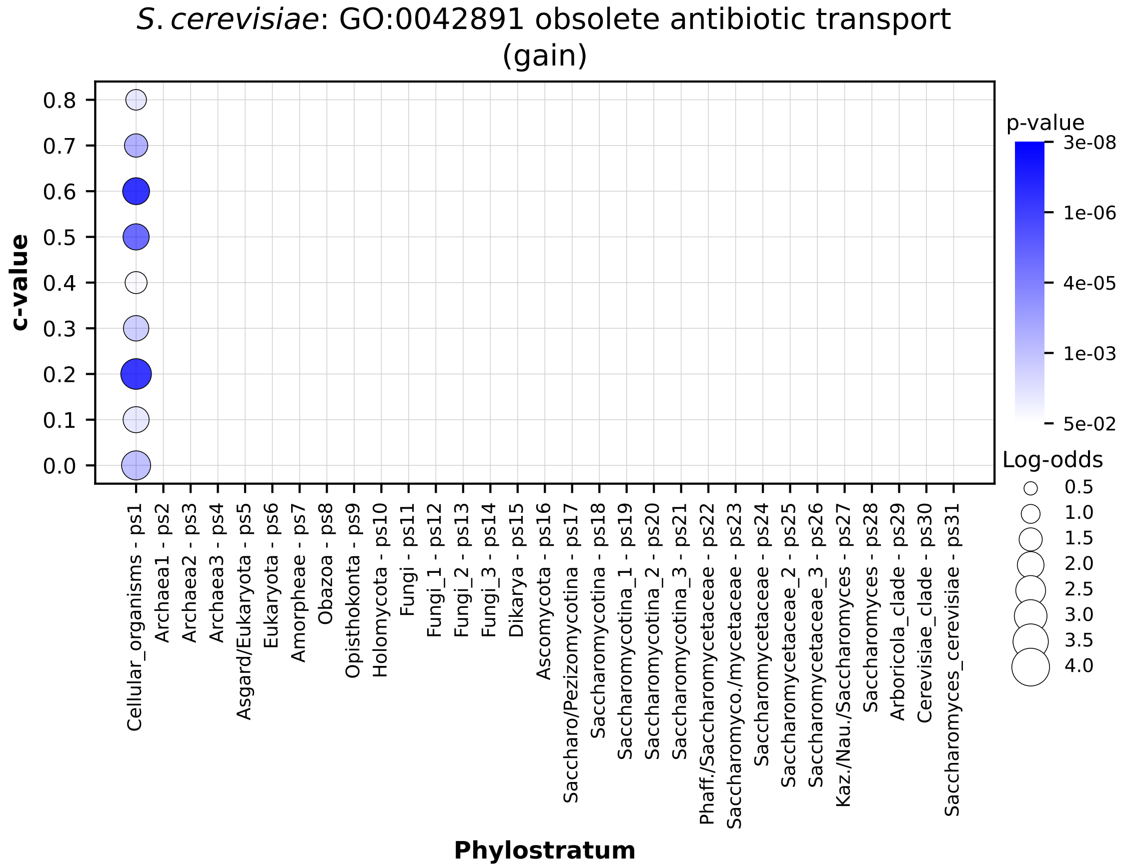
S. cerevisiae: GO:0042891 obsolete antibiotic transport
(gain)
c-value
0.8
0.7
0.6
0.5
0.4
0.3
0.2
0.1
0.0
p-value
3e-08
1e-06
4e-05
1e-03
5e-02
Log-odds
0.5
1.0
1.5
2.0
2.5
3.0
3.5
4.0
Cellular_organisms - ps1
Archaea1 - ps2
Archaea2 - ps3
Archaea3 - ps4
Asgard/Eukaryota - ps5
Eukaryota - ps6
Amorpheae - ps7
Obazoa - ps8
Opisthokonta - ps9
Holomycota - ps10
Fungi - ps11
Fungi_1 - ps12
Fungi_2 - ps13
Fungi_3 - ps14
Dikarya - ps15
Ascomycota - ps16
Saccharo/Pezizomycotina - ps17
Saccharomycotina - ps18
Saccharomycotina_1 - ps19
Saccharomycotina_2 - ps20
Saccharomycotina_3 - ps21
Phaff./Saccharomycetaceae - ps22
Saccharomyco./mycetaceae - ps23
Saccharomycetaceae - ps24
Saccharomycetaceae_2 - ps25
Saccharomycetaceae_3 - ps26
Kaz./Nau./Saccharomyces - ps27
Saccharomyces - ps28
Arboricola_clade - ps29
Cerevisiae_clade - ps30
Saccharomyces_cerevisiae - ps31
Phylostratum

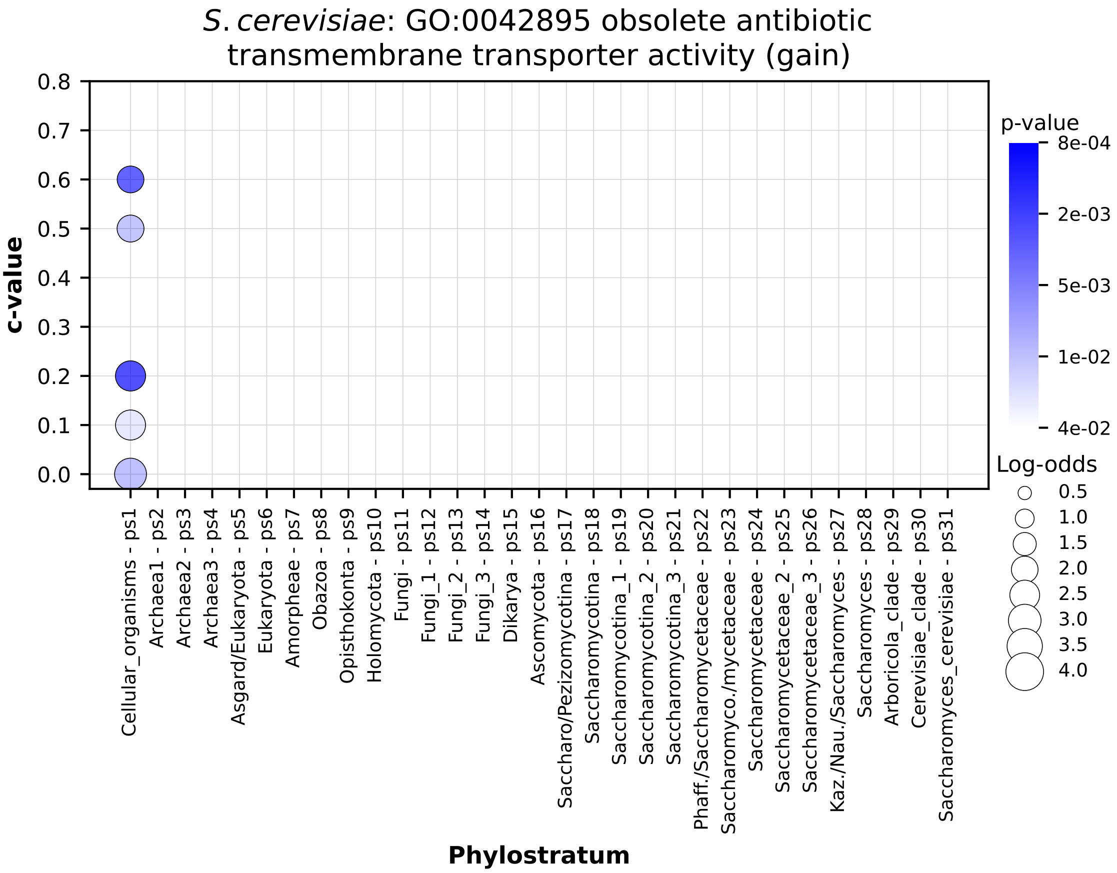
S. cerevisiae: GO:0042895 obsolete antibiotic transmembrane transporter activity (gain)
c-value
0.8
0.7
0.6
0.5
0.4
0.3
0.2
0.1
0.0
p-value
8e-04
2e-03
5e-03
1e-02
4e-02
Log-odds
0.5
1.0
1.5
2.0
2.5
3.0
3.5
4.0
Cellular_organisms - ps1
Archaea1 - ps2
Archaea2 - ps3
Archaea3 - ps4
Asgard/Eukaryota - ps5
Eukaryota - ps6
Amorpheae - ps7
Obazoa - ps8
Opisthokonta - ps9
Holomycota - ps10
Fungi - ps11
Fungi_1 - ps12
Fungi_2 - ps13
Fungi_3 - ps14
Dikarya - ps15
Ascomycota - ps16
Saccharo/Pezizomycotina - ps17
Saccharomycotina - ps18
Saccharomycotina_1 - ps19
Saccharomycotina_2 - ps20
Saccharomycotina_3 - ps21
Phaff./Saccharomycetaceae - ps22
Saccharomyco./mycetaceae - ps23
Saccharomycetaceae - ps24
Saccharomycetaceae_2 - ps25
Saccharomycetaceae_3 - ps26
Kaz./Nau./Saccharomyces - ps27
Saccharomyces - ps28
Arboricola_clade - ps29
Cerevisiae_clade - ps30
Saccharomyces_cerevisiae - ps31
Phylostratum

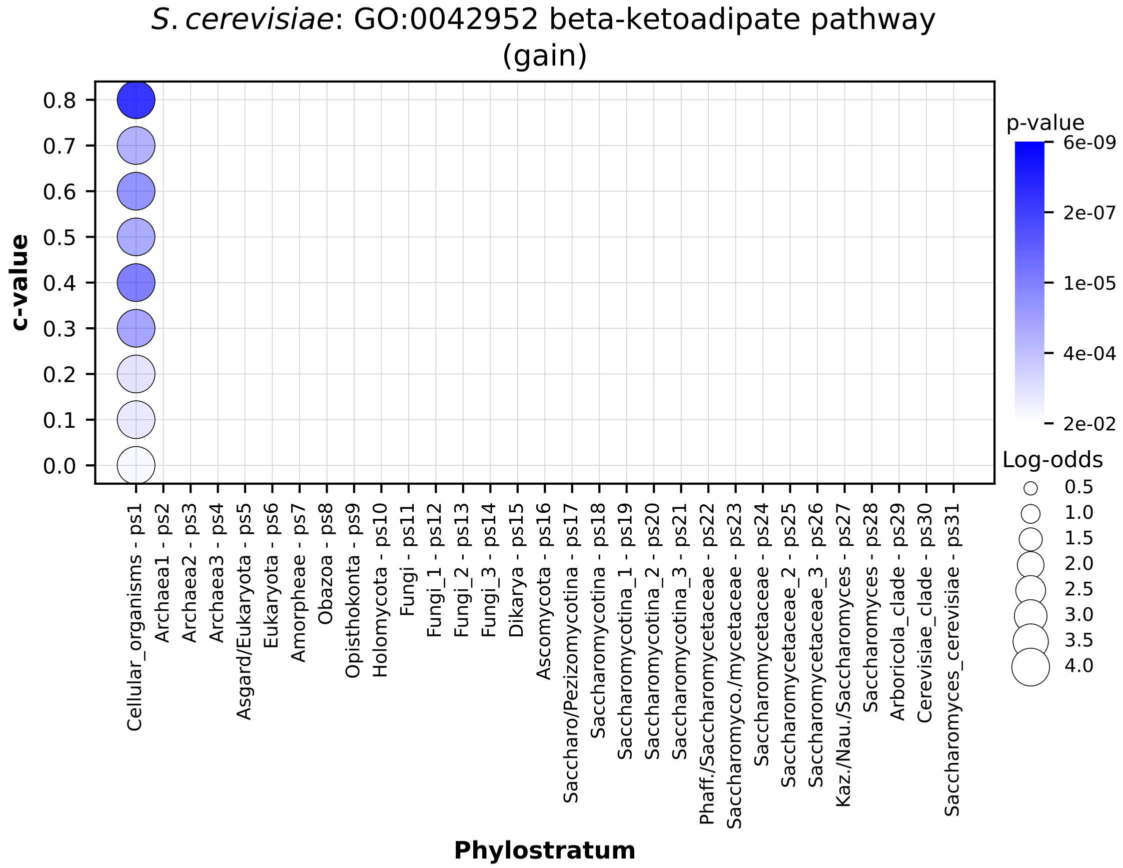

S. cerevisiae: GO:0042952 beta-ketoadipate pathway (gain)
c-value
0.8
0.7
0.6
0.5
0.4
0.3
0.2
0.1
0.0
p-value
6e-09
2e-07
1e-05
4e-04
2e-02
Log-odds
0.5
1.0
1.5
2.0
2.5
3.0
3.5
4.0
Cellular_organisms - ps1
Archaea1 - ps2
Archaea2 - ps3
Archaea3 - ps4
Asgard/Eukaryota - ps5
Eukaryota - ps6
Amorpheae - ps7
Obazoa - ps8
Opisthokonta - ps9
Holomycota - ps10
Fungi - ps11
Fungi_1 - ps12
Fungi_2 - ps13
Fungi_3 - ps14
Dikarya - ps15
Ascomycota - ps16
Saccharo/Pezizomycotina - ps17
Saccharomycotina - ps18
Saccharomycotina_1 - ps19
Saccharomycotina_2 - ps20
Saccharomycotina_3 - ps21
Phaff./Saccharomycetaceae - ps22
Saccharomyco./mycetaceae - ps23
Saccharomycetaceae - ps24
Saccharomycetaceae_2 - ps25
Saccharomycetaceae_3 - ps26
Kaz./Nau./Saccharomyces - ps27
Saccharomyces - ps28
Arboricola_clade - ps29
Cerevisiae_clade - ps30
Saccharomyces_cerevisiae - ps31
Phylostratum

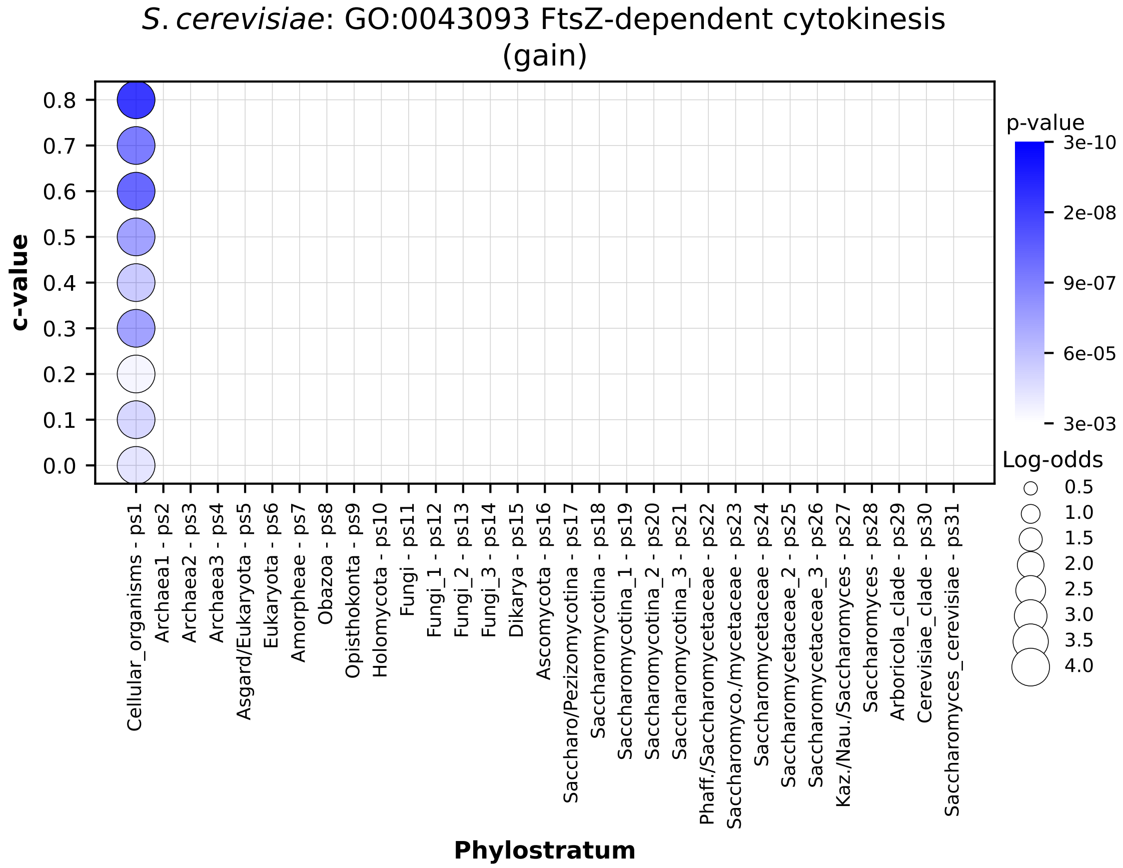
S. cerevisiae: GO:0043093 FtsZ-dependent cytokinesis
(gain)
c-value
0.8
0.7
0.6
0.5
0.4
0.3
0.2
0.1
0.0
p-value
3e-10
2e-08
9e-07
6e-05
3e-03
Log-odds
0.5
1.0
1.5
2.0
2.5
3.0
3.5
4.0
Cellular_organisms - ps1
Archaea1 - ps2
Archaea2 - ps3
Archaea3 - ps4
Asgard/Eukaryota - ps5
Eukaryota - ps6
Amorpheae - ps7
Obazoa - ps8
Opisthokonta - ps9
Holomycota - ps10
Fungi - ps11
Fungi_1 - ps12
Fungi_2 - ps13
Fungi_3 - ps14
Dikarya - ps15
Ascomycota - ps16
Saccharo/Pezizomycotina - ps17
Saccharomycotina - ps18
Saccharomycotina_1 - ps19
Saccharomycotina_2 - ps20
Saccharomycotina_3 - ps21
Phaff./Saccharomycetaceae - ps22
Saccharomyco./mycetaceae - ps23
Saccharomycetaceae - ps24
Saccharomycetaceae_2 - ps25
Saccharomycetaceae_3 - ps26
Kaz./Nau./Saccharomyces - ps27
Saccharomyces - ps28
Arboricola_clade - ps29
Cerevisiae_clade - ps30
Saccharomyces_cerevisiae - ps31
Phylostratum

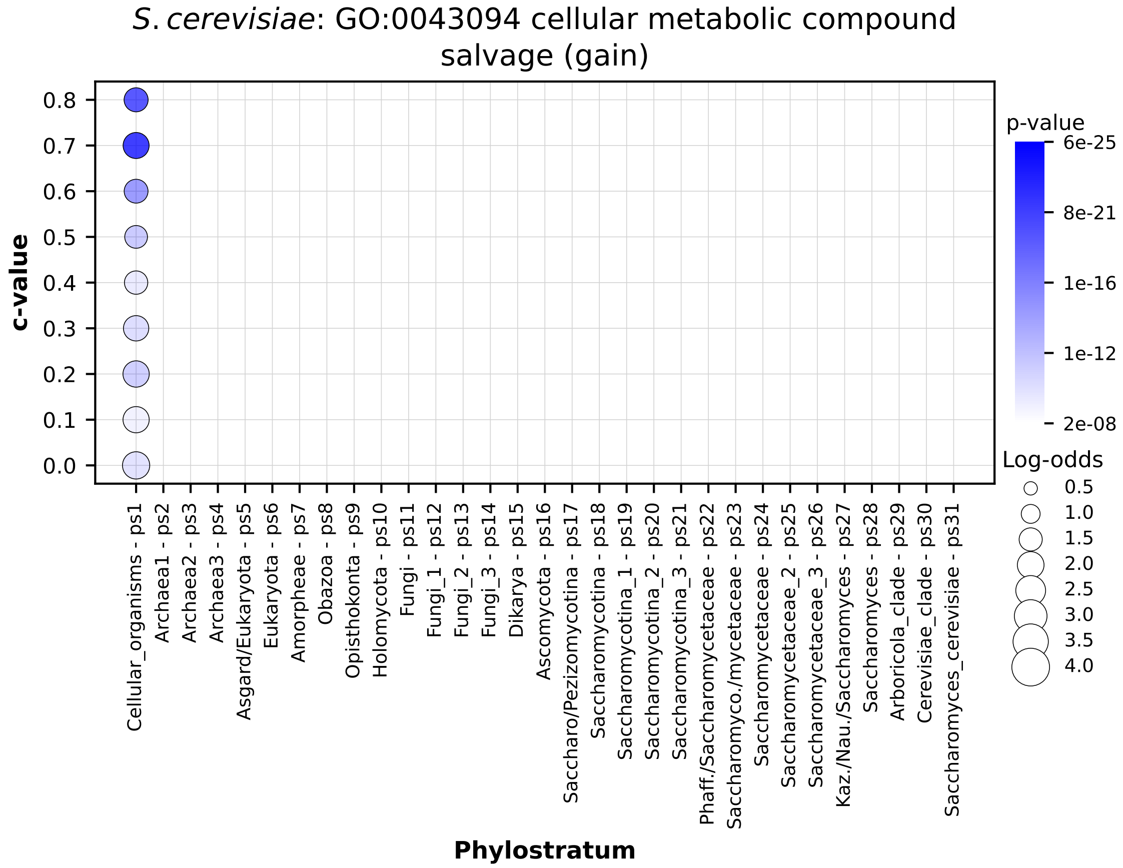

S. cerevisiae: GO:0043094 cellular metabolic compound salvage (gain)
c-value
0.0
0.1
0.2
0.3
0.4
0.5
0.6
0.7
0.8
p-value
6e-25
8e-21
1e-16
1e-12
2e-08
Log-odds
0.5
1.0
1.5
2.0
2.5
3.0
3.5
4.0
Cellular_organisms - ps1
Archaea1 - ps2
Archaea2 - ps3
Archaea3 - ps4
Asgard/Eukaryota - ps5
Eukaryota - ps6
Amorpheae - ps7
Obazoa - ps8
Opisthokonta - ps9
Holomycota - ps10
Fungi - ps11
Fungi_1 - ps12
Fungi_2 - ps13
Fungi_3 - ps14
Dikarya - ps15
Ascomycota - ps16
Saccharo/Pezizomycotina - ps17
Saccharomycotina - ps18
Saccharomycotina_1 - ps19
Saccharomycotina_2 - ps20
Saccharomycotina_3 - ps21
Phaff./Saccharomycetaceae - ps22
Saccharomyco./mycetaceae - ps23
Saccharomycetaceae - ps24
Saccharomycetaceae_2 - ps25
Saccharomycetaceae_3 - ps26
Kaz./Nau./Saccharomyces - ps27
Saccharomyces - ps28
Arboricola_clade - ps29
Cerevisiae_clade - ps30
Saccharomyces_cerevisiae - ps31
Phylostratum

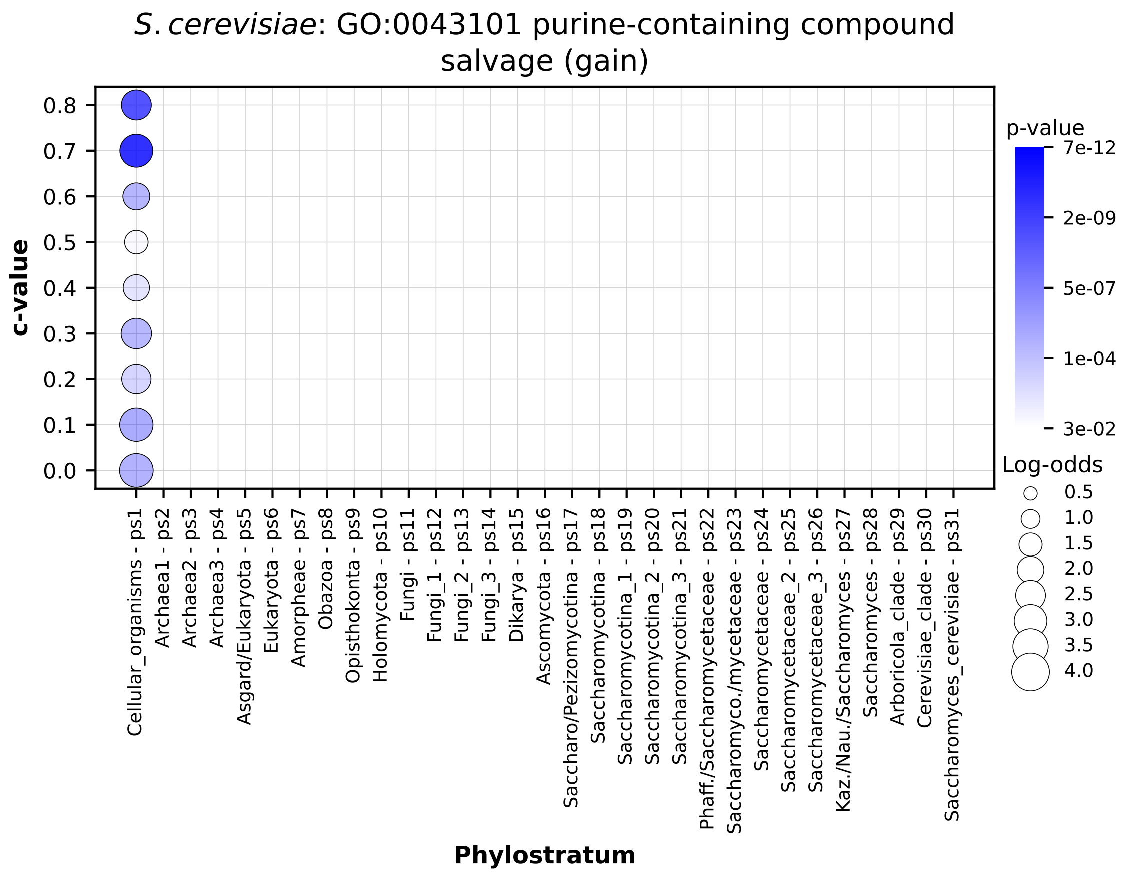
S. cerevisiae: GO:0043101 purine-containing compound salvage (gain)
c-value
0.0
0.1
0.2
0.3
0.4
0.5
0.6
0.7
0.8
p-value
7e-12
2e-09
5e-07
1e-04
3e-02
Log-odds
0.5
1.0
1.5
2.0
2.5
3.0
3.5
4.0
Cellular_organisms - ps1
Archaea1 - ps2
Archaea2 - ps3
Archaea3 - ps4
Asgard/Eukaryota - ps5
Eukaryota - ps6
Amorpheae - ps7
Obazoa - ps8
Opisthokonta - ps9
Holomycota - ps10
Fungi - ps11
Fungi_1 - ps12
Fungi_2 - ps13
Fungi_3 - ps14
Dikarya - ps15
Ascomycota - ps16
Saccharo/Pezizomycotina - ps17
Saccharomycotina - ps18
Saccharomycotina_1 - ps19
Saccharomycotina_2 - ps20
Saccharomycotina_3 - ps21
Phaff./Saccharomycetaceae - ps22
Saccharomyco./mycetaceae - ps23
Saccharomycetaceae - ps24
Saccharomycetaceae_2 - ps25
Saccharomycetaceae_3 - ps26
Kaz./Nau./Saccharomyces - ps27
Saccharomyces - ps28
Arboricola_clade - ps29
Cerevisiae_clade - ps30
Saccharomyces_cerevisiae - ps31
Phylostratum

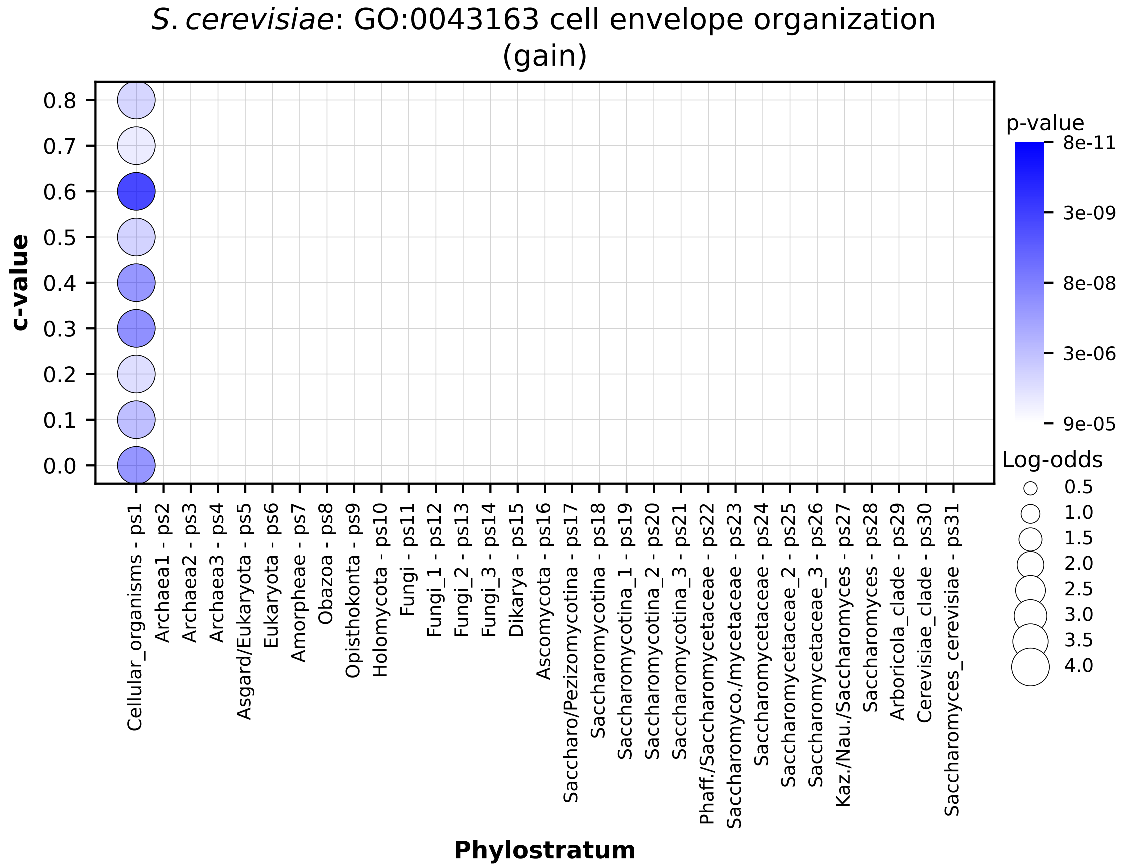

S. cerevisiae: GO:0043163 cell envelope organization (gain)
c-value
0.8
0.7
0.6
0.5
0.4
0.3
0.2
0.1
0.0
p-value
8e-11
3e-09
8e-08
3e-06
9e-05
Log-odds
0.5
1.0
1.5
2.0
2.5
3.0
3.5
4.0
Cellular_organisms - ps1
Archaea1 - ps2
Archaea2 - ps3
Archaea3 - ps4
Asgard/Eukaryota - ps5
Eukaryota - ps6
Amorpheae - ps7
Obazoa - ps8
Opisthokonta - ps9
Holomycota - ps10
Fungi - ps11
Fungi_1 - ps12
Fungi_2 - ps13
Fungi_3 - ps14
Dikarya - ps15
Ascomycota - ps16
Saccharo/Pezizomycotina - ps17
Saccharomycotina - ps18
Saccharomycotina_1 - ps19
Saccharomycotina_2 - ps20
Saccharomycotina_3 - ps21
Phaff./Saccharomycetaceae - ps22
Saccharomyco./mycetaceae - ps23
Saccharomycetaceae - ps24
Saccharomycetaceae_2 - ps25
Saccharomycetaceae_3 - ps26
Kaz./Nau./Saccharomyces - ps27
Saccharomyces - ps28
Arboricola_clade - ps29
Cerevisiae_clade - ps30
Saccharomyces_cerevisiae - ps31
Phylostratum

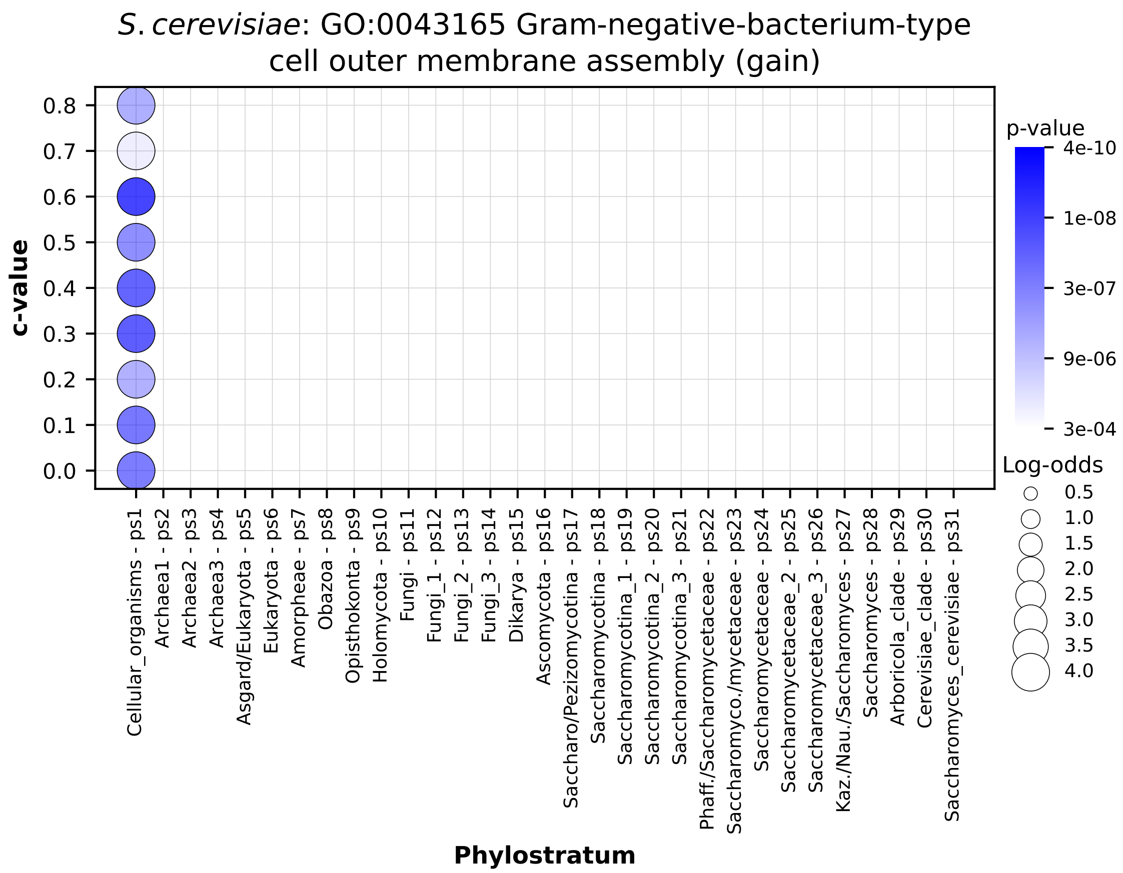
S. cerevisiae: GO:0043165 Gram-negative-bacterium-type cell outer membrane assembly (gain)
c-value
0.8
0.7
0.6
0.5
0.4
0.3
0.2
0.1
0.0
p-value
4e-10
1e-08
3e-07
9e-06
3e-04
Log-odds
0.5
1.0
1.5
2.0
2.5
3.0
3.5
4.0
Cellular_organisms - ps1
Archaea1 - ps2
Archaea2 - ps3
Archaea3 - ps4
Asgard/Eukaryota - ps5
Eukaryota - ps6
Amorpheae - ps7
Obazoa - ps8
Opisthokonta - ps9
Holomycota - ps10
Fungi - ps11
Fungi_1 - ps12
Fungi_2 - ps13
Fungi_3 - ps14
Dikarya - ps15
Ascomycota - ps16
Saccharo/Pezizomycotina - ps17
Saccharomycotina - ps18
Saccharomycotina_1 - ps19
Saccharomycotina_2 - ps20
Saccharomycotina_3 - ps21
Phaff./Saccharomycetaceae - ps22
Saccharomyco./mycetaceae - ps23
Saccharomycetaceae - ps24
Saccharomycetaceae_2 - ps25
Saccharomycetaceae_3 - ps26
Kaz./Nau./Saccharomyces - ps27
Saccharomyces - ps28
Arboricola_clade - ps29
Cerevisiae_clade - ps30
Saccharomyces_cerevisiae - ps31
Phylostratum

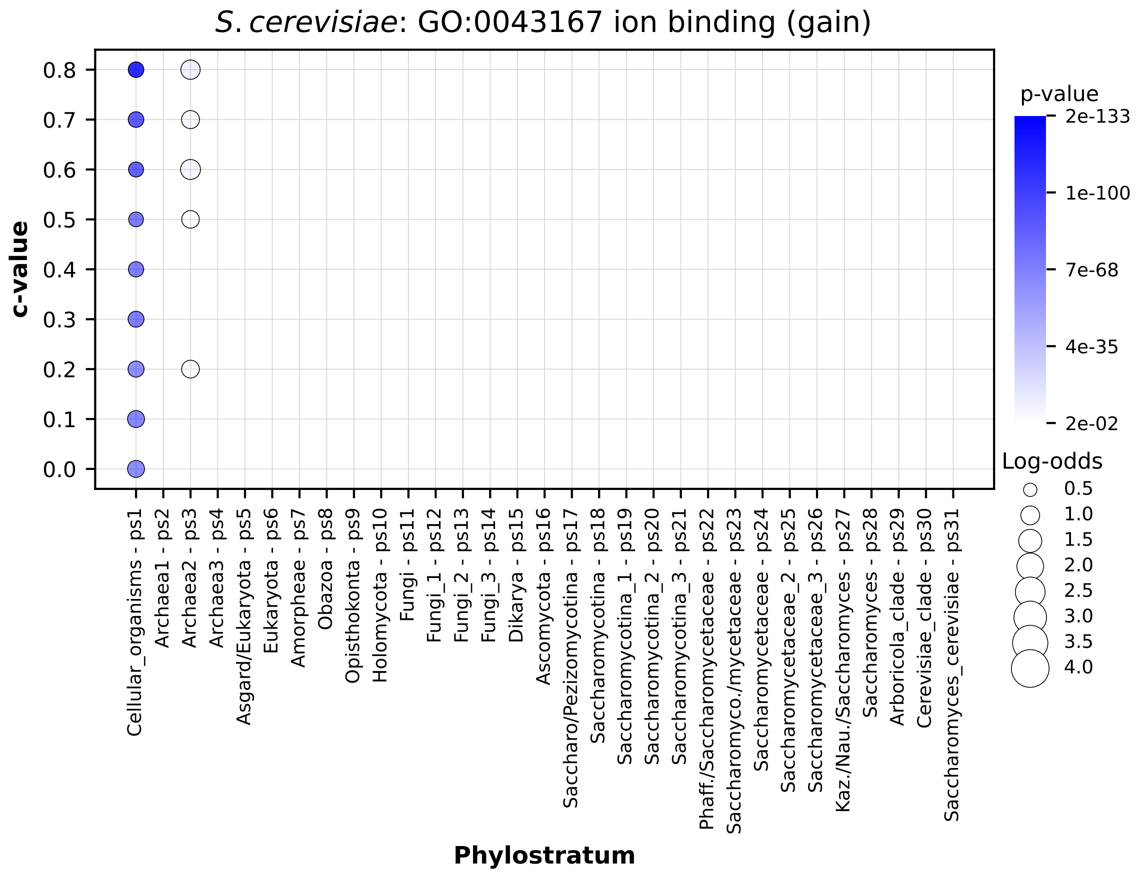

S. cerevisiae: GO:0043167 ion binding (gain)
c-value
0.8
0.7
0.6
0.5
0.4
0.3
0.2
0.1
0.0
Phylostratum
Cellular_organisms - ps1
Archaea1 - ps2
Archaea2 - ps3
Archaea3 - ps4
Asgard/Eukaryota - ps5
Eukaryota - ps6
Amorpheae - ps7
Obazoa - ps8
Opisthokonta - ps9
Holomycota - ps10
Fungi - ps11
Fungi_1 - ps12
Fungi_2 - ps13
Fungi_3 - ps14
Dikarya - ps15
Ascomycota - ps16
Saccharo/Pezizomycotina - ps17
Saccharomycotina - ps18
Saccharomycotina_1 - ps19
Saccharomycotina_2 - ps20
Saccharomycotina_3 - ps21
Phaff./Saccharomycetaceae - ps22
Saccharomyco./mycetaceae - ps23
Saccharomycetaceae - ps24
Saccharomycetaceae_2 - ps25
Saccharomycetaceae_3 - ps26
Kaz./Nau./Saccharomyces - ps27
Saccharomyces - ps28
Arboricola_clade - ps29
Cerevisiae_clade - ps30
Saccharomyces_cerevisiae - ps31
p-value
2e-133
1e-100
7e-68
4e-35
2e-02
Log-odds
0.5
1.0
1.5
2.0
2.5
3.0
3.5
4.0

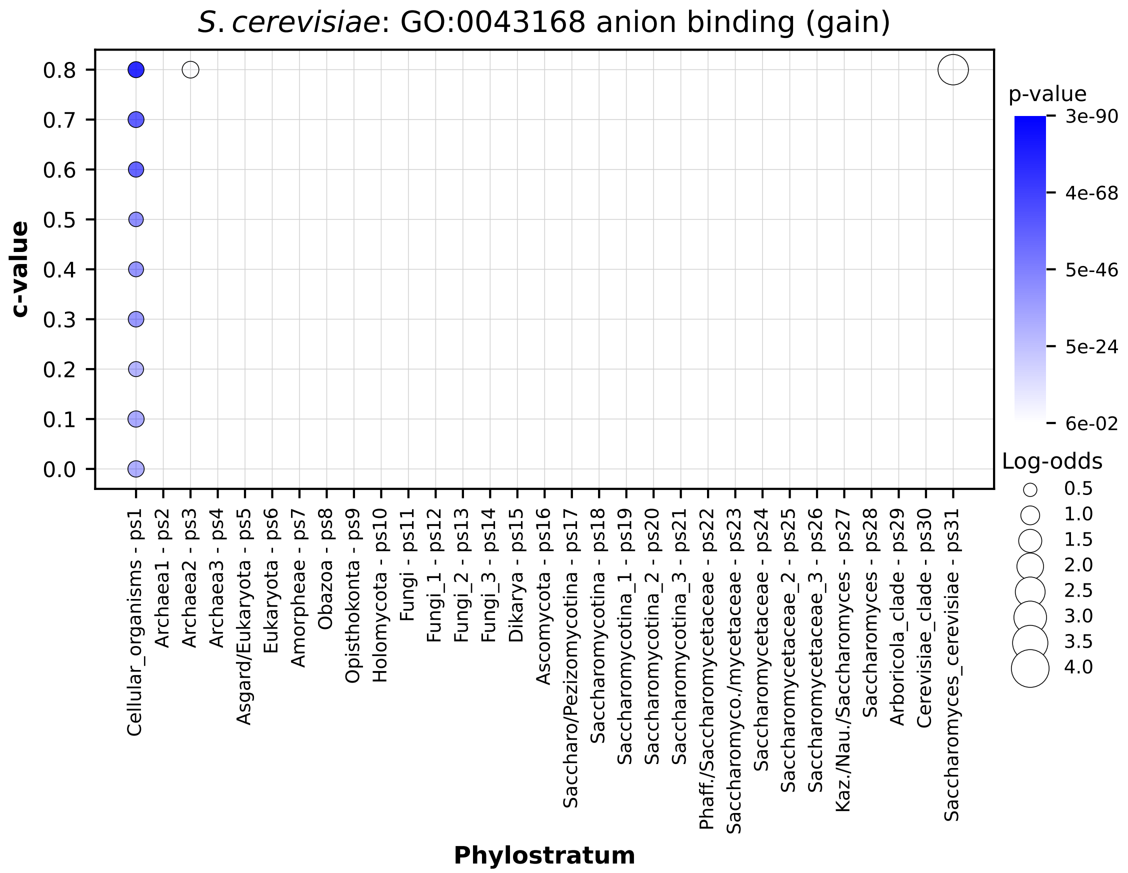
S. cerevisiae: GO:0043168 anion binding (gain)
c-value
0.0
0.1
0.2
0.3
0.4
0.5
0.6
0.7
0.8
p-value
3e-90
4e-68
5e-46
5e-24
6e-02
Log-odds
0.5
1.0
1.5
2.0
2.5
3.0
3.5
4.0
Cellular_organisms - ps1
Archaea1 - ps2
Archaea2 - ps3
Archaea3 - ps4
Asgard/Eukaryota - ps5
Eukaryota - ps6
Amorpheae - ps7
Obazoa - ps8
Opisthokonta - ps9
Holomycota - ps10
Fungi - ps11
Fungi_1 - ps12
Fungi_2 - ps13
Fungi_3 - ps14
Dikarya - ps15
Ascomycota - ps16
Saccharo/Pezizomycotina - ps17
Saccharomycotina - ps18
Saccharomycotina_1 - ps19
Saccharomycotina_2 - ps20
Saccharomycotina_3 - ps21
Phaff./Saccharomycetaceae - ps22
Saccharomyco./mycetaceae - ps23
Saccharomycetaceae - ps24
Saccharomycetaceae_2 - ps25
Saccharomycetaceae_3 - ps26
Kaz./Nau./Saccharomyces - ps27
Saccharomyces - ps28
Arboricola_clade - ps29
Cerevisiae_clade - ps30
Saccharomyces_cerevisiae - ps31
Phylostratum

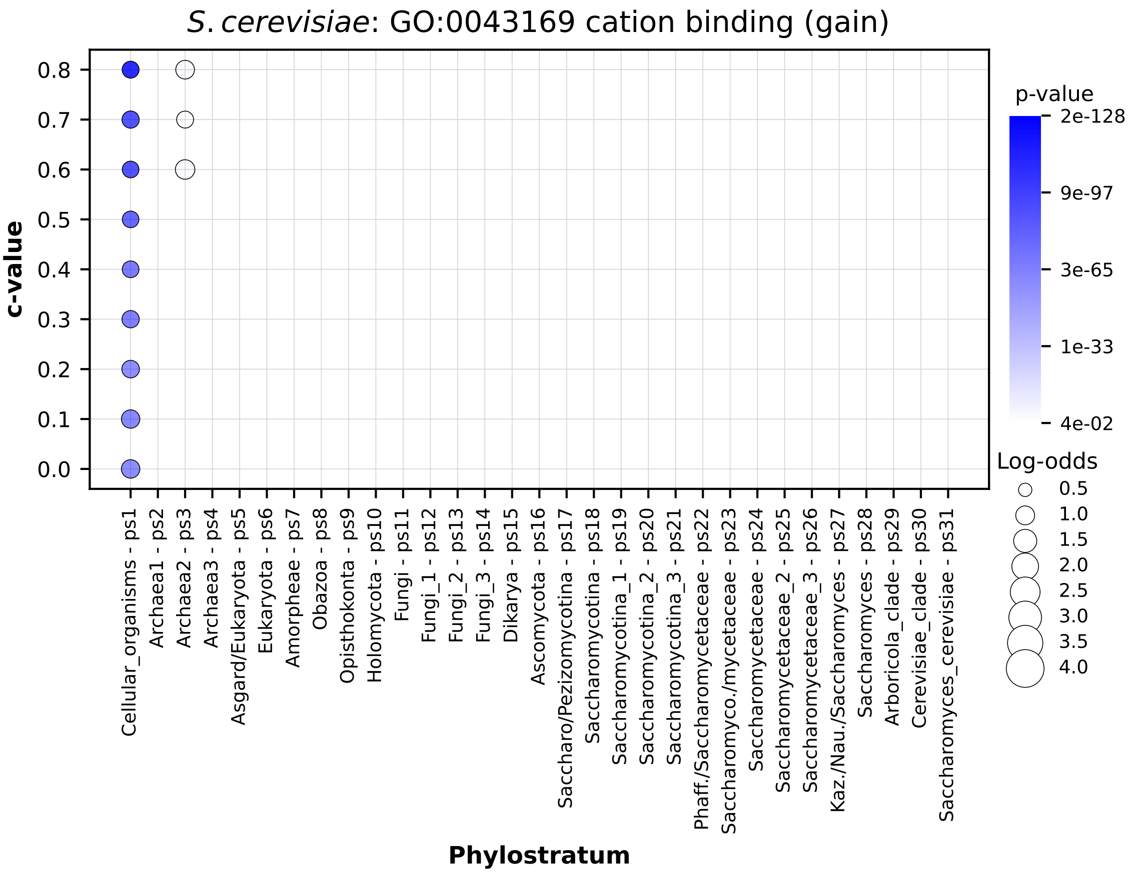

S. cerevisiae: GO:0043169 cation binding (gain)
c-value
0.0
0.1
0.2
0.3
0.4
0.5
0.6
0.7
0.8
p-value
2e-128
9e-97
3e-65
1e-33
4e-02
Log-odds
0.5
1.0
1.5
2.0
2.5
3.0
3.5
4.0
Cellular_organisms - ps1
Archaea1 - ps2
Archaea2 - ps3
Archaea3 - ps4
Asgard/Eukaryota - ps5
Eukaryota - ps6
Amorpheae - ps7
Obazoa - ps8
Opisthokonta - ps9
Holomycota - ps10
Fungi - ps11
Fungi_1 - ps12
Fungi_2 - ps13
Fungi_3 - ps14
Dikarya - ps15
Ascomycota - ps16
Saccharo/Pezizomycotina - ps17
Saccharomycotina - ps18
Saccharomycotina_1 - ps19
Saccharomycotina_2 - ps20
Saccharomycotina_3 - ps21
Phaff./Saccharomycetaceae - ps22
Saccharomyco./mycetaceae - ps23
Saccharomycetaceae - ps24
Saccharomycetaceae_2 - ps25
Saccharomycetaceae_3 - ps26
Kaz./Nau./Saccharomyces - ps27
Saccharomyces - ps28
Arboricola_clade - ps29
Cerevisiae_clade - ps30
Saccharomyces_cerevisiae - ps31
Phylostratum

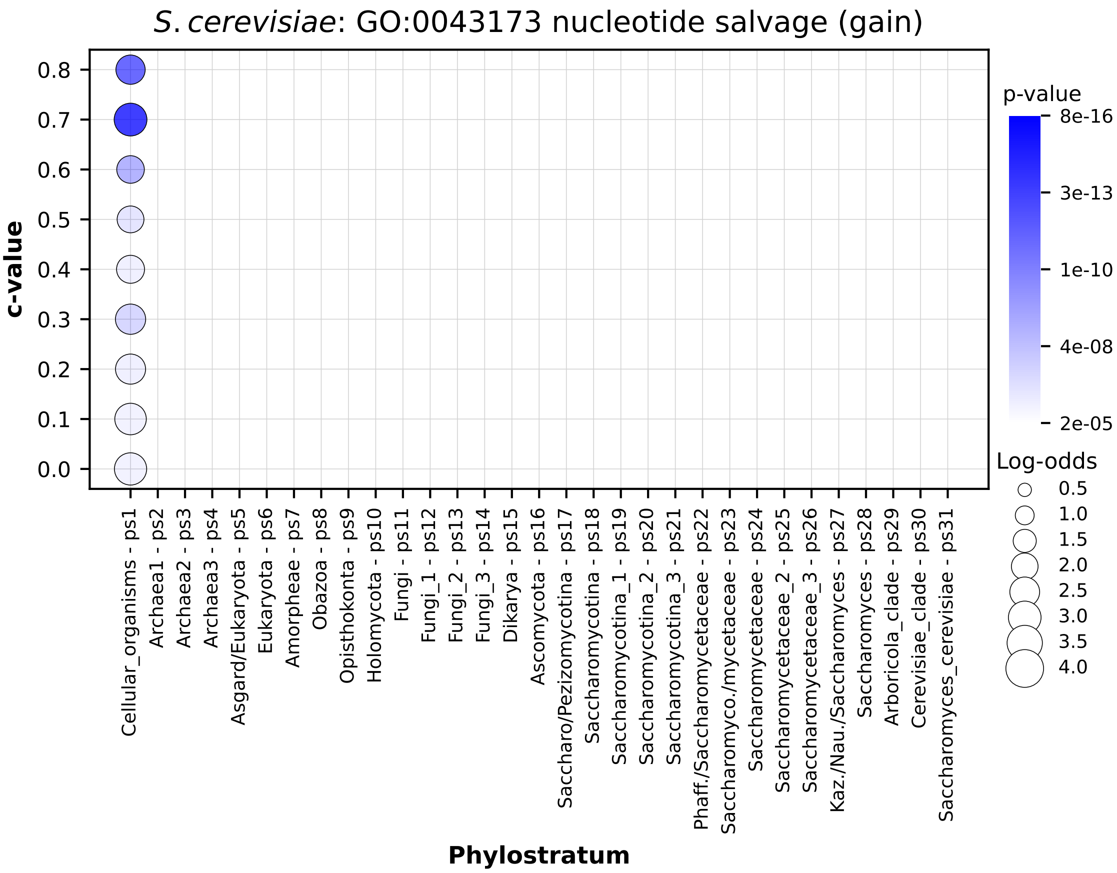
S. cerevisiae: GO:0043173 nucleotide salvage (gain)
c-value
0.8
0.7
0.6
0.5
0.4
0.3
0.2
0.1
0.0
p-value
8e-16
3e-13
1e-10
4e-08
2e-05
Log-odds
0.5
1.0
1.5
2.0
2.5
3.0
3.5
4.0
Cellular_organisms - ps1
Archaea1 - ps2
Archaea2 - ps3
Archaea3 - ps4
Asgard/Eukaryota - ps5
Eukaryota - ps6
Amorpheae - ps7
Obazoa - ps8
Opisthokonta - ps9
Holomycota - ps10
Fungi - ps11
Fungi_1 - ps12
Fungi_2 - ps13
Fungi_3 - ps14
Dikarya - ps15
Ascomycota - ps16
Saccharo/Pezizomycotina - ps17
Saccharomycotina - ps18
Saccharomycotina_1 - ps19
Saccharomycotina_2 - ps20
Saccharomycotina_3 - ps21
Phaff./Saccharomycetaceae - ps22
Saccharomyco./mycetaceae - ps23
Saccharomycetaceae - ps24
Saccharomycetaceae_2 - ps25
Saccharomycetaceae_3 - ps26
Kaz./Nau./Saccharomyces - ps27
Saccharomyces - ps28
Arboricola_clade - ps29
Cerevisiae_clade - ps30
Saccharomyces_cerevisiae - ps31
Phylostratum

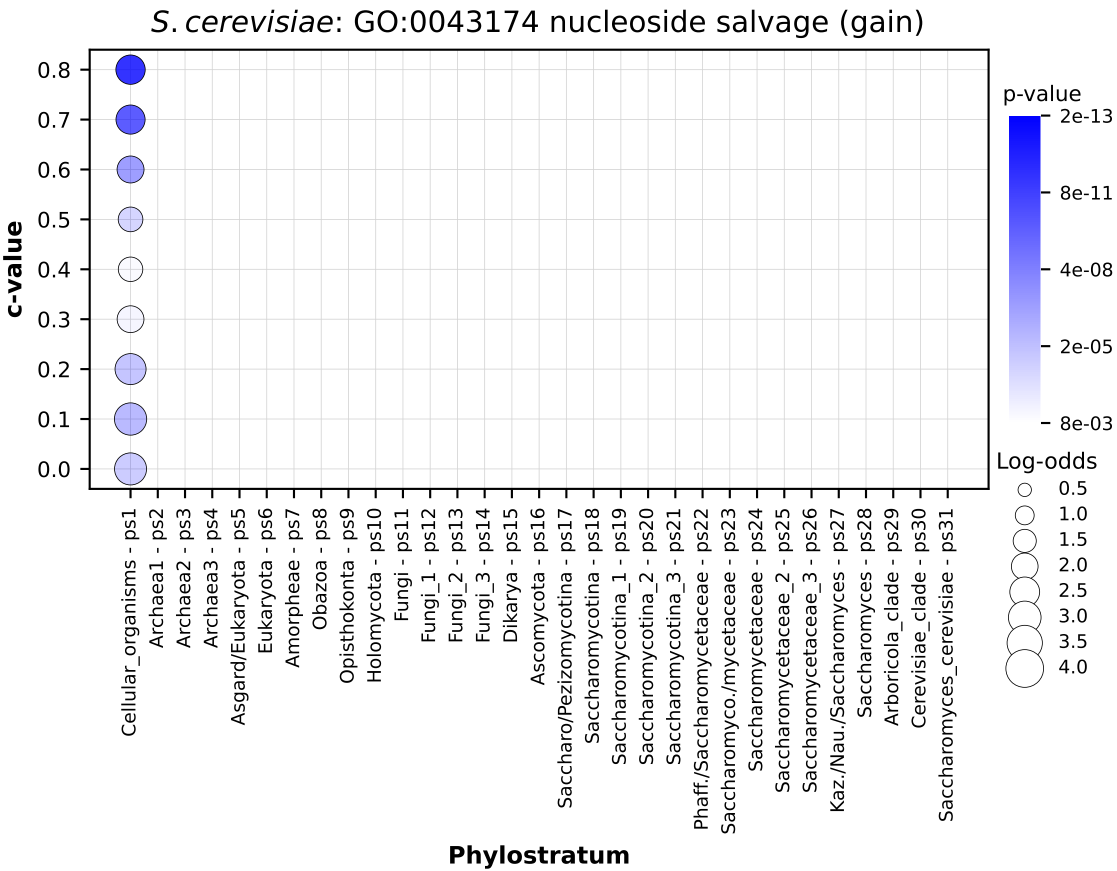

S. cerevisiae: GO:0043174 nucleoside salvage (gain)
c-value
0.8
0.7
0.6
0.5
0.4
0.3
0.2
0.1
0.0
Phylostratum
Cellular_organisms - ps1
Archaea1 - ps2
Archaea2 - ps3
Archaea3 - ps4
Asgard/Eukaryota - ps5
Eukaryota - ps6
Amorpheae - ps7
Obazoa - ps8
Opisthokonta - ps9
Holomycota - ps10
Fungi - ps11
Fungi_1 - ps12
Fungi_2 - ps13
Fungi_3 - ps14
Dikarya - ps15
Ascomycota - ps16
Saccharo/Pezizomycotina - ps17
Saccharomycotina - ps18
Saccharomycotina_1 - ps19
Saccharomycotina_2 - ps20
Saccharomycotina_3 - ps21
Phaff./Saccharomycetaceae - ps22
Saccharomyco./mycetaceae - ps23
Saccharomycetaceae - ps24
Saccharomycetaceae_2 - ps25
Saccharomycetaceae_3 - ps26
Kaz./Nau./Saccharomyces - ps27
Saccharomyces - ps28
Arboricola_clade - ps29
Cerevisiae_clade - ps30
Saccharomyces_cerevisiae - ps31
p-value
2e-13
8e-11
4e-08
2e-05
8e-03
Log-odds
0.5
1.0
1.5
2.0
2.5
3.0
3.5
4.0

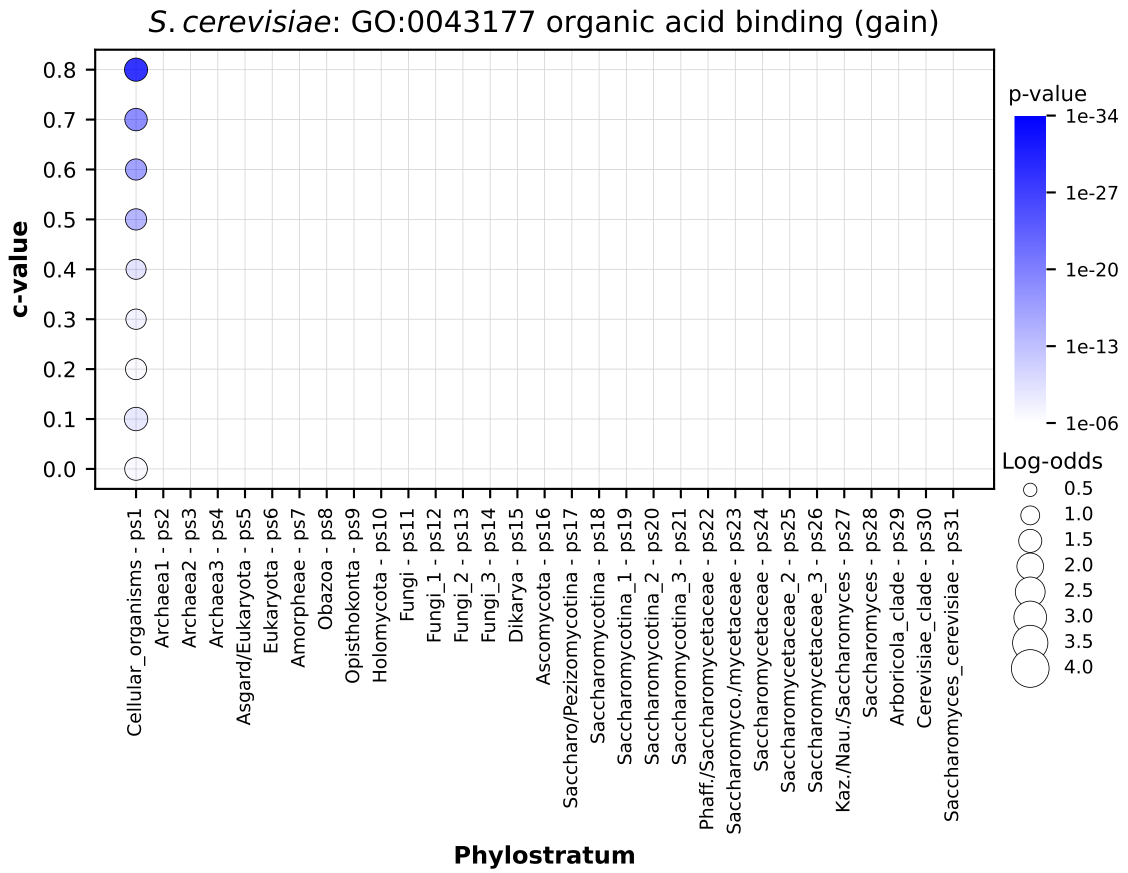

S. cerevisiae: GO:0043177 organic acid binding (gain)
c-value
0.0
0.1
0.2
0.3
0.4
0.5
0.6
0.7
0.8
p-value
1e-34
1e-27
1e-20
1e-13
1e-06
Log-odds
0.5
1.0
1.5
2.0
2.5
3.0
3.5
4.0
Cellular_organisms - ps1
Archaea1 - ps2
Archaea2 - ps3
Archaea3 - ps4
Asgard/Eukaryota - ps5
Eukaryota - ps6
Amorpheae - ps7
Obazoa - ps8
Opisthokonta - ps9
Holomycota - ps10
Fungi - ps11
Fungi_1 - ps12
Fungi_2 - ps13
Fungi_3 - ps14
Dikarya - ps15
Ascomycota - ps16
Saccharo/Pezizomycotina - ps17
Saccharomycotina - ps18
Saccharomycotina_1 - ps19
Saccharomycotina_2 - ps20
Saccharomycotina_3 - ps21
Phaff./Saccharomycetaceae - ps22
Saccharomyco./mycetaceae - ps23
Saccharomycetaceae - ps24
Saccharomycetaceae_2 - ps25
Saccharomycetaceae_3 - ps26
Kaz./Nau./Saccharomyces - ps27
Saccharomyces - ps28
Arboricola_clade - ps29
Cerevisiae_clade - ps30
Saccharomyces_cerevisiae - ps31
Phylostratum

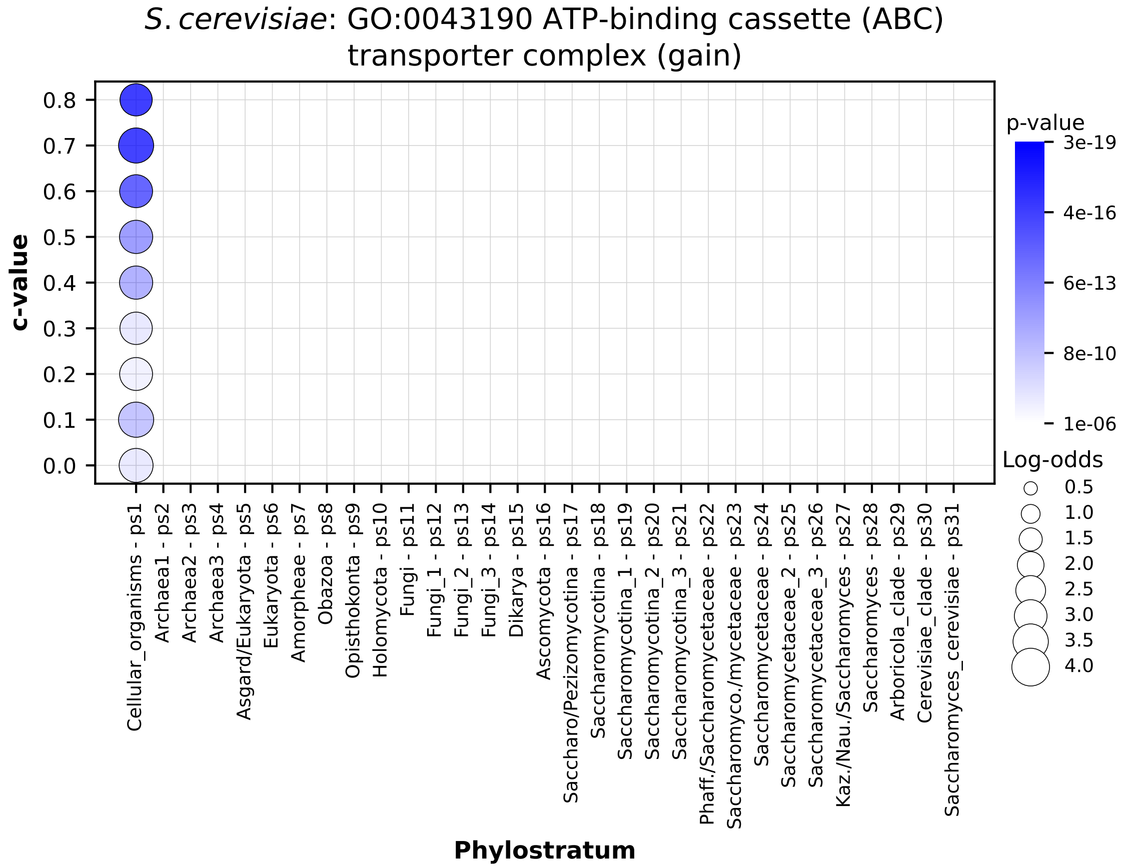

S. cerevisiae: GO:0043190 ATP-binding cassette (ABC) transporter complex (gain)
c-value
0.8
0.7
0.6
0.5
0.4
0.3
0.2
0.1
0.0
p-value
3e-19
4e-16
6e-13
8e-10
1e-06
Log-odds
0.5
1.0
1.5
2.0
2.5
3.0
3.5
4.0
Cellular_organisms - ps1
Archaea1 - ps2
Archaea2 - ps3
Archaea3 - ps4
Asgard/Eukaryota - ps5
Eukaryota - ps6
Amorpheae - ps7
Obazoa - ps8
Opisthokonta - ps9
Holomycota - ps10
Fungi - ps11
Fungi_1 - ps12
Fungi_2 - ps13
Fungi_3 - ps14
Dikarya - ps15
Ascomycota - ps16
Saccharo/Pezizomycotina - ps17
Saccharomycotina - ps18
Saccharomycotina_1 - ps19
Saccharomycotina_2 - ps20
Saccharomycotina_3 - ps21
Phaff./Saccharomycetaceae - ps22
Saccharomyco./mycetaceae - ps23
Saccharomycetaceae - ps24
Saccharomycetaceae_2 - ps25
Saccharomycetaceae_3 - ps26
Kaz./Nau./Saccharomyces - ps27
Saccharomyces - ps28
Arboricola_clade - ps29
Cerevisiae_clade - ps30
Saccharomyces_cerevisiae - ps31
Phylostratum

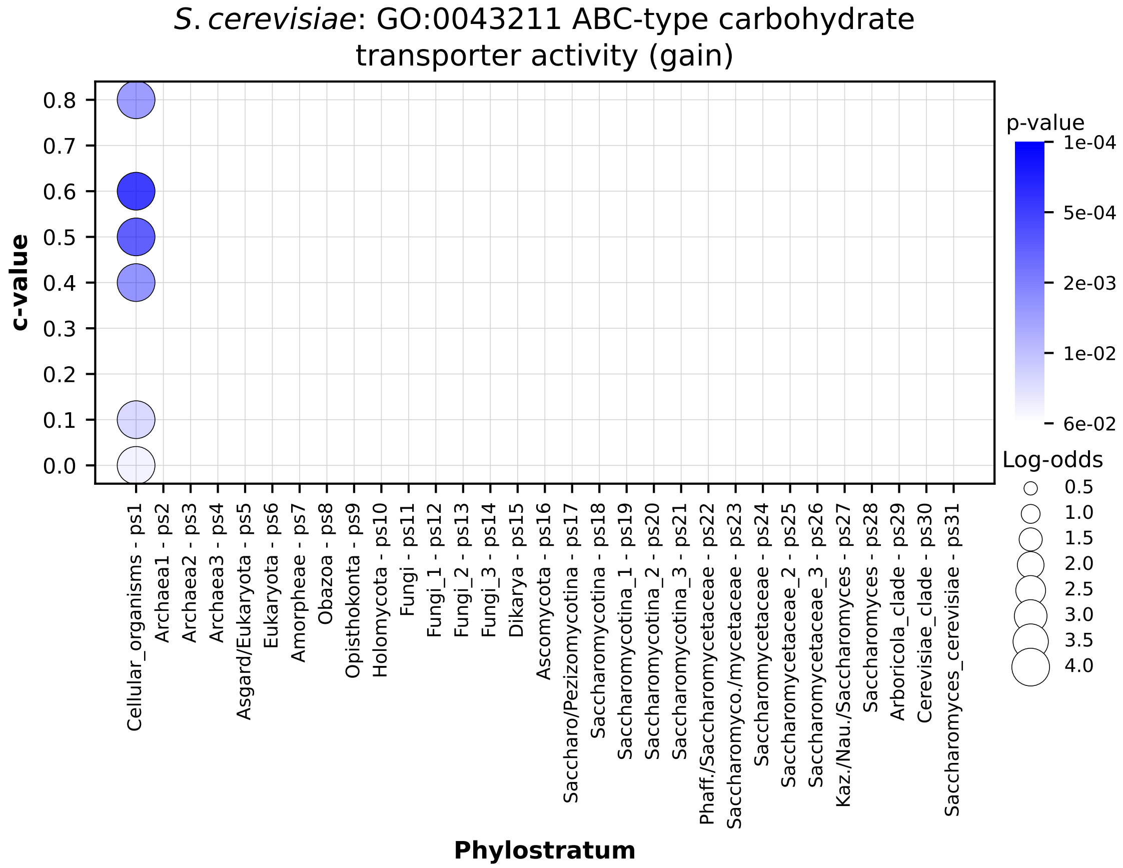

S. cerevisiae: GO:0043211 ABC-type carbohydrate transporter activity (gain)
c-value
0.8
0.7
0.6
0.5
0.4
0.3
0.2
0.1
0.0
p-value
1e-04
5e-04
2e-03
1e-02
6e-02
Log-odds
0.5
1.0
1.5
2.0
2.5
3.0
3.5
4.0
Cellular_organisms - ps1
Archaea1 - ps2
Archaea2 - ps3
Archaea3 - ps4
Asgard/Eukaryota - ps5
Eukaryota - ps6
Amorpheae - ps7
Obazoa - ps8
Opisthokonta - ps9
Holomycota - ps10
Fungi - ps11
Fungi_1 - ps12
Fungi_2 - ps13
Fungi_3 - ps14
Dikarya - ps15
Ascomycota - ps16
Saccharo/Pezizomycotina - ps17
Saccharomycotina - ps18
Saccharomycotina_1 - ps19
Saccharomycotina_2 - ps20
Saccharomycotina_3 - ps21
Phaff./Saccharomycetaceae - ps22
Saccharomyco./mycetaceae - ps23
Saccharomycetaceae - ps24
Saccharomycetaceae_2 - ps25
Saccharomycetaceae_3 - ps26
Kaz./Nau./Saccharomyces - ps27
Saccharomyces - ps28
Arboricola_clade - ps29
Cerevisiae_clade - ps30
Saccharomyces_cerevisiae - ps31
Phylostratum

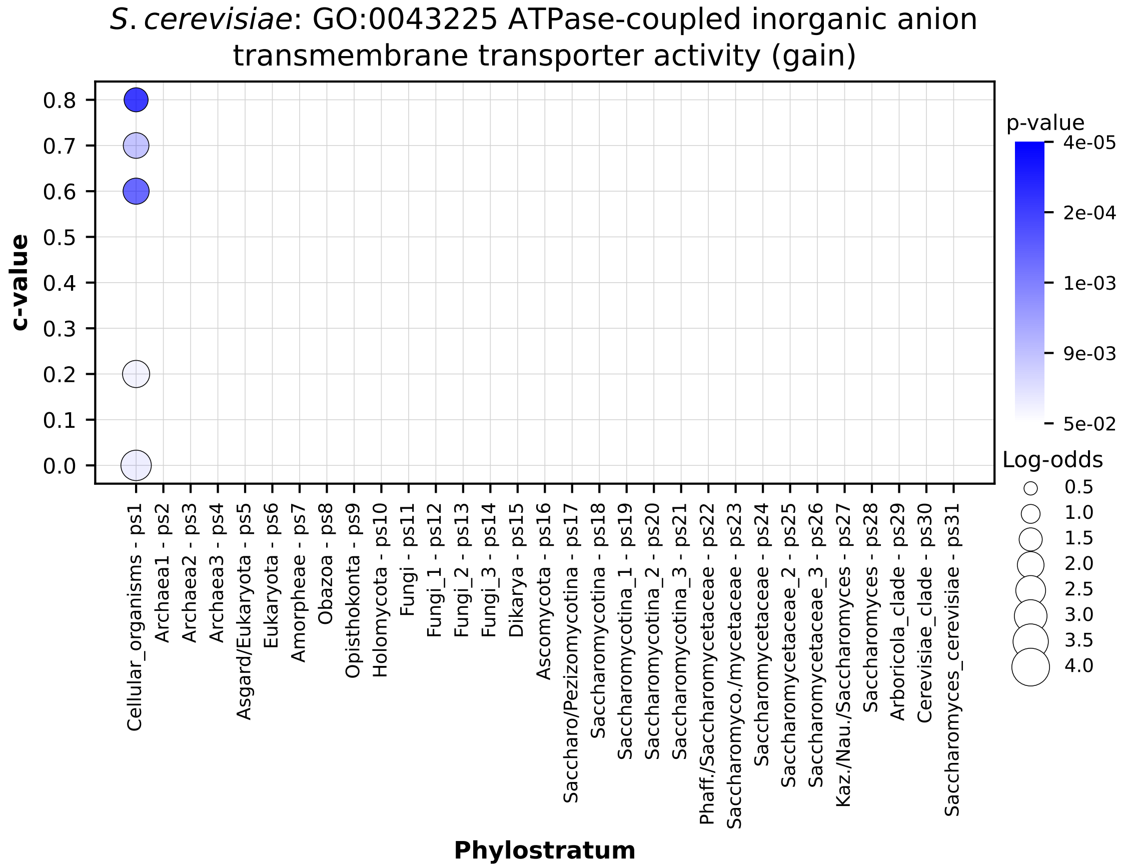
S. cerevisiae: GO:0043225 ATPase-coupled inorganic anion transmembrane transporter activity (gain)
c-value
0.8
0.7
0.6
0.5
0.4
0.3
0.2
0.1
0.0
p-value
4e-05
2e-04
1e-03
9e-03
5e-02
Log-odds
0.5
1.0
1.5
2.0
2.5
3.0
3.5
4.0
Cellular_organisms - ps1
Archaea1 - ps2
Archaea2 - ps3
Archaea3 - ps4
Asgard/Eukaryota - ps5
Eukaryota - ps6
Amorpheae - ps7
Obazoa - ps8
Opisthokonta - ps9
Holomycota - ps10
Fungi - ps11
Fungi_1 - ps12
Fungi_2 - ps13
Fungi_3 - ps14
Dikarya - ps15
Ascomycota - ps16
Saccharo/Pezizomycotina - ps17
Saccharomycotina - ps18
Saccharomycotina_1 - ps19
Saccharomycotina_2 - ps20
Saccharomycotina_3 - ps21
Phaff./Saccharomycetaceae - ps22
Saccharomyco./mycetaceae - ps23
Saccharomycetaceae - ps24
Saccharomycetaceae_2 - ps25
Saccharomycetaceae_3 - ps26
Kaz./Nau./Saccharomyces - ps27
Saccharomyces - ps28
Arboricola_clade - ps29
Cerevisiae_clade - ps30
Saccharomyces_cerevisiae - ps31
Phylostratum

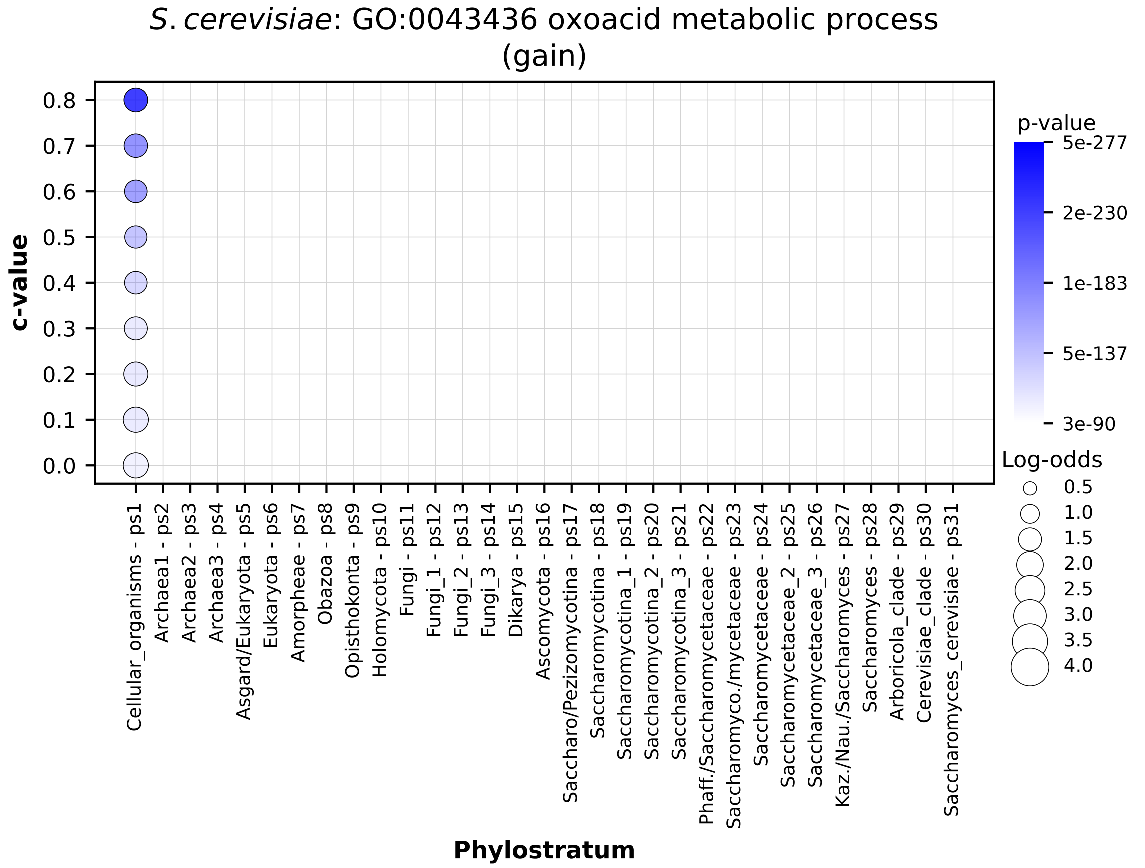

S. cerevisiae: GO:0043436 oxoacid metabolic process (gain)
c-value
0.8
0.7
0.6
0.5
0.4
0.3
0.2
0.1
0.0
p-value
5e-277
2e-230
1e-183
5e-137
3e-90
Log-odds
0.5
1.0
1.5
2.0
2.5
3.0
3.5
4.0
Cellular_organisms - ps1
Archaea1 - ps2
Archaea2 - ps3
Archaea3 - ps4
Asgard/Eukaryota - ps5
Eukaryota - ps6
Amorpheae - ps7
Obazoa - ps8
Opisthokonta - ps9
Holomycota - ps10
Fungi - ps11
Fungi_1 - ps12
Fungi_2 - ps13
Fungi_3 - ps14
Dikarya - ps15
Ascomycota - ps16
Saccharo/Pezizomycotina - ps17
Saccharomycotina - ps18
Saccharomycotina_1 - ps19
Saccharomycotina_2 - ps20
Saccharomycotina_3 - ps21
Phaff./Saccharomycetaceae - ps22
Saccharomyco./mycetaceae - ps23
Saccharomycetaceae - ps24
Saccharomycetaceae_2 - ps25
Saccharomycetaceae_3 - ps26
Kaz./Nau./Saccharomyces - ps27
Saccharomyces - ps28
Arboricola_clade - ps29
Cerevisiae_clade - ps30
Saccharomyces_cerevisiae - ps31
Phylostratum

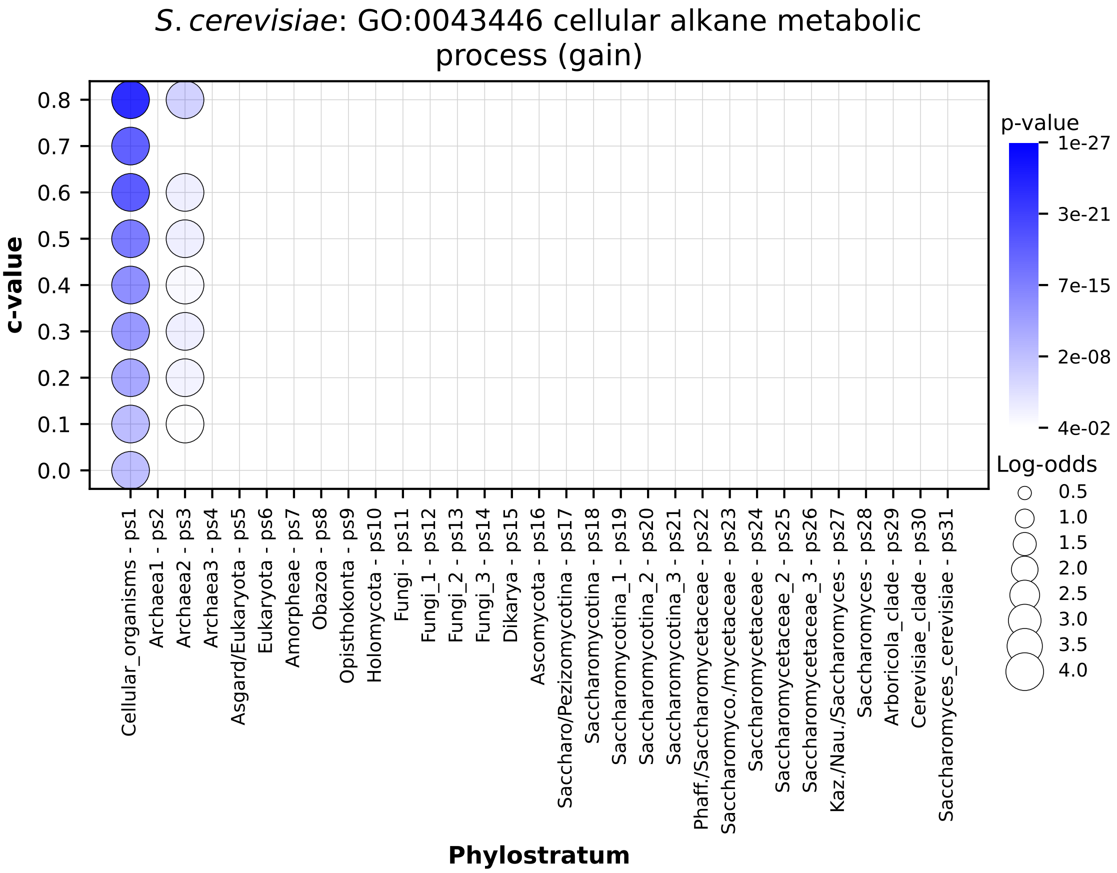
S. cerevisiae: GO:0043446 cellular alkane metabolic process (gain)
c-value
0.0
0.1
0.2
0.3
0.4
0.5
0.6
0.7
0.8
p-value
1e-27
3e-21
7e-15
2e-08
4e-02
Log-odds
0.5
1.0
1.5
2.0
2.5
3.0
3.5
4.0
Cellular_organisms - ps1
Archaea1 - ps2
Archaea2 - ps3
Archaea3 - ps4
Asgard/Eukaryota - ps5
Eukaryota - ps6
Amorpheae - ps7
Obazoa - ps8
Opisthokonta - ps9
Holomycota - ps10
Fungi - ps11
Fungi_1 - ps12
Fungi_2 - ps13
Fungi_3 - ps14
Dikarya - ps15
Ascomycota - ps16
Saccharo/Pezizomycotina - ps17
Saccharomycotina - ps18
Saccharomycotina_1 - ps19
Saccharomycotina_2 - ps20
Saccharomycotina_3 - ps21
Phaff./Saccharomycetaceae - ps22
Saccharomyco./mycetaceae - ps23
Saccharomycetaceae - ps24
Saccharomycetaceae_2 - ps25
Saccharomycetaceae_3 - ps26
Kaz./Nau./Saccharomyces - ps27
Saccharomyces - ps28
Arboricola_clade - ps29
Cerevisiae_clade - ps30
Saccharomyces_cerevisiae - ps31
Phylostratum

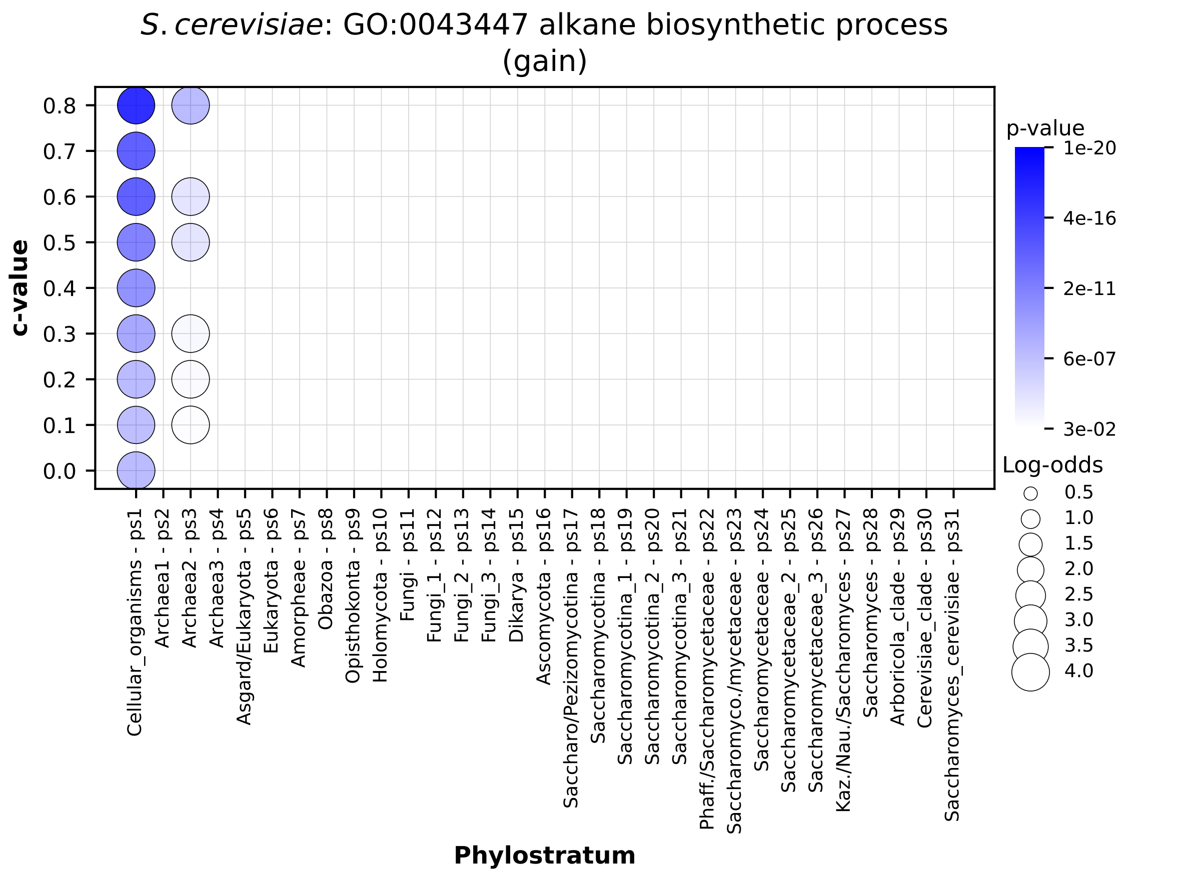

S. cerevisiae: GO:0043447 alkane biosynthetic process
(gain)
c-value
0.8
0.7
0.6
0.5
0.4
0.3
0.2
0.1
0.0
p-value
1e-20
4e-16
2e-11
6e-07
3e-02
Log-odds
0.5
1.0
1.5
2.0
2.5
3.0
3.5
4.0
Cellular_organisms - ps1
Archaea1 - ps2
Archaea2 - ps3
Archaea3 - ps4
Asgard/Eukaryota - ps5
Eukaryota - ps6
Amorpheae - ps7
Obazoa - ps8
Opisthokonta - ps9
Holomycota - ps10
Fungi - ps11
Fungi_1 - ps12
Fungi_2 - ps13
Fungi_3 - ps14
Dikarya - ps15
Ascomycota - ps16
Saccharo/Pezizomycotina - ps17
Saccharomycotina - ps18
Saccharomycotina_1 - ps19
Saccharomycotina_2 - ps20
Saccharomycotina_3 - ps21
Phaff./Saccharomycetaceae - ps22
Saccharomyco./mycetaceae - ps23
Saccharomycetaceae - ps24
Saccharomycetaceae_2 - ps25
Saccharomycetaceae_3 - ps26
Kaz./Nau./Saccharomyces - ps27
Saccharomyces - ps28
Arboricola_clade - ps29
Cerevisiae_clade - ps30
Saccharomyces_cerevisiae - ps31
Phylostratum

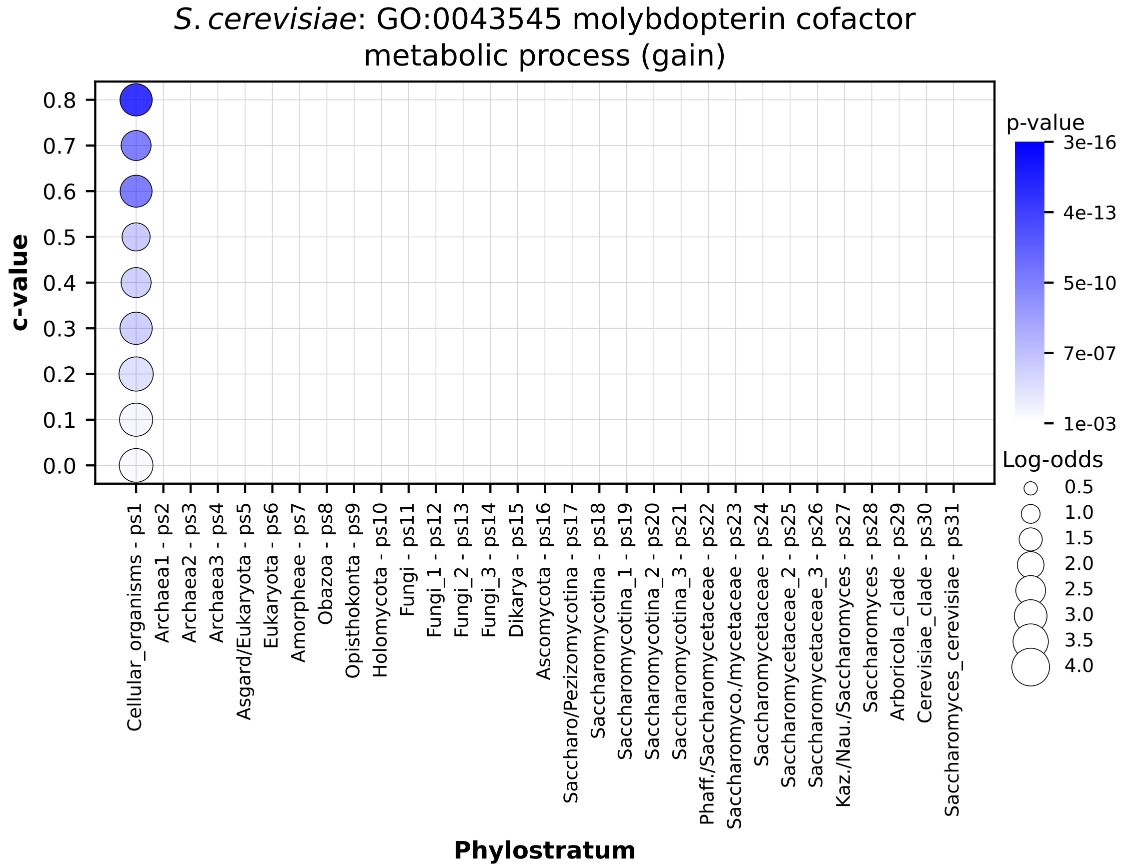

S. cerevisiae: GO:0043545 molybdopterin cofactor metabolic process (gain)
c-value
0.0
0.1
0.2
0.3
0.4
0.5
0.6
0.7
0.8
p-value
3e-16
4e-13
5e-10
7e-07
1e-03
Log-odds
0.5
1.0
1.5
2.0
2.5
3.0
3.5
4.0
Cellular_organisms - ps1
Archaea1 - ps2
Archaea2 - ps3
Archaea3 - ps4
Asgard/Eukaryota - ps5
Eukaryota - ps6
Amorpheae - ps7
Obazoa - ps8
Opisthokonta - ps9
Holomycota - ps10
Fungi - ps11
Fungi_1 - ps12
Fungi_2 - ps13
Fungi_3 - ps14
Dikarya - ps15
Ascomycota - ps16
Saccharo/Pezizomycotina - ps17
Saccharomycotina - ps18
Saccharomycotina_1 - ps19
Saccharomycotina_2 - ps20
Saccharomycotina_3 - ps21
Phaff./Saccharomycetaceae - ps22
Saccharomyco./mycetaceae - ps23
Saccharomycetaceae - ps24
Saccharomycetaceae_2 - ps25
Saccharomycetaceae_3 - ps26
Kaz./Nau./Saccharomyces - ps27
Saccharomyces - ps28
Arboricola_clade - ps29
Cerevisiae_clade - ps30
Saccharomyces_cerevisiae - ps31
Phylostratum

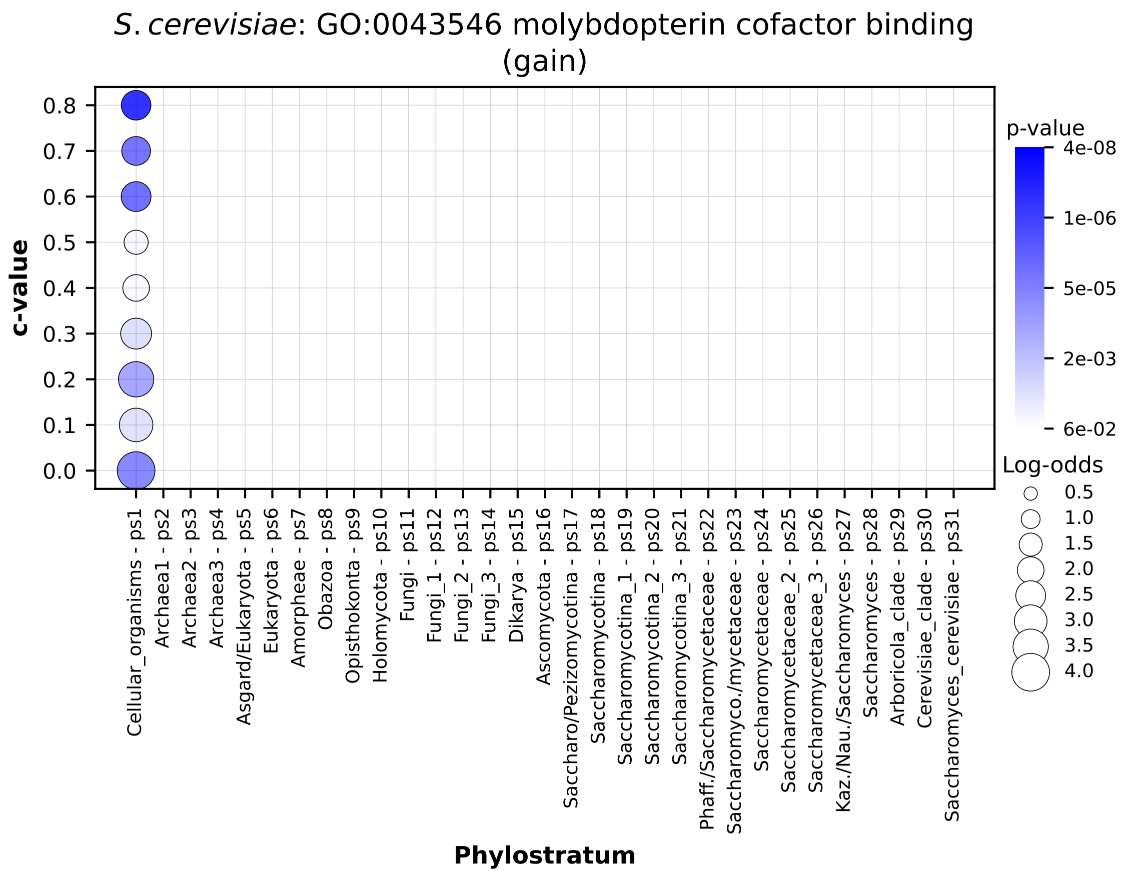

S. cerevisiae: GO:0043546 molybdopterin cofactor binding (gain)
c-value
0.8
0.7
0.6
0.5
0.4
0.3
0.2
0.1
0.0
p-value
4e-08
1e-06
5e-05
2e-03
6e-02
Log-odds
0.5
1.0
1.5
2.0
2.5
3.0
3.5
4.0
Cellular_organisms - ps1
Archaea1 - ps2
Archaea2 - ps3
Archaea3 - ps4
Asgard/Eukaryota - ps5
Eukaryota - ps6
Amorpheae - ps7
Obazoa - ps8
Opisthokonta - ps9
Holomycota - ps10
Fungi - ps11
Fungi_1 - ps12
Fungi_2 - ps13
Fungi_3 - ps14
Dikarya - ps15
Ascomycota - ps16
Saccharo/Pezizomycotina - ps17
Saccharomycotina - ps18
Saccharomycotina_1 - ps19
Saccharomycotina_2 - ps20
Saccharomycotina_3 - ps21
Phaff./Saccharomycetaceae - ps22
Saccharomyco./mycetaceae - ps23
Saccharomycetaceae - ps24
Saccharomycetaceae_2 - ps25
Saccharomycetaceae_3 - ps26
Kaz./Nau./Saccharomyces - ps27
Saccharomyces - ps28
Arboricola_clade - ps29
Cerevisiae_clade - ps30
Saccharomyces_cerevisiae - ps31
Phylostratum

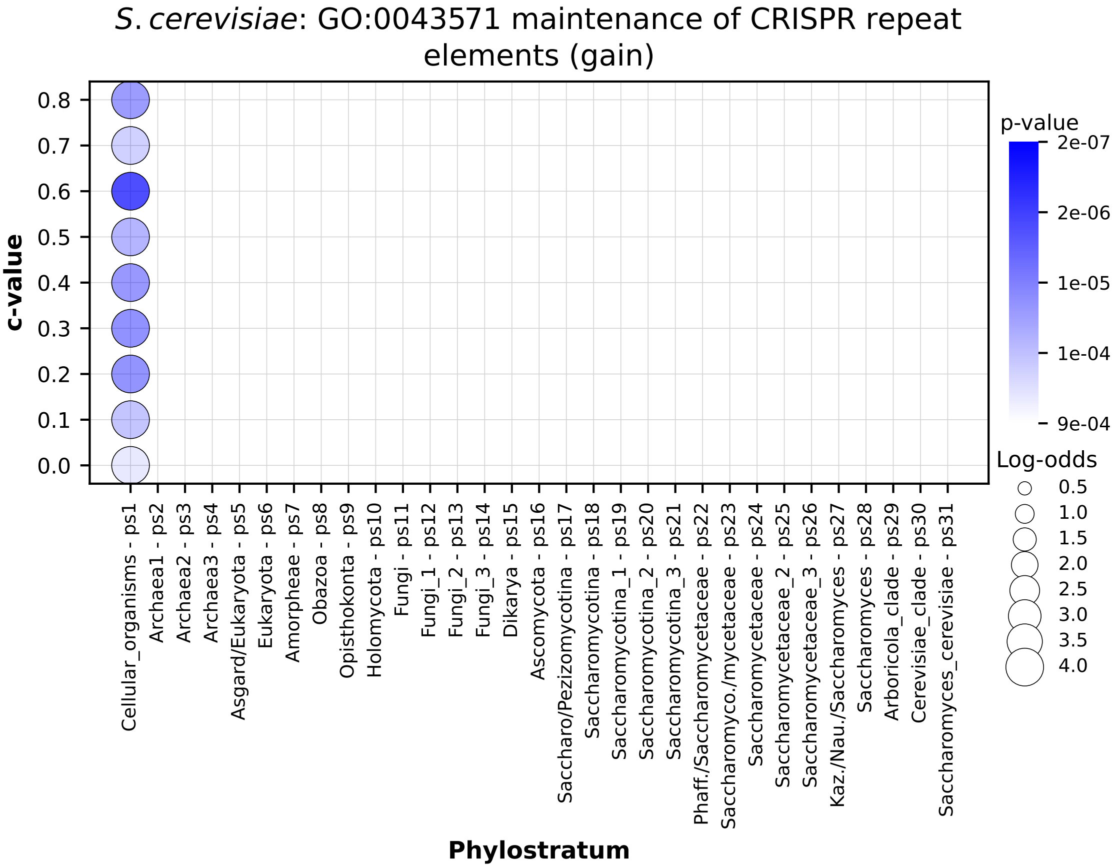

S. cerevisiae: GO:0043571 maintenance of CRISPR repeat elements (gain)
c-value
0.0
0.1
0.2
0.3
0.4
0.5
0.6
0.7
0.8
Phylostratum
Cellular_organisms - ps1
Archaea1 - ps2
Archaea2 - ps3
Archaea3 - ps4
Asgard/Eukaryota - ps5
Eukaryota - ps6
Amorpheae - ps7
Obazoa - ps8
Opisthokonta - ps9
Holomycota - ps10
Fungi - ps11
Fungi_1 - ps12
Fungi_2 - ps13
Fungi_3 - ps14
Dikarya - ps15
Ascomycota - ps16
Saccharo/Pezizomycotina - ps17
Saccharomycotina - ps18
Saccharomycotina_1 - ps19
Saccharomycotina_2 - ps20
Saccharomycotina_3 - ps21
Phaff./Saccharomycetaceae - ps22
Saccharomyco./mycetaceae - ps23
Saccharomycetaceae - ps24
Saccharomycetaceae_2 - ps25
Saccharomycetaceae_3 - ps26
Kaz./Nau./Saccharomyces - ps27
Saccharomyces - ps28
Arboricola_clade - ps29
Cerevisiae_clade - ps30
Saccharomyces_cerevisiae - ps31
p-value
2e-07
2e-06
1e-05
1e-04
9e-04
Log-odds
0.5
1.0
1.5
2.0
2.5
3.0
3.5
4.0

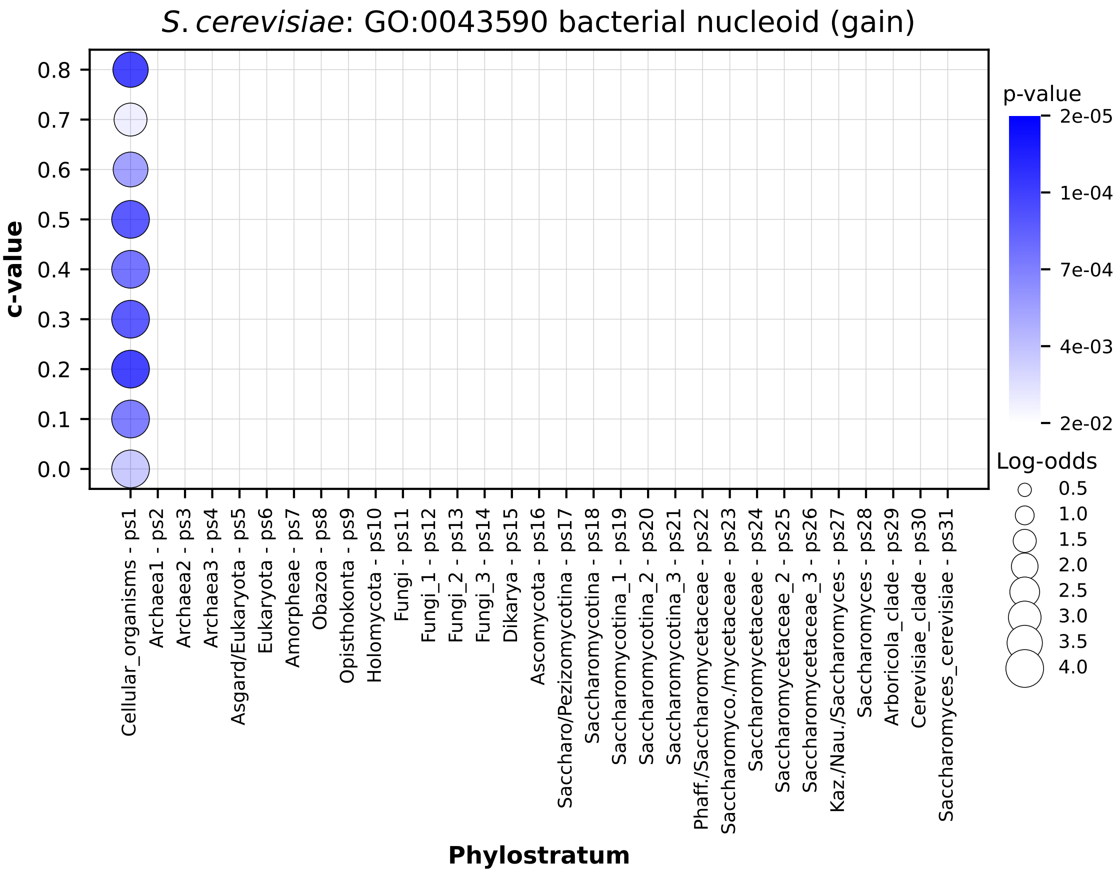

S. cerevisiae: GO:0043590 bacterial nucleoid (gain)
c-value
0.8
0.7
0.6
0.5
0.4
0.3
0.2
0.1
0.0
Phylostratum
Cellular_organisms - ps1
Archaea1 - ps2
Archaea2 - ps3
Archaea3 - ps4
Asgard/Eukaryota - ps5
Eukaryota - ps6
Amorpheae - ps7
Obazoa - ps8
Opisthokonta - ps9
Holomycota - ps10
Fungi - ps11
Fungi_1 - ps12
Fungi_2 - ps13
Fungi_3 - ps14
Dikarya - ps15
Ascomycota - ps16
Saccharo/Pezizomycotina - ps17
Saccharomycotina - ps18
Saccharomycotina_1 - ps19
Saccharomycotina_2 - ps20
Saccharomycotina_3 - ps21
Phaff./Saccharomycetaceae - ps22
Saccharomyco./mycetaceae - ps23
Saccharomycetaceae - ps24
Saccharomycetaceae_2 - ps25
Saccharomycetaceae_3 - ps26
Kaz./Nau./Saccharomyces - ps27
Saccharomyces - ps28
Arboricola_clade - ps29
Cerevisiae_clade - ps30
Saccharomyces_cerevisiae - ps31
p-value
2e-05
1e-04
7e-04
4e-03
2e-02
Log-odds
0.5
1.0
1.5
2.0
2.5
3.0
3.5
4.0

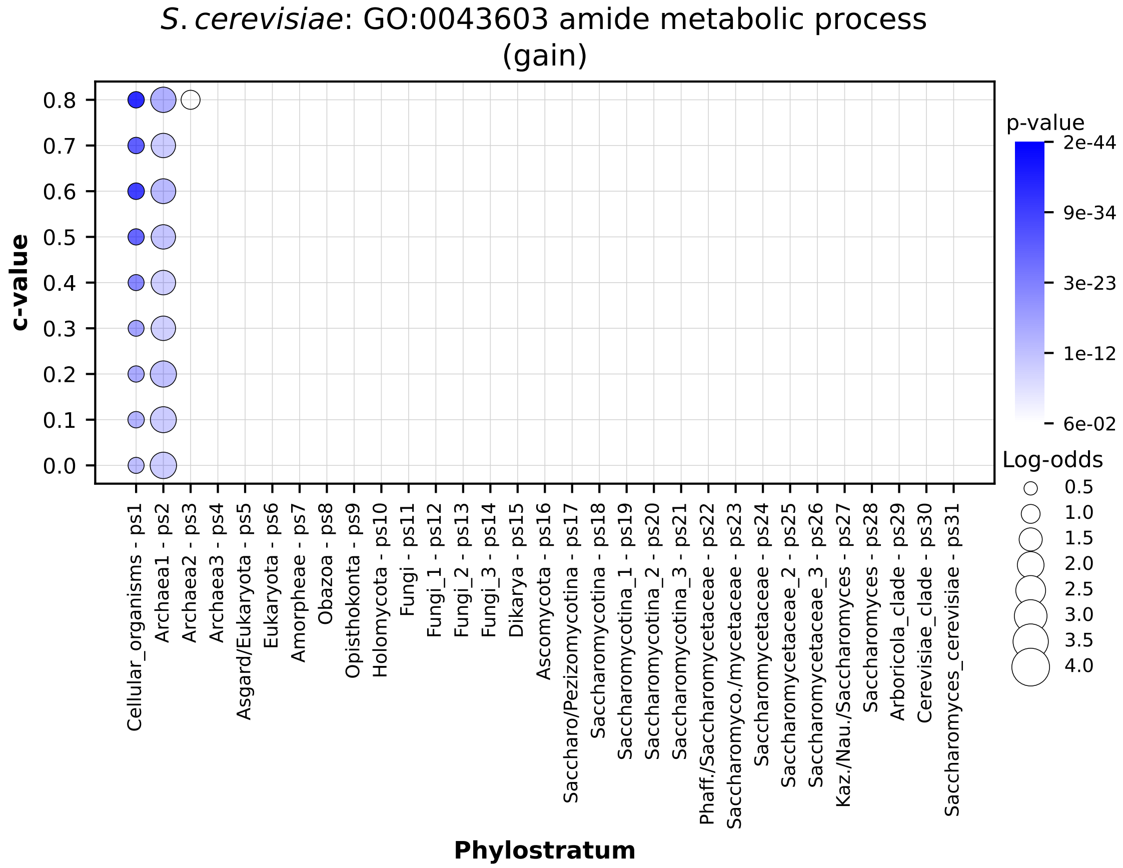

S. cerevisiae: GO:0043603 amide metabolic process
(gain)
c-value
0.8
0.7
0.6
0.5
0.4
0.3
0.2
0.1
0.0
p-value
2e-44
9e-34
3e-23
1e-12
6e-02
Log-odds
0.5
1.0
1.5
2.0
2.5
3.0
3.5
4.0
Cellular_organisms - ps1
Archaea1 - ps2
Archaea2 - ps3
Archaea3 - ps4
Asgard/Eukaryota - ps5
Eukaryota - ps6
Amorpheae - ps7
Obazoa - ps8
Opisthokonta - ps9
Holomycota - ps10
Fungi - ps11
Fungi_1 - ps12
Fungi_2 - ps13
Fungi_3 - ps14
Dikarya - ps15
Ascomycota - ps16
Saccharo/Pezizomycotina - ps17
Saccharomycotina - ps18
Saccharomycotina_1 - ps19
Saccharomycotina_2 - ps20
Saccharomycotina_3 - ps21
Phaff./Saccharomycetaceae - ps22
Saccharomyco./mycetaceae - ps23
Saccharomycetaceae - ps24
Saccharomycetaceae_2 - ps25
Saccharomycetaceae_3 - ps26
Kaz./Nau./Saccharomyces - ps27
Saccharomyces - ps28
Arboricola_clade - ps29
Cerevisiae_clade - ps30
Saccharomyces_cerevisiae - ps31
Phylostratum

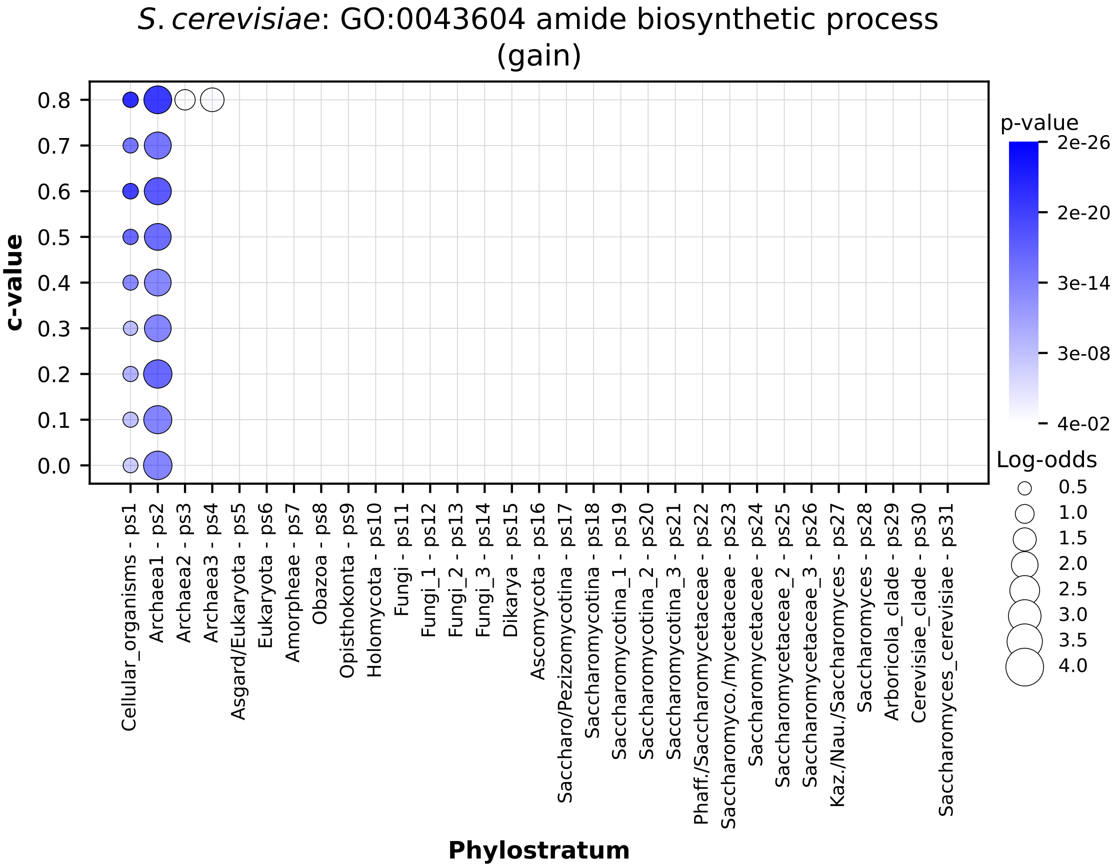

S. cerevisiae: GO:0043604 amide biosynthetic process
(gain)
c-value
0.8
0.7
0.6
0.5
0.4
0.3
0.2
0.1
0.0
p-value
2e-26
2e-20
3e-14
3e-08
4e-02
Log-odds
0.5
1.0
1.5
2.0
2.5
3.0
3.5
4.0
Cellular_organisms - ps1
Archaea1 - ps2
Archaea2 - ps3
Archaea3 - ps4
Asgard/Eukaryota - ps5
Eukaryota - ps6
Amorpheae - ps7
Obazoa - ps8
Opisthokonta - ps9
Holomycota - ps10
Fungi - ps11
Fungi_1 - ps12
Fungi_2 - ps13
Fungi_3 - ps14
Dikarya - ps15
Ascomycota - ps16
Saccharo/Pezizomycotina - ps17
Saccharomycotina - ps18
Saccharomycotina_1 - ps19
Saccharomycotina_2 - ps20
Saccharomycotina_3 - ps21
Phaff./Saccharomycetaceae - ps22
Saccharomyco./mycetaceae - ps23
Saccharomycetaceae - ps24
Saccharomycetaceae_2 - ps25
Saccharomycetaceae_3 - ps26
Kaz./Nau./Saccharomyces - ps27
Saccharomyces - ps28
Arboricola_clade - ps29
Cerevisiae_clade - ps30
Saccharomyces_cerevisiae - ps31
Phylostratum

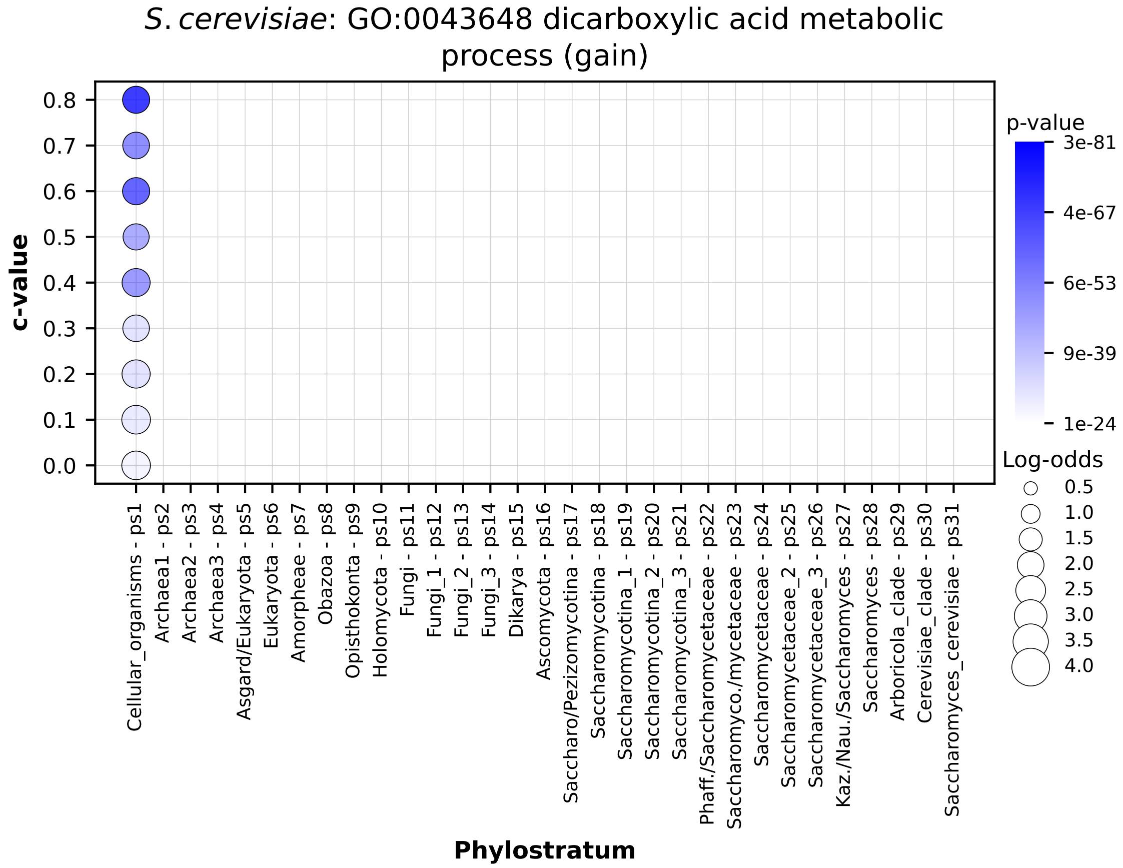
S. cerevisiae: GO:0043648 dicarboxylic acid metabolic process (gain)
c-value
0.8
0.7
0.6
0.5
0.4
0.3
0.2
0.1
0.0
p-value
3e-81
4e-67
6e-53
9e-39
1e-24
Log-odds
0.5
1.0
1.5
2.0
2.5
3.0
3.5
4.0
Cellular_organisms - ps1
Archaea1 - ps2
Archaea2 - ps3
Archaea3 - ps4
Asgard/Eukaryota - ps5
Eukaryota - ps6
Amorpheae - ps7
Obazoa - ps8
Opisthokonta - ps9
Holomycota - ps10
Fungi - ps11
Fungi_1 - ps12
Fungi_2 - ps13
Fungi_3 - ps14
Dikarya - ps15
Ascomycota - ps16
Saccharo/Pezizomycotina - ps17
Saccharomycotina - ps18
Saccharomycotina_1 - ps19
Saccharomycotina_2 - ps20
Saccharomycotina_3 - ps21
Phaff./Saccharomycetaceae - ps22
Saccharomyco./mycetaceae - ps23
Saccharomycetaceae - ps24
Saccharomycetaceae_2 - ps25
Saccharomycetaceae_3 - ps26
Kaz./Nau./Saccharomyces - ps27
Saccharomyces - ps28
Arboricola_clade - ps29
Cerevisiae_clade - ps30
Saccharomyces_cerevisiae - ps31
Phylostratum

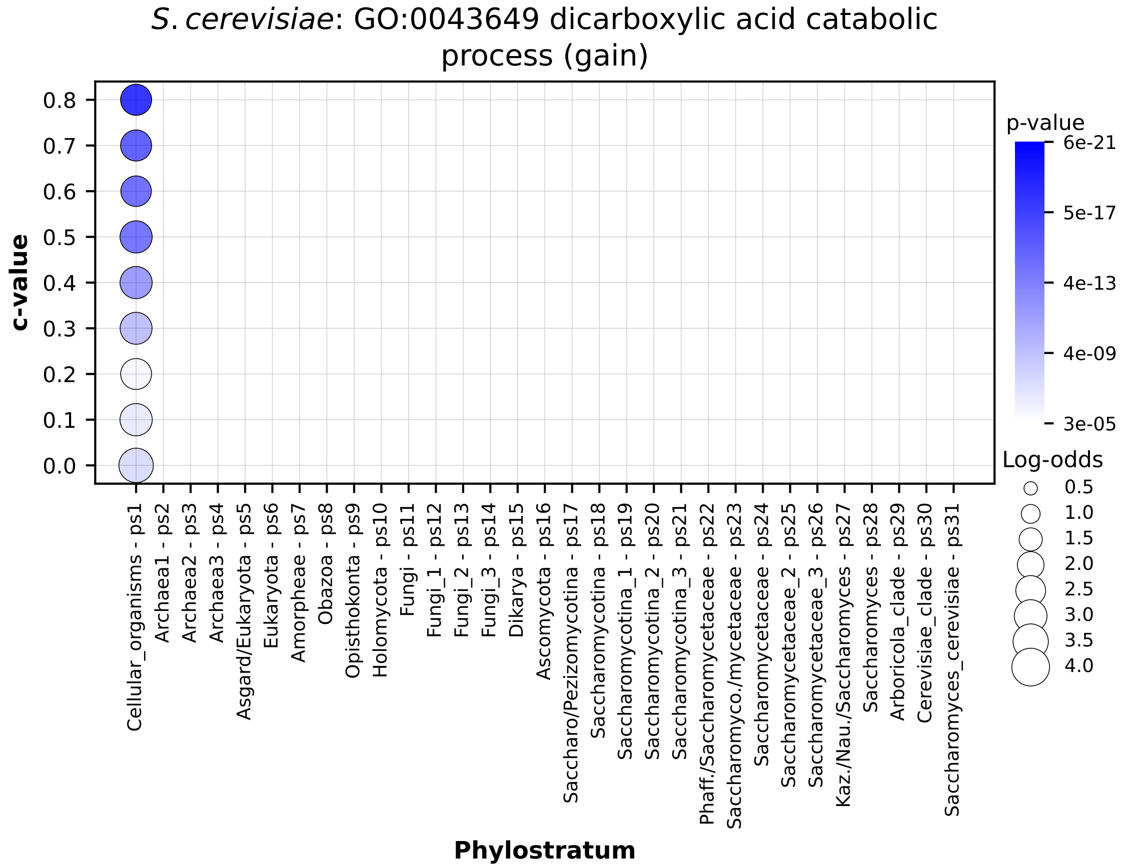

S. cerevisiae: GO:0043649 dicarboxylic acid catabolic process (gain)
c-value
0.8
0.7
0.6
0.5
0.4
0.3
0.2
0.1
0.0
p-value
6e-21
5e-17
4e-13
4e-09
3e-05
Log-odds
0.5
1.0
1.5
2.0
2.5
3.0
3.5
4.0
Cellular_organisms - ps1
Archaea1 - ps2
Archaea2 - ps3
Archaea3 - ps4
Asgard/Eukaryota - ps5
Eukaryota - ps6
Amorpheae - ps7
Obazoa - ps8
Opisthokonta - ps9
Holomycota - ps10
Fungi - ps11
Fungi_1 - ps12
Fungi_2 - ps13
Fungi_3 - ps14
Dikarya - ps15
Ascomycota - ps16
Saccharo/Pezizomycotina - ps17
Saccharomycotina - ps18
Saccharomycotina_1 - ps19
Saccharomycotina_2 - ps20
Saccharomycotina_3 - ps21
Phaff./Saccharomycetaceae - ps22
Saccharomyco./mycetaceae - ps23
Saccharomycetaceae - ps24
Saccharomycetaceae_2 - ps25
Saccharomycetaceae_3 - ps26
Kaz./Nau./Saccharomyces - ps27
Saccharomyces - ps28
Arboricola_clade - ps29
Cerevisiae_clade - ps30
Saccharomyces_cerevisiae - ps31
Phylostratum

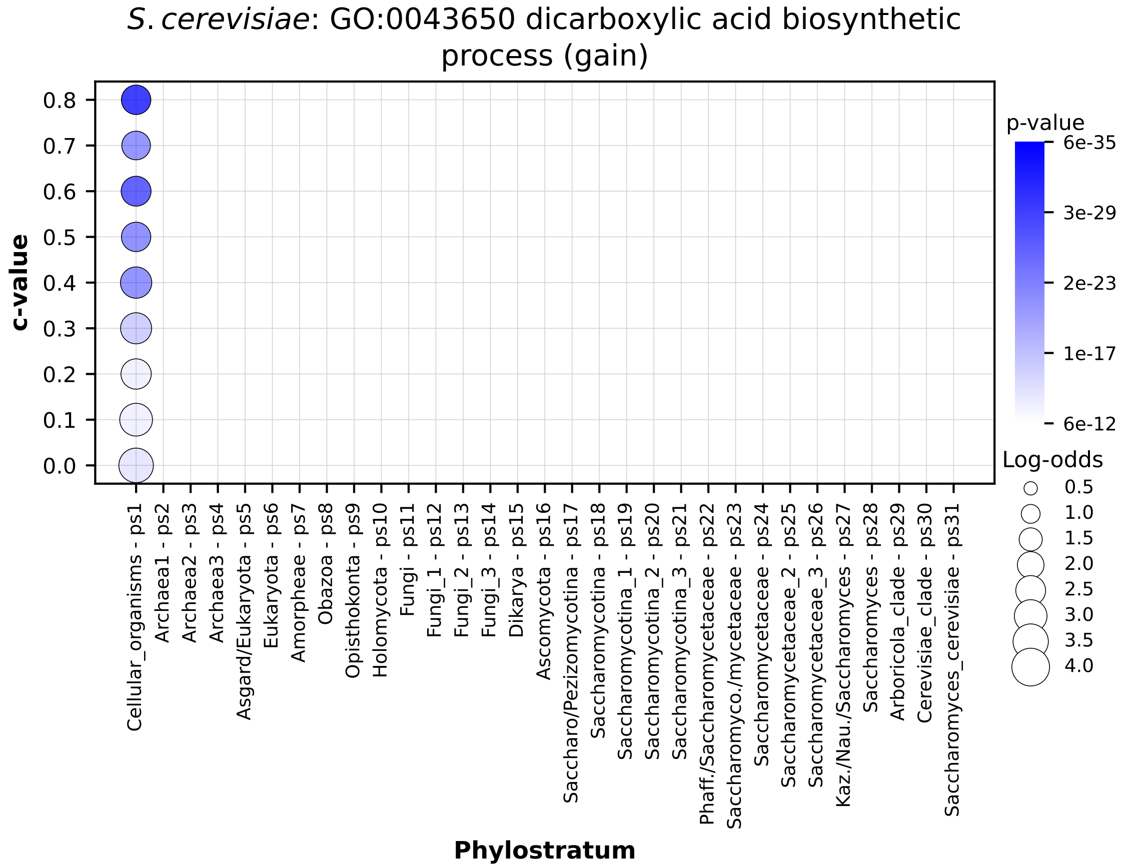
S. cerevisiae: GO:0043650 dicarboxylic acid biosynthetic process (gain)
c-value
0.0
0.1
0.2
0.3
0.4
0.5
0.6
0.7
0.8
Phylostratum
Cellular_organisms - ps1
Archaea1 - ps2
Archaea2 - ps3
Archaea3 - ps4
Asgard/Eukaryota - ps5
Eukaryota - ps6
Amorpheae - ps7
Obazoa - ps8
Opisthokonta - ps9
Holomycota - ps10
Fungi - ps11
Fungi_1 - ps12
Fungi_2 - ps13
Fungi_3 - ps14
Dikarya - ps15
Ascomycota - ps16
Saccharo/Pezizomycotina - ps17
Saccharomycotina - ps18
Saccharomycotina_1 - ps19
Saccharomycotina_2 - ps20
Saccharomycotina_3 - ps21
Phaff./Saccharomycetaceae - ps22
Saccharomyco./mycetaceae - ps23
Saccharomycetaceae - ps24
Saccharomycetaceae_2 - ps25
Saccharomycetaceae_3 - ps26
Kaz./Nau./Saccharomyces - ps27
Saccharomyces - ps28
Arboricola_clade - ps29
Cerevisiae_clade - ps30
Saccharomyces_cerevisiae - ps31
p-value
6e-35
3e-29
2e-23
1e-17
6e-12
Log-odds
0.5
1.0
1.5
2.0
2.5
3.0
3.5
4.0

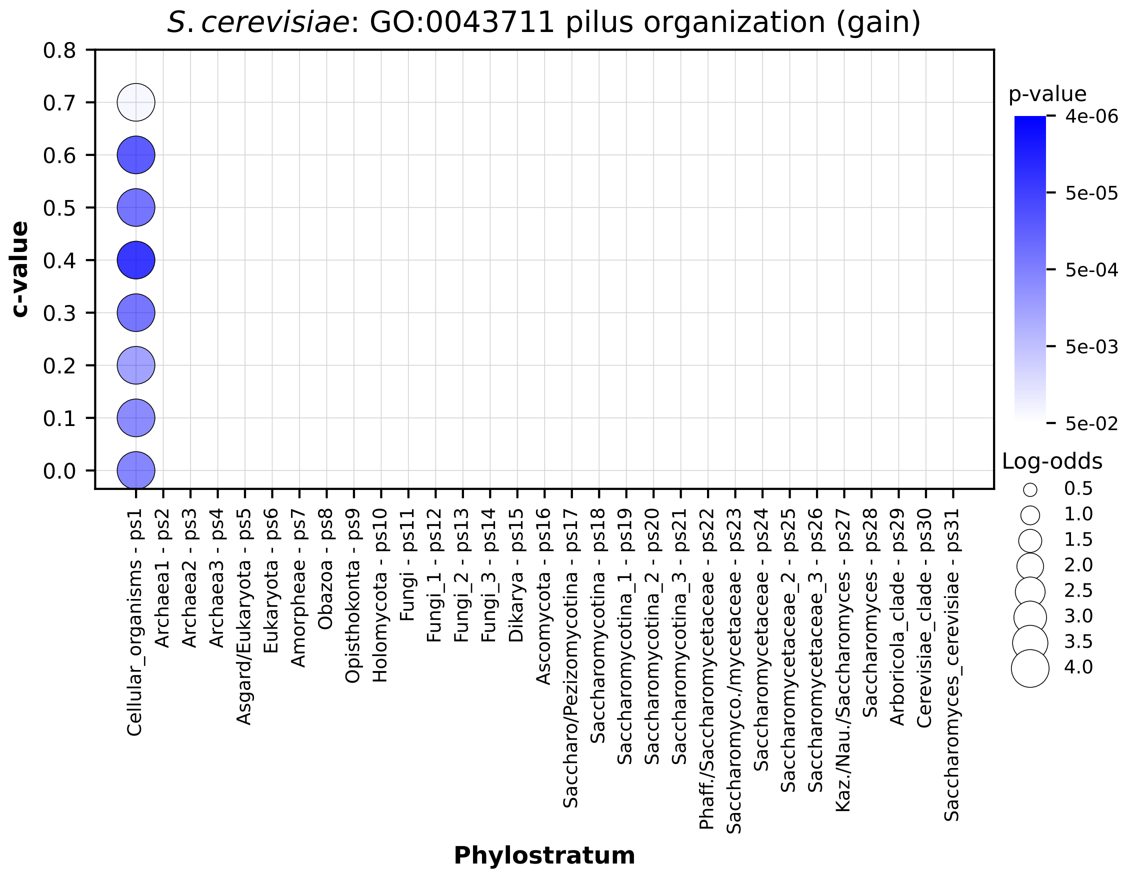
S. cerevisiae: GO:0043711 pilus organization (gain)
c-value
0.0
0.1
0.2
0.3
0.4
0.5
0.6
0.7
0.8
p-value
4e-06
5e-05
5e-04
5e-03
5e-02
Log-odds
0.5
1.0
1.5
2.0
2.5
3.0
3.5
4.0
Cellular_organisms - ps1
Archaea1 - ps2
Archaea2 - ps3
Archaea3 - ps4
Asgard/Eukaryota - ps5
Eukaryota - ps6
Amorpheae - ps7
Obazoa - ps8
Opisthokonta - ps9
Holomycota - ps10
Fungi - ps11
Fungi_1 - ps12
Fungi_2 - ps13
Fungi_3 - ps14
Dikarya - ps15
Ascomycota - ps16
Saccharo/Pezizomycotina - ps17
Saccharomycotina - ps18
Saccharomycotina_1 - ps19
Saccharomycotina_2 - ps20
Saccharomycotina_3 - ps21
Phaff./Saccharomycetaceae - ps22
Saccharomyco./mycetaceae - ps23
Saccharomycetaceae - ps24
Saccharomycetaceae_2 - ps25
Saccharomycetaceae_3 - ps26
Kaz./Nau./Saccharomyces - ps27
Saccharomyces - ps28
Arboricola_clade - ps29
Cerevisiae_clade - ps30
Saccharomyces_cerevisiae - ps31
Phylostratum

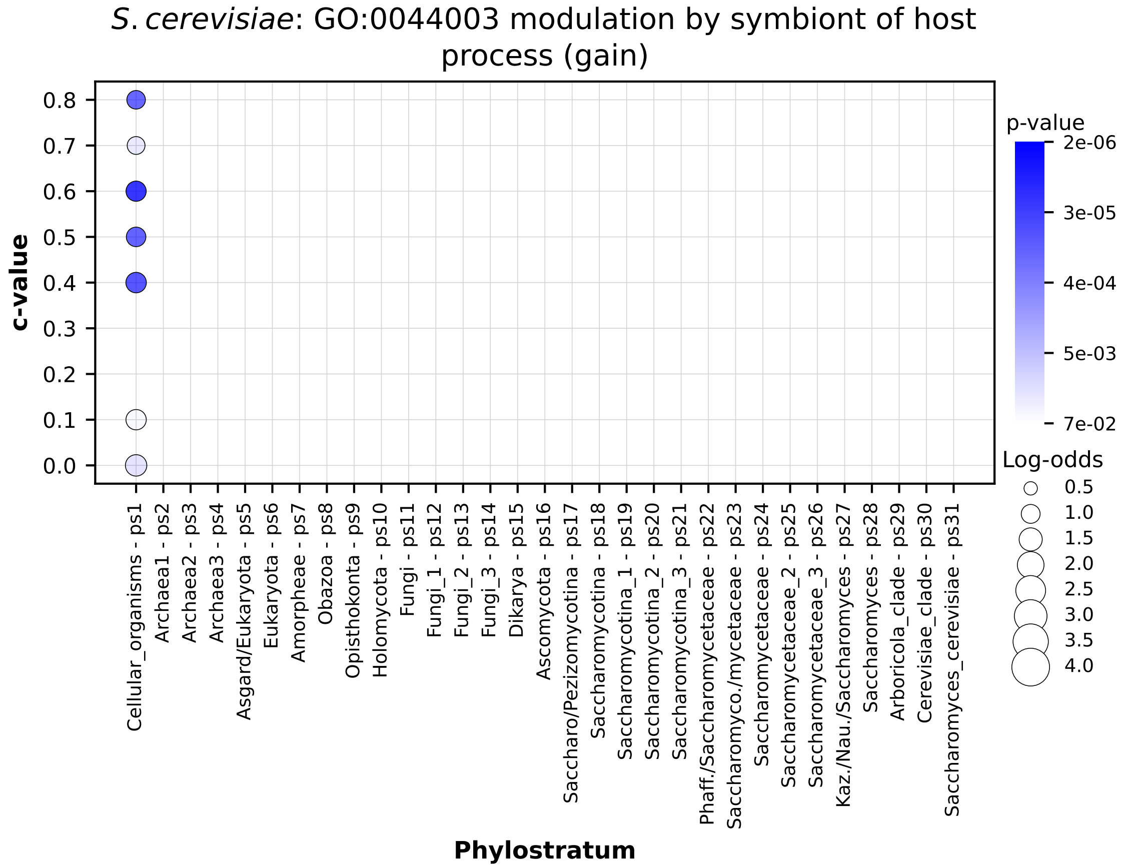
S. cerevisiae: GO:0044003 modulation by symbiont of host process (gain)
c-value
0.8
0.7
0.6
0.5
0.4
0.3
0.2
0.1
0.0
p-value
2e-06
3e-05
4e-04
5e-03
7e-02
Log-odds
0.5
1.0
1.5
2.0
2.5
3.0
3.5
4.0
Cellular_organisms - ps1
Archaea1 - ps2
Archaea2 - ps3
Archaea3 - ps4
Asgard/Eukaryota - ps5
Eukaryota - ps6
Amorpheae - ps7
Obazoa - ps8
Opisthokonta - ps9
Holomycota - ps10
Fungi - ps11
Fungi_1 - ps12
Fungi_2 - ps13
Fungi_3 - ps14
Dikarya - ps15
Ascomycota - ps16
Saccharo/Pezizomycotina - ps17
Saccharomycotina - ps18
Saccharomycotina_1 - ps19
Saccharomycotina_2 - ps20
Saccharomycotina_3 - ps21
Phaff./Saccharomycetaceae - ps22
Saccharomyco./mycetaceae - ps23
Saccharomycetaceae - ps24
Saccharomycetaceae_2 - ps25
Saccharomycetaceae_3 - ps26
Kaz./Nau./Saccharomyces - ps27
Saccharomyces - ps28
Arboricola_clade - ps29
Cerevisiae_clade - ps30
Saccharomyces_cerevisiae - ps31
Phylostratum

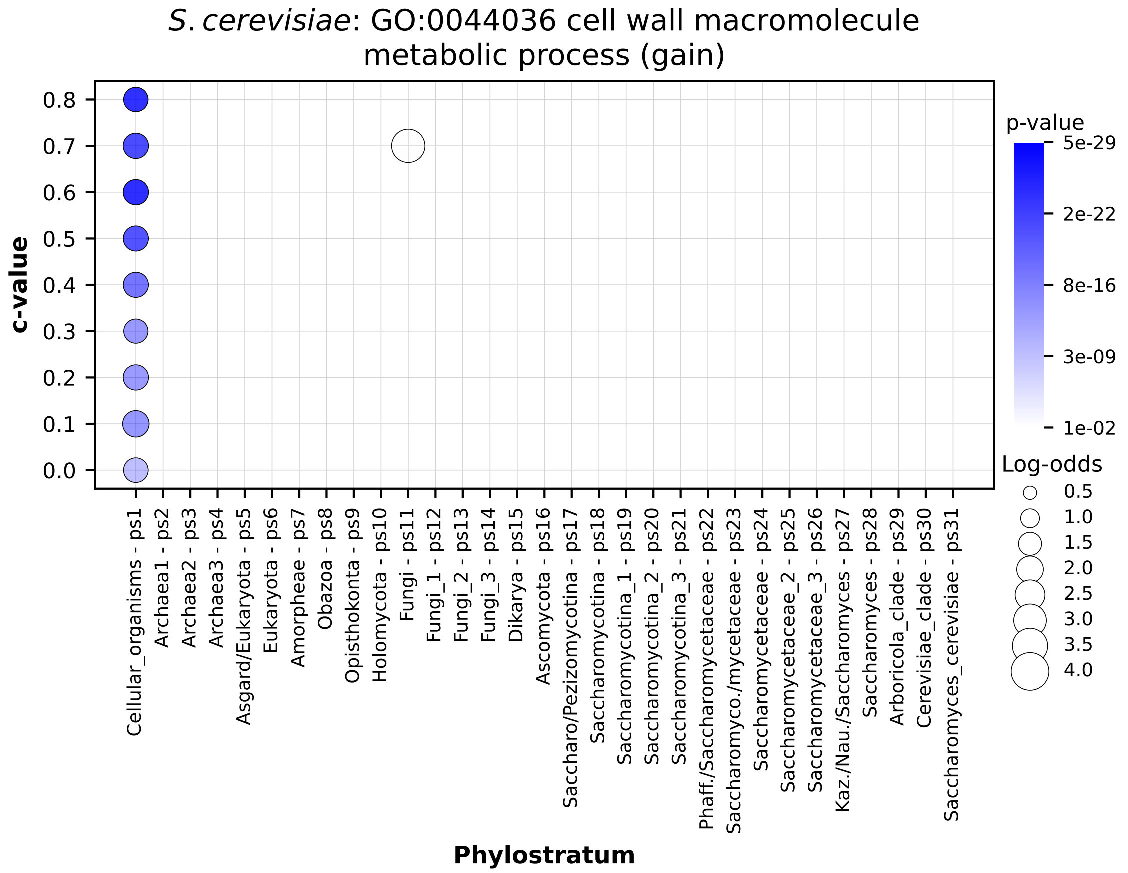

S. cerevisiae: GO:0044036 cell wall macromolecule metabolic process (gain)
c-value
0.8
0.7
0.6
0.5
0.4
0.3
0.2
0.1
0.0
p-value
5e-29
2e-22
8e-16
3e-09
1e-02
Log-odds
0.5
1.0
1.5
2.0
2.5
3.0
3.5
4.0
Cellular_organisms - ps1
Archaea1 - ps2
Archaea2 - ps3
Archaea3 - ps4
Asgard/Eukaryota - ps5
Eukaryota - ps6
Amorpheae - ps7
Obazoa - ps8
Opisthokonta - ps9
Holomycota - ps10
Fungi - ps11
Fungi_1 - ps12
Fungi_2 - ps13
Fungi_3 - ps14
Dikarya - ps15
Ascomycota - ps16
Saccharo/Pezizomycotina - ps17
Saccharomycotina - ps18
Saccharomycotina_1 - ps19
Saccharomycotina_2 - ps20
Saccharomycotina_3 - ps21
Phaff./Saccharomycetaceae - ps22
Saccharomyco./mycetaceae - ps23
Saccharomycetaceae - ps24
Saccharomycetaceae_2 - ps25
Saccharomycetaceae_3 - ps26
Kaz./Nau./Saccharomyces - ps27
Saccharomyces - ps28
Arboricola_clade - ps29
Cerevisiae_clade - ps30
Saccharomyces_cerevisiae - ps31
Phylostratum

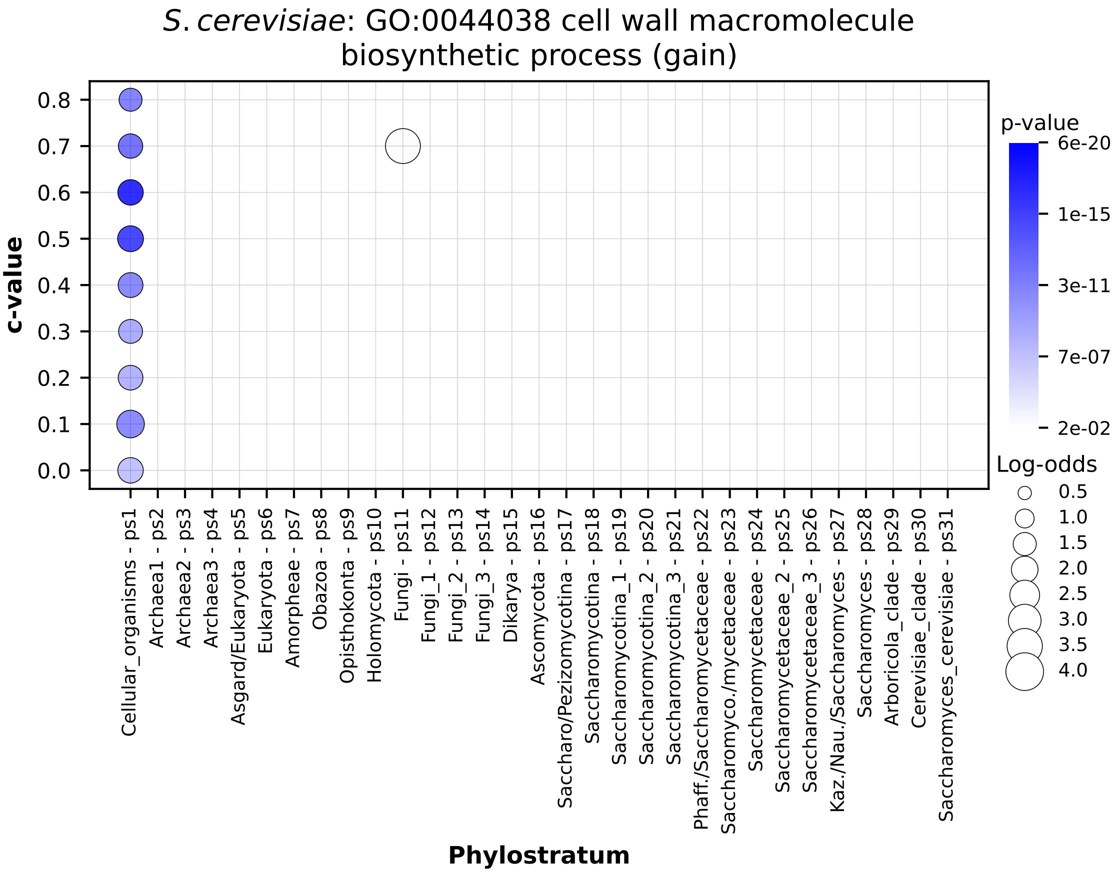

S. cerevisiae: GO:0044038 cell wall macromolecule biosynthetic process (gain)
c-value
0.8
0.7
0.6
0.5
0.4
0.3
0.2
0.1
0.0
p-value
6e-20
1e-15
3e-11
7e-07
2e-02
Log-odds
0.5
1.0
1.5
2.0
2.5
3.0
3.5
4.0
Cellular_organisms - ps1
Archaea1 - ps2
Archaea2 - ps3
Archaea3 - ps4
Asgard/Eukaryota - ps5
Eukaryota - ps6
Amorpheae - ps7
Obazoa - ps8
Opisthokonta - ps9
Holomycota - ps10
Fungi - ps11
Fungi_1 - ps12
Fungi_2 - ps13
Fungi_3 - ps14
Dikarya - ps15
Ascomycota - ps16
Saccharo/Pezizomycotina - ps17
Saccharomycotina - ps18
Saccharomycotina_1 - ps19
Saccharomycotina_2 - ps20
Saccharomycotina_3 - ps21
Phaff./Saccharomycetaceae - ps22
Saccharomyco./mycetaceae - ps23
Saccharomycetaceae - ps24
Saccharomycetaceae_2 - ps25
Saccharomycetaceae_3 - ps26
Kaz./Nau./Saccharomyces - ps27
Saccharomyces - ps28
Arboricola_clade - ps29
Cerevisiae_clade - ps30
Saccharomyces_cerevisiae - ps31
Phylostratum

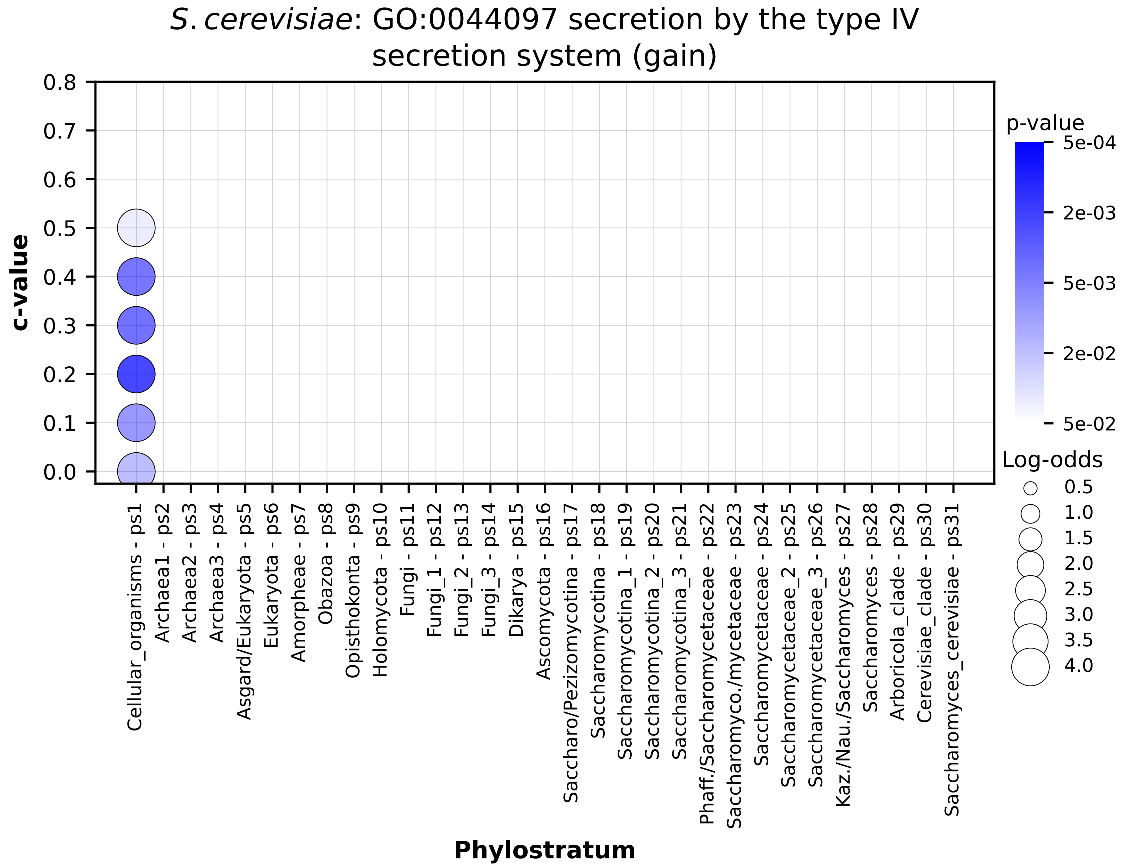

S. cerevisiae: GO:0044097 secretion by the type IV secretion system (gain)
c-value
0.8
0.7
0.6
0.5
0.4
0.3
0.2
0.1
0.0
p-value
5e-04
2e-03
5e-03
2e-02
5e-02
Log-odds
0.5
1.0
1.5
2.0
2.5
3.0
3.5
4.0
Cellular_organisms - ps1
Archaea1 - ps2
Archaea2 - ps3
Archaea3 - ps4
Asgard/Eukaryota - ps5
Eukaryota - ps6
Amorpheae - ps7
Obazoa - ps8
Opisthokonta - ps9
Holomycota - ps10
Fungi - ps11
Fungi_1 - ps12
Fungi_2 - ps13
Fungi_3 - ps14
Dikarya - ps15
Ascomycota - ps16
Saccharo/Pezizomycotina - ps17
Saccharomycotina - ps18
Saccharomycotina_1 - ps19
Saccharomycotina_2 - ps20
Saccharomycotina_3 - ps21
Phaff./Saccharomycetaceae - ps22
Saccharomyco./mycetaceae - ps23
Saccharomycetaceae - ps24
Saccharomycetaceae_2 - ps25
Saccharomycetaceae_3 - ps26
Kaz./Nau./Saccharomyces - ps27
Saccharomyces - ps28
Arboricola_clade - ps29
Cerevisiae_clade - ps30
Saccharomyces_cerevisiae - ps31
Phylostratum

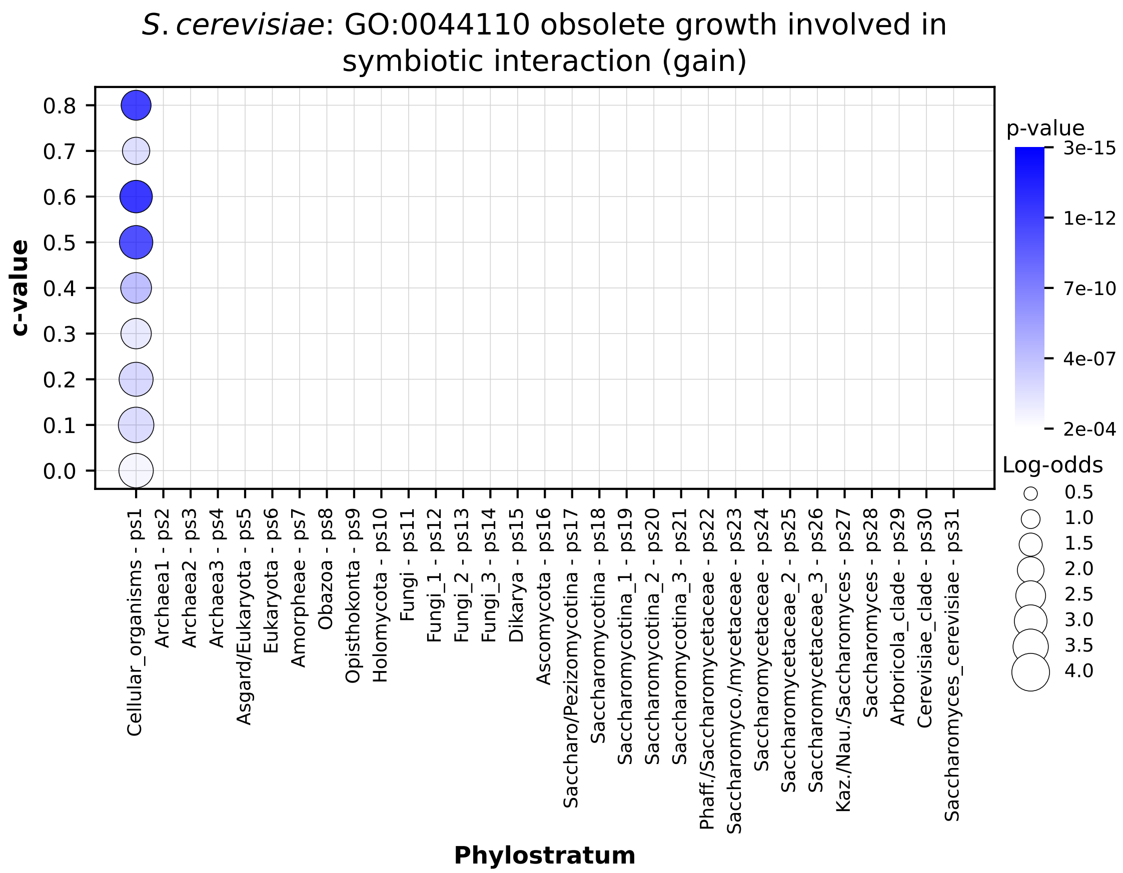
$S. cerevisiae$: GO:0044110 obsolete growth involved in symbiotic interaction (gain)
c-value
0.8
0.7
0.6
0.5
0.4
0.3
0.2
0.1
0.0
p-value
3e-15
1e-12
7e-10
4e-07
2e-04
Log-odds
0.5
1.0
1.5
2.0
2.5
3.0
3.5
4.0
Cellular_organisms - ps1
Archaea1 - ps2
Archaea2 - ps3
Archaea3 - ps4
Asgard/Eukaryota - ps5
Eukaryota - ps6
Amorpheae - ps7
Obazoa - ps8
Opisthokonta - ps9
Holomycota - ps10
Fungi - ps11
Fungi_1 - ps12
Fungi_2 - ps13
Fungi_3 - ps14
Dikarya - ps15
Ascomycota - ps16
Saccharo/Pezizomycotina - ps17
Saccharomycotina - ps18
Saccharomycotina_1 - ps19
Saccharomycotina_2 - ps20
Saccharomycotina_3 - ps21
Phaff./Saccharomycetaceae - ps22
Saccharomyco./mycetaceae - ps23
Saccharomycetaceae - ps24
Saccharomycetaceae_2 - ps25
Saccharomycetaceae_3 - ps26
Kaz./Nau./Saccharomyces - ps27
Saccharomyces - ps28
Arboricola_clade - ps29
Cerevisiae_clade - ps30
Saccharomyces_cerevisiae - ps31
Phylostratum

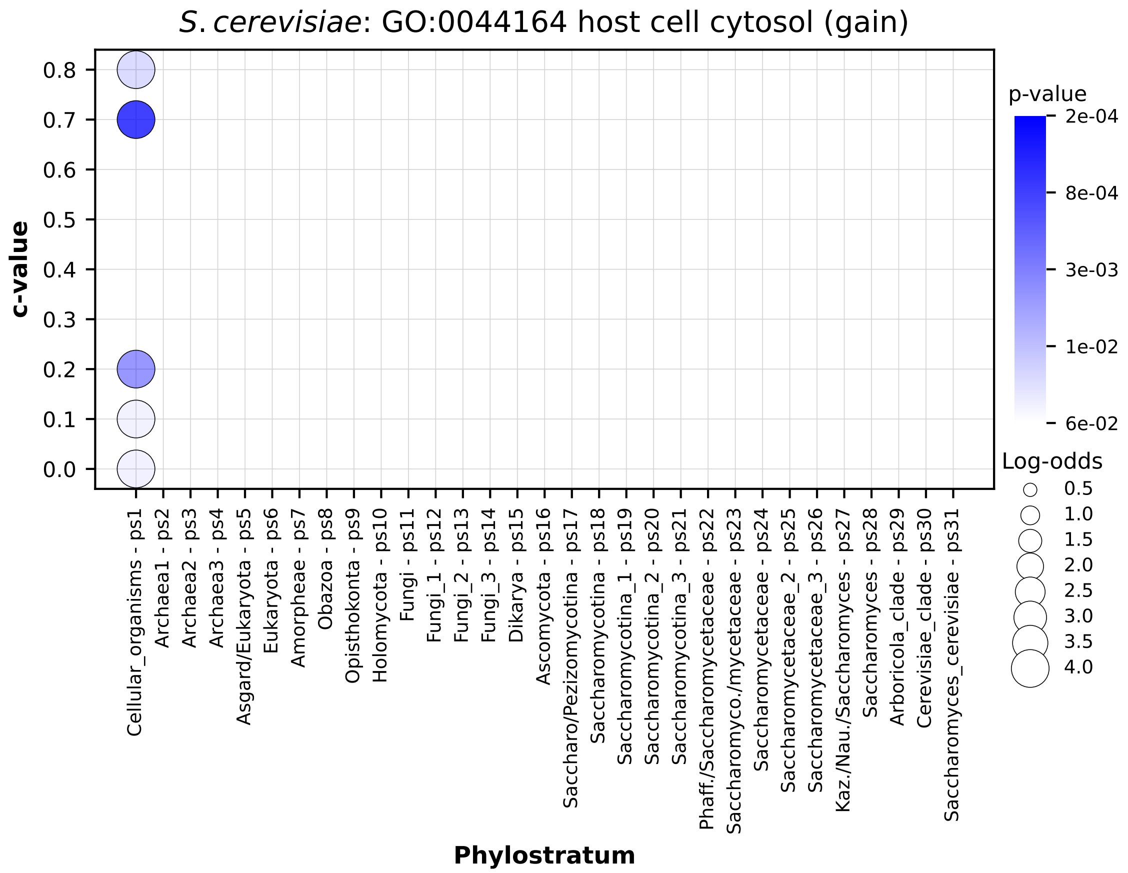

S. cerevisiae: GO:0044164 host cell cytosol (gain)
c-value
0.8
0.7
0.6
0.5
0.4
0.3
0.2
0.1
0.0
Phylostratum
Cellular_organisms - ps1
Archaea1 - ps2
Archaea2 - ps3
Archaea3 - ps4
Asgard/Eukaryota - ps5
Eukaryota - ps6
Amorpheae - ps7
Obazoa - ps8
Opisthokonta - ps9
Holomycota - ps10
Fungi - ps11
Fungi_1 - ps12
Fungi_2 - ps13
Fungi_3 - ps14
Dikarya - ps15
Ascomycota - ps16
Saccharo/Pezizomycotina - ps17
Saccharomycotina - ps18
Saccharomycotina_1 - ps19
Saccharomycotina_2 - ps20
Saccharomycotina_3 - ps21
Phaff./Saccharomycetaceae - ps22
Saccharomyco./mycetaceae - ps23
Saccharomycetaceae - ps24
Saccharomycetaceae_2 - ps25
Saccharomycetaceae_3 - ps26
Kaz./Nau./Saccharomyces - ps27
Saccharomyces - ps28
Arboricola_clade - ps29
Cerevisiae_clade - ps30
Saccharomyces_cerevisiae - ps31
p-value
2e-04
8e-04
3e-03
1e-02
6e-02
Log-odds
0.5
1.0
1.5
2.0
2.5
3.0
3.5
4.0

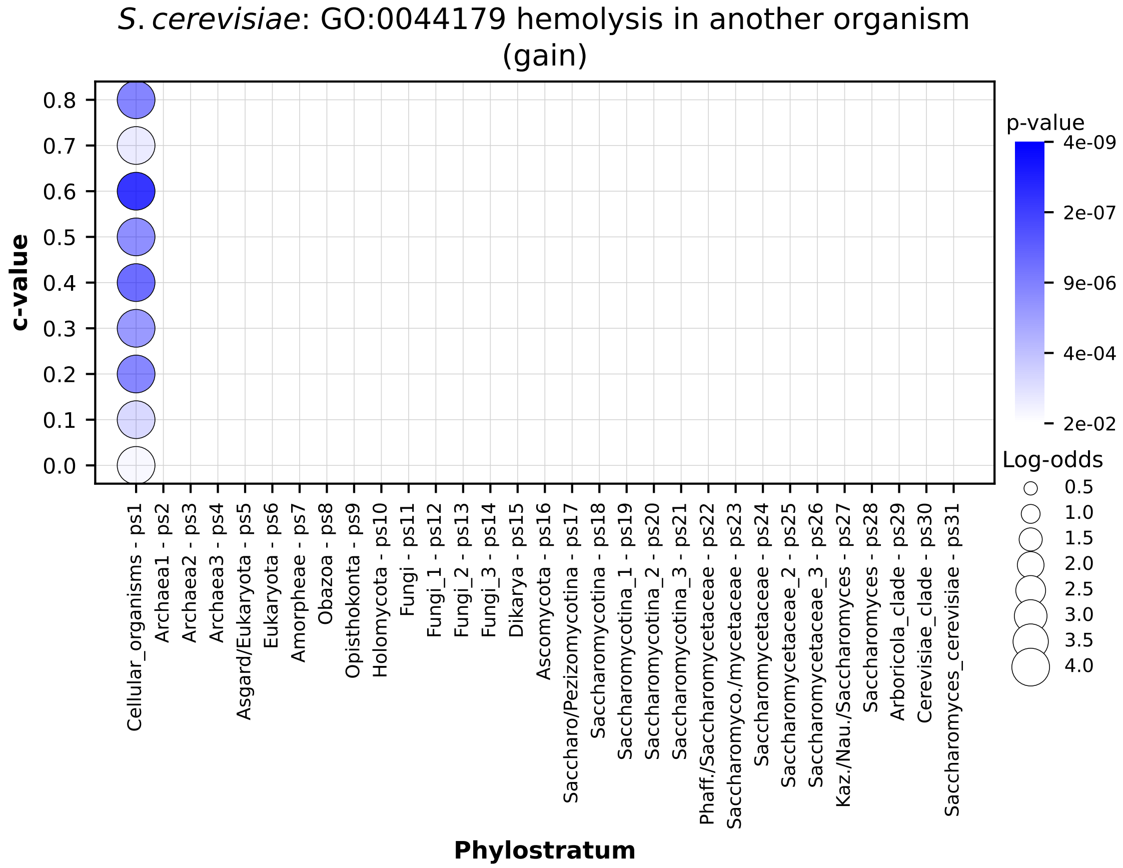

S. cerevisiae: GO:0044179 hemolysis in another organism (gain)
c-value
0.8
0.7
0.6
0.5
0.4
0.3
0.2
0.1
0.0
Phylostratum
Cellular_organisms - ps1
Archaea1 - ps2
Archaea2 - ps3
Archaea3 - ps4
Asgard/Eukaryota - ps5
Eukaryota - ps6
Amorpheae - ps7
Obazoa - ps8
Opisthokonta - ps9
Holomycota - ps10
Fungi - ps11
Fungi_1 - ps12
Fungi_2 - ps13
Fungi_3 - ps14
Dikarya - ps15
Ascomycota - ps16
Saccharo/Pezizomycotina - ps17
Saccharomycotina - ps18
Saccharomycotina_1 - ps19
Saccharomycotina_2 - ps20
Saccharomycotina_3 - ps21
Phaff./Saccharomycetaceae - ps22
Saccharomyco./mycetaceae - ps23
Saccharomycetaceae - ps24
Saccharomycetaceae_2 - ps25
Saccharomycetaceae_3 - ps26
Kaz./Nau./Saccharomyces - ps27
Saccharomyces - ps28
Arboricola_clade - ps29
Cerevisiae_clade - ps30
Saccharomyces_cerevisiae - ps31
p-value
4e-09
2e-07
9e-06
4e-04
2e-02
Log-odds
0.5
1.0
1.5
2.0
2.5
3.0
3.5
4.0

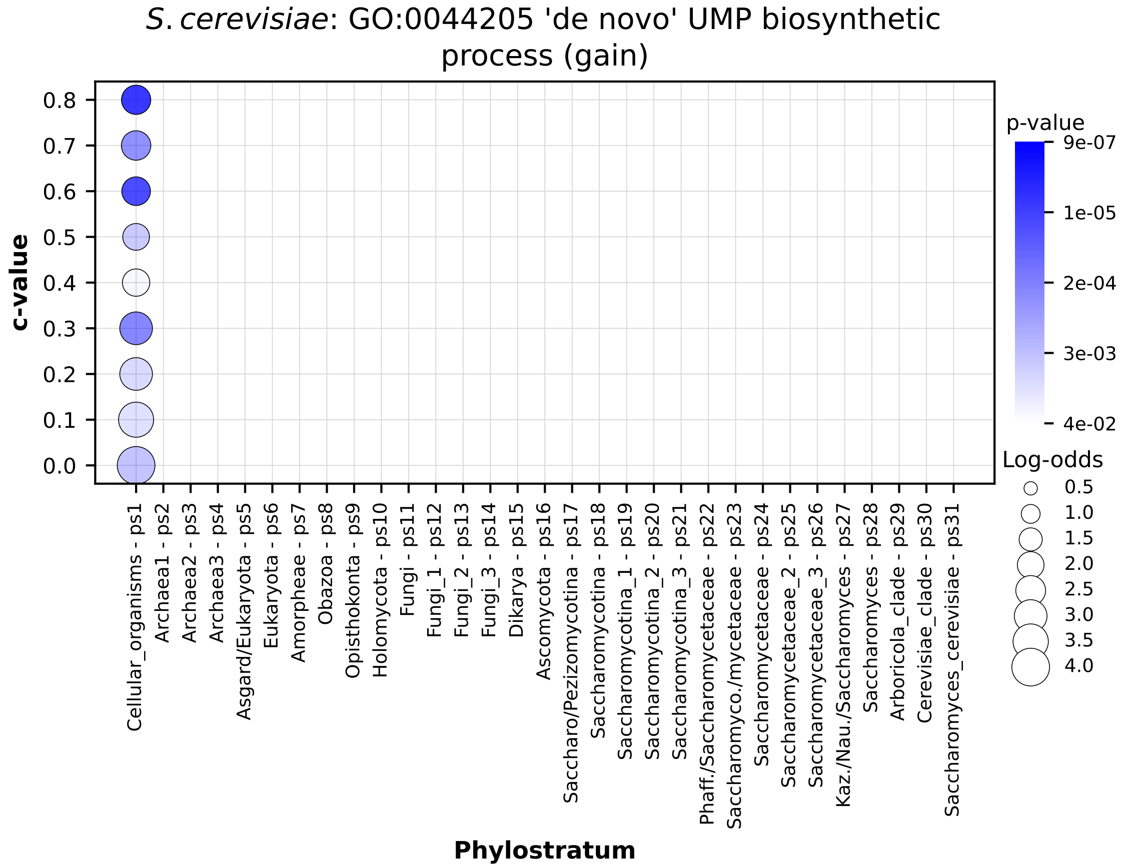
S. cerevisiae: GO:0044205 'de novo' UMP biosynthetic process (gain)
c-value
0.8
0.7
0.6
0.5
0.4
0.3
0.2
0.1
0.0
p-value
9e-07
1e-05
2e-04
3e-03
4e-02
Log-odds
0.5
1.0
1.5
2.0
2.5
3.0
3.5
4.0
Cellular_organisms - ps1
Archaea1 - ps2
Archaea2 - ps3
Archaea3 - ps4
Asgard/Eukaryota - ps5
Eukaryota - ps6
Amorpheae - ps7
Obazoa - ps8
Opisthokonta - ps9
Holomycota - ps10
Fungi - ps11
Fungi_1 - ps12
Fungi_2 - ps13
Fungi_3 - ps14
Dikarya - ps15
Ascomycota - ps16
Saccharo/Pezizomycotina - ps17
Saccharomycotina - ps18
Saccharomycotina_1 - ps19
Saccharomycotina_2 - ps20
Saccharomycotina_3 - ps21
Phaff./Saccharomycetaceae - ps22
Saccharomyco./mycetaceae - ps23
Saccharomycetaceae - ps24
Saccharomycetaceae_2 - ps25
Saccharomycetaceae_3 - ps26
Kaz./Nau./Saccharomyces - ps27
Saccharomyces - ps28
Arboricola_clade - ps29
Cerevisiae_clade - ps30
Saccharomyces_cerevisiae - ps31
Phylostratum

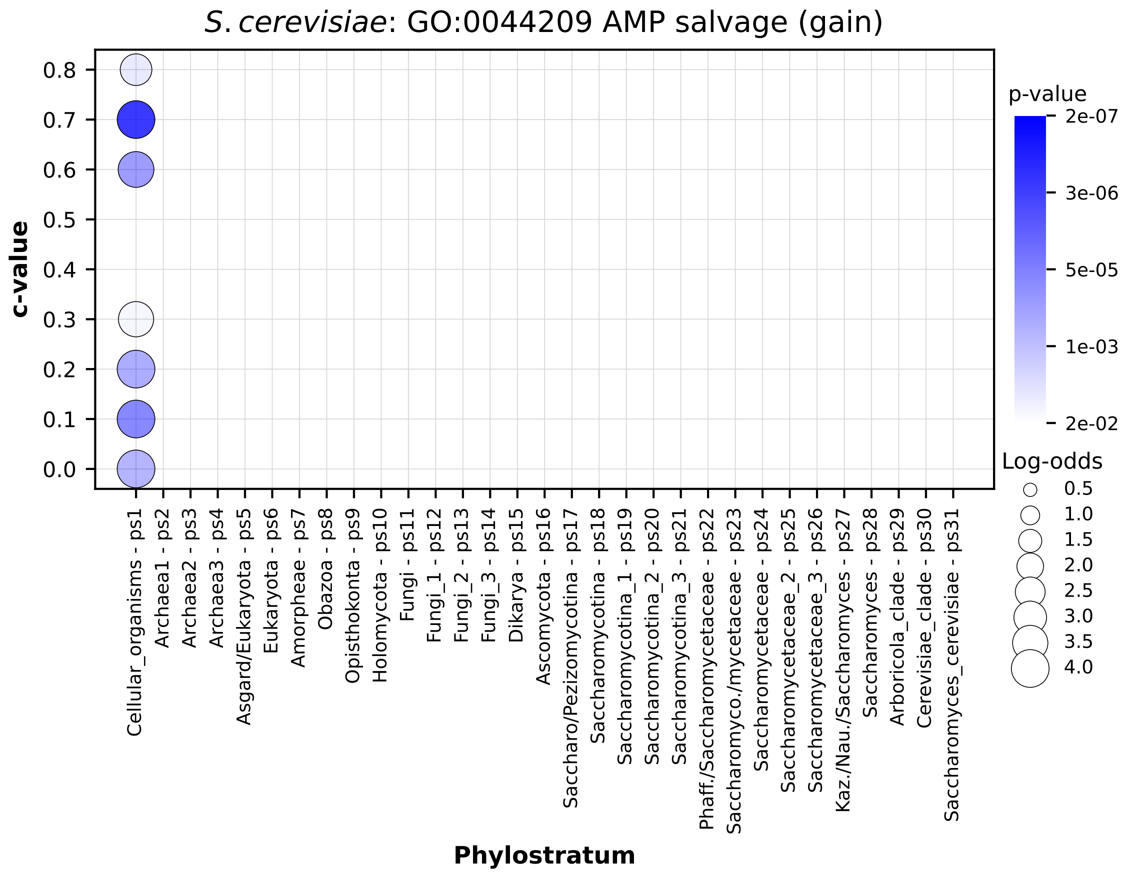
S. cerevisiae: GO:0044209 AMP salvage (gain)
c-value
0.8
0.7
0.6
0.5
0.4
0.3
0.2
0.1
0.0
p-value
2e-07
3e-06
5e-05
1e-03
2e-02
Log-odds
0.5
1.0
1.5
2.0
2.5
3.0
3.5
4.0
Cellular_organisms - ps1
Archaea1 - ps2
Archaea2 - ps3
Archaea3 - ps4
Asgard/Eukaryota - ps5
Eukaryota - ps6
Amorpheae - ps7
Obazoa - ps8
Opisthokonta - ps9
Holomycota - ps10
Fungi - ps11
Fungi_1 - ps12
Fungi_2 - ps13
Fungi_3 - ps14
Dikarya - ps15
Ascomycota - ps16
Saccharo/Pezizomycotina - ps17
Saccharomycotina - ps18
Saccharomycotina_1 - ps19
Saccharomycotina_2 - ps20
Saccharomycotina_3 - ps21
Phaff./Saccharomycetaceae - ps22
Saccharomyco./mycetaceae - ps23
Saccharomycetaceae - ps24
Saccharomycetaceae_2 - ps25
Saccharomycetaceae_3 - ps26
Kaz./Nau./Saccharomyces - ps27
Saccharomyces - ps28
Arboricola_clade - ps29
Cerevisiae_clade - ps30
Saccharomyces_cerevisiae - ps31
Phylostratum

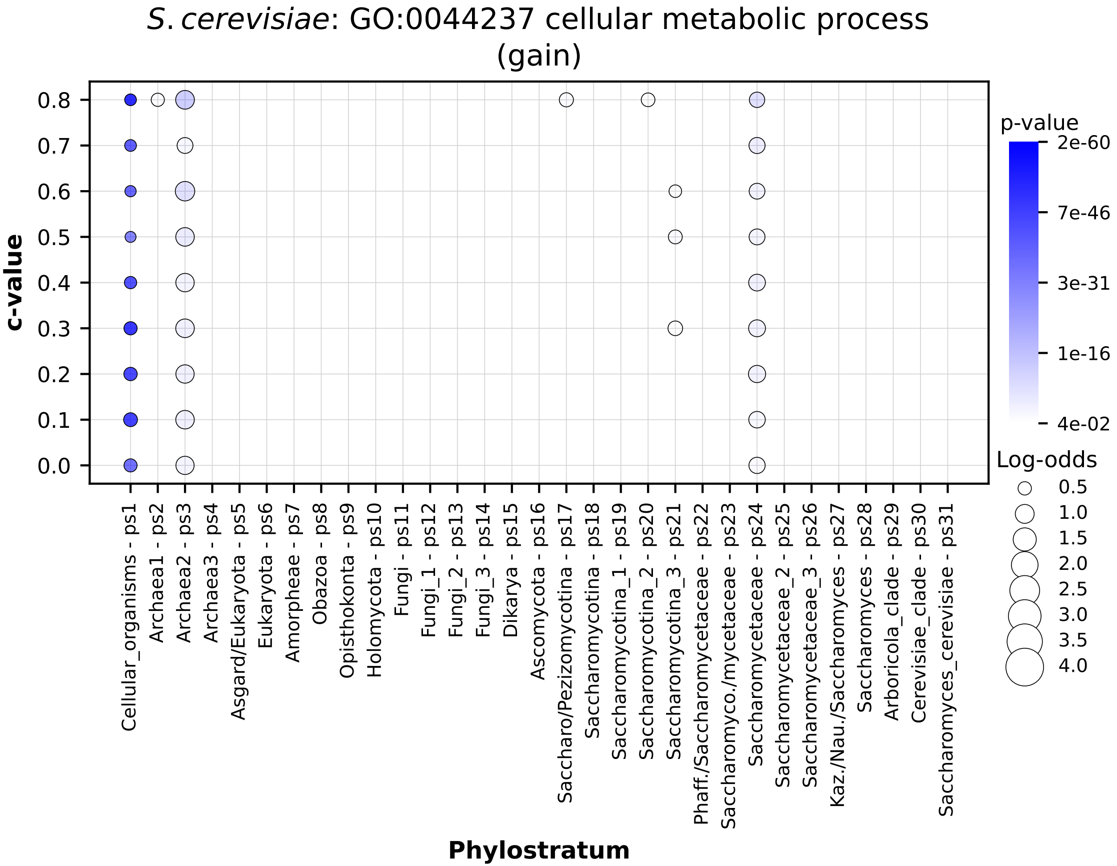

S. cerevisiae: GO:0044237 cellular metabolic process
(gain)
c-value
0.8
0.7
0.6
0.5
0.4
0.3
0.2
0.1
0.0
p-value
2e-60
7e-46
3e-31
1e-16
4e-02
Log-odds
0.5
1.0
1.5
2.0
2.5
3.0
3.5
4.0
Cellular_organisms - ps1
Archaea1 - ps2
Archaea2 - ps3
Archaea3 - ps4
Asgard/Eukaryota - ps5
Eukaryota - ps6
Amorpheae - ps7
Obazoa - ps8
Opisthokonta - ps9
Holomycota - ps10
Fungi - ps11
Fungi_1 - ps12
Fungi_2 - ps13
Fungi_3 - ps14
Dikarya - ps15
Ascomycota - ps16
Saccharo/Pezizomycotina - ps17
Saccharomycotina - ps18
Saccharomycotina_1 - ps19
Saccharomycotina_2 - ps20
Saccharomycotina_3 - ps21
Phaff./Saccharomycetaceae - ps22
Saccharomyco./mycetaceae - ps23
Saccharomycetaceae - ps24
Saccharomycetaceae_2 - ps25
Saccharomycetaceae_3 - ps26
Kaz./Nau./Saccharomyces - ps27
Saccharomyces - ps28
Arboricola_clade - ps29
Cerevisiae_clade - ps30
Saccharomyces_cerevisiae - ps31
Phylostratum

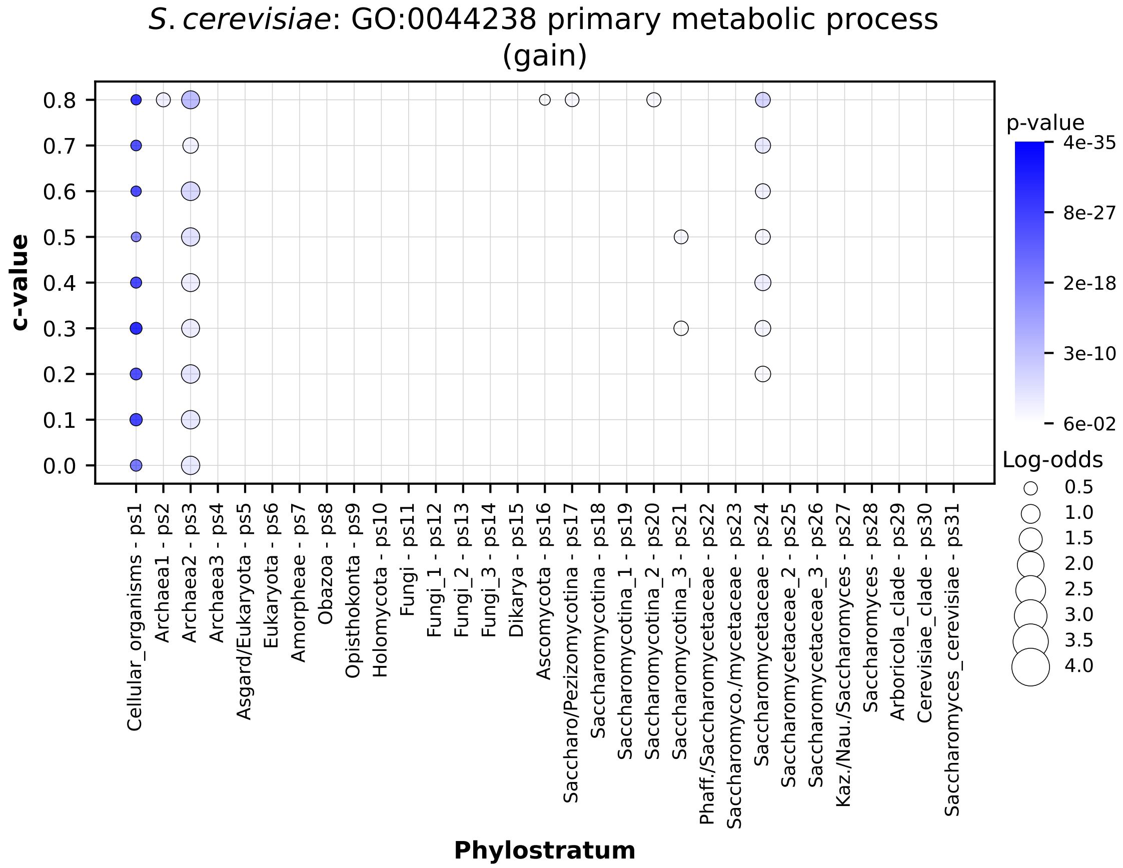
S. cerevisiae: GO:0044238 primary metabolic process
(gain)
c-value
0.0
0.1
0.2
0.3
0.4
0.5
0.6
0.7
0.8
p-value
4e-35
8e-27
2e-18
3e-10
6e-02
Log-odds
0.5
1.0
1.5
2.0
2.5
3.0
3.5
4.0
Cellular_organisms - ps1
Archaea1 - ps2
Archaea2 - ps3
Archaea3 - ps4
Asgard/Eukaryota - ps5
Eukaryota - ps6
Amorpheae - ps7
Obazoa - ps8
Opisthokonta - ps9
Holomycota - ps10
Fungi - ps11
Fungi_1 - ps12
Fungi_2 - ps13
Fungi_3 - ps14
Dikarya - ps15
Ascomycota - ps16
Saccharo/Pezizomycotina - ps17
Saccharomycotina - ps18
Saccharomycotina_1 - ps19
Saccharomycotina_2 - ps20
Saccharomycotina_3 - ps21
Phaff./Saccharomycetaceae - ps22
Saccharomyco./mycetaceae - ps23
Saccharomycetaceae - ps24
Saccharomycetaceae_2 - ps25
Saccharomycetaceae_3 - ps26
Kaz./Nau./Saccharomyces - ps27
Saccharomyces - ps28
Arboricola_clade - ps29
Cerevisiae_clade - ps30
Saccharomyces_cerevisiae - ps31
Phylostratum

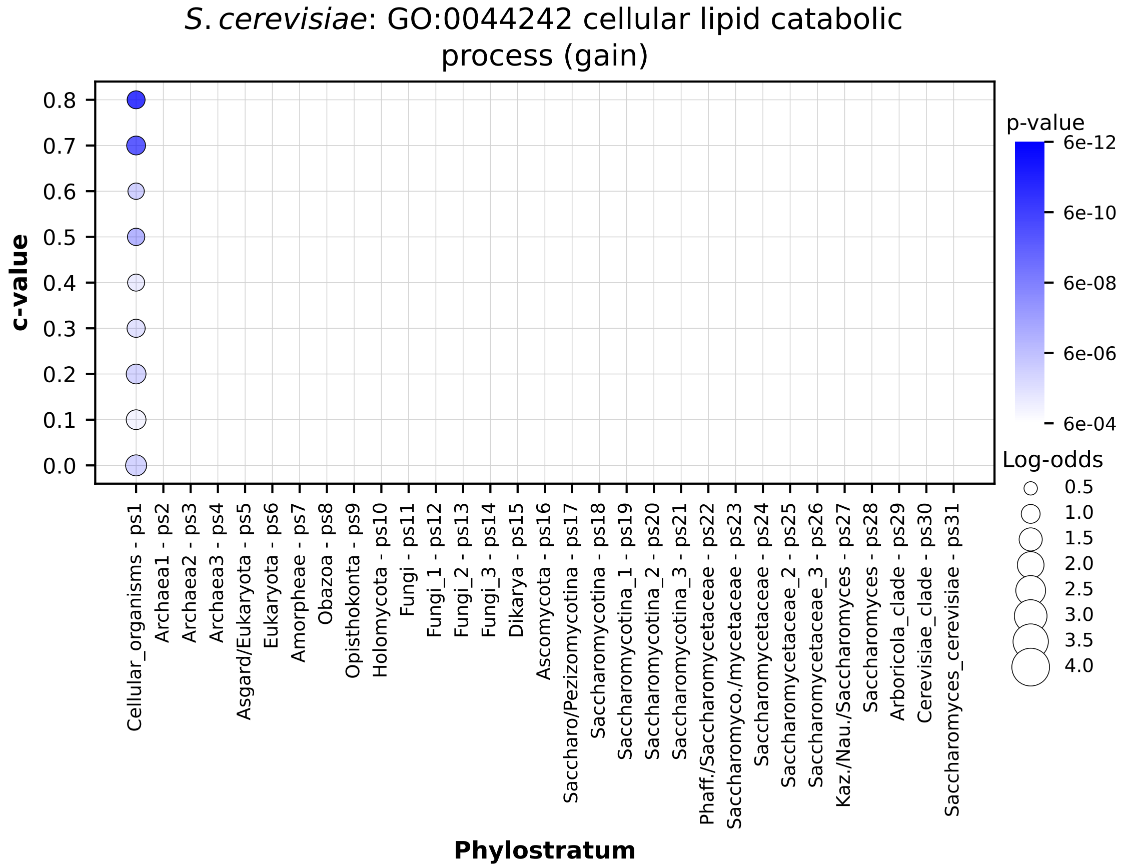
S. cerevisiae: GO:0044242 cellular lipid catabolic process (gain)
c-value
0.8
0.7
0.6
0.5
0.4
0.3
0.2
0.1
0.0
p-value
6e-12
6e-10
6e-08
6e-06
6e-04
Log-odds
0.5
1.0
1.5
2.0
2.5
3.0
3.5
4.0
Cellular_organisms - ps1
Archaea1 - ps2
Archaea2 - ps3
Archaea3 - ps4
Asgard/Eukaryota - ps5
Eukaryota - ps6
Amorpheae - ps7
Obazoa - ps8
Opisthokonta - ps9
Holomycota - ps10
Fungi - ps11
Fungi_1 - ps12
Fungi_2 - ps13
Fungi_3 - ps14
Dikarya - ps15
Ascomycota - ps16
Saccharo/Pezizomycotina - ps17
Saccharomycotina - ps18
Saccharomycotina_1 - ps19
Saccharomycotina_2 - ps20
Saccharomycotina_3 - ps21
Phaff./Saccharomycetaceae - ps22
Saccharomyco./mycetaceae - ps23
Saccharomycetaceae - ps24
Saccharomycetaceae_2 - ps25
Saccharomycetaceae_3 - ps26
Kaz./Nau./Saccharomyces - ps27
Saccharomyces - ps28
Arboricola_clade - ps29
Cerevisiae_clade - ps30
Saccharomyces_cerevisiae - ps31
Phylostratum

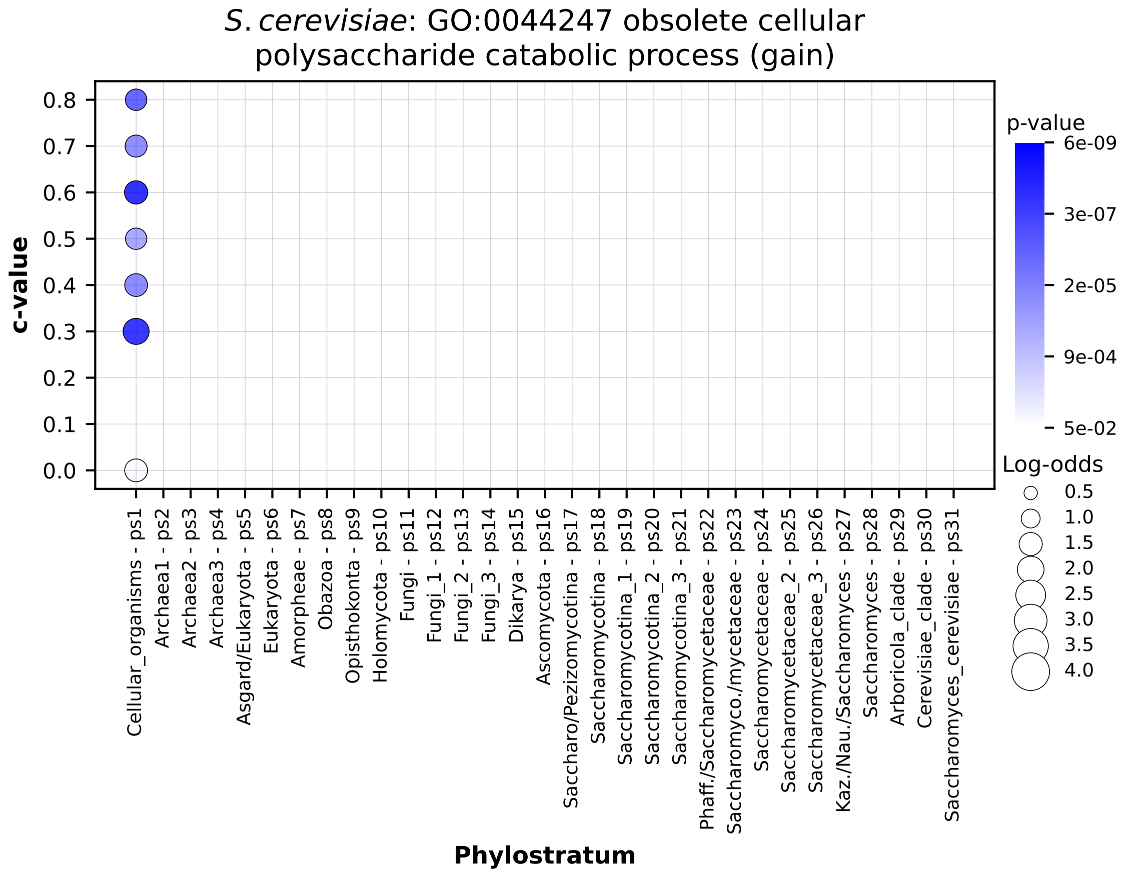

S. cerevisiae: GO:0044247 obsolete cellular polysaccharide catabolic process (gain)
c-value
0.0
0.1
0.2
0.3
0.4
0.5
0.6
0.7
0.8
Phylostratum
Cellular_organisms - ps1
Archaea1 - ps2
Archaea2 - ps3
Archaea3 - ps4
Asgard/Eukaryota - ps5
Eukaryota - ps6
Amorpheae - ps7
Obazoa - ps8
Opisthokonta - ps9
Holomycota - ps10
Fungi - ps11
Fungi_1 - ps12
Fungi_2 - ps13
Fungi_3 - ps14
Dikarya - ps15
Ascomycota - ps16
Saccharo/Pezizomycotina - ps17
Saccharomycotina - ps18
Saccharomycotina_1 - ps19
Saccharomycotina_2 - ps20
Saccharomycotina_3 - ps21
Phaff./Saccharomycetaceae - ps22
Saccharomyco./mycetaceae - ps23
Saccharomycetaceae - ps24
Saccharomycetaceae_2 - ps25
Saccharomycetaceae_3 - ps26
Kaz./Nau./Saccharomyces - ps27
Saccharomyces - ps28
Arboricola_clade - ps29
Cerevisiae_clade - ps30
Saccharomyces_cerevisiae - ps31
p-value
6e-09
3e-07
2e-05
9e-04
5e-02
Log-odds
0.5
1.0
1.5
2.0
2.5
3.0
3.5
4.0

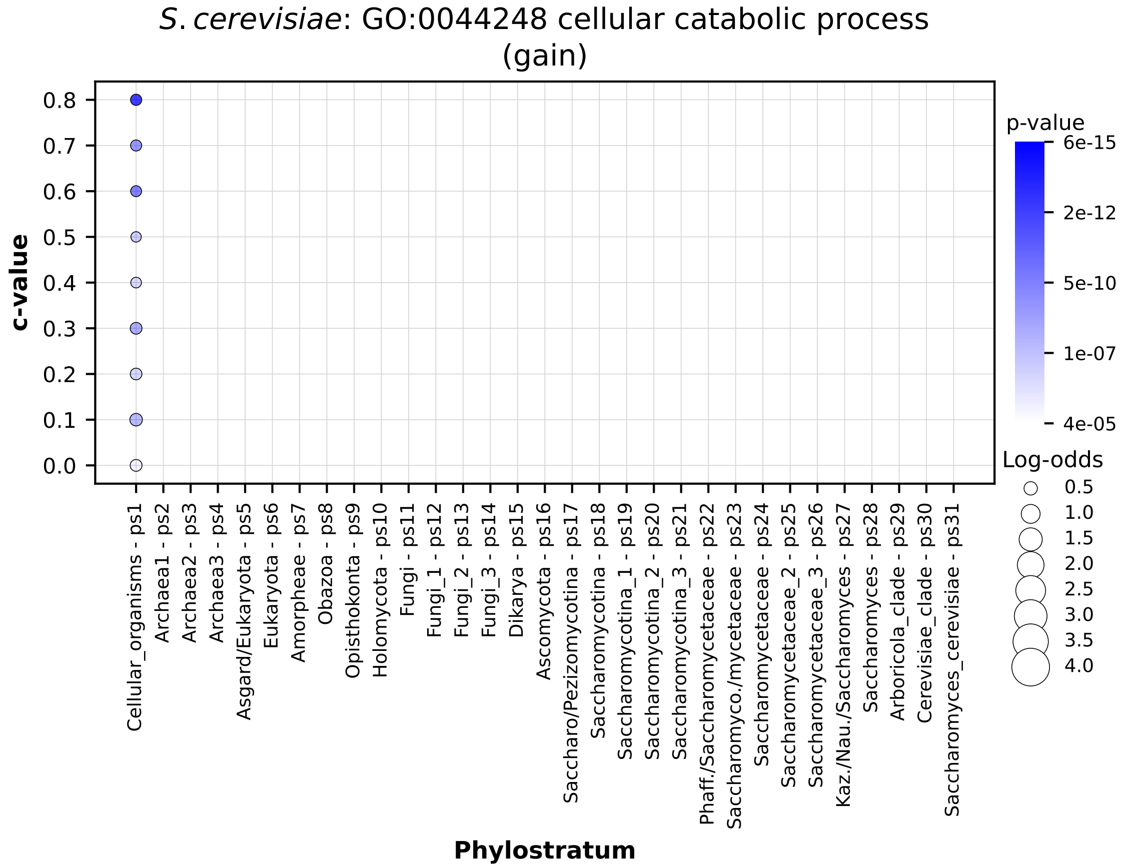

S. cerevisiae: GO:0044248 cellular catabolic process
(gain)
c-value
0.8
0.7
0.6
0.5
0.4
0.3
0.2
0.1
0.0
p-value
6e-15
2e-12
5e-10
1e-07
4e-05
Log-odds
0.5
1.0
1.5
2.0
2.5
3.0
3.5
4.0
Cellular_organisms - ps1
Archaea1 - ps2
Archaea2 - ps3
Archaea3 - ps4
Asgard/Eukaryota - ps5
Eukaryota - ps6
Amorpheae - ps7
Obazoa - ps8
Opisthokonta - ps9
Holomycota - ps10
Fungi - ps11
Fungi_1 - ps12
Fungi_2 - ps13
Fungi_3 - ps14
Dikarya - ps15
Ascomycota - ps16
Saccharo/Pezizomycotina - ps17
Saccharomycotina - ps18
Saccharomycotina_1 - ps19
Saccharomycotina_2 - ps20
Saccharomycotina_3 - ps21
Phaff./Saccharomycetaceae - ps22
Saccharomyco./mycetaceae - ps23
Saccharomycetaceae - ps24
Saccharomycetaceae_2 - ps25
Saccharomycetaceae_3 - ps26
Kaz./Nau./Saccharomyces - ps27
Saccharomyces - ps28
Arboricola_clade - ps29
Cerevisiae_clade - ps30
Saccharomyces_cerevisiae - ps31
Phylostratum

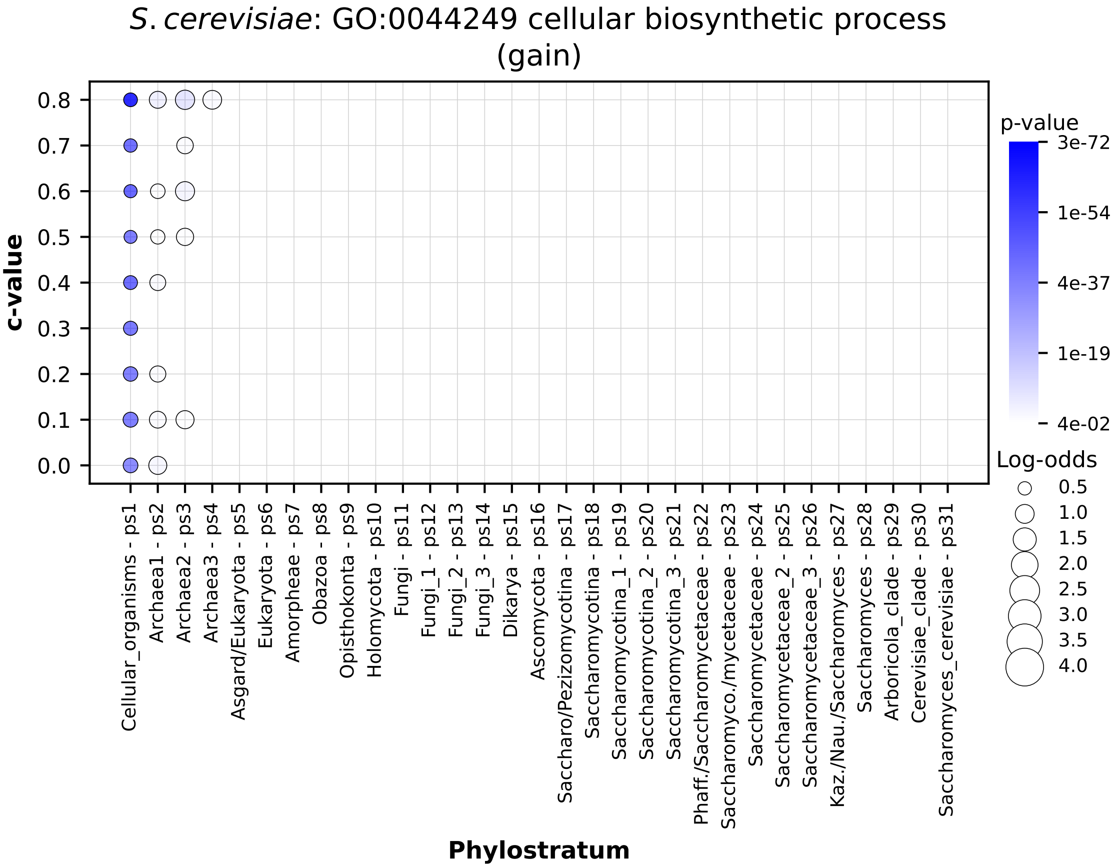

S. cerevisiae: GO:0044249 cellular biosynthetic process (gain)
c-value
0.8
0.7
0.6
0.5
0.4
0.3
0.2
0.1
0.0
Phylostratum
Cellular_organisms - ps1
Archaea1 - ps2
Archaea2 - ps3
Archaea3 - ps4
Asgard/Eukaryota - ps5
Eukaryota - ps6
Amorpheae - ps7
Obazoa - ps8
Opisthokonta - ps9
Holomycota - ps10
Fungi - ps11
Fungi_1 - ps12
Fungi_2 - ps13
Fungi_3 - ps14
Dikarya - ps15
Ascomycota - ps16
Saccharo/Pezizomycotina - ps17
Saccharomycotina - ps18
Saccharomycotina_1 - ps19
Saccharomycotina_2 - ps20
Saccharomycotina_3 - ps21
Phaff./Saccharomycetaceae - ps22
Saccharomyco./mycetaceae - ps23
Saccharomycetaceae - ps24
Saccharomycetaceae_2 - ps25
Saccharomycetaceae_3 - ps26
Kaz./Nau./Saccharomyces - ps27
Saccharomyces - ps28
Arboricola_clade - ps29
Cerevisiae_clade - ps30
Saccharomyces_cerevisiae - ps31
p-value
3e-72
1e-54
4e-37
1e-19
4e-02
Log-odds
0.5
1.0
1.5
2.0
2.5
3.0
3.5
4.0

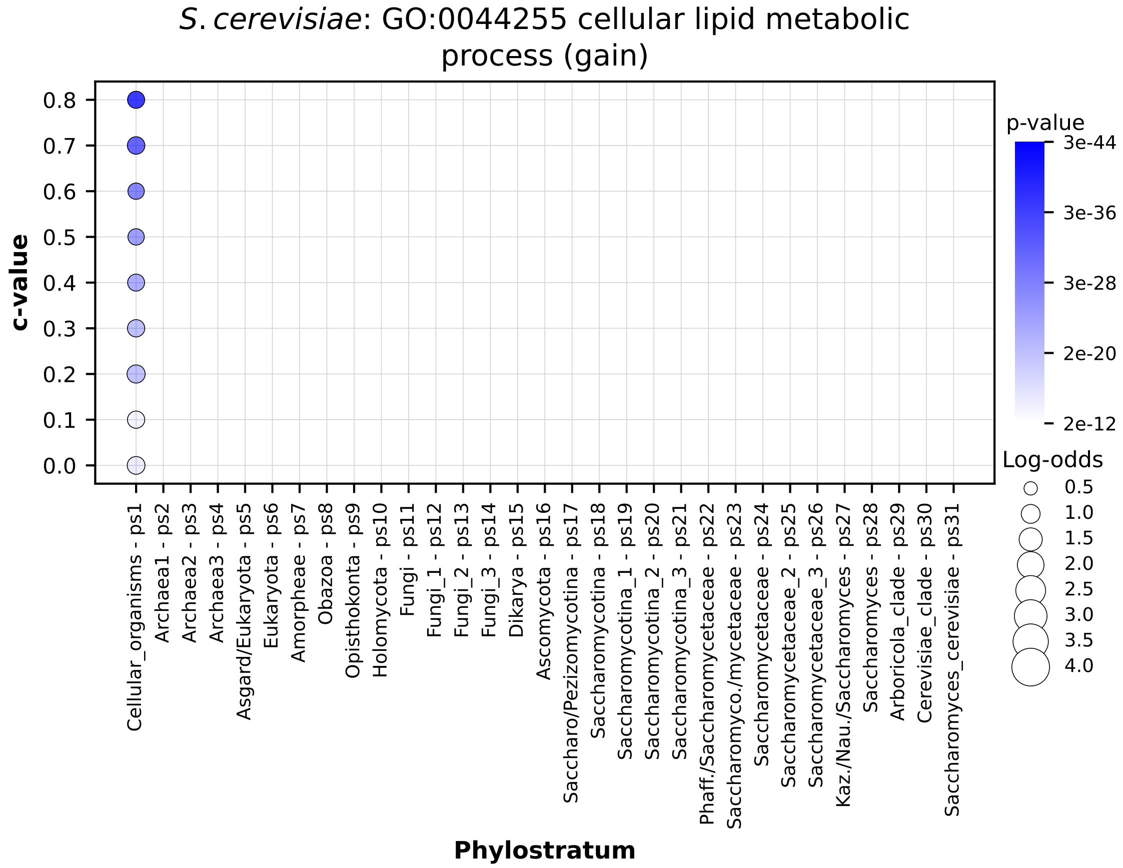

S. cerevisiae: GO:0044255 cellular lipid metabolic process (gain)
c-value
0.8
0.7
0.6
0.5
0.4
0.3
0.2
0.1
0.0
p-value
3e-44
3e-36
3e-28
2e-20
2e-12
Log-odds
0.5
1.0
1.5
2.0
2.5
3.0
3.5
4.0
Cellular_organisms - ps1
Archaea1 - ps2
Archaea2 - ps3
Archaea3 - ps4
Asgard/Eukaryota - ps5
Eukaryota - ps6
Amorpheae - ps7
Obazoa - ps8
Opisthokonta - ps9
Holomycota - ps10
Fungi - ps11
Fungi_1 - ps12
Fungi_2 - ps13
Fungi_3 - ps14
Dikarya - ps15
Ascomycota - ps16
Saccharo/Pezizomycotina - ps17
Saccharomycotina - ps18
Saccharomycotina_1 - ps19
Saccharomycotina_2 - ps20
Saccharomycotina_3 - ps21
Phaff./Saccharomycetaceae - ps22
Saccharomyco./mycetaceae - ps23
Saccharomycetaceae - ps24
Saccharomycetaceae_2 - ps25
Saccharomycetaceae_3 - ps26
Kaz./Nau./Saccharomyces - ps27
Saccharomyces - ps28
Arboricola_clade - ps29
Cerevisiae_clade - ps30
Saccharomyces_cerevisiae - ps31
Phylostratum

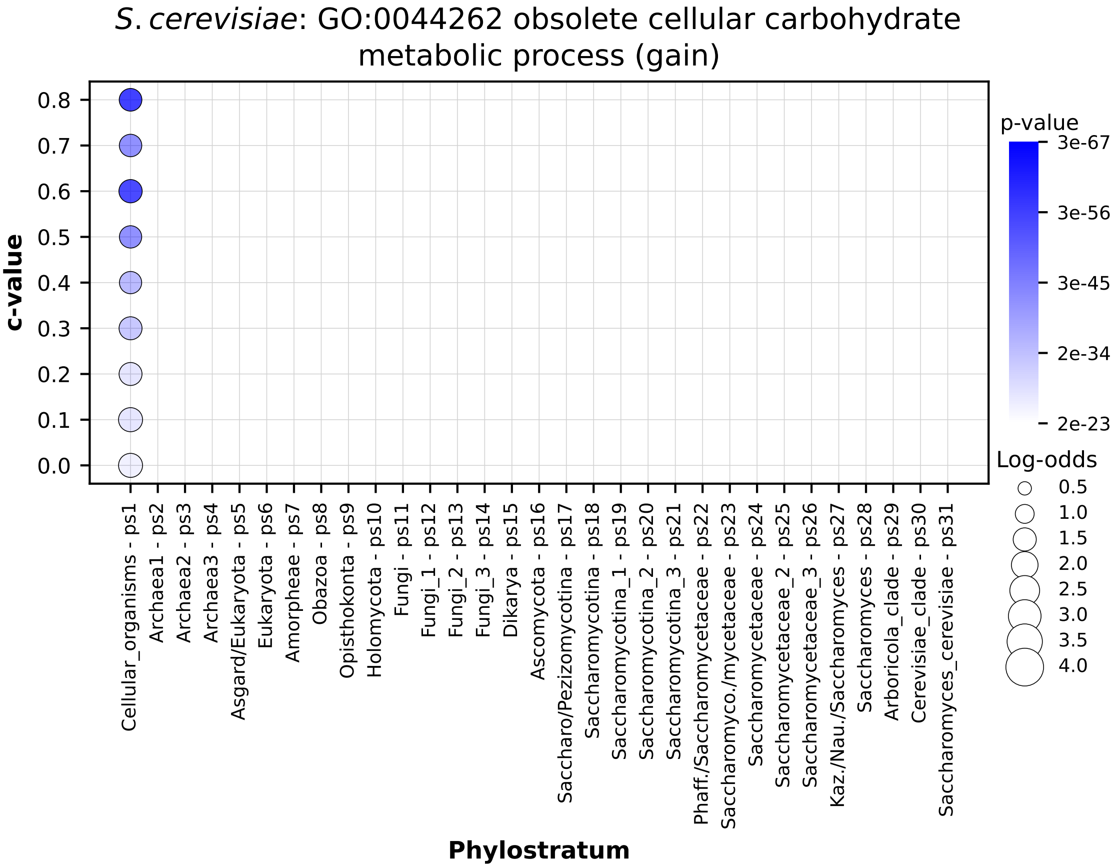

S. cerevisiae: GO:0044262 obsolete cellular carbohydrate metabolic process (gain)
c-value
0.8
0.7
0.6
0.5
0.4
0.3
0.2
0.1
0.0
Phylostratum
Cellular_organisms - ps1
Archaea1 - ps2
Archaea2 - ps3
Archaea3 - ps4
Asgard/Eukaryota - ps5
Eukaryota - ps6
Amorpheae - ps7
Obazoa - ps8
Opisthokonta - ps9
Holomycota - ps10
Fungi - ps11
Fungi_1 - ps12
Fungi_2 - ps13
Fungi_3 - ps14
Dikarya - ps15
Ascomycota - ps16
Saccharo/Pezizomycotina - ps17
Saccharomycotina - ps18
Saccharomycotina_1 - ps19
Saccharomycotina_2 - ps20
Saccharomycotina_3 - ps21
Phaff./Saccharomycetaceae - ps22
Saccharomyco./mycetaceae - ps23
Saccharomycetaceae - ps24
Saccharomycetaceae_2 - ps25
Saccharomycetaceae_3 - ps26
Kaz./Nau./Saccharomyces - ps27
Saccharomyces - ps28
Arboricola_clade - ps29
Cerevisiae_clade - ps30
Saccharomyces_cerevisiae - ps31
p-value
3e-67
3e-56
3e-45
2e-34
2e-23
Log-odds
0.5
1.0
1.5
2.0
2.5
3.0
3.5
4.0

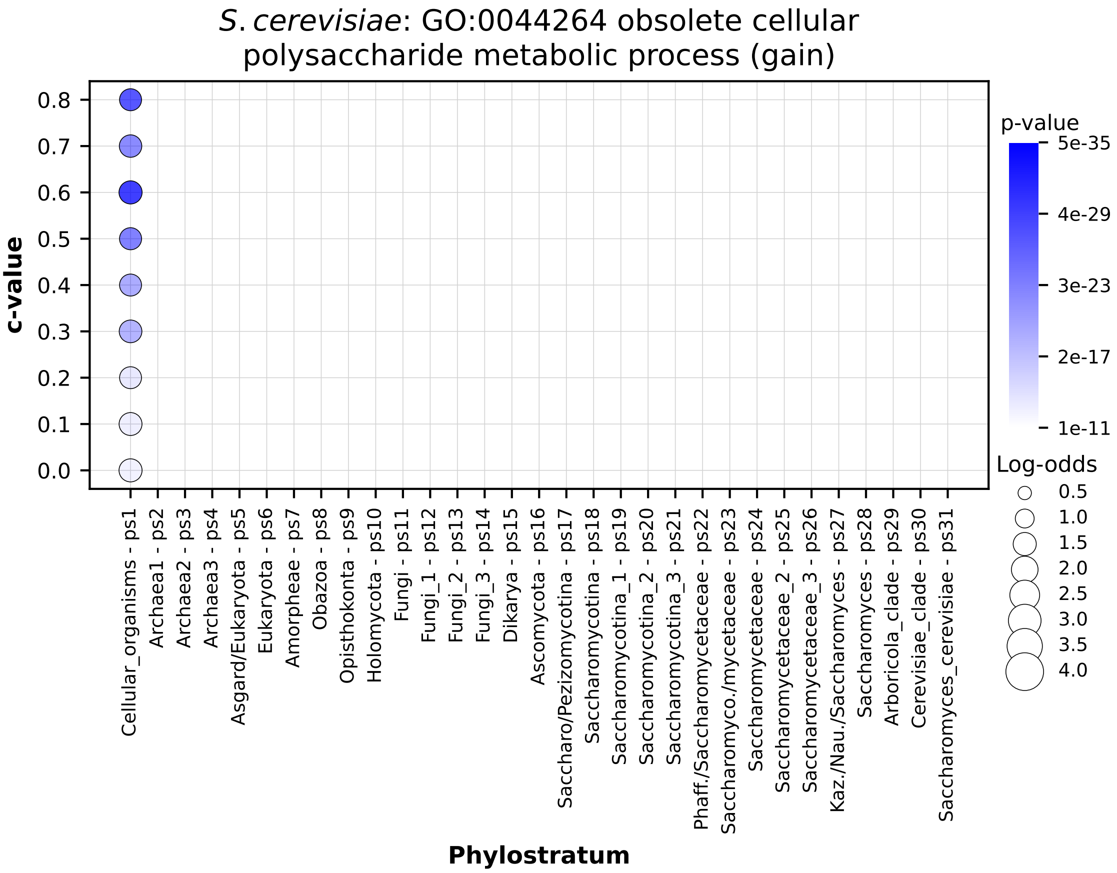

S. cerevisiae: GO:0044264 obsolete cellular polysaccharide metabolic process (gain)
c-value
0.0
0.1
0.2
0.3
0.4
0.5
0.6
0.7
0.8
Phylostratum
Cellular_organisms - ps1
Archaea1 - ps2
Archaea2 - ps3
Archaea3 - ps4
Asgard/Eukaryota - ps5
Eukaryota - ps6
Amorpheae - ps7
Obazoa - ps8
Opisthokonta - ps9
Holomycota - ps10
Fungi - ps11
Fungi_1 - ps12
Fungi_2 - ps13
Fungi_3 - ps14
Dikarya - ps15
Ascomycota - ps16
Saccharo/Pezizomycotina - ps17
Saccharomycotina - ps18
Saccharomycotina_1 - ps19
Saccharomycotina_2 - ps20
Saccharomycotina_3 - ps21
Phaff./Saccharomycetaceae - ps22
Saccharomyco./mycetaceae - ps23
Saccharomycetaceae - ps24
Saccharomycetaceae_2 - ps25
Saccharomycetaceae_3 - ps26
Kaz./Nau./Saccharomyces - ps27
Saccharomyces - ps28
Arboricola_clade - ps29
Cerevisiae_clade - ps30
Saccharomyces_cerevisiae - ps31
p-value
5e-35
4e-29
3e-23
2e-17
1e-11
Log-odds
0.5
1.0
1.5
2.0
2.5
3.0
3.5
4.0

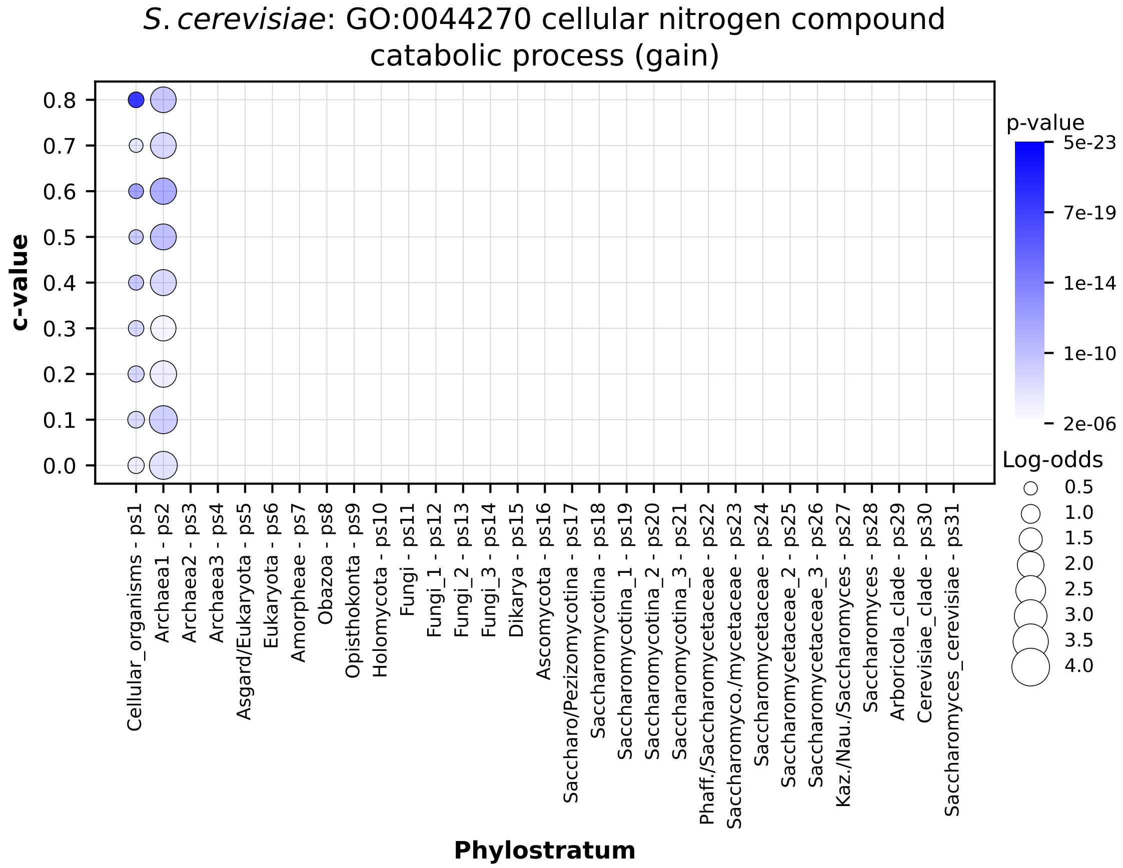

S. cerevisiae: GO:0044270 cellular nitrogen compound catabolic process (gain)
c-value
0.8
0.7
0.6
0.5
0.4
0.3
0.2
0.1
0.0
p-value
5e-23
7e-19
1e-14
1e-10
2e-06
Log-odds
0.5
1.0
1.5
2.0
2.5
3.0
3.5
4.0
Cellular_organisms - ps1
Archaea1 - ps2
Archaea2 - ps3
Archaea3 - ps4
Asgard/Eukaryota - ps5
Eukaryota - ps6
Amorpheae - ps7
Obazoa - ps8
Opisthokonta - ps9
Holomycota - ps10
Fungi - ps11
Fungi_1 - ps12
Fungi_2 - ps13
Fungi_3 - ps14
Dikarya - ps15
Ascomycota - ps16
Saccharo/Pezizomycotina - ps17
Saccharomycotina - ps18
Saccharomycotina_1 - ps19
Saccharomycotina_2 - ps20
Saccharomycotina_3 - ps21
Phaff./Saccharomycetaceae - ps22
Saccharomyco./mycetaceae - ps23
Saccharomycetaceae - ps24
Saccharomycetaceae_2 - ps25
Saccharomycetaceae_3 - ps26
Kaz./Nau./Saccharomyces - ps27
Saccharomyces - ps28
Arboricola_clade - ps29
Cerevisiae_clade - ps30
Saccharomyces_cerevisiae - ps31
Phylostratum

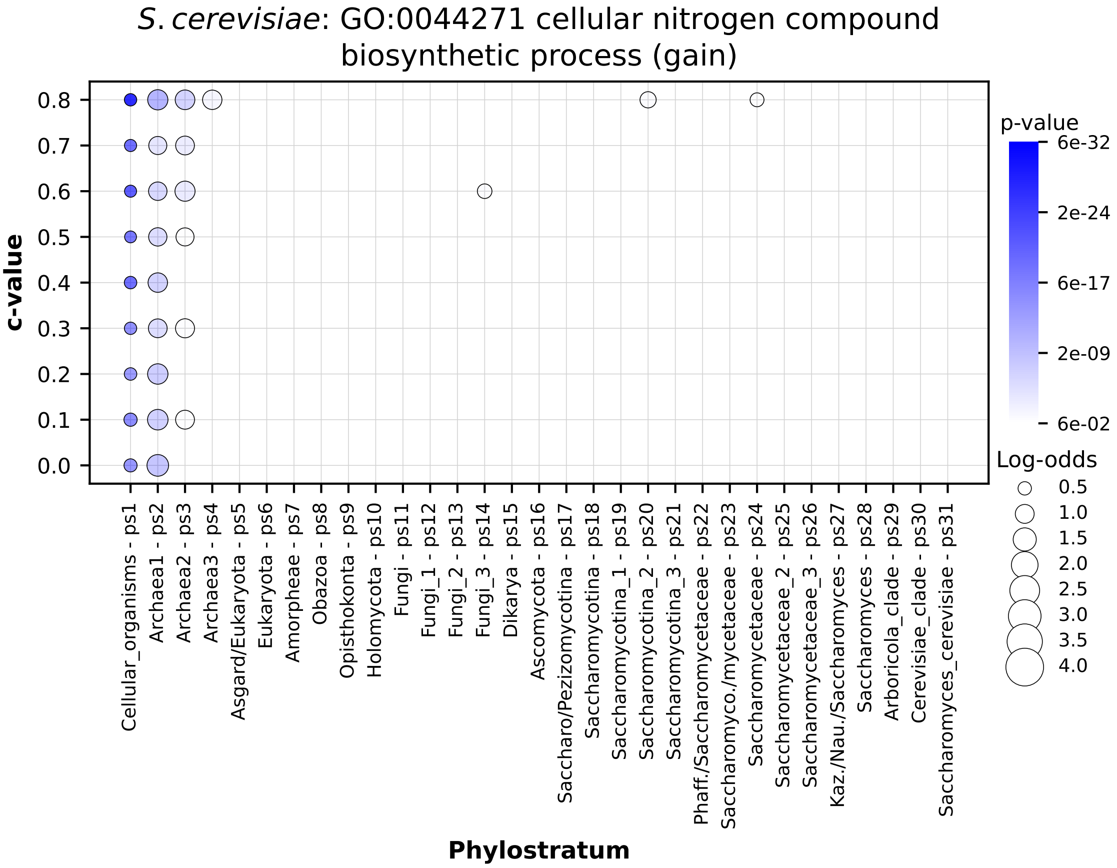
S. cerevisiae: GO:0044271 cellular nitrogen compound biosynthetic process (gain)
c-value
0.8
0.7
0.6
0.5
0.4
0.3
0.2
0.1
0.0
p-value
6e-32
2e-24
6e-17
2e-09
6e-02
Log-odds
0.5
1.0
1.5
2.0
2.5
3.0
3.5
4.0
Cellular_organisms - ps1
Archaea1 - ps2
Archaea2 - ps3
Archaea3 - ps4
Asgard/Eukaryota - ps5
Eukaryota - ps6
Amorpheae - ps7
Obazoa - ps8
Opisthokonta - ps9
Holomycota - ps10
Fungi - ps11
Fungi_1 - ps12
Fungi_2 - ps13
Fungi_3 - ps14
Dikarya - ps15
Ascomycota - ps16
Saccharo/Pezizomycotina - ps17
Saccharomycotina - ps18
Saccharomycotina_1 - ps19
Saccharomycotina_2 - ps20
Saccharomycotina_3 - ps21
Phaff./Saccharomycetaceae - ps22
Saccharomyco./mycetaceae - ps23
Saccharomycetaceae - ps24
Saccharomycetaceae_2 - ps25
Saccharomycetaceae_3 - ps26
Kaz./Nau./Saccharomyces - ps27
Saccharomyces - ps28
Arboricola_clade - ps29
Cerevisiae_clade - ps30
Saccharomyces_cerevisiae - ps31
Phylostratum

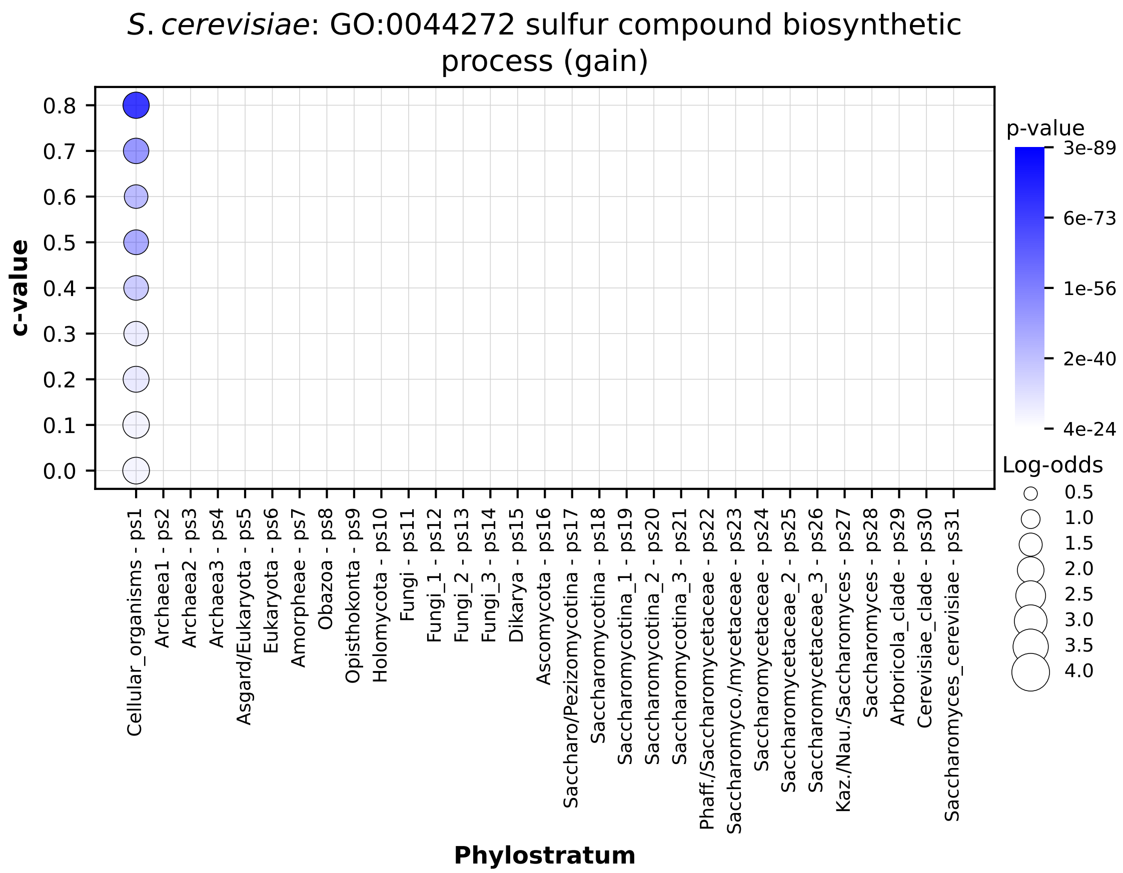

S. cerevisiae: GO:0044272 sulfur compound biosynthetic process (gain)
c-value
0.8
0.7
0.6
0.5
0.4
0.3
0.2
0.1
0.0
p-value
3e-89
6e-73
1e-56
2e-40
4e-24
Log-odds
0.5
1.0
1.5
2.0
2.5
3.0
3.5
4.0
Cellular_organisms - ps1
Archaea1 - ps2
Archaea2 - ps3
Archaea3 - ps4
Asgard/Eukaryota - ps5
Eukaryota - ps6
Amorpheae - ps7
Obazoa - ps8
Opisthokonta - ps9
Holomycota - ps10
Fungi - ps11
Fungi_1 - ps12
Fungi_2 - ps13
Fungi_3 - ps14
Dikarya - ps15
Ascomycota - ps16
Saccharo/Pezizomycotina - ps17
Saccharomycotina - ps18
Saccharomycotina_1 - ps19
Saccharomycotina_2 - ps20
Saccharomycotina_3 - ps21
Phaff./Saccharomycetaceae - ps22
Saccharomyco./mycetaceae - ps23
Saccharomycetaceae - ps24
Saccharomycetaceae_2 - ps25
Saccharomycetaceae_3 - ps26
Kaz./Nau./Saccharomyces - ps27
Saccharomyces - ps28
Arboricola_clade - ps29
Cerevisiae_clade - ps30
Saccharomyces_cerevisiae - ps31
Phylostratum

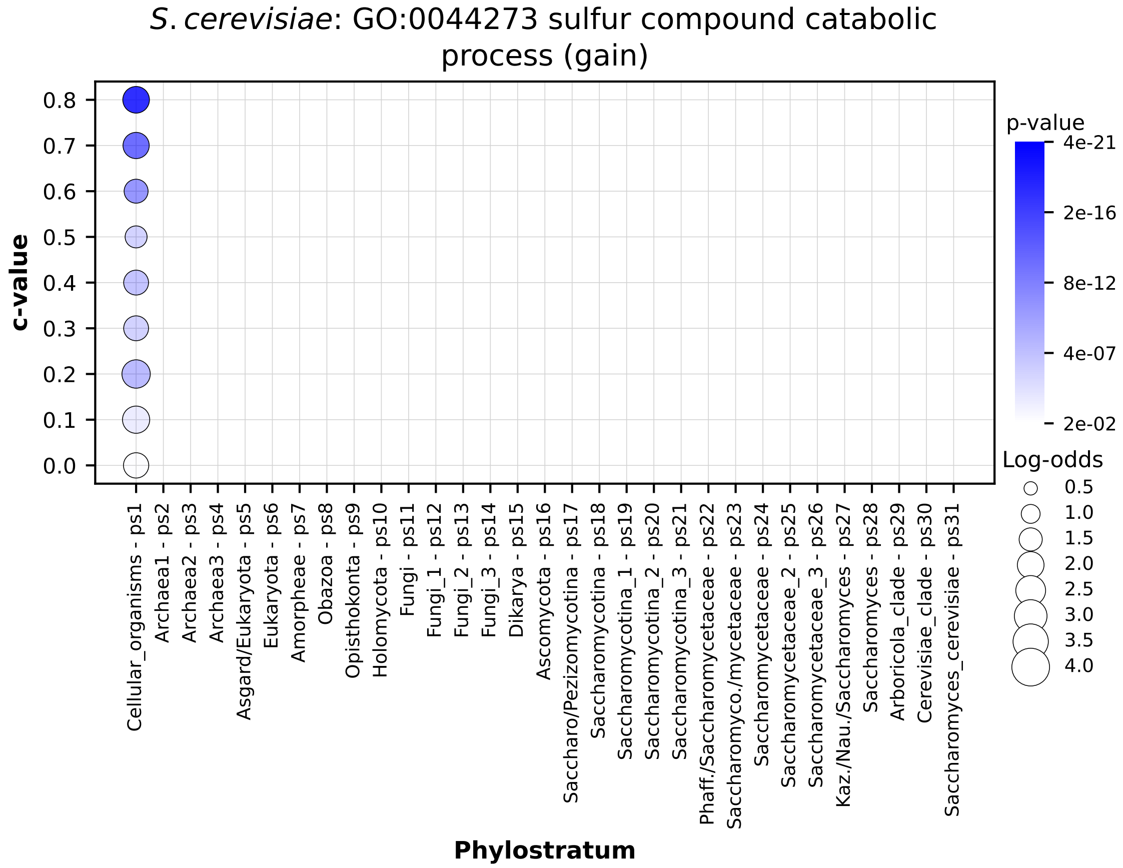

S. cerevisiae: GO:0044273 sulfur compound catabolic process (gain)
c-value
0.8
0.7
0.6
0.5
0.4
0.3
0.2
0.1
0.0
p-value
4e-21
2e-16
8e-12
4e-07
2e-02
Log-odds
0.5
1.0
1.5
2.0
2.5
3.0
3.5
4.0
Cellular_organisms - ps1
Archaea1 - ps2
Archaea2 - ps3
Archaea3 - ps4
Asgard/Eukaryota - ps5
Eukaryota - ps6
Amorpheae - ps7
Obazoa - ps8
Opisthokonta - ps9
Holomycota - ps10
Fungi - ps11
Fungi_1 - ps12
Fungi_2 - ps13
Fungi_3 - ps14
Dikarya - ps15
Ascomycota - ps16
Saccharo/Pezizomycotina - ps17
Saccharomycotina - ps18
Saccharomycotina_1 - ps19
Saccharomycotina_2 - ps20
Saccharomycotina_3 - ps21
Phaff./Saccharomycetaceae - ps22
Saccharomyco./mycetaceae - ps23
Saccharomycetaceae - ps24
Saccharomycetaceae_2 - ps25
Saccharomycetaceae_3 - ps26
Kaz./Nau./Saccharomyces - ps27
Saccharomyces - ps28
Arboricola_clade - ps29
Cerevisiae_clade - ps30
Saccharomyces_cerevisiae - ps31
Phylostratum

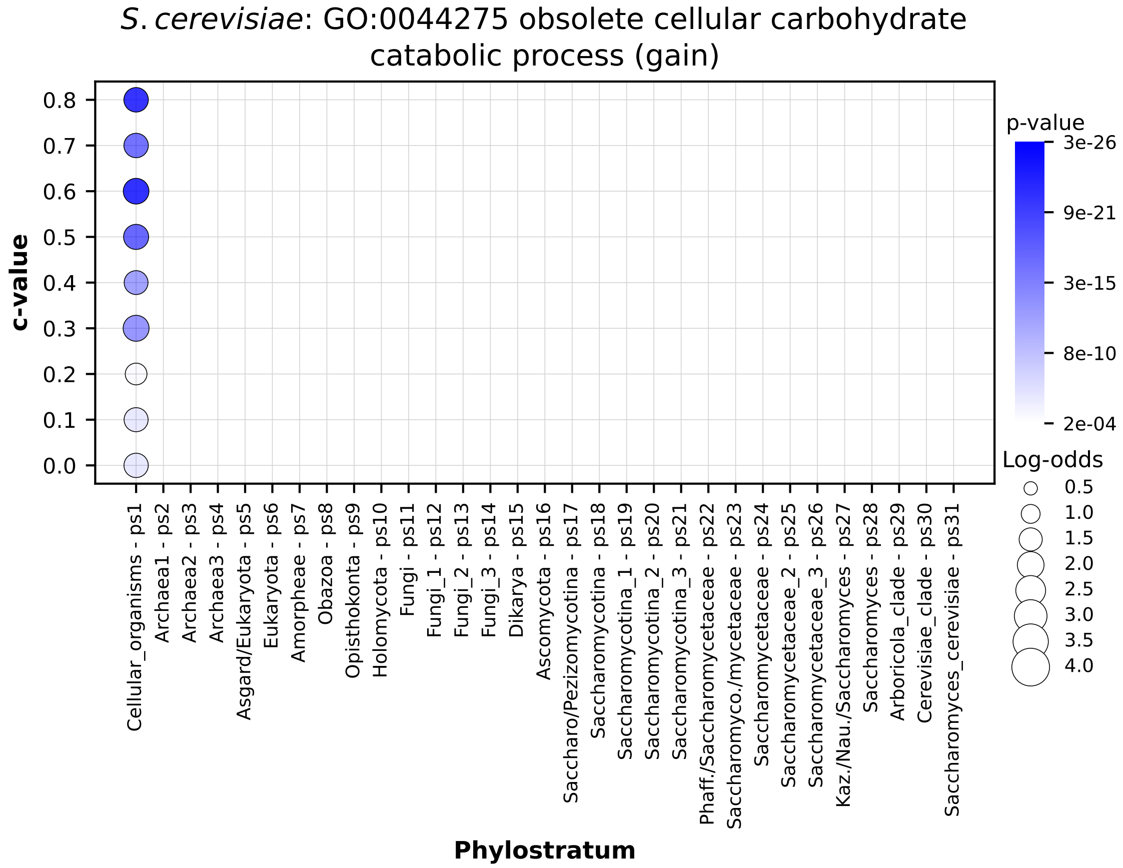

S. cerevisiae: GO:0044275 obsolete cellular carbohydrate catabolic process (gain)
c-value
0.0
0.1
0.2
0.3
0.4
0.5
0.6
0.7
0.8
p-value
3e-26
9e-21
3e-15
8e-10
2e-04
Log-odds
0.5
1.0
1.5
2.0
2.5
3.0
3.5
4.0
Cellular_organisms - ps1
Archaea1 - ps2
Archaea2 - ps3
Archaea3 - ps4
Asgard/Eukaryota - ps5
Eukaryota - ps6
Amorpheae - ps7
Obazoa - ps8
Opisthokonta - ps9
Holomycota - ps10
Fungi - ps11
Fungi_1 - ps12
Fungi_2 - ps13
Fungi_3 - ps14
Dikarya - ps15
Ascomycota - ps16
Saccharo/Pezizomycotina - ps17
Saccharomycotina - ps18
Saccharomycotina_1 - ps19
Saccharomycotina_2 - ps20
Saccharomycotina_3 - ps21
Phaff./Saccharomycetaceae - ps22
Saccharomyco./mycetaceae - ps23
Saccharomycetaceae - ps24
Saccharomycetaceae_2 - ps25
Saccharomycetaceae_3 - ps26
Kaz./Nau./Saccharomyces - ps27
Saccharomyces - ps28
Arboricola_clade - ps29
Cerevisiae_clade - ps30
Saccharomyces_cerevisiae - ps31
Phylostratum

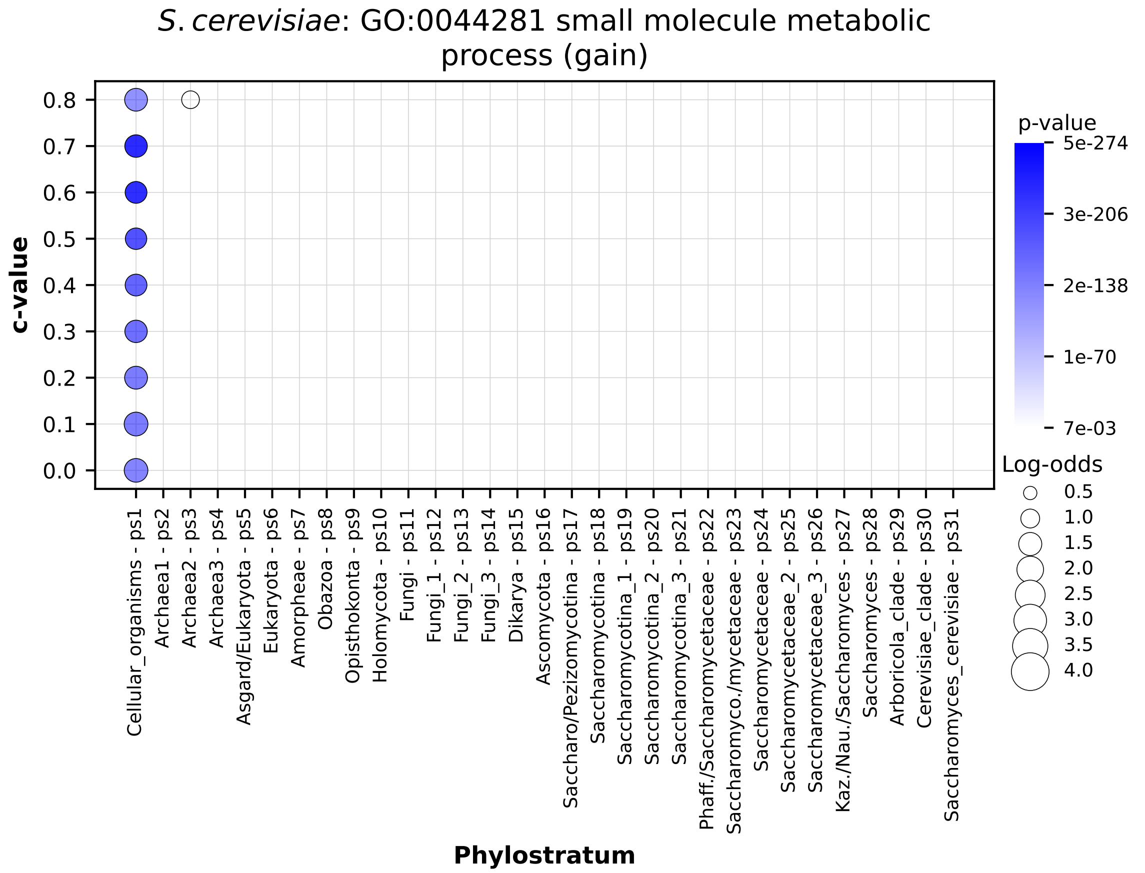
S. cerevisiae: GO:0044281 small molecule metabolic process (gain)
c-value
0.8
0.7
0.6
0.5
0.4
0.3
0.2
0.1
0.0
p-value
5e-274
3e-206
2e-138
1e-70
7e-03
Log-odds
0.5
1.0
1.5
2.0
2.5
3.0
3.5
4.0
Cellular_organisms - ps1
Archaea1 - ps2
Archaea2 - ps3
Archaea3 - ps4
Asgard/Eukaryota - ps5
Eukaryota - ps6
Amorpheae - ps7
Obazoa - ps8
Opisthokonta - ps9
Holomycota - ps10
Fungi - ps11
Fungi_1 - ps12
Fungi_2 - ps13
Fungi_3 - ps14
Dikarya - ps15
Ascomycota - ps16
Saccharo/Pezizomycotina - ps17
Saccharomycotina - ps18
Saccharomycotina_1 - ps19
Saccharomycotina_2 - ps20
Saccharomycotina_3 - ps21
Phaff./Saccharomycetaceae - ps22
Saccharomyco./mycetaceae - ps23
Saccharomycetaceae - ps24
Saccharomycetaceae_2 - ps25
Saccharomycetaceae_3 - ps26
Kaz./Nau./Saccharomyces - ps27
Saccharomyces - ps28
Arboricola_clade - ps29
Cerevisiae_clade - ps30
Saccharomyces_cerevisiae - ps31
Phylostratum

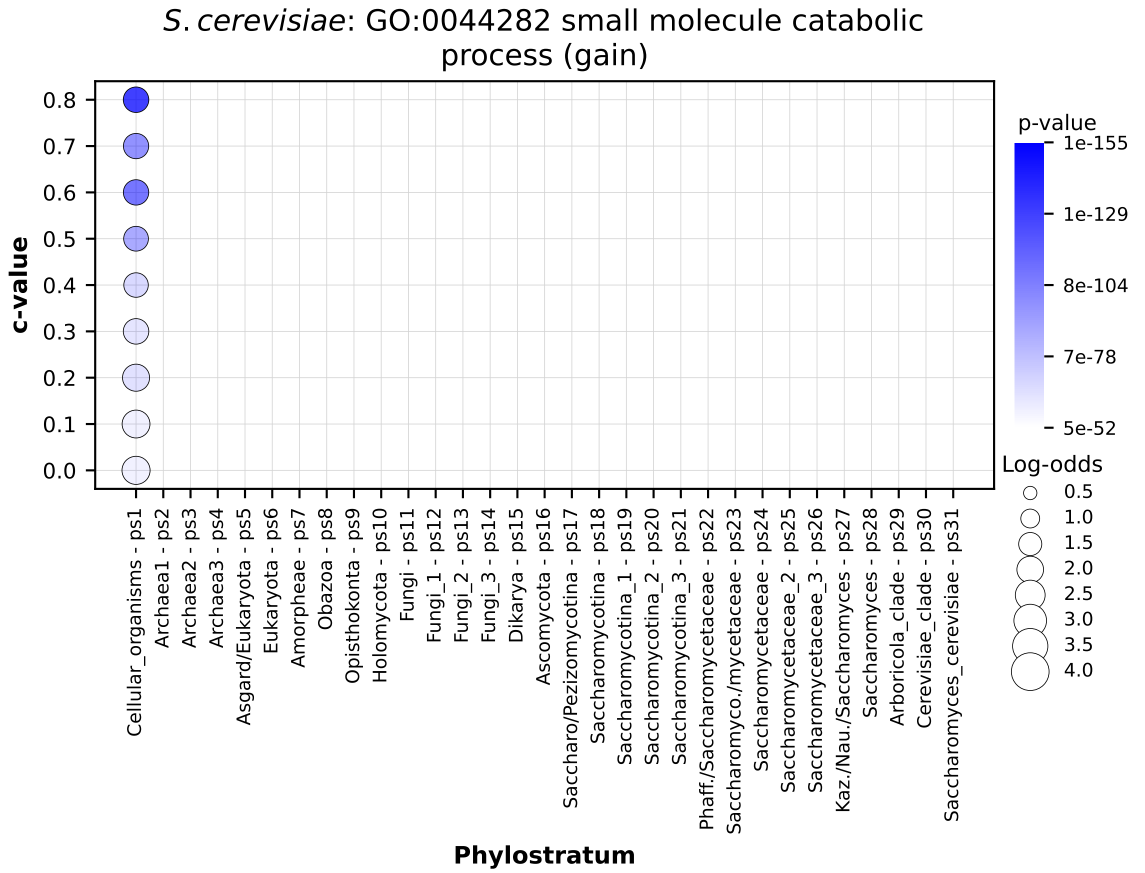

S. cerevisiae: GO:0044282 small molecule catabolic process (gain)
c-value
0.8
0.7
0.6
0.5
0.4
0.3
0.2
0.1
0.0
Phylostratum
Cellular_organisms - ps1
Archaea1 - ps2
Archaea2 - ps3
Archaea3 - ps4
Asgard/Eukaryota - ps5
Eukaryota - ps6
Amorpheae - ps7
Obazoa - ps8
Opisthokonta - ps9
Holomycota - ps10
Fungi - ps11
Fungi_1 - ps12
Fungi_2 - ps13
Fungi_3 - ps14
Dikarya - ps15
Ascomycota - ps16
Saccharo/Pezizomycotina - ps17
Saccharomycotina - ps18
Saccharomycotina_1 - ps19
Saccharomycotina_2 - ps20
Saccharomycotina_3 - ps21
Phaff./Saccharomycetaceae - ps22
Saccharomyco./mycetaceae - ps23
Saccharomycetaceae - ps24
Saccharomycetaceae_2 - ps25
Saccharomycetaceae_3 - ps26
Kaz./Nau./Saccharomyces - ps27
Saccharomyces - ps28
Arboricola_clade - ps29
Cerevisiae_clade - ps30
Saccharomyces_cerevisiae - ps31
p-value
1e-155
1e-129
8e-104
7e-78
5e-52
Log-odds
0.5
1.0
1.5
2.0
2.5
3.0
3.5
4.0

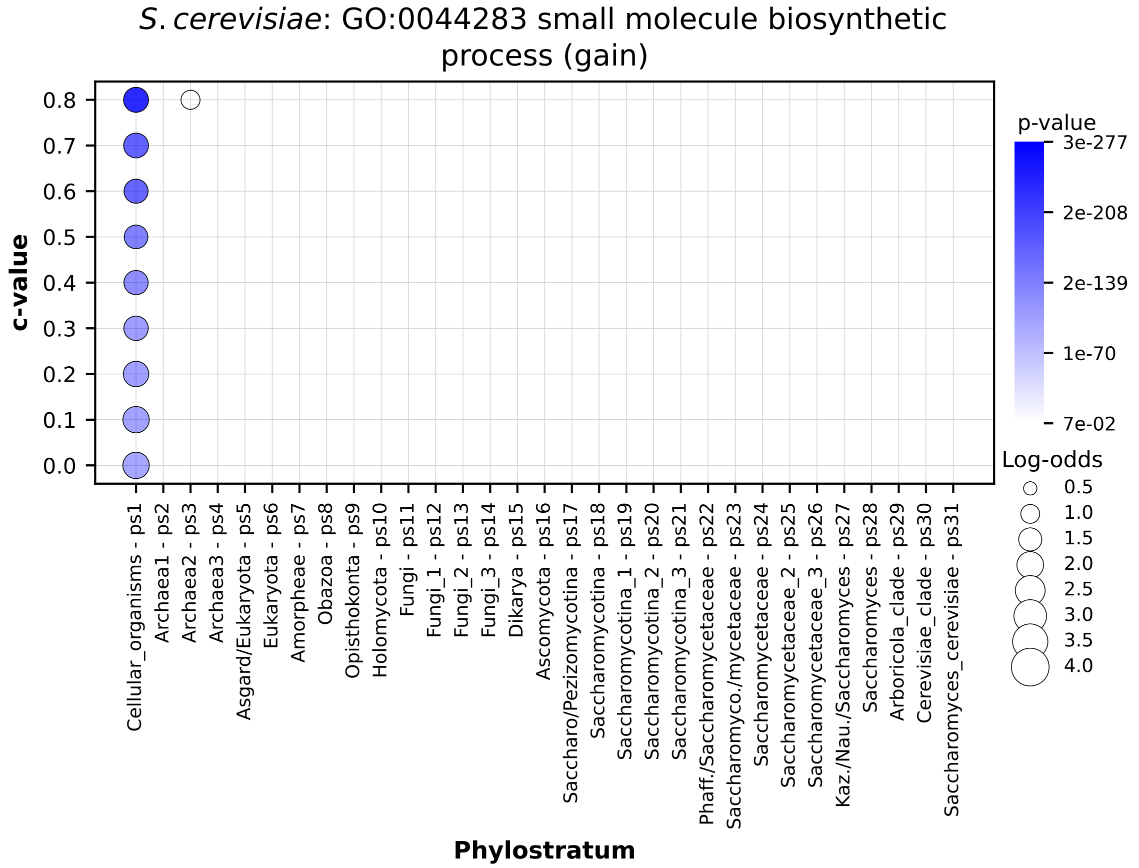
S. cerevisiae: GO:0044283 small molecule biosynthetic process (gain)
c-value
0.8
0.7
0.6
0.5
0.4
0.3
0.2
0.1
0.0
p-value
3e-277
2e-208
2e-139
1e-70
7e-02
Log-odds
0.5
1.0
1.5
2.0
2.5
3.0
3.5
4.0
Cellular_organisms - ps1
Archaea1 - ps2
Archaea2 - ps3
Archaea3 - ps4
Asgard/Eukaryota - ps5
Eukaryota - ps6
Amorpheae - ps7
Obazoa - ps8
Opisthokonta - ps9
Holomycota - ps10
Fungi - ps11
Fungi_1 - ps12
Fungi_2 - ps13
Fungi_3 - ps14
Dikarya - ps15
Ascomycota - ps16
Saccharo/Pezizomycotina - ps17
Saccharomycotina - ps18
Saccharomycotina_1 - ps19
Saccharomycotina_2 - ps20
Saccharomycotina_3 - ps21
Phaff./Saccharomycetaceae - ps22
Saccharomyco./mycetaceae - ps23
Saccharomycetaceae - ps24
Saccharomycetaceae_2 - ps25
Saccharomycetaceae_3 - ps26
Kaz./Nau./Saccharomyces - ps27
Saccharomyces - ps28
Arboricola_clade - ps29
Cerevisiae_clade - ps30
Saccharomyces_cerevisiae - ps31
Phylostratum

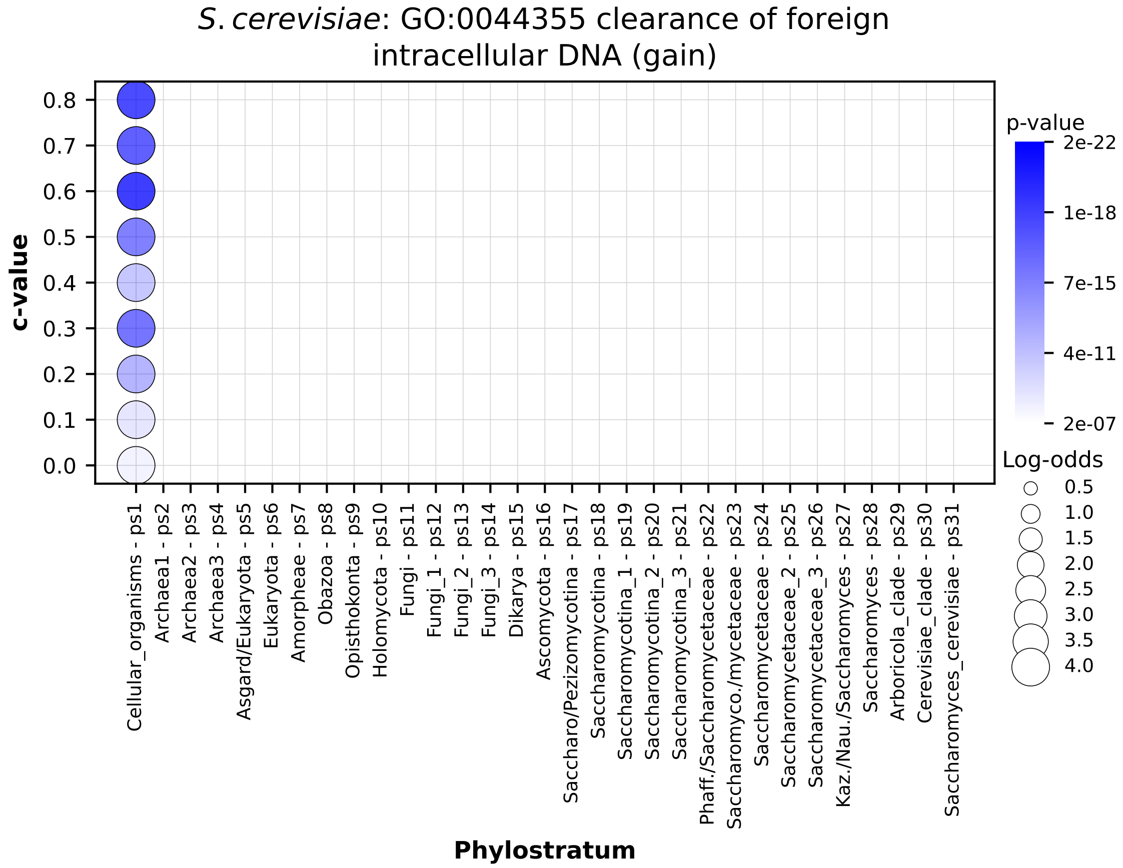

S. cerevisiae: GO:0044355 clearance of foreign intracellular DNA (gain)
c-value
0.8
0.7
0.6
0.5
0.4
0.3
0.2
0.1
0.0
p-value
2e-22
1e-18
7e-15
4e-11
2e-07
Log-odds
0.5
1.0
1.5
2.0
2.5
3.0
3.5
4.0
Cellular_organisms - ps1
Archaea1 - ps2
Archaea2 - ps3
Archaea3 - ps4
Asgard/Eukaryota - ps5
Eukaryota - ps6
Amorpheae - ps7
Obazoa - ps8
Opisthokonta - ps9
Holomycota - ps10
Fungi - ps11
Fungi_1 - ps12
Fungi_2 - ps13
Fungi_3 - ps14
Dikarya - ps15
Ascomycota - ps16
Saccharo/Pezizomycotina - ps17
Saccharomycotina - ps18
Saccharomycotina_1 - ps19
Saccharomycotina_2 - ps20
Saccharomycotina_3 - ps21
Phaff./Saccharomycetaceae - ps22
Saccharomyco./mycetaceae - ps23
Saccharomycetaceae - ps24
Saccharomycetaceae_2 - ps25
Saccharomycetaceae_3 - ps26
Kaz./Nau./Saccharomyces - ps27
Saccharomyces - ps28
Arboricola_clade - ps29
Cerevisiae_clade - ps30
Saccharomyces_cerevisiae - ps31
Phylostratum

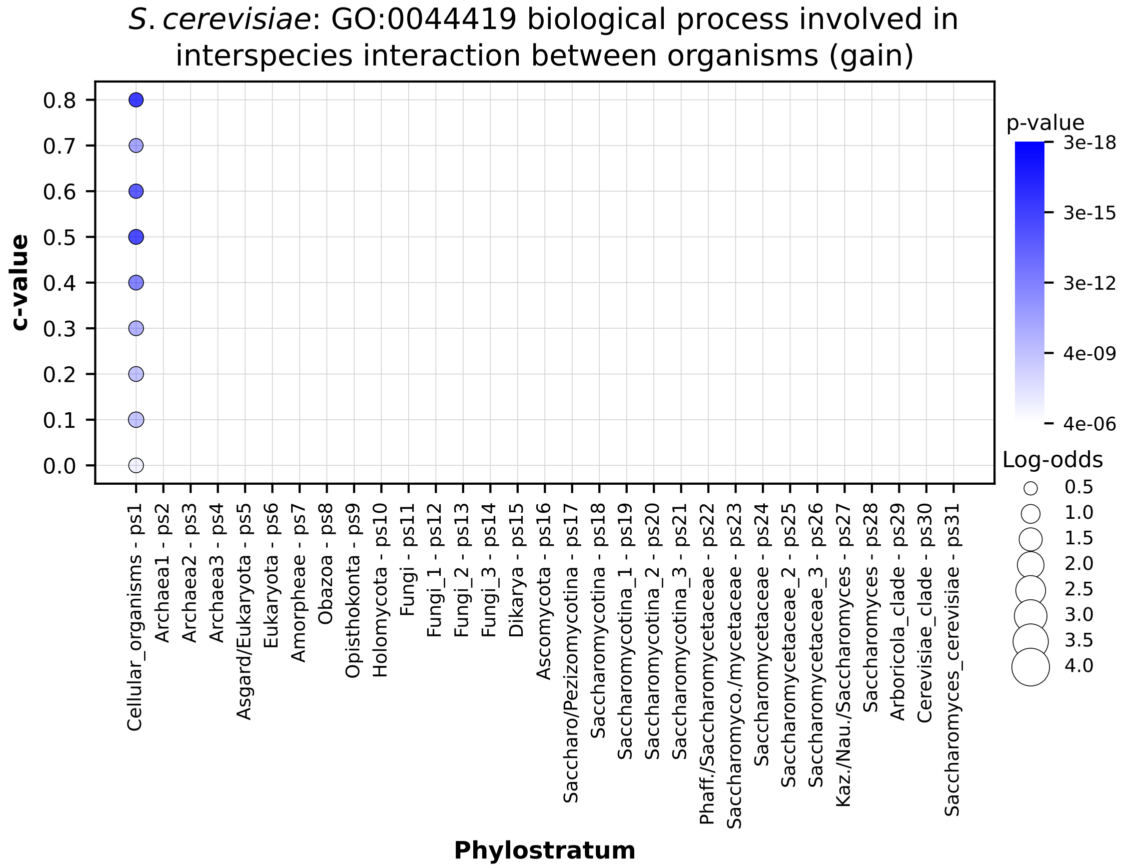

S. cerevisiae: GO:0044419 biological process involved in interspecies interaction between organisms (gain)
c-value
0.8
0.7
0.6
0.5
0.4
0.3
0.2
0.1
0.0
p-value
3e-18
3e-15
3e-12
4e-09
4e-06
Log-odds
0.5
1.0
1.5
2.0
2.5
3.0
3.5
4.0
Cellular_organisms - ps1
Archaea1 - ps2
Archaea2 - ps3
Archaea3 - ps4
Asgard/Eukaryota - ps5
Eukaryota - ps6
Amorpheae - ps7
Obazoa - ps8
Opisthokonta - ps9
Holomycota - ps10
Fungi - ps11
Fungi_1 - ps12
Fungi_2 - ps13
Fungi_3 - ps14
Dikarya - ps15
Ascomycota - ps16
Saccharo/Pezizomycotina - ps17
Saccharomycotina - ps18
Saccharomycotina_1 - ps19
Saccharomycotina_2 - ps20
Saccharomycotina_3 - ps21
Phaff./Saccharomycetaceae - ps22
Saccharomyco./mycetaceae - ps23
Saccharomycetaceae - ps24
Saccharomycetaceae_2 - ps25
Saccharomycetaceae_3 - ps26
Kaz./Nau./Saccharomyces - ps27
Saccharomyces - ps28
Arboricola_clade - ps29
Cerevisiae_clade - ps30
Saccharomyces_cerevisiae - ps31
Phylostratum

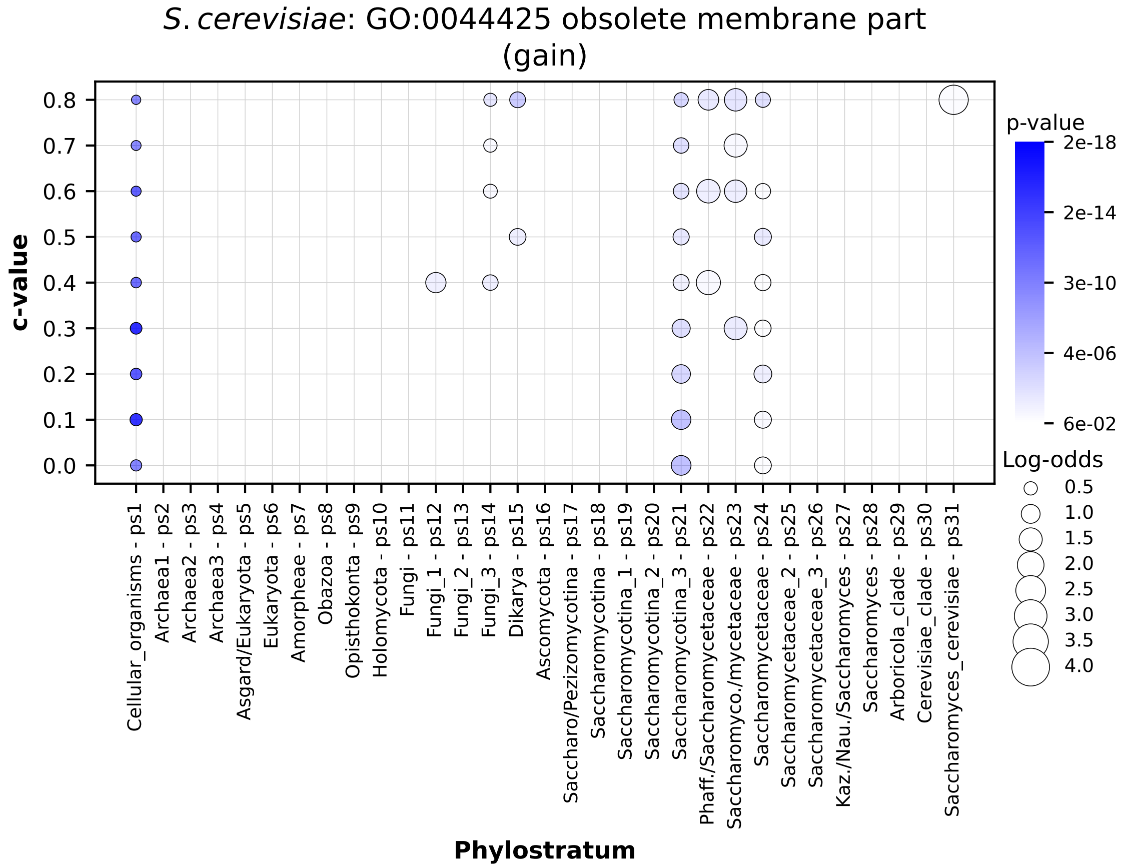

S. cerevisiae: GO:0044425 obsolete membrane part
(gain)
c-value
0.8
0.7
0.6
0.5
0.4
0.3
0.2
0.1
0.0
p-value
2e-18
2e-14
3e-10
4e-06
6e-02
Log-odds
0.5
1.0
1.5
2.0
2.5
3.0
3.5
4.0
Cellular_organisms - ps1
Archaea1 - ps2
Archaea2 - ps3
Archaea3 - ps4
Asgard/Eukaryota - ps5
Eukaryota - ps6
Amorpheae - ps7
Obazoa - ps8
Opisthokonta - ps9
Holomycota - ps10
Fungi - ps11
Fungi_1 - ps12
Fungi_2 - ps13
Fungi_3 - ps14
Dikarya - ps15
Ascomycota - ps16
Saccharo/Pezizomycotina - ps17
Saccharomycotina - ps18
Saccharomycotina_1 - ps19
Saccharomycotina_2 - ps20
Saccharomycotina_3 - ps21
Phaff./Saccharomycetaceae - ps22
Saccharomyco./mycetaceae - ps23
Saccharomycetaceae - ps24
Saccharomycetaceae_2 - ps25
Saccharomycetaceae_3 - ps26
Kaz./Nau./Saccharomyces - ps27
Saccharomyces - ps28
Arboricola_clade - ps29
Cerevisiae_clade - ps30
Saccharomyces_cerevisiae - ps31
Phylostratum

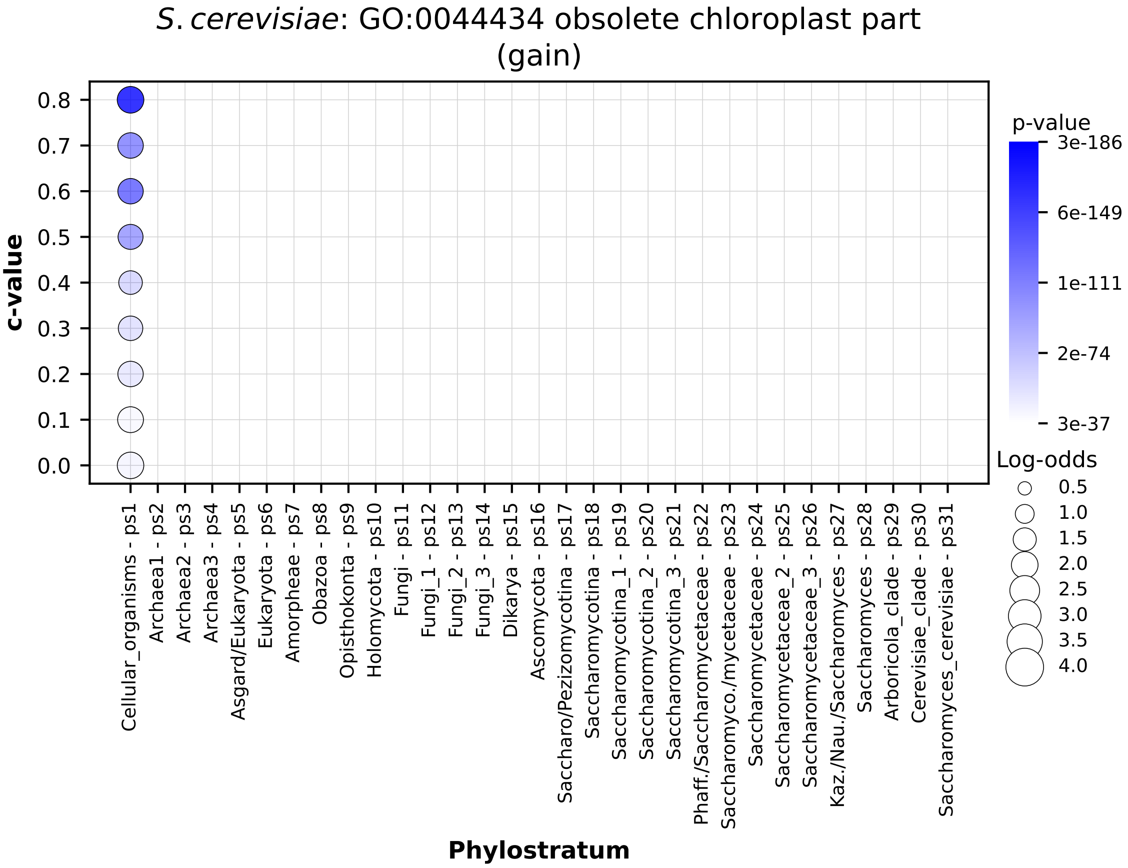
S. cerevisiae: GO:0044434 obsolete chloroplast part
(gain)
c-value
0.8
0.7
0.6
0.5
0.4
0.3
0.2
0.1
0.0
p-value
3e-186
6e-149
1e-111
2e-74
3e-37
Log-odds
0.5
1.0
1.5
2.0
2.5
3.0
3.5
4.0
Cellular_organisms - ps1
Archaea1 - ps2
Archaea2 - ps3
Archaea3 - ps4
Asgard/Eukaryota - ps5
Eukaryota - ps6
Amorpheae - ps7
Obazoa - ps8
Opisthokonta - ps9
Holomycota - ps10
Fungi - ps11
Fungi_1 - ps12
Fungi_2 - ps13
Fungi_3 - ps14
Dikarya - ps15
Ascomycota - ps16
Saccharo/Pezizomycotina - ps17
Saccharomycotina - ps18
Saccharomycotina_1 - ps19
Saccharomycotina_2 - ps20
Saccharomycotina_3 - ps21
Phaff./Saccharomycetaceae - ps22
Saccharomyco./mycetaceae - ps23
Saccharomycetaceae - ps24
Saccharomycetaceae_2 - ps25
Saccharomycetaceae_3 - ps26
Kaz./Nau./Saccharomyces - ps27
Saccharomyces - ps28
Arboricola_clade - ps29
Cerevisiae_clade - ps30
Saccharomyces_cerevisiae - ps31
Phylostratum

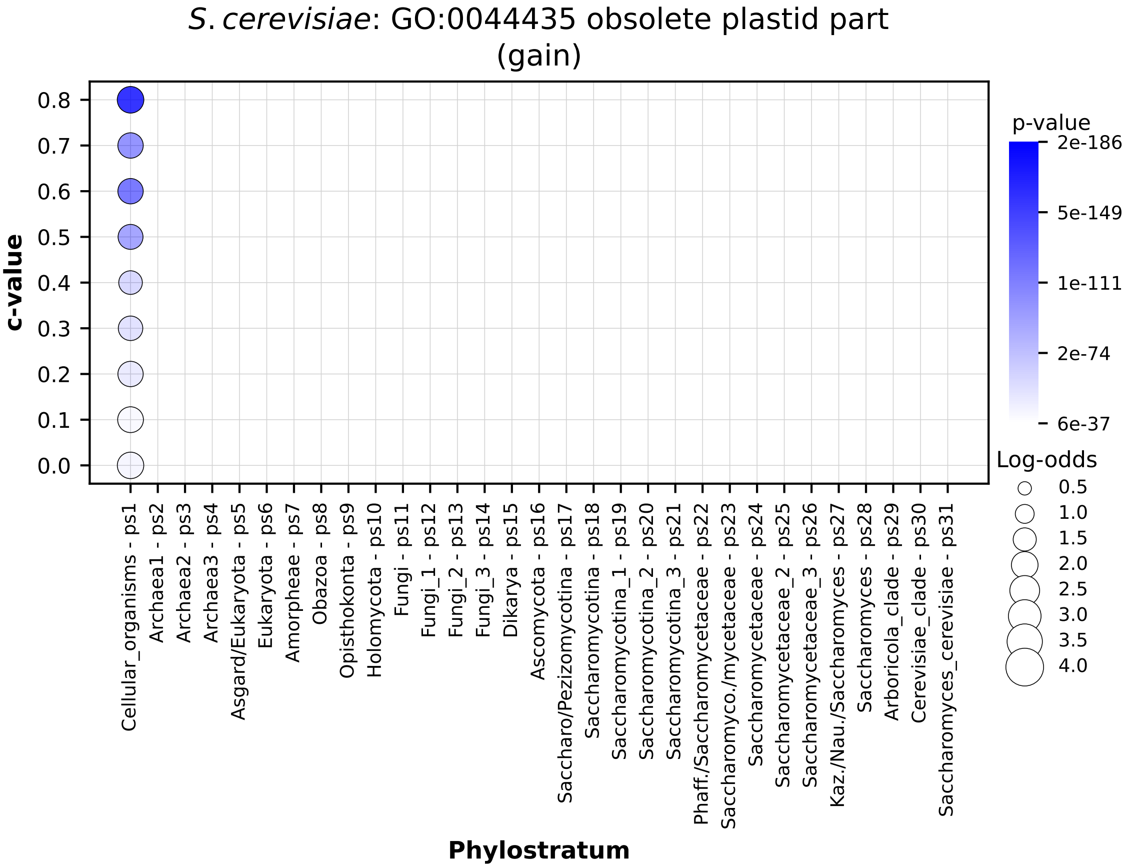
S. cerevisiae: GO:0044435 obsolete plastid part
(gain)
c-value
0.8
0.7
0.6
0.5
0.4
0.3
0.2
0.1
0.0
p-value
2e-186
5e-149
1e-111
2e-74
6e-37
Log-odds
0.5
1.0
1.5
2.0
2.5
3.0
3.5
4.0
Cellular_organisms - ps1
Archaea1 - ps2
Archaea2 - ps3
Archaea3 - ps4
Asgard/Eukaryota - ps5
Eukaryota - ps6
Amorpheae - ps7
Obazoa - ps8
Opisthokonta - ps9
Holomycota - ps10
Fungi - ps11
Fungi_1 - ps12
Fungi_2 - ps13
Fungi_3 - ps14
Dikarya - ps15
Ascomycota - ps16
Saccharo/Pezizomycotina - ps17
Saccharomycotina - ps18
Saccharomycotina_1 - ps19
Saccharomycotina_2 - ps20
Saccharomycotina_3 - ps21
Phaff./Saccharomycetaceae - ps22
Saccharomyco./mycetaceae - ps23
Saccharomycetaceae - ps24
Saccharomycetaceae_2 - ps25
Saccharomycetaceae_3 - ps26
Kaz./Nau./Saccharomyces - ps27
Saccharomyces - ps28
Arboricola_clade - ps29
Cerevisiae_clade - ps30
Saccharomyces_cerevisiae - ps31
Phylostratum

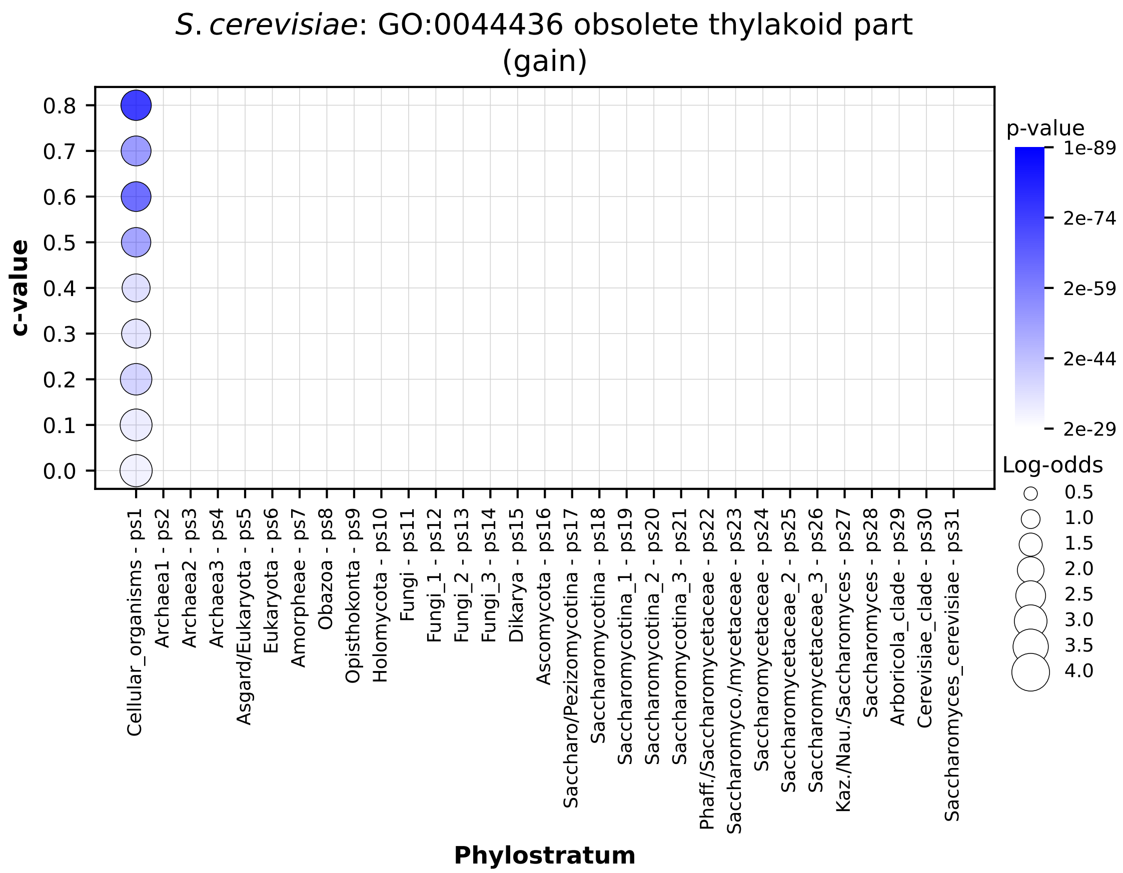
S. cerevisiae: GO:0044436 obsolete thylakoid part
(gain)
c-value
0.8
0.7
0.6
0.5
0.4
0.3
0.2
0.1
0.0
p-value
1e-89
2e-74
2e-59
2e-44
2e-29
Log-odds
0.5
1.0
1.5
2.0
2.5
3.0
3.5
4.0
Cellular_organisms - ps1
Archaea1 - ps2
Archaea2 - ps3
Archaea3 - ps4
Asgard/Eukaryota - ps5
Eukaryota - ps6
Amorpheae - ps7
Obazoa - ps8
Opisthokonta - ps9
Holomycota - ps10
Fungi - ps11
Fungi_1 - ps12
Fungi_2 - ps13
Fungi_3 - ps14
Dikarya - ps15
Ascomycota - ps16
Saccharo/Pezizomycotina - ps17
Saccharomycotina - ps18
Saccharomycotina_1 - ps19
Saccharomycotina_2 - ps20
Saccharomycotina_3 - ps21
Phaff./Saccharomycetaceae - ps22
Saccharomyco./mycetaceae - ps23
Saccharomycetaceae - ps24
Saccharomycetaceae_2 - ps25
Saccharomycetaceae_3 - ps26
Kaz./Nau./Saccharomyces - ps27
Saccharomyces - ps28
Arboricola_clade - ps29
Cerevisiae_clade - ps30
Saccharomyces_cerevisiae - ps31
Phylostratum

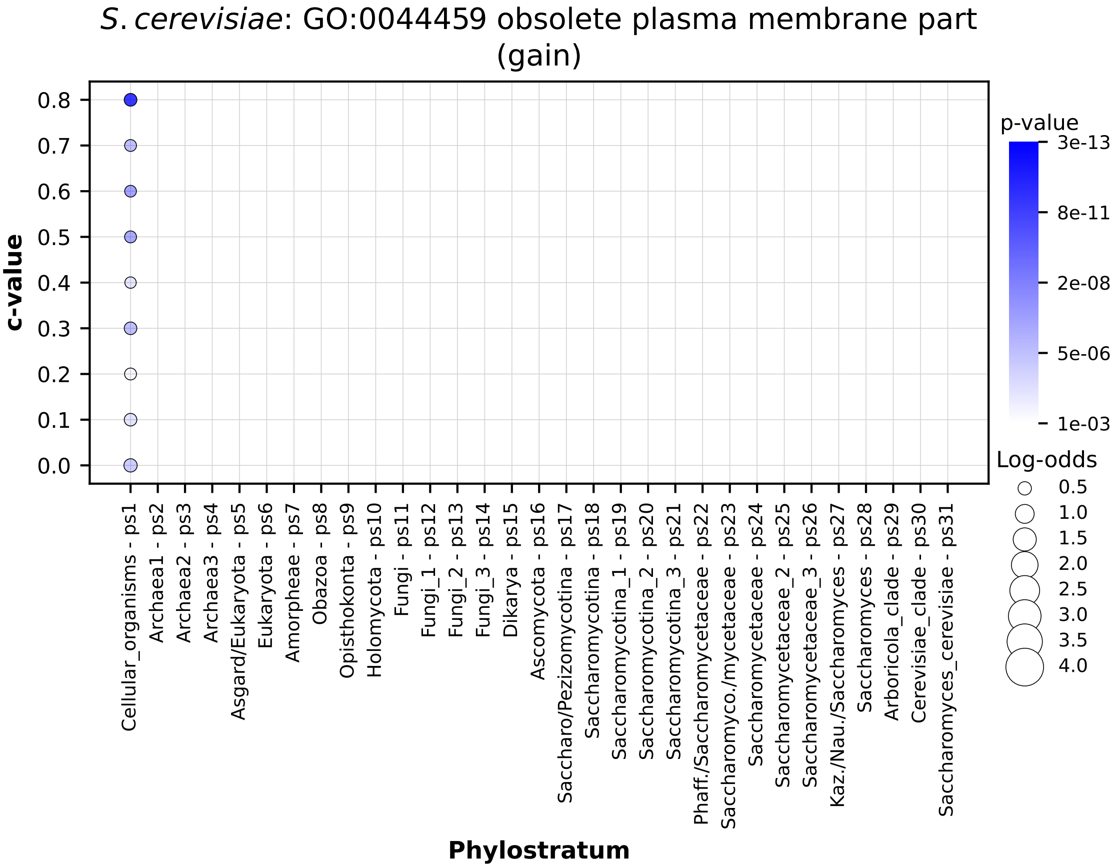
S. cerevisiae: GO:0044459 obsolete plasma membrane part (gain)
c-value
0.0
0.1
0.2
0.3
0.4
0.5
0.6
0.7
0.8
Phylostratum
Cellular_organisms - ps1
Archaea1 - ps2
Archaea2 - ps3
Archaea3 - ps4
Asgard/Eukaryota - ps5
Eukaryota - ps6
Amorpheae - ps7
Obazoa - ps8
Opisthokonta - ps9
Holomycota - ps10
Fungi - ps11
Fungi_1 - ps12
Fungi_2 - ps13
Fungi_3 - ps14
Dikarya - ps15
Ascomycota - ps16
Saccharo/Pezizomycotina - ps17
Saccharomycotina - ps18
Saccharomycotina_1 - ps19
Saccharomycotina_2 - ps20
Saccharomycotina_3 - ps21
Phaff./Saccharomycetaceae - ps22
Saccharomyco./mycetaceae - ps23
Saccharomycetaceae - ps24
Saccharomycetaceae_2 - ps25
Saccharomycetaceae_3 - ps26
Kaz./Nau./Saccharomyces - ps27
Saccharomyces - ps28
Arboricola_clade - ps29
Cerevisiae_clade - ps30
Saccharomyces_cerevisiae - ps31
p-value
3e-13
8e-11
2e-08
5e-06
1e-03
Log-odds
0.5
1.0
1.5
2.0
2.5
3.0
3.5
4.0

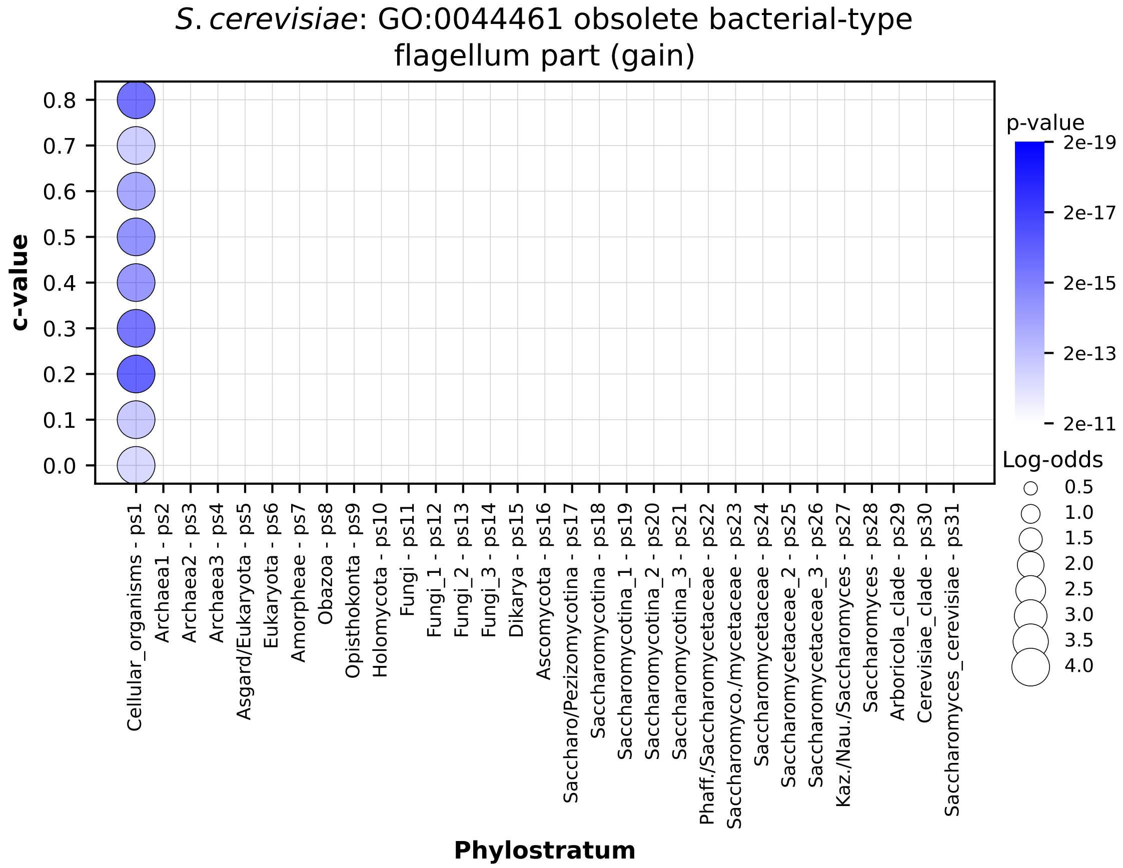
S. cerevisiae: GO:0044461 obsolete bacterial-type flagellum part (gain)
c-value
0.8
0.7
0.6
0.5
0.4
0.3
0.2
0.1
0.0
p-value
2e-19
2e-17
2e-15
2e-13
2e-11
Log-odds
0.5
1.0
1.5
2.0
2.5
3.0
3.5
4.0
Cellular_organisms - ps1
Archaea1 - ps2
Archaea2 - ps3
Archaea3 - ps4
Asgard/Eukaryota - ps5
Eukaryota - ps6
Amorpheae - ps7
Obazoa - ps8
Opisthokonta - ps9
Holomycota - ps10
Fungi - ps11
Fungi_1 - ps12
Fungi_2 - ps13
Fungi_3 - ps14
Dikarya - ps15
Ascomycota - ps16
Saccharo/Pezizomycotina - ps17
Saccharomycotina - ps18
Saccharomycotina_1 - ps19
Saccharomycotina_2 - ps20
Saccharomycotina_3 - ps21
Phaff./Saccharomycetaceae - ps22
Saccharomyco./mycetaceae - ps23
Saccharomycetaceae - ps24
Saccharomycetaceae_2 - ps25
Saccharomycetaceae_3 - ps26
Kaz./Nau./Saccharomyces - ps27
Saccharomyces - ps28
Arboricola_clade - ps29
Cerevisiae_clade - ps30
Saccharomyces_cerevisiae - ps31
Phylostratum

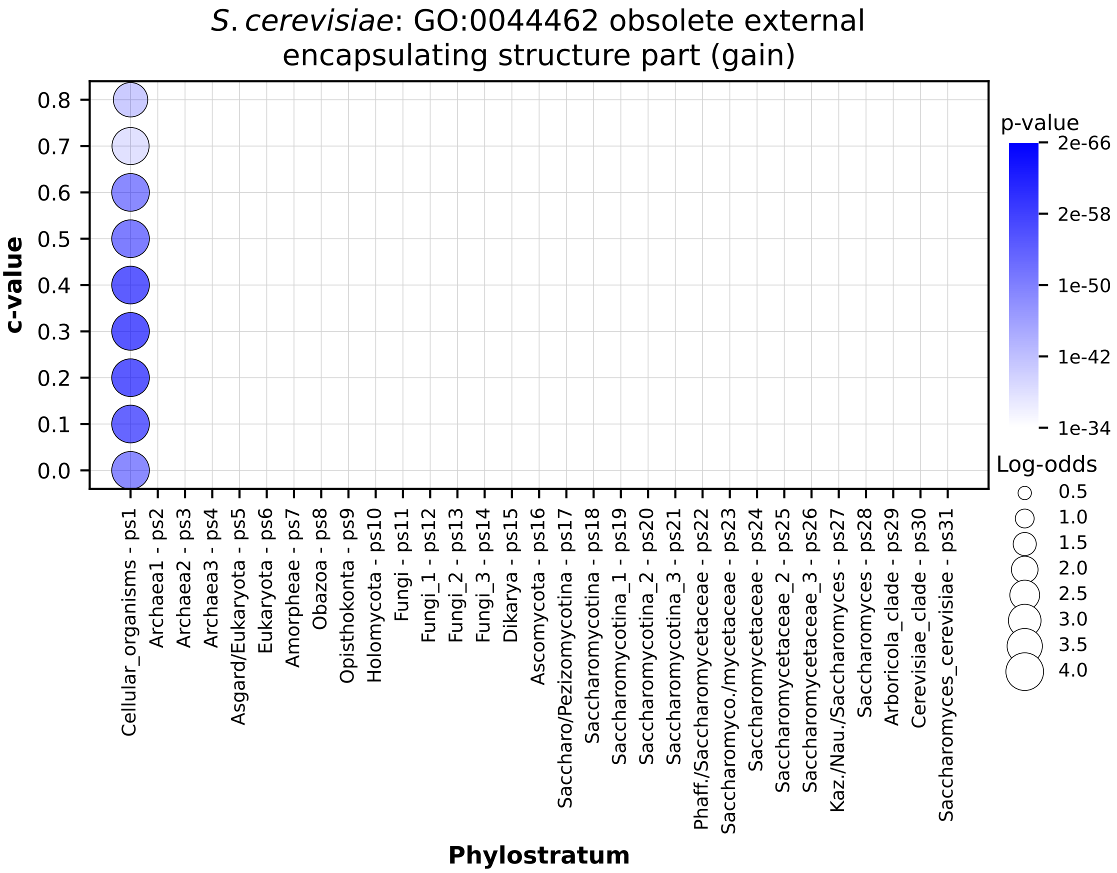

S. cerevisiae: GO:0044462 obsolete external encapsulating structure part (gain)
c-value
0.8
0.7
0.6
0.5
0.4
0.3
0.2
0.1
0.0
p-value
2e-66
2e-58
1e-50
1e-42
1e-34
Log-odds
0.5
1.0
1.5
2.0
2.5
3.0
3.5
4.0
Cellular_organisms - ps1
Archaea1 - ps2
Archaea2 - ps3
Archaea3 - ps4
Asgard/Eukaryota - ps5
Eukaryota - ps6
Amorpheae - ps7
Obazoa - ps8
Opisthokonta - ps9
Holomycota - ps10
Fungi - ps11
Fungi_1 - ps12
Fungi_2 - ps13
Fungi_3 - ps14
Dikarya - ps15
Ascomycota - ps16
Saccharo/Pezizomycotina - ps17
Saccharomycotina - ps18
Saccharomycotina_1 - ps19
Saccharomycotina_2 - ps20
Saccharomycotina_3 - ps21
Phaff./Saccharomycetaceae - ps22
Saccharomyco./mycetaceae - ps23
Saccharomycetaceae - ps24
Saccharomycetaceae_2 - ps25
Saccharomycetaceae_3 - ps26
Kaz./Nau./Saccharomyces - ps27
Saccharomyces - ps28
Arboricola_clade - ps29
Cerevisiae_clade - ps30
Saccharomyces_cerevisiae - ps31
Phylostratum

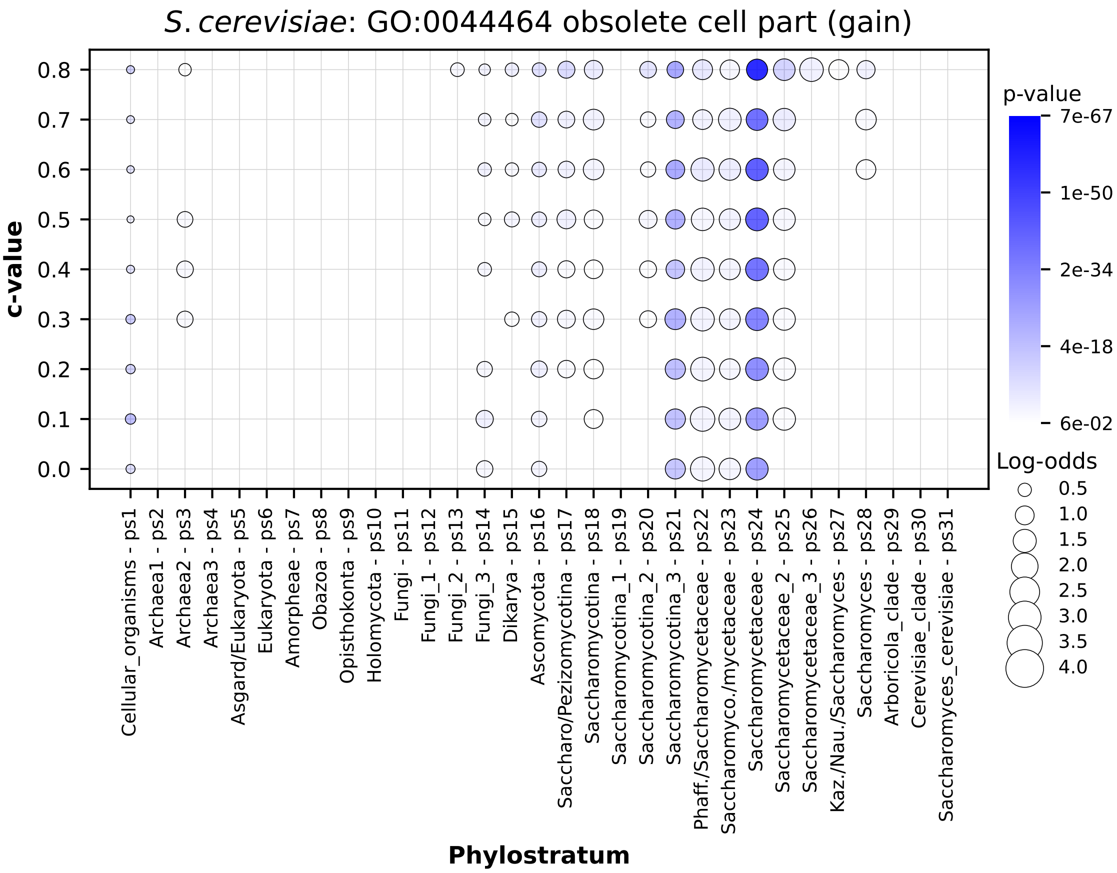
S. cerevisiae: GO:0044464 obsolete cell part (gain)
c-value
0.8
0.7
0.6
0.5
0.4
0.3
0.2
0.1
0.0
p-value
7e-67
1e-50
2e-34
4e-18
6e-02
Log-odds
0.5
1.0
1.5
2.0
2.5
3.0
3.5
4.0
Cellular_organisms - ps1
Archaea1 - ps2
Archaea2 - ps3
Archaea3 - ps4
Asgard/Eukaryota - ps5
Eukaryota - ps6
Amorpheae - ps7
Obazoa - ps8
Opisthokonta - ps9
Holomycota - ps10
Fungi - ps11
Fungi_1 - ps12
Fungi_2 - ps13
Fungi_3 - ps14
Dikarya - ps15
Ascomycota - ps16
Saccharo/Pezizomycotina - ps17
Saccharomycotina - ps18
Saccharomycotina_1 - ps19
Saccharomycotina_2 - ps20
Saccharomycotina_3 - ps21
Phaff./Saccharomycetaceae - ps22
Saccharomyco./mycetaceae - ps23
Saccharomycetaceae - ps24
Saccharomycetaceae_2 - ps25
Saccharomycetaceae_3 - ps26
Kaz./Nau./Saccharomyces - ps27
Saccharomyces - ps28
Arboricola_clade - ps29
Cerevisiae_clade - ps30
Saccharomyces_cerevisiae - ps31
Phylostratum

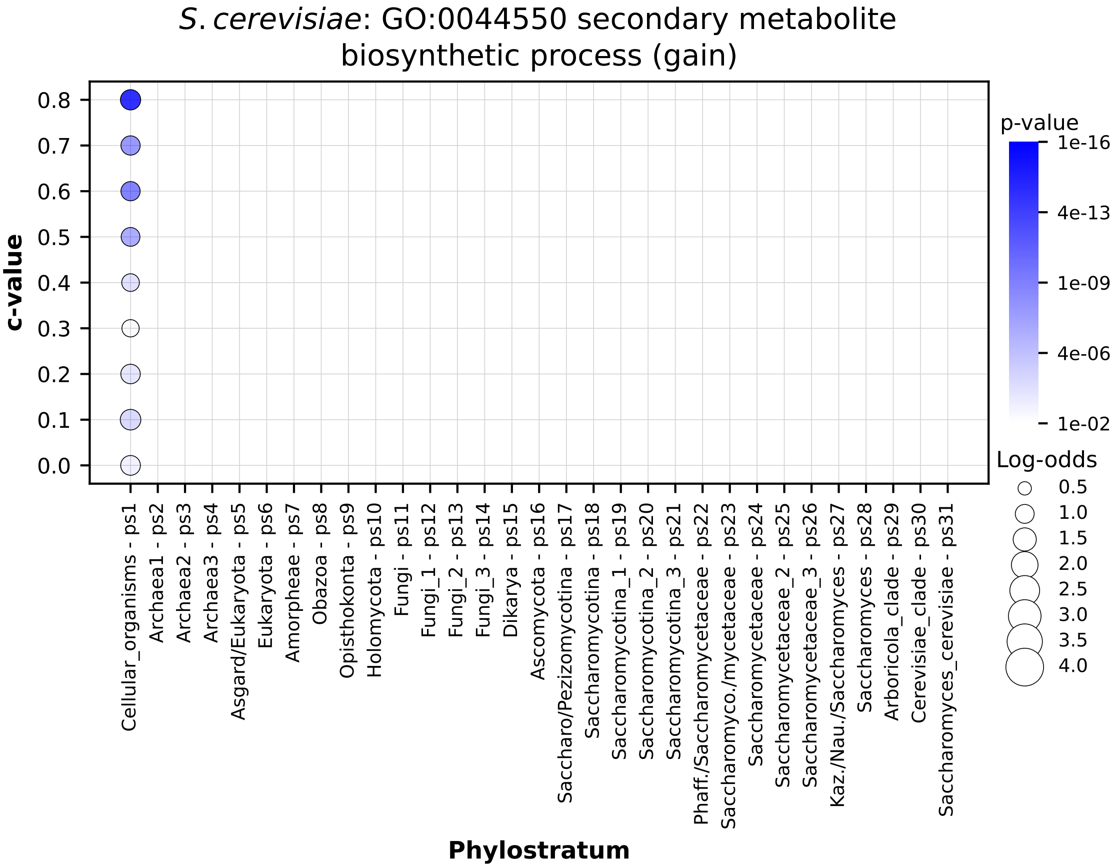
S. cerevisiae: GO:0044550 secondary metabolite biosynthetic process (gain)
c-value
0.8
0.7
0.6
0.5
0.4
0.3
0.2
0.1
0.0
p-value
1e-16
4e-13
1e-09
4e-06
1e-02
Log-odds
0.5
1.0
1.5
2.0
2.5
3.0
3.5
4.0
Cellular_organisms - ps1
Archaea1 - ps2
Archaea2 - ps3
Archaea3 - ps4
Asgard/Eukaryota - ps5
Eukaryota - ps6
Amorpheae - ps7
Obazoa - ps8
Opisthokonta - ps9
Holomycota - ps10
Fungi - ps11
Fungi_1 - ps12
Fungi_2 - ps13
Fungi_3 - ps14
Dikarya - ps15
Ascomycota - ps16
Saccharo/Pezizomycotina - ps17
Saccharomycotina - ps18
Saccharomycotina_1 - ps19
Saccharomycotina_2 - ps20
Saccharomycotina_3 - ps21
Phaff./Saccharomycetaceae - ps22
Saccharomyco./mycetaceae - ps23
Saccharomycetaceae - ps24
Saccharomycetaceae_2 - ps25
Saccharomycetaceae_3 - ps26
Kaz./Nau./Saccharomyces - ps27
Saccharomyces - ps28
Arboricola_clade - ps29
Cerevisiae_clade - ps30
Saccharomyces_cerevisiae - ps31
Phylostratum

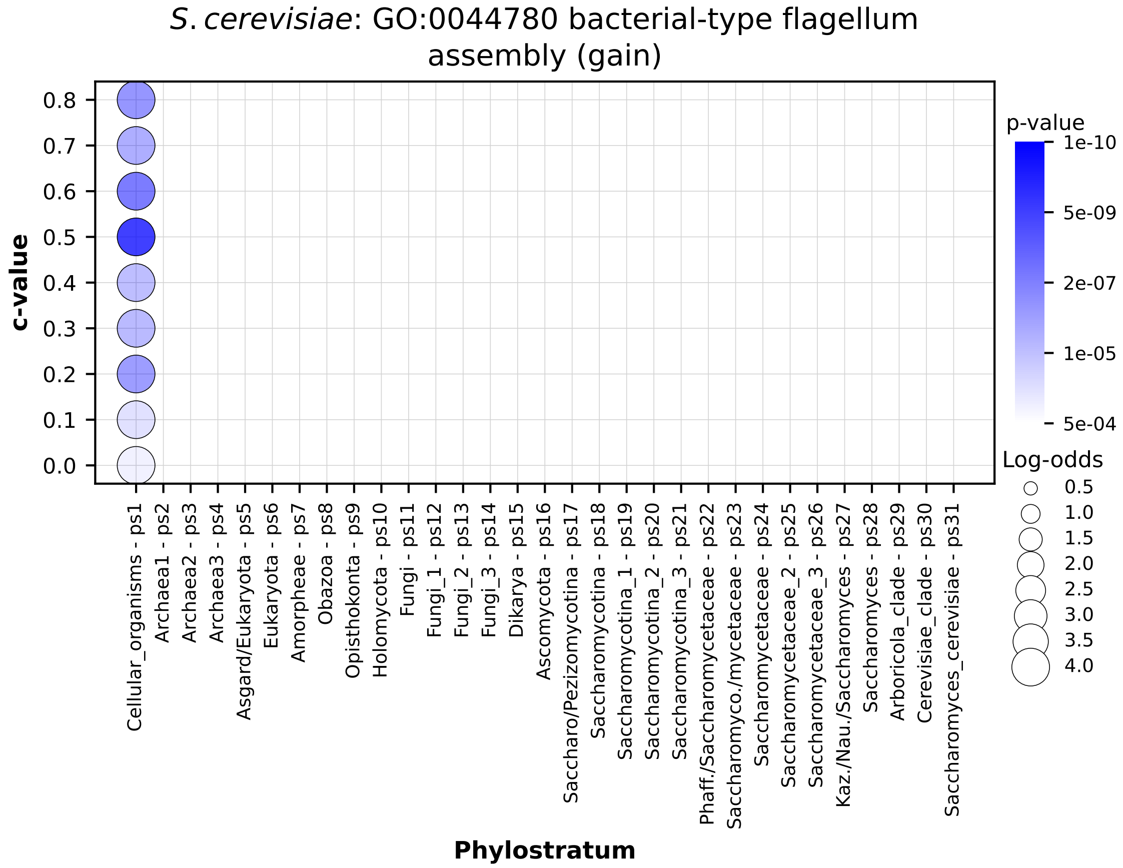

S. cerevisiae: GO:0044780 bacterial-type flagellum assembly (gain)
c-value
0.8
0.7
0.6
0.5
0.4
0.3
0.2
0.1
0.0
p-value
1e-10
5e-09
2e-07
1e-05
5e-04
Log-odds
0.5
1.0
1.5
2.0
2.5
3.0
3.5
4.0
Cellular_organisms - ps1
Archaea1 - ps2
Archaea2 - ps3
Archaea3 - ps4
Asgard/Eukaryota - ps5
Eukaryota - ps6
Amorpheae - ps7
Obazoa - ps8
Opisthokonta - ps9
Holomycota - ps10
Fungi - ps11
Fungi_1 - ps12
Fungi_2 - ps13
Fungi_3 - ps14
Dikarya - ps15
Ascomycota - ps16
Saccharo/Pezizomycotina - ps17
Saccharomycotina - ps18
Saccharomycotina_1 - ps19
Saccharomycotina_2 - ps20
Saccharomycotina_3 - ps21
Phaff./Saccharomycetaceae - ps22
Saccharomyco./mycetaceae - ps23
Saccharomycetaceae - ps24
Saccharomycetaceae_2 - ps25
Saccharomycetaceae_3 - ps26
Kaz./Nau./Saccharomyces - ps27
Saccharomyces - ps28
Arboricola_clade - ps29
Cerevisiae_clade - ps30
Saccharomyces_cerevisiae - ps31
Phylostratum

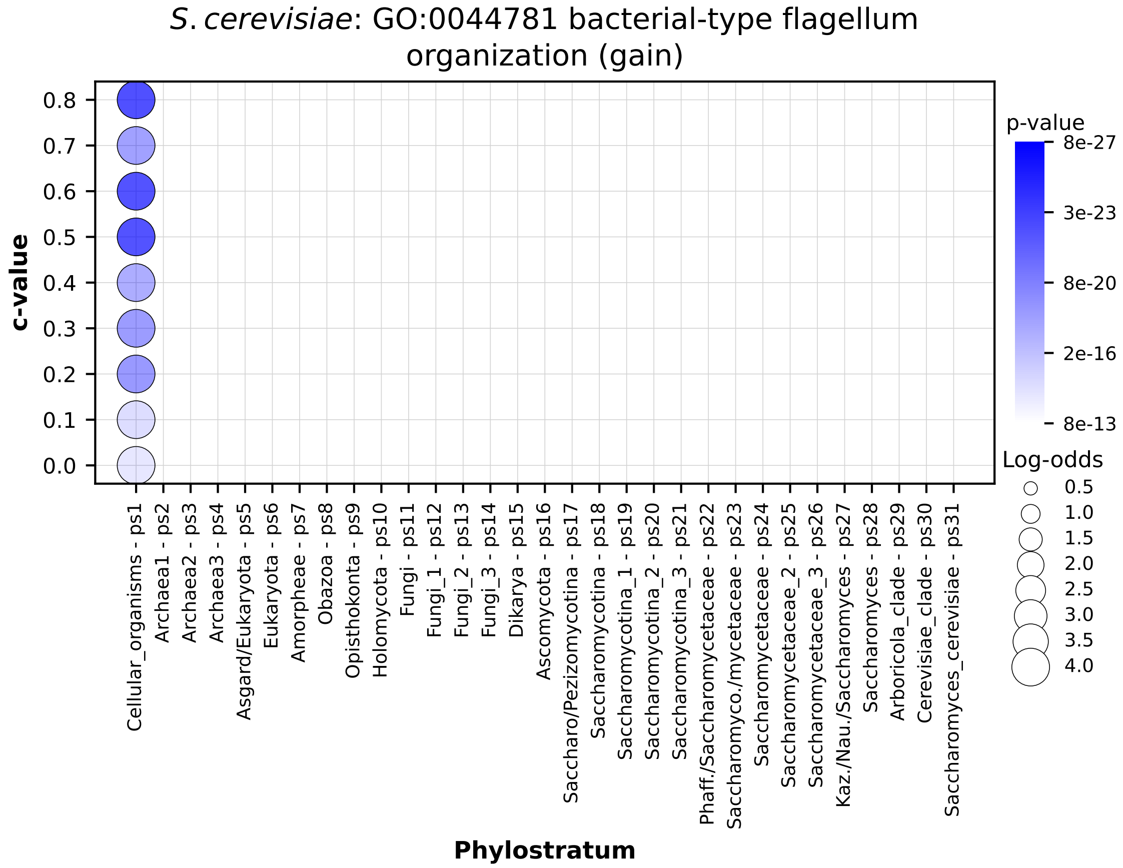
S. cerevisiae: GO:0044781 bacterial-type flagellum organization (gain)
c-value
0.8
0.7
0.6
0.5
0.4
0.3
0.2
0.1
0.0
Phylostratum
Cellular_organisms - ps1
Archaea1 - ps2
Archaea2 - ps3
Archaea3 - ps4
Asgard/Eukaryota - ps5
Eukaryota - ps6
Amorpheae - ps7
Obazoa - ps8
Opisthokonta - ps9
Holomycota - ps10
Fungi - ps11
Fungi_1 - ps12
Fungi_2 - ps13
Fungi_3 - ps14
Dikarya - ps15
Ascomycota - ps16
Saccharo/Pezizomycotina - ps17
Saccharomycotina - ps18
Saccharomycotina_1 - ps19
Saccharomycotina_2 - ps20
Saccharomycotina_3 - ps21
Phaff./Saccharomycetaceae - ps22
Saccharomyco./mycetaceae - ps23
Saccharomycetaceae - ps24
Saccharomycetaceae_2 - ps25
Saccharomycetaceae_3 - ps26
Kaz./Nau./Saccharomyces - ps27
Saccharomyces - ps28
Arboricola_clade - ps29
Cerevisiae_clade - ps30
Saccharomyces_cerevisiae - ps31
p-value
8e-27
3e-23
8e-20
2e-16
8e-13
Log-odds
0.5
1.0
1.5
2.0
2.5
3.0
3.5
4.0

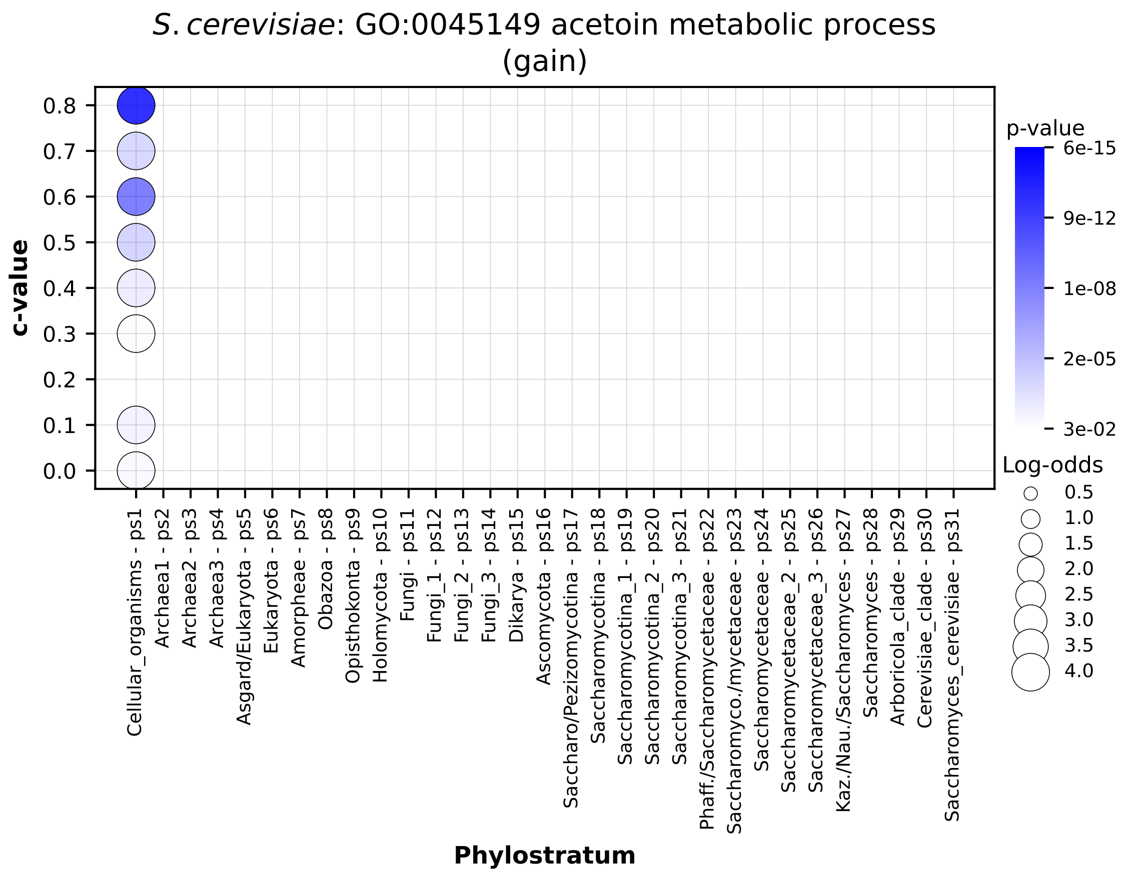
S. cerevisiae: GO:0045149 acetoin metabolic process
(gain)
c-value
0.8
0.7
0.6
0.5
0.4
0.3
0.2
0.1
0.0
p-value
6e-15
9e-12
1e-08
2e-05
3e-02
Log-odds
0.5
1.0
1.5
2.0
2.5
3.0
3.5
4.0
Cellular_organisms - ps1
Archaea1 - ps2
Archaea2 - ps3
Archaea3 - ps4
Asgard/Eukaryota - ps5
Eukaryota - ps6
Amorpheae - ps7
Obazoa - ps8
Opisthokonta - ps9
Holomycota - ps10
Fungi - ps11
Fungi_1 - ps12
Fungi_2 - ps13
Fungi_3 - ps14
Dikarya - ps15
Ascomycota - ps16
Saccharo/Pezizomycotina - ps17
Saccharomycotina - ps18
Saccharomycotina_1 - ps19
Saccharomycotina_2 - ps20
Saccharomycotina_3 - ps21
Phaff./Saccharomycetaceae - ps22
Saccharomyco./mycetaceae - ps23
Saccharomycetaceae - ps24
Saccharomycetaceae_2 - ps25
Saccharomycetaceae_3 - ps26
Kaz./Nau./Saccharomyces - ps27
Saccharomyces - ps28
Arboricola_clade - ps29
Cerevisiae_clade - ps30
Saccharomyces_cerevisiae - ps31
Phylostratum

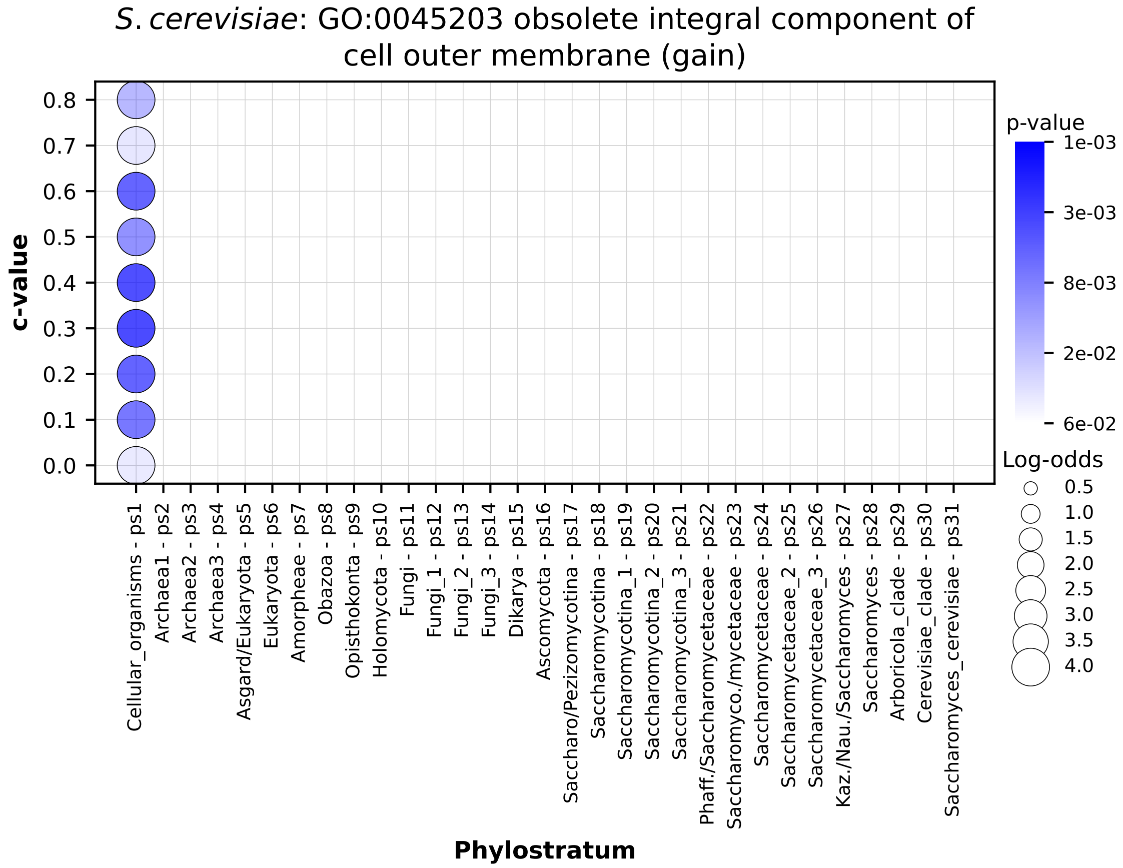

S. cerevisiae: GO:0045203 obsolete integral component of cell outer membrane (gain)
c-value
0.8
0.7
0.6
0.5
0.4
0.3
0.2
0.1
0.0
Phylostratum
Cellular_organisms - ps1
Archaea1 - ps2
Archaea2 - ps3
Archaea3 - ps4
Asgard/Eukaryota - ps5
Eukaryota - ps6
Amorpheae - ps7
Obazoa - ps8
Opisthokonta - ps9
Holomycota - ps10
Fungi - ps11
Fungi_1 - ps12
Fungi_2 - ps13
Fungi_3 - ps14
Dikarya - ps15
Ascomycota - ps16
Saccharo/Pezizomycotina - ps17
Saccharomycotina - ps18
Saccharomycotina_1 - ps19
Saccharomycotina_2 - ps20
Saccharomycotina_3 - ps21
Phaff./Saccharomycetaceae - ps22
Saccharomyco./mycetaceae - ps23
Saccharomycetaceae - ps24
Saccharomycetaceae_2 - ps25
Saccharomycetaceae_3 - ps26
Kaz./Nau./Saccharomyces - ps27
Saccharomyces - ps28
Arboricola_clade - ps29
Cerevisiae_clade - ps30
Saccharomyces_cerevisiae - ps31
p-value
1e-03
3e-03
8e-03
2e-02
6e-02
Log-odds
0.5
1.0
1.5
2.0
2.5
3.0
3.5
4.0

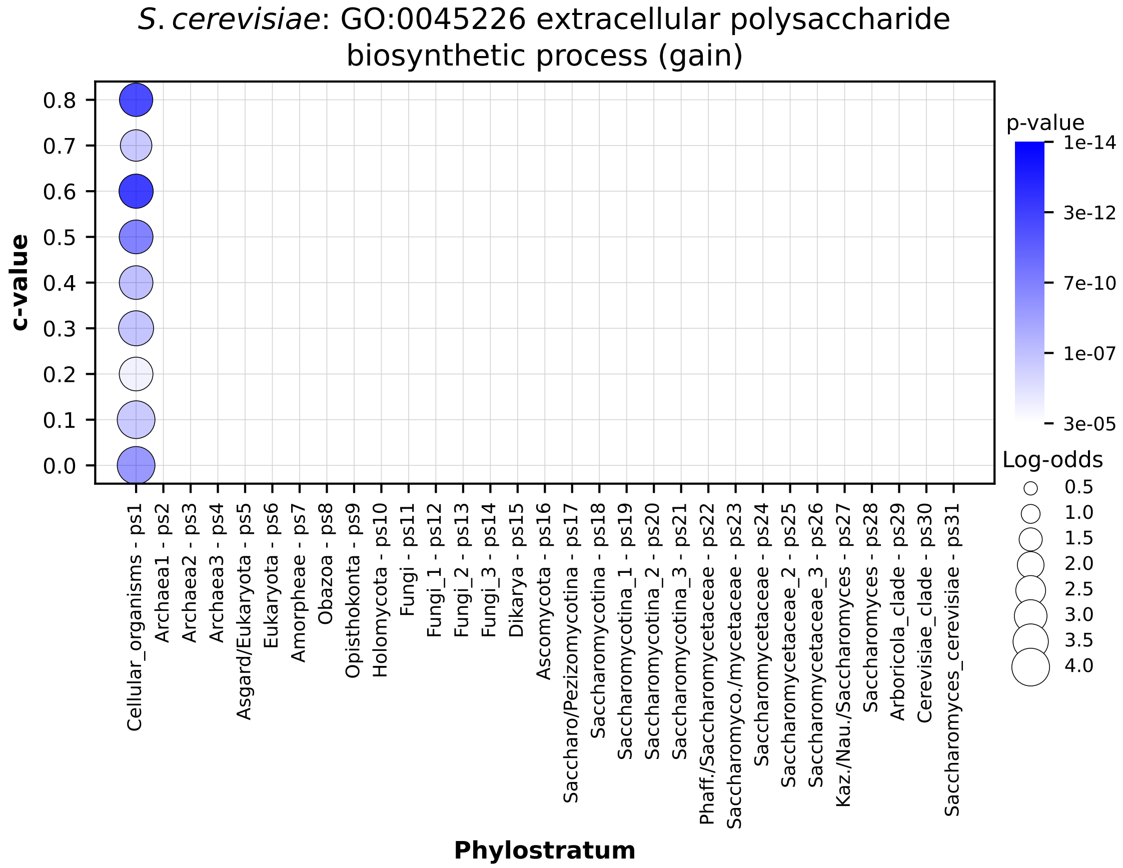

S. cerevisiae: GO:0045226 extracellular polysaccharide biosynthetic process (gain)
c-value
0.8
0.7
0.6
0.5
0.4
0.3
0.2
0.1
0.0
p-value
1e-14
3e-12
7e-10
1e-07
3e-05
Log-odds
0.5
1.0
1.5
2.0
2.5
3.0
3.5
4.0
Cellular_organisms - ps1
Archaea1 - ps2
Archaea2 - ps3
Archaea3 - ps4
Asgard/Eukaryota - ps5
Eukaryota - ps6
Amorpheae - ps7
Obazoa - ps8
Opisthokonta - ps9
Holomycota - ps10
Fungi - ps11
Fungi_1 - ps12
Fungi_2 - ps13
Fungi_3 - ps14
Dikarya - ps15
Ascomycota - ps16
Saccharo/Pezizomycotina - ps17
Saccharomycotina - ps18
Saccharomycotina_1 - ps19
Saccharomycotina_2 - ps20
Saccharomycotina_3 - ps21
Phaff./Saccharomycetaceae - ps22
Saccharomyco./mycetaceae - ps23
Saccharomycetaceae - ps24
Saccharomycetaceae_2 - ps25
Saccharomycetaceae_3 - ps26
Kaz./Nau./Saccharomyces - ps27
Saccharomyces - ps28
Arboricola_clade - ps29
Cerevisiae_clade - ps30
Saccharomyces_cerevisiae - ps31
Phylostratum

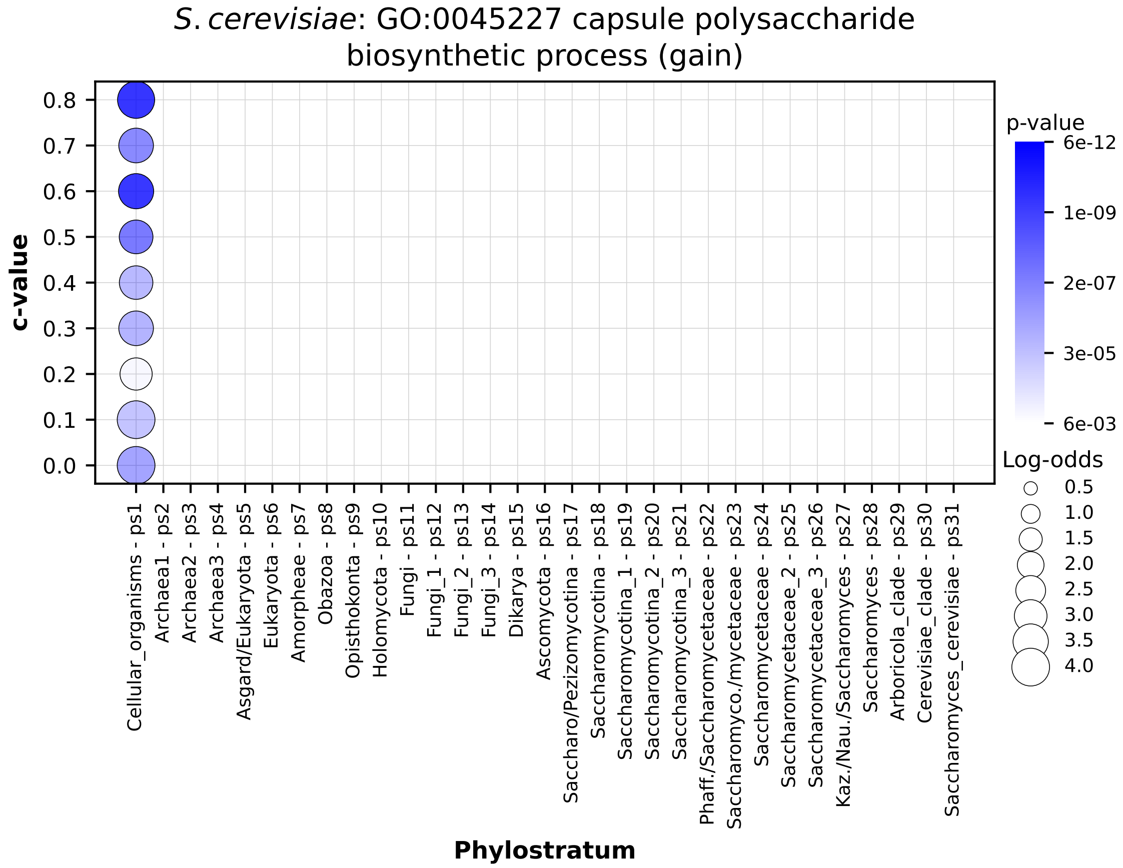
S. cerevisiae: GO:0045227 capsule polysaccharide biosynthetic process (gain)
c-value
0.8
0.7
0.6
0.5
0.4
0.3
0.2
0.1
0.0
p-value
6e-12
1e-09
2e-07
3e-05
6e-03
Log-odds
0.5
1.0
1.5
2.0
2.5
3.0
3.5
4.0
Cellular_organisms - ps1
Archaea1 - ps2
Archaea2 - ps3
Archaea3 - ps4
Asgard/Eukaryota - ps5
Eukaryota - ps6
Amorpheae - ps7
Obazoa - ps8
Opisthokonta - ps9
Holomycota - ps10
Fungi - ps11
Fungi_1 - ps12
Fungi_2 - ps13
Fungi_3 - ps14
Dikarya - ps15
Ascomycota - ps16
Saccharo/Pezizomycotina - ps17
Saccharomycotina - ps18
Saccharomycotina_1 - ps19
Saccharomycotina_2 - ps20
Saccharomycotina_3 - ps21
Phaff./Saccharomycetaceae - ps22
Saccharomyco./mycetaceae - ps23
Saccharomycetaceae - ps24
Saccharomycetaceae_2 - ps25
Saccharomycetaceae_3 - ps26
Kaz./Nau./Saccharomyces - ps27
Saccharomyces - ps28
Arboricola_clade - ps29
Cerevisiae_clade - ps30
Saccharomyces_cerevisiae - ps31
Phylostratum

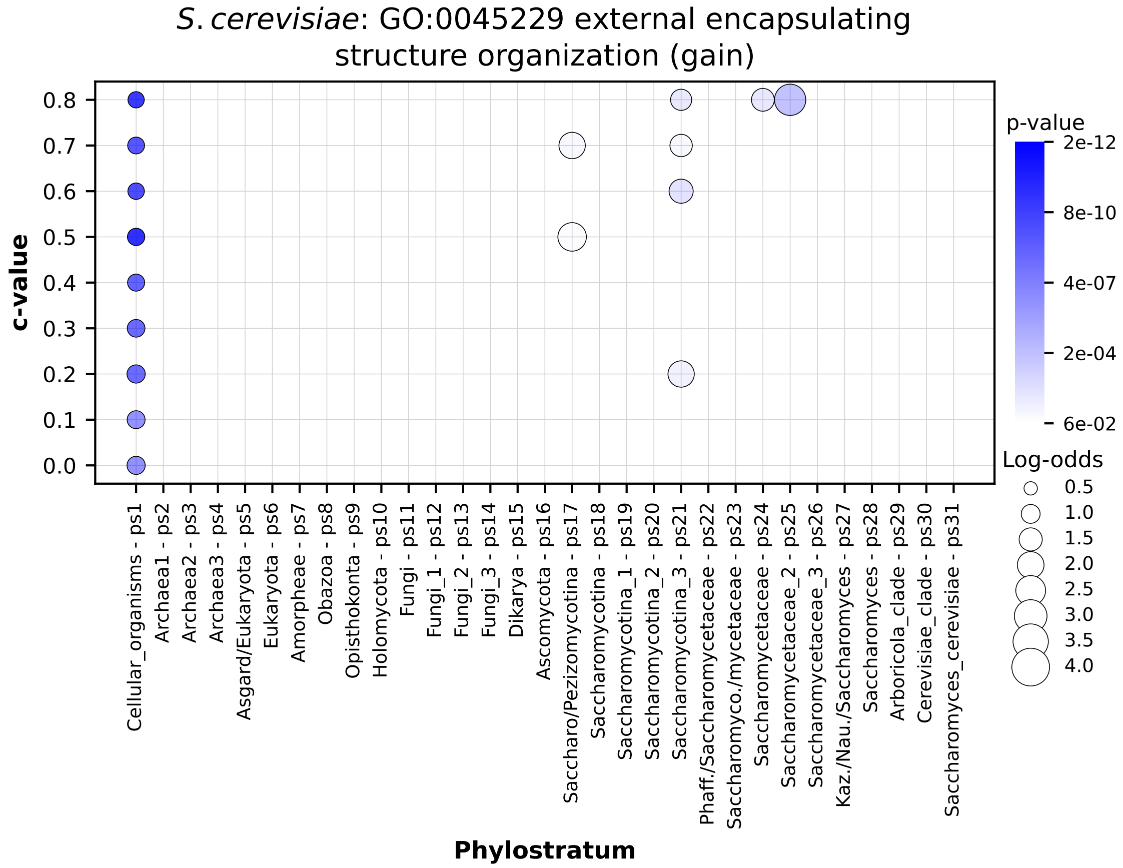
S. cerevisiae: GO:0045229 external encapsulating structure organization (gain)
c-value
0.0
0.1
0.2
0.3
0.4
0.5
0.6
0.7
0.8
p-value
2e-12
8e-10
4e-07
2e-04
6e-02
Log-odds
0.5
1.0
1.5
2.0
2.5
3.0
3.5
4.0
Cellular_organisms - ps1
Archaea1 - ps2
Archaea2 - ps3
Archaea3 - ps4
Asgard/Eukaryota - ps5
Eukaryota - ps6
Amorpheae - ps7
Obazoa - ps8
Opisthokonta - ps9
Holomycota - ps10
Fungi - ps11
Fungi_1 - ps12
Fungi_2 - ps13
Fungi_3 - ps14
Dikarya - ps15
Ascomycota - ps16
Saccharo/Pezizomycotina - ps17
Saccharomycotina - ps18
Saccharomycotina_1 - ps19
Saccharomycotina_2 - ps20
Saccharomycotina_3 - ps21
Phaff./Saccharomycetaceae - ps22
Saccharomyco./mycetaceae - ps23
Saccharomycetaceae - ps24
Saccharomycetaceae_2 - ps25
Saccharomycetaceae_3 - ps26
Kaz./Nau./Saccharomyces - ps27
Saccharomyces - ps28
Arboricola_clade - ps29
Cerevisiae_clade - ps30
Saccharomyces_cerevisiae - ps31
Phylostratum

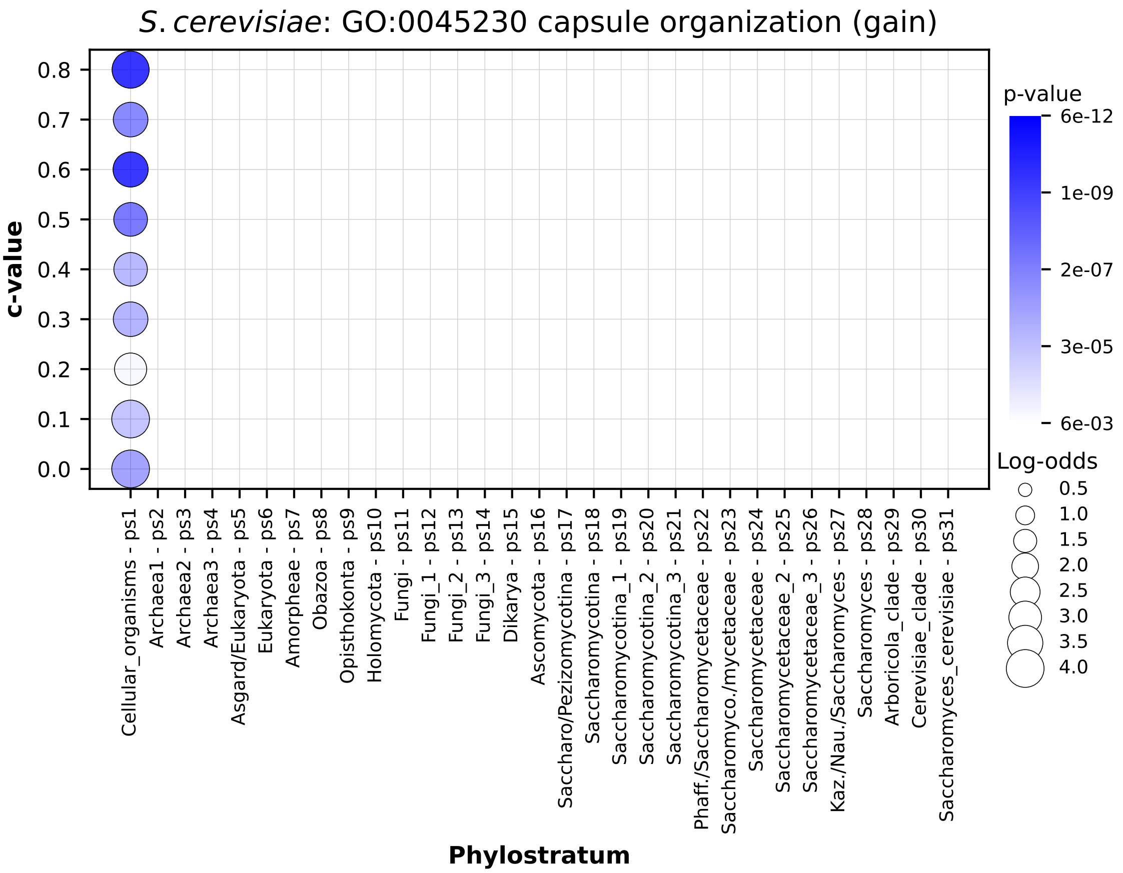

S. cerevisiae: GO:0045230 capsule organization (gain)
c-value
0.0
0.1
0.2
0.3
0.4
0.5
0.6
0.7
0.8
Phylostratum
Cellular_organisms - ps1
Archaea1 - ps2
Archaea2 - ps3
Archaea3 - ps4
Asgard/Eukaryota - ps5
Eukaryota - ps6
Amorpheae - ps7
Obazoa - ps8
Opisthokonta - ps9
Holomycota - ps10
Fungi - ps11
Fungi_1 - ps12
Fungi_2 - ps13
Fungi_3 - ps14
Dikarya - ps15
Ascomycota - ps16
Saccharo/Pezizomycotina - ps17
Saccharomycotina - ps18
Saccharomycotina_1 - ps19
Saccharomycotina_2 - ps20
Saccharomycotina_3 - ps21
Phaff./Saccharomycetaceae - ps22
Saccharomyco./mycetaceae - ps23
Saccharomycetaceae - ps24
Saccharomycetaceae_2 - ps25
Saccharomycetaceae_3 - ps26
Kaz./Nau./Saccharomyces - ps27
Saccharomyces - ps28
Arboricola_clade - ps29
Cerevisiae_clade - ps30
Saccharomyces_cerevisiae - ps31
p-value
6e-12
1e-09
2e-07
3e-05
6e-03
Log-odds
0.5
1.0
1.5
2.0
2.5
3.0
3.5
4.0

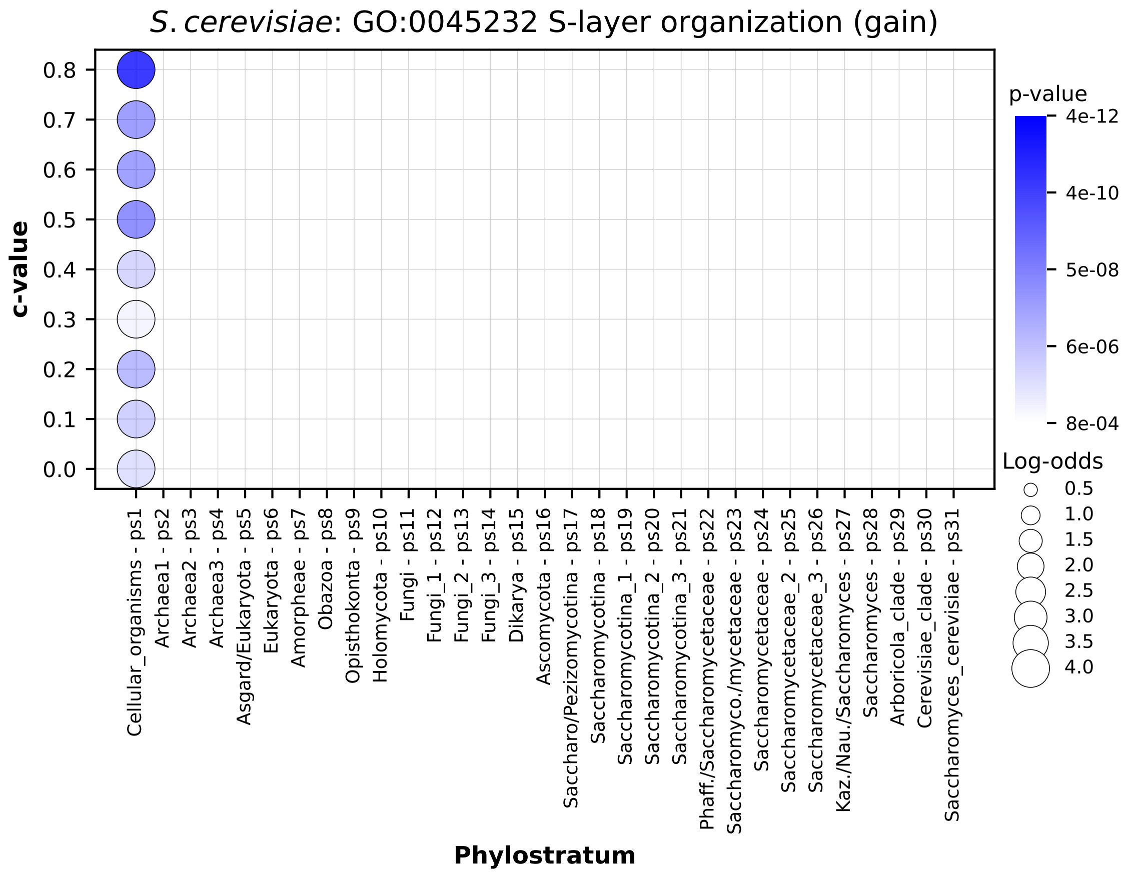

S. cerevisiae: GO:0045232 S-layer organization (gain)
c-value
0.8
0.7
0.6
0.5
0.4
0.3
0.2
0.1
0.0
p-value
4e-12
4e-10
5e-08
6e-06
8e-04
Log-odds
0.5
1.0
1.5
2.0
2.5
3.0
3.5
4.0
Cellular_organisms - ps1
Archaea1 - ps2
Archaea2 - ps3
Archaea3 - ps4
Asgard/Eukaryota - ps5
Eukaryota - ps6
Amorpheae - ps7
Obazoa - ps8
Opisthokonta - ps9
Holomycota - ps10
Fungi - ps11
Fungi_1 - ps12
Fungi_2 - ps13
Fungi_3 - ps14
Dikarya - ps15
Ascomycota - ps16
Saccharo/Pezizomycotina - ps17
Saccharomycotina - ps18
Saccharomycotina_1 - ps19
Saccharomycotina_2 - ps20
Saccharomycotina_3 - ps21
Phaff./Saccharomycetaceae - ps22
Saccharomyco./mycetaceae - ps23
Saccharomycetaceae - ps24
Saccharomycetaceae_2 - ps25
Saccharomycetaceae_3 - ps26
Kaz./Nau./Saccharomyces - ps27
Saccharomyces - ps28
Arboricola_clade - ps29
Cerevisiae_clade - ps30
Saccharomyces_cerevisiae - ps31
Phylostratum

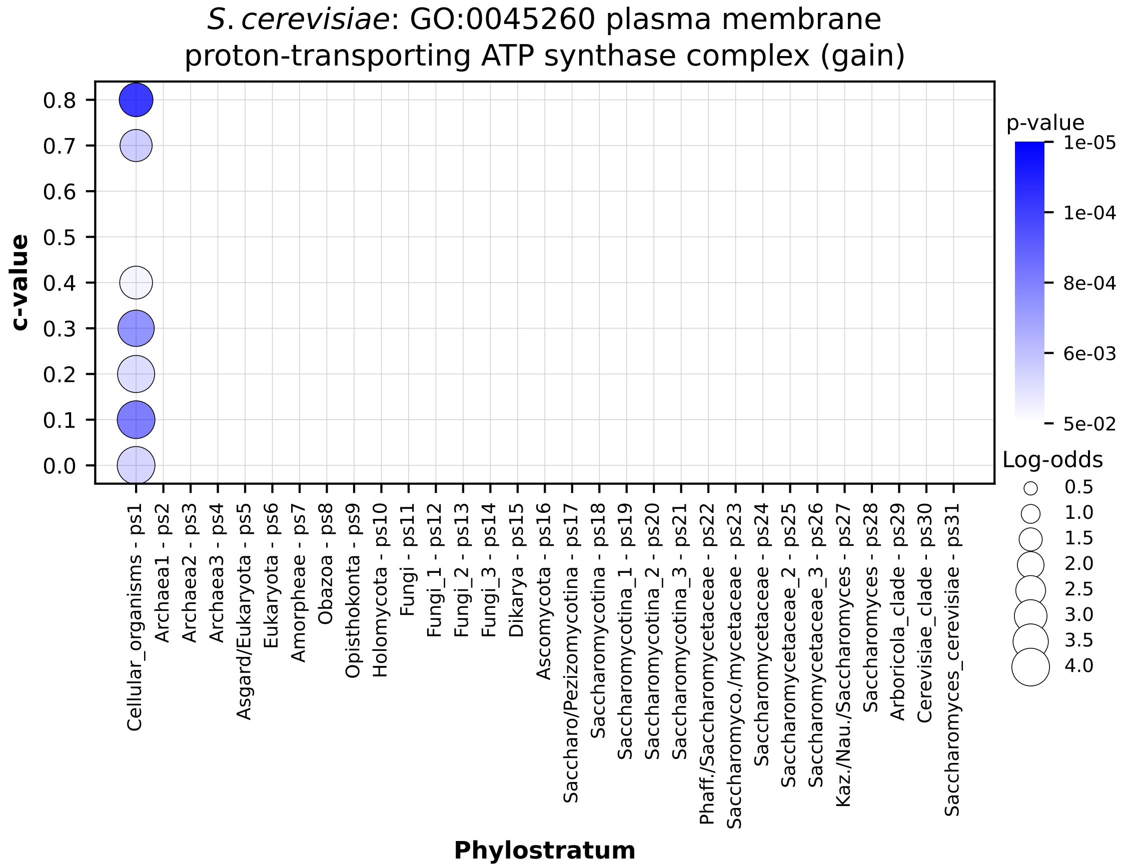

S. cerevisiae: GO:0045260 plasma membrane
proton-transporting ATP synthase complex (gain)
c-value
0.8
0.7
0.6
0.5
0.4
0.3
0.2
0.1
0.0
p-value
1e-05
1e-04
8e-04
6e-03
5e-02
Log-odds
0.5
1.0
1.5
2.0
2.5
3.0
3.5
4.0
Cellular_organisms - ps1
Archaea1 - ps2
Archaea2 - ps3
Archaea3 - ps4
Asgard/Eukaryota - ps5
Eukaryota - ps6
Amorpheae - ps7
Obazoa - ps8
Opisthokonta - ps9
Holomycota - ps10
Fungi - ps11
Fungi_1 - ps12
Fungi_2 - ps13
Fungi_3 - ps14
Dikarya - ps15
Ascomycota - ps16
Saccharo/Pezizomycotina - ps17
Saccharomycotina - ps18
Saccharomycotina_1 - ps19
Saccharomycotina_2 - ps20
Saccharomycotina_3 - ps21
Phaff./Saccharomycetaceae - ps22
Saccharomyco./mycetaceae - ps23
Saccharomycetaceae - ps24
Saccharomycetaceae_2 - ps25
Saccharomycetaceae_3 - ps26
Kaz./Nau./Saccharomyces - ps27
Saccharomyces - ps28
Arboricola_clade - ps29
Cerevisiae_clade - ps30
Saccharomyces_cerevisiae - ps31
Phylostratum

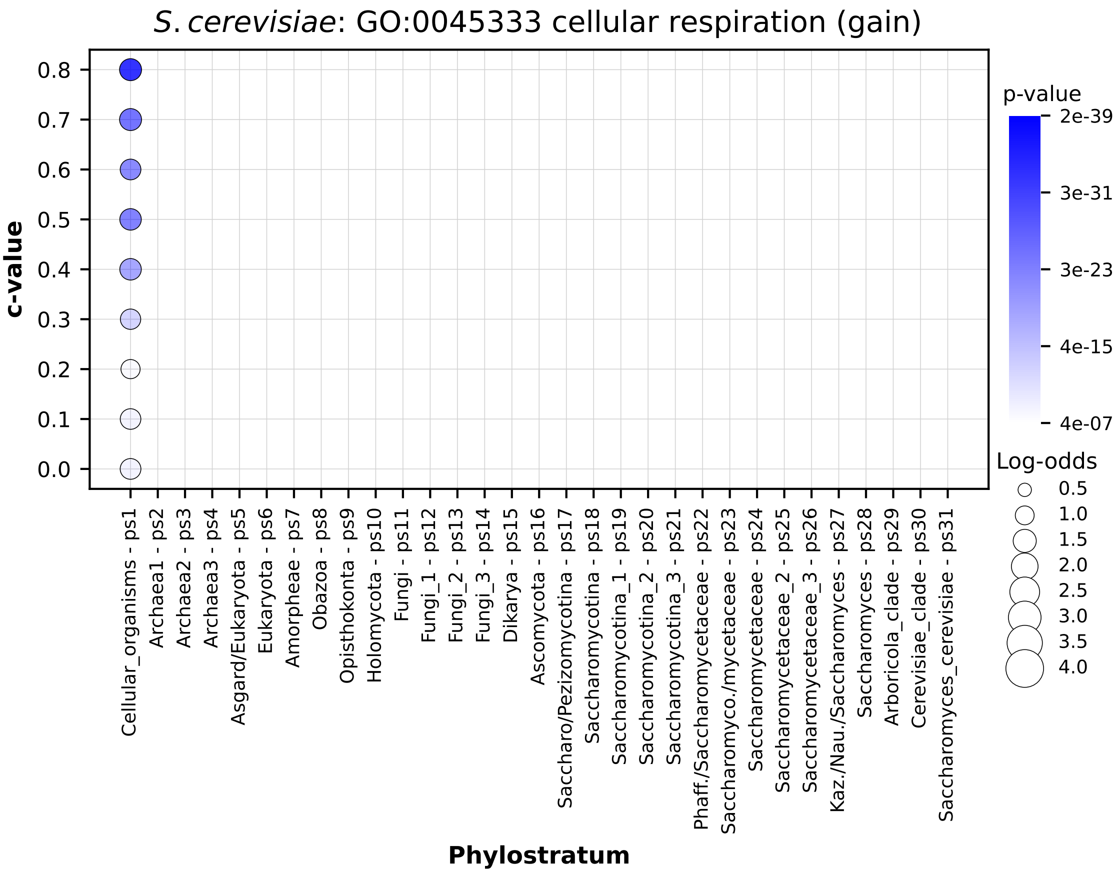

S. cerevisiae: GO:0045333 cellular respiration (gain)
c-value
0.8
0.7
0.6
0.5
0.4
0.3
0.2
0.1
0.0
p-value
2e-39
3e-31
3e-23
4e-15
4e-07
Log-odds
0.5
1.0
1.5
2.0
2.5
3.0
3.5
4.0
Cellular_organisms - ps1
Archaea1 - ps2
Archaea2 - ps3
Archaea3 - ps4
Asgard/Eukaryota - ps5
Eukaryota - ps6
Amorpheae - ps7
Obazoa - ps8
Opisthokonta - ps9
Holomycota - ps10
Fungi - ps11
Fungi_1 - ps12
Fungi_2 - ps13
Fungi_3 - ps14
Dikarya - ps15
Ascomycota - ps16
Saccharo/Pezizomycotina - ps17
Saccharomycotina - ps18
Saccharomycotina_1 - ps19
Saccharomycotina_2 - ps20
Saccharomycotina_3 - ps21
Phaff./Saccharomycetaceae - ps22
Saccharomyco./mycetaceae - ps23
Saccharomycetaceae - ps24
Saccharomycetaceae_2 - ps25
Saccharomycetaceae_3 - ps26
Kaz./Nau./Saccharomyces - ps27
Saccharomyces - ps28
Arboricola_clade - ps29
Cerevisiae_clade - ps30
Saccharomyces_cerevisiae - ps31
Phylostratum

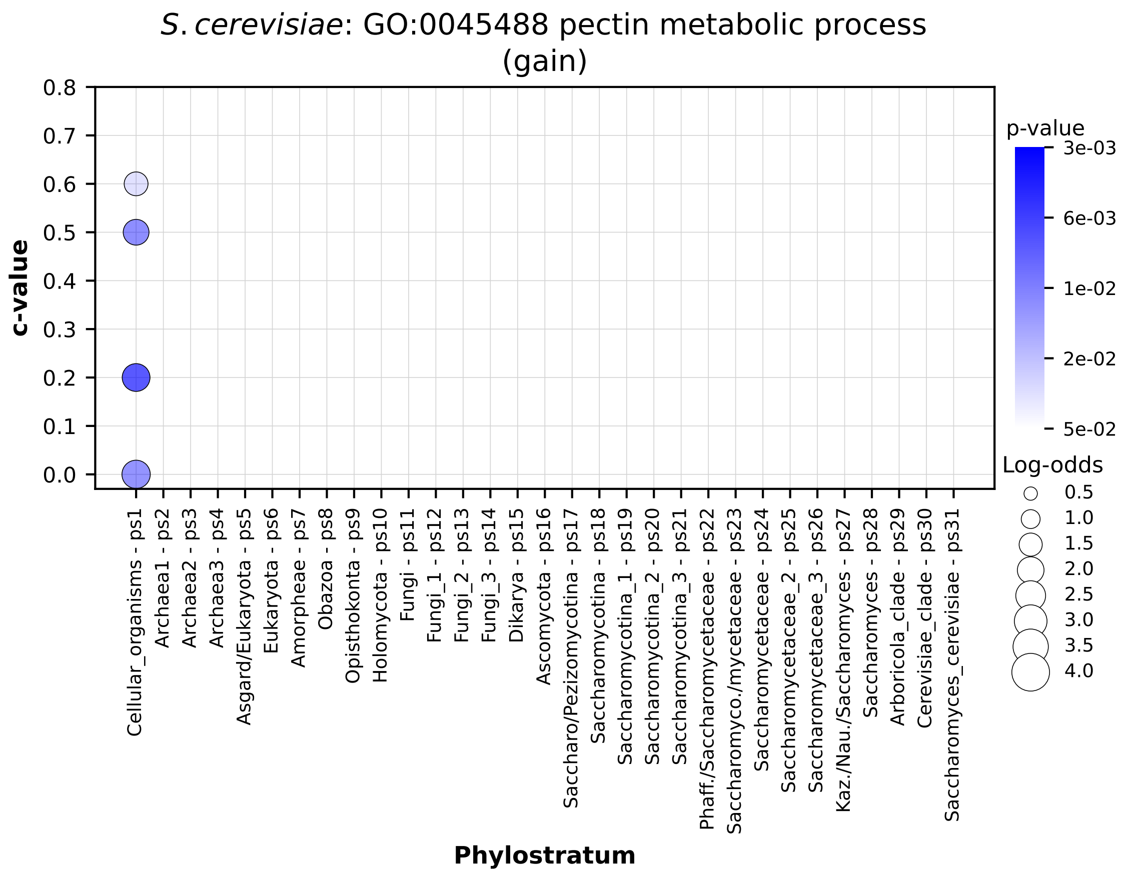

S. cerevisiae: GO:0045488 pectin metabolic process
(gain)
c-value
0.8
0.7
0.6
0.5
0.4
0.3
0.2
0.1
0.0
p-value
3e-03
6e-03
1e-02
2e-02
5e-02
Log-odds
0.5
1.0
1.5
2.0
2.5
3.0
3.5
4.0
Cellular_organisms - ps1
Archaea1 - ps2
Archaea2 - ps3
Archaea3 - ps4
Asgard/Eukaryota - ps5
Eukaryota - ps6
Amorpheae - ps7
Obazoa - ps8
Opisthokonta - ps9
Holomycota - ps10
Fungi - ps11
Fungi_1 - ps12
Fungi_2 - ps13
Fungi_3 - ps14
Dikarya - ps15
Ascomycota - ps16
Saccharo/Pezizomycotina - ps17
Saccharomycotina - ps18
Saccharomycotina_1 - ps19
Saccharomycotina_2 - ps20
Saccharomycotina_3 - ps21
Phaff./Saccharomycetaceae - ps22
Saccharomyco./mycetaceae - ps23
Saccharomycetaceae - ps24
Saccharomycetaceae_2 - ps25
Saccharomycetaceae_3 - ps26
Kaz./Nau./Saccharomyces - ps27
Saccharomyces - ps28
Arboricola_clade - ps29
Cerevisiae_clade - ps30
Saccharomyces_cerevisiae - ps31
Phylostratum

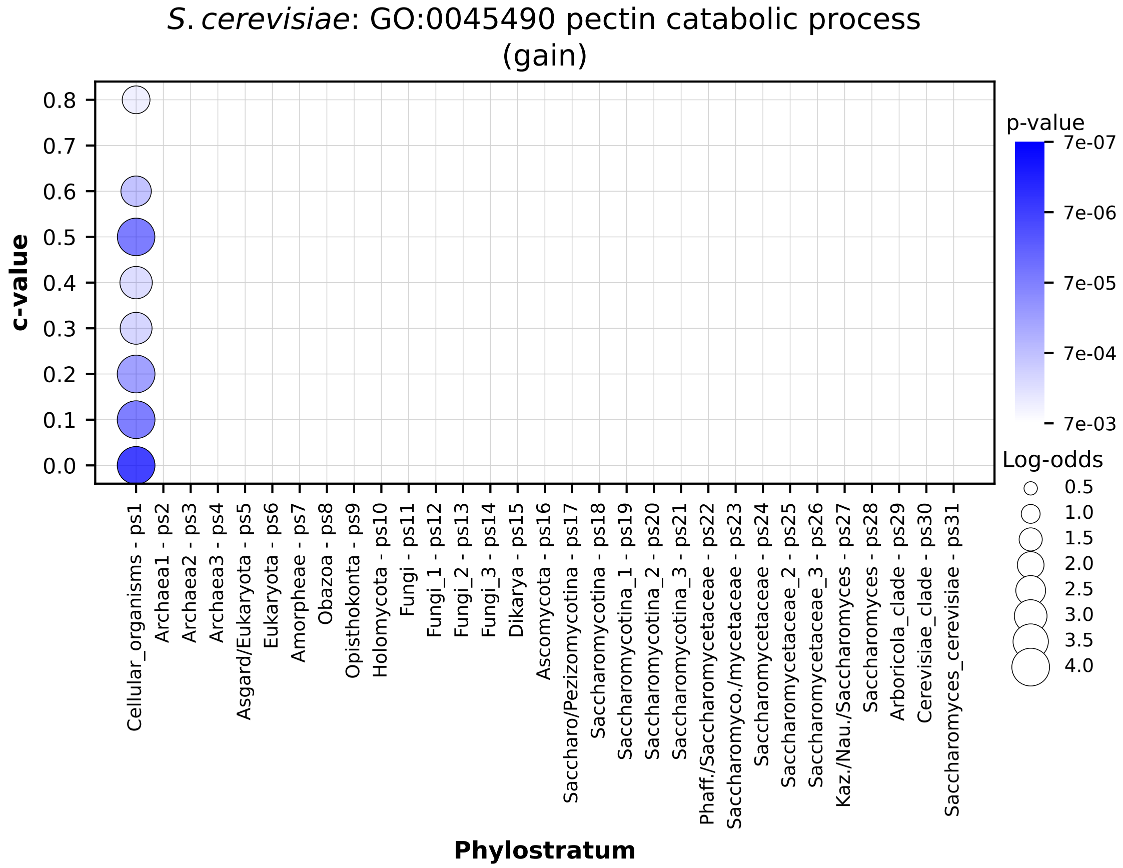

S. cerevisiae: GO:0045490 pectin catabolic process (gain)
c-value
0.8
0.7
0.6
0.5
0.4
0.3
0.2
0.1
0.0
p-value
7e-07
7e-06
7e-05
7e-04
7e-03
Log-odds
0.5
1.0
1.5
2.0
2.5
3.0
3.5
4.0
Cellular_organisms - ps1
Archaea1 - ps2
Archaea2 - ps3
Archaea3 - ps4
Asgard/Eukaryota - ps5
Eukaryota - ps6
Amorpheae - ps7
Obazoa - ps8
Opisthokonta - ps9
Holomycota - ps10
Fungi - ps11
Fungi_1 - ps12
Fungi_2 - ps13
Fungi_3 - ps14
Dikarya - ps15
Ascomycota - ps16
Saccharo/Pezizomycotina - ps17
Saccharomycotina - ps18
Saccharomycotina_1 - ps19
Saccharomycotina_2 - ps20
Saccharomycotina_3 - ps21
Phaff./Saccharomycetaceae - ps22
Saccharomyco./mycetaceae - ps23
Saccharomycetaceae - ps24
Saccharomycetaceae_2 - ps25
Saccharomycetaceae_3 - ps26
Kaz./Nau./Saccharomyces - ps27
Saccharomyces - ps28
Arboricola_clade - ps29
Cerevisiae_clade - ps30
Saccharomyces_cerevisiae - ps31
Phylostratum

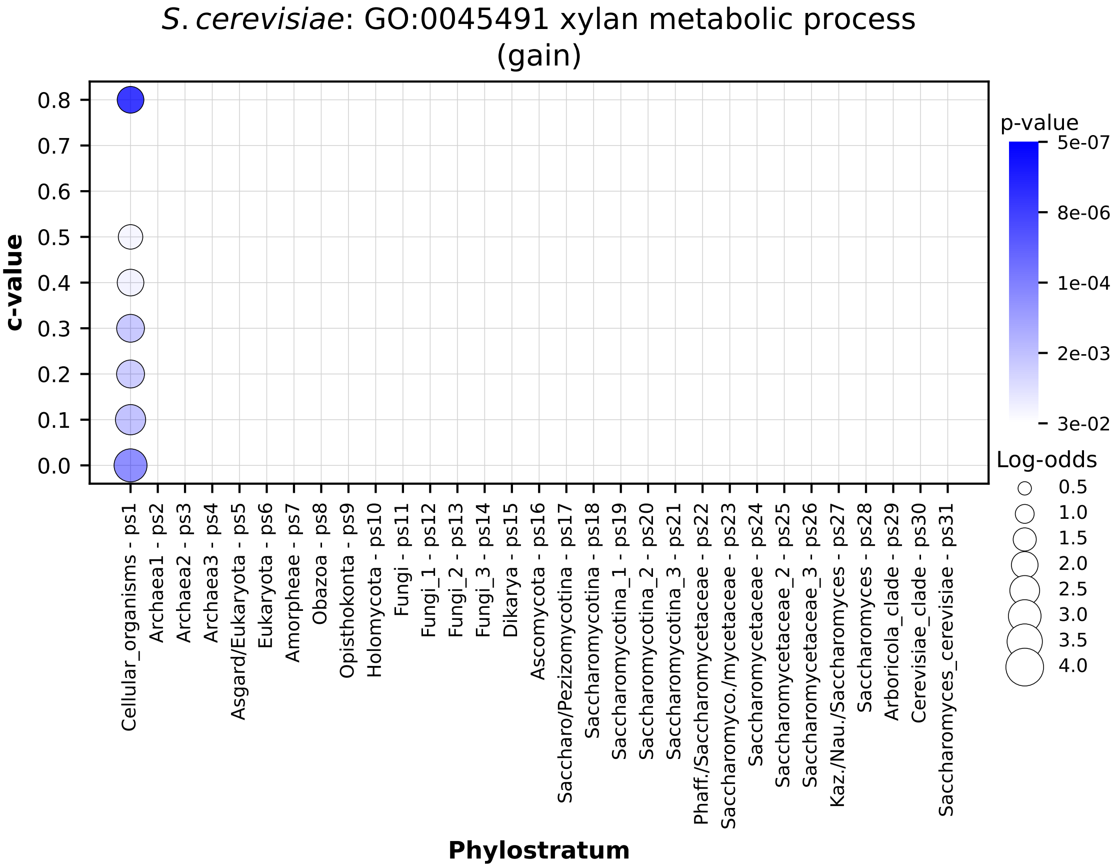

S. cerevisiae: GO:0045491 xylan metabolic process
(gain)
c-value
0.8
0.7
0.6
0.5
0.4
0.3
0.2
0.1
0.0
p-value
5e-07
8e-06
1e-04
2e-03
3e-02
Log-odds
0.5
1.0
1.5
2.0
2.5
3.0
3.5
4.0
Cellular_organisms - ps1
Archaea1 - ps2
Archaea2 - ps3
Archaea3 - ps4
Asgard/Eukaryota - ps5
Eukaryota - ps6
Amorpheae - ps7
Obazoa - ps8
Opisthokonta - ps9
Holomycota - ps10
Fungi - ps11
Fungi_1 - ps12
Fungi_2 - ps13
Fungi_3 - ps14
Dikarya - ps15
Ascomycota - ps16
Saccharo/Pezizomycotina - ps17
Saccharomycotina - ps18
Saccharomycotina_1 - ps19
Saccharomycotina_2 - ps20
Saccharomycotina_3 - ps21
Phaff./Saccharomycetaceae - ps22
Saccharomyco./mycetaceae - ps23
Saccharomycetaceae - ps24
Saccharomycetaceae_2 - ps25
Saccharomycetaceae_3 - ps26
Kaz./Nau./Saccharomyces - ps27
Saccharomyces - ps28
Arboricola_clade - ps29
Cerevisiae_clade - ps30
Saccharomyces_cerevisiae - ps31
Phylostratum

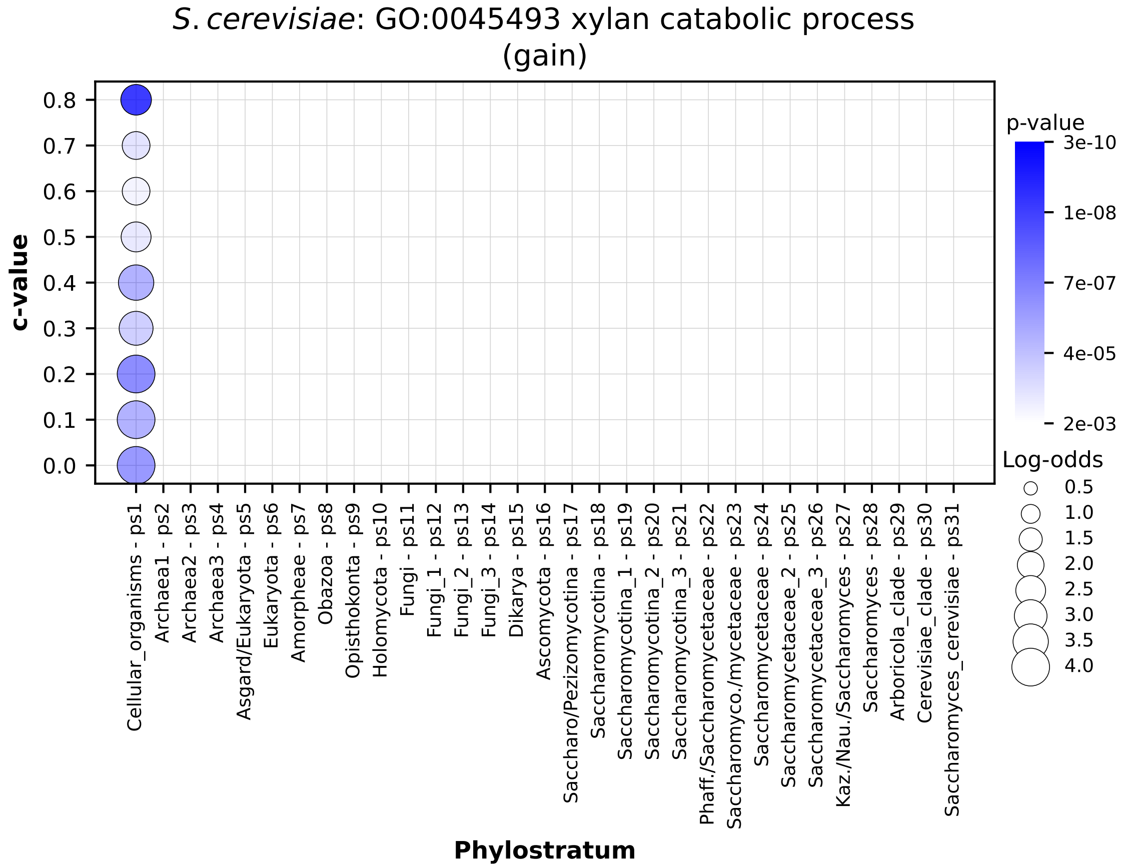

S. cerevisiae: GO:0045493 xylan catabolic process
(gain)
c-value
0.8
0.7
0.6
0.5
0.4
0.3
0.2
0.1
0.0
p-value
3e-10
1e-08
7e-07
4e-05
2e-03
Log-odds
0.5
1.0
1.5
2.0
2.5
3.0
3.5
4.0
Cellular_organisms - ps1
Archaea1 - ps2
Archaea2 - ps3
Archaea3 - ps4
Asgard/Eukaryota - ps5
Eukaryota - ps6
Amorpheae - ps7
Obazoa - ps8
Opisthokonta - ps9
Holomycota - ps10
Fungi - ps11
Fungi_1 - ps12
Fungi_2 - ps13
Fungi_3 - ps14
Dikarya - ps15
Ascomycota - ps16
Saccharo/Pezizomycotina - ps17
Saccharomycotina - ps18
Saccharomycotina_1 - ps19
Saccharomycotina_2 - ps20
Saccharomycotina_3 - ps21
Phaff./Saccharomycetaceae - ps22
Saccharomyco./mycetaceae - ps23
Saccharomycetaceae - ps24
Saccharomycetaceae_2 - ps25
Saccharomycetaceae_3 - ps26
Kaz./Nau./Saccharomyces - ps27
Saccharomyces - ps28
Arboricola_clade - ps29
Cerevisiae_clade - ps30
Saccharomyces_cerevisiae - ps31
Phylostratum

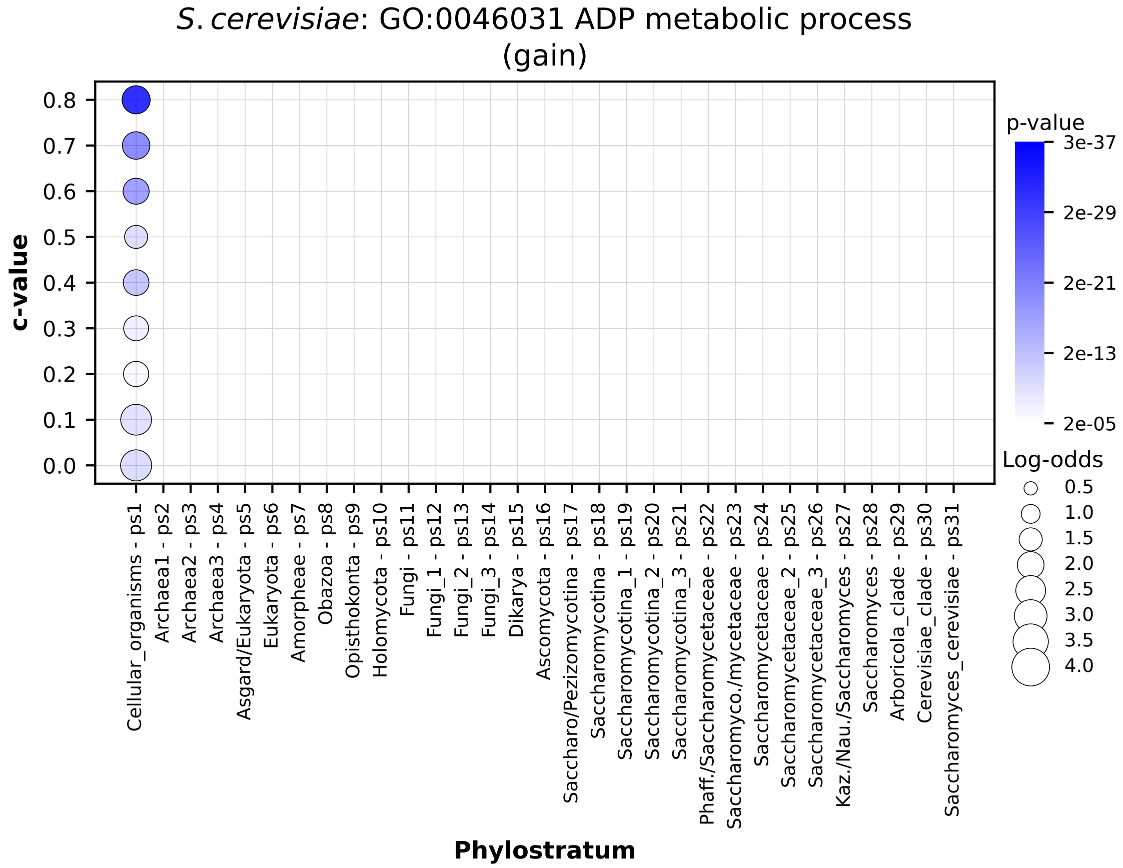
S. cerevisiae: GO:0046031 ADP metabolic process
(gain)
c-value
Phylostratum
p-value
3e-37
2e-29
2e-21
2e-13
2e-05
Log-odds
0.5
1.0
1.5
2.0
2.5
3.0
3.5
4.0
0.0
0.1
0.2
0.3
0.4
0.5
0.6
0.7
0.8
Cellular_organisms - ps1
Archaea1 - ps2
Archaea2 - ps3
Archaea3 - ps4
Asgard/Eukaryota - ps5
Eukaryota - ps6
Amorpheae - ps7
Obazoa - ps8
Opisthokonta - ps9
Holomycota - ps10
Fungi - ps11
Fungi_1 - ps12
Fungi_2 - ps13
Fungi_3 - ps14
Dikarya - ps15
Ascomycota - ps16
Saccharo/Pezizomycotina - ps17
Saccharomycotina - ps18
Saccharomycotina_1 - ps19
Saccharomycotina_2 - ps20
Saccharomycotina_3 - ps21
Phaff./Saccharomycetaceae - ps22
Saccharomyco./mycetaceae - ps23
Saccharomycetaceae - ps24
Saccharomycetaceae_2 - ps25
Saccharomycetaceae_3 - ps26
Kaz./Nau./Saccharomyces - ps27
Saccharomyces - ps28
Arboricola_clade - ps29
Cerevisiae_clade - ps30
Saccharomyces_cerevisiae - ps31

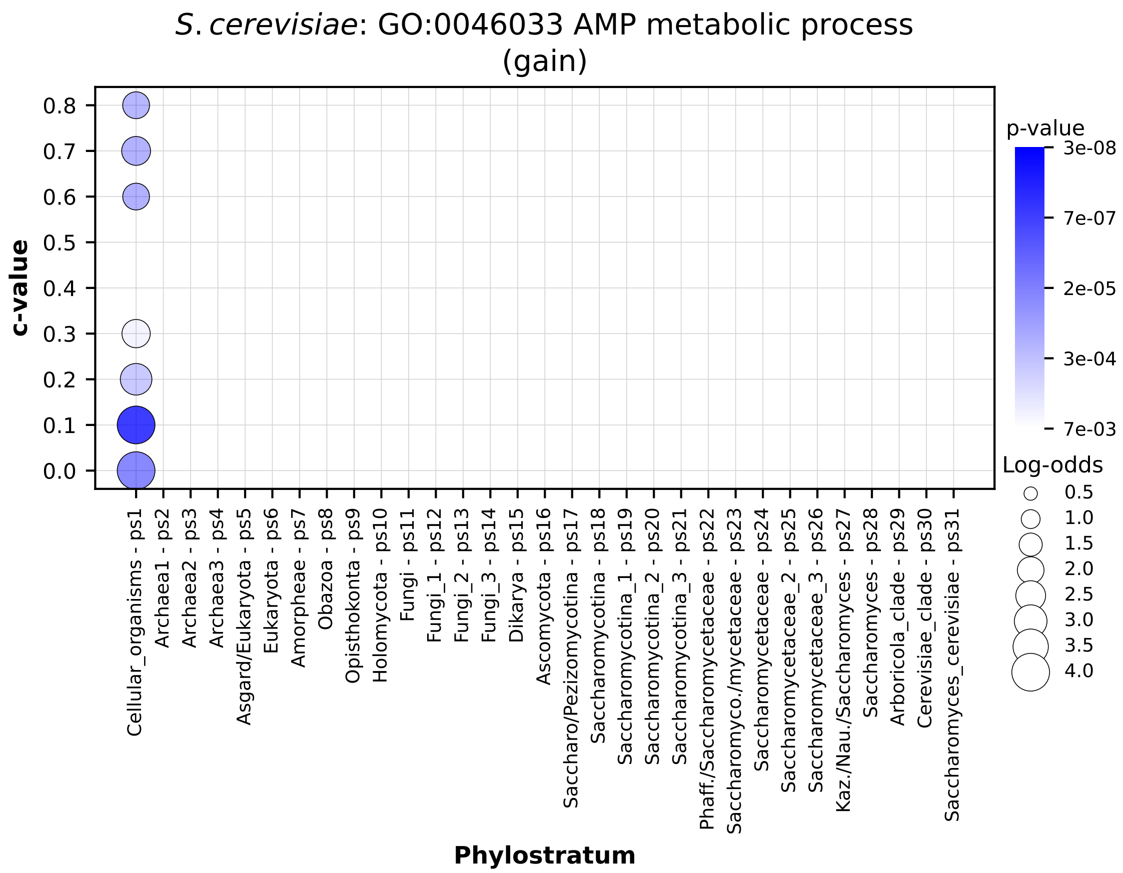

S. cerevisiae: GO:0046033 AMP metabolic process (gain)
c-value
0.8
0.7
0.6
0.5
0.4
0.3
0.2
0.1
0.0
Phylostratum
Cellular_organisms - ps1
Archaea1 - ps2
Archaea2 - ps3
Archaea3 - ps4
Asgard/Eukaryota - ps5
Eukaryota - ps6
Amorpheae - ps7
Obazoa - ps8
Opisthokonta - ps9
Holomycota - ps10
Fungi - ps11
Fungi_1 - ps12
Fungi_2 - ps13
Fungi_3 - ps14
Dikarya - ps15
Ascomycota - ps16
Saccharo/Pezizomycotina - ps17
Saccharomycotina - ps18
Saccharomycotina_1 - ps19
Saccharomycotina_2 - ps20
Saccharomycotina_3 - ps21
Phaff./Saccharomycetaceae - ps22
Saccharomyco./mycetaceae - ps23
Saccharomycetaceae - ps24
Saccharomycetaceae_2 - ps25
Saccharomycetaceae_3 - ps26
Kaz./Nau./Saccharomyces - ps27
Saccharomyces - ps28
Arboricola_clade - ps29
Cerevisiae_clade - ps30
Saccharomyces_cerevisiae - ps31
p-value
3e-08
7e-07
2e-05
3e-04
7e-03
Log-odds
0.5
1.0
1.5
2.0
2.5
3.0
3.5
4.0

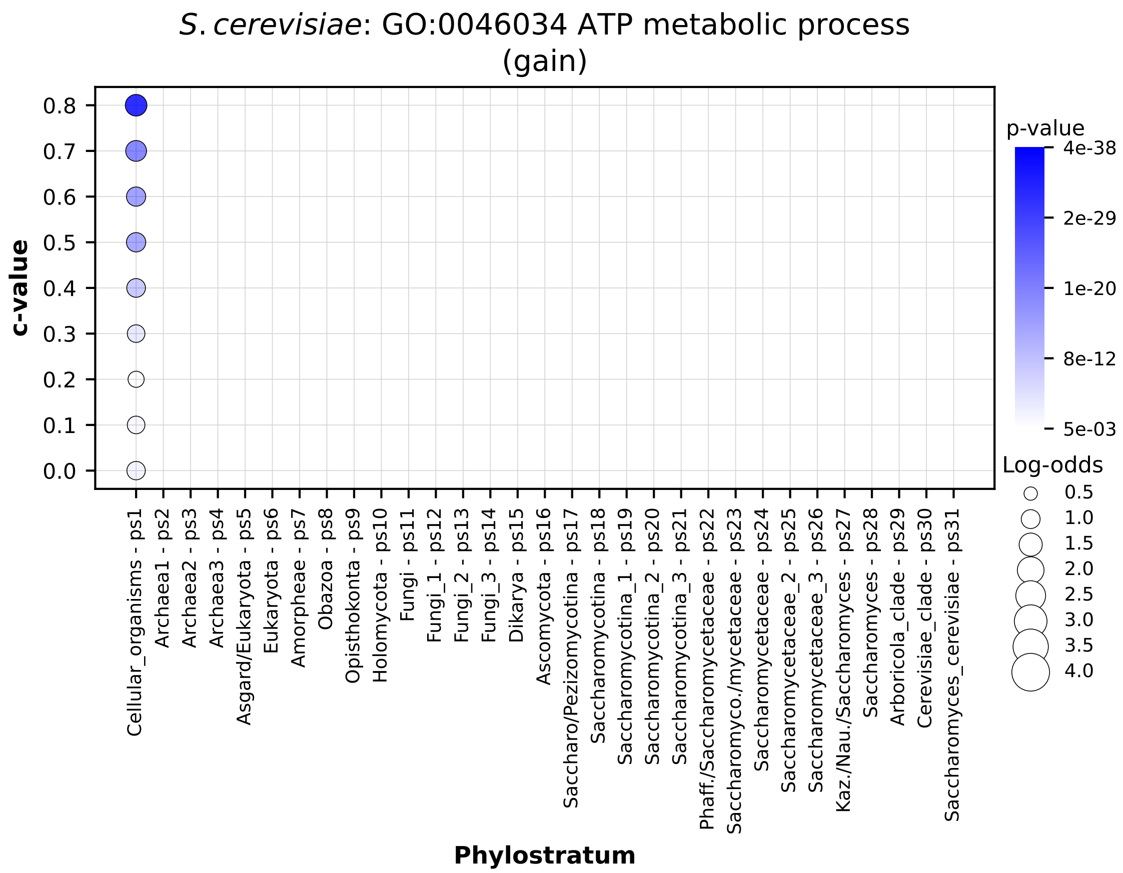

S. cerevisiae: GO:0046034 ATP metabolic process
(gain)
c-value
0.8
0.7
0.6
0.5
0.4
0.3
0.2
0.1
0.0
p-value
4e-38
2e-29
1e-20
8e-12
5e-03
Log-odds
0.5
1.0
1.5
2.0
2.5
3.0
3.5
4.0
Cellular_organisms - ps1
Archaea1 - ps2
Archaea2 - ps3
Archaea3 - ps4
Asgard/Eukaryota - ps5
Eukaryota - ps6
Amorpheae - ps7
Obazoa - ps8
Opisthokonta - ps9
Holomycota - ps10
Fungi - ps11
Fungi_1 - ps12
Fungi_2 - ps13
Fungi_3 - ps14
Dikarya - ps15
Ascomycota - ps16
Saccharo/Pezizomycotina - ps17
Saccharomycotina - ps18
Saccharomycotina_1 - ps19
Saccharomycotina_2 - ps20
Saccharomycotina_3 - ps21
Phaff./Saccharomycetaceae - ps22
Saccharomyco./mycetaceae - ps23
Saccharomycetaceae - ps24
Saccharomycetaceae_2 - ps25
Saccharomycetaceae_3 - ps26
Kaz./Nau./Saccharomyces - ps27
Saccharomyces - ps28
Arboricola_clade - ps29
Cerevisiae_clade - ps30
Saccharomyces_cerevisiae - ps31
Phylostratum

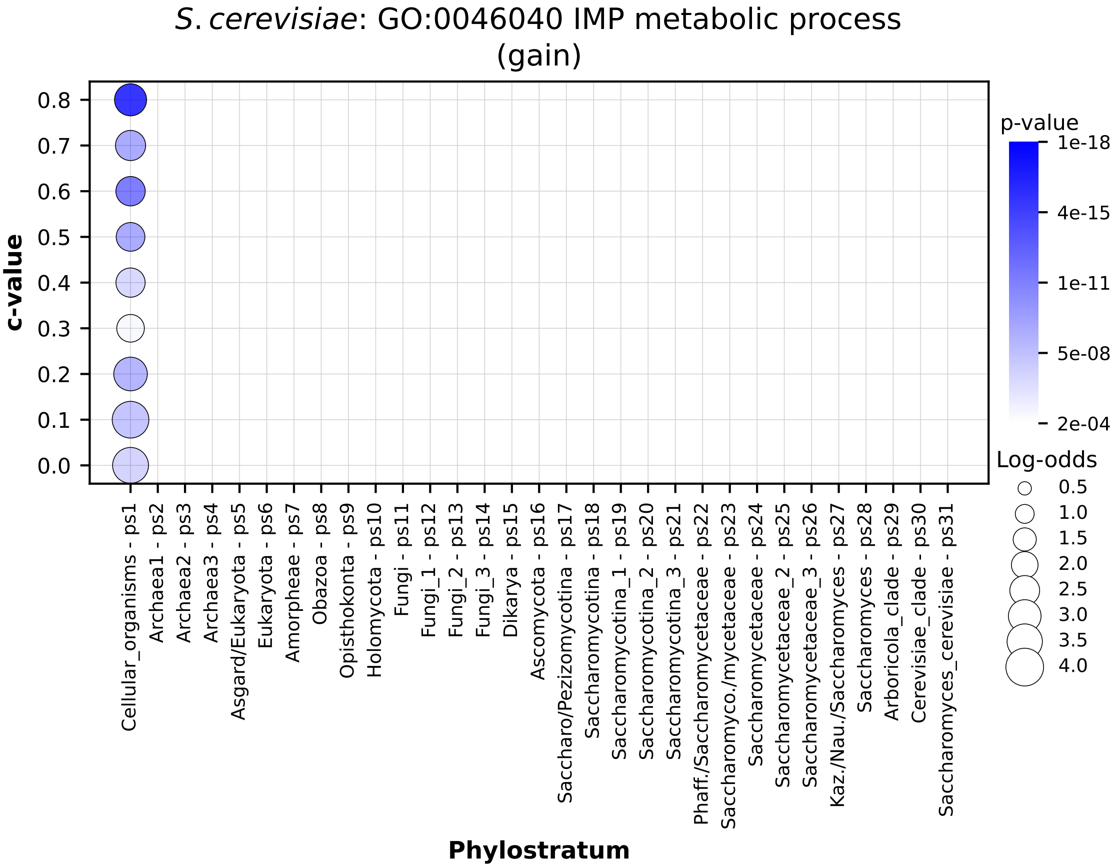
S. cerevisiae: GO:0046040 IMP metabolic process
(gain)
c-value
0.8
0.7
0.6
0.5
0.4
0.3
0.2
0.1
0.0
p-value
1e-18
4e-15
1e-11
5e-08
2e-04
Log-odds
0.5
1.0
1.5
2.0
2.5
3.0
3.5
4.0
Cellular_organisms - ps1
Archaea1 - ps2
Archaea2 - ps3
Archaea3 - ps4
Asgard/Eukaryota - ps5
Eukaryota - ps6
Amorpheae - ps7
Obazoa - ps8
Opisthokonta - ps9
Holomycota - ps10
Fungi - ps11
Fungi_1 - ps12
Fungi_2 - ps13
Fungi_3 - ps14
Dikarya - ps15
Ascomycota - ps16
Saccharo/Pezizomycotina - ps17
Saccharomycotina - ps18
Saccharomycotina_1 - ps19
Saccharomycotina_2 - ps20
Saccharomycotina_3 - ps21
Phaff./Saccharomycetaceae - ps22
Saccharomyco./mycetaceae - ps23
Saccharomycetaceae - ps24
Saccharomycetaceae_2 - ps25
Saccharomycetaceae_3 - ps26
Kaz./Nau./Saccharomyces - ps27
Saccharomyces - ps28
Arboricola_clade - ps29
Cerevisiae_clade - ps30
Saccharomyces_cerevisiae - ps31
Phylostratum

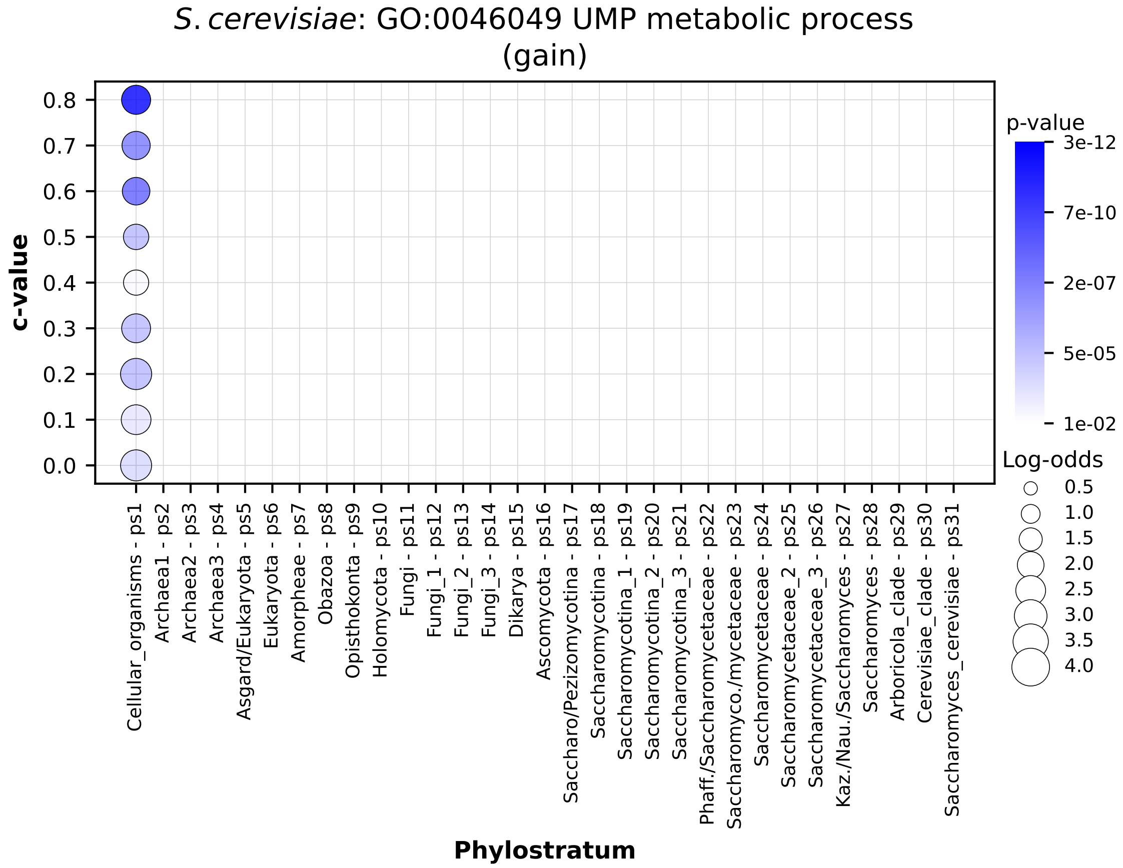

S. cerevisiae: GO:0046049 UMP metabolic process
(gain)
c-value
0.8
0.7
0.6
0.5
0.4
0.3
0.2
0.1
0.0
p-value
3e-12
7e-10
2e-07
5e-05
1e-02
Log-odds
0.5
1.0
1.5
2.0
2.5
3.0
3.5
4.0
Cellular_organisms - ps1
Archaea1 - ps2
Archaea2 - ps3
Archaea3 - ps4
Asgard/Eukaryota - ps5
Eukaryota - ps6
Amorpheae - ps7
Obazoa - ps8
Opisthokonta - ps9
Holomycota - ps10
Fungi - ps11
Fungi_1 - ps12
Fungi_2 - ps13
Fungi_3 - ps14
Dikarya - ps15
Ascomycota - ps16
Saccharo/Pezizomycotina - ps17
Saccharomycotina - ps18
Saccharomycotina_1 - ps19
Saccharomycotina_2 - ps20
Saccharomycotina_3 - ps21
Phaff./Saccharomycetaceae - ps22
Saccharomyco./mycetaceae - ps23
Saccharomycetaceae - ps24
Saccharomycetaceae_2 - ps25
Saccharomycetaceae_3 - ps26
Kaz./Nau./Saccharomyces - ps27
Saccharomyces - ps28
Arboricola_clade - ps29
Cerevisiae_clade - ps30
Saccharomyces_cerevisiae - ps31
Phylostratum

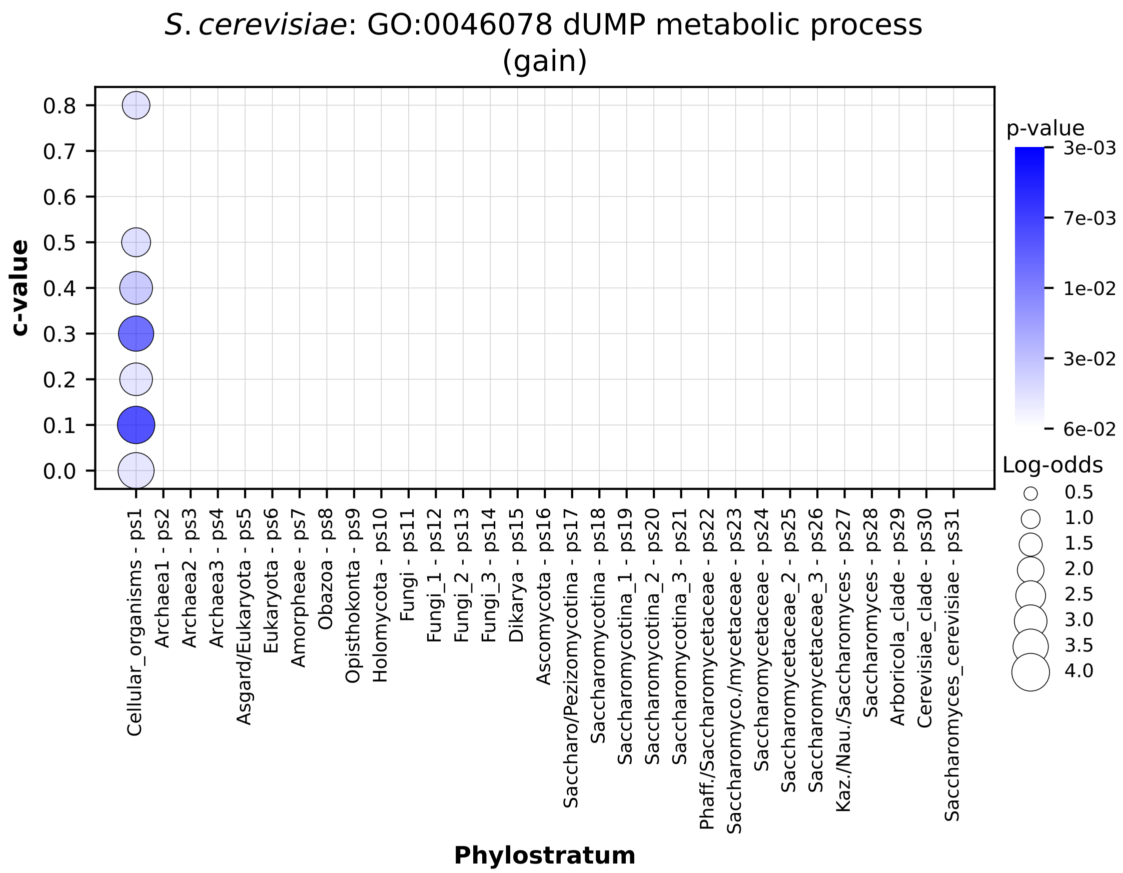
S. cerevisiae: GO:0046078 dUMP metabolic process (gain)
c-value
0.0
0.1
0.2
0.3
0.4
0.5
0.6
0.7
0.8
p-value
3e-03
7e-03
1e-02
3e-02
6e-02
Log-odds
0.5
1.0
1.5
2.0
2.5
3.0
3.5
4.0
Cellular_organisms - ps1
Archaea1 - ps2
Archaea2 - ps3
Archaea3 - ps4
Asgard/Eukaryota - ps5
Eukaryota - ps6
Amorpheae - ps7
Obazoa - ps8
Opisthokonta - ps9
Holomycota - ps10
Fungi - ps11
Fungi_1 - ps12
Fungi_2 - ps13
Fungi_3 - ps14
Dikarya - ps15
Ascomycota - ps16
Saccharo/Pezizomycotina - ps17
Saccharomycotina - ps18
Saccharomycotina_1 - ps19
Saccharomycotina_2 - ps20
Saccharomycotina_3 - ps21
Phaff./Saccharomycetaceae - ps22
Saccharomyco./mycetaceae - ps23
Saccharomycetaceae - ps24
Saccharomycetaceae_2 - ps25
Saccharomycetaceae_3 - ps26
Kaz./Nau./Saccharomyces - ps27
Saccharomyces - ps28
Arboricola_clade - ps29
Cerevisiae_clade - ps30
Saccharomyces_cerevisiae - ps31
Phylostratum

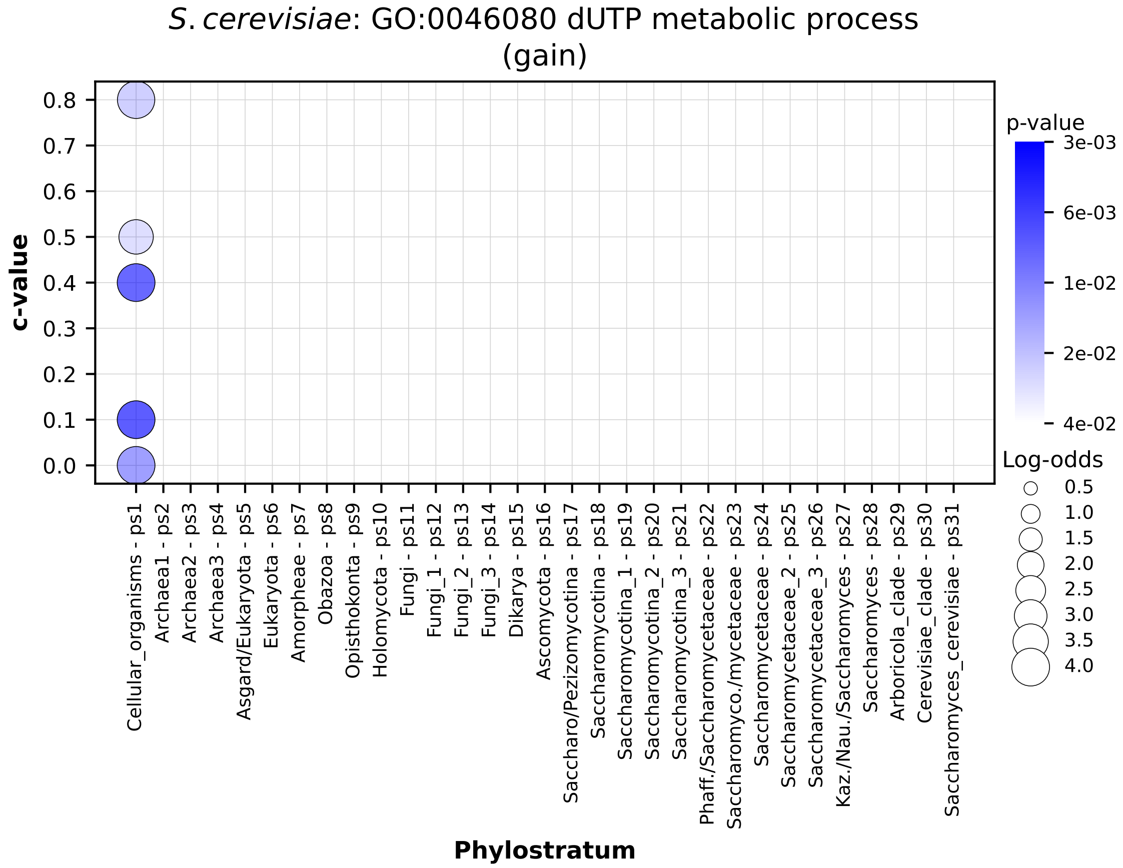

S. cerevisiae: GO:0046080 dUTP metabolic process
(gain)
c-value
0.8
0.7
0.6
0.5
0.4
0.3
0.2
0.1
0.0
Phylostratum
Cellular_organisms - ps1
Archaea1 - ps2
Archaea2 - ps3
Archaea3 - ps4
Asgard/Eukaryota - ps5
Eukaryota - ps6
Amorpheae - ps7
Obazoa - ps8
Opisthokonta - ps9
Holomycota - ps10
Fungi - ps11
Fungi_1 - ps12
Fungi_2 - ps13
Fungi_3 - ps14
Dikarya - ps15
Ascomycota - ps16
Saccharo/Pezizomycotina - ps17
Saccharomycotina - ps18
Saccharomycotina_1 - ps19
Saccharomycotina_2 - ps20
Saccharomycotina_3 - ps21
Phaff./Saccharomycetaceae - ps22
Saccharomyco./mycetaceae - ps23
Saccharomycetaceae - ps24
Saccharomycetaceae_2 - ps25
Saccharomycetaceae_3 - ps26
Kaz./Nau./Saccharomyces - ps27
Saccharomyces - ps28
Arboricola_clade - ps29
Cerevisiae_clade - ps30
Saccharomyces_cerevisiae - ps31
p-value
3e-03
6e-03
1e-02
2e-02
4e-02
Log-odds
0.5
1.0
1.5
2.0
2.5
3.0
3.5
4.0

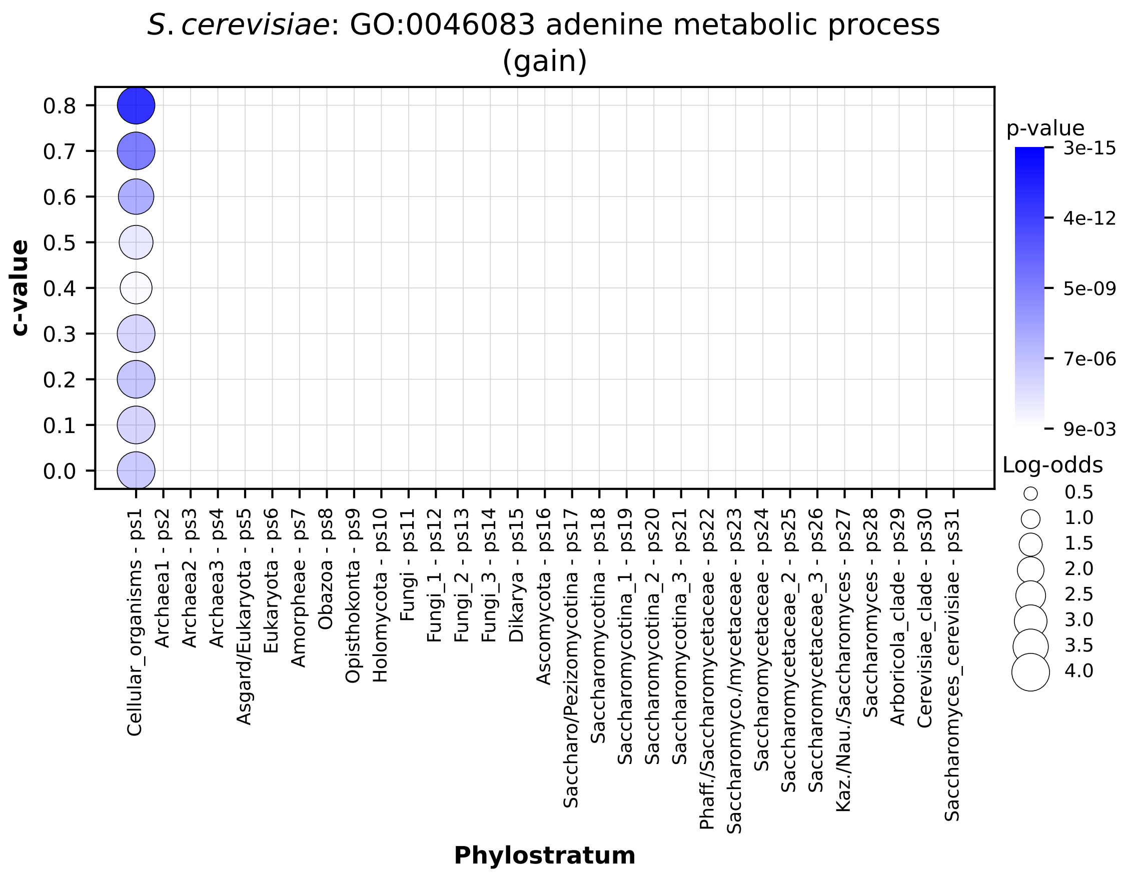

S. cerevisiae: GO:0046083 adenine metabolic process
(gain)
c-value
0.8
0.7
0.6
0.5
0.4
0.3
0.2
0.1
0.0
p-value
3e-15
4e-12
5e-09
7e-06
9e-03
Log-odds
0.5
1.0
1.5
2.0
2.5
3.0
3.5
4.0
Cellular_organisms - ps1
Archaea1 - ps2
Archaea2 - ps3
Archaea3 - ps4
Asgard/Eukaryota - ps5
Eukaryota - ps6
Amorpheae - ps7
Obazoa - ps8
Opisthokonta - ps9
Holomycota - ps10
Fungi - ps11
Fungi_1 - ps12
Fungi_2 - ps13
Fungi_3 - ps14
Dikarya - ps15
Ascomycota - ps16
Saccharo/Pezizomycotina - ps17
Saccharomycotina - ps18
Saccharomycotina_1 - ps19
Saccharomycotina_2 - ps20
Saccharomycotina_3 - ps21
Phaff./Saccharomycetaceae - ps22
Saccharomyco./mycetaceae - ps23
Saccharomycetaceae - ps24
Saccharomycetaceae_2 - ps25
Saccharomycetaceae_3 - ps26
Kaz./Nau./Saccharomyces - ps27
Saccharomyces - ps28
Arboricola_clade - ps29
Cerevisiae_clade - ps30
Saccharomyces_cerevisiae - ps31
Phylostratum

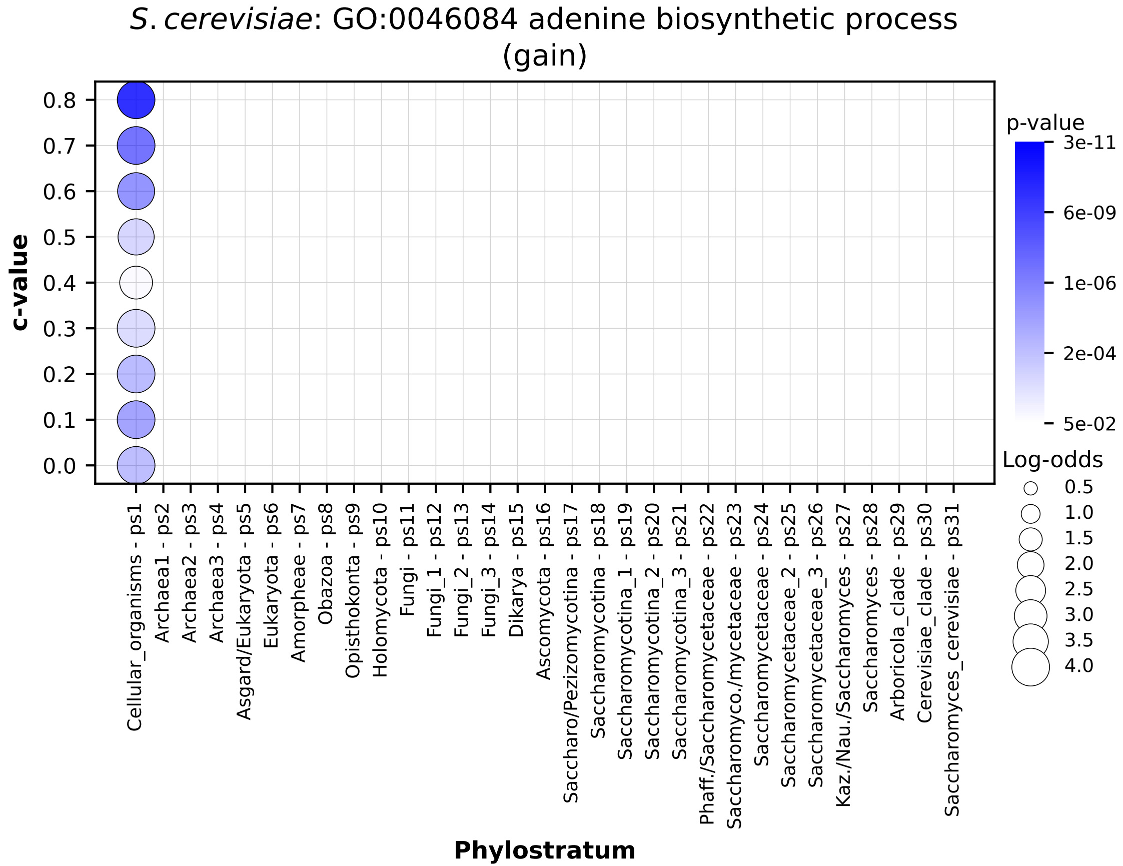

S. cerevisiae: GO:0046084 adenine biosynthetic process
(gain)
c-value
0.8
0.7
0.6
0.5
0.4
0.3
0.2
0.1
0.0
Phylostratum
Cellular_organisms - ps1
Archaea1 - ps2
Archaea2 - ps3
Archaea3 - ps4
Asgard/Eukaryota - ps5
Eukaryota - ps6
Amorpheae - ps7
Obazoa - ps8
Opisthokonta - ps9
Holomycota - ps10
Fungi - ps11
Fungi_1 - ps12
Fungi_2 - ps13
Fungi_3 - ps14
Dikarya - ps15
Ascomycota - ps16
Saccharo/Pezizomycotina - ps17
Saccharomycotina - ps18
Saccharomycotina_1 - ps19
Saccharomycotina_2 - ps20
Saccharomycotina_3 - ps21
Phaff./Saccharomycetaceae - ps22
Saccharomyco./mycetaceae - ps23
Saccharomycetaceae - ps24
Saccharomycetaceae_2 - ps25
Saccharomycetaceae_3 - ps26
Kaz./Nau./Saccharomyces - ps27
Saccharomyces - ps28
Arboricola_clade - ps29
Cerevisiae_clade - ps30
Saccharomyces_cerevisiae - ps31
p-value
3e-11
6e-09
1e-06
2e-04
5e-02
Log-odds
0.5
1.0
1.5
2.0
2.5
3.0
3.5
4.0

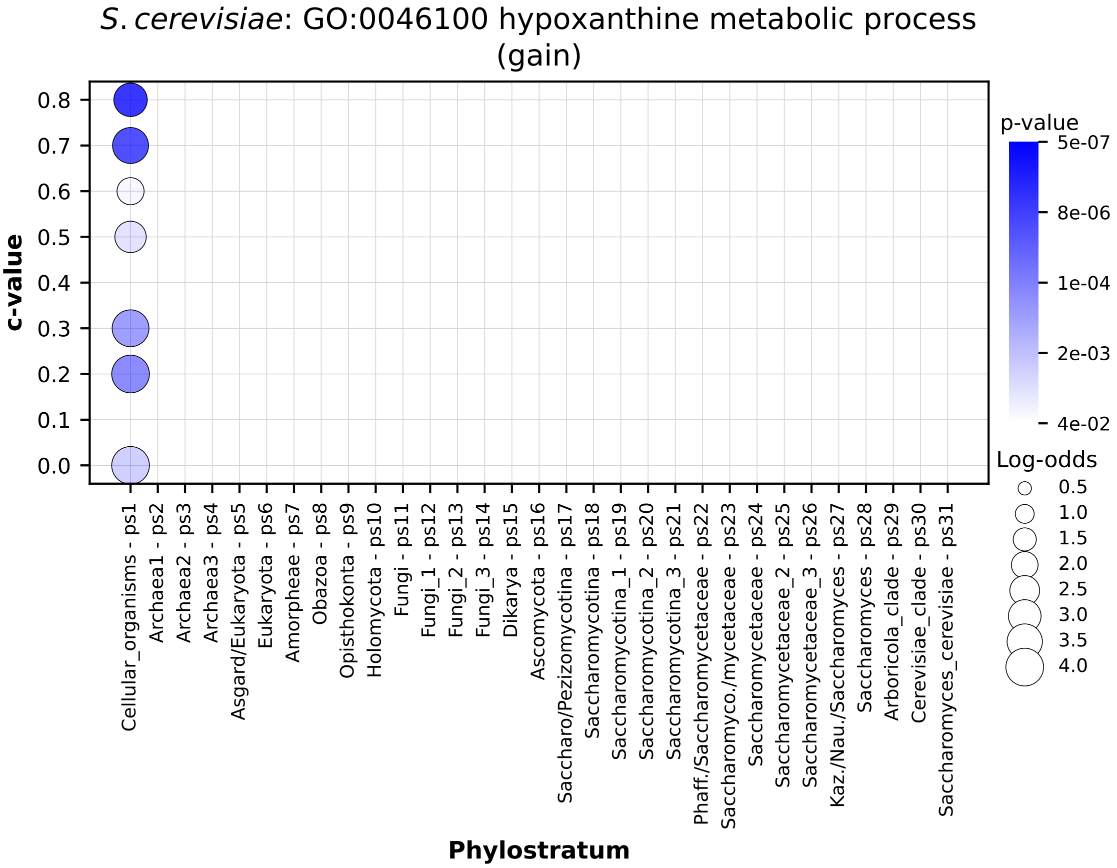
S. cerevisiae: GO:0046100 hypoxanthine metabolic process (gain)
c-value
0.8
0.7
0.6
0.5
0.4
0.3
0.2
0.1
0.0
p-value
5e-07
8e-06
1e-04
2e-03
4e-02
Log-odds
0.5
1.0
1.5
2.0
2.5
3.0
3.5
4.0
Cellular_organisms - ps1
Archaea1 - ps2
Archaea2 - ps3
Archaea3 - ps4
Asgard/Eukaryota - ps5
Eukaryota - ps6
Amorpheae - ps7
Obazoa - ps8
Opisthokonta - ps9
Holomycota - ps10
Fungi - ps11
Fungi_1 - ps12
Fungi_2 - ps13
Fungi_3 - ps14
Dikarya - ps15
Ascomycota - ps16
Saccharo/Pezizomycotina - ps17
Saccharomycotina - ps18
Saccharomycotina_1 - ps19
Saccharomycotina_2 - ps20
Saccharomycotina_3 - ps21
Phaff./Saccharomycetaceae - ps22
Saccharomyco./mycetaceae - ps23
Saccharomycetaceae - ps24
Saccharomycetaceae_2 - ps25
Saccharomycetaceae_3 - ps26
Kaz./Nau./Saccharomyces - ps27
Saccharomyces - ps28
Arboricola_clade - ps29
Cerevisiae_clade - ps30
Saccharomyces_cerevisiae - ps31
Phylostratum

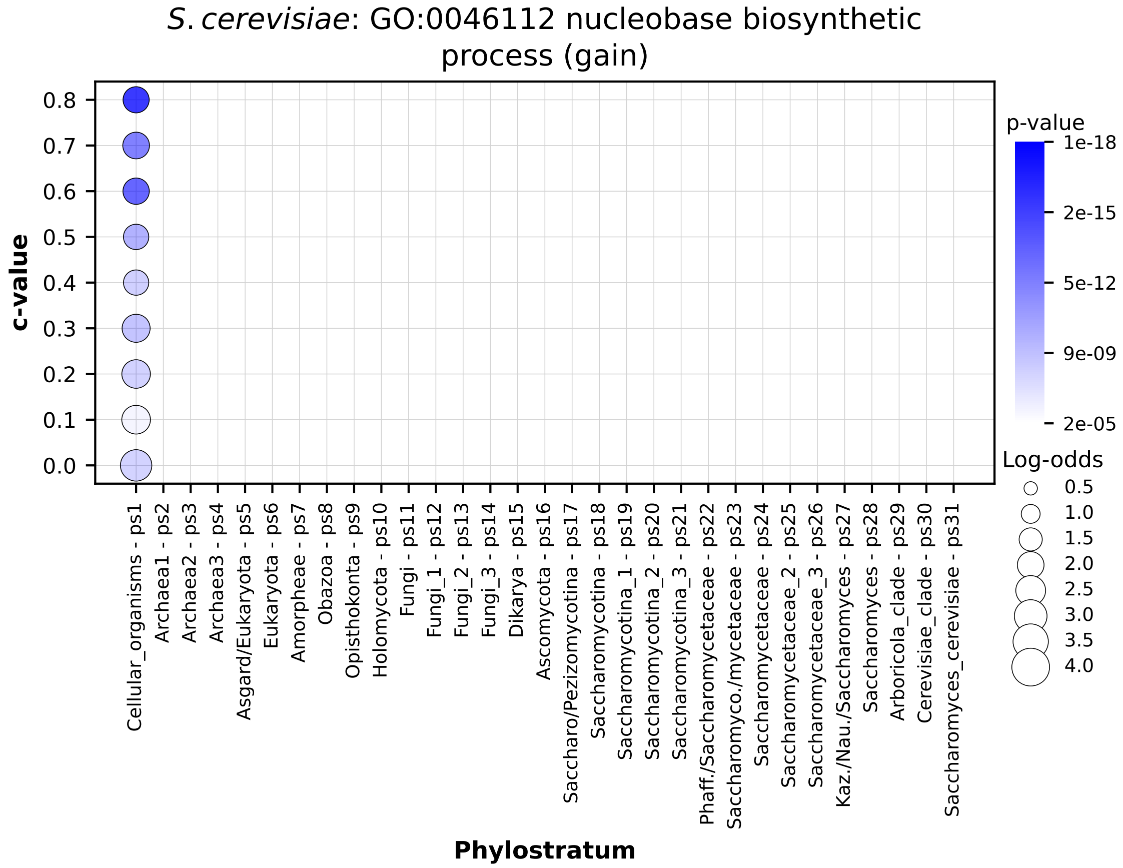

S. cerevisiae: GO:0046112 nucleobase biosynthetic process (gain)
c-value
0.8
0.7
0.6
0.5
0.4
0.3
0.2
0.1
0.0
p-value
1e-18
2e-15
5e-12
9e-09
2e-05
Log-odds
0.5
1.0
1.5
2.0
2.5
3.0
3.5
4.0
Cellular_organisms - ps1
Archaea1 - ps2
Archaea2 - ps3
Archaea3 - ps4
Asgard/Eukaryota - ps5
Eukaryota - ps6
Amorpheae - ps7
Obazoa - ps8
Opisthokonta - ps9
Holomycota - ps10
Fungi - ps11
Fungi_1 - ps12
Fungi_2 - ps13
Fungi_3 - ps14
Dikarya - ps15
Ascomycota - ps16
Saccharo/Pezizomycotina - ps17
Saccharomycotina - ps18
Saccharomycotina_1 - ps19
Saccharomycotina_2 - ps20
Saccharomycotina_3 - ps21
Phaff./Saccharomycetaceae - ps22
Saccharomyco./mycetaceae - ps23
Saccharomycetaceae - ps24
Saccharomycetaceae_2 - ps25
Saccharomycetaceae_3 - ps26
Kaz./Nau./Saccharomyces - ps27
Saccharomyces - ps28
Arboricola_clade - ps29
Cerevisiae_clade - ps30
Saccharomyces_cerevisiae - ps31
Phylostratum

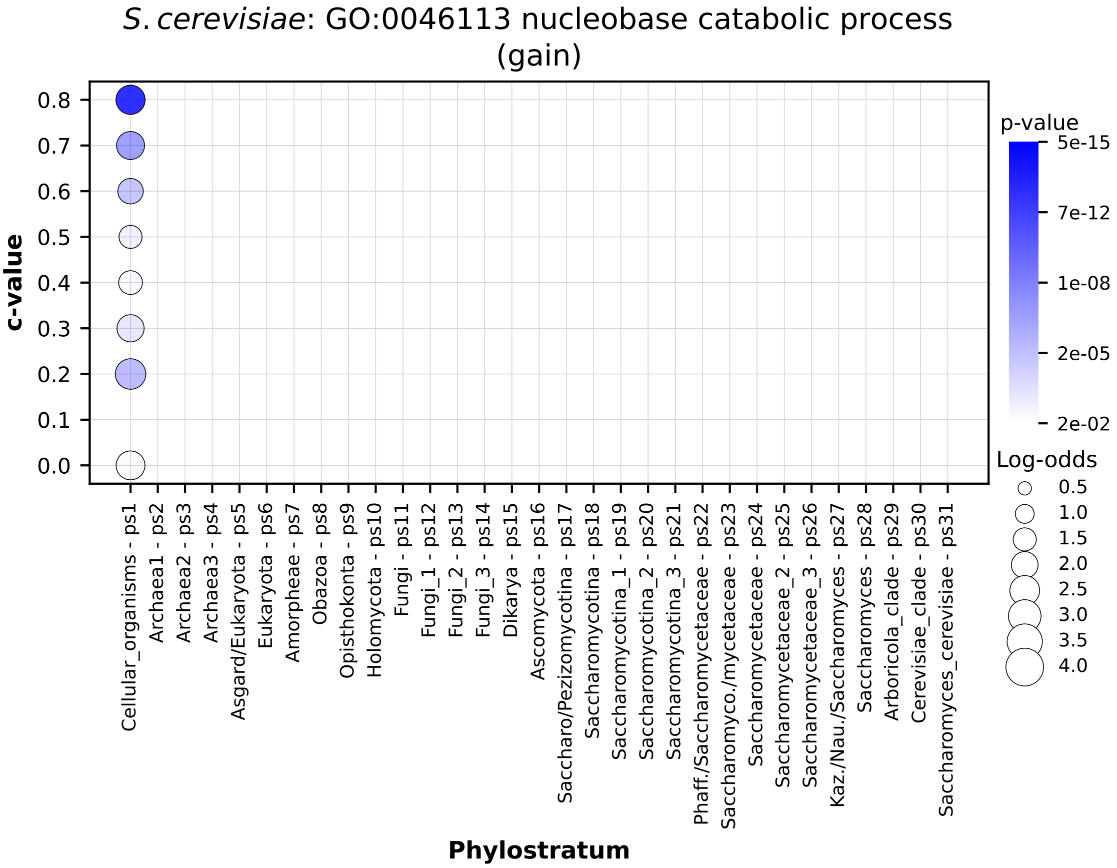

S. cerevisiae: GO:0046113 nucleobase catabolic process (gain)
c-value
0.8
0.7
0.6
0.5
0.4
0.3
0.2
0.1
0.0
p-value
5e-15
7e-12
1e-08
2e-05
2e-02
Log-odds
0.5
1.0
1.5
2.0
2.5
3.0
3.5
4.0
Cellular_organisms - ps1
Archaea1 - ps2
Archaea2 - ps3
Archaea3 - ps4
Asgard/Eukaryota - ps5
Eukaryota - ps6
Amorpheae - ps7
Obazoa - ps8
Opisthokonta - ps9
Holomycota - ps10
Fungi - ps11
Fungi_1 - ps12
Fungi_2 - ps13
Fungi_3 - ps14
Dikarya - ps15
Ascomycota - ps16
Saccharo/Pezizomycotina - ps17
Saccharomycotina - ps18
Saccharomycotina_1 - ps19
Saccharomycotina_2 - ps20
Saccharomycotina_3 - ps21
Phaff./Saccharomycetaceae - ps22
Saccharomyco./mycetaceae - ps23
Saccharomycetaceae - ps24
Saccharomycetaceae_2 - ps25
Saccharomycetaceae_3 - ps26
Kaz./Nau./Saccharomyces - ps27
Saccharomyces - ps28
Arboricola_clade - ps29
Cerevisiae_clade - ps30
Saccharomyces_cerevisiae - ps31
Phylostratum

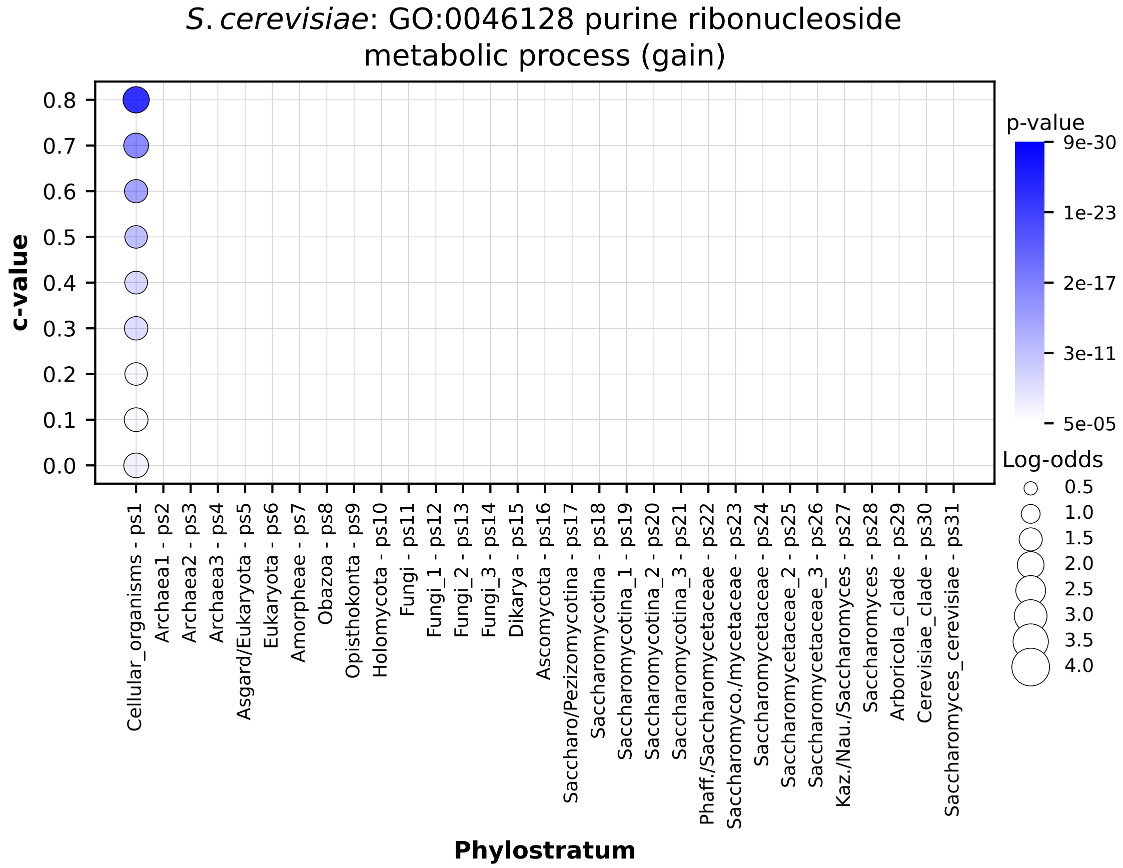
S. cerevisiae: GO:0046128 purine ribonucleoside metabolic process (gain)
c-value
0.0
0.1
0.2
0.3
0.4
0.5
0.6
0.7
0.8
Phylostratum
Cellular_organisms - ps1
Archaea1 - ps2
Archaea2 - ps3
Archaea3 - ps4
Asgard/Eukaryota - ps5
Eukaryota - ps6
Amorpheae - ps7
Obazoa - ps8
Opisthokonta - ps9
Holomycota - ps10
Fungi - ps11
Fungi_1 - ps12
Fungi_2 - ps13
Fungi_3 - ps14
Dikarya - ps15
Ascomycota - ps16
Saccharo/Pezizomycotina - ps17
Saccharomycotina - ps18
Saccharomycotina_1 - ps19
Saccharomycotina_2 - ps20
Saccharomycotina_3 - ps21
Phaff./Saccharomycetaceae - ps22
Saccharomyco./mycetaceae - ps23
Saccharomycetaceae - ps24
Saccharomycetaceae_2 - ps25
Saccharomycetaceae_3 - ps26
Kaz./Nau./Saccharomyces - ps27
Saccharomyces - ps28
Arboricola_clade - ps29
Cerevisiae_clade - ps30
Saccharomyces_cerevisiae - ps31
p-value
9e-30
1e-23
2e-17
3e-11
5e-05
Log-odds
0.5
1.0
1.5
2.0
2.5
3.0
3.5
4.0

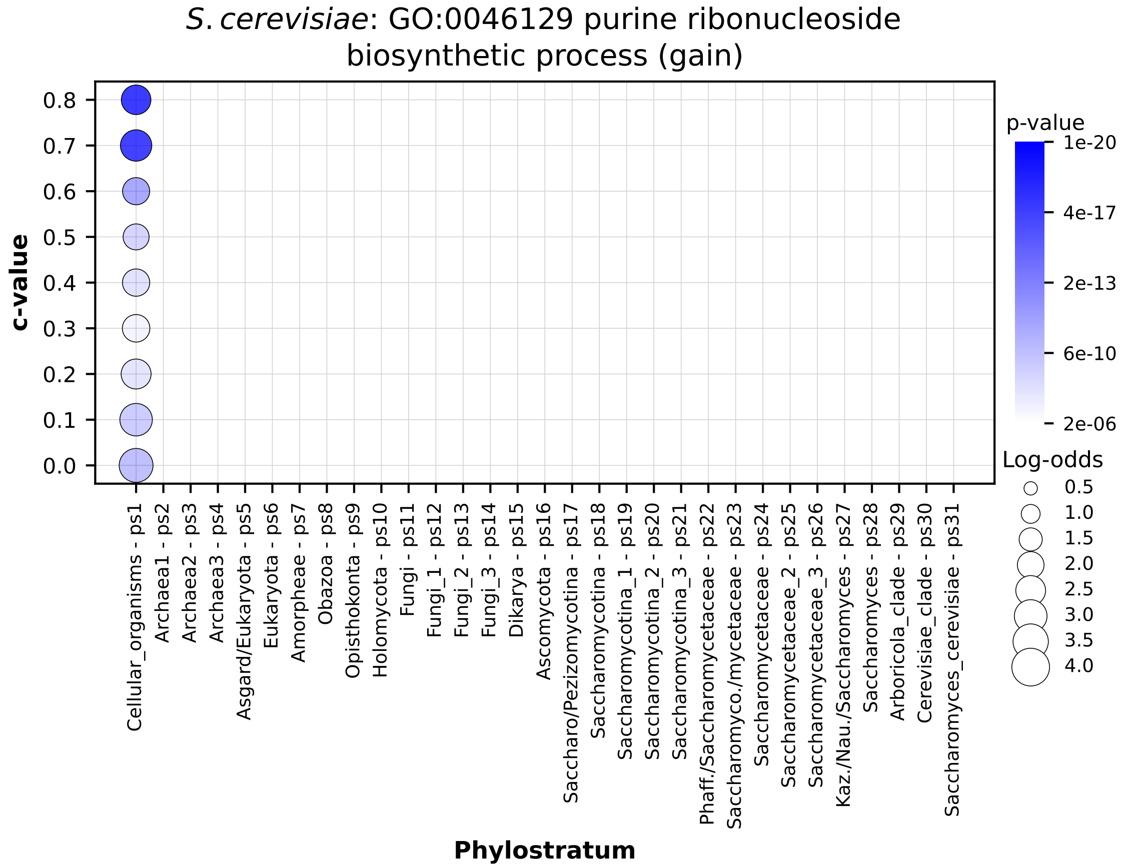

S. cerevisiae: GO:0046129 purine ribonucleoside biosynthetic process (gain)
c-value
0.8
0.7
0.6
0.5
0.4
0.3
0.2
0.1
0.0
p-value
1e-20
4e-17
2e-13
6e-10
2e-06
Log-odds
0.5
1.0
1.5
2.0
2.5
3.0
3.5
4.0
Cellular_organisms - ps1
Archaea1 - ps2
Archaea2 - ps3
Archaea3 - ps4
Asgard/Eukaryota - ps5
Eukaryota - ps6
Amorpheae - ps7
Obazoa - ps8
Opisthokonta - ps9
Holomycota - ps10
Fungi - ps11
Fungi_1 - ps12
Fungi_2 - ps13
Fungi_3 - ps14
Dikarya - ps15
Ascomycota - ps16
Saccharo/Pezizomycotina - ps17
Saccharomycotina - ps18
Saccharomycotina_1 - ps19
Saccharomycotina_2 - ps20
Saccharomycotina_3 - ps21
Phaff./Saccharomycetaceae - ps22
Saccharomyco./mycetaceae - ps23
Saccharomycetaceae - ps24
Saccharomycetaceae_2 - ps25
Saccharomycetaceae_3 - ps26
Kaz./Nau./Saccharomyces - ps27
Saccharomyces - ps28
Arboricola_clade - ps29
Cerevisiae_clade - ps30
Saccharomyces_cerevisiae - ps31
Phylostratum

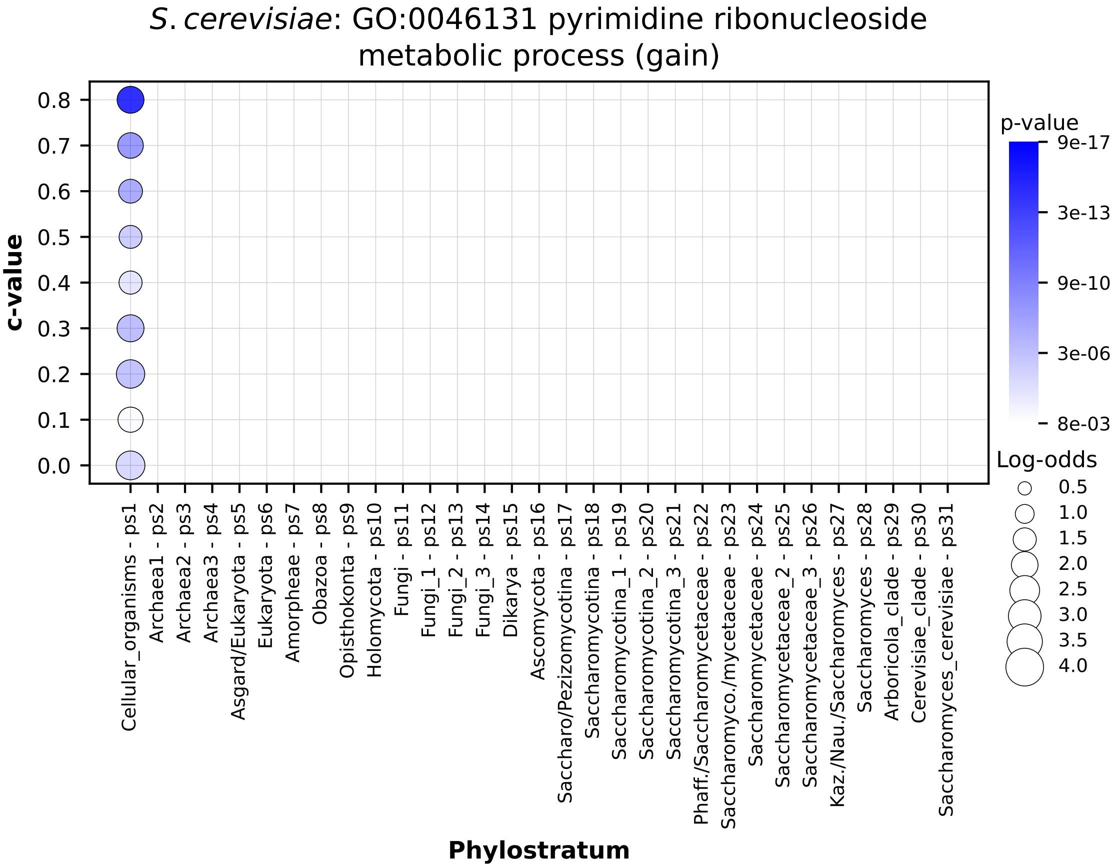

S. cerevisiae: GO:0046131 pyrimidine ribonucleoside metabolic process (gain)
c-value
0.8
0.7
0.6
0.5
0.4
0.3
0.2
0.1
0.0
p-value
9e-17
3e-13
9e-10
3e-06
8e-03
Log-odds
0.5
1.0
1.5
2.0
2.5
3.0
3.5
4.0
Cellular_organisms - ps1
Archaea1 - ps2
Archaea2 - ps3
Archaea3 - ps4
Asgard/Eukaryota - ps5
Eukaryota - ps6
Amorpheae - ps7
Obazoa - ps8
Opisthokonta - ps9
Holomycota - ps10
Fungi - ps11
Fungi_1 - ps12
Fungi_2 - ps13
Fungi_3 - ps14
Dikarya - ps15
Ascomycota - ps16
Saccharo/Pezizomycotina - ps17
Saccharomycotina - ps18
Saccharomycotina_1 - ps19
Saccharomycotina_2 - ps20
Saccharomycotina_3 - ps21
Phaff./Saccharomycetaceae - ps22
Saccharomyco./mycetaceae - ps23
Saccharomycetaceae - ps24
Saccharomycetaceae_2 - ps25
Saccharomycetaceae_3 - ps26
Kaz./Nau./Saccharomyces - ps27
Saccharomyces - ps28
Arboricola_clade - ps29
Cerevisiae_clade - ps30
Saccharomyces_cerevisiae - ps31
Phylostratum

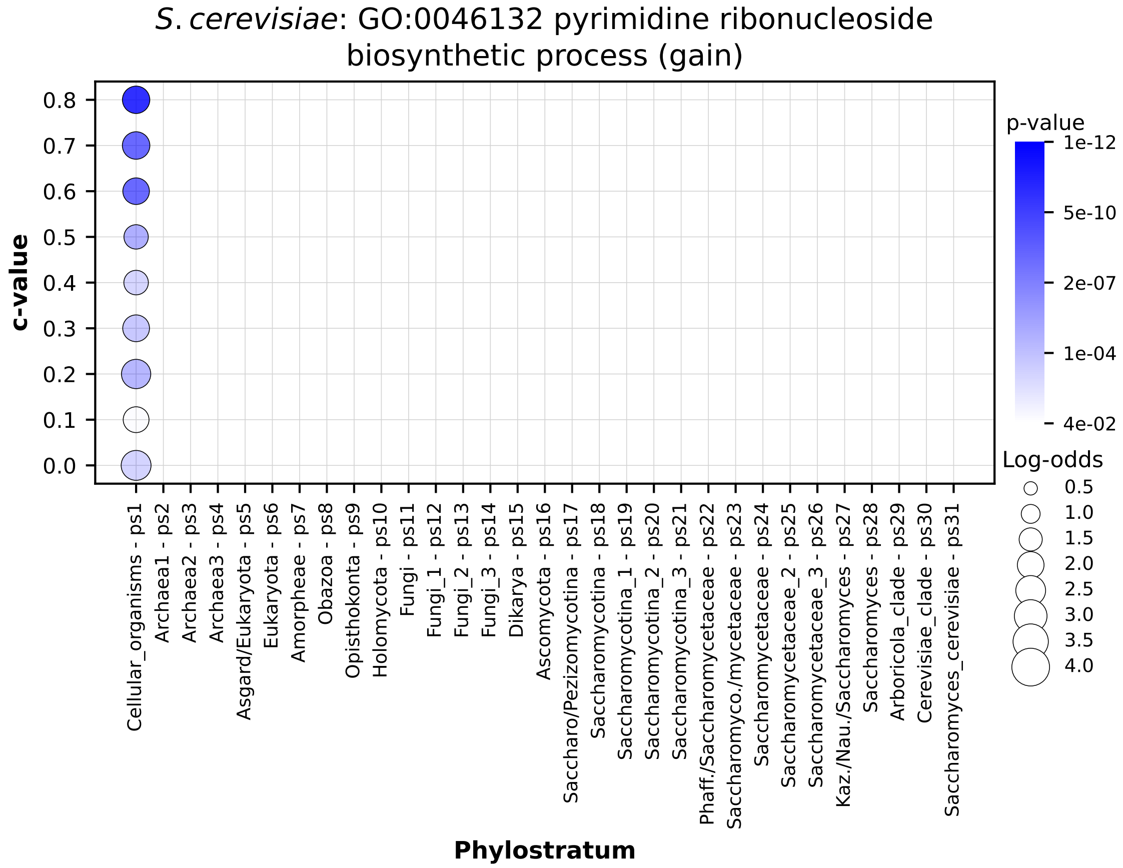

S. cerevisiae: GO:0046132 pyrimidine ribonucleoside biosynthetic process (gain)
c-value
0.0
0.1
0.2
0.3
0.4
0.5
0.6
0.7
0.8
p-value
1e-12
5e-10
2e-07
1e-04
4e-02
Log-odds
0.5
1.0
1.5
2.0
2.5
3.0
3.5
4.0
Cellular_organisms - ps1
Archaea1 - ps2
Archaea2 - ps3
Archaea3 - ps4
Asgard/Eukaryota - ps5
Eukaryota - ps6
Amorpheae - ps7
Obazoa - ps8
Opisthokonta - ps9
Holomycota - ps10
Fungi - ps11
Fungi_1 - ps12
Fungi_2 - ps13
Fungi_3 - ps14
Dikarya - ps15
Ascomycota - ps16
Saccharo/Pezizomycotina - ps17
Saccharomycotina - ps18
Saccharomycotina_1 - ps19
Saccharomycotina_2 - ps20
Saccharomycotina_3 - ps21
Phaff./Saccharomycetaceae - ps22
Saccharomyco./mycetaceae - ps23
Saccharomycetaceae - ps24
Saccharomycetaceae_2 - ps25
Saccharomycetaceae_3 - ps26
Kaz./Nau./Saccharomyces - ps27
Saccharomyces - ps28
Arboricola_clade - ps29
Cerevisiae_clade - ps30
Saccharomyces_cerevisiae - ps31
Phylostratum

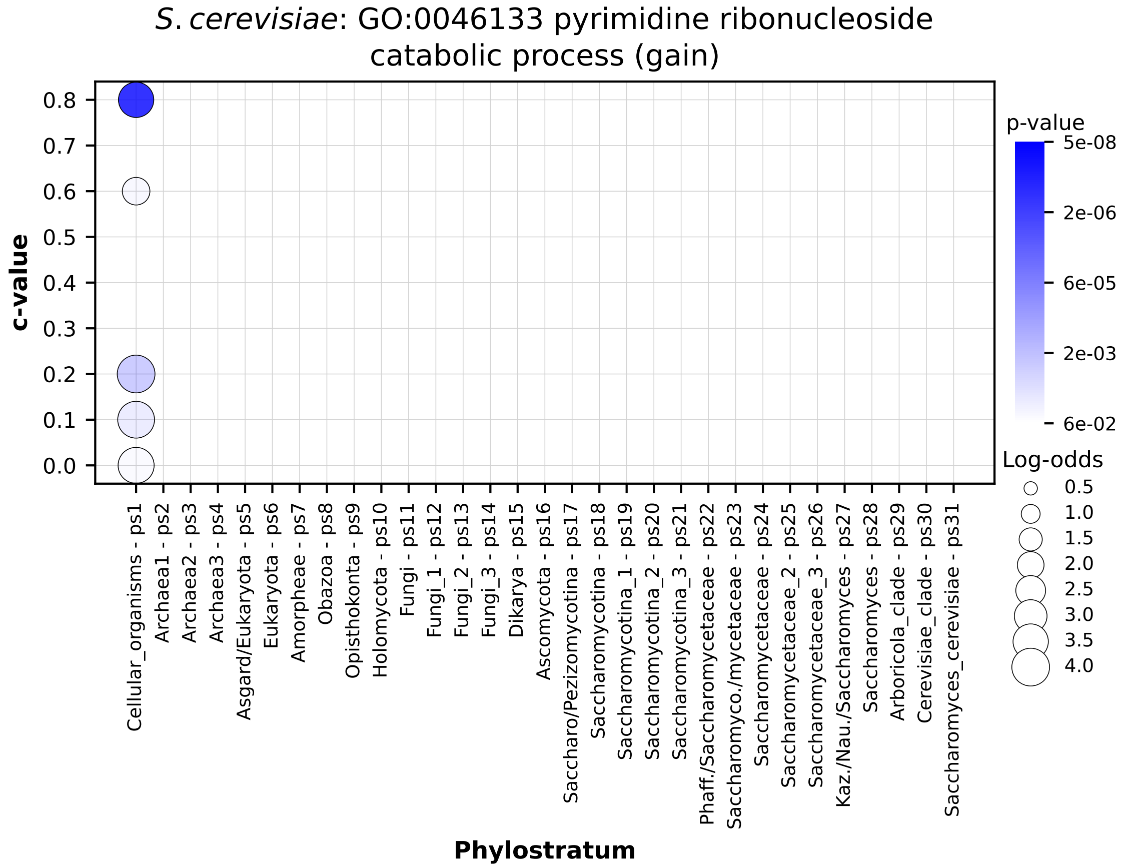

S. cerevisiae: GO:0046133 pyrimidine ribonucleoside catabolic process (gain)
c-value
0.8
0.7
0.6
0.5
0.4
0.3
0.2
0.1
0.0
p-value
5e-08
2e-06
6e-05
2e-03
6e-02
Log-odds
0.5
1.0
1.5
2.0
2.5
3.0
3.5
4.0
Cellular_organisms - ps1
Archaea1 - ps2
Archaea2 - ps3
Archaea3 - ps4
Asgard/Eukaryota - ps5
Eukaryota - ps6
Amorpheae - ps7
Obazoa - ps8
Opisthokonta - ps9
Holomycota - ps10
Fungi - ps11
Fungi_1 - ps12
Fungi_2 - ps13
Fungi_3 - ps14
Dikarya - ps15
Ascomycota - ps16
Saccharo/Pezizomycotina - ps17
Saccharomycotina - ps18
Saccharomycotina_1 - ps19
Saccharomycotina_2 - ps20
Saccharomycotina_3 - ps21
Phaff./Saccharomycetaceae - ps22
Saccharomyco./mycetaceae - ps23
Saccharomycetaceae - ps24
Saccharomycetaceae_2 - ps25
Saccharomycetaceae_3 - ps26
Kaz./Nau./Saccharomyces - ps27
Saccharomyces - ps28
Arboricola_clade - ps29
Cerevisiae_clade - ps30
Saccharomyces_cerevisiae - ps31
Phylostratum

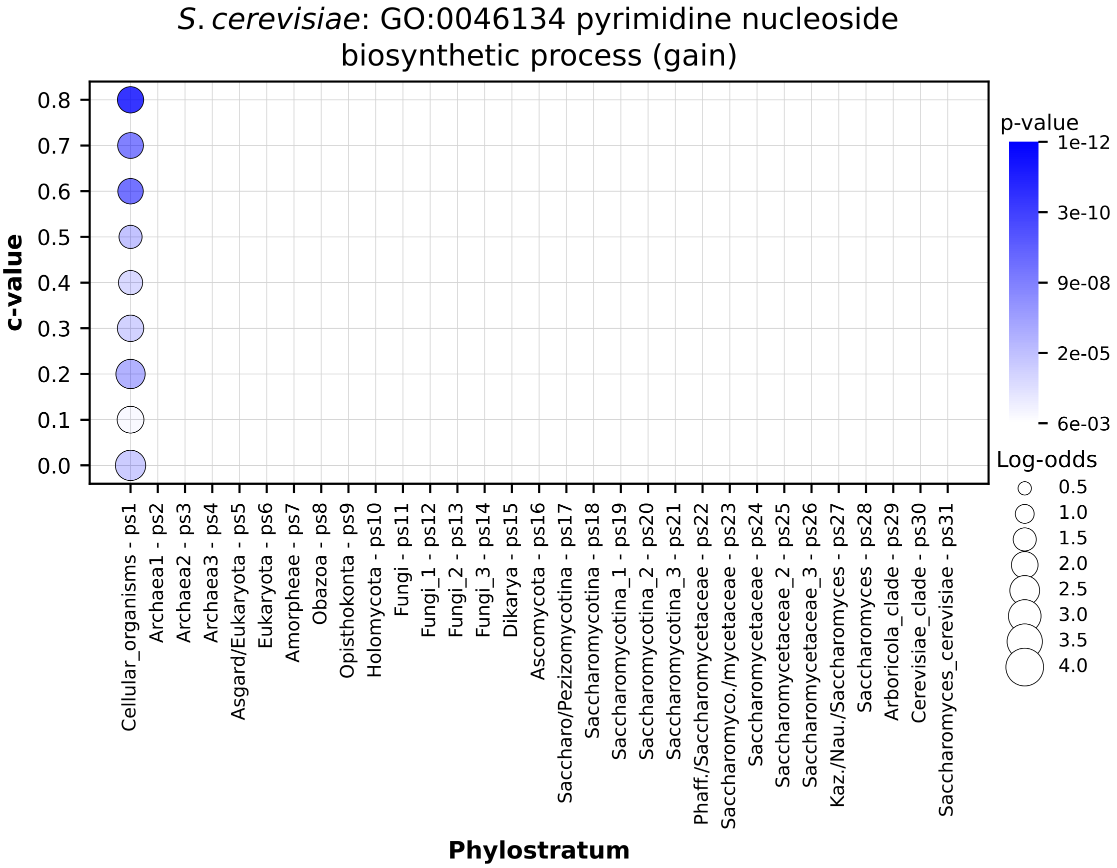

S. cerevisiae: GO:0046134 pyrimidine nucleoside biosynthetic process (gain)
c-value
0.8
0.7
0.6
0.5
0.4
0.3
0.2
0.1
0.0
p-value
1e-12
3e-10
9e-08
2e-05
6e-03
Log-odds
0.5
1.0
1.5
2.0
2.5
3.0
3.5
4.0
Cellular_organisms - ps1
Archaea1 - ps2
Archaea2 - ps3
Archaea3 - ps4
Asgard/Eukaryota - ps5
Eukaryota - ps6
Amorpheae - ps7
Obazoa - ps8
Opisthokonta - ps9
Holomycota - ps10
Fungi - ps11
Fungi_1 - ps12
Fungi_2 - ps13
Fungi_3 - ps14
Dikarya - ps15
Ascomycota - ps16
Saccharo/Pezizomycotina - ps17
Saccharomycotina - ps18
Saccharomycotina_1 - ps19
Saccharomycotina_2 - ps20
Saccharomycotina_3 - ps21
Phaff./Saccharomycetaceae - ps22
Saccharomyco./mycetaceae - ps23
Saccharomycetaceae - ps24
Saccharomycetaceae_2 - ps25
Saccharomycetaceae_3 - ps26
Kaz./Nau./Saccharomyces - ps27
Saccharomyces - ps28
Arboricola_clade - ps29
Cerevisiae_clade - ps30
Saccharomyces_cerevisiae - ps31
Phylostratum

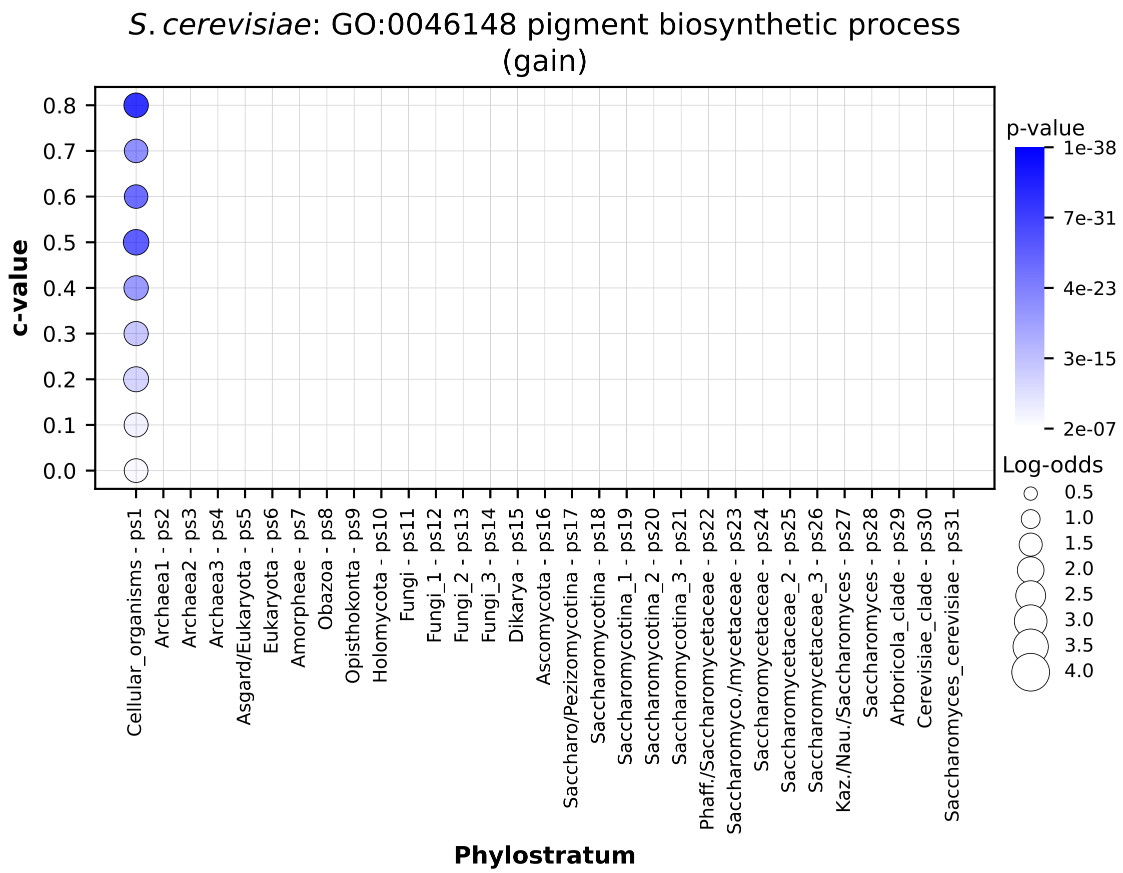

S. cerevisiae: GO:0046148 pigment biosynthetic process
(gain)
c-value
0.8
0.7
0.6
0.5
0.4
0.3
0.2
0.1
0.0
p-value
1e-38
7e-31
4e-23
3e-15
2e-07
Log-odds
0.5
1.0
1.5
2.0
2.5
3.0
3.5
4.0
Cellular_organisms - ps1
Archaea1 - ps2
Archaea2 - ps3
Archaea3 - ps4
Asgard/Eukaryota - ps5
Eukaryota - ps6
Amorpheae - ps7
Obazoa - ps8
Opisthokonta - ps9
Holomycota - ps10
Fungi - ps11
Fungi_1 - ps12
Fungi_2 - ps13
Fungi_3 - ps14
Dikarya - ps15
Ascomycota - ps16
Saccharo/Pezizomycotina - ps17
Saccharomycotina - ps18
Saccharomycotina_1 - ps19
Saccharomycotina_2 - ps20
Saccharomycotina_3 - ps21
Phaff./Saccharomycetaceae - ps22
Saccharomyco./mycetaceae - ps23
Saccharomycetaceae - ps24
Saccharomycetaceae_2 - ps25
Saccharomycetaceae_3 - ps26
Kaz./Nau./Saccharomyces - ps27
Saccharomyces - ps28
Arboricola_clade - ps29
Cerevisiae_clade - ps30
Saccharomyces_cerevisiae - ps31
Phylostratum

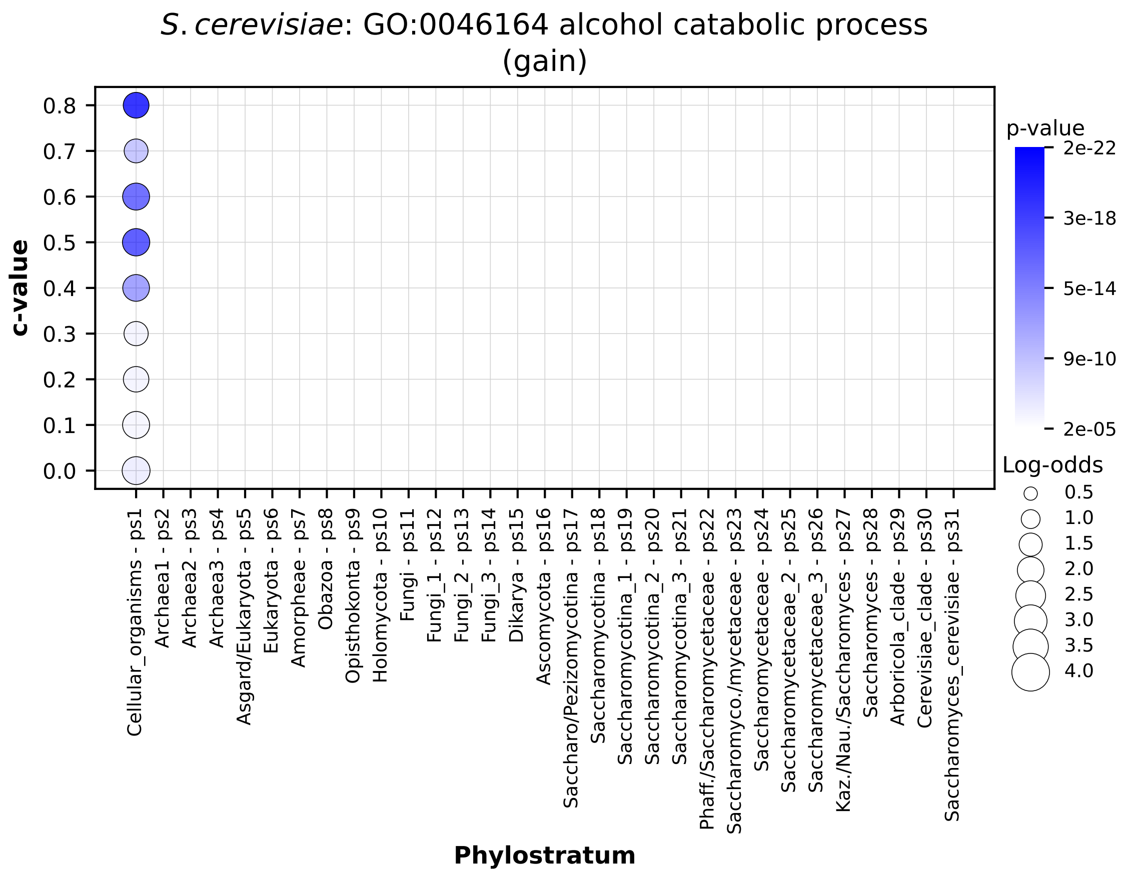
S. cerevisiae: GO:0046164 alcohol catabolic process
(gain)
c-value
0.8
0.7
0.6
0.5
0.4
0.3
0.2
0.1
0.0
p-value
2e-22
3e-18
5e-14
9e-10
2e-05
Log-odds
0.5
1.0
1.5
2.0
2.5
3.0
3.5
4.0
Cellular_organisms - ps1
Archaea1 - ps2
Archaea2 - ps3
Archaea3 - ps4
Asgard/Eukaryota - ps5
Eukaryota - ps6
Amorpheae - ps7
Obazoa - ps8
Opisthokonta - ps9
Holomycota - ps10
Fungi - ps11
Fungi_1 - ps12
Fungi_2 - ps13
Fungi_3 - ps14
Dikarya - ps15
Ascomycota - ps16
Saccharo/Pezizomycotina - ps17
Saccharomycotina - ps18
Saccharomycotina_1 - ps19
Saccharomycotina_2 - ps20
Saccharomycotina_3 - ps21
Phaff./Saccharomycetaceae - ps22
Saccharomyco./mycetaceae - ps23
Saccharomycetaceae - ps24
Saccharomycetaceae_2 - ps25
Saccharomycetaceae_3 - ps26
Kaz./Nau./Saccharomyces - ps27
Saccharomyces - ps28
Arboricola_clade - ps29
Cerevisiae_clade - ps30
Saccharomyces_cerevisiae - ps31
Phylostratum

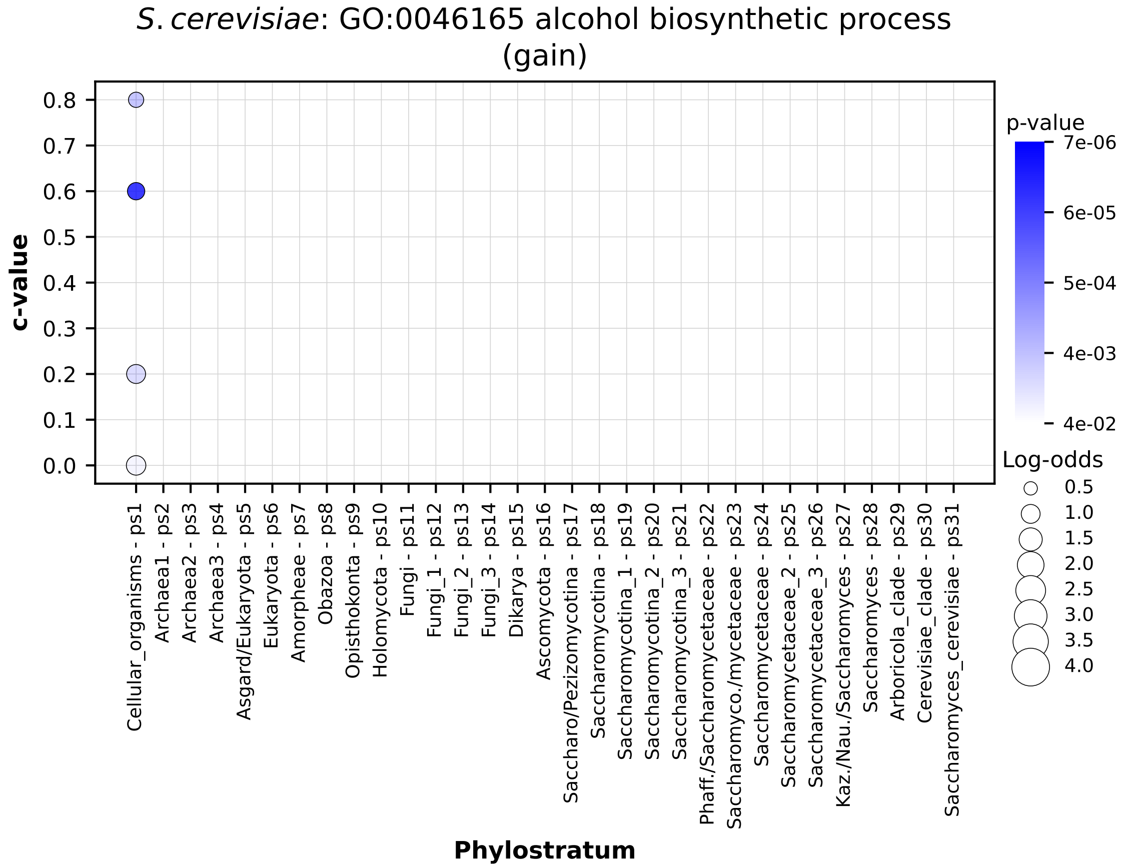

S. cerevisiae: GO:0046165 alcohol biosynthetic process (gain)
c-value
0.0
0.1
0.2
0.3
0.4
0.5
0.6
0.7
0.8
p-value
7e-06
6e-05
5e-04
4e-03
4e-02
Log-odds
0.5
1.0
1.5
2.0
2.5
3.0
3.5
4.0
Cellular_organisms - ps1
Archaea1 - ps2
Archaea2 - ps3
Archaea3 - ps4
Asgard/Eukaryota - ps5
Eukaryota - ps6
Amorpheae - ps7
Obazoa - ps8
Opisthokonta - ps9
Holomycota - ps10
Fungi - ps11
Fungi_1 - ps12
Fungi_2 - ps13
Fungi_3 - ps14
Dikarya - ps15
Ascomycota - ps16
Saccharo/Pezizomycotina - ps17
Saccharomycotina - ps18
Saccharomycotina_1 - ps19
Saccharomycotina_2 - ps20
Saccharomycotina_3 - ps21
Phaff./Saccharomycetaceae - ps22
Saccharomyco./mycetaceae - ps23
Saccharomycetaceae - ps24
Saccharomycetaceae_2 - ps25
Saccharomycetaceae_3 - ps26
Kaz./Nau./Saccharomyces - ps27
Saccharomyces - ps28
Arboricola_clade - ps29
Cerevisiae_clade - ps30
Saccharomyces_cerevisiae - ps31
Phylostratum

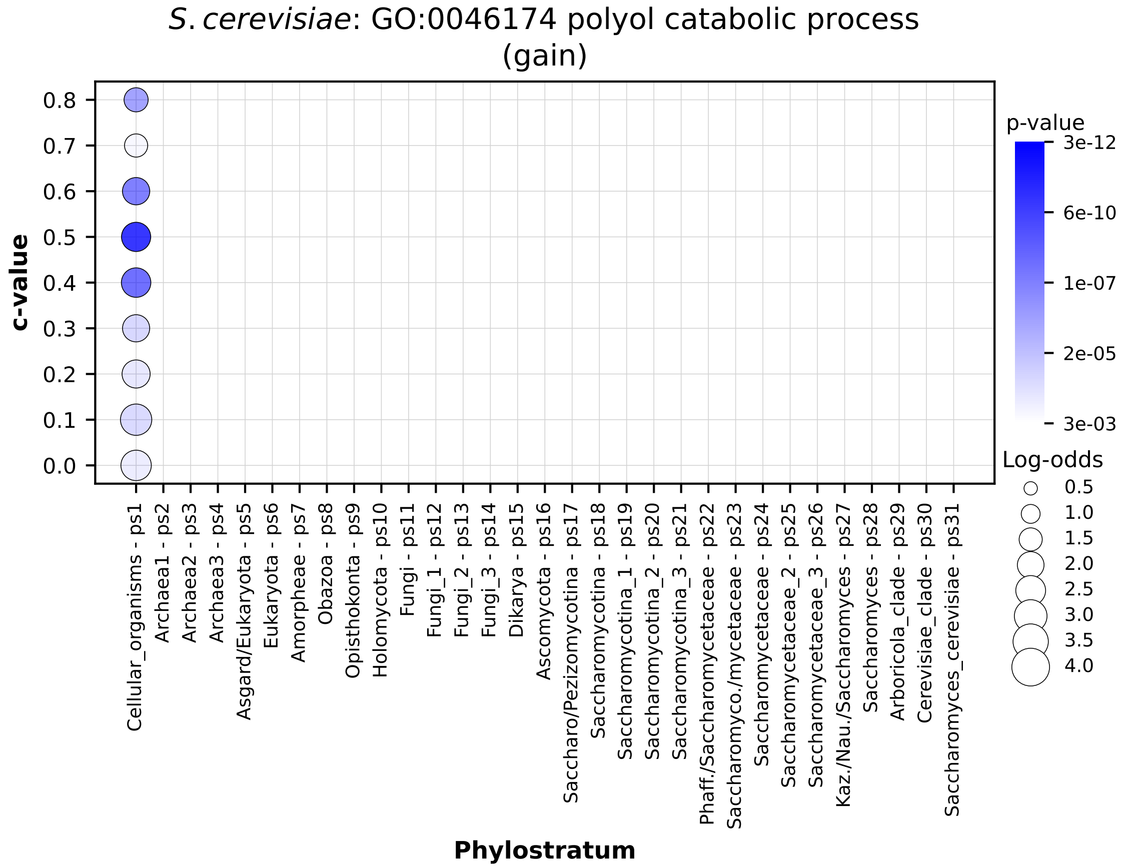
S. cerevisiae: GO:0046174 polyol catabolic process
(gain)
c-value
0.8
0.7
0.6
0.5
0.4
0.3
0.2
0.1
0.0
p-value
3e-12
6e-10
1e-07
2e-05
3e-03
Log-odds
0.5
1.0
1.5
2.0
2.5
3.0
3.5
4.0
Cellular_organisms - ps1
Archaea1 - ps2
Archaea2 - ps3
Archaea3 - ps4
Asgard/Eukaryota - ps5
Eukaryota - ps6
Amorpheae - ps7
Obazoa - ps8
Opisthokonta - ps9
Holomycota - ps10
Fungi - ps11
Fungi_1 - ps12
Fungi_2 - ps13
Fungi_3 - ps14
Dikarya - ps15
Ascomycota - ps16
Saccharo/Pezizomycotina - ps17
Saccharomycotina - ps18
Saccharomycotina_1 - ps19
Saccharomycotina_2 - ps20
Saccharomycotina_3 - ps21
Phaff./Saccharomycetaceae - ps22
Saccharomyco./mycetaceae - ps23
Saccharomycetaceae - ps24
Saccharomycetaceae_2 - ps25
Saccharomycetaceae_3 - ps26
Kaz./Nau./Saccharomyces - ps27
Saccharomyces - ps28
Arboricola_clade - ps29
Cerevisiae_clade - ps30
Saccharomyces_cerevisiae - ps31
Phylostratum

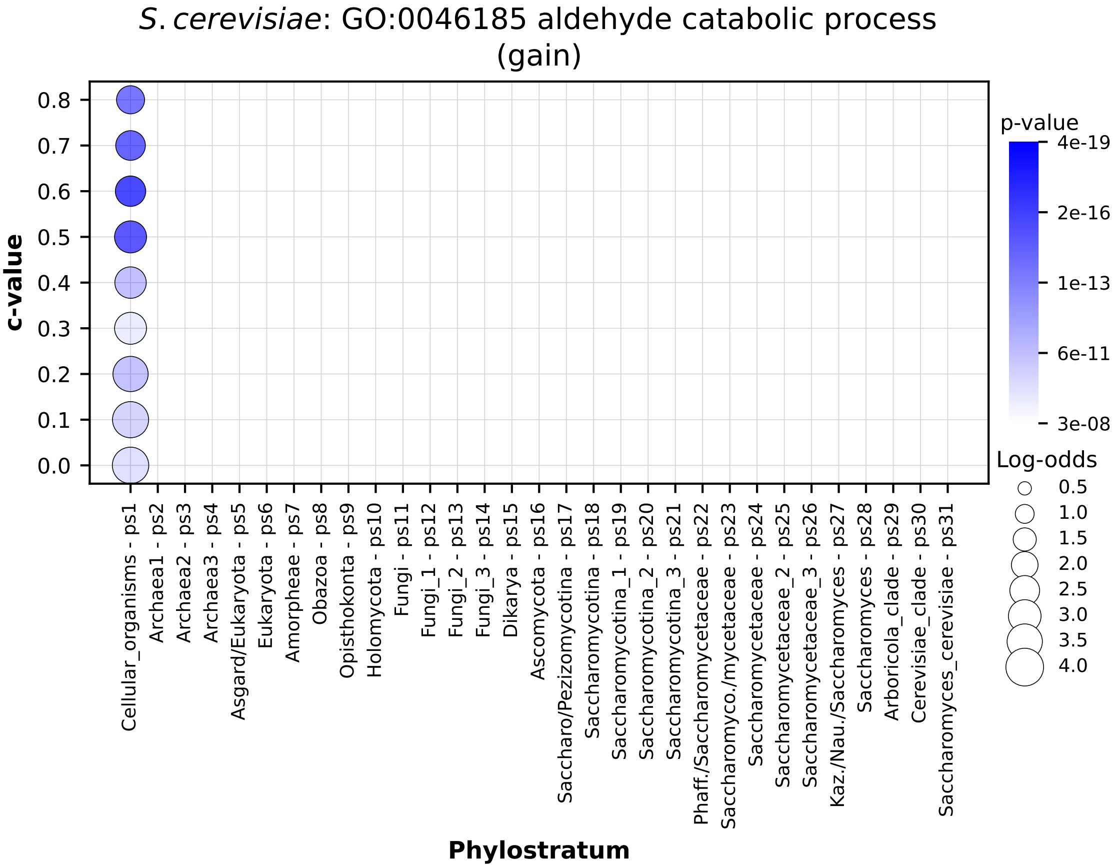

S. cerevisiae: GO:0046185 aldehyde catabolic process
(gain)
c-value
0.8
0.7
0.6
0.5
0.4
0.3
0.2
0.1
0.0
p-value
4e-19
2e-16
1e-13
6e-11
3e-08
Log-odds
0.5
1.0
1.5
2.0
2.5
3.0
3.5
4.0
Cellular_organisms - ps1
Archaea1 - ps2
Archaea2 - ps3
Archaea3 - ps4
Asgard/Eukaryota - ps5
Eukaryota - ps6
Amorpheae - ps7
Obazoa - ps8
Opisthokonta - ps9
Holomycota - ps10
Fungi - ps11
Fungi_1 - ps12
Fungi_2 - ps13
Fungi_3 - ps14
Dikarya - ps15
Ascomycota - ps16
Saccharo/Pezizomycotina - ps17
Saccharomycotina - ps18
Saccharomycotina_1 - ps19
Saccharomycotina_2 - ps20
Saccharomycotina_3 - ps21
Phaff./Saccharomycetaceae - ps22
Saccharomyco./mycetaceae - ps23
Saccharomycetaceae - ps24
Saccharomycetaceae_2 - ps25
Saccharomycetaceae_3 - ps26
Kaz./Nau./Saccharomyces - ps27
Saccharomyces - ps28
Arboricola_clade - ps29
Cerevisiae_clade - ps30
Saccharomyces_cerevisiae - ps31
Phylostratum

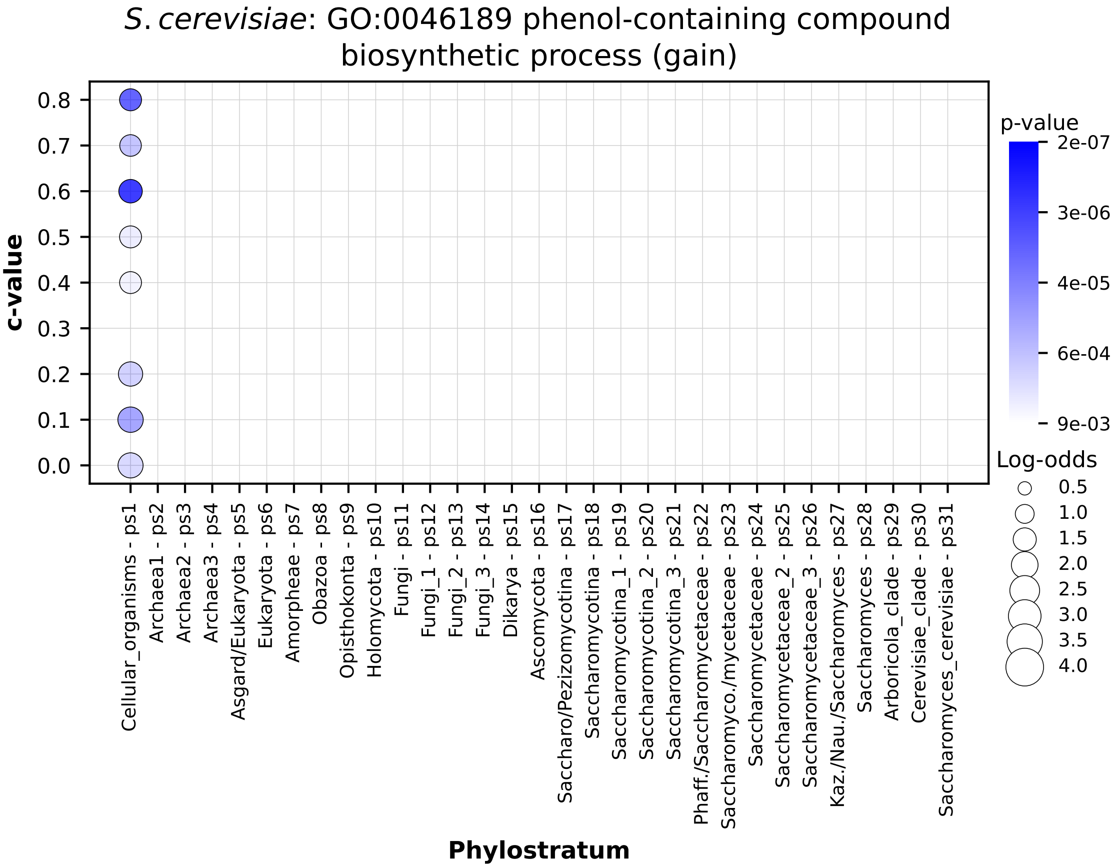
S. cerevisiae: GO:0046189 phenol-containing compound biosynthetic process (gain)
c-value
0.8
0.7
0.6
0.5
0.4
0.3
0.2
0.1
0.0
Phylostratum
Cellular_organisms - ps1
Archaea1 - ps2
Archaea2 - ps3
Archaea3 - ps4
Asgard/Eukaryota - ps5
Eukaryota - ps6
Amorpheae - ps7
Obazoa - ps8
Opisthokonta - ps9
Holomycota - ps10
Fungi - ps11
Fungi_1 - ps12
Fungi_2 - ps13
Fungi_3 - ps14
Dikarya - ps15
Ascomycota - ps16
Saccharo/Pezizomycotina - ps17
Saccharomycotina - ps18
Saccharomycotina_1 - ps19
Saccharomycotina_2 - ps20
Saccharomycotina_3 - ps21
Phaff./Saccharomycetaceae - ps22
Saccharomyco./mycetaceae - ps23
Saccharomycetaceae - ps24
Saccharomycetaceae_2 - ps25
Saccharomycetaceae_3 - ps26
Kaz./Nau./Saccharomyces - ps27
Saccharomyces - ps28
Arboricola_clade - ps29
Cerevisiae_clade - ps30
Saccharomyces_cerevisiae - ps31
p-value
2e-07
3e-06
4e-05
6e-04
9e-03
Log-odds
0.5
1.0
1.5
2.0
2.5
3.0
3.5
4.0

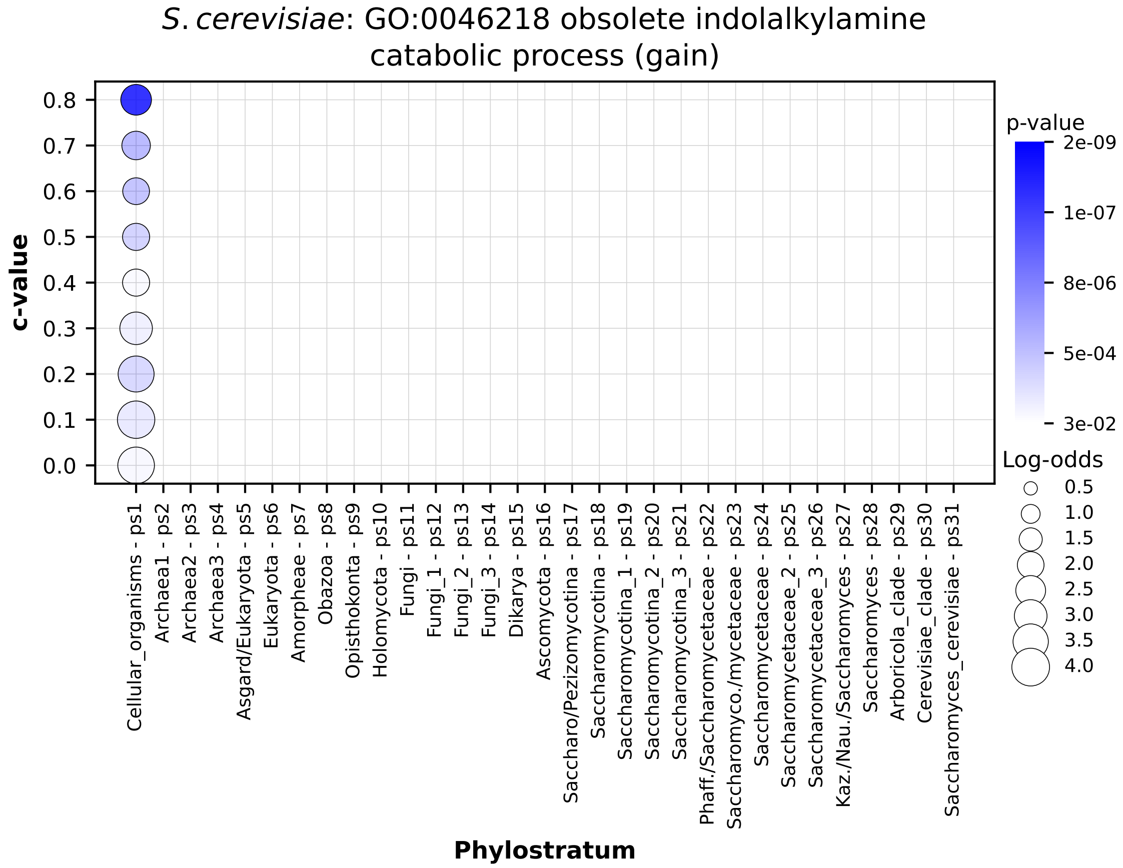

S. cerevisiae: GO:0046218 obsolete indolalkylamine catabolic process (gain)
c-value
0.8
0.7
0.6
0.5
0.4
0.3
0.2
0.1
0.0
p-value
2e-09
1e-07
8e-06
5e-04
3e-02
Log-odds
0.5
1.0
1.5
2.0
2.5
3.0
3.5
4.0
Cellular_organisms - ps1
Archaea1 - ps2
Archaea2 - ps3
Archaea3 - ps4
Asgard/Eukaryota - ps5
Eukaryota - ps6
Amorpheae - ps7
Obazoa - ps8
Opisthokonta - ps9
Holomycota - ps10
Fungi - ps11
Fungi_1 - ps12
Fungi_2 - ps13
Fungi_3 - ps14
Dikarya - ps15
Ascomycota - ps16
Saccharo/Pezizomycotina - ps17
Saccharomycotina - ps18
Saccharomycotina_1 - ps19
Saccharomycotina_2 - ps20
Saccharomycotina_3 - ps21
Phaff./Saccharomycetaceae - ps22
Saccharomyco./mycetaceae - ps23
Saccharomycetaceae - ps24
Saccharomycetaceae_2 - ps25
Saccharomycetaceae_3 - ps26
Kaz./Nau./Saccharomyces - ps27
Saccharomyces - ps28
Arboricola_clade - ps29
Cerevisiae_clade - ps30
Saccharomyces_cerevisiae - ps31
Phylostratum

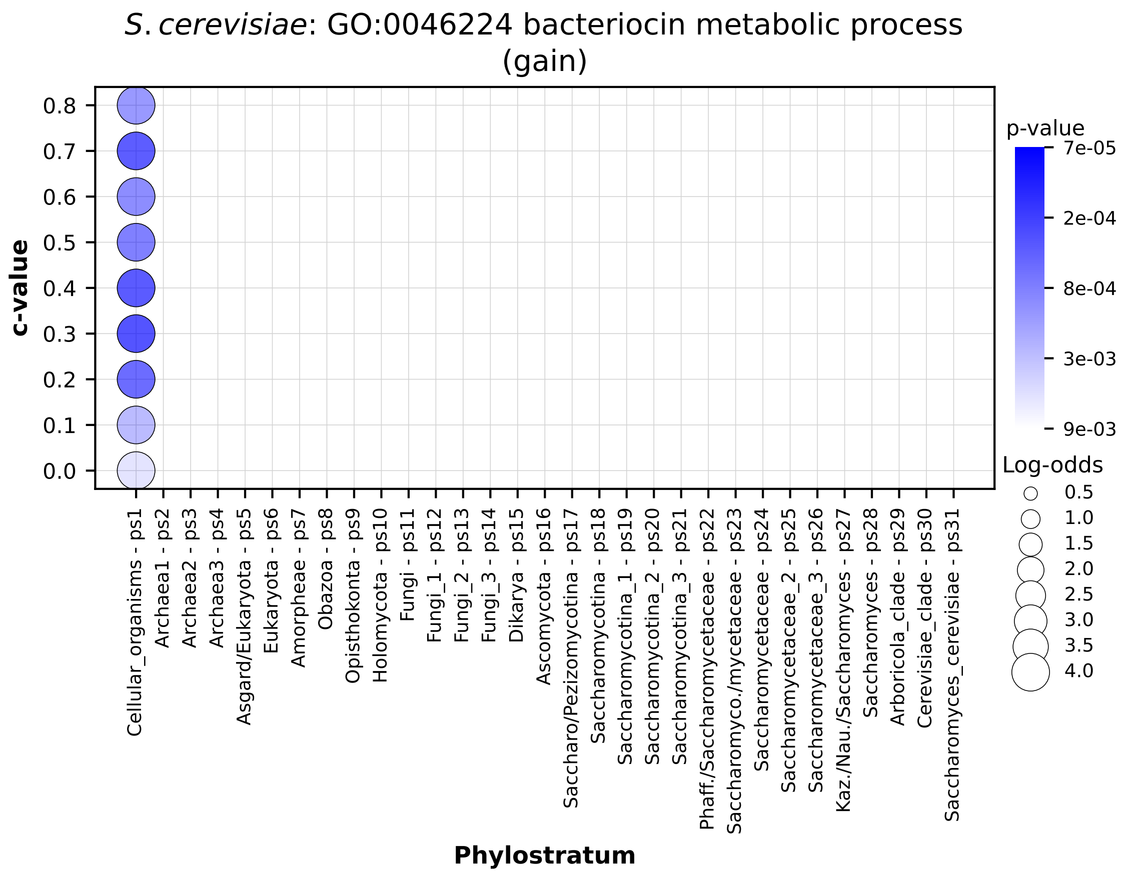
S. cerevisiae: GO:0046224 bacteriocin metabolic process
(gain)
c-value
0.8
0.7
0.6
0.5
0.4
0.3
0.2
0.1
0.0
p-value
7e-05
2e-04
8e-04
3e-03
9e-03
Log-odds
0.5
1.0
1.5
2.0
2.5
3.0
3.5
4.0
Cellular_organisms - ps1
Archaea1 - ps2
Archaea2 - ps3
Archaea3 - ps4
Asgard/Eukaryota - ps5
Eukaryota - ps6
Amorpheae - ps7
Obazoa - ps8
Opisthokonta - ps9
Holomycota - ps10
Fungi - ps11
Fungi_1 - ps12
Fungi_2 - ps13
Fungi_3 - ps14
Dikarya - ps15
Ascomycota - ps16
Saccharo/Pezizomycotina - ps17
Saccharomycotina - ps18
Saccharomycotina_1 - ps19
Saccharomycotina_2 - ps20
Saccharomycotina_3 - ps21
Phaff./Saccharomycetaceae - ps22
Saccharomyco./mycetaceae - ps23
Saccharomycetaceae - ps24
Saccharomycetaceae_2 - ps25
Saccharomycetaceae_3 - ps26
Kaz./Nau./Saccharomyces - ps27
Saccharomyces - ps28
Arboricola_clade - ps29
Cerevisiae_clade - ps30
Saccharomyces_cerevisiae - ps31
Phylostratum

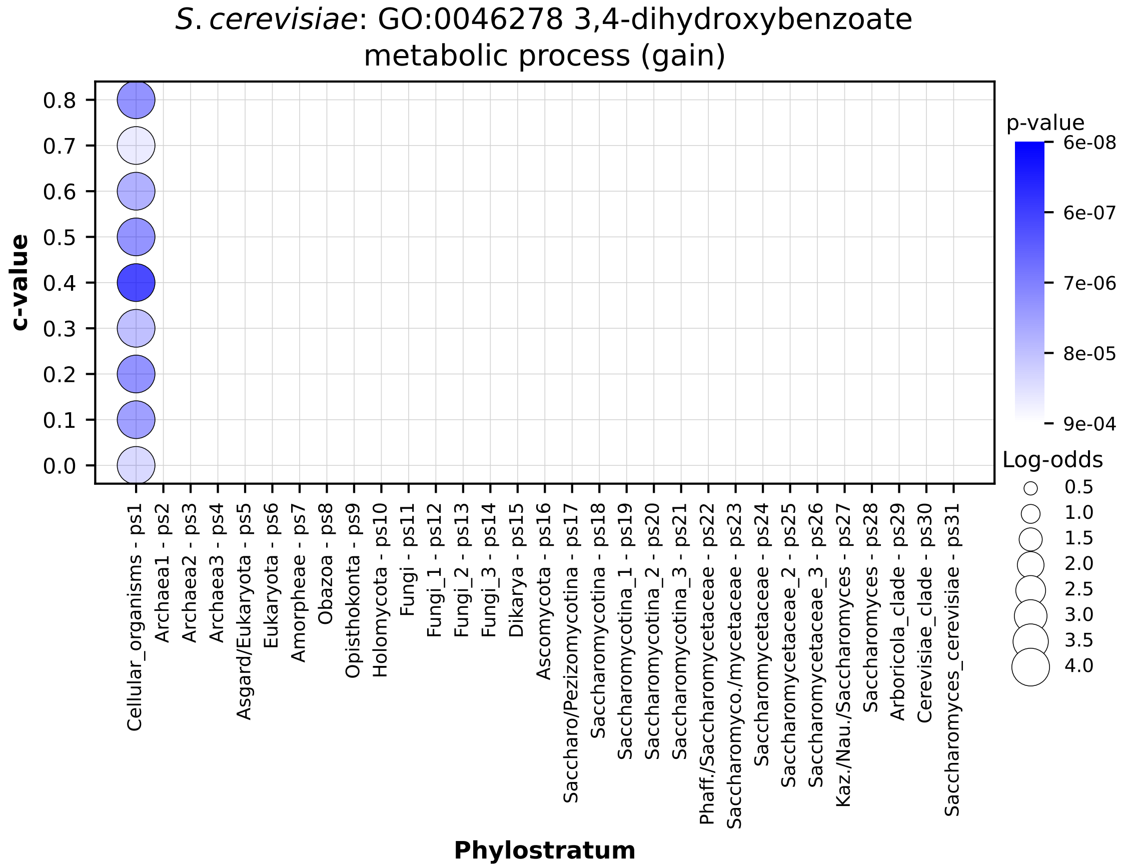

S. cerevisiae: GO:0046278 3,4-dihydroxybenzoate metabolic process (gain)
c-value
0.0
0.1
0.2
0.3
0.4
0.5
0.6
0.7
0.8
p-value
6e-08
6e-07
7e-06
8e-05
9e-04
Log-odds
0.5
1.0
1.5
2.0
2.5
3.0
3.5
4.0
Cellular_organisms - ps1
Archaea1 - ps2
Archaea2 - ps3
Archaea3 - ps4
Asgard/Eukaryota - ps5
Eukaryota - ps6
Amorpheae - ps7
Obazoa - ps8
Opisthokonta - ps9
Holomycota - ps10
Fungi - ps11
Fungi_1 - ps12
Fungi_2 - ps13
Fungi_3 - ps14
Dikarya - ps15
Ascomycota - ps16
Saccharo/Pezizomycotina - ps17
Saccharomycotina - ps18
Saccharomycotina_1 - ps19
Saccharomycotina_2 - ps20
Saccharomycotina_3 - ps21
Phaff./Saccharomycetaceae - ps22
Saccharomyco./mycetaceae - ps23
Saccharomycetaceae - ps24
Saccharomycetaceae_2 - ps25
Saccharomycetaceae_3 - ps26
Kaz./Nau./Saccharomyces - ps27
Saccharomyces - ps28
Arboricola_clade - ps29
Cerevisiae_clade - ps30
Saccharomyces_cerevisiae - ps31
Phylostratum

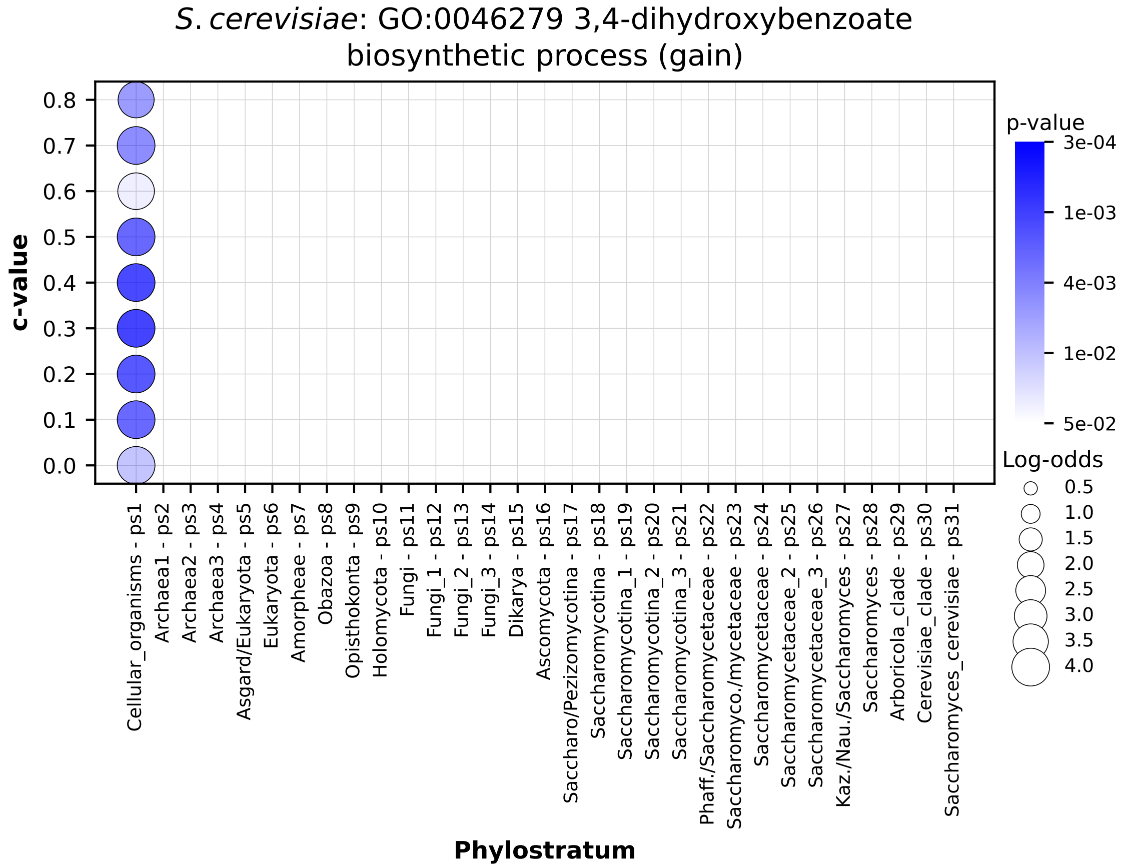
S. cerevisiae: GO:0046279 3,4-dihydroxybenzoate biosynthetic process (gain)
c-value
0.8
0.7
0.6
0.5
0.4
0.3
0.2
0.1
0.0
p-value
3e-04
1e-03
4e-03
1e-02
5e-02
Log-odds
0.5
1.0
1.5
2.0
2.5
3.0
3.5
4.0
Cellular_organisms - ps1
Archaea1 - ps2
Archaea2 - ps3
Archaea3 - ps4
Asgard/Eukaryota - ps5
Eukaryota - ps6
Amorpheae - ps7
Obazoa - ps8
Opisthokonta - ps9
Holomycota - ps10
Fungi - ps11
Fungi_1 - ps12
Fungi_2 - ps13
Fungi_3 - ps14
Dikarya - ps15
Ascomycota - ps16
Saccharo/Pezizomycotina - ps17
Saccharomycotina - ps18
Saccharomycotina_1 - ps19
Saccharomycotina_2 - ps20
Saccharomycotina_3 - ps21
Phaff./Saccharomycetaceae - ps22
Saccharomyco./mycetaceae - ps23
Saccharomycetaceae - ps24
Saccharomycetaceae_2 - ps25
Saccharomycetaceae_3 - ps26
Kaz./Nau./Saccharomyces - ps27
Saccharomyces - ps28
Arboricola_clade - ps29
Cerevisiae_clade - ps30
Saccharomyces_cerevisiae - ps31
Phylostratum

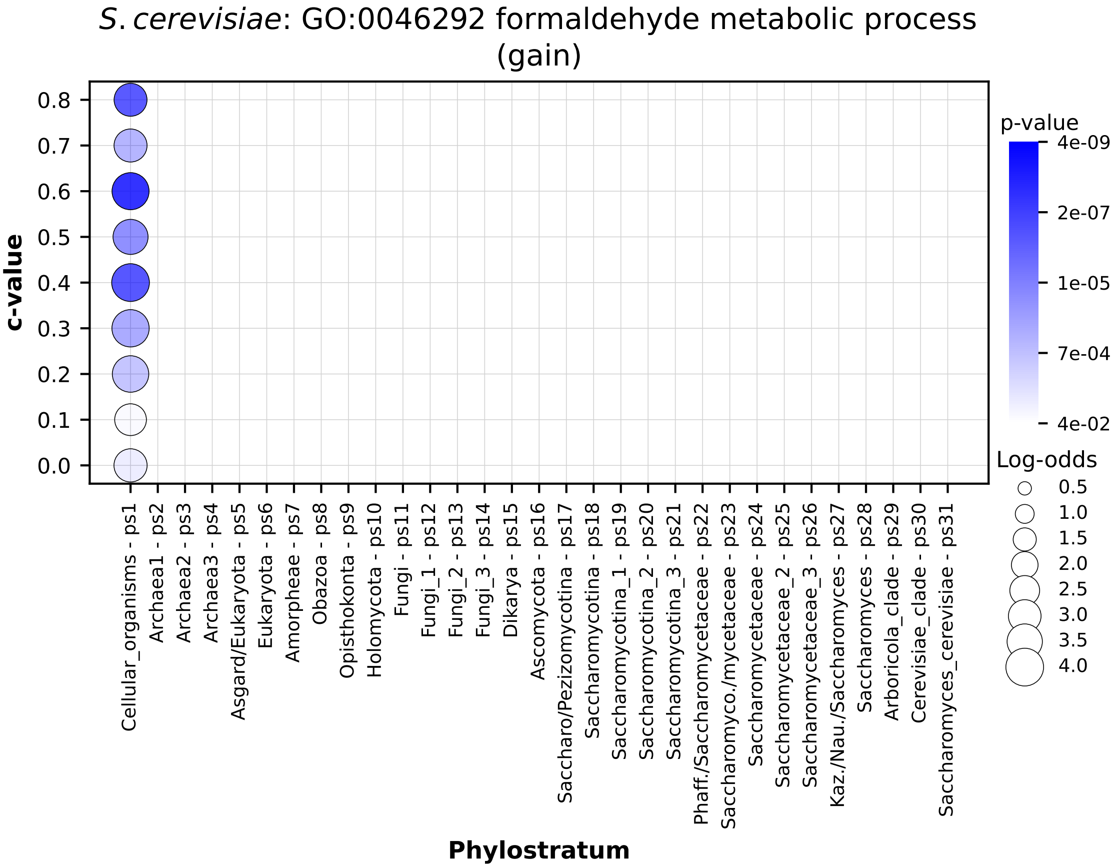

S. cerevisiae: GO:0046292 formaldehyde metabolic process
(gain)
c-value
0.0
0.1
0.2
0.3
0.4
0.5
0.6
0.7
0.8
p-value
4e-09
2e-07
1e-05
7e-04
4e-02
Log-odds
0.5
1.0
1.5
2.0
2.5
3.0
3.5
4.0
Cellular_organisms - ps1
Archaea1 - ps2
Archaea2 - ps3
Archaea3 - ps4
Asgard/Eukaryota - ps5
Eukaryota - ps6
Amorpheae - ps7
Obazoa - ps8
Opisthokonta - ps9
Holomycota - ps10
Fungi - ps11
Fungi_1 - ps12
Fungi_2 - ps13
Fungi_3 - ps14
Dikarya - ps15
Ascomycota - ps16
Saccharo/Pezizomycotina - ps17
Saccharomycotina - ps18
Saccharomycotina_1 - ps19
Saccharomycotina_2 - ps20
Saccharomycotina_3 - ps21
Phaff./Saccharomycetaceae - ps22
Saccharomyco./mycetaceae - ps23
Saccharomycetaceae - ps24
Saccharomycetaceae_2 - ps25
Saccharomycetaceae_3 - ps26
Kaz./Nau./Saccharomyces - ps27
Saccharomyces - ps28
Arboricola_clade - ps29
Cerevisiae_clade - ps30
Saccharomyces_cerevisiae - ps31
Phylostratum

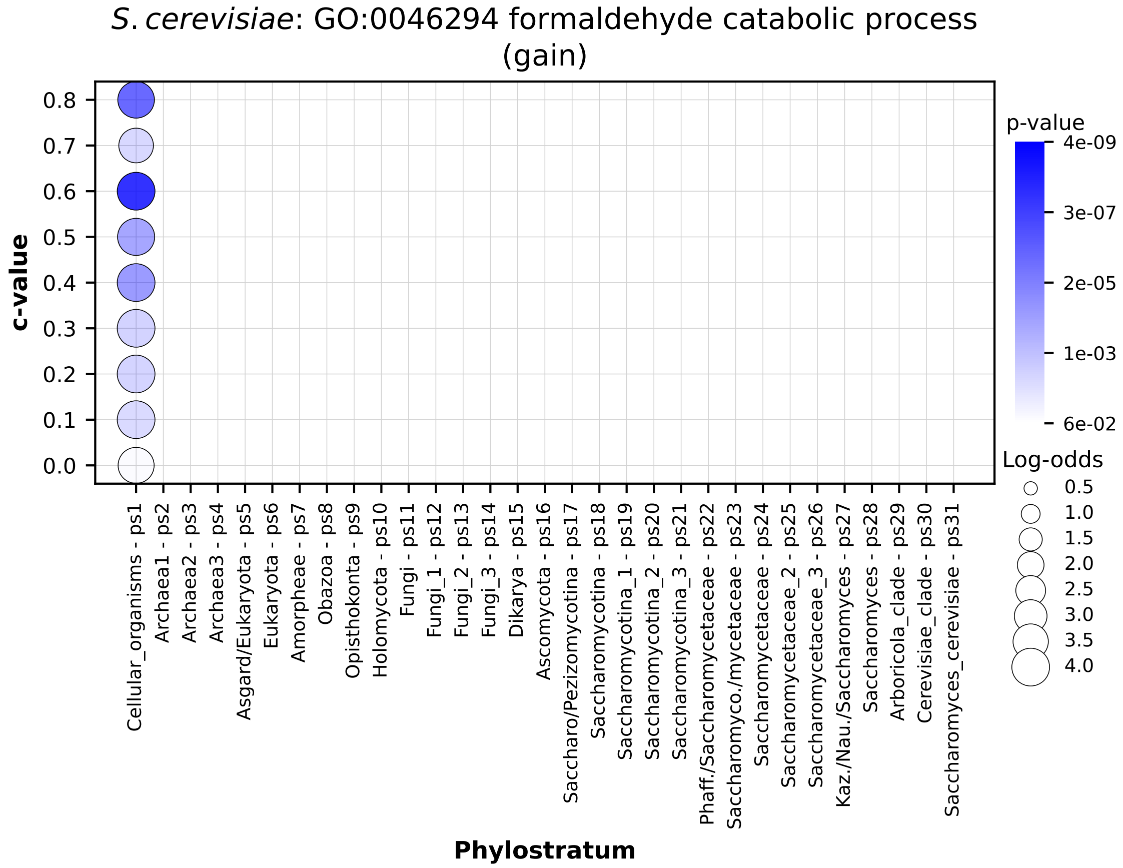

S. cerevisiae: GO:0046294 formaldehyde catabolic process (gain)
c-value
0.8
0.7
0.6
0.5
0.4
0.3
0.2
0.1
0.0
p-value
4e-09
3e-07
2e-05
1e-03
6e-02
Log-odds
0.5
1.0
1.5
2.0
2.5
3.0
3.5
4.0
Cellular_organisms - ps1
Archaea1 - ps2
Archaea2 - ps3
Archaea3 - ps4
Asgard/Eukaryota - ps5
Eukaryota - ps6
Amorpheae - ps7
Obazoa - ps8
Opisthokonta - ps9
Holomycota - ps10
Fungi - ps11
Fungi_1 - ps12
Fungi_2 - ps13
Fungi_3 - ps14
Dikarya - ps15
Ascomycota - ps16
Saccharo/Pezizomycotina - ps17
Saccharomycotina - ps18
Saccharomycotina_1 - ps19
Saccharomycotina_2 - ps20
Saccharomycotina_3 - ps21
Phaff./Saccharomycetaceae - ps22
Saccharomyco./mycetaceae - ps23
Saccharomycetaceae - ps24
Saccharomycetaceae_2 - ps25
Saccharomycetaceae_3 - ps26
Kaz./Nau./Saccharomyces - ps27
Saccharomyces - ps28
Arboricola_clade - ps29
Cerevisiae_clade - ps30
Saccharomyces_cerevisiae - ps31
Phylostratum

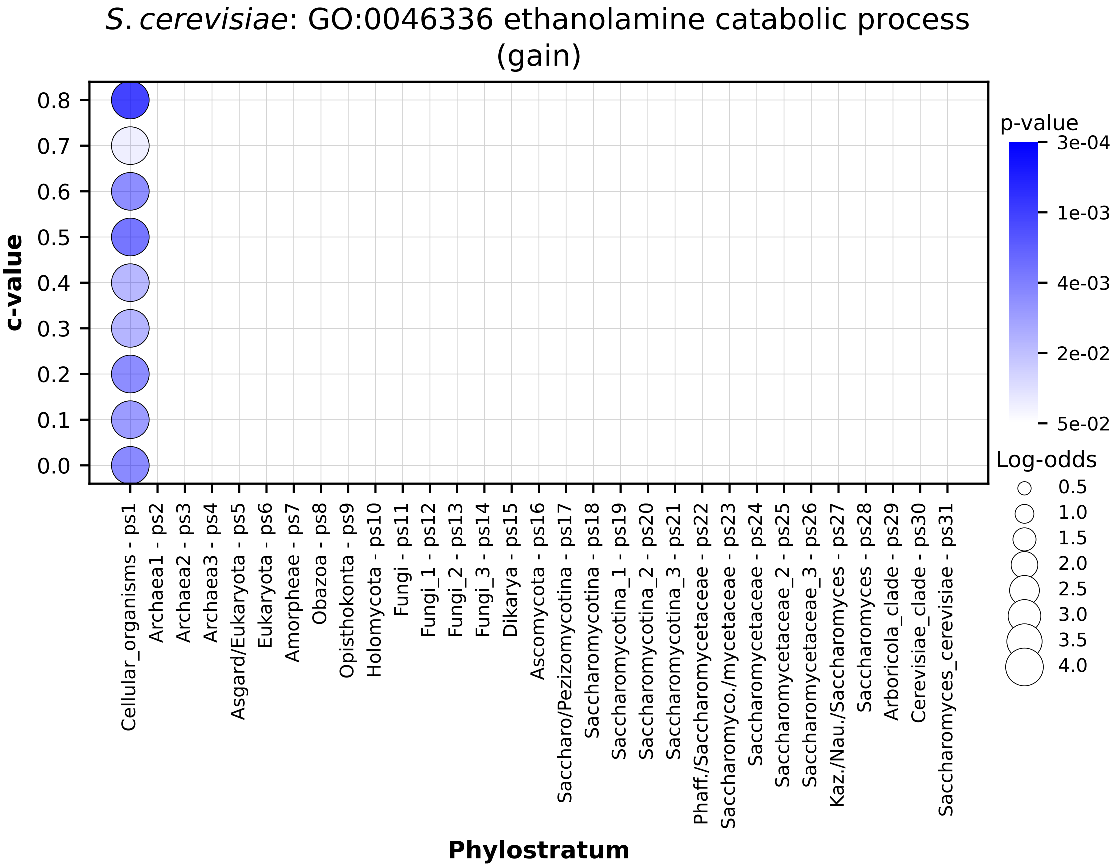

S. cerevisiae: GO:0046336 ethanolamine catabolic process (gain)
c-value
0.8
0.7
0.6
0.5
0.4
0.3
0.2
0.1
0.0
p-value
3e-04
1e-03
4e-03
2e-02
5e-02
Log-odds
0.5
1.0
1.5
2.0
2.5
3.0
3.5
4.0
Cellular_organisms - ps1
Archaea1 - ps2
Archaea2 - ps3
Archaea3 - ps4
Asgard/Eukaryota - ps5
Eukaryota - ps6
Amorpheae - ps7
Obazoa - ps8
Opisthokonta - ps9
Holomycota - ps10
Fungi - ps11
Fungi_1 - ps12
Fungi_2 - ps13
Fungi_3 - ps14
Dikarya - ps15
Ascomycota - ps16
Saccharo/Pezizomycotina - ps17
Saccharomycotina - ps18
Saccharomycotina_1 - ps19
Saccharomycotina_2 - ps20
Saccharomycotina_3 - ps21
Phaff./Saccharomycetaceae - ps22
Saccharomyco./mycetaceae - ps23
Saccharomycetaceae - ps24
Saccharomycetaceae_2 - ps25
Saccharomycetaceae_3 - ps26
Kaz./Nau./Saccharomyces - ps27
Saccharomyces - ps28
Arboricola_clade - ps29
Cerevisiae_clade - ps30
Saccharomyces_cerevisiae - ps31
Phylostratum

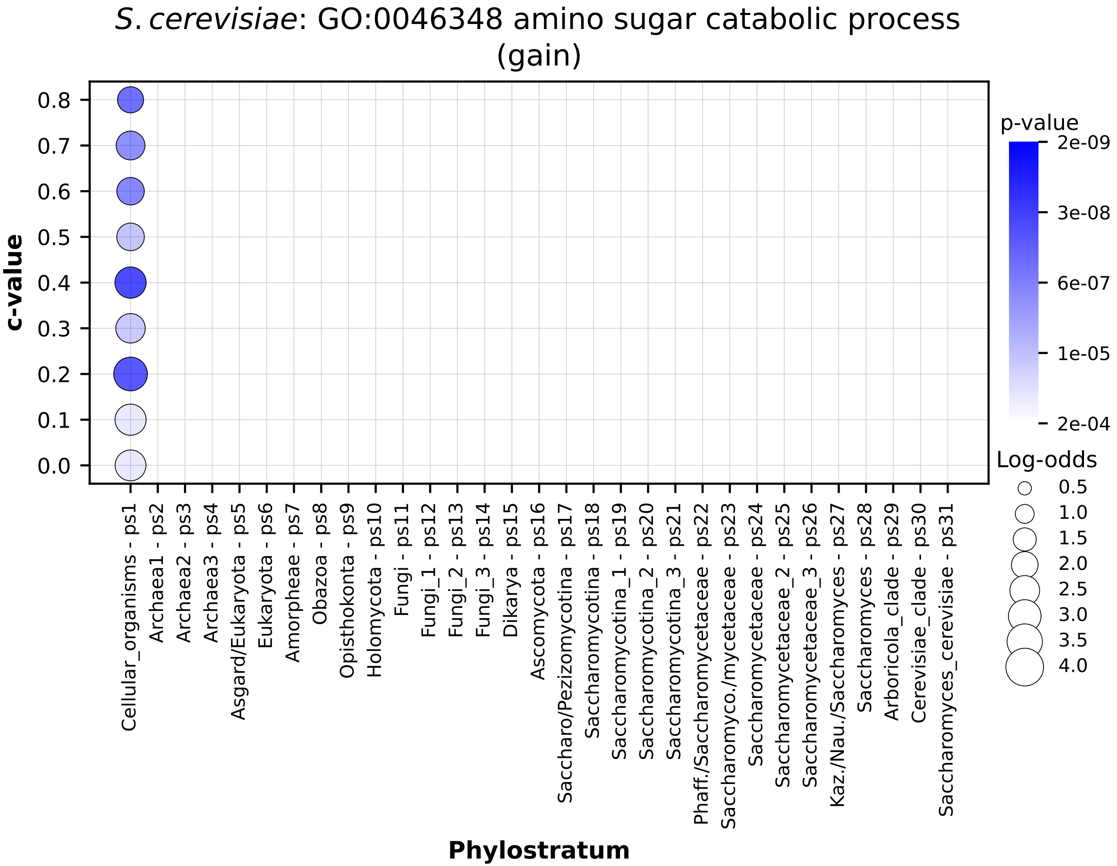

S. cerevisiae: GO:0046348 amino sugar catabolic process (gain)
c-value
0.8
0.7
0.6
0.5
0.4
0.3
0.2
0.1
0.0
p-value
2e-09
3e-08
6e-07
1e-05
2e-04
Log-odds
0.5
1.0
1.5
2.0
2.5
3.0
3.5
4.0
Cellular_organisms - ps1
Archaea1 - ps2
Archaea2 - ps3
Archaea3 - ps4
Asgard/Eukaryota - ps5
Eukaryota - ps6
Amorpheae - ps7
Obazoa - ps8
Opisthokonta - ps9
Holomycota - ps10
Fungi - ps11
Fungi_1 - ps12
Fungi_2 - ps13
Fungi_3 - ps14
Dikarya - ps15
Ascomycota - ps16
Saccharo/Pezizomycotina - ps17
Saccharomycotina - ps18
Saccharomycotina_1 - ps19
Saccharomycotina_2 - ps20
Saccharomycotina_3 - ps21
Phaff./Saccharomycetaceae - ps22
Saccharomyco./mycetaceae - ps23
Saccharomycetaceae - ps24
Saccharomycetaceae_2 - ps25
Saccharomycetaceae_3 - ps26
Kaz./Nau./Saccharomyces - ps27
Saccharomyces - ps28
Arboricola_clade - ps29
Cerevisiae_clade - ps30
Saccharomyces_cerevisiae - ps31
Phylostratum

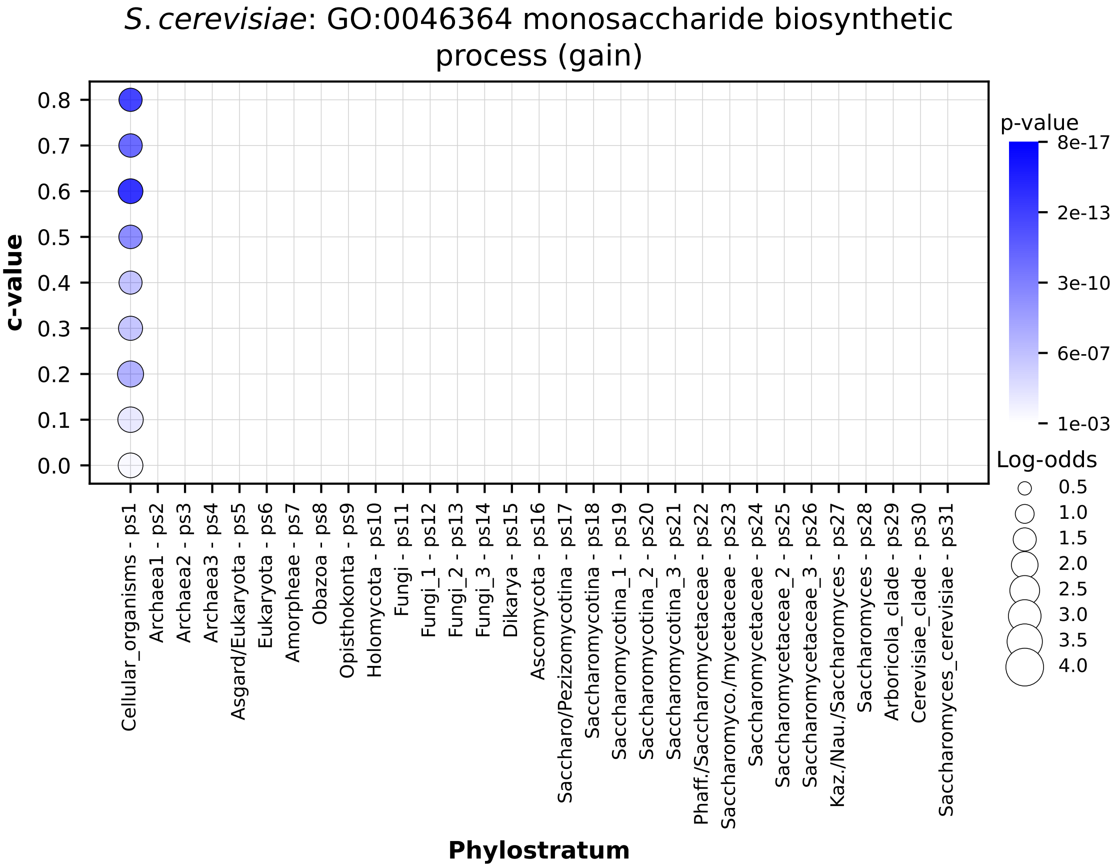

S. cerevisiae: GO:0046364 monosaccharide biosynthetic process (gain)
c-value
0.8
0.7
0.6
0.5
0.4
0.3
0.2
0.1
0.0
p-value
8e-17
2e-13
3e-10
6e-07
1e-03
Log-odds
0.5
1.0
1.5
2.0
2.5
3.0
3.5
4.0
Cellular_organisms - ps1
Archaea1 - ps2
Archaea2 - ps3
Archaea3 - ps4
Asgard/Eukaryota - ps5
Eukaryota - ps6
Amorpheae - ps7
Obazoa - ps8
Opisthokonta - ps9
Holomycota - ps10
Fungi - ps11
Fungi_1 - ps12
Fungi_2 - ps13
Fungi_3 - ps14
Dikarya - ps15
Ascomycota - ps16
Saccharo/Pezizomycotina - ps17
Saccharomycotina - ps18
Saccharomycotina_1 - ps19
Saccharomycotina_2 - ps20
Saccharomycotina_3 - ps21
Phaff./Saccharomycetaceae - ps22
Saccharomyco./mycetaceae - ps23
Saccharomycetaceae - ps24
Saccharomycetaceae_2 - ps25
Saccharomycetaceae_3 - ps26
Kaz./Nau./Saccharomyces - ps27
Saccharomyces - ps28
Arboricola_clade - ps29
Cerevisiae_clade - ps30
Saccharomyces_cerevisiae - ps31
Phylostratum

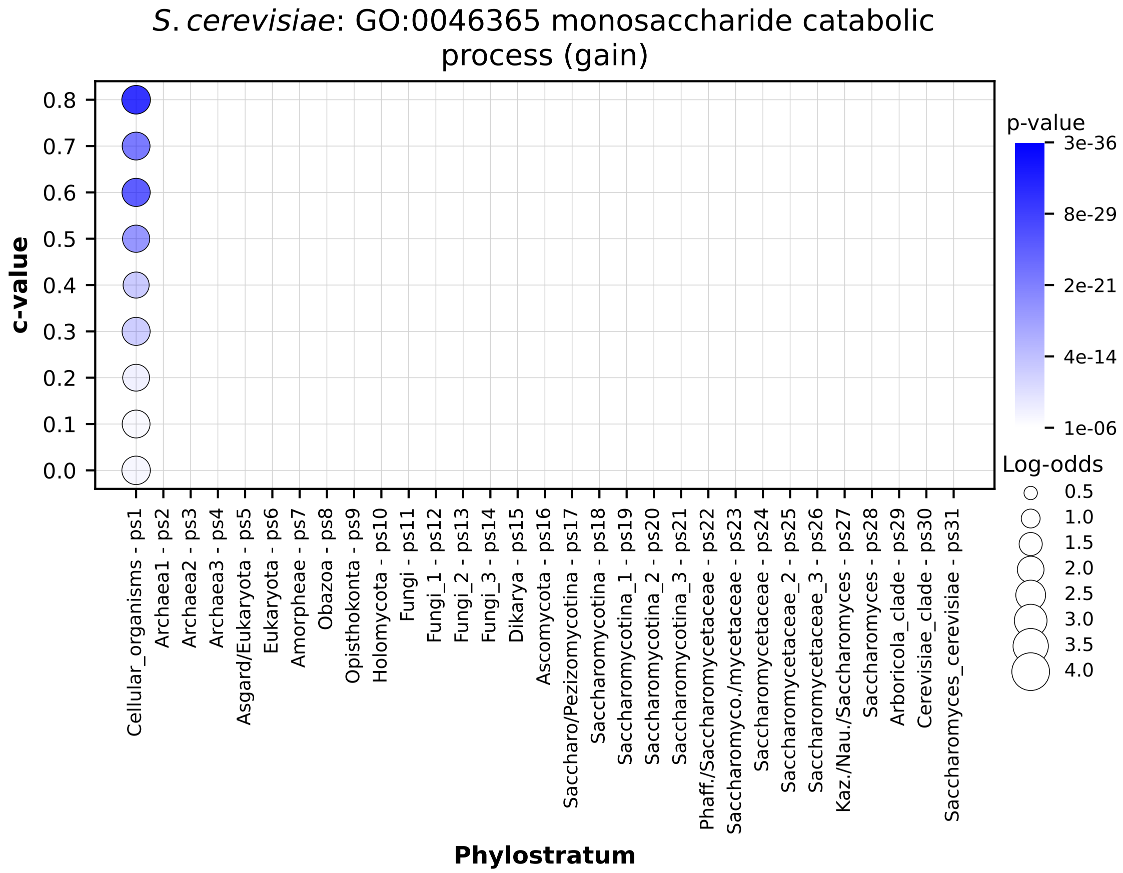

S. cerevisiae: GO:0046365 monosaccharide catabolic process (gain)
c-value
0.8
0.7
0.6
0.5
0.4
0.3
0.2
0.1
0.0
Phylostratum
Cellular_organisms - ps1
Archaea1 - ps2
Archaea2 - ps3
Archaea3 - ps4
Asgard/Eukaryota - ps5
Eukaryota - ps6
Amorpheae - ps7
Obazoa - ps8
Opisthokonta - ps9
Holomycota - ps10
Fungi - ps11
Fungi_1 - ps12
Fungi_2 - ps13
Fungi_3 - ps14
Dikarya - ps15
Ascomycota - ps16
Saccharo/Pezizomycotina - ps17
Saccharomycotina - ps18
Saccharomycotina_1 - ps19
Saccharomycotina_2 - ps20
Saccharomycotina_3 - ps21
Phaff./Saccharomycetaceae - ps22
Saccharomyco./mycetaceae - ps23
Saccharomycetaceae - ps24
Saccharomycetaceae_2 - ps25
Saccharomycetaceae_3 - ps26
Kaz./Nau./Saccharomyces - ps27
Saccharomyces - ps28
Arboricola_clade - ps29
Cerevisiae_clade - ps30
Saccharomyces_cerevisiae - ps31
p-value
3e-36
8e-29
2e-21
4e-14
1e-06
Log-odds
0.5
1.0
1.5
2.0
2.5
3.0
3.5
4.0

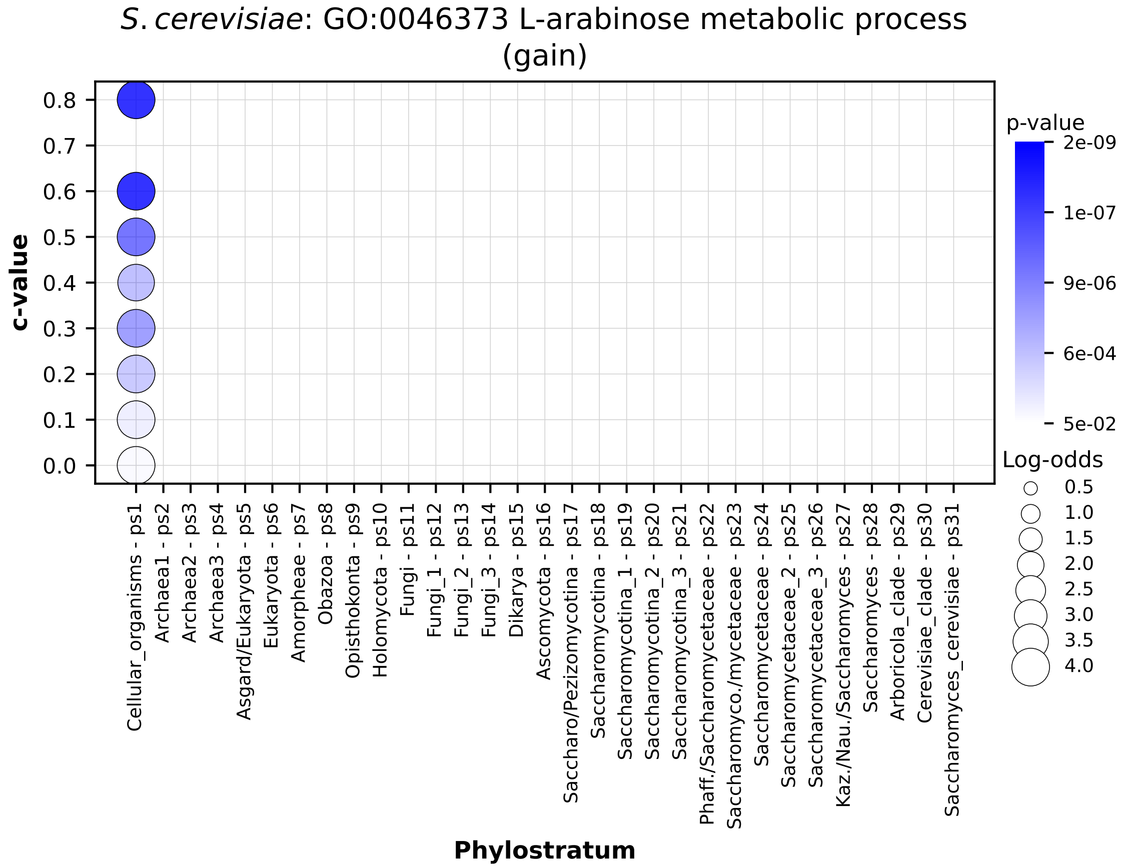

S. cerevisiae: GO:0046373 L-arabinose metabolic process (gain)
c-value
0.0
0.1
0.2
0.3
0.4
0.5
0.6
0.7
0.8
p-value
2e-09
1e-07
9e-06
6e-04
5e-02
Log-odds
0.5
1.0
1.5
2.0
2.5
3.0
3.5
4.0
Cellular_organisms - ps1
Archaea1 - ps2
Archaea2 - ps3
Archaea3 - ps4
Asgard/Eukaryota - ps5
Eukaryota - ps6
Amorpheae - ps7
Obazoa - ps8
Opisthokonta - ps9
Holomycota - ps10
Fungi - ps11
Fungi_1 - ps12
Fungi_2 - ps13
Fungi_3 - ps14
Dikarya - ps15
Ascomycota - ps16
Saccharo/Pezizomycotina - ps17
Saccharomycotina - ps18
Saccharomycotina_1 - ps19
Saccharomycotina_2 - ps20
Saccharomycotina_3 - ps21
Phaff./Saccharomycetaceae - ps22
Saccharomyco./mycetaceae - ps23
Saccharomycetaceae - ps24
Saccharomycetaceae_2 - ps25
Saccharomycetaceae_3 - ps26
Kaz./Nau./Saccharomyces - ps27
Saccharomyces - ps28
Arboricola_clade - ps29
Cerevisiae_clade - ps30
Saccharomyces_cerevisiae - ps31
Phylostratum

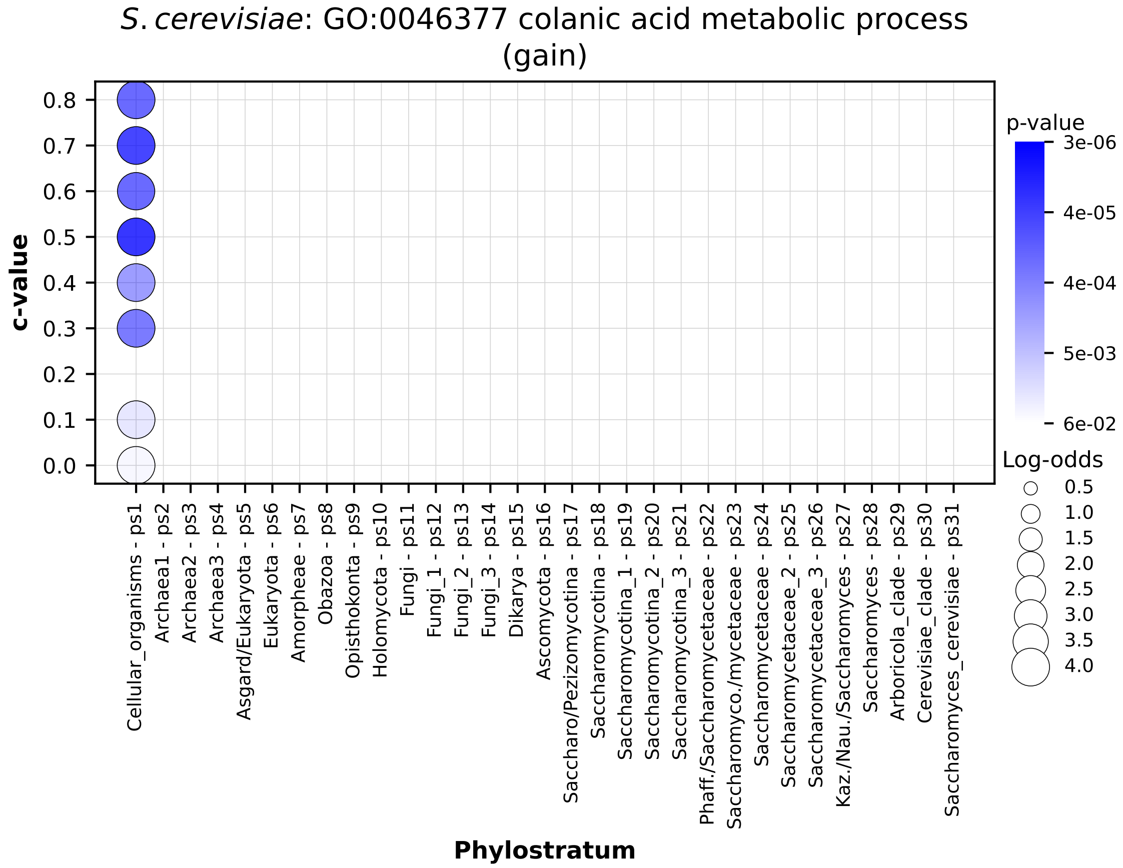

S. cerevisiae: GO:0046377 colanic acid metabolic process (gain)
c-value
0.8
0.7
0.6
0.5
0.4
0.3
0.2
0.1
0.0
p-value
3e-06
4e-05
4e-04
5e-03
6e-02
Log-odds
0.5
1.0
1.5
2.0
2.5
3.0
3.5
4.0
Cellular_organisms - ps1
Archaea1 - ps2
Archaea2 - ps3
Archaea3 - ps4
Asgard/Eukaryota - ps5
Eukaryota - ps6
Amorpheae - ps7
Obazoa - ps8
Opisthokonta - ps9
Holomycota - ps10
Fungi - ps11
Fungi_1 - ps12
Fungi_2 - ps13
Fungi_3 - ps14
Dikarya - ps15
Ascomycota - ps16
Saccharo/Pezizomycotina - ps17
Saccharomycotina - ps18
Saccharomycotina_1 - ps19
Saccharomycotina_2 - ps20
Saccharomycotina_3 - ps21
Phaff./Saccharomycetaceae - ps22
Saccharomyco./mycetaceae - ps23
Saccharomycetaceae - ps24
Saccharomycetaceae_2 - ps25
Saccharomycetaceae_3 - ps26
Kaz./Nau./Saccharomyces - ps27
Saccharomyces - ps28
Arboricola_clade - ps29
Cerevisiae_clade - ps30
Saccharomyces_cerevisiae - ps31
Phylostratum

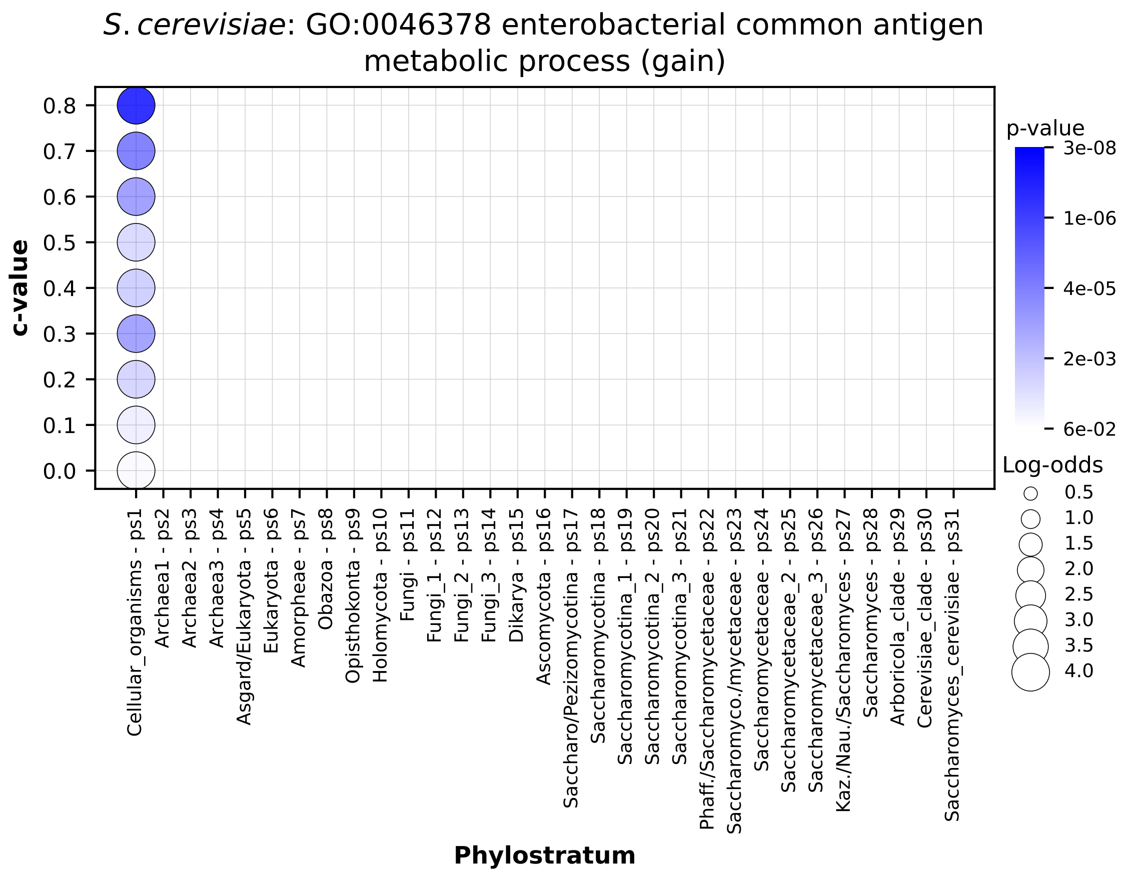
S. cerevisiae: GO:0046378 enterobacterial common antigen metabolic process (gain)
c-value
0.8
0.7
0.6
0.5
0.4
0.3
0.2
0.1
0.0
p-value
3e-08
1e-06
4e-05
2e-03
6e-02
Log-odds
0.5
1.0
1.5
2.0
2.5
3.0
3.5
4.0
Cellular_organisms - ps1
Archaea1 - ps2
Archaea2 - ps3
Archaea3 - ps4
Asgard/Eukaryota - ps5
Eukaryota - ps6
Amorpheae - ps7
Obazoa - ps8
Opisthokonta - ps9
Holomycota - ps10
Fungi - ps11
Fungi_1 - ps12
Fungi_2 - ps13
Fungi_3 - ps14
Dikarya - ps15
Ascomycota - ps16
Saccharo/Pezizomycotina - ps17
Saccharomycotina - ps18
Saccharomycotina_1 - ps19
Saccharomycotina_2 - ps20
Saccharomycotina_3 - ps21
Phaff./Saccharomycetaceae - ps22
Saccharomyco./mycetaceae - ps23
Saccharomycetaceae - ps24
Saccharomycetaceae_2 - ps25
Saccharomycetaceae_3 - ps26
Kaz./Nau./Saccharomyces - ps27
Saccharomyces - ps28
Arboricola_clade - ps29
Cerevisiae_clade - ps30
Saccharomyces_cerevisiae - ps31
Phylostratum

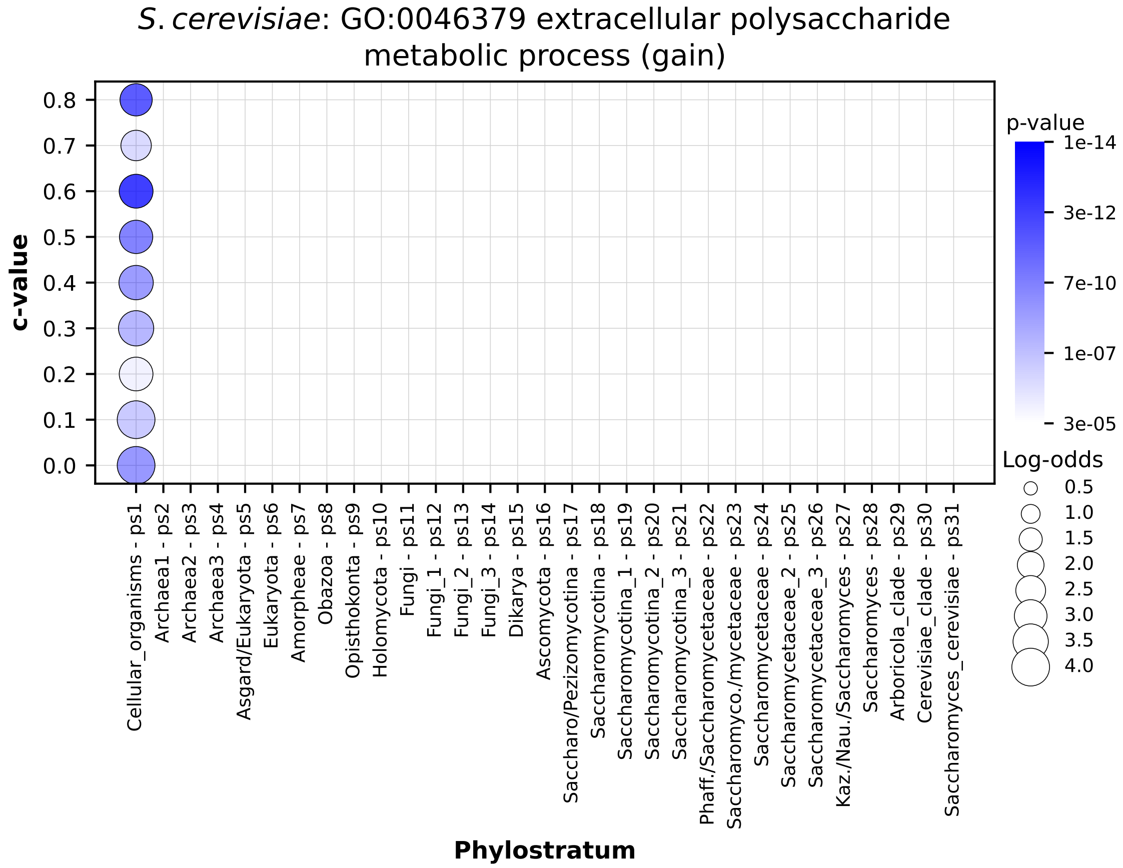

S. cerevisiae: GO:0046379 extracellular polysaccharide metabolic process (gain)
c-value
0.8
0.7
0.6
0.5
0.4
0.3
0.2
0.1
0.0
p-value
1e-14
3e-12
7e-10
1e-07
3e-05
Log-odds
0.5
1.0
1.5
2.0
2.5
3.0
3.5
4.0
Cellular_organisms - ps1
Archaea1 - ps2
Archaea2 - ps3
Archaea3 - ps4
Asgard/Eukaryota - ps5
Eukaryota - ps6
Amorpheae - ps7
Obazoa - ps8
Opisthokonta - ps9
Holomycota - ps10
Fungi - ps11
Fungi_1 - ps12
Fungi_2 - ps13
Fungi_3 - ps14
Dikarya - ps15
Ascomycota - ps16
Saccharo/Pezizomycotina - ps17
Saccharomycotina - ps18
Saccharomycotina_1 - ps19
Saccharomycotina_2 - ps20
Saccharomycotina_3 - ps21
Phaff./Saccharomycetaceae - ps22
Saccharomyco./mycetaceae - ps23
Saccharomycetaceae - ps24
Saccharomycetaceae_2 - ps25
Saccharomycetaceae_3 - ps26
Kaz./Nau./Saccharomyces - ps27
Saccharomyces - ps28
Arboricola_clade - ps29
Cerevisiae_clade - ps30
Saccharomyces_cerevisiae - ps31
Phylostratum

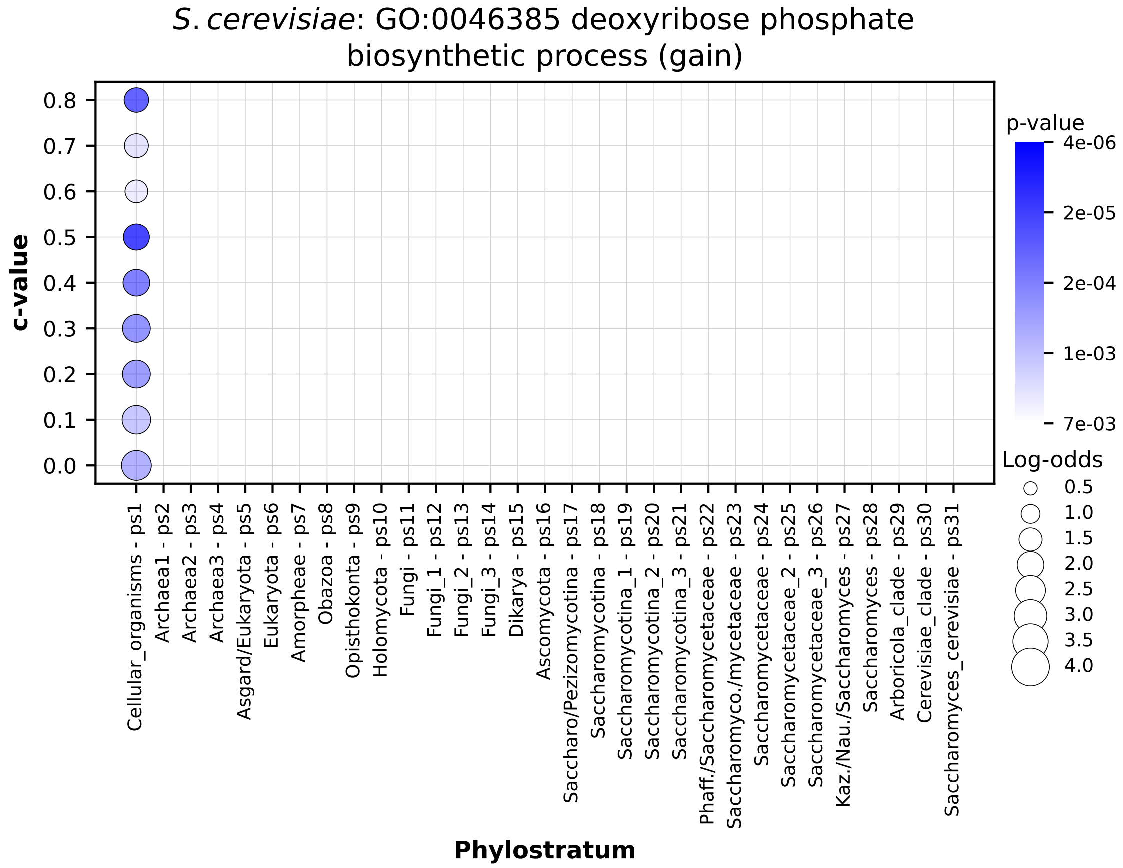
S. cerevisiae: GO:0046385 deoxyribose phosphate biosynthetic process (gain)
c-value
0.8
0.7
0.6
0.5
0.4
0.3
0.2
0.1
0.0
p-value
4e-06
2e-05
2e-04
1e-03
7e-03
Log-odds
0.5
1.0
1.5
2.0
2.5
3.0
3.5
4.0
Cellular_organisms - ps1
Archaea1 - ps2
Archaea2 - ps3
Archaea3 - ps4
Asgard/Eukaryota - ps5
Eukaryota - ps6
Amorpheae - ps7
Obazoa - ps8
Opisthokonta - ps9
Holomycota - ps10
Fungi - ps11
Fungi_1 - ps12
Fungi_2 - ps13
Fungi_3 - ps14
Dikarya - ps15
Ascomycota - ps16
Saccharo/Pezizomycotina - ps17
Saccharomycotina - ps18
Saccharomycotina_1 - ps19
Saccharomycotina_2 - ps20
Saccharomycotina_3 - ps21
Phaff./Saccharomycetaceae - ps22
Saccharomyco./mycetaceae - ps23
Saccharomycetaceae - ps24
Saccharomycetaceae_2 - ps25
Saccharomycetaceae_3 - ps26
Kaz./Nau./Saccharomyces - ps27
Saccharomyces - ps28
Arboricola_clade - ps29
Cerevisiae_clade - ps30
Saccharomyces_cerevisiae - ps31
Phylostratum

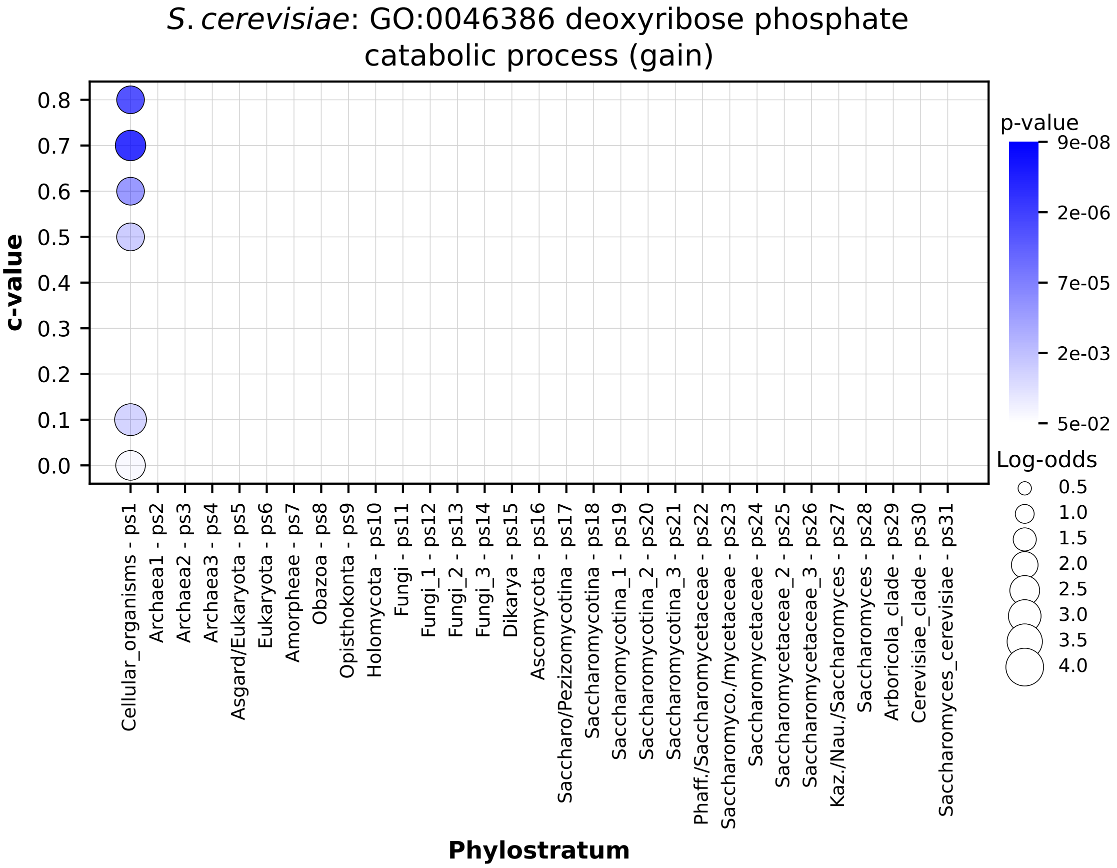
S. cerevisiae: GO:0046386 deoxyribose phosphate catabolic process (gain)
c-value
0.8
0.7
0.6
0.5
0.4
0.3
0.2
0.1
0.0
p-value
9e-08
2e-06
7e-05
2e-03
5e-02
Log-odds
0.5
1.0
1.5
2.0
2.5
3.0
3.5
4.0
Cellular_organisms - ps1
Archaea1 - ps2
Archaea2 - ps3
Archaea3 - ps4
Asgard/Eukaryota - ps5
Eukaryota - ps6
Amorpheae - ps7
Obazoa - ps8
Opisthokonta - ps9
Holomycota - ps10
Fungi - ps11
Fungi_1 - ps12
Fungi_2 - ps13
Fungi_3 - ps14
Dikarya - ps15
Ascomycota - ps16
Saccharo/Pezizomycotina - ps17
Saccharomycotina - ps18
Saccharomycotina_1 - ps19
Saccharomycotina_2 - ps20
Saccharomycotina_3 - ps21
Phaff./Saccharomycetaceae - ps22
Saccharomyco./mycetaceae - ps23
Saccharomycetaceae - ps24
Saccharomycetaceae_2 - ps25
Saccharomycetaceae_3 - ps26
Kaz./Nau./Saccharomyces - ps27
Saccharomyces - ps28
Arboricola_clade - ps29
Cerevisiae_clade - ps30
Saccharomyces_cerevisiae - ps31
Phylostratum

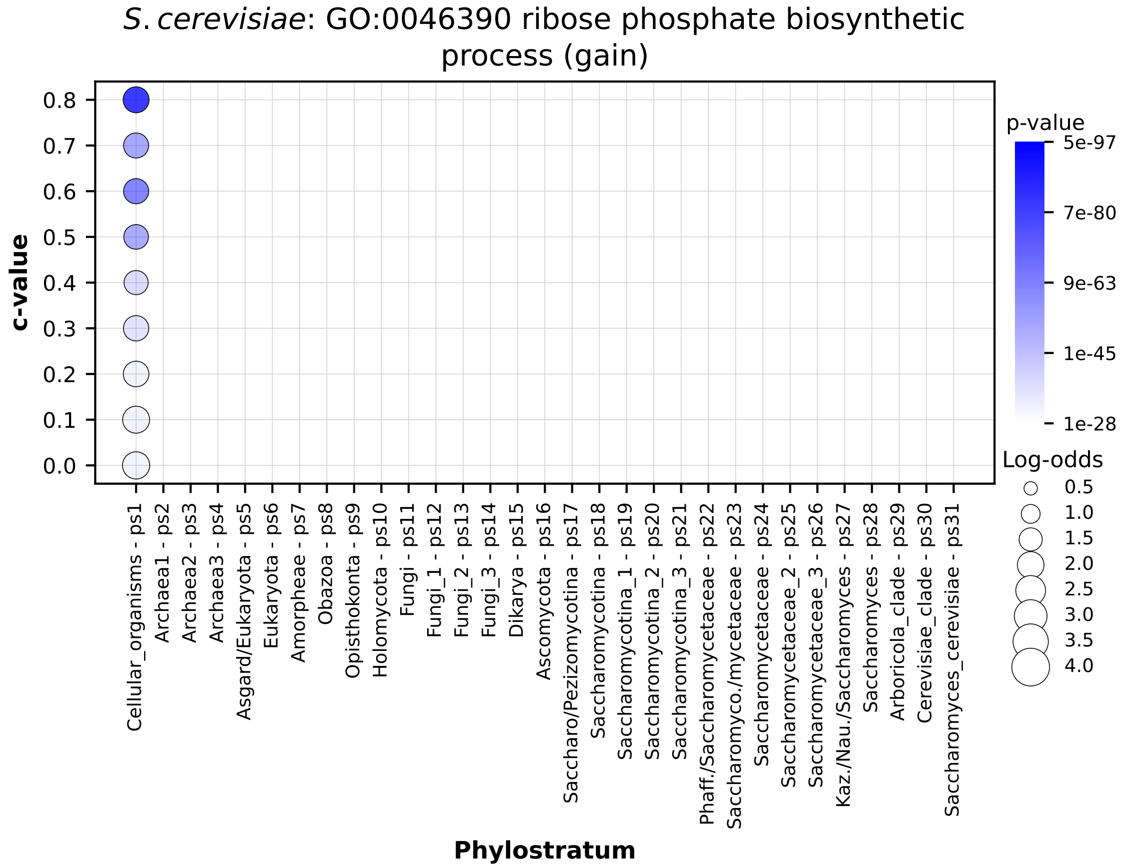

S. cerevisiae: GO:0046390 ribose phosphate biosynthetic process (gain)
c-value
0.8
0.7
0.6
0.5
0.4
0.3
0.2
0.1
0.0
Phylostratum
Cellular_organisms - ps1
Archaea1 - ps2
Archaea2 - ps3
Archaea3 - ps4
Asgard/Eukaryota - ps5
Eukaryota - ps6
Amorpheae - ps7
Obazoa - ps8
Opisthokonta - ps9
Holomycota - ps10
Fungi - ps11
Fungi_1 - ps12
Fungi_2 - ps13
Fungi_3 - ps14
Dikarya - ps15
Ascomycota - ps16
Saccharo/Pezizomycotina - ps17
Saccharomycotina - ps18
Saccharomycotina_1 - ps19
Saccharomycotina_2 - ps20
Saccharomycotina_3 - ps21
Phaff./Saccharomycetaceae - ps22
Saccharomyco./mycetaceae - ps23
Saccharomycetaceae - ps24
Saccharomycetaceae_2 - ps25
Saccharomycetaceae_3 - ps26
Kaz./Nau./Saccharomyces - ps27
Saccharomyces - ps28
Arboricola_clade - ps29
Cerevisiae_clade - ps30
Saccharomyces_cerevisiae - ps31
p-value
5e-97
7e-80
9e-63
1e-45
1e-28
Log-odds
0.5
1.0
1.5
2.0
2.5
3.0
3.5
4.0

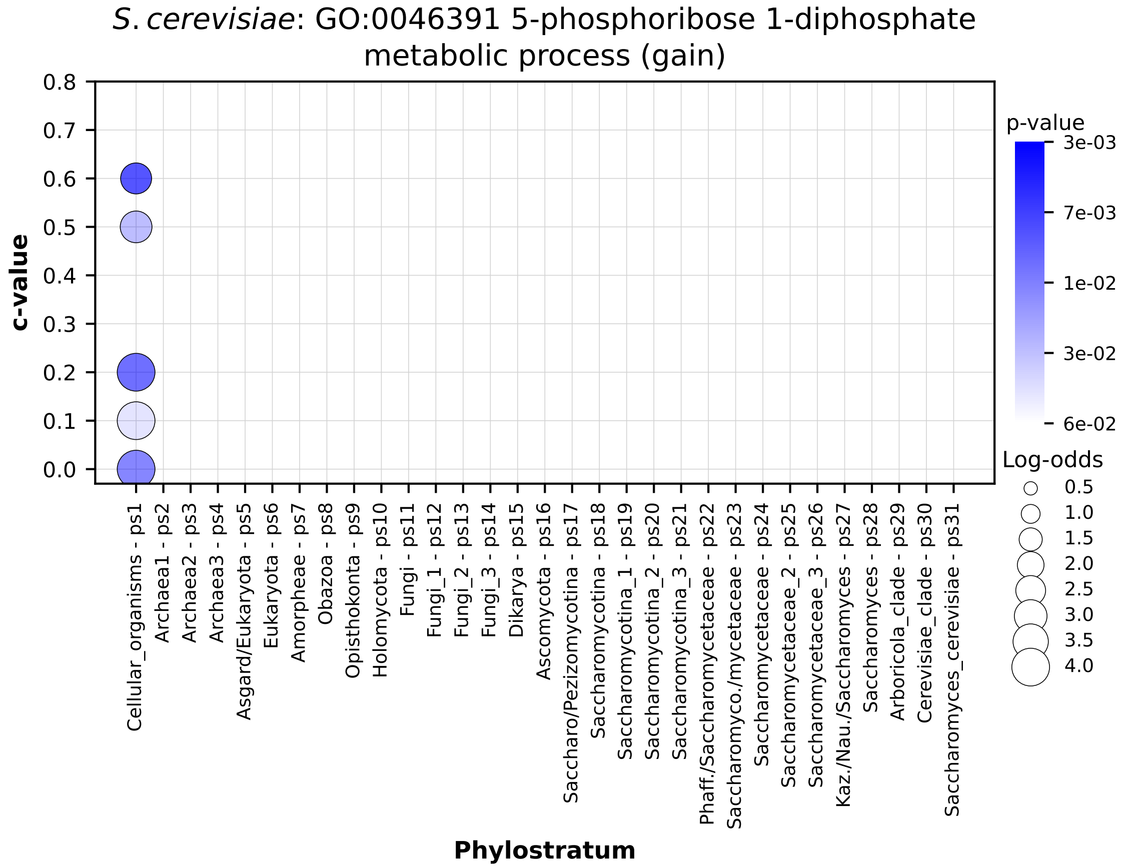

S. cerevisiae: GO:0046391 5-phosphoribose 1-diphosphate metabolic process (gain)
c-value
0.8
0.7
0.6
0.5
0.4
0.3
0.2
0.1
0.0
p-value
3e-03
7e-03
1e-02
3e-02
6e-02
Log-odds
0.5
1.0
1.5
2.0
2.5
3.0
3.5
4.0
Cellular_organisms - ps1
Archaea1 - ps2
Archaea2 - ps3
Archaea3 - ps4
Asgard/Eukaryota - ps5
Eukaryota - ps6
Amorpheae - ps7
Obazoa - ps8
Opisthokonta - ps9
Holomycota - ps10
Fungi - ps11
Fungi_1 - ps12
Fungi_2 - ps13
Fungi_3 - ps14
Dikarya - ps15
Ascomycota - ps16
Saccharo/Pezizomycotina - ps17
Saccharomycotina - ps18
Saccharomycotina_1 - ps19
Saccharomycotina_2 - ps20
Saccharomycotina_3 - ps21
Phaff./Saccharomycetaceae - ps22
Saccharomyco./mycetaceae - ps23
Saccharomycetaceae - ps24
Saccharomycetaceae_2 - ps25
Saccharomycetaceae_3 - ps26
Kaz./Nau./Saccharomyces - ps27
Saccharomyces - ps28
Arboricola_clade - ps29
Cerevisiae_clade - ps30
Saccharomyces_cerevisiae - ps31
Phylostratum

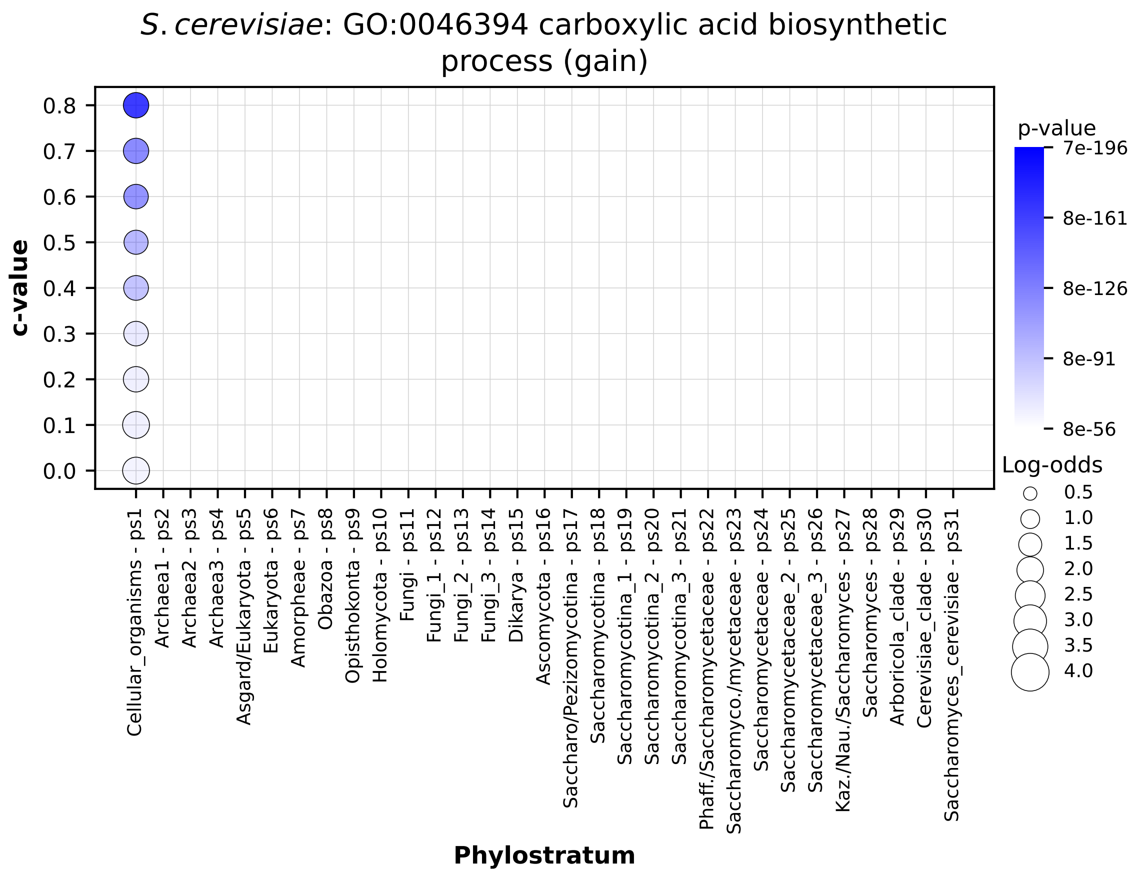

S. cerevisiae: GO:0046394 carboxylic acid biosynthetic process (gain)
c-value
0.8
0.7
0.6
0.5
0.4
0.3
0.2
0.1
0.0
p-value
7e-196
8e-161
8e-126
8e-91
8e-56
Log-odds
0.5
1.0
1.5
2.0
2.5
3.0
3.5
4.0
Cellular_organisms - ps1
Archaea1 - ps2
Archaea2 - ps3
Archaea3 - ps4
Asgard/Eukaryota - ps5
Eukaryota - ps6
Amorpheae - ps7
Obazoa - ps8
Opisthokonta - ps9
Holomycota - ps10
Fungi - ps11
Fungi_1 - ps12
Fungi_2 - ps13
Fungi_3 - ps14
Dikarya - ps15
Ascomycota - ps16
Saccharo/Pezizomycotina - ps17
Saccharomycotina - ps18
Saccharomycotina_1 - ps19
Saccharomycotina_2 - ps20
Saccharomycotina_3 - ps21
Phaff./Saccharomycetaceae - ps22
Saccharomyco./mycetaceae - ps23
Saccharomycetaceae - ps24
Saccharomycetaceae_2 - ps25
Saccharomycetaceae_3 - ps26
Kaz./Nau./Saccharomyces - ps27
Saccharomyces - ps28
Arboricola_clade - ps29
Cerevisiae_clade - ps30
Saccharomyces_cerevisiae - ps31
Phylostratum

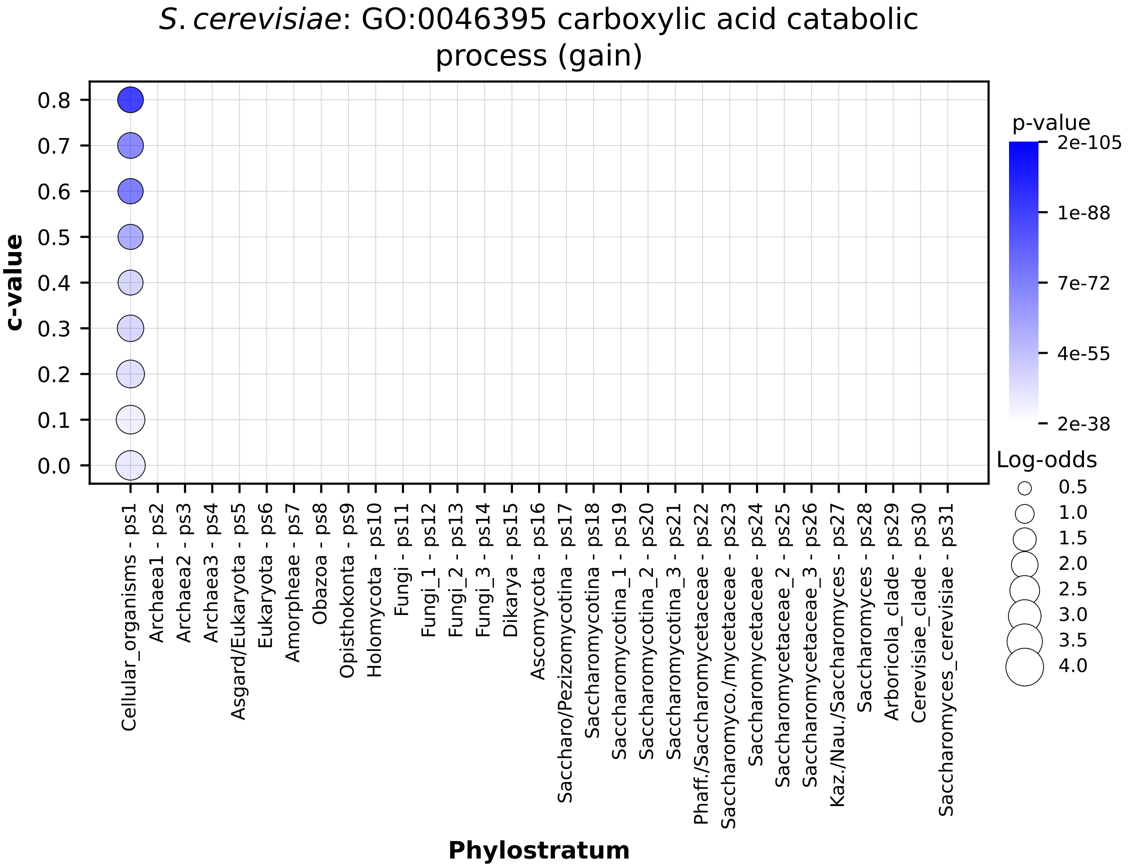
S. cerevisiae: GO:0046395 carboxylic acid catabolic process (gain)
c-value
0.8
0.7
0.6
0.5
0.4
0.3
0.2
0.1
0.0
p-value
2e-105
1e-88
7e-72
4e-55
2e-38
Log-odds
0.5
1.0
1.5
2.0
2.5
3.0
3.5
4.0
Cellular_organisms - ps1
Archaea1 - ps2
Archaea2 - ps3
Archaea3 - ps4
Asgard/Eukaryota - ps5
Eukaryota - ps6
Amorpheae - ps7
Obazoa - ps8
Opisthokonta - ps9
Holomycota - ps10
Fungi - ps11
Fungi_1 - ps12
Fungi_2 - ps13
Fungi_3 - ps14
Dikarya - ps15
Ascomycota - ps16
Saccharo/Pezizomycotina - ps17
Saccharomycotina - ps18
Saccharomycotina_1 - ps19
Saccharomycotina_2 - ps20
Saccharomycotina_3 - ps21
Phaff./Saccharomycetaceae - ps22
Saccharomyco./mycetaceae - ps23
Saccharomycetaceae - ps24
Saccharomycetaceae_2 - ps25
Saccharomycetaceae_3 - ps26
Kaz./Nau./Saccharomyces - ps27
Saccharomyces - ps28
Arboricola_clade - ps29
Cerevisiae_clade - ps30
Saccharomyces_cerevisiae - ps31
Phylostratum

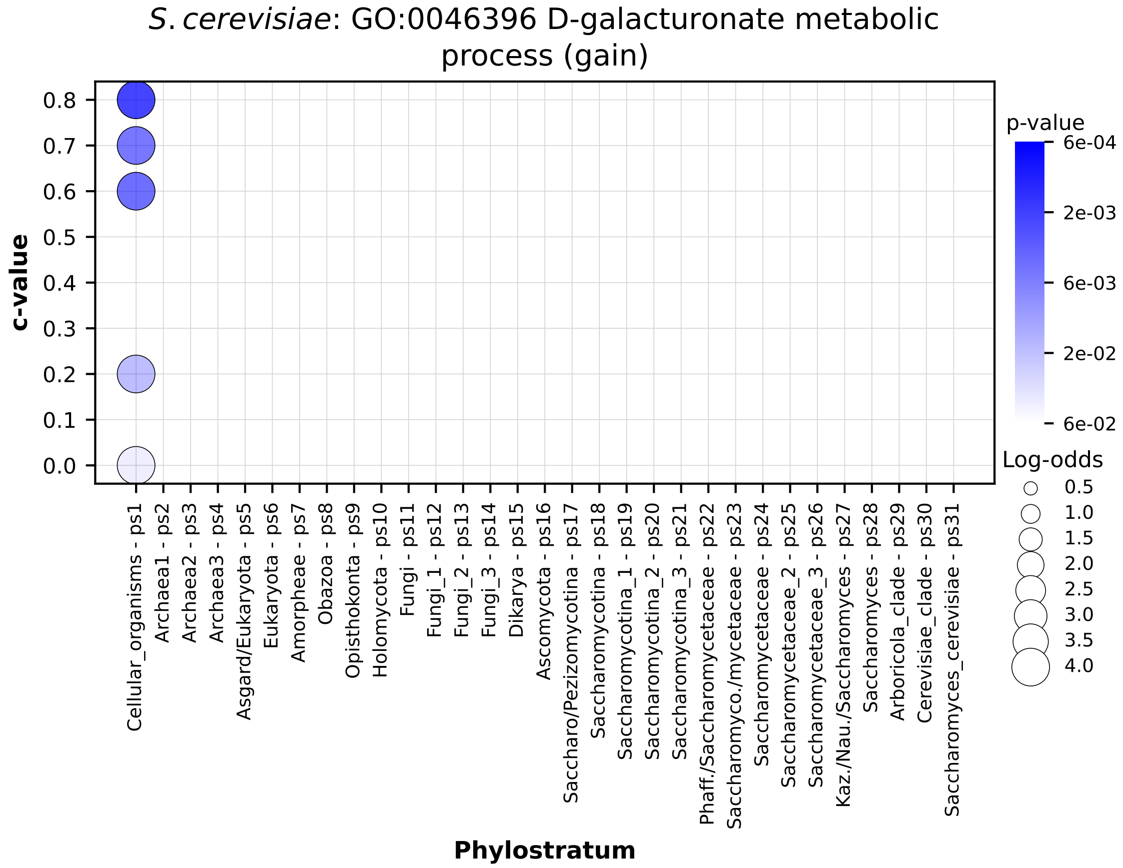

S. cerevisiae: GO:0046396 D-galacturonate metabolic process (gain)
c-value
0.8
0.7
0.6
0.5
0.4
0.3
0.2
0.1
0.0
Phylostratum
Cellular_organisms - ps1
Archaea1 - ps2
Archaea2 - ps3
Archaea3 - ps4
Asgard/Eukaryota - ps5
Eukaryota - ps6
Amorpheae - ps7
Obazoa - ps8
Opisthokonta - ps9
Holomycota - ps10
Fungi - ps11
Fungi_1 - ps12
Fungi_2 - ps13
Fungi_3 - ps14
Dikarya - ps15
Ascomycota - ps16
Saccharo/Pezizomycotina - ps17
Saccharomycotina - ps18
Saccharomycotina_1 - ps19
Saccharomycotina_2 - ps20
Saccharomycotina_3 - ps21
Phaff./Saccharomycetaceae - ps22
Saccharomyco./mycetaceae - ps23
Saccharomycetaceae - ps24
Saccharomycetaceae_2 - ps25
Saccharomycetaceae_3 - ps26
Kaz./Nau./Saccharomyces - ps27
Saccharomyces - ps28
Arboricola_clade - ps29
Cerevisiae_clade - ps30
Saccharomyces_cerevisiae - ps31
p-value
6e-04
2e-03
6e-03
2e-02
6e-02
Log-odds
0.5
1.0
1.5
2.0
2.5
3.0
3.5
4.0

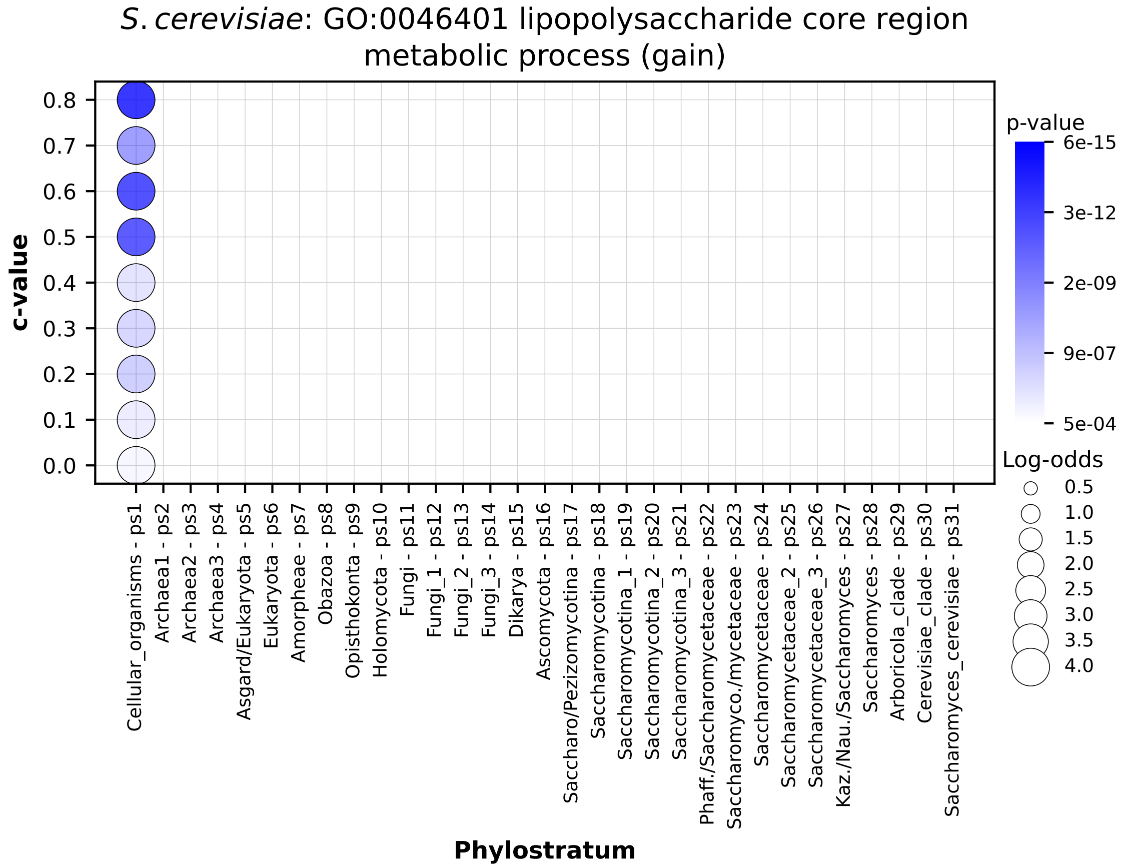

S. cerevisiae: GO:0046401 lipopolysaccharide core region metabolic process (gain)
c-value
0.8
0.7
0.6
0.5
0.4
0.3
0.2
0.1
0.0
p-value
6e-15
3e-12
2e-09
9e-07
5e-04
Log-odds
0.5
1.0
1.5
2.0
2.5
3.0
3.5
4.0
Cellular_organisms - ps1
Archaea1 - ps2
Archaea2 - ps3
Archaea3 - ps4
Asgard/Eukaryota - ps5
Eukaryota - ps6
Amorpheae - ps7
Obazoa - ps8
Opisthokonta - ps9
Holomycota - ps10
Fungi - ps11
Fungi_1 - ps12
Fungi_2 - ps13
Fungi_3 - ps14
Dikarya - ps15
Ascomycota - ps16
Saccharo/Pezizomycotina - ps17
Saccharomycotina - ps18
Saccharomycotina_1 - ps19
Saccharomycotina_2 - ps20
Saccharomycotina_3 - ps21
Phaff./Saccharomycetaceae - ps22
Saccharomyco./mycetaceae - ps23
Saccharomycetaceae - ps24
Saccharomycetaceae_2 - ps25
Saccharomycetaceae_3 - ps26
Kaz./Nau./Saccharomyces - ps27
Saccharomyces - ps28
Arboricola_clade - ps29
Cerevisiae_clade - ps30
Saccharomyces_cerevisiae - ps31
Phylostratum

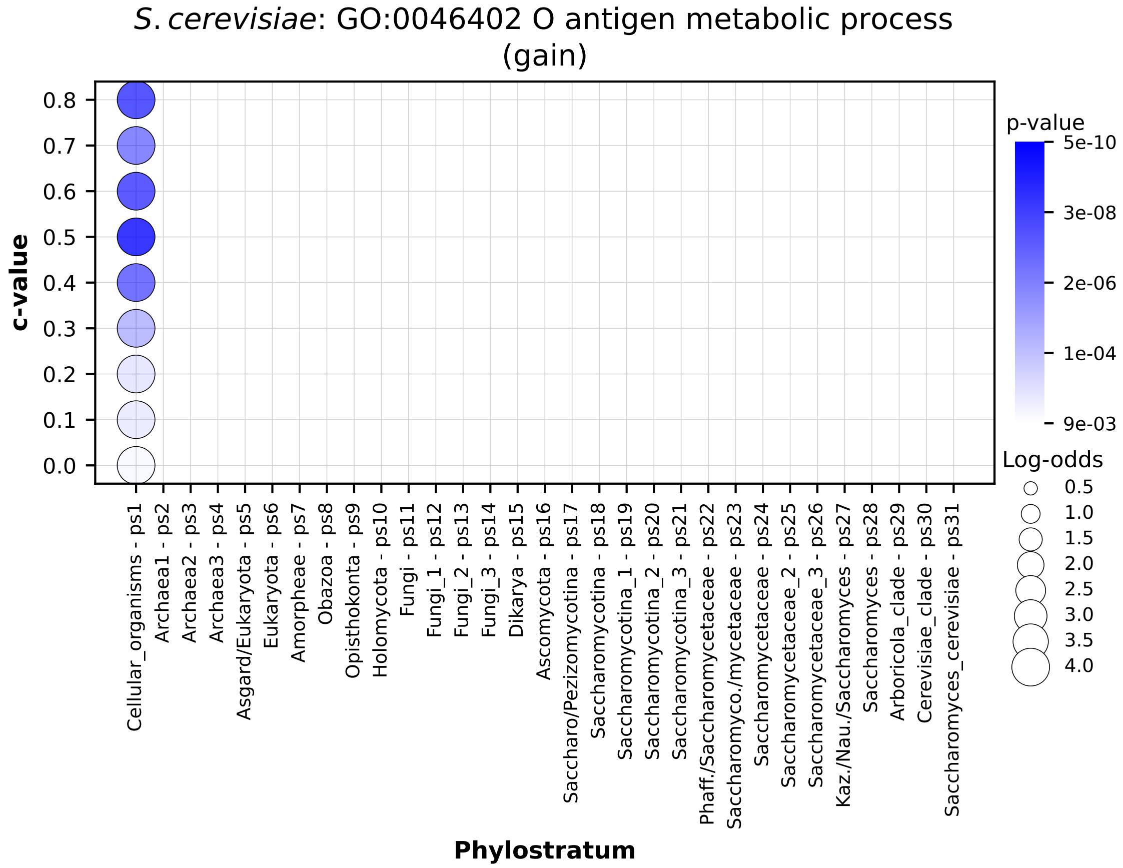

S. cerevisiae: GO:0046402 O antigen metabolic process
(gain)
c-value
0.8
0.7
0.6
0.5
0.4
0.3
0.2
0.1
0.0
p-value
5e-10
3e-08
2e-06
1e-04
9e-03
Log-odds
0.5
1.0
1.5
2.0
2.5
3.0
3.5
4.0
Cellular_organisms - ps1
Archaea1 - ps2
Archaea2 - ps3
Archaea3 - ps4
Asgard/Eukaryota - ps5
Eukaryota - ps6
Amorpheae - ps7
Obazoa - ps8
Opisthokonta - ps9
Holomycota - ps10
Fungi - ps11
Fungi_1 - ps12
Fungi_2 - ps13
Fungi_3 - ps14
Dikarya - ps15
Ascomycota - ps16
Saccharo/Pezizomycotina - ps17
Saccharomycotina - ps18
Saccharomycotina_1 - ps19
Saccharomycotina_2 - ps20
Saccharomycotina_3 - ps21
Phaff./Saccharomycetaceae - ps22
Saccharomyco./mycetaceae - ps23
Saccharomycetaceae - ps24
Saccharomycetaceae_2 - ps25
Saccharomycetaceae_3 - ps26
Kaz./Nau./Saccharomyces - ps27
Saccharomyces - ps28
Arboricola_clade - ps29
Cerevisiae_clade - ps30
Saccharomyces_cerevisiae - ps31
Phylostratum

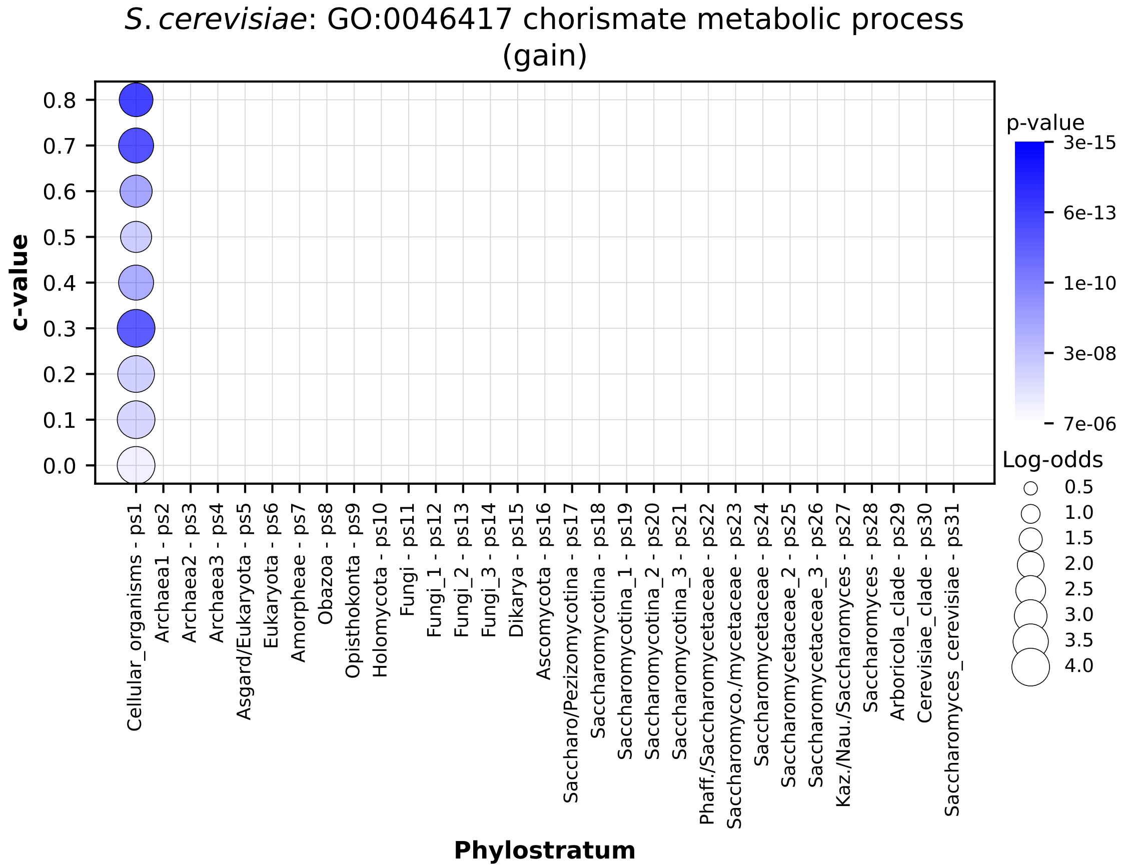

S. cerevisiae: GO:0046417 chorismate metabolic process
(gain)
c-value
0.8
0.7
0.6
0.5
0.4
0.3
0.2
0.1
0.0
p-value
3e-15
6e-13
1e-10
3e-08
7e-06
Log-odds
0.5
1.0
1.5
2.0
2.5
3.0
3.5
4.0
Cellular_organisms - ps1
Archaea1 - ps2
Archaea2 - ps3
Archaea3 - ps4
Asgard/Eukaryota - ps5
Eukaryota - ps6
Amorpheae - ps7
Obazoa - ps8
Opisthokonta - ps9
Holomycota - ps10
Fungi - ps11
Fungi_1 - ps12
Fungi_2 - ps13
Fungi_3 - ps14
Dikarya - ps15
Ascomycota - ps16
Saccharo/Pezizomycotina - ps17
Saccharomycotina - ps18
Saccharomycotina_1 - ps19
Saccharomycotina_2 - ps20
Saccharomycotina_3 - ps21
Phaff./Saccharomycetaceae - ps22
Saccharomyco./mycetaceae - ps23
Saccharomycetaceae - ps24
Saccharomycetaceae_2 - ps25
Saccharomycetaceae_3 - ps26
Kaz./Nau./Saccharomyces - ps27
Saccharomyces - ps28
Arboricola_clade - ps29
Cerevisiae_clade - ps30
Saccharomyces_cerevisiae - ps31
Phylostratum

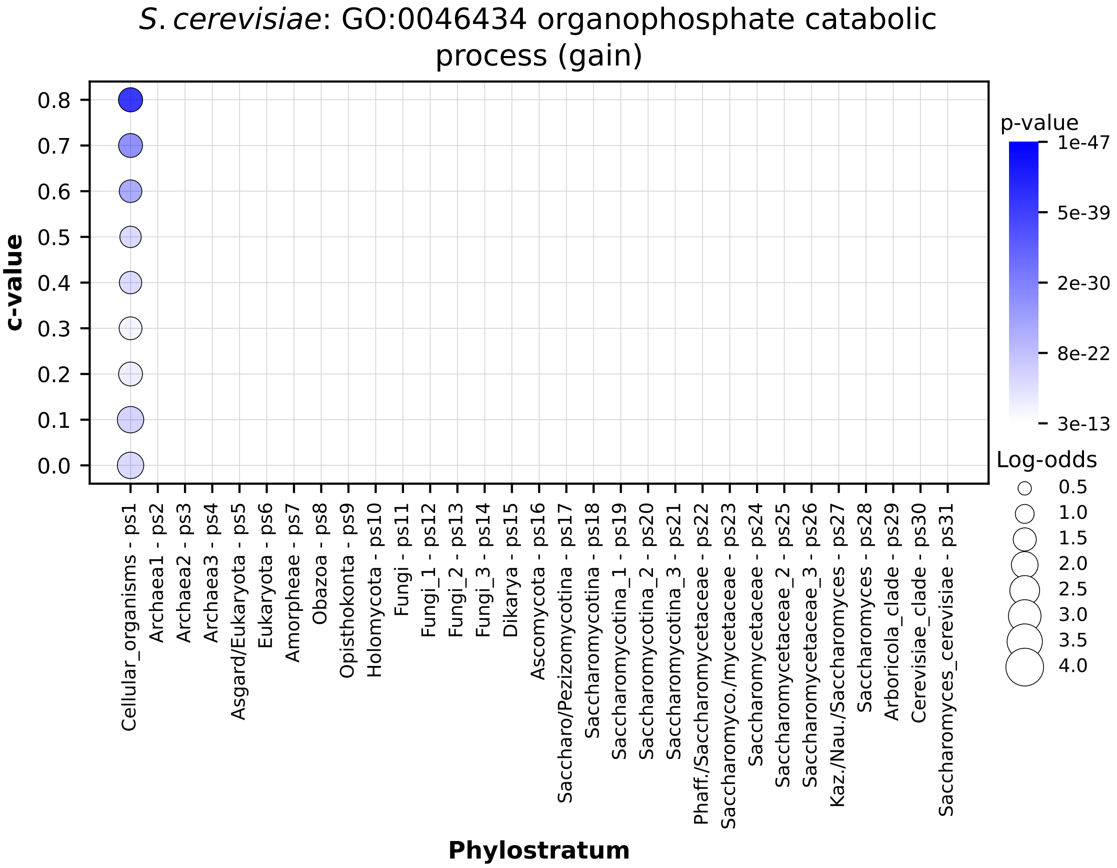

S. cerevisiae: GO:0046434 organophosphate catabolic process (gain)
c-value
0.8
0.7
0.6
0.5
0.4
0.3
0.2
0.1
0.0
p-value
1e-47
5e-39
2e-30
8e-22
3e-13
Log-odds
0.5
1.0
1.5
2.0
2.5
3.0
3.5
4.0
Cellular_organisms - ps1
Archaea1 - ps2
Archaea2 - ps3
Archaea3 - ps4
Asgard/Eukaryota - ps5
Eukaryota - ps6
Amorpheae - ps7
Obazoa - ps8
Opisthokonta - ps9
Holomycota - ps10
Fungi - ps11
Fungi_1 - ps12
Fungi_2 - ps13
Fungi_3 - ps14
Dikarya - ps15
Ascomycota - ps16
Saccharo/Pezizomycotina - ps17
Saccharomycotina - ps18
Saccharomycotina_1 - ps19
Saccharomycotina_2 - ps20
Saccharomycotina_3 - ps21
Phaff./Saccharomycetaceae - ps22
Saccharomyco./mycetaceae - ps23
Saccharomycetaceae - ps24
Saccharomycetaceae_2 - ps25
Saccharomycetaceae_3 - ps26
Kaz./Nau./Saccharomyces - ps27
Saccharomyces - ps28
Arboricola_clade - ps29
Cerevisiae_clade - ps30
Saccharomyces_cerevisiae - ps31
Phylostratum

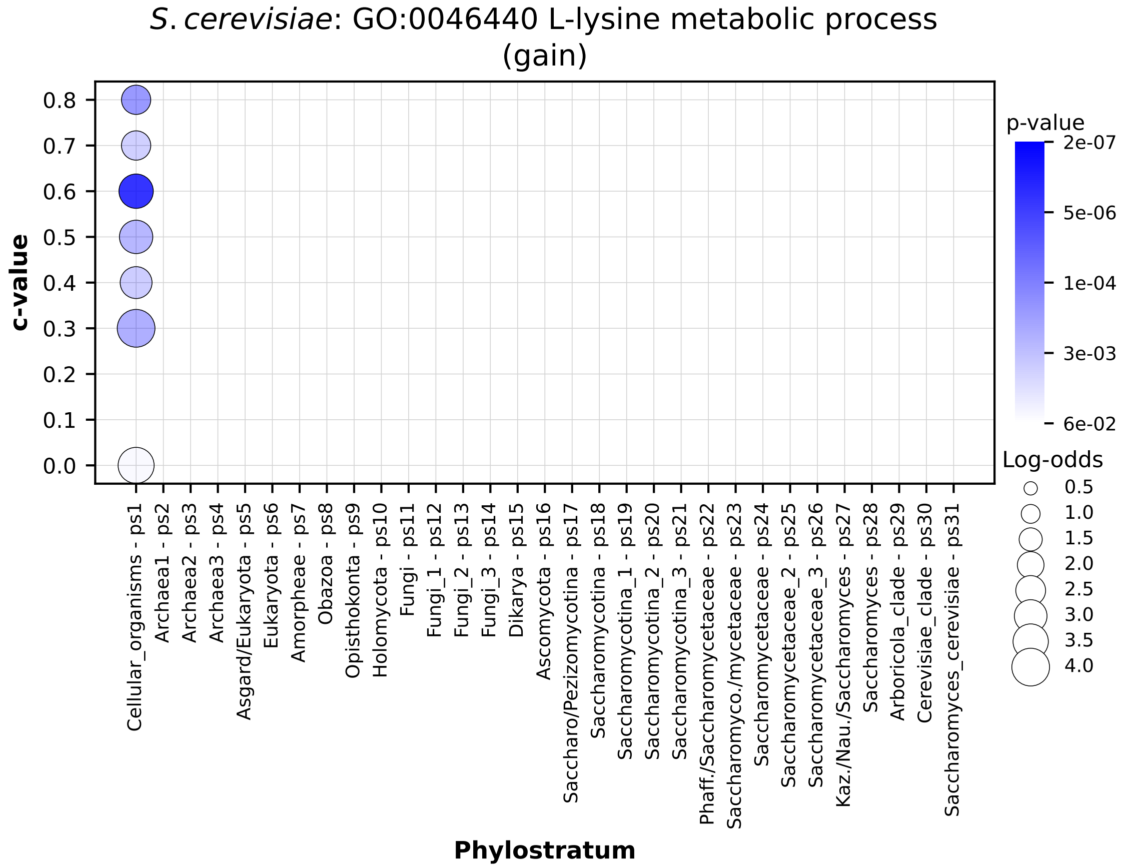
S. cerevisiae: GO:0046440 L-lysine metabolic process
(gain)
c-value
0.8
0.7
0.6
0.5
0.4
0.3
0.2
0.1
0.0
p-value
2e-07
5e-06
1e-04
3e-03
6e-02
Log-odds
0.5
1.0
1.5
2.0
2.5
3.0
3.5
4.0
Cellular_organisms - ps1
Archaea1 - ps2
Archaea2 - ps3
Archaea3 - ps4
Asgard/Eukaryota - ps5
Eukaryota - ps6
Amorpheae - ps7
Obazoa - ps8
Opisthokonta - ps9
Holomycota - ps10
Fungi - ps11
Fungi_1 - ps12
Fungi_2 - ps13
Fungi_3 - ps14
Dikarya - ps15
Ascomycota - ps16
Saccharo/Pezizomycotina - ps17
Saccharomycotina - ps18
Saccharomycotina_1 - ps19
Saccharomycotina_2 - ps20
Saccharomycotina_3 - ps21
Phaff./Saccharomycetaceae - ps22
Saccharomyco./mycetaceae - ps23
Saccharomycetaceae - ps24
Saccharomycetaceae_2 - ps25
Saccharomycetaceae_3 - ps26
Kaz./Nau./Saccharomyces - ps27
Saccharomyces - ps28
Arboricola_clade - ps29
Cerevisiae_clade - ps30
Saccharomyces_cerevisiae - ps31
Phylostratum

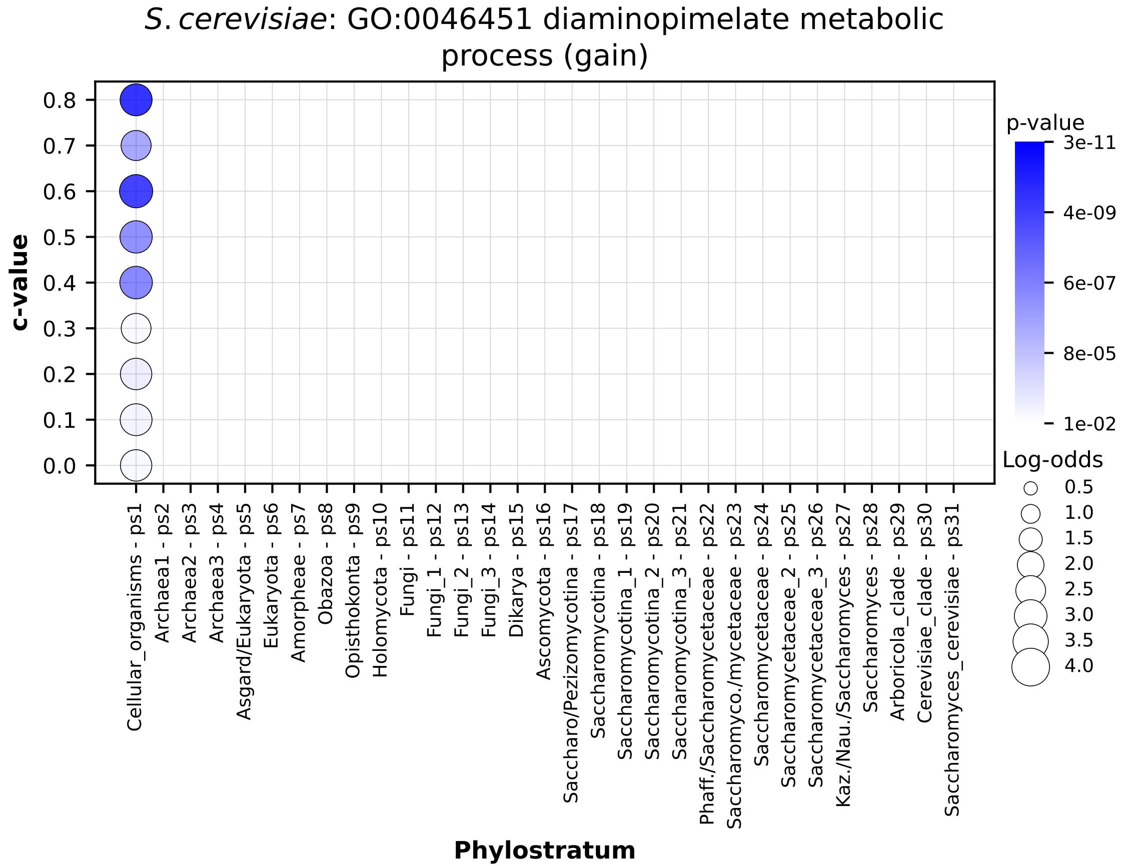

S. cerevisiae: GO:0046451 diaminopimelate metabolic process (gain)
c-value
0.8
0.7
0.6
0.5
0.4
0.3
0.2
0.1
0.0
p-value
3e-11
4e-09
6e-07
8e-05
1e-02
Log-odds
0.5
1.0
1.5
2.0
2.5
3.0
3.5
4.0
Cellular_organisms - ps1
Archaea1 - ps2
Archaea2 - ps3
Archaea3 - ps4
Asgard/Eukaryota - ps5
Eukaryota - ps6
Amorpheae - ps7
Obazoa - ps8
Opisthokonta - ps9
Holomycota - ps10
Fungi - ps11
Fungi_1 - ps12
Fungi_2 - ps13
Fungi_3 - ps14
Dikarya - ps15
Ascomycota - ps16
Saccharo/Pezizomycotina - ps17
Saccharomycotina - ps18
Saccharomycotina_1 - ps19
Saccharomycotina_2 - ps20
Saccharomycotina_3 - ps21
Phaff./Saccharomycetaceae - ps22
Saccharomyco./mycetaceae - ps23
Saccharomycetaceae - ps24
Saccharomycetaceae_2 - ps25
Saccharomycetaceae_3 - ps26
Kaz./Nau./Saccharomyces - ps27
Saccharomyces - ps28
Arboricola_clade - ps29
Cerevisiae_clade - ps30
Saccharomyces_cerevisiae - ps31
Phylostratum

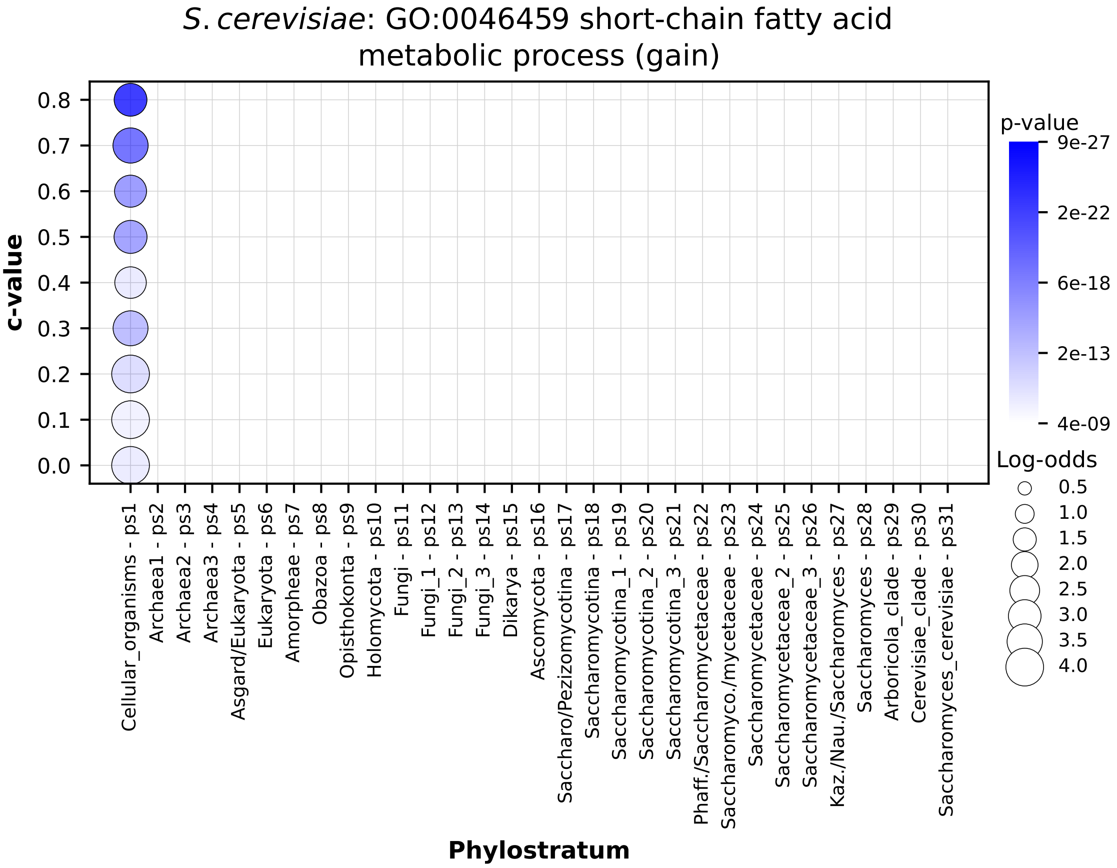

S. cerevisiae: GO:0046459 short-chain fatty acid metabolic process (gain)
c-value
0.8
0.7
0.6
0.5
0.4
0.3
0.2
0.1
0.0
p-value
9e-27
2e-22
6e-18
2e-13
4e-09
Log-odds
0.5
1.0
1.5
2.0
2.5
3.0
3.5
4.0
Cellular_organisms - ps1
Archaea1 - ps2
Archaea2 - ps3
Archaea3 - ps4
Asgard/Eukaryota - ps5
Eukaryota - ps6
Amorpheae - ps7
Obazoa - ps8
Opisthokonta - ps9
Holomycota - ps10
Fungi - ps11
Fungi_1 - ps12
Fungi_2 - ps13
Fungi_3 - ps14
Dikarya - ps15
Ascomycota - ps16
Saccharo/Pezizomycotina - ps17
Saccharomycotina - ps18
Saccharomycotina_1 - ps19
Saccharomycotina_2 - ps20
Saccharomycotina_3 - ps21
Phaff./Saccharomycetaceae - ps22
Saccharomyco./mycetaceae - ps23
Saccharomycetaceae - ps24
Saccharomycetaceae_2 - ps25
Saccharomycetaceae_3 - ps26
Kaz./Nau./Saccharomyces - ps27
Saccharomyces - ps28
Arboricola_clade - ps29
Cerevisiae_clade - ps30
Saccharomyces_cerevisiae - ps31
Phylostratum

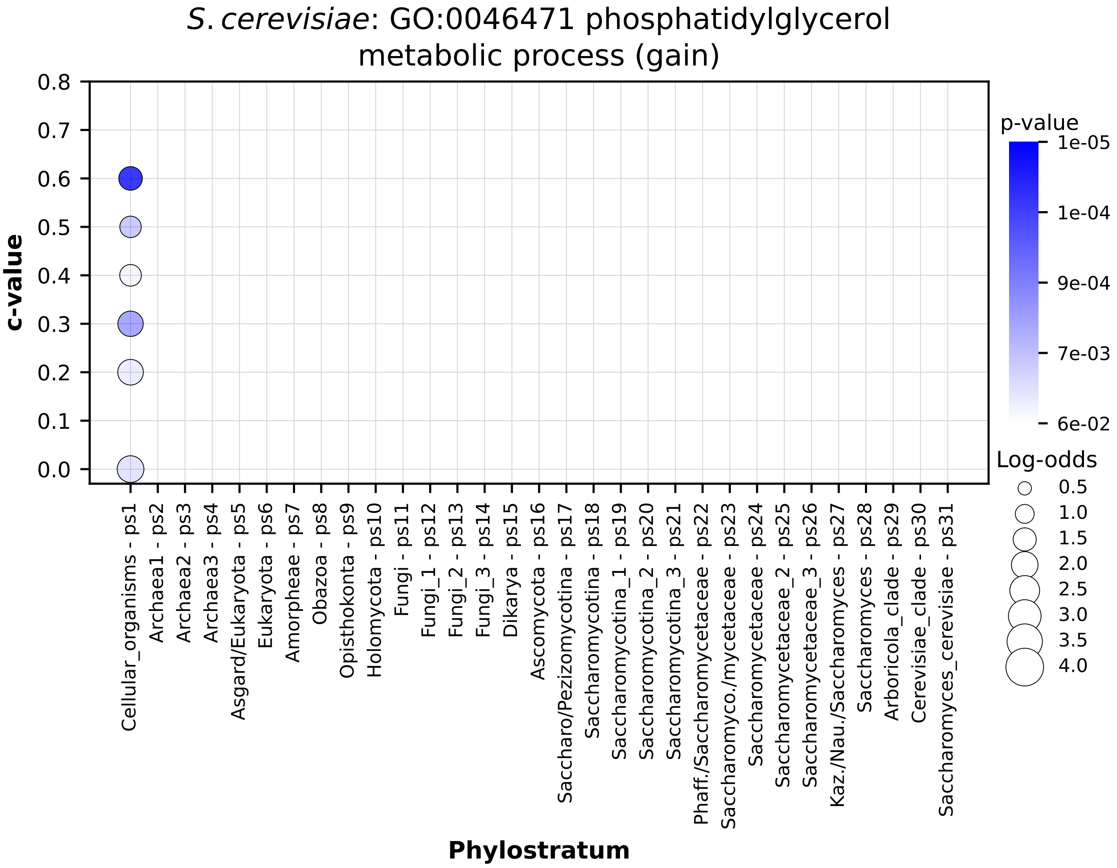

S. cerevisiae: GO:0046471 phosphatidylglycerol metabolic process (gain)
c-value
0.0
0.1
0.2
0.3
0.4
0.5
0.6
0.7
0.8
p-value
1e-05
1e-04
9e-04
7e-03
6e-02
Log-odds
0.5
1.0
1.5
2.0
2.5
3.0
3.5
4.0
Cellular_organisms - ps1
Archaea1 - ps2
Archaea2 - ps3
Archaea3 - ps4
Asgard/Eukaryota - ps5
Eukaryota - ps6
Amorpheae - ps7
Obazoa - ps8
Opisthokonta - ps9
Holomycota - ps10
Fungi - ps11
Fungi_1 - ps12
Fungi_2 - ps13
Fungi_3 - ps14
Dikarya - ps15
Ascomycota - ps16
Saccharo/Pezizomycotina - ps17
Saccharomycotina - ps18
Saccharomycotina_1 - ps19
Saccharomycotina_2 - ps20
Saccharomycotina_3 - ps21
Phaff./Saccharomycetaceae - ps22
Saccharomyco./mycetaceae - ps23
Saccharomycetaceae - ps24
Saccharomycetaceae_2 - ps25
Saccharomycetaceae_3 - ps26
Kaz./Nau./Saccharomyces - ps27
Saccharomyces - ps28
Arboricola_clade - ps29
Cerevisiae_clade - ps30
Saccharomyces_cerevisiae - ps31
Phylostratum

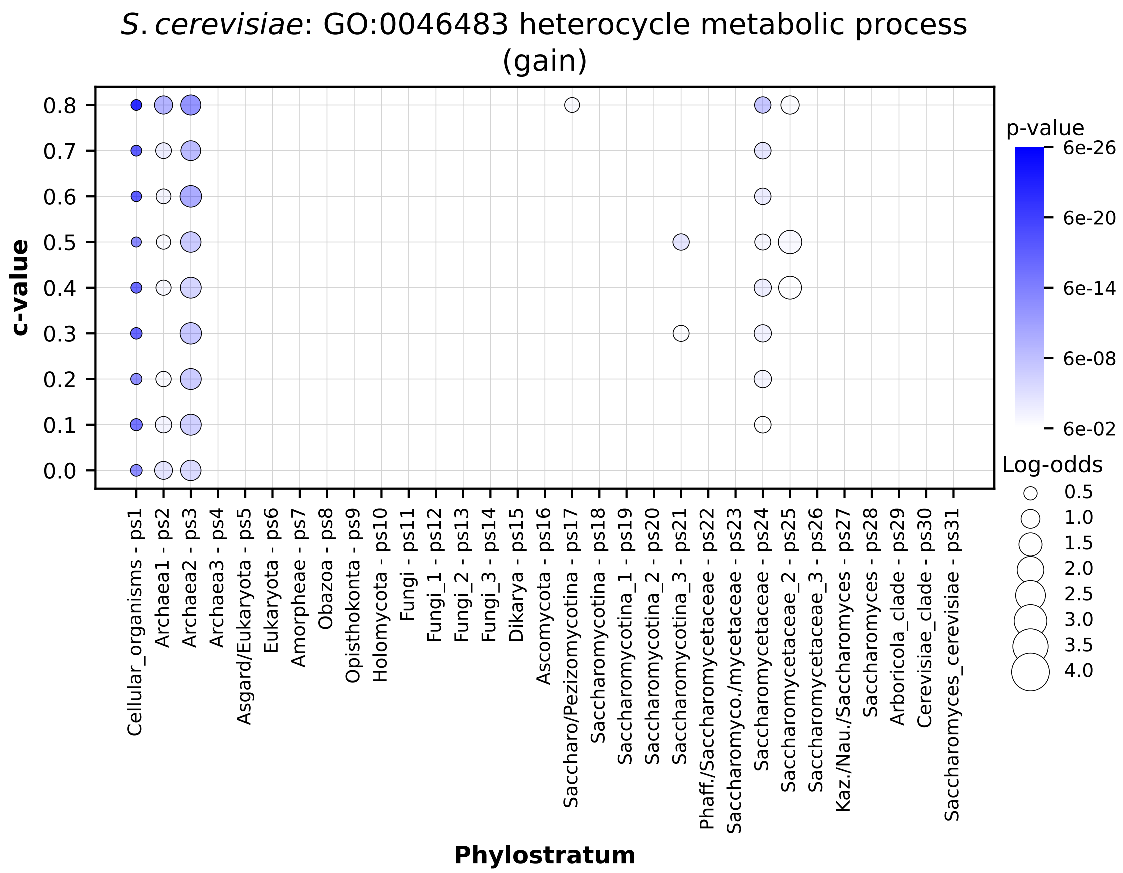

S. cerevisiae: GO:0046483 heterocycle metabolic process
(gain)
c-value
0.8
0.7
0.6
0.5
0.4
0.3
0.2
0.1
0.0
Phylostratum
Cellular_organisms - ps1
Archaea1 - ps2
Archaea2 - ps3
Archaea3 - ps4
Asgard/Eukaryota - ps5
Eukaryota - ps6
Amorpheae - ps7
Obazoa - ps8
Opisthokonta - ps9
Holomycota - ps10
Fungi - ps11
Fungi_1 - ps12
Fungi_2 - ps13
Fungi_3 - ps14
Dikarya - ps15
Ascomycota - ps16
Saccharo/Pezizomycotina - ps17
Saccharomycotina - ps18
Saccharomycotina_1 - ps19
Saccharomycotina_2 - ps20
Saccharomycotina_3 - ps21
Phaff./Saccharomycetaceae - ps22
Saccharomyco./mycetaceae - ps23
Saccharomycetaceae - ps24
Saccharomycetaceae_2 - ps25
Saccharomycetaceae_3 - ps26
Kaz./Nau./Saccharomyces - ps27
Saccharomyces - ps28
Arboricola_clade - ps29
Cerevisiae_clade - ps30
Saccharomyces_cerevisiae - ps31
p-value
6e-26
6e-20
6e-14
6e-08
6e-02
Log-odds
0.5
1.0
1.5
2.0
2.5
3.0
3.5
4.0

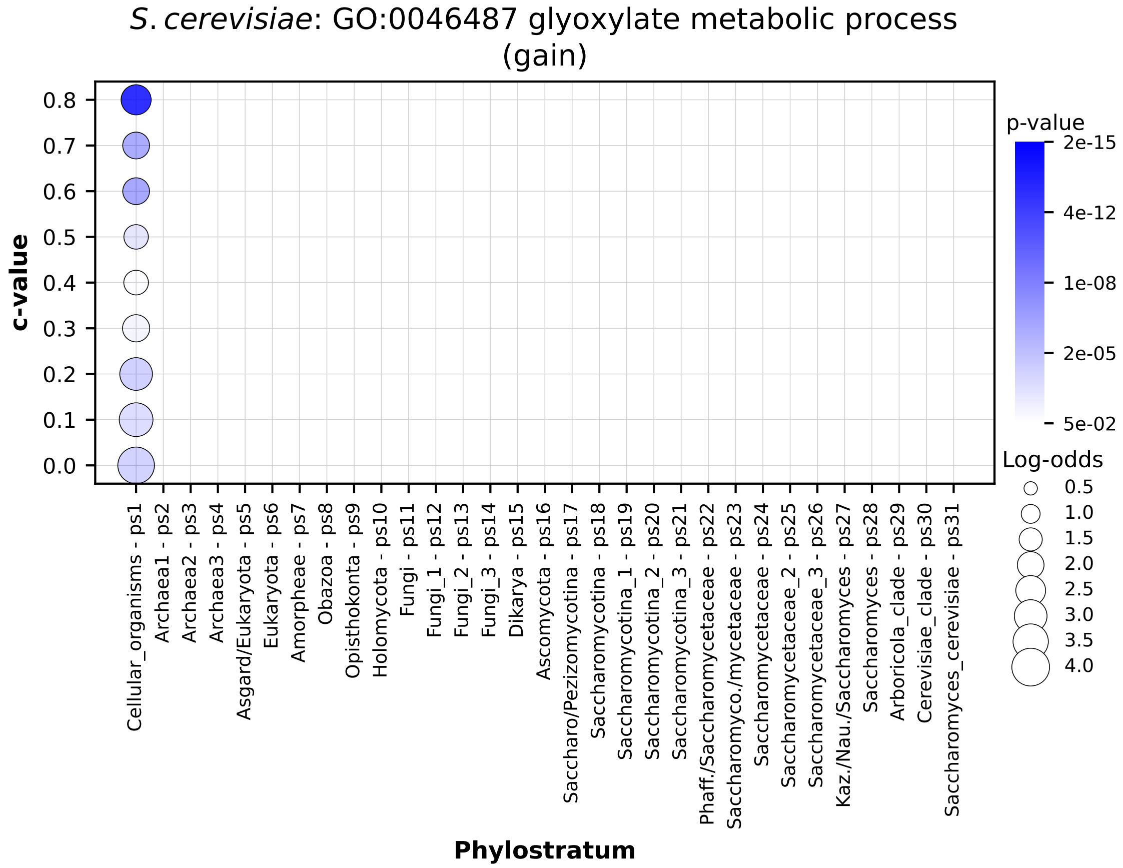

S. cerevisiae: GO:0046487 glyoxylate metabolic process
(gain)
c-value
0.8
0.7
0.6
0.5
0.4
0.3
0.2
0.1
0.0
p-value
2e-15
4e-12
1e-08
2e-05
5e-02
Log-odds
0.5
1.0
1.5
2.0
2.5
3.0
3.5
4.0
Cellular_organisms - ps1
Archaea1 - ps2
Archaea2 - ps3
Archaea3 - ps4
Asgard/Eukaryota - ps5
Eukaryota - ps6
Amorpheae - ps7
Obazoa - ps8
Opisthokonta - ps9
Holomycota - ps10
Fungi - ps11
Fungi_1 - ps12
Fungi_2 - ps13
Fungi_3 - ps14
Dikarya - ps15
Ascomycota - ps16
Saccharo/Pezizomycotina - ps17
Saccharomycotina - ps18
Saccharomycotina_1 - ps19
Saccharomycotina_2 - ps20
Saccharomycotina_3 - ps21
Phaff./Saccharomycetaceae - ps22
Saccharomyco./mycetaceae - ps23
Saccharomycetaceae - ps24
Saccharomycetaceae_2 - ps25
Saccharomycetaceae_3 - ps26
Kaz./Nau./Saccharomyces - ps27
Saccharomyces - ps28
Arboricola_clade - ps29
Cerevisiae_clade - ps30
Saccharomyces_cerevisiae - ps31
Phylostratum

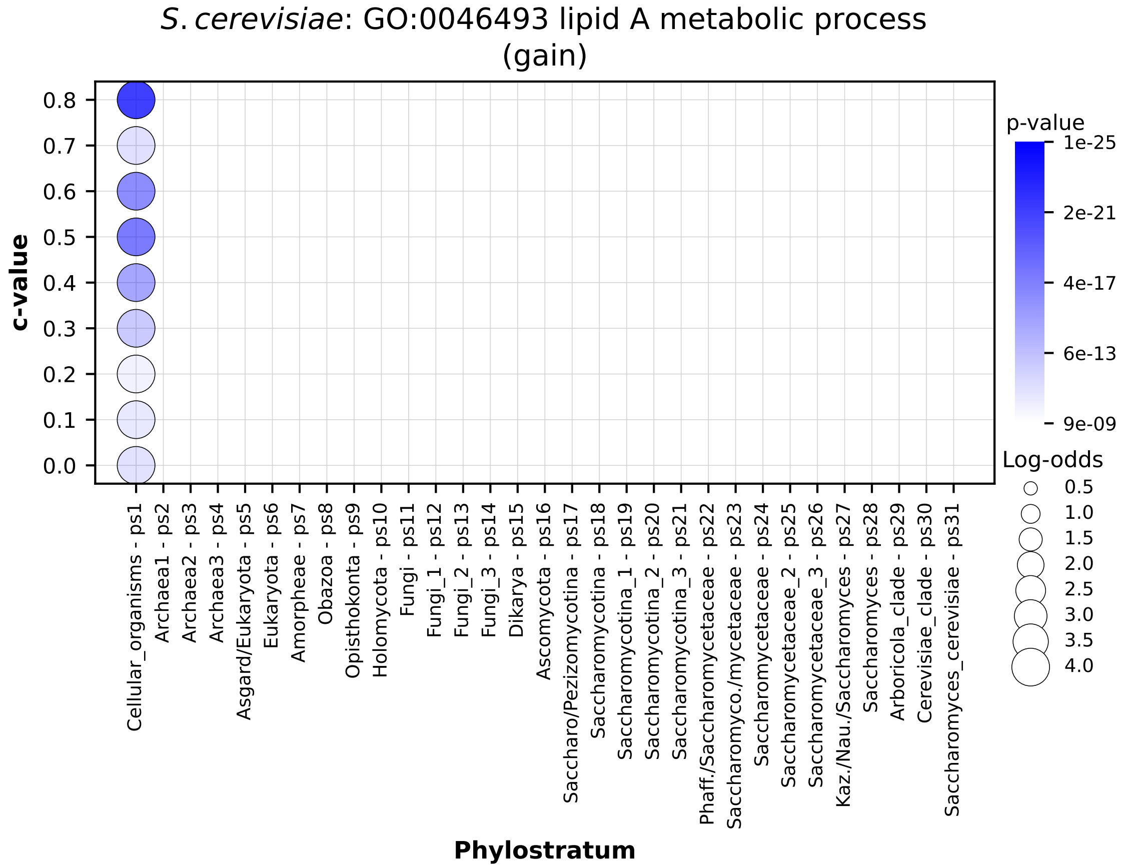

S. cerevisiae: GO:0046493 lipid A metabolic process
(gain)
c-value
0.8
0.7
0.6
0.5
0.4
0.3
0.2
0.1
0.0
p-value
1e-25
2e-21
4e-17
6e-13
9e-09
Log-odds
0.5
1.0
1.5
2.0
2.5
3.0
3.5
4.0
Cellular_organisms - ps1
Archaea1 - ps2
Archaea2 - ps3
Archaea3 - ps4
Asgard/Eukaryota - ps5
Eukaryota - ps6
Amorpheae - ps7
Obazoa - ps8
Opisthokonta - ps9
Holomycota - ps10
Fungi - ps11
Fungi_1 - ps12
Fungi_2 - ps13
Fungi_3 - ps14
Dikarya - ps15
Ascomycota - ps16
Saccharo/Pezizomycotina - ps17
Saccharomycotina - ps18
Saccharomycotina_1 - ps19
Saccharomycotina_2 - ps20
Saccharomycotina_3 - ps21
Phaff./Saccharomycetaceae - ps22
Saccharomyco./mycetaceae - ps23
Saccharomycetaceae - ps24
Saccharomycetaceae_2 - ps25
Saccharomycetaceae_3 - ps26
Kaz./Nau./Saccharomyces - ps27
Saccharomyces - ps28
Arboricola_clade - ps29
Cerevisiae_clade - ps30
Saccharomyces_cerevisiae - ps31
Phylostratum

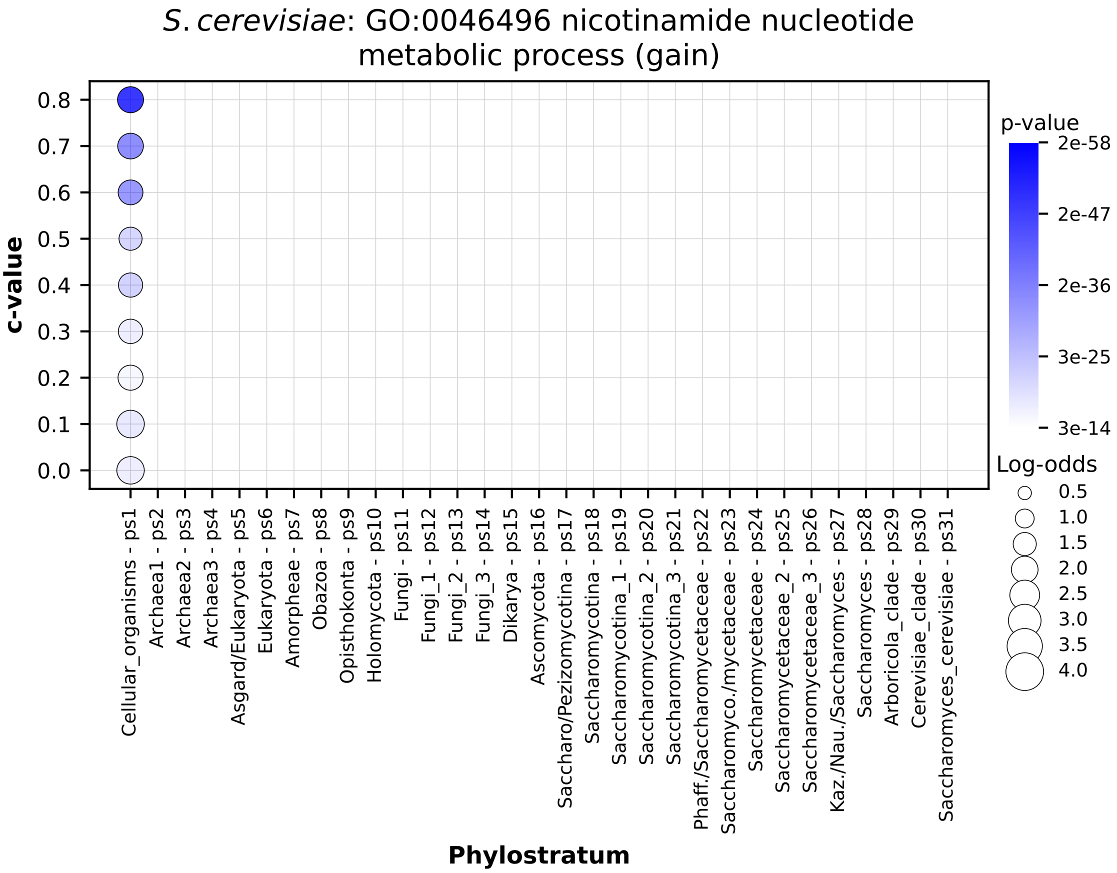

S. cerevisiae: GO:0046496 nicotinamide nucleotide metabolic process (gain)
c-value
0.8
0.7
0.6
0.5
0.4
0.3
0.2
0.1
0.0
Phylostratum
Cellular_organisms - ps1
Archaea1 - ps2
Archaea2 - ps3
Archaea3 - ps4
Asgard/Eukaryota - ps5
Eukaryota - ps6
Amorpheae - ps7
Obazoa - ps8
Opisthokonta - ps9
Holomycota - ps10
Fungi - ps11
Fungi_1 - ps12
Fungi_2 - ps13
Fungi_3 - ps14
Dikarya - ps15
Ascomycota - ps16
Saccharo/Pezizomycotina - ps17
Saccharomycotina - ps18
Saccharomycotina_1 - ps19
Saccharomycotina_2 - ps20
Saccharomycotina_3 - ps21
Phaff./Saccharomycetaceae - ps22
Saccharomyco./mycetaceae - ps23
Saccharomycetaceae - ps24
Saccharomycetaceae_2 - ps25
Saccharomycetaceae_3 - ps26
Kaz./Nau./Saccharomyces - ps27
Saccharomyces - ps28
Arboricola_clade - ps29
Cerevisiae_clade - ps30
Saccharomyces_cerevisiae - ps31
p-value
2e-58
2e-47
2e-36
3e-25
3e-14
Log-odds
0.5
1.0
1.5
2.0
2.5
3.0
3.5
4.0

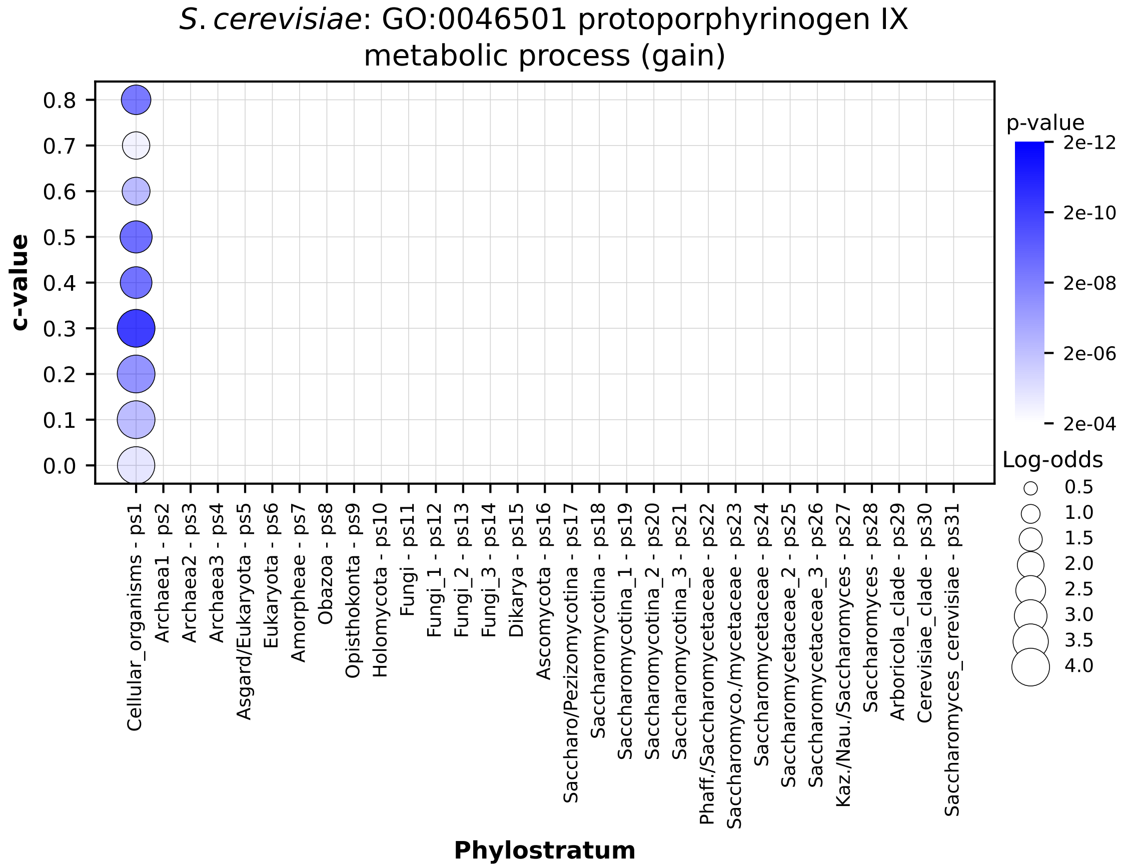

S. cerevisiae: GO:0046501 protoporphyrinogen IX metabolic process (gain)
c-value
0.0
0.1
0.2
0.3
0.4
0.5
0.6
0.7
0.8
p-value
2e-12
2e-10
2e-08
2e-06
2e-04
Log-odds
0.5
1.0
1.5
2.0
2.5
3.0
3.5
4.0
Cellular_organisms - ps1
Archaea1 - ps2
Archaea2 - ps3
Archaea3 - ps4
Asgard/Eukaryota - ps5
Eukaryota - ps6
Amorpheae - ps7
Obazoa - ps8
Opisthokonta - ps9
Holomycota - ps10
Fungi - ps11
Fungi_1 - ps12
Fungi_2 - ps13
Fungi_3 - ps14
Dikarya - ps15
Ascomycota - ps16
Saccharo/Pezizomycotina - ps17
Saccharomycotina - ps18
Saccharomycotina_1 - ps19
Saccharomycotina_2 - ps20
Saccharomycotina_3 - ps21
Phaff./Saccharomycetaceae - ps22
Saccharomyco./mycetaceae - ps23
Saccharomycetaceae - ps24
Saccharomycetaceae_2 - ps25
Saccharomycetaceae_3 - ps26
Kaz./Nau./Saccharomyces - ps27
Saccharomyces - ps28
Arboricola_clade - ps29
Cerevisiae_clade - ps30
Saccharomyces_cerevisiae - ps31
Phylostratum

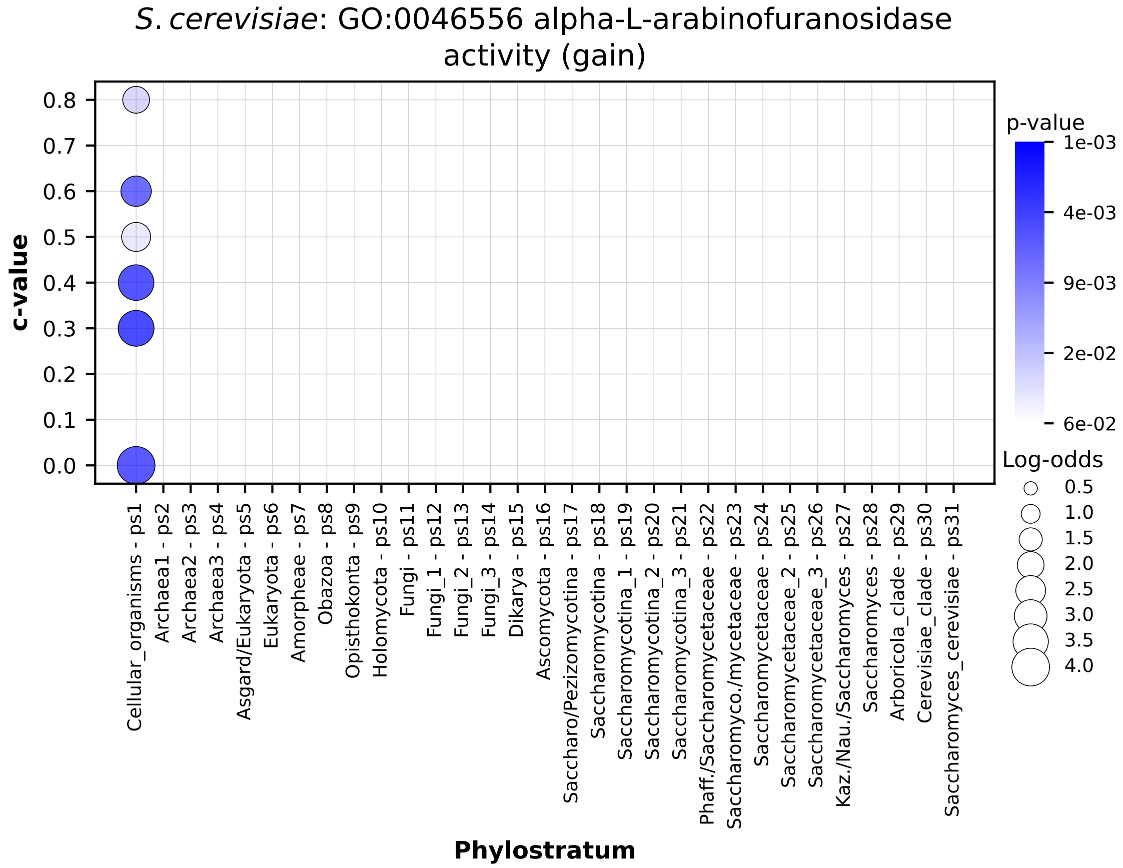

S. cerevisiae: GO:0046556 alpha-L-arabinofuranosidase activity (gain)
c-value
0.0
0.1
0.2
0.3
0.4
0.5
0.6
0.7
0.8
p-value
1e-03
4e-03
9e-03
2e-02
6e-02
Log-odds
0.5
1.0
1.5
2.0
2.5
3.0
3.5
4.0
Cellular_organisms - ps1
Archaea1 - ps2
Archaea2 - ps3
Archaea3 - ps4
Asgard/Eukaryota - ps5
Eukaryota - ps6
Amorpheae - ps7
Obazoa - ps8
Opisthokonta - ps9
Holomycota - ps10
Fungi - ps11
Fungi_1 - ps12
Fungi_2 - ps13
Fungi_3 - ps14
Dikarya - ps15
Ascomycota - ps16
Saccharo/Pezizomycotina - ps17
Saccharomycotina - ps18
Saccharomycotina_1 - ps19
Saccharomycotina_2 - ps20
Saccharomycotina_3 - ps21
Phaff./Saccharomycetaceae - ps22
Saccharomyco./mycetaceae - ps23
Saccharomycetaceae - ps24
Saccharomycetaceae_2 - ps25
Saccharomycetaceae_3 - ps26
Kaz./Nau./Saccharomyces - ps27
Saccharomyces - ps28
Arboricola_clade - ps29
Cerevisiae_clade - ps30
Saccharomyces_cerevisiae - ps31
Phylostratum

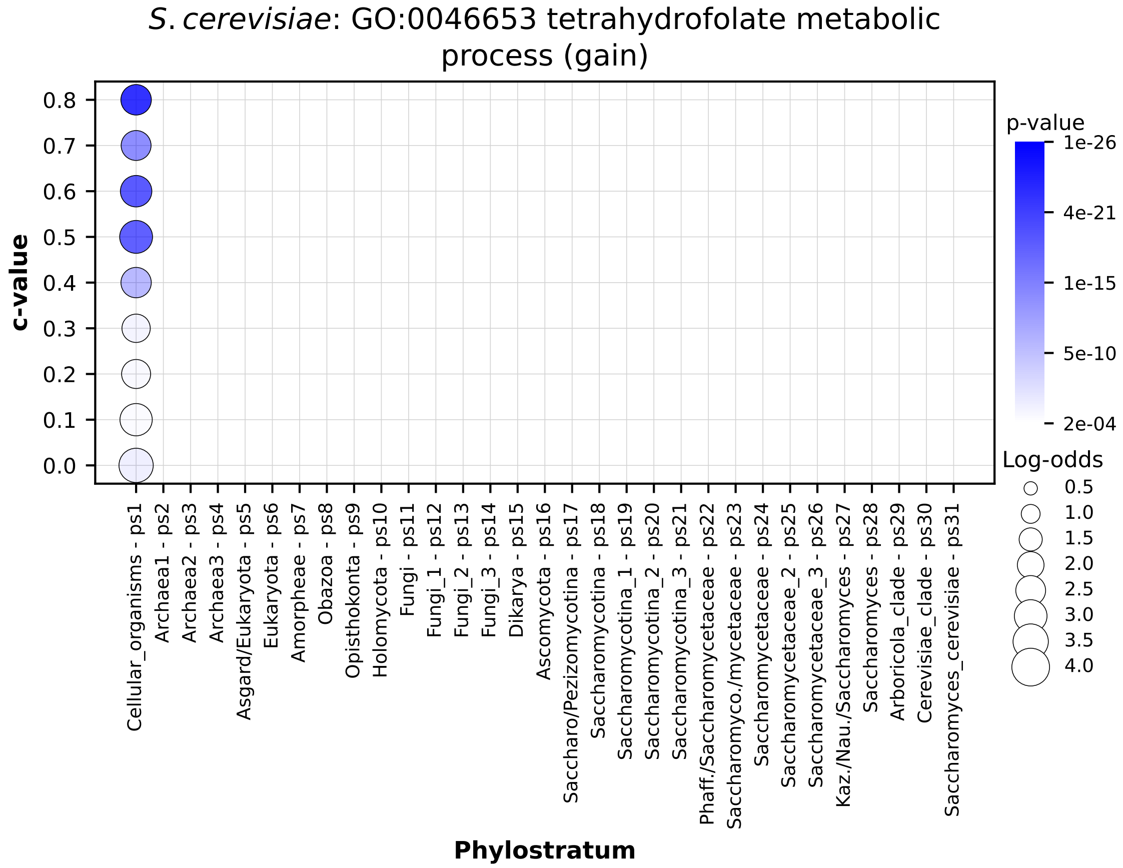

S. cerevisiae: GO:0046653 tetrahydrofolate metabolic process (gain)
c-value
0.8
0.7
0.6
0.5
0.4
0.3
0.2
0.1
0.0
Phylostratum
Cellular_organisms - ps1
Archaea1 - ps2
Archaea2 - ps3
Archaea3 - ps4
Asgard/Eukaryota - ps5
Eukaryota - ps6
Amorpheae - ps7
Obazoa - ps8
Opisthokonta - ps9
Holomycota - ps10
Fungi - ps11
Fungi_1 - ps12
Fungi_2 - ps13
Fungi_3 - ps14
Dikarya - ps15
Ascomycota - ps16
Saccharo/Pezizomycotina - ps17
Saccharomycotina - ps18
Saccharomycotina_1 - ps19
Saccharomycotina_2 - ps20
Saccharomycotina_3 - ps21
Phaff./Saccharomycetaceae - ps22
Saccharomyco./mycetaceae - ps23
Saccharomycetaceae - ps24
Saccharomycetaceae_2 - ps25
Saccharomycetaceae_3 - ps26
Kaz./Nau./Saccharomyces - ps27
Saccharomyces - ps28
Arboricola_clade - ps29
Cerevisiae_clade - ps30
Saccharomyces_cerevisiae - ps31
p-value
1e-26
4e-21
1e-15
5e-10
2e-04
Log-odds
0.5
1.0
1.5
2.0
2.5
3.0
3.5
4.0

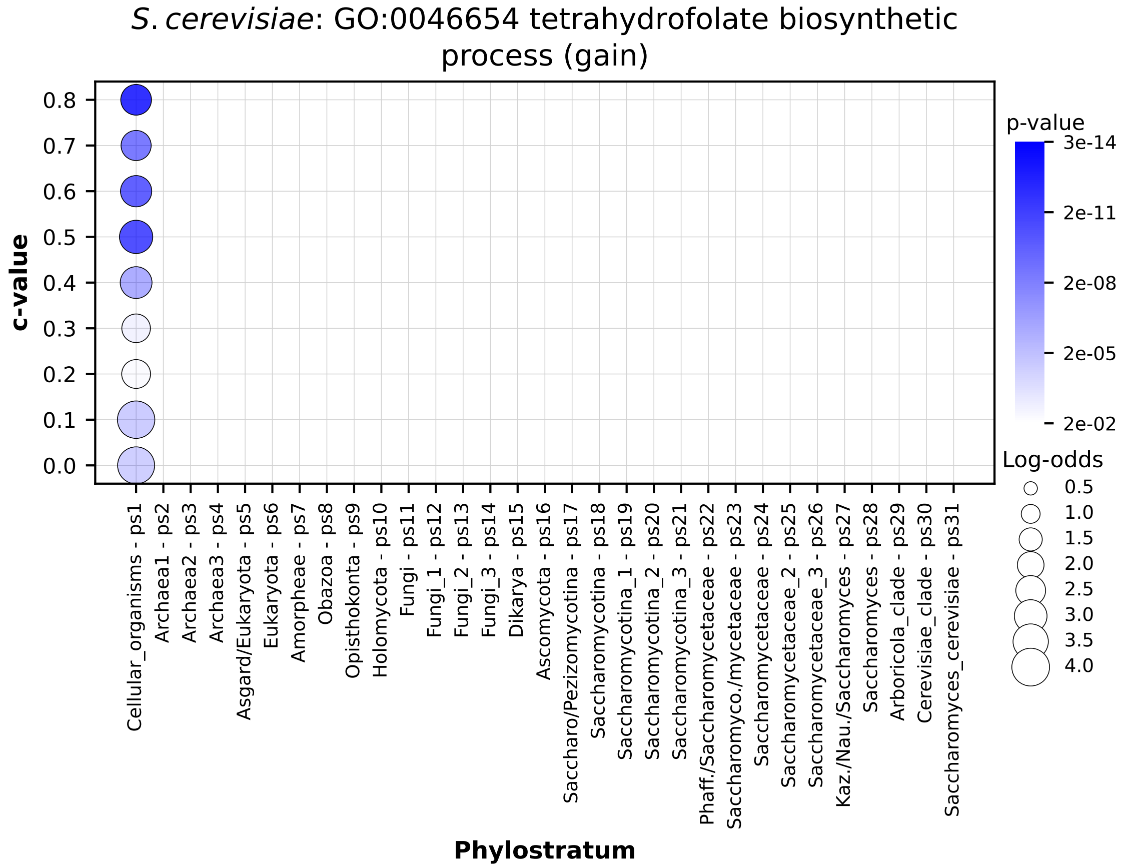

S. cerevisiae: GO:0046654 tetrahydrofolate biosynthetic process (gain)
c-value
0.8
0.7
0.6
0.5
0.4
0.3
0.2
0.1
0.0
p-value
3e-14
2e-11
2e-08
2e-05
2e-02
Log-odds
0.5
1.0
1.5
2.0
2.5
3.0
3.5
4.0
Cellular_organisms - ps1
Archaea1 - ps2
Archaea2 - ps3
Archaea3 - ps4
Asgard/Eukaryota - ps5
Eukaryota - ps6
Amorpheae - ps7
Obazoa - ps8
Opisthokonta - ps9
Holomycota - ps10
Fungi - ps11
Fungi_1 - ps12
Fungi_2 - ps13
Fungi_3 - ps14
Dikarya - ps15
Ascomycota - ps16
Saccharo/Pezizomycotina - ps17
Saccharomycotina - ps18
Saccharomycotina_1 - ps19
Saccharomycotina_2 - ps20
Saccharomycotina_3 - ps21
Phaff./Saccharomycetaceae - ps22
Saccharomyco./mycetaceae - ps23
Saccharomycetaceae - ps24
Saccharomycetaceae_2 - ps25
Saccharomycetaceae_3 - ps26
Kaz./Nau./Saccharomyces - ps27
Saccharomyces - ps28
Arboricola_clade - ps29
Cerevisiae_clade - ps30
Saccharomyces_cerevisiae - ps31
Phylostratum

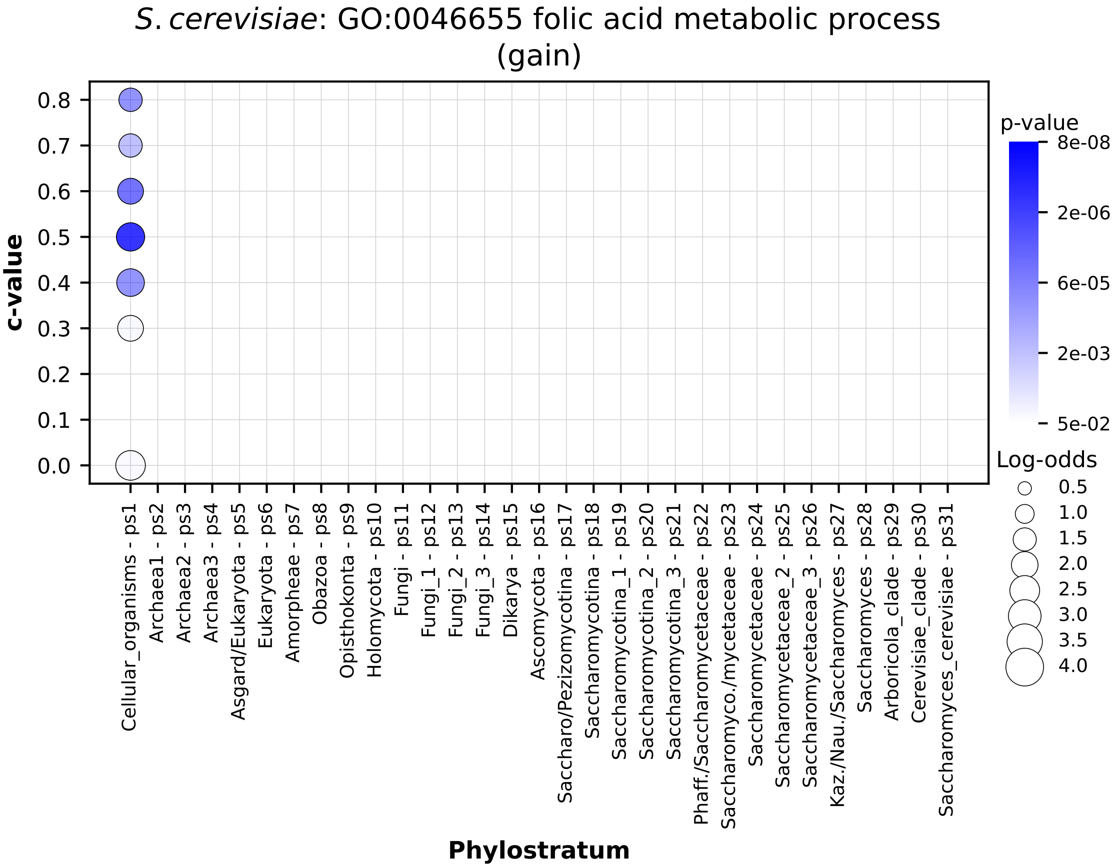

S. cerevisiae: GO:0046655 folic acid metabolic process
(gain)
c-value
0.0
0.1
0.2
0.3
0.4
0.5
0.6
0.7
0.8
p-value
8e-08
2e-06
6e-05
2e-03
5e-02
Log-odds
0.5
1.0
1.5
2.0
2.5
3.0
3.5
4.0
Cellular_organisms - ps1
Archaea1 - ps2
Archaea2 - ps3
Archaea3 - ps4
Asgard/Eukaryota - ps5
Eukaryota - ps6
Amorpheae - ps7
Obazoa - ps8
Opisthokonta - ps9
Holomycota - ps10
Fungi - ps11
Fungi_1 - ps12
Fungi_2 - ps13
Fungi_3 - ps14
Dikarya - ps15
Ascomycota - ps16
Saccharo/Pezizomycotina - ps17
Saccharomycotina - ps18
Saccharomycotina_1 - ps19
Saccharomycotina_2 - ps20
Saccharomycotina_3 - ps21
Phaff./Saccharomycetaceae - ps22
Saccharomyco./mycetaceae - ps23
Saccharomycetaceae - ps24
Saccharomycetaceae_2 - ps25
Saccharomycetaceae_3 - ps26
Kaz./Nau./Saccharomyces - ps27
Saccharomyces - ps28
Arboricola_clade - ps29
Cerevisiae_clade - ps30
Saccharomyces_cerevisiae - ps31
Phylostratum

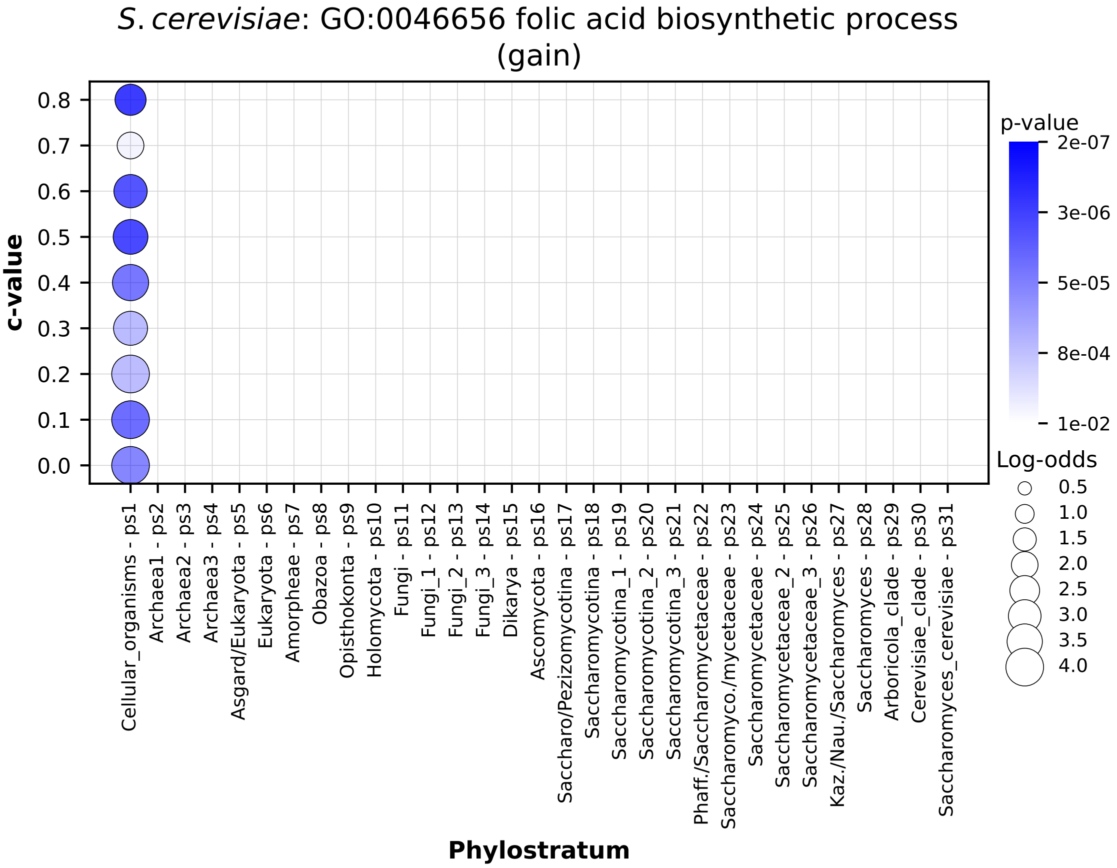
S. cerevisiae: GO:0046656 folic acid biosynthetic process
(gain)
c-value
0.8
0.7
0.6
0.5
0.4
0.3
0.2
0.1
0.0
p-value
2e-07
3e-06
5e-05
8e-04
1e-02
Log-odds
0.5
1.0
1.5
2.0
2.5
3.0
3.5
4.0
Cellular_organisms - ps1
Archaea1 - ps2
Archaea2 - ps3
Archaea3 - ps4
Asgard/Eukaryota - ps5
Eukaryota - ps6
Amorpheae - ps7
Obazoa - ps8
Opisthokonta - ps9
Holomycota - ps10
Fungi - ps11
Fungi_1 - ps12
Fungi_2 - ps13
Fungi_3 - ps14
Dikarya - ps15
Ascomycota - ps16
Saccharo/Pezizomycotina - ps17
Saccharomycotina - ps18
Saccharomycotina_1 - ps19
Saccharomycotina_2 - ps20
Saccharomycotina_3 - ps21
Phaff./Saccharomycetaceae - ps22
Saccharomyco./mycetaceae - ps23
Saccharomycetaceae - ps24
Saccharomycetaceae_2 - ps25
Saccharomycetaceae_3 - ps26
Kaz./Nau./Saccharomyces - ps27
Saccharomyces - ps28
Arboricola_clade - ps29
Cerevisiae_clade - ps30
Saccharomyces_cerevisiae - ps31
Phylostratum

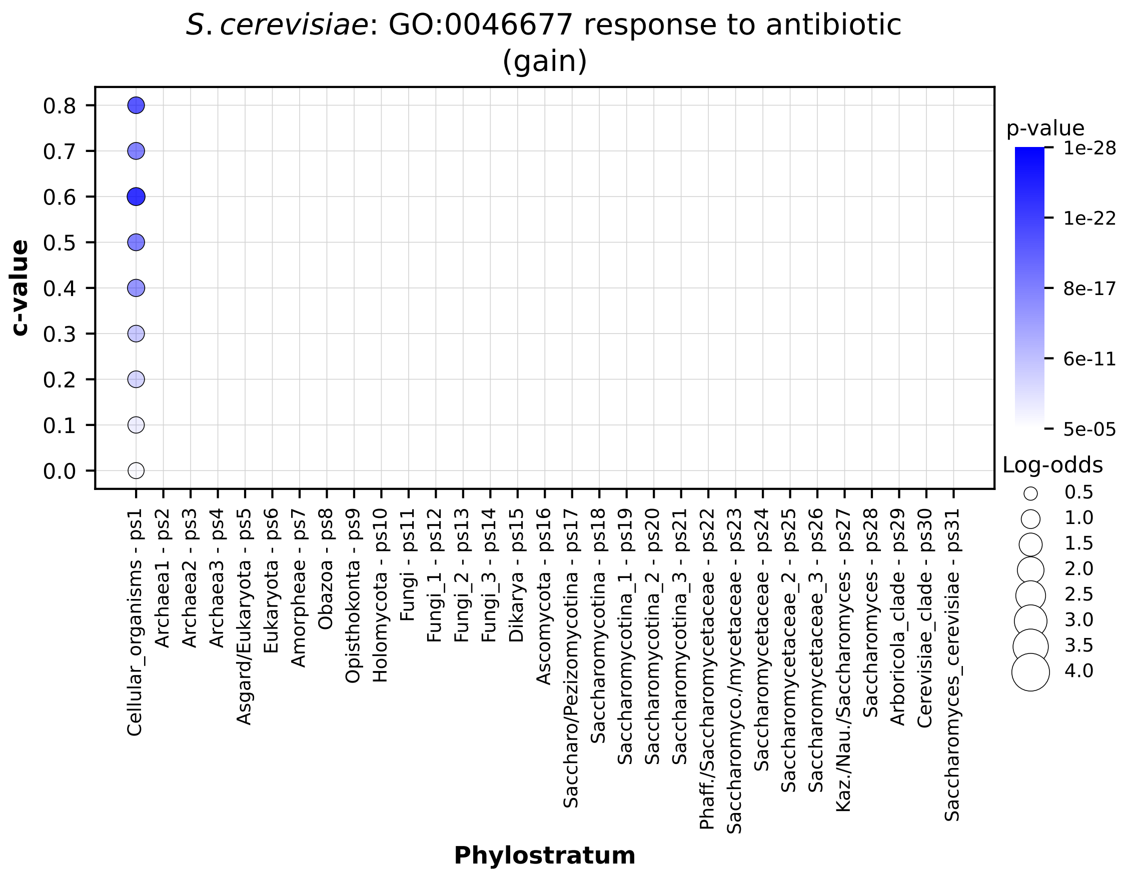

S. cerevisiae: GO:0046677 response to antibiotic
(gain)
c-value
0.0
0.1
0.2
0.3
0.4
0.5
0.6
0.7
0.8
Phylostratum
Cellular_organisms - ps1
Archaea1 - ps2
Archaea2 - ps3
Archaea3 - ps4
Asgard/Eukaryota - ps5
Eukaryota - ps6
Amorpheae - ps7
Obazoa - ps8
Opisthokonta - ps9
Holomycota - ps10
Fungi - ps11
Fungi_1 - ps12
Fungi_2 - ps13
Fungi_3 - ps14
Dikarya - ps15
Ascomycota - ps16
Saccharo/Pezizomycotina - ps17
Saccharomycotina - ps18
Saccharomycotina_1 - ps19
Saccharomycotina_2 - ps20
Saccharomycotina_3 - ps21
Phaff./Saccharomycetaceae - ps22
Saccharomyco./mycetaceae - ps23
Saccharomycetaceae - ps24
Saccharomycetaceae_2 - ps25
Saccharomycetaceae_3 - ps26
Kaz./Nau./Saccharomyces - ps27
Saccharomyces - ps28
Arboricola_clade - ps29
Cerevisiae_clade - ps30
Saccharomyces_cerevisiae - ps31
p-value
1e-28
1e-22
8e-17
6e-11
5e-05
Log-odds
0.5
1.0
1.5
2.0
2.5
3.0
3.5
4.0

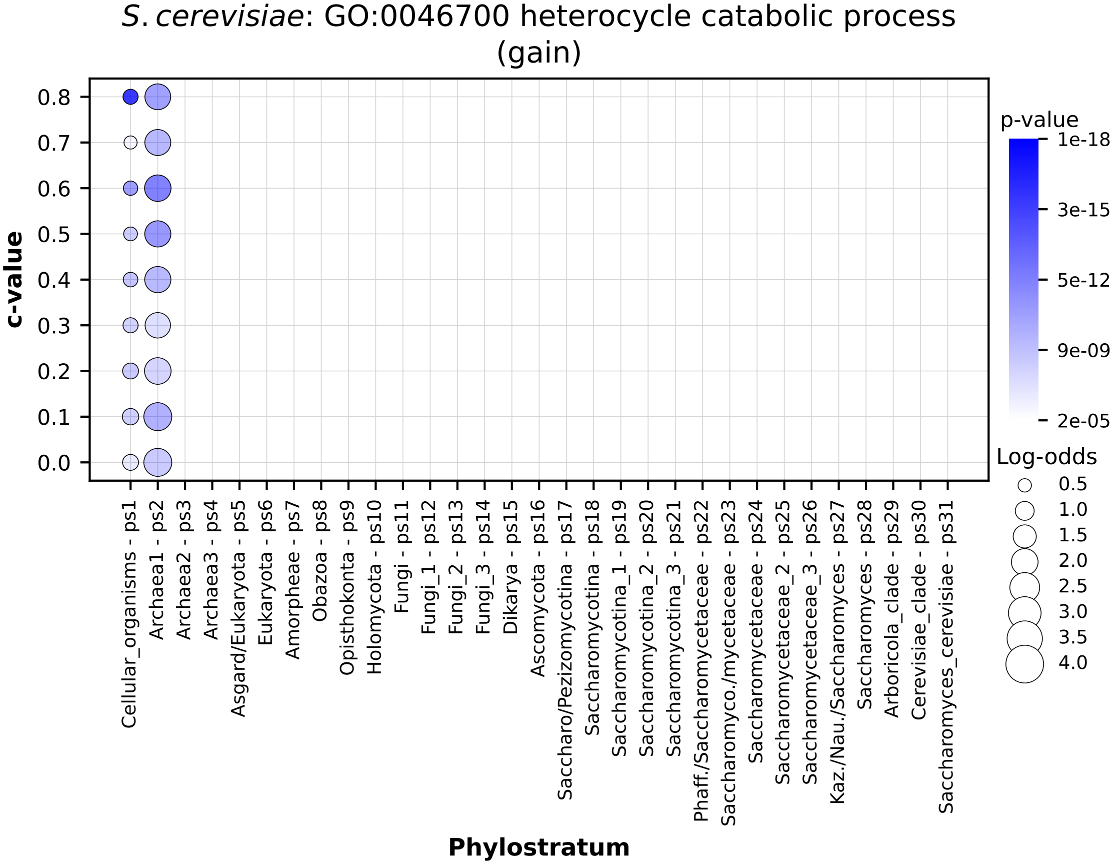

S. cerevisiae: GO:0046700 heterocycle catabolic process (gain)
c-value
0.8
0.7
0.6
0.5
0.4
0.3
0.2
0.1
0.0
p-value
1e-18
3e-15
5e-12
9e-09
2e-05
Log-odds
0.5
1.0
1.5
2.0
2.5
3.0
3.5
4.0
Cellular_organisms - ps1
Archaea1 - ps2
Archaea2 - ps3
Archaea3 - ps4
Asgard/Eukaryota - ps5
Eukaryota - ps6
Amorpheae - ps7
Obazoa - ps8
Opisthokonta - ps9
Holomycota - ps10
Fungi - ps11
Fungi_1 - ps12
Fungi_2 - ps13
Fungi_3 - ps14
Dikarya - ps15
Ascomycota - ps16
Saccharo/Pezizomycotina - ps17
Saccharomycotina - ps18
Saccharomycotina_1 - ps19
Saccharomycotina_2 - ps20
Saccharomycotina_3 - ps21
Phaff./Saccharomycetaceae - ps22
Saccharomyco./mycetaceae - ps23
Saccharomycetaceae - ps24
Saccharomycetaceae_2 - ps25
Saccharomycetaceae_3 - ps26
Kaz./Nau./Saccharomyces - ps27
Saccharomyces - ps28
Arboricola_clade - ps29
Cerevisiae_clade - ps30
Saccharomyces_cerevisiae - ps31
Phylostratum

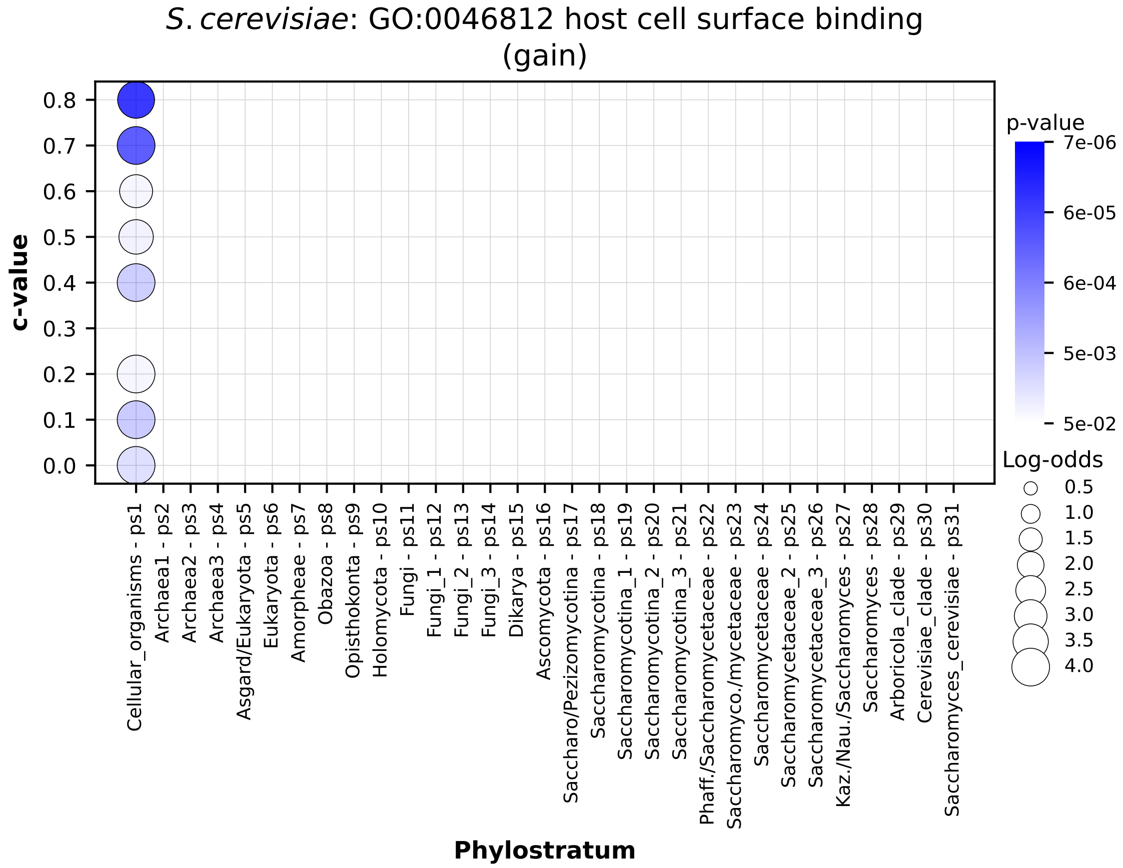
S. cerevisiae: GO:0046812 host cell surface binding
(gain)
c-value
0.8
0.7
0.6
0.5
0.4
0.3
0.2
0.1
0.0
p-value
7e-06
6e-05
6e-04
5e-03
5e-02
Log-odds
0.5
1.0
1.5
2.0
2.5
3.0
3.5
4.0
Cellular_organisms - ps1
Archaea1 - ps2
Archaea2 - ps3
Archaea3 - ps4
Asgard/Eukaryota - ps5
Eukaryota - ps6
Amorpheae - ps7
Obazoa - ps8
Opisthokonta - ps9
Holomycota - ps10
Fungi - ps11
Fungi_1 - ps12
Fungi_2 - ps13
Fungi_3 - ps14
Dikarya - ps15
Ascomycota - ps16
Saccharo/Pezizomycotina - ps17
Saccharomycotina - ps18
Saccharomycotina_1 - ps19
Saccharomycotina_2 - ps20
Saccharomycotina_3 - ps21
Phaff./Saccharomycetaceae - ps22
Saccharomyco./mycetaceae - ps23
Saccharomycetaceae - ps24
Saccharomycetaceae_2 - ps25
Saccharomycetaceae_3 - ps26
Kaz./Nau./Saccharomyces - ps27
Saccharomyces - ps28
Arboricola_clade - ps29
Cerevisiae_clade - ps30
Saccharomyces_cerevisiae - ps31
Phylostratum

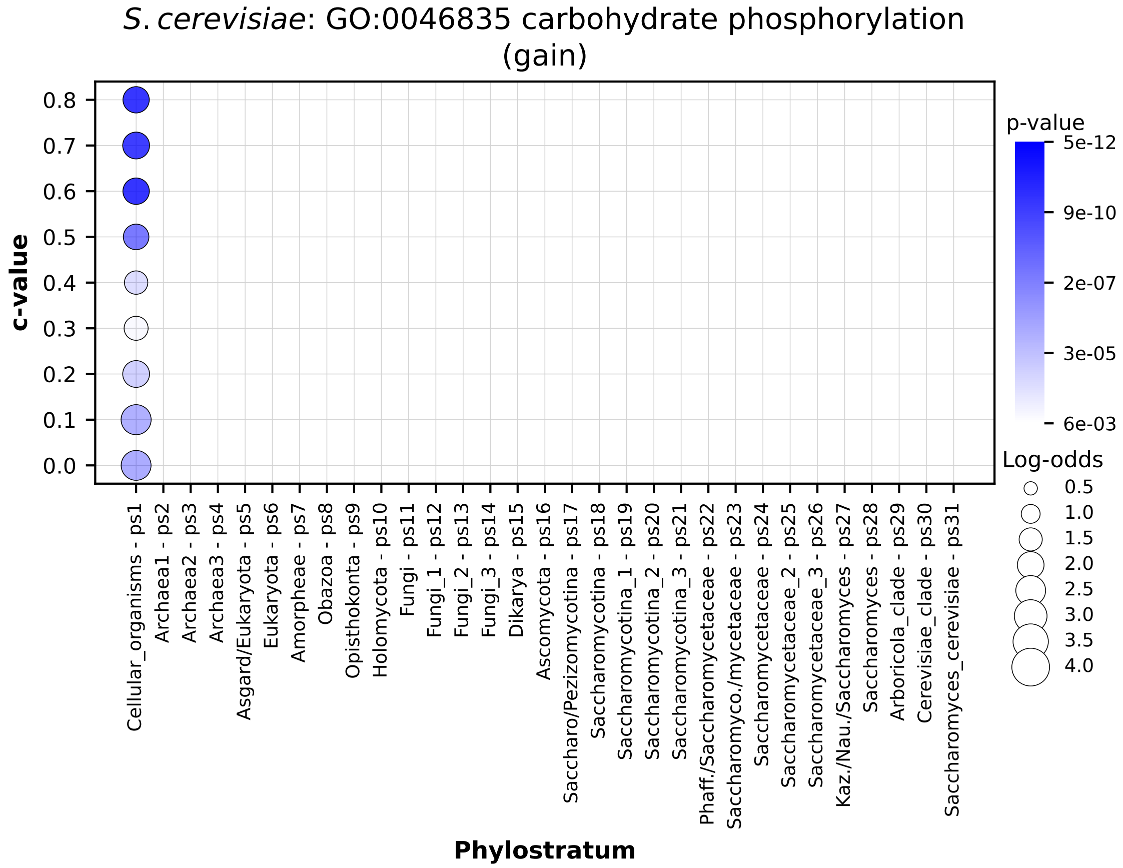

S. cerevisiae: GO:0046835 carbohydrate phosphorylation (gain)
c-value
0.8
0.7
0.6
0.5
0.4
0.3
0.2
0.1
0.0
p-value
5e-12
9e-10
2e-07
3e-05
6e-03
Log-odds
0.5
1.0
1.5
2.0
2.5
3.0
3.5
4.0
Cellular_organisms - ps1
Archaea1 - ps2
Archaea2 - ps3
Archaea3 - ps4
Asgard/Eukaryota - ps5
Eukaryota - ps6
Amorpheae - ps7
Obazoa - ps8
Opisthokonta - ps9
Holomycota - ps10
Fungi - ps11
Fungi_1 - ps12
Fungi_2 - ps13
Fungi_3 - ps14
Dikarya - ps15
Ascomycota - ps16
Saccharo/Pezizomycotina - ps17
Saccharomycotina - ps18
Saccharomycotina_1 - ps19
Saccharomycotina_2 - ps20
Saccharomycotina_3 - ps21
Phaff./Saccharomycetaceae - ps22
Saccharomyco./mycetaceae - ps23
Saccharomycetaceae - ps24
Saccharomycetaceae_2 - ps25
Saccharomycetaceae_3 - ps26
Kaz./Nau./Saccharomyces - ps27
Saccharomyces - ps28
Arboricola_clade - ps29
Cerevisiae_clade - ps30
Saccharomyces_cerevisiae - ps31
Phylostratum

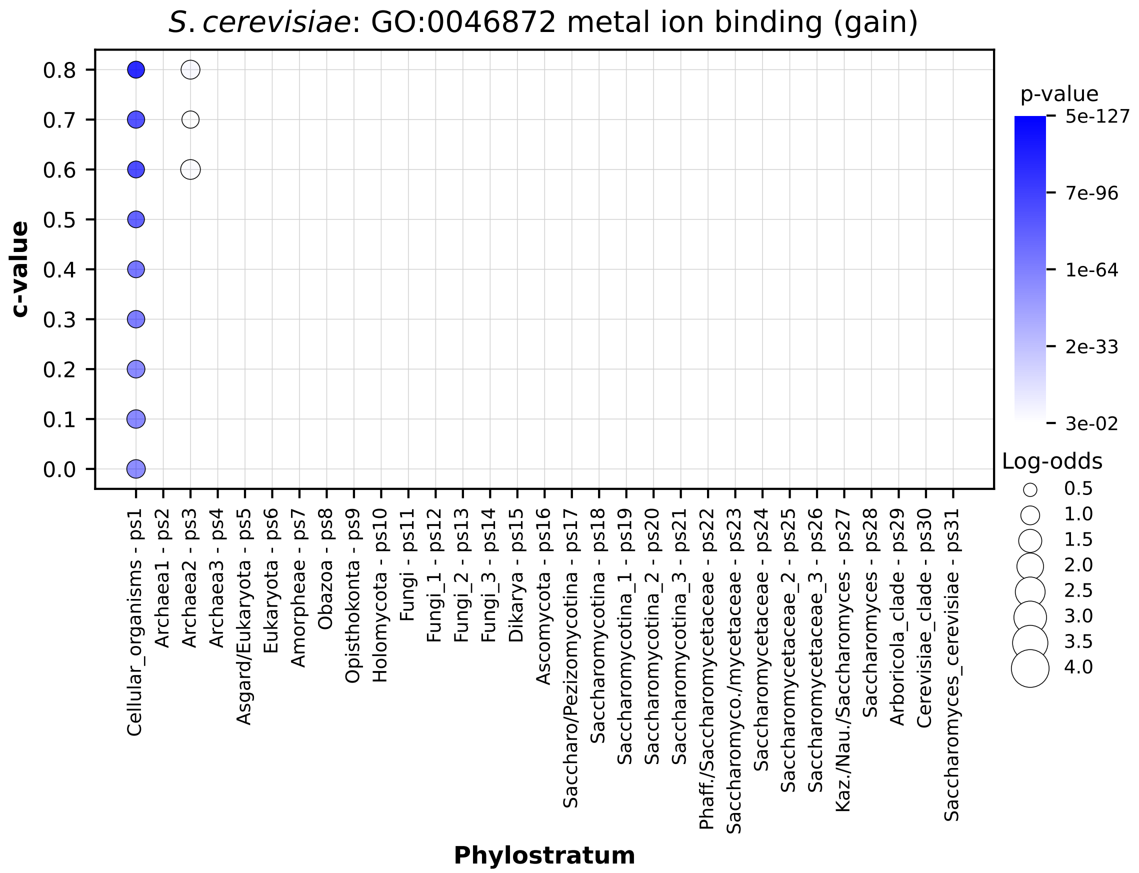

S. cerevisiae: GO:0046872 metal ion binding (gain)
c-value
0.8
0.7
0.6
0.5
0.4
0.3
0.2
0.1
0.0
p-value
5e-127
7e-96
1e-64
2e-33
3e-02
Log-odds
0.5
1.0
1.5
2.0
2.5
3.0
3.5
4.0
Cellular_organisms - ps1
Archaea1 - ps2
Archaea2 - ps3
Archaea3 - ps4
Asgard/Eukaryota - ps5
Eukaryota - ps6
Amorpheae - ps7
Obazoa - ps8
Opisthokonta - ps9
Holomycota - ps10
Fungi - ps11
Fungi_1 - ps12
Fungi_2 - ps13
Fungi_3 - ps14
Dikarya - ps15
Ascomycota - ps16
Saccharo/Pezizomycotina - ps17
Saccharomycotina - ps18
Saccharomycotina_1 - ps19
Saccharomycotina_2 - ps20
Saccharomycotina_3 - ps21
Phaff./Saccharomycetaceae - ps22
Saccharomyco./mycetaceae - ps23
Saccharomycetaceae - ps24
Saccharomycetaceae_2 - ps25
Saccharomycetaceae_3 - ps26
Kaz./Nau./Saccharomyces - ps27
Saccharomyces - ps28
Arboricola_clade - ps29
Cerevisiae_clade - ps30
Saccharomyces_cerevisiae - ps31
Phylostratum

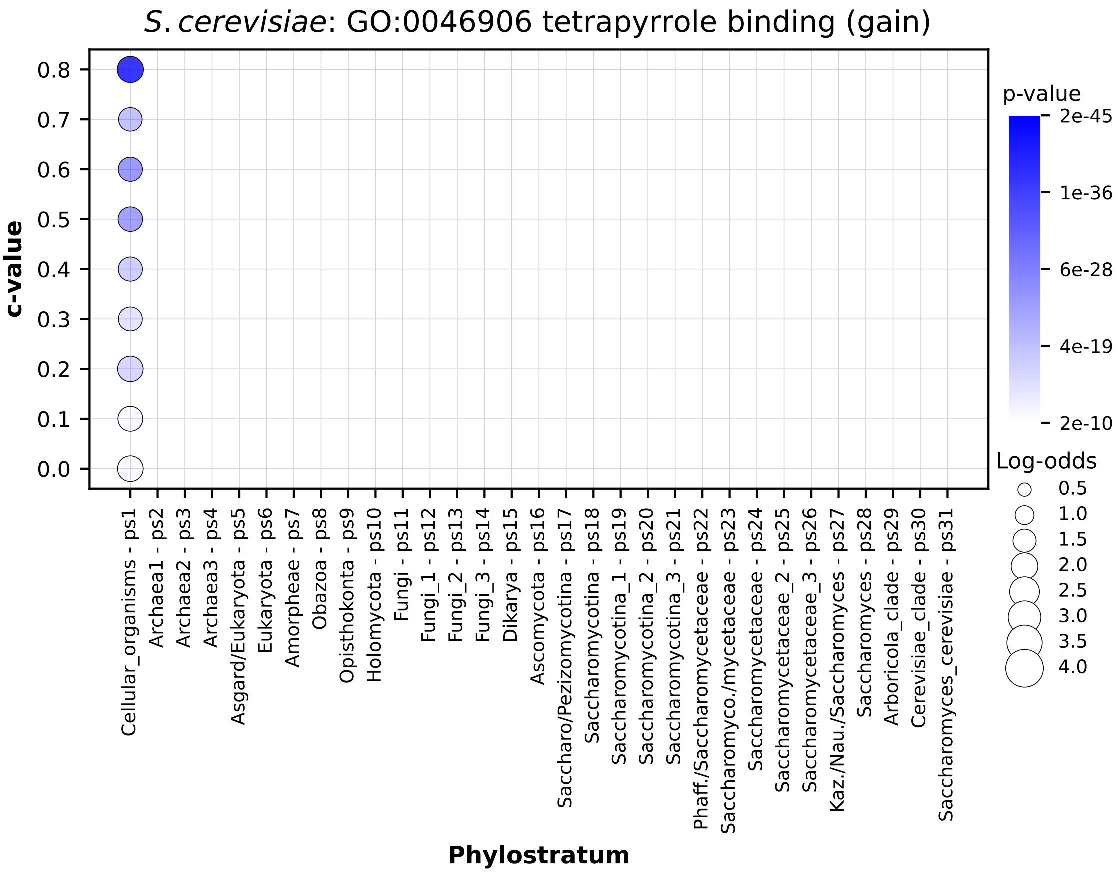

S. cerevisiae: GO:0046906 tetrapyrrole binding (gain)
c-value
0.8
0.7
0.6
0.5
0.4
0.3
0.2
0.1
0.0
p-value
2e-45
1e-36
6e-28
4e-19
2e-10
Log-odds
0.5
1.0
1.5
2.0
2.5
3.0
3.5
4.0
Cellular_organisms - ps1
Archaea1 - ps2
Archaea2 - ps3
Archaea3 - ps4
Asgard/Eukaryota - ps5
Eukaryota - ps6
Amorpheae - ps7
Obazoa - ps8
Opisthokonta - ps9
Holomycota - ps10
Fungi - ps11
Fungi_1 - ps12
Fungi_2 - ps13
Fungi_3 - ps14
Dikarya - ps15
Ascomycota - ps16
Saccharo/Pezizomycotina - ps17
Saccharomycotina - ps18
Saccharomycotina_1 - ps19
Saccharomycotina_2 - ps20
Saccharomycotina_3 - ps21
Phaff./Saccharomycetaceae - ps22
Saccharomyco./mycetaceae - ps23
Saccharomycetaceae - ps24
Saccharomycetaceae_2 - ps25
Saccharomycetaceae_3 - ps26
Kaz./Nau./Saccharomyces - ps27
Saccharomyces - ps28
Arboricola_clade - ps29
Cerevisiae_clade - ps30
Saccharomyces_cerevisiae - ps31
Phylostratum

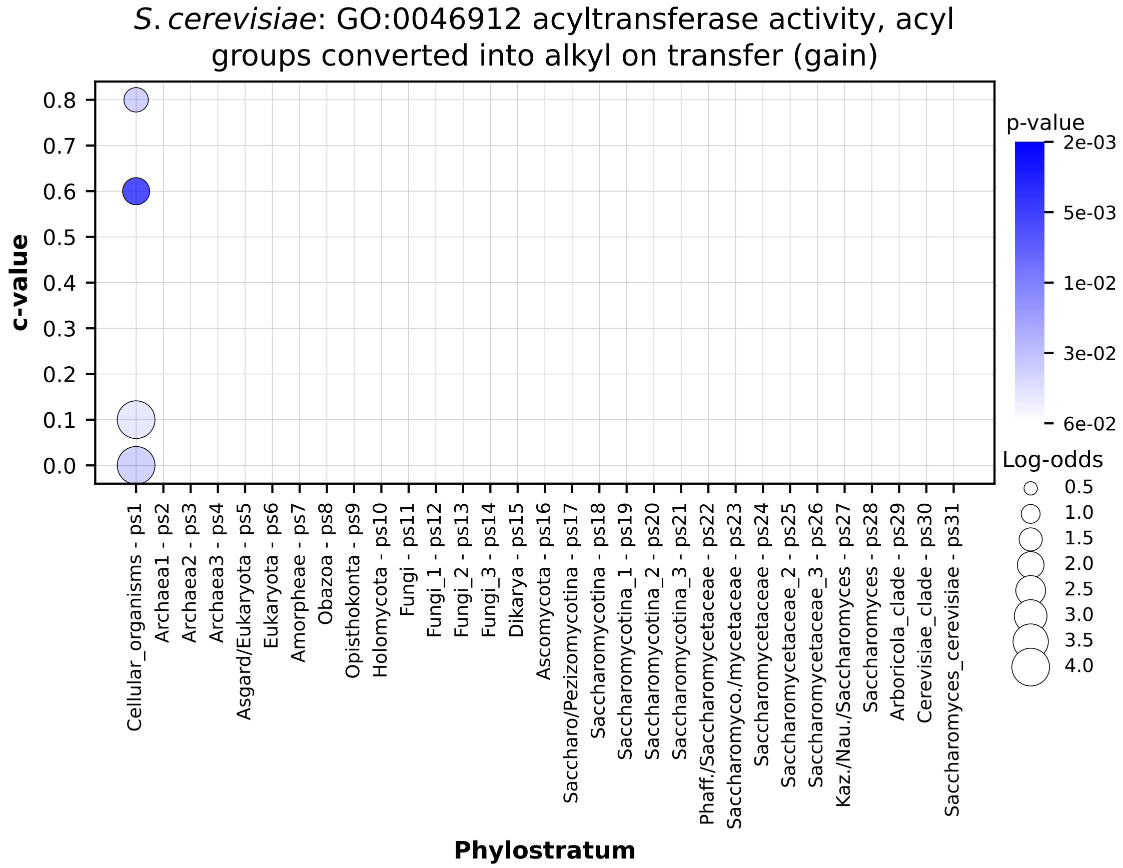

S. cerevisiae: GO:0046912 acyltransferase activity, acyl groups converted into alkyl on transfer (gain)
c-value
0.8
0.7
0.6
0.5
0.4
0.3
0.2
0.1
0.0
Phylostratum
Cellular_organisms - ps1
Archaea1 - ps2
Archaea2 - ps3
Archaea3 - ps4
Asgard/Eukaryota - ps5
Eukaryota - ps6
Amorpheae - ps7
Obazoa - ps8
Opisthokonta - ps9
Holomycota - ps10
Fungi - ps11
Fungi_1 - ps12
Fungi_2 - ps13
Fungi_3 - ps14
Dikarya - ps15
Ascomycota - ps16
Saccharo/Pezizomycotina - ps17
Saccharomycotina - ps18
Saccharomycotina_1 - ps19
Saccharomycotina_2 - ps20
Saccharomycotina_3 - ps21
Phaff./Saccharomycetaceae - ps22
Saccharomyco./mycetaceae - ps23
Saccharomycetaceae - ps24
Saccharomycetaceae_2 - ps25
Saccharomycetaceae_3 - ps26
Kaz./Nau./Saccharomyces - ps27
Saccharomyces - ps28
Arboricola_clade - ps29
Cerevisiae_clade - ps30
Saccharomyces_cerevisiae - ps31
p-value
2e-03
5e-03
1e-02
3e-02
6e-02
Log-odds
0.5
1.0
1.5
2.0
2.5
3.0
3.5
4.0

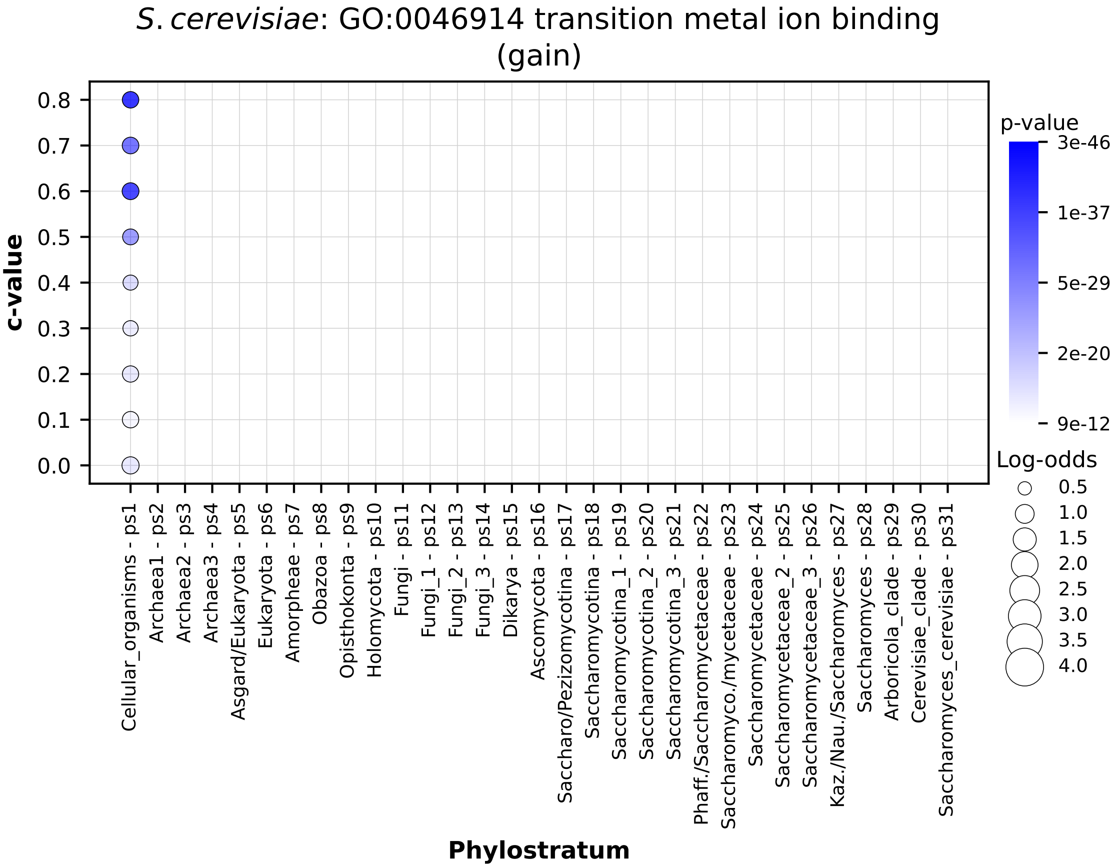

S. cerevisiae: GO:0046914 transition metal ion binding
(gain)
c-value
0.8
0.7
0.6
0.5
0.4
0.3
0.2
0.1
0.0
p-value
3e-46
1e-37
5e-29
2e-20
9e-12
Log-odds
0.5
1.0
1.5
2.0
2.5
3.0
3.5
4.0
Cellular_organisms - ps1
Archaea1 - ps2
Archaea2 - ps3
Archaea3 - ps4
Asgard/Eukaryota - ps5
Eukaryota - ps6
Amorpheae - ps7
Obazoa - ps8
Opisthokonta - ps9
Holomycota - ps10
Fungi - ps11
Fungi_1 - ps12
Fungi_2 - ps13
Fungi_3 - ps14
Dikarya - ps15
Ascomycota - ps16
Saccharo/Pezizomycotina - ps17
Saccharomycotina - ps18
Saccharomycotina_1 - ps19
Saccharomycotina_2 - ps20
Saccharomycotina_3 - ps21
Phaff./Saccharomycetaceae - ps22
Saccharomyco./mycetaceae - ps23
Saccharomycetaceae - ps24
Saccharomycetaceae_2 - ps25
Saccharomycetaceae_3 - ps26
Kaz./Nau./Saccharomyces - ps27
Saccharomyces - ps28
Arboricola_clade - ps29
Cerevisiae_clade - ps30
Saccharomyces_cerevisiae - ps31
Phylostratum

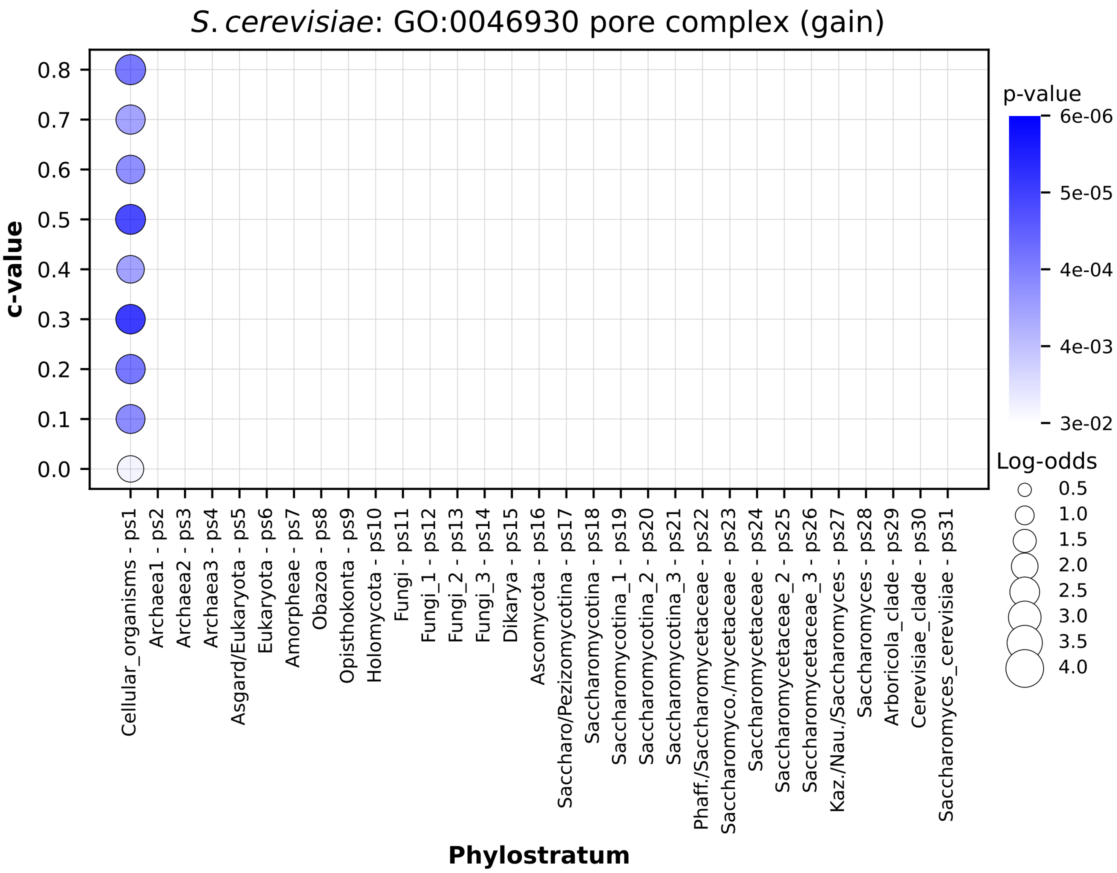

S. cerevisiae: GO:0046930 pore complex (gain)
c-value
0.8
0.7
0.6
0.5
0.4
0.3
0.2
0.1
0.0
p-value
6e-06
5e-05
4e-04
4e-03
3e-02
Log-odds
0.5
1.0
1.5
2.0
2.5
3.0
3.5
4.0
Cellular_organisms - ps1
Archaea1 - ps2
Archaea2 - ps3
Archaea3 - ps4
Asgard/Eukaryota - ps5
Eukaryota - ps6
Amorpheae - ps7
Obazoa - ps8
Opisthokonta - ps9
Holomycota - ps10
Fungi - ps11
Fungi_1 - ps12
Fungi_2 - ps13
Fungi_3 - ps14
Dikarya - ps15
Ascomycota - ps16
Saccharo/Pezizomycotina - ps17
Saccharomycotina - ps18
Saccharomycotina_1 - ps19
Saccharomycotina_2 - ps20
Saccharomycotina_3 - ps21
Phaff./Saccharomycetaceae - ps22
Saccharomyco./mycetaceae - ps23
Saccharomycetaceae - ps24
Saccharomycetaceae_2 - ps25
Saccharomycetaceae_3 - ps26
Kaz./Nau./Saccharomyces - ps27
Saccharomyces - ps28
Arboricola_clade - ps29
Cerevisiae_clade - ps30
Saccharomyces_cerevisiae - ps31
Phylostratum

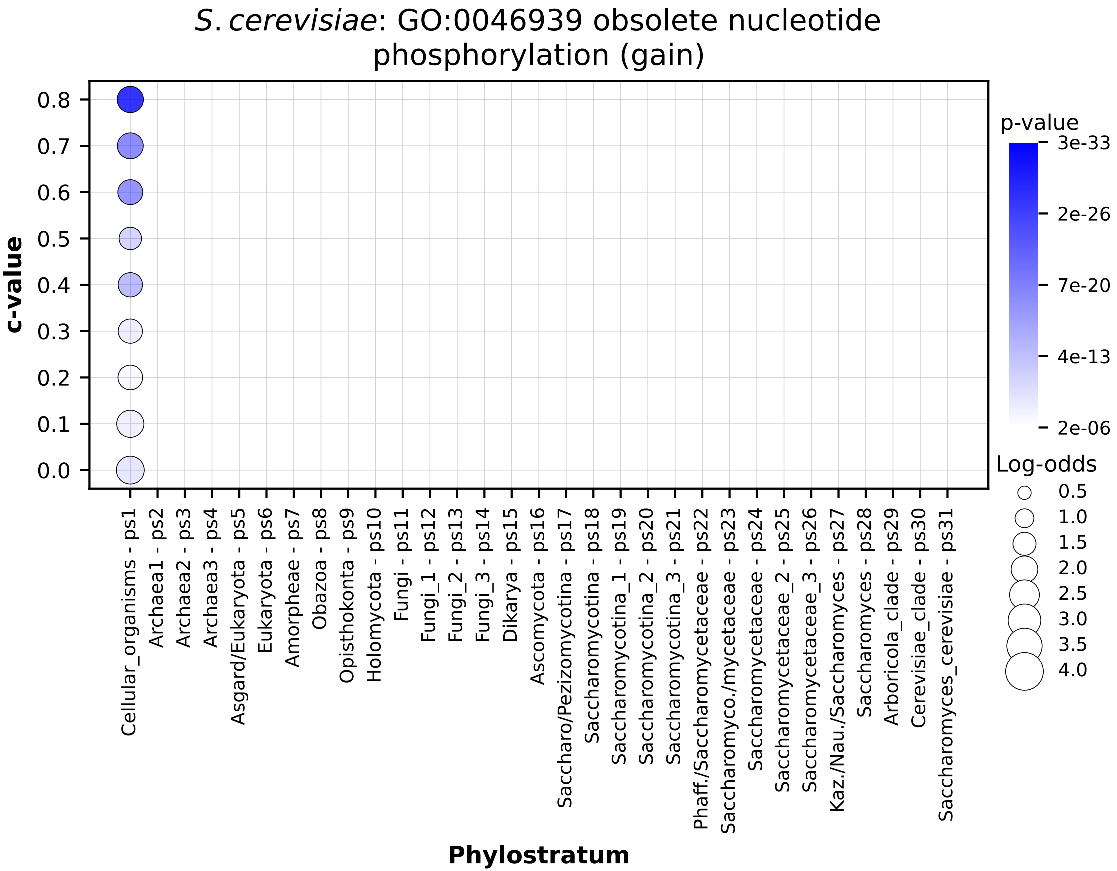
S. cerevisiae: GO:0046939 obsolete nucleotide phosphorylation (gain)
c-value
0.8
0.7
0.6
0.5
0.4
0.3
0.2
0.1
0.0
p-value
3e-33
2e-26
7e-20
4e-13
2e-06
Log-odds
0.5
1.0
1.5
2.0
2.5
3.0
3.5
4.0
Cellular_organisms - ps1
Archaea1 - ps2
Archaea2 - ps3
Archaea3 - ps4
Asgard/Eukaryota - ps5
Eukaryota - ps6
Amorpheae - ps7
Obazoa - ps8
Opisthokonta - ps9
Holomycota - ps10
Fungi - ps11
Fungi_1 - ps12
Fungi_2 - ps13
Fungi_3 - ps14
Dikarya - ps15
Ascomycota - ps16
Saccharo/Pezizomycotina - ps17
Saccharomycotina - ps18
Saccharomycotina_1 - ps19
Saccharomycotina_2 - ps20
Saccharomycotina_3 - ps21
Phaff./Saccharomycetaceae - ps22
Saccharomyco./mycetaceae - ps23
Saccharomycetaceae - ps24
Saccharomycetaceae_2 - ps25
Saccharomycetaceae_3 - ps26
Kaz./Nau./Saccharomyces - ps27
Saccharomyces - ps28
Arboricola_clade - ps29
Cerevisiae_clade - ps30
Saccharomyces_cerevisiae - ps31
Phylostratum

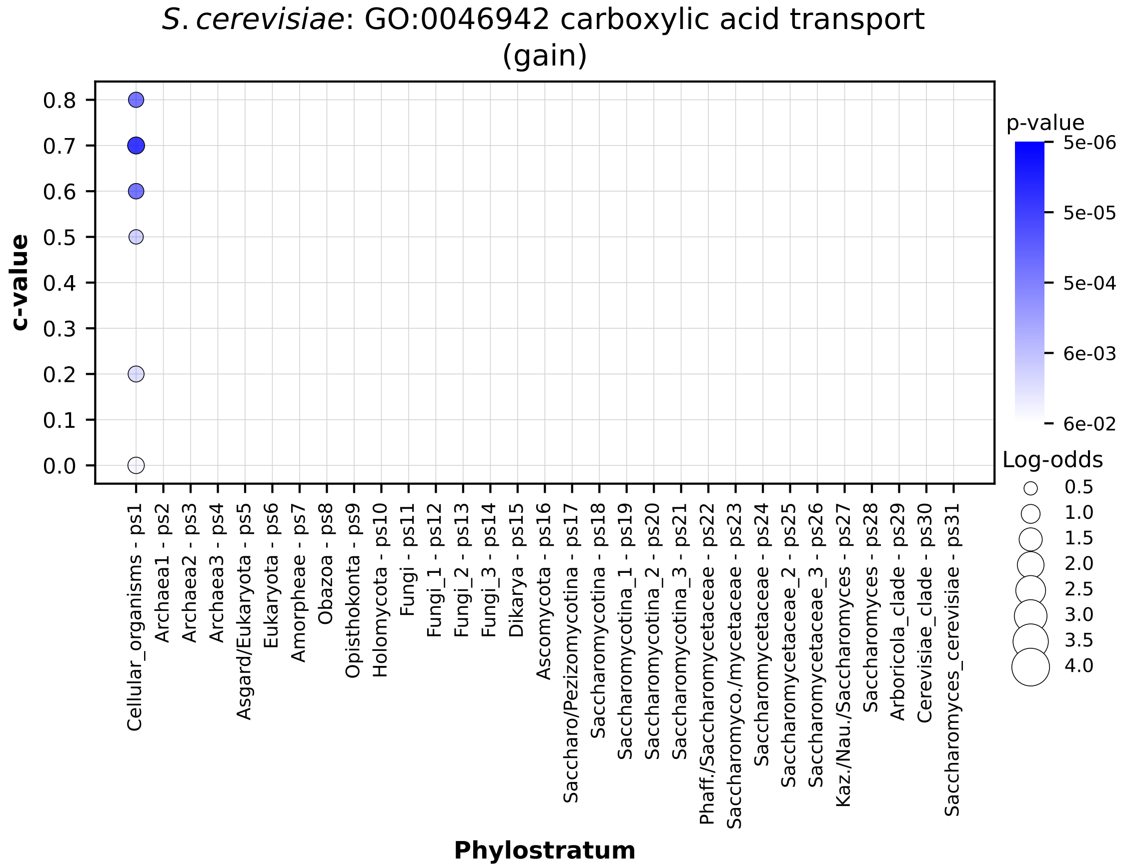

S. cerevisiae: GO:0046942 carboxylic acid transport
(gain)
c-value
0.8
0.7
0.6
0.5
0.4
0.3
0.2
0.1
0.0
Phylostratum
Cellular_organisms - ps1
Archaea1 - ps2
Archaea2 - ps3
Archaea3 - ps4
Asgard/Eukaryota - ps5
Eukaryota - ps6
Amorpheae - ps7
Obazoa - ps8
Opisthokonta - ps9
Holomycota - ps10
Fungi - ps11
Fungi_1 - ps12
Fungi_2 - ps13
Fungi_3 - ps14
Dikarya - ps15
Ascomycota - ps16
Saccharo/Pezizomycotina - ps17
Saccharomycotina - ps18
Saccharomycotina_1 - ps19
Saccharomycotina_2 - ps20
Saccharomycotina_3 - ps21
Phaff./Saccharomycetaceae - ps22
Saccharomyco./mycetaceae - ps23
Saccharomycetaceae - ps24
Saccharomycetaceae_2 - ps25
Saccharomycetaceae_3 - ps26
Kaz./Nau./Saccharomyces - ps27
Saccharomyces - ps28
Arboricola_clade - ps29
Cerevisiae_clade - ps30
Saccharomyces_cerevisiae - ps31
p-value
5e-06
5e-05
5e-04
6e-03
6e-02
Log-odds
0.5
1.0
1.5
2.0
2.5
3.0
3.5
4.0

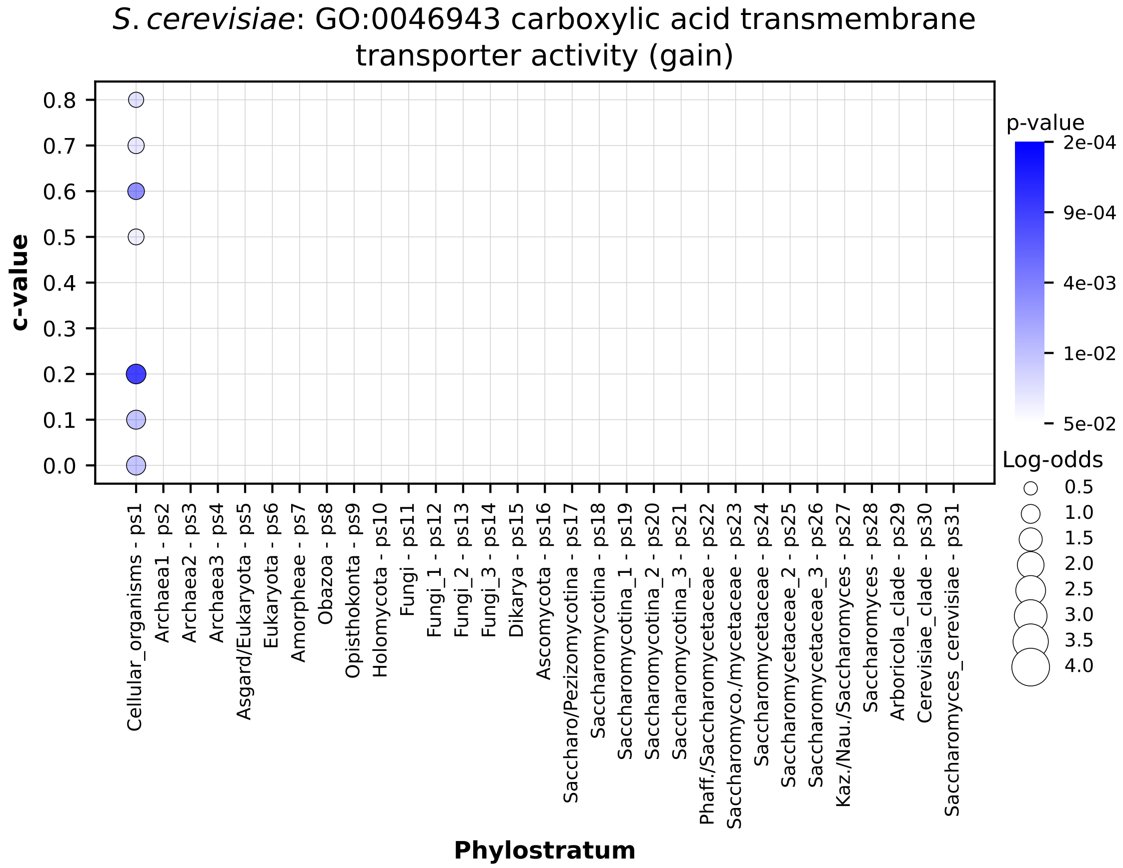
S. cerevisiae: GO:0046943 carboxylic acid transmembrane transporter activity (gain)
c-value
0.8
0.7
0.6
0.5
0.4
0.3
0.2
0.1
0.0
p-value
2e-04
9e-04
4e-03
1e-02
5e-02
Log-odds
0.5
1.0
1.5
2.0
2.5
3.0
3.5
4.0
Cellular_organisms - ps1
Archaea1 - ps2
Archaea2 - ps3
Archaea3 - ps4
Asgard/Eukaryota - ps5
Eukaryota - ps6
Amorpheae - ps7
Obazoa - ps8
Opisthokonta - ps9
Holomycota - ps10
Fungi - ps11
Fungi_1 - ps12
Fungi_2 - ps13
Fungi_3 - ps14
Dikarya - ps15
Ascomycota - ps16
Saccharo/Pezizomycotina - ps17
Saccharomycotina - ps18
Saccharomycotina_1 - ps19
Saccharomycotina_2 - ps20
Saccharomycotina_3 - ps21
Phaff./Saccharomycetaceae - ps22
Saccharomyco./mycetaceae - ps23
Saccharomycetaceae - ps24
Saccharomycetaceae_2 - ps25
Saccharomycetaceae_3 - ps26
Kaz./Nau./Saccharomyces - ps27
Saccharomyces - ps28
Arboricola_clade - ps29
Cerevisiae_clade - ps30
Saccharomyces_cerevisiae - ps31
Phylostratum

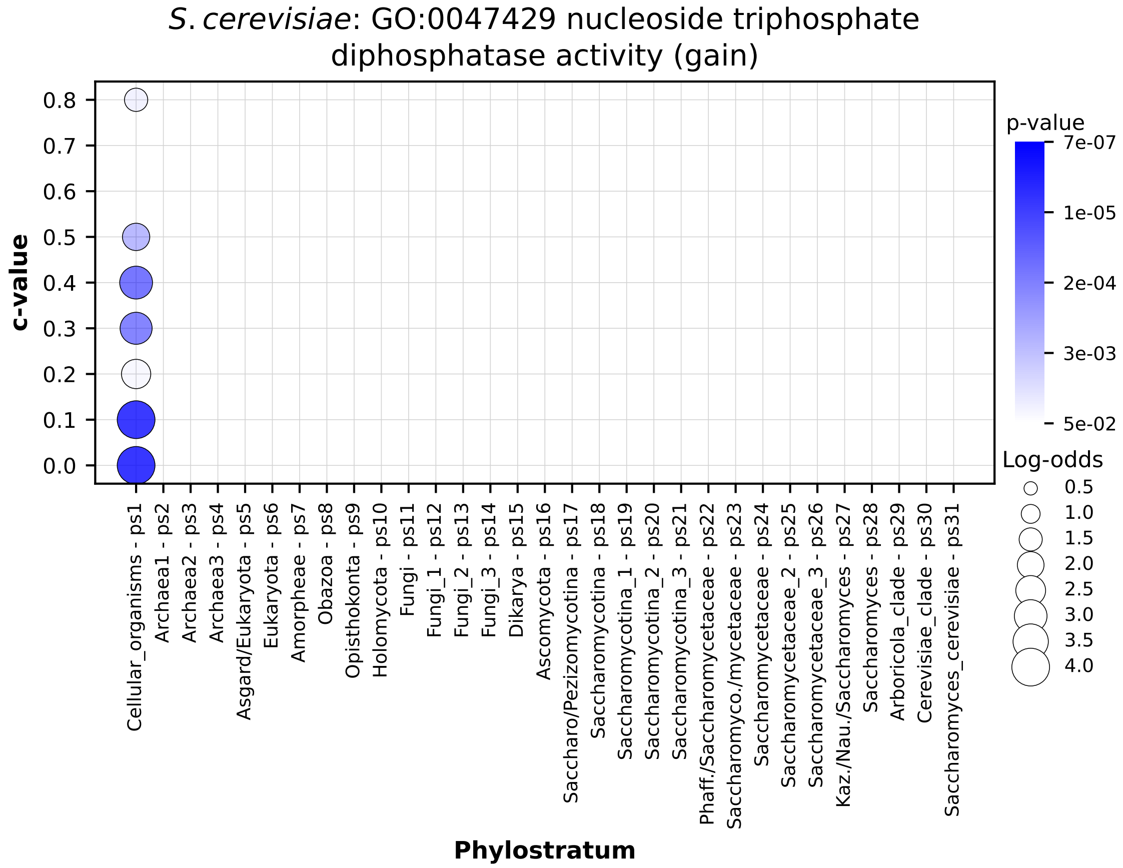

S. cerevisiae: GO:0047429 nucleoside triphosphate diphosphatase activity (gain)
c-value
0.8
0.7
0.6
0.5
0.4
0.3
0.2
0.1
0.0
p-value
7e-07
1e-05
2e-04
3e-03
5e-02
Log-odds
0.5
1.0
1.5
2.0
2.5
3.0
3.5
4.0
Cellular_organisms - ps1
Archaea1 - ps2
Archaea2 - ps3
Archaea3 - ps4
Asgard/Eukaryota - ps5
Eukaryota - ps6
Amorpheae - ps7
Obazoa - ps8
Opisthokonta - ps9
Holomycota - ps10
Fungi - ps11
Fungi_1 - ps12
Fungi_2 - ps13
Fungi_3 - ps14
Dikarya - ps15
Ascomycota - ps16
Saccharo/Pezizomycotina - ps17
Saccharomycotina - ps18
Saccharomycotina_1 - ps19
Saccharomycotina_2 - ps20
Saccharomycotina_3 - ps21
Phaff./Saccharomycetaceae - ps22
Saccharomyco./mycetaceae - ps23
Saccharomycetaceae - ps24
Saccharomycetaceae_2 - ps25
Saccharomycetaceae_3 - ps26
Kaz./Nau./Saccharomyces - ps27
Saccharomyces - ps28
Arboricola_clade - ps29
Cerevisiae_clade - ps30
Saccharomyces_cerevisiae - ps31
Phylostratum

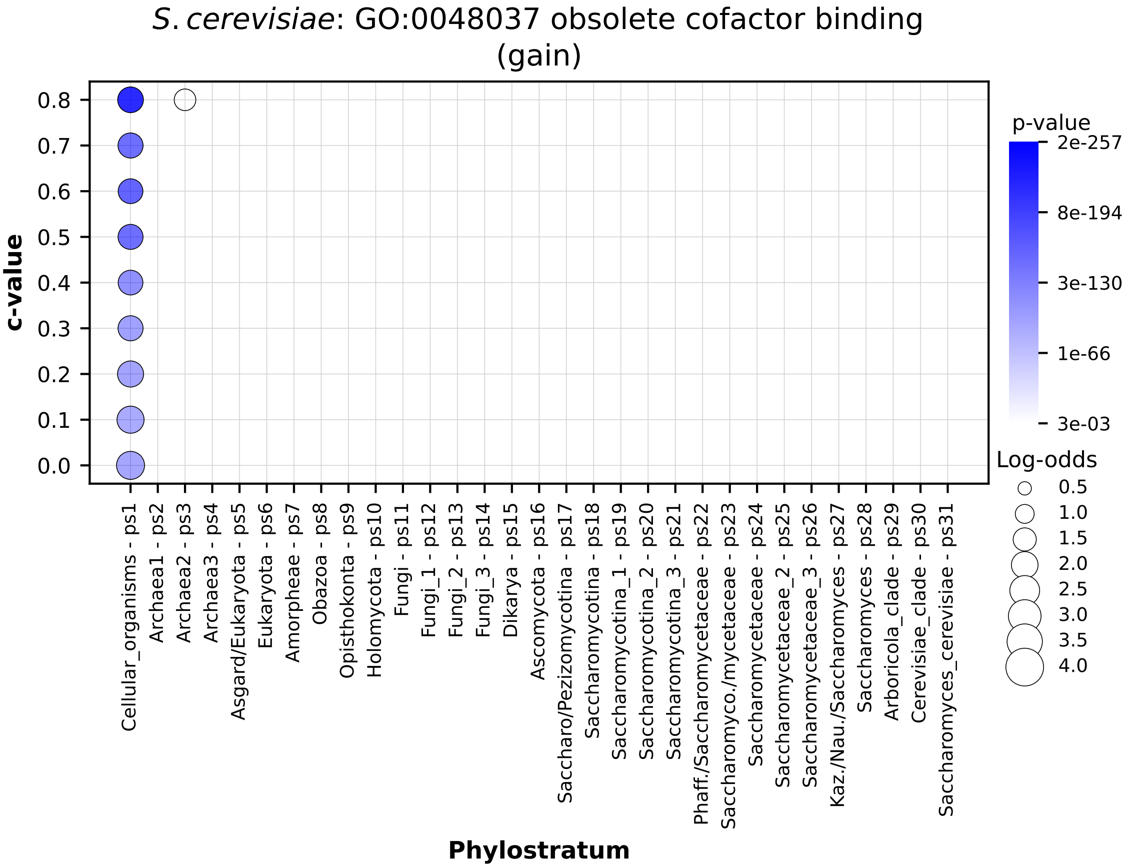

S. cerevisiae: GO:0048037 obsolete cofactor binding (gain)
c-value
0.8
0.7
0.6
0.5
0.4
0.3
0.2
0.1
0.0
p-value
2e-257
8e-194
3e-130
1e-66
3e-03
Log-odds
0.5
1.0
1.5
2.0
2.5
3.0
3.5
4.0
Cellular_organisms - ps1
Archaea1 - ps2
Archaea2 - ps3
Archaea3 - ps4
Asgard/Eukaryota - ps5
Eukaryota - ps6
Amorpheae - ps7
Obazoa - ps8
Opisthokonta - ps9
Holomycota - ps10
Fungi - ps11
Fungi_1 - ps12
Fungi_2 - ps13
Fungi_3 - ps14
Dikarya - ps15
Ascomycota - ps16
Saccharo/Pezizomycotina - ps17
Saccharomycotina - ps18
Saccharomycotina_1 - ps19
Saccharomycotina_2 - ps20
Saccharomycotina_3 - ps21
Phaff./Saccharomycetaceae - ps22
Saccharomyco./mycetaceae - ps23
Saccharomycetaceae - ps24
Saccharomycetaceae_2 - ps25
Saccharomycetaceae_3 - ps26
Kaz./Nau./Saccharomyces - ps27
Saccharomyces - ps28
Arboricola_clade - ps29
Cerevisiae_clade - ps30
Saccharomyces_cerevisiae - ps31
Phylostratum

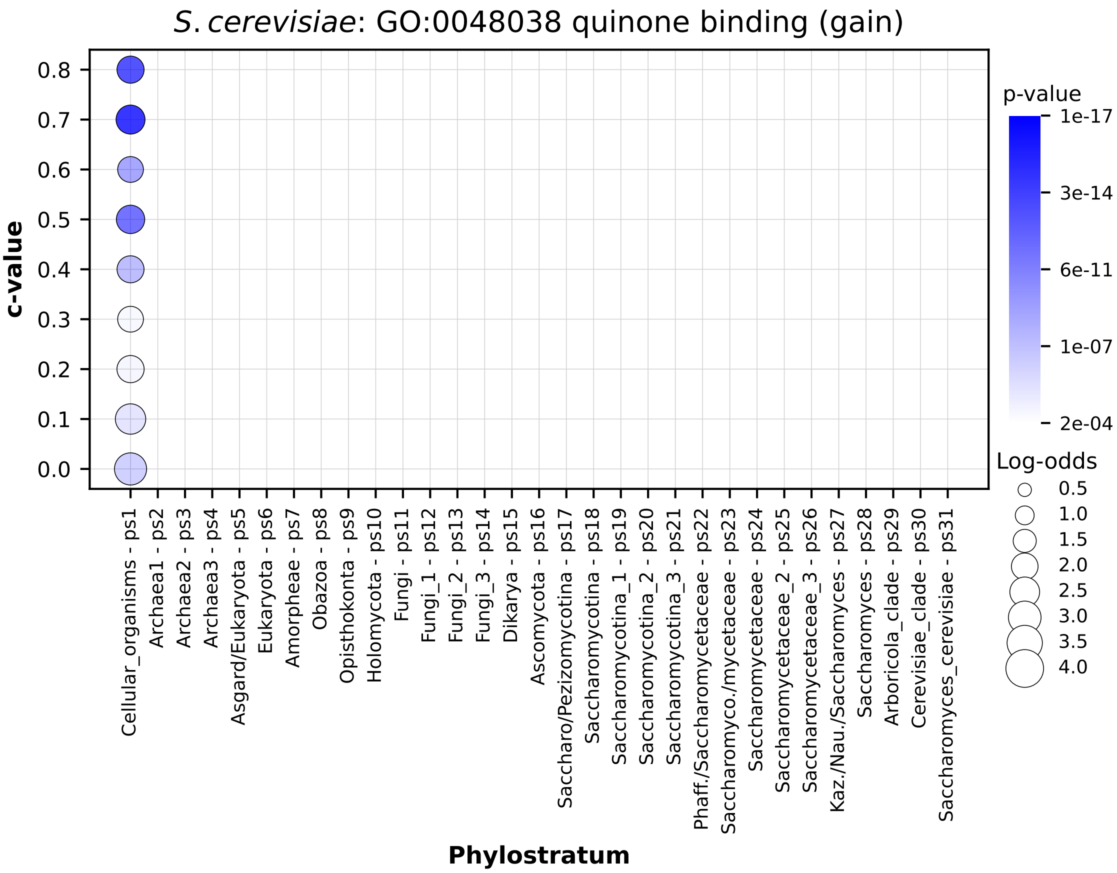

S. cerevisiae: GO:0048038 quinone binding (gain)
c-value
0.0
0.1
0.2
0.3
0.4
0.5
0.6
0.7
0.8
p-value
1e-17
3e-14
6e-11
1e-07
2e-04
Log-odds
0.5
1.0
1.5
2.0
2.5
3.0
3.5
4.0
Cellular_organisms - ps1
Archaea1 - ps2
Archaea2 - ps3
Archaea3 - ps4
Asgard/Eukaryota - ps5
Eukaryota - ps6
Amorpheae - ps7
Obazoa - ps8
Opisthokonta - ps9
Holomycota - ps10
Fungi - ps11
Fungi_1 - ps12
Fungi_2 - ps13
Fungi_3 - ps14
Dikarya - ps15
Ascomycota - ps16
Saccharo/Pezizomycotina - ps17
Saccharomycotina - ps18
Saccharomycotina_1 - ps19
Saccharomycotina_2 - ps20
Saccharomycotina_3 - ps21
Phaff./Saccharomycetaceae - ps22
Saccharomyco./mycetaceae - ps23
Saccharomycetaceae - ps24
Saccharomycetaceae_2 - ps25
Saccharomycetaceae_3 - ps26
Kaz./Nau./Saccharomyces - ps27
Saccharomyces - ps28
Arboricola_clade - ps29
Cerevisiae_clade - ps30
Saccharomyces_cerevisiae - ps31
Phylostratum

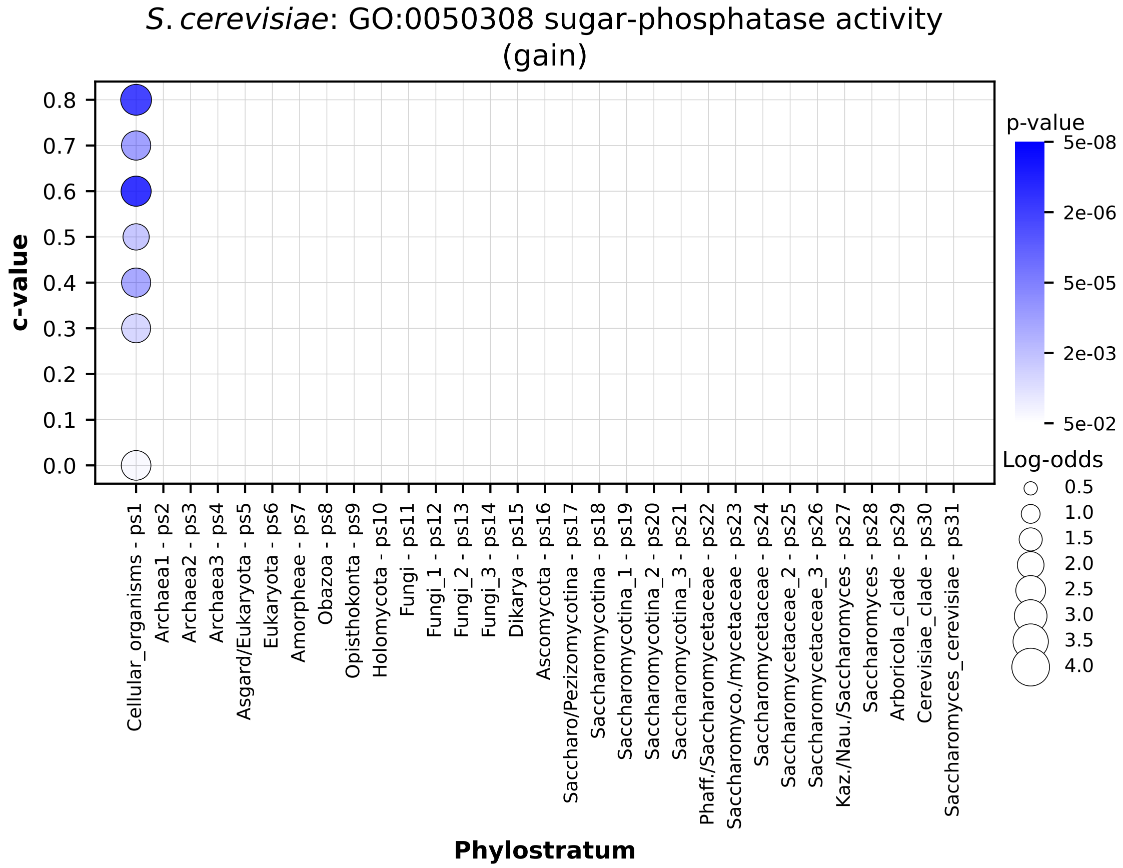

S. cerevisiae: GO:0050308 sugar-phosphatase activity (gain)
c-value
0.8
0.7
0.6
0.5
0.4
0.3
0.2
0.1
0.0
Phylostratum
Cellular_organisms - ps1
Archaea1 - ps2
Archaea2 - ps3
Archaea3 - ps4
Asgard/Eukaryota - ps5
Eukaryota - ps6
Amorpheae - ps7
Obazoa - ps8
Opisthokonta - ps9
Holomycota - ps10
Fungi - ps11
Fungi_1 - ps12
Fungi_2 - ps13
Fungi_3 - ps14
Dikarya - ps15
Ascomycota - ps16
Saccharo/Pezizomycotina - ps17
Saccharomycotina - ps18
Saccharomycotina_1 - ps19
Saccharomycotina_2 - ps20
Saccharomycotina_3 - ps21
Phaff./Saccharomycetaceae - ps22
Saccharomyco./mycetaceae - ps23
Saccharomycetaceae - ps24
Saccharomycetaceae_2 - ps25
Saccharomycetaceae_3 - ps26
Kaz./Nau./Saccharomyces - ps27
Saccharomyces - ps28
Arboricola_clade - ps29
Cerevisiae_clade - ps30
Saccharomyces_cerevisiae - ps31
p-value
5e-08
2e-06
5e-05
2e-03
5e-02
Log-odds
0.5
1.0
1.5
2.0
2.5
3.0
3.5
4.0

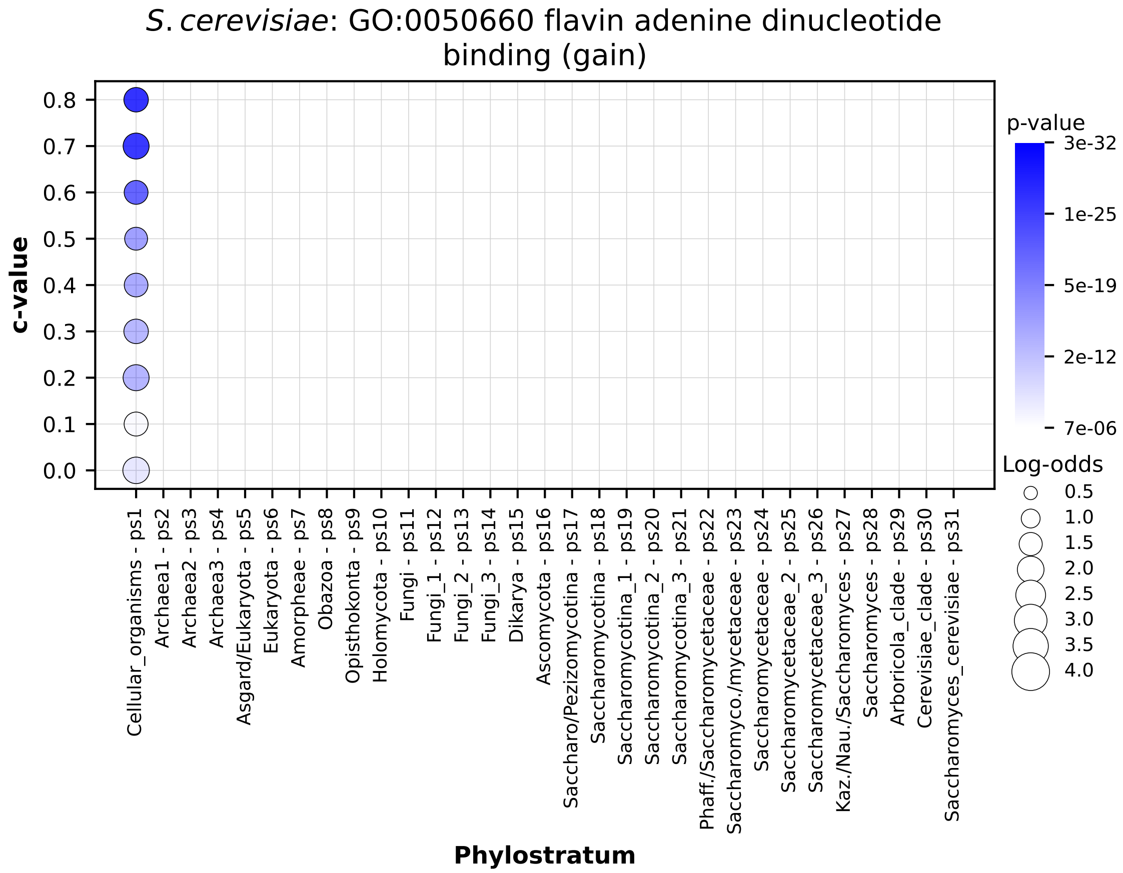
S. cerevisiae: GO:0050660 flavin adenine dinucleotide binding (gain)
c-value
0.8
0.7
0.6
0.5
0.4
0.3
0.2
0.1
0.0
p-value
3e-32
1e-25
5e-19
2e-12
7e-06
Log-odds
0.5
1.0
1.5
2.0
2.5
3.0
3.5
4.0
Cellular_organisms - ps1
Archaea1 - ps2
Archaea2 - ps3
Archaea3 - ps4
Asgard/Eukaryota - ps5
Eukaryota - ps6
Amorpheae - ps7
Obazoa - ps8
Opisthokonta - ps9
Holomycota - ps10
Fungi - ps11
Fungi_1 - ps12
Fungi_2 - ps13
Fungi_3 - ps14
Dikarya - ps15
Ascomycota - ps16
Saccharo/Pezizomycotina - ps17
Saccharomycotina - ps18
Saccharomycotina_1 - ps19
Saccharomycotina_2 - ps20
Saccharomycotina_3 - ps21
Phaff./Saccharomycetaceae - ps22
Saccharomyco./mycetaceae - ps23
Saccharomycetaceae - ps24
Saccharomycetaceae_2 - ps25
Saccharomycetaceae_3 - ps26
Kaz./Nau./Saccharomyces - ps27
Saccharomyces - ps28
Arboricola_clade - ps29
Cerevisiae_clade - ps30
Saccharomyces_cerevisiae - ps31
Phylostratum

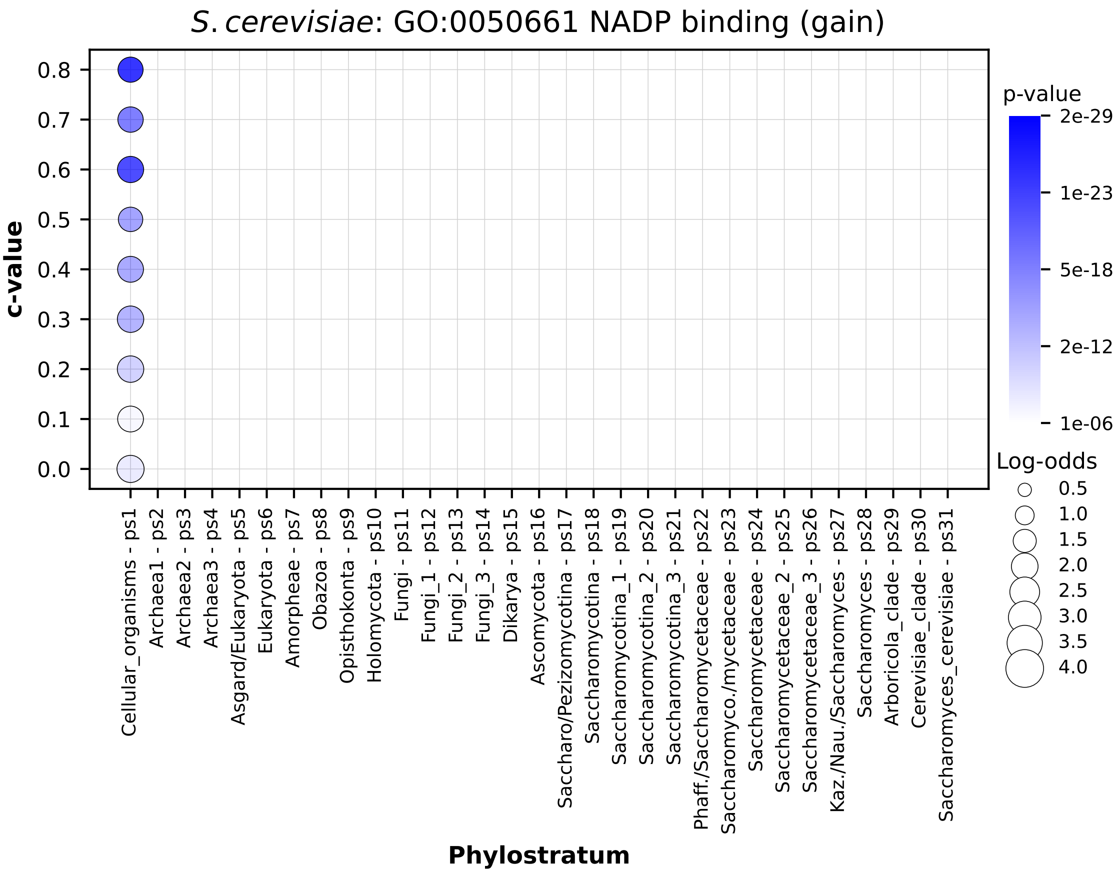

S. cerevisiae: GO:0050661 NADP binding (gain)
c-value
0.0
0.1
0.2
0.3
0.4
0.5
0.6
0.7
0.8
p-value
2e-29
1e-23
5e-18
2e-12
1e-06
Log-odds
0.5
1.0
1.5
2.0
2.5
3.0
3.5
4.0
Cellular_organisms - ps1
Archaea1 - ps2
Archaea2 - ps3
Archaea3 - ps4
Asgard/Eukaryota - ps5
Eukaryota - ps6
Amorpheae - ps7
Obazoa - ps8
Opisthokonta - ps9
Holomycota - ps10
Fungi - ps11
Fungi_1 - ps12
Fungi_2 - ps13
Fungi_3 - ps14
Dikarya - ps15
Ascomycota - ps16
Saccharo/Pezizomycotina - ps17
Saccharomycotina - ps18
Saccharomycotina_1 - ps19
Saccharomycotina_2 - ps20
Saccharomycotina_3 - ps21
Phaff./Saccharomycetaceae - ps22
Saccharomyco./mycetaceae - ps23
Saccharomycetaceae - ps24
Saccharomycetaceae_2 - ps25
Saccharomycetaceae_3 - ps26
Kaz./Nau./Saccharomyces - ps27
Saccharomyces - ps28
Arboricola_clade - ps29
Cerevisiae_clade - ps30
Saccharomyces_cerevisiae - ps31
Phylostratum

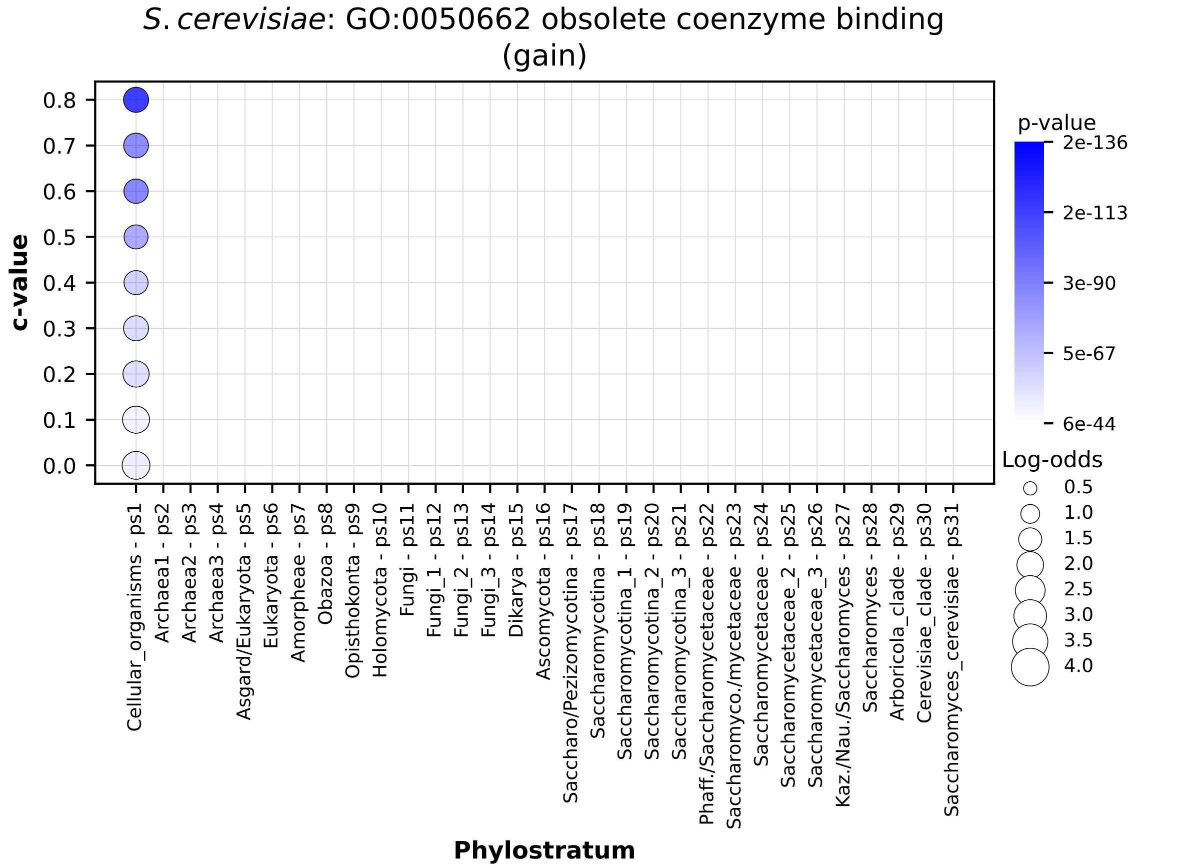

S. cerevisiae: GO:0050662 obsolete coenzyme binding
(gain)
c-value
0.8
0.7
0.6
0.5
0.4
0.3
0.2
0.1
0.0
p-value
2e-136
2e-113
3e-90
5e-67
6e-44
Log-odds
0.5
1.0
1.5
2.0
2.5
3.0
3.5
4.0
Cellular_organisms - ps1
Archaea1 - ps2
Archaea2 - ps3
Archaea3 - ps4
Asgard/Eukaryota - ps5
Eukaryota - ps6
Amorpheae - ps7
Obazoa - ps8
Opisthokonta - ps9
Holomycota - ps10
Fungi - ps11
Fungi_1 - ps12
Fungi_2 - ps13
Fungi_3 - ps14
Dikarya - ps15
Ascomycota - ps16
Saccharo/Pezizomycotina - ps17
Saccharomycotina - ps18
Saccharomycotina_1 - ps19
Saccharomycotina_2 - ps20
Saccharomycotina_3 - ps21
Phaff./Saccharomycetaceae - ps22
Saccharomyco./mycetaceae - ps23
Saccharomycetaceae - ps24
Saccharomycetaceae_2 - ps25
Saccharomycetaceae_3 - ps26
Kaz./Nau./Saccharomyces - ps27
Saccharomyces - ps28
Arboricola_clade - ps29
Cerevisiae_clade - ps30
Saccharomyces_cerevisiae - ps31
Phylostratum

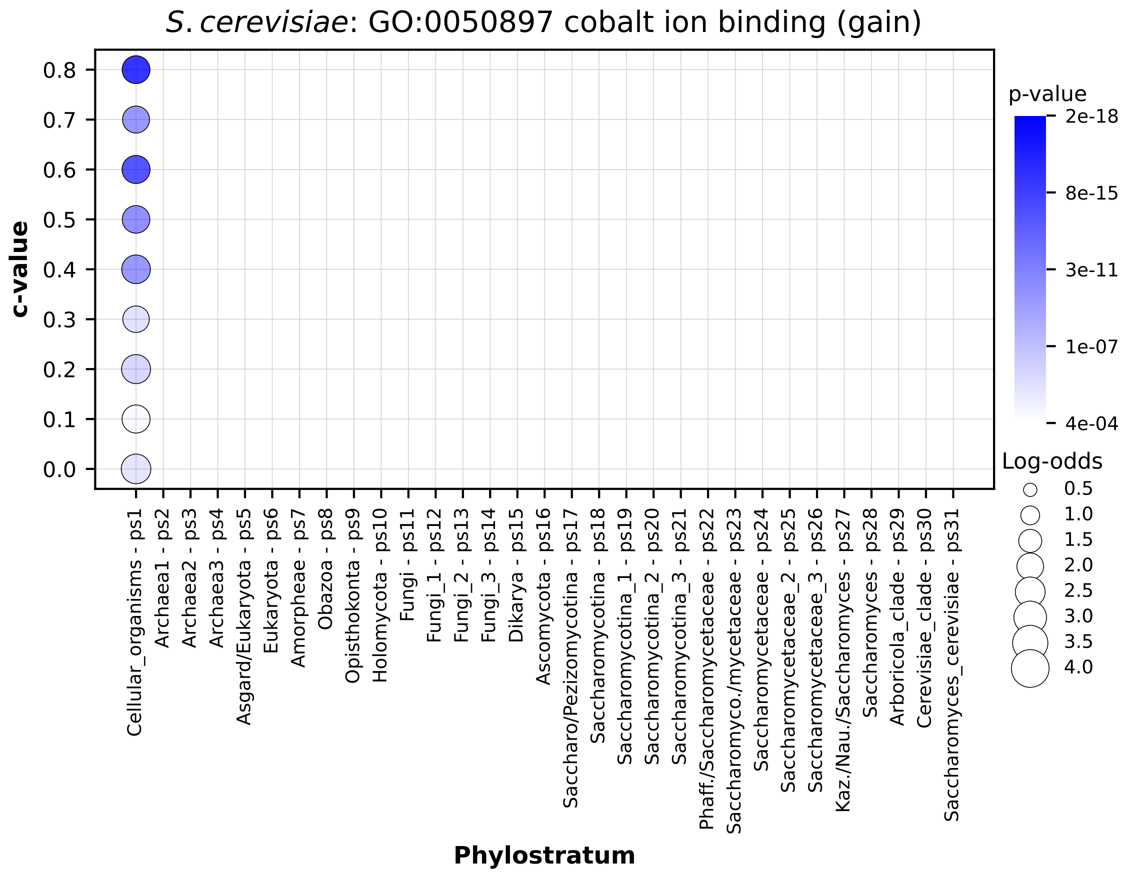
S. cerevisiae: GO:0050897 cobalt ion binding (gain)
c-value
0.8
0.7
0.6
0.5
0.4
0.3
0.2
0.1
0.0
p-value
2e-18
8e-15
3e-11
1e-07
4e-04
Log-odds
0.5
1.0
1.5
2.0
2.5
3.0
3.5
4.0
Cellular_organisms - ps1
Archaea1 - ps2
Archaea2 - ps3
Archaea3 - ps4
Asgard/Eukaryota - ps5
Eukaryota - ps6
Amorpheae - ps7
Obazoa - ps8
Opisthokonta - ps9
Holomycota - ps10
Fungi - ps11
Fungi_1 - ps12
Fungi_2 - ps13
Fungi_3 - ps14
Dikarya - ps15
Ascomycota - ps16
Saccharo/Pezizomycotina - ps17
Saccharomycotina - ps18
Saccharomycotina_1 - ps19
Saccharomycotina_2 - ps20
Saccharomycotina_3 - ps21
Phaff./Saccharomycetaceae - ps22
Saccharomyco./mycetaceae - ps23
Saccharomycetaceae - ps24
Saccharomycetaceae_2 - ps25
Saccharomycetaceae_3 - ps26
Kaz./Nau./Saccharomyces - ps27
Saccharomyces - ps28
Arboricola_clade - ps29
Cerevisiae_clade - ps30
Saccharomyces_cerevisiae - ps31
Phylostratum

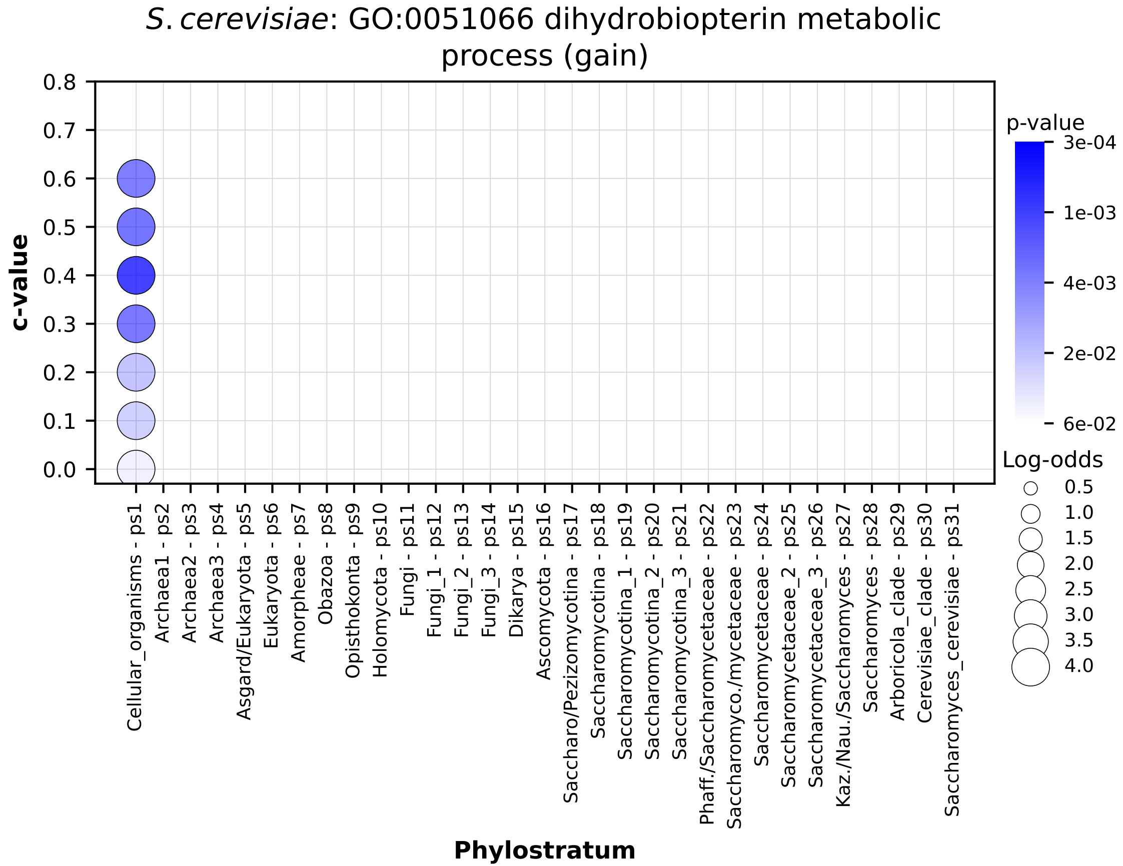

S. cerevisiae: GO:0051066 dihydrobiopterin metabolic process (gain)
c-value
0.8
0.7
0.6
0.5
0.4
0.3
0.2
0.1
0.0
p-value
3e-04
1e-03
4e-03
2e-02
6e-02
Log-odds
0.5
1.0
1.5
2.0
2.5
3.0
3.5
4.0
Cellular_organisms - ps1
Archaea1 - ps2
Archaea2 - ps3
Archaea3 - ps4
Asgard/Eukaryota - ps5
Eukaryota - ps6
Amorpheae - ps7
Obazoa - ps8
Opisthokonta - ps9
Holomycota - ps10
Fungi - ps11
Fungi_1 - ps12
Fungi_2 - ps13
Fungi_3 - ps14
Dikarya - ps15
Ascomycota - ps16
Saccharo/Pezizomycotina - ps17
Saccharomycotina - ps18
Saccharomycotina_1 - ps19
Saccharomycotina_2 - ps20
Saccharomycotina_3 - ps21
Phaff./Saccharomycetaceae - ps22
Saccharomyco./mycetaceae - ps23
Saccharomycetaceae - ps24
Saccharomycetaceae_2 - ps25
Saccharomycetaceae_3 - ps26
Kaz./Nau./Saccharomyces - ps27
Saccharomyces - ps28
Arboricola_clade - ps29
Cerevisiae_clade - ps30
Saccharomyces_cerevisiae - ps31
Phylostratum

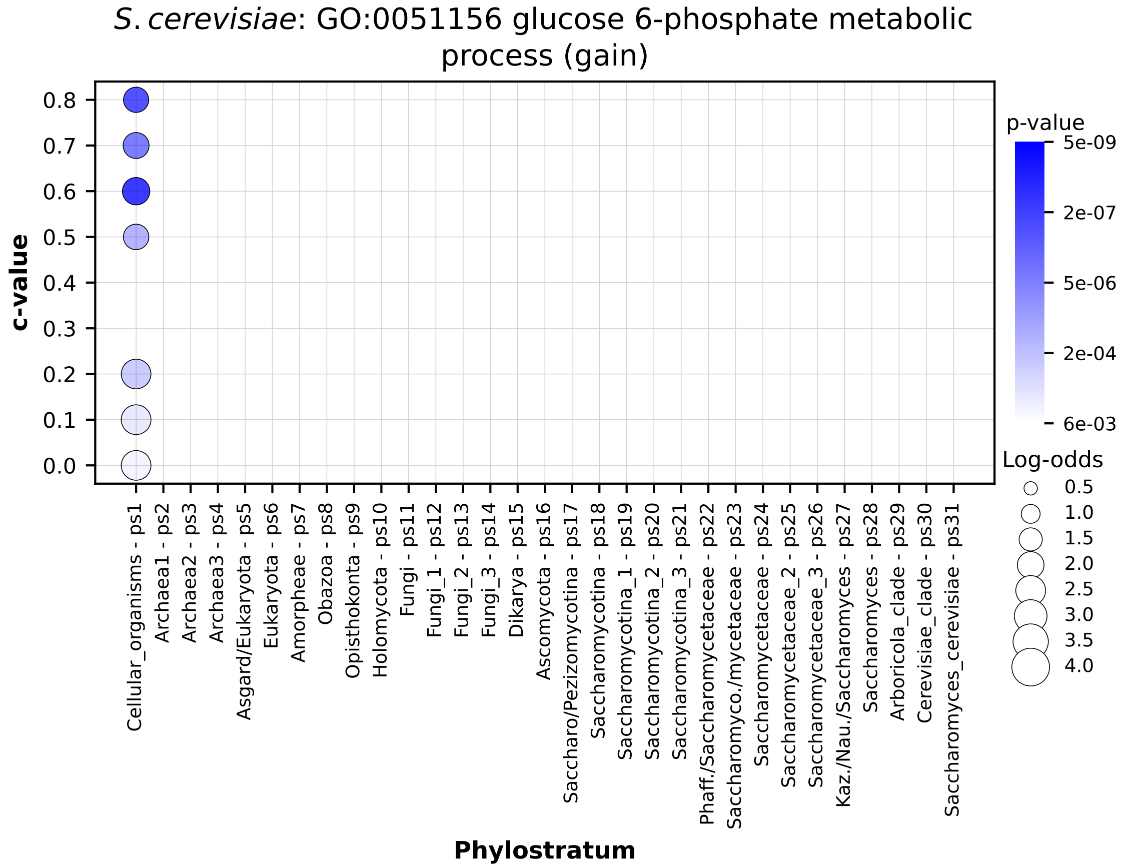
S. cerevisiae: GO:0051156 glucose 6-phosphate metabolic process (gain)
c-value
0.8
0.7
0.6
0.5
0.4
0.3
0.2
0.1
0.0
p-value
5e-09
2e-07
5e-06
2e-04
6e-03
Log-odds
0.5
1.0
1.5
2.0
2.5
3.0
3.5
4.0
Cellular_organisms - ps1
Archaea1 - ps2
Archaea2 - ps3
Archaea3 - ps4
Asgard/Eukaryota - ps5
Eukaryota - ps6
Amorpheae - ps7
Obazoa - ps8
Opisthokonta - ps9
Holomycota - ps10
Fungi - ps11
Fungi_1 - ps12
Fungi_2 - ps13
Fungi_3 - ps14
Dikarya - ps15
Ascomycota - ps16
Saccharo/Pezizomycotina - ps17
Saccharomycotina - ps18
Saccharomycotina_1 - ps19
Saccharomycotina_2 - ps20
Saccharomycotina_3 - ps21
Phaff./Saccharomycetaceae - ps22
Saccharomyco./mycetaceae - ps23
Saccharomycetaceae - ps24
Saccharomycetaceae_2 - ps25
Saccharomycetaceae_3 - ps26
Kaz./Nau./Saccharomyces - ps27
Saccharomyces - ps28
Arboricola_clade - ps29
Cerevisiae_clade - ps30
Saccharomyces_cerevisiae - ps31
Phylostratum

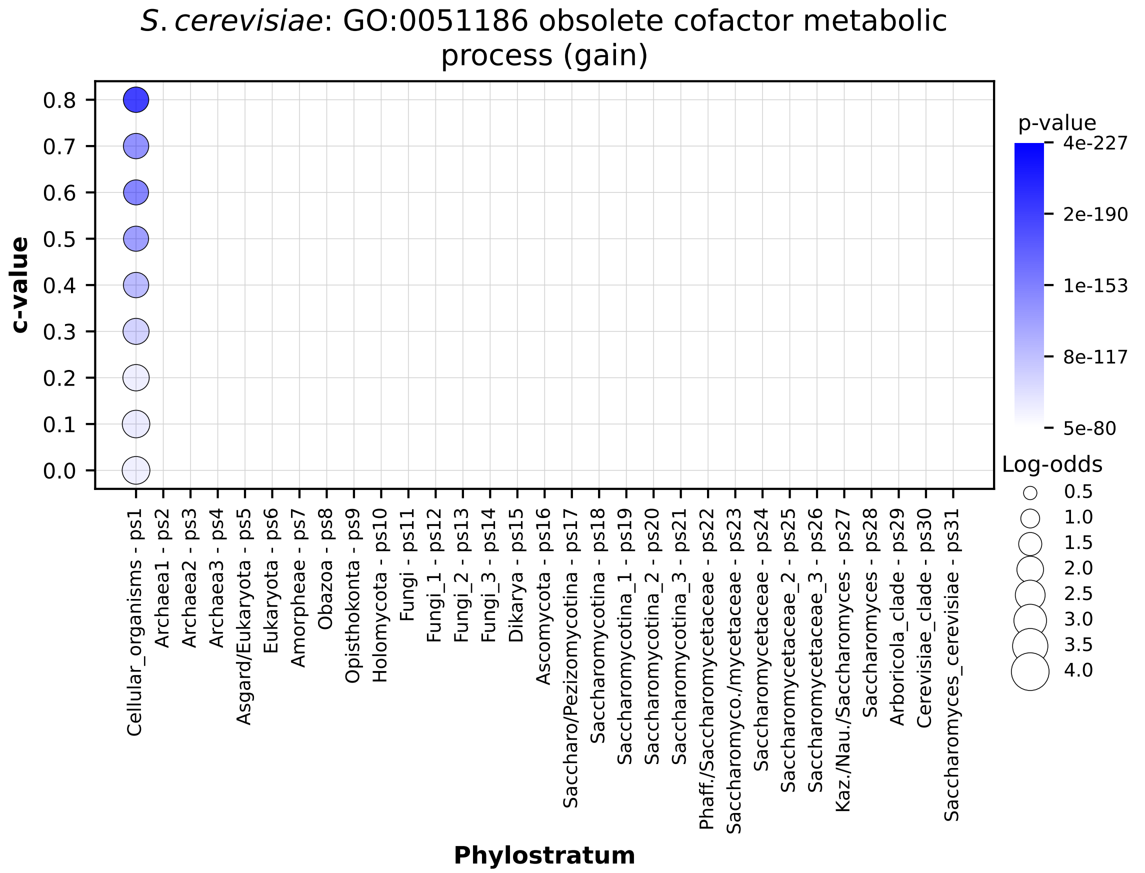

S. cerevisiae: GO:0051186 obsolete cofactor metabolic process (gain)
c-value
0.0
0.1
0.2
0.3
0.4
0.5
0.6
0.7
0.8
Phylostratum
Cellular_organisms - ps1
Archaea1 - ps2
Archaea2 - ps3
Archaea3 - ps4
Asgard/Eukaryota - ps5
Eukaryota - ps6
Amorpheae - ps7
Obazoa - ps8
Opisthokonta - ps9
Holomycota - ps10
Fungi - ps11
Fungi_1 - ps12
Fungi_2 - ps13
Fungi_3 - ps14
Dikarya - ps15
Ascomycota - ps16
Saccharo/Pezizomycotina - ps17
Saccharomycotina - ps18
Saccharomycotina_1 - ps19
Saccharomycotina_2 - ps20
Saccharomycotina_3 - ps21
Phaff./Saccharomycetaceae - ps22
Saccharomyco./mycetaceae - ps23
Saccharomycetaceae - ps24
Saccharomycetaceae_2 - ps25
Saccharomycetaceae_3 - ps26
Kaz./Nau./Saccharomyces - ps27
Saccharomyces - ps28
Arboricola_clade - ps29
Cerevisiae_clade - ps30
Saccharomyces_cerevisiae - ps31
p-value
4e-227
2e-190
1e-153
8e-117
5e-80
Log-odds
0.5
1.0
1.5
2.0
2.5
3.0
3.5
4.0

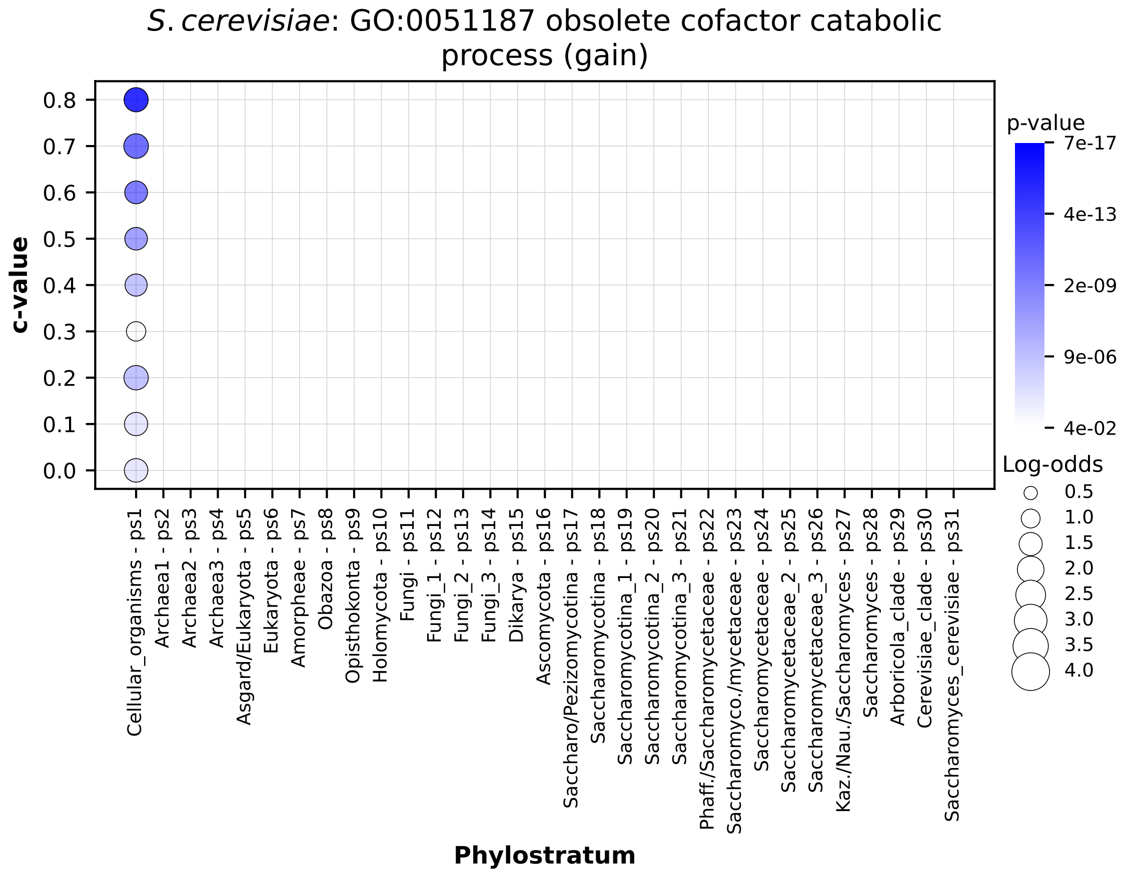

S. cerevisiae: GO:0051187 obsolete cofactor catabolic process (gain)
c-value
0.8
0.7
0.6
0.5
0.4
0.3
0.2
0.1
0.0
p-value
7e-17
4e-13
2e-09
9e-06
4e-02
Log-odds
0.5
1.0
1.5
2.0
2.5
3.0
3.5
4.0
Cellular_organisms - ps1
Archaea1 - ps2
Archaea2 - ps3
Archaea3 - ps4
Asgard/Eukaryota - ps5
Eukaryota - ps6
Amorpheae - ps7
Obazoa - ps8
Opisthokonta - ps9
Holomycota - ps10
Fungi - ps11
Fungi_1 - ps12
Fungi_2 - ps13
Fungi_3 - ps14
Dikarya - ps15
Ascomycota - ps16
Saccharo/Pezizomycotina - ps17
Saccharomycotina - ps18
Saccharomycotina_1 - ps19
Saccharomycotina_2 - ps20
Saccharomycotina_3 - ps21
Phaff./Saccharomycetaceae - ps22
Saccharomyco./mycetaceae - ps23
Saccharomycetaceae - ps24
Saccharomycetaceae_2 - ps25
Saccharomycetaceae_3 - ps26
Kaz./Nau./Saccharomyces - ps27
Saccharomyces - ps28
Arboricola_clade - ps29
Cerevisiae_clade - ps30
Saccharomyces_cerevisiae - ps31
Phylostratum

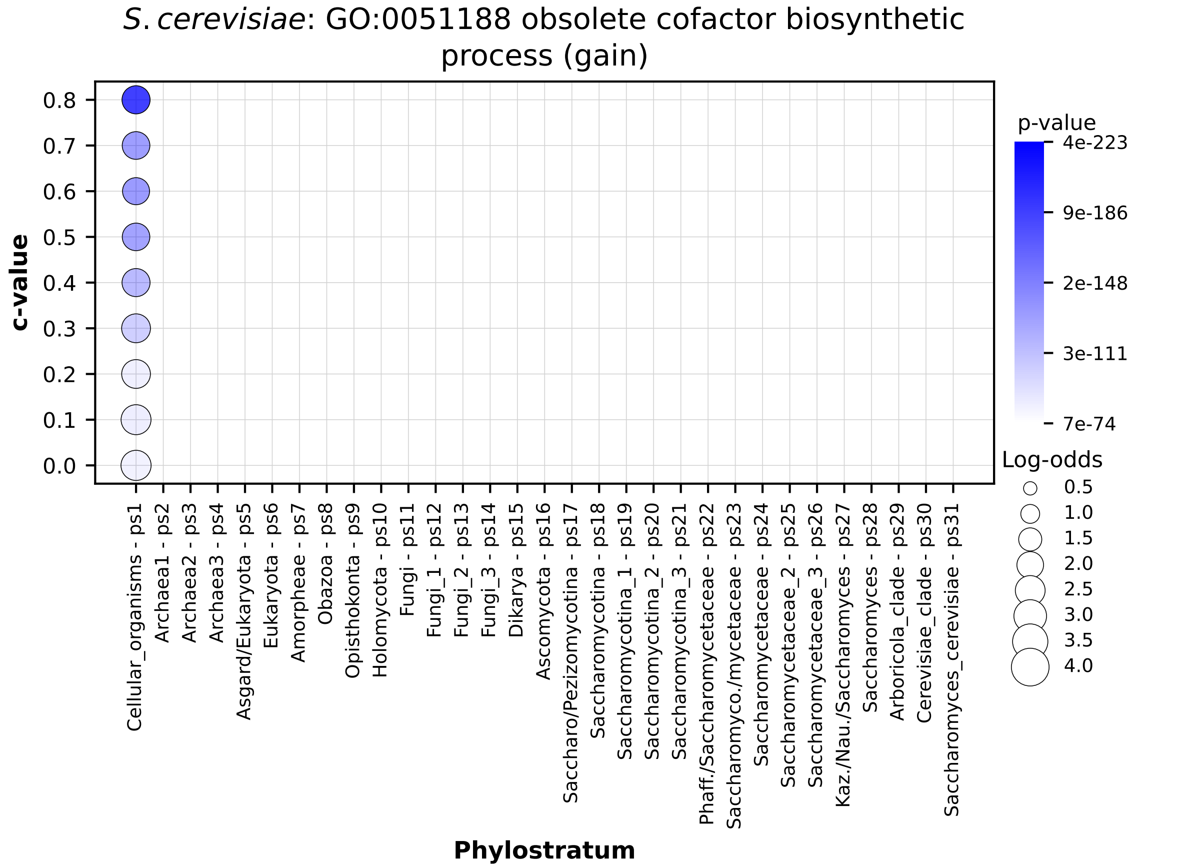
S. cerevisiae: GO:0051188 obsolete cofactor biosynthetic process (gain)
c-value
0.8
0.7
0.6
0.5
0.4
0.3
0.2
0.1
0.0
p-value
4e-223
9e-186
2e-148
3e-111
7e-74
Log-odds
0.5
1.0
1.5
2.0
2.5
3.0
3.5
4.0
Cellular_organisms - ps1
Archaea1 - ps2
Archaea2 - ps3
Archaea3 - ps4
Asgard/Eukaryota - ps5
Eukaryota - ps6
Amorpheae - ps7
Obazoa - ps8
Opisthokonta - ps9
Holomycota - ps10
Fungi - ps11
Fungi_1 - ps12
Fungi_2 - ps13
Fungi_3 - ps14
Dikarya - ps15
Ascomycota - ps16
Saccharo/Pezizomycotina - ps17
Saccharomycotina - ps18
Saccharomycotina_1 - ps19
Saccharomycotina_2 - ps20
Saccharomycotina_3 - ps21
Phaff./Saccharomycetaceae - ps22
Saccharomyco./mycetaceae - ps23
Saccharomycetaceae - ps24
Saccharomycetaceae_2 - ps25
Saccharomycetaceae_3 - ps26
Kaz./Nau./Saccharomyces - ps27
Saccharomyces - ps28
Arboricola_clade - ps29
Cerevisiae_clade - ps30
Saccharomyces_cerevisiae - ps31
Phylostratum

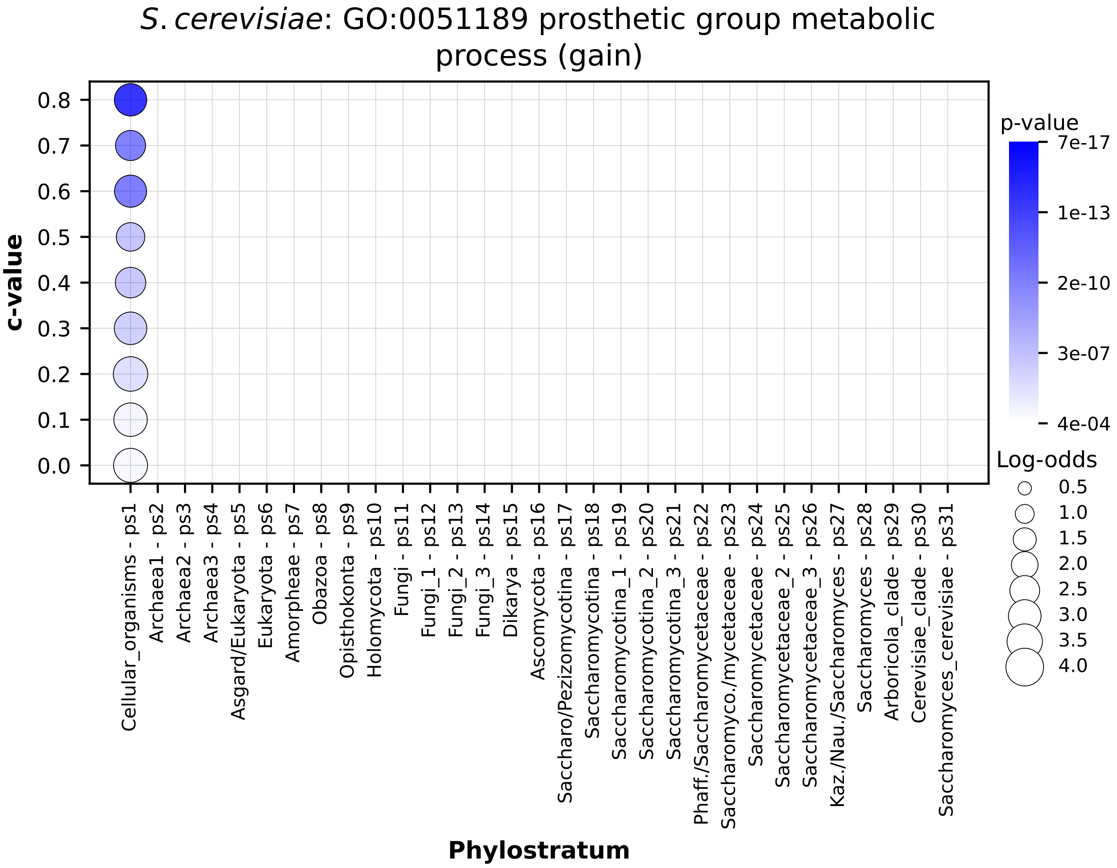
S. cerevisiae: GO:0051189 prosthetic group metabolic process (gain)
c-value
0.8
0.7
0.6
0.5
0.4
0.3
0.2
0.1
0.0
p-value
7e-17
1e-13
2e-10
3e-07
4e-04
Log-odds
0.5
1.0
1.5
2.0
2.5
3.0
3.5
4.0
Cellular_organisms - ps1
Archaea1 - ps2
Archaea2 - ps3
Archaea3 - ps4
Asgard/Eukaryota - ps5
Eukaryota - ps6
Amorpheae - ps7
Obazoa - ps8
Opisthokonta - ps9
Holomycota - ps10
Fungi - ps11
Fungi_1 - ps12
Fungi_2 - ps13
Fungi_3 - ps14
Dikarya - ps15
Ascomycota - ps16
Saccharo/Pezizomycotina - ps17
Saccharomycotina - ps18
Saccharomycotina_1 - ps19
Saccharomycotina_2 - ps20
Saccharomycotina_3 - ps21
Phaff./Saccharomycetaceae - ps22
Saccharomyco./mycetaceae - ps23
Saccharomycetaceae - ps24
Saccharomycetaceae_2 - ps25
Saccharomycetaceae_3 - ps26
Kaz./Nau./Saccharomyces - ps27
Saccharomyces - ps28
Arboricola_clade - ps29
Cerevisiae_clade - ps30
Saccharomyces_cerevisiae - ps31
Phylostratum

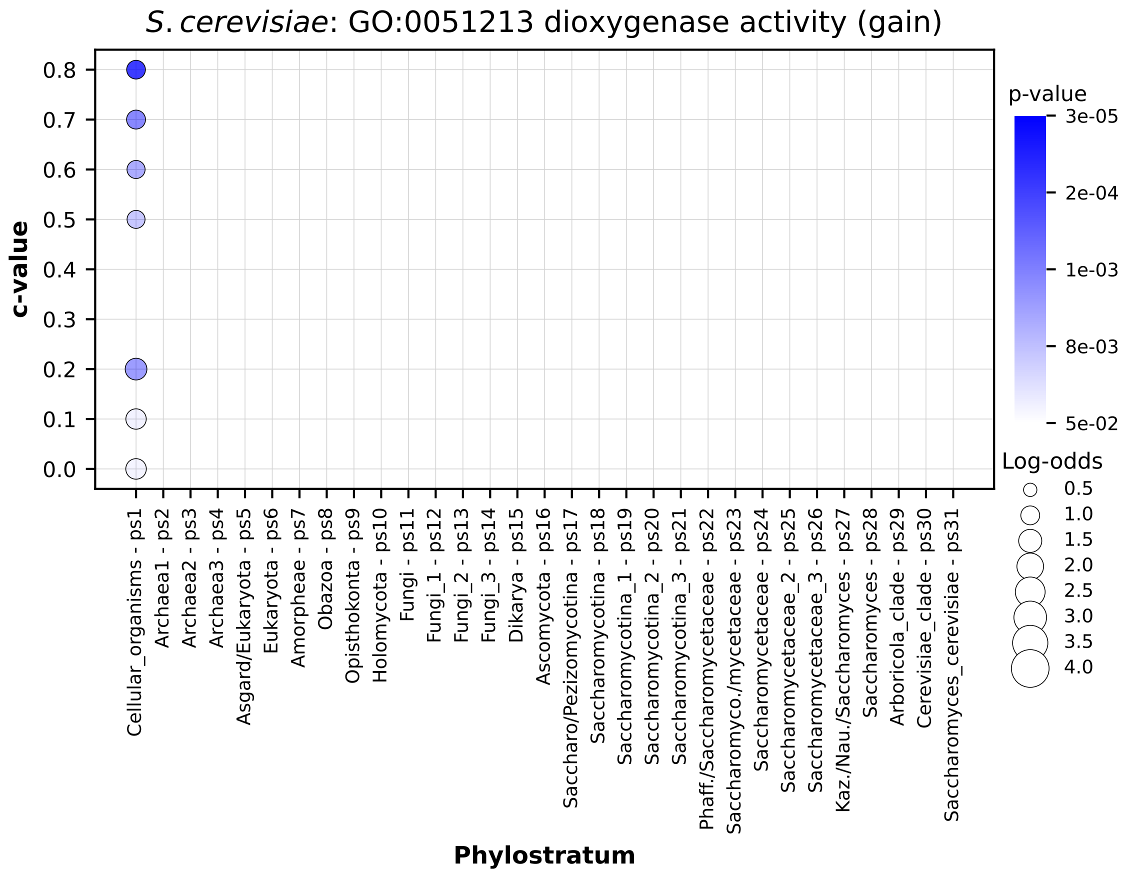
S. cerevisiae: GO:0051213 dioxygenase activity (gain)
c-value
0.0
0.1
0.2
0.3
0.4
0.5
0.6
0.7
0.8
p-value
3e-05
2e-04
1e-03
8e-03
5e-02
Log-odds
0.5
1.0
1.5
2.0
2.5
3.0
3.5
4.0
Cellular_organisms - ps1
Archaea1 - ps2
Archaea2 - ps3
Archaea3 - ps4
Asgard/Eukaryota - ps5
Eukaryota - ps6
Amorpheae - ps7
Obazoa - ps8
Opisthokonta - ps9
Holomycota - ps10
Fungi - ps11
Fungi_1 - ps12
Fungi_2 - ps13
Fungi_3 - ps14
Dikarya - ps15
Ascomycota - ps16
Saccharo/Pezizomycotina - ps17
Saccharomycotina - ps18
Saccharomycotina_1 - ps19
Saccharomycotina_2 - ps20
Saccharomycotina_3 - ps21
Phaff./Saccharomycetaceae - ps22
Saccharomyco./mycetaceae - ps23
Saccharomycetaceae - ps24
Saccharomycetaceae_2 - ps25
Saccharomycetaceae_3 - ps26
Kaz./Nau./Saccharomyces - ps27
Saccharomyces - ps28
Arboricola_clade - ps29
Cerevisiae_clade - ps30
Saccharomyces_cerevisiae - ps31
Phylostratum

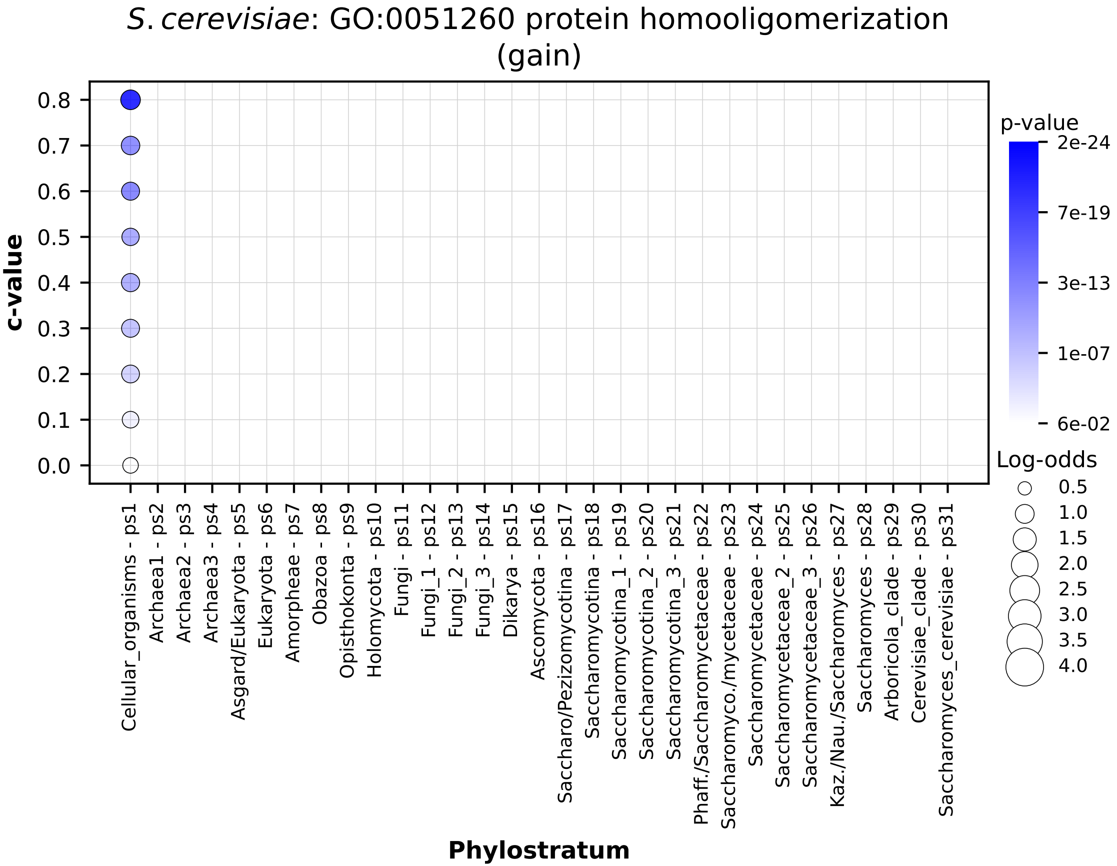

S. cerevisiae: GO:0051260 protein homooligomerization
(gain)
c-value
0.8
0.7
0.6
0.5
0.4
0.3
0.2
0.1
0.0
p-value
2e-24
7e-19
3e-13
1e-07
6e-02
Log-odds
0.5
1.0
1.5
2.0
2.5
3.0
3.5
4.0
Cellular_organisms - ps1
Archaea1 - ps2
Archaea2 - ps3
Archaea3 - ps4
Asgard/Eukaryota - ps5
Eukaryota - ps6
Amorpheae - ps7
Obazoa - ps8
Opisthokonta - ps9
Holomycota - ps10
Fungi - ps11
Fungi_1 - ps12
Fungi_2 - ps13
Fungi_3 - ps14
Dikarya - ps15
Ascomycota - ps16
Saccharo/Pezizomycotina - ps17
Saccharomycotina - ps18
Saccharomycotina_1 - ps19
Saccharomycotina_2 - ps20
Saccharomycotina_3 - ps21
Phaff./Saccharomycetaceae - ps22
Saccharomyco./mycetaceae - ps23
Saccharomycetaceae - ps24
Saccharomycetaceae_2 - ps25
Saccharomycetaceae_3 - ps26
Kaz./Nau./Saccharomyces - ps27
Saccharomyces - ps28
Arboricola_clade - ps29
Cerevisiae_clade - ps30
Saccharomyces_cerevisiae - ps31
Phylostratum

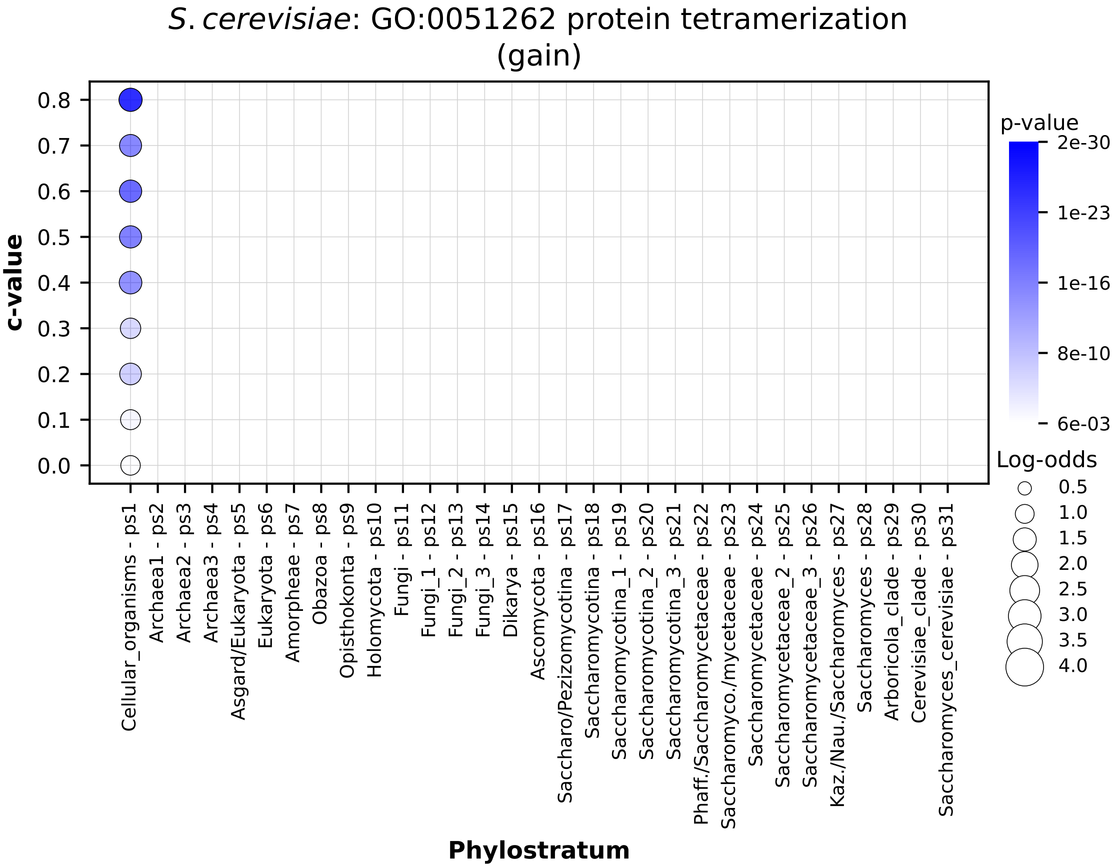
S. cerevisiae: GO:0051262 protein tetramerization
(gain)
c-value
0.8
0.7
0.6
0.5
0.4
0.3
0.2
0.1
0.0
p-value
2e-30
1e-23
1e-16
8e-10
6e-03
Log-odds
0.5
1.0
1.5
2.0
2.5
3.0
3.5
4.0
Cellular_organisms - ps1
Archaea1 - ps2
Archaea2 - ps3
Archaea3 - ps4
Asgard/Eukaryota - ps5
Eukaryota - ps6
Amorpheae - ps7
Obazoa - ps8
Opisthokonta - ps9
Holomycota - ps10
Fungi - ps11
Fungi_1 - ps12
Fungi_2 - ps13
Fungi_3 - ps14
Dikarya - ps15
Ascomycota - ps16
Saccharo/Pezizomycotina - ps17
Saccharomycotina - ps18
Saccharomycotina_1 - ps19
Saccharomycotina_2 - ps20
Saccharomycotina_3 - ps21
Phaff./Saccharomycetaceae - ps22
Saccharomyco./mycetaceae - ps23
Saccharomycetaceae - ps24
Saccharomycetaceae_2 - ps25
Saccharomycetaceae_3 - ps26
Kaz./Nau./Saccharomyces - ps27
Saccharomyces - ps28
Arboricola_clade - ps29
Cerevisiae_clade - ps30
Saccharomyces_cerevisiae - ps31
Phylostratum

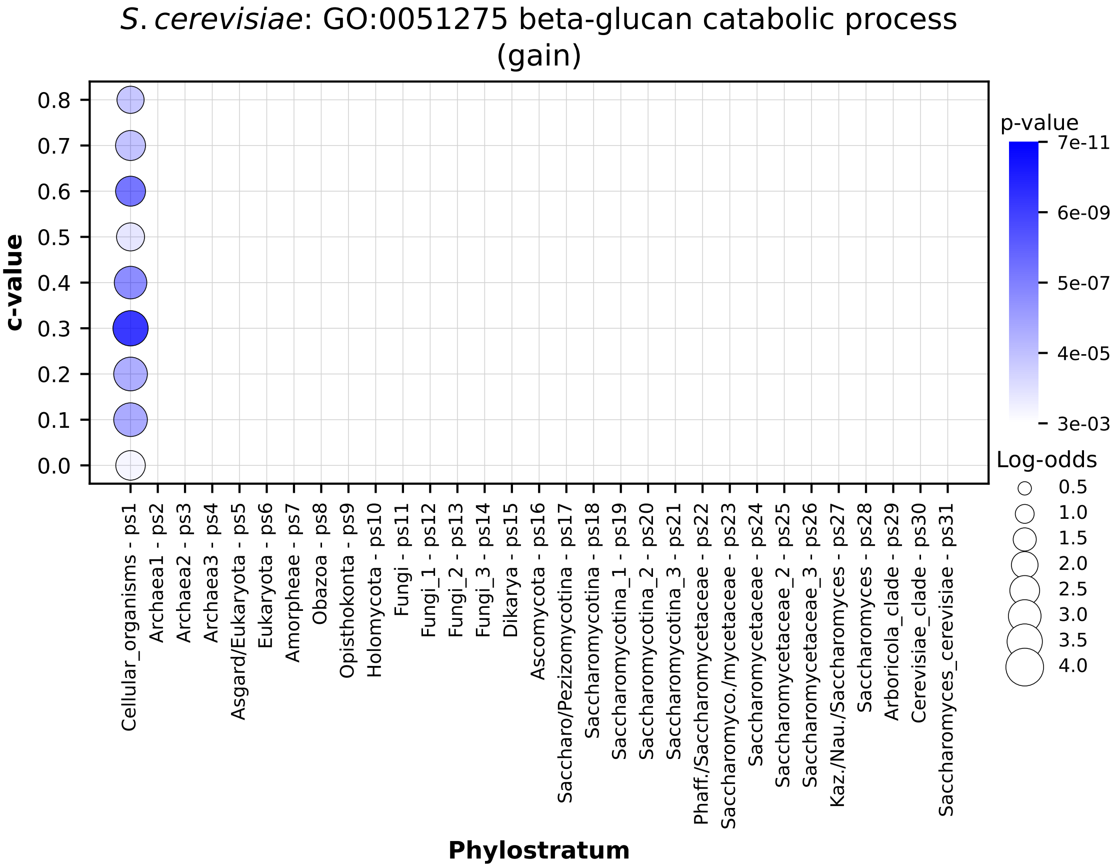
S. cerevisiae: GO:0051275 beta-glucan catabolic process
(gain)
c-value
0.8
0.7
0.6
0.5
0.4
0.3
0.2
0.1
0.0
p-value
7e-11
6e-09
5e-07
4e-05
3e-03
Log-odds
0.5
1.0
1.5
2.0
2.5
3.0
3.5
4.0
Cellular_organisms - ps1
Archaea1 - ps2
Archaea2 - ps3
Archaea3 - ps4
Asgard/Eukaryota - ps5
Eukaryota - ps6
Amorpheae - ps7
Obazoa - ps8
Opisthokonta - ps9
Holomycota - ps10
Fungi - ps11
Fungi_1 - ps12
Fungi_2 - ps13
Fungi_3 - ps14
Dikarya - ps15
Ascomycota - ps16
Saccharo/Pezizomycotina - ps17
Saccharomycotina - ps18
Saccharomycotina_1 - ps19
Saccharomycotina_2 - ps20
Saccharomycotina_3 - ps21
Phaff./Saccharomycetaceae - ps22
Saccharomyco./mycetaceae - ps23
Saccharomycetaceae - ps24
Saccharomycetaceae_2 - ps25
Saccharomycetaceae_3 - ps26
Kaz./Nau./Saccharomyces - ps27
Saccharomyces - ps28
Arboricola_clade - ps29
Cerevisiae_clade - ps30
Saccharomyces_cerevisiae - ps31
Phylostratum

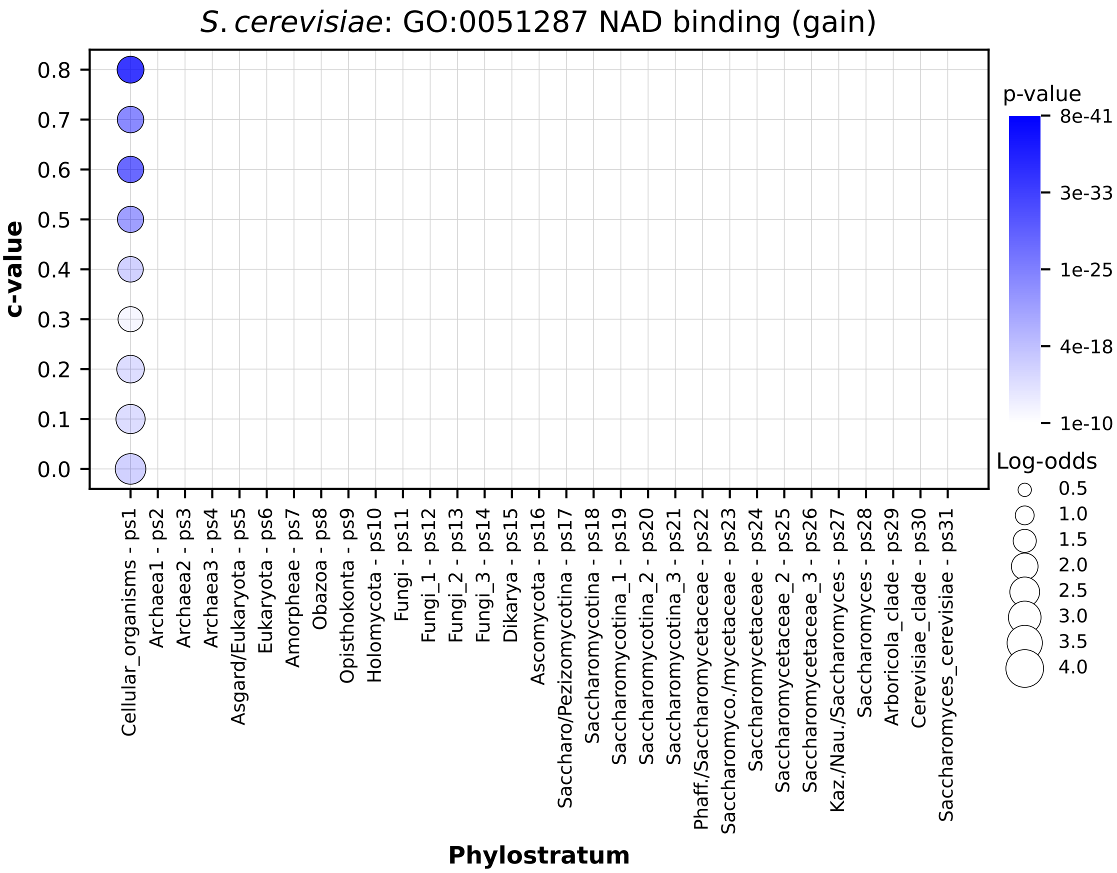

S. cerevisiae: GO:0051287 NAD binding (gain)
c-value
0.8
0.7
0.6
0.5
0.4
0.3
0.2
0.1
0.0
p-value
8e-41
3e-33
1e-25
4e-18
1e-10
Log-odds
0.5
1.0
1.5
2.0
2.5
3.0
3.5
4.0
Cellular_organisms - ps1
Archaea1 - ps2
Archaea2 - ps3
Archaea3 - ps4
Asgard/Eukaryota - ps5
Eukaryota - ps6
Amorpheae - ps7
Obazoa - ps8
Opisthokonta - ps9
Holomycota - ps10
Fungi - ps11
Fungi_1 - ps12
Fungi_2 - ps13
Fungi_3 - ps14
Dikarya - ps15
Ascomycota - ps16
Saccharo/Pezizomycotina - ps17
Saccharomycotina - ps18
Saccharomycotina_1 - ps19
Saccharomycotina_2 - ps20
Saccharomycotina_3 - ps21
Phaff./Saccharomycetaceae - ps22
Saccharomyco./mycetaceae - ps23
Saccharomycetaceae - ps24
Saccharomycetaceae_2 - ps25
Saccharomycetaceae_3 - ps26
Kaz./Nau./Saccharomyces - ps27
Saccharomyces - ps28
Arboricola_clade - ps29
Cerevisiae_clade - ps30
Saccharomyces_cerevisiae - ps31
Phylostratum

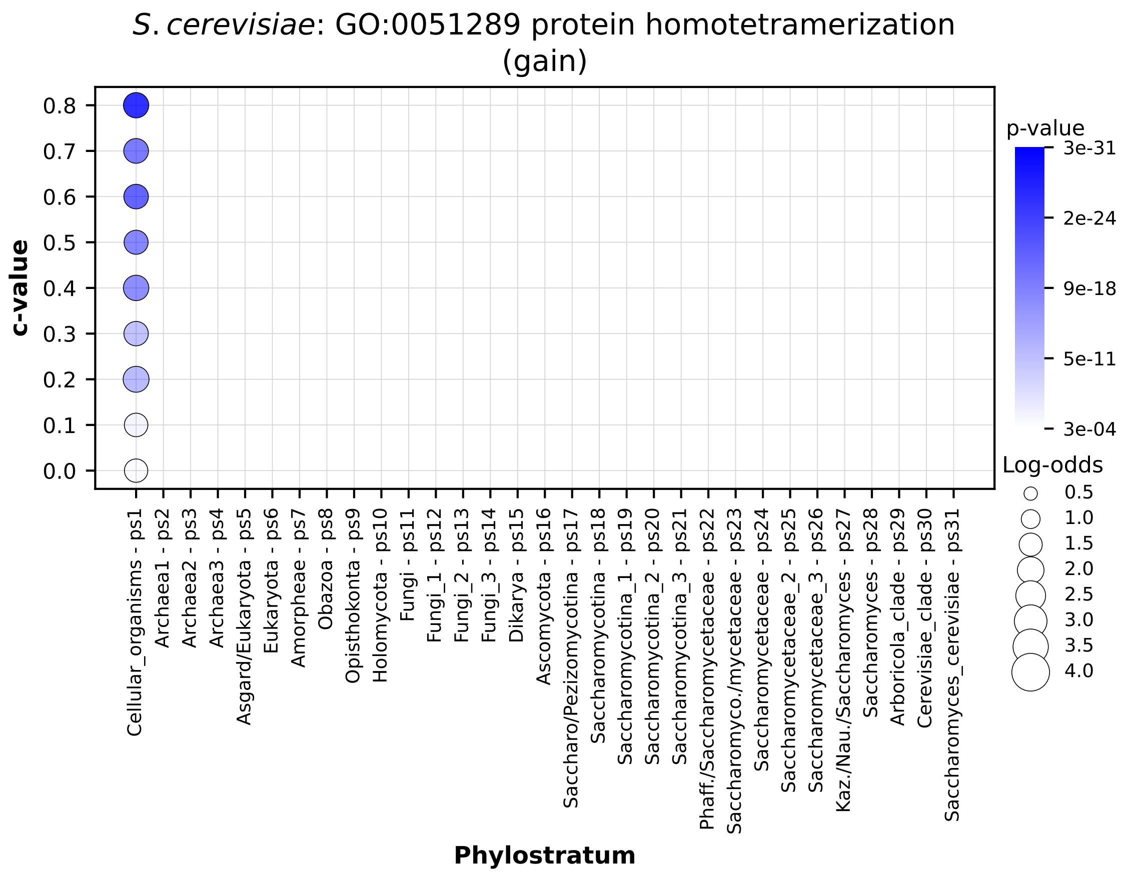
S. cerevisiae: GO:0051289 protein homotetramerization (gain)
c-value
0.8
0.7
0.6
0.5
0.4
0.3
0.2
0.1
0.0
p-value
3e-31
2e-24
9e-18
5e-11
3e-04
Log-odds
0.5
1.0
1.5
2.0
2.5
3.0
3.5
4.0
Cellular_organisms - ps1
Archaea1 - ps2
Archaea2 - ps3
Archaea3 - ps4
Asgard/Eukaryota - ps5
Eukaryota - ps6
Amorpheae - ps7
Obazoa - ps8
Opisthokonta - ps9
Holomycota - ps10
Fungi - ps11
Fungi_1 - ps12
Fungi_2 - ps13
Fungi_3 - ps14
Dikarya - ps15
Ascomycota - ps16
Saccharo/Pezizomycotina - ps17
Saccharomycotina - ps18
Saccharomycotina_1 - ps19
Saccharomycotina_2 - ps20
Saccharomycotina_3 - ps21
Phaff./Saccharomycetaceae - ps22
Saccharomyco./mycetaceae - ps23
Saccharomycetaceae - ps24
Saccharomycetaceae_2 - ps25
Saccharomycetaceae_3 - ps26
Kaz./Nau./Saccharomyces - ps27
Saccharomyces - ps28
Arboricola_clade - ps29
Cerevisiae_clade - ps30
Saccharomyces_cerevisiae - ps31
Phylostratum

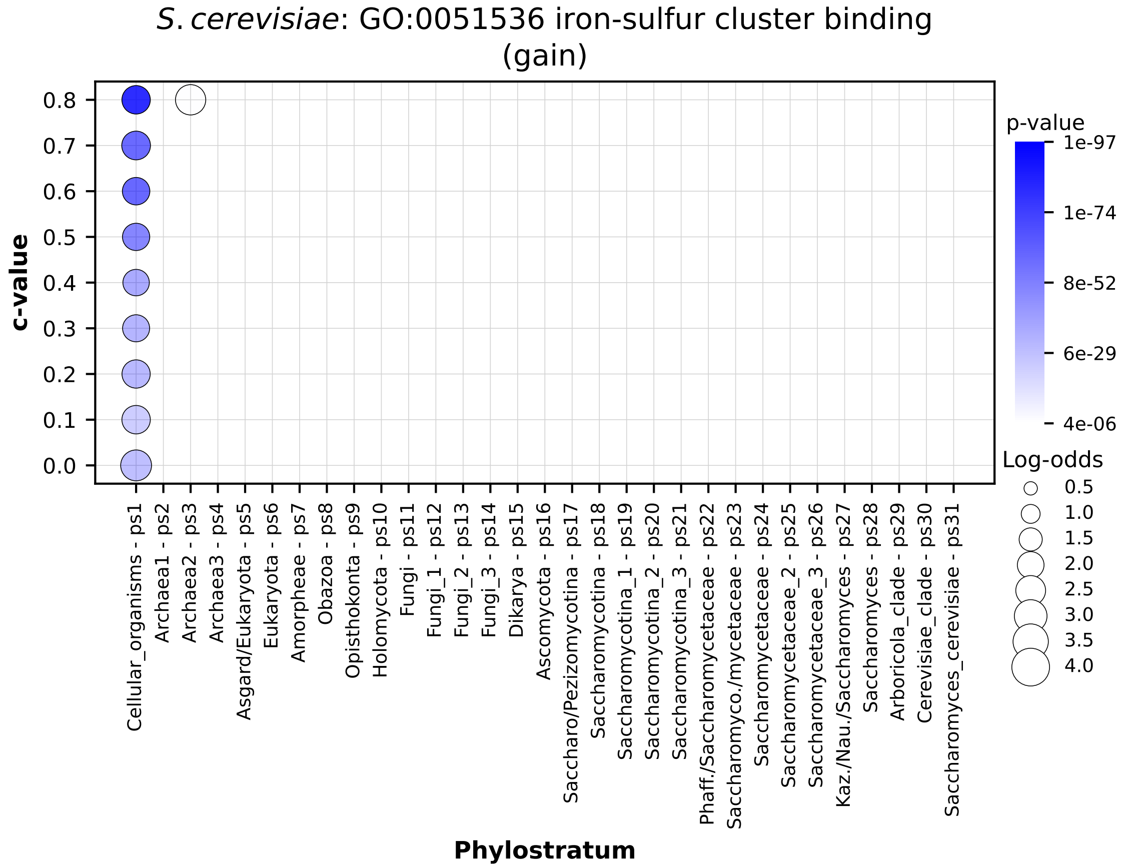

S. cerevisiae: GO:0051536 iron-sulfur cluster binding
(gain)
c-value
0.8
0.7
0.6
0.5
0.4
0.3
0.2
0.1
0.0
Cellular_organisms - ps1
Archaea1 - ps2
Archaea2 - ps3
Archaea3 - ps4
Asgard/Eukaryota - ps5
Eukaryota - ps6
Amorpheae - ps7
Obazoa - ps8
Opisthokonta - ps9
Holomycota - ps10
Fungi - ps11
Fungi_1 - ps12
Fungi_2 - ps13
Fungi_3 - ps14
Dikarya - ps15
Ascomycota - ps16
Saccharo/Pezizomycotina - ps17
Saccharomycotina - ps18
Saccharomycotina_1 - ps19
Saccharomycotina_2 - ps20
Saccharomycotina_3 - ps21
Phaff./Saccharomycetaceae - ps22
Saccharomyco./mycetaceae - ps23
Saccharomycetaceae - ps24
Saccharomycetaceae_2 - ps25
Saccharomycetaceae_3 - ps26
Kaz./Nau./Saccharomyces - ps27
Saccharomyces - ps28
Arboricola_clade - ps29
Cerevisiae_clade - ps30
Saccharomyces_cerevisiae - ps31
Phylostratum
p-value
1e-97
1e-74
8e-52
6e-29
4e-06
Log-odds
0.5
1.0
1.5
2.0
2.5
3.0
3.5
4.0

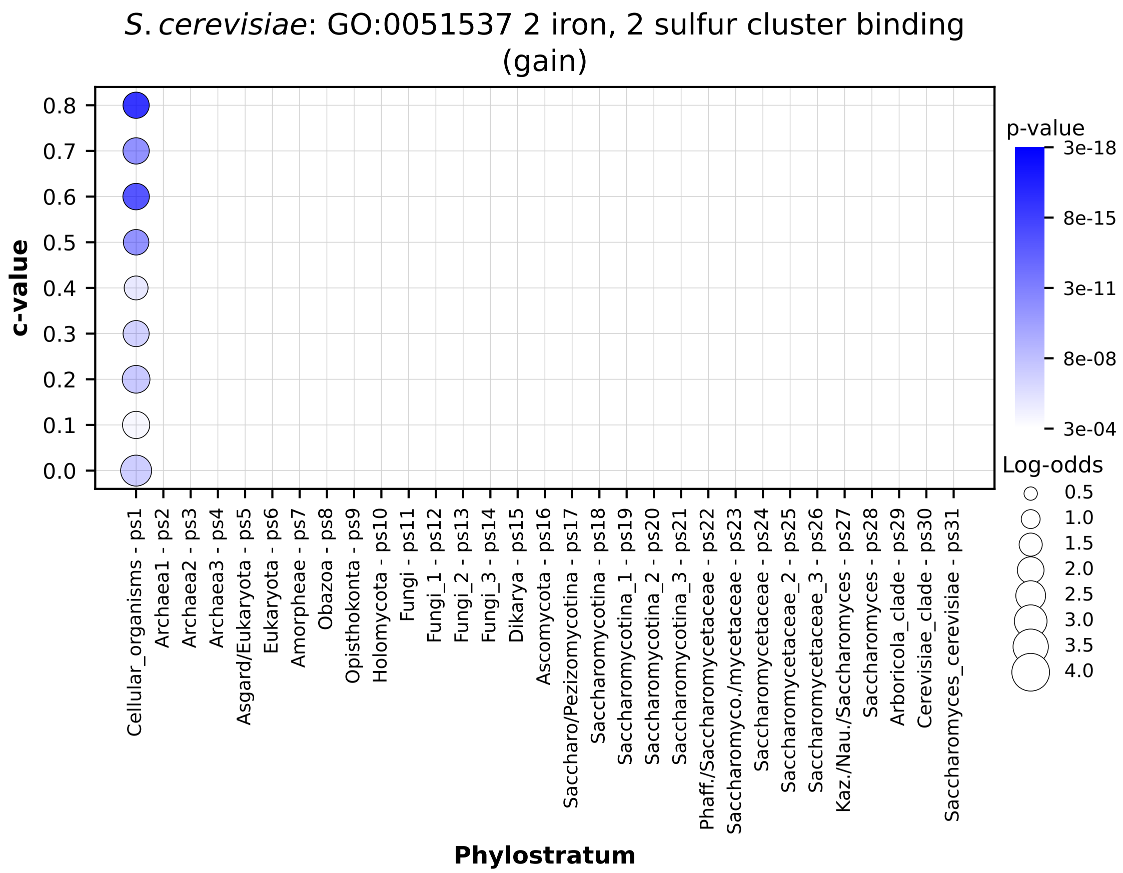

$S. cerevisiae$: GO:0051537 2 iron, 2 sulfur cluster binding (gain)
c-value
0.8
0.7
0.6
0.5
0.4
0.3
0.2
0.1
0.0
p-value
3e-18
8e-15
3e-11
8e-08
3e-04
Log-odds
0.5
1.0
1.5
2.0
2.5
3.0
3.5
4.0
Cellular_organisms - ps1
Archaea1 - ps2
Archaea2 - ps3
Archaea3 - ps4
Asgard/Eukaryota - ps5
Eukaryota - ps6
Amorpheae - ps7
Obazoa - ps8
Opisthokonta - ps9
Holomycota - ps10
Fungi - ps11
Fungi_1 - ps12
Fungi_2 - ps13
Fungi_3 - ps14
Dikarya - ps15
Ascomycota - ps16
Saccharo/Pezizomycotina - ps17
Saccharomycotina - ps18
Saccharomycotina_1 - ps19
Saccharomycotina_2 - ps20
Saccharomycotina_3 - ps21
Phaff./Saccharomycetaceae - ps22
Saccharomyco./mycetaceae - ps23
Saccharomycetaceae - ps24
Saccharomycetaceae_2 - ps25
Saccharomycetaceae_3 - ps26
Kaz./Nau./Saccharomyces - ps27
Saccharomyces - ps28
Arboricola_clade - ps29
Cerevisiae_clade - ps30
Saccharomyces_cerevisiae - ps31
Phylostratum

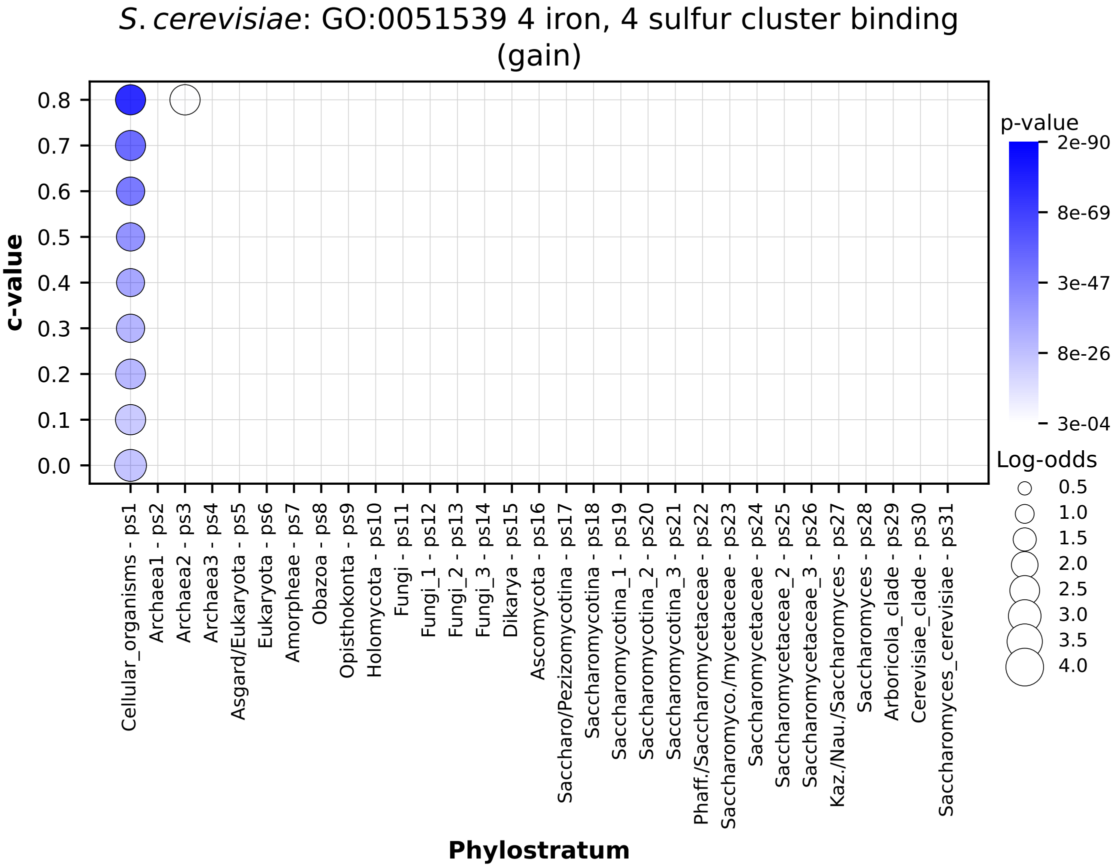
$S. cerevisiae$: GO:0051539 4 iron, 4 sulfur cluster binding
(gain)
c-value
0.8
0.7
0.6
0.5
0.4
0.3
0.2
0.1
0.0
p-value
2e-90
8e-69
3e-47
8e-26
3e-04
Log-odds
0.5
1.0
1.5
2.0
2.5
3.0
3.5
4.0
Cellular_organisms - ps1
Archaea1 - ps2
Archaea2 - ps3
Archaea3 - ps4
Asgard/Eukaryota - ps5
Eukaryota - ps6
Amorpheae - ps7
Obazoa - ps8
Opisthokonta - ps9
Holomycota - ps10
Fungi - ps11
Fungi_1 - ps12
Fungi_2 - ps13
Fungi_3 - ps14
Dikarya - ps15
Ascomycota - ps16
Saccharo/Pezizomycotina - ps17
Saccharomycotina - ps18
Saccharomycotina_1 - ps19
Saccharomycotina_2 - ps20
Saccharomycotina_3 - ps21
Phaff./Saccharomycetaceae - ps22
Saccharomyco./mycetaceae - ps23
Saccharomycetaceae - ps24
Saccharomycetaceae_2 - ps25
Saccharomycetaceae_3 - ps26
Kaz./Nau./Saccharomyces - ps27
Saccharomyces - ps28
Arboricola_clade - ps29
Cerevisiae_clade - ps30
Saccharomyces_cerevisiae - ps31
Phylostratum

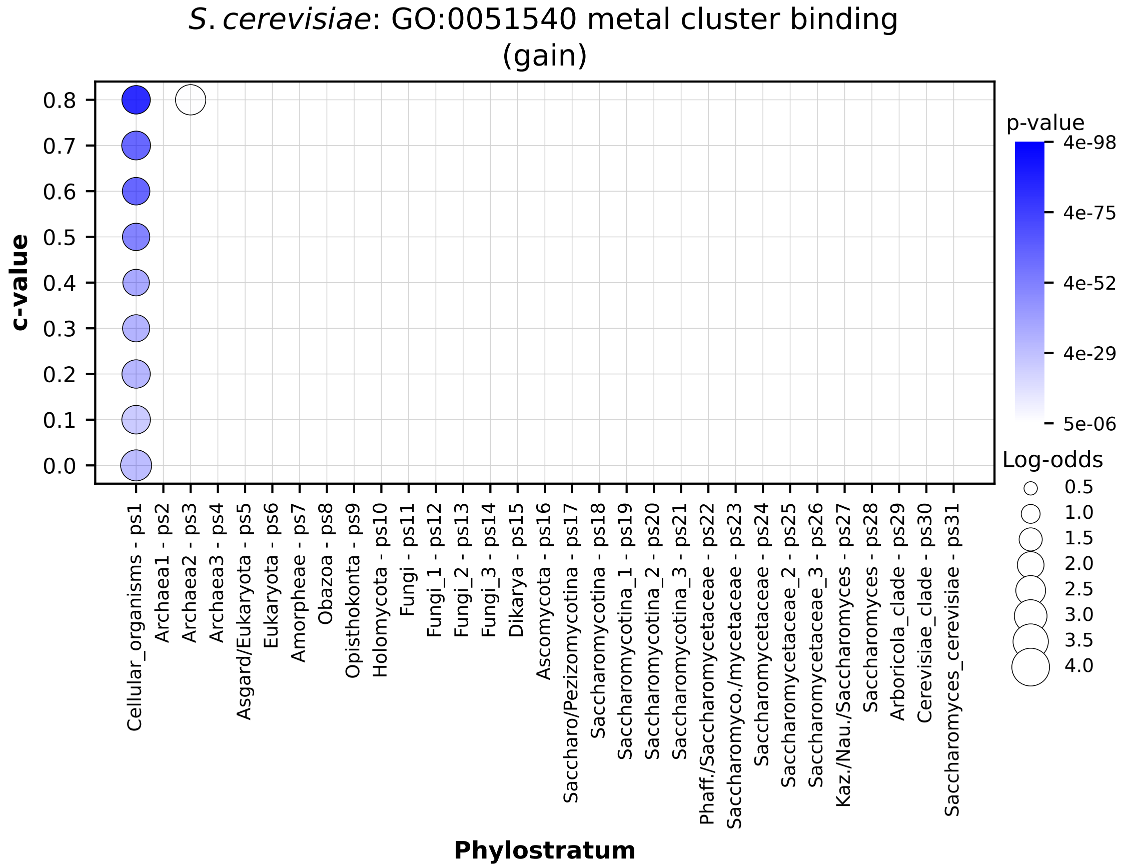
S. cerevisiae: GO:0051540 metal cluster binding
(gain)
c-value
0.8
0.7
0.6
0.5
0.4
0.3
0.2
0.1
0.0
p-value
4e-98
4e-75
4e-52
4e-29
5e-06
Log-odds
0.5
1.0
1.5
2.0
2.5
3.0
3.5
4.0
Cellular_organisms - ps1
Archaea1 - ps2
Archaea2 - ps3
Archaea3 - ps4
Asgard/Eukaryota - ps5
Eukaryota - ps6
Amorpheae - ps7
Obazoa - ps8
Opisthokonta - ps9
Holomycota - ps10
Fungi - ps11
Fungi_1 - ps12
Fungi_2 - ps13
Fungi_3 - ps14
Dikarya - ps15
Ascomycota - ps16
Saccharo/Pezizomycotina - ps17
Saccharomycotina - ps18
Saccharomycotina_1 - ps19
Saccharomycotina_2 - ps20
Saccharomycotina_3 - ps21
Phaff./Saccharomycetaceae - ps22
Saccharomyco./mycetaceae - ps23
Saccharomycetaceae - ps24
Saccharomycetaceae_2 - ps25
Saccharomycetaceae_3 - ps26
Kaz./Nau./Saccharomyces - ps27
Saccharomyces - ps28
Arboricola_clade - ps29
Cerevisiae_clade - ps30
Saccharomyces_cerevisiae - ps31
Phylostratum

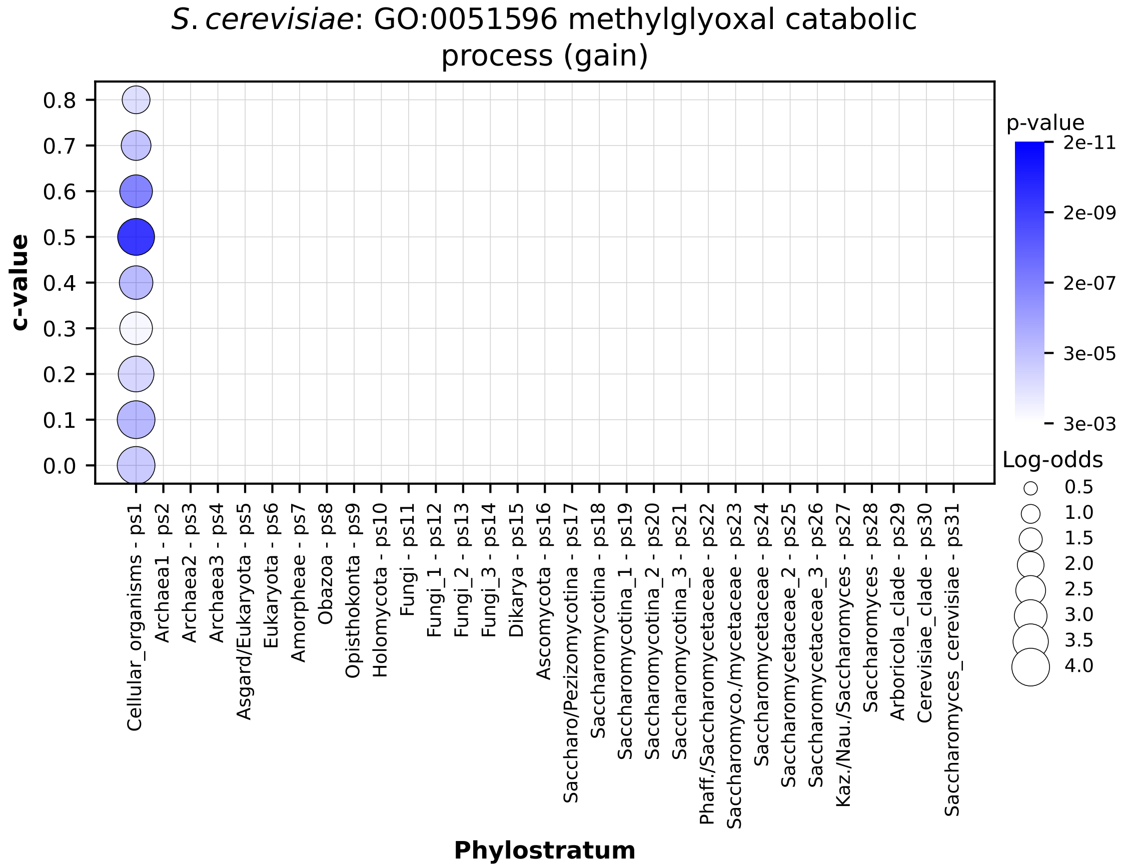

S. cerevisiae: GO:0051596 methylglyoxal catabolic process (gain)
c-value
0.8
0.7
0.6
0.5
0.4
0.3
0.2
0.1
0.0
p-value
2e-11
2e-09
2e-07
3e-05
3e-03
Log-odds
0.5
1.0
1.5
2.0
2.5
3.0
3.5
4.0
Cellular_organisms - ps1
Archaea1 - ps2
Archaea2 - ps3
Archaea3 - ps4
Asgard/Eukaryota - ps5
Eukaryota - ps6
Amorpheae - ps7
Obazoa - ps8
Opisthokonta - ps9
Holomycota - ps10
Fungi - ps11
Fungi_1 - ps12
Fungi_2 - ps13
Fungi_3 - ps14
Dikarya - ps15
Ascomycota - ps16
Saccharo/Pezizomycotina - ps17
Saccharomycotina - ps18
Saccharomycotina_1 - ps19
Saccharomycotina_2 - ps20
Saccharomycotina_3 - ps21
Phaff./Saccharomycetaceae - ps22
Saccharomyco./mycetaceae - ps23
Saccharomycetaceae - ps24
Saccharomycetaceae_2 - ps25
Saccharomycetaceae_3 - ps26
Kaz./Nau./Saccharomyces - ps27
Saccharomyces - ps28
Arboricola_clade - ps29
Cerevisiae_clade - ps30
Saccharomyces_cerevisiae - ps31
Phylostratum

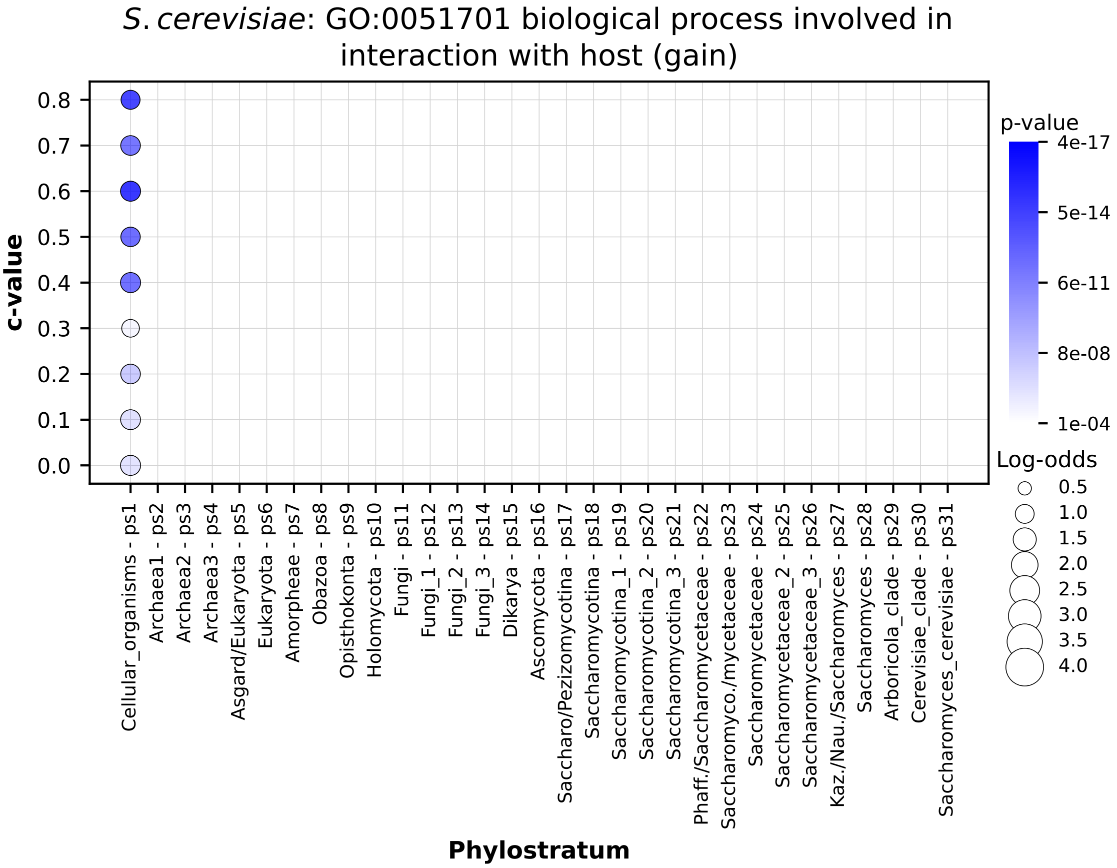

S. cerevisiae: GO:0051701 biological process involved in interaction with host (gain)
c-value
0.8
0.7
0.6
0.5
0.4
0.3
0.2
0.1
0.0
p-value
4e-17
5e-14
6e-11
8e-08
1e-04
Log-odds
0.5
1.0
1.5
2.0
2.5
3.0
3.5
4.0
Cellular_organisms - ps1
Archaea1 - ps2
Archaea2 - ps3
Archaea3 - ps4
Asgard/Eukaryota - ps5
Eukaryota - ps6
Amorpheae - ps7
Obazoa - ps8
Opisthokonta - ps9
Holomycota - ps10
Fungi - ps11
Fungi_1 - ps12
Fungi_2 - ps13
Fungi_3 - ps14
Dikarya - ps15
Ascomycota - ps16
Saccharo/Pezizomycotina - ps17
Saccharomycotina - ps18
Saccharomycotina_1 - ps19
Saccharomycotina_2 - ps20
Saccharomycotina_3 - ps21
Phaff./Saccharomycetaceae - ps22
Saccharomyco./mycetaceae - ps23
Saccharomycetaceae - ps24
Saccharomycetaceae_2 - ps25
Saccharomycetaceae_3 - ps26
Kaz./Nau./Saccharomyces - ps27
Saccharomyces - ps28
Arboricola_clade - ps29
Cerevisiae_clade - ps30
Saccharomyces_cerevisiae - ps31
Phylostratum

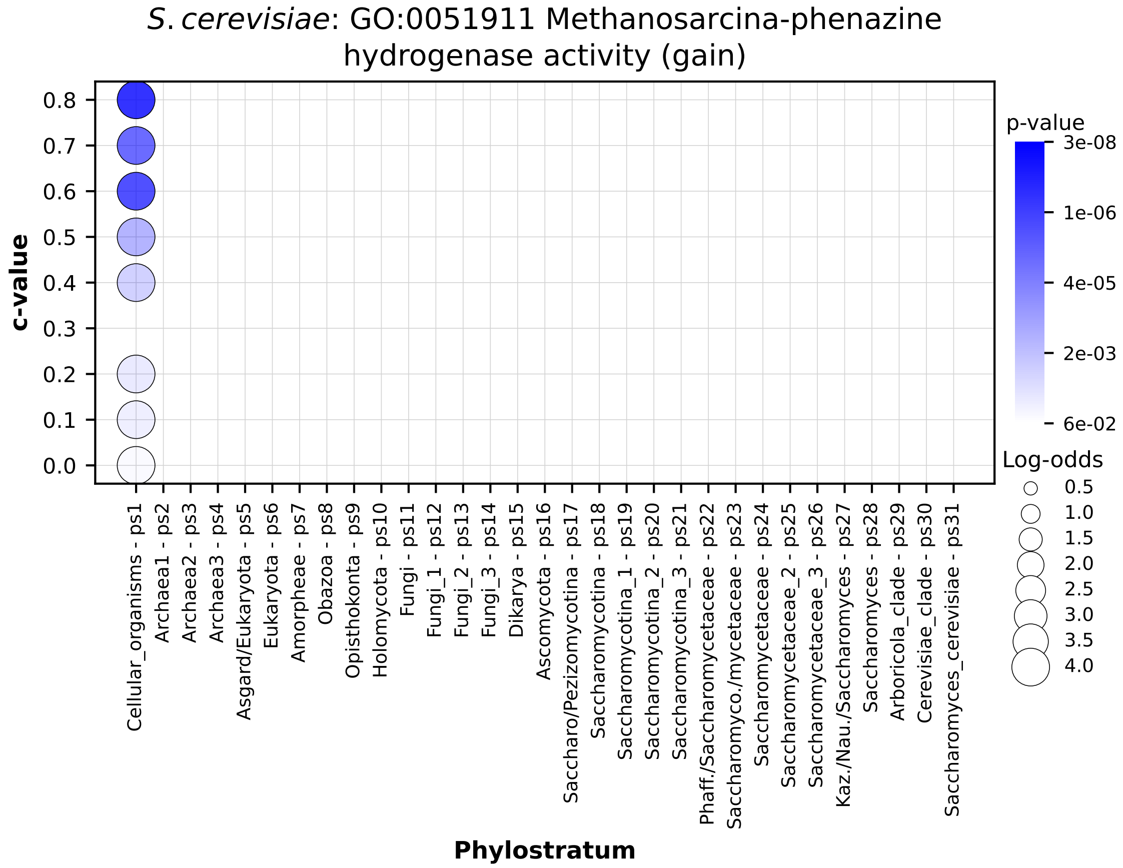

S. cerevisiae: GO:0051911 Methanosarcina-phenazine hydrogenase activity (gain)
c-value
0.8
0.7
0.6
0.5
0.4
0.3
0.2
0.1
0.0
p-value
3e-08
1e-06
4e-05
2e-03
6e-02
Log-odds
0.5
1.0
1.5
2.0
2.5
3.0
3.5
4.0
Cellular_organisms - ps1
Archaea1 - ps2
Archaea2 - ps3
Archaea3 - ps4
Asgard/Eukaryota - ps5
Eukaryota - ps6
Amorpheae - ps7
Obazoa - ps8
Opisthokonta - ps9
Holomycota - ps10
Fungi - ps11
Fungi_1 - ps12
Fungi_2 - ps13
Fungi_3 - ps14
Dikarya - ps15
Ascomycota - ps16
Saccharo/Pezizomycotina - ps17
Saccharomycotina - ps18
Saccharomycotina_1 - ps19
Saccharomycotina_2 - ps20
Saccharomycotina_3 - ps21
Phaff./Saccharomycetaceae - ps22
Saccharomyco./mycetaceae - ps23
Saccharomycetaceae - ps24
Saccharomycetaceae_2 - ps25
Saccharomycetaceae_3 - ps26
Kaz./Nau./Saccharomyces - ps27
Saccharomyces - ps28
Arboricola_clade - ps29
Cerevisiae_clade - ps30
Saccharomyces_cerevisiae - ps31
Phylostratum

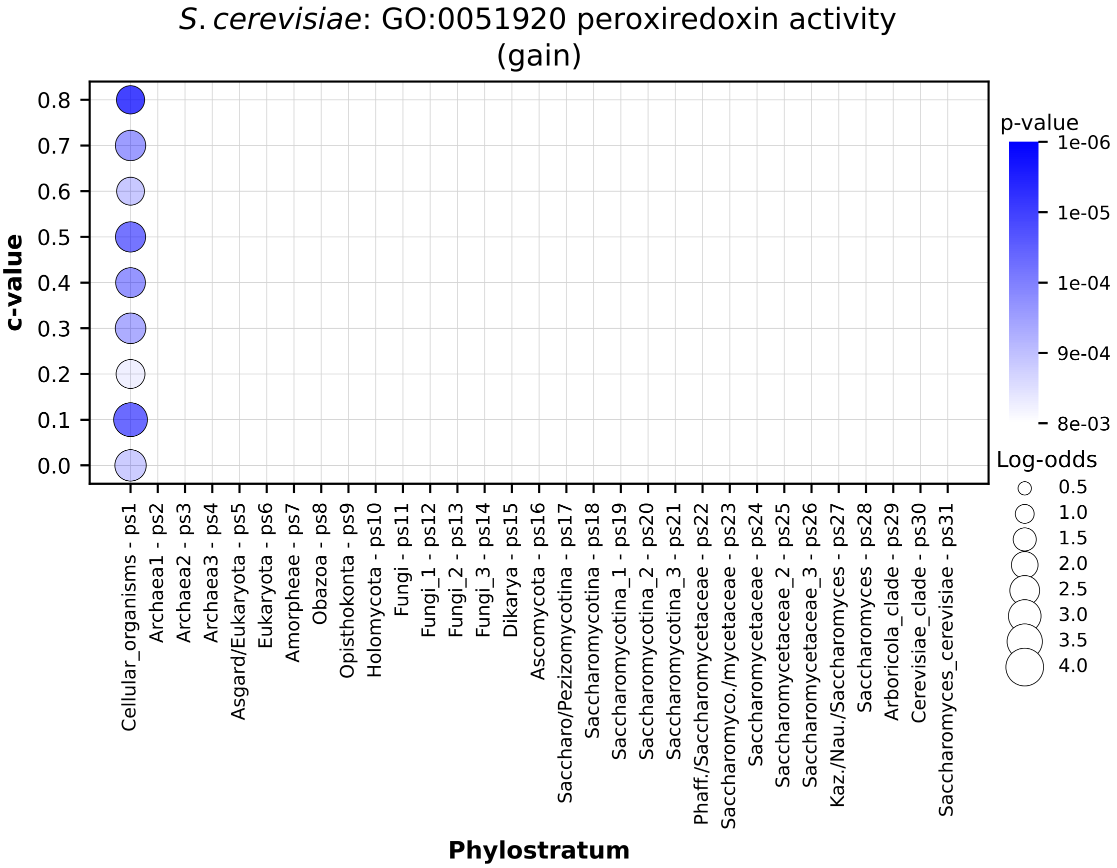

S. cerevisiae: GO:0051920 peroxiredoxin activity
(gain)
c-value
0.8
0.7
0.6
0.5
0.4
0.3
0.2
0.1
0.0
p-value
1e-06
1e-05
1e-04
9e-04
8e-03
Log-odds
0.5
1.0
1.5
2.0
2.5
3.0
3.5
4.0
Cellular_organisms - ps1
Archaea1 - ps2
Archaea2 - ps3
Archaea3 - ps4
Asgard/Eukaryota - ps5
Eukaryota - ps6
Amorpheae - ps7
Obazoa - ps8
Opisthokonta - ps9
Holomycota - ps10
Fungi - ps11
Fungi_1 - ps12
Fungi_2 - ps13
Fungi_3 - ps14
Dikarya - ps15
Ascomycota - ps16
Saccharo/Pezizomycotina - ps17
Saccharomycotina - ps18
Saccharomycotina_1 - ps19
Saccharomycotina_2 - ps20
Saccharomycotina_3 - ps21
Phaff./Saccharomycetaceae - ps22
Saccharomyco./mycetaceae - ps23
Saccharomycetaceae - ps24
Saccharomycetaceae_2 - ps25
Saccharomycetaceae_3 - ps26
Kaz./Nau./Saccharomyces - ps27
Saccharomyces - ps28
Arboricola_clade - ps29
Cerevisiae_clade - ps30
Saccharomyces_cerevisiae - ps31
Phylostratum

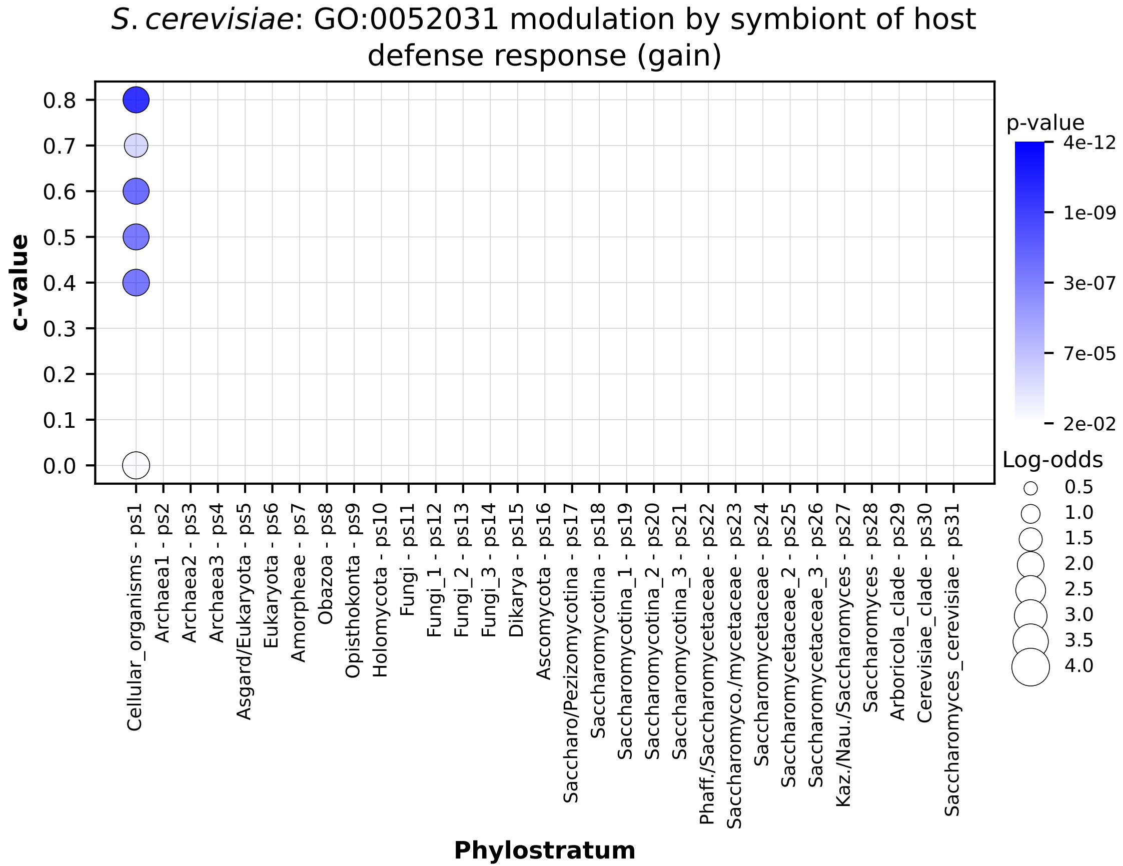
S. cerevisiae: GO:0052031 modulation by symbiont of host defense response (gain)
c-value
0.8
0.7
0.6
0.5
0.4
0.3
0.2
0.1
0.0
p-value
4e-12
1e-09
3e-07
7e-05
2e-02
Log-odds
0.5
1.0
1.5
2.0
2.5
3.0
3.5
4.0
Cellular_organisms - ps1
Archaea1 - ps2
Archaea2 - ps3
Archaea3 - ps4
Asgard/Eukaryota - ps5
Eukaryota - ps6
Amorpheae - ps7
Obazoa - ps8
Opisthokonta - ps9
Holomycota - ps10
Fungi - ps11
Fungi_1 - ps12
Fungi_2 - ps13
Fungi_3 - ps14
Dikarya - ps15
Ascomycota - ps16
Saccharo/Pezizomycotina - ps17
Saccharomycotina - ps18
Saccharomycotina_1 - ps19
Saccharomycotina_2 - ps20
Saccharomycotina_3 - ps21
Phaff./Saccharomycetaceae - ps22
Saccharomyco./mycetaceae - ps23
Saccharomycetaceae - ps24
Saccharomycetaceae_2 - ps25
Saccharomycetaceae_3 - ps26
Kaz./Nau./Saccharomyces - ps27
Saccharomyces - ps28
Arboricola_clade - ps29
Cerevisiae_clade - ps30
Saccharomyces_cerevisiae - ps31
Phylostratum

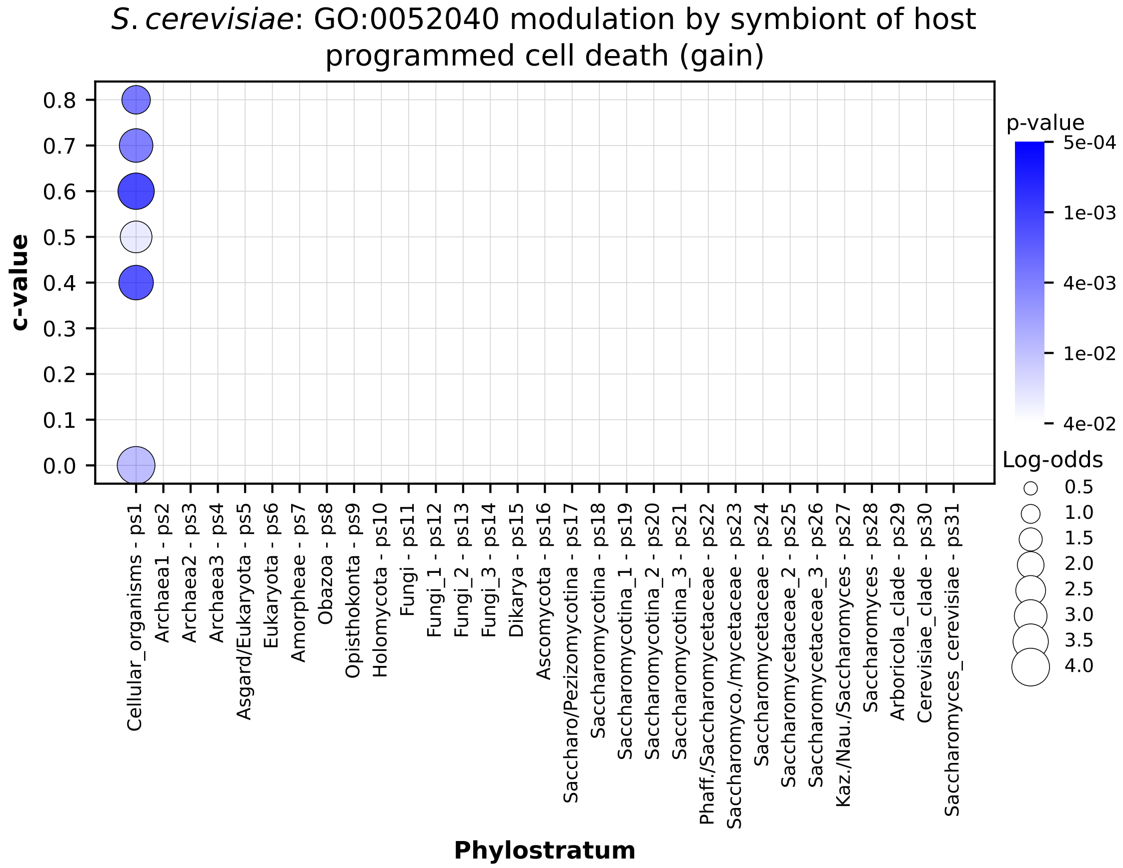
S. cerevisiae: GO:0052040 modulation by symbiont of host programmed cell death (gain)
c-value
0.8
0.7
0.6
0.5
0.4
0.3
0.2
0.1
0.0
p-value
5e-04
1e-03
4e-03
1e-02
4e-02
Log-odds
0.5
1.0
1.5
2.0
2.5
3.0
3.5
4.0
Cellular_organisms - ps1
Archaea1 - ps2
Archaea2 - ps3
Archaea3 - ps4
Asgard/Eukaryota - ps5
Eukaryota - ps6
Amorpheae - ps7
Obazoa - ps8
Opisthokonta - ps9
Holomycota - ps10
Fungi - ps11
Fungi_1 - ps12
Fungi_2 - ps13
Fungi_3 - ps14
Dikarya - ps15
Ascomycota - ps16
Saccharo/Pezizomycotina - ps17
Saccharomycotina - ps18
Saccharomycotina_1 - ps19
Saccharomycotina_2 - ps20
Saccharomycotina_3 - ps21
Phaff./Saccharomycetaceae - ps22
Saccharomyco./mycetaceae - ps23
Saccharomycetaceae - ps24
Saccharomycetaceae_2 - ps25
Saccharomycetaceae_3 - ps26
Kaz./Nau./Saccharomyces - ps27
Saccharomyces - ps28
Arboricola_clade - ps29
Cerevisiae_clade - ps30
Saccharomyces_cerevisiae - ps31
Phylostratum

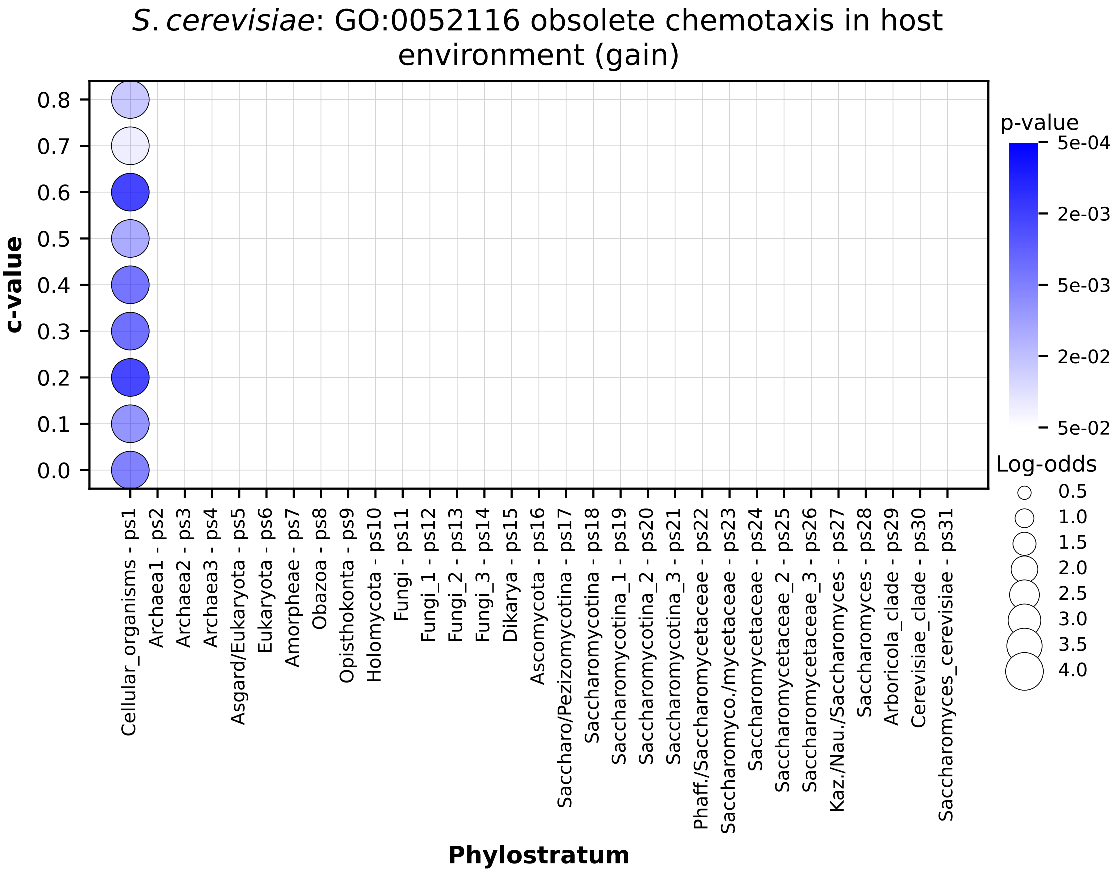
S. cerevisiae: GO:0052116 obsolete chemotaxis in host environment (gain)
c-value
0.8
0.7
0.6
0.5
0.4
0.3
0.2
0.1
0.0
p-value
5e-04
2e-03
5e-03
2e-02
5e-02
Log-odds
0.5
1.0
1.5
2.0
2.5
3.0
3.5
4.0
Cellular_organisms - ps1
Archaea1 - ps2
Archaea2 - ps3
Archaea3 - ps4
Asgard/Eukaryota - ps5
Eukaryota - ps6
Amorpheae - ps7
Obazoa - ps8
Opisthokonta - ps9
Holomycota - ps10
Fungi - ps11
Fungi_1 - ps12
Fungi_2 - ps13
Fungi_3 - ps14
Dikarya - ps15
Ascomycota - ps16
Saccharo/Pezizomycotina - ps17
Saccharomycotina - ps18
Saccharomycotina_1 - ps19
Saccharomycotina_2 - ps20
Saccharomycotina_3 - ps21
Phaff./Saccharomycetaceae - ps22
Saccharomyco./mycetaceae - ps23
Saccharomycetaceae - ps24
Saccharomycetaceae_2 - ps25
Saccharomycetaceae_3 - ps26
Kaz./Nau./Saccharomyces - ps27
Saccharomyces - ps28
Arboricola_clade - ps29
Cerevisiae_clade - ps30
Saccharomyces_cerevisiae - ps31
Phylostratum

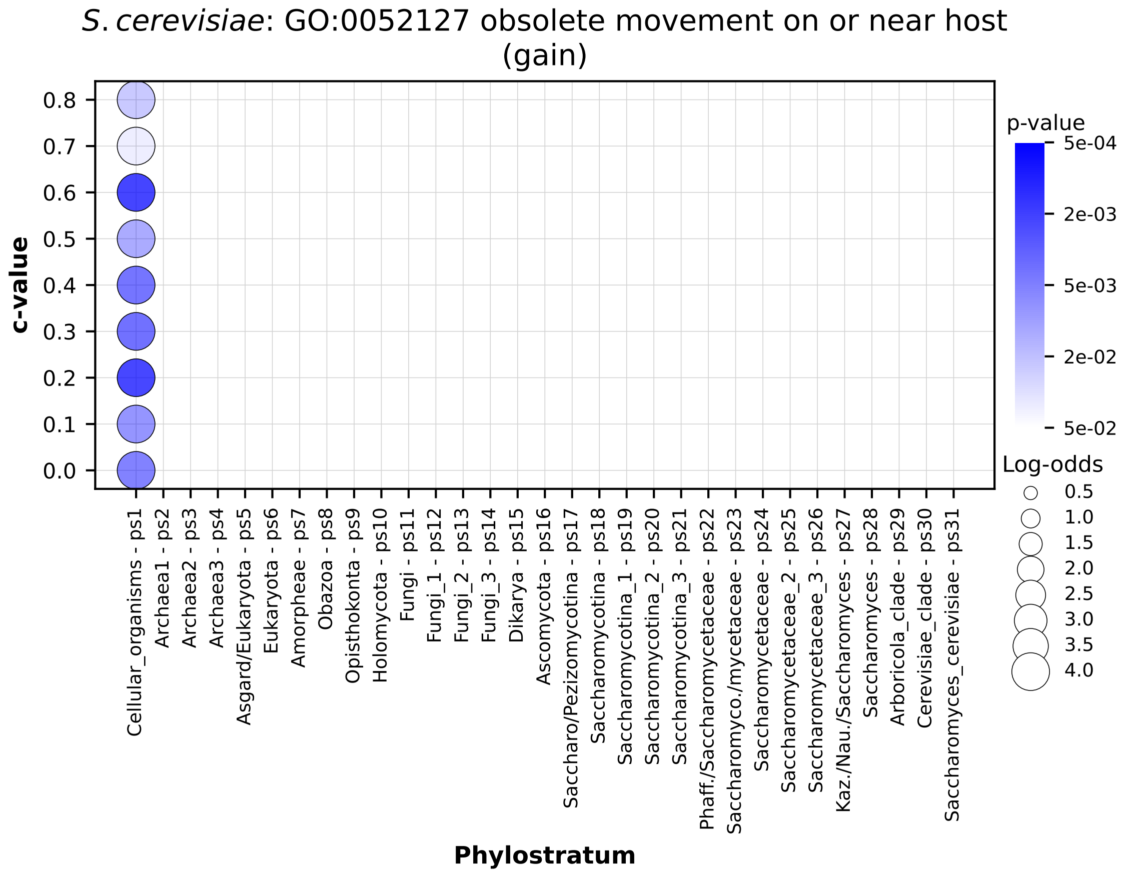
S. cerevisiae: GO:0052127 obsolete movement on or near host (gain)
c-value
0.8
0.7
0.6
0.5
0.4
0.3
0.2
0.1
0.0
p-value
5e-04
2e-03
5e-03
2e-02
5e-02
Log-odds
0.5
1.0
1.5
2.0
2.5
3.0
3.5
4.0
Cellular_organisms - ps1
Archaea1 - ps2
Archaea2 - ps3
Archaea3 - ps4
Asgard/Eukaryota - ps5
Eukaryota - ps6
Amorpheae - ps7
Obazoa - ps8
Opisthokonta - ps9
Holomycota - ps10
Fungi - ps11
Fungi_1 - ps12
Fungi_2 - ps13
Fungi_3 - ps14
Dikarya - ps15
Ascomycota - ps16
Saccharo/Pezizomycotina - ps17
Saccharomycotina - ps18
Saccharomycotina_1 - ps19
Saccharomycotina_2 - ps20
Saccharomycotina_3 - ps21
Phaff./Saccharomycetaceae - ps22
Saccharomyco./mycetaceae - ps23
Saccharomycetaceae - ps24
Saccharomycetaceae_2 - ps25
Saccharomycetaceae_3 - ps26
Kaz./Nau./Saccharomyces - ps27
Saccharomyces - ps28
Arboricola_clade - ps29
Cerevisiae_clade - ps30
Saccharomyces_cerevisiae - ps31
Phylostratum

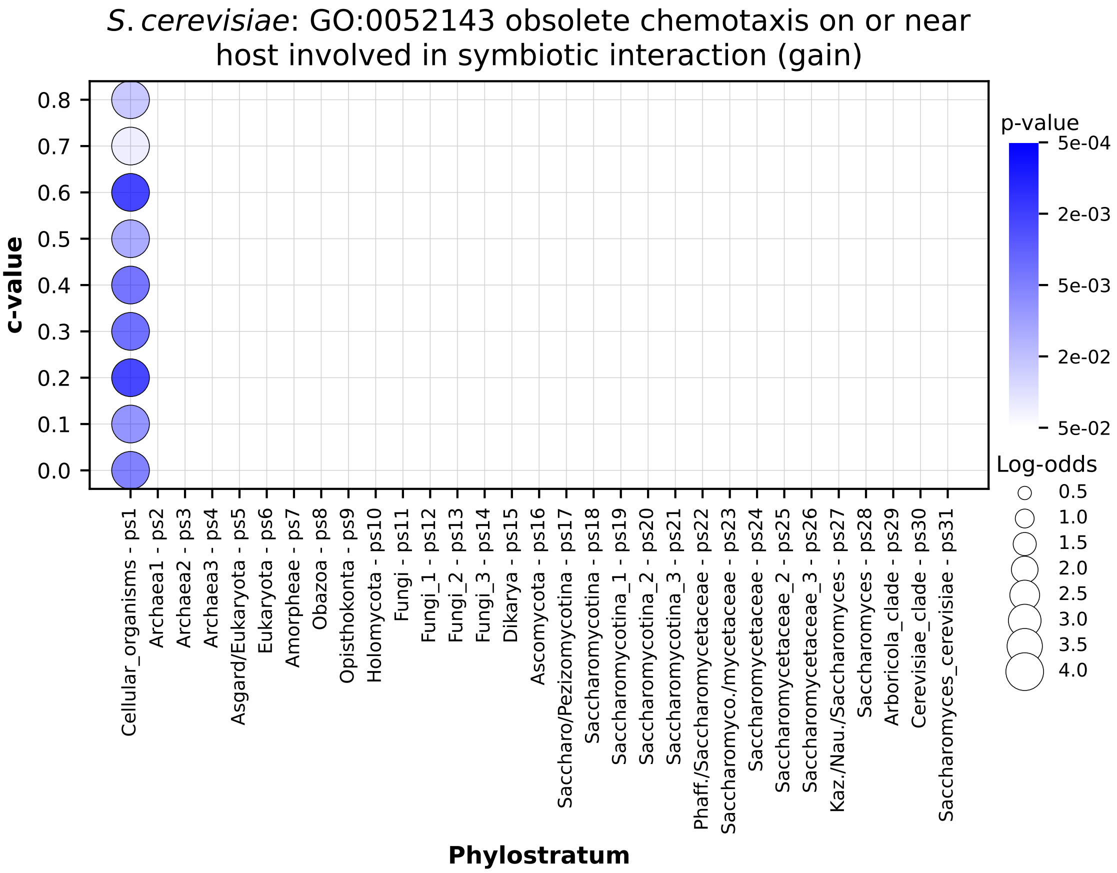

S. cerevisiae: GO:0052143 obsolete chemotaxis on or near host involved in symbiotic interaction (gain)
c-value
0.8
0.7
0.6
0.5
0.4
0.3
0.2
0.1
0.0
Phylostratum
Cellular_organisms - ps1
Archaea1 - ps2
Archaea2 - ps3
Archaea3 - ps4
Asgard/Eukaryota - ps5
Eukaryota - ps6
Amorpheae - ps7
Obazoa - ps8
Opisthokonta - ps9
Holomycota - ps10
Fungi - ps11
Fungi_1 - ps12
Fungi_2 - ps13
Fungi_3 - ps14
Dikarya - ps15
Ascomycota - ps16
Saccharo/Pezizomycotina - ps17
Saccharomycotina - ps18
Saccharomycotina_1 - ps19
Saccharomycotina_2 - ps20
Saccharomycotina_3 - ps21
Phaff./Saccharomycetaceae - ps22
Saccharomyco./mycetaceae - ps23
Saccharomycetaceae - ps24
Saccharomycetaceae_2 - ps25
Saccharomycetaceae_3 - ps26
Kaz./Nau./Saccharomyces - ps27
Saccharomyces - ps28
Arboricola_clade - ps29
Cerevisiae_clade - ps30
Saccharomyces_cerevisiae - ps31
p-value
5e-04
2e-03
5e-03
2e-02
5e-02
Log-odds
0.5
1.0
1.5
2.0
2.5
3.0
3.5
4.0

S. cerevisiae: GO:0052173 response to defenses of other organism (gain)

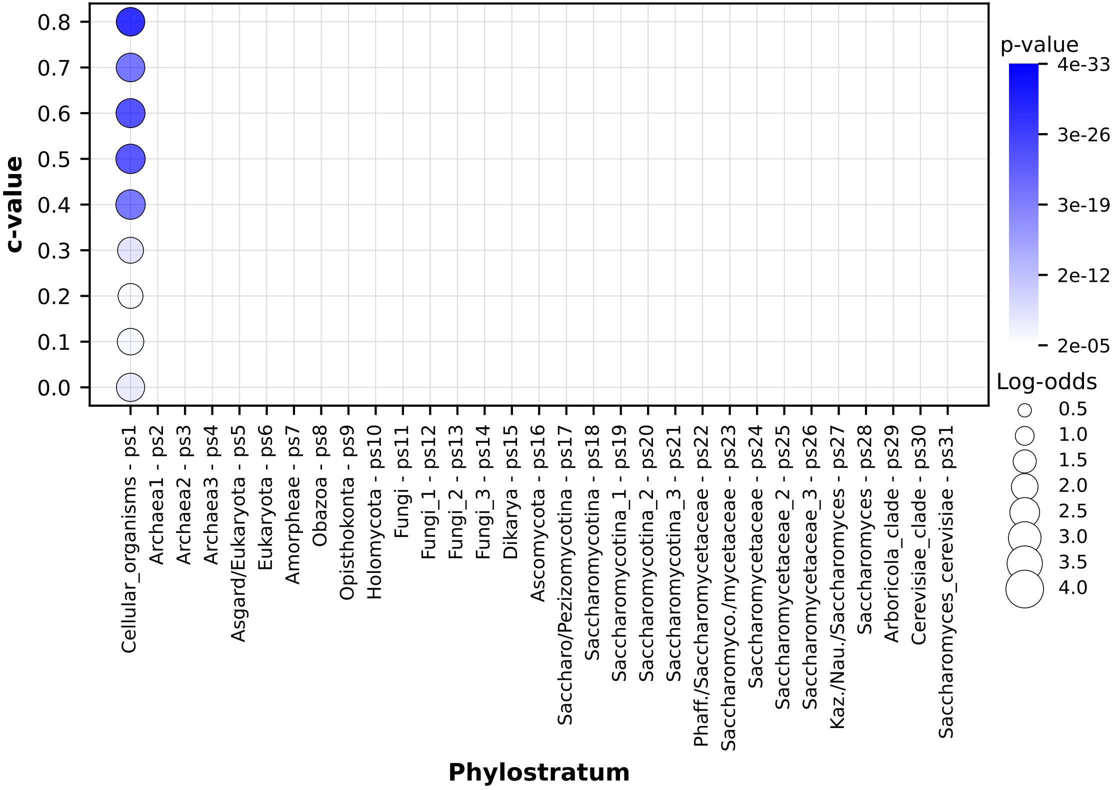

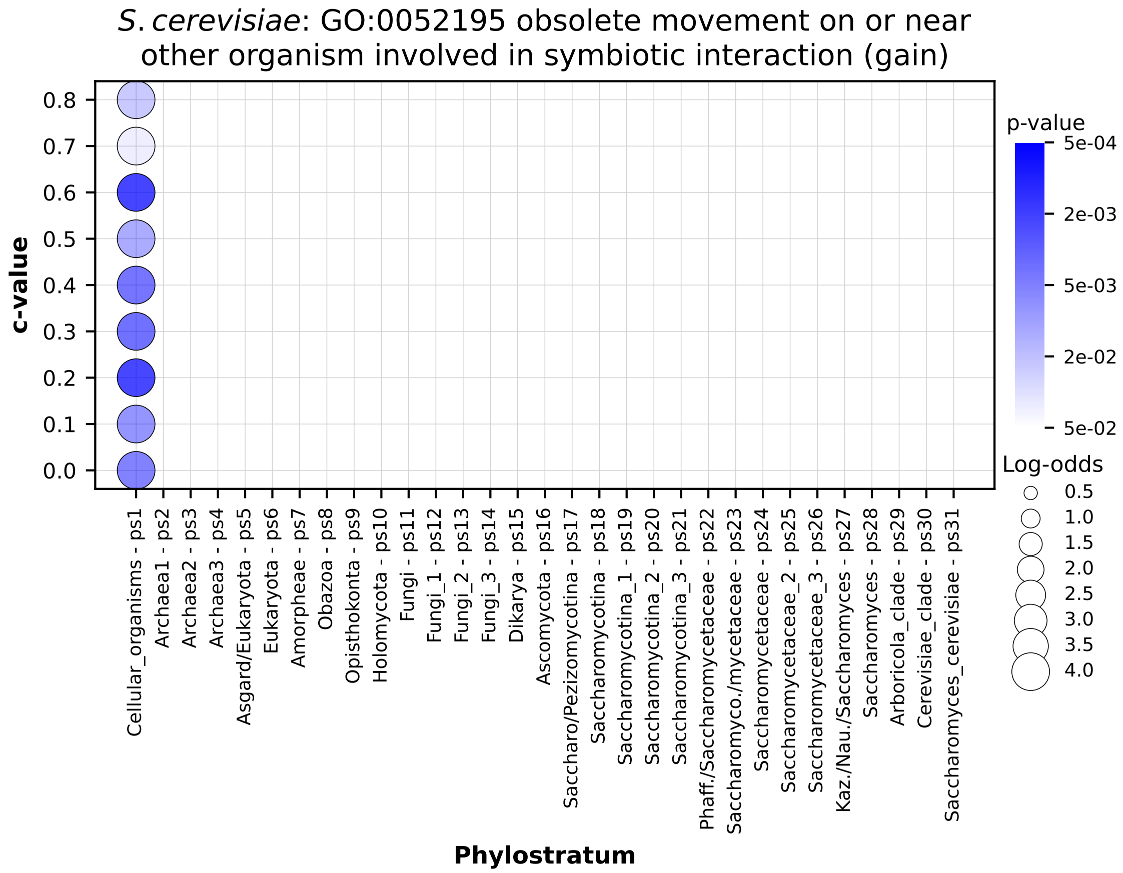

S. cerevisiae: GO:0052195 obsolete movement on or near other organism involved in symbiotic interaction (gain)
c-value
0.8
0.7
0.6
0.5
0.4
0.3
0.2
0.1
0.0
p-value
5e-04
2e-03
5e-03
2e-02
5e-02
Log-odds
0.5
1.0
1.5
2.0
2.5
3.0
3.5
4.0
Cellular_organisms - ps1
Archaea1 - ps2
Archaea2 - ps3
Archaea3 - ps4
Asgard/Eukaryota - ps5
Eukaryota - ps6
Amorpheae - ps7
Obazoa - ps8
Opisthokonta - ps9
Holomycota - ps10
Fungi - ps11
Fungi_1 - ps12
Fungi_2 - ps13
Fungi_3 - ps14
Dikarya - ps15
Ascomycota - ps16
Saccharo/Pezizomycotina - ps17
Saccharomycotina - ps18
Saccharomycotina_1 - ps19
Saccharomycotina_2 - ps20
Saccharomycotina_3 - ps21
Phaff./Saccharomycetaceae - ps22
Saccharomyco./mycetaceae - ps23
Saccharomycetaceae - ps24
Saccharomycetaceae_2 - ps25
Saccharomycetaceae_3 - ps26
Kaz./Nau./Saccharomyces - ps27
Saccharomyces - ps28
Arboricola_clade - ps29
Cerevisiae_clade - ps30
Saccharomyces_cerevisiae - ps31
Phylostratum

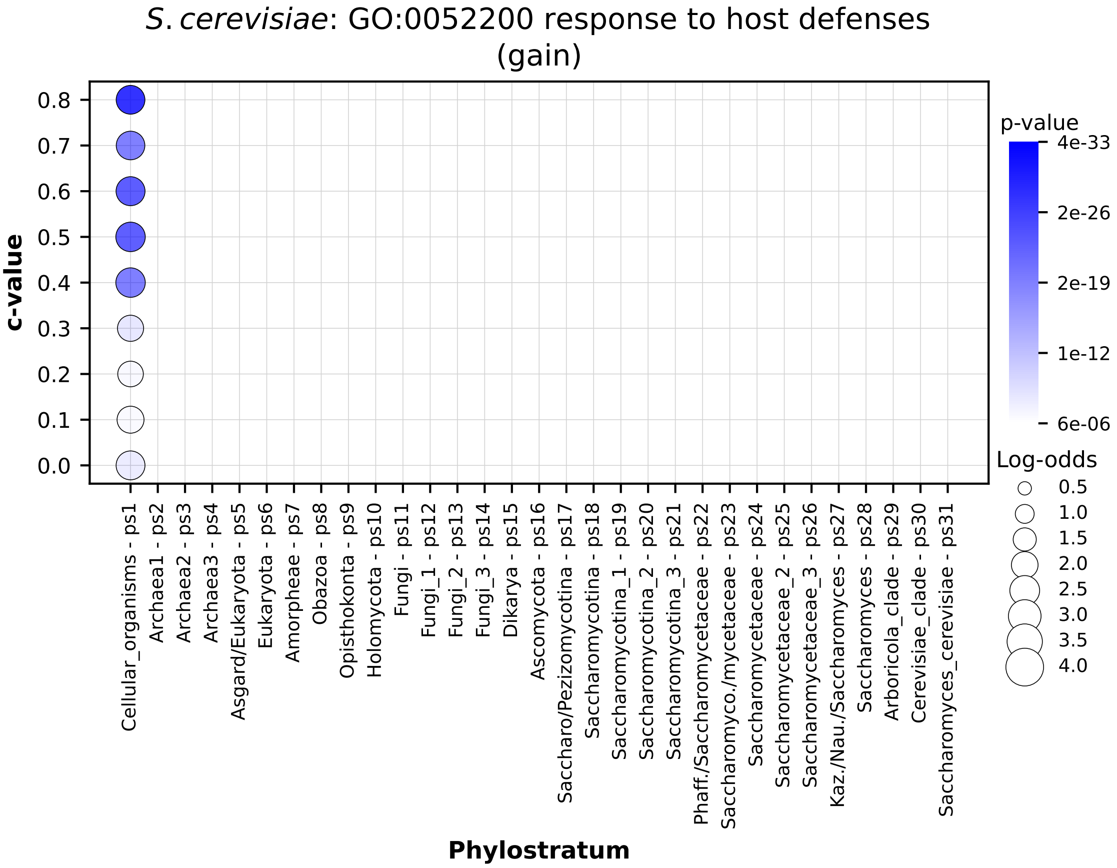

S. cerevisiae: GO:0052200 response to host defenses
(gain)
c-value
0.0
0.1
0.2
0.3
0.4
0.5
0.6
0.7
0.8
p-value
4e-33
2e-26
2e-19
1e-12
6e-06
Log-odds
0.5
1.0
1.5
2.0
2.5
3.0
3.5
4.0
Cellular_organisms - ps1
Archaea1 - ps2
Archaea2 - ps3
Archaea3 - ps4
Asgard/Eukaryota - ps5
Eukaryota - ps6
Amorpheae - ps7
Obazoa - ps8
Opisthokonta - ps9
Holomycota - ps10
Fungi - ps11
Fungi_1 - ps12
Fungi_2 - ps13
Fungi_3 - ps14
Dikarya - ps15
Ascomycota - ps16
Saccharo/Pezizomycotina - ps17
Saccharomycotina - ps18
Saccharomycotina_1 - ps19
Saccharomycotina_2 - ps20
Saccharomycotina_3 - ps21
Phaff./Saccharomycetaceae - ps22
Saccharomyco./mycetaceae - ps23
Saccharomycetaceae - ps24
Saccharomycetaceae_2 - ps25
Saccharomycetaceae_3 - ps26
Kaz./Nau./Saccharomyces - ps27
Saccharomyces - ps28
Arboricola_clade - ps29
Cerevisiae_clade - ps30
Saccharomyces_cerevisiae - ps31
Phylostratum

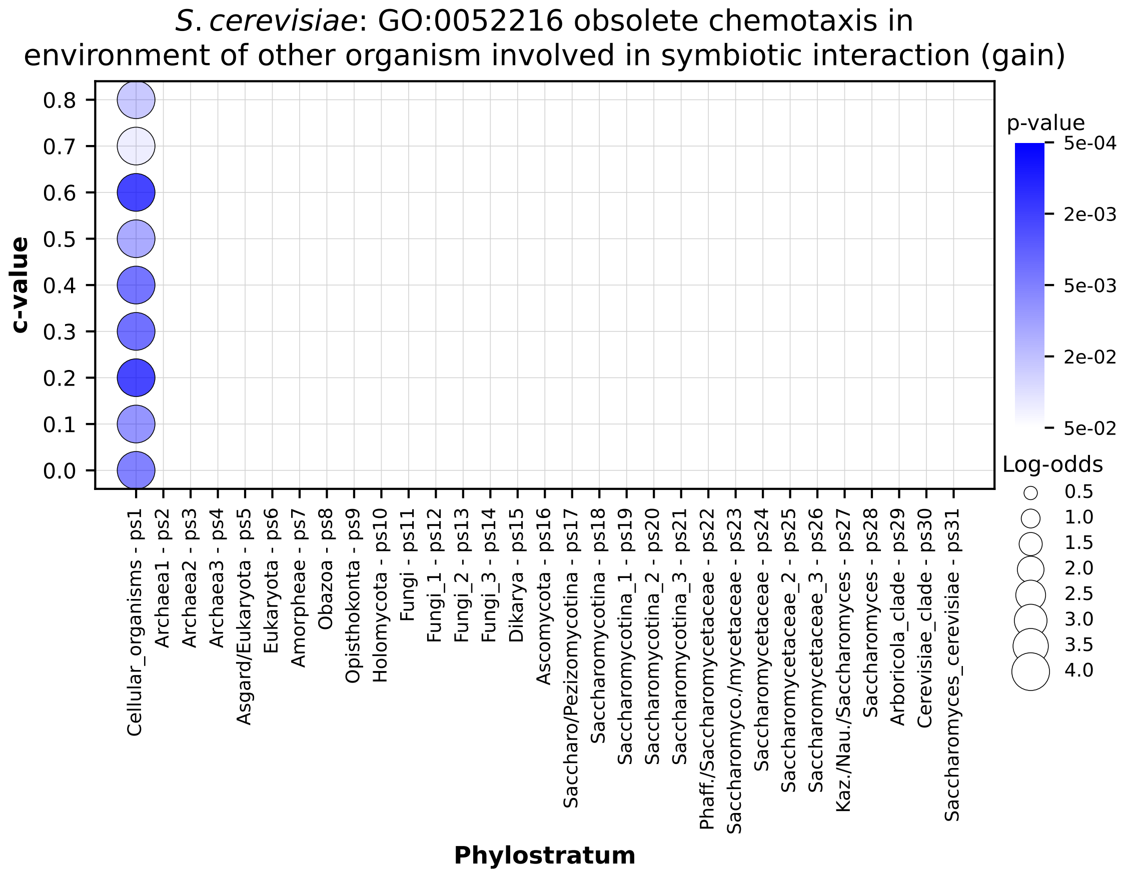

S. cerevisiae: GO:0052216 obsolete chemotaxis in environment of other organism involved in symbiotic interaction (gain)
c-value
0.8
0.7
0.6
0.5
0.4
0.3
0.2
0.1
0.0
p-value
5e-04
2e-03
5e-03
2e-02
5e-02
Log-odds
0.5
1.0
1.5
2.0
2.5
3.0
3.5
4.0
Cellular_organisms - ps1
Archaea1 - ps2
Archaea2 - ps3
Archaea3 - ps4
Asgard/Eukaryota - ps5
Eukaryota - ps6
Amorpheae - ps7
Obazoa - ps8
Opisthokonta - ps9
Holomycota - ps10
Fungi - ps11
Fungi_1 - ps12
Fungi_2 - ps13
Fungi_3 - ps14
Dikarya - ps15
Ascomycota - ps16
Saccharo/Pezizomycotina - ps17
Saccharomycotina - ps18
Saccharomycotina_1 - ps19
Saccharomycotina_2 - ps20
Saccharomycotina_3 - ps21
Phaff./Saccharomycetaceae - ps22
Saccharomyco./mycetaceae - ps23
Saccharomycetaceae - ps24
Saccharomycetaceae_2 - ps25
Saccharomycetaceae_3 - ps26
Kaz./Nau./Saccharomyces - ps27
Saccharomyces - ps28
Arboricola_clade - ps29
Cerevisiae_clade - ps30
Saccharomyces_cerevisiae - ps31
Phylostratum

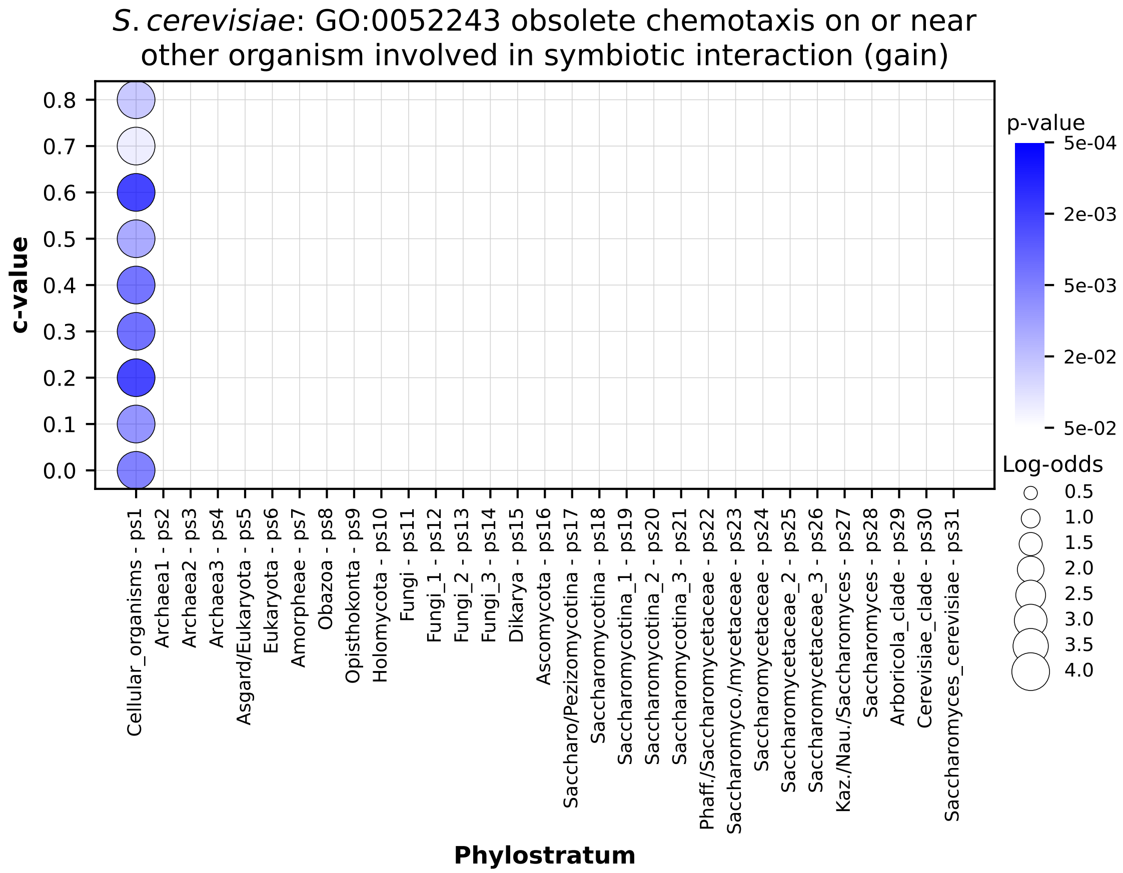
S. cerevisiae: GO:0052243 obsolete chemotaxis on or near other organism involved in symbiotic interaction (gain)
c-value
0.8
0.7
0.6
0.5
0.4
0.3
0.2
0.1
0.0
p-value
5e-04
2e-03
5e-03
2e-02
5e-02
Log-odds
0.5
1.0
1.5
2.0
2.5
3.0
3.5
4.0
Cellular_organisms - ps1
Archaea1 - ps2
Archaea2 - ps3
Archaea3 - ps4
Asgard/Eukaryota - ps5
Eukaryota - ps6
Amorpheae - ps7
Obazoa - ps8
Opisthokonta - ps9
Holomycota - ps10
Fungi - ps11
Fungi_1 - ps12
Fungi_2 - ps13
Fungi_3 - ps14
Dikarya - ps15
Ascomycota - ps16
Saccharo/Pezizomycotina - ps17
Saccharomycotina - ps18
Saccharomycotina_1 - ps19
Saccharomycotina_2 - ps20
Saccharomycotina_3 - ps21
Phaff./Saccharomycetaceae - ps22
Saccharomyco./mycetaceae - ps23
Saccharomycetaceae - ps24
Saccharomycetaceae_2 - ps25
Saccharomycetaceae_3 - ps26
Kaz./Nau./Saccharomyces - ps27
Saccharomyces - ps28
Arboricola_clade - ps29
Cerevisiae_clade - ps30
Saccharomyces_cerevisiae - ps31
Phylostratum

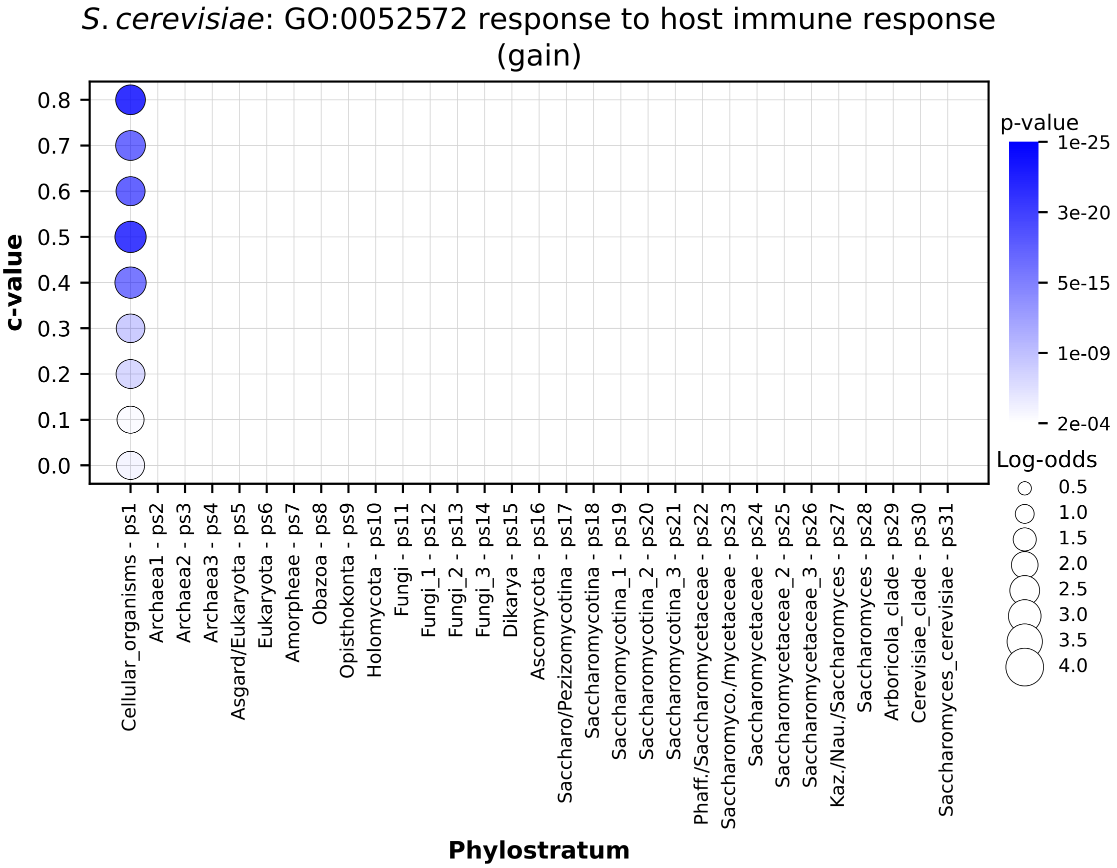

S. cerevisiae: GO:0052572 response to host immune response (gain)
c-value
0.8
0.7
0.6
0.5
0.4
0.3
0.2
0.1
0.0
p-value
1e-25
3e-20
5e-15
1e-09
2e-04
Log-odds
0.5
1.0
1.5
2.0
2.5
3.0
3.5
4.0
Cellular_organisms - ps1
Archaea1 - ps2
Archaea2 - ps3
Archaea3 - ps4
Asgard/Eukaryota - ps5
Eukaryota - ps6
Amorpheae - ps7
Obazoa - ps8
Opisthokonta - ps9
Holomycota - ps10
Fungi - ps11
Fungi_1 - ps12
Fungi_2 - ps13
Fungi_3 - ps14
Dikarya - ps15
Ascomycota - ps16
Saccharo/Pezizomycotina - ps17
Saccharomycotina - ps18
Saccharomycotina_1 - ps19
Saccharomycotina_2 - ps20
Saccharomycotina_3 - ps21
Phaff./Saccharomycetaceae - ps22
Saccharomyco./mycetaceae - ps23
Saccharomycetaceae - ps24
Saccharomycetaceae_2 - ps25
Saccharomycetaceae_3 - ps26
Kaz./Nau./Saccharomyces - ps27
Saccharomyces - ps28
Arboricola_clade - ps29
Cerevisiae_clade - ps30
Saccharomyces_cerevisiae - ps31
Phylostratum

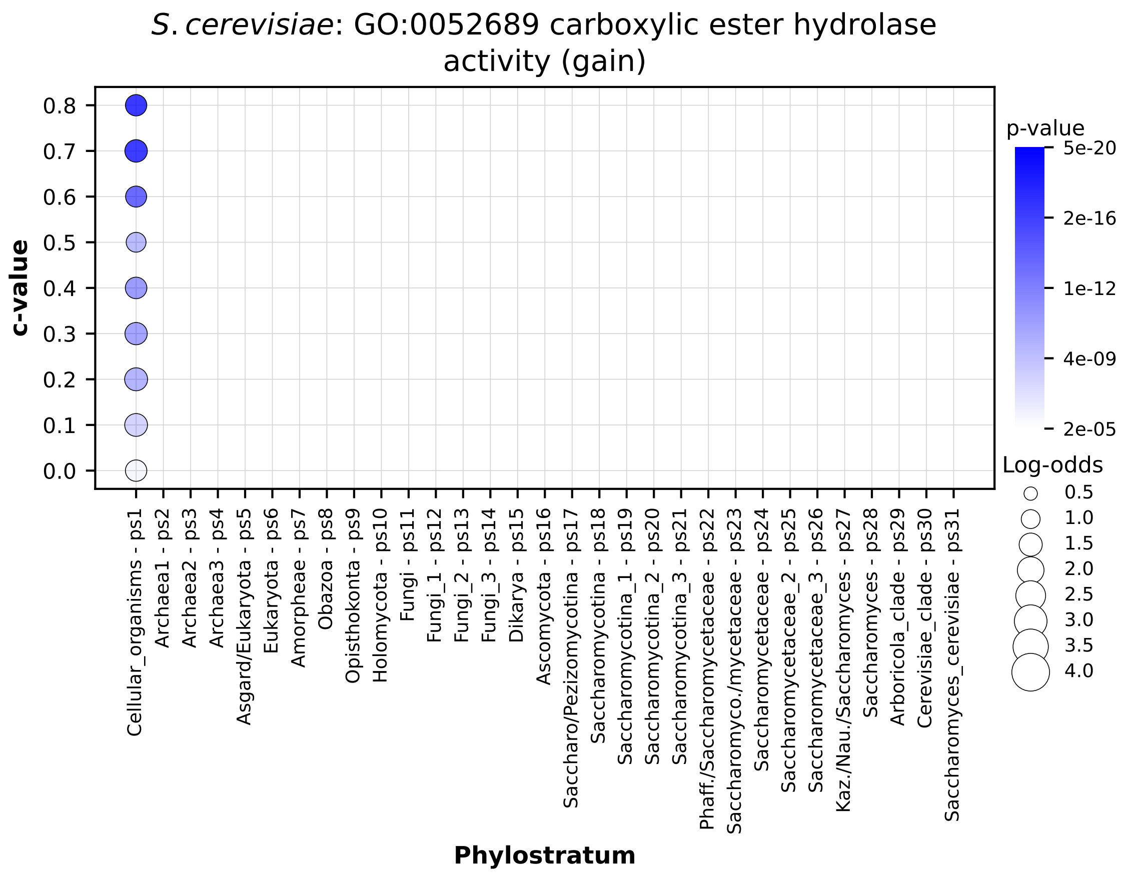

S. cerevisiae: GO:0052689 carboxylic ester hydrolase activity (gain)
c-value
0.8
0.7
0.6
0.5
0.4
0.3
0.2
0.1
0.0
p-value
5e-20
2e-16
1e-12
4e-09
2e-05
Log-odds
0.5
1.0
1.5
2.0
2.5
3.0
3.5
4.0
Cellular_organisms - ps1
Archaea1 - ps2
Archaea2 - ps3
Archaea3 - ps4
Asgard/Eukaryota - ps5
Eukaryota - ps6
Amorpheae - ps7
Obazoa - ps8
Opisthokonta - ps9
Holomycota - ps10
Fungi - ps11
Fungi_1 - ps12
Fungi_2 - ps13
Fungi_3 - ps14
Dikarya - ps15
Ascomycota - ps16
Saccharo/Pezizomycotina - ps17
Saccharomycotina - ps18
Saccharomycotina_1 - ps19
Saccharomycotina_2 - ps20
Saccharomycotina_3 - ps21
Phaff./Saccharomycetaceae - ps22
Saccharomyco./mycetaceae - ps23
Saccharomycetaceae - ps24
Saccharomycetaceae_2 - ps25
Saccharomycetaceae_3 - ps26
Kaz./Nau./Saccharomyces - ps27
Saccharomyces - ps28
Arboricola_clade - ps29
Cerevisiae_clade - ps30
Saccharomyces_cerevisiae - ps31
Phylostratum

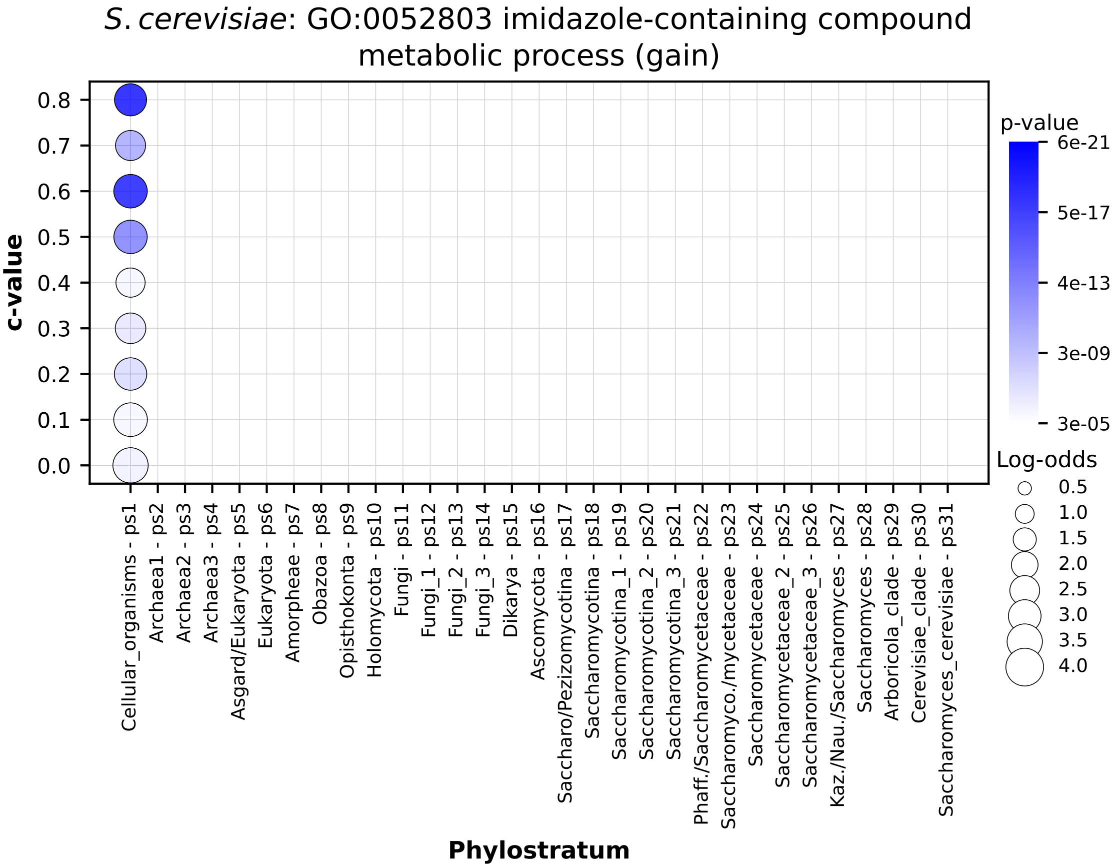
S. cerevisiae: GO:0052803 imidazole-containing compound metabolic process (gain)
c-value
0.8
0.7
0.6
0.5
0.4
0.3
0.2
0.1
0.0
p-value
6e-21
5e-17
4e-13
3e-09
3e-05
Log-odds
0.5
1.0
1.5
2.0
2.5
3.0
3.5
4.0
Cellular_organisms - ps1
Archaea1 - ps2
Archaea2 - ps3
Archaea3 - ps4
Asgard/Eukaryota - ps5
Eukaryota - ps6
Amorpheae - ps7
Obazoa - ps8
Opisthokonta - ps9
Holomycota - ps10
Fungi - ps11
Fungi_1 - ps12
Fungi_2 - ps13
Fungi_3 - ps14
Dikarya - ps15
Ascomycota - ps16
Saccharo/Pezizomycotina - ps17
Saccharomycotina - ps18
Saccharomycotina_1 - ps19
Saccharomycotina_2 - ps20
Saccharomycotina_3 - ps21
Phaff./Saccharomycetaceae - ps22
Saccharomyco./mycetaceae - ps23
Saccharomycetaceae - ps24
Saccharomycetaceae_2 - ps25
Saccharomycetaceae_3 - ps26
Kaz./Nau./Saccharomyces - ps27
Saccharomyces - ps28
Arboricola_clade - ps29
Cerevisiae_clade - ps30
Saccharomyces_cerevisiae - ps31
Phylostratum

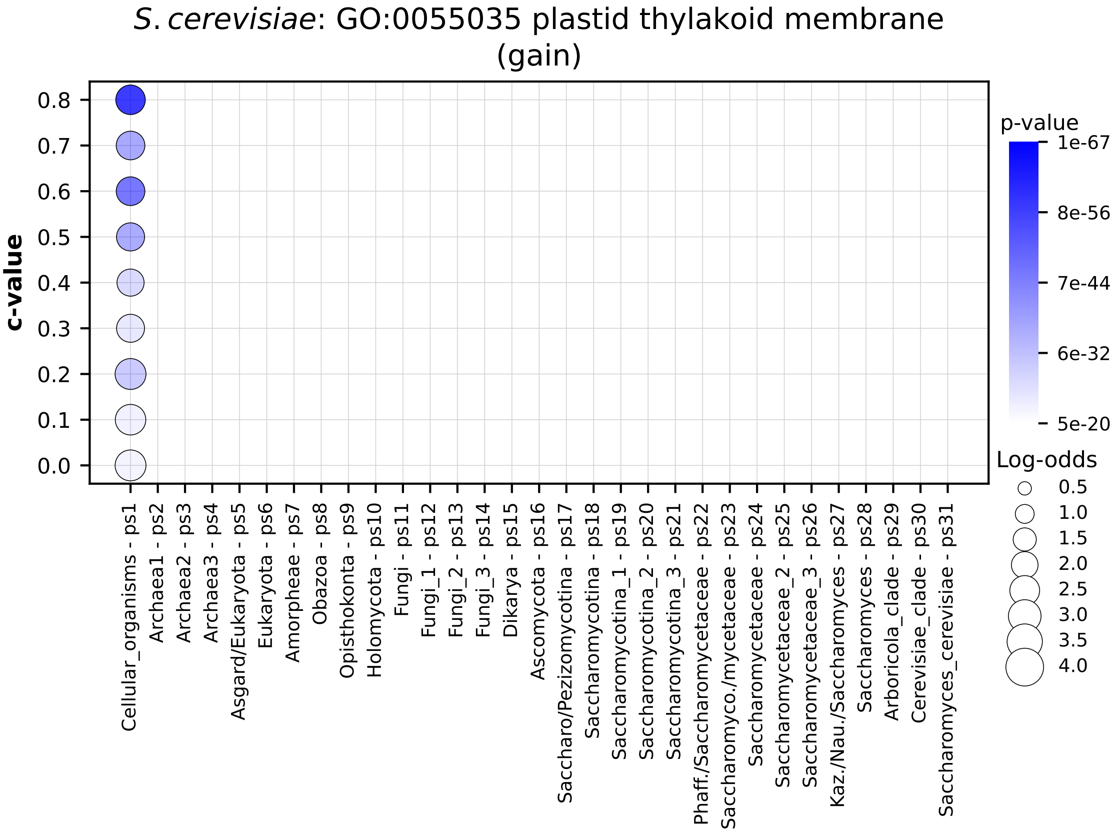

S. cerevisiae: GO:0055035 plastid thylakoid membrane
(gain)
c-value
0.8
0.7
0.6
0.5
0.4
0.3
0.2
0.1
0.0
p-value
1e-67
8e-56
7e-44
6e-32
5e-20
Log-odds
0.5
1.0
1.5
2.0
2.5
3.0
3.5
4.0
Cellular_organisms - ps1
Archaea1 - ps2
Archaea2 - ps3
Archaea3 - ps4
Asgard/Eukaryota - ps5
Eukaryota - ps6
Amorpheae - ps7
Obazoa - ps8
Opisthokonta - ps9
Holomycota - ps10
Fungi - ps11
Fungi_1 - ps12
Fungi_2 - ps13
Fungi_3 - ps14
Dikarya - ps15
Ascomycota - ps16
Saccharo/Pezizomycotina - ps17
Saccharomycotina - ps18
Saccharomycotina_1 - ps19
Saccharomycotina_2 - ps20
Saccharomycotina_3 - ps21
Phaff./Saccharomycetaceae - ps22
Saccharomyco./mycetaceae - ps23
Saccharomycetaceae - ps24
Saccharomycetaceae_2 - ps25
Saccharomycetaceae_3 - ps26
Kaz./Nau./Saccharomyces - ps27
Saccharomyces - ps28
Arboricola_clade - ps29
Cerevisiae_clade - ps30
Saccharomyces_cerevisiae - ps31
Phylostratum

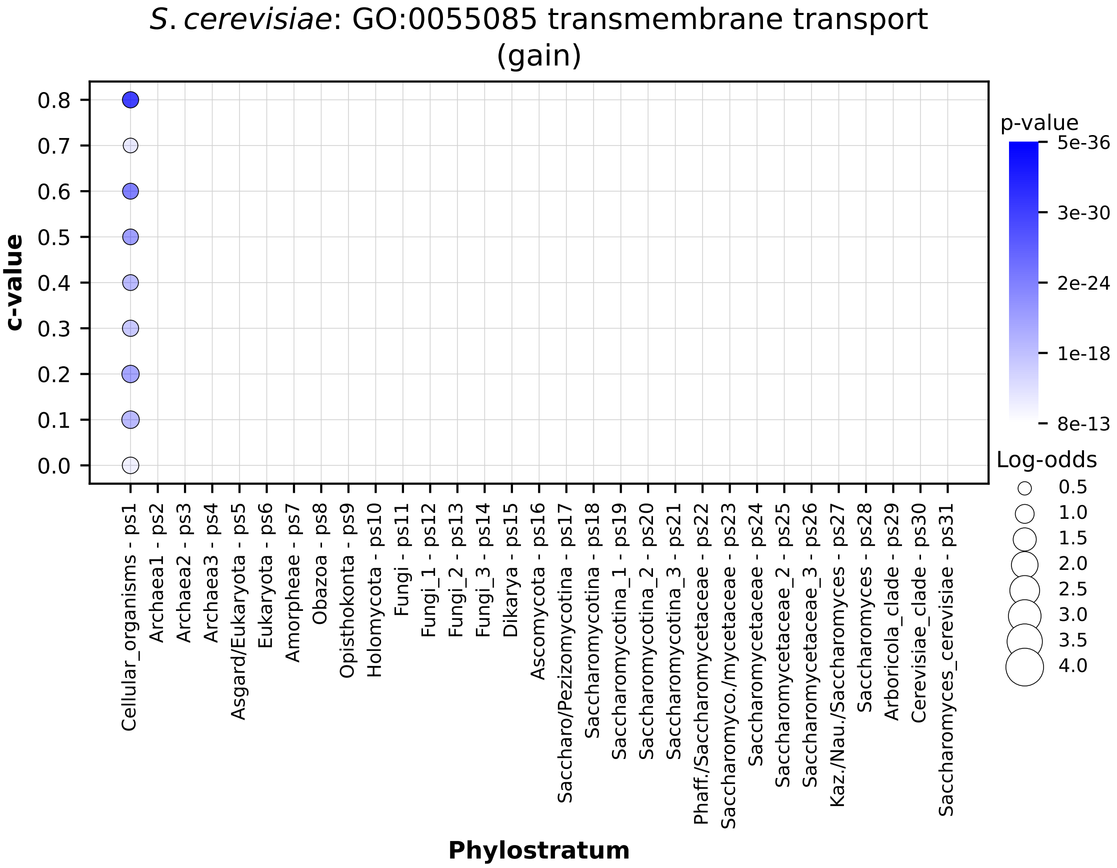

S. cerevisiae: GO:0055085 transmembrane transport
(gain)
c-value
0.8
0.7
0.6
0.5
0.4
0.3
0.2
0.1
0.0
p-value
5e-36
3e-30
2e-24
1e-18
8e-13
Log-odds
0.5
1.0
1.5
2.0
2.5
3.0
3.5
4.0
Cellular_organisms - ps1
Archaea1 - ps2
Archaea2 - ps3
Archaea3 - ps4
Asgard/Eukaryota - ps5
Eukaryota - ps6
Amorpheae - ps7
Obazoa - ps8
Opisthokonta - ps9
Holomycota - ps10
Fungi - ps11
Fungi_1 - ps12
Fungi_2 - ps13
Fungi_3 - ps14
Dikarya - ps15
Ascomycota - ps16
Saccharo/Pezizomycotina - ps17
Saccharomycotina - ps18
Saccharomycotina_1 - ps19
Saccharomycotina_2 - ps20
Saccharomycotina_3 - ps21
Phaff./Saccharomycetaceae - ps22
Saccharomyco./mycetaceae - ps23
Saccharomycetaceae - ps24
Saccharomycetaceae_2 - ps25
Saccharomycetaceae_3 - ps26
Kaz./Nau./Saccharomyces - ps27
Saccharomyces - ps28
Arboricola_clade - ps29
Cerevisiae_clade - ps30
Saccharomyces_cerevisiae - ps31
Phylostratum

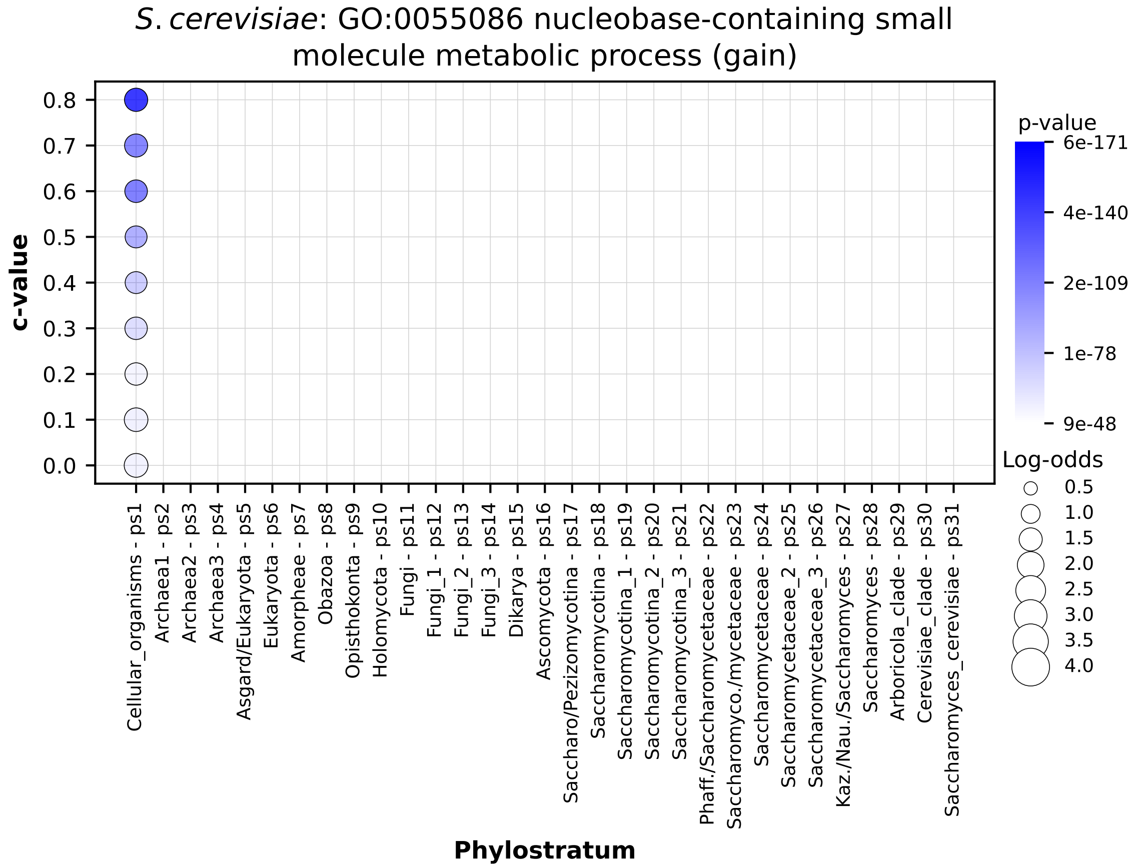

S. cerevisiae: GO:0055086 nucleobase-containing small molecule metabolic process (gain)
c-value
0.8
0.7
0.6
0.5
0.4
0.3
0.2
0.1
0.0
p-value
6e-171
4e-140
2e-109
1e-78
9e-48
Log-odds
0.5
1.0
1.5
2.0
2.5
3.0
3.5
4.0
Cellular_organisms - ps1
Archaea1 - ps2
Archaea2 - ps3
Archaea3 - ps4
Asgard/Eukaryota - ps5
Eukaryota - ps6
Amorpheae - ps7
Obazoa - ps8
Opisthokonta - ps9
Holomycota - ps10
Fungi - ps11
Fungi_1 - ps12
Fungi_2 - ps13
Fungi_3 - ps14
Dikarya - ps15
Ascomycota - ps16
Saccharo/Pezizomycotina - ps17
Saccharomycotina - ps18
Saccharomycotina_1 - ps19
Saccharomycotina_2 - ps20
Saccharomycotina_3 - ps21
Phaff./Saccharomycetaceae - ps22
Saccharomyco./mycetaceae - ps23
Saccharomycetaceae - ps24
Saccharomycetaceae_2 - ps25
Saccharomycetaceae_3 - ps26
Kaz./Nau./Saccharomyces - ps27
Saccharomyces - ps28
Arboricola_clade - ps29
Cerevisiae_clade - ps30
Saccharomyces_cerevisiae - ps31
Phylostratum

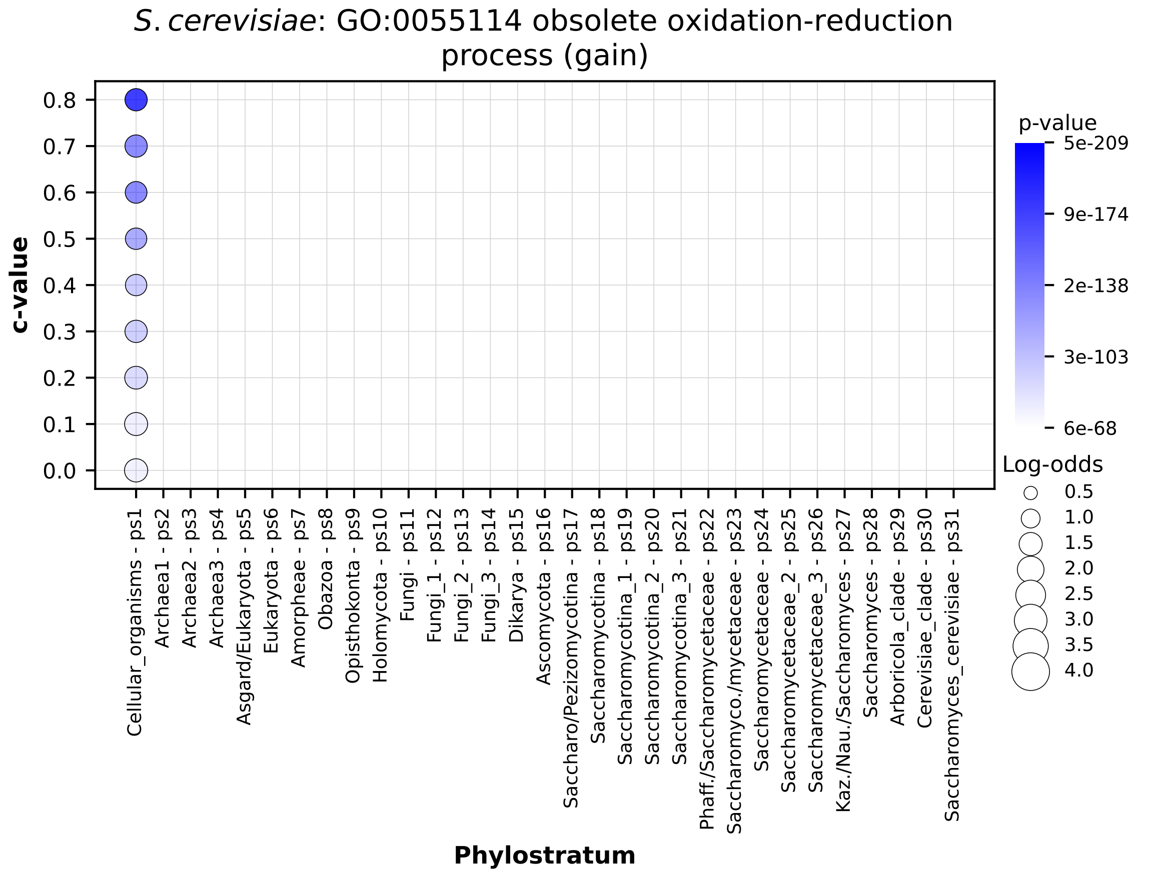

S. cerevisiae: GO:0055114 obsolete oxidation-reduction process (gain)
c-value
0.8
0.7
0.6
0.5
0.4
0.3
0.2
0.1
0.0
p-value
5e-209
9e-174
2e-138
3e-103
6e-68
Log-odds
0.5
1.0
1.5
2.0
2.5
3.0
3.5
4.0
Cellular_organisms - ps1
Archaea1 - ps2
Archaea2 - ps3
Archaea3 - ps4
Asgard/Eukaryota - ps5
Eukaryota - ps6
Amorpheae - ps7
Obazoa - ps8
Opisthokonta - ps9
Holomycota - ps10
Fungi - ps11
Fungi_1 - ps12
Fungi_2 - ps13
Fungi_3 - ps14
Dikarya - ps15
Ascomycota - ps16
Saccharo/Pezizomycotina - ps17
Saccharomycotina - ps18
Saccharomycotina_1 - ps19
Saccharomycotina_2 - ps20
Saccharomycotina_3 - ps21
Phaff./Saccharomycetaceae - ps22
Saccharomyco./mycetaceae - ps23
Saccharomycetaceae - ps24
Saccharomycetaceae_2 - ps25
Saccharomycetaceae_3 - ps26
Kaz./Nau./Saccharomyces - ps27
Saccharomyces - ps28
Arboricola_clade - ps29
Cerevisiae_clade - ps30
Saccharomyces_cerevisiae - ps31
Phylostratum

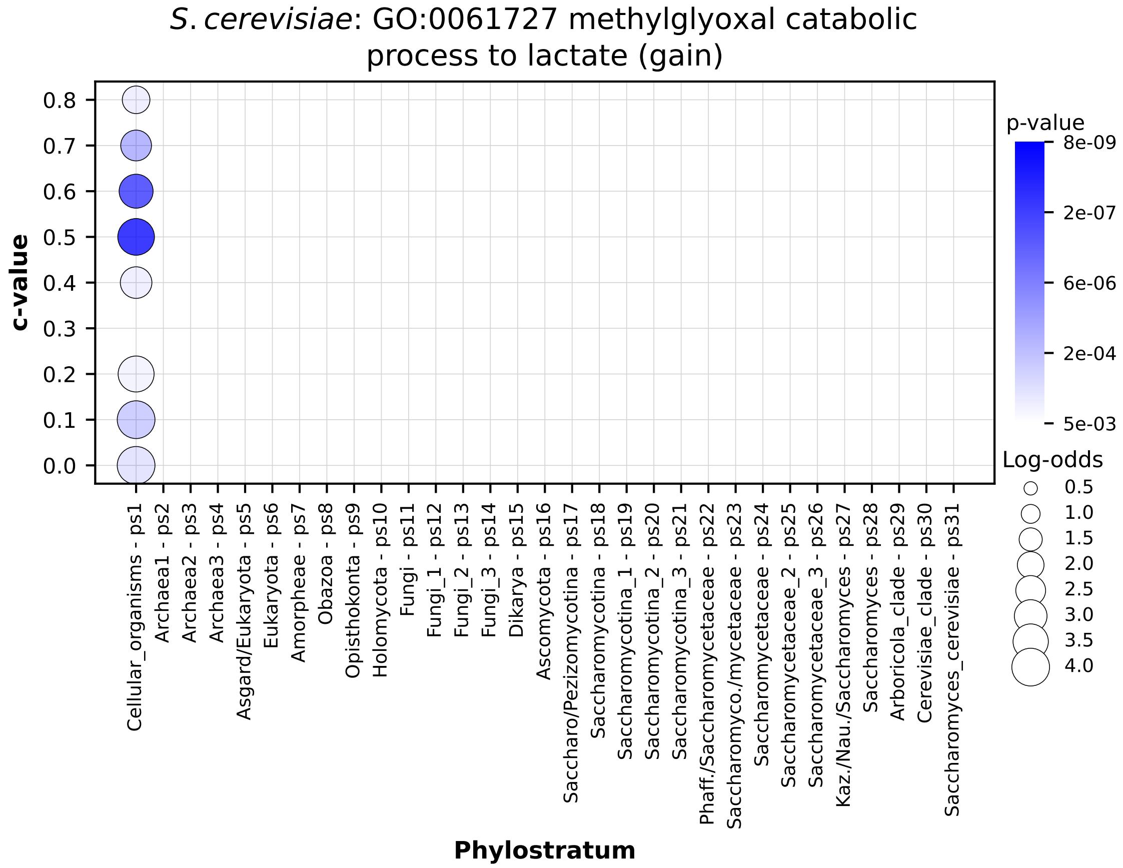
S. cerevisiae: GO:0061727 methylglyoxal catabolic process to lactate (gain)
c-value
0.8
0.7
0.6
0.5
0.4
0.3
0.2
0.1
0.0
p-value
8e-09
2e-07
6e-06
2e-04
5e-03
Log-odds
0.5
1.0
1.5
2.0
2.5
3.0
3.5
4.0
Cellular_organisms - ps1
Archaea1 - ps2
Archaea2 - ps3
Archaea3 - ps4
Asgard/Eukaryota - ps5
Eukaryota - ps6
Amorpheae - ps7
Obazoa - ps8
Opisthokonta - ps9
Holomycota - ps10
Fungi - ps11
Fungi_1 - ps12
Fungi_2 - ps13
Fungi_3 - ps14
Dikarya - ps15
Ascomycota - ps16
Saccharo/Pezizomycotina - ps17
Saccharomycotina - ps18
Saccharomycotina_1 - ps19
Saccharomycotina_2 - ps20
Saccharomycotina_3 - ps21
Phaff./Saccharomycetaceae - ps22
Saccharomyco./mycetaceae - ps23
Saccharomycetaceae - ps24
Saccharomycetaceae_2 - ps25
Saccharomycetaceae_3 - ps26
Kaz./Nau./Saccharomyces - ps27
Saccharomyces - ps28
Arboricola_clade - ps29
Cerevisiae_clade - ps30
Saccharomyces_cerevisiae - ps31
Phylostratum

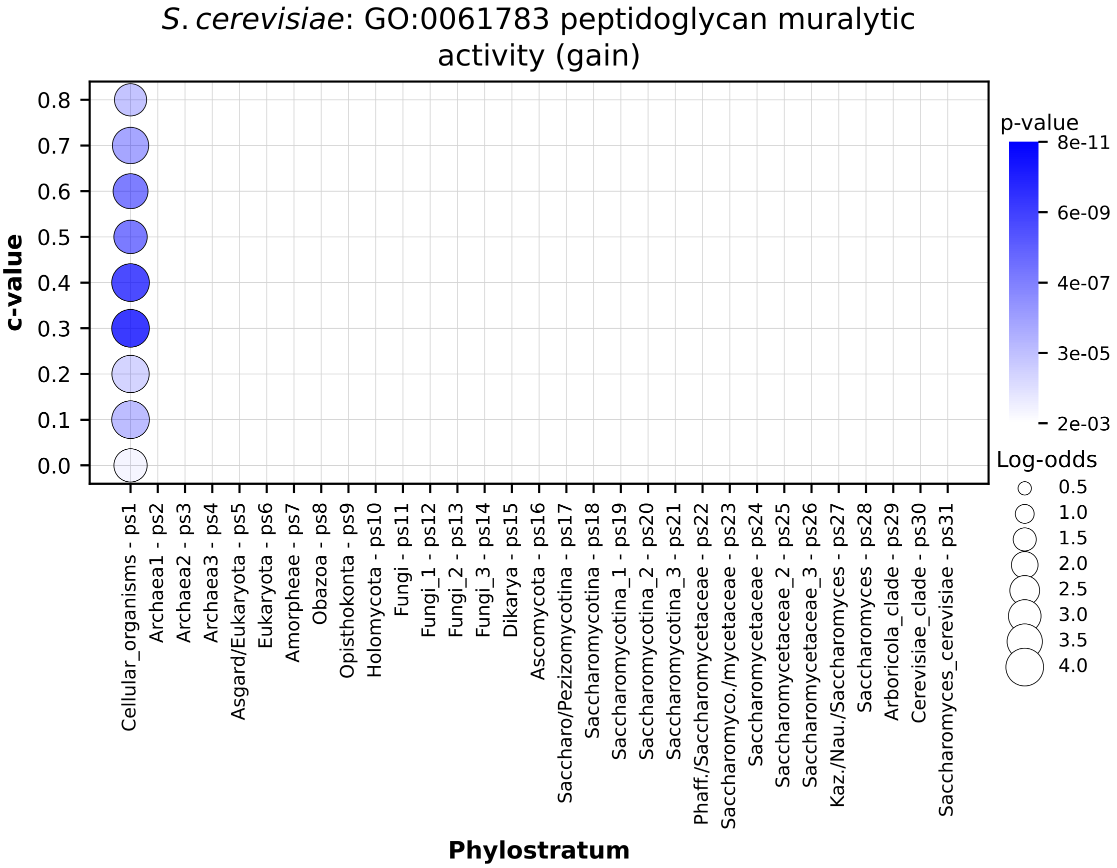

S. cerevisiae: GO:0061783 peptidoglycan muralytic activity (gain)
c-value
0.0
0.1
0.2
0.3
0.4
0.5
0.6
0.7
0.8
p-value
8e-11
6e-09
4e-07
3e-05
2e-03
Log-odds
0.5
1.0
1.5
2.0
2.5
3.0
3.5
4.0
Cellular_organisms - ps1
Archaea1 - ps2
Archaea2 - ps3
Archaea3 - ps4
Asgard/Eukaryota - ps5
Eukaryota - ps6
Amorpheae - ps7
Obazoa - ps8
Opisthokonta - ps9
Holomycota - ps10
Fungi - ps11
Fungi_1 - ps12
Fungi_2 - ps13
Fungi_3 - ps14
Dikarya - ps15
Ascomycota - ps16
Saccharo/Pezizomycotina - ps17
Saccharomycotina - ps18
Saccharomycotina_1 - ps19
Saccharomycotina_2 - ps20
Saccharomycotina_3 - ps21
Phaff./Saccharomycetaceae - ps22
Saccharomyco./mycetaceae - ps23
Saccharomycetaceae - ps24
Saccharomycetaceae_2 - ps25
Saccharomycetaceae_3 - ps26
Kaz./Nau./Saccharomyces - ps27
Saccharomyces - ps28
Arboricola_clade - ps29
Cerevisiae_clade - ps30
Saccharomyces_cerevisiae - ps31
Phylostratum

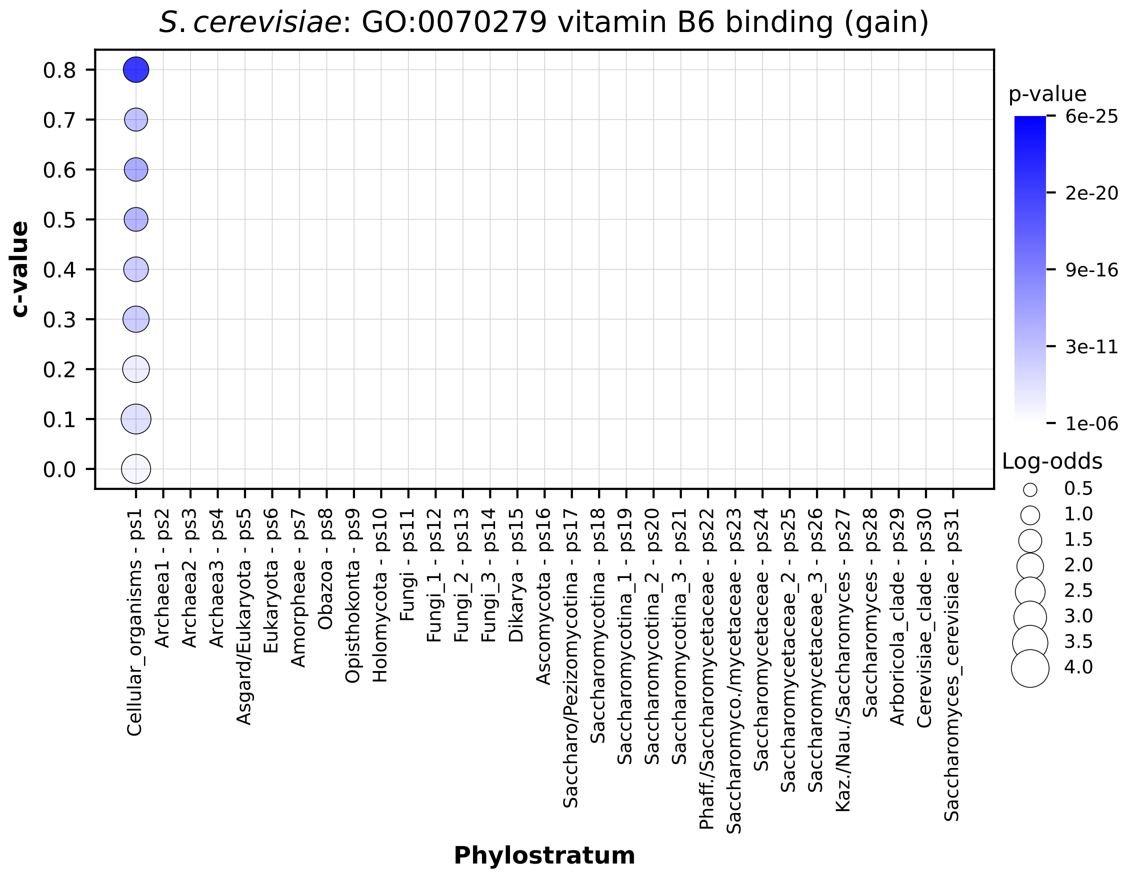
S. cerevisiae: GO:0070279 vitamin B6 binding (gain)
c-value
0.8
0.7
0.6
0.5
0.4
0.3
0.2
0.1
0.0
p-value
6e-25
2e-20
9e-16
3e-11
1e-06
Log-odds
0.5
1.0
1.5
2.0
2.5
3.0
3.5
4.0
Cellular_organisms - ps1
Archaea1 - ps2
Archaea2 - ps3
Archaea3 - ps4
Asgard/Eukaryota - ps5
Eukaryota - ps6
Amorpheae - ps7
Obazoa - ps8
Opisthokonta - ps9
Holomycota - ps10
Fungi - ps11
Fungi_1 - ps12
Fungi_2 - ps13
Fungi_3 - ps14
Dikarya - ps15
Ascomycota - ps16
Saccharo/Pezizomycotina - ps17
Saccharomycotina - ps18
Saccharomycotina_1 - ps19
Saccharomycotina_2 - ps20
Saccharomycotina_3 - ps21
Phaff./Saccharomycetaceae - ps22
Saccharomyco./mycetaceae - ps23
Saccharomycetaceae - ps24
Saccharomycetaceae_2 - ps25
Saccharomycetaceae_3 - ps26
Kaz./Nau./Saccharomyces - ps27
Saccharomyces - ps28
Arboricola_clade - ps29
Cerevisiae_clade - ps30
Saccharomyces_cerevisiae - ps31
Phylostratum

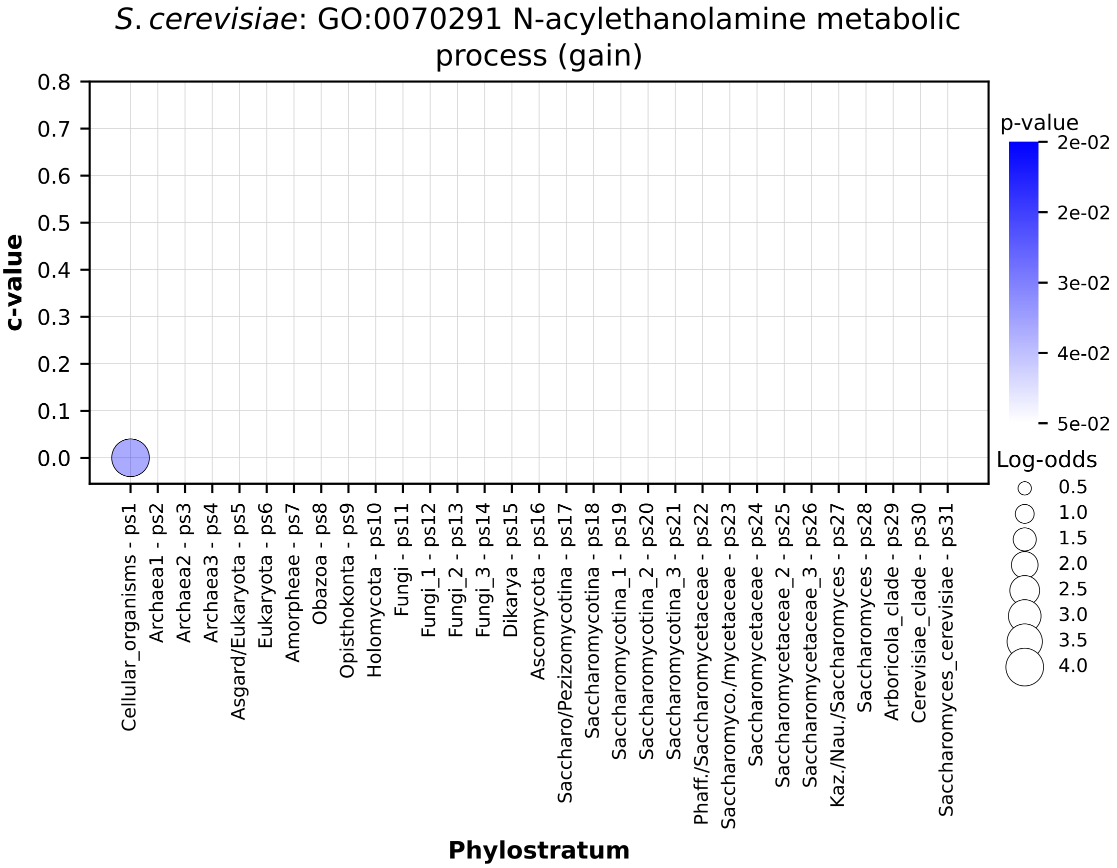

S. cerevisiae: GO:0070291 N-acylethanolamine metabolic process (gain)
c-value
0.8
0.7
0.6
0.5
0.4
0.3
0.2
0.1
0.0
Phylostratum
Cellular_organisms - ps1
Archaea1 - ps2
Archaea2 - ps3
Archaea3 - ps4
Asgard/Eukaryota - ps5
Eukaryota - ps6
Amorpheae - ps7
Obazoa - ps8
Opisthokonta - ps9
Holomycota - ps10
Fungi - ps11
Fungi_1 - ps12
Fungi_2 - ps13
Fungi_3 - ps14
Dikarya - ps15
Ascomycota - ps16
Saccharo/Pezizomycotina - ps17
Saccharomycotina - ps18
Saccharomycotina_1 - ps19
Saccharomycotina_2 - ps20
Saccharomycotina_3 - ps21
Phaff./Saccharomycetaceae - ps22
Saccharomyco./mycetaceae - ps23
Saccharomycetaceae - ps24
Saccharomycetaceae_2 - ps25
Saccharomycetaceae_3 - ps26
Kaz./Nau./Saccharomyces - ps27
Saccharomyces - ps28
Arboricola_clade - ps29
Cerevisiae_clade - ps30
Saccharomyces_cerevisiae - ps31
p-value
2e-02
2e-02
3e-02
4e-02
5e-02
Log-odds
0.5
1.0
1.5
2.0
2.5
3.0
3.5
4.0

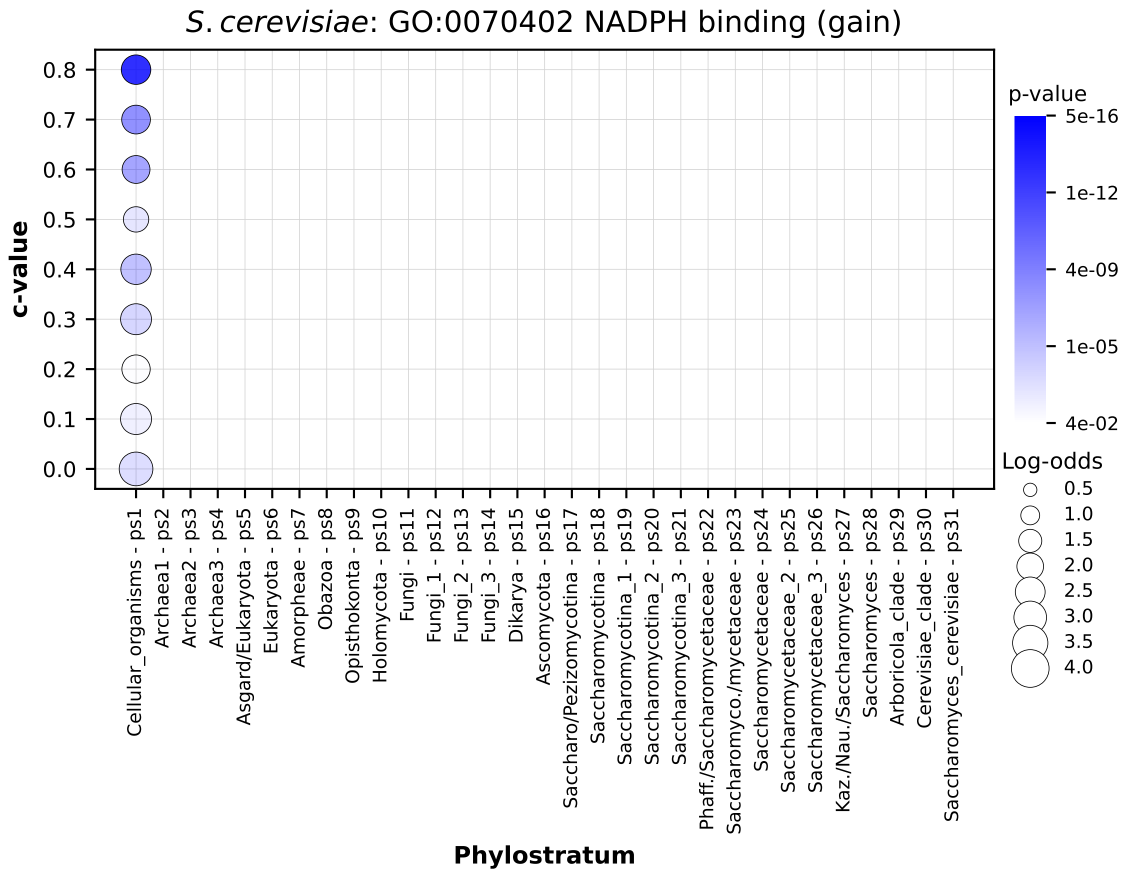

S. cerevisiae: GO:0070402 NADPH binding (gain)
c-value
0.8
0.7
0.6
0.5
0.4
0.3
0.2
0.1
0.0
p-value
5e-16
1e-12
4e-09
1e-05
4e-02
Log-odds
0.5
1.0
1.5
2.0
2.5
3.0
3.5
4.0
Cellular_organisms - ps1
Archaea1 - ps2
Archaea2 - ps3
Archaea3 - ps4
Asgard/Eukaryota - ps5
Eukaryota - ps6
Amorpheae - ps7
Obazoa - ps8
Opisthokonta - ps9
Holomycota - ps10
Fungi - ps11
Fungi_1 - ps12
Fungi_2 - ps13
Fungi_3 - ps14
Dikarya - ps15
Ascomycota - ps16
Saccharo/Pezizomycotina - ps17
Saccharomycotina - ps18
Saccharomycotina_1 - ps19
Saccharomycotina_2 - ps20
Saccharomycotina_3 - ps21
Phaff./Saccharomycetaceae - ps22
Saccharomyco./mycetaceae - ps23
Saccharomycetaceae - ps24
Saccharomycetaceae_2 - ps25
Saccharomycetaceae_3 - ps26
Kaz./Nau./Saccharomyces - ps27
Saccharomyces - ps28
Arboricola_clade - ps29
Cerevisiae_clade - ps30
Saccharomyces_cerevisiae - ps31
Phylostratum

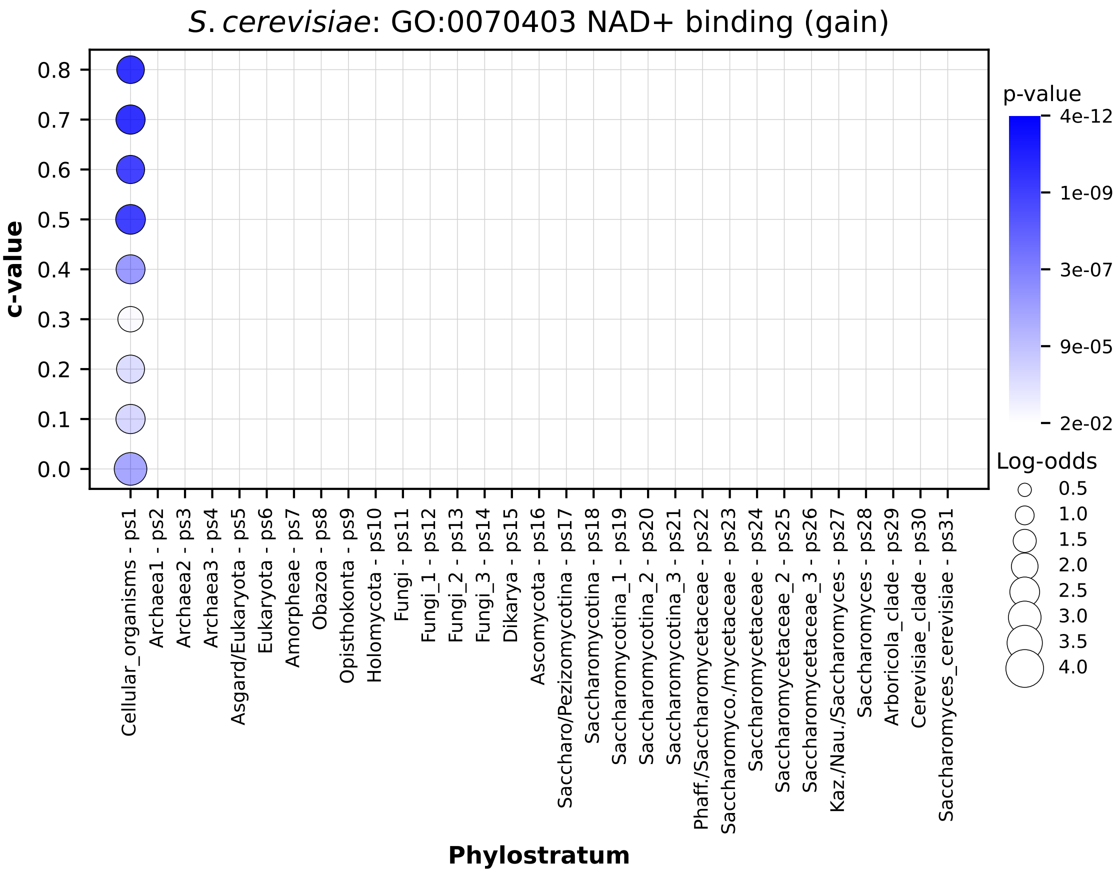

S. cerevisiae: GO:0070403 NAD+ binding (gain)
c-value
0.8
0.7
0.6
0.5
0.4
0.3
0.2
0.1
0.0
p-value
4e-12
1e-09
3e-07
9e-05
2e-02
Log-odds
0.5
1.0
1.5
2.0
2.5
3.0
3.5
4.0
Cellular_organisms - ps1
Archaea1 - ps2
Archaea2 - ps3
Archaea3 - ps4
Asgard/Eukaryota - ps5
Eukaryota - ps6
Amorpheae - ps7
Obazoa - ps8
Opisthokonta - ps9
Holomycota - ps10
Fungi - ps11
Fungi_1 - ps12
Fungi_2 - ps13
Fungi_3 - ps14
Dikarya - ps15
Ascomycota - ps16
Saccharo/Pezizomycotina - ps17
Saccharomycotina - ps18
Saccharomycotina_1 - ps19
Saccharomycotina_2 - ps20
Saccharomycotina_3 - ps21
Phaff./Saccharomycetaceae - ps22
Saccharomyco./mycetaceae - ps23
Saccharomycetaceae - ps24
Saccharomycetaceae_2 - ps25
Saccharomycetaceae_3 - ps26
Kaz./Nau./Saccharomyces - ps27
Saccharomyces - ps28
Arboricola_clade - ps29
Cerevisiae_clade - ps30
Saccharomyces_cerevisiae - ps31
Phylostratum

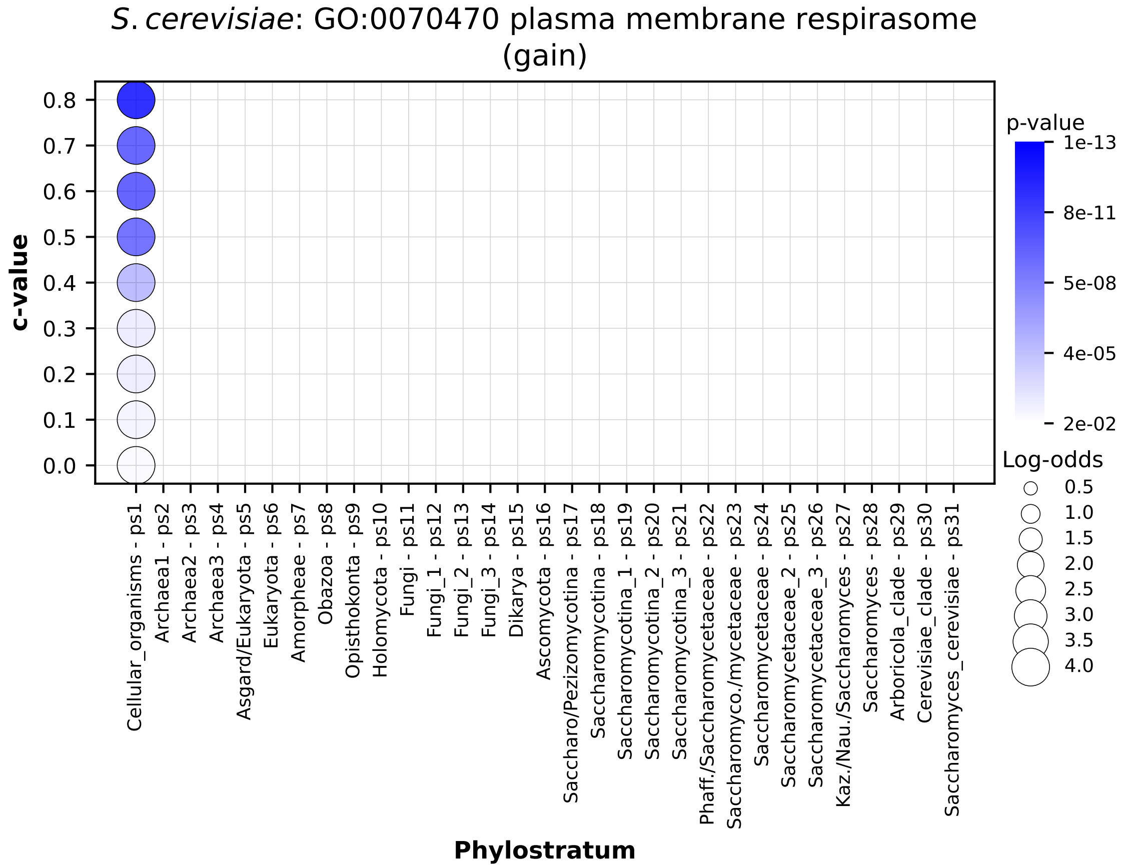

S. cerevisiae: GO:0070470 plasma membrane respirasome (gain)
c-value
0.8
0.7
0.6
0.5
0.4
0.3
0.2
0.1
0.0
p-value
1e-13
8e-11
5e-08
4e-05
2e-02
Log-odds
0.5
1.0
1.5
2.0
2.5
3.0
3.5
4.0
Cellular_organisms - ps1
Archaea1 - ps2
Archaea2 - ps3
Archaea3 - ps4
Asgard/Eukaryota - ps5
Eukaryota - ps6
Amorpheae - ps7
Obazoa - ps8
Opisthokonta - ps9
Holomycota - ps10
Fungi - ps11
Fungi_1 - ps12
Fungi_2 - ps13
Fungi_3 - ps14
Dikarya - ps15
Ascomycota - ps16
Saccharo/Pezizomycotina - ps17
Saccharomycotina - ps18
Saccharomycotina_1 - ps19
Saccharomycotina_2 - ps20
Saccharomycotina_3 - ps21
Phaff./Saccharomycetaceae - ps22
Saccharomyco./mycetaceae - ps23
Saccharomycetaceae - ps24
Saccharomycetaceae_2 - ps25
Saccharomycetaceae_3 - ps26
Kaz./Nau./Saccharomyces - ps27
Saccharomyces - ps28
Arboricola_clade - ps29
Cerevisiae_clade - ps30
Saccharomyces_cerevisiae - ps31
Phylostratum

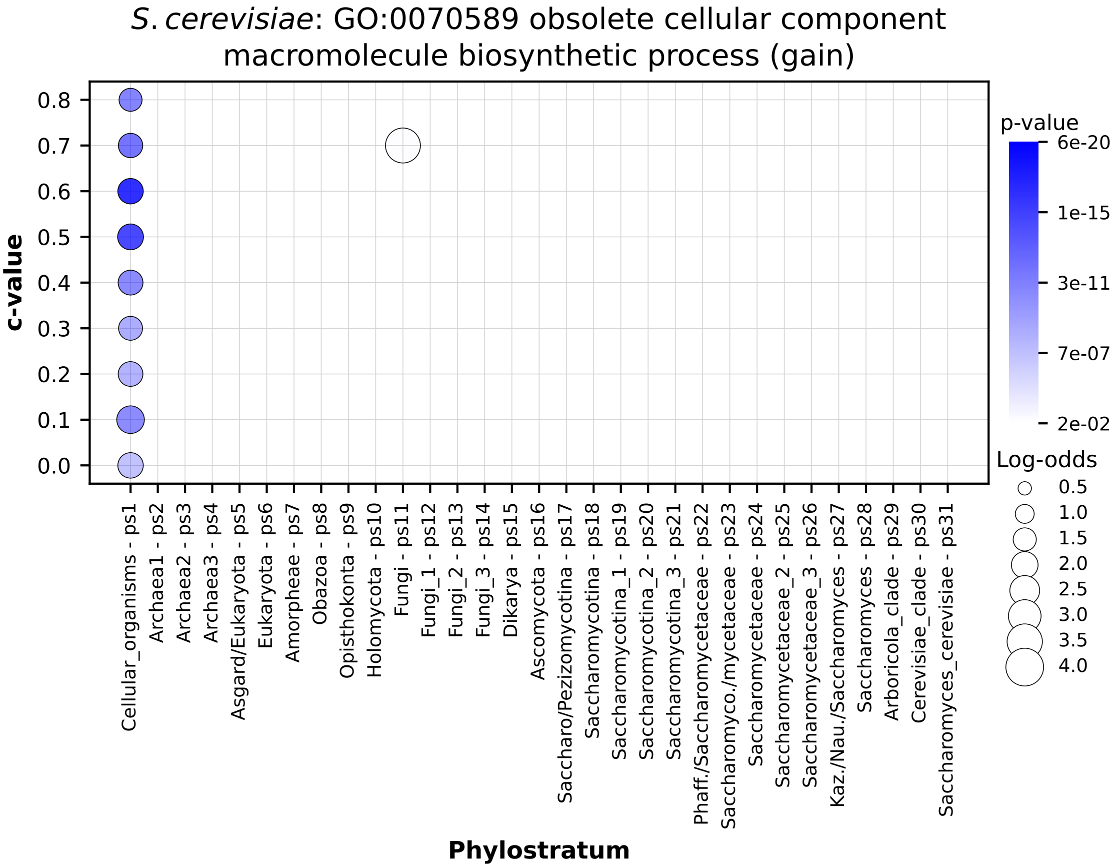
S. cerevisiae: GO:0070589 obsolete cellular component macromolecule biosynthetic process (gain)
c-value
0.0
0.1
0.2
0.3
0.4
0.5
0.6
0.7
0.8
p-value
6e-20
1e-15
3e-11
7e-07
2e-02
Log-odds
0.5
1.0
1.5
2.0
2.5
3.0
3.5
4.0
Cellular_organisms - ps1
Archaea1 - ps2
Archaea2 - ps3
Archaea3 - ps4
Asgard/Eukaryota - ps5
Eukaryota - ps6
Amorpheae - ps7
Obazoa - ps8
Opisthokonta - ps9
Holomycota - ps10
Fungi - ps11
Fungi_1 - ps12
Fungi_2 - ps13
Fungi_3 - ps14
Dikarya - ps15
Ascomycota - ps16
Saccharo/Pezizomycotina - ps17
Saccharomycotina - ps18
Saccharomycotina_1 - ps19
Saccharomycotina_2 - ps20
Saccharomycotina_3 - ps21
Phaff./Saccharomycetaceae - ps22
Saccharomyco./mycetaceae - ps23
Saccharomycetaceae - ps24
Saccharomycetaceae_2 - ps25
Saccharomycetaceae_3 - ps26
Kaz./Nau./Saccharomyces - ps27
Saccharomyces - ps28
Arboricola_clade - ps29
Cerevisiae_clade - ps30
Saccharomyces_cerevisiae - ps31
Phylostratum

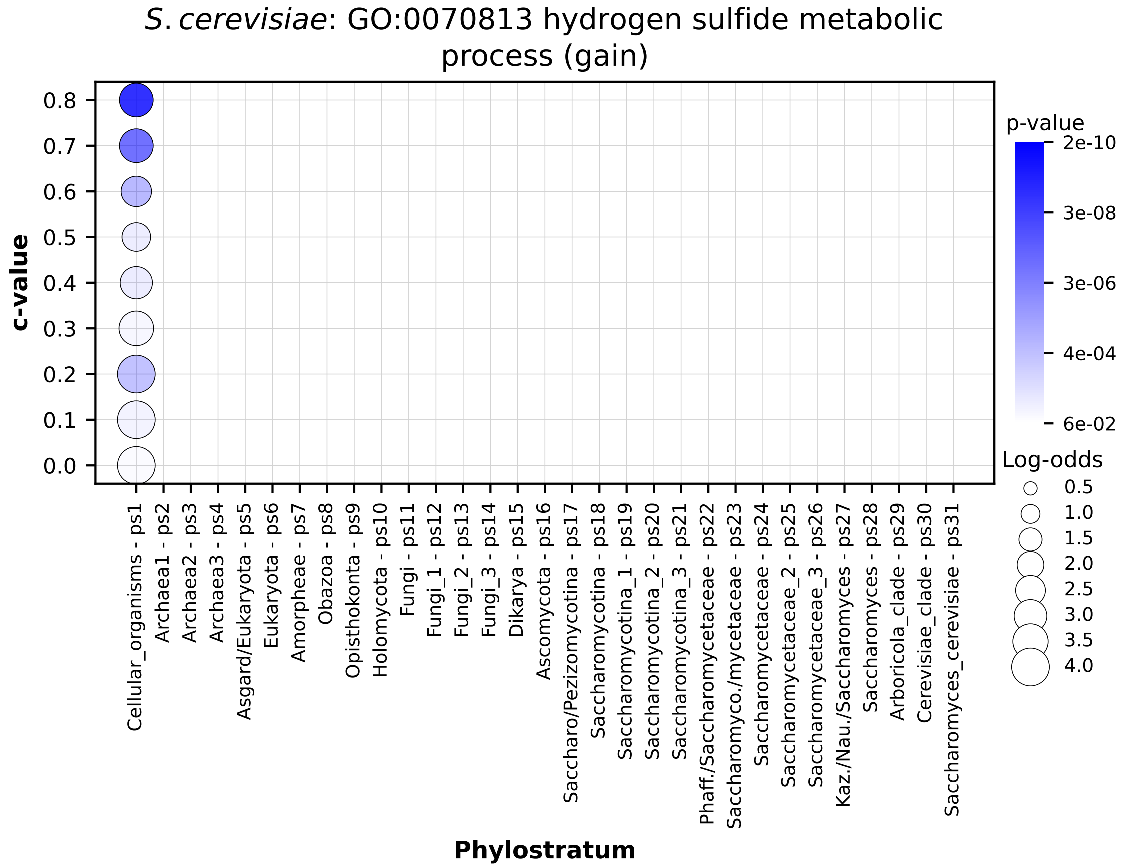
S. cerevisiae: GO:0070813 hydrogen sulfide metabolic process (gain)
c-value
0.8
0.7
0.6
0.5
0.4
0.3
0.2
0.1
0.0
p-value
2e-10
3e-08
3e-06
4e-04
6e-02
Log-odds
0.5
1.0
1.5
2.0
2.5
3.0
3.5
4.0
Cellular_organisms - ps1
Archaea1 - ps2
Archaea2 - ps3
Archaea3 - ps4
Asgard/Eukaryota - ps5
Eukaryota - ps6
Amorpheae - ps7
Obazoa - ps8
Opisthokonta - ps9
Holomycota - ps10
Fungi - ps11
Fungi_1 - ps12
Fungi_2 - ps13
Fungi_3 - ps14
Dikarya - ps15
Ascomycota - ps16
Saccharo/Pezizomycotina - ps17
Saccharomycotina - ps18
Saccharomycotina_1 - ps19
Saccharomycotina_2 - ps20
Saccharomycotina_3 - ps21
Phaff./Saccharomycetaceae - ps22
Saccharomyco./mycetaceae - ps23
Saccharomycetaceae - ps24
Saccharomycetaceae_2 - ps25
Saccharomycetaceae_3 - ps26
Kaz./Nau./Saccharomyces - ps27
Saccharomyces - ps28
Arboricola_clade - ps29
Cerevisiae_clade - ps30
Saccharomyces_cerevisiae - ps31
Phylostratum

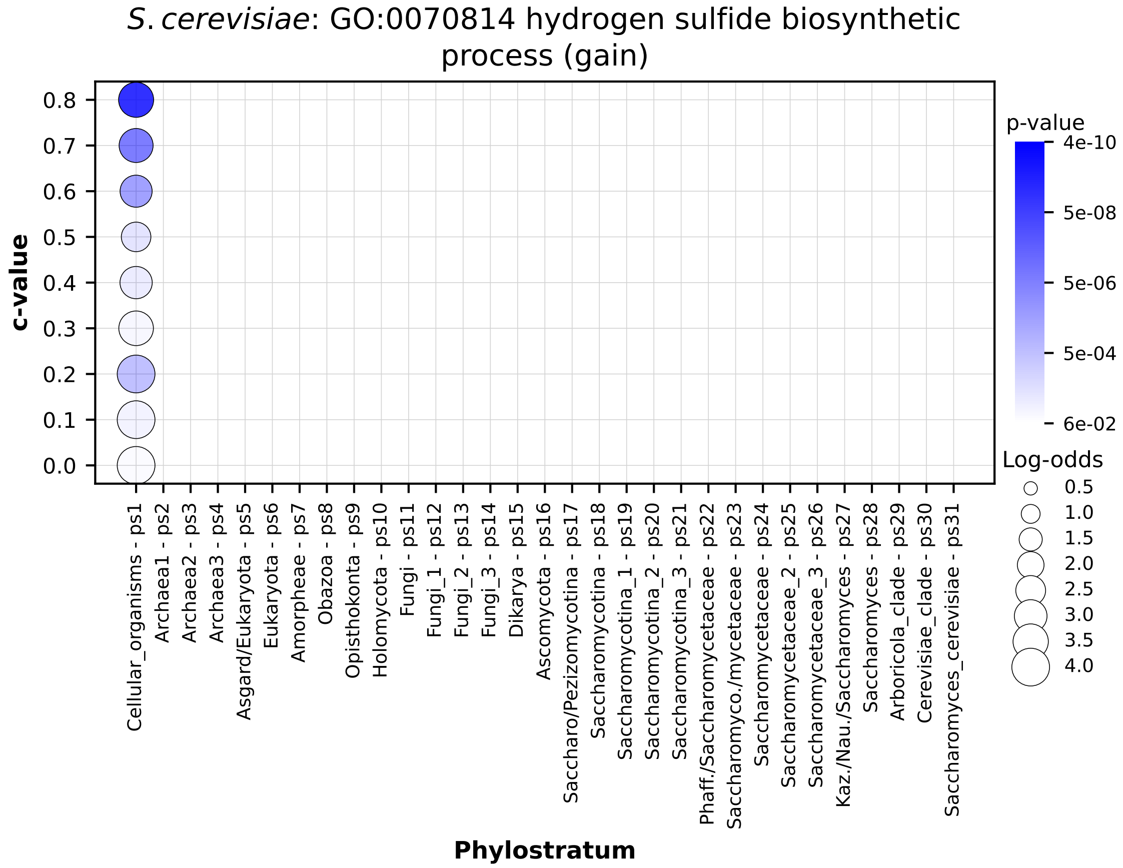

S. cerevisiae: GO:0070814 hydrogen sulfide biosynthetic process (gain)
c-value
0.0
0.1
0.2
0.3
0.4
0.5
0.6
0.7
0.8
Phylostratum
Cellular_organisms - ps1
Archaea1 - ps2
Archaea2 - ps3
Archaea3 - ps4
Asgard/Eukaryota - ps5
Eukaryota - ps6
Amorpheae - ps7
Obazoa - ps8
Opisthokonta - ps9
Holomycota - ps10
Fungi - ps11
Fungi_1 - ps12
Fungi_2 - ps13
Fungi_3 - ps14
Dikarya - ps15
Ascomycota - ps16
Saccharo/Pezizomycotina - ps17
Saccharomycotina - ps18
Saccharomycotina_1 - ps19
Saccharomycotina_2 - ps20
Saccharomycotina_3 - ps21
Phaff./Saccharomycetaceae - ps22
Saccharomyco./mycetaceae - ps23
Saccharomycetaceae - ps24
Saccharomycetaceae_2 - ps25
Saccharomycetaceae_3 - ps26
Kaz./Nau./Saccharomyces - ps27
Saccharomyces - ps28
Arboricola_clade - ps29
Cerevisiae_clade - ps30
Saccharomyces_cerevisiae - ps31
p-value
4e-10
5e-08
5e-06
5e-04
6e-02
Log-odds
0.5
1.0
1.5
2.0
2.5
3.0
3.5
4.0

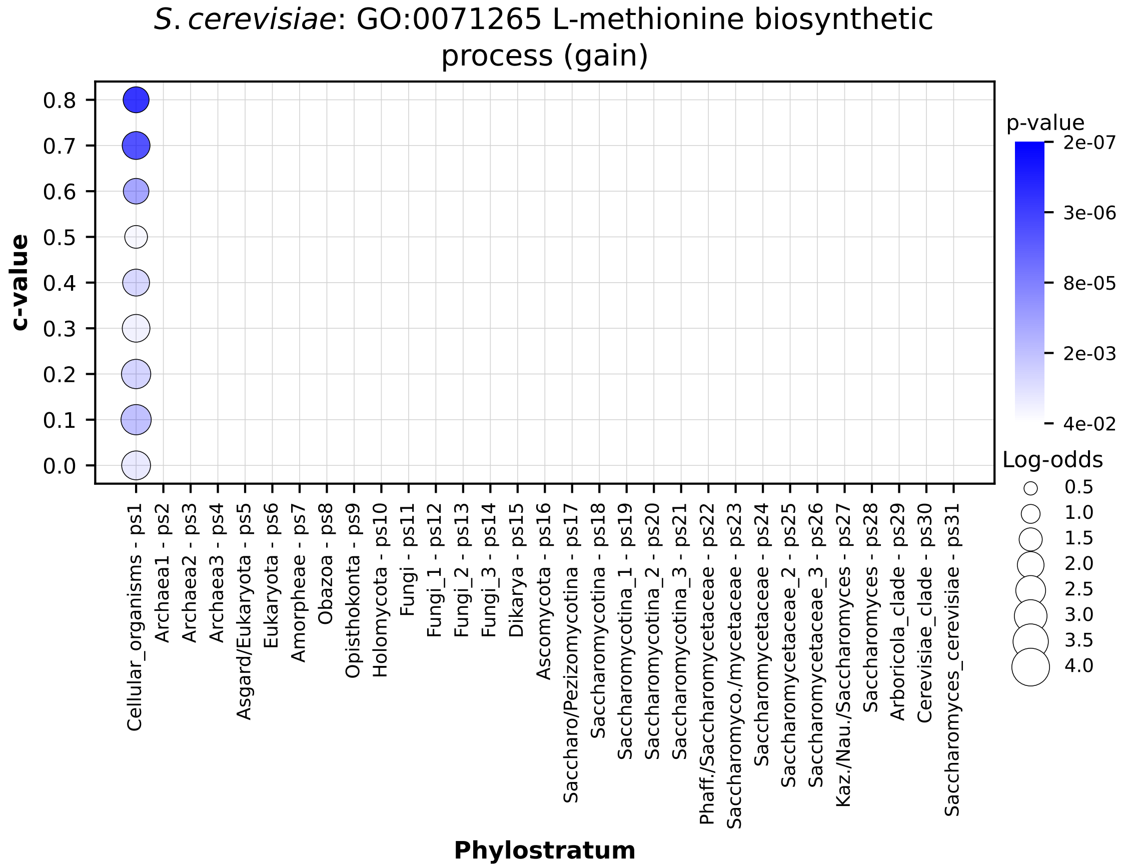

S. cerevisiae: GO:0071265 L-methionine biosynthetic process (gain)
c-value
0.8
0.7
0.6
0.5
0.4
0.3
0.2
0.1
0.0
p-value
2e-07
3e-06
8e-05
2e-03
4e-02
Log-odds
0.5
1.0
1.5
2.0
2.5
3.0
3.5
4.0
Cellular_organisms - ps1
Archaea1 - ps2
Archaea2 - ps3
Archaea3 - ps4
Asgard/Eukaryota - ps5
Eukaryota - ps6
Amorpheae - ps7
Obazoa - ps8
Opisthokonta - ps9
Holomycota - ps10
Fungi - ps11
Fungi_1 - ps12
Fungi_2 - ps13
Fungi_3 - ps14
Dikarya - ps15
Ascomycota - ps16
Saccharo/Pezizomycotina - ps17
Saccharomycotina - ps18
Saccharomycotina_1 - ps19
Saccharomycotina_2 - ps20
Saccharomycotina_3 - ps21
Phaff./Saccharomycetaceae - ps22
Saccharomyco./mycetaceae - ps23
Saccharomycetaceae - ps24
Saccharomycetaceae_2 - ps25
Saccharomycetaceae_3 - ps26
Kaz./Nau./Saccharomyces - ps27
Saccharomyces - ps28
Arboricola_clade - ps29
Cerevisiae_clade - ps30
Saccharomyces_cerevisiae - ps31
Phylostratum

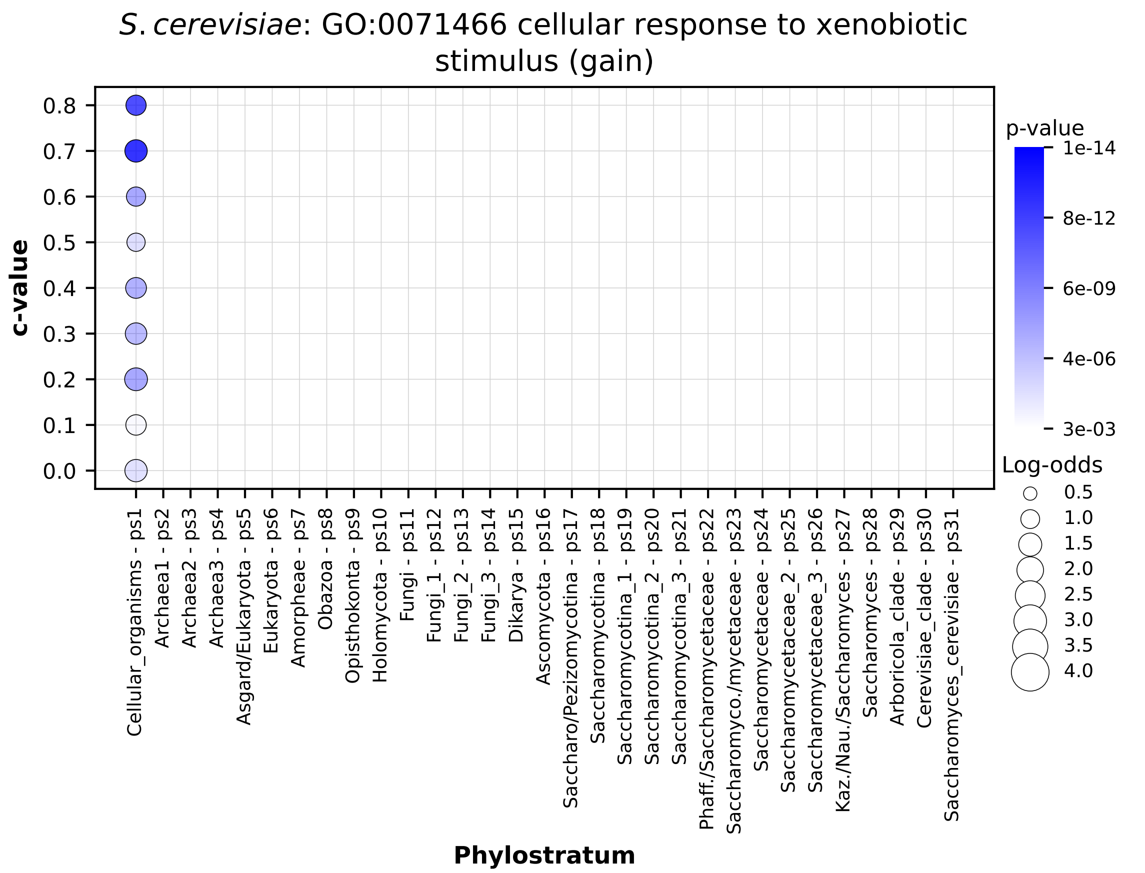

S. cerevisiae: GO:0071466 cellular response to xenobiotic stimulus (gain)
c-value
0.8
0.7
0.6
0.5
0.4
0.3
0.2
0.1
0.0
p-value
1e-14
8e-12
6e-09
4e-06
3e-03
Log-odds
0.5
1.0
1.5
2.0
2.5
3.0
3.5
4.0
Cellular_organisms - ps1
Archaea1 - ps2
Archaea2 - ps3
Archaea3 - ps4
Asgard/Eukaryota - ps5
Eukaryota - ps6
Amorpheae - ps7
Obazoa - ps8
Opisthokonta - ps9
Holomycota - ps10
Fungi - ps11
Fungi_1 - ps12
Fungi_2 - ps13
Fungi_3 - ps14
Dikarya - ps15
Ascomycota - ps16
Saccharo/Pezizomycotina - ps17
Saccharomycotina - ps18
Saccharomycotina_1 - ps19
Saccharomycotina_2 - ps20
Saccharomycotina_3 - ps21
Phaff./Saccharomycetaceae - ps22
Saccharomyco./mycetaceae - ps23
Saccharomycetaceae - ps24
Saccharomycetaceae_2 - ps25
Saccharomycetaceae_3 - ps26
Kaz./Nau./Saccharomyces - ps27
Saccharomyces - ps28
Arboricola_clade - ps29
Cerevisiae_clade - ps30
Saccharomyces_cerevisiae - ps31
Phylostratum

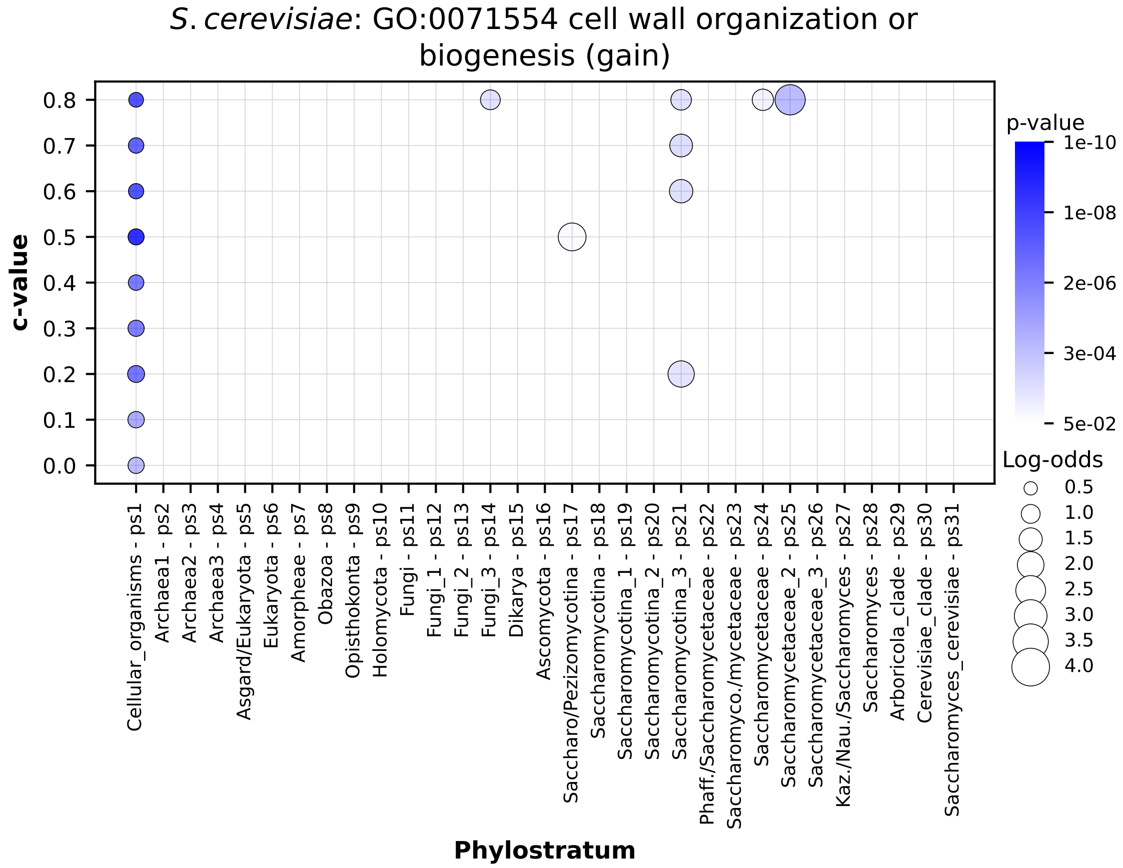

S. cerevisiae: GO:0071554 cell wall organization or biogenesis (gain)
c-value
0.0
0.1
0.2
0.3
0.4
0.5
0.6
0.7
0.8
Phylostratum
Cellular_organisms - ps1
Archaea1 - ps2
Archaea2 - ps3
Archaea3 - ps4
Asgard/Eukaryota - ps5
Eukaryota - ps6
Amorpheae - ps7
Obazoa - ps8
Opisthokonta - ps9
Holomycota - ps10
Fungi - ps11
Fungi_1 - ps12
Fungi_2 - ps13
Fungi_3 - ps14
Dikarya - ps15
Ascomycota - ps16
Saccharo/Pezizomycotina - ps17
Saccharomycotina - ps18
Saccharomycotina_1 - ps19
Saccharomycotina_2 - ps20
Saccharomycotina_3 - ps21
Phaff./Saccharomycetaceae - ps22
Saccharomyco./mycetaceae - ps23
Saccharomycetaceae - ps24
Saccharomycetaceae_2 - ps25
Saccharomycetaceae_3 - ps26
Kaz./Nau./Saccharomyces - ps27
Saccharomyces - ps28
Arboricola_clade - ps29
Cerevisiae_clade - ps30
Saccharomyces_cerevisiae - ps31
p-value
1e-10
1e-08
2e-06
3e-04
5e-02
Log-odds
0.5
1.0
1.5
2.0
2.5
3.0
3.5
4.0

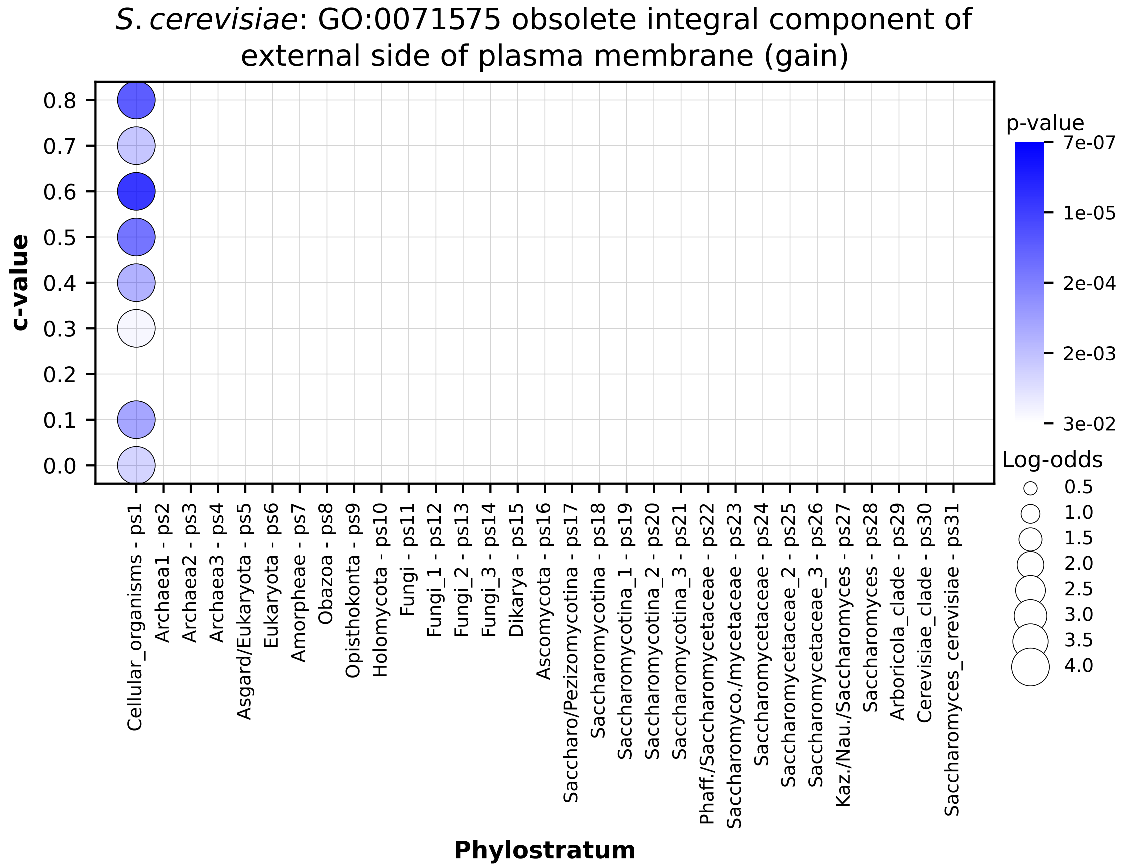

S. cerevisiae: GO:0071575 obsolete integral component of external side of plasma membrane (gain)
c-value
0.8
0.7
0.6
0.5
0.4
0.3
0.2
0.1
0.0
Phylostratum
Cellular_organisms - ps1
Archaea1 - ps2
Archaea2 - ps3
Archaea3 - ps4
Asgard/Eukaryota - ps5
Eukaryota - ps6
Amorpheae - ps7
Obazoa - ps8
Opisthokonta - ps9
Holomycota - ps10
Fungi - ps11
Fungi_1 - ps12
Fungi_2 - ps13
Fungi_3 - ps14
Dikarya - ps15
Ascomycota - ps16
Saccharo/Pezizomycotina - ps17
Saccharomycotina - ps18
Saccharomycotina_1 - ps19
Saccharomycotina_2 - ps20
Saccharomycotina_3 - ps21
Phaff./Saccharomycetaceae - ps22
Saccharomyco./mycetaceae - ps23
Saccharomycetaceae - ps24
Saccharomycetaceae_2 - ps25
Saccharomycetaceae_3 - ps26
Kaz./Nau./Saccharomyces - ps27
Saccharomyces - ps28
Arboricola_clade - ps29
Cerevisiae_clade - ps30
Saccharomyces_cerevisiae - ps31
p-value
7e-07
1e-05
2e-04
2e-03
3e-02
Log-odds
0.5
1.0
1.5
2.0
2.5
3.0
3.5
4.0

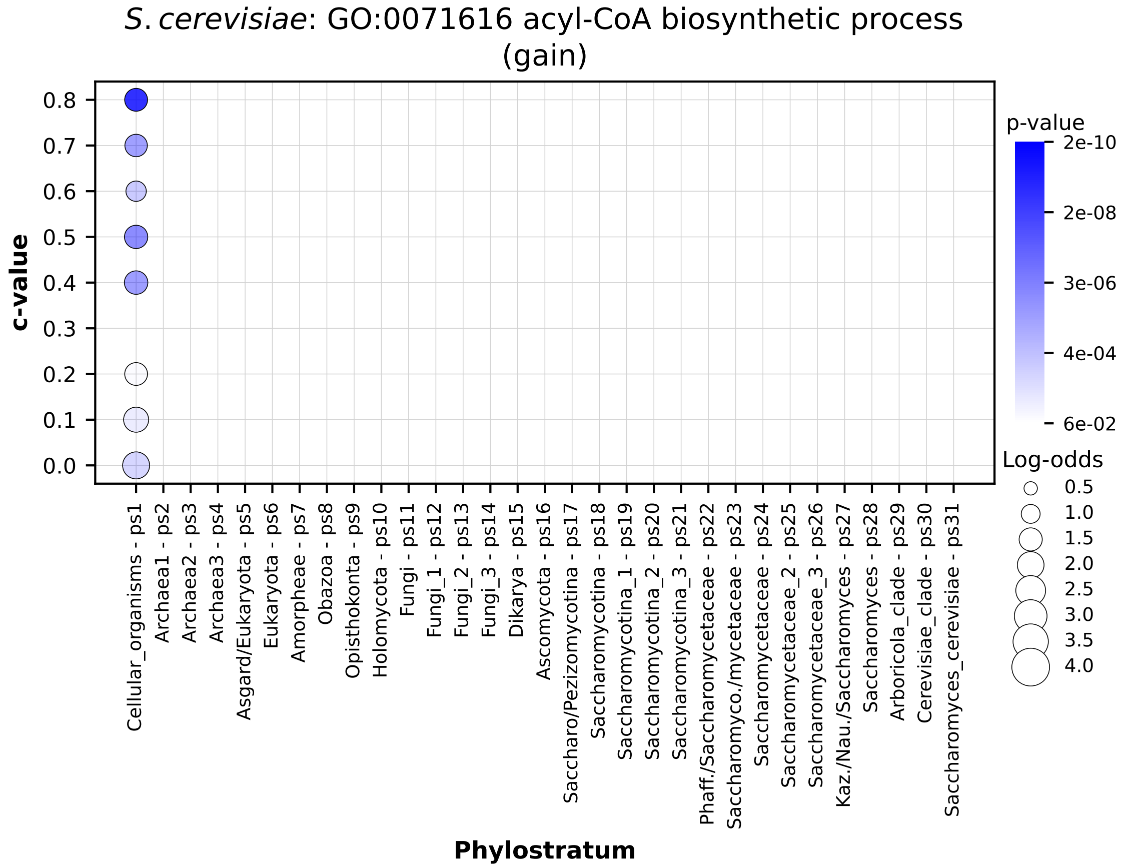

S. cerevisiae: GO:0071616 acyl-CoA biosynthetic process
(gain)
c-value
0.8
0.7
0.6
0.5
0.4
0.3
0.2
0.1
0.0
p-value
2e-10
2e-08
3e-06
4e-04
6e-02
Log-odds
0.5
1.0
1.5
2.0
2.5
3.0
3.5
4.0
Cellular_organisms - ps1
Archaea1 - ps2
Archaea2 - ps3
Archaea3 - ps4
Asgard/Eukaryota - ps5
Eukaryota - ps6
Amorpheae - ps7
Obazoa - ps8
Opisthokonta - ps9
Holomycota - ps10
Fungi - ps11
Fungi_1 - ps12
Fungi_2 - ps13
Fungi_3 - ps14
Dikarya - ps15
Ascomycota - ps16
Saccharo/Pezizomycotina - ps17
Saccharomycotina - ps18
Saccharomycotina_1 - ps19
Saccharomycotina_2 - ps20
Saccharomycotina_3 - ps21
Phaff./Saccharomycetaceae - ps22
Saccharomyco./mycetaceae - ps23
Saccharomycetaceae - ps24
Saccharomycetaceae_2 - ps25
Saccharomycetaceae_3 - ps26
Kaz./Nau./Saccharomyces - ps27
Saccharomyces - ps28
Arboricola_clade - ps29
Cerevisiae_clade - ps30
Saccharomyces_cerevisiae - ps31
Phylostratum

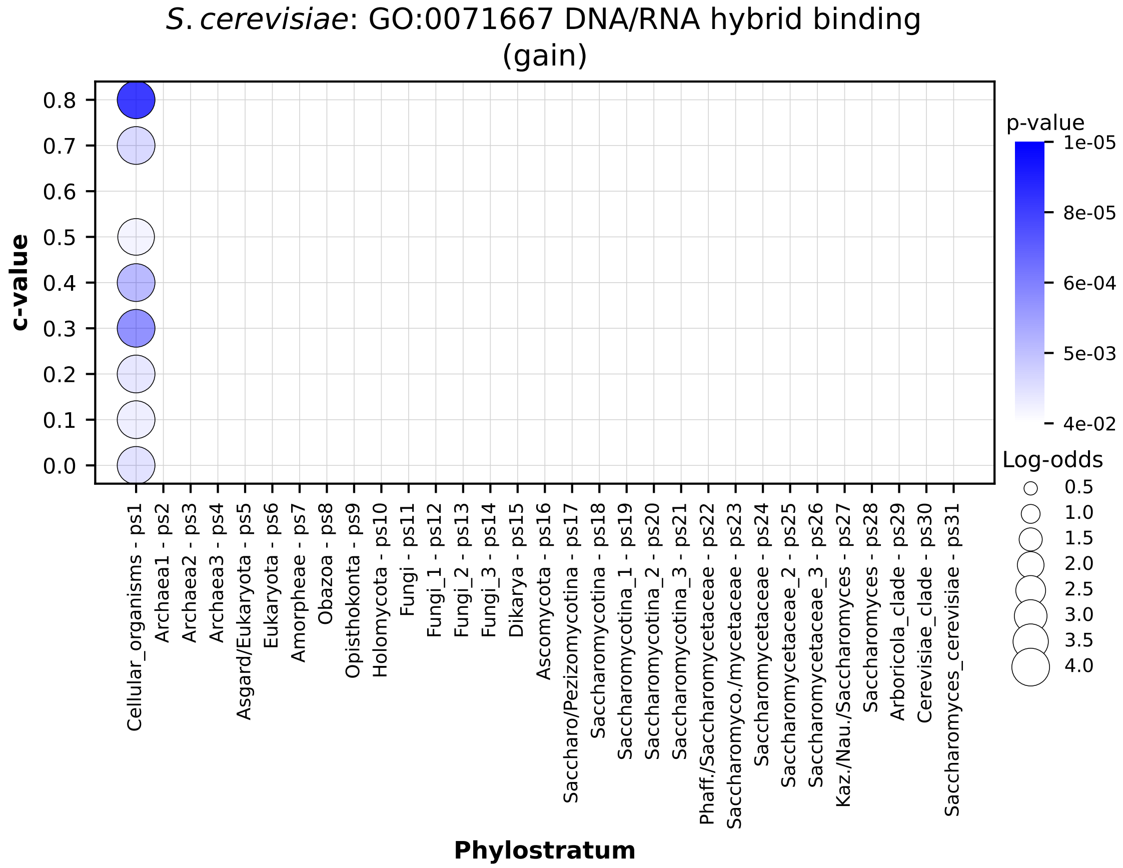
S. cerevisiae: GO:0071667 DNA/RNA hybrid binding
(gain)
c-value
0.8
0.7
0.6
0.5
0.4
0.3
0.2
0.1
0.0
p-value
1e-05
8e-05
6e-04
5e-03
4e-02
Log-odds
0.5
1.0
1.5
2.0
2.5
3.0
3.5
4.0
Cellular_organisms - ps1
Archaea1 - ps2
Archaea2 - ps3
Archaea3 - ps4
Asgard/Eukaryota - ps5
Eukaryota - ps6
Amorpheae - ps7
Obazoa - ps8
Opisthokonta - ps9
Holomycota - ps10
Fungi - ps11
Fungi_1 - ps12
Fungi_2 - ps13
Fungi_3 - ps14
Dikarya - ps15
Ascomycota - ps16
Saccharo/Pezizomycotina - ps17
Saccharomycotina - ps18
Saccharomycotina_1 - ps19
Saccharomycotina_2 - ps20
Saccharomycotina_3 - ps21
Phaff./Saccharomycetaceae - ps22
Saccharomyco./mycetaceae - ps23
Saccharomycetaceae - ps24
Saccharomycetaceae_2 - ps25
Saccharomycetaceae_3 - ps26
Kaz./Nau./Saccharomyces - ps27
Saccharomyces - ps28
Arboricola_clade - ps29
Cerevisiae_clade - ps30
Saccharomyces_cerevisiae - ps31
Phylostratum

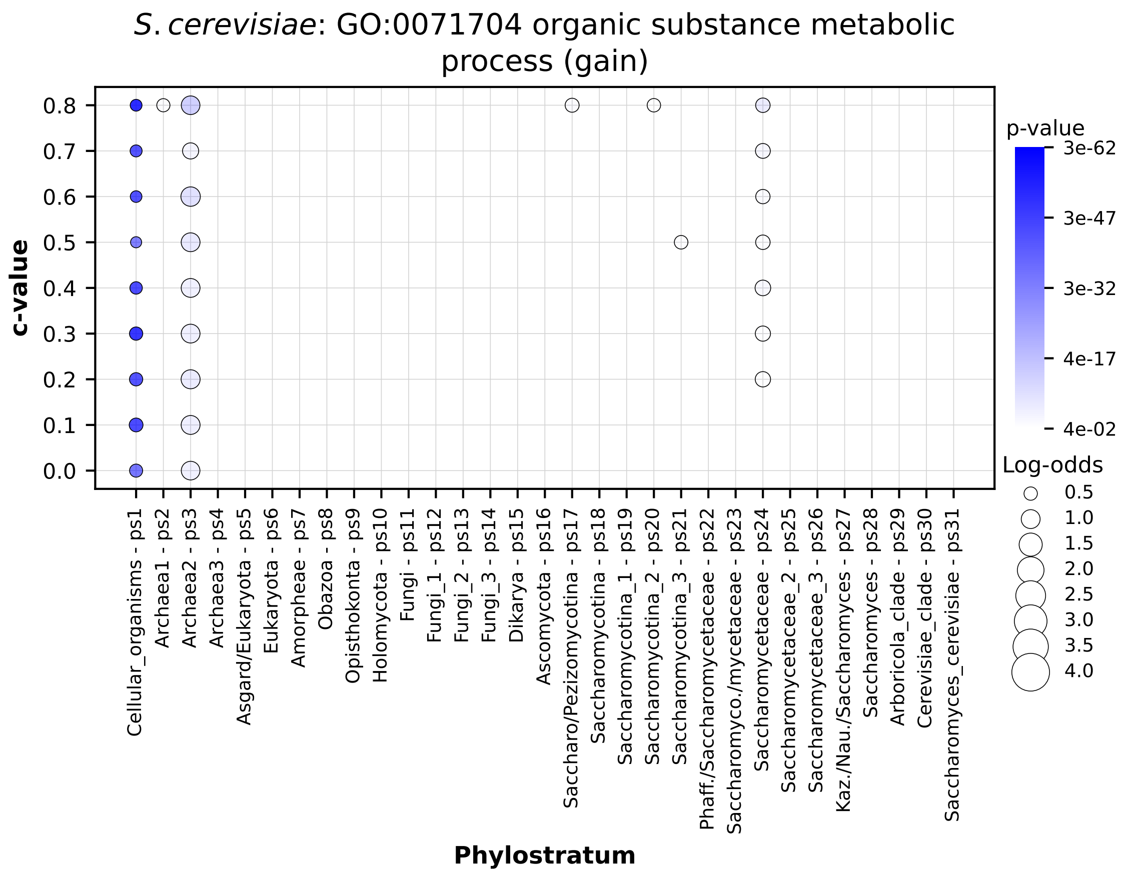
S. cerevisiae: GO:0071704 organic substance metabolic process (gain)
c-value
0.8
0.7
0.6
0.5
0.4
0.3
0.2
0.1
0.0
Phylostratum
Cellular_organisms - ps1
Archaea1 - ps2
Archaea2 - ps3
Archaea3 - ps4
Asgard/Eukaryota - ps5
Eukaryota - ps6
Amorpheae - ps7
Obazoa - ps8
Opisthokonta - ps9
Holomycota - ps10
Fungi - ps11
Fungi_1 - ps12
Fungi_2 - ps13
Fungi_3 - ps14
Dikarya - ps15
Ascomycota - ps16
Saccharo/Pezizomycotina - ps17
Saccharomycotina - ps18
Saccharomycotina_1 - ps19
Saccharomycotina_2 - ps20
Saccharomycotina_3 - ps21
Phaff./Saccharomycetaceae - ps22
Saccharomyco./mycetaceae - ps23
Saccharomycetaceae - ps24
Saccharomycetaceae_2 - ps25
Saccharomycetaceae_3 - ps26
Kaz./Nau./Saccharomyces - ps27
Saccharomyces - ps28
Arboricola_clade - ps29
Cerevisiae_clade - ps30
Saccharomyces_cerevisiae - ps31
p-value
3e-62
3e-47
3e-32
4e-17
4e-02
Log-odds
0.5
1.0
1.5
2.0
2.5
3.0
3.5
4.0

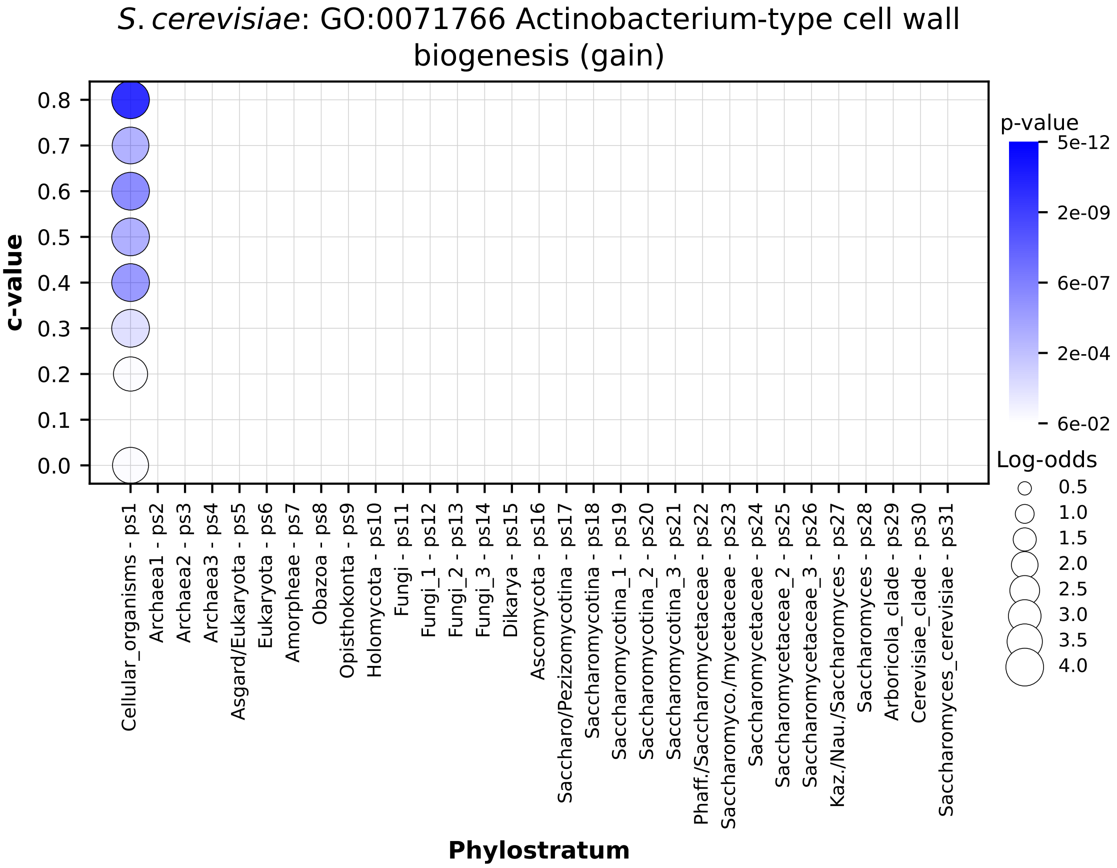

S. cerevisiae: GO:0071766 Actinobacterium-type cell wall biogenesis (gain)
c-value
0.8
0.7
0.6
0.5
0.4
0.3
0.2
0.1
0.0
p-value
5e-12
2e-09
6e-07
2e-04
6e-02
Log-odds
0.5
1.0
1.5
2.0
2.5
3.0
3.5
4.0
Cellular_organisms - ps1
Archaea1 - ps2
Archaea2 - ps3
Archaea3 - ps4
Asgard/Eukaryota - ps5
Eukaryota - ps6
Amorpheae - ps7
Obazoa - ps8
Opisthokonta - ps9
Holomycota - ps10
Fungi - ps11
Fungi_1 - ps12
Fungi_2 - ps13
Fungi_3 - ps14
Dikarya - ps15
Ascomycota - ps16
Saccharo/Pezizomycotina - ps17
Saccharomycotina - ps18
Saccharomycotina_1 - ps19
Saccharomycotina_2 - ps20
Saccharomycotina_3 - ps21
Phaff./Saccharomycetaceae - ps22
Saccharomyco./mycetaceae - ps23
Saccharomycetaceae - ps24
Saccharomycetaceae_2 - ps25
Saccharomycetaceae_3 - ps26
Kaz./Nau./Saccharomyces - ps27
Saccharomyces - ps28
Arboricola_clade - ps29
Cerevisiae_clade - ps30
Saccharomyces_cerevisiae - ps31
Phylostratum

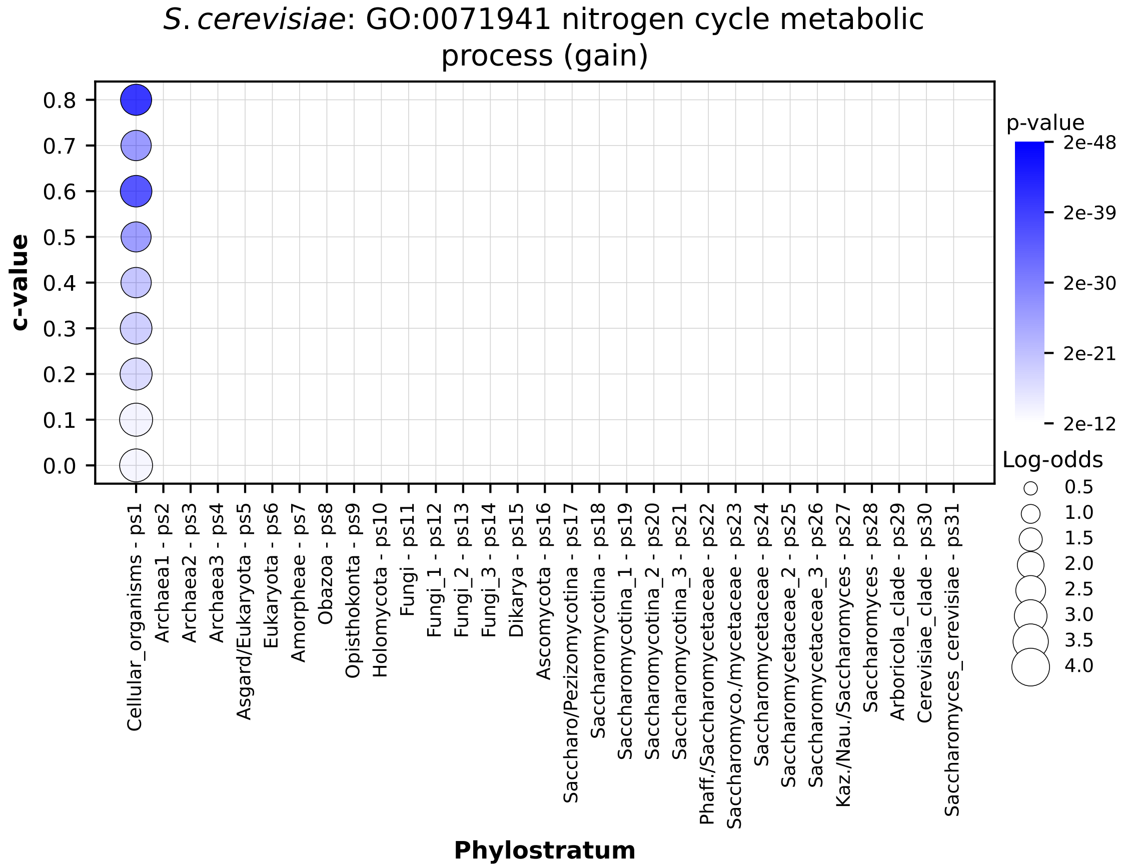

$S. cerevisiae$: GO:0071941 nitrogen cycle metabolic process (gain)
c-value
0.0
0.1
0.2
0.3
0.4
0.5
0.6
0.7
0.8
Phylostratum
Cellular_organisms - ps1
Archaea1 - ps2
Archaea2 - ps3
Archaea3 - ps4
Asgard/Eukaryota - ps5
Eukaryota - ps6
Amorpheae - ps7
Obazoa - ps8
Opisthokonta - ps9
Holomycota - ps10
Fungi - ps11
Fungi_1 - ps12
Fungi_2 - ps13
Fungi_3 - ps14
Dikarya - ps15
Ascomycota - ps16
Saccharo/Pezizomycotina - ps17
Saccharomycotina - ps18
Saccharomycotina_1 - ps19
Saccharomycotina_2 - ps20
Saccharomycotina_3 - ps21
Phaff./Saccharomycetaceae - ps22
Saccharomyco./mycetaceae - ps23
Saccharomycetaceae - ps24
Saccharomycetaceae_2 - ps25
Saccharomycetaceae_3 - ps26
Kaz./Nau./Saccharomyces - ps27
Saccharomyces - ps28
Arboricola_clade - ps29
Cerevisiae_clade - ps30
Saccharomyces_cerevisiae - ps31
p-value
2e-48
2e-39
2e-30
2e-21
2e-12
Log-odds
0.5
1.0
1.5
2.0
2.5
3.0
3.5
4.0

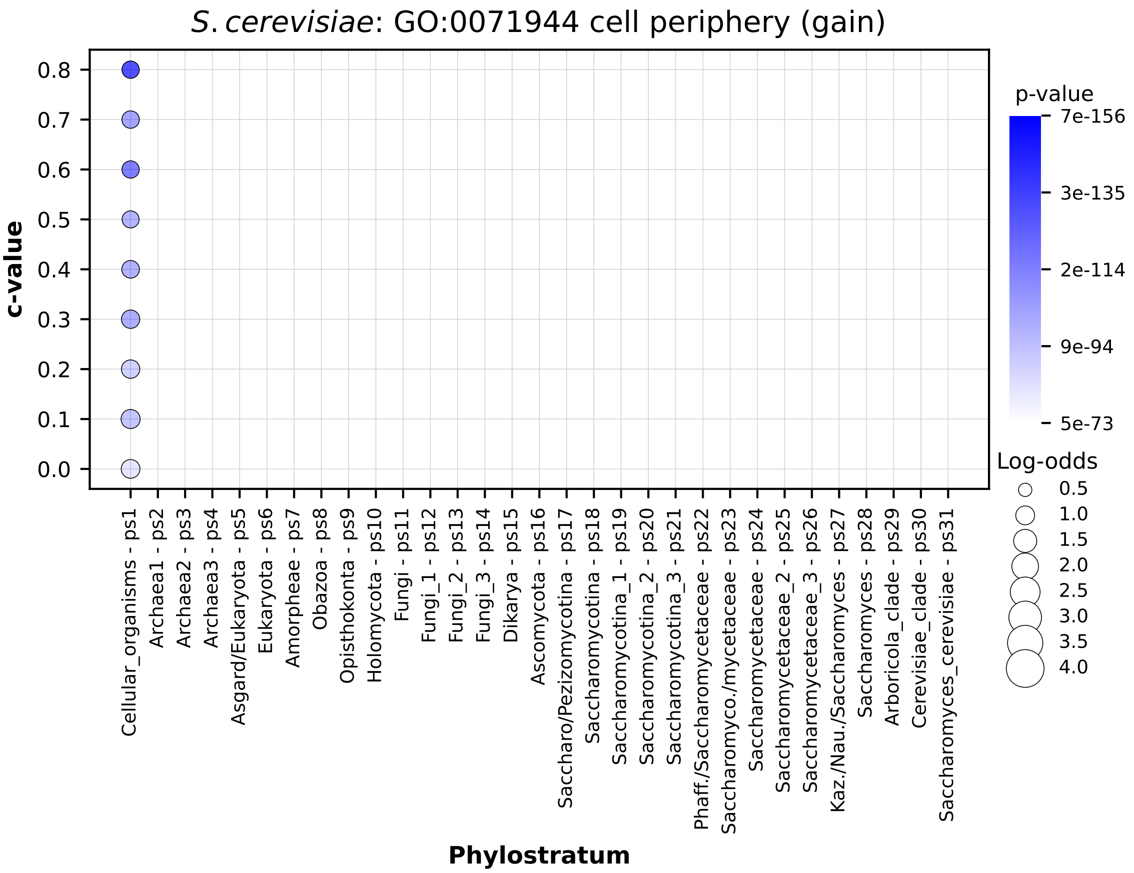

S. cerevisiae: GO:0071944 cell periphery (gain)
c-value
0.0
0.1
0.2
0.3
0.4
0.5
0.6
0.7
0.8
Phylostratum
Cellular_organisms - ps1
Archaea1 - ps2
Archaea2 - ps3
Archaea3 - ps4
Asgard/Eukaryota - ps5
Eukaryota - ps6
Amorpheae - ps7
Obazoa - ps8
Opisthokonta - ps9
Holomycota - ps10
Fungi - ps11
Fungi_1 - ps12
Fungi_2 - ps13
Fungi_3 - ps14
Dikarya - ps15
Ascomycota - ps16
Saccharo/Pezizomycotina - ps17
Saccharomycotina - ps18
Saccharomycotina_1 - ps19
Saccharomycotina_2 - ps20
Saccharomycotina_3 - ps21
Phaff./Saccharomycetaceae - ps22
Saccharomyco./mycetaceae - ps23
Saccharomycetaceae - ps24
Saccharomycetaceae_2 - ps25
Saccharomycetaceae_3 - ps26
Kaz./Nau./Saccharomyces - ps27
Saccharomyces - ps28
Arboricola_clade - ps29
Cerevisiae_clade - ps30
Saccharomyces_cerevisiae - ps31
p-value
7e-156
3e-135
2e-114
9e-94
5e-73
Log-odds
0.5
1.0
1.5
2.0
2.5
3.0
3.5
4.0

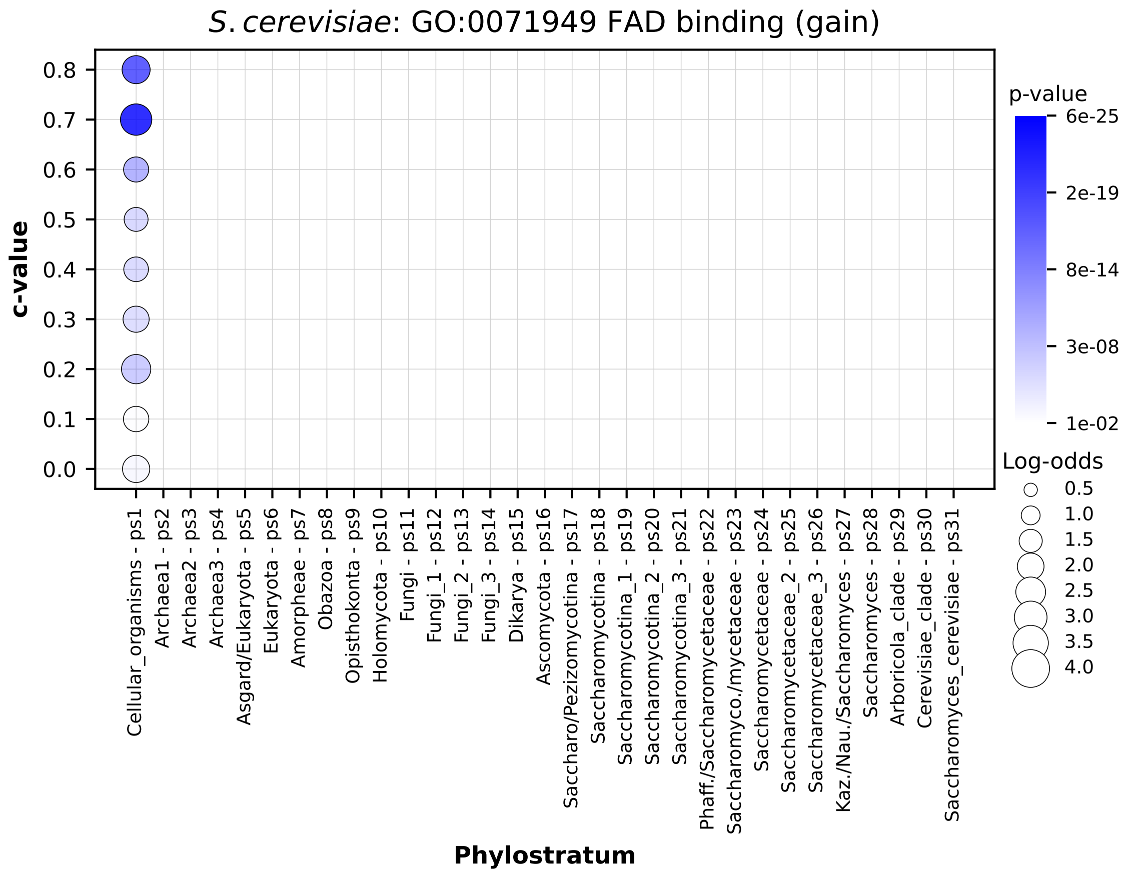

S. cerevisiae: GO:0071949 FAD binding (gain)
c-value
0.0
0.1
0.2
0.3
0.4
0.5
0.6
0.7
0.8
p-value
6e-25
2e-19
8e-14
3e-08
1e-02
Log-odds
0.5
1.0
1.5
2.0
2.5
3.0
3.5
4.0
Cellular_organisms - ps1
Archaea1 - ps2
Archaea2 - ps3
Archaea3 - ps4
Asgard/Eukaryota - ps5
Eukaryota - ps6
Amorpheae - ps7
Obazoa - ps8
Opisthokonta - ps9
Holomycota - ps10
Fungi - ps11
Fungi_1 - ps12
Fungi_2 - ps13
Fungi_3 - ps14
Dikarya - ps15
Ascomycota - ps16
Saccharo/Pezizomycotina - ps17
Saccharomycotina - ps18
Saccharomycotina_1 - ps19
Saccharomycotina_2 - ps20
Saccharomycotina_3 - ps21
Phaff./Saccharomycetaceae - ps22
Saccharomyco./mycetaceae - ps23
Saccharomycetaceae - ps24
Saccharomycetaceae_2 - ps25
Saccharomycetaceae_3 - ps26
Kaz./Nau./Saccharomyces - ps27
Saccharomyces - ps28
Arboricola_clade - ps29
Cerevisiae_clade - ps30
Saccharomyces_cerevisiae - ps31
Phylostratum

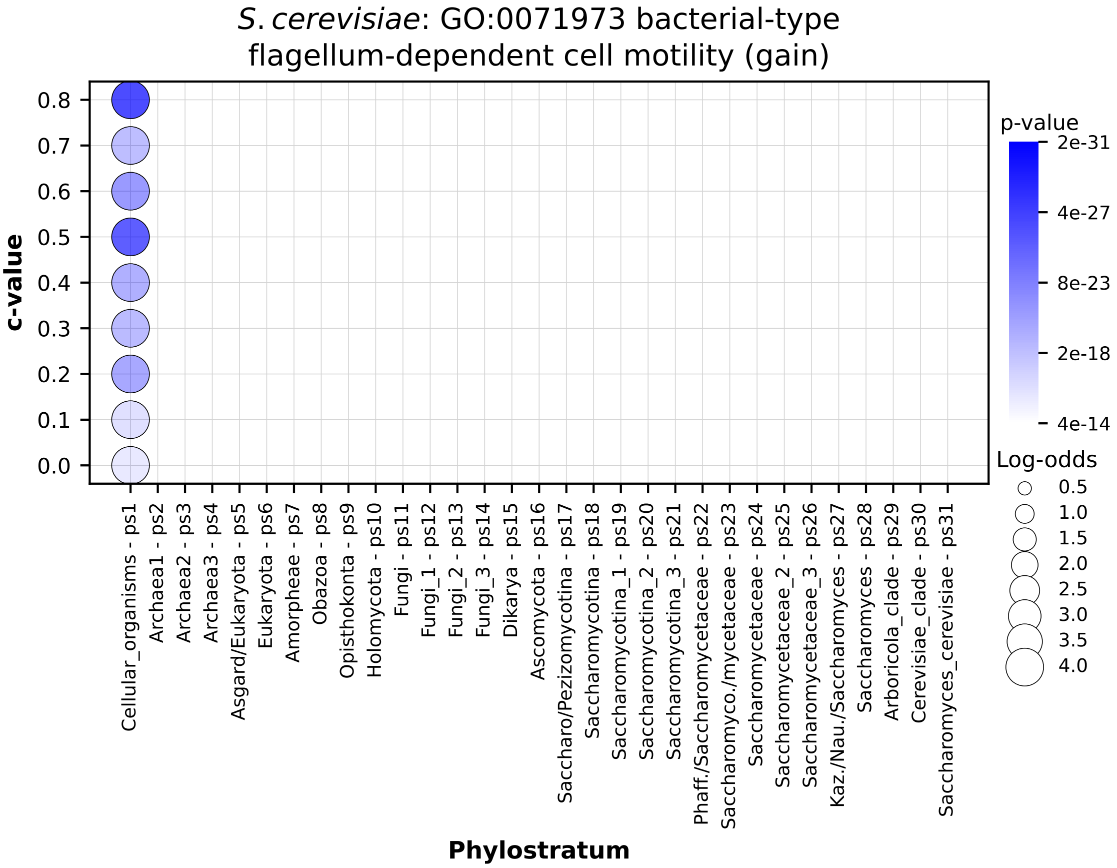
S. cerevisiae: GO:0071973 bacterial-type flagellum-dependent cell motility (gain)
c-value
0.0
0.1
0.2
0.3
0.4
0.5
0.6
0.7
0.8
p-value
2e-31
4e-27
8e-23
2e-18
4e-14
Log-odds
0.5
1.0
1.5
2.0
2.5
3.0
3.5
4.0
Cellular_organisms - ps1
Archaea1 - ps2
Archaea2 - ps3
Archaea3 - ps4
Asgard/Eukaryota - ps5
Eukaryota - ps6
Amorpheae - ps7
Obazoa - ps8
Opisthokonta - ps9
Holomycota - ps10
Fungi - ps11
Fungi_1 - ps12
Fungi_2 - ps13
Fungi_3 - ps14
Dikarya - ps15
Ascomycota - ps16
Saccharo/Pezizomycotina - ps17
Saccharomycotina - ps18
Saccharomycotina_1 - ps19
Saccharomycotina_2 - ps20
Saccharomycotina_3 - ps21
Phaff./Saccharomycetaceae - ps22
Saccharomyco./mycetaceae - ps23
Saccharomycetaceae - ps24
Saccharomycetaceae_2 - ps25
Saccharomycetaceae_3 - ps26
Kaz./Nau./Saccharomyces - ps27
Saccharomyces - ps28
Arboricola_clade - ps29
Cerevisiae_clade - ps30
Saccharomyces_cerevisiae - ps31
Phylostratum

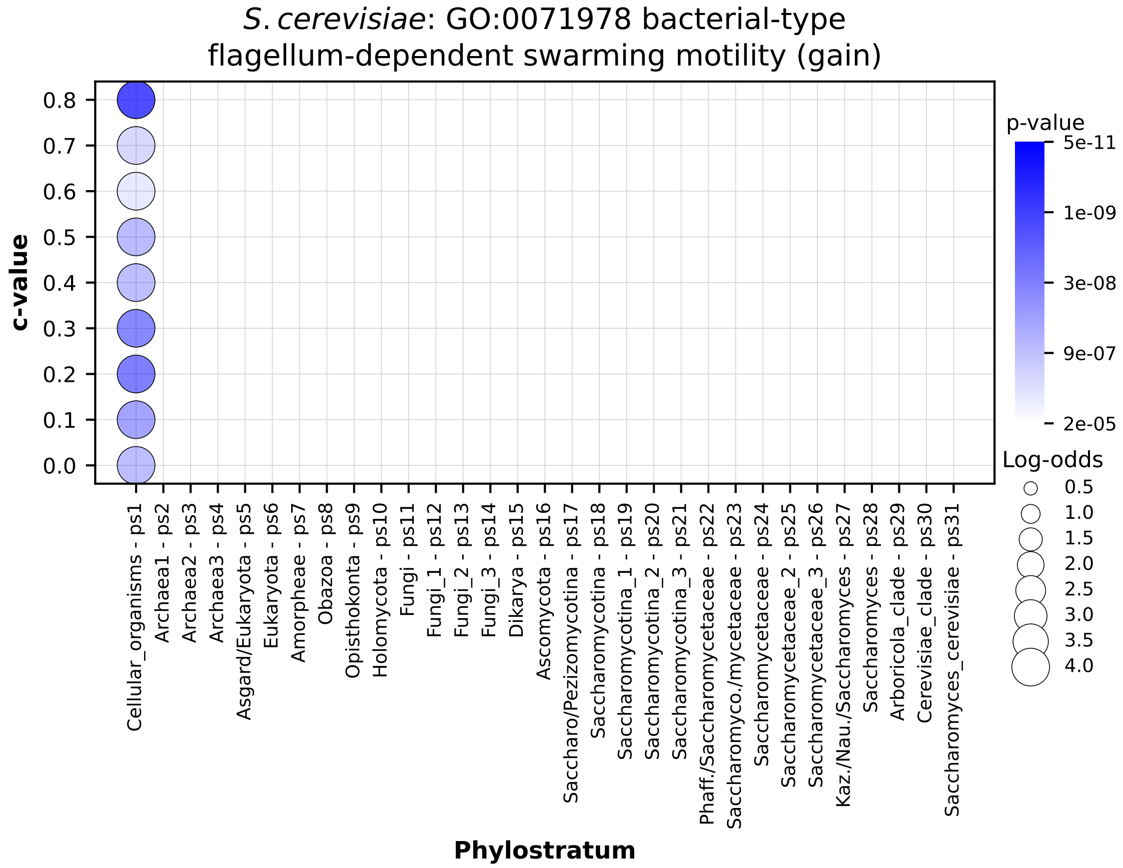

S. cerevisiae: GO:0071978 bacterial-type flagellum-dependent swarming motility (gain)
c-value
0.8
0.7
0.6
0.5
0.4
0.3
0.2
0.1
0.0
p-value
5e-11
1e-09
3e-08
9e-07
2e-05
Log-odds
0.5
1.0
1.5
2.0
2.5
3.0
3.5
4.0
Cellular_organisms - ps1
Archaea1 - ps2
Archaea2 - ps3
Archaea3 - ps4
Asgard/Eukaryota - ps5
Eukaryota - ps6
Amorpheae - ps7
Obazoa - ps8
Opisthokonta - ps9
Holomycota - ps10
Fungi - ps11
Fungi_1 - ps12
Fungi_2 - ps13
Fungi_3 - ps14
Dikarya - ps15
Ascomycota - ps16
Saccharo/Pezizomycotina - ps17
Saccharomycotina - ps18
Saccharomycotina_1 - ps19
Saccharomycotina_2 - ps20
Saccharomycotina_3 - ps21
Phaff./Saccharomycetaceae - ps22
Saccharomyco./mycetaceae - ps23
Saccharomycetaceae - ps24
Saccharomycetaceae_2 - ps25
Saccharomycetaceae_3 - ps26
Kaz./Nau./Saccharomyces - ps27
Saccharomyces - ps28
Arboricola_clade - ps29
Cerevisiae_clade - ps30
Saccharomyces_cerevisiae - ps31
Phylostratum

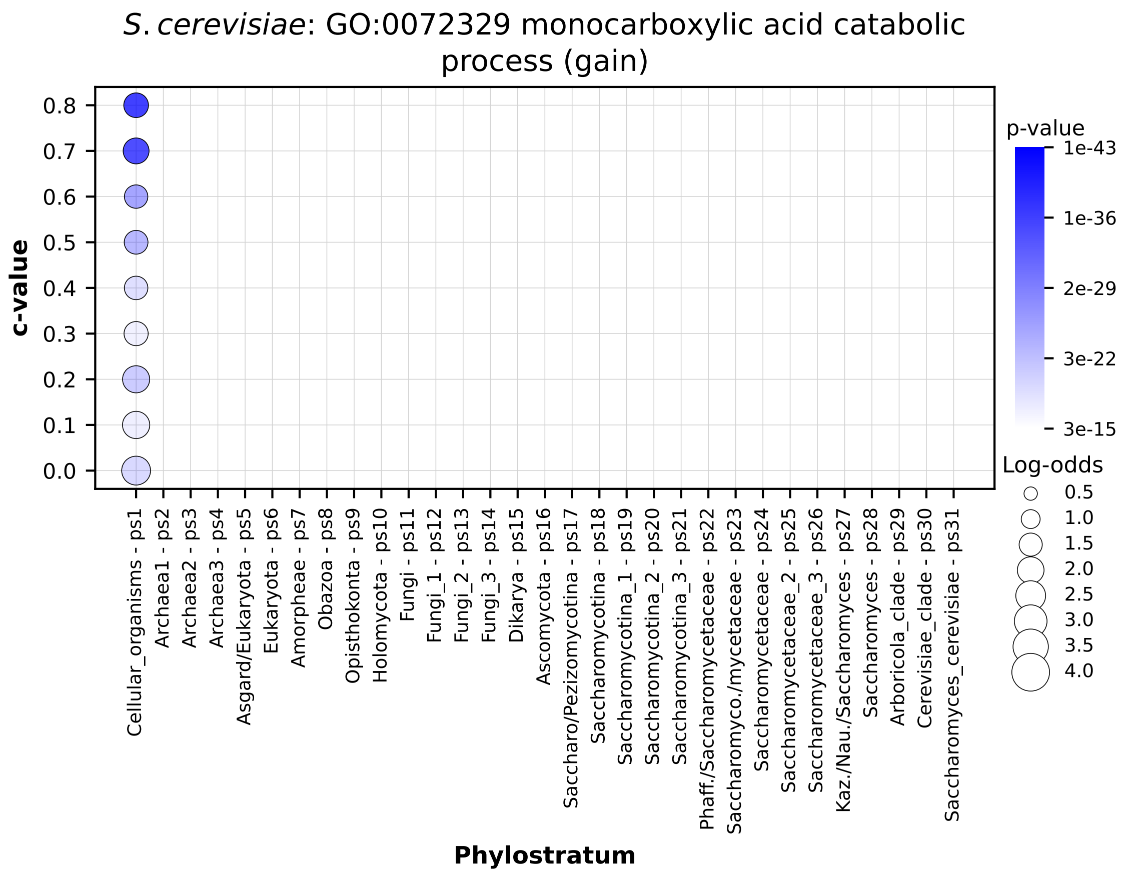

S. cerevisiae: GO:0072329 monocarboxylic acid catabolic process (gain)
c-value
0.8
0.7
0.6
0.5
0.4
0.3
0.2
0.1
0.0
p-value
1e-43
1e-36
2e-29
3e-22
3e-15
Log-odds
0.5
1.0
1.5
2.0
2.5
3.0
3.5
4.0
Cellular_organisms - ps1
Archaea1 - ps2
Archaea2 - ps3
Archaea3 - ps4
Asgard/Eukaryota - ps5
Eukaryota - ps6
Amorpheae - ps7
Obazoa - ps8
Opisthokonta - ps9
Holomycota - ps10
Fungi - ps11
Fungi_1 - ps12
Fungi_2 - ps13
Fungi_3 - ps14
Dikarya - ps15
Ascomycota - ps16
Saccharo/Pezizomycotina - ps17
Saccharomycotina - ps18
Saccharomycotina_1 - ps19
Saccharomycotina_2 - ps20
Saccharomycotina_3 - ps21
Phaff./Saccharomycetaceae - ps22
Saccharomyco./mycetaceae - ps23
Saccharomycetaceae - ps24
Saccharomycetaceae_2 - ps25
Saccharomycetaceae_3 - ps26
Kaz./Nau./Saccharomyces - ps27
Saccharomyces - ps28
Arboricola_clade - ps29
Cerevisiae_clade - ps30
Saccharomyces_cerevisiae - ps31
Phylostratum

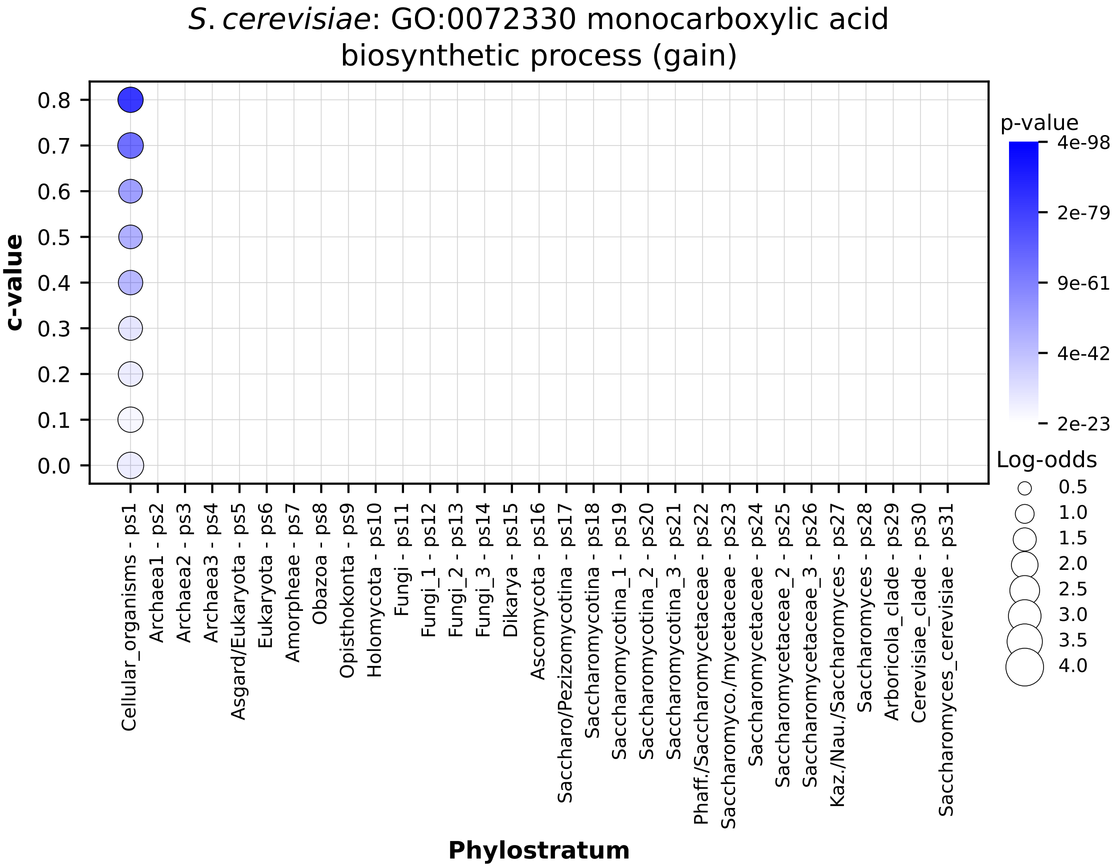

S. cerevisiae: GO:0072330 monocarboxylic acid biosynthetic process (gain)
c-value
0.8
0.7
0.6
0.5
0.4
0.3
0.2
0.1
0.0
p-value
4e-98
2e-79
9e-61
4e-42
2e-23
Log-odds
0.5
1.0
1.5
2.0
2.5
3.0
3.5
4.0
Cellular_organisms - ps1
Archaea1 - ps2
Archaea2 - ps3
Archaea3 - ps4
Asgard/Eukaryota - ps5
Eukaryota - ps6
Amorpheae - ps7
Obazoa - ps8
Opisthokonta - ps9
Holomycota - ps10
Fungi - ps11
Fungi_1 - ps12
Fungi_2 - ps13
Fungi_3 - ps14
Dikarya - ps15
Ascomycota - ps16
Saccharo/Pezizomycotina - ps17
Saccharomycotina - ps18
Saccharomycotina_1 - ps19
Saccharomycotina_2 - ps20
Saccharomycotina_3 - ps21
Phaff./Saccharomycetaceae - ps22
Saccharomyco./mycetaceae - ps23
Saccharomycetaceae - ps24
Saccharomycetaceae_2 - ps25
Saccharomycetaceae_3 - ps26
Kaz./Nau./Saccharomyces - ps27
Saccharomyces - ps28
Arboricola_clade - ps29
Cerevisiae_clade - ps30
Saccharomyces_cerevisiae - ps31
Phylostratum

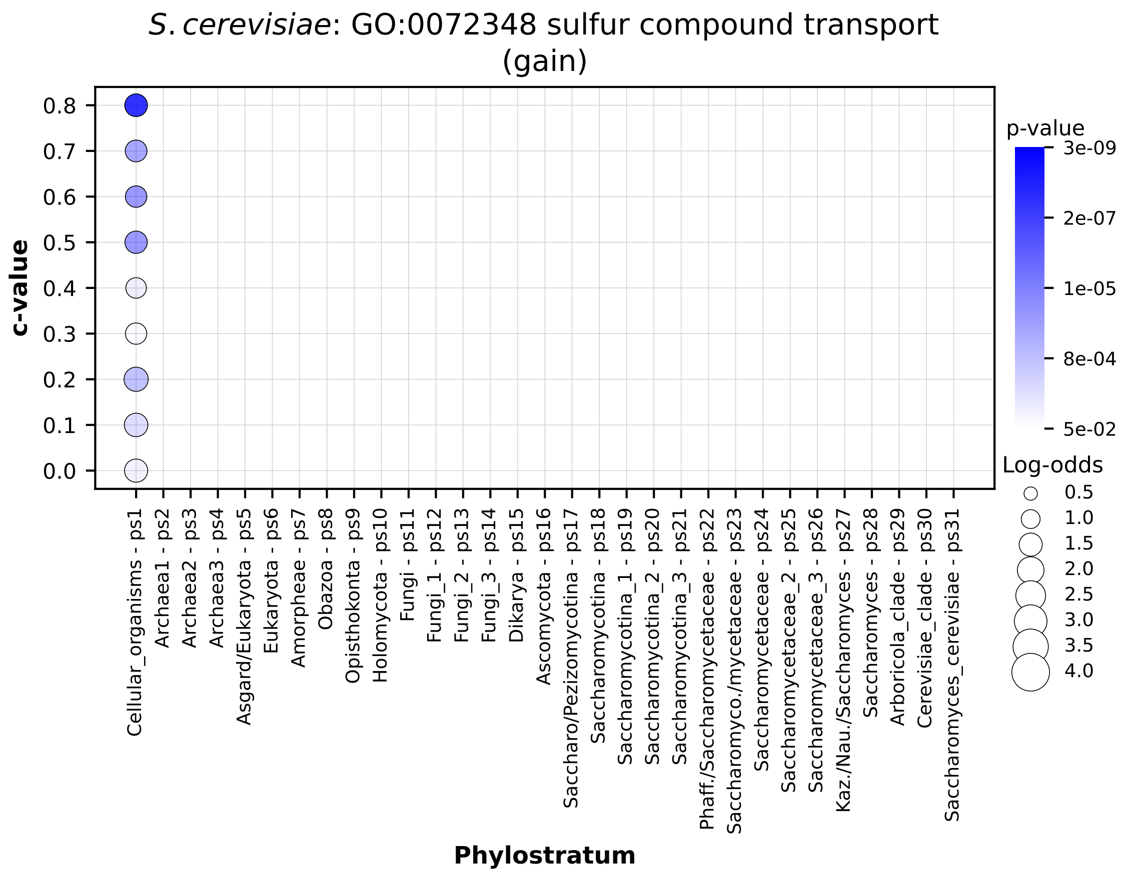
S. cerevisiae: GO:0072348 sulfur compound transport (gain)
c-value
0.8
0.7
0.6
0.5
0.4
0.3
0.2
0.1
0.0
p-value
3e-09
2e-07
1e-05
8e-04
5e-02
Log-odds
0.5
1.0
1.5
2.0
2.5
3.0
3.5
4.0
Cellular_organisms - ps1
Archaea1 - ps2
Archaea2 - ps3
Archaea3 - ps4
Asgard/Eukaryota - ps5
Eukaryota - ps6
Amorpheae - ps7
Obazoa - ps8
Opisthokonta - ps9
Holomycota - ps10
Fungi - ps11
Fungi_1 - ps12
Fungi_2 - ps13
Fungi_3 - ps14
Dikarya - ps15
Ascomycota - ps16
Saccharo/Pezizomycotina - ps17
Saccharomycotina - ps18
Saccharomycotina_1 - ps19
Saccharomycotina_2 - ps20
Saccharomycotina_3 - ps21
Phaff./Saccharomycetaceae - ps22
Saccharomyco./mycetaceae - ps23
Saccharomycetaceae - ps24
Saccharomycetaceae_2 - ps25
Saccharomycetaceae_3 - ps26
Kaz./Nau./Saccharomyces - ps27
Saccharomyces - ps28
Arboricola_clade - ps29
Cerevisiae_clade - ps30
Saccharomyces_cerevisiae - ps31
Phylostratum

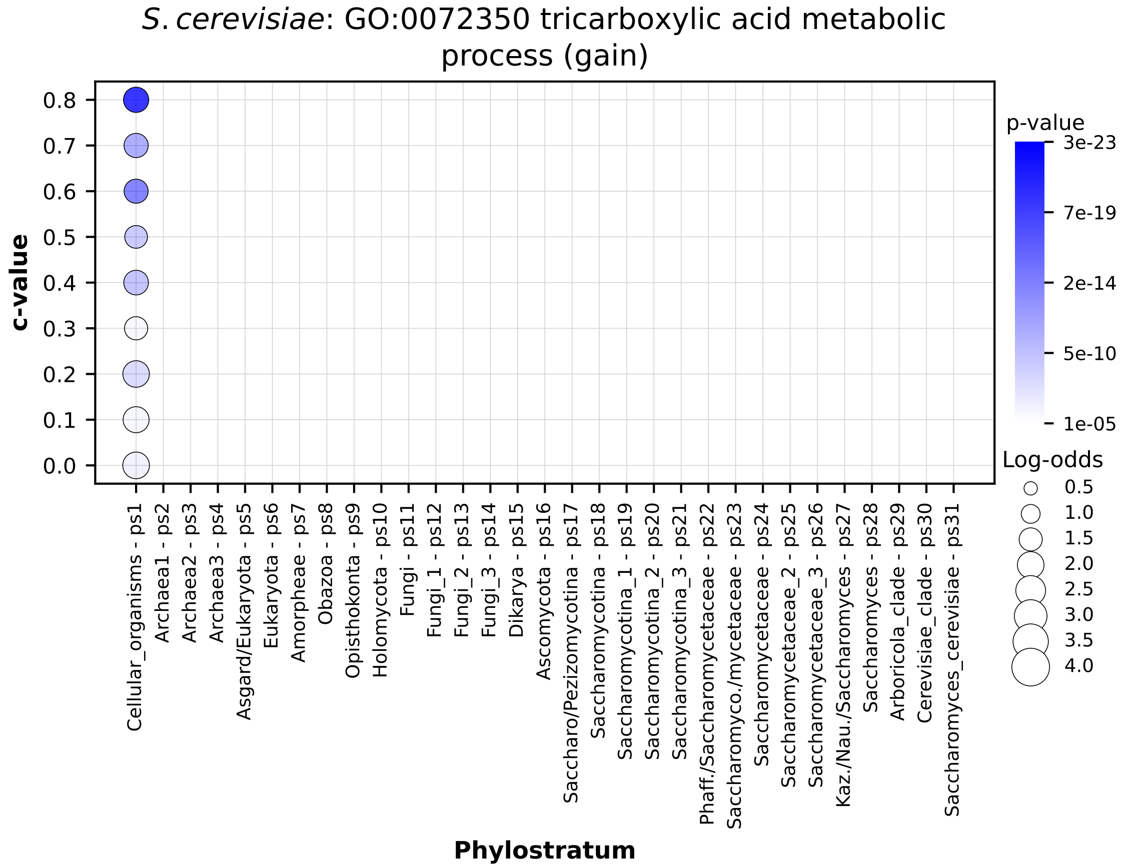

S. cerevisiae: GO:0072350 tricarboxylic acid metabolic process (gain)
c-value
0.8
0.7
0.6
0.5
0.4
0.3
0.2
0.1
0.0
p-value
3e-23
7e-19
2e-14
5e-10
1e-05
Log-odds
0.5
1.0
1.5
2.0
2.5
3.0
3.5
4.0
Cellular_organisms - ps1
Archaea1 - ps2
Archaea2 - ps3
Archaea3 - ps4
Asgard/Eukaryota - ps5
Eukaryota - ps6
Amorpheae - ps7
Obazoa - ps8
Opisthokonta - ps9
Holomycota - ps10
Fungi - ps11
Fungi_1 - ps12
Fungi_2 - ps13
Fungi_3 - ps14
Dikarya - ps15
Ascomycota - ps16
Saccharo/Pezizomycotina - ps17
Saccharomycotina - ps18
Saccharomycotina_1 - ps19
Saccharomycotina_2 - ps20
Saccharomycotina_3 - ps21
Phaff./Saccharomycetaceae - ps22
Saccharomyco./mycetaceae - ps23
Saccharomycetaceae - ps24
Saccharomycetaceae_2 - ps25
Saccharomycetaceae_3 - ps26
Kaz./Nau./Saccharomyces - ps27
Saccharomyces - ps28
Arboricola_clade - ps29
Cerevisiae_clade - ps30
Saccharomyces_cerevisiae - ps31
Phylostratum

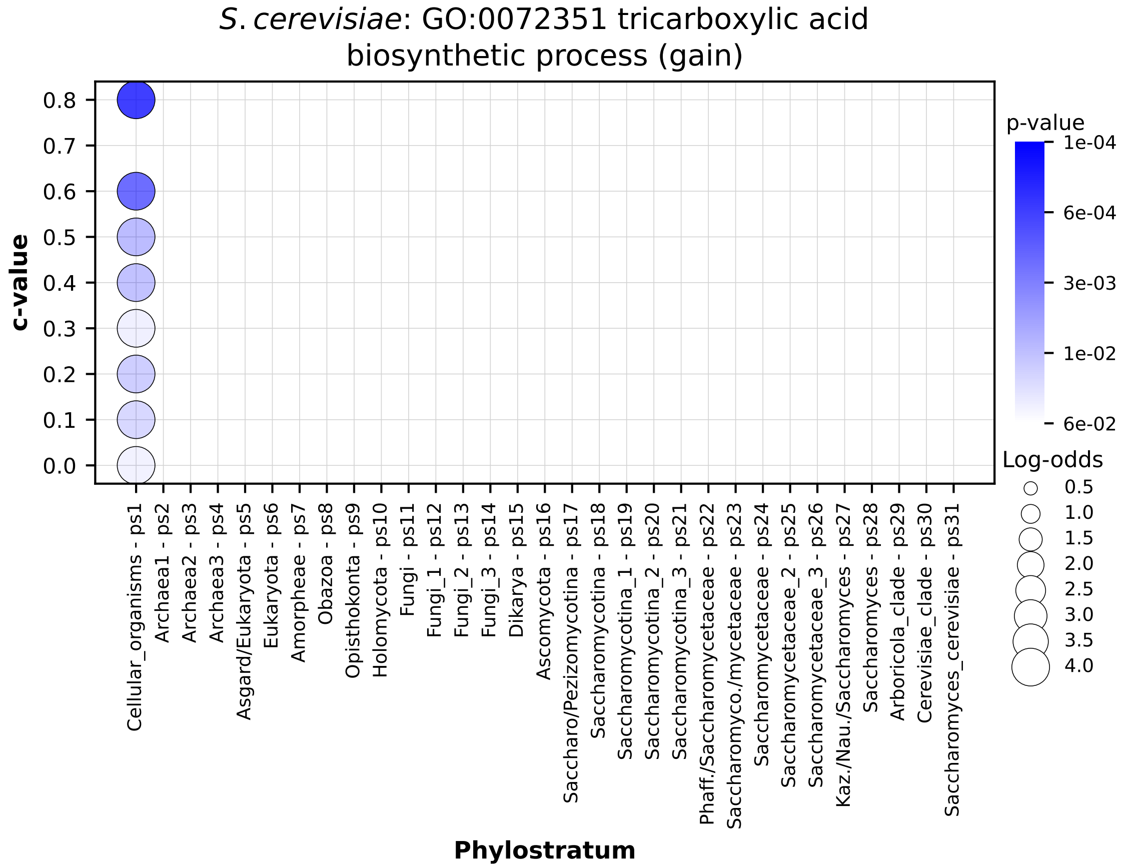

S. cerevisiae: GO:0072351 tricarboxylic acid biosynthetic process (gain)
c-value
0.8
0.7
0.6
0.5
0.4
0.3
0.2
0.1
0.0
p-value
1e-04
6e-04
3e-03
1e-02
6e-02
Log-odds
0.5
1.0
1.5
2.0
2.5
3.0
3.5
4.0
Cellular_organisms - ps1
Archaea1 - ps2
Archaea2 - ps3
Archaea3 - ps4
Asgard/Eukaryota - ps5
Eukaryota - ps6
Amorpheae - ps7
Obazoa - ps8
Opisthokonta - ps9
Holomycota - ps10
Fungi - ps11
Fungi_1 - ps12
Fungi_2 - ps13
Fungi_3 - ps14
Dikarya - ps15
Ascomycota - ps16
Saccharo/Pezizomycotina - ps17
Saccharomycotina - ps18
Saccharomycotina_1 - ps19
Saccharomycotina_2 - ps20
Saccharomycotina_3 - ps21
Phaff./Saccharomycetaceae - ps22
Saccharomyco./mycetaceae - ps23
Saccharomycetaceae - ps24
Saccharomycetaceae_2 - ps25
Saccharomycetaceae_3 - ps26
Kaz./Nau./Saccharomyces - ps27
Saccharomyces - ps28
Arboricola_clade - ps29
Cerevisiae_clade - ps30
Saccharomyces_cerevisiae - ps31
Phylostratum

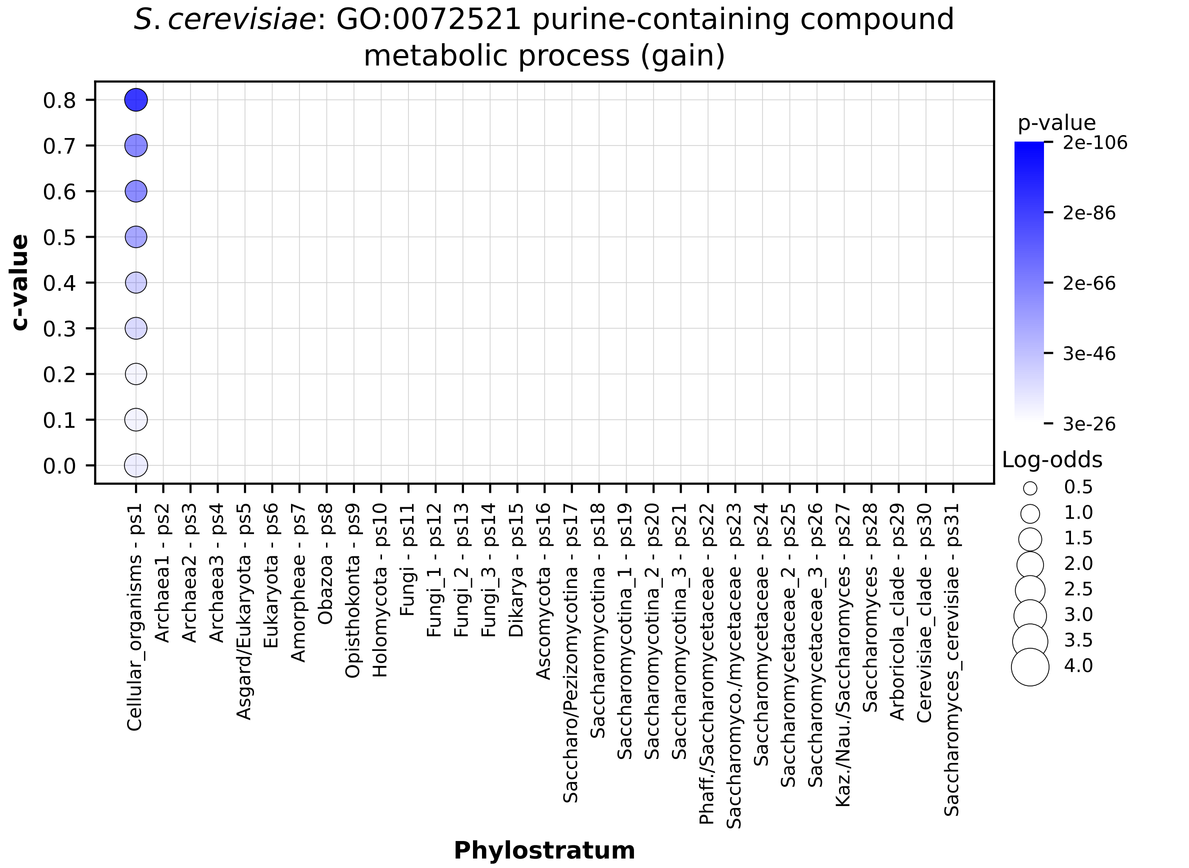

S. cerevisiae: GO:0072521 purine-containing compound metabolic process (gain)
c-value
0.8
0.7
0.6
0.5
0.4
0.3
0.2
0.1
0.0
p-value
2e-106
2e-86
2e-66
3e-46
3e-26
Log-odds
0.5
1.0
1.5
2.0
2.5
3.0
3.5
4.0
Cellular_organisms - ps1
Archaea1 - ps2
Archaea2 - ps3
Archaea3 - ps4
Asgard/Eukaryota - ps5
Eukaryota - ps6
Amorpheae - ps7
Obazoa - ps8
Opisthokonta - ps9
Holomycota - ps10
Fungi - ps11
Fungi_1 - ps12
Fungi_2 - ps13
Fungi_3 - ps14
Dikarya - ps15
Ascomycota - ps16
Saccharo/Pezizomycotina - ps17
Saccharomycotina - ps18
Saccharomycotina_1 - ps19
Saccharomycotina_2 - ps20
Saccharomycotina_3 - ps21
Phaff./Saccharomycetaceae - ps22
Saccharomyco./mycetaceae - ps23
Saccharomycetaceae - ps24
Saccharomycetaceae_2 - ps25
Saccharomycetaceae_3 - ps26
Kaz./Nau./Saccharomyces - ps27
Saccharomyces - ps28
Arboricola_clade - ps29
Cerevisiae_clade - ps30
Saccharomyces_cerevisiae - ps31
Phylostratum

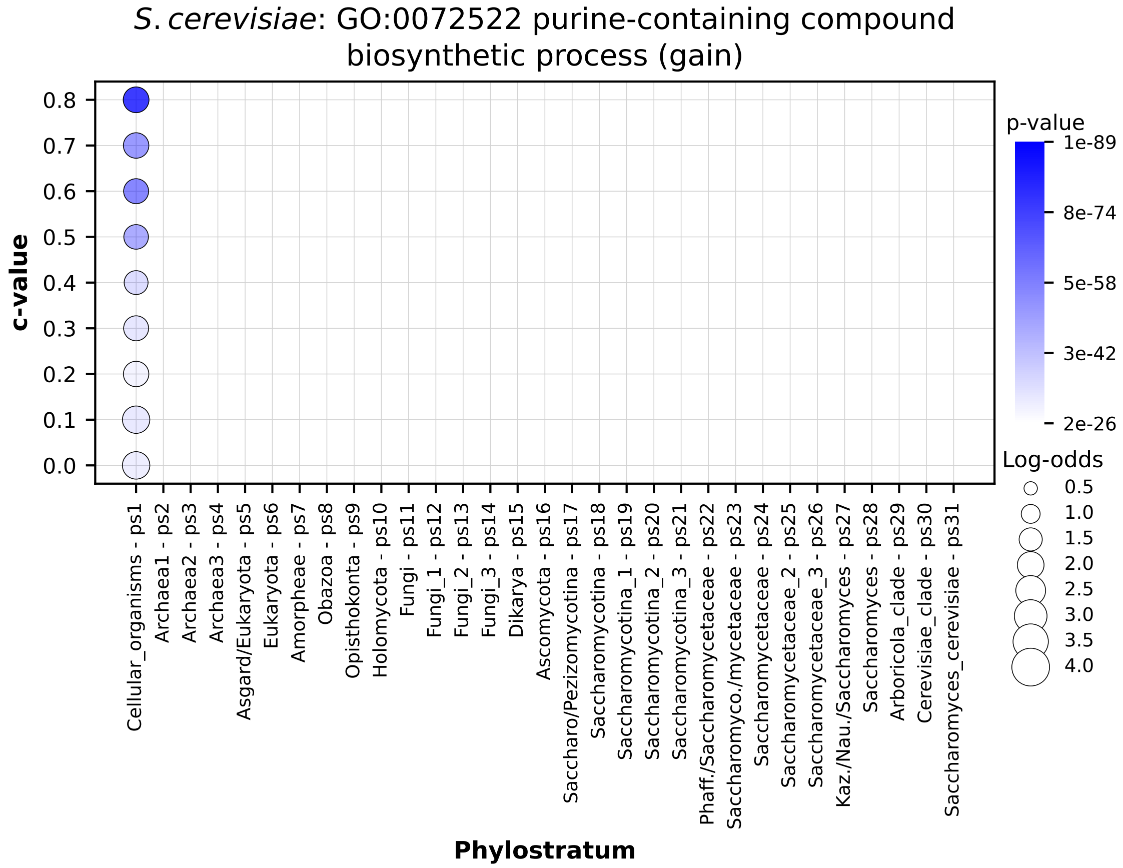

$S. cerevisiae$: GO:0072522 purine-containing compound biosynthetic process (gain)
c-value
0.8
0.7
0.6
0.5
0.4
0.3
0.2
0.1
0.0
p-value
1e-89
8e-74
5e-58
3e-42
2e-26
Log-odds
0.5
1.0
1.5
2.0
2.5
3.0
3.5
4.0
Cellular_organisms - ps1
Archaea1 - ps2
Archaea2 - ps3
Archaea3 - ps4
Asgard/Eukaryota - ps5
Eukaryota - ps6
Amorpheae - ps7
Obazoa - ps8
Opisthokonta - ps9
Holomycota - ps10
Fungi - ps11
Fungi_1 - ps12
Fungi_2 - ps13
Fungi_3 - ps14
Dikarya - ps15
Ascomycota - ps16
Saccharo/Pezizomycotina - ps17
Saccharomycotina - ps18
Saccharomycotina_1 - ps19
Saccharomycotina_2 - ps20
Saccharomycotina_3 - ps21
Phaff./Saccharomycetaceae - ps22
Saccharomyco./mycetaceae - ps23
Saccharomycetaceae - ps24
Saccharomycetaceae_2 - ps25
Saccharomycetaceae_3 - ps26
Kaz./Nau./Saccharomyces - ps27
Saccharomyces - ps28
Arboricola_clade - ps29
Cerevisiae_clade - ps30
Saccharomyces_cerevisiae - ps31
Phylostratum

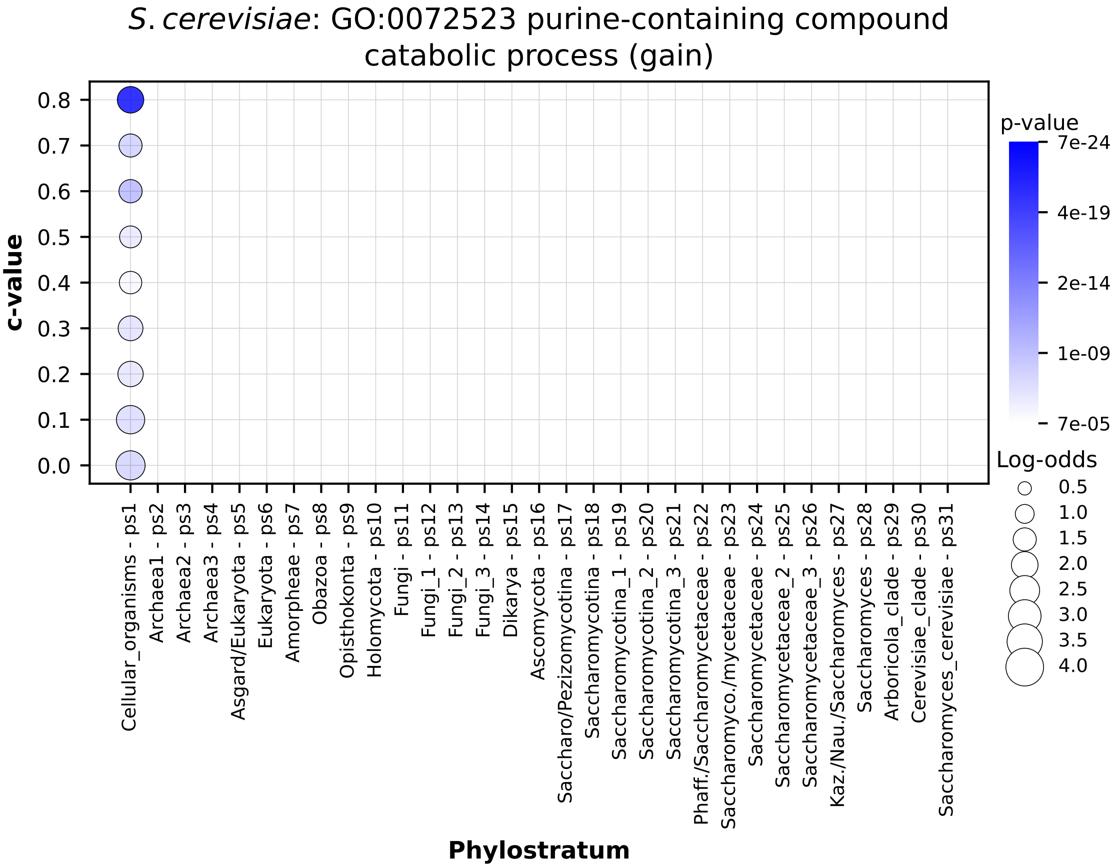

S. cerevisiae: GO:0072523 purine-containing compound catabolic process (gain)
c-value
0.8
0.7
0.6
0.5
0.4
0.3
0.2
0.1
0.0
p-value
7e-24
4e-19
2e-14
1e-09
7e-05
Log-odds
0.5
1.0
1.5
2.0
2.5
3.0
3.5
4.0
Cellular_organisms - ps1
Archaea1 - ps2
Archaea2 - ps3
Archaea3 - ps4
Asgard/Eukaryota - ps5
Eukaryota - ps6
Amorpheae - ps7
Obazoa - ps8
Opisthokonta - ps9
Holomycota - ps10
Fungi - ps11
Fungi_1 - ps12
Fungi_2 - ps13
Fungi_3 - ps14
Dikarya - ps15
Ascomycota - ps16
Saccharo/Pezizomycotina - ps17
Saccharomycotina - ps18
Saccharomycotina_1 - ps19
Saccharomycotina_2 - ps20
Saccharomycotina_3 - ps21
Phaff./Saccharomycetaceae - ps22
Saccharomyco./mycetaceae - ps23
Saccharomycetaceae - ps24
Saccharomycetaceae_2 - ps25
Saccharomycetaceae_3 - ps26
Kaz./Nau./Saccharomyces - ps27
Saccharomyces - ps28
Arboricola_clade - ps29
Cerevisiae_clade - ps30
Saccharomyces_cerevisiae - ps31
Phylostratum

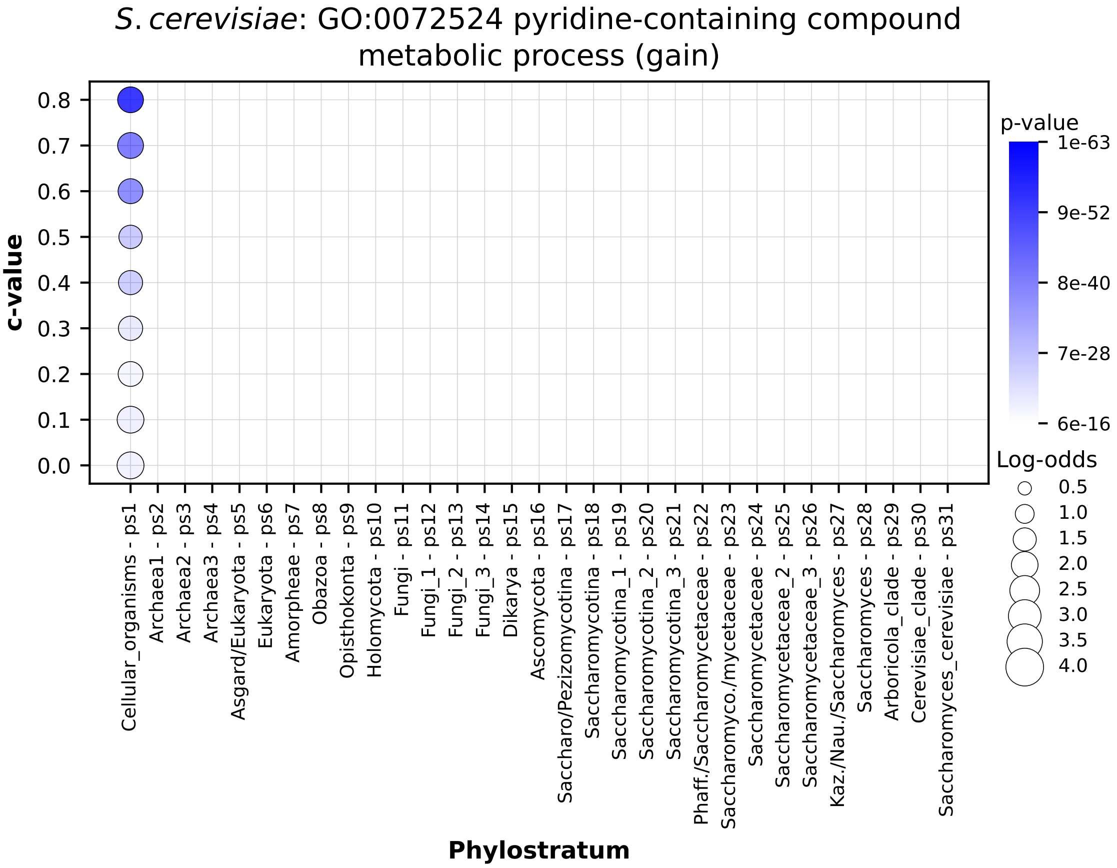

S. cerevisiae: GO:0072524 pyridine-containing compound metabolic process (gain)
c-value
0.8
0.7
0.6
0.5
0.4
0.3
0.2
0.1
0.0
p-value
1e-63
9e-52
8e-40
7e-28
6e-16
Log-odds
0.5
1.0
1.5
2.0
2.5
3.0
3.5
4.0
Cellular_organisms - ps1
Archaea1 - ps2
Archaea2 - ps3
Archaea3 - ps4
Asgard/Eukaryota - ps5
Eukaryota - ps6
Amorpheae - ps7
Obazoa - ps8
Opisthokonta - ps9
Holomycota - ps10
Fungi - ps11
Fungi_1 - ps12
Fungi_2 - ps13
Fungi_3 - ps14
Dikarya - ps15
Ascomycota - ps16
Saccharo/Pezizomycotina - ps17
Saccharomycotina - ps18
Saccharomycotina_1 - ps19
Saccharomycotina_2 - ps20
Saccharomycotina_3 - ps21
Phaff./Saccharomycetaceae - ps22
Saccharomyco./mycetaceae - ps23
Saccharomycetaceae - ps24
Saccharomycetaceae_2 - ps25
Saccharomycetaceae_3 - ps26
Kaz./Nau./Saccharomyces - ps27
Saccharomyces - ps28
Arboricola_clade - ps29
Cerevisiae_clade - ps30
Saccharomyces_cerevisiae - ps31
Phylostratum

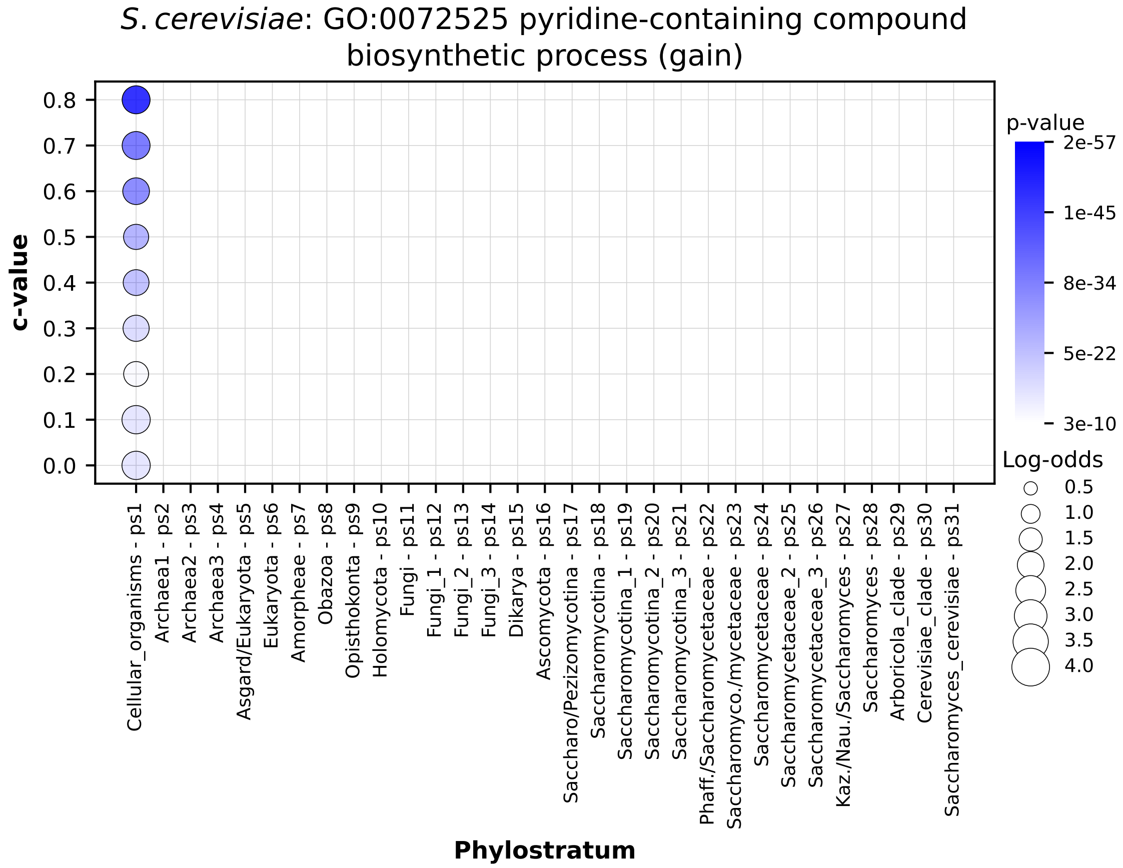

S. cerevisiae: GO:0072525 pyridine-containing compound biosynthetic process (gain)
c-value
0.8
0.7
0.6
0.5
0.4
0.3
0.2
0.1
0.0
p-value
2e-57
1e-45
8e-34
5e-22
3e-10
Log-odds
0.5
1.0
1.5
2.0
2.5
3.0
3.5
4.0
Cellular_organisms - ps1
Archaea1 - ps2
Archaea2 - ps3
Archaea3 - ps4
Asgard/Eukaryota - ps5
Eukaryota - ps6
Amorpheae - ps7
Obazoa - ps8
Opisthokonta - ps9
Holomycota - ps10
Fungi - ps11
Fungi_1 - ps12
Fungi_2 - ps13
Fungi_3 - ps14
Dikarya - ps15
Ascomycota - ps16
Saccharo/Pezizomycotina - ps17
Saccharomycotina - ps18
Saccharomycotina_1 - ps19
Saccharomycotina_2 - ps20
Saccharomycotina_3 - ps21
Phaff./Saccharomycetaceae - ps22
Saccharomyco./mycetaceae - ps23
Saccharomycetaceae - ps24
Saccharomycetaceae_2 - ps25
Saccharomycetaceae_3 - ps26
Kaz./Nau./Saccharomyces - ps27
Saccharomyces - ps28
Arboricola_clade - ps29
Cerevisiae_clade - ps30
Saccharomyces_cerevisiae - ps31
Phylostratum

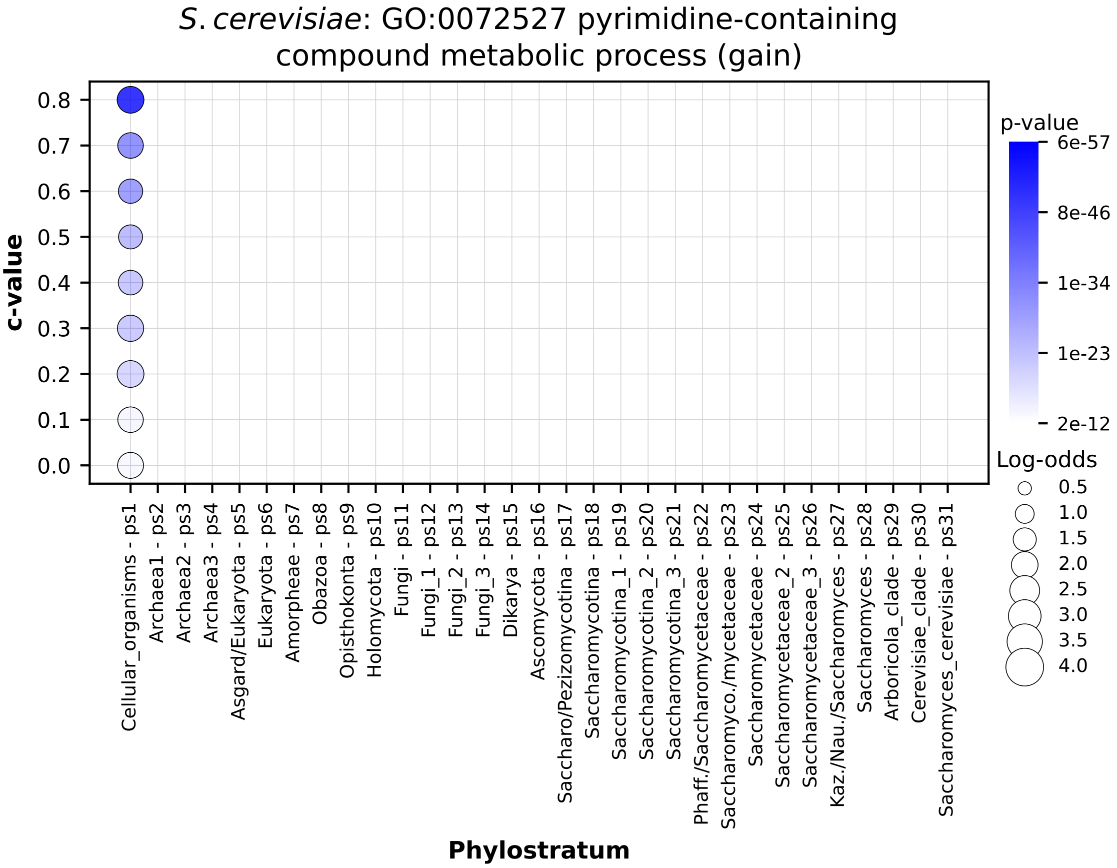

S. cerevisiae: GO:0072527 pyrimidine-containing compound metabolic process (gain)
c-value
0.0
0.1
0.2
0.3
0.4
0.5
0.6
0.7
0.8
p-value
6e-57
8e-46
1e-34
1e-23
2e-12
Log-odds
0.5
1.0
1.5
2.0
2.5
3.0
3.5
4.0
Cellular_organisms - ps1
Archaea1 - ps2
Archaea2 - ps3
Archaea3 - ps4
Asgard/Eukaryota - ps5
Eukaryota - ps6
Amorpheae - ps7
Obazoa - ps8
Opisthokonta - ps9
Holomycota - ps10
Fungi - ps11
Fungi_1 - ps12
Fungi_2 - ps13
Fungi_3 - ps14
Dikarya - ps15
Ascomycota - ps16
Saccharo/Pezizomycotina - ps17
Saccharomycotina - ps18
Saccharomycotina_1 - ps19
Saccharomycotina_2 - ps20
Saccharomycotina_3 - ps21
Phaff./Saccharomycetaceae - ps22
Saccharomyco./mycetaceae - ps23
Saccharomycetaceae - ps24
Saccharomycetaceae_2 - ps25
Saccharomycetaceae_3 - ps26
Kaz./Nau./Saccharomyces - ps27
Saccharomyces - ps28
Arboricola_clade - ps29
Cerevisiae_clade - ps30
Saccharomyces_cerevisiae - ps31
Phylostratum

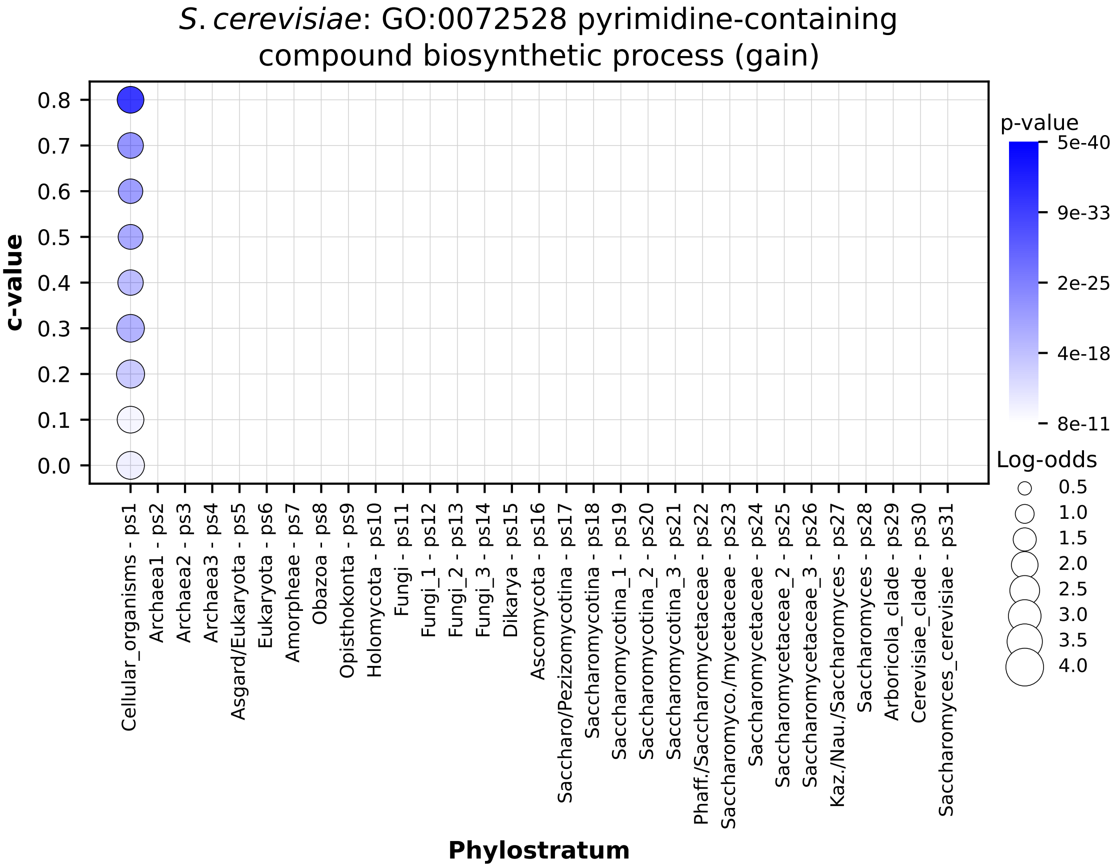
S. cerevisiae: GO:0072528 pyrimidine-containing compound biosynthetic process (gain)
c-value
0.8
0.7
0.6
0.5
0.4
0.3
0.2
0.1
0.0
Phylostratum
Cellular_organisms - ps1
Archaea1 - ps2
Archaea2 - ps3
Archaea3 - ps4
Asgard/Eukaryota - ps5
Eukaryota - ps6
Amorpheae - ps7
Obazoa - ps8
Opisthokonta - ps9
Holomycota - ps10
Fungi - ps11
Fungi_1 - ps12
Fungi_2 - ps13
Fungi_3 - ps14
Dikarya - ps15
Ascomycota - ps16
Saccharo/Pezizomycotina - ps17
Saccharomycotina - ps18
Saccharomycotina_1 - ps19
Saccharomycotina_2 - ps20
Saccharomycotina_3 - ps21
Phaff./Saccharomycetaceae - ps22
Saccharomyco./mycetaceae - ps23
Saccharomycetaceae - ps24
Saccharomycetaceae_2 - ps25
Saccharomycetaceae_3 - ps26
Kaz./Nau./Saccharomyces - ps27
Saccharomyces - ps28
Arboricola_clade - ps29
Cerevisiae_clade - ps30
Saccharomyces_cerevisiae - ps31
p-value
5e-40
9e-33
2e-25
4e-18
8e-11
Log-odds
0.5
1.0
1.5
2.0
2.5
3.0
3.5
4.0

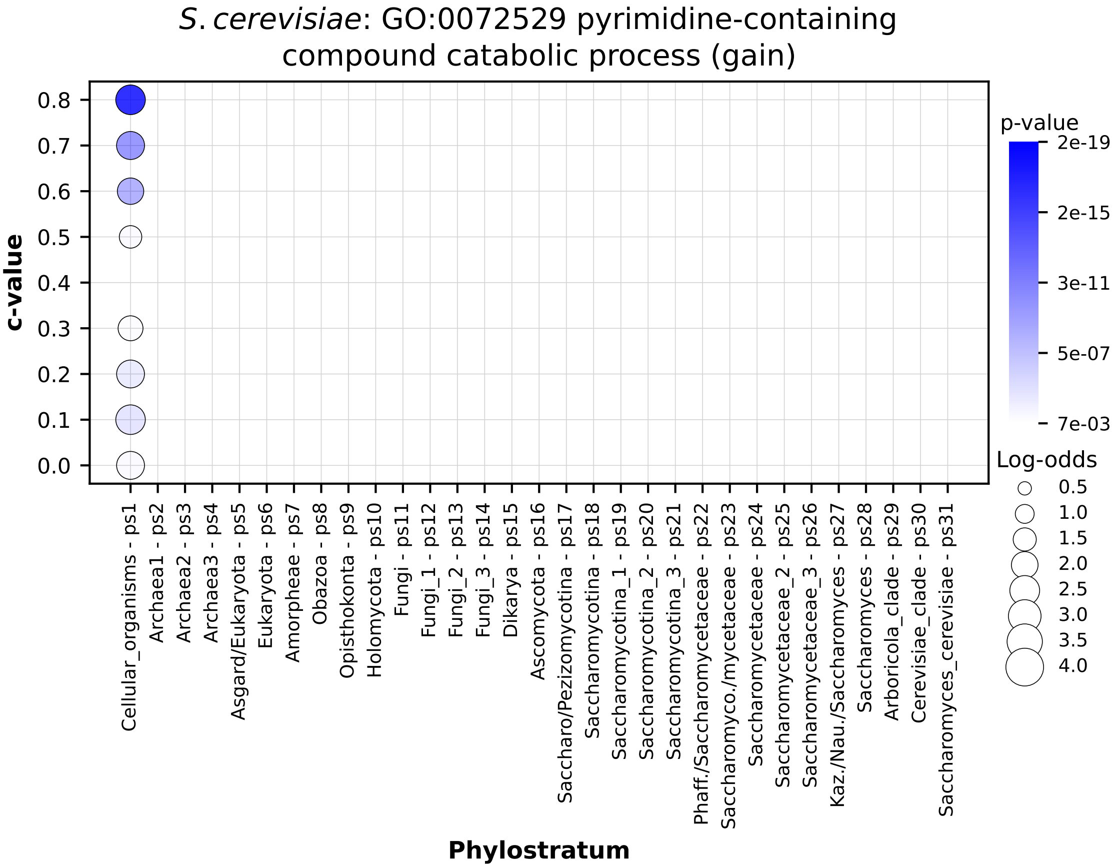

S. cerevisiae: GO:0072529 pyrimidine-containing compound catabolic process (gain)
c-value
0.8
0.7
0.6
0.5
0.4
0.3
0.2
0.1
0.0
p-value
2e-19
2e-15
3e-11
5e-07
7e-03
Log-odds
0.5
1.0
1.5
2.0
2.5
3.0
3.5
4.0
Cellular_organisms - ps1
Archaea1 - ps2
Archaea2 - ps3
Archaea3 - ps4
Asgard/Eukaryota - ps5
Eukaryota - ps6
Amorpheae - ps7
Obazoa - ps8
Opisthokonta - ps9
Holomycota - ps10
Fungi - ps11
Fungi_1 - ps12
Fungi_2 - ps13
Fungi_3 - ps14
Dikarya - ps15
Ascomycota - ps16
Saccharo/Pezizomycotina - ps17
Saccharomycotina - ps18
Saccharomycotina_1 - ps19
Saccharomycotina_2 - ps20
Saccharomycotina_3 - ps21
Phaff./Saccharomycetaceae - ps22
Saccharomyco./mycetaceae - ps23
Saccharomycetaceae - ps24
Saccharomycetaceae_2 - ps25
Saccharomycetaceae_3 - ps26
Kaz./Nau./Saccharomyces - ps27
Saccharomyces - ps28
Arboricola_clade - ps29
Cerevisiae_clade - ps30
Saccharomyces_cerevisiae - ps31
Phylostratum

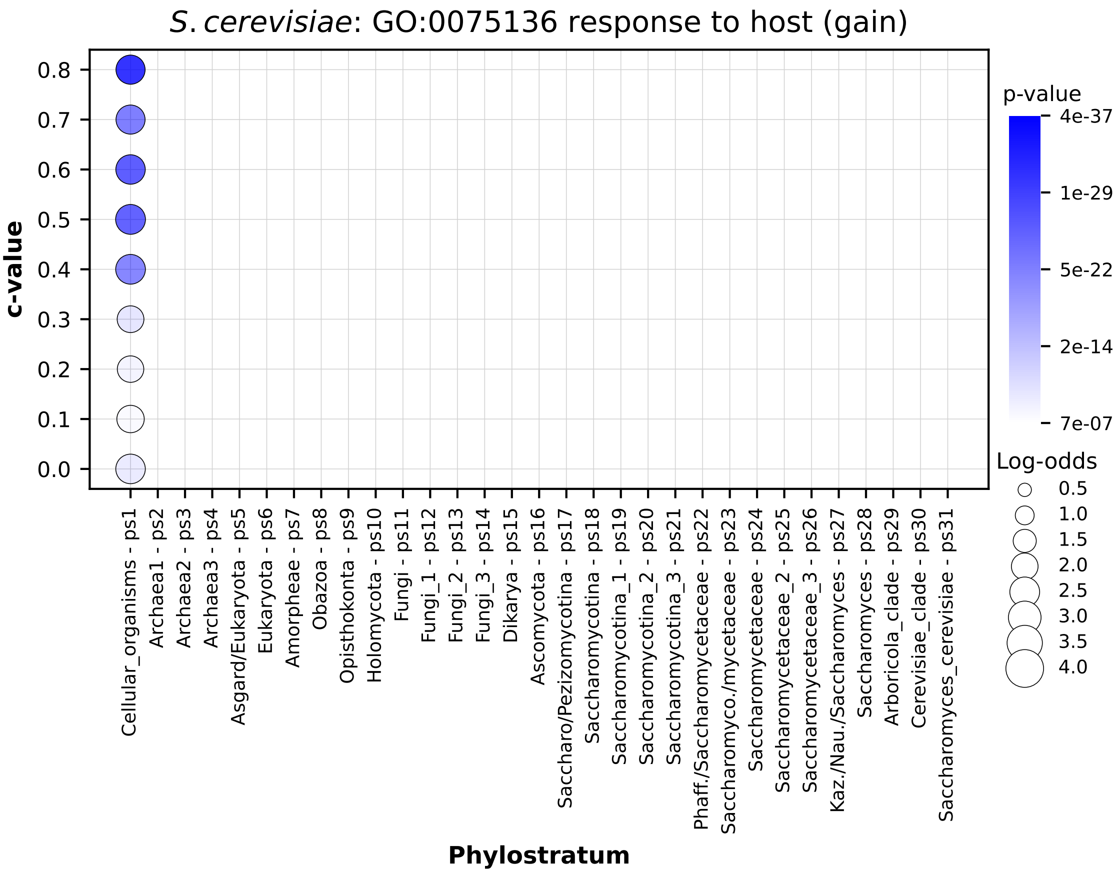
S. cerevisiae: GO:0075136 response to host (gain)
c-value
0.8
0.7
0.6
0.5
0.4
0.3
0.2
0.1
0.0
p-value
4e-37
1e-29
5e-22
2e-14
7e-07
Log-odds
0.5
1.0
1.5
2.0
2.5
3.0
3.5
4.0
Cellular_organisms - ps1
Archaea1 - ps2
Archaea2 - ps3
Archaea3 - ps4
Asgard/Eukaryota - ps5
Eukaryota - ps6
Amorpheae - ps7
Obazoa - ps8
Opisthokonta - ps9
Holomycota - ps10
Fungi - ps11
Fungi_1 - ps12
Fungi_2 - ps13
Fungi_3 - ps14
Dikarya - ps15
Ascomycota - ps16
Saccharo/Pezizomycotina - ps17
Saccharomycotina - ps18
Saccharomycotina_1 - ps19
Saccharomycotina_2 - ps20
Saccharomycotina_3 - ps21
Phaff./Saccharomycetaceae - ps22
Saccharomyco./mycetaceae - ps23
Saccharomycetaceae - ps24
Saccharomycetaceae_2 - ps25
Saccharomycetaceae_3 - ps26
Kaz./Nau./Saccharomyces - ps27
Saccharomyces - ps28
Arboricola_clade - ps29
Cerevisiae_clade - ps30
Saccharomyces_cerevisiae - ps31
Phylostratum

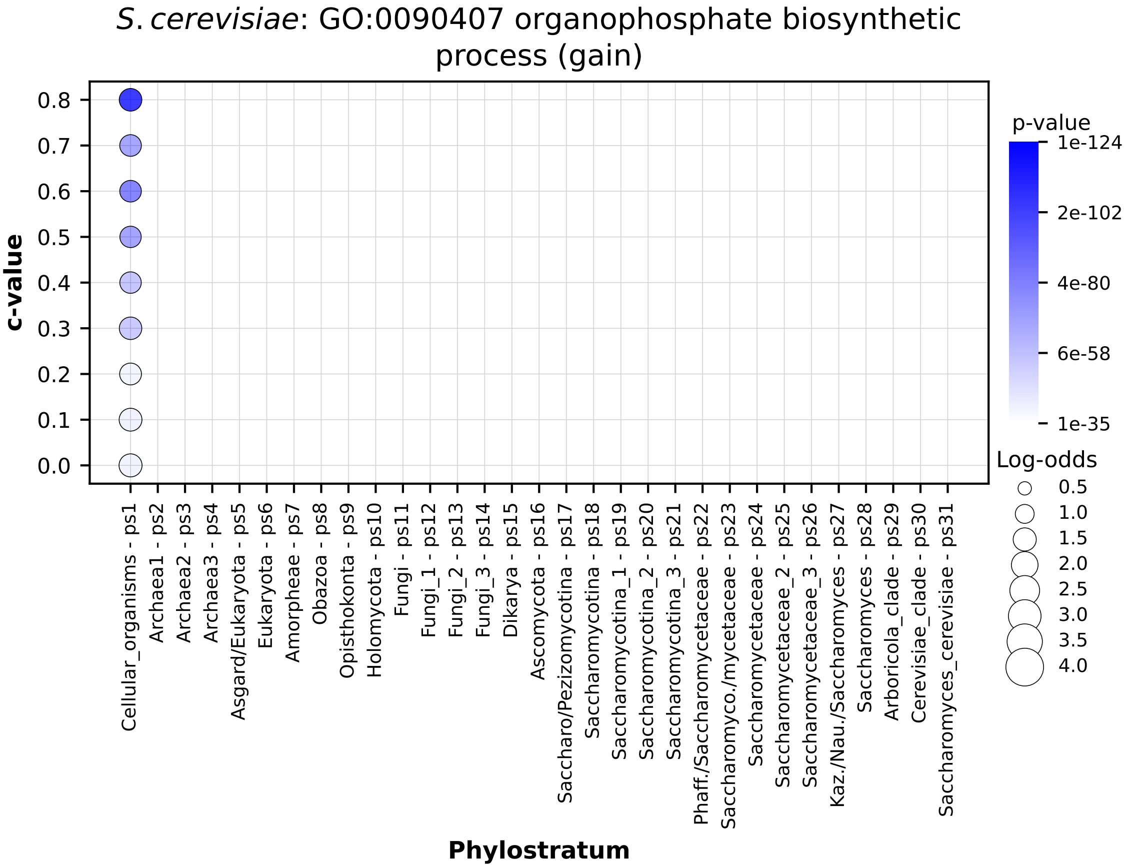

S. cerevisiae: GO:0090407 organophosphate biosynthetic process (gain)
c-value
0.0
0.1
0.2
0.3
0.4
0.5
0.6
0.7
0.8
Phylostratum
Cellular_organisms - ps1
Archaea1 - ps2
Archaea2 - ps3
Archaea3 - ps4
Asgard/Eukaryota - ps5
Eukaryota - ps6
Amorpheae - ps7
Obazoa - ps8
Opisthokonta - ps9
Holomycota - ps10
Fungi - ps11
Fungi_1 - ps12
Fungi_2 - ps13
Fungi_3 - ps14
Dikarya - ps15
Ascomycota - ps16
Saccharo/Pezizomycotina - ps17
Saccharomycotina - ps18
Saccharomycotina_1 - ps19
Saccharomycotina_2 - ps20
Saccharomycotina_3 - ps21
Phaff./Saccharomycetaceae - ps22
Saccharomyco./mycetaceae - ps23
Saccharomycetaceae - ps24
Saccharomycetaceae_2 - ps25
Saccharomycetaceae_3 - ps26
Kaz./Nau./Saccharomyces - ps27
Saccharomyces - ps28
Arboricola_clade - ps29
Cerevisiae_clade - ps30
Saccharomyces_cerevisiae - ps31
p-value
1e-124
2e-102
4e-80
6e-58
1e-35
Log-odds
0.5
1.0
1.5
2.0
2.5
3.0
3.5
4.0

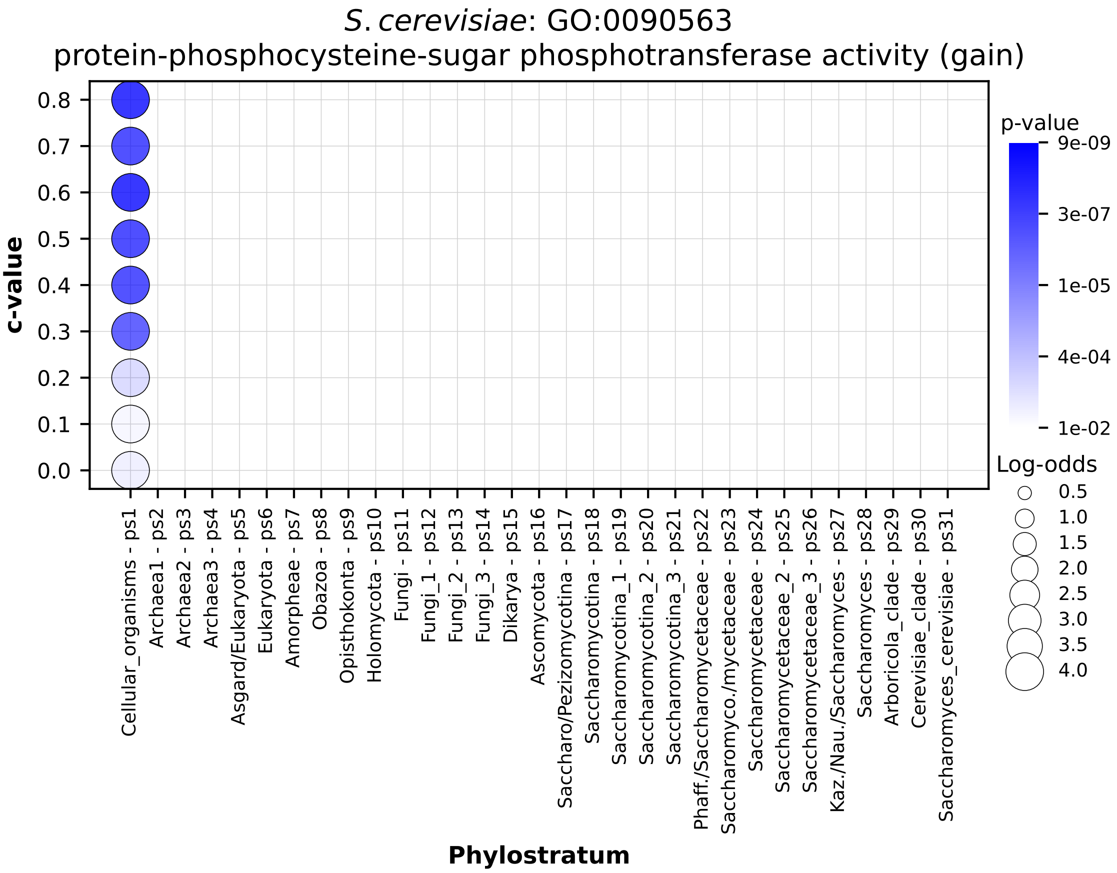

S. cerevisiae: GO:0090563
protein-phosphocysteine-sugar phosphotransferase activity (gain)
c-value
0.8
0.7
0.6
0.5
0.4
0.3
0.2
0.1
0.0
p-value
9e-09
3e-07
1e-05
4e-04
1e-02
Log-odds
0.5
1.0
1.5
2.0
2.5
3.0
3.5
4.0
Cellular_organisms - ps1
Archaea1 - ps2
Archaea2 - ps3
Archaea3 - ps4
Asgard/Eukaryota - ps5
Eukaryota - ps6
Amorpheae - ps7
Obazoa - ps8
Opisthokonta - ps9
Holomycota - ps10
Fungi - ps11
Fungi_1 - ps12
Fungi_2 - ps13
Fungi_3 - ps14
Dikarya - ps15
Ascomycota - ps16
Saccharo/Pezizomycotina - ps17
Saccharomycotina - ps18
Saccharomycotina_1 - ps19
Saccharomycotina_2 - ps20
Saccharomycotina_3 - ps21
Phaff./Saccharomycetaceae - ps22
Saccharomyco./mycetaceae - ps23
Saccharomycetaceae - ps24
Saccharomycetaceae_2 - ps25
Saccharomycetaceae_3 - ps26
Kaz./Nau./Saccharomyces - ps27
Saccharomyces - ps28
Arboricola_clade - ps29
Cerevisiae_clade - ps30
Saccharomyces_cerevisiae - ps31
Phylostratum

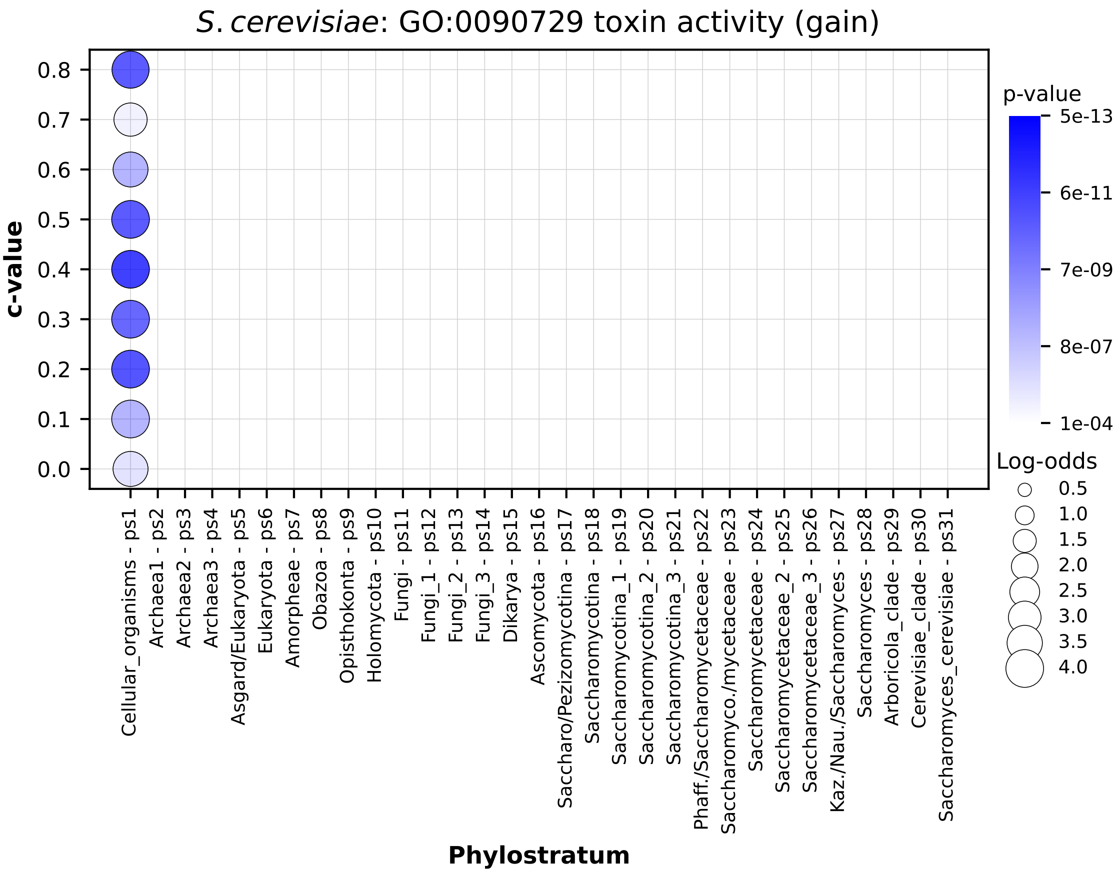

S. cerevisiae: GO:0090729 toxin activity (gain)
c-value
0.0
0.1
0.2
0.3
0.4
0.5
0.6
0.7
0.8
Phylostratum
Cellular_organisms - ps1
Archaea1 - ps2
Archaea2 - ps3
Archaea3 - ps4
Asgard/Eukaryota - ps5
Eukaryota - ps6
Amorpheae - ps7
Obazoa - ps8
Opisthokonta - ps9
Holomycota - ps10
Fungi - ps11
Fungi_1 - ps12
Fungi_2 - ps13
Fungi_3 - ps14
Dikarya - ps15
Ascomycota - ps16
Saccharo/Pezizomycotina - ps17
Saccharomycotina - ps18
Saccharomycotina_1 - ps19
Saccharomycotina_2 - ps20
Saccharomycotina_3 - ps21
Phaff./Saccharomycetaceae - ps22
Saccharomyco./mycetaceae - ps23
Saccharomycetaceae - ps24
Saccharomycetaceae_2 - ps25
Saccharomycetaceae_3 - ps26
Kaz./Nau./Saccharomyces - ps27
Saccharomyces - ps28
Arboricola_clade - ps29
Cerevisiae_clade - ps30
Saccharomyces_cerevisiae - ps31
p-value
5e-13
6e-11
7e-09
8e-07
1e-04
Log-odds
0.5
1.0
1.5
2.0
2.5
3.0
3.5
4.0

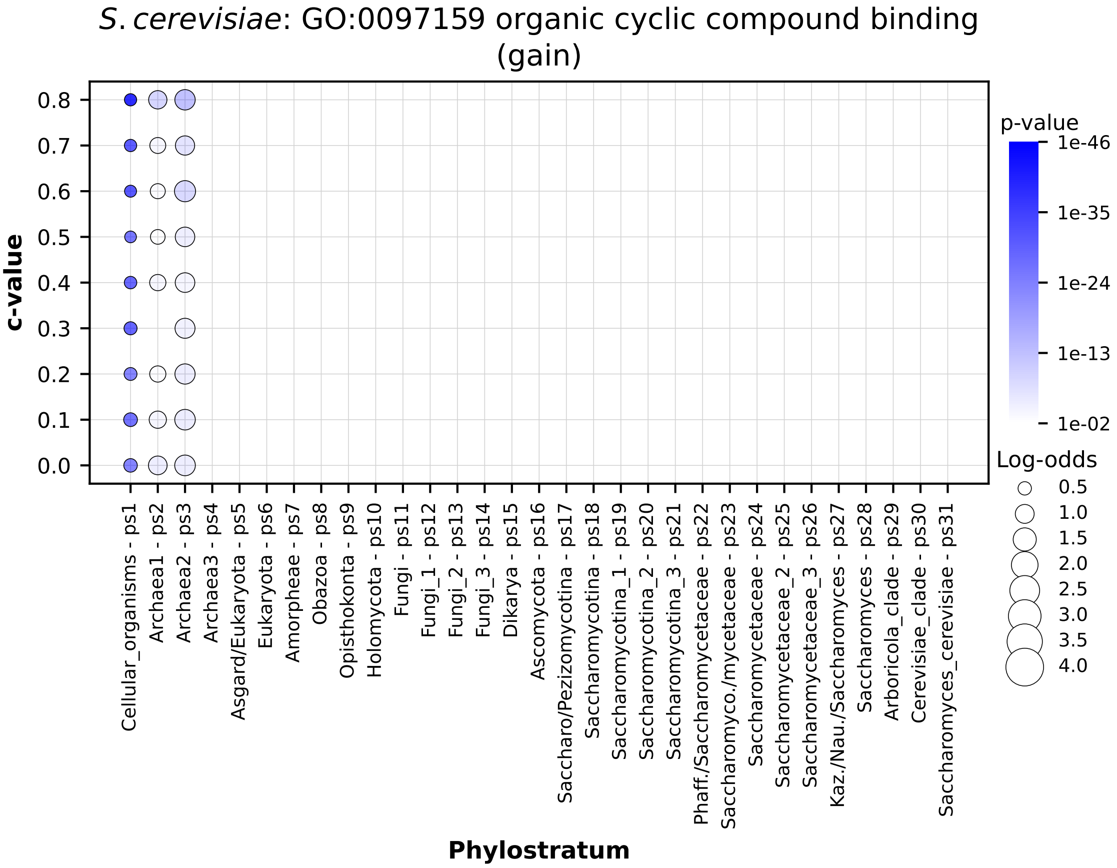
S. cerevisiae: GO:0097159 organic cyclic compound binding
(gain)
c-value
0.8
0.7
0.6
0.5
0.4
0.3
0.2
0.1
0.0
p-value
1e-46
1e-35
1e-24
1e-13
1e-02
Log-odds
0.5
1.0
1.5
2.0
2.5
3.0
3.5
4.0
Cellular_organisms - ps1
Archaea1 - ps2
Archaea2 - ps3
Archaea3 - ps4
Asgard/Eukaryota - ps5
Eukaryota - ps6
Amorpheae - ps7
Obazoa - ps8
Opisthokonta - ps9
Holomycota - ps10
Fungi - ps11
Fungi_1 - ps12
Fungi_2 - ps13
Fungi_3 - ps14
Dikarya - ps15
Ascomycota - ps16
Saccharo/Pezizomycotina - ps17
Saccharomycotina - ps18
Saccharomycotina_1 - ps19
Saccharomycotina_2 - ps20
Saccharomycotina_3 - ps21
Phaff./Saccharomycetaceae - ps22
Saccharomyco./mycetaceae - ps23
Saccharomycetaceae - ps24
Saccharomycetaceae_2 - ps25
Saccharomycetaceae_3 - ps26
Kaz./Nau./Saccharomyces - ps27
Saccharomyces - ps28
Arboricola_clade - ps29
Cerevisiae_clade - ps30
Saccharomyces_cerevisiae - ps31
Phylostratum

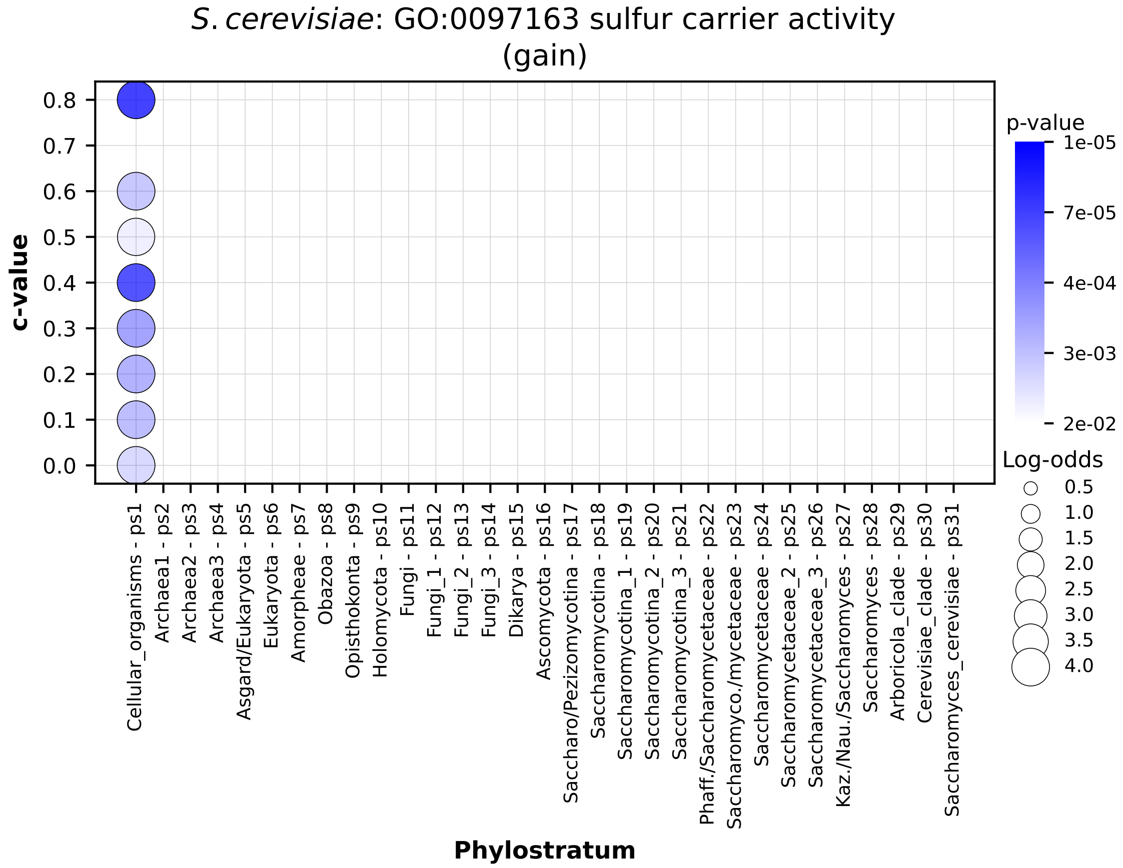

S. cerevisiae: GO:0097163 sulfur carrier activity
(gain)
c-value
0.8
0.7
0.6
0.5
0.4
0.3
0.2
0.1
0.0
p-value
1e-05
7e-05
4e-04
3e-03
2e-02
Log-odds
0.5
1.0
1.5
2.0
2.5
3.0
3.5
4.0
Cellular_organisms - ps1
Archaea1 - ps2
Archaea2 - ps3
Archaea3 - ps4
Asgard/Eukaryota - ps5
Eukaryota - ps6
Amorpheae - ps7
Obazoa - ps8
Opisthokonta - ps9
Holomycota - ps10
Fungi - ps11
Fungi_1 - ps12
Fungi_2 - ps13
Fungi_3 - ps14
Dikarya - ps15
Ascomycota - ps16
Saccharo/Pezizomycotina - ps17
Saccharomycotina - ps18
Saccharomycotina_1 - ps19
Saccharomycotina_2 - ps20
Saccharomycotina_3 - ps21
Phaff./Saccharomycetaceae - ps22
Saccharomyco./mycetaceae - ps23
Saccharomycetaceae - ps24
Saccharomycetaceae_2 - ps25
Saccharomycetaceae_3 - ps26
Kaz./Nau./Saccharomyces - ps27
Saccharomyces - ps28
Arboricola_clade - ps29
Cerevisiae_clade - ps30
Saccharomyces_cerevisiae - ps31
Phylostratum

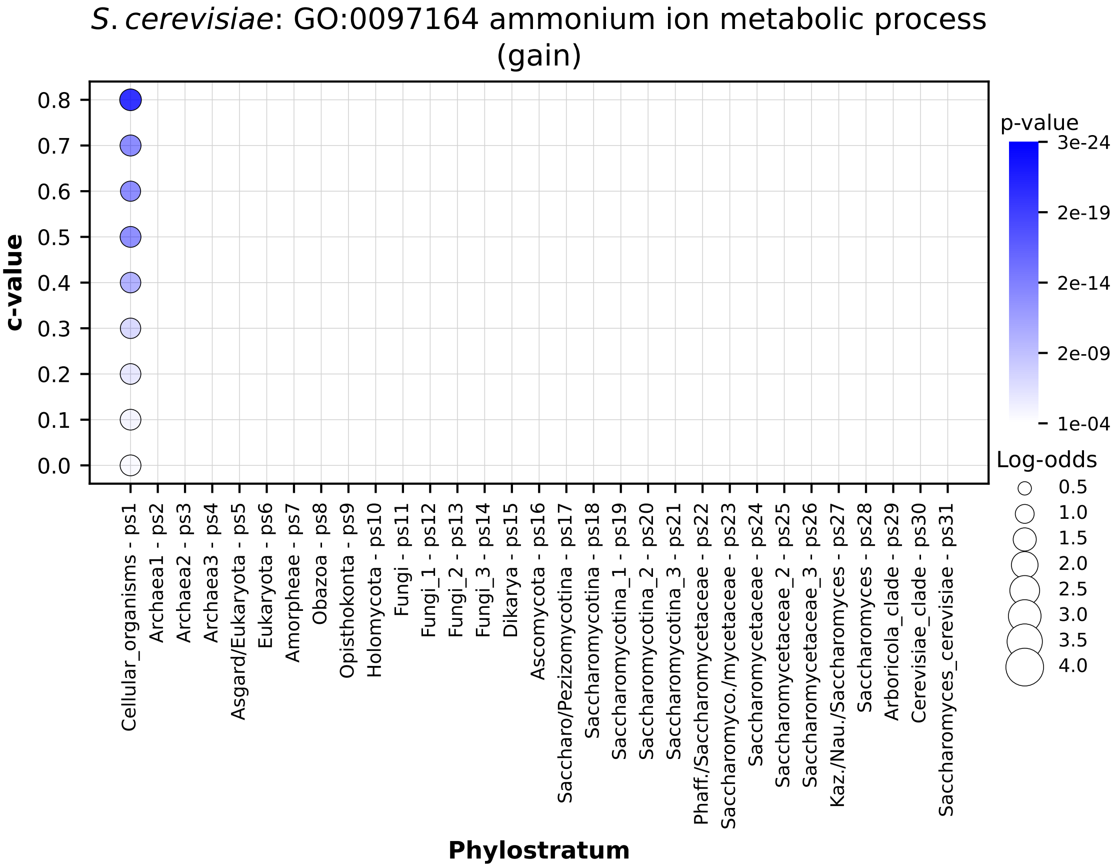

S. cerevisiae: GO:0097164 ammonium ion metabolic process
(gain)
c-value
0.8
0.7
0.6
0.5
0.4
0.3
0.2
0.1
0.0
p-value
3e-24
2e-19
2e-14
2e-09
1e-04
Log-odds
0.5
1.0
1.5
2.0
2.5
3.0
3.5
4.0
Cellular_organisms - ps1
Archaea1 - ps2
Archaea2 - ps3
Archaea3 - ps4
Asgard/Eukaryota - ps5
Eukaryota - ps6
Amorpheae - ps7
Obazoa - ps8
Opisthokonta - ps9
Holomycota - ps10
Fungi - ps11
Fungi_1 - ps12
Fungi_2 - ps13
Fungi_3 - ps14
Dikarya - ps15
Ascomycota - ps16
Saccharo/Pezizomycotina - ps17
Saccharomycotina - ps18
Saccharomycotina_1 - ps19
Saccharomycotina_2 - ps20
Saccharomycotina_3 - ps21
Phaff./Saccharomycetaceae - ps22
Saccharomyco./mycetaceae - ps23
Saccharomycetaceae - ps24
Saccharomycetaceae_2 - ps25
Saccharomycetaceae_3 - ps26
Kaz./Nau./Saccharomyces - ps27
Saccharomyces - ps28
Arboricola_clade - ps29
Cerevisiae_clade - ps30
Saccharomyces_cerevisiae - ps31
Phylostratum

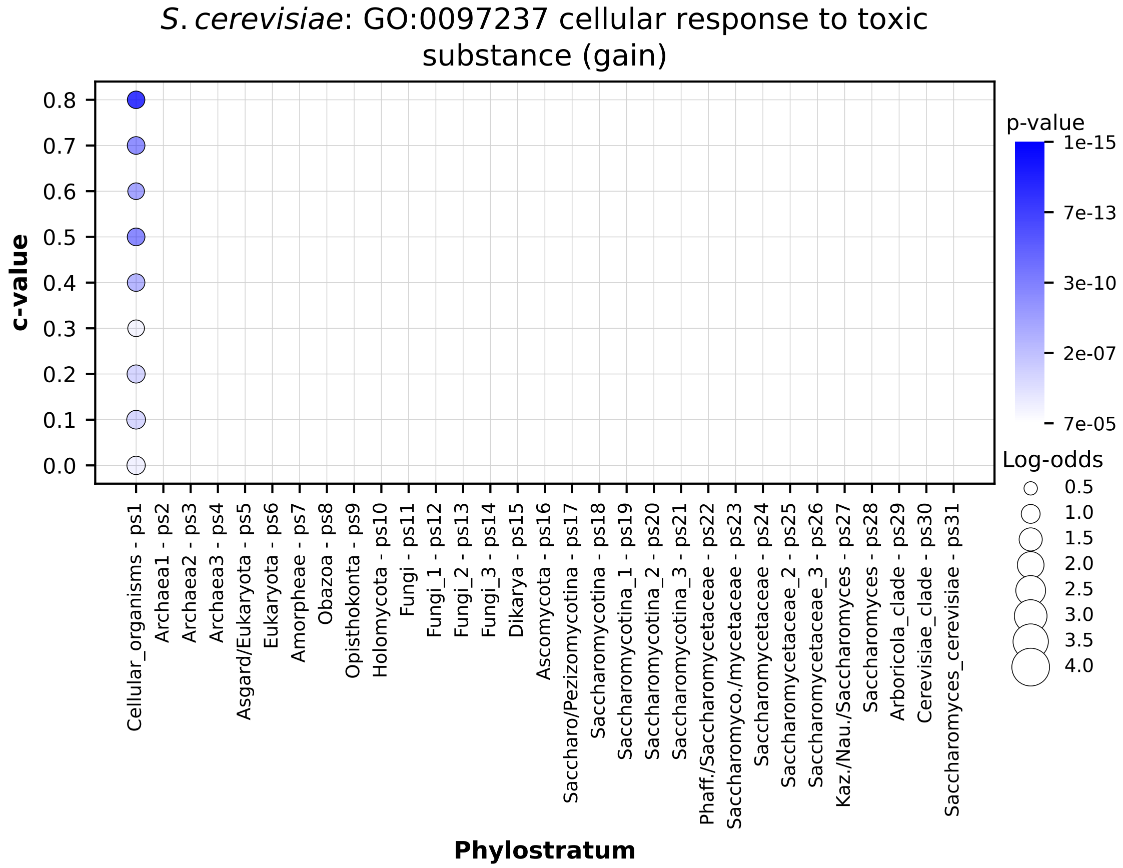

S. cerevisiae: GO:0097237 cellular response to toxic substance (gain)
c-value
0.8
0.7
0.6
0.5
0.4
0.3
0.2
0.1
0.0
p-value
1e-15
7e-13
3e-10
2e-07
7e-05
Log-odds
0.5
1.0
1.5
2.0
2.5
3.0
3.5
4.0
Cellular_organisms - ps1
Archaea1 - ps2
Archaea2 - ps3
Archaea3 - ps4
Asgard/Eukaryota - ps5
Eukaryota - ps6
Amorpheae - ps7
Obazoa - ps8
Opisthokonta - ps9
Holomycota - ps10
Fungi - ps11
Fungi_1 - ps12
Fungi_2 - ps13
Fungi_3 - ps14
Dikarya - ps15
Ascomycota - ps16
Saccharo/Pezizomycotina - ps17
Saccharomycotina - ps18
Saccharomycotina_1 - ps19
Saccharomycotina_2 - ps20
Saccharomycotina_3 - ps21
Phaff./Saccharomycetaceae - ps22
Saccharomyco./mycetaceae - ps23
Saccharomycetaceae - ps24
Saccharomycetaceae_2 - ps25
Saccharomycetaceae_3 - ps26
Kaz./Nau./Saccharomyces - ps27
Saccharomyces - ps28
Arboricola_clade - ps29
Cerevisiae_clade - ps30
Saccharomyces_cerevisiae - ps31
Phylostratum

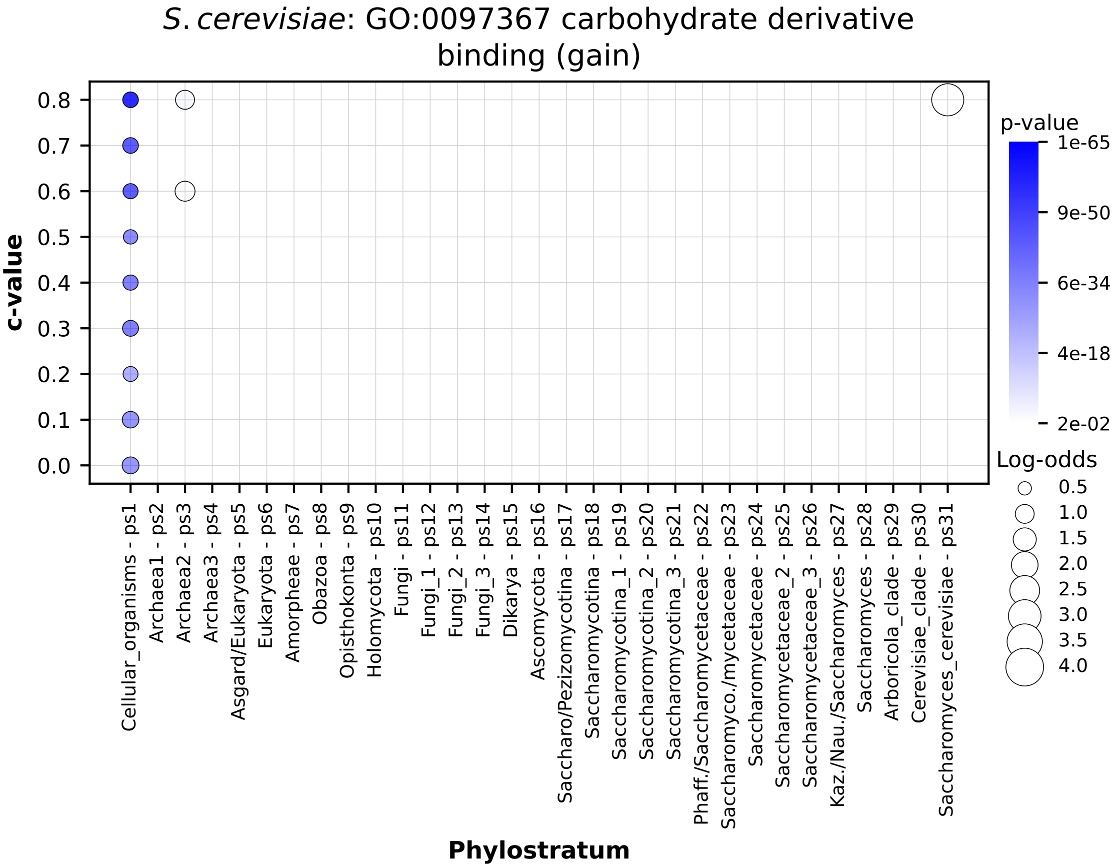
S. cerevisiae: GO:0097367 carbohydrate derivative binding (gain)
c-value
0.8
0.7
0.6
0.5
0.4
0.3
0.2
0.1
0.0
p-value
1e-65
9e-50
6e-34
4e-18
2e-02
Log-odds
0.5
1.0
1.5
2.0
2.5
3.0
3.5
4.0
Cellular_organisms - ps1
Archaea1 - ps2
Archaea2 - ps3
Archaea3 - ps4
Asgard/Eukaryota - ps5
Eukaryota - ps6
Amorpheae - ps7
Obazoa - ps8
Opisthokonta - ps9
Holomycota - ps10
Fungi - ps11
Fungi_1 - ps12
Fungi_2 - ps13
Fungi_3 - ps14
Dikarya - ps15
Ascomycota - ps16
Saccharo/Pezizomycotina - ps17
Saccharomycotina - ps18
Saccharomycotina_1 - ps19
Saccharomycotina_2 - ps20
Saccharomycotina_3 - ps21
Phaff./Saccharomycetaceae - ps22
Saccharomyco./mycetaceae - ps23
Saccharomycetaceae - ps24
Saccharomycetaceae_2 - ps25
Saccharomycetaceae_3 - ps26
Kaz./Nau./Saccharomyces - ps27
Saccharomyces - ps28
Arboricola_clade - ps29
Cerevisiae_clade - ps30
Saccharomyces_cerevisiae - ps31
Phylostratum

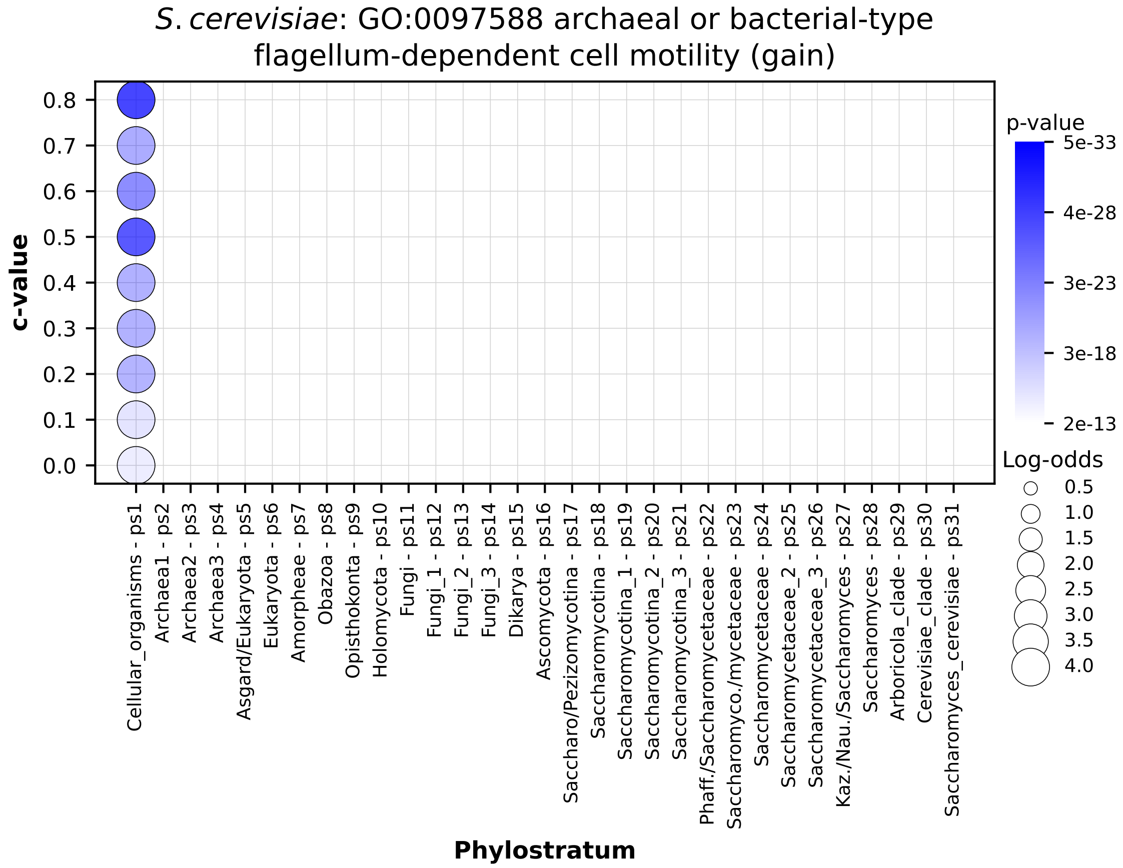
S. cerevisiae: GO:0097588 archaeal or bacterial-type flagellum-dependent cell motility (gain)
c-value
0.8
0.7
0.6
0.5
0.4
0.3
0.2
0.1
0.0
p-value
5e-33
4e-28
3e-23
3e-18
2e-13
Log-odds
0.5
1.0
1.5
2.0
2.5
3.0
3.5
4.0
Cellular_organisms - ps1
Archaea1 - ps2
Archaea2 - ps3
Archaea3 - ps4
Asgard/Eukaryota - ps5
Eukaryota - ps6
Amorpheae - ps7
Obazoa - ps8
Opisthokonta - ps9
Holomycota - ps10
Fungi - ps11
Fungi_1 - ps12
Fungi_2 - ps13
Fungi_3 - ps14
Dikarya - ps15
Ascomycota - ps16
Saccharo/Pezizomycotina - ps17
Saccharomycotina - ps18
Saccharomycotina_1 - ps19
Saccharomycotina_2 - ps20
Saccharomycotina_3 - ps21
Phaff./Saccharomycetaceae - ps22
Saccharomyco./mycetaceae - ps23
Saccharomycetaceae - ps24
Saccharomycetaceae_2 - ps25
Saccharomycetaceae_3 - ps26
Kaz./Nau./Saccharomyces - ps27
Saccharomyces - ps28
Arboricola_clade - ps29
Cerevisiae_clade - ps30
Saccharomyces_cerevisiae - ps31
Phylostratum

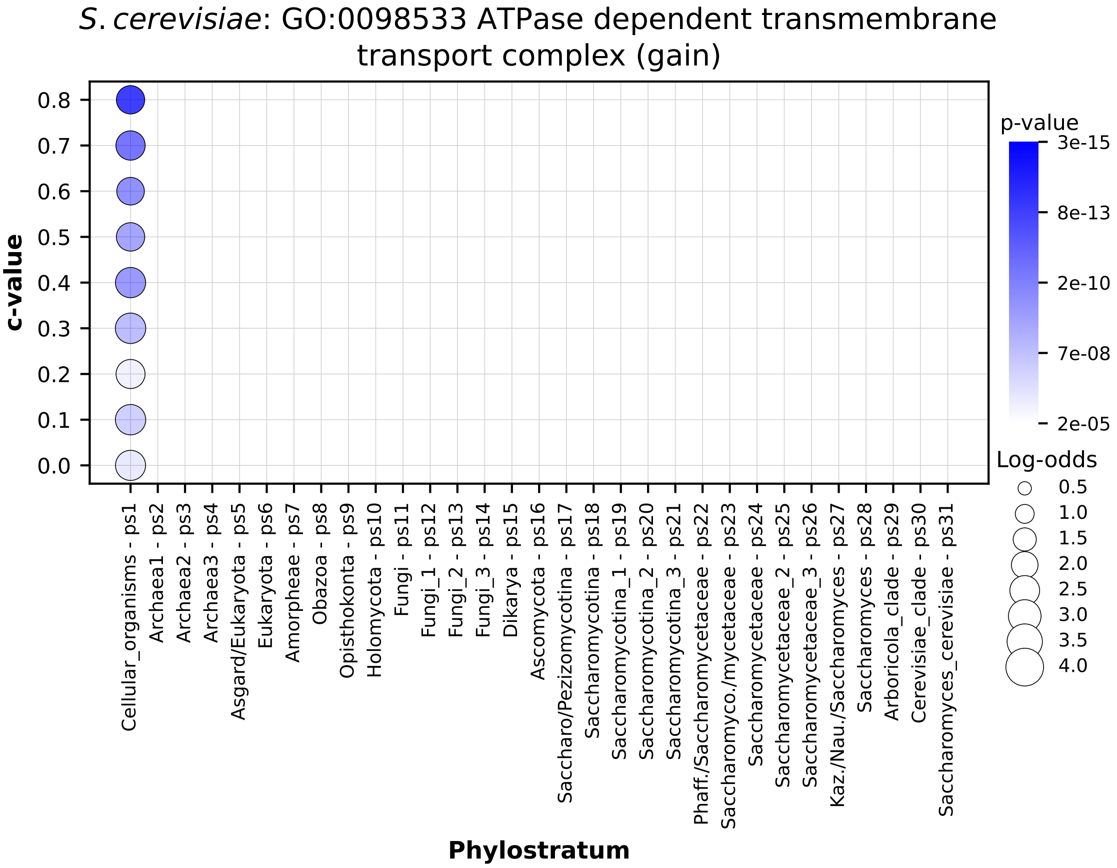
S. cerevisiae: GO:0098533 ATPase dependent transmembrane transport complex (gain)
c-value
0.8
0.7
0.6
0.5
0.4
0.3
0.2
0.1
0.0
Phylostratum
Cellular_organisms - ps1
Archaea1 - ps2
Archaea2 - ps3
Archaea3 - ps4
Asgard/Eukaryota - ps5
Eukaryota - ps6
Amorpheae - ps7
Obazoa - ps8
Opisthokonta - ps9
Holomycota - ps10
Fungi - ps11
Fungi_1 - ps12
Fungi_2 - ps13
Fungi_3 - ps14
Dikarya - ps15
Ascomycota - ps16
Saccharo/Pezizomycotina - ps17
Saccharomycotina - ps18
Saccharomycotina_1 - ps19
Saccharomycotina_2 - ps20
Saccharomycotina_3 - ps21
Phaff./Saccharomycetaceae - ps22
Saccharomyco./mycetaceae - ps23
Saccharomycetaceae - ps24
Saccharomycetaceae_2 - ps25
Saccharomycetaceae_3 - ps26
Kaz./Nau./Saccharomyces - ps27
Saccharomyces - ps28
Arboricola_clade - ps29
Cerevisiae_clade - ps30
Saccharomyces_cerevisiae - ps31
p-value
3e-15
8e-13
2e-10
7e-08
2e-05
Log-odds
0.5
1.0
1.5
2.0
2.5
3.0
3.5
4.0

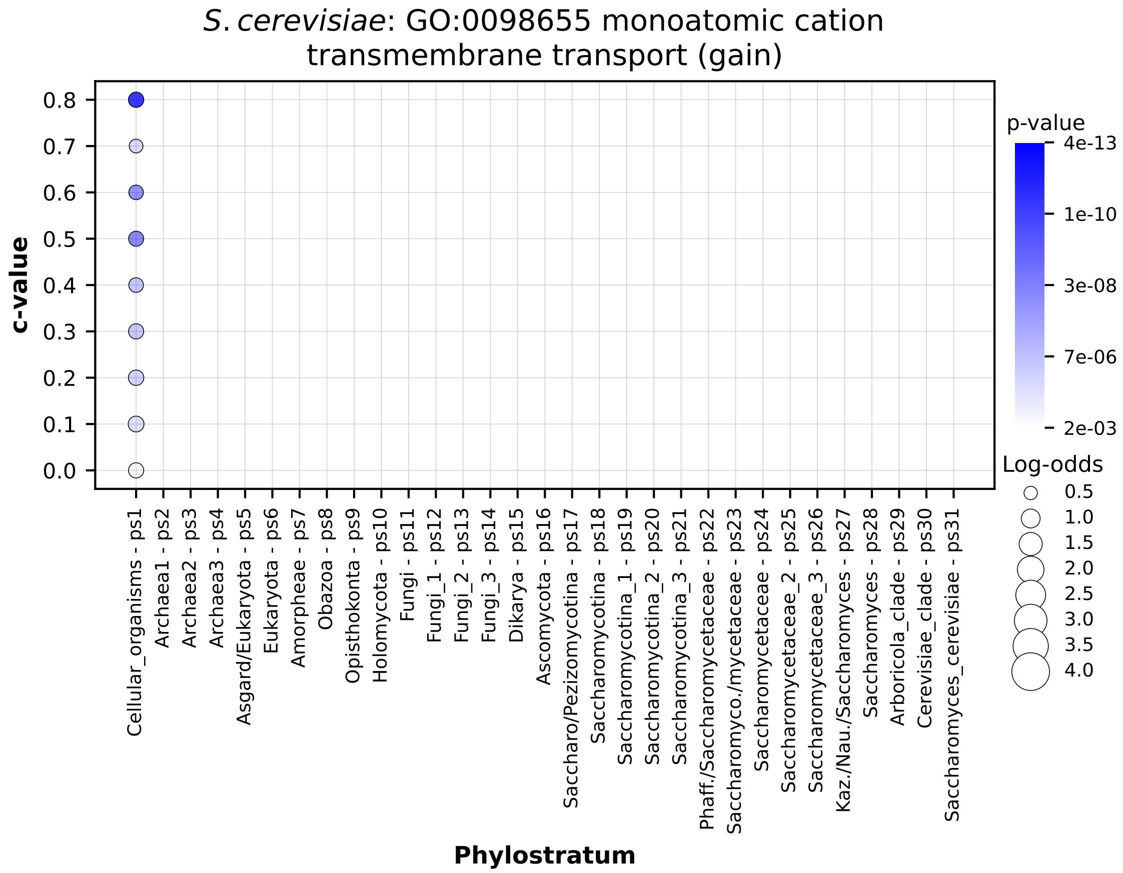

S. cerevisiae: GO:0098655 monoatomic cation transmembrane transport (gain)
c-value
0.8
0.7
0.6
0.5
0.4
0.3
0.2
0.1
0.0
p-value
4e-13
1e-10
3e-08
7e-06
2e-03
Log-odds
0.5
1.0
1.5
2.0
2.5
3.0
3.5
4.0
Cellular_organisms - ps1
Archaea1 - ps2
Archaea2 - ps3
Archaea3 - ps4
Asgard/Eukaryota - ps5
Eukaryota - ps6
Amorpheae - ps7
Obazoa - ps8
Opisthokonta - ps9
Holomycota - ps10
Fungi - ps11
Fungi_1 - ps12
Fungi_2 - ps13
Fungi_3 - ps14
Dikarya - ps15
Ascomycota - ps16
Saccharo/Pezizomycotina - ps17
Saccharomycotina - ps18
Saccharomycotina_1 - ps19
Saccharomycotina_2 - ps20
Saccharomycotina_3 - ps21
Phaff./Saccharomycetaceae - ps22
Saccharomyco./mycetaceae - ps23
Saccharomycetaceae - ps24
Saccharomycetaceae_2 - ps25
Saccharomycetaceae_3 - ps26
Kaz./Nau./Saccharomyces - ps27
Saccharomyces - ps28
Arboricola_clade - ps29
Cerevisiae_clade - ps30
Saccharomyces_cerevisiae - ps31
Phylostratum

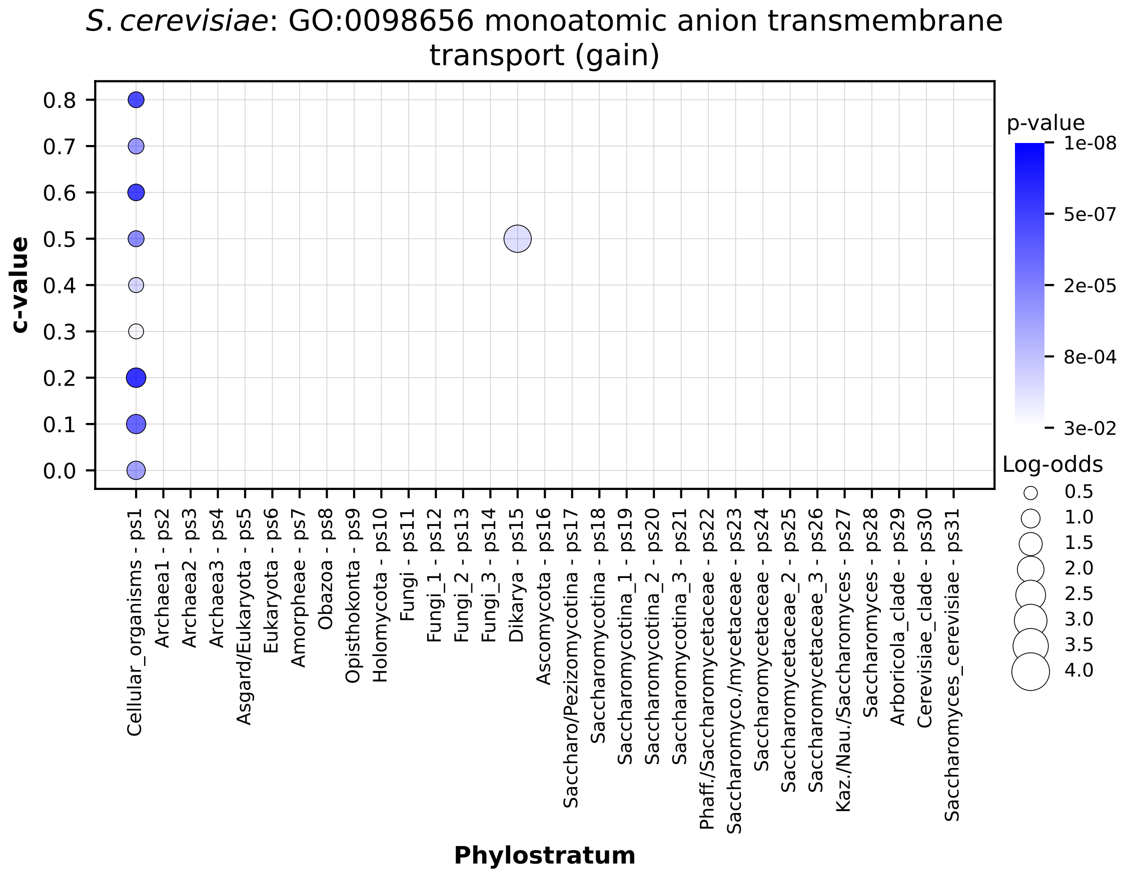
S. cerevisiae: GO:0098656 monoatomic anion transmembrane transport (gain)
c-value
0.8
0.7
0.6
0.5
0.4
0.3
0.2
0.1
0.0
Phylostratum
Cellular_organisms - ps1
Archaea1 - ps2
Archaea2 - ps3
Archaea3 - ps4
Asgard/Eukaryota - ps5
Eukaryota - ps6
Amorpheae - ps7
Obazoa - ps8
Opisthokonta - ps9
Holomycota - ps10
Fungi - ps11
Fungi_1 - ps12
Fungi_2 - ps13
Fungi_3 - ps14
Dikarya - ps15
Ascomycota - ps16
Saccharo/Pezizomycotina - ps17
Saccharomycotina - ps18
Saccharomycotina_1 - ps19
Saccharomycotina_2 - ps20
Saccharomycotina_3 - ps21
Phaff./Saccharomycetaceae - ps22
Saccharomyco./mycetaceae - ps23
Saccharomycetaceae - ps24
Saccharomycetaceae_2 - ps25
Saccharomycetaceae_3 - ps26
Kaz./Nau./Saccharomyces - ps27
Saccharomyces - ps28
Arboricola_clade - ps29
Cerevisiae_clade - ps30
Saccharomyces_cerevisiae - ps31
p-value
1e-08
5e-07
2e-05
8e-04
3e-02
Log-odds
0.5
1.0
1.5
2.0
2.5
3.0
3.5
4.0

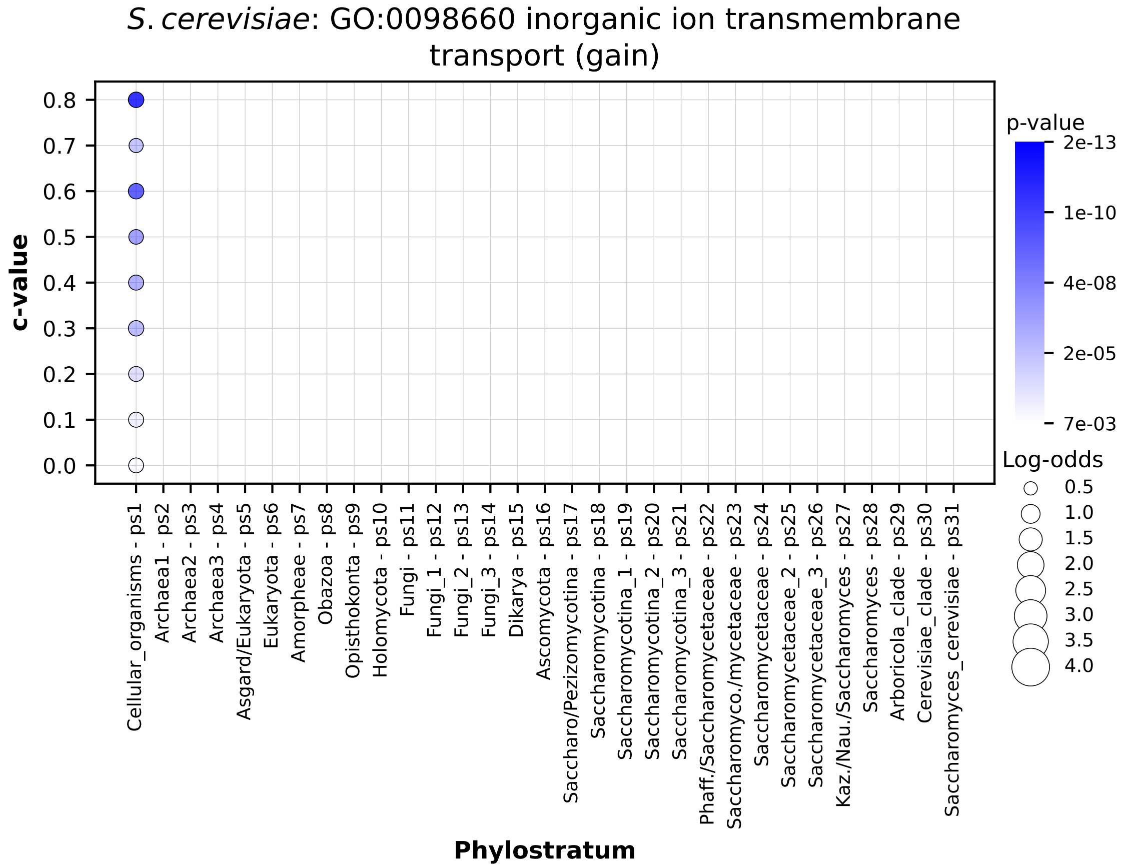
S. cerevisiae: GO:0098660 inorganic ion transmembrane transport (gain)
c-value
0.8
0.7
0.6
0.5
0.4
0.3
0.2
0.1
0.0
p-value
2e-13
1e-10
4e-08
2e-05
7e-03
Log-odds
0.5
1.0
1.5
2.0
2.5
3.0
3.5
4.0
Cellular_organisms - ps1
Archaea1 - ps2
Archaea2 - ps3
Archaea3 - ps4
Asgard/Eukaryota - ps5
Eukaryota - ps6
Amorpheae - ps7
Obazoa - ps8
Opisthokonta - ps9
Holomycota - ps10
Fungi - ps11
Fungi_1 - ps12
Fungi_2 - ps13
Fungi_3 - ps14
Dikarya - ps15
Ascomycota - ps16
Saccharo/Pezizomycotina - ps17
Saccharomycotina - ps18
Saccharomycotina_1 - ps19
Saccharomycotina_2 - ps20
Saccharomycotina_3 - ps21
Phaff./Saccharomycetaceae - ps22
Saccharomyco./mycetaceae - ps23
Saccharomycetaceae - ps24
Saccharomycetaceae_2 - ps25
Saccharomycetaceae_3 - ps26
Kaz./Nau./Saccharomyces - ps27
Saccharomyces - ps28
Arboricola_clade - ps29
Cerevisiae_clade - ps30
Saccharomyces_cerevisiae - ps31
Phylostratum

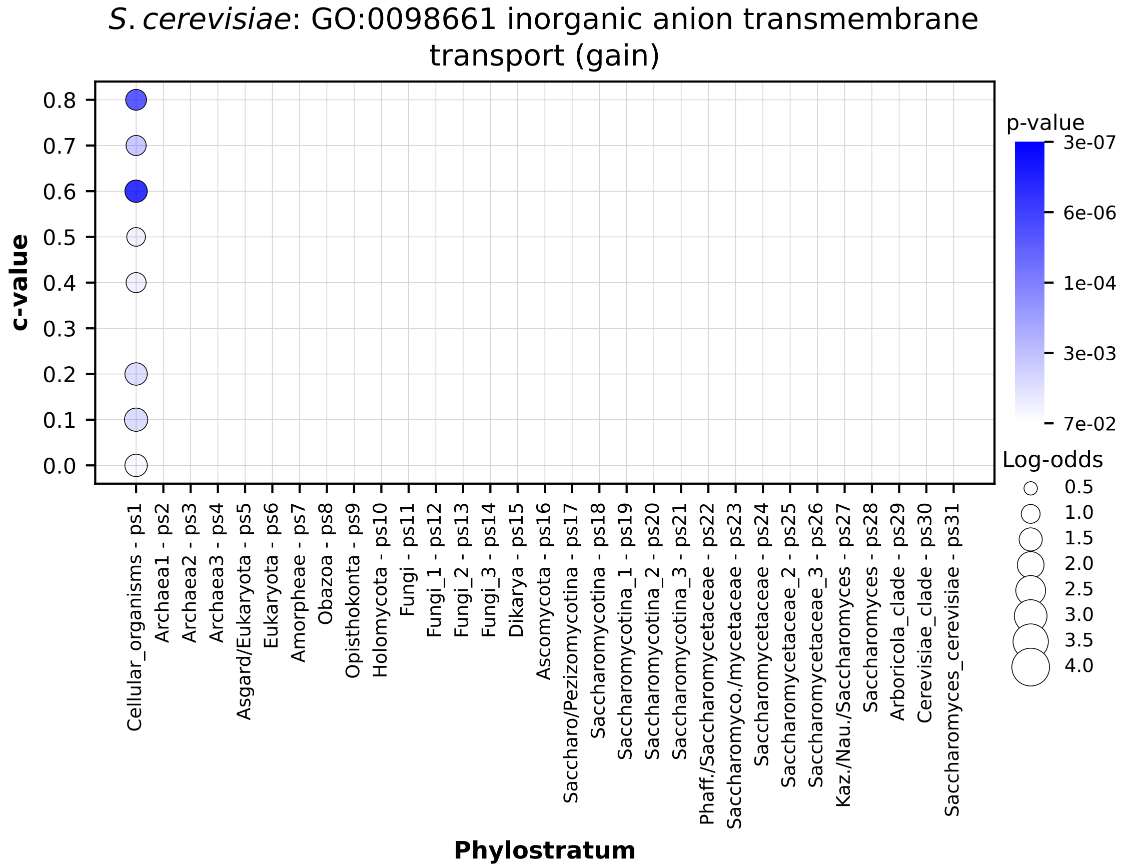
S. cerevisiae: GO:0098661 inorganic anion transmembrane transport (gain)
c-value
0.8
0.7
0.6
0.5
0.4
0.3
0.2
0.1
0.0
p-value
3e-07
6e-06
1e-04
3e-03
7e-02
Log-odds
0.5
1.0
1.5
2.0
2.5
3.0
3.5
4.0
Cellular_organisms - ps1
Archaea1 - ps2
Archaea2 - ps3
Archaea3 - ps4
Asgard/Eukaryota - ps5
Eukaryota - ps6
Amorpheae - ps7
Obazoa - ps8
Opisthokonta - ps9
Holomycota - ps10
Fungi - ps11
Fungi_1 - ps12
Fungi_2 - ps13
Fungi_3 - ps14
Dikarya - ps15
Ascomycota - ps16
Saccharo/Pezizomycotina - ps17
Saccharomycotina - ps18
Saccharomycotina_1 - ps19
Saccharomycotina_2 - ps20
Saccharomycotina_3 - ps21
Phaff./Saccharomycetaceae - ps22
Saccharomyco./mycetaceae - ps23
Saccharomycetaceae - ps24
Saccharomycetaceae_2 - ps25
Saccharomycetaceae_3 - ps26
Kaz./Nau./Saccharomyces - ps27
Saccharomyces - ps28
Arboricola_clade - ps29
Cerevisiae_clade - ps30
Saccharomyces_cerevisiae - ps31
Phylostratum

S. cerevisiae: GO:0098754 detoxification (gain)

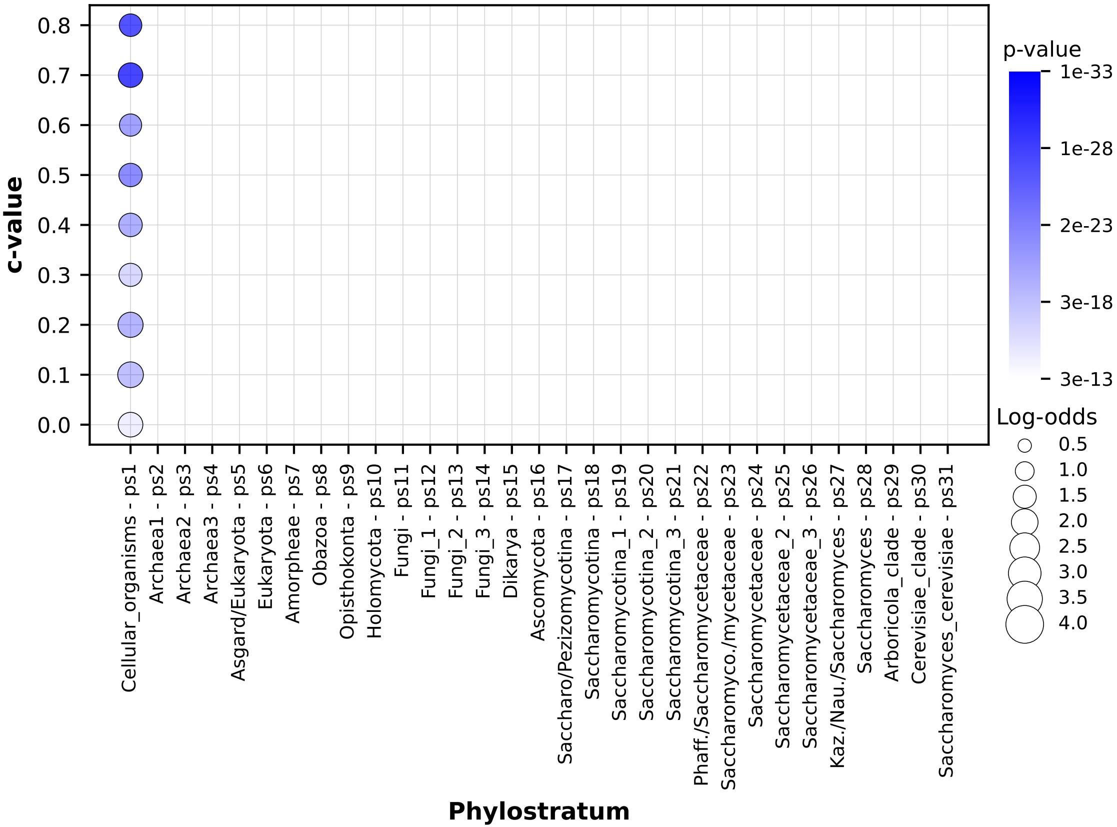

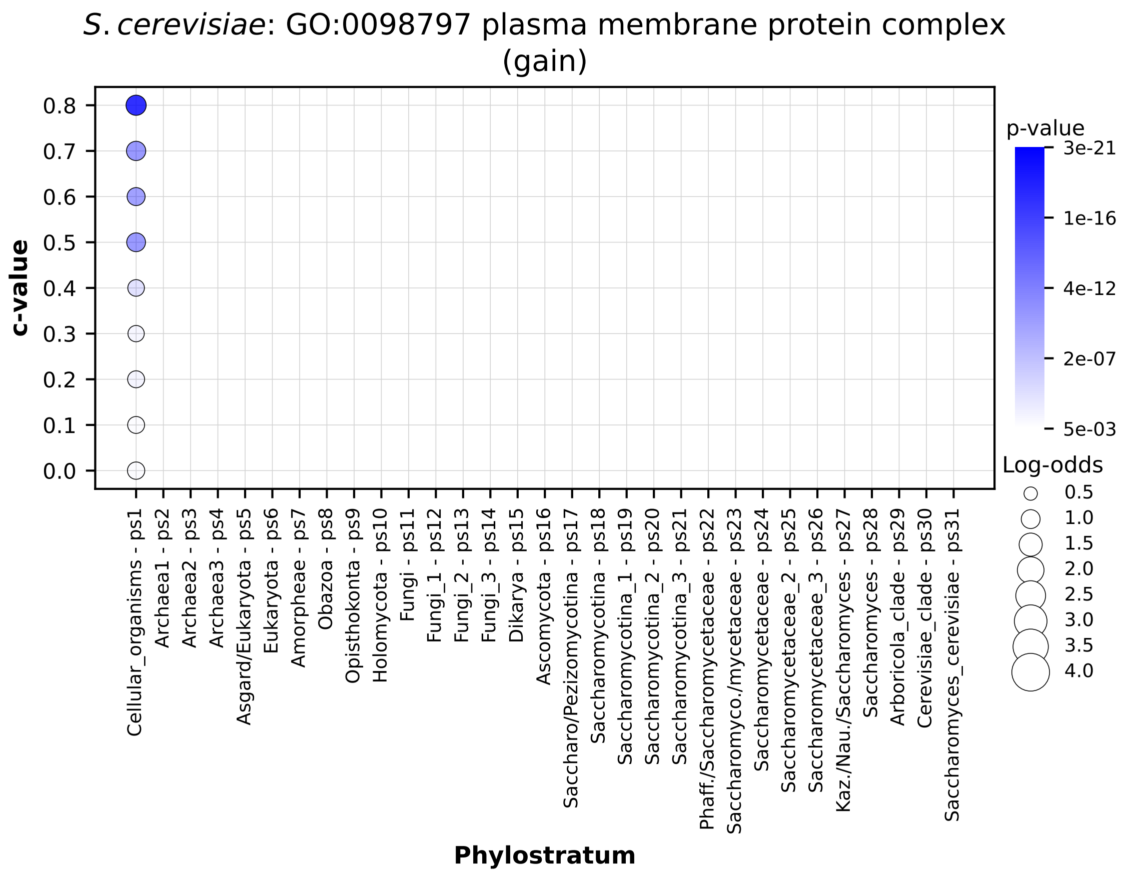

S. cerevisiae: GO:0098797 plasma membrane protein complex (gain)
c-value
0.8
0.7
0.6
0.5
0.4
0.3
0.2
0.1
0.0
p-value
3e-21
1e-16
4e-12
2e-07
5e-03
Log-odds
0.5
1.0
1.5
2.0
2.5
3.0
3.5
4.0
Cellular_organisms - ps1
Archaea1 - ps2
Archaea2 - ps3
Archaea3 - ps4
Asgard/Eukaryota - ps5
Eukaryota - ps6
Amorpheae - ps7
Obazoa - ps8
Opisthokonta - ps9
Holomycota - ps10
Fungi - ps11
Fungi_1 - ps12
Fungi_2 - ps13
Fungi_3 - ps14
Dikarya - ps15
Ascomycota - ps16
Saccharo/Pezizomycotina - ps17
Saccharomycotina - ps18
Saccharomycotina_1 - ps19
Saccharomycotina_2 - ps20
Saccharomycotina_3 - ps21
Phaff./Saccharomycetaceae - ps22
Saccharomyco./mycetaceae - ps23
Saccharomycetaceae - ps24
Saccharomycetaceae_2 - ps25
Saccharomycetaceae_3 - ps26
Kaz./Nau./Saccharomyces - ps27
Saccharomyces - ps28
Arboricola_clade - ps29
Cerevisiae_clade - ps30
Saccharomyces_cerevisiae - ps31
Phylostratum

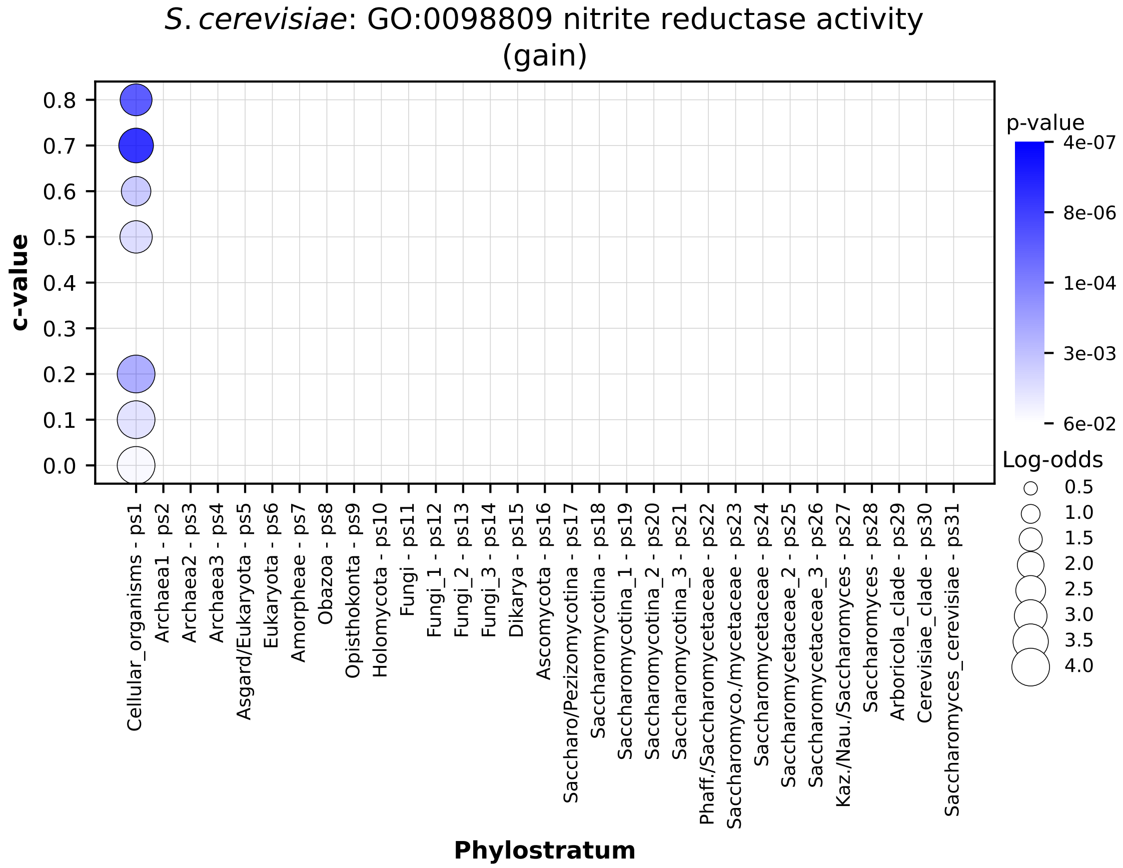

S. cerevisiae: GO:0098809 nitrite reductase activity (gain)
c-value
0.8
0.7
0.6
0.5
0.4
0.3
0.2
0.1
0.0
p-value
4e-07
8e-06
1e-04
3e-03
6e-02
Log-odds
0.5
1.0
1.5
2.0
2.5
3.0
3.5
4.0
Cellular_organisms - ps1
Archaea1 - ps2
Archaea2 - ps3
Archaea3 - ps4
Asgard/Eukaryota - ps5
Eukaryota - ps6
Amorpheae - ps7
Obazoa - ps8
Opisthokonta - ps9
Holomycota - ps10
Fungi - ps11
Fungi_1 - ps12
Fungi_2 - ps13
Fungi_3 - ps14
Dikarya - ps15
Ascomycota - ps16
Saccharo/Pezizomycotina - ps17
Saccharomycotina - ps18
Saccharomycotina_1 - ps19
Saccharomycotina_2 - ps20
Saccharomycotina_3 - ps21
Phaff./Saccharomycetaceae - ps22
Saccharomyco./mycetaceae - ps23
Saccharomycetaceae - ps24
Saccharomycetaceae_2 - ps25
Saccharomycetaceae_3 - ps26
Kaz./Nau./Saccharomyces - ps27
Saccharomyces - ps28
Arboricola_clade - ps29
Cerevisiae_clade - ps30
Saccharomyces_cerevisiae - ps31
Phylostratum

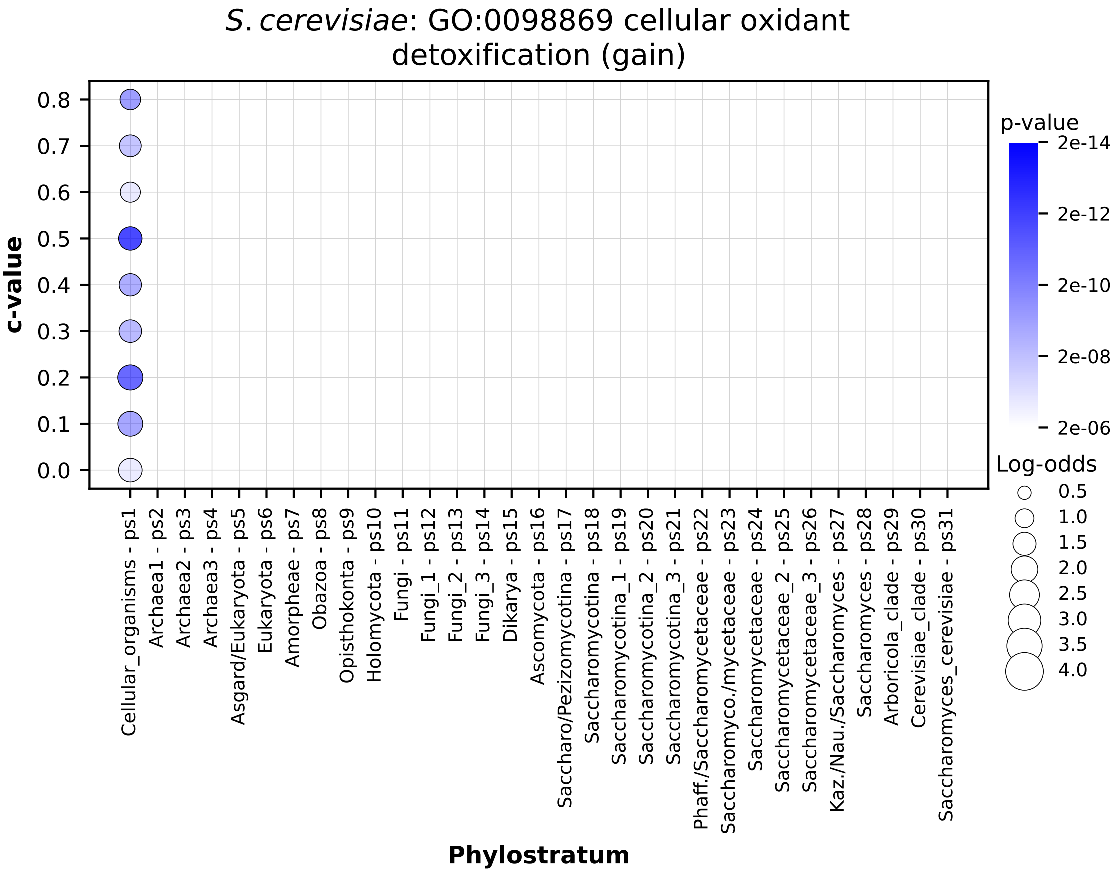
S. cerevisiae: GO:0098869 cellular oxidant detoxification (gain)
c-value
0.8
0.7
0.6
0.5
0.4
0.3
0.2
0.1
0.0
p-value
2e-14
2e-12
2e-10
2e-08
2e-06
Log-odds
0.5
1.0
1.5
2.0
2.5
3.0
3.5
4.0
Cellular_organisms - ps1
Archaea1 - ps2
Archaea2 - ps3
Archaea3 - ps4
Asgard/Eukaryota - ps5
Eukaryota - ps6
Amorpheae - ps7
Obazoa - ps8
Opisthokonta - ps9
Holomycota - ps10
Fungi - ps11
Fungi_1 - ps12
Fungi_2 - ps13
Fungi_3 - ps14
Dikarya - ps15
Ascomycota - ps16
Saccharo/Pezizomycotina - ps17
Saccharomycotina - ps18
Saccharomycotina_1 - ps19
Saccharomycotina_2 - ps20
Saccharomycotina_3 - ps21
Phaff./Saccharomycetaceae - ps22
Saccharomyco./mycetaceae - ps23
Saccharomycetaceae - ps24
Saccharomycetaceae_2 - ps25
Saccharomycetaceae_3 - ps26
Kaz./Nau./Saccharomyces - ps27
Saccharomyces - ps28
Arboricola_clade - ps29
Cerevisiae_clade - ps30
Saccharomyces_cerevisiae - ps31
Phylostratum

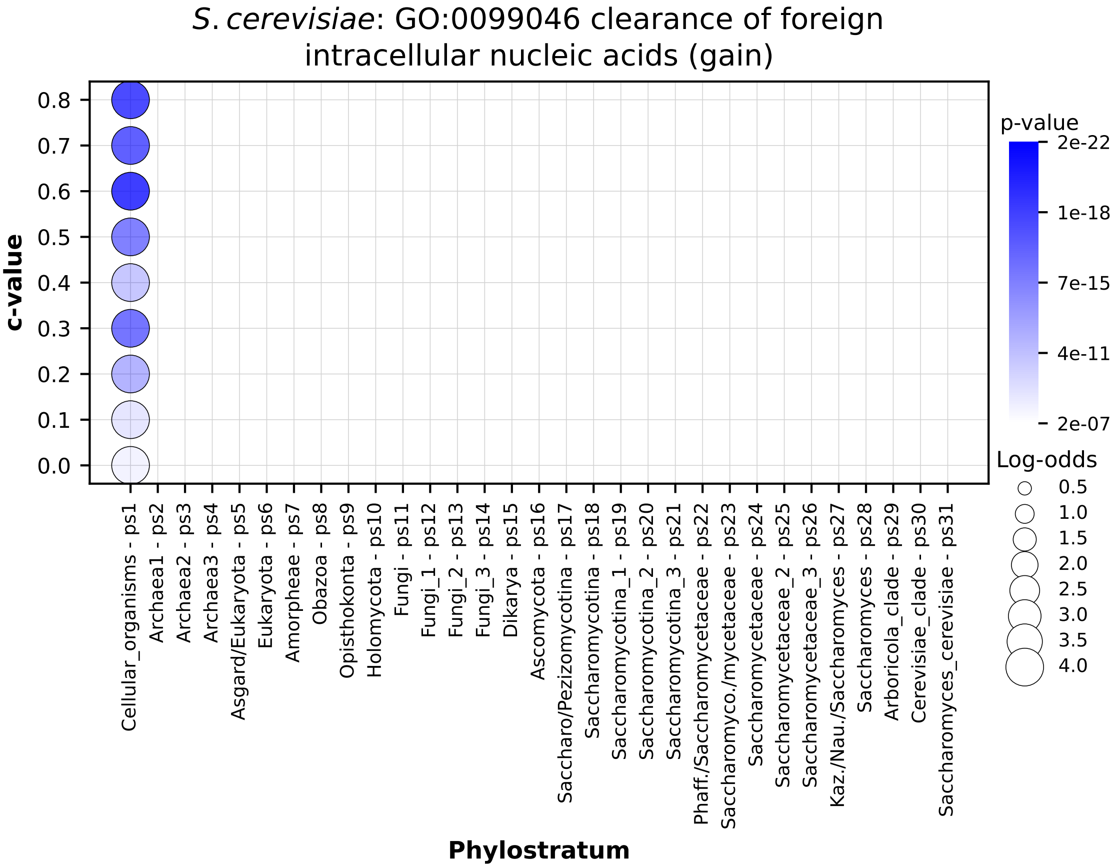

S. cerevisiae: GO:0099046 clearance of foreign intracellular nucleic acids (gain)
c-value
0.8
0.7
0.6
0.5
0.4
0.3
0.2
0.1
0.0
p-value
2e-22
1e-18
7e-15
4e-11
2e-07
Log-odds
0.5
1.0
1.5
2.0
2.5
3.0
3.5
4.0
Cellular_organisms - ps1
Archaea1 - ps2
Archaea2 - ps3
Archaea3 - ps4
Asgard/Eukaryota - ps5
Eukaryota - ps6
Amorpheae - ps7
Obazoa - ps8
Opisthokonta - ps9
Holomycota - ps10
Fungi - ps11
Fungi_1 - ps12
Fungi_2 - ps13
Fungi_3 - ps14
Dikarya - ps15
Ascomycota - ps16
Saccharo/Pezizomycotina - ps17
Saccharomycotina - ps18
Saccharomycotina_1 - ps19
Saccharomycotina_2 - ps20
Saccharomycotina_3 - ps21
Phaff./Saccharomycetaceae - ps22
Saccharomyco./mycetaceae - ps23
Saccharomycetaceae - ps24
Saccharomycetaceae_2 - ps25
Saccharomycetaceae_3 - ps26
Kaz./Nau./Saccharomyces - ps27
Saccharomyces - ps28
Arboricola_clade - ps29
Cerevisiae_clade - ps30
Saccharomyces_cerevisiae - ps31
Phylostratum

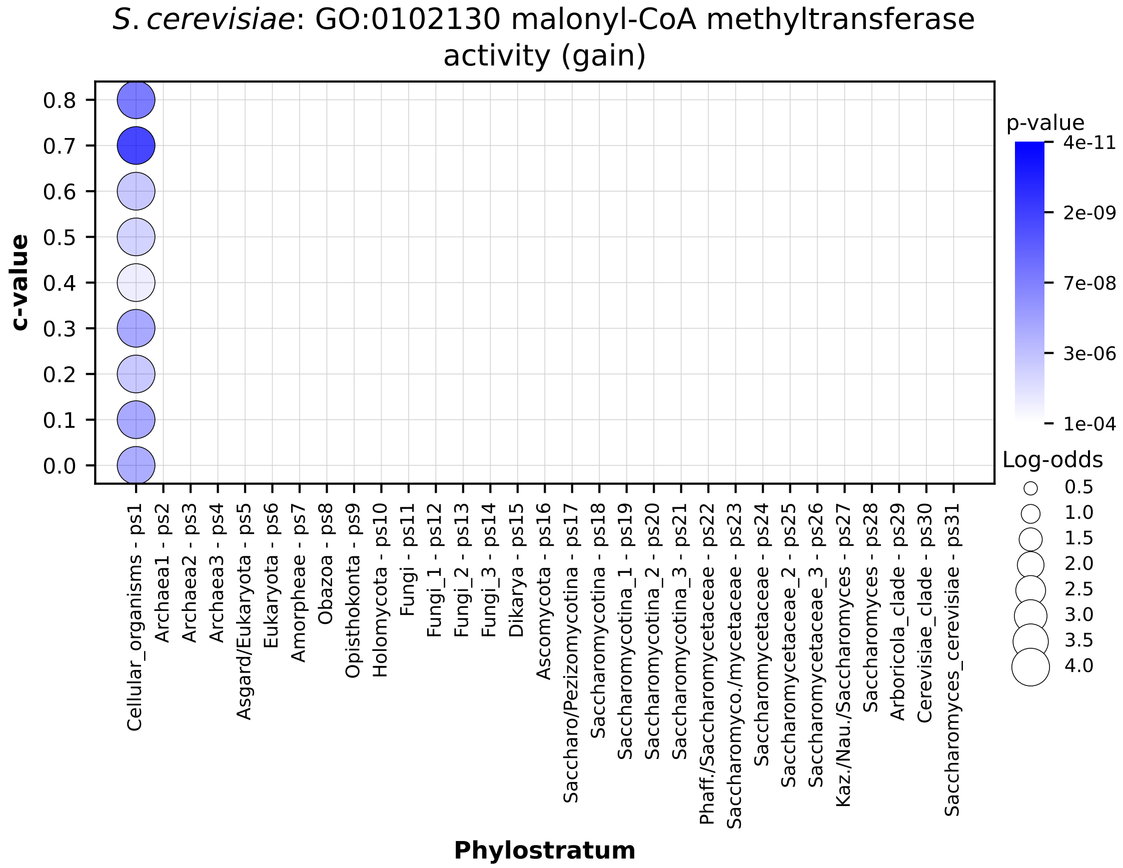

S. cerevisiae: GO:0102130 malonyl-CoA methyltransferase activity (gain)
c-value
0.8
0.7
0.6
0.5
0.4
0.3
0.2
0.1
0.0
p-value
4e-11
2e-09
7e-08
3e-06
1e-04
Log-odds
0.5
1.0
1.5
2.0
2.5
3.0
3.5
4.0
Cellular_organisms - ps1
Archaea1 - ps2
Archaea2 - ps3
Archaea3 - ps4
Asgard/Eukaryota - ps5
Eukaryota - ps6
Amorpheae - ps7
Obazoa - ps8
Opisthokonta - ps9
Holomycota - ps10
Fungi - ps11
Fungi_1 - ps12
Fungi_2 - ps13
Fungi_3 - ps14
Dikarya - ps15
Ascomycota - ps16
Saccharo/Pezizomycotina - ps17
Saccharomycotina - ps18
Saccharomycotina_1 - ps19
Saccharomycotina_2 - ps20
Saccharomycotina_3 - ps21
Phaff./Saccharomycetaceae - ps22
Saccharomyco./mycetaceae - ps23
Saccharomycetaceae - ps24
Saccharomycetaceae_2 - ps25
Saccharomycetaceae_3 - ps26
Kaz./Nau./Saccharomyces - ps27
Saccharomyces - ps28
Arboricola_clade - ps29
Cerevisiae_clade - ps30
Saccharomyces_cerevisiae - ps31
Phylostratum

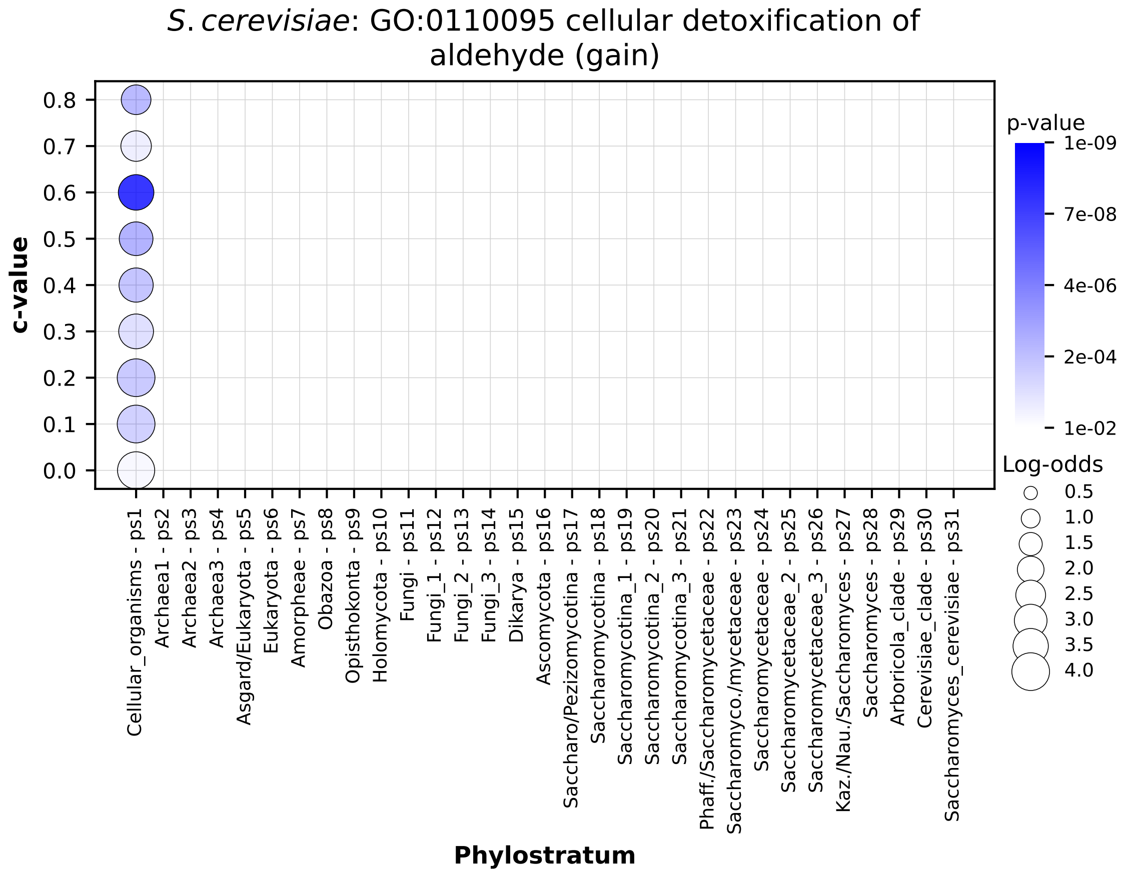
S. cerevisiae: GO:0110095 cellular detoxification of aldehyde (gain)
c-value
0.8
0.7
0.6
0.5
0.4
0.3
0.2
0.1
0.0
p-value
1e-09
7e-08
4e-06
2e-04
1e-02
Log-odds
0.5
1.0
1.5
2.0
2.5
3.0
3.5
4.0
Cellular_organisms - ps1
Archaea1 - ps2
Archaea2 - ps3
Archaea3 - ps4
Asgard/Eukaryota - ps5
Eukaryota - ps6
Amorpheae - ps7
Obazoa - ps8
Opisthokonta - ps9
Holomycota - ps10
Fungi - ps11
Fungi_1 - ps12
Fungi_2 - ps13
Fungi_3 - ps14
Dikarya - ps15
Ascomycota - ps16
Saccharo/Pezizomycotina - ps17
Saccharomycotina - ps18
Saccharomycotina_1 - ps19
Saccharomycotina_2 - ps20
Saccharomycotina_3 - ps21
Phaff./Saccharomycetaceae - ps22
Saccharomyco./mycetaceae - ps23
Saccharomycetaceae - ps24
Saccharomycetaceae_2 - ps25
Saccharomycetaceae_3 - ps26
Kaz./Nau./Saccharomyces - ps27
Saccharomyces - ps28
Arboricola_clade - ps29
Cerevisiae_clade - ps30
Saccharomyces_cerevisiae - ps31
Phylostratum

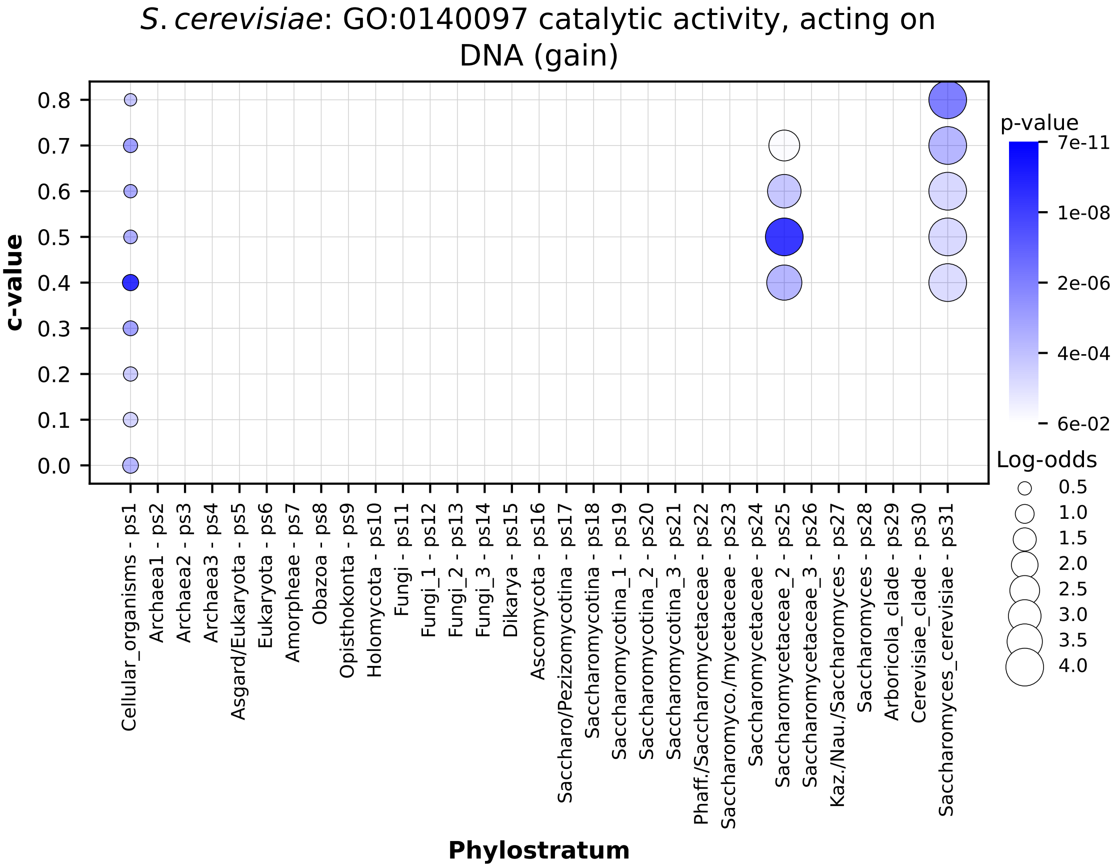

S. cerevisiae: GO:0140097 catalytic activity, acting on DNA (gain)
c-value
0.0
0.1
0.2
0.3
0.4
0.5
0.6
0.7
0.8
Phylostratum
Cellular_organisms - ps1
Archaea1 - ps2
Archaea2 - ps3
Archaea3 - ps4
Asgard/Eukaryota - ps5
Eukaryota - ps6
Amorpheae - ps7
Obazoa - ps8
Opisthokonta - ps9
Holomycota - ps10
Fungi - ps11
Fungi_1 - ps12
Fungi_2 - ps13
Fungi_3 - ps14
Dikarya - ps15
Ascomycota - ps16
Saccharo/Pezizomycotina - ps17
Saccharomycotina - ps18
Saccharomycotina_1 - ps19
Saccharomycotina_2 - ps20
Saccharomycotina_3 - ps21
Phaff./Saccharomycetaceae - ps22
Saccharomyco./mycetaceae - ps23
Saccharomycetaceae - ps24
Saccharomycetaceae_2 - ps25
Saccharomycetaceae_3 - ps26
Kaz./Nau./Saccharomyces - ps27
Saccharomyces - ps28
Arboricola_clade - ps29
Cerevisiae_clade - ps30
Saccharomyces_cerevisiae - ps31
p-value
7e-11
1e-08
2e-06
4e-04
6e-02
Log-odds
0.5
1.0
1.5
2.0
2.5
3.0
3.5
4.0

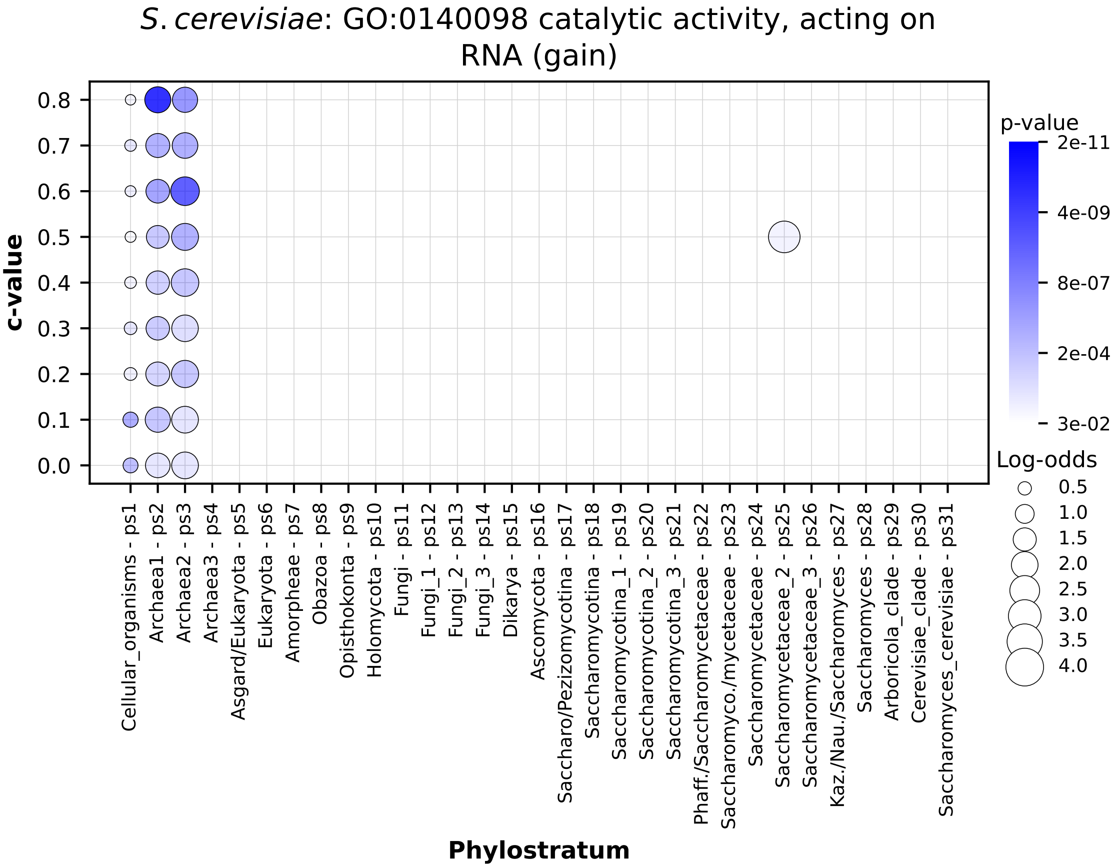

S. cerevisiae: GO:0140098 catalytic activity, acting on RNA (gain)
c-value
0.8
0.7
0.6
0.5
0.4
0.3
0.2
0.1
0.0
p-value
2e-11
4e-09
8e-07
2e-04
3e-02
Log-odds
0.5
1.0
1.5
2.0
2.5
3.0
3.5
4.0
Cellular_organisms - ps1
Archaea1 - ps2
Archaea2 - ps3
Archaea3 - ps4
Asgard/Eukaryota - ps5
Eukaryota - ps6
Amorpheae - ps7
Obazoa - ps8
Opisthokonta - ps9
Holomycota - ps10
Fungi - ps11
Fungi_1 - ps12
Fungi_2 - ps13
Fungi_3 - ps14
Dikarya - ps15
Ascomycota - ps16
Saccharo/Pezizomycotina - ps17
Saccharomycotina - ps18
Saccharomycotina_1 - ps19
Saccharomycotina_2 - ps20
Saccharomycotina_3 - ps21
Phaff./Saccharomycetaceae - ps22
Saccharomyco./mycetaceae - ps23
Saccharomycetaceae - ps24
Saccharomycetaceae_2 - ps25
Saccharomycetaceae_3 - ps26
Kaz./Nau./Saccharomyces - ps27
Saccharomyces - ps28
Arboricola_clade - ps29
Cerevisiae_clade - ps30
Saccharomyces_cerevisiae - ps31
Phylostratum

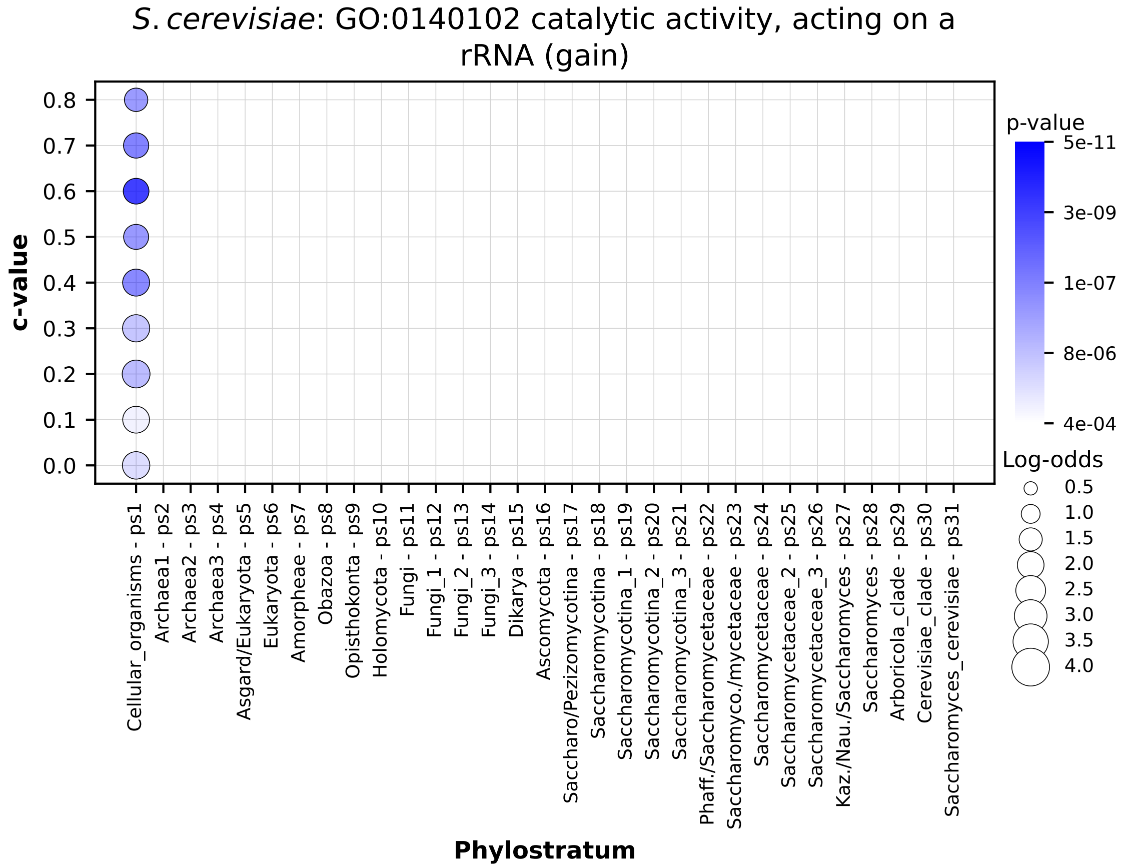

S. cerevisiae: GO:0140102 catalytic activity, acting on a rRNA (gain)
c-value
0.8
0.7
0.6
0.5
0.4
0.3
0.2
0.1
0.0
p-value
5e-11
3e-09
1e-07
8e-06
4e-04
Log-odds
0.5
1.0
1.5
2.0
2.5
3.0
3.5
4.0
Cellular_organisms - ps1
Archaea1 - ps2
Archaea2 - ps3
Archaea3 - ps4
Asgard/Eukaryota - ps5
Eukaryota - ps6
Amorpheae - ps7
Obazoa - ps8
Opisthokonta - ps9
Holomycota - ps10
Fungi - ps11
Fungi_1 - ps12
Fungi_2 - ps13
Fungi_3 - ps14
Dikarya - ps15
Ascomycota - ps16
Saccharo/Pezizomycotina - ps17
Saccharomycotina - ps18
Saccharomycotina_1 - ps19
Saccharomycotina_2 - ps20
Saccharomycotina_3 - ps21
Phaff./Saccharomycetaceae - ps22
Saccharomyco./mycetaceae - ps23
Saccharomycetaceae - ps24
Saccharomycetaceae_2 - ps25
Saccharomycetaceae_3 - ps26
Kaz./Nau./Saccharomyces - ps27
Saccharomyces - ps28
Arboricola_clade - ps29
Cerevisiae_clade - ps30
Saccharomyces_cerevisiae - ps31
Phylostratum

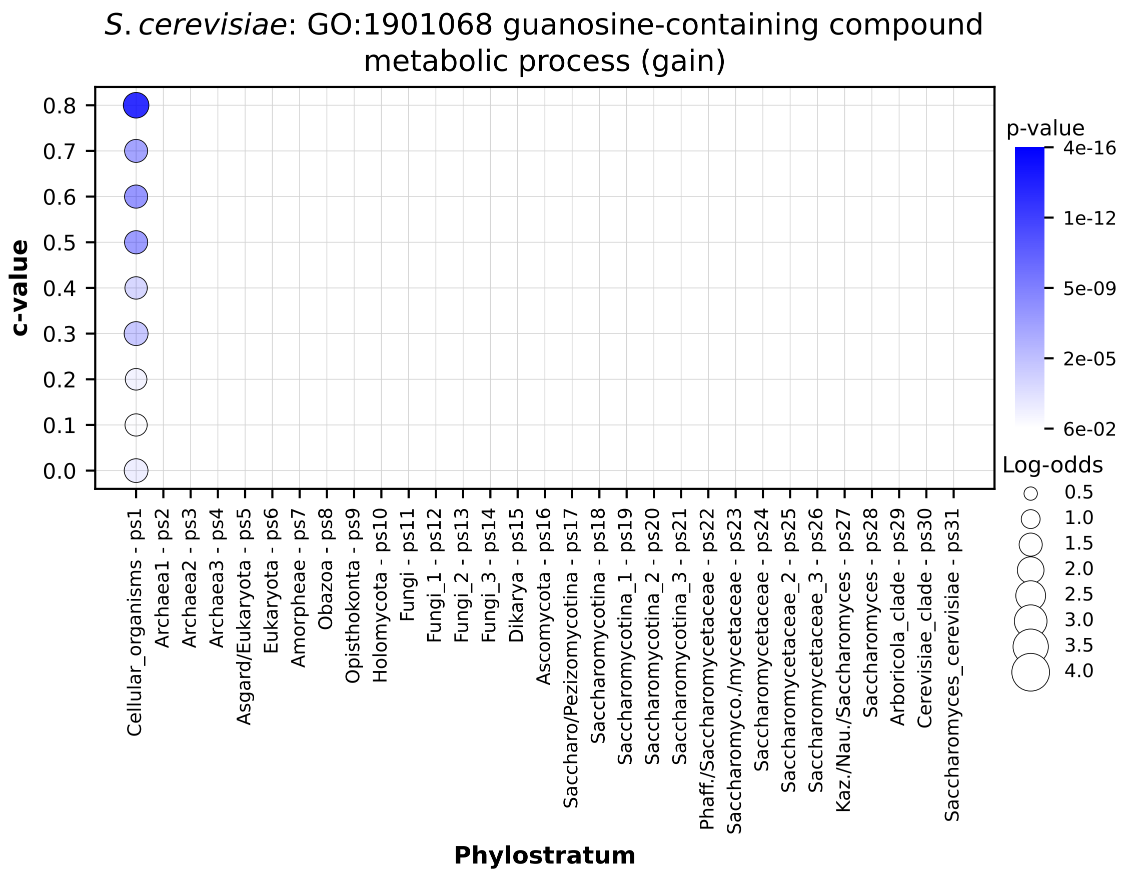

S. cerevisiae: GO:1901068 guanosine-containing compound metabolic process (gain)
c-value
0.0
0.1
0.2
0.3
0.4
0.5
0.6
0.7
0.8
p-value
4e-16
1e-12
5e-09
2e-05
6e-02
Log-odds
0.5
1.0
1.5
2.0
2.5
3.0
3.5
4.0
Cellular_organisms - ps1
Archaea1 - ps2
Archaea2 - ps3
Archaea3 - ps4
Asgard/Eukaryota - ps5
Eukaryota - ps6
Amorpheae - ps7
Obazoa - ps8
Opisthokonta - ps9
Holomycota - ps10
Fungi - ps11
Fungi_1 - ps12
Fungi_2 - ps13
Fungi_3 - ps14
Dikarya - ps15
Ascomycota - ps16
Saccharo/Pezizomycotina - ps17
Saccharomycotina - ps18
Saccharomycotina_1 - ps19
Saccharomycotina_2 - ps20
Saccharomycotina_3 - ps21
Phaff./Saccharomycetaceae - ps22
Saccharomyco./mycetaceae - ps23
Saccharomycetaceae - ps24
Saccharomycetaceae_2 - ps25
Saccharomycetaceae_3 - ps26
Kaz./Nau./Saccharomyces - ps27
Saccharomyces - ps28
Arboricola_clade - ps29
Cerevisiae_clade - ps30
Saccharomyces_cerevisiae - ps31
Phylostratum

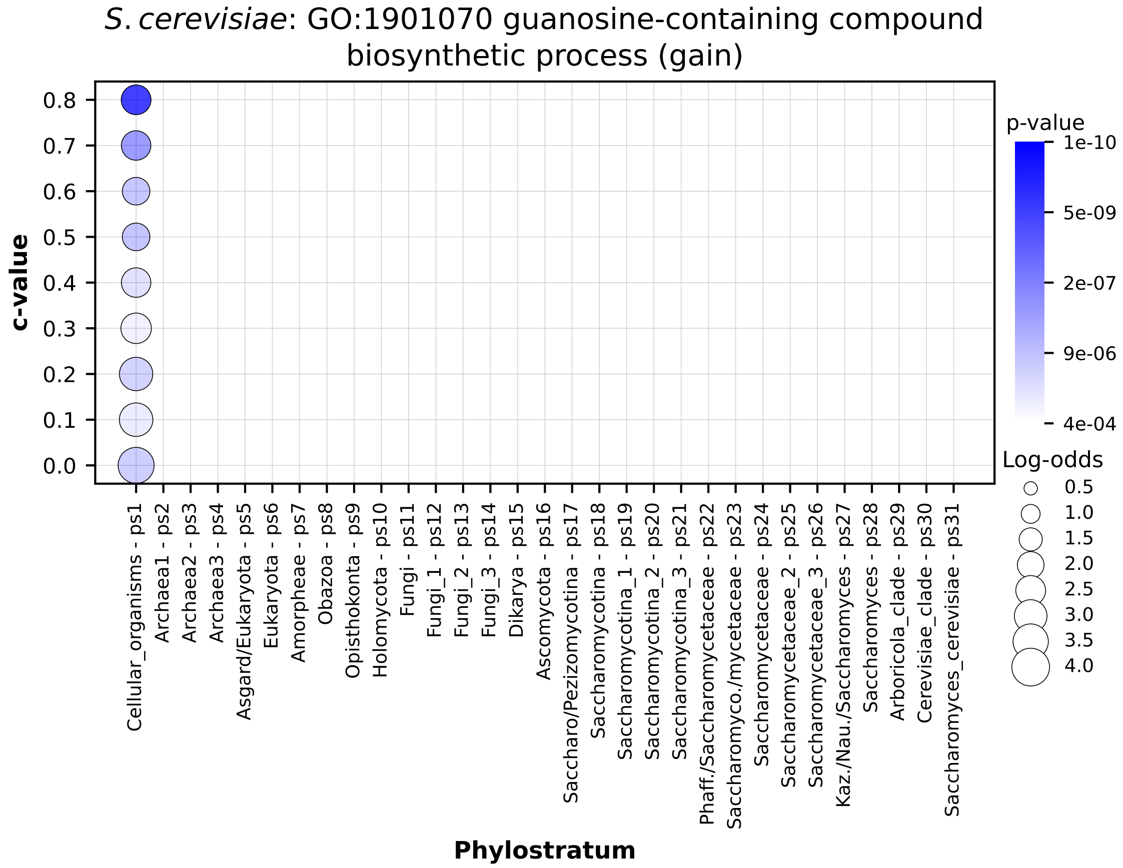

S. cerevisiae: GO:1901070 guanosine-containing compound biosynthetic process (gain)
c-value
0.8
0.7
0.6
0.5
0.4
0.3
0.2
0.1
0.0
p-value
1e-10
5e-09
2e-07
9e-06
4e-04
Log-odds
0.5
1.0
1.5
2.0
2.5
3.0
3.5
4.0
Cellular_organisms - ps1
Archaea1 - ps2
Archaea2 - ps3
Archaea3 - ps4
Asgard/Eukaryota - ps5
Eukaryota - ps6
Amorpheae - ps7
Obazoa - ps8
Opisthokonta - ps9
Holomycota - ps10
Fungi - ps11
Fungi_1 - ps12
Fungi_2 - ps13
Fungi_3 - ps14
Dikarya - ps15
Ascomycota - ps16
Saccharo/Pezizomycotina - ps17
Saccharomycotina - ps18
Saccharomycotina_1 - ps19
Saccharomycotina_2 - ps20
Saccharomycotina_3 - ps21
Phaff./Saccharomycetaceae - ps22
Saccharomyco./mycetaceae - ps23
Saccharomycetaceae - ps24
Saccharomycetaceae_2 - ps25
Saccharomycetaceae_3 - ps26
Kaz./Nau./Saccharomyces - ps27
Saccharomyces - ps28
Arboricola_clade - ps29
Cerevisiae_clade - ps30
Saccharomyces_cerevisiae - ps31
Phylostratum

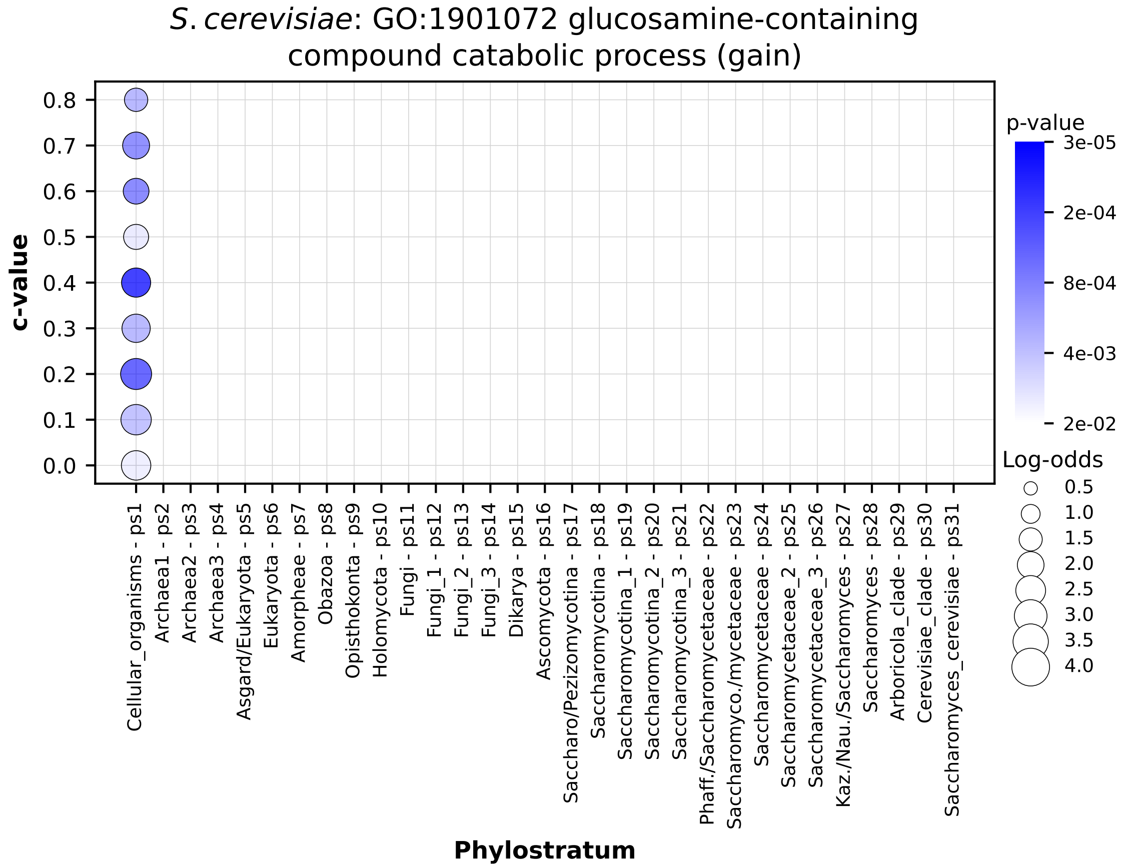
S. cerevisiae: GO:1901072 glucosamine-containing compound catabolic process (gain)
c-value
0.0
0.1
0.2
0.3
0.4
0.5
0.6
0.7
0.8
p-value
3e-05
2e-04
8e-04
4e-03
2e-02
Log-odds
0.5
1.0
1.5
2.0
2.5
3.0
3.5
4.0
Cellular_organisms - ps1
Archaea1 - ps2
Archaea2 - ps3
Archaea3 - ps4
Asgard/Eukaryota - ps5
Eukaryota - ps6
Amorpheae - ps7
Obazoa - ps8
Opisthokonta - ps9
Holomycota - ps10
Fungi - ps11
Fungi_1 - ps12
Fungi_2 - ps13
Fungi_3 - ps14
Dikarya - ps15
Ascomycota - ps16
Saccharo/Pezizomycotina - ps17
Saccharomycotina - ps18
Saccharomycotina_1 - ps19
Saccharomycotina_2 - ps20
Saccharomycotina_3 - ps21
Phaff./Saccharomycetaceae - ps22
Saccharomyco./mycetaceae - ps23
Saccharomycetaceae - ps24
Saccharomycetaceae_2 - ps25
Saccharomycetaceae_3 - ps26
Kaz./Nau./Saccharomyces - ps27
Saccharomyces - ps28
Arboricola_clade - ps29
Cerevisiae_clade - ps30
Saccharomyces_cerevisiae - ps31
Phylostratum

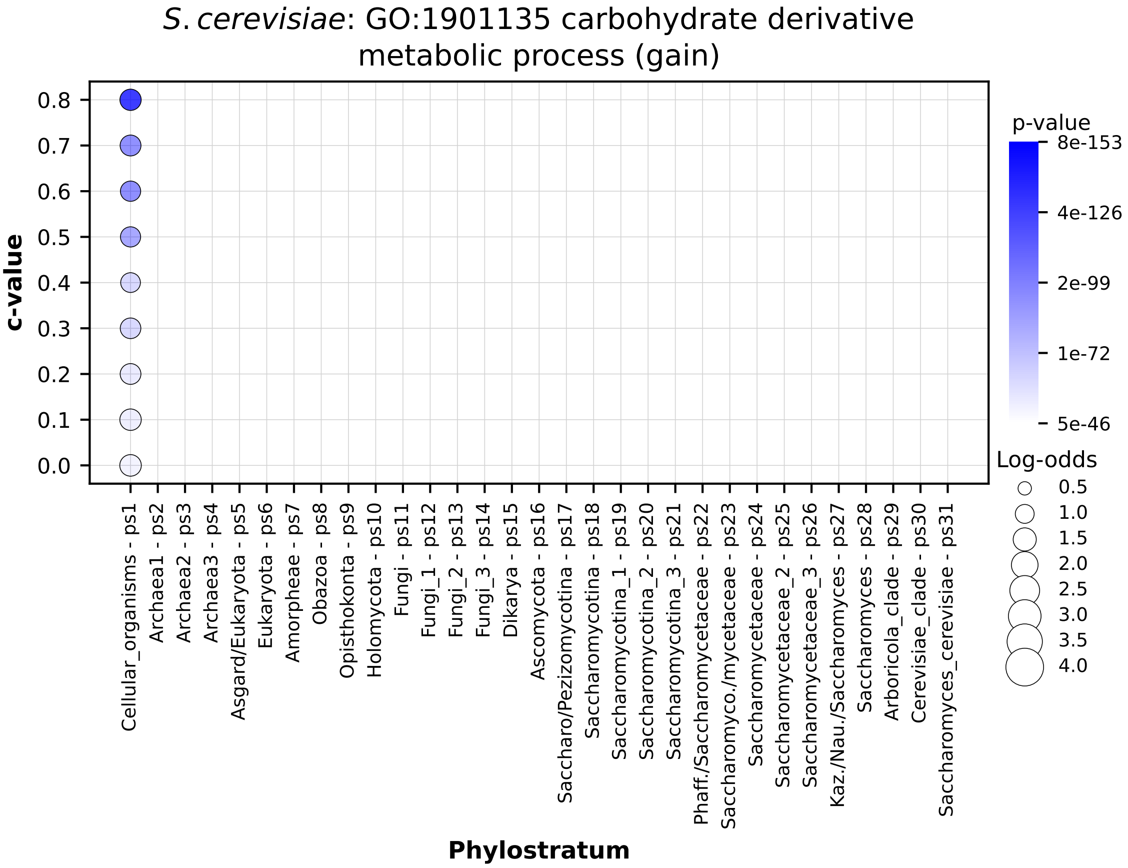

S. cerevisiae: GO:1901135 carbohydrate derivative metabolic process (gain)
c-value
0.8
0.7
0.6
0.5
0.4
0.3
0.2
0.1
0.0
p-value
8e-153
4e-126
2e-99
1e-72
5e-46
Log-odds
0.5
1.0
1.5
2.0
2.5
3.0
3.5
4.0
Cellular_organisms - ps1
Archaea1 - ps2
Archaea2 - ps3
Archaea3 - ps4
Asgard/Eukaryota - ps5
Eukaryota - ps6
Amorpheae - ps7
Obazoa - ps8
Opisthokonta - ps9
Holomycota - ps10
Fungi - ps11
Fungi_1 - ps12
Fungi_2 - ps13
Fungi_3 - ps14
Dikarya - ps15
Ascomycota - ps16
Saccharo/Pezizomycotina - ps17
Saccharomycotina - ps18
Saccharomycotina_1 - ps19
Saccharomycotina_2 - ps20
Saccharomycotina_3 - ps21
Phaff./Saccharomycetaceae - ps22
Saccharomyco./mycetaceae - ps23
Saccharomycetaceae - ps24
Saccharomycetaceae_2 - ps25
Saccharomycetaceae_3 - ps26
Kaz./Nau./Saccharomyces - ps27
Saccharomyces - ps28
Arboricola_clade - ps29
Cerevisiae_clade - ps30
Saccharomyces_cerevisiae - ps31
Phylostratum

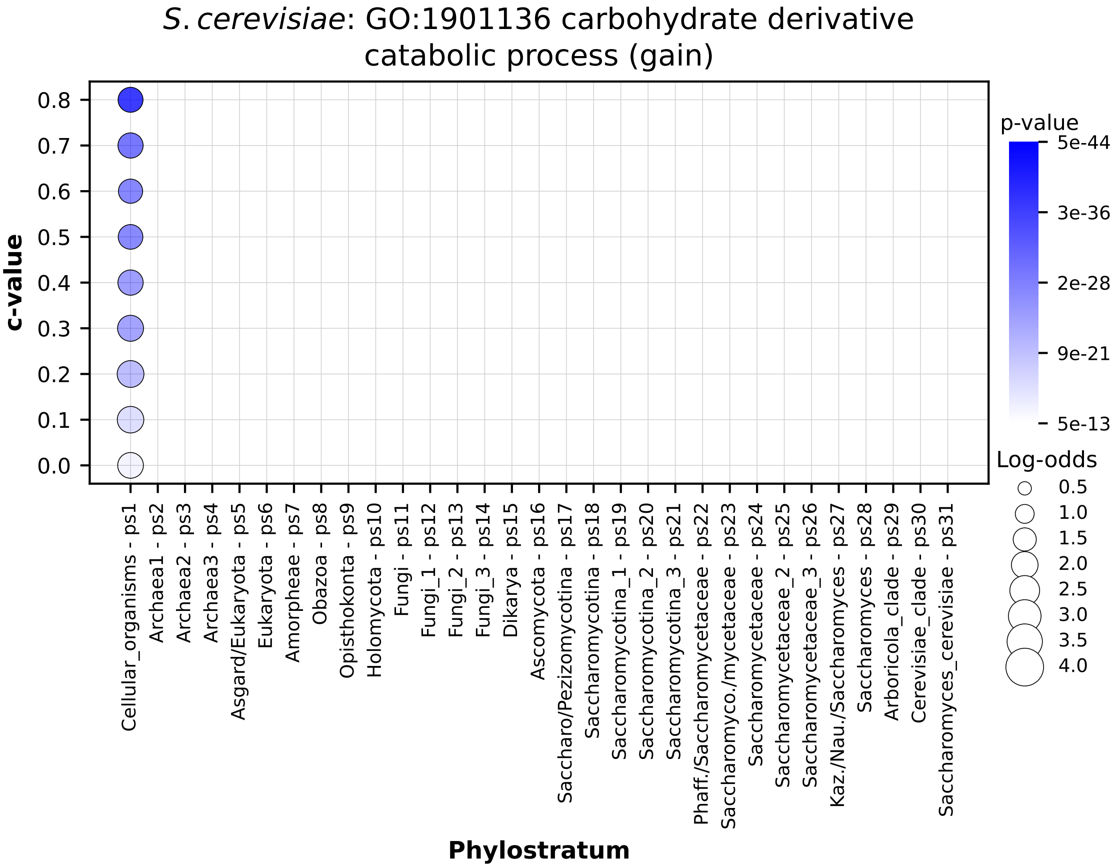

S. cerevisiae: GO:1901136 carbohydrate derivative catabolic process (gain)
c-value
0.8
0.7
0.6
0.5
0.4
0.3
0.2
0.1
0.0
p-value
5e-44
3e-36
2e-28
9e-21
5e-13
Log-odds
0.5
1.0
1.5
2.0
2.5
3.0
3.5
4.0
Cellular_organisms - ps1
Archaea1 - ps2
Archaea2 - ps3
Archaea3 - ps4
Asgard/Eukaryota - ps5
Eukaryota - ps6
Amorpheae - ps7
Obazoa - ps8
Opisthokonta - ps9
Holomycota - ps10
Fungi - ps11
Fungi_1 - ps12
Fungi_2 - ps13
Fungi_3 - ps14
Dikarya - ps15
Ascomycota - ps16
Saccharo/Pezizomycotina - ps17
Saccharomycotina - ps18
Saccharomycotina_1 - ps19
Saccharomycotina_2 - ps20
Saccharomycotina_3 - ps21
Phaff./Saccharomycetaceae - ps22
Saccharomyco./mycetaceae - ps23
Saccharomycetaceae - ps24
Saccharomycetaceae_2 - ps25
Saccharomycetaceae_3 - ps26
Kaz./Nau./Saccharomyces - ps27
Saccharomyces - ps28
Arboricola_clade - ps29
Cerevisiae_clade - ps30
Saccharomyces_cerevisiae - ps31
Phylostratum

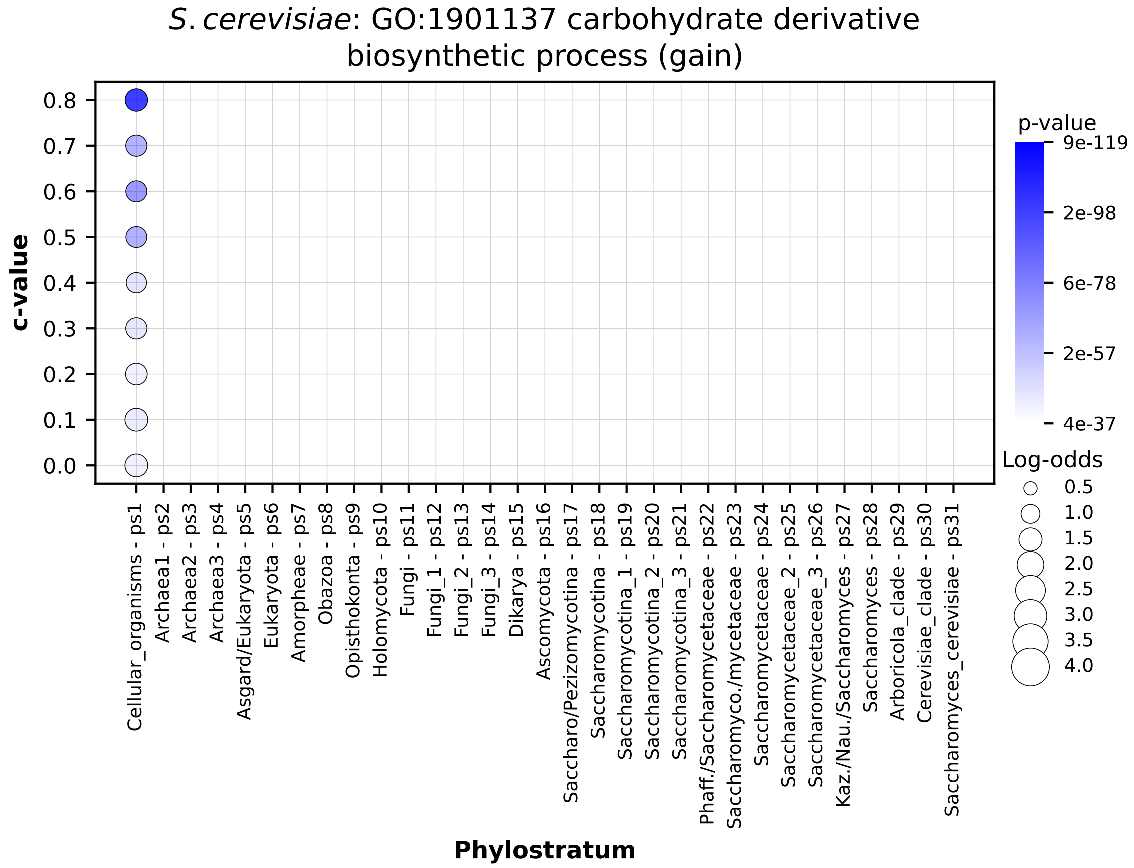
S. cerevisiae: GO:1901137 carbohydrate derivative biosynthetic process (gain)
c-value
0.8
0.7
0.6
0.5
0.4
0.3
0.2
0.1
0.0
p-value
9e-119
2e-98
6e-78
2e-57
4e-37
Log-odds
0.5
1.0
1.5
2.0
2.5
3.0
3.5
4.0
Cellular_organisms - ps1
Archaea1 - ps2
Archaea2 - ps3
Archaea3 - ps4
Asgard/Eukaryota - ps5
Eukaryota - ps6
Amorpheae - ps7
Obazoa - ps8
Opisthokonta - ps9
Holomycota - ps10
Fungi - ps11
Fungi_1 - ps12
Fungi_2 - ps13
Fungi_3 - ps14
Dikarya - ps15
Ascomycota - ps16
Saccharo/Pezizomycotina - ps17
Saccharomycotina - ps18
Saccharomycotina_1 - ps19
Saccharomycotina_2 - ps20
Saccharomycotina_3 - ps21
Phaff./Saccharomycetaceae - ps22
Saccharomyco./mycetaceae - ps23
Saccharomycetaceae - ps24
Saccharomycetaceae_2 - ps25
Saccharomycetaceae_3 - ps26
Kaz./Nau./Saccharomyces - ps27
Saccharomyces - ps28
Arboricola_clade - ps29
Cerevisiae_clade - ps30
Saccharomyces_cerevisiae - ps31
Phylostratum

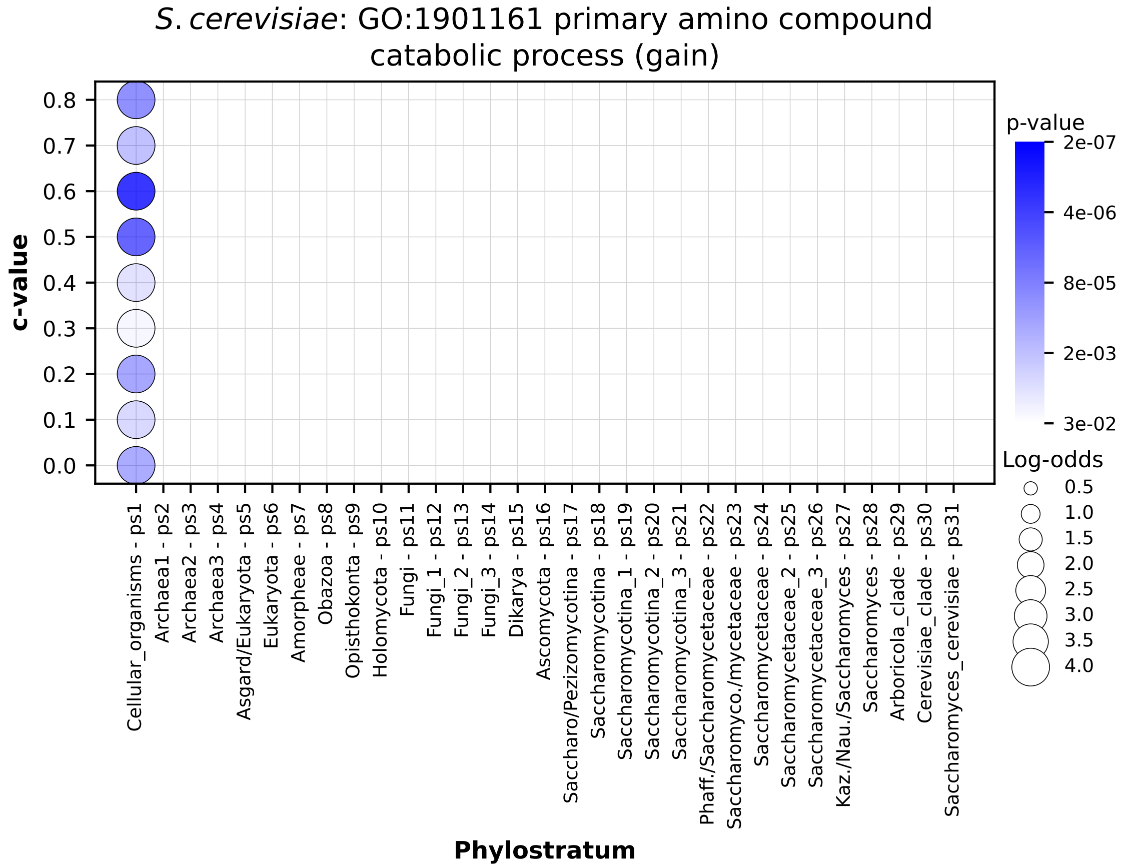

S. cerevisiae: GO:1901161 primary amino compound catabolic process (gain)
c-value
0.0
0.1
0.2
0.3
0.4
0.5
0.6
0.7
0.8
p-value
2e-07
4e-06
8e-05
2e-03
3e-02
Log-odds
0.5
1.0
1.5
2.0
2.5
3.0
3.5
4.0
Cellular_organisms - ps1
Archaea1 - ps2
Archaea2 - ps3
Archaea3 - ps4
Asgard/Eukaryota - ps5
Eukaryota - ps6
Amorpheae - ps7
Obazoa - ps8
Opisthokonta - ps9
Holomycota - ps10
Fungi - ps11
Fungi_1 - ps12
Fungi_2 - ps13
Fungi_3 - ps14
Dikarya - ps15
Ascomycota - ps16
Saccharo/Pezizomycotina - ps17
Saccharomycotina - ps18
Saccharomycotina_1 - ps19
Saccharomycotina_2 - ps20
Saccharomycotina_3 - ps21
Phaff./Saccharomycetaceae - ps22
Saccharomyco./mycetaceae - ps23
Saccharomycetaceae - ps24
Saccharomycetaceae_2 - ps25
Saccharomycetaceae_3 - ps26
Kaz./Nau./Saccharomyces - ps27
Saccharomyces - ps28
Arboricola_clade - ps29
Cerevisiae_clade - ps30
Saccharomyces_cerevisiae - ps31
Phylostratum

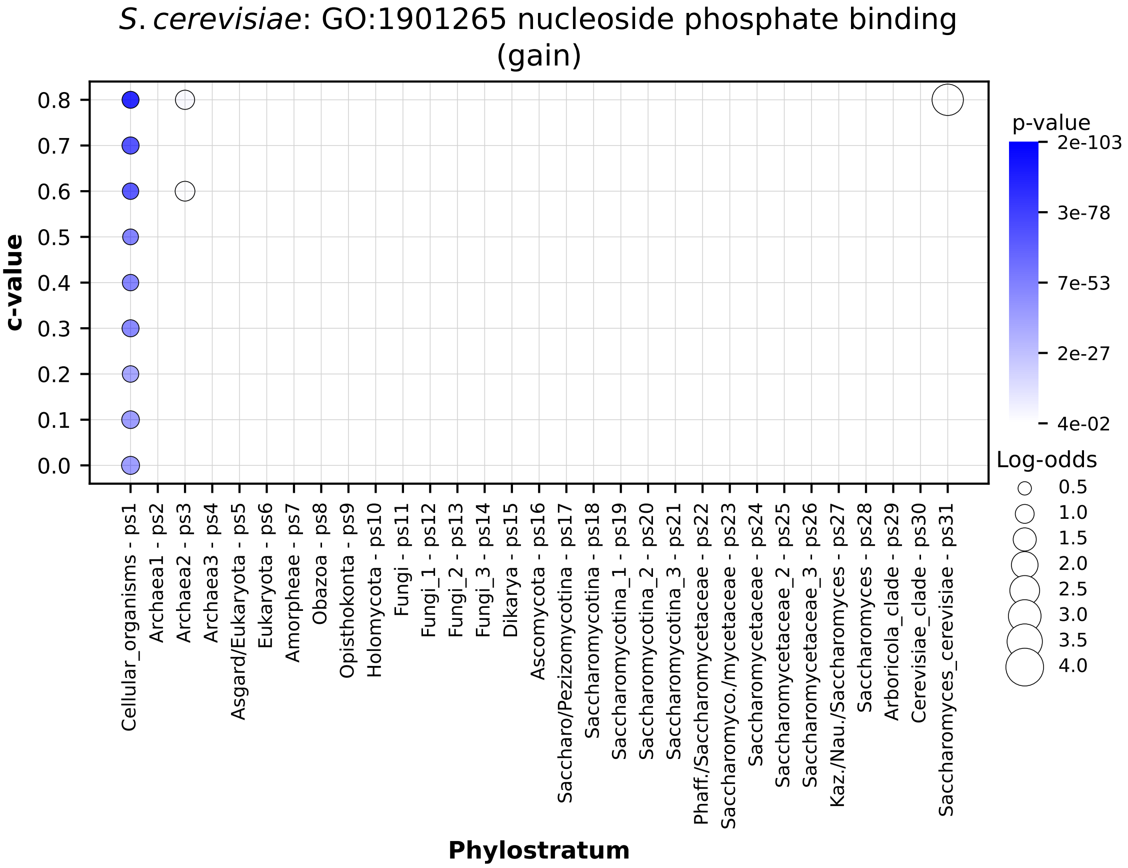

S. cerevisiae: GO:1901265 nucleoside phosphate binding
(gain)
c-value
0.8
0.7
0.6
0.5
0.4
0.3
0.2
0.1
0.0
p-value
2e-103
3e-78
7e-53
2e-27
4e-02
Log-odds
0.5
1.0
1.5
2.0
2.5
3.0
3.5
4.0
Cellular_organisms - ps1
Archaea1 - ps2
Archaea2 - ps3
Archaea3 - ps4
Asgard/Eukaryota - ps5
Eukaryota - ps6
Amorpheae - ps7
Obazoa - ps8
Opisthokonta - ps9
Holomycota - ps10
Fungi - ps11
Fungi_1 - ps12
Fungi_2 - ps13
Fungi_3 - ps14
Dikarya - ps15
Ascomycota - ps16
Saccharo/Pezizomycotina - ps17
Saccharomycotina - ps18
Saccharomycotina_1 - ps19
Saccharomycotina_2 - ps20
Saccharomycotina_3 - ps21
Phaff./Saccharomycetaceae - ps22
Saccharomyco./mycetaceae - ps23
Saccharomycetaceae - ps24
Saccharomycetaceae_2 - ps25
Saccharomycetaceae_3 - ps26
Kaz./Nau./Saccharomyces - ps27
Saccharomyces - ps28
Arboricola_clade - ps29
Cerevisiae_clade - ps30
Saccharomyces_cerevisiae - ps31
Phylostratum

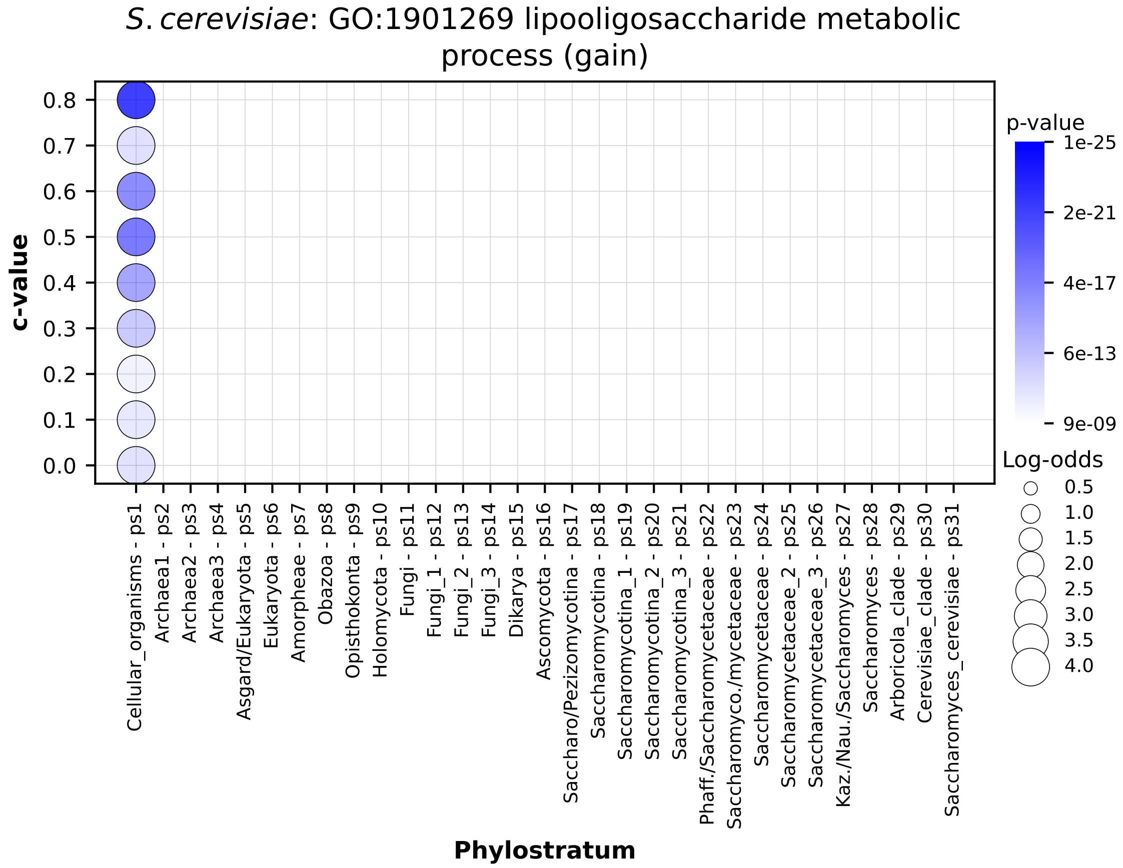
S. cerevisiae: GO:1901269 lipooligosaccharide metabolic process (gain)
c-value
0.8
0.7
0.6
0.5
0.4
0.3
0.2
0.1
0.0
p-value
1e-25
2e-21
4e-17
6e-13
9e-09
Log-odds
0.5
1.0
1.5
2.0
2.5
3.0
3.5
4.0
Cellular_organisms - ps1
Archaea1 - ps2
Archaea2 - ps3
Archaea3 - ps4
Asgard/Eukaryota - ps5
Eukaryota - ps6
Amorpheae - ps7
Obazoa - ps8
Opisthokonta - ps9
Holomycota - ps10
Fungi - ps11
Fungi_1 - ps12
Fungi_2 - ps13
Fungi_3 - ps14
Dikarya - ps15
Ascomycota - ps16
Saccharo/Pezizomycotina - ps17
Saccharomycotina - ps18
Saccharomycotina_1 - ps19
Saccharomycotina_2 - ps20
Saccharomycotina_3 - ps21
Phaff./Saccharomycetaceae - ps22
Saccharomyco./mycetaceae - ps23
Saccharomycetaceae - ps24
Saccharomycetaceae_2 - ps25
Saccharomycetaceae_3 - ps26
Kaz./Nau./Saccharomyces - ps27
Saccharomyces - ps28
Arboricola_clade - ps29
Cerevisiae_clade - ps30
Saccharomyces_cerevisiae - ps31
Phylostratum

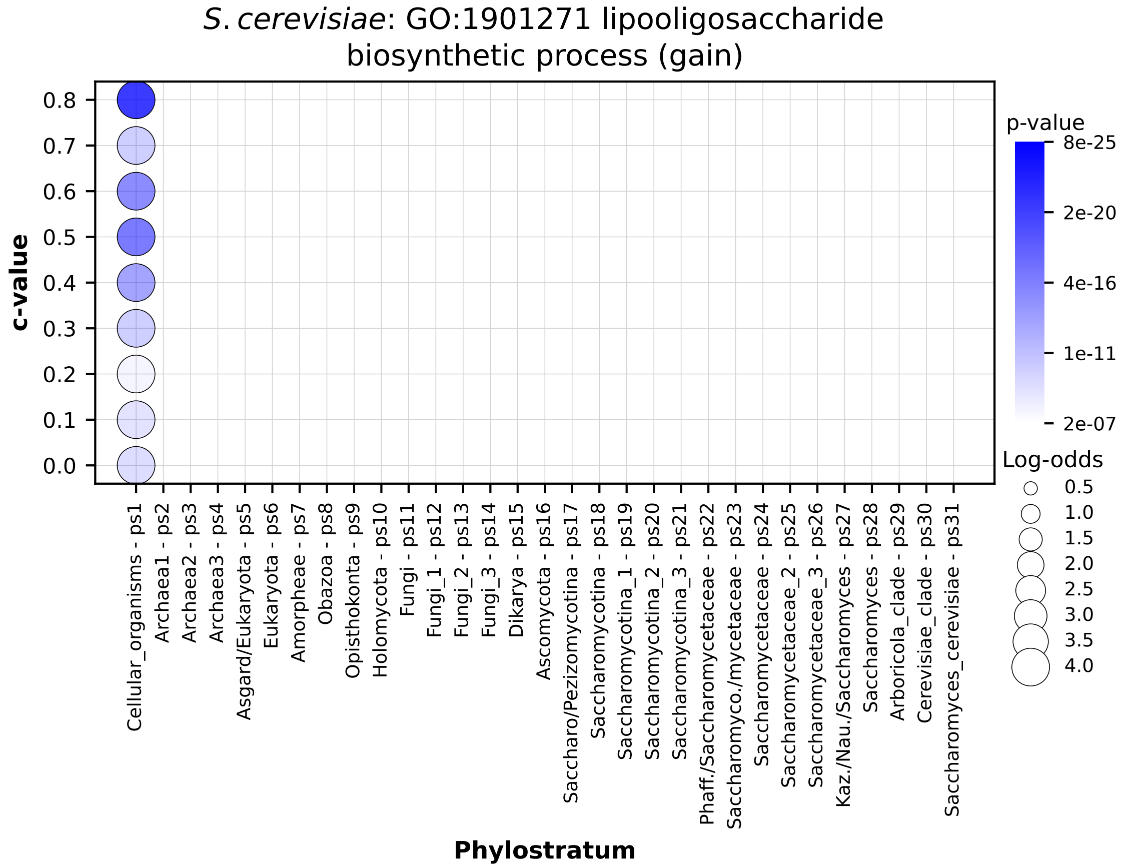

S. cerevisiae: GO:1901271 lipooligosaccharide biosynthetic process (gain)
c-value
0.8
0.7
0.6
0.5
0.4
0.3
0.2
0.1
0.0
p-value
8e-25
2e-20
4e-16
1e-11
2e-07
Log-odds
0.5
1.0
1.5
2.0
2.5
3.0
3.5
4.0
Cellular_organisms - ps1
Archaea1 - ps2
Archaea2 - ps3
Archaea3 - ps4
Asgard/Eukaryota - ps5
Eukaryota - ps6
Amorpheae - ps7
Obazoa - ps8
Opisthokonta - ps9
Holomycota - ps10
Fungi - ps11
Fungi_1 - ps12
Fungi_2 - ps13
Fungi_3 - ps14
Dikarya - ps15
Ascomycota - ps16
Saccharo/Pezizomycotina - ps17
Saccharomycotina - ps18
Saccharomycotina_1 - ps19
Saccharomycotina_2 - ps20
Saccharomycotina_3 - ps21
Phaff./Saccharomycetaceae - ps22
Saccharomyco./mycetaceae - ps23
Saccharomycetaceae - ps24
Saccharomycetaceae_2 - ps25
Saccharomycetaceae_3 - ps26
Kaz./Nau./Saccharomyces - ps27
Saccharomyces - ps28
Arboricola_clade - ps29
Cerevisiae_clade - ps30
Saccharomyces_cerevisiae - ps31
Phylostratum

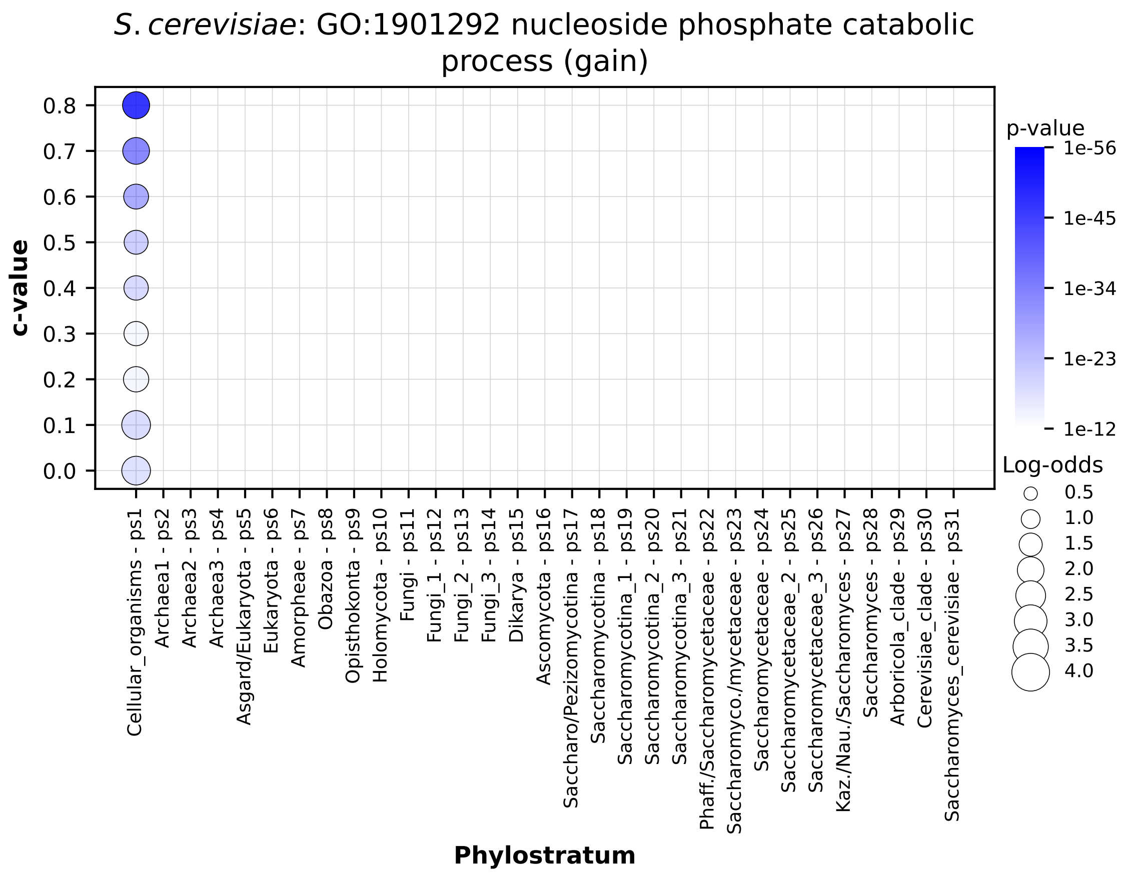
S. cerevisiae: GO:1901292 nucleoside phosphate catabolic process (gain)
c-value
0.8
0.7
0.6
0.5
0.4
0.3
0.2
0.1
0.0
p-value
1e-56
1e-45
1e-34
1e-23
1e-12
Log-odds
0.5
1.0
1.5
2.0
2.5
3.0
3.5
4.0
Cellular_organisms - ps1
Archaea1 - ps2
Archaea2 - ps3
Archaea3 - ps4
Asgard/Eukaryota - ps5
Eukaryota - ps6
Amorpheae - ps7
Obazoa - ps8
Opisthokonta - ps9
Holomycota - ps10
Fungi - ps11
Fungi_1 - ps12
Fungi_2 - ps13
Fungi_3 - ps14
Dikarya - ps15
Ascomycota - ps16
Saccharo/Pezizomycotina - ps17
Saccharomycotina - ps18
Saccharomycotina_1 - ps19
Saccharomycotina_2 - ps20
Saccharomycotina_3 - ps21
Phaff./Saccharomycetaceae - ps22
Saccharomyco./mycetaceae - ps23
Saccharomycetaceae - ps24
Saccharomycetaceae_2 - ps25
Saccharomycetaceae_3 - ps26
Kaz./Nau./Saccharomyces - ps27
Saccharomyces - ps28
Arboricola_clade - ps29
Cerevisiae_clade - ps30
Saccharomyces_cerevisiae - ps31
Phylostratum

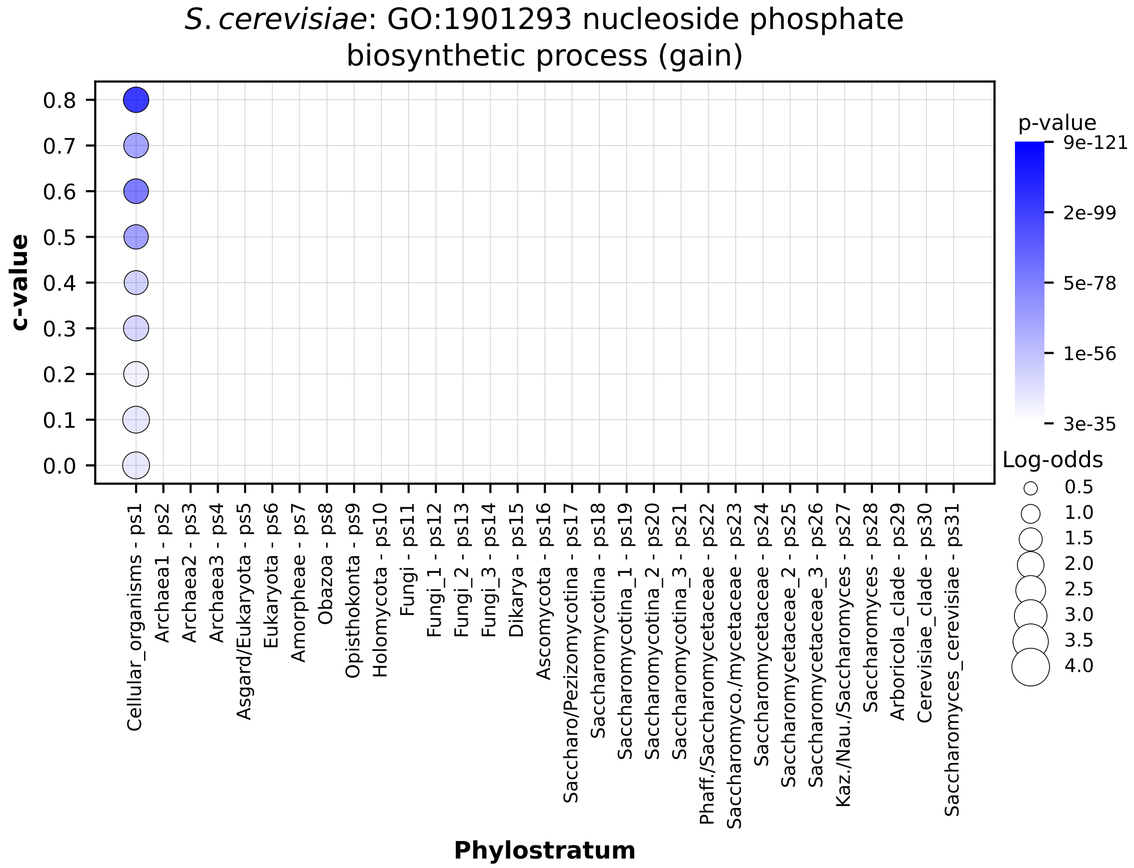
S. cerevisiae: GO:1901293 nucleoside phosphate biosynthetic process (gain)
c-value
0.8
0.7
0.6
0.5
0.4
0.3
0.2
0.1
0.0
p-value
9e-121
2e-99
5e-78
1e-56
3e-35
Log-odds
0.5
1.0
1.5
2.0
2.5
3.0
3.5
4.0
Cellular_organisms - ps1
Archaea1 - ps2
Archaea2 - ps3
Archaea3 - ps4
Asgard/Eukaryota - ps5
Eukaryota - ps6
Amorpheae - ps7
Obazoa - ps8
Opisthokonta - ps9
Holomycota - ps10
Fungi - ps11
Fungi_1 - ps12
Fungi_2 - ps13
Fungi_3 - ps14
Dikarya - ps15
Ascomycota - ps16
Saccharo/Pezizomycotina - ps17
Saccharomycotina - ps18
Saccharomycotina_1 - ps19
Saccharomycotina_2 - ps20
Saccharomycotina_3 - ps21
Phaff./Saccharomycetaceae - ps22
Saccharomyco./mycetaceae - ps23
Saccharomycetaceae - ps24
Saccharomycetaceae_2 - ps25
Saccharomycetaceae_3 - ps26
Kaz./Nau./Saccharomyces - ps27
Saccharomyces - ps28
Arboricola_clade - ps29
Cerevisiae_clade - ps30
Saccharomyces_cerevisiae - ps31
Phylostratum

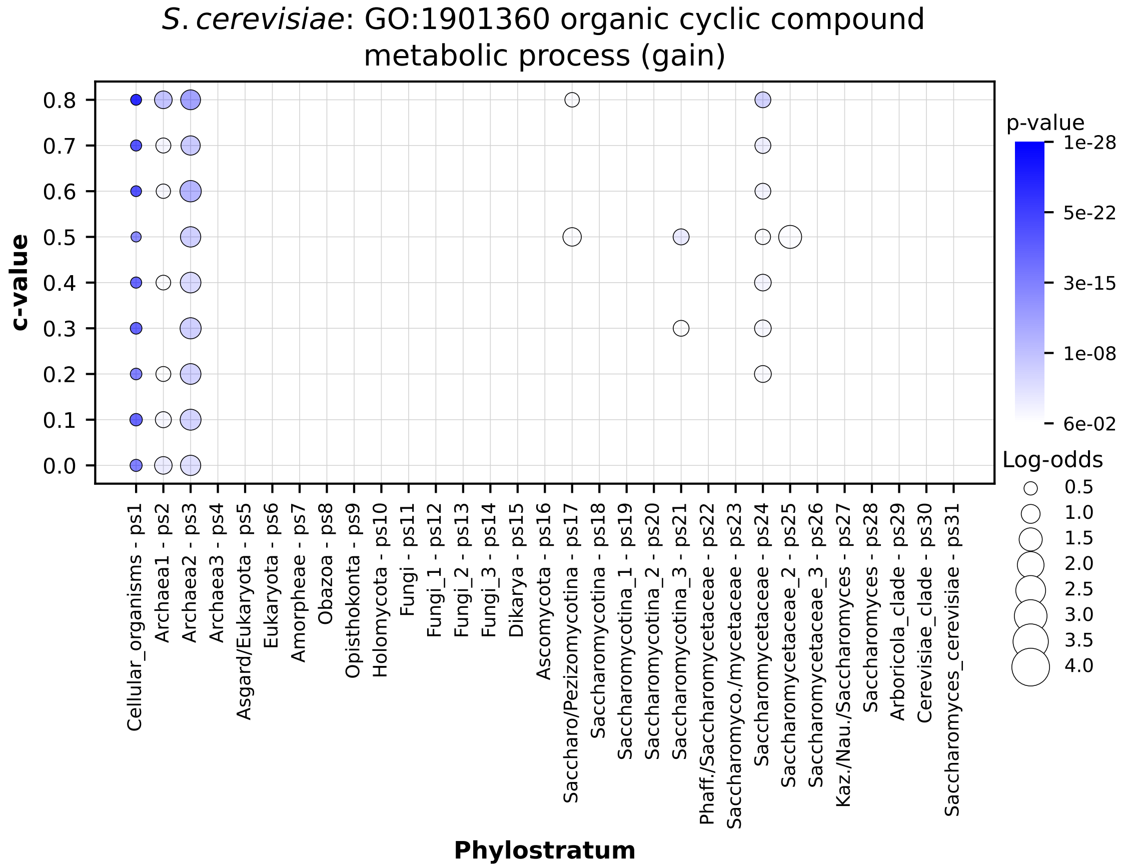
S. cerevisiae: GO:1901360 organic cyclic compound metabolic process (gain)
c-value
0.8
0.7
0.6
0.5
0.4
0.3
0.2
0.1
0.0
p-value
1e-28
5e-22
3e-15
1e-08
6e-02
Log-odds
0.5
1.0
1.5
2.0
2.5
3.0
3.5
4.0
Cellular_organisms - ps1
Archaea1 - ps2
Archaea2 - ps3
Archaea3 - ps4
Asgard/Eukaryota - ps5
Eukaryota - ps6
Amorpheae - ps7
Obazoa - ps8
Opisthokonta - ps9
Holomycota - ps10
Fungi - ps11
Fungi_1 - ps12
Fungi_2 - ps13
Fungi_3 - ps14
Dikarya - ps15
Ascomycota - ps16
Saccharo/Pezizomycotina - ps17
Saccharomycotina - ps18
Saccharomycotina_1 - ps19
Saccharomycotina_2 - ps20
Saccharomycotina_3 - ps21
Phaff./Saccharomycetaceae - ps22
Saccharomyco./mycetaceae - ps23
Saccharomycetaceae - ps24
Saccharomycetaceae_2 - ps25
Saccharomycetaceae_3 - ps26
Kaz./Nau./Saccharomyces - ps27
Saccharomyces - ps28
Arboricola_clade - ps29
Cerevisiae_clade - ps30
Saccharomyces_cerevisiae - ps31
Phylostratum

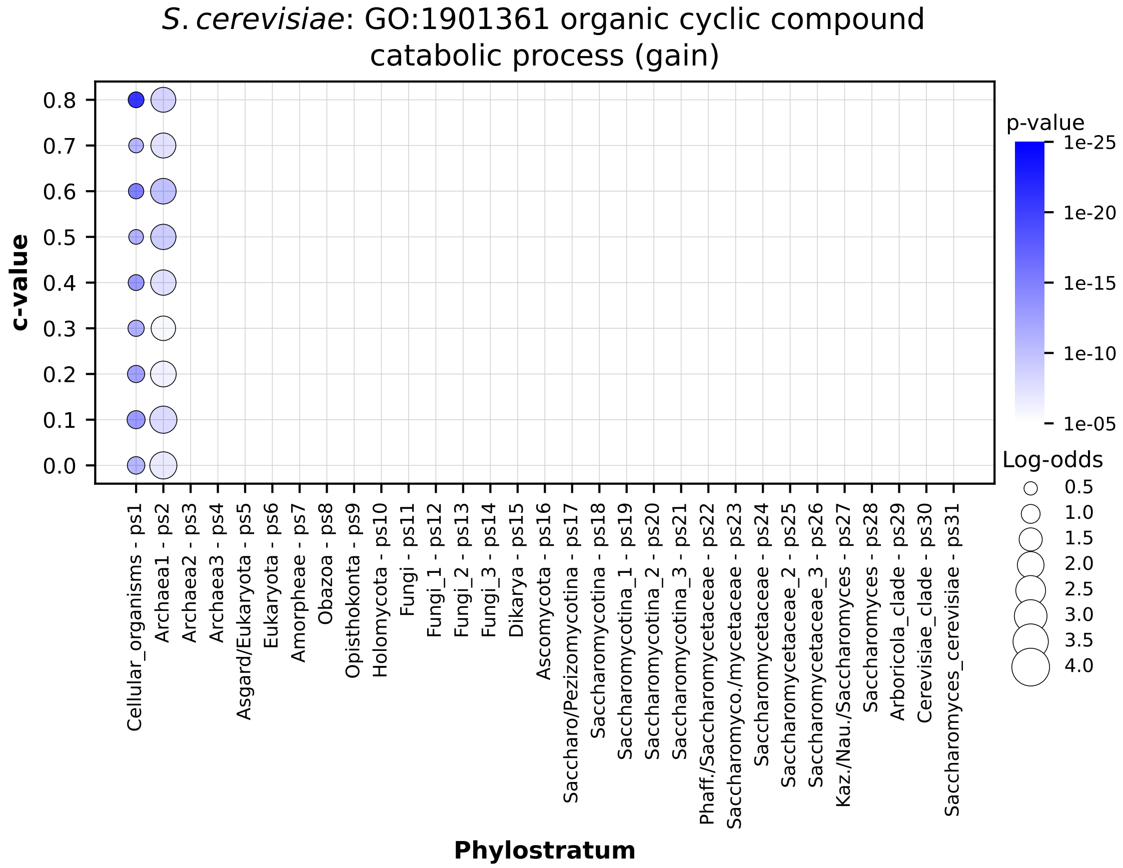

S. cerevisiae: GO:1901361 organic cyclic compound catabolic process (gain)
c-value
0.8
0.7
0.6
0.5
0.4
0.3
0.2
0.1
0.0
p-value
1e-25
1e-20
1e-15
1e-10
1e-05
Log-odds
0.5
1.0
1.5
2.0
2.5
3.0
3.5
4.0
Cellular_organisms - ps1
Archaea1 - ps2
Archaea2 - ps3
Archaea3 - ps4
Asgard/Eukaryota - ps5
Eukaryota - ps6
Amorpheae - ps7
Obazoa - ps8
Opisthokonta - ps9
Holomycota - ps10
Fungi - ps11
Fungi_1 - ps12
Fungi_2 - ps13
Fungi_3 - ps14
Dikarya - ps15
Ascomycota - ps16
Saccharo/Pezizomycotina - ps17
Saccharomycotina - ps18
Saccharomycotina_1 - ps19
Saccharomycotina_2 - ps20
Saccharomycotina_3 - ps21
Phaff./Saccharomycetaceae - ps22
Saccharomyco./mycetaceae - ps23
Saccharomycetaceae - ps24
Saccharomycetaceae_2 - ps25
Saccharomycetaceae_3 - ps26
Kaz./Nau./Saccharomyces - ps27
Saccharomyces - ps28
Arboricola_clade - ps29
Cerevisiae_clade - ps30
Saccharomyces_cerevisiae - ps31
Phylostratum

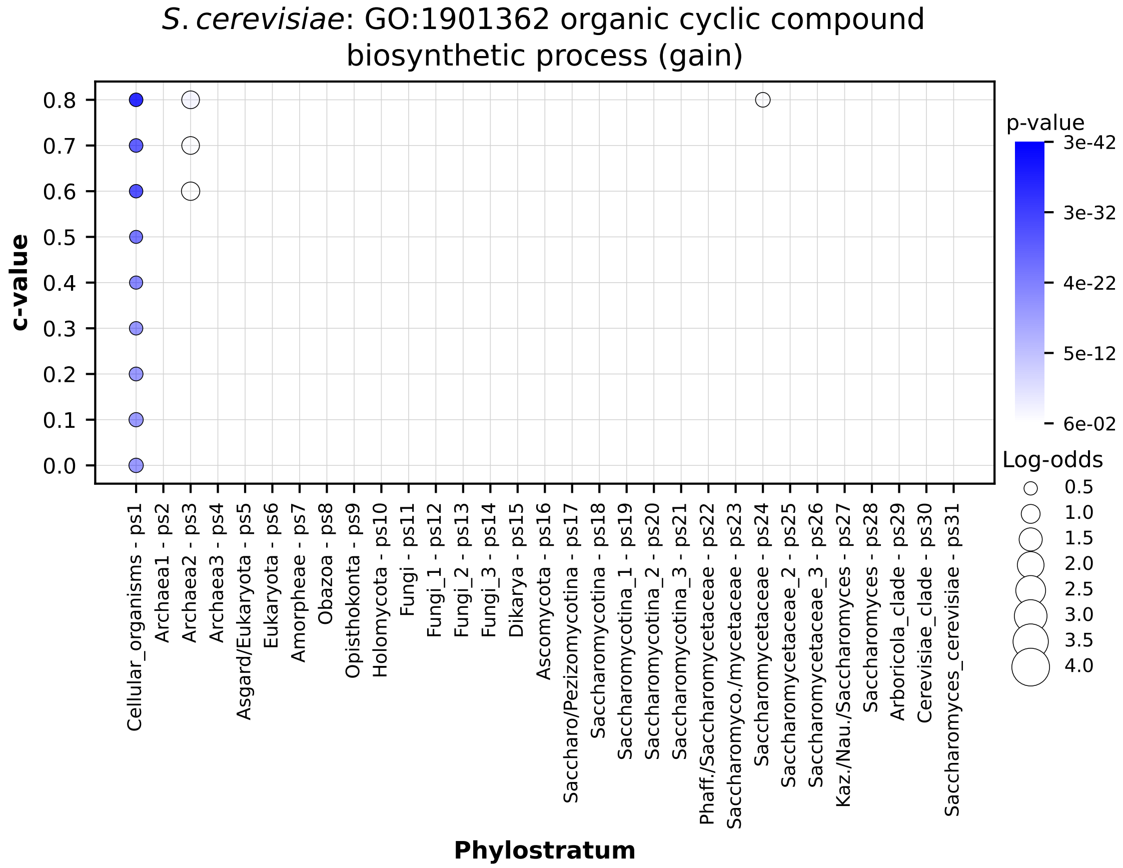
S. cerevisiae: GO:1901362 organic cyclic compound biosynthetic process (gain)
c-value
0.0
0.1
0.2
0.3
0.4
0.5
0.6
0.7
0.8
p-value
3e-42
3e-32
4e-22
5e-12
6e-02
Log-odds
0.5
1.0
1.5
2.0
2.5
3.0
3.5
4.0
Cellular_organisms - ps1
Archaea1 - ps2
Archaea2 - ps3
Archaea3 - ps4
Asgard/Eukaryota - ps5
Eukaryota - ps6
Amorpheae - ps7
Obazoa - ps8
Opisthokonta - ps9
Holomycota - ps10
Fungi - ps11
Fungi_1 - ps12
Fungi_2 - ps13
Fungi_3 - ps14
Dikarya - ps15
Ascomycota - ps16
Saccharo/Pezizomycotina - ps17
Saccharomycotina - ps18
Saccharomycotina_1 - ps19
Saccharomycotina_2 - ps20
Saccharomycotina_3 - ps21
Phaff./Saccharomycetaceae - ps22
Saccharomyco./mycetaceae - ps23
Saccharomycetaceae - ps24
Saccharomycetaceae_2 - ps25
Saccharomycetaceae_3 - ps26
Kaz./Nau./Saccharomyces - ps27
Saccharomyces - ps28
Arboricola_clade - ps29
Cerevisiae_clade - ps30
Saccharomyces_cerevisiae - ps31
Phylostratum

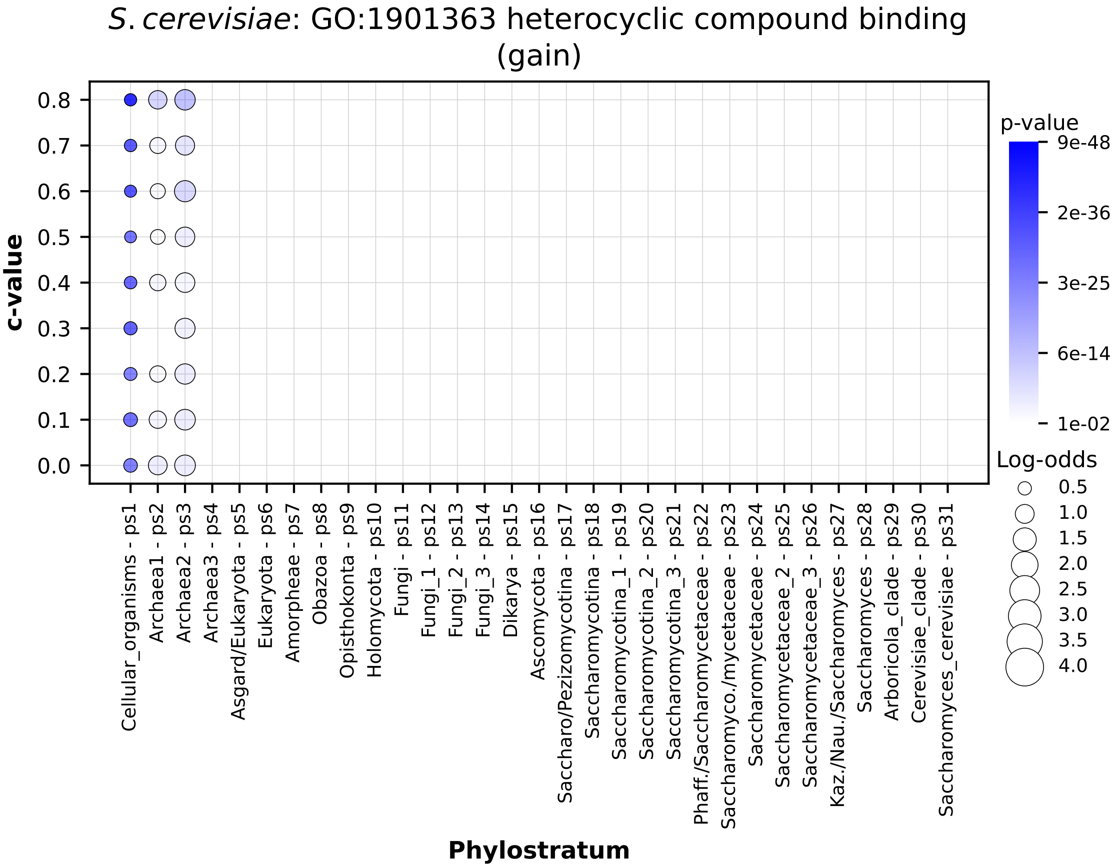

S. cerevisiae: GO:1901363 heterocyclic compound binding (gain)
c-value
0.8
0.7
0.6
0.5
0.4
0.3
0.2
0.1
0.0
p-value
9e-48
2e-36
3e-25
6e-14
1e-02
Log-odds
0.5
1.0
1.5
2.0
2.5
3.0
3.5
4.0
Cellular_organisms - ps1
Archaea1 - ps2
Archaea2 - ps3
Archaea3 - ps4
Asgard/Eukaryota - ps5
Eukaryota - ps6
Amorpheae - ps7
Obazoa - ps8
Opisthokonta - ps9
Holomycota - ps10
Fungi - ps11
Fungi_1 - ps12
Fungi_2 - ps13
Fungi_3 - ps14
Dikarya - ps15
Ascomycota - ps16
Saccharo/Pezizomycotina - ps17
Saccharomycotina - ps18
Saccharomycotina_1 - ps19
Saccharomycotina_2 - ps20
Saccharomycotina_3 - ps21
Phaff./Saccharomycetaceae - ps22
Saccharomyco./mycetaceae - ps23
Saccharomycetaceae - ps24
Saccharomycetaceae_2 - ps25
Saccharomycetaceae_3 - ps26
Kaz./Nau./Saccharomyces - ps27
Saccharomyces - ps28
Arboricola_clade - ps29
Cerevisiae_clade - ps30
Saccharomyces_cerevisiae - ps31
Phylostratum

S. cerevisiae: GO:1901564 organonitrogen compound metabolic process (gain)

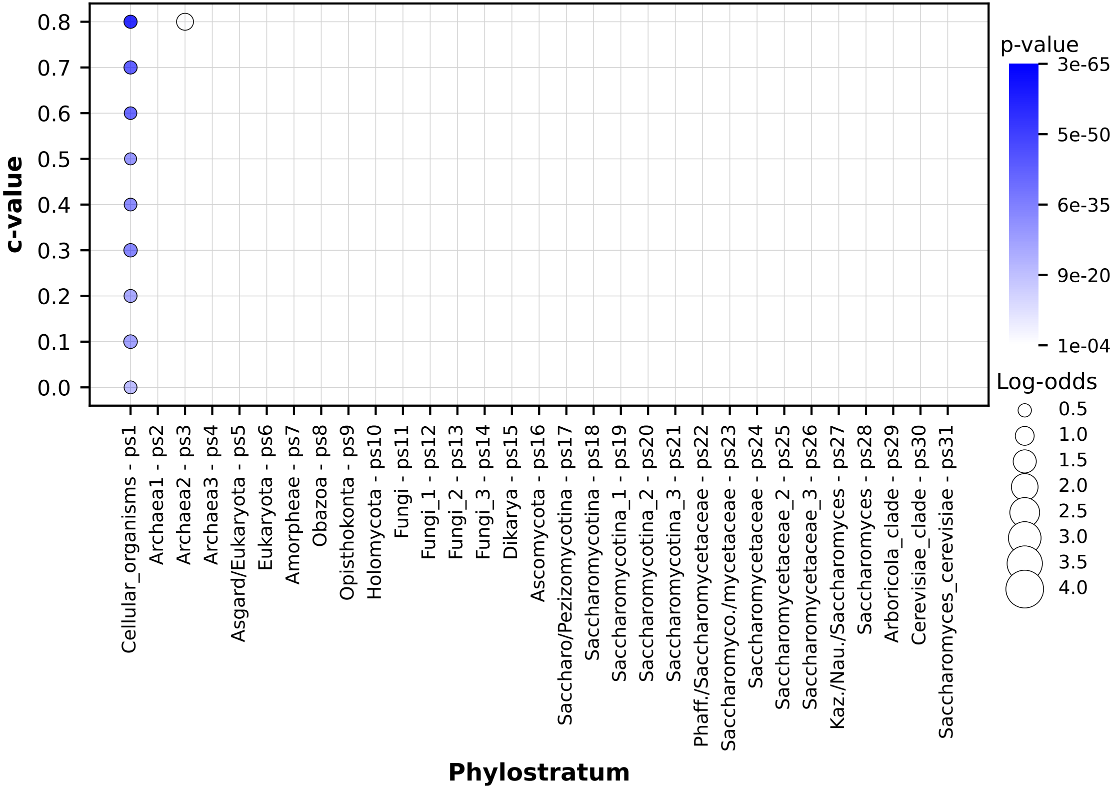

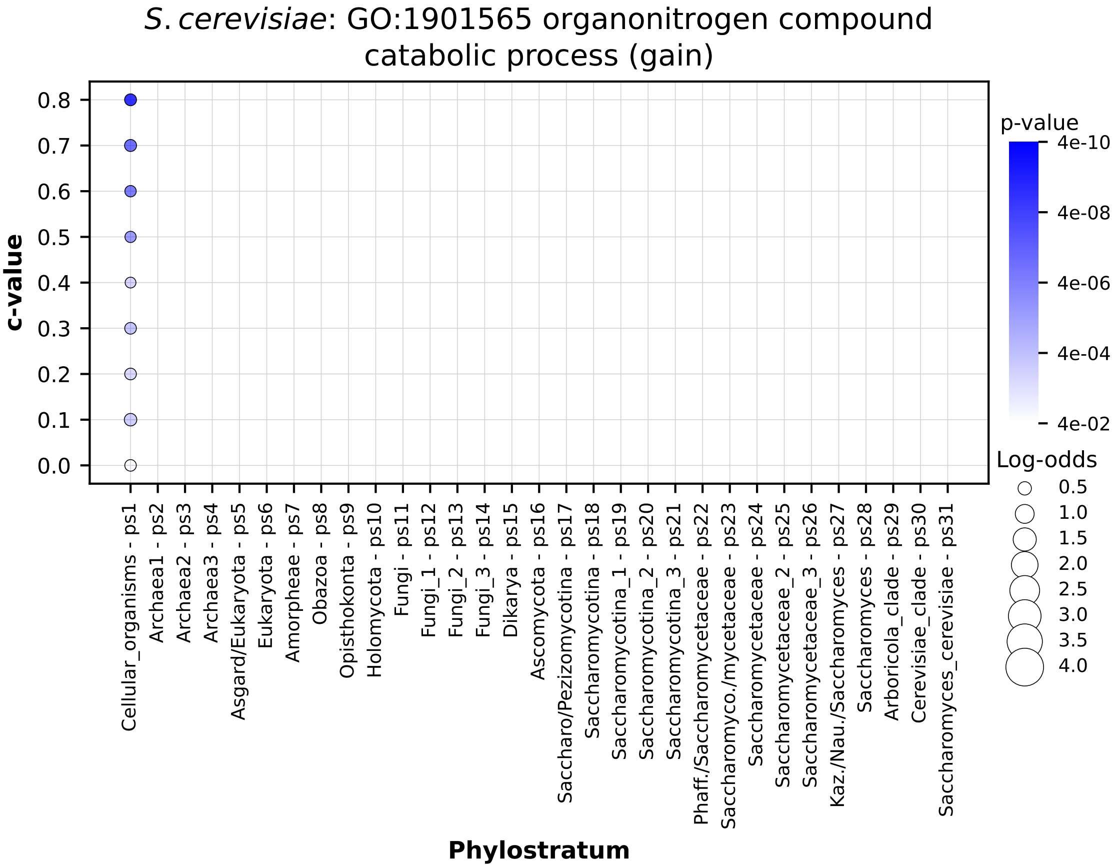

S. cerevisiae: GO:1901565 organonitrogen compound catabolic process (gain)
c-value
0.8
0.7
0.6
0.5
0.4
0.3
0.2
0.1
0.0
p-value
4e-10
4e-08
4e-06
4e-04
4e-02
Log-odds
0.5
1.0
1.5
2.0
2.5
3.0
3.5
4.0
Cellular_organisms - ps1
Archaea1 - ps2
Archaea2 - ps3
Archaea3 - ps4
Asgard/Eukaryota - ps5
Eukaryota - ps6
Amorpheae - ps7
Obazoa - ps8
Opisthokonta - ps9
Holomycota - ps10
Fungi - ps11
Fungi_1 - ps12
Fungi_2 - ps13
Fungi_3 - ps14
Dikarya - ps15
Ascomycota - ps16
Saccharo/Pezizomycotina - ps17
Saccharomycotina - ps18
Saccharomycotina_1 - ps19
Saccharomycotina_2 - ps20
Saccharomycotina_3 - ps21
Phaff./Saccharomycetaceae - ps22
Saccharomyco./mycetaceae - ps23
Saccharomycetaceae - ps24
Saccharomycetaceae_2 - ps25
Saccharomycetaceae_3 - ps26
Kaz./Nau./Saccharomyces - ps27
Saccharomyces - ps28
Arboricola_clade - ps29
Cerevisiae_clade - ps30
Saccharomyces_cerevisiae - ps31
Phylostratum

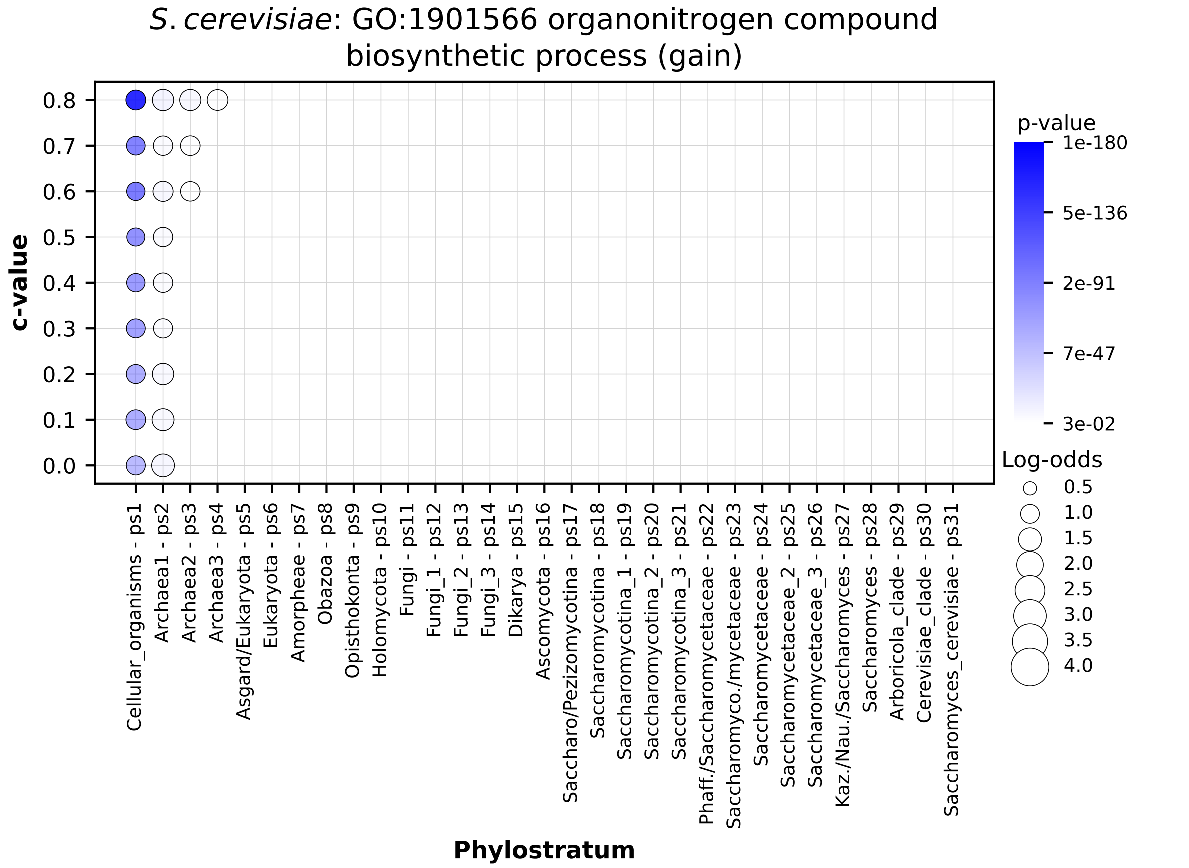

S. cerevisiae: GO:1901566 organonitrogen compound biosynthetic process (gain)
c-value
0.8
0.7
0.6
0.5
0.4
0.3
0.2
0.1
0.0
p-value
1e-180
5e-136
2e-91
7e-47
3e-02
Log-odds
0.5
1.0
1.5
2.0
2.5
3.0
3.5
4.0
Cellular_organisms - ps1
Archaea1 - ps2
Archaea2 - ps3
Archaea3 - ps4
Asgard/Eukaryota - ps5
Eukaryota - ps6
Amorpheae - ps7
Obazoa - ps8
Opisthokonta - ps9
Holomycota - ps10
Fungi - ps11
Fungi_1 - ps12
Fungi_2 - ps13
Fungi_3 - ps14
Dikarya - ps15
Ascomycota - ps16
Saccharo/Pezizomycotina - ps17
Saccharomycotina - ps18
Saccharomycotina_1 - ps19
Saccharomycotina_2 - ps20
Saccharomycotina_3 - ps21
Phaff./Saccharomycetaceae - ps22
Saccharomyco./mycetaceae - ps23
Saccharomycetaceae - ps24
Saccharomycetaceae_2 - ps25
Saccharomycetaceae_3 - ps26
Kaz./Nau./Saccharomyces - ps27
Saccharomyces - ps28
Arboricola_clade - ps29
Cerevisiae_clade - ps30
Saccharomyces_cerevisiae - ps31
Phylostratum

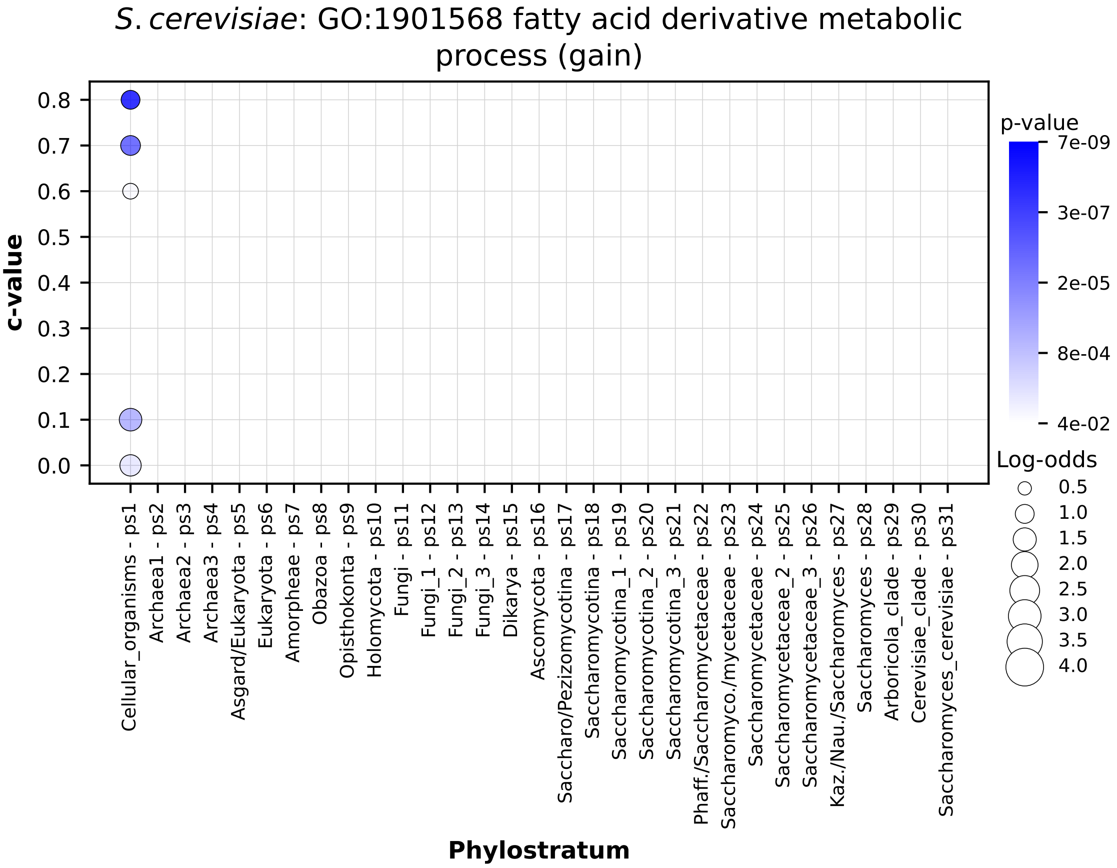

S. cerevisiae: GO:1901568 fatty acid derivative metabolic process (gain)
c-value
0.8
0.7
0.6
0.5
0.4
0.3
0.2
0.1
0.0
p-value
7e-09
3e-07
2e-05
8e-04
4e-02
Log-odds
0.5
1.0
1.5
2.0
2.5
3.0
3.5
4.0
Cellular_organisms - ps1
Archaea1 - ps2
Archaea2 - ps3
Archaea3 - ps4
Asgard/Eukaryota - ps5
Eukaryota - ps6
Amorpheae - ps7
Obazoa - ps8
Opisthokonta - ps9
Holomycota - ps10
Fungi - ps11
Fungi_1 - ps12
Fungi_2 - ps13
Fungi_3 - ps14
Dikarya - ps15
Ascomycota - ps16
Saccharo/Pezizomycotina - ps17
Saccharomycotina - ps18
Saccharomycotina_1 - ps19
Saccharomycotina_2 - ps20
Saccharomycotina_3 - ps21
Phaff./Saccharomycetaceae - ps22
Saccharomyco./mycetaceae - ps23
Saccharomycetaceae - ps24
Saccharomycetaceae_2 - ps25
Saccharomycetaceae_3 - ps26
Kaz./Nau./Saccharomyces - ps27
Saccharomyces - ps28
Arboricola_clade - ps29
Cerevisiae_clade - ps30
Saccharomyces_cerevisiae - ps31
Phylostratum

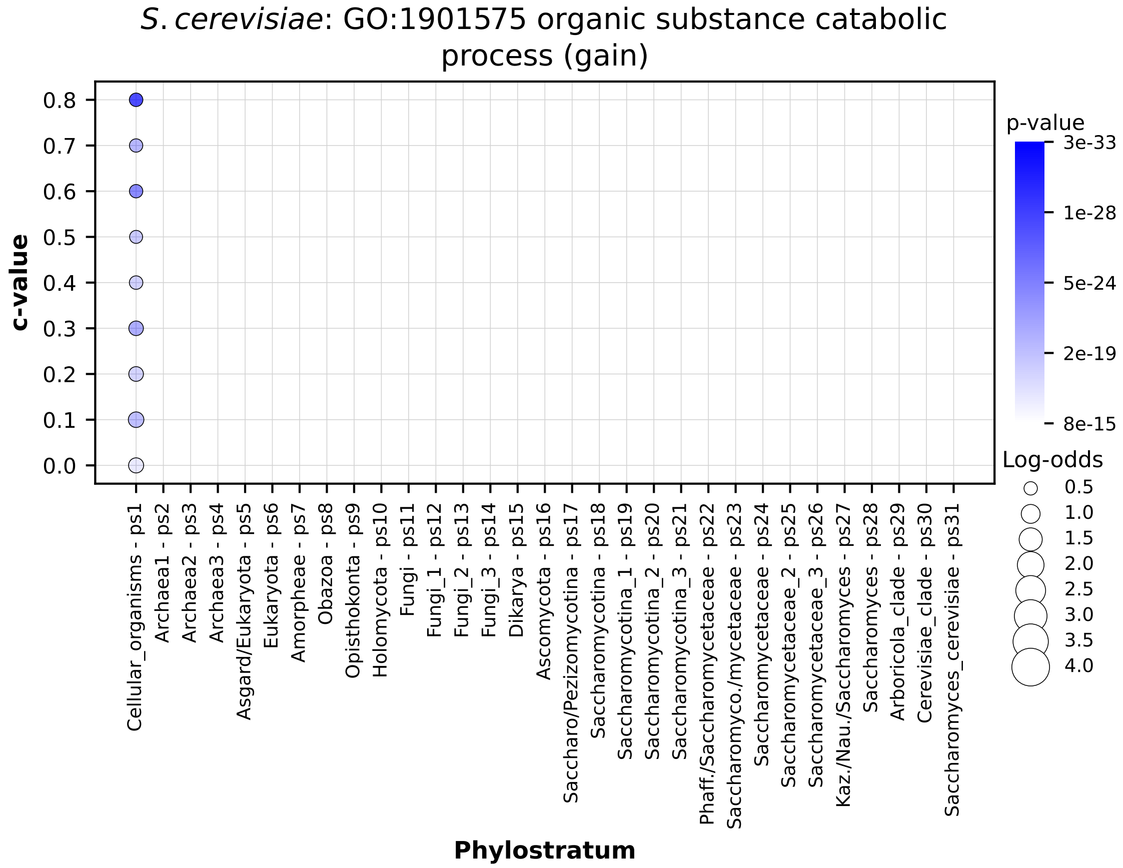

S. cerevisiae: GO:1901575 organic substance catabolic process (gain)
c-value
0.8
0.7
0.6
0.5
0.4
0.3
0.2
0.1
0.0
p-value
3e-33
1e-28
5e-24
2e-19
8e-15
Log-odds
0.5
1.0
1.5
2.0
2.5
3.0
3.5
4.0
Cellular_organisms - ps1
Archaea1 - ps2
Archaea2 - ps3
Archaea3 - ps4
Asgard/Eukaryota - ps5
Eukaryota - ps6
Amorpheae - ps7
Obazoa - ps8
Opisthokonta - ps9
Holomycota - ps10
Fungi - ps11
Fungi_1 - ps12
Fungi_2 - ps13
Fungi_3 - ps14
Dikarya - ps15
Ascomycota - ps16
Saccharo/Pezizomycotina - ps17
Saccharomycotina - ps18
Saccharomycotina_1 - ps19
Saccharomycotina_2 - ps20
Saccharomycotina_3 - ps21
Phaff./Saccharomycetaceae - ps22
Saccharomyco./mycetaceae - ps23
Saccharomycetaceae - ps24
Saccharomycetaceae_2 - ps25
Saccharomycetaceae_3 - ps26
Kaz./Nau./Saccharomyces - ps27
Saccharomyces - ps28
Arboricola_clade - ps29
Cerevisiae_clade - ps30
Saccharomyces_cerevisiae - ps31
Phylostratum

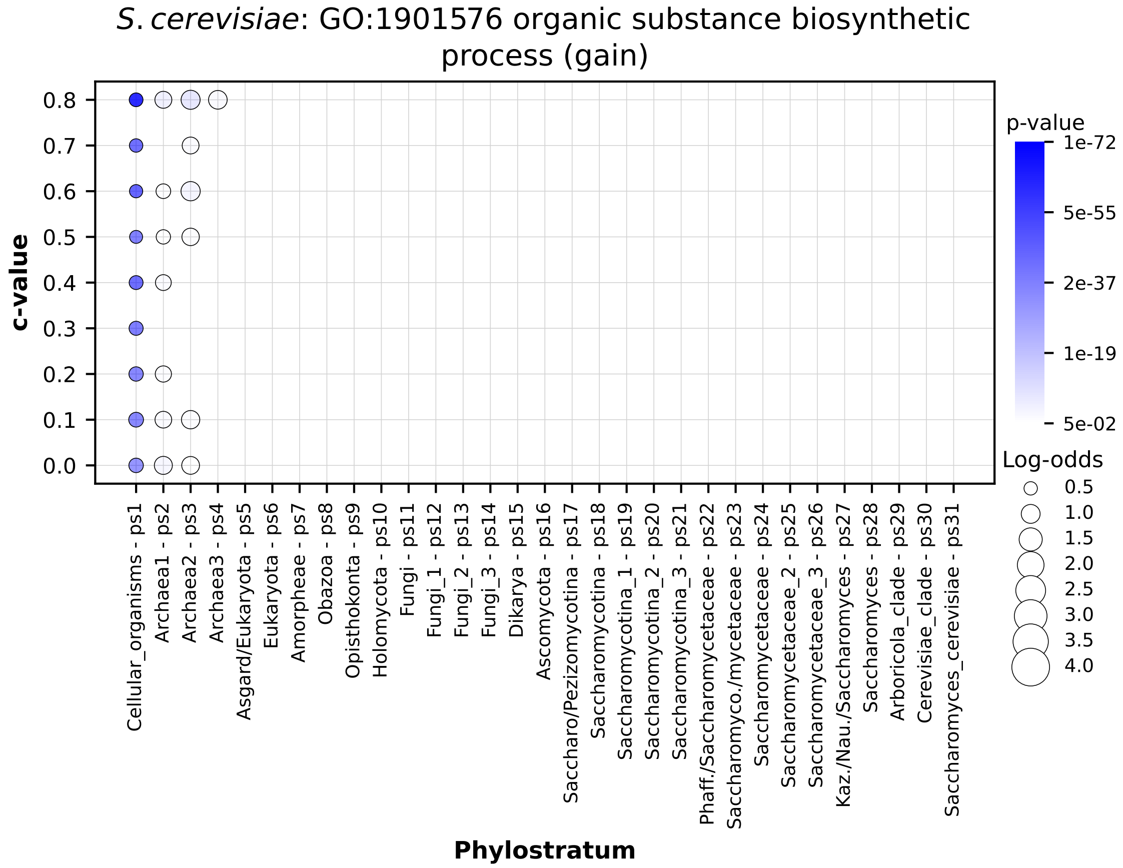

S. cerevisiae: GO:1901576 organic substance biosynthetic process (gain)
c-value
0.8
0.7
0.6
0.5
0.4
0.3
0.2
0.1
0.0
p-value
1e-72
5e-55
2e-37
1e-19
5e-02
Log-odds
0.5
1.0
1.5
2.0
2.5
3.0
3.5
4.0
Cellular_organisms - ps1
Archaea1 - ps2
Archaea2 - ps3
Archaea3 - ps4
Asgard/Eukaryota - ps5
Eukaryota - ps6
Amorpheae - ps7
Obazoa - ps8
Opisthokonta - ps9
Holomycota - ps10
Fungi - ps11
Fungi_1 - ps12
Fungi_2 - ps13
Fungi_3 - ps14
Dikarya - ps15
Ascomycota - ps16
Saccharo/Pezizomycotina - ps17
Saccharomycotina - ps18
Saccharomycotina_1 - ps19
Saccharomycotina_2 - ps20
Saccharomycotina_3 - ps21
Phaff./Saccharomycetaceae - ps22
Saccharomyco./mycetaceae - ps23
Saccharomycetaceae - ps24
Saccharomycetaceae_2 - ps25
Saccharomycetaceae_3 - ps26
Kaz./Nau./Saccharomyces - ps27
Saccharomyces - ps28
Arboricola_clade - ps29
Cerevisiae_clade - ps30
Saccharomyces_cerevisiae - ps31
Phylostratum

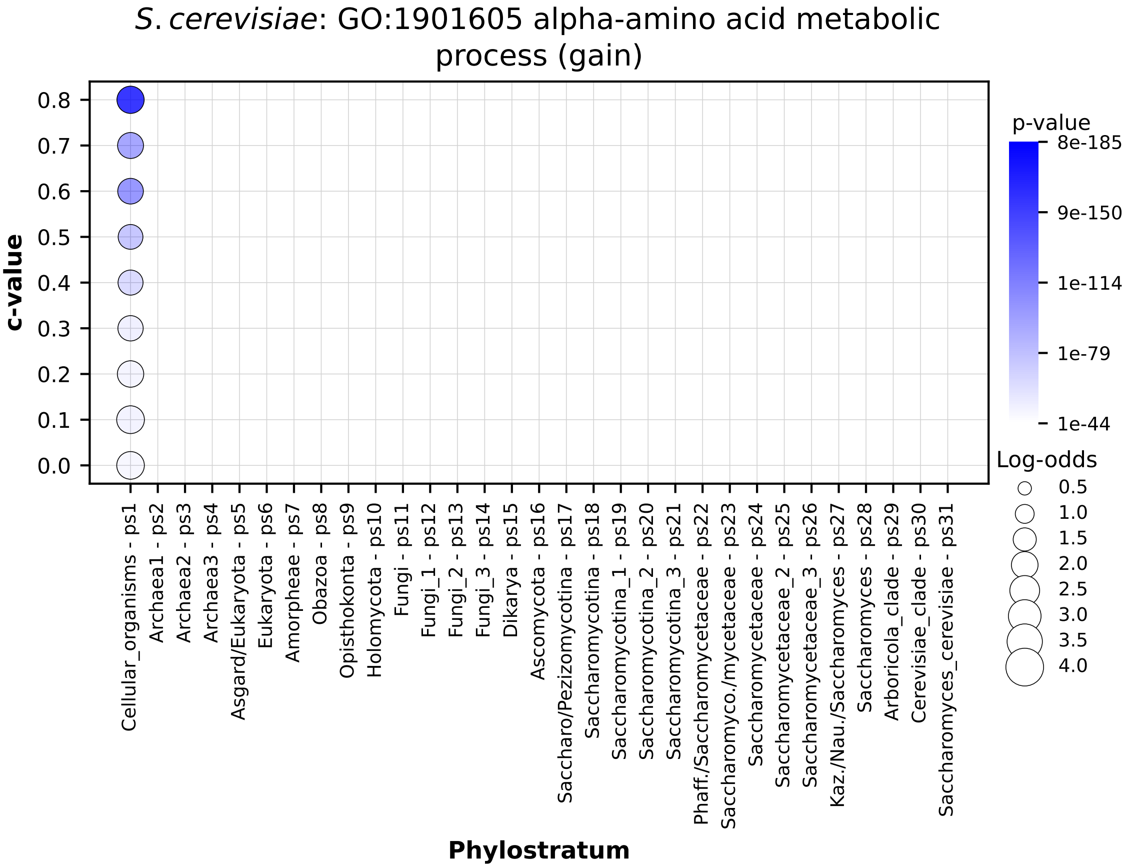

S. cerevisiae: GO:1901605 alpha-amino acid metabolic process (gain)
c-value
0.8
0.7
0.6
0.5
0.4
0.3
0.2
0.1
0.0
p-value
8e-185
9e-150
1e-114
1e-79
1e-44
Log-odds
0.5
1.0
1.5
2.0
2.5
3.0
3.5
4.0
Cellular_organisms - ps1
Archaea1 - ps2
Archaea2 - ps3
Archaea3 - ps4
Asgard/Eukaryota - ps5
Eukaryota - ps6
Amorpheae - ps7
Obazoa - ps8
Opisthokonta - ps9
Holomycota - ps10
Fungi - ps11
Fungi_1 - ps12
Fungi_2 - ps13
Fungi_3 - ps14
Dikarya - ps15
Ascomycota - ps16
Saccharo/Pezizomycotina - ps17
Saccharomycotina - ps18
Saccharomycotina_1 - ps19
Saccharomycotina_2 - ps20
Saccharomycotina_3 - ps21
Phaff./Saccharomycetaceae - ps22
Saccharomyco./mycetaceae - ps23
Saccharomycetaceae - ps24
Saccharomycetaceae_2 - ps25
Saccharomycetaceae_3 - ps26
Kaz./Nau./Saccharomyces - ps27
Saccharomyces - ps28
Arboricola_clade - ps29
Cerevisiae_clade - ps30
Saccharomyces_cerevisiae - ps31
Phylostratum

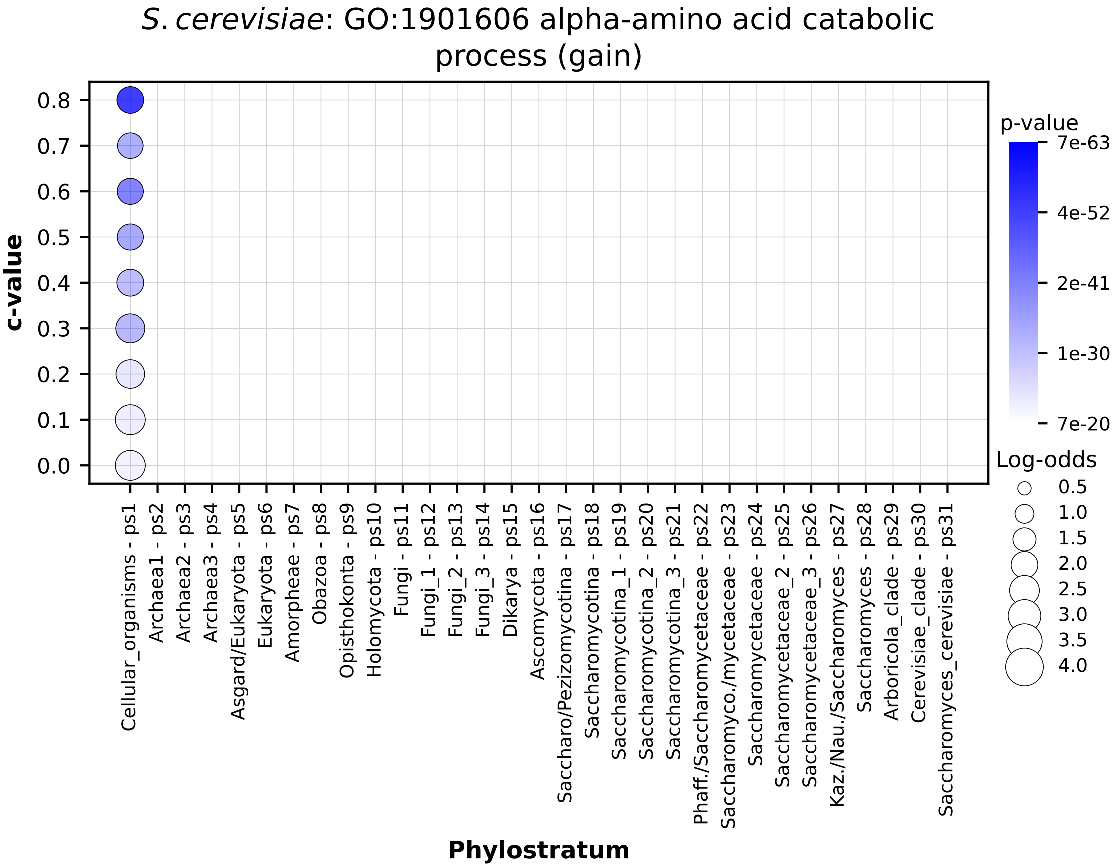

S. cerevisiae: GO:1901606 alpha-amino acid catabolic process (gain)
c-value
0.8
0.7
0.6
0.5
0.4
0.3
0.2
0.1
0.0
p-value
7e-63
4e-52
2e-41
1e-30
7e-20
Log-odds
0.5
1.0
1.5
2.0
2.5
3.0
3.5
4.0
Cellular_organisms - ps1
Archaea1 - ps2
Archaea2 - ps3
Archaea3 - ps4
Asgard/Eukaryota - ps5
Eukaryota - ps6
Amorpheae - ps7
Obazoa - ps8
Opisthokonta - ps9
Holomycota - ps10
Fungi - ps11
Fungi_1 - ps12
Fungi_2 - ps13
Fungi_3 - ps14
Dikarya - ps15
Ascomycota - ps16
Saccharo/Pezizomycotina - ps17
Saccharomycotina - ps18
Saccharomycotina_1 - ps19
Saccharomycotina_2 - ps20
Saccharomycotina_3 - ps21
Phaff./Saccharomycetaceae - ps22
Saccharomyco./mycetaceae - ps23
Saccharomycetaceae - ps24
Saccharomycetaceae_2 - ps25
Saccharomycetaceae_3 - ps26
Kaz./Nau./Saccharomyces - ps27
Saccharomyces - ps28
Arboricola_clade - ps29
Cerevisiae_clade - ps30
Saccharomyces_cerevisiae - ps31
Phylostratum

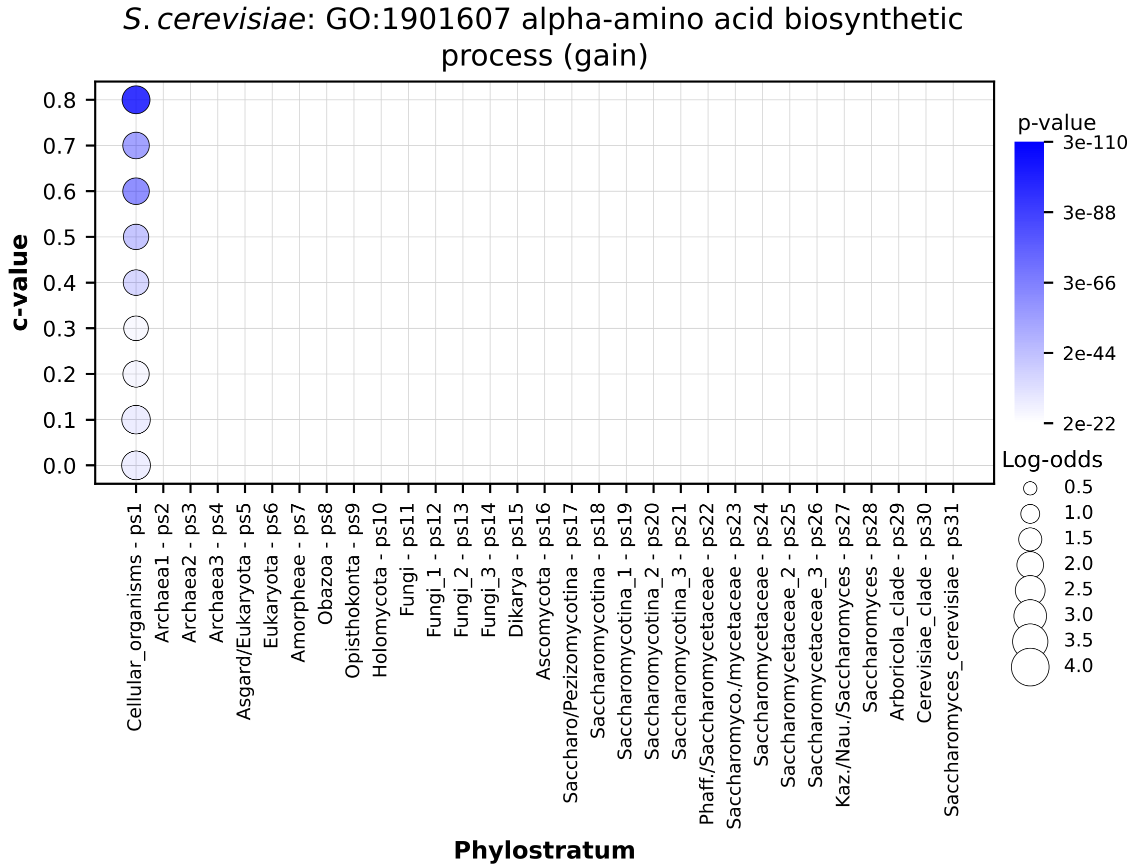
S. cerevisiae: GO:1901607 alpha-amino acid biosynthetic process (gain)
c-value
0.8
0.7
0.6
0.5
0.4
0.3
0.2
0.1
0.0
p-value
3e-110
3e-88
3e-66
2e-44
2e-22
Log-odds
0.5
1.0
1.5
2.0
2.5
3.0
3.5
4.0
Cellular_organisms - ps1
Archaea1 - ps2
Archaea2 - ps3
Archaea3 - ps4
Asgard/Eukaryota - ps5
Eukaryota - ps6
Amorpheae - ps7
Obazoa - ps8
Opisthokonta - ps9
Holomycota - ps10
Fungi - ps11
Fungi_1 - ps12
Fungi_2 - ps13
Fungi_3 - ps14
Dikarya - ps15
Ascomycota - ps16
Saccharo/Pezizomycotina - ps17
Saccharomycotina - ps18
Saccharomycotina_1 - ps19
Saccharomycotina_2 - ps20
Saccharomycotina_3 - ps21
Phaff./Saccharomycetaceae - ps22
Saccharomyco./mycetaceae - ps23
Saccharomycetaceae - ps24
Saccharomycetaceae_2 - ps25
Saccharomycetaceae_3 - ps26
Kaz./Nau./Saccharomyces - ps27
Saccharomyces - ps28
Arboricola_clade - ps29
Cerevisiae_clade - ps30
Saccharomyces_cerevisiae - ps31
Phylostratum

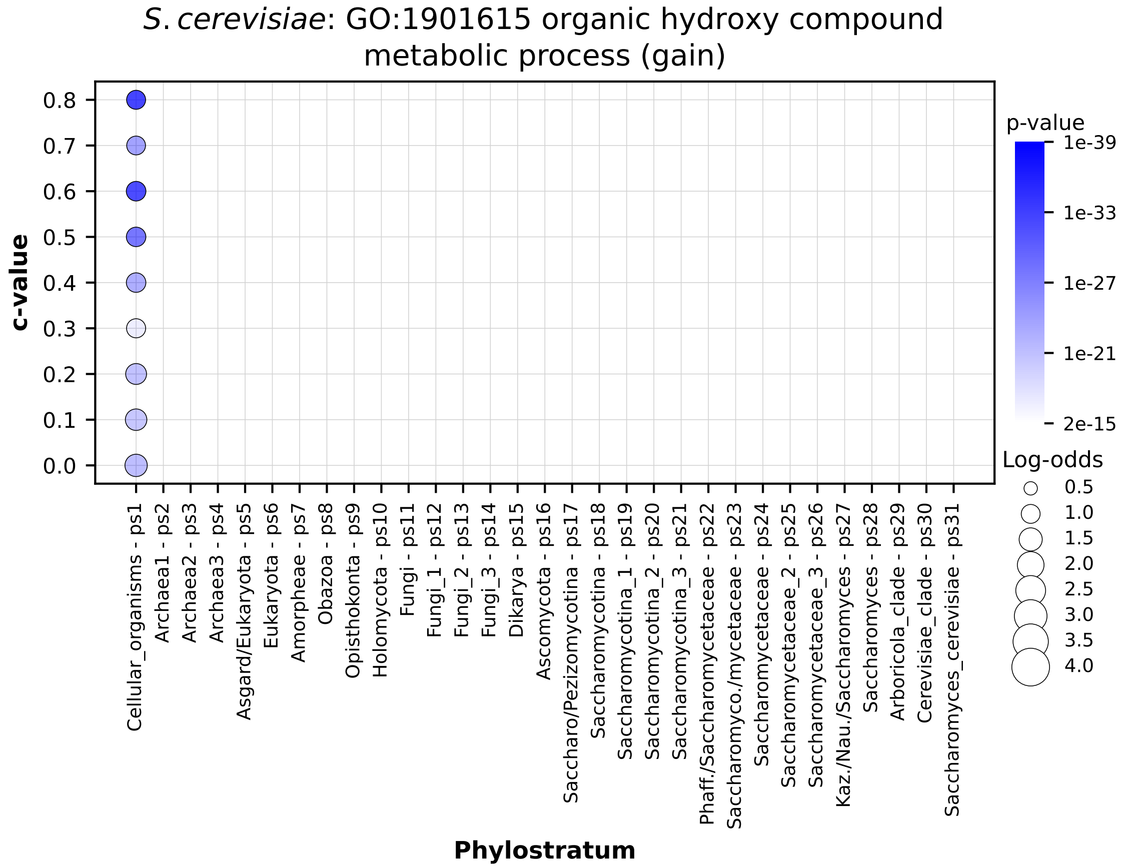

S. cerevisiae: GO:1901615 organic hydroxy compound metabolic process (gain)
c-value
0.0
0.1
0.2
0.3
0.4
0.5
0.6
0.7
0.8
p-value
1e-39
1e-33
1e-27
1e-21
2e-15
Log-odds
0.5
1.0
1.5
2.0
2.5
3.0
3.5
4.0
Cellular_organisms - ps1
Archaea1 - ps2
Archaea2 - ps3
Archaea3 - ps4
Asgard/Eukaryota - ps5
Eukaryota - ps6
Amorpheae - ps7
Obazoa - ps8
Opisthokonta - ps9
Holomycota - ps10
Fungi - ps11
Fungi_1 - ps12
Fungi_2 - ps13
Fungi_3 - ps14
Dikarya - ps15
Ascomycota - ps16
Saccharo/Pezizomycotina - ps17
Saccharomycotina - ps18
Saccharomycotina_1 - ps19
Saccharomycotina_2 - ps20
Saccharomycotina_3 - ps21
Phaff./Saccharomycetaceae - ps22
Saccharomyco./mycetaceae - ps23
Saccharomycetaceae - ps24
Saccharomycetaceae_2 - ps25
Saccharomycetaceae_3 - ps26
Kaz./Nau./Saccharomyces - ps27
Saccharomyces - ps28
Arboricola_clade - ps29
Cerevisiae_clade - ps30
Saccharomyces_cerevisiae - ps31
Phylostratum

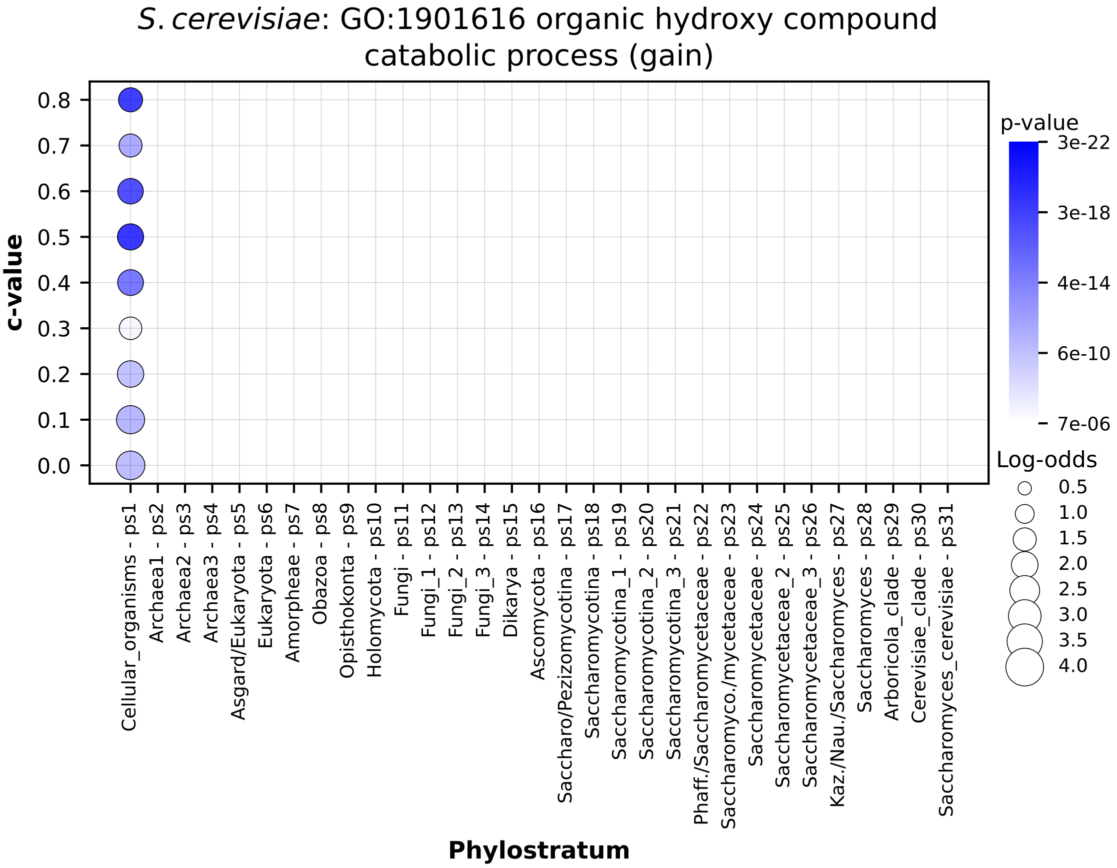
S. cerevisiae: GO:1901616 organic hydroxy compound catabolic process (gain)
c-value
0.8
0.7
0.6
0.5
0.4
0.3
0.2
0.1
0.0
p-value
3e-22
3e-18
4e-14
6e-10
7e-06
Log-odds
0.5
1.0
1.5
2.0
2.5
3.0
3.5
4.0
Cellular_organisms - ps1
Archaea1 - ps2
Archaea2 - ps3
Archaea3 - ps4
Asgard/Eukaryota - ps5
Eukaryota - ps6
Amorpheae - ps7
Obazoa - ps8
Opisthokonta - ps9
Holomycota - ps10
Fungi - ps11
Fungi_1 - ps12
Fungi_2 - ps13
Fungi_3 - ps14
Dikarya - ps15
Ascomycota - ps16
Saccharo/Pezizomycotina - ps17
Saccharomycotina - ps18
Saccharomycotina_1 - ps19
Saccharomycotina_2 - ps20
Saccharomycotina_3 - ps21
Phaff./Saccharomycetaceae - ps22
Saccharomyco./mycetaceae - ps23
Saccharomycetaceae - ps24
Saccharomycetaceae_2 - ps25
Saccharomycetaceae_3 - ps26
Kaz./Nau./Saccharomyces - ps27
Saccharomyces - ps28
Arboricola_clade - ps29
Cerevisiae_clade - ps30
Saccharomyces_cerevisiae - ps31
Phylostratum

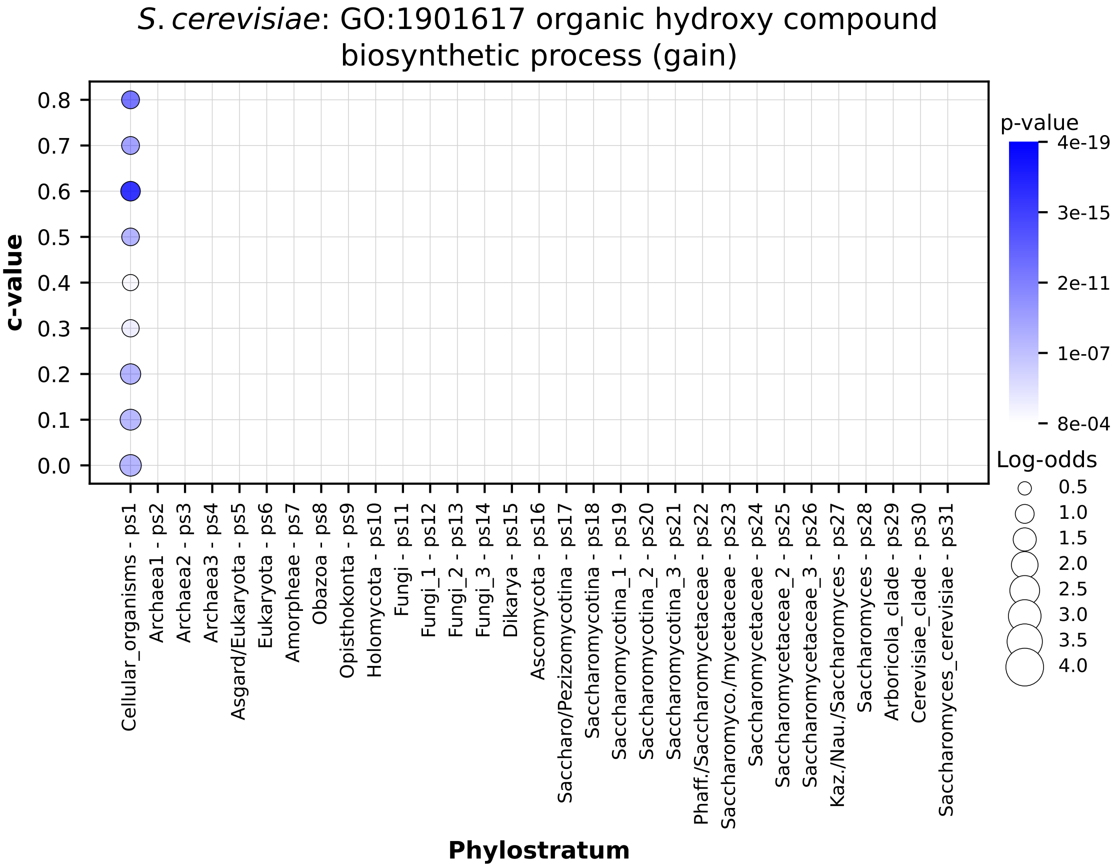
S. cerevisiae: GO:1901617 organic hydroxy compound biosynthetic process (gain)
c-value
0.8
0.7
0.6
0.5
0.4
0.3
0.2
0.1
0.0
Phylostratum
Cellular_organisms - ps1
Archaea1 - ps2
Archaea2 - ps3
Archaea3 - ps4
Asgard/Eukaryota - ps5
Eukaryota - ps6
Amorpheae - ps7
Obazoa - ps8
Opisthokonta - ps9
Holomycota - ps10
Fungi - ps11
Fungi_1 - ps12
Fungi_2 - ps13
Fungi_3 - ps14
Dikarya - ps15
Ascomycota - ps16
Saccharo/Pezizomycotina - ps17
Saccharomycotina - ps18
Saccharomycotina_1 - ps19
Saccharomycotina_2 - ps20
Saccharomycotina_3 - ps21
Phaff./Saccharomycetaceae - ps22
Saccharomyco./mycetaceae - ps23
Saccharomycetaceae - ps24
Saccharomycetaceae_2 - ps25
Saccharomycetaceae_3 - ps26
Kaz./Nau./Saccharomyces - ps27
Saccharomyces - ps28
Arboricola_clade - ps29
Cerevisiae_clade - ps30
Saccharomyces_cerevisiae - ps31
p-value
4e-19
3e-15
2e-11
1e-07
8e-04
Log-odds
0.5
1.0
1.5
2.0
2.5
3.0
3.5
4.0

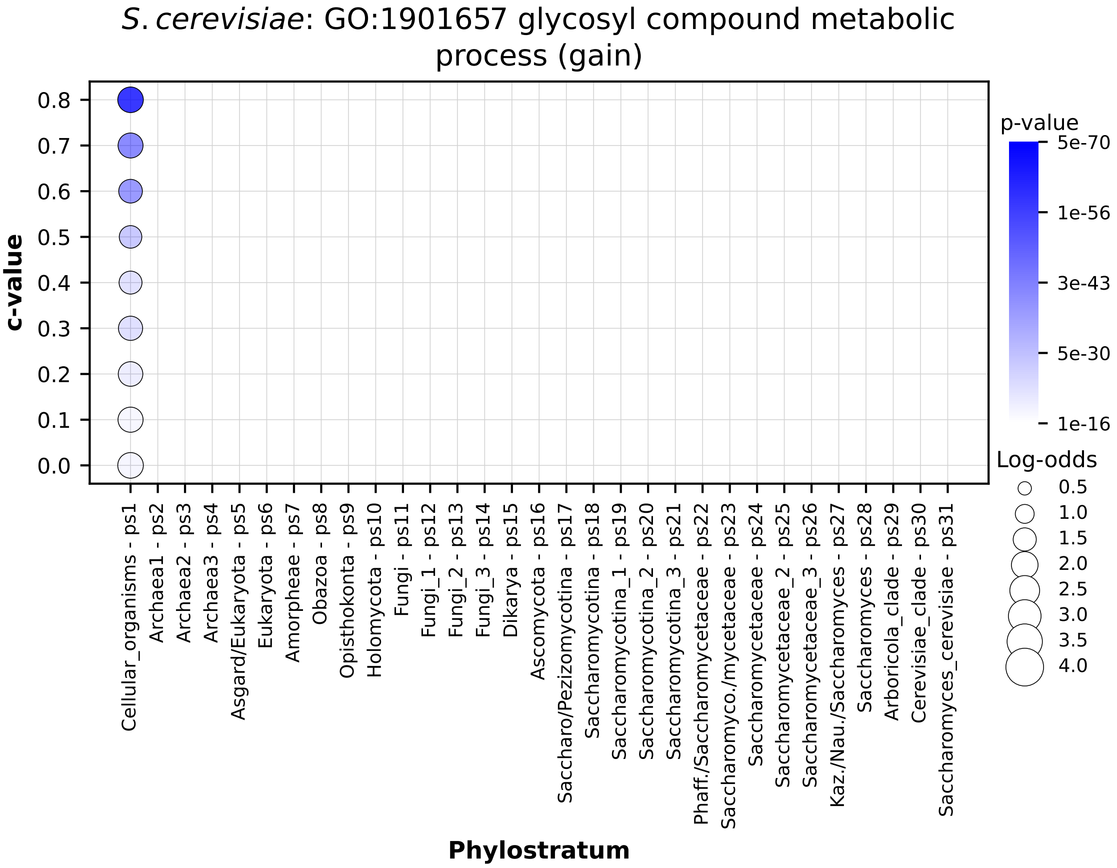
S. cerevisiae: GO:1901657 glycosyl compound metabolic process (gain)
c-value
0.8
0.7
0.6
0.5
0.4
0.3
0.2
0.1
0.0
p-value
5e-70
1e-56
3e-43
5e-30
1e-16
Log-odds
0.5
1.0
1.5
2.0
2.5
3.0
3.5
4.0
Cellular_organisms - ps1
Archaea1 - ps2
Archaea2 - ps3
Archaea3 - ps4
Asgard/Eukaryota - ps5
Eukaryota - ps6
Amorpheae - ps7
Obazoa - ps8
Opisthokonta - ps9
Holomycota - ps10
Fungi - ps11
Fungi_1 - ps12
Fungi_2 - ps13
Fungi_3 - ps14
Dikarya - ps15
Ascomycota - ps16
Saccharo/Pezizomycotina - ps17
Saccharomycotina - ps18
Saccharomycotina_1 - ps19
Saccharomycotina_2 - ps20
Saccharomycotina_3 - ps21
Phaff./Saccharomycetaceae - ps22
Saccharomyco./mycetaceae - ps23
Saccharomycetaceae - ps24
Saccharomycetaceae_2 - ps25
Saccharomycetaceae_3 - ps26
Kaz./Nau./Saccharomyces - ps27
Saccharomyces - ps28
Arboricola_clade - ps29
Cerevisiae_clade - ps30
Saccharomyces_cerevisiae - ps31
Phylostratum

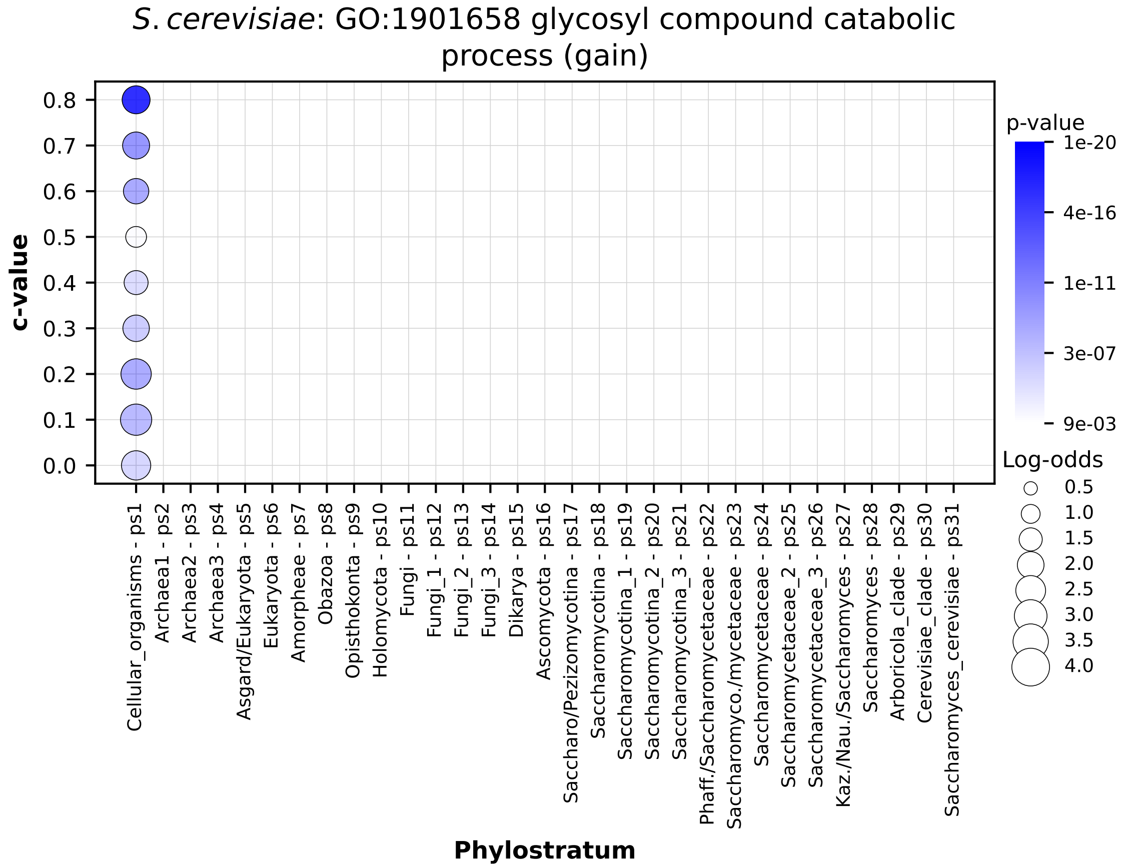

S. cerevisiae: GO:1901658 glycosyl compound catabolic process (gain)
c-value
0.8
0.7
0.6
0.5
0.4
0.3
0.2
0.1
0.0
p-value
1e-20
4e-16
1e-11
3e-07
9e-03
Log-odds
0.5
1.0
1.5
2.0
2.5
3.0
3.5
4.0
Cellular_organisms - ps1
Archaea1 - ps2
Archaea2 - ps3
Archaea3 - ps4
Asgard/Eukaryota - ps5
Eukaryota - ps6
Amorpheae - ps7
Obazoa - ps8
Opisthokonta - ps9
Holomycota - ps10
Fungi - ps11
Fungi_1 - ps12
Fungi_2 - ps13
Fungi_3 - ps14
Dikarya - ps15
Ascomycota - ps16
Saccharo/Pezizomycotina - ps17
Saccharomycotina - ps18
Saccharomycotina_1 - ps19
Saccharomycotina_2 - ps20
Saccharomycotina_3 - ps21
Phaff./Saccharomycetaceae - ps22
Saccharomyco./mycetaceae - ps23
Saccharomycetaceae - ps24
Saccharomycetaceae_2 - ps25
Saccharomycetaceae_3 - ps26
Kaz./Nau./Saccharomyces - ps27
Saccharomyces - ps28
Arboricola_clade - ps29
Cerevisiae_clade - ps30
Saccharomyces_cerevisiae - ps31
Phylostratum

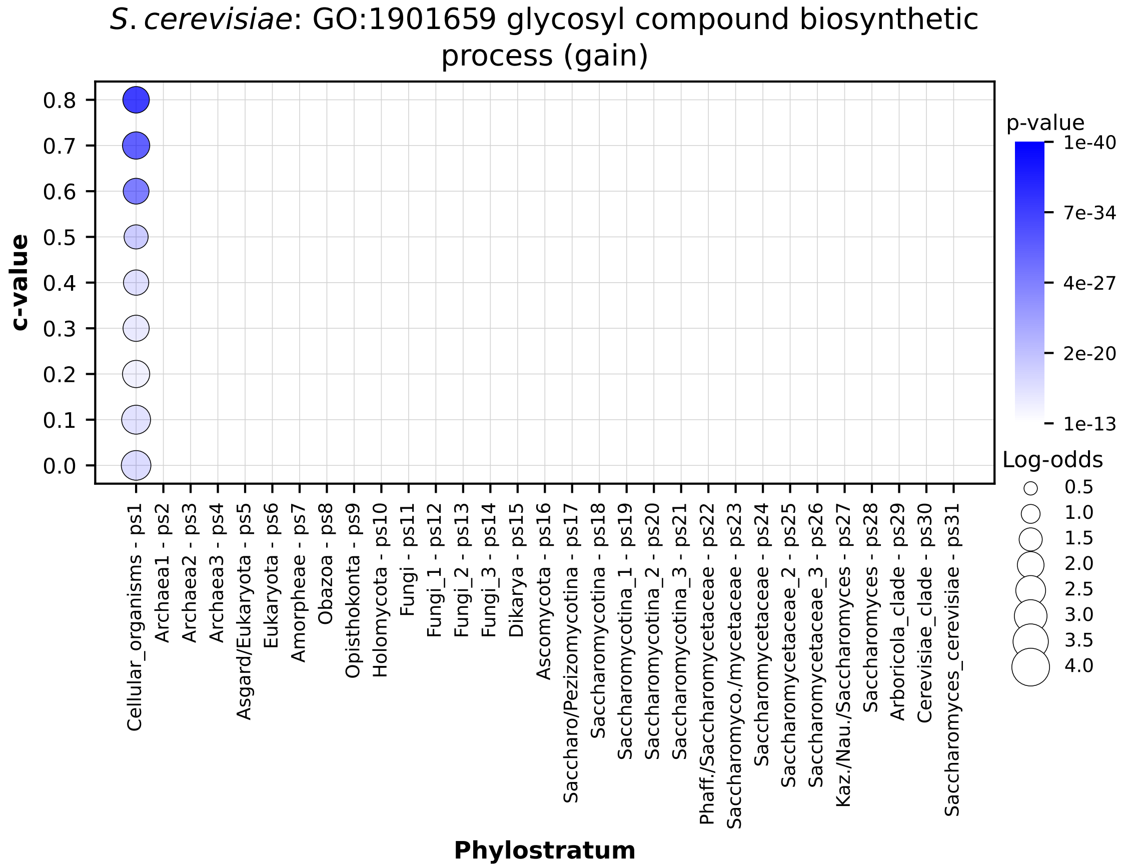

S. cerevisiae: GO:1901659 glycosyl compound biosynthetic process (gain)
c-value
0.8
0.7
0.6
0.5
0.4
0.3
0.2
0.1
0.0
p-value
1e-40
7e-34
4e-27
2e-20
1e-13
Log-odds
0.5
1.0
1.5
2.0
2.5
3.0
3.5
4.0
Cellular_organisms - ps1
Archaea1 - ps2
Archaea2 - ps3
Archaea3 - ps4
Asgard/Eukaryota - ps5
Eukaryota - ps6
Amorpheae - ps7
Obazoa - ps8
Opisthokonta - ps9
Holomycota - ps10
Fungi - ps11
Fungi_1 - ps12
Fungi_2 - ps13
Fungi_3 - ps14
Dikarya - ps15
Ascomycota - ps16
Saccharo/Pezizomycotina - ps17
Saccharomycotina - ps18
Saccharomycotina_1 - ps19
Saccharomycotina_2 - ps20
Saccharomycotina_3 - ps21
Phaff./Saccharomycetaceae - ps22
Saccharomyco./mycetaceae - ps23
Saccharomycetaceae - ps24
Saccharomycetaceae_2 - ps25
Saccharomycetaceae_3 - ps26
Kaz./Nau./Saccharomyces - ps27
Saccharomyces - ps28
Arboricola_clade - ps29
Cerevisiae_clade - ps30
Saccharomyces_cerevisiae - ps31
Phylostratum

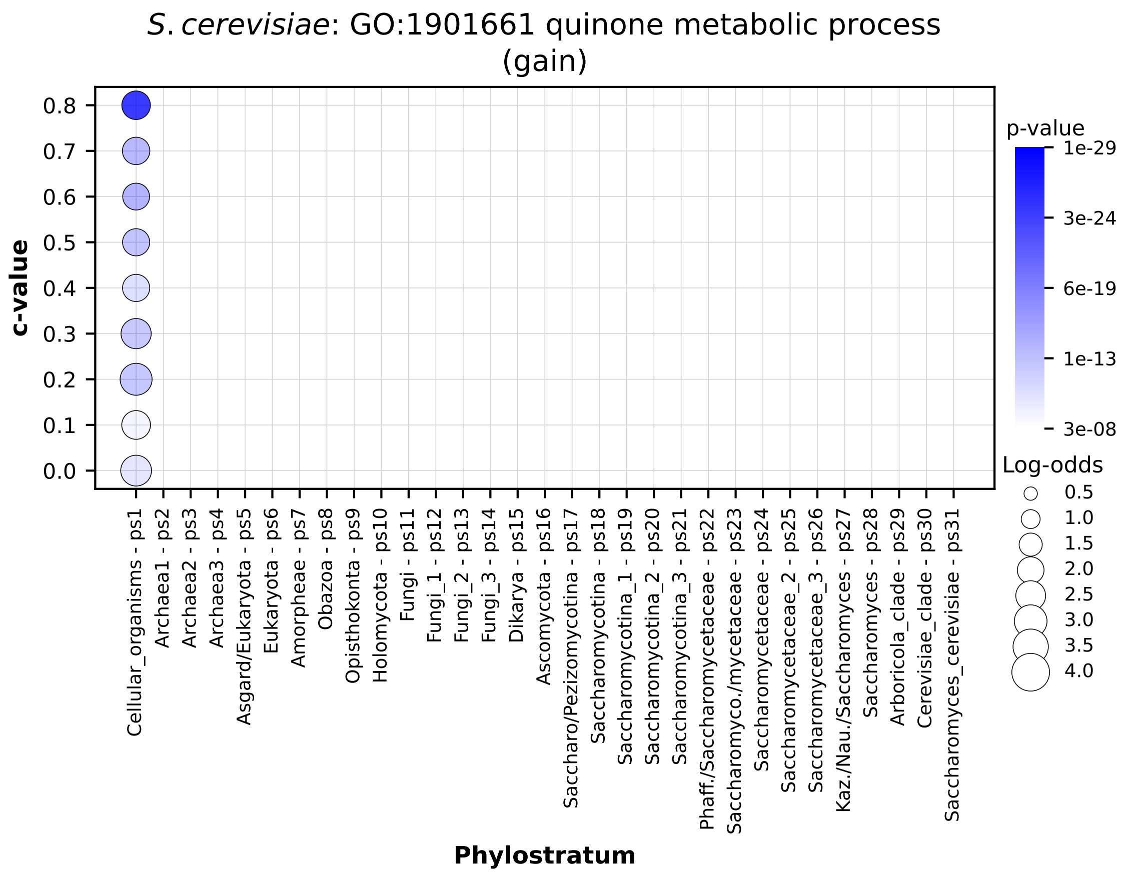

S. cerevisiae: GO:1901661 quinone metabolic process (gain)
c-value
0.8
0.7
0.6
0.5
0.4
0.3
0.2
0.1
0.0
p-value
1e-29
3e-24
6e-19
1e-13
3e-08
Log-odds
0.5
1.0
1.5
2.0
2.5
3.0
3.5
4.0
Cellular_organisms - ps1
Archaea1 - ps2
Archaea2 - ps3
Archaea3 - ps4
Asgard/Eukaryota - ps5
Eukaryota - ps6
Amorpheae - ps7
Obazoa - ps8
Opisthokonta - ps9
Holomycota - ps10
Fungi - ps11
Fungi_1 - ps12
Fungi_2 - ps13
Fungi_3 - ps14
Dikarya - ps15
Ascomycota - ps16
Saccharo/Pezizomycotina - ps17
Saccharomycotina - ps18
Saccharomycotina_1 - ps19
Saccharomycotina_2 - ps20
Saccharomycotina_3 - ps21
Phaff./Saccharomycetaceae - ps22
Saccharomyco./mycetaceae - ps23
Saccharomycetaceae - ps24
Saccharomycetaceae_2 - ps25
Saccharomycetaceae_3 - ps26
Kaz./Nau./Saccharomyces - ps27
Saccharomyces - ps28
Arboricola_clade - ps29
Cerevisiae_clade - ps30
Saccharomyces_cerevisiae - ps31
Phylostratum

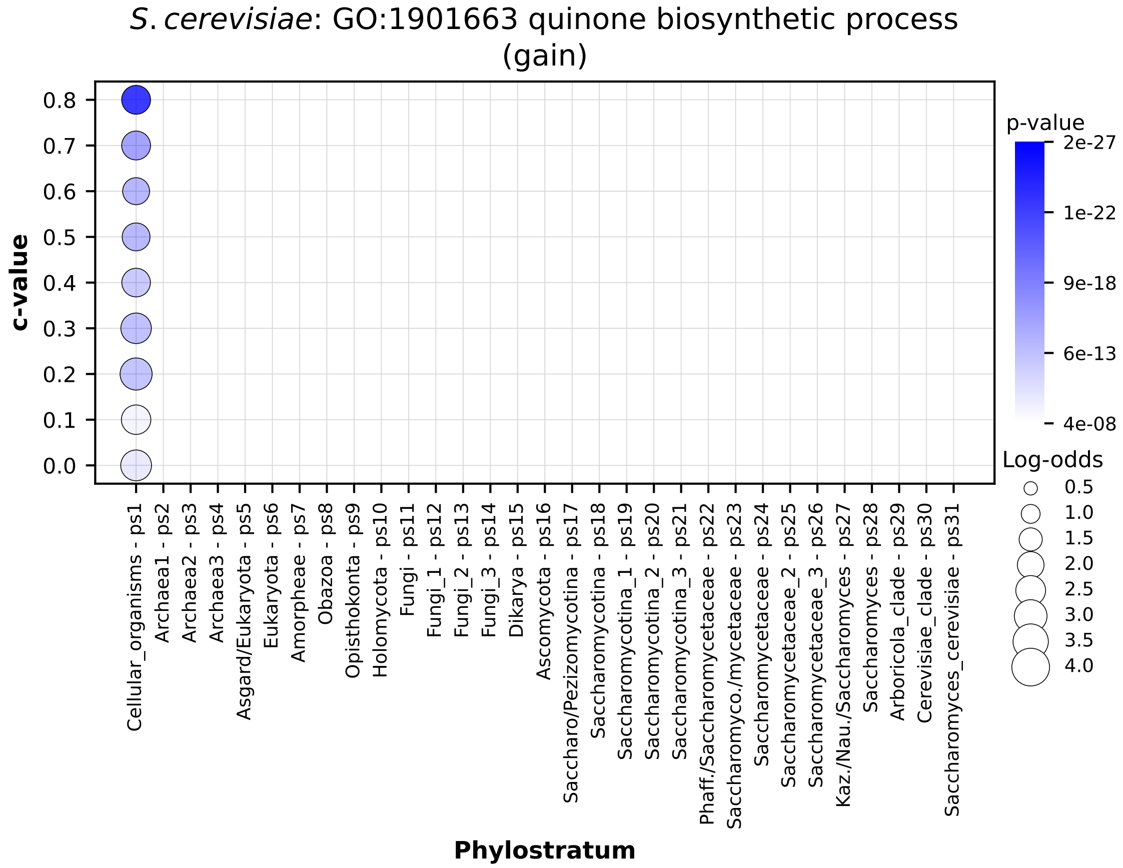
S. cerevisiae: GO:1901663 quinone biosynthetic process
(gain)
c-value
0.0
0.1
0.2
0.3
0.4
0.5
0.6
0.7
0.8
Phylostratum
Cellular_organisms - ps1
Archaea1 - ps2
Archaea2 - ps3
Archaea3 - ps4
Asgard/Eukaryota - ps5
Eukaryota - ps6
Amorpheae - ps7
Obazoa - ps8
Opisthokonta - ps9
Holomycota - ps10
Fungi - ps11
Fungi_1 - ps12
Fungi_2 - ps13
Fungi_3 - ps14
Dikarya - ps15
Ascomycota - ps16
Saccharo/Pezizomycotina - ps17
Saccharomycotina - ps18
Saccharomycotina_1 - ps19
Saccharomycotina_2 - ps20
Saccharomycotina_3 - ps21
Phaff./Saccharomycetaceae - ps22
Saccharomyco./mycetaceae - ps23
Saccharomycetaceae - ps24
Saccharomycetaceae_2 - ps25
Saccharomycetaceae_3 - ps26
Kaz./Nau./Saccharomyces - ps27
Saccharomyces - ps28
Arboricola_clade - ps29
Cerevisiae_clade - ps30
Saccharomyces_cerevisiae - ps31
p-value
2e-27
1e-22
9e-18
6e-13
4e-08
Log-odds
0.5
1.0
1.5
2.0
2.5
3.0
3.5
4.0

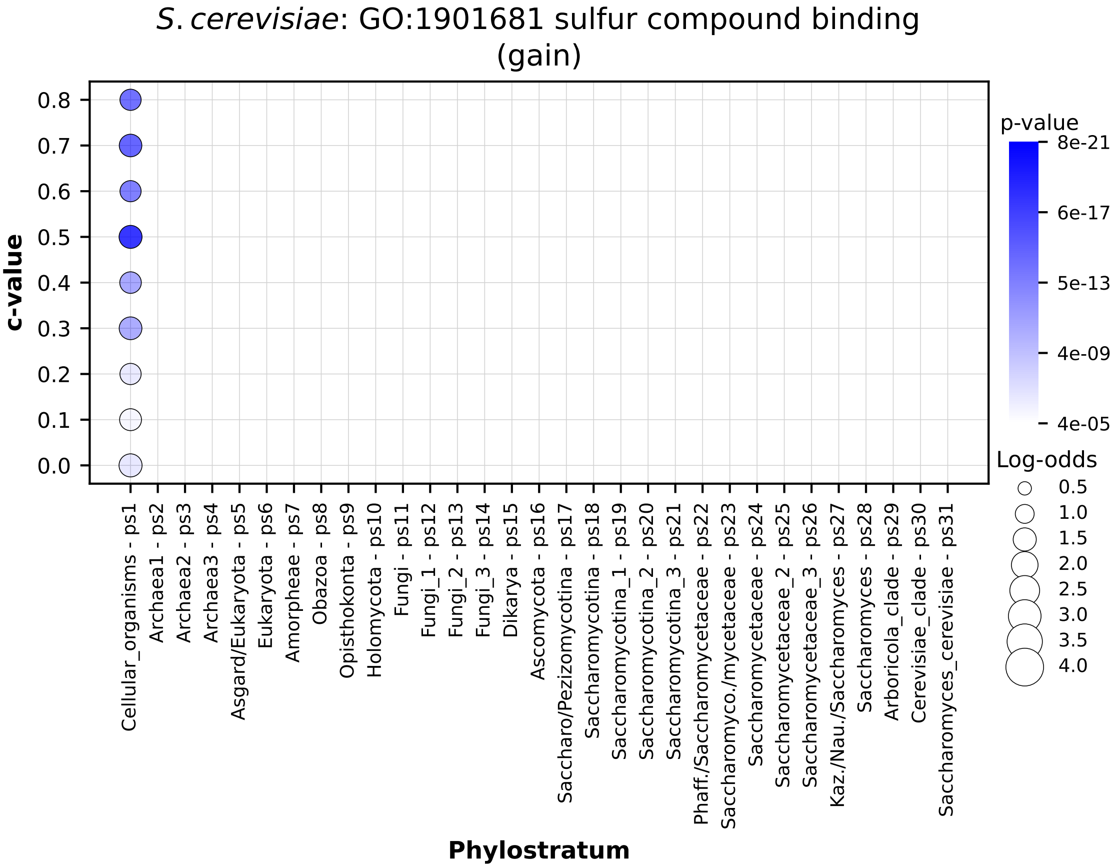
S. cerevisiae: GO:1901681 sulfur compound binding
(gain)
c-value
0.8
0.7
0.6
0.5
0.4
0.3
0.2
0.1
0.0
p-value
8e-21
6e-17
5e-13
4e-09
4e-05
Log-odds
0.5
1.0
1.5
2.0
2.5
3.0
3.5
4.0
Cellular_organisms - ps1
Archaea1 - ps2
Archaea2 - ps3
Archaea3 - ps4
Asgard/Eukaryota - ps5
Eukaryota - ps6
Amorpheae - ps7
Obazoa - ps8
Opisthokonta - ps9
Holomycota - ps10
Fungi - ps11
Fungi_1 - ps12
Fungi_2 - ps13
Fungi_3 - ps14
Dikarya - ps15
Ascomycota - ps16
Saccharo/Pezizomycotina - ps17
Saccharomycotina - ps18
Saccharomycotina_1 - ps19
Saccharomycotina_2 - ps20
Saccharomycotina_3 - ps21
Phaff./Saccharomycetaceae - ps22
Saccharomyco./mycetaceae - ps23
Saccharomycetaceae - ps24
Saccharomycetaceae_2 - ps25
Saccharomycetaceae_3 - ps26
Kaz./Nau./Saccharomyces - ps27
Saccharomyces - ps28
Arboricola_clade - ps29
Cerevisiae_clade - ps30
Saccharomyces_cerevisiae - ps31
Phylostratum

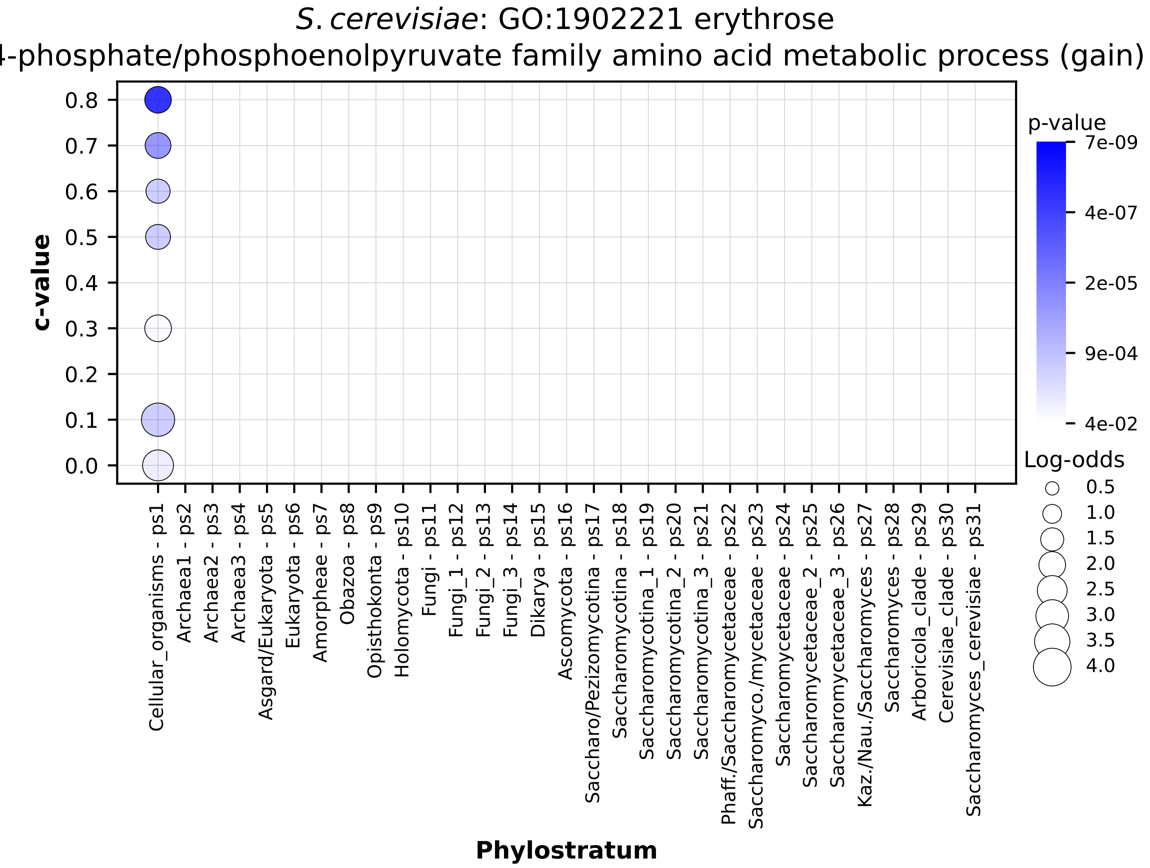

S. cerevisiae: GO:1902221 erythrose
4-phosphate/phosphoenolpyruvate family amino acid metabolic process (gain)
c-value
0.8
0.7
0.6
0.5
0.4
0.3
0.2
0.1
0.0
p-value
7e-09
4e-07
2e-05
9e-04
4e-02
Log-odds
0.5
1.0
1.5
2.0
2.5
3.0
3.5
4.0
Cellular_organisms - ps1
Archaea1 - ps2
Archaea2 - ps3
Archaea3 - ps4
Asgard/Eukaryota - ps5
Eukaryota - ps6
Amorpheae - ps7
Obazoa - ps8
Opisthokonta - ps9
Holomycota - ps10
Fungi - ps11
Fungi_1 - ps12
Fungi_2 - ps13
Fungi_3 - ps14
Dikarya - ps15
Ascomycota - ps16
Saccharo/Pezizomycotina - ps17
Saccharomycotina - ps18
Saccharomycotina_1 - ps19
Saccharomycotina_2 - ps20
Saccharomycotina_3 - ps21
Phaff./Saccharomycetaceae - ps22
Saccharomyco./mycetaceae - ps23
Saccharomycetaceae - ps24
Saccharomycetaceae_2 - ps25
Saccharomycetaceae_3 - ps26
Kaz./Nau./Saccharomyces - ps27
Saccharomyces - ps28
Arboricola_clade - ps29
Cerevisiae_clade - ps30
Saccharomyces_cerevisiae - ps31
Phylostratum

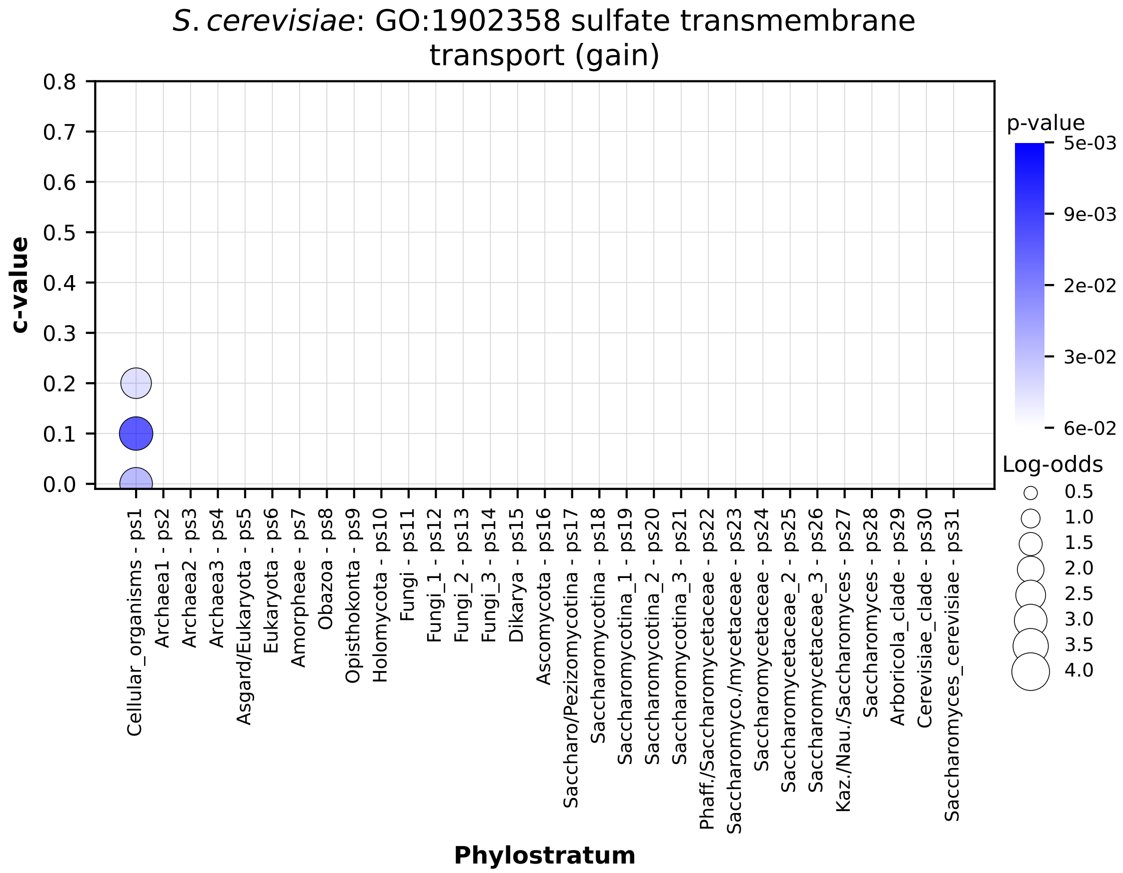

S. cerevisiae: GO:1902358 sulfate transmembrane transport (gain)
c-value
0.8
0.7
0.6
0.5
0.4
0.3
0.2
0.1
0.0
p-value
5e-03
9e-03
2e-02
3e-02
6e-02
Log-odds
0.5
1.0
1.5
2.0
2.5
3.0
3.5
4.0
Cellular_organisms - ps1
Archaea1 - ps2
Archaea2 - ps3
Archaea3 - ps4
Asgard/Eukaryota - ps5
Eukaryota - ps6
Amorpheae - ps7
Obazoa - ps8
Opisthokonta - ps9
Holomycota - ps10
Fungi - ps11
Fungi_1 - ps12
Fungi_2 - ps13
Fungi_3 - ps14
Dikarya - ps15
Ascomycota - ps16
Saccharo/Pezizomycotina - ps17
Saccharomycotina - ps18
Saccharomycotina_1 - ps19
Saccharomycotina_2 - ps20
Saccharomycotina_3 - ps21
Phaff./Saccharomycetaceae - ps22
Saccharomyco./mycetaceae - ps23
Saccharomycetaceae - ps24
Saccharomycetaceae_2 - ps25
Saccharomycetaceae_3 - ps26
Kaz./Nau./Saccharomyces - ps27
Saccharomyces - ps28
Arboricola_clade - ps29
Cerevisiae_clade - ps30
Saccharomyces_cerevisiae - ps31
Phylostratum

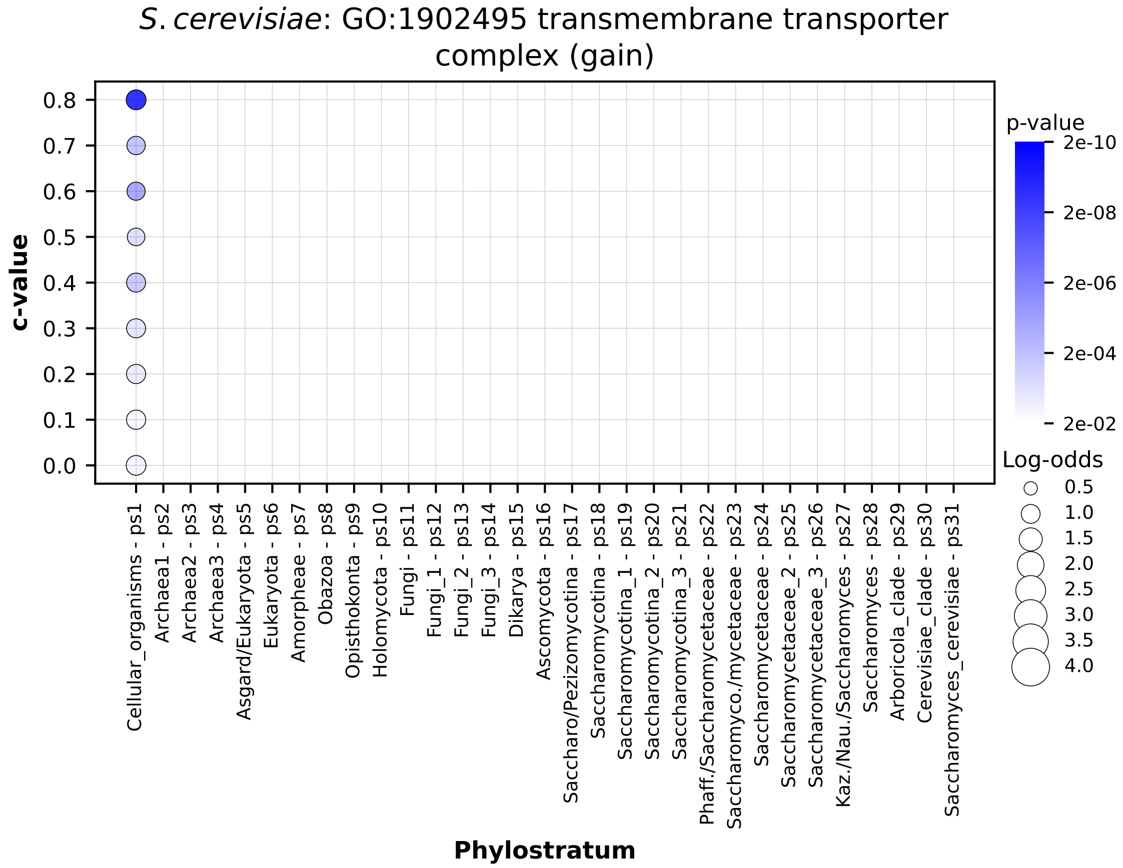

S. cerevisiae: GO:1902495 transmembrane transporter complex (gain)
c-value
0.8
0.7
0.6
0.5
0.4
0.3
0.2
0.1
0.0
Phylostratum
Cellular_organisms - ps1
Archaea1 - ps2
Archaea2 - ps3
Archaea3 - ps4
Asgard/Eukaryota - ps5
Eukaryota - ps6
Amorpheae - ps7
Obazoa - ps8
Opisthokonta - ps9
Holomycota - ps10
Fungi - ps11
Fungi_1 - ps12
Fungi_2 - ps13
Fungi_3 - ps14
Dikarya - ps15
Ascomycota - ps16
Saccharo/Pezizomycotina - ps17
Saccharomycotina - ps18
Saccharomycotina_1 - ps19
Saccharomycotina_2 - ps20
Saccharomycotina_3 - ps21
Phaff./Saccharomycetaceae - ps22
Saccharomyco./mycetaceae - ps23
Saccharomycetaceae - ps24
Saccharomycetaceae_2 - ps25
Saccharomycetaceae_3 - ps26
Kaz./Nau./Saccharomyces - ps27
Saccharomyces - ps28
Arboricola_clade - ps29
Cerevisiae_clade - ps30
Saccharomyces_cerevisiae - ps31
p-value
2e-10
2e-08
2e-06
2e-04
2e-02
Log-odds
0.5
1.0
1.5
2.0
2.5
3.0
3.5
4.0

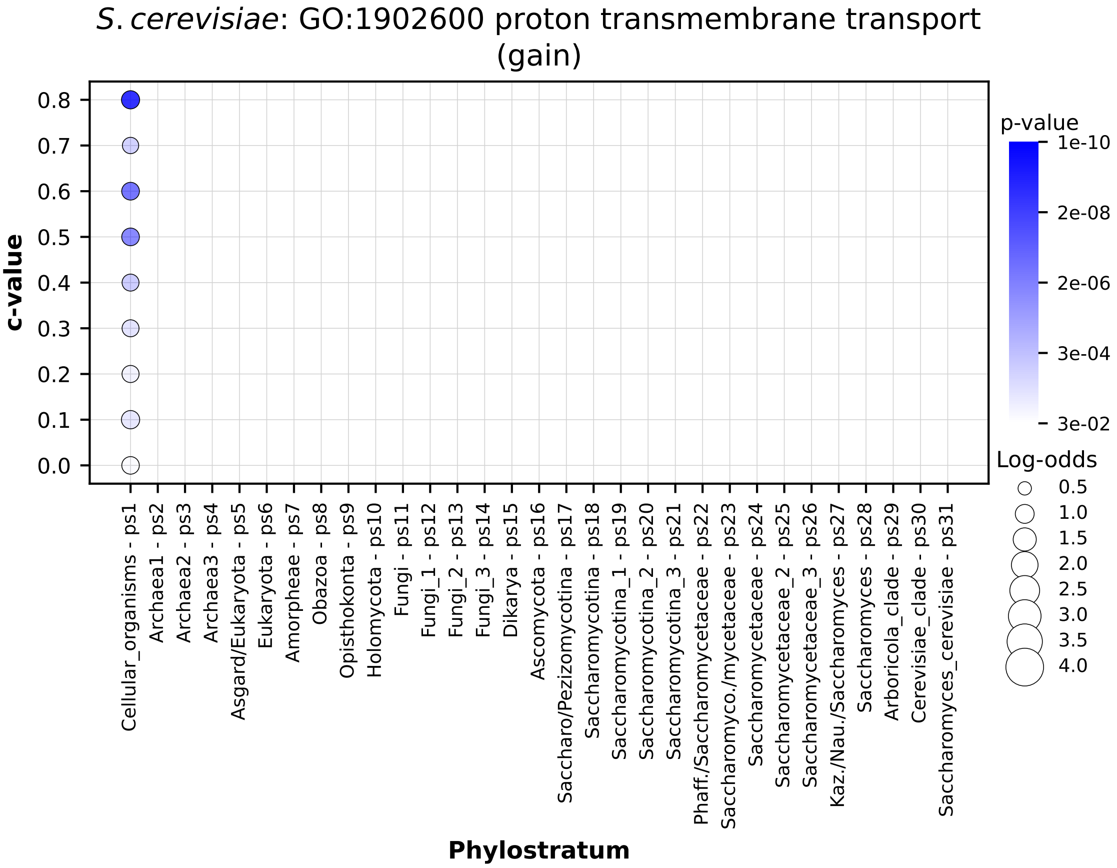

S. cerevisiae: GO:1902600 proton transmembrane transport
(gain)
c-value
0.8
0.7
0.6
0.5
0.4
0.3
0.2
0.1
0.0
p-value
1e-10
2e-08
2e-06
3e-04
3e-02
Log-odds
0.5
1.0
1.5
2.0
2.5
3.0
3.5
4.0
Cellular_organisms - ps1
Archaea1 - ps2
Archaea2 - ps3
Archaea3 - ps4
Asgard/Eukaryota - ps5
Eukaryota - ps6
Amorpheae - ps7
Obazoa - ps8
Opisthokonta - ps9
Holomycota - ps10
Fungi - ps11
Fungi_1 - ps12
Fungi_2 - ps13
Fungi_3 - ps14
Dikarya - ps15
Ascomycota - ps16
Saccharo/Pezizomycotina - ps17
Saccharomycotina - ps18
Saccharomycotina_1 - ps19
Saccharomycotina_2 - ps20
Saccharomycotina_3 - ps21
Phaff./Saccharomycetaceae - ps22
Saccharomyco./mycetaceae - ps23
Saccharomycetaceae - ps24
Saccharomycetaceae_2 - ps25
Saccharomycetaceae_3 - ps26
Kaz./Nau./Saccharomyces - ps27
Saccharomyces - ps28
Arboricola_clade - ps29
Cerevisiae_clade - ps30
Saccharomyces_cerevisiae - ps31
Phylostratum

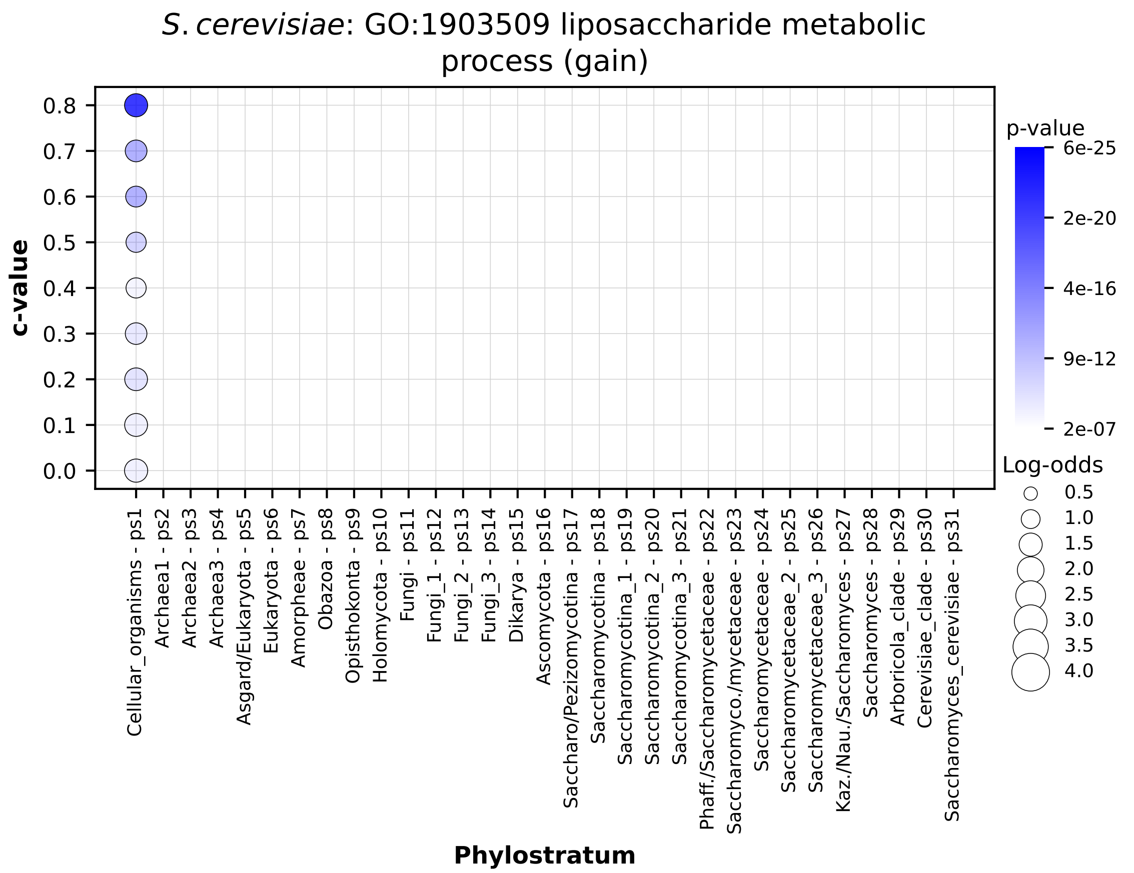

S. cerevisiae: GO:1903509 liposaccharide metabolic process (gain)
c-value
0.8
0.7
0.6
0.5
0.4
0.3
0.2
0.1
0.0
p-value
6e-25
2e-20
4e-16
9e-12
2e-07
Log-odds
0.5
1.0
1.5
2.0
2.5
3.0
3.5
4.0
Cellular_organisms - ps1
Archaea1 - ps2
Archaea2 - ps3
Archaea3 - ps4
Asgard/Eukaryota - ps5
Eukaryota - ps6
Amorpheae - ps7
Obazoa - ps8
Opisthokonta - ps9
Holomycota - ps10
Fungi - ps11
Fungi_1 - ps12
Fungi_2 - ps13
Fungi_3 - ps14
Dikarya - ps15
Ascomycota - ps16
Saccharo/Pezizomycotina - ps17
Saccharomycotina - ps18
Saccharomycotina_1 - ps19
Saccharomycotina_2 - ps20
Saccharomycotina_3 - ps21
Phaff./Saccharomycetaceae - ps22
Saccharomyco./mycetaceae - ps23
Saccharomycetaceae - ps24
Saccharomycetaceae_2 - ps25
Saccharomycetaceae_3 - ps26
Kaz./Nau./Saccharomyces - ps27
Saccharomyces - ps28
Arboricola_clade - ps29
Cerevisiae_clade - ps30
Saccharomyces_cerevisiae - ps31
Phylostratum

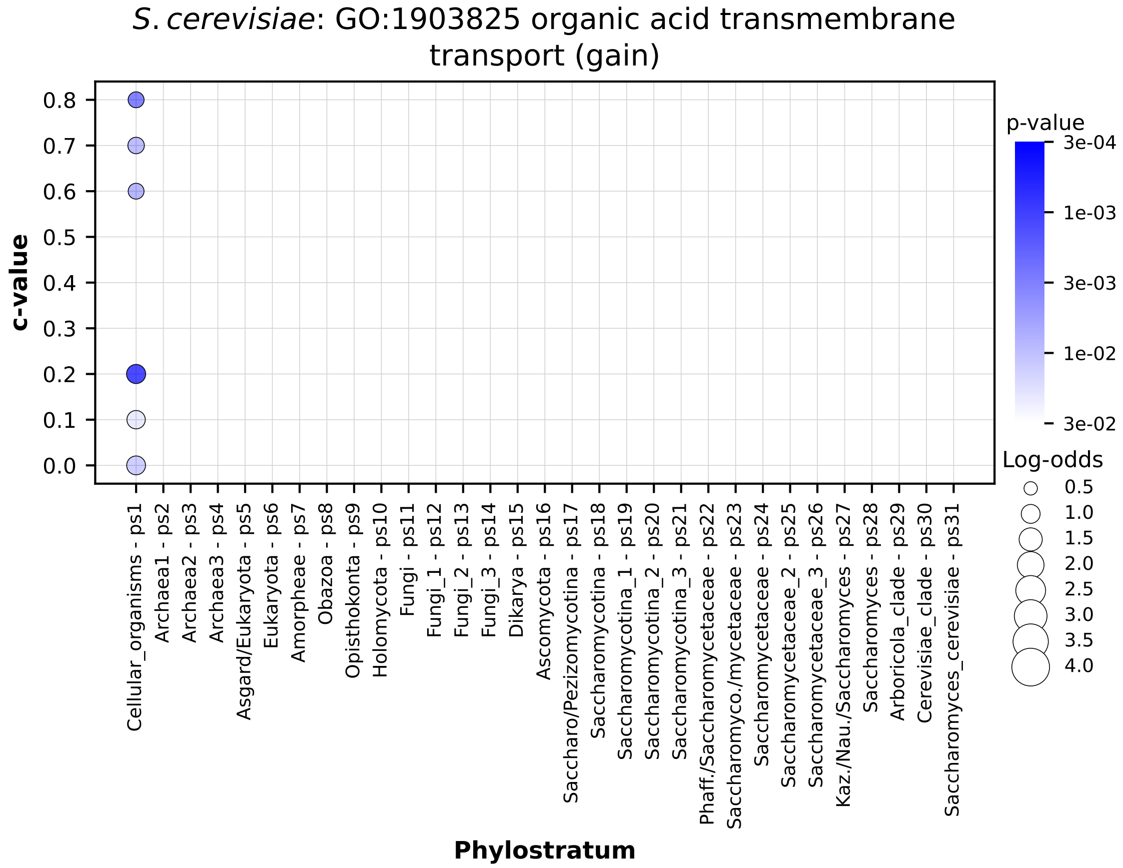
S. cerevisiae: GO:1903825 organic acid transmembrane transport (gain)
c-value
0.8
0.7
0.6
0.5
0.4
0.3
0.2
0.1
0.0
Phylostratum
Cellular_organisms - ps1
Archaea1 - ps2
Archaea2 - ps3
Archaea3 - ps4
Asgard/Eukaryota - ps5
Eukaryota - ps6
Amorpheae - ps7
Obazoa - ps8
Opisthokonta - ps9
Holomycota - ps10
Fungi - ps11
Fungi_1 - ps12
Fungi_2 - ps13
Fungi_3 - ps14
Dikarya - ps15
Ascomycota - ps16
Saccharo/Pezizomycotina - ps17
Saccharomycotina - ps18
Saccharomycotina_1 - ps19
Saccharomycotina_2 - ps20
Saccharomycotina_3 - ps21
Phaff./Saccharomycetaceae - ps22
Saccharomyco./mycetaceae - ps23
Saccharomycetaceae - ps24
Saccharomycetaceae_2 - ps25
Saccharomycetaceae_3 - ps26
Kaz./Nau./Saccharomyces - ps27
Saccharomyces - ps28
Arboricola_clade - ps29
Cerevisiae_clade - ps30
Saccharomyces_cerevisiae - ps31
p-value
3e-04
1e-03
3e-03
1e-02
3e-02
Log-odds
0.5
1.0
1.5
2.0
2.5
3.0
3.5
4.0

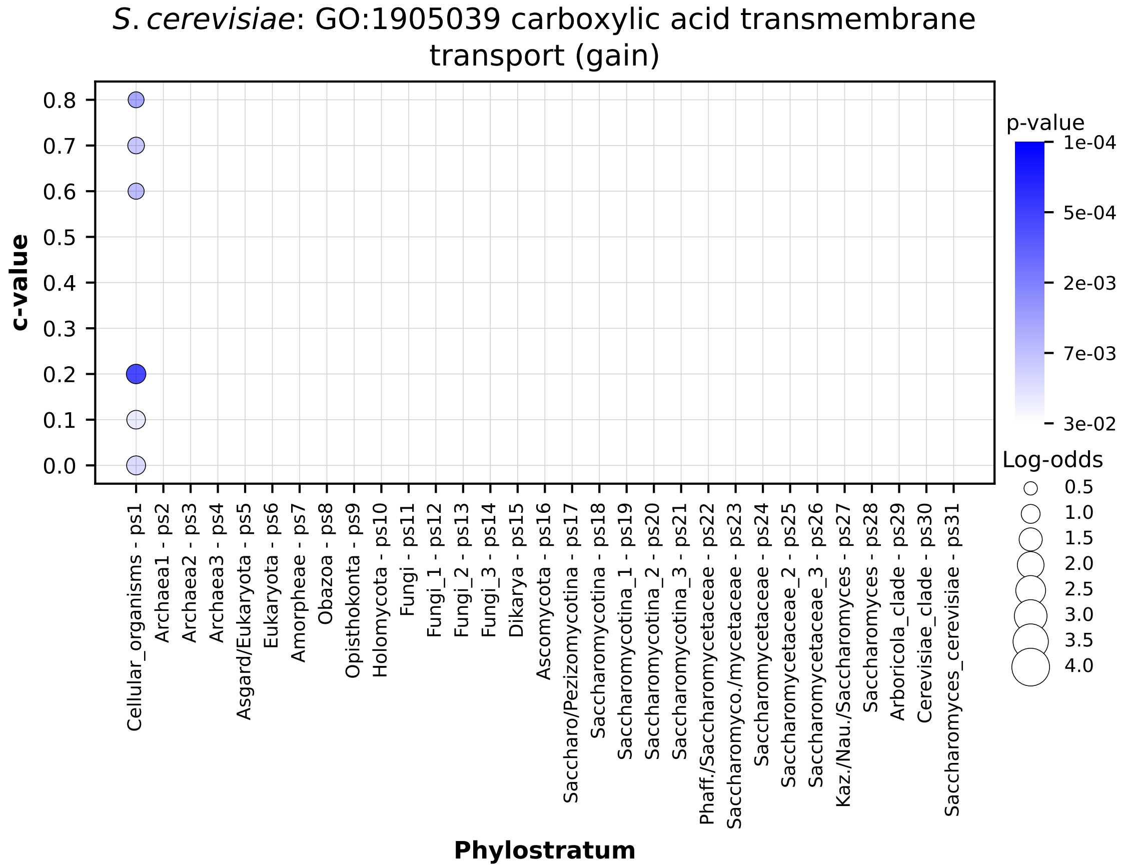

S. cerevisiae: GO:1905039 carboxylic acid transmembrane transport (gain)
c-value
0.8
0.7
0.6
0.5
0.4
0.3
0.2
0.1
0.0
p-value
1e-04
5e-04
2e-03
7e-03
3e-02
Log-odds
0.5
1.0
1.5
2.0
2.5
3.0
3.5
4.0
Cellular_organisms - ps1
Archaea1 - ps2
Archaea2 - ps3
Archaea3 - ps4
Asgard/Eukaryota - ps5
Eukaryota - ps6
Amorpheae - ps7
Obazoa - ps8
Opisthokonta - ps9
Holomycota - ps10
Fungi - ps11
Fungi_1 - ps12
Fungi_2 - ps13
Fungi_3 - ps14
Dikarya - ps15
Ascomycota - ps16
Saccharo/Pezizomycotina - ps17
Saccharomycotina - ps18
Saccharomycotina_1 - ps19
Saccharomycotina_2 - ps20
Saccharomycotina_3 - ps21
Phaff./Saccharomycetaceae - ps22
Saccharomyco./mycetaceae - ps23
Saccharomycetaceae - ps24
Saccharomycetaceae_2 - ps25
Saccharomycetaceae_3 - ps26
Kaz./Nau./Saccharomyces - ps27
Saccharomyces - ps28
Arboricola_clade - ps29
Cerevisiae_clade - ps30
Saccharomyces_cerevisiae - ps31
Phylostratum

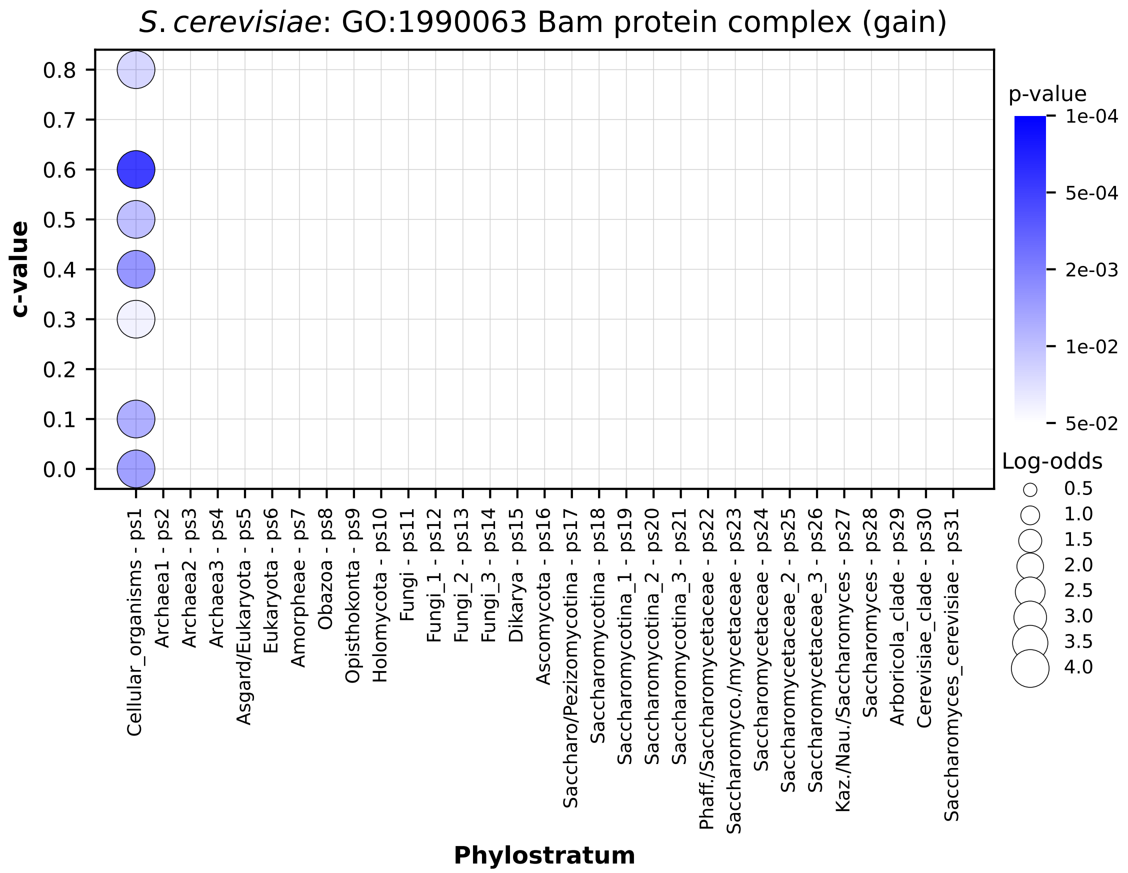

S. cerevisiae: GO:1990063 Bam protein complex (gain)
c-value
0.8
0.7
0.6
0.5
0.4
0.3
0.2
0.1
0.0
p-value
1e-04
5e-04
2e-03
1e-02
5e-02
Log-odds
0.5
1.0
1.5
2.0
2.5
3.0
3.5
4.0
Cellular_organisms - ps1
Archaea1 - ps2
Archaea2 - ps3
Archaea3 - ps4
Asgard/Eukaryota - ps5
Eukaryota - ps6
Amorpheae - ps7
Obazoa - ps8
Opisthokonta - ps9
Holomycota - ps10
Fungi - ps11
Fungi_1 - ps12
Fungi_2 - ps13
Fungi_3 - ps14
Dikarya - ps15
Ascomycota - ps16
Saccharo/Pezizomycotina - ps17
Saccharomycotina - ps18
Saccharomycotina_1 - ps19
Saccharomycotina_2 - ps20
Saccharomycotina_3 - ps21
Phaff./Saccharomycetaceae - ps22
Saccharomyco./mycetaceae - ps23
Saccharomycetaceae - ps24
Saccharomycetaceae_2 - ps25
Saccharomycetaceae_3 - ps26
Kaz./Nau./Saccharomyces - ps27
Saccharomyces - ps28
Arboricola_clade - ps29
Cerevisiae_clade - ps30
Saccharomyces_cerevisiae - ps31
Phylostratum

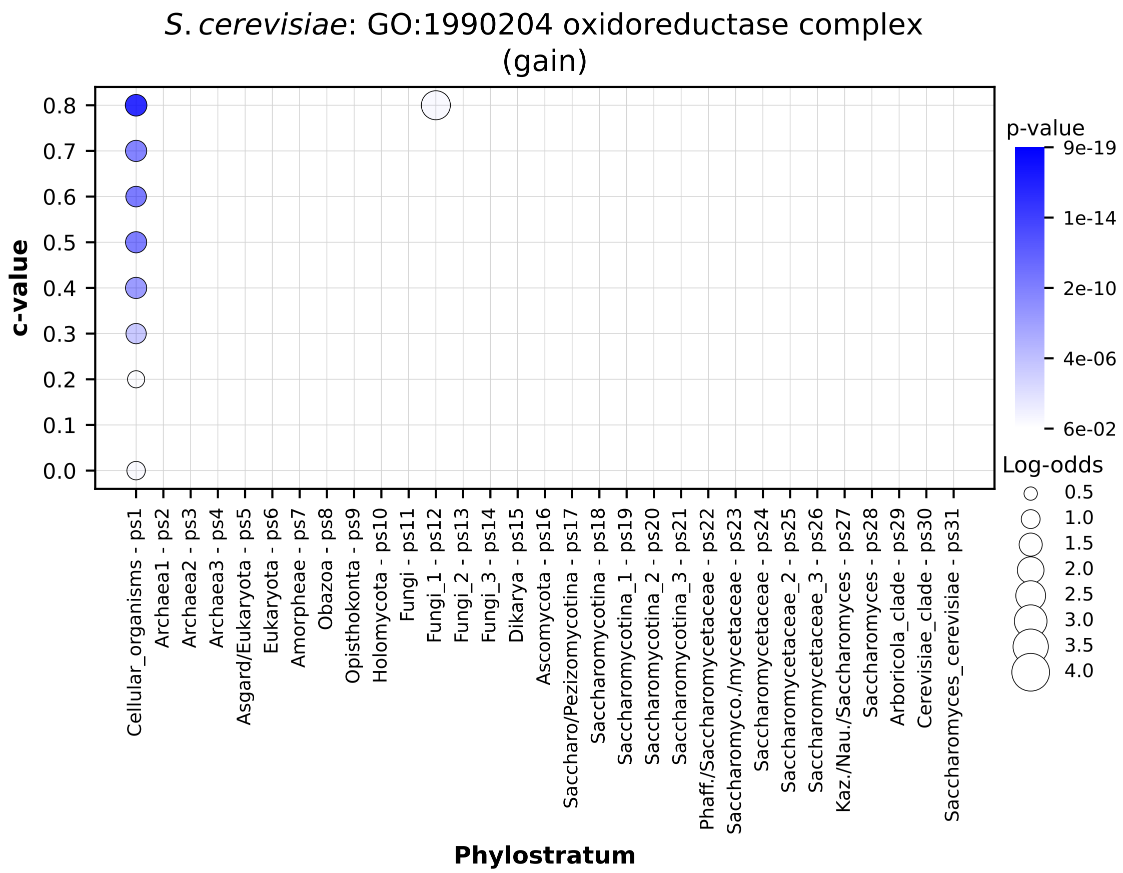

S. cerevisiae: GO:1990204 oxidoreductase complex
(gain)
c-value
0.8
0.7
0.6
0.5
0.4
0.3
0.2
0.1
0.0
p-value
9e-19
1e-14
2e-10
4e-06
6e-02
Log-odds
0.5
1.0
1.5
2.0
2.5
3.0
3.5
4.0
Cellular_organisms - ps1
Archaea1 - ps2
Archaea2 - ps3
Archaea3 - ps4
Asgard/Eukaryota - ps5
Eukaryota - ps6
Amorpheae - ps7
Obazoa - ps8
Opisthokonta - ps9
Holomycota - ps10
Fungi - ps11
Fungi_1 - ps12
Fungi_2 - ps13
Fungi_3 - ps14
Dikarya - ps15
Ascomycota - ps16
Saccharo/Pezizomycotina - ps17
Saccharomycotina - ps18
Saccharomycotina_1 - ps19
Saccharomycotina_2 - ps20
Saccharomycotina_3 - ps21
Phaff./Saccharomycetaceae - ps22
Saccharomyco./mycetaceae - ps23
Saccharomycetaceae - ps24
Saccharomycetaceae_2 - ps25
Saccharomycetaceae_3 - ps26
Kaz./Nau./Saccharomyces - ps27
Saccharomyces - ps28
Arboricola_clade - ps29
Cerevisiae_clade - ps30
Saccharomyces_cerevisiae - ps31
Phylostratum

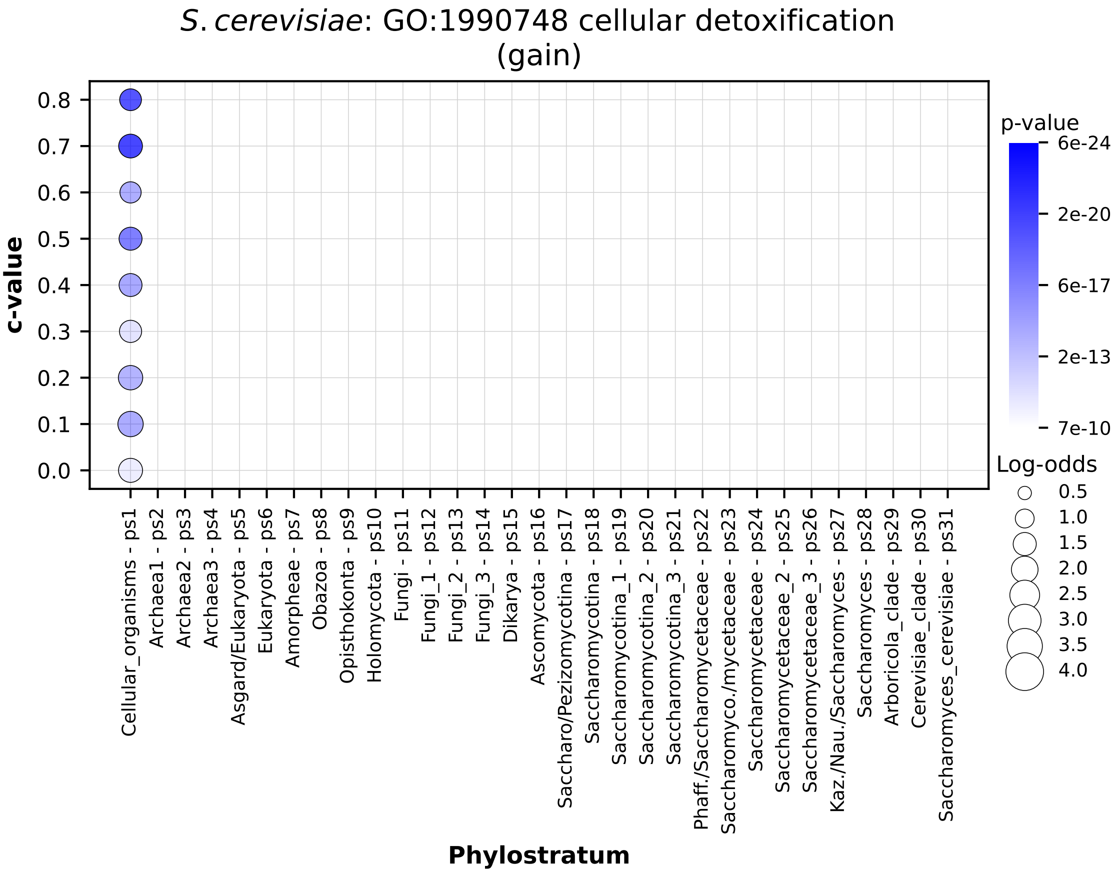

S. cerevisiae: GO:1990748 cellular detoxification
(gain)
c-value
0.0
0.1
0.2
0.3
0.4
0.5
0.6
0.7
0.8
Phylostratum
Cellular_organisms - ps1
Archaea1 - ps2
Archaea2 - ps3
Archaea3 - ps4
Asgard/Eukaryota - ps5
Eukaryota - ps6
Amorpheae - ps7
Obazoa - ps8
Opisthokonta - ps9
Holomycota - ps10
Fungi - ps11
Fungi_1 - ps12
Fungi_2 - ps13
Fungi_3 - ps14
Dikarya - ps15
Ascomycota - ps16
Saccharo/Pezizomycotina - ps17
Saccharomycotina - ps18
Saccharomycotina_1 - ps19
Saccharomycotina_2 - ps20
Saccharomycotina_3 - ps21
Phaff./Saccharomycetaceae - ps22
Saccharomyco./mycetaceae - ps23
Saccharomycetaceae - ps24
Saccharomycetaceae_2 - ps25
Saccharomycetaceae_3 - ps26
Kaz./Nau./Saccharomyces - ps27
Saccharomyces - ps28
Arboricola_clade - ps29
Cerevisiae_clade - ps30
Saccharomyces_cerevisiae - ps31
p-value
6e-24
2e-20
6e-17
2e-13
7e-10
Log-odds
0.5
1.0
1.5
2.0
2.5
3.0
3.5
4.0

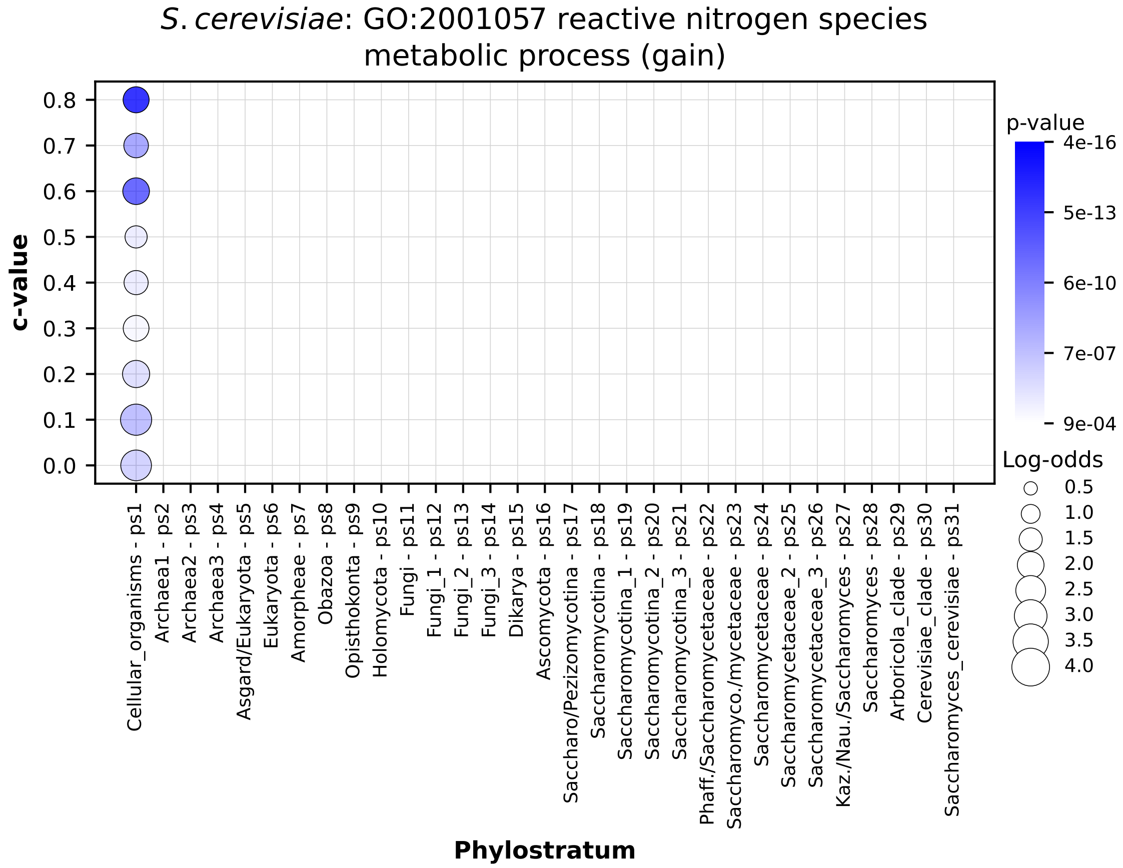

S. cerevisiae: GO:2001057 reactive nitrogen species metabolic process (gain)
c-value
0.8
0.7
0.6
0.5
0.4
0.3
0.2
0.1
0.0
p-value
4e-16
5e-13
6e-10
7e-07
9e-04
Log-odds
0.5
1.0
1.5
2.0
2.5
3.0
3.5
4.0
Cellular_organisms - ps1
Archaea1 - ps2
Archaea2 - ps3
Archaea3 - ps4
Asgard/Eukaryota - ps5
Eukaryota - ps6
Amorpheae - ps7
Obazoa - ps8
Opisthokonta - ps9
Holomycota - ps10
Fungi - ps11
Fungi_1 - ps12
Fungi_2 - ps13
Fungi_3 - ps14
Dikarya - ps15
Ascomycota - ps16
Saccharo/Pezizomycotina - ps17
Saccharomycotina - ps18
Saccharomycotina_1 - ps19
Saccharomycotina_2 - ps20
Saccharomycotina_3 - ps21
Phaff./Saccharomycetaceae - ps22
Saccharomyco./mycetaceae - ps23
Saccharomycetaceae - ps24
Saccharomycetaceae_2 - ps25
Saccharomycetaceae_3 - ps26
Kaz./Nau./Saccharomyces - ps27
Saccharomyces - ps28
Arboricola_clade - ps29
Cerevisiae_clade - ps30
Saccharomyces_cerevisiae - ps31
Phylostratum

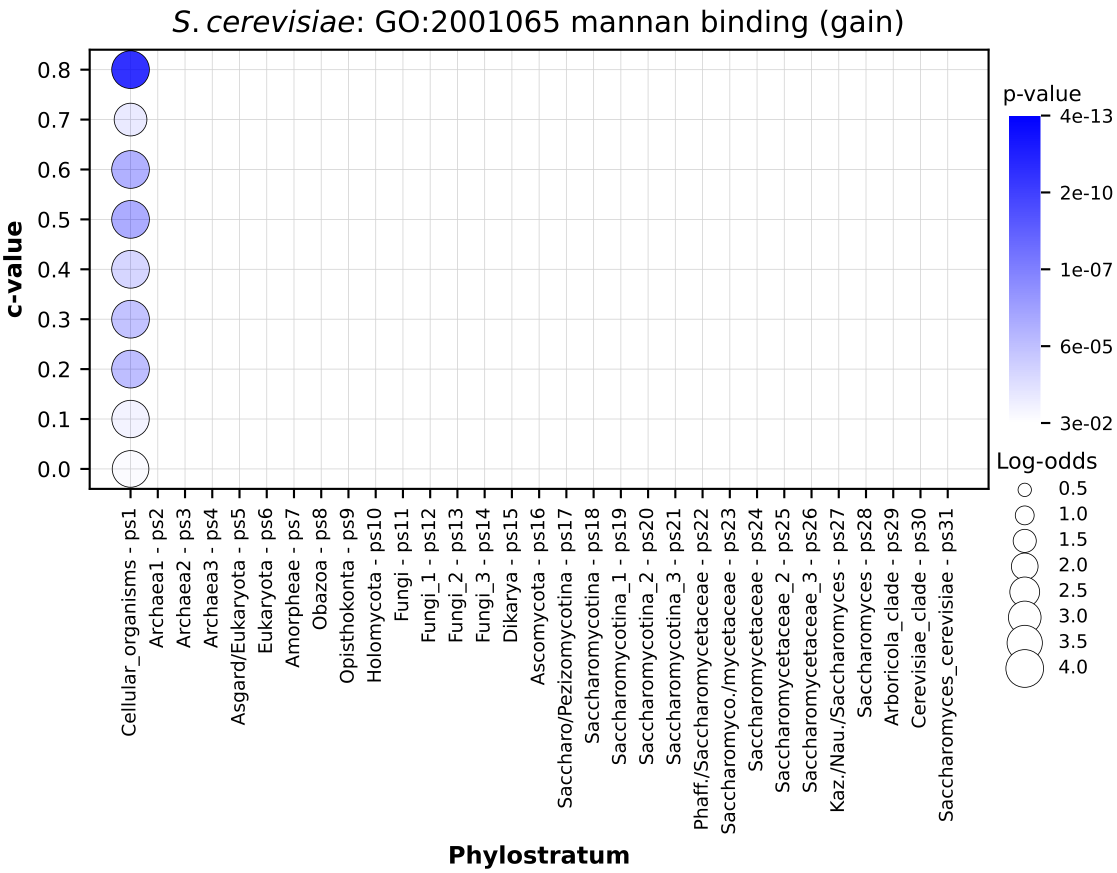

S. cerevisiae: GO:2001065 mannan binding (gain)
c-value
0.8
0.7
0.6
0.5
0.4
0.3
0.2
0.1
0.0
p-value
4e-13
2e-10
1e-07
6e-05
3e-02
Log-odds
0.5
1.0
1.5
2.0
2.5
3.0
3.5
4.0
Cellular_organisms - ps1
Archaea1 - ps2
Archaea2 - ps3
Archaea3 - ps4
Asgard/Eukaryota - ps5
Eukaryota - ps6
Amorpheae - ps7
Obazoa - ps8
Opisthokonta - ps9
Holomycota - ps10
Fungi - ps11
Fungi_1 - ps12
Fungi_2 - ps13
Fungi_3 - ps14
Dikarya - ps15
Ascomycota - ps16
Saccharo/Pezizomycotina - ps17
Saccharomycotina - ps18
Saccharomycotina_1 - ps19
Saccharomycotina_2 - ps20
Saccharomycotina_3 - ps21
Phaff./Saccharomycetaceae - ps22
Saccharomyco./mycetaceae - ps23
Saccharomycetaceae - ps24
Saccharomycetaceae_2 - ps25
Saccharomycetaceae_3 - ps26
Kaz./Nau./Saccharomyces - ps27
Saccharomyces - ps28
Arboricola_clade - ps29
Cerevisiae_clade - ps30
Saccharomyces_cerevisiae - ps31
Phylostratum

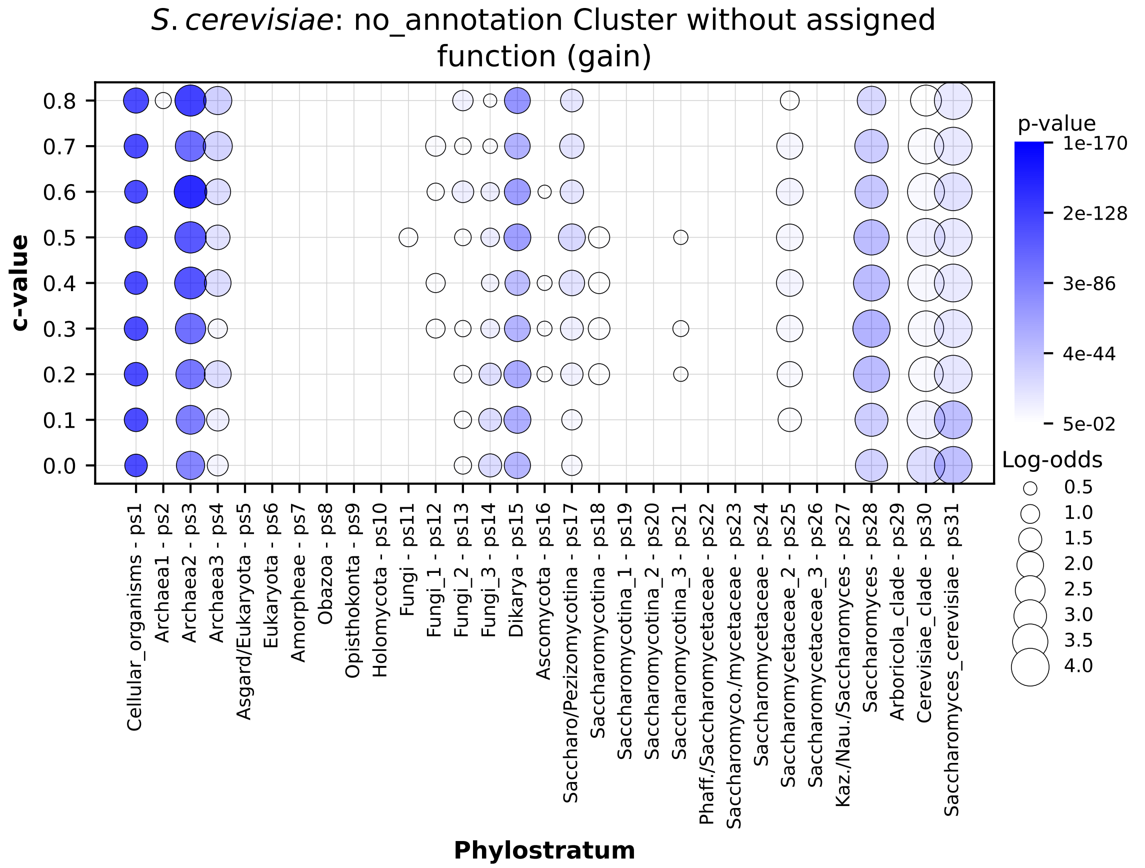
S. cerevisiae: no_annotation Cluster without assigned function (gain)
c-value
0.8
0.7
0.6
0.5
0.4
0.3
0.2
0.1
0.0
p-value
1e-170
2e-128
3e-86
4e-44
5e-02
Log-odds
0.5
1.0
1.5
2.0
2.5
3.0
3.5
4.0
Cellular_organisms - ps1
Archaea1 - ps2
Archaea2 - ps3
Archaea3 - ps4
Asgard/Eukaryota - ps5
Eukaryota - ps6
Amorpheae - ps7
Obazoa - ps8
Opisthokonta - ps9
Holomycota - ps10
Fungi - ps11
Fungi_1 - ps12
Fungi_2 - ps13
Fungi_3 - ps14
Dikarya - ps15
Ascomycota - ps16
Saccharo/Pezizomycotina - ps17
Saccharomycotina - ps18
Saccharomycotina_1 - ps19
Saccharomycotina_2 - ps20
Saccharomycotina_3 - ps21
Phaff./Saccharomycetaceae - ps22
Saccharomyco./mycetaceae - ps23
Saccharomycetaceae - ps24
Saccharomycetaceae_2 - ps25
Saccharomycetaceae_3 - ps26
Kaz./Nau./Saccharomyces - ps27
Saccharomyces - ps28
Arboricola_clade - ps29
Cerevisiae_clade - ps30
Saccharomyces_cerevisiae - ps31
Phylostratum

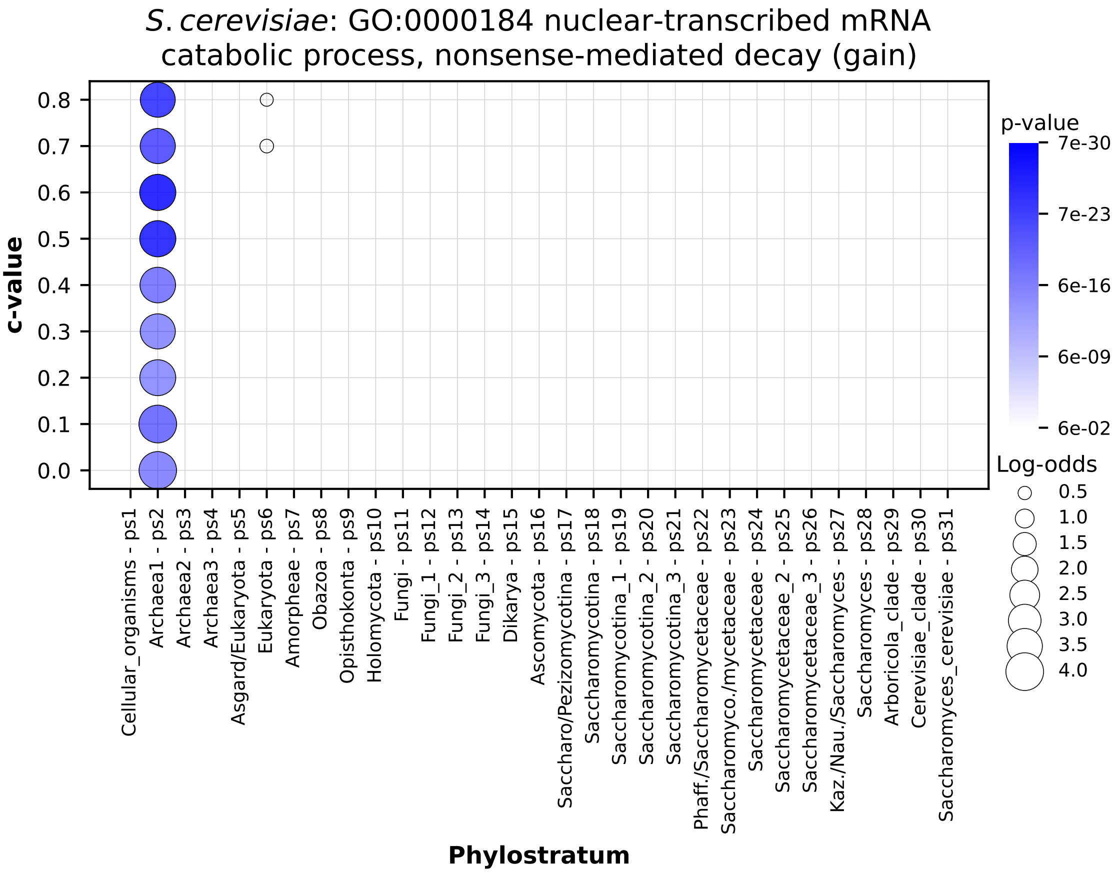

S. cerevisiae: GO:0000184 nuclear-transcribed mRNA catabolic process, nonsense-mediated decay (gain)
c-value
0.8
0.7
0.6
0.5
0.4
0.3
0.2
0.1
0.0
p-value
7e-30
7e-23
6e-16
6e-09
6e-02
Log-odds
0.5
1.0
1.5
2.0
2.5
3.0
3.5
4.0
Cellular_organisms - ps1
Archaea1 - ps2
Archaea2 - ps3
Archaea3 - ps4
Asgard/Eukaryota - ps5
Eukaryota - ps6
Amorpheae - ps7
Obazoa - ps8
Opisthokonta - ps9
Holomycota - ps10
Fungi - ps11
Fungi_1 - ps12
Fungi_2 - ps13
Fungi_3 - ps14
Dikarya - ps15
Ascomycota - ps16
Saccharo/Pezizomycotina - ps17
Saccharomycotina - ps18
Saccharomycotina_1 - ps19
Saccharomycotina_2 - ps20
Saccharomycotina_3 - ps21
Phaff./Saccharomycetaceae - ps22
Saccharomyco./mycetaceae - ps23
Saccharomycetaceae - ps24
Saccharomycetaceae_2 - ps25
Saccharomycetaceae_3 - ps26
Kaz./Nau./Saccharomyces - ps27
Saccharomyces - ps28
Arboricola_clade - ps29
Cerevisiae_clade - ps30
Saccharomyces_cerevisiae - ps31
Phylostratum

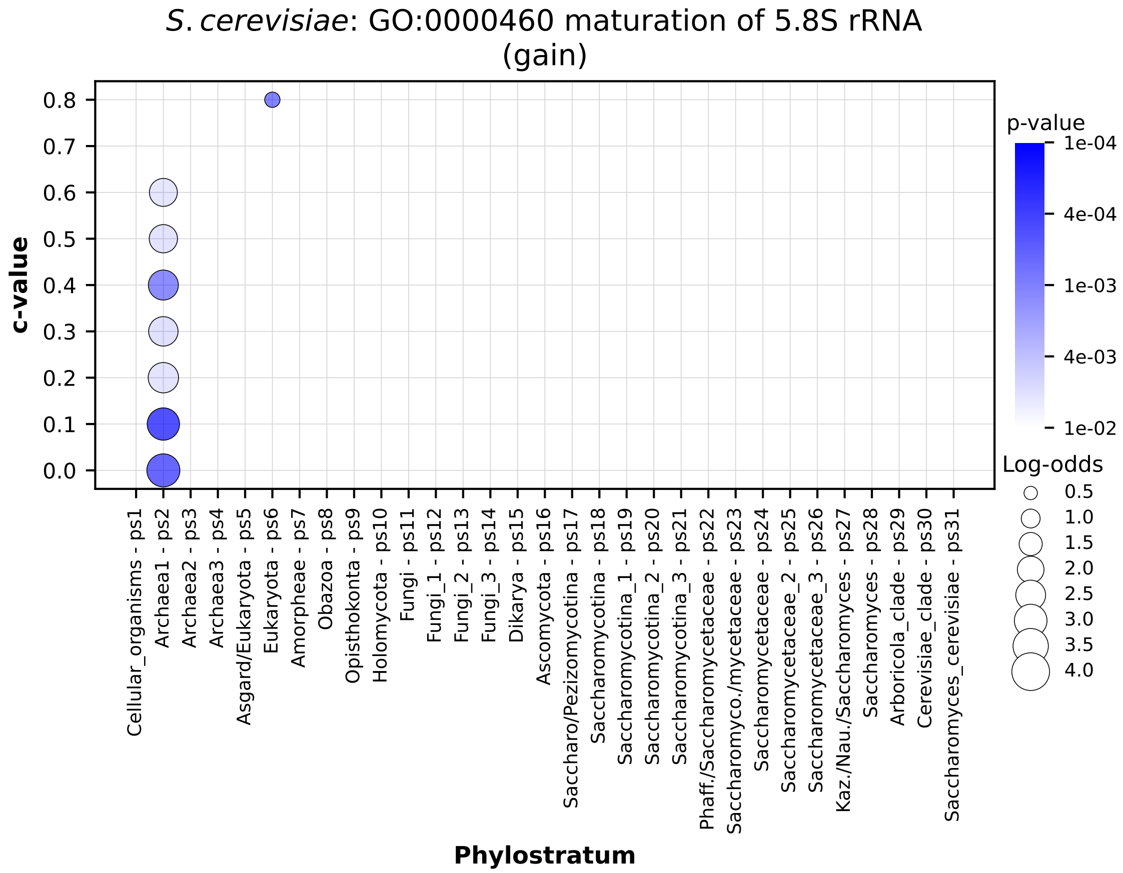

S. cerevisiae: GO:0000460 maturation of 5.8S rRNA
(gain)
c-value
0.0
0.1
0.2
0.3
0.4
0.5
0.6
0.7
0.8
p-value
1e-04
4e-04
1e-03
4e-03
1e-02
Log-odds
0.5
1.0
1.5
2.0
2.5
3.0
3.5
4.0
Cellular_organisms - ps1
Archaea1 - ps2
Archaea2 - ps3
Archaea3 - ps4
Asgard/Eukaryota - ps5
Eukaryota - ps6
Amorpheae - ps7
Obazoa - ps8
Opisthokonta - ps9
Holomycota - ps10
Fungi - ps11
Fungi_1 - ps12
Fungi_2 - ps13
Fungi_3 - ps14
Dikarya - ps15
Ascomycota - ps16
Saccharo/Pezizomycotina - ps17
Saccharomycotina - ps18
Saccharomycotina_1 - ps19
Saccharomycotina_2 - ps20
Saccharomycotina_3 - ps21
Phaff./Saccharomycetaceae - ps22
Saccharomyco./mycetaceae - ps23
Saccharomycetaceae - ps24
Saccharomycetaceae_2 - ps25
Saccharomycetaceae_3 - ps26
Kaz./Nau./Saccharomyces - ps27
Saccharomyces - ps28
Arboricola_clade - ps29
Cerevisiae_clade - ps30
Saccharomyces_cerevisiae - ps31
Phylostratum

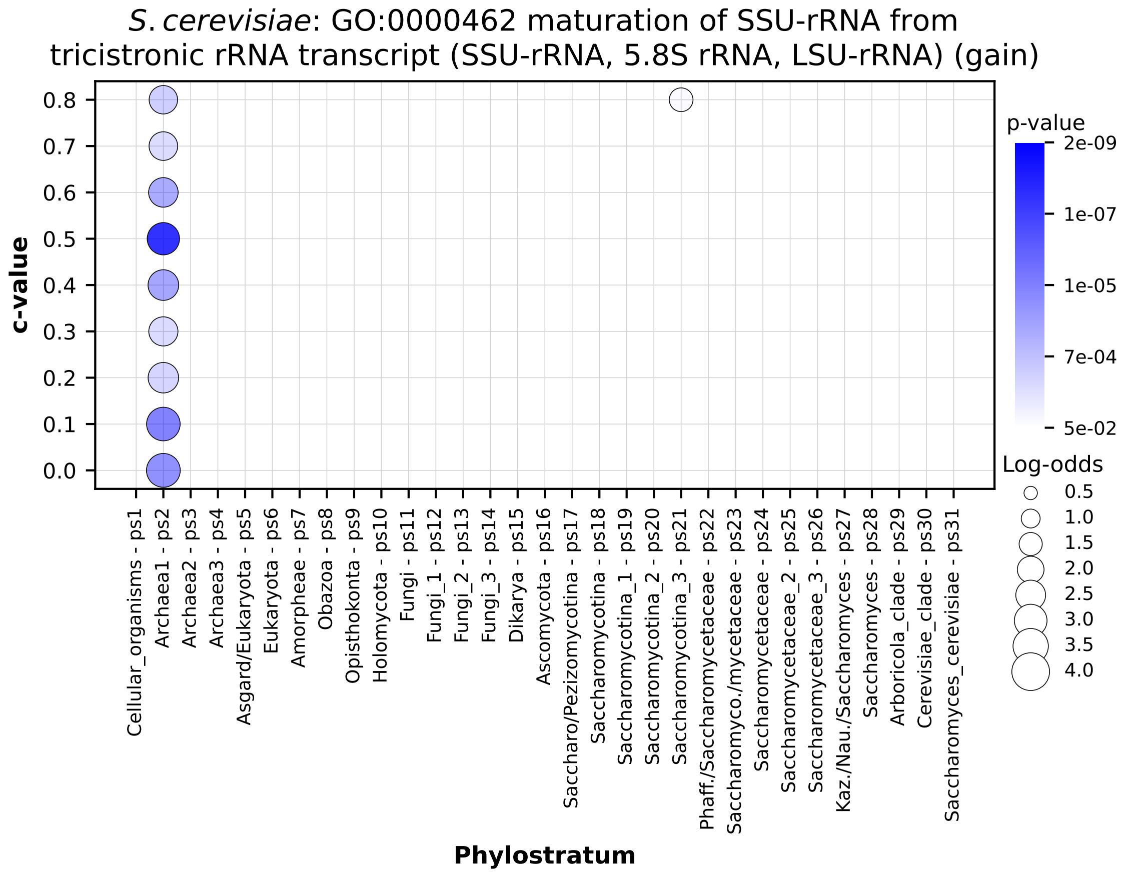

S. cerevisiae: GO:0000462 maturation of SSU-rRNA from tricistronic rRNA transcript (SSU-rRNA, 5.8S rRNA, LSU-rRNA) (gain)
c-value
0.0
0.1
0.2
0.3
0.4
0.5
0.6
0.7
0.8
Phylostratum
Cellular_organisms - ps1
Archaea1 - ps2
Archaea2 - ps3
Archaea3 - ps4
Asgard/Eukaryota - ps5
Eukaryota - ps6
Amorpheae - ps7
Obazoa - ps8
Opisthokonta - ps9
Holomycota - ps10
Fungi - ps11
Fungi_1 - ps12
Fungi_2 - ps13
Fungi_3 - ps14
Dikarya - ps15
Ascomycota - ps16
Saccharo/Pezizomycotina - ps17
Saccharomycotina - ps18
Saccharomycotina_1 - ps19
Saccharomycotina_2 - ps20
Saccharomycotina_3 - ps21
Phaff./Saccharomycetaceae - ps22
Saccharomyco./mycetaceae - ps23
Saccharomycetaceae - ps24
Saccharomycetaceae_2 - ps25
Saccharomycetaceae_3 - ps26
Kaz./Nau./Saccharomyces - ps27
Saccharomyces - ps28
Arboricola_clade - ps29
Cerevisiae_clade - ps30
Saccharomyces_cerevisiae - ps31
p-value
2e-09
1e-07
1e-05
7e-04
5e-02
Log-odds
0.5
1.0
1.5
2.0
2.5
3.0
3.5
4.0

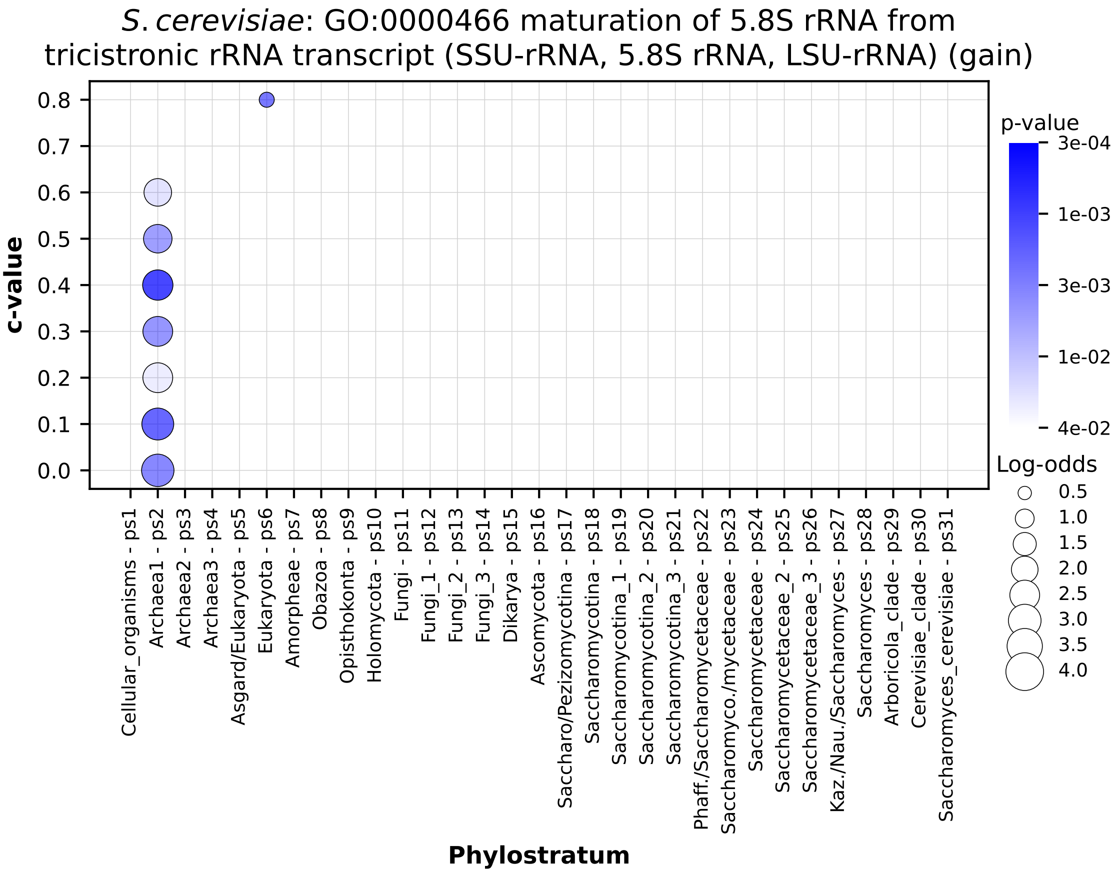

S. cerevisiae: GO:0000466 maturation of 5.8S rRNA from
tricistronic rRNA transcript (SSU-rRNA, 5.8S rRNA, LSU-rRNA) (gain)
c-value
0.0
0.1
0.2
0.3
0.4
0.5
0.6
0.7
0.8
p-value
3e-04
1e-03
3e-03
1e-02
4e-02
Log-odds
0.5
1.0
1.5
2.0
2.5
3.0
3.5
4.0
Cellular_organisms - ps1
Archaea1 - ps2
Archaea2 - ps3
Archaea3 - ps4
Asgard/Eukaryota - ps5
Eukaryota - ps6
Amorpheae - ps7
Obazoa - ps8
Opisthokonta - ps9
Holomycota - ps10
Fungi - ps11
Fungi_1 - ps12
Fungi_2 - ps13
Fungi_3 - ps14
Dikarya - ps15
Ascomycota - ps16
Saccharo/Pezizomycotina - ps17
Saccharomycotina - ps18
Saccharomycotina_1 - ps19
Saccharomycotina_2 - ps20
Saccharomycotina_3 - ps21
Phaff./Saccharomycetaceae - ps22
Saccharomyco./mycetaceae - ps23
Saccharomycetaceae - ps24
Saccharomycetaceae_2 - ps25
Saccharomycetaceae_3 - ps26
Kaz./Nau./Saccharomyces - ps27
Saccharomyces - ps28
Arboricola_clade - ps29
Cerevisiae_clade - ps30
Saccharomyces_cerevisiae - ps31
Phylostratum

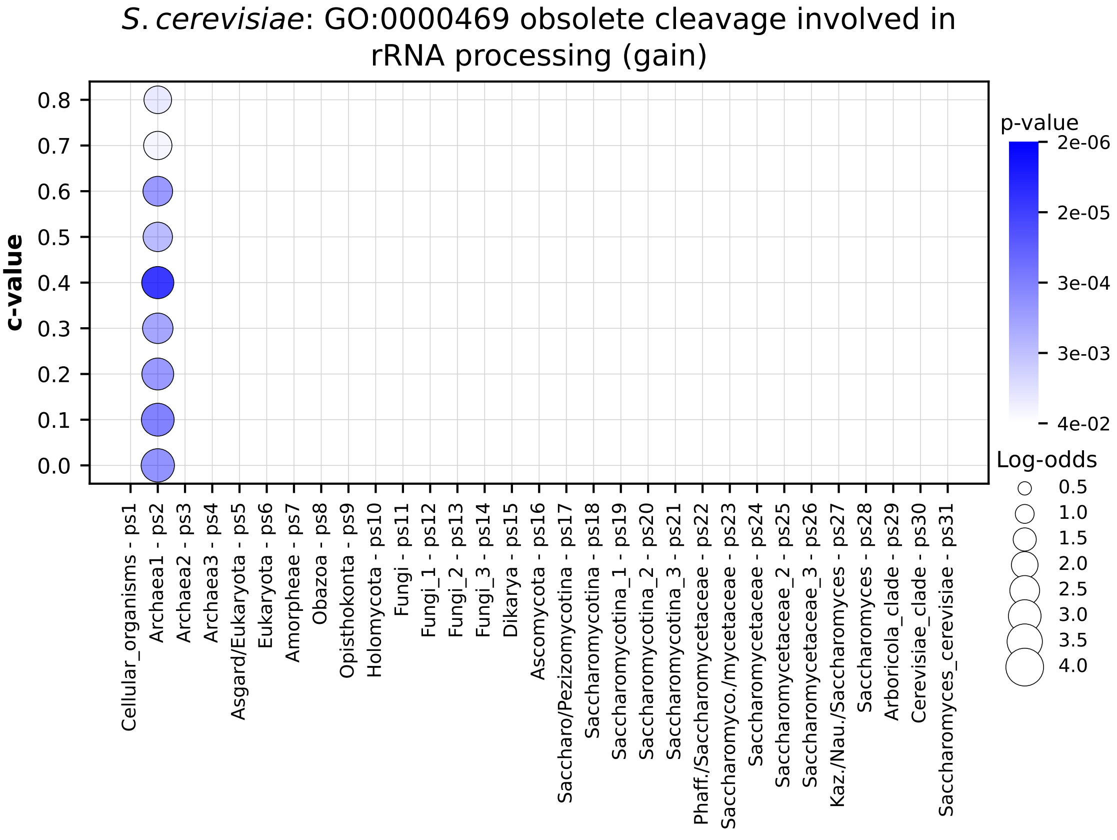

S. cerevisiae: GO:0000469 obsolete cleavage involved in rRNA processing (gain)
c-value
0.8
0.7
0.6
0.5
0.4
0.3
0.2
0.1
0.0
Phylostratum
Cellular_organisms - ps1
Archaea1 - ps2
Archaea2 - ps3
Archaea3 - ps4
Asgard/Eukaryota - ps5
Eukaryota - ps6
Amorpheae - ps7
Obazoa - ps8
Opisthokonta - ps9
Holomycota - ps10
Fungi - ps11
Fungi_1 - ps12
Fungi_2 - ps13
Fungi_3 - ps14
Dikarya - ps15
Ascomycota - ps16
Saccharo/Pezizomycotina - ps17
Saccharomycotina - ps18
Saccharomycotina_1 - ps19
Saccharomycotina_2 - ps20
Saccharomycotina_3 - ps21
Phaff./Saccharomycetaceae - ps22
Saccharomyco./mycetaceae - ps23
Saccharomycetaceae - ps24
Saccharomycetaceae_2 - ps25
Saccharomycetaceae_3 - ps26
Kaz./Nau./Saccharomyces - ps27
Saccharomyces - ps28
Arboricola_clade - ps29
Cerevisiae_clade - ps30
Saccharomyces_cerevisiae - ps31
p-value
2e-06
2e-05
3e-04
3e-03
4e-02
Log-odds
0.5
1.0
1.5
2.0
2.5
3.0
3.5
4.0

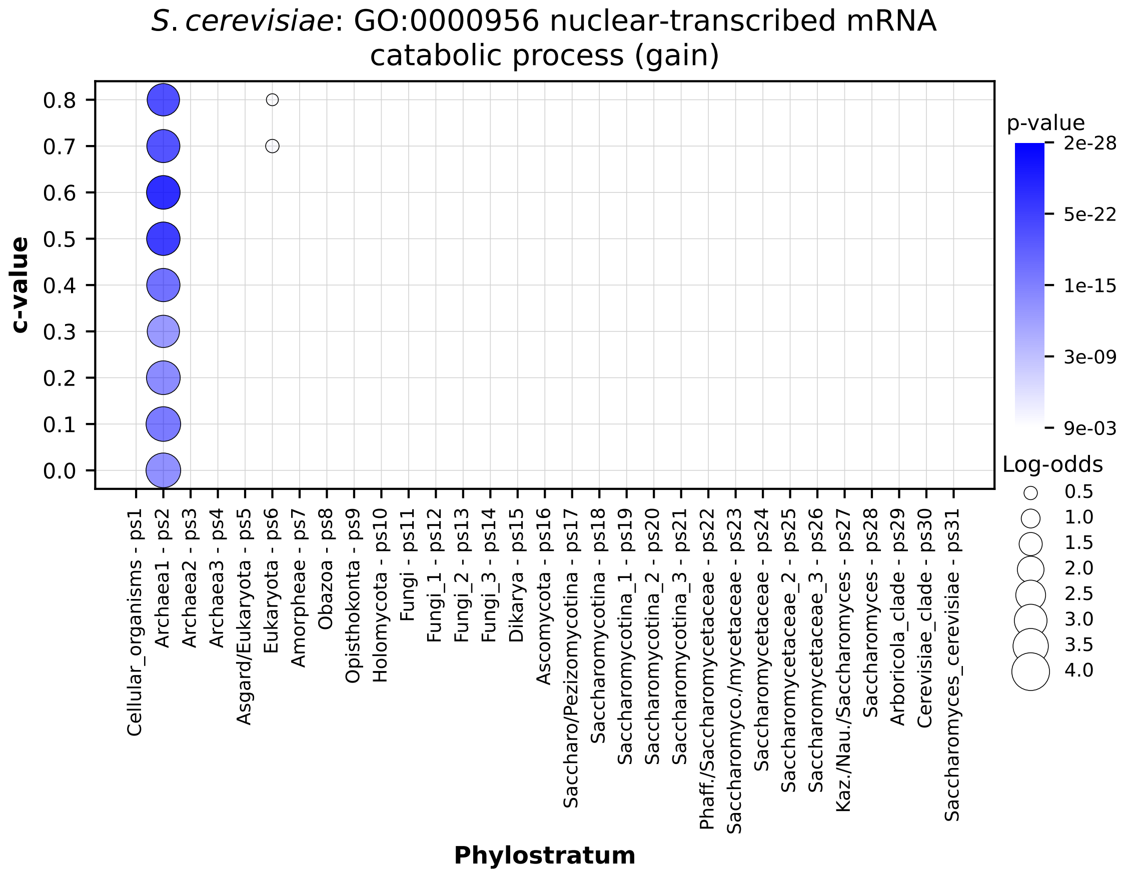

S. cerevisiae: GO:0000956 nuclear-transcribed mRNA catabolic process (gain)
c-value
0.8
0.7
0.6
0.5
0.4
0.3
0.2
0.1
0.0
p-value
2e-28
5e-22
1e-15
3e-09
9e-03
Log-odds
0.5
1.0
1.5
2.0
2.5
3.0
3.5
4.0
Cellular_organisms - ps1
Archaea1 - ps2
Archaea2 - ps3
Archaea3 - ps4
Asgard/Eukaryota - ps5
Eukaryota - ps6
Amorpheae - ps7
Obazoa - ps8
Opisthokonta - ps9
Holomycota - ps10
Fungi - ps11
Fungi_1 - ps12
Fungi_2 - ps13
Fungi_3 - ps14
Dikarya - ps15
Ascomycota - ps16
Saccharo/Pezizomycotina - ps17
Saccharomycotina - ps18
Saccharomycotina_1 - ps19
Saccharomycotina_2 - ps20
Saccharomycotina_3 - ps21
Phaff./Saccharomycetaceae - ps22
Saccharomyco./mycetaceae - ps23
Saccharomycetaceae - ps24
Saccharomycetaceae_2 - ps25
Saccharomycetaceae_3 - ps26
Kaz./Nau./Saccharomyces - ps27
Saccharomyces - ps28
Arboricola_clade - ps29
Cerevisiae_clade - ps30
Saccharomyces_cerevisiae - ps31
Phylostratum

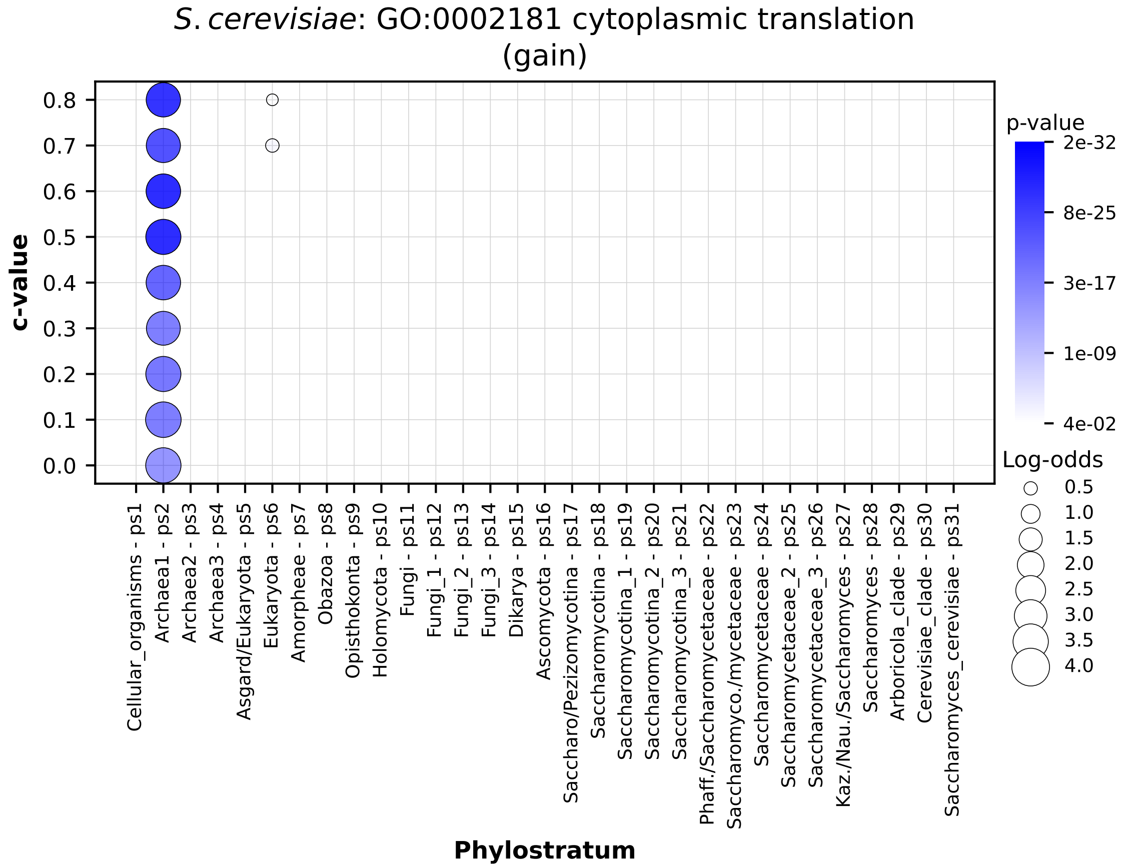

S. cerevisiae: GO:0002181 cytoplasmic translation
(gain)
c-value
0.0
0.1
0.2
0.3
0.4
0.5
0.6
0.7
0.8
Phylostratum
Cellular_organisms - ps1
Archaea1 - ps2
Archaea2 - ps3
Archaea3 - ps4
Asgard/Eukaryota - ps5
Eukaryota - ps6
Amorpheae - ps7
Obazoa - ps8
Opisthokonta - ps9
Holomycota - ps10
Fungi - ps11
Fungi_1 - ps12
Fungi_2 - ps13
Fungi_3 - ps14
Dikarya - ps15
Ascomycota - ps16
Saccharo/Pezizomycotina - ps17
Saccharomycotina - ps18
Saccharomycotina_1 - ps19
Saccharomycotina_2 - ps20
Saccharomycotina_3 - ps21
Phaff./Saccharomycetaceae - ps22
Saccharomyco./mycetaceae - ps23
Saccharomycetaceae - ps24
Saccharomycetaceae_2 - ps25
Saccharomycetaceae_3 - ps26
Kaz./Nau./Saccharomyces - ps27
Saccharomyces - ps28
Arboricola_clade - ps29
Cerevisiae_clade - ps30
Saccharomyces_cerevisiae - ps31
p-value
2e-32
8e-25
3e-17
1e-09
4e-02
Log-odds
0.5
1.0
1.5
2.0
2.5
3.0
3.5
4.0

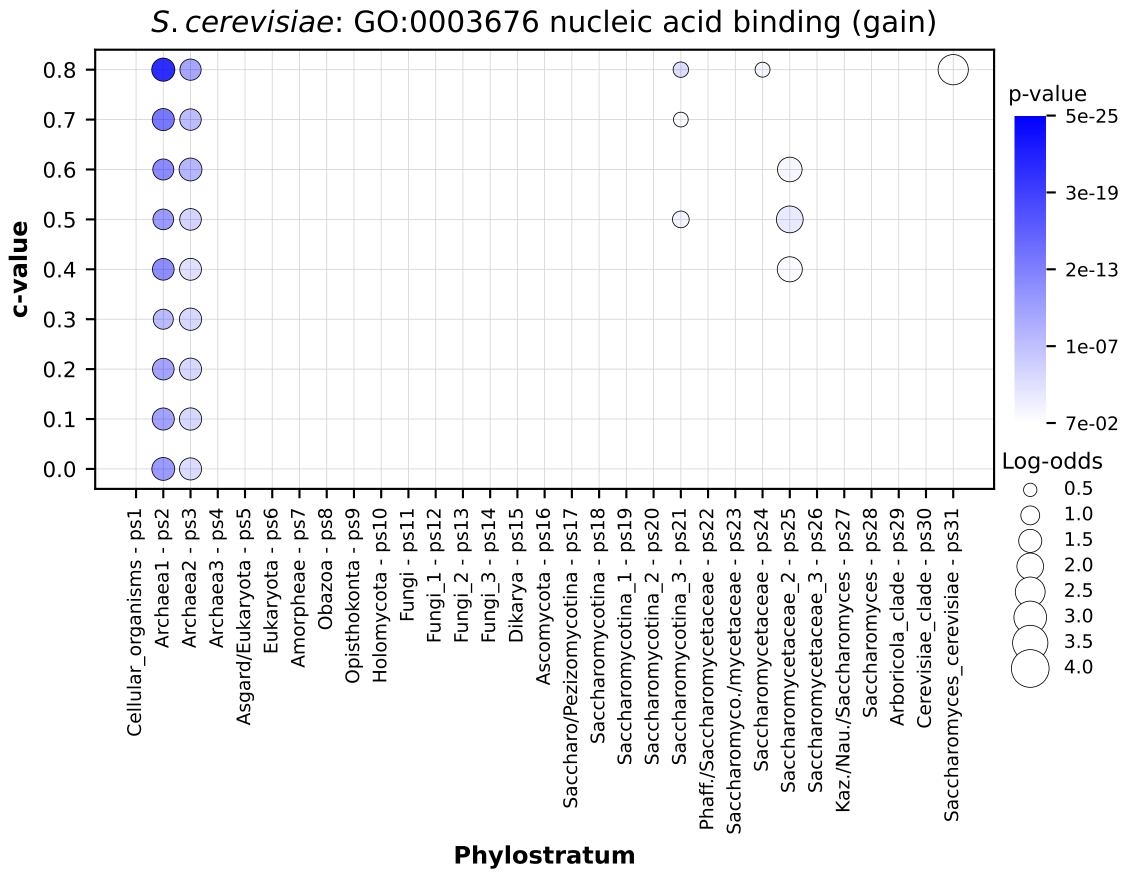

S. cerevisiae: GO:0003676 nucleic acid binding (gain)
c-value
0.8
0.7
0.6
0.5
0.4
0.3
0.2
0.1
0.0
p-value
5e-25
3e-19
2e-13
1e-07
7e-02
Log-odds
0.5
1.0
1.5
2.0
2.5
3.0
3.5
4.0
Cellular_organisms - ps1
Archaea1 - ps2
Archaea2 - ps3
Archaea3 - ps4
Asgard/Eukaryota - ps5
Eukaryota - ps6
Amorpheae - ps7
Obazoa - ps8
Opisthokonta - ps9
Holomycota - ps10
Fungi - ps11
Fungi_1 - ps12
Fungi_2 - ps13
Fungi_3 - ps14
Dikarya - ps15
Ascomycota - ps16
Saccharo/Pezizomycotina - ps17
Saccharomycotina - ps18
Saccharomycotina_1 - ps19
Saccharomycotina_2 - ps20
Saccharomycotina_3 - ps21
Phaff./Saccharomycetaceae - ps22
Saccharomyco./mycetaceae - ps23
Saccharomycetaceae - ps24
Saccharomycetaceae_2 - ps25
Saccharomycetaceae_3 - ps26
Kaz./Nau./Saccharomyces - ps27
Saccharomyces - ps28
Arboricola_clade - ps29
Cerevisiae_clade - ps30
Saccharomyces_cerevisiae - ps31
Phylostratum

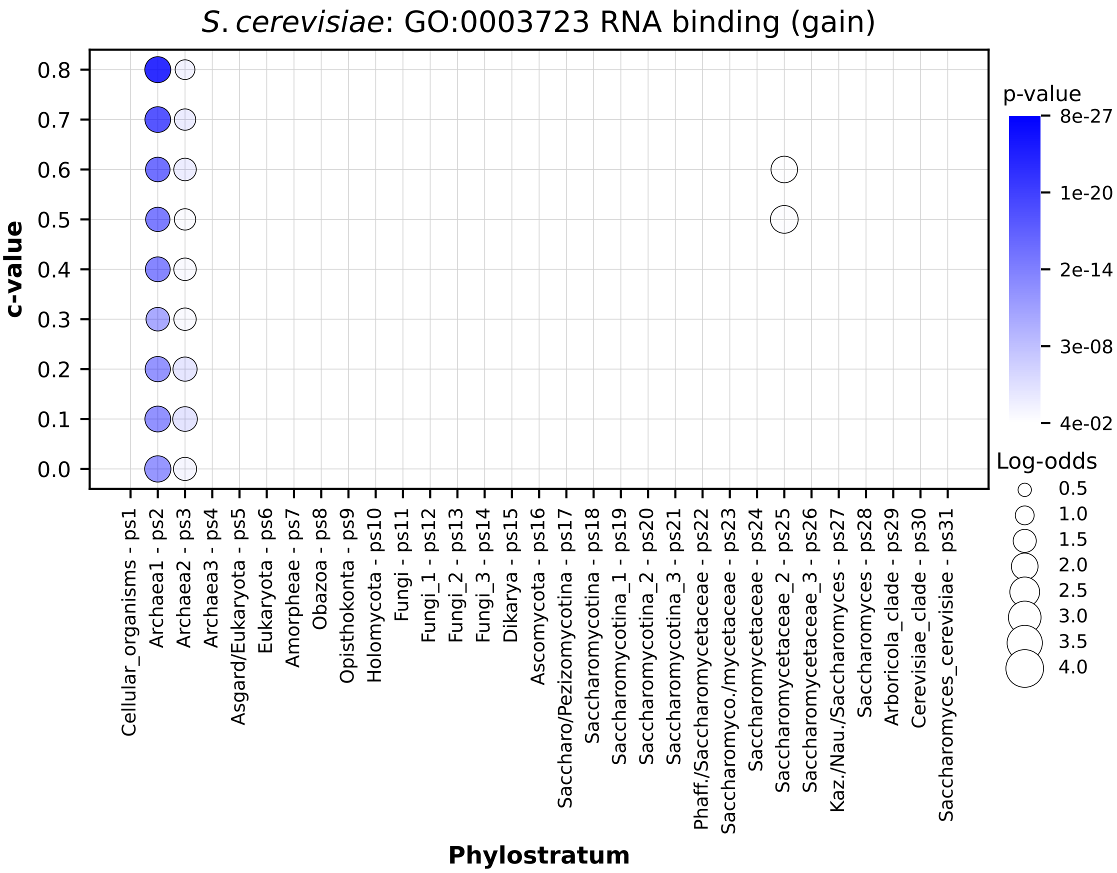

S. cerevisiae: GO:0003723 RNA binding (gain)
c-value
0.8
0.7
0.6
0.5
0.4
0.3
0.2
0.1
0.0
p-value
8e-27
1e-20
2e-14
3e-08
4e-02
Log-odds
0.5
1.0
1.5
2.0
2.5
3.0
3.5
4.0
Cellular_organisms - ps1
Archaea1 - ps2
Archaea2 - ps3
Archaea3 - ps4
Asgard/Eukaryota - ps5
Eukaryota - ps6
Amorpheae - ps7
Obazoa - ps8
Opisthokonta - ps9
Holomycota - ps10
Fungi - ps11
Fungi_1 - ps12
Fungi_2 - ps13
Fungi_3 - ps14
Dikarya - ps15
Ascomycota - ps16
Saccharo/Pezizomycotina - ps17
Saccharomycotina - ps18
Saccharomycotina_1 - ps19
Saccharomycotina_2 - ps20
Saccharomycotina_3 - ps21
Phaff./Saccharomycetaceae - ps22
Saccharomyco./mycetaceae - ps23
Saccharomycetaceae - ps24
Saccharomycetaceae_2 - ps25
Saccharomycetaceae_3 - ps26
Kaz./Nau./Saccharomyces - ps27
Saccharomyces - ps28
Arboricola_clade - ps29
Cerevisiae_clade - ps30
Saccharomyces_cerevisiae - ps31
Phylostratum

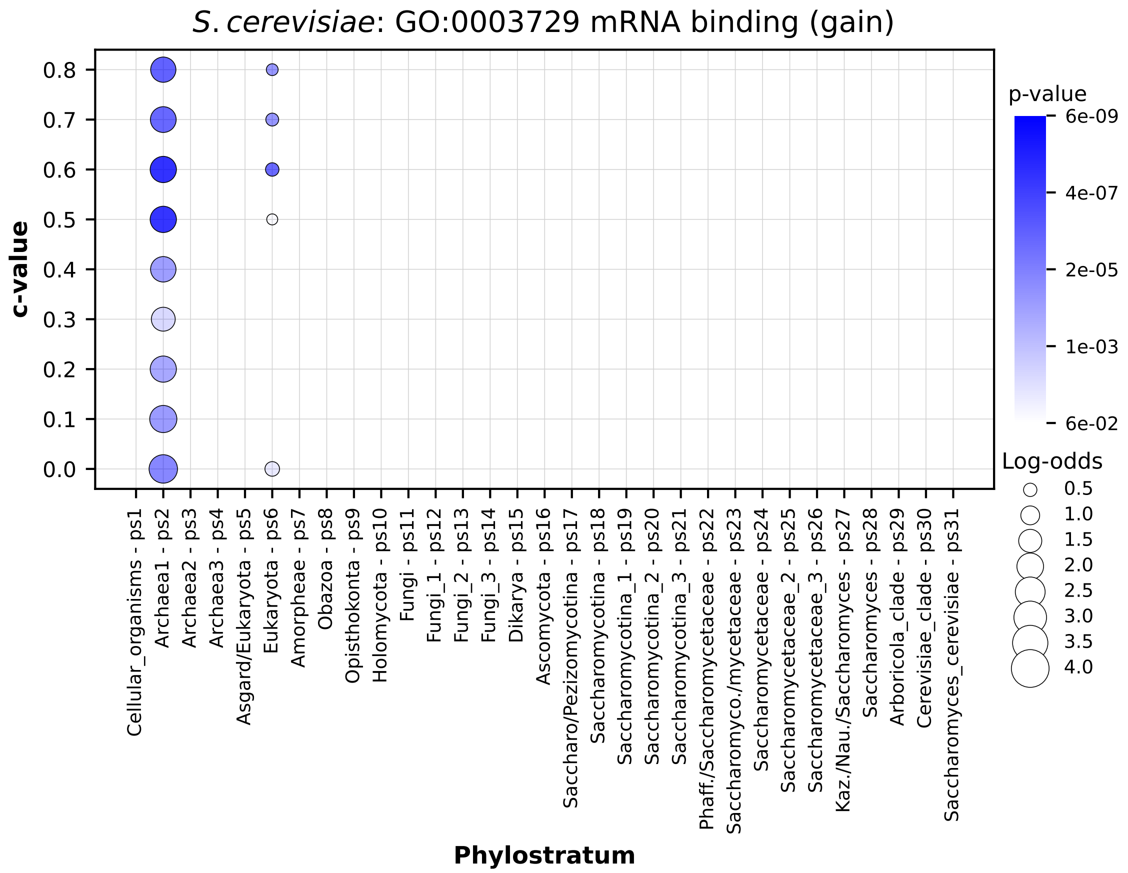

S. cerevisiae: GO:0003729 mRNA binding (gain)
c-value
0.0
0.1
0.2
0.3
0.4
0.5
0.6
0.7
0.8
p-value
6e-09
4e-07
2e-05
1e-03
6e-02
Log-odds
0.5
1.0
1.5
2.0
2.5
3.0
3.5
4.0
Cellular_organisms - ps1
Archaea1 - ps2
Archaea2 - ps3
Archaea3 - ps4
Asgard/Eukaryota - ps5
Eukaryota - ps6
Amorpheae - ps7
Obazoa - ps8
Opisthokonta - ps9
Holomycota - ps10
Fungi - ps11
Fungi_1 - ps12
Fungi_2 - ps13
Fungi_3 - ps14
Dikarya - ps15
Ascomycota - ps16
Saccharo/Pezizomycotina - ps17
Saccharomycotina - ps18
Saccharomycotina_1 - ps19
Saccharomycotina_2 - ps20
Saccharomycotina_3 - ps21
Phaff./Saccharomycetaceae - ps22
Saccharomyco./mycetaceae - ps23
Saccharomycetaceae - ps24
Saccharomycetaceae_2 - ps25
Saccharomycetaceae_3 - ps26
Kaz./Nau./Saccharomyces - ps27
Saccharomyces - ps28
Arboricola_clade - ps29
Cerevisiae_clade - ps30
Saccharomyces_cerevisiae - ps31
Phylostratum

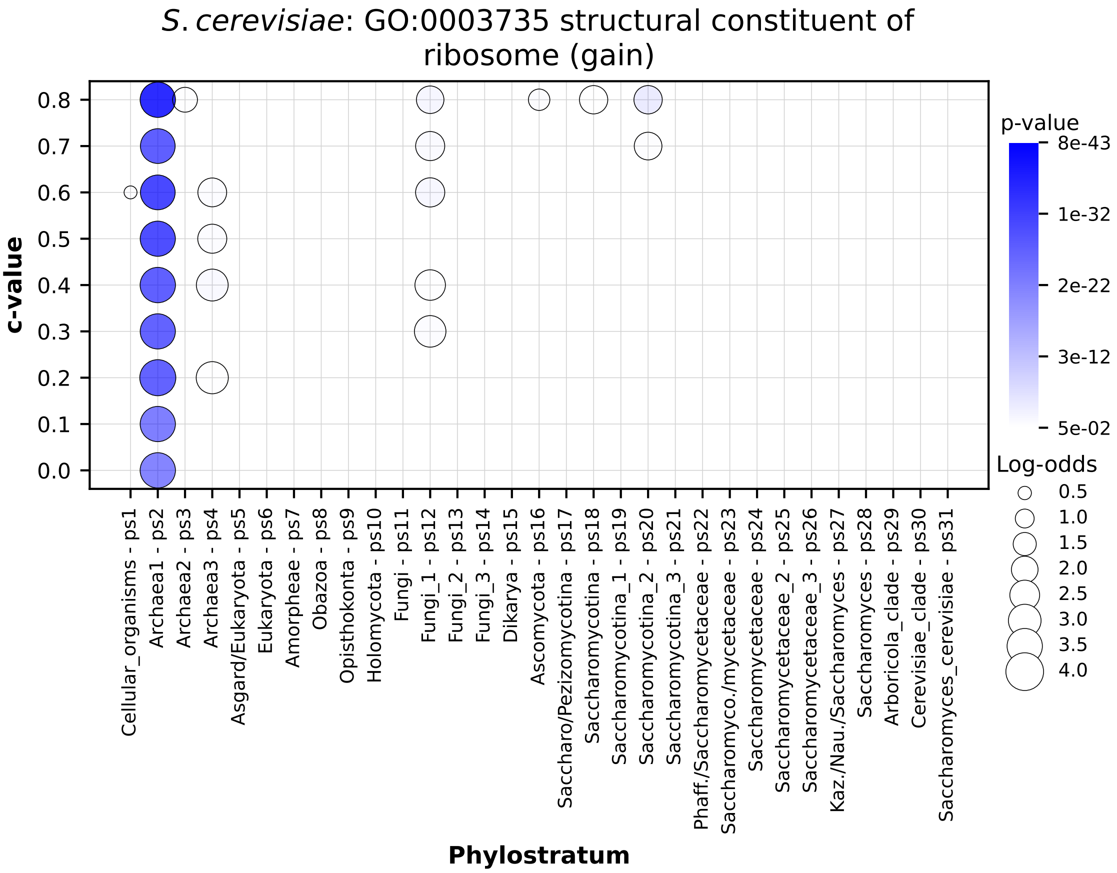

S. cerevisiae: GO:0003735 structural constituent of ribosome (gain)
c-value
0.8
0.7
0.6
0.5
0.4
0.3
0.2
0.1
0.0
Phylostratum
Cellular_organisms - ps1
Archaea1 - ps2
Archaea2 - ps3
Archaea3 - ps4
Asgard/Eukaryota - ps5
Eukaryota - ps6
Amorpheae - ps7
Obazoa - ps8
Opisthokonta - ps9
Holomycota - ps10
Fungi - ps11
Fungi_1 - ps12
Fungi_2 - ps13
Fungi_3 - ps14
Dikarya - ps15
Ascomycota - ps16
Saccharo/Pezizomycotina - ps17
Saccharomycotina - ps18
Saccharomycotina_1 - ps19
Saccharomycotina_2 - ps20
Saccharomycotina_3 - ps21
Phaff./Saccharomycetaceae - ps22
Saccharomyco./mycetaceae - ps23
Saccharomycetaceae - ps24
Saccharomycetaceae_2 - ps25
Saccharomycetaceae_3 - ps26
Kaz./Nau./Saccharomyces - ps27
Saccharomyces - ps28
Arboricola_clade - ps29
Cerevisiae_clade - ps30
Saccharomyces_cerevisiae - ps31
p-value
8e-43
1e-32
2e-22
3e-12
5e-02
Log-odds
0.5
1.0
1.5
2.0
2.5
3.0
3.5
4.0

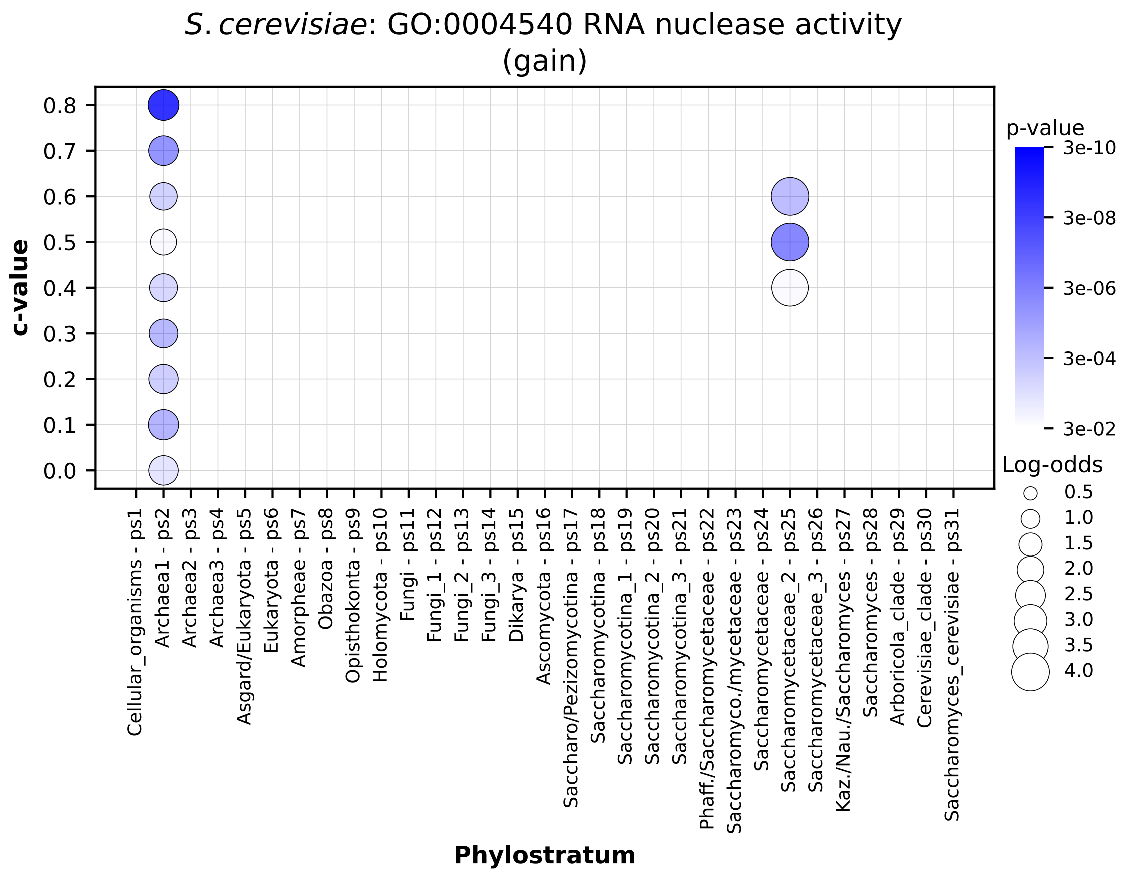
S. cerevisiae: GO:0004540 RNA nuclease activity
(gain)
c-value
0.8
0.7
0.6
0.5
0.4
0.3
0.2
0.1
0.0
p-value
3e-10
3e-08
3e-06
3e-04
3e-02
Log-odds
0.5
1.0
1.5
2.0
2.5
3.0
3.5
4.0
Cellular_organisms - ps1
Archaea1 - ps2
Archaea2 - ps3
Archaea3 - ps4
Asgard/Eukaryota - ps5
Eukaryota - ps6
Amorpheae - ps7
Obazoa - ps8
Opisthokonta - ps9
Holomycota - ps10
Fungi - ps11
Fungi_1 - ps12
Fungi_2 - ps13
Fungi_3 - ps14
Dikarya - ps15
Ascomycota - ps16
Saccharo/Pezizomycotina - ps17
Saccharomycotina - ps18
Saccharomycotina_1 - ps19
Saccharomycotina_2 - ps20
Saccharomycotina_3 - ps21
Phaff./Saccharomycetaceae - ps22
Saccharomyco./mycetaceae - ps23
Saccharomycetaceae - ps24
Saccharomycetaceae_2 - ps25
Saccharomycetaceae_3 - ps26
Kaz./Nau./Saccharomyces - ps27
Saccharomyces - ps28
Arboricola_clade - ps29
Cerevisiae_clade - ps30
Saccharomyces_cerevisiae - ps31
Phylostratum

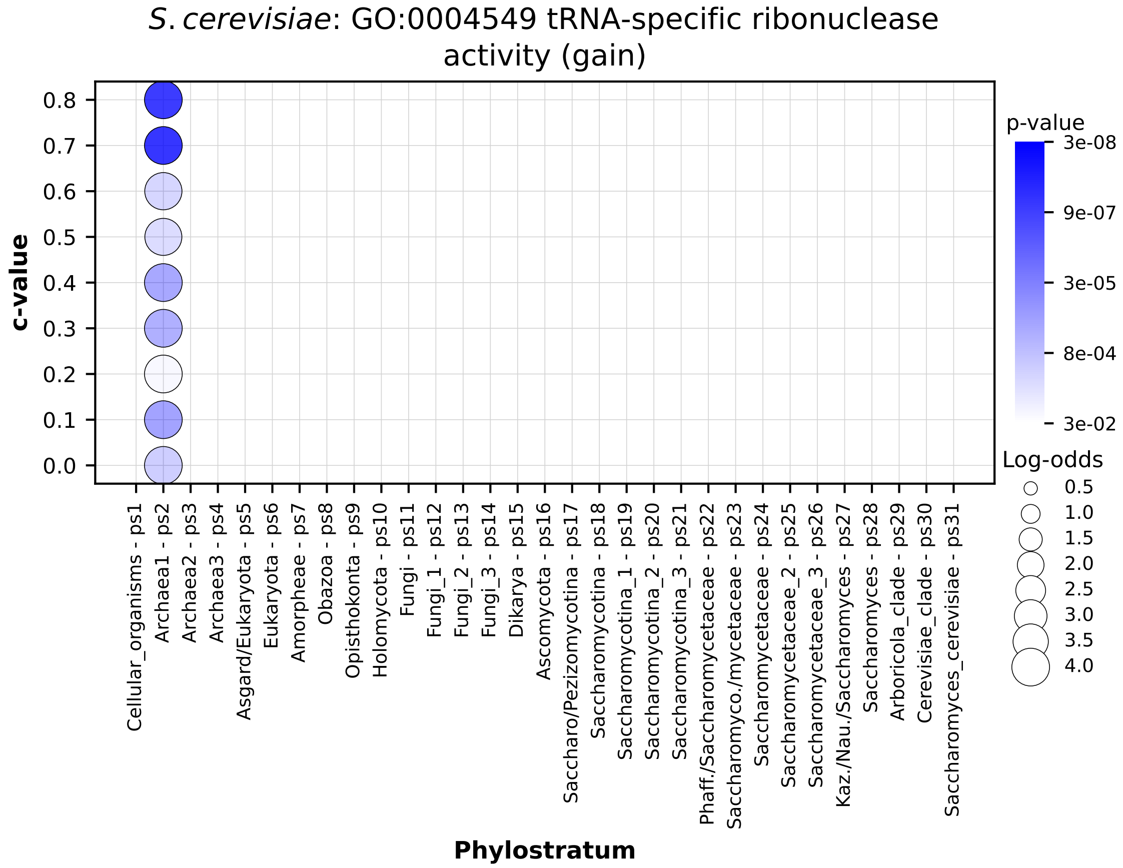

S. cerevisiae: GO:0004549 tRNA-specific ribonuclease activity (gain)
c-value
0.8
0.7
0.6
0.5
0.4
0.3
0.2
0.1
0.0
p-value
3e-08
9e-07
3e-05
8e-04
3e-02
Log-odds
0.5
1.0
1.5
2.0
2.5
3.0
3.5
4.0
Cellular_organisms - ps1
Archaea1 - ps2
Archaea2 - ps3
Archaea3 - ps4
Asgard/Eukaryota - ps5
Eukaryota - ps6
Amorpheae - ps7
Obazoa - ps8
Opisthokonta - ps9
Holomycota - ps10
Fungi - ps11
Fungi_1 - ps12
Fungi_2 - ps13
Fungi_3 - ps14
Dikarya - ps15
Ascomycota - ps16
Saccharo/Pezizomycotina - ps17
Saccharomycotina - ps18
Saccharomycotina_1 - ps19
Saccharomycotina_2 - ps20
Saccharomycotina_3 - ps21
Phaff./Saccharomycetaceae - ps22
Saccharomyco./mycetaceae - ps23
Saccharomycetaceae - ps24
Saccharomycetaceae_2 - ps25
Saccharomycetaceae_3 - ps26
Kaz./Nau./Saccharomyces - ps27
Saccharomyces - ps28
Arboricola_clade - ps29
Cerevisiae_clade - ps30
Saccharomyces_cerevisiae - ps31
Phylostratum

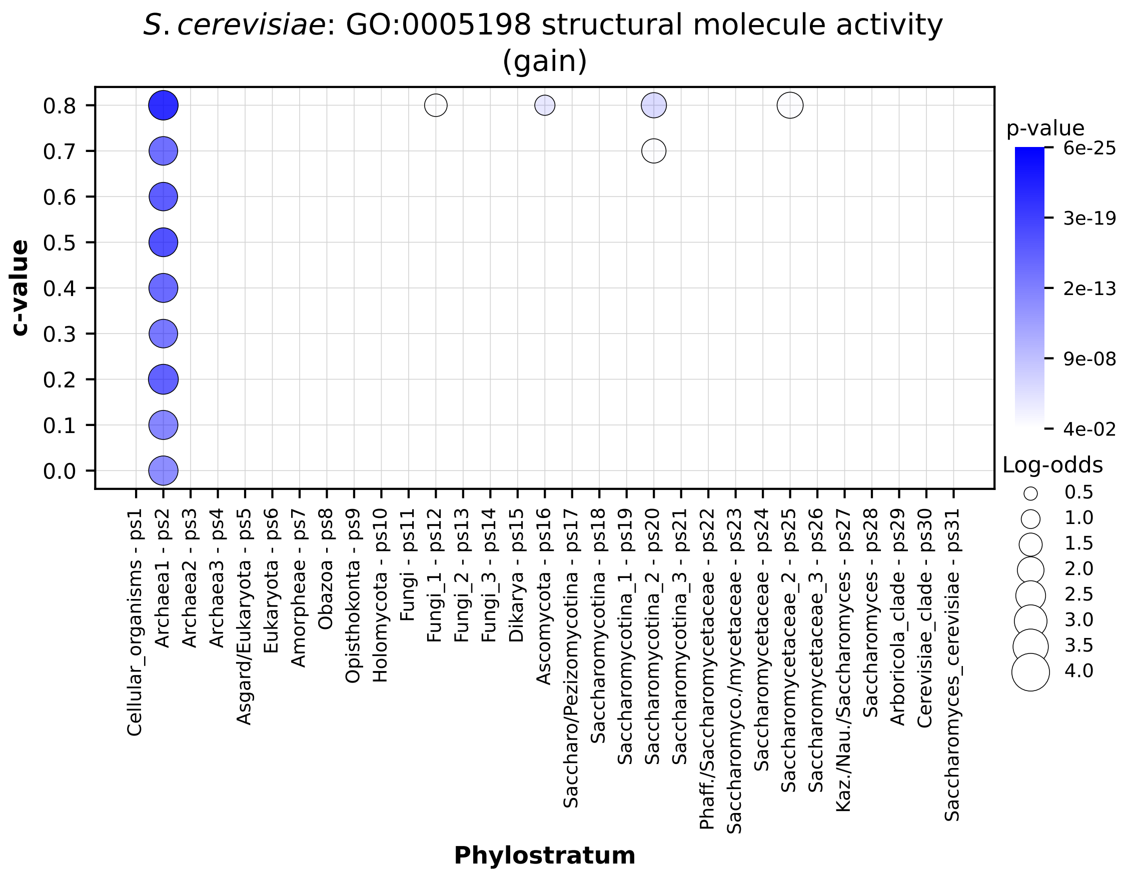

S. cerevisiae: GO:0005198 structural molecule activity (gain)
c-value
0.8
0.7
0.6
0.5
0.4
0.3
0.2
0.1
0.0
p-value
6e-25
3e-19
2e-13
9e-08
4e-02
Log-odds
0.5
1.0
1.5
2.0
2.5
3.0
3.5
4.0
Cellular_organisms - ps1
Archaea1 - ps2
Archaea2 - ps3
Archaea3 - ps4
Asgard/Eukaryota - ps5
Eukaryota - ps6
Amorpheae - ps7
Obazoa - ps8
Opisthokonta - ps9
Holomycota - ps10
Fungi - ps11
Fungi_1 - ps12
Fungi_2 - ps13
Fungi_3 - ps14
Dikarya - ps15
Ascomycota - ps16
Saccharo/Pezizomycotina - ps17
Saccharomycotina - ps18
Saccharomycotina_1 - ps19
Saccharomycotina_2 - ps20
Saccharomycotina_3 - ps21
Phaff./Saccharomycetaceae - ps22
Saccharomyco./mycetaceae - ps23
Saccharomycetaceae - ps24
Saccharomycetaceae_2 - ps25
Saccharomycetaceae_3 - ps26
Kaz./Nau./Saccharomyces - ps27
Saccharomyces - ps28
Arboricola_clade - ps29
Cerevisiae_clade - ps30
Saccharomyces_cerevisiae - ps31
Phylostratum

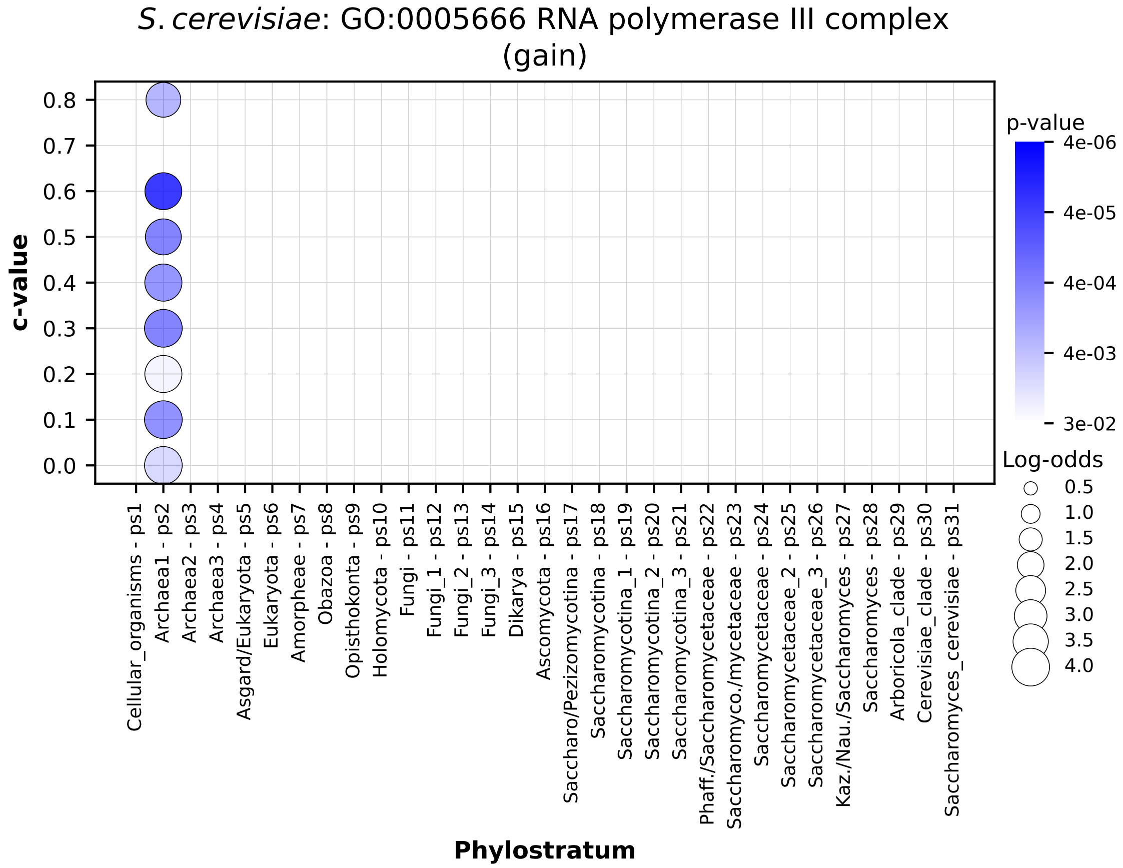

S. cerevisiae: GO:0005666 RNA polymerase III complex
(gain)
c-value
0.0
0.1
0.2
0.3
0.4
0.5
0.6
0.7
0.8
p-value
4e-06
4e-05
4e-04
4e-03
3e-02
Log-odds
0.5
1.0
1.5
2.0
2.5
3.0
3.5
4.0
Cellular_organisms - ps1
Archaea1 - ps2
Archaea2 - ps3
Archaea3 - ps4
Asgard/Eukaryota - ps5
Eukaryota - ps6
Amorpheae - ps7
Obazoa - ps8
Opisthokonta - ps9
Holomycota - ps10
Fungi - ps11
Fungi_1 - ps12
Fungi_2 - ps13
Fungi_3 - ps14
Dikarya - ps15
Ascomycota - ps16
Saccharo/Pezizomycotina - ps17
Saccharomycotina - ps18
Saccharomycotina_1 - ps19
Saccharomycotina_2 - ps20
Saccharomycotina_3 - ps21
Phaff./Saccharomycetaceae - ps22
Saccharomyco./mycetaceae - ps23
Saccharomycetaceae - ps24
Saccharomycetaceae_2 - ps25
Saccharomycetaceae_3 - ps26
Kaz./Nau./Saccharomyces - ps27
Saccharomyces - ps28
Arboricola_clade - ps29
Cerevisiae_clade - ps30
Saccharomyces_cerevisiae - ps31
Phylostratum

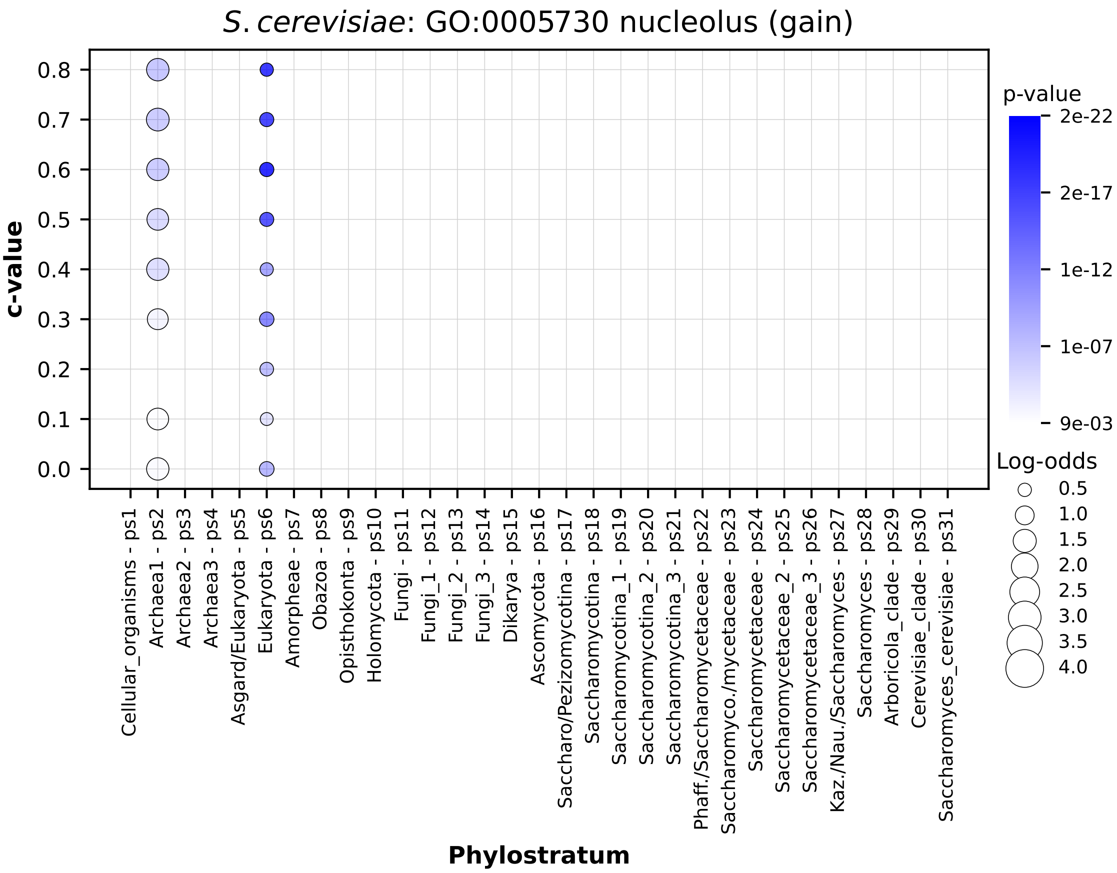

S. cerevisiae: GO:0005730 nucleolus (gain)
c-value
0.8
0.7
0.6
0.5
0.4
0.3
0.2
0.1
0.0
p-value
2e-22
2e-17
1e-12
1e-07
9e-03
Log-odds
0.5
1.0
1.5
2.0
2.5
3.0
3.5
4.0
Cellular_organisms - ps1
Archaea1 - ps2
Archaea2 - ps3
Archaea3 - ps4
Asgard/Eukaryota - ps5
Eukaryota - ps6
Amorpheae - ps7
Obazoa - ps8
Opisthokonta - ps9
Holomycota - ps10
Fungi - ps11
Fungi_1 - ps12
Fungi_2 - ps13
Fungi_3 - ps14
Dikarya - ps15
Ascomycota - ps16
Saccharo/Pezizomycotina - ps17
Saccharomycotina - ps18
Saccharomycotina_1 - ps19
Saccharomycotina_2 - ps20
Saccharomycotina_3 - ps21
Phaff./Saccharomycetaceae - ps22
Saccharomyco./mycetaceae - ps23
Saccharomycetaceae - ps24
Saccharomycetaceae_2 - ps25
Saccharomycetaceae_3 - ps26
Kaz./Nau./Saccharomyces - ps27
Saccharomyces - ps28
Arboricola_clade - ps29
Cerevisiae_clade - ps30
Saccharomyces_cerevisiae - ps31
Phylostratum

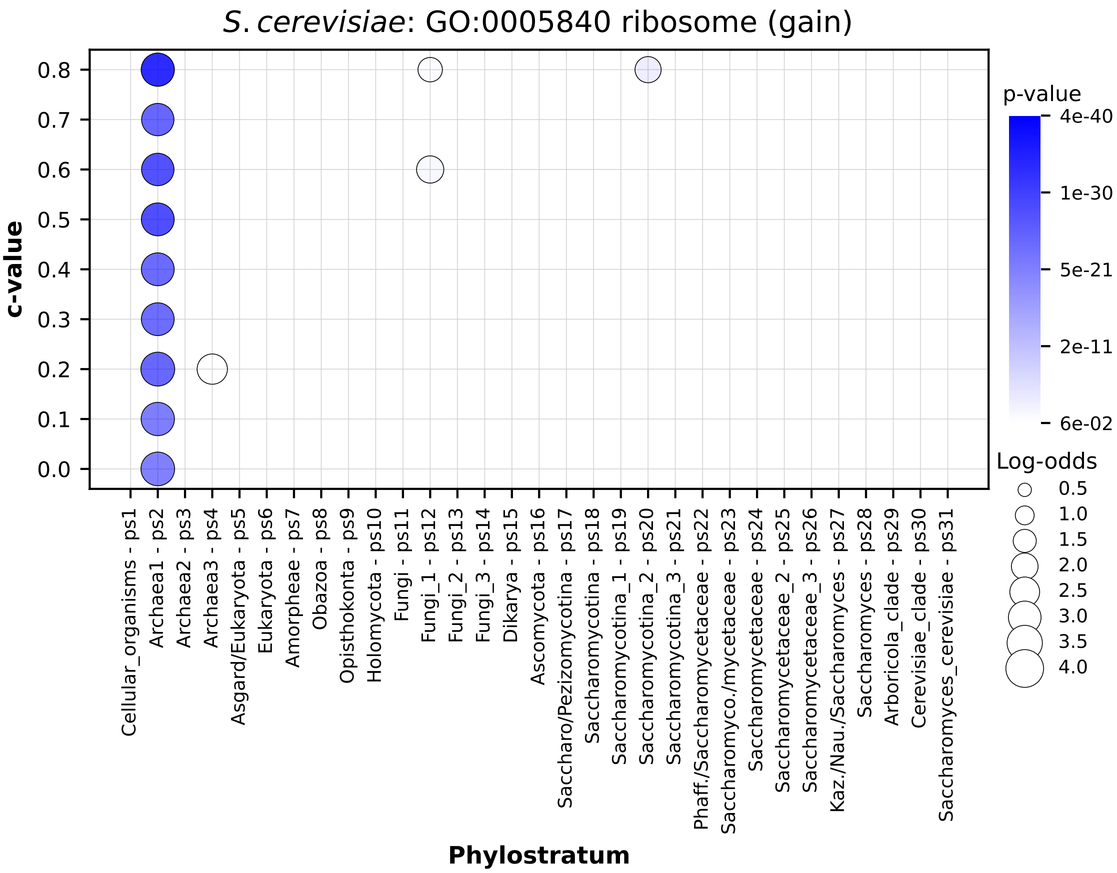

S. cerevisiae: GO:0005840 ribosome (gain)
c-value
0.8
0.7
0.6
0.5
0.4
0.3
0.2
0.1
0.0
p-value
4e-40
1e-30
5e-21
2e-11
6e-02
Log-odds
0.5
1.0
1.5
2.0
2.5
3.0
3.5
4.0
Cellular_organisms - ps1
Archaea1 - ps2
Archaea2 - ps3
Archaea3 - ps4
Asgard/Eukaryota - ps5
Eukaryota - ps6
Amorpheae - ps7
Obazoa - ps8
Opisthokonta - ps9
Holomycota - ps10
Fungi - ps11
Fungi_1 - ps12
Fungi_2 - ps13
Fungi_3 - ps14
Dikarya - ps15
Ascomycota - ps16
Saccharo/Pezizomycotina - ps17
Saccharomycotina - ps18
Saccharomycotina_1 - ps19
Saccharomycotina_2 - ps20
Saccharomycotina_3 - ps21
Phaff./Saccharomycetaceae - ps22
Saccharomyco./mycetaceae - ps23
Saccharomycetaceae - ps24
Saccharomycetaceae_2 - ps25
Saccharomycetaceae_3 - ps26
Kaz./Nau./Saccharomyces - ps27
Saccharomyces - ps28
Arboricola_clade - ps29
Cerevisiae_clade - ps30
Saccharomyces_cerevisiae - ps31
Phylostratum

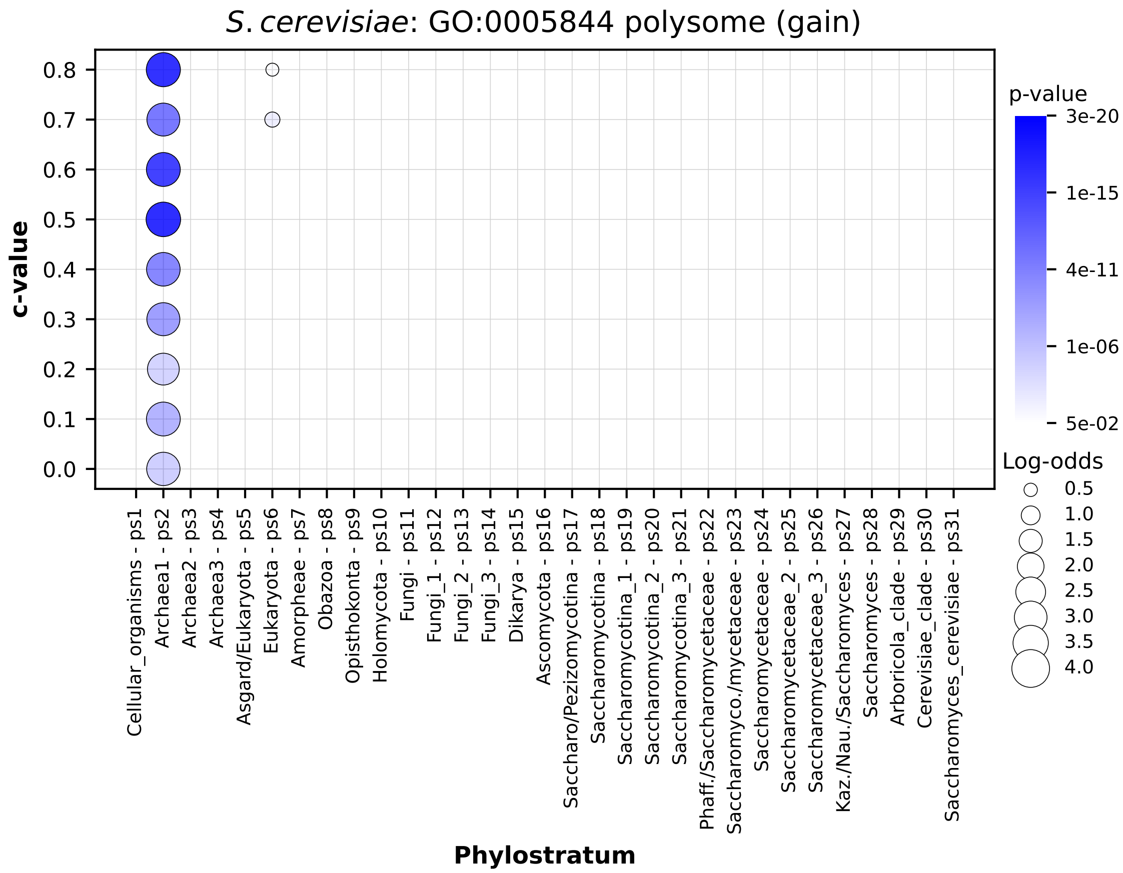

S. cerevisiae: GO:0005844 polysome (gain)
c-value
0.8
0.7
0.6
0.5
0.4
0.3
0.2
0.1
0.0
p-value
3e-20
1e-15
4e-11
1e-06
5e-02
Log-odds
0.5
1.0
1.5
2.0
2.5
3.0
3.5
4.0
Cellular_organisms - ps1
Archaea1 - ps2
Archaea2 - ps3
Archaea3 - ps4
Asgard/Eukaryota - ps5
Eukaryota - ps6
Amorpheae - ps7
Obazoa - ps8
Opisthokonta - ps9
Holomycota - ps10
Fungi - ps11
Fungi_1 - ps12
Fungi_2 - ps13
Fungi_3 - ps14
Dikarya - ps15
Ascomycota - ps16
Saccharo/Pezizomycotina - ps17
Saccharomycotina - ps18
Saccharomycotina_1 - ps19
Saccharomycotina_2 - ps20
Saccharomycotina_3 - ps21
Phaff./Saccharomycetaceae - ps22
Saccharomyco./mycetaceae - ps23
Saccharomycetaceae - ps24
Saccharomycetaceae_2 - ps25
Saccharomycetaceae_3 - ps26
Kaz./Nau./Saccharomyces - ps27
Saccharomyces - ps28
Arboricola_clade - ps29
Cerevisiae_clade - ps30
Saccharomyces_cerevisiae - ps31
Phylostratum

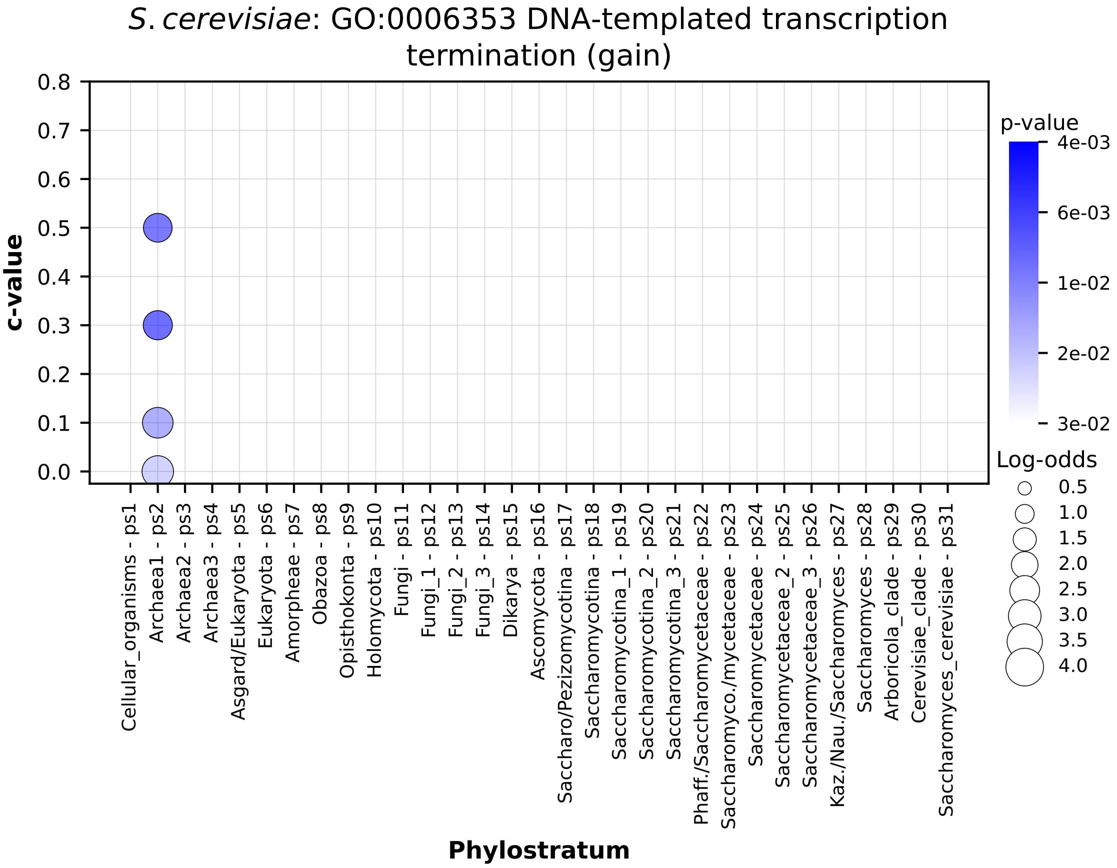

S. cerevisiae: GO:0006353 DNA-templated transcription termination (gain)
c-value
0.8
0.7
0.6
0.5
0.4
0.3
0.2
0.1
0.0
p-value
4e-03
6e-03
1e-02
2e-02
3e-02
Log-odds
0.5
1.0
1.5
2.0
2.5
3.0
3.5
4.0
Cellular_organisms - ps1
Archaea1 - ps2
Archaea2 - ps3
Archaea3 - ps4
Asgard/Eukaryota - ps5
Eukaryota - ps6
Amorpheae - ps7
Obazoa - ps8
Opisthokonta - ps9
Holomycota - ps10
Fungi - ps11
Fungi_1 - ps12
Fungi_2 - ps13
Fungi_3 - ps14
Dikarya - ps15
Ascomycota - ps16
Saccharo/Pezizomycotina - ps17
Saccharomycotina - ps18
Saccharomycotina_1 - ps19
Saccharomycotina_2 - ps20
Saccharomycotina_3 - ps21
Phaff./Saccharomycetaceae - ps22
Saccharomyco./mycetaceae - ps23
Saccharomycetaceae - ps24
Saccharomycetaceae_2 - ps25
Saccharomycetaceae_3 - ps26
Kaz./Nau./Saccharomyces - ps27
Saccharomyces - ps28
Arboricola_clade - ps29
Cerevisiae_clade - ps30
Saccharomyces_cerevisiae - ps31
Phylostratum

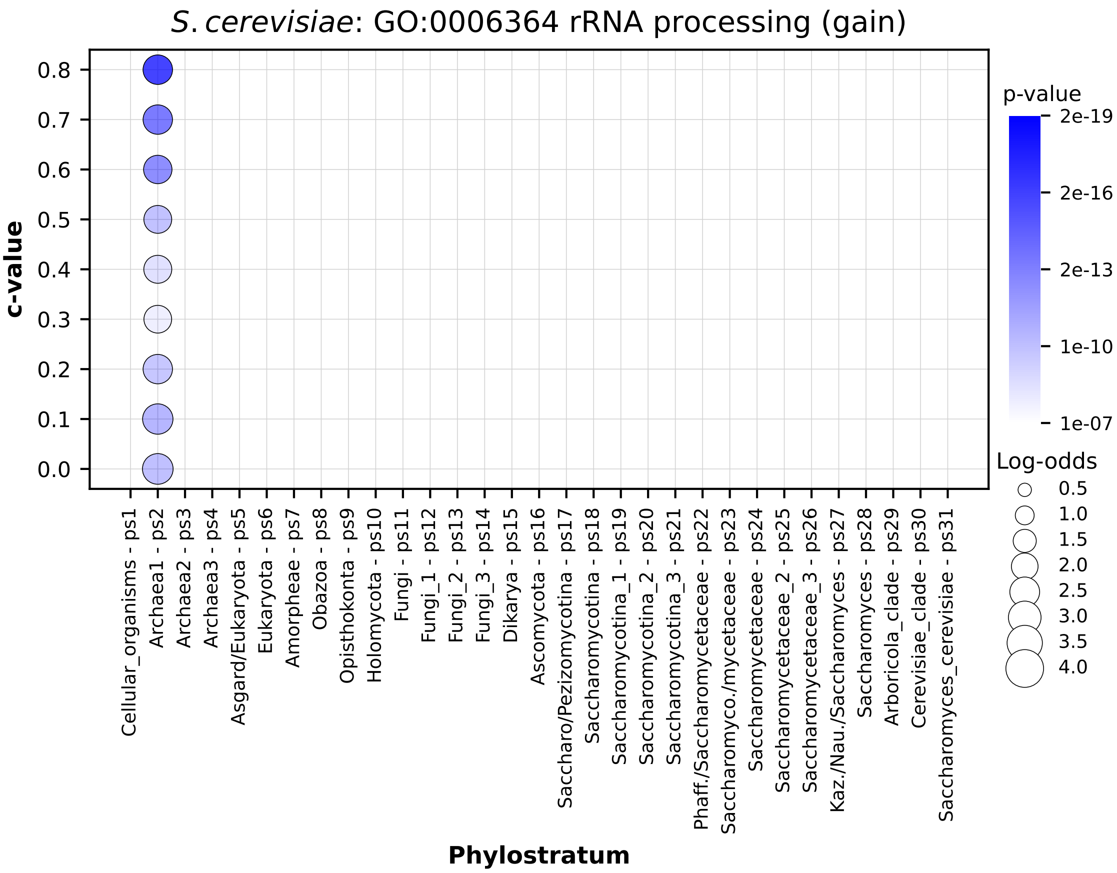

S. cerevisiae: GO:0006364 rRNA processing (gain)
c-value
0.0
0.1
0.2
0.3
0.4
0.5
0.6
0.7
0.8
p-value
2e-19
2e-16
2e-13
1e-10
1e-07
Log-odds
0.5
1.0
1.5
2.0
2.5
3.0
3.5
4.0
Cellular_organisms - ps1
Archaea1 - ps2
Archaea2 - ps3
Archaea3 - ps4
Asgard/Eukaryota - ps5
Eukaryota - ps6
Amorpheae - ps7
Obazoa - ps8
Opisthokonta - ps9
Holomycota - ps10
Fungi - ps11
Fungi_1 - ps12
Fungi_2 - ps13
Fungi_3 - ps14
Dikarya - ps15
Ascomycota - ps16
Saccharo/Pezizomycotina - ps17
Saccharomycotina - ps18
Saccharomycotina_1 - ps19
Saccharomycotina_2 - ps20
Saccharomycotina_3 - ps21
Phaff./Saccharomycetaceae - ps22
Saccharomyco./mycetaceae - ps23
Saccharomycetaceae - ps24
Saccharomycetaceae_2 - ps25
Saccharomycetaceae_3 - ps26
Kaz./Nau./Saccharomyces - ps27
Saccharomyces - ps28
Arboricola_clade - ps29
Cerevisiae_clade - ps30
Saccharomyces_cerevisiae - ps31
Phylostratum

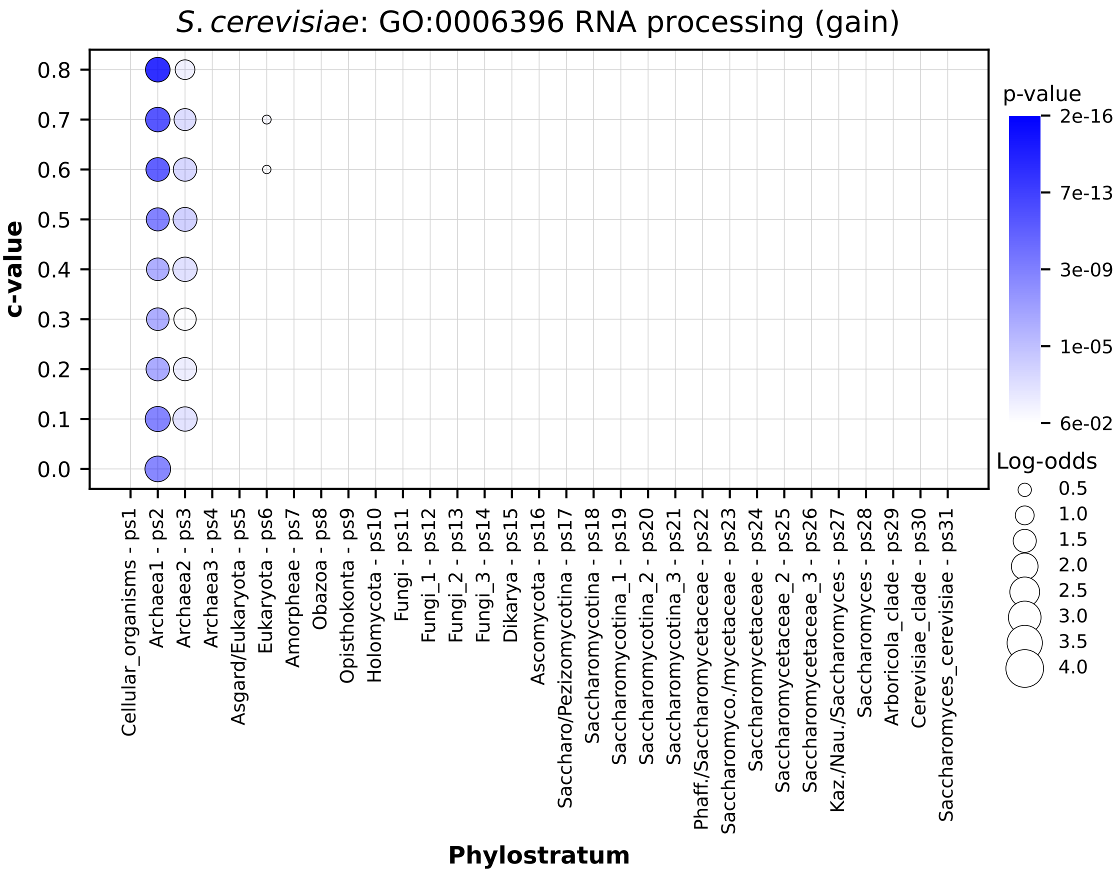

S. cerevisiae: GO:0006396 RNA processing (gain)
c-value
0.8
0.7
0.6
0.5
0.4
0.3
0.2
0.1
0.0
p-value
2e-16
7e-13
3e-09
1e-05
6e-02
Log-odds
0.5
1.0
1.5
2.0
2.5
3.0
3.5
4.0
Cellular_organisms - ps1
Archaea1 - ps2
Archaea2 - ps3
Archaea3 - ps4
Asgard/Eukaryota - ps5
Eukaryota - ps6
Amorpheae - ps7
Obazoa - ps8
Opisthokonta - ps9
Holomycota - ps10
Fungi - ps11
Fungi_1 - ps12
Fungi_2 - ps13
Fungi_3 - ps14
Dikarya - ps15
Ascomycota - ps16
Saccharo/Pezizomycotina - ps17
Saccharomycotina - ps18
Saccharomycotina_1 - ps19
Saccharomycotina_2 - ps20
Saccharomycotina_3 - ps21
Phaff./Saccharomycetaceae - ps22
Saccharomyco./mycetaceae - ps23
Saccharomycetaceae - ps24
Saccharomycetaceae_2 - ps25
Saccharomycetaceae_3 - ps26
Kaz./Nau./Saccharomyces - ps27
Saccharomyces - ps28
Arboricola_clade - ps29
Cerevisiae_clade - ps30
Saccharomyces_cerevisiae - ps31
Phylostratum

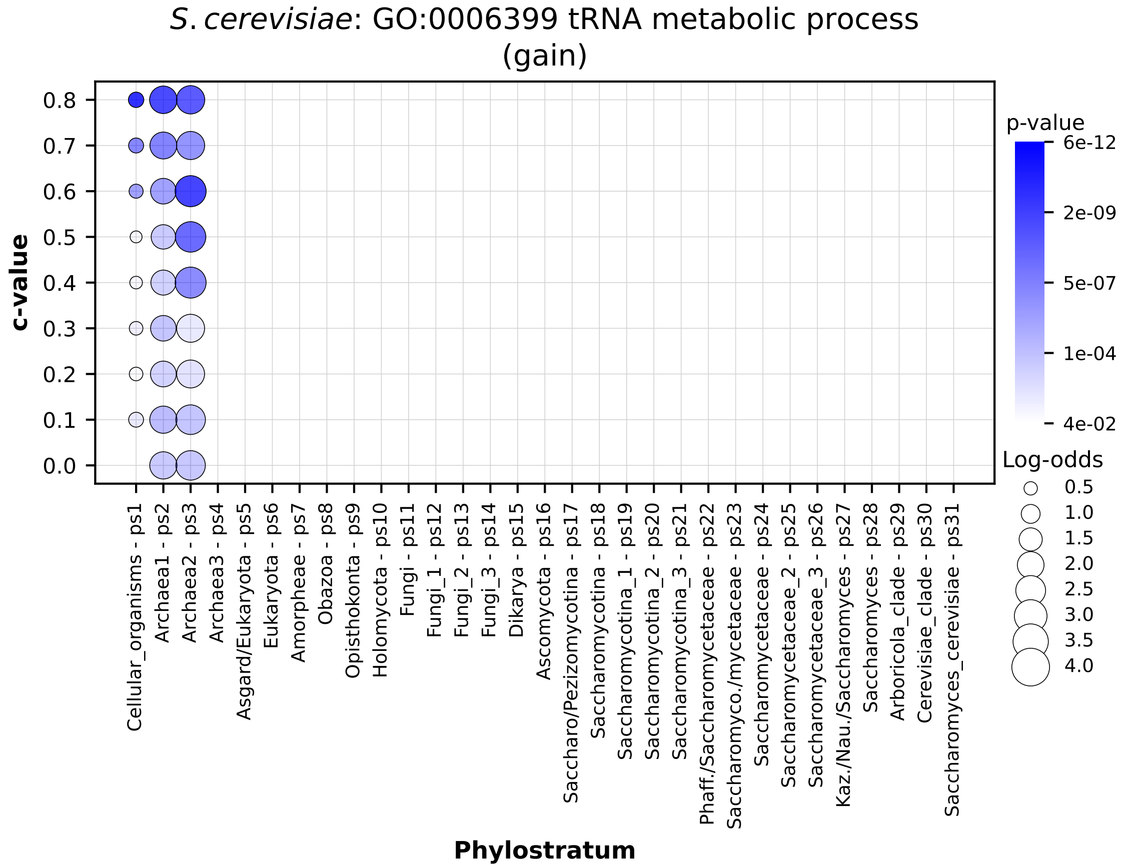

S. cerevisiae: GO:0006399 tRNA metabolic process
(gain)
c-value
0.8
0.7
0.6
0.5
0.4
0.3
0.2
0.1
0.0
Phylostratum
Cellular_organisms - ps1
Archaea1 - ps2
Archaea2 - ps3
Archaea3 - ps4
Asgard/Eukaryota - ps5
Eukaryota - ps6
Amorpheae - ps7
Obazoa - ps8
Opisthokonta - ps9
Holomycota - ps10
Fungi - ps11
Fungi_1 - ps12
Fungi_2 - ps13
Fungi_3 - ps14
Dikarya - ps15
Ascomycota - ps16
Saccharo/Pezizomycotina - ps17
Saccharomycotina - ps18
Saccharomycotina_1 - ps19
Saccharomycotina_2 - ps20
Saccharomycotina_3 - ps21
Phaff./Saccharomycetaceae - ps22
Saccharomyco./mycetaceae - ps23
Saccharomycetaceae - ps24
Saccharomycetaceae_2 - ps25
Saccharomycetaceae_3 - ps26
Kaz./Nau./Saccharomyces - ps27
Saccharomyces - ps28
Arboricola_clade - ps29
Cerevisiae_clade - ps30
Saccharomyces_cerevisiae - ps31
p-value
6e-12
2e-09
5e-07
1e-04
4e-02
Log-odds
0.5
1.0
1.5
2.0
2.5
3.0
3.5
4.0

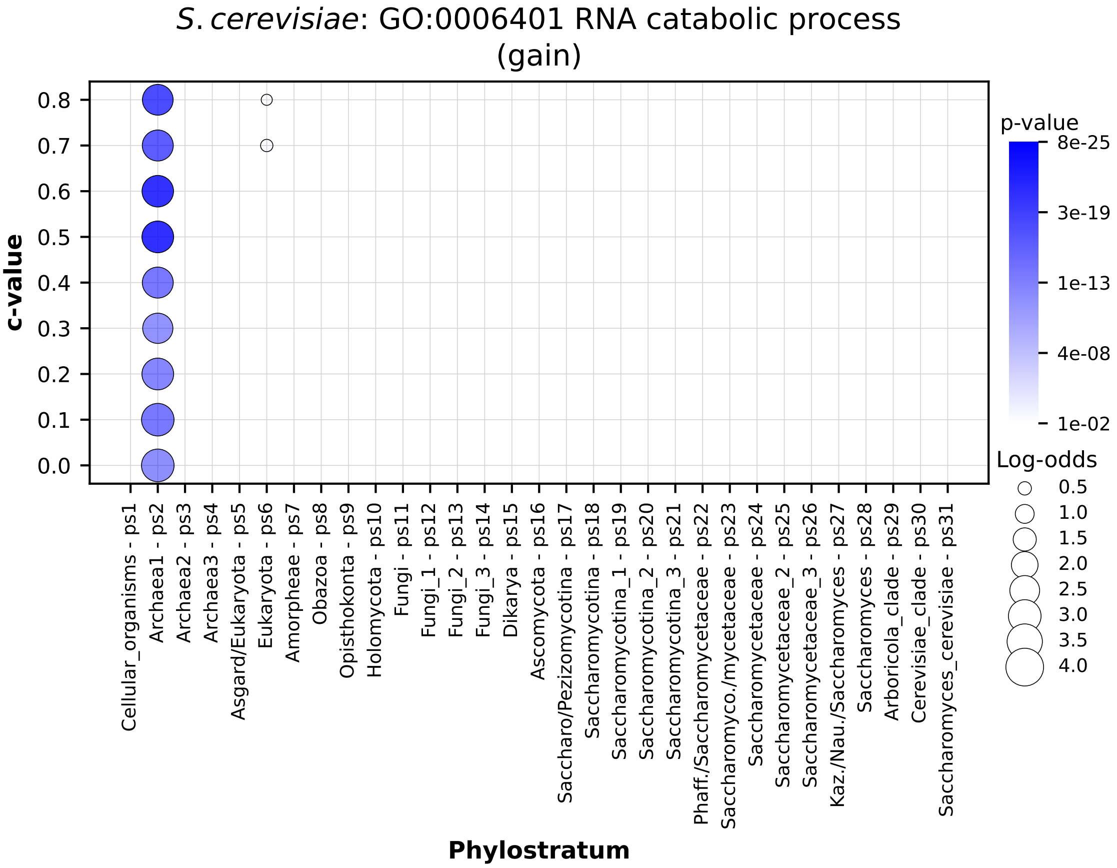

S. cerevisiae: GO:0006401 RNA catabolic process
(gain)
c-value
0.8
0.7
0.6
0.5
0.4
0.3
0.2
0.1
0.0
p-value
8e-25
3e-19
1e-13
4e-08
1e-02
Log-odds
0.5
1.0
1.5
2.0
2.5
3.0
3.5
4.0
Cellular_organisms - ps1
Archaea1 - ps2
Archaea2 - ps3
Archaea3 - ps4
Asgard/Eukaryota - ps5
Eukaryota - ps6
Amorpheae - ps7
Obazoa - ps8
Opisthokonta - ps9
Holomycota - ps10
Fungi - ps11
Fungi_1 - ps12
Fungi_2 - ps13
Fungi_3 - ps14
Dikarya - ps15
Ascomycota - ps16
Saccharo/Pezizomycotina - ps17
Saccharomycotina - ps18
Saccharomycotina_1 - ps19
Saccharomycotina_2 - ps20
Saccharomycotina_3 - ps21
Phaff./Saccharomycetaceae - ps22
Saccharomyco./mycetaceae - ps23
Saccharomycetaceae - ps24
Saccharomycetaceae_2 - ps25
Saccharomycetaceae_3 - ps26
Kaz./Nau./Saccharomyces - ps27
Saccharomyces - ps28
Arboricola_clade - ps29
Cerevisiae_clade - ps30
Saccharomyces_cerevisiae - ps31
Phylostratum

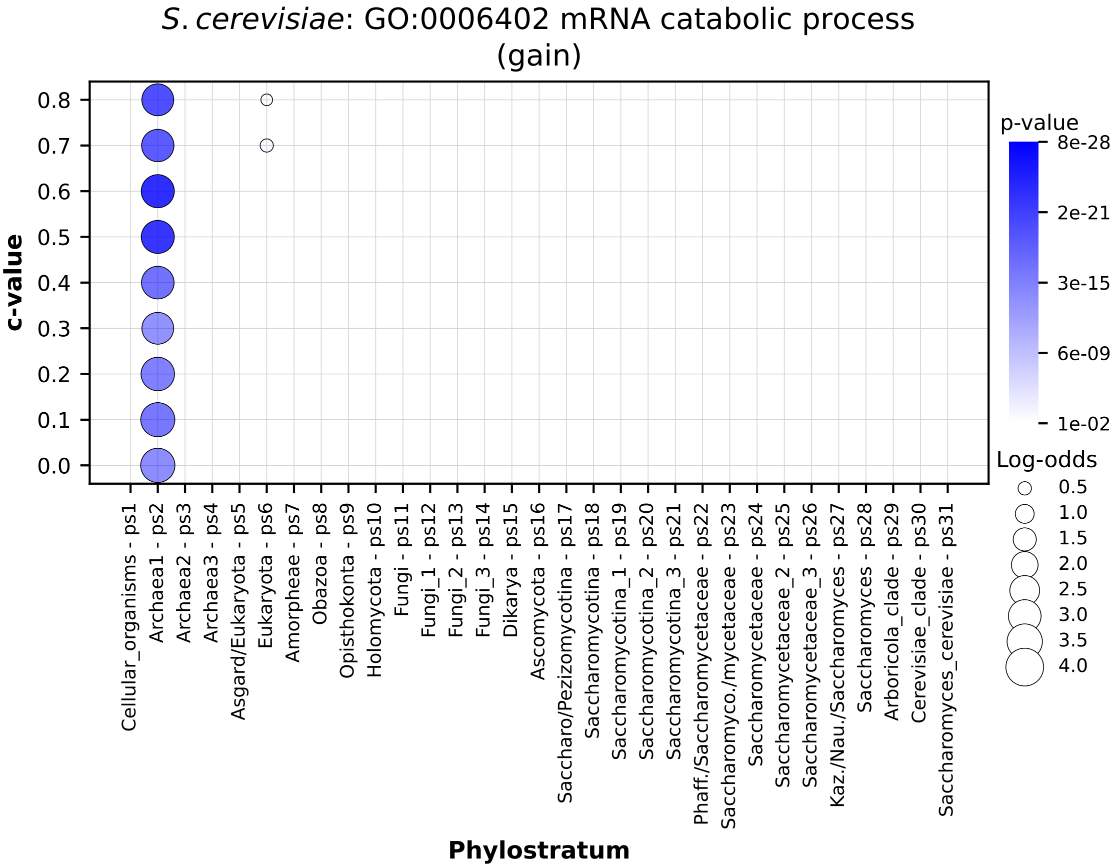

S. cerevisiae: GO:0006402 mRNA catabolic process
(gain)
c-value
0.8
0.7
0.6
0.5
0.4
0.3
0.2
0.1
0.0
p-value
8e-28
2e-21
3e-15
6e-09
1e-02
Log-odds
0.5
1.0
1.5
2.0
2.5
3.0
3.5
4.0
Cellular_organisms - ps1
Archaea1 - ps2
Archaea2 - ps3
Archaea3 - ps4
Asgard/Eukaryota - ps5
Eukaryota - ps6
Amorpheae - ps7
Obazoa - ps8
Opisthokonta - ps9
Holomycota - ps10
Fungi - ps11
Fungi_1 - ps12
Fungi_2 - ps13
Fungi_3 - ps14
Dikarya - ps15
Ascomycota - ps16
Saccharo/Pezizomycotina - ps17
Saccharomycotina - ps18
Saccharomycotina_1 - ps19
Saccharomycotina_2 - ps20
Saccharomycotina_3 - ps21
Phaff./Saccharomycetaceae - ps22
Saccharomyco./mycetaceae - ps23
Saccharomycetaceae - ps24
Saccharomycetaceae_2 - ps25
Saccharomycetaceae_3 - ps26
Kaz./Nau./Saccharomyces - ps27
Saccharomyces - ps28
Arboricola_clade - ps29
Cerevisiae_clade - ps30
Saccharomyces_cerevisiae - ps31
Phylostratum

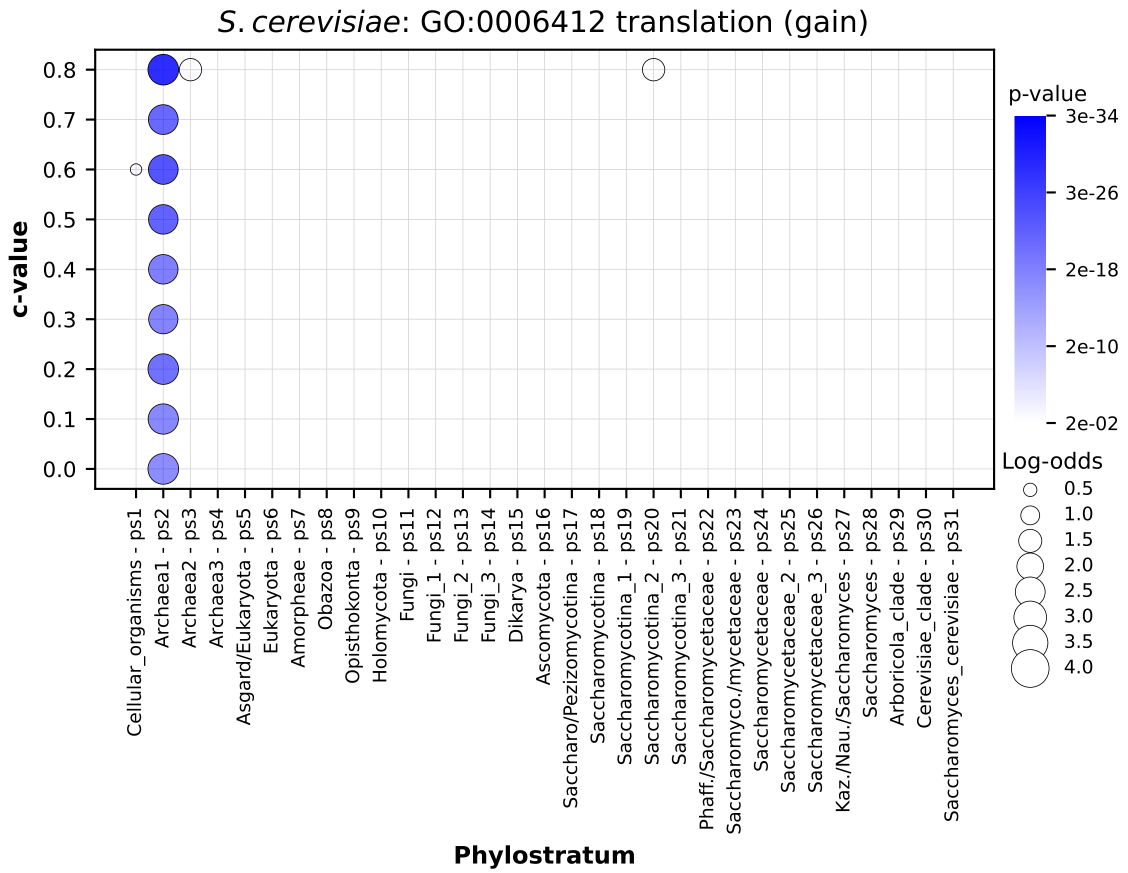

S. cerevisiae: GO:0006412 translation (gain)
c-value
0.8
0.7
0.6
0.5
0.4
0.3
0.2
0.1
0.0
p-value
3e-34
3e-26
2e-18
2e-10
2e-02
Log-odds
0.5
1.0
1.5
2.0
2.5
3.0
3.5
4.0
Cellular_organisms - ps1
Archaea1 - ps2
Archaea2 - ps3
Archaea3 - ps4
Asgard/Eukaryota - ps5
Eukaryota - ps6
Amorpheae - ps7
Obazoa - ps8
Opisthokonta - ps9
Holomycota - ps10
Fungi - ps11
Fungi_1 - ps12
Fungi_2 - ps13
Fungi_3 - ps14
Dikarya - ps15
Ascomycota - ps16
Saccharo/Pezizomycotina - ps17
Saccharomycotina - ps18
Saccharomycotina_1 - ps19
Saccharomycotina_2 - ps20
Saccharomycotina_3 - ps21
Phaff./Saccharomycetaceae - ps22
Saccharomyco./mycetaceae - ps23
Saccharomycetaceae - ps24
Saccharomycetaceae_2 - ps25
Saccharomycetaceae_3 - ps26
Kaz./Nau./Saccharomyces - ps27
Saccharomyces - ps28
Arboricola_clade - ps29
Cerevisiae_clade - ps30
Saccharomyces_cerevisiae - ps31
Phylostratum

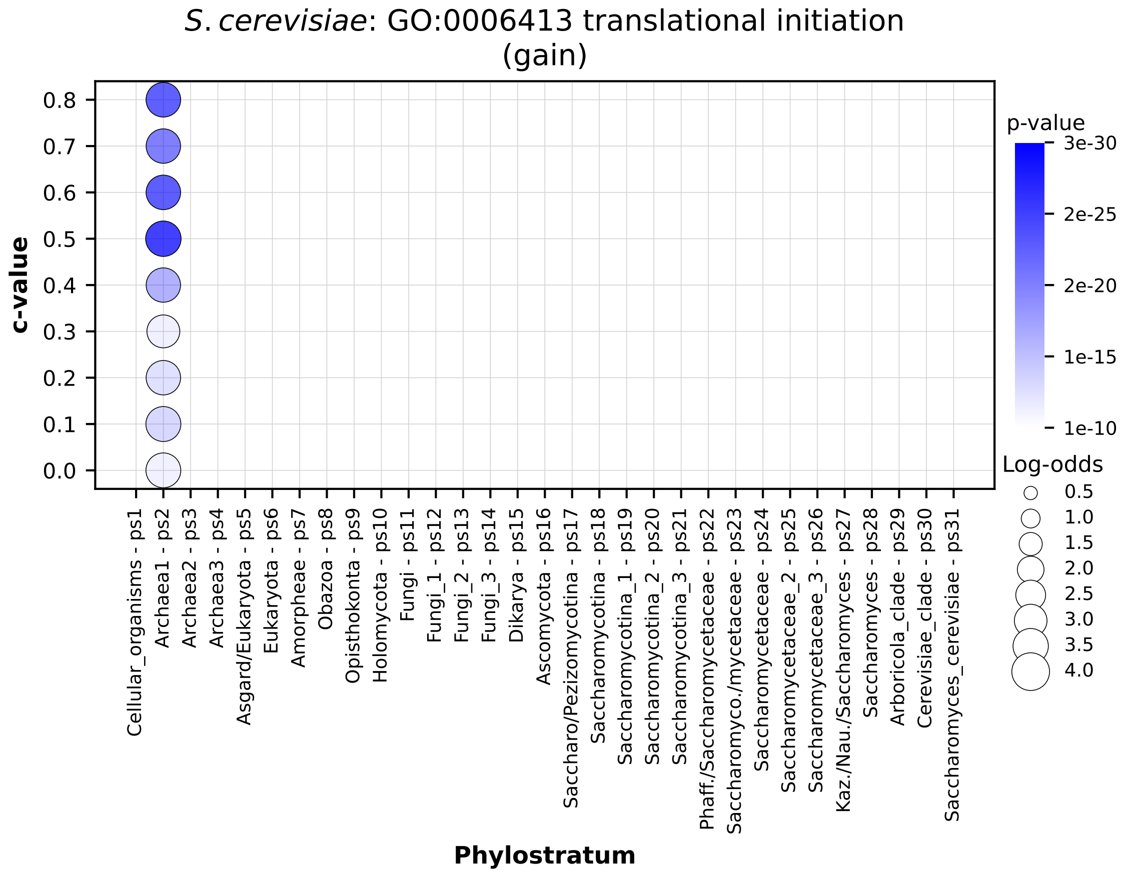

S. cerevisiae: GO:0006413 translational initiation
(gain)
c-value
0.8
0.7
0.6
0.5
0.4
0.3
0.2
0.1
0.0
p-value
3e-30
2e-25
2e-20
1e-15
1e-10
Log-odds
0.5
1.0
1.5
2.0
2.5
3.0
3.5
4.0
Cellular_organisms - ps1
Archaea1 - ps2
Archaea2 - ps3
Archaea3 - ps4
Asgard/Eukaryota - ps5
Eukaryota - ps6
Amorpheae - ps7
Obazoa - ps8
Opisthokonta - ps9
Holomycota - ps10
Fungi - ps11
Fungi_1 - ps12
Fungi_2 - ps13
Fungi_3 - ps14
Dikarya - ps15
Ascomycota - ps16
Saccharo/Pezizomycotina - ps17
Saccharomycotina - ps18
Saccharomycotina_1 - ps19
Saccharomycotina_2 - ps20
Saccharomycotina_3 - ps21
Phaff./Saccharomycetaceae - ps22
Saccharomyco./mycetaceae - ps23
Saccharomycetaceae - ps24
Saccharomycetaceae_2 - ps25
Saccharomycetaceae_3 - ps26
Kaz./Nau./Saccharomyces - ps27
Saccharomyces - ps28
Arboricola_clade - ps29
Cerevisiae_clade - ps30
Saccharomyces_cerevisiae - ps31
Phylostratum

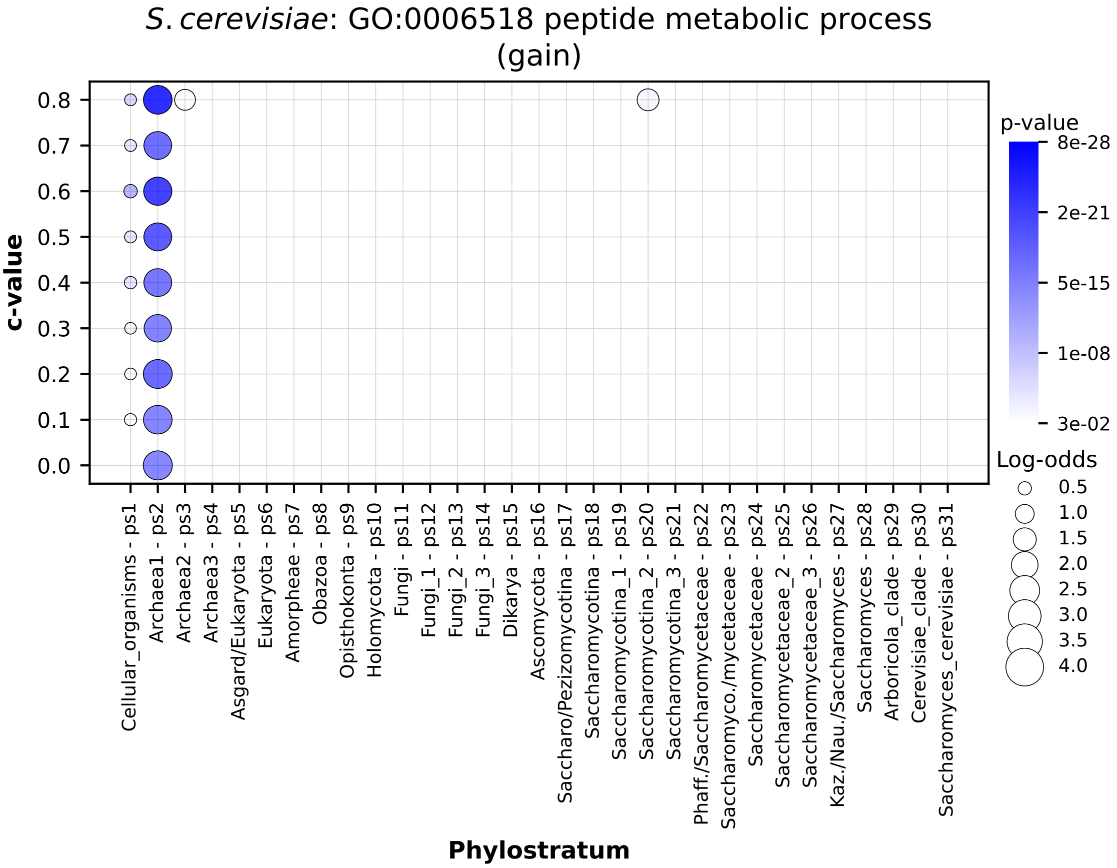

S. cerevisiae: GO:0006518 peptide metabolic process (gain)
c-value
0.0
0.1
0.2
0.3
0.4
0.5
0.6
0.7
0.8
Phylostratum
Cellular_organisms - ps1
Archaea1 - ps2
Archaea2 - ps3
Archaea3 - ps4
Asgard/Eukaryota - ps5
Eukaryota - ps6
Amorpheae - ps7
Obazoa - ps8
Opisthokonta - ps9
Holomycota - ps10
Fungi - ps11
Fungi_1 - ps12
Fungi_2 - ps13
Fungi_3 - ps14
Dikarya - ps15
Ascomycota - ps16
Saccharo/Pezizomycotina - ps17
Saccharomycotina - ps18
Saccharomycotina_1 - ps19
Saccharomycotina_2 - ps20
Saccharomycotina_3 - ps21
Phaff./Saccharomycetaceae - ps22
Saccharomyco./mycetaceae - ps23
Saccharomycetaceae - ps24
Saccharomycetaceae_2 - ps25
Saccharomycetaceae_3 - ps26
Kaz./Nau./Saccharomyces - ps27
Saccharomyces - ps28
Arboricola_clade - ps29
Cerevisiae_clade - ps30
Saccharomyces_cerevisiae - ps31
p-value
8e-28
2e-21
5e-15
1e-08
3e-02
Log-odds
0.5
1.0
1.5
2.0
2.5
3.0
3.5
4.0

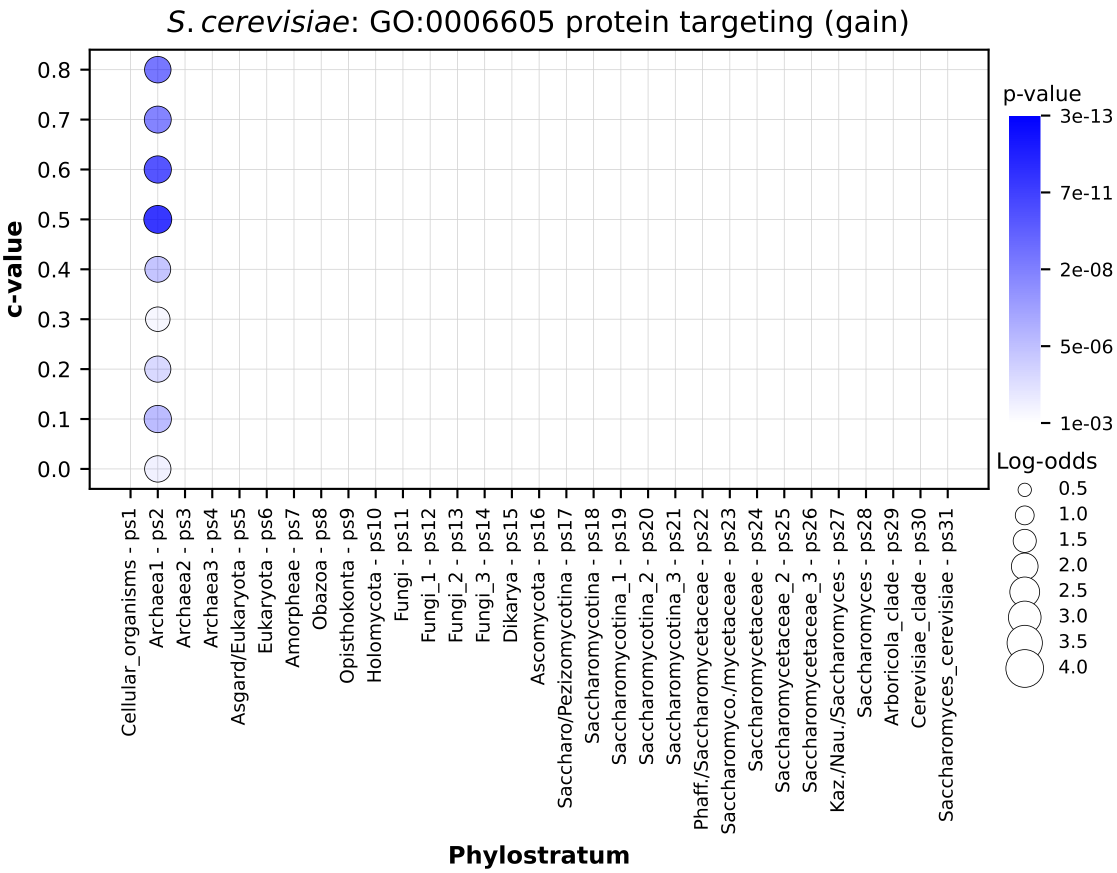

S. cerevisiae: GO:0006605 protein targeting (gain)
c-value
0.8
0.7
0.6
0.5
0.4
0.3
0.2
0.1
0.0
p-value
3e-13
7e-11
2e-08
5e-06
1e-03
Log-odds
0.5
1.0
1.5
2.0
2.5
3.0
3.5
4.0
Cellular_organisms - ps1
Archaea1 - ps2
Archaea2 - ps3
Archaea3 - ps4
Asgard/Eukaryota - ps5
Eukaryota - ps6
Amorpheae - ps7
Obazoa - ps8
Opisthokonta - ps9
Holomycota - ps10
Fungi - ps11
Fungi_1 - ps12
Fungi_2 - ps13
Fungi_3 - ps14
Dikarya - ps15
Ascomycota - ps16
Saccharo/Pezizomycotina - ps17
Saccharomycotina - ps18
Saccharomycotina_1 - ps19
Saccharomycotina_2 - ps20
Saccharomycotina_3 - ps21
Phaff./Saccharomycetaceae - ps22
Saccharomyco./mycetaceae - ps23
Saccharomycetaceae - ps24
Saccharomycetaceae_2 - ps25
Saccharomycetaceae_3 - ps26
Kaz./Nau./Saccharomyces - ps27
Saccharomyces - ps28
Arboricola_clade - ps29
Cerevisiae_clade - ps30
Saccharomyces_cerevisiae - ps31
Phylostratum

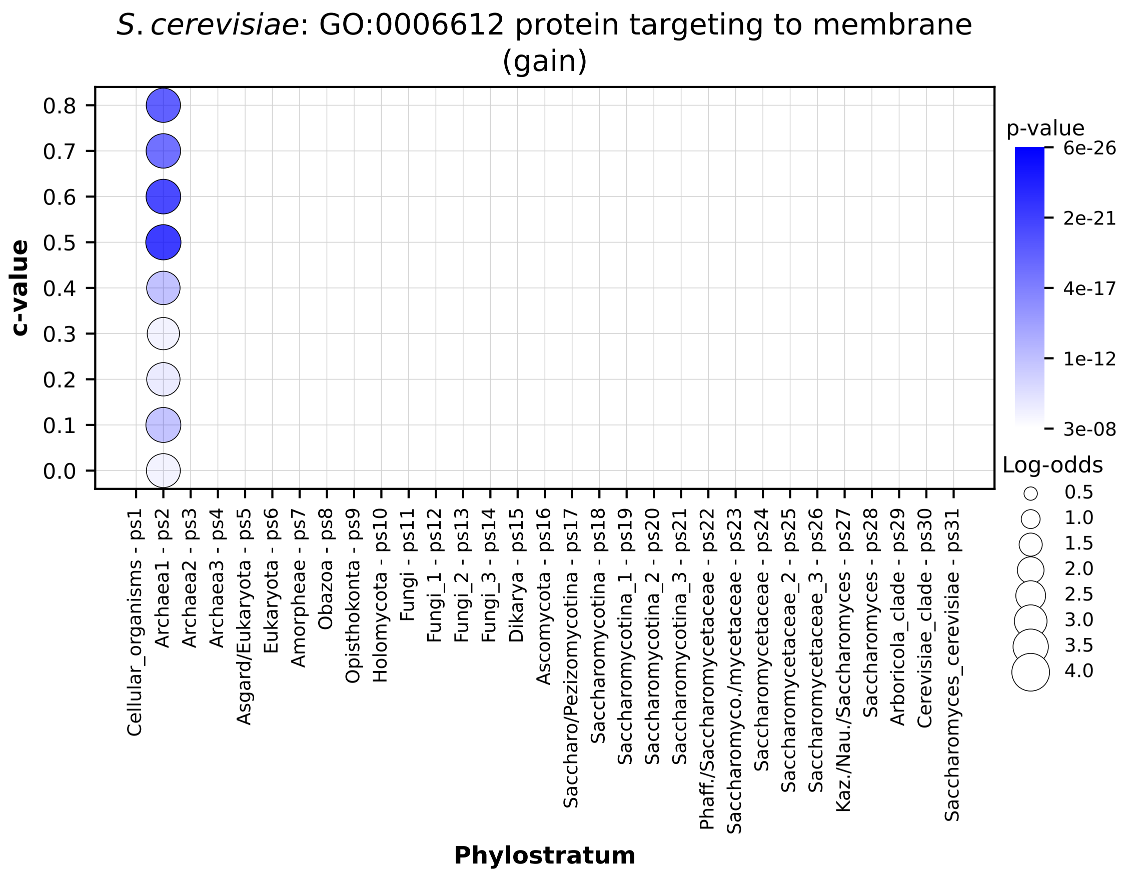

S. cerevisiae: GO:0006612 protein targeting to membrane
(gain)
c-value
0.8
0.7
0.6
0.5
0.4
0.3
0.2
0.1
0.0
p-value
6e-26
2e-21
4e-17
1e-12
3e-08
Log-odds
0.5
1.0
1.5
2.0
2.5
3.0
3.5
4.0
Cellular_organisms - ps1
Archaea1 - ps2
Archaea2 - ps3
Archaea3 - ps4
Asgard/Eukaryota - ps5
Eukaryota - ps6
Amorpheae - ps7
Obazoa - ps8
Opisthokonta - ps9
Holomycota - ps10
Fungi - ps11
Fungi_1 - ps12
Fungi_2 - ps13
Fungi_3 - ps14
Dikarya - ps15
Ascomycota - ps16
Saccharo/Pezizomycotina - ps17
Saccharomycotina - ps18
Saccharomycotina_1 - ps19
Saccharomycotina_2 - ps20
Saccharomycotina_3 - ps21
Phaff./Saccharomycetaceae - ps22
Saccharomyco./mycetaceae - ps23
Saccharomycetaceae - ps24
Saccharomycetaceae_2 - ps25
Saccharomycetaceae_3 - ps26
Kaz./Nau./Saccharomyces - ps27
Saccharomyces - ps28
Arboricola_clade - ps29
Cerevisiae_clade - ps30
Saccharomyces_cerevisiae - ps31
Phylostratum

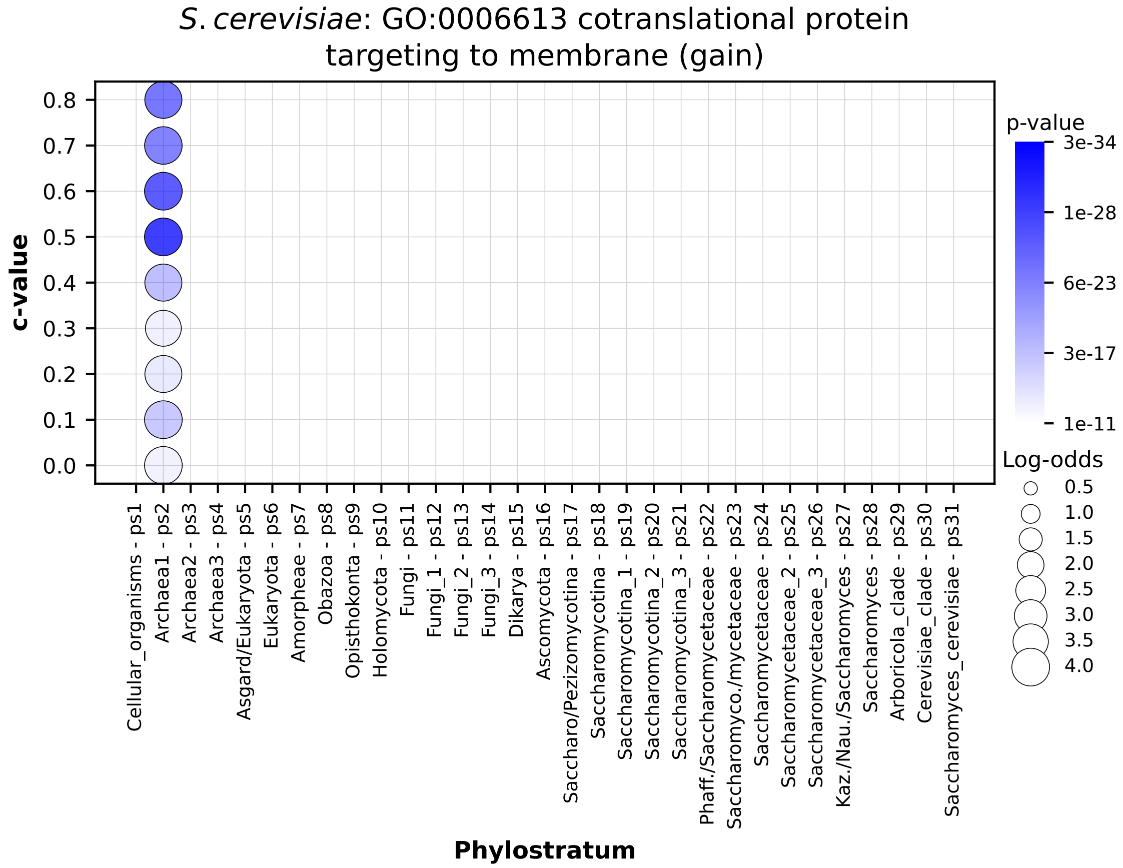

S. cerevisiae: GO:0006613 cotranslational protein targeting to membrane (gain)
c-value
0.8
0.7
0.6
0.5
0.4
0.3
0.2
0.1
0.0
Phylostratum
Cellular_organisms - ps1
Archaea1 - ps2
Archaea2 - ps3
Archaea3 - ps4
Asgard/Eukaryota - ps5
Eukaryota - ps6
Amorpheae - ps7
Obazoa - ps8
Opisthokonta - ps9
Holomycota - ps10
Fungi - ps11
Fungi_1 - ps12
Fungi_2 - ps13
Fungi_3 - ps14
Dikarya - ps15
Ascomycota - ps16
Saccharo/Pezizomycotina - ps17
Saccharomycotina - ps18
Saccharomycotina_1 - ps19
Saccharomycotina_2 - ps20
Saccharomycotina_3 - ps21
Phaff./Saccharomycetaceae - ps22
Saccharomyco./mycetaceae - ps23
Saccharomycetaceae - ps24
Saccharomycetaceae_2 - ps25
Saccharomycetaceae_3 - ps26
Kaz./Nau./Saccharomyces - ps27
Saccharomyces - ps28
Arboricola_clade - ps29
Cerevisiae_clade - ps30
Saccharomyces_cerevisiae - ps31
p-value
3e-34
1e-28
6e-23
3e-17
1e-11
Log-odds
0.5
1.0
1.5
2.0
2.5
3.0
3.5
4.0

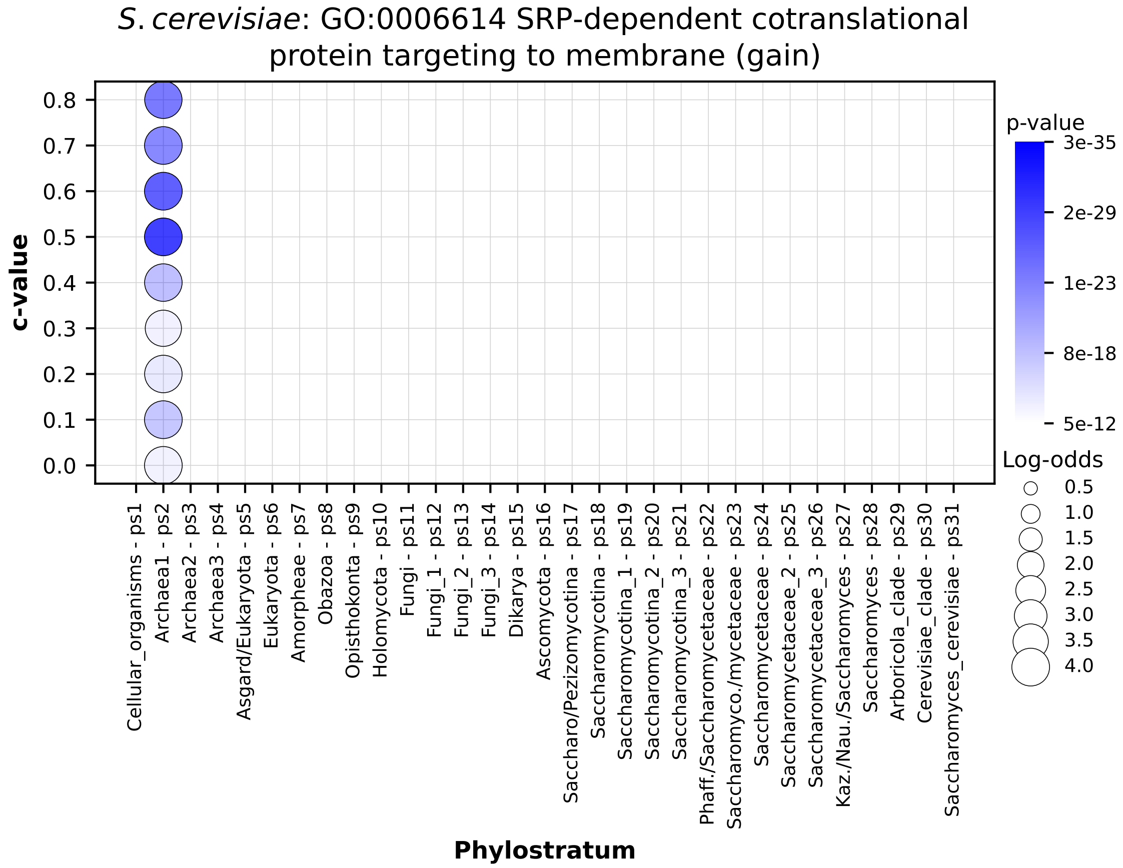

S. cerevisiae: GO:0006614 SRP-dependent cotranslational protein targeting to membrane (gain)
c-value
0.8
0.7
0.6
0.5
0.4
0.3
0.2
0.1
0.0
p-value
3e-35
2e-29
1e-23
8e-18
5e-12
Log-odds
0.5
1.0
1.5
2.0
2.5
3.0
3.5
4.0
Cellular_organisms - ps1
Archaea1 - ps2
Archaea2 - ps3
Archaea3 - ps4
Asgard/Eukaryota - ps5
Eukaryota - ps6
Amorpheae - ps7
Obazoa - ps8
Opisthokonta - ps9
Holomycota - ps10
Fungi - ps11
Fungi_1 - ps12
Fungi_2 - ps13
Fungi_3 - ps14
Dikarya - ps15
Ascomycota - ps16
Saccharo/Pezizomycotina - ps17
Saccharomycotina - ps18
Saccharomycotina_1 - ps19
Saccharomycotina_2 - ps20
Saccharomycotina_3 - ps21
Phaff./Saccharomycetaceae - ps22
Saccharomyco./mycetaceae - ps23
Saccharomycetaceae - ps24
Saccharomycetaceae_2 - ps25
Saccharomycetaceae_3 - ps26
Kaz./Nau./Saccharomyces - ps27
Saccharomyces - ps28
Arboricola_clade - ps29
Cerevisiae_clade - ps30
Saccharomyces_cerevisiae - ps31
Phylostratum

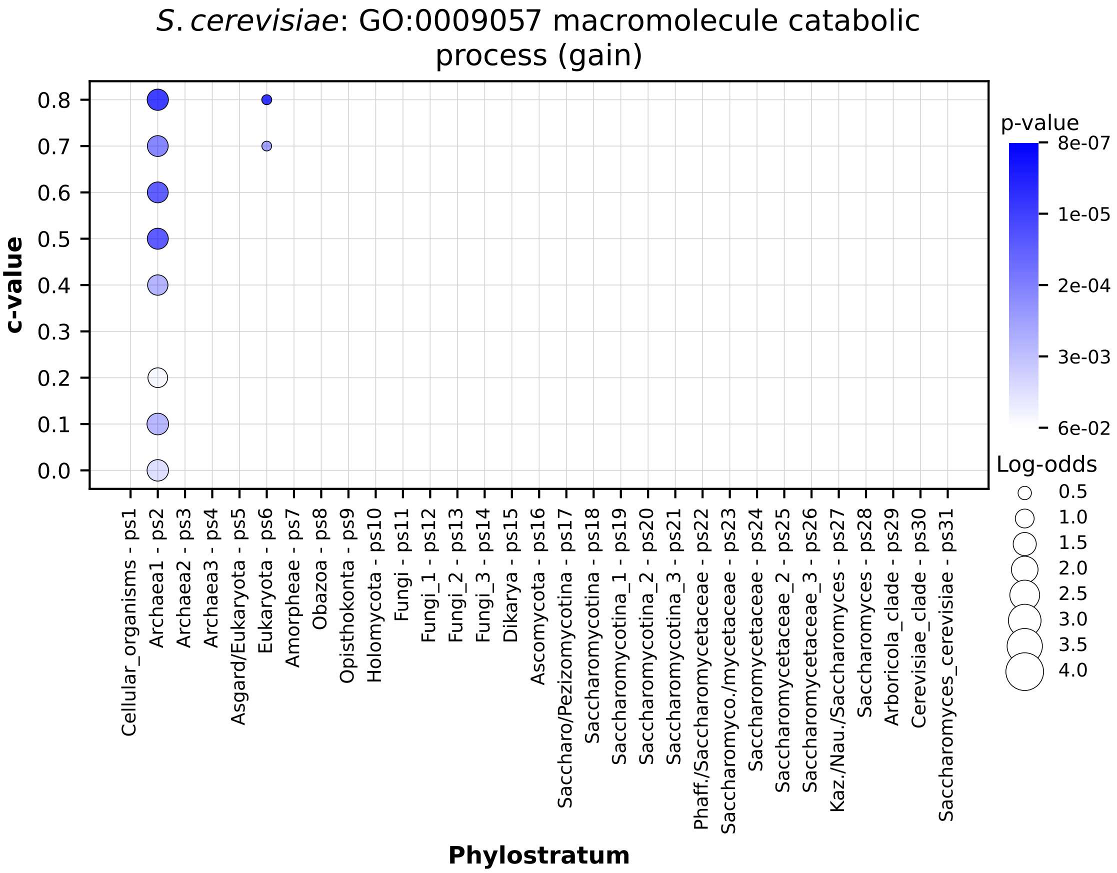

S. cerevisiae: GO:0009057 macromolecule catabolic process (gain)
c-value
0.8
0.7
0.6
0.5
0.4
0.3
0.2
0.1
0.0
p-value
8e-07
1e-05
2e-04
3e-03
6e-02
Log-odds
0.5
1.0
1.5
2.0
2.5
3.0
3.5
4.0
Cellular_organisms - ps1
Archaea1 - ps2
Archaea2 - ps3
Archaea3 - ps4
Asgard/Eukaryota - ps5
Eukaryota - ps6
Amorpheae - ps7
Obazoa - ps8
Opisthokonta - ps9
Holomycota - ps10
Fungi - ps11
Fungi_1 - ps12
Fungi_2 - ps13
Fungi_3 - ps14
Dikarya - ps15
Ascomycota - ps16
Saccharo/Pezizomycotina - ps17
Saccharomycotina - ps18
Saccharomycotina_1 - ps19
Saccharomycotina_2 - ps20
Saccharomycotina_3 - ps21
Phaff./Saccharomycetaceae - ps22
Saccharomyco./mycetaceae - ps23
Saccharomycetaceae - ps24
Saccharomycetaceae_2 - ps25
Saccharomycetaceae_3 - ps26
Kaz./Nau./Saccharomyces - ps27
Saccharomyces - ps28
Arboricola_clade - ps29
Cerevisiae_clade - ps30
Saccharomyces_cerevisiae - ps31
Phylostratum

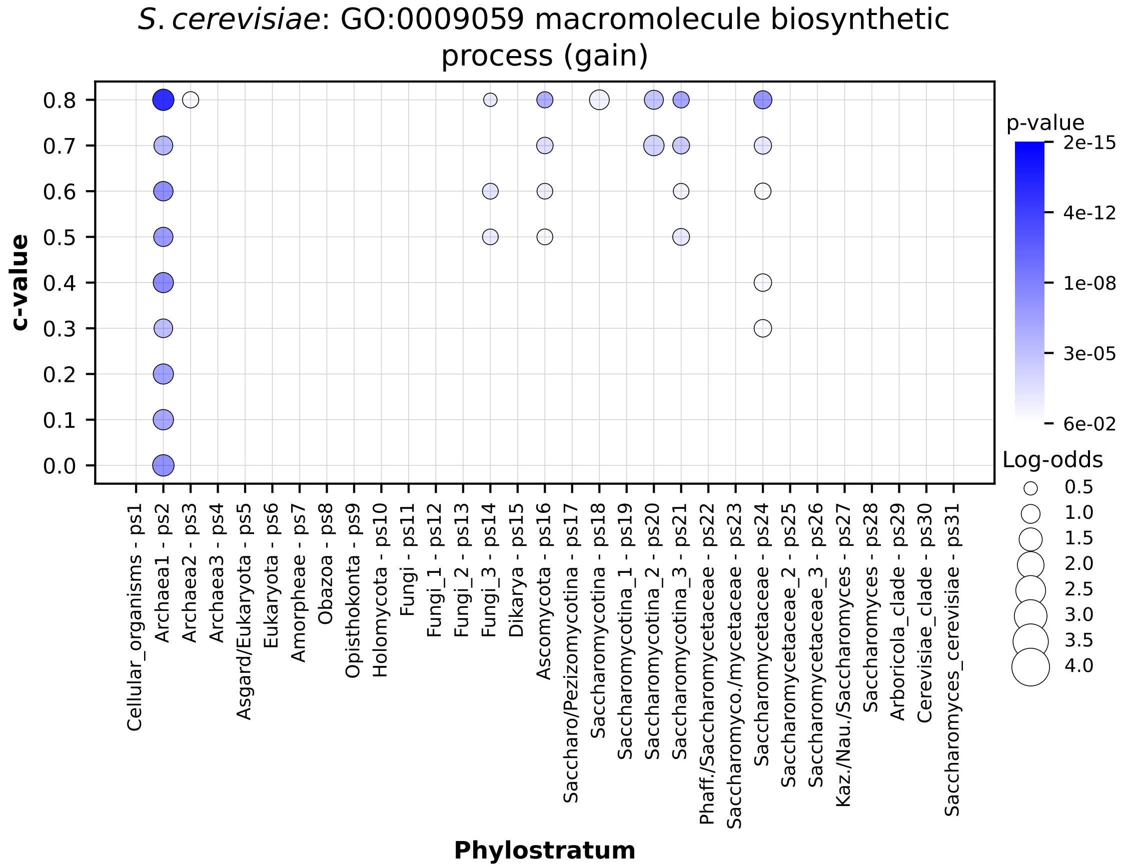

S. cerevisiae: GO:0009059 macromolecule biosynthetic process (gain)
c-value
0.8
0.7
0.6
0.5
0.4
0.3
0.2
0.1
0.0
p-value
2e-15
4e-12
1e-08
3e-05
6e-02
Log-odds
0.5
1.0
1.5
2.0
2.5
3.0
3.5
4.0
Cellular_organisms - ps1
Archaea1 - ps2
Archaea2 - ps3
Archaea3 - ps4
Asgard/Eukaryota - ps5
Eukaryota - ps6
Amorpheae - ps7
Obazoa - ps8
Opisthokonta - ps9
Holomycota - ps10
Fungi - ps11
Fungi_1 - ps12
Fungi_2 - ps13
Fungi_3 - ps14
Dikarya - ps15
Ascomycota - ps16
Saccharo/Pezizomycotina - ps17
Saccharomycotina - ps18
Saccharomycotina_1 - ps19
Saccharomycotina_2 - ps20
Saccharomycotina_3 - ps21
Phaff./Saccharomycetaceae - ps22
Saccharomyco./mycetaceae - ps23
Saccharomycetaceae - ps24
Saccharomycetaceae_2 - ps25
Saccharomycetaceae_3 - ps26
Kaz./Nau./Saccharomyces - ps27
Saccharomyces - ps28
Arboricola_clade - ps29
Cerevisiae_clade - ps30
Saccharomyces_cerevisiae - ps31
Phylostratum

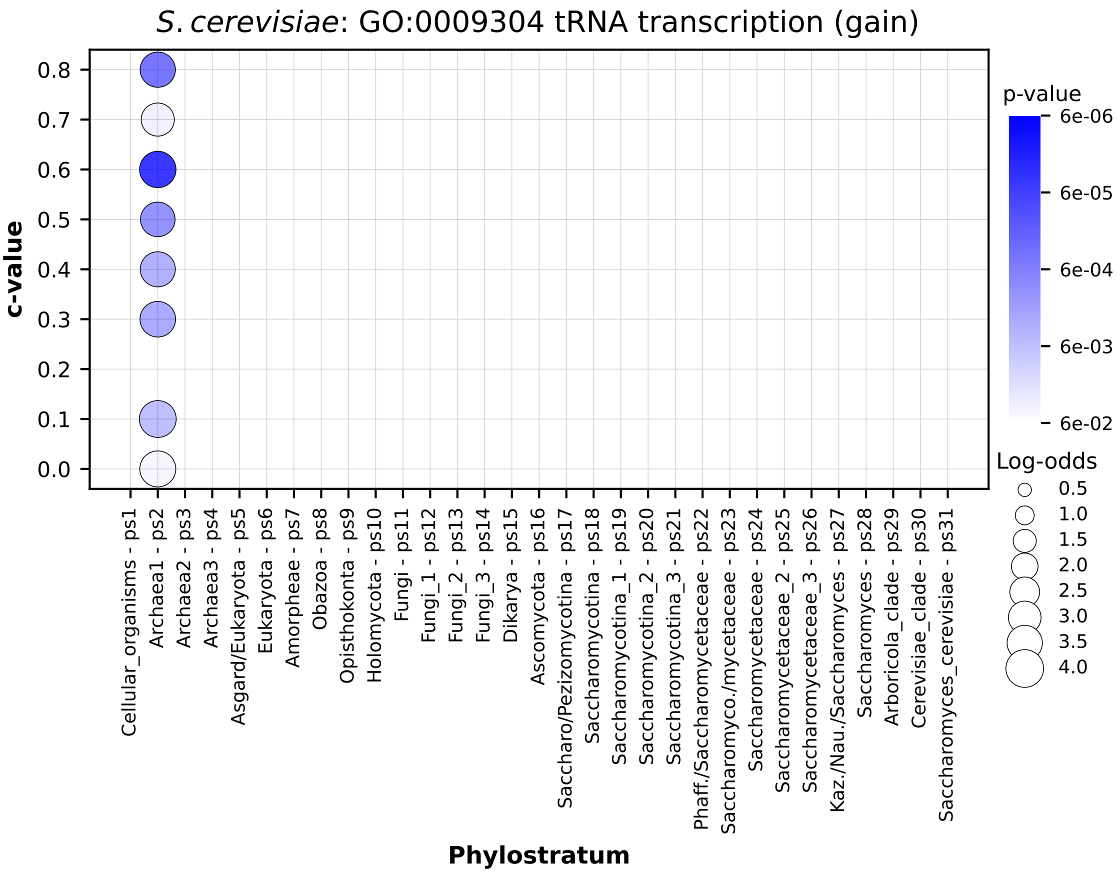

S. cerevisiae: GO:0009304 tRNA transcription (gain)
c-value
0.8
0.7
0.6
0.5
0.4
0.3
0.2
0.1
0.0
p-value
6e-06
6e-05
6e-04
6e-03
6e-02
Log-odds
0.5
1.0
1.5
2.0
2.5
3.0
3.5
4.0
Cellular_organisms - ps1
Archaea1 - ps2
Archaea2 - ps3
Archaea3 - ps4
Asgard/Eukaryota - ps5
Eukaryota - ps6
Amorpheae - ps7
Obazoa - ps8
Opisthokonta - ps9
Holomycota - ps10
Fungi - ps11
Fungi_1 - ps12
Fungi_2 - ps13
Fungi_3 - ps14
Dikarya - ps15
Ascomycota - ps16
Saccharo/Pezizomycotina - ps17
Saccharomycotina - ps18
Saccharomycotina_1 - ps19
Saccharomycotina_2 - ps20
Saccharomycotina_3 - ps21
Phaff./Saccharomycetaceae - ps22
Saccharomyco./mycetaceae - ps23
Saccharomycetaceae - ps24
Saccharomycetaceae_2 - ps25
Saccharomycetaceae_3 - ps26
Kaz./Nau./Saccharomyces - ps27
Saccharomyces - ps28
Arboricola_clade - ps29
Cerevisiae_clade - ps30
Saccharomyces_cerevisiae - ps31
Phylostratum

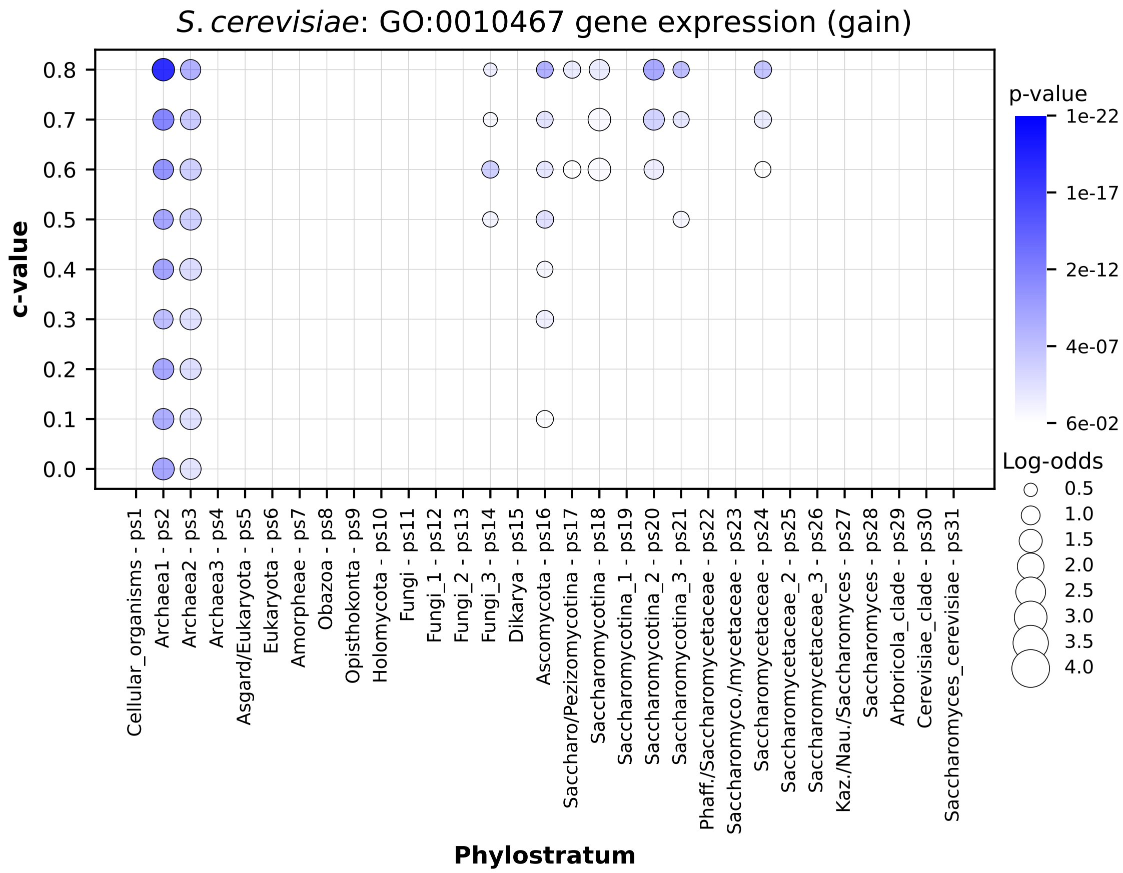

S. cerevisiae: GO:0010467 gene expression (gain)
c-value
0.8
0.7
0.6
0.5
0.4
0.3
0.2
0.1
0.0
p-value
1e-22
1e-17
2e-12
4e-07
6e-02
Log-odds
0.5
1.0
1.5
2.0
2.5
3.0
3.5
4.0
Cellular_organisms - ps1
Archaea1 - ps2
Archaea2 - ps3
Archaea3 - ps4
Asgard/Eukaryota - ps5
Eukaryota - ps6
Amorpheae - ps7
Obazoa - ps8
Opisthokonta - ps9
Holomycota - ps10
Fungi - ps11
Fungi_1 - ps12
Fungi_2 - ps13
Fungi_3 - ps14
Dikarya - ps15
Ascomycota - ps16
Saccharo/Pezizomycotina - ps17
Saccharomycotina - ps18
Saccharomycotina_1 - ps19
Saccharomycotina_2 - ps20
Saccharomycotina_3 - ps21
Phaff./Saccharomycetaceae - ps22
Saccharomyco./mycetaceae - ps23
Saccharomycetaceae - ps24
Saccharomycetaceae_2 - ps25
Saccharomycetaceae_3 - ps26
Kaz./Nau./Saccharomyces - ps27
Saccharomyces - ps28
Arboricola_clade - ps29
Cerevisiae_clade - ps30
Saccharomyces_cerevisiae - ps31
Phylostratum

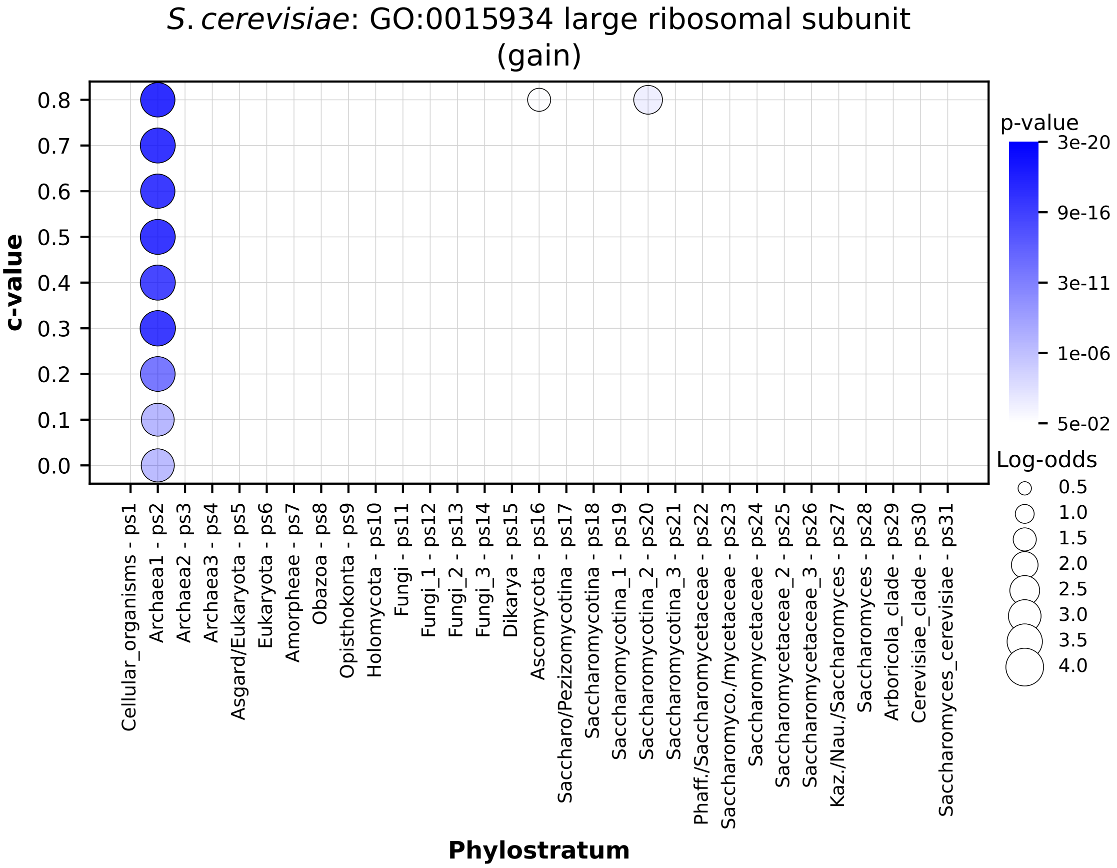
S. cerevisiae: GO:0015934 large ribosomal subunit
(gain)
c-value
0.8
0.7
0.6
0.5
0.4
0.3
0.2
0.1
0.0
p-value
3e-20
9e-16
3e-11
1e-06
5e-02
Log-odds
0.5
1.0
1.5
2.0
2.5
3.0
3.5
4.0
Cellular_organisms - ps1
Archaea1 - ps2
Archaea2 - ps3
Archaea3 - ps4
Asgard/Eukaryota - ps5
Eukaryota - ps6
Amorpheae - ps7
Obazoa - ps8
Opisthokonta - ps9
Holomycota - ps10
Fungi - ps11
Fungi_1 - ps12
Fungi_2 - ps13
Fungi_3 - ps14
Dikarya - ps15
Ascomycota - ps16
Saccharo/Pezizomycotina - ps17
Saccharomycotina - ps18
Saccharomycotina_1 - ps19
Saccharomycotina_2 - ps20
Saccharomycotina_3 - ps21
Phaff./Saccharomycetaceae - ps22
Saccharomyco./mycetaceae - ps23
Saccharomycetaceae - ps24
Saccharomycetaceae_2 - ps25
Saccharomycetaceae_3 - ps26
Kaz./Nau./Saccharomyces - ps27
Saccharomyces - ps28
Arboricola_clade - ps29
Cerevisiae_clade - ps30
Saccharomyces_cerevisiae - ps31
Phylostratum

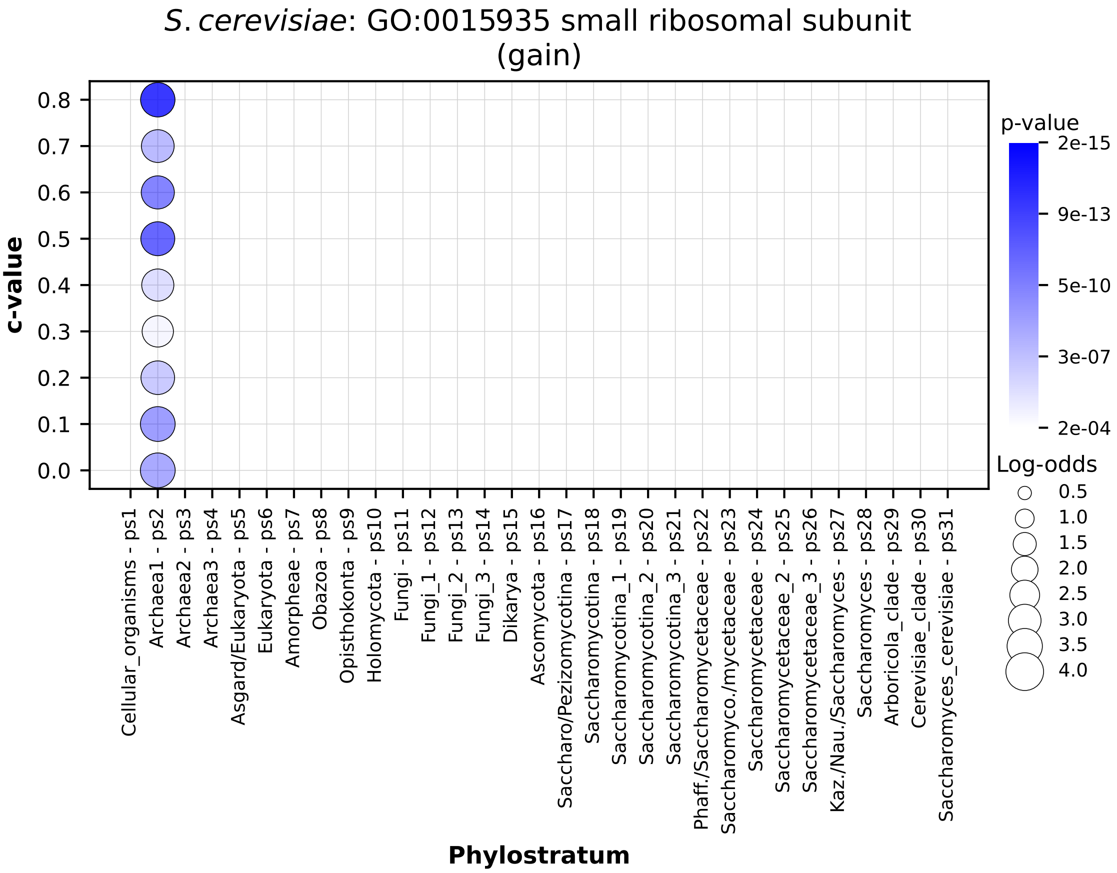

S. cerevisiae: GO:0015935 small ribosomal subunit
(gain)
c-value
0.8
0.7
0.6
0.5
0.4
0.3
0.2
0.1
0.0
p-value
2e-15
9e-13
5e-10
3e-07
2e-04
Log-odds
0.5
1.0
1.5
2.0
2.5
3.0
3.5
4.0
Cellular_organisms - ps1
Archaea1 - ps2
Archaea2 - ps3
Archaea3 - ps4
Asgard/Eukaryota - ps5
Eukaryota - ps6
Amorpheae - ps7
Obazoa - ps8
Opisthokonta - ps9
Holomycota - ps10
Fungi - ps11
Fungi_1 - ps12
Fungi_2 - ps13
Fungi_3 - ps14
Dikarya - ps15
Ascomycota - ps16
Saccharo/Pezizomycotina - ps17
Saccharomycotina - ps18
Saccharomycotina_1 - ps19
Saccharomycotina_2 - ps20
Saccharomycotina_3 - ps21
Phaff./Saccharomycetaceae - ps22
Saccharomyco./mycetaceae - ps23
Saccharomycetaceae - ps24
Saccharomycetaceae_2 - ps25
Saccharomycetaceae_3 - ps26
Kaz./Nau./Saccharomyces - ps27
Saccharomyces - ps28
Arboricola_clade - ps29
Cerevisiae_clade - ps30
Saccharomyces_cerevisiae - ps31
Phylostratum

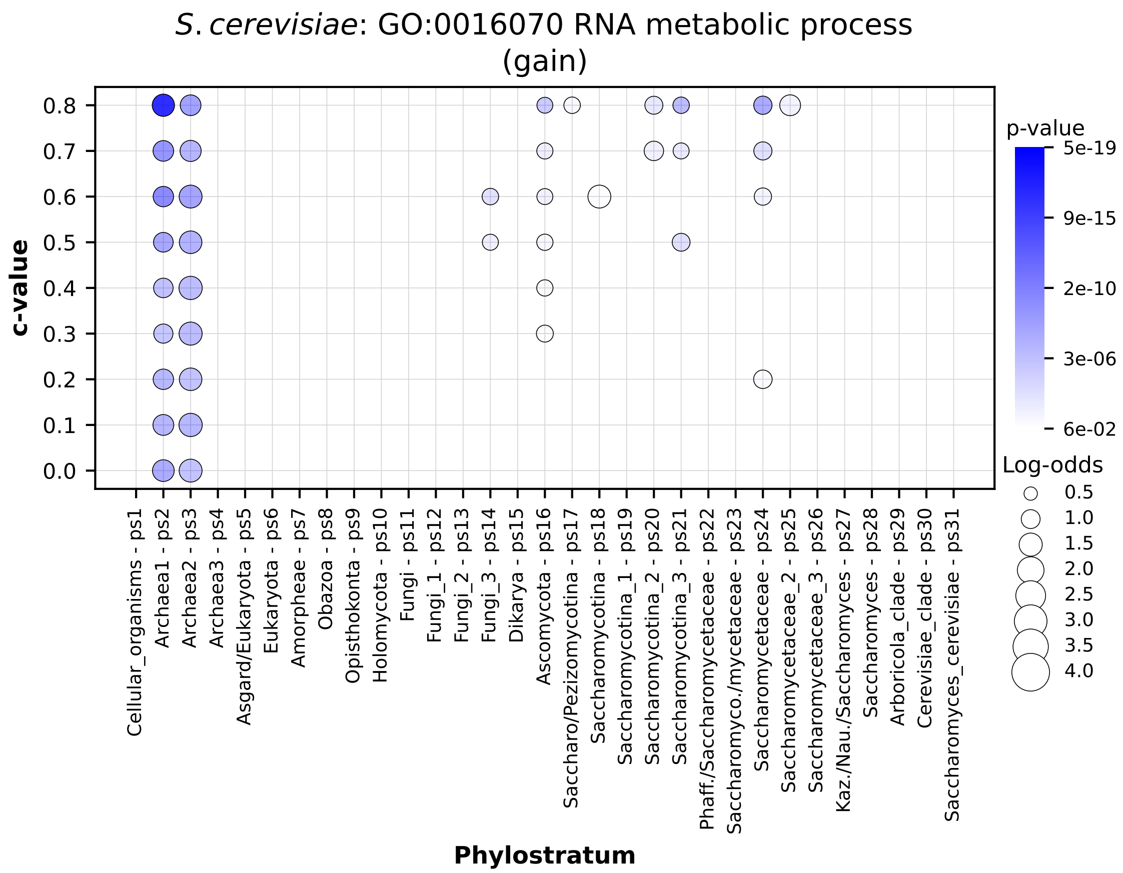
S. cerevisiae: GO:0016070 RNA metabolic process
(gain)
c-value
0.8
0.7
0.6
0.5
0.4
0.3
0.2
0.1
0.0
p-value
5e-19
9e-15
2e-10
3e-06
6e-02
Log-odds
0.5
1.0
1.5
2.0
2.5
3.0
3.5
4.0
Cellular_organisms - ps1
Archaea1 - ps2
Archaea2 - ps3
Archaea3 - ps4
Asgard/Eukaryota - ps5
Eukaryota - ps6
Amorpheae - ps7
Obazoa - ps8
Opisthokonta - ps9
Holomycota - ps10
Fungi - ps11
Fungi_1 - ps12
Fungi_2 - ps13
Fungi_3 - ps14
Dikarya - ps15
Ascomycota - ps16
Saccharo/Pezizomycotina - ps17
Saccharomycotina - ps18
Saccharomycotina_1 - ps19
Saccharomycotina_2 - ps20
Saccharomycotina_3 - ps21
Phaff./Saccharomycetaceae - ps22
Saccharomyco./mycetaceae - ps23
Saccharomycetaceae - ps24
Saccharomycetaceae_2 - ps25
Saccharomycetaceae_3 - ps26
Kaz./Nau./Saccharomyces - ps27
Saccharomyces - ps28
Arboricola_clade - ps29
Cerevisiae_clade - ps30
Saccharomyces_cerevisiae - ps31
Phylostratum

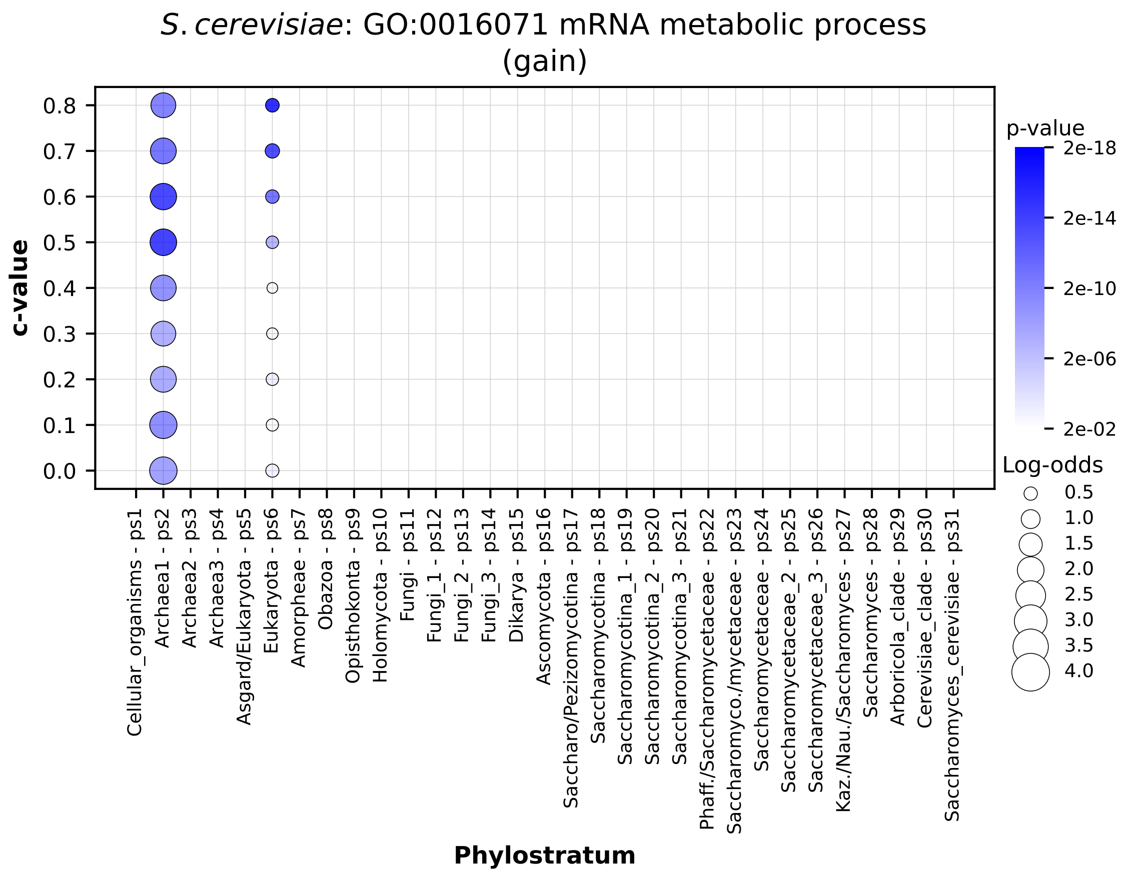

S. cerevisiae: GO:0016071 mRNA metabolic process
(gain)
c-value
0.0
0.1
0.2
0.3
0.4
0.5
0.6
0.7
0.8
p-value
2e-18
2e-14
2e-10
2e-06
2e-02
Log-odds
0.5
1.0
1.5
2.0
2.5
3.0
3.5
4.0
Cellular_organisms - ps1
Archaea1 - ps2
Archaea2 - ps3
Archaea3 - ps4
Asgard/Eukaryota - ps5
Eukaryota - ps6
Amorpheae - ps7
Obazoa - ps8
Opisthokonta - ps9
Holomycota - ps10
Fungi - ps11
Fungi_1 - ps12
Fungi_2 - ps13
Fungi_3 - ps14
Dikarya - ps15
Ascomycota - ps16
Saccharo/Pezizomycotina - ps17
Saccharomycotina - ps18
Saccharomycotina_1 - ps19
Saccharomycotina_2 - ps20
Saccharomycotina_3 - ps21
Phaff./Saccharomycetaceae - ps22
Saccharomyco./mycetaceae - ps23
Saccharomycetaceae - ps24
Saccharomycetaceae_2 - ps25
Saccharomycetaceae_3 - ps26
Kaz./Nau./Saccharomyces - ps27
Saccharomyces - ps28
Arboricola_clade - ps29
Cerevisiae_clade - ps30
Saccharomyces_cerevisiae - ps31
Phylostratum

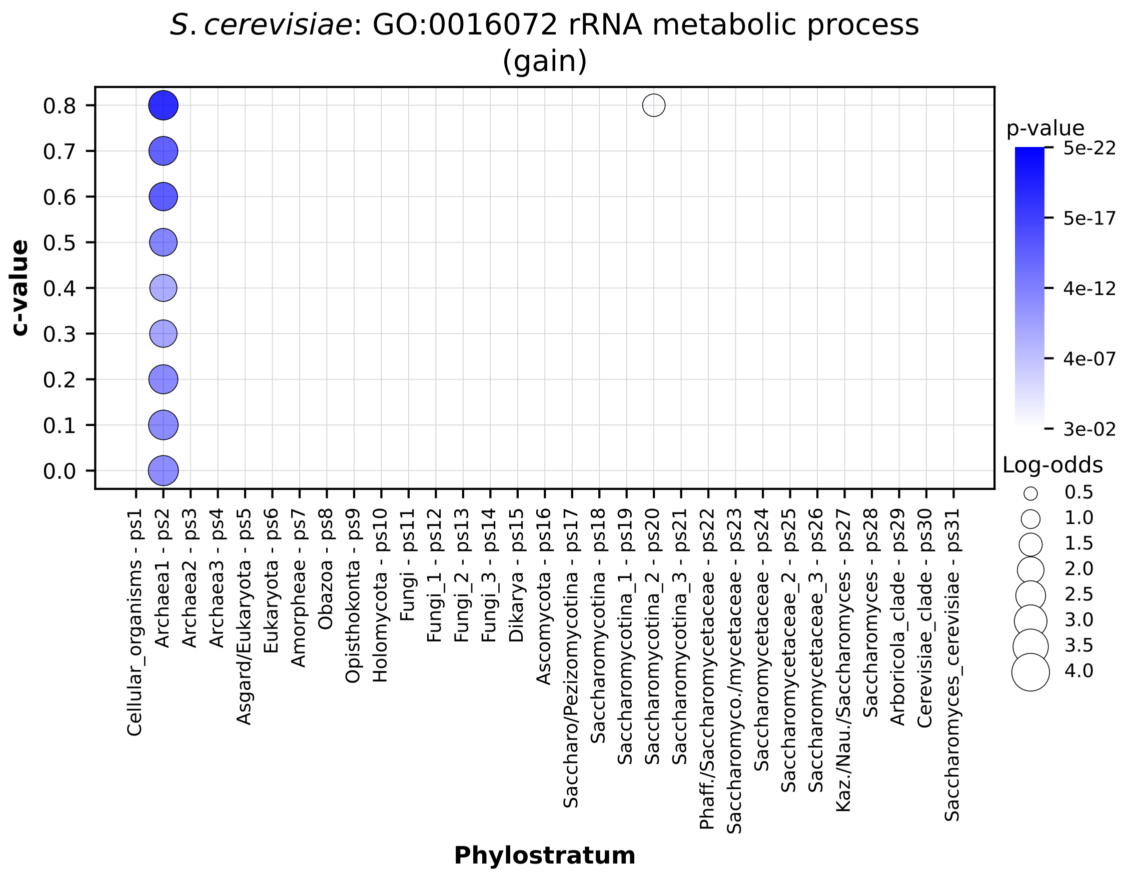

S. cerevisiae: GO:0016072 rRNA metabolic process
(gain)
c-value
0.0
0.1
0.2
0.3
0.4
0.5
0.6
0.7
0.8
Phylostratum
Cellular_organisms - ps1
Archaea1 - ps2
Archaea2 - ps3
Archaea3 - ps4
Asgard/Eukaryota - ps5
Eukaryota - ps6
Amorpheae - ps7
Obazoa - ps8
Opisthokonta - ps9
Holomycota - ps10
Fungi - ps11
Fungi_1 - ps12
Fungi_2 - ps13
Fungi_3 - ps14
Dikarya - ps15
Ascomycota - ps16
Saccharo/Pezizomycotina - ps17
Saccharomycotina - ps18
Saccharomycotina_1 - ps19
Saccharomycotina_2 - ps20
Saccharomycotina_3 - ps21
Phaff./Saccharomycetaceae - ps22
Saccharomyco./mycetaceae - ps23
Saccharomycetaceae - ps24
Saccharomycetaceae_2 - ps25
Saccharomycetaceae_3 - ps26
Kaz./Nau./Saccharomyces - ps27
Saccharomyces - ps28
Arboricola_clade - ps29
Cerevisiae_clade - ps30
Saccharomyces_cerevisiae - ps31
p-value
5e-22
5e-17
4e-12
4e-07
3e-02
Log-odds
0.5
1.0
1.5
2.0
2.5
3.0
3.5
4.0

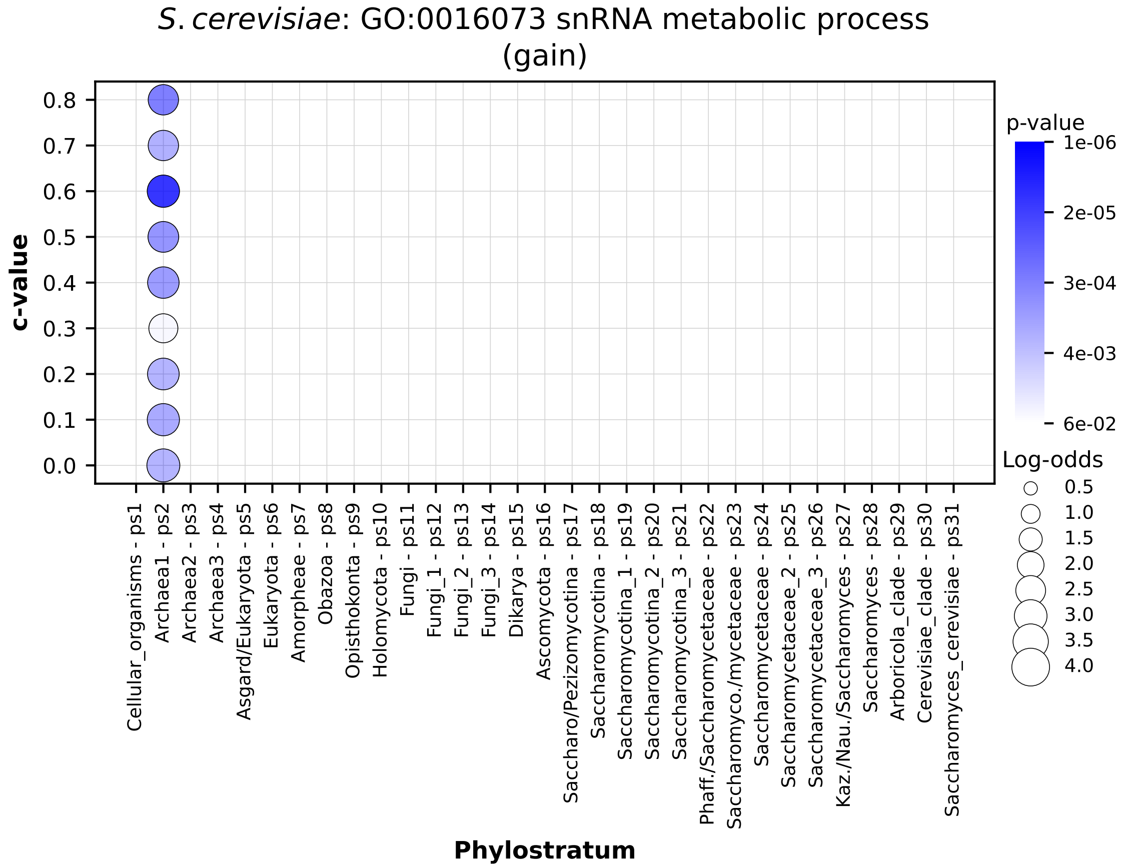

S. cerevisiae: GO:0016073 snRNA metabolic process (gain)
c-value
0.8
0.7
0.6
0.5
0.4
0.3
0.2
0.1
0.0
p-value
1e-06
2e-05
3e-04
4e-03
6e-02
Log-odds
0.5
1.0
1.5
2.0
2.5
3.0
3.5
4.0
Cellular_organisms - ps1
Archaea1 - ps2
Archaea2 - ps3
Archaea3 - ps4
Asgard/Eukaryota - ps5
Eukaryota - ps6
Amorpheae - ps7
Obazoa - ps8
Opisthokonta - ps9
Holomycota - ps10
Fungi - ps11
Fungi_1 - ps12
Fungi_2 - ps13
Fungi_3 - ps14
Dikarya - ps15
Ascomycota - ps16
Saccharo/Pezizomycotina - ps17
Saccharomycotina - ps18
Saccharomycotina_1 - ps19
Saccharomycotina_2 - ps20
Saccharomycotina_3 - ps21
Phaff./Saccharomycetaceae - ps22
Saccharomyco./mycetaceae - ps23
Saccharomycetaceae - ps24
Saccharomycetaceae_2 - ps25
Saccharomycetaceae_3 - ps26
Kaz./Nau./Saccharomyces - ps27
Saccharomyces - ps28
Arboricola_clade - ps29
Cerevisiae_clade - ps30
Saccharomyces_cerevisiae - ps31
Phylostratum

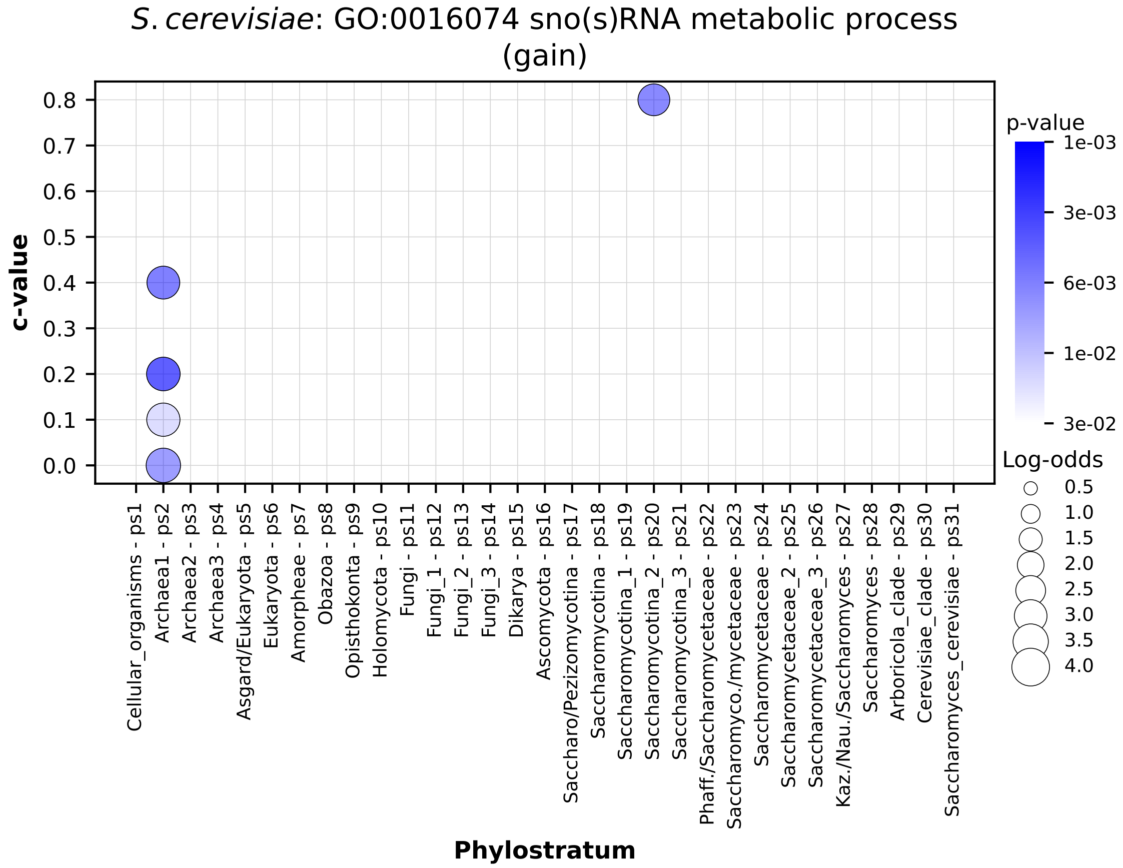

S. cerevisiae: GO:0016074 sno(s)RNA metabolic process (gain)
c-value
0.8
0.7
0.6
0.5
0.4
0.3
0.2
0.1
0.0
p-value
1e-03
3e-03
6e-03
1e-02
3e-02
Log-odds
0.5
1.0
1.5
2.0
2.5
3.0
3.5
4.0
Cellular_organisms - ps1
Archaea1 - ps2
Archaea2 - ps3
Archaea3 - ps4
Asgard/Eukaryota - ps5
Eukaryota - ps6
Amorpheae - ps7
Obazoa - ps8
Opisthokonta - ps9
Holomycota - ps10
Fungi - ps11
Fungi_1 - ps12
Fungi_2 - ps13
Fungi_3 - ps14
Dikarya - ps15
Ascomycota - ps16
Saccharo/Pezizomycotina - ps17
Saccharomycotina - ps18
Saccharomycotina_1 - ps19
Saccharomycotina_2 - ps20
Saccharomycotina_3 - ps21
Phaff./Saccharomycetaceae - ps22
Saccharomyco./mycetaceae - ps23
Saccharomycetaceae - ps24
Saccharomycetaceae_2 - ps25
Saccharomycetaceae_3 - ps26
Kaz./Nau./Saccharomyces - ps27
Saccharomyces - ps28
Arboricola_clade - ps29
Cerevisiae_clade - ps30
Saccharomyces_cerevisiae - ps31
Phylostratum

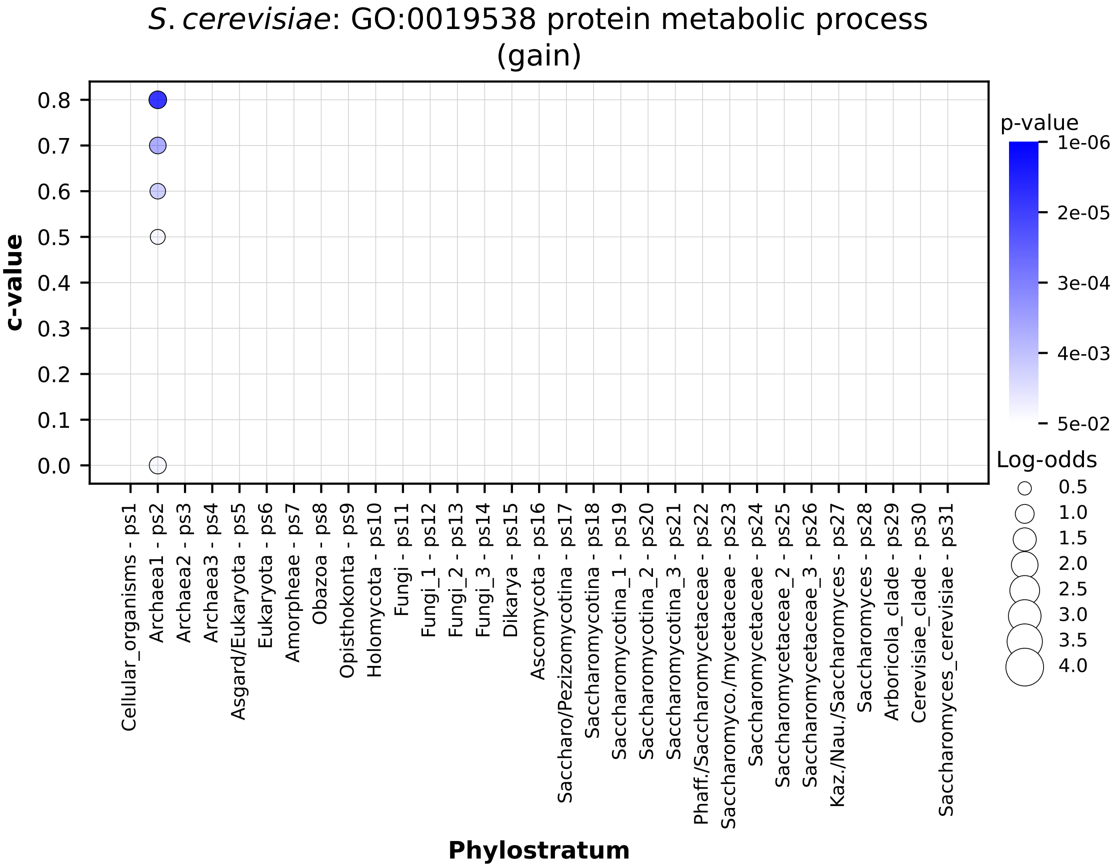

S. cerevisiae: GO:0019538 protein metabolic process
(gain)
c-value
0.0
0.1
0.2
0.3
0.4
0.5
0.6
0.7
0.8
p-value
1e-06
2e-05
3e-04
4e-03
5e-02
Log-odds
0.5
1.0
1.5
2.0
2.5
3.0
3.5
4.0
Cellular_organisms - ps1
Archaea1 - ps2
Archaea2 - ps3
Archaea3 - ps4
Asgard/Eukaryota - ps5
Eukaryota - ps6
Amorpheae - ps7
Obazoa - ps8
Opisthokonta - ps9
Holomycota - ps10
Fungi - ps11
Fungi_1 - ps12
Fungi_2 - ps13
Fungi_3 - ps14
Dikarya - ps15
Ascomycota - ps16
Saccharo/Pezizomycotina - ps17
Saccharomycotina - ps18
Saccharomycotina_1 - ps19
Saccharomycotina_2 - ps20
Saccharomycotina_3 - ps21
Phaff./Saccharomycetaceae - ps22
Saccharomyco./mycetaceae - ps23
Saccharomycetaceae - ps24
Saccharomycetaceae_2 - ps25
Saccharomycetaceae_3 - ps26
Kaz./Nau./Saccharomyces - ps27
Saccharomyces - ps28
Arboricola_clade - ps29
Cerevisiae_clade - ps30
Saccharomyces_cerevisiae - ps31
Phylostratum

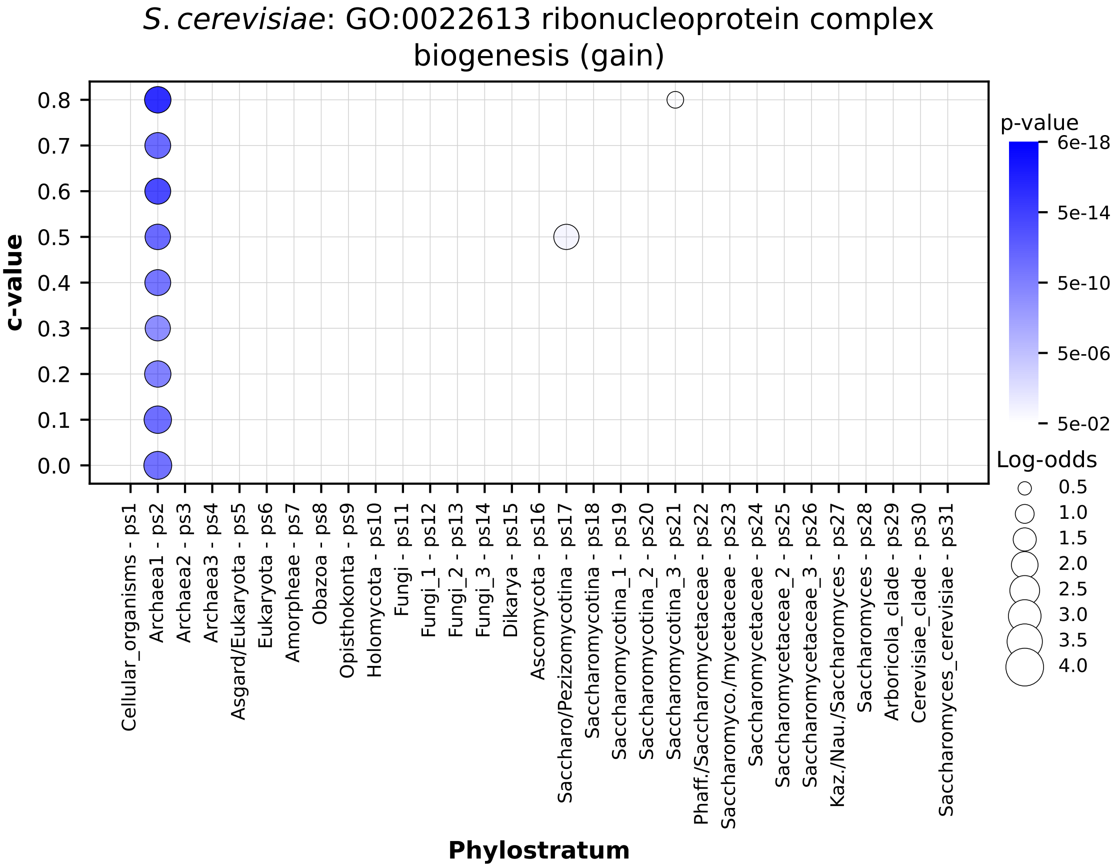

S. cerevisiae: GO:0022613 ribonucleoprotein complex biogenesis (gain)
c-value
0.8
0.7
0.6
0.5
0.4
0.3
0.2
0.1
0.0
Phylostratum
Cellular_organisms - ps1
Archaea1 - ps2
Archaea2 - ps3
Archaea3 - ps4
Asgard/Eukaryota - ps5
Eukaryota - ps6
Amorpheae - ps7
Obazoa - ps8
Opisthokonta - ps9
Holomycota - ps10
Fungi - ps11
Fungi_1 - ps12
Fungi_2 - ps13
Fungi_3 - ps14
Dikarya - ps15
Ascomycota - ps16
Saccharo/Pezizomycotina - ps17
Saccharomycotina - ps18
Saccharomycotina_1 - ps19
Saccharomycotina_2 - ps20
Saccharomycotina_3 - ps21
Phaff./Saccharomycetaceae - ps22
Saccharomyco./mycetaceae - ps23
Saccharomycetaceae - ps24
Saccharomycetaceae_2 - ps25
Saccharomycetaceae_3 - ps26
Kaz./Nau./Saccharomyces - ps27
Saccharomyces - ps28
Arboricola_clade - ps29
Cerevisiae_clade - ps30
Saccharomyces_cerevisiae - ps31
p-value
6e-18
5e-14
5e-10
5e-06
5e-02
Log-odds
0.5
1.0
1.5
2.0
2.5
3.0
3.5
4.0

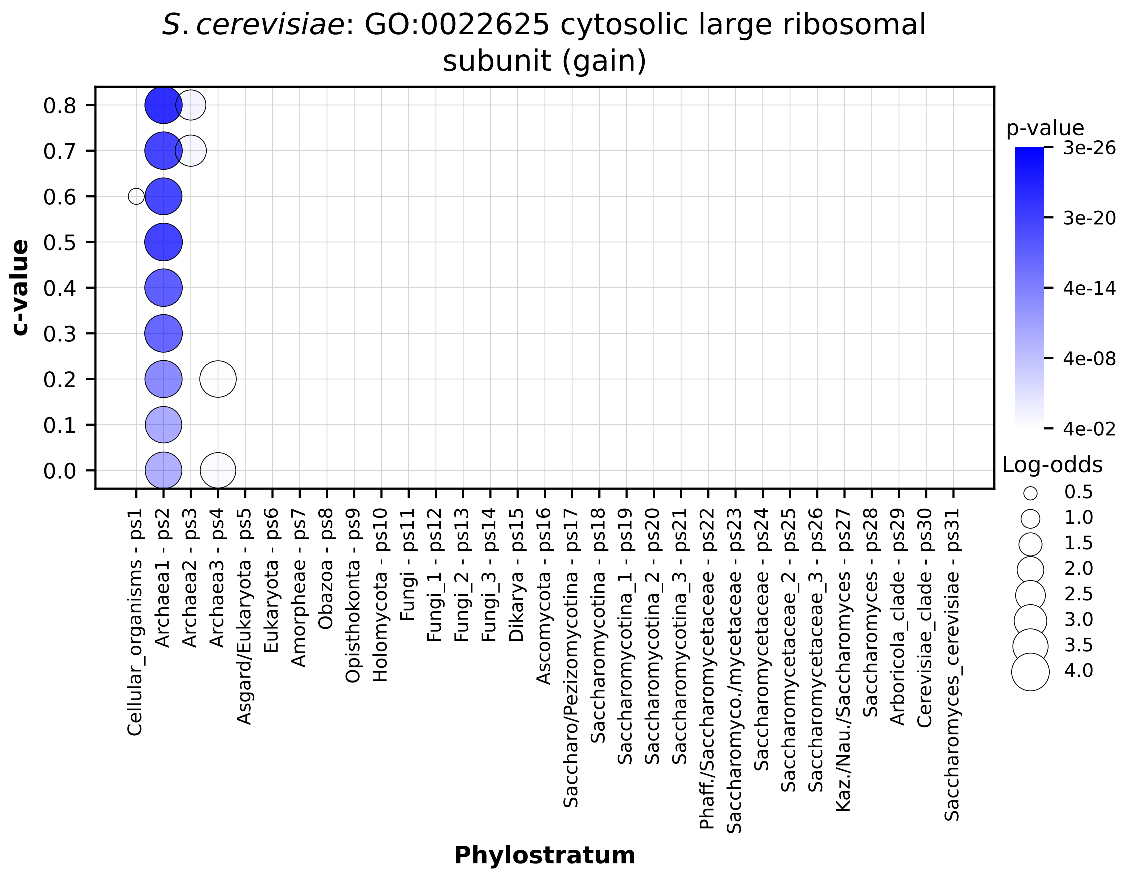

S. cerevisiae: GO:0022625 cytosolic large ribosomal subunit (gain)
c-value
0.8
0.7
0.6
0.5
0.4
0.3
0.2
0.1
0.0
p-value
3e-26
3e-20
4e-14
4e-08
4e-02
Log-odds
0.5
1.0
1.5
2.0
2.5
3.0
3.5
4.0
Cellular_organisms - ps1
Archaea1 - ps2
Archaea2 - ps3
Archaea3 - ps4
Asgard/Eukaryota - ps5
Eukaryota - ps6
Amorpheae - ps7
Obazoa - ps8
Opisthokonta - ps9
Holomycota - ps10
Fungi - ps11
Fungi_1 - ps12
Fungi_2 - ps13
Fungi_3 - ps14
Dikarya - ps15
Ascomycota - ps16
Saccharo/Pezizomycotina - ps17
Saccharomycotina - ps18
Saccharomycotina_1 - ps19
Saccharomycotina_2 - ps20
Saccharomycotina_3 - ps21
Phaff./Saccharomycetaceae - ps22
Saccharomyco./mycetaceae - ps23
Saccharomycetaceae - ps24
Saccharomycetaceae_2 - ps25
Saccharomycetaceae_3 - ps26
Kaz./Nau./Saccharomyces - ps27
Saccharomyces - ps28
Arboricola_clade - ps29
Cerevisiae_clade - ps30
Saccharomyces_cerevisiae - ps31
Phylostratum

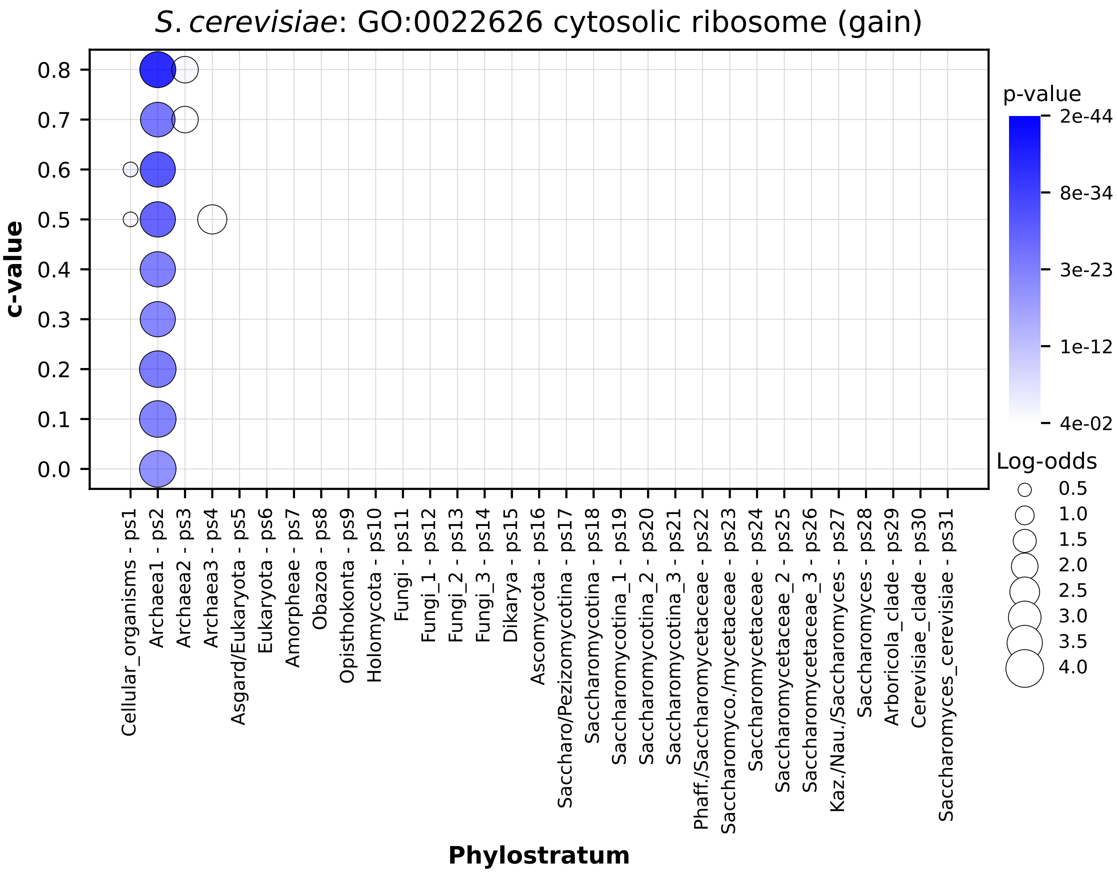

S. cerevisiae: GO:0022626 cytosolic ribosome (gain)
c-value
0.8
0.7
0.6
0.5
0.4
0.3
0.2
0.1
0.0
p-value
2e-44
8e-34
3e-23
1e-12
4e-02
Log-odds
0.5
1.0
1.5
2.0
2.5
3.0
3.5
4.0
Cellular_organisms - ps1
Archaea1 - ps2
Archaea2 - ps3
Archaea3 - ps4
Asgard/Eukaryota - ps5
Eukaryota - ps6
Amorpheae - ps7
Obazoa - ps8
Opisthokonta - ps9
Holomycota - ps10
Fungi - ps11
Fungi_1 - ps12
Fungi_2 - ps13
Fungi_3 - ps14
Dikarya - ps15
Ascomycota - ps16
Saccharo/Pezizomycotina - ps17
Saccharomycotina - ps18
Saccharomycotina_1 - ps19
Saccharomycotina_2 - ps20
Saccharomycotina_3 - ps21
Phaff./Saccharomycetaceae - ps22
Saccharomyco./mycetaceae - ps23
Saccharomycetaceae - ps24
Saccharomycetaceae_2 - ps25
Saccharomycetaceae_3 - ps26
Kaz./Nau./Saccharomyces - ps27
Saccharomyces - ps28
Arboricola_clade - ps29
Cerevisiae_clade - ps30
Saccharomyces_cerevisiae - ps31
Phylostratum

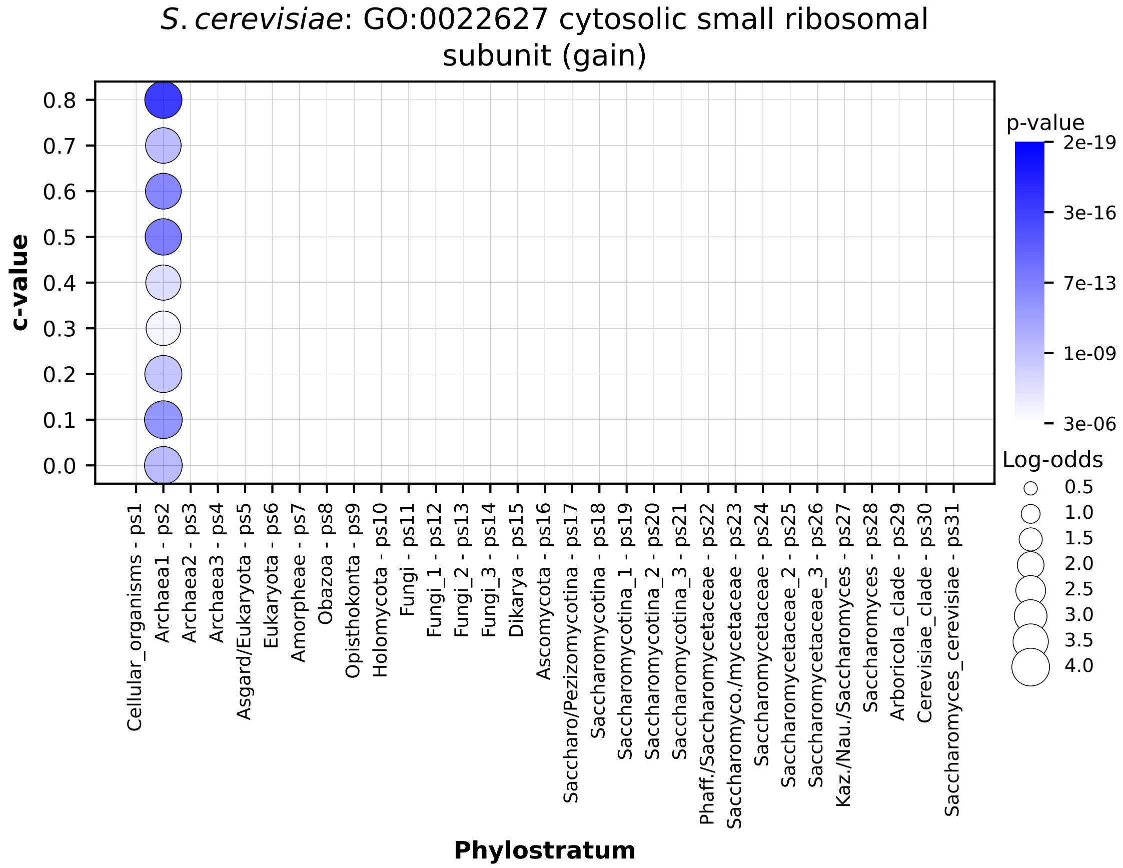
S. cerevisiae: GO:0022627 cytosolic small ribosomal subunit (gain)
c-value
0.8
0.7
0.6
0.5
0.4
0.3
0.2
0.1
0.0
p-value
2e-19
3e-16
7e-13
1e-09
3e-06
Log-odds
0.5
1.0
1.5
2.0
2.5
3.0
3.5
4.0
Cellular_organisms - ps1
Archaea1 - ps2
Archaea2 - ps3
Archaea3 - ps4
Asgard/Eukaryota - ps5
Eukaryota - ps6
Amorpheae - ps7
Obazoa - ps8
Opisthokonta - ps9
Holomycota - ps10
Fungi - ps11
Fungi_1 - ps12
Fungi_2 - ps13
Fungi_3 - ps14
Dikarya - ps15
Ascomycota - ps16
Saccharo/Pezizomycotina - ps17
Saccharomycotina - ps18
Saccharomycotina_1 - ps19
Saccharomycotina_2 - ps20
Saccharomycotina_3 - ps21
Phaff./Saccharomycetaceae - ps22
Saccharomyco./mycetaceae - ps23
Saccharomycetaceae - ps24
Saccharomycetaceae_2 - ps25
Saccharomycetaceae_3 - ps26
Kaz./Nau./Saccharomyces - ps27
Saccharomyces - ps28
Arboricola_clade - ps29
Cerevisiae_clade - ps30
Saccharomyces_cerevisiae - ps31
Phylostratum

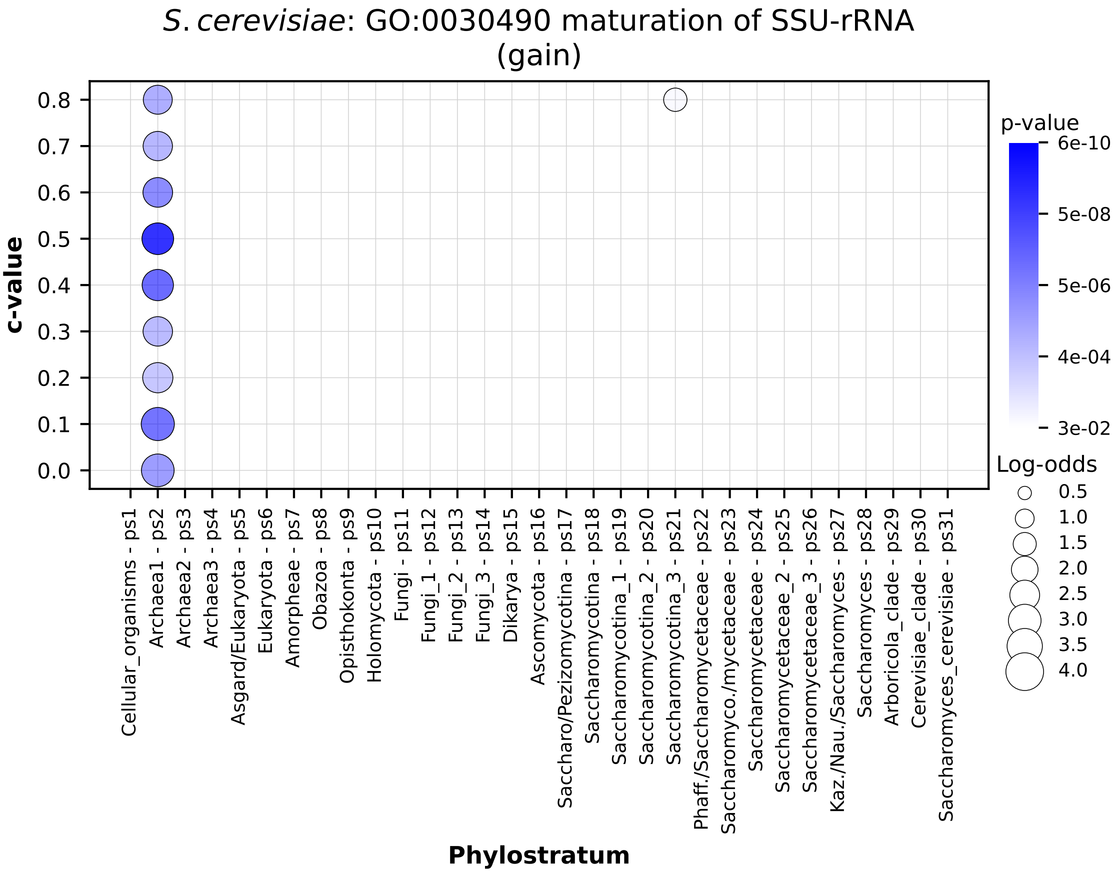

S. cerevisiae: GO:0030490 maturation of SSU-rRNA
(gain)
c-value
0.8
0.7
0.6
0.5
0.4
0.3
0.2
0.1
0.0
p-value
6e-10
5e-08
5e-06
4e-04
3e-02
Log-odds
0.5
1.0
1.5
2.0
2.5
3.0
3.5
4.0
Cellular_organisms - ps1
Archaea1 - ps2
Archaea2 - ps3
Archaea3 - ps4
Asgard/Eukaryota - ps5
Eukaryota - ps6
Amorpheae - ps7
Obazoa - ps8
Opisthokonta - ps9
Holomycota - ps10
Fungi - ps11
Fungi_1 - ps12
Fungi_2 - ps13
Fungi_3 - ps14
Dikarya - ps15
Ascomycota - ps16
Saccharo/Pezizomycotina - ps17
Saccharomycotina - ps18
Saccharomycotina_1 - ps19
Saccharomycotina_2 - ps20
Saccharomycotina_3 - ps21
Phaff./Saccharomycetaceae - ps22
Saccharomyco./mycetaceae - ps23
Saccharomycetaceae - ps24
Saccharomycetaceae_2 - ps25
Saccharomycetaceae_3 - ps26
Kaz./Nau./Saccharomyces - ps27
Saccharomyces - ps28
Arboricola_clade - ps29
Cerevisiae_clade - ps30
Saccharomyces_cerevisiae - ps31
Phylostratum

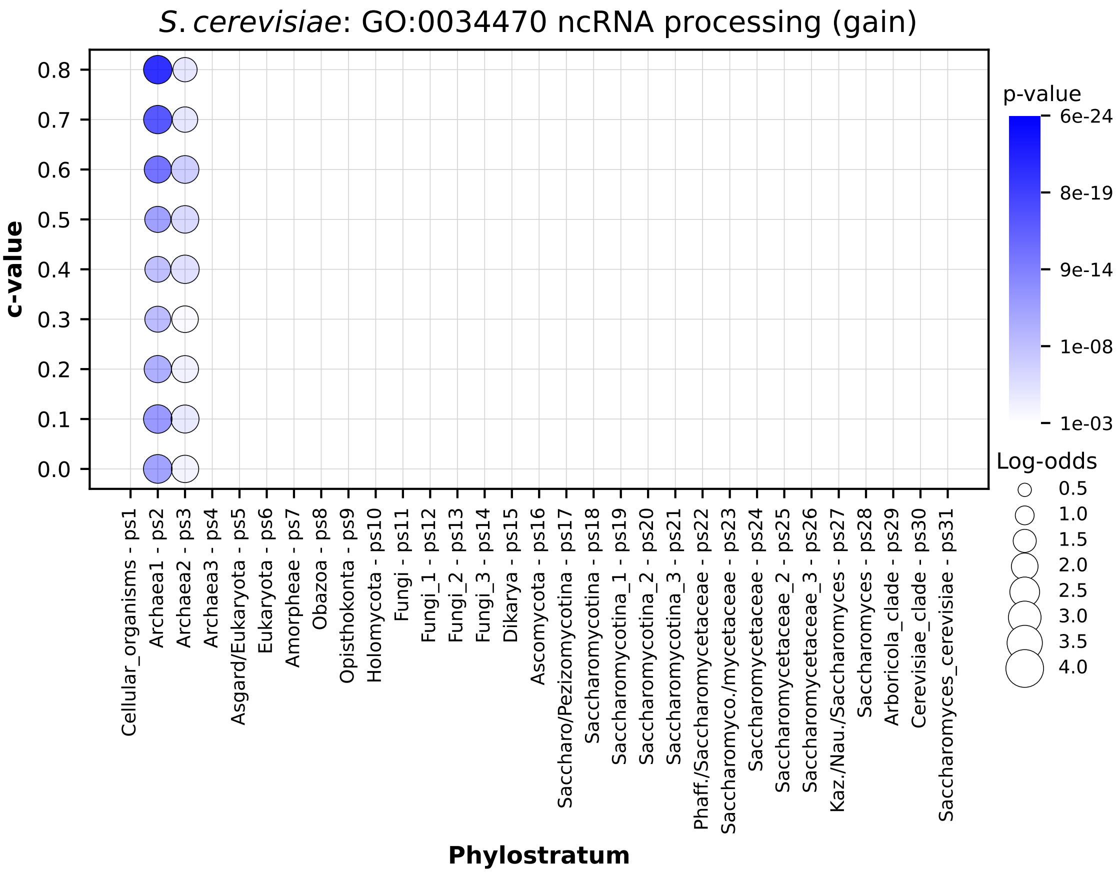

S. cerevisiae: GO:0034470 ncRNA processing (gain)
c-value
0.8
0.7
0.6
0.5
0.4
0.3
0.2
0.1
0.0
p-value
6e-24
8e-19
9e-14
1e-08
1e-03
Log-odds
0.5
1.0
1.5
2.0
2.5
3.0
3.5
4.0
Cellular_organisms - ps1
Archaea1 - ps2
Archaea2 - ps3
Archaea3 - ps4
Asgard/Eukaryota - ps5
Eukaryota - ps6
Amorpheae - ps7
Obazoa - ps8
Opisthokonta - ps9
Holomycota - ps10
Fungi - ps11
Fungi_1 - ps12
Fungi_2 - ps13
Fungi_3 - ps14
Dikarya - ps15
Ascomycota - ps16
Saccharo/Pezizomycotina - ps17
Saccharomycotina - ps18
Saccharomycotina_1 - ps19
Saccharomycotina_2 - ps20
Saccharomycotina_3 - ps21
Phaff./Saccharomycetaceae - ps22
Saccharomyco./mycetaceae - ps23
Saccharomycetaceae - ps24
Saccharomycetaceae_2 - ps25
Saccharomycetaceae_3 - ps26
Kaz./Nau./Saccharomyces - ps27
Saccharomyces - ps28
Arboricola_clade - ps29
Cerevisiae_clade - ps30
Saccharomyces_cerevisiae - ps31
Phylostratum

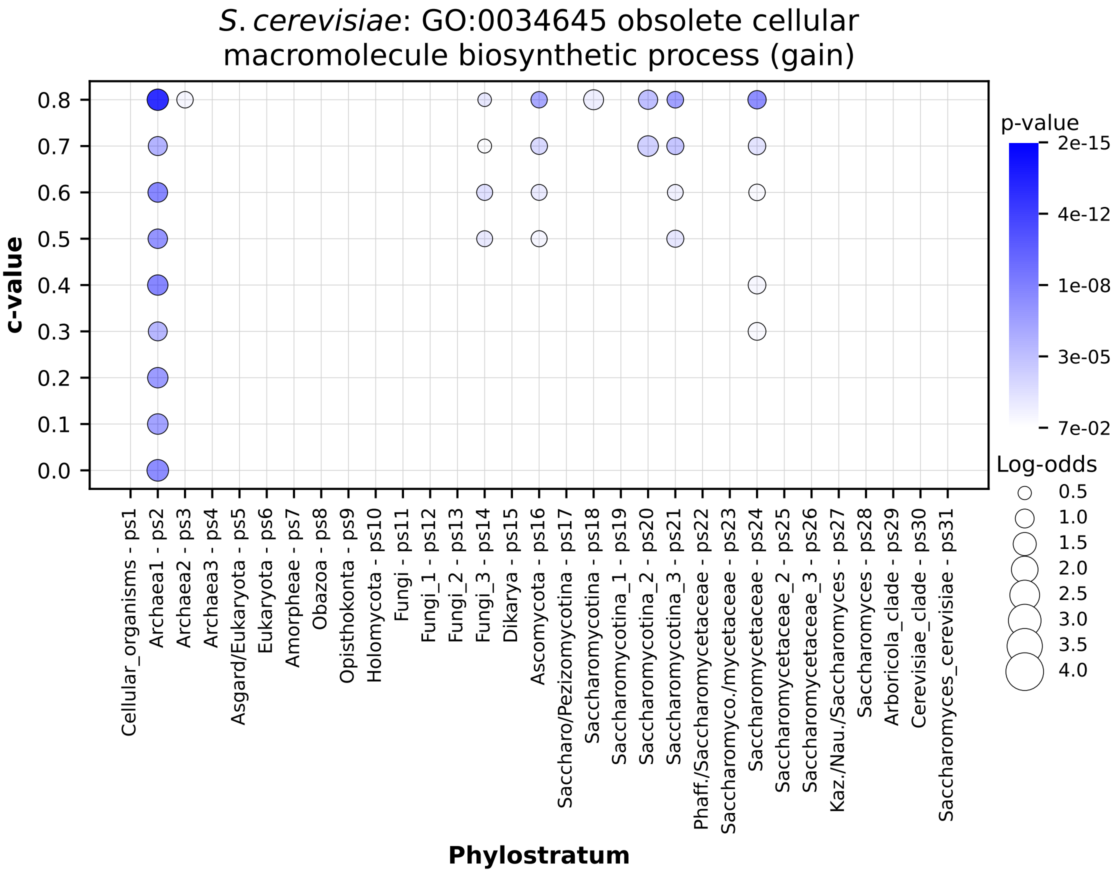

S. cerevisiae: GO:0034645 obsolete cellular macromolecule biosynthetic process (gain)
c-value
0.8
0.7
0.6
0.5
0.4
0.3
0.2
0.1
0.0
p-value
2e-15
4e-12
1e-08
3e-05
7e-02
Log-odds
0.5
1.0
1.5
2.0
2.5
3.0
3.5
4.0
Cellular_organisms - ps1
Archaea1 - ps2
Archaea2 - ps3
Archaea3 - ps4
Asgard/Eukaryota - ps5
Eukaryota - ps6
Amorpheae - ps7
Obazoa - ps8
Opisthokonta - ps9
Holomycota - ps10
Fungi - ps11
Fungi_1 - ps12
Fungi_2 - ps13
Fungi_3 - ps14
Dikarya - ps15
Ascomycota - ps16
Saccharo/Pezizomycotina - ps17
Saccharomycotina - ps18
Saccharomycotina_1 - ps19
Saccharomycotina_2 - ps20
Saccharomycotina_3 - ps21
Phaff./Saccharomycetaceae - ps22
Saccharomyco./mycetaceae - ps23
Saccharomycetaceae - ps24
Saccharomycetaceae_2 - ps25
Saccharomycetaceae_3 - ps26
Kaz./Nau./Saccharomyces - ps27
Saccharomyces - ps28
Arboricola_clade - ps29
Cerevisiae_clade - ps30
Saccharomyces_cerevisiae - ps31
Phylostratum

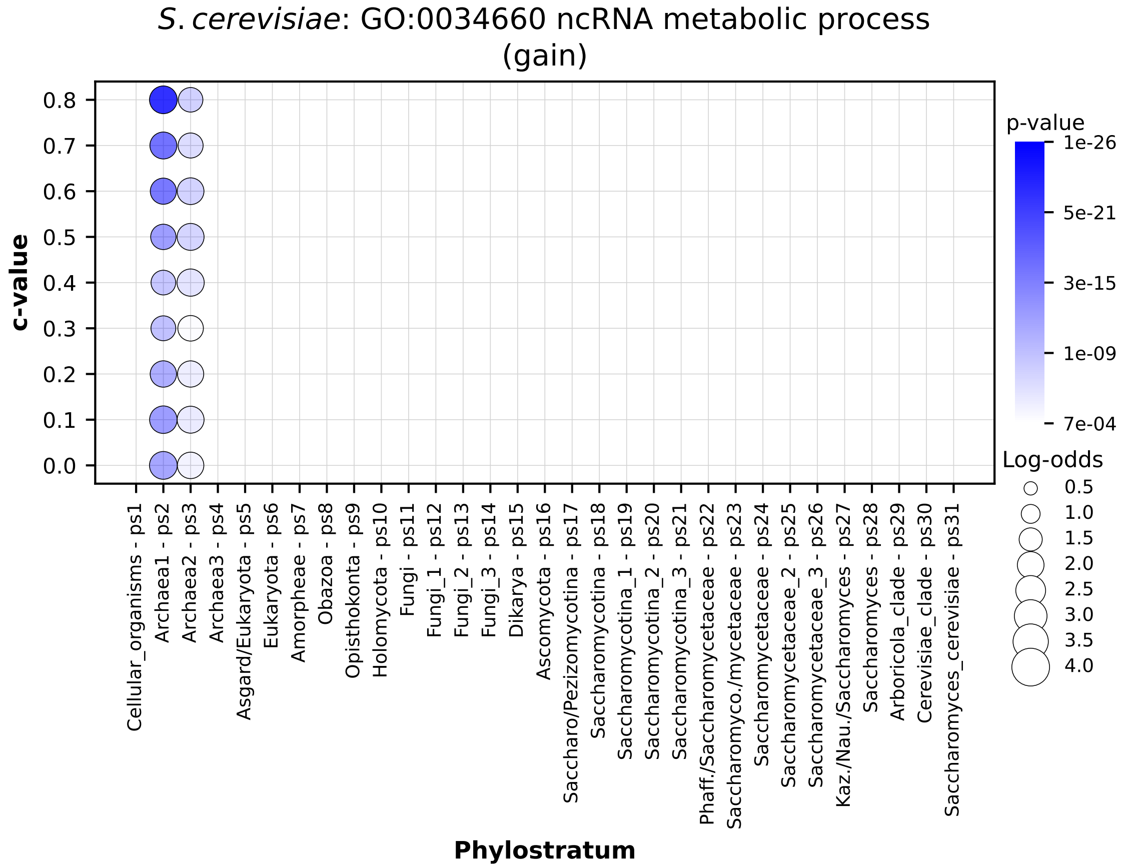

S. cerevisiae: GO:0034660 ncRNA metabolic process
(gain)
c-value
0.8
0.7
0.6
0.5
0.4
0.3
0.2
0.1
0.0
p-value
1e-26
5e-21
3e-15
1e-09
7e-04
Log-odds
0.5
1.0
1.5
2.0
2.5
3.0
3.5
4.0
Cellular_organisms - ps1
Archaea1 - ps2
Archaea2 - ps3
Archaea3 - ps4
Asgard/Eukaryota - ps5
Eukaryota - ps6
Amorpheae - ps7
Obazoa - ps8
Opisthokonta - ps9
Holomycota - ps10
Fungi - ps11
Fungi_1 - ps12
Fungi_2 - ps13
Fungi_3 - ps14
Dikarya - ps15
Ascomycota - ps16
Saccharo/Pezizomycotina - ps17
Saccharomycotina - ps18
Saccharomycotina_1 - ps19
Saccharomycotina_2 - ps20
Saccharomycotina_3 - ps21
Phaff./Saccharomycetaceae - ps22
Saccharomyco./mycetaceae - ps23
Saccharomycetaceae - ps24
Saccharomycetaceae_2 - ps25
Saccharomycetaceae_3 - ps26
Kaz./Nau./Saccharomyces - ps27
Saccharomyces - ps28
Arboricola_clade - ps29
Cerevisiae_clade - ps30
Saccharomyces_cerevisiae - ps31
Phylostratum

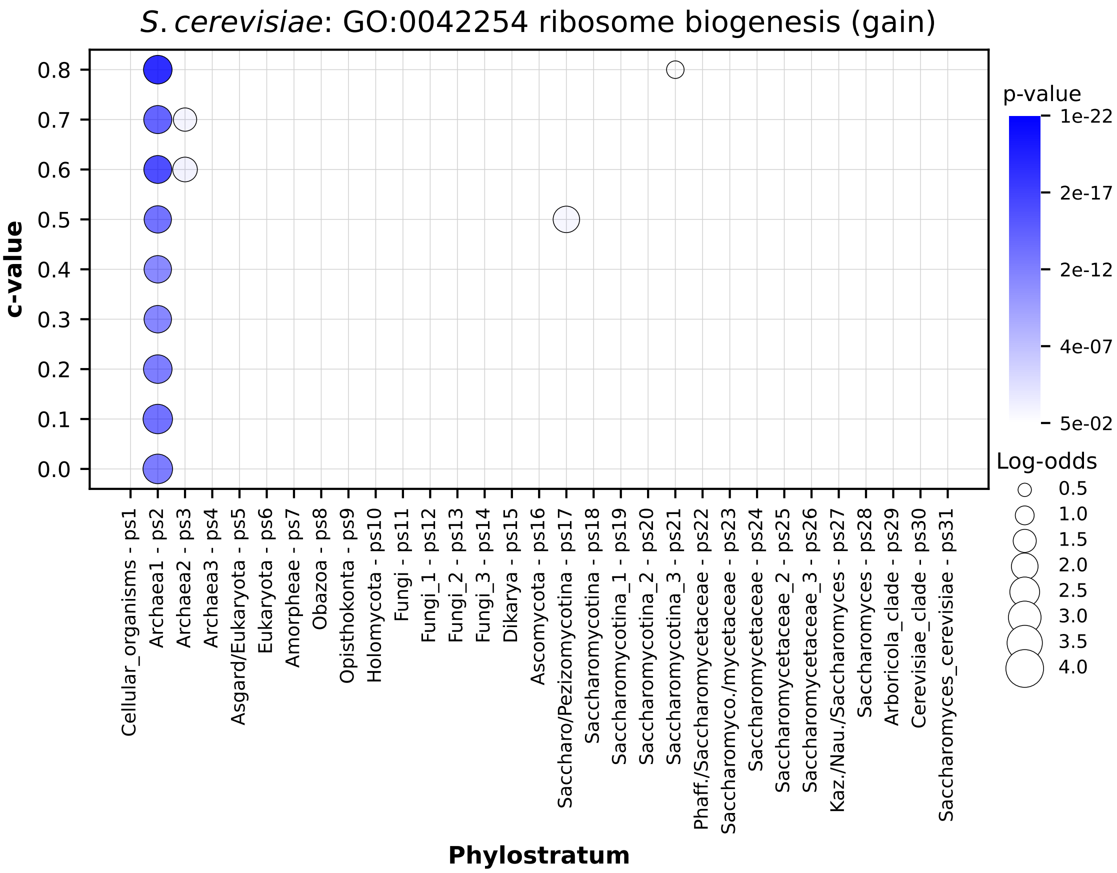

S. cerevisiae: GO:0042254 ribosome biogenesis (gain)
c-value
0.8
0.7
0.6
0.5
0.4
0.3
0.2
0.1
0.0
Phylostratum
Cellular_organisms - ps1
Archaea1 - ps2
Archaea2 - ps3
Archaea3 - ps4
Asgard/Eukaryota - ps5
Eukaryota - ps6
Amorpheae - ps7
Obazoa - ps8
Opisthokonta - ps9
Holomycota - ps10
Fungi - ps11
Fungi_1 - ps12
Fungi_2 - ps13
Fungi_3 - ps14
Dikarya - ps15
Ascomycota - ps16
Saccharo/Pezizomycotina - ps17
Saccharomycotina - ps18
Saccharomycotina_1 - ps19
Saccharomycotina_2 - ps20
Saccharomycotina_3 - ps21
Phaff./Saccharomycetaceae - ps22
Saccharomyco./mycetaceae - ps23
Saccharomycetaceae - ps24
Saccharomycetaceae_2 - ps25
Saccharomycetaceae_3 - ps26
Kaz./Nau./Saccharomyces - ps27
Saccharomyces - ps28
Arboricola_clade - ps29
Cerevisiae_clade - ps30
Saccharomyces_cerevisiae - ps31
p-value
1e-22
2e-17
2e-12
4e-07
5e-02
Log-odds
0.5
1.0
1.5
2.0
2.5
3.0
3.5
4.0

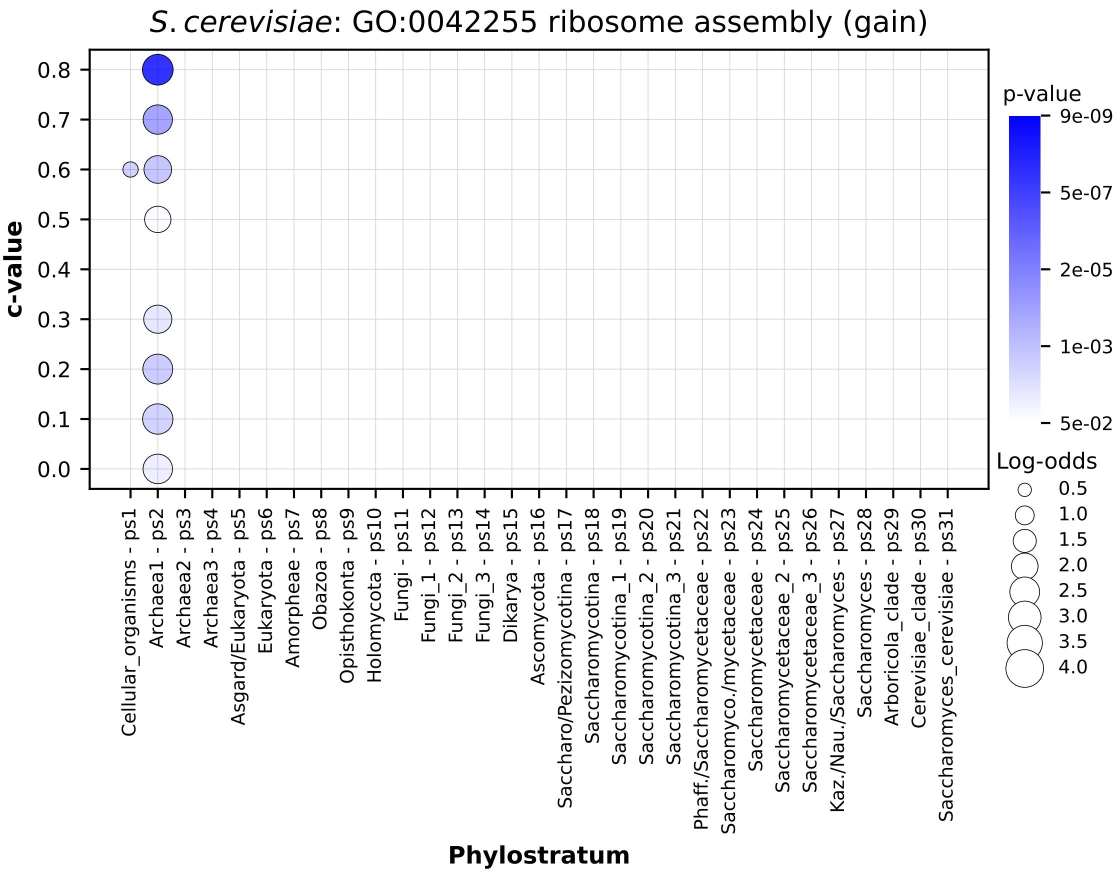
S. cerevisiae: GO:0042255 ribosome assembly (gain)
c-value
0.0
0.1
0.2
0.3
0.4
0.5
0.6
0.7
0.8
Phylostratum
Cellular_organisms - ps1
Archaea1 - ps2
Archaea2 - ps3
Archaea3 - ps4
Asgard/Eukaryota - ps5
Eukaryota - ps6
Amorpheae - ps7
Obazoa - ps8
Opisthokonta - ps9
Holomycota - ps10
Fungi - ps11
Fungi_1 - ps12
Fungi_2 - ps13
Fungi_3 - ps14
Dikarya - ps15
Ascomycota - ps16
Saccharo/Pezizomycotina - ps17
Saccharomycotina - ps18
Saccharomycotina_1 - ps19
Saccharomycotina_2 - ps20
Saccharomycotina_3 - ps21
Phaff./Saccharomycetaceae - ps22
Saccharomyco./mycetaceae - ps23
Saccharomycetaceae - ps24
Saccharomycetaceae_2 - ps25
Saccharomycetaceae_3 - ps26
Kaz./Nau./Saccharomyces - ps27
Saccharomyces - ps28
Arboricola_clade - ps29
Cerevisiae_clade - ps30
Saccharomyces_cerevisiae - ps31
p-value
9e-09
5e-07
2e-05
1e-03
5e-02
Log-odds
0.5
1.0
1.5
2.0
2.5
3.0
3.5
4.0

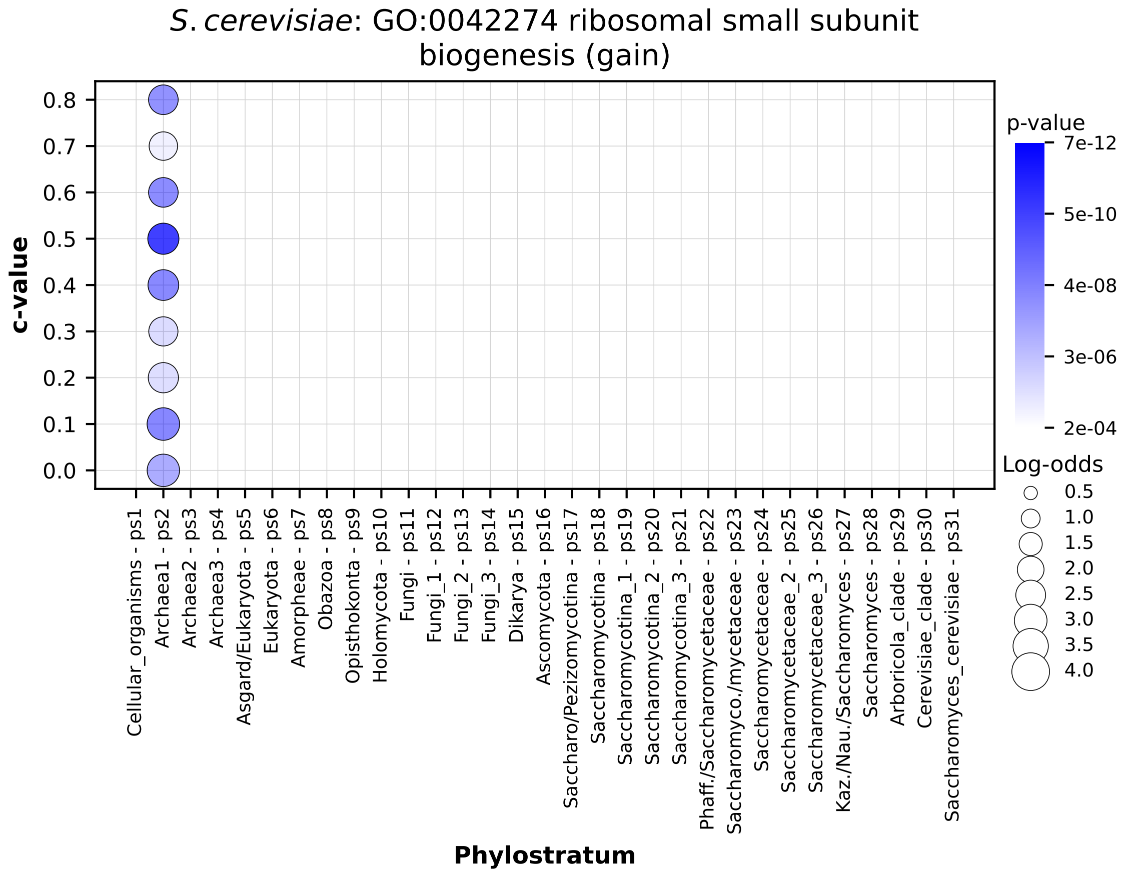

S. cerevisiae: GO:0042274 ribosomal small subunit biogenesis (gain)
c-value
0.8
0.7
0.6
0.5
0.4
0.3
0.2
0.1
0.0
Phylostratum
Cellular_organisms - ps1
Archaea1 - ps2
Archaea2 - ps3
Archaea3 - ps4
Asgard/Eukaryota - ps5
Eukaryota - ps6
Amorpheae - ps7
Obazoa - ps8
Opisthokonta - ps9
Holomycota - ps10
Fungi - ps11
Fungi_1 - ps12
Fungi_2 - ps13
Fungi_3 - ps14
Dikarya - ps15
Ascomycota - ps16
Saccharo/Pezizomycotina - ps17
Saccharomycotina - ps18
Saccharomycotina_1 - ps19
Saccharomycotina_2 - ps20
Saccharomycotina_3 - ps21
Phaff./Saccharomycetaceae - ps22
Saccharomyco./mycetaceae - ps23
Saccharomycetaceae - ps24
Saccharomycetaceae_2 - ps25
Saccharomycetaceae_3 - ps26
Kaz./Nau./Saccharomyces - ps27
Saccharomyces - ps28
Arboricola_clade - ps29
Cerevisiae_clade - ps30
Saccharomyces_cerevisiae - ps31
p-value
7e-12
5e-10
4e-08
3e-06
2e-04
Log-odds
0.5
1.0
1.5
2.0
2.5
3.0
3.5
4.0

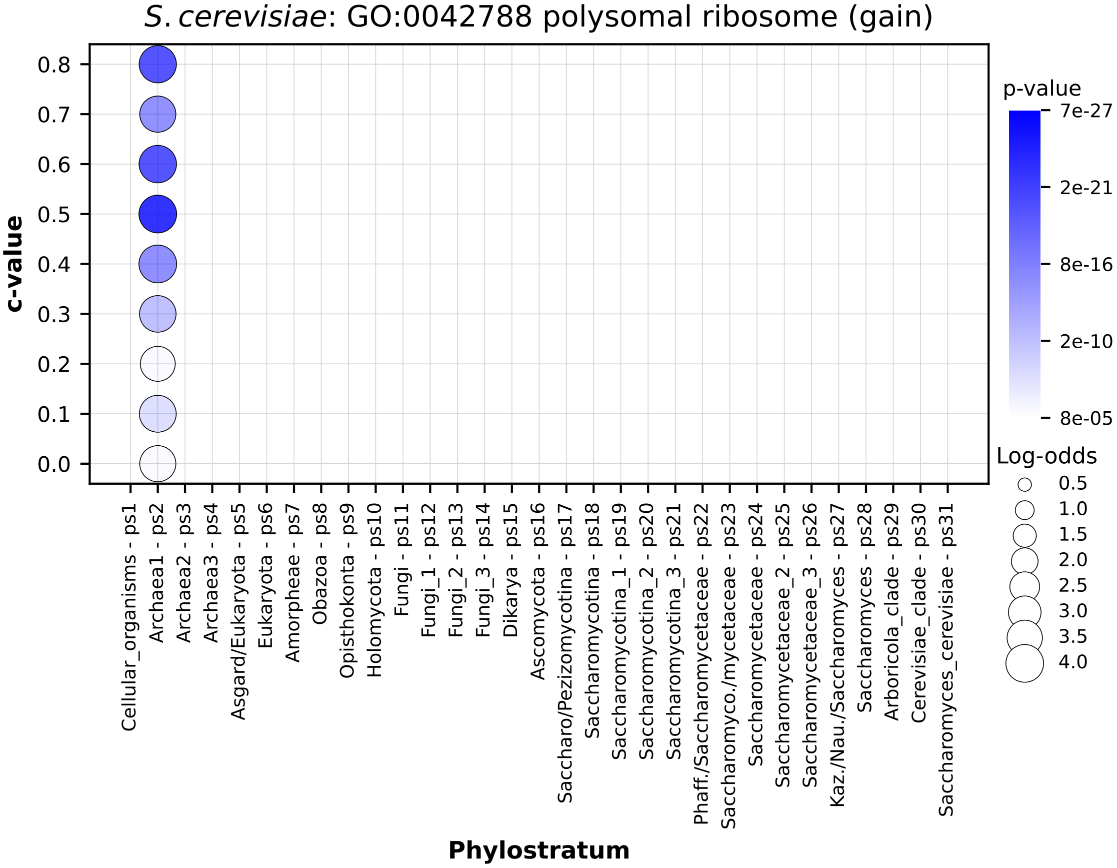

S. cerevisiae: GO:0042788 polysomal ribosome (gain)
c-value
0.0
0.1
0.2
0.3
0.4
0.5
0.6
0.7
0.8
Phylostratum
Cellular_organisms - ps1
Archaea1 - ps2
Archaea2 - ps3
Archaea3 - ps4
Asgard/Eukaryota - ps5
Eukaryota - ps6
Amorpheae - ps7
Obazoa - ps8
Opisthokonta - ps9
Holomycota - ps10
Fungi - ps11
Fungi_1 - ps12
Fungi_2 - ps13
Fungi_3 - ps14
Dikarya - ps15
Ascomycota - ps16
Saccharo/Pezizomycotina - ps17
Saccharomycotina - ps18
Saccharomycotina_1 - ps19
Saccharomycotina_2 - ps20
Saccharomycotina_3 - ps21
Phaff./Saccharomycetaceae - ps22
Saccharomyco./mycetaceae - ps23
Saccharomycetaceae - ps24
Saccharomycetaceae_2 - ps25
Saccharomycetaceae_3 - ps26
Kaz./Nau./Saccharomyces - ps27
Saccharomyces - ps28
Arboricola_clade - ps29
Cerevisiae_clade - ps30
Saccharomyces_cerevisiae - ps31
p-value
7e-27
2e-21
8e-16
2e-10
8e-05
Log-odds
0.5
1.0
1.5
2.0
2.5
3.0
3.5
4.0

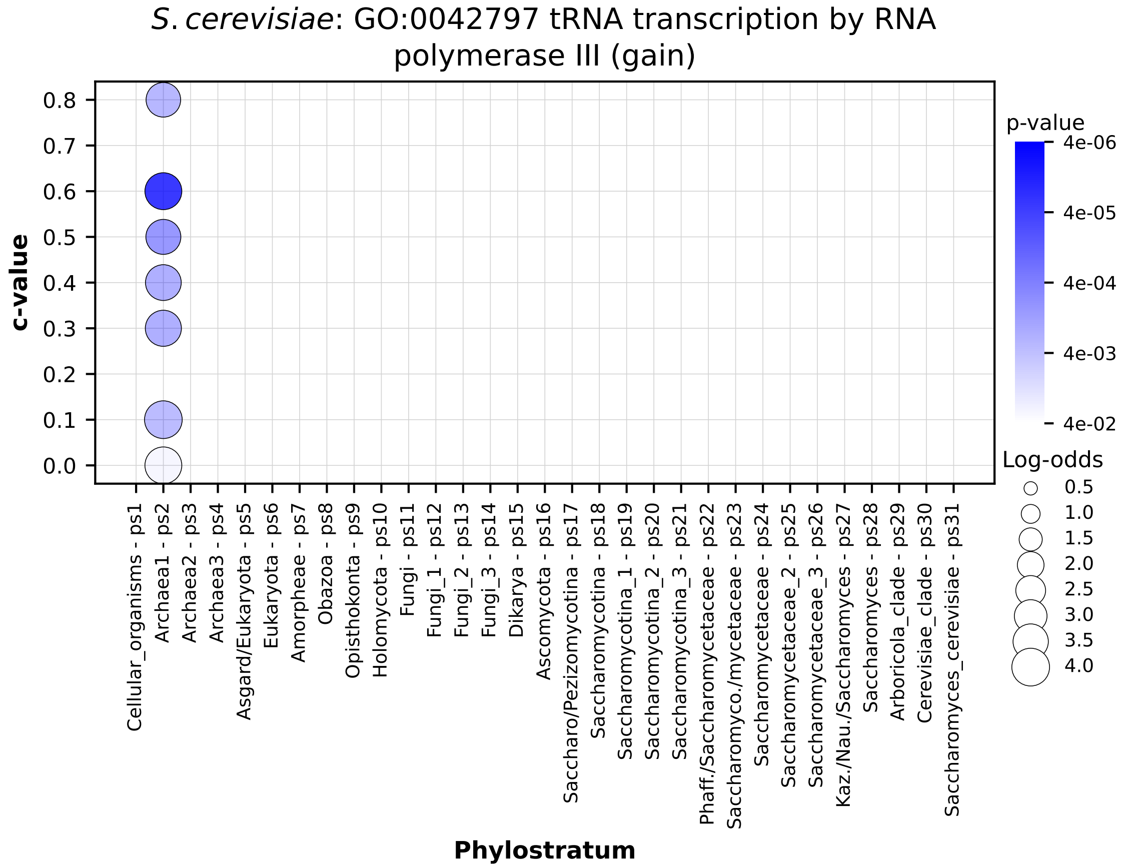

S. cerevisiae: GO:0042797 tRNA transcription by RNA polymerase III (gain)
c-value
0.8
0.7
0.6
0.5
0.4
0.3
0.2
0.1
0.0
p-value
4e-06
4e-05
4e-04
4e-03
4e-02
Log-odds
0.5
1.0
1.5
2.0
2.5
3.0
3.5
4.0
Cellular_organisms - ps1
Archaea1 - ps2
Archaea2 - ps3
Archaea3 - ps4
Asgard/Eukaryota - ps5
Eukaryota - ps6
Amorpheae - ps7
Obazoa - ps8
Opisthokonta - ps9
Holomycota - ps10
Fungi - ps11
Fungi_1 - ps12
Fungi_2 - ps13
Fungi_3 - ps14
Dikarya - ps15
Ascomycota - ps16
Saccharo/Pezizomycotina - ps17
Saccharomycotina - ps18
Saccharomycotina_1 - ps19
Saccharomycotina_2 - ps20
Saccharomycotina_3 - ps21
Phaff./Saccharomycetaceae - ps22
Saccharomyco./mycetaceae - ps23
Saccharomycetaceae - ps24
Saccharomycetaceae_2 - ps25
Saccharomycetaceae_3 - ps26
Kaz./Nau./Saccharomyces - ps27
Saccharomyces - ps28
Arboricola_clade - ps29
Cerevisiae_clade - ps30
Saccharomyces_cerevisiae - ps31
Phylostratum

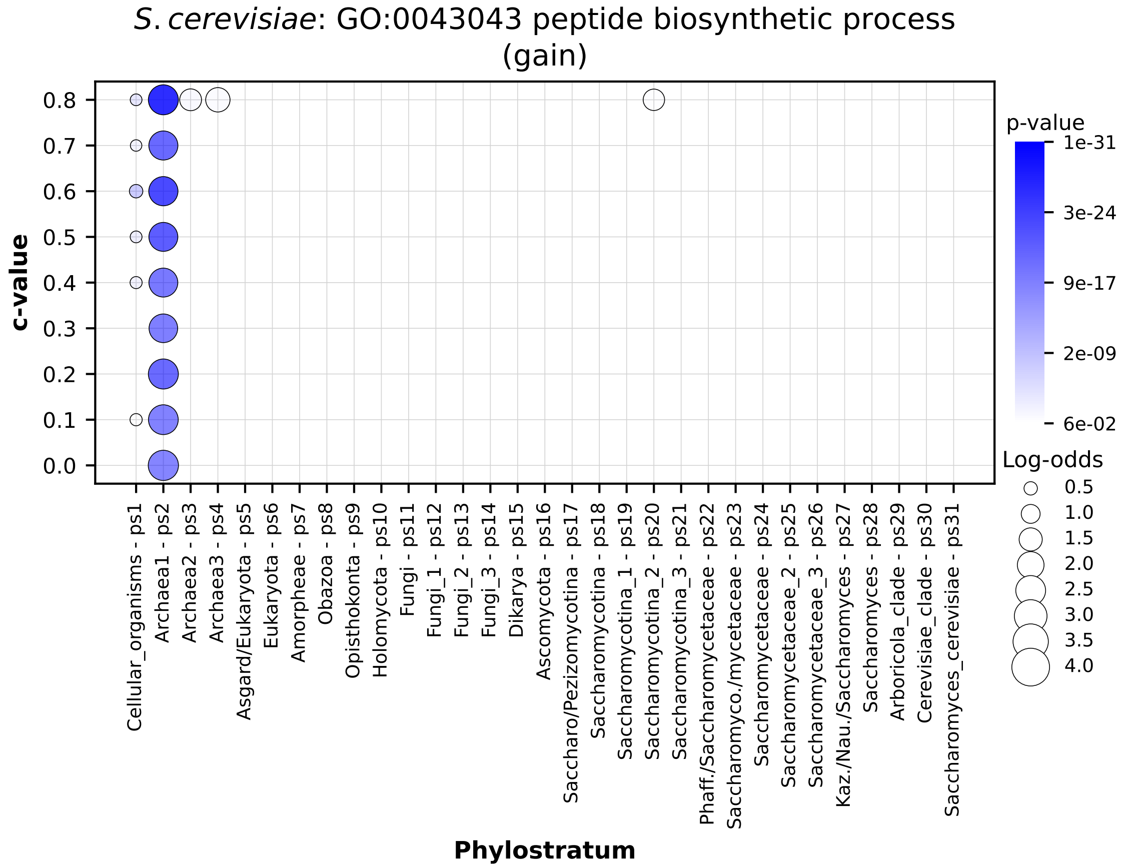
S. cerevisiae: GO:0043043 peptide biosynthetic process
(gain)
c-value
0.8
0.7
0.6
0.5
0.4
0.3
0.2
0.1
0.0
p-value
1e-31
3e-24
9e-17
2e-09
6e-02
Log-odds
0.5
1.0
1.5
2.0
2.5
3.0
3.5
4.0
Cellular_organisms - ps1
Archaea1 - ps2
Archaea2 - ps3
Archaea3 - ps4
Asgard/Eukaryota - ps5
Eukaryota - ps6
Amorpheae - ps7
Obazoa - ps8
Opisthokonta - ps9
Holomycota - ps10
Fungi - ps11
Fungi_1 - ps12
Fungi_2 - ps13
Fungi_3 - ps14
Dikarya - ps15
Ascomycota - ps16
Saccharo/Pezizomycotina - ps17
Saccharomycotina - ps18
Saccharomycotina_1 - ps19
Saccharomycotina_2 - ps20
Saccharomycotina_3 - ps21
Phaff./Saccharomycetaceae - ps22
Saccharomyco./mycetaceae - ps23
Saccharomycetaceae - ps24
Saccharomycetaceae_2 - ps25
Saccharomycetaceae_3 - ps26
Kaz./Nau./Saccharomyces - ps27
Saccharomyces - ps28
Arboricola_clade - ps29
Cerevisiae_clade - ps30
Saccharomyces_cerevisiae - ps31
Phylostratum

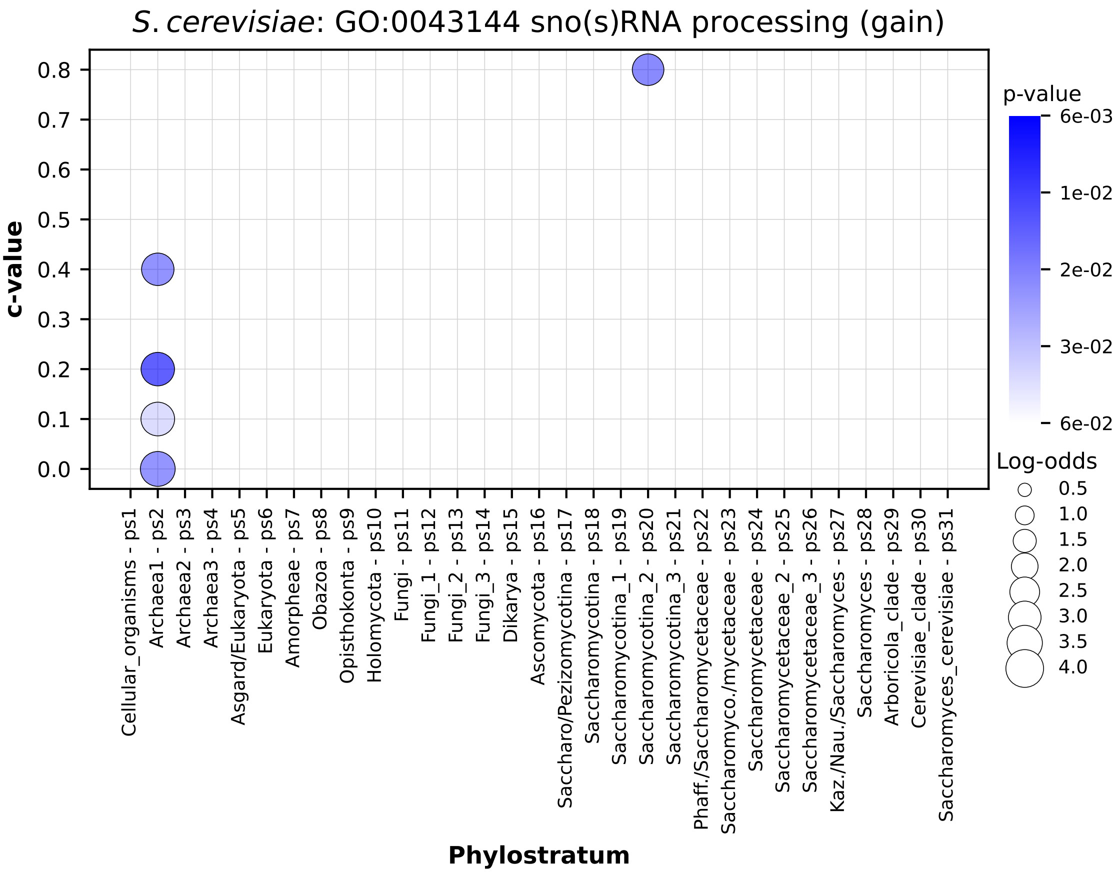

S. cerevisiae: GO:0043144 sno(s)RNA processing (gain)
c-value
0.8
0.7
0.6
0.5
0.4
0.3
0.2
0.1
0.0
p-value
6e-03
1e-02
2e-02
3e-02
6e-02
Log-odds
0.5
1.0
1.5
2.0
2.5
3.0
3.5
4.0
Cellular_organisms - ps1
Archaea1 - ps2
Archaea2 - ps3
Archaea3 - ps4
Asgard/Eukaryota - ps5
Eukaryota - ps6
Amorpheae - ps7
Obazoa - ps8
Opisthokonta - ps9
Holomycota - ps10
Fungi - ps11
Fungi_1 - ps12
Fungi_2 - ps13
Fungi_3 - ps14
Dikarya - ps15
Ascomycota - ps16
Saccharo/Pezizomycotina - ps17
Saccharomycotina - ps18
Saccharomycotina_1 - ps19
Saccharomycotina_2 - ps20
Saccharomycotina_3 - ps21
Phaff./Saccharomycetaceae - ps22
Saccharomyco./mycetaceae - ps23
Saccharomycetaceae - ps24
Saccharomycetaceae_2 - ps25
Saccharomycetaceae_3 - ps26
Kaz./Nau./Saccharomyces - ps27
Saccharomyces - ps28
Arboricola_clade - ps29
Cerevisiae_clade - ps30
Saccharomyces_cerevisiae - ps31
Phylostratum

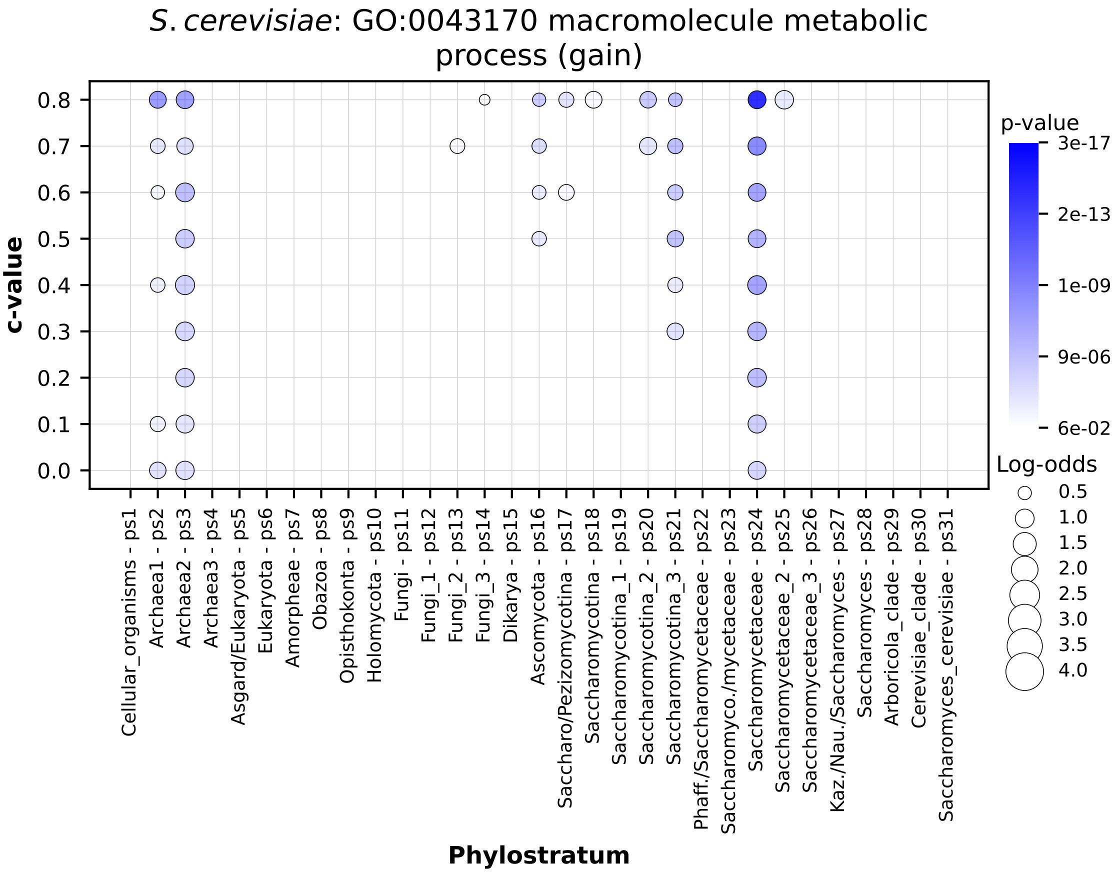

S. cerevisiae: GO:0043170 macromolecule metabolic process (gain)
c-value
0.8
0.7
0.6
0.5
0.4
0.3
0.2
0.1
0.0
p-value
3e-17
2e-13
1e-09
9e-06
6e-02
Log-odds
0.5
1.0
1.5
2.0
2.5
3.0
3.5
4.0
Cellular_organisms - ps1
Archaea1 - ps2
Archaea2 - ps3
Archaea3 - ps4
Asgard/Eukaryota - ps5
Eukaryota - ps6
Amorpheae - ps7
Obazoa - ps8
Opisthokonta - ps9
Holomycota - ps10
Fungi - ps11
Fungi_1 - ps12
Fungi_2 - ps13
Fungi_3 - ps14
Dikarya - ps15
Ascomycota - ps16
Saccharo/Pezizomycotina - ps17
Saccharomycotina - ps18
Saccharomycotina_1 - ps19
Saccharomycotina_2 - ps20
Saccharomycotina_3 - ps21
Phaff./Saccharomycetaceae - ps22
Saccharomyco./mycetaceae - ps23
Saccharomycetaceae - ps24
Saccharomycetaceae_2 - ps25
Saccharomycetaceae_3 - ps26
Kaz./Nau./Saccharomyces - ps27
Saccharomyces - ps28
Arboricola_clade - ps29
Cerevisiae_clade - ps30
Saccharomyces_cerevisiae - ps31
Phylostratum

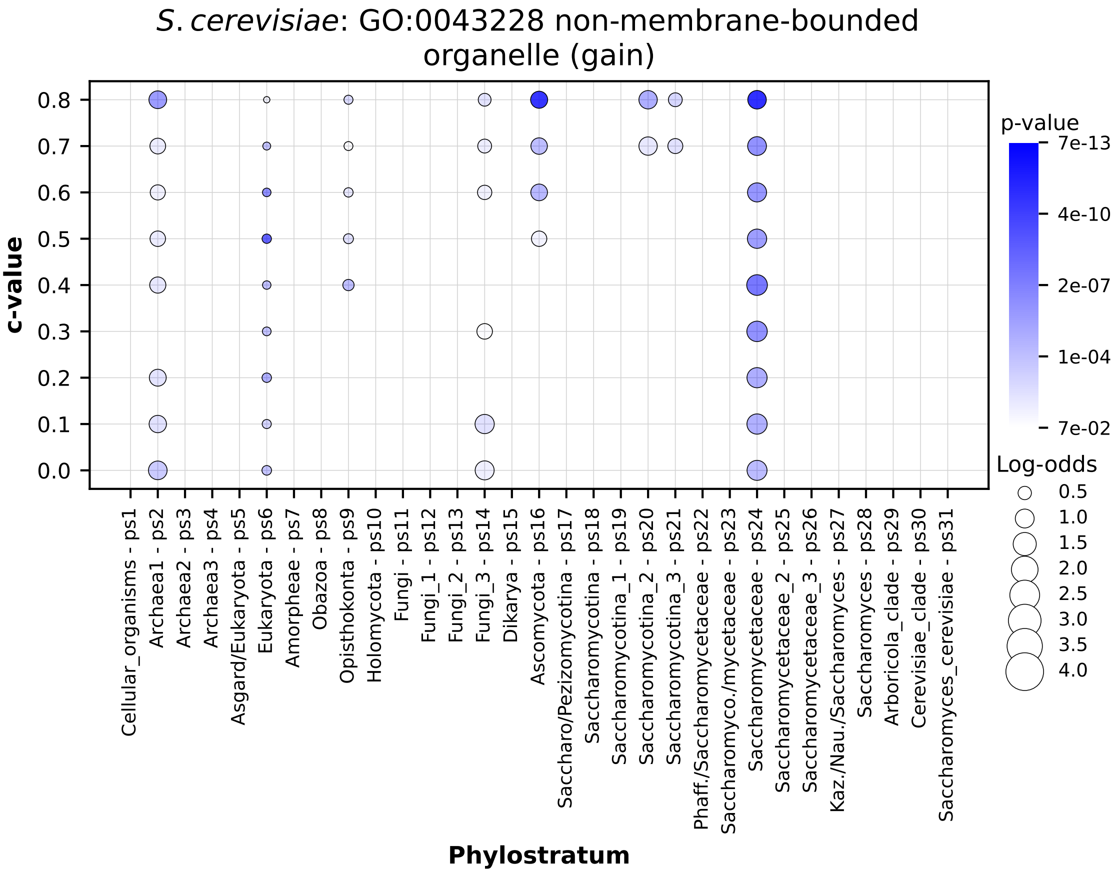

S. cerevisiae: GO:0043228 non-membrane-bounded organelle (gain)
c-value
0.8
0.7
0.6
0.5
0.4
0.3
0.2
0.1
0.0
p-value
7e-13
4e-10
2e-07
1e-04
7e-02
Log-odds
0.5
1.0
1.5
2.0
2.5
3.0
3.5
4.0
Cellular_organisms - ps1
Archaea1 - ps2
Archaea2 - ps3
Archaea3 - ps4
Asgard/Eukaryota - ps5
Eukaryota - ps6
Amorpheae - ps7
Obazoa - ps8
Opisthokonta - ps9
Holomycota - ps10
Fungi - ps11
Fungi_1 - ps12
Fungi_2 - ps13
Fungi_3 - ps14
Dikarya - ps15
Ascomycota - ps16
Saccharo/Pezizomycotina - ps17
Saccharomycotina - ps18
Saccharomycotina_1 - ps19
Saccharomycotina_2 - ps20
Saccharomycotina_3 - ps21
Phaff./Saccharomycetaceae - ps22
Saccharomyco./mycetaceae - ps23
Saccharomycetaceae - ps24
Saccharomycetaceae_2 - ps25
Saccharomycetaceae_3 - ps26
Kaz./Nau./Saccharomyces - ps27
Saccharomyces - ps28
Arboricola_clade - ps29
Cerevisiae_clade - ps30
Saccharomyces_cerevisiae - ps31
Phylostratum

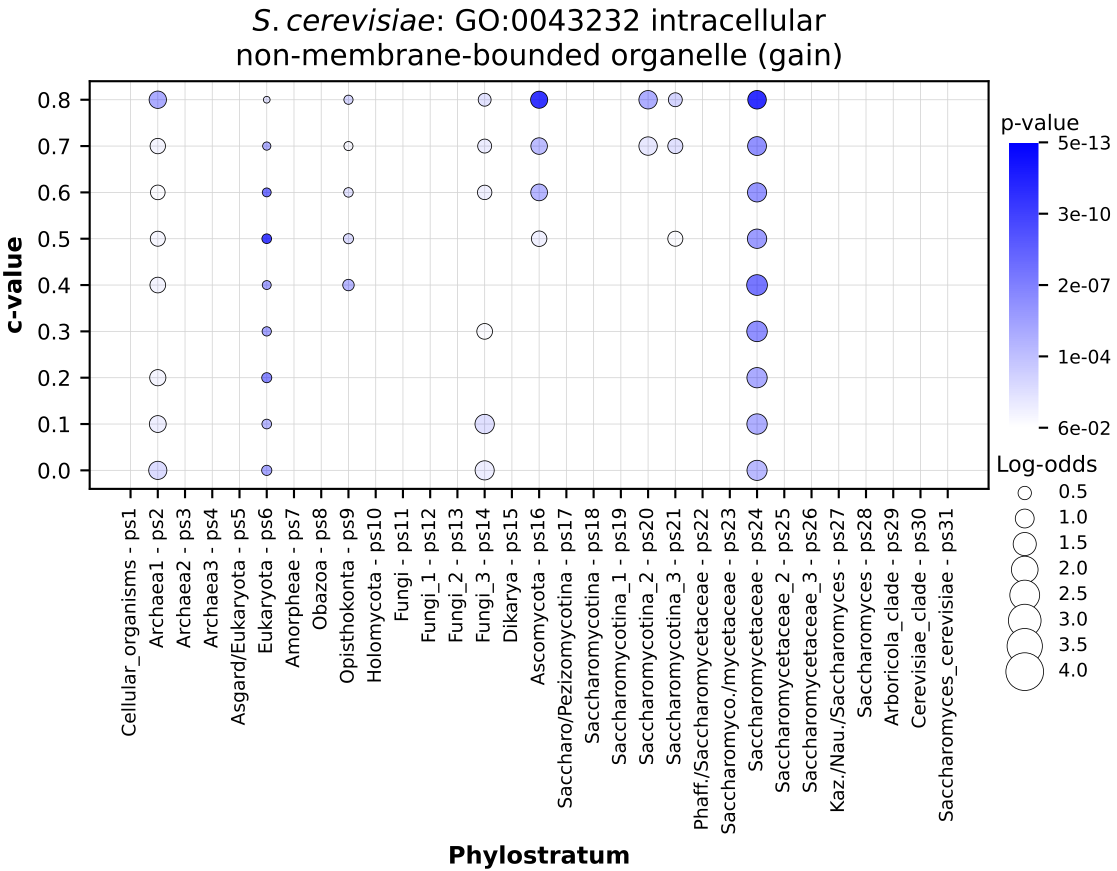

S. cerevisiae: GO:0043232 intracellular non-membrane-bounded organelle (gain)
c-value
0.8
0.7
0.6
0.5
0.4
0.3
0.2
0.1
0.0
p-value
5e-13
3e-10
2e-07
1e-04
6e-02
Log-odds
0.5
1.0
1.5
2.0
2.5
3.0
3.5
4.0
Cellular_organisms - ps1
Archaea1 - ps2
Archaea2 - ps3
Archaea3 - ps4
Asgard/Eukaryota - ps5
Eukaryota - ps6
Amorpheae - ps7
Obazoa - ps8
Opisthokonta - ps9
Holomycota - ps10
Fungi - ps11
Fungi_1 - ps12
Fungi_2 - ps13
Fungi_3 - ps14
Dikarya - ps15
Ascomycota - ps16
Saccharo/Pezizomycotina - ps17
Saccharomycotina - ps18
Saccharomycotina_1 - ps19
Saccharomycotina_2 - ps20
Saccharomycotina_3 - ps21
Phaff./Saccharomycetaceae - ps22
Saccharomyco./mycetaceae - ps23
Saccharomycetaceae - ps24
Saccharomycetaceae_2 - ps25
Saccharomycetaceae_3 - ps26
Kaz./Nau./Saccharomyces - ps27
Saccharomyces - ps28
Arboricola_clade - ps29
Cerevisiae_clade - ps30
Saccharomyces_cerevisiae - ps31
Phylostratum

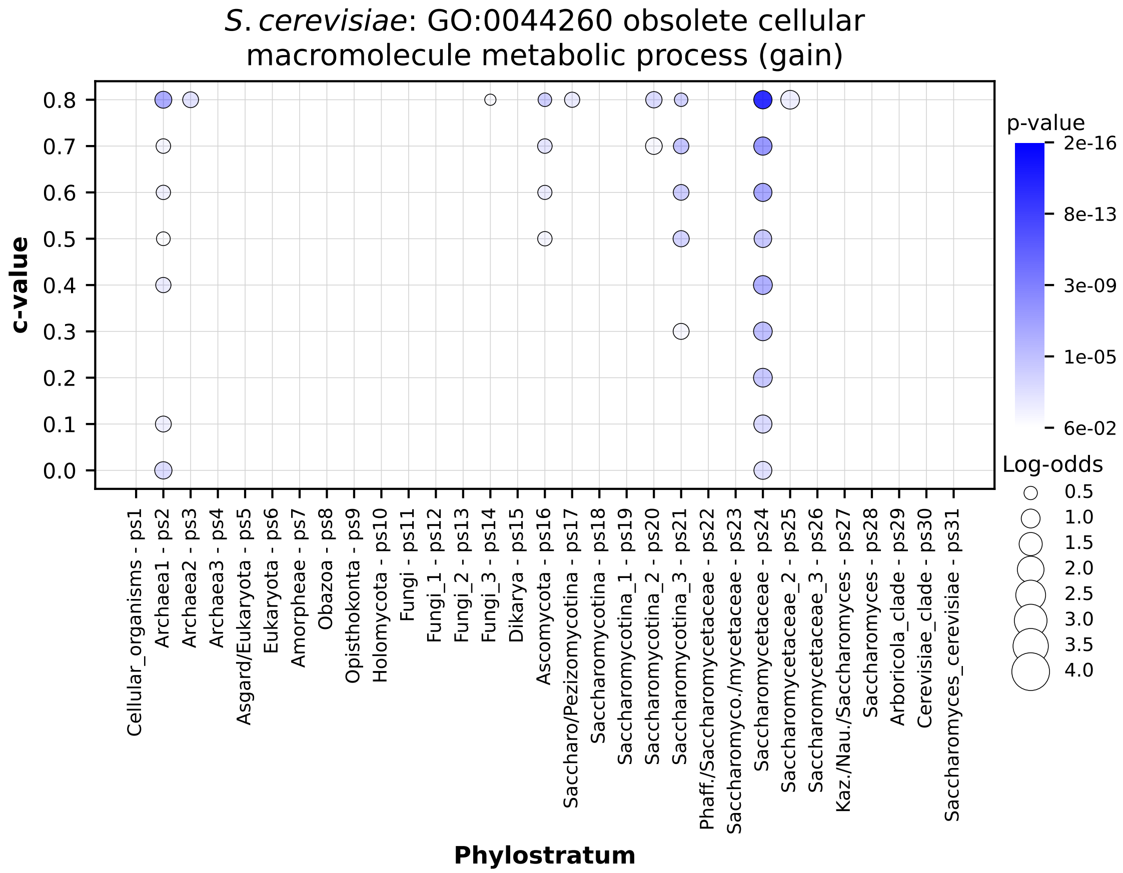
S. cerevisiae: GO:0044260 obsolete cellular macromolecule metabolic process (gain)
c-value
0.0
0.1
0.2
0.3
0.4
0.5
0.6
0.7
0.8
Phylostratum
Cellular_organisms - ps1
Archaea1 - ps2
Archaea2 - ps3
Archaea3 - ps4
Asgard/Eukaryota - ps5
Eukaryota - ps6
Amorpheae - ps7
Obazoa - ps8
Opisthokonta - ps9
Holomycota - ps10
Fungi - ps11
Fungi_1 - ps12
Fungi_2 - ps13
Fungi_3 - ps14
Dikarya - ps15
Ascomycota - ps16
Saccharo/Pezizomycotina - ps17
Saccharomycotina - ps18
Saccharomycotina_1 - ps19
Saccharomycotina_2 - ps20
Saccharomycotina_3 - ps21
Phaff./Saccharomycetaceae - ps22
Saccharomyco./mycetaceae - ps23
Saccharomycetaceae - ps24
Saccharomycetaceae_2 - ps25
Saccharomycetaceae_3 - ps26
Kaz./Nau./Saccharomyces - ps27
Saccharomyces - ps28
Arboricola_clade - ps29
Cerevisiae_clade - ps30
Saccharomyces_cerevisiae - ps31
p-value
2e-16
8e-13
3e-09
1e-05
6e-02
Log-odds
0.5
1.0
1.5
2.0
2.5
3.0
3.5
4.0

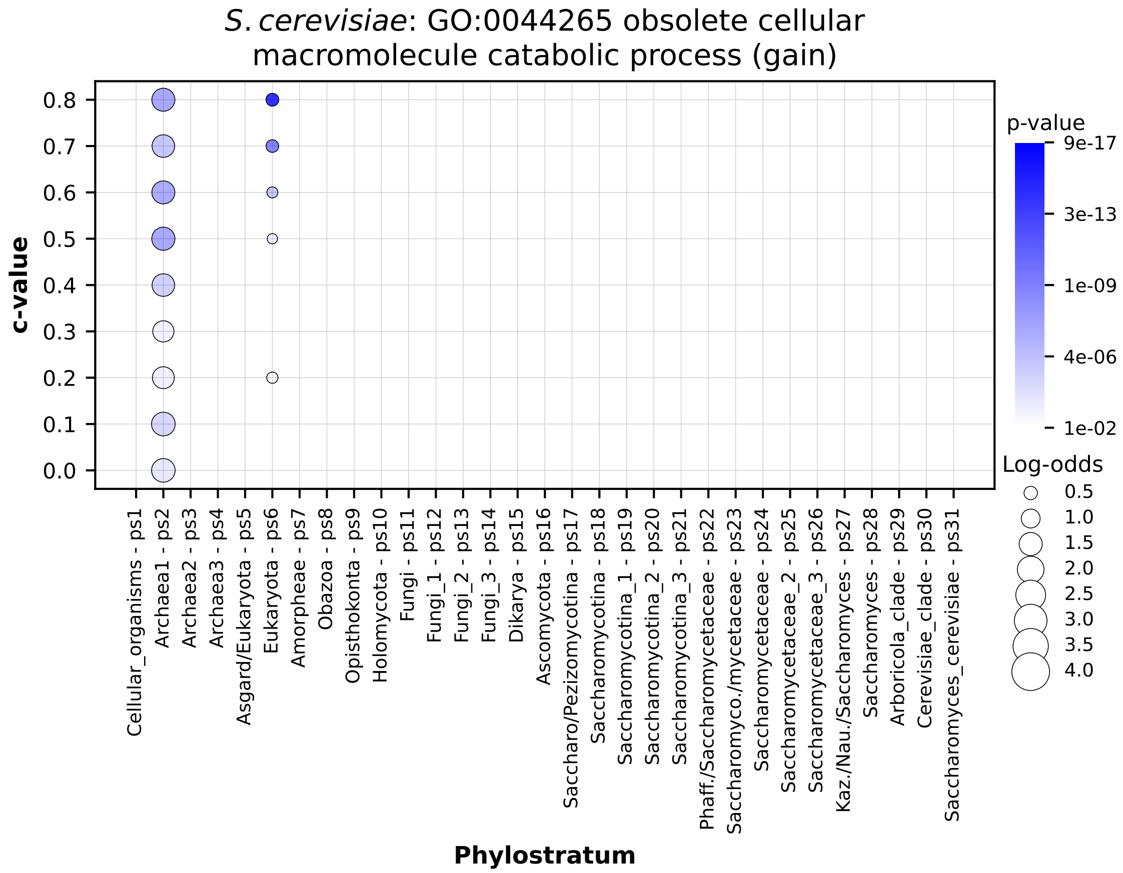

S. cerevisiae: GO:0044265 obsolete cellular macromolecule catabolic process (gain)
c-value
0.8
0.7
0.6
0.5
0.4
0.3
0.2
0.1
0.0
p-value
9e-17
3e-13
1e-09
4e-06
1e-02
Log-odds
0.5
1.0
1.5
2.0
2.5
3.0
3.5
4.0
Cellular_organisms - ps1
Archaea1 - ps2
Archaea2 - ps3
Archaea3 - ps4
Asgard/Eukaryota - ps5
Eukaryota - ps6
Amorpheae - ps7
Obazoa - ps8
Opisthokonta - ps9
Holomycota - ps10
Fungi - ps11
Fungi_1 - ps12
Fungi_2 - ps13
Fungi_3 - ps14
Dikarya - ps15
Ascomycota - ps16
Saccharo/Pezizomycotina - ps17
Saccharomycotina - ps18
Saccharomycotina_1 - ps19
Saccharomycotina_2 - ps20
Saccharomycotina_3 - ps21
Phaff./Saccharomycetaceae - ps22
Saccharomyco./mycetaceae - ps23
Saccharomycetaceae - ps24
Saccharomycetaceae_2 - ps25
Saccharomycetaceae_3 - ps26
Kaz./Nau./Saccharomyces - ps27
Saccharomyces - ps28
Arboricola_clade - ps29
Cerevisiae_clade - ps30
Saccharomyces_cerevisiae - ps31
Phylostratum

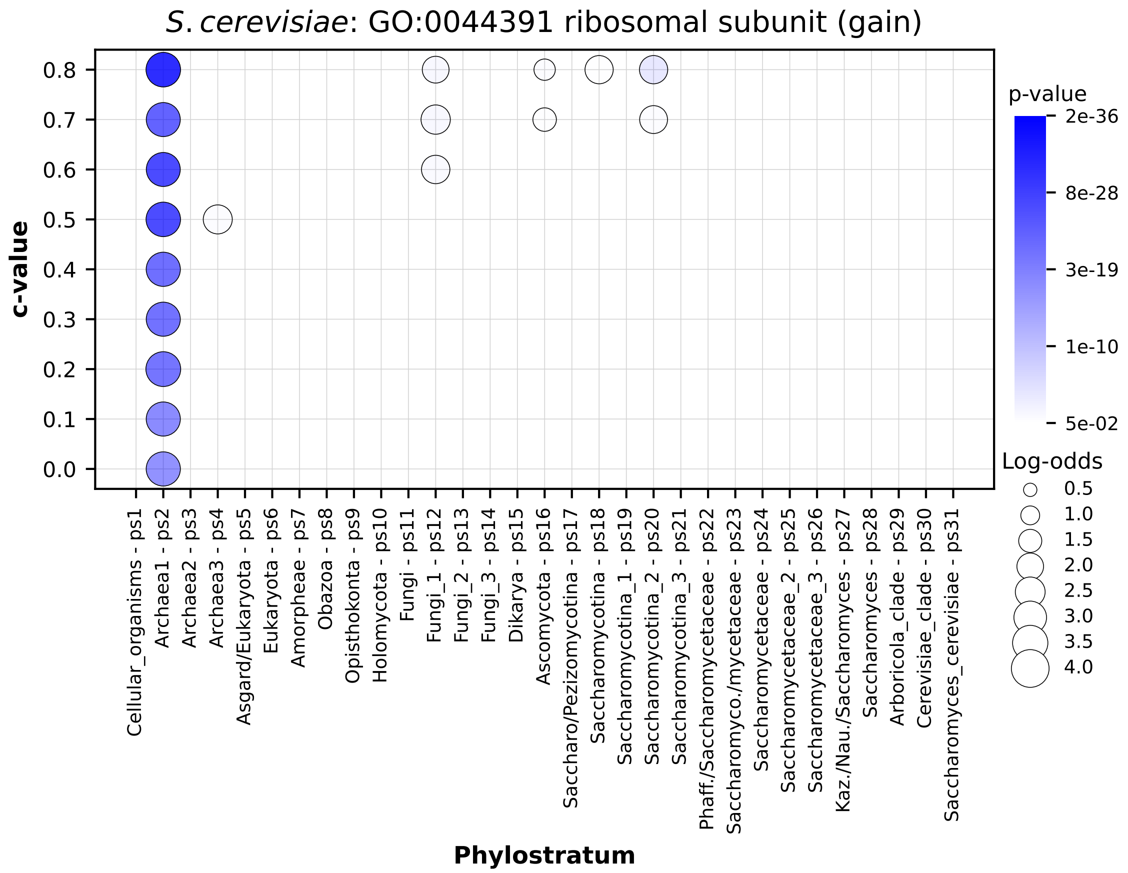

S. cerevisiae: GO:0044391 ribosomal subunit (gain)
c-value
0.8
0.7
0.6
0.5
0.4
0.3
0.2
0.1
0.0
p-value
2e-36
8e-28
3e-19
1e-10
5e-02
Log-odds
0.5
1.0
1.5
2.0
2.5
3.0
3.5
4.0
Cellular_organisms - ps1
Archaea1 - ps2
Archaea2 - ps3
Archaea3 - ps4
Asgard/Eukaryota - ps5
Eukaryota - ps6
Amorpheae - ps7
Obazoa - ps8
Opisthokonta - ps9
Holomycota - ps10
Fungi - ps11
Fungi_1 - ps12
Fungi_2 - ps13
Fungi_3 - ps14
Dikarya - ps15
Ascomycota - ps16
Saccharo/Pezizomycotina - ps17
Saccharomycotina - ps18
Saccharomycotina_1 - ps19
Saccharomycotina_2 - ps20
Saccharomycotina_3 - ps21
Phaff./Saccharomycetaceae - ps22
Saccharomyco./mycetaceae - ps23
Saccharomycetaceae - ps24
Saccharomycetaceae_2 - ps25
Saccharomycetaceae_3 - ps26
Kaz./Nau./Saccharomyces - ps27
Saccharomyces - ps28
Arboricola_clade - ps29
Cerevisiae_clade - ps30
Saccharomyces_cerevisiae - ps31
Phylostratum

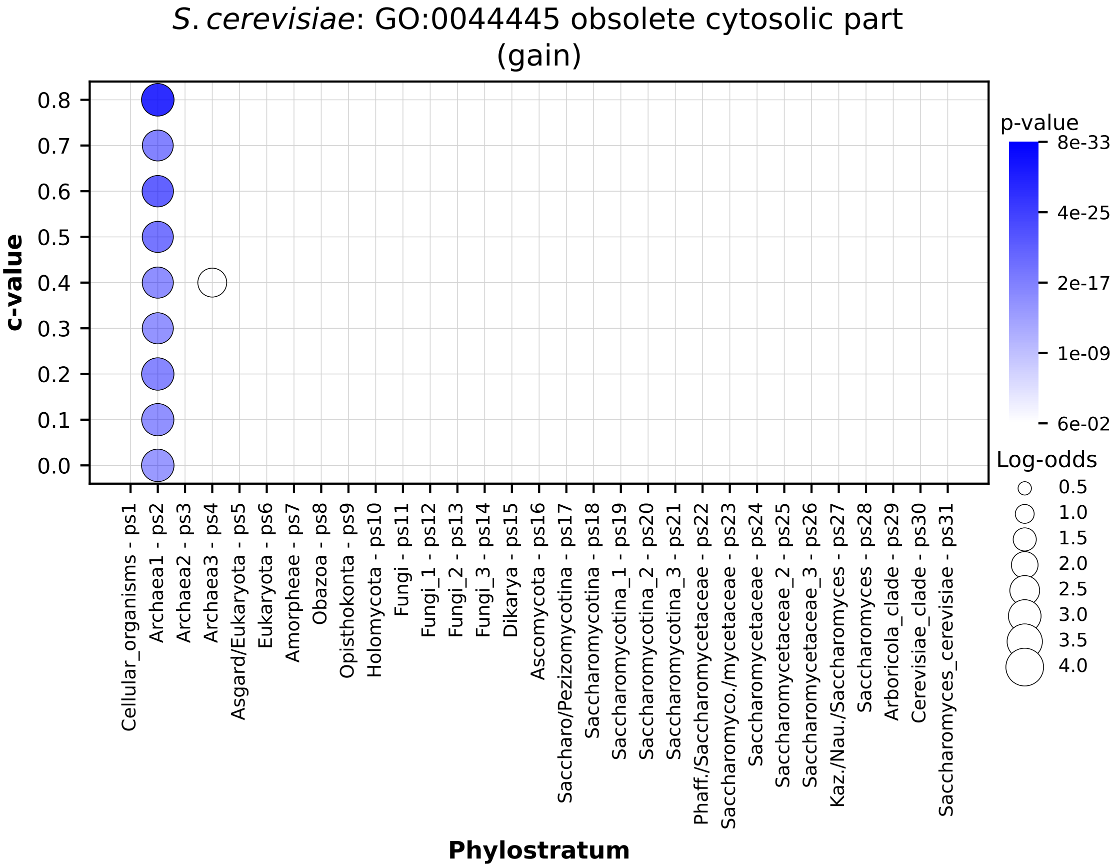

S. cerevisiae: GO:0044445 obsolete cytosolic part
(gain)
c-value
0.0
0.1
0.2
0.3
0.4
0.5
0.6
0.7
0.8
p-value
8e-33
4e-25
2e-17
1e-09
6e-02
Log-odds
0.5
1.0
1.5
2.0
2.5
3.0
3.5
4.0
Cellular_organisms - ps1
Archaea1 - ps2
Archaea2 - ps3
Archaea3 - ps4
Asgard/Eukaryota - ps5
Eukaryota - ps6
Amorpheae - ps7
Obazoa - ps8
Opisthokonta - ps9
Holomycota - ps10
Fungi - ps11
Fungi_1 - ps12
Fungi_2 - ps13
Fungi_3 - ps14
Dikarya - ps15
Ascomycota - ps16
Saccharo/Pezizomycotina - ps17
Saccharomycotina - ps18
Saccharomycotina_1 - ps19
Saccharomycotina_2 - ps20
Saccharomycotina_3 - ps21
Phaff./Saccharomycetaceae - ps22
Saccharomyco./mycetaceae - ps23
Saccharomycetaceae - ps24
Saccharomycetaceae_2 - ps25
Saccharomycetaceae_3 - ps26
Kaz./Nau./Saccharomyces - ps27
Saccharomyces - ps28
Arboricola_clade - ps29
Cerevisiae_clade - ps30
Saccharomyces_cerevisiae - ps31
Phylostratum

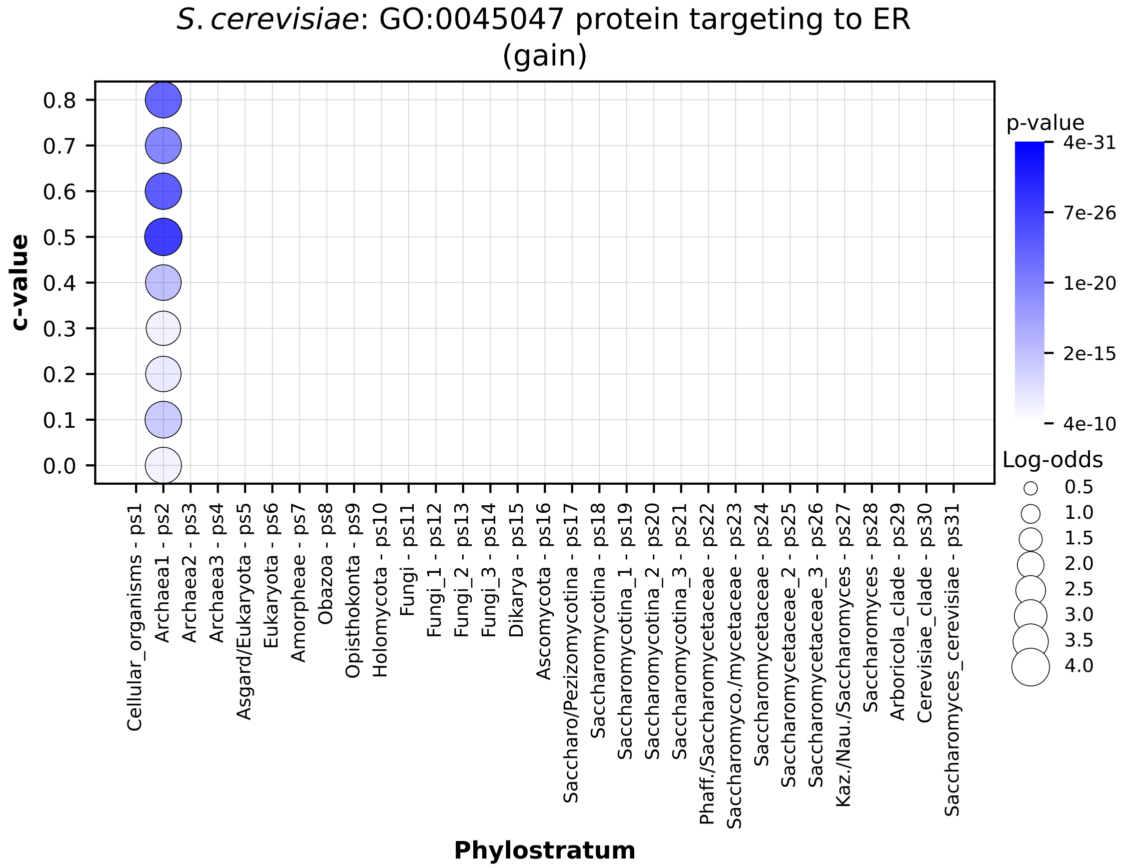
S. cerevisiae: GO:0045047 protein targeting to ER
(gain)
c-value
0.8
0.7
0.6
0.5
0.4
0.3
0.2
0.1
0.0
p-value
4e-31
7e-26
1e-20
2e-15
4e-10
Log-odds
0.5
1.0
1.5
2.0
2.5
3.0
3.5
4.0
Cellular_organisms - ps1
Archaea1 - ps2
Archaea2 - ps3
Archaea3 - ps4
Asgard/Eukaryota - ps5
Eukaryota - ps6
Amorpheae - ps7
Obazoa - ps8
Opisthokonta - ps9
Holomycota - ps10
Fungi - ps11
Fungi_1 - ps12
Fungi_2 - ps13
Fungi_3 - ps14
Dikarya - ps15
Ascomycota - ps16
Saccharo/Pezizomycotina - ps17
Saccharomycotina - ps18
Saccharomycotina_1 - ps19
Saccharomycotina_2 - ps20
Saccharomycotina_3 - ps21
Phaff./Saccharomycetaceae - ps22
Saccharomyco./mycetaceae - ps23
Saccharomycetaceae - ps24
Saccharomycetaceae_2 - ps25
Saccharomycetaceae_3 - ps26
Kaz./Nau./Saccharomyces - ps27
Saccharomyces - ps28
Arboricola_clade - ps29
Cerevisiae_clade - ps30
Saccharomyces_cerevisiae - ps31
Phylostratum

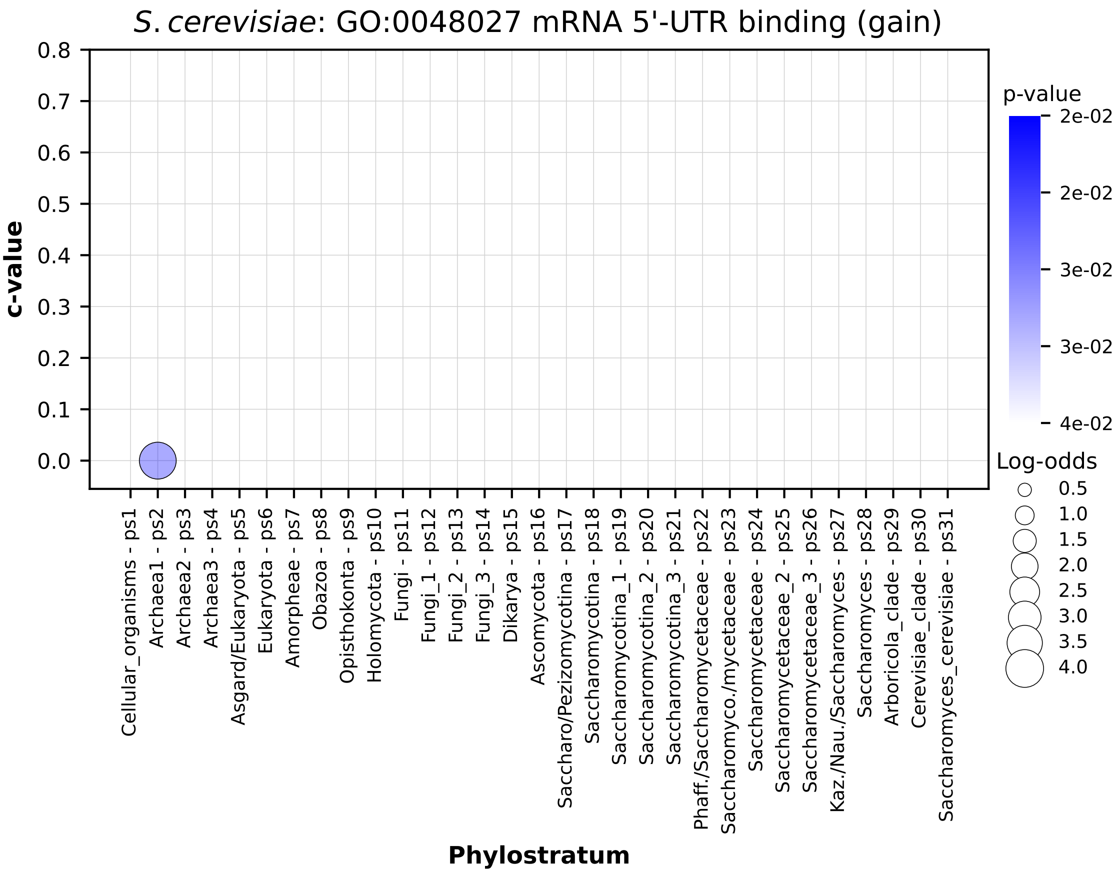
S. cerevisiae: GO:0048027 mRNA 5'-UTR binding (gain)
c-value
0.8
0.7
0.6
0.5
0.4
0.3
0.2
0.1
0.0
p-value
2e-02
2e-02
3e-02
3e-02
4e-02
Log-odds
0.5
1.0
1.5
2.0
2.5
3.0
3.5
4.0
Cellular_organisms - ps1
Archaea1 - ps2
Archaea2 - ps3
Archaea3 - ps4
Asgard/Eukaryota - ps5
Eukaryota - ps6
Amorpheae - ps7
Obazoa - ps8
Opisthokonta - ps9
Holomycota - ps10
Fungi - ps11
Fungi_1 - ps12
Fungi_2 - ps13
Fungi_3 - ps14
Dikarya - ps15
Ascomycota - ps16
Saccharo/Pezizomycotina - ps17
Saccharomycotina - ps18
Saccharomycotina_1 - ps19
Saccharomycotina_2 - ps20
Saccharomycotina_3 - ps21
Phaff./Saccharomycetaceae - ps22
Saccharomyco./mycetaceae - ps23
Saccharomycetaceae - ps24
Saccharomycetaceae_2 - ps25
Saccharomycetaceae_3 - ps26
Kaz./Nau./Saccharomyces - ps27
Saccharomyces - ps28
Arboricola_clade - ps29
Cerevisiae_clade - ps30
Saccharomyces_cerevisiae - ps31
Phylostratum

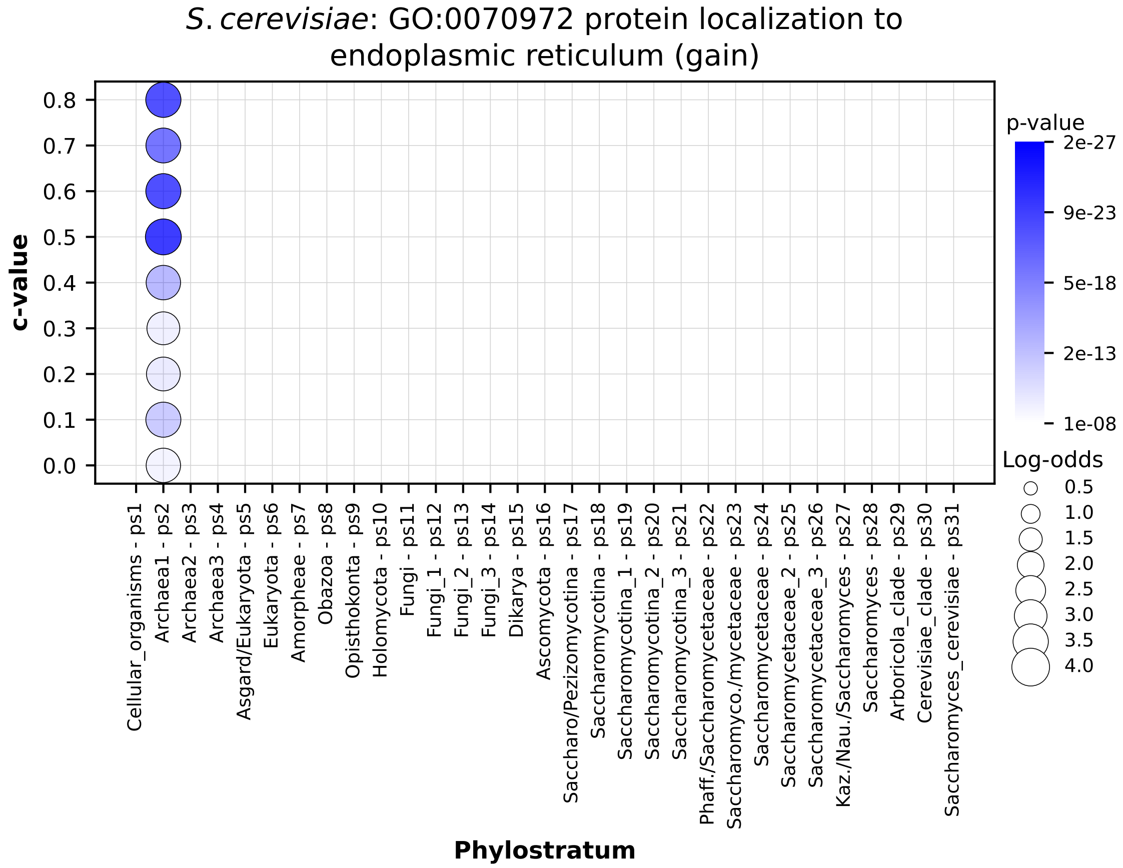

S. cerevisiae: GO:0070972 protein localization to endoplasmic reticulum (gain)
c-value
0.8
0.7
0.6
0.5
0.4
0.3
0.2
0.1
0.0
p-value
2e-27
9e-23
5e-18
2e-13
1e-08
Log-odds
0.5
1.0
1.5
2.0
2.5
3.0
3.5
4.0
Cellular_organisms - ps1
Archaea1 - ps2
Archaea2 - ps3
Archaea3 - ps4
Asgard/Eukaryota - ps5
Eukaryota - ps6
Amorpheae - ps7
Obazoa - ps8
Opisthokonta - ps9
Holomycota - ps10
Fungi - ps11
Fungi_1 - ps12
Fungi_2 - ps13
Fungi_3 - ps14
Dikarya - ps15
Ascomycota - ps16
Saccharo/Pezizomycotina - ps17
Saccharomycotina - ps18
Saccharomycotina_1 - ps19
Saccharomycotina_2 - ps20
Saccharomycotina_3 - ps21
Phaff./Saccharomycetaceae - ps22
Saccharomyco./mycetaceae - ps23
Saccharomycetaceae - ps24
Saccharomycetaceae_2 - ps25
Saccharomycetaceae_3 - ps26
Kaz./Nau./Saccharomyces - ps27
Saccharomyces - ps28
Arboricola_clade - ps29
Cerevisiae_clade - ps30
Saccharomyces_cerevisiae - ps31
Phylostratum

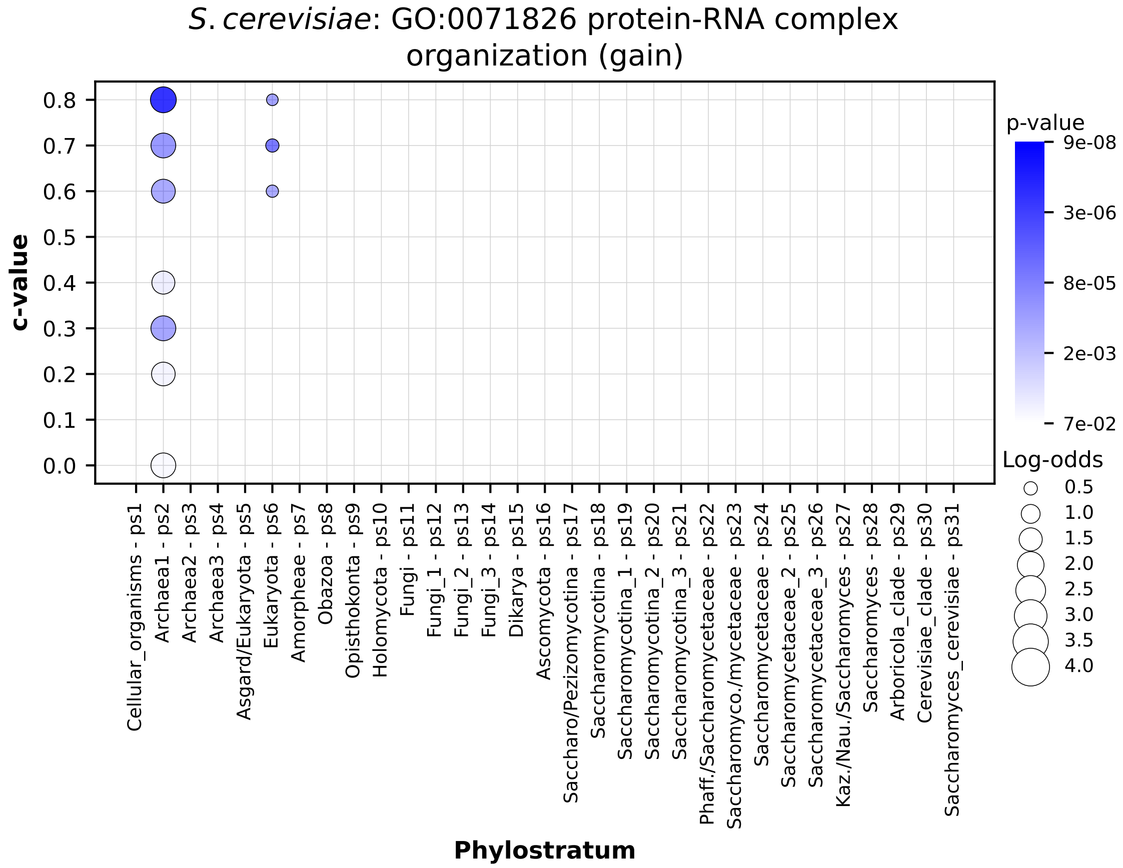

S. cerevisiae: GO:0071826 protein-RNA complex organization (gain)
c-value
0.8
0.7
0.6
0.5
0.4
0.3
0.2
0.1
0.0
Phylostratum
Cellular_organisms - ps1
Archaea1 - ps2
Archaea2 - ps3
Archaea3 - ps4
Asgard/Eukaryota - ps5
Eukaryota - ps6
Amorpheae - ps7
Obazoa - ps8
Opisthokonta - ps9
Holomycota - ps10
Fungi - ps11
Fungi_1 - ps12
Fungi_2 - ps13
Fungi_3 - ps14
Dikarya - ps15
Ascomycota - ps16
Saccharo/Pezizomycotina - ps17
Saccharomycotina - ps18
Saccharomycotina_1 - ps19
Saccharomycotina_2 - ps20
Saccharomycotina_3 - ps21
Phaff./Saccharomycetaceae - ps22
Saccharomyco./mycetaceae - ps23
Saccharomycetaceae - ps24
Saccharomycetaceae_2 - ps25
Saccharomycetaceae_3 - ps26
Kaz./Nau./Saccharomyces - ps27
Saccharomyces - ps28
Arboricola_clade - ps29
Cerevisiae_clade - ps30
Saccharomyces_cerevisiae - ps31
p-value
9e-08
3e-06
8e-05
2e-03
7e-02
Log-odds
0.5
1.0
1.5
2.0
2.5
3.0
3.5
4.0

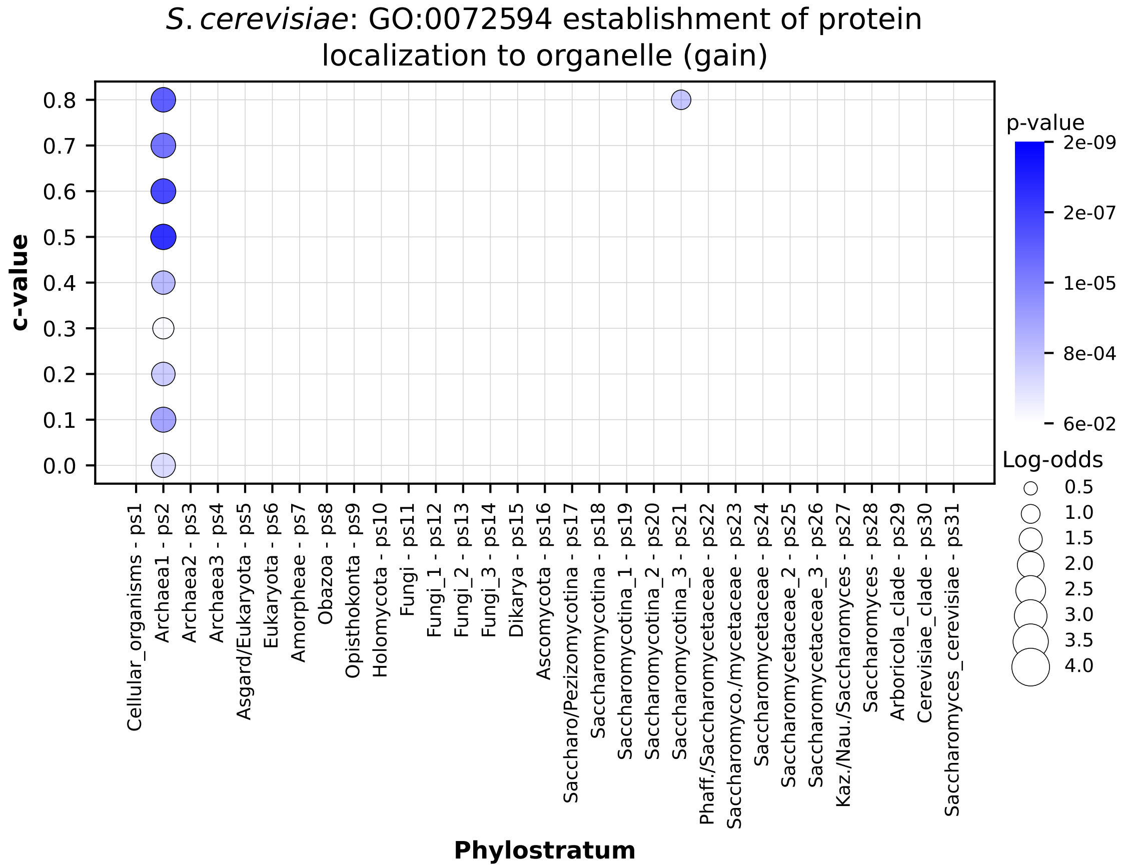
S. cerevisiae: GO:0072594 establishment of protein localization to organelle (gain)
c-value
0.8
0.7
0.6
0.5
0.4
0.3
0.2
0.1
0.0
p-value
2e-09
2e-07
1e-05
8e-04
6e-02
Log-odds
0.5
1.0
1.5
2.0
2.5
3.0
3.5
4.0
Cellular_organisms - ps1
Archaea1 - ps2
Archaea2 - ps3
Archaea3 - ps4
Asgard/Eukaryota - ps5
Eukaryota - ps6
Amorpheae - ps7
Obazoa - ps8
Opisthokonta - ps9
Holomycota - ps10
Fungi - ps11
Fungi_1 - ps12
Fungi_2 - ps13
Fungi_3 - ps14
Dikarya - ps15
Ascomycota - ps16
Saccharo/Pezizomycotina - ps17
Saccharomycotina - ps18
Saccharomycotina_1 - ps19
Saccharomycotina_2 - ps20
Saccharomycotina_3 - ps21
Phaff./Saccharomycetaceae - ps22
Saccharomyco./mycetaceae - ps23
Saccharomycetaceae - ps24
Saccharomycetaceae_2 - ps25
Saccharomycetaceae_3 - ps26
Kaz./Nau./Saccharomyces - ps27
Saccharomyces - ps28
Arboricola_clade - ps29
Cerevisiae_clade - ps30
Saccharomyces_cerevisiae - ps31
Phylostratum

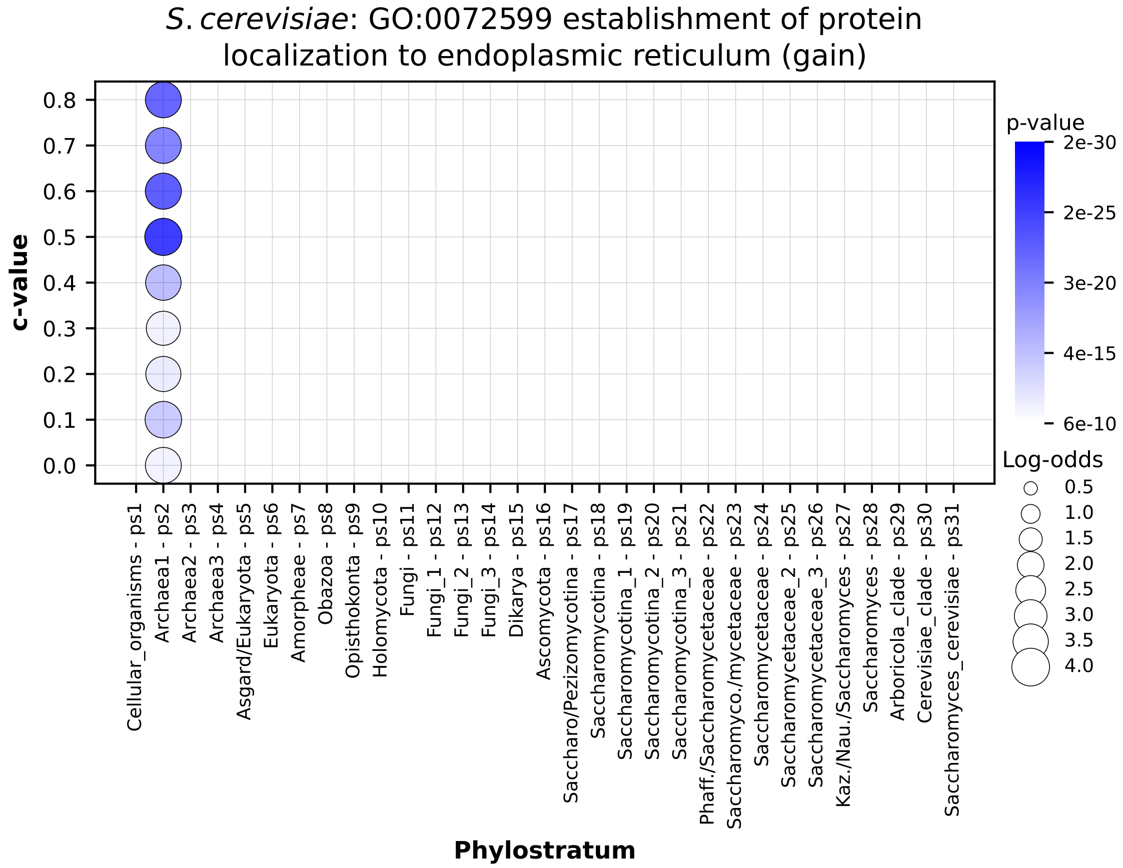

S. cerevisiae: GO:0072599 establishment of protein localization to endoplasmic reticulum (gain)
c-value
0.8
0.7
0.6
0.5
0.4
0.3
0.2
0.1
0.0
p-value
2e-30
2e-25
3e-20
4e-15
6e-10
Log-odds
0.5
1.0
1.5
2.0
2.5
3.0
3.5
4.0
Cellular_organisms - ps1
Archaea1 - ps2
Archaea2 - ps3
Archaea3 - ps4
Asgard/Eukaryota - ps5
Eukaryota - ps6
Amorpheae - ps7
Obazoa - ps8
Opisthokonta - ps9
Holomycota - ps10
Fungi - ps11
Fungi_1 - ps12
Fungi_2 - ps13
Fungi_3 - ps14
Dikarya - ps15
Ascomycota - ps16
Saccharo/Pezizomycotina - ps17
Saccharomycotina - ps18
Saccharomycotina_1 - ps19
Saccharomycotina_2 - ps20
Saccharomycotina_3 - ps21
Phaff./Saccharomycetaceae - ps22
Saccharomyco./mycetaceae - ps23
Saccharomycetaceae - ps24
Saccharomycetaceae_2 - ps25
Saccharomycetaceae_3 - ps26
Kaz./Nau./Saccharomyces - ps27
Saccharomyces - ps28
Arboricola_clade - ps29
Cerevisiae_clade - ps30
Saccharomyces_cerevisiae - ps31
Phylostratum

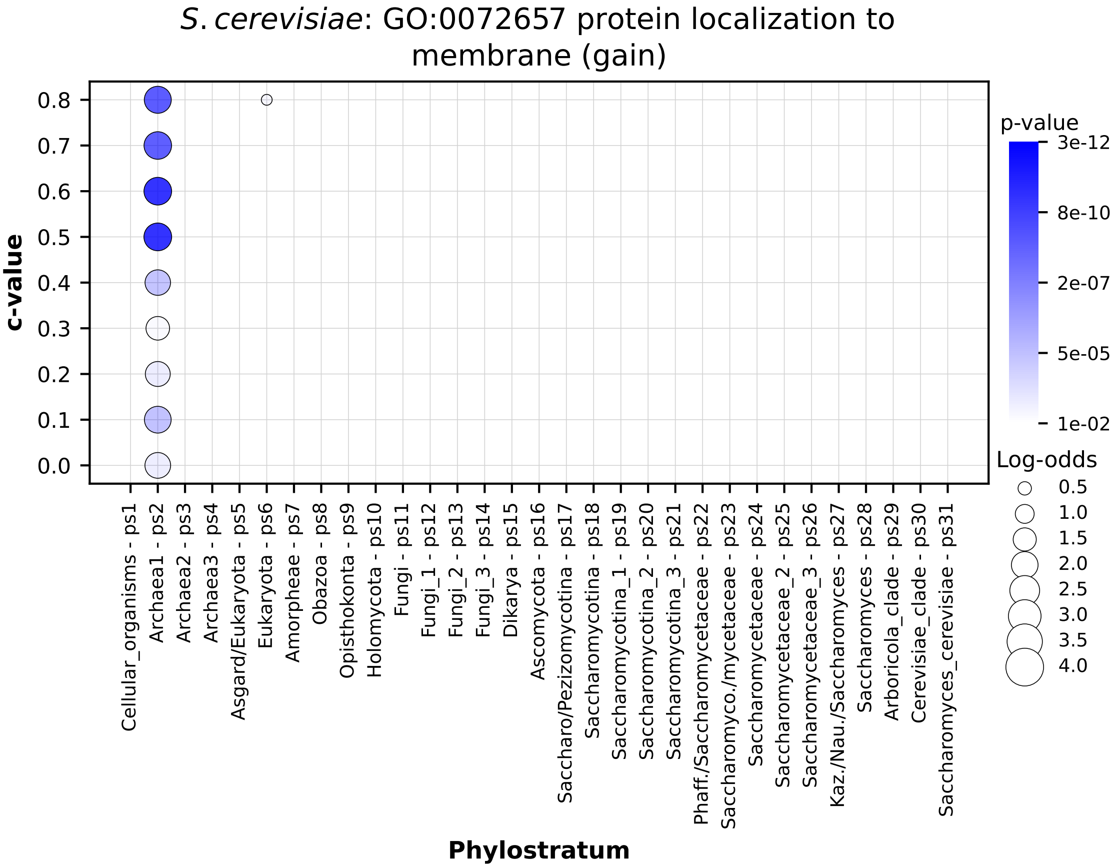

S. cerevisiae: GO:0072657 protein localization to membrane (gain)
c-value
0.8
0.7
0.6
0.5
0.4
0.3
0.2
0.1
0.0
p-value
3e-12
8e-10
2e-07
5e-05
1e-02
Log-odds
0.5
1.0
1.5
2.0
2.5
3.0
3.5
4.0
Cellular_organisms - ps1
Archaea1 - ps2
Archaea2 - ps3
Archaea3 - ps4
Asgard/Eukaryota - ps5
Eukaryota - ps6
Amorpheae - ps7
Obazoa - ps8
Opisthokonta - ps9
Holomycota - ps10
Fungi - ps11
Fungi_1 - ps12
Fungi_2 - ps13
Fungi_3 - ps14
Dikarya - ps15
Ascomycota - ps16
Saccharo/Pezizomycotina - ps17
Saccharomycotina - ps18
Saccharomycotina_1 - ps19
Saccharomycotina_2 - ps20
Saccharomycotina_3 - ps21
Phaff./Saccharomycetaceae - ps22
Saccharomyco./mycetaceae - ps23
Saccharomycetaceae - ps24
Saccharomycetaceae_2 - ps25
Saccharomycetaceae_3 - ps26
Kaz./Nau./Saccharomyces - ps27
Saccharomyces - ps28
Arboricola_clade - ps29
Cerevisiae_clade - ps30
Saccharomyces_cerevisiae - ps31
Phylostratum

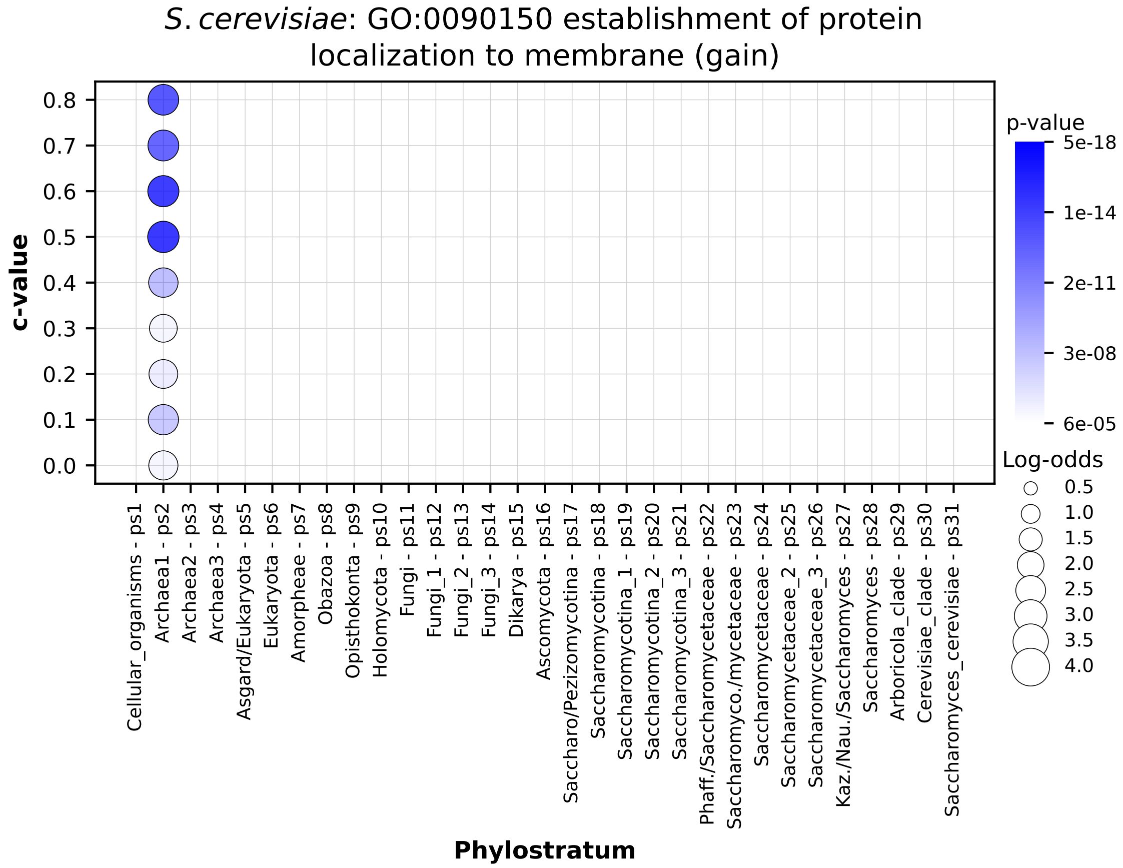
S. cerevisiae: GO:0090150 establishment of protein localization to membrane (gain)
c-value
0.8
0.7
0.6
0.5
0.4
0.3
0.2
0.1
0.0
p-value
5e-18
1e-14
2e-11
3e-08
6e-05
Log-odds
0.5
1.0
1.5
2.0
2.5
3.0
3.5
4.0
Cellular_organisms - ps1
Archaea1 - ps2
Archaea2 - ps3
Archaea3 - ps4
Asgard/Eukaryota - ps5
Eukaryota - ps6
Amorpheae - ps7
Obazoa - ps8
Opisthokonta - ps9
Holomycota - ps10
Fungi - ps11
Fungi_1 - ps12
Fungi_2 - ps13
Fungi_3 - ps14
Dikarya - ps15
Ascomycota - ps16
Saccharo/Pezizomycotina - ps17
Saccharomycotina - ps18
Saccharomycotina_1 - ps19
Saccharomycotina_2 - ps20
Saccharomycotina_3 - ps21
Phaff./Saccharomycetaceae - ps22
Saccharomyco./mycetaceae - ps23
Saccharomycetaceae - ps24
Saccharomycetaceae_2 - ps25
Saccharomycetaceae_3 - ps26
Kaz./Nau./Saccharomyces - ps27
Saccharomyces - ps28
Arboricola_clade - ps29
Cerevisiae_clade - ps30
Saccharomyces_cerevisiae - ps31
Phylostratum

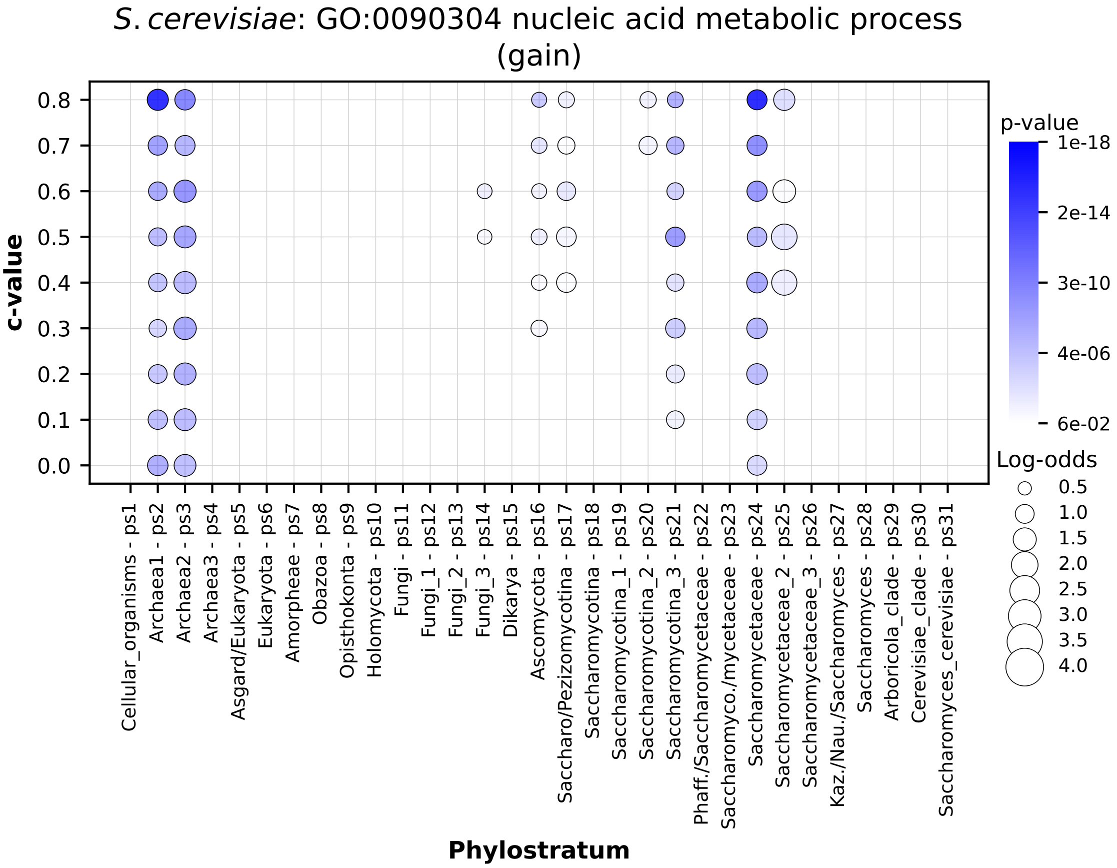
S. cerevisiae: GO:0090304 nucleic acid metabolic process (gain)
c-value
0.8
0.7
0.6
0.5
0.4
0.3
0.2
0.1
0.0
p-value
1e-18
2e-14
3e-10
4e-06
6e-02
Log-odds
0.5
1.0
1.5
2.0
2.5
3.0
3.5
4.0
Cellular_organisms - ps1
Archaea1 - ps2
Archaea2 - ps3
Archaea3 - ps4
Asgard/Eukaryota - ps5
Eukaryota - ps6
Amorpheae - ps7
Obazoa - ps8
Opisthokonta - ps9
Holomycota - ps10
Fungi - ps11
Fungi_1 - ps12
Fungi_2 - ps13
Fungi_3 - ps14
Dikarya - ps15
Ascomycota - ps16
Saccharo/Pezizomycotina - ps17
Saccharomycotina - ps18
Saccharomycotina_1 - ps19
Saccharomycotina_2 - ps20
Saccharomycotina_3 - ps21
Phaff./Saccharomycetaceae - ps22
Saccharomyco./mycetaceae - ps23
Saccharomycetaceae - ps24
Saccharomycetaceae_2 - ps25
Saccharomycetaceae_3 - ps26
Kaz./Nau./Saccharomyces - ps27
Saccharomyces - ps28
Arboricola_clade - ps29
Cerevisiae_clade - ps30
Saccharomyces_cerevisiae - ps31
Phylostratum

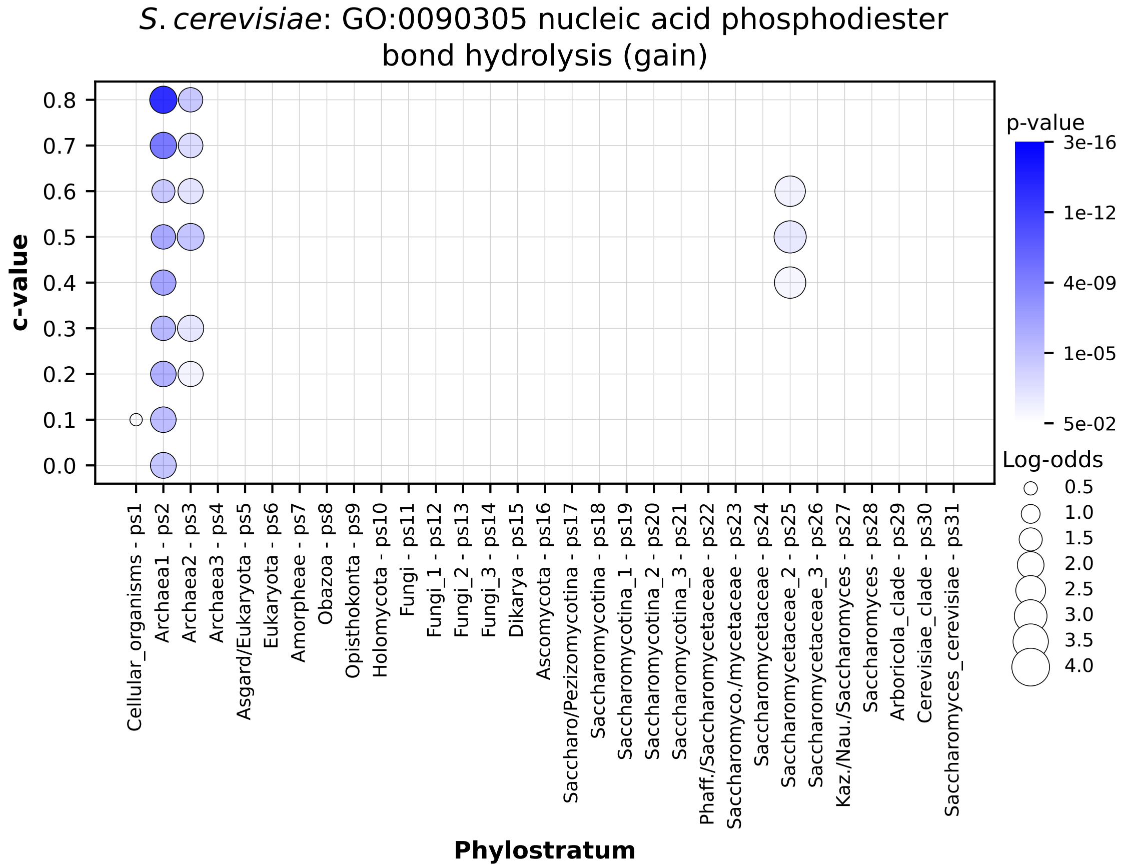

S. cerevisiae: GO:0090305 nucleic acid phosphodiester bond hydrolysis (gain)
c-value
0.8
0.7
0.6
0.5
0.4
0.3
0.2
0.1
0.0
p-value
3e-16
1e-12
4e-09
1e-05
5e-02
Log-odds
0.5
1.0
1.5
2.0
2.5
3.0
3.5
4.0
Cellular_organisms - ps1
Archaea1 - ps2
Archaea2 - ps3
Archaea3 - ps4
Asgard/Eukaryota - ps5
Eukaryota - ps6
Amorpheae - ps7
Obazoa - ps8
Opisthokonta - ps9
Holomycota - ps10
Fungi - ps11
Fungi_1 - ps12
Fungi_2 - ps13
Fungi_3 - ps14
Dikarya - ps15
Ascomycota - ps16
Saccharo/Pezizomycotina - ps17
Saccharomycotina - ps18
Saccharomycotina_1 - ps19
Saccharomycotina_2 - ps20
Saccharomycotina_3 - ps21
Phaff./Saccharomycetaceae - ps22
Saccharomyco./mycetaceae - ps23
Saccharomycetaceae - ps24
Saccharomycetaceae_2 - ps25
Saccharomycetaceae_3 - ps26
Kaz./Nau./Saccharomyces - ps27
Saccharomyces - ps28
Arboricola_clade - ps29
Cerevisiae_clade - ps30
Saccharomyces_cerevisiae - ps31
Phylostratum

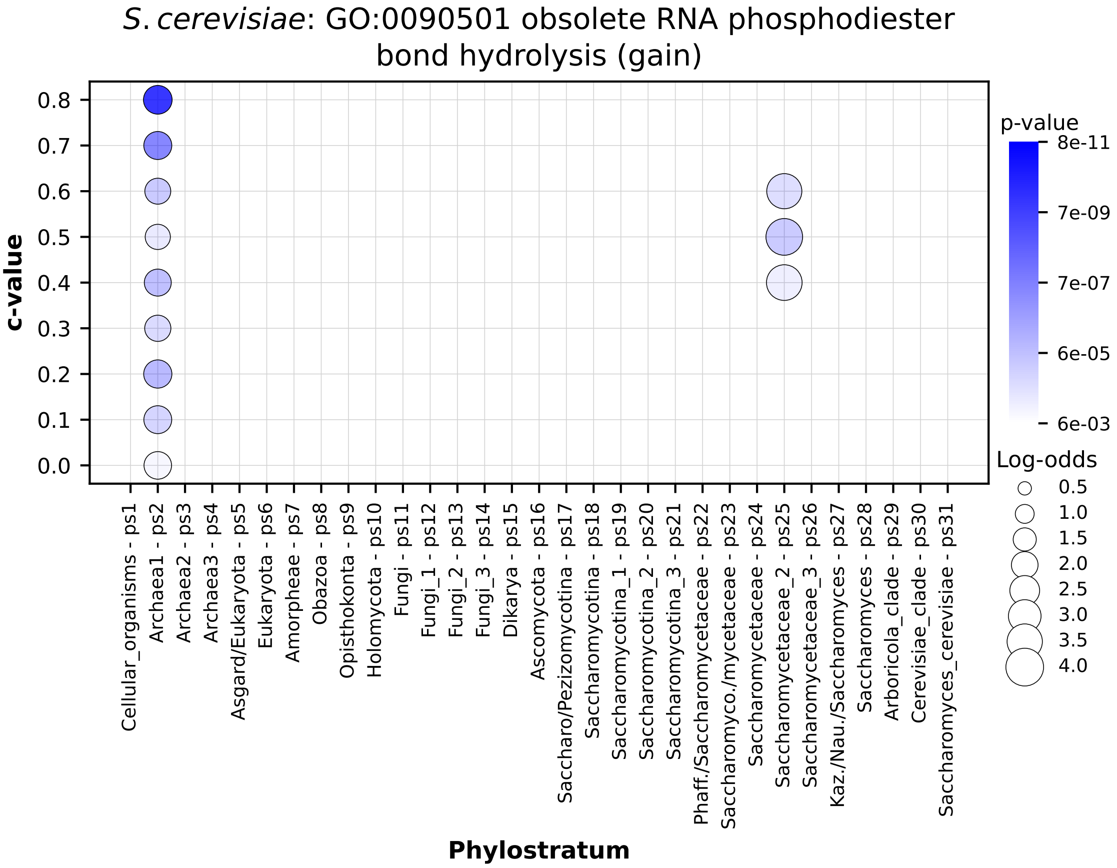

S. cerevisiae: GO:0090501 obsolete RNA phosphodiester bond hydrolysis (gain)
c-value
0.8
0.7
0.6
0.5
0.4
0.3
0.2
0.1
0.0
p-value
8e-11
7e-09
7e-07
6e-05
6e-03
Log-odds
0.5
1.0
1.5
2.0
2.5
3.0
3.5
4.0
Cellular_organisms - ps1
Archaea1 - ps2
Archaea2 - ps3
Archaea3 - ps4
Asgard/Eukaryota - ps5
Eukaryota - ps6
Amorpheae - ps7
Obazoa - ps8
Opisthokonta - ps9
Holomycota - ps10
Fungi - ps11
Fungi_1 - ps12
Fungi_2 - ps13
Fungi_3 - ps14
Dikarya - ps15
Ascomycota - ps16
Saccharo/Pezizomycotina - ps17
Saccharomycotina - ps18
Saccharomycotina_1 - ps19
Saccharomycotina_2 - ps20
Saccharomycotina_3 - ps21
Phaff./Saccharomycetaceae - ps22
Saccharomyco./mycetaceae - ps23
Saccharomycetaceae - ps24
Saccharomycetaceae_2 - ps25
Saccharomycetaceae_3 - ps26
Kaz./Nau./Saccharomyces - ps27
Saccharomyces - ps28
Arboricola_clade - ps29
Cerevisiae_clade - ps30
Saccharomyces_cerevisiae - ps31
Phylostratum

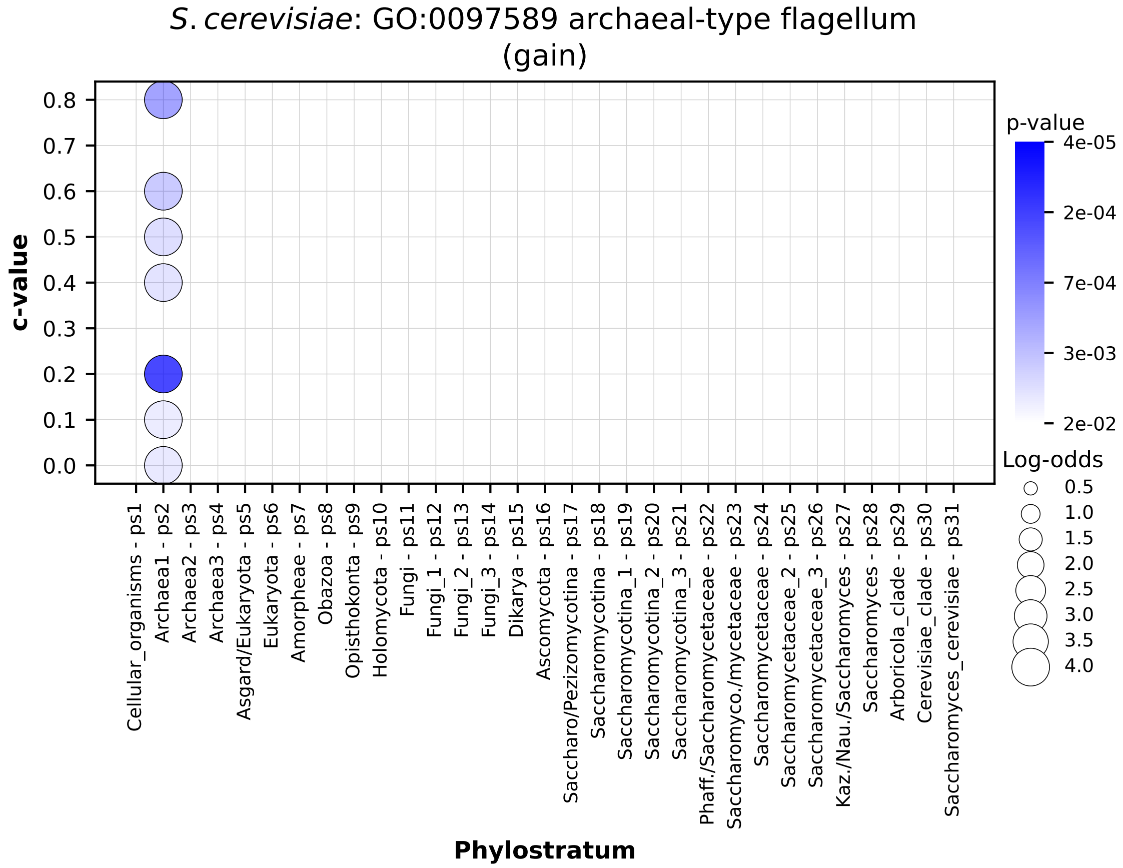

S. cerevisiae: GO:0097589 archaeal-type flagellum
(gain)
c-value
0.8
0.7
0.6
0.5
0.4
0.3
0.2
0.1
0.0
Phylostratum
Cellular_organisms - ps1
Archaea1 - ps2
Archaea2 - ps3
Archaea3 - ps4
Asgard/Eukaryota - ps5
Eukaryota - ps6
Amorpheae - ps7
Obazoa - ps8
Opisthokonta - ps9
Holomycota - ps10
Fungi - ps11
Fungi_1 - ps12
Fungi_2 - ps13
Fungi_3 - ps14
Dikarya - ps15
Ascomycota - ps16
Saccharo/Pezizomycotina - ps17
Saccharomycotina - ps18
Saccharomycotina_1 - ps19
Saccharomycotina_2 - ps20
Saccharomycotina_3 - ps21
Phaff./Saccharomycetaceae - ps22
Saccharomyco./mycetaceae - ps23
Saccharomycetaceae - ps24
Saccharomycetaceae_2 - ps25
Saccharomycetaceae_3 - ps26
Kaz./Nau./Saccharomyces - ps27
Saccharomyces - ps28
Arboricola_clade - ps29
Cerevisiae_clade - ps30
Saccharomyces_cerevisiae - ps31
p-value
4e-05
2e-04
7e-04
3e-03
2e-02
Log-odds
0.5
1.0
1.5
2.0
2.5
3.0
3.5
4.0

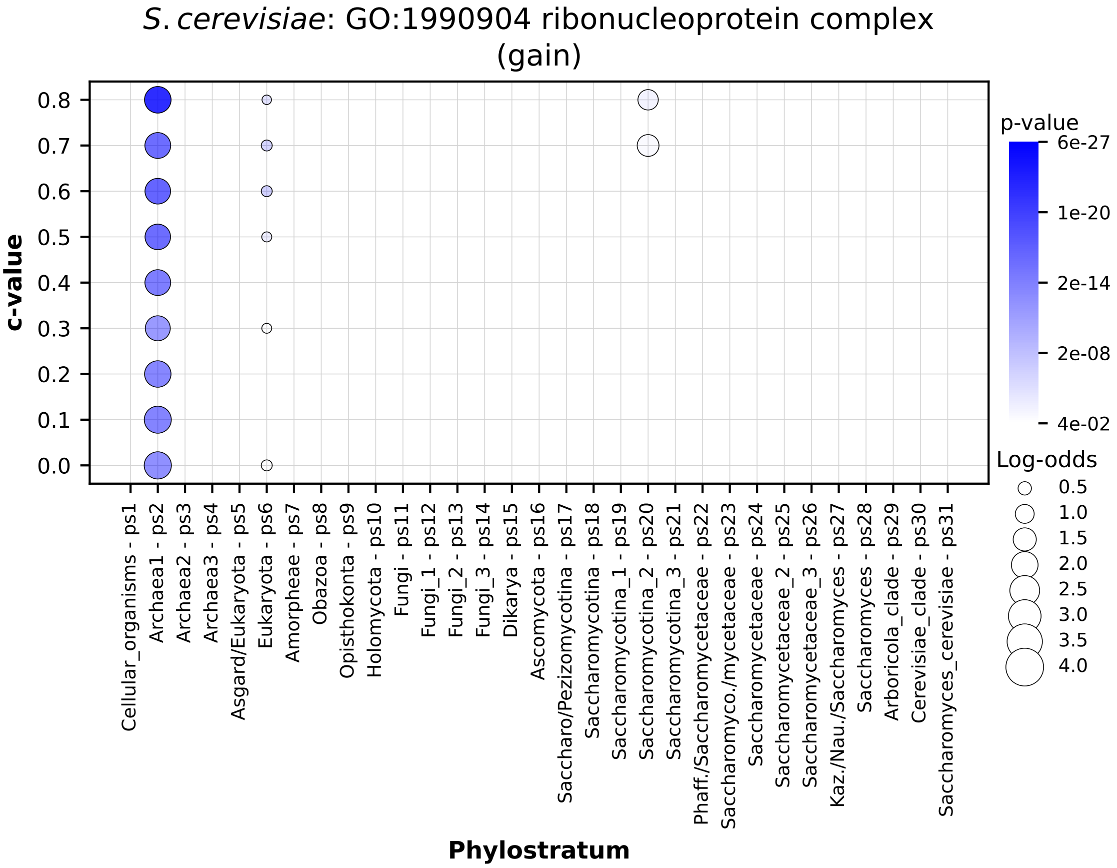

S. cerevisiae: GO:1990904 ribonucleoprotein complex (gain)
c-value
0.8
0.7
0.6
0.5
0.4
0.3
0.2
0.1
0.0
p-value
6e-27
1e-20
2e-14
2e-08
4e-02
Log-odds
0.5
1.0
1.5
2.0
2.5
3.0
3.5
4.0
Cellular_organisms - ps1
Archaea1 - ps2
Archaea2 - ps3
Archaea3 - ps4
Asgard/Eukaryota - ps5
Eukaryota - ps6
Amorpheae - ps7
Obazoa - ps8
Opisthokonta - ps9
Holomycota - ps10
Fungi - ps11
Fungi_1 - ps12
Fungi_2 - ps13
Fungi_3 - ps14
Dikarya - ps15
Ascomycota - ps16
Saccharo/Pezizomycotina - ps17
Saccharomycotina - ps18
Saccharomycotina_1 - ps19
Saccharomycotina_2 - ps20
Saccharomycotina_3 - ps21
Phaff./Saccharomycetaceae - ps22
Saccharomyco./mycetaceae - ps23
Saccharomycetaceae - ps24
Saccharomycetaceae_2 - ps25
Saccharomycetaceae_3 - ps26
Kaz./Nau./Saccharomyces - ps27
Saccharomyces - ps28
Arboricola_clade - ps29
Cerevisiae_clade - ps30
Saccharomyces_cerevisiae - ps31
Phylostratum

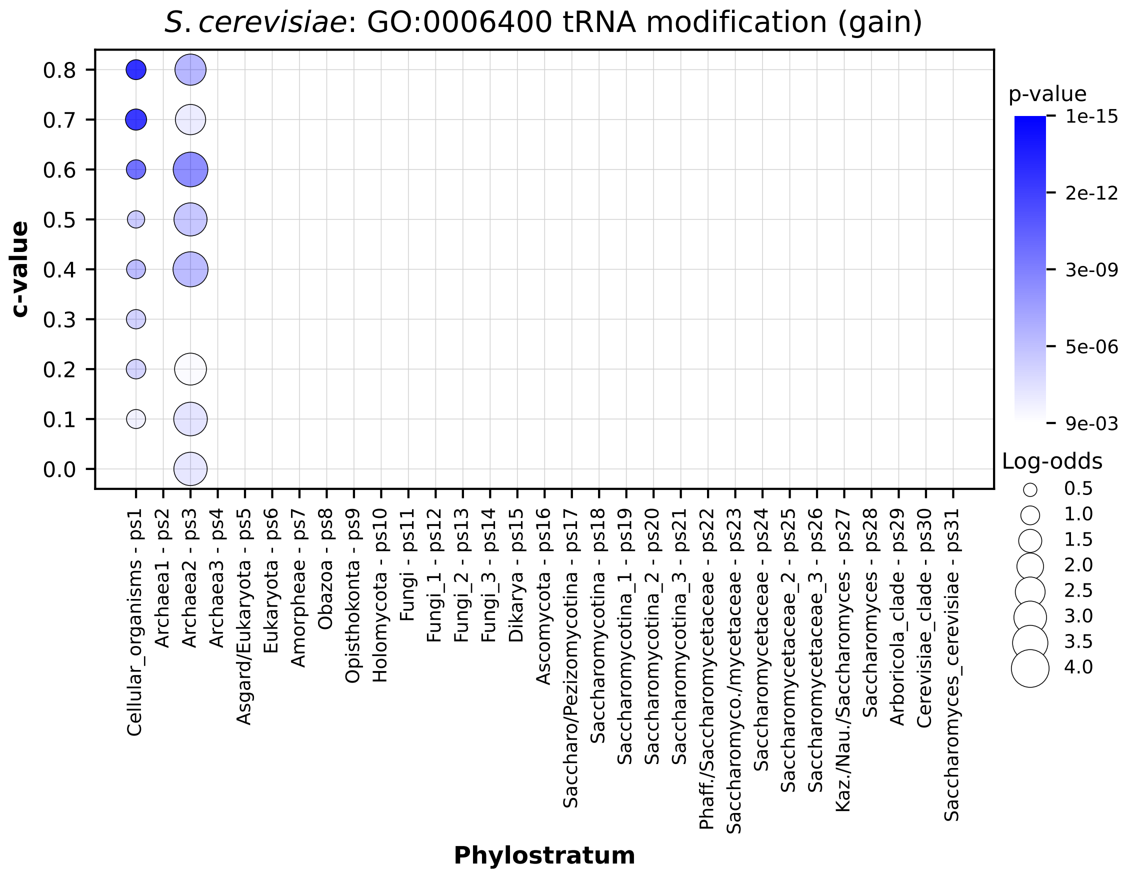
S. cerevisiae: GO:0006400 tRNA modification (gain)
c-value
0.8
0.7
0.6
0.5
0.4
0.3
0.2
0.1
0.0
Phylostratum
Cellular_organisms - ps1
Archaea1 - ps2
Archaea2 - ps3
Archaea3 - ps4
Asgard/Eukaryota - ps5
Eukaryota - ps6
Amorpheae - ps7
Obazoa - ps8
Opisthokonta - ps9
Holomycota - ps10
Fungi - ps11
Fungi_1 - ps12
Fungi_2 - ps13
Fungi_3 - ps14
Dikarya - ps15
Ascomycota - ps16
Saccharo/Pezizomycotina - ps17
Saccharomycotina - ps18
Saccharomycotina_1 - ps19
Saccharomycotina_2 - ps20
Saccharomycotina_3 - ps21
Phaff./Saccharomycetaceae - ps22
Saccharomyco./mycetaceae - ps23
Saccharomycetaceae - ps24
Saccharomycetaceae_2 - ps25
Saccharomycetaceae_3 - ps26
Kaz./Nau./Saccharomyces - ps27
Saccharomyces - ps28
Arboricola_clade - ps29
Cerevisiae_clade - ps30
Saccharomyces_cerevisiae - ps31
p-value
1e-15
2e-12
3e-09
5e-06
9e-03
Log-odds
0.5
1.0
1.5
2.0
2.5
3.0
3.5
4.0

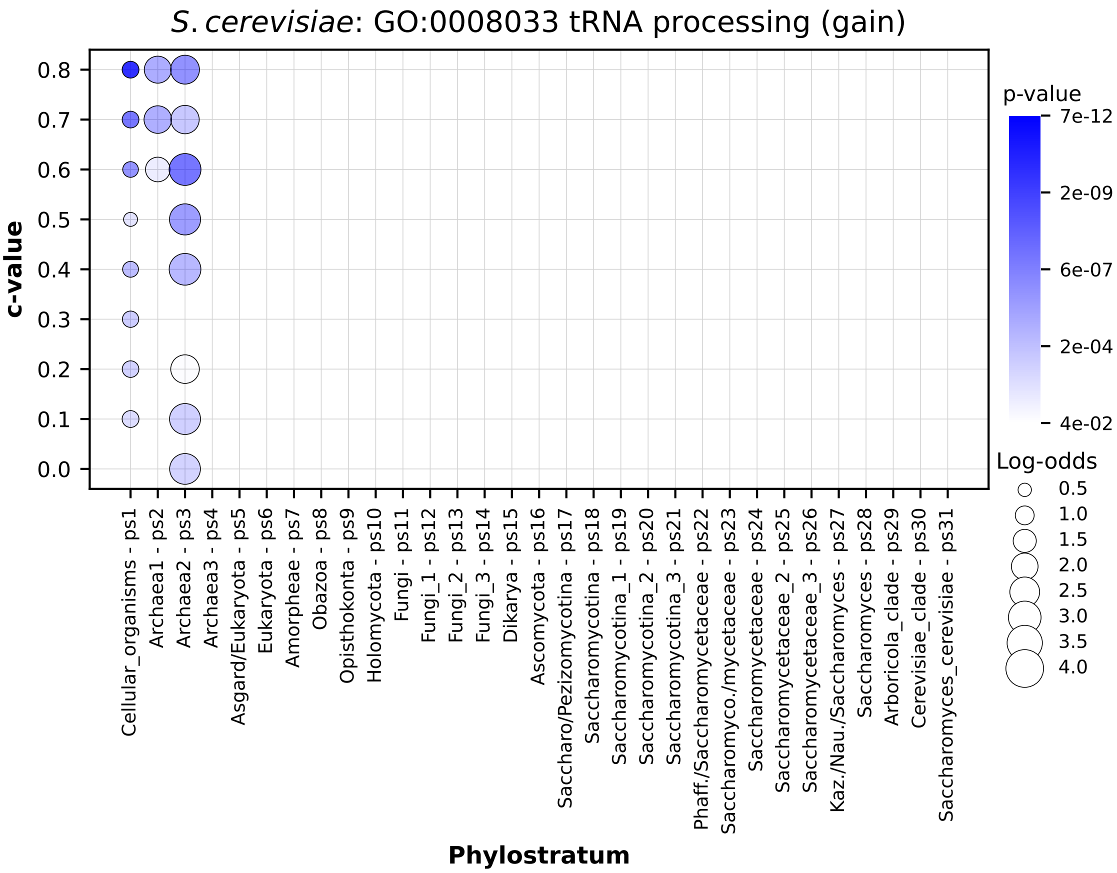

S. cerevisiae: GO:0008033 tRNA processing (gain)
c-value
0.8
0.7
0.6
0.5
0.4
0.3
0.2
0.1
0.0
p-value
7e-12
2e-09
6e-07
2e-04
4e-02
Log-odds
0.5
1.0
1.5
2.0
2.5
3.0
3.5
4.0
Cellular_organisms - ps1
Archaea1 - ps2
Archaea2 - ps3
Archaea3 - ps4
Asgard/Eukaryota - ps5
Eukaryota - ps6
Amorpheae - ps7
Obazoa - ps8
Opisthokonta - ps9
Holomycota - ps10
Fungi - ps11
Fungi_1 - ps12
Fungi_2 - ps13
Fungi_3 - ps14
Dikarya - ps15
Ascomycota - ps16
Saccharo/Pezizomycotina - ps17
Saccharomycotina - ps18
Saccharomycotina_1 - ps19
Saccharomycotina_2 - ps20
Saccharomycotina_3 - ps21
Phaff./Saccharomycetaceae - ps22
Saccharomyco./mycetaceae - ps23
Saccharomycetaceae - ps24
Saccharomycetaceae_2 - ps25
Saccharomycetaceae_3 - ps26
Kaz./Nau./Saccharomyces - ps27
Saccharomyces - ps28
Arboricola_clade - ps29
Cerevisiae_clade - ps30
Saccharomyces_cerevisiae - ps31
Phylostratum

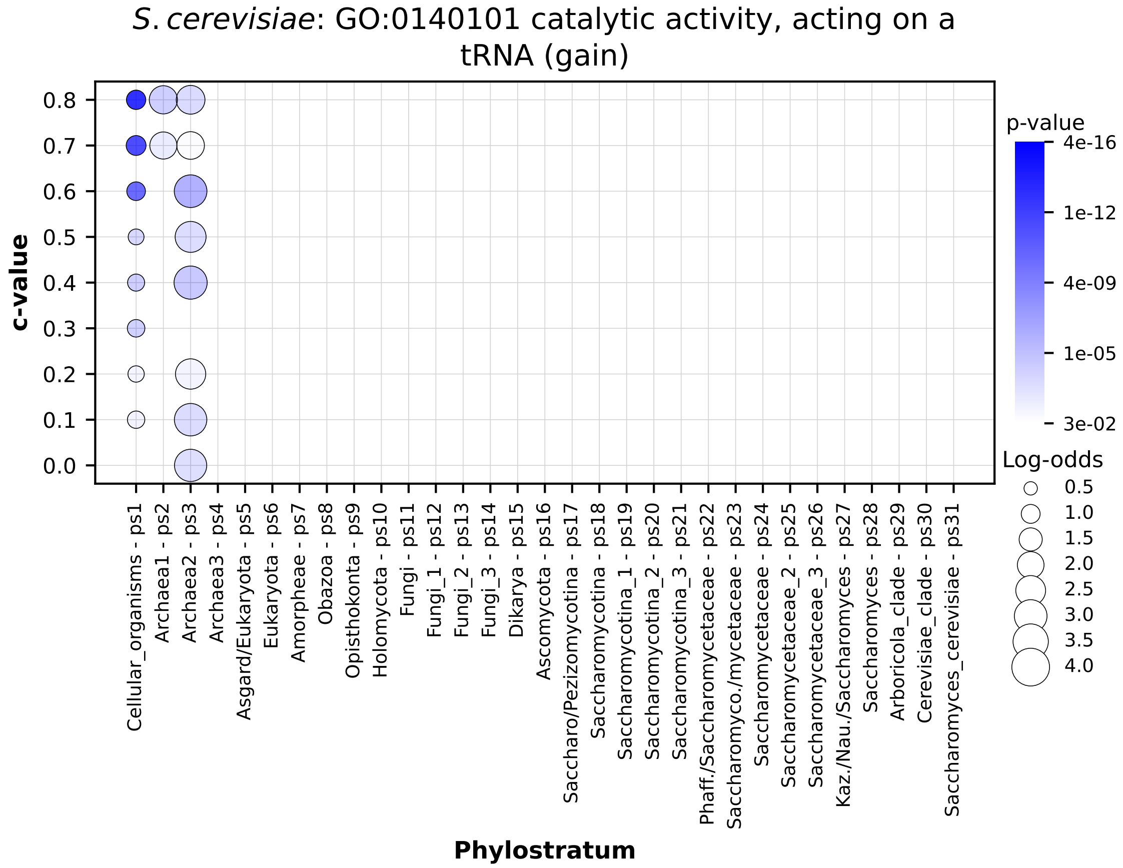

S. cerevisiae: GO:0140101 catalytic activity, acting on a tRNA (gain)
c-value
0.8
0.7
0.6
0.5
0.4
0.3
0.2
0.1
0.0
p-value
4e-16
1e-12
4e-09
1e-05
3e-02
Log-odds
0.5
1.0
1.5
2.0
2.5
3.0
3.5
4.0
Cellular_organisms - ps1
Archaea1 - ps2
Archaea2 - ps3
Archaea3 - ps4
Asgard/Eukaryota - ps5
Eukaryota - ps6
Amorpheae - ps7
Obazoa - ps8
Opisthokonta - ps9
Holomycota - ps10
Fungi - ps11
Fungi_1 - ps12
Fungi_2 - ps13
Fungi_3 - ps14
Dikarya - ps15
Ascomycota - ps16
Saccharo/Pezizomycotina - ps17
Saccharomycotina - ps18
Saccharomycotina_1 - ps19
Saccharomycotina_2 - ps20
Saccharomycotina_3 - ps21
Phaff./Saccharomycetaceae - ps22
Saccharomyco./mycetaceae - ps23
Saccharomycetaceae - ps24
Saccharomycetaceae_2 - ps25
Saccharomycetaceae_3 - ps26
Kaz./Nau./Saccharomyces - ps27
Saccharomyces - ps28
Arboricola_clade - ps29
Cerevisiae_clade - ps30
Saccharomyces_cerevisiae - ps31
Phylostratum

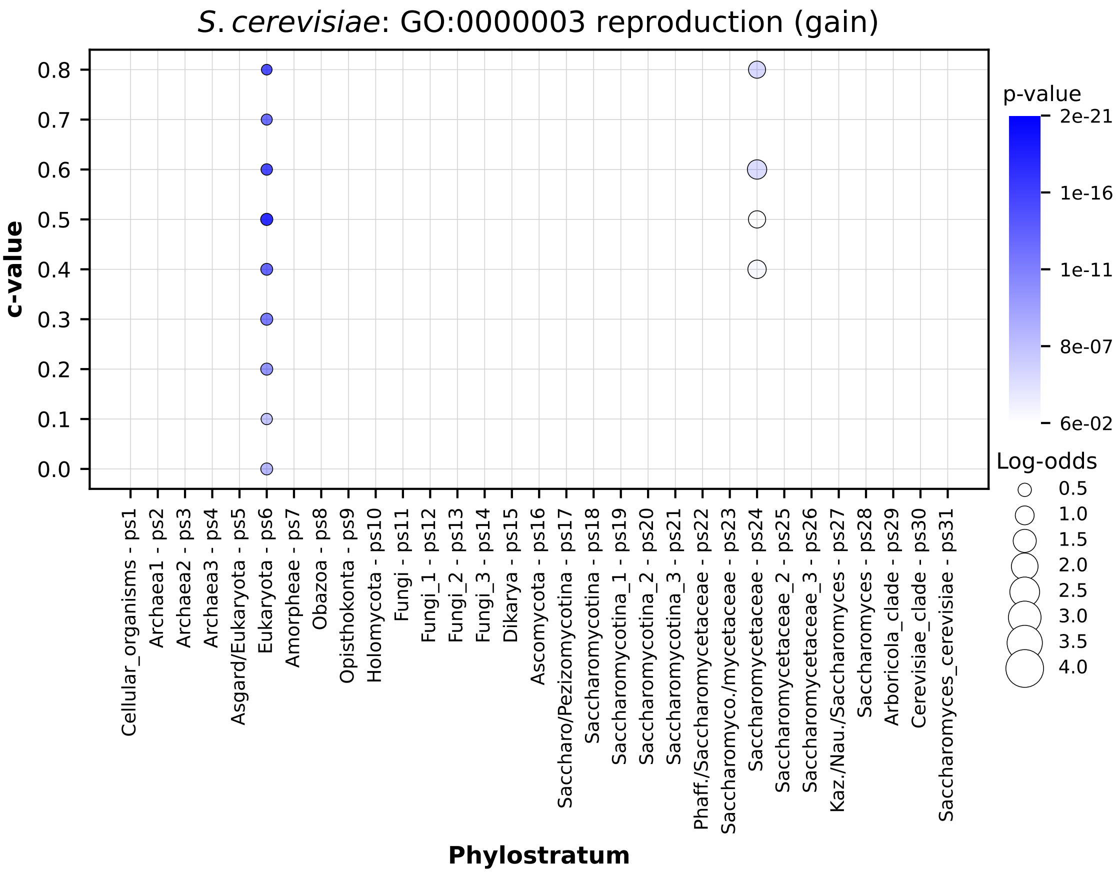
S. cerevisiae: GO:0000003 reproduction (gain)
c-value
0.8
0.7
0.6
0.5
0.4
0.3
0.2
0.1
0.0
Phylostratum
Cellular_organisms - ps1
Archaea1 - ps2
Archaea2 - ps3
Archaea3 - ps4
Asgard/Eukaryota - ps5
Eukaryota - ps6
Amorpheae - ps7
Obazoa - ps8
Opisthokonta - ps9
Holomycota - ps10
Fungi - ps11
Fungi_1 - ps12
Fungi_2 - ps13
Fungi_3 - ps14
Dikarya - ps15
Ascomycota - ps16
Saccharo/Pezizomycotina - ps17
Saccharomycotina - ps18
Saccharomycotina_1 - ps19
Saccharomycotina_2 - ps20
Saccharomycotina_3 - ps21
Phaff./Saccharomycetaceae - ps22
Saccharomyco./mycetaceae - ps23
Saccharomycetaceae - ps24
Saccharomycetaceae_2 - ps25
Saccharomycetaceae_3 - ps26
Kaz./Nau./Saccharomyces - ps27
Saccharomyces - ps28
Arboricola_clade - ps29
Cerevisiae_clade - ps30
Saccharomyces_cerevisiae - ps31
p-value
2e-21
1e-16
1e-11
8e-07
6e-02
Log-odds
0.5
1.0
1.5
2.0
2.5
3.0
3.5
4.0

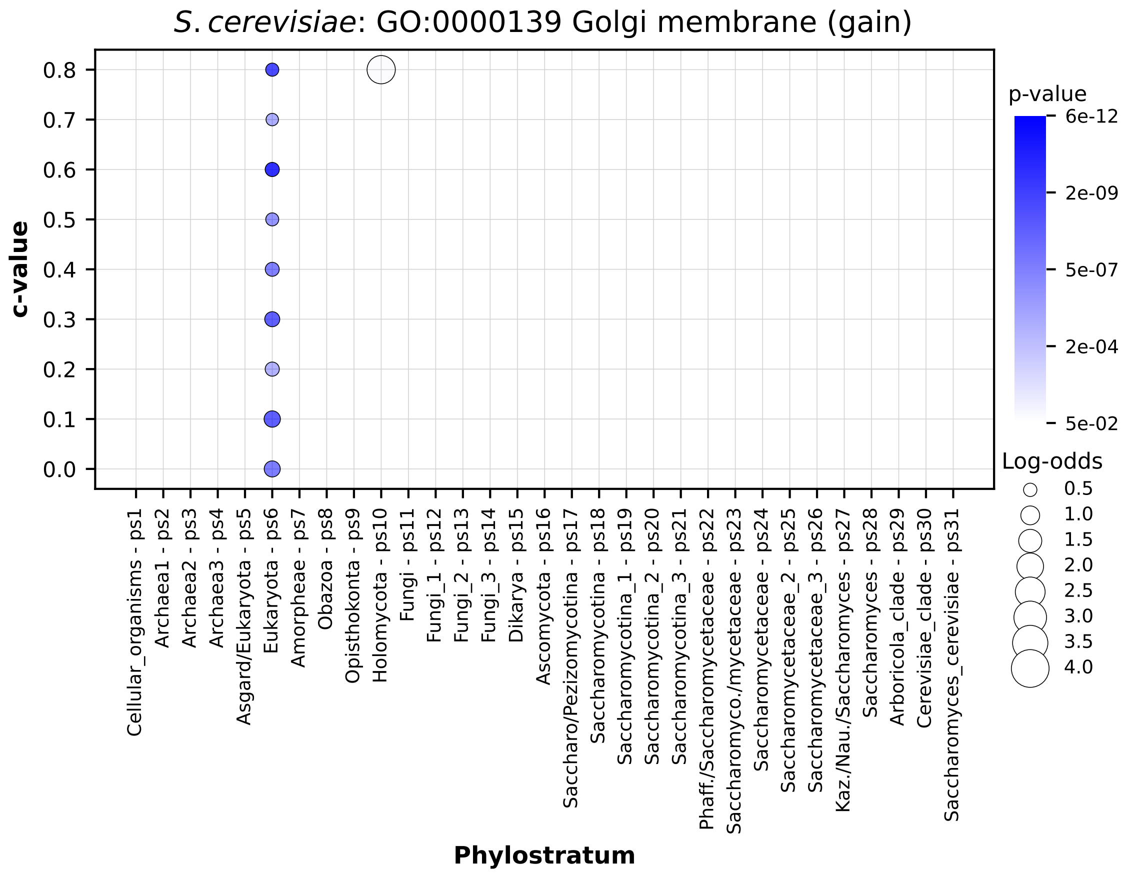

S. cerevisiae: GO:0000139 Golgi membrane (gain)
c-value
0.8
0.7
0.6
0.5
0.4
0.3
0.2
0.1
0.0
p-value
6e-12
2e-09
5e-07
2e-04
5e-02
Log-odds
0.5
1.0
1.5
2.0
2.5
3.0
3.5
4.0
Cellular_organisms - ps1
Archaea1 - ps2
Archaea2 - ps3
Archaea3 - ps4
Asgard/Eukaryota - ps5
Eukaryota - ps6
Amorpheae - ps7
Obazoa - ps8
Opisthokonta - ps9
Holomycota - ps10
Fungi - ps11
Fungi_1 - ps12
Fungi_2 - ps13
Fungi_3 - ps14
Dikarya - ps15
Ascomycota - ps16
Saccharo/Pezizomycotina - ps17
Saccharomycotina - ps18
Saccharomycotina_1 - ps19
Saccharomycotina_2 - ps20
Saccharomycotina_3 - ps21
Phaff./Saccharomycetaceae - ps22
Saccharomyco./mycetaceae - ps23
Saccharomycetaceae - ps24
Saccharomycetaceae_2 - ps25
Saccharomycetaceae_3 - ps26
Kaz./Nau./Saccharomyces - ps27
Saccharomyces - ps28
Arboricola_clade - ps29
Cerevisiae_clade - ps30
Saccharomyces_cerevisiae - ps31
Phylostratum

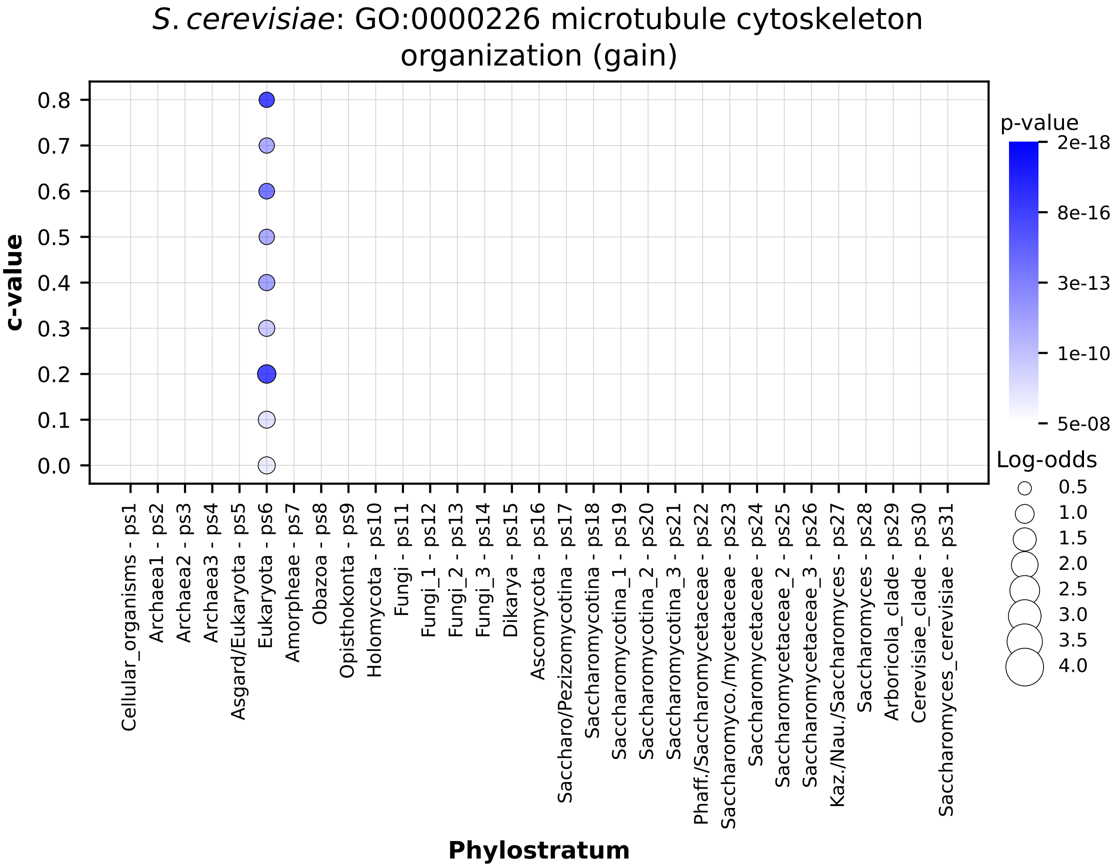

S. cerevisiae: GO:0000226 microtubule cytoskeleton organization (gain)
c-value
0.8
0.7
0.6
0.5
0.4
0.3
0.2
0.1
0.0
p-value
2e-18
8e-16
3e-13
1e-10
5e-08
Log-odds
0.5
1.0
1.5
2.0
2.5
3.0
3.5
4.0
Cellular_organisms - ps1
Archaea1 - ps2
Archaea2 - ps3
Archaea3 - ps4
Asgard/Eukaryota - ps5
Eukaryota - ps6
Amorpheae - ps7
Obazoa - ps8
Opisthokonta - ps9
Holomycota - ps10
Fungi - ps11
Fungi_1 - ps12
Fungi_2 - ps13
Fungi_3 - ps14
Dikarya - ps15
Ascomycota - ps16
Saccharo/Pezizomycotina - ps17
Saccharomycotina - ps18
Saccharomycotina_1 - ps19
Saccharomycotina_2 - ps20
Saccharomycotina_3 - ps21
Phaff./Saccharomycetaceae - ps22
Saccharomyco./mycetaceae - ps23
Saccharomycetaceae - ps24
Saccharomycetaceae_2 - ps25
Saccharomycetaceae_3 - ps26
Kaz./Nau./Saccharomyces - ps27
Saccharomyces - ps28
Arboricola_clade - ps29
Cerevisiae_clade - ps30
Saccharomyces_cerevisiae - ps31
Phylostratum

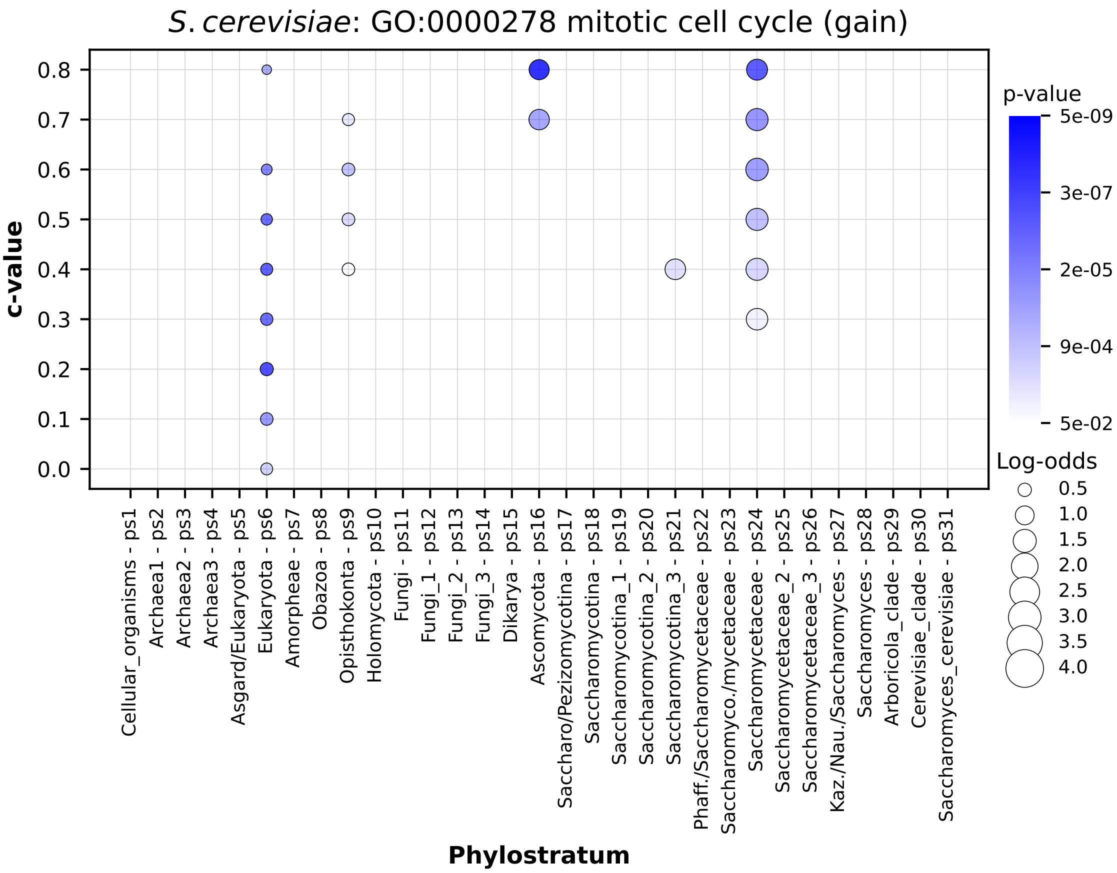

S. cerevisiae: GO:0000278 mitotic cell cycle (gain)
c-value
0.8
0.7
0.6
0.5
0.4
0.3
0.2
0.1
0.0
p-value
5e-09
3e-07
2e-05
9e-04
5e-02
Log-odds
0.5
1.0
1.5
2.0
2.5
3.0
3.5
4.0
Cellular_organisms - ps1
Archaea1 - ps2
Archaea2 - ps3
Archaea3 - ps4
Asgard/Eukaryota - ps5
Eukaryota - ps6
Amorpheae - ps7
Obazoa - ps8
Opisthokonta - ps9
Holomycota - ps10
Fungi - ps11
Fungi_1 - ps12
Fungi_2 - ps13
Fungi_3 - ps14
Dikarya - ps15
Ascomycota - ps16
Saccharo/Pezizomycotina - ps17
Saccharomycotina - ps18
Saccharomycotina_1 - ps19
Saccharomycotina_2 - ps20
Saccharomycotina_3 - ps21
Phaff./Saccharomycetaceae - ps22
Saccharomyco./mycetaceae - ps23
Saccharomycetaceae - ps24
Saccharomycetaceae_2 - ps25
Saccharomycetaceae_3 - ps26
Kaz./Nau./Saccharomyces - ps27
Saccharomyces - ps28
Arboricola_clade - ps29
Cerevisiae_clade - ps30
Saccharomyces_cerevisiae - ps31
Phylostratum

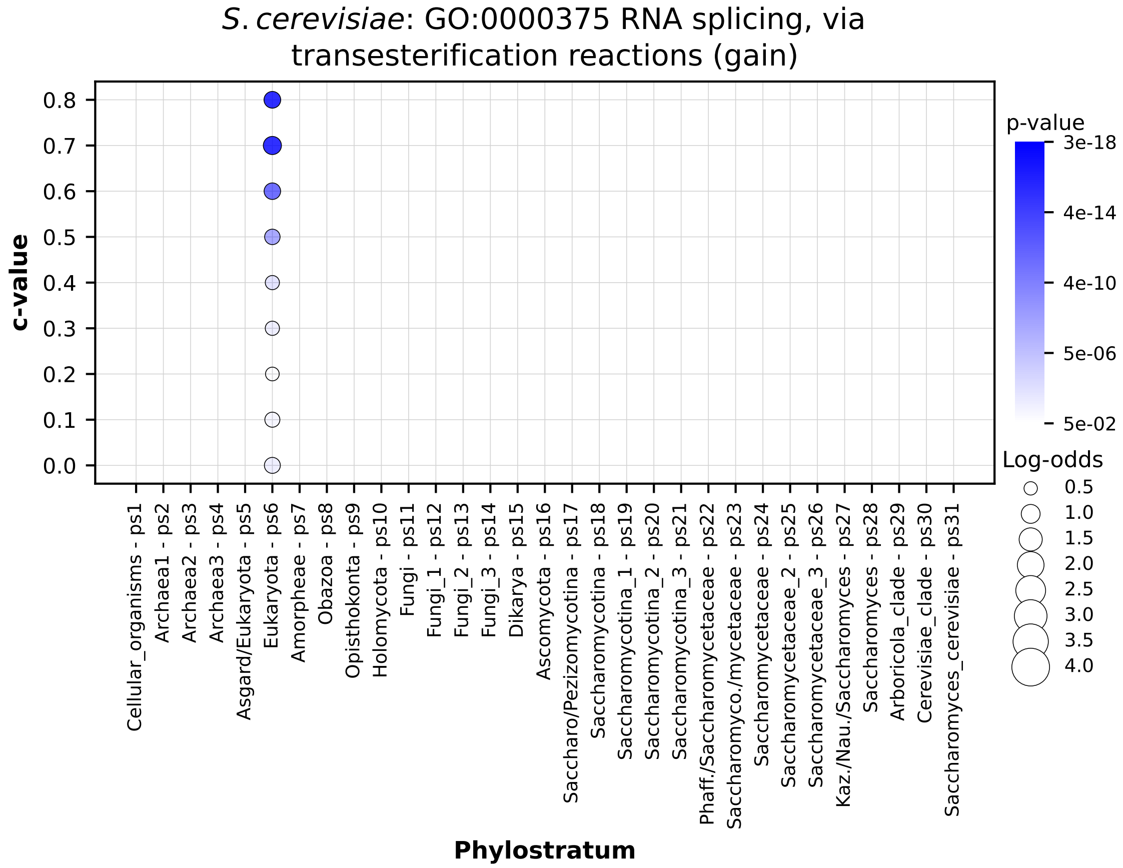

S. cerevisiae: GO:0000375 RNA splicing, via transesterification reactions (gain)
c-value
0.8
0.7
0.6
0.5
0.4
0.3
0.2
0.1
0.0
p-value
3e-18
4e-14
4e-10
5e-06
5e-02
Log-odds
0.5
1.0
1.5
2.0
2.5
3.0
3.5
4.0
Cellular_organisms - ps1
Archaea1 - ps2
Archaea2 - ps3
Archaea3 - ps4
Asgard/Eukaryota - ps5
Eukaryota - ps6
Amorpheae - ps7
Obazoa - ps8
Opisthokonta - ps9
Holomycota - ps10
Fungi - ps11
Fungi_1 - ps12
Fungi_2 - ps13
Fungi_3 - ps14
Dikarya - ps15
Ascomycota - ps16
Saccharo/Pezizomycotina - ps17
Saccharomycotina - ps18
Saccharomycotina_1 - ps19
Saccharomycotina_2 - ps20
Saccharomycotina_3 - ps21
Phaff./Saccharomycetaceae - ps22
Saccharomyco./mycetaceae - ps23
Saccharomycetaceae - ps24
Saccharomycetaceae_2 - ps25
Saccharomycetaceae_3 - ps26
Kaz./Nau./Saccharomyces - ps27
Saccharomyces - ps28
Arboricola_clade - ps29
Cerevisiae_clade - ps30
Saccharomyces_cerevisiae - ps31
Phylostratum

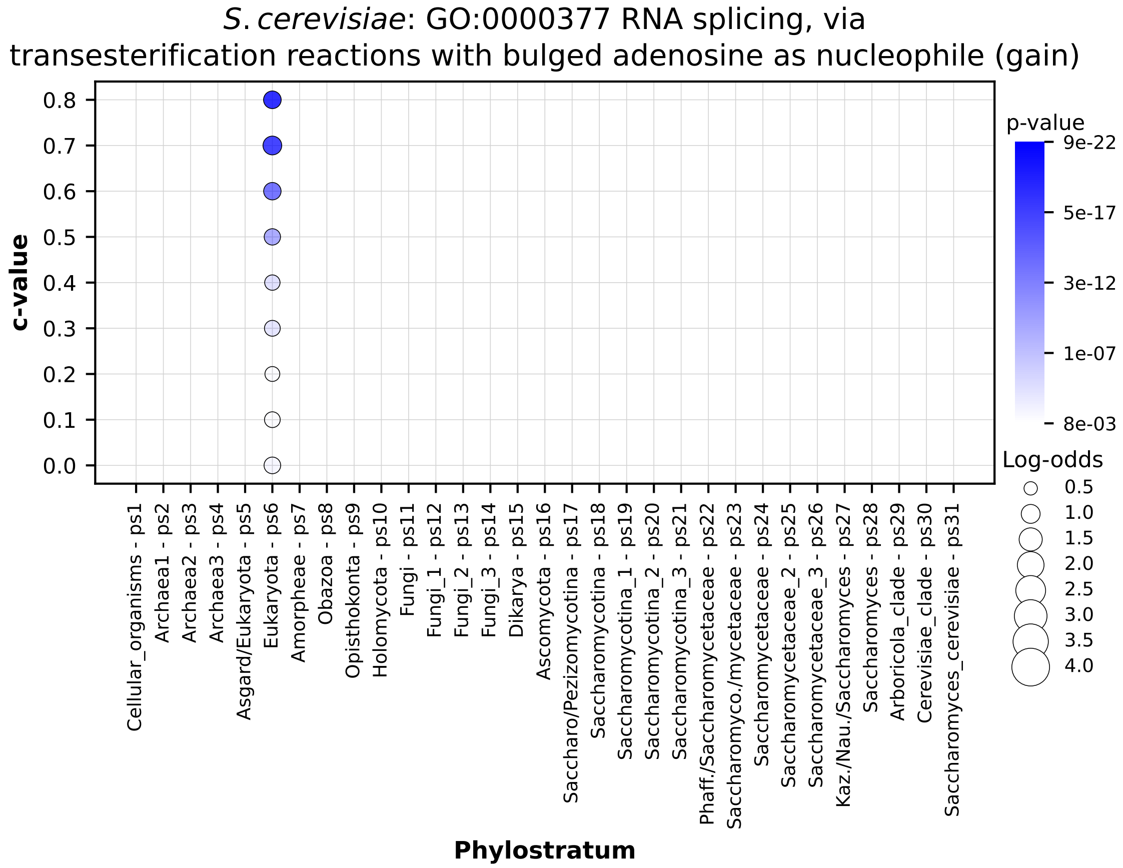
S. cerevisiae: GO:0000377 RNA splicing, via transesterification reactions with bulged adenosine as nucleophile (gain)
c-value
0.0
0.1
0.2
0.3
0.4
0.5
0.6
0.7
0.8
p-value
9e-22
5e-17
3e-12
1e-07
8e-03
Log-odds
0.5
1.0
1.5
2.0
2.5
3.0
3.5
4.0
Cellular_organisms - ps1
Archaea1 - ps2
Archaea2 - ps3
Archaea3 - ps4
Asgard/Eukaryota - ps5
Eukaryota - ps6
Amorpheae - ps7
Obazoa - ps8
Opisthokonta - ps9
Holomycota - ps10
Fungi - ps11
Fungi_1 - ps12
Fungi_2 - ps13
Fungi_3 - ps14
Dikarya - ps15
Ascomycota - ps16
Saccharo/Pezizomycotina - ps17
Saccharomycotina - ps18
Saccharomycotina_1 - ps19
Saccharomycotina_2 - ps20
Saccharomycotina_3 - ps21
Phaff./Saccharomycetaceae - ps22
Saccharomyco./mycetaceae - ps23
Saccharomycetaceae - ps24
Saccharomycetaceae_2 - ps25
Saccharomycetaceae_3 - ps26
Kaz./Nau./Saccharomyces - ps27
Saccharomyces - ps28
Arboricola_clade - ps29
Cerevisiae_clade - ps30
Saccharomyces_cerevisiae - ps31
Phylostratum

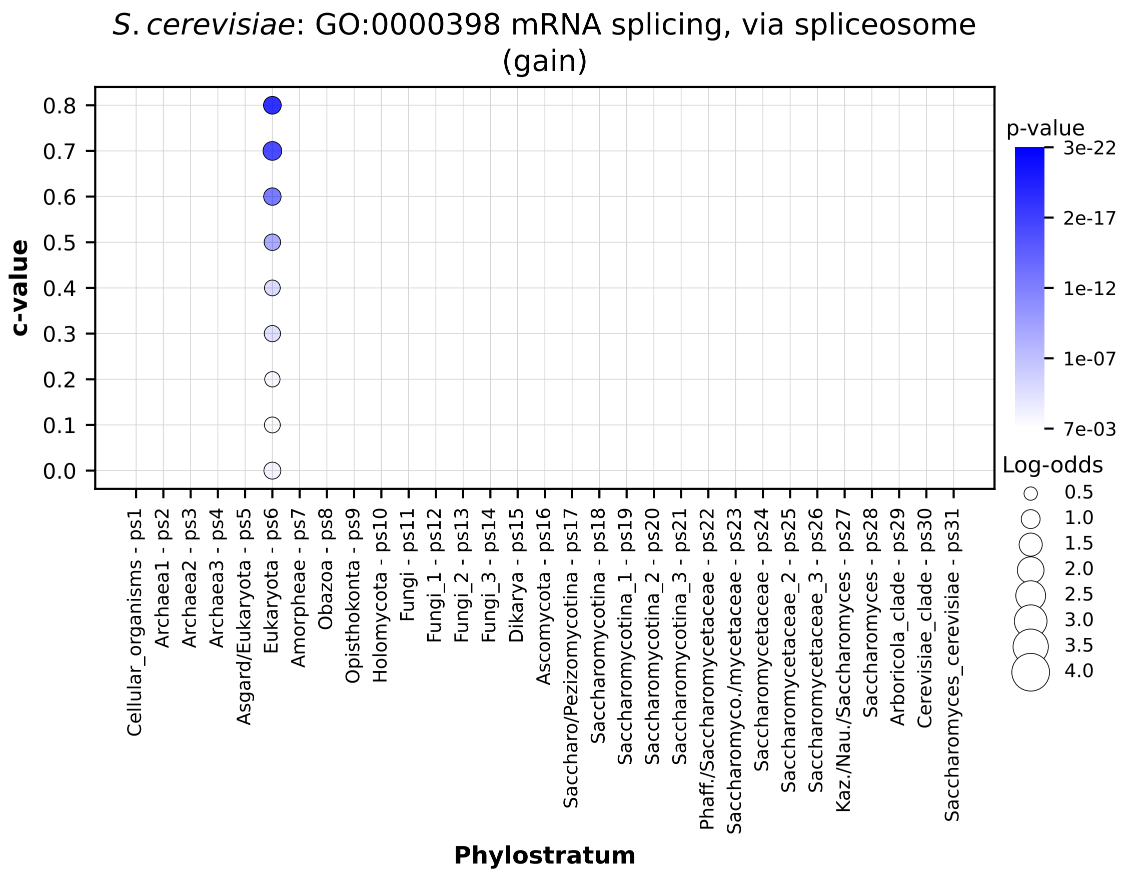

S. cerevisiae: GO:0000398 mRNA splicing, via spliceosome (gain)
c-value
0.8
0.7
0.6
0.5
0.4
0.3
0.2
0.1
0.0
Phylostratum
Cellular_organisms - ps1
Archaea1 - ps2
Archaea2 - ps3
Archaea3 - ps4
Asgard/Eukaryota - ps5
Eukaryota - ps6
Amorpheae - ps7
Obazoa - ps8
Opisthokonta - ps9
Holomycota - ps10
Fungi - ps11
Fungi_1 - ps12
Fungi_2 - ps13
Fungi_3 - ps14
Dikarya - ps15
Ascomycota - ps16
Saccharo/Pezizomycotina - ps17
Saccharomycotina - ps18
Saccharomycotina_1 - ps19
Saccharomycotina_2 - ps20
Saccharomycotina_3 - ps21
Phaff./Saccharomycetaceae - ps22
Saccharomyco./mycetaceae - ps23
Saccharomycetaceae - ps24
Saccharomycetaceae_2 - ps25
Saccharomycetaceae_3 - ps26
Kaz./Nau./Saccharomyces - ps27
Saccharomyces - ps28
Arboricola_clade - ps29
Cerevisiae_clade - ps30
Saccharomyces_cerevisiae - ps31
p-value
3e-22
2e-17
1e-12
1e-07
7e-03
Log-odds
0.5
1.0
1.5
2.0
2.5
3.0
3.5
4.0

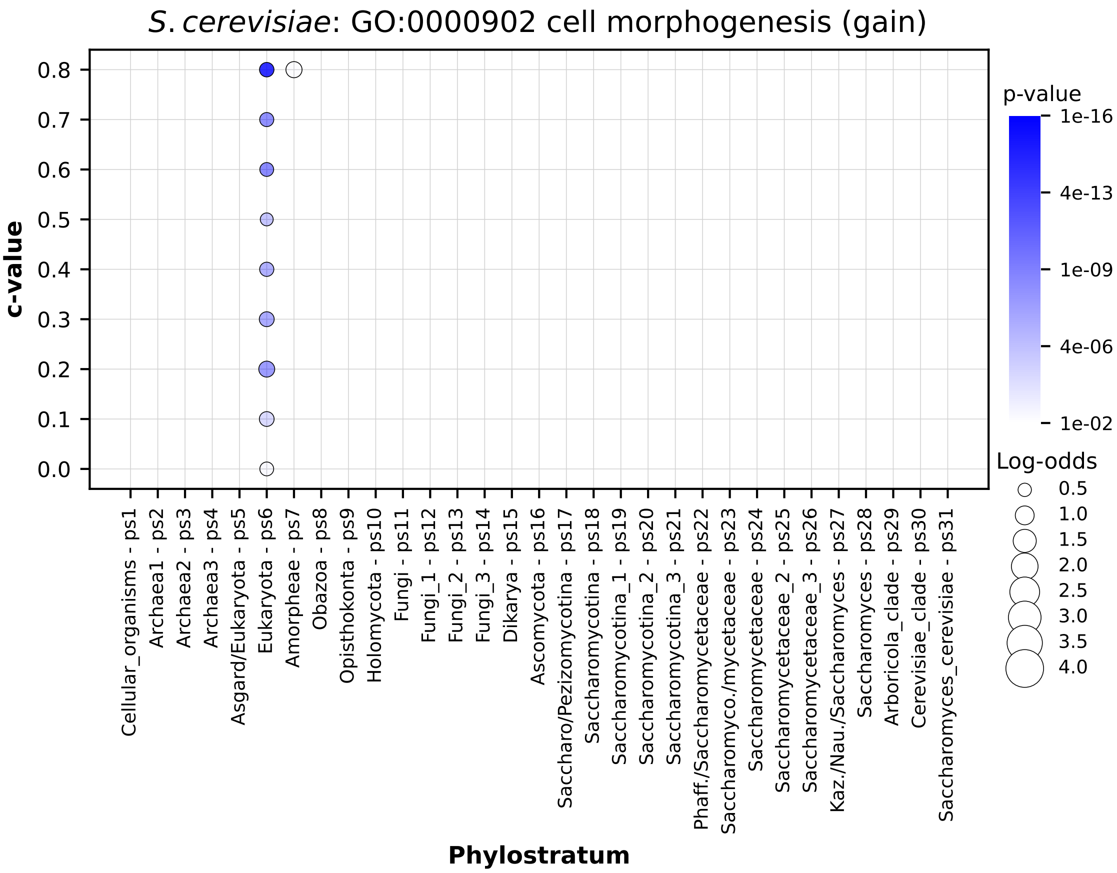

S. cerevisiae: GO:0000902 cell morphogenesis (gain)
c-value
0.8
0.7
0.6
0.5
0.4
0.3
0.2
0.1
0.0
Phylostratum
Cellular_organisms - ps1
Archaea1 - ps2
Archaea2 - ps3
Archaea3 - ps4
Asgard/Eukaryota - ps5
Eukaryota - ps6
Amorpheae - ps7
Obazoa - ps8
Opisthokonta - ps9
Holomycota - ps10
Fungi - ps11
Fungi_1 - ps12
Fungi_2 - ps13
Fungi_3 - ps14
Dikarya - ps15
Ascomycota - ps16
Saccharo/Pezizomycotina - ps17
Saccharomycotina - ps18
Saccharomycotina_1 - ps19
Saccharomycotina_2 - ps20
Saccharomycotina_3 - ps21
Phaff./Saccharomycetaceae - ps22
Saccharomyco./mycetaceae - ps23
Saccharomycetaceae - ps24
Saccharomycetaceae_2 - ps25
Saccharomycetaceae_3 - ps26
Kaz./Nau./Saccharomyces - ps27
Saccharomyces - ps28
Arboricola_clade - ps29
Cerevisiae_clade - ps30
Saccharomyces_cerevisiae - ps31
p-value
1e-16
4e-13
1e-09
4e-06
1e-02
Log-odds
0.5
1.0
1.5
2.0
2.5
3.0
3.5
4.0

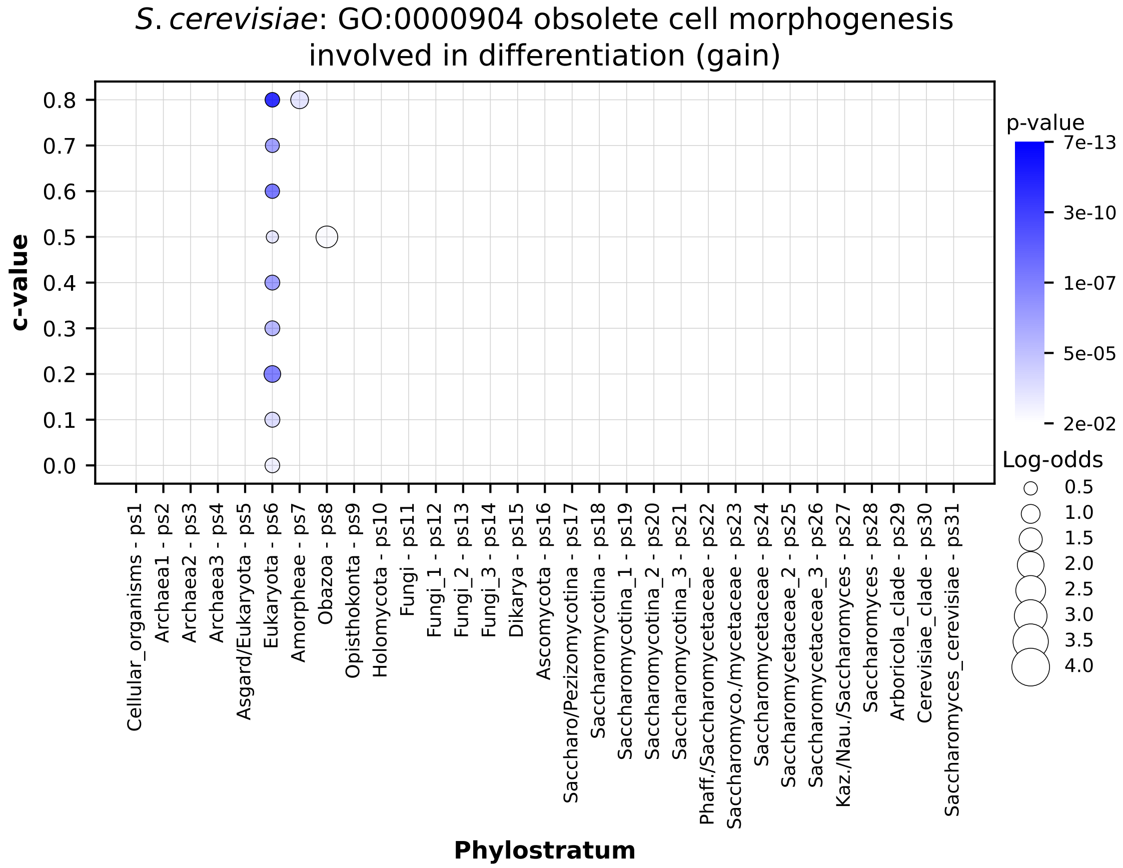

S. cerevisiae: GO:0000904 obsolete cell morphogenesis involved in differentiation (gain)
c-value
0.8
0.7
0.6
0.5
0.4
0.3
0.2
0.1
0.0
p-value
7e-13
3e-10
1e-07
5e-05
2e-02
Log-odds
0.5
1.0
1.5
2.0
2.5
3.0
3.5
4.0
Cellular_organisms - ps1
Archaea1 - ps2
Archaea2 - ps3
Archaea3 - ps4
Asgard/Eukaryota - ps5
Eukaryota - ps6
Amorpheae - ps7
Obazoa - ps8
Opisthokonta - ps9
Holomycota - ps10
Fungi - ps11
Fungi_1 - ps12
Fungi_2 - ps13
Fungi_3 - ps14
Dikarya - ps15
Ascomycota - ps16
Saccharo/Pezizomycotina - ps17
Saccharomycotina - ps18
Saccharomycotina_1 - ps19
Saccharomycotina_2 - ps20
Saccharomycotina_3 - ps21
Phaff./Saccharomycetaceae - ps22
Saccharomyco./mycetaceae - ps23
Saccharomycetaceae - ps24
Saccharomycetaceae_2 - ps25
Saccharomycetaceae_3 - ps26
Kaz./Nau./Saccharomyces - ps27
Saccharomyces - ps28
Arboricola_clade - ps29
Cerevisiae_clade - ps30
Saccharomyces_cerevisiae - ps31
Phylostratum

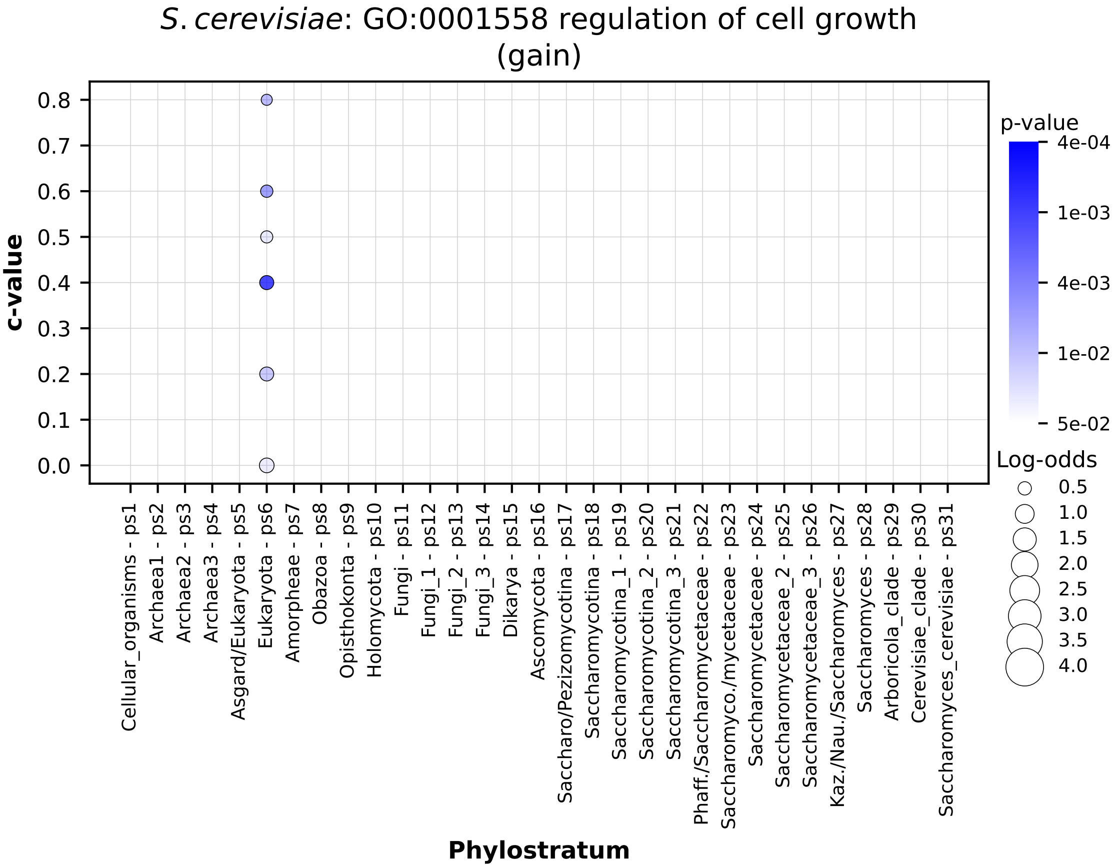

S. cerevisiae: GO:0001558 regulation of cell growth
(gain)
c-value
0.8
0.7
0.6
0.5
0.4
0.3
0.2
0.1
0.0
p-value
4e-04
1e-03
4e-03
1e-02
5e-02
Log-odds
0.5
1.0
1.5
2.0
2.5
3.0
3.5
4.0
Cellular_organisms - ps1
Archaea1 - ps2
Archaea2 - ps3
Archaea3 - ps4
Asgard/Eukaryota - ps5
Eukaryota - ps6
Amorpheae - ps7
Obazoa - ps8
Opisthokonta - ps9
Holomycota - ps10
Fungi - ps11
Fungi_1 - ps12
Fungi_2 - ps13
Fungi_3 - ps14
Dikarya - ps15
Ascomycota - ps16
Saccharo/Pezizomycotina - ps17
Saccharomycotina - ps18
Saccharomycotina_1 - ps19
Saccharomycotina_2 - ps20
Saccharomycotina_3 - ps21
Phaff./Saccharomycetaceae - ps22
Saccharomyco./mycetaceae - ps23
Saccharomycetaceae - ps24
Saccharomycetaceae_2 - ps25
Saccharomycetaceae_3 - ps26
Kaz./Nau./Saccharomyces - ps27
Saccharomyces - ps28
Arboricola_clade - ps29
Cerevisiae_clade - ps30
Saccharomyces_cerevisiae - ps31
Phylostratum

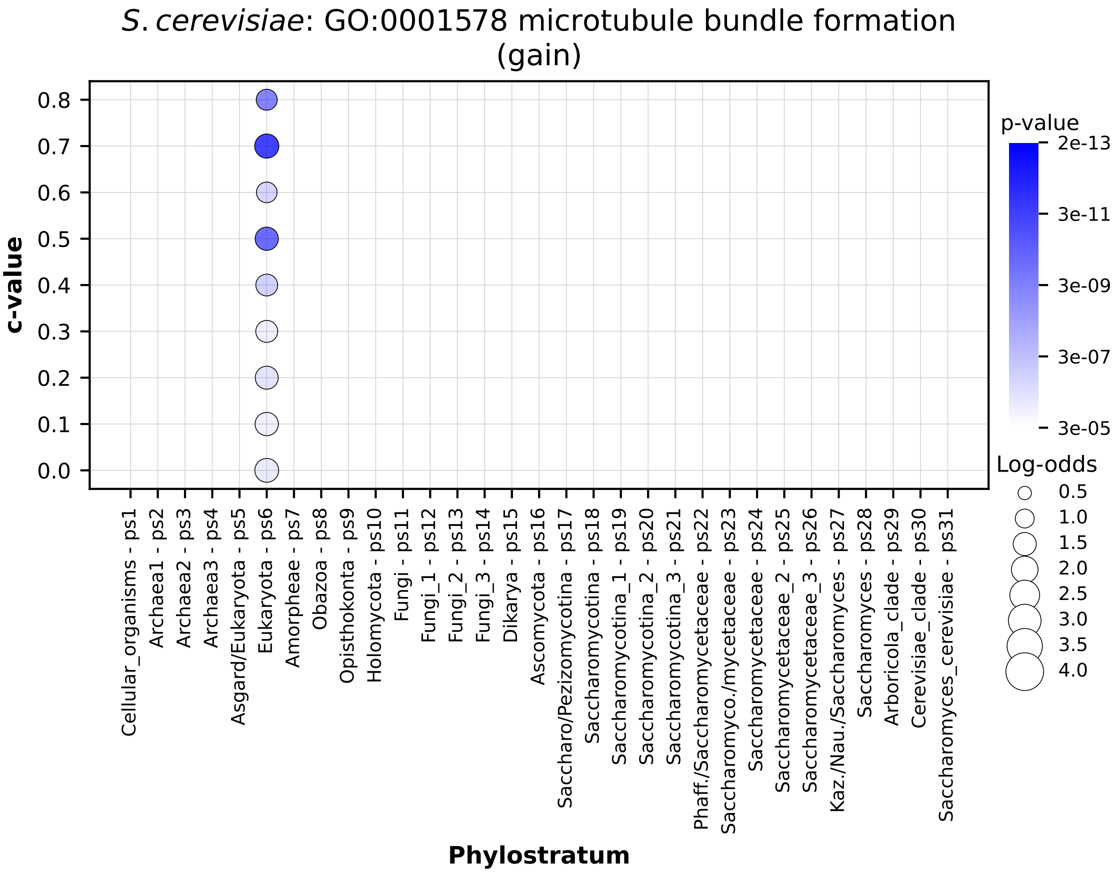
S. cerevisiae: GO:0001578 microtubule bundle formation (gain)
c-value
0.8
0.7
0.6
0.5
0.4
0.3
0.2
0.1
0.0
Phylostratum
Cellular_organisms - ps1
Archaea1 - ps2
Archaea2 - ps3
Archaea3 - ps4
Asgard/Eukaryota - ps5
Eukaryota - ps6
Amorpheae - ps7
Obazoa - ps8
Opisthokonta - ps9
Holomycota - ps10
Fungi - ps11
Fungi_1 - ps12
Fungi_2 - ps13
Fungi_3 - ps14
Dikarya - ps15
Ascomycota - ps16
Saccharo/Pezizomycotina - ps17
Saccharomycotina - ps18
Saccharomycotina_1 - ps19
Saccharomycotina_2 - ps20
Saccharomycotina_3 - ps21
Phaff./Saccharomycetaceae - ps22
Saccharomyco./mycetaceae - ps23
Saccharomycetaceae - ps24
Saccharomycetaceae_2 - ps25
Saccharomycetaceae_3 - ps26
Kaz./Nau./Saccharomyces - ps27
Saccharomyces - ps28
Arboricola_clade - ps29
Cerevisiae_clade - ps30
Saccharomyces_cerevisiae - ps31
p-value
2e-13
3e-11
3e-09
3e-07
3e-05
Log-odds
0.5
1.0
1.5
2.0
2.5
3.0
3.5
4.0

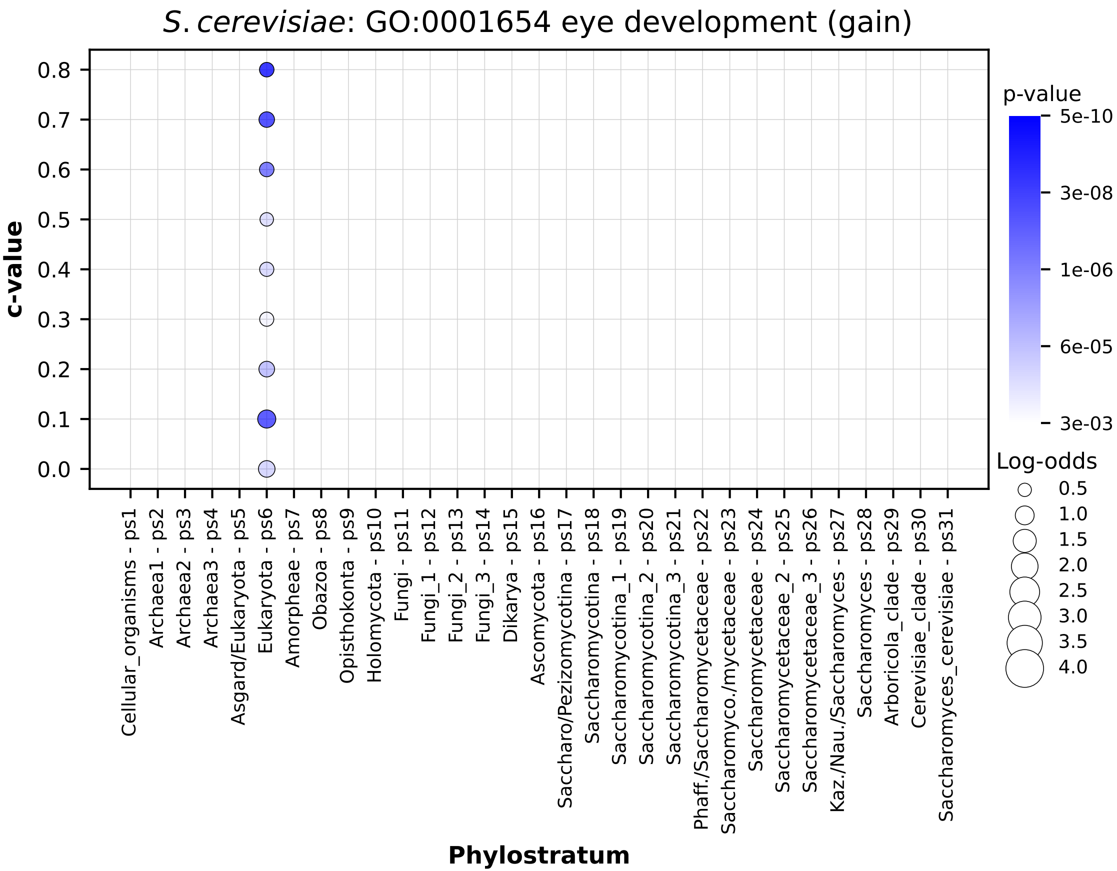

S. cerevisiae: GO:0001654 eye development (gain)
c-value
0.0
0.1
0.2
0.3
0.4
0.5
0.6
0.7
0.8
p-value
5e-10
3e-08
1e-06
6e-05
3e-03
Log-odds
0.5
1.0
1.5
2.0
2.5
3.0
3.5
4.0
Cellular_organisms - ps1
Archaea1 - ps2
Archaea2 - ps3
Archaea3 - ps4
Asgard/Eukaryota - ps5
Eukaryota - ps6
Amorpheae - ps7
Obazoa - ps8
Opisthokonta - ps9
Holomycota - ps10
Fungi - ps11
Fungi_1 - ps12
Fungi_2 - ps13
Fungi_3 - ps14
Dikarya - ps15
Ascomycota - ps16
Saccharo/Pezizomycotina - ps17
Saccharomycotina - ps18
Saccharomycotina_1 - ps19
Saccharomycotina_2 - ps20
Saccharomycotina_3 - ps21
Phaff./Saccharomycetaceae - ps22
Saccharomyco./mycetaceae - ps23
Saccharomycetaceae - ps24
Saccharomycetaceae_2 - ps25
Saccharomycetaceae_3 - ps26
Kaz./Nau./Saccharomyces - ps27
Saccharomyces - ps28
Arboricola_clade - ps29
Cerevisiae_clade - ps30
Saccharomyces_cerevisiae - ps31
Phylostratum

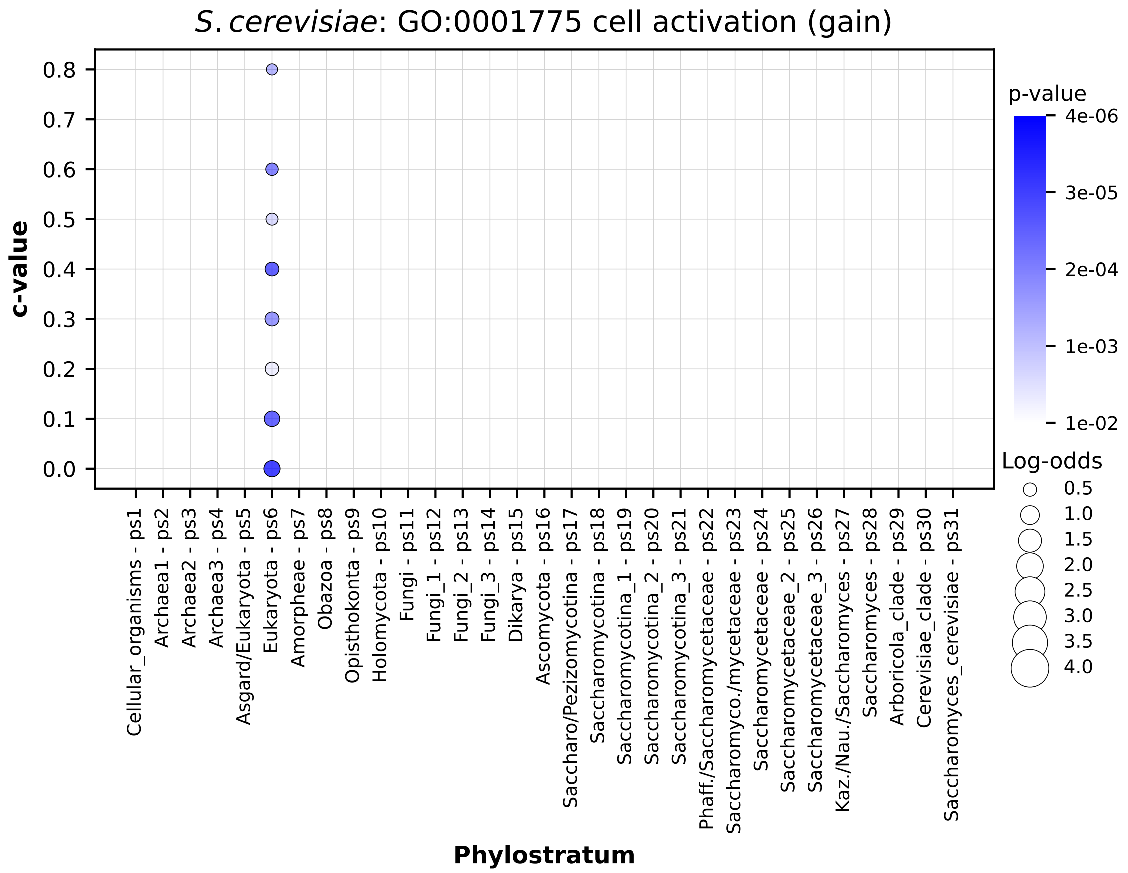

S. cerevisiae: GO:0001775 cell activation (gain)
c-value
0.8
0.7
0.6
0.5
0.4
0.3
0.2
0.1
0.0
p-value
4e-06
3e-05
2e-04
1e-03
1e-02
Log-odds
0.5
1.0
1.5
2.0
2.5
3.0
3.5
4.0
Cellular_organisms - ps1
Archaea1 - ps2
Archaea2 - ps3
Archaea3 - ps4
Asgard/Eukaryota - ps5
Eukaryota - ps6
Amorpheae - ps7
Obazoa - ps8
Opisthokonta - ps9
Holomycota - ps10
Fungi - ps11
Fungi_1 - ps12
Fungi_2 - ps13
Fungi_3 - ps14
Dikarya - ps15
Ascomycota - ps16
Saccharo/Pezizomycotina - ps17
Saccharomycotina - ps18
Saccharomycotina_1 - ps19
Saccharomycotina_2 - ps20
Saccharomycotina_3 - ps21
Phaff./Saccharomycetaceae - ps22
Saccharomyco./mycetaceae - ps23
Saccharomycetaceae - ps24
Saccharomycetaceae_2 - ps25
Saccharomycetaceae_3 - ps26
Kaz./Nau./Saccharomyces - ps27
Saccharomyces - ps28
Arboricola_clade - ps29
Cerevisiae_clade - ps30
Saccharomyces_cerevisiae - ps31
Phylostratum

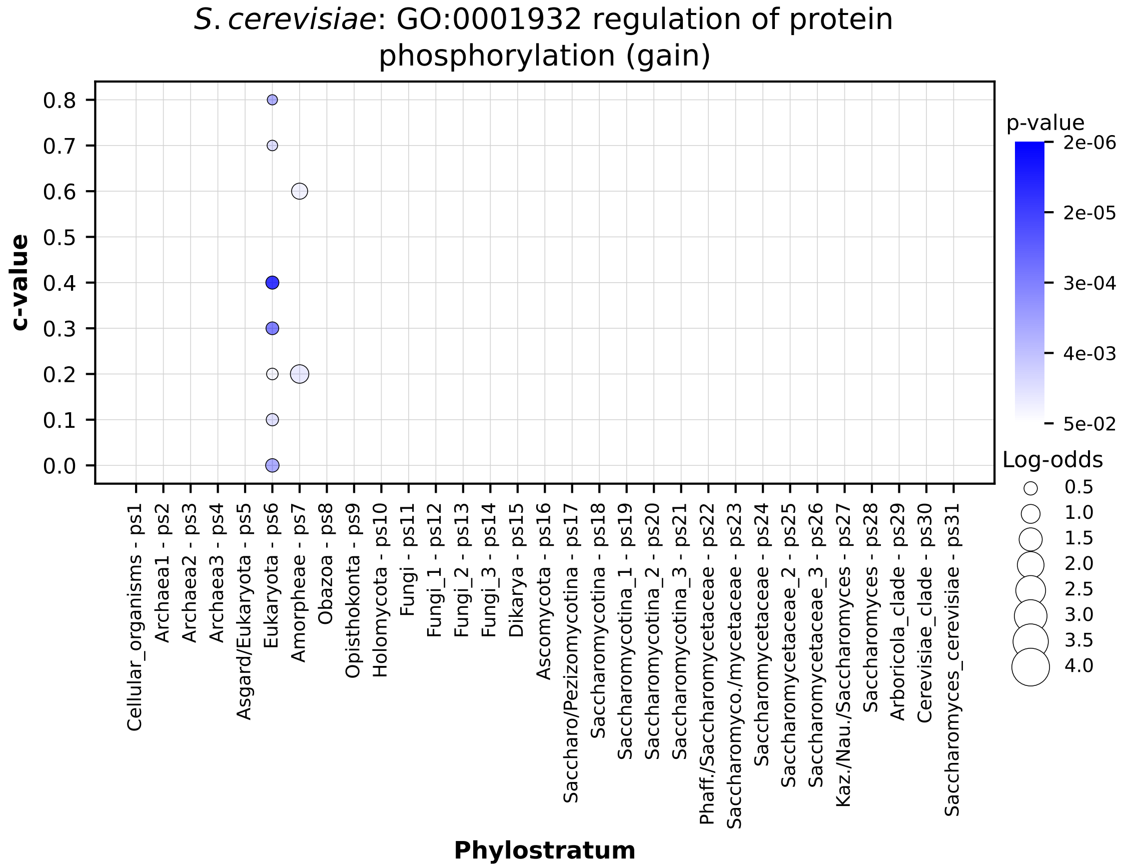

S. cerevisiae: GO:0001932 regulation of protein phosphorylation (gain)
c-value
0.8
0.7
0.6
0.5
0.4
0.3
0.2
0.1
0.0
Phylostratum
Cellular_organisms - ps1
Archaea1 - ps2
Archaea2 - ps3
Archaea3 - ps4
Asgard/Eukaryota - ps5
Eukaryota - ps6
Amorpheae - ps7
Obazoa - ps8
Opisthokonta - ps9
Holomycota - ps10
Fungi - ps11
Fungi_1 - ps12
Fungi_2 - ps13
Fungi_3 - ps14
Dikarya - ps15
Ascomycota - ps16
Saccharo/Pezizomycotina - ps17
Saccharomycotina - ps18
Saccharomycotina_1 - ps19
Saccharomycotina_2 - ps20
Saccharomycotina_3 - ps21
Phaff./Saccharomycetaceae - ps22
Saccharomyco./mycetaceae - ps23
Saccharomycetaceae - ps24
Saccharomycetaceae_2 - ps25
Saccharomycetaceae_3 - ps26
Kaz./Nau./Saccharomyces - ps27
Saccharomyces - ps28
Arboricola_clade - ps29
Cerevisiae_clade - ps30
Saccharomyces_cerevisiae - ps31
p-value
2e-06
2e-05
3e-04
4e-03
5e-02
Log-odds
0.5
1.0
1.5
2.0
2.5
3.0
3.5
4.0

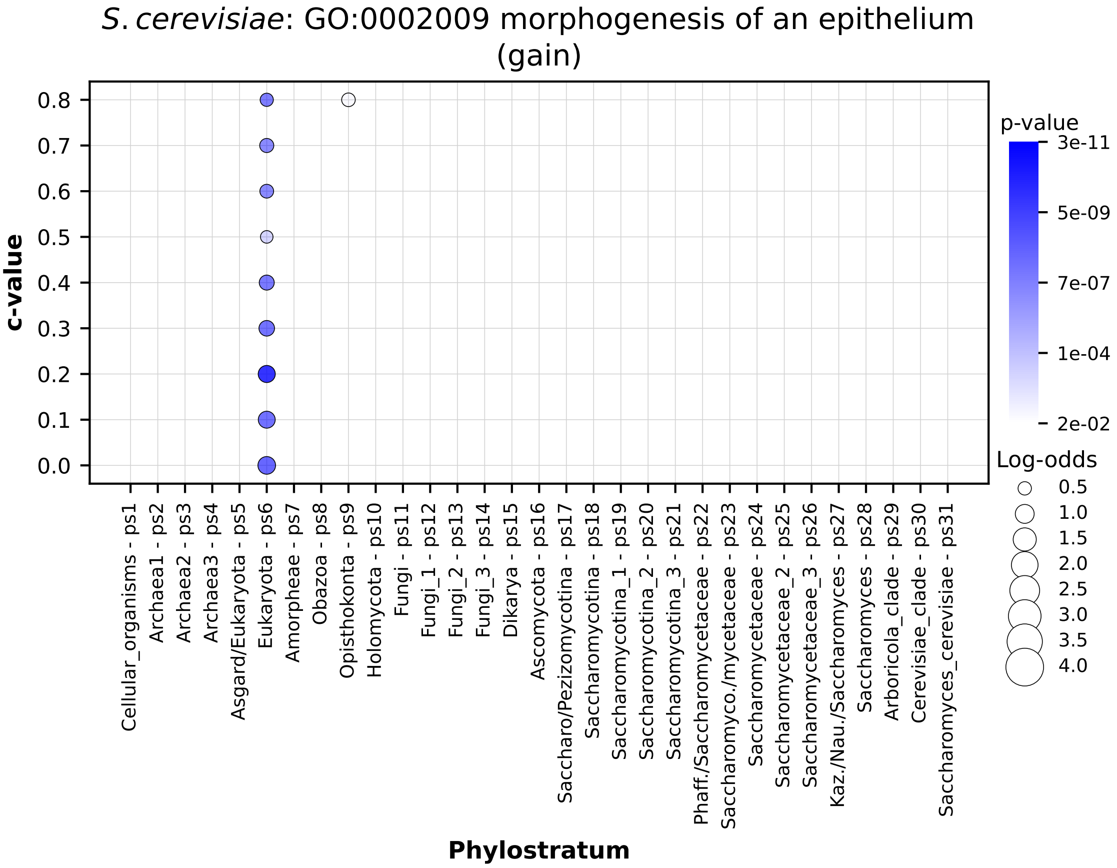

S. cerevisiae: GO:0002009 morphogenesis of an epithelium (gain)
c-value
0.8
0.7
0.6
0.5
0.4
0.3
0.2
0.1
0.0
p-value
3e-11
5e-09
7e-07
1e-04
2e-02
Log-odds
0.5
1.0
1.5
2.0
2.5
3.0
3.5
4.0
Cellular_organisms - ps1
Archaea1 - ps2
Archaea2 - ps3
Archaea3 - ps4
Asgard/Eukaryota - ps5
Eukaryota - ps6
Amorpheae - ps7
Obazoa - ps8
Opisthokonta - ps9
Holomycota - ps10
Fungi - ps11
Fungi_1 - ps12
Fungi_2 - ps13
Fungi_3 - ps14
Dikarya - ps15
Ascomycota - ps16
Saccharo/Pezizomycotina - ps17
Saccharomycotina - ps18
Saccharomycotina_1 - ps19
Saccharomycotina_2 - ps20
Saccharomycotina_3 - ps21
Phaff./Saccharomycetaceae - ps22
Saccharomyco./mycetaceae - ps23
Saccharomycetaceae - ps24
Saccharomycetaceae_2 - ps25
Saccharomycetaceae_3 - ps26
Kaz./Nau./Saccharomyces - ps27
Saccharomyces - ps28
Arboricola_clade - ps29
Cerevisiae_clade - ps30
Saccharomyces_cerevisiae - ps31
Phylostratum

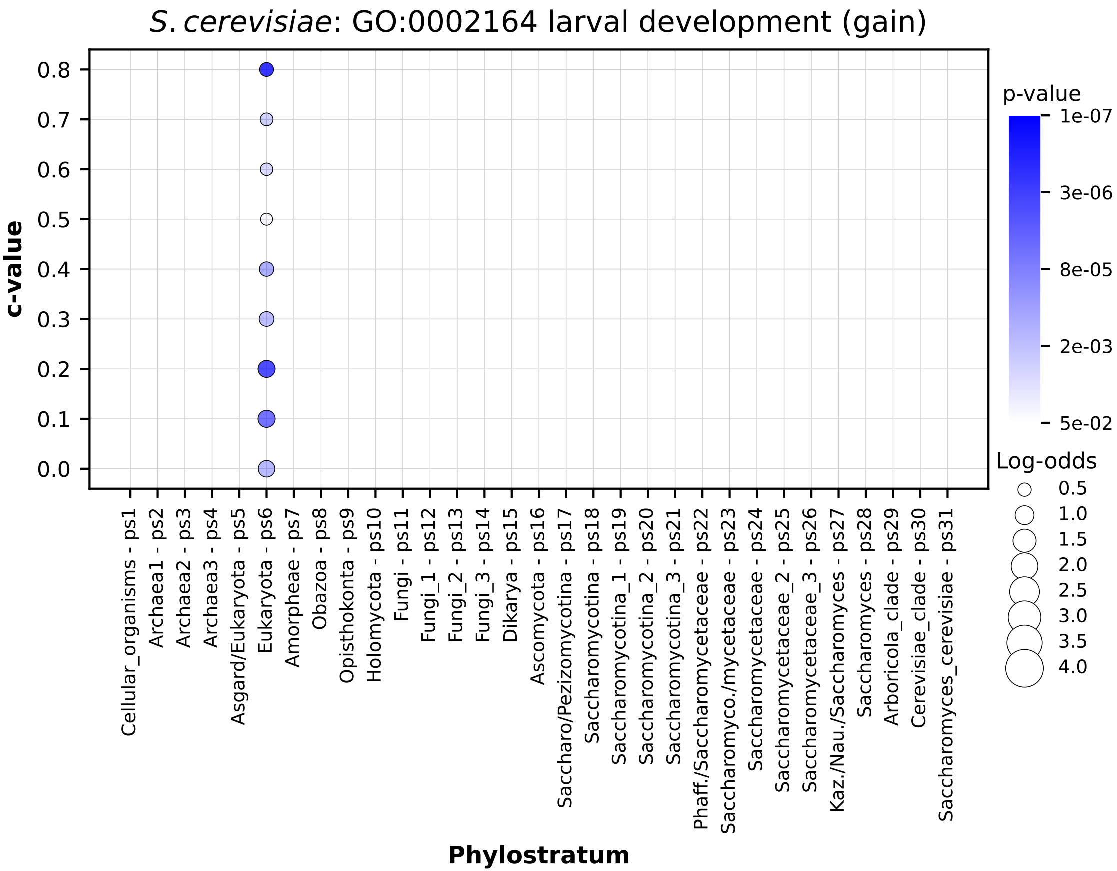
S. cerevisiae: GO:0002164 larval development (gain)
c-value
0.0
0.1
0.2
0.3
0.4
0.5
0.6
0.7
0.8
Phylostratum
Cellular_organisms - ps1
Archaea1 - ps2
Archaea2 - ps3
Archaea3 - ps4
Asgard/Eukaryota - ps5
Eukaryota - ps6
Amorpheae - ps7
Obazoa - ps8
Opisthokonta - ps9
Holomycota - ps10
Fungi - ps11
Fungi_1 - ps12
Fungi_2 - ps13
Fungi_3 - ps14
Dikarya - ps15
Ascomycota - ps16
Saccharo/Pezizomycotina - ps17
Saccharomycotina - ps18
Saccharomycotina_1 - ps19
Saccharomycotina_2 - ps20
Saccharomycotina_3 - ps21
Phaff./Saccharomycetaceae - ps22
Saccharomyco./mycetaceae - ps23
Saccharomycetaceae - ps24
Saccharomycetaceae_2 - ps25
Saccharomycetaceae_3 - ps26
Kaz./Nau./Saccharomyces - ps27
Saccharomyces - ps28
Arboricola_clade - ps29
Cerevisiae_clade - ps30
Saccharomyces_cerevisiae - ps31
p-value
1e-07
3e-06
8e-05
2e-03
5e-02
Log-odds
0.5
1.0
1.5
2.0
2.5
3.0
3.5
4.0

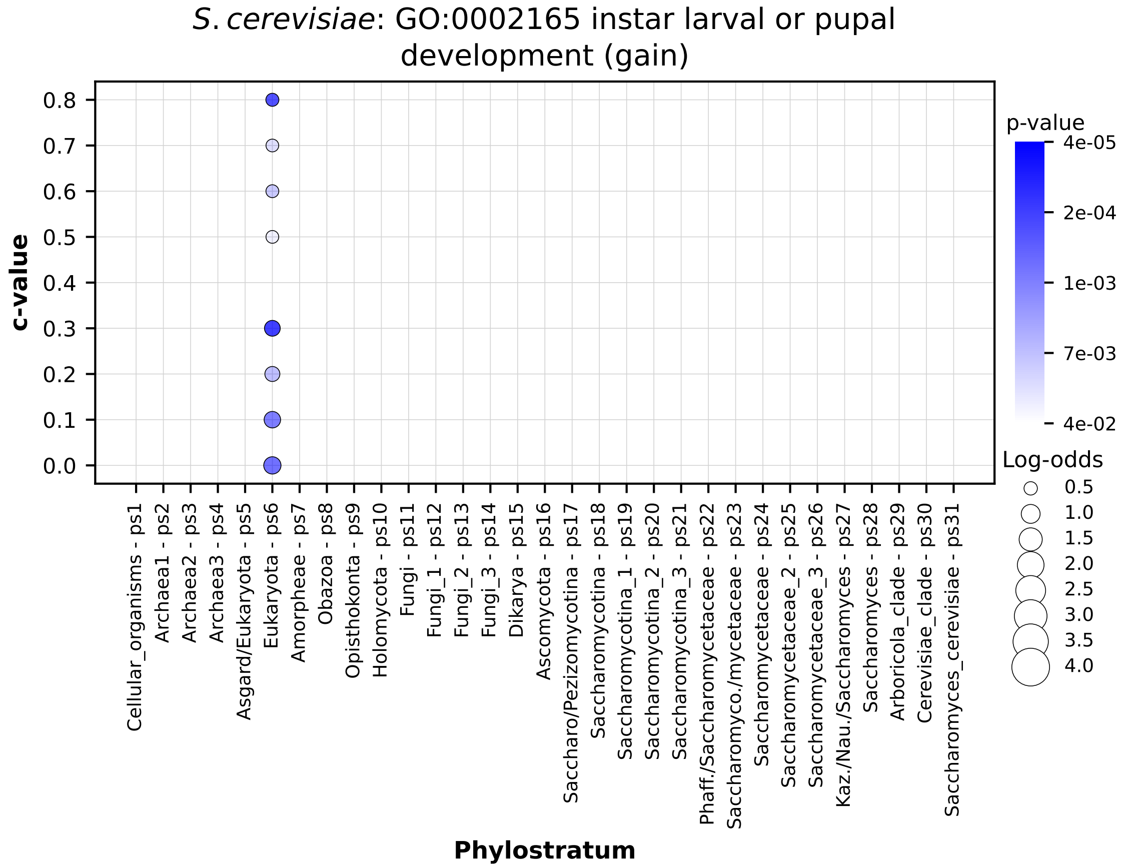

S. cerevisiae: GO:0002165 instar larval or pupal development (gain)
c-value
0.8
0.7
0.6
0.5
0.4
0.3
0.2
0.1
0.0
p-value
4e-05
2e-04
1e-03
7e-03
4e-02
Log-odds
0.5
1.0
1.5
2.0
2.5
3.0
3.5
4.0
Cellular_organisms - ps1
Archaea1 - ps2
Archaea2 - ps3
Archaea3 - ps4
Asgard/Eukaryota - ps5
Eukaryota - ps6
Amorpheae - ps7
Obazoa - ps8
Opisthokonta - ps9
Holomycota - ps10
Fungi - ps11
Fungi_1 - ps12
Fungi_2 - ps13
Fungi_3 - ps14
Dikarya - ps15
Ascomycota - ps16
Saccharo/Pezizomycotina - ps17
Saccharomycotina - ps18
Saccharomycotina_1 - ps19
Saccharomycotina_2 - ps20
Saccharomycotina_3 - ps21
Phaff./Saccharomycetaceae - ps22
Saccharomyco./mycetaceae - ps23
Saccharomycetaceae - ps24
Saccharomycetaceae_2 - ps25
Saccharomycetaceae_3 - ps26
Kaz./Nau./Saccharomyces - ps27
Saccharomyces - ps28
Arboricola_clade - ps29
Cerevisiae_clade - ps30
Saccharomyces_cerevisiae - ps31
Phylostratum

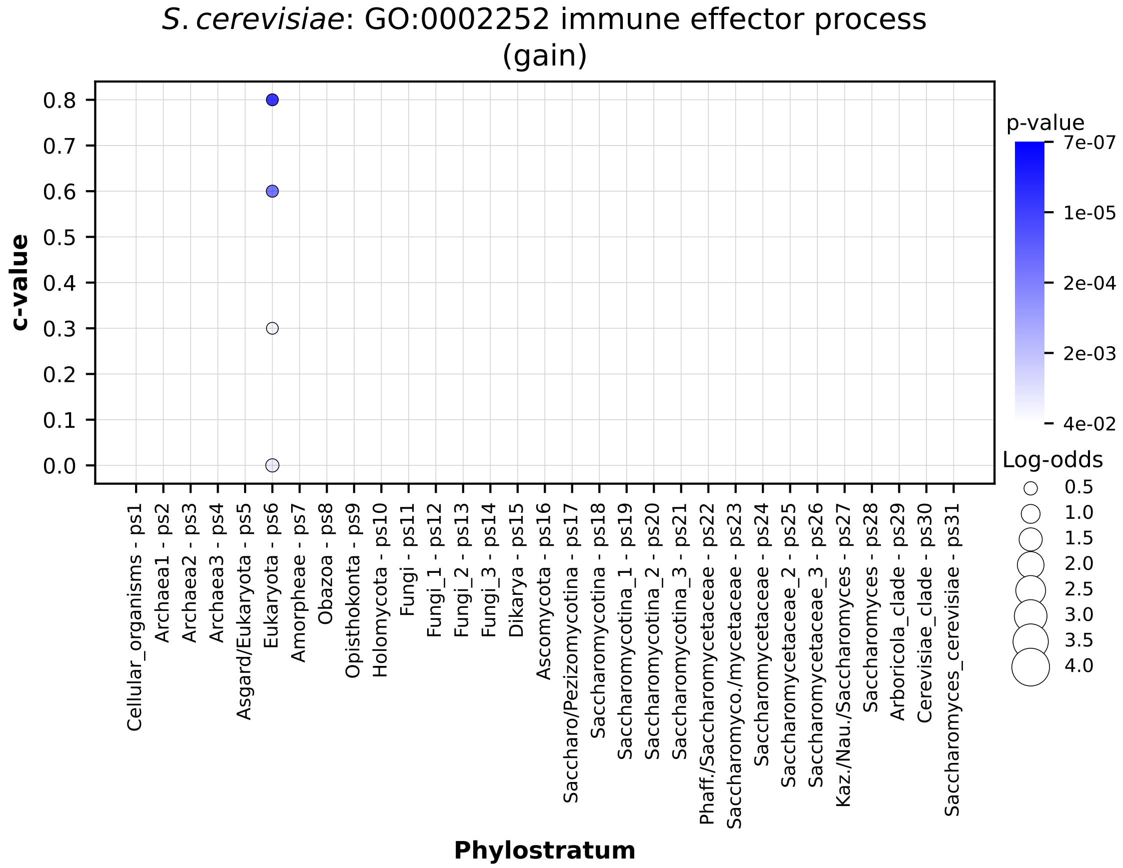

S. cerevisiae: GO:0002252 immune effector process
(gain)
c-value
0.8
0.7
0.6
0.5
0.4
0.3
0.2
0.1
0.0
p-value
7e-07
1e-05
2e-04
2e-03
4e-02
Log-odds
0.5
1.0
1.5
2.0
2.5
3.0
3.5
4.0
Cellular_organisms - ps1
Archaea1 - ps2
Archaea2 - ps3
Archaea3 - ps4
Asgard/Eukaryota - ps5
Eukaryota - ps6
Amorpheae - ps7
Obazoa - ps8
Opisthokonta - ps9
Holomycota - ps10
Fungi - ps11
Fungi_1 - ps12
Fungi_2 - ps13
Fungi_3 - ps14
Dikarya - ps15
Ascomycota - ps16
Saccharo/Pezizomycotina - ps17
Saccharomycotina - ps18
Saccharomycotina_1 - ps19
Saccharomycotina_2 - ps20
Saccharomycotina_3 - ps21
Phaff./Saccharomycetaceae - ps22
Saccharomyco./mycetaceae - ps23
Saccharomycetaceae - ps24
Saccharomycetaceae_2 - ps25
Saccharomycetaceae_3 - ps26
Kaz./Nau./Saccharomyces - ps27
Saccharomyces - ps28
Arboricola_clade - ps29
Cerevisiae_clade - ps30
Saccharomyces_cerevisiae - ps31
Phylostratum

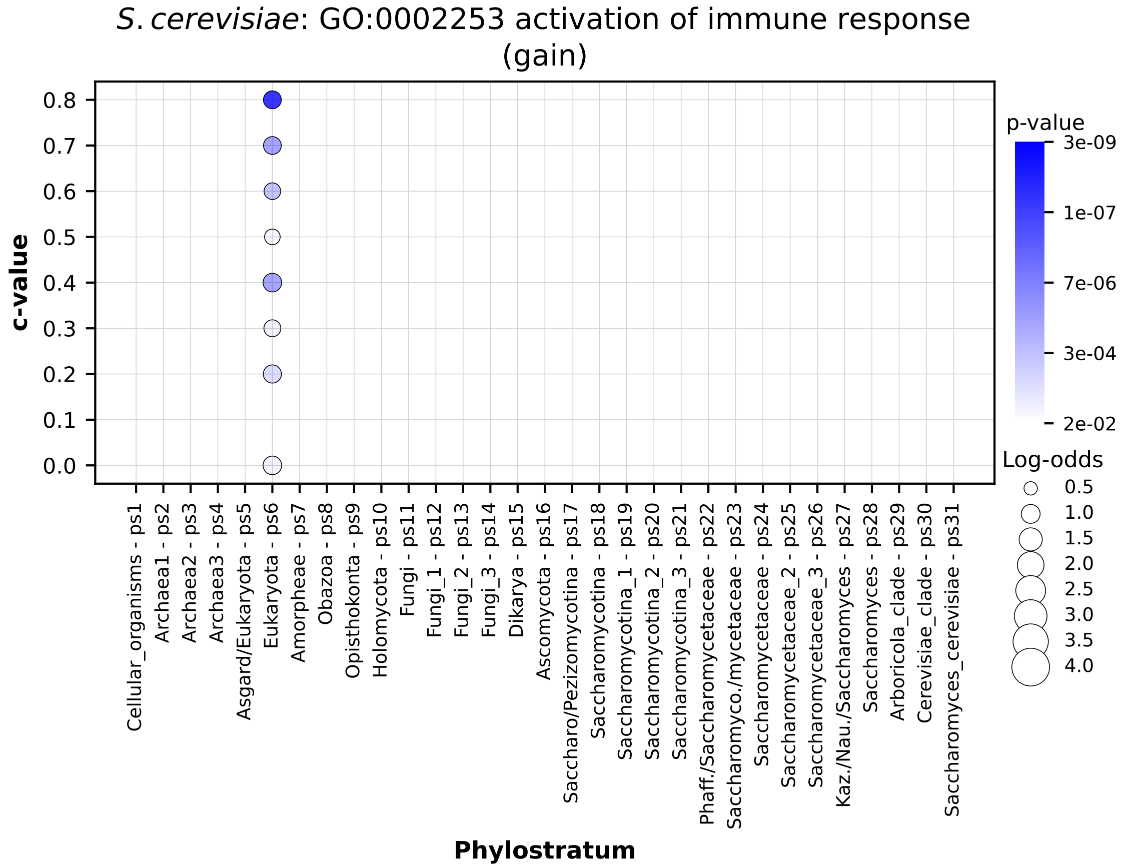

S. cerevisiae: GO:0002253 activation of immune response (gain)
c-value
0.8
0.7
0.6
0.5
0.4
0.3
0.2
0.1
0.0
p-value
3e-09
1e-07
7e-06
3e-04
2e-02
Log-odds
0.5
1.0
1.5
2.0
2.5
3.0
3.5
4.0
Cellular_organisms - ps1
Archaea1 - ps2
Archaea2 - ps3
Archaea3 - ps4
Asgard/Eukaryota - ps5
Eukaryota - ps6
Amorpheae - ps7
Obazoa - ps8
Opisthokonta - ps9
Holomycota - ps10
Fungi - ps11
Fungi_1 - ps12
Fungi_2 - ps13
Fungi_3 - ps14
Dikarya - ps15
Ascomycota - ps16
Saccharo/Pezizomycotina - ps17
Saccharomycotina - ps18
Saccharomycotina_1 - ps19
Saccharomycotina_2 - ps20
Saccharomycotina_3 - ps21
Phaff./Saccharomycetaceae - ps22
Saccharomyco./mycetaceae - ps23
Saccharomycetaceae - ps24
Saccharomycetaceae_2 - ps25
Saccharomycetaceae_3 - ps26
Kaz./Nau./Saccharomyces - ps27
Saccharomyces - ps28
Arboricola_clade - ps29
Cerevisiae_clade - ps30
Saccharomyces_cerevisiae - ps31
Phylostratum

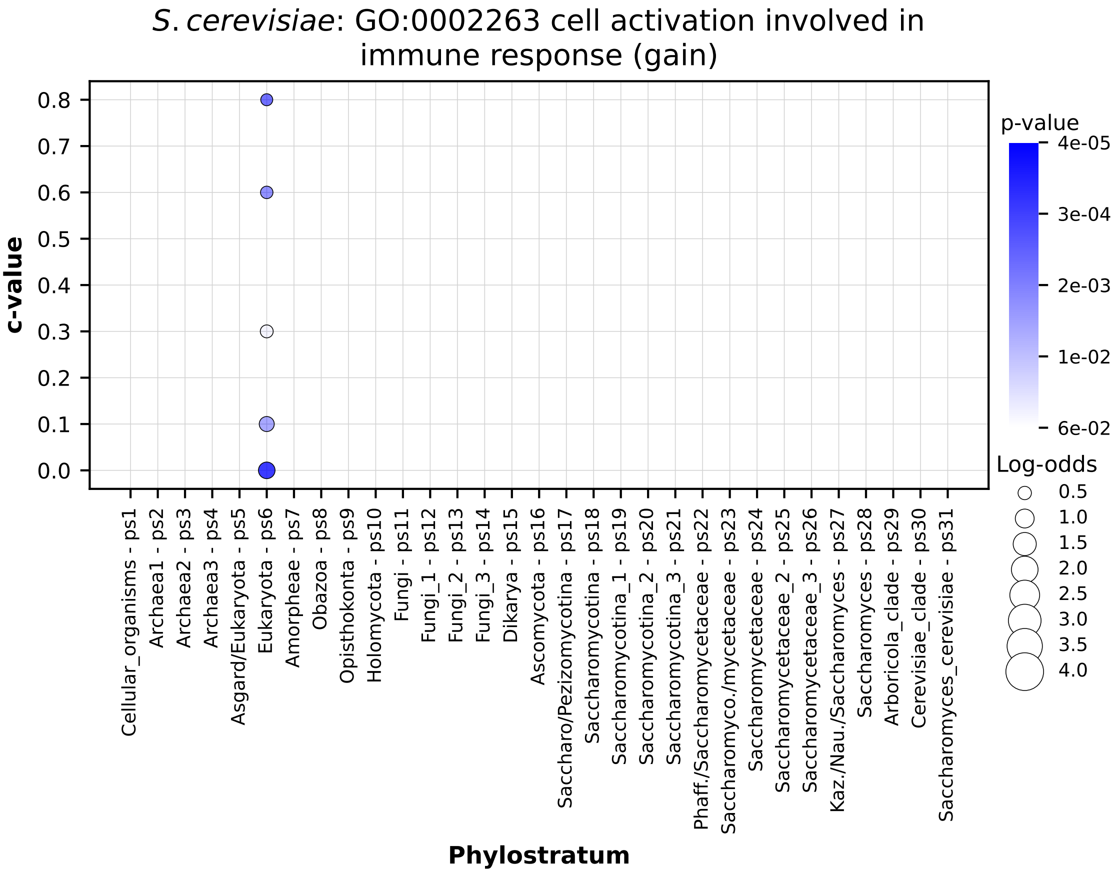

S. cerevisiae: GO:0002263 cell activation involved in immune response (gain)
c-value
0.8
0.7
0.6
0.5
0.4
0.3
0.2
0.1
0.0
p-value
4e-05
3e-04
2e-03
1e-02
6e-02
Log-odds
0.5
1.0
1.5
2.0
2.5
3.0
3.5
4.0
Cellular_organisms - ps1
Archaea1 - ps2
Archaea2 - ps3
Archaea3 - ps4
Asgard/Eukaryota - ps5
Eukaryota - ps6
Amorpheae - ps7
Obazoa - ps8
Opisthokonta - ps9
Holomycota - ps10
Fungi - ps11
Fungi_1 - ps12
Fungi_2 - ps13
Fungi_3 - ps14
Dikarya - ps15
Ascomycota - ps16
Saccharo/Pezizomycotina - ps17
Saccharomycotina - ps18
Saccharomycotina_1 - ps19
Saccharomycotina_2 - ps20
Saccharomycotina_3 - ps21
Phaff./Saccharomycetaceae - ps22
Saccharomyco./mycetaceae - ps23
Saccharomycetaceae - ps24
Saccharomycetaceae_2 - ps25
Saccharomycetaceae_3 - ps26
Kaz./Nau./Saccharomyces - ps27
Saccharomyces - ps28
Arboricola_clade - ps29
Cerevisiae_clade - ps30
Saccharomyces_cerevisiae - ps31
Phylostratum

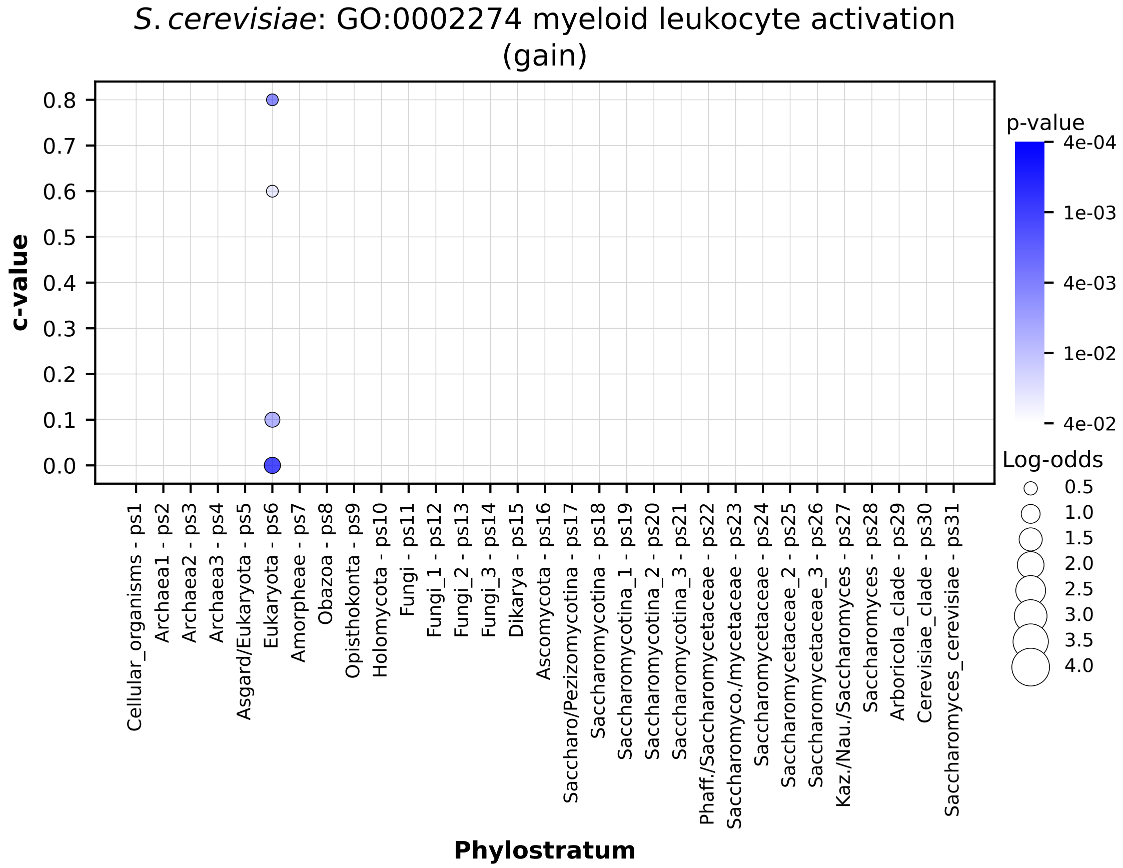

S. cerevisiae: GO:0002274 myeloid leukocyte activation (gain)
c-value
0.0
0.1
0.2
0.3
0.4
0.5
0.6
0.7
0.8
p-value
4e-04
1e-03
4e-03
1e-02
4e-02
Log-odds
0.5
1.0
1.5
2.0
2.5
3.0
3.5
4.0
Cellular_organisms - ps1
Archaea1 - ps2
Archaea2 - ps3
Archaea3 - ps4
Asgard/Eukaryota - ps5
Eukaryota - ps6
Amorpheae - ps7
Obazoa - ps8
Opisthokonta - ps9
Holomycota - ps10
Fungi - ps11
Fungi_1 - ps12
Fungi_2 - ps13
Fungi_3 - ps14
Dikarya - ps15
Ascomycota - ps16
Saccharo/Pezizomycotina - ps17
Saccharomycotina - ps18
Saccharomycotina_1 - ps19
Saccharomycotina_2 - ps20
Saccharomycotina_3 - ps21
Phaff./Saccharomycetaceae - ps22
Saccharomyco./mycetaceae - ps23
Saccharomycetaceae - ps24
Saccharomycetaceae_2 - ps25
Saccharomycetaceae_3 - ps26
Kaz./Nau./Saccharomyces - ps27
Saccharomyces - ps28
Arboricola_clade - ps29
Cerevisiae_clade - ps30
Saccharomyces_cerevisiae - ps31
Phylostratum

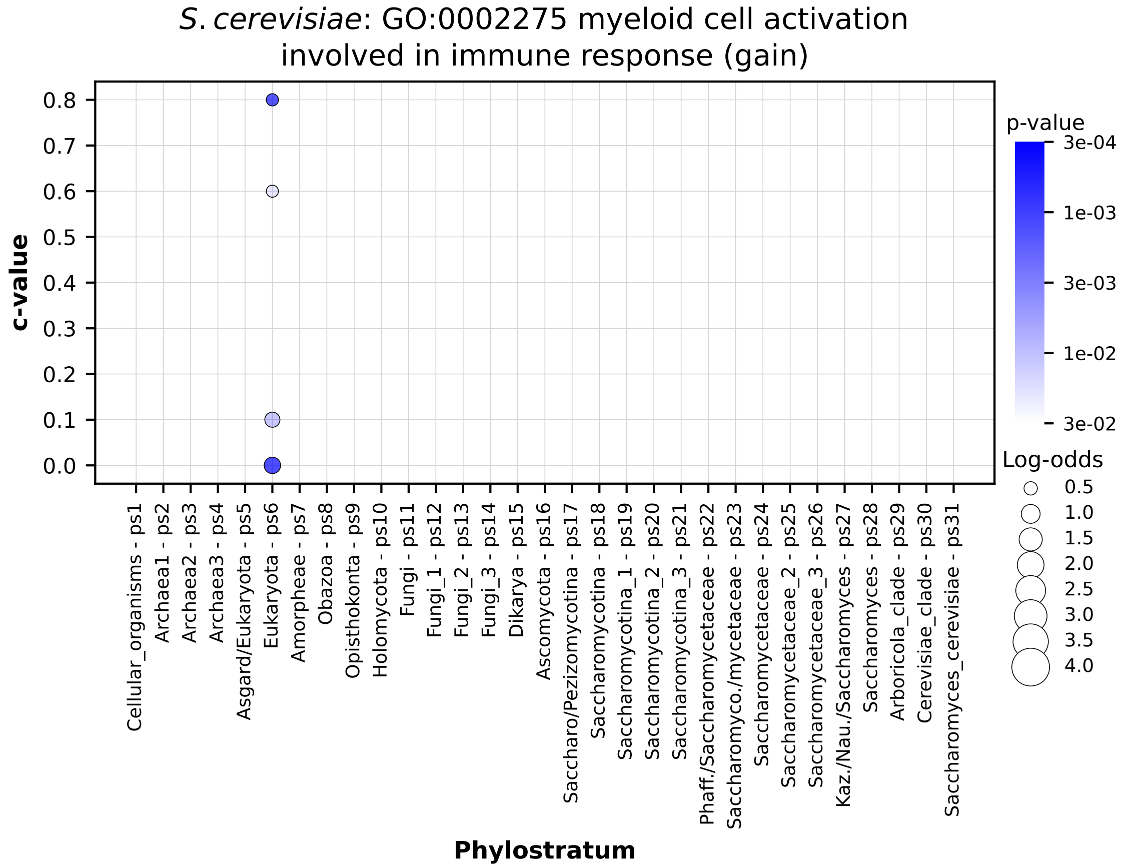

S. cerevisiae: GO:0002275 myeloid cell activation involved in immune response (gain)
c-value
0.8
0.7
0.6
0.5
0.4
0.3
0.2
0.1
0.0
p-value
3e-04
1e-03
3e-03
1e-02
3e-02
Log-odds
0.5
1.0
1.5
2.0
2.5
3.0
3.5
4.0
Cellular_organisms - ps1
Archaea1 - ps2
Archaea2 - ps3
Archaea3 - ps4
Asgard/Eukaryota - ps5
Eukaryota - ps6
Amorpheae - ps7
Obazoa - ps8
Opisthokonta - ps9
Holomycota - ps10
Fungi - ps11
Fungi_1 - ps12
Fungi_2 - ps13
Fungi_3 - ps14
Dikarya - ps15
Ascomycota - ps16
Saccharo/Pezizomycotina - ps17
Saccharomycotina - ps18
Saccharomycotina_1 - ps19
Saccharomycotina_2 - ps20
Saccharomycotina_3 - ps21
Phaff./Saccharomycetaceae - ps22
Saccharomyco./mycetaceae - ps23
Saccharomycetaceae - ps24
Saccharomycetaceae_2 - ps25
Saccharomycetaceae_3 - ps26
Kaz./Nau./Saccharomyces - ps27
Saccharomyces - ps28
Arboricola_clade - ps29
Cerevisiae_clade - ps30
Saccharomyces_cerevisiae - ps31
Phylostratum

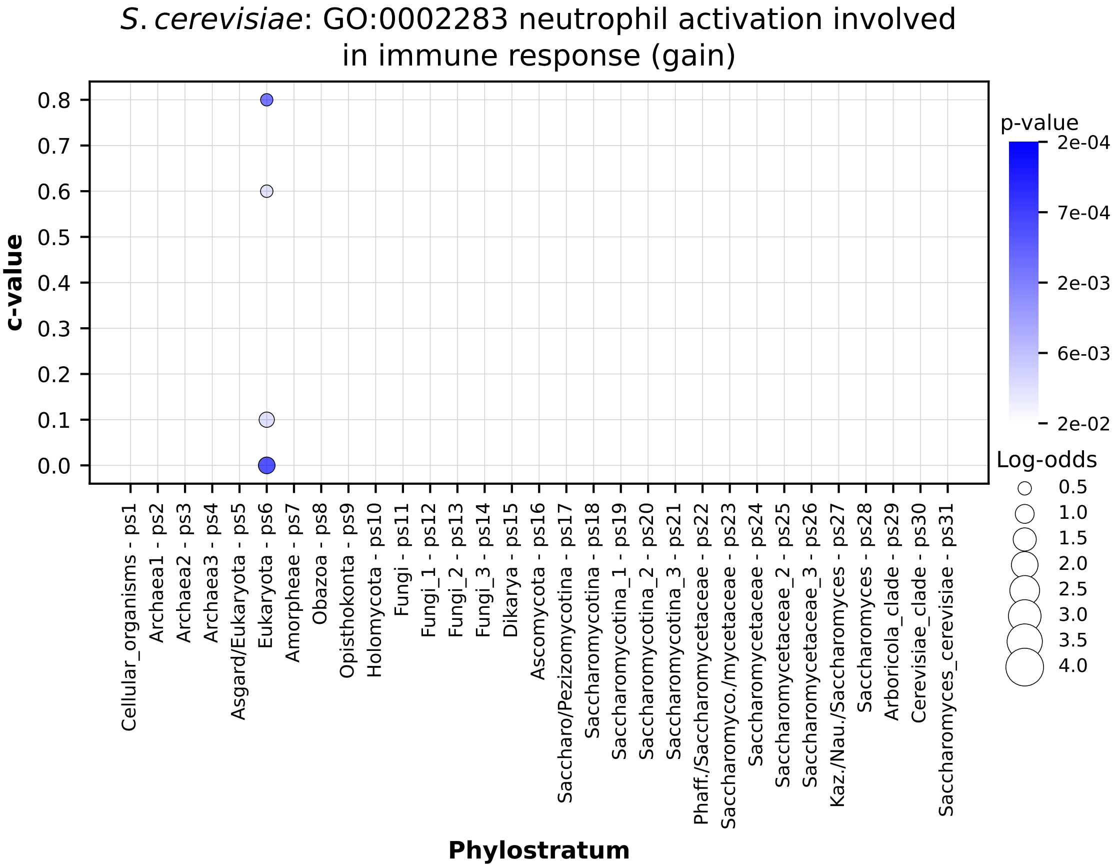

S. cerevisiae: GO:0002283 neutrophil activation involved in immune response (gain)
c-value
0.8
0.7
0.6
0.5
0.4
0.3
0.2
0.1
0.0
p-value
2e-04
7e-04
2e-03
6e-03
2e-02
Log-odds
0.5
1.0
1.5
2.0
2.5
3.0
3.5
4.0
Cellular_organisms - ps1
Archaea1 - ps2
Archaea2 - ps3
Archaea3 - ps4
Asgard/Eukaryota - ps5
Eukaryota - ps6
Amorpheae - ps7
Obazoa - ps8
Opisthokonta - ps9
Holomycota - ps10
Fungi - ps11
Fungi_1 - ps12
Fungi_2 - ps13
Fungi_3 - ps14
Dikarya - ps15
Ascomycota - ps16
Saccharo/Pezizomycotina - ps17
Saccharomycotina - ps18
Saccharomycotina_1 - ps19
Saccharomycotina_2 - ps20
Saccharomycotina_3 - ps21
Phaff./Saccharomycetaceae - ps22
Saccharomyco./mycetaceae - ps23
Saccharomycetaceae - ps24
Saccharomycetaceae_2 - ps25
Saccharomycetaceae_3 - ps26
Kaz./Nau./Saccharomyces - ps27
Saccharomyces - ps28
Arboricola_clade - ps29
Cerevisiae_clade - ps30
Saccharomyces_cerevisiae - ps31
Phylostratum

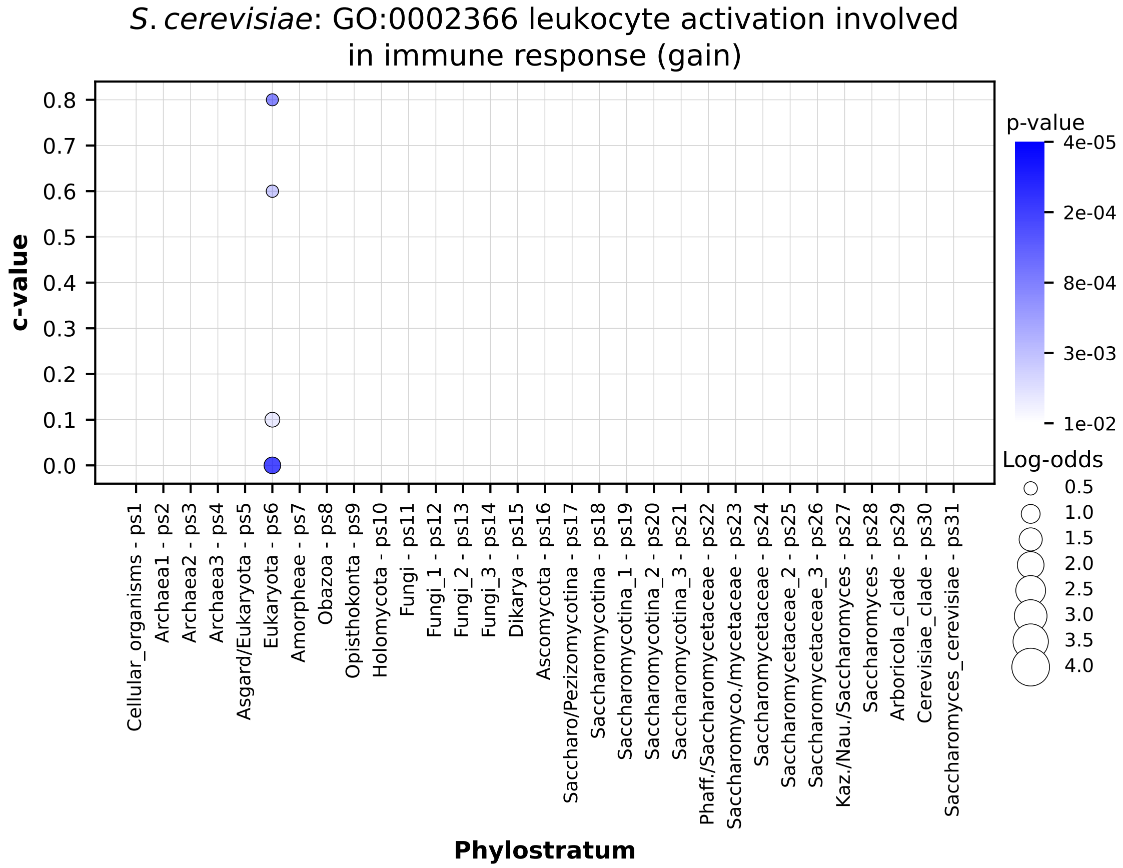

S. cerevisiae: GO:0002366 leukocyte activation involved in immune response (gain)
c-value
0.8
0.7
0.6
0.5
0.4
0.3
0.2
0.1
0.0
p-value
4e-05
2e-04
8e-04
3e-03
1e-02
Log-odds
0.5
1.0
1.5
2.0
2.5
3.0
3.5
4.0
Cellular_organisms - ps1
Archaea1 - ps2
Archaea2 - ps3
Archaea3 - ps4
Asgard/Eukaryota - ps5
Eukaryota - ps6
Amorpheae - ps7
Obazoa - ps8
Opisthokonta - ps9
Holomycota - ps10
Fungi - ps11
Fungi_1 - ps12
Fungi_2 - ps13
Fungi_3 - ps14
Dikarya - ps15
Ascomycota - ps16
Saccharo/Pezizomycotina - ps17
Saccharomycotina - ps18
Saccharomycotina_1 - ps19
Saccharomycotina_2 - ps20
Saccharomycotina_3 - ps21
Phaff./Saccharomycetaceae - ps22
Saccharomyco./mycetaceae - ps23
Saccharomycetaceae - ps24
Saccharomycetaceae_2 - ps25
Saccharomycetaceae_3 - ps26
Kaz./Nau./Saccharomyces - ps27
Saccharomyces - ps28
Arboricola_clade - ps29
Cerevisiae_clade - ps30
Saccharomyces_cerevisiae - ps31
Phylostratum

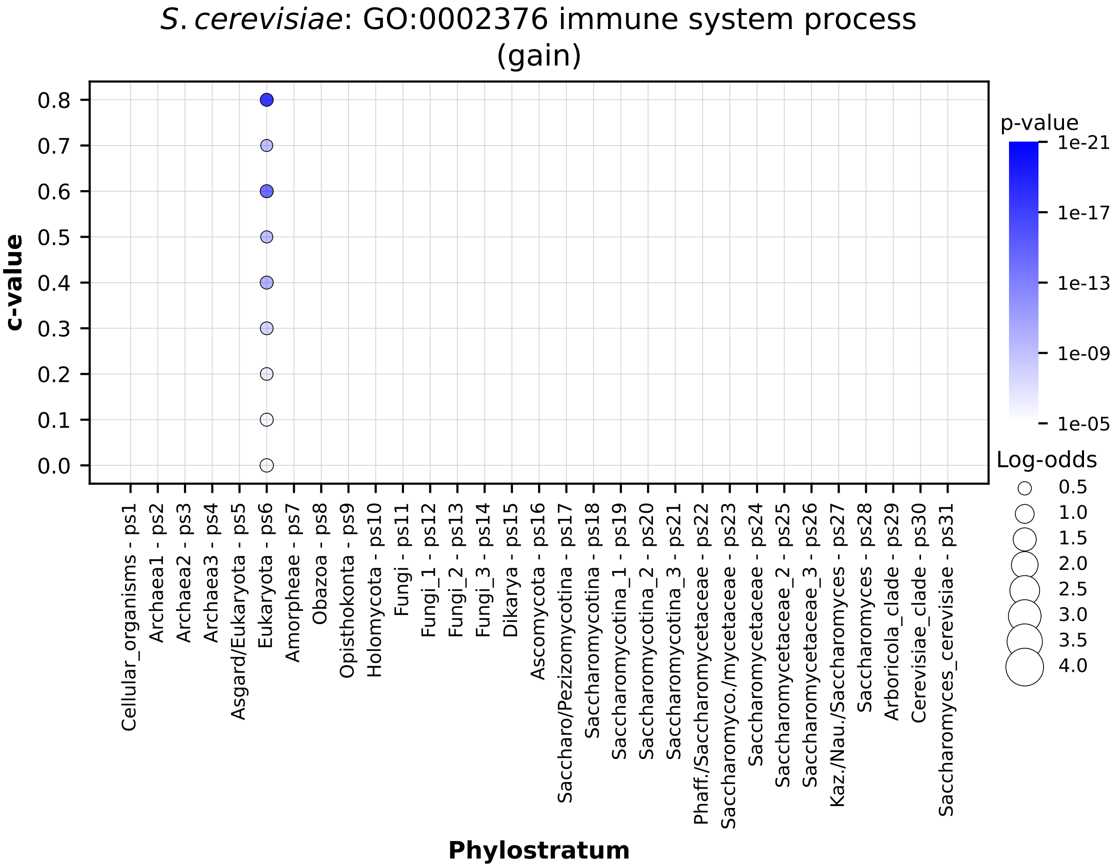

S. cerevisiae: GO:0002376 immune system process (gain)
c-value
0.8
0.7
0.6
0.5
0.4
0.3
0.2
0.1
0.0
p-value
1e-21
1e-17
1e-13
1e-09
1e-05
Log-odds
0.5
1.0
1.5
2.0
2.5
3.0
3.5
4.0
Cellular_organisms - ps1
Archaea1 - ps2
Archaea2 - ps3
Archaea3 - ps4
Asgard/Eukaryota - ps5
Eukaryota - ps6
Amorpheae - ps7
Obazoa - ps8
Opisthokonta - ps9
Holomycota - ps10
Fungi - ps11
Fungi_1 - ps12
Fungi_2 - ps13
Fungi_3 - ps14
Dikarya - ps15
Ascomycota - ps16
Saccharo/Pezizomycotina - ps17
Saccharomycotina - ps18
Saccharomycotina_1 - ps19
Saccharomycotina_2 - ps20
Saccharomycotina_3 - ps21
Phaff./Saccharomycetaceae - ps22
Saccharomyco./mycetaceae - ps23
Saccharomycetaceae - ps24
Saccharomycetaceae_2 - ps25
Saccharomycetaceae_3 - ps26
Kaz./Nau./Saccharomyces - ps27
Saccharomyces - ps28
Arboricola_clade - ps29
Cerevisiae_clade - ps30
Saccharomyces_cerevisiae - ps31
Phylostratum

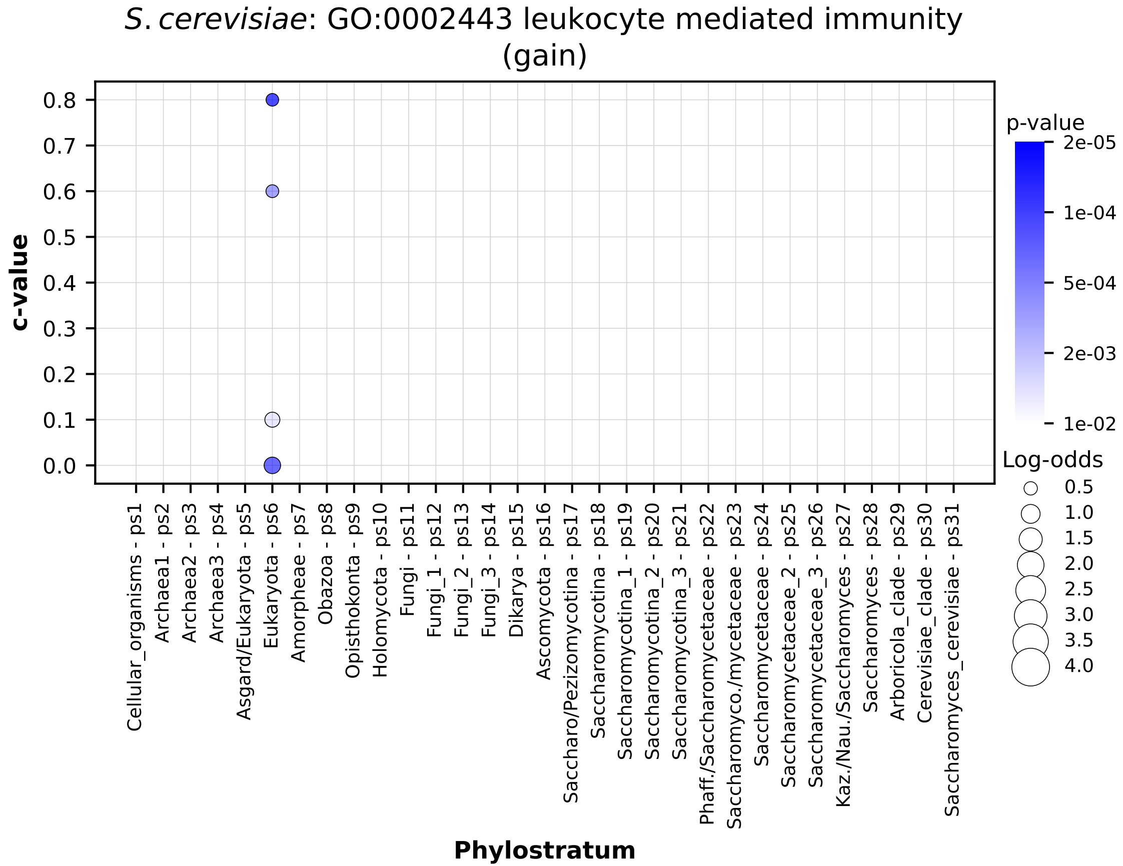

S. cerevisiae: GO:0002443 leukocyte mediated immunity
(gain)
c-value
0.8
0.7
0.6
0.5
0.4
0.3
0.2
0.1
0.0
p-value
2e-05
1e-04
5e-04
2e-03
1e-02
Log-odds
0.5
1.0
1.5
2.0
2.5
3.0
3.5
4.0
Cellular_organisms - ps1
Archaea1 - ps2
Archaea2 - ps3
Archaea3 - ps4
Asgard/Eukaryota - ps5
Eukaryota - ps6
Amorpheae - ps7
Obazoa - ps8
Opisthokonta - ps9
Holomycota - ps10
Fungi - ps11
Fungi_1 - ps12
Fungi_2 - ps13
Fungi_3 - ps14
Dikarya - ps15
Ascomycota - ps16
Saccharo/Pezizomycotina - ps17
Saccharomycotina - ps18
Saccharomycotina_1 - ps19
Saccharomycotina_2 - ps20
Saccharomycotina_3 - ps21
Phaff./Saccharomycetaceae - ps22
Saccharomyco./mycetaceae - ps23
Saccharomycetaceae - ps24
Saccharomycetaceae_2 - ps25
Saccharomycetaceae_3 - ps26
Kaz./Nau./Saccharomyces - ps27
Saccharomyces - ps28
Arboricola_clade - ps29
Cerevisiae_clade - ps30
Saccharomyces_cerevisiae - ps31
Phylostratum

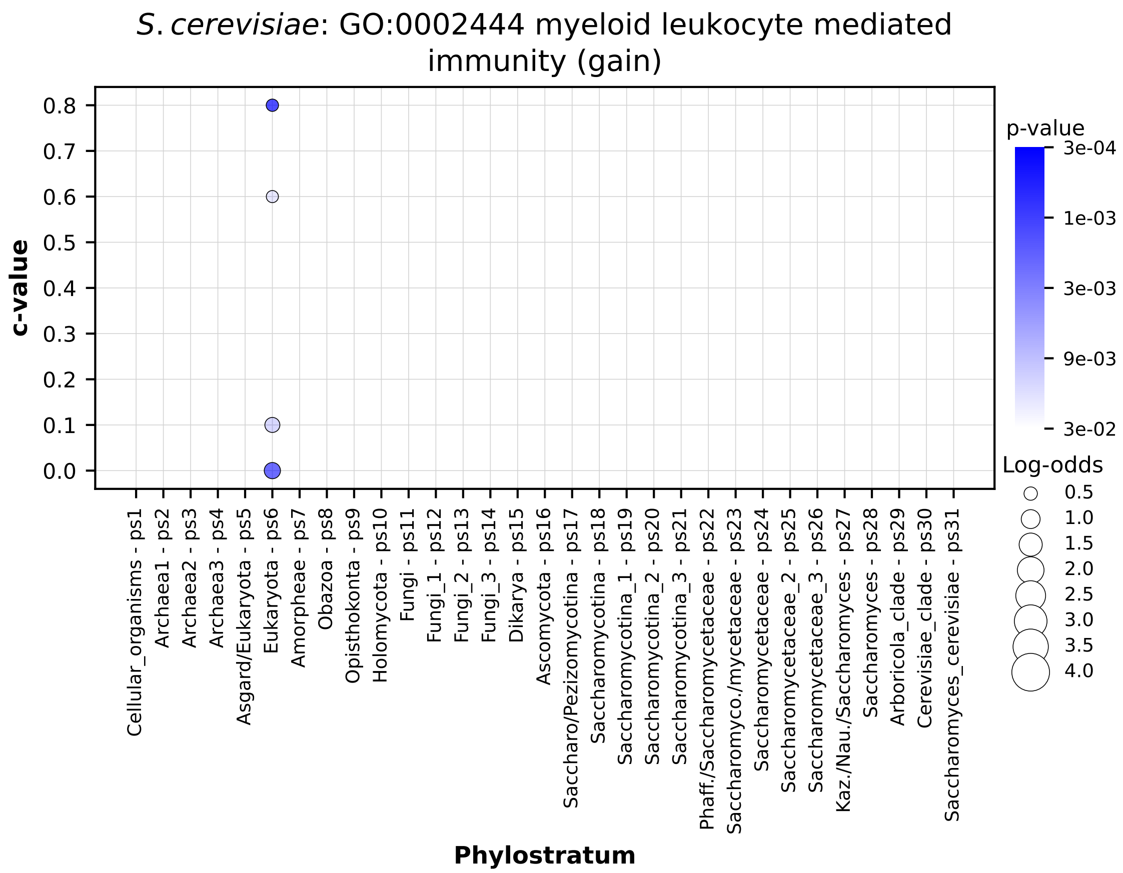

S. cerevisiae: GO:0002444 myeloid leukocyte mediated immunity (gain)
c-value
0.0
0.1
0.2
0.3
0.4
0.5
0.6
0.7
0.8
p-value
3e-04
1e-03
3e-03
9e-03
3e-02
Log-odds
0.5
1.0
1.5
2.0
2.5
3.0
3.5
4.0
Cellular_organisms - ps1
Archaea1 - ps2
Archaea2 - ps3
Archaea3 - ps4
Asgard/Eukaryota - ps5
Eukaryota - ps6
Amorpheae - ps7
Obazoa - ps8
Opisthokonta - ps9
Holomycota - ps10
Fungi - ps11
Fungi_1 - ps12
Fungi_2 - ps13
Fungi_3 - ps14
Dikarya - ps15
Ascomycota - ps16
Saccharo/Pezizomycotina - ps17
Saccharomycotina - ps18
Saccharomycotina_1 - ps19
Saccharomycotina_2 - ps20
Saccharomycotina_3 - ps21
Phaff./Saccharomycetaceae - ps22
Saccharomyco./mycetaceae - ps23
Saccharomycetaceae - ps24
Saccharomycetaceae_2 - ps25
Saccharomycetaceae_3 - ps26
Kaz./Nau./Saccharomyces - ps27
Saccharomyces - ps28
Arboricola_clade - ps29
Cerevisiae_clade - ps30
Saccharomyces_cerevisiae - ps31
Phylostratum

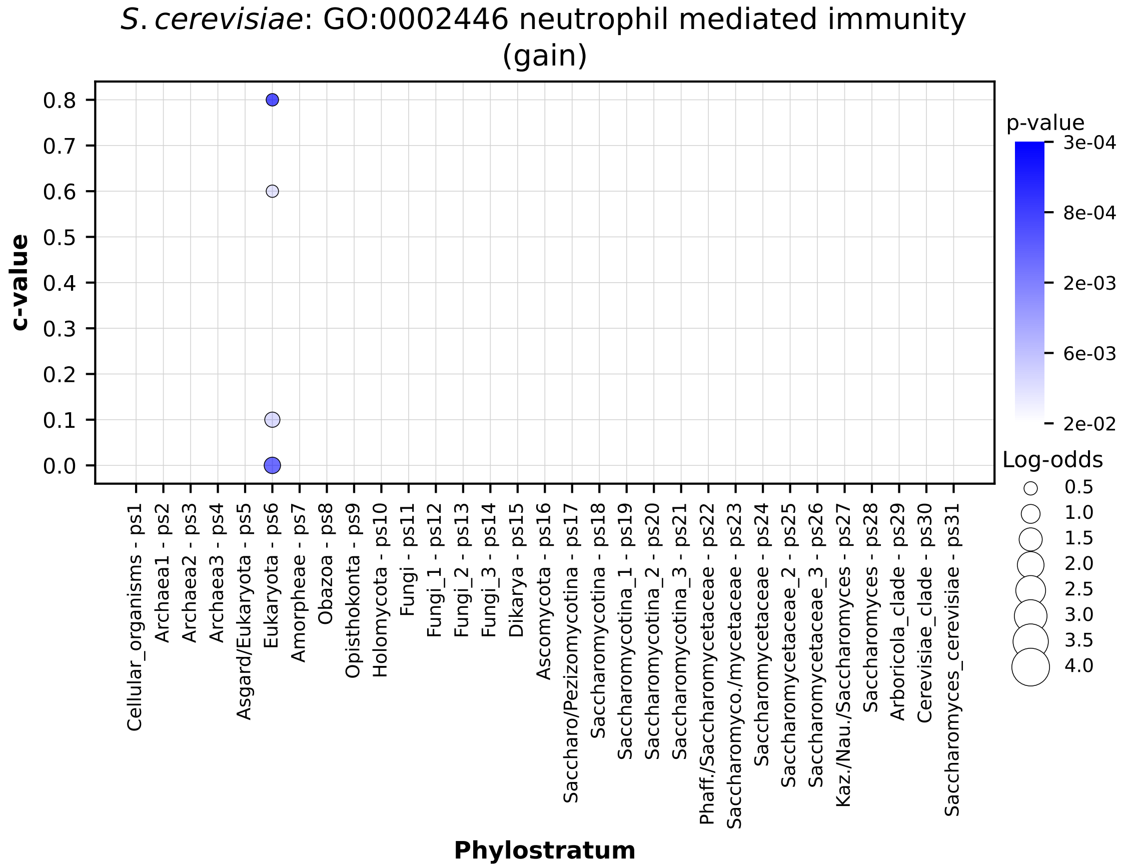

S. cerevisiae: GO:0002446 neutrophil mediated immunity
(gain)
c-value
0.0
0.1
0.2
0.3
0.4
0.5
0.6
0.7
0.8
p-value
3e-04
8e-04
2e-03
6e-03
2e-02
Log-odds
0.5
1.0
1.5
2.0
2.5
3.0
3.5
4.0
Cellular_organisms - ps1
Archaea1 - ps2
Archaea2 - ps3
Archaea3 - ps4
Asgard/Eukaryota - ps5
Eukaryota - ps6
Amorpheae - ps7
Obazoa - ps8
Opisthokonta - ps9
Holomycota - ps10
Fungi - ps11
Fungi_1 - ps12
Fungi_2 - ps13
Fungi_3 - ps14
Dikarya - ps15
Ascomycota - ps16
Saccharo/Pezizomycotina - ps17
Saccharomycotina - ps18
Saccharomycotina_1 - ps19
Saccharomycotina_2 - ps20
Saccharomycotina_3 - ps21
Phaff./Saccharomycetaceae - ps22
Saccharomyco./mycetaceae - ps23
Saccharomycetaceae - ps24
Saccharomycetaceae_2 - ps25
Saccharomycetaceae_3 - ps26
Kaz./Nau./Saccharomyces - ps27
Saccharomyces - ps28
Arboricola_clade - ps29
Cerevisiae_clade - ps30
Saccharomyces_cerevisiae - ps31
Phylostratum

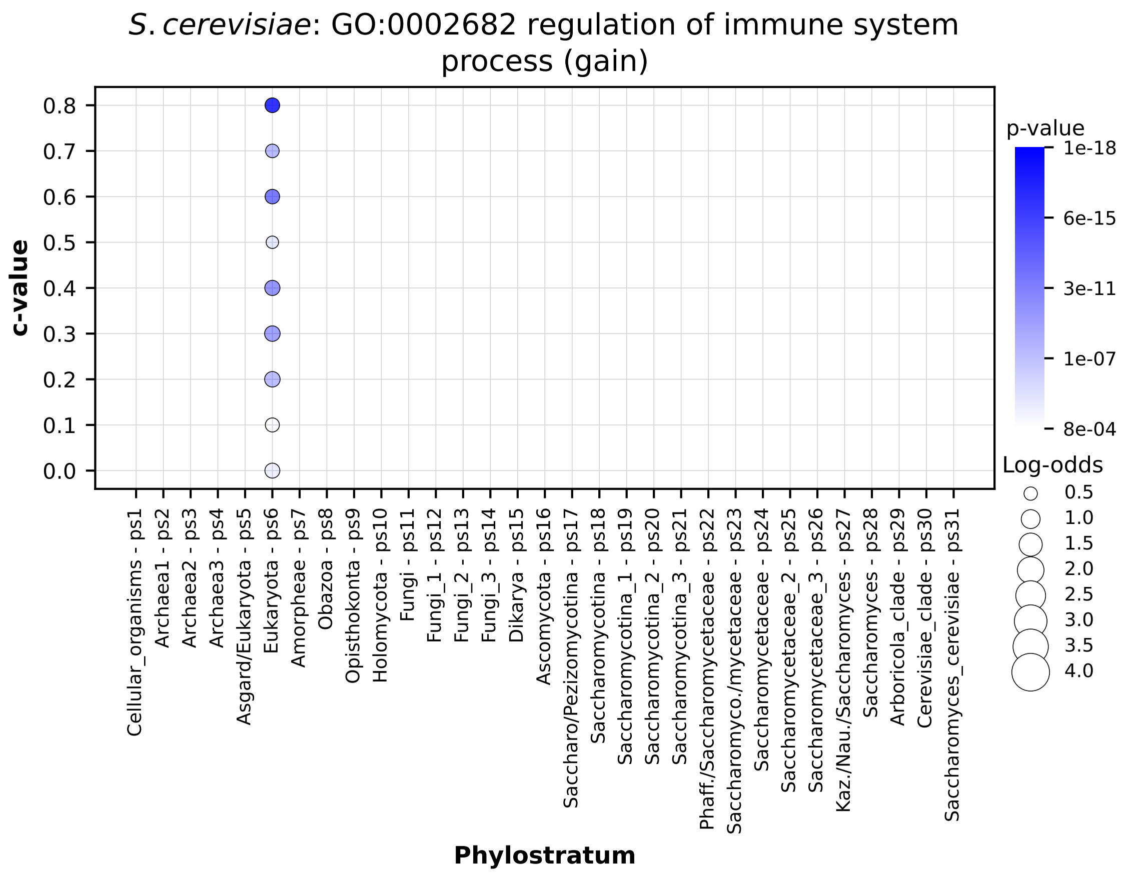

S. cerevisiae: GO:0002682 regulation of immune system process (gain)
c-value
0.8
0.7
0.6
0.5
0.4
0.3
0.2
0.1
0.0
p-value
1e-18
6e-15
3e-11
1e-07
8e-04
Log-odds
0.5
1.0
1.5
2.0
2.5
3.0
3.5
4.0
Cellular_organisms - ps1
Archaea1 - ps2
Archaea2 - ps3
Archaea3 - ps4
Asgard/Eukaryota - ps5
Eukaryota - ps6
Amorpheae - ps7
Obazoa - ps8
Opisthokonta - ps9
Holomycota - ps10
Fungi - ps11
Fungi_1 - ps12
Fungi_2 - ps13
Fungi_3 - ps14
Dikarya - ps15
Ascomycota - ps16
Saccharo/Pezizomycotina - ps17
Saccharomycotina - ps18
Saccharomycotina_1 - ps19
Saccharomycotina_2 - ps20
Saccharomycotina_3 - ps21
Phaff./Saccharomycetaceae - ps22
Saccharomyco./mycetaceae - ps23
Saccharomycetaceae - ps24
Saccharomycetaceae_2 - ps25
Saccharomycetaceae_3 - ps26
Kaz./Nau./Saccharomyces - ps27
Saccharomyces - ps28
Arboricola_clade - ps29
Cerevisiae_clade - ps30
Saccharomyces_cerevisiae - ps31
Phylostratum

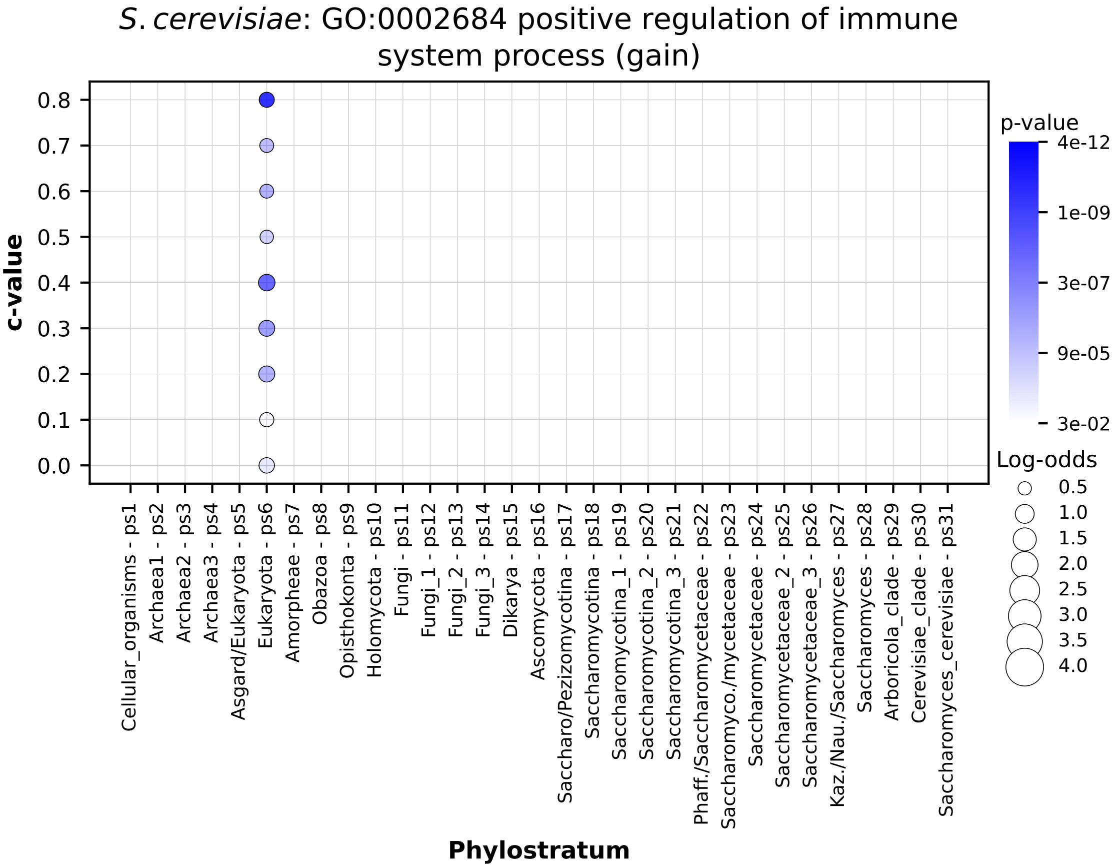

S. cerevisiae: GO:0002684 positive regulation of immune system process (gain)
c-value
0.0
0.1
0.2
0.3
0.4
0.5
0.6
0.7
0.8
p-value
4e-12
1e-09
3e-07
9e-05
3e-02
Log-odds
0.5
1.0
1.5
2.0
2.5
3.0
3.5
4.0
Cellular_organisms - ps1
Archaea1 - ps2
Archaea2 - ps3
Archaea3 - ps4
Asgard/Eukaryota - ps5
Eukaryota - ps6
Amorpheae - ps7
Obazoa - ps8
Opisthokonta - ps9
Holomycota - ps10
Fungi - ps11
Fungi_1 - ps12
Fungi_2 - ps13
Fungi_3 - ps14
Dikarya - ps15
Ascomycota - ps16
Saccharo/Pezizomycotina - ps17
Saccharomycotina - ps18
Saccharomycotina_1 - ps19
Saccharomycotina_2 - ps20
Saccharomycotina_3 - ps21
Phaff./Saccharomycetaceae - ps22
Saccharomyco./mycetaceae - ps23
Saccharomycetaceae - ps24
Saccharomycetaceae_2 - ps25
Saccharomycetaceae_3 - ps26
Kaz./Nau./Saccharomyces - ps27
Saccharomyces - ps28
Arboricola_clade - ps29
Cerevisiae_clade - ps30
Saccharomyces_cerevisiae - ps31
Phylostratum

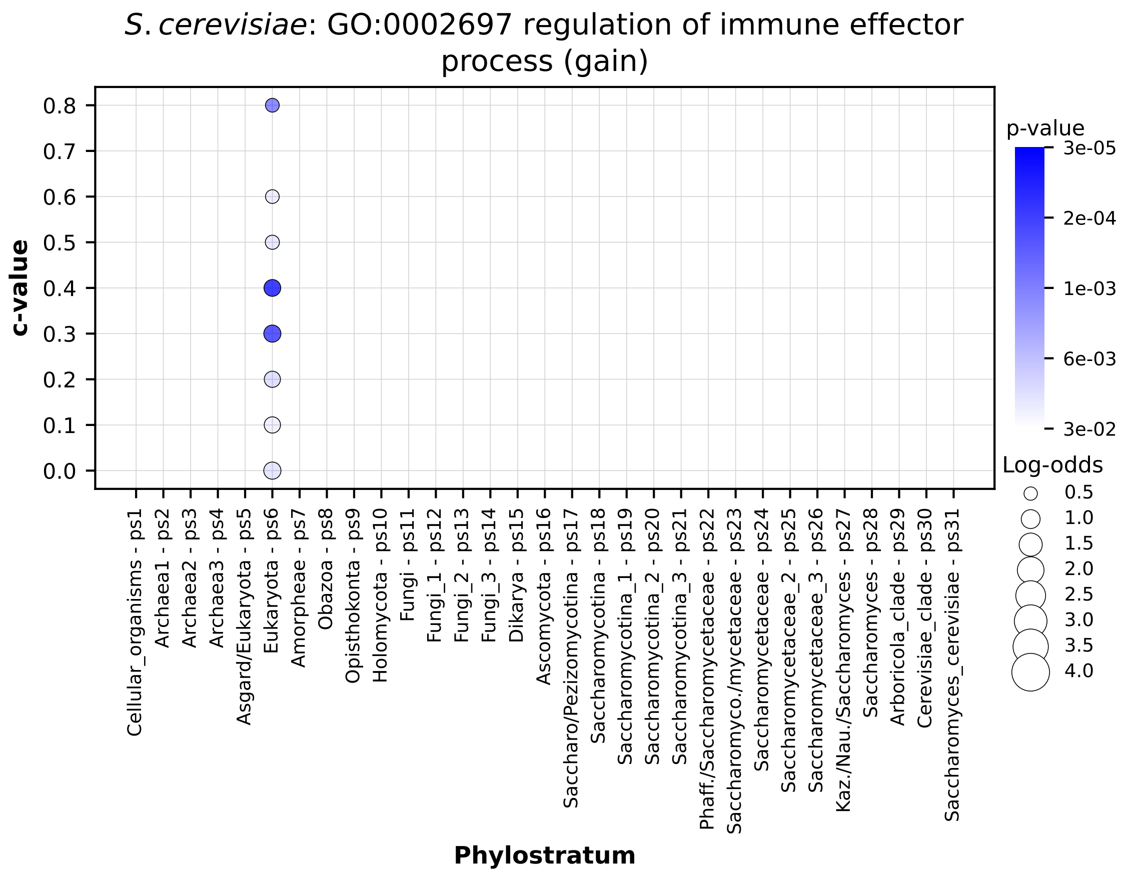

S. cerevisiae: GO:0002697 regulation of immune effector process (gain)
c-value
0.0
0.1
0.2
0.3
0.4
0.5
0.6
0.7
0.8
Phylostratum
Cellular_organisms - ps1
Archaea1 - ps2
Archaea2 - ps3
Archaea3 - ps4
Asgard/Eukaryota - ps5
Eukaryota - ps6
Amorpheae - ps7
Obazoa - ps8
Opisthokonta - ps9
Holomycota - ps10
Fungi - ps11
Fungi_1 - ps12
Fungi_2 - ps13
Fungi_3 - ps14
Dikarya - ps15
Ascomycota - ps16
Saccharo/Pezizomycotina - ps17
Saccharomycotina - ps18
Saccharomycotina_1 - ps19
Saccharomycotina_2 - ps20
Saccharomycotina_3 - ps21
Phaff./Saccharomycetaceae - ps22
Saccharomyco./mycetaceae - ps23
Saccharomycetaceae - ps24
Saccharomycetaceae_2 - ps25
Saccharomycetaceae_3 - ps26
Kaz./Nau./Saccharomyces - ps27
Saccharomyces - ps28
Arboricola_clade - ps29
Cerevisiae_clade - ps30
Saccharomyces_cerevisiae - ps31
p-value
3e-05
2e-04
1e-03
6e-03
3e-02
Log-odds
0.5
1.0
1.5
2.0
2.5
3.0
3.5
4.0

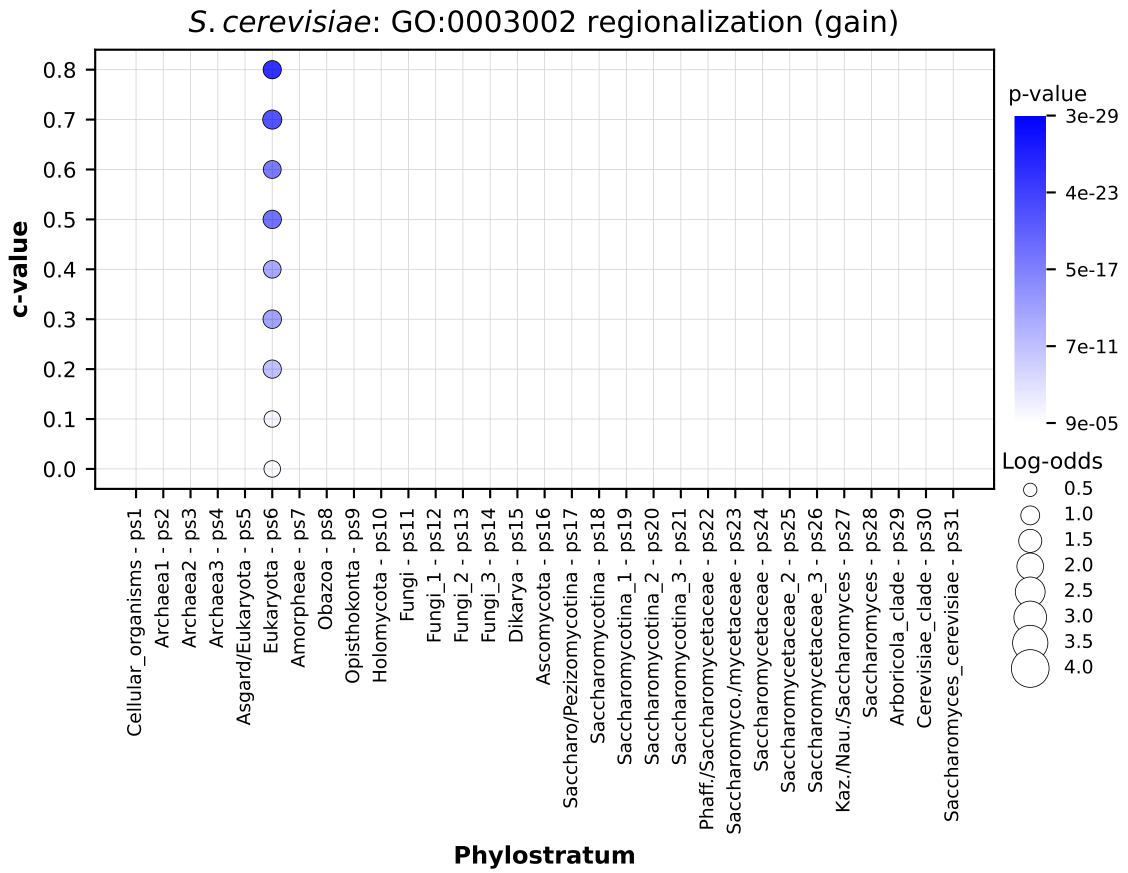

S. cerevisiae: GO:0003002 regionalization (gain)
c-value
0.8
0.7
0.6
0.5
0.4
0.3
0.2
0.1
0.0
p-value
3e-29
4e-23
5e-17
7e-11
9e-05
Log-odds
0.5
1.0
1.5
2.0
2.5
3.0
3.5
4.0
Cellular_organisms - ps1
Archaea1 - ps2
Archaea2 - ps3
Archaea3 - ps4
Asgard/Eukaryota - ps5
Eukaryota - ps6
Amorpheae - ps7
Obazoa - ps8
Opisthokonta - ps9
Holomycota - ps10
Fungi - ps11
Fungi_1 - ps12
Fungi_2 - ps13
Fungi_3 - ps14
Dikarya - ps15
Ascomycota - ps16
Saccharo/Pezizomycotina - ps17
Saccharomycotina - ps18
Saccharomycotina_1 - ps19
Saccharomycotina_2 - ps20
Saccharomycotina_3 - ps21
Phaff./Saccharomycetaceae - ps22
Saccharomyco./mycetaceae - ps23
Saccharomycetaceae - ps24
Saccharomycetaceae_2 - ps25
Saccharomycetaceae_3 - ps26
Kaz./Nau./Saccharomyces - ps27
Saccharomyces - ps28
Arboricola_clade - ps29
Cerevisiae_clade - ps30
Saccharomyces_cerevisiae - ps31
Phylostratum

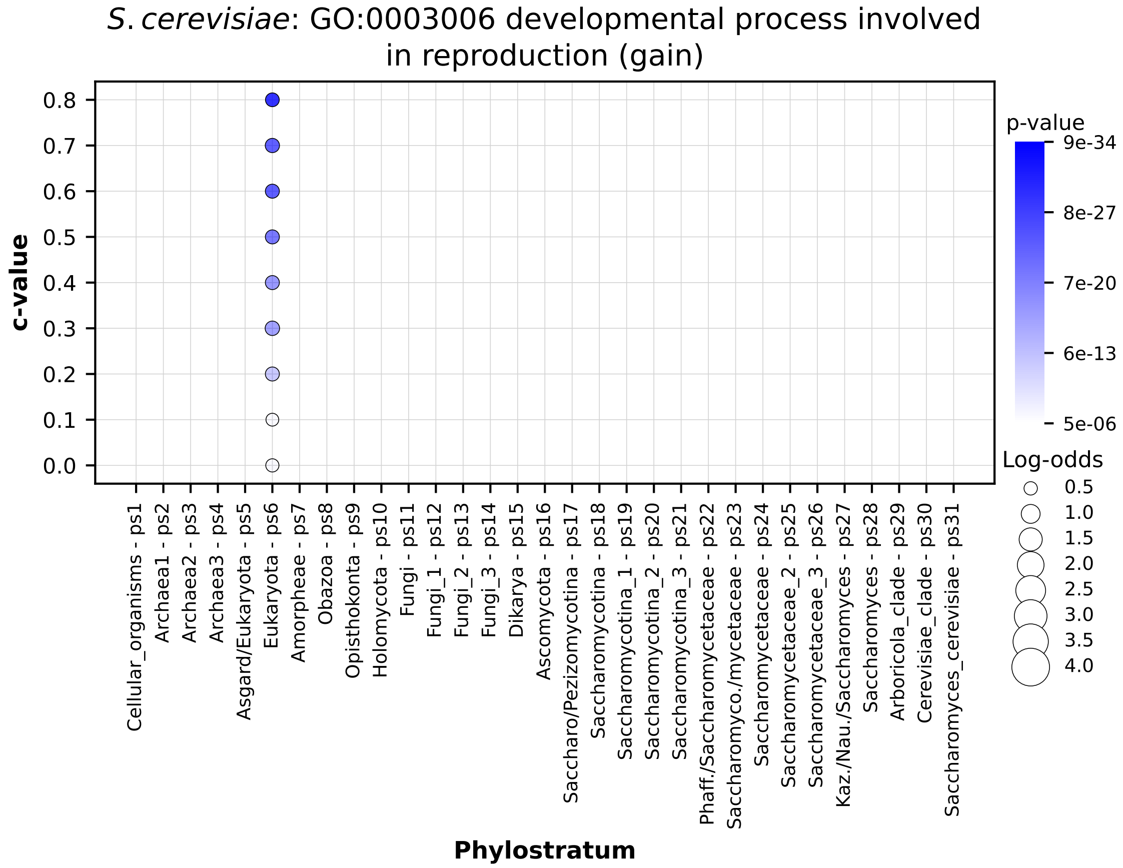

S. cerevisiae: GO:0003006 developmental process involved in reproduction (gain)
c-value
0.0
0.1
0.2
0.3
0.4
0.5
0.6
0.7
0.8
p-value
9e-34
8e-27
7e-20
6e-13
5e-06
Log-odds
0.5
1.0
1.5
2.0
2.5
3.0
3.5
4.0
Cellular_organisms - ps1
Archaea1 - ps2
Archaea2 - ps3
Archaea3 - ps4
Asgard/Eukaryota - ps5
Eukaryota - ps6
Amorpheae - ps7
Obazoa - ps8
Opisthokonta - ps9
Holomycota - ps10
Fungi - ps11
Fungi_1 - ps12
Fungi_2 - ps13
Fungi_3 - ps14
Dikarya - ps15
Ascomycota - ps16
Saccharo/Pezizomycotina - ps17
Saccharomycotina - ps18
Saccharomycotina_1 - ps19
Saccharomycotina_2 - ps20
Saccharomycotina_3 - ps21
Phaff./Saccharomycetaceae - ps22
Saccharomyco./mycetaceae - ps23
Saccharomycetaceae - ps24
Saccharomycetaceae_2 - ps25
Saccharomycetaceae_3 - ps26
Kaz./Nau./Saccharomyces - ps27
Saccharomyces - ps28
Arboricola_clade - ps29
Cerevisiae_clade - ps30
Saccharomyces_cerevisiae - ps31
Phylostratum

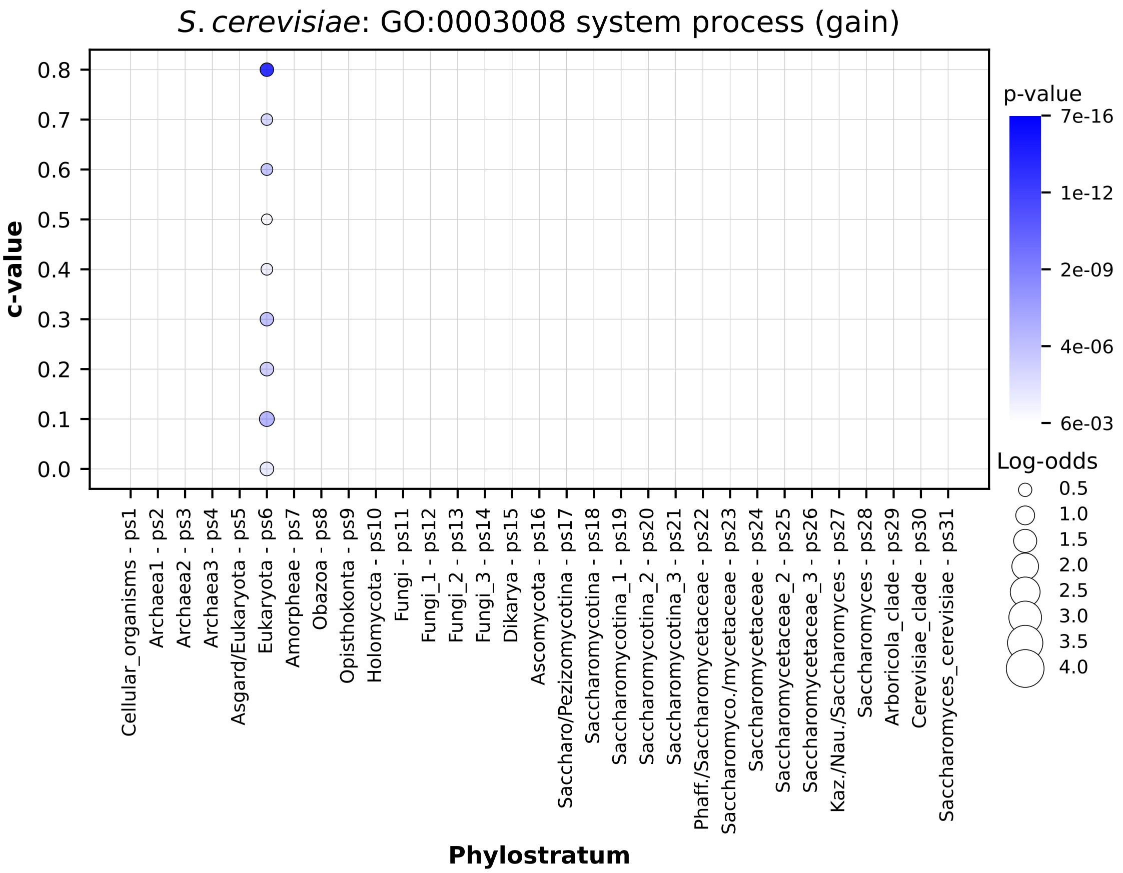

S. cerevisiae: GO:0003008 system process (gain)
c-value
0.0
0.1
0.2
0.3
0.4
0.5
0.6
0.7
0.8
p-value
7e-16
1e-12
2e-09
4e-06
6e-03
Log-odds
0.5
1.0
1.5
2.0
2.5
3.0
3.5
4.0
Cellular_organisms - ps1
Archaea1 - ps2
Archaea2 - ps3
Archaea3 - ps4
Asgard/Eukaryota - ps5
Eukaryota - ps6
Amorpheae - ps7
Obazoa - ps8
Opisthokonta - ps9
Holomycota - ps10
Fungi - ps11
Fungi_1 - ps12
Fungi_2 - ps13
Fungi_3 - ps14
Dikarya - ps15
Ascomycota - ps16
Saccharo/Pezizomycotina - ps17
Saccharomycotina - ps18
Saccharomycotina_1 - ps19
Saccharomycotina_2 - ps20
Saccharomycotina_3 - ps21
Phaff./Saccharomycetaceae - ps22
Saccharomyco./mycetaceae - ps23
Saccharomycetaceae - ps24
Saccharomycetaceae_2 - ps25
Saccharomycetaceae_3 - ps26
Kaz./Nau./Saccharomyces - ps27
Saccharomyces - ps28
Arboricola_clade - ps29
Cerevisiae_clade - ps30
Saccharomyces_cerevisiae - ps31
Phylostratum

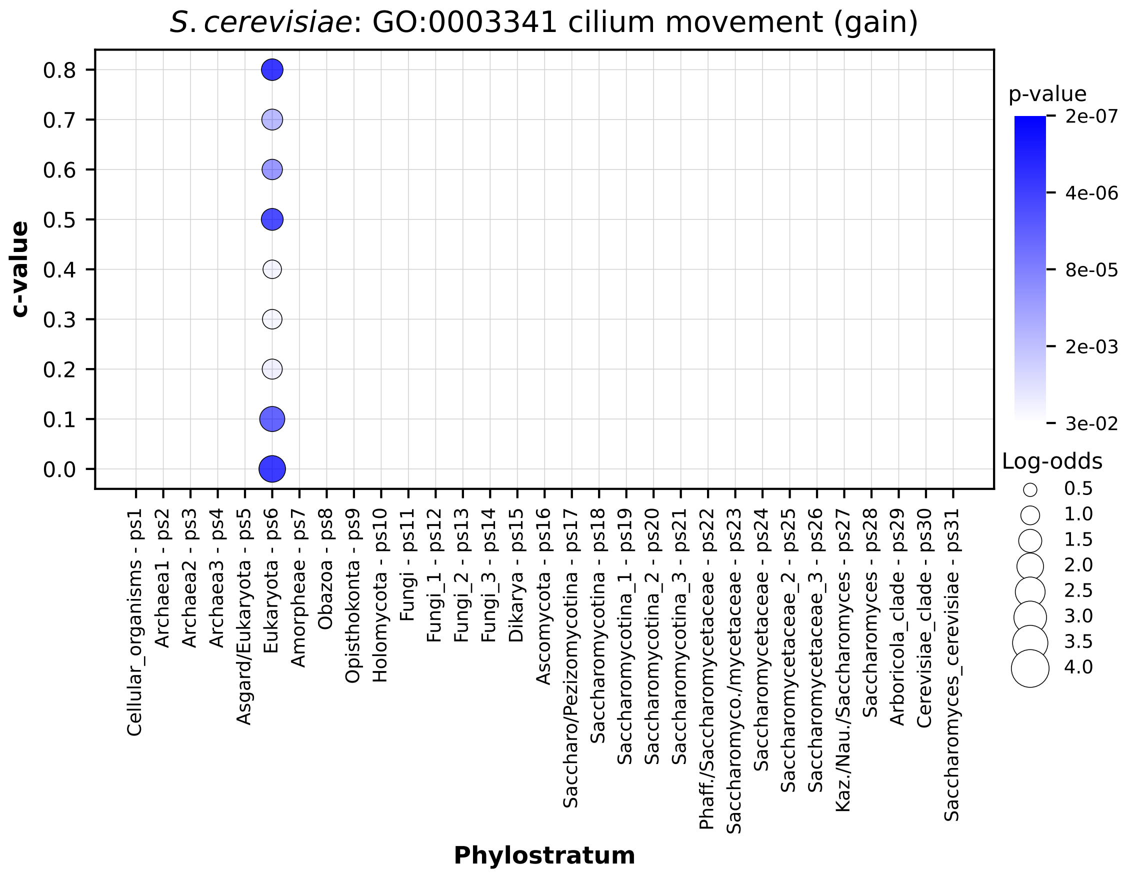
S. cerevisiae: GO:0003341 cilium movement (gain)
c-value
0.8
0.7
0.6
0.5
0.4
0.3
0.2
0.1
0.0
p-value
2e-07
4e-06
8e-05
2e-03
3e-02
Log-odds
0.5
1.0
1.5
2.0
2.5
3.0
3.5
4.0
Cellular_organisms - ps1
Archaea1 - ps2
Archaea2 - ps3
Archaea3 - ps4
Asgard/Eukaryota - ps5
Eukaryota - ps6
Amorpheae - ps7
Obazoa - ps8
Opisthokonta - ps9
Holomycota - ps10
Fungi - ps11
Fungi_1 - ps12
Fungi_2 - ps13
Fungi_3 - ps14
Dikarya - ps15
Ascomycota - ps16
Saccharo/Pezizomycotina - ps17
Saccharomycotina - ps18
Saccharomycotina_1 - ps19
Saccharomycotina_2 - ps20
Saccharomycotina_3 - ps21
Phaff./Saccharomycetaceae - ps22
Saccharomyco./mycetaceae - ps23
Saccharomycetaceae - ps24
Saccharomycetaceae_2 - ps25
Saccharomycetaceae_3 - ps26
Kaz./Nau./Saccharomyces - ps27
Saccharomyces - ps28
Arboricola_clade - ps29
Cerevisiae_clade - ps30
Saccharomyces_cerevisiae - ps31
Phylostratum

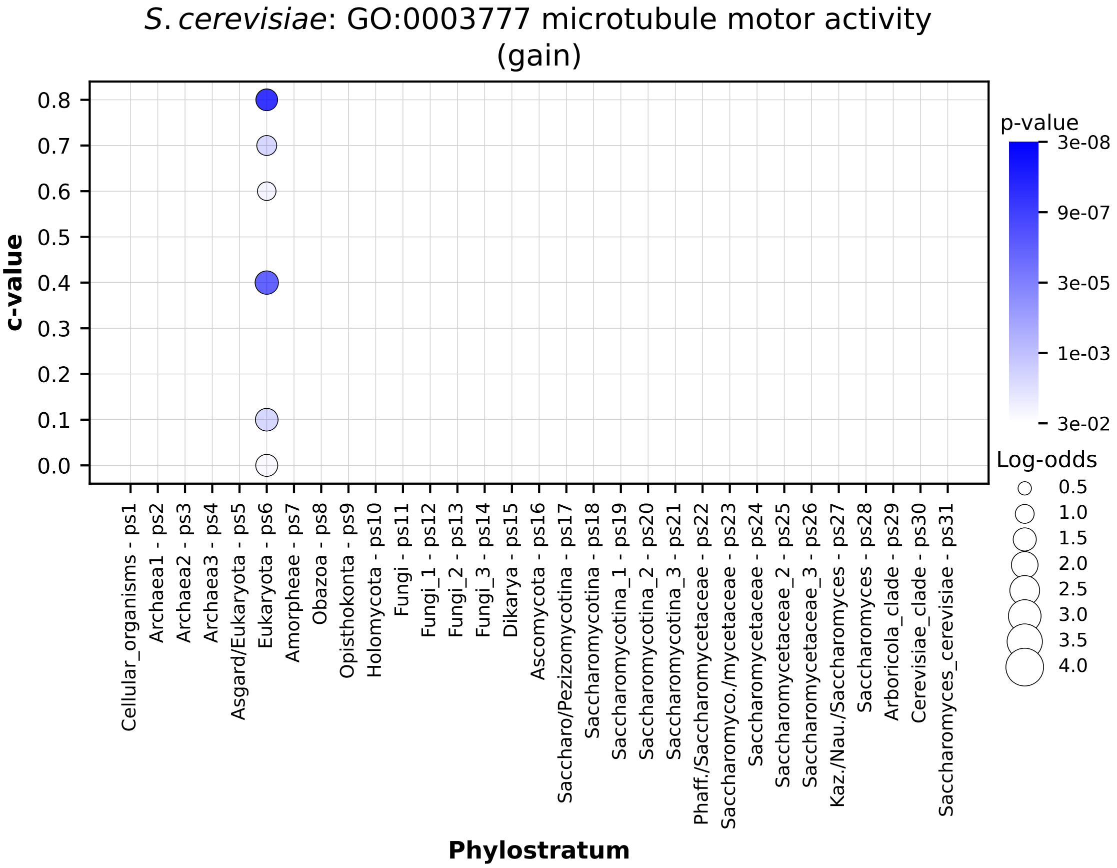
S. cerevisiae: GO:0003777 microtubule motor activity
(gain)
c-value
0.8
0.7
0.6
0.5
0.4
0.3
0.2
0.1
0.0
p-value
3e-08
9e-07
3e-05
1e-03
3e-02
Log-odds
0.5
1.0
1.5
2.0
2.5
3.0
3.5
4.0
Cellular_organisms - ps1
Archaea1 - ps2
Archaea2 - ps3
Archaea3 - ps4
Asgard/Eukaryota - ps5
Eukaryota - ps6
Amorpheae - ps7
Obazoa - ps8
Opisthokonta - ps9
Holomycota - ps10
Fungi - ps11
Fungi_1 - ps12
Fungi_2 - ps13
Fungi_3 - ps14
Dikarya - ps15
Ascomycota - ps16
Saccharo/Pezizomycotina - ps17
Saccharomycotina - ps18
Saccharomycotina_1 - ps19
Saccharomycotina_2 - ps20
Saccharomycotina_3 - ps21
Phaff./Saccharomycetaceae - ps22
Saccharomyco./mycetaceae - ps23
Saccharomycetaceae - ps24
Saccharomycetaceae_2 - ps25
Saccharomycetaceae_3 - ps26
Kaz./Nau./Saccharomyces - ps27
Saccharomyces - ps28
Arboricola_clade - ps29
Cerevisiae_clade - ps30
Saccharomyces_cerevisiae - ps31
Phylostratum

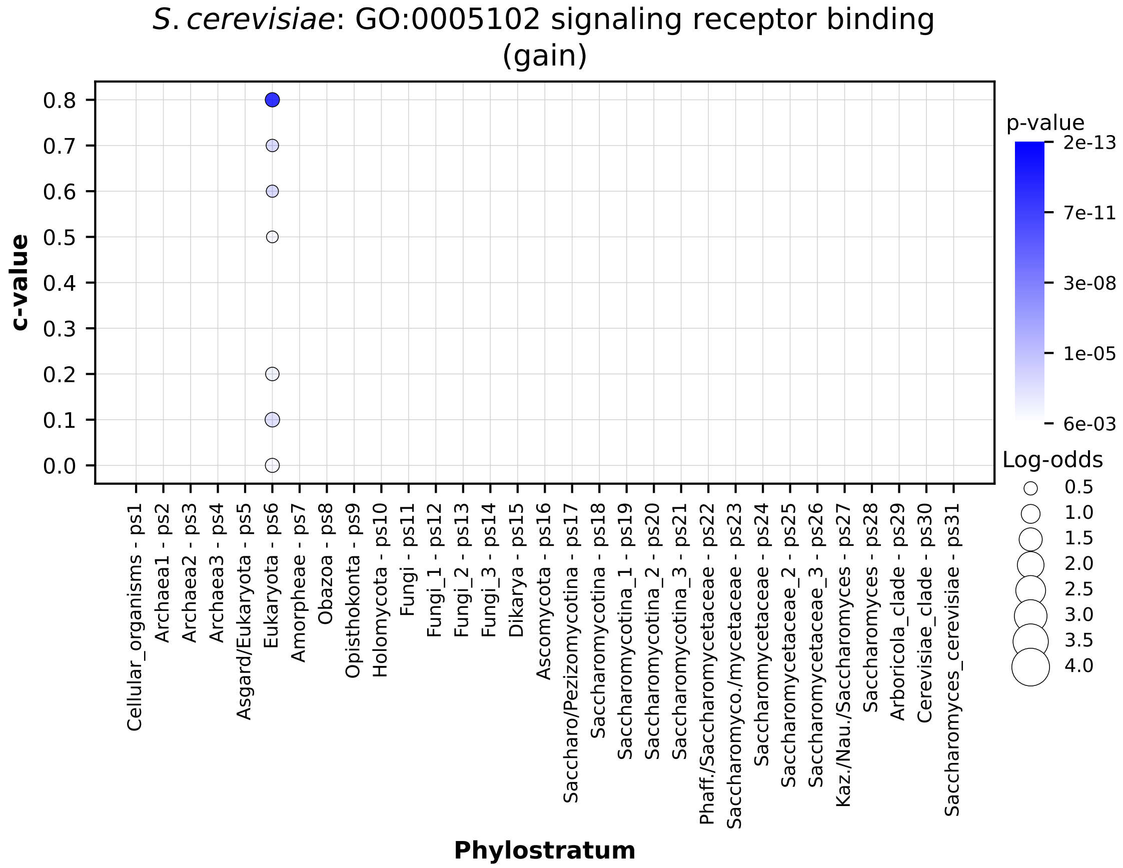

S. cerevisiae: GO:0005102 signaling receptor binding
(gain)
c-value
0.8
0.7
0.6
0.5
0.4
0.3
0.2
0.1
0.0
p-value
2e-13
7e-11
3e-08
1e-05
6e-03
Log-odds
0.5
1.0
1.5
2.0
2.5
3.0
3.5
4.0
Cellular_organisms - ps1
Archaea1 - ps2
Archaea2 - ps3
Archaea3 - ps4
Asgard/Eukaryota - ps5
Eukaryota - ps6
Amorpheae - ps7
Obazoa - ps8
Opisthokonta - ps9
Holomycota - ps10
Fungi - ps11
Fungi_1 - ps12
Fungi_2 - ps13
Fungi_3 - ps14
Dikarya - ps15
Ascomycota - ps16
Saccharo/Pezizomycotina - ps17
Saccharomycotina - ps18
Saccharomycotina_1 - ps19
Saccharomycotina_2 - ps20
Saccharomycotina_3 - ps21
Phaff./Saccharomycetaceae - ps22
Saccharomyco./mycetaceae - ps23
Saccharomycetaceae - ps24
Saccharomycetaceae_2 - ps25
Saccharomycetaceae_3 - ps26
Kaz./Nau./Saccharomyces - ps27
Saccharomyces - ps28
Arboricola_clade - ps29
Cerevisiae_clade - ps30
Saccharomyces_cerevisiae - ps31
Phylostratum

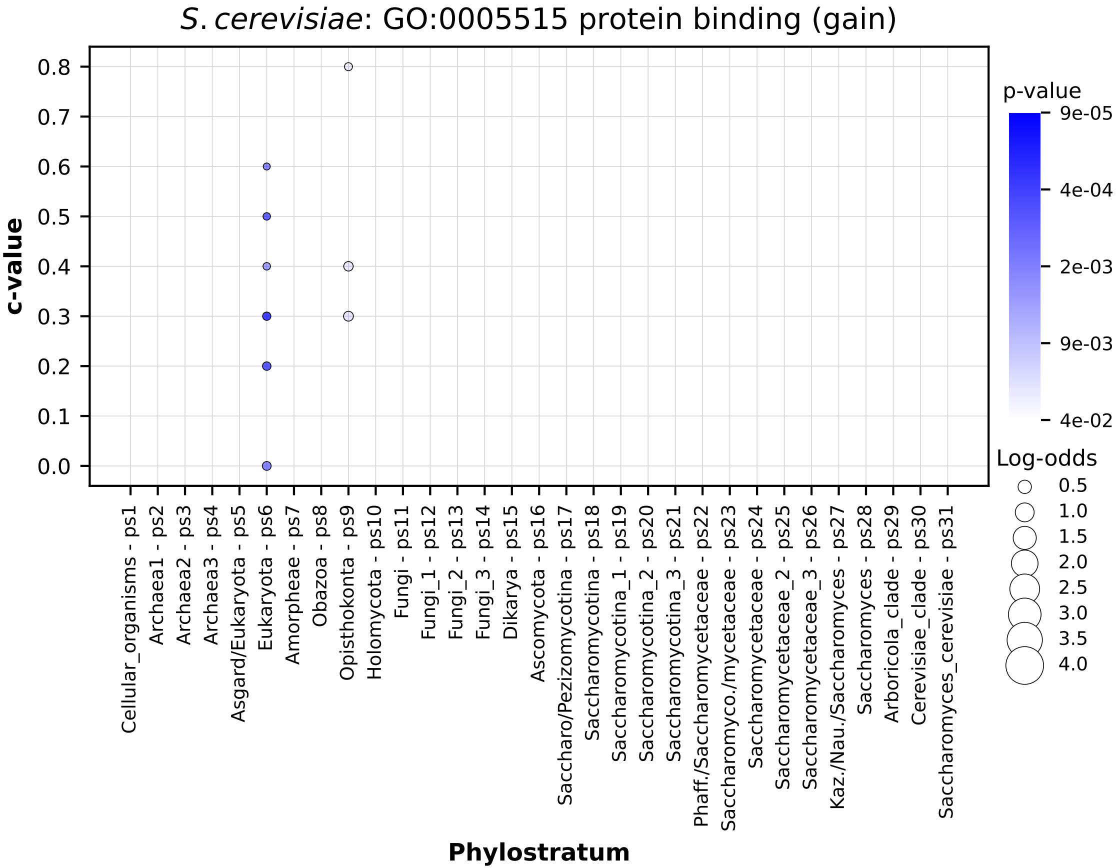

S. cerevisiae: GO:0005515 protein binding (gain)
c-value
0.0
0.1
0.2
0.3
0.4
0.5
0.6
0.7
0.8
Phylostratum
Cellular_organisms - ps1
Archaea1 - ps2
Archaea2 - ps3
Archaea3 - ps4
Asgard/Eukaryota - ps5
Eukaryota - ps6
Amorpheae - ps7
Obazoa - ps8
Opisthokonta - ps9
Holomycota - ps10
Fungi - ps11
Fungi_1 - ps12
Fungi_2 - ps13
Fungi_3 - ps14
Dikarya - ps15
Ascomycota - ps16
Saccharo/Pezizomycotina - ps17
Saccharomycotina - ps18
Saccharomycotina_1 - ps19
Saccharomycotina_2 - ps20
Saccharomycotina_3 - ps21
Phaff./Saccharomycetaceae - ps22
Saccharomyco./mycetaceae - ps23
Saccharomycetaceae - ps24
Saccharomycetaceae_2 - ps25
Saccharomycetaceae_3 - ps26
Kaz./Nau./Saccharomyces - ps27
Saccharomyces - ps28
Arboricola_clade - ps29
Cerevisiae_clade - ps30
Saccharomyces_cerevisiae - ps31
p-value
9e-05
4e-04
2e-03
9e-03
4e-02
Log-odds
0.5
1.0
1.5
2.0
2.5
3.0
3.5
4.0

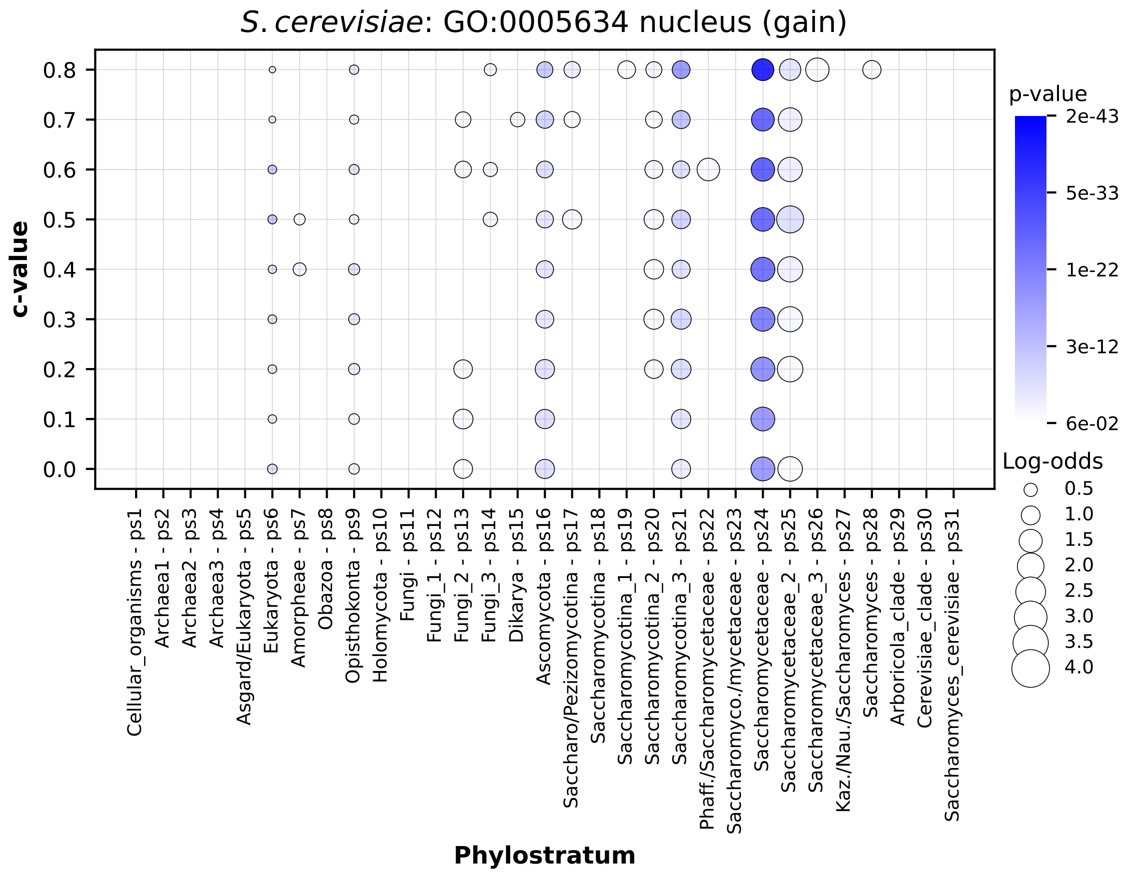

S. cerevisiae: GO:0005634 nucleus (gain)
c-value
0.8
0.7
0.6
0.5
0.4
0.3
0.2
0.1
0.0
p-value
2e-43
5e-33
1e-22
3e-12
6e-02
Log-odds
0.5
1.0
1.5
2.0
2.5
3.0
3.5
4.0
Cellular_organisms - ps1
Archaea1 - ps2
Archaea2 - ps3
Archaea3 - ps4
Asgard/Eukaryota - ps5
Eukaryota - ps6
Amorpheae - ps7
Obazoa - ps8
Opisthokonta - ps9
Holomycota - ps10
Fungi - ps11
Fungi_1 - ps12
Fungi_2 - ps13
Fungi_3 - ps14
Dikarya - ps15
Ascomycota - ps16
Saccharo/Pezizomycotina - ps17
Saccharomycotina - ps18
Saccharomycotina_1 - ps19
Saccharomycotina_2 - ps20
Saccharomycotina_3 - ps21
Phaff./Saccharomycetaceae - ps22
Saccharomyco./mycetaceae - ps23
Saccharomycetaceae - ps24
Saccharomycetaceae_2 - ps25
Saccharomycetaceae_3 - ps26
Kaz./Nau./Saccharomyces - ps27
Saccharomyces - ps28
Arboricola_clade - ps29
Cerevisiae_clade - ps30
Saccharomyces_cerevisiae - ps31
Phylostratum

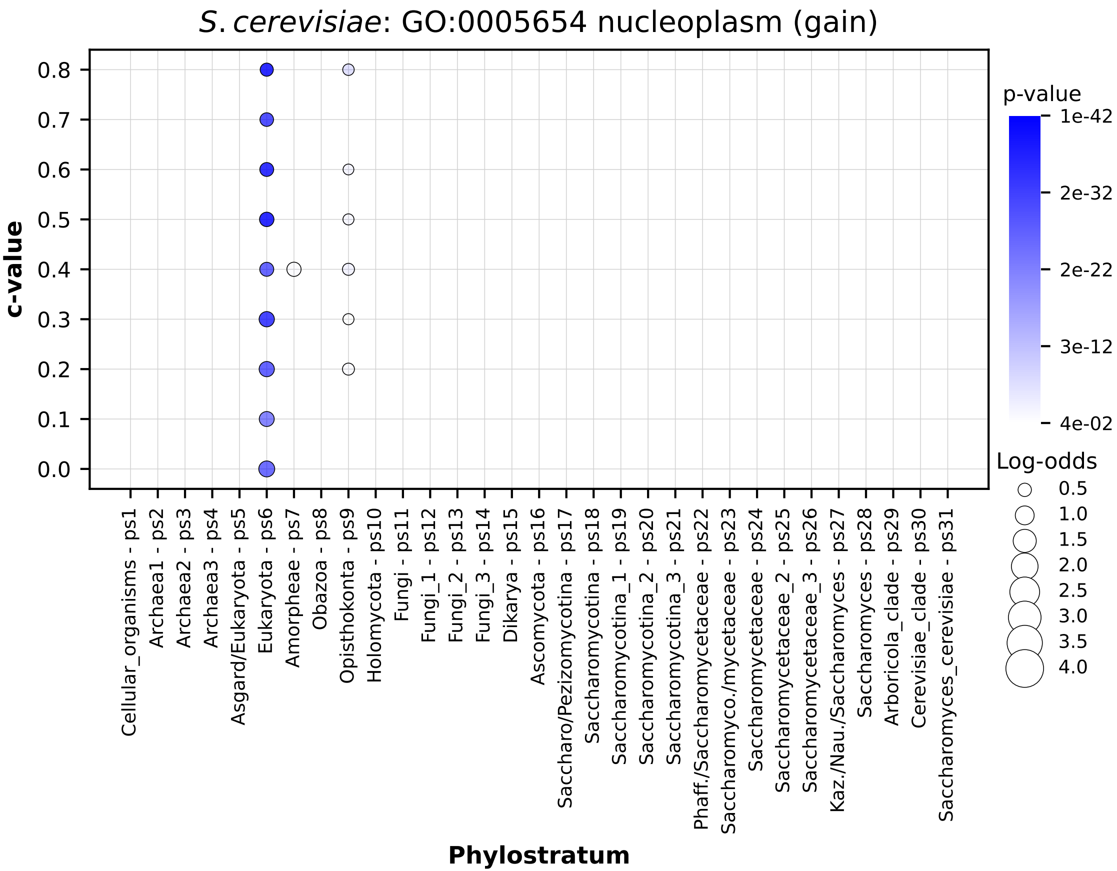

S. cerevisiae: GO:0005654 nucleoplasm (gain)
c-value
0.0
0.1
0.2
0.3
0.4
0.5
0.6
0.7
0.8
p-value
1e-42
2e-32
2e-22
3e-12
4e-02
Log-odds
0.5
1.0
1.5
2.0
2.5
3.0
3.5
4.0
Cellular_organisms - ps1
Archaea1 - ps2
Archaea2 - ps3
Archaea3 - ps4
Asgard/Eukaryota - ps5
Eukaryota - ps6
Amorpheae - ps7
Obazoa - ps8
Opisthokonta - ps9
Holomycota - ps10
Fungi - ps11
Fungi_1 - ps12
Fungi_2 - ps13
Fungi_3 - ps14
Dikarya - ps15
Ascomycota - ps16
Saccharo/Pezizomycotina - ps17
Saccharomycotina - ps18
Saccharomycotina_1 - ps19
Saccharomycotina_2 - ps20
Saccharomycotina_3 - ps21
Phaff./Saccharomycetaceae - ps22
Saccharomyco./mycetaceae - ps23
Saccharomycetaceae - ps24
Saccharomycetaceae_2 - ps25
Saccharomycetaceae_3 - ps26
Kaz./Nau./Saccharomyces - ps27
Saccharomyces - ps28
Arboricola_clade - ps29
Cerevisiae_clade - ps30
Saccharomyces_cerevisiae - ps31
Phylostratum

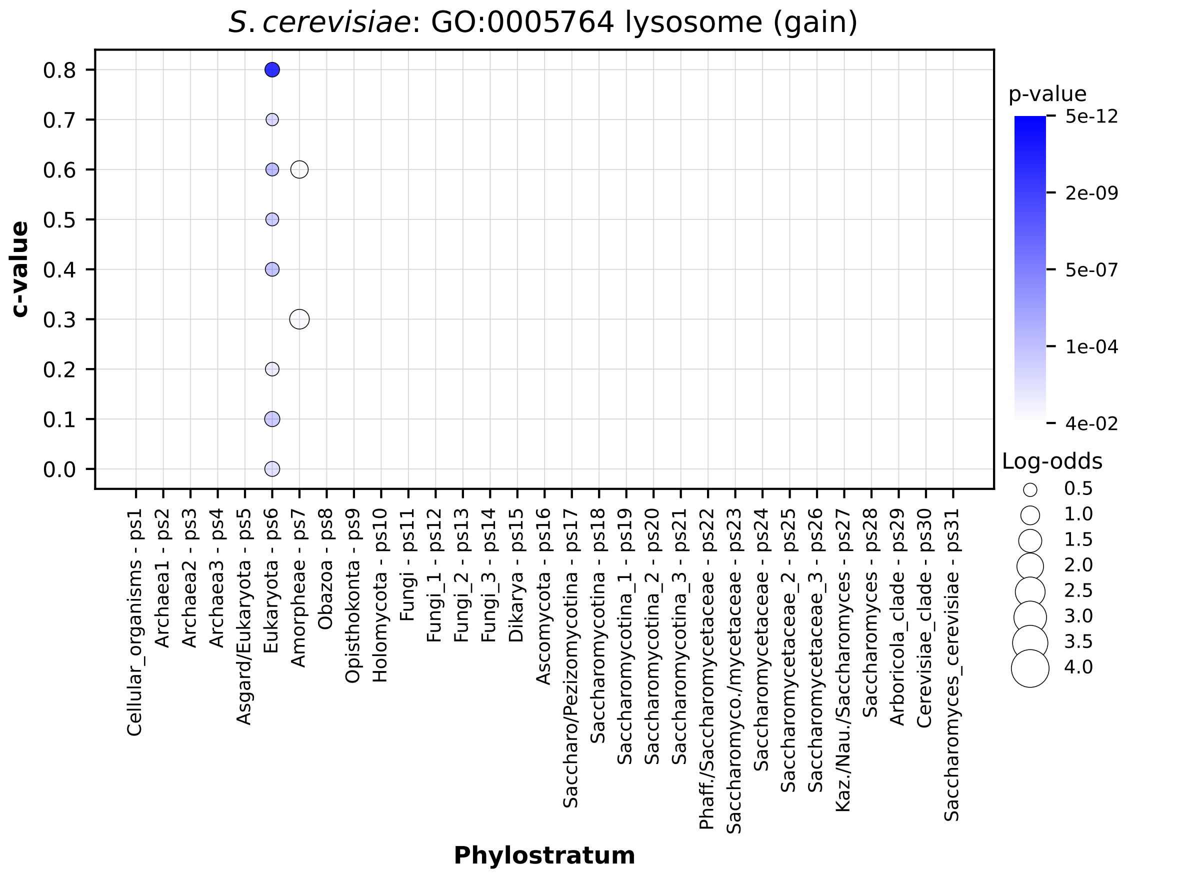

S. cerevisiae: GO:0005764 lysosome (gain)
c-value
0.8
0.7
0.6
0.5
0.4
0.3
0.2
0.1
0.0
p-value
5e-12
2e-09
5e-07
1e-04
4e-02
Log-odds
0.5
1.0
1.5
2.0
2.5
3.0
3.5
4.0
Cellular_organisms - ps1
Archaea1 - ps2
Archaea2 - ps3
Archaea3 - ps4
Asgard/Eukaryota - ps5
Eukaryota - ps6
Amorpheae - ps7
Obazoa - ps8
Opisthokonta - ps9
Holomycota - ps10
Fungi - ps11
Fungi_1 - ps12
Fungi_2 - ps13
Fungi_3 - ps14
Dikarya - ps15
Ascomycota - ps16
Saccharo/Pezizomycotina - ps17
Saccharomycotina - ps18
Saccharomycotina_1 - ps19
Saccharomycotina_2 - ps20
Saccharomycotina_3 - ps21
Phaff./Saccharomycetaceae - ps22
Saccharomyco./mycetaceae - ps23
Saccharomycetaceae - ps24
Saccharomycetaceae_2 - ps25
Saccharomycetaceae_3 - ps26
Kaz./Nau./Saccharomyces - ps27
Saccharomyces - ps28
Arboricola_clade - ps29
Cerevisiae_clade - ps30
Saccharomyces_cerevisiae - ps31
Phylostratum

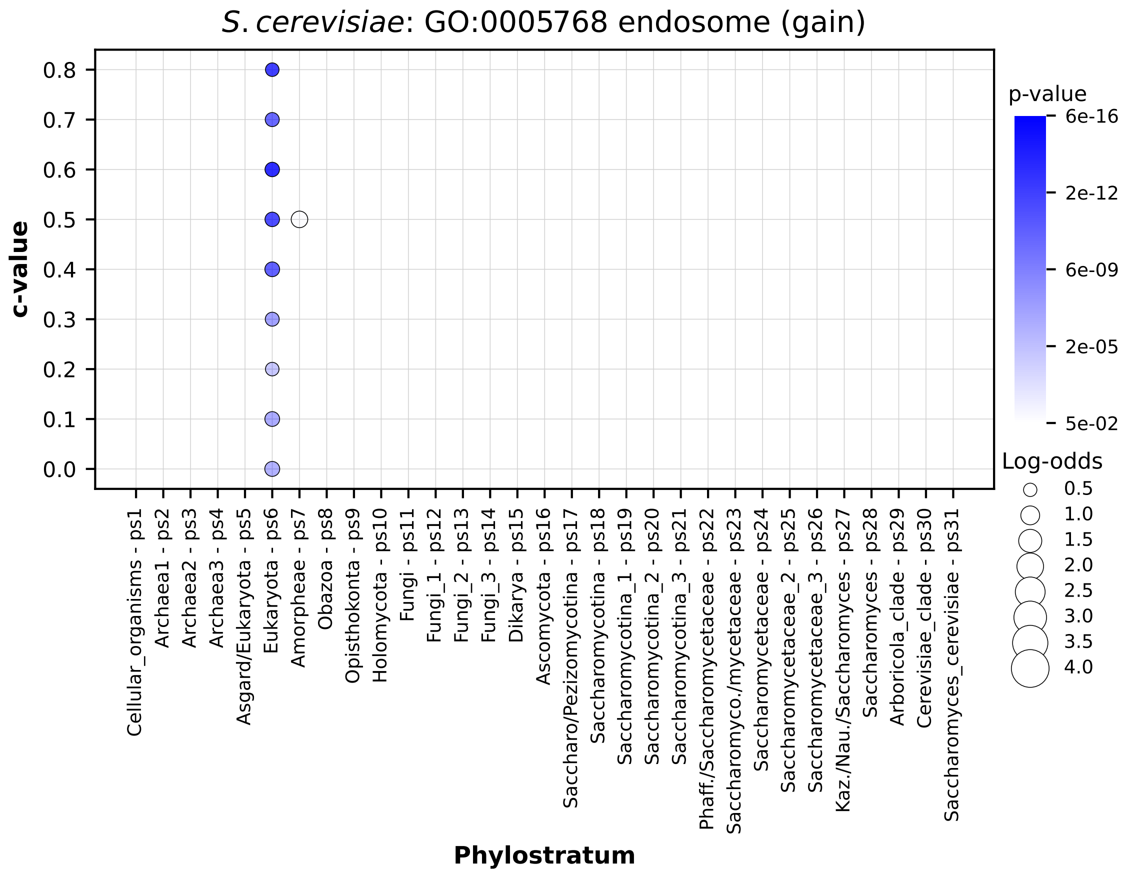
S. cerevisiae: GO:0005768 endosome (gain)
c-value
0.0
0.1
0.2
0.3
0.4
0.5
0.6
0.7
0.8
Phylostratum
Cellular_organisms - ps1
Archaea1 - ps2
Archaea2 - ps3
Archaea3 - ps4
Asgard/Eukaryota - ps5
Eukaryota - ps6
Amorpheae - ps7
Obazoa - ps8
Opisthokonta - ps9
Holomycota - ps10
Fungi - ps11
Fungi_1 - ps12
Fungi_2 - ps13
Fungi_3 - ps14
Dikarya - ps15
Ascomycota - ps16
Saccharo/Pezizomycotina - ps17
Saccharomycotina - ps18
Saccharomycotina_1 - ps19
Saccharomycotina_2 - ps20
Saccharomycotina_3 - ps21
Phaff./Saccharomycetaceae - ps22
Saccharomyco./mycetaceae - ps23
Saccharomycetaceae - ps24
Saccharomycetaceae_2 - ps25
Saccharomycetaceae_3 - ps26
Kaz./Nau./Saccharomyces - ps27
Saccharomyces - ps28
Arboricola_clade - ps29
Cerevisiae_clade - ps30
Saccharomyces_cerevisiae - ps31
p-value
6e-16
2e-12
6e-09
2e-05
5e-02
Log-odds
0.5
1.0
1.5
2.0
2.5
3.0
3.5
4.0

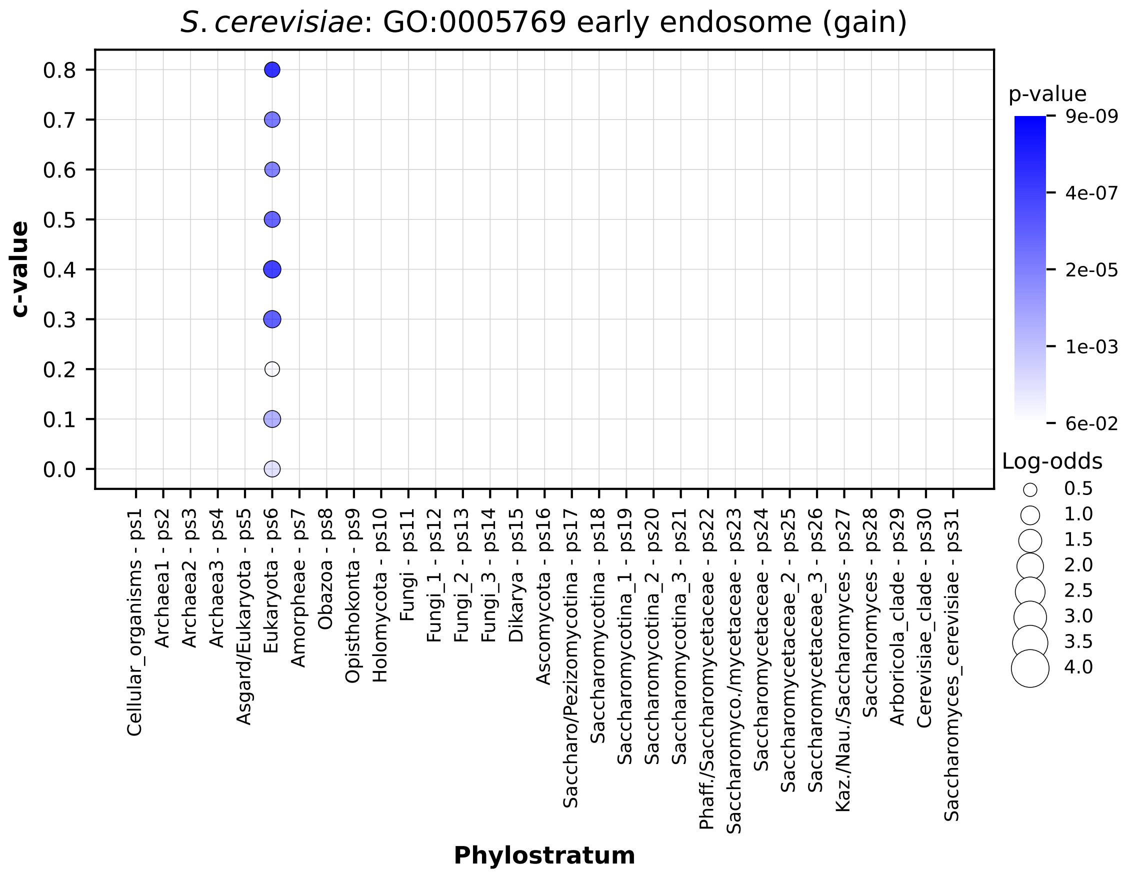

S. cerevisiae: GO:0005769 early endosome (gain)
c-value
0.8
0.7
0.6
0.5
0.4
0.3
0.2
0.1
0.0
p-value
9e-09
4e-07
2e-05
1e-03
6e-02
Log-odds
0.5
1.0
1.5
2.0
2.5
3.0
3.5
4.0
Cellular_organisms - ps1
Archaea1 - ps2
Archaea2 - ps3
Archaea3 - ps4
Asgard/Eukaryota - ps5
Eukaryota - ps6
Amorpheae - ps7
Obazoa - ps8
Opisthokonta - ps9
Holomycota - ps10
Fungi - ps11
Fungi_1 - ps12
Fungi_2 - ps13
Fungi_3 - ps14
Dikarya - ps15
Ascomycota - ps16
Saccharo/Pezizomycotina - ps17
Saccharomycotina - ps18
Saccharomycotina_1 - ps19
Saccharomycotina_2 - ps20
Saccharomycotina_3 - ps21
Phaff./Saccharomycetaceae - ps22
Saccharomyco./mycetaceae - ps23
Saccharomycetaceae - ps24
Saccharomycetaceae_2 - ps25
Saccharomycetaceae_3 - ps26
Kaz./Nau./Saccharomyces - ps27
Saccharomyces - ps28
Arboricola_clade - ps29
Cerevisiae_clade - ps30
Saccharomyces_cerevisiae - ps31
Phylostratum

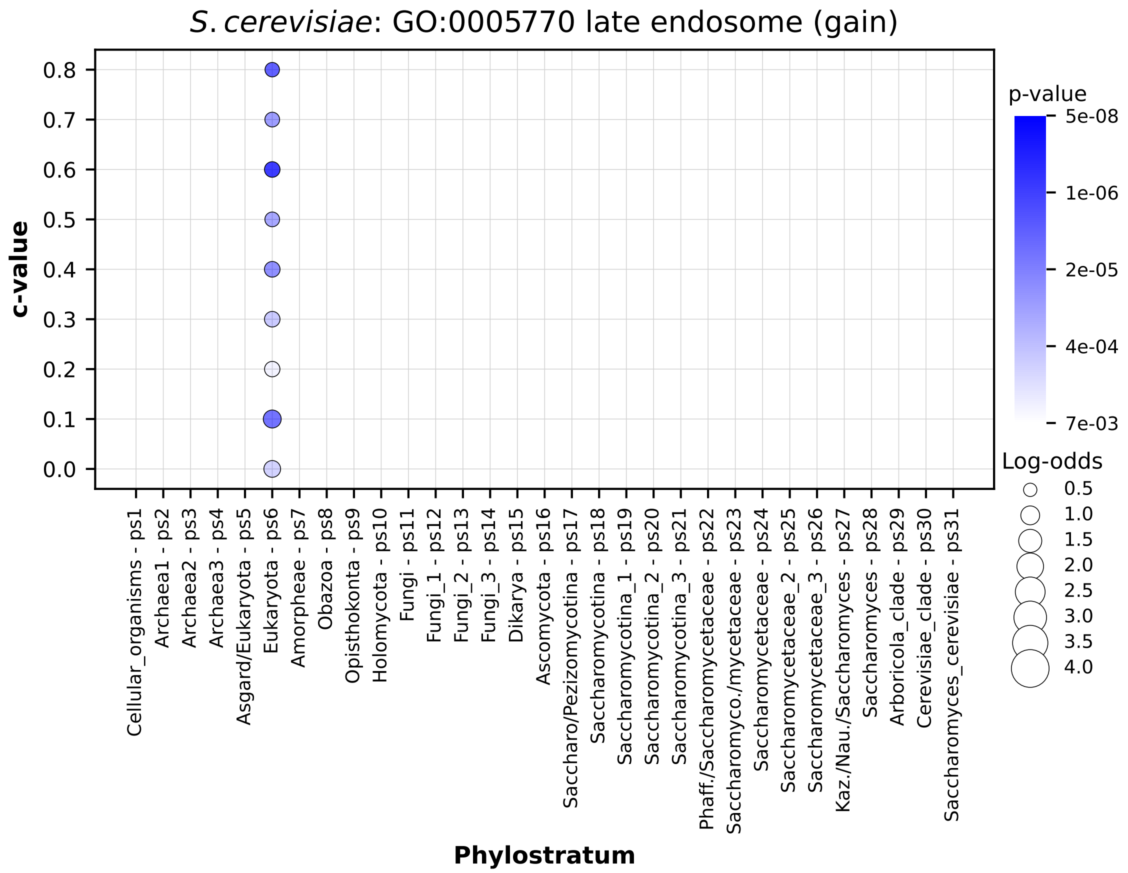
S. cerevisiae: GO:0005770 late endosome (gain)
c-value
0.0
0.1
0.2
0.3
0.4
0.5
0.6
0.7
0.8
p-value
5e-08
1e-06
2e-05
4e-04
7e-03
Log-odds
0.5
1.0
1.5
2.0
2.5
3.0
3.5
4.0
Cellular_organisms - ps1
Archaea1 - ps2
Archaea2 - ps3
Archaea3 - ps4
Asgard/Eukaryota - ps5
Eukaryota - ps6
Amorpheae - ps7
Obazoa - ps8
Opisthokonta - ps9
Holomycota - ps10
Fungi - ps11
Fungi_1 - ps12
Fungi_2 - ps13
Fungi_3 - ps14
Dikarya - ps15
Ascomycota - ps16
Saccharo/Pezizomycotina - ps17
Saccharomycotina - ps18
Saccharomycotina_1 - ps19
Saccharomycotina_2 - ps20
Saccharomycotina_3 - ps21
Phaff./Saccharomycetaceae - ps22
Saccharomyco./mycetaceae - ps23
Saccharomycetaceae - ps24
Saccharomycetaceae_2 - ps25
Saccharomycetaceae_3 - ps26
Kaz./Nau./Saccharomyces - ps27
Saccharomyces - ps28
Arboricola_clade - ps29
Cerevisiae_clade - ps30
Saccharomyces_cerevisiae - ps31
Phylostratum

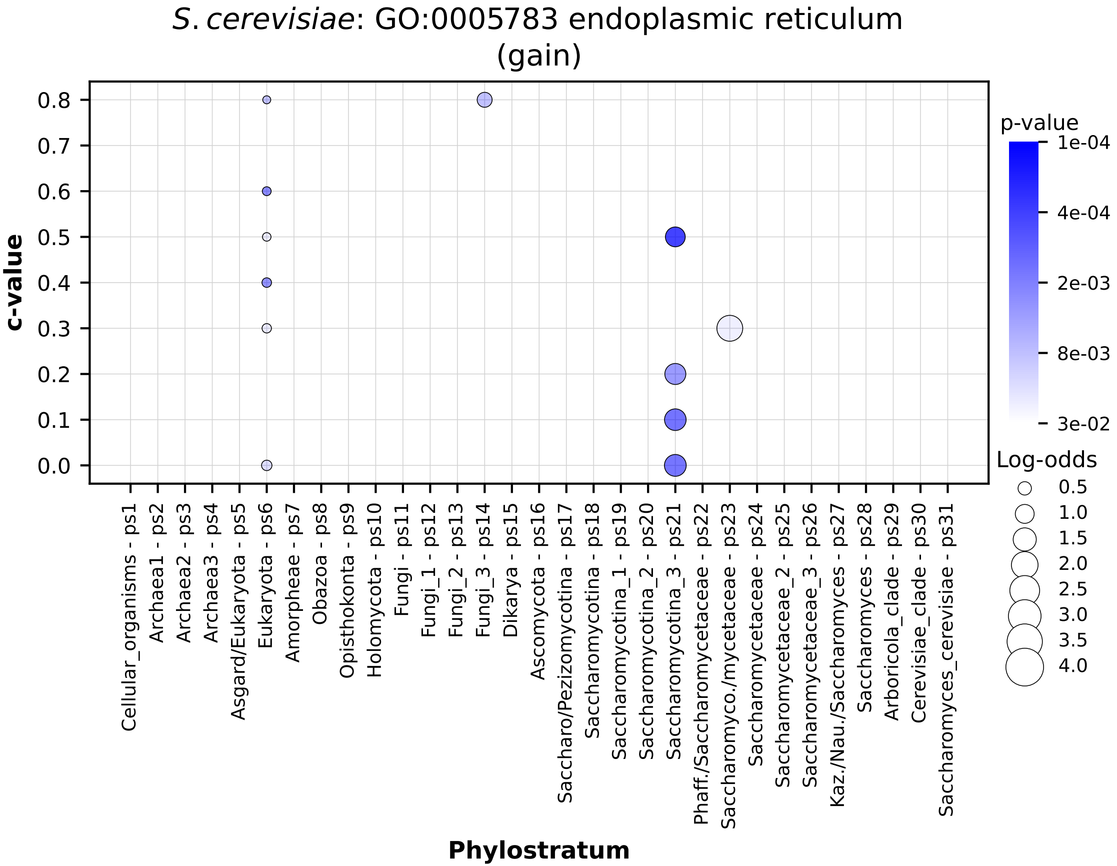

S. cerevisiae: GO:0005783 endoplasmic reticulum
(gain)
c-value
0.8
0.7
0.6
0.5
0.4
0.3
0.2
0.1
0.0
p-value
1e-04
4e-04
2e-03
8e-03
3e-02
Log-odds
0.5
1.0
1.5
2.0
2.5
3.0
3.5
4.0
Cellular_organisms - ps1
Archaea1 - ps2
Archaea2 - ps3
Archaea3 - ps4
Asgard/Eukaryota - ps5
Eukaryota - ps6
Amorpheae - ps7
Obazoa - ps8
Opisthokonta - ps9
Holomycota - ps10
Fungi - ps11
Fungi_1 - ps12
Fungi_2 - ps13
Fungi_3 - ps14
Dikarya - ps15
Ascomycota - ps16
Saccharo/Pezizomycotina - ps17
Saccharomycotina - ps18
Saccharomycotina_1 - ps19
Saccharomycotina_2 - ps20
Saccharomycotina_3 - ps21
Phaff./Saccharomycetaceae - ps22
Saccharomyco./mycetaceae - ps23
Saccharomycetaceae - ps24
Saccharomycetaceae_2 - ps25
Saccharomycetaceae_3 - ps26
Kaz./Nau./Saccharomyces - ps27
Saccharomyces - ps28
Arboricola_clade - ps29
Cerevisiae_clade - ps30
Saccharomyces_cerevisiae - ps31
Phylostratum

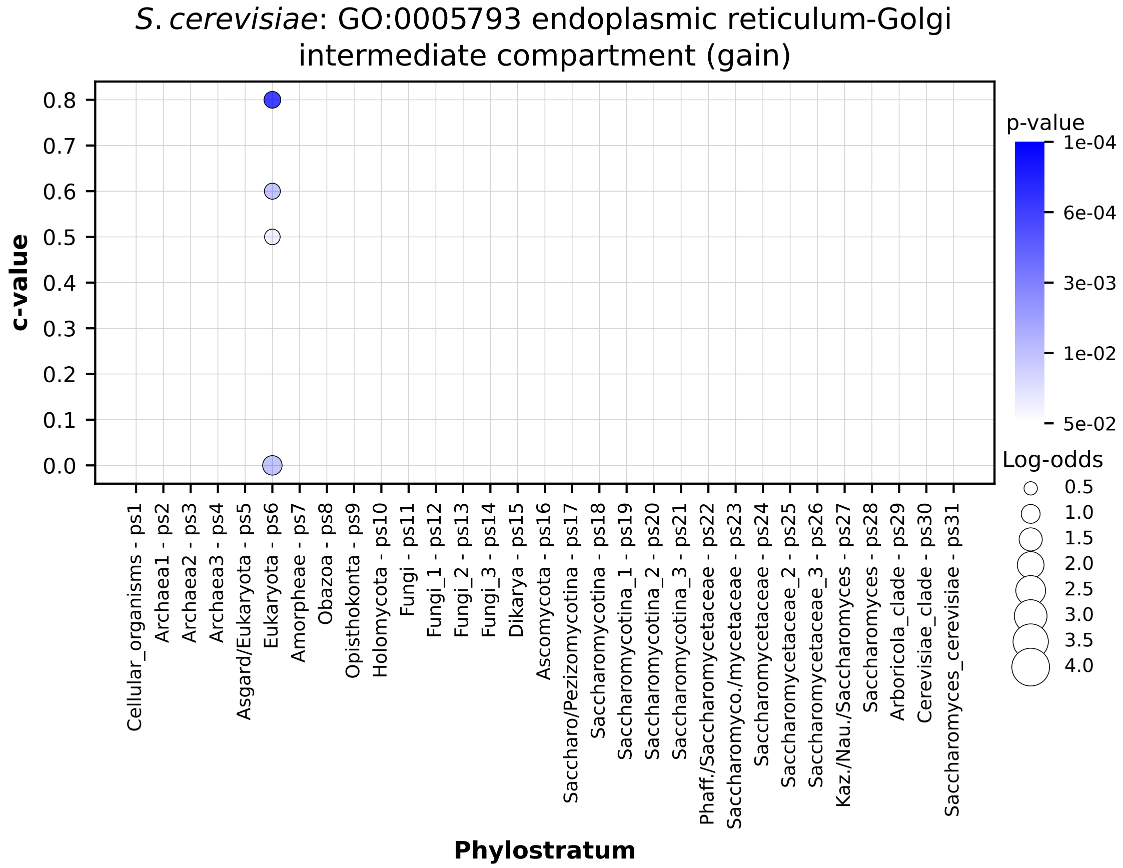

S. cerevisiae: GO:0005793 endoplasmic reticulum-Golgi intermediate compartment (gain)
c-value
0.0
0.1
0.2
0.3
0.4
0.5
0.6
0.7
0.8
p-value
1e-04
6e-04
3e-03
1e-02
5e-02
Log-odds
0.5
1.0
1.5
2.0
2.5
3.0
3.5
4.0
Cellular_organisms - ps1
Archaea1 - ps2
Archaea2 - ps3
Archaea3 - ps4
Asgard/Eukaryota - ps5
Eukaryota - ps6
Amorpheae - ps7
Obazoa - ps8
Opisthokonta - ps9
Holomycota - ps10
Fungi - ps11
Fungi_1 - ps12
Fungi_2 - ps13
Fungi_3 - ps14
Dikarya - ps15
Ascomycota - ps16
Saccharo/Pezizomycotina - ps17
Saccharomycotina - ps18
Saccharomycotina_1 - ps19
Saccharomycotina_2 - ps20
Saccharomycotina_3 - ps21
Phaff./Saccharomycetaceae - ps22
Saccharomyco./mycetaceae - ps23
Saccharomycetaceae - ps24
Saccharomycetaceae_2 - ps25
Saccharomycetaceae_3 - ps26
Kaz./Nau./Saccharomyces - ps27
Saccharomyces - ps28
Arboricola_clade - ps29
Cerevisiae_clade - ps30
Saccharomyces_cerevisiae - ps31
Phylostratum

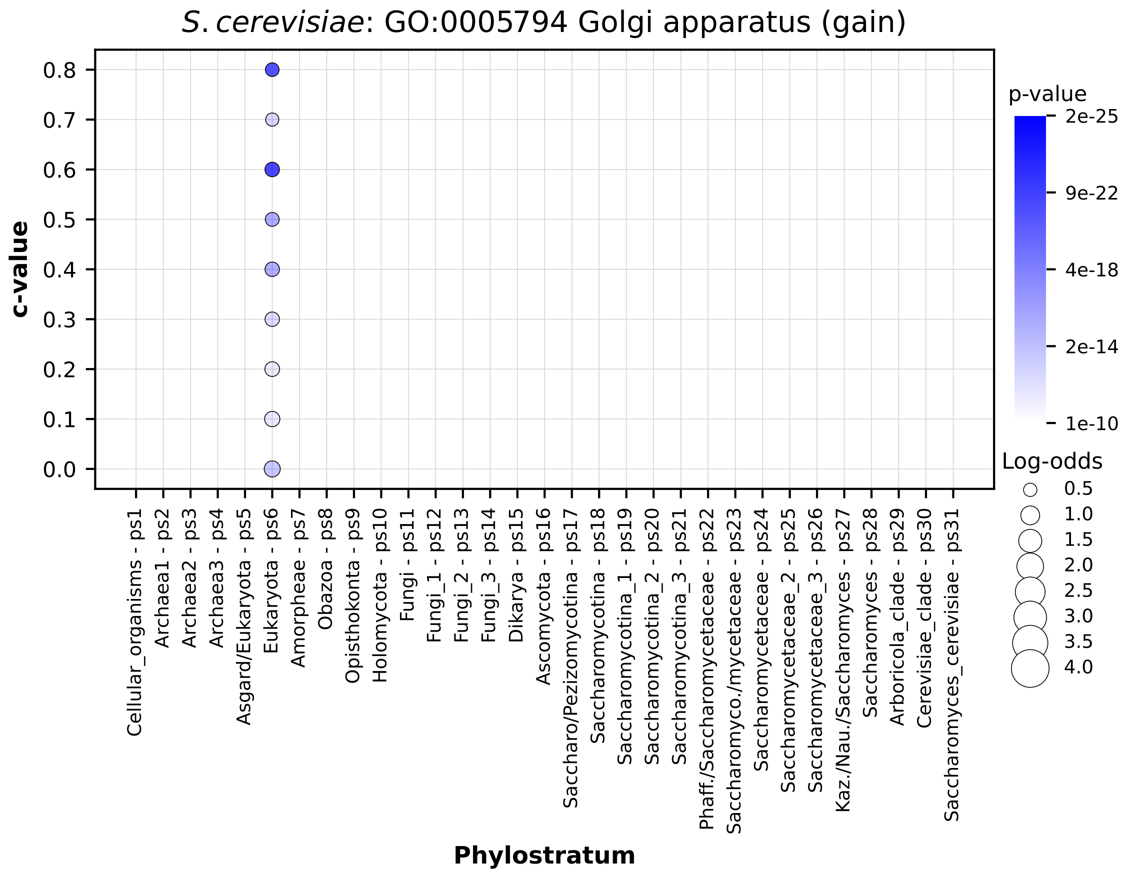
S. cerevisiae: GO:0005794 Golgi apparatus (gain)
c-value
0.8
0.7
0.6
0.5
0.4
0.3
0.2
0.1
0.0
Phylostratum
Cellular_organisms - ps1
Archaea1 - ps2
Archaea2 - ps3
Archaea3 - ps4
Asgard/Eukaryota - ps5
Eukaryota - ps6
Amorpheae - ps7
Obazoa - ps8
Opisthokonta - ps9
Holomycota - ps10
Fungi - ps11
Fungi_1 - ps12
Fungi_2 - ps13
Fungi_3 - ps14
Dikarya - ps15
Ascomycota - ps16
Saccharo/Pezizomycotina - ps17
Saccharomycotina - ps18
Saccharomycotina_1 - ps19
Saccharomycotina_2 - ps20
Saccharomycotina_3 - ps21
Phaff./Saccharomycetaceae - ps22
Saccharomyco./mycetaceae - ps23
Saccharomycetaceae - ps24
Saccharomycetaceae_2 - ps25
Saccharomycetaceae_3 - ps26
Kaz./Nau./Saccharomyces - ps27
Saccharomyces - ps28
Arboricola_clade - ps29
Cerevisiae_clade - ps30
Saccharomyces_cerevisiae - ps31
p-value
2e-25
9e-22
4e-18
2e-14
1e-10
Log-odds
0.5
1.0
1.5
2.0
2.5
3.0
3.5
4.0

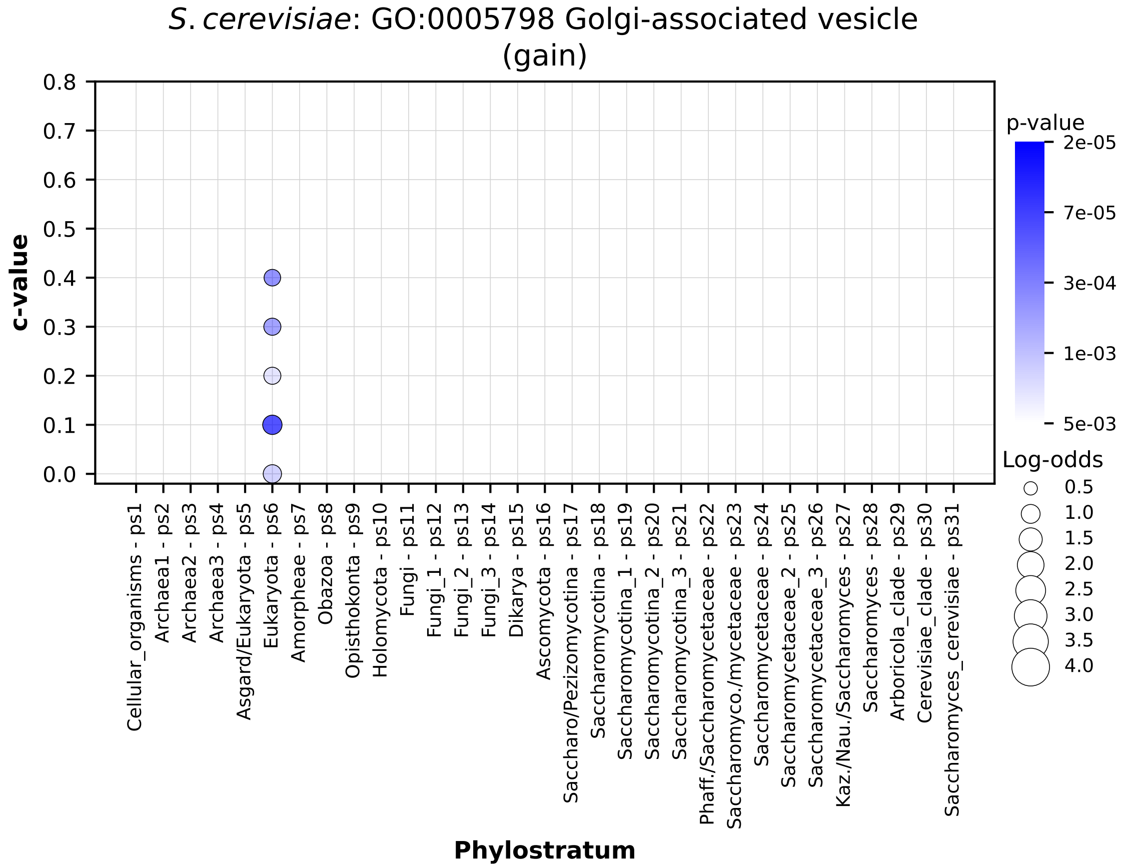
S. cerevisiae: GO:0005798 Golgi-associated vesicle
(gain)
c-value
0.8
0.7
0.6
0.5
0.4
0.3
0.2
0.1
0.0
p-value
2e-05
7e-05
3e-04
1e-03
5e-03
Log-odds
0.5
1.0
1.5
2.0
2.5
3.0
3.5
4.0
Cellular_organisms - ps1
Archaea1 - ps2
Archaea2 - ps3
Archaea3 - ps4
Asgard/Eukaryota - ps5
Eukaryota - ps6
Amorpheae - ps7
Obazoa - ps8
Opisthokonta - ps9
Holomycota - ps10
Fungi - ps11
Fungi_1 - ps12
Fungi_2 - ps13
Fungi_3 - ps14
Dikarya - ps15
Ascomycota - ps16
Saccharo/Pezizomycotina - ps17
Saccharomycotina - ps18
Saccharomycotina_1 - ps19
Saccharomycotina_2 - ps20
Saccharomycotina_3 - ps21
Phaff./Saccharomycetaceae - ps22
Saccharomyco./mycetaceae - ps23
Saccharomycetaceae - ps24
Saccharomycetaceae_2 - ps25
Saccharomycetaceae_3 - ps26
Kaz./Nau./Saccharomyces - ps27
Saccharomyces - ps28
Arboricola_clade - ps29
Cerevisiae_clade - ps30
Saccharomyces_cerevisiae - ps31
Phylostratum

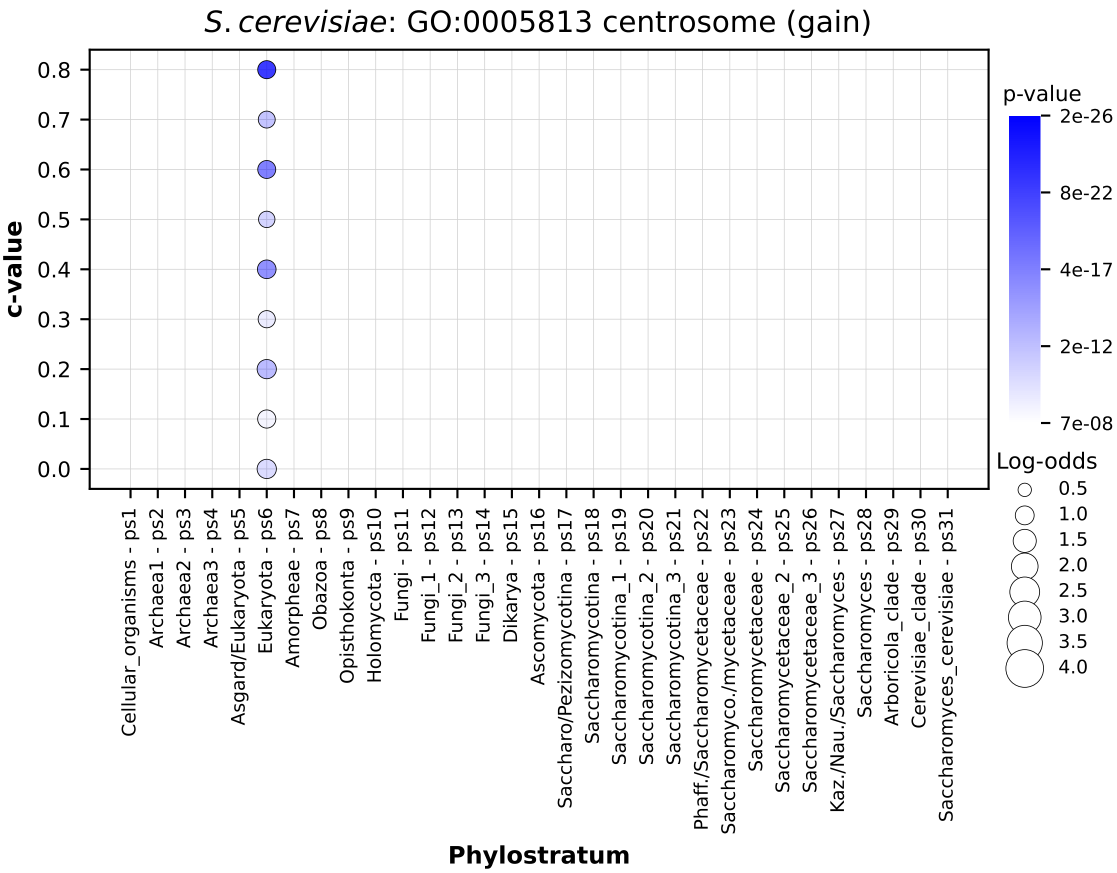

S. cerevisiae: GO:0005813 centrosome (gain)
c-value
0.8
0.7
0.6
0.5
0.4
0.3
0.2
0.1
0.0
p-value
2e-26
8e-22
4e-17
2e-12
7e-08
Log-odds
0.5
1.0
1.5
2.0
2.5
3.0
3.5
4.0
Cellular_organisms - ps1
Archaea1 - ps2
Archaea2 - ps3
Archaea3 - ps4
Asgard/Eukaryota - ps5
Eukaryota - ps6
Amorpheae - ps7
Obazoa - ps8
Opisthokonta - ps9
Holomycota - ps10
Fungi - ps11
Fungi_1 - ps12
Fungi_2 - ps13
Fungi_3 - ps14
Dikarya - ps15
Ascomycota - ps16
Saccharo/Pezizomycotina - ps17
Saccharomycotina - ps18
Saccharomycotina_1 - ps19
Saccharomycotina_2 - ps20
Saccharomycotina_3 - ps21
Phaff./Saccharomycetaceae - ps22
Saccharomyco./mycetaceae - ps23
Saccharomycetaceae - ps24
Saccharomycetaceae_2 - ps25
Saccharomycetaceae_3 - ps26
Kaz./Nau./Saccharomyces - ps27
Saccharomyces - ps28
Arboricola_clade - ps29
Cerevisiae_clade - ps30
Saccharomyces_cerevisiae - ps31
Phylostratum

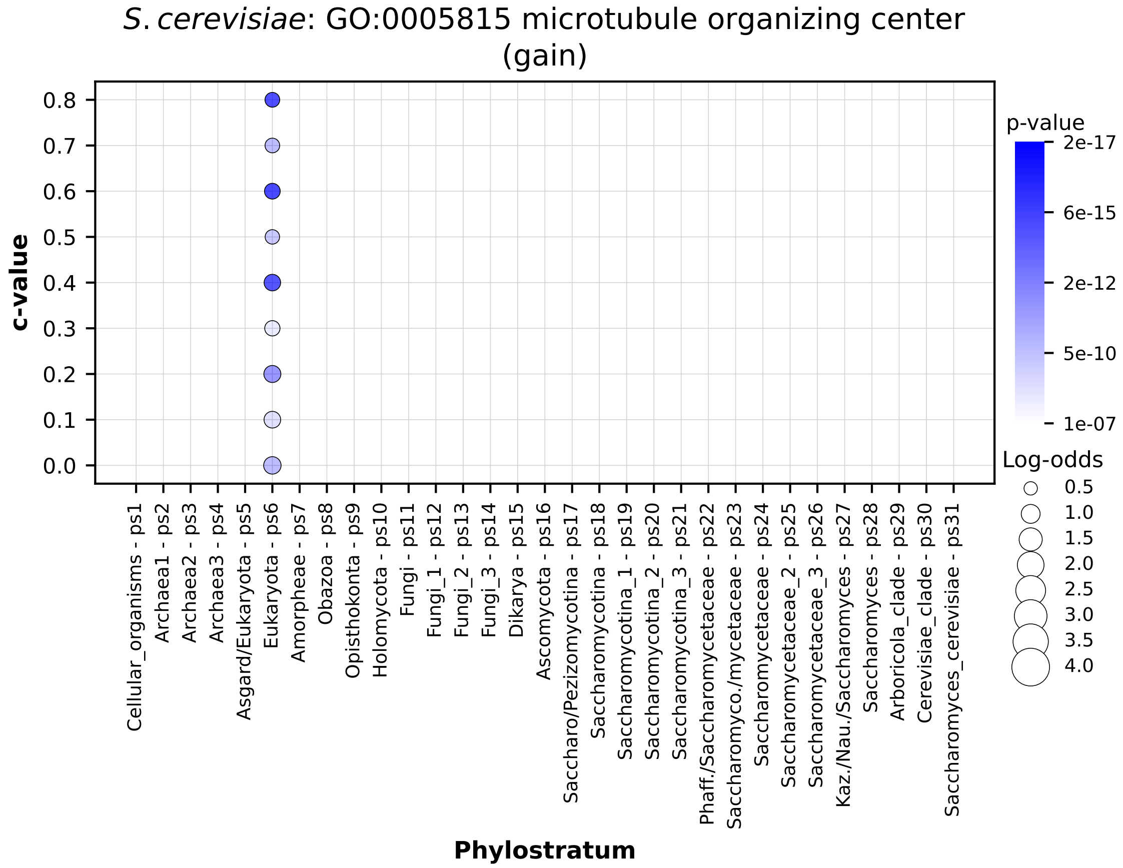

S. cerevisiae: GO:0005815 microtubule organizing center (gain)
c-value
0.8
0.7
0.6
0.5
0.4
0.3
0.2
0.1
0.0
Phylostratum
Cellular_organisms - ps1
Archaea1 - ps2
Archaea2 - ps3
Archaea3 - ps4
Asgard/Eukaryota - ps5
Eukaryota - ps6
Amorpheae - ps7
Obazoa - ps8
Opisthokonta - ps9
Holomycota - ps10
Fungi - ps11
Fungi_1 - ps12
Fungi_2 - ps13
Fungi_3 - ps14
Dikarya - ps15
Ascomycota - ps16
Saccharo/Pezizomycotina - ps17
Saccharomycotina - ps18
Saccharomycotina_1 - ps19
Saccharomycotina_2 - ps20
Saccharomycotina_3 - ps21
Phaff./Saccharomycetaceae - ps22
Saccharomyco./mycetaceae - ps23
Saccharomycetaceae - ps24
Saccharomycetaceae_2 - ps25
Saccharomycetaceae_3 - ps26
Kaz./Nau./Saccharomyces - ps27
Saccharomyces - ps28
Arboricola_clade - ps29
Cerevisiae_clade - ps30
Saccharomyces_cerevisiae - ps31
p-value
2e-17
6e-15
2e-12
5e-10
1e-07
Log-odds
0.5
1.0
1.5
2.0
2.5
3.0
3.5
4.0

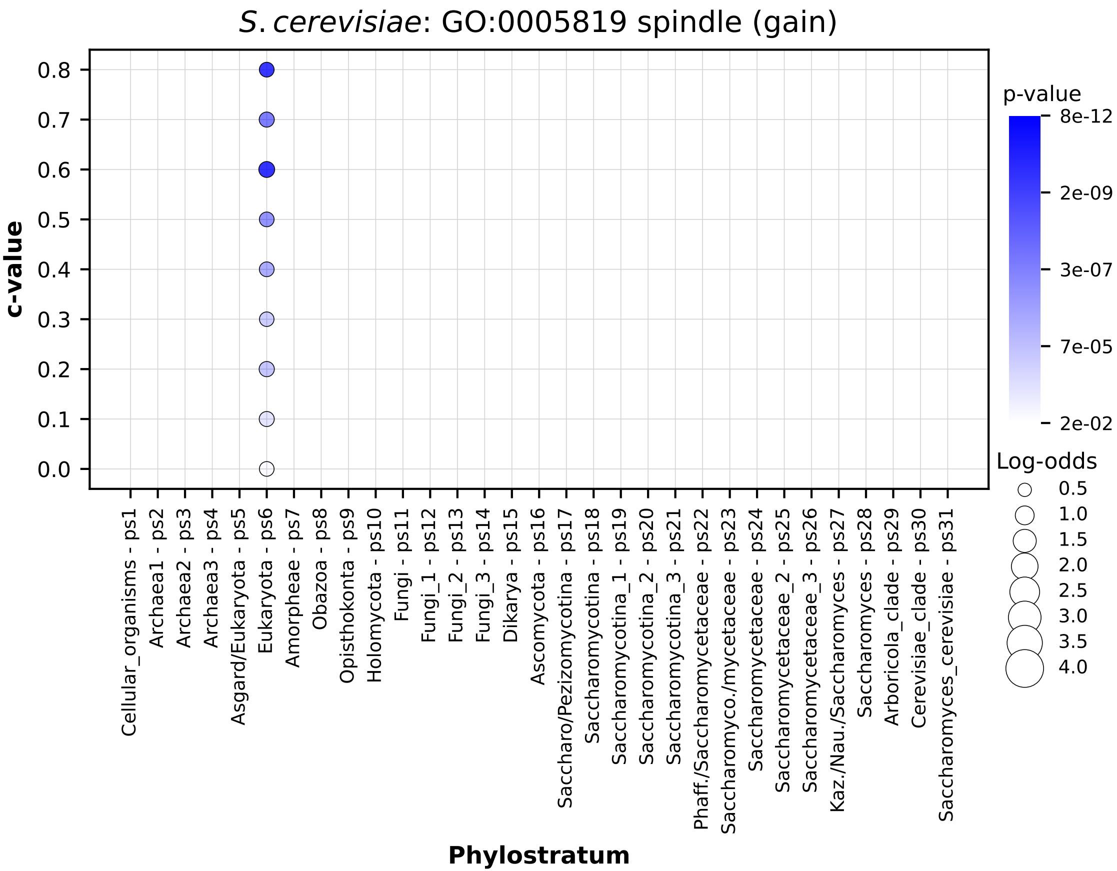

S. cerevisiae: GO:0005819 spindle (gain)
c-value
0.0
0.1
0.2
0.3
0.4
0.5
0.6
0.7
0.8
p-value
8e-12
2e-09
3e-07
7e-05
2e-02
Log-odds
0.5
1.0
1.5
2.0
2.5
3.0
3.5
4.0
Cellular_organisms - ps1
Archaea1 - ps2
Archaea2 - ps3
Archaea3 - ps4
Asgard/Eukaryota - ps5
Eukaryota - ps6
Amorpheae - ps7
Obazoa - ps8
Opisthokonta - ps9
Holomycota - ps10
Fungi - ps11
Fungi_1 - ps12
Fungi_2 - ps13
Fungi_3 - ps14
Dikarya - ps15
Ascomycota - ps16
Saccharo/Pezizomycotina - ps17
Saccharomycotina - ps18
Saccharomycotina_1 - ps19
Saccharomycotina_2 - ps20
Saccharomycotina_3 - ps21
Phaff./Saccharomycetaceae - ps22
Saccharomyco./mycetaceae - ps23
Saccharomycetaceae - ps24
Saccharomycetaceae_2 - ps25
Saccharomycetaceae_3 - ps26
Kaz./Nau./Saccharomyces - ps27
Saccharomyces - ps28
Arboricola_clade - ps29
Cerevisiae_clade - ps30
Saccharomyces_cerevisiae - ps31
Phylostratum

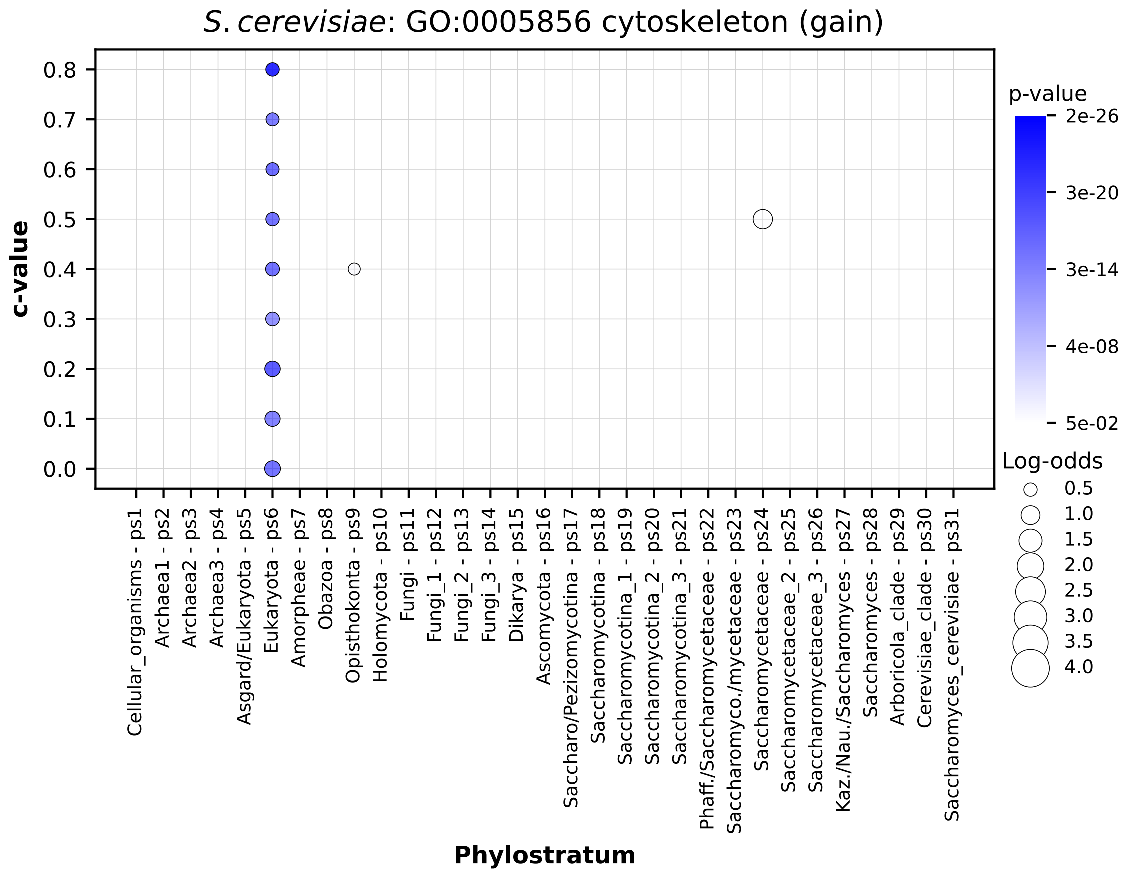

S. cerevisiae: GO:0005856 cytoskeleton (gain)
c-value
0.0
0.1
0.2
0.3
0.4
0.5
0.6
0.7
0.8
p-value
2e-26
3e-20
3e-14
4e-08
5e-02
Log-odds
0.5
1.0
1.5
2.0
2.5
3.0
3.5
4.0
Cellular_organisms - ps1
Archaea1 - ps2
Archaea2 - ps3
Archaea3 - ps4
Asgard/Eukaryota - ps5
Eukaryota - ps6
Amorpheae - ps7
Obazoa - ps8
Opisthokonta - ps9
Holomycota - ps10
Fungi - ps11
Fungi_1 - ps12
Fungi_2 - ps13
Fungi_3 - ps14
Dikarya - ps15
Ascomycota - ps16
Saccharo/Pezizomycotina - ps17
Saccharomycotina - ps18
Saccharomycotina_1 - ps19
Saccharomycotina_2 - ps20
Saccharomycotina_3 - ps21
Phaff./Saccharomycetaceae - ps22
Saccharomyco./mycetaceae - ps23
Saccharomycetaceae - ps24
Saccharomycetaceae_2 - ps25
Saccharomycetaceae_3 - ps26
Kaz./Nau./Saccharomyces - ps27
Saccharomyces - ps28
Arboricola_clade - ps29
Cerevisiae_clade - ps30
Saccharomyces_cerevisiae - ps31
Phylostratum

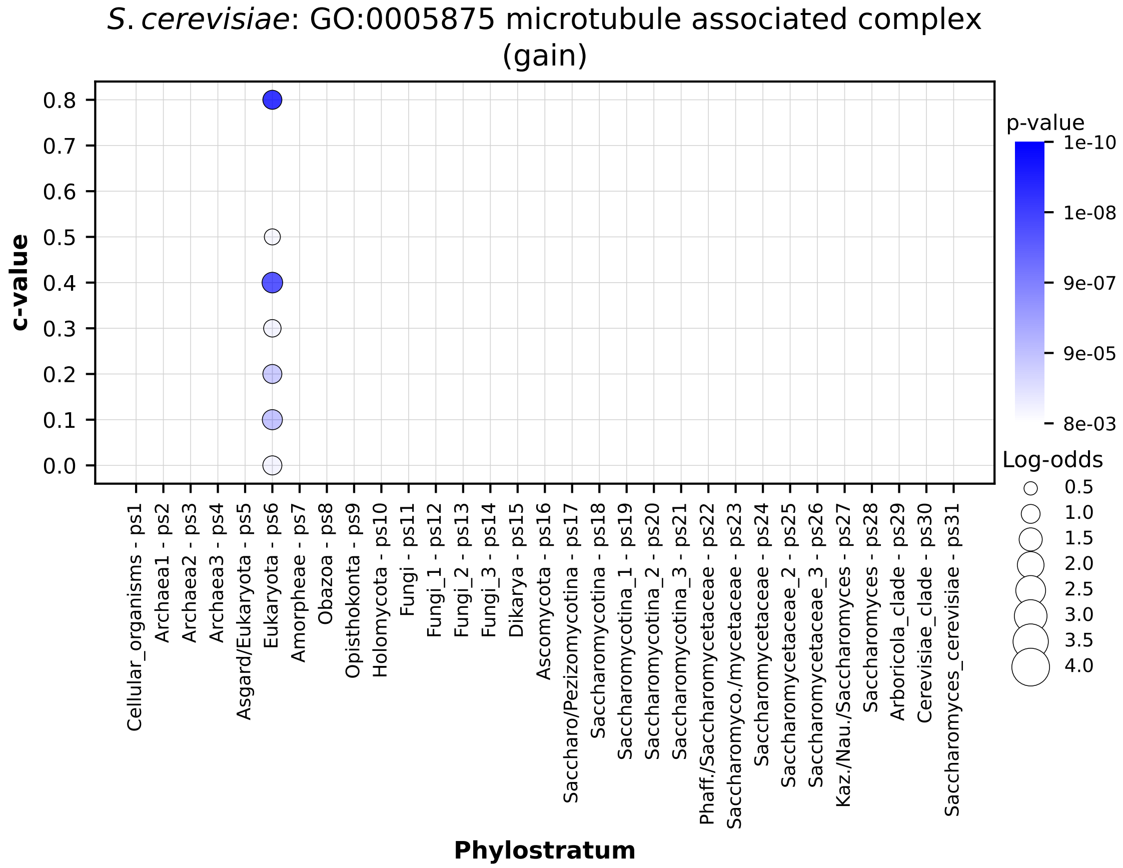

S. cerevisiae: GO:0005875 microtubule associated complex (gain)
c-value
0.8
0.7
0.6
0.5
0.4
0.3
0.2
0.1
0.0
p-value
1e-10
1e-08
9e-07
9e-05
8e-03
Log-odds
0.5
1.0
1.5
2.0
2.5
3.0
3.5
4.0
Cellular_organisms - ps1
Archaea1 - ps2
Archaea2 - ps3
Archaea3 - ps4
Asgard/Eukaryota - ps5
Eukaryota - ps6
Amorpheae - ps7
Obazoa - ps8
Opisthokonta - ps9
Holomycota - ps10
Fungi - ps11
Fungi_1 - ps12
Fungi_2 - ps13
Fungi_3 - ps14
Dikarya - ps15
Ascomycota - ps16
Saccharo/Pezizomycotina - ps17
Saccharomycotina - ps18
Saccharomycotina_1 - ps19
Saccharomycotina_2 - ps20
Saccharomycotina_3 - ps21
Phaff./Saccharomycetaceae - ps22
Saccharomyco./mycetaceae - ps23
Saccharomycetaceae - ps24
Saccharomycetaceae_2 - ps25
Saccharomycetaceae_3 - ps26
Kaz./Nau./Saccharomyces - ps27
Saccharomyces - ps28
Arboricola_clade - ps29
Cerevisiae_clade - ps30
Saccharomyces_cerevisiae - ps31
Phylostratum

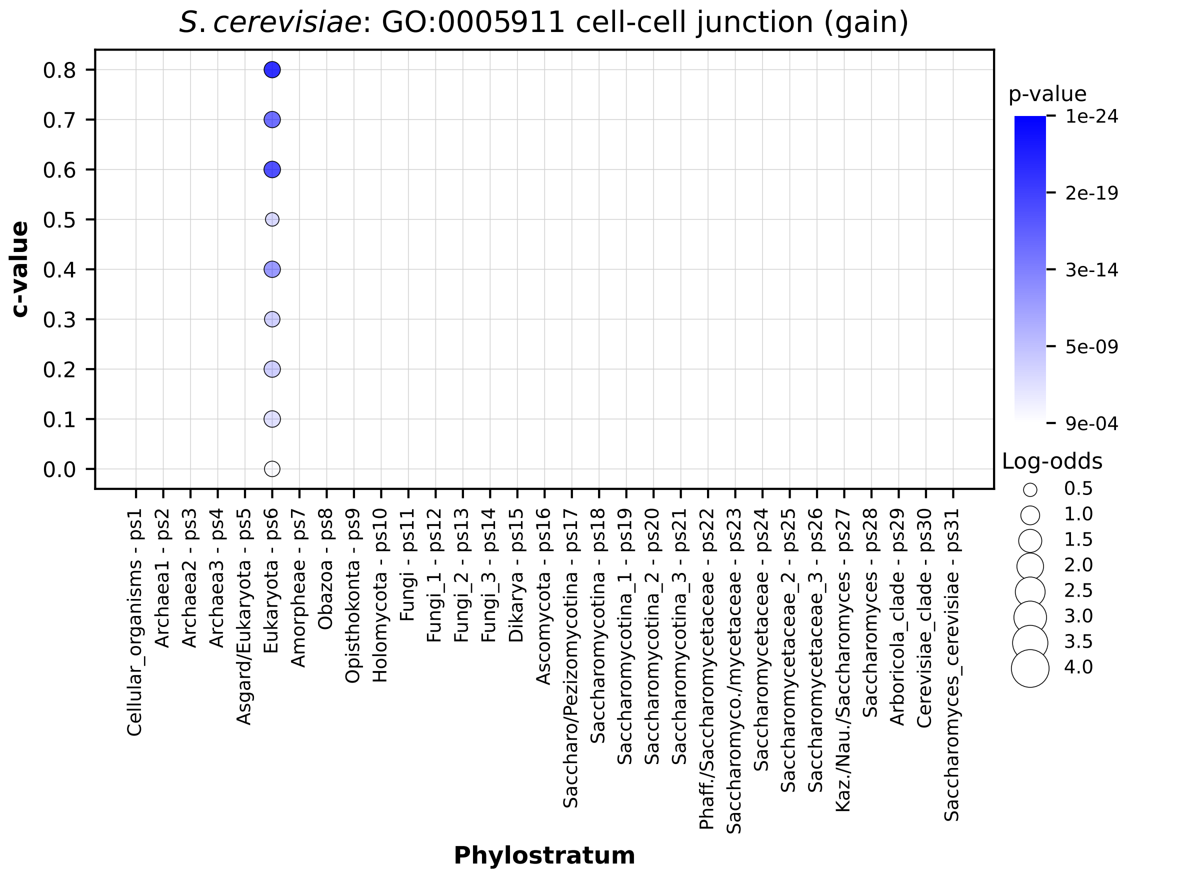
S. cerevisiae: GO:0005911 cell-cell junction (gain)
c-value
0.8
0.7
0.6
0.5
0.4
0.3
0.2
0.1
0.0
p-value
1e-24
2e-19
3e-14
5e-09
9e-04
Log-odds
0.5
1.0
1.5
2.0
2.5
3.0
3.5
4.0
Cellular_organisms - ps1
Archaea1 - ps2
Archaea2 - ps3
Archaea3 - ps4
Asgard/Eukaryota - ps5
Eukaryota - ps6
Amorpheae - ps7
Obazoa - ps8
Opisthokonta - ps9
Holomycota - ps10
Fungi - ps11
Fungi_1 - ps12
Fungi_2 - ps13
Fungi_3 - ps14
Dikarya - ps15
Ascomycota - ps16
Saccharo/Pezizomycotina - ps17
Saccharomycotina - ps18
Saccharomycotina_1 - ps19
Saccharomycotina_2 - ps20
Saccharomycotina_3 - ps21
Phaff./Saccharomycetaceae - ps22
Saccharomyco./mycetaceae - ps23
Saccharomycetaceae - ps24
Saccharomycetaceae_2 - ps25
Saccharomycetaceae_3 - ps26
Kaz./Nau./Saccharomyces - ps27
Saccharomyces - ps28
Arboricola_clade - ps29
Cerevisiae_clade - ps30
Saccharomyces_cerevisiae - ps31
Phylostratum

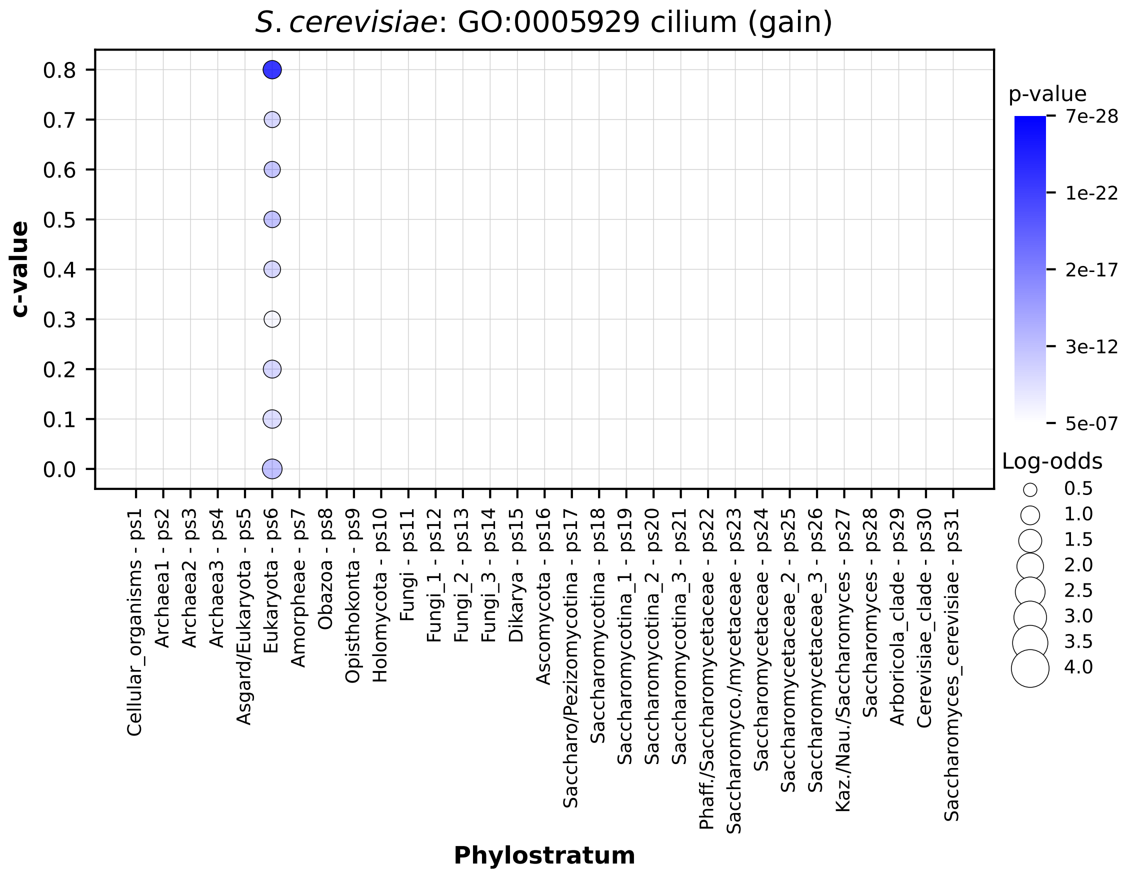

S. cerevisiae: GO:0005929 cilium (gain)
c-value
0.0
0.1
0.2
0.3
0.4
0.5
0.6
0.7
0.8
Phylostratum
Cellular_organisms - ps1
Archaea1 - ps2
Archaea2 - ps3
Archaea3 - ps4
Asgard/Eukaryota - ps5
Eukaryota - ps6
Amorpheae - ps7
Obazoa - ps8
Opisthokonta - ps9
Holomycota - ps10
Fungi - ps11
Fungi_1 - ps12
Fungi_2 - ps13
Fungi_3 - ps14
Dikarya - ps15
Ascomycota - ps16
Saccharo/Pezizomycotina - ps17
Saccharomycotina - ps18
Saccharomycotina_1 - ps19
Saccharomycotina_2 - ps20
Saccharomycotina_3 - ps21
Phaff./Saccharomycetaceae - ps22
Saccharomyco./mycetaceae - ps23
Saccharomycetaceae - ps24
Saccharomycetaceae_2 - ps25
Saccharomycetaceae_3 - ps26
Kaz./Nau./Saccharomyces - ps27
Saccharomyces - ps28
Arboricola_clade - ps29
Cerevisiae_clade - ps30
Saccharomyces_cerevisiae - ps31
p-value
7e-28
1e-22
2e-17
3e-12
5e-07
Log-odds
0.5
1.0
1.5
2.0
2.5
3.0
3.5
4.0

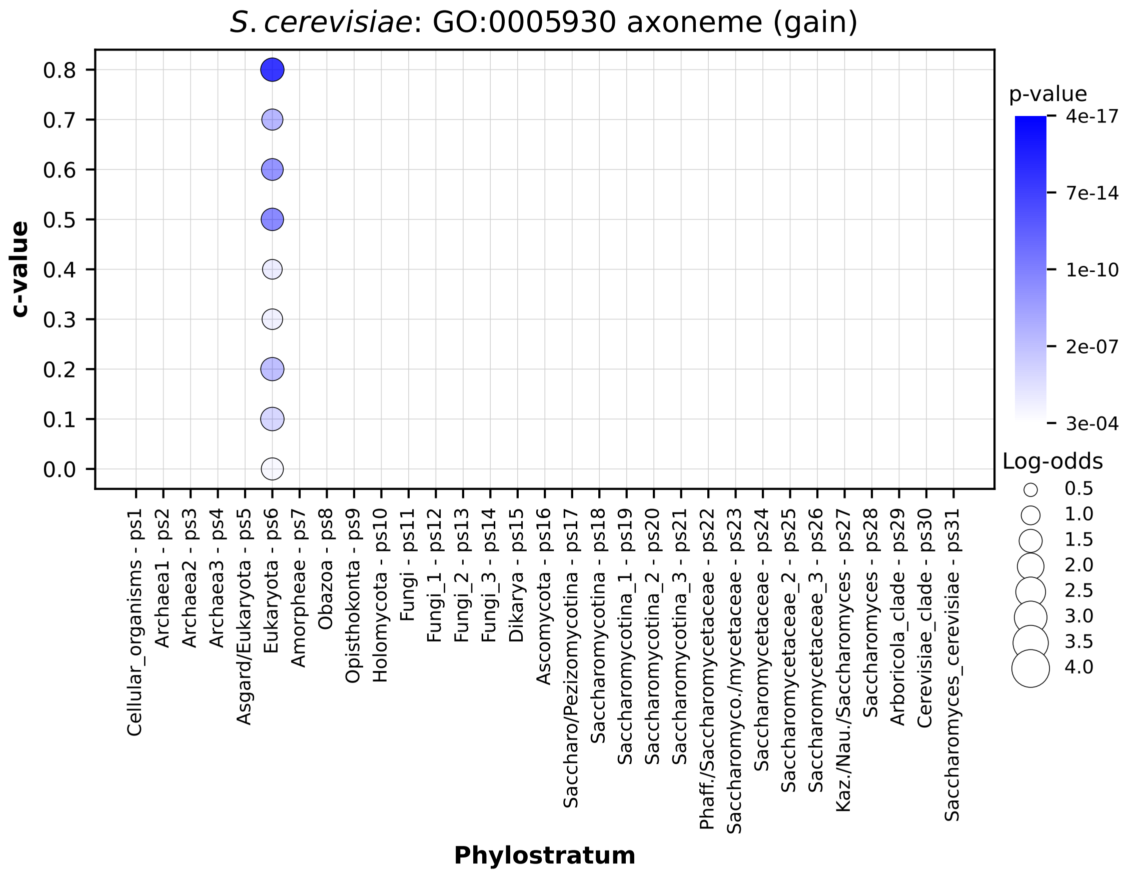

S. cerevisiae: GO:0005930 axoneme (gain)
c-value
0.8
0.7
0.6
0.5
0.4
0.3
0.2
0.1
0.0
p-value
4e-17
7e-14
1e-10
2e-07
3e-04
Log-odds
0.5
1.0
1.5
2.0
2.5
3.0
3.5
4.0
Cellular_organisms - ps1
Archaea1 - ps2
Archaea2 - ps3
Archaea3 - ps4
Asgard/Eukaryota - ps5
Eukaryota - ps6
Amorpheae - ps7
Obazoa - ps8
Opisthokonta - ps9
Holomycota - ps10
Fungi - ps11
Fungi_1 - ps12
Fungi_2 - ps13
Fungi_3 - ps14
Dikarya - ps15
Ascomycota - ps16
Saccharo/Pezizomycotina - ps17
Saccharomycotina - ps18
Saccharomycotina_1 - ps19
Saccharomycotina_2 - ps20
Saccharomycotina_3 - ps21
Phaff./Saccharomycetaceae - ps22
Saccharomyco./mycetaceae - ps23
Saccharomycetaceae - ps24
Saccharomycetaceae_2 - ps25
Saccharomycetaceae_3 - ps26
Kaz./Nau./Saccharomyces - ps27
Saccharomyces - ps28
Arboricola_clade - ps29
Cerevisiae_clade - ps30
Saccharomyces_cerevisiae - ps31
Phylostratum

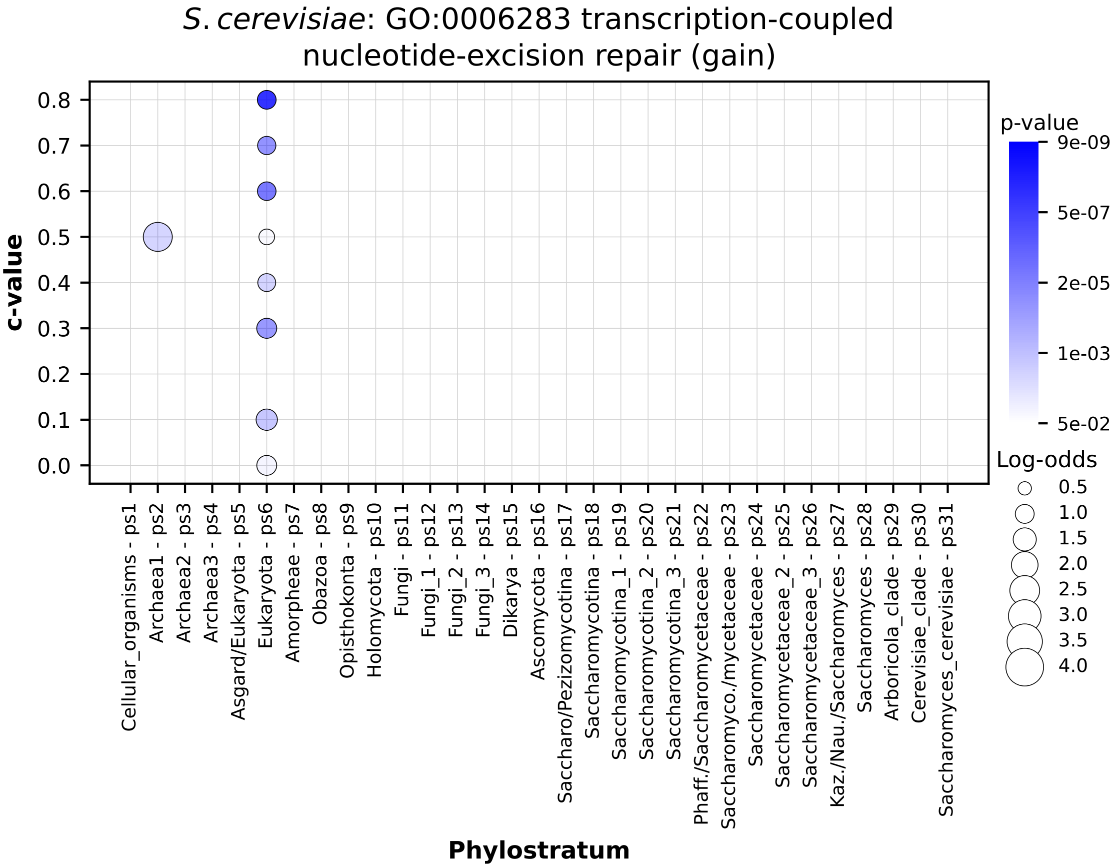

S. cerevisiae: GO:0006283 transcription-coupled nucleotide-excision repair (gain)
c-value
0.8
0.7
0.6
0.5
0.4
0.3
0.2
0.1
0.0
p-value
9e-09
5e-07
2e-05
1e-03
5e-02
Log-odds
0.5
1.0
1.5
2.0
2.5
3.0
3.5
4.0
Cellular_organisms - ps1
Archaea1 - ps2
Archaea2 - ps3
Archaea3 - ps4
Asgard/Eukaryota - ps5
Eukaryota - ps6
Amorpheae - ps7
Obazoa - ps8
Opisthokonta - ps9
Holomycota - ps10
Fungi - ps11
Fungi_1 - ps12
Fungi_2 - ps13
Fungi_3 - ps14
Dikarya - ps15
Ascomycota - ps16
Saccharo/Pezizomycotina - ps17
Saccharomycotina - ps18
Saccharomycotina_1 - ps19
Saccharomycotina_2 - ps20
Saccharomycotina_3 - ps21
Phaff./Saccharomycetaceae - ps22
Saccharomyco./mycetaceae - ps23
Saccharomycetaceae - ps24
Saccharomycetaceae_2 - ps25
Saccharomycetaceae_3 - ps26
Kaz./Nau./Saccharomyces - ps27
Saccharomyces - ps28
Arboricola_clade - ps29
Cerevisiae_clade - ps30
Saccharomyces_cerevisiae - ps31
Phylostratum

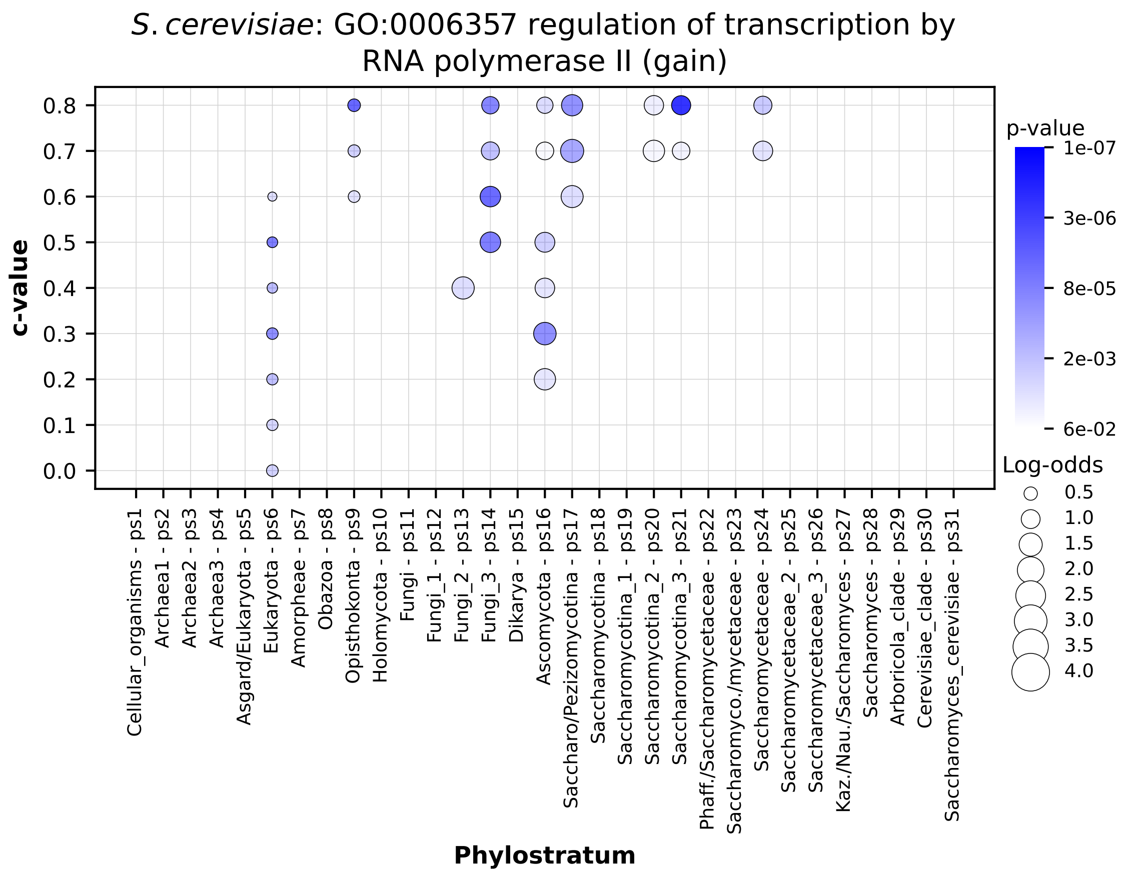

S. cerevisiae: GO:0006357 regulation of transcription by RNA polymerase II (gain)
c-value
0.8
0.7
0.6
0.5
0.4
0.3
0.2
0.1
0.0
Phylostratum
Cellular_organisms - ps1
Archaea1 - ps2
Archaea2 - ps3
Archaea3 - ps4
Asgard/Eukaryota - ps5
Eukaryota - ps6
Amorpheae - ps7
Obazoa - ps8
Opisthokonta - ps9
Holomycota - ps10
Fungi - ps11
Fungi_1 - ps12
Fungi_2 - ps13
Fungi_3 - ps14
Dikarya - ps15
Ascomycota - ps16
Saccharo/Pezizomycotina - ps17
Saccharomycotina - ps18
Saccharomycotina_1 - ps19
Saccharomycotina_2 - ps20
Saccharomycotina_3 - ps21
Phaff./Saccharomycetaceae - ps22
Saccharomyco./mycetaceae - ps23
Saccharomycetaceae - ps24
Saccharomycetaceae_2 - ps25
Saccharomycetaceae_3 - ps26
Kaz./Nau./Saccharomyces - ps27
Saccharomyces - ps28
Arboricola_clade - ps29
Cerevisiae_clade - ps30
Saccharomyces_cerevisiae - ps31
p-value
1e-07
3e-06
8e-05
2e-03
6e-02
Log-odds
0.5
1.0
1.5
2.0
2.5
3.0
3.5
4.0

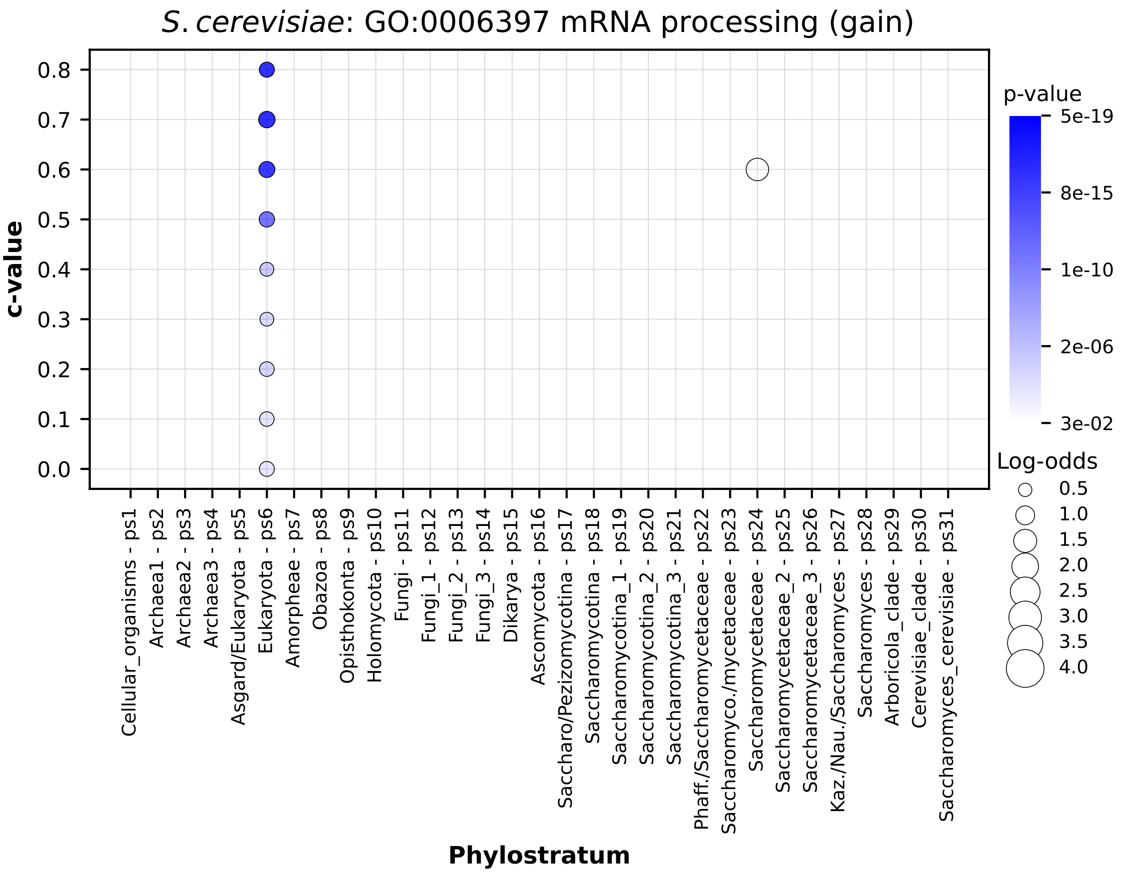

S. cerevisiae: GO:0006397 mRNA processing (gain)
c-value
0.0
0.1
0.2
0.3
0.4
0.5
0.6
0.7
0.8
p-value
5e-19
8e-15
1e-10
2e-06
3e-02
Log-odds
0.5
1.0
1.5
2.0
2.5
3.0
3.5
4.0
Cellular_organisms - ps1
Archaea1 - ps2
Archaea2 - ps3
Archaea3 - ps4
Asgard/Eukaryota - ps5
Eukaryota - ps6
Amorpheae - ps7
Obazoa - ps8
Opisthokonta - ps9
Holomycota - ps10
Fungi - ps11
Fungi_1 - ps12
Fungi_2 - ps13
Fungi_3 - ps14
Dikarya - ps15
Ascomycota - ps16
Saccharo/Pezizomycotina - ps17
Saccharomycotina - ps18
Saccharomycotina_1 - ps19
Saccharomycotina_2 - ps20
Saccharomycotina_3 - ps21
Phaff./Saccharomycetaceae - ps22
Saccharomyco./mycetaceae - ps23
Saccharomycetaceae - ps24
Saccharomycetaceae_2 - ps25
Saccharomycetaceae_3 - ps26
Kaz./Nau./Saccharomyces - ps27
Saccharomyces - ps28
Arboricola_clade - ps29
Cerevisiae_clade - ps30
Saccharomyces_cerevisiae - ps31
Phylostratum

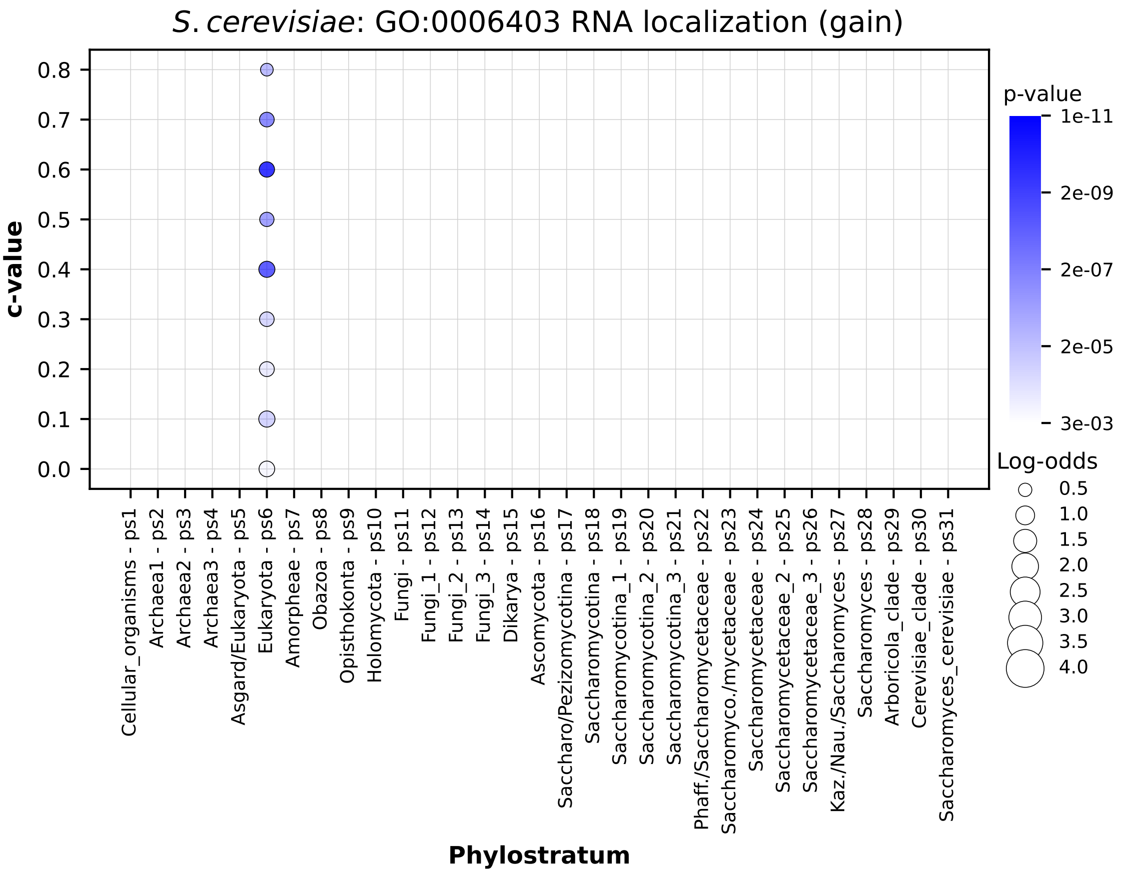

S. cerevisiae: GO:0006403 RNA localization (gain)
c-value
0.0
0.1
0.2
0.3
0.4
0.5
0.6
0.7
0.8
p-value
1e-11
2e-09
2e-07
2e-05
3e-03
Log-odds
0.5
1.0
1.5
2.0
2.5
3.0
3.5
4.0
Cellular_organisms - ps1
Archaea1 - ps2
Archaea2 - ps3
Archaea3 - ps4
Asgard/Eukaryota - ps5
Eukaryota - ps6
Amorpheae - ps7
Obazoa - ps8
Opisthokonta - ps9
Holomycota - ps10
Fungi - ps11
Fungi_1 - ps12
Fungi_2 - ps13
Fungi_3 - ps14
Dikarya - ps15
Ascomycota - ps16
Saccharo/Pezizomycotina - ps17
Saccharomycotina - ps18
Saccharomycotina_1 - ps19
Saccharomycotina_2 - ps20
Saccharomycotina_3 - ps21
Phaff./Saccharomycetaceae - ps22
Saccharomyco./mycetaceae - ps23
Saccharomycetaceae - ps24
Saccharomycetaceae_2 - ps25
Saccharomycetaceae_3 - ps26
Kaz./Nau./Saccharomyces - ps27
Saccharomyces - ps28
Arboricola_clade - ps29
Cerevisiae_clade - ps30
Saccharomyces_cerevisiae - ps31
Phylostratum

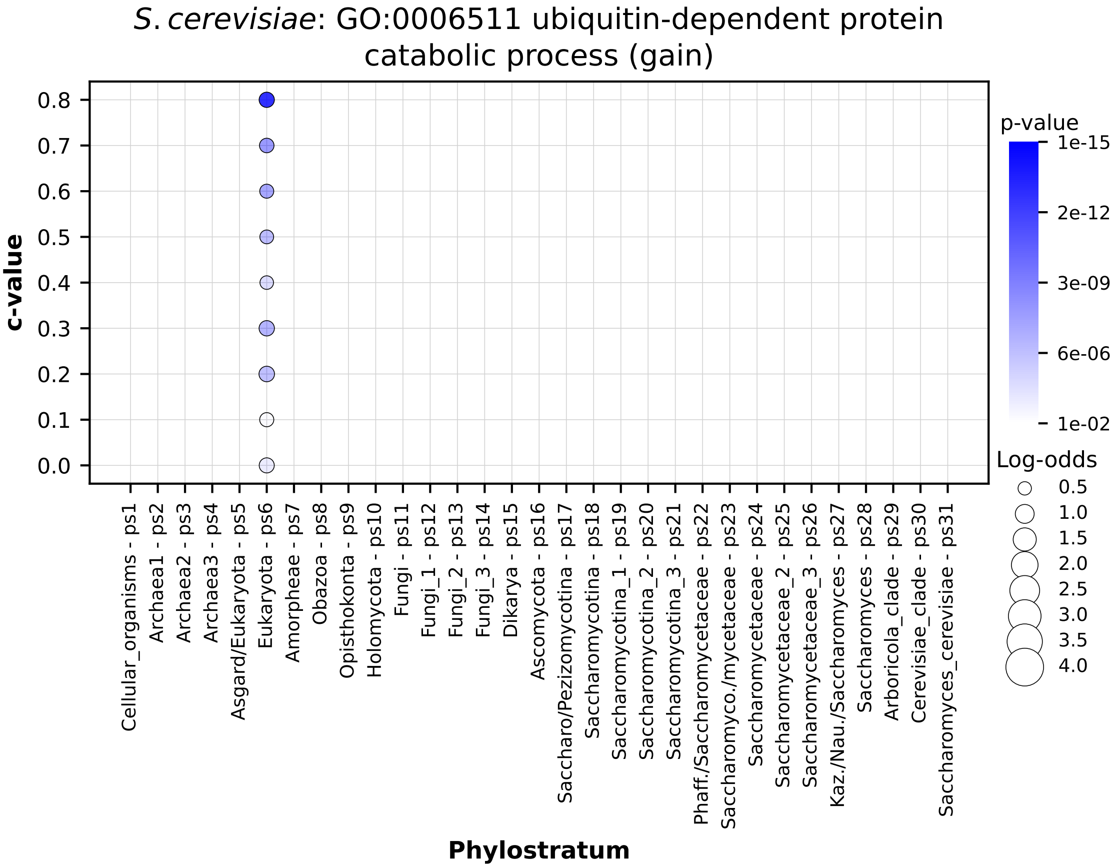

S. cerevisiae: GO:0006511 ubiquitin-dependent protein catabolic process (gain)
c-value
0.8
0.7
0.6
0.5
0.4
0.3
0.2
0.1
0.0
p-value
1e-15
2e-12
3e-09
6e-06
1e-02
Log-odds
0.5
1.0
1.5
2.0
2.5
3.0
3.5
4.0
Cellular_organisms - ps1
Archaea1 - ps2
Archaea2 - ps3
Archaea3 - ps4
Asgard/Eukaryota - ps5
Eukaryota - ps6
Amorpheae - ps7
Obazoa - ps8
Opisthokonta - ps9
Holomycota - ps10
Fungi - ps11
Fungi_1 - ps12
Fungi_2 - ps13
Fungi_3 - ps14
Dikarya - ps15
Ascomycota - ps16
Saccharo/Pezizomycotina - ps17
Saccharomycotina - ps18
Saccharomycotina_1 - ps19
Saccharomycotina_2 - ps20
Saccharomycotina_3 - ps21
Phaff./Saccharomycetaceae - ps22
Saccharomyco./mycetaceae - ps23
Saccharomycetaceae - ps24
Saccharomycetaceae_2 - ps25
Saccharomycetaceae_3 - ps26
Kaz./Nau./Saccharomyces - ps27
Saccharomyces - ps28
Arboricola_clade - ps29
Cerevisiae_clade - ps30
Saccharomyces_cerevisiae - ps31
Phylostratum

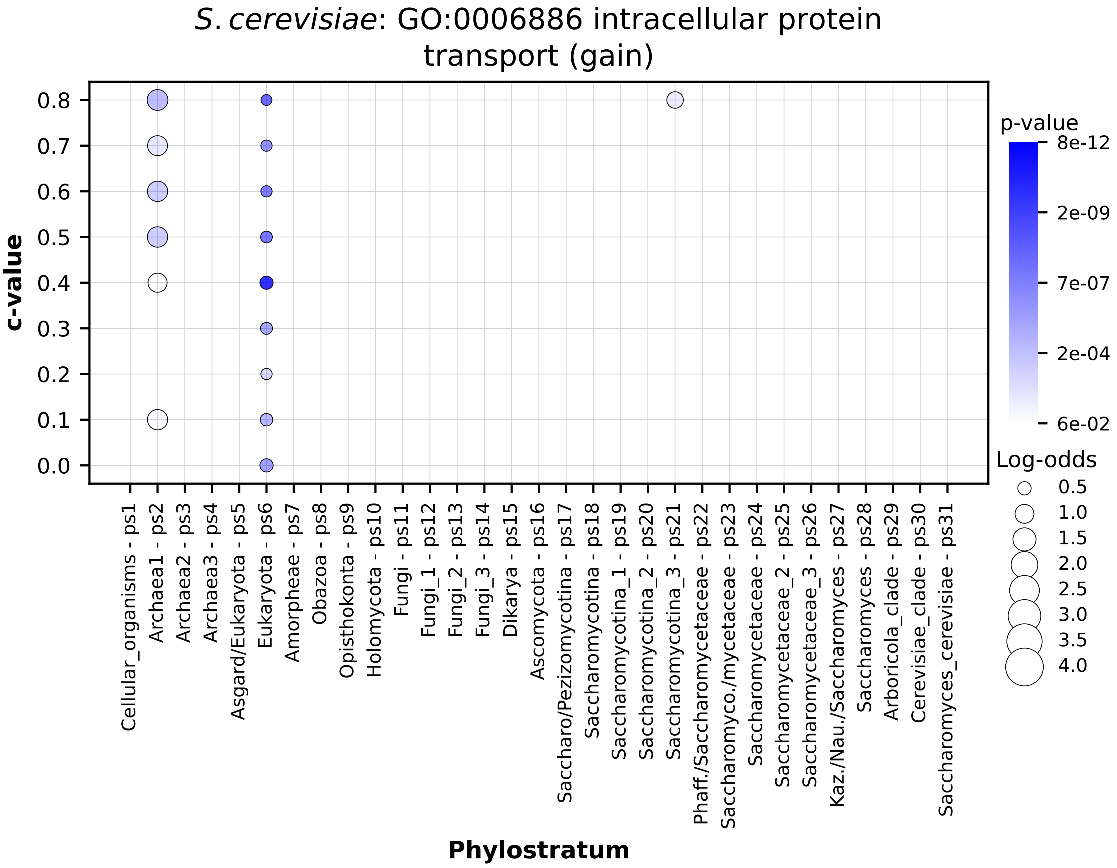

S. cerevisiae: GO:0006886 intracellular protein transport (gain)
c-value
0.0
0.1
0.2
0.3
0.4
0.5
0.6
0.7
0.8
p-value
8e-12
2e-09
7e-07
2e-04
6e-02
Log-odds
0.5
1.0
1.5
2.0
2.5
3.0
3.5
4.0
Cellular_organisms - ps1
Archaea1 - ps2
Archaea2 - ps3
Archaea3 - ps4
Asgard/Eukaryota - ps5
Eukaryota - ps6
Amorpheae - ps7
Obazoa - ps8
Opisthokonta - ps9
Holomycota - ps10
Fungi - ps11
Fungi_1 - ps12
Fungi_2 - ps13
Fungi_3 - ps14
Dikarya - ps15
Ascomycota - ps16
Saccharo/Pezizomycotina - ps17
Saccharomycotina - ps18
Saccharomycotina_1 - ps19
Saccharomycotina_2 - ps20
Saccharomycotina_3 - ps21
Phaff./Saccharomycetaceae - ps22
Saccharomyco./mycetaceae - ps23
Saccharomycetaceae - ps24
Saccharomycetaceae_2 - ps25
Saccharomycetaceae_3 - ps26
Kaz./Nau./Saccharomyces - ps27
Saccharomyces - ps28
Arboricola_clade - ps29
Cerevisiae_clade - ps30
Saccharomyces_cerevisiae - ps31
Phylostratum

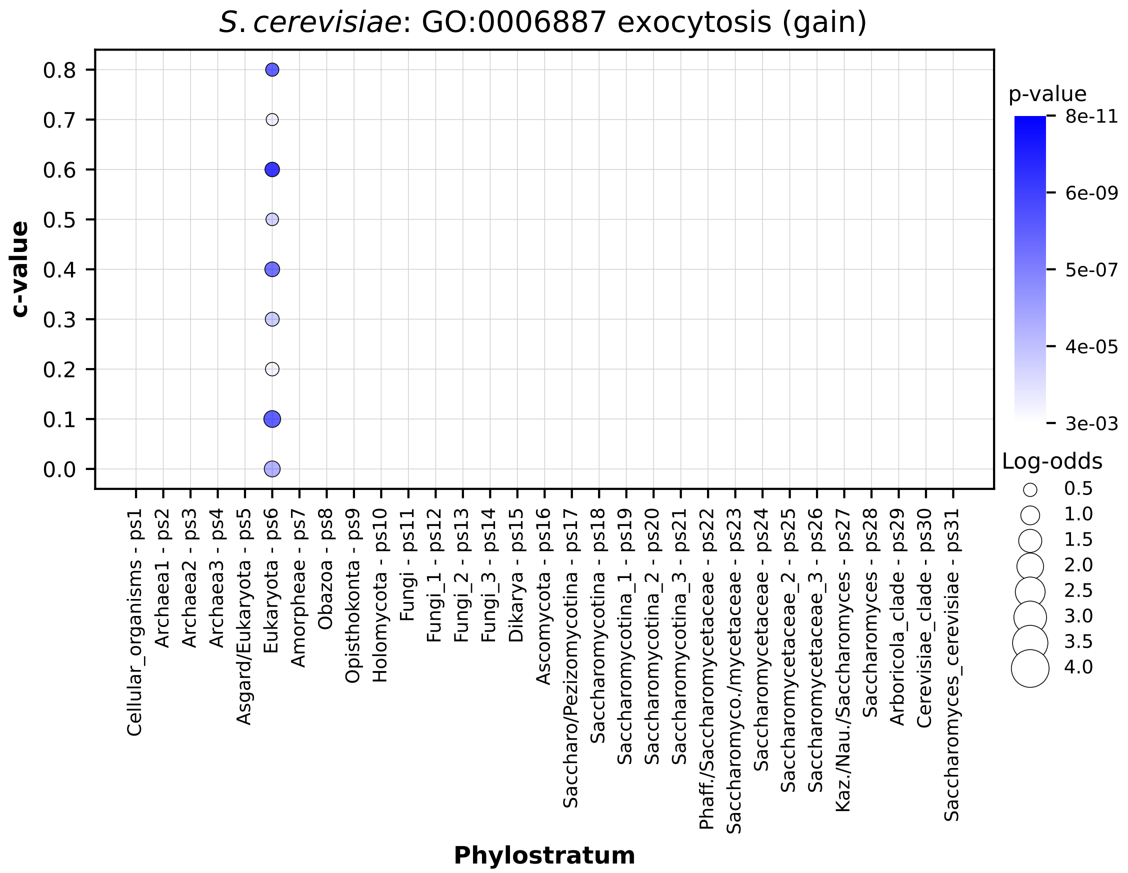

S. cerevisiae: GO:0006887 exocytosis (gain)
c-value
0.8
0.7
0.6
0.5
0.4
0.3
0.2
0.1
0.0
p-value
8e-11
6e-09
5e-07
4e-05
3e-03
Log-odds
0.5
1.0
1.5
2.0
2.5
3.0
3.5
4.0
Cellular_organisms - ps1
Archaea1 - ps2
Archaea2 - ps3
Archaea3 - ps4
Asgard/Eukaryota - ps5
Eukaryota - ps6
Amorpheae - ps7
Obazoa - ps8
Opisthokonta - ps9
Holomycota - ps10
Fungi - ps11
Fungi_1 - ps12
Fungi_2 - ps13
Fungi_3 - ps14
Dikarya - ps15
Ascomycota - ps16
Saccharo/Pezizomycotina - ps17
Saccharomycotina - ps18
Saccharomycotina_1 - ps19
Saccharomycotina_2 - ps20
Saccharomycotina_3 - ps21
Phaff./Saccharomycetaceae - ps22
Saccharomyco./mycetaceae - ps23
Saccharomycetaceae - ps24
Saccharomycetaceae_2 - ps25
Saccharomycetaceae_3 - ps26
Kaz./Nau./Saccharomyces - ps27
Saccharomyces - ps28
Arboricola_clade - ps29
Cerevisiae_clade - ps30
Saccharomyces_cerevisiae - ps31
Phylostratum

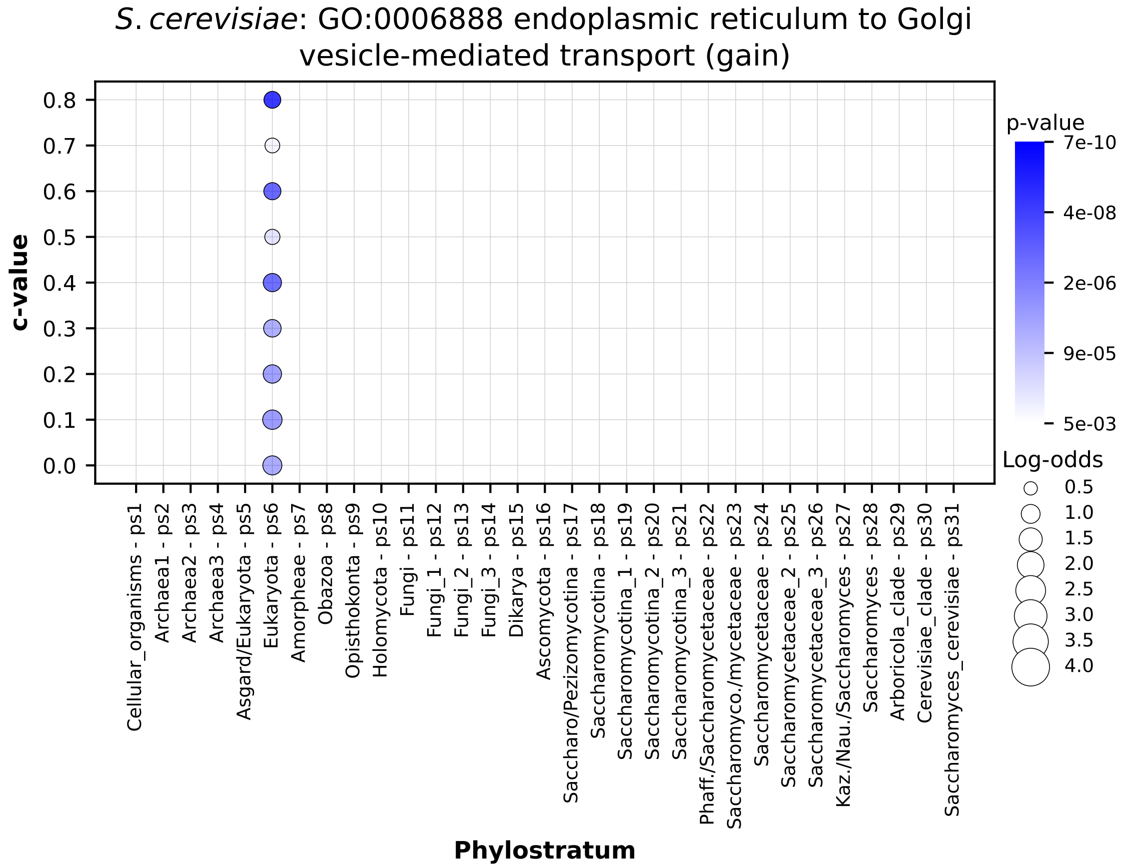

S. cerevisiae: GO:0006888 endoplasmic reticulum to Golgi vesicle-mediated transport (gain)
c-value
0.8
0.7
0.6
0.5
0.4
0.3
0.2
0.1
0.0
p-value
7e-10
4e-08
2e-06
9e-05
5e-03
Log-odds
0.5
1.0
1.5
2.0
2.5
3.0
3.5
4.0
Cellular_organisms - ps1
Archaea1 - ps2
Archaea2 - ps3
Archaea3 - ps4
Asgard/Eukaryota - ps5
Eukaryota - ps6
Amorpheae - ps7
Obazoa - ps8
Opisthokonta - ps9
Holomycota - ps10
Fungi - ps11
Fungi_1 - ps12
Fungi_2 - ps13
Fungi_3 - ps14
Dikarya - ps15
Ascomycota - ps16
Saccharo/Pezizomycotina - ps17
Saccharomycotina - ps18
Saccharomycotina_1 - ps19
Saccharomycotina_2 - ps20
Saccharomycotina_3 - ps21
Phaff./Saccharomycetaceae - ps22
Saccharomyco./mycetaceae - ps23
Saccharomycetaceae - ps24
Saccharomycetaceae_2 - ps25
Saccharomycetaceae_3 - ps26
Kaz./Nau./Saccharomyces - ps27
Saccharomyces - ps28
Arboricola_clade - ps29
Cerevisiae_clade - ps30
Saccharomyces_cerevisiae - ps31
Phylostratum

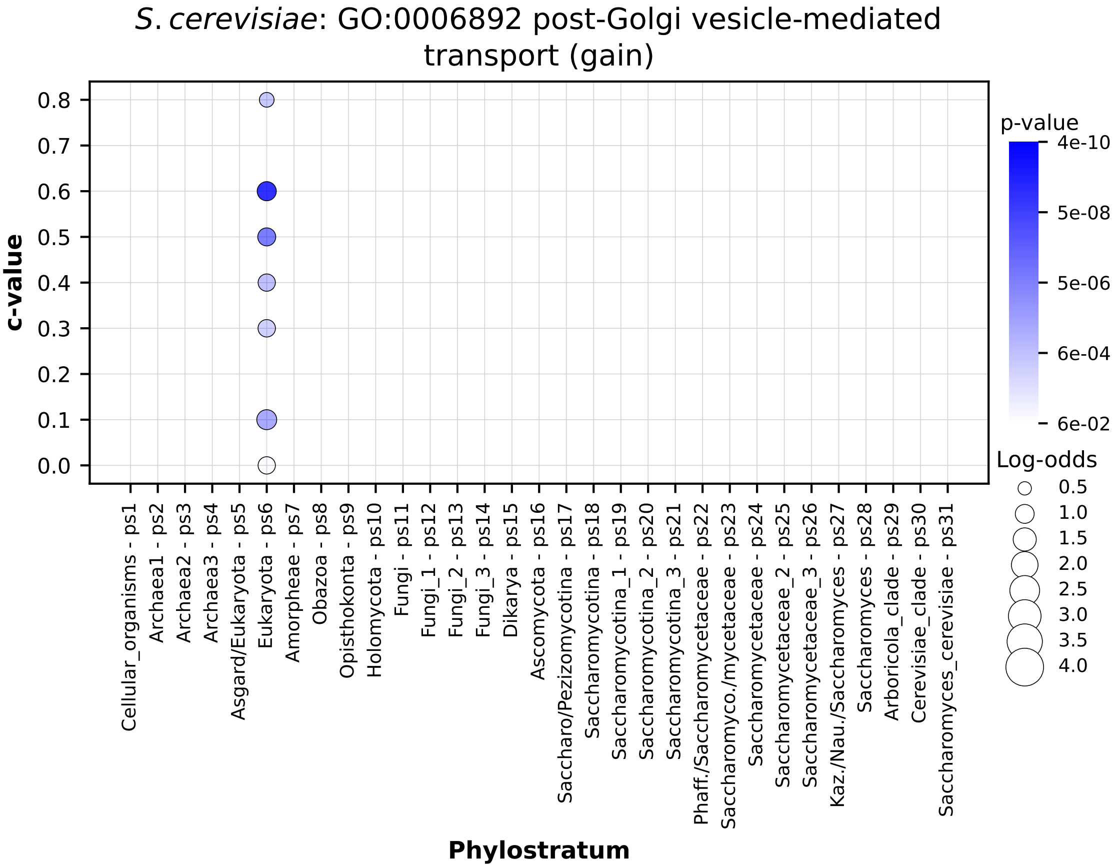

S. cerevisiae: GO:0006892 post-Golgi vesicle-mediated transport (gain)
c-value
0.8
0.7
0.6
0.5
0.4
0.3
0.2
0.1
0.0
p-value
4e-10
5e-08
5e-06
6e-04
6e-02
Log-odds
0.5
1.0
1.5
2.0
2.5
3.0
3.5
4.0
Cellular_organisms - ps1
Archaea1 - ps2
Archaea2 - ps3
Archaea3 - ps4
Asgard/Eukaryota - ps5
Eukaryota - ps6
Amorpheae - ps7
Obazoa - ps8
Opisthokonta - ps9
Holomycota - ps10
Fungi - ps11
Fungi_1 - ps12
Fungi_2 - ps13
Fungi_3 - ps14
Dikarya - ps15
Ascomycota - ps16
Saccharo/Pezizomycotina - ps17
Saccharomycotina - ps18
Saccharomycotina_1 - ps19
Saccharomycotina_2 - ps20
Saccharomycotina_3 - ps21
Phaff./Saccharomycetaceae - ps22
Saccharomyco./mycetaceae - ps23
Saccharomycetaceae - ps24
Saccharomycetaceae_2 - ps25
Saccharomycetaceae_3 - ps26
Kaz./Nau./Saccharomyces - ps27
Saccharomyces - ps28
Arboricola_clade - ps29
Cerevisiae_clade - ps30
Saccharomyces_cerevisiae - ps31
Phylostratum

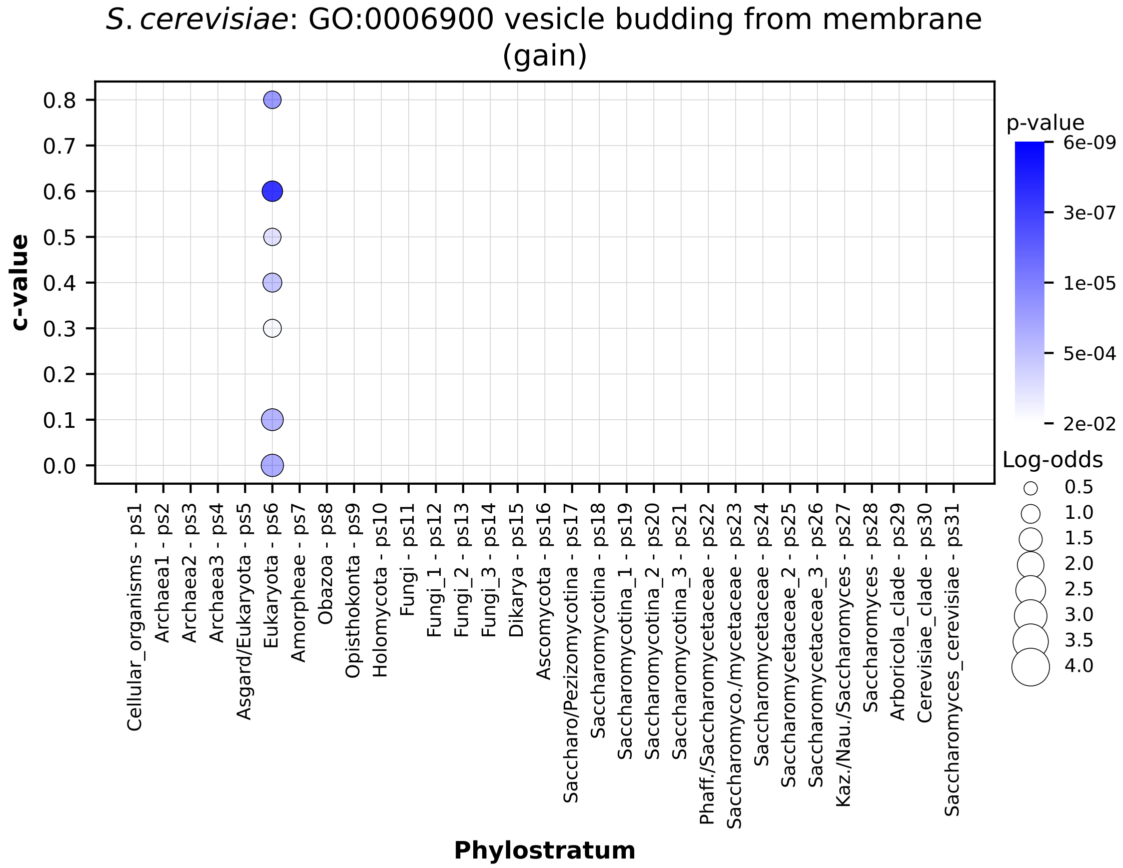

S. cerevisiae: GO:0006900 vesicle budding from membrane (gain)
c-value
0.8
0.7
0.6
0.5
0.4
0.3
0.2
0.1
0.0
p-value
6e-09
3e-07
1e-05
5e-04
2e-02
Log-odds
0.5
1.0
1.5
2.0
2.5
3.0
3.5
4.0
Cellular_organisms - ps1
Archaea1 - ps2
Archaea2 - ps3
Archaea3 - ps4
Asgard/Eukaryota - ps5
Eukaryota - ps6
Amorpheae - ps7
Obazoa - ps8
Opisthokonta - ps9
Holomycota - ps10
Fungi - ps11
Fungi_1 - ps12
Fungi_2 - ps13
Fungi_3 - ps14
Dikarya - ps15
Ascomycota - ps16
Saccharo/Pezizomycotina - ps17
Saccharomycotina - ps18
Saccharomycotina_1 - ps19
Saccharomycotina_2 - ps20
Saccharomycotina_3 - ps21
Phaff./Saccharomycetaceae - ps22
Saccharomyco./mycetaceae - ps23
Saccharomycetaceae - ps24
Saccharomycetaceae_2 - ps25
Saccharomycetaceae_3 - ps26
Kaz./Nau./Saccharomyces - ps27
Saccharomyces - ps28
Arboricola_clade - ps29
Cerevisiae_clade - ps30
Saccharomyces_cerevisiae - ps31
Phylostratum

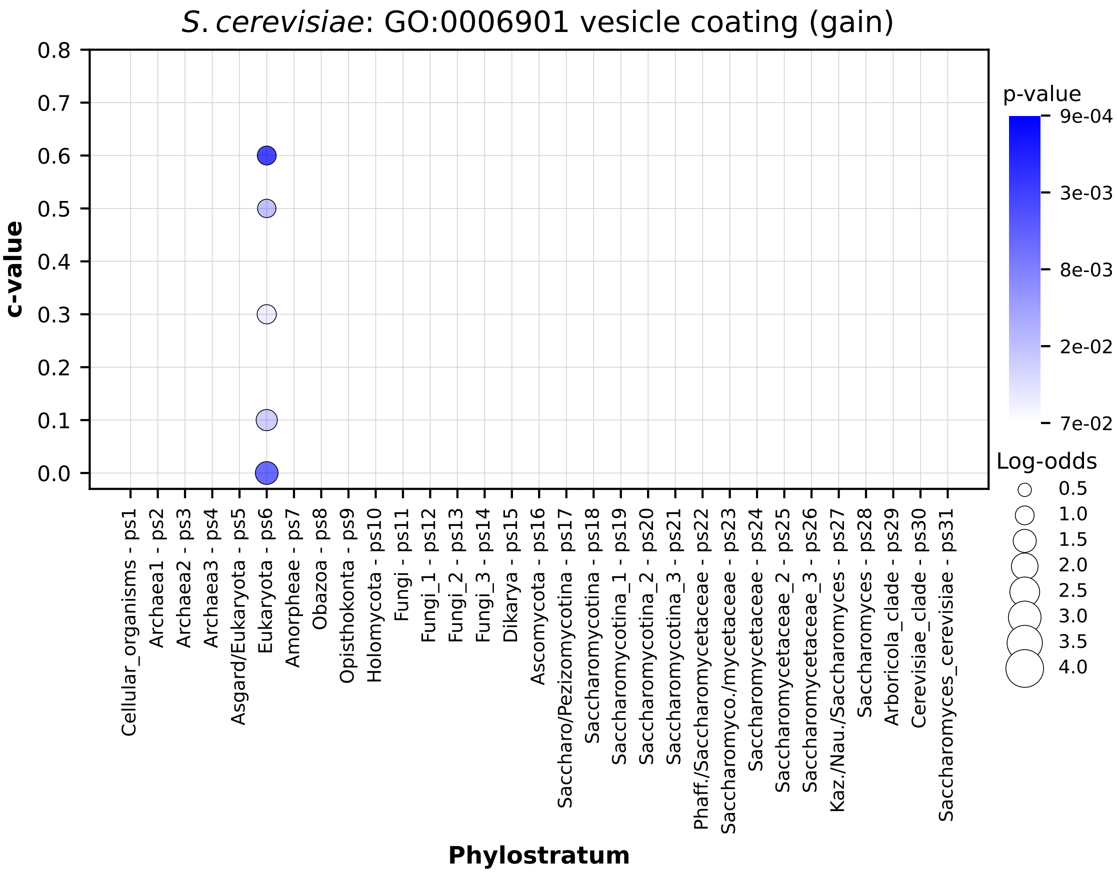

S. cerevisiae: GO:0006901 vesicle coating (gain)
c-value
0.8
0.7
0.6
0.5
0.4
0.3
0.2
0.1
0.0
p-value
9e-04
3e-03
8e-03
2e-02
7e-02
Log-odds
0.5
1.0
1.5
2.0
2.5
3.0
3.5
4.0
Cellular_organisms - ps1
Archaea1 - ps2
Archaea2 - ps3
Archaea3 - ps4
Asgard/Eukaryota - ps5
Eukaryota - ps6
Amorpheae - ps7
Obazoa - ps8
Opisthokonta - ps9
Holomycota - ps10
Fungi - ps11
Fungi_1 - ps12
Fungi_2 - ps13
Fungi_3 - ps14
Dikarya - ps15
Ascomycota - ps16
Saccharo/Pezizomycotina - ps17
Saccharomycotina - ps18
Saccharomycotina_1 - ps19
Saccharomycotina_2 - ps20
Saccharomycotina_3 - ps21
Phaff./Saccharomycetaceae - ps22
Saccharomyco./mycetaceae - ps23
Saccharomycetaceae - ps24
Saccharomycetaceae_2 - ps25
Saccharomycetaceae_3 - ps26
Kaz./Nau./Saccharomyces - ps27
Saccharomyces - ps28
Arboricola_clade - ps29
Cerevisiae_clade - ps30
Saccharomyces_cerevisiae - ps31
Phylostratum

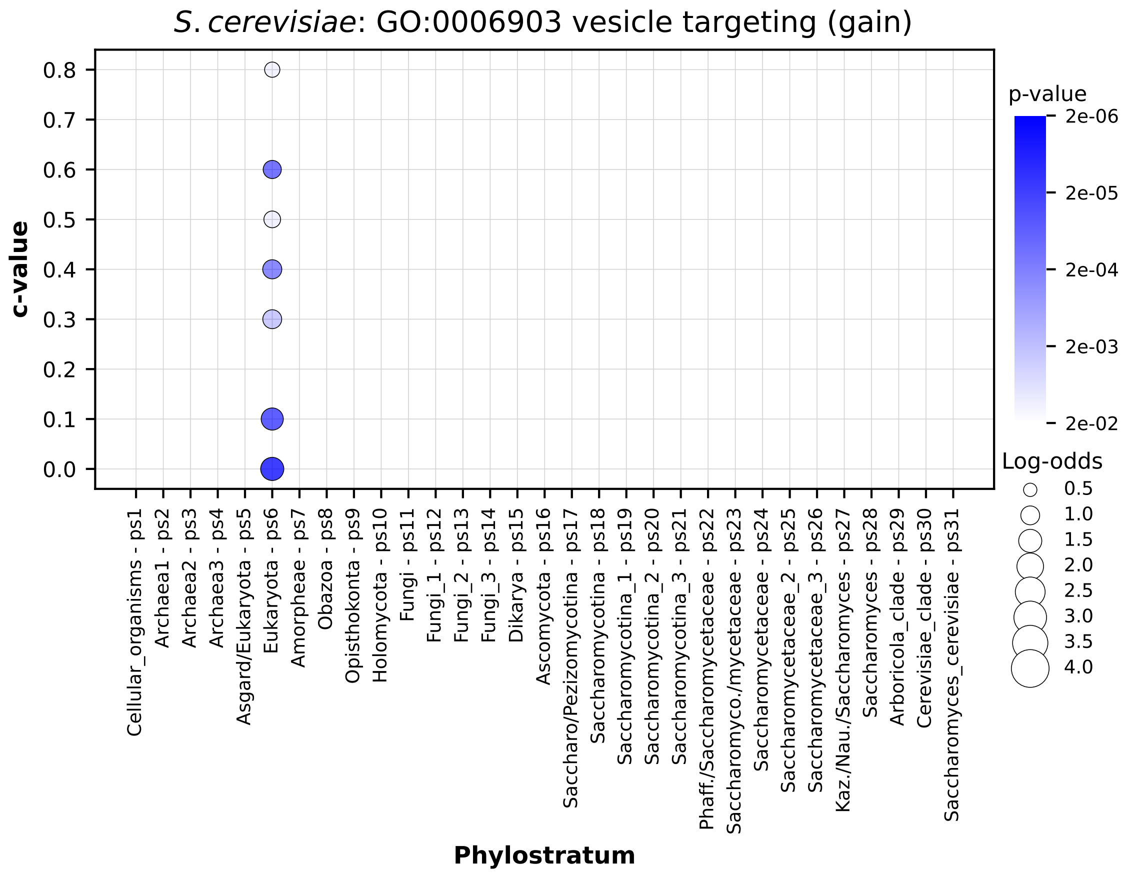
S. cerevisiae: GO:0006903 vesicle targeting (gain)
c-value
0.8
0.7
0.6
0.5
0.4
0.3
0.2
0.1
0.0
p-value
2e-06
2e-05
2e-04
2e-03
2e-02
Log-odds
0.5
1.0
1.5
2.0
2.5
3.0
3.5
4.0
Cellular_organisms - ps1
Archaea1 - ps2
Archaea2 - ps3
Archaea3 - ps4
Asgard/Eukaryota - ps5
Eukaryota - ps6
Amorpheae - ps7
Obazoa - ps8
Opisthokonta - ps9
Holomycota - ps10
Fungi - ps11
Fungi_1 - ps12
Fungi_2 - ps13
Fungi_3 - ps14
Dikarya - ps15
Ascomycota - ps16
Saccharo/Pezizomycotina - ps17
Saccharomycotina - ps18
Saccharomycotina_1 - ps19
Saccharomycotina_2 - ps20
Saccharomycotina_3 - ps21
Phaff./Saccharomycetaceae - ps22
Saccharomyco./mycetaceae - ps23
Saccharomycetaceae - ps24
Saccharomycetaceae_2 - ps25
Saccharomycetaceae_3 - ps26
Kaz./Nau./Saccharomyces - ps27
Saccharomyces - ps28
Arboricola_clade - ps29
Cerevisiae_clade - ps30
Saccharomyces_cerevisiae - ps31
Phylostratum

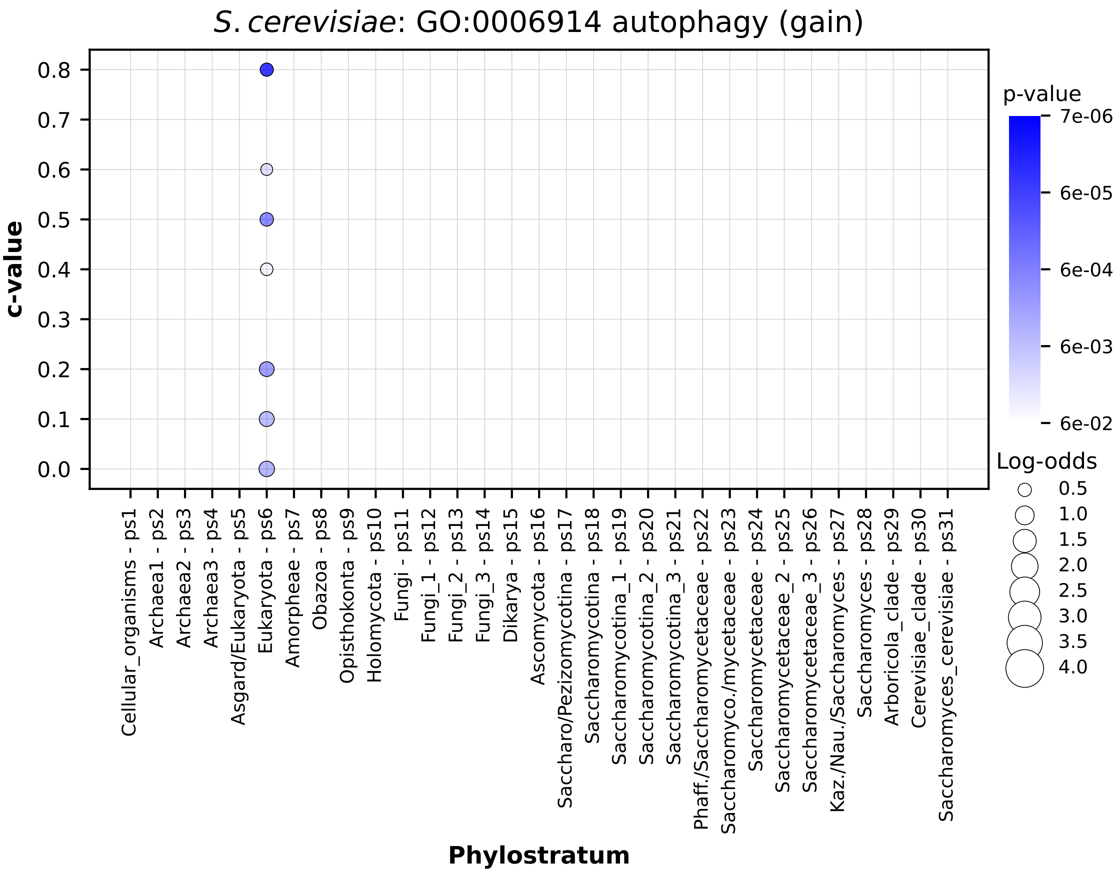
S. cerevisiae: GO:0006914 autophagy (gain)
c-value
0.0
0.1
0.2
0.3
0.4
0.5
0.6
0.7
0.8
p-value
7e-06
6e-05
6e-04
6e-03
6e-02
Log-odds
0.5
1.0
1.5
2.0
2.5
3.0
3.5
4.0
Cellular_organisms - ps1
Archaea1 - ps2
Archaea2 - ps3
Archaea3 - ps4
Asgard/Eukaryota - ps5
Eukaryota - ps6
Amorpheae - ps7
Obazoa - ps8
Opisthokonta - ps9
Holomycota - ps10
Fungi - ps11
Fungi_1 - ps12
Fungi_2 - ps13
Fungi_3 - ps14
Dikarya - ps15
Ascomycota - ps16
Saccharo/Pezizomycotina - ps17
Saccharomycotina - ps18
Saccharomycotina_1 - ps19
Saccharomycotina_2 - ps20
Saccharomycotina_3 - ps21
Phaff./Saccharomycetaceae - ps22
Saccharomyco./mycetaceae - ps23
Saccharomycetaceae - ps24
Saccharomycetaceae_2 - ps25
Saccharomycetaceae_3 - ps26
Kaz./Nau./Saccharomyces - ps27
Saccharomyces - ps28
Arboricola_clade - ps29
Cerevisiae_clade - ps30
Saccharomyces_cerevisiae - ps31
Phylostratum

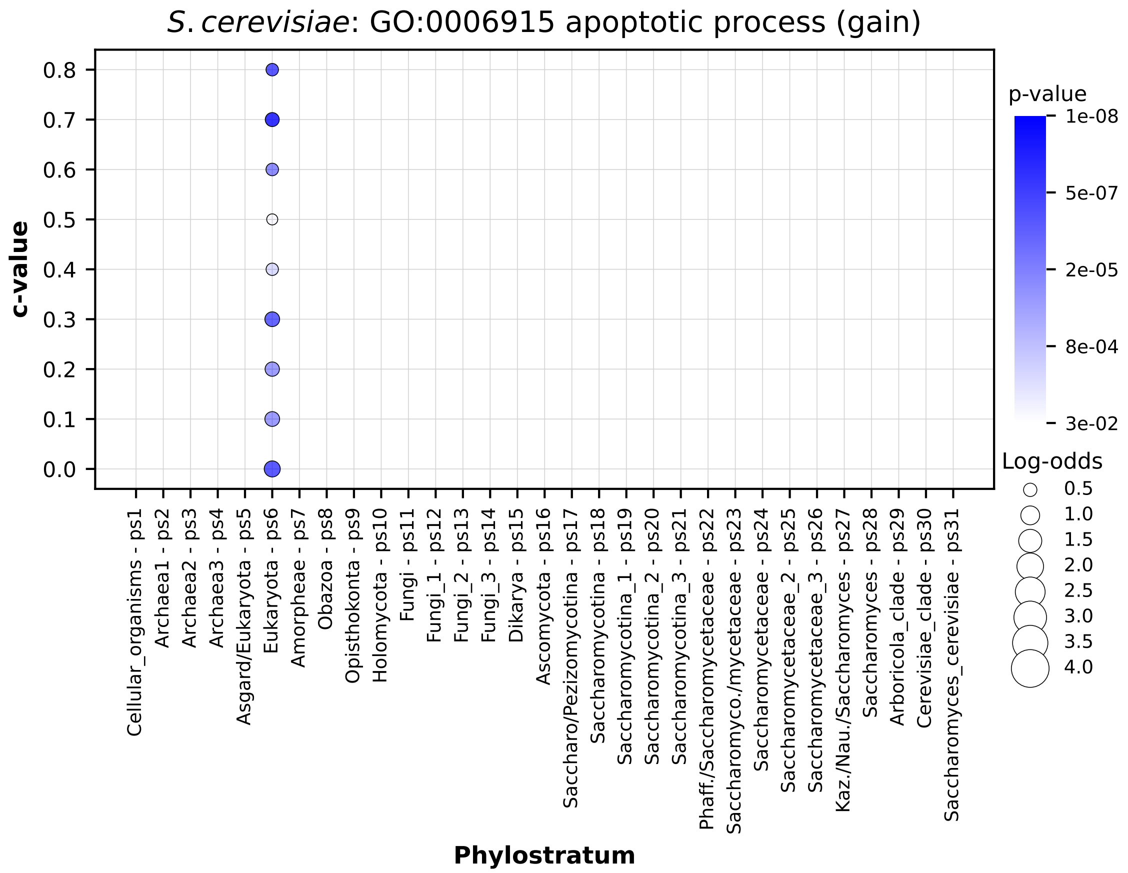

S. cerevisiae: GO:0006915 apoptotic process (gain)
c-value
0.8
0.7
0.6
0.5
0.4
0.3
0.2
0.1
0.0
p-value
1e-08
5e-07
2e-05
8e-04
3e-02
Log-odds
0.5
1.0
1.5
2.0
2.5
3.0
3.5
4.0
Cellular_organisms - ps1
Archaea1 - ps2
Archaea2 - ps3
Archaea3 - ps4
Asgard/Eukaryota - ps5
Eukaryota - ps6
Amorpheae - ps7
Obazoa - ps8
Opisthokonta - ps9
Holomycota - ps10
Fungi - ps11
Fungi_1 - ps12
Fungi_2 - ps13
Fungi_3 - ps14
Dikarya - ps15
Ascomycota - ps16
Saccharo/Pezizomycotina - ps17
Saccharomycotina - ps18
Saccharomycotina_1 - ps19
Saccharomycotina_2 - ps20
Saccharomycotina_3 - ps21
Phaff./Saccharomycetaceae - ps22
Saccharomyco./mycetaceae - ps23
Saccharomycetaceae - ps24
Saccharomycetaceae_2 - ps25
Saccharomycetaceae_3 - ps26
Kaz./Nau./Saccharomyces - ps27
Saccharomyces - ps28
Arboricola_clade - ps29
Cerevisiae_clade - ps30
Saccharomyces_cerevisiae - ps31
Phylostratum

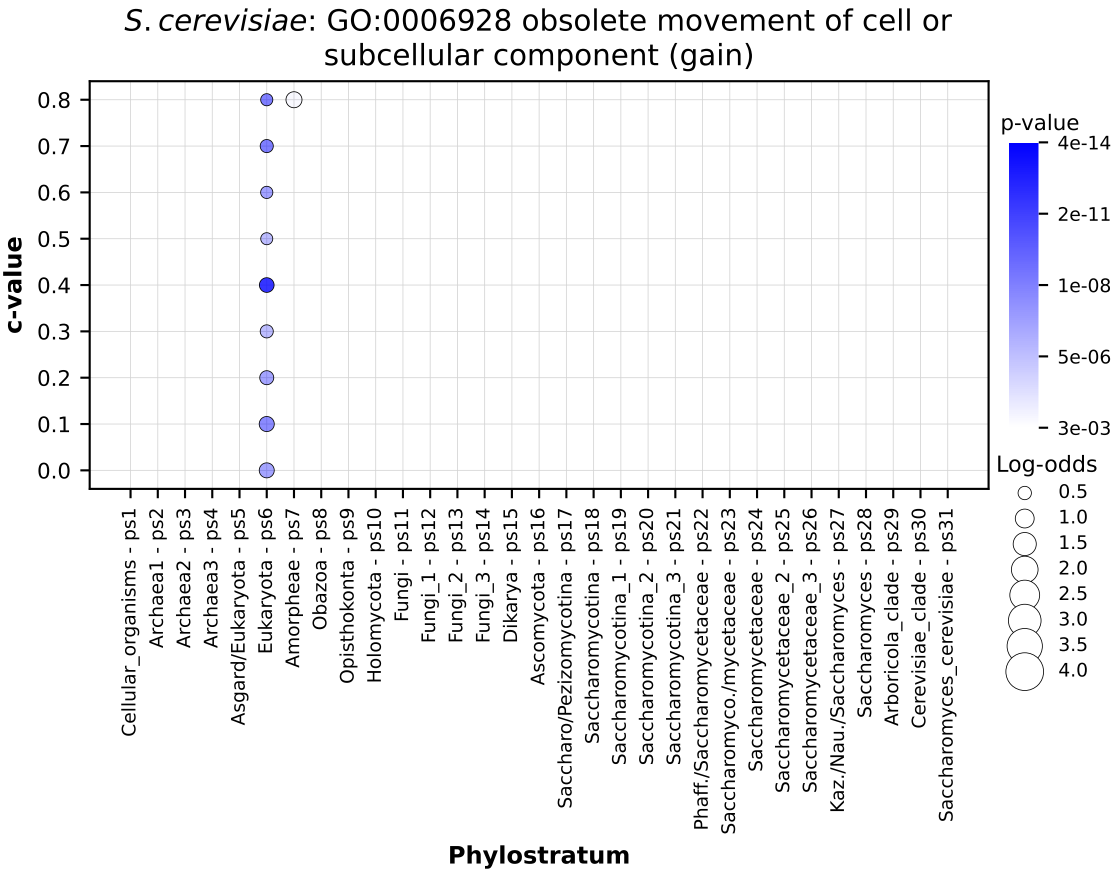

S. cerevisiae: GO:0006928 obsolete movement of cell or subcellular component (gain)
c-value
0.8
0.7
0.6
0.5
0.4
0.3
0.2
0.1
0.0
p-value
4e-14
2e-11
1e-08
5e-06
3e-03
Log-odds
0.5
1.0
1.5
2.0
2.5
3.0
3.5
4.0
Cellular_organisms - ps1
Archaea1 - ps2
Archaea2 - ps3
Archaea3 - ps4
Asgard/Eukaryota - ps5
Eukaryota - ps6
Amorpheae - ps7
Obazoa - ps8
Opisthokonta - ps9
Holomycota - ps10
Fungi - ps11
Fungi_1 - ps12
Fungi_2 - ps13
Fungi_3 - ps14
Dikarya - ps15
Ascomycota - ps16
Saccharo/Pezizomycotina - ps17
Saccharomycotina - ps18
Saccharomycotina_1 - ps19
Saccharomycotina_2 - ps20
Saccharomycotina_3 - ps21
Phaff./Saccharomycetaceae - ps22
Saccharomyco./mycetaceae - ps23
Saccharomycetaceae - ps24
Saccharomycetaceae_2 - ps25
Saccharomycetaceae_3 - ps26
Kaz./Nau./Saccharomyces - ps27
Saccharomyces - ps28
Arboricola_clade - ps29
Cerevisiae_clade - ps30
Saccharomyces_cerevisiae - ps31
Phylostratum

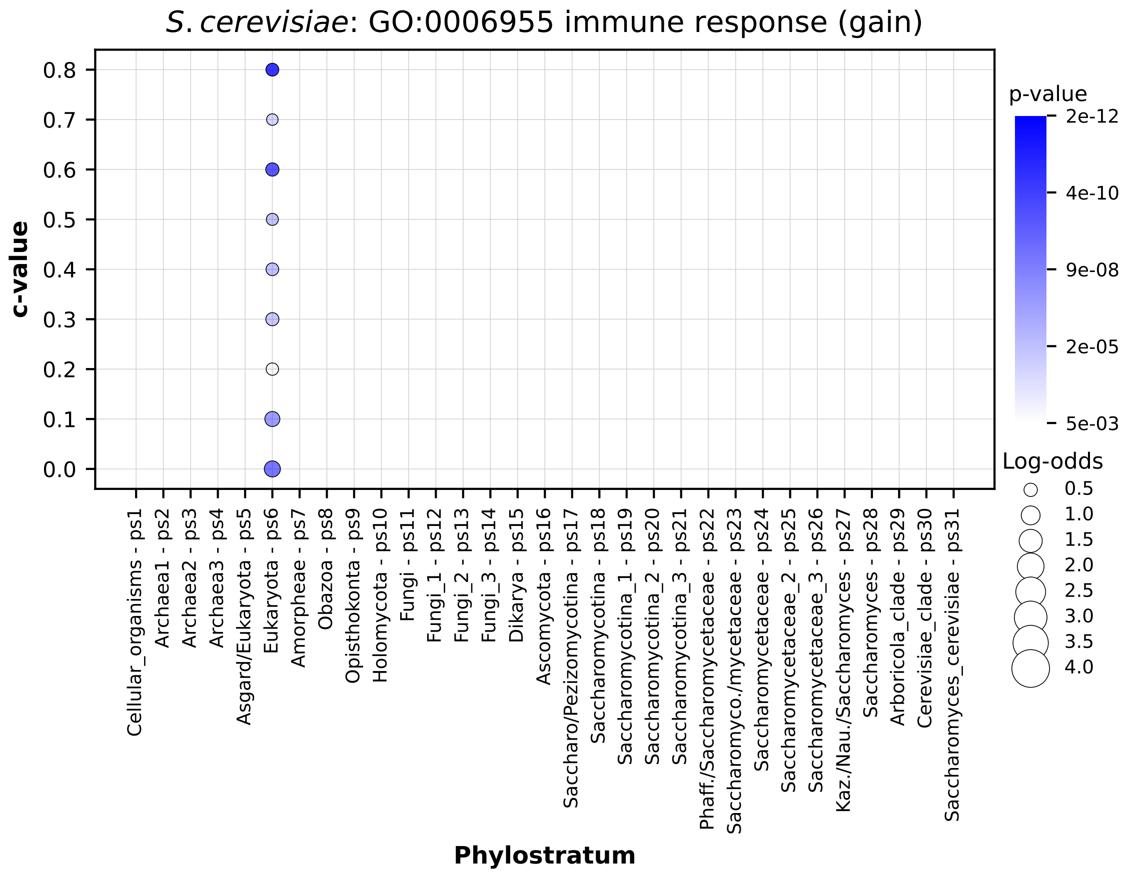

S. cerevisiae: GO:0006955 immune response (gain)
c-value
0.0
0.1
0.2
0.3
0.4
0.5
0.6
0.7
0.8
p-value
2e-12
4e-10
9e-08
2e-05
5e-03
Log-odds
0.5
1.0
1.5
2.0
2.5
3.0
3.5
4.0
Cellular_organisms - ps1
Archaea1 - ps2
Archaea2 - ps3
Archaea3 - ps4
Asgard/Eukaryota - ps5
Eukaryota - ps6
Amorpheae - ps7
Obazoa - ps8
Opisthokonta - ps9
Holomycota - ps10
Fungi - ps11
Fungi_1 - ps12
Fungi_2 - ps13
Fungi_3 - ps14
Dikarya - ps15
Ascomycota - ps16
Saccharo/Pezizomycotina - ps17
Saccharomycotina - ps18
Saccharomycotina_1 - ps19
Saccharomycotina_2 - ps20
Saccharomycotina_3 - ps21
Phaff./Saccharomycetaceae - ps22
Saccharomyco./mycetaceae - ps23
Saccharomycetaceae - ps24
Saccharomycetaceae_2 - ps25
Saccharomycetaceae_3 - ps26
Kaz./Nau./Saccharomyces - ps27
Saccharomyces - ps28
Arboricola_clade - ps29
Cerevisiae_clade - ps30
Saccharomyces_cerevisiae - ps31
Phylostratum

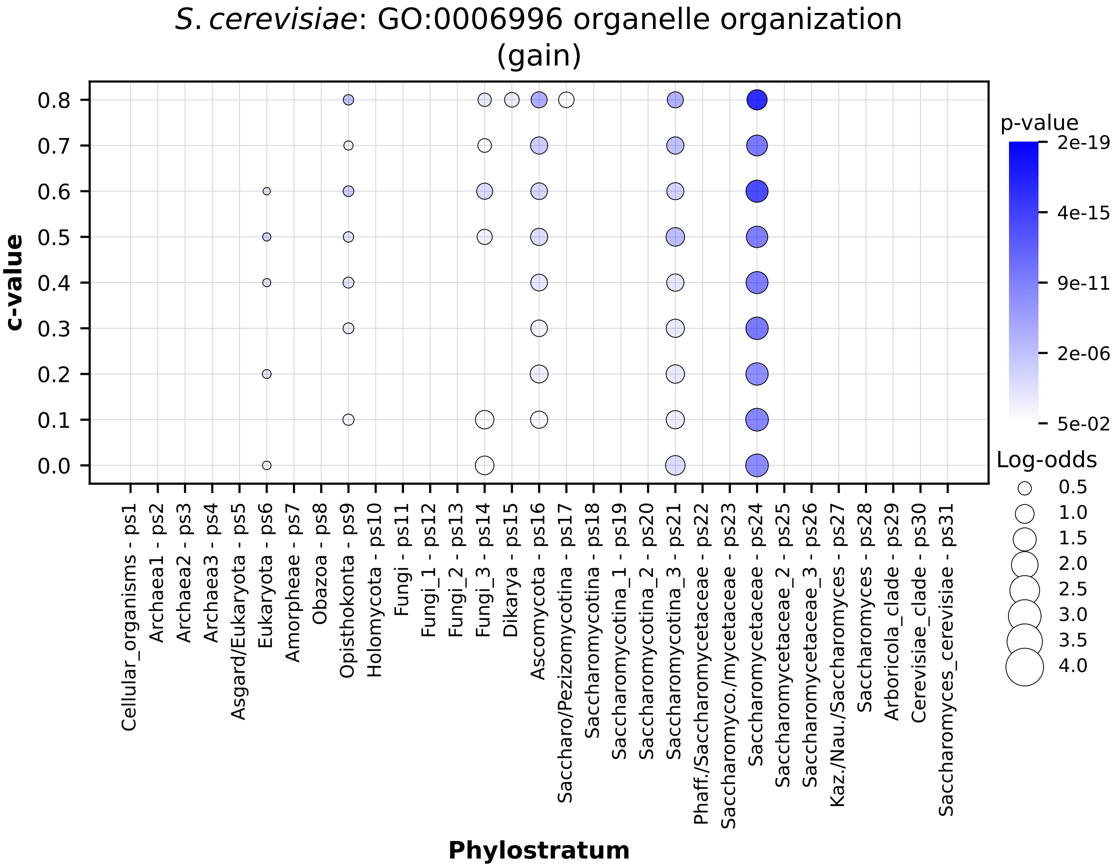

$S. cerevisiae$: GO:0006996 organelle organization
(gain)
c-value
0.8
0.7
0.6
0.5
0.4
0.3
0.2
0.1
0.0
Phylostratum
Cellular_organisms - ps1
Archaea1 - ps2
Archaea2 - ps3
Archaea3 - ps4
Asgard/Eukaryota - ps5
Eukaryota - ps6
Amorpheae - ps7
Obazoa - ps8
Opisthokonta - ps9
Holomycota - ps10
Fungi - ps11
Fungi_1 - ps12
Fungi_2 - ps13
Fungi_3 - ps14
Dikarya - ps15
Ascomycota - ps16
Saccharo/Pezizomycotina - ps17
Saccharomycotina - ps18
Saccharomycotina_1 - ps19
Saccharomycotina_2 - ps20
Saccharomycotina_3 - ps21
Phaff./Saccharomycetaceae - ps22
Saccharomyco./mycetaceae - ps23
Saccharomycetaceae - ps24
Saccharomycetaceae_2 - ps25
Saccharomycetaceae_3 - ps26
Kaz./Nau./Saccharomyces - ps27
Saccharomyces - ps28
Arboricola_clade - ps29
Cerevisiae_clade - ps30
Saccharomyces_cerevisiae - ps31
p-value
2e-19
4e-15
9e-11
2e-06
5e-02
Log-odds
0.5
1.0
1.5
2.0
2.5
3.0
3.5
4.0

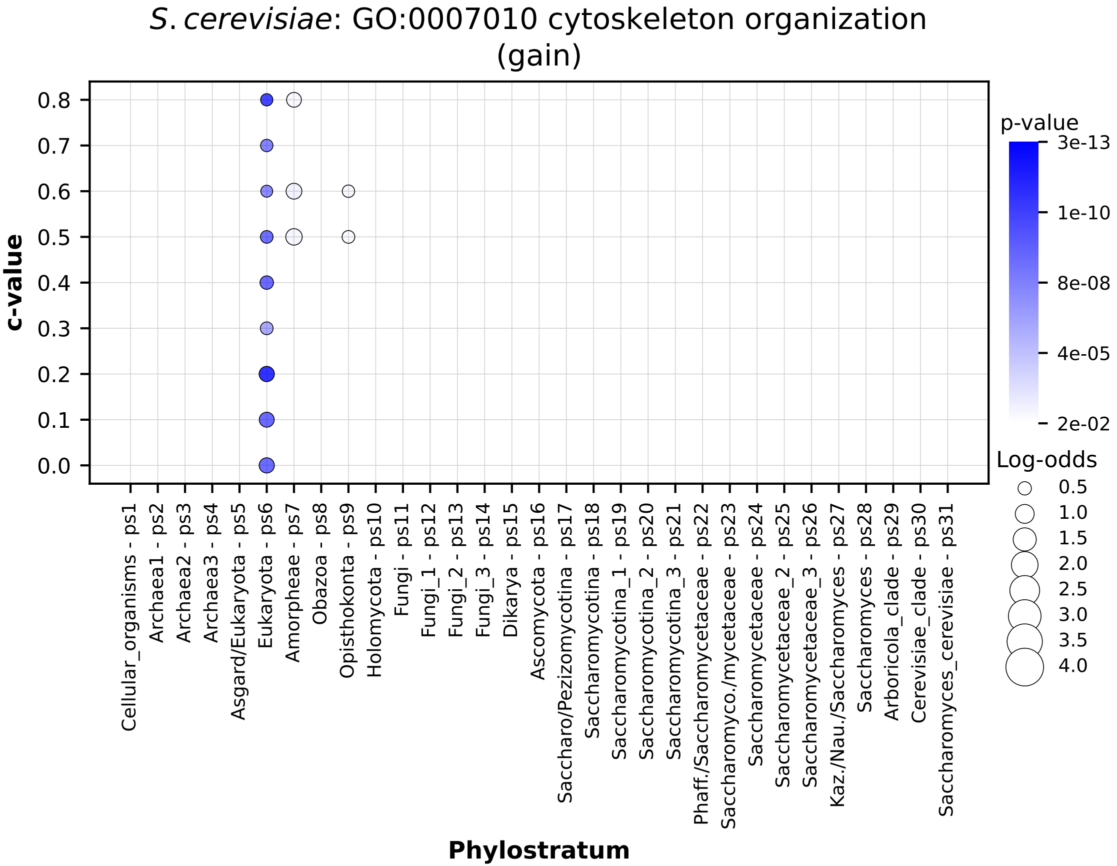

S. cerevisiae: GO:0007010 cytoskeleton organization
(gain)
c-value
0.8
0.7
0.6
0.5
0.4
0.3
0.2
0.1
0.0
p-value
3e-13
1e-10
8e-08
4e-05
2e-02
Log-odds
0.5
1.0
1.5
2.0
2.5
3.0
3.5
4.0
Cellular_organisms - ps1
Archaea1 - ps2
Archaea2 - ps3
Archaea3 - ps4
Asgard/Eukaryota - ps5
Eukaryota - ps6
Amorpheae - ps7
Obazoa - ps8
Opisthokonta - ps9
Holomycota - ps10
Fungi - ps11
Fungi_1 - ps12
Fungi_2 - ps13
Fungi_3 - ps14
Dikarya - ps15
Ascomycota - ps16
Saccharo/Pezizomycotina - ps17
Saccharomycotina - ps18
Saccharomycotina_1 - ps19
Saccharomycotina_2 - ps20
Saccharomycotina_3 - ps21
Phaff./Saccharomycetaceae - ps22
Saccharomyco./mycetaceae - ps23
Saccharomycetaceae - ps24
Saccharomycetaceae_2 - ps25
Saccharomycetaceae_3 - ps26
Kaz./Nau./Saccharomyces - ps27
Saccharomyces - ps28
Arboricola_clade - ps29
Cerevisiae_clade - ps30
Saccharomyces_cerevisiae - ps31
Phylostratum

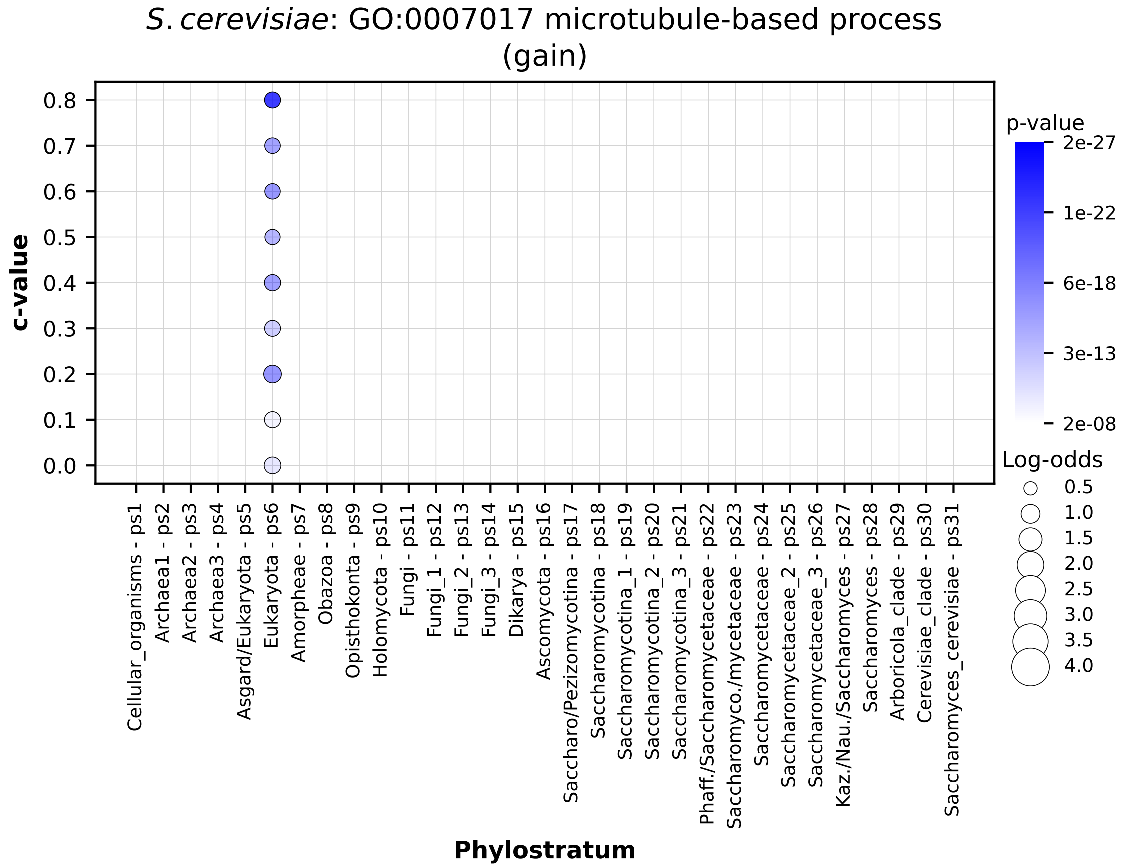

S. cerevisiae: GO:0007017 microtubule-based process
(gain)
c-value
0.0
0.1
0.2
0.3
0.4
0.5
0.6
0.7
0.8
p-value
2e-27
1e-22
6e-18
3e-13
2e-08
Log-odds
0.5
1.0
1.5
2.0
2.5
3.0
3.5
4.0
Cellular_organisms - ps1
Archaea1 - ps2
Archaea2 - ps3
Archaea3 - ps4
Asgard/Eukaryota - ps5
Eukaryota - ps6
Amorpheae - ps7
Obazoa - ps8
Opisthokonta - ps9
Holomycota - ps10
Fungi - ps11
Fungi_1 - ps12
Fungi_2 - ps13
Fungi_3 - ps14
Dikarya - ps15
Ascomycota - ps16
Saccharo/Pezizomycotina - ps17
Saccharomycotina - ps18
Saccharomycotina_1 - ps19
Saccharomycotina_2 - ps20
Saccharomycotina_3 - ps21
Phaff./Saccharomycetaceae - ps22
Saccharomyco./mycetaceae - ps23
Saccharomycetaceae - ps24
Saccharomycetaceae_2 - ps25
Saccharomycetaceae_3 - ps26
Kaz./Nau./Saccharomyces - ps27
Saccharomyces - ps28
Arboricola_clade - ps29
Cerevisiae_clade - ps30
Saccharomyces_cerevisiae - ps31
Phylostratum

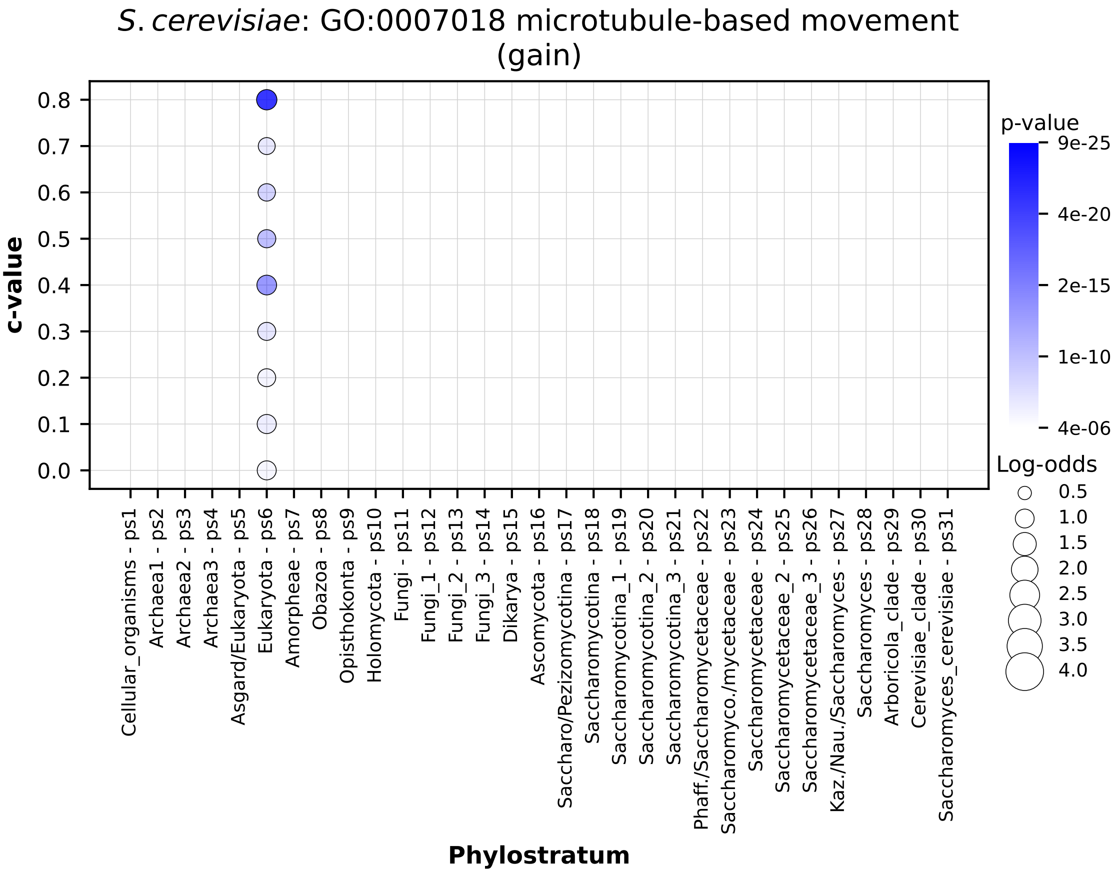

S. cerevisiae: GO:0007018 microtubule-based movement (gain)
c-value
0.8
0.7
0.6
0.5
0.4
0.3
0.2
0.1
0.0
p-value
9e-25
4e-20
2e-15
1e-10
4e-06
Log-odds
0.5
1.0
1.5
2.0
2.5
3.0
3.5
4.0
Cellular_organisms - ps1
Archaea1 - ps2
Archaea2 - ps3
Archaea3 - ps4
Asgard/Eukaryota - ps5
Eukaryota - ps6
Amorpheae - ps7
Obazoa - ps8
Opisthokonta - ps9
Holomycota - ps10
Fungi - ps11
Fungi_1 - ps12
Fungi_2 - ps13
Fungi_3 - ps14
Dikarya - ps15
Ascomycota - ps16
Saccharo/Pezizomycotina - ps17
Saccharomycotina - ps18
Saccharomycotina_1 - ps19
Saccharomycotina_2 - ps20
Saccharomycotina_3 - ps21
Phaff./Saccharomycetaceae - ps22
Saccharomyco./mycetaceae - ps23
Saccharomycetaceae - ps24
Saccharomycetaceae_2 - ps25
Saccharomycetaceae_3 - ps26
Kaz./Nau./Saccharomyces - ps27
Saccharomyces - ps28
Arboricola_clade - ps29
Cerevisiae_clade - ps30
Saccharomyces_cerevisiae - ps31
Phylostratum

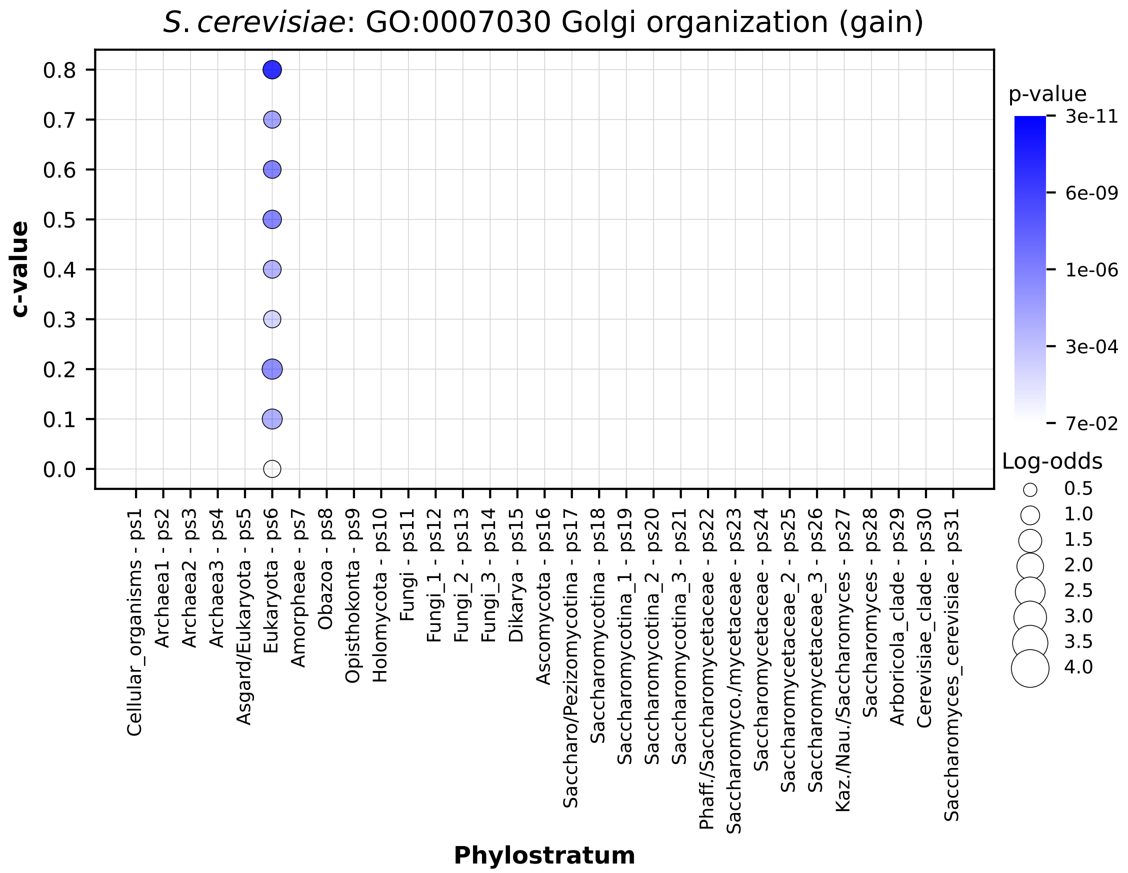

S. cerevisiae: GO:0007030 Golgi organization (gain)
c-value
0.0
0.1
0.2
0.3
0.4
0.5
0.6
0.7
0.8
p-value
3e-11
6e-09
1e-06
3e-04
7e-02
Log-odds
0.5
1.0
1.5
2.0
2.5
3.0
3.5
4.0
Cellular_organisms - ps1
Archaea1 - ps2
Archaea2 - ps3
Archaea3 - ps4
Asgard/Eukaryota - ps5
Eukaryota - ps6
Amorpheae - ps7
Obazoa - ps8
Opisthokonta - ps9
Holomycota - ps10
Fungi - ps11
Fungi_1 - ps12
Fungi_2 - ps13
Fungi_3 - ps14
Dikarya - ps15
Ascomycota - ps16
Saccharo/Pezizomycotina - ps17
Saccharomycotina - ps18
Saccharomycotina_1 - ps19
Saccharomycotina_2 - ps20
Saccharomycotina_3 - ps21
Phaff./Saccharomycetaceae - ps22
Saccharomyco./mycetaceae - ps23
Saccharomycetaceae - ps24
Saccharomycetaceae_2 - ps25
Saccharomycetaceae_3 - ps26
Kaz./Nau./Saccharomyces - ps27
Saccharomyces - ps28
Arboricola_clade - ps29
Cerevisiae_clade - ps30
Saccharomyces_cerevisiae - ps31
Phylostratum

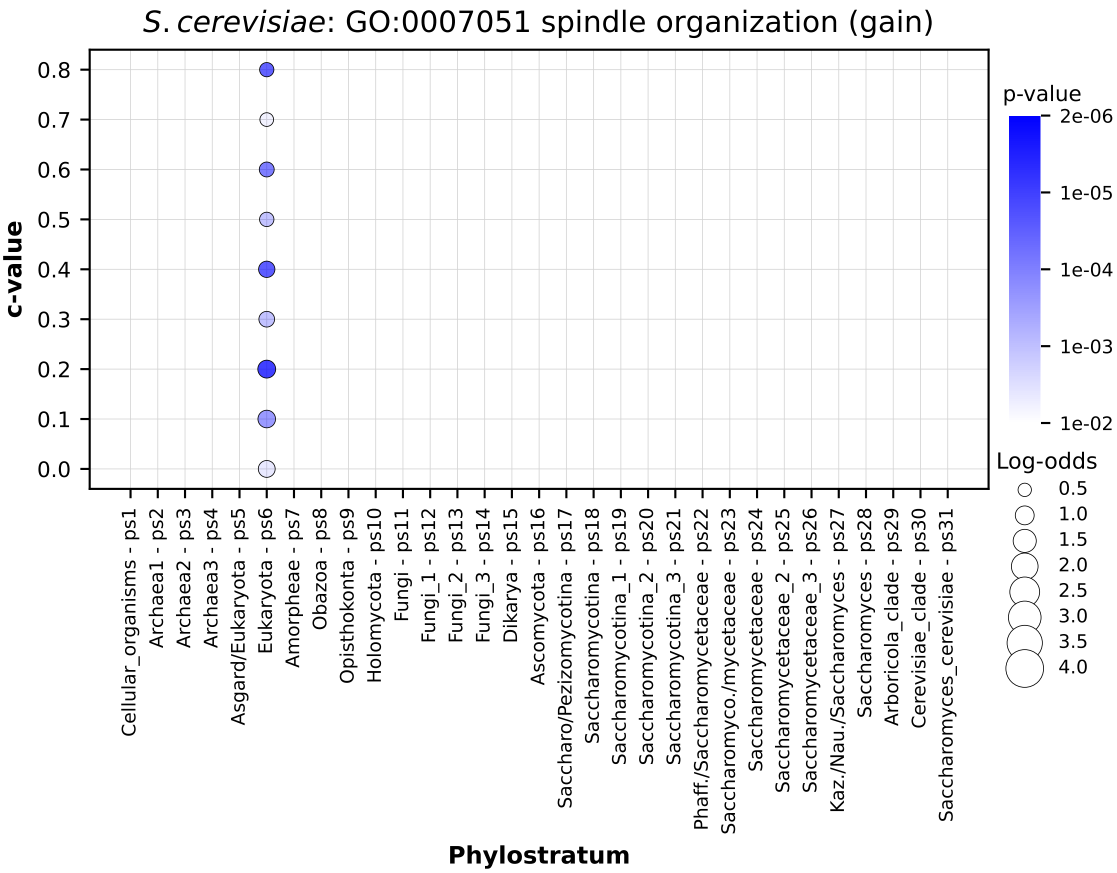
S. cerevisiae: GO:0007051 spindle organization (gain)
c-value
0.0
0.1
0.2
0.3
0.4
0.5
0.6
0.7
0.8
Phylostratum
Cellular_organisms - ps1
Archaea1 - ps2
Archaea2 - ps3
Archaea3 - ps4
Asgard/Eukaryota - ps5
Eukaryota - ps6
Amorpheae - ps7
Obazoa - ps8
Opisthokonta - ps9
Holomycota - ps10
Fungi - ps11
Fungi_1 - ps12
Fungi_2 - ps13
Fungi_3 - ps14
Dikarya - ps15
Ascomycota - ps16
Saccharo/Pezizomycotina - ps17
Saccharomycotina - ps18
Saccharomycotina_1 - ps19
Saccharomycotina_2 - ps20
Saccharomycotina_3 - ps21
Phaff./Saccharomycetaceae - ps22
Saccharomyco./mycetaceae - ps23
Saccharomycetaceae - ps24
Saccharomycetaceae_2 - ps25
Saccharomycetaceae_3 - ps26
Kaz./Nau./Saccharomyces - ps27
Saccharomyces - ps28
Arboricola_clade - ps29
Cerevisiae_clade - ps30
Saccharomyces_cerevisiae - ps31
p-value
2e-06
1e-05
1e-04
1e-03
1e-02
Log-odds
0.5
1.0
1.5
2.0
2.5
3.0
3.5
4.0

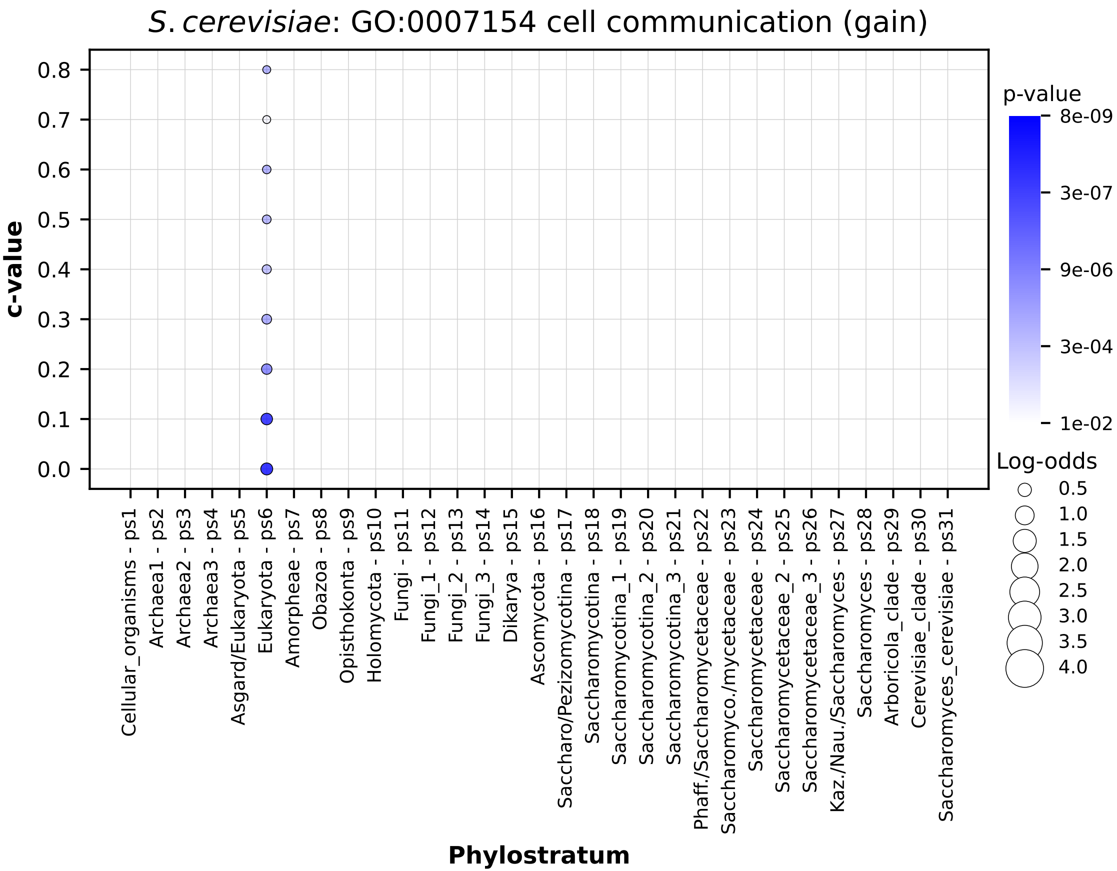

S. cerevisiae: GO:0007154 cell communication (gain)
c-value
0.0
0.1
0.2
0.3
0.4
0.5
0.6
0.7
0.8
p-value
8e-09
3e-07
9e-06
3e-04
1e-02
Log-odds
0.5
1.0
1.5
2.0
2.5
3.0
3.5
4.0
Cellular_organisms - ps1
Archaea1 - ps2
Archaea2 - ps3
Archaea3 - ps4
Asgard/Eukaryota - ps5
Eukaryota - ps6
Amorpheae - ps7
Obazoa - ps8
Opisthokonta - ps9
Holomycota - ps10
Fungi - ps11
Fungi_1 - ps12
Fungi_2 - ps13
Fungi_3 - ps14
Dikarya - ps15
Ascomycota - ps16
Saccharo/Pezizomycotina - ps17
Saccharomycotina - ps18
Saccharomycotina_1 - ps19
Saccharomycotina_2 - ps20
Saccharomycotina_3 - ps21
Phaff./Saccharomycetaceae - ps22
Saccharomyco./mycetaceae - ps23
Saccharomycetaceae - ps24
Saccharomycetaceae_2 - ps25
Saccharomycetaceae_3 - ps26
Kaz./Nau./Saccharomyces - ps27
Saccharomyces - ps28
Arboricola_clade - ps29
Cerevisiae_clade - ps30
Saccharomyces_cerevisiae - ps31
Phylostratum

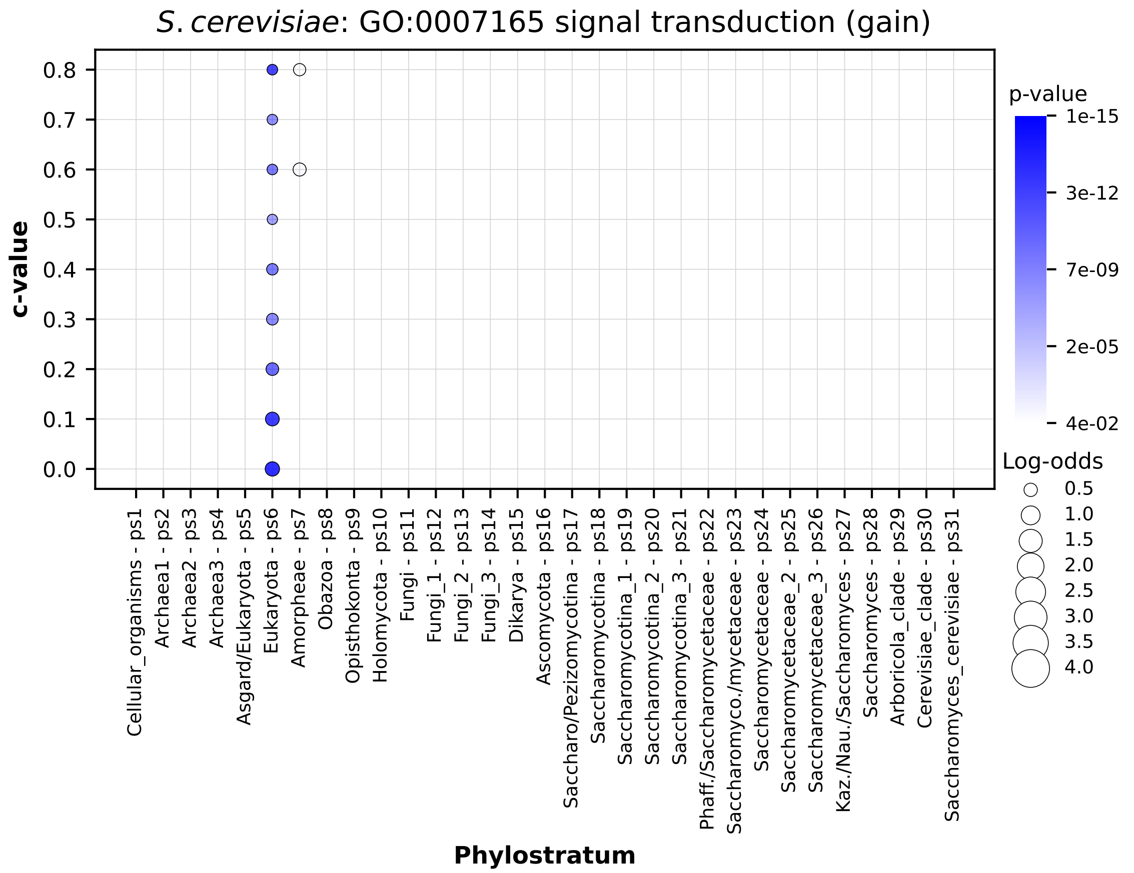
S. cerevisiae: GO:0007165 signal transduction (gain)
c-value
0.0
0.1
0.2
0.3
0.4
0.5
0.6
0.7
0.8
Phylostratum
Cellular_organisms - ps1
Archaea1 - ps2
Archaea2 - ps3
Archaea3 - ps4
Asgard/Eukaryota - ps5
Eukaryota - ps6
Amorpheae - ps7
Obazoa - ps8
Opisthokonta - ps9
Holomycota - ps10
Fungi - ps11
Fungi_1 - ps12
Fungi_2 - ps13
Fungi_3 - ps14
Dikarya - ps15
Ascomycota - ps16
Saccharo/Pezizomycotina - ps17
Saccharomycotina - ps18
Saccharomycotina_1 - ps19
Saccharomycotina_2 - ps20
Saccharomycotina_3 - ps21
Phaff./Saccharomycetaceae - ps22
Saccharomyco./mycetaceae - ps23
Saccharomycetaceae - ps24
Saccharomycetaceae_2 - ps25
Saccharomycetaceae_3 - ps26
Kaz./Nau./Saccharomyces - ps27
Saccharomyces - ps28
Arboricola_clade - ps29
Cerevisiae_clade - ps30
Saccharomyces_cerevisiae - ps31
p-value
1e-15
3e-12
7e-09
2e-05
4e-02
Log-odds
0.5
1.0
1.5
2.0
2.5
3.0
3.5
4.0

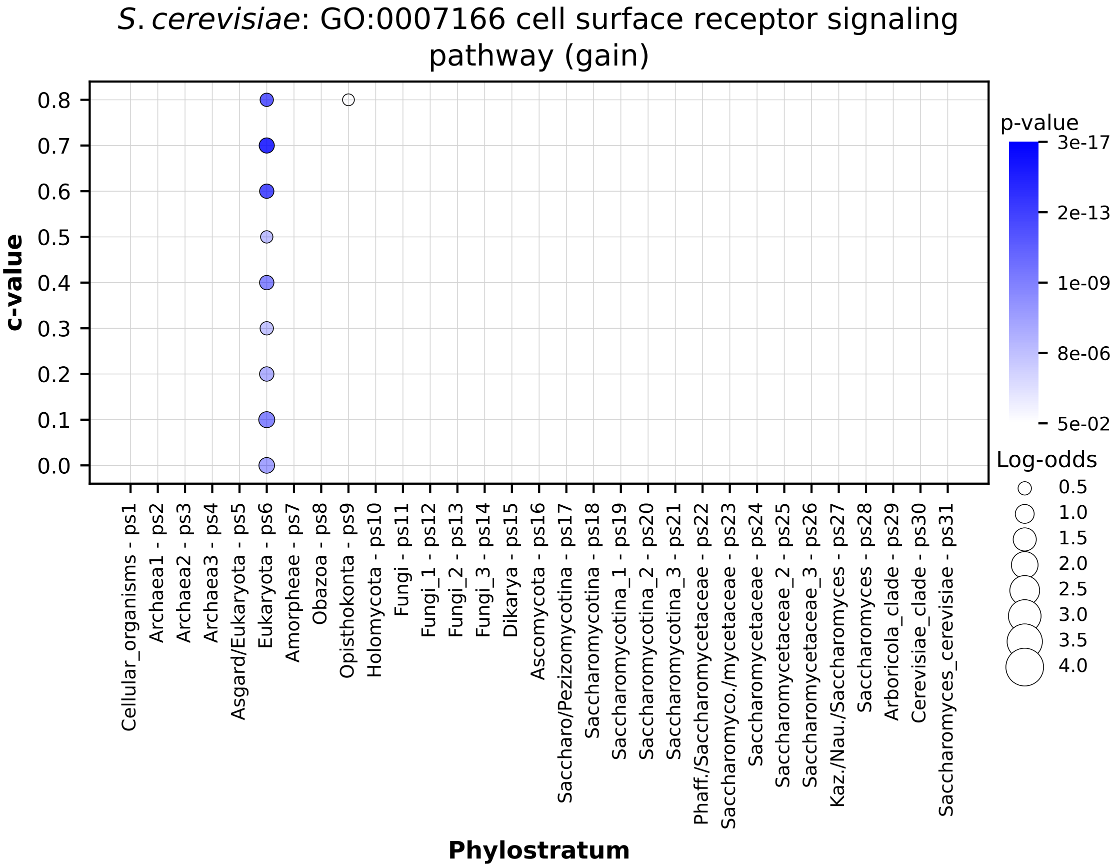

S. cerevisiae: GO:0007166 cell surface receptor signaling pathway (gain)
c-value
0.8
0.7
0.6
0.5
0.4
0.3
0.2
0.1
0.0
Phylostratum
Cellular_organisms - ps1
Archaea1 - ps2
Archaea2 - ps3
Archaea3 - ps4
Asgard/Eukaryota - ps5
Eukaryota - ps6
Amorpheae - ps7
Obazoa - ps8
Opisthokonta - ps9
Holomycota - ps10
Fungi - ps11
Fungi_1 - ps12
Fungi_2 - ps13
Fungi_3 - ps14
Dikarya - ps15
Ascomycota - ps16
Saccharo/Pezizomycotina - ps17
Saccharomycotina - ps18
Saccharomycotina_1 - ps19
Saccharomycotina_2 - ps20
Saccharomycotina_3 - ps21
Phaff./Saccharomycetaceae - ps22
Saccharomyco./mycetaceae - ps23
Saccharomycetaceae - ps24
Saccharomycetaceae_2 - ps25
Saccharomycetaceae_3 - ps26
Kaz./Nau./Saccharomyces - ps27
Saccharomyces - ps28
Arboricola_clade - ps29
Cerevisiae_clade - ps30
Saccharomyces_cerevisiae - ps31
p-value
3e-17
2e-13
1e-09
8e-06
5e-02
Log-odds
0.5
1.0
1.5
2.0
2.5
3.0
3.5
4.0

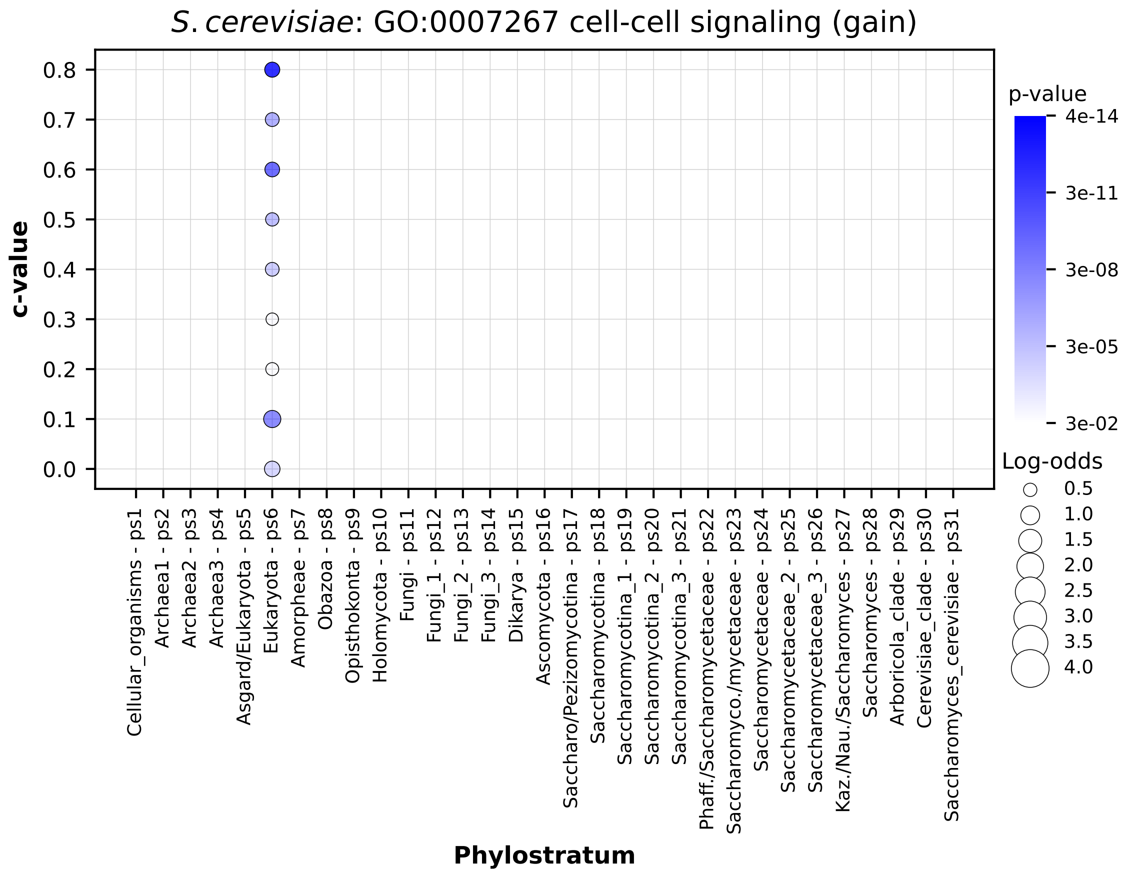

S. cerevisiae: GO:0007267 cell-cell signaling (gain)
c-value
0.0
0.1
0.2
0.3
0.4
0.5
0.6
0.7
0.8
p-value
4e-14
3e-11
3e-08
3e-05
3e-02
Log-odds
0.5
1.0
1.5
2.0
2.5
3.0
3.5
4.0
Cellular_organisms - ps1
Archaea1 - ps2
Archaea2 - ps3
Archaea3 - ps4
Asgard/Eukaryota - ps5
Eukaryota - ps6
Amorpheae - ps7
Obazoa - ps8
Opisthokonta - ps9
Holomycota - ps10
Fungi - ps11
Fungi_1 - ps12
Fungi_2 - ps13
Fungi_3 - ps14
Dikarya - ps15
Ascomycota - ps16
Saccharo/Pezizomycotina - ps17
Saccharomycotina - ps18
Saccharomycotina_1 - ps19
Saccharomycotina_2 - ps20
Saccharomycotina_3 - ps21
Phaff./Saccharomycetaceae - ps22
Saccharomyco./mycetaceae - ps23
Saccharomycetaceae - ps24
Saccharomycetaceae_2 - ps25
Saccharomycetaceae_3 - ps26
Kaz./Nau./Saccharomyces - ps27
Saccharomyces - ps28
Arboricola_clade - ps29
Cerevisiae_clade - ps30
Saccharomyces_cerevisiae - ps31
Phylostratum

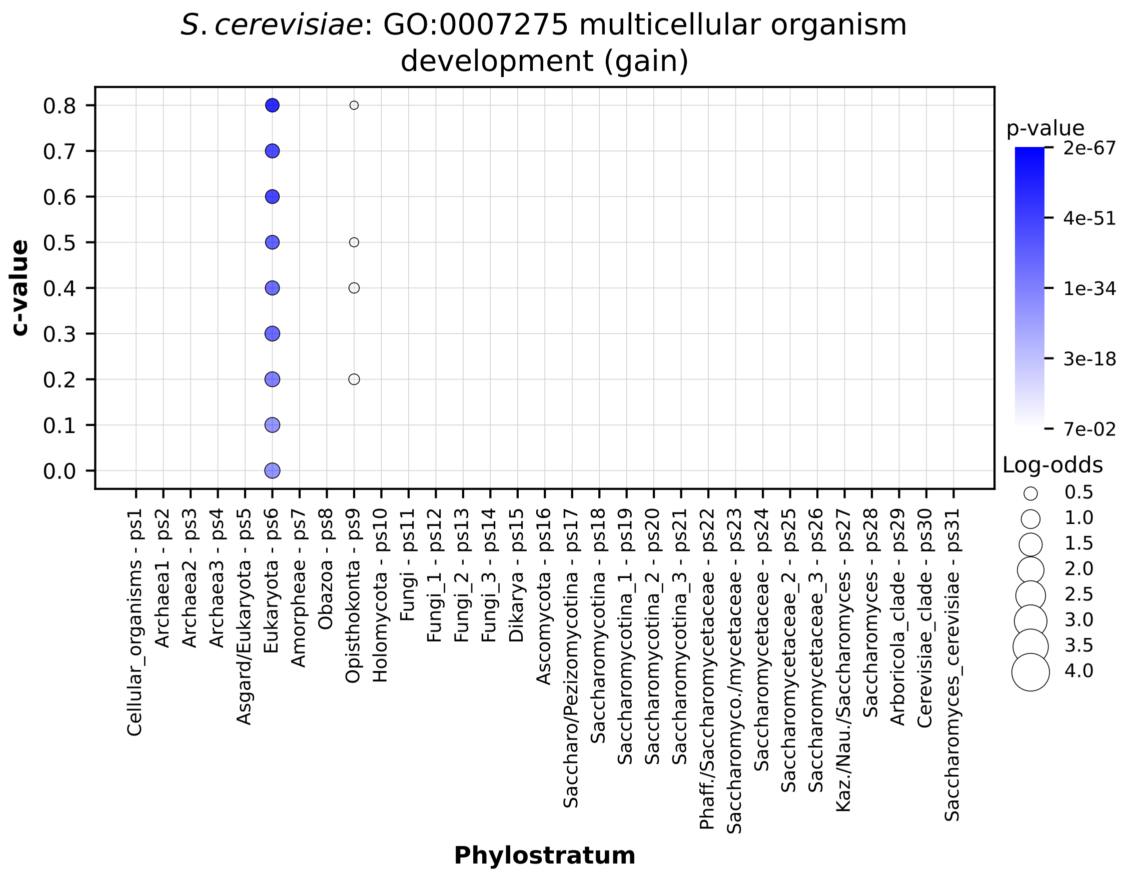

S. cerevisiae: GO:0007275 multicellular organism development (gain)
c-value
0.8
0.7
0.6
0.5
0.4
0.3
0.2
0.1
0.0
p-value
2e-67
4e-51
1e-34
3e-18
7e-02
Log-odds
0.5
1.0
1.5
2.0
2.5
3.0
3.5
4.0
Cellular_organisms - ps1
Archaea1 - ps2
Archaea2 - ps3
Archaea3 - ps4
Asgard/Eukaryota - ps5
Eukaryota - ps6
Amorpheae - ps7
Obazoa - ps8
Opisthokonta - ps9
Holomycota - ps10
Fungi - ps11
Fungi_1 - ps12
Fungi_2 - ps13
Fungi_3 - ps14
Dikarya - ps15
Ascomycota - ps16
Saccharo/Pezizomycotina - ps17
Saccharomycotina - ps18
Saccharomycotina_1 - ps19
Saccharomycotina_2 - ps20
Saccharomycotina_3 - ps21
Phaff./Saccharomycetaceae - ps22
Saccharomyco./mycetaceae - ps23
Saccharomycetaceae - ps24
Saccharomycetaceae_2 - ps25
Saccharomycetaceae_3 - ps26
Kaz./Nau./Saccharomyces - ps27
Saccharomyces - ps28
Arboricola_clade - ps29
Cerevisiae_clade - ps30
Saccharomyces_cerevisiae - ps31
Phylostratum

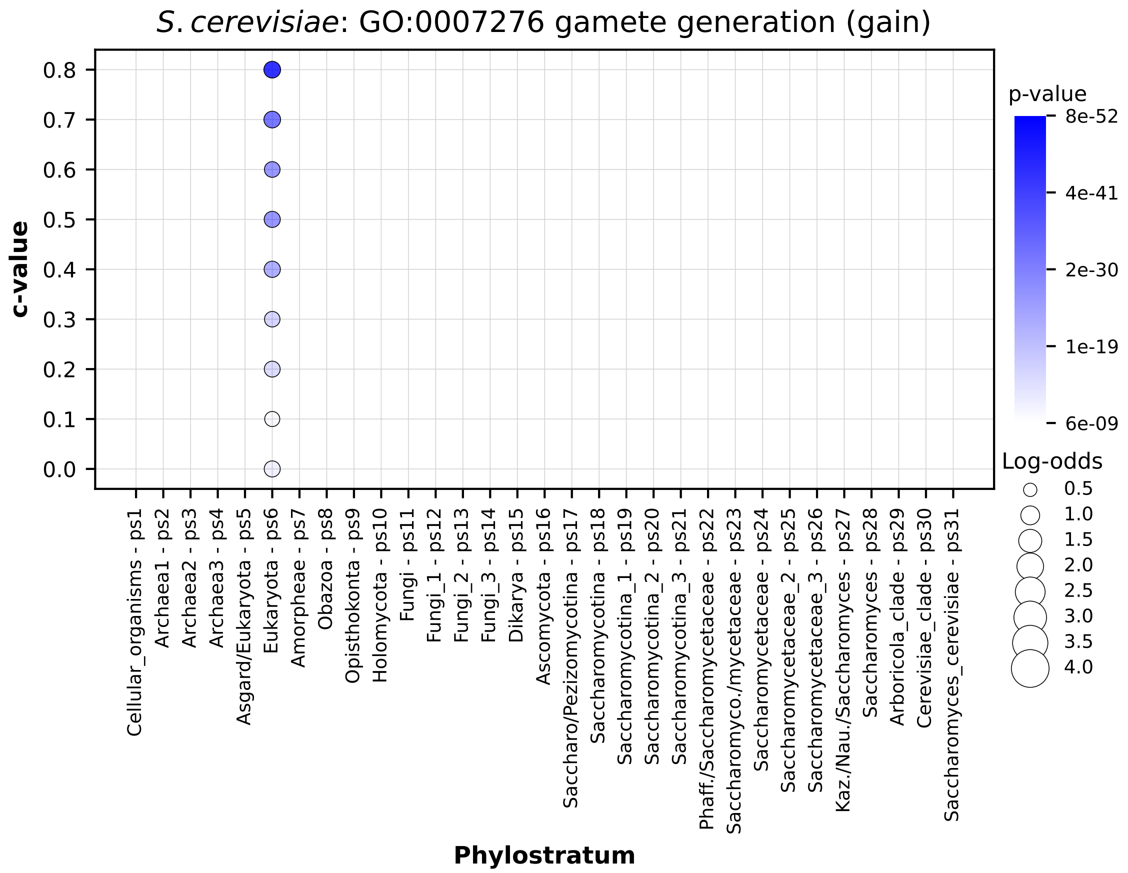

S. cerevisiae: GO:0007276 gamete generation (gain)
c-value
0.8
0.7
0.6
0.5
0.4
0.3
0.2
0.1
0.0
p-value
8e-52
4e-41
2e-30
1e-19
6e-09
Log-odds
0.5
1.0
1.5
2.0
2.5
3.0
3.5
4.0
Cellular_organisms - ps1
Archaea1 - ps2
Archaea2 - ps3
Archaea3 - ps4
Asgard/Eukaryota - ps5
Eukaryota - ps6
Amorpheae - ps7
Obazoa - ps8
Opisthokonta - ps9
Holomycota - ps10
Fungi - ps11
Fungi_1 - ps12
Fungi_2 - ps13
Fungi_3 - ps14
Dikarya - ps15
Ascomycota - ps16
Saccharo/Pezizomycotina - ps17
Saccharomycotina - ps18
Saccharomycotina_1 - ps19
Saccharomycotina_2 - ps20
Saccharomycotina_3 - ps21
Phaff./Saccharomycetaceae - ps22
Saccharomyco./mycetaceae - ps23
Saccharomycetaceae - ps24
Saccharomycetaceae_2 - ps25
Saccharomycetaceae_3 - ps26
Kaz./Nau./Saccharomyces - ps27
Saccharomyces - ps28
Arboricola_clade - ps29
Cerevisiae_clade - ps30
Saccharomyces_cerevisiae - ps31
Phylostratum

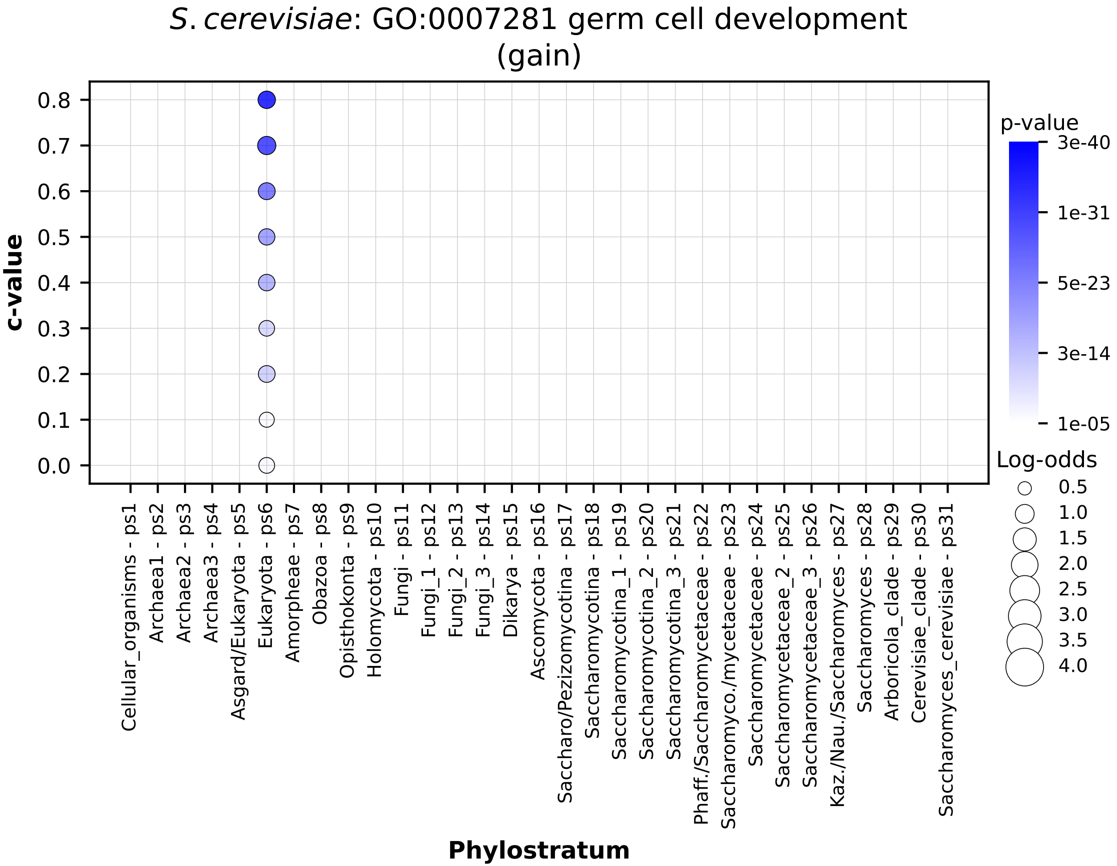

S. cerevisiae: GO:0007281 germ cell development
(gain)
c-value
0.8
0.7
0.6
0.5
0.4
0.3
0.2
0.1
0.0
p-value
3e-40
1e-31
5e-23
3e-14
1e-05
Log-odds
0.5
1.0
1.5
2.0
2.5
3.0
3.5
4.0
Cellular_organisms - ps1
Archaea1 - ps2
Archaea2 - ps3
Archaea3 - ps4
Asgard/Eukaryota - ps5
Eukaryota - ps6
Amorpheae - ps7
Obazoa - ps8
Opisthokonta - ps9
Holomycota - ps10
Fungi - ps11
Fungi_1 - ps12
Fungi_2 - ps13
Fungi_3 - ps14
Dikarya - ps15
Ascomycota - ps16
Saccharo/Pezizomycotina - ps17
Saccharomycotina - ps18
Saccharomycotina_1 - ps19
Saccharomycotina_2 - ps20
Saccharomycotina_3 - ps21
Phaff./Saccharomycetaceae - ps22
Saccharomyco./mycetaceae - ps23
Saccharomycetaceae - ps24
Saccharomycetaceae_2 - ps25
Saccharomycetaceae_3 - ps26
Kaz./Nau./Saccharomyces - ps27
Saccharomyces - ps28
Arboricola_clade - ps29
Cerevisiae_clade - ps30
Saccharomyces_cerevisiae - ps31
Phylostratum

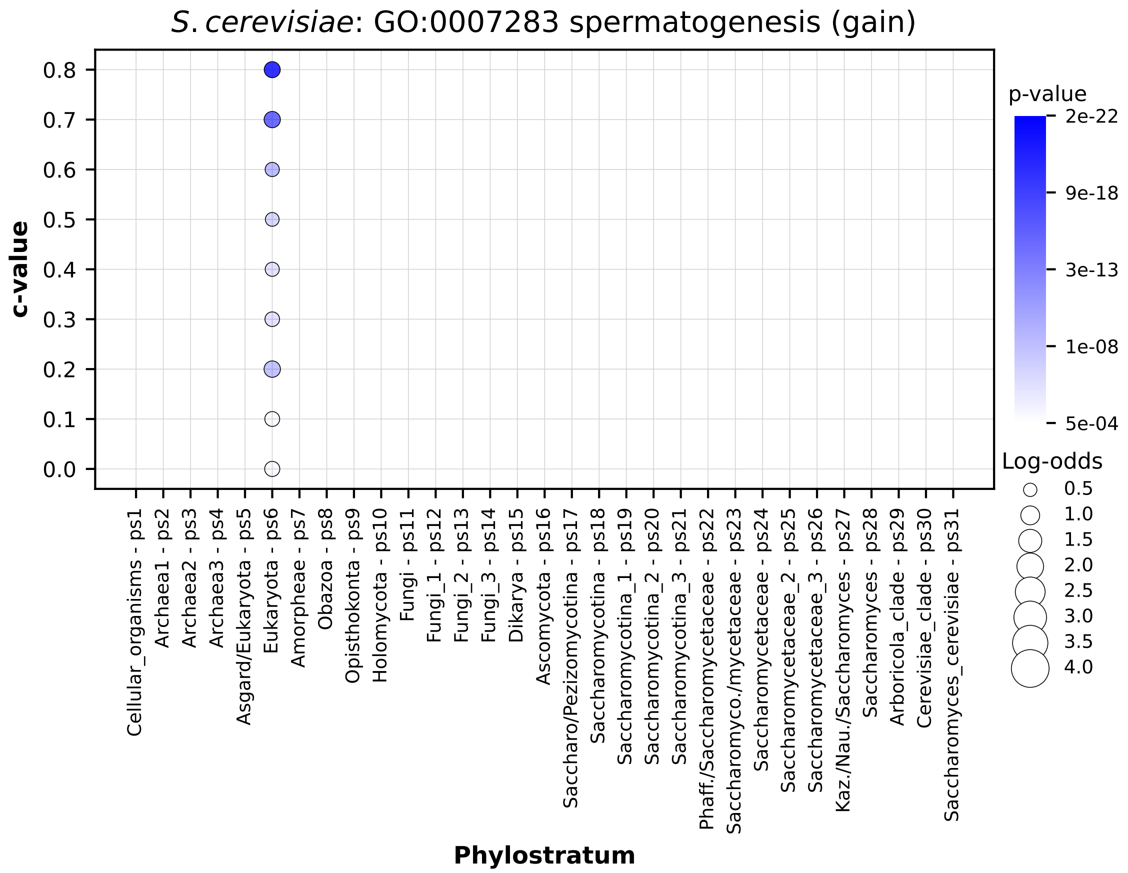

S. cerevisiae: GO:0007283 spermatogenesis (gain)
c-value
0.8
0.7
0.6
0.5
0.4
0.3
0.2
0.1
0.0
p-value
2e-22
9e-18
3e-13
1e-08
5e-04
Log-odds
0.5
1.0
1.5
2.0
2.5
3.0
3.5
4.0
Cellular_organisms - ps1
Archaea1 - ps2
Archaea2 - ps3
Archaea3 - ps4
Asgard/Eukaryota - ps5
Eukaryota - ps6
Amorpheae - ps7
Obazoa - ps8
Opisthokonta - ps9
Holomycota - ps10
Fungi - ps11
Fungi_1 - ps12
Fungi_2 - ps13
Fungi_3 - ps14
Dikarya - ps15
Ascomycota - ps16
Saccharo/Pezizomycotina - ps17
Saccharomycotina - ps18
Saccharomycotina_1 - ps19
Saccharomycotina_2 - ps20
Saccharomycotina_3 - ps21
Phaff./Saccharomycetaceae - ps22
Saccharomyco./mycetaceae - ps23
Saccharomycetaceae - ps24
Saccharomycetaceae_2 - ps25
Saccharomycetaceae_3 - ps26
Kaz./Nau./Saccharomyces - ps27
Saccharomyces - ps28
Arboricola_clade - ps29
Cerevisiae_clade - ps30
Saccharomyces_cerevisiae - ps31
Phylostratum

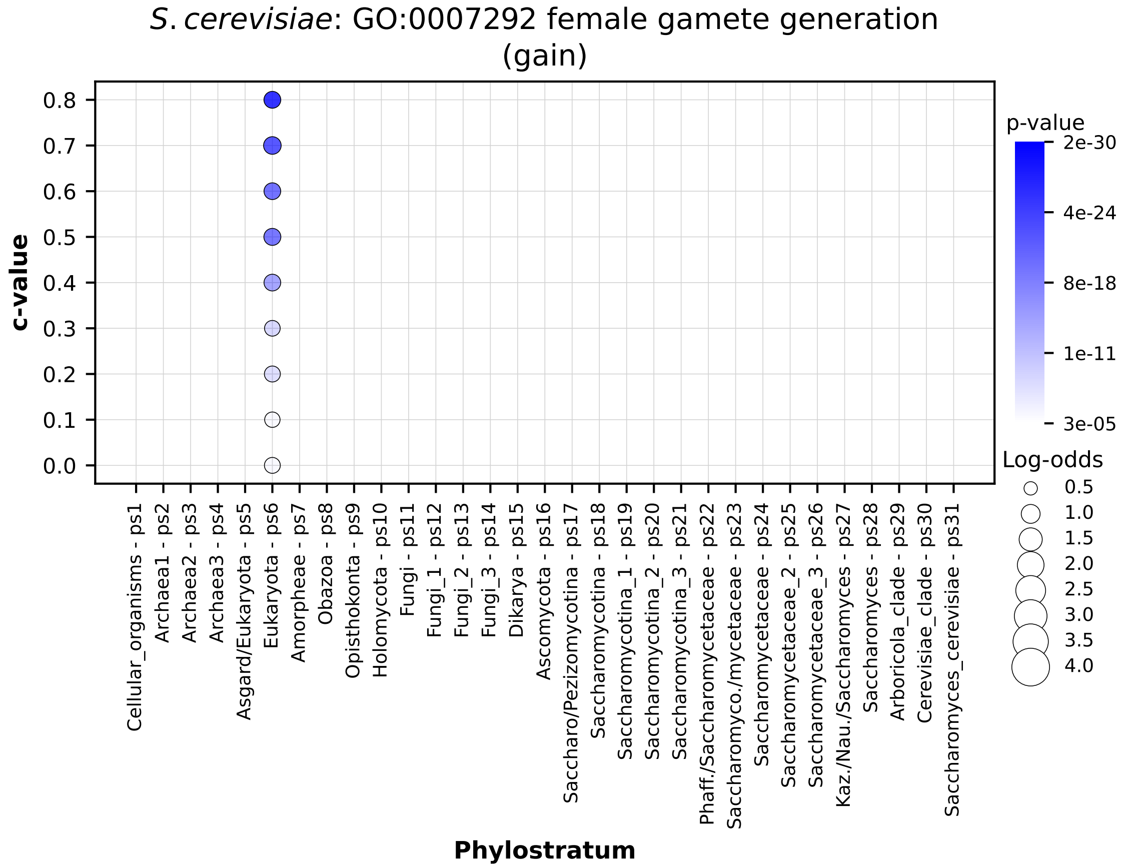

S. cerevisiae: GO:0007292 female gamete generation
(gain)
c-value
0.0
0.1
0.2
0.3
0.4
0.5
0.6
0.7
0.8
p-value
2e-30
4e-24
8e-18
1e-11
3e-05
Log-odds
0.5
1.0
1.5
2.0
2.5
3.0
3.5
4.0
Cellular_organisms - ps1
Archaea1 - ps2
Archaea2 - ps3
Archaea3 - ps4
Asgard/Eukaryota - ps5
Eukaryota - ps6
Amorpheae - ps7
Obazoa - ps8
Opisthokonta - ps9
Holomycota - ps10
Fungi - ps11
Fungi_1 - ps12
Fungi_2 - ps13
Fungi_3 - ps14
Dikarya - ps15
Ascomycota - ps16
Saccharo/Pezizomycotina - ps17
Saccharomycotina - ps18
Saccharomycotina_1 - ps19
Saccharomycotina_2 - ps20
Saccharomycotina_3 - ps21
Phaff./Saccharomycetaceae - ps22
Saccharomyco./mycetaceae - ps23
Saccharomycetaceae - ps24
Saccharomycetaceae_2 - ps25
Saccharomycetaceae_3 - ps26
Kaz./Nau./Saccharomyces - ps27
Saccharomyces - ps28
Arboricola_clade - ps29
Cerevisiae_clade - ps30
Saccharomyces_cerevisiae - ps31
Phylostratum

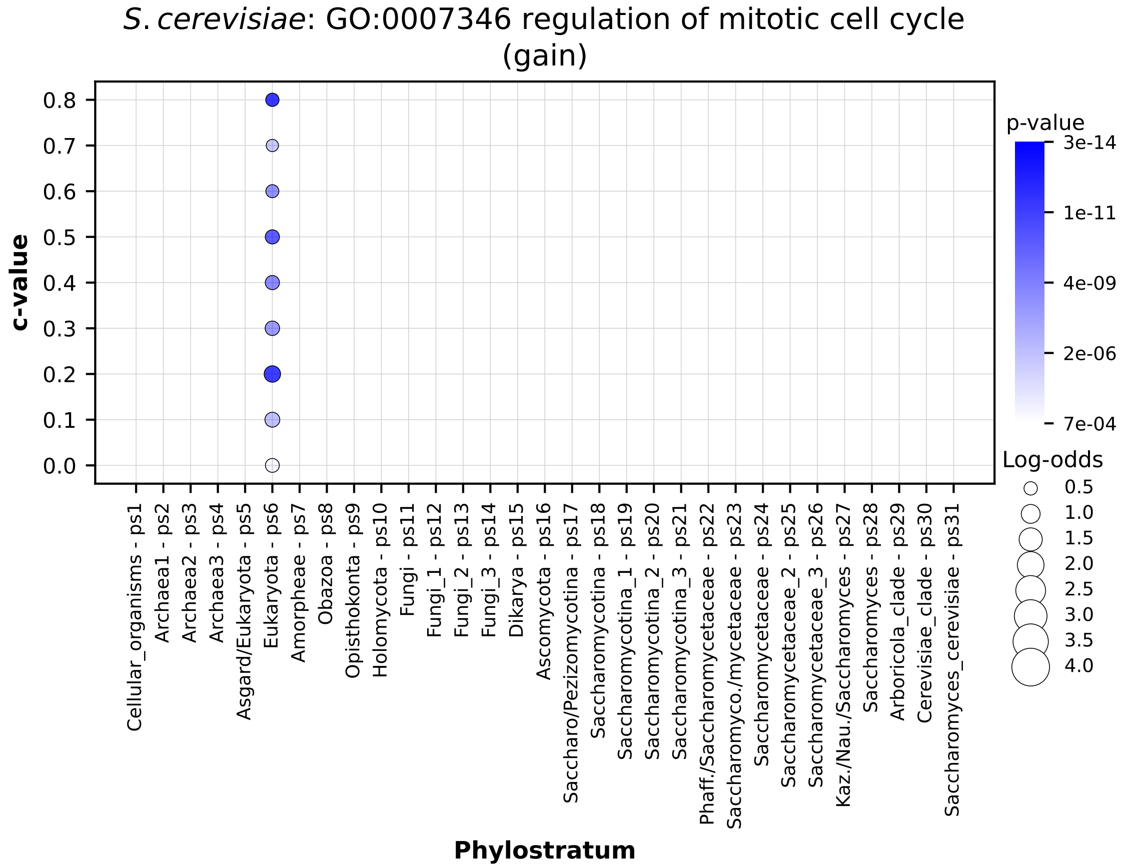

S. cerevisiae: GO:0007346 regulation of mitotic cell cycle (gain)
c-value
0.8
0.7
0.6
0.5
0.4
0.3
0.2
0.1
0.0
Phylostratum
Cellular_organisms - ps1
Archaea1 - ps2
Archaea2 - ps3
Archaea3 - ps4
Asgard/Eukaryota - ps5
Eukaryota - ps6
Amorpheae - ps7
Obazoa - ps8
Opisthokonta - ps9
Holomycota - ps10
Fungi - ps11
Fungi_1 - ps12
Fungi_2 - ps13
Fungi_3 - ps14
Dikarya - ps15
Ascomycota - ps16
Saccharo/Pezizomycotina - ps17
Saccharomycotina - ps18
Saccharomycotina_1 - ps19
Saccharomycotina_2 - ps20
Saccharomycotina_3 - ps21
Phaff./Saccharomycetaceae - ps22
Saccharomyco./mycetaceae - ps23
Saccharomycetaceae - ps24
Saccharomycetaceae_2 - ps25
Saccharomycetaceae_3 - ps26
Kaz./Nau./Saccharomyces - ps27
Saccharomyces - ps28
Arboricola_clade - ps29
Cerevisiae_clade - ps30
Saccharomyces_cerevisiae - ps31
p-value
3e-14
1e-11
4e-09
2e-06
7e-04
Log-odds
0.5
1.0
1.5
2.0
2.5
3.0
3.5
4.0

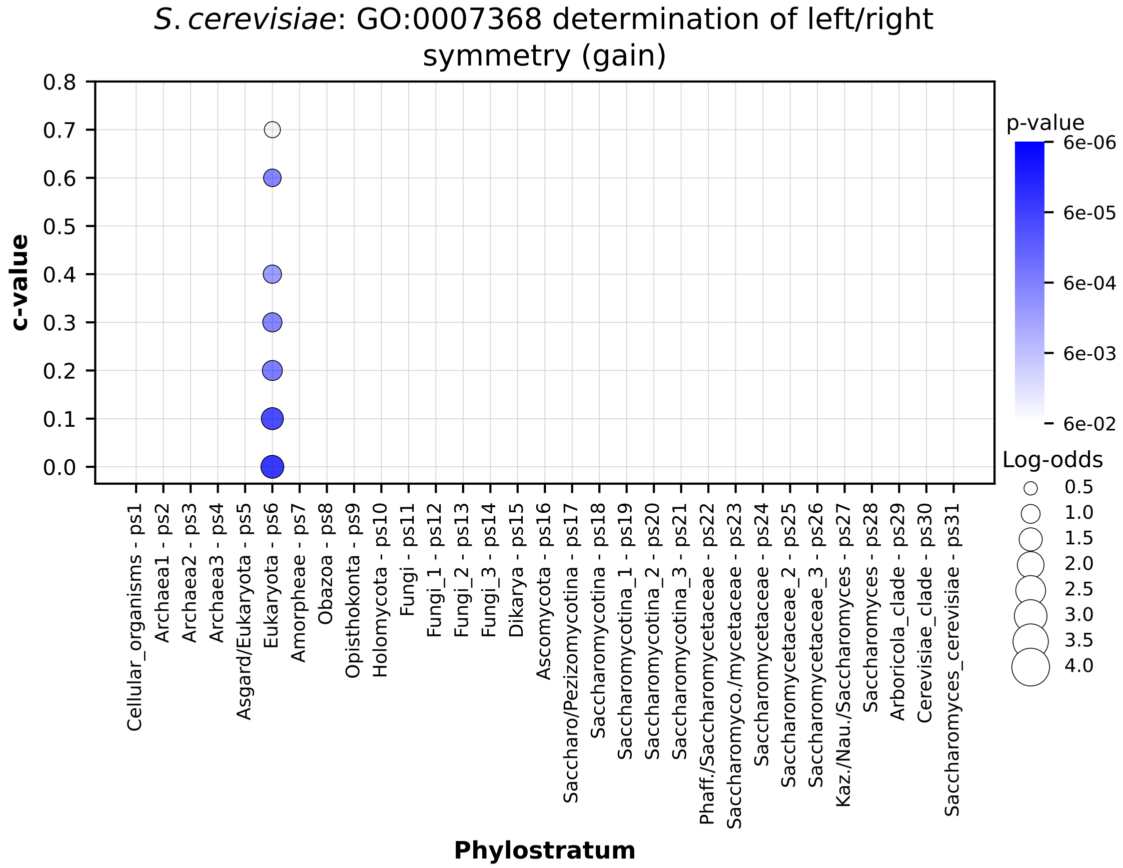

S. cerevisiae: GO:0007368 determination of left/right symmetry (gain)
c-value
0.8
0.7
0.6
0.5
0.4
0.3
0.2
0.1
0.0
p-value
6e-06
6e-05
6e-04
6e-03
6e-02
Log-odds
0.5
1.0
1.5
2.0
2.5
3.0
3.5
4.0
Cellular_organisms - ps1
Archaea1 - ps2
Archaea2 - ps3
Archaea3 - ps4
Asgard/Eukaryota - ps5
Eukaryota - ps6
Amorpheae - ps7
Obazoa - ps8
Opisthokonta - ps9
Holomycota - ps10
Fungi - ps11
Fungi_1 - ps12
Fungi_2 - ps13
Fungi_3 - ps14
Dikarya - ps15
Ascomycota - ps16
Saccharo/Pezizomycotina - ps17
Saccharomycotina - ps18
Saccharomycotina_1 - ps19
Saccharomycotina_2 - ps20
Saccharomycotina_3 - ps21
Phaff./Saccharomycetaceae - ps22
Saccharomyco./mycetaceae - ps23
Saccharomycetaceae - ps24
Saccharomycetaceae_2 - ps25
Saccharomycetaceae_3 - ps26
Kaz./Nau./Saccharomyces - ps27
Saccharomyces - ps28
Arboricola_clade - ps29
Cerevisiae_clade - ps30
Saccharomyces_cerevisiae - ps31
Phylostratum

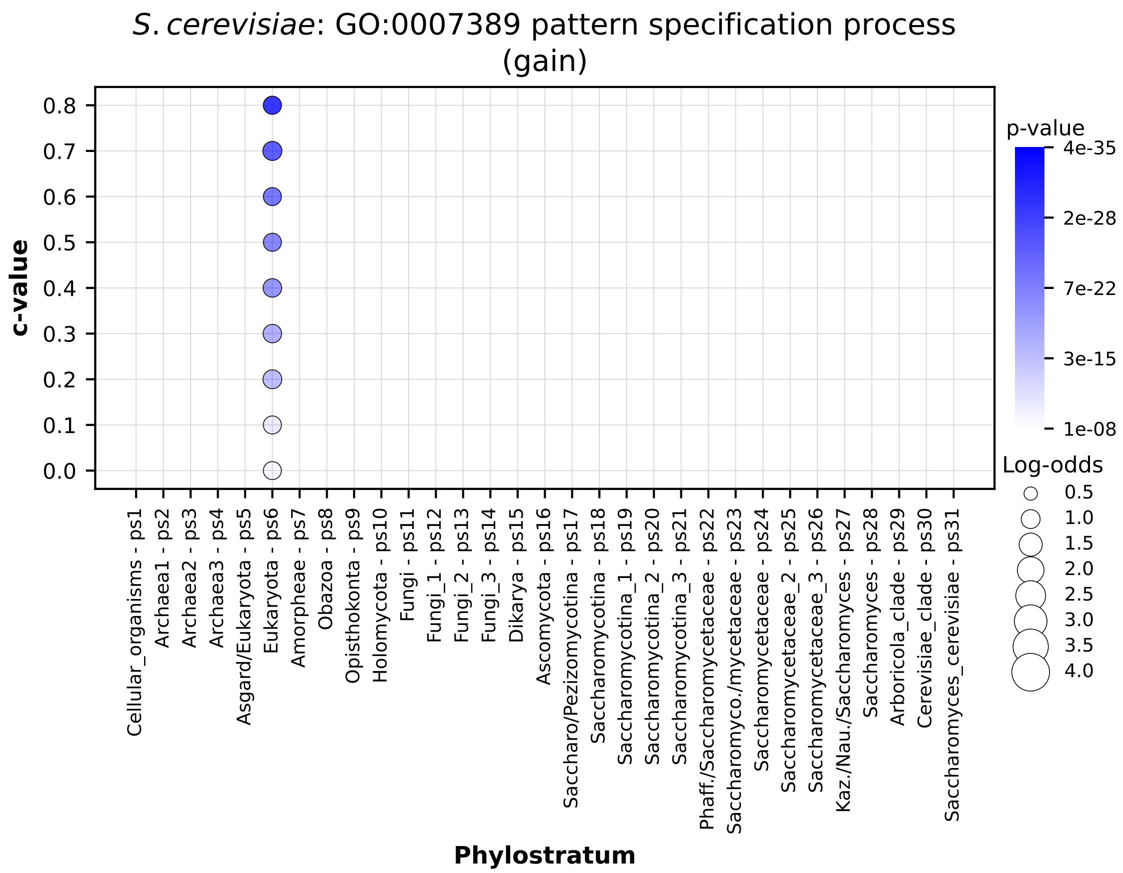

S. cerevisiae: GO:0007389 pattern specification process
(gain)
c-value
0.8
0.7
0.6
0.5
0.4
0.3
0.2
0.1
0.0
p-value
4e-35
2e-28
7e-22
3e-15
1e-08
Log-odds
0.5
1.0
1.5
2.0
2.5
3.0
3.5
4.0
Cellular_organisms - ps1
Archaea1 - ps2
Archaea2 - ps3
Archaea3 - ps4
Asgard/Eukaryota - ps5
Eukaryota - ps6
Amorpheae - ps7
Obazoa - ps8
Opisthokonta - ps9
Holomycota - ps10
Fungi - ps11
Fungi_1 - ps12
Fungi_2 - ps13
Fungi_3 - ps14
Dikarya - ps15
Ascomycota - ps16
Saccharo/Pezizomycotina - ps17
Saccharomycotina - ps18
Saccharomycotina_1 - ps19
Saccharomycotina_2 - ps20
Saccharomycotina_3 - ps21
Phaff./Saccharomycetaceae - ps22
Saccharomyco./mycetaceae - ps23
Saccharomycetaceae - ps24
Saccharomycetaceae_2 - ps25
Saccharomycetaceae_3 - ps26
Kaz./Nau./Saccharomyces - ps27
Saccharomyces - ps28
Arboricola_clade - ps29
Cerevisiae_clade - ps30
Saccharomyces_cerevisiae - ps31
Phylostratum

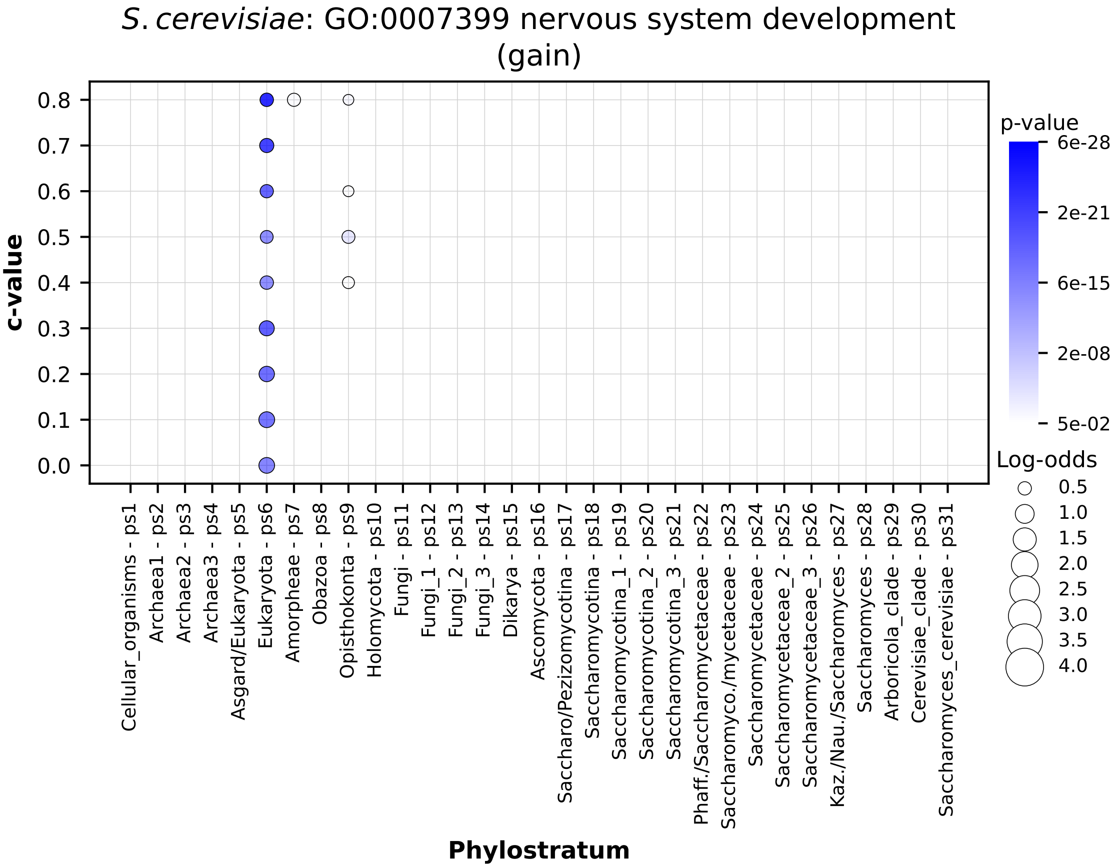
S. cerevisiae: GO:0007399 nervous system development (gain)
c-value
0.0
0.1
0.2
0.3
0.4
0.5
0.6
0.7
0.8
p-value
6e-28
2e-21
6e-15
2e-08
5e-02
Log-odds
0.5
1.0
1.5
2.0
2.5
3.0
3.5
4.0
Cellular_organisms - ps1
Archaea1 - ps2
Archaea2 - ps3
Archaea3 - ps4
Asgard/Eukaryota - ps5
Eukaryota - ps6
Amorpheae - ps7
Obazoa - ps8
Opisthokonta - ps9
Holomycota - ps10
Fungi - ps11
Fungi_1 - ps12
Fungi_2 - ps13
Fungi_3 - ps14
Dikarya - ps15
Ascomycota - ps16
Saccharo/Pezizomycotina - ps17
Saccharomycotina - ps18
Saccharomycotina_1 - ps19
Saccharomycotina_2 - ps20
Saccharomycotina_3 - ps21
Phaff./Saccharomycetaceae - ps22
Saccharomyco./mycetaceae - ps23
Saccharomycetaceae - ps24
Saccharomycetaceae_2 - ps25
Saccharomycetaceae_3 - ps26
Kaz./Nau./Saccharomyces - ps27
Saccharomyces - ps28
Arboricola_clade - ps29
Cerevisiae_clade - ps30
Saccharomyces_cerevisiae - ps31
Phylostratum

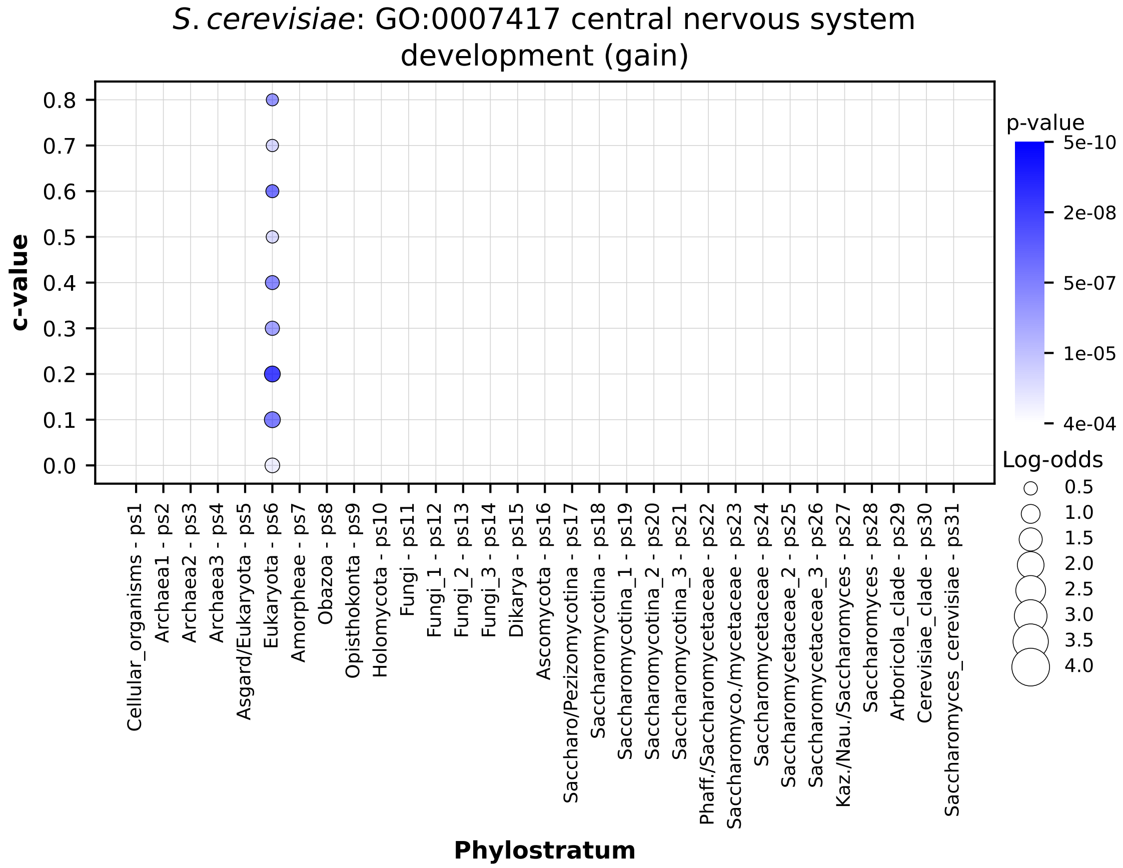

S. cerevisiae: GO:0007417 central nervous system development (gain)
c-value
0.8
0.7
0.6
0.5
0.4
0.3
0.2
0.1
0.0
p-value
5e-10
2e-08
5e-07
1e-05
4e-04
Log-odds
0.5
1.0
1.5
2.0
2.5
3.0
3.5
4.0
Cellular_organisms - ps1
Archaea1 - ps2
Archaea2 - ps3
Archaea3 - ps4
Asgard/Eukaryota - ps5
Eukaryota - ps6
Amorpheae - ps7
Obazoa - ps8
Opisthokonta - ps9
Holomycota - ps10
Fungi - ps11
Fungi_1 - ps12
Fungi_2 - ps13
Fungi_3 - ps14
Dikarya - ps15
Ascomycota - ps16
Saccharo/Pezizomycotina - ps17
Saccharomycotina - ps18
Saccharomycotina_1 - ps19
Saccharomycotina_2 - ps20
Saccharomycotina_3 - ps21
Phaff./Saccharomycetaceae - ps22
Saccharomyco./mycetaceae - ps23
Saccharomycetaceae - ps24
Saccharomycetaceae_2 - ps25
Saccharomycetaceae_3 - ps26
Kaz./Nau./Saccharomyces - ps27
Saccharomyces - ps28
Arboricola_clade - ps29
Cerevisiae_clade - ps30
Saccharomyces_cerevisiae - ps31
Phylostratum

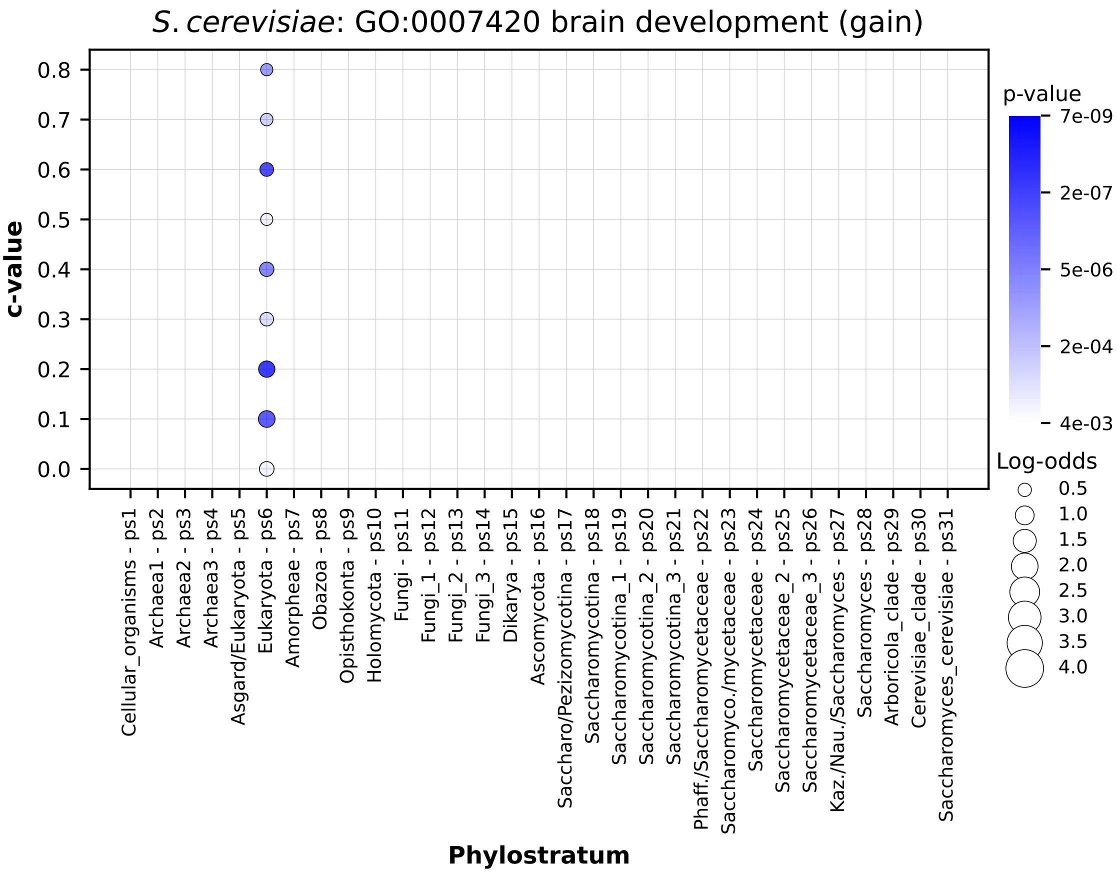

S. cerevisiae: GO:0007420 brain development (gain)
c-value
0.0
0.1
0.2
0.3
0.4
0.5
0.6
0.7
0.8
p-value
7e-09
2e-07
5e-06
2e-04
4e-03
Log-odds
0.5
1.0
1.5
2.0
2.5
3.0
3.5
4.0
Cellular_organisms - ps1
Archaea1 - ps2
Archaea2 - ps3
Archaea3 - ps4
Asgard/Eukaryota - ps5
Eukaryota - ps6
Amorpheae - ps7
Obazoa - ps8
Opisthokonta - ps9
Holomycota - ps10
Fungi - ps11
Fungi_1 - ps12
Fungi_2 - ps13
Fungi_3 - ps14
Dikarya - ps15
Ascomycota - ps16
Saccharo/Pezizomycotina - ps17
Saccharomycotina - ps18
Saccharomycotina_1 - ps19
Saccharomycotina_2 - ps20
Saccharomycotina_3 - ps21
Phaff./Saccharomycetaceae - ps22
Saccharomyco./mycetaceae - ps23
Saccharomycetaceae - ps24
Saccharomycetaceae_2 - ps25
Saccharomycetaceae_3 - ps26
Kaz./Nau./Saccharomyces - ps27
Saccharomyces - ps28
Arboricola_clade - ps29
Cerevisiae_clade - ps30
Saccharomyces_cerevisiae - ps31
Phylostratum

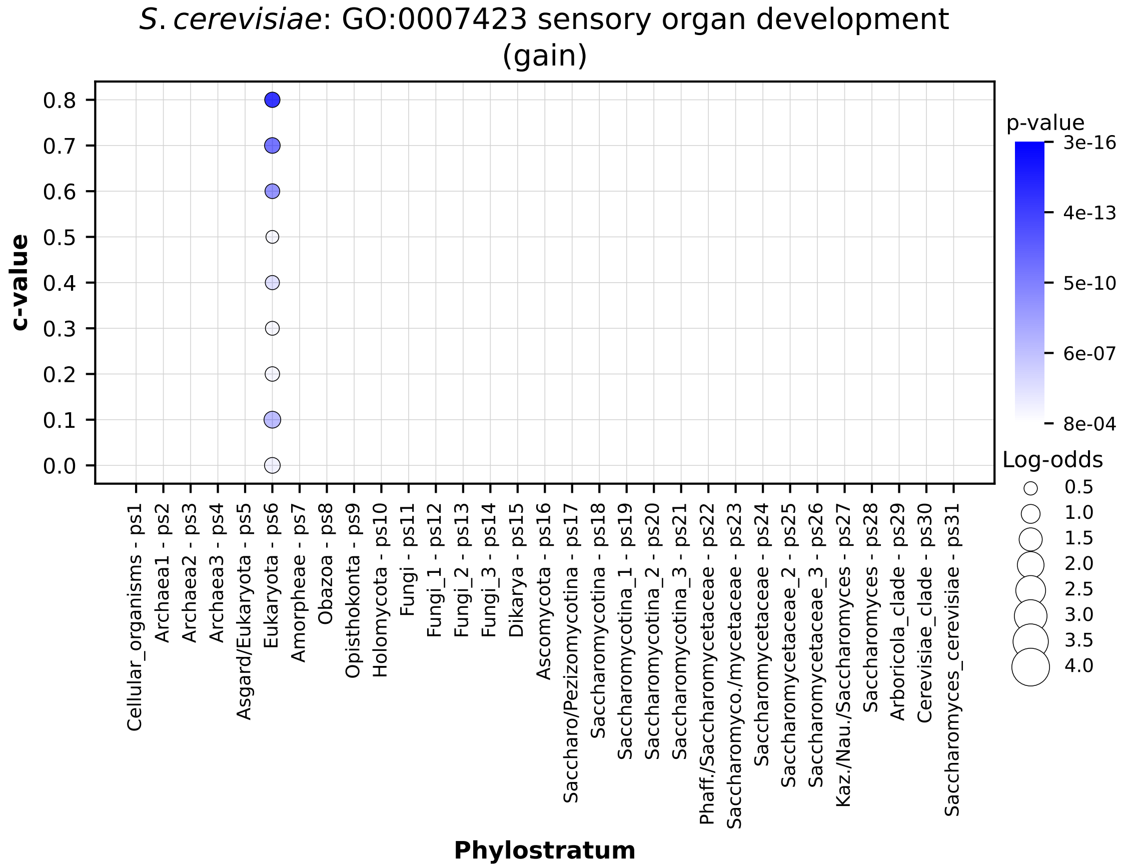

S. cerevisiae: GO:0007423 sensory organ development (gain)
c-value
0.8
0.7
0.6
0.5
0.4
0.3
0.2
0.1
0.0
p-value
3e-16
4e-13
5e-10
6e-07
8e-04
Log-odds
0.5
1.0
1.5
2.0
2.5
3.0
3.5
4.0
Cellular_organisms - ps1
Archaea1 - ps2
Archaea2 - ps3
Archaea3 - ps4
Asgard/Eukaryota - ps5
Eukaryota - ps6
Amorpheae - ps7
Obazoa - ps8
Opisthokonta - ps9
Holomycota - ps10
Fungi - ps11
Fungi_1 - ps12
Fungi_2 - ps13
Fungi_3 - ps14
Dikarya - ps15
Ascomycota - ps16
Saccharo/Pezizomycotina - ps17
Saccharomycotina - ps18
Saccharomycotina_1 - ps19
Saccharomycotina_2 - ps20
Saccharomycotina_3 - ps21
Phaff./Saccharomycetaceae - ps22
Saccharomyco./mycetaceae - ps23
Saccharomycetaceae - ps24
Saccharomycetaceae_2 - ps25
Saccharomycetaceae_3 - ps26
Kaz./Nau./Saccharomyces - ps27
Saccharomyces - ps28
Arboricola_clade - ps29
Cerevisiae_clade - ps30
Saccharomyces_cerevisiae - ps31
Phylostratum

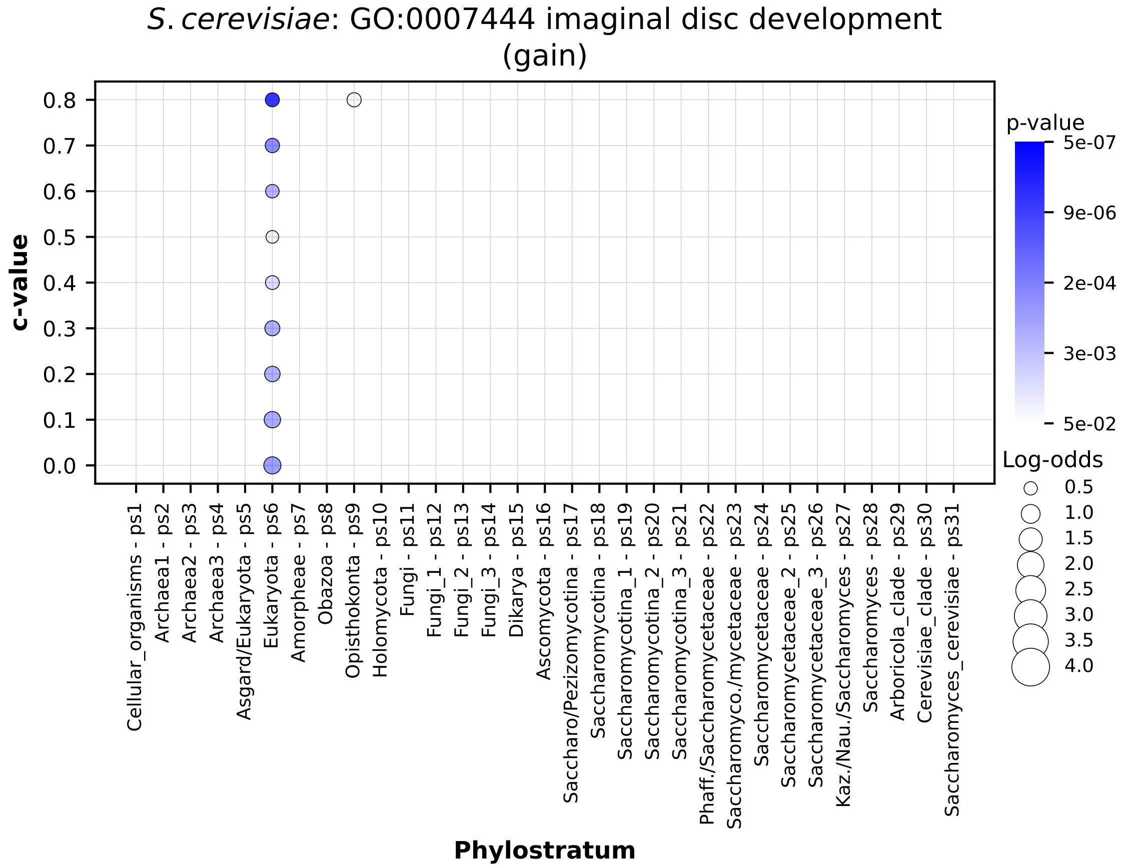

S. cerevisiae: GO:0007444 imaginal disc development
(gain)
c-value
0.8
0.7
0.6
0.5
0.4
0.3
0.2
0.1
0.0
p-value
5e-07
9e-06
2e-04
3e-03
5e-02
Log-odds
0.5
1.0
1.5
2.0
2.5
3.0
3.5
4.0
Cellular_organisms - ps1
Archaea1 - ps2
Archaea2 - ps3
Archaea3 - ps4
Asgard/Eukaryota - ps5
Eukaryota - ps6
Amorpheae - ps7
Obazoa - ps8
Opisthokonta - ps9
Holomycota - ps10
Fungi - ps11
Fungi_1 - ps12
Fungi_2 - ps13
Fungi_3 - ps14
Dikarya - ps15
Ascomycota - ps16
Saccharo/Pezizomycotina - ps17
Saccharomycotina - ps18
Saccharomycotina_1 - ps19
Saccharomycotina_2 - ps20
Saccharomycotina_3 - ps21
Phaff./Saccharomycetaceae - ps22
Saccharomyco./mycetaceae - ps23
Saccharomycetaceae - ps24
Saccharomycetaceae_2 - ps25
Saccharomycetaceae_3 - ps26
Kaz./Nau./Saccharomyces - ps27
Saccharomyces - ps28
Arboricola_clade - ps29
Cerevisiae_clade - ps30
Saccharomyces_cerevisiae - ps31
Phylostratum

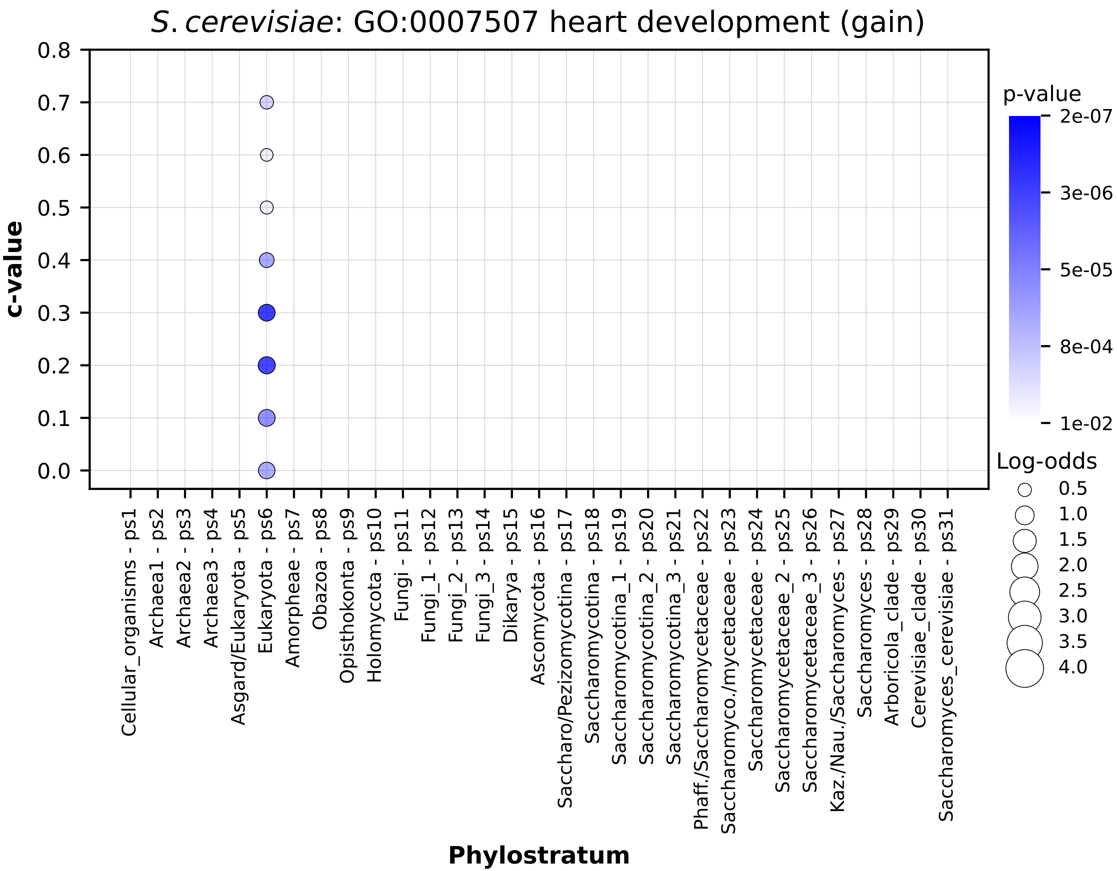

S. cerevisiae: GO:0007507 heart development (gain)
c-value
0.8
0.7
0.6
0.5
0.4
0.3
0.2
0.1
0.0
p-value
2e-07
3e-06
5e-05
8e-04
1e-02
Log-odds
0.5
1.0
1.5
2.0
2.5
3.0
3.5
4.0
Cellular_organisms - ps1
Archaea1 - ps2
Archaea2 - ps3
Archaea3 - ps4
Asgard/Eukaryota - ps5
Eukaryota - ps6
Amorpheae - ps7
Obazoa - ps8
Opisthokonta - ps9
Holomycota - ps10
Fungi - ps11
Fungi_1 - ps12
Fungi_2 - ps13
Fungi_3 - ps14
Dikarya - ps15
Ascomycota - ps16
Saccharo/Pezizomycotina - ps17
Saccharomycotina - ps18
Saccharomycotina_1 - ps19
Saccharomycotina_2 - ps20
Saccharomycotina_3 - ps21
Phaff./Saccharomycetaceae - ps22
Saccharomyco./mycetaceae - ps23
Saccharomycetaceae - ps24
Saccharomycetaceae_2 - ps25
Saccharomycetaceae_3 - ps26
Kaz./Nau./Saccharomyces - ps27
Saccharomyces - ps28
Arboricola_clade - ps29
Cerevisiae_clade - ps30
Saccharomyces_cerevisiae - ps31
Phylostratum

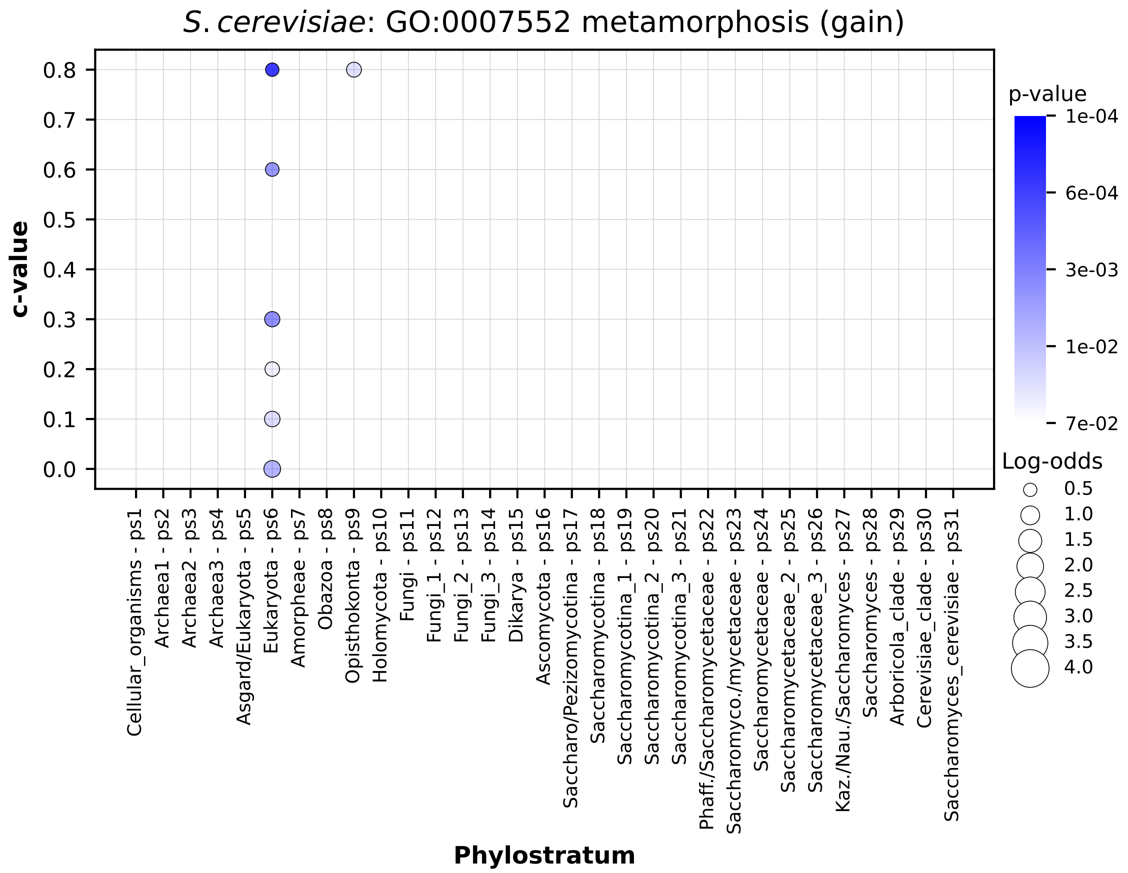

S. cerevisiae: GO:0007552 metamorphosis (gain)
c-value
0.0
0.1
0.2
0.3
0.4
0.5
0.6
0.7
0.8
p-value
1e-04
6e-04
3e-03
1e-02
7e-02
Log-odds
0.5
1.0
1.5
2.0
2.5
3.0
3.5
4.0
Cellular_organisms - ps1
Archaea1 - ps2
Archaea2 - ps3
Archaea3 - ps4
Asgard/Eukaryota - ps5
Eukaryota - ps6
Amorpheae - ps7
Obazoa - ps8
Opisthokonta - ps9
Holomycota - ps10
Fungi - ps11
Fungi_1 - ps12
Fungi_2 - ps13
Fungi_3 - ps14
Dikarya - ps15
Ascomycota - ps16
Saccharo/Pezizomycotina - ps17
Saccharomycotina - ps18
Saccharomycotina_1 - ps19
Saccharomycotina_2 - ps20
Saccharomycotina_3 - ps21
Phaff./Saccharomycetaceae - ps22
Saccharomyco./mycetaceae - ps23
Saccharomycetaceae - ps24
Saccharomycetaceae_2 - ps25
Saccharomycetaceae_3 - ps26
Kaz./Nau./Saccharomyces - ps27
Saccharomyces - ps28
Arboricola_clade - ps29
Cerevisiae_clade - ps30
Saccharomyces_cerevisiae - ps31
Phylostratum

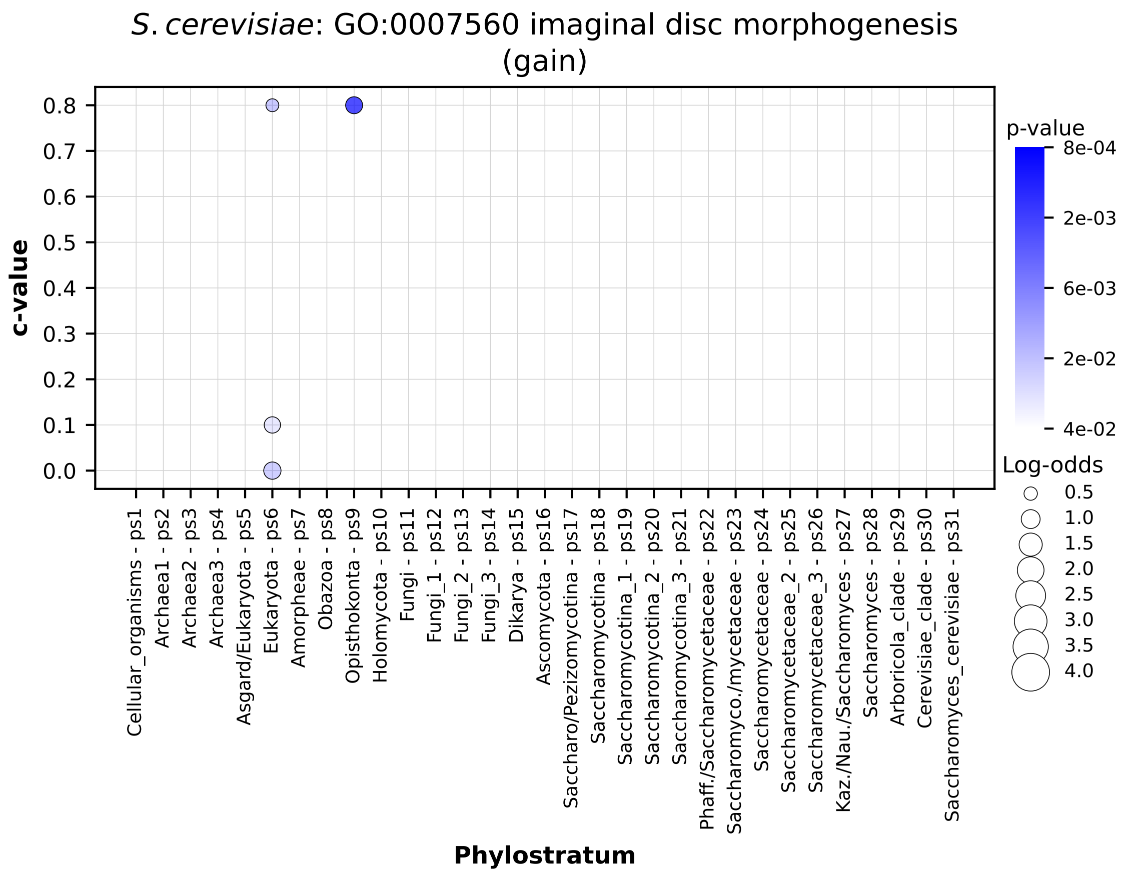

S. cerevisiae: GO:0007560 imaginal disc morphogenesis (gain)
c-value
0.0
0.1
0.2
0.3
0.4
0.5
0.6
0.7
0.8
Phylostratum
Cellular_organisms - ps1
Archaea1 - ps2
Archaea2 - ps3
Archaea3 - ps4
Asgard/Eukaryota - ps5
Eukaryota - ps6
Amorpheae - ps7
Obazoa - ps8
Opisthokonta - ps9
Holomycota - ps10
Fungi - ps11
Fungi_1 - ps12
Fungi_2 - ps13
Fungi_3 - ps14
Dikarya - ps15
Ascomycota - ps16
Saccharo/Pezizomycotina - ps17
Saccharomycotina - ps18
Saccharomycotina_1 - ps19
Saccharomycotina_2 - ps20
Saccharomycotina_3 - ps21
Phaff./Saccharomycetaceae - ps22
Saccharomyco./mycetaceae - ps23
Saccharomycetaceae - ps24
Saccharomycetaceae_2 - ps25
Saccharomycetaceae_3 - ps26
Kaz./Nau./Saccharomyces - ps27
Saccharomyces - ps28
Arboricola_clade - ps29
Cerevisiae_clade - ps30
Saccharomyces_cerevisiae - ps31
p-value
8e-04
2e-03
6e-03
2e-02
4e-02
Log-odds
0.5
1.0
1.5
2.0
2.5
3.0
3.5
4.0

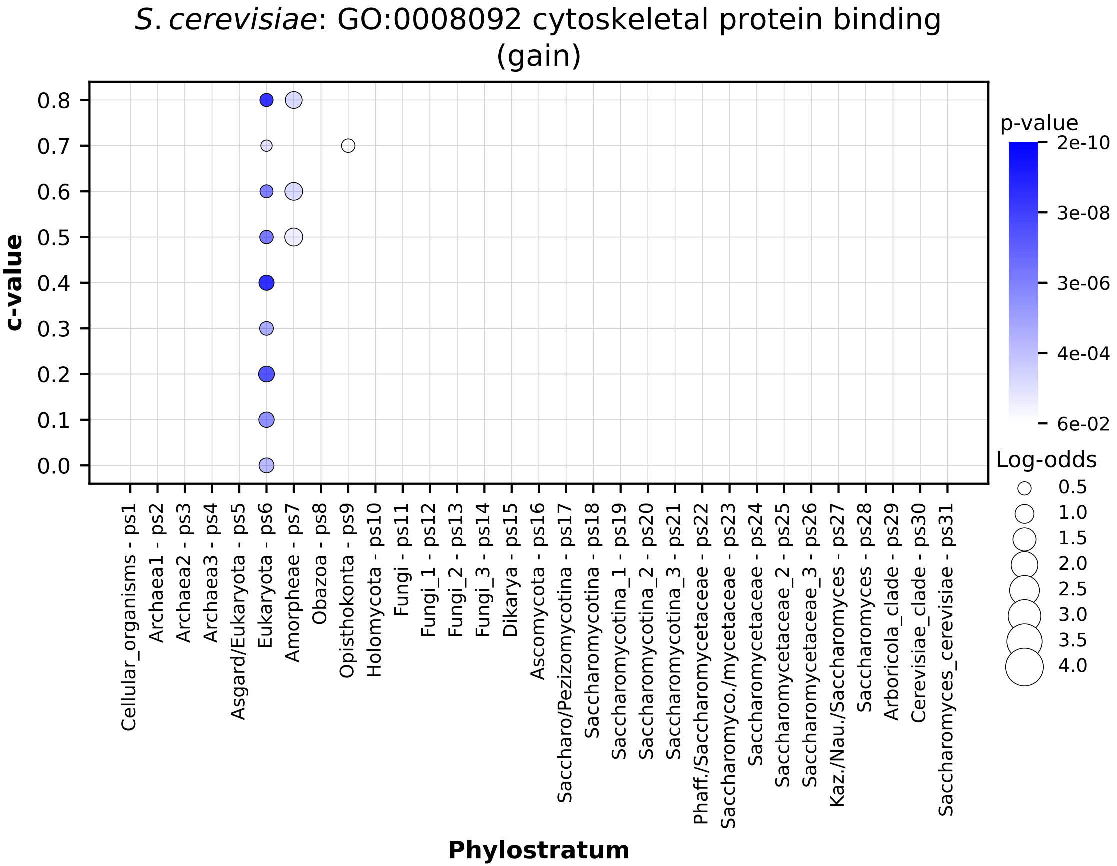

S. cerevisiae: GO:0008092 cytoskeletal protein binding (gain)
c-value
0.8
0.7
0.6
0.5
0.4
0.3
0.2
0.1
0.0
p-value
2e-10
3e-08
3e-06
4e-04
6e-02
Log-odds
0.5
1.0
1.5
2.0
2.5
3.0
3.5
4.0
Cellular_organisms - ps1
Archaea1 - ps2
Archaea2 - ps3
Archaea3 - ps4
Asgard/Eukaryota - ps5
Eukaryota - ps6
Amorpheae - ps7
Obazoa - ps8
Opisthokonta - ps9
Holomycota - ps10
Fungi - ps11
Fungi_1 - ps12
Fungi_2 - ps13
Fungi_3 - ps14
Dikarya - ps15
Ascomycota - ps16
Saccharo/Pezizomycotina - ps17
Saccharomycotina - ps18
Saccharomycotina_1 - ps19
Saccharomycotina_2 - ps20
Saccharomycotina_3 - ps21
Phaff./Saccharomycetaceae - ps22
Saccharomyco./mycetaceae - ps23
Saccharomycetaceae - ps24
Saccharomycetaceae_2 - ps25
Saccharomycetaceae_3 - ps26
Kaz./Nau./Saccharomyces - ps27
Saccharomyces - ps28
Arboricola_clade - ps29
Cerevisiae_clade - ps30
Saccharomyces_cerevisiae - ps31
Phylostratum

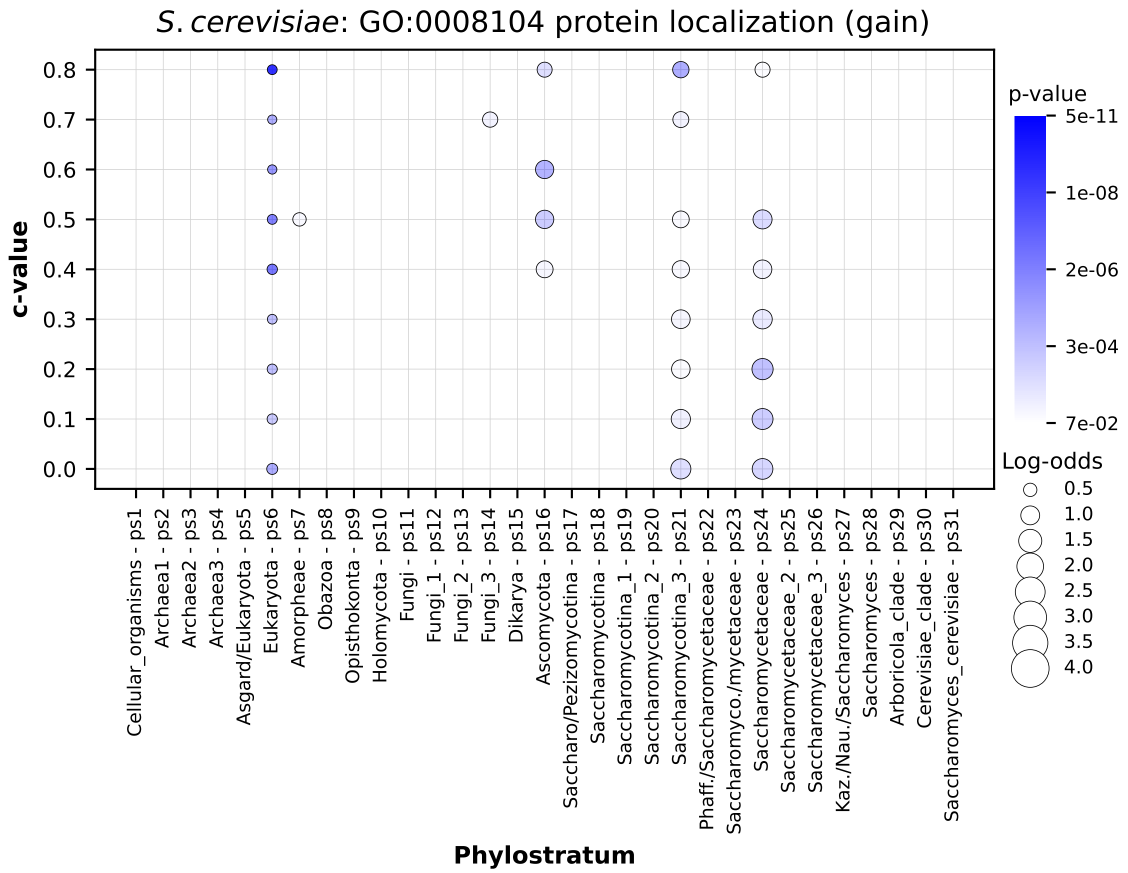

S. cerevisiae: GO:0008104 protein localization (gain)
c-value
0.8
0.7
0.6
0.5
0.4
0.3
0.2
0.1
0.0
p-value
5e-11
1e-08
2e-06
3e-04
7e-02
Log-odds
0.5
1.0
1.5
2.0
2.5
3.0
3.5
4.0
Cellular_organisms - ps1
Archaea1 - ps2
Archaea2 - ps3
Archaea3 - ps4
Asgard/Eukaryota - ps5
Eukaryota - ps6
Amorpheae - ps7
Obazoa - ps8
Opisthokonta - ps9
Holomycota - ps10
Fungi - ps11
Fungi_1 - ps12
Fungi_2 - ps13
Fungi_3 - ps14
Dikarya - ps15
Ascomycota - ps16
Saccharo/Pezizomycotina - ps17
Saccharomycotina - ps18
Saccharomycotina_1 - ps19
Saccharomycotina_2 - ps20
Saccharomycotina_3 - ps21
Phaff./Saccharomycetaceae - ps22
Saccharomyco./mycetaceae - ps23
Saccharomycetaceae - ps24
Saccharomycetaceae_2 - ps25
Saccharomycetaceae_3 - ps26
Kaz./Nau./Saccharomyces - ps27
Saccharomyces - ps28
Arboricola_clade - ps29
Cerevisiae_clade - ps30
Saccharomyces_cerevisiae - ps31
Phylostratum

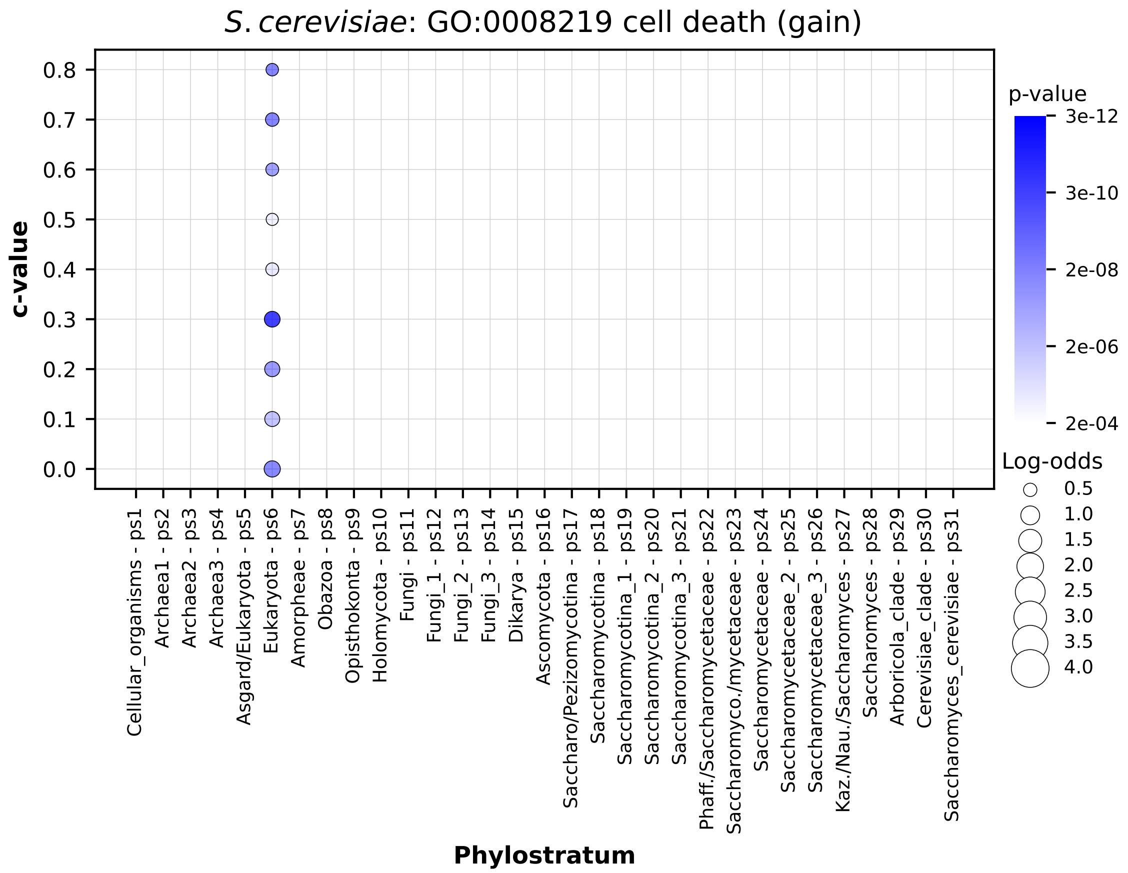

S. cerevisiae: GO:0008219 cell death (gain)
c-value
0.0
0.1
0.2
0.3
0.4
0.5
0.6
0.7
0.8
Phylostratum
Cellular_organisms - ps1
Archaea1 - ps2
Archaea2 - ps3
Archaea3 - ps4
Asgard/Eukaryota - ps5
Eukaryota - ps6
Amorpheae - ps7
Obazoa - ps8
Opisthokonta - ps9
Holomycota - ps10
Fungi - ps11
Fungi_1 - ps12
Fungi_2 - ps13
Fungi_3 - ps14
Dikarya - ps15
Ascomycota - ps16
Saccharo/Pezizomycotina - ps17
Saccharomycotina - ps18
Saccharomycotina_1 - ps19
Saccharomycotina_2 - ps20
Saccharomycotina_3 - ps21
Phaff./Saccharomycetaceae - ps22
Saccharomyco./mycetaceae - ps23
Saccharomycetaceae - ps24
Saccharomycetaceae_2 - ps25
Saccharomycetaceae_3 - ps26
Kaz./Nau./Saccharomyces - ps27
Saccharomyces - ps28
Arboricola_clade - ps29
Cerevisiae_clade - ps30
Saccharomyces_cerevisiae - ps31
p-value
3e-12
3e-10
2e-08
2e-06
2e-04
Log-odds
0.5
1.0
1.5
2.0
2.5
3.0
3.5
4.0

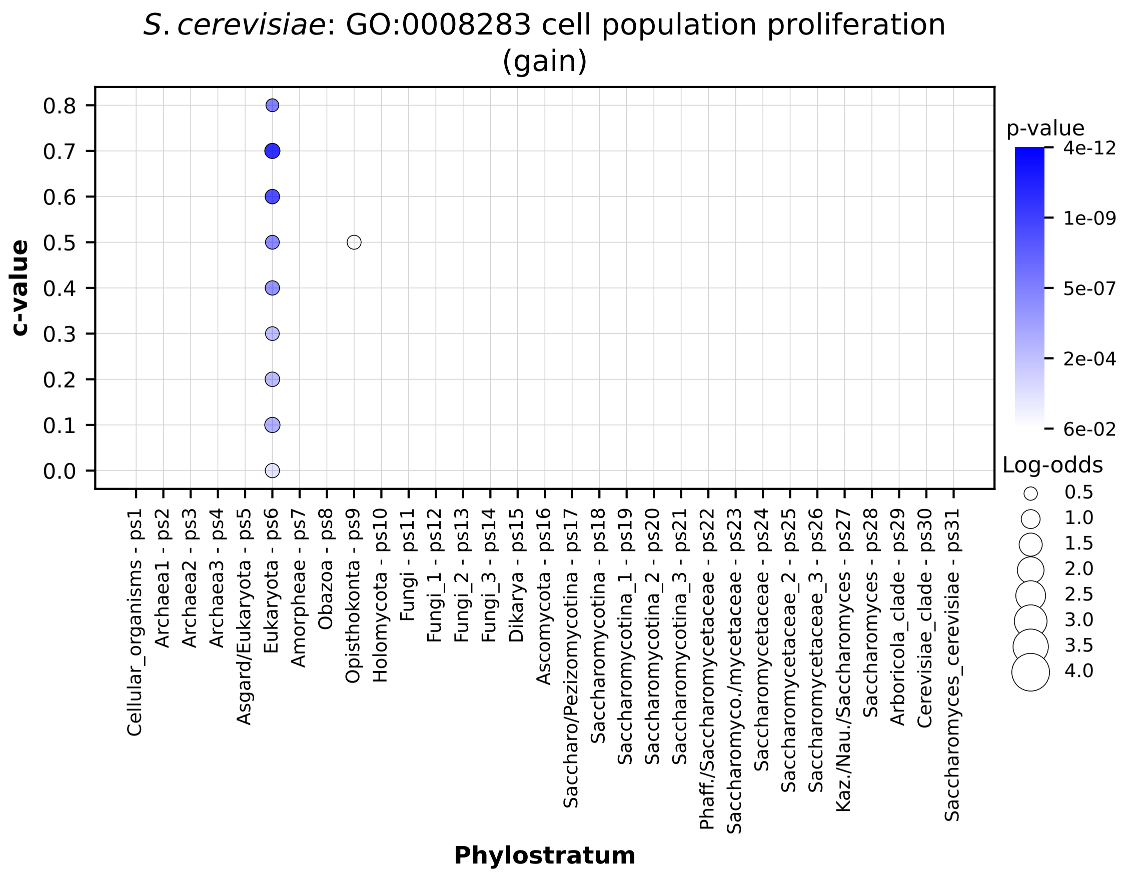

S. cerevisiae: GO:0008283 cell population proliferation (gain)
c-value
0.8
0.7
0.6
0.5
0.4
0.3
0.2
0.1
0.0
Phylostratum
Cellular_organisms - ps1
Archaea1 - ps2
Archaea2 - ps3
Archaea3 - ps4
Asgard/Eukaryota - ps5
Eukaryota - ps6
Amorpheae - ps7
Obazoa - ps8
Opisthokonta - ps9
Holomycota - ps10
Fungi - ps11
Fungi_1 - ps12
Fungi_2 - ps13
Fungi_3 - ps14
Dikarya - ps15
Ascomycota - ps16
Saccharo/Pezizomycotina - ps17
Saccharomycotina - ps18
Saccharomycotina_1 - ps19
Saccharomycotina_2 - ps20
Saccharomycotina_3 - ps21
Phaff./Saccharomycetaceae - ps22
Saccharomyco./mycetaceae - ps23
Saccharomycetaceae - ps24
Saccharomycetaceae_2 - ps25
Saccharomycetaceae_3 - ps26
Kaz./Nau./Saccharomyces - ps27
Saccharomyces - ps28
Arboricola_clade - ps29
Cerevisiae_clade - ps30
Saccharomyces_cerevisiae - ps31
p-value
4e-12
1e-09
5e-07
2e-04
6e-02
Log-odds
0.5
1.0
1.5
2.0
2.5
3.0
3.5
4.0

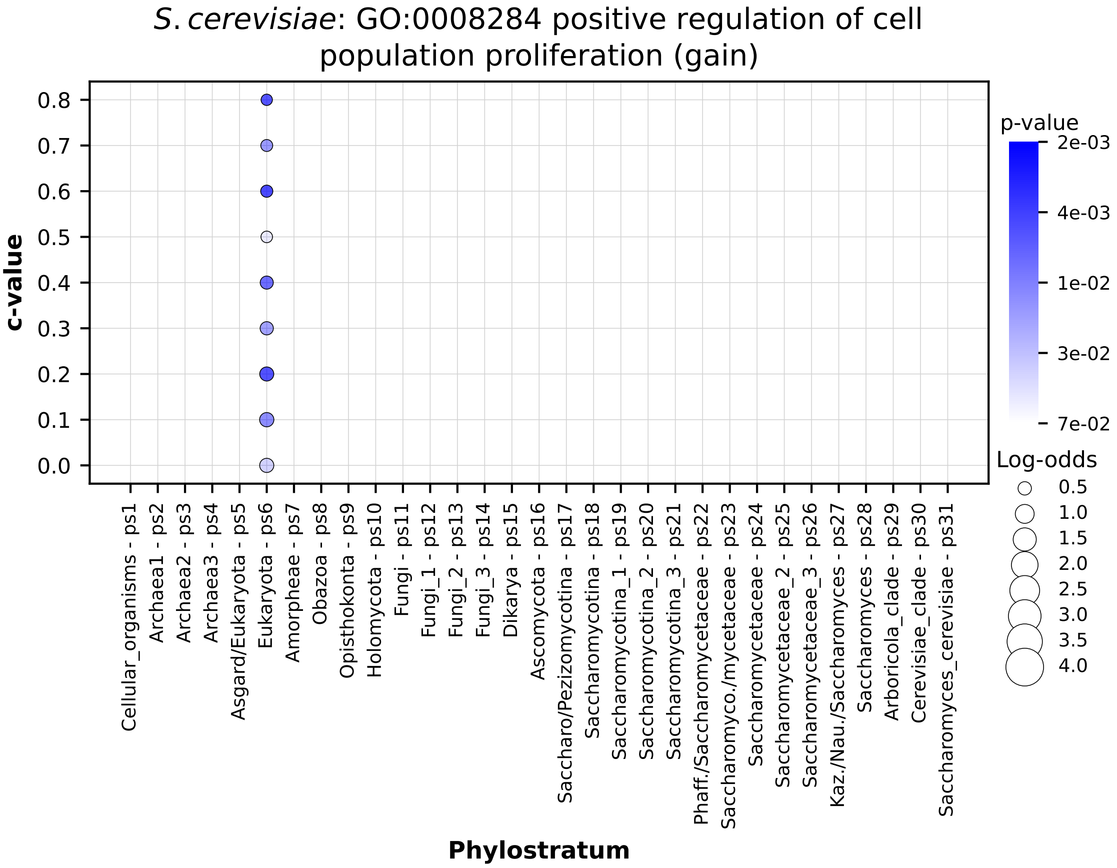

S. cerevisiae: GO:0008284 positive regulation of cell population proliferation (gain)
c-value
0.8
0.7
0.6
0.5
0.4
0.3
0.2
0.1
0.0
p-value
2e-03
4e-03
1e-02
3e-02
7e-02
Log-odds
0.5
1.0
1.5
2.0
2.5
3.0
3.5
4.0
Cellular_organisms - ps1
Archaea1 - ps2
Archaea2 - ps3
Archaea3 - ps4
Asgard/Eukaryota - ps5
Eukaryota - ps6
Amorpheae - ps7
Obazoa - ps8
Opisthokonta - ps9
Holomycota - ps10
Fungi - ps11
Fungi_1 - ps12
Fungi_2 - ps13
Fungi_3 - ps14
Dikarya - ps15
Ascomycota - ps16
Saccharo/Pezizomycotina - ps17
Saccharomycotina - ps18
Saccharomycotina_1 - ps19
Saccharomycotina_2 - ps20
Saccharomycotina_3 - ps21
Phaff./Saccharomycetaceae - ps22
Saccharomyco./mycetaceae - ps23
Saccharomycetaceae - ps24
Saccharomycetaceae_2 - ps25
Saccharomycetaceae_3 - ps26
Kaz./Nau./Saccharomyces - ps27
Saccharomyces - ps28
Arboricola_clade - ps29
Cerevisiae_clade - ps30
Saccharomyces_cerevisiae - ps31
Phylostratum

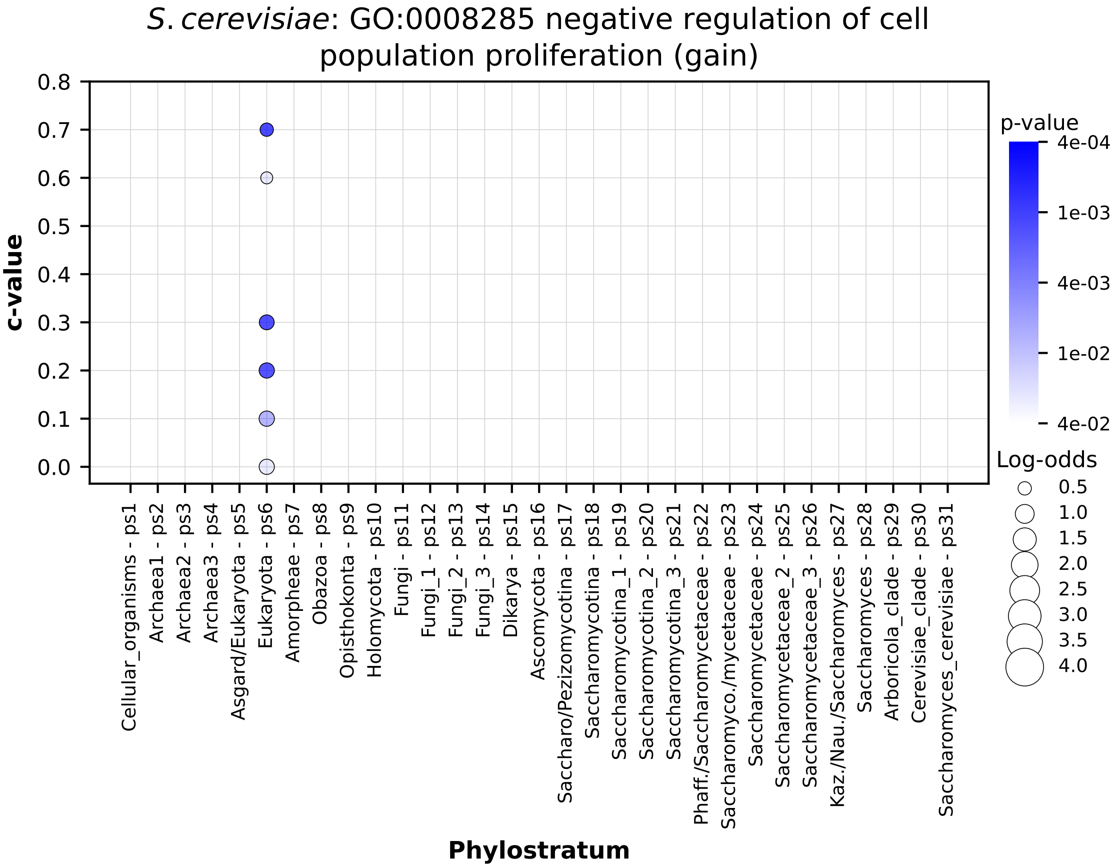
S. cerevisiae: GO:0008285 negative regulation of cell population proliferation (gain)
c-value
0.8
0.7
0.6
0.5
0.4
0.3
0.2
0.1
0.0
p-value
4e-04
1e-03
4e-03
1e-02
4e-02
Log-odds
0.5
1.0
1.5
2.0
2.5
3.0
3.5
4.0
Cellular_organisms - ps1
Archaea1 - ps2
Archaea2 - ps3
Archaea3 - ps4
Asgard/Eukaryota - ps5
Eukaryota - ps6
Amorpheae - ps7
Obazoa - ps8
Opisthokonta - ps9
Holomycota - ps10
Fungi - ps11
Fungi_1 - ps12
Fungi_2 - ps13
Fungi_3 - ps14
Dikarya - ps15
Ascomycota - ps16
Saccharo/Pezizomycotina - ps17
Saccharomycotina - ps18
Saccharomycotina_1 - ps19
Saccharomycotina_2 - ps20
Saccharomycotina_3 - ps21
Phaff./Saccharomycetaceae - ps22
Saccharomyco./mycetaceae - ps23
Saccharomycetaceae - ps24
Saccharomycetaceae_2 - ps25
Saccharomycetaceae_3 - ps26
Kaz./Nau./Saccharomyces - ps27
Saccharomyces - ps28
Arboricola_clade - ps29
Cerevisiae_clade - ps30
Saccharomyces_cerevisiae - ps31
Phylostratum

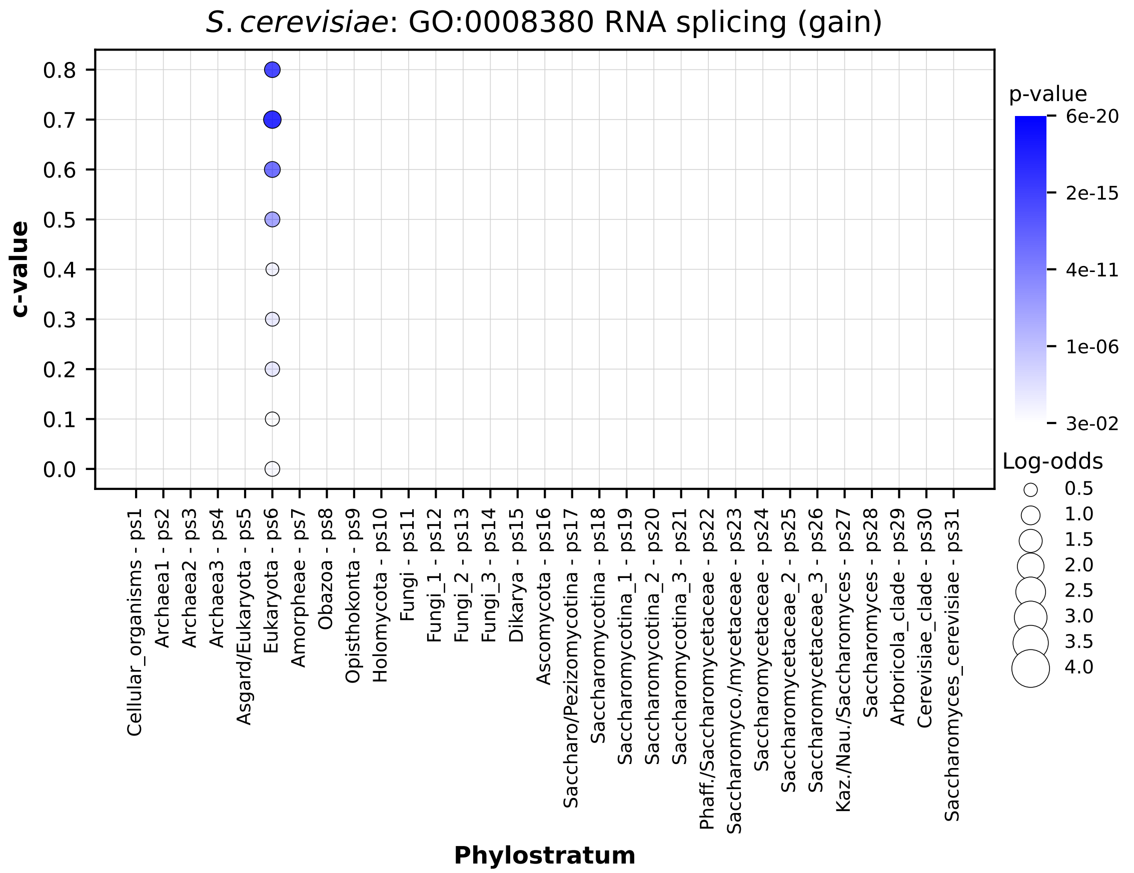
S. cerevisiae: GO:0008380 RNA splicing (gain)
c-value
0.8
0.7
0.6
0.5
0.4
0.3
0.2
0.1
0.0
Phylostratum
Cellular_organisms - ps1
Archaea1 - ps2
Archaea2 - ps3
Archaea3 - ps4
Asgard/Eukaryota - ps5
Eukaryota - ps6
Amorpheae - ps7
Obazoa - ps8
Opisthokonta - ps9
Holomycota - ps10
Fungi - ps11
Fungi_1 - ps12
Fungi_2 - ps13
Fungi_3 - ps14
Dikarya - ps15
Ascomycota - ps16
Saccharo/Pezizomycotina - ps17
Saccharomycotina - ps18
Saccharomycotina_1 - ps19
Saccharomycotina_2 - ps20
Saccharomycotina_3 - ps21
Phaff./Saccharomycetaceae - ps22
Saccharomyco./mycetaceae - ps23
Saccharomycetaceae - ps24
Saccharomycetaceae_2 - ps25
Saccharomycetaceae_3 - ps26
Kaz./Nau./Saccharomyces - ps27
Saccharomyces - ps28
Arboricola_clade - ps29
Cerevisiae_clade - ps30
Saccharomyces_cerevisiae - ps31
p-value
6e-20
2e-15
4e-11
1e-06
3e-02
Log-odds
0.5
1.0
1.5
2.0
2.5
3.0
3.5
4.0

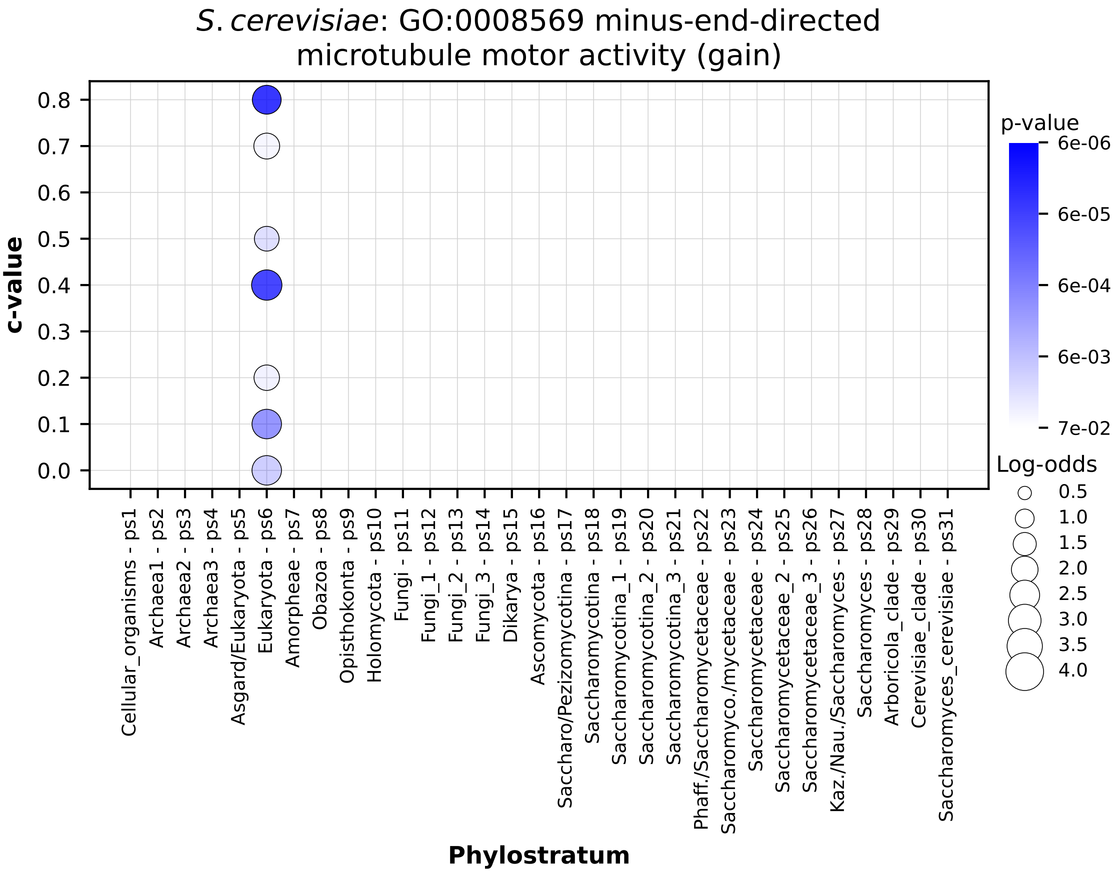

S. cerevisiae: GO:0008569 minus-end-directed microtubule motor activity (gain)
c-value
0.8
0.7
0.6
0.5
0.4
0.3
0.2
0.1
0.0
p-value
6e-06
6e-05
6e-04
6e-03
7e-02
Log-odds
0.5
1.0
1.5
2.0
2.5
3.0
3.5
4.0
Cellular_organisms - ps1
Archaea1 - ps2
Archaea2 - ps3
Archaea3 - ps4
Asgard/Eukaryota - ps5
Eukaryota - ps6
Amorpheae - ps7
Obazoa - ps8
Opisthokonta - ps9
Holomycota - ps10
Fungi - ps11
Fungi_1 - ps12
Fungi_2 - ps13
Fungi_3 - ps14
Dikarya - ps15
Ascomycota - ps16
Saccharo/Pezizomycotina - ps17
Saccharomycotina - ps18
Saccharomycotina_1 - ps19
Saccharomycotina_2 - ps20
Saccharomycotina_3 - ps21
Phaff./Saccharomycetaceae - ps22
Saccharomyco./mycetaceae - ps23
Saccharomycetaceae - ps24
Saccharomycetaceae_2 - ps25
Saccharomycetaceae_3 - ps26
Kaz./Nau./Saccharomyces - ps27
Saccharomyces - ps28
Arboricola_clade - ps29
Cerevisiae_clade - ps30
Saccharomyces_cerevisiae - ps31
Phylostratum

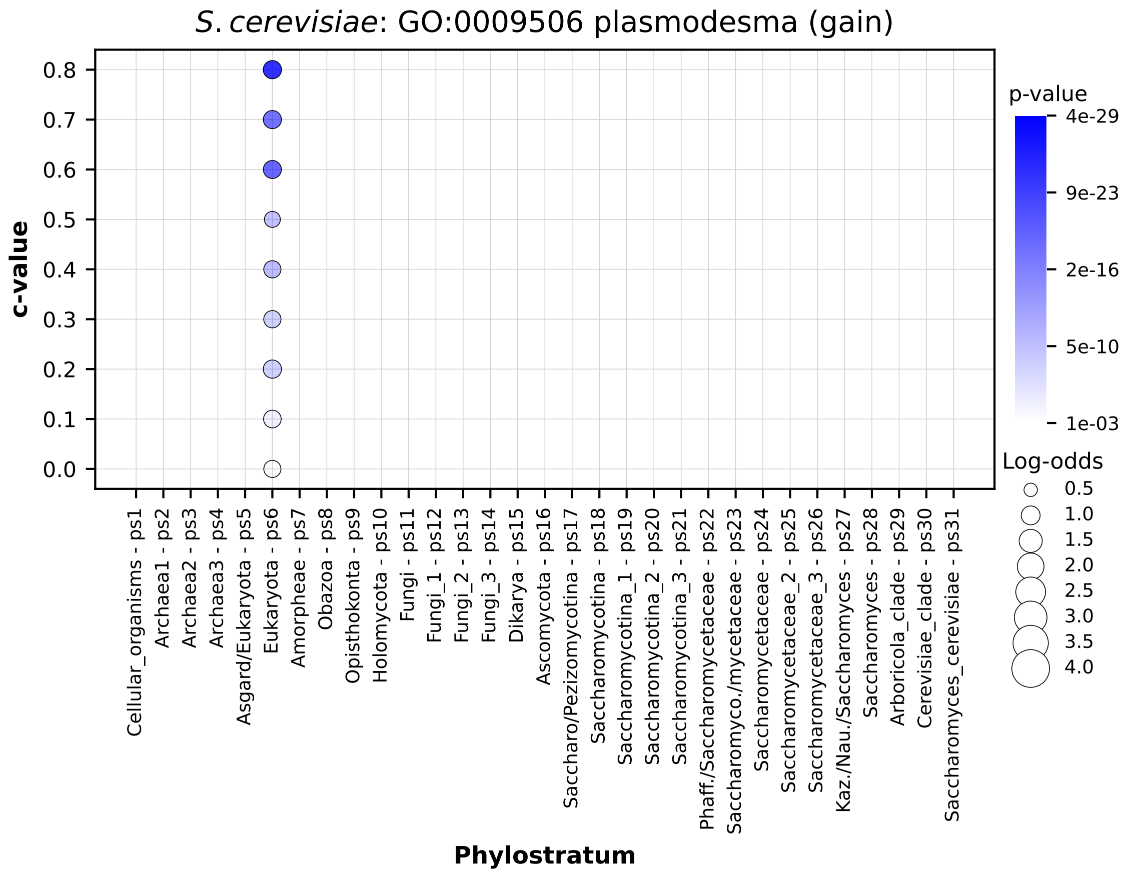

S. cerevisiae: GO:0009506 plasmodesma (gain)
c-value
0.8
0.7
0.6
0.5
0.4
0.3
0.2
0.1
0.0
p-value
4e-29
9e-23
2e-16
5e-10
1e-03
Log-odds
0.5
1.0
1.5
2.0
2.5
3.0
3.5
4.0
Cellular_organisms - ps1
Archaea1 - ps2
Archaea2 - ps3
Archaea3 - ps4
Asgard/Eukaryota - ps5
Eukaryota - ps6
Amorpheae - ps7
Obazoa - ps8
Opisthokonta - ps9
Holomycota - ps10
Fungi - ps11
Fungi_1 - ps12
Fungi_2 - ps13
Fungi_3 - ps14
Dikarya - ps15
Ascomycota - ps16
Saccharo/Pezizomycotina - ps17
Saccharomycotina - ps18
Saccharomycotina_1 - ps19
Saccharomycotina_2 - ps20
Saccharomycotina_3 - ps21
Phaff./Saccharomycetaceae - ps22
Saccharomyco./mycetaceae - ps23
Saccharomycetaceae - ps24
Saccharomycetaceae_2 - ps25
Saccharomycetaceae_3 - ps26
Kaz./Nau./Saccharomyces - ps27
Saccharomyces - ps28
Arboricola_clade - ps29
Cerevisiae_clade - ps30
Saccharomyces_cerevisiae - ps31
Phylostratum

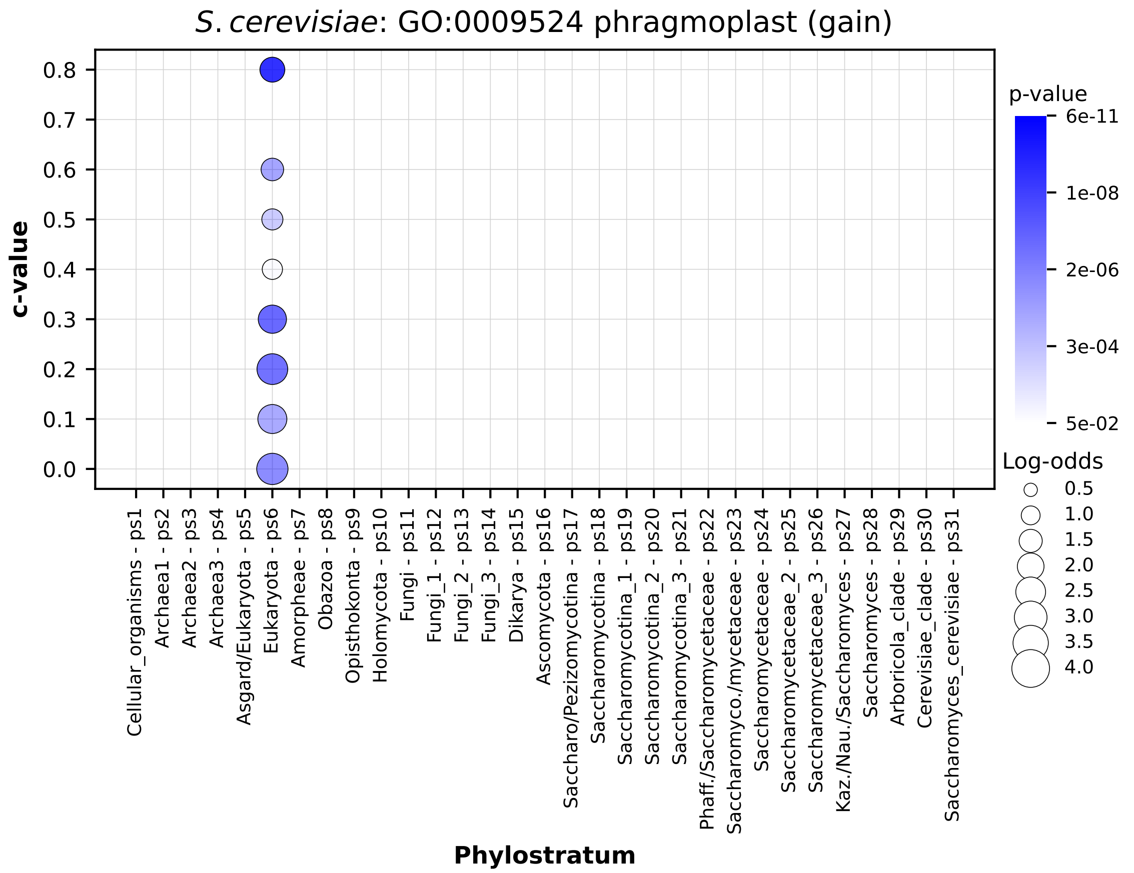
S. cerevisiae: GO:0009524 phragmoplast (gain)
c-value
0.8
0.7
0.6
0.5
0.4
0.3
0.2
0.1
0.0
p-value
6e-11
1e-08
2e-06
3e-04
5e-02
Log-odds
0.5
1.0
1.5
2.0
2.5
3.0
3.5
4.0
Cellular_organisms - ps1
Archaea1 - ps2
Archaea2 - ps3
Archaea3 - ps4
Asgard/Eukaryota - ps5
Eukaryota - ps6
Amorpheae - ps7
Obazoa - ps8
Opisthokonta - ps9
Holomycota - ps10
Fungi - ps11
Fungi_1 - ps12
Fungi_2 - ps13
Fungi_3 - ps14
Dikarya - ps15
Ascomycota - ps16
Saccharo/Pezizomycotina - ps17
Saccharomycotina - ps18
Saccharomycotina_1 - ps19
Saccharomycotina_2 - ps20
Saccharomycotina_3 - ps21
Phaff./Saccharomycetaceae - ps22
Saccharomyco./mycetaceae - ps23
Saccharomycetaceae - ps24
Saccharomycetaceae_2 - ps25
Saccharomycetaceae_3 - ps26
Kaz./Nau./Saccharomyces - ps27
Saccharomyces - ps28
Arboricola_clade - ps29
Cerevisiae_clade - ps30
Saccharomyces_cerevisiae - ps31
Phylostratum

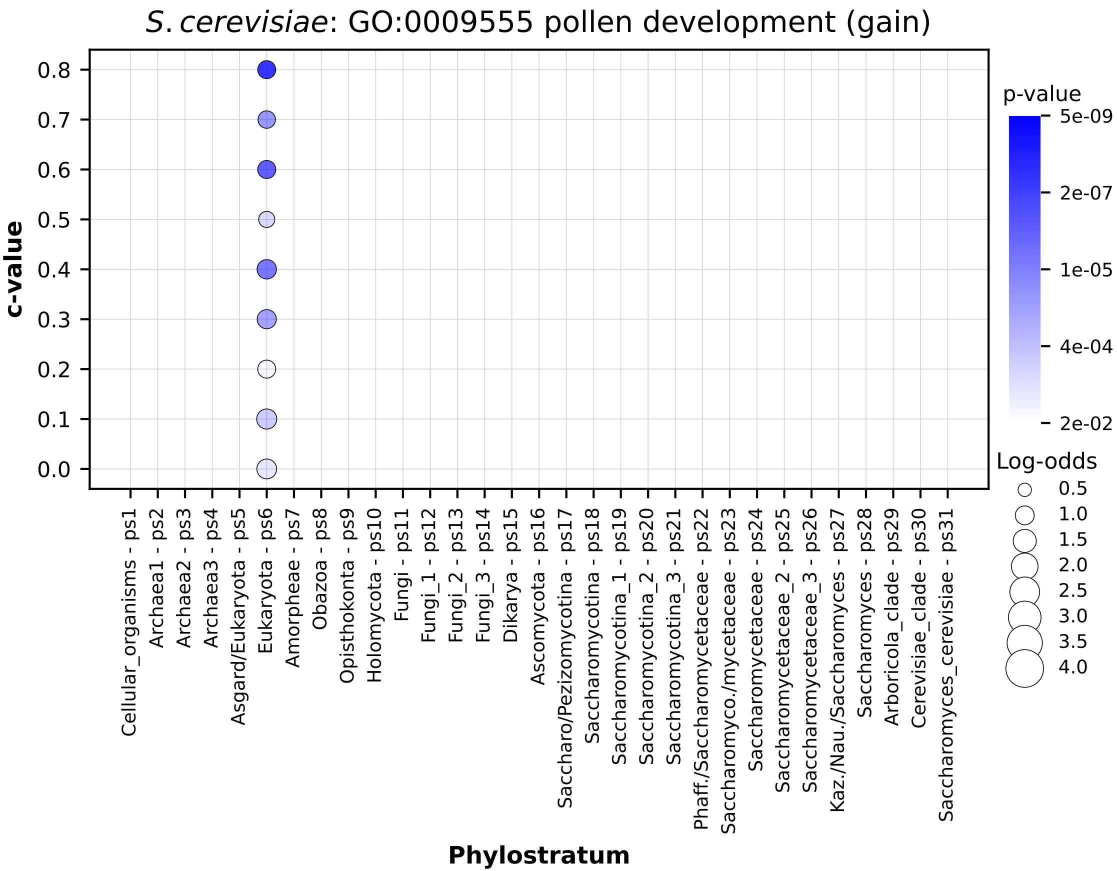

S. cerevisiae: GO:0009555 pollen development (gain)
c-value
0.8
0.7
0.6
0.5
0.4
0.3
0.2
0.1
0.0
Phylostratum
Cellular_organisms - ps1
Archaea1 - ps2
Archaea2 - ps3
Archaea3 - ps4
Asgard/Eukaryota - ps5
Eukaryota - ps6
Amorpheae - ps7
Obazoa - ps8
Opisthokonta - ps9
Holomycota - ps10
Fungi - ps11
Fungi_1 - ps12
Fungi_2 - ps13
Fungi_3 - ps14
Dikarya - ps15
Ascomycota - ps16
Saccharo/Pezizomycotina - ps17
Saccharomycotina - ps18
Saccharomycotina_1 - ps19
Saccharomycotina_2 - ps20
Saccharomycotina_3 - ps21
Phaff./Saccharomycetaceae - ps22
Saccharomyco./mycetaceae - ps23
Saccharomycetaceae - ps24
Saccharomycetaceae_2 - ps25
Saccharomycetaceae_3 - ps26
Kaz./Nau./Saccharomyces - ps27
Saccharomyces - ps28
Arboricola_clade - ps29
Cerevisiae_clade - ps30
Saccharomyces_cerevisiae - ps31
p-value
5e-09
2e-07
1e-05
4e-04
2e-02
Log-odds
0.5
1.0
1.5
2.0
2.5
3.0
3.5
4.0

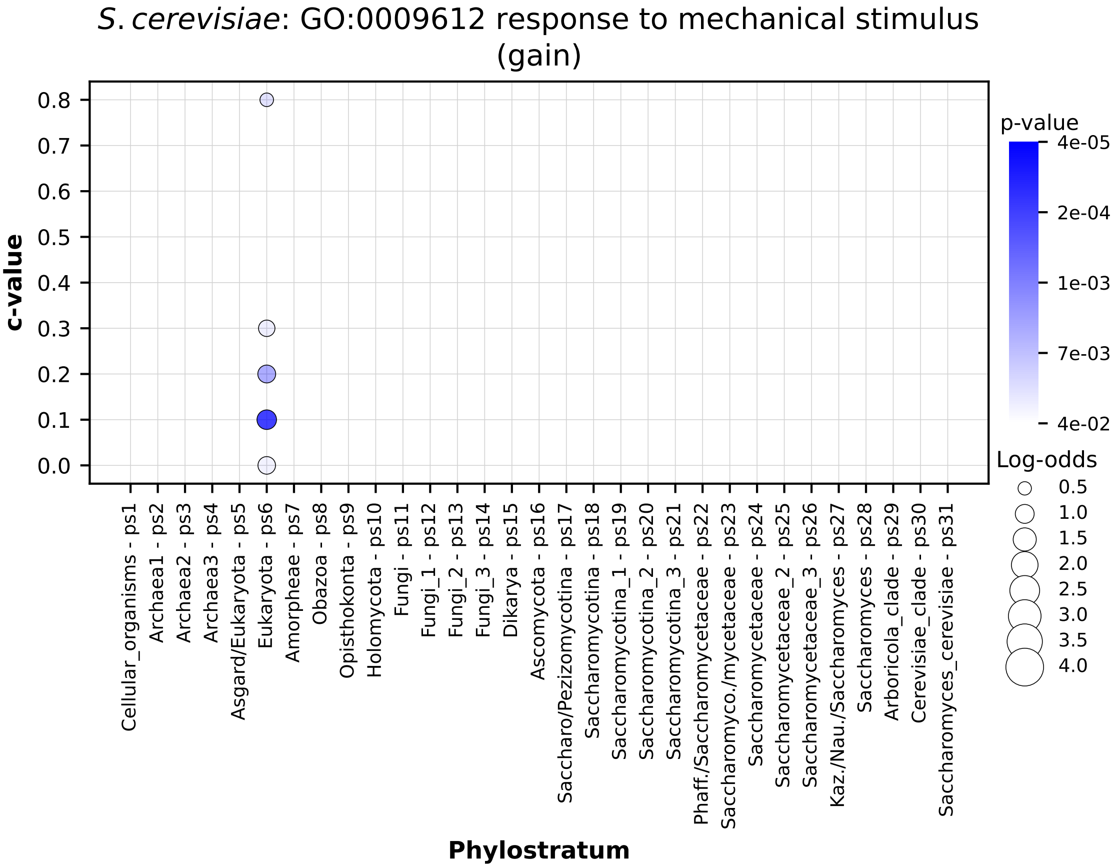

S. cerevisiae: GO:0009612 response to mechanical stimulus (gain)
c-value
0.8
0.7
0.6
0.5
0.4
0.3
0.2
0.1
0.0
p-value
4e-05
2e-04
1e-03
7e-03
4e-02
Log-odds
0.5
1.0
1.5
2.0
2.5
3.0
3.5
4.0
Cellular_organisms - ps1
Archaea1 - ps2
Archaea2 - ps3
Archaea3 - ps4
Asgard/Eukaryota - ps5
Eukaryota - ps6
Amorpheae - ps7
Obazoa - ps8
Opisthokonta - ps9
Holomycota - ps10
Fungi - ps11
Fungi_1 - ps12
Fungi_2 - ps13
Fungi_3 - ps14
Dikarya - ps15
Ascomycota - ps16
Saccharo/Pezizomycotina - ps17
Saccharomycotina - ps18
Saccharomycotina_1 - ps19
Saccharomycotina_2 - ps20
Saccharomycotina_3 - ps21
Phaff./Saccharomycetaceae - ps22
Saccharomyco./mycetaceae - ps23
Saccharomycetaceae - ps24
Saccharomycetaceae_2 - ps25
Saccharomycetaceae_3 - ps26
Kaz./Nau./Saccharomyces - ps27
Saccharomyces - ps28
Arboricola_clade - ps29
Cerevisiae_clade - ps30
Saccharomyces_cerevisiae - ps31
Phylostratum

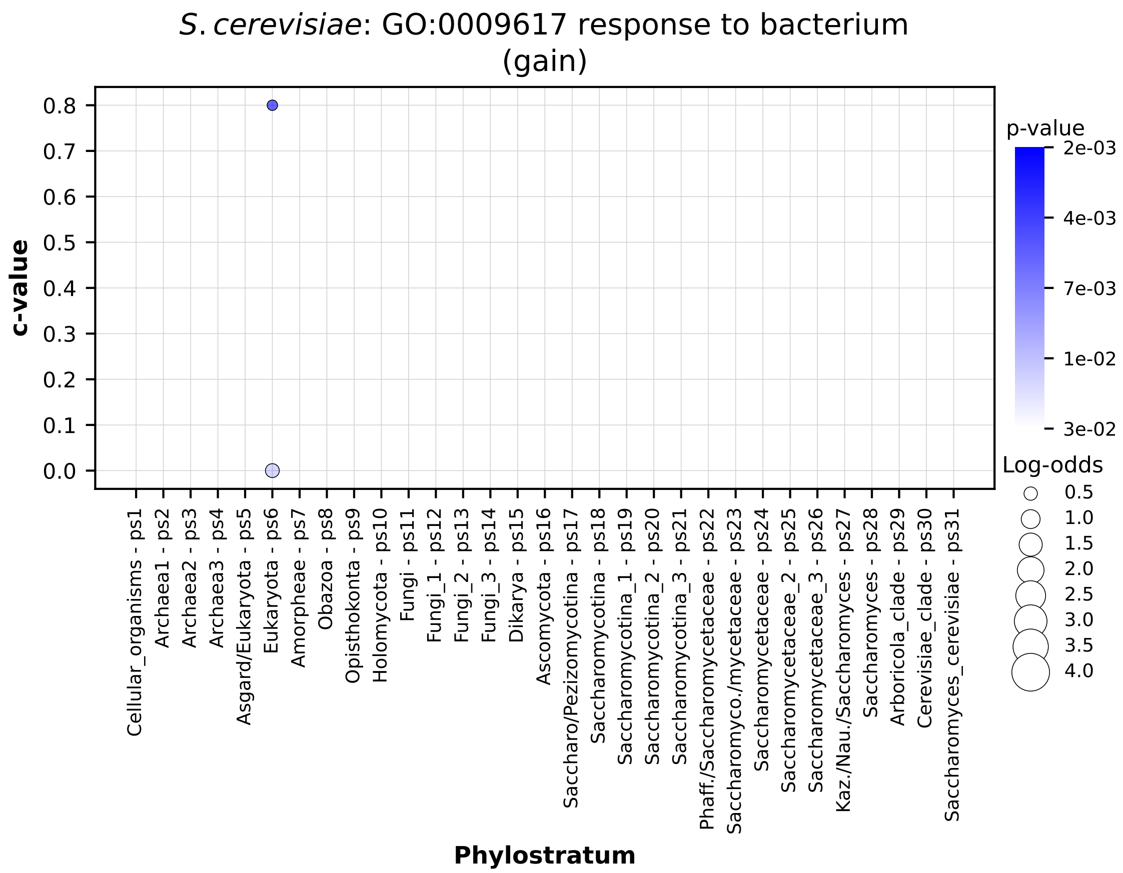

S. cerevisiae: GO:0009617 response to bacterium
(gain)
c-value
0.8
0.7
0.6
0.5
0.4
0.3
0.2
0.1
0.0
p-value
2e-03
4e-03
7e-03
1e-02
3e-02
Log-odds
0.5
1.0
1.5
2.0
2.5
3.0
3.5
4.0
Cellular_organisms - ps1
Archaea1 - ps2
Archaea2 - ps3
Archaea3 - ps4
Asgard/Eukaryota - ps5
Eukaryota - ps6
Amorpheae - ps7
Obazoa - ps8
Opisthokonta - ps9
Holomycota - ps10
Fungi - ps11
Fungi_1 - ps12
Fungi_2 - ps13
Fungi_3 - ps14
Dikarya - ps15
Ascomycota - ps16
Saccharo/Pezizomycotina - ps17
Saccharomycotina - ps18
Saccharomycotina_1 - ps19
Saccharomycotina_2 - ps20
Saccharomycotina_3 - ps21
Phaff./Saccharomycetaceae - ps22
Saccharomyco./mycetaceae - ps23
Saccharomycetaceae - ps24
Saccharomycetaceae_2 - ps25
Saccharomycetaceae_3 - ps26
Kaz./Nau./Saccharomyces - ps27
Saccharomyces - ps28
Arboricola_clade - ps29
Cerevisiae_clade - ps30
Saccharomyces_cerevisiae - ps31
Phylostratum

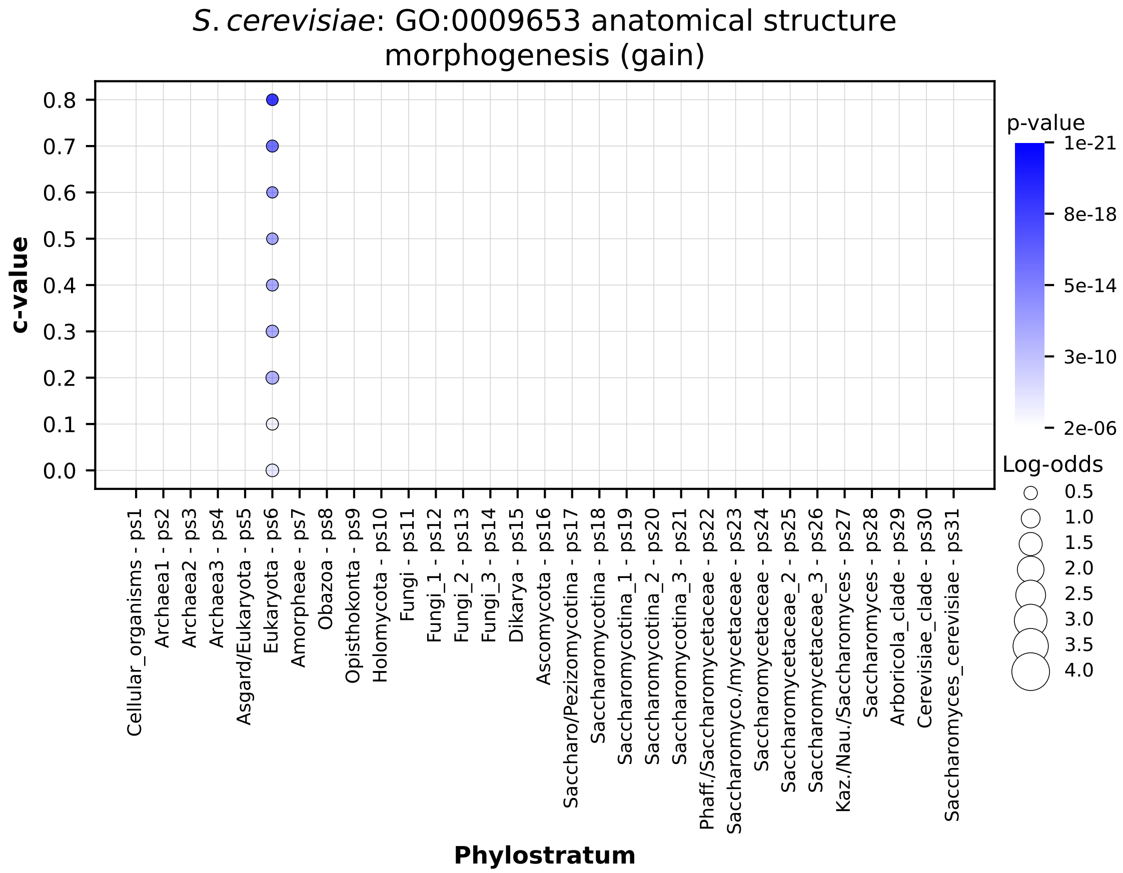

S. cerevisiae: GO:0009653 anatomical structure morphogenesis (gain)
c-value
0.8
0.7
0.6
0.5
0.4
0.3
0.2
0.1
0.0
p-value
1e-21
8e-18
5e-14
3e-10
2e-06
Log-odds
0.5
1.0
1.5
2.0
2.5
3.0
3.5
4.0
Cellular_organisms - ps1
Archaea1 - ps2
Archaea2 - ps3
Archaea3 - ps4
Asgard/Eukaryota - ps5
Eukaryota - ps6
Amorpheae - ps7
Obazoa - ps8
Opisthokonta - ps9
Holomycota - ps10
Fungi - ps11
Fungi_1 - ps12
Fungi_2 - ps13
Fungi_3 - ps14
Dikarya - ps15
Ascomycota - ps16
Saccharo/Pezizomycotina - ps17
Saccharomycotina - ps18
Saccharomycotina_1 - ps19
Saccharomycotina_2 - ps20
Saccharomycotina_3 - ps21
Phaff./Saccharomycetaceae - ps22
Saccharomyco./mycetaceae - ps23
Saccharomycetaceae - ps24
Saccharomycetaceae_2 - ps25
Saccharomycetaceae_3 - ps26
Kaz./Nau./Saccharomyces - ps27
Saccharomyces - ps28
Arboricola_clade - ps29
Cerevisiae_clade - ps30
Saccharomyces_cerevisiae - ps31
Phylostratum

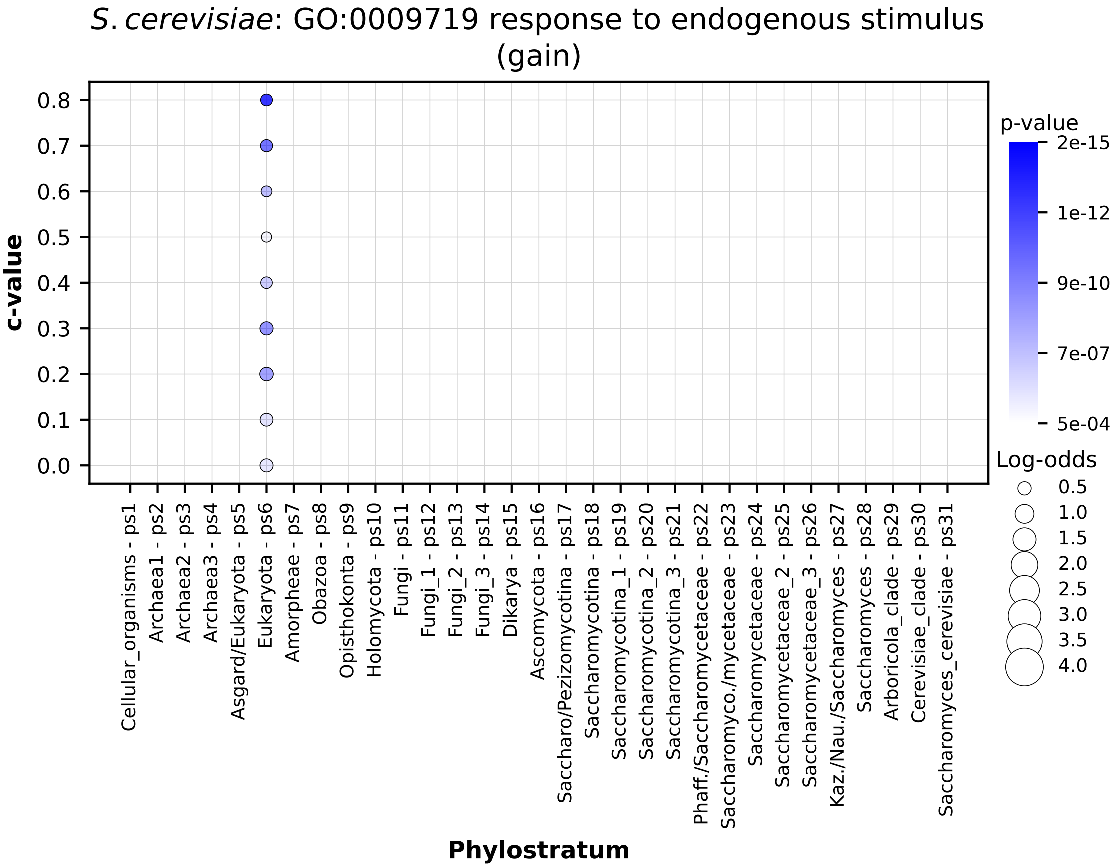

S. cerevisiae: GO:0009719 response to endogenous stimulus (gain)
c-value
0.0
0.1
0.2
0.3
0.4
0.5
0.6
0.7
0.8
p-value
2e-15
1e-12
9e-10
7e-07
5e-04
Log-odds
0.5
1.0
1.5
2.0
2.5
3.0
3.5
4.0
Cellular_organisms - ps1
Archaea1 - ps2
Archaea2 - ps3
Archaea3 - ps4
Asgard/Eukaryota - ps5
Eukaryota - ps6
Amorpheae - ps7
Obazoa - ps8
Opisthokonta - ps9
Holomycota - ps10
Fungi - ps11
Fungi_1 - ps12
Fungi_2 - ps13
Fungi_3 - ps14
Dikarya - ps15
Ascomycota - ps16
Saccharo/Pezizomycotina - ps17
Saccharomycotina - ps18
Saccharomycotina_1 - ps19
Saccharomycotina_2 - ps20
Saccharomycotina_3 - ps21
Phaff./Saccharomycetaceae - ps22
Saccharomyco./mycetaceae - ps23
Saccharomycetaceae - ps24
Saccharomycetaceae_2 - ps25
Saccharomycetaceae_3 - ps26
Kaz./Nau./Saccharomyces - ps27
Saccharomyces - ps28
Arboricola_clade - ps29
Cerevisiae_clade - ps30
Saccharomyces_cerevisiae - ps31
Phylostratum

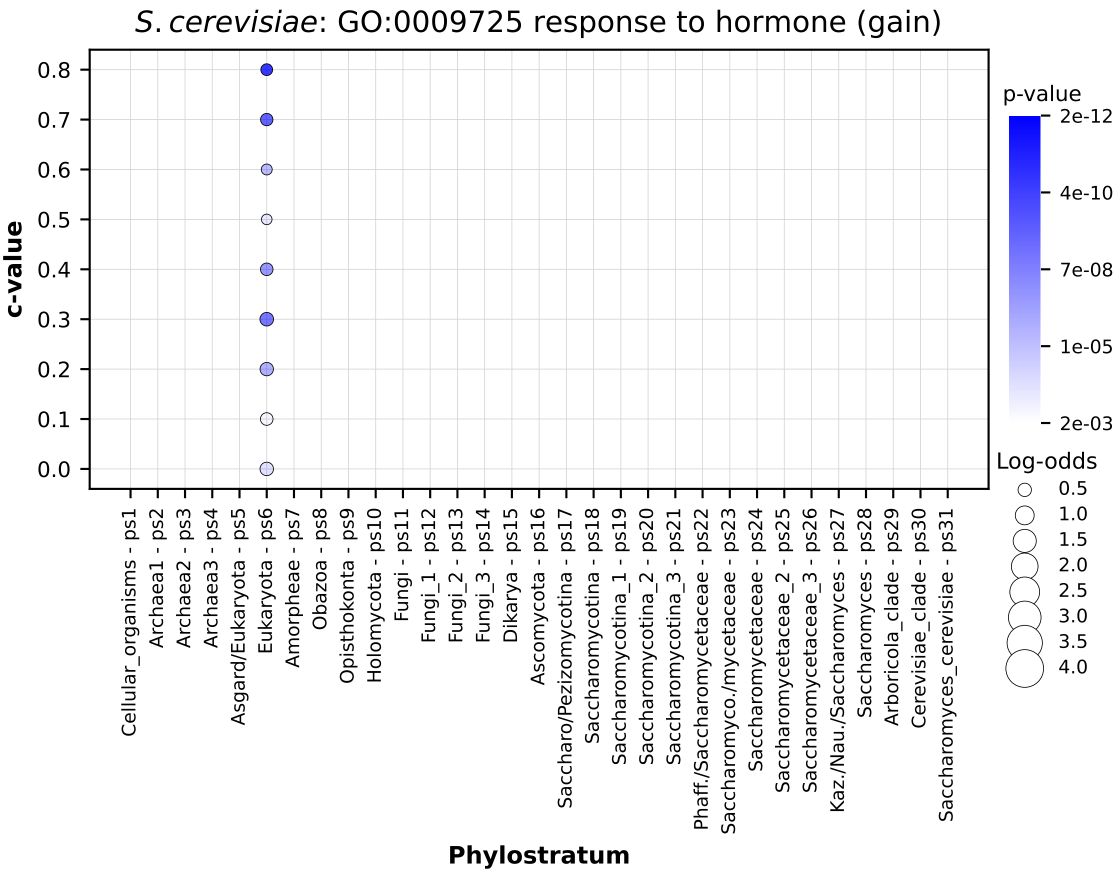
S. cerevisiae: GO:0009725 response to hormone (gain)
c-value
0.0
0.1
0.2
0.3
0.4
0.5
0.6
0.7
0.8
p-value
2e-12
4e-10
7e-08
1e-05
2e-03
Log-odds
0.5
1.0
1.5
2.0
2.5
3.0
3.5
4.0
Cellular_organisms - ps1
Archaea1 - ps2
Archaea2 - ps3
Archaea3 - ps4
Asgard/Eukaryota - ps5
Eukaryota - ps6
Amorpheae - ps7
Obazoa - ps8
Opisthokonta - ps9
Holomycota - ps10
Fungi - ps11
Fungi_1 - ps12
Fungi_2 - ps13
Fungi_3 - ps14
Dikarya - ps15
Ascomycota - ps16
Saccharo/Pezizomycotina - ps17
Saccharomycotina - ps18
Saccharomycotina_1 - ps19
Saccharomycotina_2 - ps20
Saccharomycotina_3 - ps21
Phaff./Saccharomycetaceae - ps22
Saccharomyco./mycetaceae - ps23
Saccharomycetaceae - ps24
Saccharomycetaceae_2 - ps25
Saccharomycetaceae_3 - ps26
Kaz./Nau./Saccharomyces - ps27
Saccharomyces - ps28
Arboricola_clade - ps29
Cerevisiae_clade - ps30
Saccharomyces_cerevisiae - ps31
Phylostratum

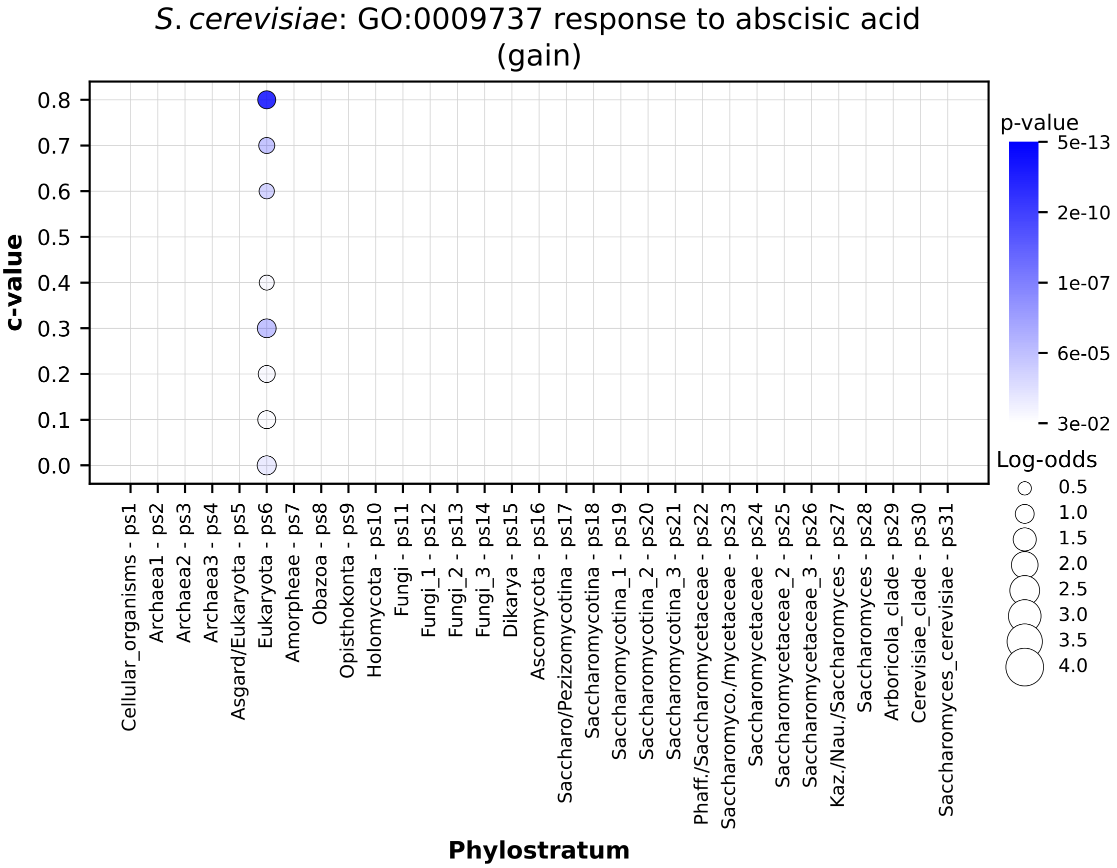

S. cerevisiae: GO:0009737 response to abscisic acid
(gain)
c-value
0.0
0.1
0.2
0.3
0.4
0.5
0.6
0.7
0.8
p-value
5e-13
2e-10
1e-07
6e-05
3e-02
Log-odds
0.5
1.0
1.5
2.0
2.5
3.0
3.5
4.0
Cellular_organisms - ps1
Archaea1 - ps2
Archaea2 - ps3
Archaea3 - ps4
Asgard/Eukaryota - ps5
Eukaryota - ps6
Amorpheae - ps7
Obazoa - ps8
Opisthokonta - ps9
Holomycota - ps10
Fungi - ps11
Fungi_1 - ps12
Fungi_2 - ps13
Fungi_3 - ps14
Dikarya - ps15
Ascomycota - ps16
Saccharo/Pezizomycotina - ps17
Saccharomycotina - ps18
Saccharomycotina_1 - ps19
Saccharomycotina_2 - ps20
Saccharomycotina_3 - ps21
Phaff./Saccharomycetaceae - ps22
Saccharomyco./mycetaceae - ps23
Saccharomycetaceae - ps24
Saccharomycetaceae_2 - ps25
Saccharomycetaceae_3 - ps26
Kaz./Nau./Saccharomyces - ps27
Saccharomyces - ps28
Arboricola_clade - ps29
Cerevisiae_clade - ps30
Saccharomyces_cerevisiae - ps31
Phylostratum

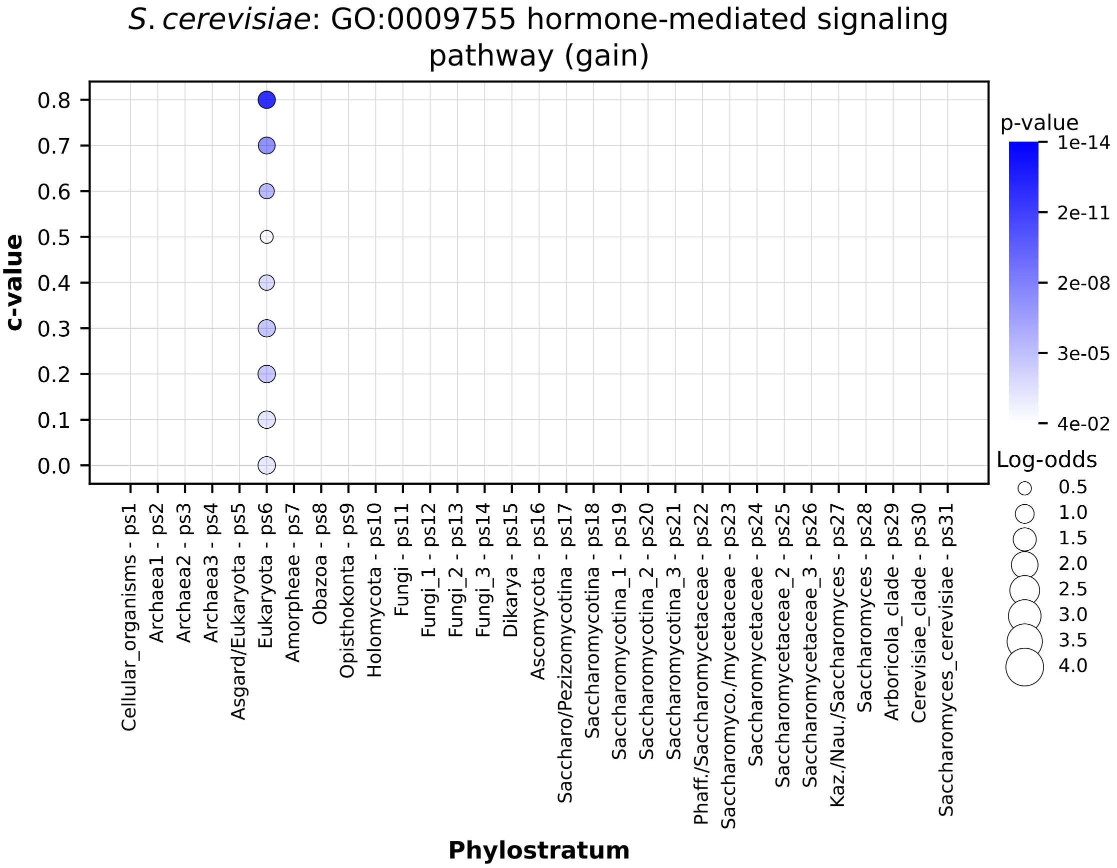

S. cerevisiae: GO:0009755 hormone-mediated signaling pathway (gain)
c-value
0.8
0.7
0.6
0.5
0.4
0.3
0.2
0.1
0.0
p-value
1e-14
2e-11
2e-08
3e-05
4e-02
Log-odds
0.5
1.0
1.5
2.0
2.5
3.0
3.5
4.0
Cellular_organisms - ps1
Archaea1 - ps2
Archaea2 - ps3
Archaea3 - ps4
Asgard/Eukaryota - ps5
Eukaryota - ps6
Amorpheae - ps7
Obazoa - ps8
Opisthokonta - ps9
Holomycota - ps10
Fungi - ps11
Fungi_1 - ps12
Fungi_2 - ps13
Fungi_3 - ps14
Dikarya - ps15
Ascomycota - ps16
Saccharo/Pezizomycotina - ps17
Saccharomycotina - ps18
Saccharomycotina_1 - ps19
Saccharomycotina_2 - ps20
Saccharomycotina_3 - ps21
Phaff./Saccharomycetaceae - ps22
Saccharomyco./mycetaceae - ps23
Saccharomycetaceae - ps24
Saccharomycetaceae_2 - ps25
Saccharomycetaceae_3 - ps26
Kaz./Nau./Saccharomyces - ps27
Saccharomyces - ps28
Arboricola_clade - ps29
Cerevisiae_clade - ps30
Saccharomyces_cerevisiae - ps31
Phylostratum

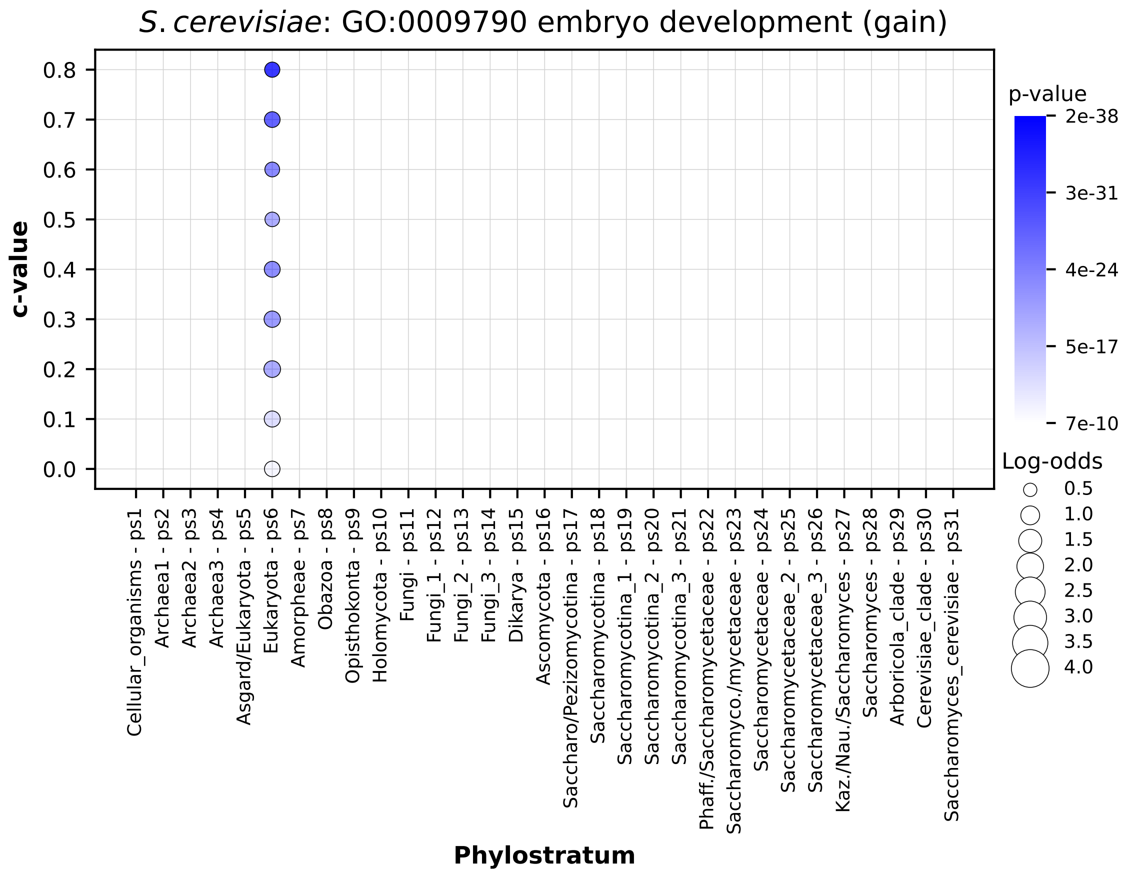
S. cerevisiae: GO:0009790 embryo development (gain)
c-value
0.0
0.1
0.2
0.3
0.4
0.5
0.6
0.7
0.8
Phylostratum
Cellular_organisms - ps1
Archaea1 - ps2
Archaea2 - ps3
Archaea3 - ps4
Asgard/Eukaryota - ps5
Eukaryota - ps6
Amorpheae - ps7
Obazoa - ps8
Opisthokonta - ps9
Holomycota - ps10
Fungi - ps11
Fungi_1 - ps12
Fungi_2 - ps13
Fungi_3 - ps14
Dikarya - ps15
Ascomycota - ps16
Saccharo/Pezizomycotina - ps17
Saccharomycotina - ps18
Saccharomycotina_1 - ps19
Saccharomycotina_2 - ps20
Saccharomycotina_3 - ps21
Phaff./Saccharomycetaceae - ps22
Saccharomyco./mycetaceae - ps23
Saccharomycetaceae - ps24
Saccharomycetaceae_2 - ps25
Saccharomycetaceae_3 - ps26
Kaz./Nau./Saccharomyces - ps27
Saccharomyces - ps28
Arboricola_clade - ps29
Cerevisiae_clade - ps30
Saccharomyces_cerevisiae - ps31
p-value
2e-38
3e-31
4e-24
5e-17
7e-10
Log-odds
0.5
1.0
1.5
2.0
2.5
3.0
3.5
4.0

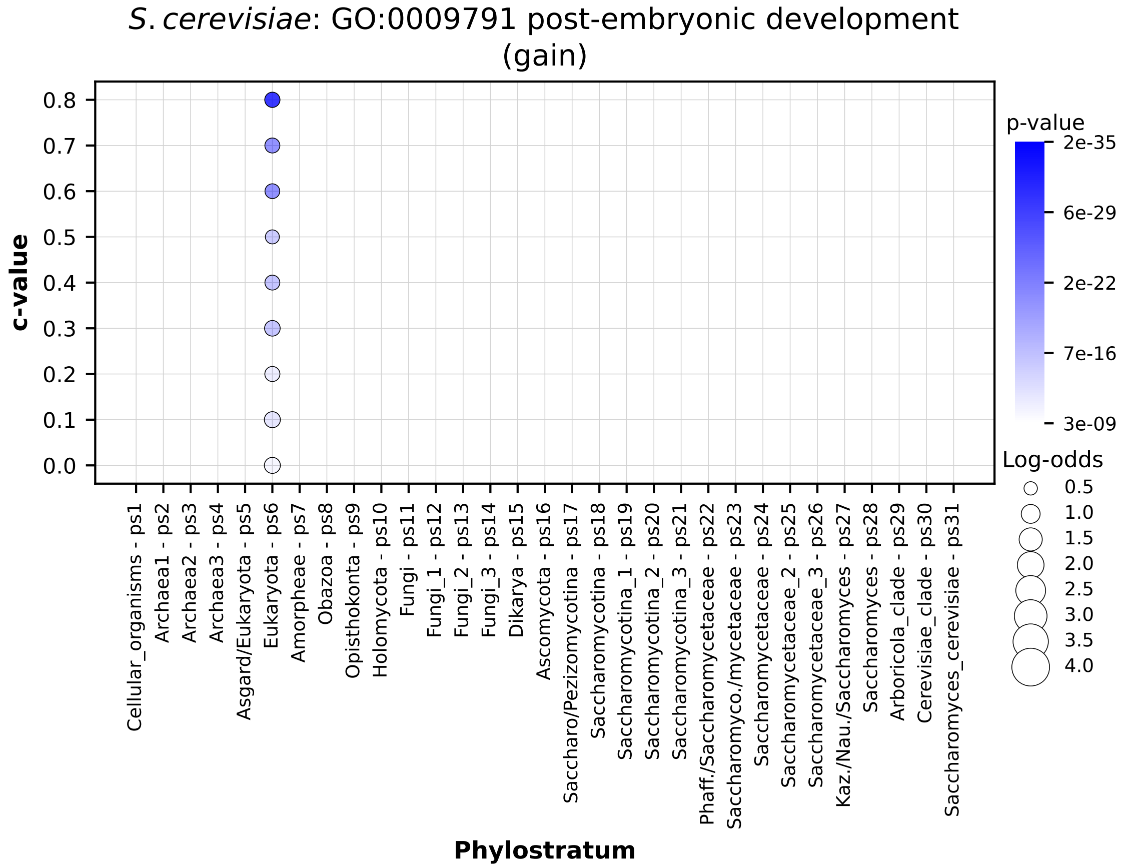

S. cerevisiae: GO:0009791 post-embryonic development (gain)
c-value
0.8
0.7
0.6
0.5
0.4
0.3
0.2
0.1
0.0
p-value
2e-35
6e-29
2e-22
7e-16
3e-09
Log-odds
0.5
1.0
1.5
2.0
2.5
3.0
3.5
4.0
Cellular_organisms - ps1
Archaea1 - ps2
Archaea2 - ps3
Archaea3 - ps4
Asgard/Eukaryota - ps5
Eukaryota - ps6
Amorpheae - ps7
Obazoa - ps8
Opisthokonta - ps9
Holomycota - ps10
Fungi - ps11
Fungi_1 - ps12
Fungi_2 - ps13
Fungi_3 - ps14
Dikarya - ps15
Ascomycota - ps16
Saccharo/Pezizomycotina - ps17
Saccharomycotina - ps18
Saccharomycotina_1 - ps19
Saccharomycotina_2 - ps20
Saccharomycotina_3 - ps21
Phaff./Saccharomycetaceae - ps22
Saccharomyco./mycetaceae - ps23
Saccharomycetaceae - ps24
Saccharomycetaceae_2 - ps25
Saccharomycetaceae_3 - ps26
Kaz./Nau./Saccharomyces - ps27
Saccharomyces - ps28
Arboricola_clade - ps29
Cerevisiae_clade - ps30
Saccharomyces_cerevisiae - ps31
Phylostratum

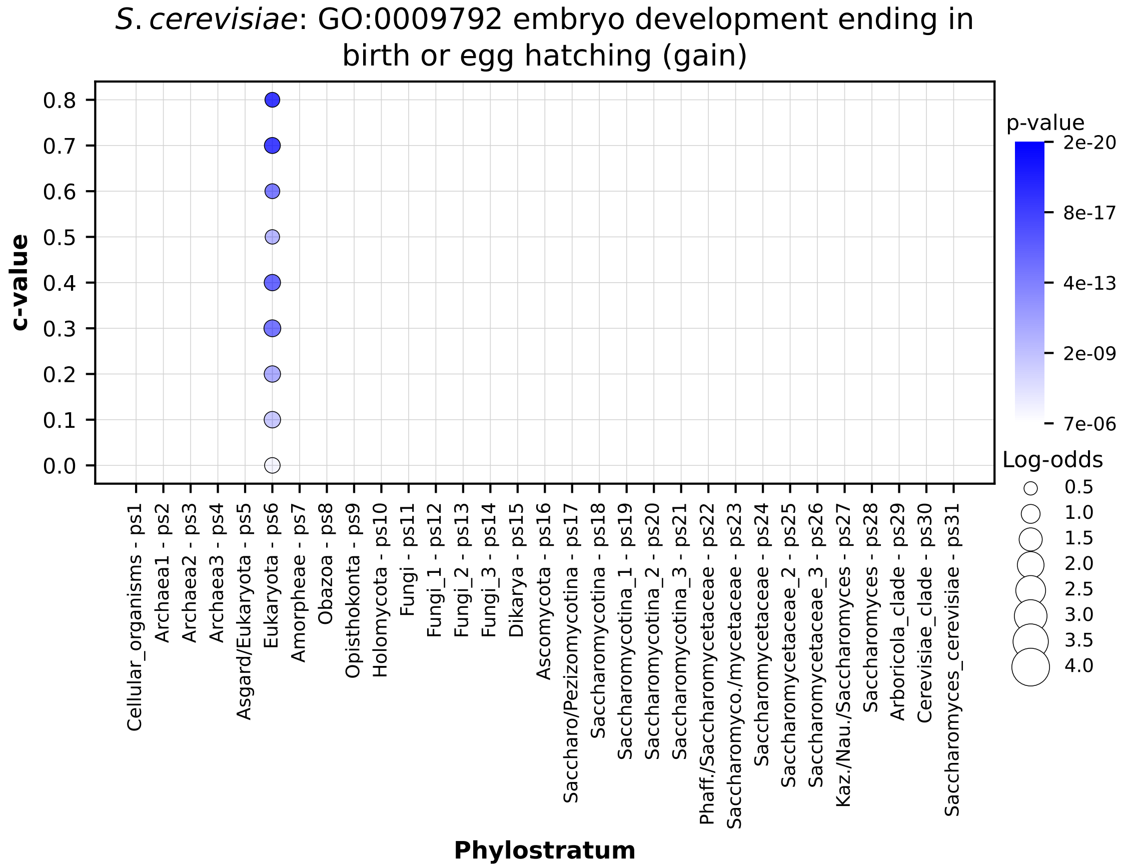

S. cerevisiae: GO:0009792 embryo development ending in birth or egg hatching (gain)
c-value
0.8
0.7
0.6
0.5
0.4
0.3
0.2
0.1
0.0
p-value
2e-20
8e-17
4e-13
2e-09
7e-06
Log-odds
0.5
1.0
1.5
2.0
2.5
3.0
3.5
4.0
Cellular_organisms - ps1
Archaea1 - ps2
Archaea2 - ps3
Archaea3 - ps4
Asgard/Eukaryota - ps5
Eukaryota - ps6
Amorpheae - ps7
Obazoa - ps8
Opisthokonta - ps9
Holomycota - ps10
Fungi - ps11
Fungi_1 - ps12
Fungi_2 - ps13
Fungi_3 - ps14
Dikarya - ps15
Ascomycota - ps16
Saccharo/Pezizomycotina - ps17
Saccharomycotina - ps18
Saccharomycotina_1 - ps19
Saccharomycotina_2 - ps20
Saccharomycotina_3 - ps21
Phaff./Saccharomycetaceae - ps22
Saccharomyco./mycetaceae - ps23
Saccharomycetaceae - ps24
Saccharomycetaceae_2 - ps25
Saccharomycetaceae_3 - ps26
Kaz./Nau./Saccharomyces - ps27
Saccharomyces - ps28
Arboricola_clade - ps29
Cerevisiae_clade - ps30
Saccharomyces_cerevisiae - ps31
Phylostratum

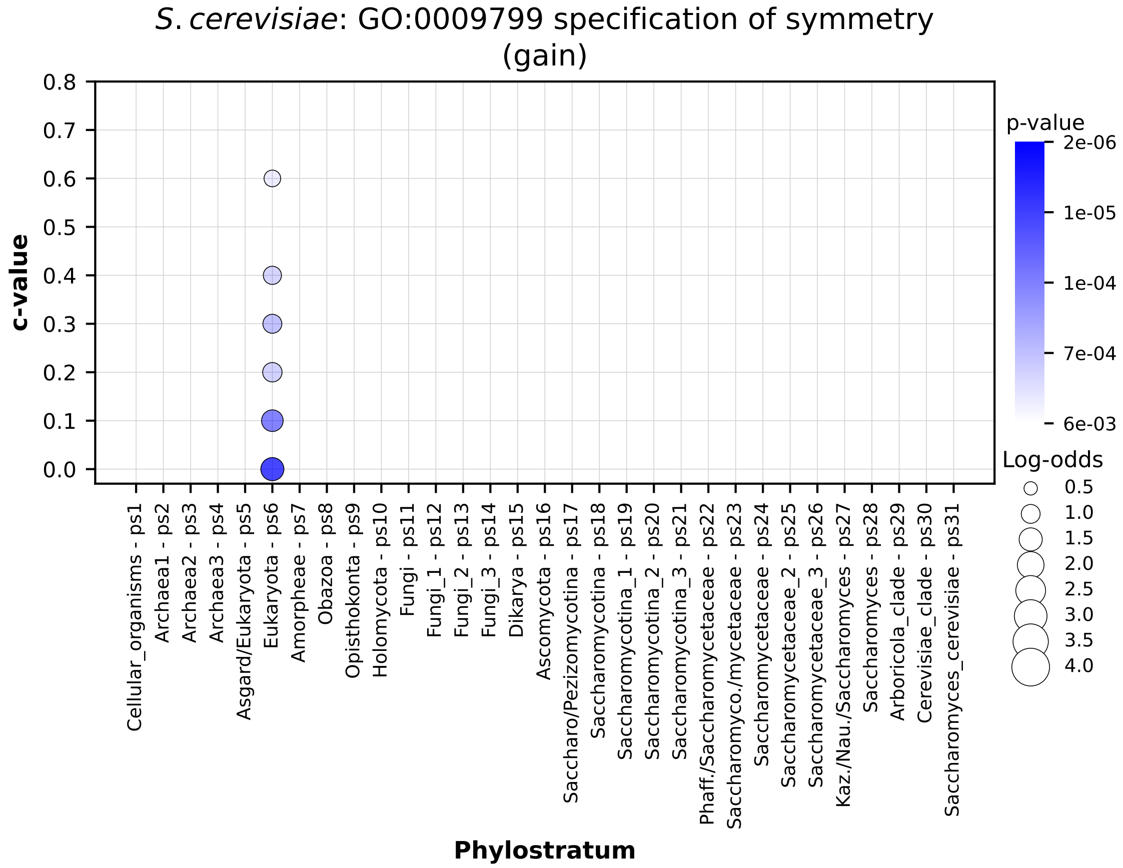

S. cerevisiae: GO:0009799 specification of symmetry (gain)
c-value
0.8
0.7
0.6
0.5
0.4
0.3
0.2
0.1
0.0
p-value
2e-06
1e-05
1e-04
7e-04
6e-03
Log-odds
0.5
1.0
1.5
2.0
2.5
3.0
3.5
4.0
Cellular_organisms - ps1
Archaea1 - ps2
Archaea2 - ps3
Archaea3 - ps4
Asgard/Eukaryota - ps5
Eukaryota - ps6
Amorpheae - ps7
Obazoa - ps8
Opisthokonta - ps9
Holomycota - ps10
Fungi - ps11
Fungi_1 - ps12
Fungi_2 - ps13
Fungi_3 - ps14
Dikarya - ps15
Ascomycota - ps16
Saccharo/Pezizomycotina - ps17
Saccharomycotina - ps18
Saccharomycotina_1 - ps19
Saccharomycotina_2 - ps20
Saccharomycotina_3 - ps21
Phaff./Saccharomycetaceae - ps22
Saccharomyco./mycetaceae - ps23
Saccharomycetaceae - ps24
Saccharomycetaceae_2 - ps25
Saccharomycetaceae_3 - ps26
Kaz./Nau./Saccharomyces - ps27
Saccharomyces - ps28
Arboricola_clade - ps29
Cerevisiae_clade - ps30
Saccharomyces_cerevisiae - ps31
Phylostratum

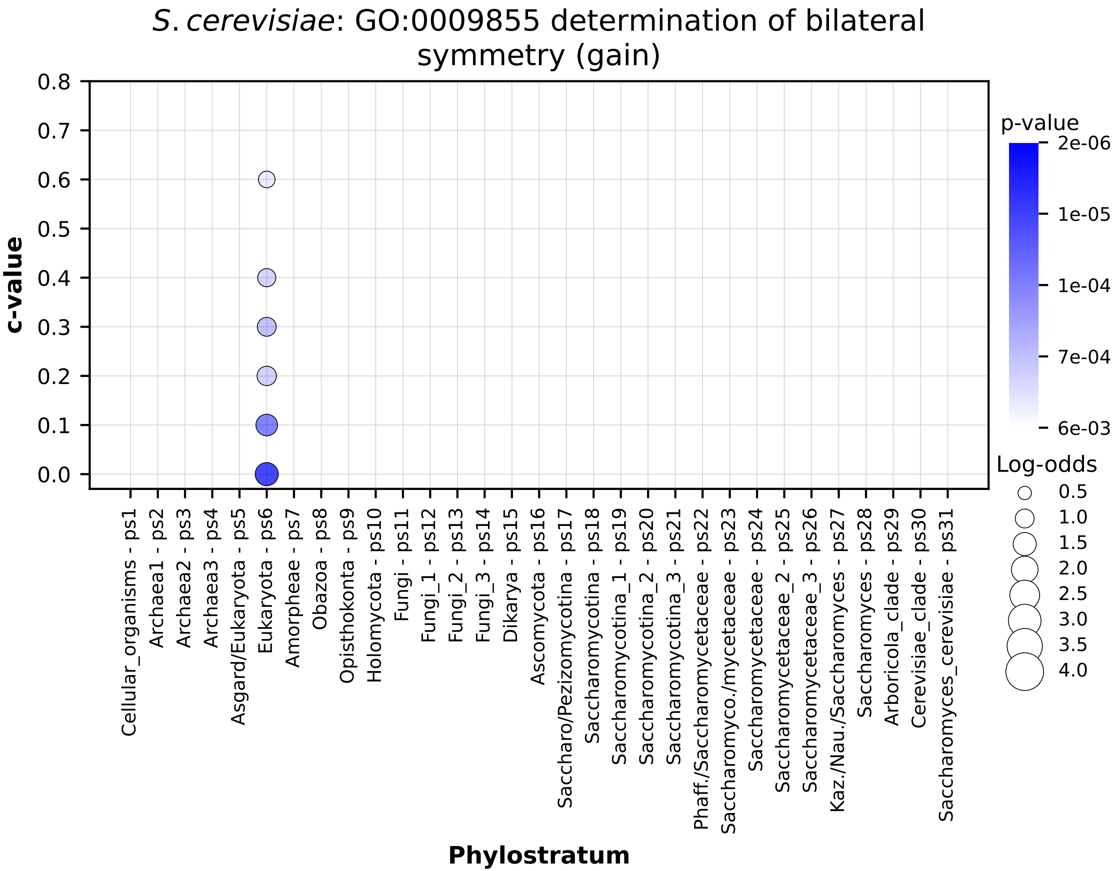
S. cerevisiae: GO:0009855 determination of bilateral symmetry (gain)
c-value
0.8
0.7
0.6
0.5
0.4
0.3
0.2
0.1
0.0
Phylostratum
Cellular_organisms - ps1
Archaea1 - ps2
Archaea2 - ps3
Archaea3 - ps4
Asgard/Eukaryota - ps5
Eukaryota - ps6
Amorpheae - ps7
Obazoa - ps8
Opisthokonta - ps9
Holomycota - ps10
Fungi - ps11
Fungi_1 - ps12
Fungi_2 - ps13
Fungi_3 - ps14
Dikarya - ps15
Ascomycota - ps16
Saccharo/Pezizomycotina - ps17
Saccharomycotina - ps18
Saccharomycotina_1 - ps19
Saccharomycotina_2 - ps20
Saccharomycotina_3 - ps21
Phaff./Saccharomycetaceae - ps22
Saccharomyco./mycetaceae - ps23
Saccharomycetaceae - ps24
Saccharomycetaceae_2 - ps25
Saccharomycetaceae_3 - ps26
Kaz./Nau./Saccharomyces - ps27
Saccharomyces - ps28
Arboricola_clade - ps29
Cerevisiae_clade - ps30
Saccharomyces_cerevisiae - ps31
p-value
2e-06
1e-05
1e-04
7e-04
6e-03
Log-odds
0.5
1.0
1.5
2.0
2.5
3.0
3.5
4.0

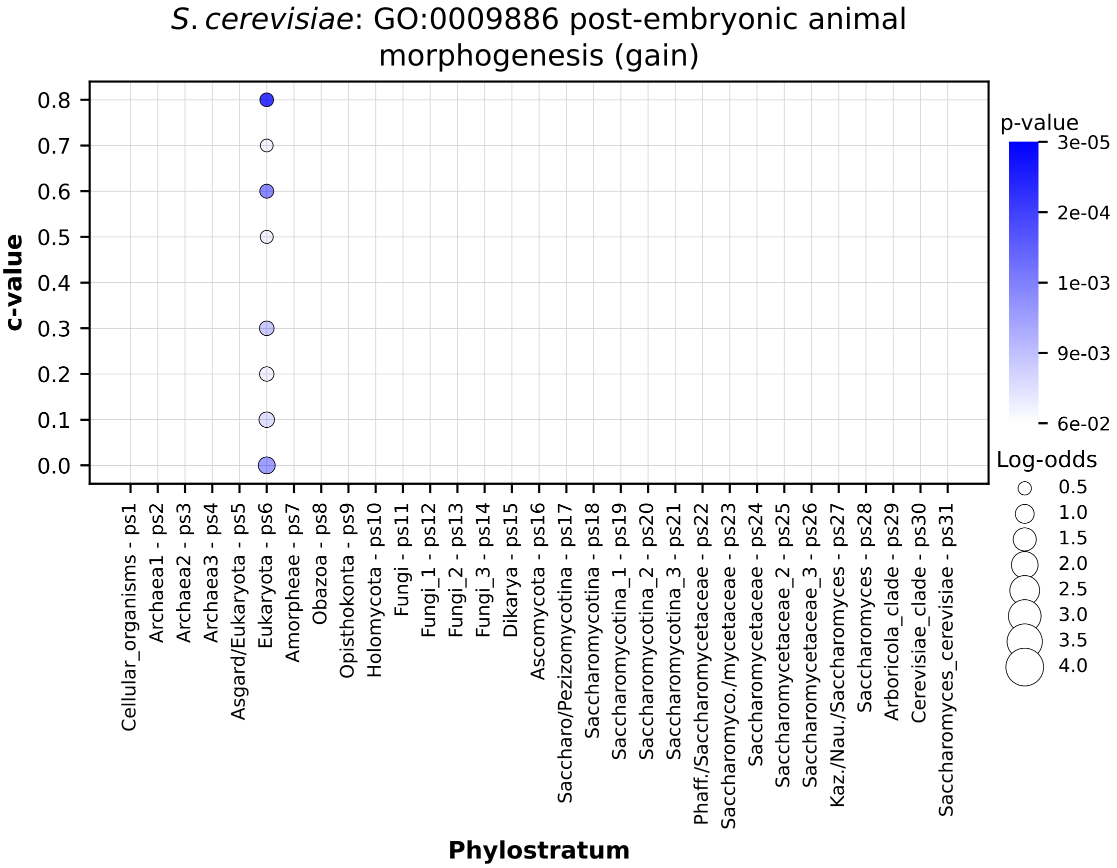
S. cerevisiae: GO:0009886 post-embryonic animal morphogenesis (gain)
c-value
0.8
0.7
0.6
0.5
0.4
0.3
0.2
0.1
0.0
p-value
3e-05
2e-04
1e-03
9e-03
6e-02
Log-odds
0.5
1.0
1.5
2.0
2.5
3.0
3.5
4.0
Cellular_organisms - ps1
Archaea1 - ps2
Archaea2 - ps3
Archaea3 - ps4
Asgard/Eukaryota - ps5
Eukaryota - ps6
Amorpheae - ps7
Obazoa - ps8
Opisthokonta - ps9
Holomycota - ps10
Fungi - ps11
Fungi_1 - ps12
Fungi_2 - ps13
Fungi_3 - ps14
Dikarya - ps15
Ascomycota - ps16
Saccharo/Pezizomycotina - ps17
Saccharomycotina - ps18
Saccharomycotina_1 - ps19
Saccharomycotina_2 - ps20
Saccharomycotina_3 - ps21
Phaff./Saccharomycetaceae - ps22
Saccharomyco./mycetaceae - ps23
Saccharomycetaceae - ps24
Saccharomycetaceae_2 - ps25
Saccharomycetaceae_3 - ps26
Kaz./Nau./Saccharomyces - ps27
Saccharomyces - ps28
Arboricola_clade - ps29
Cerevisiae_clade - ps30
Saccharomyces_cerevisiae - ps31
Phylostratum

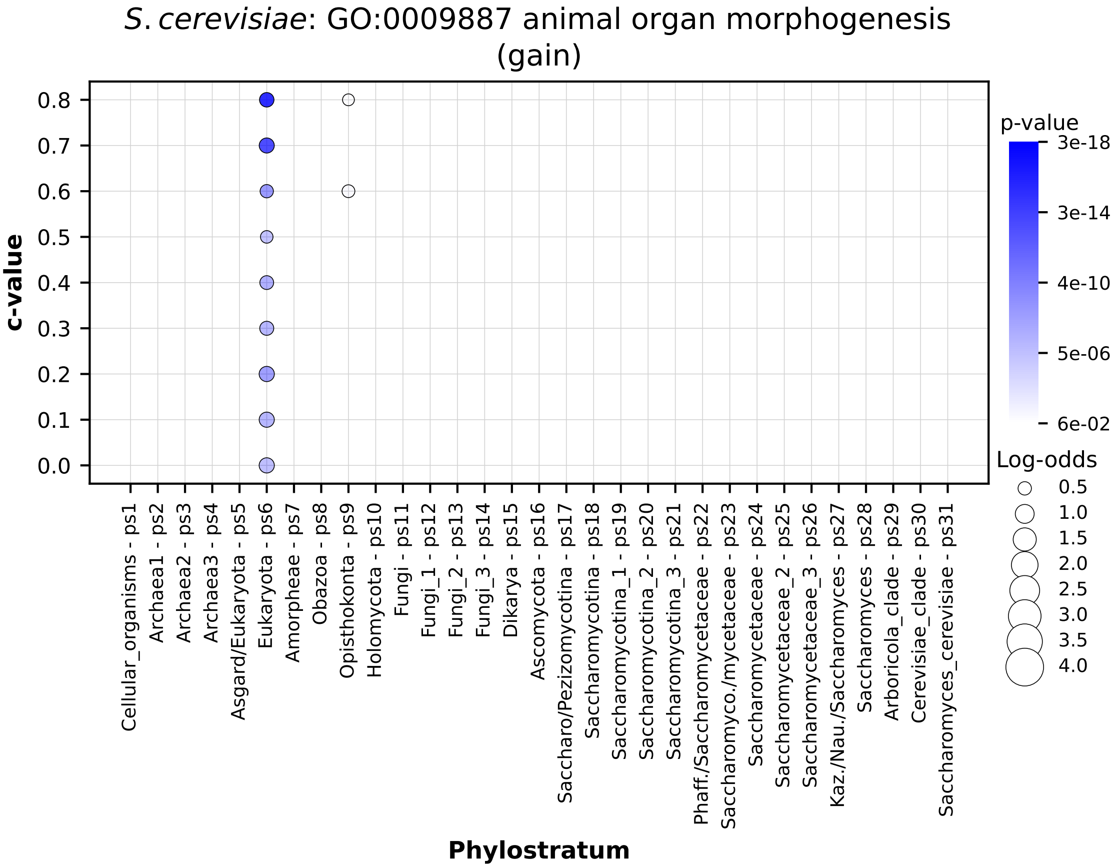

S. cerevisiae: GO:0009887 animal organ morphogenesis
(gain)
c-value
0.8
0.7
0.6
0.5
0.4
0.3
0.2
0.1
0.0
Phylostratum
Cellular_organisms - ps1
Archaea1 - ps2
Archaea2 - ps3
Archaea3 - ps4
Asgard/Eukaryota - ps5
Eukaryota - ps6
Amorpheae - ps7
Obazoa - ps8
Opisthokonta - ps9
Holomycota - ps10
Fungi - ps11
Fungi_1 - ps12
Fungi_2 - ps13
Fungi_3 - ps14
Dikarya - ps15
Ascomycota - ps16
Saccharo/Pezizomycotina - ps17
Saccharomycotina - ps18
Saccharomycotina_1 - ps19
Saccharomycotina_2 - ps20
Saccharomycotina_3 - ps21
Phaff./Saccharomycetaceae - ps22
Saccharomyco./mycetaceae - ps23
Saccharomycetaceae - ps24
Saccharomycetaceae_2 - ps25
Saccharomycetaceae_3 - ps26
Kaz./Nau./Saccharomyces - ps27
Saccharomyces - ps28
Arboricola_clade - ps29
Cerevisiae_clade - ps30
Saccharomyces_cerevisiae - ps31
p-value
3e-18
3e-14
4e-10
5e-06
6e-02
Log-odds
0.5
1.0
1.5
2.0
2.5
3.0
3.5
4.0

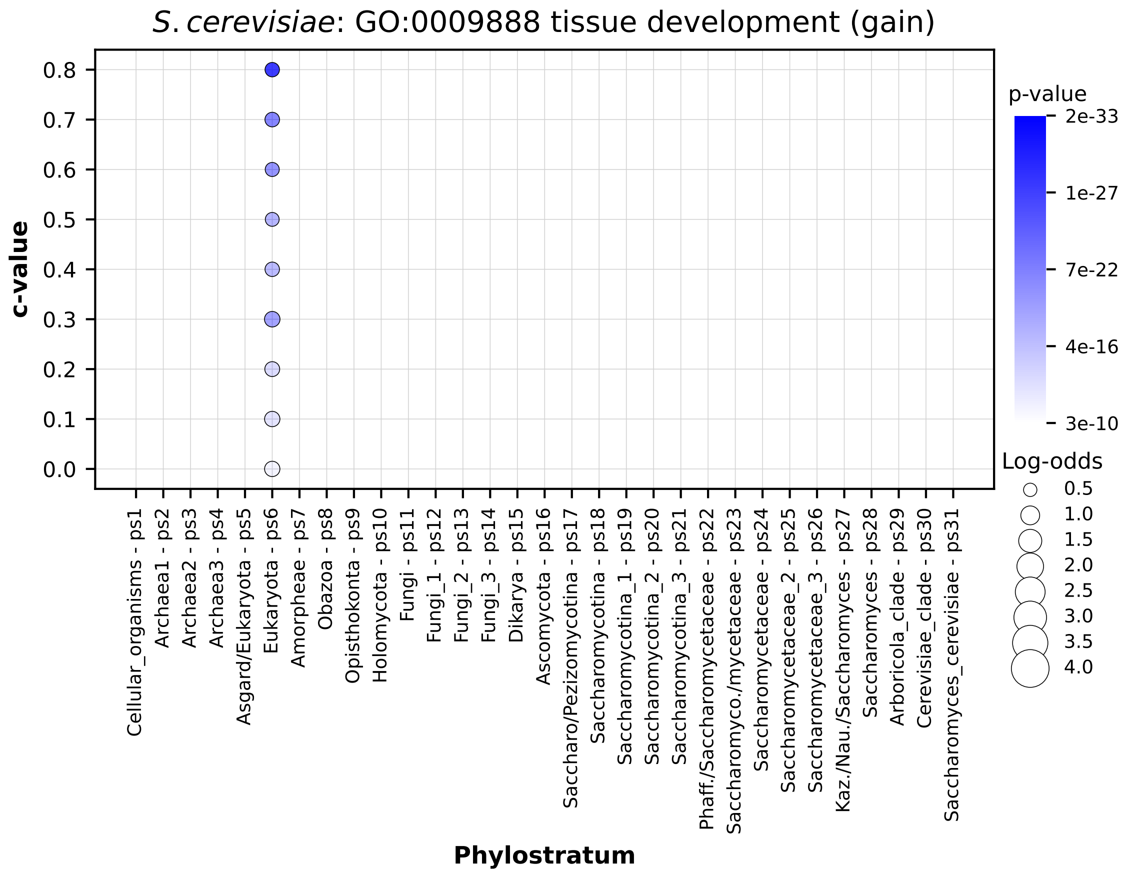
S. cerevisiae: GO:0009888 tissue development (gain)
c-value
0.0
0.1
0.2
0.3
0.4
0.5
0.6
0.7
0.8
p-value
2e-33
1e-27
7e-22
4e-16
3e-10
Log-odds
0.5
1.0
1.5
2.0
2.5
3.0
3.5
4.0
Cellular_organisms - ps1
Archaea1 - ps2
Archaea2 - ps3
Archaea3 - ps4
Asgard/Eukaryota - ps5
Eukaryota - ps6
Amorpheae - ps7
Obazoa - ps8
Opisthokonta - ps9
Holomycota - ps10
Fungi - ps11
Fungi_1 - ps12
Fungi_2 - ps13
Fungi_3 - ps14
Dikarya - ps15
Ascomycota - ps16
Saccharo/Pezizomycotina - ps17
Saccharomycotina - ps18
Saccharomycotina_1 - ps19
Saccharomycotina_2 - ps20
Saccharomycotina_3 - ps21
Phaff./Saccharomycetaceae - ps22
Saccharomyco./mycetaceae - ps23
Saccharomycetaceae - ps24
Saccharomycetaceae_2 - ps25
Saccharomycetaceae_3 - ps26
Kaz./Nau./Saccharomyces - ps27
Saccharomyces - ps28
Arboricola_clade - ps29
Cerevisiae_clade - ps30
Saccharomyces_cerevisiae - ps31
Phylostratum

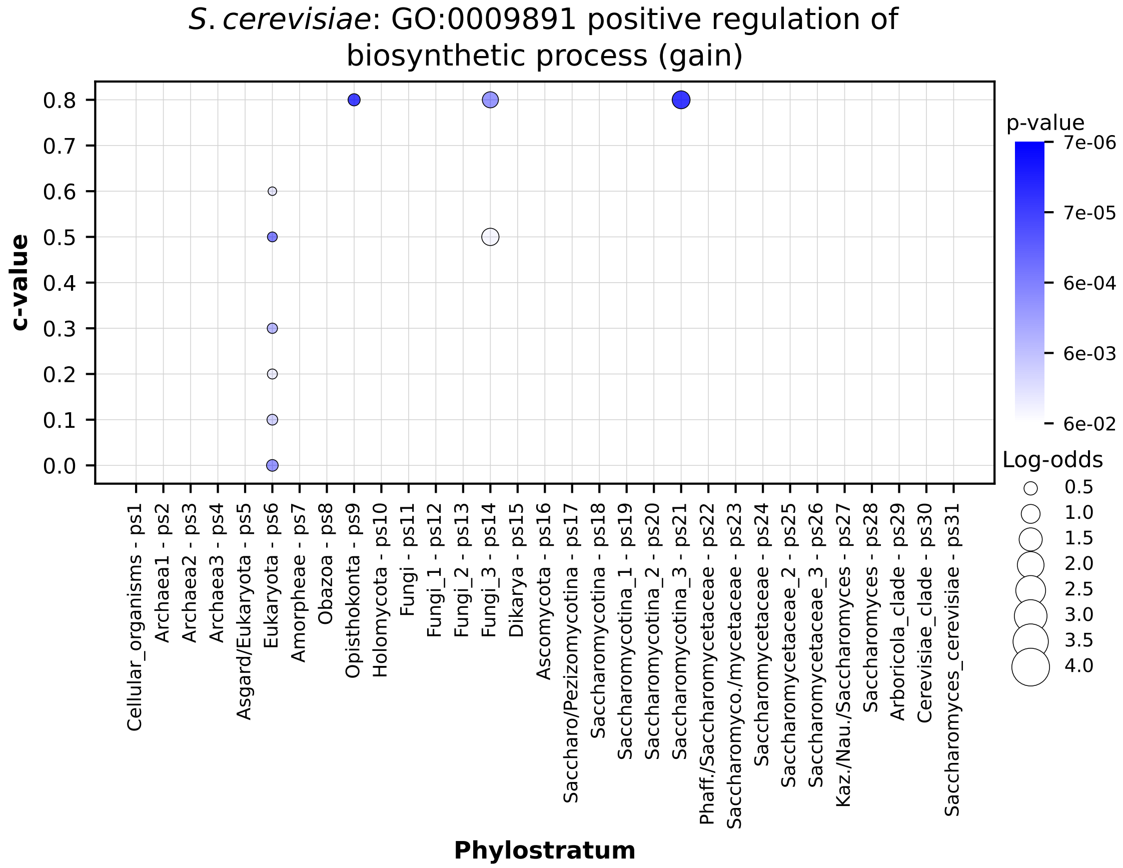

S. cerevisiae: GO:0009891 positive regulation of biosynthetic process (gain)
c-value
0.0
0.1
0.2
0.3
0.4
0.5
0.6
0.7
0.8
p-value
7e-06
7e-05
6e-04
6e-03
6e-02
Log-odds
0.5
1.0
1.5
2.0
2.5
3.0
3.5
4.0
Cellular_organisms - ps1
Archaea1 - ps2
Archaea2 - ps3
Archaea3 - ps4
Asgard/Eukaryota - ps5
Eukaryota - ps6
Amorpheae - ps7
Obazoa - ps8
Opisthokonta - ps9
Holomycota - ps10
Fungi - ps11
Fungi_1 - ps12
Fungi_2 - ps13
Fungi_3 - ps14
Dikarya - ps15
Ascomycota - ps16
Saccharo/Pezizomycotina - ps17
Saccharomycotina - ps18
Saccharomycotina_1 - ps19
Saccharomycotina_2 - ps20
Saccharomycotina_3 - ps21
Phaff./Saccharomycetaceae - ps22
Saccharomyco./mycetaceae - ps23
Saccharomycetaceae - ps24
Saccharomycetaceae_2 - ps25
Saccharomycetaceae_3 - ps26
Kaz./Nau./Saccharomyces - ps27
Saccharomyces - ps28
Arboricola_clade - ps29
Cerevisiae_clade - ps30
Saccharomyces_cerevisiae - ps31
Phylostratum

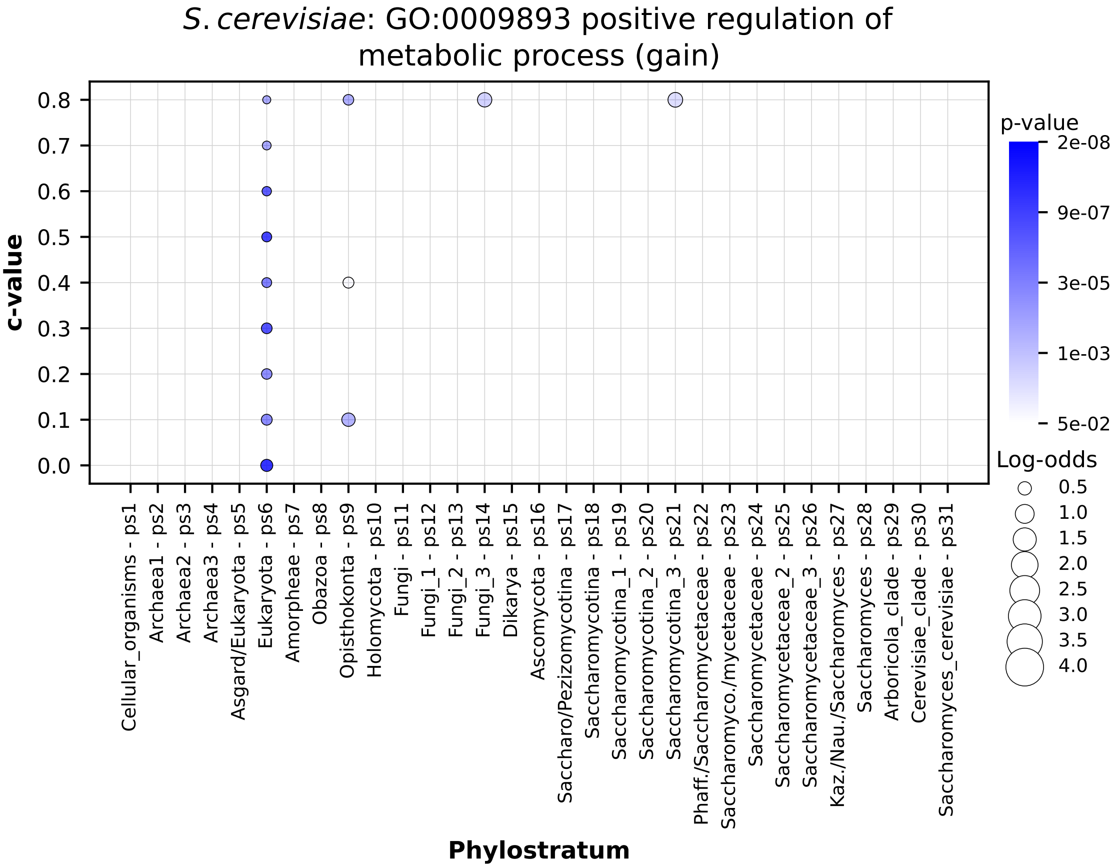

S. cerevisiae: GO:0009893 positive regulation of metabolic process (gain)
c-value
0.0
0.1
0.2
0.3
0.4
0.5
0.6
0.7
0.8
Phylostratum
Cellular_organisms - ps1
Archaea1 - ps2
Archaea2 - ps3
Archaea3 - ps4
Asgard/Eukaryota - ps5
Eukaryota - ps6
Amorpheae - ps7
Obazoa - ps8
Opisthokonta - ps9
Holomycota - ps10
Fungi - ps11
Fungi_1 - ps12
Fungi_2 - ps13
Fungi_3 - ps14
Dikarya - ps15
Ascomycota - ps16
Saccharo/Pezizomycotina - ps17
Saccharomycotina - ps18
Saccharomycotina_1 - ps19
Saccharomycotina_2 - ps20
Saccharomycotina_3 - ps21
Phaff./Saccharomycetaceae - ps22
Saccharomyco./mycetaceae - ps23
Saccharomycetaceae - ps24
Saccharomycetaceae_2 - ps25
Saccharomycetaceae_3 - ps26
Kaz./Nau./Saccharomyces - ps27
Saccharomyces - ps28
Arboricola_clade - ps29
Cerevisiae_clade - ps30
Saccharomyces_cerevisiae - ps31
p-value
2e-08
9e-07
3e-05
1e-03
5e-02
Log-odds
0.5
1.0
1.5
2.0
2.5
3.0
3.5
4.0

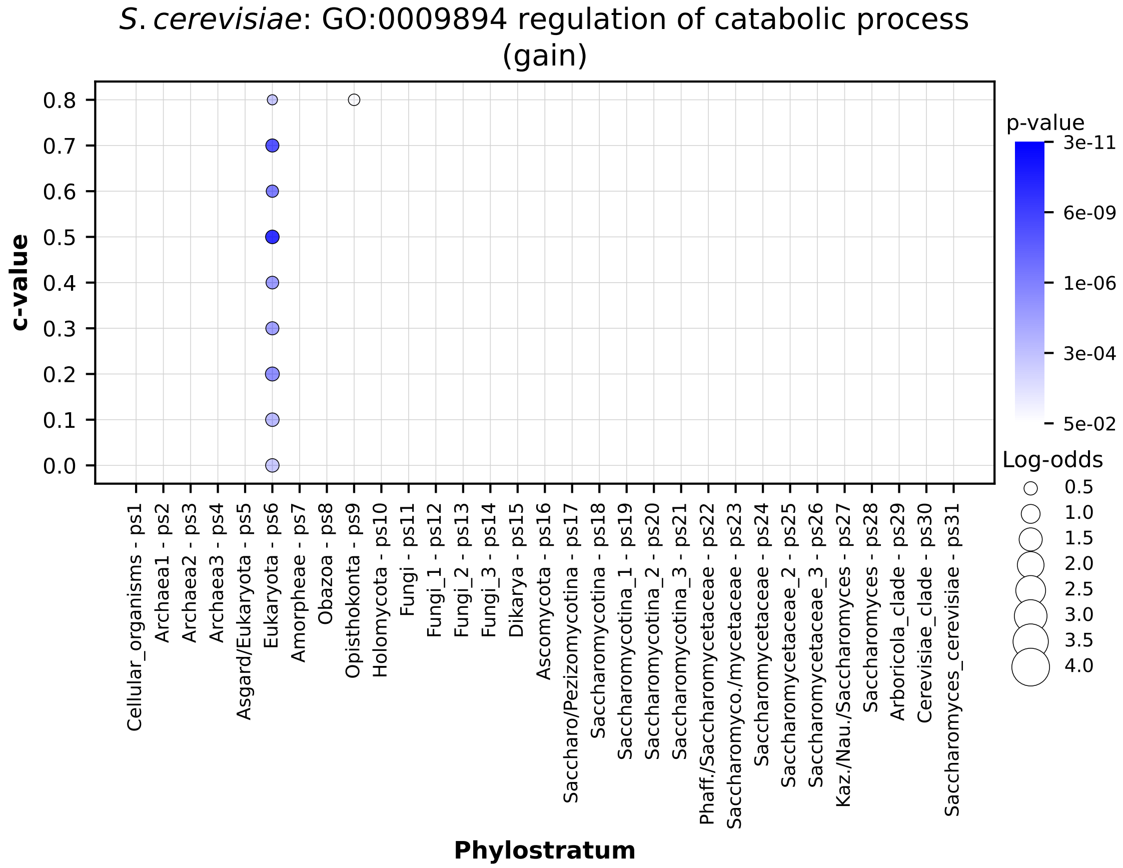

S. cerevisiae: GO:0009894 regulation of catabolic process (gain)
c-value
0.0
0.1
0.2
0.3
0.4
0.5
0.6
0.7
0.8
Phylostratum
Cellular_organisms - ps1
Archaea1 - ps2
Archaea2 - ps3
Archaea3 - ps4
Asgard/Eukaryota - ps5
Eukaryota - ps6
Amorpheae - ps7
Obazoa - ps8
Opisthokonta - ps9
Holomycota - ps10
Fungi - ps11
Fungi_1 - ps12
Fungi_2 - ps13
Fungi_3 - ps14
Dikarya - ps15
Ascomycota - ps16
Saccharo/Pezizomycotina - ps17
Saccharomycotina - ps18
Saccharomycotina_1 - ps19
Saccharomycotina_2 - ps20
Saccharomycotina_3 - ps21
Phaff./Saccharomycetaceae - ps22
Saccharomyco./mycetaceae - ps23
Saccharomycetaceae - ps24
Saccharomycetaceae_2 - ps25
Saccharomycetaceae_3 - ps26
Kaz./Nau./Saccharomyces - ps27
Saccharomyces - ps28
Arboricola_clade - ps29
Cerevisiae_clade - ps30
Saccharomyces_cerevisiae - ps31
p-value
3e-11
6e-09
1e-06
3e-04
5e-02
Log-odds
0.5
1.0
1.5
2.0
2.5
3.0
3.5
4.0

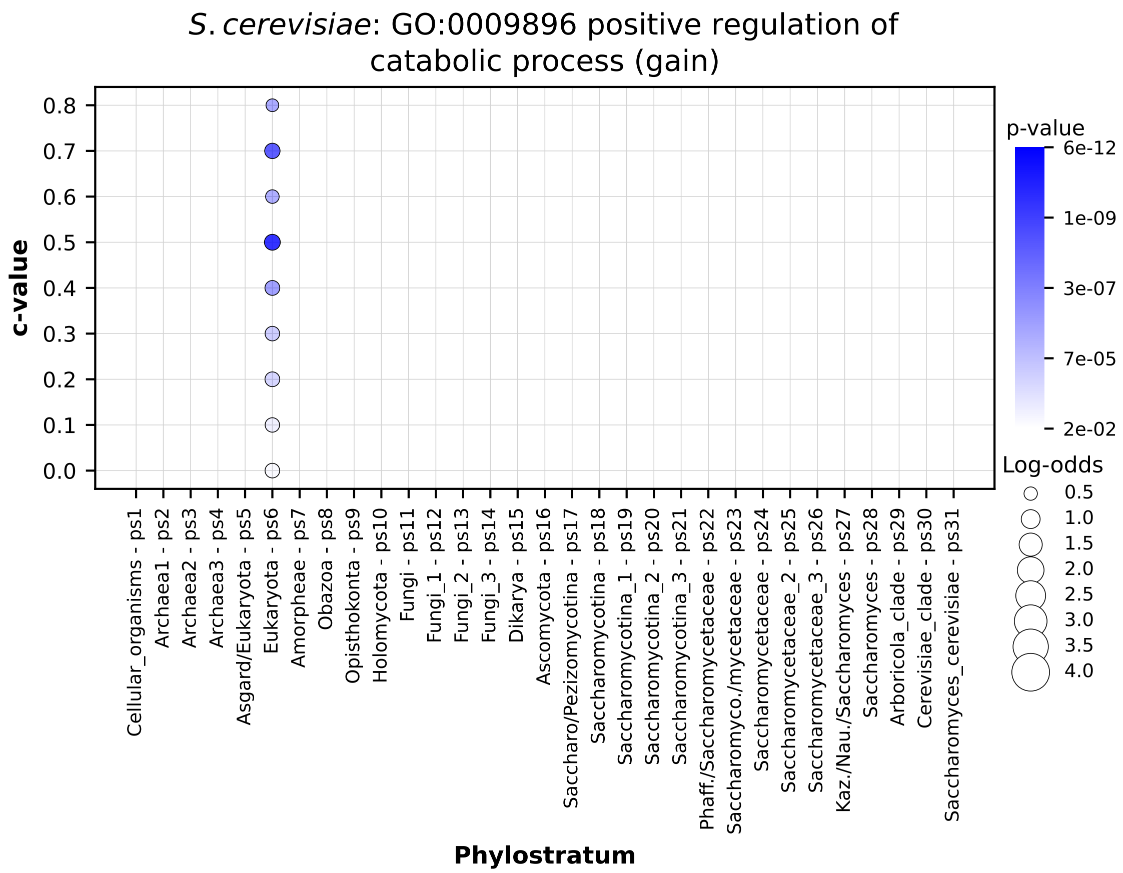

S. cerevisiae: GO:0009896 positive regulation of catabolic process (gain)
c-value
0.8
0.7
0.6
0.5
0.4
0.3
0.2
0.1
0.0
p-value
6e-12
1e-09
3e-07
7e-05
2e-02
Log-odds
0.5
1.0
1.5
2.0
2.5
3.0
3.5
4.0
Cellular_organisms - ps1
Archaea1 - ps2
Archaea2 - ps3
Archaea3 - ps4
Asgard/Eukaryota - ps5
Eukaryota - ps6
Amorpheae - ps7
Obazoa - ps8
Opisthokonta - ps9
Holomycota - ps10
Fungi - ps11
Fungi_1 - ps12
Fungi_2 - ps13
Fungi_3 - ps14
Dikarya - ps15
Ascomycota - ps16
Saccharo/Pezizomycotina - ps17
Saccharomycotina - ps18
Saccharomycotina_1 - ps19
Saccharomycotina_2 - ps20
Saccharomycotina_3 - ps21
Phaff./Saccharomycetaceae - ps22
Saccharomyco./mycetaceae - ps23
Saccharomycetaceae - ps24
Saccharomycetaceae_2 - ps25
Saccharomycetaceae_3 - ps26
Kaz./Nau./Saccharomyces - ps27
Saccharomyces - ps28
Arboricola_clade - ps29
Cerevisiae_clade - ps30
Saccharomyces_cerevisiae - ps31
Phylostratum

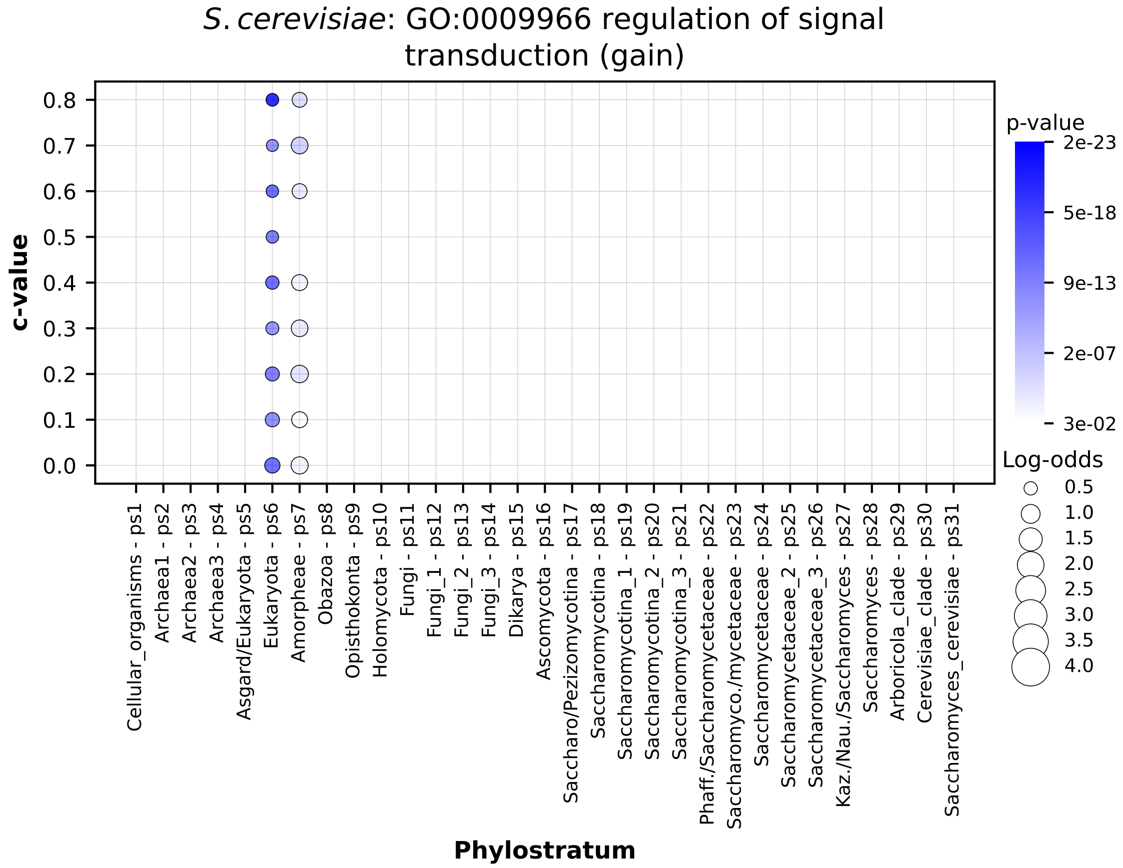
S. cerevisiae: GO:0009966 regulation of signal transduction (gain)
c-value
0.8
0.7
0.6
0.5
0.4
0.3
0.2
0.1
0.0
Phylostratum
Cellular_organisms - ps1
Archaea1 - ps2
Archaea2 - ps3
Archaea3 - ps4
Asgard/Eukaryota - ps5
Eukaryota - ps6
Amorpheae - ps7
Obazoa - ps8
Opisthokonta - ps9
Holomycota - ps10
Fungi - ps11
Fungi_1 - ps12
Fungi_2 - ps13
Fungi_3 - ps14
Dikarya - ps15
Ascomycota - ps16
Saccharo/Pezizomycotina - ps17
Saccharomycotina - ps18
Saccharomycotina_1 - ps19
Saccharomycotina_2 - ps20
Saccharomycotina_3 - ps21
Phaff./Saccharomycetaceae - ps22
Saccharomyco./mycetaceae - ps23
Saccharomycetaceae - ps24
Saccharomycetaceae_2 - ps25
Saccharomycetaceae_3 - ps26
Kaz./Nau./Saccharomyces - ps27
Saccharomyces - ps28
Arboricola_clade - ps29
Cerevisiae_clade - ps30
Saccharomyces_cerevisiae - ps31
p-value
2e-23
5e-18
9e-13
2e-07
3e-02
Log-odds
0.5
1.0
1.5
2.0
2.5
3.0
3.5
4.0

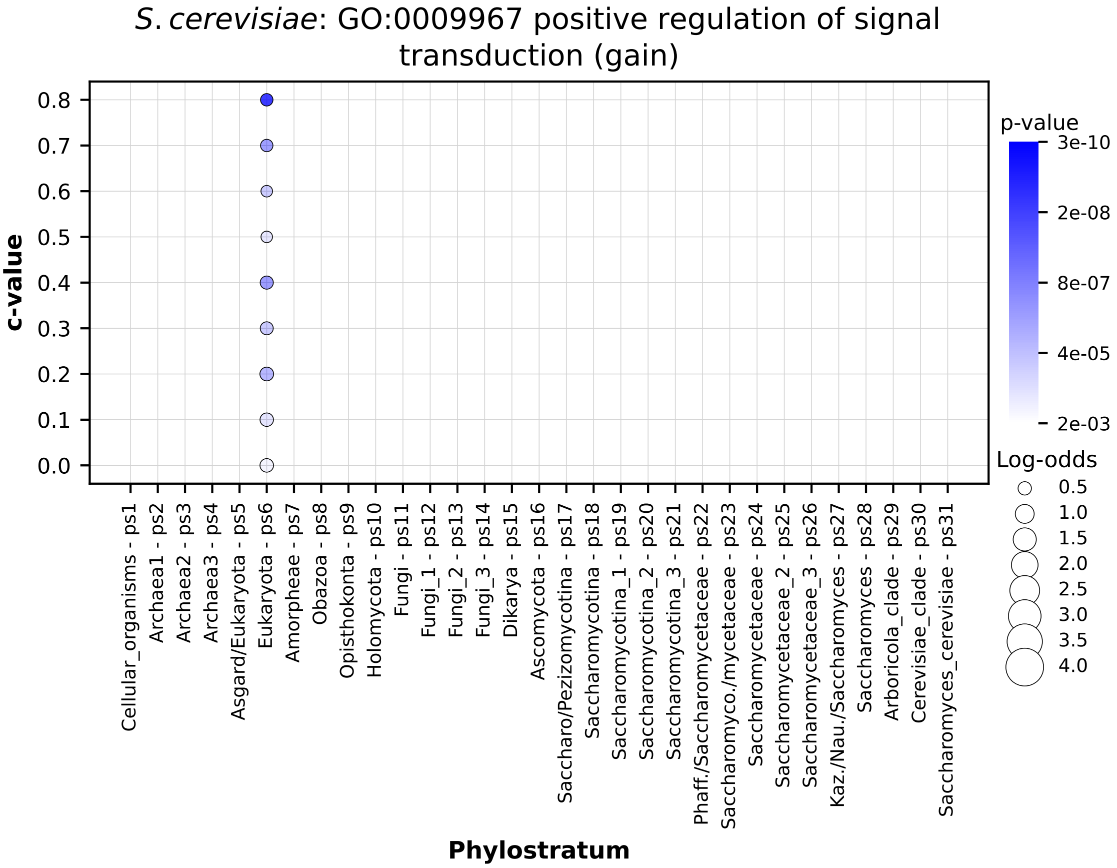

S. cerevisiae: GO:0009967 positive regulation of signal transduction (gain)
c-value
0.0
0.1
0.2
0.3
0.4
0.5
0.6
0.7
0.8
p-value
3e-10
2e-08
8e-07
4e-05
2e-03
Log-odds
0.5
1.0
1.5
2.0
2.5
3.0
3.5
4.0
Cellular_organisms - ps1
Archaea1 - ps2
Archaea2 - ps3
Archaea3 - ps4
Asgard/Eukaryota - ps5
Eukaryota - ps6
Amorpheae - ps7
Obazoa - ps8
Opisthokonta - ps9
Holomycota - ps10
Fungi - ps11
Fungi_1 - ps12
Fungi_2 - ps13
Fungi_3 - ps14
Dikarya - ps15
Ascomycota - ps16
Saccharo/Pezizomycotina - ps17
Saccharomycotina - ps18
Saccharomycotina_1 - ps19
Saccharomycotina_2 - ps20
Saccharomycotina_3 - ps21
Phaff./Saccharomycetaceae - ps22
Saccharomyco./mycetaceae - ps23
Saccharomycetaceae - ps24
Saccharomycetaceae_2 - ps25
Saccharomycetaceae_3 - ps26
Kaz./Nau./Saccharomyces - ps27
Saccharomyces - ps28
Arboricola_clade - ps29
Cerevisiae_clade - ps30
Saccharomyces_cerevisiae - ps31
Phylostratum

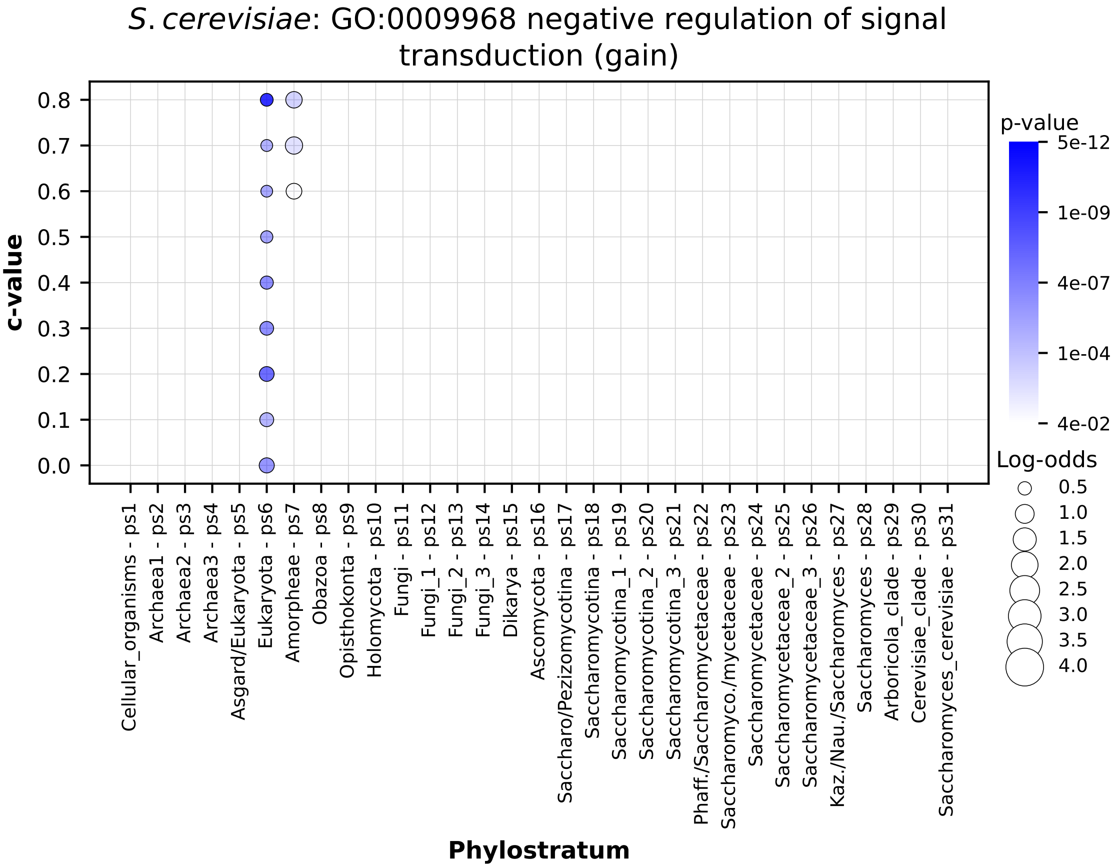

S. cerevisiae: GO:0009968 negative regulation of signal transduction (gain)
c-value
0.8
0.7
0.6
0.5
0.4
0.3
0.2
0.1
0.0
p-value
5e-12
1e-09
4e-07
1e-04
4e-02
Log-odds
0.5
1.0
1.5
2.0
2.5
3.0
3.5
4.0
Cellular_organisms - ps1
Archaea1 - ps2
Archaea2 - ps3
Archaea3 - ps4
Asgard/Eukaryota - ps5
Eukaryota - ps6
Amorpheae - ps7
Obazoa - ps8
Opisthokonta - ps9
Holomycota - ps10
Fungi - ps11
Fungi_1 - ps12
Fungi_2 - ps13
Fungi_3 - ps14
Dikarya - ps15
Ascomycota - ps16
Saccharo/Pezizomycotina - ps17
Saccharomycotina - ps18
Saccharomycotina_1 - ps19
Saccharomycotina_2 - ps20
Saccharomycotina_3 - ps21
Phaff./Saccharomycetaceae - ps22
Saccharomyco./mycetaceae - ps23
Saccharomycetaceae - ps24
Saccharomycetaceae_2 - ps25
Saccharomycetaceae_3 - ps26
Kaz./Nau./Saccharomyces - ps27
Saccharomyces - ps28
Arboricola_clade - ps29
Cerevisiae_clade - ps30
Saccharomyces_cerevisiae - ps31
Phylostratum

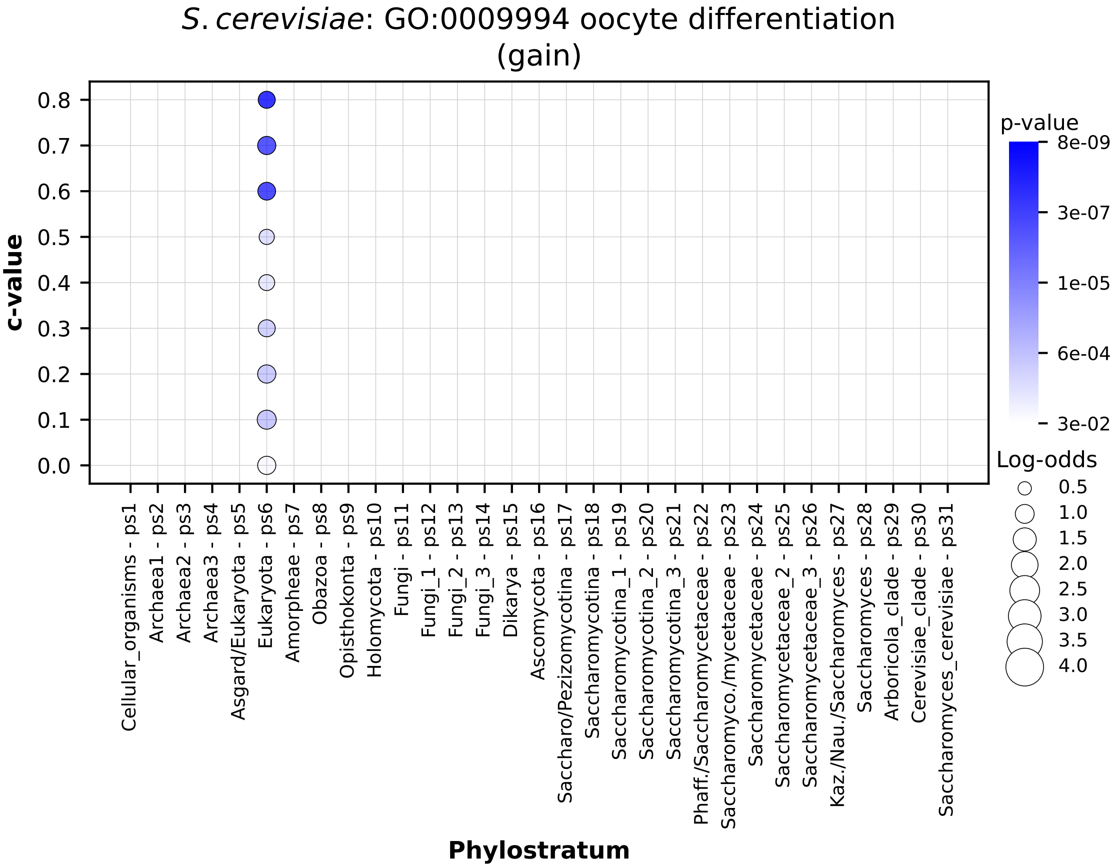

S. cerevisiae: GO:0009994 oocyte differentiation
(gain)
c-value
0.8
0.7
0.6
0.5
0.4
0.3
0.2
0.1
0.0
p-value
8e-09
3e-07
1e-05
6e-04
3e-02
Log-odds
0.5
1.0
1.5
2.0
2.5
3.0
3.5
4.0
Cellular_organisms - ps1
Archaea1 - ps2
Archaea2 - ps3
Archaea3 - ps4
Asgard/Eukaryota - ps5
Eukaryota - ps6
Amorpheae - ps7
Obazoa - ps8
Opisthokonta - ps9
Holomycota - ps10
Fungi - ps11
Fungi_1 - ps12
Fungi_2 - ps13
Fungi_3 - ps14
Dikarya - ps15
Ascomycota - ps16
Saccharo/Pezizomycotina - ps17
Saccharomycotina - ps18
Saccharomycotina_1 - ps19
Saccharomycotina_2 - ps20
Saccharomycotina_3 - ps21
Phaff./Saccharomycetaceae - ps22
Saccharomyco./mycetaceae - ps23
Saccharomycetaceae - ps24
Saccharomycetaceae_2 - ps25
Saccharomycetaceae_3 - ps26
Kaz./Nau./Saccharomyces - ps27
Saccharomyces - ps28
Arboricola_clade - ps29
Cerevisiae_clade - ps30
Saccharomyces_cerevisiae - ps31
Phylostratum

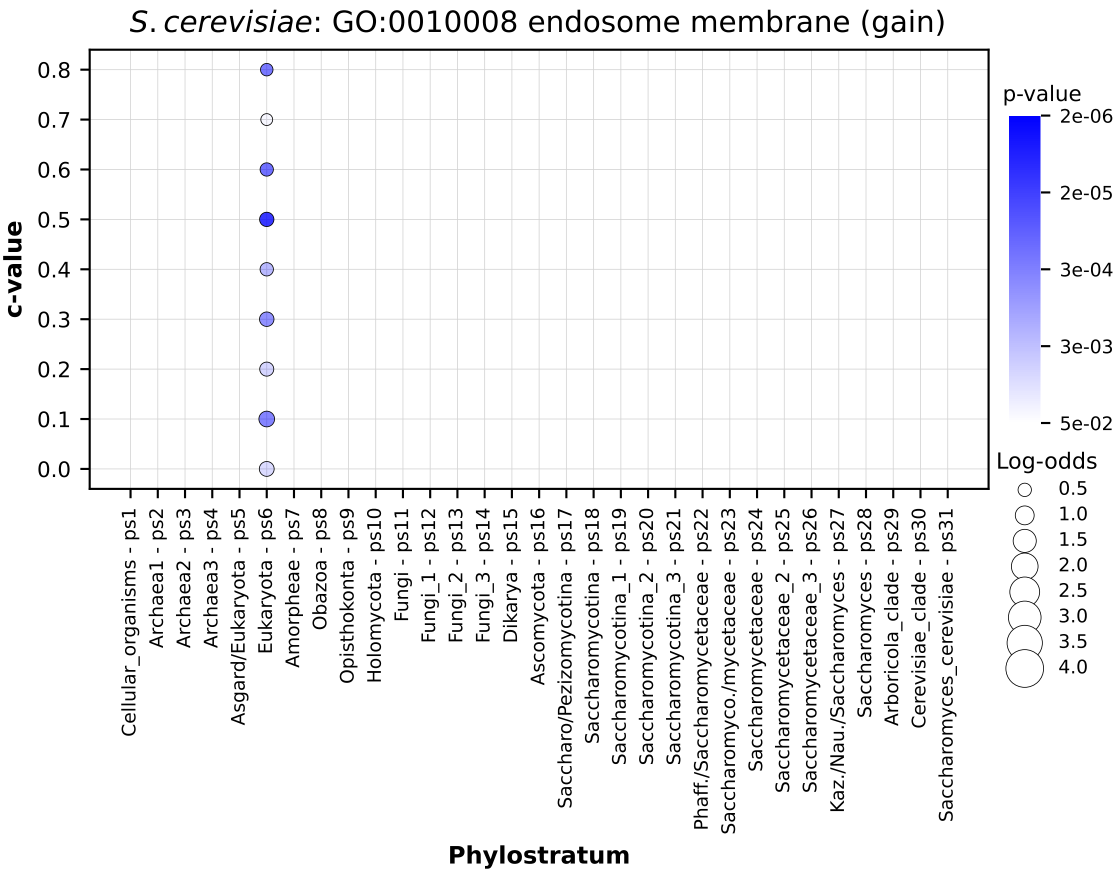

S. cerevisiae: GO:0010008 endosome membrane (gain)
c-value
0.0
0.1
0.2
0.3
0.4
0.5
0.6
0.7
0.8
Phylostratum
Cellular_organisms - ps1
Archaea1 - ps2
Archaea2 - ps3
Archaea3 - ps4
Asgard/Eukaryota - ps5
Eukaryota - ps6
Amorpheae - ps7
Obazoa - ps8
Opisthokonta - ps9
Holomycota - ps10
Fungi - ps11
Fungi_1 - ps12
Fungi_2 - ps13
Fungi_3 - ps14
Dikarya - ps15
Ascomycota - ps16
Saccharo/Pezizomycotina - ps17
Saccharomycotina - ps18
Saccharomycotina_1 - ps19
Saccharomycotina_2 - ps20
Saccharomycotina_3 - ps21
Phaff./Saccharomycetaceae - ps22
Saccharomyco./mycetaceae - ps23
Saccharomycetaceae - ps24
Saccharomycetaceae_2 - ps25
Saccharomycetaceae_3 - ps26
Kaz./Nau./Saccharomyces - ps27
Saccharomyces - ps28
Arboricola_clade - ps29
Cerevisiae_clade - ps30
Saccharomyces_cerevisiae - ps31
p-value
2e-06
2e-05
3e-04
3e-03
5e-02
Log-odds
0.5
1.0
1.5
2.0
2.5
3.0
3.5
4.0

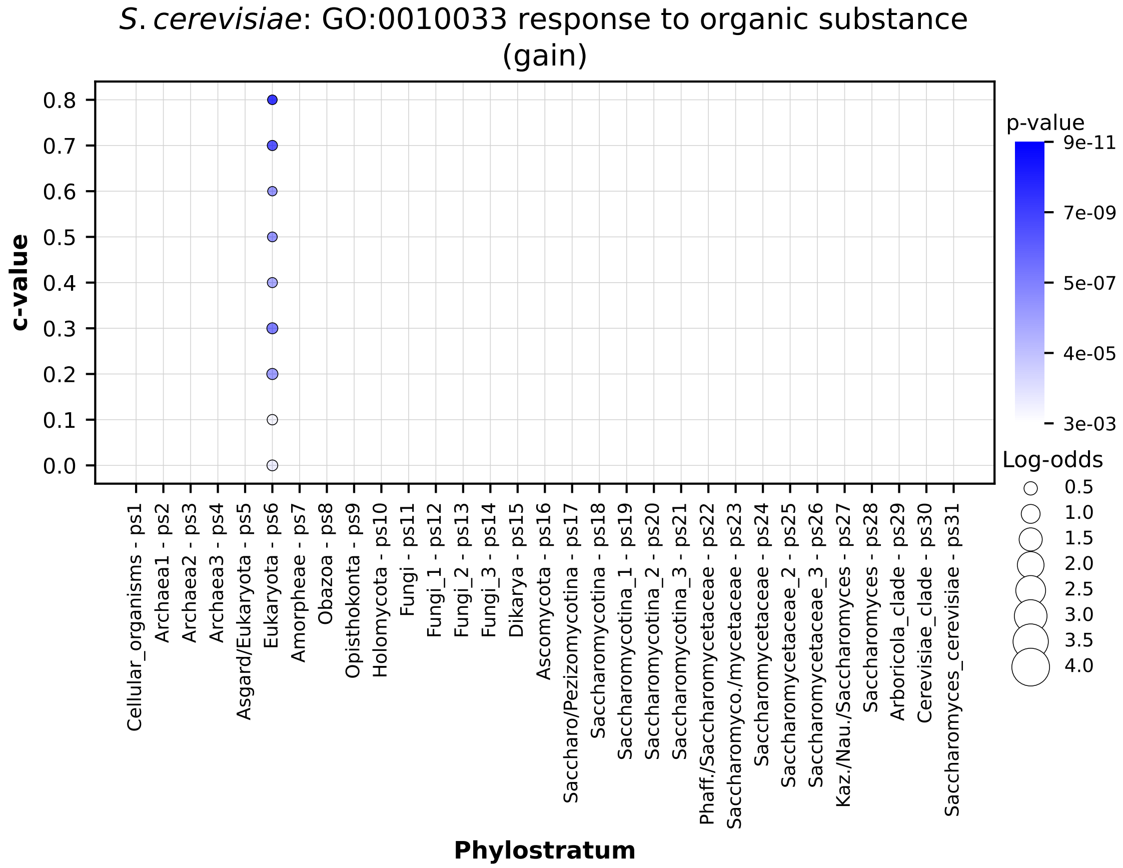

S. cerevisiae: GO:0010033 response to organic substance
(gain)
c-value
0.8
0.7
0.6
0.5
0.4
0.3
0.2
0.1
0.0
p-value
9e-11
7e-09
5e-07
4e-05
3e-03
Log-odds
0.5
1.0
1.5
2.0
2.5
3.0
3.5
4.0
Cellular_organisms - ps1
Archaea1 - ps2
Archaea2 - ps3
Archaea3 - ps4
Asgard/Eukaryota - ps5
Eukaryota - ps6
Amorpheae - ps7
Obazoa - ps8
Opisthokonta - ps9
Holomycota - ps10
Fungi - ps11
Fungi_1 - ps12
Fungi_2 - ps13
Fungi_3 - ps14
Dikarya - ps15
Ascomycota - ps16
Saccharo/Pezizomycotina - ps17
Saccharomycotina - ps18
Saccharomycotina_1 - ps19
Saccharomycotina_2 - ps20
Saccharomycotina_3 - ps21
Phaff./Saccharomycetaceae - ps22
Saccharomyco./mycetaceae - ps23
Saccharomycetaceae - ps24
Saccharomycetaceae_2 - ps25
Saccharomycetaceae_3 - ps26
Kaz./Nau./Saccharomyces - ps27
Saccharomyces - ps28
Arboricola_clade - ps29
Cerevisiae_clade - ps30
Saccharomyces_cerevisiae - ps31
Phylostratum

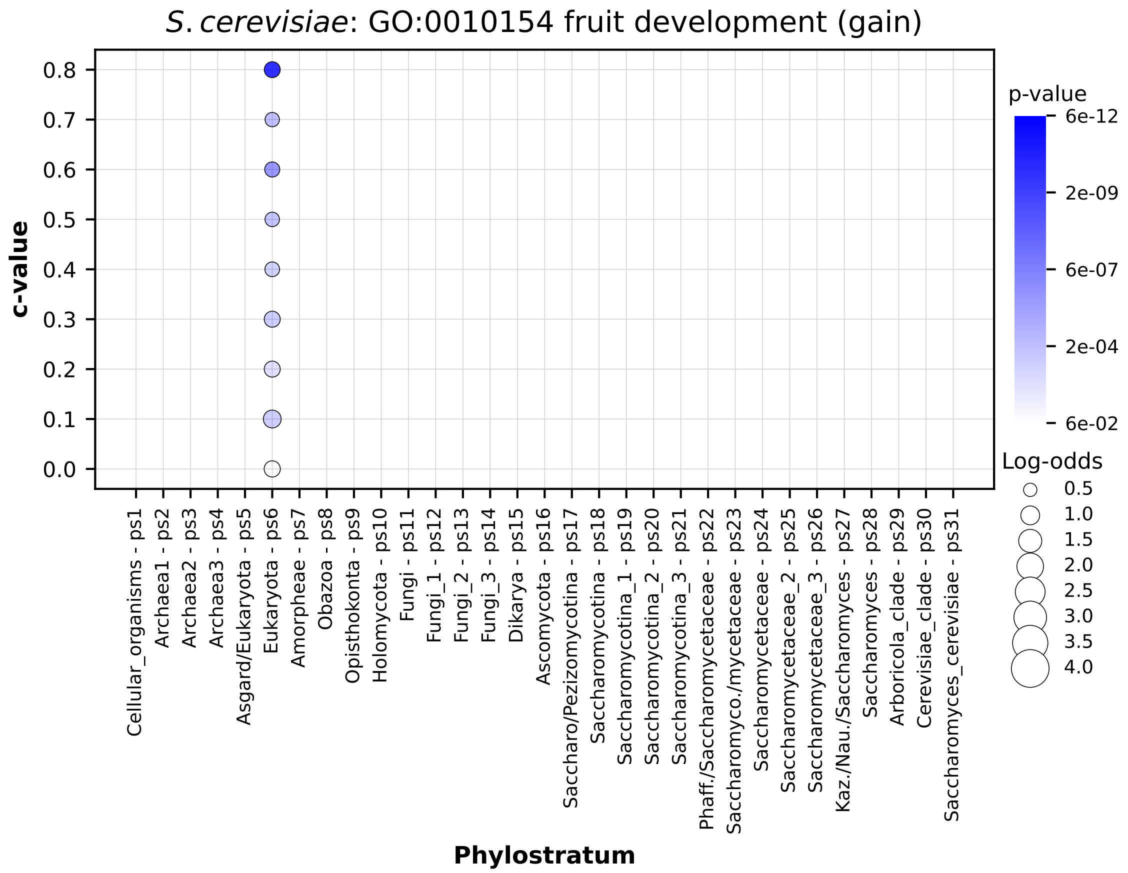

S. cerevisiae: GO:0010154 fruit development (gain)
c-value
0.8
0.7
0.6
0.5
0.4
0.3
0.2
0.1
0.0
Phylostratum
Cellular_organisms - ps1
Archaea1 - ps2
Archaea2 - ps3
Archaea3 - ps4
Asgard/Eukaryota - ps5
Eukaryota - ps6
Amorpheae - ps7
Obazoa - ps8
Opisthokonta - ps9
Holomycota - ps10
Fungi - ps11
Fungi_1 - ps12
Fungi_2 - ps13
Fungi_3 - ps14
Dikarya - ps15
Ascomycota - ps16
Saccharo/Pezizomycotina - ps17
Saccharomycotina - ps18
Saccharomycotina_1 - ps19
Saccharomycotina_2 - ps20
Saccharomycotina_3 - ps21
Phaff./Saccharomycetaceae - ps22
Saccharomyco./mycetaceae - ps23
Saccharomycetaceae - ps24
Saccharomycetaceae_2 - ps25
Saccharomycetaceae_3 - ps26
Kaz./Nau./Saccharomyces - ps27
Saccharomyces - ps28
Arboricola_clade - ps29
Cerevisiae_clade - ps30
Saccharomyces_cerevisiae - ps31
p-value
6e-12
2e-09
6e-07
2e-04
6e-02
Log-odds
0.5
1.0
1.5
2.0
2.5
3.0
3.5
4.0

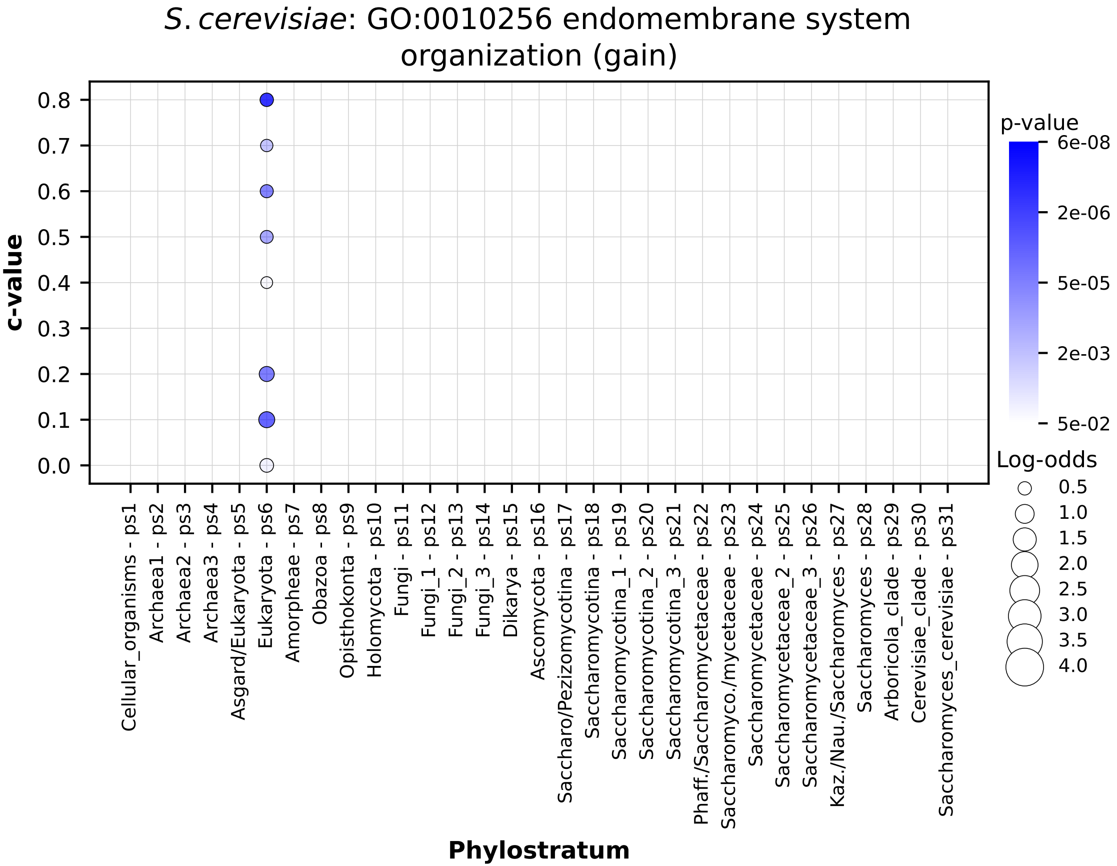

S. cerevisiae: GO:0010256 endomembrane system organization (gain)
c-value
0.0
0.1
0.2
0.3
0.4
0.5
0.6
0.7
0.8
Phylostratum
Cellular_organisms - ps1
Archaea1 - ps2
Archaea2 - ps3
Archaea3 - ps4
Asgard/Eukaryota - ps5
Eukaryota - ps6
Amorpheae - ps7
Obazoa - ps8
Opisthokonta - ps9
Holomycota - ps10
Fungi - ps11
Fungi_1 - ps12
Fungi_2 - ps13
Fungi_3 - ps14
Dikarya - ps15
Ascomycota - ps16
Saccharo/Pezizomycotina - ps17
Saccharomycotina - ps18
Saccharomycotina_1 - ps19
Saccharomycotina_2 - ps20
Saccharomycotina_3 - ps21
Phaff./Saccharomycetaceae - ps22
Saccharomyco./mycetaceae - ps23
Saccharomycetaceae - ps24
Saccharomycetaceae_2 - ps25
Saccharomycetaceae_3 - ps26
Kaz./Nau./Saccharomyces - ps27
Saccharomyces - ps28
Arboricola_clade - ps29
Cerevisiae_clade - ps30
Saccharomyces_cerevisiae - ps31
p-value
6e-08
2e-06
5e-05
2e-03
5e-02
Log-odds
0.5
1.0
1.5
2.0
2.5
3.0
3.5
4.0

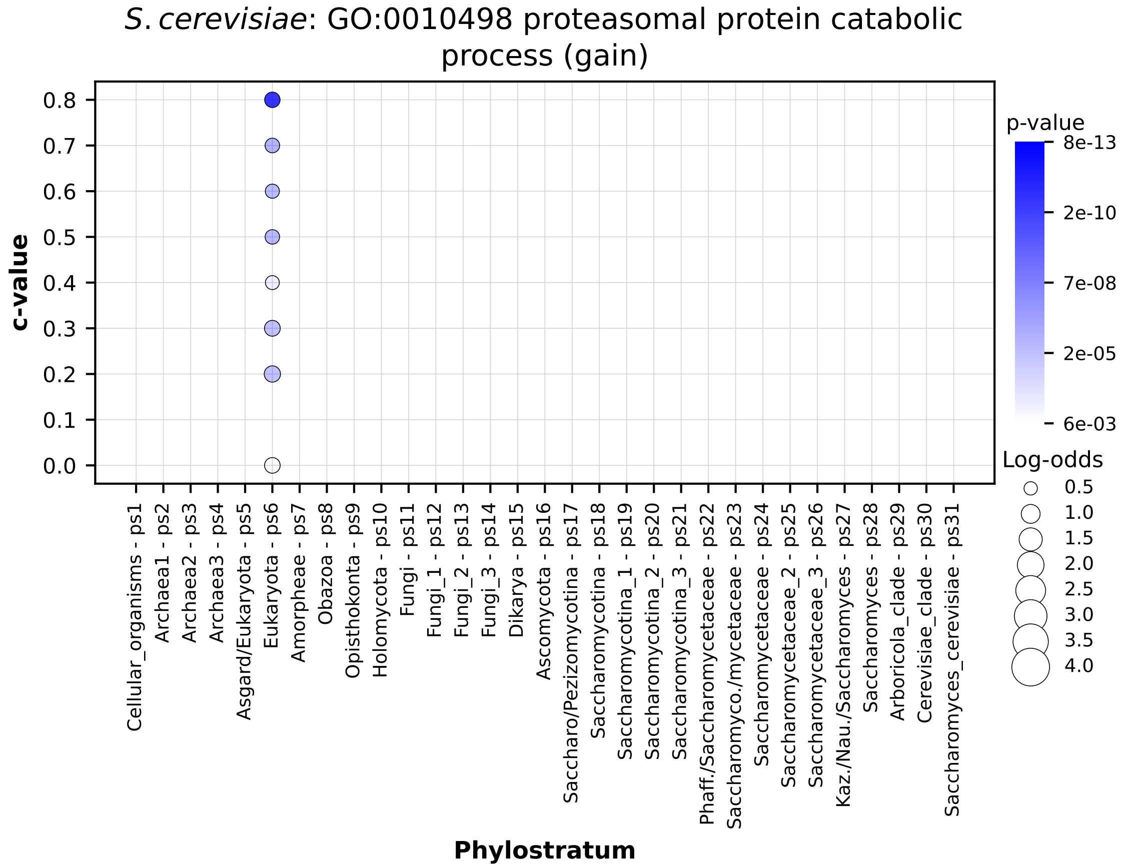

S. cerevisiae: GO:0010498 proteasomal protein catabolic process (gain)
c-value
0.8
0.7
0.6
0.5
0.4
0.3
0.2
0.1
0.0
p-value
8e-13
2e-10
7e-08
2e-05
6e-03
Log-odds
0.5
1.0
1.5
2.0
2.5
3.0
3.5
4.0
Cellular_organisms - ps1
Archaea1 - ps2
Archaea2 - ps3
Archaea3 - ps4
Asgard/Eukaryota - ps5
Eukaryota - ps6
Amorpheae - ps7
Obazoa - ps8
Opisthokonta - ps9
Holomycota - ps10
Fungi - ps11
Fungi_1 - ps12
Fungi_2 - ps13
Fungi_3 - ps14
Dikarya - ps15
Ascomycota - ps16
Saccharo/Pezizomycotina - ps17
Saccharomycotina - ps18
Saccharomycotina_1 - ps19
Saccharomycotina_2 - ps20
Saccharomycotina_3 - ps21
Phaff./Saccharomycetaceae - ps22
Saccharomyco./mycetaceae - ps23
Saccharomycetaceae - ps24
Saccharomycetaceae_2 - ps25
Saccharomycetaceae_3 - ps26
Kaz./Nau./Saccharomyces - ps27
Saccharomyces - ps28
Arboricola_clade - ps29
Cerevisiae_clade - ps30
Saccharomyces_cerevisiae - ps31
Phylostratum

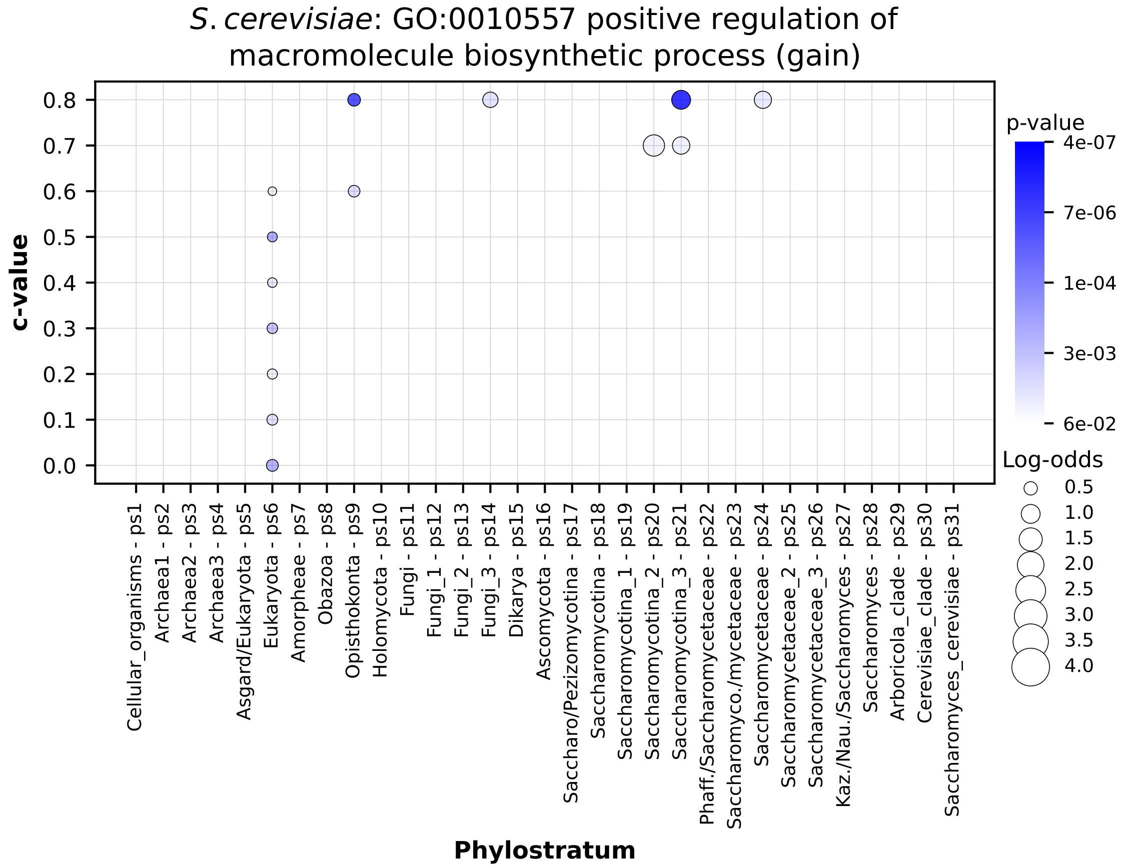

S. cerevisiae: GO:0010557 positive regulation of macromolecule biosynthetic process (gain)
c-value
0.0
0.1
0.2
0.3
0.4
0.5
0.6
0.7
0.8
p-value
4e-07
7e-06
1e-04
3e-03
6e-02
Log-odds
0.5
1.0
1.5
2.0
2.5
3.0
3.5
4.0
Cellular_organisms - ps1
Archaea1 - ps2
Archaea2 - ps3
Archaea3 - ps4
Asgard/Eukaryota - ps5
Eukaryota - ps6
Amorpheae - ps7
Obazoa - ps8
Opisthokonta - ps9
Holomycota - ps10
Fungi - ps11
Fungi_1 - ps12
Fungi_2 - ps13
Fungi_3 - ps14
Dikarya - ps15
Ascomycota - ps16
Saccharo/Pezizomycotina - ps17
Saccharomycotina - ps18
Saccharomycotina_1 - ps19
Saccharomycotina_2 - ps20
Saccharomycotina_3 - ps21
Phaff./Saccharomycetaceae - ps22
Saccharomyco./mycetaceae - ps23
Saccharomycetaceae - ps24
Saccharomycetaceae_2 - ps25
Saccharomycetaceae_3 - ps26
Kaz./Nau./Saccharomyces - ps27
Saccharomyces - ps28
Arboricola_clade - ps29
Cerevisiae_clade - ps30
Saccharomyces_cerevisiae - ps31
Phylostratum

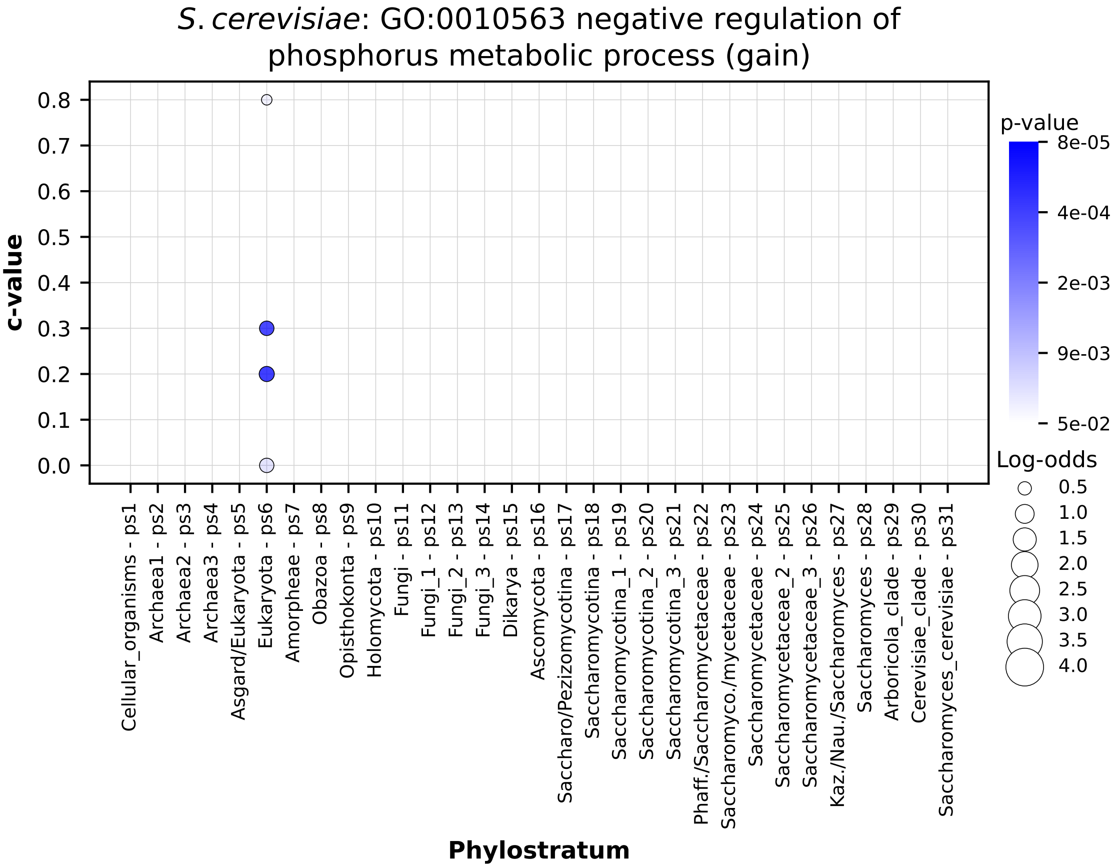

S. cerevisiae: GO:0010563 negative regulation of phosphorus metabolic process (gain)
c-value
0.8
0.7
0.6
0.5
0.4
0.3
0.2
0.1
0.0
p-value
8e-05
4e-04
2e-03
9e-03
5e-02
Log-odds
0.5
1.0
1.5
2.0
2.5
3.0
3.5
4.0
Cellular_organisms - ps1
Archaea1 - ps2
Archaea2 - ps3
Archaea3 - ps4
Asgard/Eukaryota - ps5
Eukaryota - ps6
Amorpheae - ps7
Obazoa - ps8
Opisthokonta - ps9
Holomycota - ps10
Fungi - ps11
Fungi_1 - ps12
Fungi_2 - ps13
Fungi_3 - ps14
Dikarya - ps15
Ascomycota - ps16
Saccharo/Pezizomycotina - ps17
Saccharomycotina - ps18
Saccharomycotina_1 - ps19
Saccharomycotina_2 - ps20
Saccharomycotina_3 - ps21
Phaff./Saccharomycetaceae - ps22
Saccharomyco./mycetaceae - ps23
Saccharomycetaceae - ps24
Saccharomycetaceae_2 - ps25
Saccharomycetaceae_3 - ps26
Kaz./Nau./Saccharomyces - ps27
Saccharomyces - ps28
Arboricola_clade - ps29
Cerevisiae_clade - ps30
Saccharomyces_cerevisiae - ps31
Phylostratum

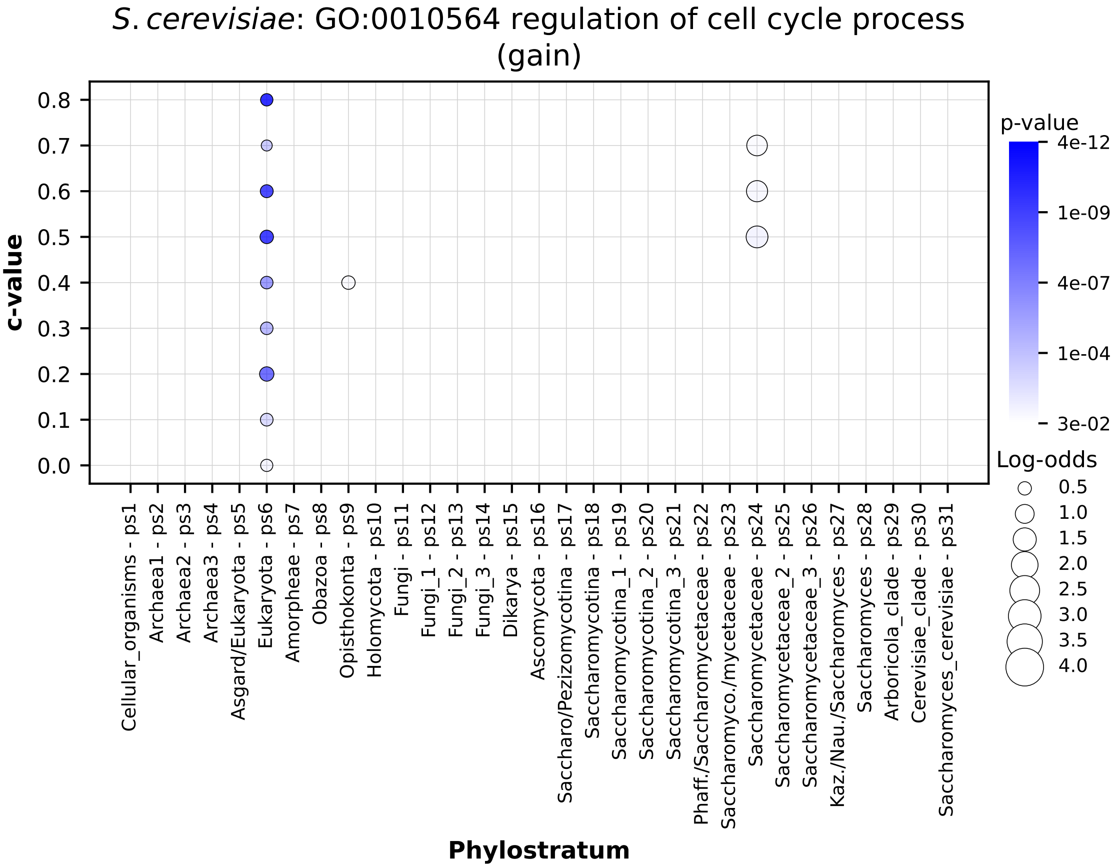

$S. cerevisiae$: GO:0010564 regulation of cell cycle process
(gain)
c-value
0.8
0.7
0.6
0.5
0.4
0.3
0.2
0.1
0.0
Phylostratum
Cellular_organisms - ps1
Archaea1 - ps2
Archaea2 - ps3
Archaea3 - ps4
Asgard/Eukaryota - ps5
Eukaryota - ps6
Amorpheae - ps7
Obazoa - ps8
Opisthokonta - ps9
Holomycota - ps10
Fungi - ps11
Fungi_1 - ps12
Fungi_2 - ps13
Fungi_3 - ps14
Dikarya - ps15
Ascomycota - ps16
Saccharo/Pezizomycotina - ps17
Saccharomycotina - ps18
Saccharomycotina_1 - ps19
Saccharomycotina_2 - ps20
Saccharomycotina_3 - ps21
Phaff./Saccharomycetaceae - ps22
Saccharomyco./mycetaceae - ps23
Saccharomycetaceae - ps24
Saccharomycetaceae_2 - ps25
Saccharomycetaceae_3 - ps26
Kaz./Nau./Saccharomyces - ps27
Saccharomyces - ps28
Arboricola_clade - ps29
Cerevisiae_clade - ps30
Saccharomyces_cerevisiae - ps31
p-value
4e-12
1e-09
4e-07
1e-04
3e-02
Log-odds
0.5
1.0
1.5
2.0
2.5
3.0
3.5
4.0

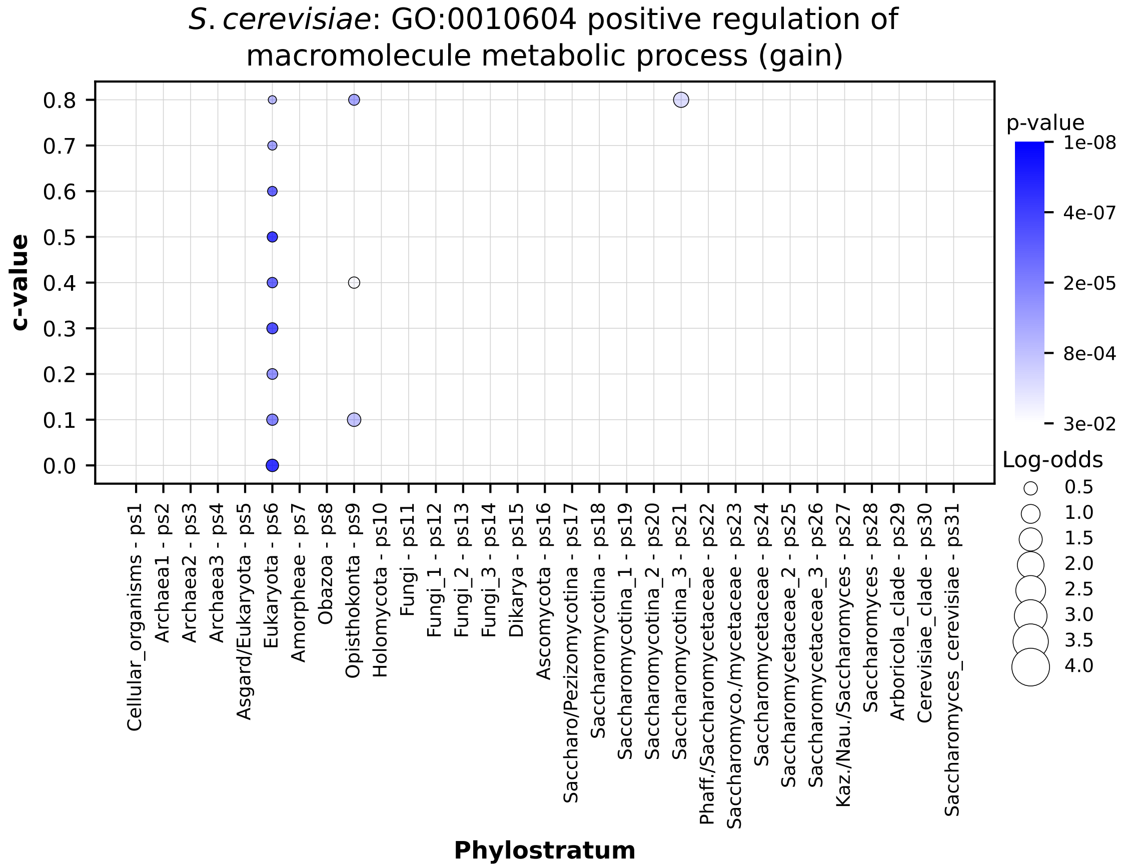
S. cerevisiae: GO:0010604 positive regulation of macromolecule metabolic process (gain)
c-value
0.8
0.7
0.6
0.5
0.4
0.3
0.2
0.1
0.0
p-value
1e-08
4e-07
2e-05
8e-04
3e-02
Log-odds
0.5
1.0
1.5
2.0
2.5
3.0
3.5
4.0
Cellular_organisms - ps1
Archaea1 - ps2
Archaea2 - ps3
Archaea3 - ps4
Asgard/Eukaryota - ps5
Eukaryota - ps6
Amorpheae - ps7
Obazoa - ps8
Opisthokonta - ps9
Holomycota - ps10
Fungi - ps11
Fungi_1 - ps12
Fungi_2 - ps13
Fungi_3 - ps14
Dikarya - ps15
Ascomycota - ps16
Saccharo/Pezizomycotina - ps17
Saccharomycotina - ps18
Saccharomycotina_1 - ps19
Saccharomycotina_2 - ps20
Saccharomycotina_3 - ps21
Phaff./Saccharomycetaceae - ps22
Saccharomyco./mycetaceae - ps23
Saccharomycetaceae - ps24
Saccharomycetaceae_2 - ps25
Saccharomycetaceae_3 - ps26
Kaz./Nau./Saccharomyces - ps27
Saccharomyces - ps28
Arboricola_clade - ps29
Cerevisiae_clade - ps30
Saccharomyces_cerevisiae - ps31
Phylostratum

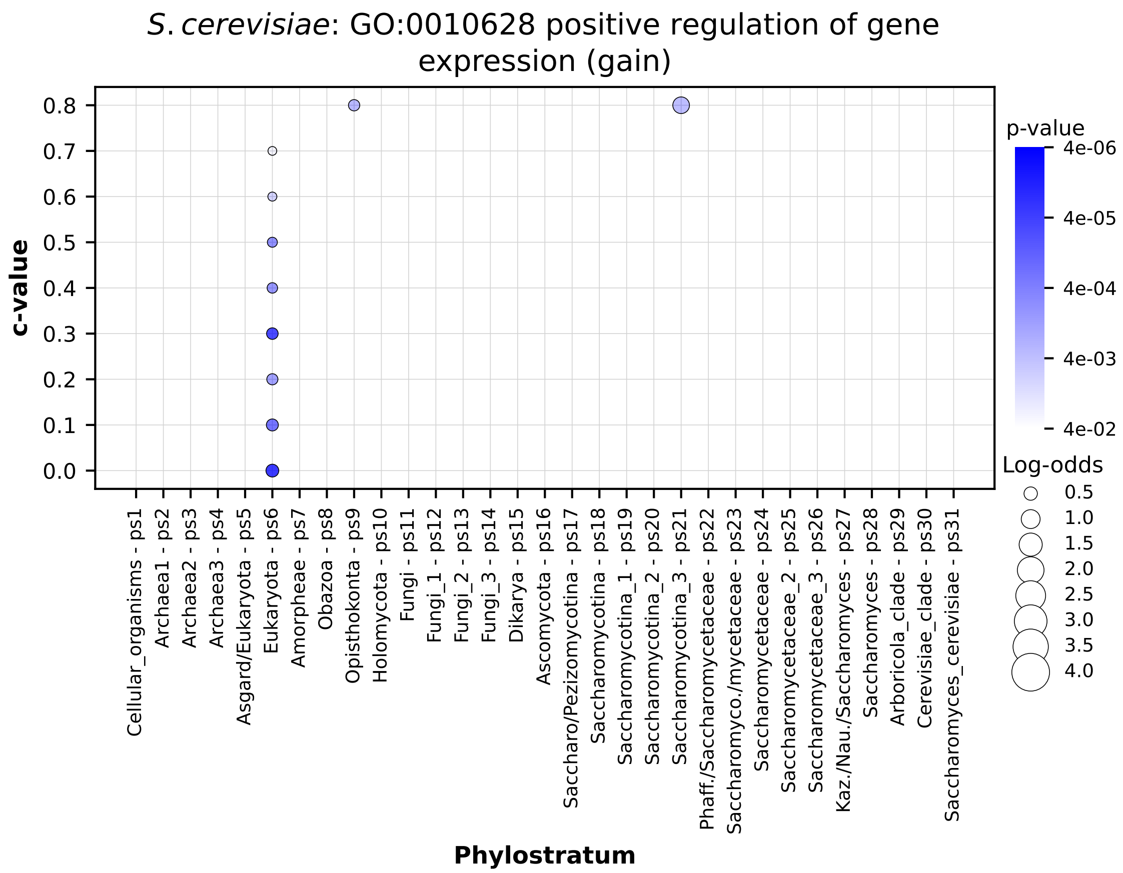

S. cerevisiae: GO:0010628 positive regulation of gene expression (gain)
c-value
0.8
0.7
0.6
0.5
0.4
0.3
0.2
0.1
0.0
p-value
4e-06
4e-05
4e-04
4e-03
4e-02
Log-odds
0.5
1.0
1.5
2.0
2.5
3.0
3.5
4.0
Cellular_organisms - ps1
Archaea1 - ps2
Archaea2 - ps3
Archaea3 - ps4
Asgard/Eukaryota - ps5
Eukaryota - ps6
Amorpheae - ps7
Obazoa - ps8
Opisthokonta - ps9
Holomycota - ps10
Fungi - ps11
Fungi_1 - ps12
Fungi_2 - ps13
Fungi_3 - ps14
Dikarya - ps15
Ascomycota - ps16
Saccharo/Pezizomycotina - ps17
Saccharomycotina - ps18
Saccharomycotina_1 - ps19
Saccharomycotina_2 - ps20
Saccharomycotina_3 - ps21
Phaff./Saccharomycetaceae - ps22
Saccharomyco./mycetaceae - ps23
Saccharomycetaceae - ps24
Saccharomycetaceae_2 - ps25
Saccharomycetaceae_3 - ps26
Kaz./Nau./Saccharomyces - ps27
Saccharomyces - ps28
Arboricola_clade - ps29
Cerevisiae_clade - ps30
Saccharomyces_cerevisiae - ps31
Phylostratum

S. cerevisiae: GO:0010638 positive regulation of organelle organization (gain)

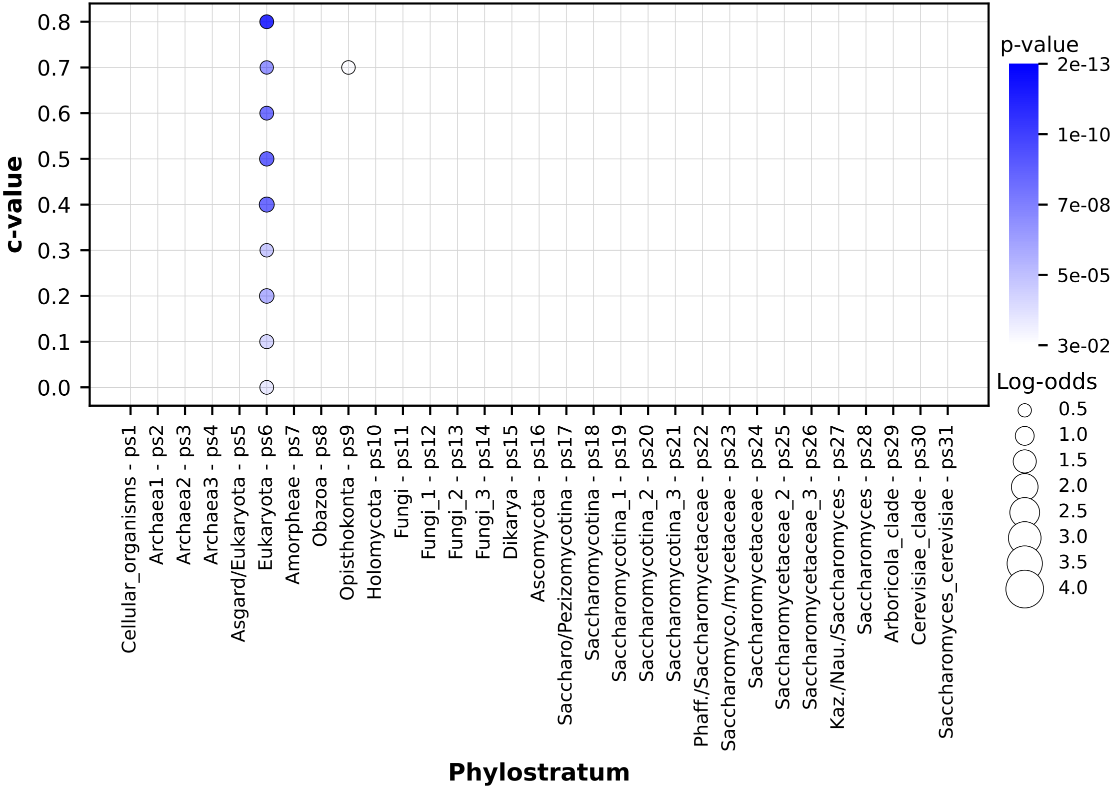

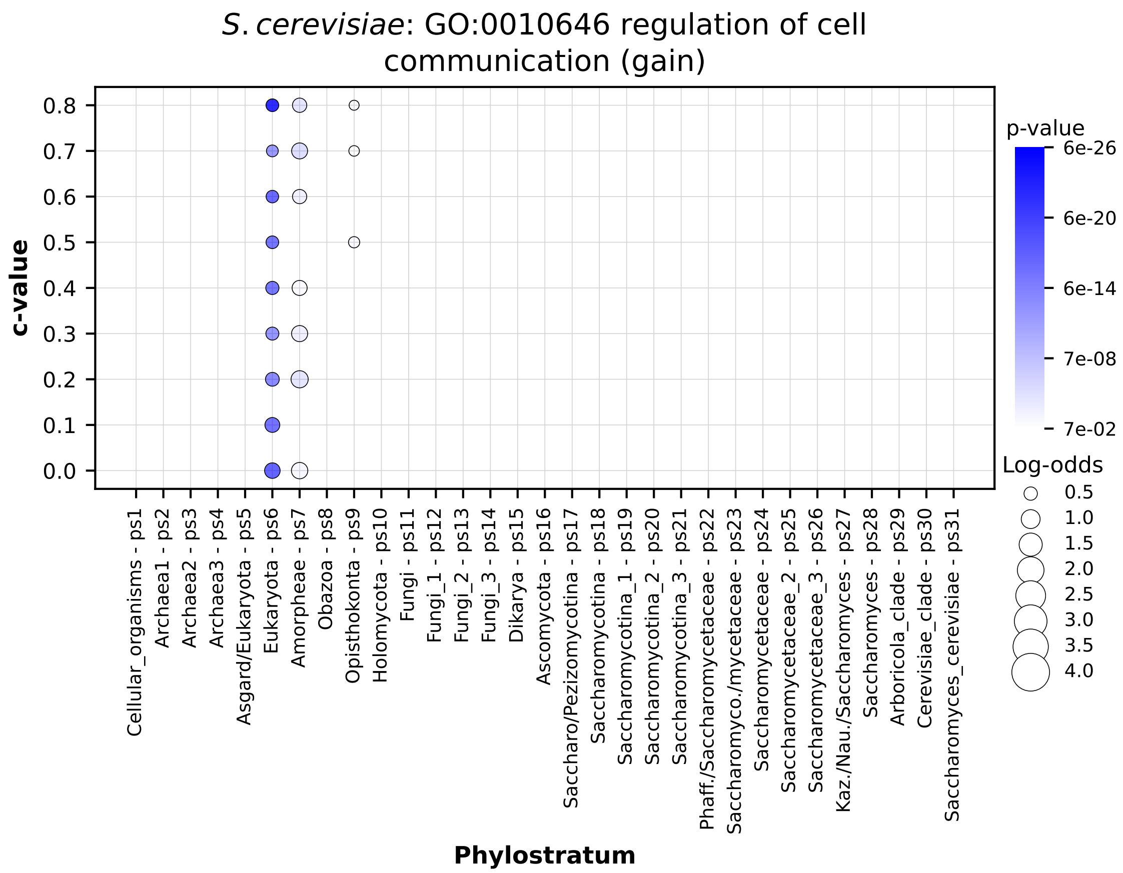

S. cerevisiae: GO:0010646 regulation of cell communication (gain)
c-value
0.8
0.7
0.6
0.5
0.4
0.3
0.2
0.1
0.0
p-value
6e-26
6e-20
6e-14
7e-08
7e-02
Log-odds
0.5
1.0
1.5
2.0
2.5
3.0
3.5
4.0
Cellular_organisms - ps1
Archaea1 - ps2
Archaea2 - ps3
Archaea3 - ps4
Asgard/Eukaryota - ps5
Eukaryota - ps6
Amorpheae - ps7
Obazoa - ps8
Opisthokonta - ps9
Holomycota - ps10
Fungi - ps11
Fungi_1 - ps12
Fungi_2 - ps13
Fungi_3 - ps14
Dikarya - ps15
Ascomycota - ps16
Saccharo/Pezizomycotina - ps17
Saccharomycotina - ps18
Saccharomycotina_1 - ps19
Saccharomycotina_2 - ps20
Saccharomycotina_3 - ps21
Phaff./Saccharomycetaceae - ps22
Saccharomyco./mycetaceae - ps23
Saccharomycetaceae - ps24
Saccharomycetaceae_2 - ps25
Saccharomycetaceae_3 - ps26
Kaz./Nau./Saccharomyces - ps27
Saccharomyces - ps28
Arboricola_clade - ps29
Cerevisiae_clade - ps30
Saccharomyces_cerevisiae - ps31
Phylostratum

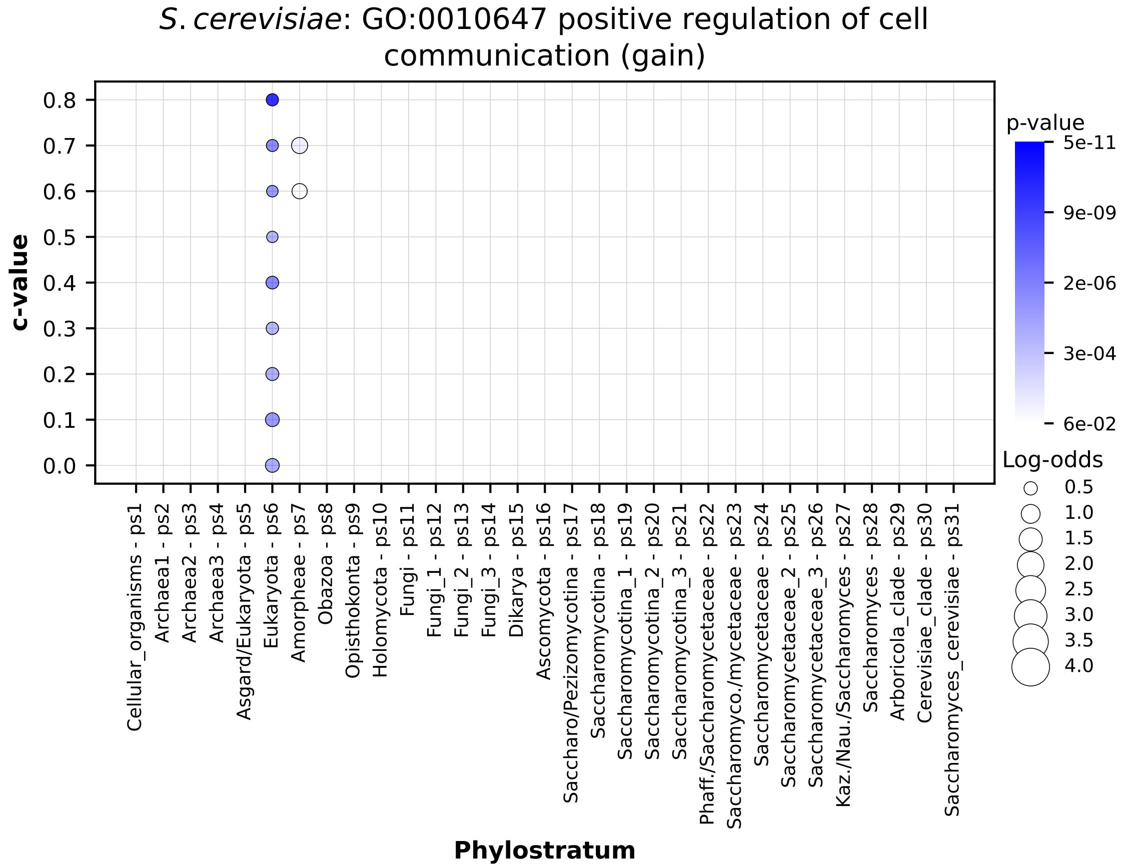

S. cerevisiae: GO:0010647 positive regulation of cell communication (gain)
c-value
0.8
0.7
0.6
0.5
0.4
0.3
0.2
0.1
0.0
p-value
5e-11
9e-09
2e-06
3e-04
6e-02
Log-odds
0.5
1.0
1.5
2.0
2.5
3.0
3.5
4.0
Cellular_organisms - ps1
Archaea1 - ps2
Archaea2 - ps3
Archaea3 - ps4
Asgard/Eukaryota - ps5
Eukaryota - ps6
Amorpheae - ps7
Obazoa - ps8
Opisthokonta - ps9
Holomycota - ps10
Fungi - ps11
Fungi_1 - ps12
Fungi_2 - ps13
Fungi_3 - ps14
Dikarya - ps15
Ascomycota - ps16
Saccharo/Pezizomycotina - ps17
Saccharomycotina - ps18
Saccharomycotina_1 - ps19
Saccharomycotina_2 - ps20
Saccharomycotina_3 - ps21
Phaff./Saccharomycetaceae - ps22
Saccharomyco./mycetaceae - ps23
Saccharomycetaceae - ps24
Saccharomycetaceae_2 - ps25
Saccharomycetaceae_3 - ps26
Kaz./Nau./Saccharomyces - ps27
Saccharomyces - ps28
Arboricola_clade - ps29
Cerevisiae_clade - ps30
Saccharomyces_cerevisiae - ps31
Phylostratum

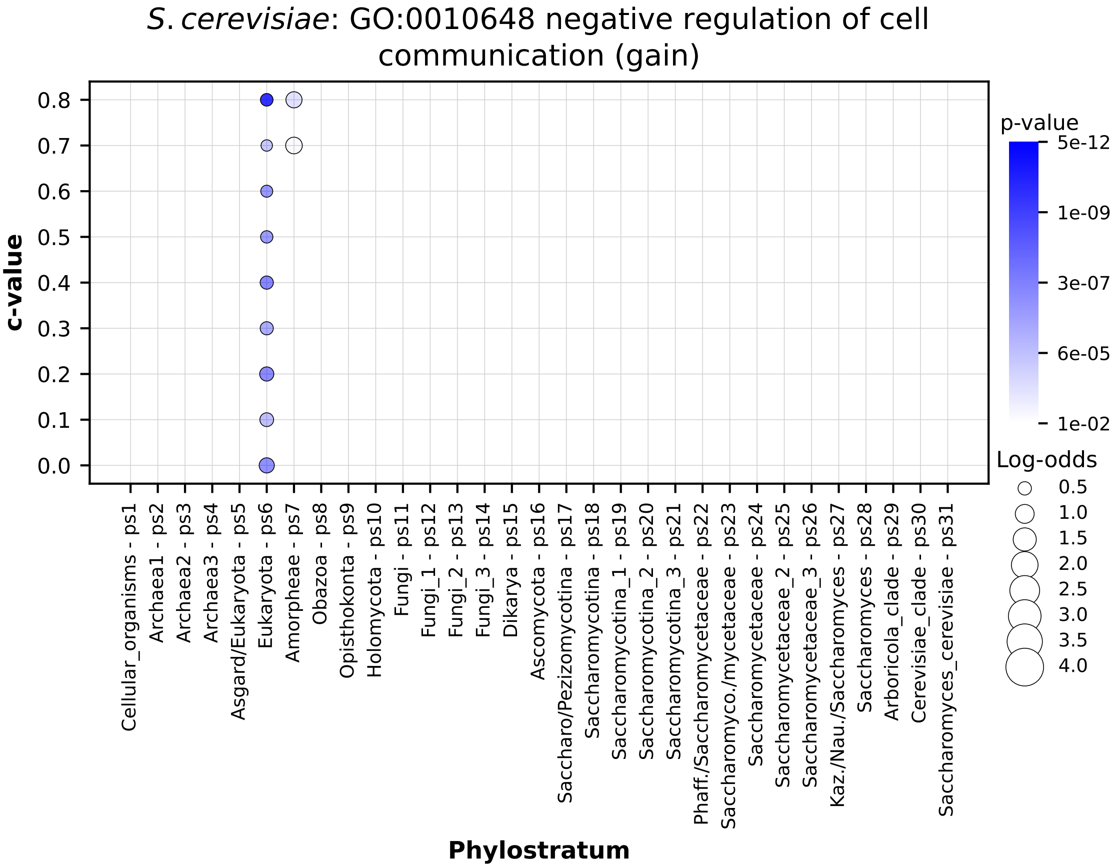

S. cerevisiae: GO:0010648 negative regulation of cell communication (gain)
c-value
0.8
0.7
0.6
0.5
0.4
0.3
0.2
0.1
0.0
p-value
5e-12
1e-09
3e-07
6e-05
1e-02
Log-odds
0.5
1.0
1.5
2.0
2.5
3.0
3.5
4.0
Cellular_organisms - ps1
Archaea1 - ps2
Archaea2 - ps3
Archaea3 - ps4
Asgard/Eukaryota - ps5
Eukaryota - ps6
Amorpheae - ps7
Obazoa - ps8
Opisthokonta - ps9
Holomycota - ps10
Fungi - ps11
Fungi_1 - ps12
Fungi_2 - ps13
Fungi_3 - ps14
Dikarya - ps15
Ascomycota - ps16
Saccharo/Pezizomycotina - ps17
Saccharomycotina - ps18
Saccharomycotina_1 - ps19
Saccharomycotina_2 - ps20
Saccharomycotina_3 - ps21
Phaff./Saccharomycetaceae - ps22
Saccharomyco./mycetaceae - ps23
Saccharomycetaceae - ps24
Saccharomycetaceae_2 - ps25
Saccharomycetaceae_3 - ps26
Kaz./Nau./Saccharomyces - ps27
Saccharomyces - ps28
Arboricola_clade - ps29
Cerevisiae_clade - ps30
Saccharomyces_cerevisiae - ps31
Phylostratum

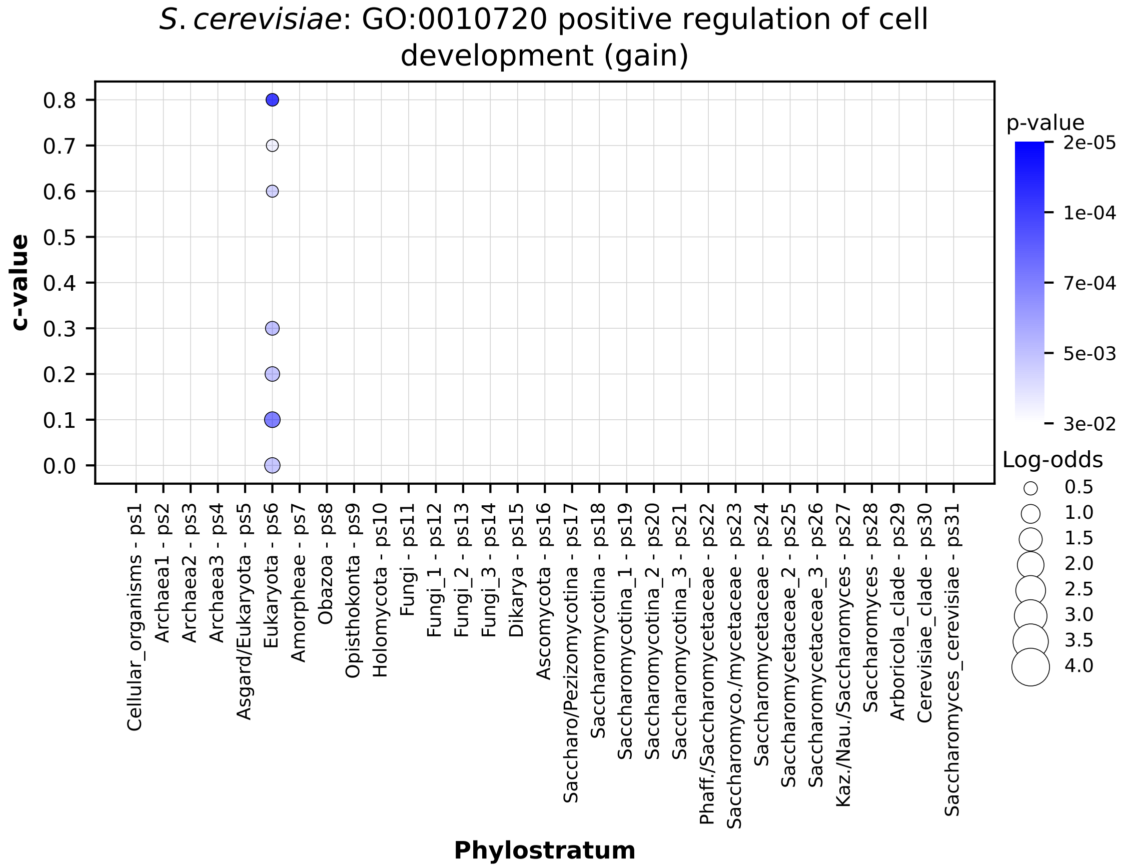
S. cerevisiae: GO:0010720 positive regulation of cell development (gain)
c-value
0.8
0.7
0.6
0.5
0.4
0.3
0.2
0.1
0.0
p-value
2e-05
1e-04
7e-04
5e-03
3e-02
Log-odds
0.5
1.0
1.5
2.0
2.5
3.0
3.5
4.0
Cellular_organisms - ps1
Archaea1 - ps2
Archaea2 - ps3
Archaea3 - ps4
Asgard/Eukaryota - ps5
Eukaryota - ps6
Amorpheae - ps7
Obazoa - ps8
Opisthokonta - ps9
Holomycota - ps10
Fungi - ps11
Fungi_1 - ps12
Fungi_2 - ps13
Fungi_3 - ps14
Dikarya - ps15
Ascomycota - ps16
Saccharo/Pezizomycotina - ps17
Saccharomycotina - ps18
Saccharomycotina_1 - ps19
Saccharomycotina_2 - ps20
Saccharomycotina_3 - ps21
Phaff./Saccharomycetaceae - ps22
Saccharomyco./mycetaceae - ps23
Saccharomycetaceae - ps24
Saccharomycetaceae_2 - ps25
Saccharomycetaceae_3 - ps26
Kaz./Nau./Saccharomyces - ps27
Saccharomyces - ps28
Arboricola_clade - ps29
Cerevisiae_clade - ps30
Saccharomyces_cerevisiae - ps31
Phylostratum

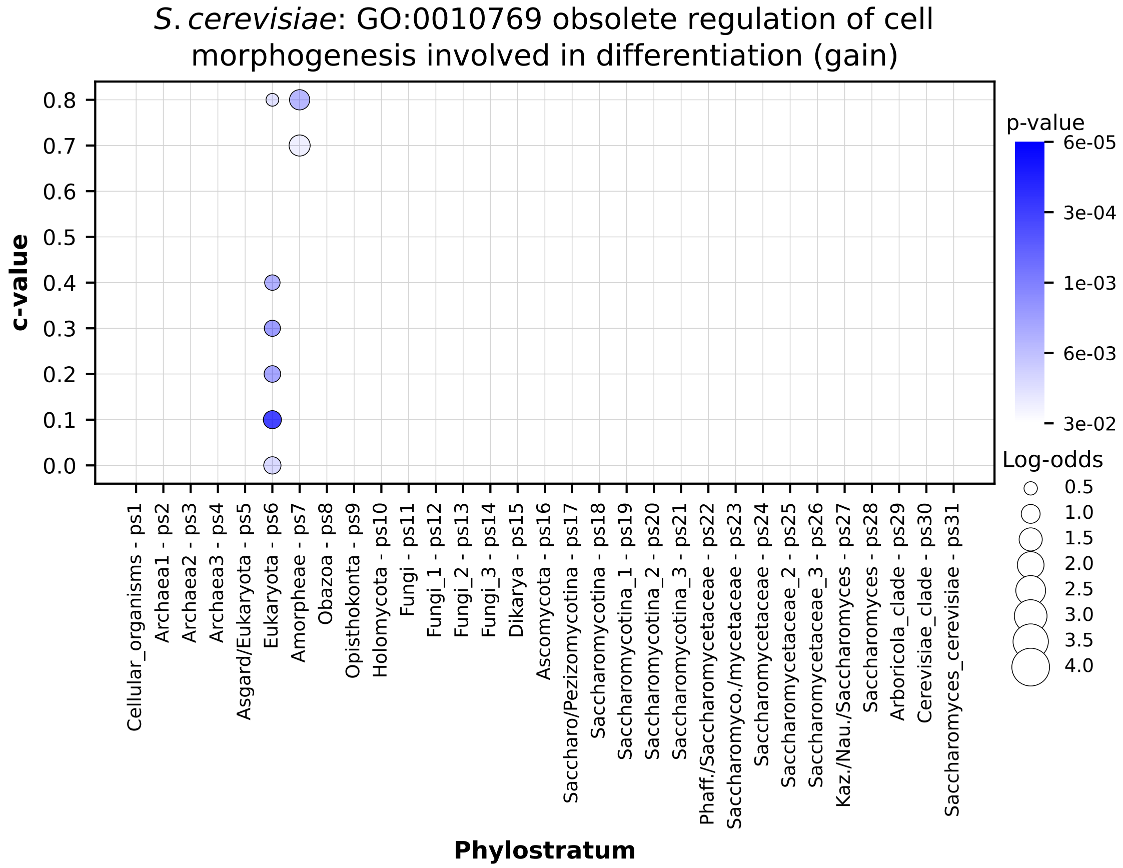

S. cerevisiae: GO:0010769 obsolete regulation of cell morphogenesis involved in differentiation (gain)
c-value
0.8
0.7
0.6
0.5
0.4
0.3
0.2
0.1
0.0
p-value
6e-05
3e-04
1e-03
6e-03
3e-02
Log-odds
0.5
1.0
1.5
2.0
2.5
3.0
3.5
4.0
Cellular_organisms - ps1
Archaea1 - ps2
Archaea2 - ps3
Archaea3 - ps4
Asgard/Eukaryota - ps5
Eukaryota - ps6
Amorpheae - ps7
Obazoa - ps8
Opisthokonta - ps9
Holomycota - ps10
Fungi - ps11
Fungi_1 - ps12
Fungi_2 - ps13
Fungi_3 - ps14
Dikarya - ps15
Ascomycota - ps16
Saccharo/Pezizomycotina - ps17
Saccharomycotina - ps18
Saccharomycotina_1 - ps19
Saccharomycotina_2 - ps20
Saccharomycotina_3 - ps21
Phaff./Saccharomycetaceae - ps22
Saccharomyco./mycetaceae - ps23
Saccharomycetaceae - ps24
Saccharomycetaceae_2 - ps25
Saccharomycetaceae_3 - ps26
Kaz./Nau./Saccharomyces - ps27
Saccharomyces - ps28
Arboricola_clade - ps29
Cerevisiae_clade - ps30
Saccharomyces_cerevisiae - ps31
Phylostratum

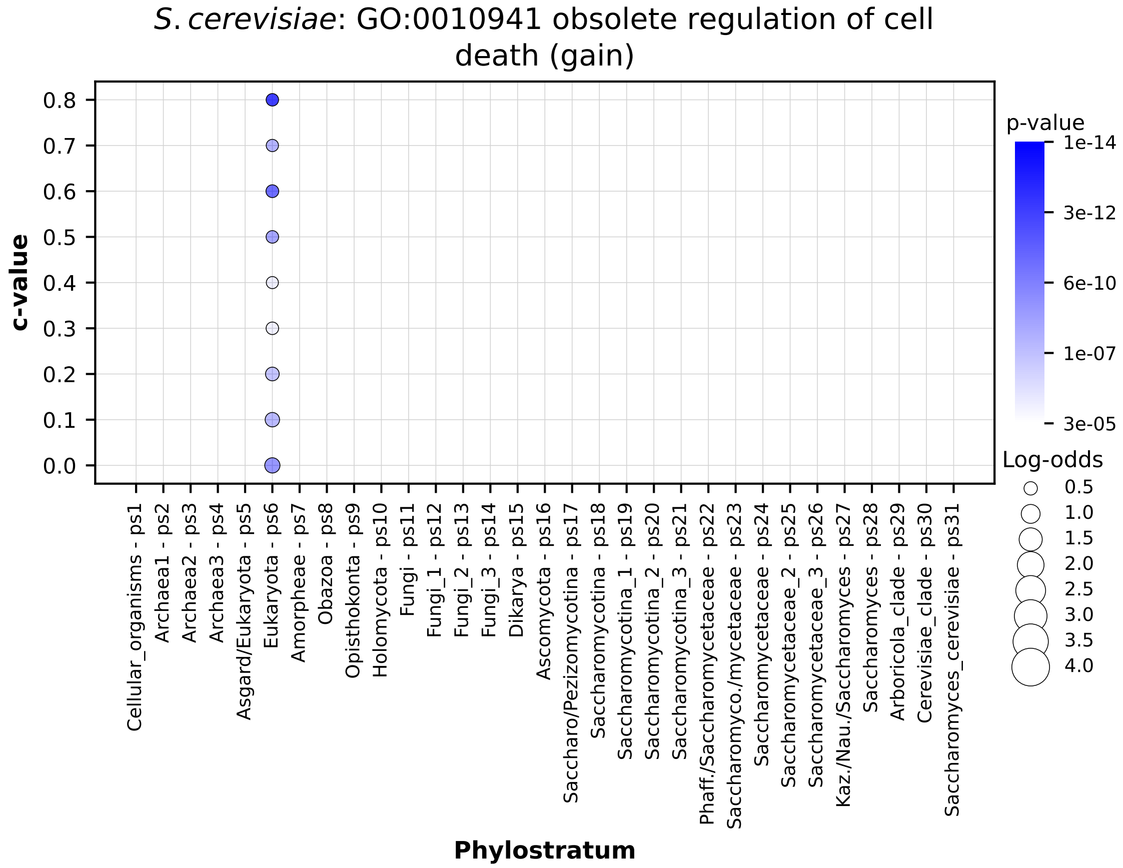

S. cerevisiae: GO:0010941 obsolete regulation of cell death (gain)
c-value
0.8
0.7
0.6
0.5
0.4
0.3
0.2
0.1
0.0
p-value
1e-14
3e-12
6e-10
1e-07
3e-05
Log-odds
0.5
1.0
1.5
2.0
2.5
3.0
3.5
4.0
Cellular_organisms - ps1
Archaea1 - ps2
Archaea2 - ps3
Archaea3 - ps4
Asgard/Eukaryota - ps5
Eukaryota - ps6
Amorpheae - ps7
Obazoa - ps8
Opisthokonta - ps9
Holomycota - ps10
Fungi - ps11
Fungi_1 - ps12
Fungi_2 - ps13
Fungi_3 - ps14
Dikarya - ps15
Ascomycota - ps16
Saccharo/Pezizomycotina - ps17
Saccharomycotina - ps18
Saccharomycotina_1 - ps19
Saccharomycotina_2 - ps20
Saccharomycotina_3 - ps21
Phaff./Saccharomycetaceae - ps22
Saccharomyco./mycetaceae - ps23
Saccharomycetaceae - ps24
Saccharomycetaceae_2 - ps25
Saccharomycetaceae_3 - ps26
Kaz./Nau./Saccharomyces - ps27
Saccharomyces - ps28
Arboricola_clade - ps29
Cerevisiae_clade - ps30
Saccharomyces_cerevisiae - ps31
Phylostratum

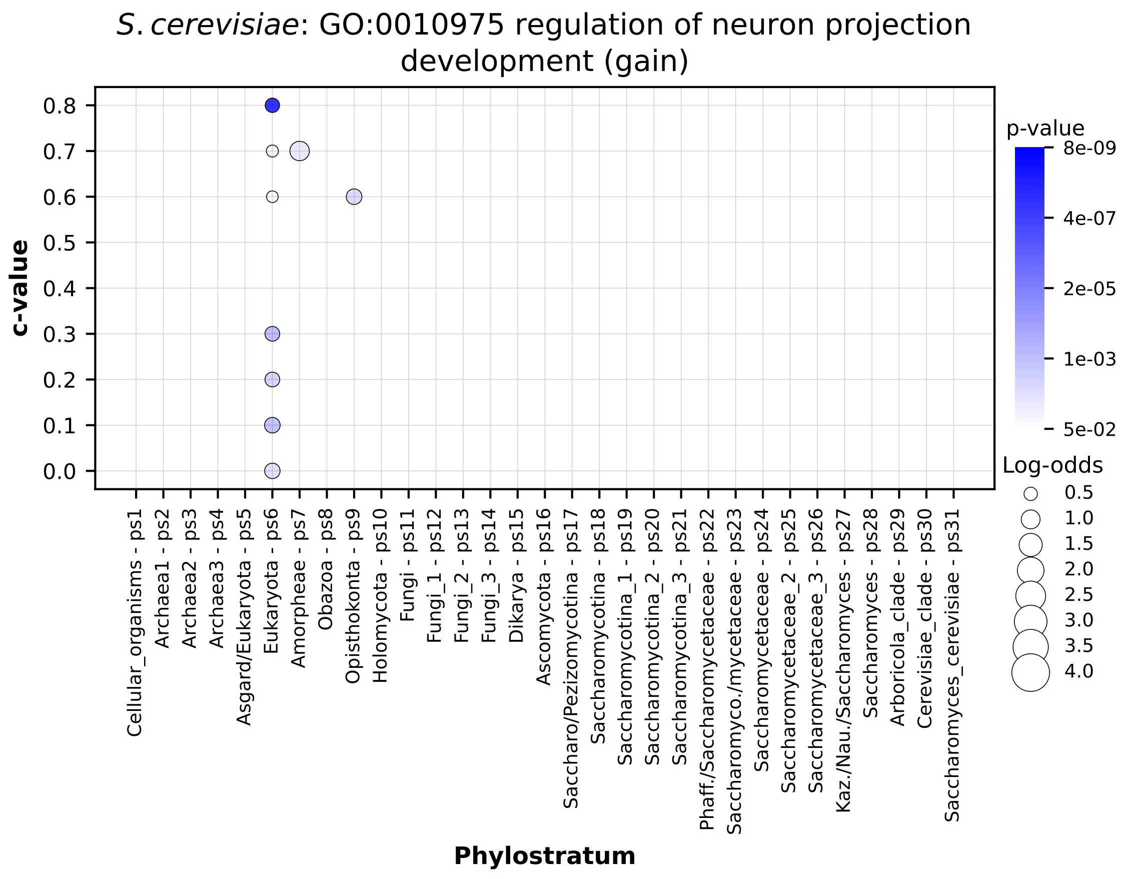

S. cerevisiae: GO:0010975 regulation of neuron projection development (gain)
c-value
0.0
0.1
0.2
0.3
0.4
0.5
0.6
0.7
0.8
p-value
8e-09
4e-07
2e-05
1e-03
5e-02
Log-odds
0.5
1.0
1.5
2.0
2.5
3.0
3.5
4.0
Cellular_organisms - ps1
Archaea1 - ps2
Archaea2 - ps3
Archaea3 - ps4
Asgard/Eukaryota - ps5
Eukaryota - ps6
Amorpheae - ps7
Obazoa - ps8
Opisthokonta - ps9
Holomycota - ps10
Fungi - ps11
Fungi_1 - ps12
Fungi_2 - ps13
Fungi_3 - ps14
Dikarya - ps15
Ascomycota - ps16
Saccharo/Pezizomycotina - ps17
Saccharomycotina - ps18
Saccharomycotina_1 - ps19
Saccharomycotina_2 - ps20
Saccharomycotina_3 - ps21
Phaff./Saccharomycetaceae - ps22
Saccharomyco./mycetaceae - ps23
Saccharomycetaceae - ps24
Saccharomycetaceae_2 - ps25
Saccharomycetaceae_3 - ps26
Kaz./Nau./Saccharomyces - ps27
Saccharomyces - ps28
Arboricola_clade - ps29
Cerevisiae_clade - ps30
Saccharomyces_cerevisiae - ps31
Phylostratum

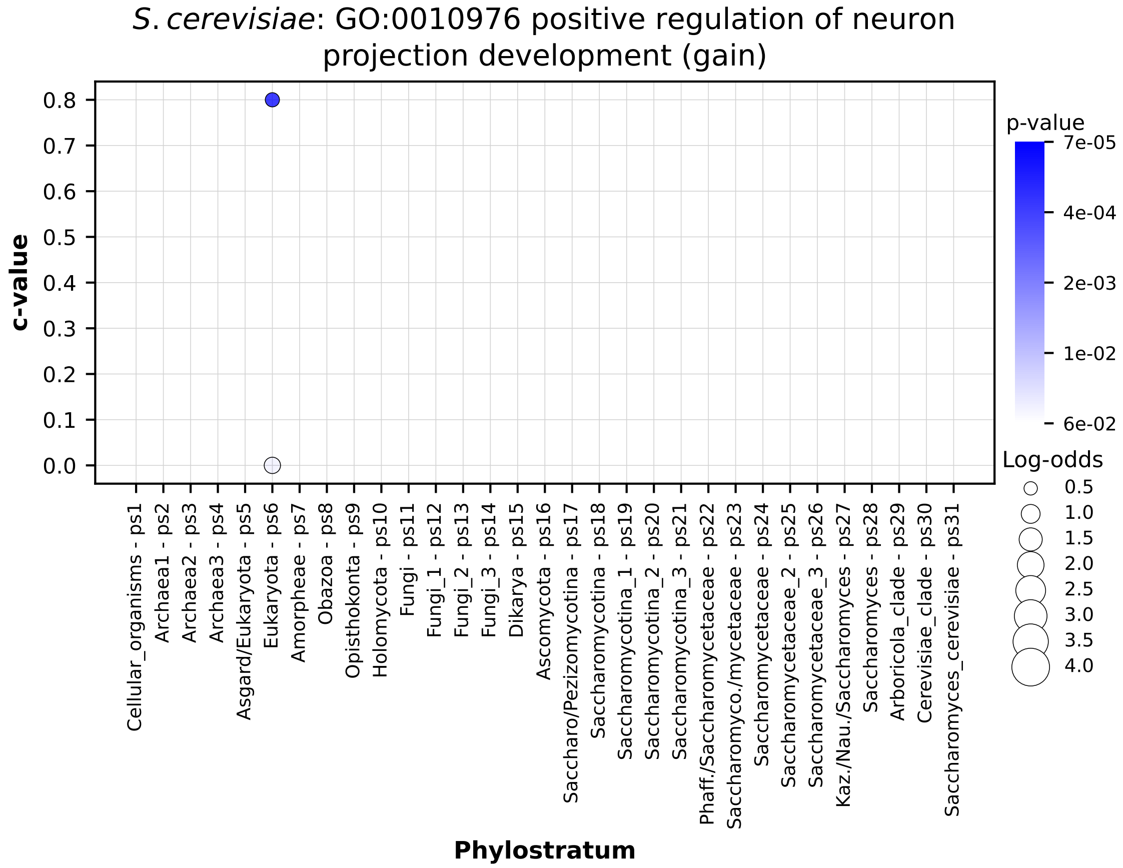

S. cerevisiae: GO:0010976 positive regulation of neuron projection development (gain)
c-value
0.8
0.7
0.6
0.5
0.4
0.3
0.2
0.1
0.0
p-value
7e-05
4e-04
2e-03
1e-02
6e-02
Log-odds
0.5
1.0
1.5
2.0
2.5
3.0
3.5
4.0
Cellular_organisms - ps1
Archaea1 - ps2
Archaea2 - ps3
Archaea3 - ps4
Asgard/Eukaryota - ps5
Eukaryota - ps6
Amorpheae - ps7
Obazoa - ps8
Opisthokonta - ps9
Holomycota - ps10
Fungi - ps11
Fungi_1 - ps12
Fungi_2 - ps13
Fungi_3 - ps14
Dikarya - ps15
Ascomycota - ps16
Saccharo/Pezizomycotina - ps17
Saccharomycotina - ps18
Saccharomycotina_1 - ps19
Saccharomycotina_2 - ps20
Saccharomycotina_3 - ps21
Phaff./Saccharomycetaceae - ps22
Saccharomyco./mycetaceae - ps23
Saccharomycetaceae - ps24
Saccharomycetaceae_2 - ps25
Saccharomycetaceae_3 - ps26
Kaz./Nau./Saccharomyces - ps27
Saccharomyces - ps28
Arboricola_clade - ps29
Cerevisiae_clade - ps30
Saccharomyces_cerevisiae - ps31
Phylostratum

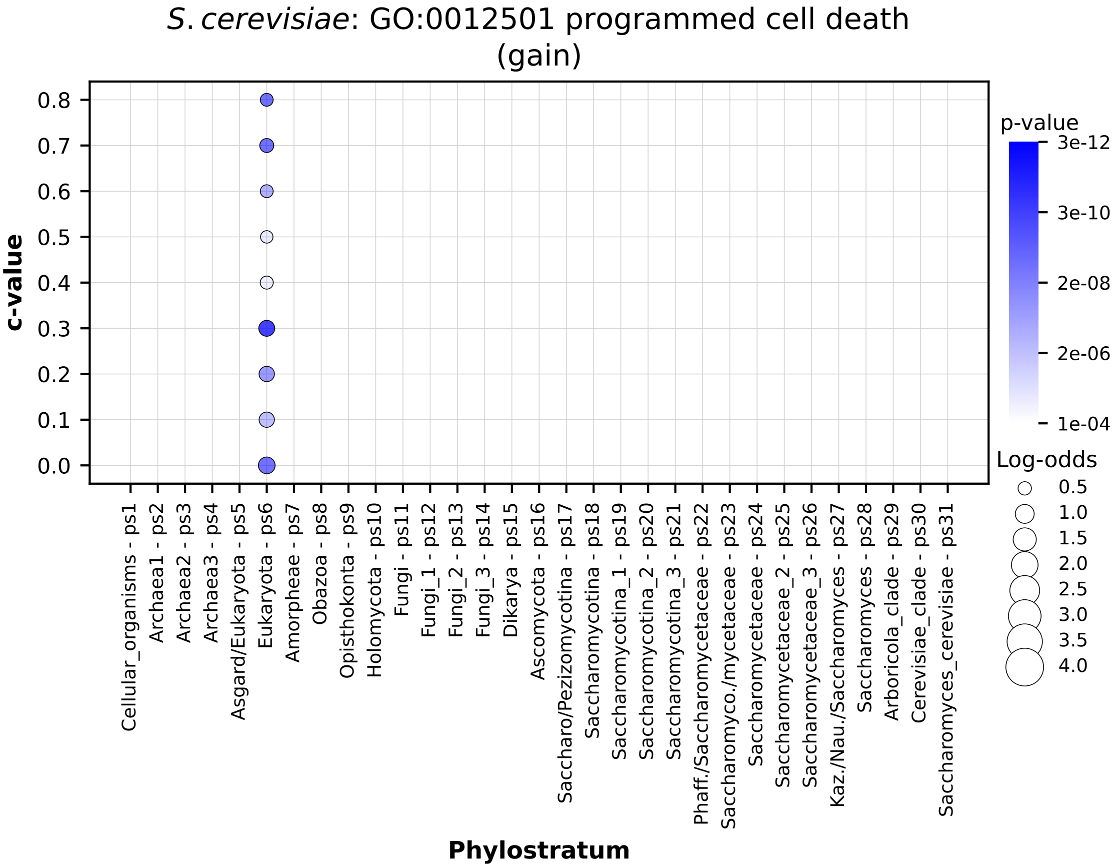
S. cerevisiae: GO:0012501 programmed cell death
(gain)
c-value
0.8
0.7
0.6
0.5
0.4
0.3
0.2
0.1
0.0
p-value
3e-12
3e-10
2e-08
2e-06
1e-04
Log-odds
0.5
1.0
1.5
2.0
2.5
3.0
3.5
4.0
Cellular_organisms - ps1
Archaea1 - ps2
Archaea2 - ps3
Archaea3 - ps4
Asgard/Eukaryota - ps5
Eukaryota - ps6
Amorpheae - ps7
Obazoa - ps8
Opisthokonta - ps9
Holomycota - ps10
Fungi - ps11
Fungi_1 - ps12
Fungi_2 - ps13
Fungi_3 - ps14
Dikarya - ps15
Ascomycota - ps16
Saccharo/Pezizomycotina - ps17
Saccharomycotina - ps18
Saccharomycotina_1 - ps19
Saccharomycotina_2 - ps20
Saccharomycotina_3 - ps21
Phaff./Saccharomycetaceae - ps22
Saccharomyco./mycetaceae - ps23
Saccharomycetaceae - ps24
Saccharomycetaceae_2 - ps25
Saccharomycetaceae_3 - ps26
Kaz./Nau./Saccharomyces - ps27
Saccharomyces - ps28
Arboricola_clade - ps29
Cerevisiae_clade - ps30
Saccharomyces_cerevisiae - ps31
Phylostratum

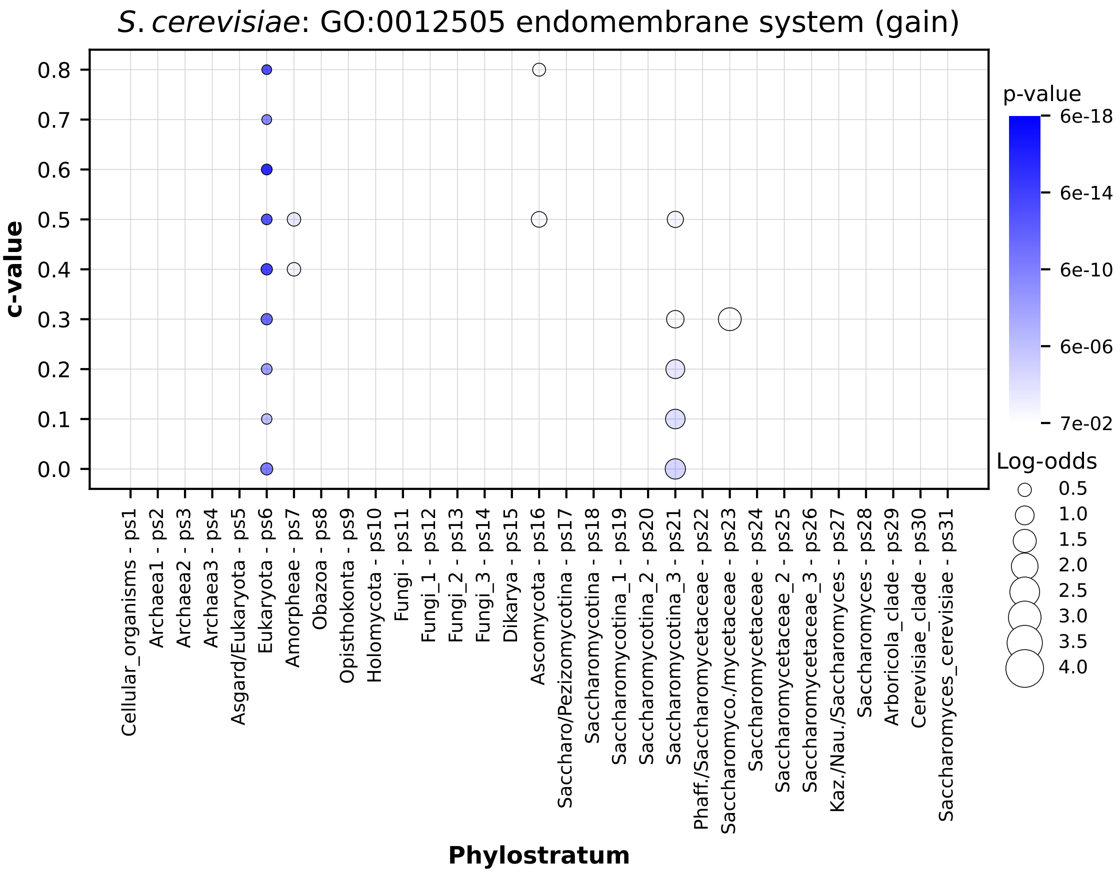

S. cerevisiae: GO:0012505 endomembrane system (gain)
c-value
0.8
0.7
0.6
0.5
0.4
0.3
0.2
0.1
0.0
p-value
6e-18
6e-14
6e-10
6e-06
7e-02
Log-odds
0.5
1.0
1.5
2.0
2.5
3.0
3.5
4.0
Cellular_organisms - ps1
Archaea1 - ps2
Archaea2 - ps3
Archaea3 - ps4
Asgard/Eukaryota - ps5
Eukaryota - ps6
Amorpheae - ps7
Obazoa - ps8
Opisthokonta - ps9
Holomycota - ps10
Fungi - ps11
Fungi_1 - ps12
Fungi_2 - ps13
Fungi_3 - ps14
Dikarya - ps15
Ascomycota - ps16
Saccharo/Pezizomycotina - ps17
Saccharomycotina - ps18
Saccharomycotina_1 - ps19
Saccharomycotina_2 - ps20
Saccharomycotina_3 - ps21
Phaff./Saccharomycetaceae - ps22
Saccharomyco./mycetaceae - ps23
Saccharomycetaceae - ps24
Saccharomycetaceae_2 - ps25
Saccharomycetaceae_3 - ps26
Kaz./Nau./Saccharomyces - ps27
Saccharomyces - ps28
Arboricola_clade - ps29
Cerevisiae_clade - ps30
Saccharomyces_cerevisiae - ps31
Phylostratum

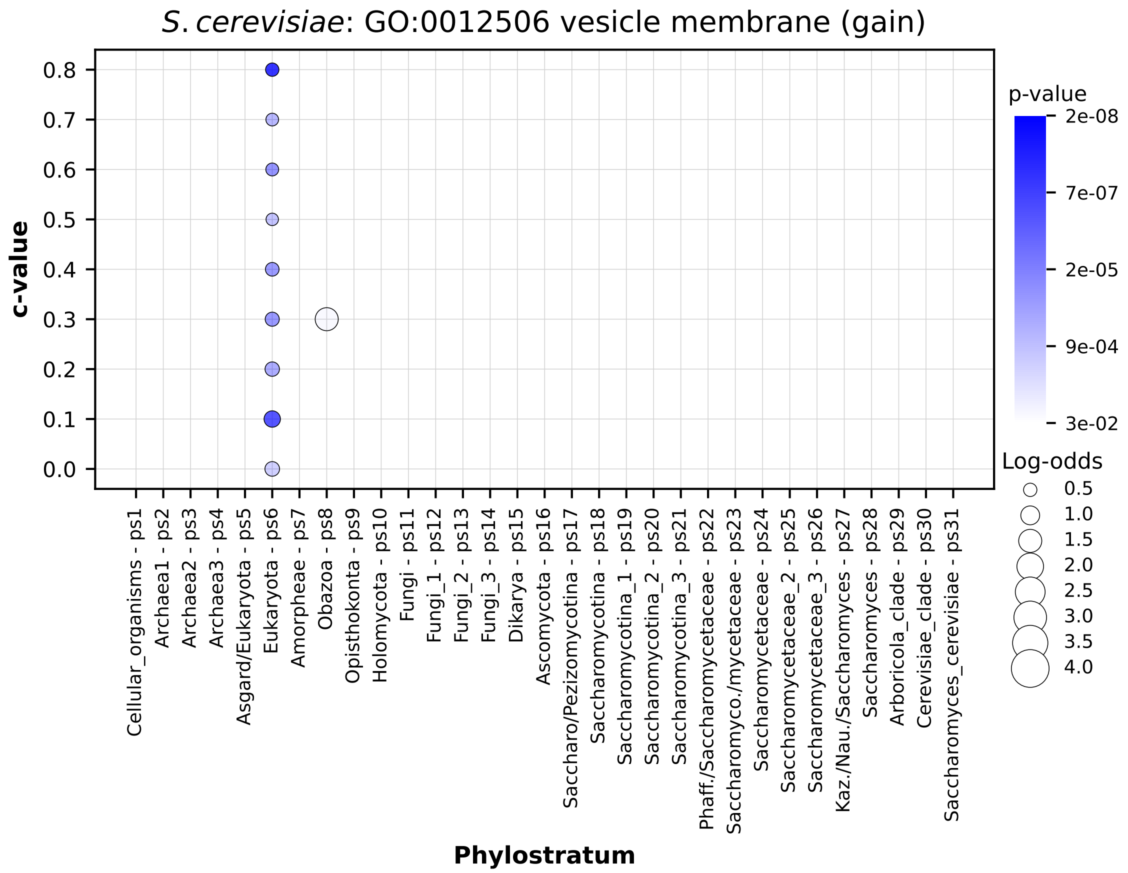

S. cerevisiae: GO:0012506 vesicle membrane (gain)
c-value
0.8
0.7
0.6
0.5
0.4
0.3
0.2
0.1
0.0
p-value
2e-08
7e-07
2e-05
9e-04
3e-02
Log-odds
0.5
1.0
1.5
2.0
2.5
3.0
3.5
4.0
Cellular_organisms - ps1
Archaea1 - ps2
Archaea2 - ps3
Archaea3 - ps4
Asgard/Eukaryota - ps5
Eukaryota - ps6
Amorpheae - ps7
Obazoa - ps8
Opisthokonta - ps9
Holomycota - ps10
Fungi - ps11
Fungi_1 - ps12
Fungi_2 - ps13
Fungi_3 - ps14
Dikarya - ps15
Ascomycota - ps16
Saccharo/Pezizomycotina - ps17
Saccharomycotina - ps18
Saccharomycotina_1 - ps19
Saccharomycotina_2 - ps20
Saccharomycotina_3 - ps21
Phaff./Saccharomycetaceae - ps22
Saccharomyco./mycetaceae - ps23
Saccharomycetaceae - ps24
Saccharomycetaceae_2 - ps25
Saccharomycetaceae_3 - ps26
Kaz./Nau./Saccharomyces - ps27
Saccharomyces - ps28
Arboricola_clade - ps29
Cerevisiae_clade - ps30
Saccharomyces_cerevisiae - ps31
Phylostratum

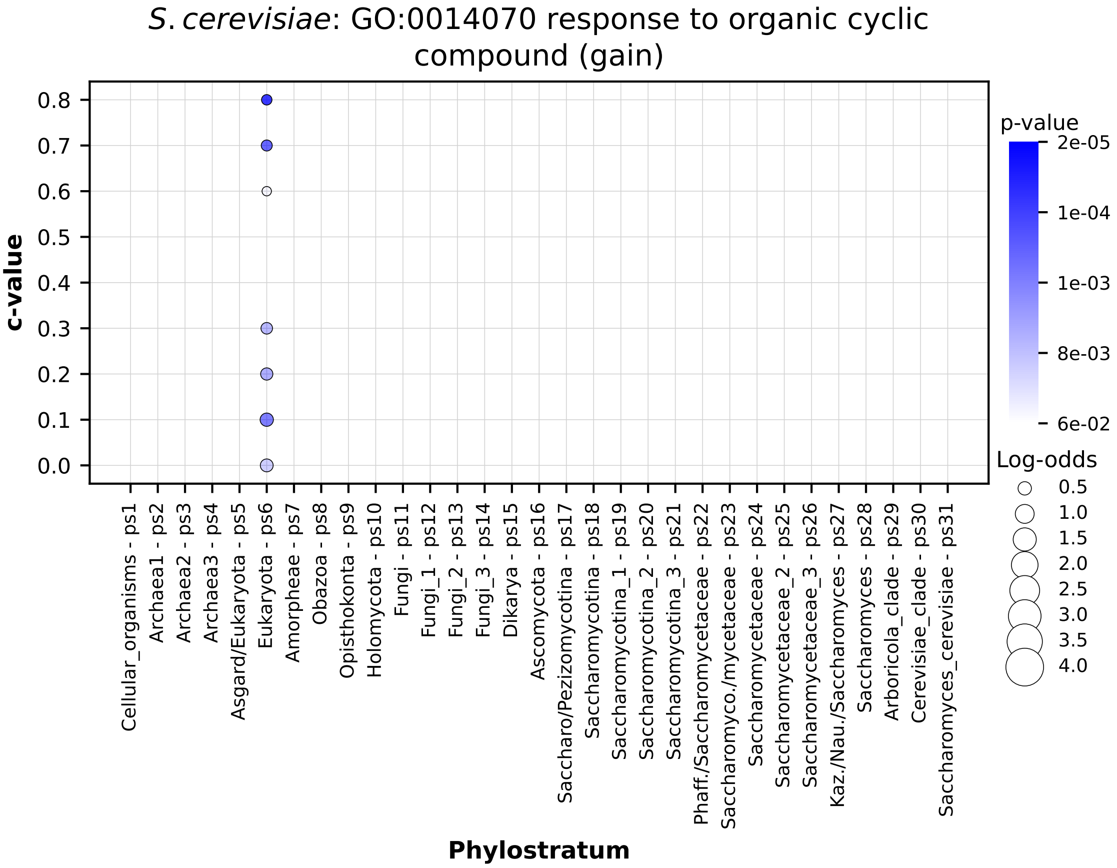

S. cerevisiae: GO:0014070 response to organic cyclic compound (gain)
c-value
0.0
0.1
0.2
0.3
0.4
0.5
0.6
0.7
0.8
p-value
2e-05
1e-04
1e-03
8e-03
6e-02
Log-odds
0.5
1.0
1.5
2.0
2.5
3.0
3.5
4.0
Cellular_organisms - ps1
Archaea1 - ps2
Archaea2 - ps3
Archaea3 - ps4
Asgard/Eukaryota - ps5
Eukaryota - ps6
Amorpheae - ps7
Obazoa - ps8
Opisthokonta - ps9
Holomycota - ps10
Fungi - ps11
Fungi_1 - ps12
Fungi_2 - ps13
Fungi_3 - ps14
Dikarya - ps15
Ascomycota - ps16
Saccharo/Pezizomycotina - ps17
Saccharomycotina - ps18
Saccharomycotina_1 - ps19
Saccharomycotina_2 - ps20
Saccharomycotina_3 - ps21
Phaff./Saccharomycetaceae - ps22
Saccharomyco./mycetaceae - ps23
Saccharomycetaceae - ps24
Saccharomycetaceae_2 - ps25
Saccharomycetaceae_3 - ps26
Kaz./Nau./Saccharomyces - ps27
Saccharomyces - ps28
Arboricola_clade - ps29
Cerevisiae_clade - ps30
Saccharomyces_cerevisiae - ps31
Phylostratum

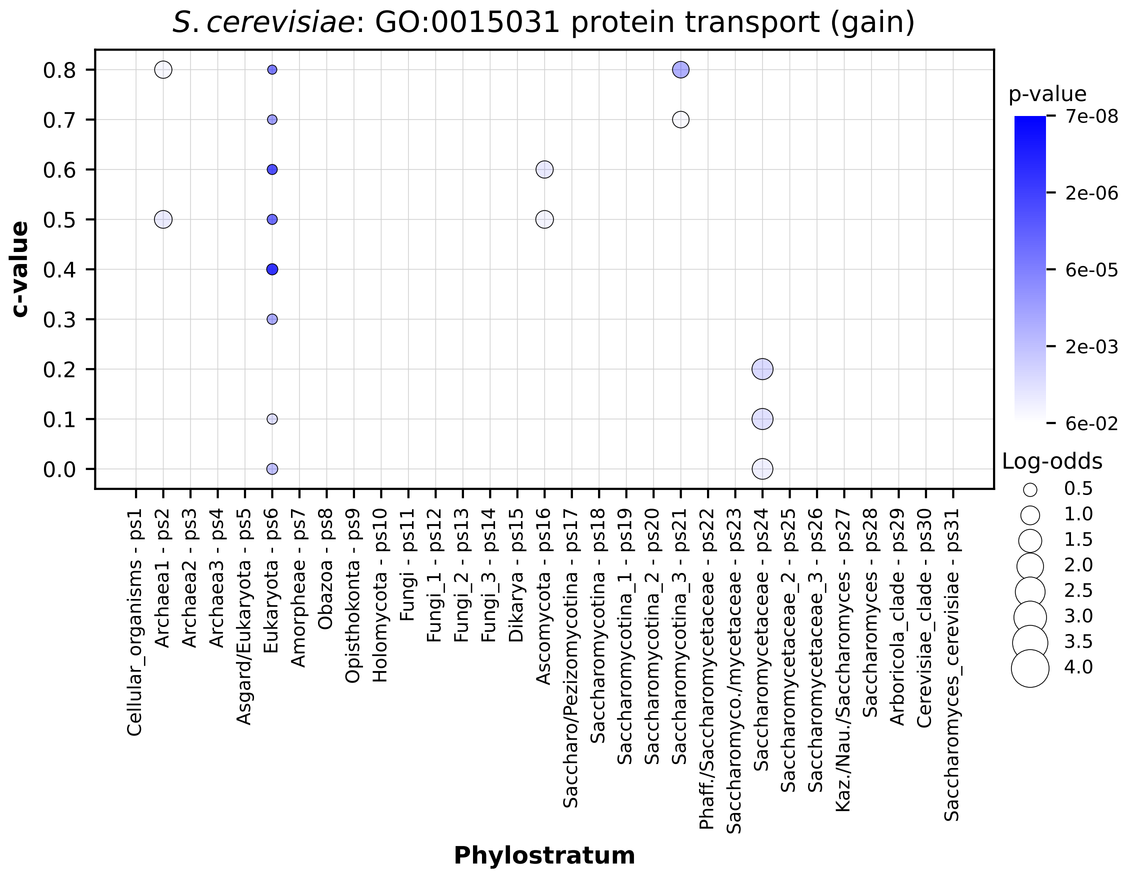
S. cerevisiae: GO:0015031 protein transport (gain)
c-value
0.8
0.7
0.6
0.5
0.4
0.3
0.2
0.1
0.0
p-value
7e-08
2e-06
6e-05
2e-03
6e-02
Log-odds
0.5
1.0
1.5
2.0
2.5
3.0
3.5
4.0
Cellular_organisms - ps1
Archaea1 - ps2
Archaea2 - ps3
Archaea3 - ps4
Asgard/Eukaryota - ps5
Eukaryota - ps6
Amorpheae - ps7
Obazoa - ps8
Opisthokonta - ps9
Holomycota - ps10
Fungi - ps11
Fungi_1 - ps12
Fungi_2 - ps13
Fungi_3 - ps14
Dikarya - ps15
Ascomycota - ps16
Saccharo/Pezizomycotina - ps17
Saccharomycotina - ps18
Saccharomycotina_1 - ps19
Saccharomycotina_2 - ps20
Saccharomycotina_3 - ps21
Phaff./Saccharomycetaceae - ps22
Saccharomyco./mycetaceae - ps23
Saccharomycetaceae - ps24
Saccharomycetaceae_2 - ps25
Saccharomycetaceae_3 - ps26
Kaz./Nau./Saccharomyces - ps27
Saccharomyces - ps28
Arboricola_clade - ps29
Cerevisiae_clade - ps30
Saccharomyces_cerevisiae - ps31
Phylostratum

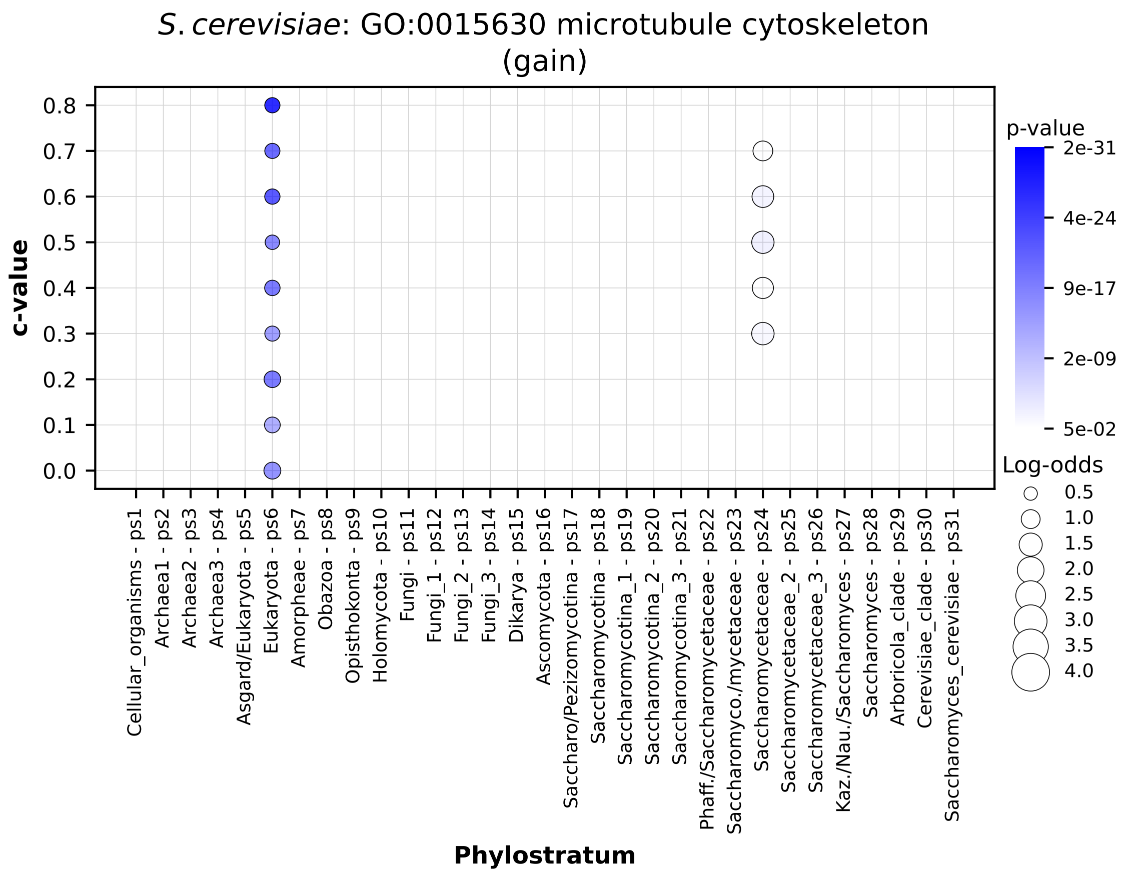

$S. cerevisiae$: GO:0015630 microtubule cytoskeleton
(gain)
c-value
0.0
0.1
0.2
0.3
0.4
0.5
0.6
0.7
0.8
p-value
2e-31
4e-24
9e-17
2e-09
5e-02
Log-odds
0.5
1.0
1.5
2.0
2.5
3.0
3.5
4.0
Cellular_organisms - ps1
Archaea1 - ps2
Archaea2 - ps3
Archaea3 - ps4
Asgard/Eukaryota - ps5
Eukaryota - ps6
Amorpheae - ps7
Obazoa - ps8
Opisthokonta - ps9
Holomycota - ps10
Fungi - ps11
Fungi_1 - ps12
Fungi_2 - ps13
Fungi_3 - ps14
Dikarya - ps15
Ascomycota - ps16
Saccharo/Pezizomycotina - ps17
Saccharomycotina - ps18
Saccharomycotina_1 - ps19
Saccharomycotina_2 - ps20
Saccharomycotina_3 - ps21
Phaff./Saccharomycetaceae - ps22
Saccharomyco./mycetaceae - ps23
Saccharomycetaceae - ps24
Saccharomycetaceae_2 - ps25
Saccharomycetaceae_3 - ps26
Kaz./Nau./Saccharomyces - ps27
Saccharomyces - ps28
Arboricola_clade - ps29
Cerevisiae_clade - ps30
Saccharomyces_cerevisiae - ps31
Phylostratum

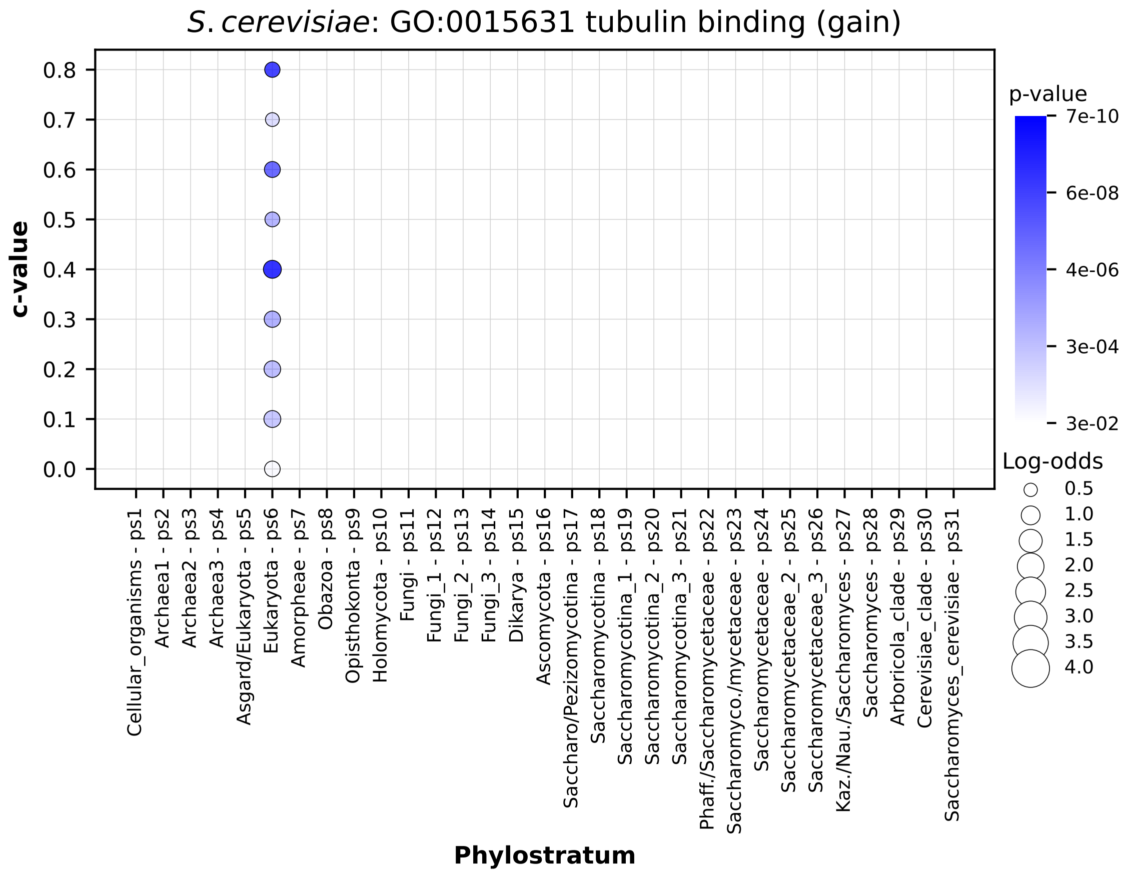

S. cerevisiae: GO:0015631 tubulin binding (gain)
c-value
0.0
0.1
0.2
0.3
0.4
0.5
0.6
0.7
0.8
p-value
7e-10
6e-08
4e-06
3e-04
3e-02
Log-odds
0.5
1.0
1.5
2.0
2.5
3.0
3.5
4.0
Cellular_organisms - ps1
Archaea1 - ps2
Archaea2 - ps3
Archaea3 - ps4
Asgard/Eukaryota - ps5
Eukaryota - ps6
Amorpheae - ps7
Obazoa - ps8
Opisthokonta - ps9
Holomycota - ps10
Fungi - ps11
Fungi_1 - ps12
Fungi_2 - ps13
Fungi_3 - ps14
Dikarya - ps15
Ascomycota - ps16
Saccharo/Pezizomycotina - ps17
Saccharomycotina - ps18
Saccharomycotina_1 - ps19
Saccharomycotina_2 - ps20
Saccharomycotina_3 - ps21
Phaff./Saccharomycetaceae - ps22
Saccharomyco./mycetaceae - ps23
Saccharomycetaceae - ps24
Saccharomycetaceae_2 - ps25
Saccharomycetaceae_3 - ps26
Kaz./Nau./Saccharomyces - ps27
Saccharomyces - ps28
Arboricola_clade - ps29
Cerevisiae_clade - ps30
Saccharomyces_cerevisiae - ps31
Phylostratum

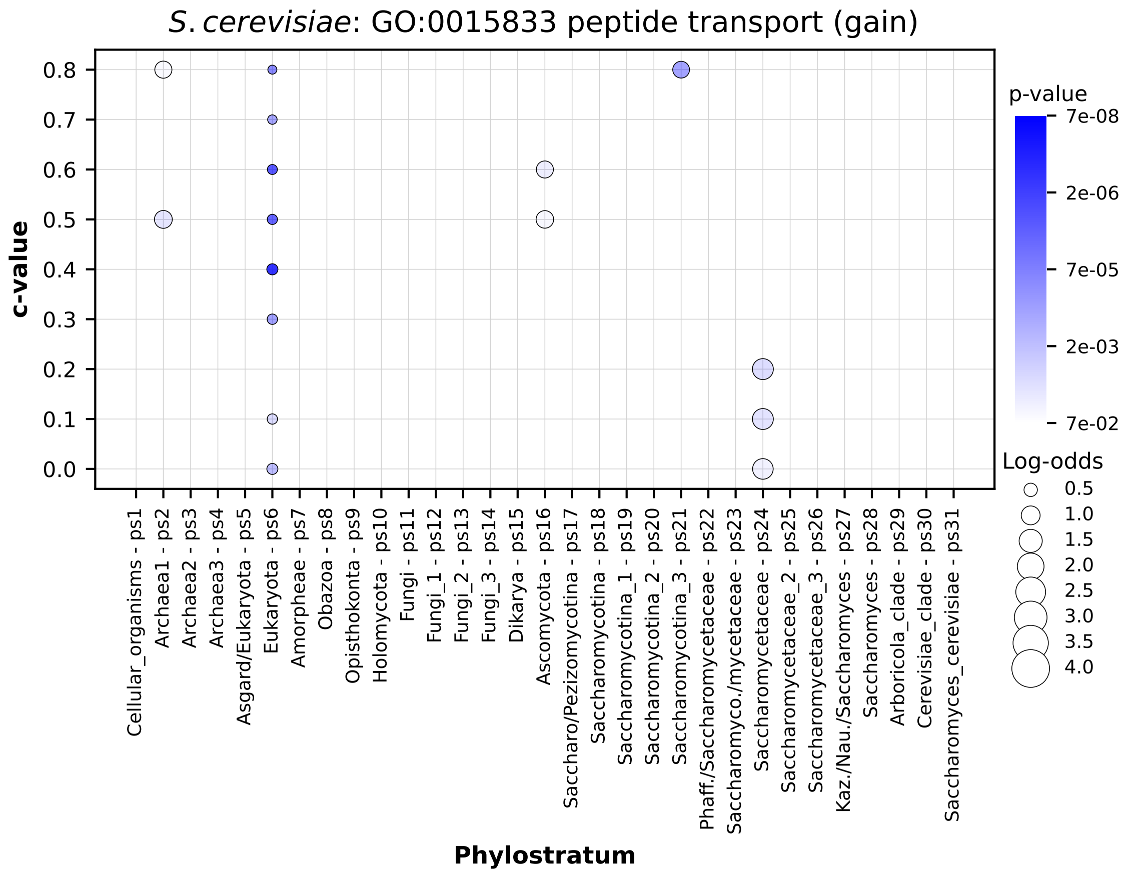

S. cerevisiae: GO:0015833 peptide transport (gain)
c-value
0.0
0.1
0.2
0.3
0.4
0.5
0.6
0.7
0.8
p-value
7e-08
2e-06
7e-05
2e-03
7e-02
Log-odds
0.5
1.0
1.5
2.0
2.5
3.0
3.5
4.0
Cellular_organisms - ps1
Archaea1 - ps2
Archaea2 - ps3
Archaea3 - ps4
Asgard/Eukaryota - ps5
Eukaryota - ps6
Amorpheae - ps7
Obazoa - ps8
Opisthokonta - ps9
Holomycota - ps10
Fungi - ps11
Fungi_1 - ps12
Fungi_2 - ps13
Fungi_3 - ps14
Dikarya - ps15
Ascomycota - ps16
Saccharo/Pezizomycotina - ps17
Saccharomycotina - ps18
Saccharomycotina_1 - ps19
Saccharomycotina_2 - ps20
Saccharomycotina_3 - ps21
Phaff./Saccharomycetaceae - ps22
Saccharomyco./mycetaceae - ps23
Saccharomycetaceae - ps24
Saccharomycetaceae_2 - ps25
Saccharomycetaceae_3 - ps26
Kaz./Nau./Saccharomyces - ps27
Saccharomyces - ps28
Arboricola_clade - ps29
Cerevisiae_clade - ps30
Saccharomyces_cerevisiae - ps31
Phylostratum

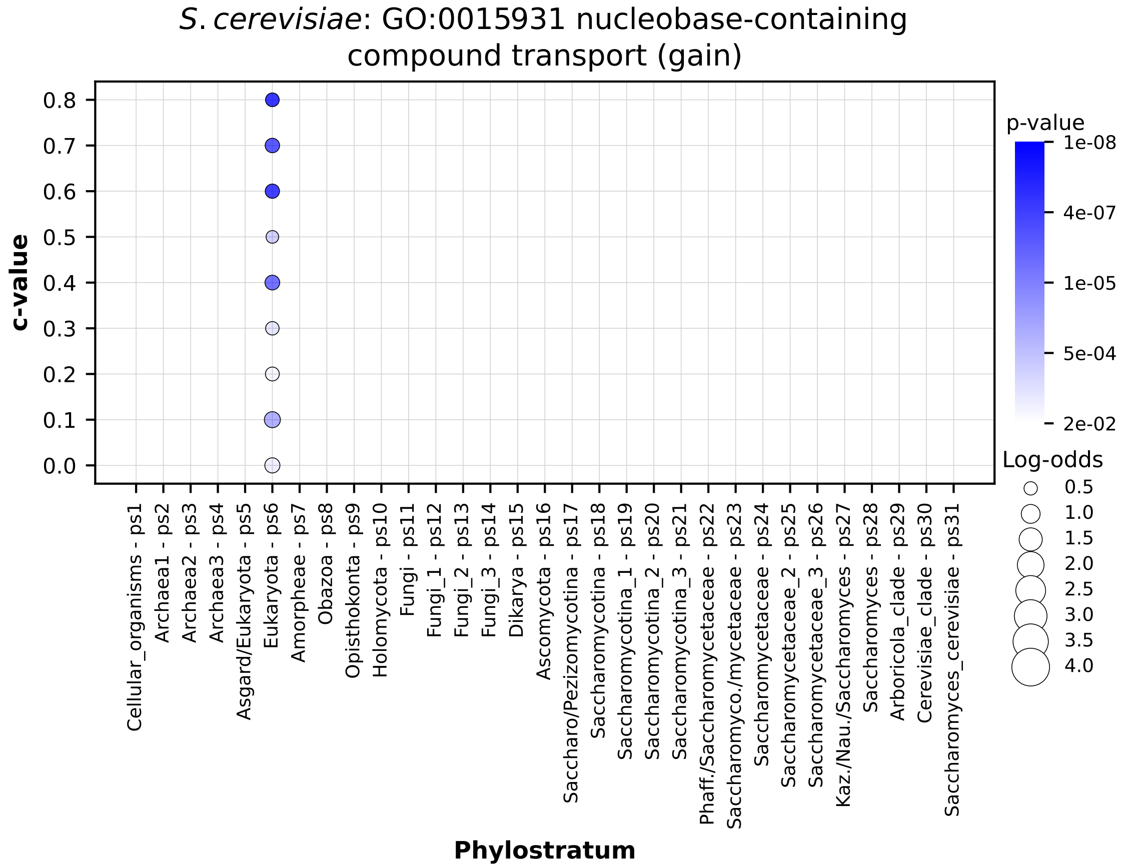
S. cerevisiae: GO:0015931 nucleobase-containing compound transport (gain)
c-value
0.0
0.1
0.2
0.3
0.4
0.5
0.6
0.7
0.8
p-value
1e-08
4e-07
1e-05
5e-04
2e-02
Log-odds
0.5
1.0
1.5
2.0
2.5
3.0
3.5
4.0
Cellular_organisms - ps1
Archaea1 - ps2
Archaea2 - ps3
Archaea3 - ps4
Asgard/Eukaryota - ps5
Eukaryota - ps6
Amorpheae - ps7
Obazoa - ps8
Opisthokonta - ps9
Holomycota - ps10
Fungi - ps11
Fungi_1 - ps12
Fungi_2 - ps13
Fungi_3 - ps14
Dikarya - ps15
Ascomycota - ps16
Saccharo/Pezizomycotina - ps17
Saccharomycotina - ps18
Saccharomycotina_1 - ps19
Saccharomycotina_2 - ps20
Saccharomycotina_3 - ps21
Phaff./Saccharomycetaceae - ps22
Saccharomyco./mycetaceae - ps23
Saccharomycetaceae - ps24
Saccharomycetaceae_2 - ps25
Saccharomycetaceae_3 - ps26
Kaz./Nau./Saccharomyces - ps27
Saccharomyces - ps28
Arboricola_clade - ps29
Cerevisiae_clade - ps30
Saccharomyces_cerevisiae - ps31
Phylostratum

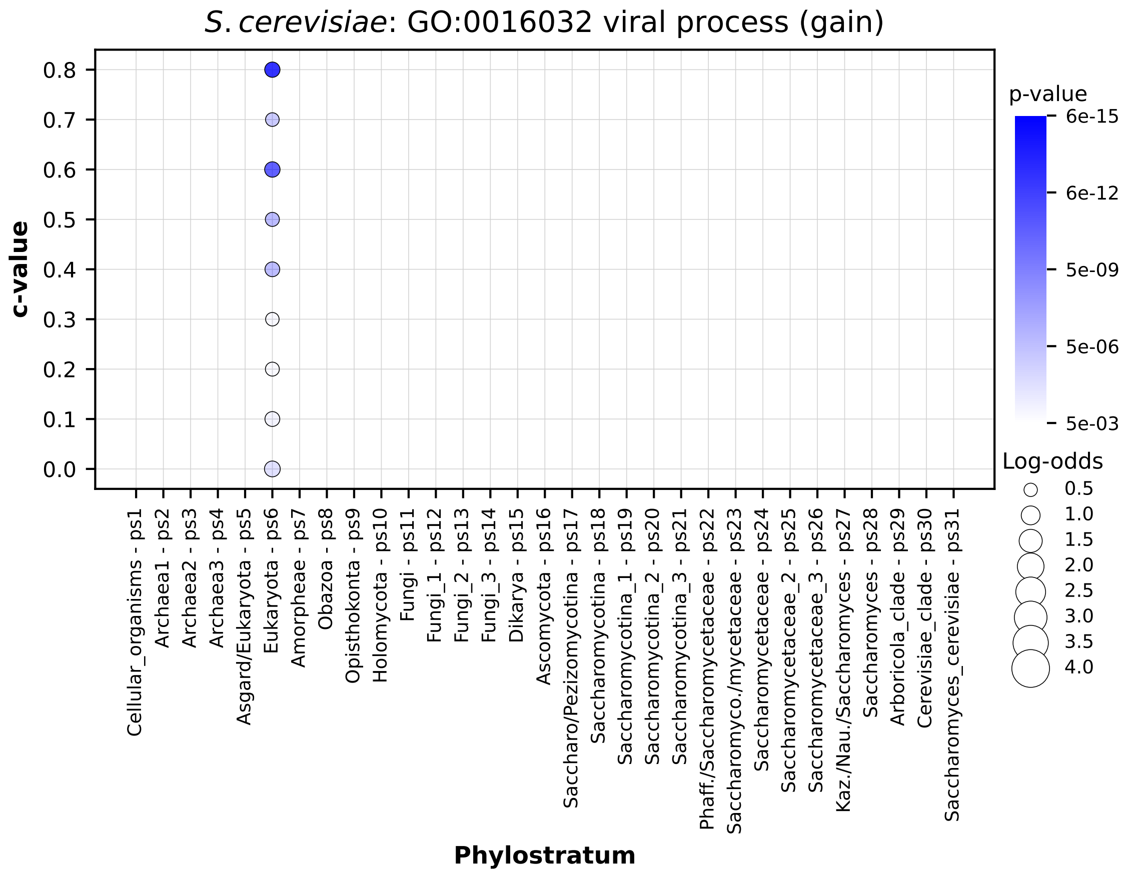
S. cerevisiae: GO:0016032 viral process (gain)
c-value
0.0
0.1
0.2
0.3
0.4
0.5
0.6
0.7
0.8
p-value
6e-15
6e-12
5e-09
5e-06
5e-03
Log-odds
0.5
1.0
1.5
2.0
2.5
3.0
3.5
4.0
Cellular_organisms - ps1
Archaea1 - ps2
Archaea2 - ps3
Archaea3 - ps4
Asgard/Eukaryota - ps5
Eukaryota - ps6
Amorpheae - ps7
Obazoa - ps8
Opisthokonta - ps9
Holomycota - ps10
Fungi - ps11
Fungi_1 - ps12
Fungi_2 - ps13
Fungi_3 - ps14
Dikarya - ps15
Ascomycota - ps16
Saccharo/Pezizomycotina - ps17
Saccharomycotina - ps18
Saccharomycotina_1 - ps19
Saccharomycotina_2 - ps20
Saccharomycotina_3 - ps21
Phaff./Saccharomycetaceae - ps22
Saccharomyco./mycetaceae - ps23
Saccharomycetaceae - ps24
Saccharomycetaceae_2 - ps25
Saccharomycetaceae_3 - ps26
Kaz./Nau./Saccharomyces - ps27
Saccharomyces - ps28
Arboricola_clade - ps29
Cerevisiae_clade - ps30
Saccharomyces_cerevisiae - ps31
Phylostratum

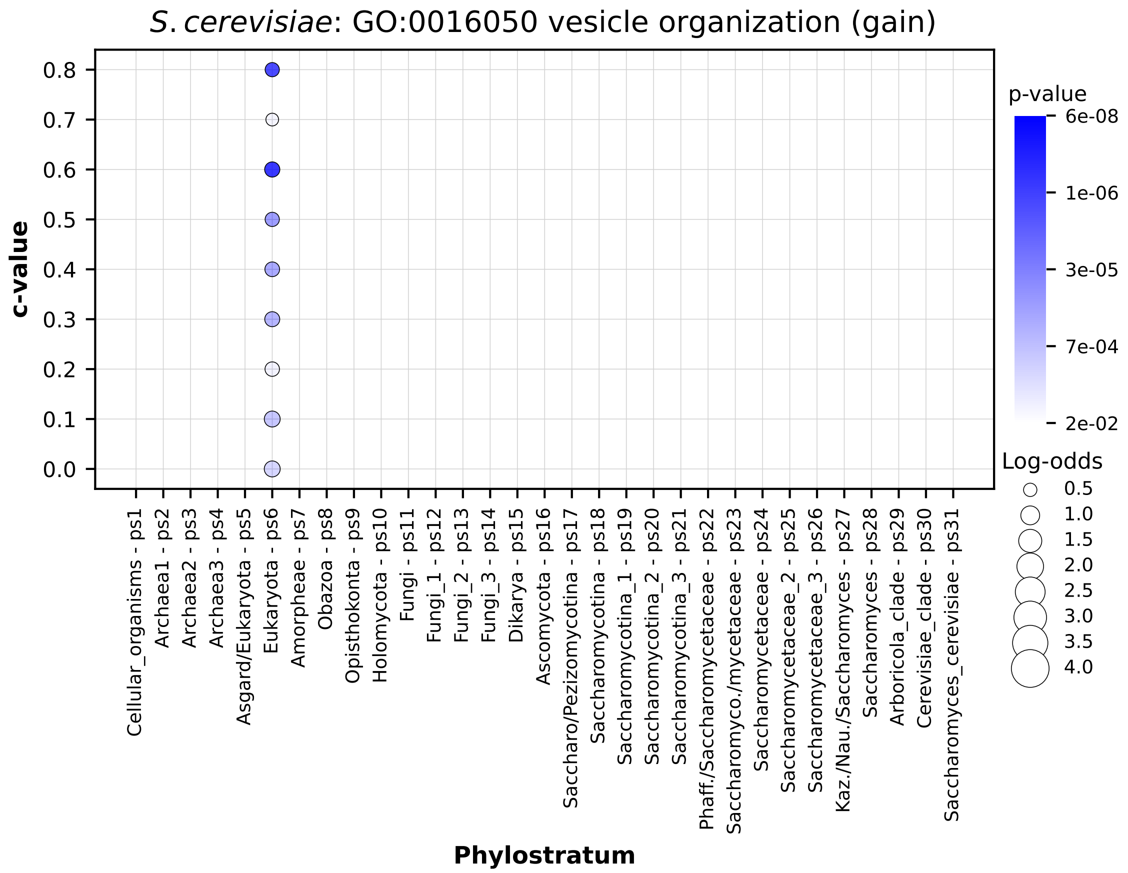

S. cerevisiae: GO:0016050 vesicle organization (gain)
c-value
0.0
0.1
0.2
0.3
0.4
0.5
0.6
0.7
0.8
Phylostratum
Cellular_organisms - ps1
Archaea1 - ps2
Archaea2 - ps3
Archaea3 - ps4
Asgard/Eukaryota - ps5
Eukaryota - ps6
Amorpheae - ps7
Obazoa - ps8
Opisthokonta - ps9
Holomycota - ps10
Fungi - ps11
Fungi_1 - ps12
Fungi_2 - ps13
Fungi_3 - ps14
Dikarya - ps15
Ascomycota - ps16
Saccharo/Pezizomycotina - ps17
Saccharomycotina - ps18
Saccharomycotina_1 - ps19
Saccharomycotina_2 - ps20
Saccharomycotina_3 - ps21
Phaff./Saccharomycetaceae - ps22
Saccharomyco./mycetaceae - ps23
Saccharomycetaceae - ps24
Saccharomycetaceae_2 - ps25
Saccharomycetaceae_3 - ps26
Kaz./Nau./Saccharomyces - ps27
Saccharomyces - ps28
Arboricola_clade - ps29
Cerevisiae_clade - ps30
Saccharomyces_cerevisiae - ps31
p-value
6e-08
1e-06
3e-05
7e-04
2e-02
Log-odds
0.5
1.0
1.5
2.0
2.5
3.0
3.5
4.0

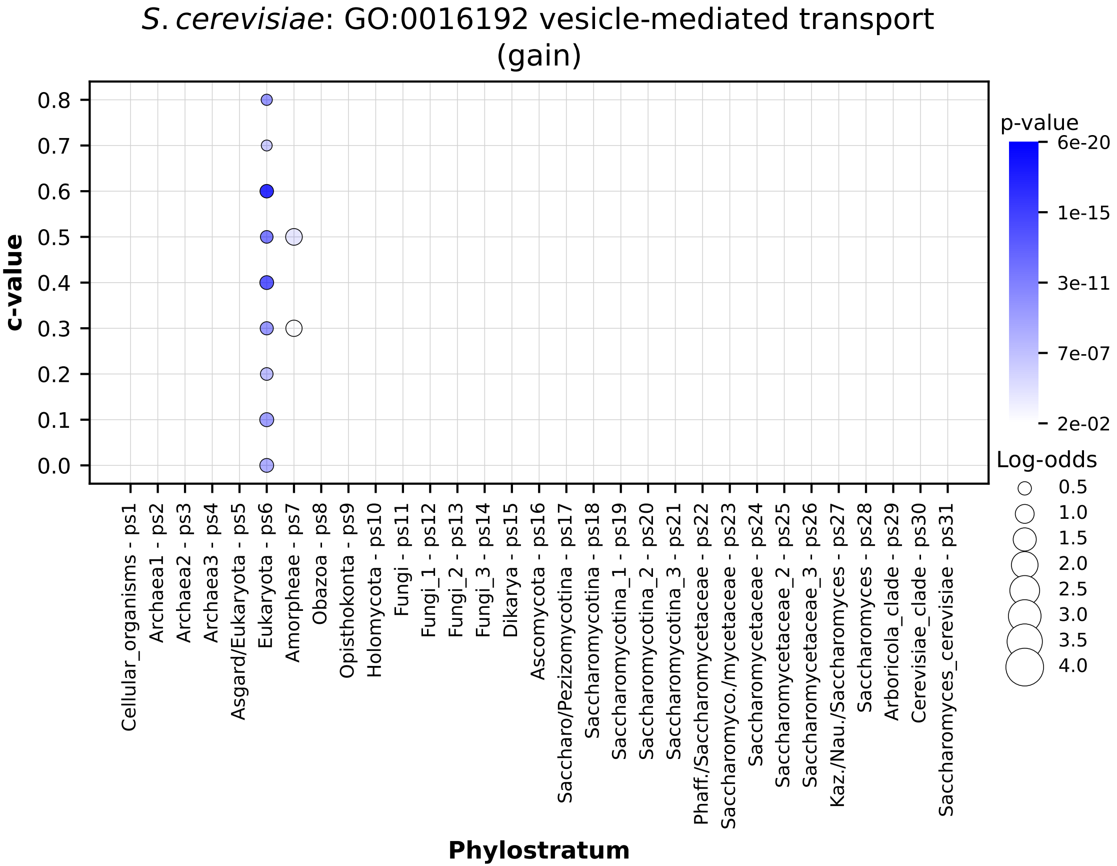

S. cerevisiae: GO:0016192 vesicle-mediated transport
(gain)
c-value
0.8
0.7
0.6
0.5
0.4
0.3
0.2
0.1
0.0
Phylostratum
Cellular_organisms - ps1
Archaea1 - ps2
Archaea2 - ps3
Archaea3 - ps4
Asgard/Eukaryota - ps5
Eukaryota - ps6
Amorpheae - ps7
Obazoa - ps8
Opisthokonta - ps9
Holomycota - ps10
Fungi - ps11
Fungi_1 - ps12
Fungi_2 - ps13
Fungi_3 - ps14
Dikarya - ps15
Ascomycota - ps16
Saccharo/Pezizomycotina - ps17
Saccharomycotina - ps18
Saccharomycotina_1 - ps19
Saccharomycotina_2 - ps20
Saccharomycotina_3 - ps21
Phaff./Saccharomycetaceae - ps22
Saccharomyco./mycetaceae - ps23
Saccharomycetaceae - ps24
Saccharomycetaceae_2 - ps25
Saccharomycetaceae_3 - ps26
Kaz./Nau./Saccharomyces - ps27
Saccharomyces - ps28
Arboricola_clade - ps29
Cerevisiae_clade - ps30
Saccharomyces_cerevisiae - ps31
p-value
6e-20
1e-15
3e-11
7e-07
2e-02
Log-odds
0.5
1.0
1.5
2.0
2.5
3.0
3.5
4.0

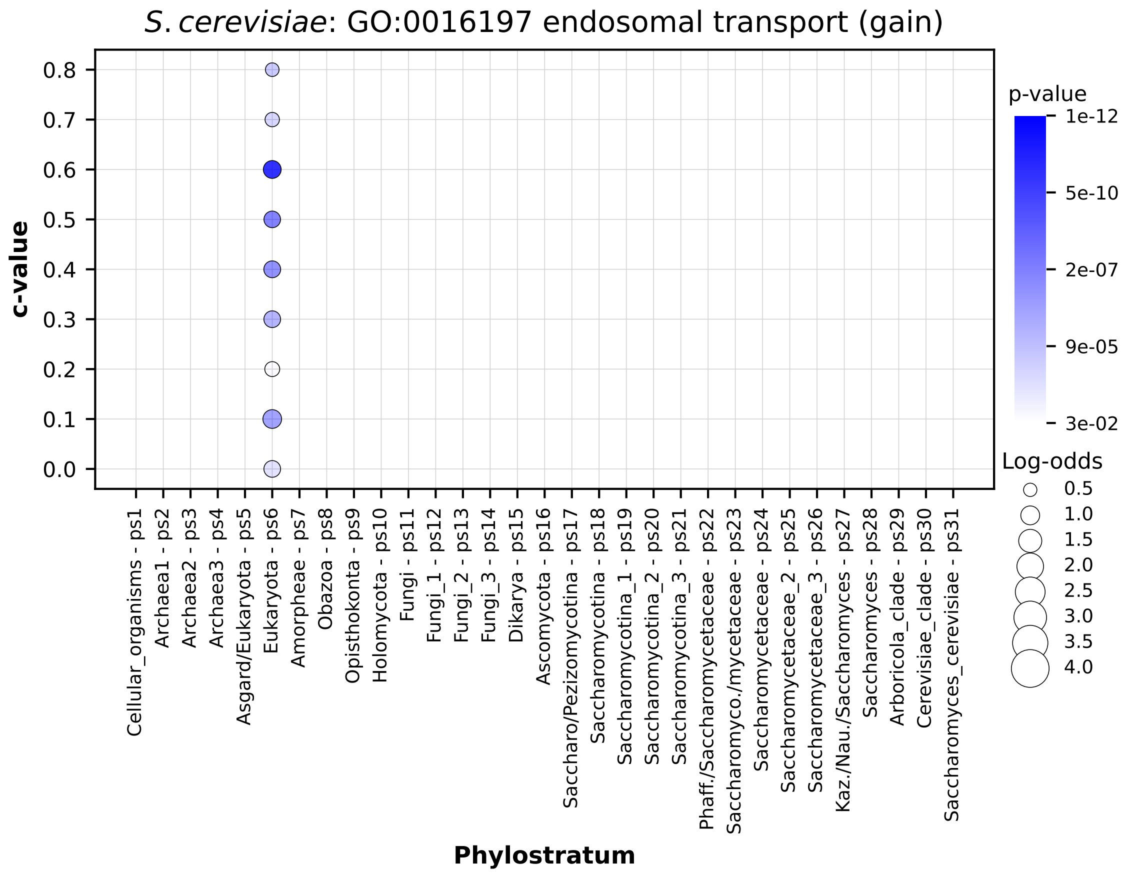

S. cerevisiae: GO:0016197 endosomal transport (gain)
c-value
0.8
0.7
0.6
0.5
0.4
0.3
0.2
0.1
0.0
Phylostratum
Cellular_organisms - ps1
Archaea1 - ps2
Archaea2 - ps3
Archaea3 - ps4
Asgard/Eukaryota - ps5
Eukaryota - ps6
Amorpheae - ps7
Obazoa - ps8
Opisthokonta - ps9
Holomycota - ps10
Fungi - ps11
Fungi_1 - ps12
Fungi_2 - ps13
Fungi_3 - ps14
Dikarya - ps15
Ascomycota - ps16
Saccharo/Pezizomycotina - ps17
Saccharomycotina - ps18
Saccharomycotina_1 - ps19
Saccharomycotina_2 - ps20
Saccharomycotina_3 - ps21
Phaff./Saccharomycetaceae - ps22
Saccharomyco./mycetaceae - ps23
Saccharomycetaceae - ps24
Saccharomycetaceae_2 - ps25
Saccharomycetaceae_3 - ps26
Kaz./Nau./Saccharomyces - ps27
Saccharomyces - ps28
Arboricola_clade - ps29
Cerevisiae_clade - ps30
Saccharomyces_cerevisiae - ps31
p-value
1e-12
5e-10
2e-07
9e-05
3e-02
Log-odds
0.5
1.0
1.5
2.0
2.5
3.0
3.5
4.0

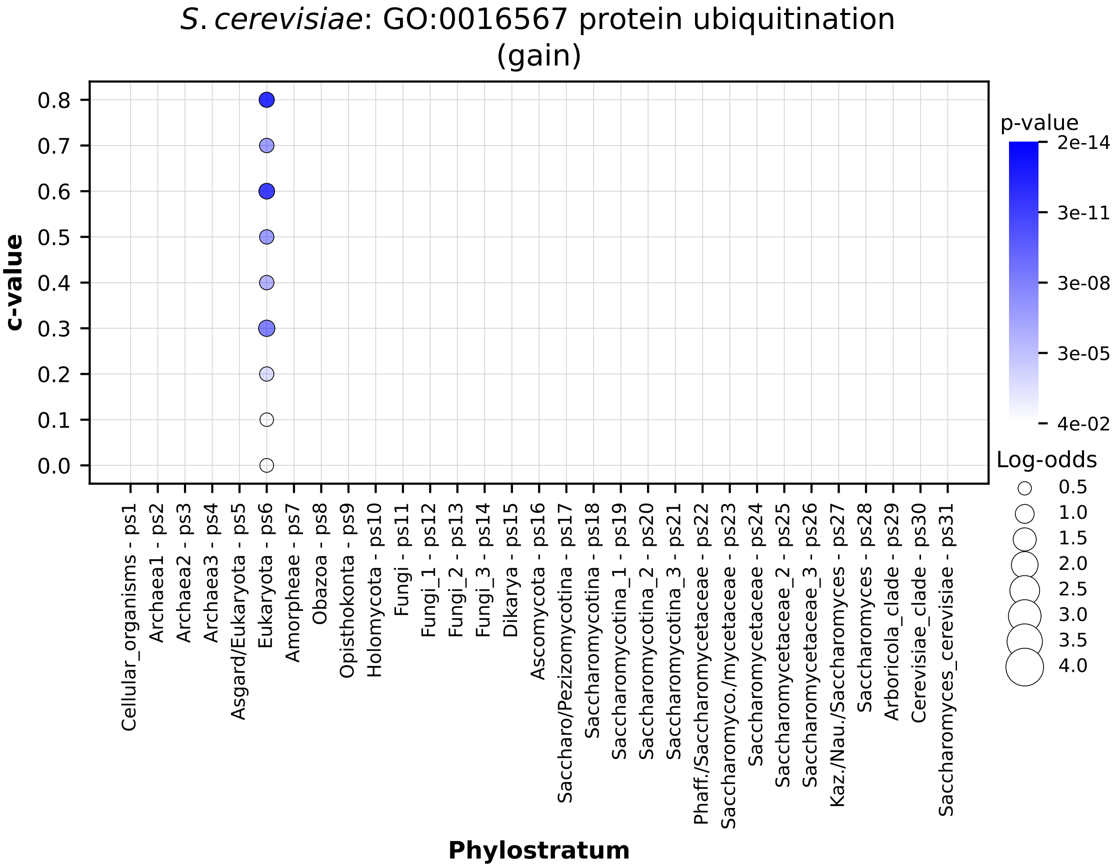

S. cerevisiae: GO:0016567 protein ubiquitination
(gain)
c-value
0.0
0.1
0.2
0.3
0.4
0.5
0.6
0.7
0.8
p-value
2e-14
3e-11
3e-08
3e-05
4e-02
Log-odds
0.5
1.0
1.5
2.0
2.5
3.0
3.5
4.0
Cellular_organisms - ps1
Archaea1 - ps2
Archaea2 - ps3
Archaea3 - ps4
Asgard/Eukaryota - ps5
Eukaryota - ps6
Amorpheae - ps7
Obazoa - ps8
Opisthokonta - ps9
Holomycota - ps10
Fungi - ps11
Fungi_1 - ps12
Fungi_2 - ps13
Fungi_3 - ps14
Dikarya - ps15
Ascomycota - ps16
Saccharo/Pezizomycotina - ps17
Saccharomycotina - ps18
Saccharomycotina_1 - ps19
Saccharomycotina_2 - ps20
Saccharomycotina_3 - ps21
Phaff./Saccharomycetaceae - ps22
Saccharomyco./mycetaceae - ps23
Saccharomycetaceae - ps24
Saccharomycetaceae_2 - ps25
Saccharomycetaceae_3 - ps26
Kaz./Nau./Saccharomyces - ps27
Saccharomyces - ps28
Arboricola_clade - ps29
Cerevisiae_clade - ps30
Saccharomyces_cerevisiae - ps31
Phylostratum

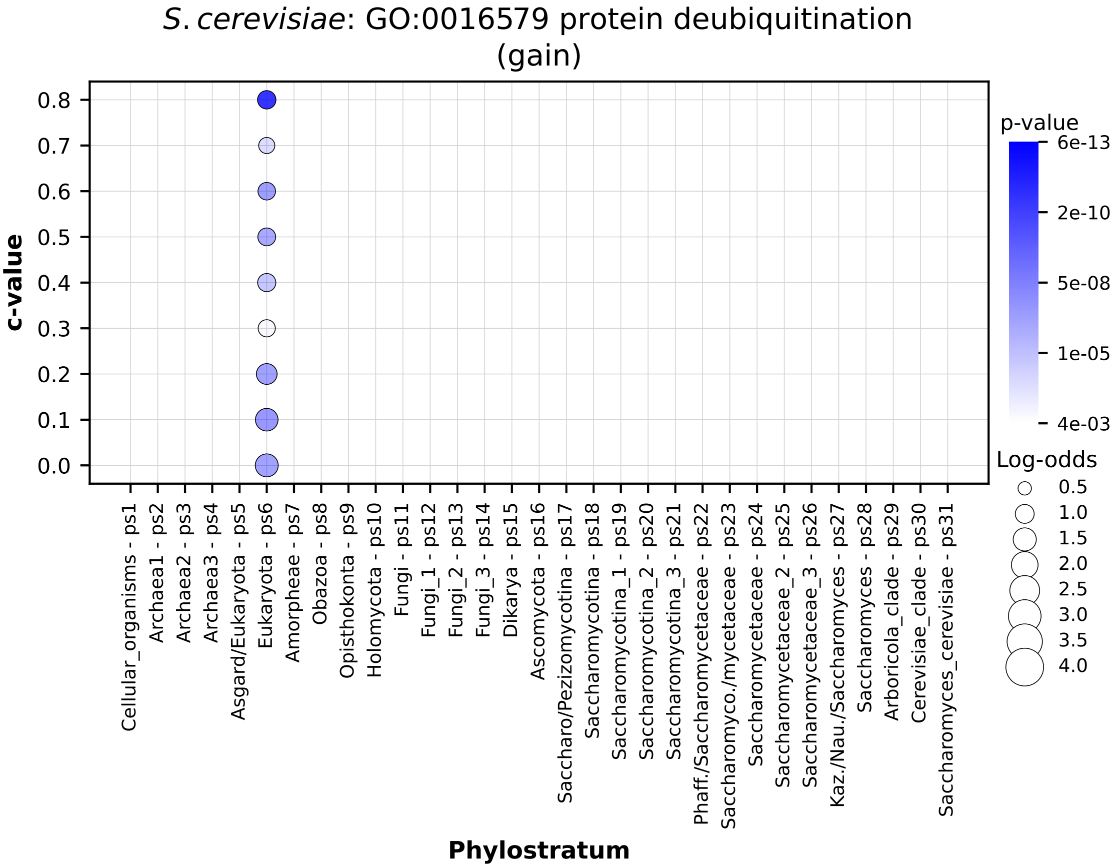

S. cerevisiae: GO:0016579 protein deubiquitination
(gain)
c-value
0.8
0.7
0.6
0.5
0.4
0.3
0.2
0.1
0.0
p-value
6e-13
2e-10
5e-08
1e-05
4e-03
Log-odds
0.5
1.0
1.5
2.0
2.5
3.0
3.5
4.0
Cellular_organisms - ps1
Archaea1 - ps2
Archaea2 - ps3
Archaea3 - ps4
Asgard/Eukaryota - ps5
Eukaryota - ps6
Amorpheae - ps7
Obazoa - ps8
Opisthokonta - ps9
Holomycota - ps10
Fungi - ps11
Fungi_1 - ps12
Fungi_2 - ps13
Fungi_3 - ps14
Dikarya - ps15
Ascomycota - ps16
Saccharo/Pezizomycotina - ps17
Saccharomycotina - ps18
Saccharomycotina_1 - ps19
Saccharomycotina_2 - ps20
Saccharomycotina_3 - ps21
Phaff./Saccharomycetaceae - ps22
Saccharomyco./mycetaceae - ps23
Saccharomycetaceae - ps24
Saccharomycetaceae_2 - ps25
Saccharomycetaceae_3 - ps26
Kaz./Nau./Saccharomyces - ps27
Saccharomyces - ps28
Arboricola_clade - ps29
Cerevisiae_clade - ps30
Saccharomyces_cerevisiae - ps31
Phylostratum

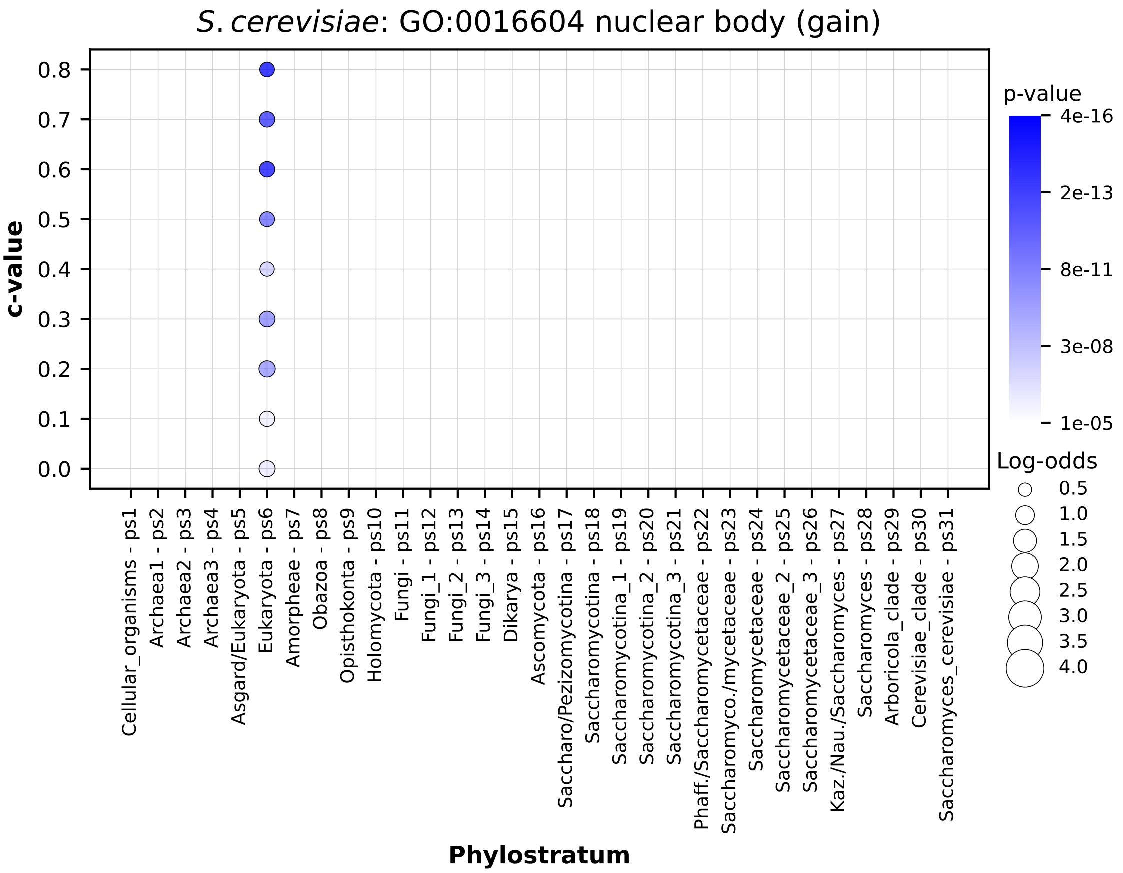

S. cerevisiae: GO:0016604 nuclear body (gain)
c-value
0.0
0.1
0.2
0.3
0.4
0.5
0.6
0.7
0.8
Phylostratum
Cellular_organisms - ps1
Archaea1 - ps2
Archaea2 - ps3
Archaea3 - ps4
Asgard/Eukaryota - ps5
Eukaryota - ps6
Amorpheae - ps7
Obazoa - ps8
Opisthokonta - ps9
Holomycota - ps10
Fungi - ps11
Fungi_1 - ps12
Fungi_2 - ps13
Fungi_3 - ps14
Dikarya - ps15
Ascomycota - ps16
Saccharo/Pezizomycotina - ps17
Saccharomycotina - ps18
Saccharomycotina_1 - ps19
Saccharomycotina_2 - ps20
Saccharomycotina_3 - ps21
Phaff./Saccharomycetaceae - ps22
Saccharomyco./mycetaceae - ps23
Saccharomycetaceae - ps24
Saccharomycetaceae_2 - ps25
Saccharomycetaceae_3 - ps26
Kaz./Nau./Saccharomyces - ps27
Saccharomyces - ps28
Arboricola_clade - ps29
Cerevisiae_clade - ps30
Saccharomyces_cerevisiae - ps31
p-value
4e-16
2e-13
8e-11
3e-08
1e-05
Log-odds
0.5
1.0
1.5
2.0
2.5
3.0
3.5
4.0

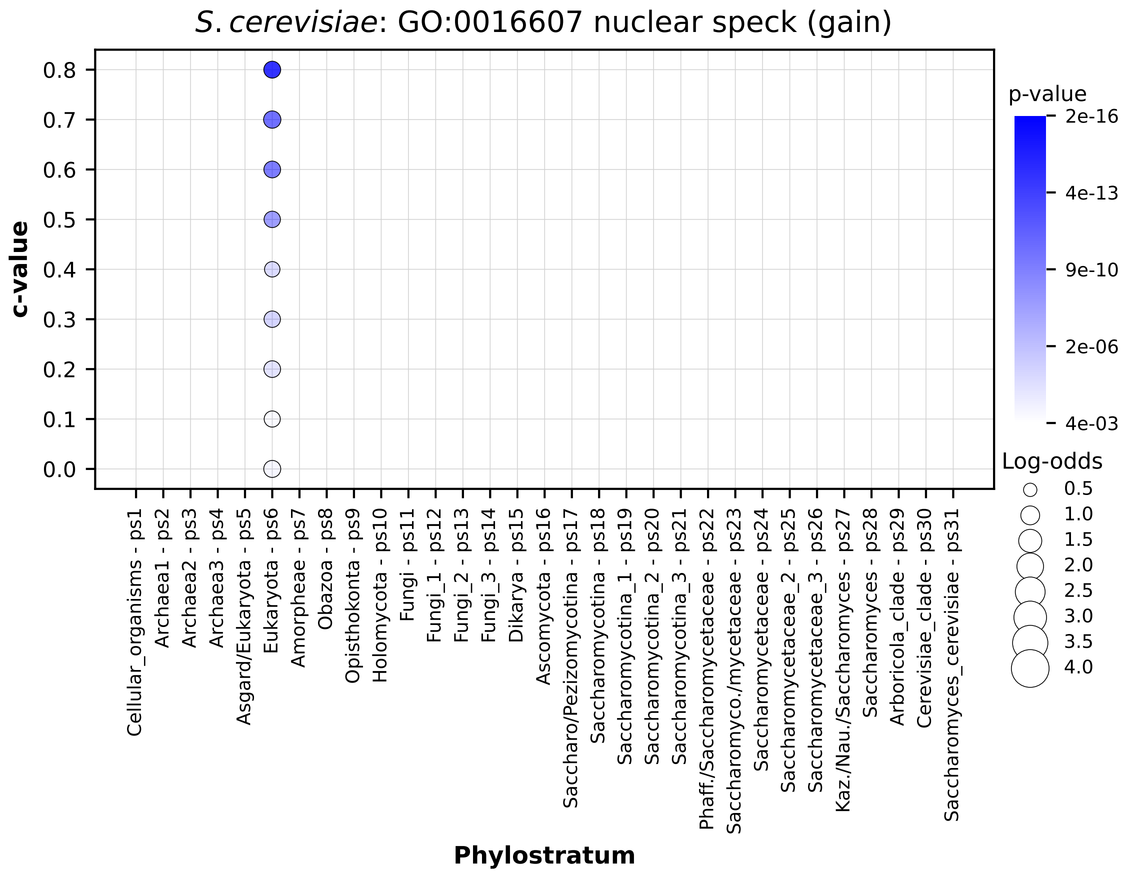
S. cerevisiae: GO:0016607 nuclear speck (gain)
c-value
0.8
0.7
0.6
0.5
0.4
0.3
0.2
0.1
0.0
p-value
2e-16
4e-13
9e-10
2e-06
4e-03
Log-odds
0.5
1.0
1.5
2.0
2.5
3.0
3.5
4.0
Cellular_organisms - ps1
Archaea1 - ps2
Archaea2 - ps3
Archaea3 - ps4
Asgard/Eukaryota - ps5
Eukaryota - ps6
Amorpheae - ps7
Obazoa - ps8
Opisthokonta - ps9
Holomycota - ps10
Fungi - ps11
Fungi_1 - ps12
Fungi_2 - ps13
Fungi_3 - ps14
Dikarya - ps15
Ascomycota - ps16
Saccharo/Pezizomycotina - ps17
Saccharomycotina - ps18
Saccharomycotina_1 - ps19
Saccharomycotina_2 - ps20
Saccharomycotina_3 - ps21
Phaff./Saccharomycetaceae - ps22
Saccharomyco./mycetaceae - ps23
Saccharomycetaceae - ps24
Saccharomycetaceae_2 - ps25
Saccharomycetaceae_3 - ps26
Kaz./Nau./Saccharomyces - ps27
Saccharomyces - ps28
Arboricola_clade - ps29
Cerevisiae_clade - ps30
Saccharomyces_cerevisiae - ps31
Phylostratum

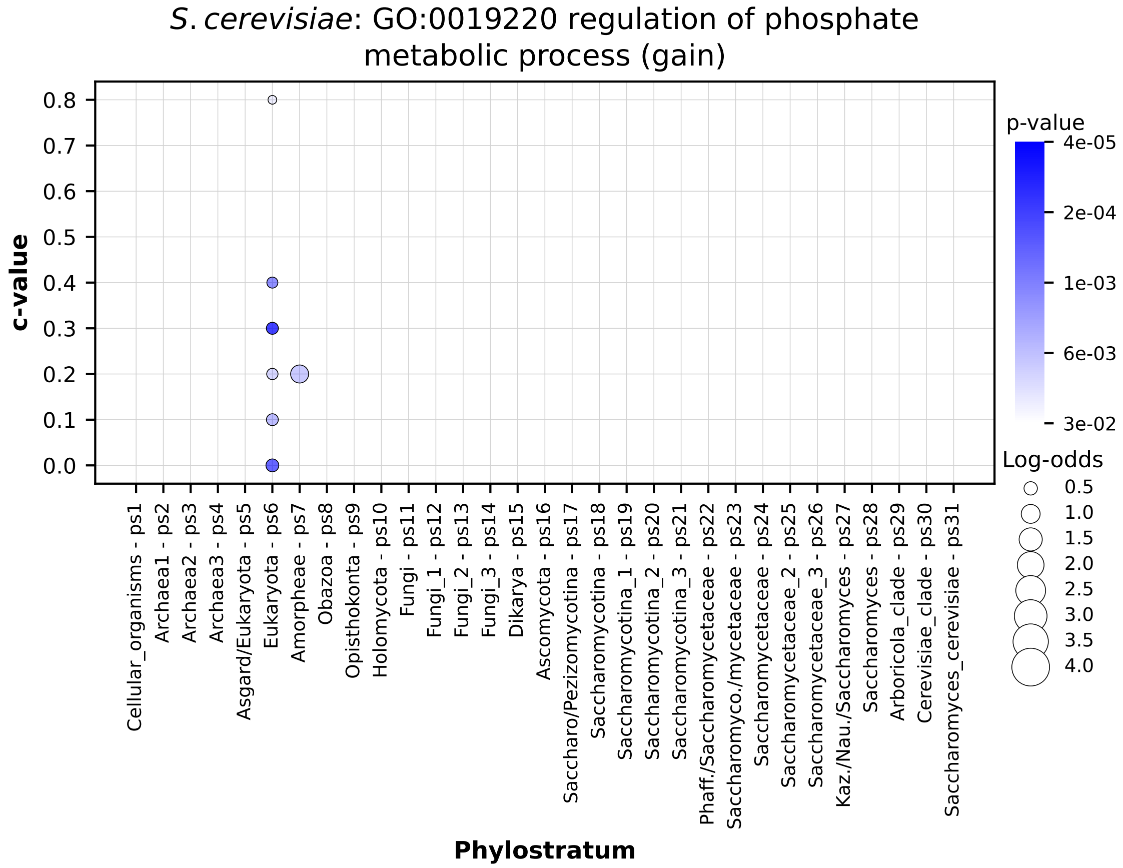

S. cerevisiae: GO:0019220 regulation of phosphate metabolic process (gain)
c-value
0.0
0.1
0.2
0.3
0.4
0.5
0.6
0.7
0.8
Phylostratum
Cellular_organisms - ps1
Archaea1 - ps2
Archaea2 - ps3
Archaea3 - ps4
Asgard/Eukaryota - ps5
Eukaryota - ps6
Amorpheae - ps7
Obazoa - ps8
Opisthokonta - ps9
Holomycota - ps10
Fungi - ps11
Fungi_1 - ps12
Fungi_2 - ps13
Fungi_3 - ps14
Dikarya - ps15
Ascomycota - ps16
Saccharo/Pezizomycotina - ps17
Saccharomycotina - ps18
Saccharomycotina_1 - ps19
Saccharomycotina_2 - ps20
Saccharomycotina_3 - ps21
Phaff./Saccharomycetaceae - ps22
Saccharomyco./mycetaceae - ps23
Saccharomycetaceae - ps24
Saccharomycetaceae_2 - ps25
Saccharomycetaceae_3 - ps26
Kaz./Nau./Saccharomyces - ps27
Saccharomyces - ps28
Arboricola_clade - ps29
Cerevisiae_clade - ps30
Saccharomyces_cerevisiae - ps31
p-value
4e-05
2e-04
1e-03
6e-03
3e-02
Log-odds
0.5
1.0
1.5
2.0
2.5
3.0
3.5
4.0

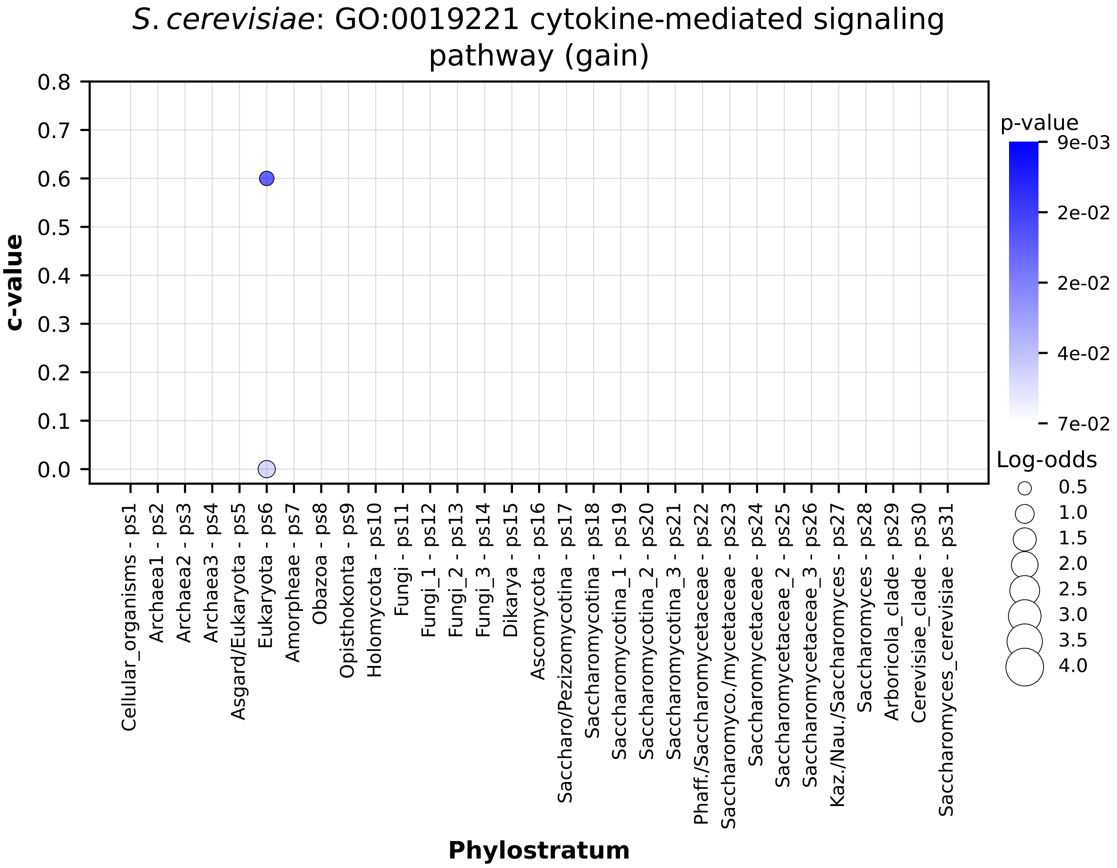
S. cerevisiae: GO:0019221 cytokine-mediated signaling pathway (gain)
c-value
0.8
0.7
0.6
0.5
0.4
0.3
0.2
0.1
0.0
p-value
9e-03
2e-02
2e-02
4e-02
7e-02
Log-odds
0.5
1.0
1.5
2.0
2.5
3.0
3.5
4.0
Cellular_organisms - ps1
Archaea1 - ps2
Archaea2 - ps3
Archaea3 - ps4
Asgard/Eukaryota - ps5
Eukaryota - ps6
Amorpheae - ps7
Obazoa - ps8
Opisthokonta - ps9
Holomycota - ps10
Fungi - ps11
Fungi_1 - ps12
Fungi_2 - ps13
Fungi_3 - ps14
Dikarya - ps15
Ascomycota - ps16
Saccharo/Pezizomycotina - ps17
Saccharomycotina - ps18
Saccharomycotina_1 - ps19
Saccharomycotina_2 - ps20
Saccharomycotina_3 - ps21
Phaff./Saccharomycetaceae - ps22
Saccharomyco./mycetaceae - ps23
Saccharomycetaceae - ps24
Saccharomycetaceae_2 - ps25
Saccharomycetaceae_3 - ps26
Kaz./Nau./Saccharomyces - ps27
Saccharomyces - ps28
Arboricola_clade - ps29
Cerevisiae_clade - ps30
Saccharomyces_cerevisiae - ps31
Phylostratum

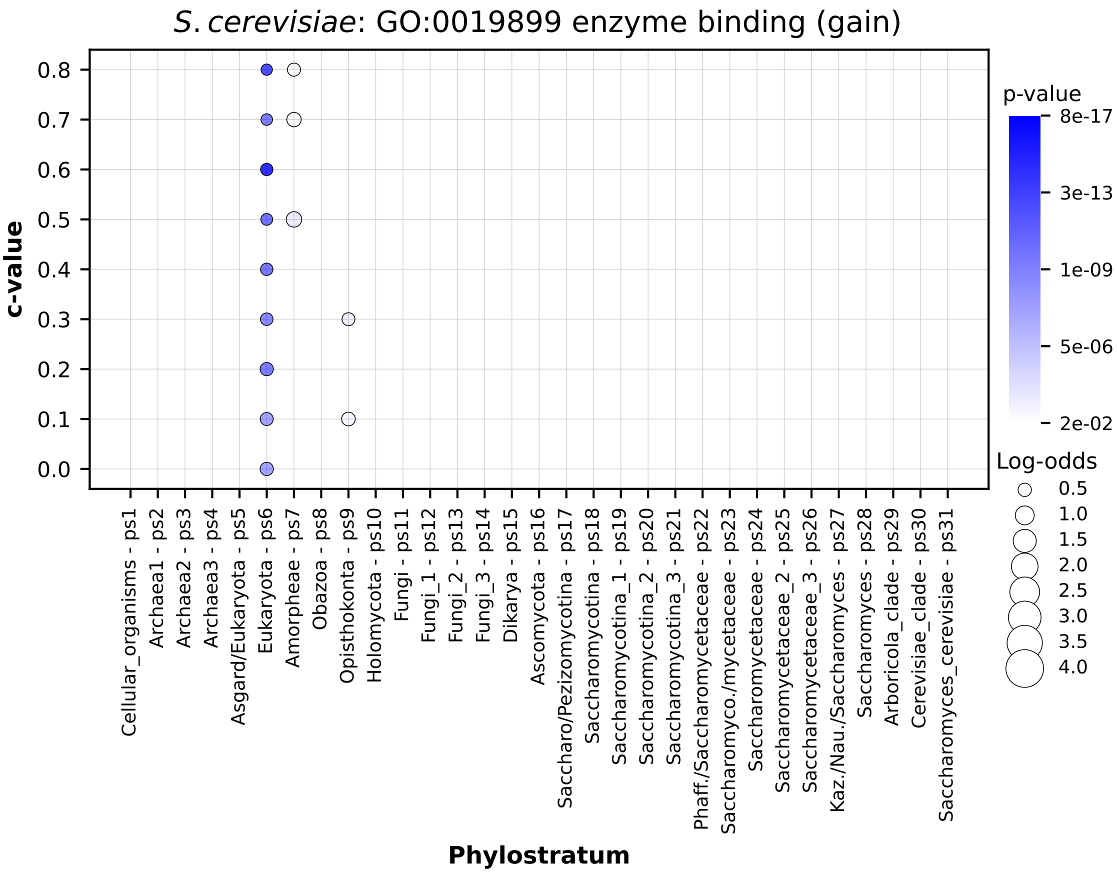
S. cerevisiae: GO:0019899 enzyme binding (gain)
c-value
0.8
0.7
0.6
0.5
0.4
0.3
0.2
0.1
0.0
p-value
8e-17
3e-13
1e-09
5e-06
2e-02
Log-odds
0.5
1.0
1.5
2.0
2.5
3.0
3.5
4.0
Cellular_organisms - ps1
Archaea1 - ps2
Archaea2 - ps3
Archaea3 - ps4
Asgard/Eukaryota - ps5
Eukaryota - ps6
Amorpheae - ps7
Obazoa - ps8
Opisthokonta - ps9
Holomycota - ps10
Fungi - ps11
Fungi_1 - ps12
Fungi_2 - ps13
Fungi_3 - ps14
Dikarya - ps15
Ascomycota - ps16
Saccharo/Pezizomycotina - ps17
Saccharomycotina - ps18
Saccharomycotina_1 - ps19
Saccharomycotina_2 - ps20
Saccharomycotina_3 - ps21
Phaff./Saccharomycetaceae - ps22
Saccharomyco./mycetaceae - ps23
Saccharomycetaceae - ps24
Saccharomycetaceae_2 - ps25
Saccharomycetaceae_3 - ps26
Kaz./Nau./Saccharomyces - ps27
Saccharomyces - ps28
Arboricola_clade - ps29
Cerevisiae_clade - ps30
Saccharomyces_cerevisiae - ps31
Phylostratum

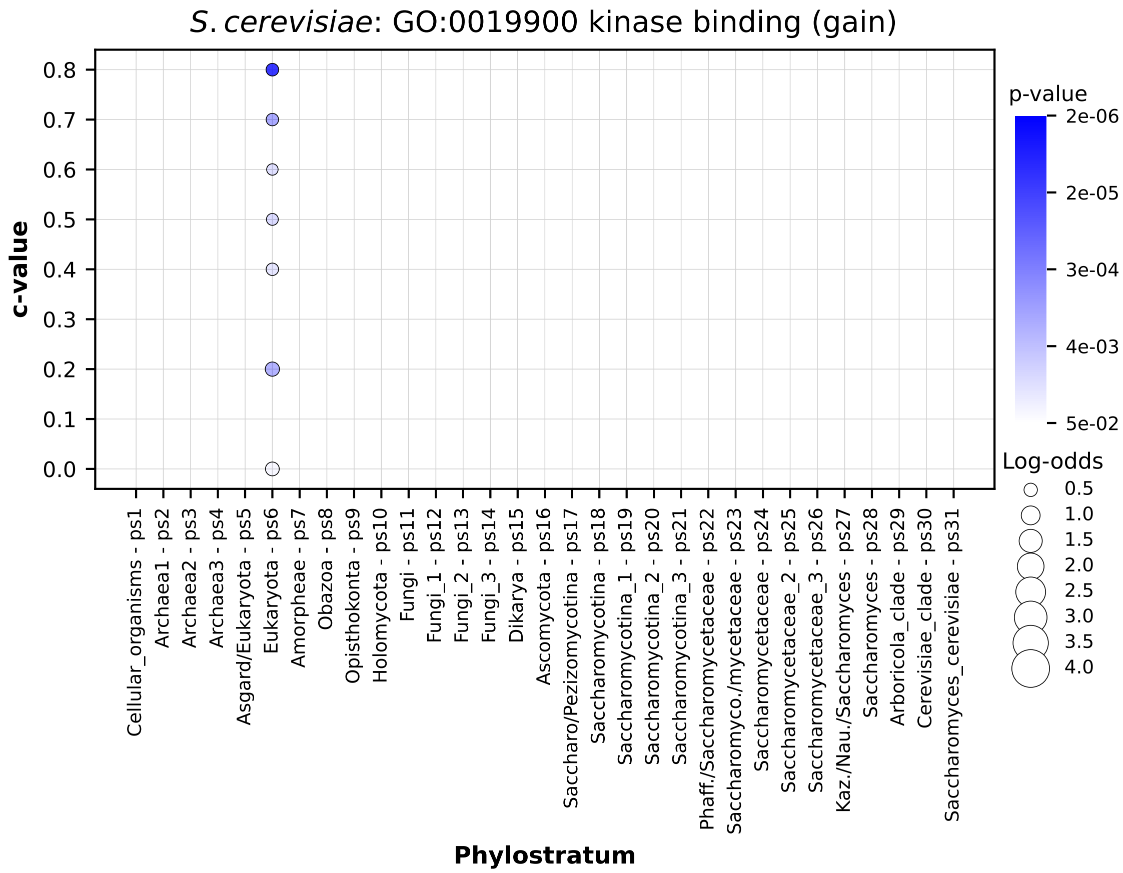

S. cerevisiae: GO:0019900 kinase binding (gain)
c-value
0.0
0.1
0.2
0.3
0.4
0.5
0.6
0.7
0.8
p-value
2e-06
2e-05
3e-04
4e-03
5e-02
Log-odds
0.5
1.0
1.5
2.0
2.5
3.0
3.5
4.0
Cellular_organisms - ps1
Archaea1 - ps2
Archaea2 - ps3
Archaea3 - ps4
Asgard/Eukaryota - ps5
Eukaryota - ps6
Amorpheae - ps7
Obazoa - ps8
Opisthokonta - ps9
Holomycota - ps10
Fungi - ps11
Fungi_1 - ps12
Fungi_2 - ps13
Fungi_3 - ps14
Dikarya - ps15
Ascomycota - ps16
Saccharo/Pezizomycotina - ps17
Saccharomycotina - ps18
Saccharomycotina_1 - ps19
Saccharomycotina_2 - ps20
Saccharomycotina_3 - ps21
Phaff./Saccharomycetaceae - ps22
Saccharomyco./mycetaceae - ps23
Saccharomycetaceae - ps24
Saccharomycetaceae_2 - ps25
Saccharomycetaceae_3 - ps26
Kaz./Nau./Saccharomyces - ps27
Saccharomyces - ps28
Arboricola_clade - ps29
Cerevisiae_clade - ps30
Saccharomyces_cerevisiae - ps31
Phylostratum

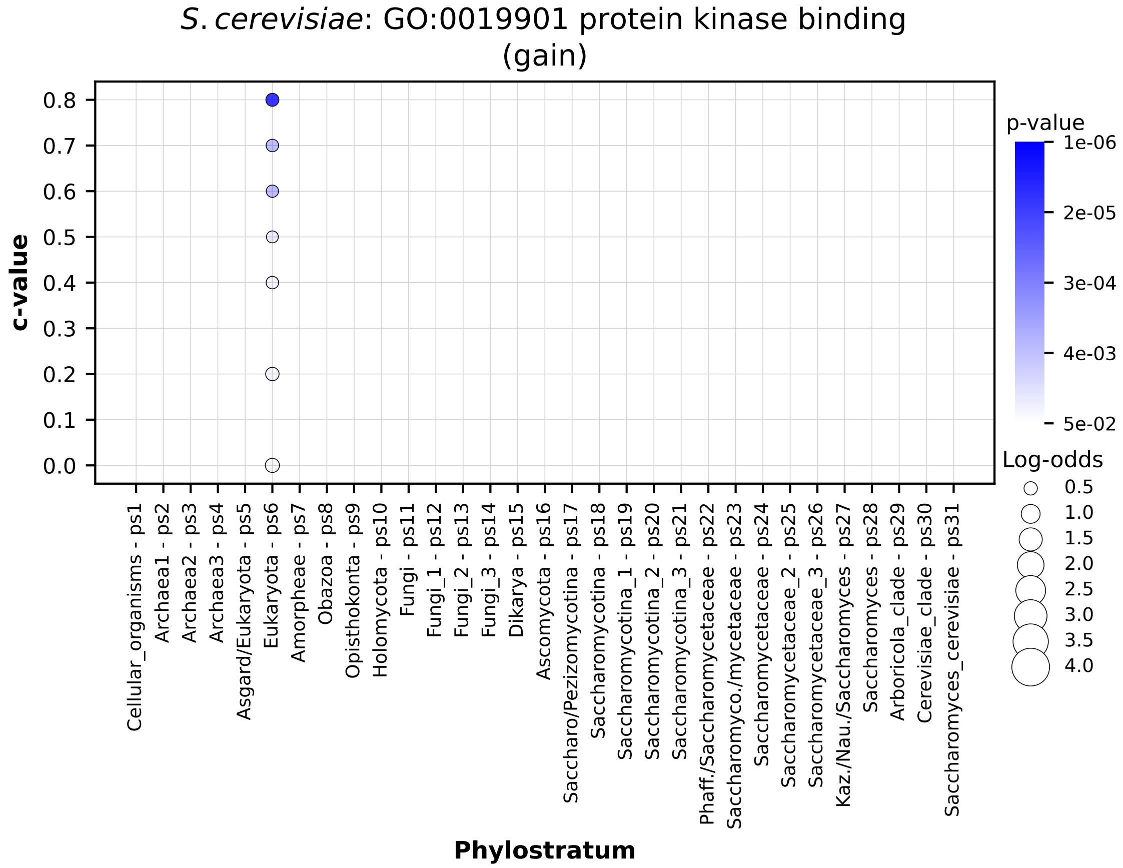

S. cerevisiae: GO:0019901 protein kinase binding
(gain)
c-value
0.8
0.7
0.6
0.5
0.4
0.3
0.2
0.1
0.0
p-value
1e-06
2e-05
3e-04
4e-03
5e-02
Log-odds
0.5
1.0
1.5
2.0
2.5
3.0
3.5
4.0
Cellular_organisms - ps1
Archaea1 - ps2
Archaea2 - ps3
Archaea3 - ps4
Asgard/Eukaryota - ps5
Eukaryota - ps6
Amorpheae - ps7
Obazoa - ps8
Opisthokonta - ps9
Holomycota - ps10
Fungi - ps11
Fungi_1 - ps12
Fungi_2 - ps13
Fungi_3 - ps14
Dikarya - ps15
Ascomycota - ps16
Saccharo/Pezizomycotina - ps17
Saccharomycotina - ps18
Saccharomycotina_1 - ps19
Saccharomycotina_2 - ps20
Saccharomycotina_3 - ps21
Phaff./Saccharomycetaceae - ps22
Saccharomyco./mycetaceae - ps23
Saccharomycetaceae - ps24
Saccharomycetaceae_2 - ps25
Saccharomycetaceae_3 - ps26
Kaz./Nau./Saccharomyces - ps27
Saccharomyces - ps28
Arboricola_clade - ps29
Cerevisiae_clade - ps30
Saccharomyces_cerevisiae - ps31
Phylostratum

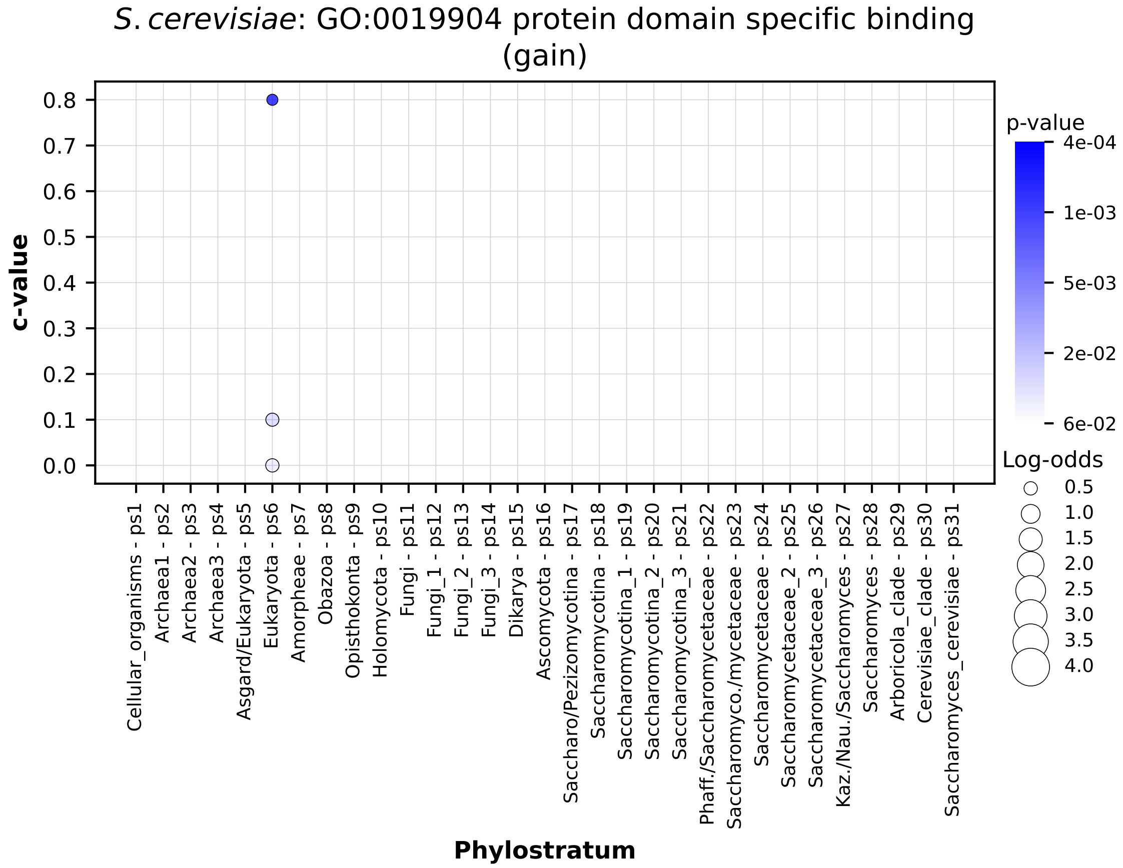

S. cerevisiae: GO:0019904 protein domain specific binding (gain)
c-value
0.0
0.1
0.2
0.3
0.4
0.5
0.6
0.7
0.8
p-value
4e-04
1e-03
5e-03
2e-02
6e-02
Log-odds
0.5
1.0
1.5
2.0
2.5
3.0
3.5
4.0
Cellular_organisms - ps1
Archaea1 - ps2
Archaea2 - ps3
Archaea3 - ps4
Asgard/Eukaryota - ps5
Eukaryota - ps6
Amorpheae - ps7
Obazoa - ps8
Opisthokonta - ps9
Holomycota - ps10
Fungi - ps11
Fungi_1 - ps12
Fungi_2 - ps13
Fungi_3 - ps14
Dikarya - ps15
Ascomycota - ps16
Saccharo/Pezizomycotina - ps17
Saccharomycotina - ps18
Saccharomycotina_1 - ps19
Saccharomycotina_2 - ps20
Saccharomycotina_3 - ps21
Phaff./Saccharomycetaceae - ps22
Saccharomyco./mycetaceae - ps23
Saccharomycetaceae - ps24
Saccharomycetaceae_2 - ps25
Saccharomycetaceae_3 - ps26
Kaz./Nau./Saccharomyces - ps27
Saccharomyces - ps28
Arboricola_clade - ps29
Cerevisiae_clade - ps30
Saccharomyces_cerevisiae - ps31
Phylostratum

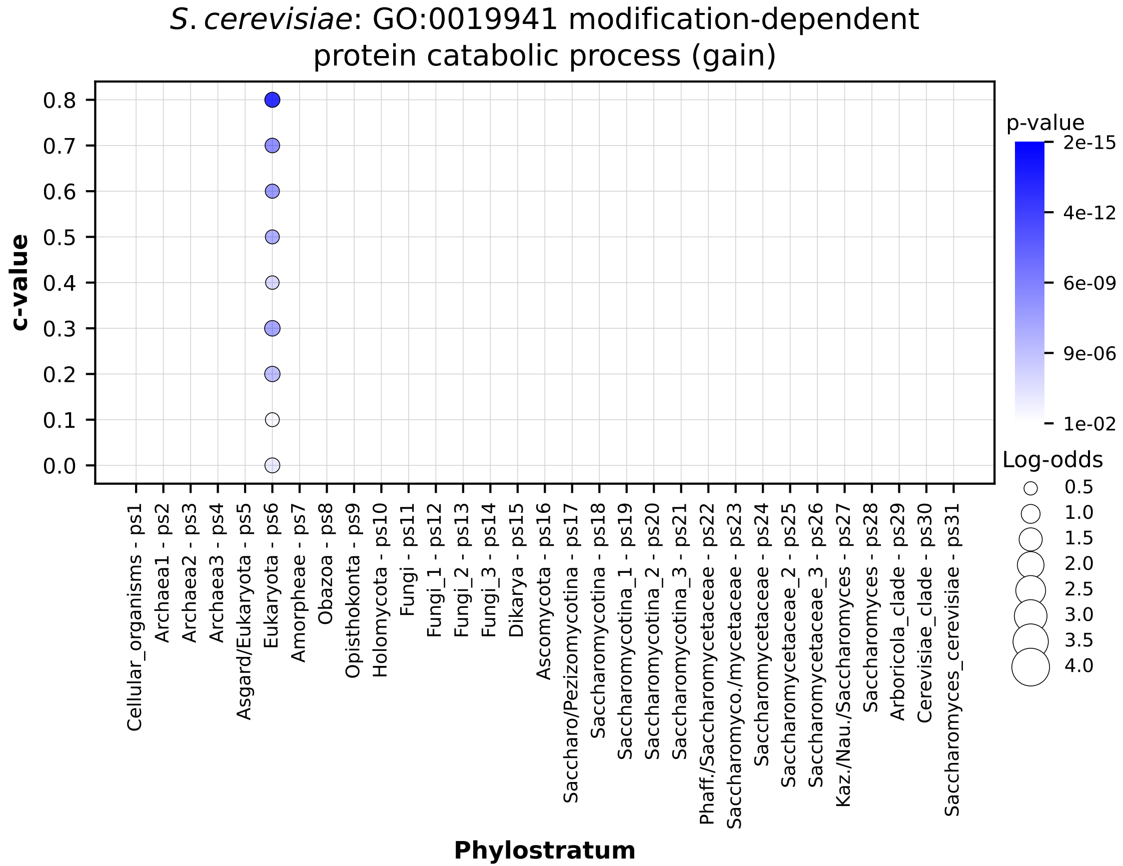

S. cerevisiae: GO:0019941 modification-dependent protein catabolic process (gain)
c-value
0.8
0.7
0.6
0.5
0.4
0.3
0.2
0.1
0.0
p-value
2e-15
4e-12
6e-09
9e-06
1e-02
Log-odds
0.5
1.0
1.5
2.0
2.5
3.0
3.5
4.0
Cellular_organisms - ps1
Archaea1 - ps2
Archaea2 - ps3
Archaea3 - ps4
Asgard/Eukaryota - ps5
Eukaryota - ps6
Amorpheae - ps7
Obazoa - ps8
Opisthokonta - ps9
Holomycota - ps10
Fungi - ps11
Fungi_1 - ps12
Fungi_2 - ps13
Fungi_3 - ps14
Dikarya - ps15
Ascomycota - ps16
Saccharo/Pezizomycotina - ps17
Saccharomycotina - ps18
Saccharomycotina_1 - ps19
Saccharomycotina_2 - ps20
Saccharomycotina_3 - ps21
Phaff./Saccharomycetaceae - ps22
Saccharomyco./mycetaceae - ps23
Saccharomycetaceae - ps24
Saccharomycetaceae_2 - ps25
Saccharomycetaceae_3 - ps26
Kaz./Nau./Saccharomyces - ps27
Saccharomyces - ps28
Arboricola_clade - ps29
Cerevisiae_clade - ps30
Saccharomyces_cerevisiae - ps31
Phylostratum

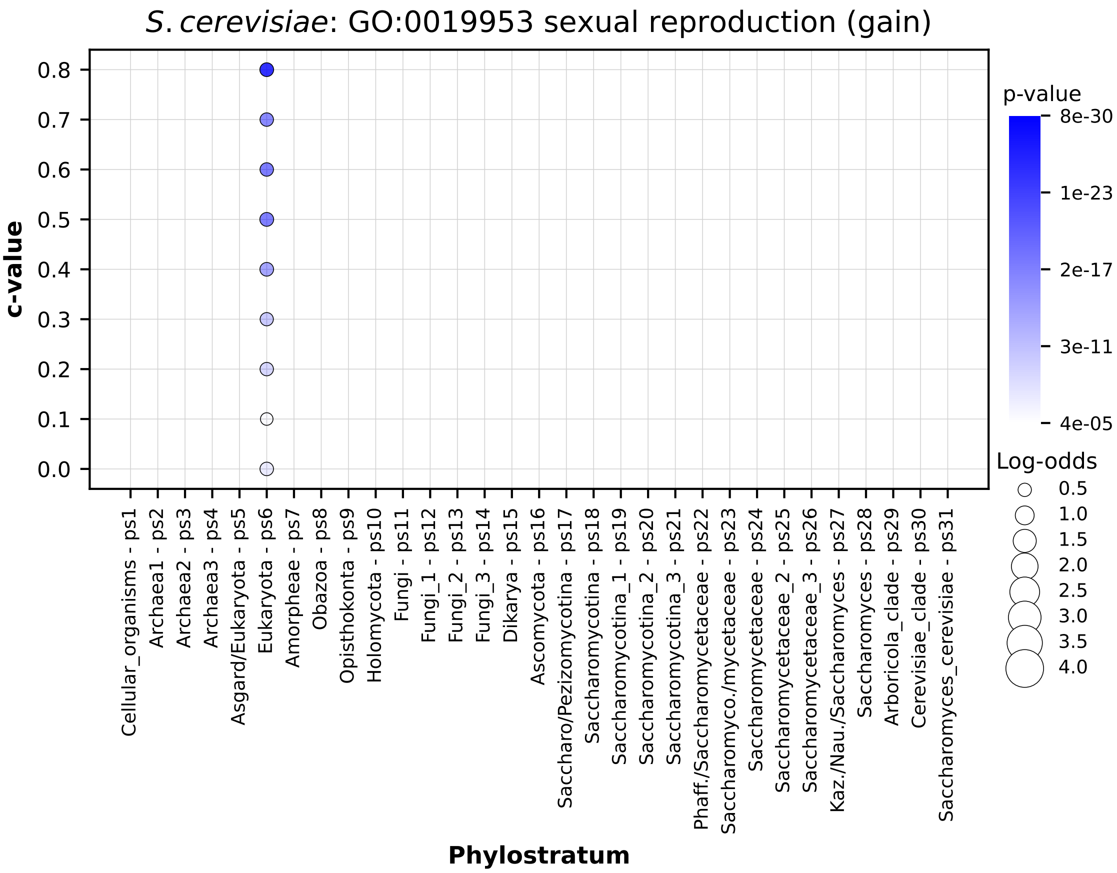
S. cerevisiae: GO:0019953 sexual reproduction (gain)
c-value
0.8
0.7
0.6
0.5
0.4
0.3
0.2
0.1
0.0
p-value
8e-30
1e-23
2e-17
3e-11
4e-05
Log-odds
0.5
1.0
1.5
2.0
2.5
3.0
3.5
4.0
Cellular_organisms - ps1
Archaea1 - ps2
Archaea2 - ps3
Archaea3 - ps4
Asgard/Eukaryota - ps5
Eukaryota - ps6
Amorpheae - ps7
Obazoa - ps8
Opisthokonta - ps9
Holomycota - ps10
Fungi - ps11
Fungi_1 - ps12
Fungi_2 - ps13
Fungi_3 - ps14
Dikarya - ps15
Ascomycota - ps16
Saccharo/Pezizomycotina - ps17
Saccharomycotina - ps18
Saccharomycotina_1 - ps19
Saccharomycotina_2 - ps20
Saccharomycotina_3 - ps21
Phaff./Saccharomycetaceae - ps22
Saccharomyco./mycetaceae - ps23
Saccharomycetaceae - ps24
Saccharomycetaceae_2 - ps25
Saccharomycetaceae_3 - ps26
Kaz./Nau./Saccharomyces - ps27
Saccharomyces - ps28
Arboricola_clade - ps29
Cerevisiae_clade - ps30
Saccharomyces_cerevisiae - ps31
Phylostratum

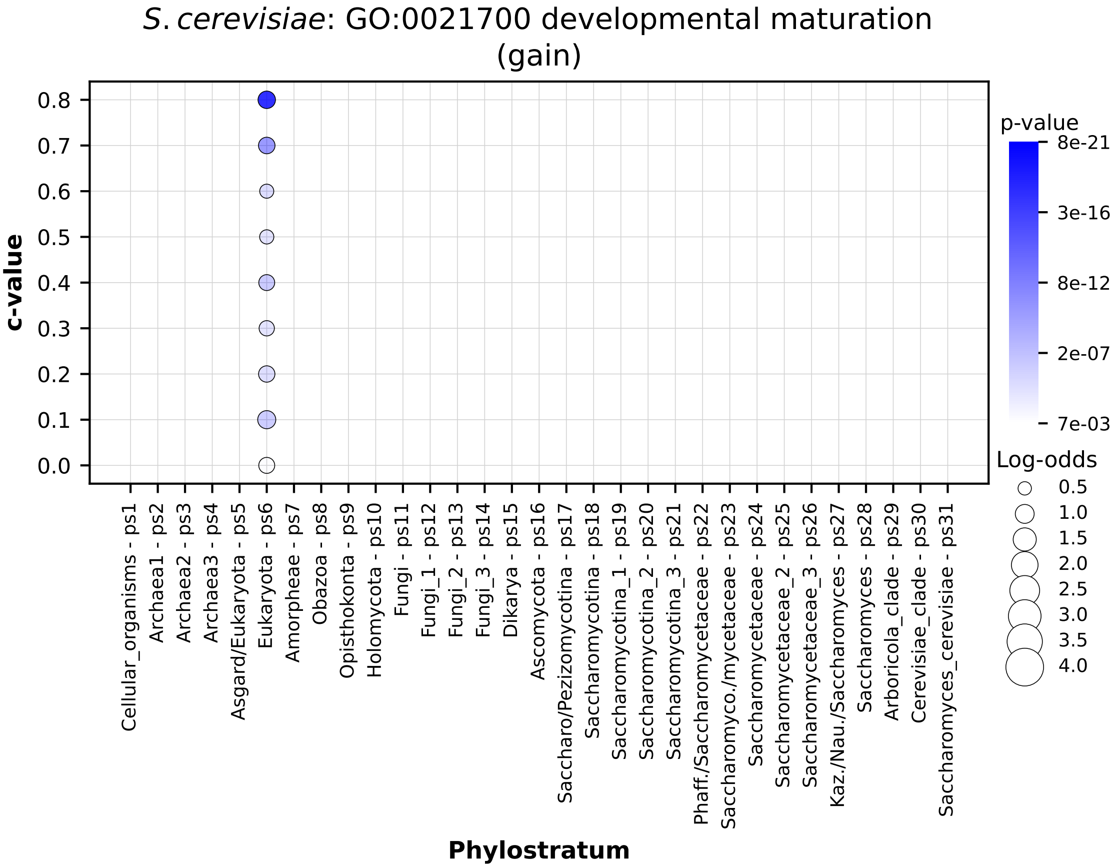

S. cerevisiae: GO:0021700 developmental maturation (gain)
c-value
0.0
0.1
0.2
0.3
0.4
0.5
0.6
0.7
0.8
Phylostratum
Cellular_organisms - ps1
Archaea1 - ps2
Archaea2 - ps3
Archaea3 - ps4
Asgard/Eukaryota - ps5
Eukaryota - ps6
Amorpheae - ps7
Obazoa - ps8
Opisthokonta - ps9
Holomycota - ps10
Fungi - ps11
Fungi_1 - ps12
Fungi_2 - ps13
Fungi_3 - ps14
Dikarya - ps15
Ascomycota - ps16
Saccharo/Pezizomycotina - ps17
Saccharomycotina - ps18
Saccharomycotina_1 - ps19
Saccharomycotina_2 - ps20
Saccharomycotina_3 - ps21
Phaff./Saccharomycetaceae - ps22
Saccharomyco./mycetaceae - ps23
Saccharomycetaceae - ps24
Saccharomycetaceae_2 - ps25
Saccharomycetaceae_3 - ps26
Kaz./Nau./Saccharomyces - ps27
Saccharomyces - ps28
Arboricola_clade - ps29
Cerevisiae_clade - ps30
Saccharomyces_cerevisiae - ps31
p-value
8e-21
3e-16
8e-12
2e-07
7e-03
Log-odds
0.5
1.0
1.5
2.0
2.5
3.0
3.5
4.0

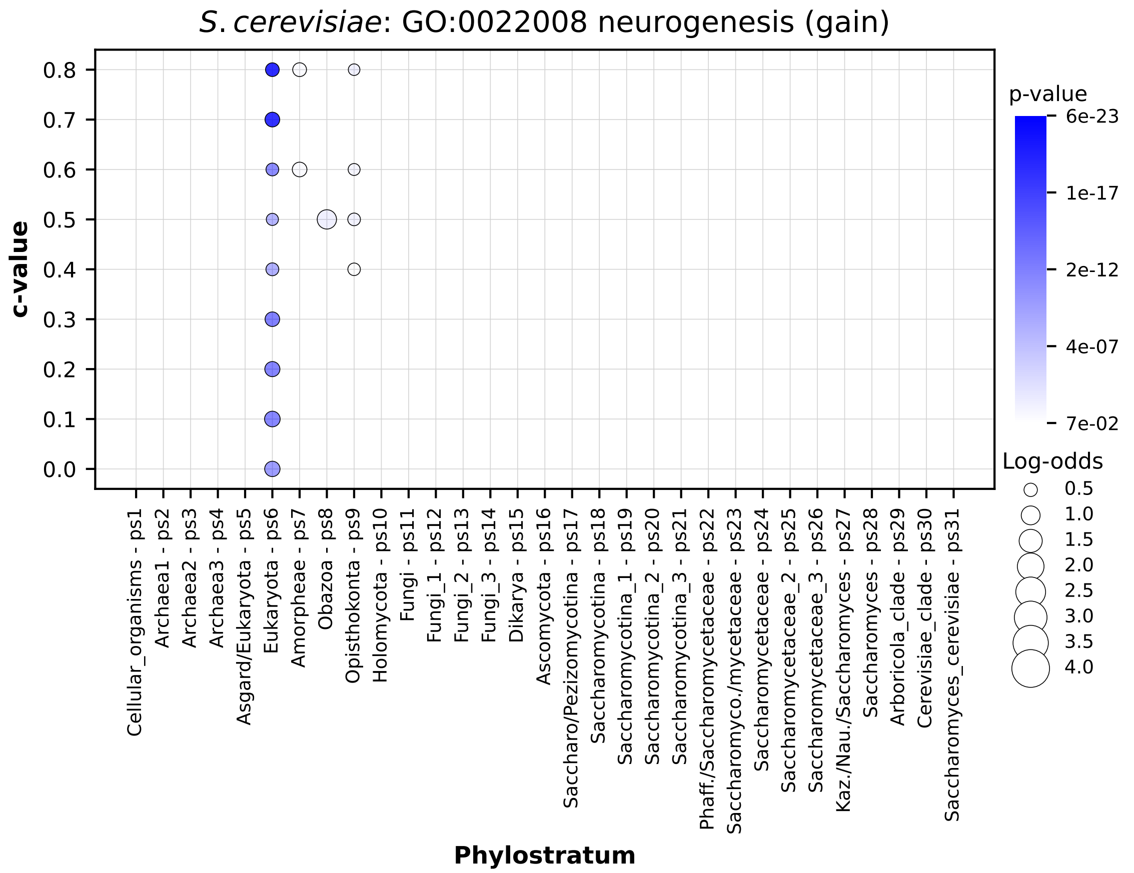

S. cerevisiae: GO:0022008 neurogenesis (gain)
c-value
0.0
0.1
0.2
0.3
0.4
0.5
0.6
0.7
0.8
Phylostratum
Cellular_organisms - ps1
Archaea1 - ps2
Archaea2 - ps3
Archaea3 - ps4
Asgard/Eukaryota - ps5
Eukaryota - ps6
Amorpheae - ps7
Obazoa - ps8
Opisthokonta - ps9
Holomycota - ps10
Fungi - ps11
Fungi_1 - ps12
Fungi_2 - ps13
Fungi_3 - ps14
Dikarya - ps15
Ascomycota - ps16
Saccharo/Pezizomycotina - ps17
Saccharomycotina - ps18
Saccharomycotina_1 - ps19
Saccharomycotina_2 - ps20
Saccharomycotina_3 - ps21
Phaff./Saccharomycetaceae - ps22
Saccharomyco./mycetaceae - ps23
Saccharomycetaceae - ps24
Saccharomycetaceae_2 - ps25
Saccharomycetaceae_3 - ps26
Kaz./Nau./Saccharomyces - ps27
Saccharomyces - ps28
Arboricola_clade - ps29
Cerevisiae_clade - ps30
Saccharomyces_cerevisiae - ps31
p-value
6e-23
1e-17
2e-12
4e-07
7e-02
Log-odds
0.5
1.0
1.5
2.0
2.5
3.0
3.5
4.0

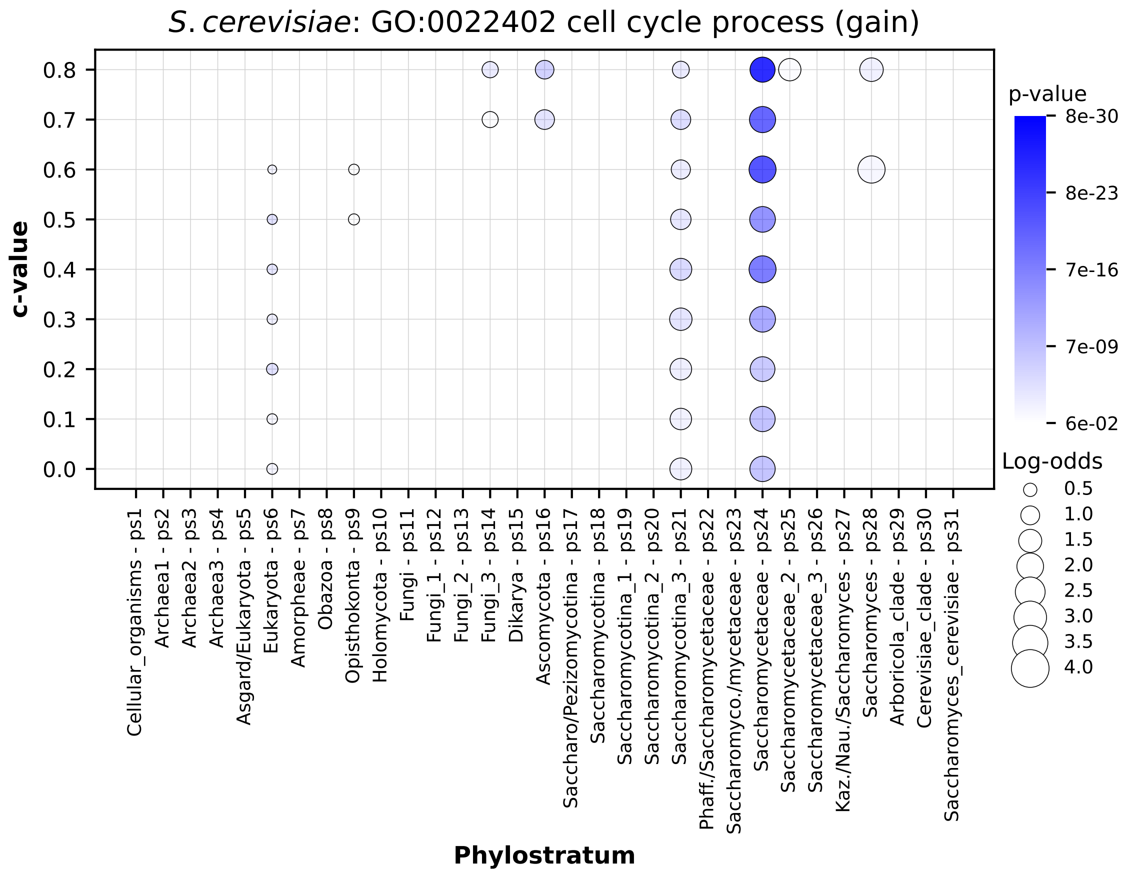

S. cerevisiae: GO:0022402 cell cycle process (gain)
c-value
0.0
0.1
0.2
0.3
0.4
0.5
0.6
0.7
0.8
Phylostratum
Cellular_organisms - ps1
Archaea1 - ps2
Archaea2 - ps3
Archaea3 - ps4
Asgard/Eukaryota - ps5
Eukaryota - ps6
Amorpheae - ps7
Obazoa - ps8
Opisthokonta - ps9
Holomycota - ps10
Fungi - ps11
Fungi_1 - ps12
Fungi_2 - ps13
Fungi_3 - ps14
Dikarya - ps15
Ascomycota - ps16
Saccharo/Pezizomycotina - ps17
Saccharomycotina - ps18
Saccharomycotina_1 - ps19
Saccharomycotina_2 - ps20
Saccharomycotina_3 - ps21
Phaff./Saccharomycetaceae - ps22
Saccharomyco./mycetaceae - ps23
Saccharomycetaceae - ps24
Saccharomycetaceae_2 - ps25
Saccharomycetaceae_3 - ps26
Kaz./Nau./Saccharomyces - ps27
Saccharomyces - ps28
Arboricola_clade - ps29
Cerevisiae_clade - ps30
Saccharomyces_cerevisiae - ps31
p-value
8e-30
8e-23
7e-16
7e-09
6e-02
Log-odds
0.5
1.0
1.5
2.0
2.5
3.0
3.5
4.0

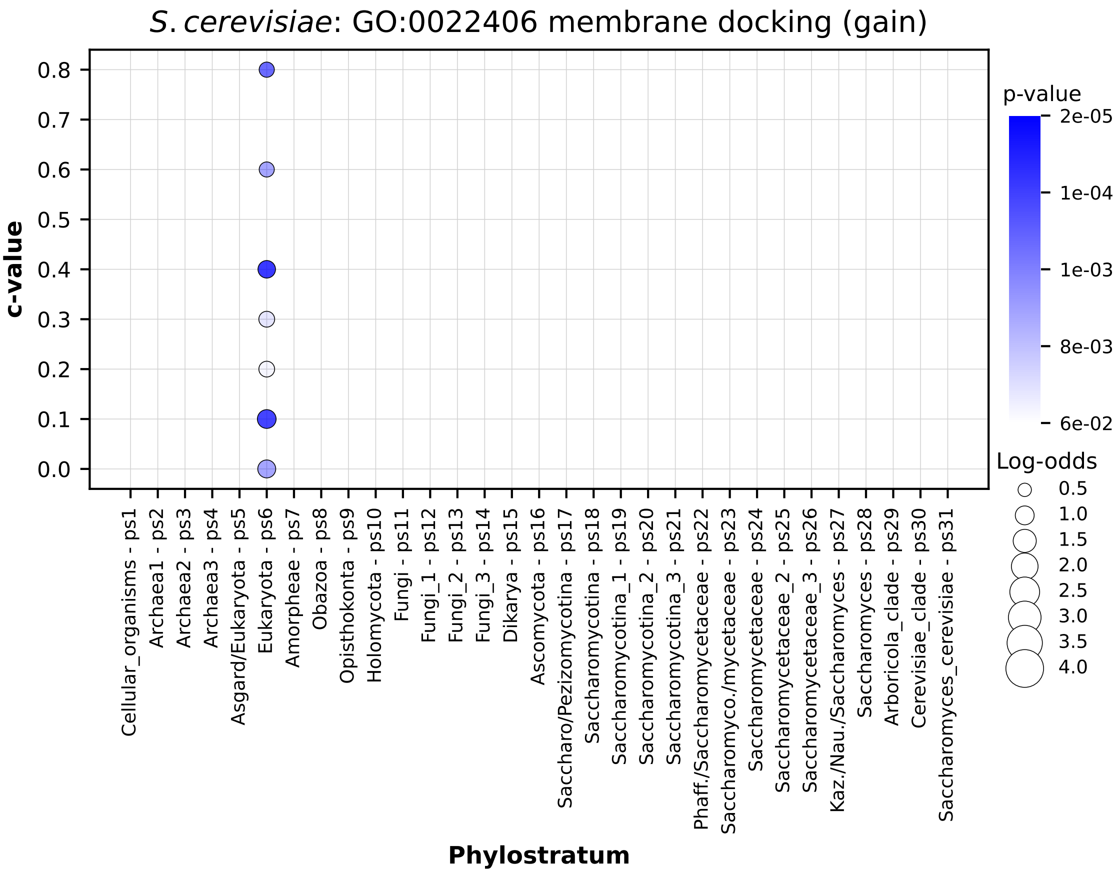

S. cerevisiae: GO:0022406 membrane docking (gain)
c-value
0.8
0.7
0.6
0.5
0.4
0.3
0.2
0.1
0.0
p-value
2e-05
1e-04
1e-03
8e-03
6e-02
Log-odds
0.5
1.0
1.5
2.0
2.5
3.0
3.5
4.0
Cellular_organisms - ps1
Archaea1 - ps2
Archaea2 - ps3
Archaea3 - ps4
Asgard/Eukaryota - ps5
Eukaryota - ps6
Amorpheae - ps7
Obazoa - ps8
Opisthokonta - ps9
Holomycota - ps10
Fungi - ps11
Fungi_1 - ps12
Fungi_2 - ps13
Fungi_3 - ps14
Dikarya - ps15
Ascomycota - ps16
Saccharo/Pezizomycotina - ps17
Saccharomycotina - ps18
Saccharomycotina_1 - ps19
Saccharomycotina_2 - ps20
Saccharomycotina_3 - ps21
Phaff./Saccharomycetaceae - ps22
Saccharomyco./mycetaceae - ps23
Saccharomycetaceae - ps24
Saccharomycetaceae_2 - ps25
Saccharomycetaceae_3 - ps26
Kaz./Nau./Saccharomyces - ps27
Saccharomyces - ps28
Arboricola_clade - ps29
Cerevisiae_clade - ps30
Saccharomyces_cerevisiae - ps31
Phylostratum

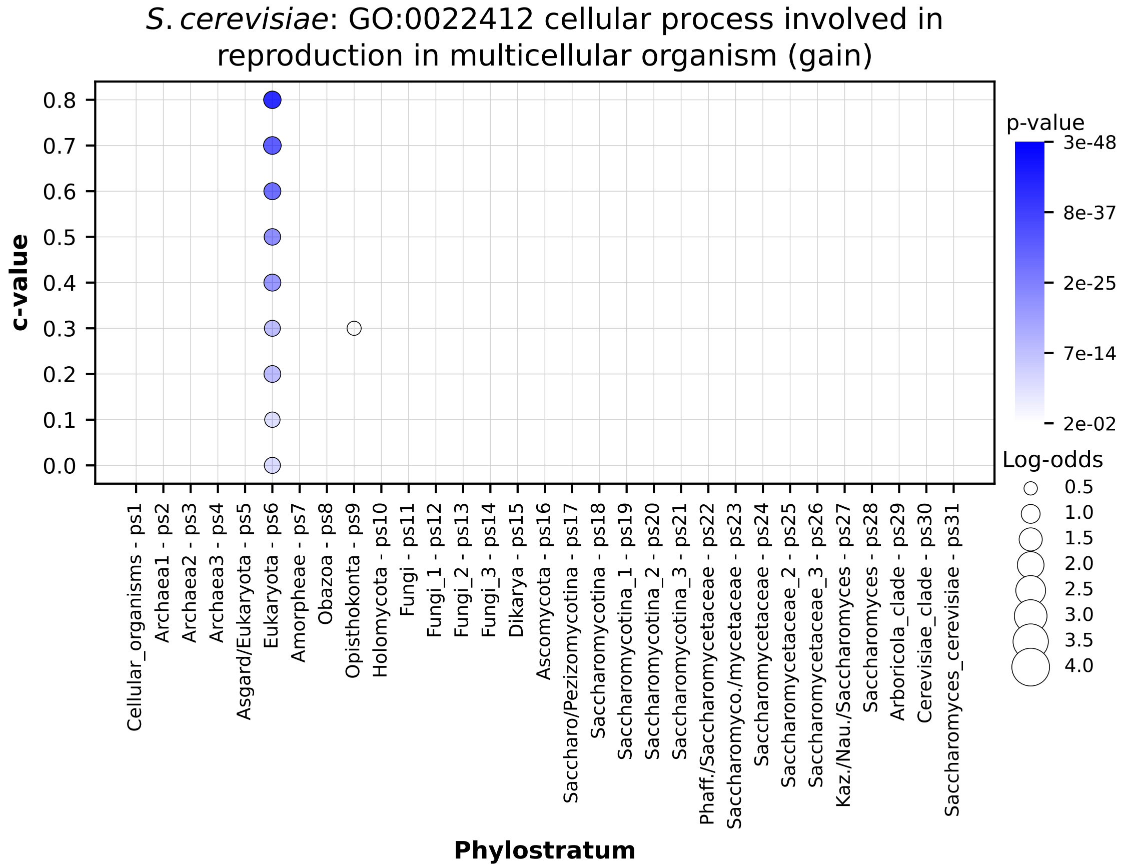

S. cerevisiae: GO:0022412 cellular process involved in reproduction in multicellular organism (gain)
c-value
0.8
0.7
0.6
0.5
0.4
0.3
0.2
0.1
0.0
p-value
3e-48
8e-37
2e-25
7e-14
2e-02
Log-odds
0.5
1.0
1.5
2.0
2.5
3.0
3.5
4.0
Cellular_organisms - ps1
Archaea1 - ps2
Archaea2 - ps3
Archaea3 - ps4
Asgard/Eukaryota - ps5
Eukaryota - ps6
Amorpheae - ps7
Obazoa - ps8
Opisthokonta - ps9
Holomycota - ps10
Fungi - ps11
Fungi_1 - ps12
Fungi_2 - ps13
Fungi_3 - ps14
Dikarya - ps15
Ascomycota - ps16
Saccharo/Pezizomycotina - ps17
Saccharomycotina - ps18
Saccharomycotina_1 - ps19
Saccharomycotina_2 - ps20
Saccharomycotina_3 - ps21
Phaff./Saccharomycetaceae - ps22
Saccharomyco./mycetaceae - ps23
Saccharomycetaceae - ps24
Saccharomycetaceae_2 - ps25
Saccharomycetaceae_3 - ps26
Kaz./Nau./Saccharomyces - ps27
Saccharomyces - ps28
Arboricola_clade - ps29
Cerevisiae_clade - ps30
Saccharomyces_cerevisiae - ps31
Phylostratum

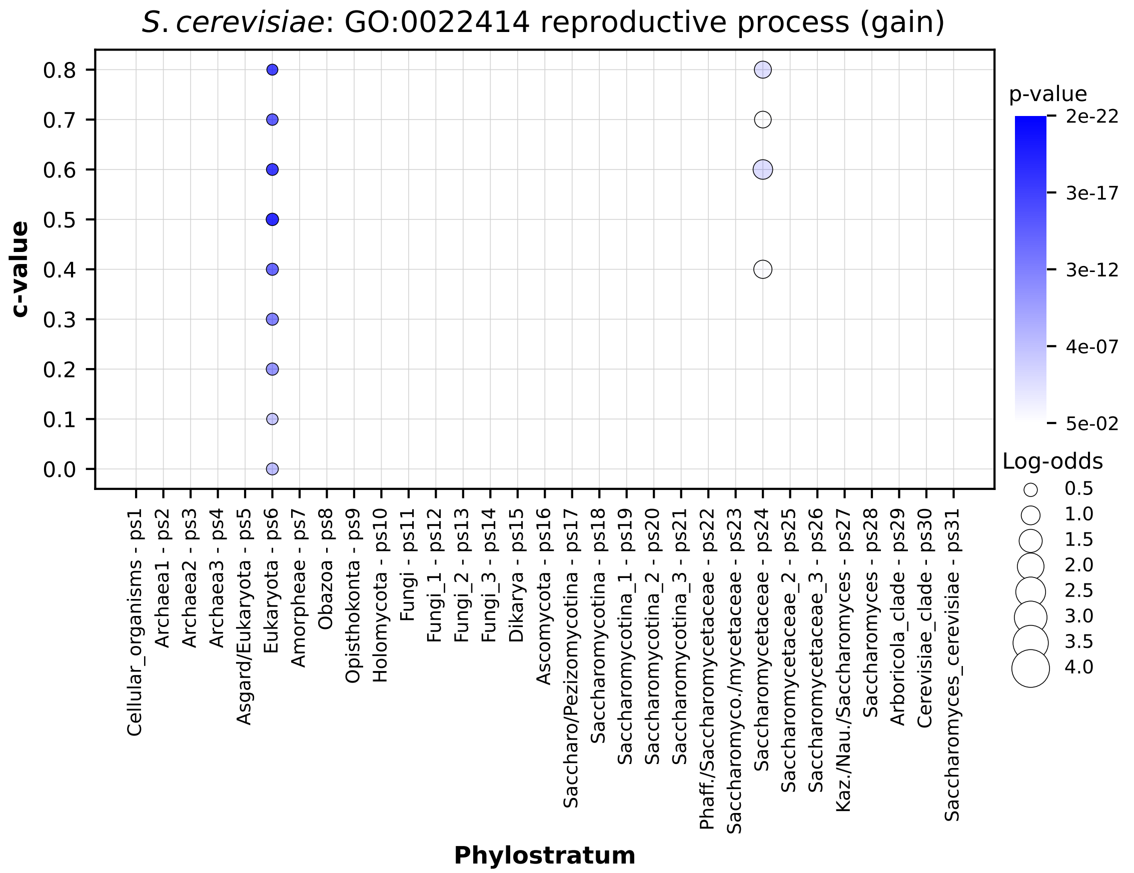

S. cerevisiae: GO:0022414 reproductive process (gain)
c-value
0.8
0.7
0.6
0.5
0.4
0.3
0.2
0.1
0.0
p-value
2e-22
3e-17
3e-12
4e-07
5e-02
Log-odds
0.5
1.0
1.5
2.0
2.5
3.0
3.5
4.0
Cellular_organisms - ps1
Archaea1 - ps2
Archaea2 - ps3
Archaea3 - ps4
Asgard/Eukaryota - ps5
Eukaryota - ps6
Amorpheae - ps7
Obazoa - ps8
Opisthokonta - ps9
Holomycota - ps10
Fungi - ps11
Fungi_1 - ps12
Fungi_2 - ps13
Fungi_3 - ps14
Dikarya - ps15
Ascomycota - ps16
Saccharo/Pezizomycotina - ps17
Saccharomycotina - ps18
Saccharomycotina_1 - ps19
Saccharomycotina_2 - ps20
Saccharomycotina_3 - ps21
Phaff./Saccharomycetaceae - ps22
Saccharomyco./mycetaceae - ps23
Saccharomycetaceae - ps24
Saccharomycetaceae_2 - ps25
Saccharomycetaceae_3 - ps26
Kaz./Nau./Saccharomyces - ps27
Saccharomyces - ps28
Arboricola_clade - ps29
Cerevisiae_clade - ps30
Saccharomyces_cerevisiae - ps31
Phylostratum

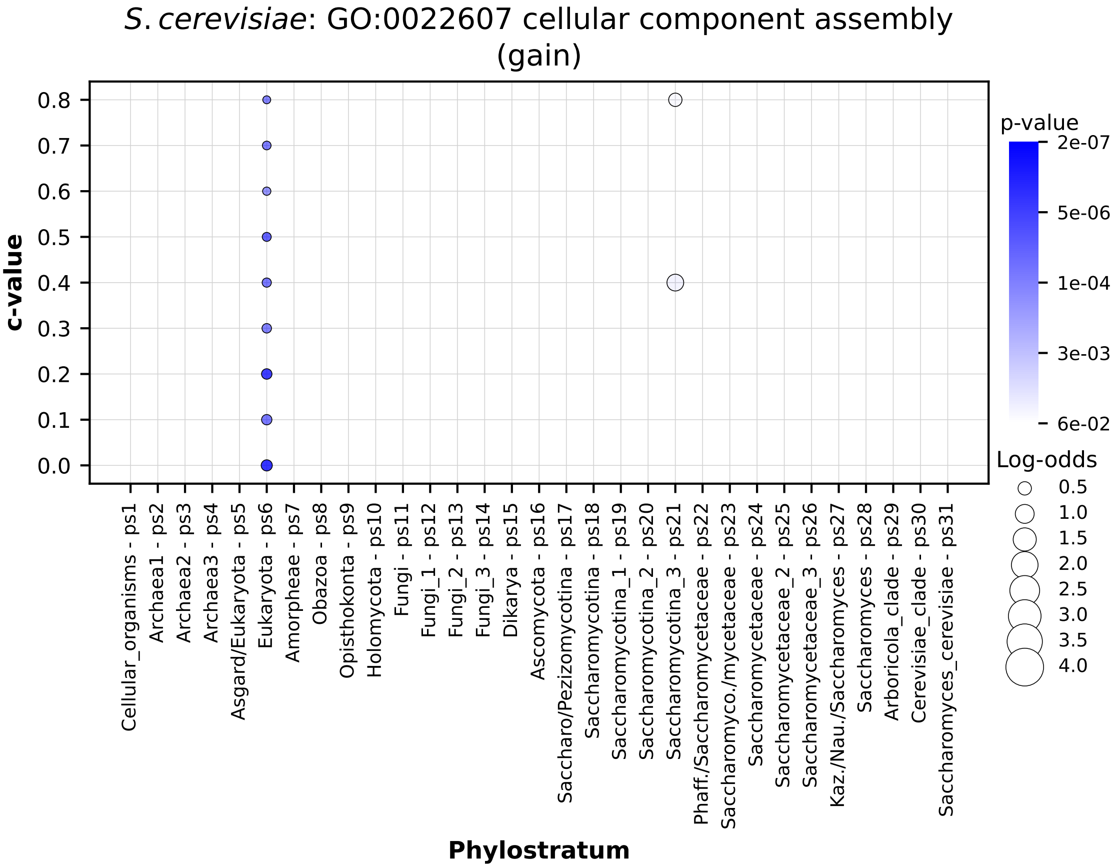

S. cerevisiae: GO:0022607 cellular component assembly (gain)
c-value
0.8
0.7
0.6
0.5
0.4
0.3
0.2
0.1
0.0
p-value
2e-07
5e-06
1e-04
3e-03
6e-02
Log-odds
0.5
1.0
1.5
2.0
2.5
3.0
3.5
4.0
Cellular_organisms - ps1
Archaea1 - ps2
Archaea2 - ps3
Archaea3 - ps4
Asgard/Eukaryota - ps5
Eukaryota - ps6
Amorpheae - ps7
Obazoa - ps8
Opisthokonta - ps9
Holomycota - ps10
Fungi - ps11
Fungi_1 - ps12
Fungi_2 - ps13
Fungi_3 - ps14
Dikarya - ps15
Ascomycota - ps16
Saccharo/Pezizomycotina - ps17
Saccharomycotina - ps18
Saccharomycotina_1 - ps19
Saccharomycotina_2 - ps20
Saccharomycotina_3 - ps21
Phaff./Saccharomycetaceae - ps22
Saccharomyco./mycetaceae - ps23
Saccharomycetaceae - ps24
Saccharomycetaceae_2 - ps25
Saccharomycetaceae_3 - ps26
Kaz./Nau./Saccharomyces - ps27
Saccharomyces - ps28
Arboricola_clade - ps29
Cerevisiae_clade - ps30
Saccharomyces_cerevisiae - ps31
Phylostratum

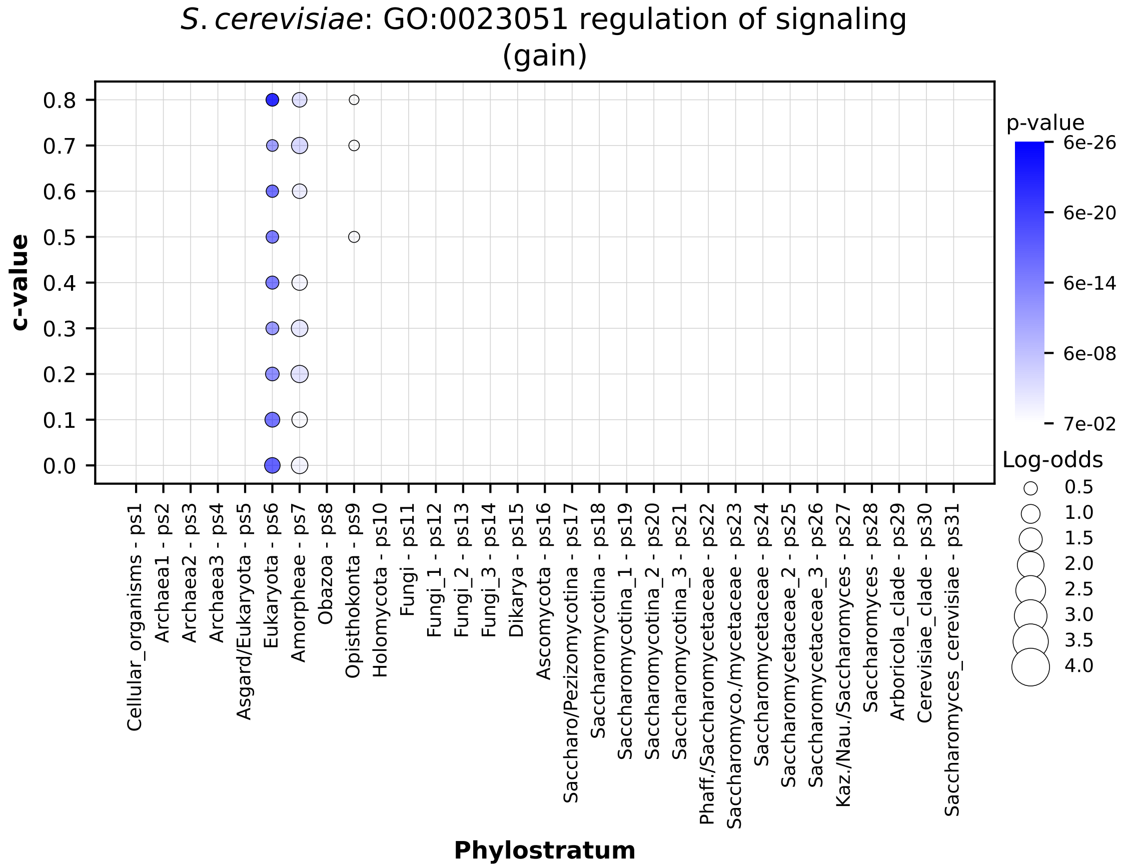

S. cerevisiae: GO:0023051 regulation of signaling
(gain)
c-value
0.8
0.7
0.6
0.5
0.4
0.3
0.2
0.1
0.0
Phylostratum
Cellular_organisms - ps1
Archaea1 - ps2
Archaea2 - ps3
Archaea3 - ps4
Asgard/Eukaryota - ps5
Eukaryota - ps6
Amorpheae - ps7
Obazoa - ps8
Opisthokonta - ps9
Holomycota - ps10
Fungi - ps11
Fungi_1 - ps12
Fungi_2 - ps13
Fungi_3 - ps14
Dikarya - ps15
Ascomycota - ps16
Saccharo/Pezizomycotina - ps17
Saccharomycotina - ps18
Saccharomycotina_1 - ps19
Saccharomycotina_2 - ps20
Saccharomycotina_3 - ps21
Phaff./Saccharomycetaceae - ps22
Saccharomyco./mycetaceae - ps23
Saccharomycetaceae - ps24
Saccharomycetaceae_2 - ps25
Saccharomycetaceae_3 - ps26
Kaz./Nau./Saccharomyces - ps27
Saccharomyces - ps28
Arboricola_clade - ps29
Cerevisiae_clade - ps30
Saccharomyces_cerevisiae - ps31
p-value
6e-26
6e-20
6e-14
6e-08
7e-02
Log-odds
0.5
1.0
1.5
2.0
2.5
3.0
3.5
4.0

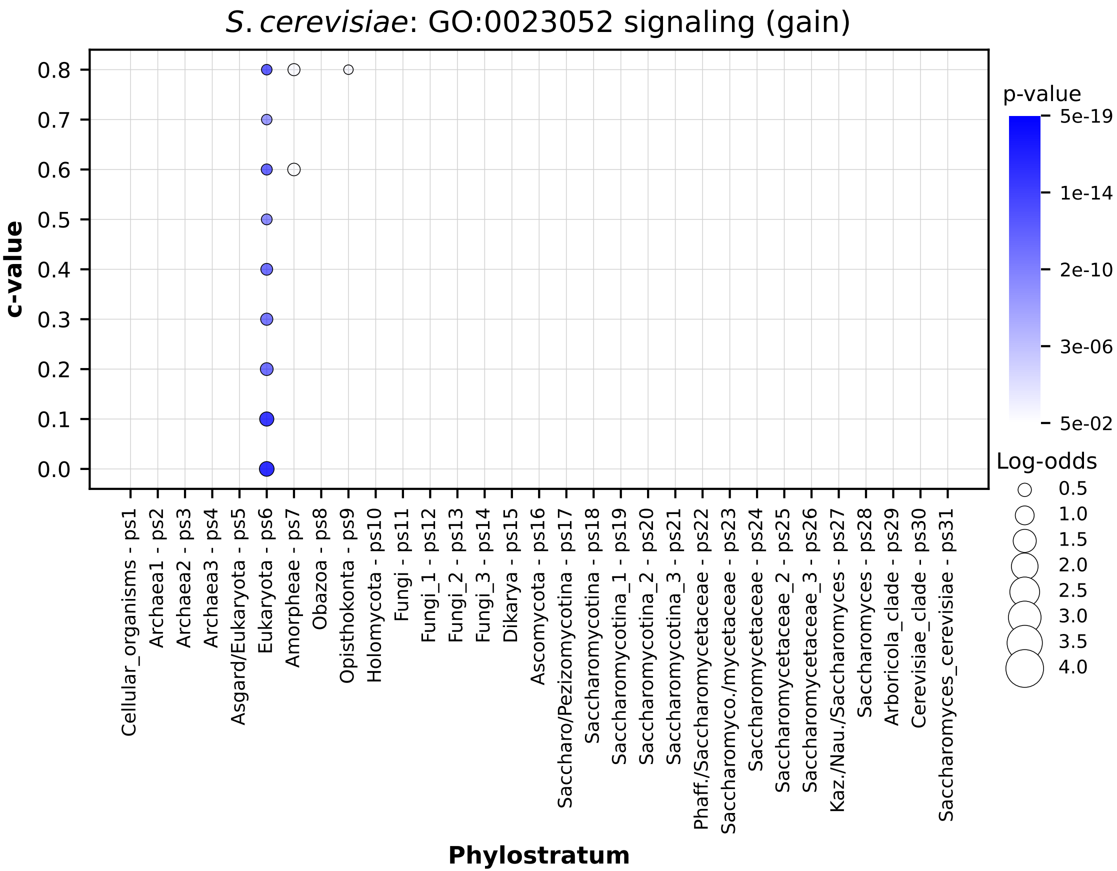

S. cerevisiae: GO:0023052 signaling (gain)
c-value
0.8
0.7
0.6
0.5
0.4
0.3
0.2
0.1
0.0
p-value
5e-19
1e-14
2e-10
3e-06
5e-02
Log-odds
0.5
1.0
1.5
2.0
2.5
3.0
3.5
4.0
Cellular_organisms - ps1
Archaea1 - ps2
Archaea2 - ps3
Archaea3 - ps4
Asgard/Eukaryota - ps5
Eukaryota - ps6
Amorpheae - ps7
Obazoa - ps8
Opisthokonta - ps9
Holomycota - ps10
Fungi - ps11
Fungi_1 - ps12
Fungi_2 - ps13
Fungi_3 - ps14
Dikarya - ps15
Ascomycota - ps16
Saccharo/Pezizomycotina - ps17
Saccharomycotina - ps18
Saccharomycotina_1 - ps19
Saccharomycotina_2 - ps20
Saccharomycotina_3 - ps21
Phaff./Saccharomycetaceae - ps22
Saccharomyco./mycetaceae - ps23
Saccharomycetaceae - ps24
Saccharomycetaceae_2 - ps25
Saccharomycetaceae_3 - ps26
Kaz./Nau./Saccharomyces - ps27
Saccharomyces - ps28
Arboricola_clade - ps29
Cerevisiae_clade - ps30
Saccharomyces_cerevisiae - ps31
Phylostratum

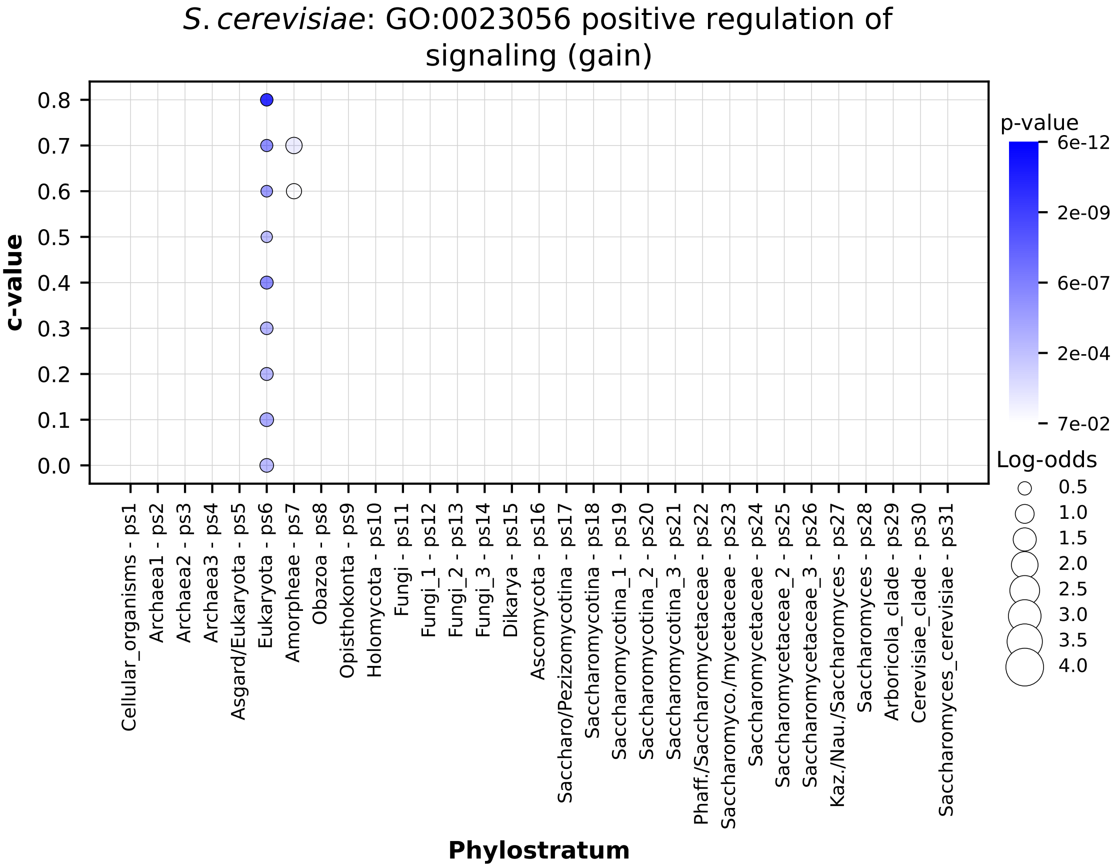

S. cerevisiae: GO:0023056 positive regulation of signaling (gain)
c-value
0.0
0.1
0.2
0.3
0.4
0.5
0.6
0.7
0.8
Phylostratum
Cellular_organisms - ps1
Archaea1 - ps2
Archaea2 - ps3
Archaea3 - ps4
Asgard/Eukaryota - ps5
Eukaryota - ps6
Amorpheae - ps7
Obazoa - ps8
Opisthokonta - ps9
Holomycota - ps10
Fungi - ps11
Fungi_1 - ps12
Fungi_2 - ps13
Fungi_3 - ps14
Dikarya - ps15
Ascomycota - ps16
Saccharo/Pezizomycotina - ps17
Saccharomycotina - ps18
Saccharomycotina_1 - ps19
Saccharomycotina_2 - ps20
Saccharomycotina_3 - ps21
Phaff./Saccharomycetaceae - ps22
Saccharomyco./mycetaceae - ps23
Saccharomycetaceae - ps24
Saccharomycetaceae_2 - ps25
Saccharomycetaceae_3 - ps26
Kaz./Nau./Saccharomyces - ps27
Saccharomyces - ps28
Arboricola_clade - ps29
Cerevisiae_clade - ps30
Saccharomyces_cerevisiae - ps31
p-value
6e-12
2e-09
6e-07
2e-04
7e-02
Log-odds
0.5
1.0
1.5
2.0
2.5
3.0
3.5
4.0

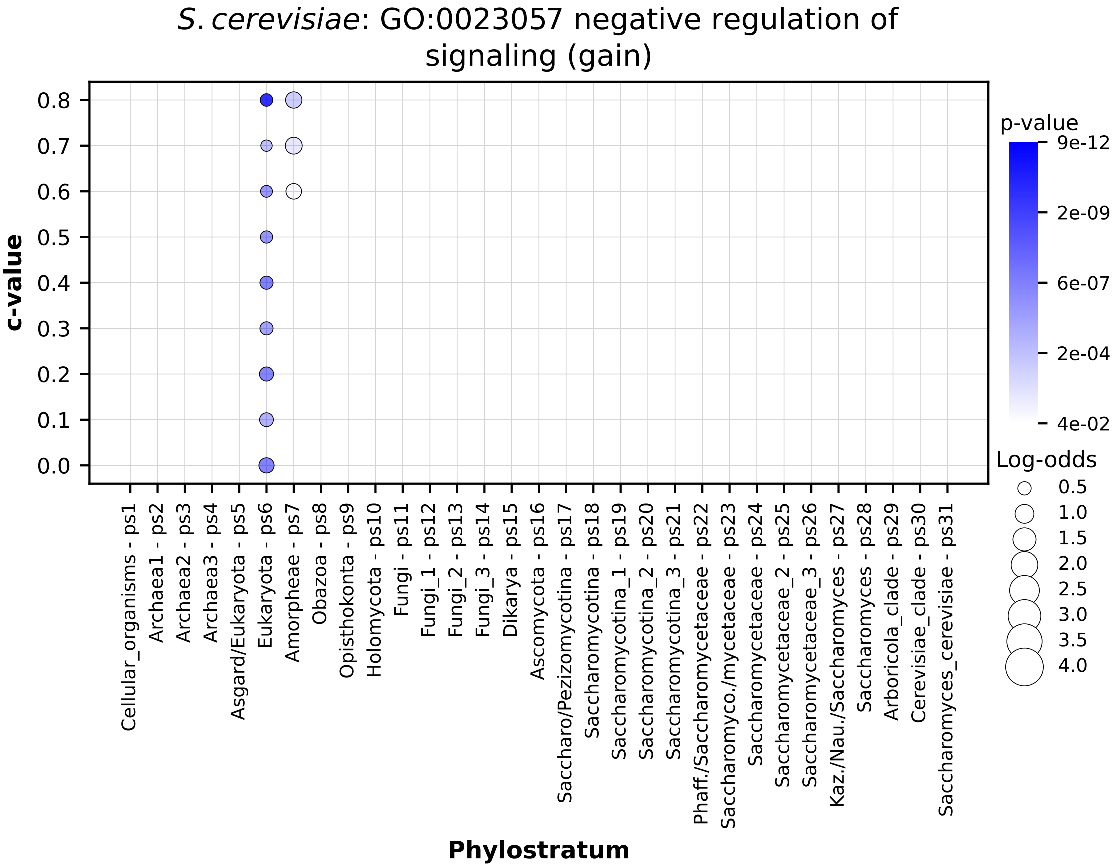

S. cerevisiae: GO:0023057 negative regulation of signaling (gain)
c-value
0.8
0.7
0.6
0.5
0.4
0.3
0.2
0.1
0.0
Phylostratum
Cellular_organisms - ps1
Archaea1 - ps2
Archaea2 - ps3
Archaea3 - ps4
Asgard/Eukaryota - ps5
Eukaryota - ps6
Amorpheae - ps7
Obazoa - ps8
Opisthokonta - ps9
Holomycota - ps10
Fungi - ps11
Fungi_1 - ps12
Fungi_2 - ps13
Fungi_3 - ps14
Dikarya - ps15
Ascomycota - ps16
Saccharo/Pezizomycotina - ps17
Saccharomycotina - ps18
Saccharomycotina_1 - ps19
Saccharomycotina_2 - ps20
Saccharomycotina_3 - ps21
Phaff./Saccharomycetaceae - ps22
Saccharomyco./mycetaceae - ps23
Saccharomycetaceae - ps24
Saccharomycetaceae_2 - ps25
Saccharomycetaceae_3 - ps26
Kaz./Nau./Saccharomyces - ps27
Saccharomyces - ps28
Arboricola_clade - ps29
Cerevisiae_clade - ps30
Saccharomyces_cerevisiae - ps31
p-value
9e-12
2e-09
6e-07
2e-04
4e-02
Log-odds
0.5
1.0
1.5
2.0
2.5
3.0
3.5
4.0

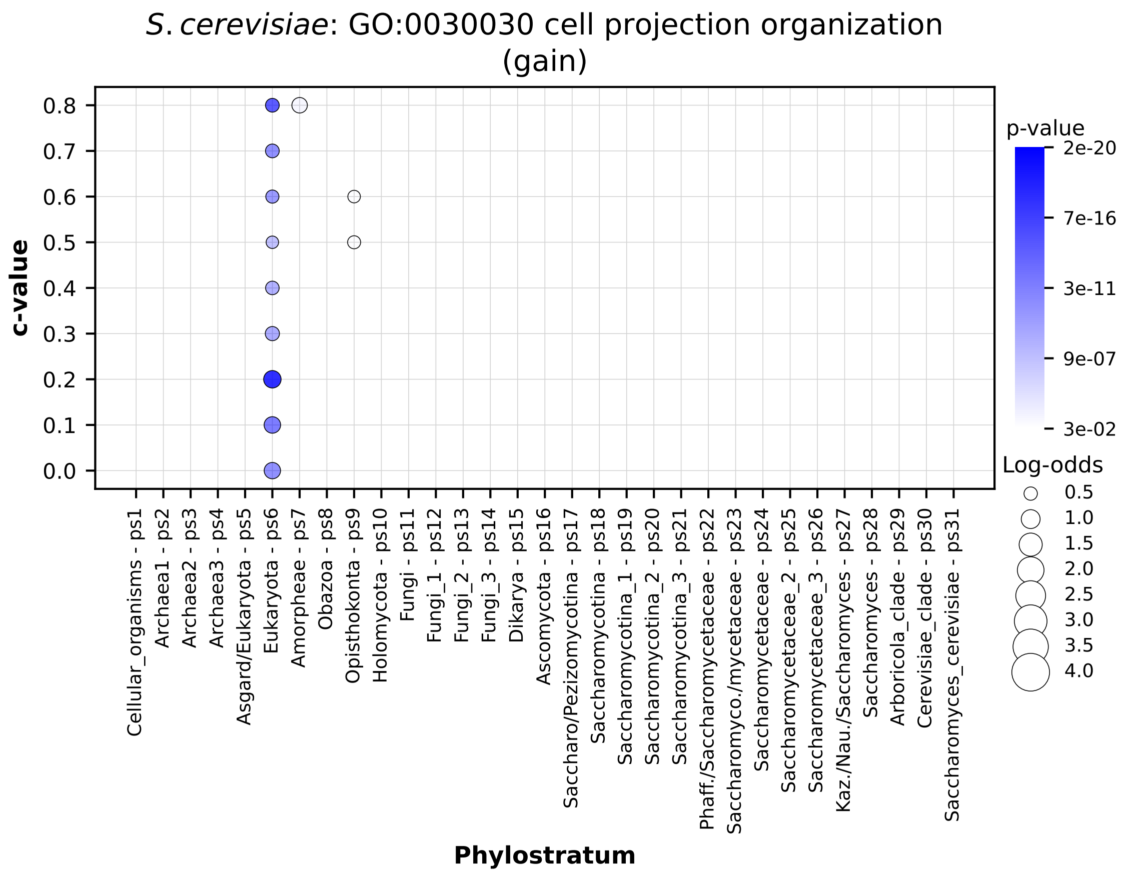

S. cerevisiae: GO:0030030 cell projection organization
(gain)
c-value
0.8
0.7
0.6
0.5
0.4
0.3
0.2
0.1
0.0
p-value
2e-20
7e-16
3e-11
9e-07
3e-02
Log-odds
0.5
1.0
1.5
2.0
2.5
3.0
3.5
4.0
Cellular_organisms - ps1
Archaea1 - ps2
Archaea2 - ps3
Archaea3 - ps4
Asgard/Eukaryota - ps5
Eukaryota - ps6
Amorpheae - ps7
Obazoa - ps8
Opisthokonta - ps9
Holomycota - ps10
Fungi - ps11
Fungi_1 - ps12
Fungi_2 - ps13
Fungi_3 - ps14
Dikarya - ps15
Ascomycota - ps16
Saccharo/Pezizomycotina - ps17
Saccharomycotina - ps18
Saccharomycotina_1 - ps19
Saccharomycotina_2 - ps20
Saccharomycotina_3 - ps21
Phaff./Saccharomycetaceae - ps22
Saccharomyco./mycetaceae - ps23
Saccharomycetaceae - ps24
Saccharomycetaceae_2 - ps25
Saccharomycetaceae_3 - ps26
Kaz./Nau./Saccharomyces - ps27
Saccharomyces - ps28
Arboricola_clade - ps29
Cerevisiae_clade - ps30
Saccharomyces_cerevisiae - ps31
Phylostratum

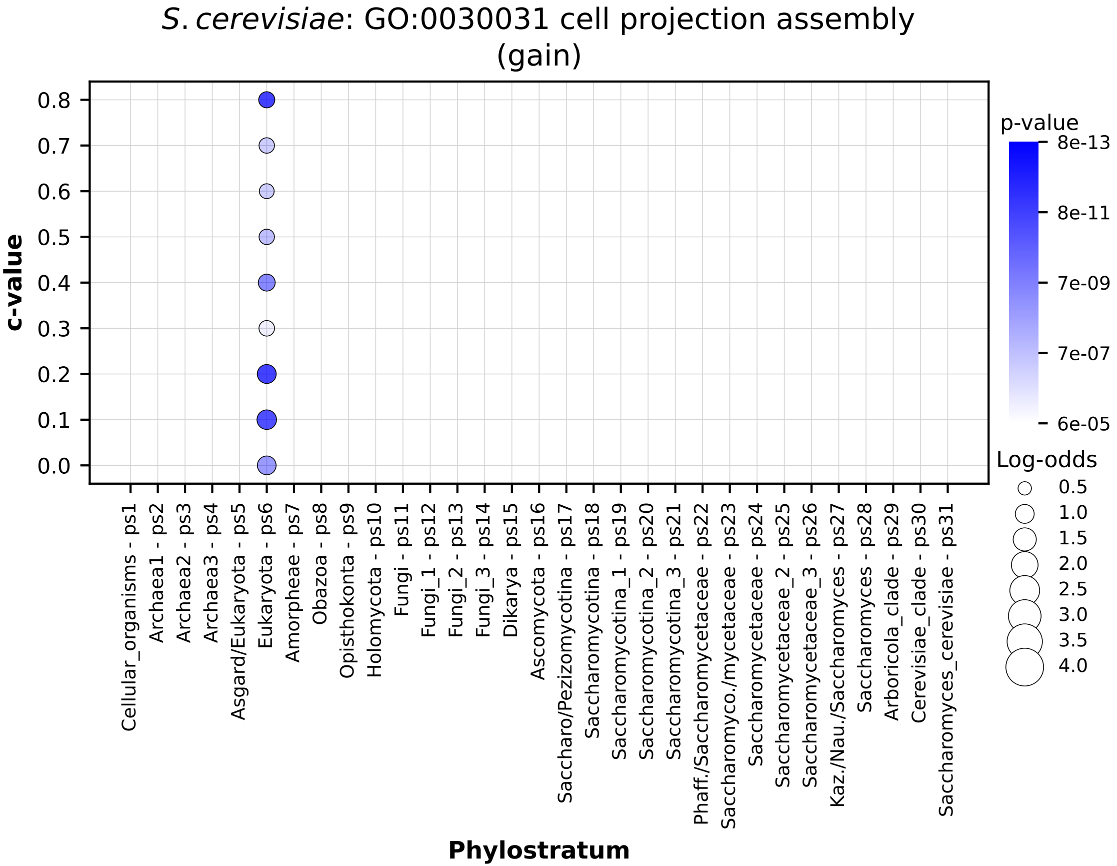

S. cerevisiae: GO:0030031 cell projection assembly
(gain)
c-value
0.8
0.7
0.6
0.5
0.4
0.3
0.2
0.1
0.0
p-value
8e-13
8e-11
7e-09
7e-07
6e-05
Log-odds
0.5
1.0
1.5
2.0
2.5
3.0
3.5
4.0
Cellular_organisms - ps1
Archaea1 - ps2
Archaea2 - ps3
Archaea3 - ps4
Asgard/Eukaryota - ps5
Eukaryota - ps6
Amorpheae - ps7
Obazoa - ps8
Opisthokonta - ps9
Holomycota - ps10
Fungi - ps11
Fungi_1 - ps12
Fungi_2 - ps13
Fungi_3 - ps14
Dikarya - ps15
Ascomycota - ps16
Saccharo/Pezizomycotina - ps17
Saccharomycotina - ps18
Saccharomycotina_1 - ps19
Saccharomycotina_2 - ps20
Saccharomycotina_3 - ps21
Phaff./Saccharomycetaceae - ps22
Saccharomyco./mycetaceae - ps23
Saccharomycetaceae - ps24
Saccharomycetaceae_2 - ps25
Saccharomycetaceae_3 - ps26
Kaz./Nau./Saccharomyces - ps27
Saccharomyces - ps28
Arboricola_clade - ps29
Cerevisiae_clade - ps30
Saccharomyces_cerevisiae - ps31
Phylostratum

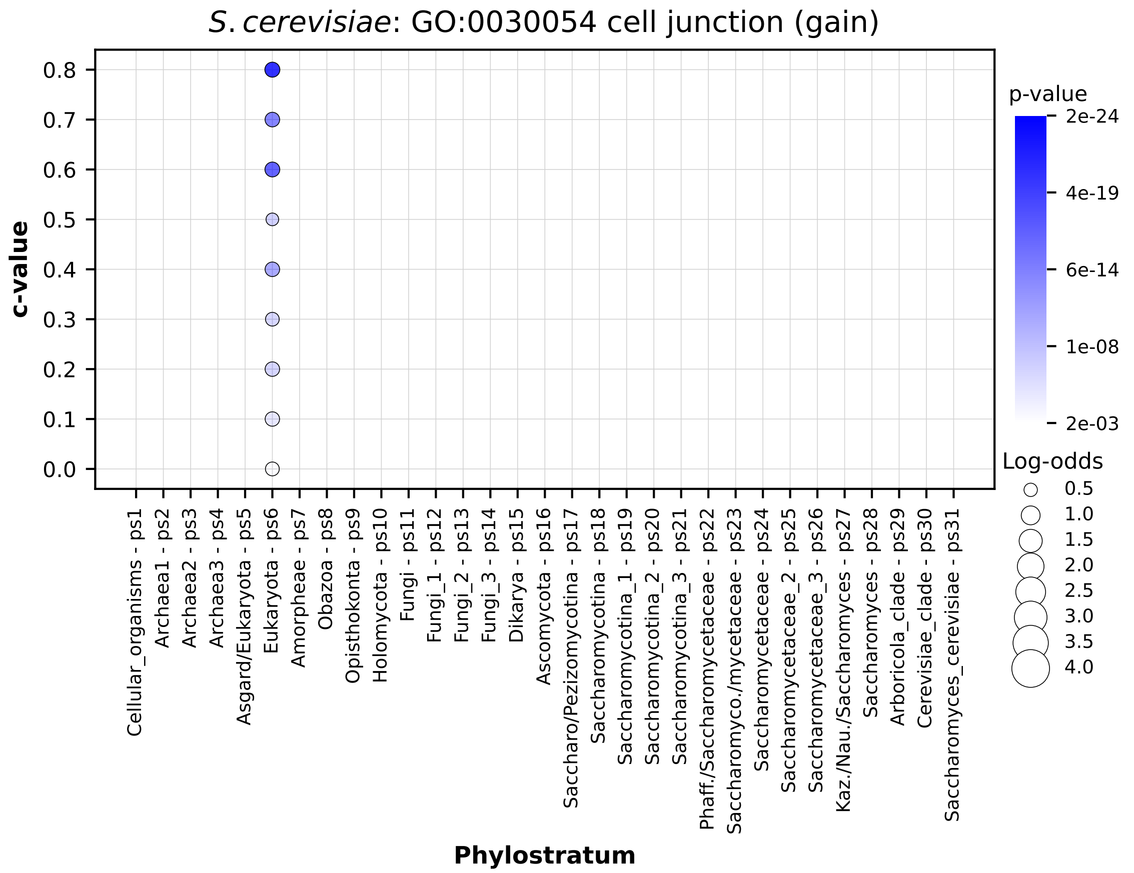

S. cerevisiae: GO:0030054 cell junction (gain)
c-value
0.0
0.1
0.2
0.3
0.4
0.5
0.6
0.7
0.8
Phylostratum
Cellular_organisms - ps1
Archaea1 - ps2
Archaea2 - ps3
Archaea3 - ps4
Asgard/Eukaryota - ps5
Eukaryota - ps6
Amorpheae - ps7
Obazoa - ps8
Opisthokonta - ps9
Holomycota - ps10
Fungi - ps11
Fungi_1 - ps12
Fungi_2 - ps13
Fungi_3 - ps14
Dikarya - ps15
Ascomycota - ps16
Saccharo/Pezizomycotina - ps17
Saccharomycotina - ps18
Saccharomycotina_1 - ps19
Saccharomycotina_2 - ps20
Saccharomycotina_3 - ps21
Phaff./Saccharomycetaceae - ps22
Saccharomyco./mycetaceae - ps23
Saccharomycetaceae - ps24
Saccharomycetaceae_2 - ps25
Saccharomycetaceae_3 - ps26
Kaz./Nau./Saccharomyces - ps27
Saccharomyces - ps28
Arboricola_clade - ps29
Cerevisiae_clade - ps30
Saccharomyces_cerevisiae - ps31
p-value
2e-24
4e-19
6e-14
1e-08
2e-03
Log-odds
0.5
1.0
1.5
2.0
2.5
3.0
3.5
4.0

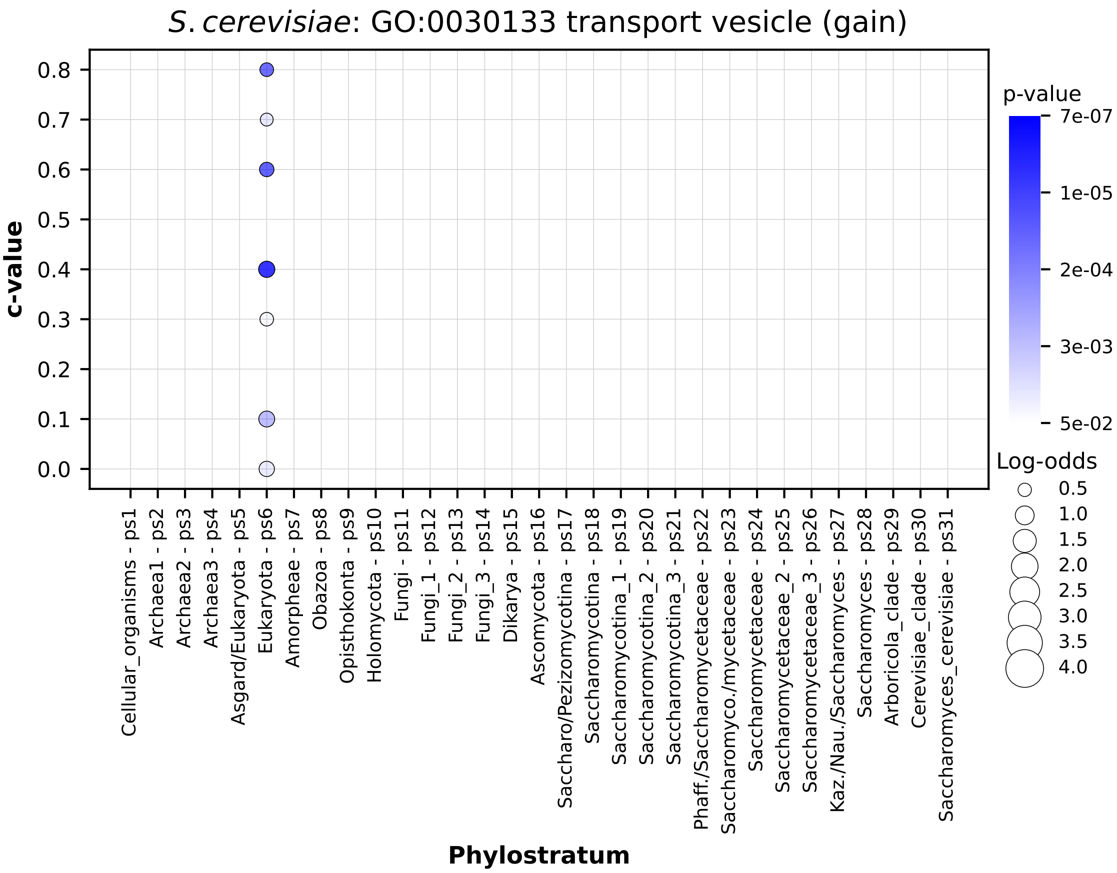

S. cerevisiae: GO:0030133 transport vesicle (gain)
c-value
0.0
0.1
0.2
0.3
0.4
0.5
0.6
0.7
0.8
p-value
7e-07
1e-05
2e-04
3e-03
5e-02
Log-odds
0.5
1.0
1.5
2.0
2.5
3.0
3.5
4.0
Cellular_organisms - ps1
Archaea1 - ps2
Archaea2 - ps3
Archaea3 - ps4
Asgard/Eukaryota - ps5
Eukaryota - ps6
Amorpheae - ps7
Obazoa - ps8
Opisthokonta - ps9
Holomycota - ps10
Fungi - ps11
Fungi_1 - ps12
Fungi_2 - ps13
Fungi_3 - ps14
Dikarya - ps15
Ascomycota - ps16
Saccharo/Pezizomycotina - ps17
Saccharomycotina - ps18
Saccharomycotina_1 - ps19
Saccharomycotina_2 - ps20
Saccharomycotina_3 - ps21
Phaff./Saccharomycetaceae - ps22
Saccharomyco./mycetaceae - ps23
Saccharomycetaceae - ps24
Saccharomycetaceae_2 - ps25
Saccharomycetaceae_3 - ps26
Kaz./Nau./Saccharomyces - ps27
Saccharomyces - ps28
Arboricola_clade - ps29
Cerevisiae_clade - ps30
Saccharomyces_cerevisiae - ps31
Phylostratum

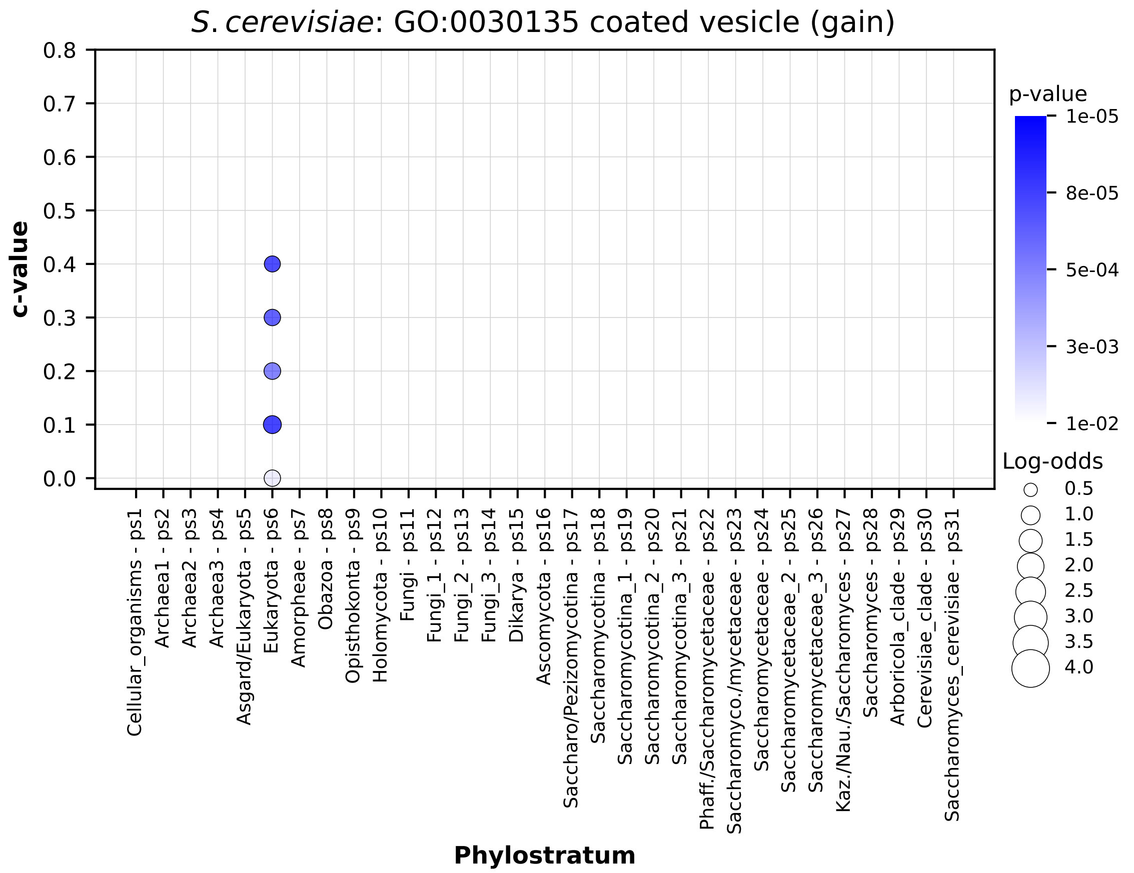

S. cerevisiae: GO:0030135 coated vesicle (gain)
c-value
0.8
0.7
0.6
0.5
0.4
0.3
0.2
0.1
0.0
p-value
1e-05
8e-05
5e-04
3e-03
1e-02
Log-odds
0.5
1.0
1.5
2.0
2.5
3.0
3.5
4.0
Cellular_organisms - ps1
Archaea1 - ps2
Archaea2 - ps3
Archaea3 - ps4
Asgard/Eukaryota - ps5
Eukaryota - ps6
Amorpheae - ps7
Obazoa - ps8
Opisthokonta - ps9
Holomycota - ps10
Fungi - ps11
Fungi_1 - ps12
Fungi_2 - ps13
Fungi_3 - ps14
Dikarya - ps15
Ascomycota - ps16
Saccharo/Pezizomycotina - ps17
Saccharomycotina - ps18
Saccharomycotina_1 - ps19
Saccharomycotina_2 - ps20
Saccharomycotina_3 - ps21
Phaff./Saccharomycetaceae - ps22
Saccharomyco./mycetaceae - ps23
Saccharomycetaceae - ps24
Saccharomycetaceae_2 - ps25
Saccharomycetaceae_3 - ps26
Kaz./Nau./Saccharomyces - ps27
Saccharomyces - ps28
Arboricola_clade - ps29
Cerevisiae_clade - ps30
Saccharomyces_cerevisiae - ps31
Phylostratum

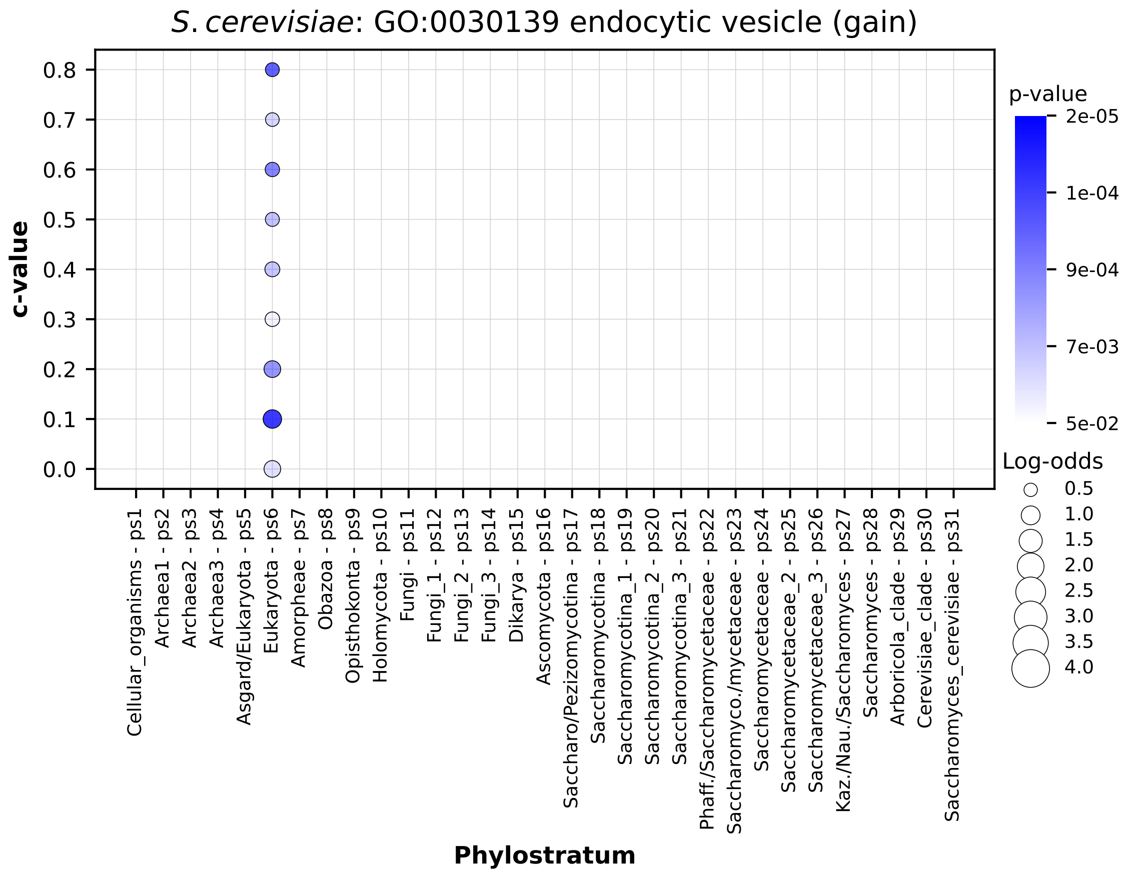

S. cerevisiae: GO:0030139 endocytic vesicle (gain)
c-value
0.0
0.1
0.2
0.3
0.4
0.5
0.6
0.7
0.8
p-value
2e-05
1e-04
9e-04
7e-03
5e-02
Log-odds
0.5
1.0
1.5
2.0
2.5
3.0
3.5
4.0
Cellular_organisms - ps1
Archaea1 - ps2
Archaea2 - ps3
Archaea3 - ps4
Asgard/Eukaryota - ps5
Eukaryota - ps6
Amorpheae - ps7
Obazoa - ps8
Opisthokonta - ps9
Holomycota - ps10
Fungi - ps11
Fungi_1 - ps12
Fungi_2 - ps13
Fungi_3 - ps14
Dikarya - ps15
Ascomycota - ps16
Saccharo/Pezizomycotina - ps17
Saccharomycotina - ps18
Saccharomycotina_1 - ps19
Saccharomycotina_2 - ps20
Saccharomycotina_3 - ps21
Phaff./Saccharomycetaceae - ps22
Saccharomyco./mycetaceae - ps23
Saccharomycetaceae - ps24
Saccharomycetaceae_2 - ps25
Saccharomycetaceae_3 - ps26
Kaz./Nau./Saccharomyces - ps27
Saccharomyces - ps28
Arboricola_clade - ps29
Cerevisiae_clade - ps30
Saccharomyces_cerevisiae - ps31
Phylostratum

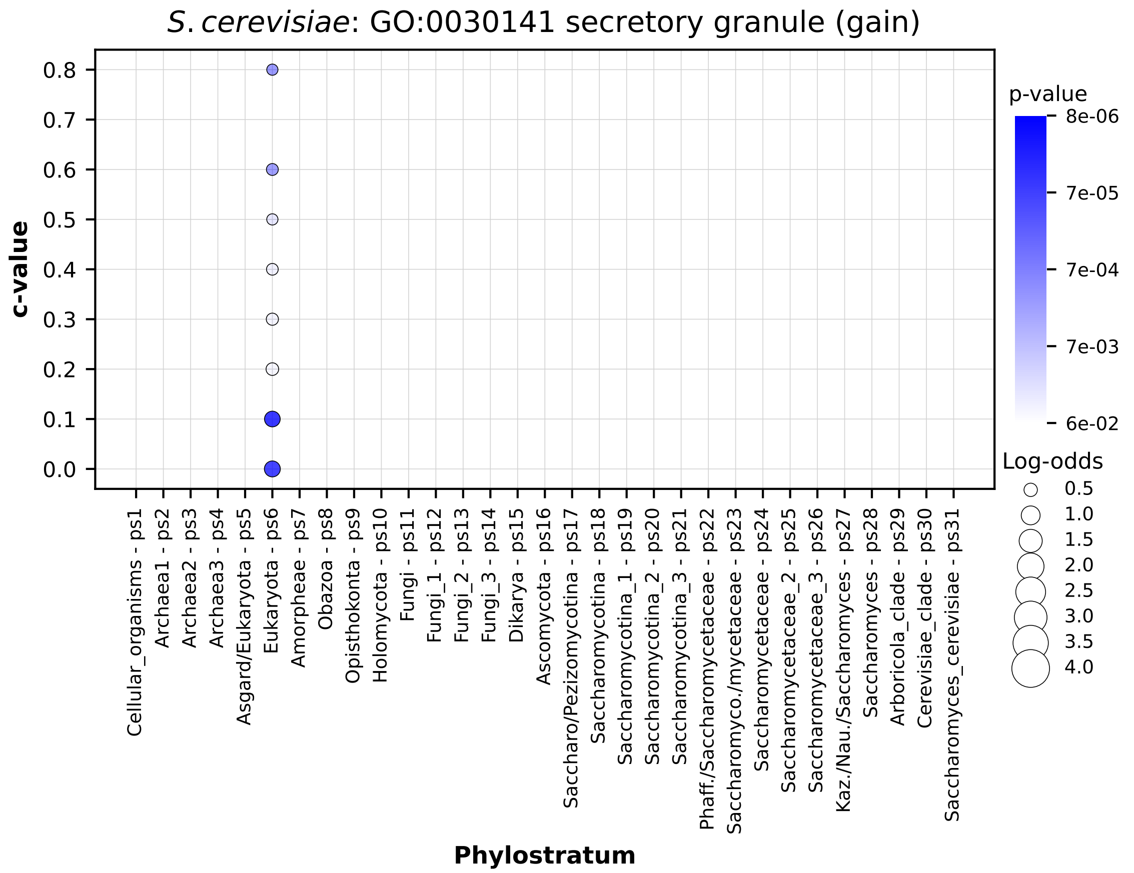

S. cerevisiae: GO:0030141 secretory granule (gain)
c-value
0.0
0.1
0.2
0.3
0.4
0.5
0.6
0.7
0.8
p-value
8e-06
7e-05
7e-04
7e-03
6e-02
Log-odds
0.5
1.0
1.5
2.0
2.5
3.0
3.5
4.0
Cellular_organisms - ps1
Archaea1 - ps2
Archaea2 - ps3
Archaea3 - ps4
Asgard/Eukaryota - ps5
Eukaryota - ps6
Amorpheae - ps7
Obazoa - ps8
Opisthokonta - ps9
Holomycota - ps10
Fungi - ps11
Fungi_1 - ps12
Fungi_2 - ps13
Fungi_3 - ps14
Dikarya - ps15
Ascomycota - ps16
Saccharo/Pezizomycotina - ps17
Saccharomycotina - ps18
Saccharomycotina_1 - ps19
Saccharomycotina_2 - ps20
Saccharomycotina_3 - ps21
Phaff./Saccharomycetaceae - ps22
Saccharomyco./mycetaceae - ps23
Saccharomycetaceae - ps24
Saccharomycetaceae_2 - ps25
Saccharomycetaceae_3 - ps26
Kaz./Nau./Saccharomyces - ps27
Saccharomyces - ps28
Arboricola_clade - ps29
Cerevisiae_clade - ps30
Saccharomyces_cerevisiae - ps31
Phylostratum

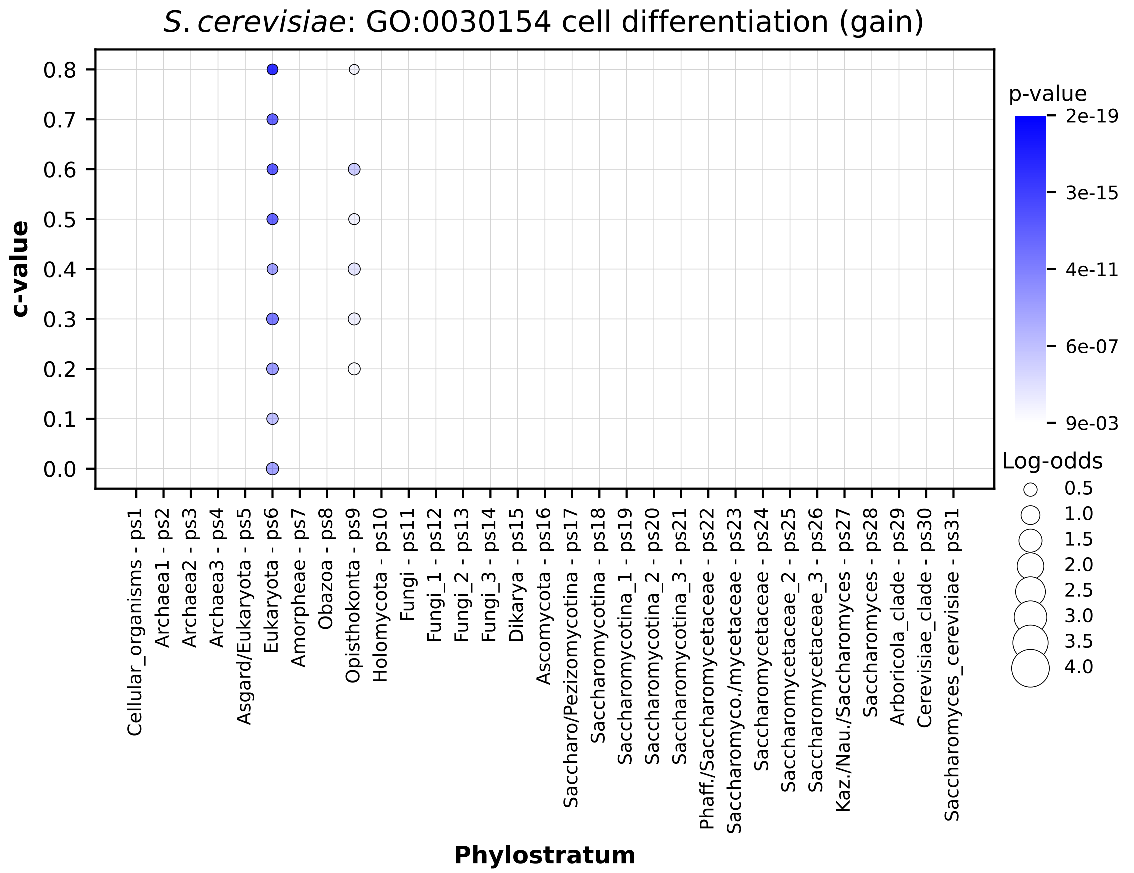
S. cerevisiae: GO:0030154 cell differentiation (gain)
c-value
0.8
0.7
0.6
0.5
0.4
0.3
0.2
0.1
0.0
p-value
2e-19
3e-15
4e-11
6e-07
9e-03
Log-odds
0.5
1.0
1.5
2.0
2.5
3.0
3.5
4.0
Cellular_organisms - ps1
Archaea1 - ps2
Archaea2 - ps3
Archaea3 - ps4
Asgard/Eukaryota - ps5
Eukaryota - ps6
Amorpheae - ps7
Obazoa - ps8
Opisthokonta - ps9
Holomycota - ps10
Fungi - ps11
Fungi_1 - ps12
Fungi_2 - ps13
Fungi_3 - ps14
Dikarya - ps15
Ascomycota - ps16
Saccharo/Pezizomycotina - ps17
Saccharomycotina - ps18
Saccharomycotina_1 - ps19
Saccharomycotina_2 - ps20
Saccharomycotina_3 - ps21
Phaff./Saccharomycetaceae - ps22
Saccharomyco./mycetaceae - ps23
Saccharomycetaceae - ps24
Saccharomycetaceae_2 - ps25
Saccharomycetaceae_3 - ps26
Kaz./Nau./Saccharomyces - ps27
Saccharomyces - ps28
Arboricola_clade - ps29
Cerevisiae_clade - ps30
Saccharomyces_cerevisiae - ps31
Phylostratum

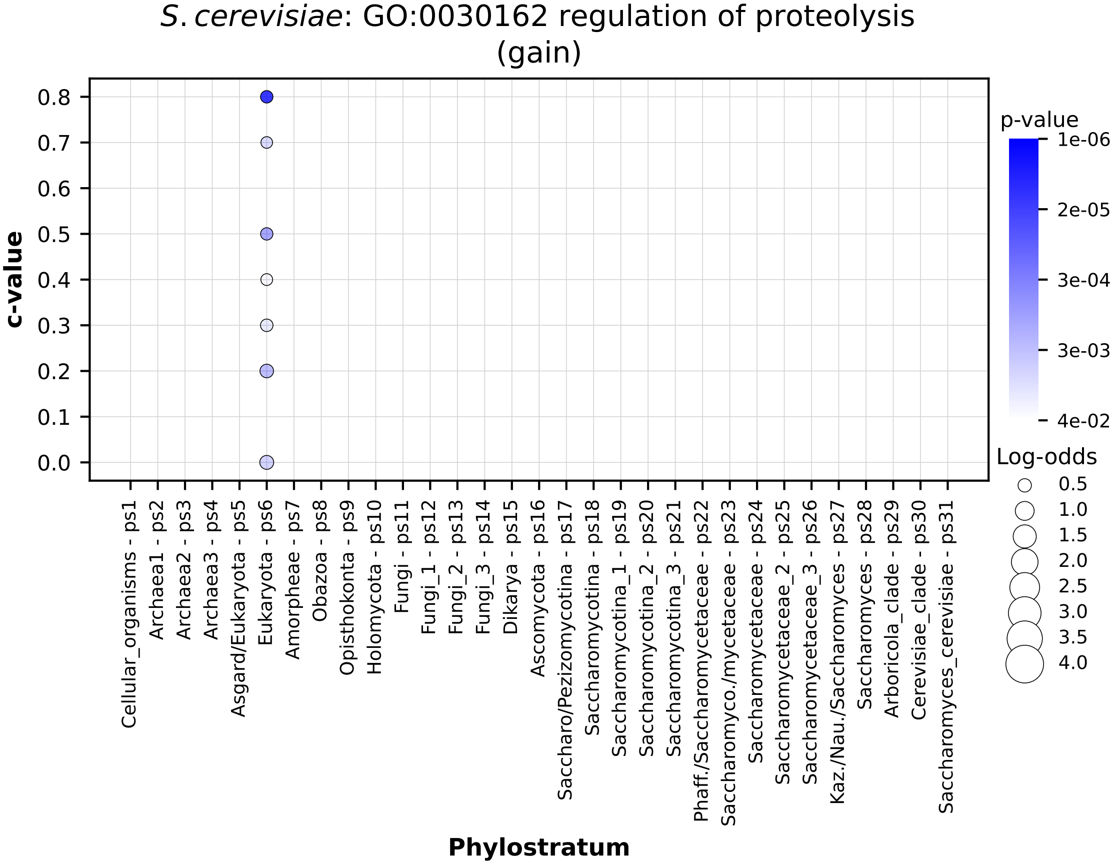

S. cerevisiae: GO:0030162 regulation of proteolysis (gain)
c-value
0.8
0.7
0.6
0.5
0.4
0.3
0.2
0.1
0.0
p-value
1e-06
2e-05
3e-04
3e-03
4e-02
Log-odds
0.5
1.0
1.5
2.0
2.5
3.0
3.5
4.0
Cellular_organisms - ps1
Archaea1 - ps2
Archaea2 - ps3
Archaea3 - ps4
Asgard/Eukaryota - ps5
Eukaryota - ps6
Amorpheae - ps7
Obazoa - ps8
Opisthokonta - ps9
Holomycota - ps10
Fungi - ps11
Fungi_1 - ps12
Fungi_2 - ps13
Fungi_3 - ps14
Dikarya - ps15
Ascomycota - ps16
Saccharo/Pezizomycotina - ps17
Saccharomycotina - ps18
Saccharomycotina_1 - ps19
Saccharomycotina_2 - ps20
Saccharomycotina_3 - ps21
Phaff./Saccharomycetaceae - ps22
Saccharomyco./mycetaceae - ps23
Saccharomycetaceae - ps24
Saccharomycetaceae_2 - ps25
Saccharomycetaceae_3 - ps26
Kaz./Nau./Saccharomyces - ps27
Saccharomyces - ps28
Arboricola_clade - ps29
Cerevisiae_clade - ps30
Saccharomyces_cerevisiae - ps31
Phylostratum

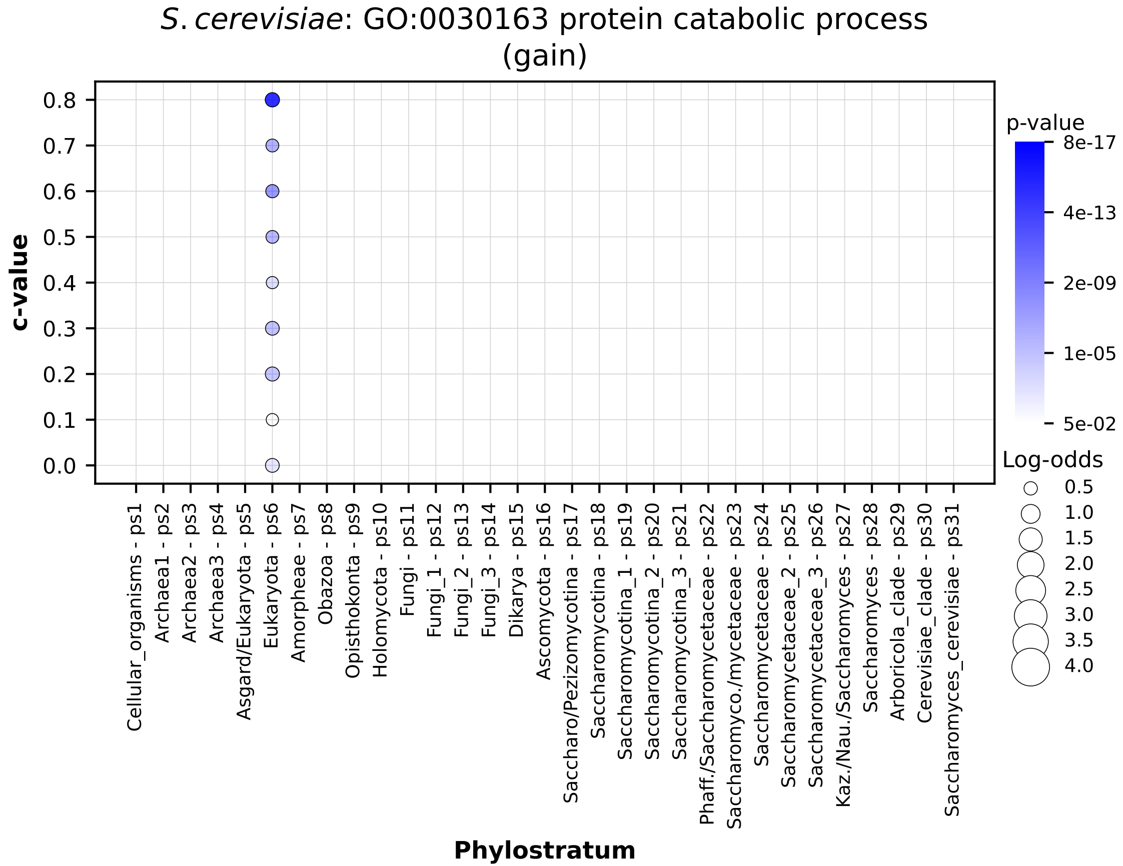

S. cerevisiae: GO:0030163 protein catabolic process (gain)
c-value
0.0
0.1
0.2
0.3
0.4
0.5
0.6
0.7
0.8
Phylostratum
Cellular_organisms - ps1
Archaea1 - ps2
Archaea2 - ps3
Archaea3 - ps4
Asgard/Eukaryota - ps5
Eukaryota - ps6
Amorpheae - ps7
Obazoa - ps8
Opisthokonta - ps9
Holomycota - ps10
Fungi - ps11
Fungi_1 - ps12
Fungi_2 - ps13
Fungi_3 - ps14
Dikarya - ps15
Ascomycota - ps16
Saccharo/Pezizomycotina - ps17
Saccharomycotina - ps18
Saccharomycotina_1 - ps19
Saccharomycotina_2 - ps20
Saccharomycotina_3 - ps21
Phaff./Saccharomycetaceae - ps22
Saccharomyco./mycetaceae - ps23
Saccharomycetaceae - ps24
Saccharomycetaceae_2 - ps25
Saccharomycetaceae_3 - ps26
Kaz./Nau./Saccharomyces - ps27
Saccharomyces - ps28
Arboricola_clade - ps29
Cerevisiae_clade - ps30
Saccharomyces_cerevisiae - ps31
p-value
8e-17
4e-13
2e-09
1e-05
5e-02
Log-odds
0.5
1.0
1.5
2.0
2.5
3.0
3.5
4.0

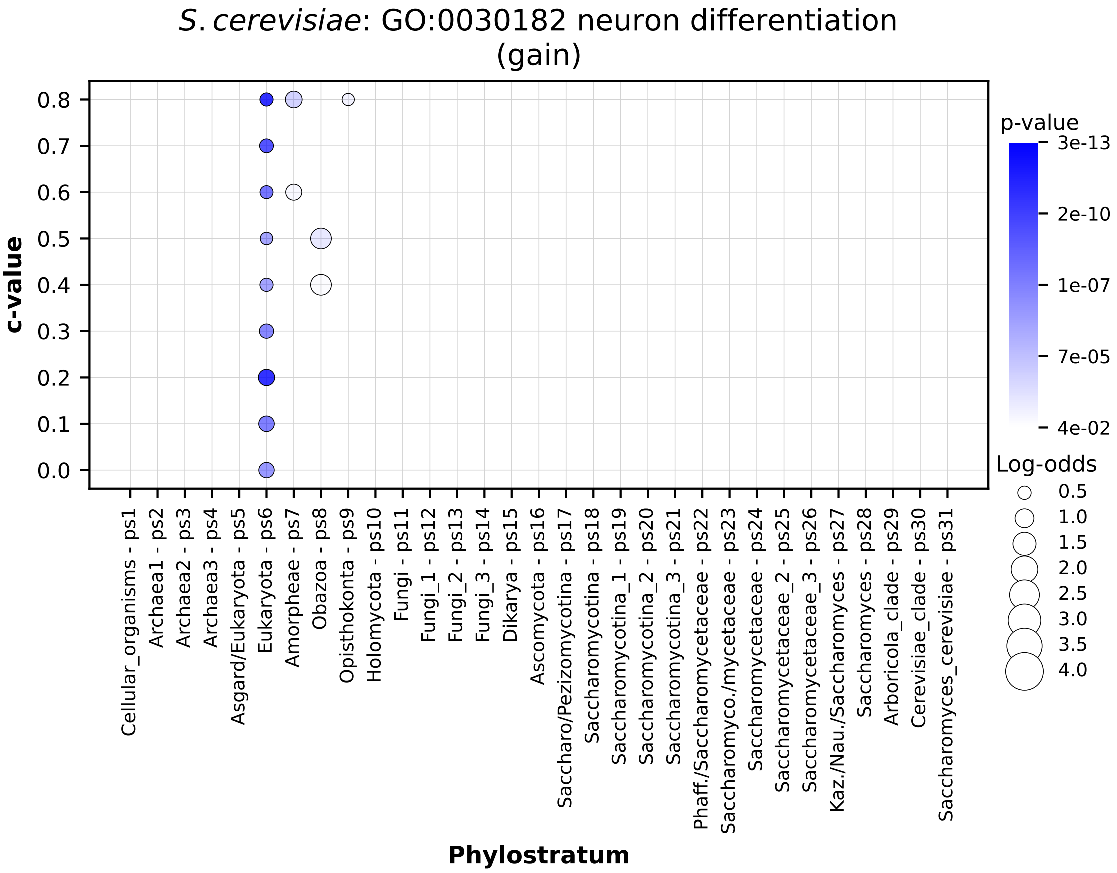

S. cerevisiae: GO:0030182 neuron differentiation
(gain)
c-value
0.0
0.1
0.2
0.3
0.4
0.5
0.6
0.7
0.8
Phylostratum
Cellular_organisms - ps1
Archaea1 - ps2
Archaea2 - ps3
Archaea3 - ps4
Asgard/Eukaryota - ps5
Eukaryota - ps6
Amorpheae - ps7
Obazoa - ps8
Opisthokonta - ps9
Holomycota - ps10
Fungi - ps11
Fungi_1 - ps12
Fungi_2 - ps13
Fungi_3 - ps14
Dikarya - ps15
Ascomycota - ps16
Saccharo/Pezizomycotina - ps17
Saccharomycotina - ps18
Saccharomycotina_1 - ps19
Saccharomycotina_2 - ps20
Saccharomycotina_3 - ps21
Phaff./Saccharomycetaceae - ps22
Saccharomyco./mycetaceae - ps23
Saccharomycetaceae - ps24
Saccharomycetaceae_2 - ps25
Saccharomycetaceae_3 - ps26
Kaz./Nau./Saccharomyces - ps27
Saccharomyces - ps28
Arboricola_clade - ps29
Cerevisiae_clade - ps30
Saccharomyces_cerevisiae - ps31
p-value
3e-13
2e-10
1e-07
7e-05
4e-02
Log-odds
0.5
1.0
1.5
2.0
2.5
3.0
3.5
4.0

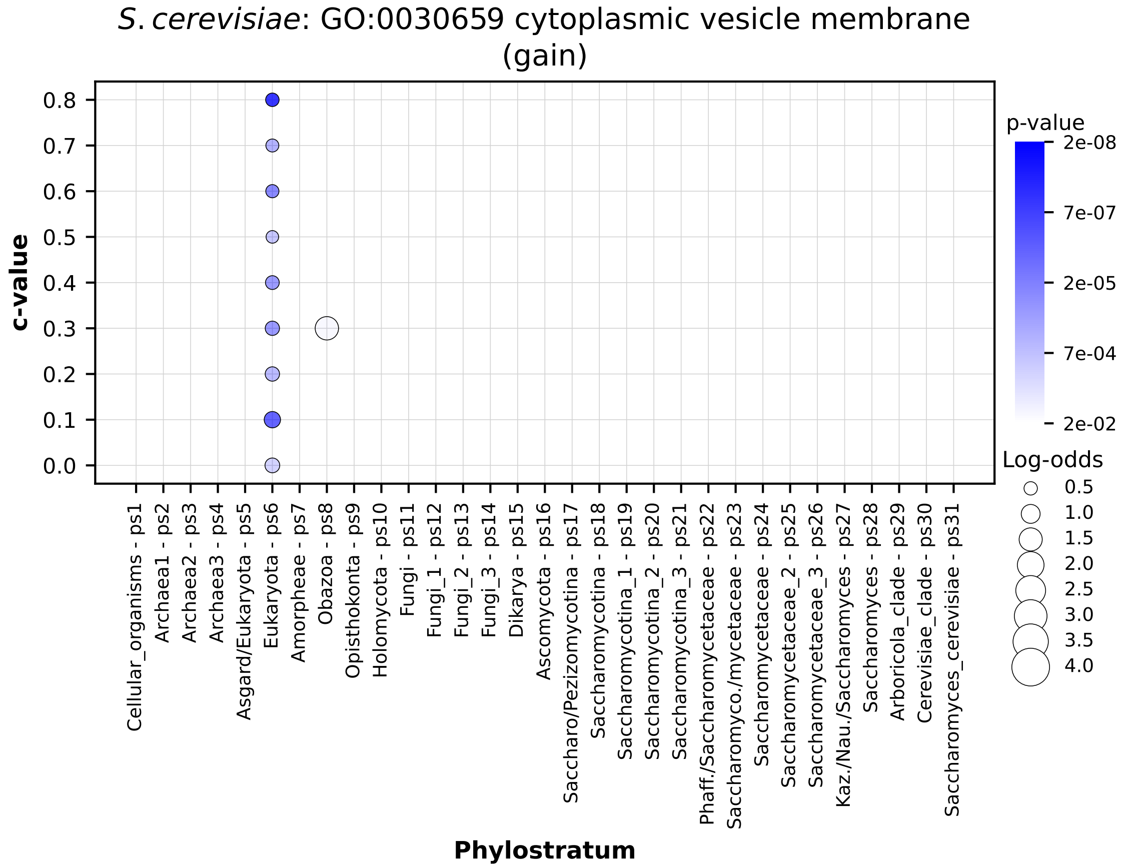

S. cerevisiae: GO:0030659 cytoplasmic vesicle membrane (gain)
c-value
0.8
0.7
0.6
0.5
0.4
0.3
0.2
0.1
0.0
p-value
2e-08
7e-07
2e-05
7e-04
2e-02
Log-odds
0.5
1.0
1.5
2.0
2.5
3.0
3.5
4.0
Cellular_organisms - ps1
Archaea1 - ps2
Archaea2 - ps3
Archaea3 - ps4
Asgard/Eukaryota - ps5
Eukaryota - ps6
Amorpheae - ps7
Obazoa - ps8
Opisthokonta - ps9
Holomycota - ps10
Fungi - ps11
Fungi_1 - ps12
Fungi_2 - ps13
Fungi_3 - ps14
Dikarya - ps15
Ascomycota - ps16
Saccharo/Pezizomycotina - ps17
Saccharomycotina - ps18
Saccharomycotina_1 - ps19
Saccharomycotina_2 - ps20
Saccharomycotina_3 - ps21
Phaff./Saccharomycetaceae - ps22
Saccharomyco./mycetaceae - ps23
Saccharomycetaceae - ps24
Saccharomycetaceae_2 - ps25
Saccharomycetaceae_3 - ps26
Kaz./Nau./Saccharomyces - ps27
Saccharomyces - ps28
Arboricola_clade - ps29
Cerevisiae_clade - ps30
Saccharomyces_cerevisiae - ps31
Phylostratum

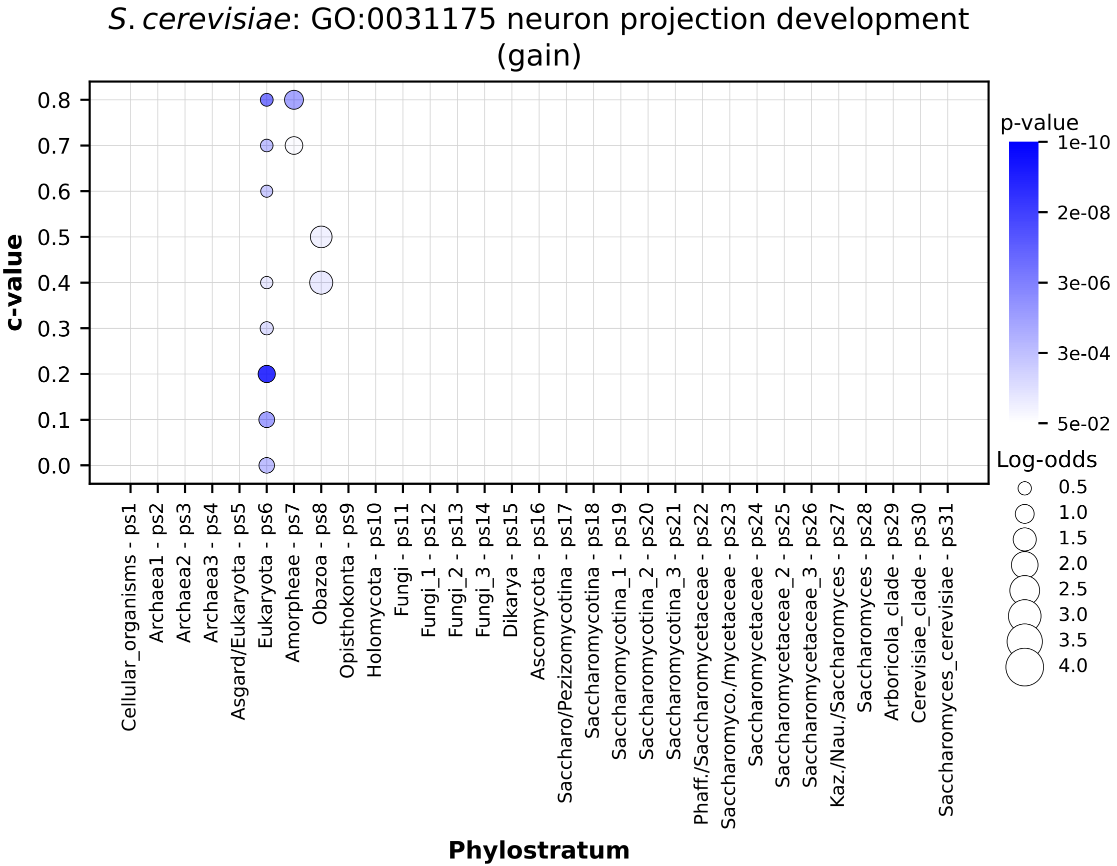

S. cerevisiae: GO:0031175 neuron projection development
(gain)
c-value
0.8
0.7
0.6
0.5
0.4
0.3
0.2
0.1
0.0
p-value
1e-10
2e-08
3e-06
3e-04
5e-02
Log-odds
0.5
1.0
1.5
2.0
2.5
3.0
3.5
4.0
Cellular_organisms - ps1
Archaea1 - ps2
Archaea2 - ps3
Archaea3 - ps4
Asgard/Eukaryota - ps5
Eukaryota - ps6
Amorpheae - ps7
Obazoa - ps8
Opisthokonta - ps9
Holomycota - ps10
Fungi - ps11
Fungi_1 - ps12
Fungi_2 - ps13
Fungi_3 - ps14
Dikarya - ps15
Ascomycota - ps16
Saccharo/Pezizomycotina - ps17
Saccharomycotina - ps18
Saccharomycotina_1 - ps19
Saccharomycotina_2 - ps20
Saccharomycotina_3 - ps21
Phaff./Saccharomycetaceae - ps22
Saccharomyco./mycetaceae - ps23
Saccharomycetaceae - ps24
Saccharomycetaceae_2 - ps25
Saccharomycetaceae_3 - ps26
Kaz./Nau./Saccharomyces - ps27
Saccharomyces - ps28
Arboricola_clade - ps29
Cerevisiae_clade - ps30
Saccharomyces_cerevisiae - ps31
Phylostratum

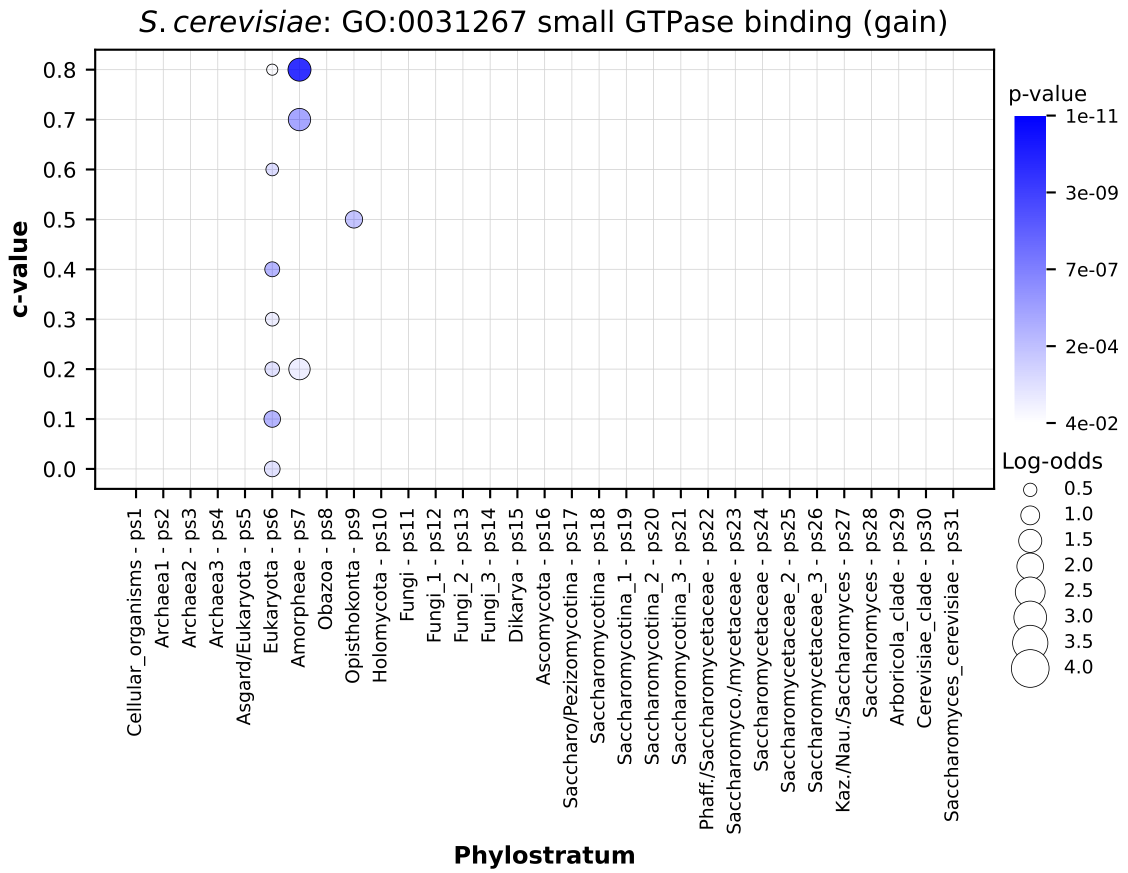

S. cerevisiae: GO:0031267 small GTPase binding (gain)
c-value
0.8
0.7
0.6
0.5
0.4
0.3
0.2
0.1
0.0
p-value
1e-11
3e-09
7e-07
2e-04
4e-02
Log-odds
0.5
1.0
1.5
2.0
2.5
3.0
3.5
4.0
Cellular_organisms - ps1
Archaea1 - ps2
Archaea2 - ps3
Archaea3 - ps4
Asgard/Eukaryota - ps5
Eukaryota - ps6
Amorpheae - ps7
Obazoa - ps8
Opisthokonta - ps9
Holomycota - ps10
Fungi - ps11
Fungi_1 - ps12
Fungi_2 - ps13
Fungi_3 - ps14
Dikarya - ps15
Ascomycota - ps16
Saccharo/Pezizomycotina - ps17
Saccharomycotina - ps18
Saccharomycotina_1 - ps19
Saccharomycotina_2 - ps20
Saccharomycotina_3 - ps21
Phaff./Saccharomycetaceae - ps22
Saccharomyco./mycetaceae - ps23
Saccharomycetaceae - ps24
Saccharomycetaceae_2 - ps25
Saccharomycetaceae_3 - ps26
Kaz./Nau./Saccharomyces - ps27
Saccharomyces - ps28
Arboricola_clade - ps29
Cerevisiae_clade - ps30
Saccharomyces_cerevisiae - ps31
Phylostratum

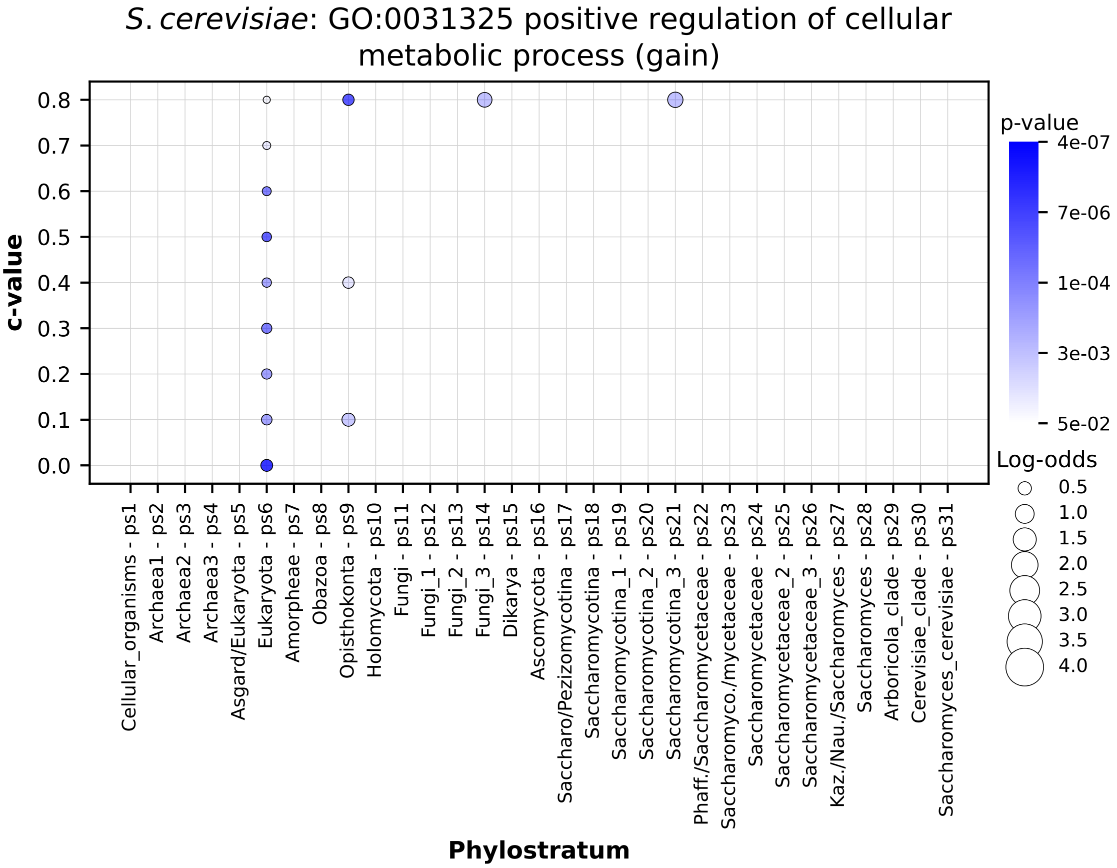

S. cerevisiae: GO:0031325 positive regulation of cellular metabolic process (gain)
c-value
0.0
0.1
0.2
0.3
0.4
0.5
0.6
0.7
0.8
Phylostratum
Cellular_organisms - ps1
Archaea1 - ps2
Archaea2 - ps3
Archaea3 - ps4
Asgard/Eukaryota - ps5
Eukaryota - ps6
Amorpheae - ps7
Obazoa - ps8
Opisthokonta - ps9
Holomycota - ps10
Fungi - ps11
Fungi_1 - ps12
Fungi_2 - ps13
Fungi_3 - ps14
Dikarya - ps15
Ascomycota - ps16
Saccharo/Pezizomycotina - ps17
Saccharomycotina - ps18
Saccharomycotina_1 - ps19
Saccharomycotina_2 - ps20
Saccharomycotina_3 - ps21
Phaff./Saccharomycetaceae - ps22
Saccharomyco./mycetaceae - ps23
Saccharomycetaceae - ps24
Saccharomycetaceae_2 - ps25
Saccharomycetaceae_3 - ps26
Kaz./Nau./Saccharomyces - ps27
Saccharomyces - ps28
Arboricola_clade - ps29
Cerevisiae_clade - ps30
Saccharomyces_cerevisiae - ps31
p-value
4e-07
7e-06
1e-04
3e-03
5e-02
Log-odds
0.5
1.0
1.5
2.0
2.5
3.0
3.5
4.0

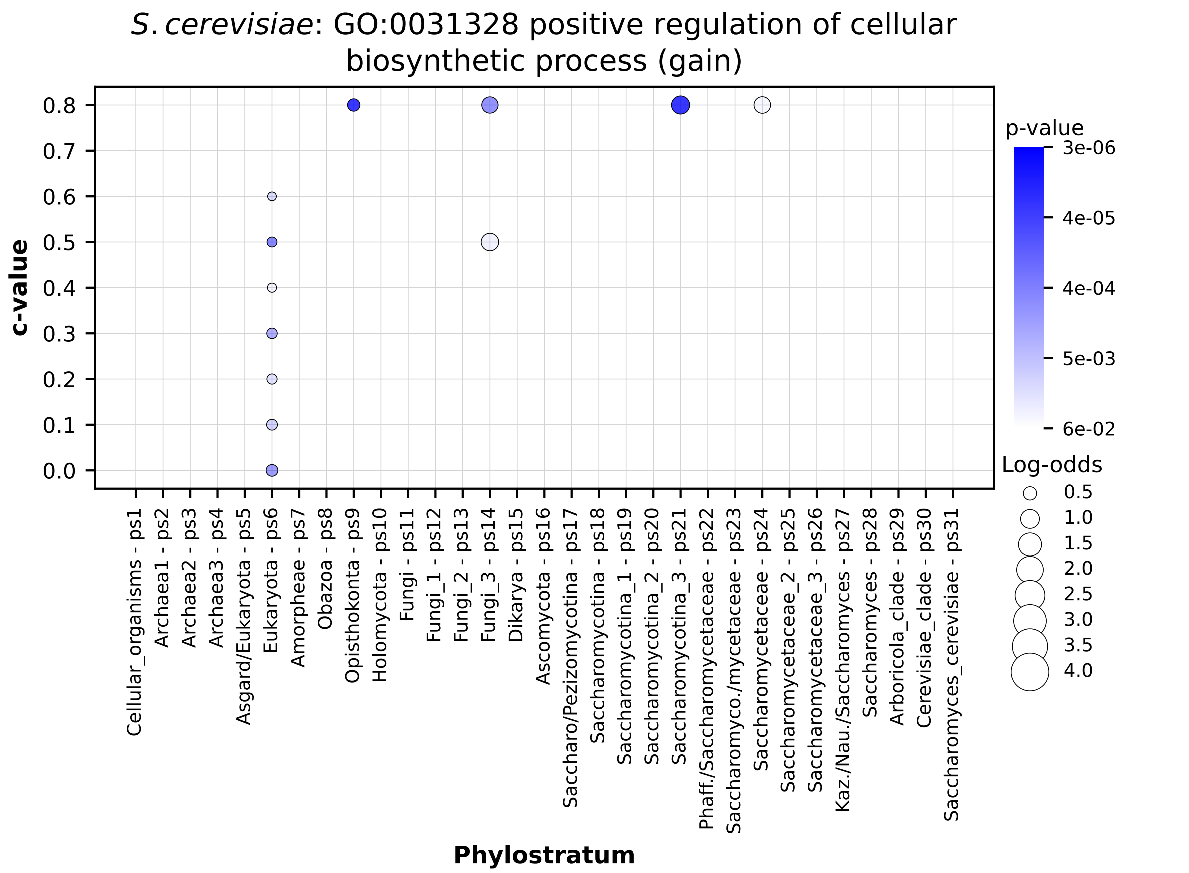

S. cerevisiae: GO:0031328 positive regulation of cellular biosynthetic process (gain)
c-value
0.0
0.1
0.2
0.3
0.4
0.5
0.6
0.7
0.8
p-value
3e-06
4e-05
4e-04
5e-03
6e-02
Log-odds
0.5
1.0
1.5
2.0
2.5
3.0
3.5
4.0
Cellular_organisms - ps1
Archaea1 - ps2
Archaea2 - ps3
Archaea3 - ps4
Asgard/Eukaryota - ps5
Eukaryota - ps6
Amorpheae - ps7
Obazoa - ps8
Opisthokonta - ps9
Holomycota - ps10
Fungi - ps11
Fungi_1 - ps12
Fungi_2 - ps13
Fungi_3 - ps14
Dikarya - ps15
Ascomycota - ps16
Saccharo/Pezizomycotina - ps17
Saccharomycotina - ps18
Saccharomycotina_1 - ps19
Saccharomycotina_2 - ps20
Saccharomycotina_3 - ps21
Phaff./Saccharomycetaceae - ps22
Saccharomyco./mycetaceae - ps23
Saccharomycetaceae - ps24
Saccharomycetaceae_2 - ps25
Saccharomycetaceae_3 - ps26
Kaz./Nau./Saccharomyces - ps27
Saccharomyces - ps28
Arboricola_clade - ps29
Cerevisiae_clade - ps30
Saccharomyces_cerevisiae - ps31
Phylostratum

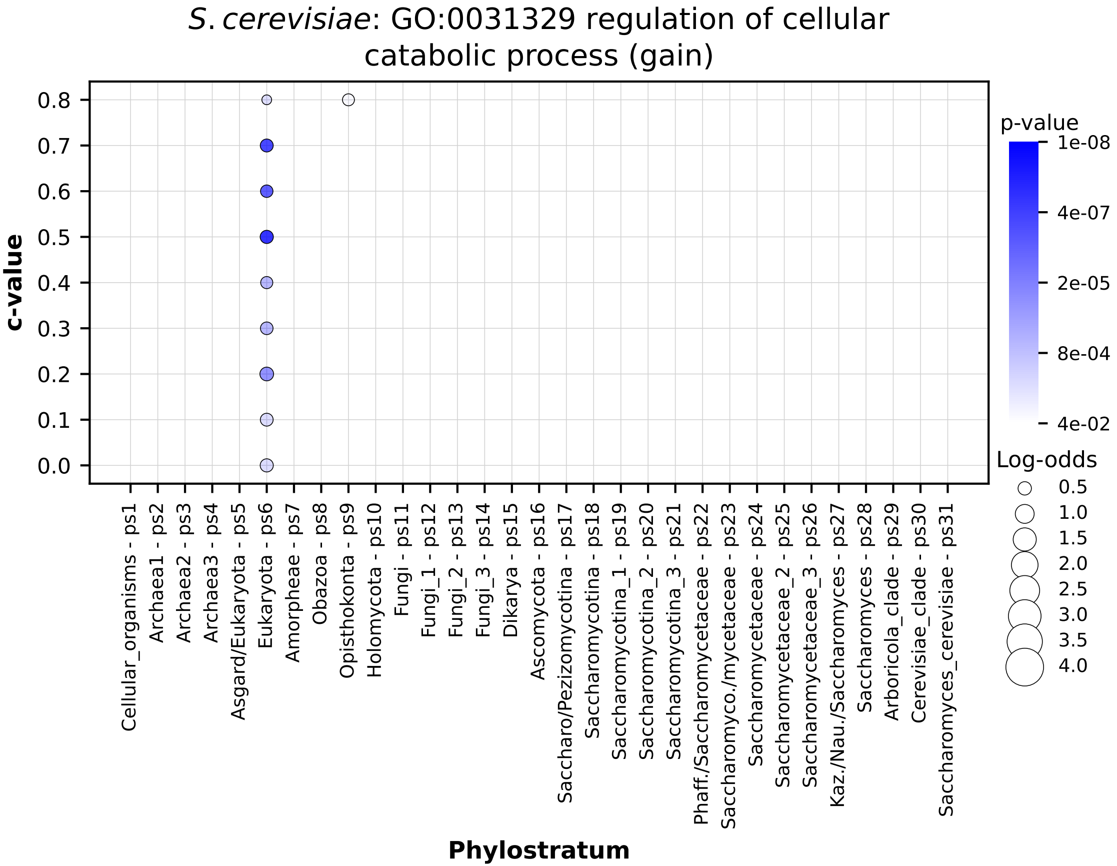

S. cerevisiae: GO:0031329 regulation of cellular catabolic process (gain)
c-value
0.8
0.7
0.6
0.5
0.4
0.3
0.2
0.1
0.0
p-value
1e-08
4e-07
2e-05
8e-04
4e-02
Log-odds
0.5
1.0
1.5
2.0
2.5
3.0
3.5
4.0
Cellular_organisms - ps1
Archaea1 - ps2
Archaea2 - ps3
Archaea3 - ps4
Asgard/Eukaryota - ps5
Eukaryota - ps6
Amorpheae - ps7
Obazoa - ps8
Opisthokonta - ps9
Holomycota - ps10
Fungi - ps11
Fungi_1 - ps12
Fungi_2 - ps13
Fungi_3 - ps14
Dikarya - ps15
Ascomycota - ps16
Saccharo/Pezizomycotina - ps17
Saccharomycotina - ps18
Saccharomycotina_1 - ps19
Saccharomycotina_2 - ps20
Saccharomycotina_3 - ps21
Phaff./Saccharomycetaceae - ps22
Saccharomyco./mycetaceae - ps23
Saccharomycetaceae - ps24
Saccharomycetaceae_2 - ps25
Saccharomycetaceae_3 - ps26
Kaz./Nau./Saccharomyces - ps27
Saccharomyces - ps28
Arboricola_clade - ps29
Cerevisiae_clade - ps30
Saccharomyces_cerevisiae - ps31
Phylostratum

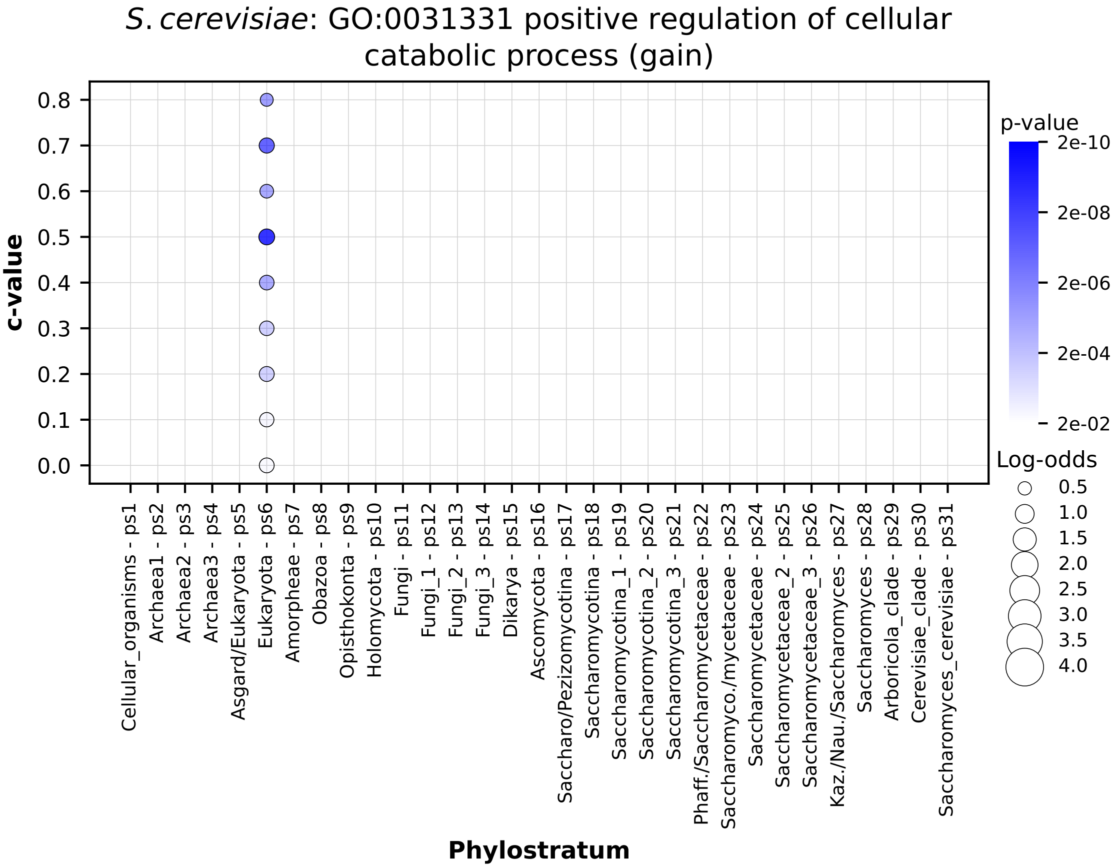
S. cerevisiae: GO:0031331 positive regulation of cellular catabolic process (gain)
c-value
0.8
0.7
0.6
0.5
0.4
0.3
0.2
0.1
0.0
p-value
2e-10
2e-08
2e-06
2e-04
2e-02
Log-odds
0.5
1.0
1.5
2.0
2.5
3.0
3.5
4.0
Cellular_organisms - ps1
Archaea1 - ps2
Archaea2 - ps3
Archaea3 - ps4
Asgard/Eukaryota - ps5
Eukaryota - ps6
Amorpheae - ps7
Obazoa - ps8
Opisthokonta - ps9
Holomycota - ps10
Fungi - ps11
Fungi_1 - ps12
Fungi_2 - ps13
Fungi_3 - ps14
Dikarya - ps15
Ascomycota - ps16
Saccharo/Pezizomycotina - ps17
Saccharomycotina - ps18
Saccharomycotina_1 - ps19
Saccharomycotina_2 - ps20
Saccharomycotina_3 - ps21
Phaff./Saccharomycetaceae - ps22
Saccharomyco./mycetaceae - ps23
Saccharomycetaceae - ps24
Saccharomycetaceae_2 - ps25
Saccharomycetaceae_3 - ps26
Kaz./Nau./Saccharomyces - ps27
Saccharomyces - ps28
Arboricola_clade - ps29
Cerevisiae_clade - ps30
Saccharomyces_cerevisiae - ps31
Phylostratum

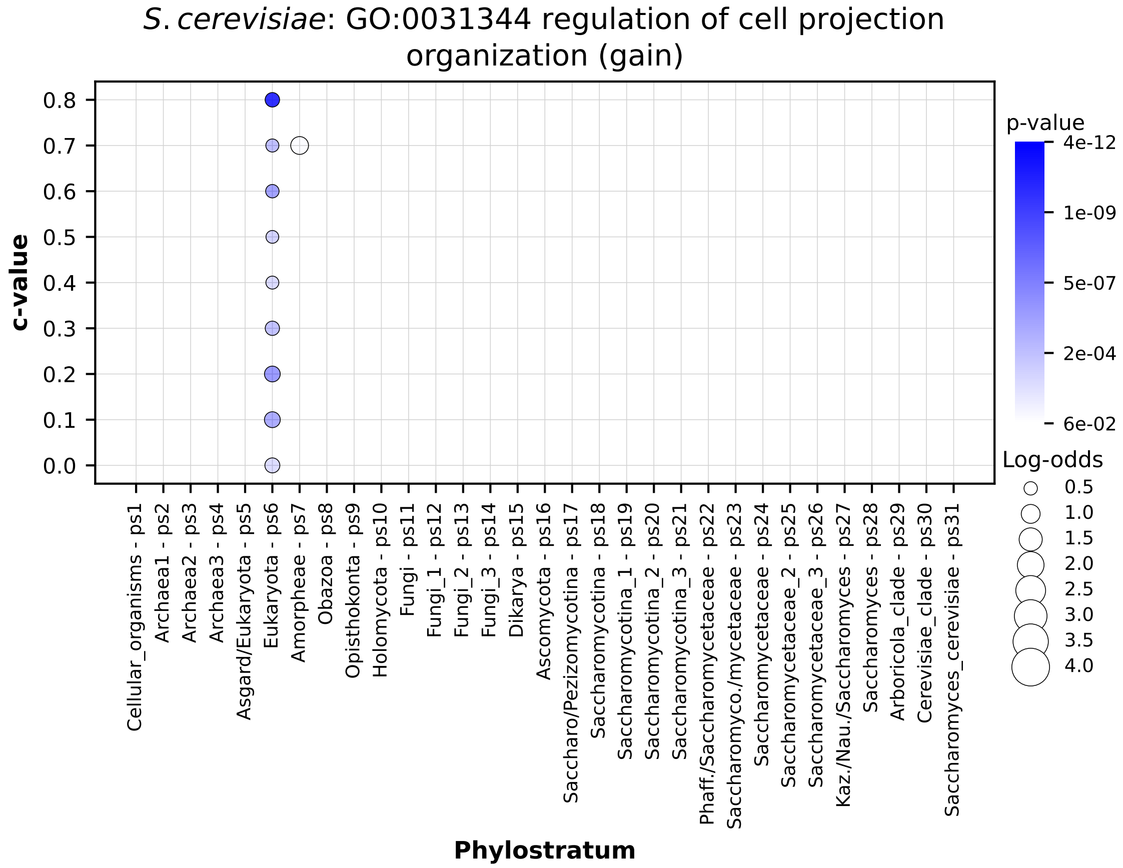

S. cerevisiae: GO:0031344 regulation of cell projection organization (gain)
c-value
0.8
0.7
0.6
0.5
0.4
0.3
0.2
0.1
0.0
p-value
4e-12
1e-09
5e-07
2e-04
6e-02
Log-odds
0.5
1.0
1.5
2.0
2.5
3.0
3.5
4.0
Cellular_organisms - ps1
Archaea1 - ps2
Archaea2 - ps3
Archaea3 - ps4
Asgard/Eukaryota - ps5
Eukaryota - ps6
Amorpheae - ps7
Obazoa - ps8
Opisthokonta - ps9
Holomycota - ps10
Fungi - ps11
Fungi_1 - ps12
Fungi_2 - ps13
Fungi_3 - ps14
Dikarya - ps15
Ascomycota - ps16
Saccharo/Pezizomycotina - ps17
Saccharomycotina - ps18
Saccharomycotina_1 - ps19
Saccharomycotina_2 - ps20
Saccharomycotina_3 - ps21
Phaff./Saccharomycetaceae - ps22
Saccharomyco./mycetaceae - ps23
Saccharomycetaceae - ps24
Saccharomycetaceae_2 - ps25
Saccharomycetaceae_3 - ps26
Kaz./Nau./Saccharomyces - ps27
Saccharomyces - ps28
Arboricola_clade - ps29
Cerevisiae_clade - ps30
Saccharomyces_cerevisiae - ps31
Phylostratum

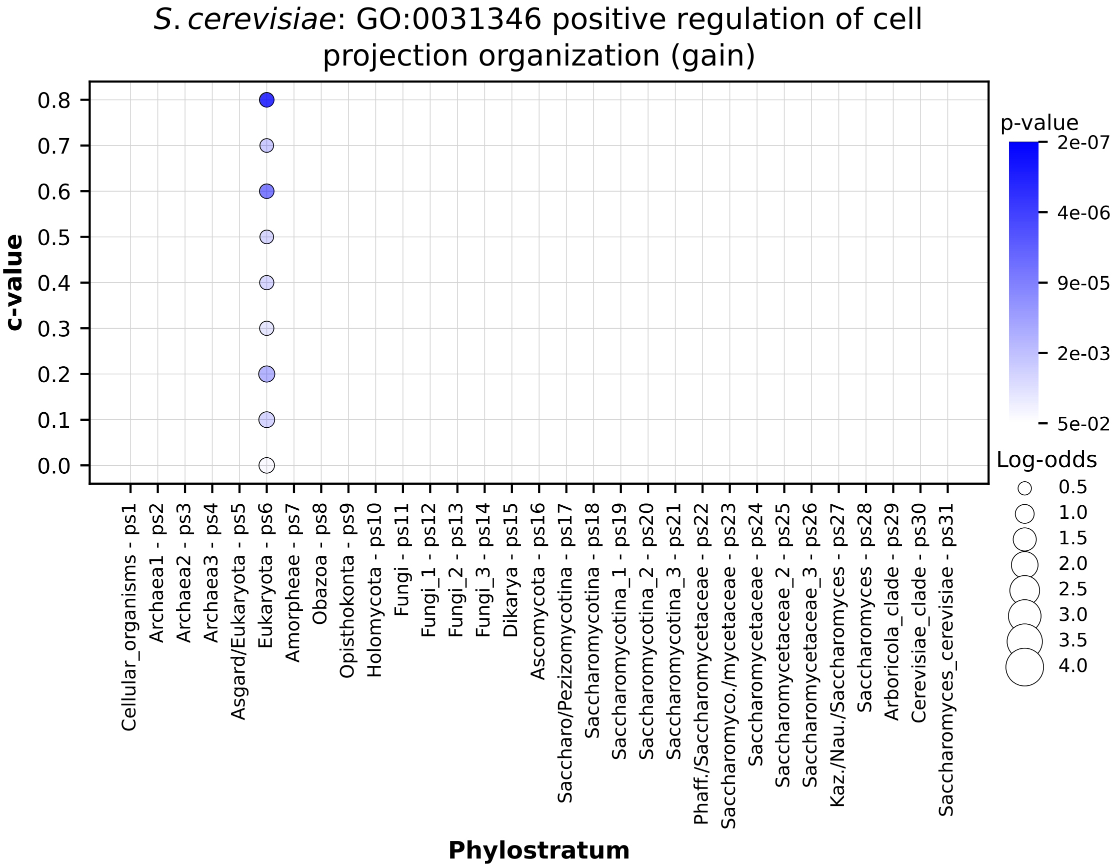

S. cerevisiae: GO:0031346 positive regulation of cell projection organization (gain)
c-value
0.8
0.7
0.6
0.5
0.4
0.3
0.2
0.1
0.0
p-value
2e-07
4e-06
9e-05
2e-03
5e-02
Log-odds
0.5
1.0
1.5
2.0
2.5
3.0
3.5
4.0
Cellular_organisms - ps1
Archaea1 - ps2
Archaea2 - ps3
Archaea3 - ps4
Asgard/Eukaryota - ps5
Eukaryota - ps6
Amorpheae - ps7
Obazoa - ps8
Opisthokonta - ps9
Holomycota - ps10
Fungi - ps11
Fungi_1 - ps12
Fungi_2 - ps13
Fungi_3 - ps14
Dikarya - ps15
Ascomycota - ps16
Saccharo/Pezizomycotina - ps17
Saccharomycotina - ps18
Saccharomycotina_1 - ps19
Saccharomycotina_2 - ps20
Saccharomycotina_3 - ps21
Phaff./Saccharomycetaceae - ps22
Saccharomyco./mycetaceae - ps23
Saccharomycetaceae - ps24
Saccharomycetaceae_2 - ps25
Saccharomycetaceae_3 - ps26
Kaz./Nau./Saccharomyces - ps27
Saccharomyces - ps28
Arboricola_clade - ps29
Cerevisiae_clade - ps30
Saccharomyces_cerevisiae - ps31
Phylostratum

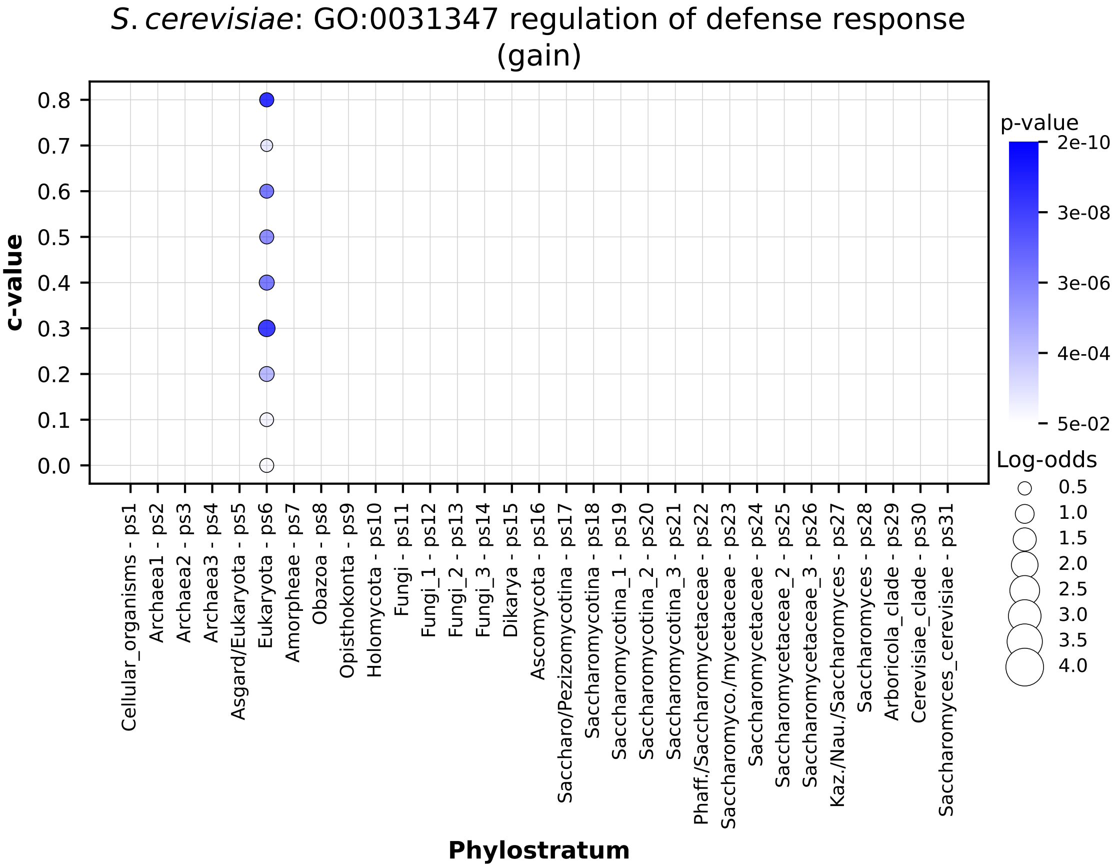

S. cerevisiae: GO:0031347 regulation of defense response
(gain)
c-value
0.8
0.7
0.6
0.5
0.4
0.3
0.2
0.1
0.0
p-value
2e-10
3e-08
3e-06
4e-04
5e-02
Log-odds
0.5
1.0
1.5
2.0
2.5
3.0
3.5
4.0
Cellular_organisms - ps1
Archaea1 - ps2
Archaea2 - ps3
Archaea3 - ps4
Asgard/Eukaryota - ps5
Eukaryota - ps6
Amorpheae - ps7
Obazoa - ps8
Opisthokonta - ps9
Holomycota - ps10
Fungi - ps11
Fungi_1 - ps12
Fungi_2 - ps13
Fungi_3 - ps14
Dikarya - ps15
Ascomycota - ps16
Saccharo/Pezizomycotina - ps17
Saccharomycotina - ps18
Saccharomycotina_1 - ps19
Saccharomycotina_2 - ps20
Saccharomycotina_3 - ps21
Phaff./Saccharomycetaceae - ps22
Saccharomyco./mycetaceae - ps23
Saccharomycetaceae - ps24
Saccharomycetaceae_2 - ps25
Saccharomycetaceae_3 - ps26
Kaz./Nau./Saccharomyces - ps27
Saccharomyces - ps28
Arboricola_clade - ps29
Cerevisiae_clade - ps30
Saccharomyces_cerevisiae - ps31
Phylostratum

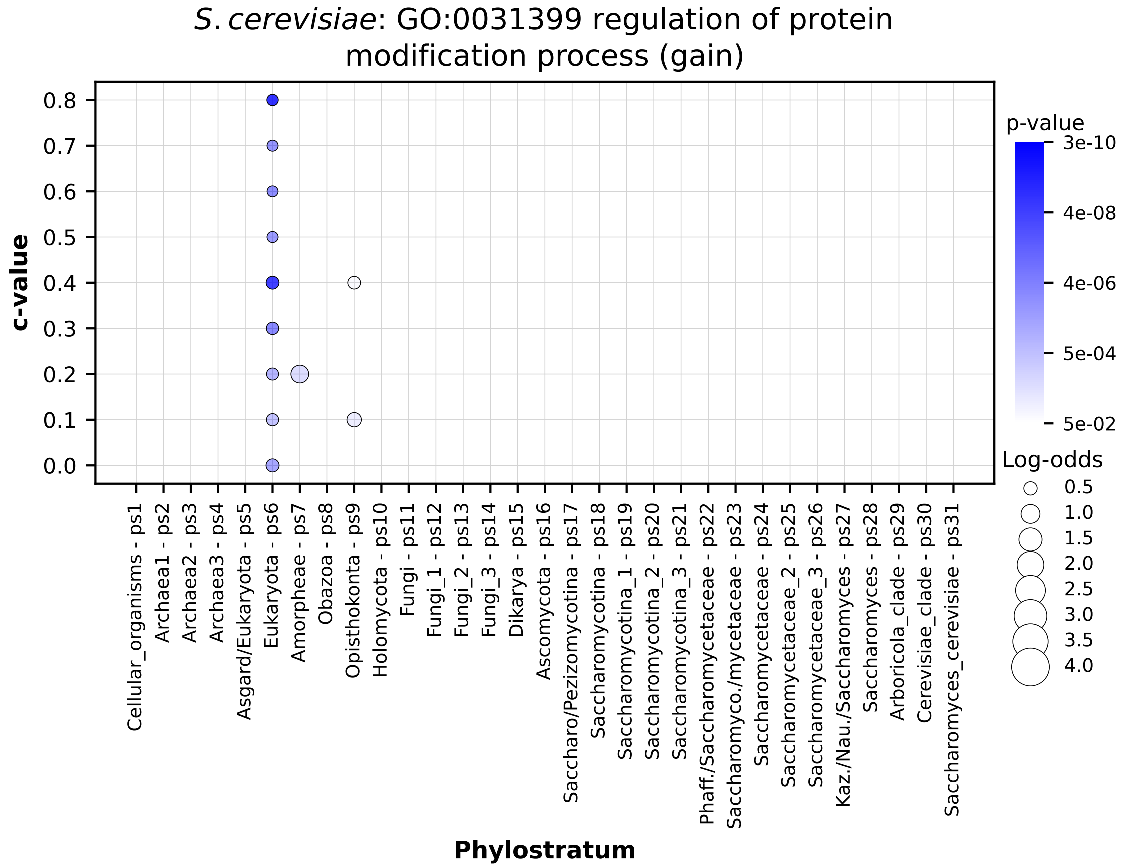

S. cerevisiae: GO:0031399 regulation of protein modification process (gain)
c-value
0.8
0.7
0.6
0.5
0.4
0.3
0.2
0.1
0.0
Phylostratum
Cellular_organisms - ps1
Archaea1 - ps2
Archaea2 - ps3
Archaea3 - ps4
Asgard/Eukaryota - ps5
Eukaryota - ps6
Amorpheae - ps7
Obazoa - ps8
Opisthokonta - ps9
Holomycota - ps10
Fungi - ps11
Fungi_1 - ps12
Fungi_2 - ps13
Fungi_3 - ps14
Dikarya - ps15
Ascomycota - ps16
Saccharo/Pezizomycotina - ps17
Saccharomycotina - ps18
Saccharomycotina_1 - ps19
Saccharomycotina_2 - ps20
Saccharomycotina_3 - ps21
Phaff./Saccharomycetaceae - ps22
Saccharomyco./mycetaceae - ps23
Saccharomycetaceae - ps24
Saccharomycetaceae_2 - ps25
Saccharomycetaceae_3 - ps26
Kaz./Nau./Saccharomyces - ps27
Saccharomyces - ps28
Arboricola_clade - ps29
Cerevisiae_clade - ps30
Saccharomyces_cerevisiae - ps31
p-value
3e-10
4e-08
4e-06
5e-04
5e-02
Log-odds
0.5
1.0
1.5
2.0
2.5
3.0
3.5
4.0

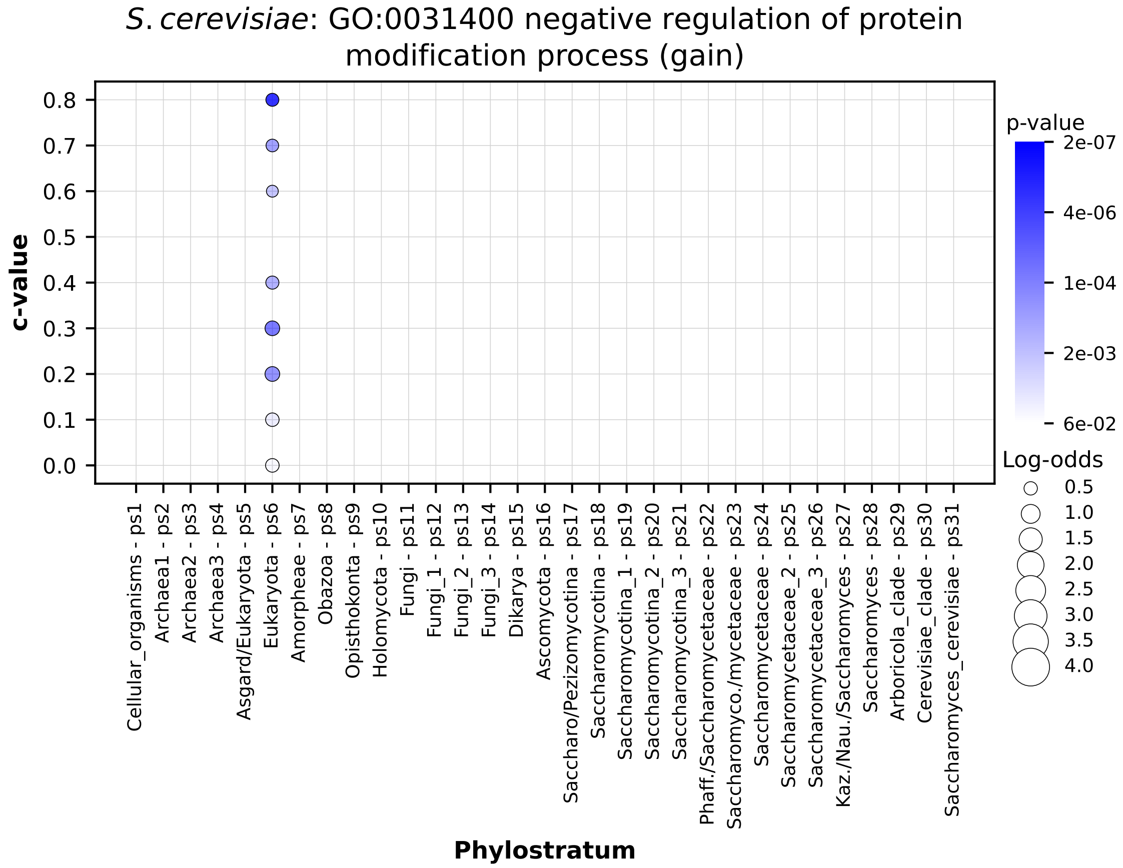

S. cerevisiae: GO:0031400 negative regulation of protein modification process (gain)
c-value
0.0
0.1
0.2
0.3
0.4
0.5
0.6
0.7
0.8
p-value
2e-07
4e-06
1e-04
2e-03
6e-02
Log-odds
0.5
1.0
1.5
2.0
2.5
3.0
3.5
4.0
Cellular_organisms - ps1
Archaea1 - ps2
Archaea2 - ps3
Archaea3 - ps4
Asgard/Eukaryota - ps5
Eukaryota - ps6
Amorpheae - ps7
Obazoa - ps8
Opisthokonta - ps9
Holomycota - ps10
Fungi - ps11
Fungi_1 - ps12
Fungi_2 - ps13
Fungi_3 - ps14
Dikarya - ps15
Ascomycota - ps16
Saccharo/Pezizomycotina - ps17
Saccharomycotina - ps18
Saccharomycotina_1 - ps19
Saccharomycotina_2 - ps20
Saccharomycotina_3 - ps21
Phaff./Saccharomycetaceae - ps22
Saccharomyco./mycetaceae - ps23
Saccharomycetaceae - ps24
Saccharomycetaceae_2 - ps25
Saccharomycetaceae_3 - ps26
Kaz./Nau./Saccharomyces - ps27
Saccharomyces - ps28
Arboricola_clade - ps29
Cerevisiae_clade - ps30
Saccharomyces_cerevisiae - ps31
Phylostratum

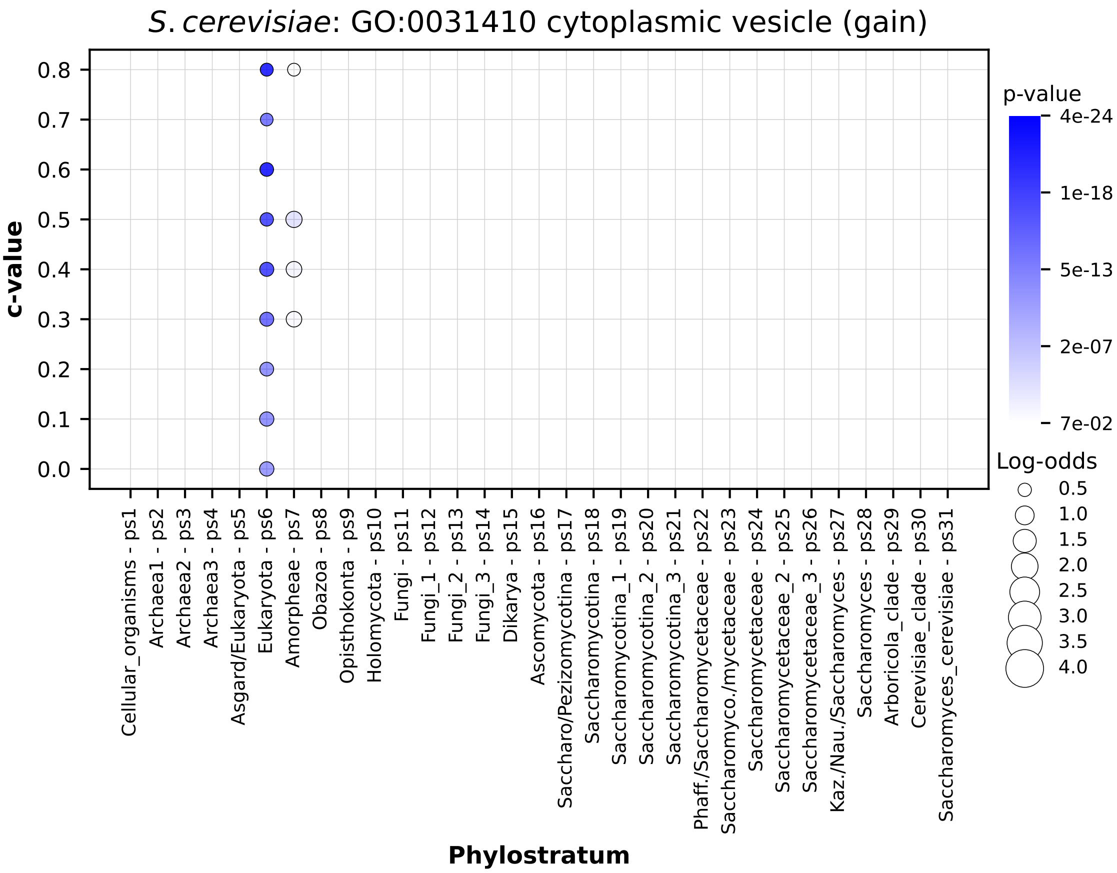

S. cerevisiae: GO:0031410 cytoplasmic vesicle (gain)
c-value
0.0
0.1
0.2
0.3
0.4
0.5
0.6
0.7
0.8
p-value
4e-24
1e-18
5e-13
2e-07
7e-02
Log-odds
0.5
1.0
1.5
2.0
2.5
3.0
3.5
4.0
Cellular_organisms - ps1
Archaea1 - ps2
Archaea2 - ps3
Archaea3 - ps4
Asgard/Eukaryota - ps5
Eukaryota - ps6
Amorpheae - ps7
Obazoa - ps8
Opisthokonta - ps9
Holomycota - ps10
Fungi - ps11
Fungi_1 - ps12
Fungi_2 - ps13
Fungi_3 - ps14
Dikarya - ps15
Ascomycota - ps16
Saccharo/Pezizomycotina - ps17
Saccharomycotina - ps18
Saccharomycotina_1 - ps19
Saccharomycotina_2 - ps20
Saccharomycotina_3 - ps21
Phaff./Saccharomycetaceae - ps22
Saccharomyco./mycetaceae - ps23
Saccharomycetaceae - ps24
Saccharomycetaceae_2 - ps25
Saccharomycetaceae_3 - ps26
Kaz./Nau./Saccharomyces - ps27
Saccharomyces - ps28
Arboricola_clade - ps29
Cerevisiae_clade - ps30
Saccharomyces_cerevisiae - ps31
Phylostratum

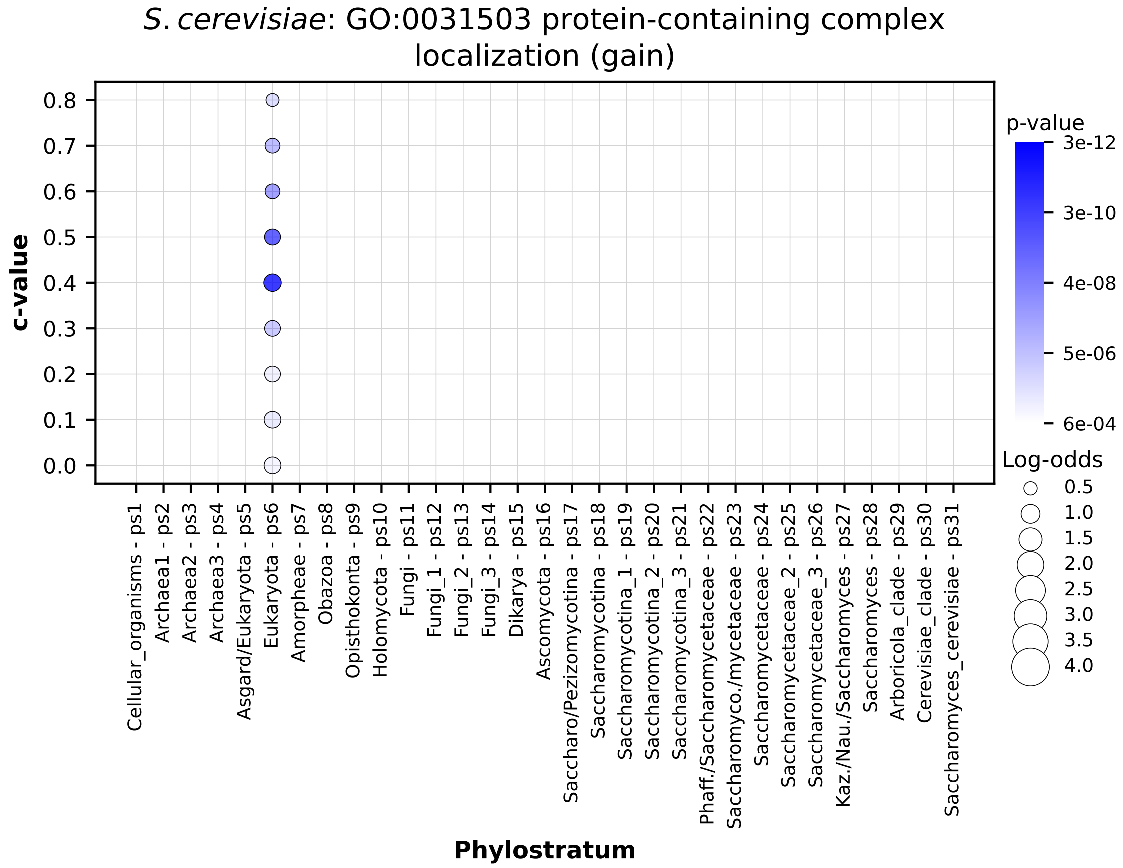

S. cerevisiae: GO:0031503 protein-containing complex localization (gain)
c-value
0.8
0.7
0.6
0.5
0.4
0.3
0.2
0.1
0.0
p-value
3e-12
3e-10
4e-08
5e-06
6e-04
Log-odds
0.5
1.0
1.5
2.0
2.5
3.0
3.5
4.0
Cellular_organisms - ps1
Archaea1 - ps2
Archaea2 - ps3
Archaea3 - ps4
Asgard/Eukaryota - ps5
Eukaryota - ps6
Amorpheae - ps7
Obazoa - ps8
Opisthokonta - ps9
Holomycota - ps10
Fungi - ps11
Fungi_1 - ps12
Fungi_2 - ps13
Fungi_3 - ps14
Dikarya - ps15
Ascomycota - ps16
Saccharo/Pezizomycotina - ps17
Saccharomycotina - ps18
Saccharomycotina_1 - ps19
Saccharomycotina_2 - ps20
Saccharomycotina_3 - ps21
Phaff./Saccharomycetaceae - ps22
Saccharomyco./mycetaceae - ps23
Saccharomycetaceae - ps24
Saccharomycetaceae_2 - ps25
Saccharomycetaceae_3 - ps26
Kaz./Nau./Saccharomyces - ps27
Saccharomyces - ps28
Arboricola_clade - ps29
Cerevisiae_clade - ps30
Saccharomyces_cerevisiae - ps31
Phylostratum

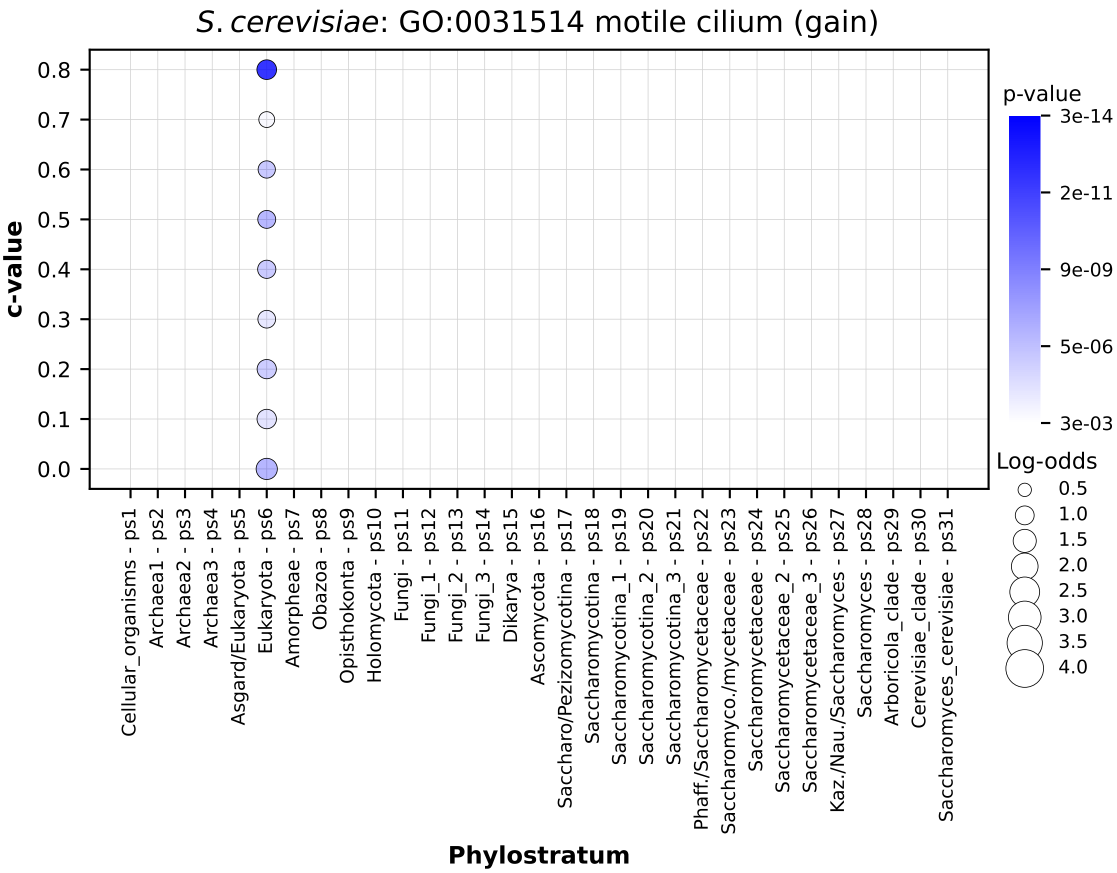

S. cerevisiae: GO:0031514 motile cilium (gain)
c-value
0.0
0.1
0.2
0.3
0.4
0.5
0.6
0.7
0.8
p-value
3e-14
2e-11
9e-09
5e-06
3e-03
Log-odds
0.5
1.0
1.5
2.0
2.5
3.0
3.5
4.0
Cellular_organisms - ps1
Archaea1 - ps2
Archaea2 - ps3
Archaea3 - ps4
Asgard/Eukaryota - ps5
Eukaryota - ps6
Amorpheae - ps7
Obazoa - ps8
Opisthokonta - ps9
Holomycota - ps10
Fungi - ps11
Fungi_1 - ps12
Fungi_2 - ps13
Fungi_3 - ps14
Dikarya - ps15
Ascomycota - ps16
Saccharo/Pezizomycotina - ps17
Saccharomycotina - ps18
Saccharomycotina_1 - ps19
Saccharomycotina_2 - ps20
Saccharomycotina_3 - ps21
Phaff./Saccharomycetaceae - ps22
Saccharomyco./mycetaceae - ps23
Saccharomycetaceae - ps24
Saccharomycetaceae_2 - ps25
Saccharomycetaceae_3 - ps26
Kaz./Nau./Saccharomyces - ps27
Saccharomyces - ps28
Arboricola_clade - ps29
Cerevisiae_clade - ps30
Saccharomyces_cerevisiae - ps31
Phylostratum

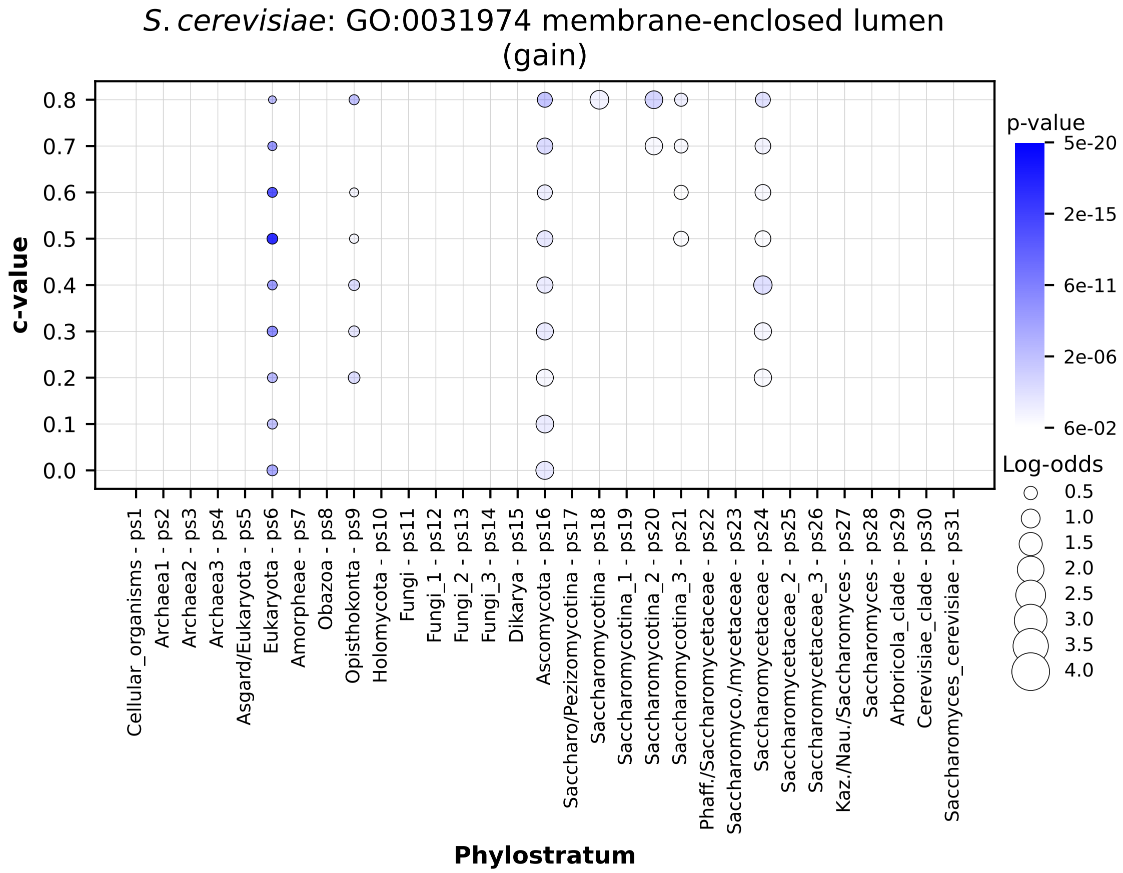

S. cerevisiae: GO:0031974 membrane-enclosed lumen
(gain)
c-value
0.8
0.7
0.6
0.5
0.4
0.3
0.2
0.1
0.0
p-value
5e-20
2e-15
6e-11
2e-06
6e-02
Log-odds
0.5
1.0
1.5
2.0
2.5
3.0
3.5
4.0
Cellular_organisms - ps1
Archaea1 - ps2
Archaea2 - ps3
Archaea3 - ps4
Asgard/Eukaryota - ps5
Eukaryota - ps6
Amorpheae - ps7
Obazoa - ps8
Opisthokonta - ps9
Holomycota - ps10
Fungi - ps11
Fungi_1 - ps12
Fungi_2 - ps13
Fungi_3 - ps14
Dikarya - ps15
Ascomycota - ps16
Saccharo/Pezizomycotina - ps17
Saccharomycotina - ps18
Saccharomycotina_1 - ps19
Saccharomycotina_2 - ps20
Saccharomycotina_3 - ps21
Phaff./Saccharomycetaceae - ps22
Saccharomyco./mycetaceae - ps23
Saccharomycetaceae - ps24
Saccharomycetaceae_2 - ps25
Saccharomycetaceae_3 - ps26
Kaz./Nau./Saccharomyces - ps27
Saccharomyces - ps28
Arboricola_clade - ps29
Cerevisiae_clade - ps30
Saccharomyces_cerevisiae - ps31
Phylostratum

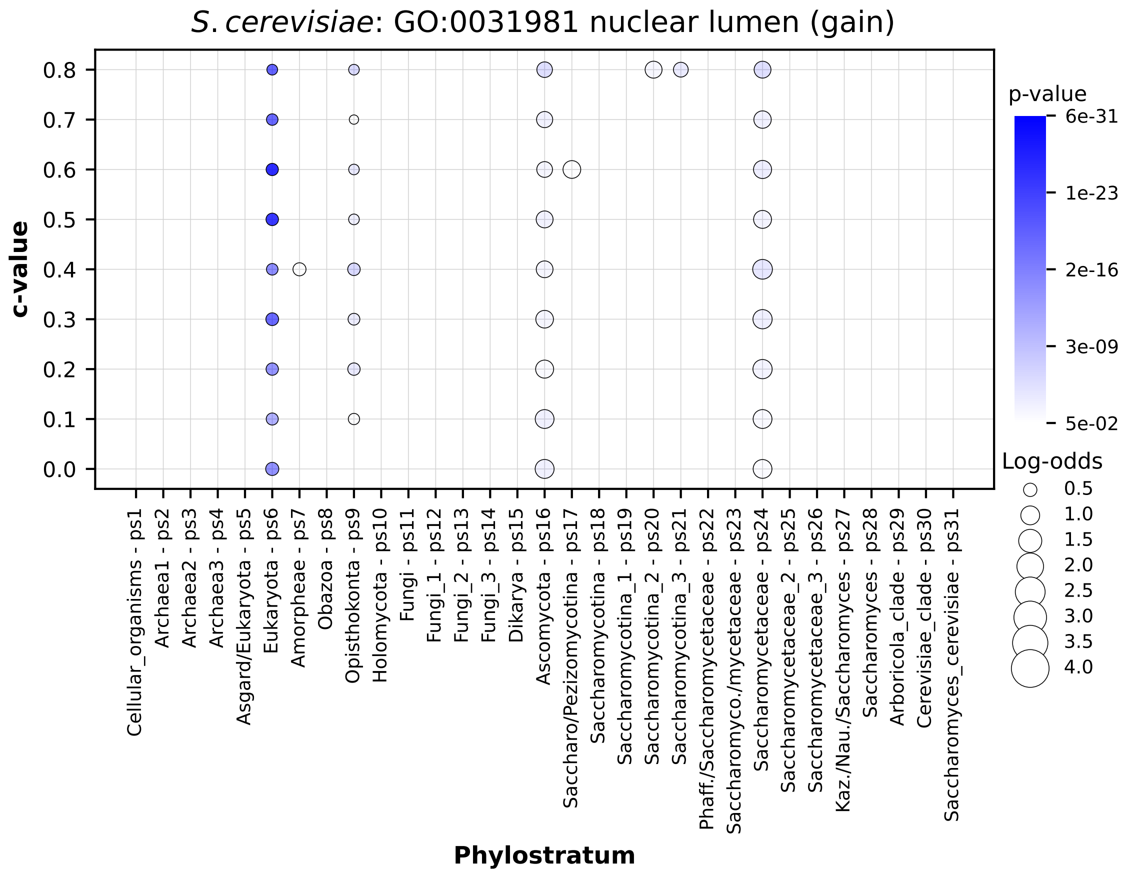

S. cerevisiae: GO:0031981 nuclear lumen (gain)
c-value
0.8
0.7
0.6
0.5
0.4
0.3
0.2
0.1
0.0
p-value
6e-31
1e-23
2e-16
3e-09
5e-02
Log-odds
0.5
1.0
1.5
2.0
2.5
3.0
3.5
4.0
Cellular_organisms - ps1
Archaea1 - ps2
Archaea2 - ps3
Archaea3 - ps4
Asgard/Eukaryota - ps5
Eukaryota - ps6
Amorpheae - ps7
Obazoa - ps8
Opisthokonta - ps9
Holomycota - ps10
Fungi - ps11
Fungi_1 - ps12
Fungi_2 - ps13
Fungi_3 - ps14
Dikarya - ps15
Ascomycota - ps16
Saccharo/Pezizomycotina - ps17
Saccharomycotina - ps18
Saccharomycotina_1 - ps19
Saccharomycotina_2 - ps20
Saccharomycotina_3 - ps21
Phaff./Saccharomycetaceae - ps22
Saccharomyco./mycetaceae - ps23
Saccharomycetaceae - ps24
Saccharomycetaceae_2 - ps25
Saccharomycetaceae_3 - ps26
Kaz./Nau./Saccharomyces - ps27
Saccharomyces - ps28
Arboricola_clade - ps29
Cerevisiae_clade - ps30
Saccharomyces_cerevisiae - ps31
Phylostratum

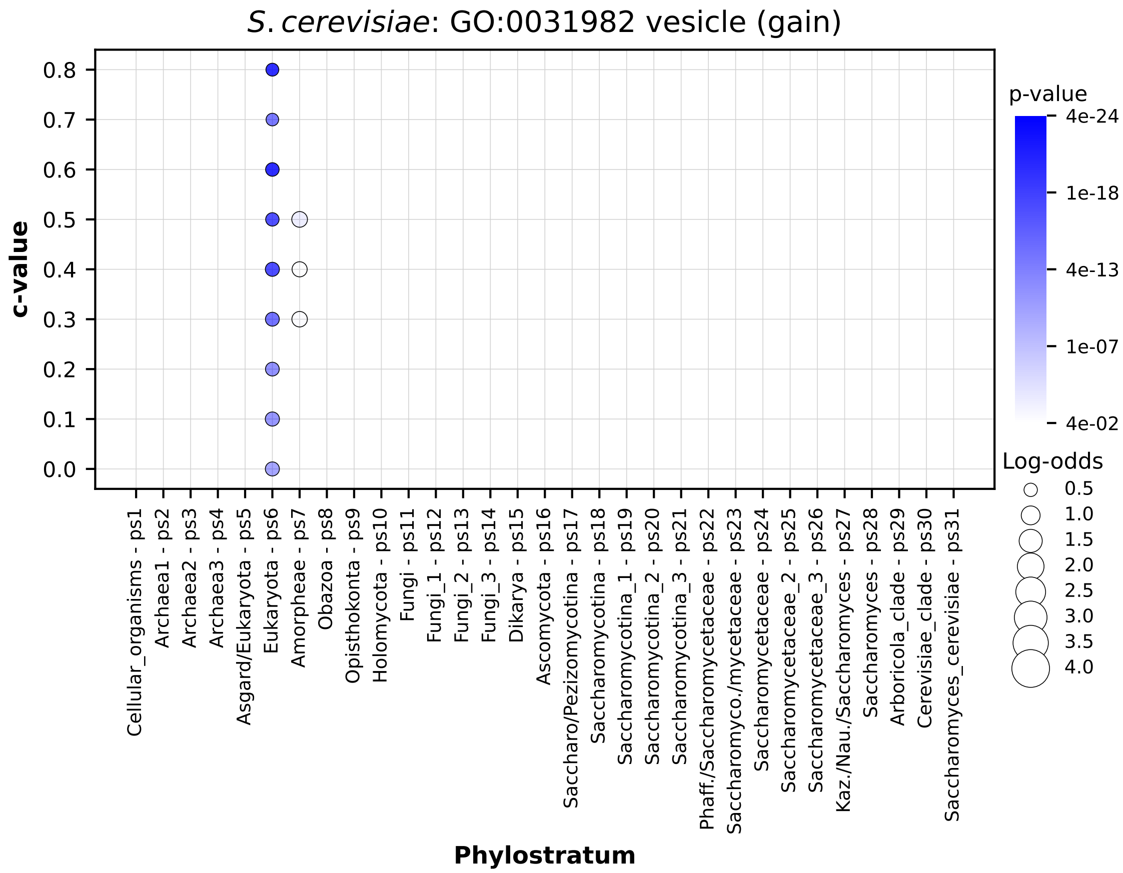

S. cerevisiae: GO:0031982 vesicle (gain)
c-value
0.0
0.1
0.2
0.3
0.4
0.5
0.6
0.7
0.8
p-value
4e-24
1e-18
4e-13
1e-07
4e-02
Log-odds
0.5
1.0
1.5
2.0
2.5
3.0
3.5
4.0
Cellular_organisms - ps1
Archaea1 - ps2
Archaea2 - ps3
Archaea3 - ps4
Asgard/Eukaryota - ps5
Eukaryota - ps6
Amorpheae - ps7
Obazoa - ps8
Opisthokonta - ps9
Holomycota - ps10
Fungi - ps11
Fungi_1 - ps12
Fungi_2 - ps13
Fungi_3 - ps14
Dikarya - ps15
Ascomycota - ps16
Saccharo/Pezizomycotina - ps17
Saccharomycotina - ps18
Saccharomycotina_1 - ps19
Saccharomycotina_2 - ps20
Saccharomycotina_3 - ps21
Phaff./Saccharomycetaceae - ps22
Saccharomyco./mycetaceae - ps23
Saccharomycetaceae - ps24
Saccharomycetaceae_2 - ps25
Saccharomycetaceae_3 - ps26
Kaz./Nau./Saccharomyces - ps27
Saccharomyces - ps28
Arboricola_clade - ps29
Cerevisiae_clade - ps30
Saccharomyces_cerevisiae - ps31
Phylostratum

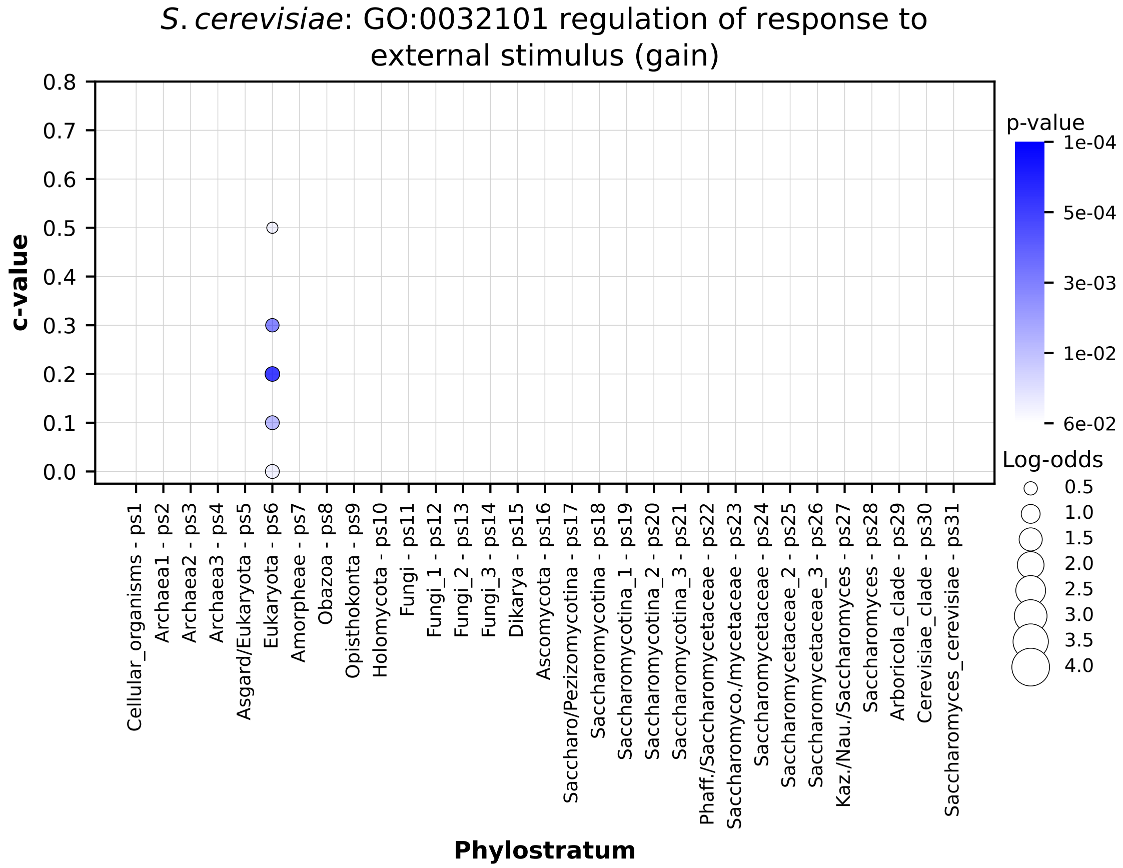

S. cerevisiae: GO:0032101 regulation of response to external stimulus (gain)
c-value
0.8
0.7
0.6
0.5
0.4
0.3
0.2
0.1
0.0
p-value
1e-04
5e-04
3e-03
1e-02
6e-02
Log-odds
0.5
1.0
1.5
2.0
2.5
3.0
3.5
4.0
Cellular_organisms - ps1
Archaea1 - ps2
Archaea2 - ps3
Archaea3 - ps4
Asgard/Eukaryota - ps5
Eukaryota - ps6
Amorpheae - ps7
Obazoa - ps8
Opisthokonta - ps9
Holomycota - ps10
Fungi - ps11
Fungi_1 - ps12
Fungi_2 - ps13
Fungi_3 - ps14
Dikarya - ps15
Ascomycota - ps16
Saccharo/Pezizomycotina - ps17
Saccharomycotina - ps18
Saccharomycotina_1 - ps19
Saccharomycotina_2 - ps20
Saccharomycotina_3 - ps21
Phaff./Saccharomycetaceae - ps22
Saccharomyco./mycetaceae - ps23
Saccharomycetaceae - ps24
Saccharomycetaceae_2 - ps25
Saccharomycetaceae_3 - ps26
Kaz./Nau./Saccharomyces - ps27
Saccharomyces - ps28
Arboricola_clade - ps29
Cerevisiae_clade - ps30
Saccharomyces_cerevisiae - ps31
Phylostratum

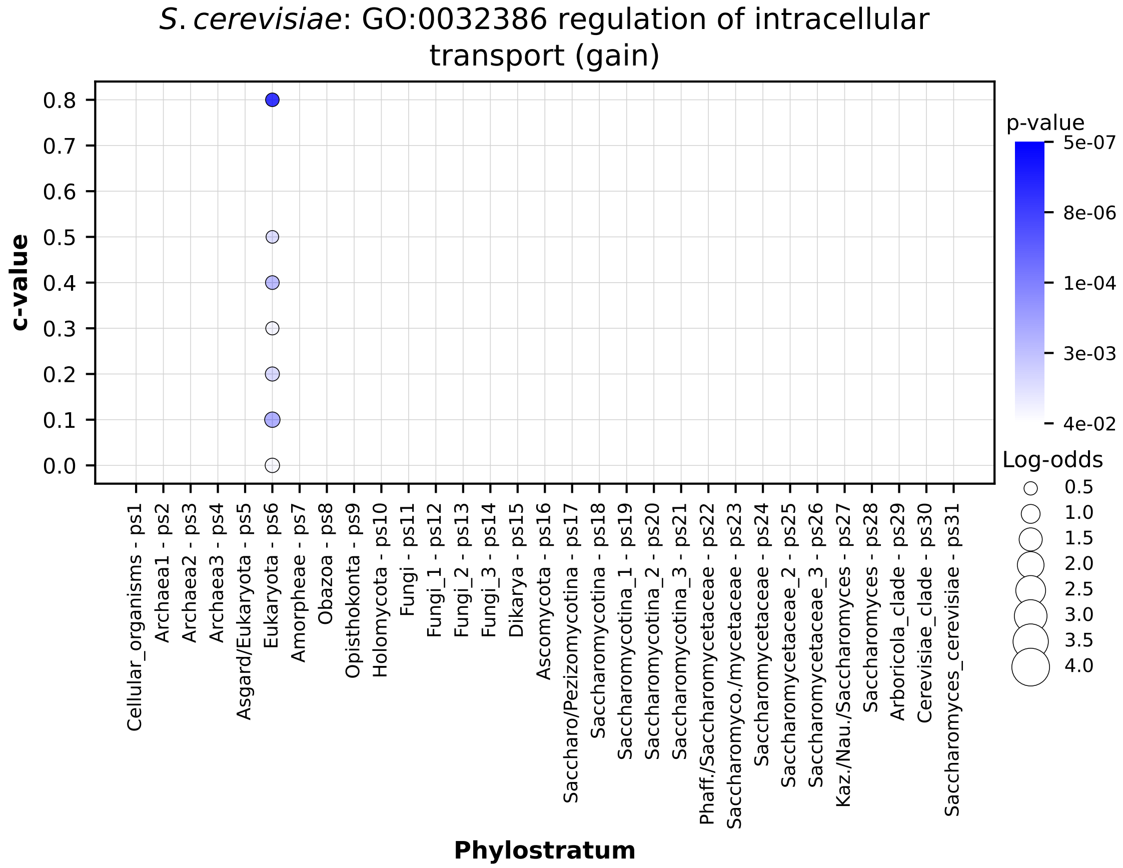

S. cerevisiae: GO:0032386 regulation of intracellular transport (gain)
c-value
0.8
0.7
0.6
0.5
0.4
0.3
0.2
0.1
0.0
p-value
5e-07
8e-06
1e-04
3e-03
4e-02
Log-odds
0.5
1.0
1.5
2.0
2.5
3.0
3.5
4.0
Cellular_organisms - ps1
Archaea1 - ps2
Archaea2 - ps3
Archaea3 - ps4
Asgard/Eukaryota - ps5
Eukaryota - ps6
Amorpheae - ps7
Obazoa - ps8
Opisthokonta - ps9
Holomycota - ps10
Fungi - ps11
Fungi_1 - ps12
Fungi_2 - ps13
Fungi_3 - ps14
Dikarya - ps15
Ascomycota - ps16
Saccharo/Pezizomycotina - ps17
Saccharomycotina - ps18
Saccharomycotina_1 - ps19
Saccharomycotina_2 - ps20
Saccharomycotina_3 - ps21
Phaff./Saccharomycetaceae - ps22
Saccharomyco./mycetaceae - ps23
Saccharomycetaceae - ps24
Saccharomycetaceae_2 - ps25
Saccharomycetaceae_3 - ps26
Kaz./Nau./Saccharomyces - ps27
Saccharomyces - ps28
Arboricola_clade - ps29
Cerevisiae_clade - ps30
Saccharomyces_cerevisiae - ps31
Phylostratum

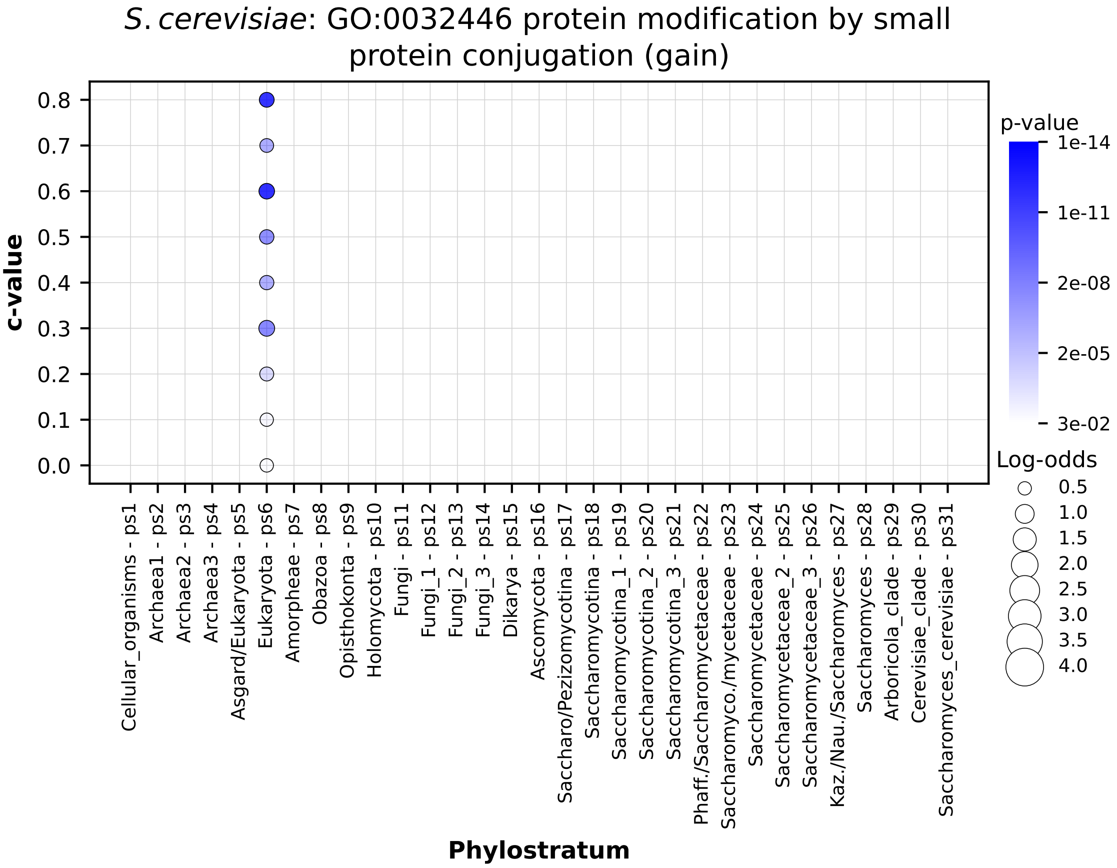
S. cerevisiae: GO:0032446 protein modification by small protein conjugation (gain)
c-value
0.8
0.7
0.6
0.5
0.4
0.3
0.2
0.1
0.0
p-value
1e-14
1e-11
2e-08
2e-05
3e-02
Log-odds
0.5
1.0
1.5
2.0
2.5
3.0
3.5
4.0
Cellular_organisms - ps1
Archaea1 - ps2
Archaea2 - ps3
Archaea3 - ps4
Asgard/Eukaryota - ps5
Eukaryota - ps6
Amorpheae - ps7
Obazoa - ps8
Opisthokonta - ps9
Holomycota - ps10
Fungi - ps11
Fungi_1 - ps12
Fungi_2 - ps13
Fungi_3 - ps14
Dikarya - ps15
Ascomycota - ps16
Saccharo/Pezizomycotina - ps17
Saccharomycotina - ps18
Saccharomycotina_1 - ps19
Saccharomycotina_2 - ps20
Saccharomycotina_3 - ps21
Phaff./Saccharomycetaceae - ps22
Saccharomyco./mycetaceae - ps23
Saccharomycetaceae - ps24
Saccharomycetaceae_2 - ps25
Saccharomycetaceae_3 - ps26
Kaz./Nau./Saccharomyces - ps27
Saccharomyces - ps28
Arboricola_clade - ps29
Cerevisiae_clade - ps30
Saccharomyces_cerevisiae - ps31
Phylostratum

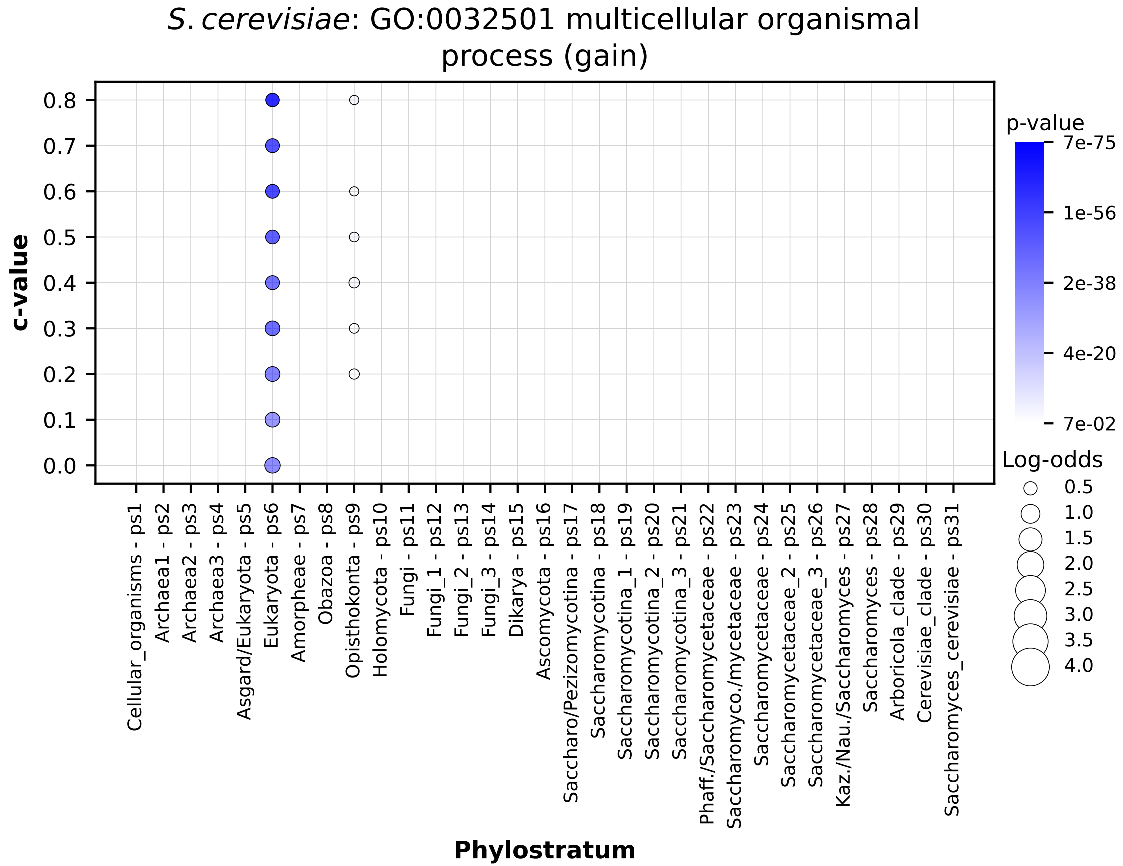

S. cerevisiae: GO:0032501 multicellular organismal process (gain)
c-value
0.8
0.7
0.6
0.5
0.4
0.3
0.2
0.1
0.0
p-value
7e-75
1e-56
2e-38
4e-20
7e-02
Log-odds
0.5
1.0
1.5
2.0
2.5
3.0
3.5
4.0
Cellular_organisms - ps1
Archaea1 - ps2
Archaea2 - ps3
Archaea3 - ps4
Asgard/Eukaryota - ps5
Eukaryota - ps6
Amorpheae - ps7
Obazoa - ps8
Opisthokonta - ps9
Holomycota - ps10
Fungi - ps11
Fungi_1 - ps12
Fungi_2 - ps13
Fungi_3 - ps14
Dikarya - ps15
Ascomycota - ps16
Saccharo/Pezizomycotina - ps17
Saccharomycotina - ps18
Saccharomycotina_1 - ps19
Saccharomycotina_2 - ps20
Saccharomycotina_3 - ps21
Phaff./Saccharomycetaceae - ps22
Saccharomyco./mycetaceae - ps23
Saccharomycetaceae - ps24
Saccharomycetaceae_2 - ps25
Saccharomycetaceae_3 - ps26
Kaz./Nau./Saccharomyces - ps27
Saccharomyces - ps28
Arboricola_clade - ps29
Cerevisiae_clade - ps30
Saccharomyces_cerevisiae - ps31
Phylostratum

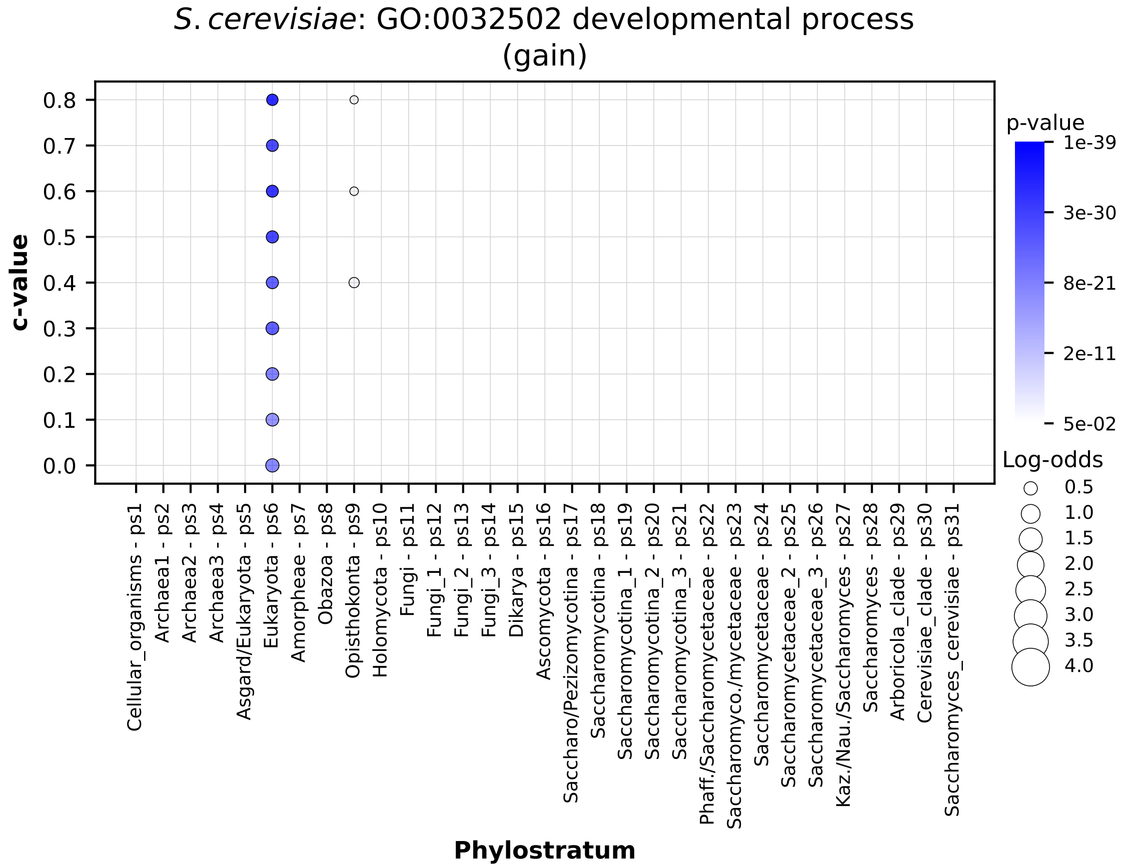

S. cerevisiae: GO:0032502 developmental process (gain)
c-value
0.8
0.7
0.6
0.5
0.4
0.3
0.2
0.1
0.0
p-value
1e-39
3e-30
8e-21
2e-11
5e-02
Log-odds
0.5
1.0
1.5
2.0
2.5
3.0
3.5
4.0
Cellular_organisms - ps1
Archaea1 - ps2
Archaea2 - ps3
Archaea3 - ps4
Asgard/Eukaryota - ps5
Eukaryota - ps6
Amorpheae - ps7
Obazoa - ps8
Opisthokonta - ps9
Holomycota - ps10
Fungi - ps11
Fungi_1 - ps12
Fungi_2 - ps13
Fungi_3 - ps14
Dikarya - ps15
Ascomycota - ps16
Saccharo/Pezizomycotina - ps17
Saccharomycotina - ps18
Saccharomycotina_1 - ps19
Saccharomycotina_2 - ps20
Saccharomycotina_3 - ps21
Phaff./Saccharomycetaceae - ps22
Saccharomyco./mycetaceae - ps23
Saccharomycetaceae - ps24
Saccharomycetaceae_2 - ps25
Saccharomycetaceae_3 - ps26
Kaz./Nau./Saccharomyces - ps27
Saccharomyces - ps28
Arboricola_clade - ps29
Cerevisiae_clade - ps30
Saccharomyces_cerevisiae - ps31
Phylostratum

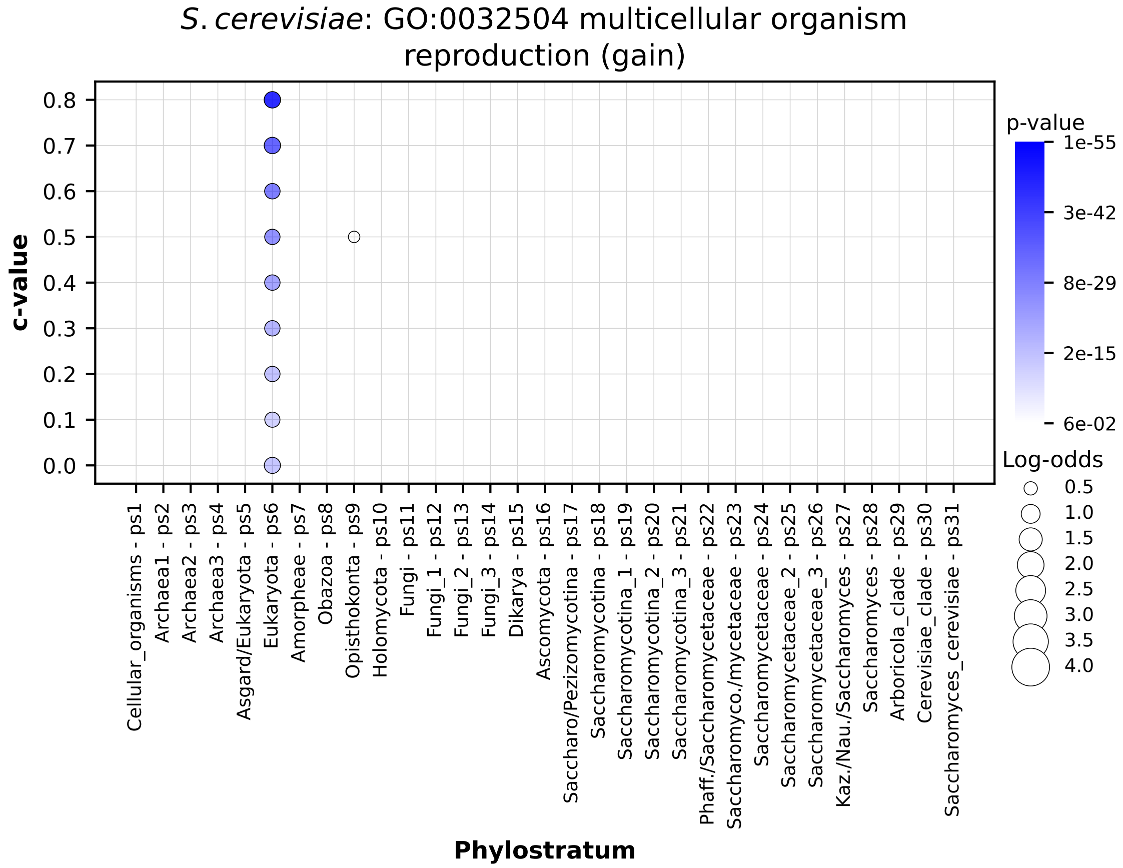
S. cerevisiae: GO:0032504 multicellular organism reproduction (gain)
c-value
0.0
0.1
0.2
0.3
0.4
0.5
0.6
0.7
0.8
Phylostratum
Cellular_organisms - ps1
Archaea1 - ps2
Archaea2 - ps3
Archaea3 - ps4
Asgard/Eukaryota - ps5
Eukaryota - ps6
Amorpheae - ps7
Obazoa - ps8
Opisthokonta - ps9
Holomycota - ps10
Fungi - ps11
Fungi_1 - ps12
Fungi_2 - ps13
Fungi_3 - ps14
Dikarya - ps15
Ascomycota - ps16
Saccharo/Pezizomycotina - ps17
Saccharomycotina - ps18
Saccharomycotina_1 - ps19
Saccharomycotina_2 - ps20
Saccharomycotina_3 - ps21
Phaff./Saccharomycetaceae - ps22
Saccharomyco./mycetaceae - ps23
Saccharomycetaceae - ps24
Saccharomycetaceae_2 - ps25
Saccharomycetaceae_3 - ps26
Kaz./Nau./Saccharomyces - ps27
Saccharomyces - ps28
Arboricola_clade - ps29
Cerevisiae_clade - ps30
Saccharomyces_cerevisiae - ps31
p-value
1e-55
3e-42
8e-29
2e-15
6e-02
Log-odds
0.5
1.0
1.5
2.0
2.5
3.0
3.5
4.0

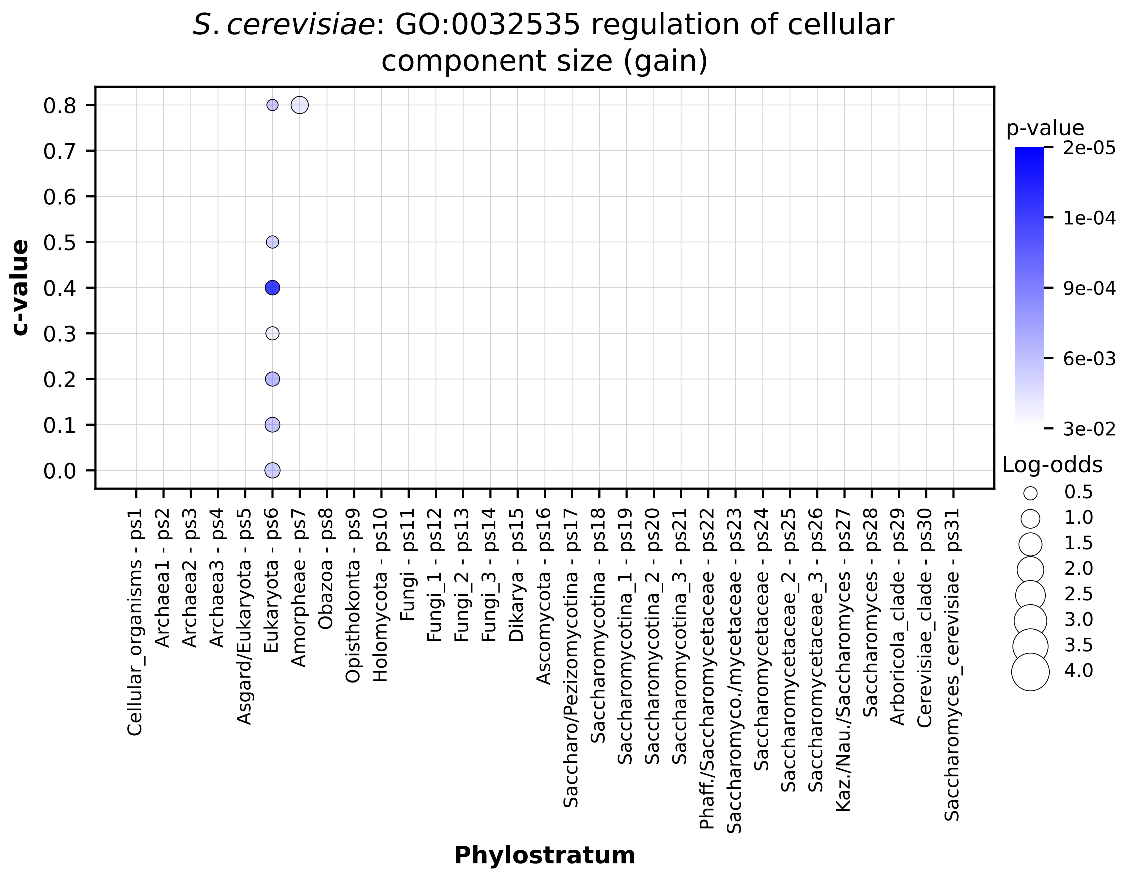

S. cerevisiae: GO:0032535 regulation of cellular component size (gain)
c-value
0.0
0.1
0.2
0.3
0.4
0.5
0.6
0.7
0.8
Phylostratum
Cellular_organisms - ps1
Archaea1 - ps2
Archaea2 - ps3
Archaea3 - ps4
Asgard/Eukaryota - ps5
Eukaryota - ps6
Amorpheae - ps7
Obazoa - ps8
Opisthokonta - ps9
Holomycota - ps10
Fungi - ps11
Fungi_1 - ps12
Fungi_2 - ps13
Fungi_3 - ps14
Dikarya - ps15
Ascomycota - ps16
Saccharo/Pezizomycotina - ps17
Saccharomycotina - ps18
Saccharomycotina_1 - ps19
Saccharomycotina_2 - ps20
Saccharomycotina_3 - ps21
Phaff./Saccharomycetaceae - ps22
Saccharomyco./mycetaceae - ps23
Saccharomycetaceae - ps24
Saccharomycetaceae_2 - ps25
Saccharomycetaceae_3 - ps26
Kaz./Nau./Saccharomyces - ps27
Saccharomyces - ps28
Arboricola_clade - ps29
Cerevisiae_clade - ps30
Saccharomyces_cerevisiae - ps31
p-value
2e-05
1e-04
9e-04
6e-03
3e-02
Log-odds
0.5
1.0
1.5
2.0
2.5
3.0
3.5
4.0

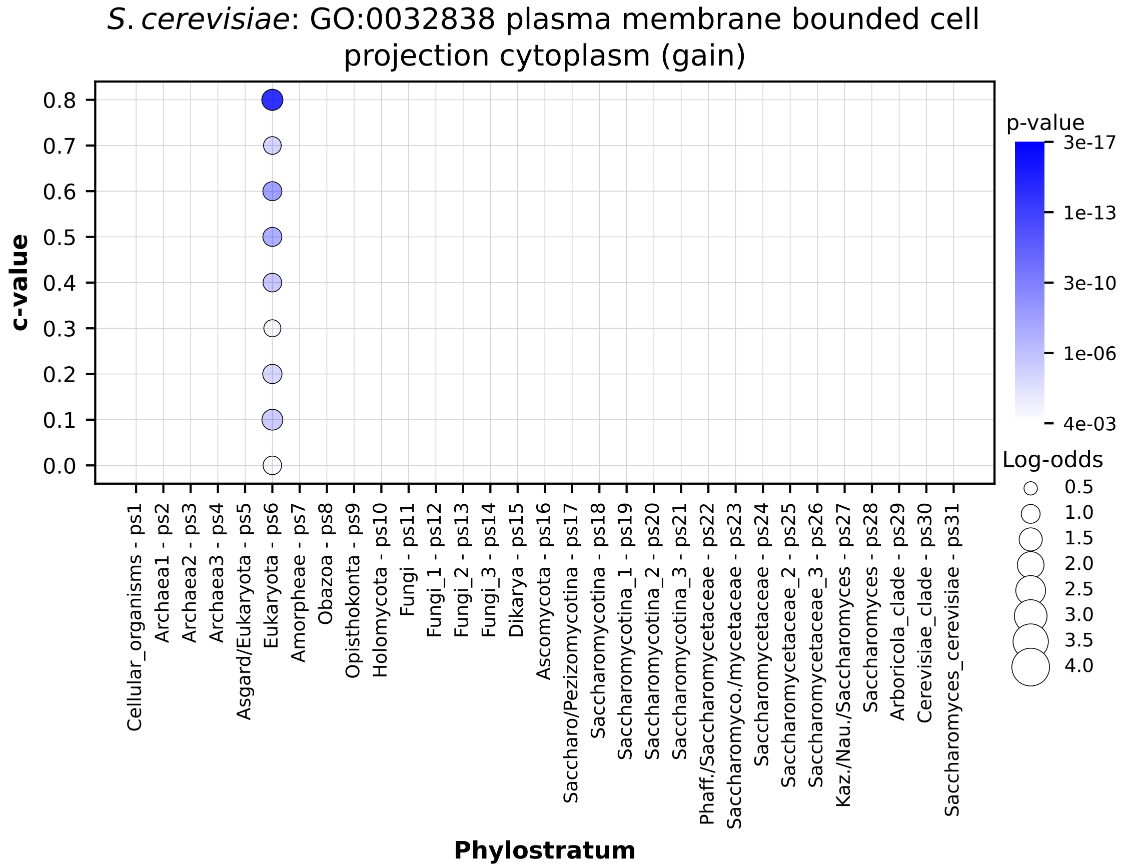

S. cerevisiae: GO:0032838 plasma membrane bounded cell projection cytoplasm (gain)
c-value
0.8
0.7
0.6
0.5
0.4
0.3
0.2
0.1
0.0
p-value
3e-17
1e-13
3e-10
1e-06
4e-03
Log-odds
0.5
1.0
1.5
2.0
2.5
3.0
3.5
4.0
Cellular_organisms - ps1
Archaea1 - ps2
Archaea2 - ps3
Archaea3 - ps4
Asgard/Eukaryota - ps5
Eukaryota - ps6
Amorpheae - ps7
Obazoa - ps8
Opisthokonta - ps9
Holomycota - ps10
Fungi - ps11
Fungi_1 - ps12
Fungi_2 - ps13
Fungi_3 - ps14
Dikarya - ps15
Ascomycota - ps16
Saccharo/Pezizomycotina - ps17
Saccharomycotina - ps18
Saccharomycotina_1 - ps19
Saccharomycotina_2 - ps20
Saccharomycotina_3 - ps21
Phaff./Saccharomycetaceae - ps22
Saccharomyco./mycetaceae - ps23
Saccharomycetaceae - ps24
Saccharomycetaceae_2 - ps25
Saccharomycetaceae_3 - ps26
Kaz./Nau./Saccharomyces - ps27
Saccharomyces - ps28
Arboricola_clade - ps29
Cerevisiae_clade - ps30
Saccharomyces_cerevisiae - ps31
Phylostratum

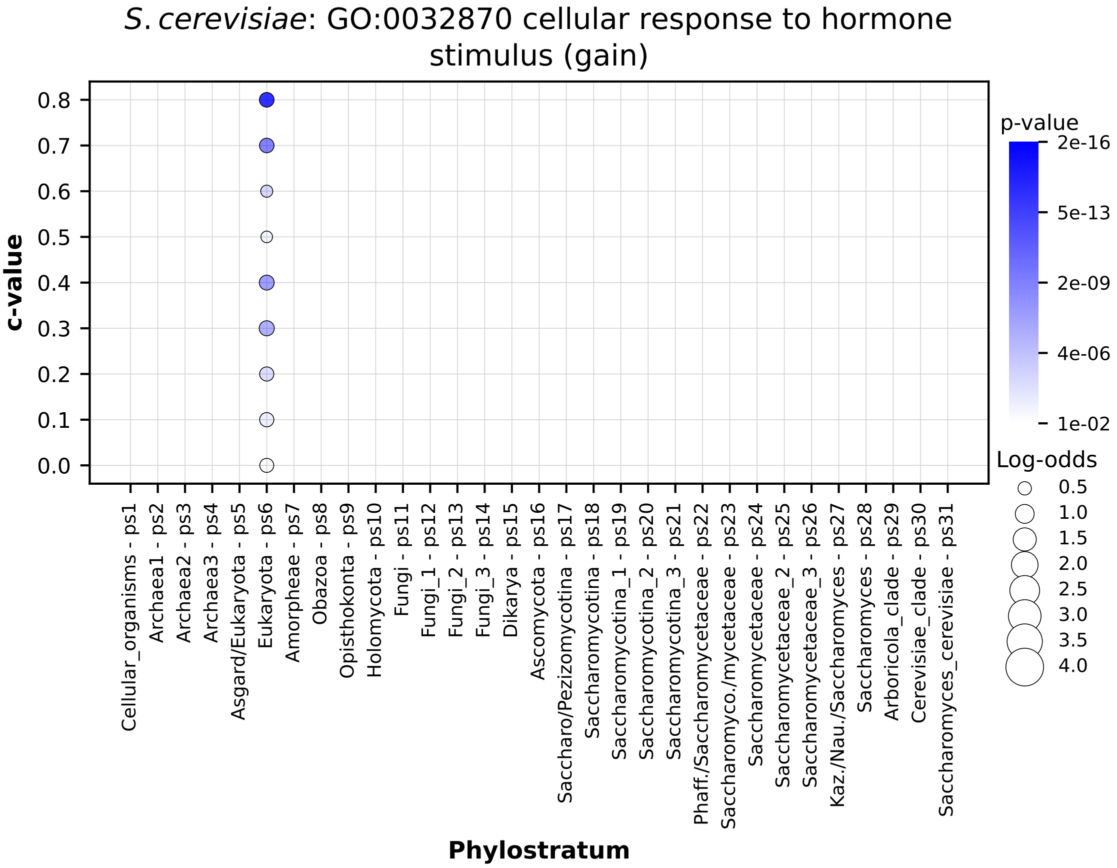

S. cerevisiae: GO:0032870 cellular response to hormone stimulus (gain)
c-value
0.0
0.1
0.2
0.3
0.4
0.5
0.6
0.7
0.8
p-value
2e-16
5e-13
2e-09
4e-06
1e-02
Log-odds
0.5
1.0
1.5
2.0
2.5
3.0
3.5
4.0
Cellular_organisms - ps1
Archaea1 - ps2
Archaea2 - ps3
Archaea3 - ps4
Asgard/Eukaryota - ps5
Eukaryota - ps6
Amorpheae - ps7
Obazoa - ps8
Opisthokonta - ps9
Holomycota - ps10
Fungi - ps11
Fungi_1 - ps12
Fungi_2 - ps13
Fungi_3 - ps14
Dikarya - ps15
Ascomycota - ps16
Saccharo/Pezizomycotina - ps17
Saccharomycotina - ps18
Saccharomycotina_1 - ps19
Saccharomycotina_2 - ps20
Saccharomycotina_3 - ps21
Phaff./Saccharomycetaceae - ps22
Saccharomyco./mycetaceae - ps23
Saccharomycetaceae - ps24
Saccharomycetaceae_2 - ps25
Saccharomycetaceae_3 - ps26
Kaz./Nau./Saccharomyces - ps27
Saccharomyces - ps28
Arboricola_clade - ps29
Cerevisiae_clade - ps30
Saccharomyces_cerevisiae - ps31
Phylostratum

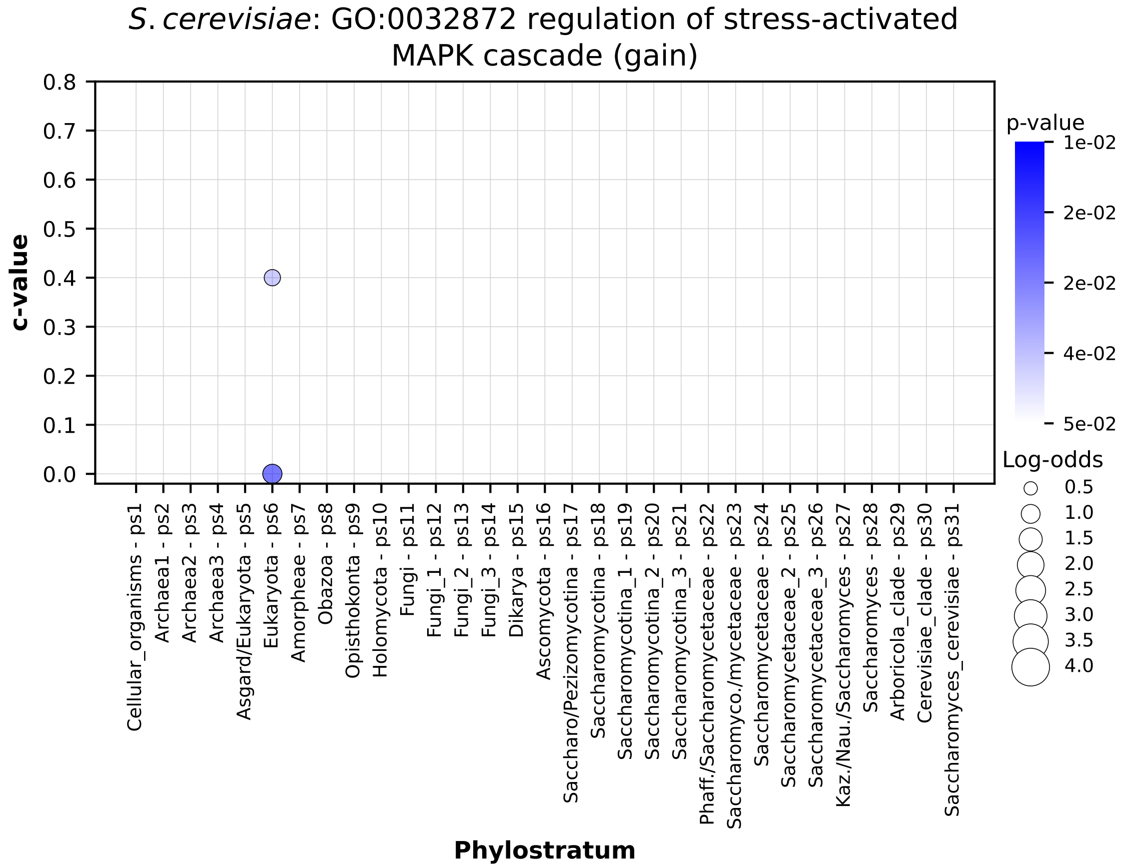

S. cerevisiae: GO:0032872 regulation of stress-activated MAPK cascade (gain)
c-value
0.8
0.7
0.6
0.5
0.4
0.3
0.2
0.1
0.0
Phylostratum
Cellular_organisms - ps1
Archaea1 - ps2
Archaea2 - ps3
Archaea3 - ps4
Asgard/Eukaryota - ps5
Eukaryota - ps6
Amorpheae - ps7
Obazoa - ps8
Opisthokonta - ps9
Holomycota - ps10
Fungi - ps11
Fungi_1 - ps12
Fungi_2 - ps13
Fungi_3 - ps14
Dikarya - ps15
Ascomycota - ps16
Saccharo/Pezizomycotina - ps17
Saccharomycotina - ps18
Saccharomycotina_1 - ps19
Saccharomycotina_2 - ps20
Saccharomycotina_3 - ps21
Phaff./Saccharomycetaceae - ps22
Saccharomyco./mycetaceae - ps23
Saccharomycetaceae - ps24
Saccharomycetaceae_2 - ps25
Saccharomycetaceae_3 - ps26
Kaz./Nau./Saccharomyces - ps27
Saccharomyces - ps28
Arboricola_clade - ps29
Cerevisiae_clade - ps30
Saccharomyces_cerevisiae - ps31
p-value
1e-02
2e-02
2e-02
4e-02
5e-02
Log-odds
0.5
1.0
1.5
2.0
2.5
3.0
3.5
4.0

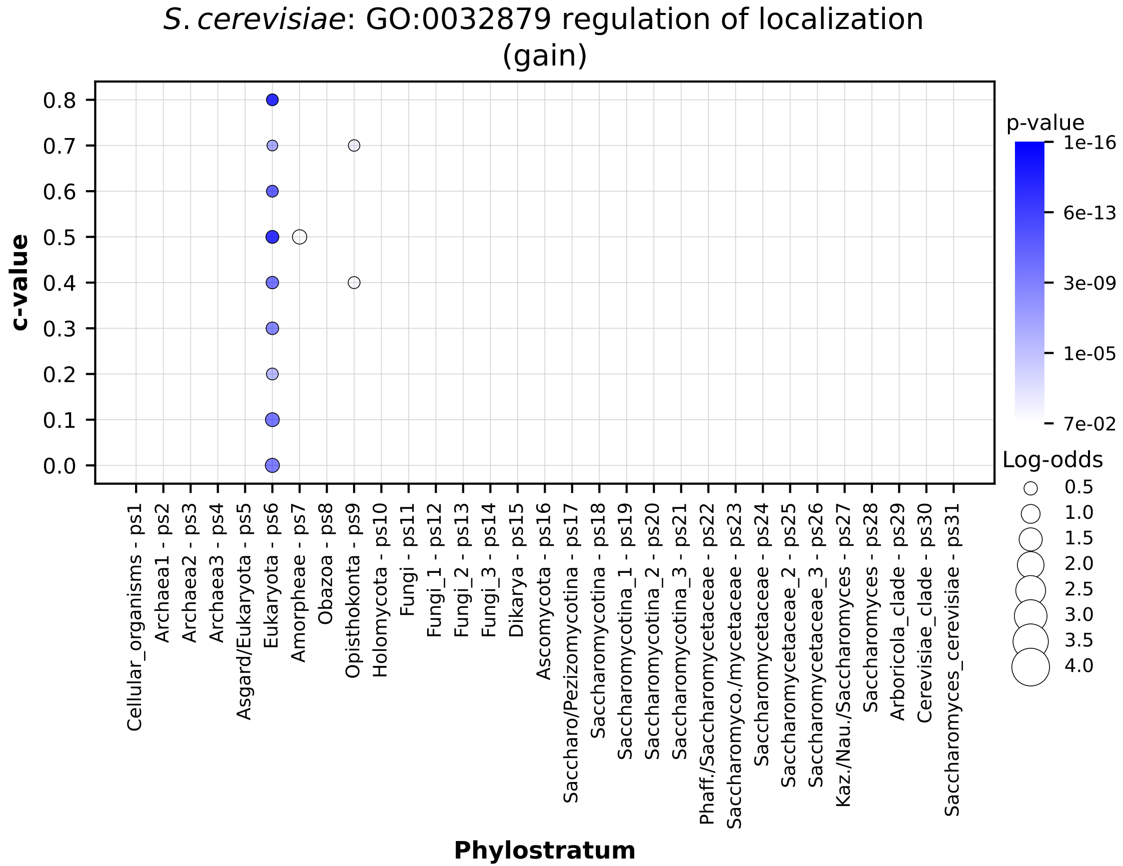

S. cerevisiae: GO:0032879 regulation of localization
(gain)
c-value
0.8
0.7
0.6
0.5
0.4
0.3
0.2
0.1
0.0
p-value
1e-16
6e-13
3e-09
1e-05
7e-02
Log-odds
0.5
1.0
1.5
2.0
2.5
3.0
3.5
4.0
Cellular_organisms - ps1
Archaea1 - ps2
Archaea2 - ps3
Archaea3 - ps4
Asgard/Eukaryota - ps5
Eukaryota - ps6
Amorpheae - ps7
Obazoa - ps8
Opisthokonta - ps9
Holomycota - ps10
Fungi - ps11
Fungi_1 - ps12
Fungi_2 - ps13
Fungi_3 - ps14
Dikarya - ps15
Ascomycota - ps16
Saccharo/Pezizomycotina - ps17
Saccharomycotina - ps18
Saccharomycotina_1 - ps19
Saccharomycotina_2 - ps20
Saccharomycotina_3 - ps21
Phaff./Saccharomycetaceae - ps22
Saccharomyco./mycetaceae - ps23
Saccharomycetaceae - ps24
Saccharomycetaceae_2 - ps25
Saccharomycetaceae_3 - ps26
Kaz./Nau./Saccharomyces - ps27
Saccharomyces - ps28
Arboricola_clade - ps29
Cerevisiae_clade - ps30
Saccharomyces_cerevisiae - ps31
Phylostratum

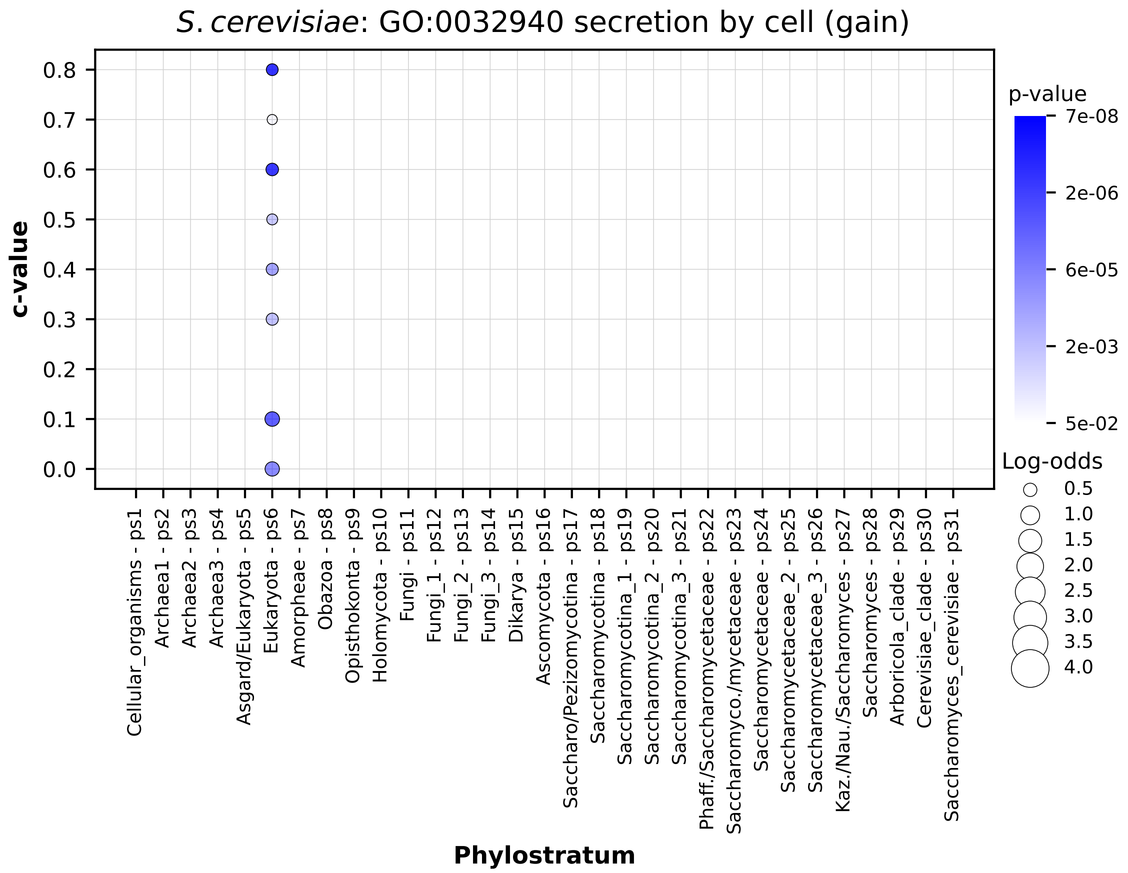

S. cerevisiae: GO:0032940 secretion by cell (gain)
c-value
0.0
0.1
0.2
0.3
0.4
0.5
0.6
0.7
0.8
p-value
7e-08
2e-06
6e-05
2e-03
5e-02
Log-odds
0.5
1.0
1.5
2.0
2.5
3.0
3.5
4.0
Cellular_organisms - ps1
Archaea1 - ps2
Archaea2 - ps3
Archaea3 - ps4
Asgard/Eukaryota - ps5
Eukaryota - ps6
Amorpheae - ps7
Obazoa - ps8
Opisthokonta - ps9
Holomycota - ps10
Fungi - ps11
Fungi_1 - ps12
Fungi_2 - ps13
Fungi_3 - ps14
Dikarya - ps15
Ascomycota - ps16
Saccharo/Pezizomycotina - ps17
Saccharomycotina - ps18
Saccharomycotina_1 - ps19
Saccharomycotina_2 - ps20
Saccharomycotina_3 - ps21
Phaff./Saccharomycetaceae - ps22
Saccharomyco./mycetaceae - ps23
Saccharomycetaceae - ps24
Saccharomycetaceae_2 - ps25
Saccharomycetaceae_3 - ps26
Kaz./Nau./Saccharomyces - ps27
Saccharomyces - ps28
Arboricola_clade - ps29
Cerevisiae_clade - ps30
Saccharomyces_cerevisiae - ps31
Phylostratum

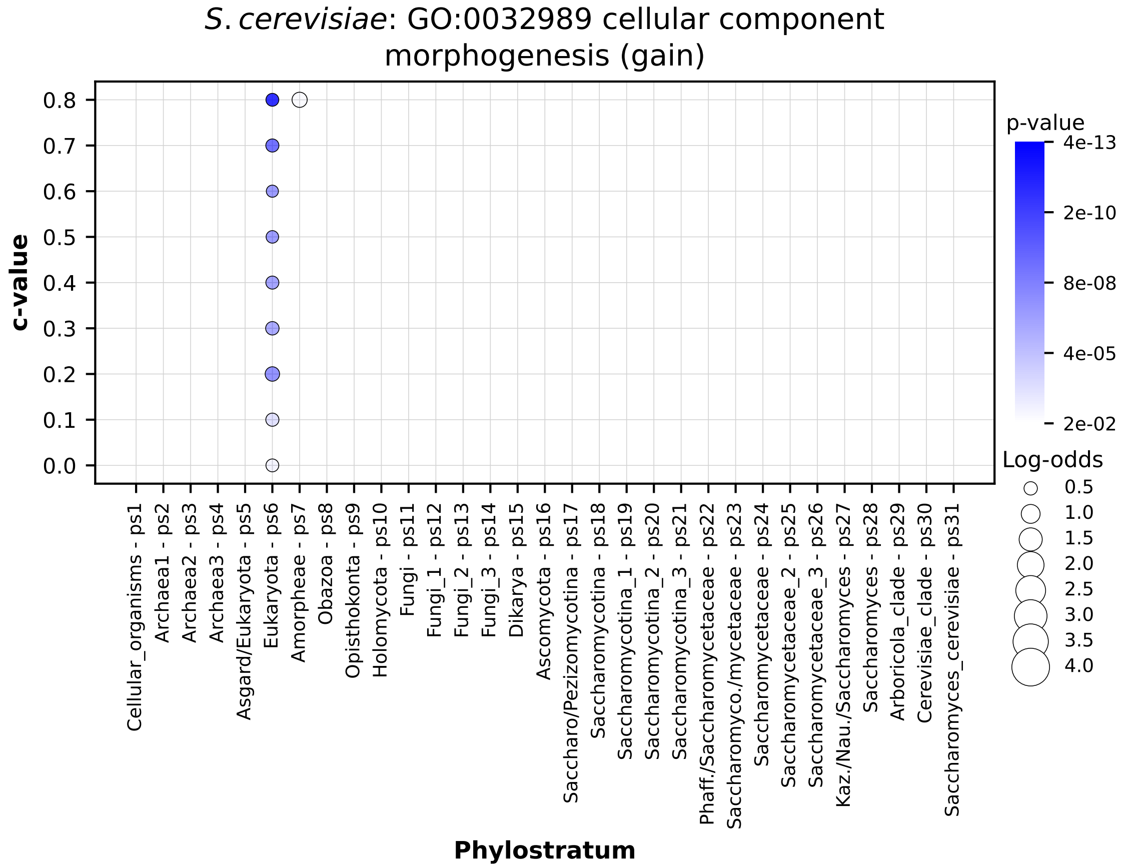

S. cerevisiae: GO:0032989 cellular component morphogenesis (gain)
c-value
0.0
0.1
0.2
0.3
0.4
0.5
0.6
0.7
0.8
Phylostratum
Cellular_organisms - ps1
Archaea1 - ps2
Archaea2 - ps3
Archaea3 - ps4
Asgard/Eukaryota - ps5
Eukaryota - ps6
Amorpheae - ps7
Obazoa - ps8
Opisthokonta - ps9
Holomycota - ps10
Fungi - ps11
Fungi_1 - ps12
Fungi_2 - ps13
Fungi_3 - ps14
Dikarya - ps15
Ascomycota - ps16
Saccharo/Pezizomycotina - ps17
Saccharomycotina - ps18
Saccharomycotina_1 - ps19
Saccharomycotina_2 - ps20
Saccharomycotina_3 - ps21
Phaff./Saccharomycetaceae - ps22
Saccharomyco./mycetaceae - ps23
Saccharomycetaceae - ps24
Saccharomycetaceae_2 - ps25
Saccharomycetaceae_3 - ps26
Kaz./Nau./Saccharomyces - ps27
Saccharomyces - ps28
Arboricola_clade - ps29
Cerevisiae_clade - ps30
Saccharomyces_cerevisiae - ps31
p-value
4e-13
2e-10
8e-08
4e-05
2e-02
Log-odds
0.5
1.0
1.5
2.0
2.5
3.0
3.5
4.0

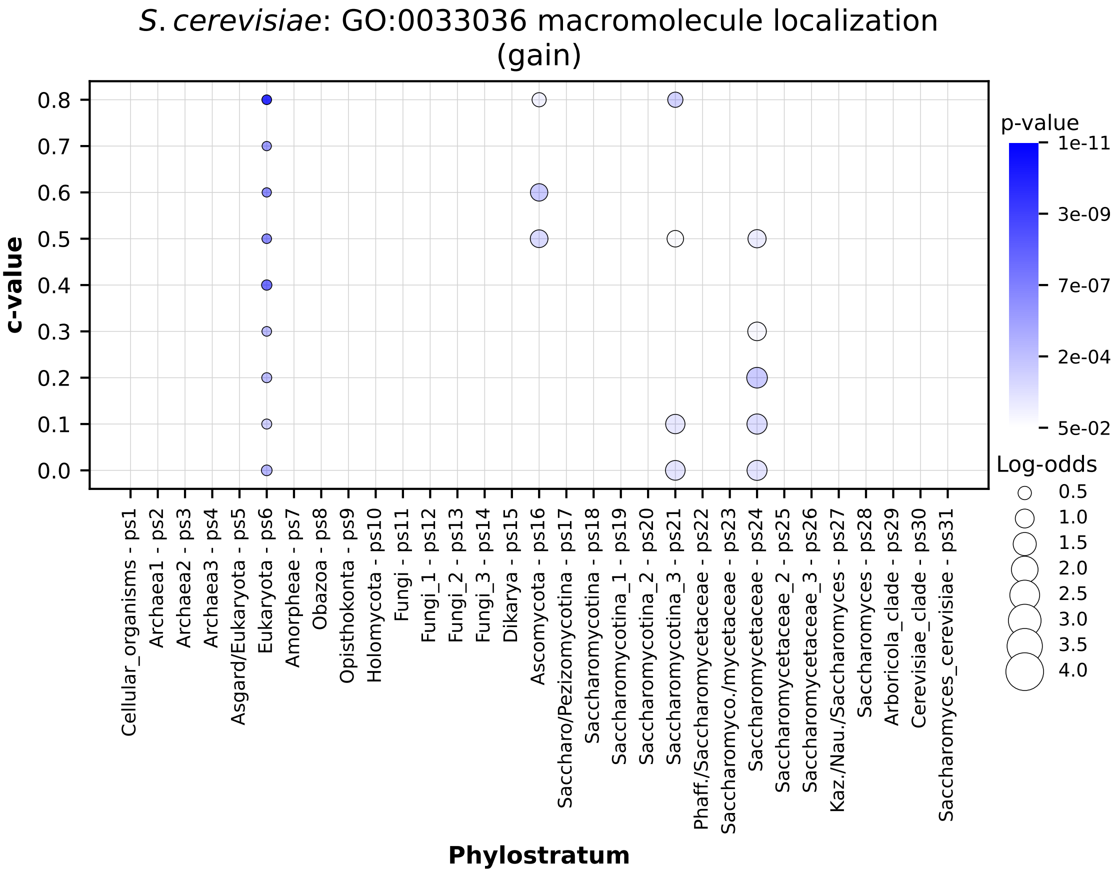

S. cerevisiae: GO:0033036 macromolecule localization
(gain)
c-value
0.8
0.7
0.6
0.5
0.4
0.3
0.2
0.1
0.0
Phylostratum
Cellular_organisms - ps1
Archaea1 - ps2
Archaea2 - ps3
Archaea3 - ps4
Asgard/Eukaryota - ps5
Eukaryota - ps6
Amorpheae - ps7
Obazoa - ps8
Opisthokonta - ps9
Holomycota - ps10
Fungi - ps11
Fungi_1 - ps12
Fungi_2 - ps13
Fungi_3 - ps14
Dikarya - ps15
Ascomycota - ps16
Saccharo/Pezizomycotina - ps17
Saccharomycotina - ps18
Saccharomycotina_1 - ps19
Saccharomycotina_2 - ps20
Saccharomycotina_3 - ps21
Phaff./Saccharomycetaceae - ps22
Saccharomyco./mycetaceae - ps23
Saccharomycetaceae - ps24
Saccharomycetaceae_2 - ps25
Saccharomycetaceae_3 - ps26
Kaz./Nau./Saccharomyces - ps27
Saccharomyces - ps28
Arboricola_clade - ps29
Cerevisiae_clade - ps30
Saccharomyces_cerevisiae - ps31
p-value
1e-11
3e-09
7e-07
2e-04
5e-02
Log-odds
0.5
1.0
1.5
2.0
2.5
3.0
3.5
4.0

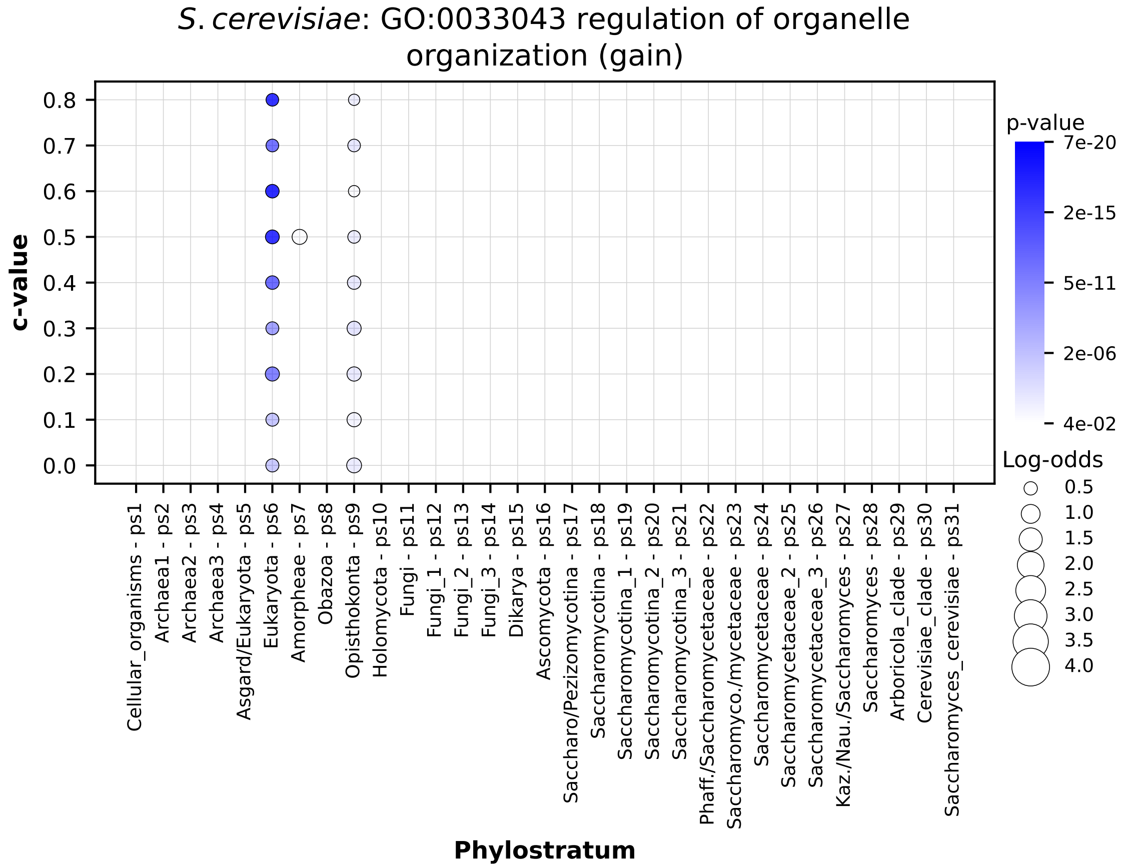

S. cerevisiae: GO:0033043 regulation of organelle organization (gain)
c-value
0.8
0.7
0.6
0.5
0.4
0.3
0.2
0.1
0.0
p-value
7e-20
2e-15
5e-11
2e-06
4e-02
Log-odds
0.5
1.0
1.5
2.0
2.5
3.0
3.5
4.0
Cellular_organisms - ps1
Archaea1 - ps2
Archaea2 - ps3
Archaea3 - ps4
Asgard/Eukaryota - ps5
Eukaryota - ps6
Amorpheae - ps7
Obazoa - ps8
Opisthokonta - ps9
Holomycota - ps10
Fungi - ps11
Fungi_1 - ps12
Fungi_2 - ps13
Fungi_3 - ps14
Dikarya - ps15
Ascomycota - ps16
Saccharo/Pezizomycotina - ps17
Saccharomycotina - ps18
Saccharomycotina_1 - ps19
Saccharomycotina_2 - ps20
Saccharomycotina_3 - ps21
Phaff./Saccharomycetaceae - ps22
Saccharomyco./mycetaceae - ps23
Saccharomycetaceae - ps24
Saccharomycetaceae_2 - ps25
Saccharomycetaceae_3 - ps26
Kaz./Nau./Saccharomyces - ps27
Saccharomyces - ps28
Arboricola_clade - ps29
Cerevisiae_clade - ps30
Saccharomyces_cerevisiae - ps31
Phylostratum

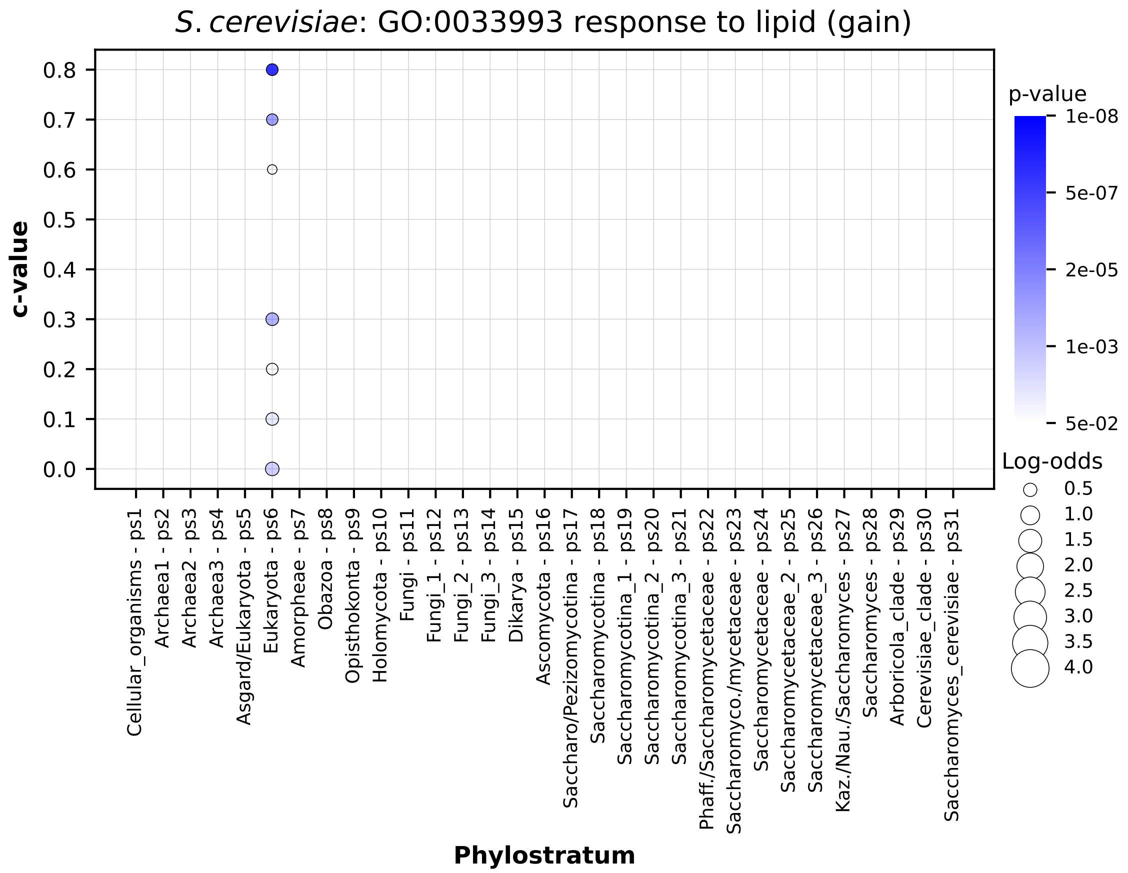

S. cerevisiae: GO:0033993 response to lipid (gain)
c-value
0.8
0.7
0.6
0.5
0.4
0.3
0.2
0.1
0.0
p-value
1e-08
5e-07
2e-05
1e-03
5e-02
Log-odds
0.5
1.0
1.5
2.0
2.5
3.0
3.5
4.0
Cellular_organisms - ps1
Archaea1 - ps2
Archaea2 - ps3
Archaea3 - ps4
Asgard/Eukaryota - ps5
Eukaryota - ps6
Amorpheae - ps7
Obazoa - ps8
Opisthokonta - ps9
Holomycota - ps10
Fungi - ps11
Fungi_1 - ps12
Fungi_2 - ps13
Fungi_3 - ps14
Dikarya - ps15
Ascomycota - ps16
Saccharo/Pezizomycotina - ps17
Saccharomycotina - ps18
Saccharomycotina_1 - ps19
Saccharomycotina_2 - ps20
Saccharomycotina_3 - ps21
Phaff./Saccharomycetaceae - ps22
Saccharomyco./mycetaceae - ps23
Saccharomycetaceae - ps24
Saccharomycetaceae_2 - ps25
Saccharomycetaceae_3 - ps26
Kaz./Nau./Saccharomyces - ps27
Saccharomyces - ps28
Arboricola_clade - ps29
Cerevisiae_clade - ps30
Saccharomyces_cerevisiae - ps31
Phylostratum

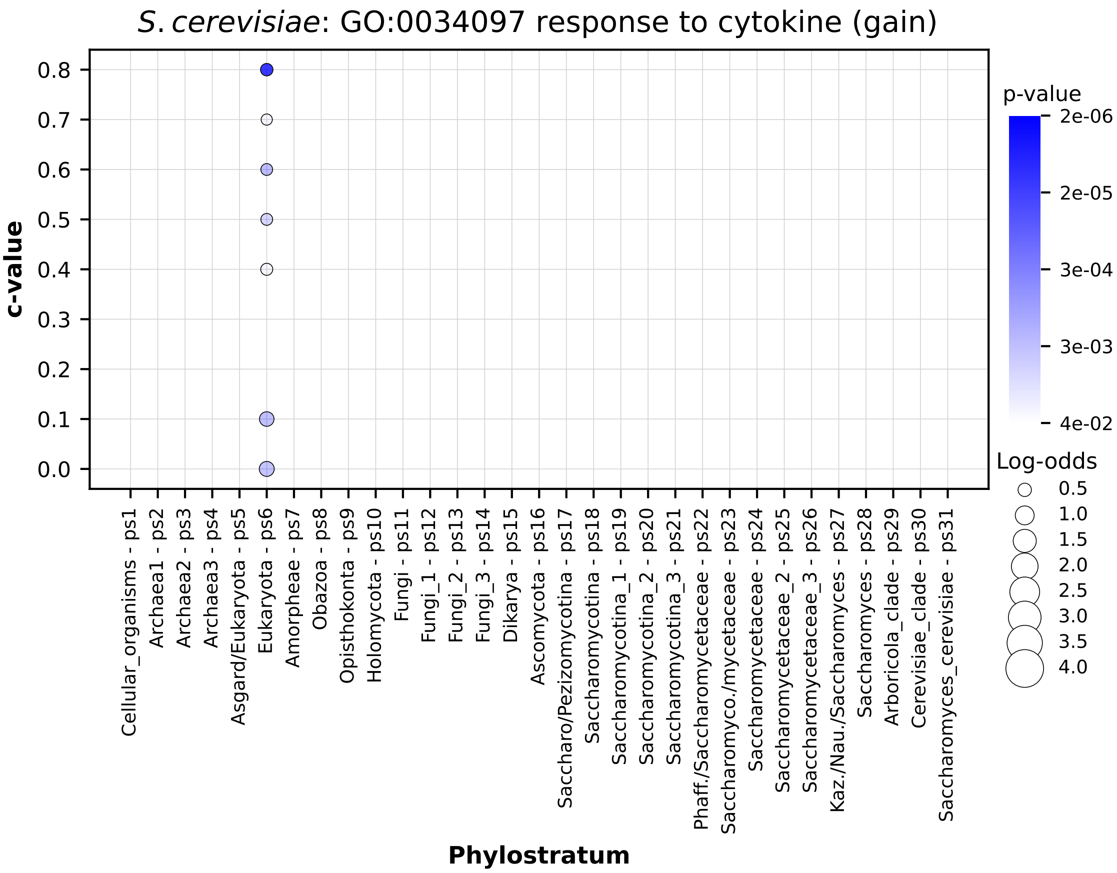

S. cerevisiae: GO:0034097 response to cytokine (gain)
c-value
0.8
0.7
0.6
0.5
0.4
0.3
0.2
0.1
0.0
p-value
2e-06
2e-05
3e-04
3e-03
4e-02
Log-odds
0.5
1.0
1.5
2.0
2.5
3.0
3.5
4.0
Cellular_organisms - ps1
Archaea1 - ps2
Archaea2 - ps3
Archaea3 - ps4
Asgard/Eukaryota - ps5
Eukaryota - ps6
Amorpheae - ps7
Obazoa - ps8
Opisthokonta - ps9
Holomycota - ps10
Fungi - ps11
Fungi_1 - ps12
Fungi_2 - ps13
Fungi_3 - ps14
Dikarya - ps15
Ascomycota - ps16
Saccharo/Pezizomycotina - ps17
Saccharomycotina - ps18
Saccharomycotina_1 - ps19
Saccharomycotina_2 - ps20
Saccharomycotina_3 - ps21
Phaff./Saccharomycetaceae - ps22
Saccharomyco./mycetaceae - ps23
Saccharomycetaceae - ps24
Saccharomycetaceae_2 - ps25
Saccharomycetaceae_3 - ps26
Kaz./Nau./Saccharomyces - ps27
Saccharomyces - ps28
Arboricola_clade - ps29
Cerevisiae_clade - ps30
Saccharomyces_cerevisiae - ps31
Phylostratum

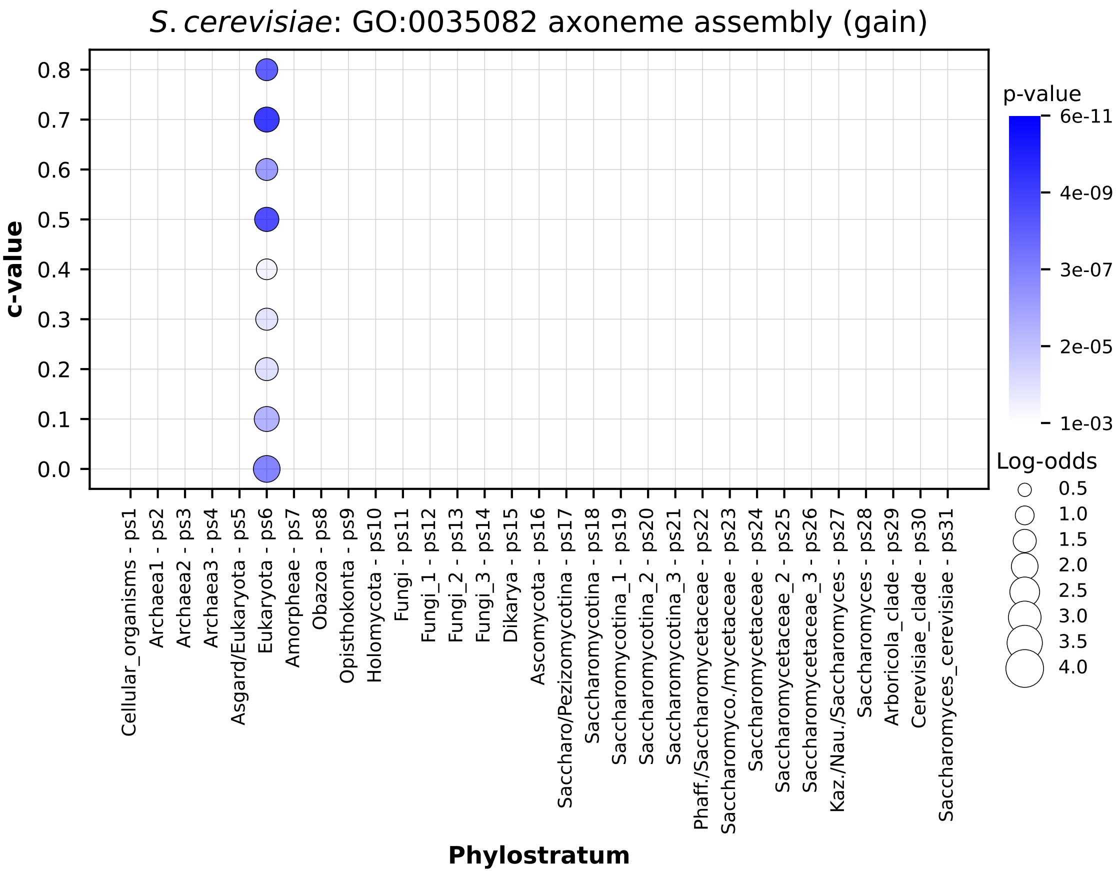

S. cerevisiae: GO:0035082 axoneme assembly (gain)
c-value
0.0
0.1
0.2
0.3
0.4
0.5
0.6
0.7
0.8
p-value
6e-11
4e-09
3e-07
2e-05
1e-03
Log-odds
0.5
1.0
1.5
2.0
2.5
3.0
3.5
4.0
Cellular_organisms - ps1
Archaea1 - ps2
Archaea2 - ps3
Archaea3 - ps4
Asgard/Eukaryota - ps5
Eukaryota - ps6
Amorpheae - ps7
Obazoa - ps8
Opisthokonta - ps9
Holomycota - ps10
Fungi - ps11
Fungi_1 - ps12
Fungi_2 - ps13
Fungi_3 - ps14
Dikarya - ps15
Ascomycota - ps16
Saccharo/Pezizomycotina - ps17
Saccharomycotina - ps18
Saccharomycotina_1 - ps19
Saccharomycotina_2 - ps20
Saccharomycotina_3 - ps21
Phaff./Saccharomycetaceae - ps22
Saccharomyco./mycetaceae - ps23
Saccharomycetaceae - ps24
Saccharomycetaceae_2 - ps25
Saccharomycetaceae_3 - ps26
Kaz./Nau./Saccharomyces - ps27
Saccharomyces - ps28
Arboricola_clade - ps29
Cerevisiae_clade - ps30
Saccharomyces_cerevisiae - ps31
Phylostratum

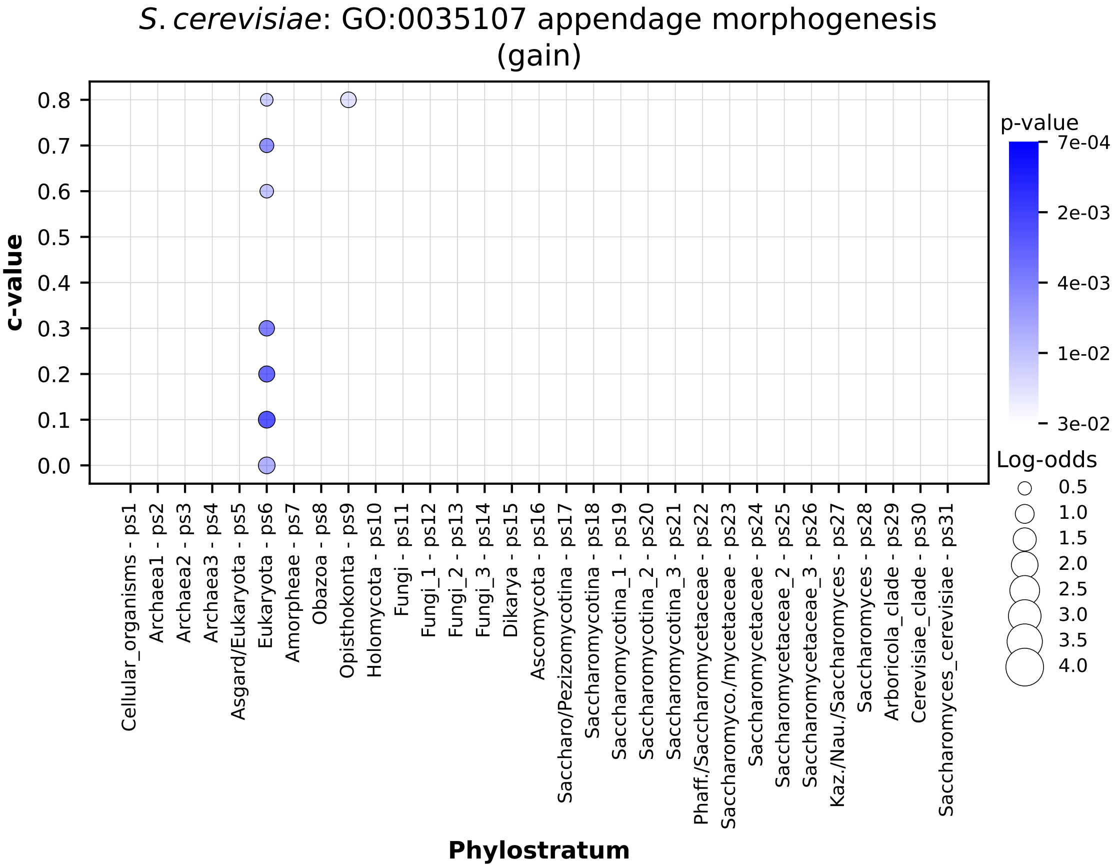

S. cerevisiae: GO:0035107 appendage morphogenesis
(gain)
c-value
0.0
0.1
0.2
0.3
0.4
0.5
0.6
0.7
0.8
p-value
7e-04
2e-03
4e-03
1e-02
3e-02
Log-odds
0.5
1.0
1.5
2.0
2.5
3.0
3.5
4.0
Cellular_organisms - ps1
Archaea1 - ps2
Archaea2 - ps3
Archaea3 - ps4
Asgard/Eukaryota - ps5
Eukaryota - ps6
Amorpheae - ps7
Obazoa - ps8
Opisthokonta - ps9
Holomycota - ps10
Fungi - ps11
Fungi_1 - ps12
Fungi_2 - ps13
Fungi_3 - ps14
Dikarya - ps15
Ascomycota - ps16
Saccharo/Pezizomycotina - ps17
Saccharomycotina - ps18
Saccharomycotina_1 - ps19
Saccharomycotina_2 - ps20
Saccharomycotina_3 - ps21
Phaff./Saccharomycetaceae - ps22
Saccharomyco./mycetaceae - ps23
Saccharomycetaceae - ps24
Saccharomycetaceae_2 - ps25
Saccharomycetaceae_3 - ps26
Kaz./Nau./Saccharomyces - ps27
Saccharomyces - ps28
Arboricola_clade - ps29
Cerevisiae_clade - ps30
Saccharomyces_cerevisiae - ps31
Phylostratum

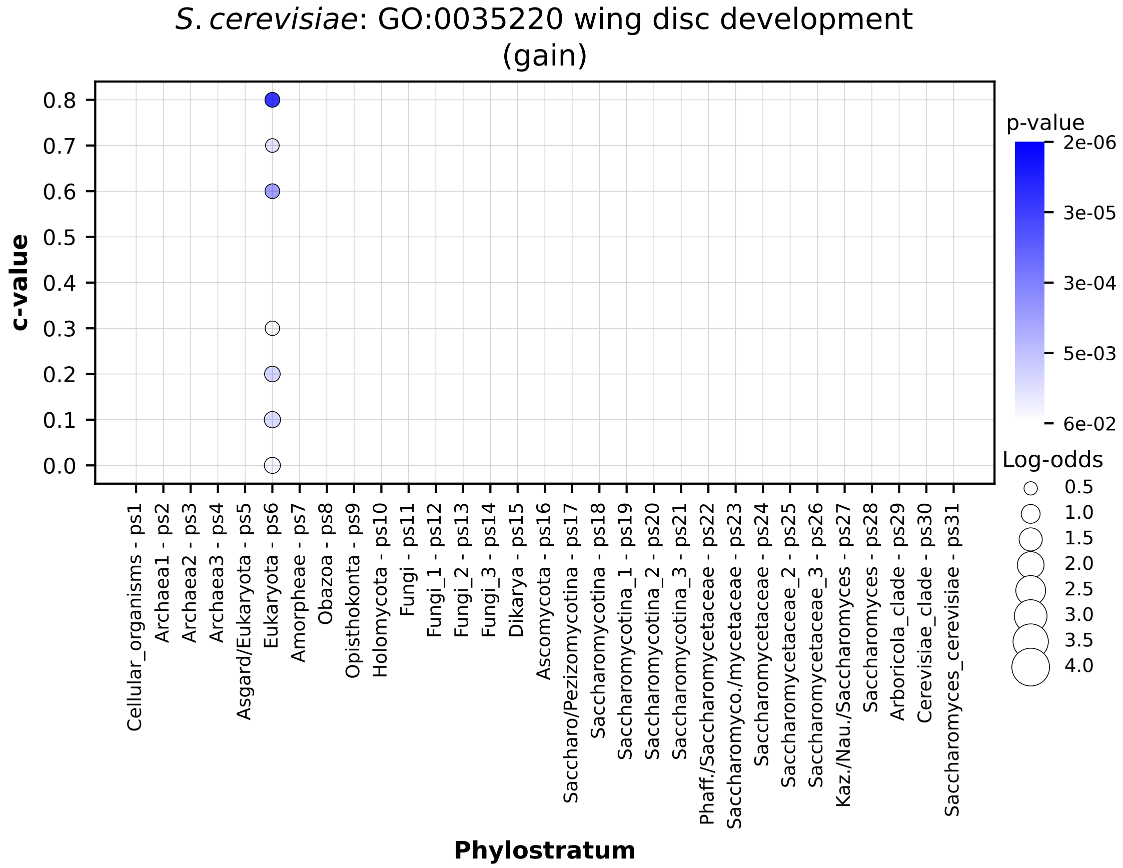
S. cerevisiae: GO:0035220 wing disc development
(gain)
c-value
0.8
0.7
0.6
0.5
0.4
0.3
0.2
0.1
0.0
p-value
2e-06
3e-05
3e-04
5e-03
6e-02
Log-odds
0.5
1.0
1.5
2.0
2.5
3.0
3.5
4.0
Cellular_organisms - ps1
Archaea1 - ps2
Archaea2 - ps3
Archaea3 - ps4
Asgard/Eukaryota - ps5
Eukaryota - ps6
Amorpheae - ps7
Obazoa - ps8
Opisthokonta - ps9
Holomycota - ps10
Fungi - ps11
Fungi_1 - ps12
Fungi_2 - ps13
Fungi_3 - ps14
Dikarya - ps15
Ascomycota - ps16
Saccharo/Pezizomycotina - ps17
Saccharomycotina - ps18
Saccharomycotina_1 - ps19
Saccharomycotina_2 - ps20
Saccharomycotina_3 - ps21
Phaff./Saccharomycetaceae - ps22
Saccharomyco./mycetaceae - ps23
Saccharomycetaceae - ps24
Saccharomycetaceae_2 - ps25
Saccharomycetaceae_3 - ps26
Kaz./Nau./Saccharomyces - ps27
Saccharomyces - ps28
Arboricola_clade - ps29
Cerevisiae_clade - ps30
Saccharomyces_cerevisiae - ps31
Phylostratum

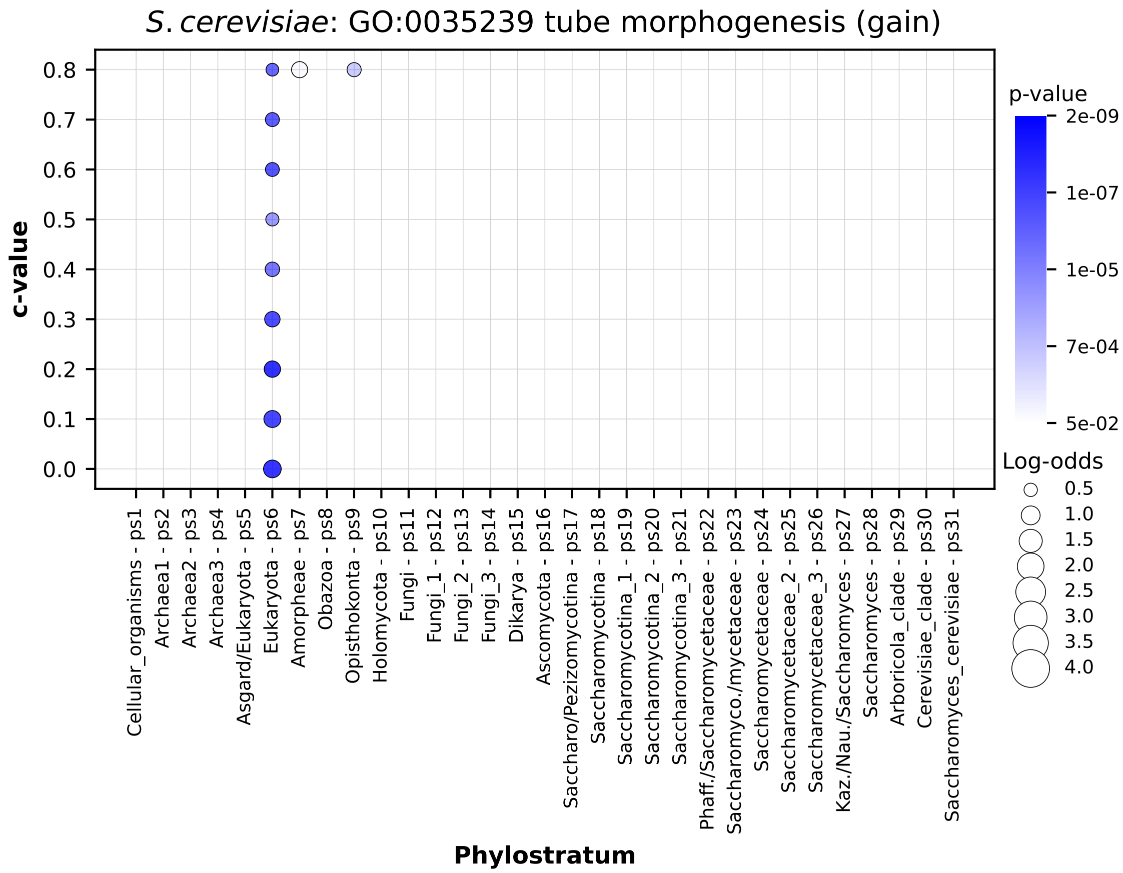
S. cerevisiae: GO:0035239 tube morphogenesis (gain)
c-value
0.0
0.1
0.2
0.3
0.4
0.5
0.6
0.7
0.8
p-value
2e-09
1e-07
1e-05
7e-04
5e-02
Log-odds
0.5
1.0
1.5
2.0
2.5
3.0
3.5
4.0
Cellular_organisms - ps1
Archaea1 - ps2
Archaea2 - ps3
Archaea3 - ps4
Asgard/Eukaryota - ps5
Eukaryota - ps6
Amorpheae - ps7
Obazoa - ps8
Opisthokonta - ps9
Holomycota - ps10
Fungi - ps11
Fungi_1 - ps12
Fungi_2 - ps13
Fungi_3 - ps14
Dikarya - ps15
Ascomycota - ps16
Saccharo/Pezizomycotina - ps17
Saccharomycotina - ps18
Saccharomycotina_1 - ps19
Saccharomycotina_2 - ps20
Saccharomycotina_3 - ps21
Phaff./Saccharomycetaceae - ps22
Saccharomyco./mycetaceae - ps23
Saccharomycetaceae - ps24
Saccharomycetaceae_2 - ps25
Saccharomycetaceae_3 - ps26
Kaz./Nau./Saccharomyces - ps27
Saccharomyces - ps28
Arboricola_clade - ps29
Cerevisiae_clade - ps30
Saccharomyces_cerevisiae - ps31
Phylostratum

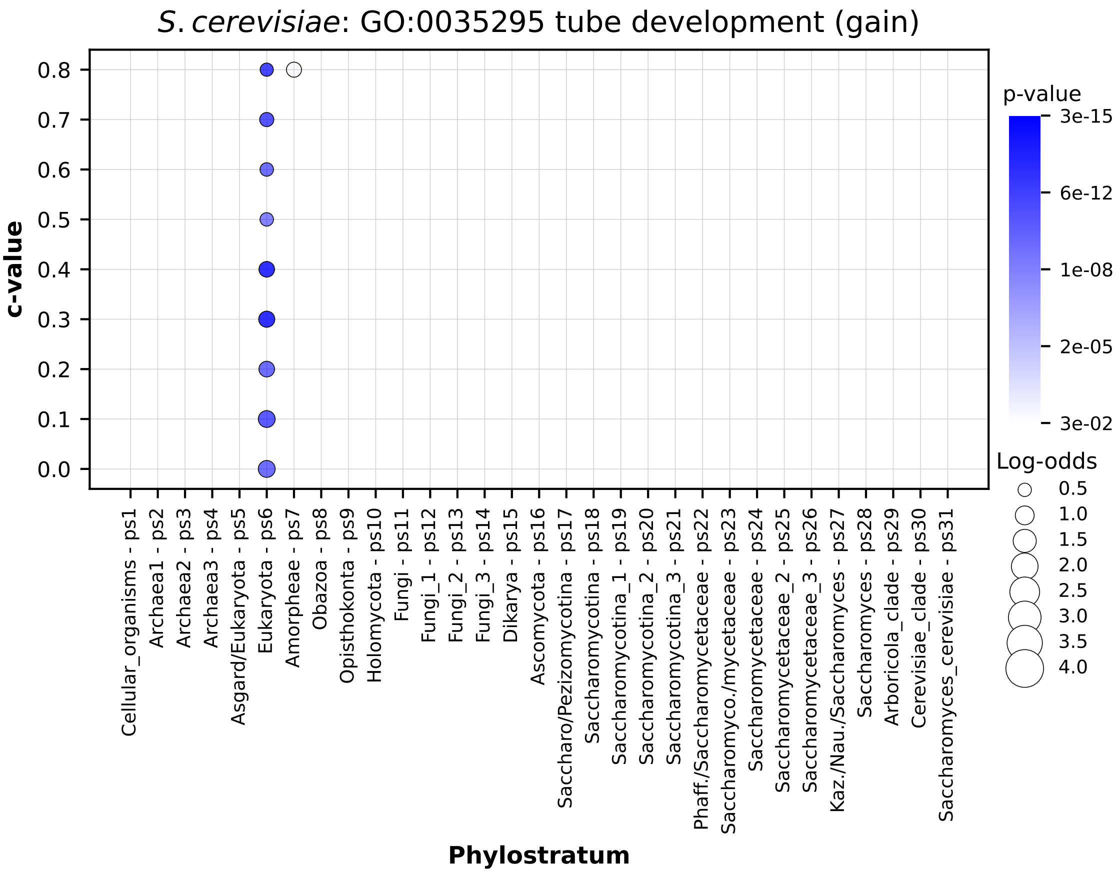

S. cerevisiae: GO:0035295 tube development (gain)
c-value
0.8
0.7
0.6
0.5
0.4
0.3
0.2
0.1
0.0
p-value
3e-15
6e-12
1e-08
2e-05
3e-02
Log-odds
0.5
1.0
1.5
2.0
2.5
3.0
3.5
4.0
Cellular_organisms - ps1
Archaea1 - ps2
Archaea2 - ps3
Archaea3 - ps4
Asgard/Eukaryota - ps5
Eukaryota - ps6
Amorpheae - ps7
Obazoa - ps8
Opisthokonta - ps9
Holomycota - ps10
Fungi - ps11
Fungi_1 - ps12
Fungi_2 - ps13
Fungi_3 - ps14
Dikarya - ps15
Ascomycota - ps16
Saccharo/Pezizomycotina - ps17
Saccharomycotina - ps18
Saccharomycotina_1 - ps19
Saccharomycotina_2 - ps20
Saccharomycotina_3 - ps21
Phaff./Saccharomycetaceae - ps22
Saccharomyco./mycetaceae - ps23
Saccharomycetaceae - ps24
Saccharomycetaceae_2 - ps25
Saccharomycetaceae_3 - ps26
Kaz./Nau./Saccharomyces - ps27
Saccharomyces - ps28
Arboricola_clade - ps29
Cerevisiae_clade - ps30
Saccharomyces_cerevisiae - ps31
Phylostratum

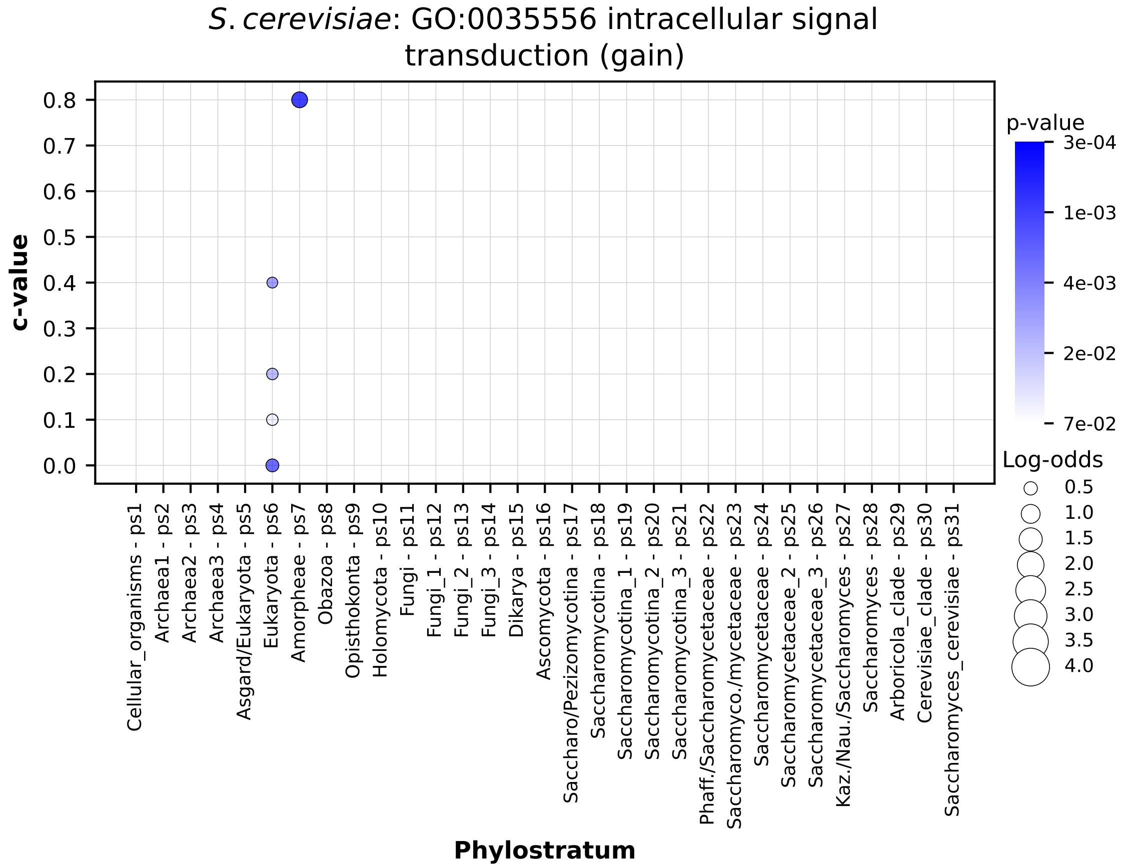

S. cerevisiae: GO:0035556 intracellular signal transduction (gain)
c-value
0.0
0.1
0.2
0.3
0.4
0.5
0.6
0.7
0.8
p-value
3e-04
1e-03
4e-03
2e-02
7e-02
Log-odds
0.5
1.0
1.5
2.0
2.5
3.0
3.5
4.0
Cellular_organisms - ps1
Archaea1 - ps2
Archaea2 - ps3
Archaea3 - ps4
Asgard/Eukaryota - ps5
Eukaryota - ps6
Amorpheae - ps7
Obazoa - ps8
Opisthokonta - ps9
Holomycota - ps10
Fungi - ps11
Fungi_1 - ps12
Fungi_2 - ps13
Fungi_3 - ps14
Dikarya - ps15
Ascomycota - ps16
Saccharo/Pezizomycotina - ps17
Saccharomycotina - ps18
Saccharomycotina_1 - ps19
Saccharomycotina_2 - ps20
Saccharomycotina_3 - ps21
Phaff./Saccharomycetaceae - ps22
Saccharomyco./mycetaceae - ps23
Saccharomycetaceae - ps24
Saccharomycetaceae_2 - ps25
Saccharomycetaceae_3 - ps26
Kaz./Nau./Saccharomyces - ps27
Saccharomyces - ps28
Arboricola_clade - ps29
Cerevisiae_clade - ps30
Saccharomyces_cerevisiae - ps31
Phylostratum

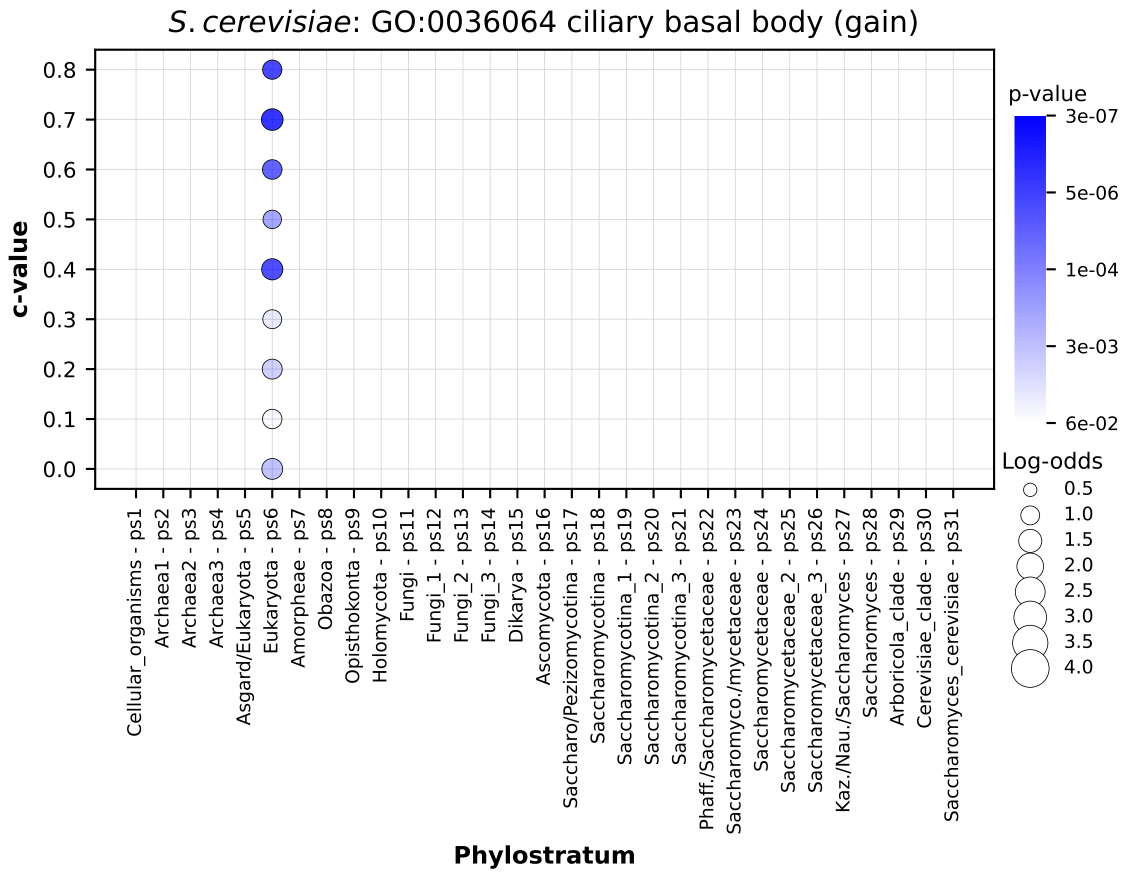

S. cerevisiae: GO:0036064 ciliary basal body (gain)
c-value
0.8
0.7
0.6
0.5
0.4
0.3
0.2
0.1
0.0
p-value
3e-07
5e-06
1e-04
3e-03
6e-02
Log-odds
0.5
1.0
1.5
2.0
2.5
3.0
3.5
4.0
Cellular_organisms - ps1
Archaea1 - ps2
Archaea2 - ps3
Archaea3 - ps4
Asgard/Eukaryota - ps5
Eukaryota - ps6
Amorpheae - ps7
Obazoa - ps8
Opisthokonta - ps9
Holomycota - ps10
Fungi - ps11
Fungi_1 - ps12
Fungi_2 - ps13
Fungi_3 - ps14
Dikarya - ps15
Ascomycota - ps16
Saccharo/Pezizomycotina - ps17
Saccharomycotina - ps18
Saccharomycotina_1 - ps19
Saccharomycotina_2 - ps20
Saccharomycotina_3 - ps21
Phaff./Saccharomycetaceae - ps22
Saccharomyco./mycetaceae - ps23
Saccharomycetaceae - ps24
Saccharomycetaceae_2 - ps25
Saccharomycetaceae_3 - ps26
Kaz./Nau./Saccharomyces - ps27
Saccharomyces - ps28
Arboricola_clade - ps29
Cerevisiae_clade - ps30
Saccharomyces_cerevisiae - ps31
Phylostratum

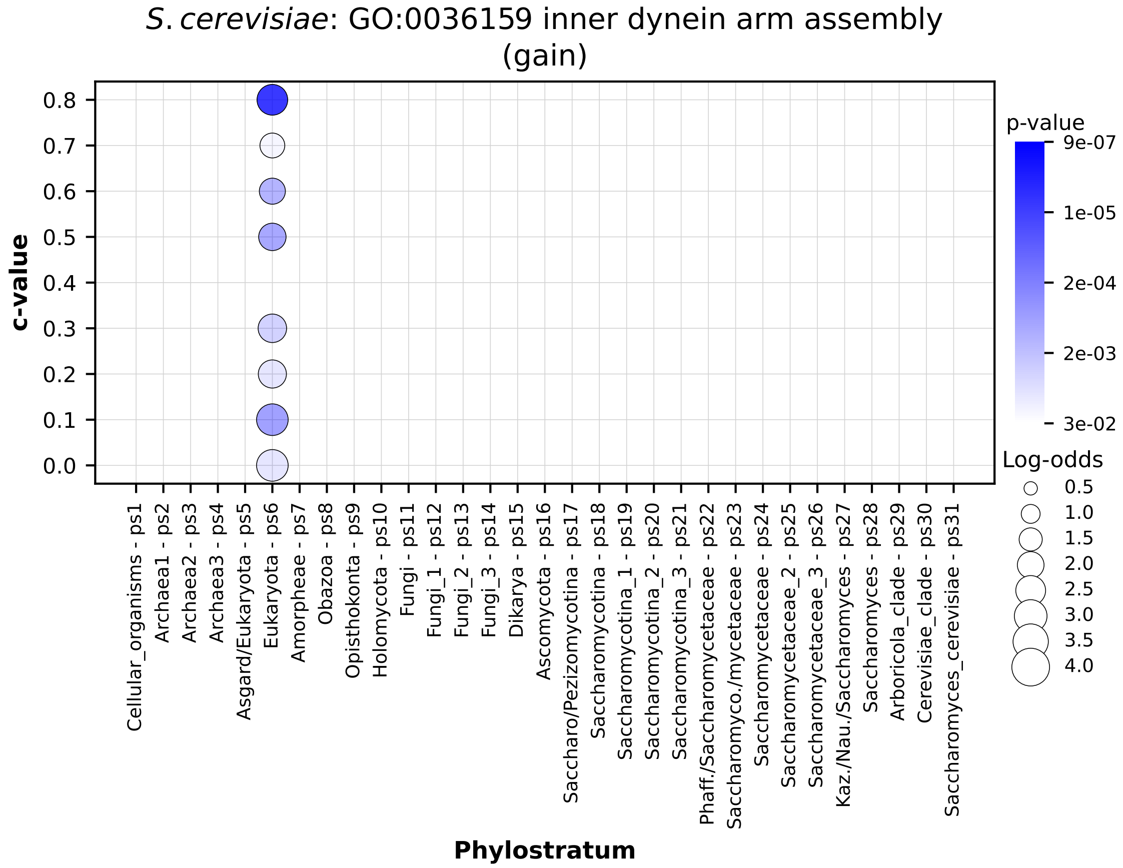

S. cerevisiae: GO:0036159 inner dynein arm assembly (gain)
c-value
0.8
0.7
0.6
0.5
0.4
0.3
0.2
0.1
0.0
p-value
9e-07
1e-05
2e-04
2e-03
3e-02
Log-odds
0.5
1.0
1.5
2.0
2.5
3.0
3.5
4.0
Cellular_organisms - ps1
Archaea1 - ps2
Archaea2 - ps3
Archaea3 - ps4
Asgard/Eukaryota - ps5
Eukaryota - ps6
Amorpheae - ps7
Obazoa - ps8
Opisthokonta - ps9
Holomycota - ps10
Fungi - ps11
Fungi_1 - ps12
Fungi_2 - ps13
Fungi_3 - ps14
Dikarya - ps15
Ascomycota - ps16
Saccharo/Pezizomycotina - ps17
Saccharomycotina - ps18
Saccharomycotina_1 - ps19
Saccharomycotina_2 - ps20
Saccharomycotina_3 - ps21
Phaff./Saccharomycetaceae - ps22
Saccharomyco./mycetaceae - ps23
Saccharomycetaceae - ps24
Saccharomycetaceae_2 - ps25
Saccharomycetaceae_3 - ps26
Kaz./Nau./Saccharomyces - ps27
Saccharomyces - ps28
Arboricola_clade - ps29
Cerevisiae_clade - ps30
Saccharomyces_cerevisiae - ps31
Phylostratum

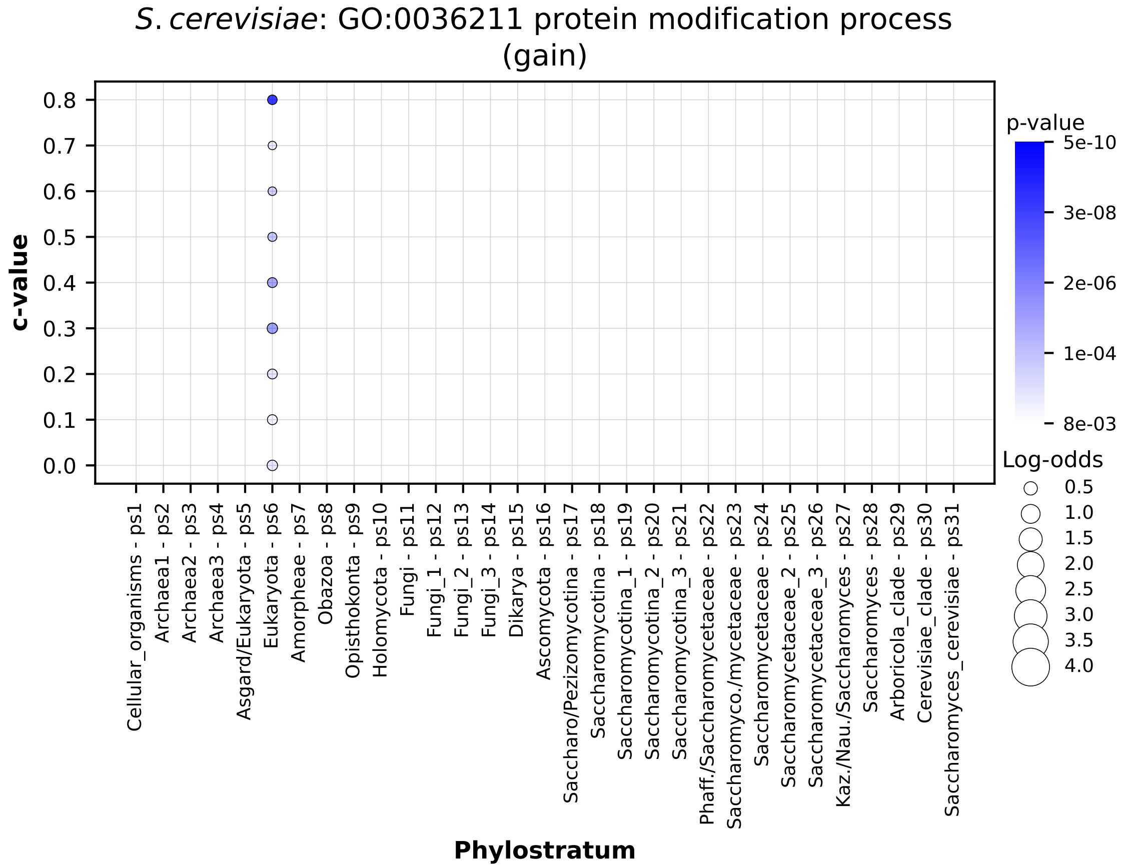

S. cerevisiae: GO:0036211 protein modification process
(gain)
c-value
0.8
0.7
0.6
0.5
0.4
0.3
0.2
0.1
0.0
p-value
5e-10
3e-08
2e-06
1e-04
8e-03
Log-odds
0.5
1.0
1.5
2.0
2.5
3.0
3.5
4.0
Cellular_organisms - ps1
Archaea1 - ps2
Archaea2 - ps3
Archaea3 - ps4
Asgard/Eukaryota - ps5
Eukaryota - ps6
Amorpheae - ps7
Obazoa - ps8
Opisthokonta - ps9
Holomycota - ps10
Fungi - ps11
Fungi_1 - ps12
Fungi_2 - ps13
Fungi_3 - ps14
Dikarya - ps15
Ascomycota - ps16
Saccharo/Pezizomycotina - ps17
Saccharomycotina - ps18
Saccharomycotina_1 - ps19
Saccharomycotina_2 - ps20
Saccharomycotina_3 - ps21
Phaff./Saccharomycetaceae - ps22
Saccharomyco./mycetaceae - ps23
Saccharomycetaceae - ps24
Saccharomycetaceae_2 - ps25
Saccharomycetaceae_3 - ps26
Kaz./Nau./Saccharomyces - ps27
Saccharomyces - ps28
Arboricola_clade - ps29
Cerevisiae_clade - ps30
Saccharomyces_cerevisiae - ps31
Phylostratum

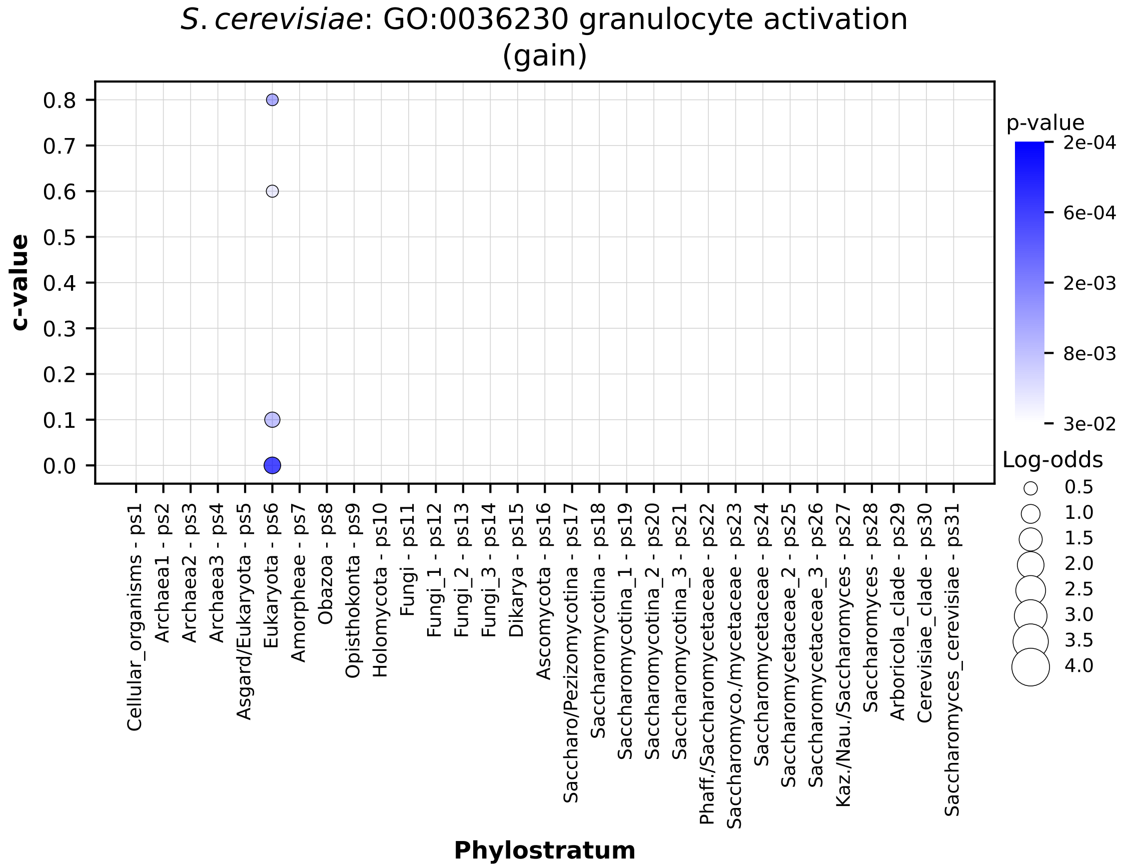

S. cerevisiae: GO:0036230 granulocyte activation
(gain)
c-value
0.8
0.7
0.6
0.5
0.4
0.3
0.2
0.1
0.0
p-value
2e-04
6e-04
2e-03
8e-03
3e-02
Log-odds
0.5
1.0
1.5
2.0
2.5
3.0
3.5
4.0
Cellular_organisms - ps1
Archaea1 - ps2
Archaea2 - ps3
Archaea3 - ps4
Asgard/Eukaryota - ps5
Eukaryota - ps6
Amorpheae - ps7
Obazoa - ps8
Opisthokonta - ps9
Holomycota - ps10
Fungi - ps11
Fungi_1 - ps12
Fungi_2 - ps13
Fungi_3 - ps14
Dikarya - ps15
Ascomycota - ps16
Saccharo/Pezizomycotina - ps17
Saccharomycotina - ps18
Saccharomycotina_1 - ps19
Saccharomycotina_2 - ps20
Saccharomycotina_3 - ps21
Phaff./Saccharomycetaceae - ps22
Saccharomyco./mycetaceae - ps23
Saccharomycetaceae - ps24
Saccharomycetaceae_2 - ps25
Saccharomycetaceae_3 - ps26
Kaz./Nau./Saccharomyces - ps27
Saccharomyces - ps28
Arboricola_clade - ps29
Cerevisiae_clade - ps30
Saccharomyces_cerevisiae - ps31
Phylostratum

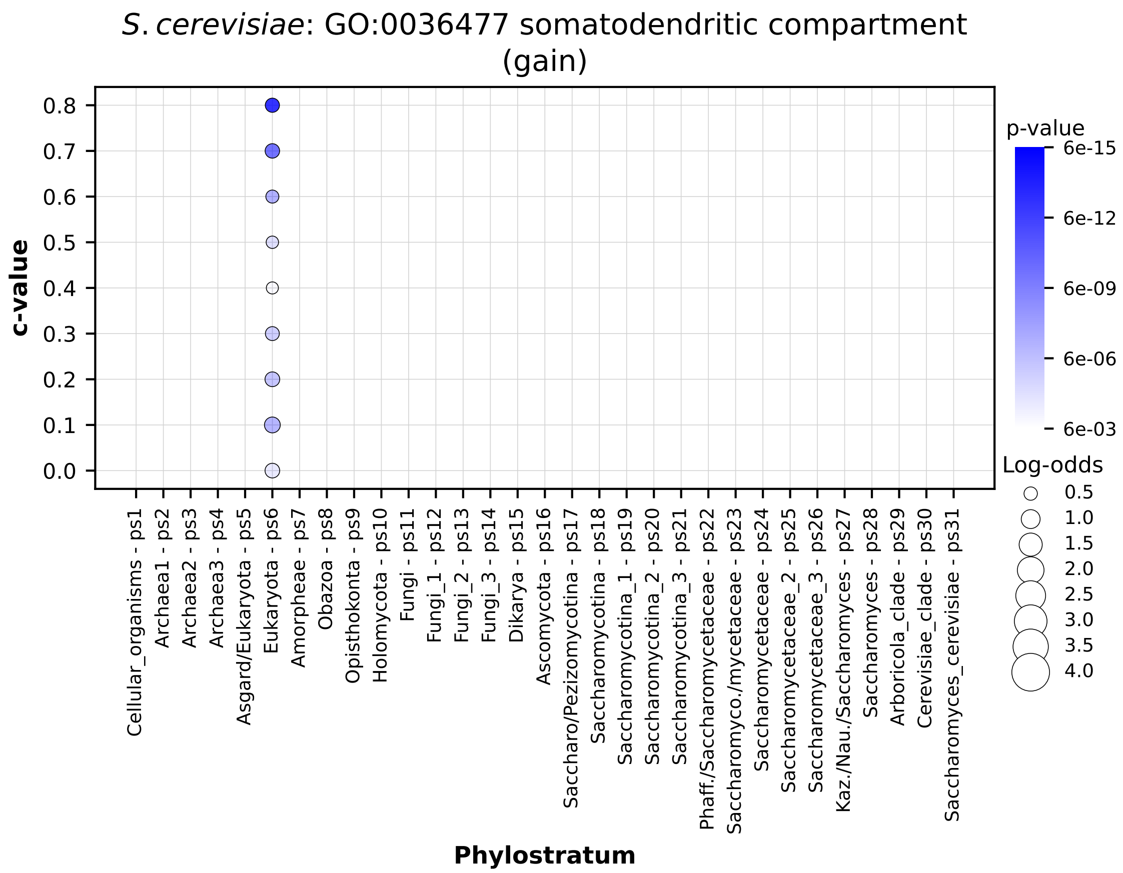

S. cerevisiae: GO:0036477 somatodendritic compartment
(gain)
c-value
0.8
0.7
0.6
0.5
0.4
0.3
0.2
0.1
0.0
p-value
6e-15
6e-12
6e-09
6e-06
6e-03
Log-odds
0.5
1.0
1.5
2.0
2.5
3.0
3.5
4.0
Cellular_organisms - ps1
Archaea1 - ps2
Archaea2 - ps3
Archaea3 - ps4
Asgard/Eukaryota - ps5
Eukaryota - ps6
Amorpheae - ps7
Obazoa - ps8
Opisthokonta - ps9
Holomycota - ps10
Fungi - ps11
Fungi_1 - ps12
Fungi_2 - ps13
Fungi_3 - ps14
Dikarya - ps15
Ascomycota - ps16
Saccharo/Pezizomycotina - ps17
Saccharomycotina - ps18
Saccharomycotina_1 - ps19
Saccharomycotina_2 - ps20
Saccharomycotina_3 - ps21
Phaff./Saccharomycetaceae - ps22
Saccharomyco./mycetaceae - ps23
Saccharomycetaceae - ps24
Saccharomycetaceae_2 - ps25
Saccharomycetaceae_3 - ps26
Kaz./Nau./Saccharomyces - ps27
Saccharomyces - ps28
Arboricola_clade - ps29
Cerevisiae_clade - ps30
Saccharomyces_cerevisiae - ps31
Phylostratum

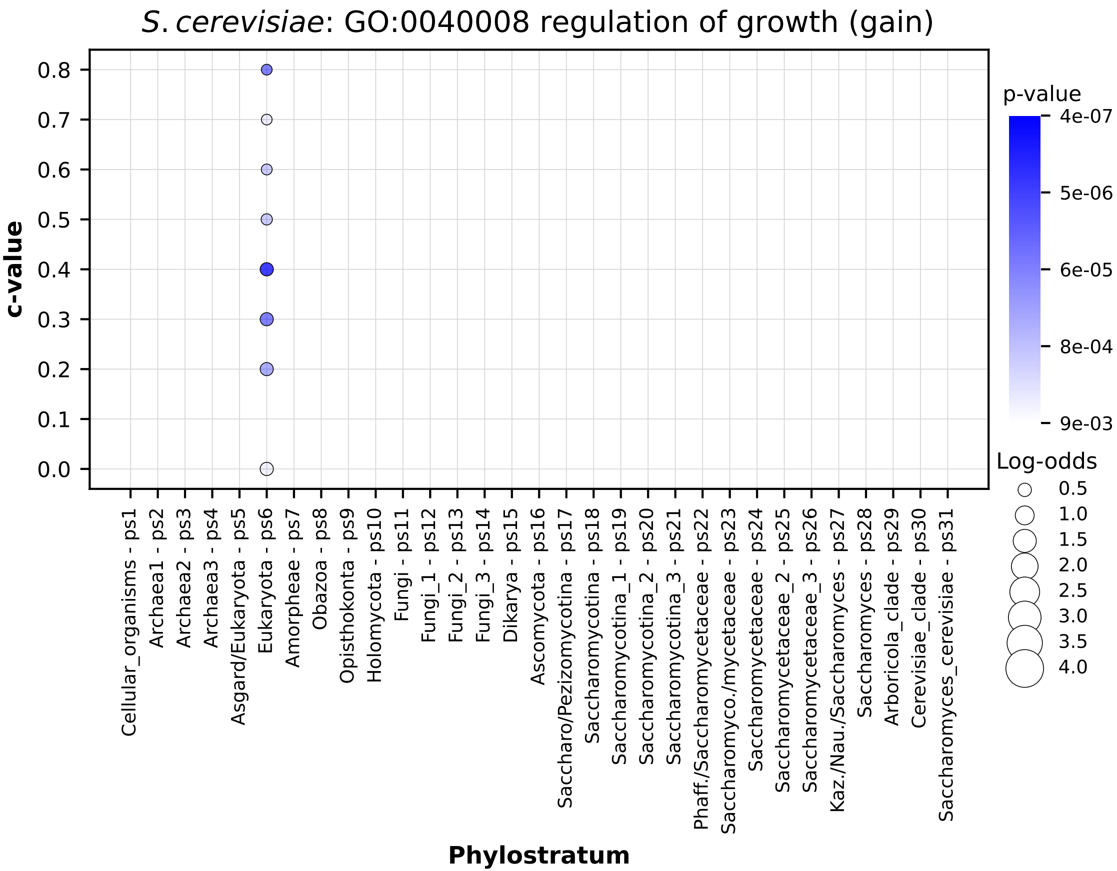
S. cerevisiae: GO:0040008 regulation of growth (gain)
c-value
0.0
0.1
0.2
0.3
0.4
0.5
0.6
0.7
0.8
Phylostratum
Cellular_organisms - ps1
Archaea1 - ps2
Archaea2 - ps3
Archaea3 - ps4
Asgard/Eukaryota - ps5
Eukaryota - ps6
Amorpheae - ps7
Obazoa - ps8
Opisthokonta - ps9
Holomycota - ps10
Fungi - ps11
Fungi_1 - ps12
Fungi_2 - ps13
Fungi_3 - ps14
Dikarya - ps15
Ascomycota - ps16
Saccharo/Pezizomycotina - ps17
Saccharomycotina - ps18
Saccharomycotina_1 - ps19
Saccharomycotina_2 - ps20
Saccharomycotina_3 - ps21
Phaff./Saccharomycetaceae - ps22
Saccharomyco./mycetaceae - ps23
Saccharomycetaceae - ps24
Saccharomycetaceae_2 - ps25
Saccharomycetaceae_3 - ps26
Kaz./Nau./Saccharomyces - ps27
Saccharomyces - ps28
Arboricola_clade - ps29
Cerevisiae_clade - ps30
Saccharomyces_cerevisiae - ps31
p-value
4e-07
5e-06
6e-05
8e-04
9e-03
Log-odds
0.5
1.0
1.5
2.0
2.5
3.0
3.5
4.0

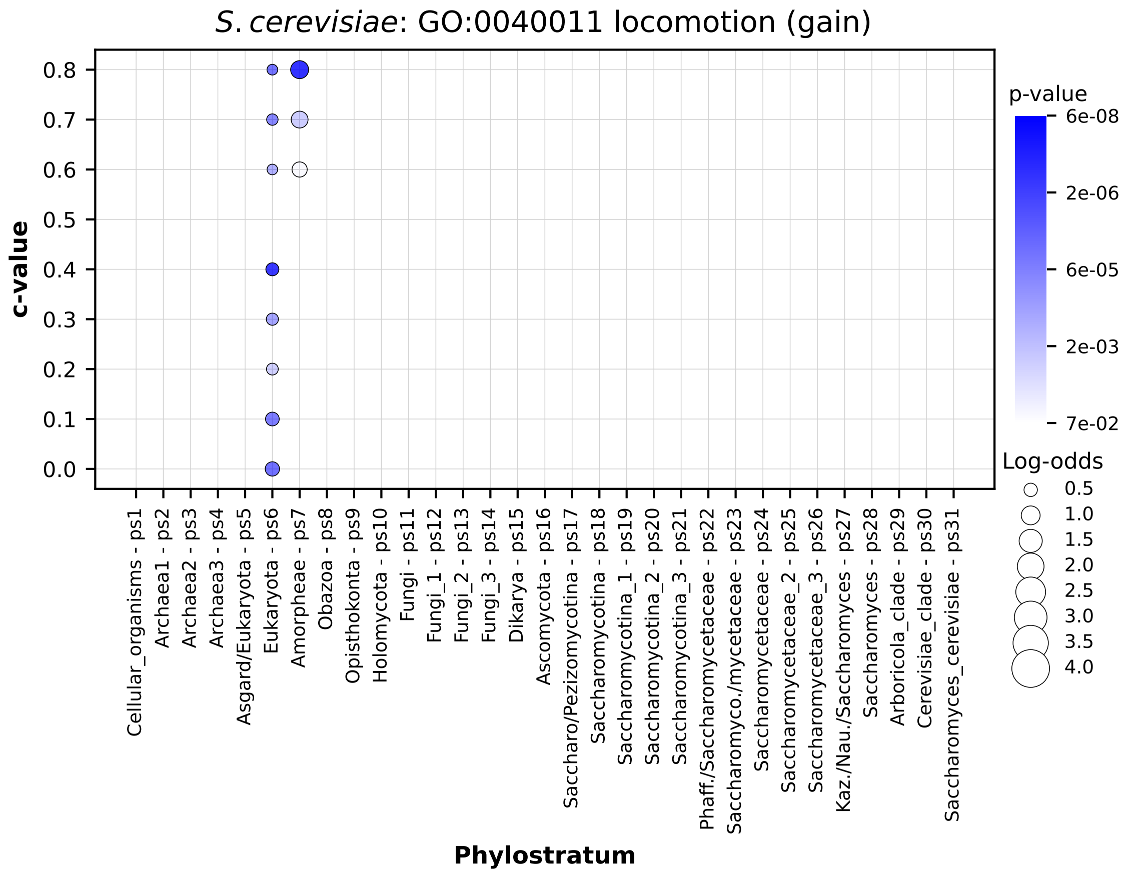
S. cerevisiae: GO:0040011 locomotion (gain)
c-value
0.8
0.7
0.6
0.5
0.4
0.3
0.2
0.1
0.0
p-value
6e-08
2e-06
6e-05
2e-03
7e-02
Log-odds
0.5
1.0
1.5
2.0
2.5
3.0
3.5
4.0
Cellular_organisms - ps1
Archaea1 - ps2
Archaea2 - ps3
Archaea3 - ps4
Asgard/Eukaryota - ps5
Eukaryota - ps6
Amorpheae - ps7
Obazoa - ps8
Opisthokonta - ps9
Holomycota - ps10
Fungi - ps11
Fungi_1 - ps12
Fungi_2 - ps13
Fungi_3 - ps14
Dikarya - ps15
Ascomycota - ps16
Saccharo/Pezizomycotina - ps17
Saccharomycotina - ps18
Saccharomycotina_1 - ps19
Saccharomycotina_2 - ps20
Saccharomycotina_3 - ps21
Phaff./Saccharomycetaceae - ps22
Saccharomyco./mycetaceae - ps23
Saccharomycetaceae - ps24
Saccharomycetaceae_2 - ps25
Saccharomycetaceae_3 - ps26
Kaz./Nau./Saccharomyces - ps27
Saccharomyces - ps28
Arboricola_clade - ps29
Cerevisiae_clade - ps30
Saccharomyces_cerevisiae - ps31
Phylostratum

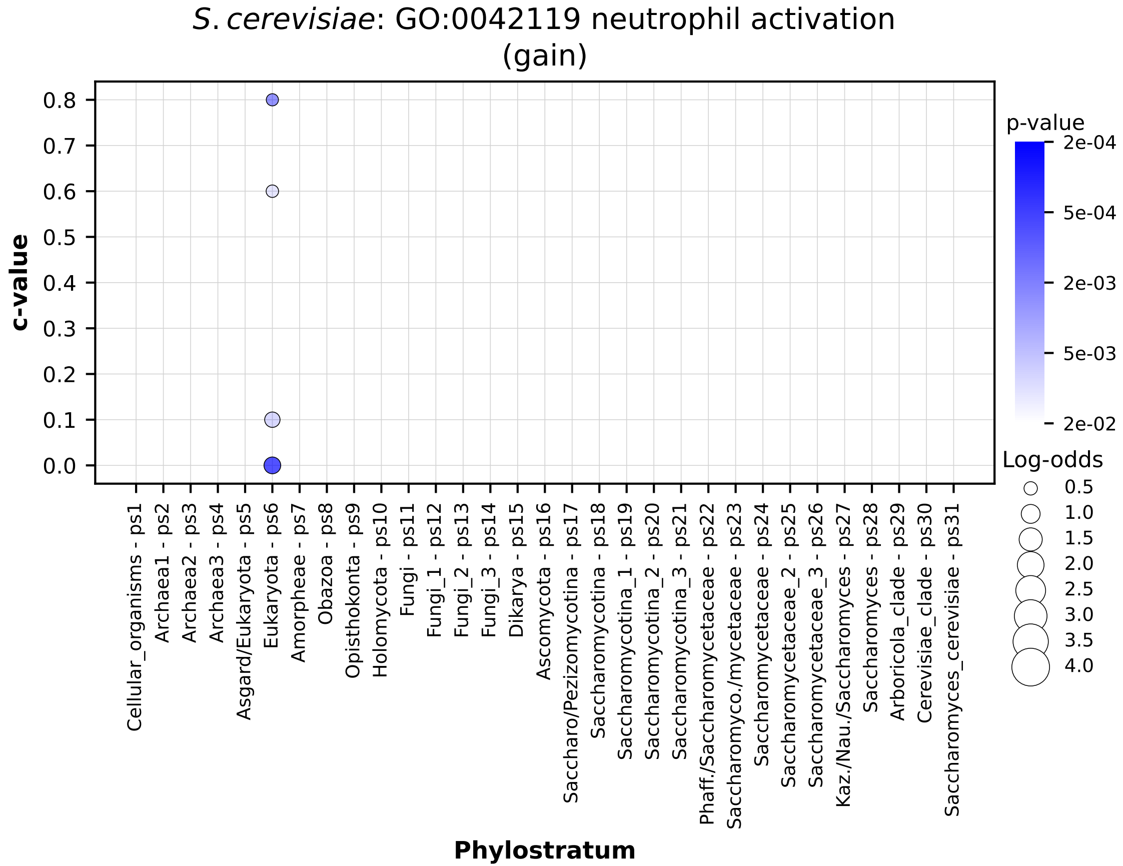
S. cerevisiae: GO:0042119 neutrophil activation
(gain)
c-value
0.0
0.1
0.2
0.3
0.4
0.5
0.6
0.7
0.8
p-value
2e-04
5e-04
2e-03
5e-03
2e-02
Log-odds
0.5
1.0
1.5
2.0
2.5
3.0
3.5
4.0
Cellular_organisms - ps1
Archaea1 - ps2
Archaea2 - ps3
Archaea3 - ps4
Asgard/Eukaryota - ps5
Eukaryota - ps6
Amorpheae - ps7
Obazoa - ps8
Opisthokonta - ps9
Holomycota - ps10
Fungi - ps11
Fungi_1 - ps12
Fungi_2 - ps13
Fungi_3 - ps14
Dikarya - ps15
Ascomycota - ps16
Saccharo/Pezizomycotina - ps17
Saccharomycotina - ps18
Saccharomycotina_1 - ps19
Saccharomycotina_2 - ps20
Saccharomycotina_3 - ps21
Phaff./Saccharomycetaceae - ps22
Saccharomyco./mycetaceae - ps23
Saccharomycetaceae - ps24
Saccharomycetaceae_2 - ps25
Saccharomycetaceae_3 - ps26
Kaz./Nau./Saccharomyces - ps27
Saccharomyces - ps28
Arboricola_clade - ps29
Cerevisiae_clade - ps30
Saccharomyces_cerevisiae - ps31
Phylostratum

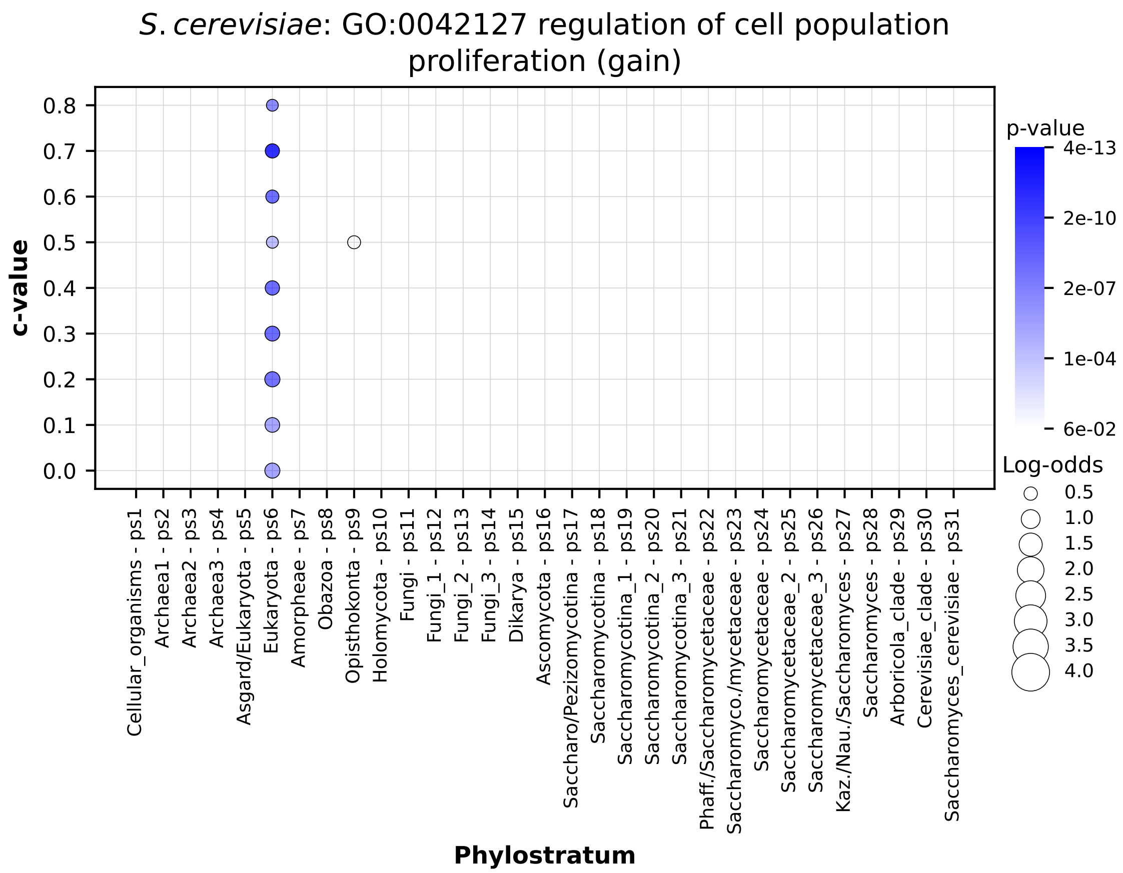

S. cerevisiae: GO:0042127 regulation of cell population proliferation (gain)
c-value
0.0
0.1
0.2
0.3
0.4
0.5
0.6
0.7
0.8
p-value
4e-13
2e-10
2e-07
1e-04
6e-02
Log-odds
0.5
1.0
1.5
2.0
2.5
3.0
3.5
4.0
Cellular_organisms - ps1
Archaea1 - ps2
Archaea2 - ps3
Archaea3 - ps4
Asgard/Eukaryota - ps5
Eukaryota - ps6
Amorpheae - ps7
Obazoa - ps8
Opisthokonta - ps9
Holomycota - ps10
Fungi - ps11
Fungi_1 - ps12
Fungi_2 - ps13
Fungi_3 - ps14
Dikarya - ps15
Ascomycota - ps16
Saccharo/Pezizomycotina - ps17
Saccharomycotina - ps18
Saccharomycotina_1 - ps19
Saccharomycotina_2 - ps20
Saccharomycotina_3 - ps21
Phaff./Saccharomycetaceae - ps22
Saccharomyco./mycetaceae - ps23
Saccharomycetaceae - ps24
Saccharomycetaceae_2 - ps25
Saccharomycetaceae_3 - ps26
Kaz./Nau./Saccharomyces - ps27
Saccharomyces - ps28
Arboricola_clade - ps29
Cerevisiae_clade - ps30
Saccharomyces_cerevisiae - ps31
Phylostratum

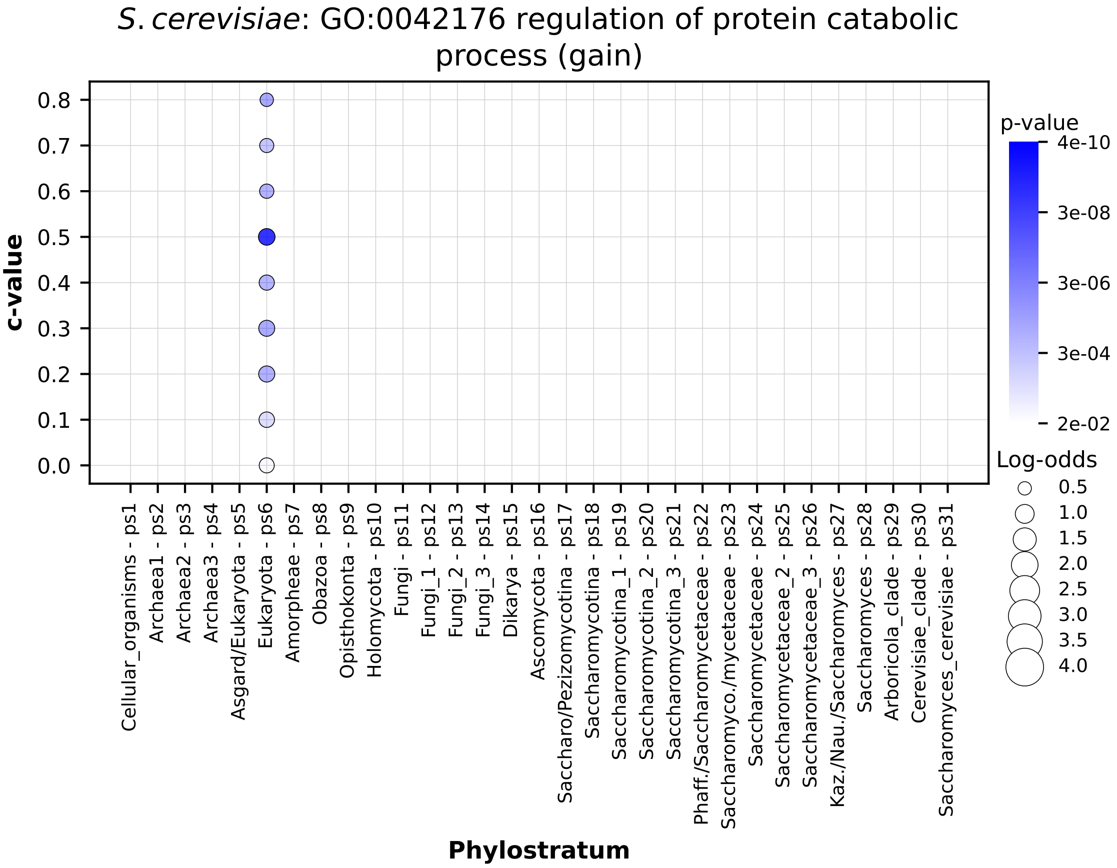

S. cerevisiae: GO:0042176 regulation of protein catabolic process (gain)
c-value
0.0
0.1
0.2
0.3
0.4
0.5
0.6
0.7
0.8
p-value
4e-10
3e-08
3e-06
3e-04
2e-02
Log-odds
0.5
1.0
1.5
2.0
2.5
3.0
3.5
4.0
Cellular_organisms - ps1
Archaea1 - ps2
Archaea2 - ps3
Archaea3 - ps4
Asgard/Eukaryota - ps5
Eukaryota - ps6
Amorpheae - ps7
Obazoa - ps8
Opisthokonta - ps9
Holomycota - ps10
Fungi - ps11
Fungi_1 - ps12
Fungi_2 - ps13
Fungi_3 - ps14
Dikarya - ps15
Ascomycota - ps16
Saccharo/Pezizomycotina - ps17
Saccharomycotina - ps18
Saccharomycotina_1 - ps19
Saccharomycotina_2 - ps20
Saccharomycotina_3 - ps21
Phaff./Saccharomycetaceae - ps22
Saccharomyco./mycetaceae - ps23
Saccharomycetaceae - ps24
Saccharomycetaceae_2 - ps25
Saccharomycetaceae_3 - ps26
Kaz./Nau./Saccharomyces - ps27
Saccharomyces - ps28
Arboricola_clade - ps29
Cerevisiae_clade - ps30
Saccharomyces_cerevisiae - ps31
Phylostratum

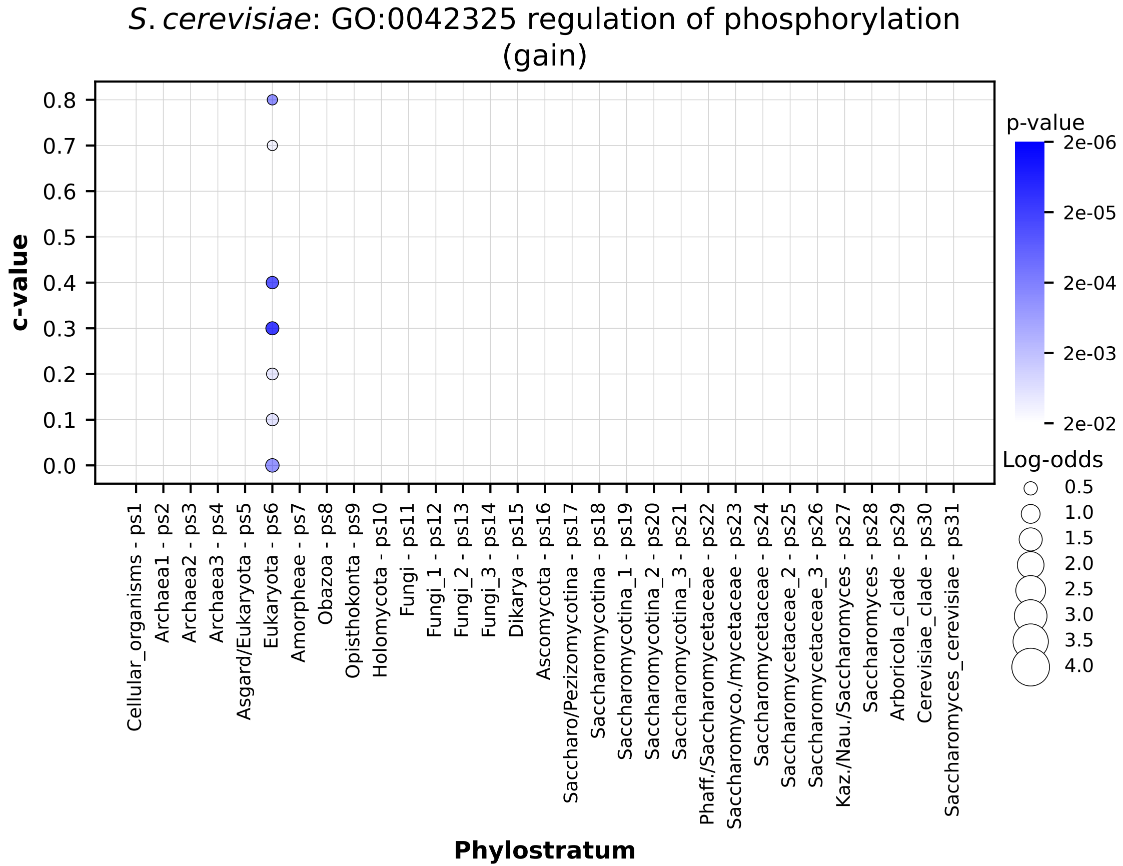

S. cerevisiae: GO:0042325 regulation of phosphorylation (gain)
c-value
0.8
0.7
0.6
0.5
0.4
0.3
0.2
0.1
0.0
p-value
2e-06
2e-05
2e-04
2e-03
2e-02
Log-odds
0.5
1.0
1.5
2.0
2.5
3.0
3.5
4.0
Cellular_organisms - ps1
Archaea1 - ps2
Archaea2 - ps3
Archaea3 - ps4
Asgard/Eukaryota - ps5
Eukaryota - ps6
Amorpheae - ps7
Obazoa - ps8
Opisthokonta - ps9
Holomycota - ps10
Fungi - ps11
Fungi_1 - ps12
Fungi_2 - ps13
Fungi_3 - ps14
Dikarya - ps15
Ascomycota - ps16
Saccharo/Pezizomycotina - ps17
Saccharomycotina - ps18
Saccharomycotina_1 - ps19
Saccharomycotina_2 - ps20
Saccharomycotina_3 - ps21
Phaff./Saccharomycetaceae - ps22
Saccharomyco./mycetaceae - ps23
Saccharomycetaceae - ps24
Saccharomycetaceae_2 - ps25
Saccharomycetaceae_3 - ps26
Kaz./Nau./Saccharomyces - ps27
Saccharomyces - ps28
Arboricola_clade - ps29
Cerevisiae_clade - ps30
Saccharomyces_cerevisiae - ps31
Phylostratum

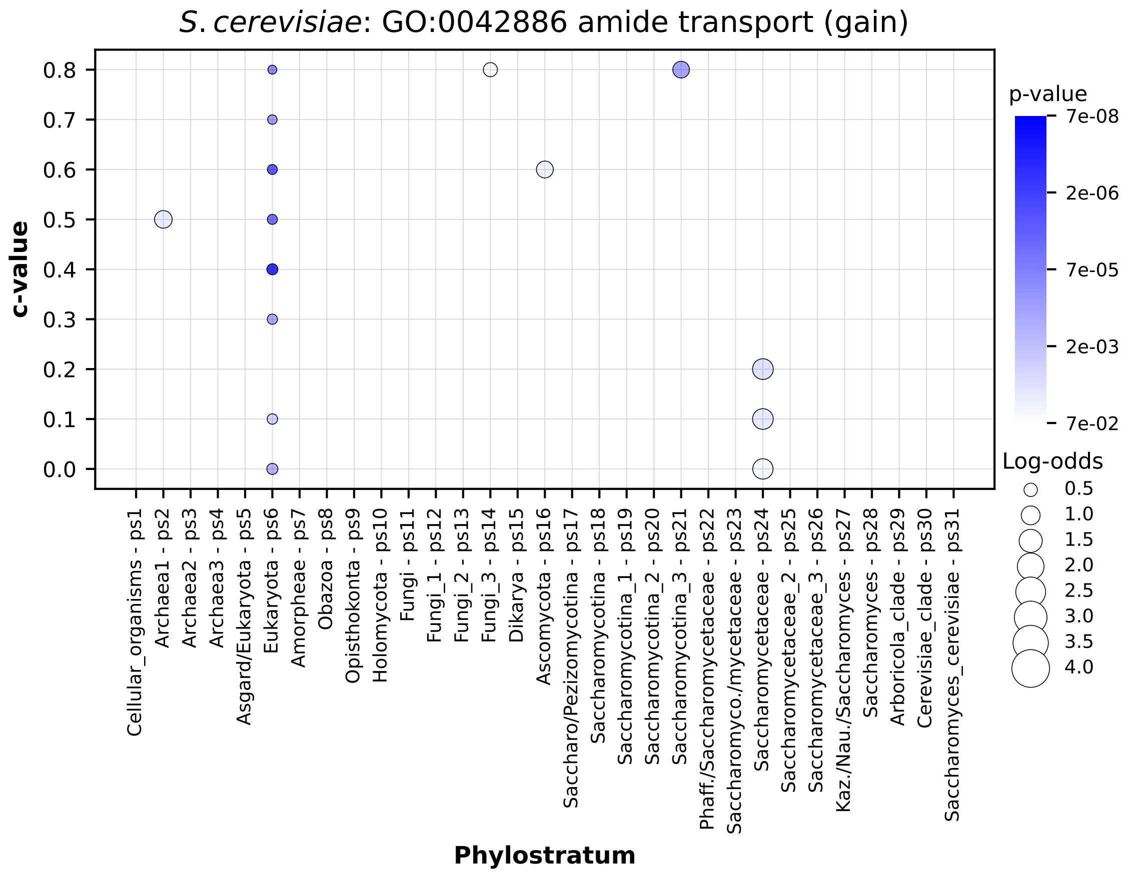

S. cerevisiae: GO:0042886 amide transport (gain)
c-value
0.0
0.1
0.2
0.3
0.4
0.5
0.6
0.7
0.8
p-value
7e-08
2e-06
7e-05
2e-03
7e-02
Log-odds
0.5
1.0
1.5
2.0
2.5
3.0
3.5
4.0
Cellular_organisms - ps1
Archaea1 - ps2
Archaea2 - ps3
Archaea3 - ps4
Asgard/Eukaryota - ps5
Eukaryota - ps6
Amorpheae - ps7
Obazoa - ps8
Opisthokonta - ps9
Holomycota - ps10
Fungi - ps11
Fungi_1 - ps12
Fungi_2 - ps13
Fungi_3 - ps14
Dikarya - ps15
Ascomycota - ps16
Saccharo/Pezizomycotina - ps17
Saccharomycotina - ps18
Saccharomycotina_1 - ps19
Saccharomycotina_2 - ps20
Saccharomycotina_3 - ps21
Phaff./Saccharomycetaceae - ps22
Saccharomyco./mycetaceae - ps23
Saccharomycetaceae - ps24
Saccharomycetaceae_2 - ps25
Saccharomycetaceae_3 - ps26
Kaz./Nau./Saccharomyces - ps27
Saccharomyces - ps28
Arboricola_clade - ps29
Cerevisiae_clade - ps30
Saccharomyces_cerevisiae - ps31
Phylostratum

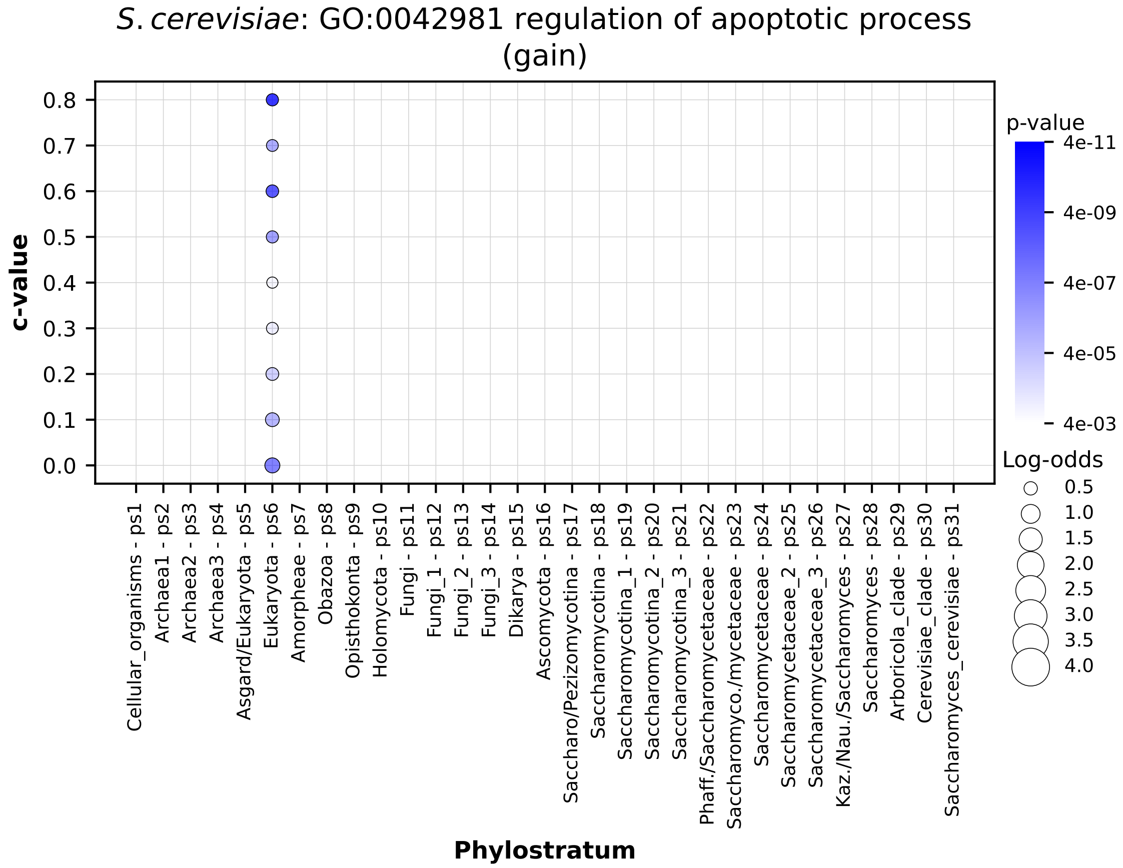

$S. cerevisiae$: GO:0042981 regulation of apoptotic process (gain)
c-value
0.0
0.1
0.2
0.3
0.4
0.5
0.6
0.7
0.8
p-value
4e-11
4e-09
4e-07
4e-05
4e-03
Log-odds
0.5
1.0
1.5
2.0
2.5
3.0
3.5
4.0
Cellular_organisms - ps1
Archaea1 - ps2
Archaea2 - ps3
Archaea3 - ps4
Asgard/Eukaryota - ps5
Eukaryota - ps6
Amorpheae - ps7
Obazoa - ps8
Opisthokonta - ps9
Holomycota - ps10
Fungi - ps11
Fungi_1 - ps12
Fungi_2 - ps13
Fungi_3 - ps14
Dikarya - ps15
Ascomycota - ps16
Saccharo/Pezizomycotina - ps17
Saccharomycotina - ps18
Saccharomycotina_1 - ps19
Saccharomycotina_2 - ps20
Saccharomycotina_3 - ps21
Phaff./Saccharomycetaceae - ps22
Saccharomyco./mycetaceae - ps23
Saccharomycetaceae - ps24
Saccharomycetaceae_2 - ps25
Saccharomycetaceae_3 - ps26
Kaz./Nau./Saccharomyces - ps27
Saccharomyces - ps28
Arboricola_clade - ps29
Cerevisiae_clade - ps30
Saccharomyces_cerevisiae - ps31
Phylostratum

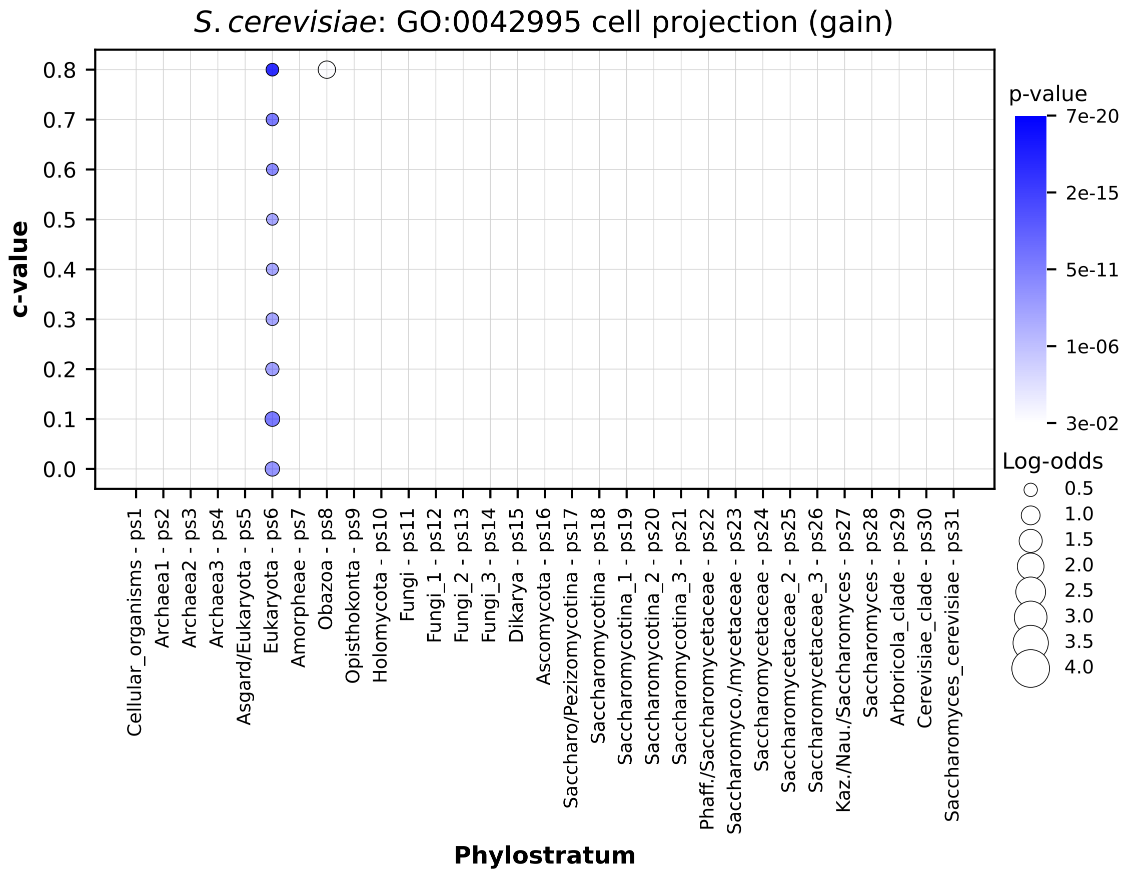

S. cerevisiae: GO:0042995 cell projection (gain)
c-value
0.0
0.1
0.2
0.3
0.4
0.5
0.6
0.7
0.8
p-value
7e-20
2e-15
5e-11
1e-06
3e-02
Log-odds
0.5
1.0
1.5
2.0
2.5
3.0
3.5
4.0
Cellular_organisms - ps1
Archaea1 - ps2
Archaea2 - ps3
Archaea3 - ps4
Asgard/Eukaryota - ps5
Eukaryota - ps6
Amorpheae - ps7
Obazoa - ps8
Opisthokonta - ps9
Holomycota - ps10
Fungi - ps11
Fungi_1 - ps12
Fungi_2 - ps13
Fungi_3 - ps14
Dikarya - ps15
Ascomycota - ps16
Saccharo/Pezizomycotina - ps17
Saccharomycotina - ps18
Saccharomycotina_1 - ps19
Saccharomycotina_2 - ps20
Saccharomycotina_3 - ps21
Phaff./Saccharomycetaceae - ps22
Saccharomyco./mycetaceae - ps23
Saccharomycetaceae - ps24
Saccharomycetaceae_2 - ps25
Saccharomycetaceae_3 - ps26
Kaz./Nau./Saccharomyces - ps27
Saccharomyces - ps28
Arboricola_clade - ps29
Cerevisiae_clade - ps30
Saccharomyces_cerevisiae - ps31
Phylostratum

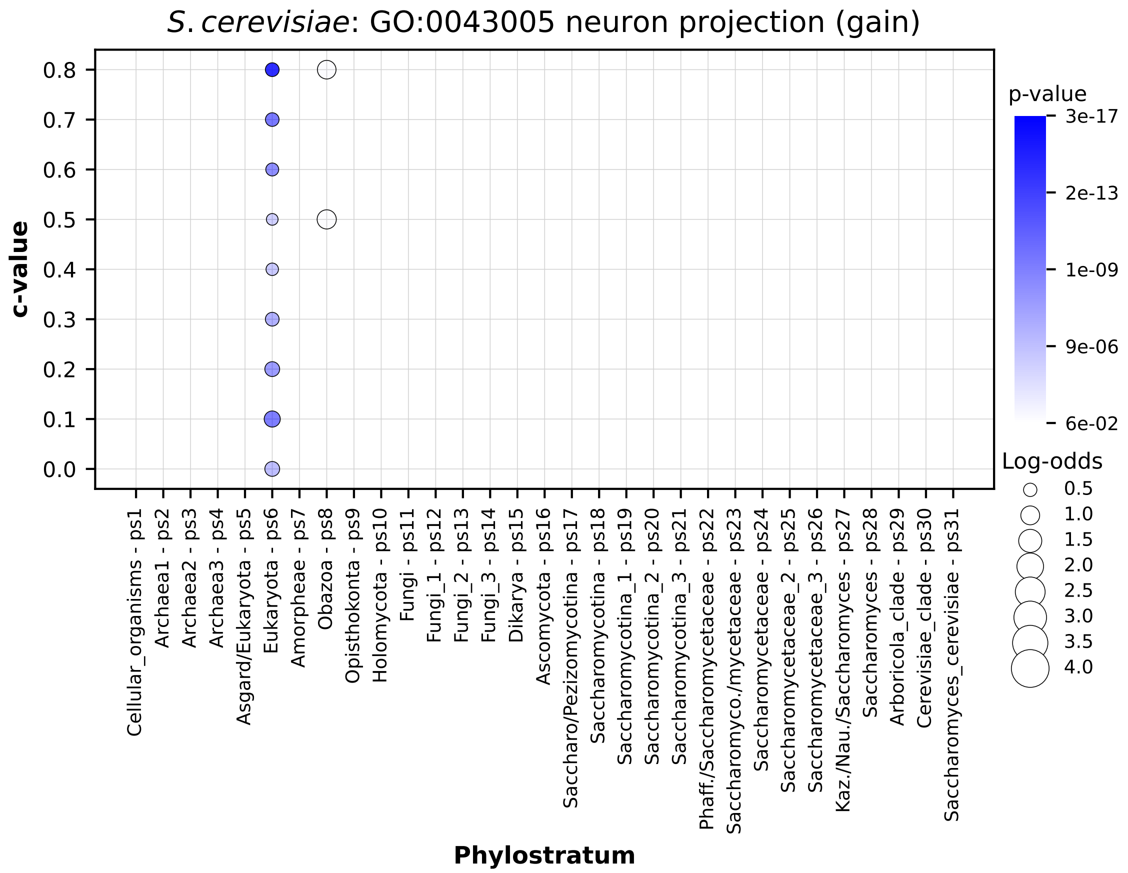

S. cerevisiae: GO:0043005 neuron projection (gain)
c-value
0.8
0.7
0.6
0.5
0.4
0.3
0.2
0.1
0.0
p-value
3e-17
2e-13
1e-09
9e-06
6e-02
Log-odds
0.5
1.0
1.5
2.0
2.5
3.0
3.5
4.0
Cellular_organisms - ps1
Archaea1 - ps2
Archaea2 - ps3
Archaea3 - ps4
Asgard/Eukaryota - ps5
Eukaryota - ps6
Amorpheae - ps7
Obazoa - ps8
Opisthokonta - ps9
Holomycota - ps10
Fungi - ps11
Fungi_1 - ps12
Fungi_2 - ps13
Fungi_3 - ps14
Dikarya - ps15
Ascomycota - ps16
Saccharo/Pezizomycotina - ps17
Saccharomycotina - ps18
Saccharomycotina_1 - ps19
Saccharomycotina_2 - ps20
Saccharomycotina_3 - ps21
Phaff./Saccharomycetaceae - ps22
Saccharomyco./mycetaceae - ps23
Saccharomycetaceae - ps24
Saccharomycetaceae_2 - ps25
Saccharomycetaceae_3 - ps26
Kaz./Nau./Saccharomyces - ps27
Saccharomyces - ps28
Arboricola_clade - ps29
Cerevisiae_clade - ps30
Saccharomyces_cerevisiae - ps31
Phylostratum

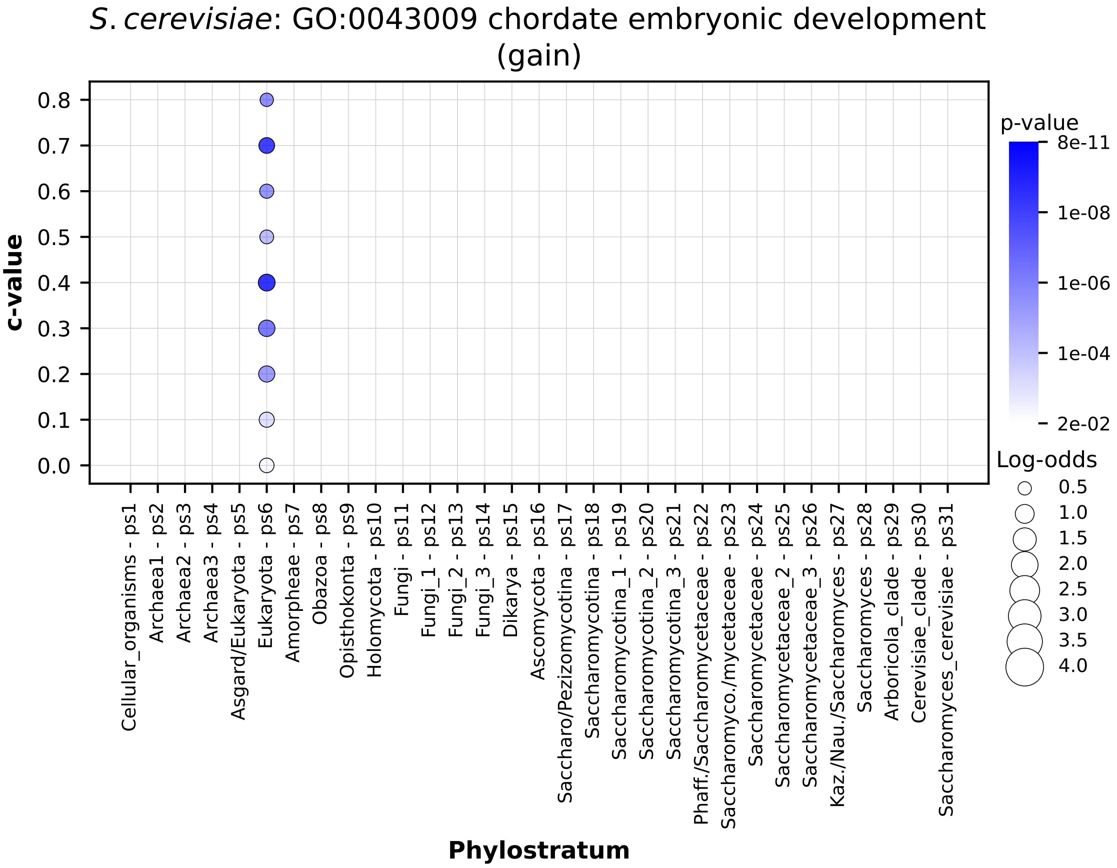

S. cerevisiae: GO:0043009 chordate embryonic development (gain)
c-value
0.0
0.1
0.2
0.3
0.4
0.5
0.6
0.7
0.8
p-value
8e-11
1e-08
1e-06
1e-04
2e-02
Log-odds
0.5
1.0
1.5
2.0
2.5
3.0
3.5
4.0
Cellular_organisms - ps1
Archaea1 - ps2
Archaea2 - ps3
Archaea3 - ps4
Asgard/Eukaryota - ps5
Eukaryota - ps6
Amorpheae - ps7
Obazoa - ps8
Opisthokonta - ps9
Holomycota - ps10
Fungi - ps11
Fungi_1 - ps12
Fungi_2 - ps13
Fungi_3 - ps14
Dikarya - ps15
Ascomycota - ps16
Saccharo/Pezizomycotina - ps17
Saccharomycotina - ps18
Saccharomycotina_1 - ps19
Saccharomycotina_2 - ps20
Saccharomycotina_3 - ps21
Phaff./Saccharomycetaceae - ps22
Saccharomyco./mycetaceae - ps23
Saccharomycetaceae - ps24
Saccharomycetaceae_2 - ps25
Saccharomycetaceae_3 - ps26
Kaz./Nau./Saccharomyces - ps27
Saccharomyces - ps28
Arboricola_clade - ps29
Cerevisiae_clade - ps30
Saccharomyces_cerevisiae - ps31
Phylostratum

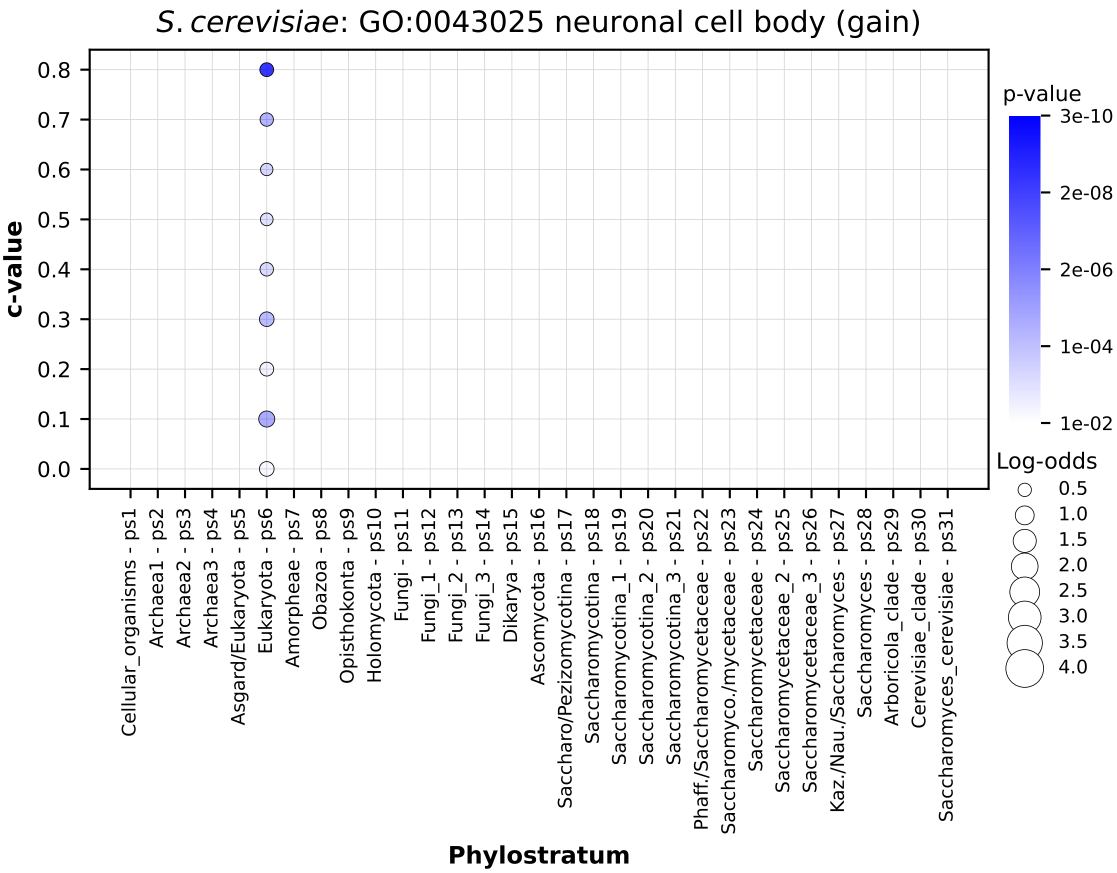

S. cerevisiae: GO:0043025 neuronal cell body (gain)
c-value
0.8
0.7
0.6
0.5
0.4
0.3
0.2
0.1
0.0
p-value
3e-10
2e-08
2e-06
1e-04
1e-02
Log-odds
0.5
1.0
1.5
2.0
2.5
3.0
3.5
4.0
Cellular_organisms - ps1
Archaea1 - ps2
Archaea2 - ps3
Archaea3 - ps4
Asgard/Eukaryota - ps5
Eukaryota - ps6
Amorpheae - ps7
Obazoa - ps8
Opisthokonta - ps9
Holomycota - ps10
Fungi - ps11
Fungi_1 - ps12
Fungi_2 - ps13
Fungi_3 - ps14
Dikarya - ps15
Ascomycota - ps16
Saccharo/Pezizomycotina - ps17
Saccharomycotina - ps18
Saccharomycotina_1 - ps19
Saccharomycotina_2 - ps20
Saccharomycotina_3 - ps21
Phaff./Saccharomycetaceae - ps22
Saccharomyco./mycetaceae - ps23
Saccharomycetaceae - ps24
Saccharomycetaceae_2 - ps25
Saccharomycetaceae_3 - ps26
Kaz./Nau./Saccharomyces - ps27
Saccharomyces - ps28
Arboricola_clade - ps29
Cerevisiae_clade - ps30
Saccharomyces_cerevisiae - ps31
Phylostratum

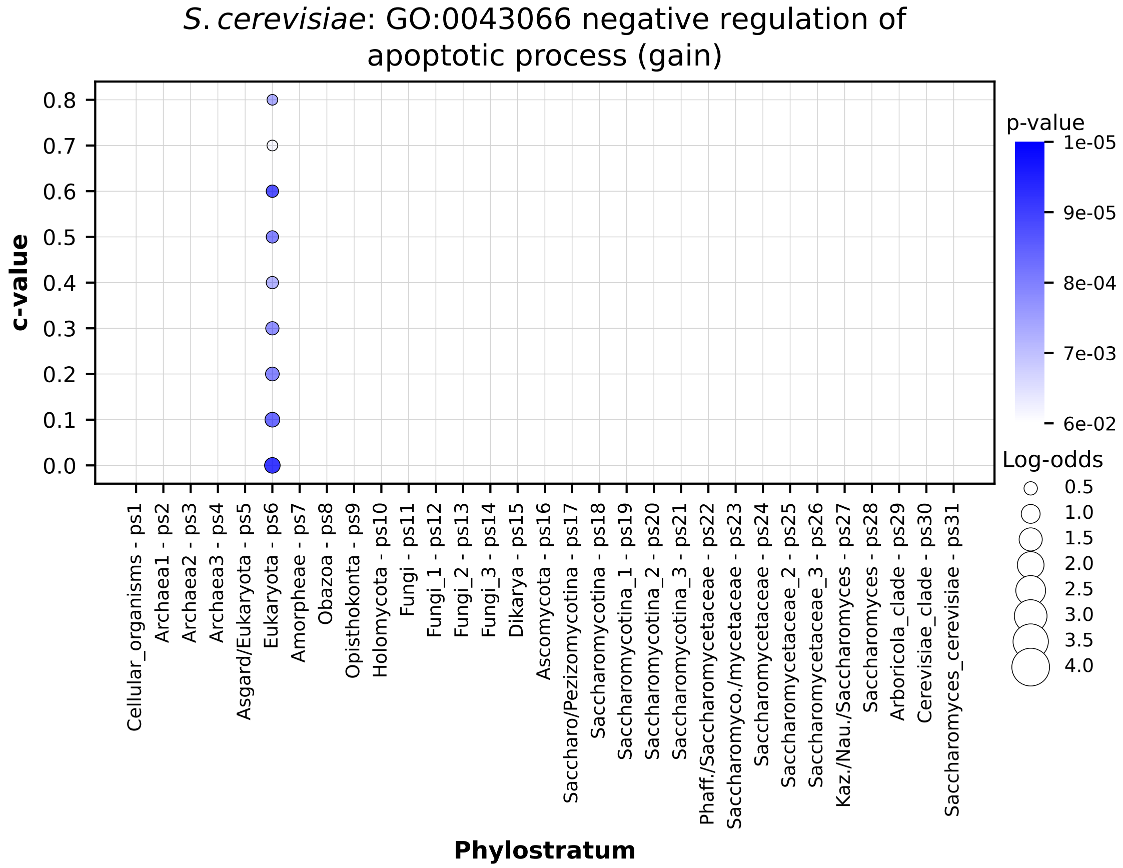

S. cerevisiae: GO:0043066 negative regulation of apoptotic process (gain)
c-value
0.0
0.1
0.2
0.3
0.4
0.5
0.6
0.7
0.8
Phylostratum
Cellular_organisms - ps1
Archaea1 - ps2
Archaea2 - ps3
Archaea3 - ps4
Asgard/Eukaryota - ps5
Eukaryota - ps6
Amorpheae - ps7
Obazoa - ps8
Opisthokonta - ps9
Holomycota - ps10
Fungi - ps11
Fungi_1 - ps12
Fungi_2 - ps13
Fungi_3 - ps14
Dikarya - ps15
Ascomycota - ps16
Saccharo/Pezizomycotina - ps17
Saccharomycotina - ps18
Saccharomycotina_1 - ps19
Saccharomycotina_2 - ps20
Saccharomycotina_3 - ps21
Phaff./Saccharomycetaceae - ps22
Saccharomyco./mycetaceae - ps23
Saccharomycetaceae - ps24
Saccharomycetaceae_2 - ps25
Saccharomycetaceae_3 - ps26
Kaz./Nau./Saccharomyces - ps27
Saccharomyces - ps28
Arboricola_clade - ps29
Cerevisiae_clade - ps30
Saccharomyces_cerevisiae - ps31
p-value
1e-05
9e-05
8e-04
7e-03
6e-02
Log-odds
0.5
1.0
1.5
2.0
2.5
3.0
3.5
4.0

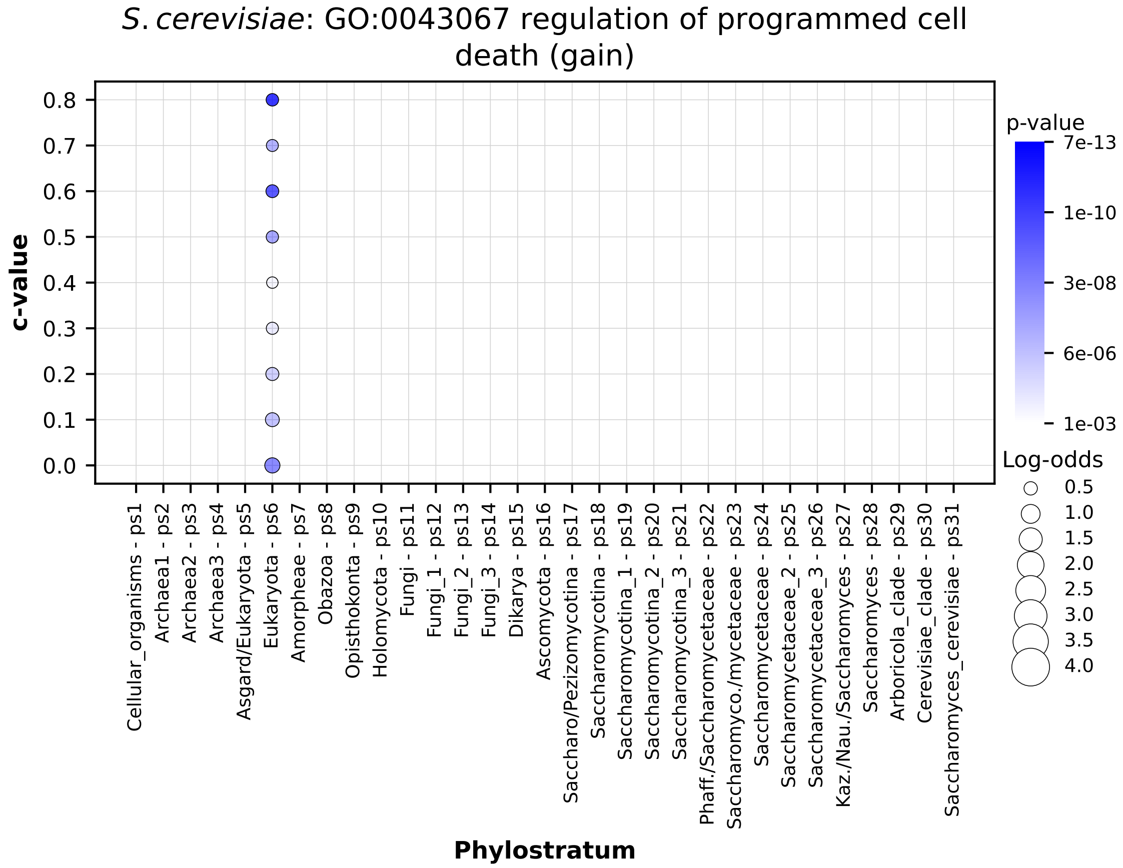

S. cerevisiae: GO:0043067 regulation of programmed cell death (gain)
c-value
0.0
0.1
0.2
0.3
0.4
0.5
0.6
0.7
0.8
p-value
7e-13
1e-10
3e-08
6e-06
1e-03
Log-odds
0.5
1.0
1.5
2.0
2.5
3.0
3.5
4.0
Cellular_organisms - ps1
Archaea1 - ps2
Archaea2 - ps3
Archaea3 - ps4
Asgard/Eukaryota - ps5
Eukaryota - ps6
Amorpheae - ps7
Obazoa - ps8
Opisthokonta - ps9
Holomycota - ps10
Fungi - ps11
Fungi_1 - ps12
Fungi_2 - ps13
Fungi_3 - ps14
Dikarya - ps15
Ascomycota - ps16
Saccharo/Pezizomycotina - ps17
Saccharomycotina - ps18
Saccharomycotina_1 - ps19
Saccharomycotina_2 - ps20
Saccharomycotina_3 - ps21
Phaff./Saccharomycetaceae - ps22
Saccharomyco./mycetaceae - ps23
Saccharomycetaceae - ps24
Saccharomycetaceae_2 - ps25
Saccharomycetaceae_3 - ps26
Kaz./Nau./Saccharomyces - ps27
Saccharomyces - ps28
Arboricola_clade - ps29
Cerevisiae_clade - ps30
Saccharomyces_cerevisiae - ps31
Phylostratum

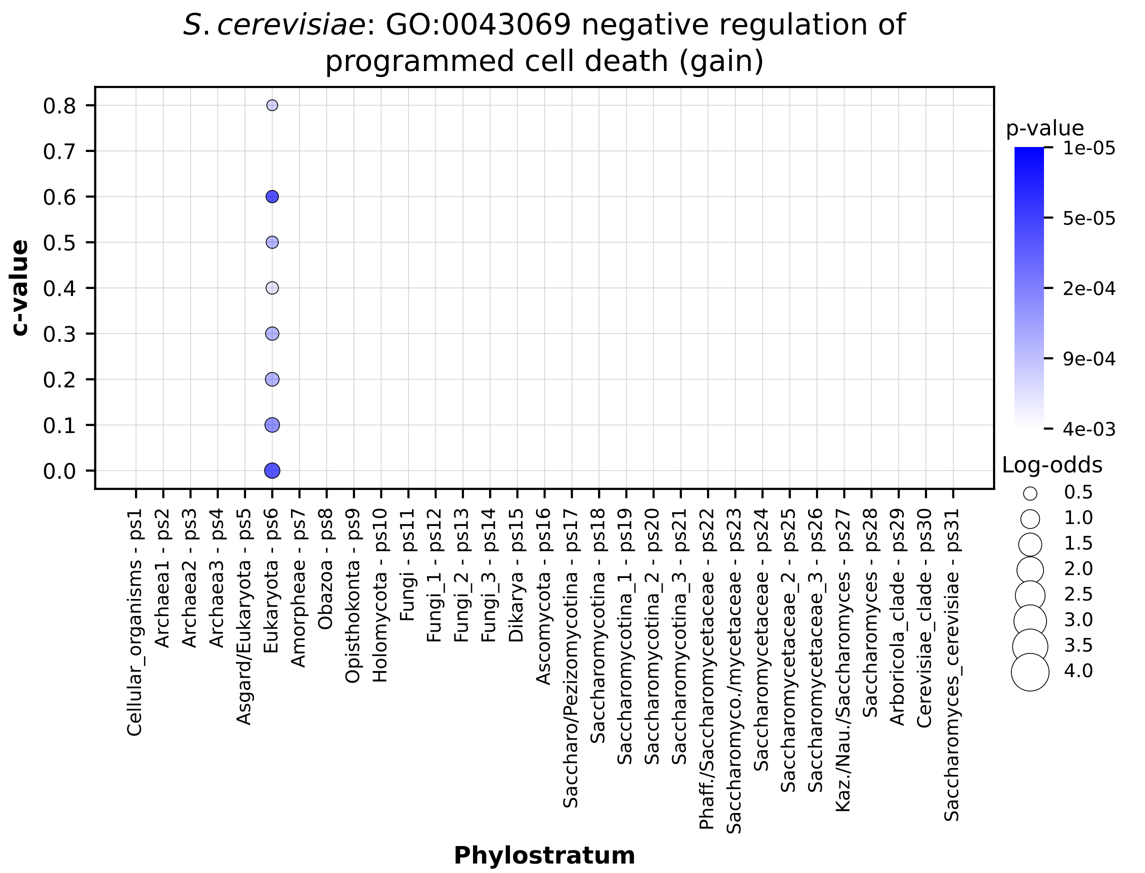

S. cerevisiae: GO:0043069 negative regulation of programmed cell death (gain)
c-value
0.8
0.7
0.6
0.5
0.4
0.3
0.2
0.1
0.0
p-value
1e-05
5e-05
2e-04
9e-04
4e-03
Log-odds
0.5
1.0
1.5
2.0
2.5
3.0
3.5
4.0
Cellular_organisms - ps1
Archaea1 - ps2
Archaea2 - ps3
Archaea3 - ps4
Asgard/Eukaryota - ps5
Eukaryota - ps6
Amorpheae - ps7
Obazoa - ps8
Opisthokonta - ps9
Holomycota - ps10
Fungi - ps11
Fungi_1 - ps12
Fungi_2 - ps13
Fungi_3 - ps14
Dikarya - ps15
Ascomycota - ps16
Saccharo/Pezizomycotina - ps17
Saccharomycotina - ps18
Saccharomycotina_1 - ps19
Saccharomycotina_2 - ps20
Saccharomycotina_3 - ps21
Phaff./Saccharomycetaceae - ps22
Saccharomyco./mycetaceae - ps23
Saccharomycetaceae - ps24
Saccharomycetaceae_2 - ps25
Saccharomycetaceae_3 - ps26
Kaz./Nau./Saccharomyces - ps27
Saccharomyces - ps28
Arboricola_clade - ps29
Cerevisiae_clade - ps30
Saccharomyces_cerevisiae - ps31
Phylostratum

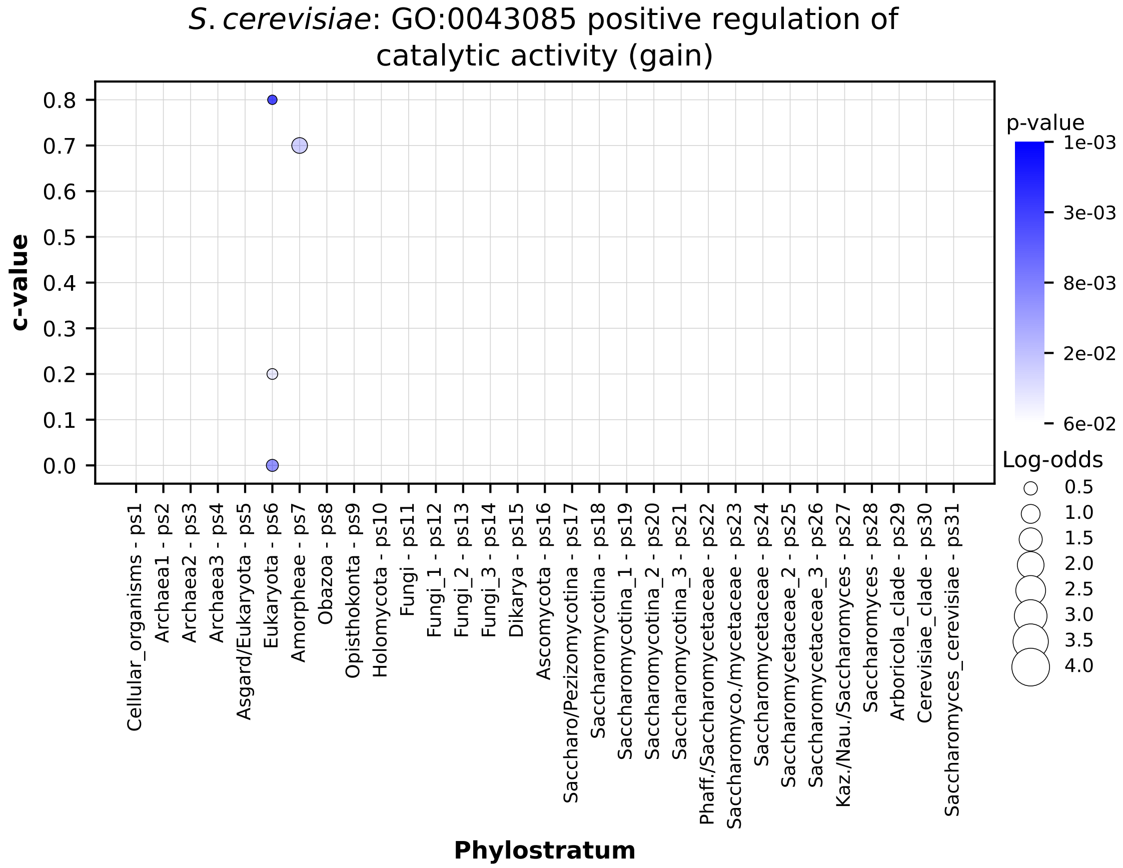

S. cerevisiae: GO:0043085 positive regulation of catalytic activity (gain)
c-value
0.8
0.7
0.6
0.5
0.4
0.3
0.2
0.1
0.0
p-value
1e-03
3e-03
8e-03
2e-02
6e-02
Log-odds
0.5
1.0
1.5
2.0
2.5
3.0
3.5
4.0
Cellular_organisms - ps1
Archaea1 - ps2
Archaea2 - ps3
Archaea3 - ps4
Asgard/Eukaryota - ps5
Eukaryota - ps6
Amorpheae - ps7
Obazoa - ps8
Opisthokonta - ps9
Holomycota - ps10
Fungi - ps11
Fungi_1 - ps12
Fungi_2 - ps13
Fungi_3 - ps14
Dikarya - ps15
Ascomycota - ps16
Saccharo/Pezizomycotina - ps17
Saccharomycotina - ps18
Saccharomycotina_1 - ps19
Saccharomycotina_2 - ps20
Saccharomycotina_3 - ps21
Phaff./Saccharomycetaceae - ps22
Saccharomyco./mycetaceae - ps23
Saccharomycetaceae - ps24
Saccharomycetaceae_2 - ps25
Saccharomycetaceae_3 - ps26
Kaz./Nau./Saccharomyces - ps27
Saccharomyces - ps28
Arboricola_clade - ps29
Cerevisiae_clade - ps30
Saccharomyces_cerevisiae - ps31
Phylostratum

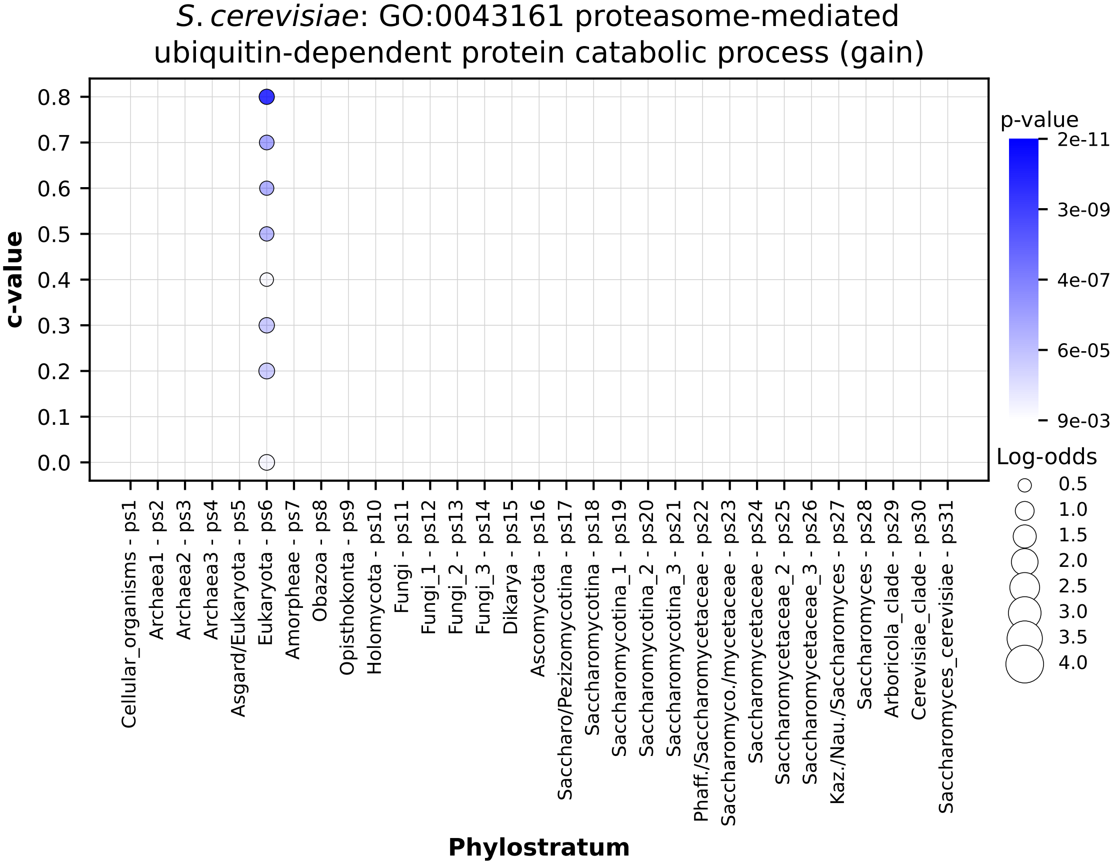

S. cerevisiae: GO:0043161 proteasome-mediated ubiquitin-dependent protein catabolic process (gain)
c-value
0.0
0.1
0.2
0.3
0.4
0.5
0.6
0.7
0.8
p-value
2e-11
3e-09
4e-07
6e-05
9e-03
Log-odds
0.5
1.0
1.5
2.0
2.5
3.0
3.5
4.0
Cellular_organisms - ps1
Archaea1 - ps2
Archaea2 - ps3
Archaea3 - ps4
Asgard/Eukaryota - ps5
Eukaryota - ps6
Amorpheae - ps7
Obazoa - ps8
Opisthokonta - ps9
Holomycota - ps10
Fungi - ps11
Fungi_1 - ps12
Fungi_2 - ps13
Fungi_3 - ps14
Dikarya - ps15
Ascomycota - ps16
Saccharo/Pezizomycotina - ps17
Saccharomycotina - ps18
Saccharomycotina_1 - ps19
Saccharomycotina_2 - ps20
Saccharomycotina_3 - ps21
Phaff./Saccharomycetaceae - ps22
Saccharomyco./mycetaceae - ps23
Saccharomycetaceae - ps24
Saccharomycetaceae_2 - ps25
Saccharomycetaceae_3 - ps26
Kaz./Nau./Saccharomyces - ps27
Saccharomyces - ps28
Arboricola_clade - ps29
Cerevisiae_clade - ps30
Saccharomyces_cerevisiae - ps31
Phylostratum

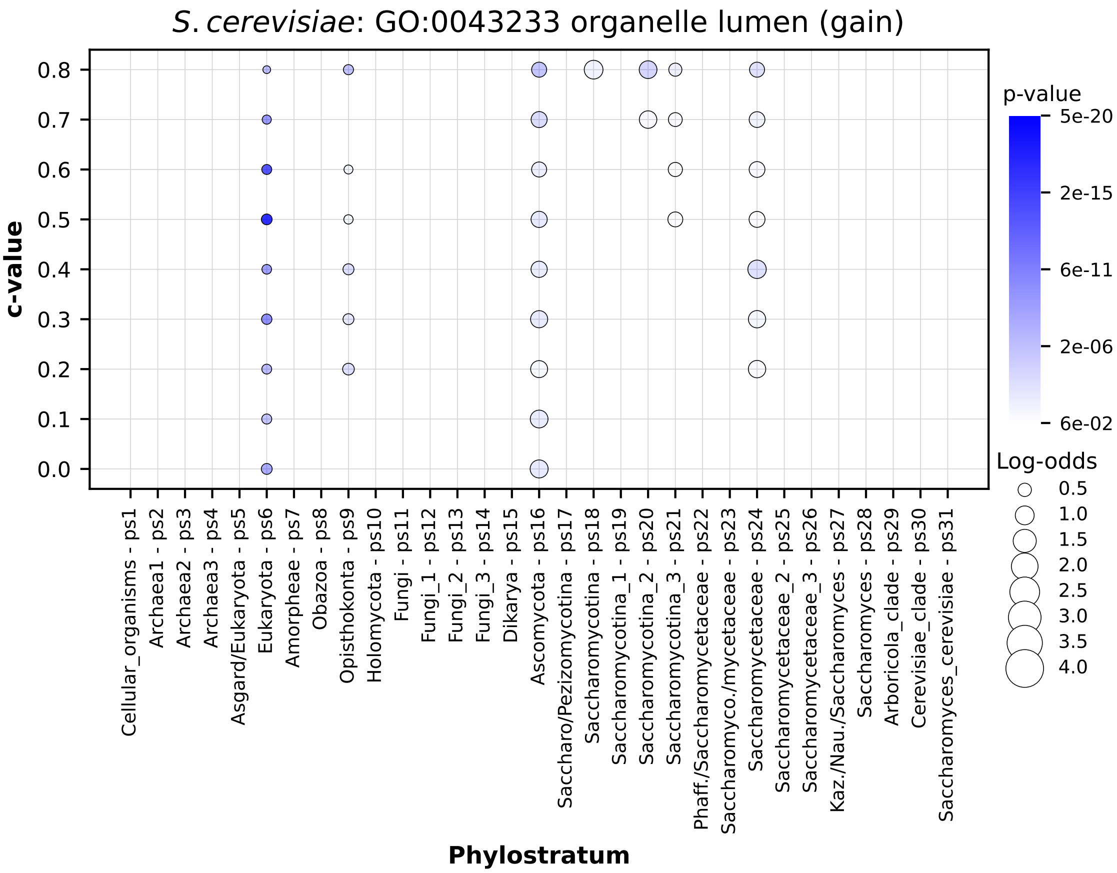
S. cerevisiae: GO:0043233 organelle lumen (gain)
c-value
0.0
0.1
0.2
0.3
0.4
0.5
0.6
0.7
0.8
Phylostratum
Cellular_organisms - ps1
Archaea1 - ps2
Archaea2 - ps3
Archaea3 - ps4
Asgard/Eukaryota - ps5
Eukaryota - ps6
Amorpheae - ps7
Obazoa - ps8
Opisthokonta - ps9
Holomycota - ps10
Fungi - ps11
Fungi_1 - ps12
Fungi_2 - ps13
Fungi_3 - ps14
Dikarya - ps15
Ascomycota - ps16
Saccharo/Pezizomycotina - ps17
Saccharomycotina - ps18
Saccharomycotina_1 - ps19
Saccharomycotina_2 - ps20
Saccharomycotina_3 - ps21
Phaff./Saccharomycetaceae - ps22
Saccharomyco./mycetaceae - ps23
Saccharomycetaceae - ps24
Saccharomycetaceae_2 - ps25
Saccharomycetaceae_3 - ps26
Kaz./Nau./Saccharomyces - ps27
Saccharomyces - ps28
Arboricola_clade - ps29
Cerevisiae_clade - ps30
Saccharomyces_cerevisiae - ps31
p-value
5e-20
2e-15
6e-11
2e-06
6e-02
Log-odds
0.5
1.0
1.5
2.0
2.5
3.0
3.5
4.0

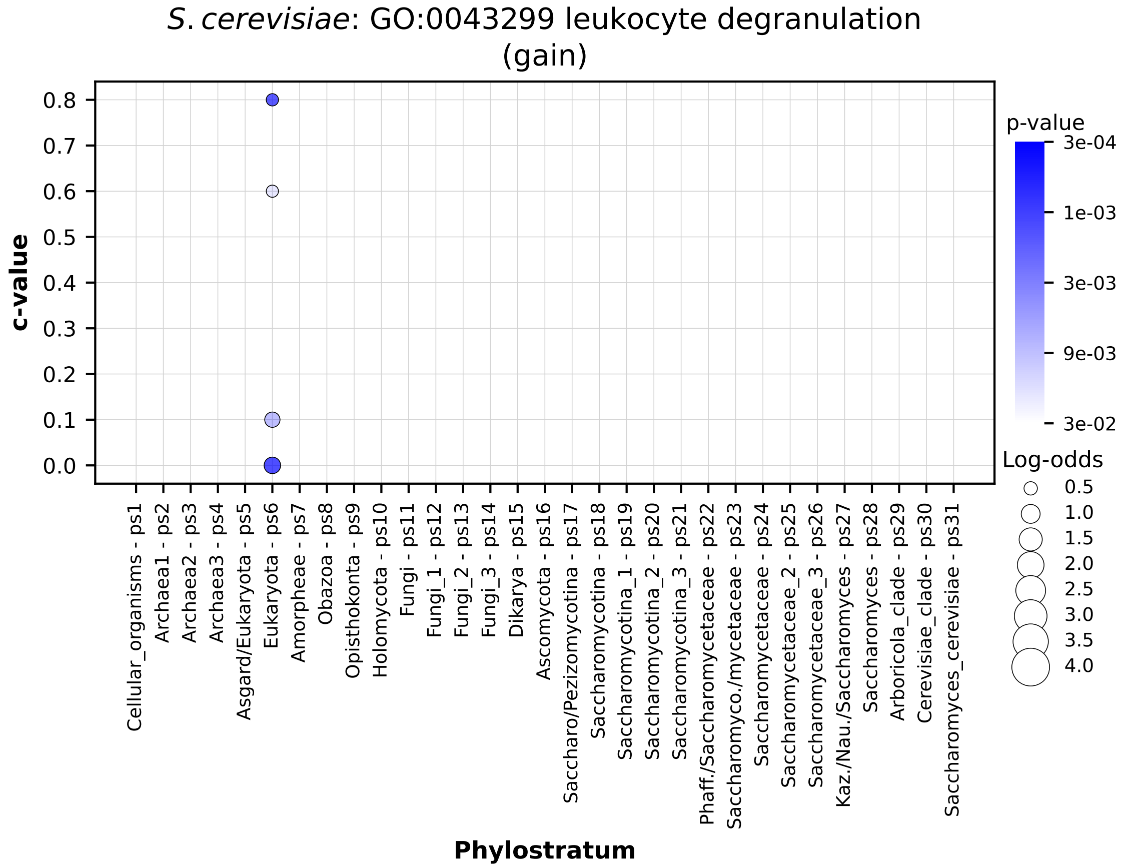
S. cerevisiae: GO:0043299 leukocyte degranulation
(gain)
c-value
0.8
0.7
0.6
0.5
0.4
0.3
0.2
0.1
0.0
p-value
3e-04
1e-03
3e-03
9e-03
3e-02
Log-odds
0.5
1.0
1.5
2.0
2.5
3.0
3.5
4.0
Cellular_organisms - ps1
Archaea1 - ps2
Archaea2 - ps3
Archaea3 - ps4
Asgard/Eukaryota - ps5
Eukaryota - ps6
Amorpheae - ps7
Obazoa - ps8
Opisthokonta - ps9
Holomycota - ps10
Fungi - ps11
Fungi_1 - ps12
Fungi_2 - ps13
Fungi_3 - ps14
Dikarya - ps15
Ascomycota - ps16
Saccharo/Pezizomycotina - ps17
Saccharomycotina - ps18
Saccharomycotina_1 - ps19
Saccharomycotina_2 - ps20
Saccharomycotina_3 - ps21
Phaff./Saccharomycetaceae - ps22
Saccharomyco./mycetaceae - ps23
Saccharomycetaceae - ps24
Saccharomycetaceae_2 - ps25
Saccharomycetaceae_3 - ps26
Kaz./Nau./Saccharomyces - ps27
Saccharomyces - ps28
Arboricola_clade - ps29
Cerevisiae_clade - ps30
Saccharomyces_cerevisiae - ps31
Phylostratum

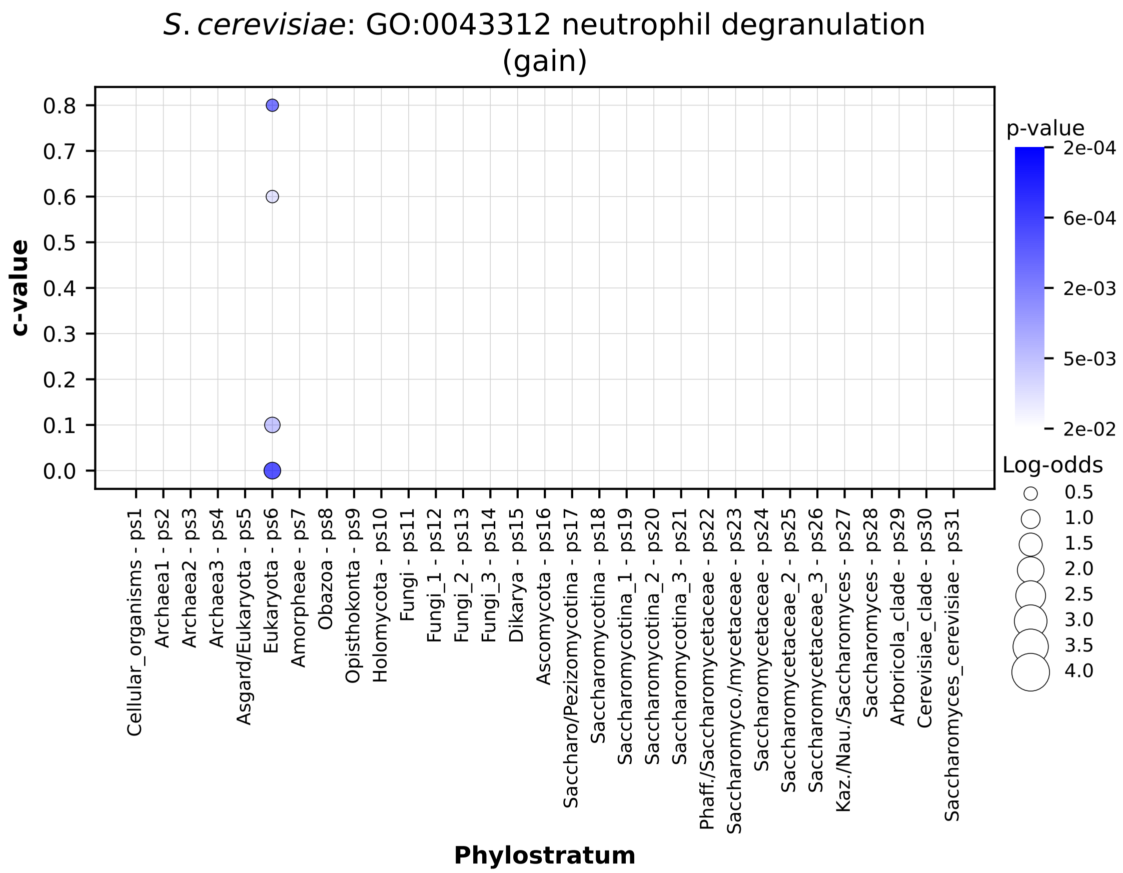

S. cerevisiae: GO:0043312 neutrophil degranulation (gain)
c-value
0.0
0.1
0.2
0.3
0.4
0.5
0.6
0.7
0.8
p-value
2e-04
6e-04
2e-03
5e-03
2e-02
Log-odds
0.5
1.0
1.5
2.0
2.5
3.0
3.5
4.0
Cellular_organisms - ps1
Archaea1 - ps2
Archaea2 - ps3
Archaea3 - ps4
Asgard/Eukaryota - ps5
Eukaryota - ps6
Amorpheae - ps7
Obazoa - ps8
Opisthokonta - ps9
Holomycota - ps10
Fungi - ps11
Fungi_1 - ps12
Fungi_2 - ps13
Fungi_3 - ps14
Dikarya - ps15
Ascomycota - ps16
Saccharo/Pezizomycotina - ps17
Saccharomycotina - ps18
Saccharomycotina_1 - ps19
Saccharomycotina_2 - ps20
Saccharomycotina_3 - ps21
Phaff./Saccharomycetaceae - ps22
Saccharomyco./mycetaceae - ps23
Saccharomycetaceae - ps24
Saccharomycetaceae_2 - ps25
Saccharomycetaceae_3 - ps26
Kaz./Nau./Saccharomyces - ps27
Saccharomyces - ps28
Arboricola_clade - ps29
Cerevisiae_clade - ps30
Saccharomyces_cerevisiae - ps31
Phylostratum

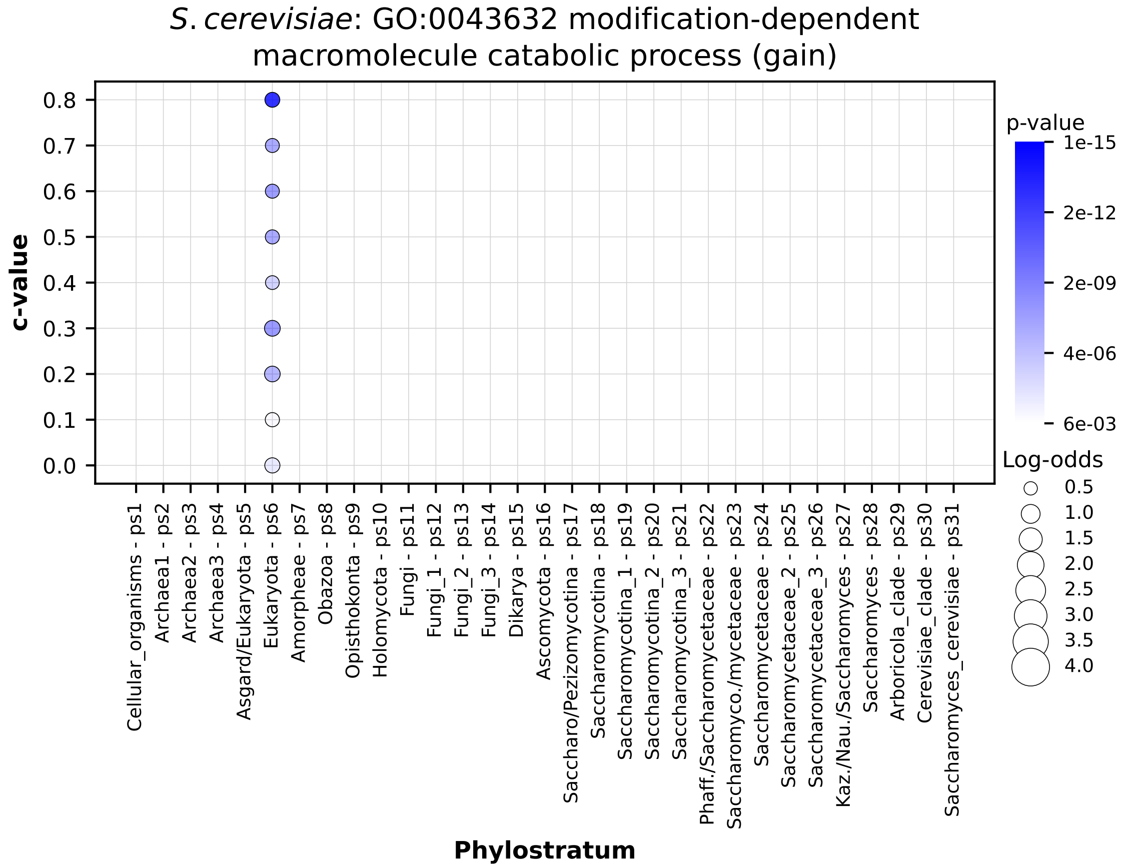

S. cerevisiae: GO:0043632 modification-dependent macromolecule catabolic process (gain)
c-value
0.8
0.7
0.6
0.5
0.4
0.3
0.2
0.1
0.0
p-value
1e-15
2e-12
2e-09
4e-06
6e-03
Log-odds
0.5
1.0
1.5
2.0
2.5
3.0
3.5
4.0
Cellular_organisms - ps1
Archaea1 - ps2
Archaea2 - ps3
Archaea3 - ps4
Asgard/Eukaryota - ps5
Eukaryota - ps6
Amorpheae - ps7
Obazoa - ps8
Opisthokonta - ps9
Holomycota - ps10
Fungi - ps11
Fungi_1 - ps12
Fungi_2 - ps13
Fungi_3 - ps14
Dikarya - ps15
Ascomycota - ps16
Saccharo/Pezizomycotina - ps17
Saccharomycotina - ps18
Saccharomycotina_1 - ps19
Saccharomycotina_2 - ps20
Saccharomycotina_3 - ps21
Phaff./Saccharomycetaceae - ps22
Saccharomyco./mycetaceae - ps23
Saccharomycetaceae - ps24
Saccharomycetaceae_2 - ps25
Saccharomycetaceae_3 - ps26
Kaz./Nau./Saccharomyces - ps27
Saccharomyces - ps28
Arboricola_clade - ps29
Cerevisiae_clade - ps30
Saccharomyces_cerevisiae - ps31
Phylostratum

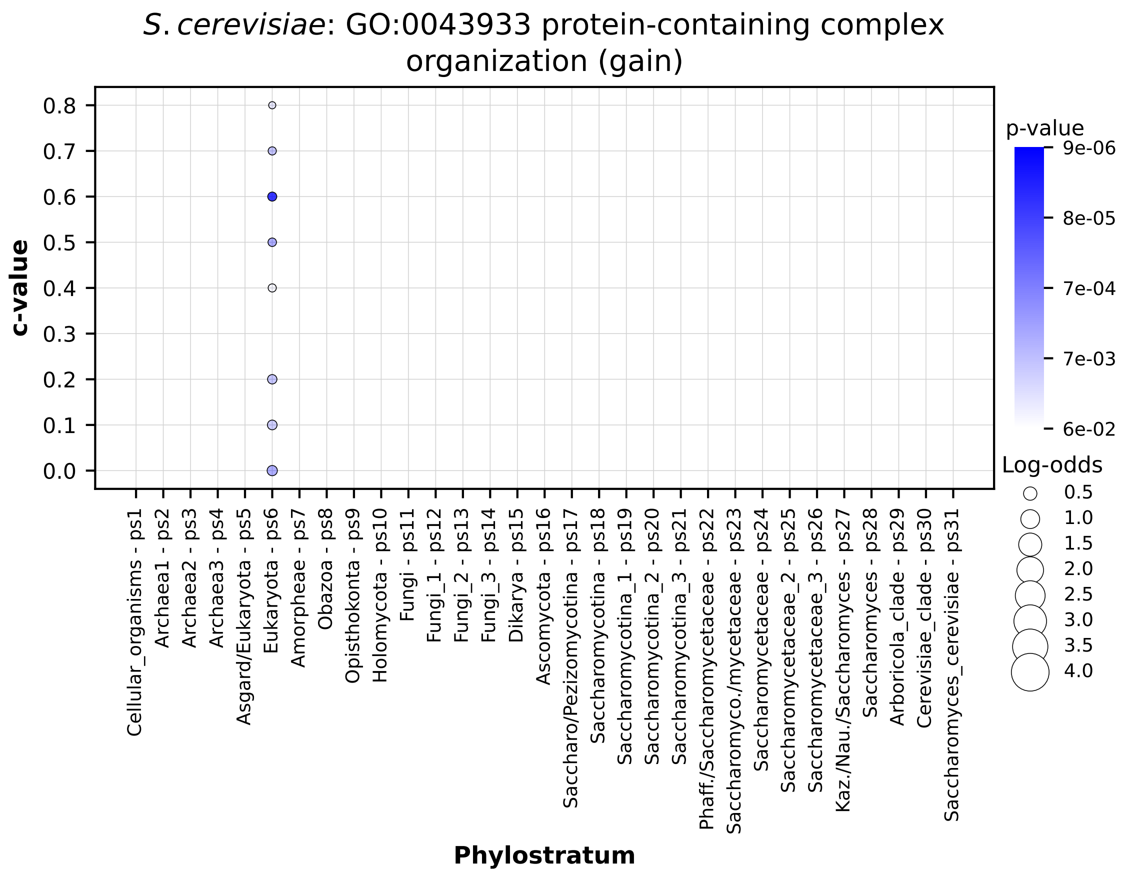

S. cerevisiae: GO:0043933 protein-containing complex organization (gain)
c-value
0.0
0.1
0.2
0.3
0.4
0.5
0.6
0.7
0.8
Phylostratum
Cellular_organisms - ps1
Archaea1 - ps2
Archaea2 - ps3
Archaea3 - ps4
Asgard/Eukaryota - ps5
Eukaryota - ps6
Amorpheae - ps7
Obazoa - ps8
Opisthokonta - ps9
Holomycota - ps10
Fungi - ps11
Fungi_1 - ps12
Fungi_2 - ps13
Fungi_3 - ps14
Dikarya - ps15
Ascomycota - ps16
Saccharo/Pezizomycotina - ps17
Saccharomycotina - ps18
Saccharomycotina_1 - ps19
Saccharomycotina_2 - ps20
Saccharomycotina_3 - ps21
Phaff./Saccharomycetaceae - ps22
Saccharomyco./mycetaceae - ps23
Saccharomycetaceae - ps24
Saccharomycetaceae_2 - ps25
Saccharomycetaceae_3 - ps26
Kaz./Nau./Saccharomyces - ps27
Saccharomyces - ps28
Arboricola_clade - ps29
Cerevisiae_clade - ps30
Saccharomyces_cerevisiae - ps31
p-value
9e-06
8e-05
7e-04
7e-03
6e-02
Log-odds
0.5
1.0
1.5
2.0
2.5
3.0
3.5
4.0

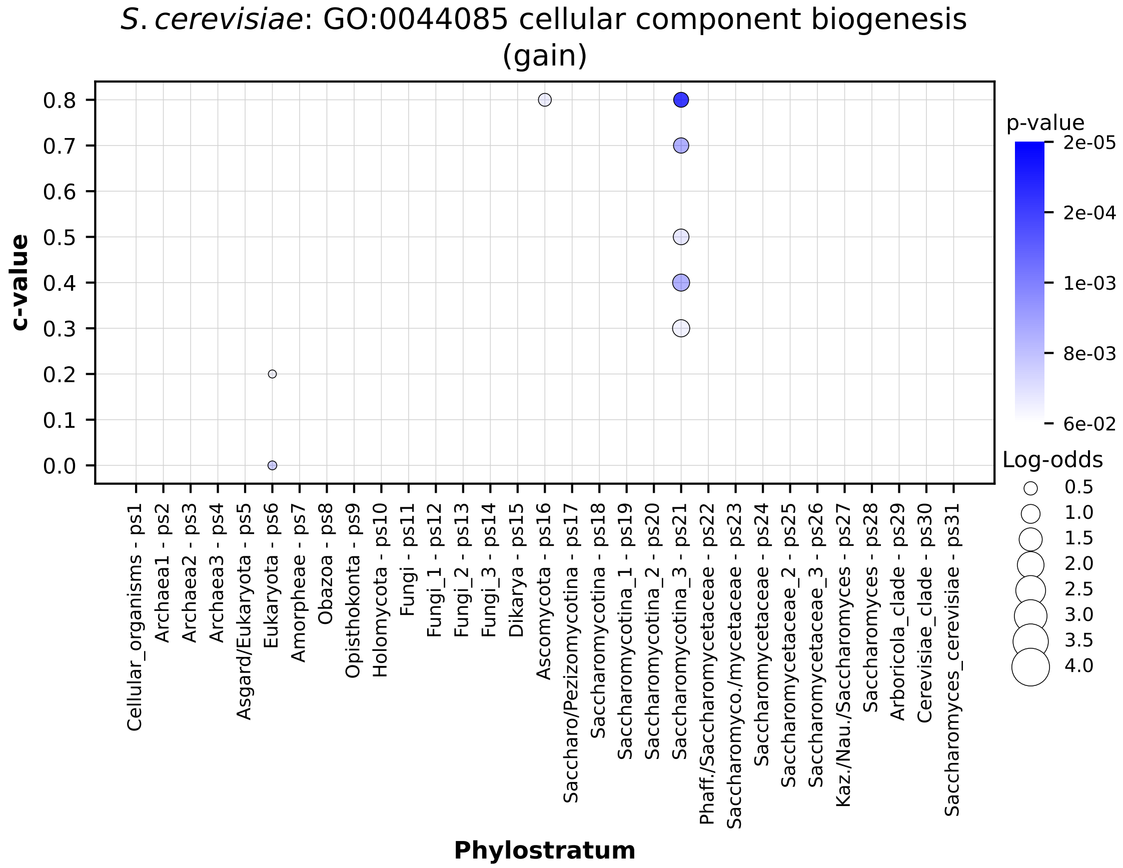
S. cerevisiae: GO:0044085 cellular component biogenesis
(gain)
c-value
0.0
0.1
0.2
0.3
0.4
0.5
0.6
0.7
0.8
p-value
2e-05
2e-04
1e-03
8e-03
6e-02
Log-odds
0.5
1.0
1.5
2.0
2.5
3.0
3.5
4.0
Cellular_organisms - ps1
Archaea1 - ps2
Archaea2 - ps3
Archaea3 - ps4
Asgard/Eukaryota - ps5
Eukaryota - ps6
Amorpheae - ps7
Obazoa - ps8
Opisthokonta - ps9
Holomycota - ps10
Fungi - ps11
Fungi_1 - ps12
Fungi_2 - ps13
Fungi_3 - ps14
Dikarya - ps15
Ascomycota - ps16
Saccharo/Pezizomycotina - ps17
Saccharomycotina - ps18
Saccharomycotina_1 - ps19
Saccharomycotina_2 - ps20
Saccharomycotina_3 - ps21
Phaff./Saccharomycetaceae - ps22
Saccharomyco./mycetaceae - ps23
Saccharomycetaceae - ps24
Saccharomycetaceae_2 - ps25
Saccharomycetaceae_3 - ps26
Kaz./Nau./Saccharomyces - ps27
Saccharomyces - ps28
Arboricola_clade - ps29
Cerevisiae_clade - ps30
Saccharomyces_cerevisiae - ps31
Phylostratum

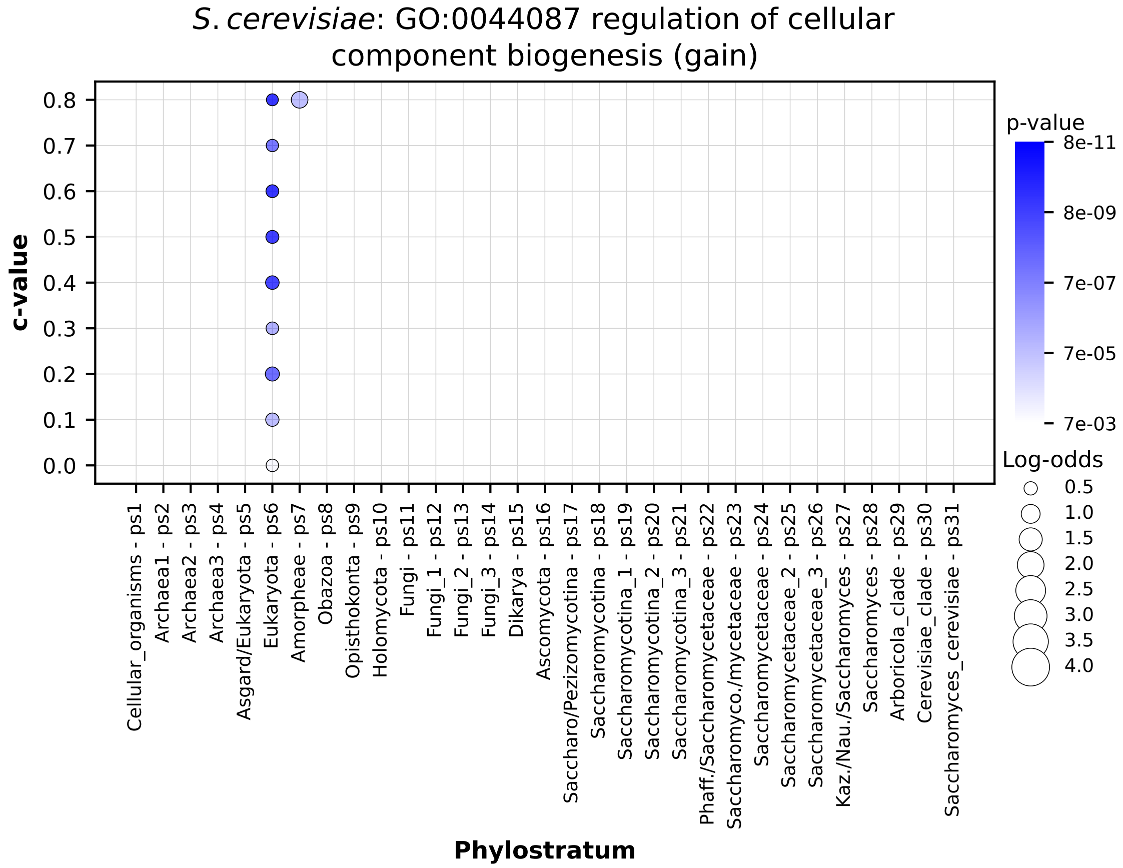
S. cerevisiae: GO:0044087 regulation of cellular component biogenesis (gain)
c-value
0.0
0.1
0.2
0.3
0.4
0.5
0.6
0.7
0.8
Phylostratum
Cellular_organisms - ps1
Archaea1 - ps2
Archaea2 - ps3
Archaea3 - ps4
Asgard/Eukaryota - ps5
Eukaryota - ps6
Amorpheae - ps7
Obazoa - ps8
Opisthokonta - ps9
Holomycota - ps10
Fungi - ps11
Fungi_1 - ps12
Fungi_2 - ps13
Fungi_3 - ps14
Dikarya - ps15
Ascomycota - ps16
Saccharo/Pezizomycotina - ps17
Saccharomycotina - ps18
Saccharomycotina_1 - ps19
Saccharomycotina_2 - ps20
Saccharomycotina_3 - ps21
Phaff./Saccharomycetaceae - ps22
Saccharomyco./mycetaceae - ps23
Saccharomycetaceae - ps24
Saccharomycetaceae_2 - ps25
Saccharomycetaceae_3 - ps26
Kaz./Nau./Saccharomyces - ps27
Saccharomyces - ps28
Arboricola_clade - ps29
Cerevisiae_clade - ps30
Saccharomyces_cerevisiae - ps31
p-value
8e-11
8e-09
7e-07
7e-05
7e-03
Log-odds
0.5
1.0
1.5
2.0
2.5
3.0
3.5
4.0

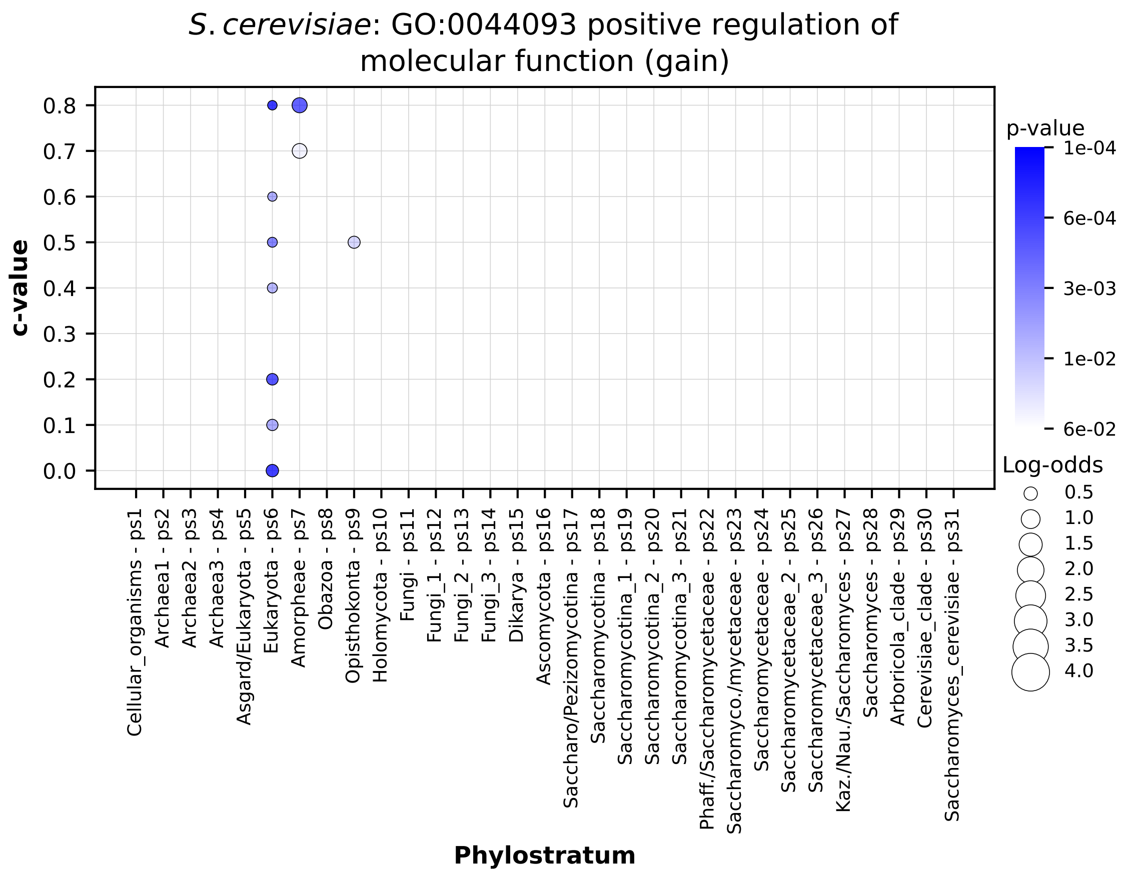

S. cerevisiae: GO:0044093 positive regulation of molecular function (gain)
c-value
0.8
0.7
0.6
0.5
0.4
0.3
0.2
0.1
0.0
p-value
1e-04
6e-04
3e-03
1e-02
6e-02
Log-odds
0.5
1.0
1.5
2.0
2.5
3.0
3.5
4.0
Cellular_organisms - ps1
Archaea1 - ps2
Archaea2 - ps3
Archaea3 - ps4
Asgard/Eukaryota - ps5
Eukaryota - ps6
Amorpheae - ps7
Obazoa - ps8
Opisthokonta - ps9
Holomycota - ps10
Fungi - ps11
Fungi_1 - ps12
Fungi_2 - ps13
Fungi_3 - ps14
Dikarya - ps15
Ascomycota - ps16
Saccharo/Pezizomycotina - ps17
Saccharomycotina - ps18
Saccharomycotina_1 - ps19
Saccharomycotina_2 - ps20
Saccharomycotina_3 - ps21
Phaff./Saccharomycetaceae - ps22
Saccharomyco./mycetaceae - ps23
Saccharomycetaceae - ps24
Saccharomycetaceae_2 - ps25
Saccharomycetaceae_3 - ps26
Kaz./Nau./Saccharomyces - ps27
Saccharomyces - ps28
Arboricola_clade - ps29
Cerevisiae_clade - ps30
Saccharomyces_cerevisiae - ps31
Phylostratum

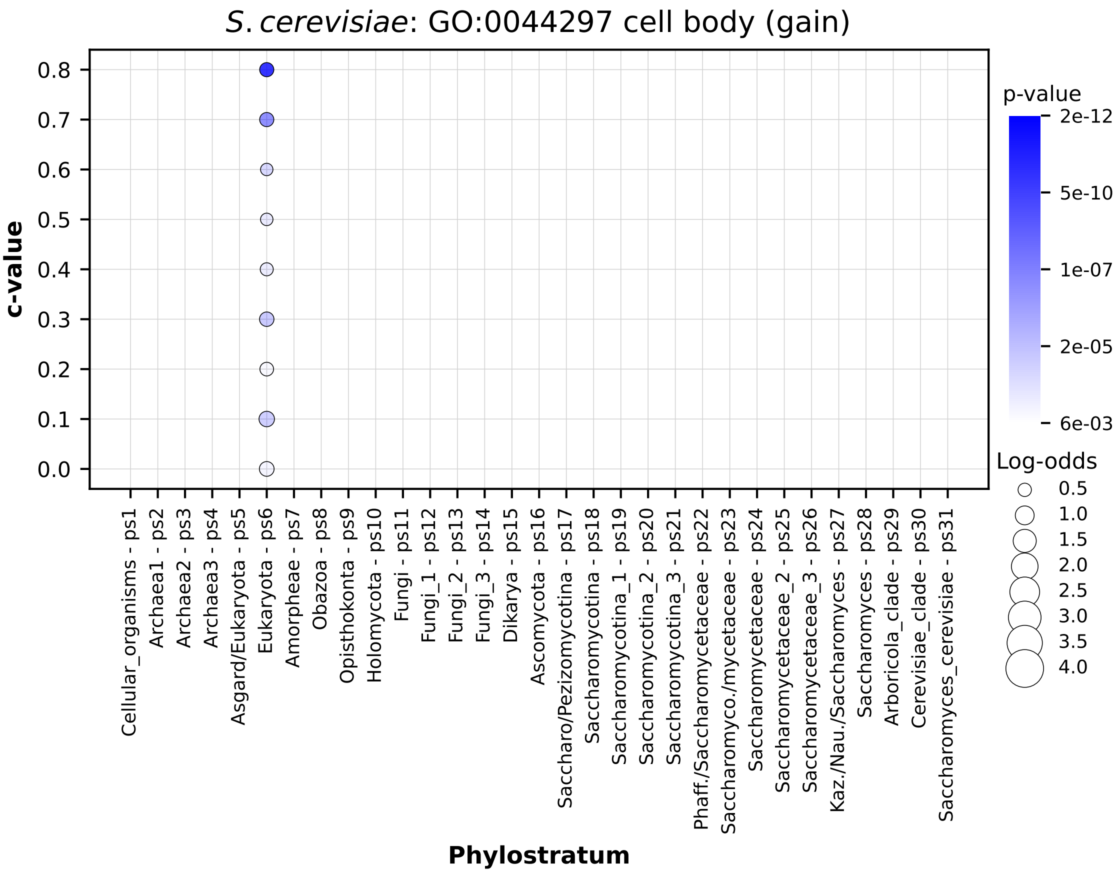

S. cerevisiae: GO:0044297 cell body (gain)
c-value
0.0
0.1
0.2
0.3
0.4
0.5
0.6
0.7
0.8
p-value
2e-12
5e-10
1e-07
2e-05
6e-03
Log-odds
0.5
1.0
1.5
2.0
2.5
3.0
3.5
4.0
Cellular_organisms - ps1
Archaea1 - ps2
Archaea2 - ps3
Archaea3 - ps4
Asgard/Eukaryota - ps5
Eukaryota - ps6
Amorpheae - ps7
Obazoa - ps8
Opisthokonta - ps9
Holomycota - ps10
Fungi - ps11
Fungi_1 - ps12
Fungi_2 - ps13
Fungi_3 - ps14
Dikarya - ps15
Ascomycota - ps16
Saccharo/Pezizomycotina - ps17
Saccharomycotina - ps18
Saccharomycotina_1 - ps19
Saccharomycotina_2 - ps20
Saccharomycotina_3 - ps21
Phaff./Saccharomycetaceae - ps22
Saccharomyco./mycetaceae - ps23
Saccharomycetaceae - ps24
Saccharomycetaceae_2 - ps25
Saccharomycetaceae_3 - ps26
Kaz./Nau./Saccharomyces - ps27
Saccharomyces - ps28
Arboricola_clade - ps29
Cerevisiae_clade - ps30
Saccharomyces_cerevisiae - ps31
Phylostratum

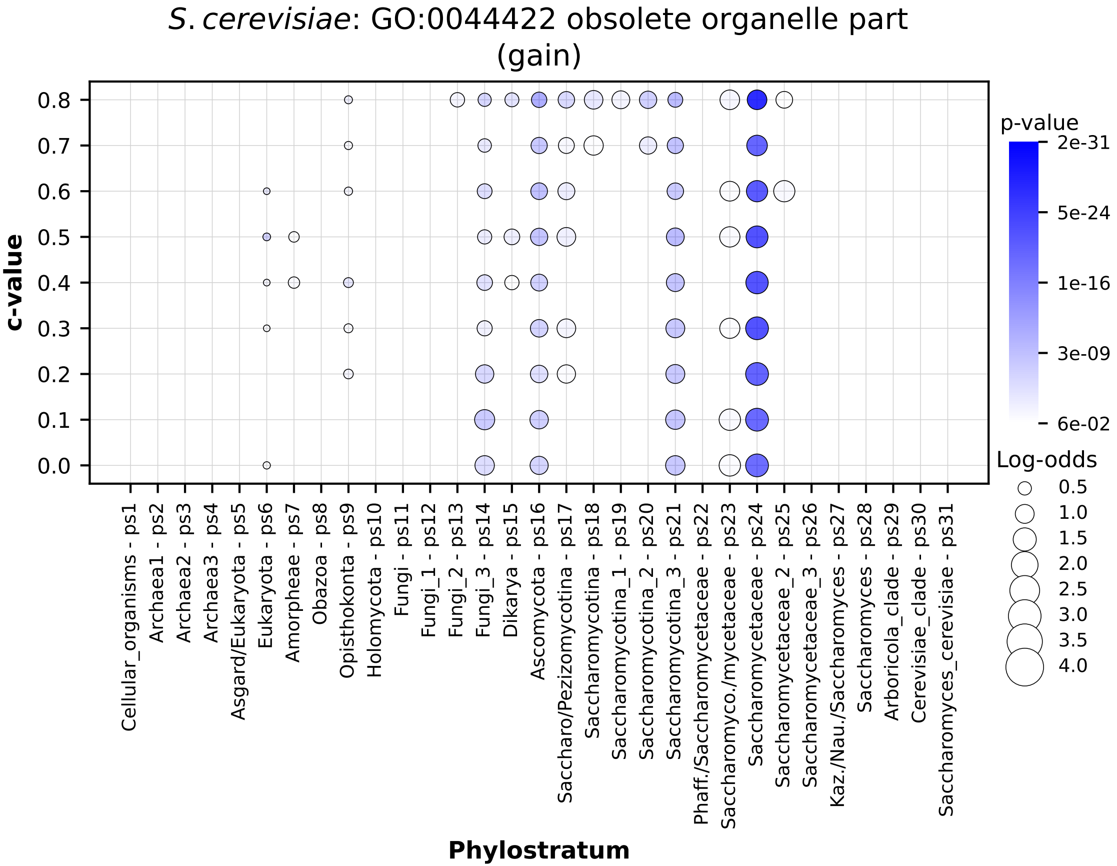

S. cerevisiae: GO:0044422 obsolete organelle part
(gain)
c-value
0.8
0.7
0.6
0.5
0.4
0.3
0.2
0.1
0.0
p-value
2e-31
5e-24
1e-16
3e-09
6e-02
Log-odds
0.5
1.0
1.5
2.0
2.5
3.0
3.5
4.0
Cellular_organisms - ps1
Archaea1 - ps2
Archaea2 - ps3
Archaea3 - ps4
Asgard/Eukaryota - ps5
Eukaryota - ps6
Amorpheae - ps7
Obazoa - ps8
Opisthokonta - ps9
Holomycota - ps10
Fungi - ps11
Fungi_1 - ps12
Fungi_2 - ps13
Fungi_3 - ps14
Dikarya - ps15
Ascomycota - ps16
Saccharo/Pezizomycotina - ps17
Saccharomycotina - ps18
Saccharomycotina_1 - ps19
Saccharomycotina_2 - ps20
Saccharomycotina_3 - ps21
Phaff./Saccharomycetaceae - ps22
Saccharomyco./mycetaceae - ps23
Saccharomycetaceae - ps24
Saccharomycetaceae_2 - ps25
Saccharomycetaceae_3 - ps26
Kaz./Nau./Saccharomyces - ps27
Saccharomyces - ps28
Arboricola_clade - ps29
Cerevisiae_clade - ps30
Saccharomyces_cerevisiae - ps31
Phylostratum

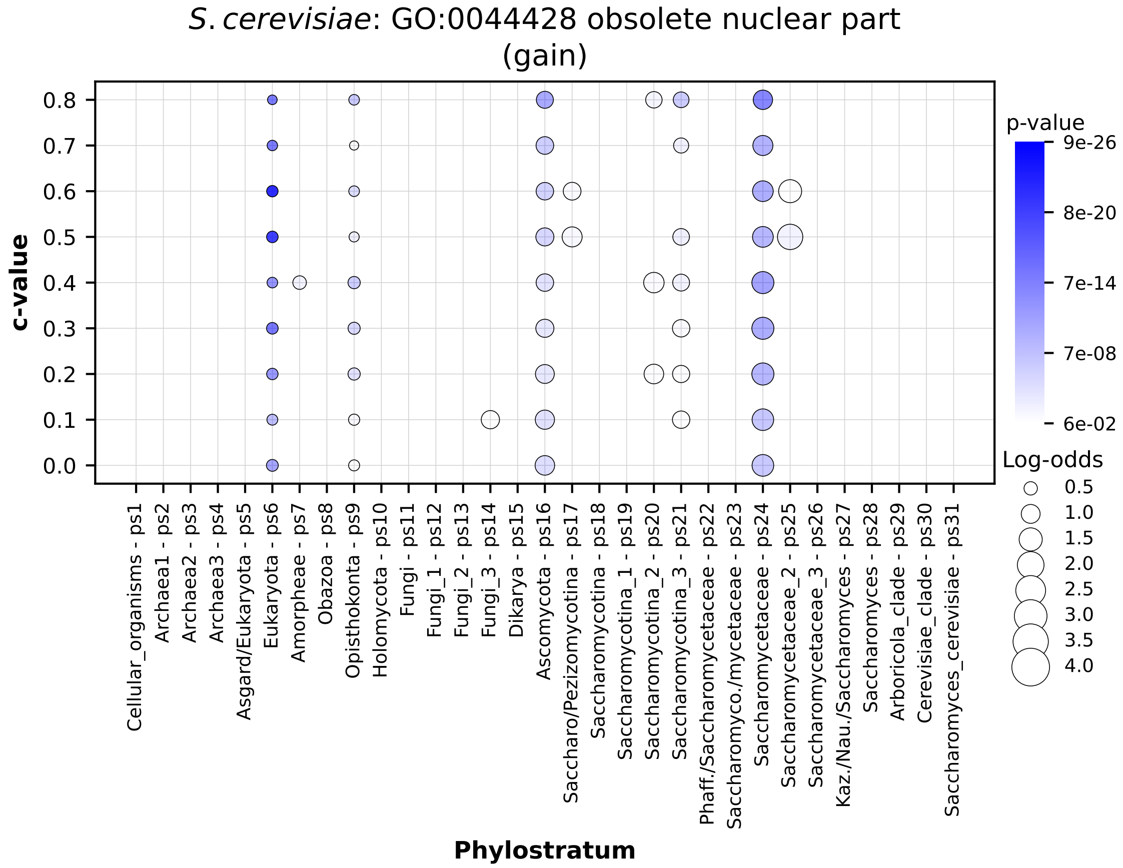

S. cerevisiae: GO:0044428 obsolete nuclear part
(gain)
c-value
0.8
0.7
0.6
0.5
0.4
0.3
0.2
0.1
0.0
p-value
9e-26
8e-20
7e-14
7e-08
6e-02
Log-odds
0.5
1.0
1.5
2.0
2.5
3.0
3.5
4.0
Cellular_organisms - ps1
Archaea1 - ps2
Archaea2 - ps3
Archaea3 - ps4
Asgard/Eukaryota - ps5
Eukaryota - ps6
Amorpheae - ps7
Obazoa - ps8
Opisthokonta - ps9
Holomycota - ps10
Fungi - ps11
Fungi_1 - ps12
Fungi_2 - ps13
Fungi_3 - ps14
Dikarya - ps15
Ascomycota - ps16
Saccharo/Pezizomycotina - ps17
Saccharomycotina - ps18
Saccharomycotina_1 - ps19
Saccharomycotina_2 - ps20
Saccharomycotina_3 - ps21
Phaff./Saccharomycetaceae - ps22
Saccharomyco./mycetaceae - ps23
Saccharomycetaceae - ps24
Saccharomycetaceae_2 - ps25
Saccharomycetaceae_3 - ps26
Kaz./Nau./Saccharomyces - ps27
Saccharomyces - ps28
Arboricola_clade - ps29
Cerevisiae_clade - ps30
Saccharomyces_cerevisiae - ps31
Phylostratum

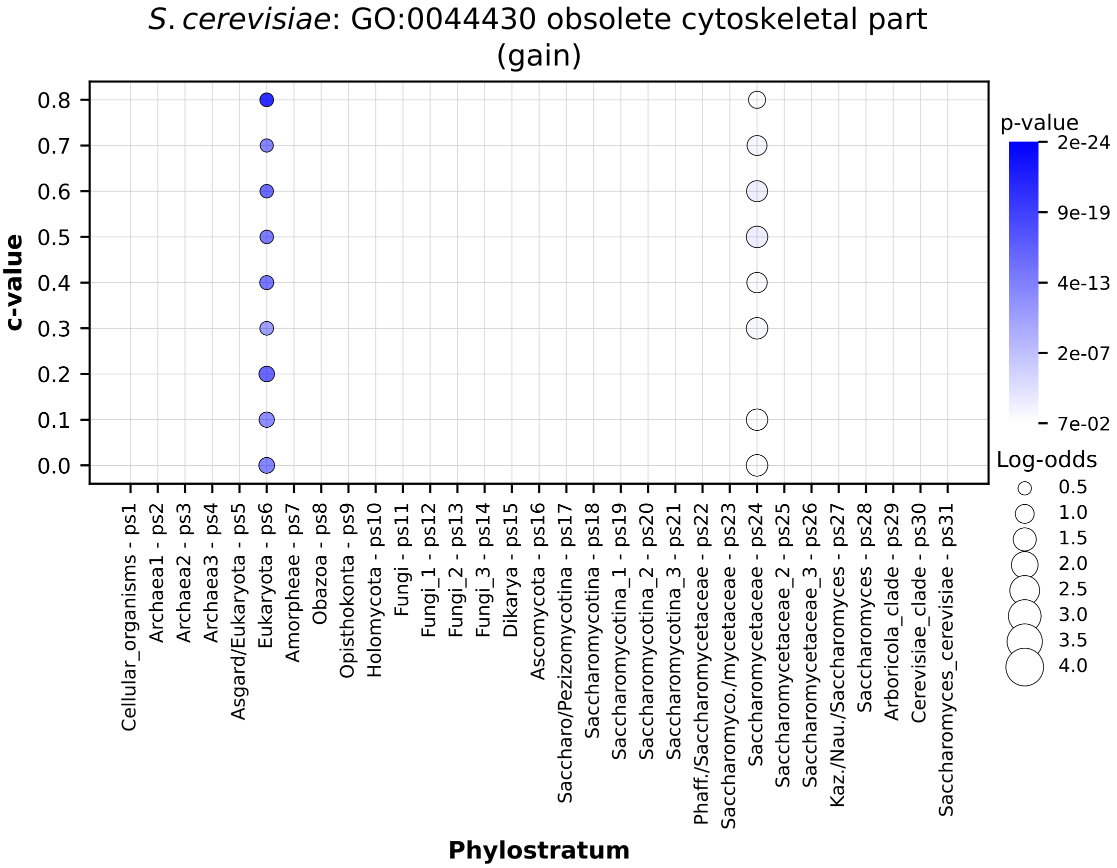
S. cerevisiae: GO:0044430 obsolete cytoskeletal part
(gain)
c-value
0.8
0.7
0.6
0.5
0.4
0.3
0.2
0.1
0.0
p-value
2e-24
9e-19
4e-13
2e-07
7e-02
Log-odds
0.5
1.0
1.5
2.0
2.5
3.0
3.5
4.0
Cellular_organisms - ps1
Archaea1 - ps2
Archaea2 - ps3
Archaea3 - ps4
Asgard/Eukaryota - ps5
Eukaryota - ps6
Amorpheae - ps7
Obazoa - ps8
Opisthokonta - ps9
Holomycota - ps10
Fungi - ps11
Fungi_1 - ps12
Fungi_2 - ps13
Fungi_3 - ps14
Dikarya - ps15
Ascomycota - ps16
Saccharo/Pezizomycotina - ps17
Saccharomycotina - ps18
Saccharomycotina_1 - ps19
Saccharomycotina_2 - ps20
Saccharomycotina_3 - ps21
Phaff./Saccharomycetaceae - ps22
Saccharomyco./mycetaceae - ps23
Saccharomycetaceae - ps24
Saccharomycetaceae_2 - ps25
Saccharomycetaceae_3 - ps26
Kaz./Nau./Saccharomyces - ps27
Saccharomyces - ps28
Arboricola_clade - ps29
Cerevisiae_clade - ps30
Saccharomyces_cerevisiae - ps31
Phylostratum

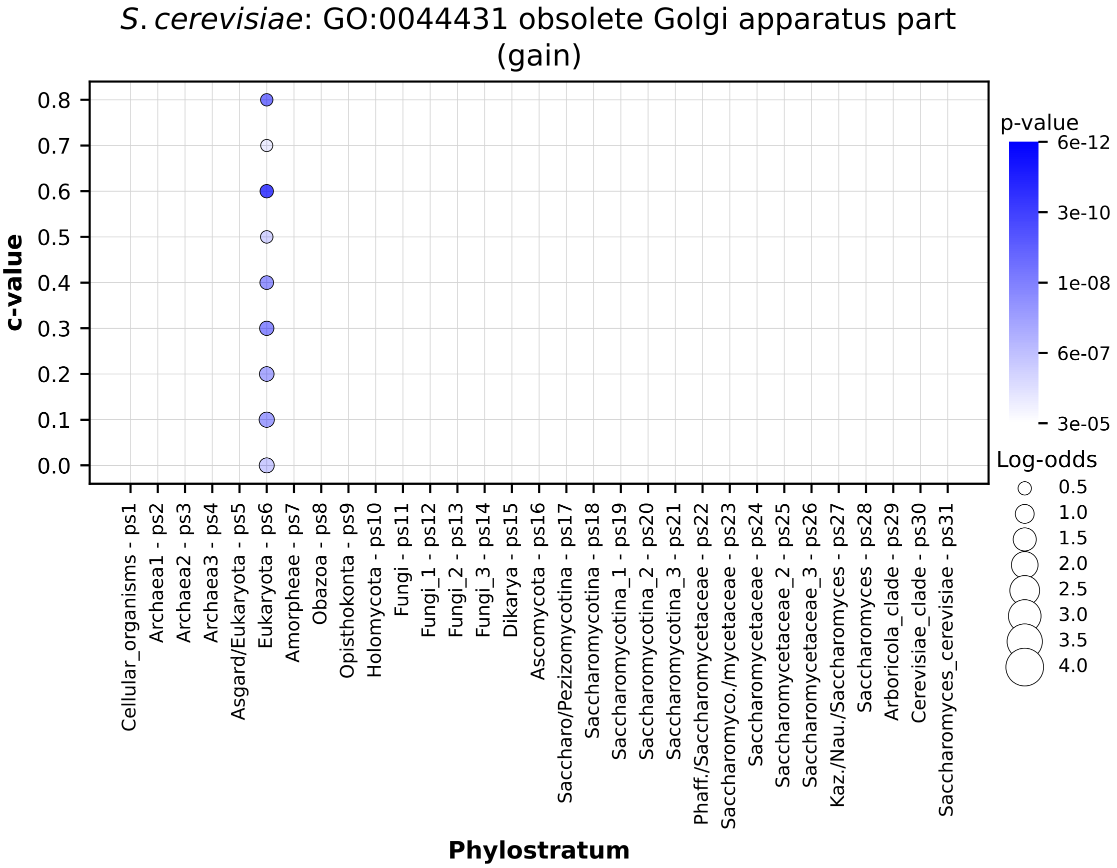

S. cerevisiae: GO:0044431 obsolete Golgi apparatus part
(gain)
c-value
0.8
0.7
0.6
0.5
0.4
0.3
0.2
0.1
0.0
p-value
6e-12
3e-10
1e-08
6e-07
3e-05
Log-odds
0.5
1.0
1.5
2.0
2.5
3.0
3.5
4.0
Cellular_organisms - ps1
Archaea1 - ps2
Archaea2 - ps3
Archaea3 - ps4
Asgard/Eukaryota - ps5
Eukaryota - ps6
Amorpheae - ps7
Obazoa - ps8
Opisthokonta - ps9
Holomycota - ps10
Fungi - ps11
Fungi_1 - ps12
Fungi_2 - ps13
Fungi_3 - ps14
Dikarya - ps15
Ascomycota - ps16
Saccharo/Pezizomycotina - ps17
Saccharomycotina - ps18
Saccharomycotina_1 - ps19
Saccharomycotina_2 - ps20
Saccharomycotina_3 - ps21
Phaff./Saccharomycetaceae - ps22
Saccharomyco./mycetaceae - ps23
Saccharomycetaceae - ps24
Saccharomycetaceae_2 - ps25
Saccharomycetaceae_3 - ps26
Kaz./Nau./Saccharomyces - ps27
Saccharomyces - ps28
Arboricola_clade - ps29
Cerevisiae_clade - ps30
Saccharomyces_cerevisiae - ps31
Phylostratum

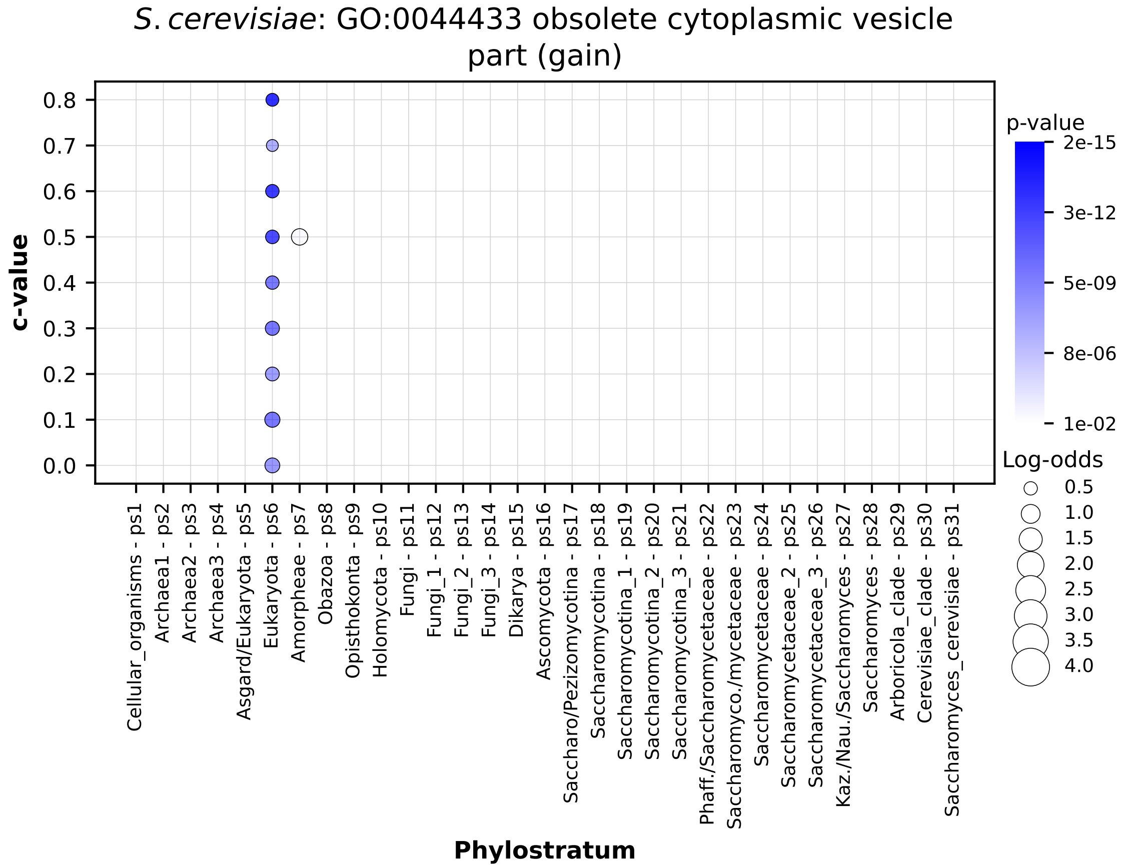

S. cerevisiae: GO:0044433 obsolete cytoplasmic vesicle part (gain)
c-value
0.8
0.7
0.6
0.5
0.4
0.3
0.2
0.1
0.0
p-value
2e-15
3e-12
5e-09
8e-06
1e-02
Log-odds
0.5
1.0
1.5
2.0
2.5
3.0
3.5
4.0
Cellular_organisms - ps1
Archaea1 - ps2
Archaea2 - ps3
Archaea3 - ps4
Asgard/Eukaryota - ps5
Eukaryota - ps6
Amorpheae - ps7
Obazoa - ps8
Opisthokonta - ps9
Holomycota - ps10
Fungi - ps11
Fungi_1 - ps12
Fungi_2 - ps13
Fungi_3 - ps14
Dikarya - ps15
Ascomycota - ps16
Saccharo/Pezizomycotina - ps17
Saccharomycotina - ps18
Saccharomycotina_1 - ps19
Saccharomycotina_2 - ps20
Saccharomycotina_3 - ps21
Phaff./Saccharomycetaceae - ps22
Saccharomyco./mycetaceae - ps23
Saccharomycetaceae - ps24
Saccharomycetaceae_2 - ps25
Saccharomycetaceae_3 - ps26
Kaz./Nau./Saccharomyces - ps27
Saccharomyces - ps28
Arboricola_clade - ps29
Cerevisiae_clade - ps30
Saccharomyces_cerevisiae - ps31
Phylostratum

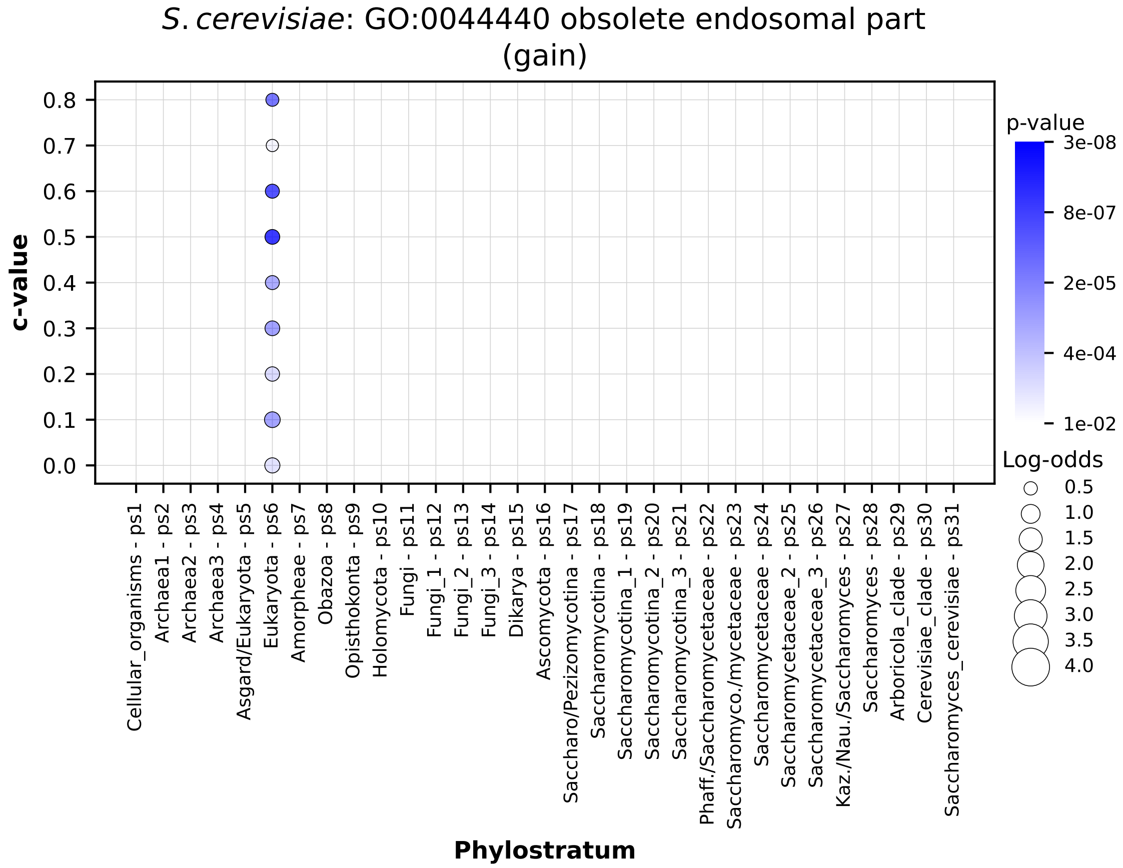

S. cerevisiae: GO:0044440 obsolete endosomal part
(gain)
c-value
0.8
0.7
0.6
0.5
0.4
0.3
0.2
0.1
0.0
p-value
3e-08
8e-07
2e-05
4e-04
1e-02
Log-odds
0.5
1.0
1.5
2.0
2.5
3.0
3.5
4.0
Cellular_organisms - ps1
Archaea1 - ps2
Archaea2 - ps3
Archaea3 - ps4
Asgard/Eukaryota - ps5
Eukaryota - ps6
Amorpheae - ps7
Obazoa - ps8
Opisthokonta - ps9
Holomycota - ps10
Fungi - ps11
Fungi_1 - ps12
Fungi_2 - ps13
Fungi_3 - ps14
Dikarya - ps15
Ascomycota - ps16
Saccharo/Pezizomycotina - ps17
Saccharomycotina - ps18
Saccharomycotina_1 - ps19
Saccharomycotina_2 - ps20
Saccharomycotina_3 - ps21
Phaff./Saccharomycetaceae - ps22
Saccharomyco./mycetaceae - ps23
Saccharomycetaceae - ps24
Saccharomycetaceae_2 - ps25
Saccharomycetaceae_3 - ps26
Kaz./Nau./Saccharomyces - ps27
Saccharomyces - ps28
Arboricola_clade - ps29
Cerevisiae_clade - ps30
Saccharomyces_cerevisiae - ps31
Phylostratum

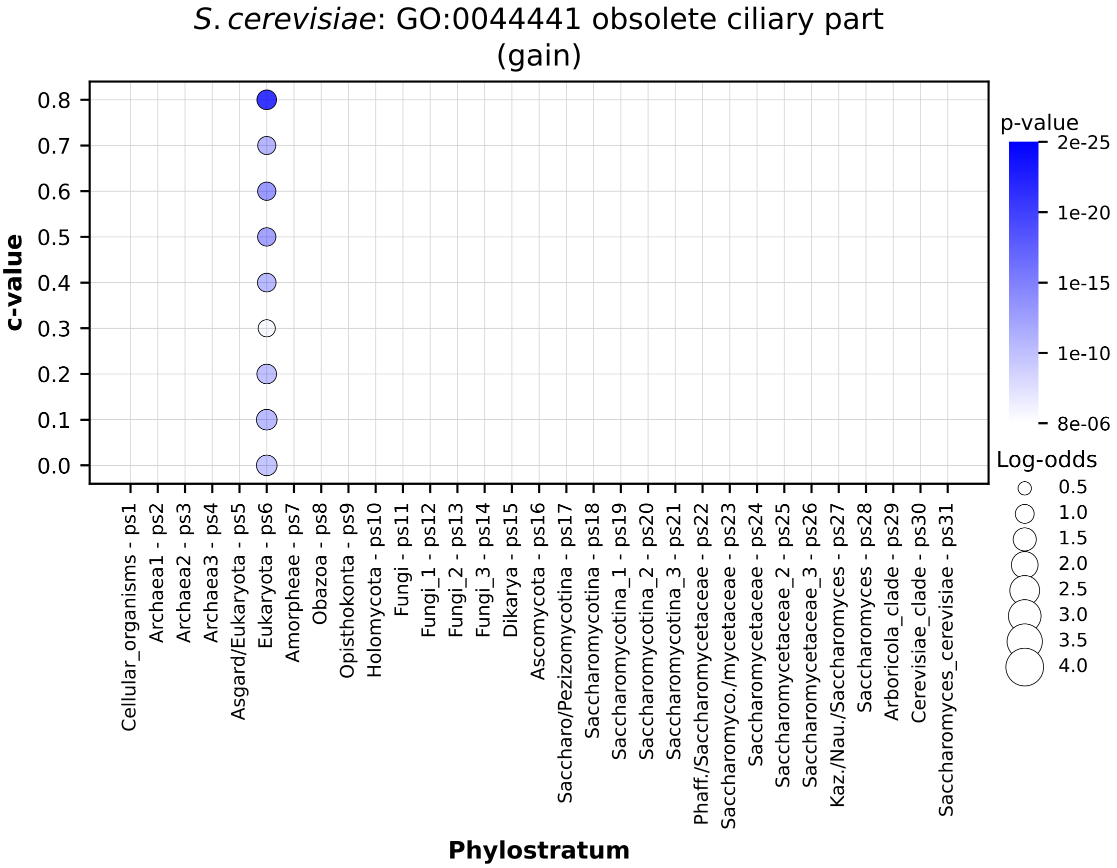

S. cerevisiae: GO:0044441 obsolete ciliary part
(gain)
c-value
0.8
0.7
0.6
0.5
0.4
0.3
0.2
0.1
0.0
p-value
2e-25
1e-20
1e-15
1e-10
8e-06
Log-odds
0.5
1.0
1.5
2.0
2.5
3.0
3.5
4.0
Cellular_organisms - ps1
Archaea1 - ps2
Archaea2 - ps3
Archaea3 - ps4
Asgard/Eukaryota - ps5
Eukaryota - ps6
Amorpheae - ps7
Obazoa - ps8
Opisthokonta - ps9
Holomycota - ps10
Fungi - ps11
Fungi_1 - ps12
Fungi_2 - ps13
Fungi_3 - ps14
Dikarya - ps15
Ascomycota - ps16
Saccharo/Pezizomycotina - ps17
Saccharomycotina - ps18
Saccharomycotina_1 - ps19
Saccharomycotina_2 - ps20
Saccharomycotina_3 - ps21
Phaff./Saccharomycetaceae - ps22
Saccharomyco./mycetaceae - ps23
Saccharomycetaceae - ps24
Saccharomycetaceae_2 - ps25
Saccharomycetaceae_3 - ps26
Kaz./Nau./Saccharomyces - ps27
Saccharomyces - ps28
Arboricola_clade - ps29
Cerevisiae_clade - ps30
Saccharomyces_cerevisiae - ps31
Phylostratum

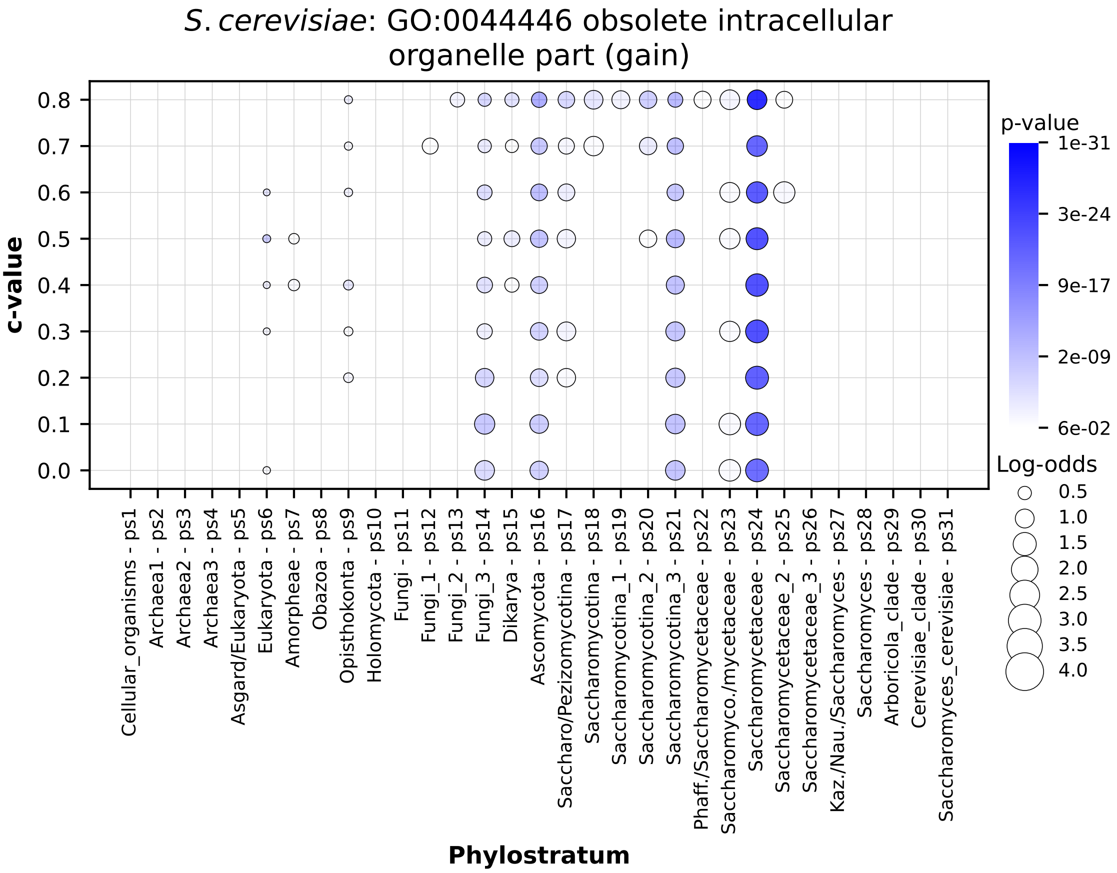

S. cerevisiae: GO:0044446 obsolete intracellular organelle part (gain)
c-value
0.8
0.7
0.6
0.5
0.4
0.3
0.2
0.1
0.0
p-value
1e-31
3e-24
9e-17
2e-09
6e-02
Log-odds
0.5
1.0
1.5
2.0
2.5
3.0
3.5
4.0
Cellular_organisms - ps1
Archaea1 - ps2
Archaea2 - ps3
Archaea3 - ps4
Asgard/Eukaryota - ps5
Eukaryota - ps6
Amorpheae - ps7
Obazoa - ps8
Opisthokonta - ps9
Holomycota - ps10
Fungi - ps11
Fungi_1 - ps12
Fungi_2 - ps13
Fungi_3 - ps14
Dikarya - ps15
Ascomycota - ps16
Saccharo/Pezizomycotina - ps17
Saccharomycotina - ps18
Saccharomycotina_1 - ps19
Saccharomycotina_2 - ps20
Saccharomycotina_3 - ps21
Phaff./Saccharomycetaceae - ps22
Saccharomyco./mycetaceae - ps23
Saccharomycetaceae - ps24
Saccharomycetaceae_2 - ps25
Saccharomycetaceae_3 - ps26
Kaz./Nau./Saccharomyces - ps27
Saccharomyces - ps28
Arboricola_clade - ps29
Cerevisiae_clade - ps30
Saccharomyces_cerevisiae - ps31
Phylostratum

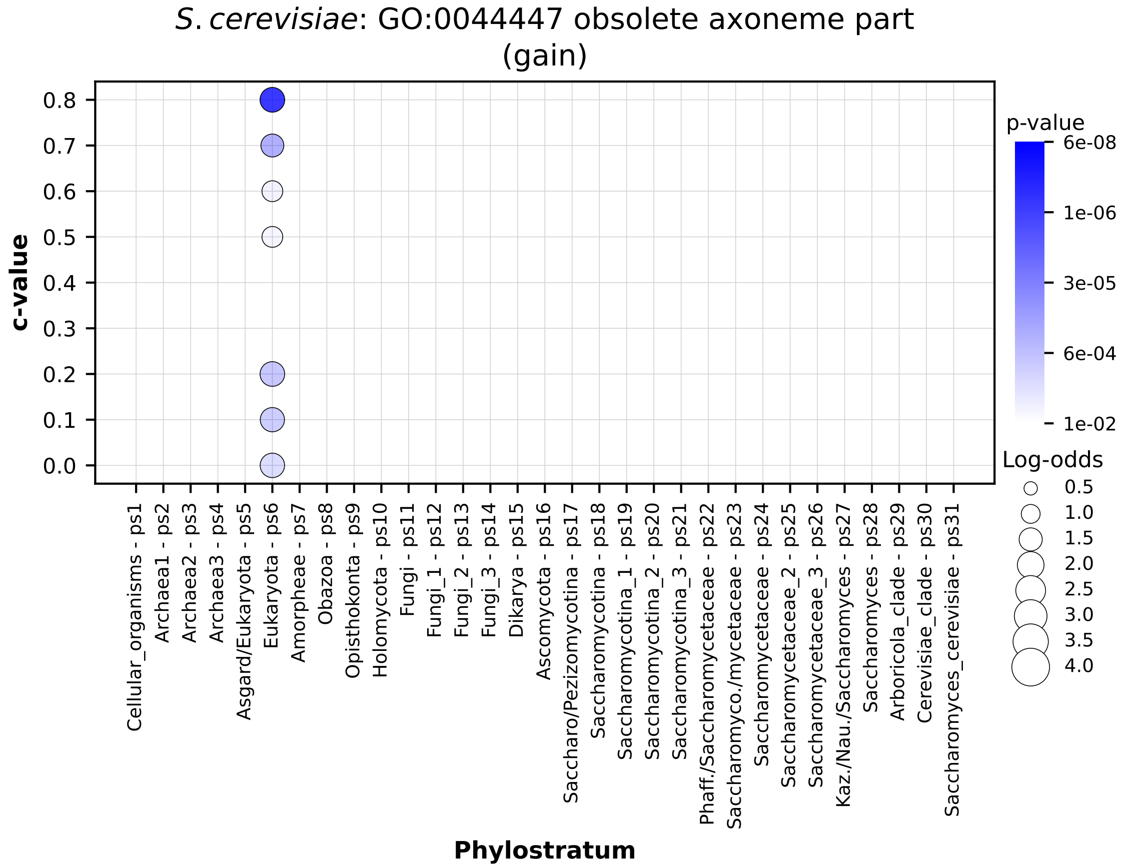

S. cerevisiae: GO:0044447 obsolete axoneme part
(gain)
c-value
0.8
0.7
0.6
0.5
0.4
0.3
0.2
0.1
0.0
p-value
6e-08
1e-06
3e-05
6e-04
1e-02
Log-odds
0.5
1.0
1.5
2.0
2.5
3.0
3.5
4.0
Cellular_organisms - ps1
Archaea1 - ps2
Archaea2 - ps3
Archaea3 - ps4
Asgard/Eukaryota - ps5
Eukaryota - ps6
Amorpheae - ps7
Obazoa - ps8
Opisthokonta - ps9
Holomycota - ps10
Fungi - ps11
Fungi_1 - ps12
Fungi_2 - ps13
Fungi_3 - ps14
Dikarya - ps15
Ascomycota - ps16
Saccharo/Pezizomycotina - ps17
Saccharomycotina - ps18
Saccharomycotina_1 - ps19
Saccharomycotina_2 - ps20
Saccharomycotina_3 - ps21
Phaff./Saccharomycetaceae - ps22
Saccharomyco./mycetaceae - ps23
Saccharomycetaceae - ps24
Saccharomycetaceae_2 - ps25
Saccharomycetaceae_3 - ps26
Kaz./Nau./Saccharomyces - ps27
Saccharomyces - ps28
Arboricola_clade - ps29
Cerevisiae_clade - ps30
Saccharomyces_cerevisiae - ps31
Phylostratum

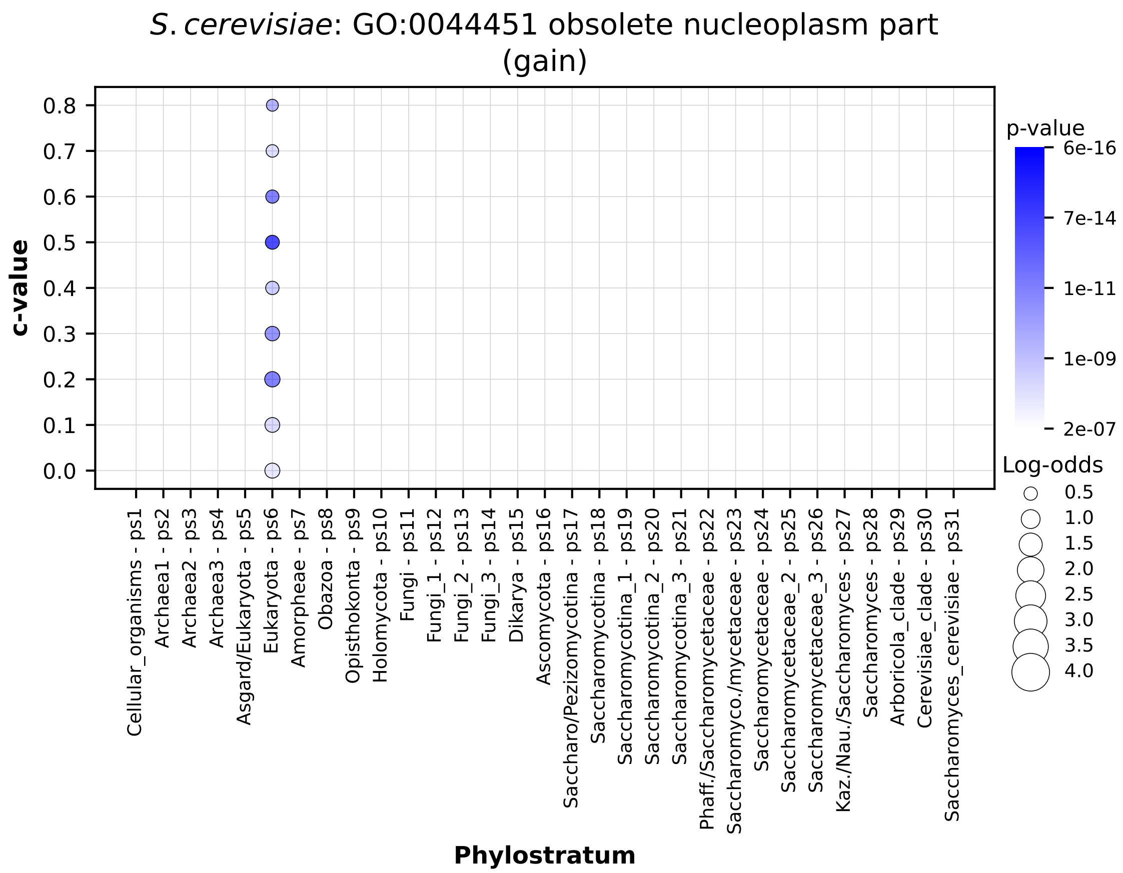

S. cerevisiae: GO:0044451 obsolete nucleoplasm part (gain)
c-value
0.0
0.1
0.2
0.3
0.4
0.5
0.6
0.7
0.8
p-value
6e-16
7e-14
1e-11
1e-09
2e-07
Log-odds
0.5
1.0
1.5
2.0
2.5
3.0
3.5
4.0
Cellular_organisms - ps1
Archaea1 - ps2
Archaea2 - ps3
Archaea3 - ps4
Asgard/Eukaryota - ps5
Eukaryota - ps6
Amorpheae - ps7
Obazoa - ps8
Opisthokonta - ps9
Holomycota - ps10
Fungi - ps11
Fungi_1 - ps12
Fungi_2 - ps13
Fungi_3 - ps14
Dikarya - ps15
Ascomycota - ps16
Saccharo/Pezizomycotina - ps17
Saccharomycotina - ps18
Saccharomycotina_1 - ps19
Saccharomycotina_2 - ps20
Saccharomycotina_3 - ps21
Phaff./Saccharomycetaceae - ps22
Saccharomyco./mycetaceae - ps23
Saccharomycetaceae - ps24
Saccharomycetaceae_2 - ps25
Saccharomycetaceae_3 - ps26
Kaz./Nau./Saccharomyces - ps27
Saccharomyces - ps28
Arboricola_clade - ps29
Cerevisiae_clade - ps30
Saccharomyces_cerevisiae - ps31
Phylostratum

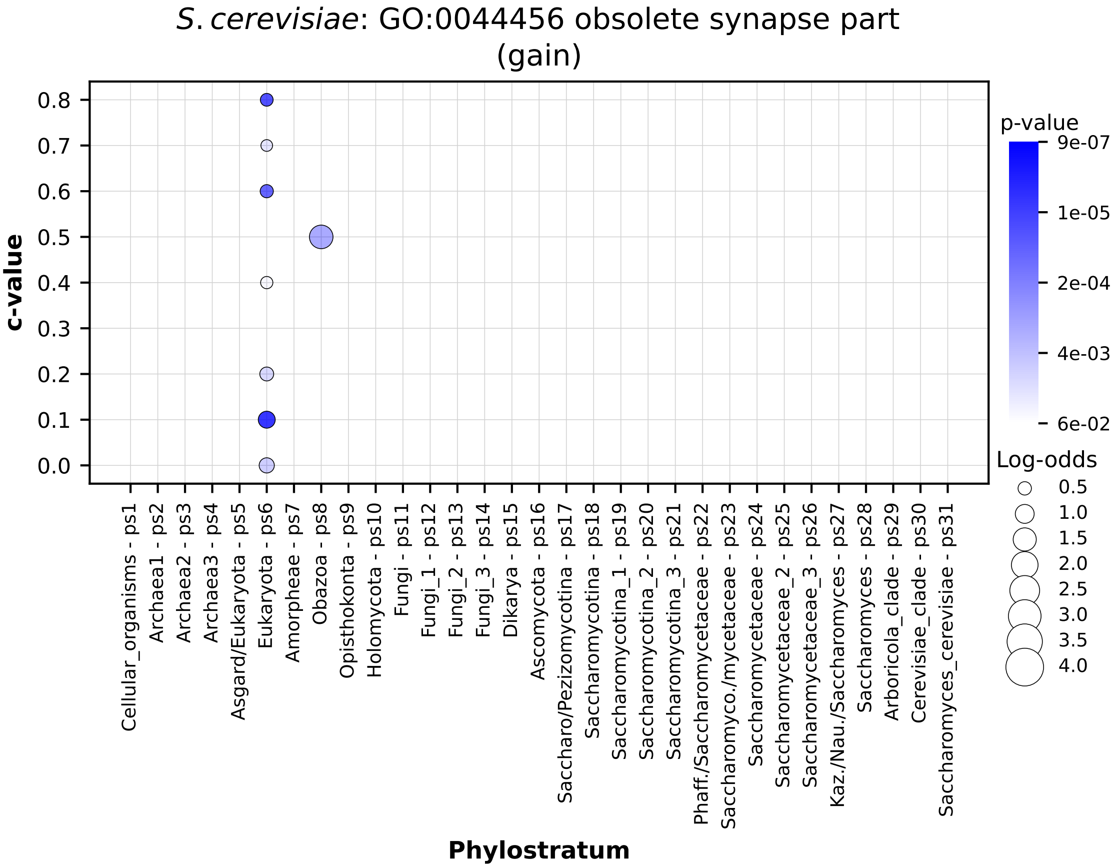

S. cerevisiae: GO:0044456 obsolete synapse part
(gain)
c-value
0.8
0.7
0.6
0.5
0.4
0.3
0.2
0.1
0.0
p-value
9e-07
1e-05
2e-04
4e-03
6e-02
Log-odds
0.5
1.0
1.5
2.0
2.5
3.0
3.5
4.0
Cellular_organisms - ps1
Archaea1 - ps2
Archaea2 - ps3
Archaea3 - ps4
Asgard/Eukaryota - ps5
Eukaryota - ps6
Amorpheae - ps7
Obazoa - ps8
Opisthokonta - ps9
Holomycota - ps10
Fungi - ps11
Fungi_1 - ps12
Fungi_2 - ps13
Fungi_3 - ps14
Dikarya - ps15
Ascomycota - ps16
Saccharo/Pezizomycotina - ps17
Saccharomycotina - ps18
Saccharomycotina_1 - ps19
Saccharomycotina_2 - ps20
Saccharomycotina_3 - ps21
Phaff./Saccharomycetaceae - ps22
Saccharomyco./mycetaceae - ps23
Saccharomycetaceae - ps24
Saccharomycetaceae_2 - ps25
Saccharomycetaceae_3 - ps26
Kaz./Nau./Saccharomyces - ps27
Saccharomyces - ps28
Arboricola_clade - ps29
Cerevisiae_clade - ps30
Saccharomyces_cerevisiae - ps31
Phylostratum

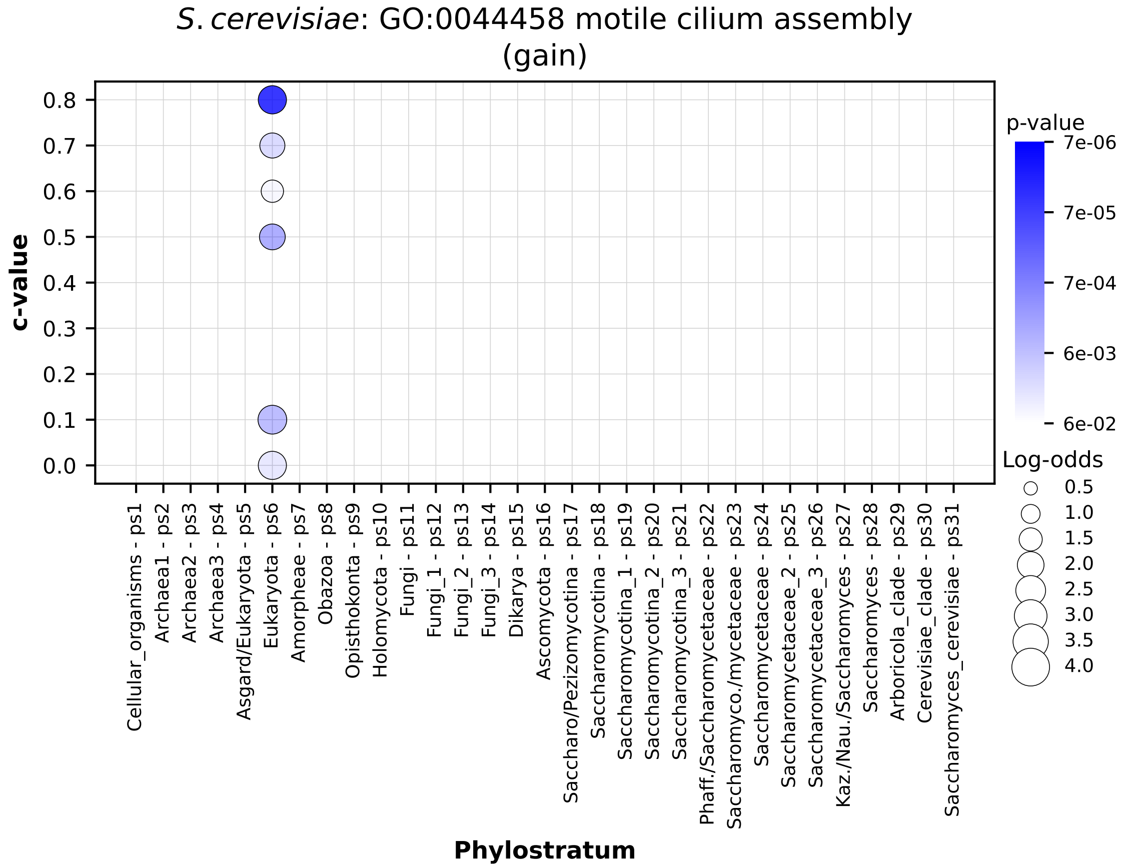

S. cerevisiae: GO:0044458 motile cilium assembly (gain)
c-value
0.8
0.7
0.6
0.5
0.4
0.3
0.2
0.1
0.0
p-value
7e-06
7e-05
7e-04
6e-03
6e-02
Log-odds
0.5
1.0
1.5
2.0
2.5
3.0
3.5
4.0
Cellular_organisms - ps1
Archaea1 - ps2
Archaea2 - ps3
Archaea3 - ps4
Asgard/Eukaryota - ps5
Eukaryota - ps6
Amorpheae - ps7
Obazoa - ps8
Opisthokonta - ps9
Holomycota - ps10
Fungi - ps11
Fungi_1 - ps12
Fungi_2 - ps13
Fungi_3 - ps14
Dikarya - ps15
Ascomycota - ps16
Saccharo/Pezizomycotina - ps17
Saccharomycotina - ps18
Saccharomycotina_1 - ps19
Saccharomycotina_2 - ps20
Saccharomycotina_3 - ps21
Phaff./Saccharomycetaceae - ps22
Saccharomyco./mycetaceae - ps23
Saccharomycetaceae - ps24
Saccharomycetaceae_2 - ps25
Saccharomycetaceae_3 - ps26
Kaz./Nau./Saccharomyces - ps27
Saccharomyces - ps28
Arboricola_clade - ps29
Cerevisiae_clade - ps30
Saccharomyces_cerevisiae - ps31
Phylostratum

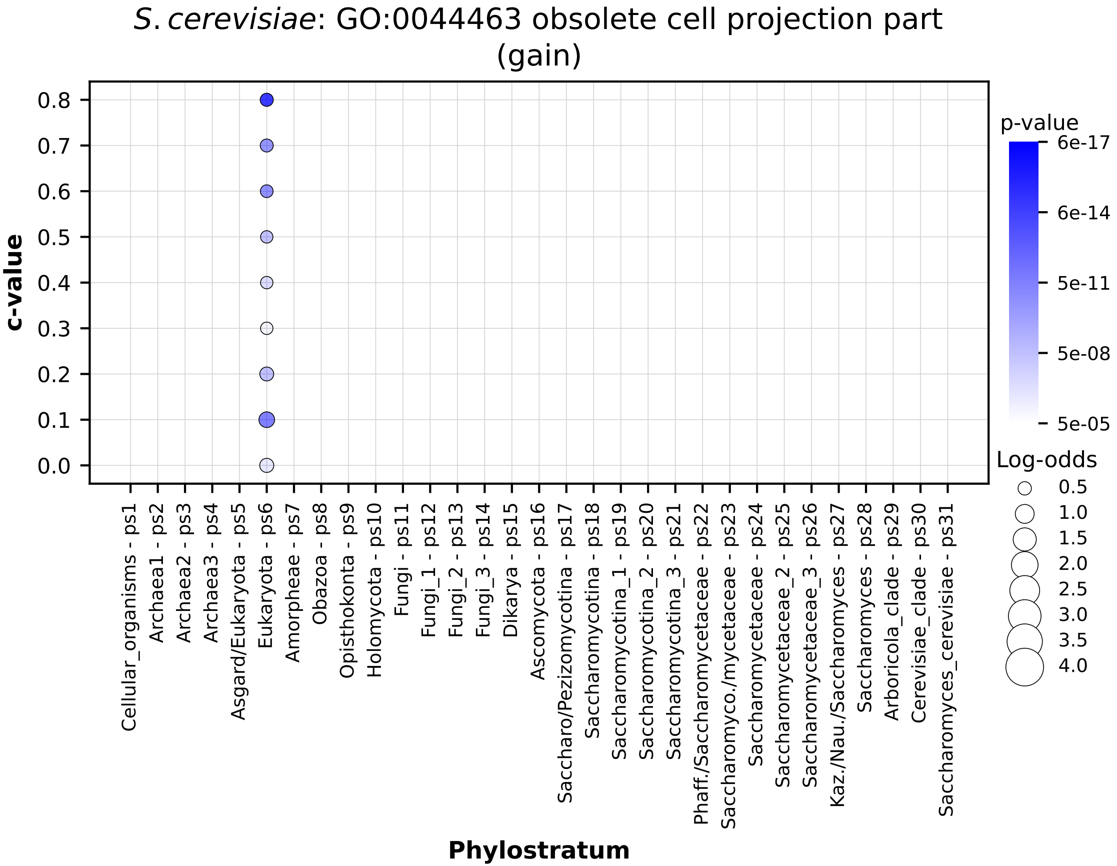
S. cerevisiae: GO:0044463 obsolete cell projection part
(gain)
c-value
0.8
0.7
0.6
0.5
0.4
0.3
0.2
0.1
0.0
p-value
6e-17
6e-14
5e-11
5e-08
5e-05
Log-odds
0.5
1.0
1.5
2.0
2.5
3.0
3.5
4.0
Cellular_organisms - ps1
Archaea1 - ps2
Archaea2 - ps3
Archaea3 - ps4
Asgard/Eukaryota - ps5
Eukaryota - ps6
Amorpheae - ps7
Obazoa - ps8
Opisthokonta - ps9
Holomycota - ps10
Fungi - ps11
Fungi_1 - ps12
Fungi_2 - ps13
Fungi_3 - ps14
Dikarya - ps15
Ascomycota - ps16
Saccharo/Pezizomycotina - ps17
Saccharomycotina - ps18
Saccharomycotina_1 - ps19
Saccharomycotina_2 - ps20
Saccharomycotina_3 - ps21
Phaff./Saccharomycetaceae - ps22
Saccharomyco./mycetaceae - ps23
Saccharomycetaceae - ps24
Saccharomycetaceae_2 - ps25
Saccharomycetaceae_3 - ps26
Kaz./Nau./Saccharomyces - ps27
Saccharomyces - ps28
Arboricola_clade - ps29
Cerevisiae_clade - ps30
Saccharomyces_cerevisiae - ps31
Phylostratum

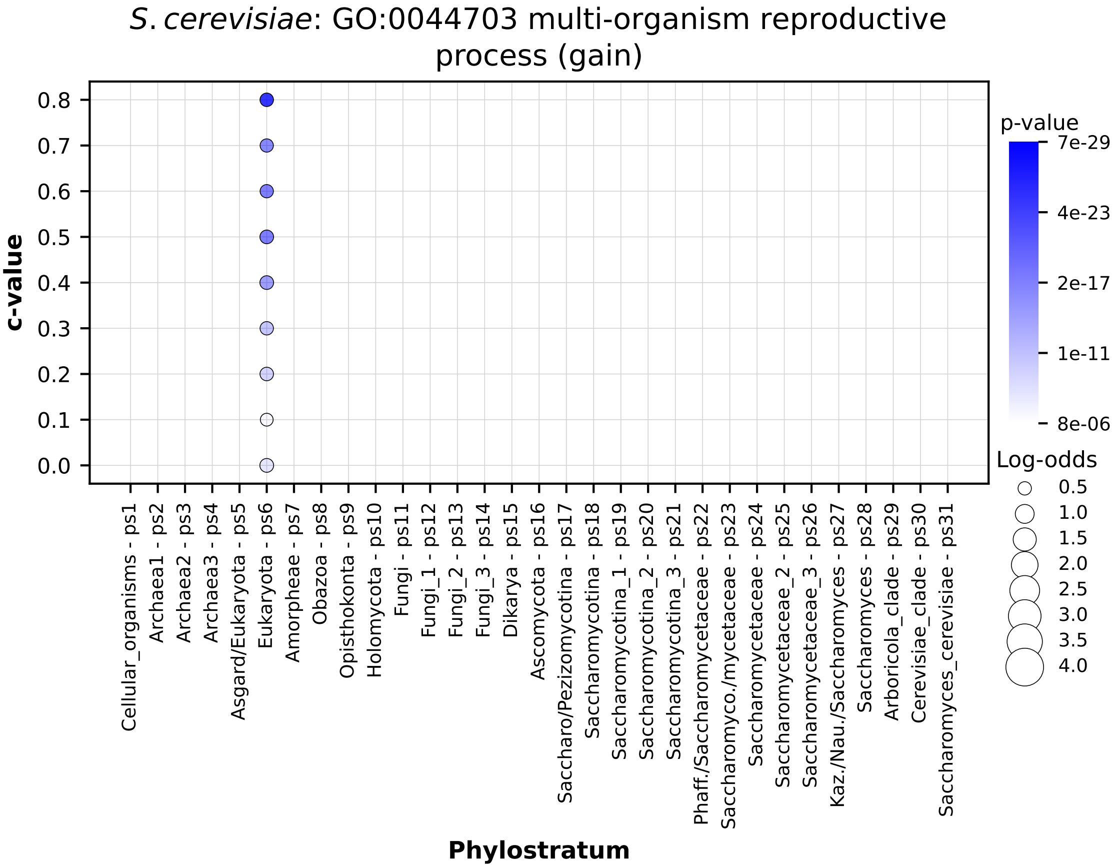
S. cerevisiae: GO:0044703 multi-organism reproductive process (gain)
c-value
0.0
0.1
0.2
0.3
0.4
0.5
0.6
0.7
0.8
p-value
7e-29
4e-23
2e-17
1e-11
8e-06
Log-odds
0.5
1.0
1.5
2.0
2.5
3.0
3.5
4.0
Cellular_organisms - ps1
Archaea1 - ps2
Archaea2 - ps3
Archaea3 - ps4
Asgard/Eukaryota - ps5
Eukaryota - ps6
Amorpheae - ps7
Obazoa - ps8
Opisthokonta - ps9
Holomycota - ps10
Fungi - ps11
Fungi_1 - ps12
Fungi_2 - ps13
Fungi_3 - ps14
Dikarya - ps15
Ascomycota - ps16
Saccharo/Pezizomycotina - ps17
Saccharomycotina - ps18
Saccharomycotina_1 - ps19
Saccharomycotina_2 - ps20
Saccharomycotina_3 - ps21
Phaff./Saccharomycetaceae - ps22
Saccharomyco./mycetaceae - ps23
Saccharomycetaceae - ps24
Saccharomycetaceae_2 - ps25
Saccharomycetaceae_3 - ps26
Kaz./Nau./Saccharomyces - ps27
Saccharomyces - ps28
Arboricola_clade - ps29
Cerevisiae_clade - ps30
Saccharomyces_cerevisiae - ps31
Phylostratum

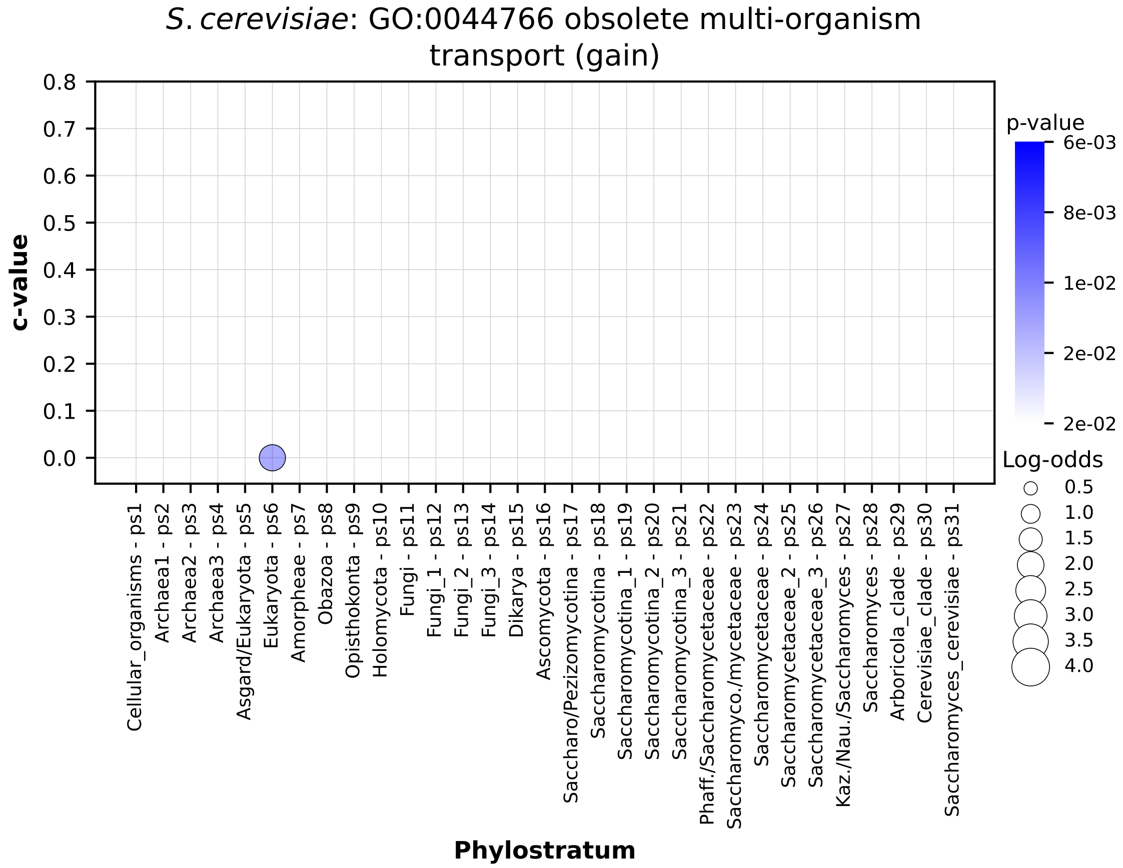

S. cerevisiae: GO:0044766 obsolete multi-organism transport (gain)
c-value
0.8
0.7
0.6
0.5
0.4
0.3
0.2
0.1
0.0
p-value
6e-03
8e-03
1e-02
2e-02
2e-02
Log-odds
0.5
1.0
1.5
2.0
2.5
3.0
3.5
4.0
Cellular_organisms - ps1
Archaea1 - ps2
Archaea2 - ps3
Archaea3 - ps4
Asgard/Eukaryota - ps5
Eukaryota - ps6
Amorpheae - ps7
Obazoa - ps8
Opisthokonta - ps9
Holomycota - ps10
Fungi - ps11
Fungi_1 - ps12
Fungi_2 - ps13
Fungi_3 - ps14
Dikarya - ps15
Ascomycota - ps16
Saccharo/Pezizomycotina - ps17
Saccharomycotina - ps18
Saccharomycotina_1 - ps19
Saccharomycotina_2 - ps20
Saccharomycotina_3 - ps21
Phaff./Saccharomycetaceae - ps22
Saccharomyco./mycetaceae - ps23
Saccharomycetaceae - ps24
Saccharomycetaceae_2 - ps25
Saccharomycetaceae_3 - ps26
Kaz./Nau./Saccharomyces - ps27
Saccharomyces - ps28
Arboricola_clade - ps29
Cerevisiae_clade - ps30
Saccharomyces_cerevisiae - ps31
Phylostratum

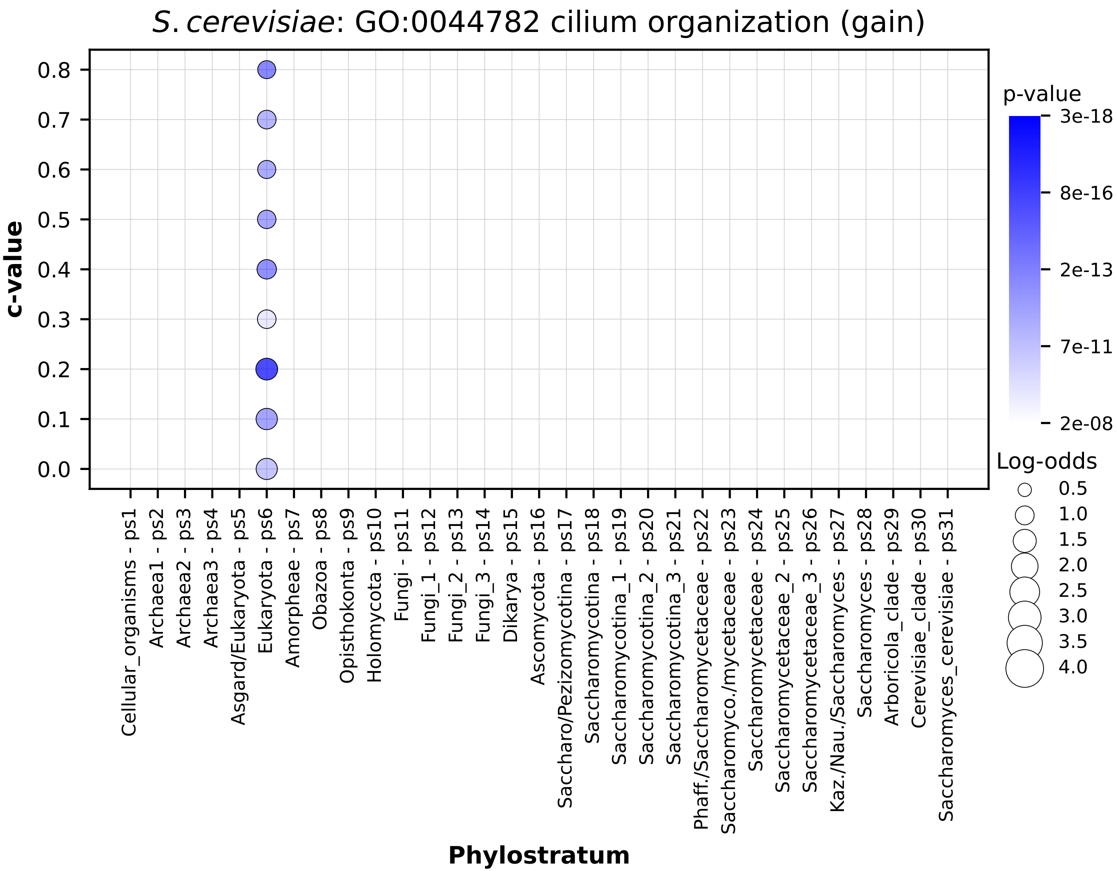

S. cerevisiae: GO:0044782 cilium organization (gain)
c-value
0.8
0.7
0.6
0.5
0.4
0.3
0.2
0.1
0.0
p-value
3e-18
8e-16
2e-13
7e-11
2e-08
Log-odds
0.5
1.0
1.5
2.0
2.5
3.0
3.5
4.0
Cellular_organisms - ps1
Archaea1 - ps2
Archaea2 - ps3
Archaea3 - ps4
Asgard/Eukaryota - ps5
Eukaryota - ps6
Amorpheae - ps7
Obazoa - ps8
Opisthokonta - ps9
Holomycota - ps10
Fungi - ps11
Fungi_1 - ps12
Fungi_2 - ps13
Fungi_3 - ps14
Dikarya - ps15
Ascomycota - ps16
Saccharo/Pezizomycotina - ps17
Saccharomycotina - ps18
Saccharomycotina_1 - ps19
Saccharomycotina_2 - ps20
Saccharomycotina_3 - ps21
Phaff./Saccharomycetaceae - ps22
Saccharomyco./mycetaceae - ps23
Saccharomycetaceae - ps24
Saccharomycetaceae_2 - ps25
Saccharomycetaceae_3 - ps26
Kaz./Nau./Saccharomyces - ps27
Saccharomyces - ps28
Arboricola_clade - ps29
Cerevisiae_clade - ps30
Saccharomyces_cerevisiae - ps31
Phylostratum

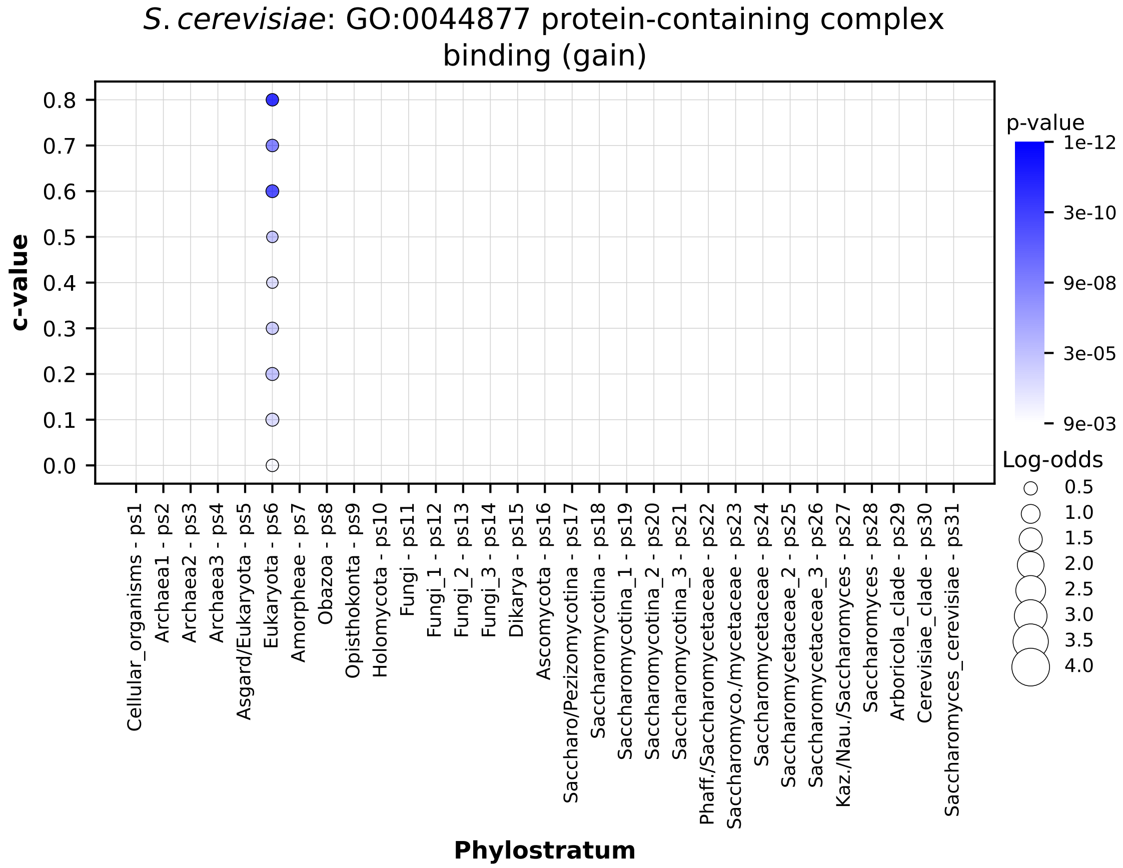

S. cerevisiae: GO:0044877 protein-containing complex binding (gain)
c-value
0.8
0.7
0.6
0.5
0.4
0.3
0.2
0.1
0.0
p-value
1e-12
3e-10
9e-08
3e-05
9e-03
Log-odds
0.5
1.0
1.5
2.0
2.5
3.0
3.5
4.0
Cellular_organisms - ps1
Archaea1 - ps2
Archaea2 - ps3
Archaea3 - ps4
Asgard/Eukaryota - ps5
Eukaryota - ps6
Amorpheae - ps7
Obazoa - ps8
Opisthokonta - ps9
Holomycota - ps10
Fungi - ps11
Fungi_1 - ps12
Fungi_2 - ps13
Fungi_3 - ps14
Dikarya - ps15
Ascomycota - ps16
Saccharo/Pezizomycotina - ps17
Saccharomycotina - ps18
Saccharomycotina_1 - ps19
Saccharomycotina_2 - ps20
Saccharomycotina_3 - ps21
Phaff./Saccharomycetaceae - ps22
Saccharomyco./mycetaceae - ps23
Saccharomycetaceae - ps24
Saccharomycetaceae_2 - ps25
Saccharomycetaceae_3 - ps26
Kaz./Nau./Saccharomyces - ps27
Saccharomyces - ps28
Arboricola_clade - ps29
Cerevisiae_clade - ps30
Saccharomyces_cerevisiae - ps31
Phylostratum

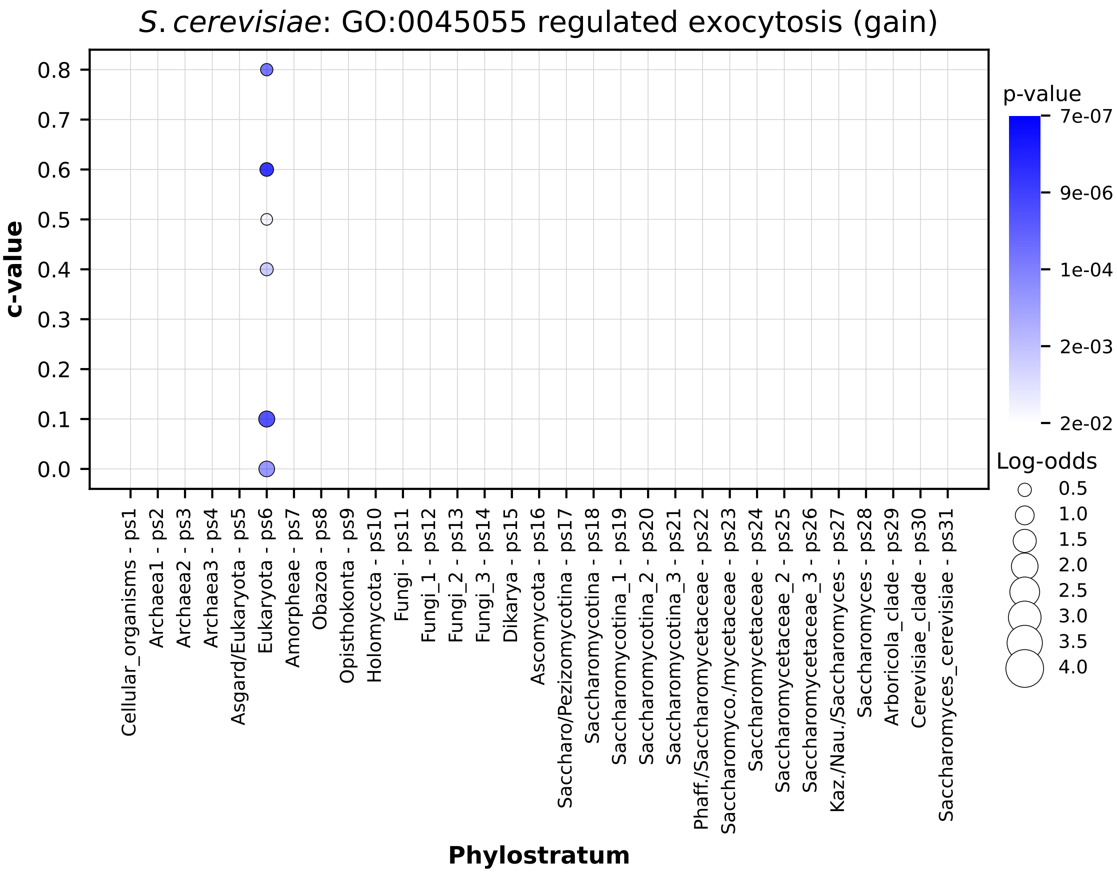

S. cerevisiae: GO:0045055 regulated exocytosis (gain)
c-value
0.0
0.1
0.2
0.3
0.4
0.5
0.6
0.7
0.8
p-value
7e-07
9e-06
1e-04
2e-03
2e-02
Log-odds
0.5
1.0
1.5
2.0
2.5
3.0
3.5
4.0
Cellular_organisms - ps1
Archaea1 - ps2
Archaea2 - ps3
Archaea3 - ps4
Asgard/Eukaryota - ps5
Eukaryota - ps6
Amorpheae - ps7
Obazoa - ps8
Opisthokonta - ps9
Holomycota - ps10
Fungi - ps11
Fungi_1 - ps12
Fungi_2 - ps13
Fungi_3 - ps14
Dikarya - ps15
Ascomycota - ps16
Saccharo/Pezizomycotina - ps17
Saccharomycotina - ps18
Saccharomycotina_1 - ps19
Saccharomycotina_2 - ps20
Saccharomycotina_3 - ps21
Phaff./Saccharomycetaceae - ps22
Saccharomyco./mycetaceae - ps23
Saccharomycetaceae - ps24
Saccharomycetaceae_2 - ps25
Saccharomycetaceae_3 - ps26
Kaz./Nau./Saccharomyces - ps27
Saccharomyces - ps28
Arboricola_clade - ps29
Cerevisiae_clade - ps30
Saccharomyces_cerevisiae - ps31
Phylostratum

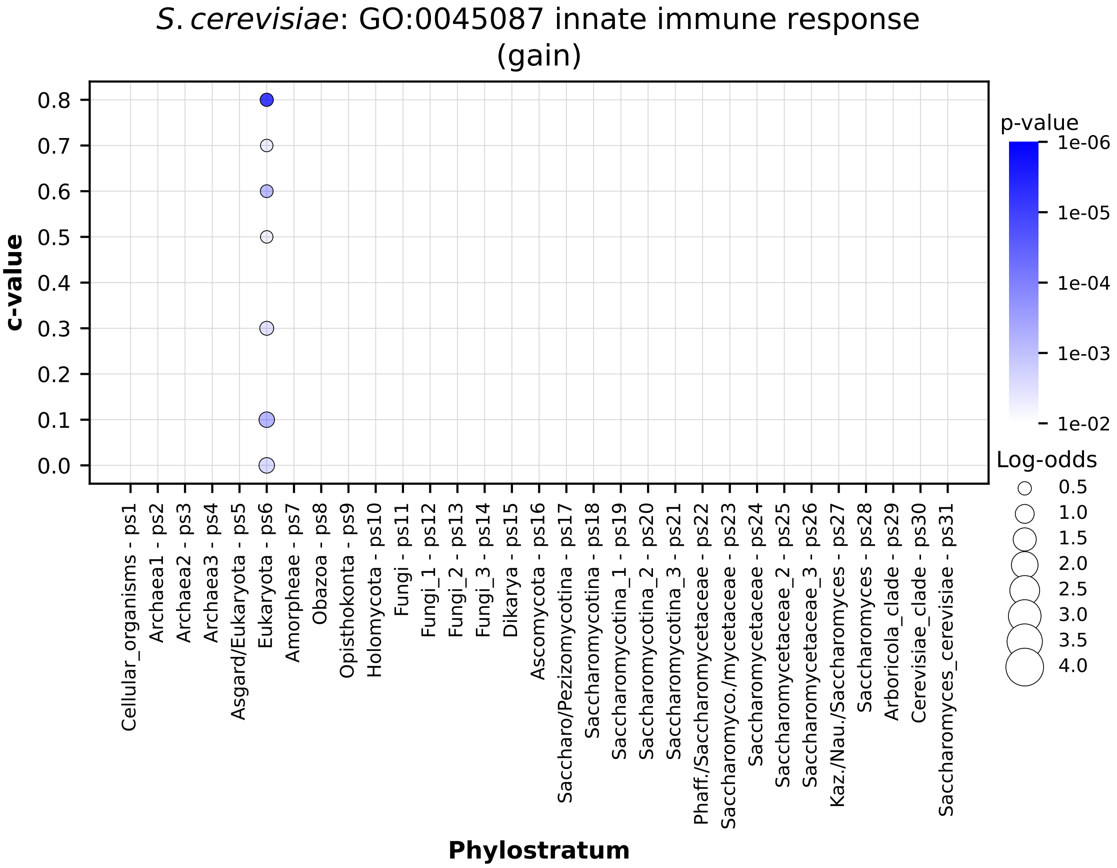

S. cerevisiae: GO:0045087 innate immune response (gain)
c-value
0.0
0.1
0.2
0.3
0.4
0.5
0.6
0.7
0.8
p-value
1e-06
1e-05
1e-04
1e-03
1e-02
Log-odds
0.5
1.0
1.5
2.0
2.5
3.0
3.5
4.0
Cellular_organisms - ps1
Archaea1 - ps2
Archaea2 - ps3
Archaea3 - ps4
Asgard/Eukaryota - ps5
Eukaryota - ps6
Amorpheae - ps7
Obazoa - ps8
Opisthokonta - ps9
Holomycota - ps10
Fungi - ps11
Fungi_1 - ps12
Fungi_2 - ps13
Fungi_3 - ps14
Dikarya - ps15
Ascomycota - ps16
Saccharo/Pezizomycotina - ps17
Saccharomycotina - ps18
Saccharomycotina_1 - ps19
Saccharomycotina_2 - ps20
Saccharomycotina_3 - ps21
Phaff./Saccharomycetaceae - ps22
Saccharomyco./mycetaceae - ps23
Saccharomycetaceae - ps24
Saccharomycetaceae_2 - ps25
Saccharomycetaceae_3 - ps26
Kaz./Nau./Saccharomyces - ps27
Saccharomyces - ps28
Arboricola_clade - ps29
Cerevisiae_clade - ps30
Saccharomyces_cerevisiae - ps31
Phylostratum

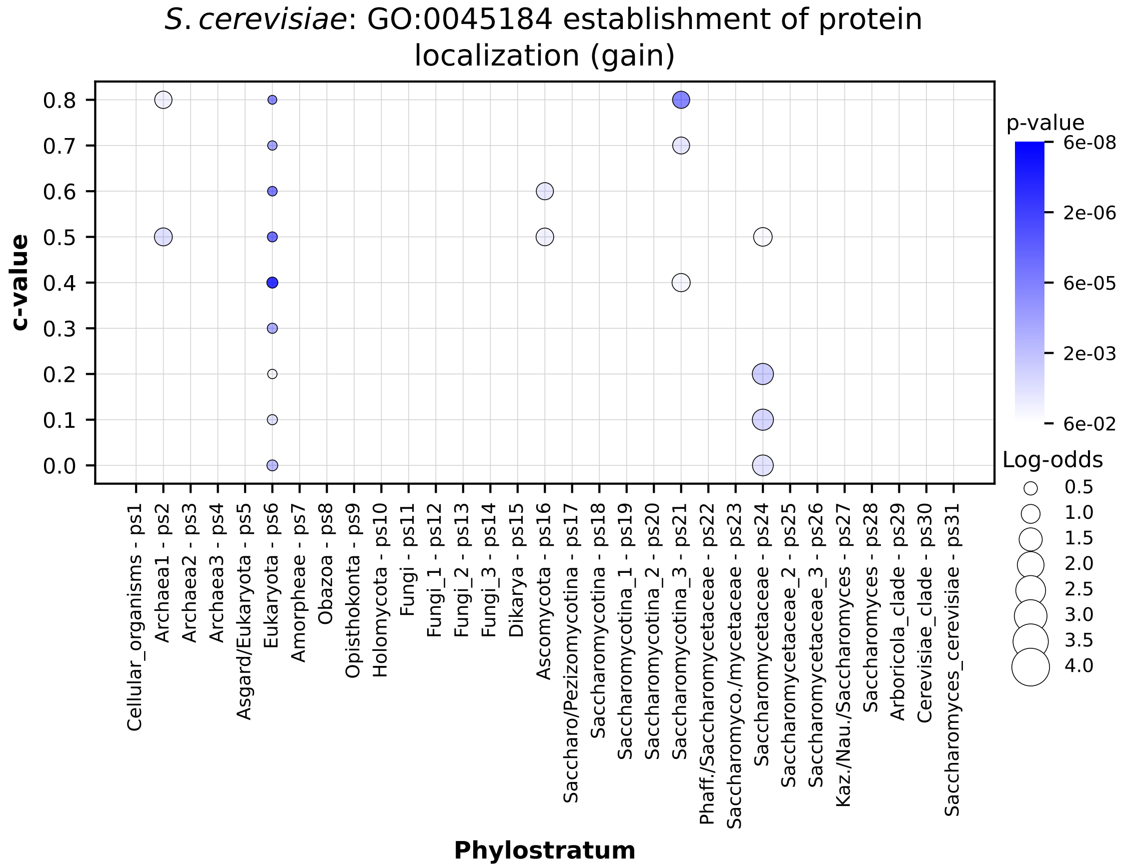

S. cerevisiae: GO:0045184 establishment of protein localization (gain)
c-value
0.8
0.7
0.6
0.5
0.4
0.3
0.2
0.1
0.0
Phylostratum
Cellular_organisms - ps1
Archaea1 - ps2
Archaea2 - ps3
Archaea3 - ps4
Asgard/Eukaryota - ps5
Eukaryota - ps6
Amorpheae - ps7
Obazoa - ps8
Opisthokonta - ps9
Holomycota - ps10
Fungi - ps11
Fungi_1 - ps12
Fungi_2 - ps13
Fungi_3 - ps14
Dikarya - ps15
Ascomycota - ps16
Saccharo/Pezizomycotina - ps17
Saccharomycotina - ps18
Saccharomycotina_1 - ps19
Saccharomycotina_2 - ps20
Saccharomycotina_3 - ps21
Phaff./Saccharomycetaceae - ps22
Saccharomyco./mycetaceae - ps23
Saccharomycetaceae - ps24
Saccharomycetaceae_2 - ps25
Saccharomycetaceae_3 - ps26
Kaz./Nau./Saccharomyces - ps27
Saccharomyces - ps28
Arboricola_clade - ps29
Cerevisiae_clade - ps30
Saccharomyces_cerevisiae - ps31
p-value
6e-08
2e-06
6e-05
2e-03
6e-02
Log-odds
0.5
1.0
1.5
2.0
2.5
3.0
3.5
4.0

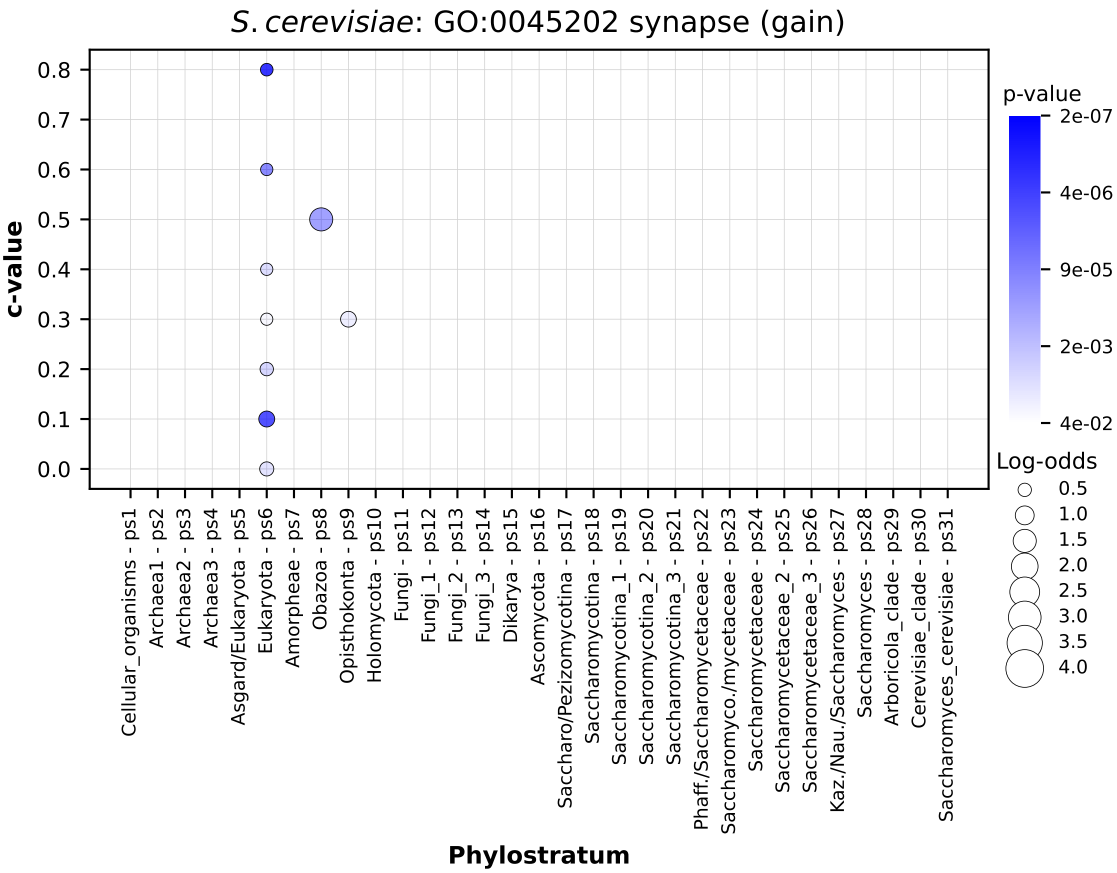

S. cerevisiae: GO:0045202 synapse (gain)
c-value
0.8
0.7
0.6
0.5
0.4
0.3
0.2
0.1
0.0
p-value
2e-07
4e-06
9e-05
2e-03
4e-02
Log-odds
0.5
1.0
1.5
2.0
2.5
3.0
3.5
4.0
Cellular_organisms - ps1
Archaea1 - ps2
Archaea2 - ps3
Archaea3 - ps4
Asgard/Eukaryota - ps5
Eukaryota - ps6
Amorpheae - ps7
Obazoa - ps8
Opisthokonta - ps9
Holomycota - ps10
Fungi - ps11
Fungi_1 - ps12
Fungi_2 - ps13
Fungi_3 - ps14
Dikarya - ps15
Ascomycota - ps16
Saccharo/Pezizomycotina - ps17
Saccharomycotina - ps18
Saccharomycotina_1 - ps19
Saccharomycotina_2 - ps20
Saccharomycotina_3 - ps21
Phaff./Saccharomycetaceae - ps22
Saccharomyco./mycetaceae - ps23
Saccharomycetaceae - ps24
Saccharomycetaceae_2 - ps25
Saccharomycetaceae_3 - ps26
Kaz./Nau./Saccharomyces - ps27
Saccharomyces - ps28
Arboricola_clade - ps29
Cerevisiae_clade - ps30
Saccharomyces_cerevisiae - ps31
Phylostratum

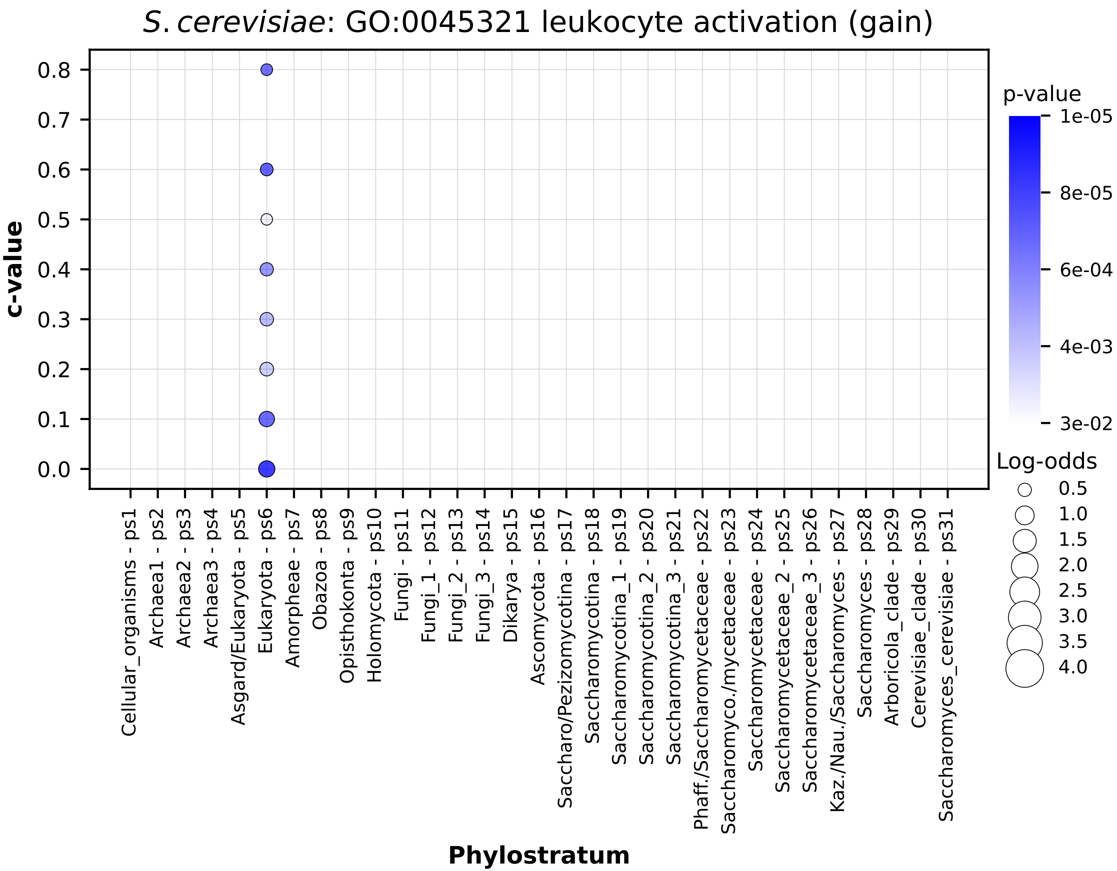

S. cerevisiae: GO:0045321 leukocyte activation (gain)
c-value
0.8
0.7
0.6
0.5
0.4
0.3
0.2
0.1
0.0
p-value
1e-05
8e-05
6e-04
4e-03
3e-02
Log-odds
0.5
1.0
1.5
2.0
2.5
3.0
3.5
4.0
Cellular_organisms - ps1
Archaea1 - ps2
Archaea2 - ps3
Archaea3 - ps4
Asgard/Eukaryota - ps5
Eukaryota - ps6
Amorpheae - ps7
Obazoa - ps8
Opisthokonta - ps9
Holomycota - ps10
Fungi - ps11
Fungi_1 - ps12
Fungi_2 - ps13
Fungi_3 - ps14
Dikarya - ps15
Ascomycota - ps16
Saccharo/Pezizomycotina - ps17
Saccharomycotina - ps18
Saccharomycotina_1 - ps19
Saccharomycotina_2 - ps20
Saccharomycotina_3 - ps21
Phaff./Saccharomycetaceae - ps22
Saccharomyco./mycetaceae - ps23
Saccharomycetaceae - ps24
Saccharomycetaceae_2 - ps25
Saccharomycetaceae_3 - ps26
Kaz./Nau./Saccharomyces - ps27
Saccharomyces - ps28
Arboricola_clade - ps29
Cerevisiae_clade - ps30
Saccharomyces_cerevisiae - ps31
Phylostratum

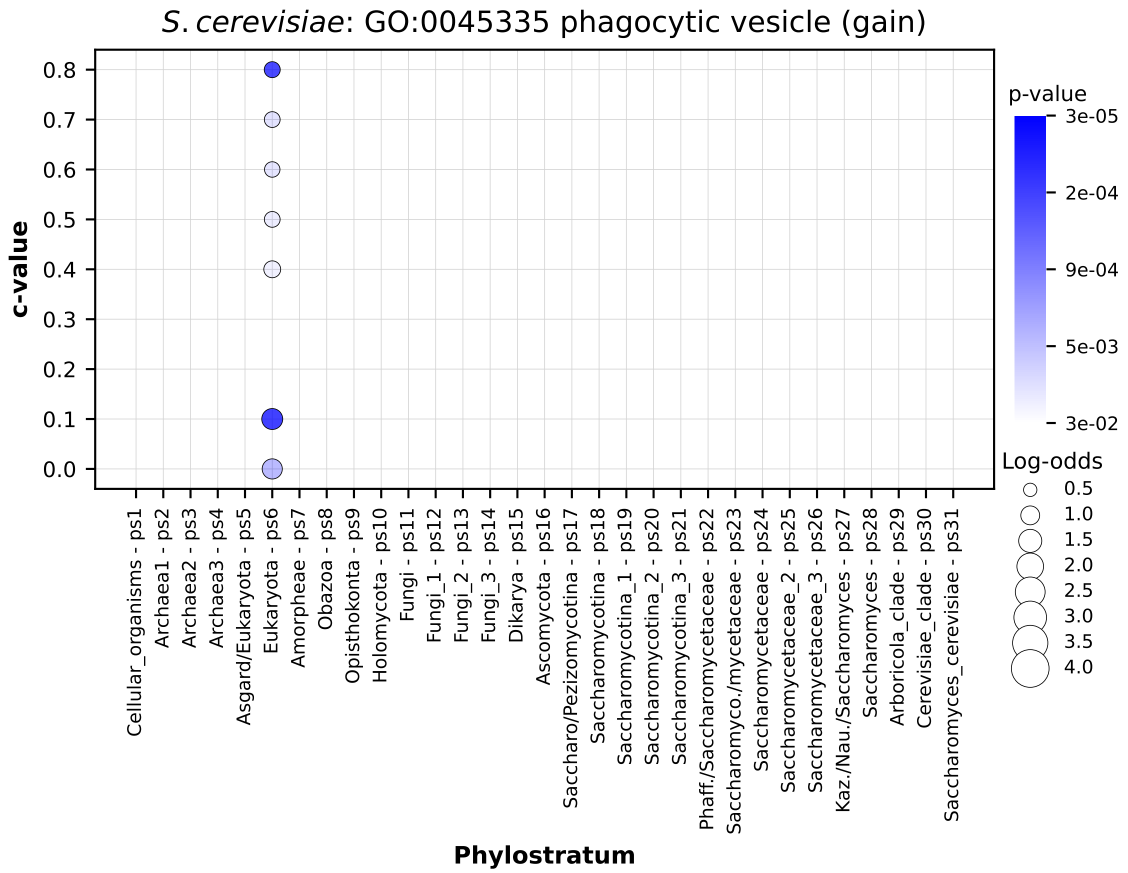

S. cerevisiae: GO:0045335 phagocytic vesicle (gain)
c-value
0.8
0.7
0.6
0.5
0.4
0.3
0.2
0.1
0.0
Phylostratum
Cellular_organisms - ps1
Archaea1 - ps2
Archaea2 - ps3
Archaea3 - ps4
Asgard/Eukaryota - ps5
Eukaryota - ps6
Amorpheae - ps7
Obazoa - ps8
Opisthokonta - ps9
Holomycota - ps10
Fungi - ps11
Fungi_1 - ps12
Fungi_2 - ps13
Fungi_3 - ps14
Dikarya - ps15
Ascomycota - ps16
Saccharo/Pezizomycotina - ps17
Saccharomycotina - ps18
Saccharomycotina_1 - ps19
Saccharomycotina_2 - ps20
Saccharomycotina_3 - ps21
Phaff./Saccharomycetaceae - ps22
Saccharomyco./mycetaceae - ps23
Saccharomycetaceae - ps24
Saccharomycetaceae_2 - ps25
Saccharomycetaceae_3 - ps26
Kaz./Nau./Saccharomyces - ps27
Saccharomyces - ps28
Arboricola_clade - ps29
Cerevisiae_clade - ps30
Saccharomyces_cerevisiae - ps31
p-value
3e-05
2e-04
9e-04
5e-03
3e-02
Log-odds
0.5
1.0
1.5
2.0
2.5
3.0
3.5
4.0

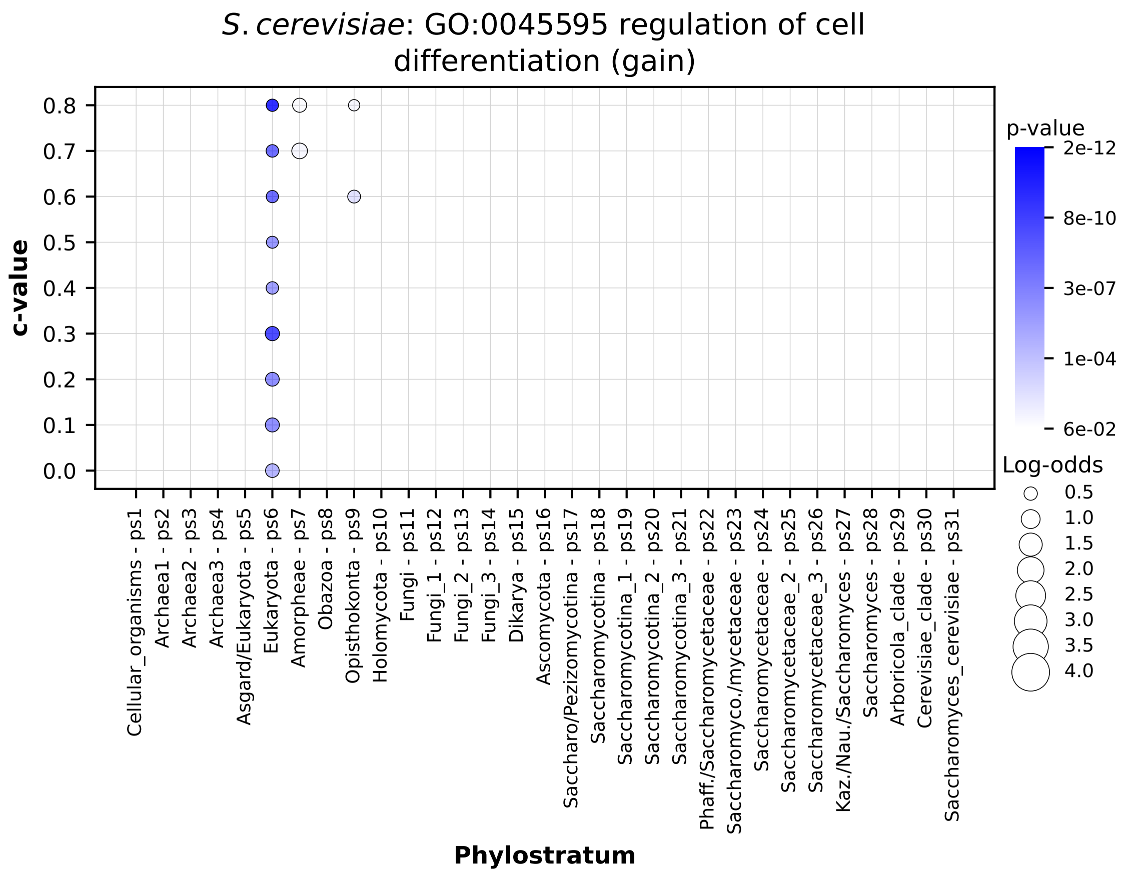

S. cerevisiae: GO:0045595 regulation of cell differentiation (gain)
c-value
0.8
0.7
0.6
0.5
0.4
0.3
0.2
0.1
0.0
p-value
2e-12
8e-10
3e-07
1e-04
6e-02
Log-odds
0.5
1.0
1.5
2.0
2.5
3.0
3.5
4.0
Cellular_organisms - ps1
Archaea1 - ps2
Archaea2 - ps3
Archaea3 - ps4
Asgard/Eukaryota - ps5
Eukaryota - ps6
Amorpheae - ps7
Obazoa - ps8
Opisthokonta - ps9
Holomycota - ps10
Fungi - ps11
Fungi_1 - ps12
Fungi_2 - ps13
Fungi_3 - ps14
Dikarya - ps15
Ascomycota - ps16
Saccharo/Pezizomycotina - ps17
Saccharomycotina - ps18
Saccharomycotina_1 - ps19
Saccharomycotina_2 - ps20
Saccharomycotina_3 - ps21
Phaff./Saccharomycetaceae - ps22
Saccharomyco./mycetaceae - ps23
Saccharomycetaceae - ps24
Saccharomycetaceae_2 - ps25
Saccharomycetaceae_3 - ps26
Kaz./Nau./Saccharomyces - ps27
Saccharomyces - ps28
Arboricola_clade - ps29
Cerevisiae_clade - ps30
Saccharomyces_cerevisiae - ps31
Phylostratum

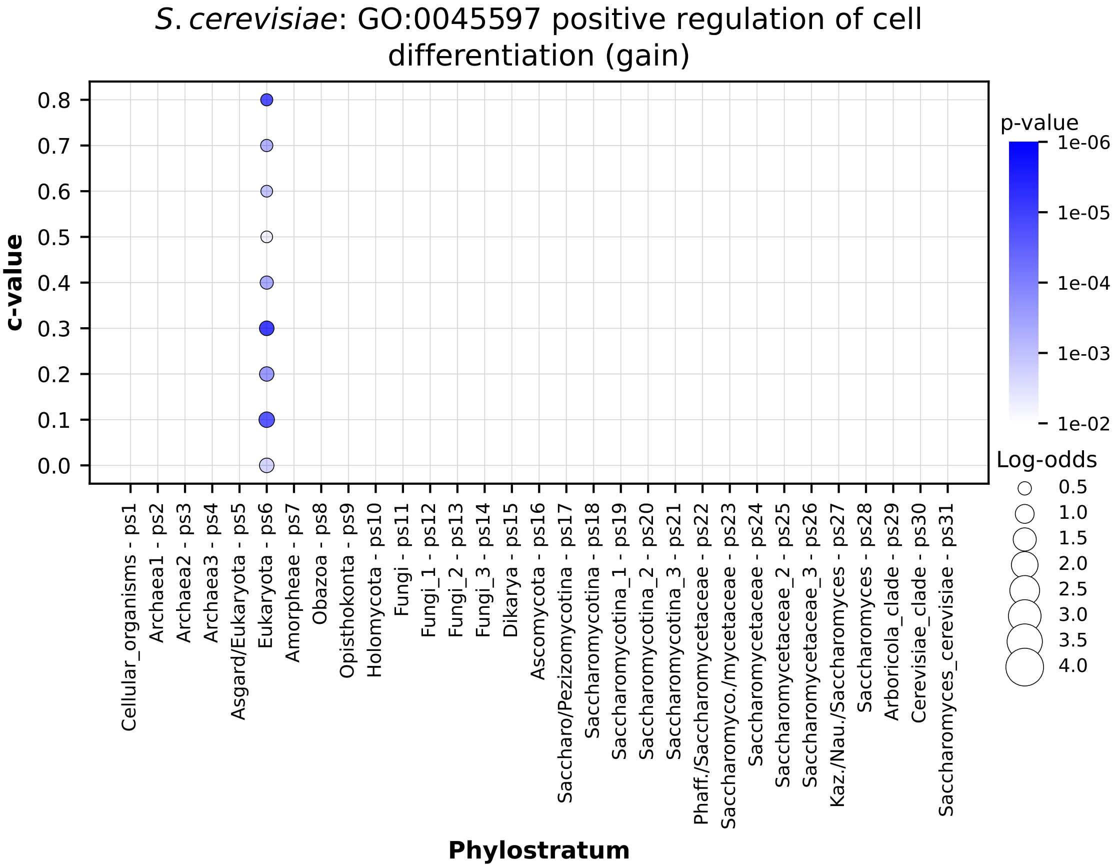

S. cerevisiae: GO:0045597 positive regulation of cell differentiation (gain)
c-value
0.8
0.7
0.6
0.5
0.4
0.3
0.2
0.1
0.0
p-value
1e-06
1e-05
1e-04
1e-03
1e-02
Log-odds
0.5
1.0
1.5
2.0
2.5
3.0
3.5
4.0
Cellular_organisms - ps1
Archaea1 - ps2
Archaea2 - ps3
Archaea3 - ps4
Asgard/Eukaryota - ps5
Eukaryota - ps6
Amorpheae - ps7
Obazoa - ps8
Opisthokonta - ps9
Holomycota - ps10
Fungi - ps11
Fungi_1 - ps12
Fungi_2 - ps13
Fungi_3 - ps14
Dikarya - ps15
Ascomycota - ps16
Saccharo/Pezizomycotina - ps17
Saccharomycotina - ps18
Saccharomycotina_1 - ps19
Saccharomycotina_2 - ps20
Saccharomycotina_3 - ps21
Phaff./Saccharomycetaceae - ps22
Saccharomyco./mycetaceae - ps23
Saccharomycetaceae - ps24
Saccharomycetaceae_2 - ps25
Saccharomycetaceae_3 - ps26
Kaz./Nau./Saccharomyces - ps27
Saccharomyces - ps28
Arboricola_clade - ps29
Cerevisiae_clade - ps30
Saccharomyces_cerevisiae - ps31
Phylostratum

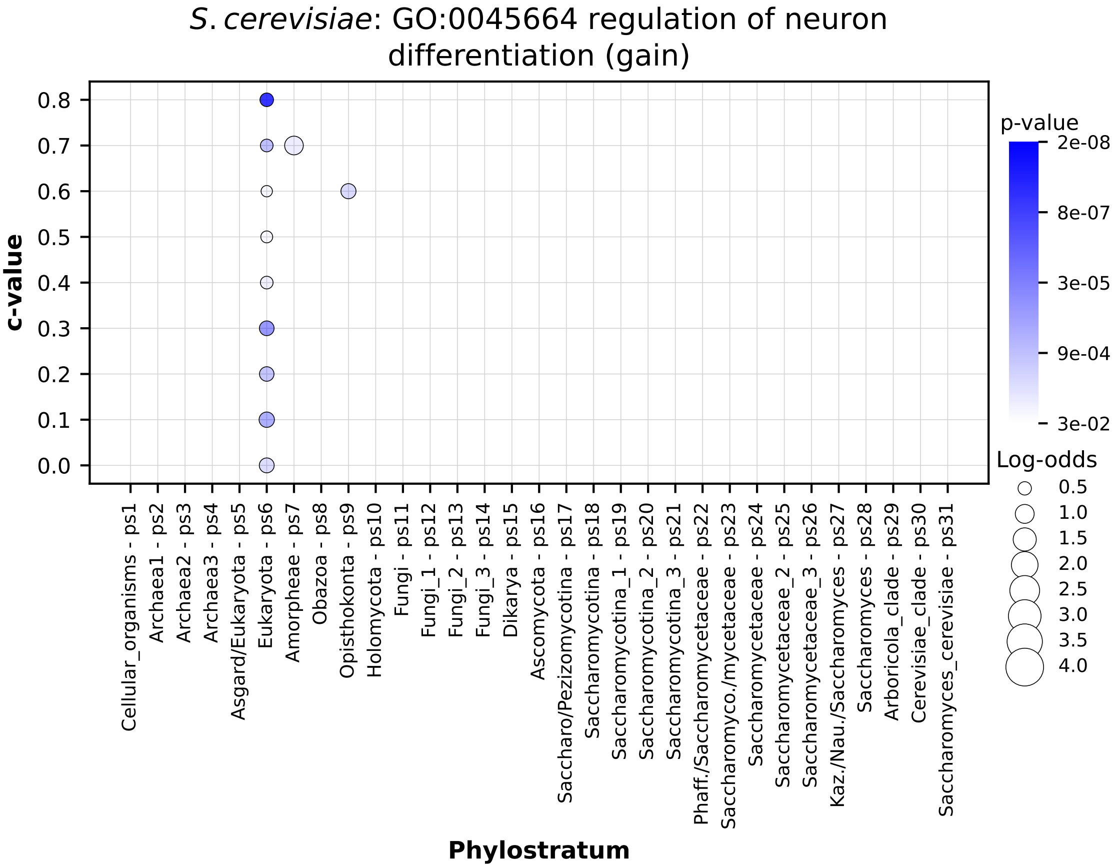

S. cerevisiae: GO:0045664 regulation of neuron differentiation (gain)
c-value
0.0
0.1
0.2
0.3
0.4
0.5
0.6
0.7
0.8
p-value
2e-08
8e-07
3e-05
9e-04
3e-02
Log-odds
0.5
1.0
1.5
2.0
2.5
3.0
3.5
4.0
Cellular_organisms - ps1
Archaea1 - ps2
Archaea2 - ps3
Archaea3 - ps4
Asgard/Eukaryota - ps5
Eukaryota - ps6
Amorpheae - ps7
Obazoa - ps8
Opisthokonta - ps9
Holomycota - ps10
Fungi - ps11
Fungi_1 - ps12
Fungi_2 - ps13
Fungi_3 - ps14
Dikarya - ps15
Ascomycota - ps16
Saccharo/Pezizomycotina - ps17
Saccharomycotina - ps18
Saccharomycotina_1 - ps19
Saccharomycotina_2 - ps20
Saccharomycotina_3 - ps21
Phaff./Saccharomycetaceae - ps22
Saccharomyco./mycetaceae - ps23
Saccharomycetaceae - ps24
Saccharomycetaceae_2 - ps25
Saccharomycetaceae_3 - ps26
Kaz./Nau./Saccharomyces - ps27
Saccharomyces - ps28
Arboricola_clade - ps29
Cerevisiae_clade - ps30
Saccharomyces_cerevisiae - ps31
Phylostratum

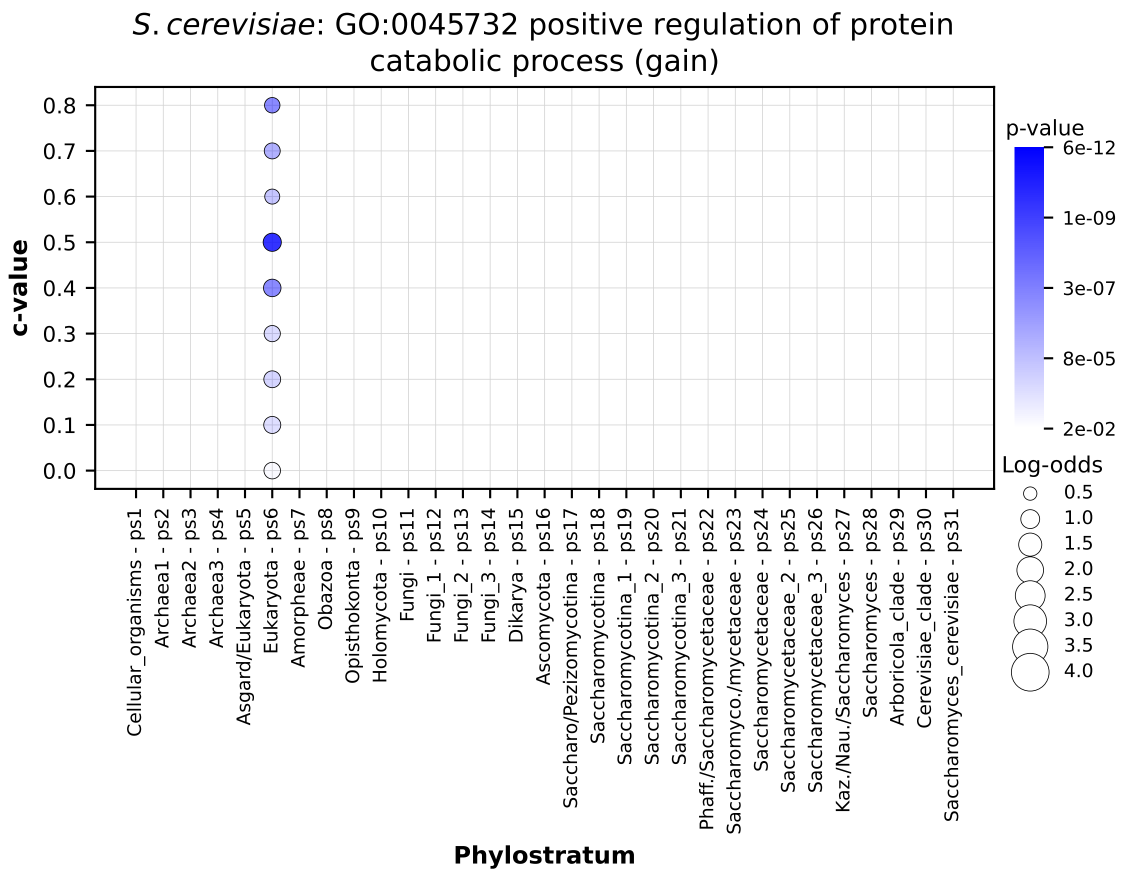

S. cerevisiae: GO:0045732 positive regulation of protein catabolic process (gain)
c-value
0.8
0.7
0.6
0.5
0.4
0.3
0.2
0.1
0.0
p-value
6e-12
1e-09
3e-07
8e-05
2e-02
Log-odds
0.5
1.0
1.5
2.0
2.5
3.0
3.5
4.0
Cellular_organisms - ps1
Archaea1 - ps2
Archaea2 - ps3
Archaea3 - ps4
Asgard/Eukaryota - ps5
Eukaryota - ps6
Amorpheae - ps7
Obazoa - ps8
Opisthokonta - ps9
Holomycota - ps10
Fungi - ps11
Fungi_1 - ps12
Fungi_2 - ps13
Fungi_3 - ps14
Dikarya - ps15
Ascomycota - ps16
Saccharo/Pezizomycotina - ps17
Saccharomycotina - ps18
Saccharomycotina_1 - ps19
Saccharomycotina_2 - ps20
Saccharomycotina_3 - ps21
Phaff./Saccharomycetaceae - ps22
Saccharomyco./mycetaceae - ps23
Saccharomycetaceae - ps24
Saccharomycetaceae_2 - ps25
Saccharomycetaceae_3 - ps26
Kaz./Nau./Saccharomyces - ps27
Saccharomyces - ps28
Arboricola_clade - ps29
Cerevisiae_clade - ps30
Saccharomyces_cerevisiae - ps31
Phylostratum

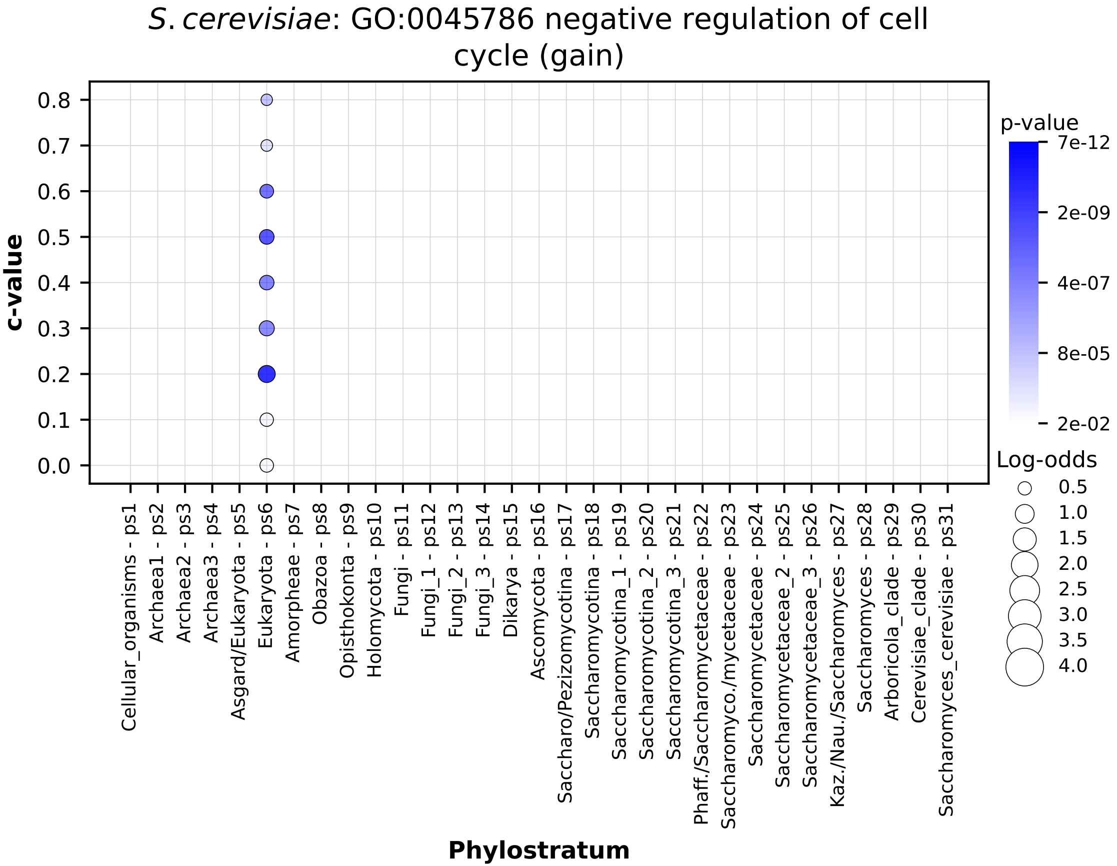

S. cerevisiae: GO:0045786 negative regulation of cell cycle (gain)
c-value
0.8
0.7
0.6
0.5
0.4
0.3
0.2
0.1
0.0
p-value
7e-12
2e-09
4e-07
8e-05
2e-02
Log-odds
0.5
1.0
1.5
2.0
2.5
3.0
3.5
4.0
Cellular_organisms - ps1
Archaea1 - ps2
Archaea2 - ps3
Archaea3 - ps4
Asgard/Eukaryota - ps5
Eukaryota - ps6
Amorpheae - ps7
Obazoa - ps8
Opisthokonta - ps9
Holomycota - ps10
Fungi - ps11
Fungi_1 - ps12
Fungi_2 - ps13
Fungi_3 - ps14
Dikarya - ps15
Ascomycota - ps16
Saccharo/Pezizomycotina - ps17
Saccharomycotina - ps18
Saccharomycotina_1 - ps19
Saccharomycotina_2 - ps20
Saccharomycotina_3 - ps21
Phaff./Saccharomycetaceae - ps22
Saccharomyco./mycetaceae - ps23
Saccharomycetaceae - ps24
Saccharomycetaceae_2 - ps25
Saccharomycetaceae_3 - ps26
Kaz./Nau./Saccharomyces - ps27
Saccharomyces - ps28
Arboricola_clade - ps29
Cerevisiae_clade - ps30
Saccharomyces_cerevisiae - ps31
Phylostratum

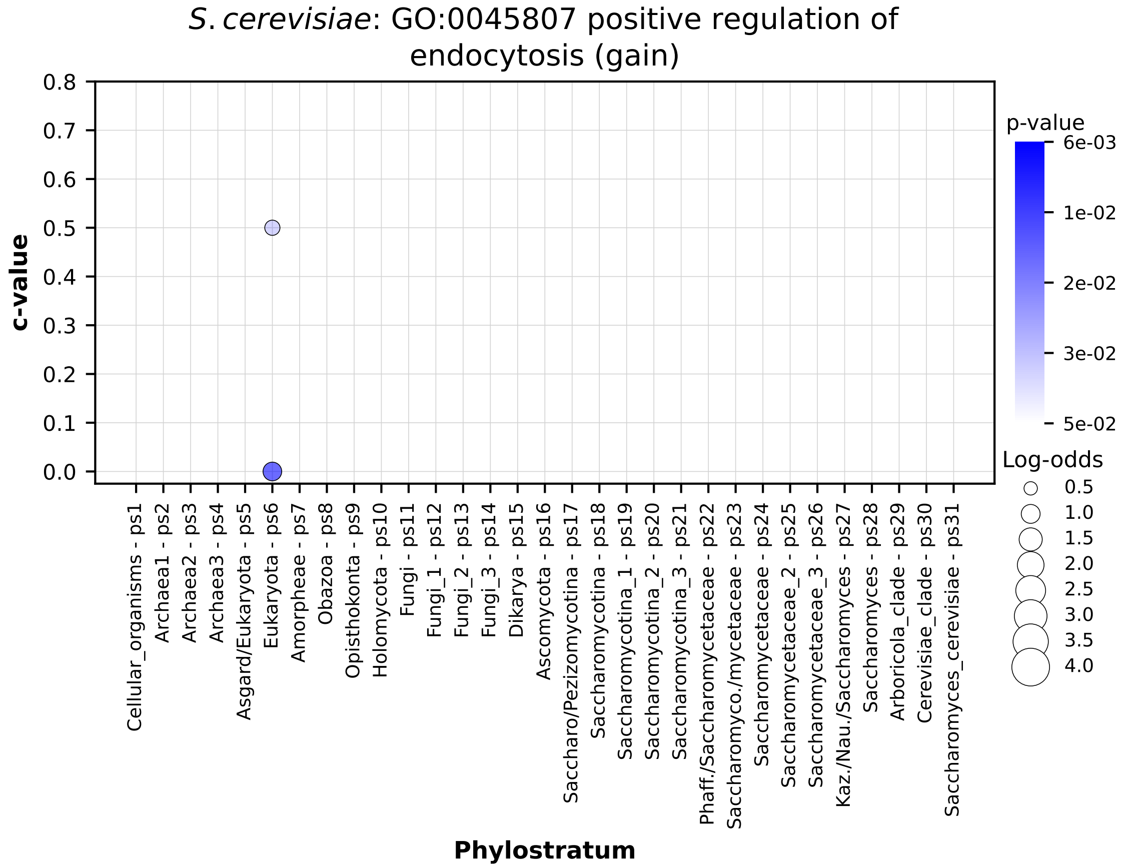

S. cerevisiae: GO:0045807 positive regulation of endocytosis (gain)
c-value
0.8
0.7
0.6
0.5
0.4
0.3
0.2
0.1
0.0
Phylostratum
Cellular_organisms - ps1
Archaea1 - ps2
Archaea2 - ps3
Archaea3 - ps4
Asgard/Eukaryota - ps5
Eukaryota - ps6
Amorpheae - ps7
Obazoa - ps8
Opisthokonta - ps9
Holomycota - ps10
Fungi - ps11
Fungi_1 - ps12
Fungi_2 - ps13
Fungi_3 - ps14
Dikarya - ps15
Ascomycota - ps16
Saccharo/Pezizomycotina - ps17
Saccharomycotina - ps18
Saccharomycotina_1 - ps19
Saccharomycotina_2 - ps20
Saccharomycotina_3 - ps21
Phaff./Saccharomycetaceae - ps22
Saccharomyco./mycetaceae - ps23
Saccharomycetaceae - ps24
Saccharomycetaceae_2 - ps25
Saccharomycetaceae_3 - ps26
Kaz./Nau./Saccharomyces - ps27
Saccharomyces - ps28
Arboricola_clade - ps29
Cerevisiae_clade - ps30
Saccharomyces_cerevisiae - ps31
p-value
6e-03
1e-02
2e-02
3e-02
5e-02
Log-odds
0.5
1.0
1.5
2.0
2.5
3.0
3.5
4.0

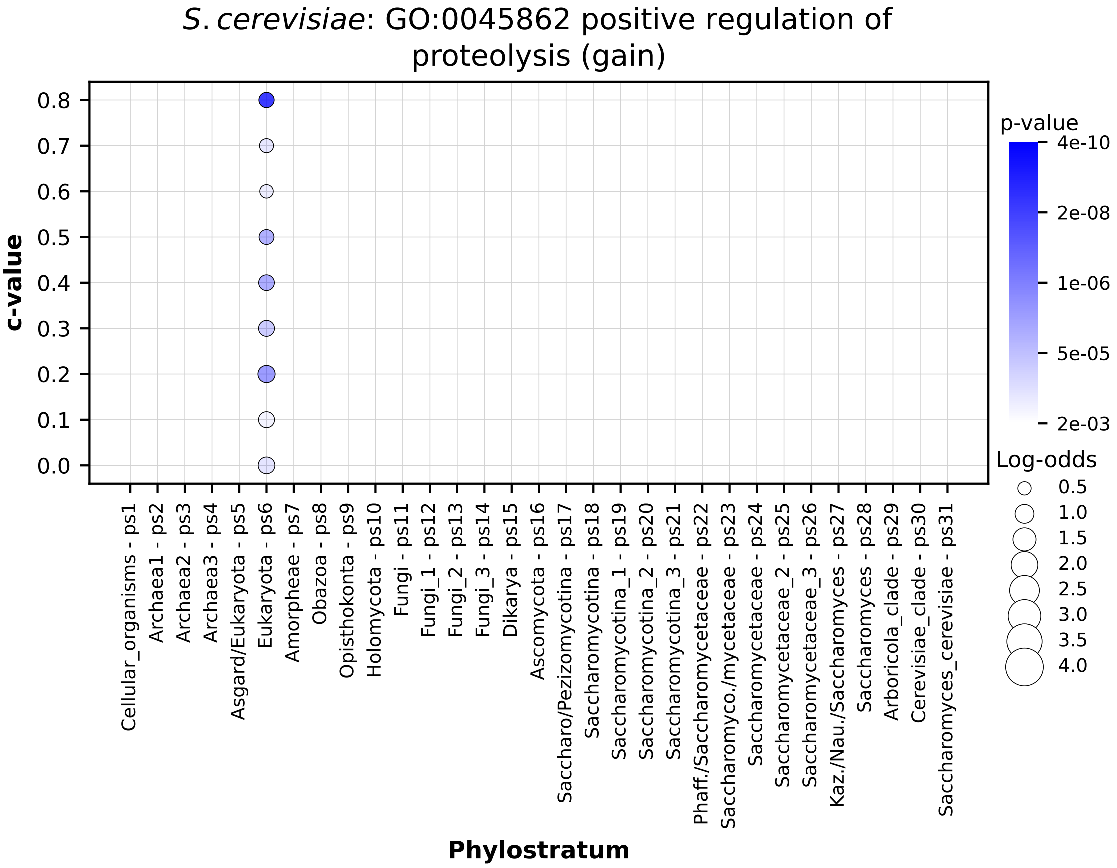

S. cerevisiae: GO:0045862 positive regulation of proteolysis (gain)
c-value
0.8
0.7
0.6
0.5
0.4
0.3
0.2
0.1
0.0
Phylostratum
Cellular_organisms - ps1
Archaea1 - ps2
Archaea2 - ps3
Archaea3 - ps4
Asgard/Eukaryota - ps5
Eukaryota - ps6
Amorpheae - ps7
Obazoa - ps8
Opisthokonta - ps9
Holomycota - ps10
Fungi - ps11
Fungi_1 - ps12
Fungi_2 - ps13
Fungi_3 - ps14
Dikarya - ps15
Ascomycota - ps16
Saccharo/Pezizomycotina - ps17
Saccharomycotina - ps18
Saccharomycotina_1 - ps19
Saccharomycotina_2 - ps20
Saccharomycotina_3 - ps21
Phaff./Saccharomycetaceae - ps22
Saccharomyco./mycetaceae - ps23
Saccharomycetaceae - ps24
Saccharomycetaceae_2 - ps25
Saccharomycetaceae_3 - ps26
Kaz./Nau./Saccharomyces - ps27
Saccharomyces - ps28
Arboricola_clade - ps29
Cerevisiae_clade - ps30
Saccharomyces_cerevisiae - ps31
p-value
4e-10
2e-08
1e-06
5e-05
2e-03
Log-odds
0.5
1.0
1.5
2.0
2.5
3.0
3.5
4.0

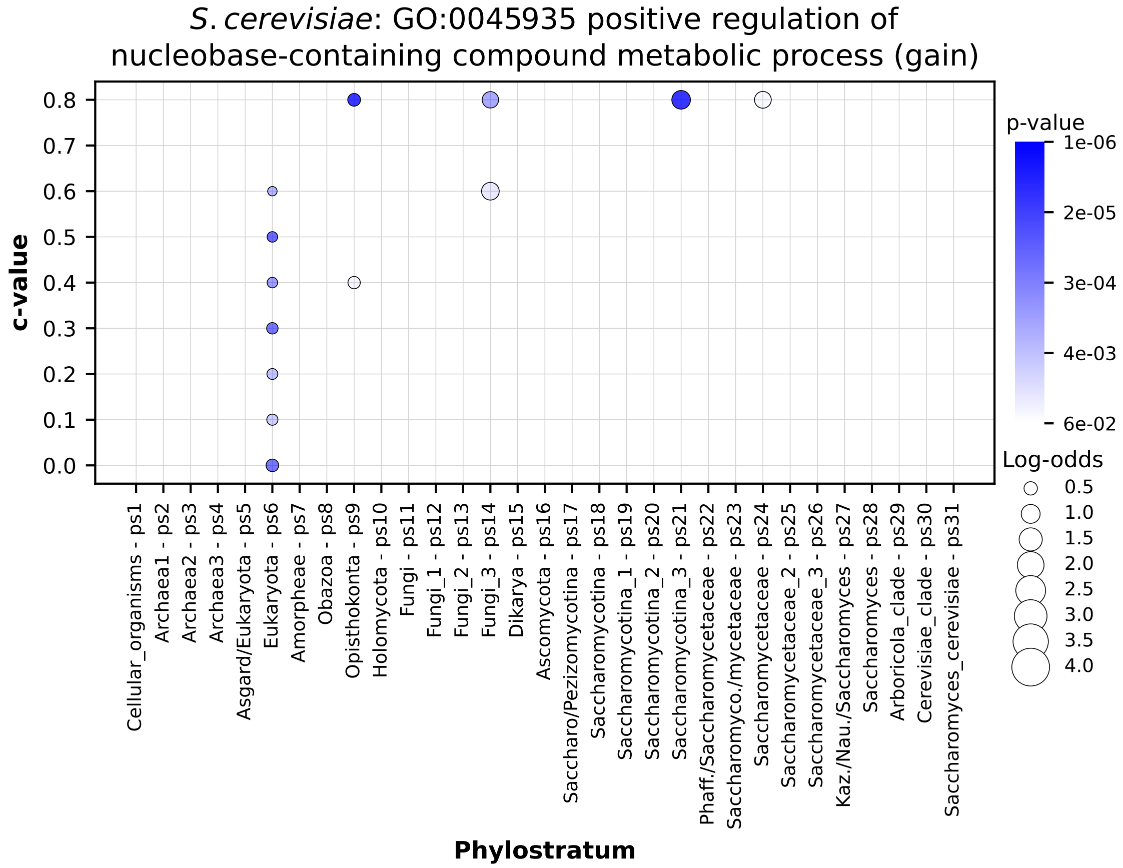

S. cerevisiae: GO:0045935 positive regulation of nucleobase-containing compound metabolic process (gain)
c-value
0.8
0.7
0.6
0.5
0.4
0.3
0.2
0.1
0.0
p-value
1e-06
2e-05
3e-04
4e-03
6e-02
Log-odds
0.5
1.0
1.5
2.0
2.5
3.0
3.5
4.0
Cellular_organisms - ps1
Archaea1 - ps2
Archaea2 - ps3
Archaea3 - ps4
Asgard/Eukaryota - ps5
Eukaryota - ps6
Amorpheae - ps7
Obazoa - ps8
Opisthokonta - ps9
Holomycota - ps10
Fungi - ps11
Fungi_1 - ps12
Fungi_2 - ps13
Fungi_3 - ps14
Dikarya - ps15
Ascomycota - ps16
Saccharo/Pezizomycotina - ps17
Saccharomycotina - ps18
Saccharomycotina_1 - ps19
Saccharomycotina_2 - ps20
Saccharomycotina_3 - ps21
Phaff./Saccharomycetaceae - ps22
Saccharomyco./mycetaceae - ps23
Saccharomycetaceae - ps24
Saccharomycetaceae_2 - ps25
Saccharomycetaceae_3 - ps26
Kaz./Nau./Saccharomyces - ps27
Saccharomyces - ps28
Arboricola_clade - ps29
Cerevisiae_clade - ps30
Saccharomyces_cerevisiae - ps31
Phylostratum

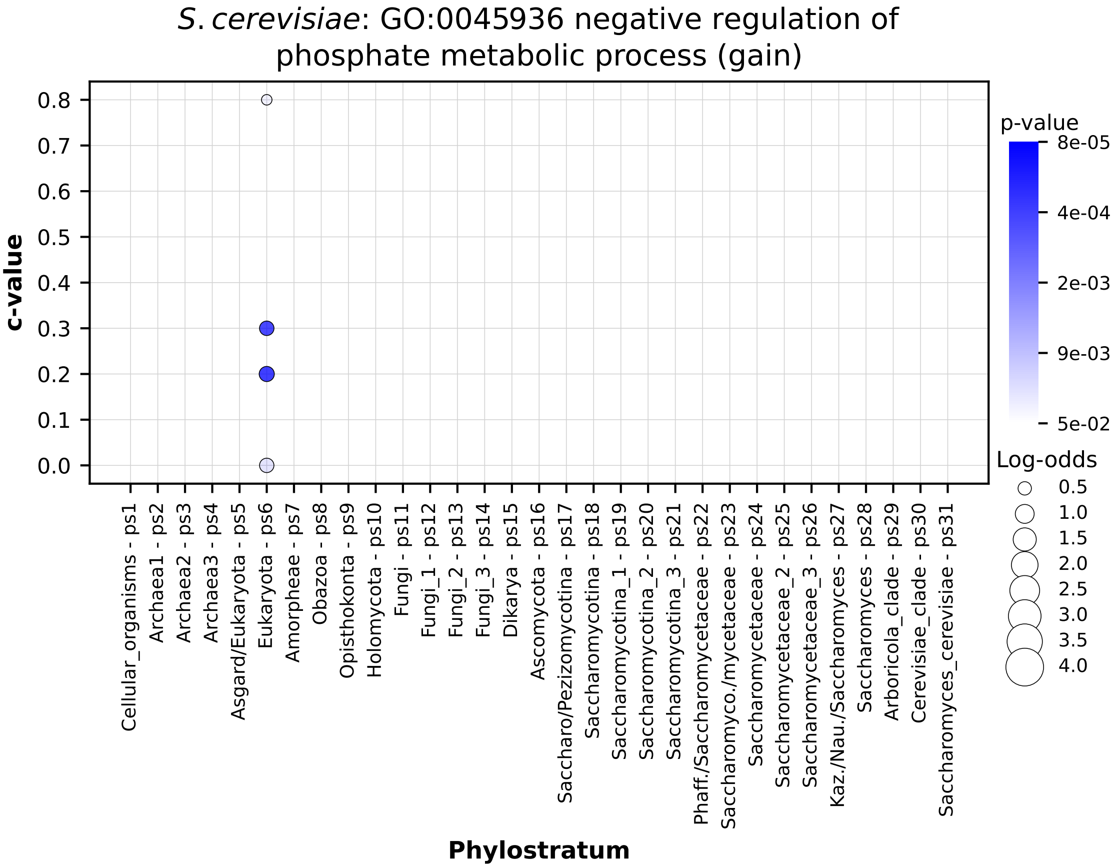
S. cerevisiae: GO:0045936 negative regulation of phosphate metabolic process (gain)
c-value
0.0
0.1
0.2
0.3
0.4
0.5
0.6
0.7
0.8
p-value
8e-05
4e-04
2e-03
9e-03
5e-02
Log-odds
0.5
1.0
1.5
2.0
2.5
3.0
3.5
4.0
Cellular_organisms - ps1
Archaea1 - ps2
Archaea2 - ps3
Archaea3 - ps4
Asgard/Eukaryota - ps5
Eukaryota - ps6
Amorpheae - ps7
Obazoa - ps8
Opisthokonta - ps9
Holomycota - ps10
Fungi - ps11
Fungi_1 - ps12
Fungi_2 - ps13
Fungi_3 - ps14
Dikarya - ps15
Ascomycota - ps16
Saccharo/Pezizomycotina - ps17
Saccharomycotina - ps18
Saccharomycotina_1 - ps19
Saccharomycotina_2 - ps20
Saccharomycotina_3 - ps21
Phaff./Saccharomycetaceae - ps22
Saccharomyco./mycetaceae - ps23
Saccharomycetaceae - ps24
Saccharomycetaceae_2 - ps25
Saccharomycetaceae_3 - ps26
Kaz./Nau./Saccharomyces - ps27
Saccharomyces - ps28
Arboricola_clade - ps29
Cerevisiae_clade - ps30
Saccharomyces_cerevisiae - ps31
Phylostratum

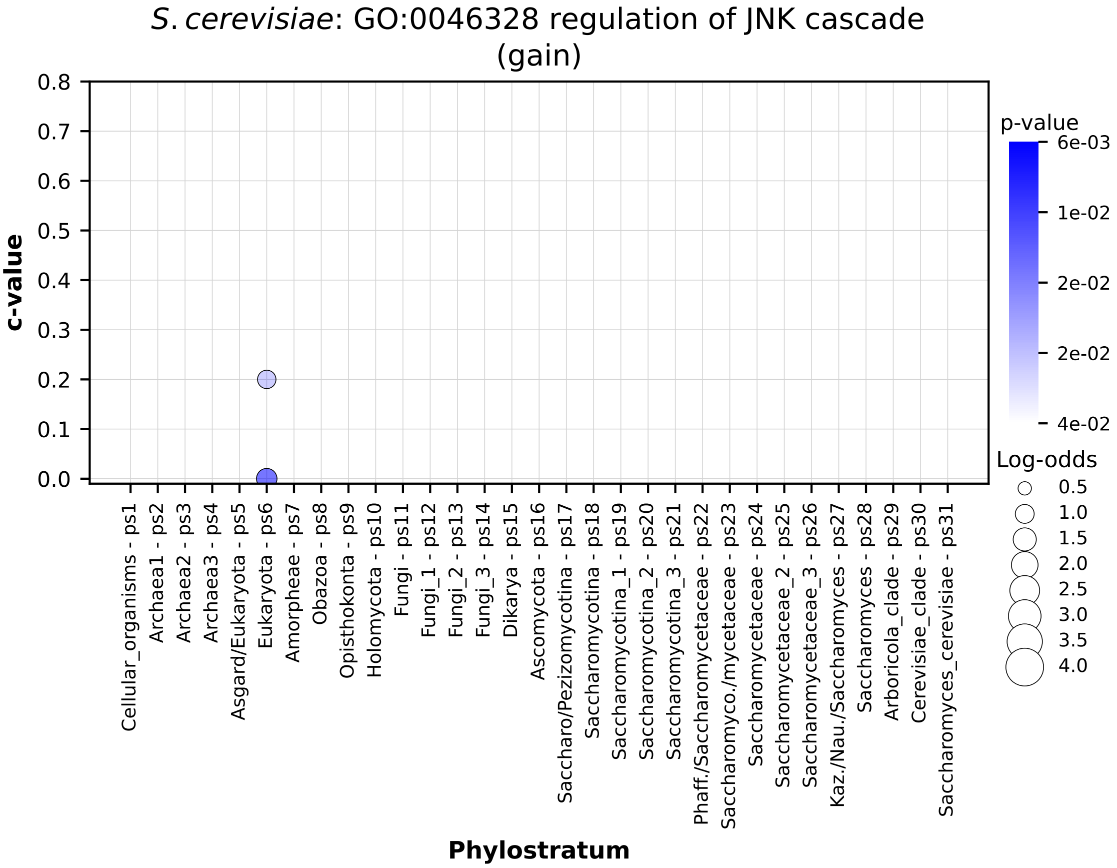

$S. cerevisiae$: GO:0046328 regulation of JNK cascade
(gain)
c-value
0.8
0.7
0.6
0.5
0.4
0.3
0.2
0.1
0.0
p-value
6e-03
1e-02
2e-02
2e-02
4e-02
Log-odds
0.5
1.0
1.5
2.0
2.5
3.0
3.5
4.0
Cellular_organisms - ps1
Archaea1 - ps2
Archaea2 - ps3
Archaea3 - ps4
Asgard/Eukaryota - ps5
Eukaryota - ps6
Amorpheae - ps7
Obazoa - ps8
Opisthokonta - ps9
Holomycota - ps10
Fungi - ps11
Fungi_1 - ps12
Fungi_2 - ps13
Fungi_3 - ps14
Dikarya - ps15
Ascomycota - ps16
Saccharo/Pezizomycotina - ps17
Saccharomycotina - ps18
Saccharomycotina_1 - ps19
Saccharomycotina_2 - ps20
Saccharomycotina_3 - ps21
Phaff./Saccharomycetaceae - ps22
Saccharomyco./mycetaceae - ps23
Saccharomycetaceae - ps24
Saccharomycetaceae_2 - ps25
Saccharomycetaceae_3 - ps26
Kaz./Nau./Saccharomyces - ps27
Saccharomyces - ps28
Arboricola_clade - ps29
Cerevisiae_clade - ps30
Saccharomyces_cerevisiae - ps31
Phylostratum

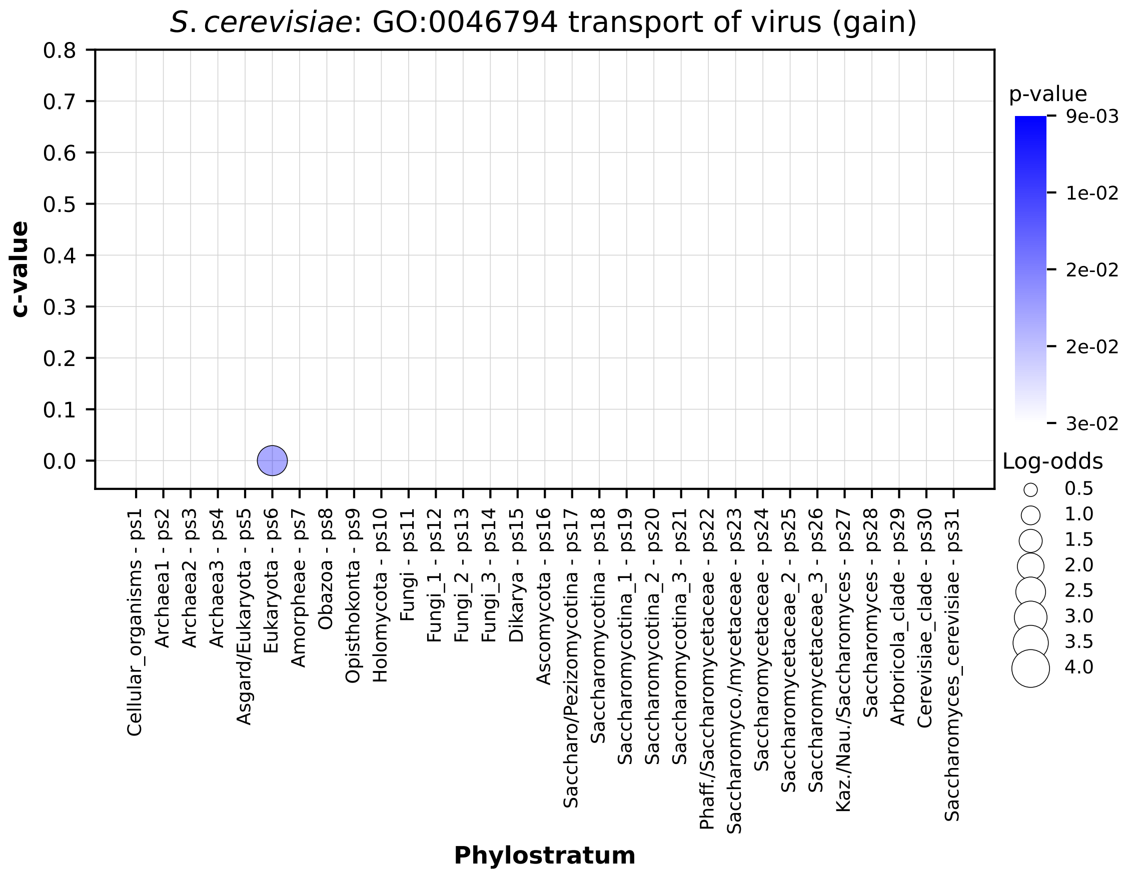

S. cerevisiae: GO:0046794 transport of virus (gain)
c-value
0.8
0.7
0.6
0.5
0.4
0.3
0.2
0.1
0.0
p-value
9e-03
1e-02
2e-02
2e-02
3e-02
Log-odds
0.5
1.0
1.5
2.0
2.5
3.0
3.5
4.0
Cellular_organisms - ps1
Archaea1 - ps2
Archaea2 - ps3
Archaea3 - ps4
Asgard/Eukaryota - ps5
Eukaryota - ps6
Amorpheae - ps7
Obazoa - ps8
Opisthokonta - ps9
Holomycota - ps10
Fungi - ps11
Fungi_1 - ps12
Fungi_2 - ps13
Fungi_3 - ps14
Dikarya - ps15
Ascomycota - ps16
Saccharo/Pezizomycotina - ps17
Saccharomycotina - ps18
Saccharomycotina_1 - ps19
Saccharomycotina_2 - ps20
Saccharomycotina_3 - ps21
Phaff./Saccharomycetaceae - ps22
Saccharomyco./mycetaceae - ps23
Saccharomycetaceae - ps24
Saccharomycetaceae_2 - ps25
Saccharomycetaceae_3 - ps26
Kaz./Nau./Saccharomyces - ps27
Saccharomyces - ps28
Arboricola_clade - ps29
Cerevisiae_clade - ps30
Saccharomyces_cerevisiae - ps31
Phylostratum

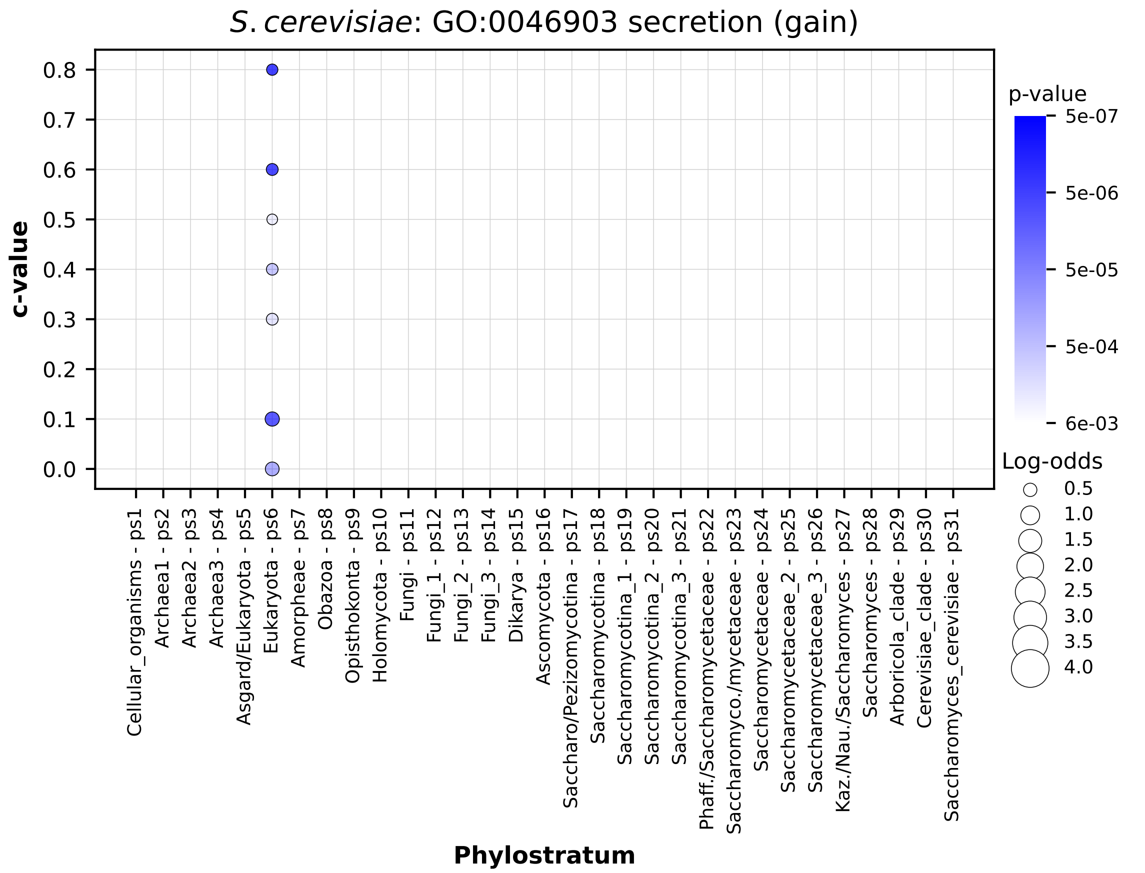

S. cerevisiae: GO:0046903 secretion (gain)
c-value
0.0
0.1
0.2
0.3
0.4
0.5
0.6
0.7
0.8
p-value
5e-07
5e-06
5e-05
5e-04
6e-03
Log-odds
0.5
1.0
1.5
2.0
2.5
3.0
3.5
4.0
Cellular_organisms - ps1
Archaea1 - ps2
Archaea2 - ps3
Archaea3 - ps4
Asgard/Eukaryota - ps5
Eukaryota - ps6
Amorpheae - ps7
Obazoa - ps8
Opisthokonta - ps9
Holomycota - ps10
Fungi - ps11
Fungi_1 - ps12
Fungi_2 - ps13
Fungi_3 - ps14
Dikarya - ps15
Ascomycota - ps16
Saccharo/Pezizomycotina - ps17
Saccharomycotina - ps18
Saccharomycotina_1 - ps19
Saccharomycotina_2 - ps20
Saccharomycotina_3 - ps21
Phaff./Saccharomycetaceae - ps22
Saccharomyco./mycetaceae - ps23
Saccharomycetaceae - ps24
Saccharomycetaceae_2 - ps25
Saccharomycetaceae_3 - ps26
Kaz./Nau./Saccharomyces - ps27
Saccharomyces - ps28
Arboricola_clade - ps29
Cerevisiae_clade - ps30
Saccharomyces_cerevisiae - ps31
Phylostratum

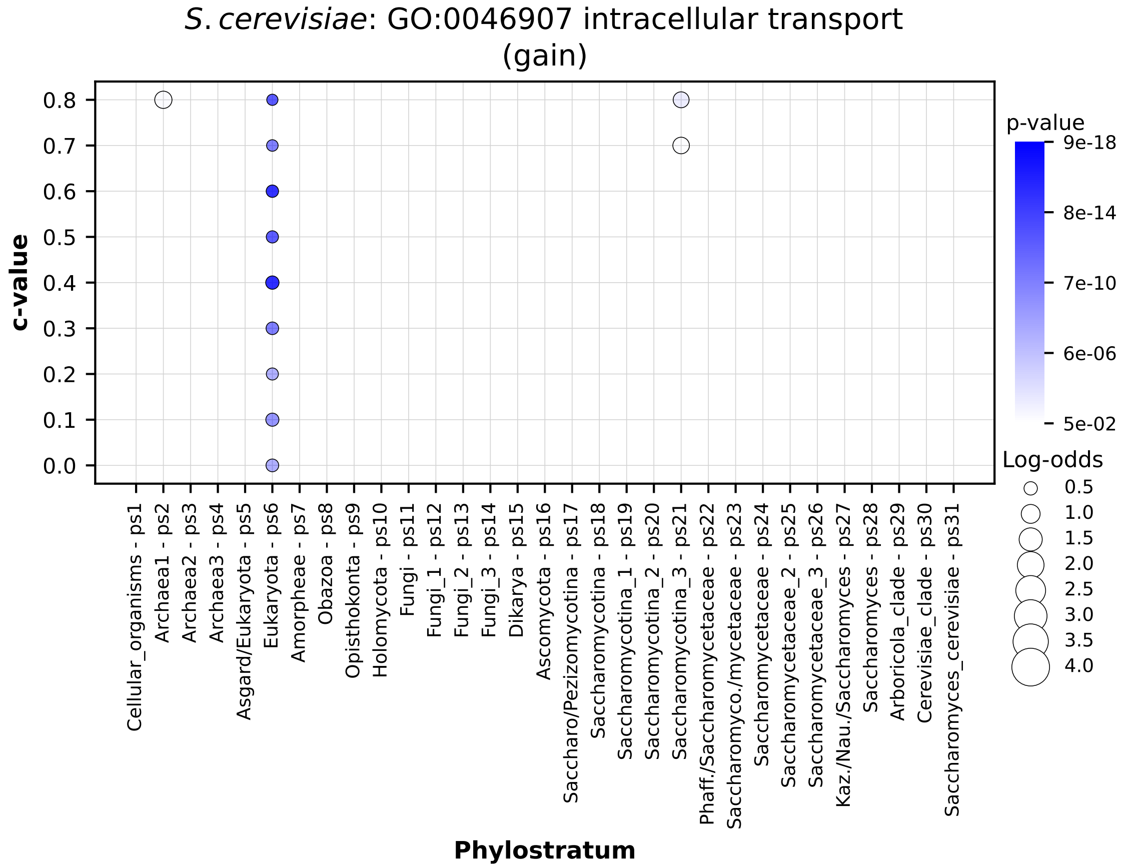

S. cerevisiae: GO:0046907 intracellular transport
(gain)
c-value
0.0
0.1
0.2
0.3
0.4
0.5
0.6
0.7
0.8
p-value
9e-18
8e-14
7e-10
6e-06
5e-02
Log-odds
0.5
1.0
1.5
2.0
2.5
3.0
3.5
4.0
Cellular_organisms - ps1
Archaea1 - ps2
Archaea2 - ps3
Archaea3 - ps4
Asgard/Eukaryota - ps5
Eukaryota - ps6
Amorpheae - ps7
Obazoa - ps8
Opisthokonta - ps9
Holomycota - ps10
Fungi - ps11
Fungi_1 - ps12
Fungi_2 - ps13
Fungi_3 - ps14
Dikarya - ps15
Ascomycota - ps16
Saccharo/Pezizomycotina - ps17
Saccharomycotina - ps18
Saccharomycotina_1 - ps19
Saccharomycotina_2 - ps20
Saccharomycotina_3 - ps21
Phaff./Saccharomycetaceae - ps22
Saccharomyco./mycetaceae - ps23
Saccharomycetaceae - ps24
Saccharomycetaceae_2 - ps25
Saccharomycetaceae_3 - ps26
Kaz./Nau./Saccharomyces - ps27
Saccharomyces - ps28
Arboricola_clade - ps29
Cerevisiae_clade - ps30
Saccharomyces_cerevisiae - ps31
Phylostratum

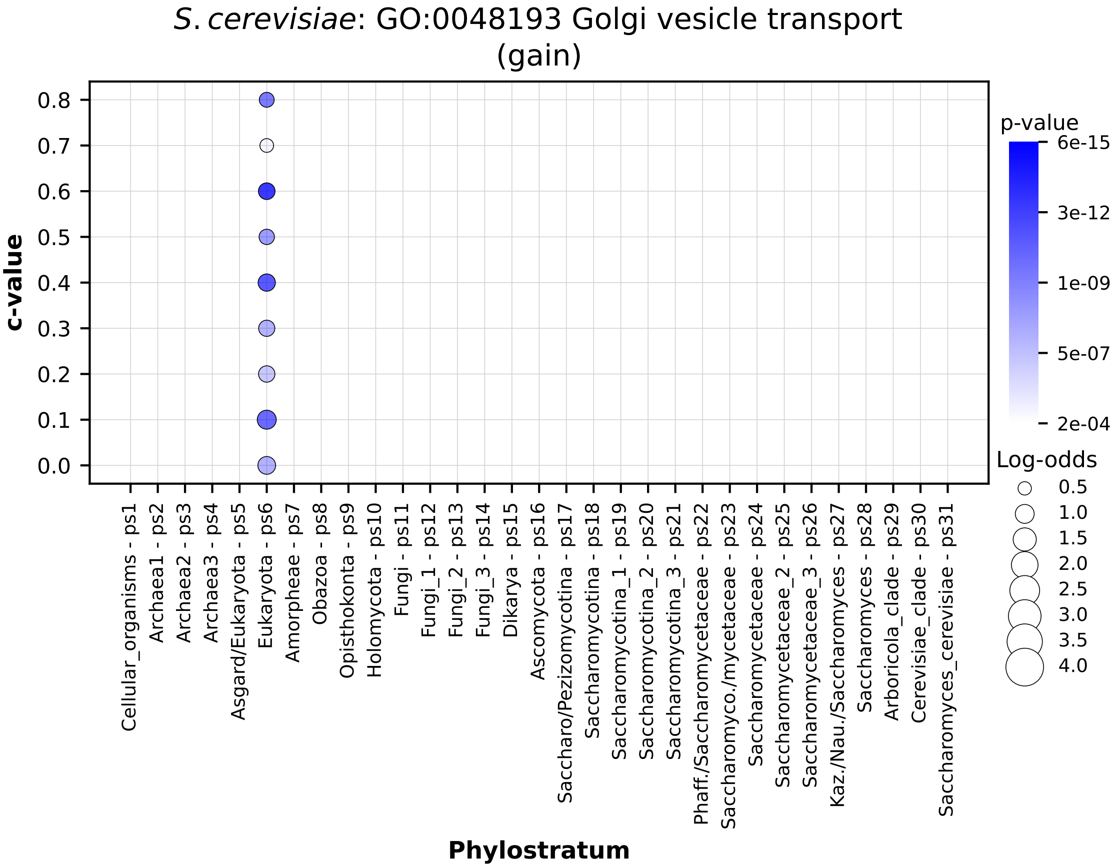

S. cerevisiae: GO:0048193 Golgi vesicle transport
(gain)
c-value
0.8
0.7
0.6
0.5
0.4
0.3
0.2
0.1
0.0
p-value
6e-15
3e-12
1e-09
5e-07
2e-04
Log-odds
0.5
1.0
1.5
2.0
2.5
3.0
3.5
4.0
Cellular_organisms - ps1
Archaea1 - ps2
Archaea2 - ps3
Archaea3 - ps4
Asgard/Eukaryota - ps5
Eukaryota - ps6
Amorpheae - ps7
Obazoa - ps8
Opisthokonta - ps9
Holomycota - ps10
Fungi - ps11
Fungi_1 - ps12
Fungi_2 - ps13
Fungi_3 - ps14
Dikarya - ps15
Ascomycota - ps16
Saccharo/Pezizomycotina - ps17
Saccharomycotina - ps18
Saccharomycotina_1 - ps19
Saccharomycotina_2 - ps20
Saccharomycotina_3 - ps21
Phaff./Saccharomycetaceae - ps22
Saccharomyco./mycetaceae - ps23
Saccharomycetaceae - ps24
Saccharomycetaceae_2 - ps25
Saccharomycetaceae_3 - ps26
Kaz./Nau./Saccharomyces - ps27
Saccharomyces - ps28
Arboricola_clade - ps29
Cerevisiae_clade - ps30
Saccharomyces_cerevisiae - ps31
Phylostratum

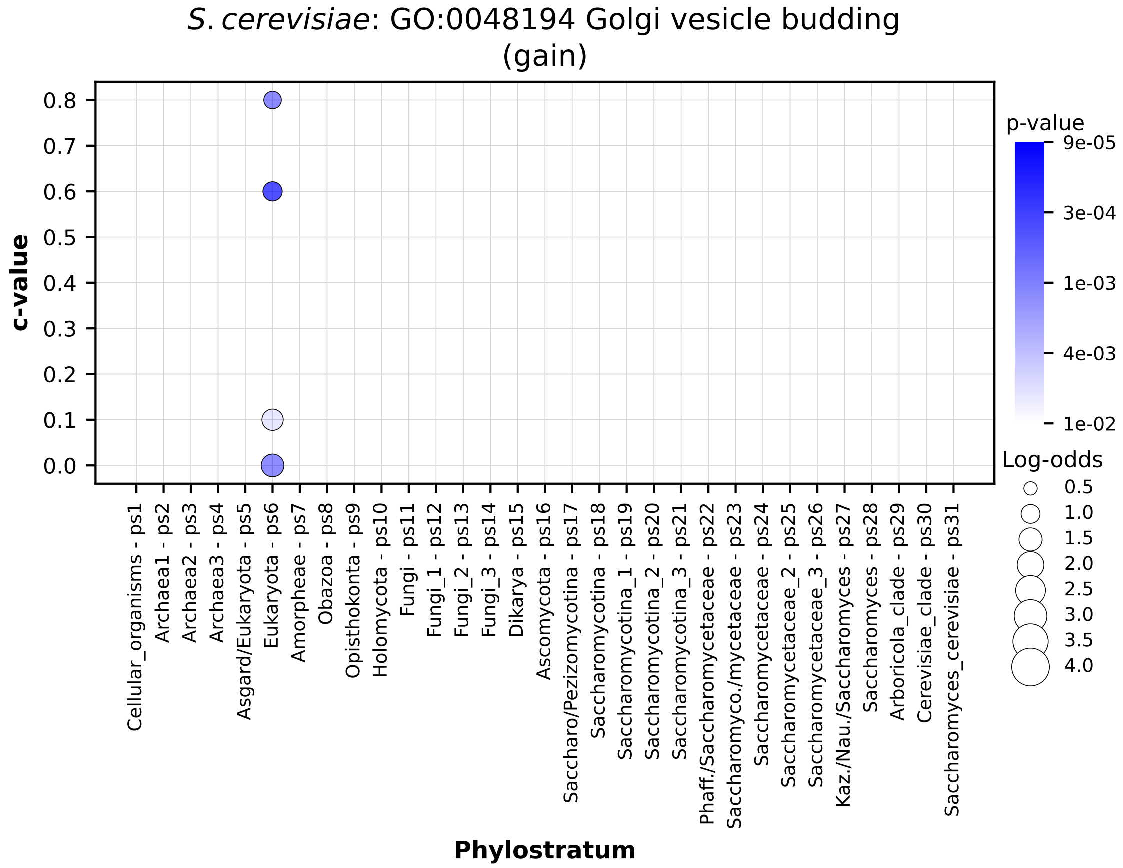

S. cerevisiae: GO:0048194 Golgi vesicle budding
(gain)
c-value
0.0
0.1
0.2
0.3
0.4
0.5
0.6
0.7
0.8
p-value
9e-05
3e-04
1e-03
4e-03
1e-02
Log-odds
0.5
1.0
1.5
2.0
2.5
3.0
3.5
4.0
Cellular_organisms - ps1
Archaea1 - ps2
Archaea2 - ps3
Archaea3 - ps4
Asgard/Eukaryota - ps5
Eukaryota - ps6
Amorpheae - ps7
Obazoa - ps8
Opisthokonta - ps9
Holomycota - ps10
Fungi - ps11
Fungi_1 - ps12
Fungi_2 - ps13
Fungi_3 - ps14
Dikarya - ps15
Ascomycota - ps16
Saccharo/Pezizomycotina - ps17
Saccharomycotina - ps18
Saccharomycotina_1 - ps19
Saccharomycotina_2 - ps20
Saccharomycotina_3 - ps21
Phaff./Saccharomycetaceae - ps22
Saccharomyco./mycetaceae - ps23
Saccharomycetaceae - ps24
Saccharomycetaceae_2 - ps25
Saccharomycetaceae_3 - ps26
Kaz./Nau./Saccharomyces - ps27
Saccharomyces - ps28
Arboricola_clade - ps29
Cerevisiae_clade - ps30
Saccharomyces_cerevisiae - ps31
Phylostratum

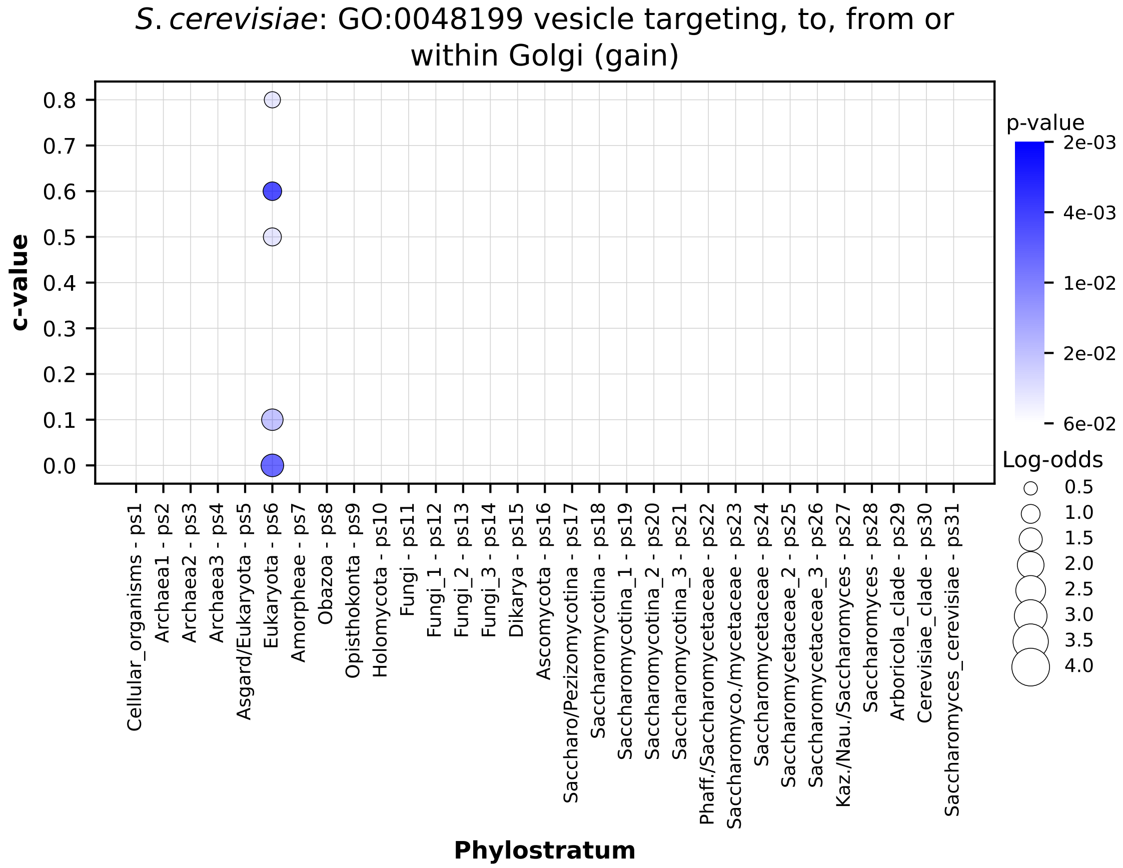
S. cerevisiae: GO:0048199 vesicle targeting, to, from or within Golgi (gain)
c-value
0.8
0.7
0.6
0.5
0.4
0.3
0.2
0.1
0.0
p-value
2e-03
4e-03
1e-02
2e-02
6e-02
Log-odds
0.5
1.0
1.5
2.0
2.5
3.0
3.5
4.0
Cellular_organisms - ps1
Archaea1 - ps2
Archaea2 - ps3
Archaea3 - ps4
Asgard/Eukaryota - ps5
Eukaryota - ps6
Amorpheae - ps7
Obazoa - ps8
Opisthokonta - ps9
Holomycota - ps10
Fungi - ps11
Fungi_1 - ps12
Fungi_2 - ps13
Fungi_3 - ps14
Dikarya - ps15
Ascomycota - ps16
Saccharo/Pezizomycotina - ps17
Saccharomycotina - ps18
Saccharomycotina_1 - ps19
Saccharomycotina_2 - ps20
Saccharomycotina_3 - ps21
Phaff./Saccharomycetaceae - ps22
Saccharomyco./mycetaceae - ps23
Saccharomycetaceae - ps24
Saccharomycetaceae_2 - ps25
Saccharomycetaceae_3 - ps26
Kaz./Nau./Saccharomyces - ps27
Saccharomyces - ps28
Arboricola_clade - ps29
Cerevisiae_clade - ps30
Saccharomyces_cerevisiae - ps31
Phylostratum

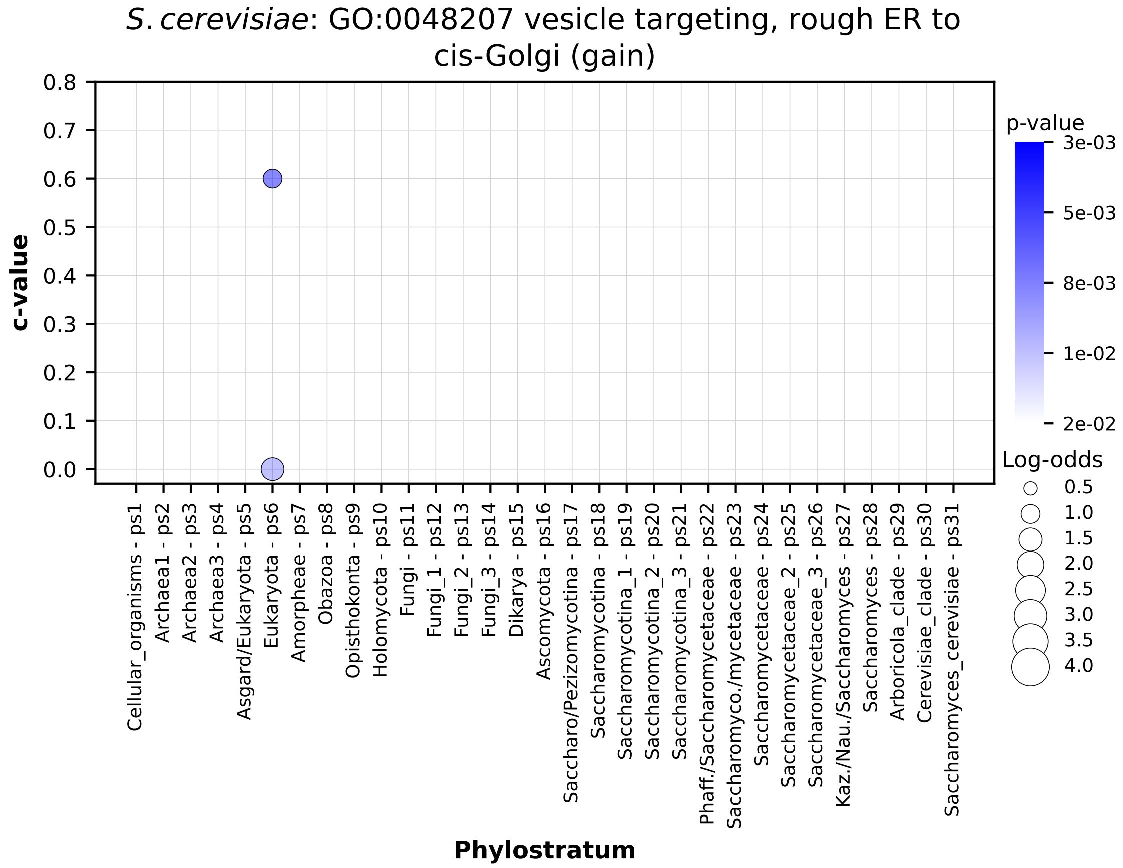

S. cerevisiae: GO:0048207 vesicle targeting, rough ER to cis-Golgi (gain)
c-value
0.8
0.7
0.6
0.5
0.4
0.3
0.2
0.1
0.0
Phylostratum
Cellular_organisms - ps1
Archaea1 - ps2
Archaea2 - ps3
Archaea3 - ps4
Asgard/Eukaryota - ps5
Eukaryota - ps6
Amorpheae - ps7
Obazoa - ps8
Opisthokonta - ps9
Holomycota - ps10
Fungi - ps11
Fungi_1 - ps12
Fungi_2 - ps13
Fungi_3 - ps14
Dikarya - ps15
Ascomycota - ps16
Saccharo/Pezizomycotina - ps17
Saccharomycotina - ps18
Saccharomycotina_1 - ps19
Saccharomycotina_2 - ps20
Saccharomycotina_3 - ps21
Phaff./Saccharomycetaceae - ps22
Saccharomyco./mycetaceae - ps23
Saccharomycetaceae - ps24
Saccharomycetaceae_2 - ps25
Saccharomycetaceae_3 - ps26
Kaz./Nau./Saccharomyces - ps27
Saccharomyces - ps28
Arboricola_clade - ps29
Cerevisiae_clade - ps30
Saccharomyces_cerevisiae - ps31
p-value
3e-03
5e-03
8e-03
1e-02
2e-02
Log-odds
0.5
1.0
1.5
2.0
2.5
3.0
3.5
4.0

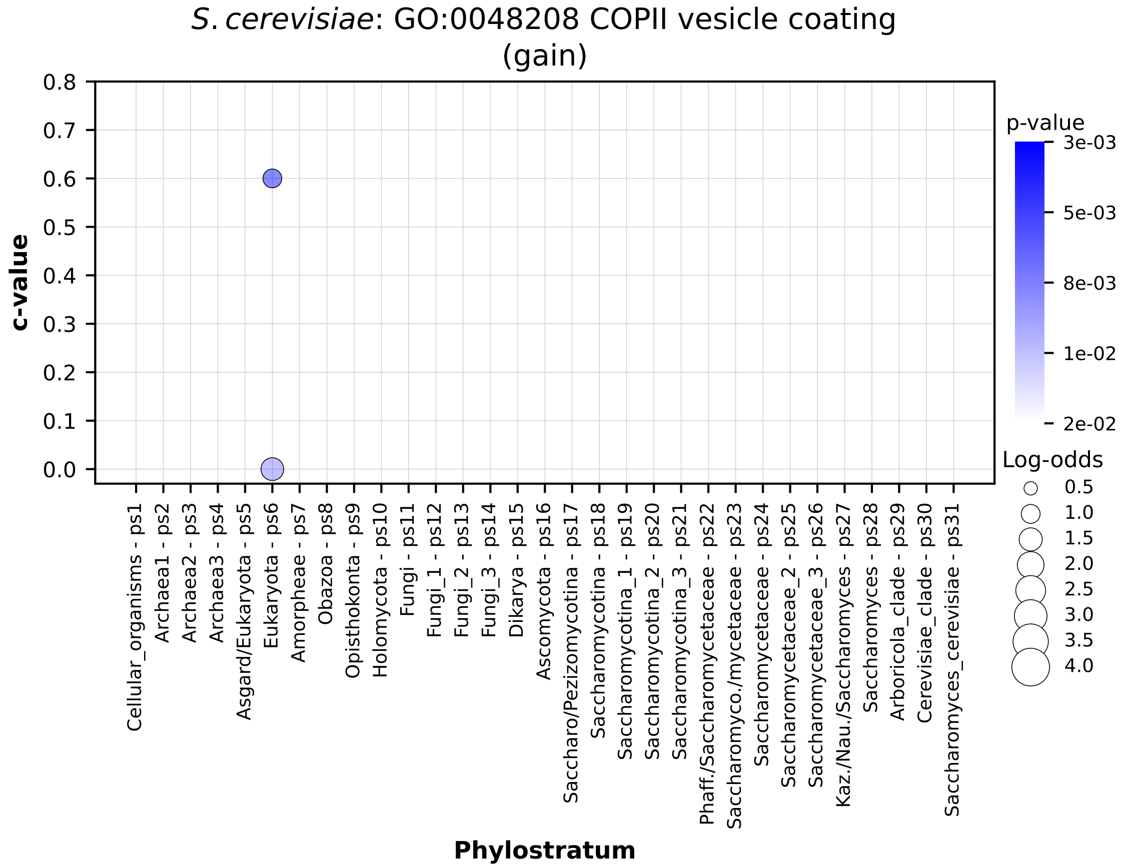

S. cerevisiae: GO:0048208 COPII vesicle coating (gain)
c-value
0.0
0.1
0.2
0.3
0.4
0.5
0.6
0.7
0.8
Phylostratum
Cellular_organisms - ps1
Archaea1 - ps2
Archaea2 - ps3
Archaea3 - ps4
Asgard/Eukaryota - ps5
Eukaryota - ps6
Amorpheae - ps7
Obazoa - ps8
Opisthokonta - ps9
Holomycota - ps10
Fungi - ps11
Fungi_1 - ps12
Fungi_2 - ps13
Fungi_3 - ps14
Dikarya - ps15
Ascomycota - ps16
Saccharo/Pezizomycotina - ps17
Saccharomycotina - ps18
Saccharomycotina_1 - ps19
Saccharomycotina_2 - ps20
Saccharomycotina_3 - ps21
Phaff./Saccharomycetaceae - ps22
Saccharomyco./mycetaceae - ps23
Saccharomycetaceae - ps24
Saccharomycetaceae_2 - ps25
Saccharomycetaceae_3 - ps26
Kaz./Nau./Saccharomyces - ps27
Saccharomyces - ps28
Arboricola_clade - ps29
Cerevisiae_clade - ps30
Saccharomyces_cerevisiae - ps31
p-value
3e-03
5e-03
8e-03
1e-02
2e-02
Log-odds
0.5
1.0
1.5
2.0
2.5
3.0
3.5
4.0

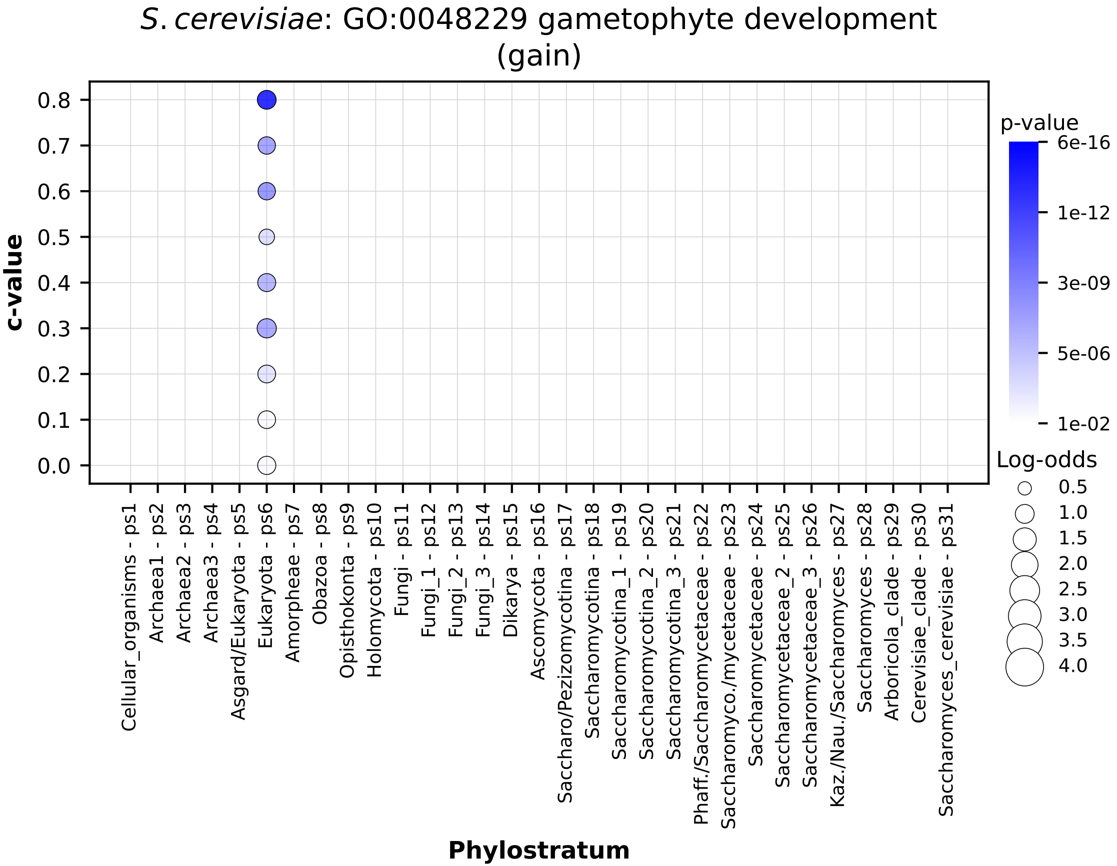

S. cerevisiae: GO:0048229 gametophyte development
(gain)
c-value
0.0
0.1
0.2
0.3
0.4
0.5
0.6
0.7
0.8
p-value
6e-16
1e-12
3e-09
5e-06
1e-02
Log-odds
0.5
1.0
1.5
2.0
2.5
3.0
3.5
4.0
Cellular_organisms - ps1
Archaea1 - ps2
Archaea2 - ps3
Archaea3 - ps4
Asgard/Eukaryota - ps5
Eukaryota - ps6
Amorpheae - ps7
Obazoa - ps8
Opisthokonta - ps9
Holomycota - ps10
Fungi - ps11
Fungi_1 - ps12
Fungi_2 - ps13
Fungi_3 - ps14
Dikarya - ps15
Ascomycota - ps16
Saccharo/Pezizomycotina - ps17
Saccharomycotina - ps18
Saccharomycotina_1 - ps19
Saccharomycotina_2 - ps20
Saccharomycotina_3 - ps21
Phaff./Saccharomycetaceae - ps22
Saccharomyco./mycetaceae - ps23
Saccharomycetaceae - ps24
Saccharomycetaceae_2 - ps25
Saccharomycetaceae_3 - ps26
Kaz./Nau./Saccharomyces - ps27
Saccharomyces - ps28
Arboricola_clade - ps29
Cerevisiae_clade - ps30
Saccharomyces_cerevisiae - ps31
Phylostratum

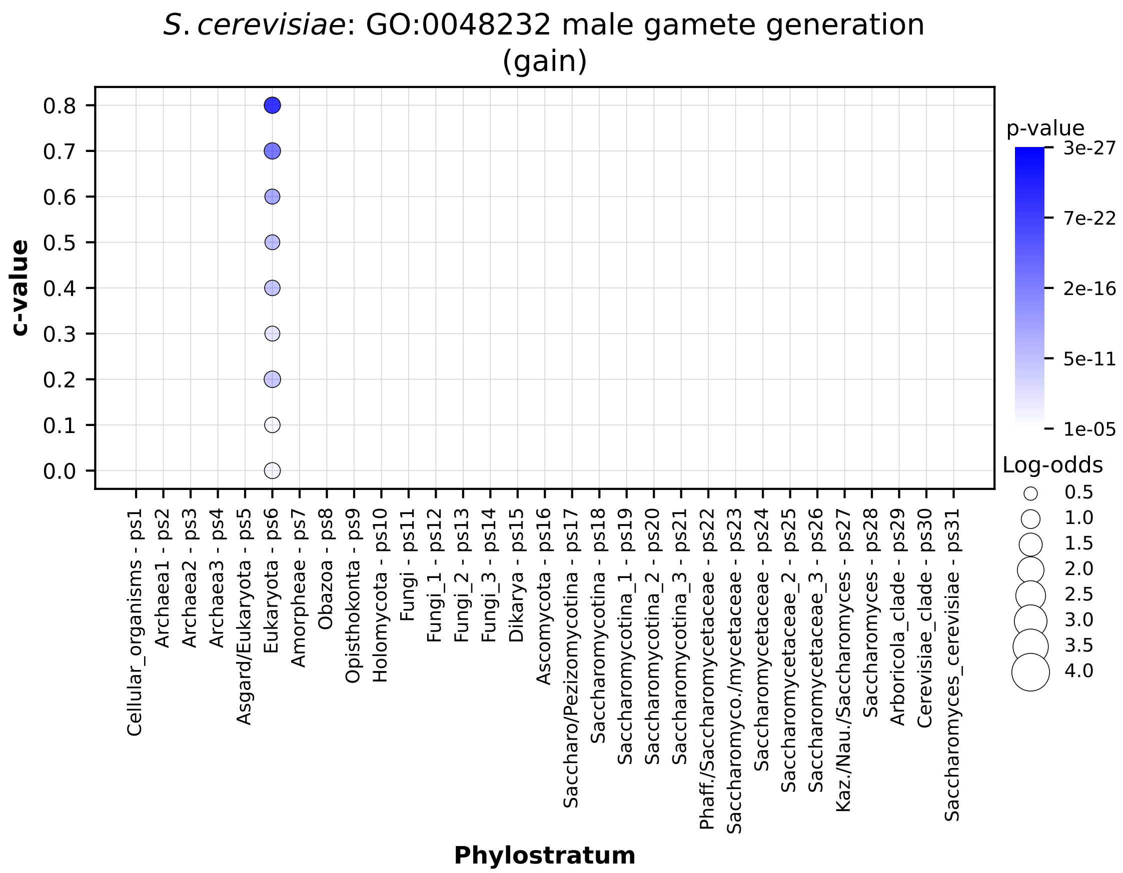

S. cerevisiae: GO:0048232 male gamete generation
(gain)
c-value
0.8
0.7
0.6
0.5
0.4
0.3
0.2
0.1
0.0
p-value
3e-27
7e-22
2e-16
5e-11
1e-05
Log-odds
0.5
1.0
1.5
2.0
2.5
3.0
3.5
4.0
Cellular_organisms - ps1
Archaea1 - ps2
Archaea2 - ps3
Archaea3 - ps4
Asgard/Eukaryota - ps5
Eukaryota - ps6
Amorpheae - ps7
Obazoa - ps8
Opisthokonta - ps9
Holomycota - ps10
Fungi - ps11
Fungi_1 - ps12
Fungi_2 - ps13
Fungi_3 - ps14
Dikarya - ps15
Ascomycota - ps16
Saccharo/Pezizomycotina - ps17
Saccharomycotina - ps18
Saccharomycotina_1 - ps19
Saccharomycotina_2 - ps20
Saccharomycotina_3 - ps21
Phaff./Saccharomycetaceae - ps22
Saccharomyco./mycetaceae - ps23
Saccharomycetaceae - ps24
Saccharomycetaceae_2 - ps25
Saccharomycetaceae_3 - ps26
Kaz./Nau./Saccharomyces - ps27
Saccharomyces - ps28
Arboricola_clade - ps29
Cerevisiae_clade - ps30
Saccharomyces_cerevisiae - ps31
Phylostratum

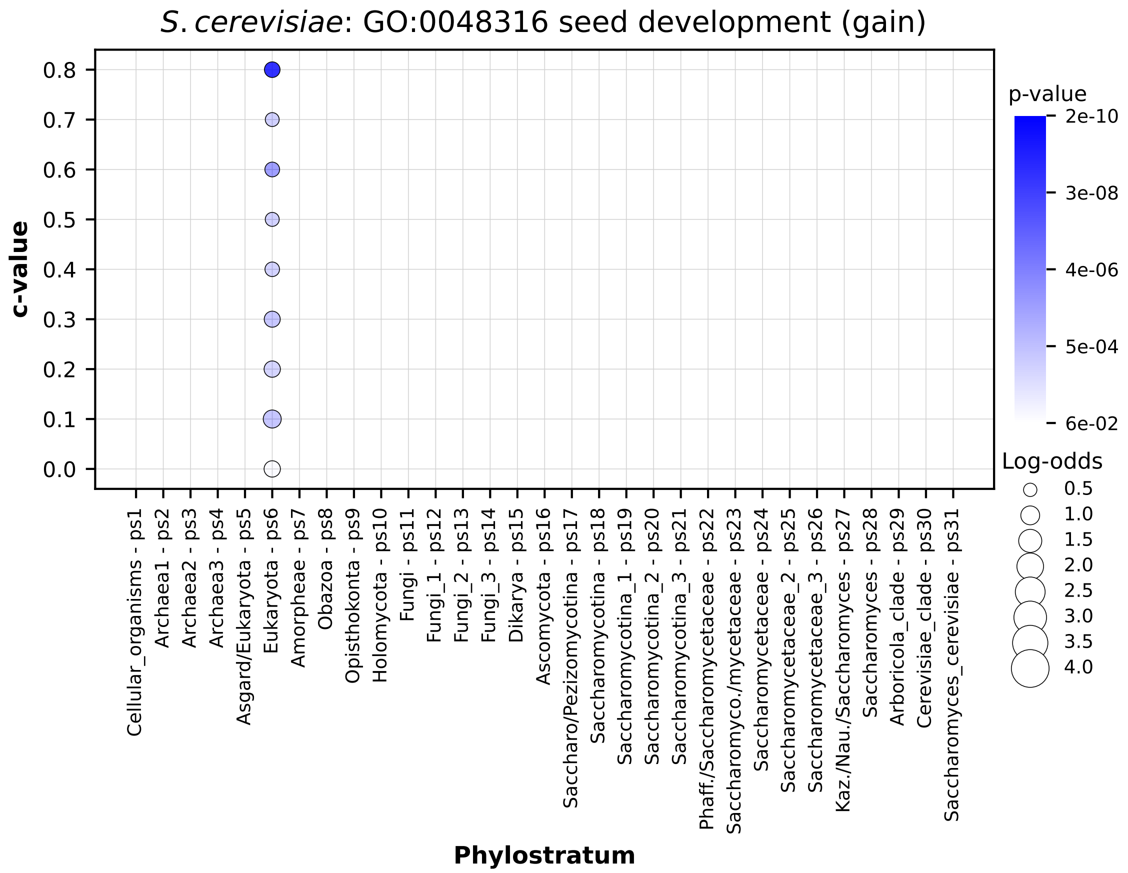

S. cerevisiae: GO:0048316 seed development (gain)
c-value
0.8
0.7
0.6
0.5
0.4
0.3
0.2
0.1
0.0
p-value
2e-10
3e-08
4e-06
5e-04
6e-02
Log-odds
0.5
1.0
1.5
2.0
2.5
3.0
3.5
4.0
Cellular_organisms - ps1
Archaea1 - ps2
Archaea2 - ps3
Archaea3 - ps4
Asgard/Eukaryota - ps5
Eukaryota - ps6
Amorpheae - ps7
Obazoa - ps8
Opisthokonta - ps9
Holomycota - ps10
Fungi - ps11
Fungi_1 - ps12
Fungi_2 - ps13
Fungi_3 - ps14
Dikarya - ps15
Ascomycota - ps16
Saccharo/Pezizomycotina - ps17
Saccharomycotina - ps18
Saccharomycotina_1 - ps19
Saccharomycotina_2 - ps20
Saccharomycotina_3 - ps21
Phaff./Saccharomycetaceae - ps22
Saccharomyco./mycetaceae - ps23
Saccharomycetaceae - ps24
Saccharomycetaceae_2 - ps25
Saccharomycetaceae_3 - ps26
Kaz./Nau./Saccharomyces - ps27
Saccharomyces - ps28
Arboricola_clade - ps29
Cerevisiae_clade - ps30
Saccharomyces_cerevisiae - ps31
Phylostratum

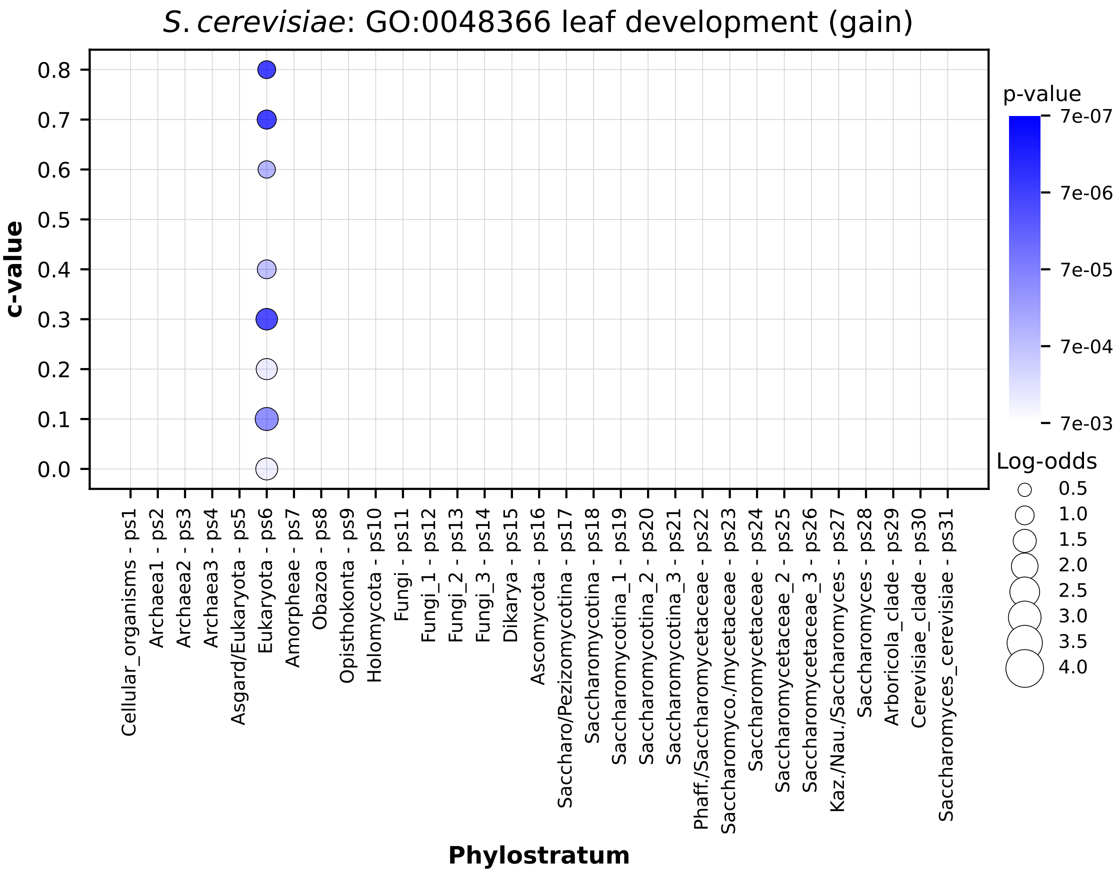

S. cerevisiae: GO:0048366 leaf development (gain)
c-value
0.0
0.1
0.2
0.3
0.4
0.5
0.6
0.7
0.8
Phylostratum
Cellular_organisms - ps1
Archaea1 - ps2
Archaea2 - ps3
Archaea3 - ps4
Asgard/Eukaryota - ps5
Eukaryota - ps6
Amorpheae - ps7
Obazoa - ps8
Opisthokonta - ps9
Holomycota - ps10
Fungi - ps11
Fungi_1 - ps12
Fungi_2 - ps13
Fungi_3 - ps14
Dikarya - ps15
Ascomycota - ps16
Saccharo/Pezizomycotina - ps17
Saccharomycotina - ps18
Saccharomycotina_1 - ps19
Saccharomycotina_2 - ps20
Saccharomycotina_3 - ps21
Phaff./Saccharomycetaceae - ps22
Saccharomyco./mycetaceae - ps23
Saccharomycetaceae - ps24
Saccharomycetaceae_2 - ps25
Saccharomycetaceae_3 - ps26
Kaz./Nau./Saccharomyces - ps27
Saccharomyces - ps28
Arboricola_clade - ps29
Cerevisiae_clade - ps30
Saccharomyces_cerevisiae - ps31
p-value
7e-07
7e-06
7e-05
7e-04
7e-03
Log-odds
0.5
1.0
1.5
2.0
2.5
3.0
3.5
4.0

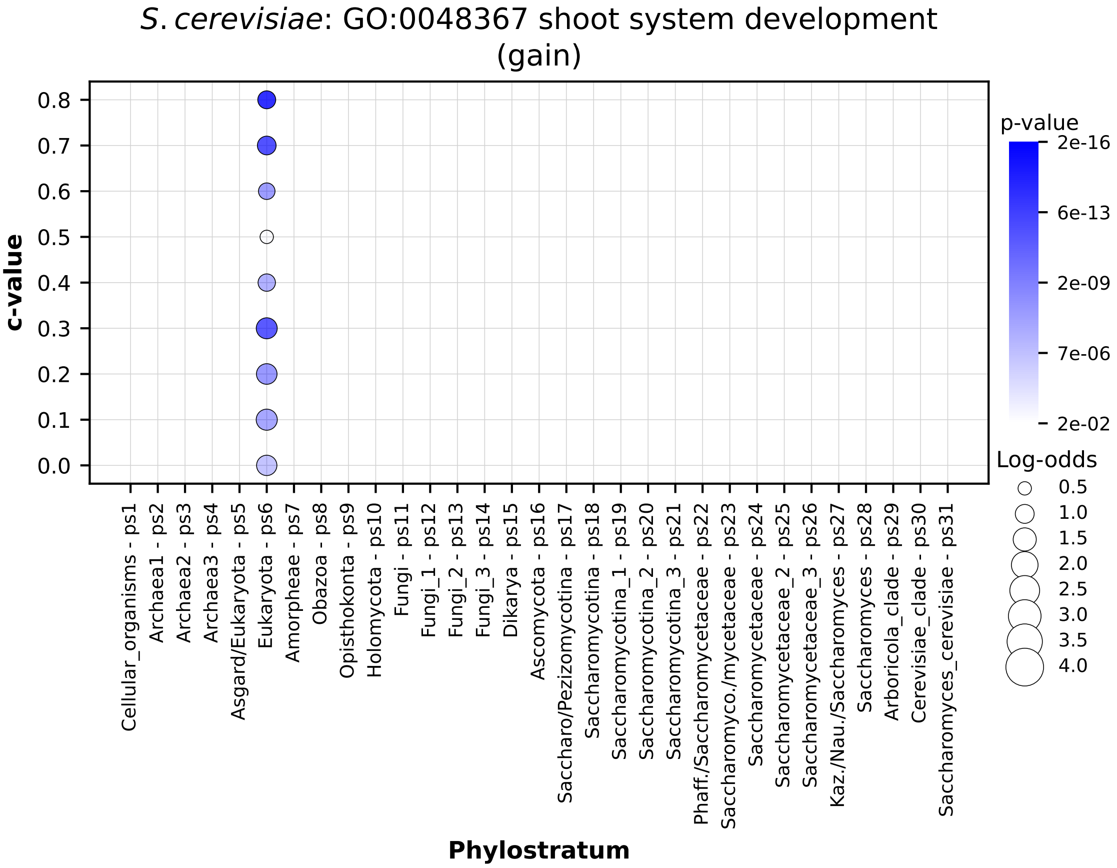

S. cerevisiae: GO:0048367 shoot system development
(gain)
c-value
0.8
0.7
0.6
0.5
0.4
0.3
0.2
0.1
0.0
p-value
2e-16
6e-13
2e-09
7e-06
2e-02
Log-odds
0.5
1.0
1.5
2.0
2.5
3.0
3.5
4.0
Cellular_organisms - ps1
Archaea1 - ps2
Archaea2 - ps3
Archaea3 - ps4
Asgard/Eukaryota - ps5
Eukaryota - ps6
Amorpheae - ps7
Obazoa - ps8
Opisthokonta - ps9
Holomycota - ps10
Fungi - ps11
Fungi_1 - ps12
Fungi_2 - ps13
Fungi_3 - ps14
Dikarya - ps15
Ascomycota - ps16
Saccharo/Pezizomycotina - ps17
Saccharomycotina - ps18
Saccharomycotina_1 - ps19
Saccharomycotina_2 - ps20
Saccharomycotina_3 - ps21
Phaff./Saccharomycetaceae - ps22
Saccharomyco./mycetaceae - ps23
Saccharomycetaceae - ps24
Saccharomycetaceae_2 - ps25
Saccharomycetaceae_3 - ps26
Kaz./Nau./Saccharomyces - ps27
Saccharomyces - ps28
Arboricola_clade - ps29
Cerevisiae_clade - ps30
Saccharomyces_cerevisiae - ps31
Phylostratum

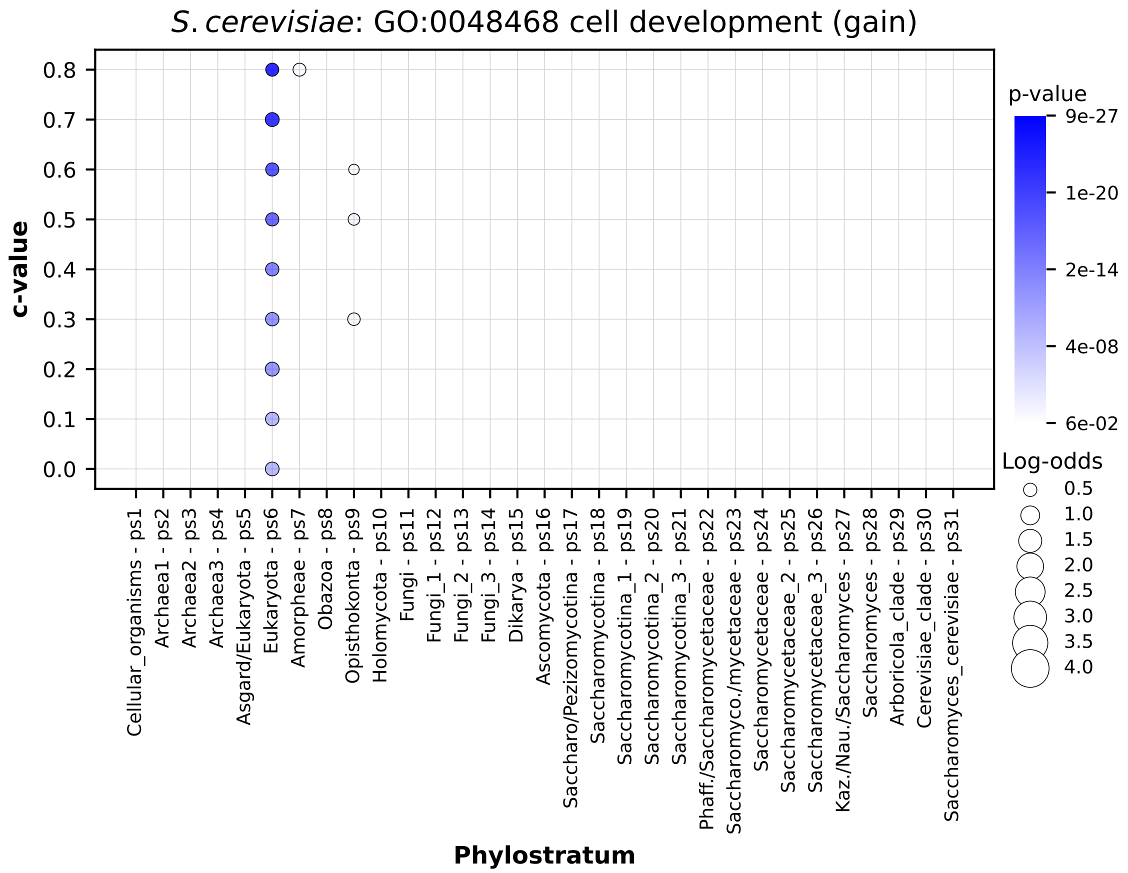

S. cerevisiae: GO:0048468 cell development (gain)
c-value
0.0
0.1
0.2
0.3
0.4
0.5
0.6
0.7
0.8
p-value
9e-27
1e-20
2e-14
4e-08
6e-02
Log-odds
0.5
1.0
1.5
2.0
2.5
3.0
3.5
4.0
Cellular_organisms - ps1
Archaea1 - ps2
Archaea2 - ps3
Archaea3 - ps4
Asgard/Eukaryota - ps5
Eukaryota - ps6
Amorpheae - ps7
Obazoa - ps8
Opisthokonta - ps9
Holomycota - ps10
Fungi - ps11
Fungi_1 - ps12
Fungi_2 - ps13
Fungi_3 - ps14
Dikarya - ps15
Ascomycota - ps16
Saccharo/Pezizomycotina - ps17
Saccharomycotina - ps18
Saccharomycotina_1 - ps19
Saccharomycotina_2 - ps20
Saccharomycotina_3 - ps21
Phaff./Saccharomycetaceae - ps22
Saccharomyco./mycetaceae - ps23
Saccharomycetaceae - ps24
Saccharomycetaceae_2 - ps25
Saccharomycetaceae_3 - ps26
Kaz./Nau./Saccharomyces - ps27
Saccharomyces - ps28
Arboricola_clade - ps29
Cerevisiae_clade - ps30
Saccharomyces_cerevisiae - ps31
Phylostratum

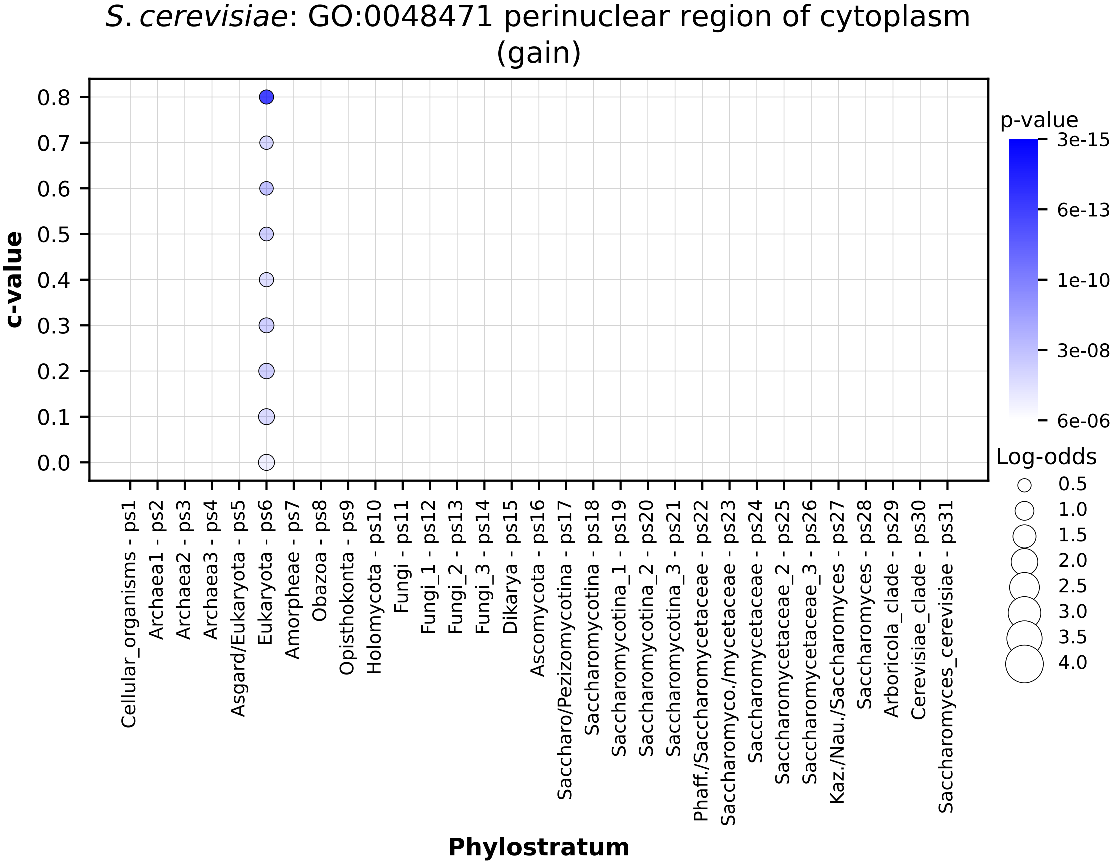

S. cerevisiae: GO:0048471 perinuclear region of cytoplasm (gain)
c-value
0.8
0.7
0.6
0.5
0.4
0.3
0.2
0.1
0.0
p-value
3e-15
6e-13
1e-10
3e-08
6e-06
Log-odds
0.5
1.0
1.5
2.0
2.5
3.0
3.5
4.0
Cellular_organisms - ps1
Archaea1 - ps2
Archaea2 - ps3
Archaea3 - ps4
Asgard/Eukaryota - ps5
Eukaryota - ps6
Amorpheae - ps7
Obazoa - ps8
Opisthokonta - ps9
Holomycota - ps10
Fungi - ps11
Fungi_1 - ps12
Fungi_2 - ps13
Fungi_3 - ps14
Dikarya - ps15
Ascomycota - ps16
Saccharo/Pezizomycotina - ps17
Saccharomycotina - ps18
Saccharomycotina_1 - ps19
Saccharomycotina_2 - ps20
Saccharomycotina_3 - ps21
Phaff./Saccharomycetaceae - ps22
Saccharomyco./mycetaceae - ps23
Saccharomycetaceae - ps24
Saccharomycetaceae_2 - ps25
Saccharomycetaceae_3 - ps26
Kaz./Nau./Saccharomyces - ps27
Saccharomyces - ps28
Arboricola_clade - ps29
Cerevisiae_clade - ps30
Saccharomyces_cerevisiae - ps31
Phylostratum

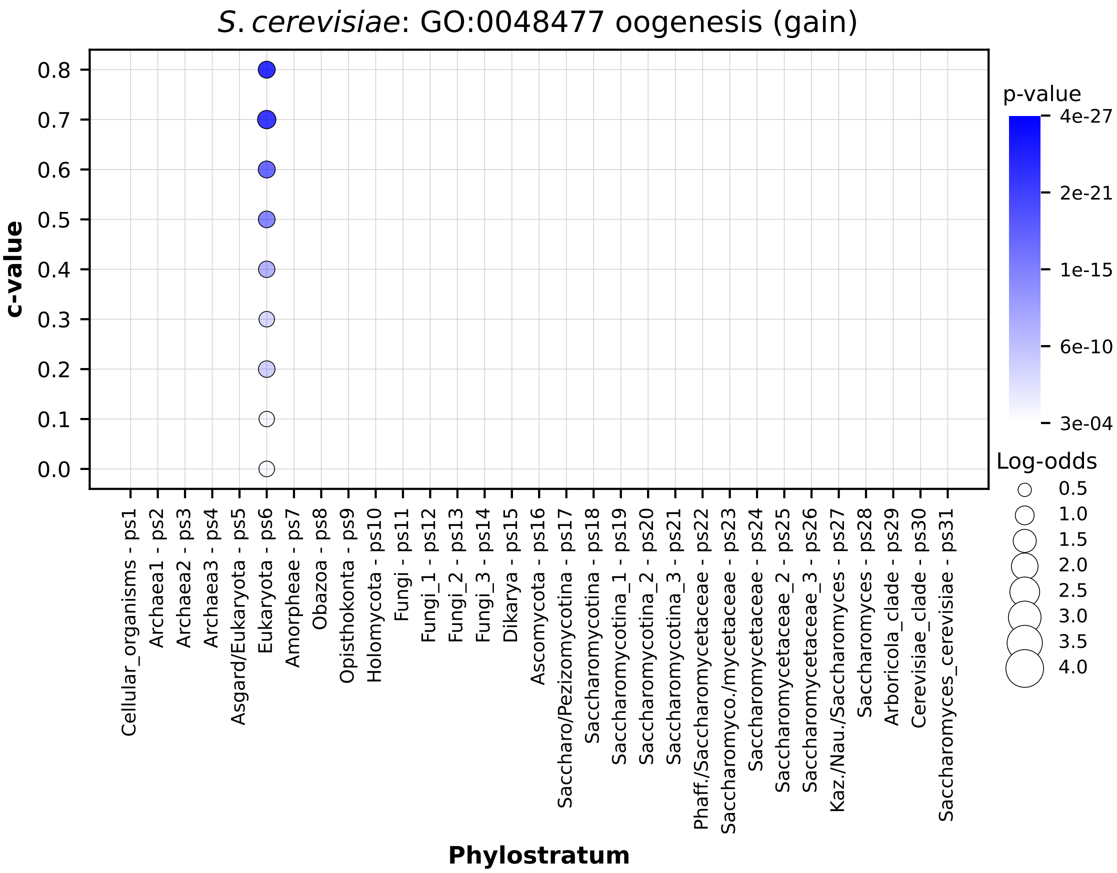

S. cerevisiae: GO:0048477 oogenesis (gain)
c-value
0.0
0.1
0.2
0.3
0.4
0.5
0.6
0.7
0.8
Phylostratum
Cellular_organisms - ps1
Archaea1 - ps2
Archaea2 - ps3
Archaea3 - ps4
Asgard/Eukaryota - ps5
Eukaryota - ps6
Amorpheae - ps7
Obazoa - ps8
Opisthokonta - ps9
Holomycota - ps10
Fungi - ps11
Fungi_1 - ps12
Fungi_2 - ps13
Fungi_3 - ps14
Dikarya - ps15
Ascomycota - ps16
Saccharo/Pezizomycotina - ps17
Saccharomycotina - ps18
Saccharomycotina_1 - ps19
Saccharomycotina_2 - ps20
Saccharomycotina_3 - ps21
Phaff./Saccharomycetaceae - ps22
Saccharomyco./mycetaceae - ps23
Saccharomycetaceae - ps24
Saccharomycetaceae_2 - ps25
Saccharomycetaceae_3 - ps26
Kaz./Nau./Saccharomyces - ps27
Saccharomyces - ps28
Arboricola_clade - ps29
Cerevisiae_clade - ps30
Saccharomyces_cerevisiae - ps31
p-value
4e-27
2e-21
1e-15
6e-10
3e-04
Log-odds
0.5
1.0
1.5
2.0
2.5
3.0
3.5
4.0

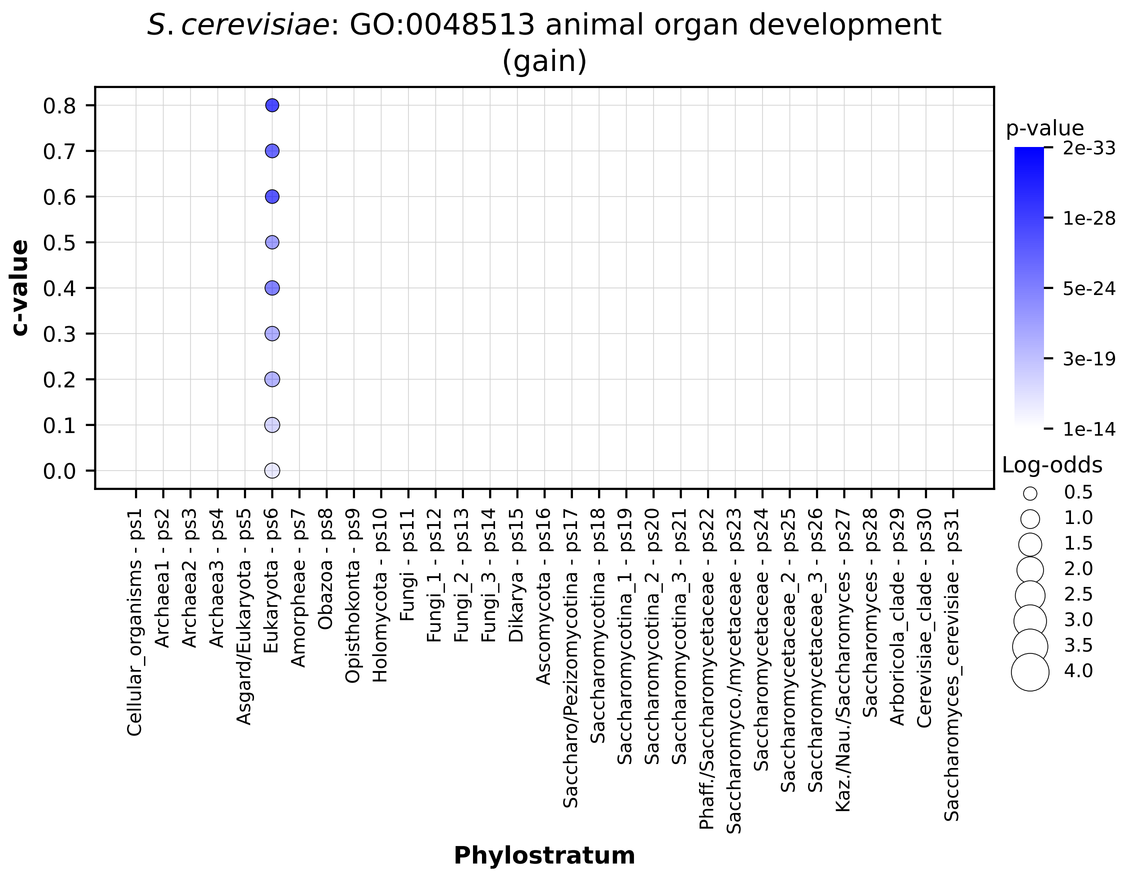

S. cerevisiae: GO:0048513 animal organ development (gain)
c-value
0.0
0.1
0.2
0.3
0.4
0.5
0.6
0.7
0.8
Phylostratum
Cellular_organisms - ps1
Archaea1 - ps2
Archaea2 - ps3
Archaea3 - ps4
Asgard/Eukaryota - ps5
Eukaryota - ps6
Amorpheae - ps7
Obazoa - ps8
Opisthokonta - ps9
Holomycota - ps10
Fungi - ps11
Fungi_1 - ps12
Fungi_2 - ps13
Fungi_3 - ps14
Dikarya - ps15
Ascomycota - ps16
Saccharo/Pezizomycotina - ps17
Saccharomycotina - ps18
Saccharomycotina_1 - ps19
Saccharomycotina_2 - ps20
Saccharomycotina_3 - ps21
Phaff./Saccharomycetaceae - ps22
Saccharomyco./mycetaceae - ps23
Saccharomycetaceae - ps24
Saccharomycetaceae_2 - ps25
Saccharomycetaceae_3 - ps26
Kaz./Nau./Saccharomyces - ps27
Saccharomyces - ps28
Arboricola_clade - ps29
Cerevisiae_clade - ps30
Saccharomyces_cerevisiae - ps31
p-value
2e-33
1e-28
5e-24
3e-19
1e-14
Log-odds
0.5
1.0
1.5
2.0
2.5
3.0
3.5
4.0

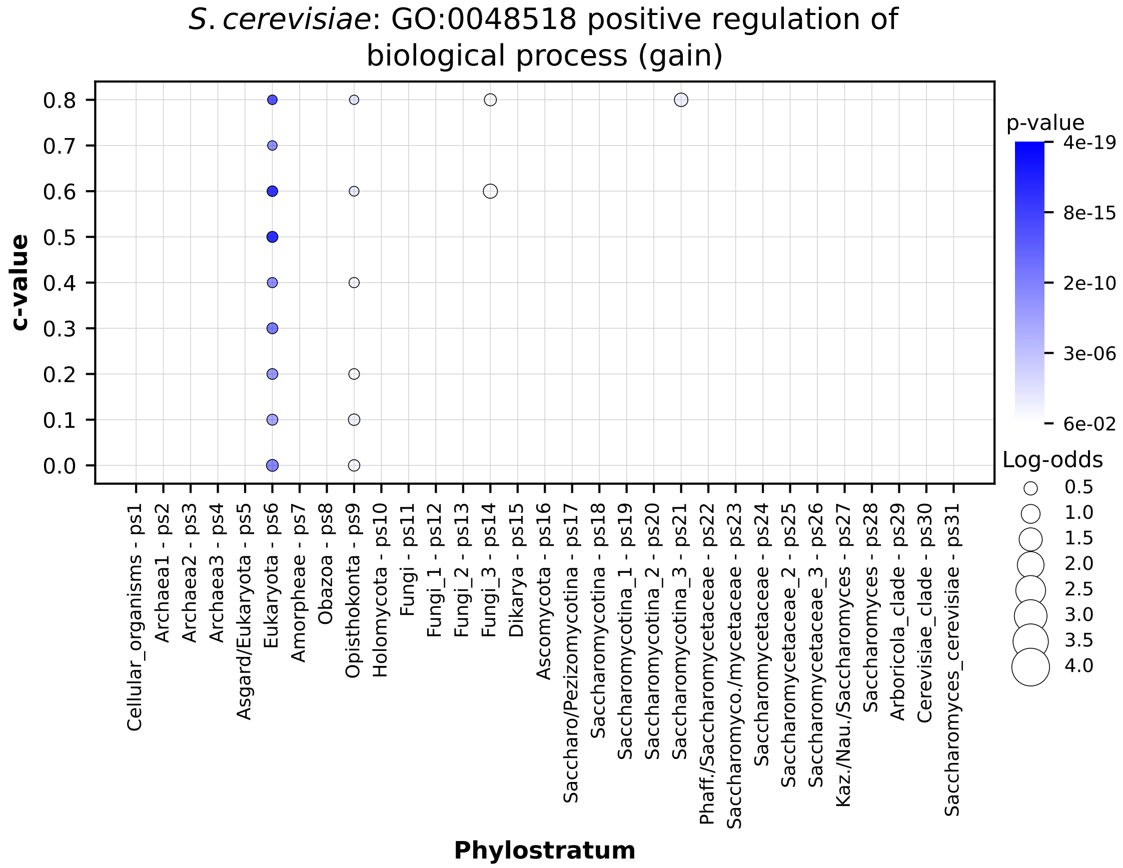
S. cerevisiae: GO:0048518 positive regulation of biological process (gain)
c-value
0.8
0.7
0.6
0.5
0.4
0.3
0.2
0.1
0.0
p-value
4e-19
8e-15
2e-10
3e-06
6e-02
Log-odds
0.5
1.0
1.5
2.0
2.5
3.0
3.5
4.0
Cellular_organisms - ps1
Archaea1 - ps2
Archaea2 - ps3
Archaea3 - ps4
Asgard/Eukaryota - ps5
Eukaryota - ps6
Amorpheae - ps7
Obazoa - ps8
Opisthokonta - ps9
Holomycota - ps10
Fungi - ps11
Fungi_1 - ps12
Fungi_2 - ps13
Fungi_3 - ps14
Dikarya - ps15
Ascomycota - ps16
Saccharo/Pezizomycotina - ps17
Saccharomycotina - ps18
Saccharomycotina_1 - ps19
Saccharomycotina_2 - ps20
Saccharomycotina_3 - ps21
Phaff./Saccharomycetaceae - ps22
Saccharomyco./mycetaceae - ps23
Saccharomycetaceae - ps24
Saccharomycetaceae_2 - ps25
Saccharomycetaceae_3 - ps26
Kaz./Nau./Saccharomyces - ps27
Saccharomyces - ps28
Arboricola_clade - ps29
Cerevisiae_clade - ps30
Saccharomyces_cerevisiae - ps31
Phylostratum

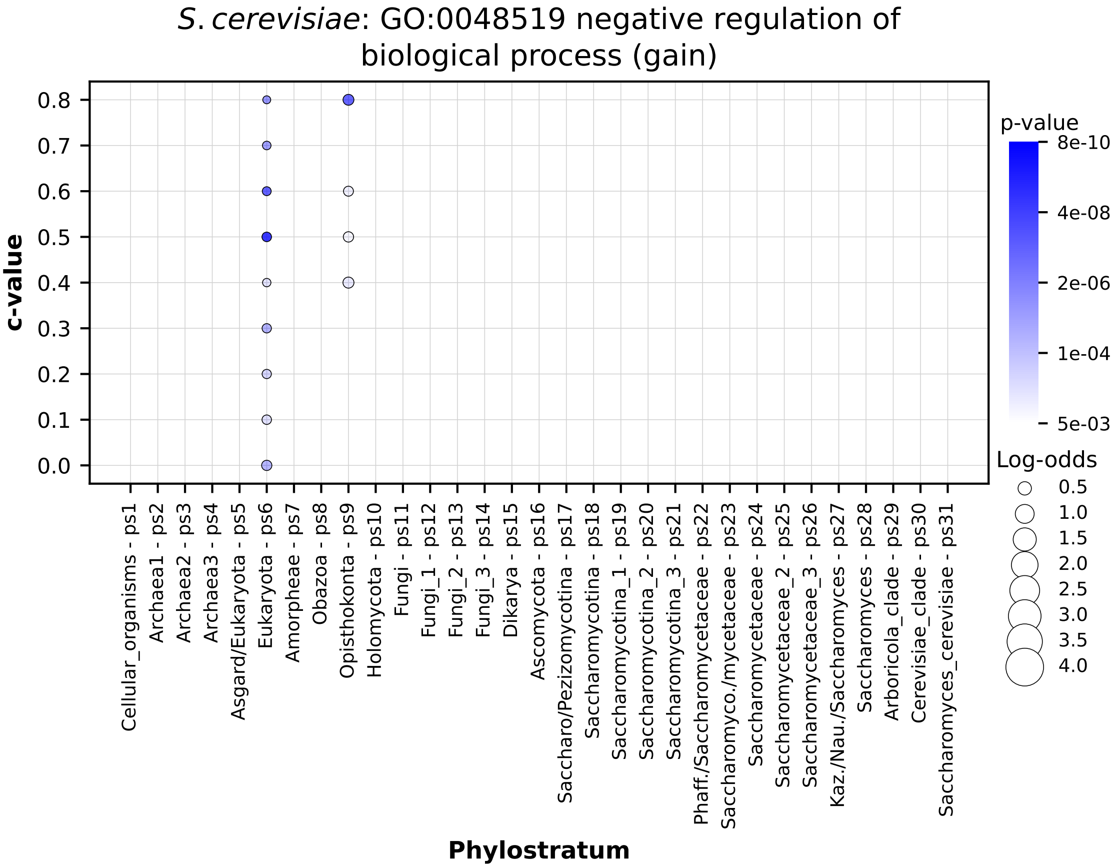

S. cerevisiae: GO:0048519 negative regulation of biological process (gain)
c-value
0.8
0.7
0.6
0.5
0.4
0.3
0.2
0.1
0.0
p-value
8e-10
4e-08
2e-06
1e-04
5e-03
Log-odds
0.5
1.0
1.5
2.0
2.5
3.0
3.5
4.0
Cellular_organisms - ps1
Archaea1 - ps2
Archaea2 - ps3
Archaea3 - ps4
Asgard/Eukaryota - ps5
Eukaryota - ps6
Amorpheae - ps7
Obazoa - ps8
Opisthokonta - ps9
Holomycota - ps10
Fungi - ps11
Fungi_1 - ps12
Fungi_2 - ps13
Fungi_3 - ps14
Dikarya - ps15
Ascomycota - ps16
Saccharo/Pezizomycotina - ps17
Saccharomycotina - ps18
Saccharomycotina_1 - ps19
Saccharomycotina_2 - ps20
Saccharomycotina_3 - ps21
Phaff./Saccharomycetaceae - ps22
Saccharomyco./mycetaceae - ps23
Saccharomycetaceae - ps24
Saccharomycetaceae_2 - ps25
Saccharomycetaceae_3 - ps26
Kaz./Nau./Saccharomyces - ps27
Saccharomyces - ps28
Arboricola_clade - ps29
Cerevisiae_clade - ps30
Saccharomyces_cerevisiae - ps31
Phylostratum

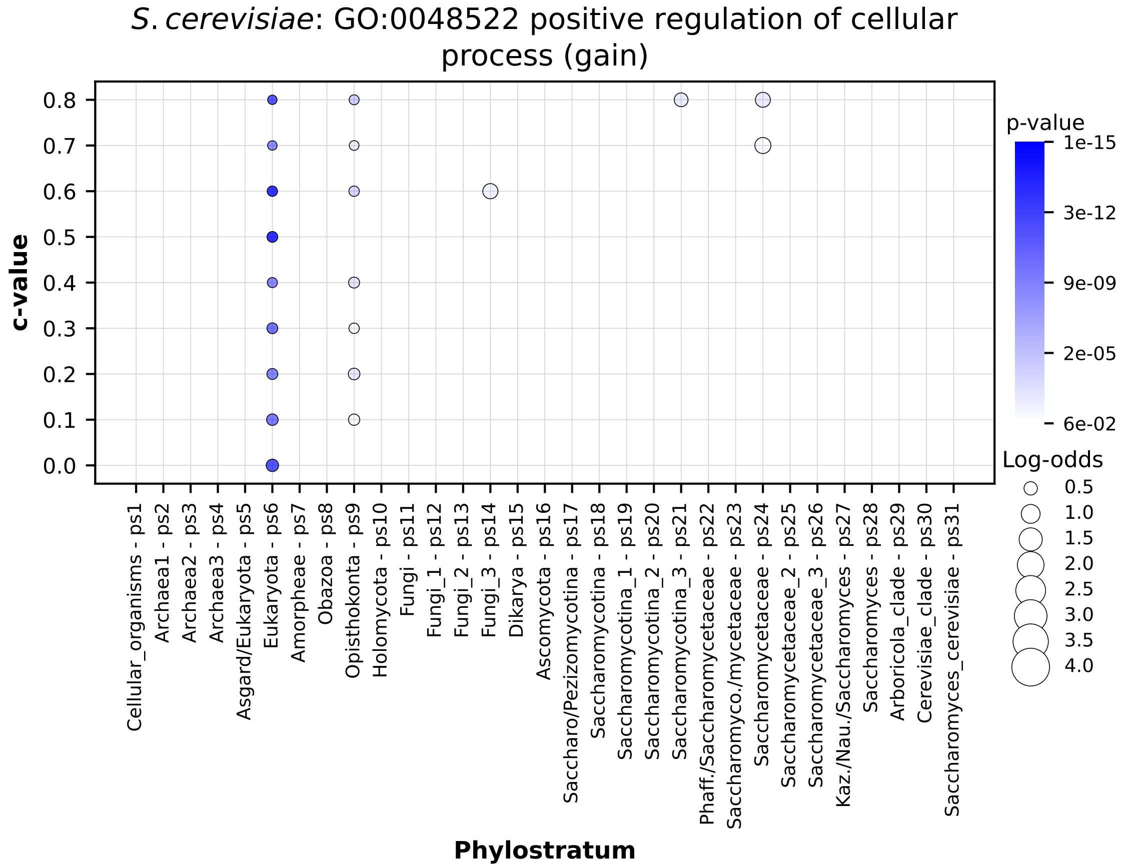

S. cerevisiae: GO:0048522 positive regulation of cellular process (gain)
c-value
0.8
0.7
0.6
0.5
0.4
0.3
0.2
0.1
0.0
Phylostratum
Cellular_organisms - ps1
Archaea1 - ps2
Archaea2 - ps3
Archaea3 - ps4
Asgard/Eukaryota - ps5
Eukaryota - ps6
Amorpheae - ps7
Obazoa - ps8
Opisthokonta - ps9
Holomycota - ps10
Fungi - ps11
Fungi_1 - ps12
Fungi_2 - ps13
Fungi_3 - ps14
Dikarya - ps15
Ascomycota - ps16
Saccharo/Pezizomycotina - ps17
Saccharomycotina - ps18
Saccharomycotina_1 - ps19
Saccharomycotina_2 - ps20
Saccharomycotina_3 - ps21
Phaff./Saccharomycetaceae - ps22
Saccharomyco./mycetaceae - ps23
Saccharomycetaceae - ps24
Saccharomycetaceae_2 - ps25
Saccharomycetaceae_3 - ps26
Kaz./Nau./Saccharomyces - ps27
Saccharomyces - ps28
Arboricola_clade - ps29
Cerevisiae_clade - ps30
Saccharomyces_cerevisiae - ps31
p-value
1e-15
3e-12
9e-09
2e-05
6e-02
Log-odds
0.5
1.0
1.5
2.0
2.5
3.0
3.5
4.0

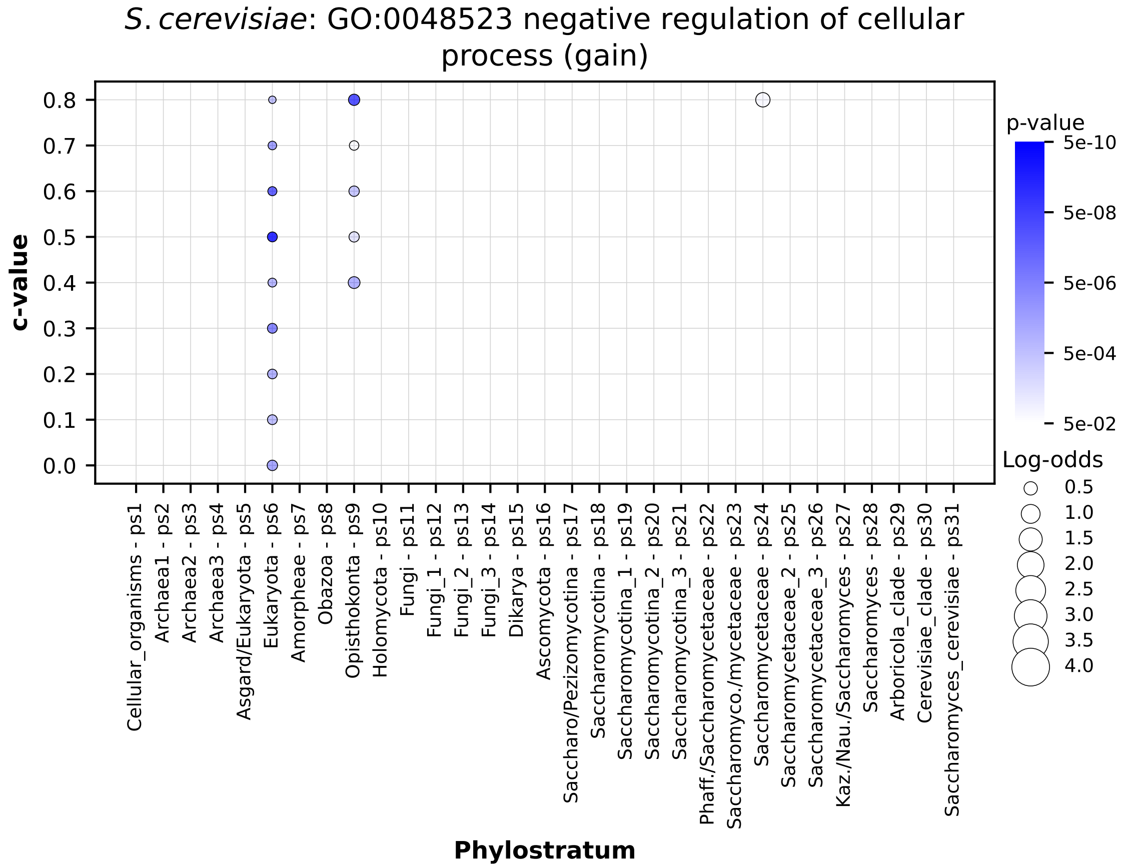

S. cerevisiae: GO:0048523 negative regulation of cellular process (gain)
c-value
0.0
0.1
0.2
0.3
0.4
0.5
0.6
0.7
0.8
Phylostratum
Cellular_organisms - ps1
Archaea1 - ps2
Archaea2 - ps3
Archaea3 - ps4
Asgard/Eukaryota - ps5
Eukaryota - ps6
Amorpheae - ps7
Obazoa - ps8
Opisthokonta - ps9
Holomycota - ps10
Fungi - ps11
Fungi_1 - ps12
Fungi_2 - ps13
Fungi_3 - ps14
Dikarya - ps15
Ascomycota - ps16
Saccharo/Pezizomycotina - ps17
Saccharomycotina - ps18
Saccharomycotina_1 - ps19
Saccharomycotina_2 - ps20
Saccharomycotina_3 - ps21
Phaff./Saccharomycetaceae - ps22
Saccharomyco./mycetaceae - ps23
Saccharomycetaceae - ps24
Saccharomycetaceae_2 - ps25
Saccharomycetaceae_3 - ps26
Kaz./Nau./Saccharomyces - ps27
Saccharomyces - ps28
Arboricola_clade - ps29
Cerevisiae_clade - ps30
Saccharomyces_cerevisiae - ps31
p-value
5e-10
5e-08
5e-06
5e-04
5e-02
Log-odds
0.5
1.0
1.5
2.0
2.5
3.0
3.5
4.0

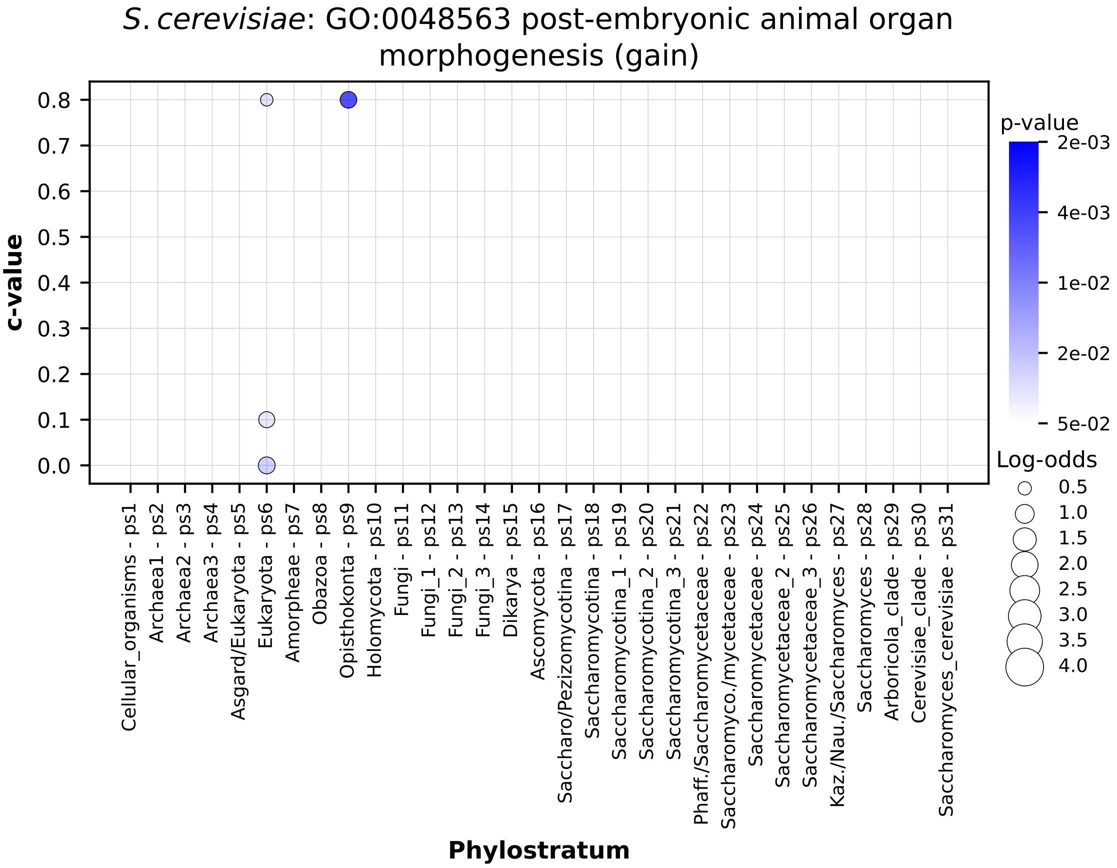

S. cerevisiae: GO:0048563 post-embryonic animal organ morphogenesis (gain)
c-value
0.8
0.7
0.6
0.5
0.4
0.3
0.2
0.1
0.0
p-value
2e-03
4e-03
1e-02
2e-02
5e-02
Log-odds
0.5
1.0
1.5
2.0
2.5
3.0
3.5
4.0
Cellular_organisms - ps1
Archaea1 - ps2
Archaea2 - ps3
Archaea3 - ps4
Asgard/Eukaryota - ps5
Eukaryota - ps6
Amorpheae - ps7
Obazoa - ps8
Opisthokonta - ps9
Holomycota - ps10
Fungi - ps11
Fungi_1 - ps12
Fungi_2 - ps13
Fungi_3 - ps14
Dikarya - ps15
Ascomycota - ps16
Saccharo/Pezizomycotina - ps17
Saccharomycotina - ps18
Saccharomycotina_1 - ps19
Saccharomycotina_2 - ps20
Saccharomycotina_3 - ps21
Phaff./Saccharomycetaceae - ps22
Saccharomyco./mycetaceae - ps23
Saccharomycetaceae - ps24
Saccharomycetaceae_2 - ps25
Saccharomycetaceae_3 - ps26
Kaz./Nau./Saccharomyces - ps27
Saccharomyces - ps28
Arboricola_clade - ps29
Cerevisiae_clade - ps30
Saccharomyces_cerevisiae - ps31
Phylostratum

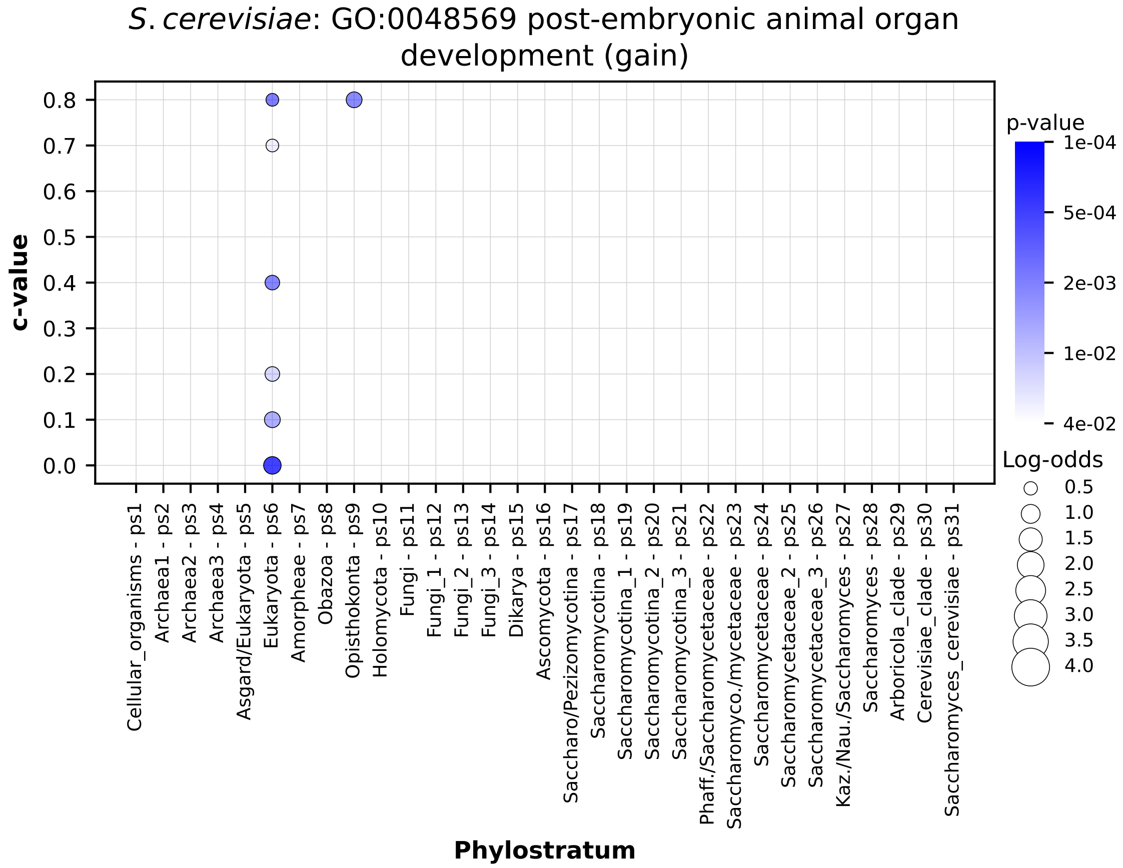

S. cerevisiae: GO:0048569 post-embryonic animal organ development (gain)
c-value
0.8
0.7
0.6
0.5
0.4
0.3
0.2
0.1
0.0
p-value
1e-04
5e-04
2e-03
1e-02
4e-02
Log-odds
0.5
1.0
1.5
2.0
2.5
3.0
3.5
4.0
Cellular_organisms - ps1
Archaea1 - ps2
Archaea2 - ps3
Archaea3 - ps4
Asgard/Eukaryota - ps5
Eukaryota - ps6
Amorpheae - ps7
Obazoa - ps8
Opisthokonta - ps9
Holomycota - ps10
Fungi - ps11
Fungi_1 - ps12
Fungi_2 - ps13
Fungi_3 - ps14
Dikarya - ps15
Ascomycota - ps16
Saccharo/Pezizomycotina - ps17
Saccharomycotina - ps18
Saccharomycotina_1 - ps19
Saccharomycotina_2 - ps20
Saccharomycotina_3 - ps21
Phaff./Saccharomycetaceae - ps22
Saccharomyco./mycetaceae - ps23
Saccharomycetaceae - ps24
Saccharomycetaceae_2 - ps25
Saccharomycetaceae_3 - ps26
Kaz./Nau./Saccharomyces - ps27
Saccharomyces - ps28
Arboricola_clade - ps29
Cerevisiae_clade - ps30
Saccharomyces_cerevisiae - ps31
Phylostratum

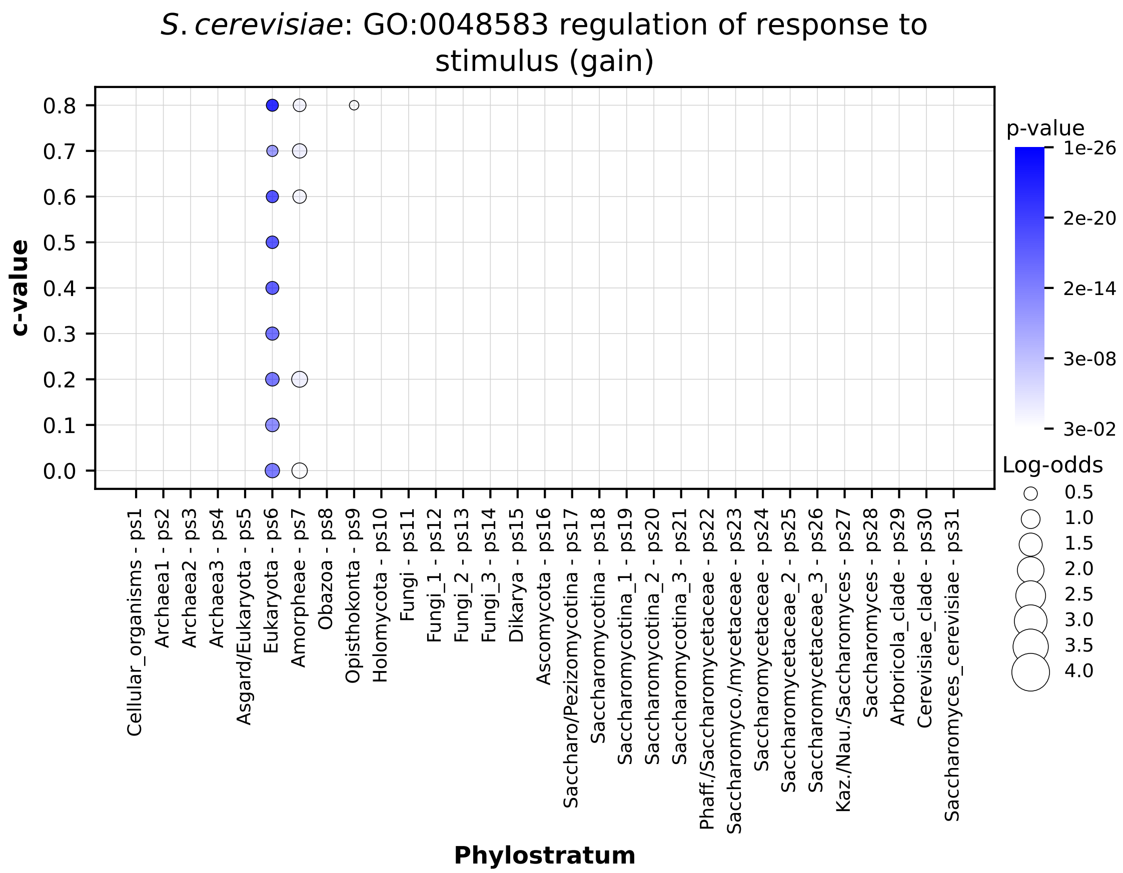

S. cerevisiae: GO:0048583 regulation of response to stimulus (gain)
c-value
0.8
0.7
0.6
0.5
0.4
0.3
0.2
0.1
0.0
Phylostratum
Cellular_organisms - ps1
Archaea1 - ps2
Archaea2 - ps3
Archaea3 - ps4
Asgard/Eukaryota - ps5
Eukaryota - ps6
Amorpheae - ps7
Obazoa - ps8
Opisthokonta - ps9
Holomycota - ps10
Fungi - ps11
Fungi_1 - ps12
Fungi_2 - ps13
Fungi_3 - ps14
Dikarya - ps15
Ascomycota - ps16
Saccharo/Pezizomycotina - ps17
Saccharomycotina - ps18
Saccharomycotina_1 - ps19
Saccharomycotina_2 - ps20
Saccharomycotina_3 - ps21
Phaff./Saccharomycetaceae - ps22
Saccharomyco./mycetaceae - ps23
Saccharomycetaceae - ps24
Saccharomycetaceae_2 - ps25
Saccharomycetaceae_3 - ps26
Kaz./Nau./Saccharomyces - ps27
Saccharomyces - ps28
Arboricola_clade - ps29
Cerevisiae_clade - ps30
Saccharomyces_cerevisiae - ps31
p-value
1e-26
2e-20
2e-14
3e-08
3e-02
Log-odds
0.5
1.0
1.5
2.0
2.5
3.0
3.5
4.0

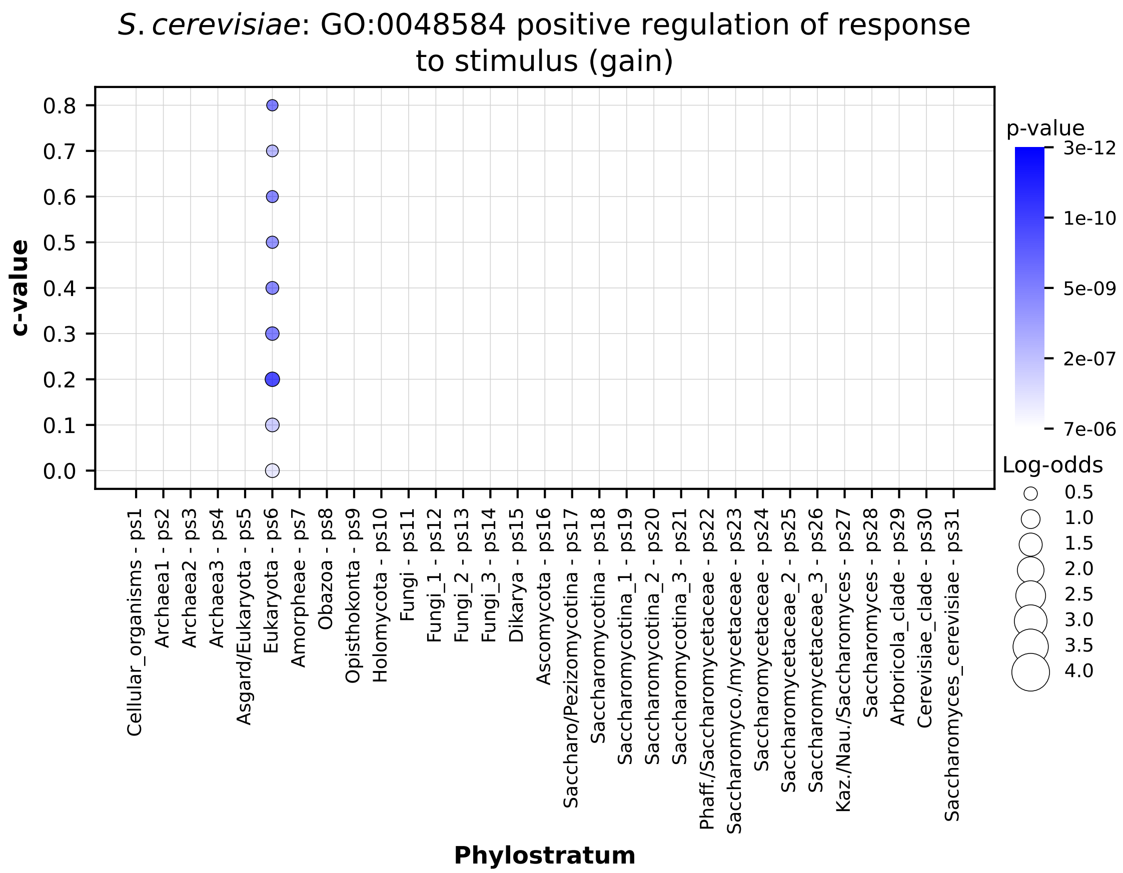
S. cerevisiae: GO:0048584 positive regulation of response to stimulus (gain)
c-value
Phylostratum
p-value
3e-12
1e-10
5e-09
2e-07
7e-06
Log-odds
0.5
1.0
1.5
2.0
2.5
3.0
3.5
4.0
Cellular_organisms - ps1
Archaea1 - ps2
Archaea2 - ps3
Archaea3 - ps4
Asgard/Eukaryota - ps5
Eukaryota - ps6
Amorpheae - ps7
Obazoa - ps8
Opisthokonta - ps9
Holomycota - ps10
Fungi - ps11
Fungi_1 - ps12
Fungi_2 - ps13
Fungi_3 - ps14
Dikarya - ps15
Ascomycota - ps16
Saccharo/Pezizomycotina - ps17
Saccharomycotina - ps18
Saccharomycotina_1 - ps19
Saccharomycotina_2 - ps20
Saccharomycotina_3 - ps21
Phaff./Saccharomycetaceae - ps22
Saccharomyco./mycetaceae - ps23
Saccharomycetaceae - ps24
Saccharomycetaceae_2 - ps25
Saccharomycetaceae_3 - ps26
Kaz./Nau./Saccharomyces - ps27
Saccharomyces - ps28
Arboricola_clade - ps29
Cerevisiae_clade - ps30
Saccharomyces_cerevisiae - ps31

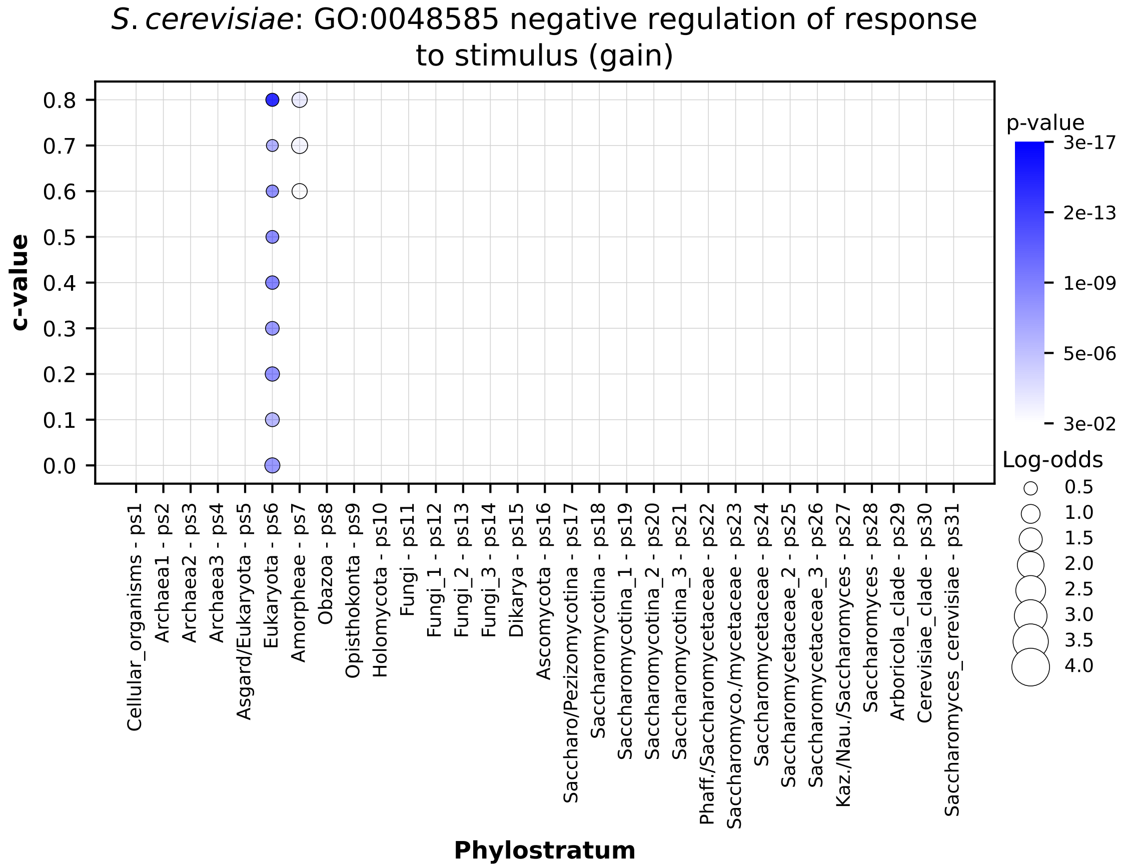

S. cerevisiae: GO:0048585 negative regulation of response to stimulus (gain)
c-value
0.8
0.7
0.6
0.5
0.4
0.3
0.2
0.1
0.0
p-value
3e-17
2e-13
1e-09
5e-06
3e-02
Log-odds
0.5
1.0
1.5
2.0
2.5
3.0
3.5
4.0
Cellular_organisms - ps1
Archaea1 - ps2
Archaea2 - ps3
Archaea3 - ps4
Asgard/Eukaryota - ps5
Eukaryota - ps6
Amorpheae - ps7
Obazoa - ps8
Opisthokonta - ps9
Holomycota - ps10
Fungi - ps11
Fungi_1 - ps12
Fungi_2 - ps13
Fungi_3 - ps14
Dikarya - ps15
Ascomycota - ps16
Saccharo/Pezizomycotina - ps17
Saccharomycotina - ps18
Saccharomycotina_1 - ps19
Saccharomycotina_2 - ps20
Saccharomycotina_3 - ps21
Phaff./Saccharomycetaceae - ps22
Saccharomyco./mycetaceae - ps23
Saccharomycetaceae - ps24
Saccharomycetaceae_2 - ps25
Saccharomycetaceae_3 - ps26
Kaz./Nau./Saccharomyces - ps27
Saccharomyces - ps28
Arboricola_clade - ps29
Cerevisiae_clade - ps30
Saccharomyces_cerevisiae - ps31
Phylostratum

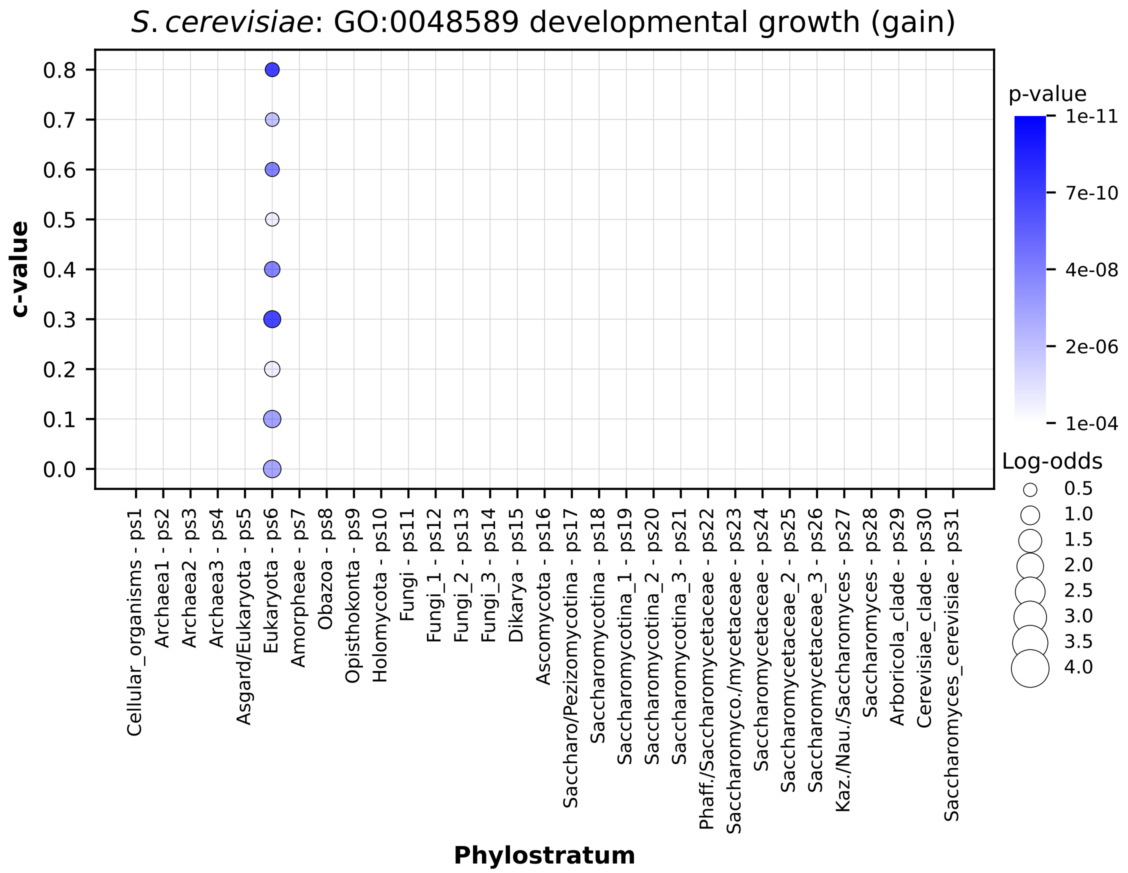
S. cerevisiae: GO:0048589 developmental growth (gain)
c-value
0.8
0.7
0.6
0.5
0.4
0.3
0.2
0.1
0.0
p-value
1e-11
7e-10
4e-08
2e-06
1e-04
Log-odds
0.5
1.0
1.5
2.0
2.5
3.0
3.5
4.0
Cellular_organisms - ps1
Archaea1 - ps2
Archaea2 - ps3
Archaea3 - ps4
Asgard/Eukaryota - ps5
Eukaryota - ps6
Amorpheae - ps7
Obazoa - ps8
Opisthokonta - ps9
Holomycota - ps10
Fungi - ps11
Fungi_1 - ps12
Fungi_2 - ps13
Fungi_3 - ps14
Dikarya - ps15
Ascomycota - ps16
Saccharo/Pezizomycotina - ps17
Saccharomycotina - ps18
Saccharomycotina_1 - ps19
Saccharomycotina_2 - ps20
Saccharomycotina_3 - ps21
Phaff./Saccharomycetaceae - ps22
Saccharomyco./mycetaceae - ps23
Saccharomycetaceae - ps24
Saccharomycetaceae_2 - ps25
Saccharomycetaceae_3 - ps26
Kaz./Nau./Saccharomyces - ps27
Saccharomyces - ps28
Arboricola_clade - ps29
Cerevisiae_clade - ps30
Saccharomyces_cerevisiae - ps31
Phylostratum

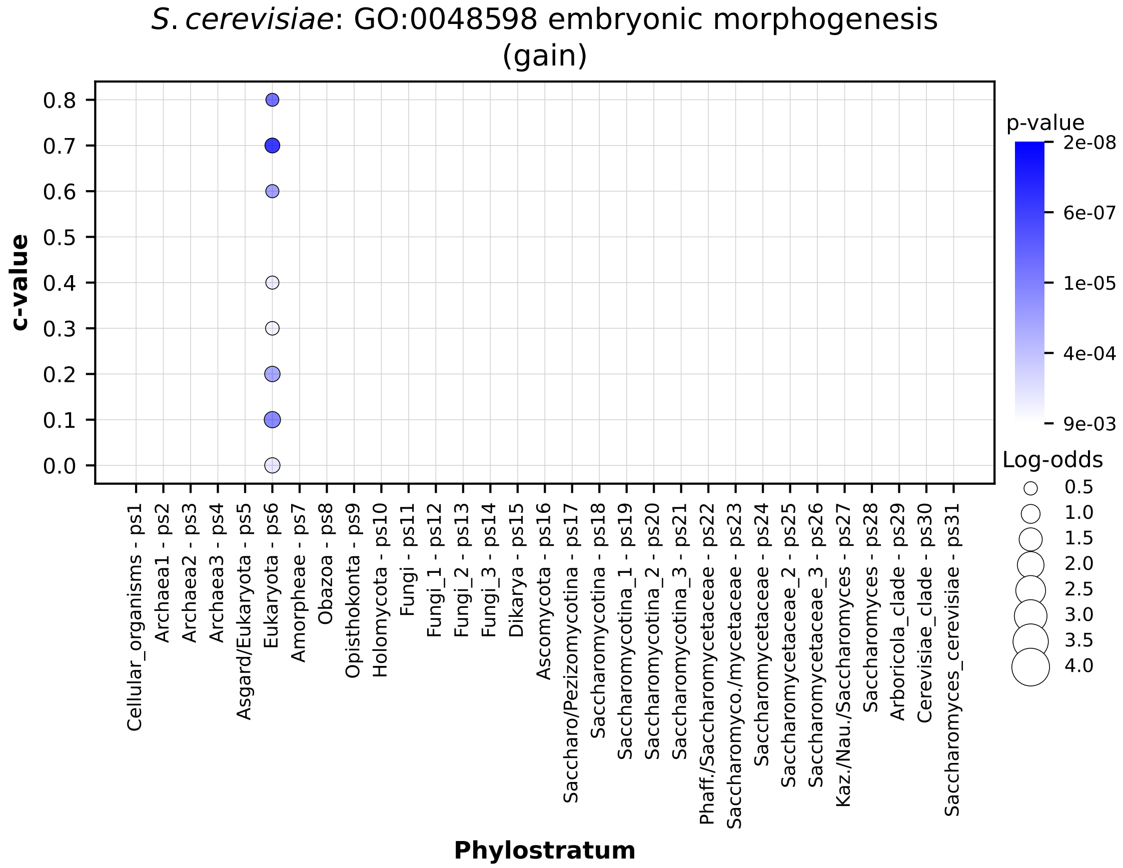

S. cerevisiae: GO:0048598 embryonic morphogenesis
(gain)
c-value
0.8
0.7
0.6
0.5
0.4
0.3
0.2
0.1
0.0
p-value
2e-08
6e-07
1e-05
4e-04
9e-03
Log-odds
0.5
1.0
1.5
2.0
2.5
3.0
3.5
4.0
Cellular_organisms - ps1
Archaea1 - ps2
Archaea2 - ps3
Archaea3 - ps4
Asgard/Eukaryota - ps5
Eukaryota - ps6
Amorpheae - ps7
Obazoa - ps8
Opisthokonta - ps9
Holomycota - ps10
Fungi - ps11
Fungi_1 - ps12
Fungi_2 - ps13
Fungi_3 - ps14
Dikarya - ps15
Ascomycota - ps16
Saccharo/Pezizomycotina - ps17
Saccharomycotina - ps18
Saccharomycotina_1 - ps19
Saccharomycotina_2 - ps20
Saccharomycotina_3 - ps21
Phaff./Saccharomycetaceae - ps22
Saccharomyco./mycetaceae - ps23
Saccharomycetaceae - ps24
Saccharomycetaceae_2 - ps25
Saccharomycetaceae_3 - ps26
Kaz./Nau./Saccharomyces - ps27
Saccharomyces - ps28
Arboricola_clade - ps29
Cerevisiae_clade - ps30
Saccharomyces_cerevisiae - ps31
Phylostratum

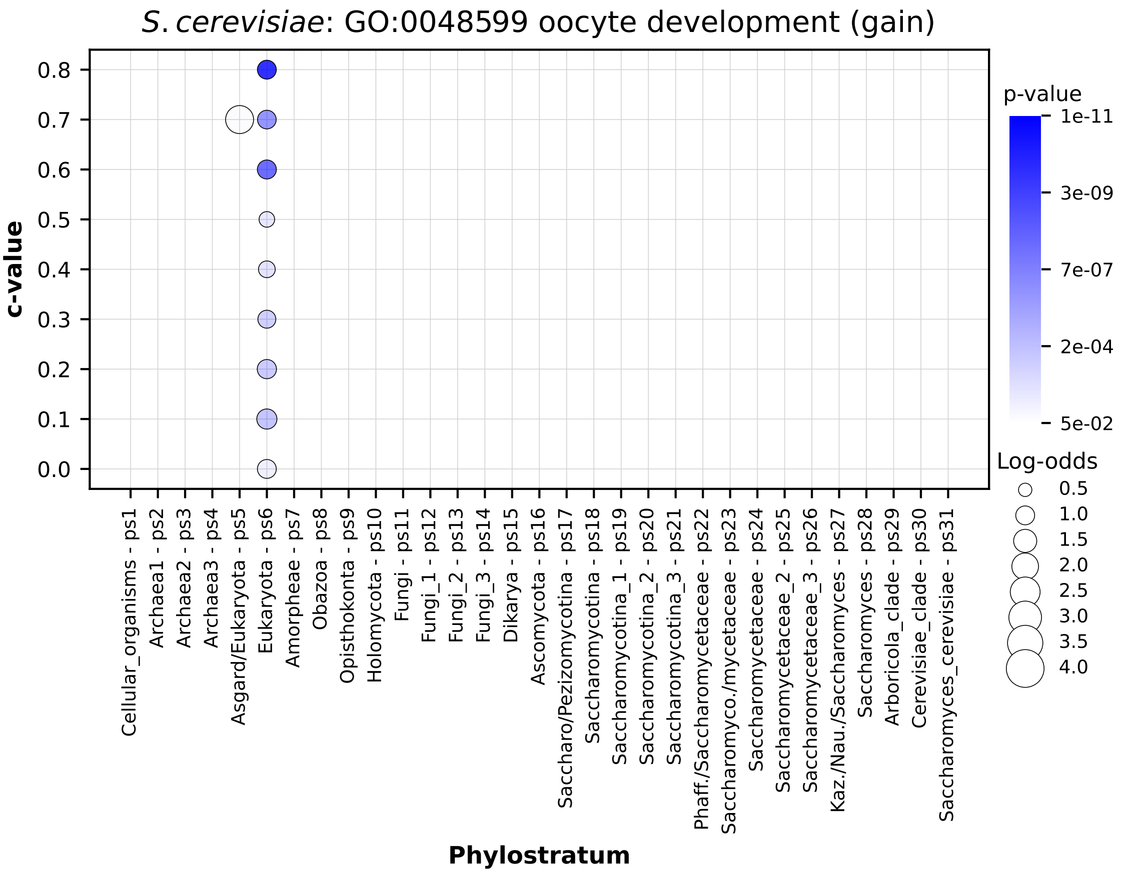
S. cerevisiae: GO:0048599 oocyte development (gain)
c-value
0.8
0.7
0.6
0.5
0.4
0.3
0.2
0.1
0.0
Phylostratum
Cellular_organisms - ps1
Archaea1 - ps2
Archaea2 - ps3
Archaea3 - ps4
Asgard/Eukaryota - ps5
Eukaryota - ps6
Amorpheae - ps7
Obazoa - ps8
Opisthokonta - ps9
Holomycota - ps10
Fungi - ps11
Fungi_1 - ps12
Fungi_2 - ps13
Fungi_3 - ps14
Dikarya - ps15
Ascomycota - ps16
Saccharo/Pezizomycotina - ps17
Saccharomycotina - ps18
Saccharomycotina_1 - ps19
Saccharomycotina_2 - ps20
Saccharomycotina_3 - ps21
Phaff./Saccharomycetaceae - ps22
Saccharomyco./mycetaceae - ps23
Saccharomycetaceae - ps24
Saccharomycetaceae_2 - ps25
Saccharomycetaceae_3 - ps26
Kaz./Nau./Saccharomyces - ps27
Saccharomyces - ps28
Arboricola_clade - ps29
Cerevisiae_clade - ps30
Saccharomyces_cerevisiae - ps31
p-value
1e-11
3e-09
7e-07
2e-04
5e-02
Log-odds
0.5
1.0
1.5
2.0
2.5
3.0
3.5
4.0

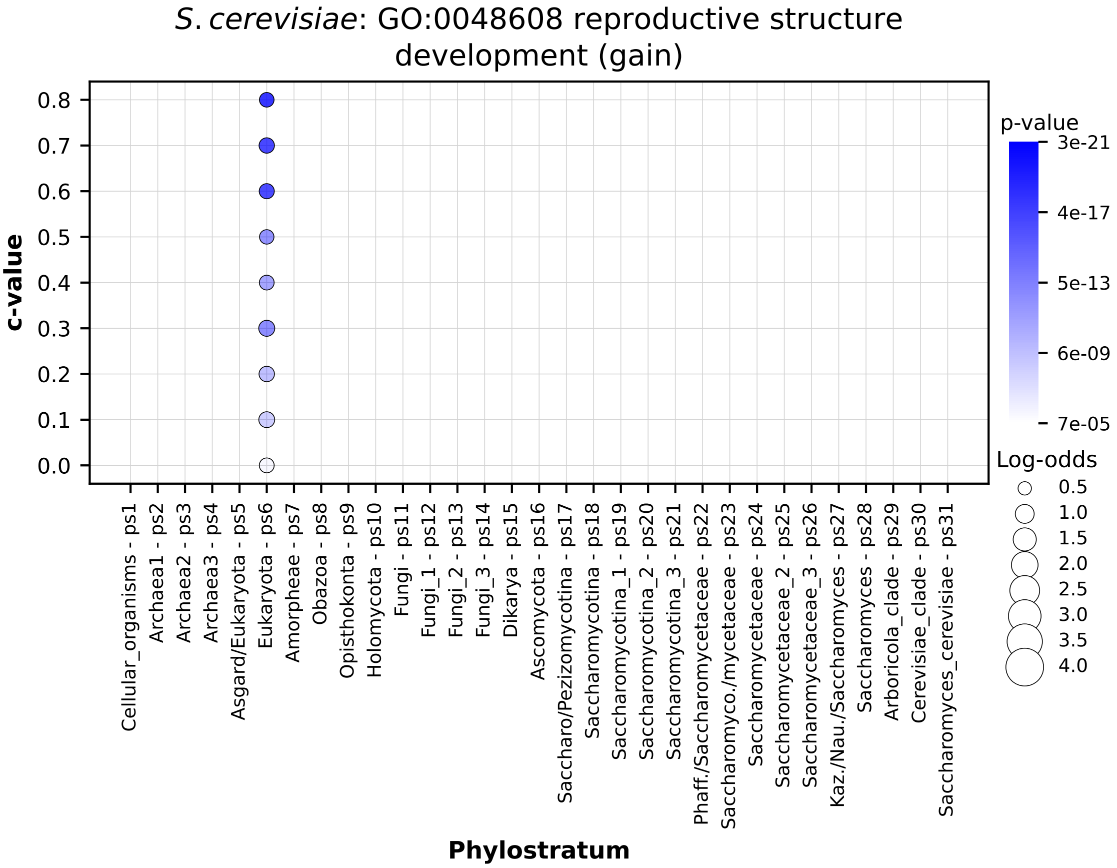

S. cerevisiae: GO:0048608 reproductive structure development (gain)
c-value
0.8
0.7
0.6
0.5
0.4
0.3
0.2
0.1
0.0
p-value
3e-21
4e-17
5e-13
6e-09
7e-05
Log-odds
0.5
1.0
1.5
2.0
2.5
3.0
3.5
4.0
Cellular_organisms - ps1
Archaea1 - ps2
Archaea2 - ps3
Archaea3 - ps4
Asgard/Eukaryota - ps5
Eukaryota - ps6
Amorpheae - ps7
Obazoa - ps8
Opisthokonta - ps9
Holomycota - ps10
Fungi - ps11
Fungi_1 - ps12
Fungi_2 - ps13
Fungi_3 - ps14
Dikarya - ps15
Ascomycota - ps16
Saccharo/Pezizomycotina - ps17
Saccharomycotina - ps18
Saccharomycotina_1 - ps19
Saccharomycotina_2 - ps20
Saccharomycotina_3 - ps21
Phaff./Saccharomycetaceae - ps22
Saccharomyco./mycetaceae - ps23
Saccharomycetaceae - ps24
Saccharomycetaceae_2 - ps25
Saccharomycetaceae_3 - ps26
Kaz./Nau./Saccharomyces - ps27
Saccharomyces - ps28
Arboricola_clade - ps29
Cerevisiae_clade - ps30
Saccharomyces_cerevisiae - ps31
Phylostratum

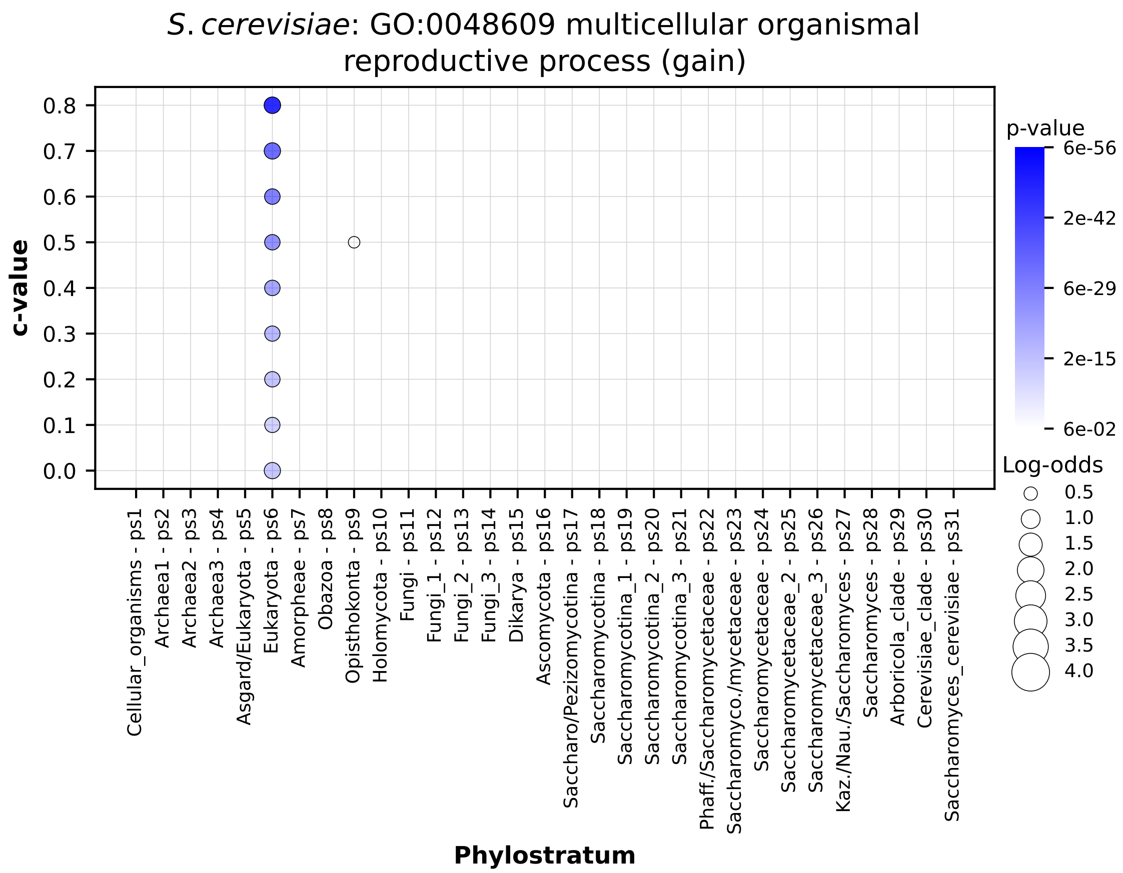

S. cerevisiae: GO:0048609 multicellular organismal reproductive process (gain)
c-value
0.8
0.7
0.6
0.5
0.4
0.3
0.2
0.1
0.0
p-value
6e-56
2e-42
6e-29
2e-15
6e-02
Log-odds
0.5
1.0
1.5
2.0
2.5
3.0
3.5
4.0
Cellular_organisms - ps1
Archaea1 - ps2
Archaea2 - ps3
Archaea3 - ps4
Asgard/Eukaryota - ps5
Eukaryota - ps6
Amorpheae - ps7
Obazoa - ps8
Opisthokonta - ps9
Holomycota - ps10
Fungi - ps11
Fungi_1 - ps12
Fungi_2 - ps13
Fungi_3 - ps14
Dikarya - ps15
Ascomycota - ps16
Saccharo/Pezizomycotina - ps17
Saccharomycotina - ps18
Saccharomycotina_1 - ps19
Saccharomycotina_2 - ps20
Saccharomycotina_3 - ps21
Phaff./Saccharomycetaceae - ps22
Saccharomyco./mycetaceae - ps23
Saccharomycetaceae - ps24
Saccharomycetaceae_2 - ps25
Saccharomycetaceae_3 - ps26
Kaz./Nau./Saccharomyces - ps27
Saccharomyces - ps28
Arboricola_clade - ps29
Cerevisiae_clade - ps30
Saccharomyces_cerevisiae - ps31
Phylostratum

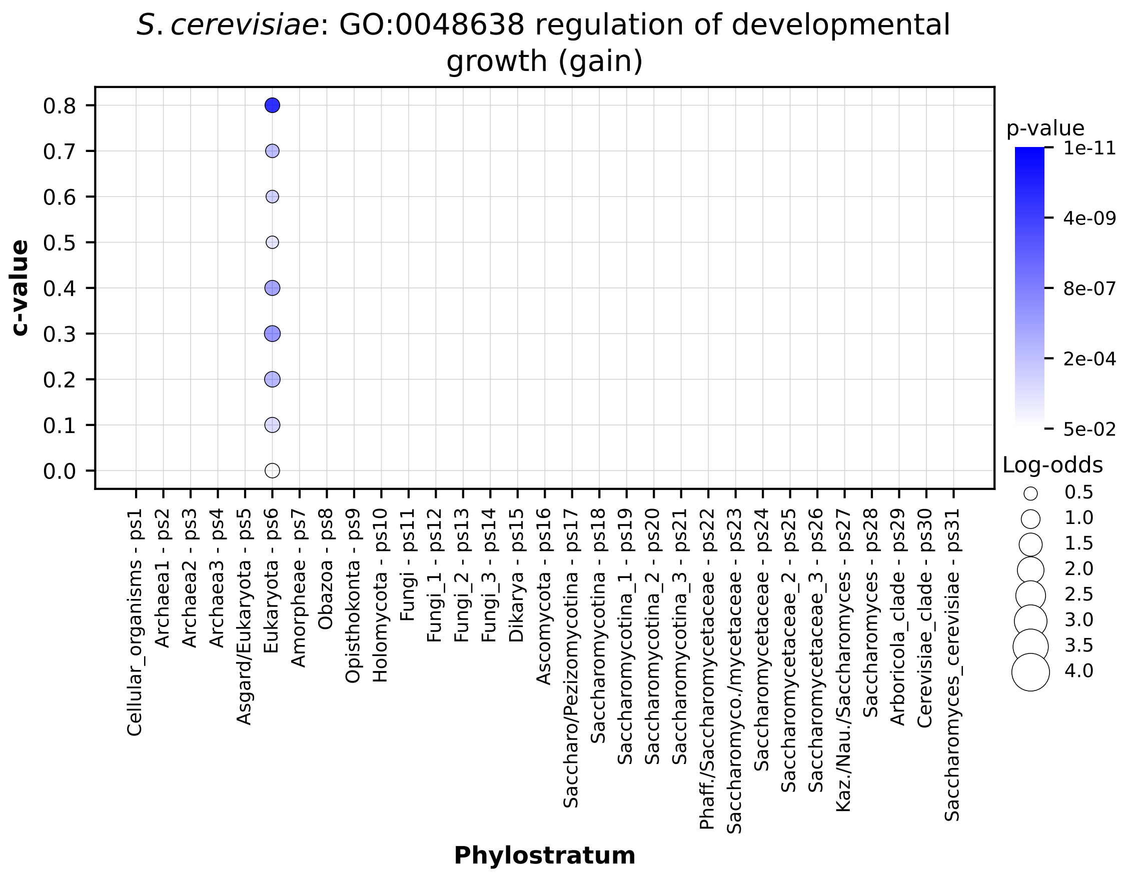
S. cerevisiae: GO:0048638 regulation of developmental growth (gain)
c-value
0.0
0.1
0.2
0.3
0.4
0.5
0.6
0.7
0.8
p-value
1e-11
4e-09
8e-07
2e-04
5e-02
Log-odds
0.5
1.0
1.5
2.0
2.5
3.0
3.5
4.0
Cellular_organisms - ps1
Archaea1 - ps2
Archaea2 - ps3
Archaea3 - ps4
Asgard/Eukaryota - ps5
Eukaryota - ps6
Amorpheae - ps7
Obazoa - ps8
Opisthokonta - ps9
Holomycota - ps10
Fungi - ps11
Fungi_1 - ps12
Fungi_2 - ps13
Fungi_3 - ps14
Dikarya - ps15
Ascomycota - ps16
Saccharo/Pezizomycotina - ps17
Saccharomycotina - ps18
Saccharomycotina_1 - ps19
Saccharomycotina_2 - ps20
Saccharomycotina_3 - ps21
Phaff./Saccharomycetaceae - ps22
Saccharomyco./mycetaceae - ps23
Saccharomycetaceae - ps24
Saccharomycetaceae_2 - ps25
Saccharomycetaceae_3 - ps26
Kaz./Nau./Saccharomyces - ps27
Saccharomyces - ps28
Arboricola_clade - ps29
Cerevisiae_clade - ps30
Saccharomyces_cerevisiae - ps31
Phylostratum

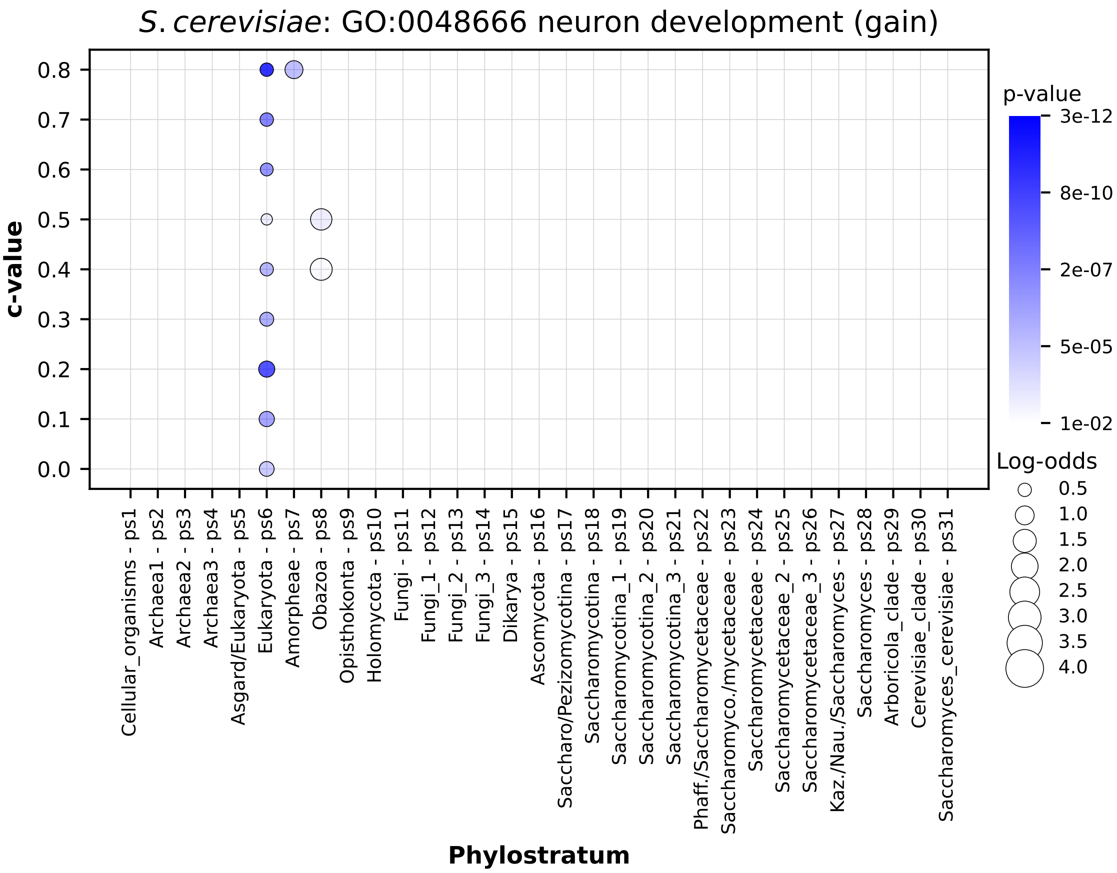

S. cerevisiae: GO:0048666 neuron development (gain)
c-value
0.8
0.7
0.6
0.5
0.4
0.3
0.2
0.1
0.0
p-value
3e-12
8e-10
2e-07
5e-05
1e-02
Log-odds
0.5
1.0
1.5
2.0
2.5
3.0
3.5
4.0
Cellular_organisms - ps1
Archaea1 - ps2
Archaea2 - ps3
Archaea3 - ps4
Asgard/Eukaryota - ps5
Eukaryota - ps6
Amorpheae - ps7
Obazoa - ps8
Opisthokonta - ps9
Holomycota - ps10
Fungi - ps11
Fungi_1 - ps12
Fungi_2 - ps13
Fungi_3 - ps14
Dikarya - ps15
Ascomycota - ps16
Saccharo/Pezizomycotina - ps17
Saccharomycotina - ps18
Saccharomycotina_1 - ps19
Saccharomycotina_2 - ps20
Saccharomycotina_3 - ps21
Phaff./Saccharomycetaceae - ps22
Saccharomyco./mycetaceae - ps23
Saccharomycetaceae - ps24
Saccharomycetaceae_2 - ps25
Saccharomycetaceae_3 - ps26
Kaz./Nau./Saccharomyces - ps27
Saccharomyces - ps28
Arboricola_clade - ps29
Cerevisiae_clade - ps30
Saccharomyces_cerevisiae - ps31
Phylostratum

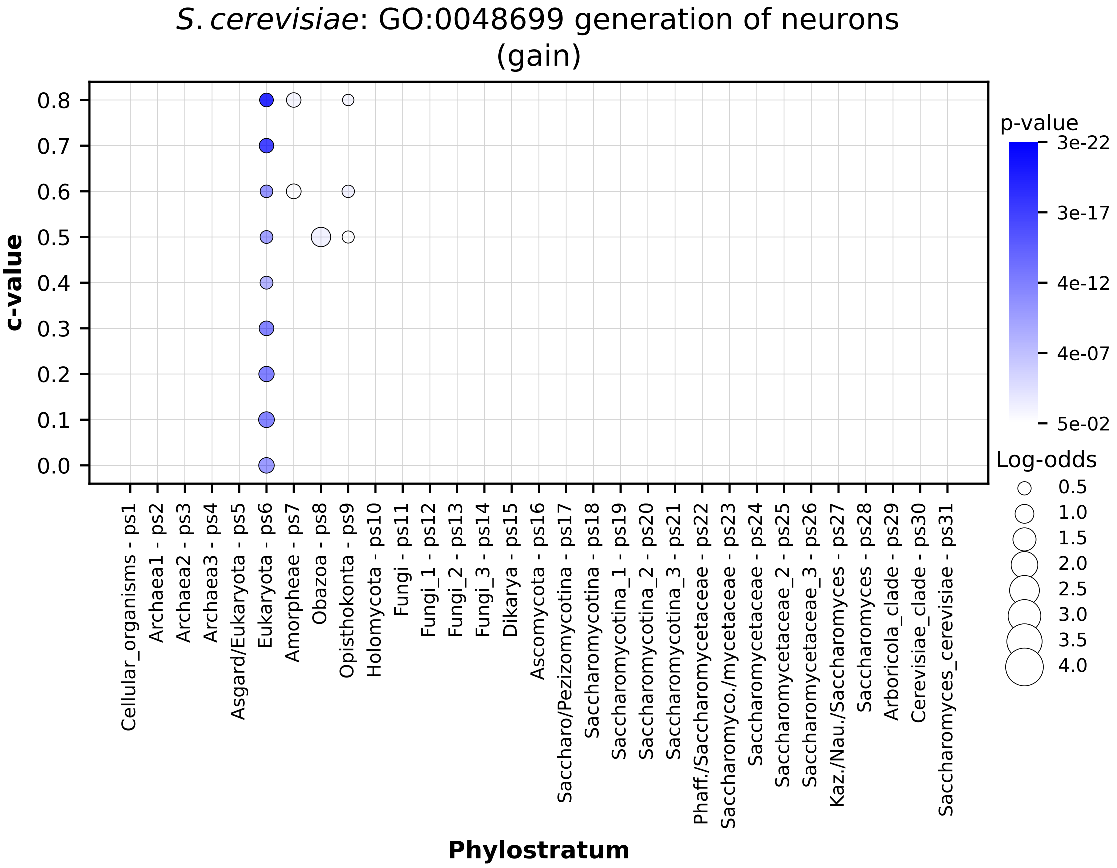
S. cerevisiae: GO:0048699 generation of neurons
(gain)
c-value
0.8
0.7
0.6
0.5
0.4
0.3
0.2
0.1
0.0
p-value
3e-22
3e-17
4e-12
4e-07
5e-02
Log-odds
0.5
1.0
1.5
2.0
2.5
3.0
3.5
4.0
Cellular_organisms - ps1
Archaea1 - ps2
Archaea2 - ps3
Archaea3 - ps4
Asgard/Eukaryota - ps5
Eukaryota - ps6
Amorpheae - ps7
Obazoa - ps8
Opisthokonta - ps9
Holomycota - ps10
Fungi - ps11
Fungi_1 - ps12
Fungi_2 - ps13
Fungi_3 - ps14
Dikarya - ps15
Ascomycota - ps16
Saccharo/Pezizomycotina - ps17
Saccharomycotina - ps18
Saccharomycotina_1 - ps19
Saccharomycotina_2 - ps20
Saccharomycotina_3 - ps21
Phaff./Saccharomycetaceae - ps22
Saccharomyco./mycetaceae - ps23
Saccharomycetaceae - ps24
Saccharomycetaceae_2 - ps25
Saccharomycetaceae_3 - ps26
Kaz./Nau./Saccharomyces - ps27
Saccharomyces - ps28
Arboricola_clade - ps29
Cerevisiae_clade - ps30
Saccharomyces_cerevisiae - ps31
Phylostratum

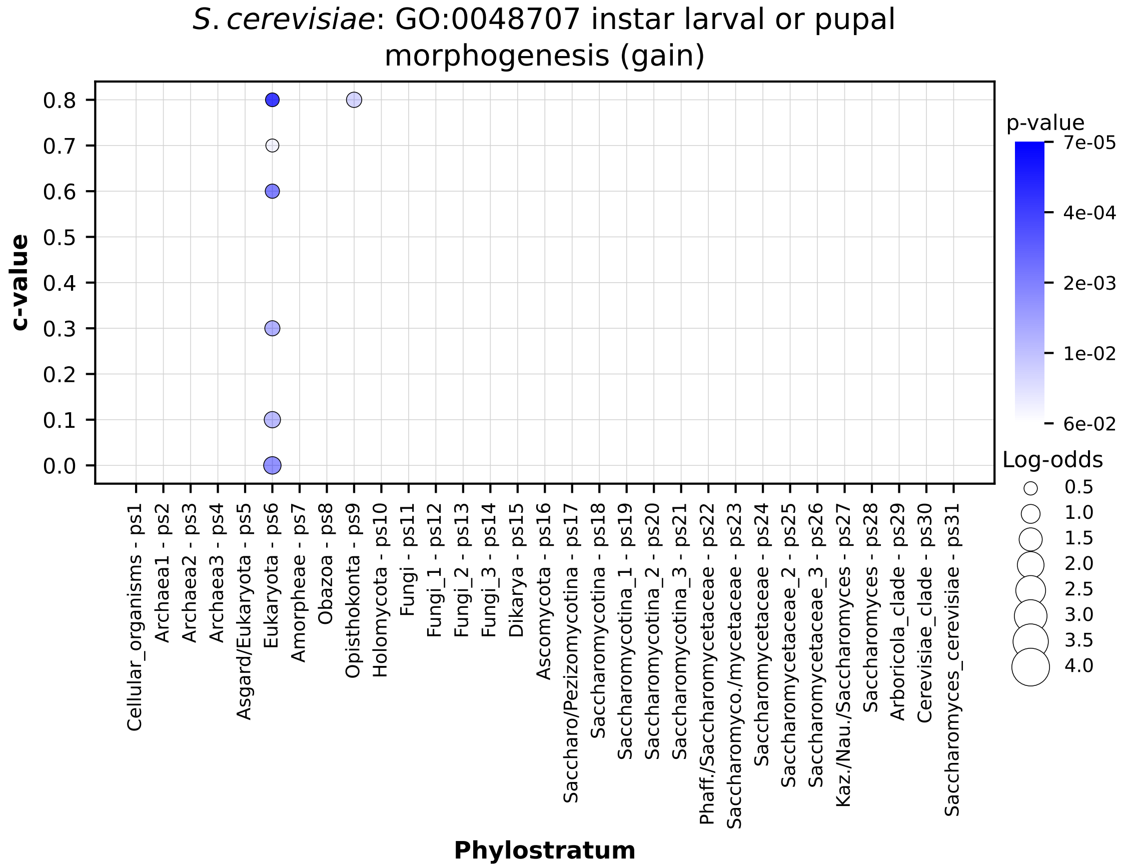

S. cerevisiae: GO:0048707 instar larval or pupal morphogenesis (gain)
c-value
0.8
0.7
0.6
0.5
0.4
0.3
0.2
0.1
0.0
p-value
7e-05
4e-04
2e-03
1e-02
6e-02
Log-odds
0.5
1.0
1.5
2.0
2.5
3.0
3.5
4.0
Cellular_organisms - ps1
Archaea1 - ps2
Archaea2 - ps3
Archaea3 - ps4
Asgard/Eukaryota - ps5
Eukaryota - ps6
Amorpheae - ps7
Obazoa - ps8
Opisthokonta - ps9
Holomycota - ps10
Fungi - ps11
Fungi_1 - ps12
Fungi_2 - ps13
Fungi_3 - ps14
Dikarya - ps15
Ascomycota - ps16
Saccharo/Pezizomycotina - ps17
Saccharomycotina - ps18
Saccharomycotina_1 - ps19
Saccharomycotina_2 - ps20
Saccharomycotina_3 - ps21
Phaff./Saccharomycetaceae - ps22
Saccharomyco./mycetaceae - ps23
Saccharomycetaceae - ps24
Saccharomycetaceae_2 - ps25
Saccharomycetaceae_3 - ps26
Kaz./Nau./Saccharomyces - ps27
Saccharomyces - ps28
Arboricola_clade - ps29
Cerevisiae_clade - ps30
Saccharomyces_cerevisiae - ps31
Phylostratum

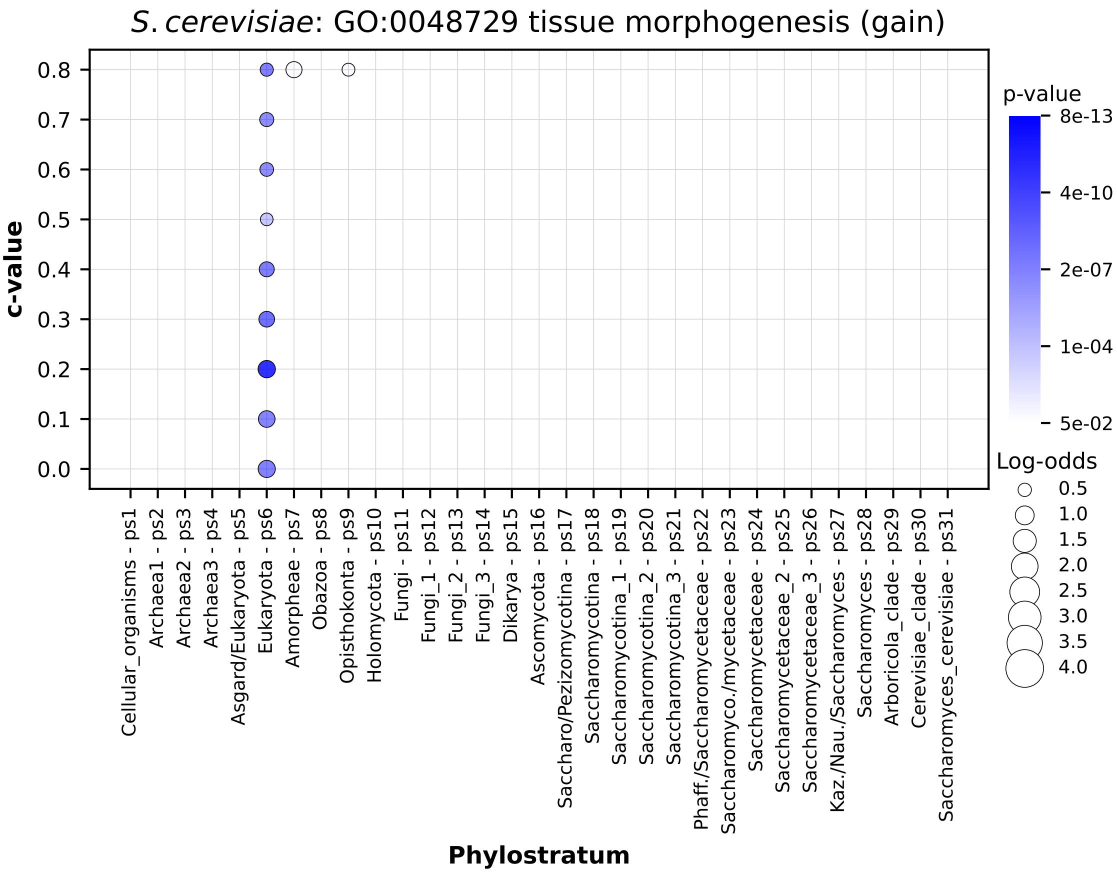

S. cerevisiae: GO:0048729 tissue morphogenesis (gain)
c-value
0.0
0.1
0.2
0.3
0.4
0.5
0.6
0.7
0.8
Phylostratum
Cellular_organisms - ps1
Archaea1 - ps2
Archaea2 - ps3
Archaea3 - ps4
Asgard/Eukaryota - ps5
Eukaryota - ps6
Amorpheae - ps7
Obazoa - ps8
Opisthokonta - ps9
Holomycota - ps10
Fungi - ps11
Fungi_1 - ps12
Fungi_2 - ps13
Fungi_3 - ps14
Dikarya - ps15
Ascomycota - ps16
Saccharo/Pezizomycotina - ps17
Saccharomycotina - ps18
Saccharomycotina_1 - ps19
Saccharomycotina_2 - ps20
Saccharomycotina_3 - ps21
Phaff./Saccharomycetaceae - ps22
Saccharomyco./mycetaceae - ps23
Saccharomycetaceae - ps24
Saccharomycetaceae_2 - ps25
Saccharomycetaceae_3 - ps26
Kaz./Nau./Saccharomyces - ps27
Saccharomyces - ps28
Arboricola_clade - ps29
Cerevisiae_clade - ps30
Saccharomyces_cerevisiae - ps31
p-value
8e-13
4e-10
2e-07
1e-04
5e-02
Log-odds
0.5
1.0
1.5
2.0
2.5
3.0
3.5
4.0

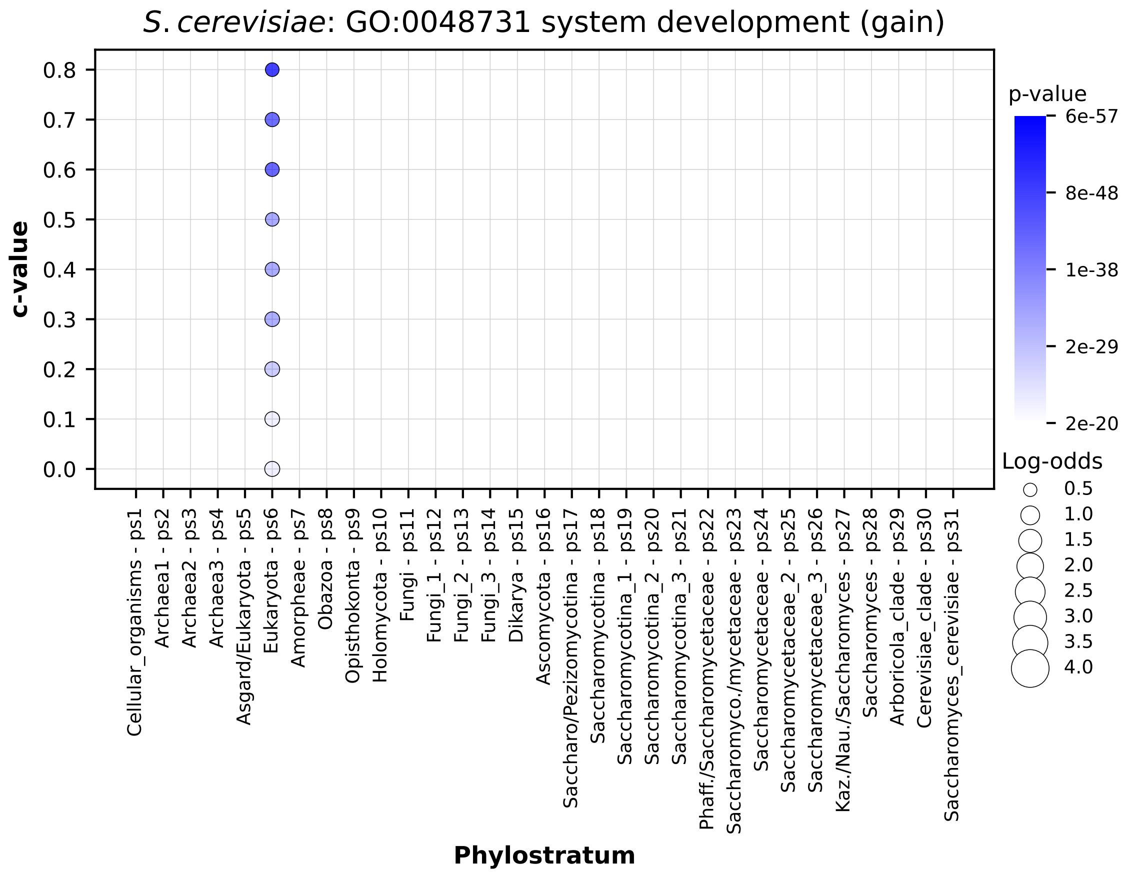

S. cerevisiae: GO:0048731 system development (gain)
c-value
0.8
0.7
0.6
0.5
0.4
0.3
0.2
0.1
0.0
p-value
6e-57
8e-48
1e-38
2e-29
2e-20
Log-odds
0.5
1.0
1.5
2.0
2.5
3.0
3.5
4.0
Cellular_organisms - ps1
Archaea1 - ps2
Archaea2 - ps3
Archaea3 - ps4
Asgard/Eukaryota - ps5
Eukaryota - ps6
Amorpheae - ps7
Obazoa - ps8
Opisthokonta - ps9
Holomycota - ps10
Fungi - ps11
Fungi_1 - ps12
Fungi_2 - ps13
Fungi_3 - ps14
Dikarya - ps15
Ascomycota - ps16
Saccharo/Pezizomycotina - ps17
Saccharomycotina - ps18
Saccharomycotina_1 - ps19
Saccharomycotina_2 - ps20
Saccharomycotina_3 - ps21
Phaff./Saccharomycetaceae - ps22
Saccharomyco./mycetaceae - ps23
Saccharomycetaceae - ps24
Saccharomycetaceae_2 - ps25
Saccharomycetaceae_3 - ps26
Kaz./Nau./Saccharomyces - ps27
Saccharomyces - ps28
Arboricola_clade - ps29
Cerevisiae_clade - ps30
Saccharomyces_cerevisiae - ps31
Phylostratum

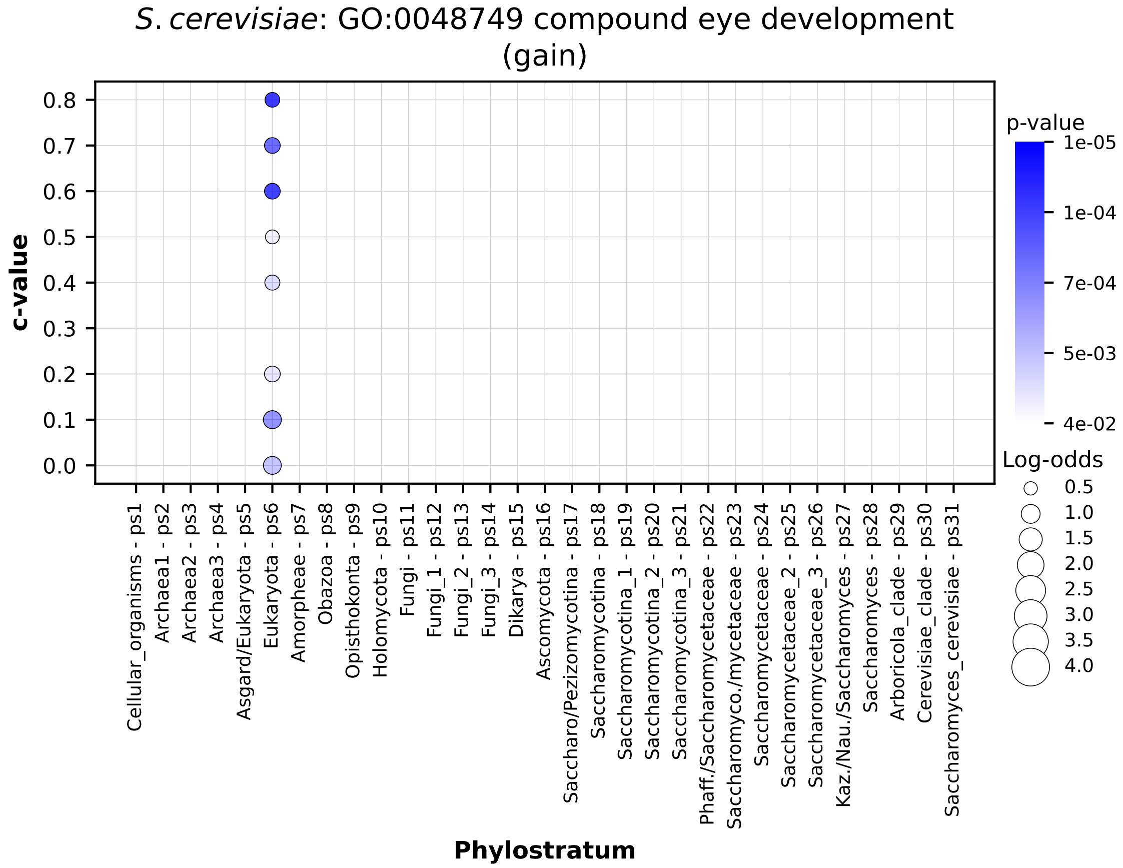

S. cerevisiae: GO:0048749 compound eye development (gain)
c-value
0.8
0.7
0.6
0.5
0.4
0.3
0.2
0.1
0.0
p-value
1e-05
1e-04
7e-04
5e-03
4e-02
Log-odds
0.5
1.0
1.5
2.0
2.5
3.0
3.5
4.0
Cellular_organisms - ps1
Archaea1 - ps2
Archaea2 - ps3
Archaea3 - ps4
Asgard/Eukaryota - ps5
Eukaryota - ps6
Amorpheae - ps7
Obazoa - ps8
Opisthokonta - ps9
Holomycota - ps10
Fungi - ps11
Fungi_1 - ps12
Fungi_2 - ps13
Fungi_3 - ps14
Dikarya - ps15
Ascomycota - ps16
Saccharo/Pezizomycotina - ps17
Saccharomycotina - ps18
Saccharomycotina_1 - ps19
Saccharomycotina_2 - ps20
Saccharomycotina_3 - ps21
Phaff./Saccharomycetaceae - ps22
Saccharomyco./mycetaceae - ps23
Saccharomycetaceae - ps24
Saccharomycetaceae_2 - ps25
Saccharomycetaceae_3 - ps26
Kaz./Nau./Saccharomyces - ps27
Saccharomyces - ps28
Arboricola_clade - ps29
Cerevisiae_clade - ps30
Saccharomyces_cerevisiae - ps31
Phylostratum

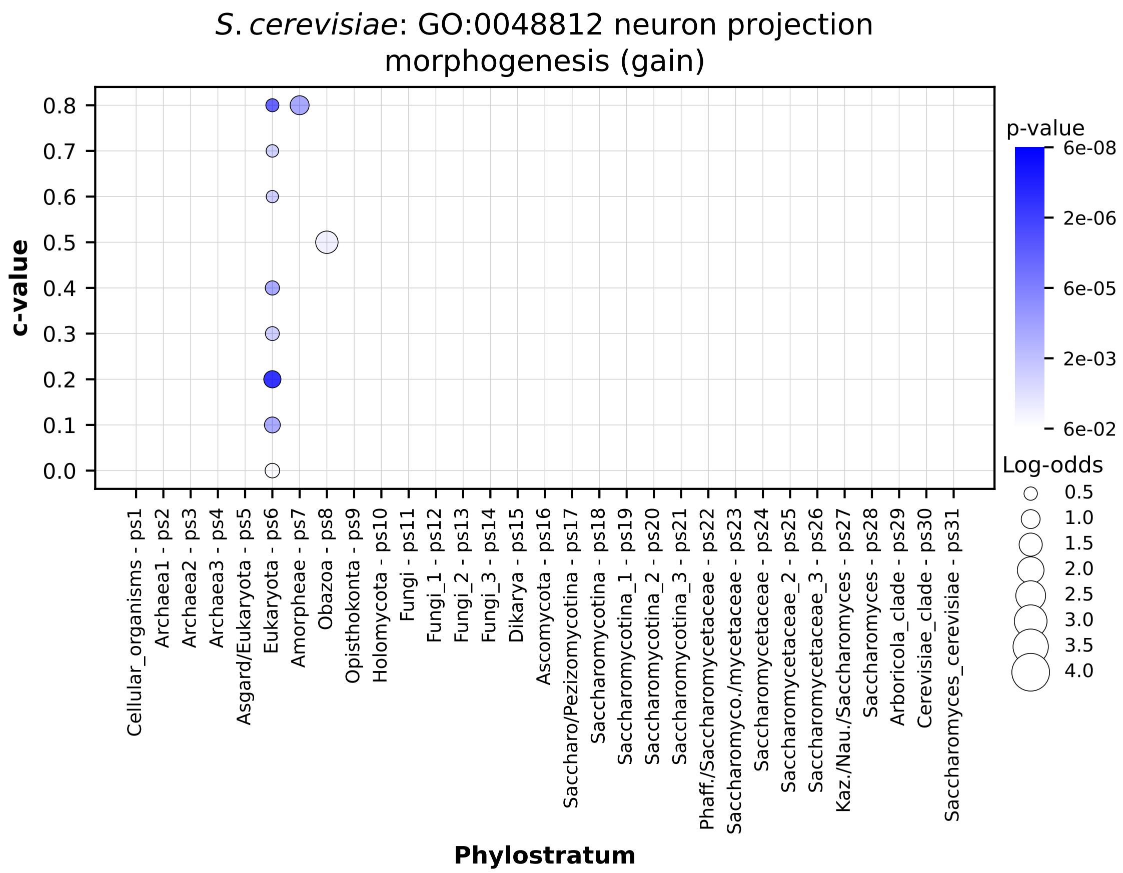

S. cerevisiae: GO:0048812 neuron projection morphogenesis (gain)
c-value
0.0
0.1
0.2
0.3
0.4
0.5
0.6
0.7
0.8
p-value
6e-08
2e-06
6e-05
2e-03
6e-02
Log-odds
0.5
1.0
1.5
2.0
2.5
3.0
3.5
4.0
Cellular_organisms - ps1
Archaea1 - ps2
Archaea2 - ps3
Archaea3 - ps4
Asgard/Eukaryota - ps5
Eukaryota - ps6
Amorpheae - ps7
Obazoa - ps8
Opisthokonta - ps9
Holomycota - ps10
Fungi - ps11
Fungi_1 - ps12
Fungi_2 - ps13
Fungi_3 - ps14
Dikarya - ps15
Ascomycota - ps16
Saccharo/Pezizomycotina - ps17
Saccharomycotina - ps18
Saccharomycotina_1 - ps19
Saccharomycotina_2 - ps20
Saccharomycotina_3 - ps21
Phaff./Saccharomycetaceae - ps22
Saccharomyco./mycetaceae - ps23
Saccharomycetaceae - ps24
Saccharomycetaceae_2 - ps25
Saccharomycetaceae_3 - ps26
Kaz./Nau./Saccharomyces - ps27
Saccharomyces - ps28
Arboricola_clade - ps29
Cerevisiae_clade - ps30
Saccharomyces_cerevisiae - ps31
Phylostratum

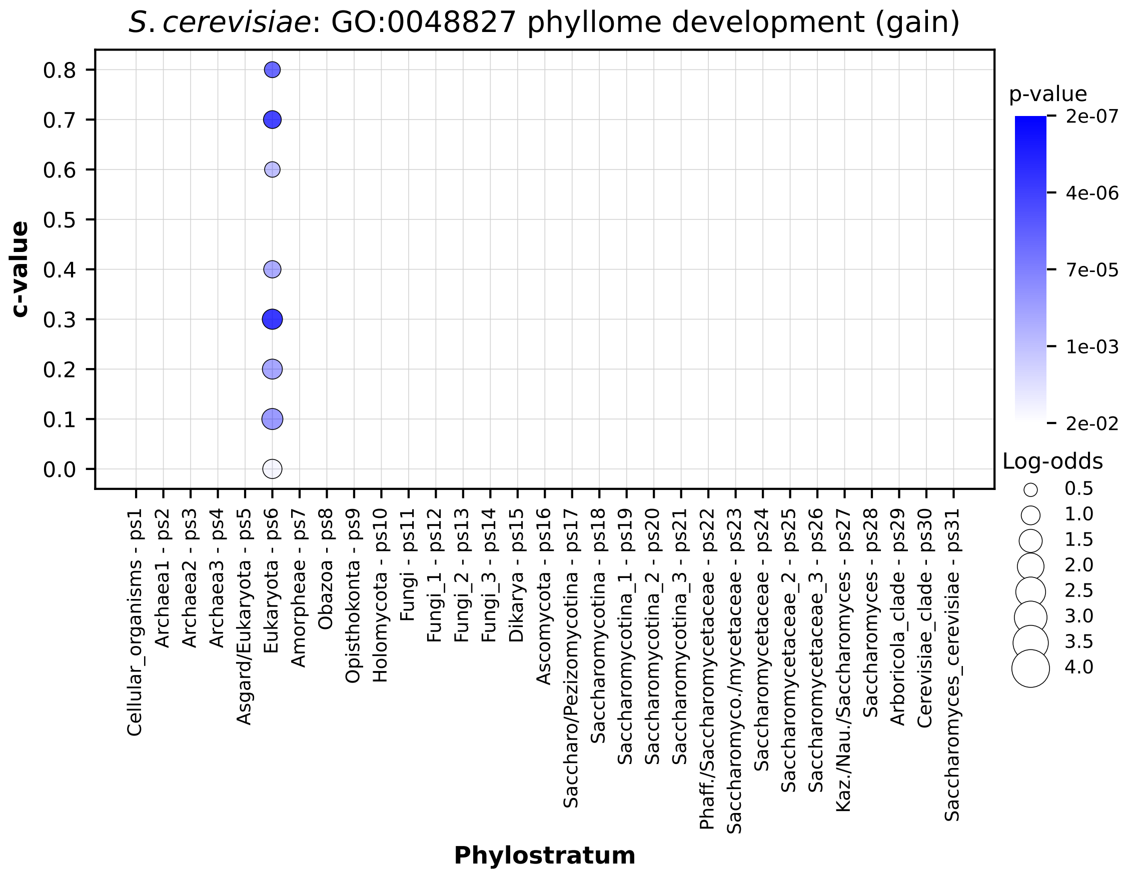

S. cerevisiae: GO:0048827 phyllome development (gain)
c-value
0.8
0.7
0.6
0.5
0.4
0.3
0.2
0.1
0.0
p-value
2e-07
4e-06
7e-05
1e-03
2e-02
Log-odds
0.5
1.0
1.5
2.0
2.5
3.0
3.5
4.0
Cellular_organisms - ps1
Archaea1 - ps2
Archaea2 - ps3
Archaea3 - ps4
Asgard/Eukaryota - ps5
Eukaryota - ps6
Amorpheae - ps7
Obazoa - ps8
Opisthokonta - ps9
Holomycota - ps10
Fungi - ps11
Fungi_1 - ps12
Fungi_2 - ps13
Fungi_3 - ps14
Dikarya - ps15
Ascomycota - ps16
Saccharo/Pezizomycotina - ps17
Saccharomycotina - ps18
Saccharomycotina_1 - ps19
Saccharomycotina_2 - ps20
Saccharomycotina_3 - ps21
Phaff./Saccharomycetaceae - ps22
Saccharomyco./mycetaceae - ps23
Saccharomycetaceae - ps24
Saccharomycetaceae_2 - ps25
Saccharomycetaceae_3 - ps26
Kaz./Nau./Saccharomyces - ps27
Saccharomyces - ps28
Arboricola_clade - ps29
Cerevisiae_clade - ps30
Saccharomyces_cerevisiae - ps31
Phylostratum

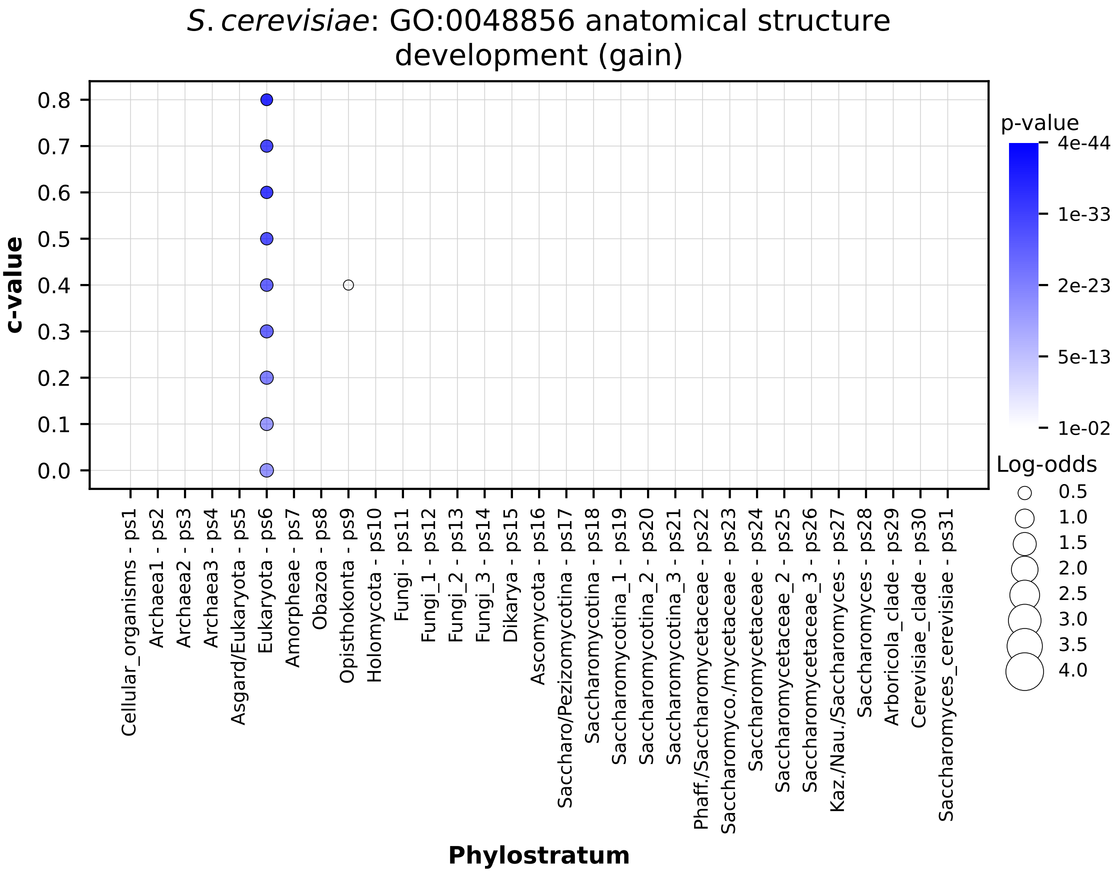

S. cerevisiae: GO:0048856 anatomical structure development (gain)
c-value
0.8
0.7
0.6
0.5
0.4
0.3
0.2
0.1
0.0
p-value
4e-44
1e-33
2e-23
5e-13
1e-02
Log-odds
0.5
1.0
1.5
2.0
2.5
3.0
3.5
4.0
Cellular_organisms - ps1
Archaea1 - ps2
Archaea2 - ps3
Archaea3 - ps4
Asgard/Eukaryota - ps5
Eukaryota - ps6
Amorpheae - ps7
Obazoa - ps8
Opisthokonta - ps9
Holomycota - ps10
Fungi - ps11
Fungi_1 - ps12
Fungi_2 - ps13
Fungi_3 - ps14
Dikarya - ps15
Ascomycota - ps16
Saccharo/Pezizomycotina - ps17
Saccharomycotina - ps18
Saccharomycotina_1 - ps19
Saccharomycotina_2 - ps20
Saccharomycotina_3 - ps21
Phaff./Saccharomycetaceae - ps22
Saccharomyco./mycetaceae - ps23
Saccharomycetaceae - ps24
Saccharomycetaceae_2 - ps25
Saccharomycetaceae_3 - ps26
Kaz./Nau./Saccharomyces - ps27
Saccharomyces - ps28
Arboricola_clade - ps29
Cerevisiae_clade - ps30
Saccharomyces_cerevisiae - ps31
Phylostratum

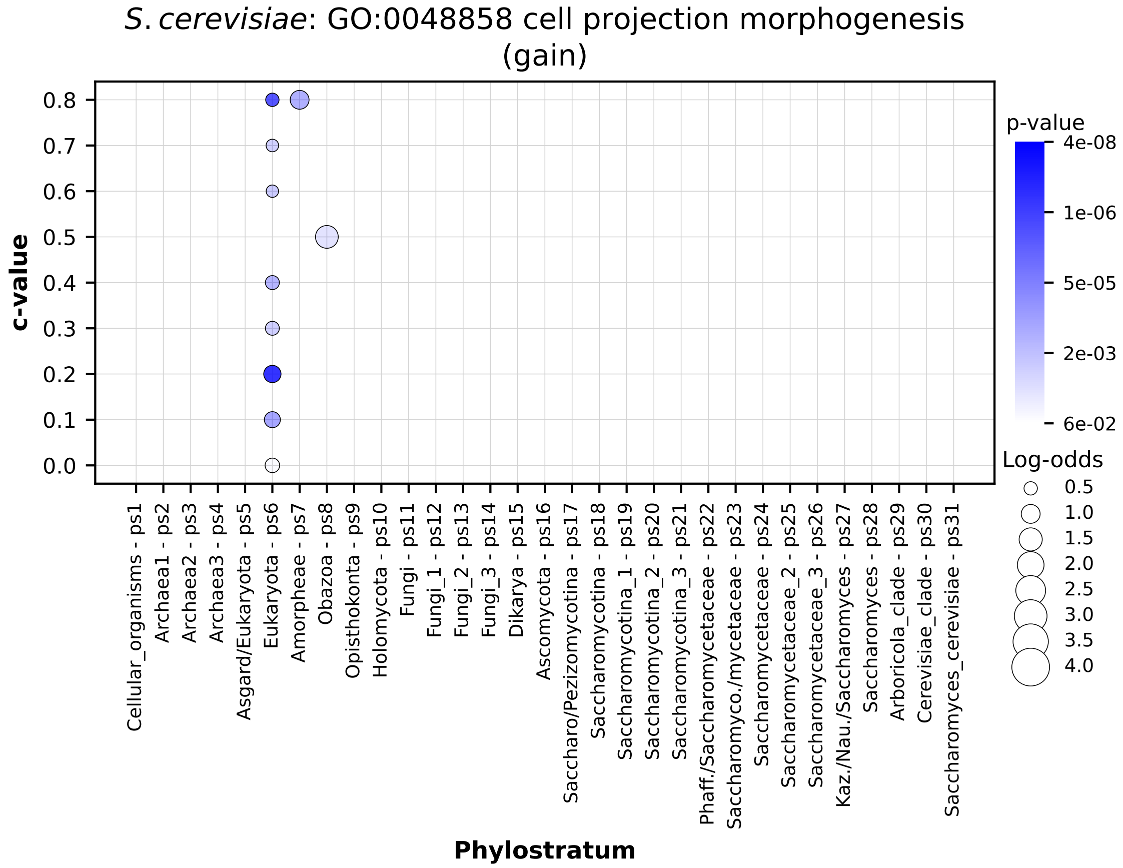

S. cerevisiae: GO:0048858 cell projection morphogenesis
(gain)
c-value
0.8
0.7
0.6
0.5
0.4
0.3
0.2
0.1
0.0
p-value
4e-08
1e-06
5e-05
2e-03
6e-02
Log-odds
0.5
1.0
1.5
2.0
2.5
3.0
3.5
4.0
Cellular_organisms - ps1
Archaea1 - ps2
Archaea2 - ps3
Archaea3 - ps4
Asgard/Eukaryota - ps5
Eukaryota - ps6
Amorpheae - ps7
Obazoa - ps8
Opisthokonta - ps9
Holomycota - ps10
Fungi - ps11
Fungi_1 - ps12
Fungi_2 - ps13
Fungi_3 - ps14
Dikarya - ps15
Ascomycota - ps16
Saccharo/Pezizomycotina - ps17
Saccharomycotina - ps18
Saccharomycotina_1 - ps19
Saccharomycotina_2 - ps20
Saccharomycotina_3 - ps21
Phaff./Saccharomycetaceae - ps22
Saccharomyco./mycetaceae - ps23
Saccharomycetaceae - ps24
Saccharomycetaceae_2 - ps25
Saccharomycetaceae_3 - ps26
Kaz./Nau./Saccharomyces - ps27
Saccharomyces - ps28
Arboricola_clade - ps29
Cerevisiae_clade - ps30
Saccharomyces_cerevisiae - ps31
Phylostratum

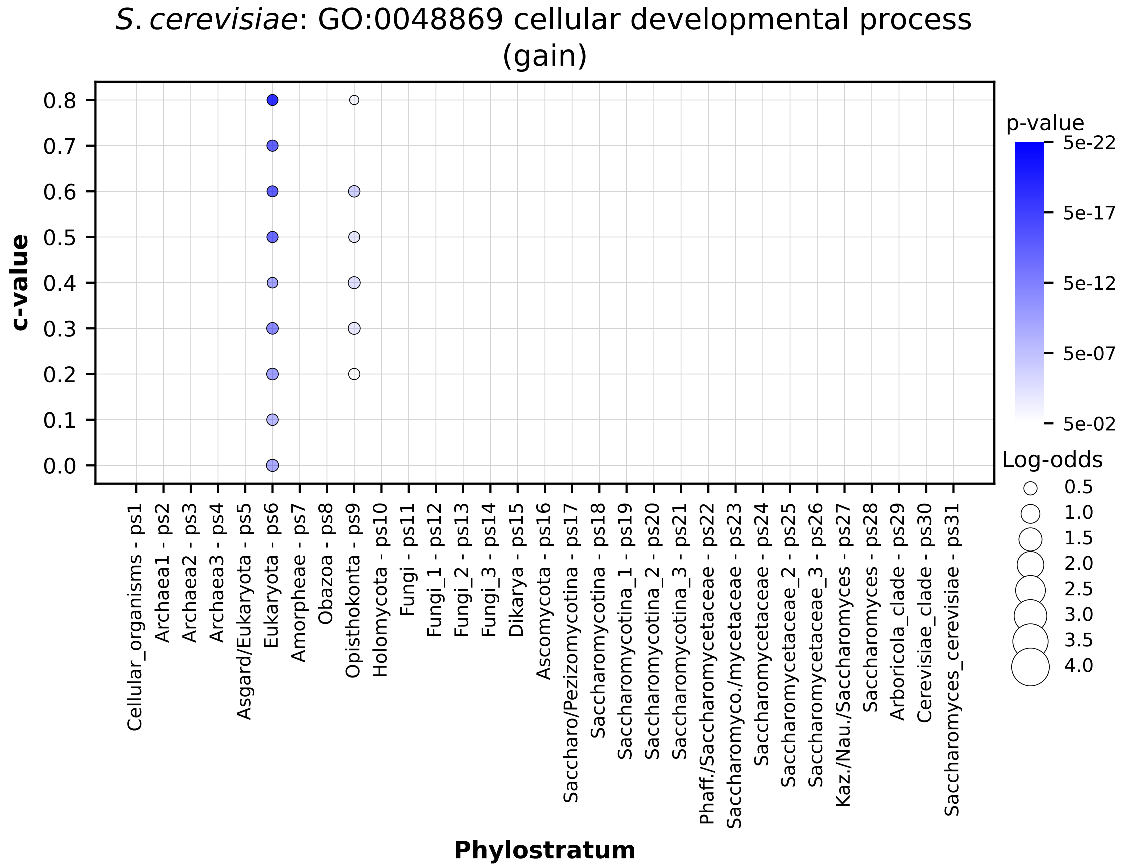

S. cerevisiae: GO:0048869 cellular developmental process (gain)
c-value
0.0
0.1
0.2
0.3
0.4
0.5
0.6
0.7
0.8
p-value
5e-22
5e-17
5e-12
5e-07
5e-02
Log-odds
0.5
1.0
1.5
2.0
2.5
3.0
3.5
4.0
Cellular_organisms - ps1
Archaea1 - ps2
Archaea2 - ps3
Archaea3 - ps4
Asgard/Eukaryota - ps5
Eukaryota - ps6
Amorpheae - ps7
Obazoa - ps8
Opisthokonta - ps9
Holomycota - ps10
Fungi - ps11
Fungi_1 - ps12
Fungi_2 - ps13
Fungi_3 - ps14
Dikarya - ps15
Ascomycota - ps16
Saccharo/Pezizomycotina - ps17
Saccharomycotina - ps18
Saccharomycotina_1 - ps19
Saccharomycotina_2 - ps20
Saccharomycotina_3 - ps21
Phaff./Saccharomycetaceae - ps22
Saccharomyco./mycetaceae - ps23
Saccharomycetaceae - ps24
Saccharomycetaceae_2 - ps25
Saccharomycetaceae_3 - ps26
Kaz./Nau./Saccharomyces - ps27
Saccharomyces - ps28
Arboricola_clade - ps29
Cerevisiae_clade - ps30
Saccharomyces_cerevisiae - ps31
Phylostratum

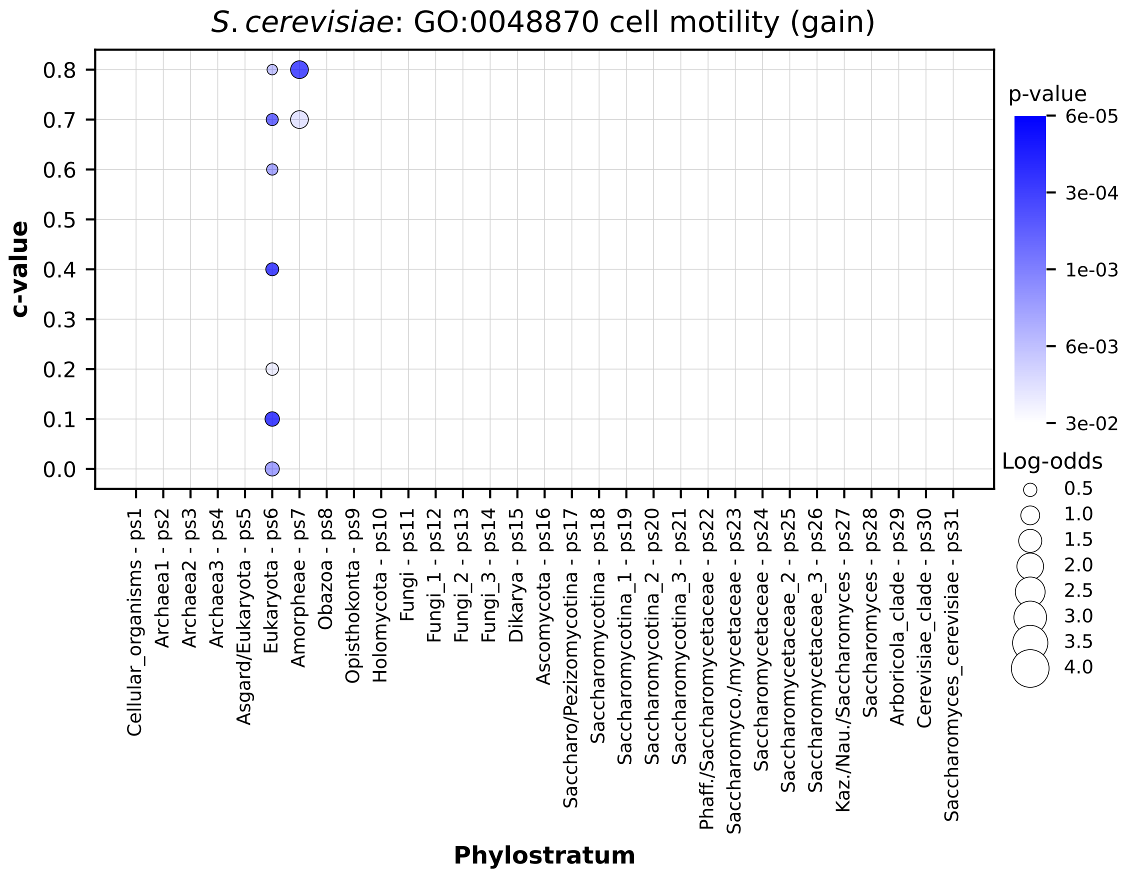

S. cerevisiae: GO:0048870 cell motility (gain)
c-value
0.8
0.7
0.6
0.5
0.4
0.3
0.2
0.1
0.0
p-value
6e-05
3e-04
1e-03
6e-03
3e-02
Log-odds
0.5
1.0
1.5
2.0
2.5
3.0
3.5
4.0
Cellular_organisms - ps1
Archaea1 - ps2
Archaea2 - ps3
Archaea3 - ps4
Asgard/Eukaryota - ps5
Eukaryota - ps6
Amorpheae - ps7
Obazoa - ps8
Opisthokonta - ps9
Holomycota - ps10
Fungi - ps11
Fungi_1 - ps12
Fungi_2 - ps13
Fungi_3 - ps14
Dikarya - ps15
Ascomycota - ps16
Saccharo/Pezizomycotina - ps17
Saccharomycotina - ps18
Saccharomycotina_1 - ps19
Saccharomycotina_2 - ps20
Saccharomycotina_3 - ps21
Phaff./Saccharomycetaceae - ps22
Saccharomyco./mycetaceae - ps23
Saccharomycetaceae - ps24
Saccharomycetaceae_2 - ps25
Saccharomycetaceae_3 - ps26
Kaz./Nau./Saccharomyces - ps27
Saccharomyces - ps28
Arboricola_clade - ps29
Cerevisiae_clade - ps30
Saccharomyces_cerevisiae - ps31
Phylostratum

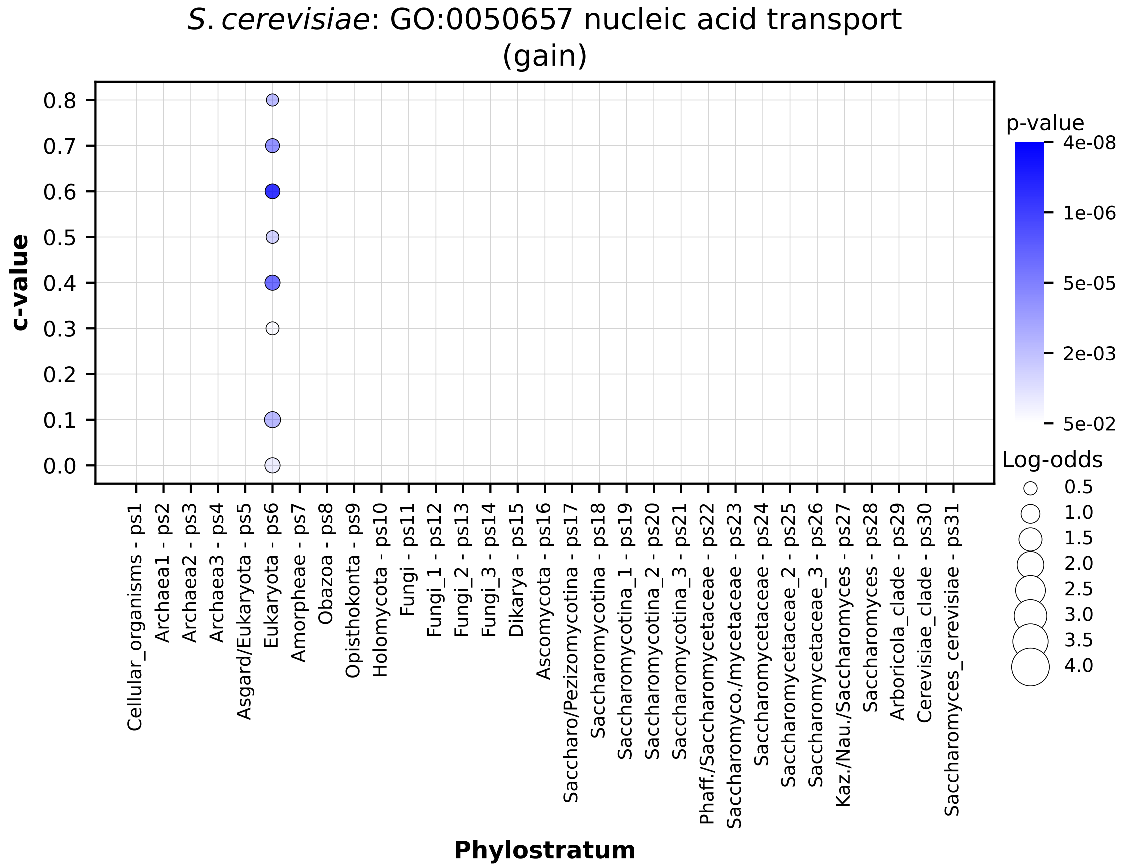
S. cerevisiae: GO:0050657 nucleic acid transport
(gain)
c-value
0.8
0.7
0.6
0.5
0.4
0.3
0.2
0.1
0.0
p-value
4e-08
1e-06
5e-05
2e-03
5e-02
Log-odds
0.5
1.0
1.5
2.0
2.5
3.0
3.5
4.0
Cellular_organisms - ps1
Archaea1 - ps2
Archaea2 - ps3
Archaea3 - ps4
Asgard/Eukaryota - ps5
Eukaryota - ps6
Amorpheae - ps7
Obazoa - ps8
Opisthokonta - ps9
Holomycota - ps10
Fungi - ps11
Fungi_1 - ps12
Fungi_2 - ps13
Fungi_3 - ps14
Dikarya - ps15
Ascomycota - ps16
Saccharo/Pezizomycotina - ps17
Saccharomycotina - ps18
Saccharomycotina_1 - ps19
Saccharomycotina_2 - ps20
Saccharomycotina_3 - ps21
Phaff./Saccharomycetaceae - ps22
Saccharomyco./mycetaceae - ps23
Saccharomycetaceae - ps24
Saccharomycetaceae_2 - ps25
Saccharomycetaceae_3 - ps26
Kaz./Nau./Saccharomyces - ps27
Saccharomyces - ps28
Arboricola_clade - ps29
Cerevisiae_clade - ps30
Saccharomyces_cerevisiae - ps31
Phylostratum

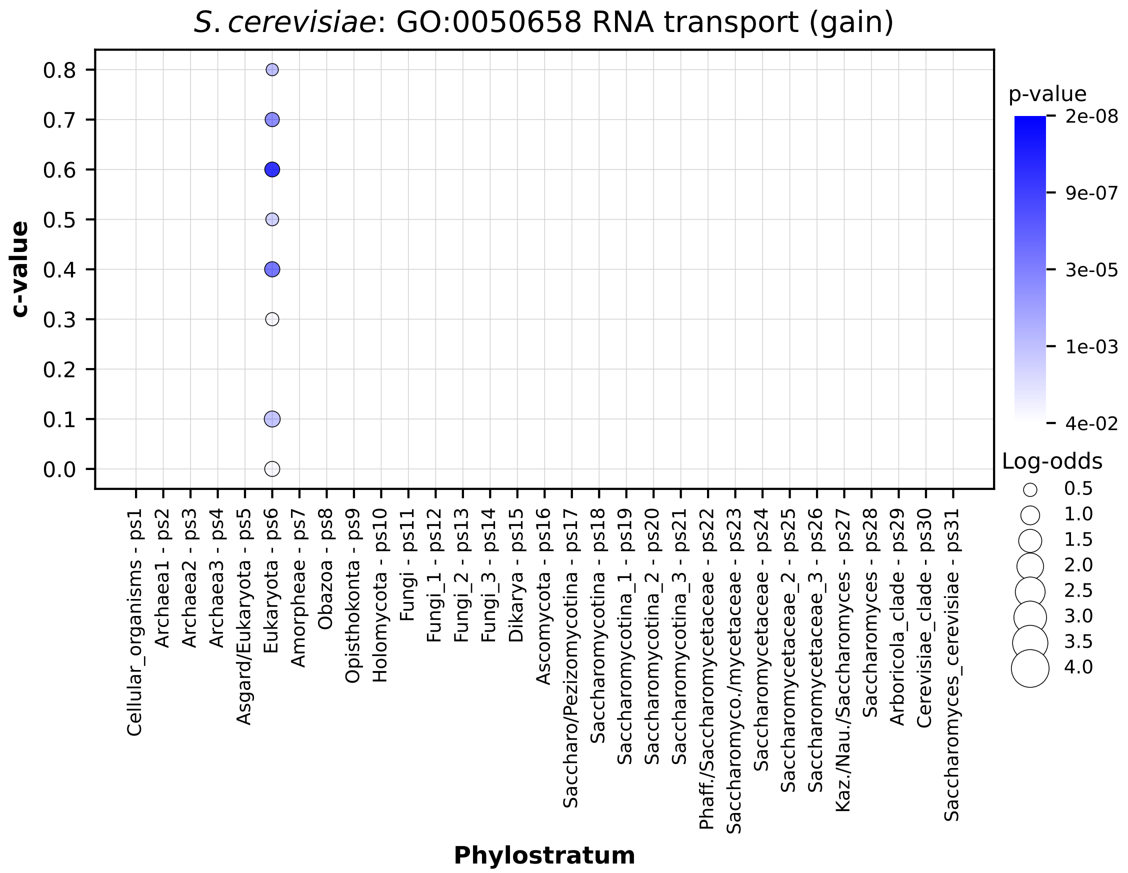
S. cerevisiae: GO:0050658 RNA transport (gain)
c-value
0.8
0.7
0.6
0.5
0.4
0.3
0.2
0.1
0.0
Phylostratum
Cellular_organisms - ps1
Archaea1 - ps2
Archaea2 - ps3
Archaea3 - ps4
Asgard/Eukaryota - ps5
Eukaryota - ps6
Amorpheae - ps7
Obazoa - ps8
Opisthokonta - ps9
Holomycota - ps10
Fungi - ps11
Fungi_1 - ps12
Fungi_2 - ps13
Fungi_3 - ps14
Dikarya - ps15
Ascomycota - ps16
Saccharo/Pezizomycotina - ps17
Saccharomycotina - ps18
Saccharomycotina_1 - ps19
Saccharomycotina_2 - ps20
Saccharomycotina_3 - ps21
Phaff./Saccharomycetaceae - ps22
Saccharomyco./mycetaceae - ps23
Saccharomycetaceae - ps24
Saccharomycetaceae_2 - ps25
Saccharomycetaceae_3 - ps26
Kaz./Nau./Saccharomyces - ps27
Saccharomyces - ps28
Arboricola_clade - ps29
Cerevisiae_clade - ps30
Saccharomyces_cerevisiae - ps31
p-value
2e-08
9e-07
3e-05
1e-03
4e-02
Log-odds
0.5
1.0
1.5
2.0
2.5
3.0
3.5
4.0

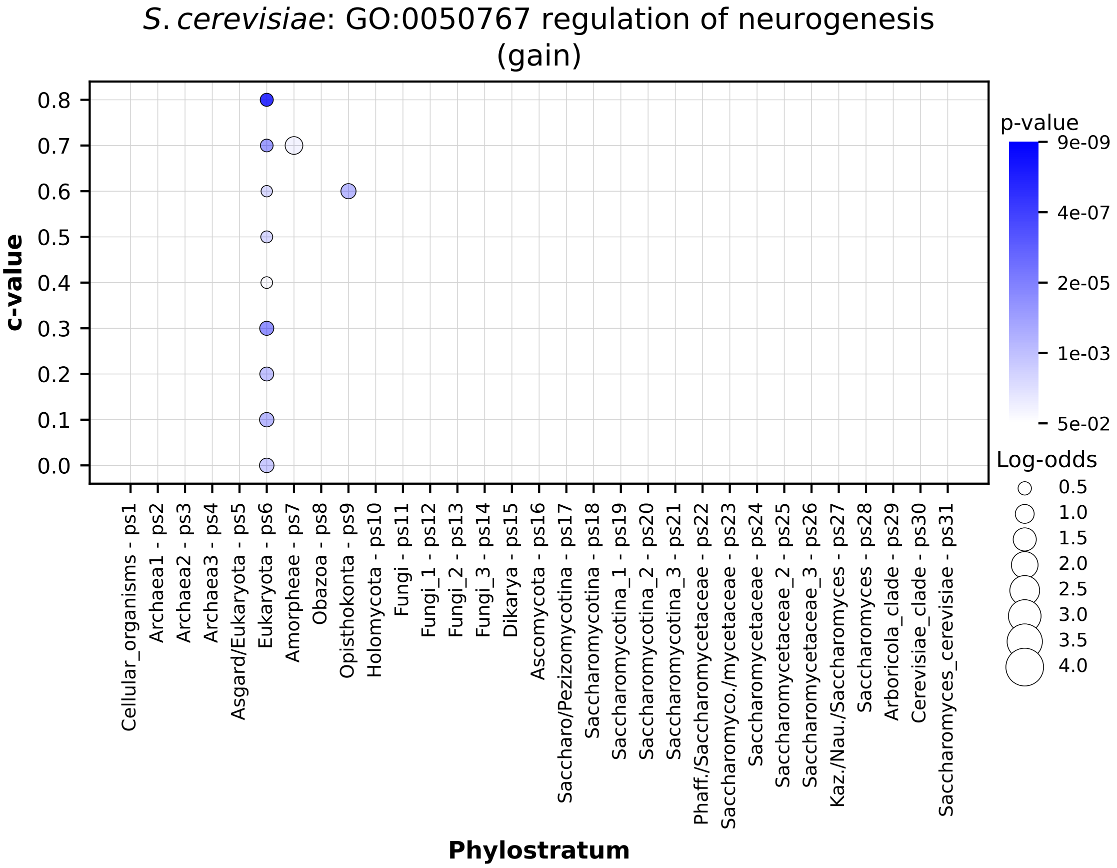

S. cerevisiae: GO:0050767 regulation of neurogenesis
(gain)
c-value
0.8
0.7
0.6
0.5
0.4
0.3
0.2
0.1
0.0
p-value
9e-09
4e-07
2e-05
1e-03
5e-02
Log-odds
0.5
1.0
1.5
2.0
2.5
3.0
3.5
4.0
Cellular_organisms - ps1
Archaea1 - ps2
Archaea2 - ps3
Archaea3 - ps4
Asgard/Eukaryota - ps5
Eukaryota - ps6
Amorpheae - ps7
Obazoa - ps8
Opisthokonta - ps9
Holomycota - ps10
Fungi - ps11
Fungi_1 - ps12
Fungi_2 - ps13
Fungi_3 - ps14
Dikarya - ps15
Ascomycota - ps16
Saccharo/Pezizomycotina - ps17
Saccharomycotina - ps18
Saccharomycotina_1 - ps19
Saccharomycotina_2 - ps20
Saccharomycotina_3 - ps21
Phaff./Saccharomycetaceae - ps22
Saccharomyco./mycetaceae - ps23
Saccharomycetaceae - ps24
Saccharomycetaceae_2 - ps25
Saccharomycetaceae_3 - ps26
Kaz./Nau./Saccharomyces - ps27
Saccharomyces - ps28
Arboricola_clade - ps29
Cerevisiae_clade - ps30
Saccharomyces_cerevisiae - ps31
Phylostratum

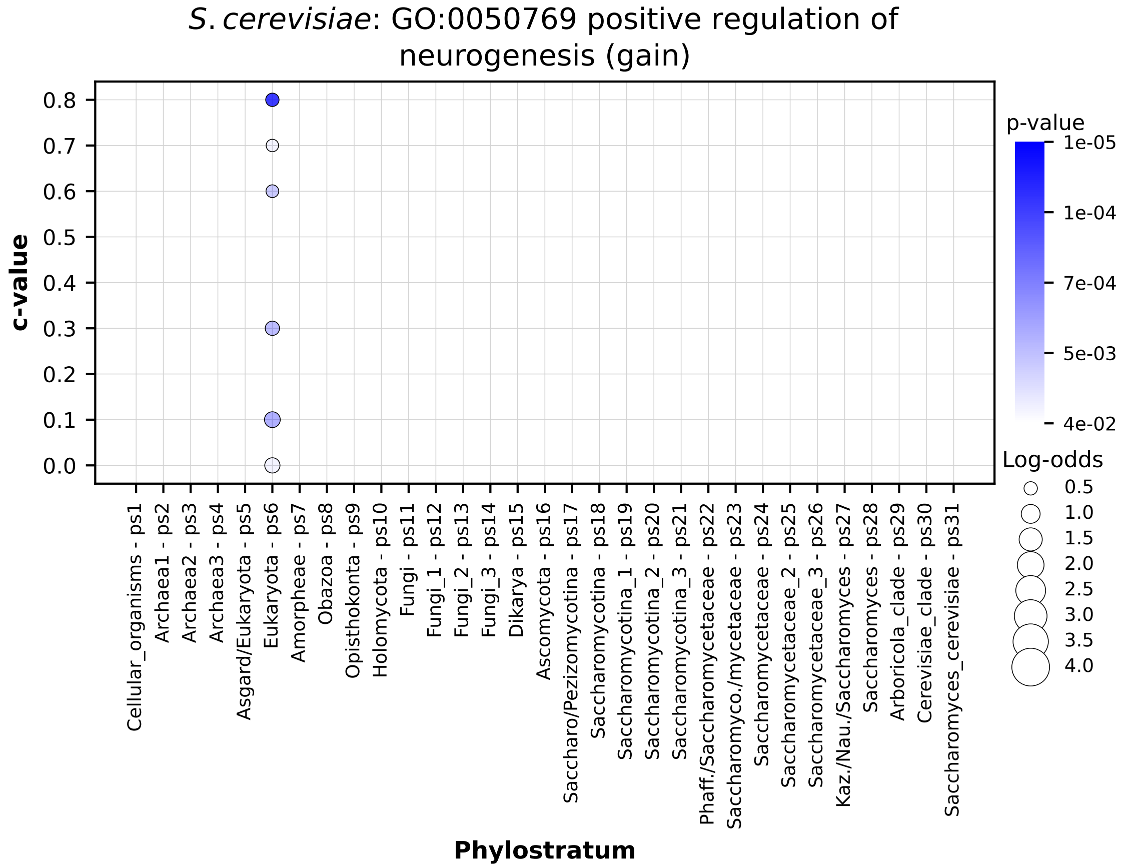

S. cerevisiae: GO:0050769 positive regulation of neurogenesis (gain)
c-value
0.8
0.7
0.6
0.5
0.4
0.3
0.2
0.1
0.0
p-value
1e-05
1e-04
7e-04
5e-03
4e-02
Log-odds
0.5
1.0
1.5
2.0
2.5
3.0
3.5
4.0
Cellular_organisms - ps1
Archaea1 - ps2
Archaea2 - ps3
Archaea3 - ps4
Asgard/Eukaryota - ps5
Eukaryota - ps6
Amorpheae - ps7
Obazoa - ps8
Opisthokonta - ps9
Holomycota - ps10
Fungi - ps11
Fungi_1 - ps12
Fungi_2 - ps13
Fungi_3 - ps14
Dikarya - ps15
Ascomycota - ps16
Saccharo/Pezizomycotina - ps17
Saccharomycotina - ps18
Saccharomycotina_1 - ps19
Saccharomycotina_2 - ps20
Saccharomycotina_3 - ps21
Phaff./Saccharomycetaceae - ps22
Saccharomyco./mycetaceae - ps23
Saccharomycetaceae - ps24
Saccharomycetaceae_2 - ps25
Saccharomycetaceae_3 - ps26
Kaz./Nau./Saccharomyces - ps27
Saccharomyces - ps28
Arboricola_clade - ps29
Cerevisiae_clade - ps30
Saccharomyces_cerevisiae - ps31
Phylostratum

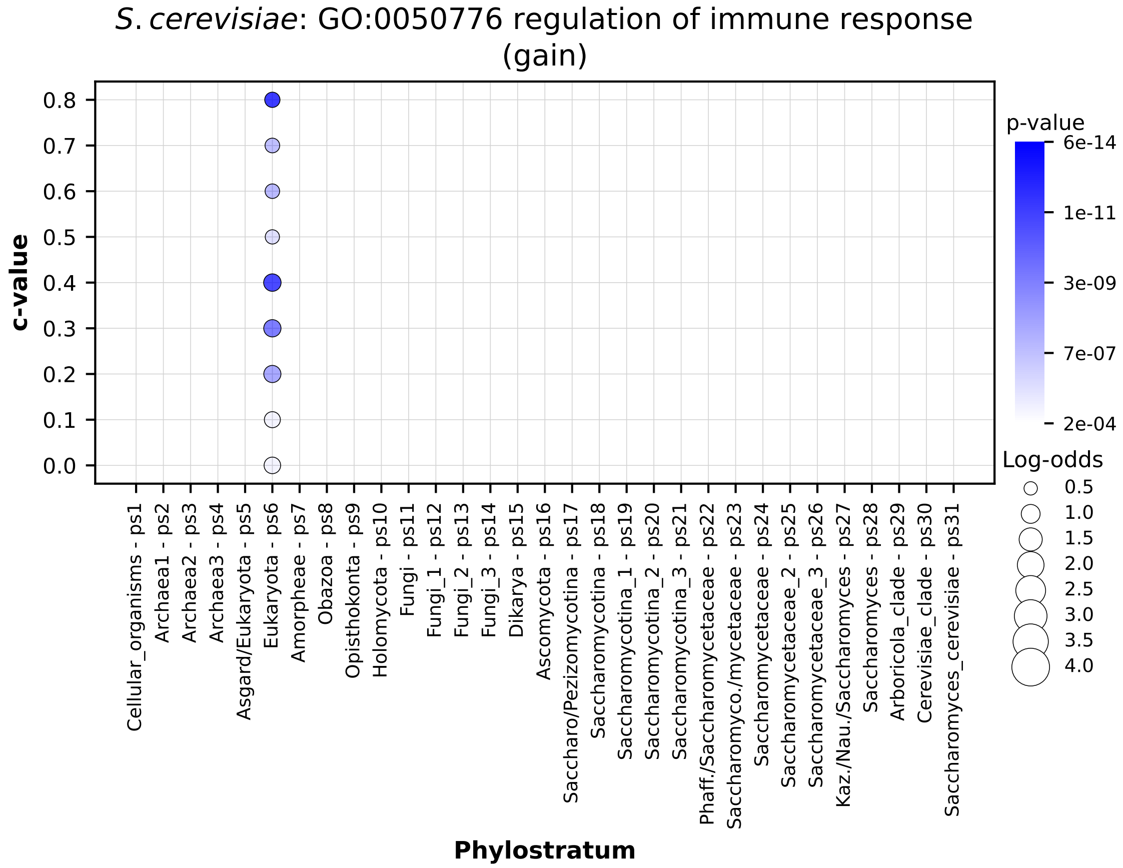

S. cerevisiae: GO:0050776 regulation of immune response (gain)
c-value
0.8
0.7
0.6
0.5
0.4
0.3
0.2
0.1
0.0
p-value
6e-14
1e-11
3e-09
7e-07
2e-04
Log-odds
0.5
1.0
1.5
2.0
2.5
3.0
3.5
4.0
Cellular_organisms - ps1
Archaea1 - ps2
Archaea2 - ps3
Archaea3 - ps4
Asgard/Eukaryota - ps5
Eukaryota - ps6
Amorpheae - ps7
Obazoa - ps8
Opisthokonta - ps9
Holomycota - ps10
Fungi - ps11
Fungi_1 - ps12
Fungi_2 - ps13
Fungi_3 - ps14
Dikarya - ps15
Ascomycota - ps16
Saccharo/Pezizomycotina - ps17
Saccharomycotina - ps18
Saccharomycotina_1 - ps19
Saccharomycotina_2 - ps20
Saccharomycotina_3 - ps21
Phaff./Saccharomycetaceae - ps22
Saccharomyco./mycetaceae - ps23
Saccharomycetaceae - ps24
Saccharomycetaceae_2 - ps25
Saccharomycetaceae_3 - ps26
Kaz./Nau./Saccharomyces - ps27
Saccharomyces - ps28
Arboricola_clade - ps29
Cerevisiae_clade - ps30
Saccharomyces_cerevisiae - ps31
Phylostratum

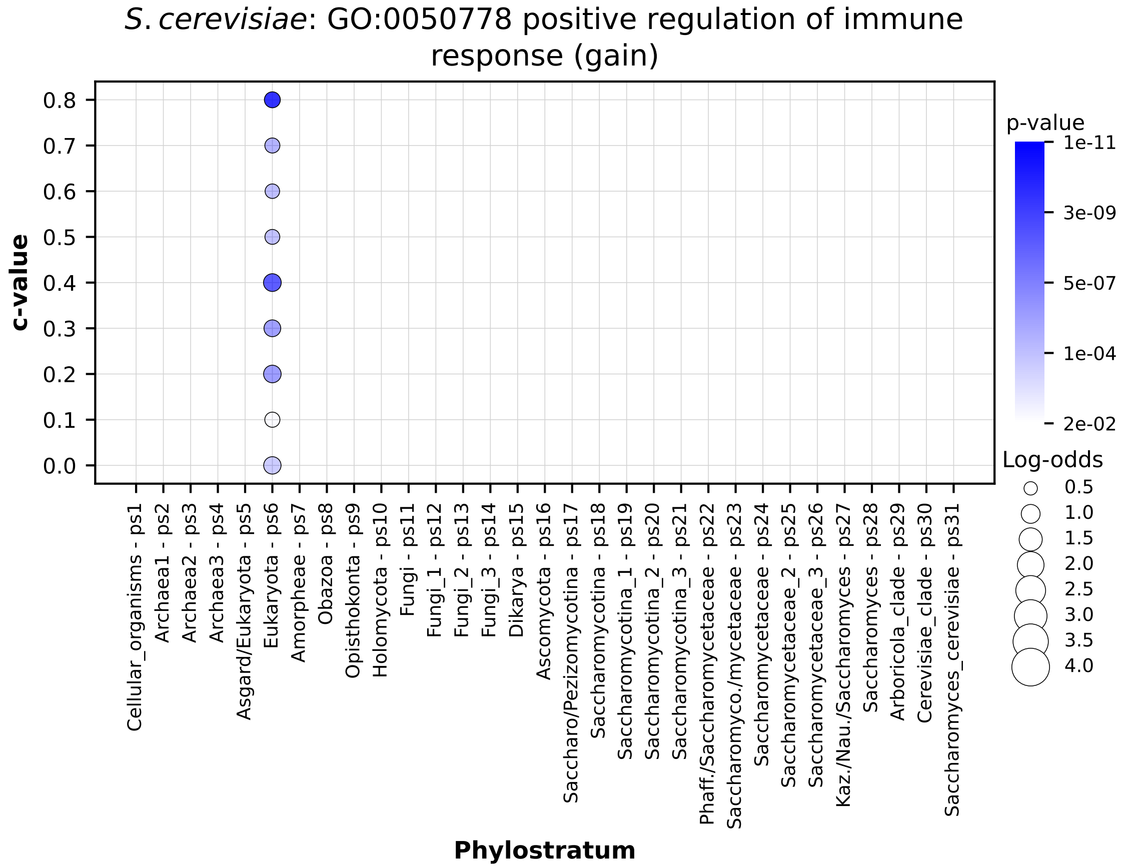

S. cerevisiae: GO:0050778 positive regulation of immune response (gain)
c-value
0.8
0.7
0.6
0.5
0.4
0.3
0.2
0.1
0.0
p-value
1e-11
3e-09
5e-07
1e-04
2e-02
Log-odds
0.5
1.0
1.5
2.0
2.5
3.0
3.5
4.0
Cellular_organisms - ps1
Archaea1 - ps2
Archaea2 - ps3
Archaea3 - ps4
Asgard/Eukaryota - ps5
Eukaryota - ps6
Amorpheae - ps7
Obazoa - ps8
Opisthokonta - ps9
Holomycota - ps10
Fungi - ps11
Fungi_1 - ps12
Fungi_2 - ps13
Fungi_3 - ps14
Dikarya - ps15
Ascomycota - ps16
Saccharo/Pezizomycotina - ps17
Saccharomycotina - ps18
Saccharomycotina_1 - ps19
Saccharomycotina_2 - ps20
Saccharomycotina_3 - ps21
Phaff./Saccharomycetaceae - ps22
Saccharomyco./mycetaceae - ps23
Saccharomycetaceae - ps24
Saccharomycetaceae_2 - ps25
Saccharomycetaceae_3 - ps26
Kaz./Nau./Saccharomyces - ps27
Saccharomyces - ps28
Arboricola_clade - ps29
Cerevisiae_clade - ps30
Saccharomyces_cerevisiae - ps31
Phylostratum

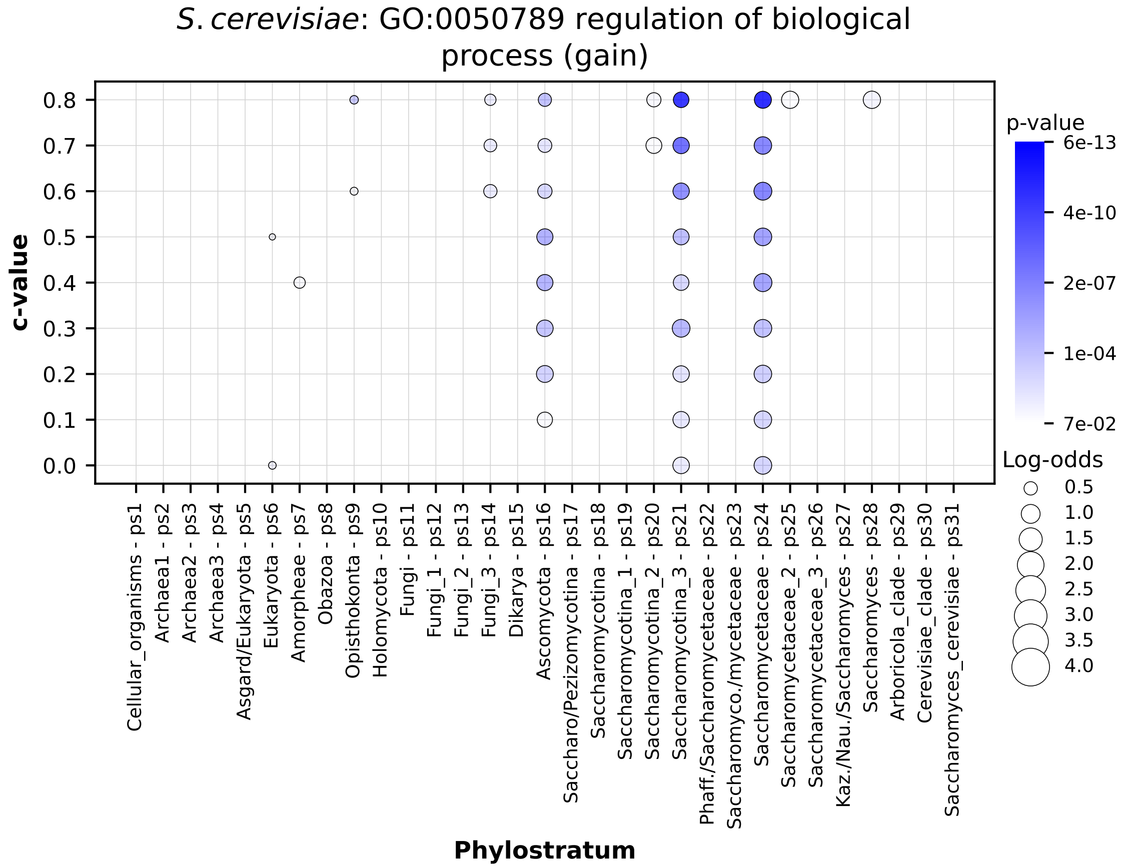
S. cerevisiae: GO:0050789 regulation of biological process (gain)
c-value
0.8
0.7
0.6
0.5
0.4
0.3
0.2
0.1
0.0
p-value
6e-13
4e-10
2e-07
1e-04
7e-02
Log-odds
0.5
1.0
1.5
2.0
2.5
3.0
3.5
4.0
Cellular_organisms - ps1
Archaea1 - ps2
Archaea2 - ps3
Archaea3 - ps4
Asgard/Eukaryota - ps5
Eukaryota - ps6
Amorpheae - ps7
Obazoa - ps8
Opisthokonta - ps9
Holomycota - ps10
Fungi - ps11
Fungi_1 - ps12
Fungi_2 - ps13
Fungi_3 - ps14
Dikarya - ps15
Ascomycota - ps16
Saccharo/Pezizomycotina - ps17
Saccharomycotina - ps18
Saccharomycotina_1 - ps19
Saccharomycotina_2 - ps20
Saccharomycotina_3 - ps21
Phaff./Saccharomycetaceae - ps22
Saccharomyco./mycetaceae - ps23
Saccharomycetaceae - ps24
Saccharomycetaceae_2 - ps25
Saccharomycetaceae_3 - ps26
Kaz./Nau./Saccharomyces - ps27
Saccharomyces - ps28
Arboricola_clade - ps29
Cerevisiae_clade - ps30
Saccharomyces_cerevisiae - ps31
Phylostratum

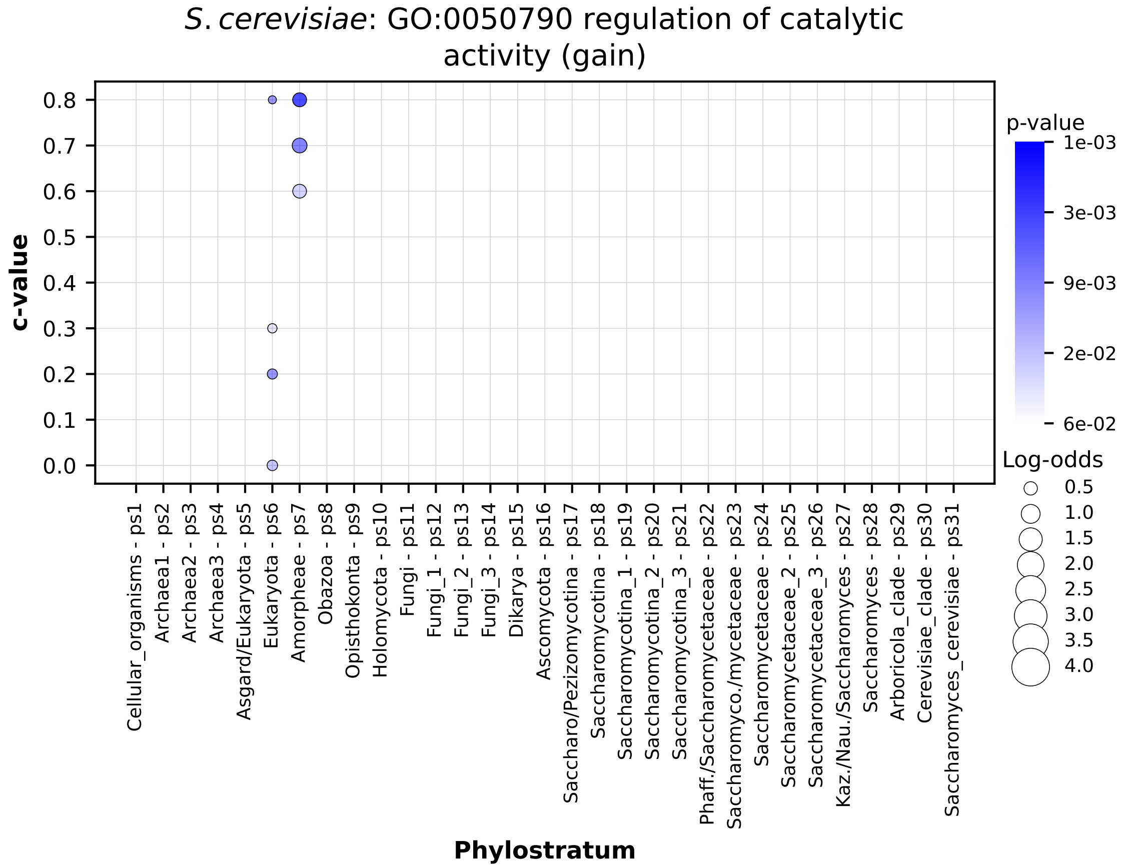

S. cerevisiae: GO:0050790 regulation of catalytic activity (gain)
c-value
0.8
0.7
0.6
0.5
0.4
0.3
0.2
0.1
0.0
p-value
1e-03
3e-03
9e-03
2e-02
6e-02
Log-odds
0.5
1.0
1.5
2.0
2.5
3.0
3.5
4.0
Cellular_organisms - ps1
Archaea1 - ps2
Archaea2 - ps3
Archaea3 - ps4
Asgard/Eukaryota - ps5
Eukaryota - ps6
Amorpheae - ps7
Obazoa - ps8
Opisthokonta - ps9
Holomycota - ps10
Fungi - ps11
Fungi_1 - ps12
Fungi_2 - ps13
Fungi_3 - ps14
Dikarya - ps15
Ascomycota - ps16
Saccharo/Pezizomycotina - ps17
Saccharomycotina - ps18
Saccharomycotina_1 - ps19
Saccharomycotina_2 - ps20
Saccharomycotina_3 - ps21
Phaff./Saccharomycetaceae - ps22
Saccharomyco./mycetaceae - ps23
Saccharomycetaceae - ps24
Saccharomycetaceae_2 - ps25
Saccharomycetaceae_3 - ps26
Kaz./Nau./Saccharomyces - ps27
Saccharomyces - ps28
Arboricola_clade - ps29
Cerevisiae_clade - ps30
Saccharomyces_cerevisiae - ps31
Phylostratum

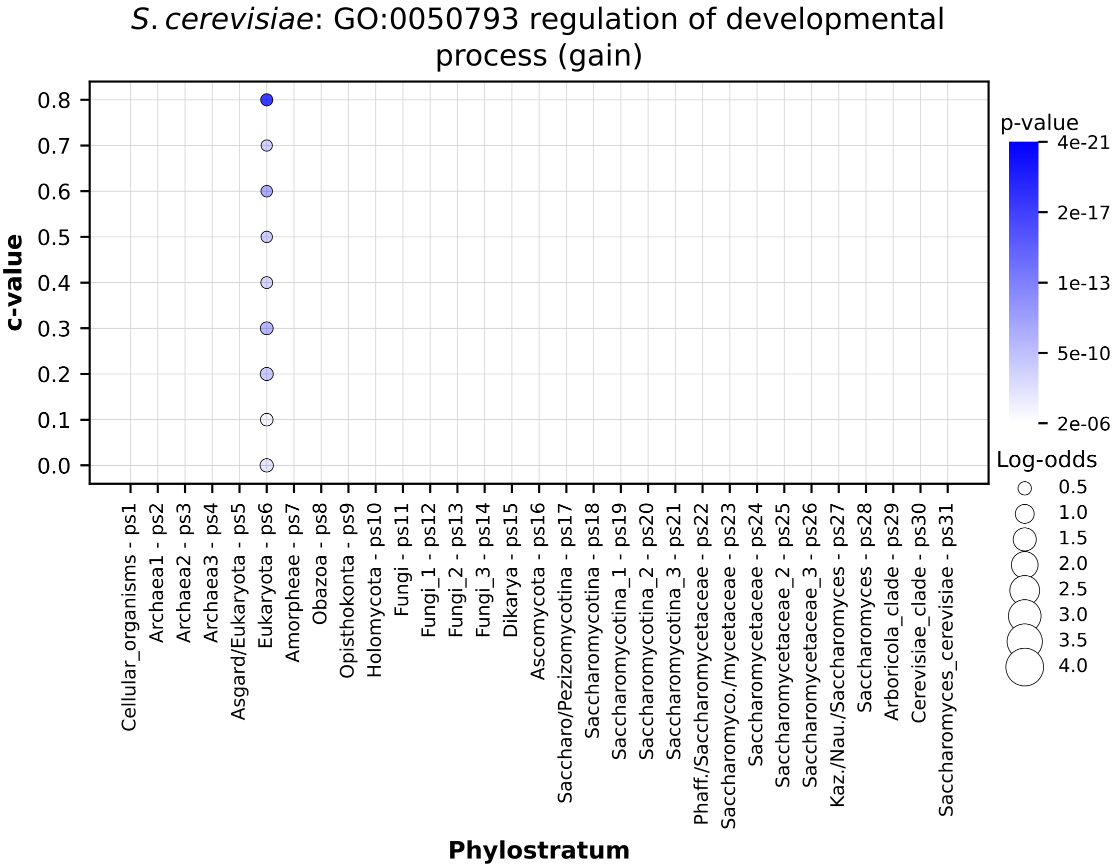

S. cerevisiae: GO:0050793 regulation of developmental process (gain)
c-value
0.8
0.7
0.6
0.5
0.4
0.3
0.2
0.1
0.0
p-value
4e-21
2e-17
1e-13
5e-10
2e-06
Log-odds
0.5
1.0
1.5
2.0
2.5
3.0
3.5
4.0
Cellular_organisms - ps1
Archaea1 - ps2
Archaea2 - ps3
Archaea3 - ps4
Asgard/Eukaryota - ps5
Eukaryota - ps6
Amorpheae - ps7
Obazoa - ps8
Opisthokonta - ps9
Holomycota - ps10
Fungi - ps11
Fungi_1 - ps12
Fungi_2 - ps13
Fungi_3 - ps14
Dikarya - ps15
Ascomycota - ps16
Saccharo/Pezizomycotina - ps17
Saccharomycotina - ps18
Saccharomycotina_1 - ps19
Saccharomycotina_2 - ps20
Saccharomycotina_3 - ps21
Phaff./Saccharomycetaceae - ps22
Saccharomyco./mycetaceae - ps23
Saccharomycetaceae - ps24
Saccharomycetaceae_2 - ps25
Saccharomycetaceae_3 - ps26
Kaz./Nau./Saccharomyces - ps27
Saccharomyces - ps28
Arboricola_clade - ps29
Cerevisiae_clade - ps30
Saccharomyces_cerevisiae - ps31
Phylostratum

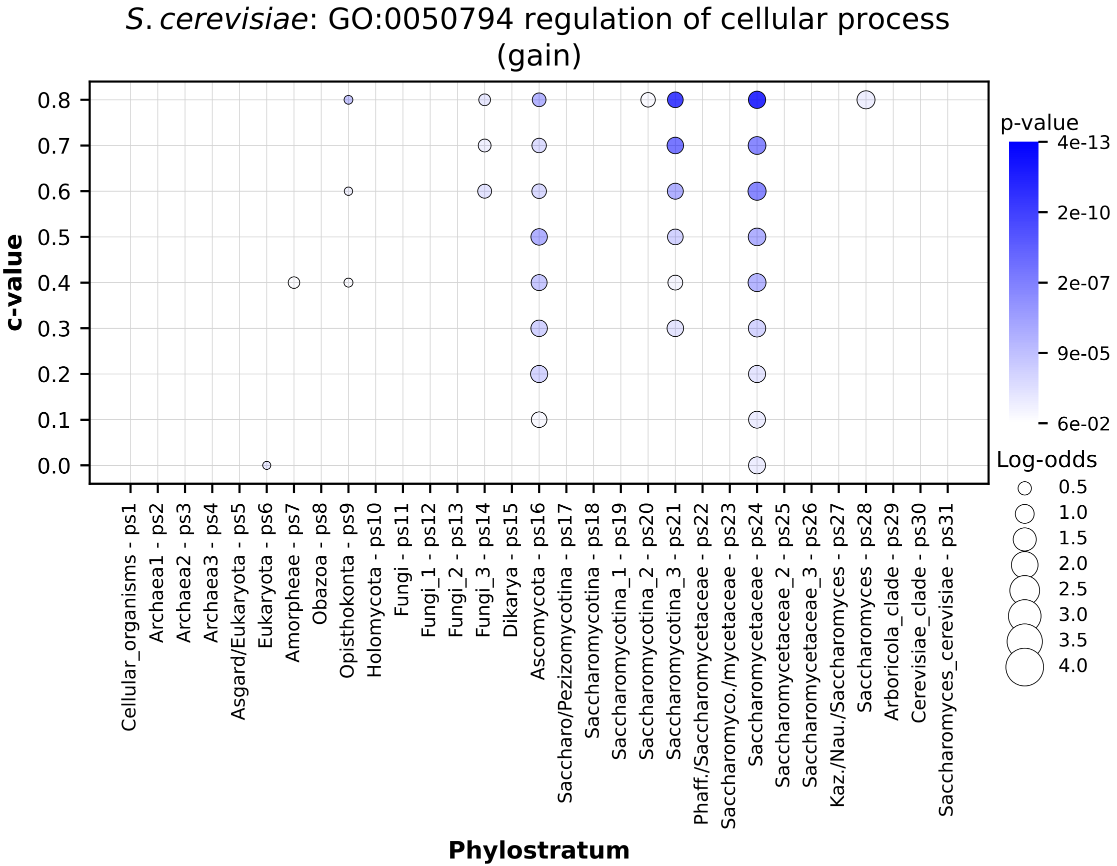

S. cerevisiae: GO:0050794 regulation of cellular process (gain)
c-value
0.8
0.7
0.6
0.5
0.4
0.3
0.2
0.1
0.0
p-value
4e-13
2e-10
2e-07
9e-05
6e-02
Log-odds
0.5
1.0
1.5
2.0
2.5
3.0
3.5
4.0
Cellular_organisms - ps1
Archaea1 - ps2
Archaea2 - ps3
Archaea3 - ps4
Asgard/Eukaryota - ps5
Eukaryota - ps6
Amorpheae - ps7
Obazoa - ps8
Opisthokonta - ps9
Holomycota - ps10
Fungi - ps11
Fungi_1 - ps12
Fungi_2 - ps13
Fungi_3 - ps14
Dikarya - ps15
Ascomycota - ps16
Saccharo/Pezizomycotina - ps17
Saccharomycotina - ps18
Saccharomycotina_1 - ps19
Saccharomycotina_2 - ps20
Saccharomycotina_3 - ps21
Phaff./Saccharomycetaceae - ps22
Saccharomyco./mycetaceae - ps23
Saccharomycetaceae - ps24
Saccharomycetaceae_2 - ps25
Saccharomycetaceae_3 - ps26
Kaz./Nau./Saccharomyces - ps27
Saccharomyces - ps28
Arboricola_clade - ps29
Cerevisiae_clade - ps30
Saccharomyces_cerevisiae - ps31
Phylostratum

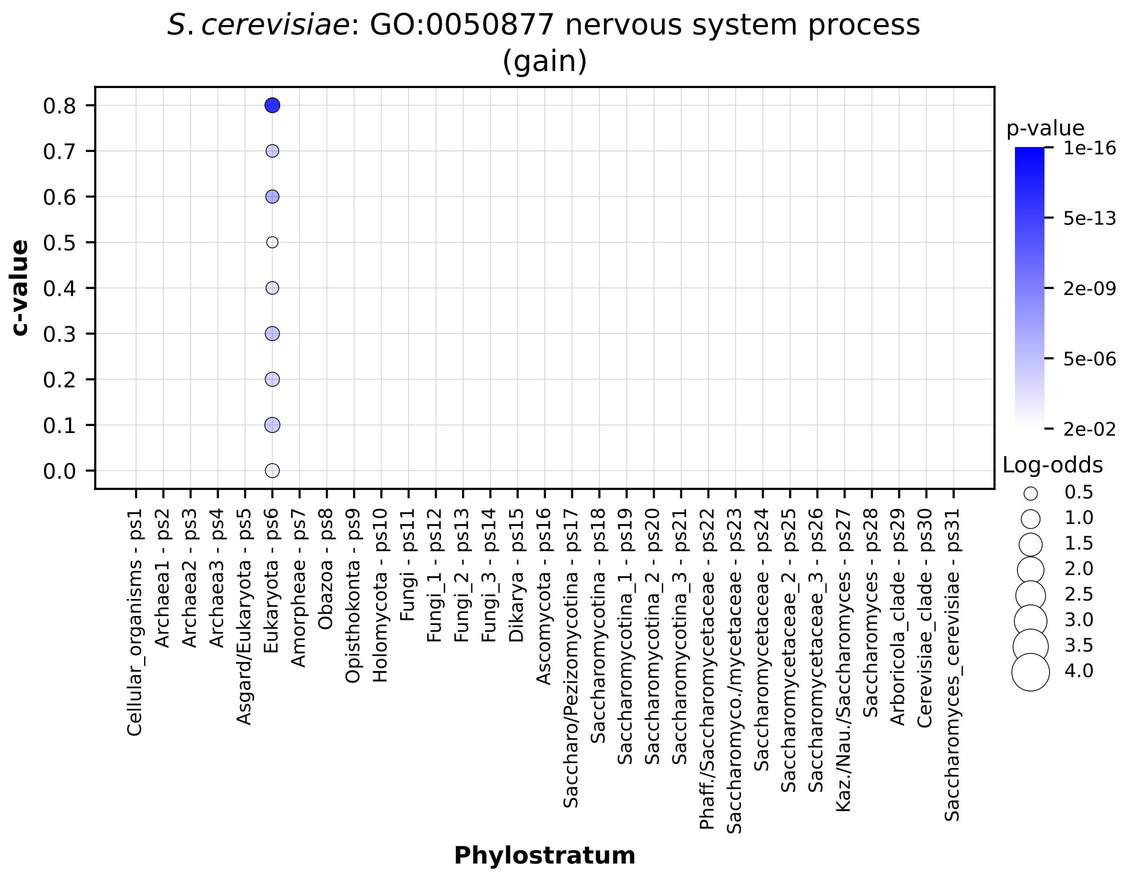

S. cerevisiae: GO:0050877 nervous system process
(gain)
c-value
0.8
0.7
0.6
0.5
0.4
0.3
0.2
0.1
0.0
p-value
1e-16
5e-13
2e-09
5e-06
2e-02
Log-odds
0.5
1.0
1.5
2.0
2.5
3.0
3.5
4.0
Cellular_organisms - ps1
Archaea1 - ps2
Archaea2 - ps3
Archaea3 - ps4
Asgard/Eukaryota - ps5
Eukaryota - ps6
Amorpheae - ps7
Obazoa - ps8
Opisthokonta - ps9
Holomycota - ps10
Fungi - ps11
Fungi_1 - ps12
Fungi_2 - ps13
Fungi_3 - ps14
Dikarya - ps15
Ascomycota - ps16
Saccharo/Pezizomycotina - ps17
Saccharomycotina - ps18
Saccharomycotina_1 - ps19
Saccharomycotina_2 - ps20
Saccharomycotina_3 - ps21
Phaff./Saccharomycetaceae - ps22
Saccharomyco./mycetaceae - ps23
Saccharomycetaceae - ps24
Saccharomycetaceae_2 - ps25
Saccharomycetaceae_3 - ps26
Kaz./Nau./Saccharomyces - ps27
Saccharomyces - ps28
Arboricola_clade - ps29
Cerevisiae_clade - ps30
Saccharomyces_cerevisiae - ps31
Phylostratum

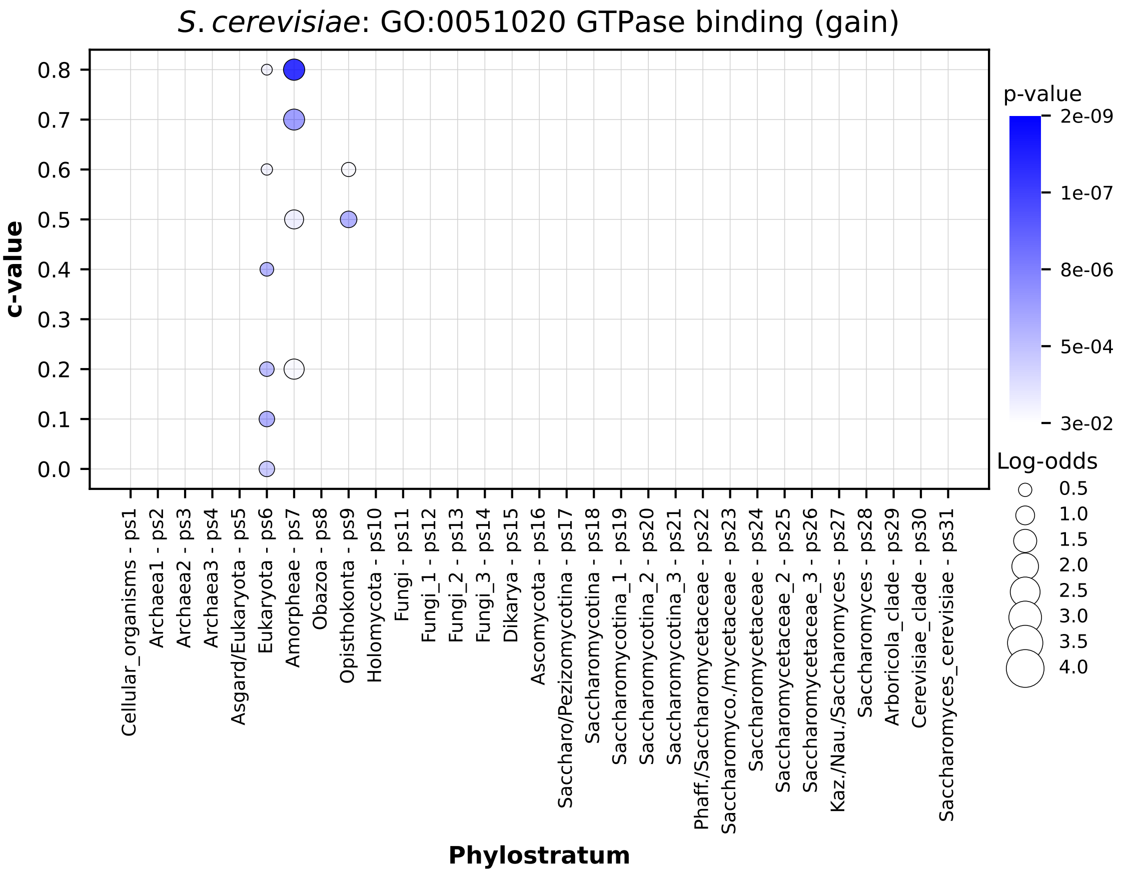

S. cerevisiae: GO:0051020 GTPase binding (gain)
c-value
0.8
0.7
0.6
0.5
0.4
0.3
0.2
0.1
0.0
p-value
2e-09
1e-07
8e-06
5e-04
3e-02
Log-odds
0.5
1.0
1.5
2.0
2.5
3.0
3.5
4.0
Cellular_organisms - ps1
Archaea1 - ps2
Archaea2 - ps3
Archaea3 - ps4
Asgard/Eukaryota - ps5
Eukaryota - ps6
Amorpheae - ps7
Obazoa - ps8
Opisthokonta - ps9
Holomycota - ps10
Fungi - ps11
Fungi_1 - ps12
Fungi_2 - ps13
Fungi_3 - ps14
Dikarya - ps15
Ascomycota - ps16
Saccharo/Pezizomycotina - ps17
Saccharomycotina - ps18
Saccharomycotina_1 - ps19
Saccharomycotina_2 - ps20
Saccharomycotina_3 - ps21
Phaff./Saccharomycetaceae - ps22
Saccharomyco./mycetaceae - ps23
Saccharomycetaceae - ps24
Saccharomycetaceae_2 - ps25
Saccharomycetaceae_3 - ps26
Kaz./Nau./Saccharomyces - ps27
Saccharomyces - ps28
Arboricola_clade - ps29
Cerevisiae_clade - ps30
Saccharomyces_cerevisiae - ps31
Phylostratum

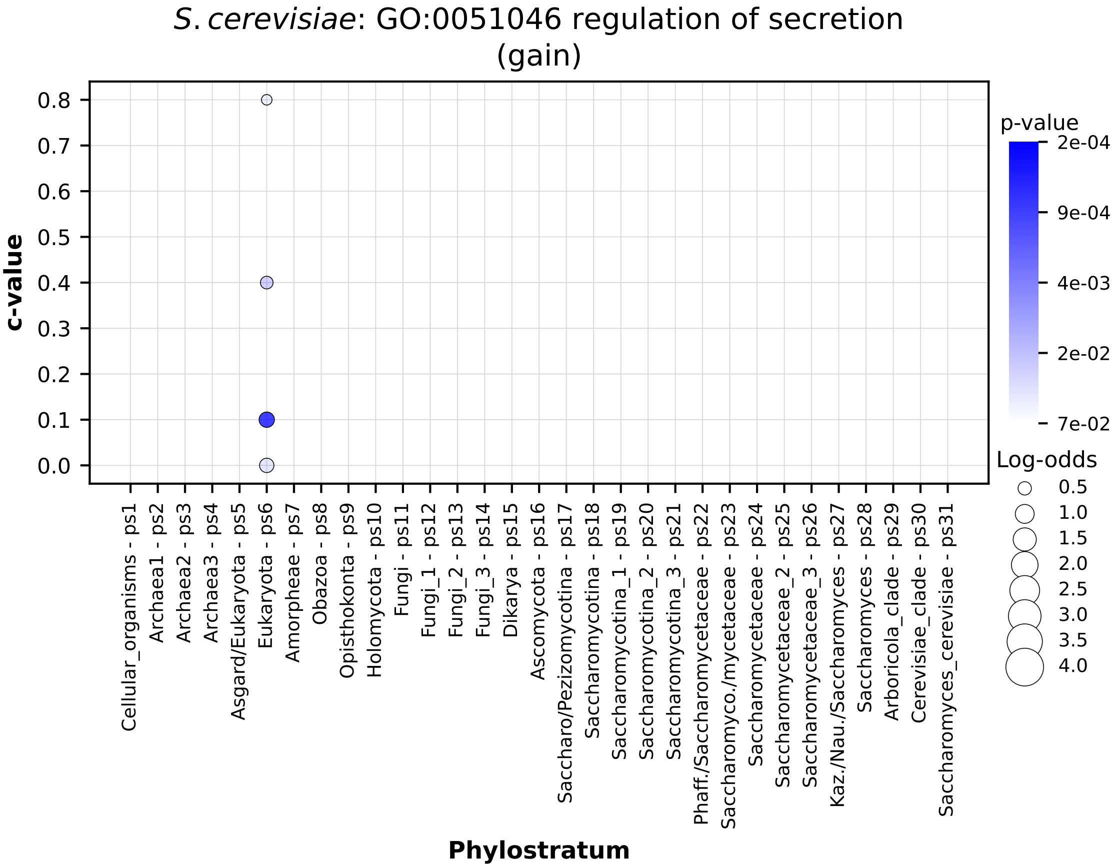
S. cerevisiae: GO:0051046 regulation of secretion (gain)
c-value
0.0
0.1
0.2
0.3
0.4
0.5
0.6
0.7
0.8
p-value
2e-04
9e-04
4e-03
2e-02
7e-02
Log-odds
0.5
1.0
1.5
2.0
2.5
3.0
3.5
4.0
Cellular_organisms - ps1
Archaea1 - ps2
Archaea2 - ps3
Archaea3 - ps4
Asgard/Eukaryota - ps5
Eukaryota - ps6
Amorpheae - ps7
Obazoa - ps8
Opisthokonta - ps9
Holomycota - ps10
Fungi - ps11
Fungi_1 - ps12
Fungi_2 - ps13
Fungi_3 - ps14
Dikarya - ps15
Ascomycota - ps16
Saccharo/Pezizomycotina - ps17
Saccharomycotina - ps18
Saccharomycotina_1 - ps19
Saccharomycotina_2 - ps20
Saccharomycotina_3 - ps21
Phaff./Saccharomycetaceae - ps22
Saccharomyco./mycetaceae - ps23
Saccharomycetaceae - ps24
Saccharomycetaceae_2 - ps25
Saccharomycetaceae_3 - ps26
Kaz./Nau./Saccharomyces - ps27
Saccharomyces - ps28
Arboricola_clade - ps29
Cerevisiae_clade - ps30
Saccharomyces_cerevisiae - ps31
Phylostratum

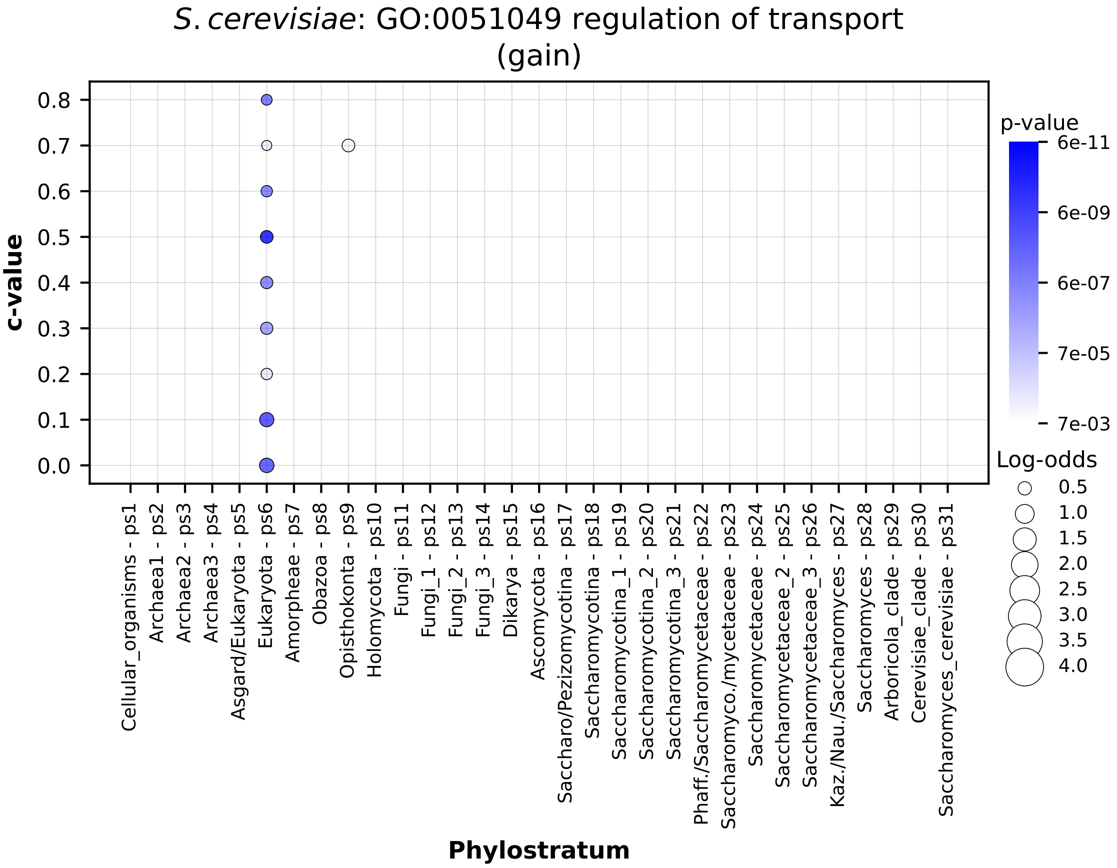

S. cerevisiae: GO:0051049 regulation of transport
(gain)
c-value
0.8
0.7
0.6
0.5
0.4
0.3
0.2
0.1
0.0
Phylostratum
Cellular_organisms - ps1
Archaea1 - ps2
Archaea2 - ps3
Archaea3 - ps4
Asgard/Eukaryota - ps5
Eukaryota - ps6
Amorpheae - ps7
Obazoa - ps8
Opisthokonta - ps9
Holomycota - ps10
Fungi - ps11
Fungi_1 - ps12
Fungi_2 - ps13
Fungi_3 - ps14
Dikarya - ps15
Ascomycota - ps16
Saccharo/Pezizomycotina - ps17
Saccharomycotina - ps18
Saccharomycotina_1 - ps19
Saccharomycotina_2 - ps20
Saccharomycotina_3 - ps21
Phaff./Saccharomycetaceae - ps22
Saccharomyco./mycetaceae - ps23
Saccharomycetaceae - ps24
Saccharomycetaceae_2 - ps25
Saccharomycetaceae_3 - ps26
Kaz./Nau./Saccharomyces - ps27
Saccharomyces - ps28
Arboricola_clade - ps29
Cerevisiae_clade - ps30
Saccharomyces_cerevisiae - ps31
p-value
6e-11
6e-09
6e-07
7e-05
7e-03
Log-odds
0.5
1.0
1.5
2.0
2.5
3.0
3.5
4.0

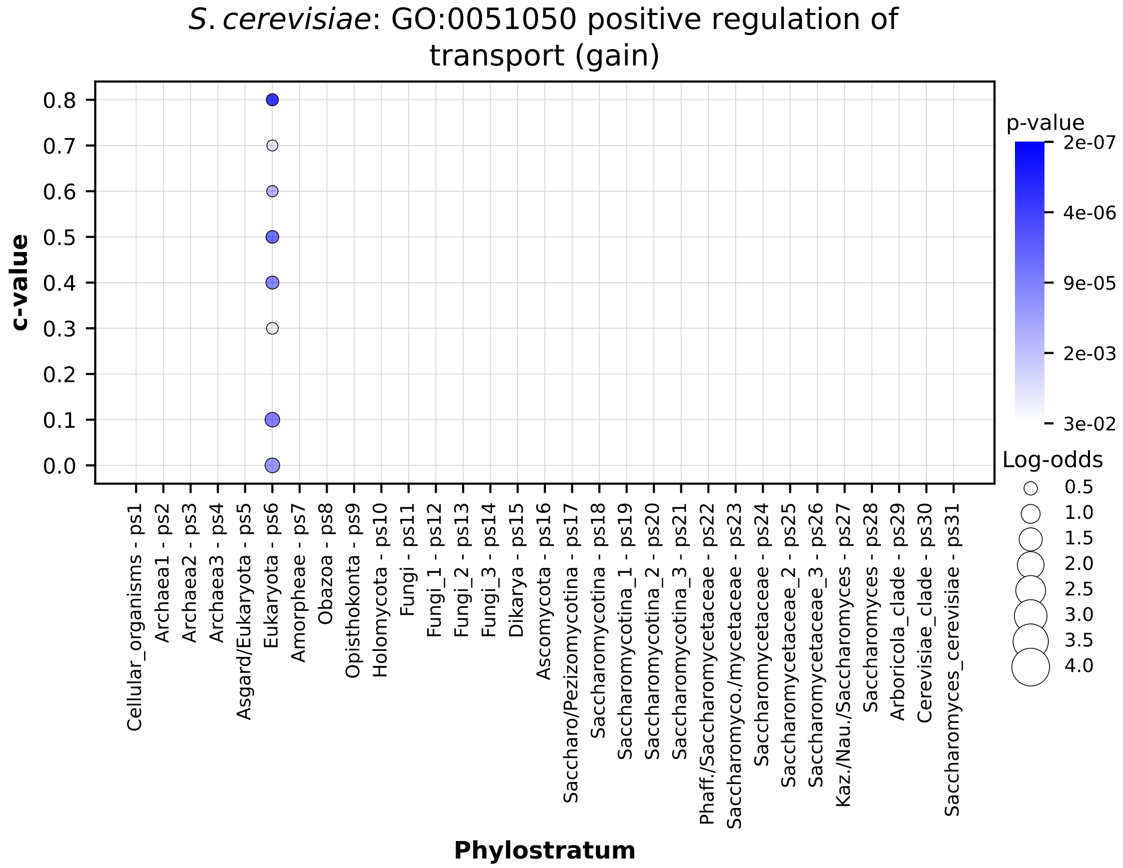
S. cerevisiae: GO:0051050 positive regulation of transport (gain)
c-value
Phylostratum
p-value
2e-07
4e-06
9e-05
2e-03
3e-02
Log-odds
0.5
1.0
1.5
2.0
2.5
3.0
3.5
4.0
Cellular_organisms - ps1
Archaea1 - ps2
Archaea2 - ps3
Archaea3 - ps4
Asgard/Eukaryota - ps5
Eukaryota - ps6
Amorpheae - ps7
Obazoa - ps8
Opisthokonta - ps9
Holomycota - ps10
Fungi - ps11
Fungi_1 - ps12
Fungi_2 - ps13
Fungi_3 - ps14
Dikarya - ps15
Ascomycota - ps16
Saccharo/Pezizomycotina - ps17
Saccharomycotina - ps18
Saccharomycotina_1 - ps19
Saccharomycotina_2 - ps20
Saccharomycotina_3 - ps21
Phaff./Saccharomycetaceae - ps22
Saccharomyco./mycetaceae - ps23
Saccharomycetaceae - ps24
Saccharomycetaceae_2 - ps25
Saccharomycetaceae_3 - ps26
Kaz./Nau./Saccharomyces - ps27
Saccharomyces - ps28
Arboricola_clade - ps29
Cerevisiae_clade - ps30
Saccharomyces_cerevisiae - ps31

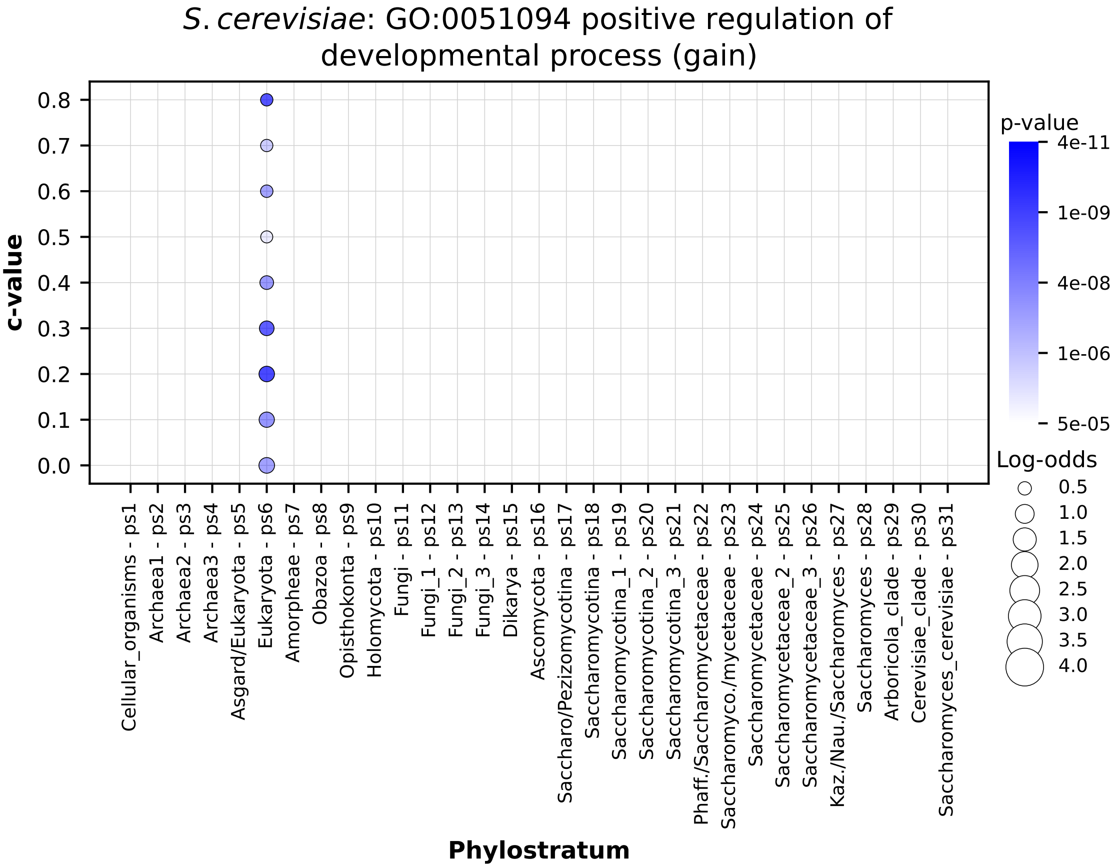

S. cerevisiae: GO:0051094 positive regulation of developmental process (gain)
c-value
0.8
0.7
0.6
0.5
0.4
0.3
0.2
0.1
0.0
Phylostratum
Cellular_organisms - ps1
Archaea1 - ps2
Archaea2 - ps3
Archaea3 - ps4
Asgard/Eukaryota - ps5
Eukaryota - ps6
Amorpheae - ps7
Obazoa - ps8
Opisthokonta - ps9
Holomycota - ps10
Fungi - ps11
Fungi_1 - ps12
Fungi_2 - ps13
Fungi_3 - ps14
Dikarya - ps15
Ascomycota - ps16
Saccharo/Pezizomycotina - ps17
Saccharomycotina - ps18
Saccharomycotina_1 - ps19
Saccharomycotina_2 - ps20
Saccharomycotina_3 - ps21
Phaff./Saccharomycetaceae - ps22
Saccharomyco./mycetaceae - ps23
Saccharomycetaceae - ps24
Saccharomycetaceae_2 - ps25
Saccharomycetaceae_3 - ps26
Kaz./Nau./Saccharomyces - ps27
Saccharomyces - ps28
Arboricola_clade - ps29
Cerevisiae_clade - ps30
Saccharomyces_cerevisiae - ps31
p-value
4e-11
1e-09
4e-08
1e-06
5e-05
Log-odds
0.5
1.0
1.5
2.0
2.5
3.0
3.5
4.0

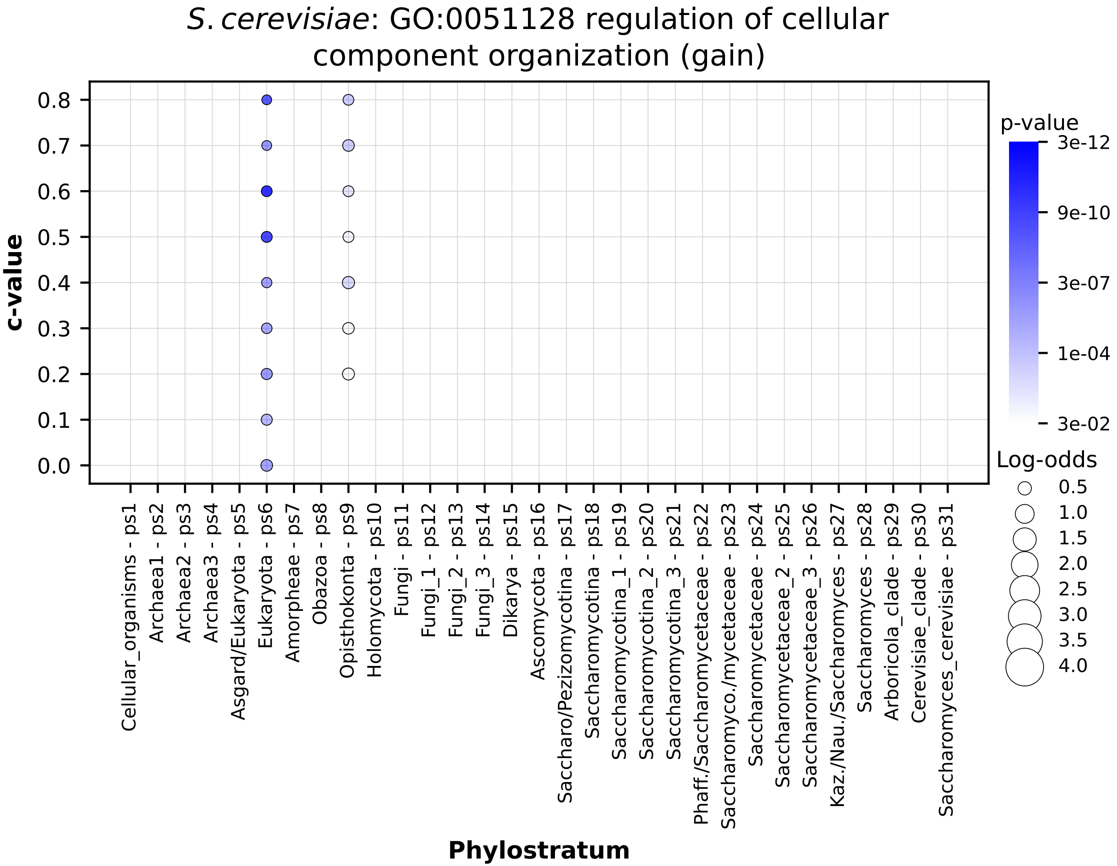

S. cerevisiae: GO:0051128 regulation of cellular component organization (gain)
c-value
0.8
0.7
0.6
0.5
0.4
0.3
0.2
0.1
0.0
Cellular_organisms - ps1
Archaea1 - ps2
Archaea2 - ps3
Archaea3 - ps4
Asgard/Eukaryota - ps5
Eukaryota - ps6
Amorpheae - ps7
Obazoa - ps8
Opisthokonta - ps9
Holomycota - ps10
Fungi - ps11
Fungi_1 - ps12
Fungi_2 - ps13
Fungi_3 - ps14
Dikarya - ps15
Ascomycota - ps16
Saccharo/Pezizomycotina - ps17
Saccharomycotina - ps18
Saccharomycotina_1 - ps19
Saccharomycotina_2 - ps20
Saccharomycotina_3 - ps21
Phaff./Saccharomycetaceae - ps22
Saccharomyco./mycetaceae - ps23
Saccharomycetaceae - ps24
Saccharomycetaceae_2 - ps25
Saccharomycetaceae_3 - ps26
Kaz./Nau./Saccharomyces - ps27
Saccharomyces - ps28
Arboricola_clade - ps29
Cerevisiae_clade - ps30
Saccharomyces_cerevisiae - ps31
Phylostratum
p-value
3e-12
9e-10
3e-07
1e-04
3e-02
Log-odds
0.5
1.0
1.5
2.0
2.5
3.0
3.5
4.0

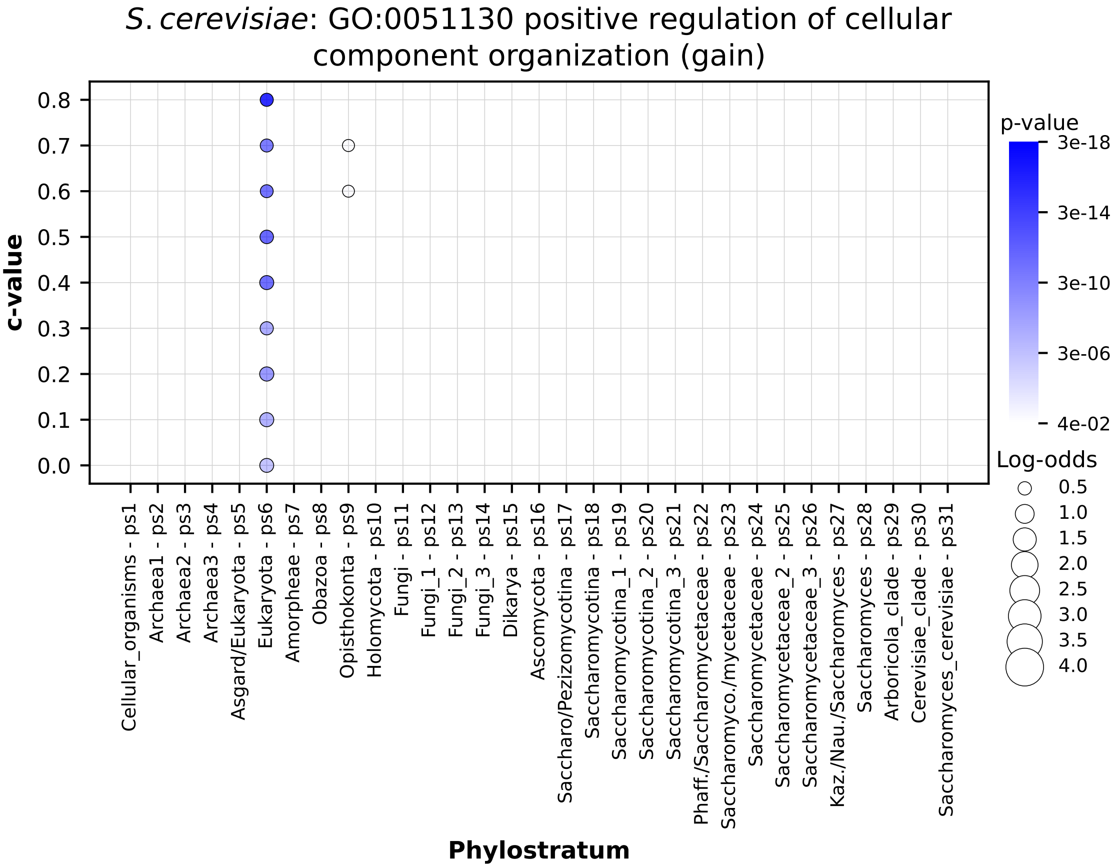

S. cerevisiae: GO:0051130 positive regulation of cellular component organization (gain)
c-value
0.0
0.1
0.2
0.3
0.4
0.5
0.6
0.7
0.8
Phylostratum
Cellular_organisms - ps1
Archaea1 - ps2
Archaea2 - ps3
Archaea3 - ps4
Asgard/Eukaryota - ps5
Eukaryota - ps6
Amorpheae - ps7
Obazoa - ps8
Opisthokonta - ps9
Holomycota - ps10
Fungi - ps11
Fungi_1 - ps12
Fungi_2 - ps13
Fungi_3 - ps14
Dikarya - ps15
Ascomycota - ps16
Saccharo/Pezizomycotina - ps17
Saccharomycotina - ps18
Saccharomycotina_1 - ps19
Saccharomycotina_2 - ps20
Saccharomycotina_3 - ps21
Phaff./Saccharomycetaceae - ps22
Saccharomyco./mycetaceae - ps23
Saccharomycetaceae - ps24
Saccharomycetaceae_2 - ps25
Saccharomycetaceae_3 - ps26
Kaz./Nau./Saccharomyces - ps27
Saccharomyces - ps28
Arboricola_clade - ps29
Cerevisiae_clade - ps30
Saccharomyces_cerevisiae - ps31
p-value
3e-18
3e-14
3e-10
3e-06
4e-02
Log-odds
0.5
1.0
1.5
2.0
2.5
3.0
3.5
4.0

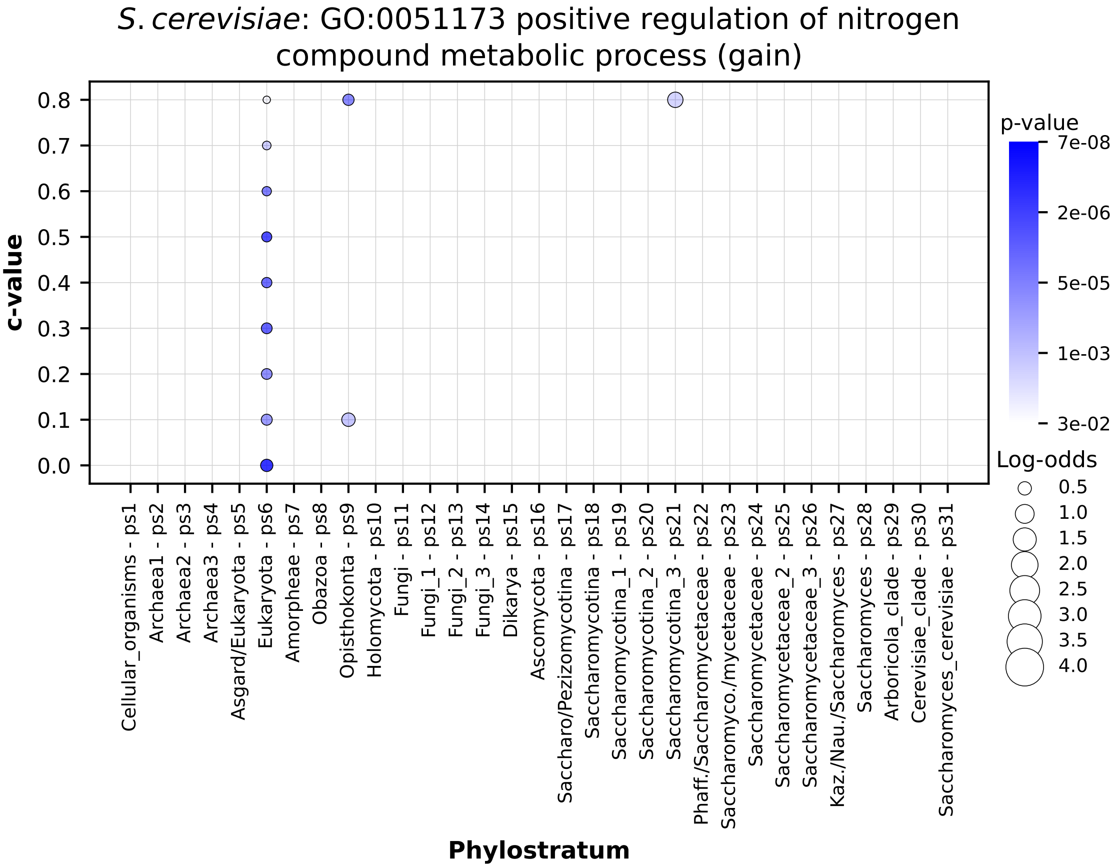

S. cerevisiae: GO:0051173 positive regulation of nitrogen compound metabolic process (gain)
c-value
0.8
0.7
0.6
0.5
0.4
0.3
0.2
0.1
0.0
p-value
7e-08
2e-06
5e-05
1e-03
3e-02
Log-odds
0.5
1.0
1.5
2.0
2.5
3.0
3.5
4.0
Cellular_organisms - ps1
Archaea1 - ps2
Archaea2 - ps3
Archaea3 - ps4
Asgard/Eukaryota - ps5
Eukaryota - ps6
Amorpheae - ps7
Obazoa - ps8
Opisthokonta - ps9
Holomycota - ps10
Fungi - ps11
Fungi_1 - ps12
Fungi_2 - ps13
Fungi_3 - ps14
Dikarya - ps15
Ascomycota - ps16
Saccharo/Pezizomycotina - ps17
Saccharomycotina - ps18
Saccharomycotina_1 - ps19
Saccharomycotina_2 - ps20
Saccharomycotina_3 - ps21
Phaff./Saccharomycetaceae - ps22
Saccharomyco./mycetaceae - ps23
Saccharomycetaceae - ps24
Saccharomycetaceae_2 - ps25
Saccharomycetaceae_3 - ps26
Kaz./Nau./Saccharomyces - ps27
Saccharomyces - ps28
Arboricola_clade - ps29
Cerevisiae_clade - ps30
Saccharomyces_cerevisiae - ps31
Phylostratum

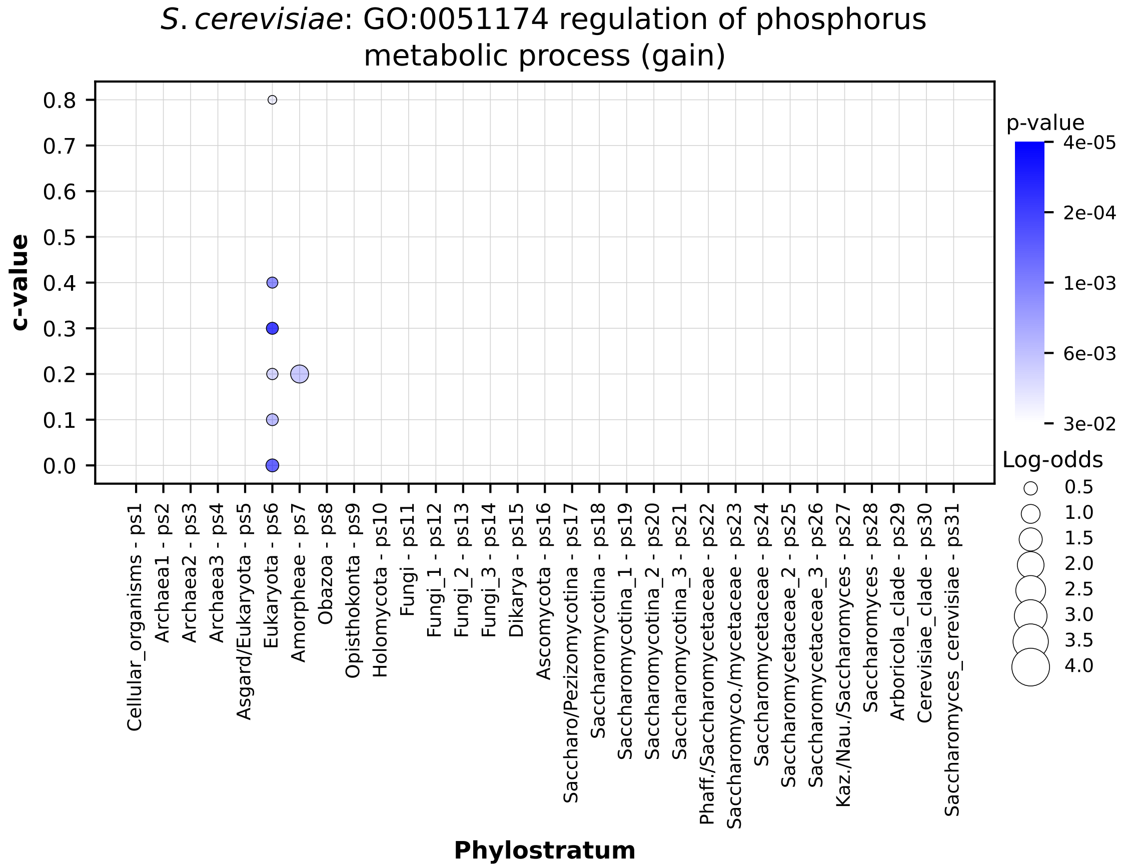

S. cerevisiae: GO:0051174 regulation of phosphorus metabolic process (gain)
c-value
0.8
0.7
0.6
0.5
0.4
0.3
0.2
0.1
0.0
p-value
4e-05
2e-04
1e-03
6e-03
3e-02
Log-odds
0.5
1.0
1.5
2.0
2.5
3.0
3.5
4.0
Cellular_organisms - ps1
Archaea1 - ps2
Archaea2 - ps3
Archaea3 - ps4
Asgard/Eukaryota - ps5
Eukaryota - ps6
Amorpheae - ps7
Obazoa - ps8
Opisthokonta - ps9
Holomycota - ps10
Fungi - ps11
Fungi_1 - ps12
Fungi_2 - ps13
Fungi_3 - ps14
Dikarya - ps15
Ascomycota - ps16
Saccharo/Pezizomycotina - ps17
Saccharomycotina - ps18
Saccharomycotina_1 - ps19
Saccharomycotina_2 - ps20
Saccharomycotina_3 - ps21
Phaff./Saccharomycetaceae - ps22
Saccharomyco./mycetaceae - ps23
Saccharomycetaceae - ps24
Saccharomycetaceae_2 - ps25
Saccharomycetaceae_3 - ps26
Kaz./Nau./Saccharomyces - ps27
Saccharomyces - ps28
Arboricola_clade - ps29
Cerevisiae_clade - ps30
Saccharomyces_cerevisiae - ps31
Phylostratum

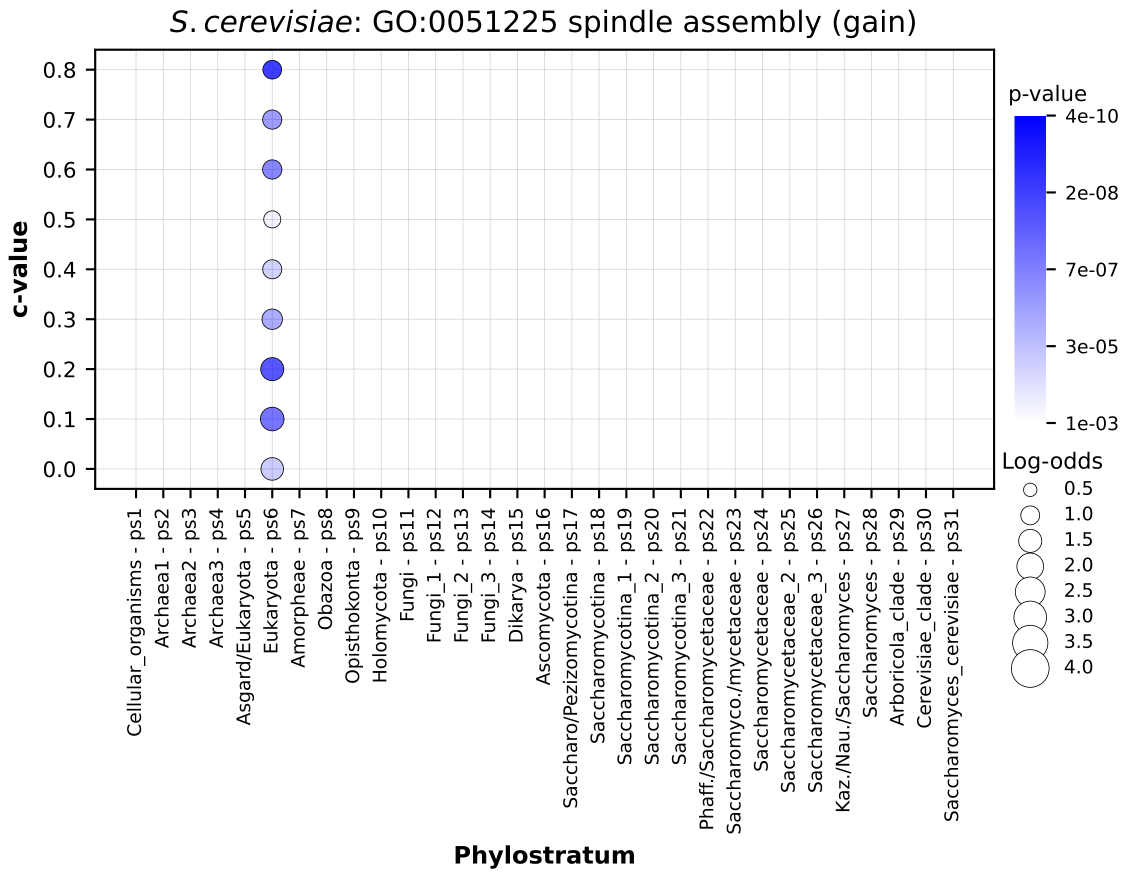

S. cerevisiae: GO:0051225 spindle assembly (gain)
c-value
0.8
0.7
0.6
0.5
0.4
0.3
0.2
0.1
0.0
p-value
4e-10
2e-08
7e-07
3e-05
1e-03
Log-odds
0.5
1.0
1.5
2.0
2.5
3.0
3.5
4.0
Cellular_organisms - ps1
Archaea1 - ps2
Archaea2 - ps3
Archaea3 - ps4
Asgard/Eukaryota - ps5
Eukaryota - ps6
Amorpheae - ps7
Obazoa - ps8
Opisthokonta - ps9
Holomycota - ps10
Fungi - ps11
Fungi_1 - ps12
Fungi_2 - ps13
Fungi_3 - ps14
Dikarya - ps15
Ascomycota - ps16
Saccharo/Pezizomycotina - ps17
Saccharomycotina - ps18
Saccharomycotina_1 - ps19
Saccharomycotina_2 - ps20
Saccharomycotina_3 - ps21
Phaff./Saccharomycetaceae - ps22
Saccharomyco./mycetaceae - ps23
Saccharomycetaceae - ps24
Saccharomycetaceae_2 - ps25
Saccharomycetaceae_3 - ps26
Kaz./Nau./Saccharomyces - ps27
Saccharomyces - ps28
Arboricola_clade - ps29
Cerevisiae_clade - ps30
Saccharomyces_cerevisiae - ps31
Phylostratum

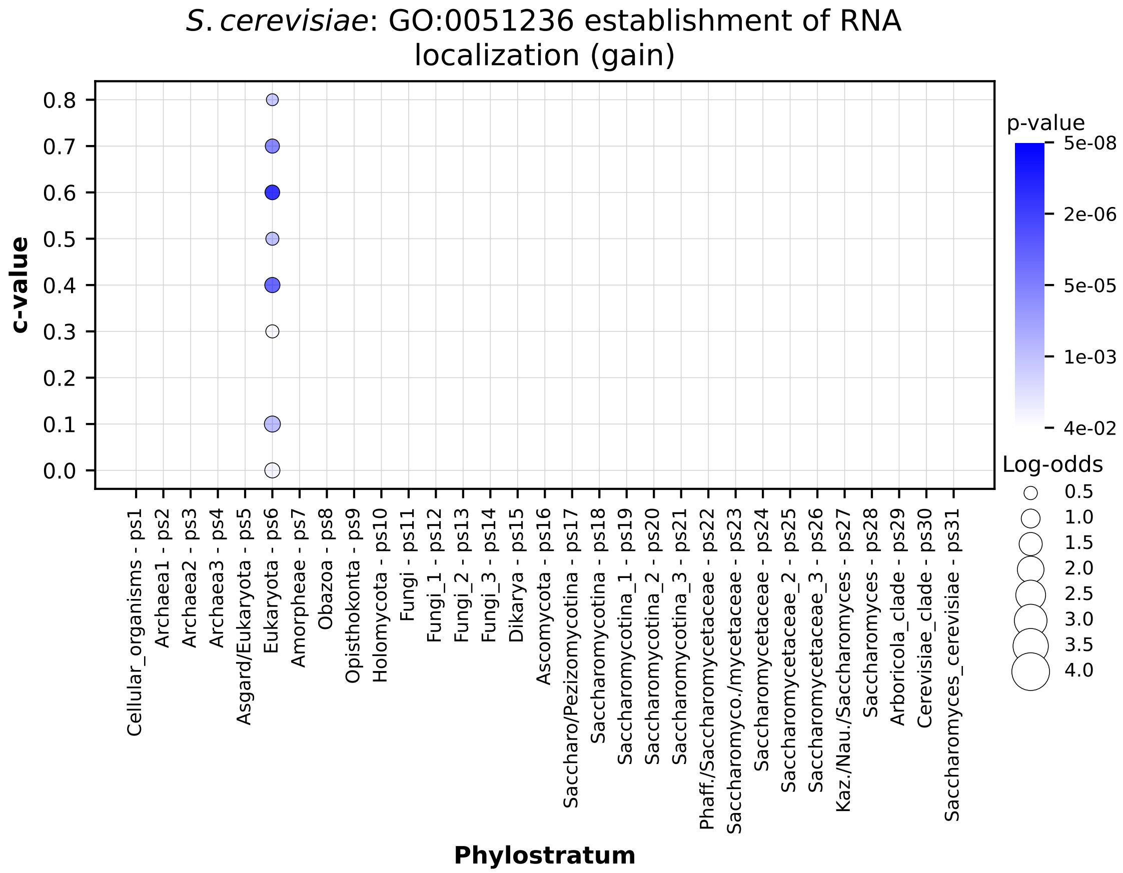

S. cerevisiae: GO:0051236 establishment of RNA localization (gain)
c-value
0.8
0.7
0.6
0.5
0.4
0.3
0.2
0.1
0.0
p-value
5e-08
2e-06
5e-05
1e-03
4e-02
Log-odds
0.5
1.0
1.5
2.0
2.5
3.0
3.5
4.0
Cellular_organisms - ps1
Archaea1 - ps2
Archaea2 - ps3
Archaea3 - ps4
Asgard/Eukaryota - ps5
Eukaryota - ps6
Amorpheae - ps7
Obazoa - ps8
Opisthokonta - ps9
Holomycota - ps10
Fungi - ps11
Fungi_1 - ps12
Fungi_2 - ps13
Fungi_3 - ps14
Dikarya - ps15
Ascomycota - ps16
Saccharo/Pezizomycotina - ps17
Saccharomycotina - ps18
Saccharomycotina_1 - ps19
Saccharomycotina_2 - ps20
Saccharomycotina_3 - ps21
Phaff./Saccharomycetaceae - ps22
Saccharomyco./mycetaceae - ps23
Saccharomycetaceae - ps24
Saccharomycetaceae_2 - ps25
Saccharomycetaceae_3 - ps26
Kaz./Nau./Saccharomyces - ps27
Saccharomyces - ps28
Arboricola_clade - ps29
Cerevisiae_clade - ps30
Saccharomyces_cerevisiae - ps31
Phylostratum

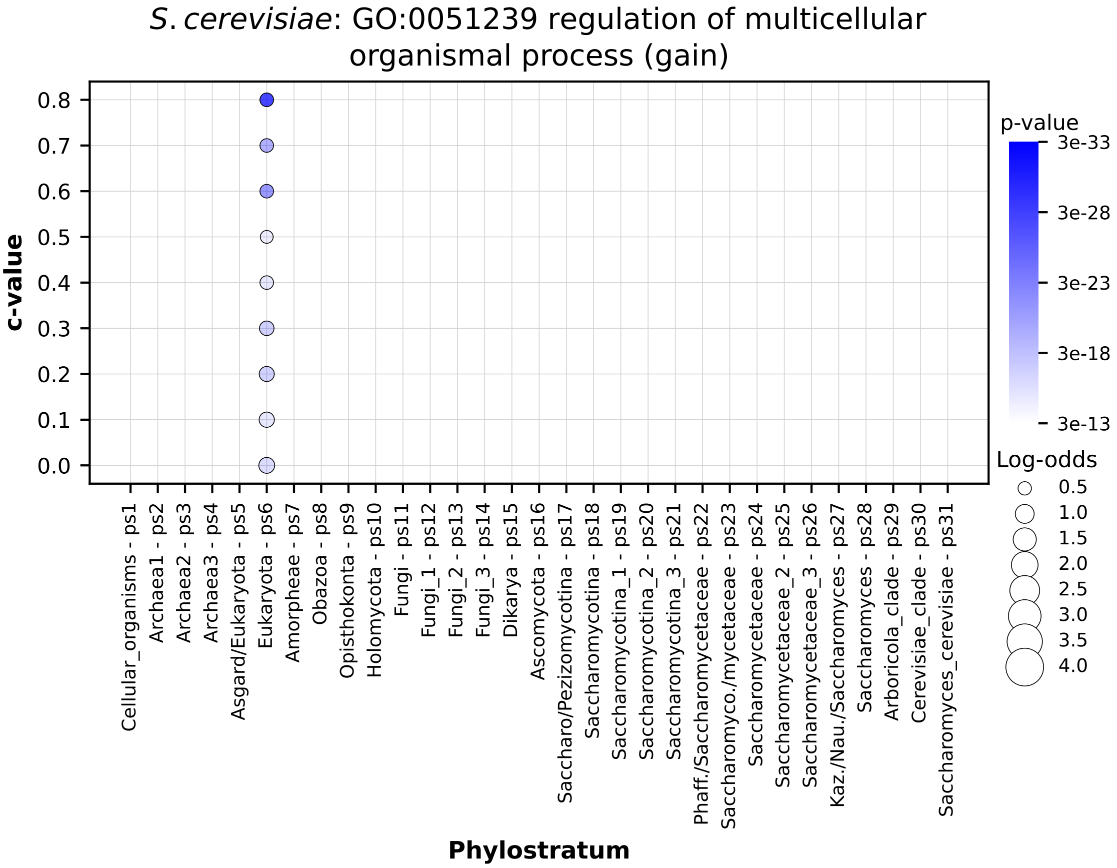

S. cerevisiae: GO:0051239 regulation of multicellular organismal process (gain)
c-value
0.0
0.1
0.2
0.3
0.4
0.5
0.6
0.7
0.8
p-value
3e-33
3e-28
3e-23
3e-18
3e-13
Log-odds
0.5
1.0
1.5
2.0
2.5
3.0
3.5
4.0
Cellular_organisms - ps1
Archaea1 - ps2
Archaea2 - ps3
Archaea3 - ps4
Asgard/Eukaryota - ps5
Eukaryota - ps6
Amorpheae - ps7
Obazoa - ps8
Opisthokonta - ps9
Holomycota - ps10
Fungi - ps11
Fungi_1 - ps12
Fungi_2 - ps13
Fungi_3 - ps14
Dikarya - ps15
Ascomycota - ps16
Saccharo/Pezizomycotina - ps17
Saccharomycotina - ps18
Saccharomycotina_1 - ps19
Saccharomycotina_2 - ps20
Saccharomycotina_3 - ps21
Phaff./Saccharomycetaceae - ps22
Saccharomyco./mycetaceae - ps23
Saccharomycetaceae - ps24
Saccharomycetaceae_2 - ps25
Saccharomycetaceae_3 - ps26
Kaz./Nau./Saccharomyces - ps27
Saccharomyces - ps28
Arboricola_clade - ps29
Cerevisiae_clade - ps30
Saccharomyces_cerevisiae - ps31
Phylostratum

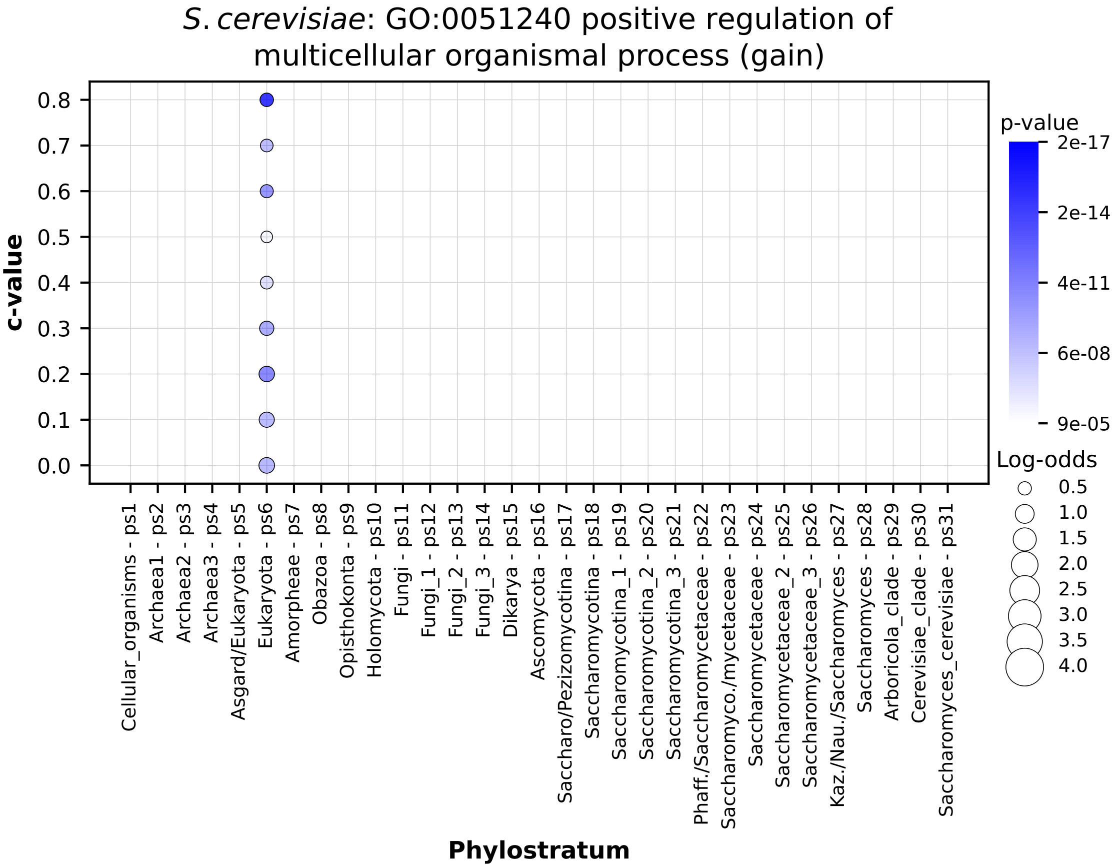

S. cerevisiae: GO:0051240 positive regulation of multicellular organismal process (gain)
c-value
0.8
0.7
0.6
0.5
0.4
0.3
0.2
0.1
0.0
Phylostratum
Cellular_organisms - ps1
Archaea1 - ps2
Archaea2 - ps3
Archaea3 - ps4
Asgard/Eukaryota - ps5
Eukaryota - ps6
Amorpheae - ps7
Obazoa - ps8
Opisthokonta - ps9
Holomycota - ps10
Fungi - ps11
Fungi_1 - ps12
Fungi_2 - ps13
Fungi_3 - ps14
Dikarya - ps15
Ascomycota - ps16
Saccharo/Pezizomycotina - ps17
Saccharomycotina - ps18
Saccharomycotina_1 - ps19
Saccharomycotina_2 - ps20
Saccharomycotina_3 - ps21
Phaff./Saccharomycetaceae - ps22
Saccharomyco./mycetaceae - ps23
Saccharomycetaceae - ps24
Saccharomycetaceae_2 - ps25
Saccharomycetaceae_3 - ps26
Kaz./Nau./Saccharomyces - ps27
Saccharomyces - ps28
Arboricola_clade - ps29
Cerevisiae_clade - ps30
Saccharomyces_cerevisiae - ps31
p-value
2e-17
2e-14
4e-11
6e-08
9e-05
Log-odds
0.5
1.0
1.5
2.0
2.5
3.0
3.5
4.0

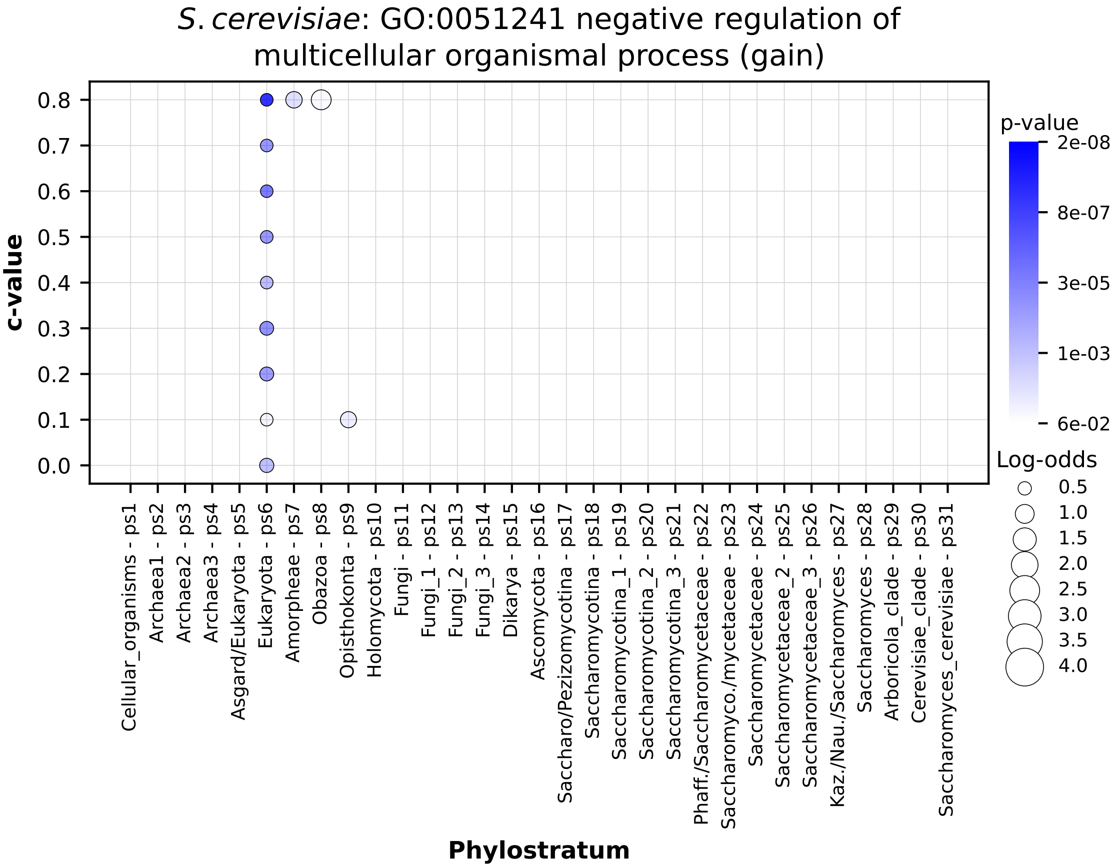

S. cerevisiae: GO:0051241 negative regulation of multicellular organismal process (gain)
c-value
0.8
0.7
0.6
0.5
0.4
0.3
0.2
0.1
0.0
p-value
2e-08
8e-07
3e-05
1e-03
6e-02
Log-odds
0.5
1.0
1.5
2.0
2.5
3.0
3.5
4.0
Cellular_organisms - ps1
Archaea1 - ps2
Archaea2 - ps3
Archaea3 - ps4
Asgard/Eukaryota - ps5
Eukaryota - ps6
Amorpheae - ps7
Obazoa - ps8
Opisthokonta - ps9
Holomycota - ps10
Fungi - ps11
Fungi_1 - ps12
Fungi_2 - ps13
Fungi_3 - ps14
Dikarya - ps15
Ascomycota - ps16
Saccharo/Pezizomycotina - ps17
Saccharomycotina - ps18
Saccharomycotina_1 - ps19
Saccharomycotina_2 - ps20
Saccharomycotina_3 - ps21
Phaff./Saccharomycetaceae - ps22
Saccharomyco./mycetaceae - ps23
Saccharomycetaceae - ps24
Saccharomycetaceae_2 - ps25
Saccharomycetaceae_3 - ps26
Kaz./Nau./Saccharomyces - ps27
Saccharomyces - ps28
Arboricola_clade - ps29
Cerevisiae_clade - ps30
Saccharomyces_cerevisiae - ps31
Phylostratum

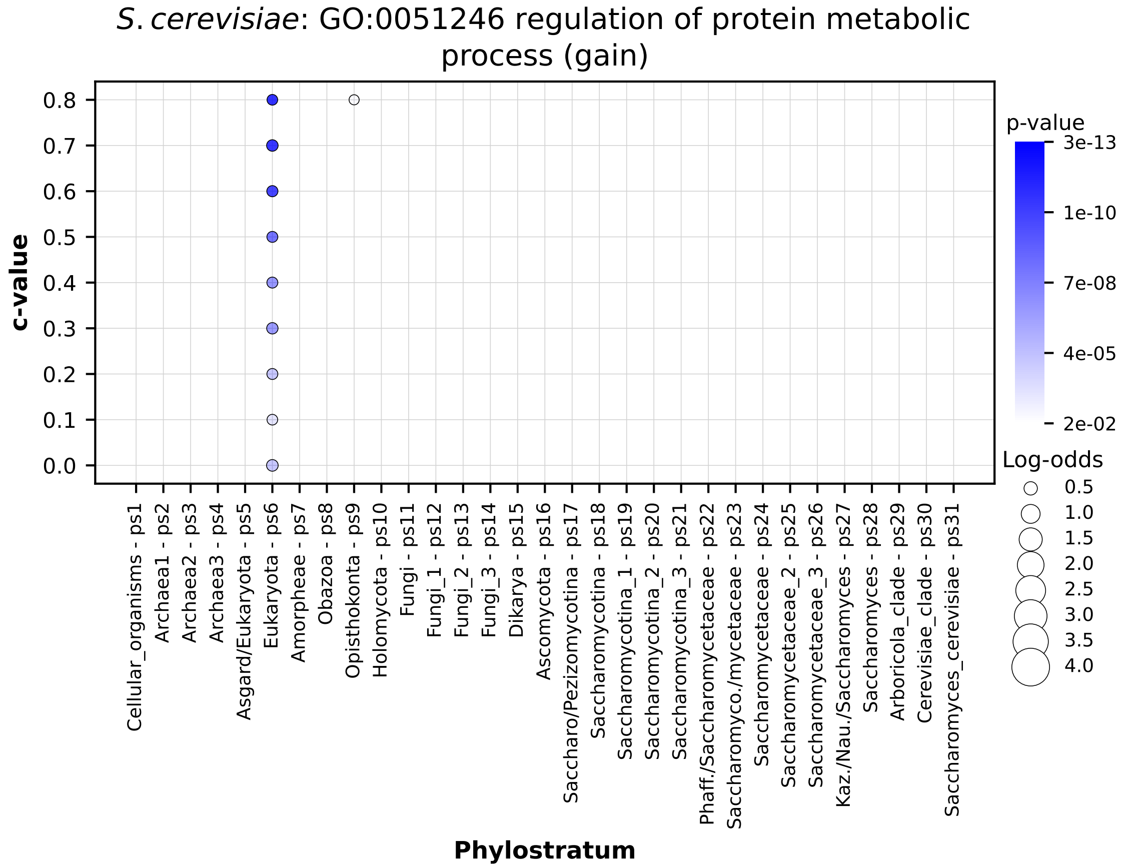

S. cerevisiae: GO:0051246 regulation of protein metabolic process (gain)
c-value
0.0
0.1
0.2
0.3
0.4
0.5
0.6
0.7
0.8
p-value
3e-13
1e-10
7e-08
4e-05
2e-02
Log-odds
0.5
1.0
1.5
2.0
2.5
3.0
3.5
4.0
Cellular_organisms - ps1
Archaea1 - ps2
Archaea2 - ps3
Archaea3 - ps4
Asgard/Eukaryota - ps5
Eukaryota - ps6
Amorpheae - ps7
Obazoa - ps8
Opisthokonta - ps9
Holomycota - ps10
Fungi - ps11
Fungi_1 - ps12
Fungi_2 - ps13
Fungi_3 - ps14
Dikarya - ps15
Ascomycota - ps16
Saccharo/Pezizomycotina - ps17
Saccharomycotina - ps18
Saccharomycotina_1 - ps19
Saccharomycotina_2 - ps20
Saccharomycotina_3 - ps21
Phaff./Saccharomycetaceae - ps22
Saccharomyco./mycetaceae - ps23
Saccharomycetaceae - ps24
Saccharomycetaceae_2 - ps25
Saccharomycetaceae_3 - ps26
Kaz./Nau./Saccharomyces - ps27
Saccharomyces - ps28
Arboricola_clade - ps29
Cerevisiae_clade - ps30
Saccharomyces_cerevisiae - ps31
Phylostratum

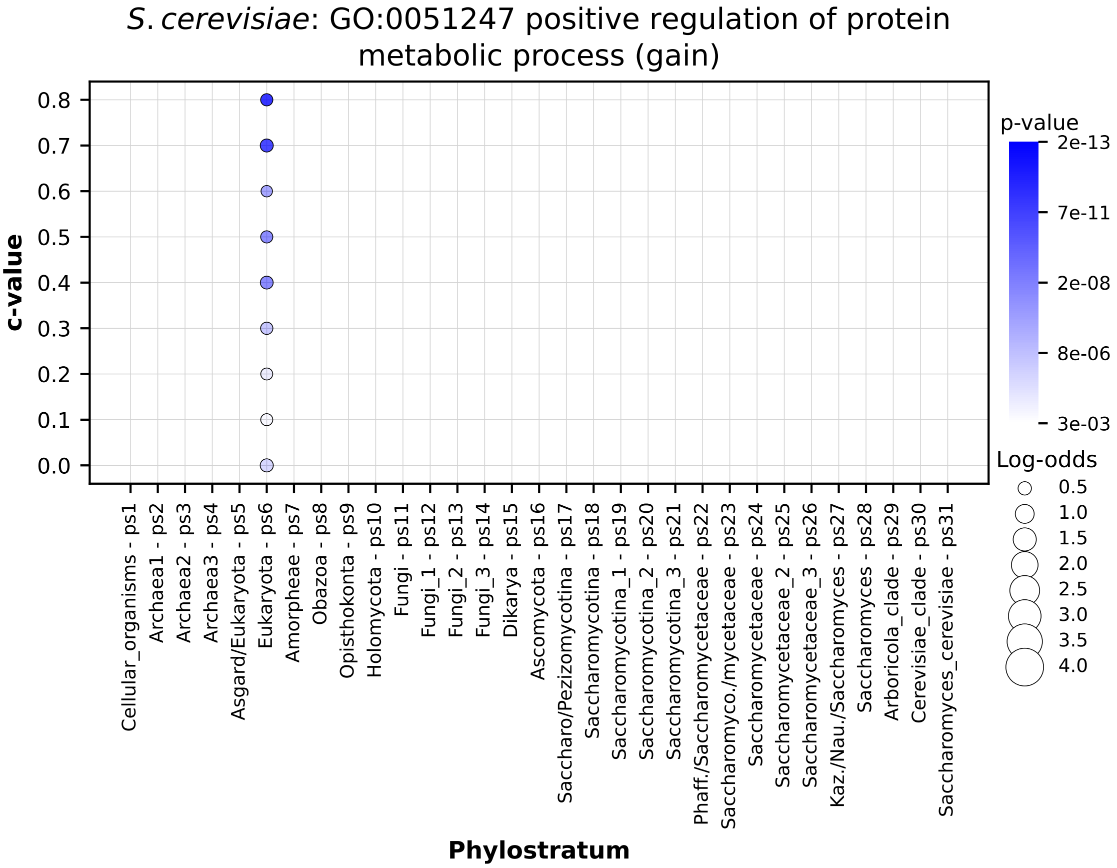

S. cerevisiae: GO:0051247 positive regulation of protein metabolic process (gain)
c-value
0.8
0.7
0.6
0.5
0.4
0.3
0.2
0.1
0.0
p-value
2e-13
7e-11
2e-08
8e-06
3e-03
Log-odds
0.5
1.0
1.5
2.0
2.5
3.0
3.5
4.0
Cellular_organisms - ps1
Archaea1 - ps2
Archaea2 - ps3
Archaea3 - ps4
Asgard/Eukaryota - ps5
Eukaryota - ps6
Amorpheae - ps7
Obazoa - ps8
Opisthokonta - ps9
Holomycota - ps10
Fungi - ps11
Fungi_1 - ps12
Fungi_2 - ps13
Fungi_3 - ps14
Dikarya - ps15
Ascomycota - ps16
Saccharo/Pezizomycotina - ps17
Saccharomycotina - ps18
Saccharomycotina_1 - ps19
Saccharomycotina_2 - ps20
Saccharomycotina_3 - ps21
Phaff./Saccharomycetaceae - ps22
Saccharomyco./mycetaceae - ps23
Saccharomycetaceae - ps24
Saccharomycetaceae_2 - ps25
Saccharomycetaceae_3 - ps26
Kaz./Nau./Saccharomyces - ps27
Saccharomyces - ps28
Arboricola_clade - ps29
Cerevisiae_clade - ps30
Saccharomyces_cerevisiae - ps31
Phylostratum

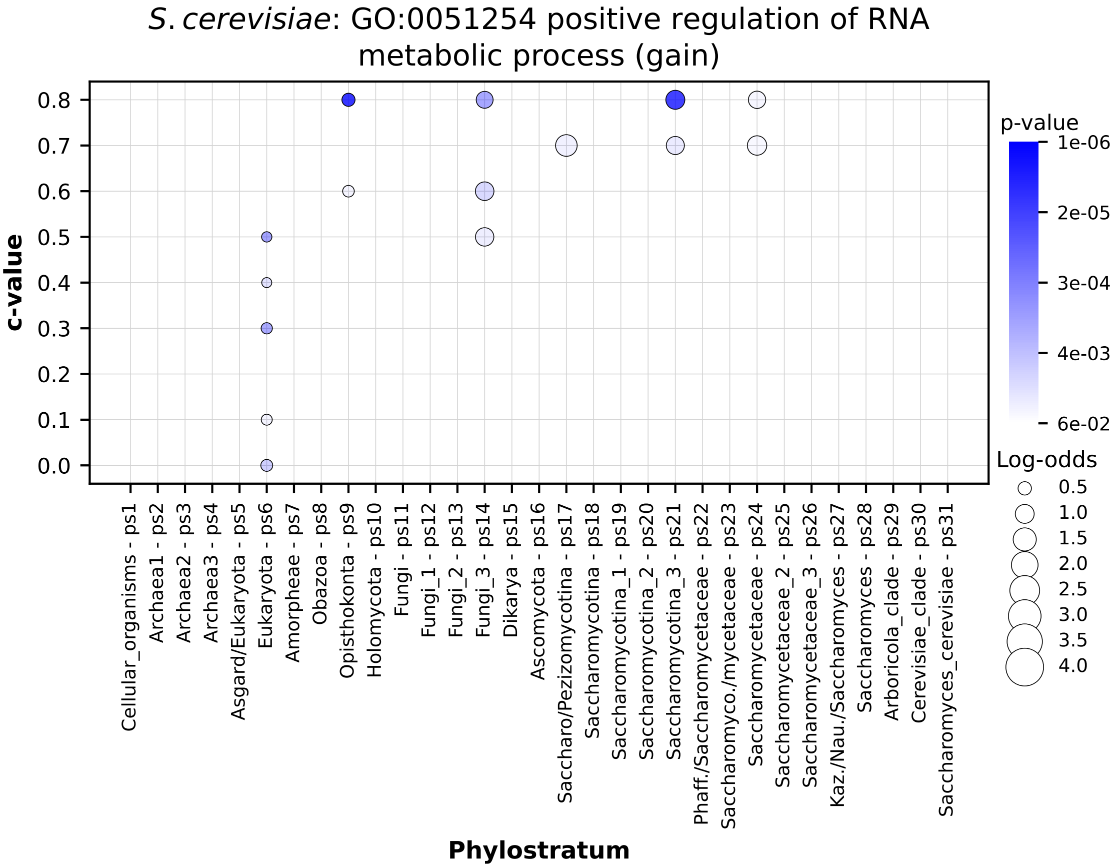

S. cerevisiae: GO:0051254 positive regulation of RNA metabolic process (gain)
c-value
0.0
0.1
0.2
0.3
0.4
0.5
0.6
0.7
0.8
Phylostratum
Cellular_organisms - ps1
Archaea1 - ps2
Archaea2 - ps3
Archaea3 - ps4
Asgard/Eukaryota - ps5
Eukaryota - ps6
Amorpheae - ps7
Obazoa - ps8
Opisthokonta - ps9
Holomycota - ps10
Fungi - ps11
Fungi_1 - ps12
Fungi_2 - ps13
Fungi_3 - ps14
Dikarya - ps15
Ascomycota - ps16
Saccharo/Pezizomycotina - ps17
Saccharomycotina - ps18
Saccharomycotina_1 - ps19
Saccharomycotina_2 - ps20
Saccharomycotina_3 - ps21
Phaff./Saccharomycetaceae - ps22
Saccharomyco./mycetaceae - ps23
Saccharomycetaceae - ps24
Saccharomycetaceae_2 - ps25
Saccharomycetaceae_3 - ps26
Kaz./Nau./Saccharomyces - ps27
Saccharomyces - ps28
Arboricola_clade - ps29
Cerevisiae_clade - ps30
Saccharomyces_cerevisiae - ps31
p-value
1e-06
2e-05
3e-04
4e-03
6e-02
Log-odds
0.5
1.0
1.5
2.0
2.5
3.0
3.5
4.0

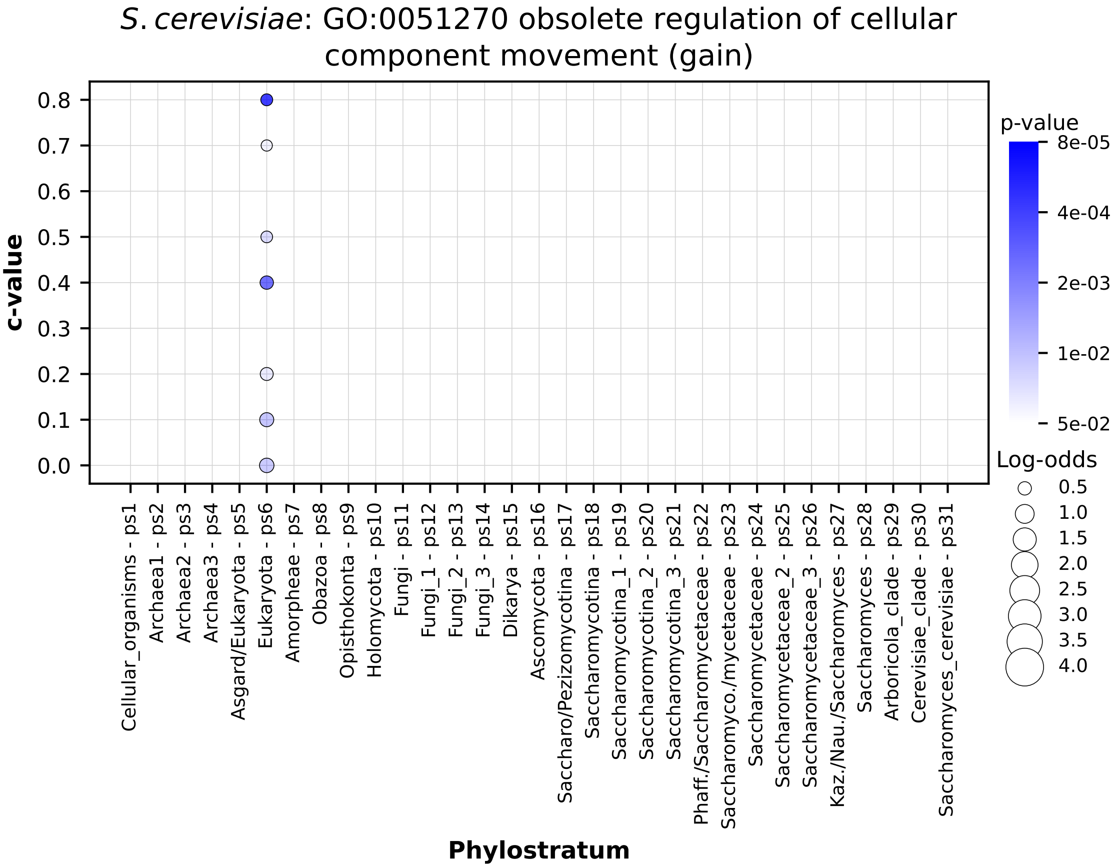

S. cerevisiae: GO:0051270 obsolete regulation of cellular component movement (gain)
c-value
0.8
0.7
0.6
0.5
0.4
0.3
0.2
0.1
0.0
p-value
8e-05
4e-04
2e-03
1e-02
5e-02
Log-odds
0.5
1.0
1.5
2.0
2.5
3.0
3.5
4.0
Cellular_organisms - ps1
Archaea1 - ps2
Archaea2 - ps3
Archaea3 - ps4
Asgard/Eukaryota - ps5
Eukaryota - ps6
Amorpheae - ps7
Obazoa - ps8
Opisthokonta - ps9
Holomycota - ps10
Fungi - ps11
Fungi_1 - ps12
Fungi_2 - ps13
Fungi_3 - ps14
Dikarya - ps15
Ascomycota - ps16
Saccharo/Pezizomycotina - ps17
Saccharomycotina - ps18
Saccharomycotina_1 - ps19
Saccharomycotina_2 - ps20
Saccharomycotina_3 - ps21
Phaff./Saccharomycetaceae - ps22
Saccharomyco./mycetaceae - ps23
Saccharomycetaceae - ps24
Saccharomycetaceae_2 - ps25
Saccharomycetaceae_3 - ps26
Kaz./Nau./Saccharomyces - ps27
Saccharomyces - ps28
Arboricola_clade - ps29
Cerevisiae_clade - ps30
Saccharomyces_cerevisiae - ps31
Phylostratum

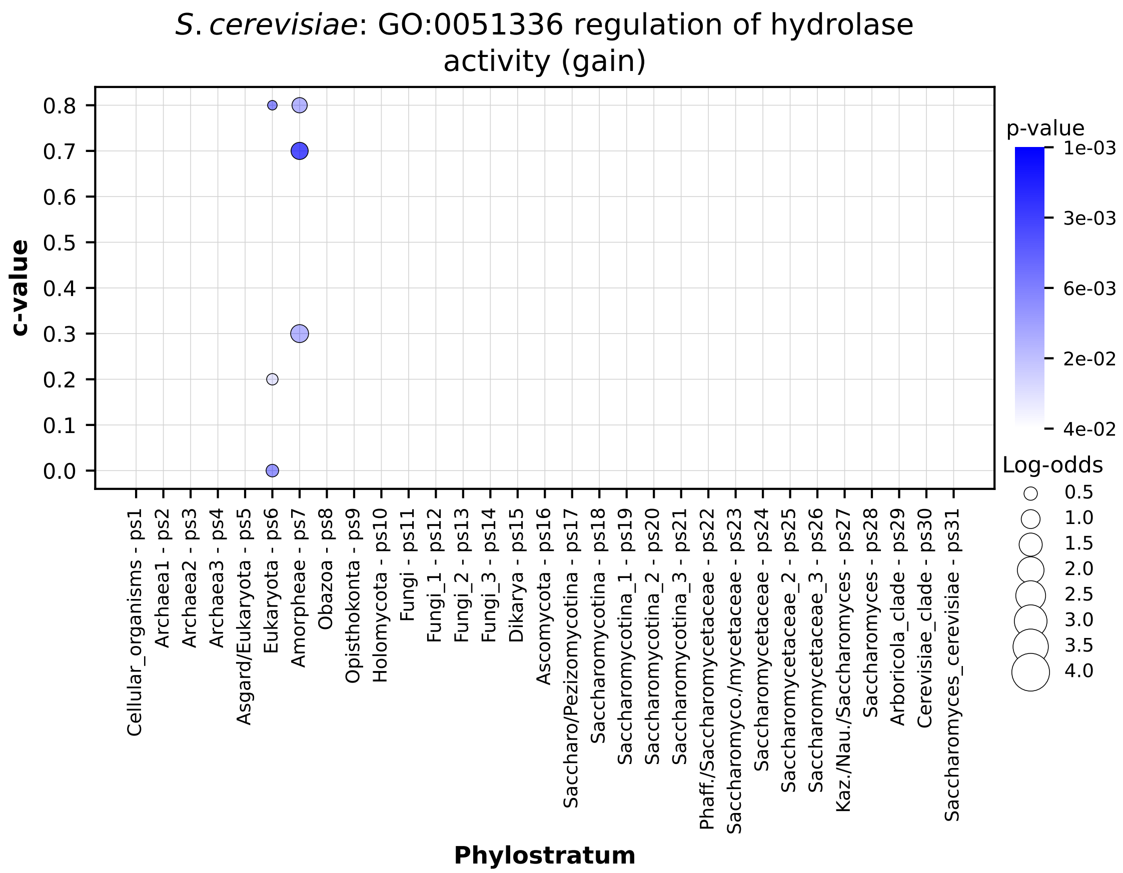
S. cerevisiae: GO:0051336 regulation of hydrolase activity (gain)
c-value
0.8
0.7
0.6
0.5
0.4
0.3
0.2
0.1
0.0
p-value
1e-03
3e-03
6e-03
2e-02
4e-02
Log-odds
0.5
1.0
1.5
2.0
2.5
3.0
3.5
4.0
Cellular_organisms - ps1
Archaea1 - ps2
Archaea2 - ps3
Archaea3 - ps4
Asgard/Eukaryota - ps5
Eukaryota - ps6
Amorpheae - ps7
Obazoa - ps8
Opisthokonta - ps9
Holomycota - ps10
Fungi - ps11
Fungi_1 - ps12
Fungi_2 - ps13
Fungi_3 - ps14
Dikarya - ps15
Ascomycota - ps16
Saccharo/Pezizomycotina - ps17
Saccharomycotina - ps18
Saccharomycotina_1 - ps19
Saccharomycotina_2 - ps20
Saccharomycotina_3 - ps21
Phaff./Saccharomycetaceae - ps22
Saccharomyco./mycetaceae - ps23
Saccharomycetaceae - ps24
Saccharomycetaceae_2 - ps25
Saccharomycetaceae_3 - ps26
Kaz./Nau./Saccharomyces - ps27
Saccharomyces - ps28
Arboricola_clade - ps29
Cerevisiae_clade - ps30
Saccharomyces_cerevisiae - ps31
Phylostratum

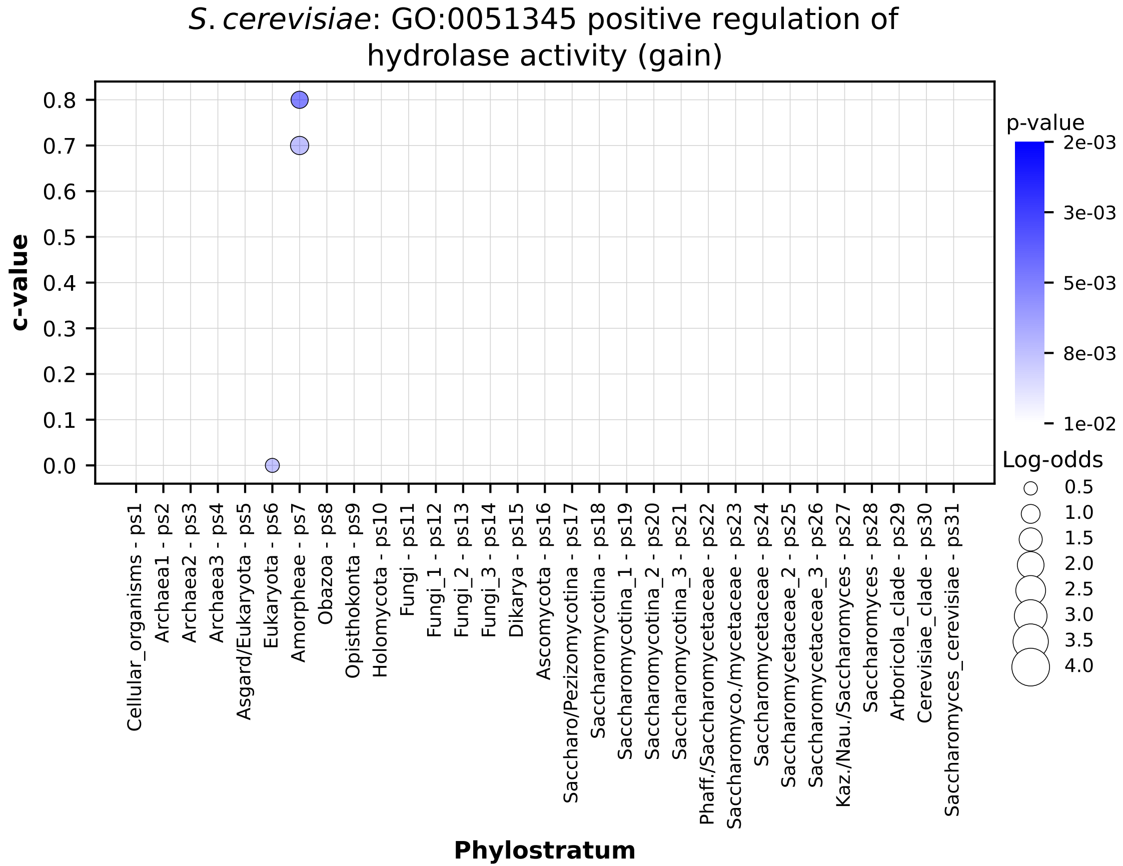

S. cerevisiae: GO:0051345 positive regulation of hydrolase activity (gain)
c-value
0.8
0.7
0.6
0.5
0.4
0.3
0.2
0.1
0.0
Phylostratum
Cellular_organisms - ps1
Archaea1 - ps2
Archaea2 - ps3
Archaea3 - ps4
Asgard/Eukaryota - ps5
Eukaryota - ps6
Amorpheae - ps7
Obazoa - ps8
Opisthokonta - ps9
Holomycota - ps10
Fungi - ps11
Fungi_1 - ps12
Fungi_2 - ps13
Fungi_3 - ps14
Dikarya - ps15
Ascomycota - ps16
Saccharo/Pezizomycotina - ps17
Saccharomycotina - ps18
Saccharomycotina_1 - ps19
Saccharomycotina_2 - ps20
Saccharomycotina_3 - ps21
Phaff./Saccharomycetaceae - ps22
Saccharomyco./mycetaceae - ps23
Saccharomycetaceae - ps24
Saccharomycetaceae_2 - ps25
Saccharomycetaceae_3 - ps26
Kaz./Nau./Saccharomyces - ps27
Saccharomyces - ps28
Arboricola_clade - ps29
Cerevisiae_clade - ps30
Saccharomyces_cerevisiae - ps31
p-value
2e-03
3e-03
5e-03
8e-03
1e-02
Log-odds
0.5
1.0
1.5
2.0
2.5
3.0
3.5
4.0

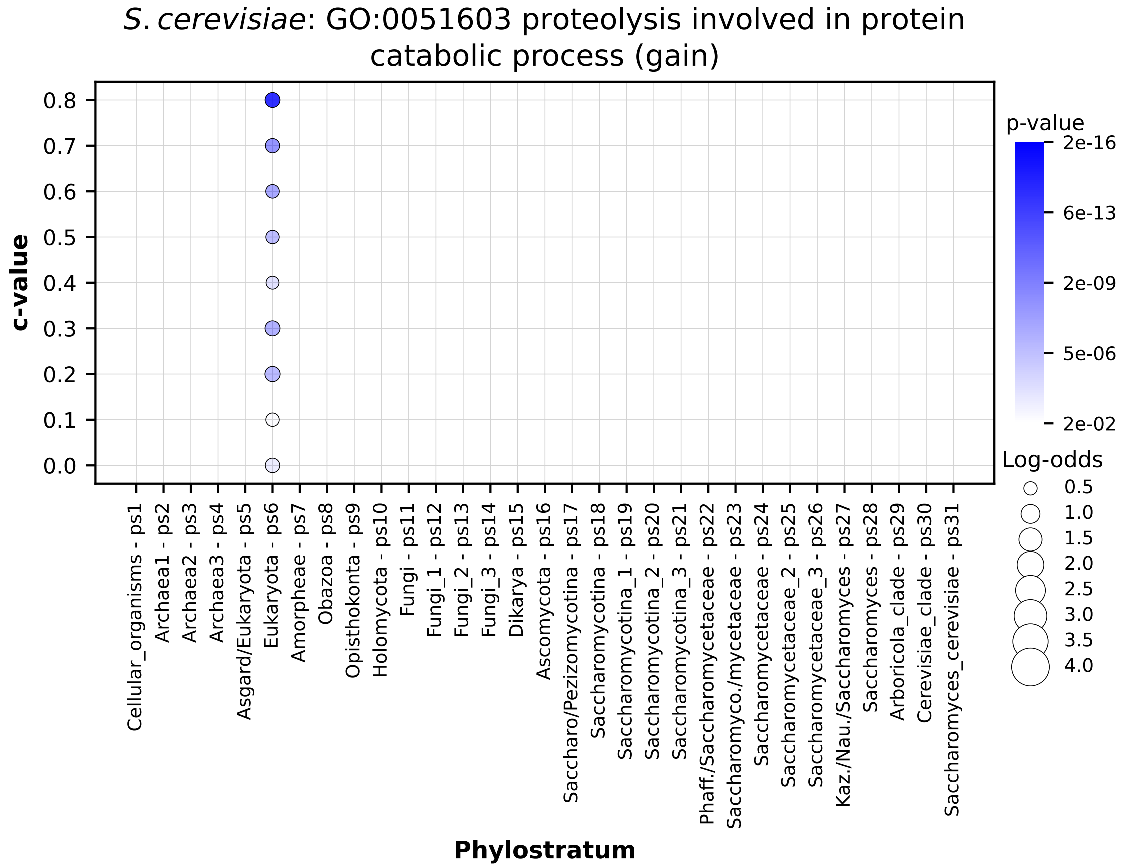

S. cerevisiae: GO:0051603 proteolysis involved in protein catabolic process (gain)
c-value
0.8
0.7
0.6
0.5
0.4
0.3
0.2
0.1
0.0
p-value
2e-16
6e-13
2e-09
5e-06
2e-02
Log-odds
0.5
1.0
1.5
2.0
2.5
3.0
3.5
4.0
Cellular_organisms - ps1
Archaea1 - ps2
Archaea2 - ps3
Archaea3 - ps4
Asgard/Eukaryota - ps5
Eukaryota - ps6
Amorpheae - ps7
Obazoa - ps8
Opisthokonta - ps9
Holomycota - ps10
Fungi - ps11
Fungi_1 - ps12
Fungi_2 - ps13
Fungi_3 - ps14
Dikarya - ps15
Ascomycota - ps16
Saccharo/Pezizomycotina - ps17
Saccharomycotina - ps18
Saccharomycotina_1 - ps19
Saccharomycotina_2 - ps20
Saccharomycotina_3 - ps21
Phaff./Saccharomycetaceae - ps22
Saccharomyco./mycetaceae - ps23
Saccharomycetaceae - ps24
Saccharomycetaceae_2 - ps25
Saccharomycetaceae_3 - ps26
Kaz./Nau./Saccharomyces - ps27
Saccharomyces - ps28
Arboricola_clade - ps29
Cerevisiae_clade - ps30
Saccharomyces_cerevisiae - ps31
Phylostratum

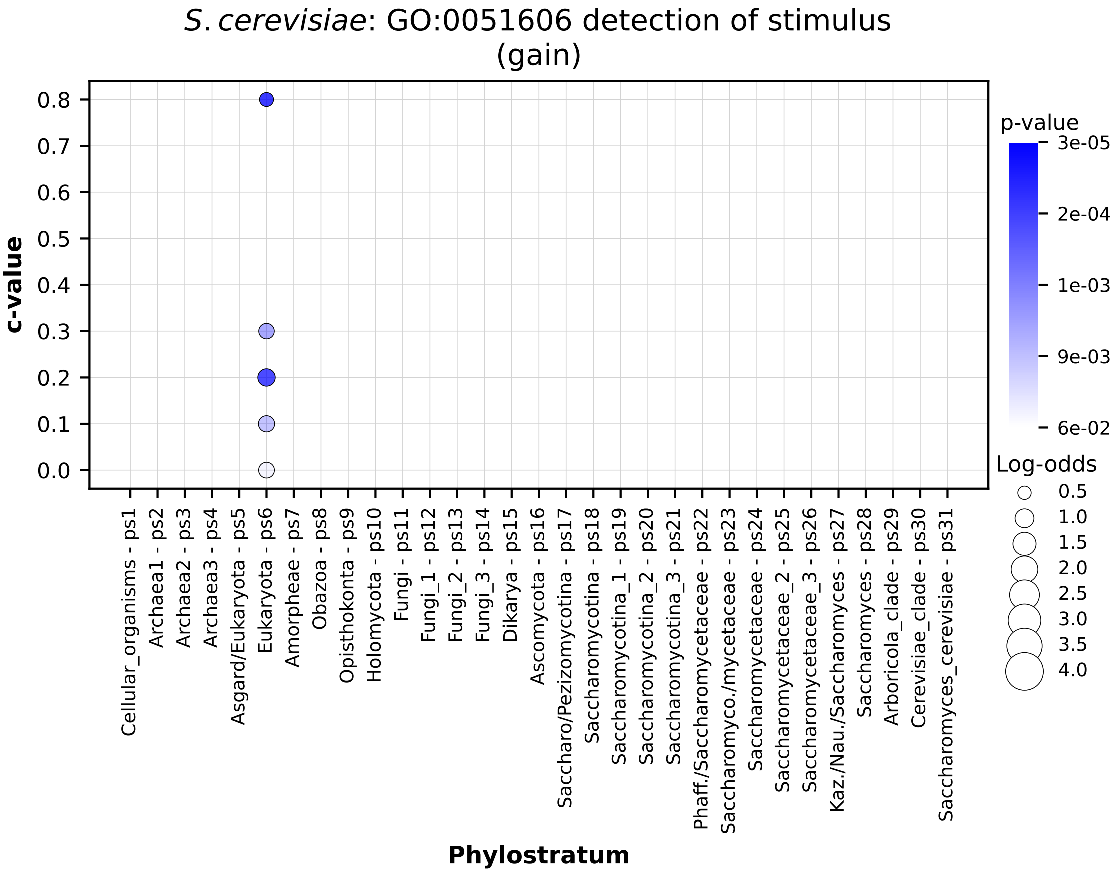
S. cerevisiae: GO:0051606 detection of stimulus (gain)
c-value
0.8
0.7
0.6
0.5
0.4
0.3
0.2
0.1
0.0
p-value
3e-05
2e-04
1e-03
9e-03
6e-02
Log-odds
0.5
1.0
1.5
2.0
2.5
3.0
3.5
4.0
Cellular_organisms - ps1
Archaea1 - ps2
Archaea2 - ps3
Archaea3 - ps4
Asgard/Eukaryota - ps5
Eukaryota - ps6
Amorpheae - ps7
Obazoa - ps8
Opisthokonta - ps9
Holomycota - ps10
Fungi - ps11
Fungi_1 - ps12
Fungi_2 - ps13
Fungi_3 - ps14
Dikarya - ps15
Ascomycota - ps16
Saccharo/Pezizomycotina - ps17
Saccharomycotina - ps18
Saccharomycotina_1 - ps19
Saccharomycotina_2 - ps20
Saccharomycotina_3 - ps21
Phaff./Saccharomycetaceae - ps22
Saccharomyco./mycetaceae - ps23
Saccharomycetaceae - ps24
Saccharomycetaceae_2 - ps25
Saccharomycetaceae_3 - ps26
Kaz./Nau./Saccharomyces - ps27
Saccharomyces - ps28
Arboricola_clade - ps29
Cerevisiae_clade - ps30
Saccharomyces_cerevisiae - ps31
Phylostratum

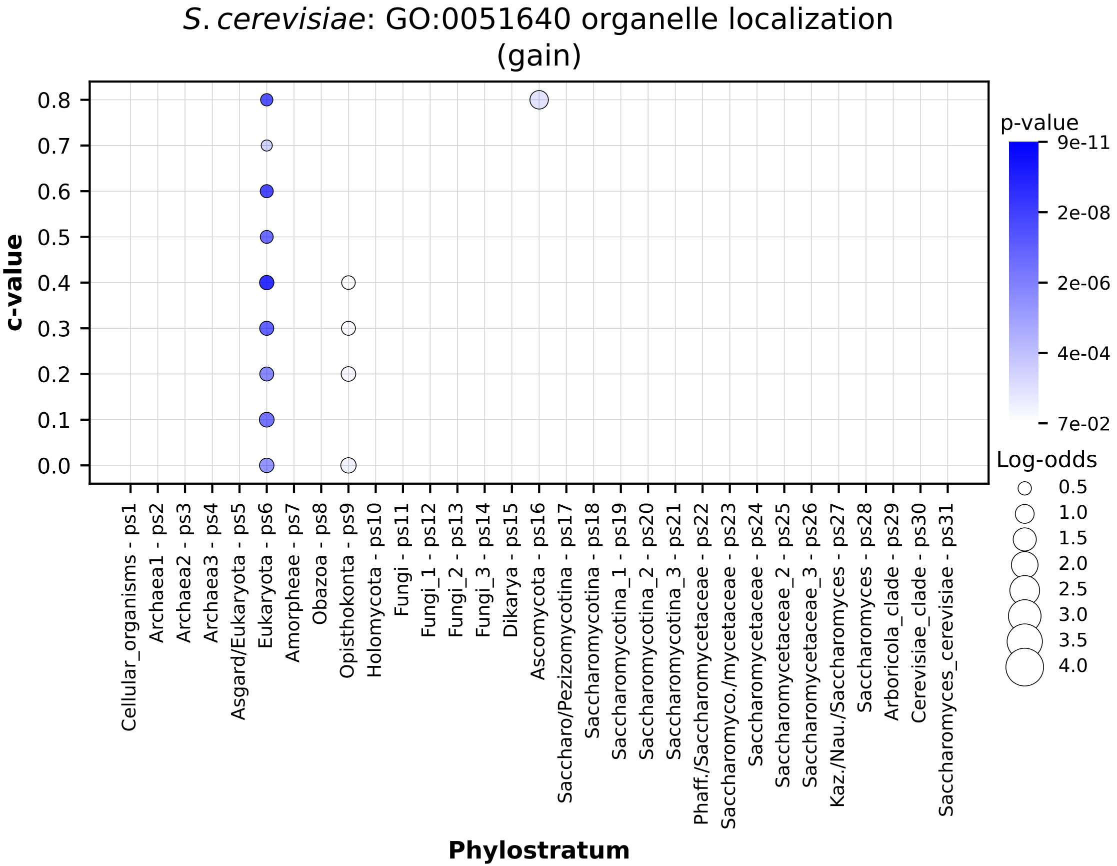

S. cerevisiae: GO:0051640 organelle localization
(gain)
c-value
0.8
0.7
0.6
0.5
0.4
0.3
0.2
0.1
0.0
p-value
9e-11
2e-08
2e-06
4e-04
7e-02
Log-odds
0.5
1.0
1.5
2.0
2.5
3.0
3.5
4.0
Cellular_organisms - ps1
Archaea1 - ps2
Archaea2 - ps3
Archaea3 - ps4
Asgard/Eukaryota - ps5
Eukaryota - ps6
Amorpheae - ps7
Obazoa - ps8
Opisthokonta - ps9
Holomycota - ps10
Fungi - ps11
Fungi_1 - ps12
Fungi_2 - ps13
Fungi_3 - ps14
Dikarya - ps15
Ascomycota - ps16
Saccharo/Pezizomycotina - ps17
Saccharomycotina - ps18
Saccharomycotina_1 - ps19
Saccharomycotina_2 - ps20
Saccharomycotina_3 - ps21
Phaff./Saccharomycetaceae - ps22
Saccharomyco./mycetaceae - ps23
Saccharomycetaceae - ps24
Saccharomycetaceae_2 - ps25
Saccharomycetaceae_3 - ps26
Kaz./Nau./Saccharomyces - ps27
Saccharomyces - ps28
Arboricola_clade - ps29
Cerevisiae_clade - ps30
Saccharomyces_cerevisiae - ps31
Phylostratum

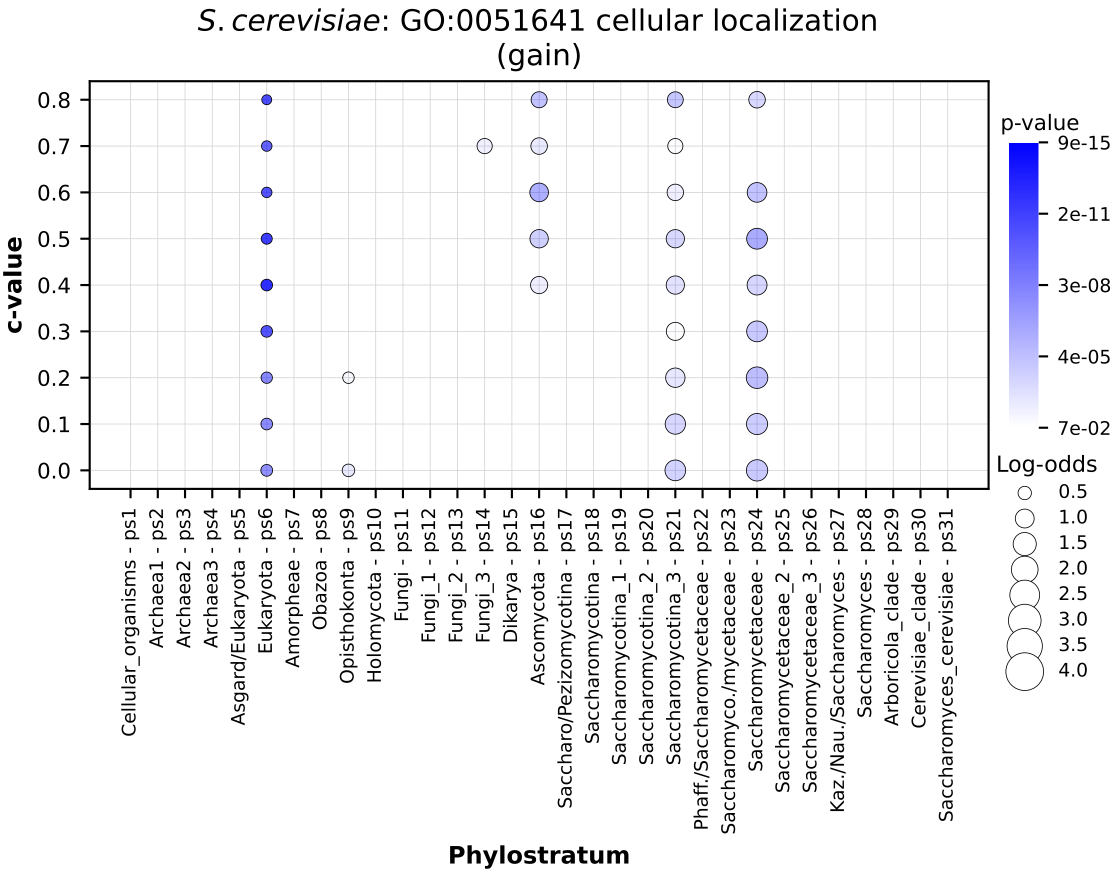

S. cerevisiae: GO:0051641 cellular localization
(gain)
c-value
0.8
0.7
0.6
0.5
0.4
0.3
0.2
0.1
0.0
p-value
9e-15
2e-11
3e-08
4e-05
7e-02
Log-odds
0.5
1.0
1.5
2.0
2.5
3.0
3.5
4.0
Cellular_organisms - ps1
Archaea1 - ps2
Archaea2 - ps3
Archaea3 - ps4
Asgard/Eukaryota - ps5
Eukaryota - ps6
Amorpheae - ps7
Obazoa - ps8
Opisthokonta - ps9
Holomycota - ps10
Fungi - ps11
Fungi_1 - ps12
Fungi_2 - ps13
Fungi_3 - ps14
Dikarya - ps15
Ascomycota - ps16
Saccharo/Pezizomycotina - ps17
Saccharomycotina - ps18
Saccharomycotina_1 - ps19
Saccharomycotina_2 - ps20
Saccharomycotina_3 - ps21
Phaff./Saccharomycetaceae - ps22
Saccharomyco./mycetaceae - ps23
Saccharomycetaceae - ps24
Saccharomycetaceae_2 - ps25
Saccharomycetaceae_3 - ps26
Kaz./Nau./Saccharomyces - ps27
Saccharomyces - ps28
Arboricola_clade - ps29
Cerevisiae_clade - ps30
Saccharomyces_cerevisiae - ps31
Phylostratum

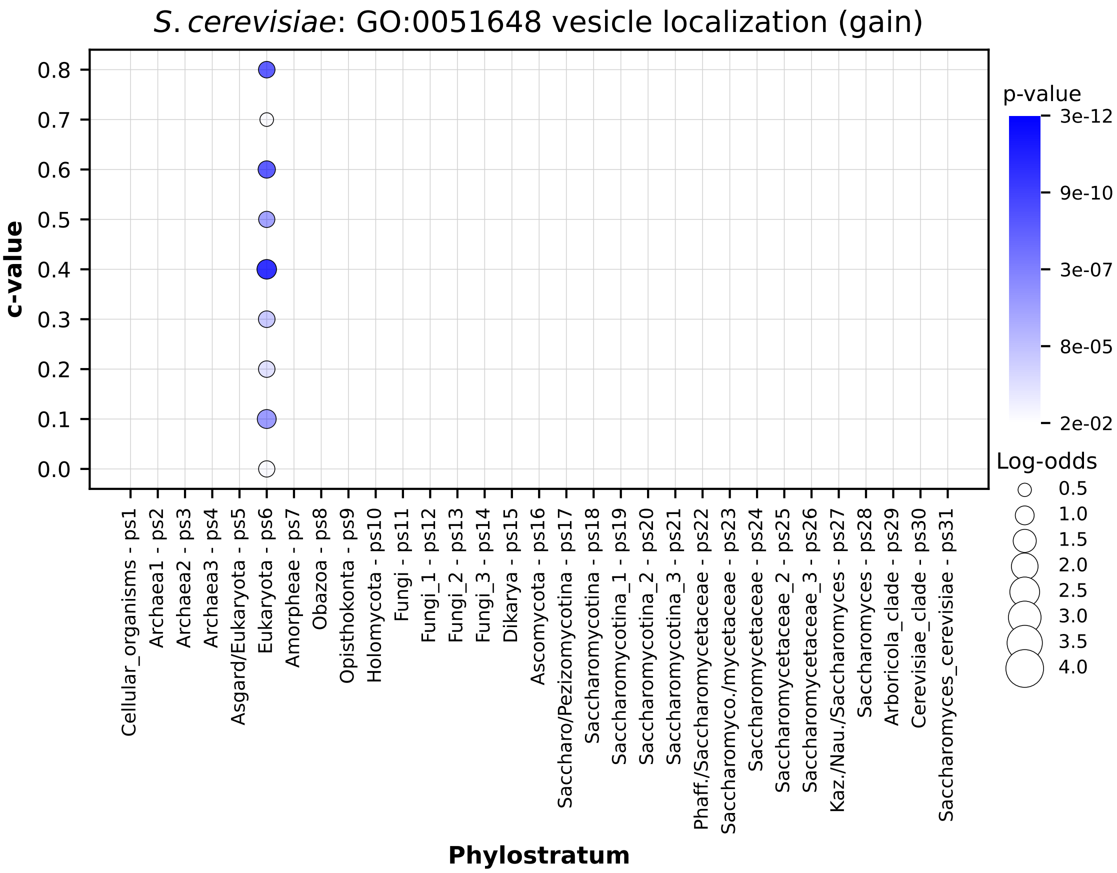

S. cerevisiae: GO:0051648 vesicle localization (gain)
c-value
0.8
0.7
0.6
0.5
0.4
0.3
0.2
0.1
0.0
p-value
3e-12
9e-10
3e-07
8e-05
2e-02
Log-odds
0.5
1.0
1.5
2.0
2.5
3.0
3.5
4.0
Cellular_organisms - ps1
Archaea1 - ps2
Archaea2 - ps3
Archaea3 - ps4
Asgard/Eukaryota - ps5
Eukaryota - ps6
Amorpheae - ps7
Obazoa - ps8
Opisthokonta - ps9
Holomycota - ps10
Fungi - ps11
Fungi_1 - ps12
Fungi_2 - ps13
Fungi_3 - ps14
Dikarya - ps15
Ascomycota - ps16
Saccharo/Pezizomycotina - ps17
Saccharomycotina - ps18
Saccharomycotina_1 - ps19
Saccharomycotina_2 - ps20
Saccharomycotina_3 - ps21
Phaff./Saccharomycetaceae - ps22
Saccharomyco./mycetaceae - ps23
Saccharomycetaceae - ps24
Saccharomycetaceae_2 - ps25
Saccharomycetaceae_3 - ps26
Kaz./Nau./Saccharomyces - ps27
Saccharomyces - ps28
Arboricola_clade - ps29
Cerevisiae_clade - ps30
Saccharomyces_cerevisiae - ps31
Phylostratum

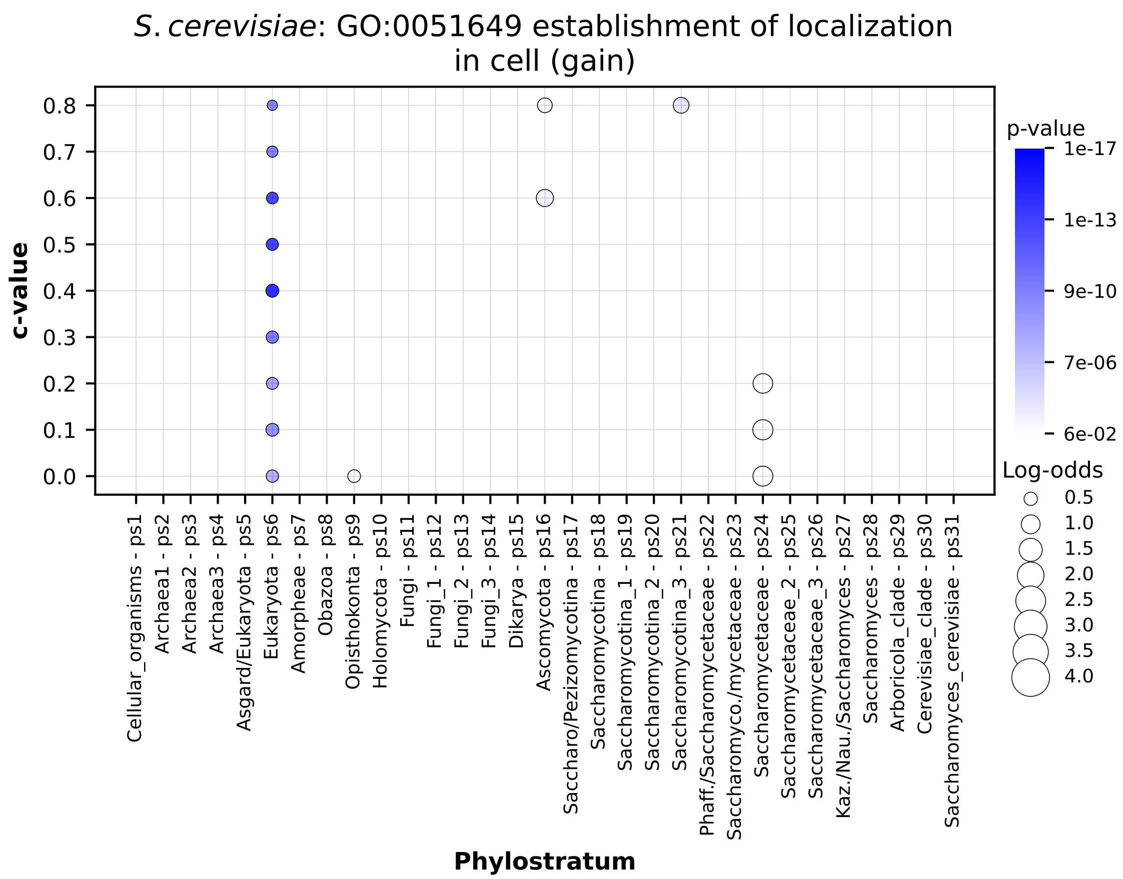
S. cerevisiae: GO:0051649 establishment of localization in cell (gain)
c-value
0.8
0.7
0.6
0.5
0.4
0.3
0.2
0.1
0.0
p-value
1e-17
1e-13
9e-10
7e-06
6e-02
Log-odds
0.5
1.0
1.5
2.0
2.5
3.0
3.5
4.0
Cellular_organisms - ps1
Archaea1 - ps2
Archaea2 - ps3
Archaea3 - ps4
Asgard/Eukaryota - ps5
Eukaryota - ps6
Amorpheae - ps7
Obazoa - ps8
Opisthokonta - ps9
Holomycota - ps10
Fungi - ps11
Fungi_1 - ps12
Fungi_2 - ps13
Fungi_3 - ps14
Dikarya - ps15
Ascomycota - ps16
Saccharo/Pezizomycotina - ps17
Saccharomycotina - ps18
Saccharomycotina_1 - ps19
Saccharomycotina_2 - ps20
Saccharomycotina_3 - ps21
Phaff./Saccharomycetaceae - ps22
Saccharomyco./mycetaceae - ps23
Saccharomycetaceae - ps24
Saccharomycetaceae_2 - ps25
Saccharomycetaceae_3 - ps26
Kaz./Nau./Saccharomyces - ps27
Saccharomyces - ps28
Arboricola_clade - ps29
Cerevisiae_clade - ps30
Saccharomyces_cerevisiae - ps31
Phylostratum

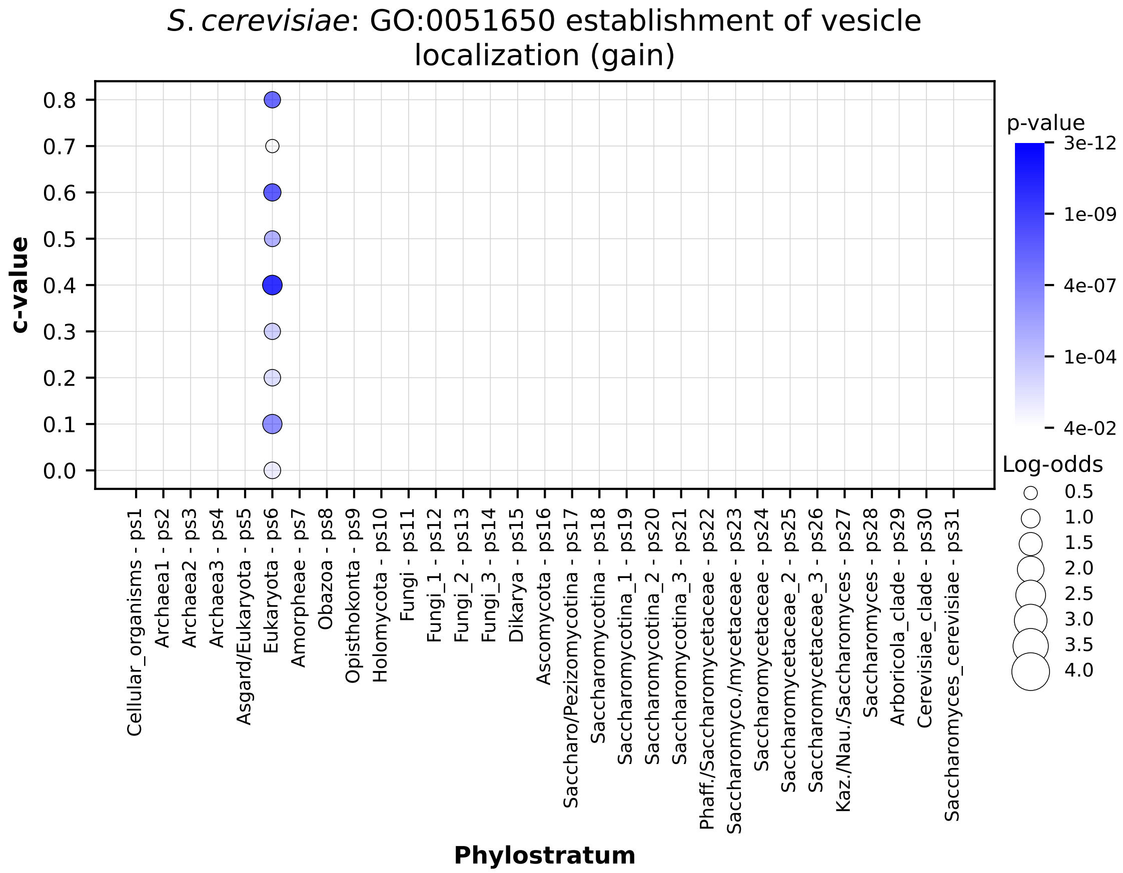

S. cerevisiae: GO:0051650 establishment of vesicle localization (gain)
c-value
0.8
0.7
0.6
0.5
0.4
0.3
0.2
0.1
0.0
p-value
3e-12
1e-09
4e-07
1e-04
4e-02
Log-odds
0.5
1.0
1.5
2.0
2.5
3.0
3.5
4.0
Cellular_organisms - ps1
Archaea1 - ps2
Archaea2 - ps3
Archaea3 - ps4
Asgard/Eukaryota - ps5
Eukaryota - ps6
Amorpheae - ps7
Obazoa - ps8
Opisthokonta - ps9
Holomycota - ps10
Fungi - ps11
Fungi_1 - ps12
Fungi_2 - ps13
Fungi_3 - ps14
Dikarya - ps15
Ascomycota - ps16
Saccharo/Pezizomycotina - ps17
Saccharomycotina - ps18
Saccharomycotina_1 - ps19
Saccharomycotina_2 - ps20
Saccharomycotina_3 - ps21
Phaff./Saccharomycetaceae - ps22
Saccharomyco./mycetaceae - ps23
Saccharomycetaceae - ps24
Saccharomycetaceae_2 - ps25
Saccharomycetaceae_3 - ps26
Kaz./Nau./Saccharomyces - ps27
Saccharomyces - ps28
Arboricola_clade - ps29
Cerevisiae_clade - ps30
Saccharomyces_cerevisiae - ps31
Phylostratum

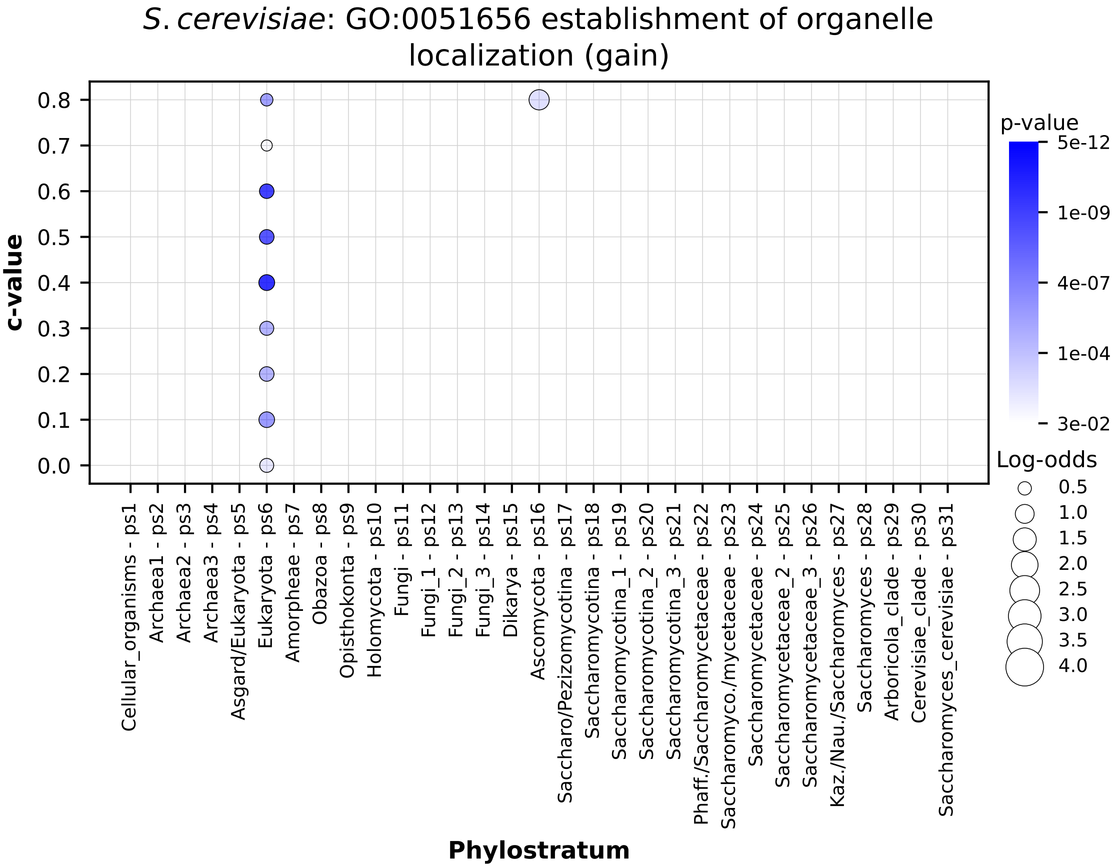

S. cerevisiae: GO:0051656 establishment of organelle localization (gain)
c-value
0.0
0.1
0.2
0.3
0.4
0.5
0.6
0.7
0.8
Phylostratum
Cellular_organisms - ps1
Archaea1 - ps2
Archaea2 - ps3
Archaea3 - ps4
Asgard/Eukaryota - ps5
Eukaryota - ps6
Amorpheae - ps7
Obazoa - ps8
Opisthokonta - ps9
Holomycota - ps10
Fungi - ps11
Fungi_1 - ps12
Fungi_2 - ps13
Fungi_3 - ps14
Dikarya - ps15
Ascomycota - ps16
Saccharo/Pezizomycotina - ps17
Saccharomycotina - ps18
Saccharomycotina_1 - ps19
Saccharomycotina_2 - ps20
Saccharomycotina_3 - ps21
Phaff./Saccharomycetaceae - ps22
Saccharomyco./mycetaceae - ps23
Saccharomycetaceae - ps24
Saccharomycetaceae_2 - ps25
Saccharomycetaceae_3 - ps26
Kaz./Nau./Saccharomyces - ps27
Saccharomyces - ps28
Arboricola_clade - ps29
Cerevisiae_clade - ps30
Saccharomyces_cerevisiae - ps31
p-value
5e-12
1e-09
4e-07
1e-04
3e-02
Log-odds
0.5
1.0
1.5
2.0
2.5
3.0
3.5
4.0

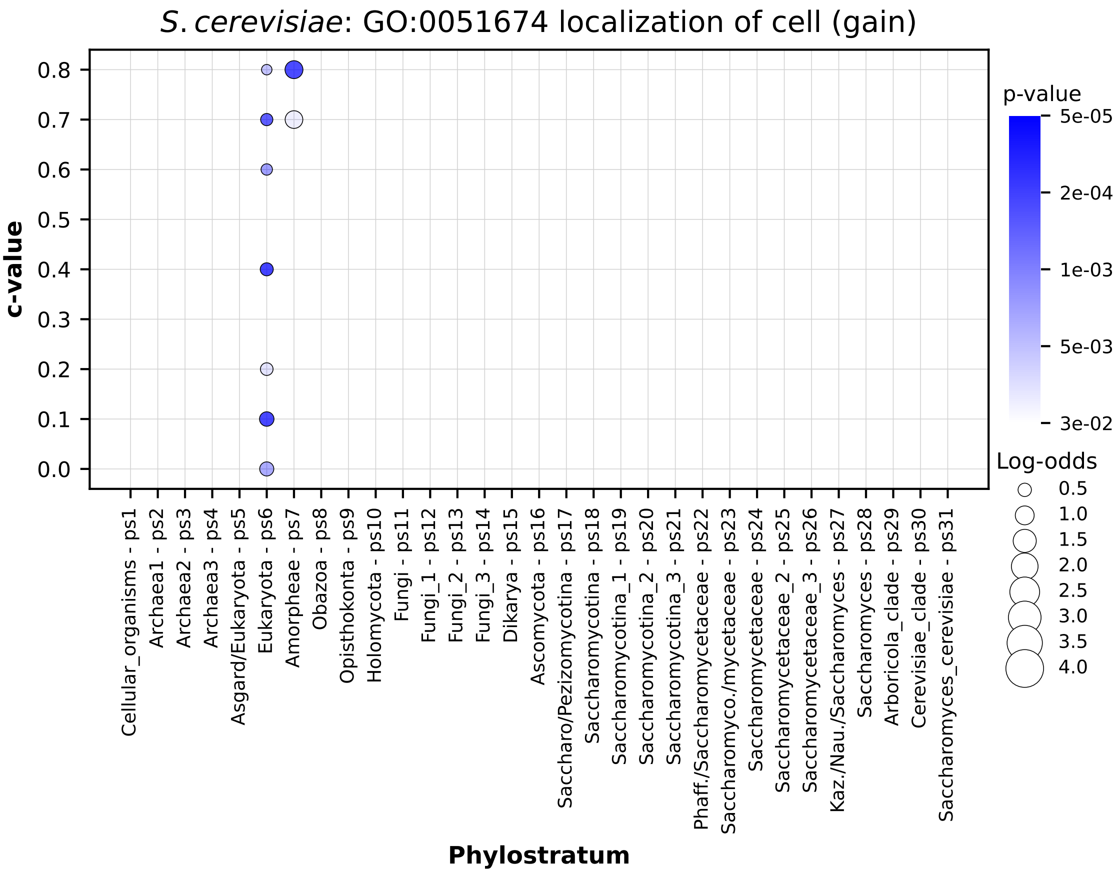
S. cerevisiae: GO:0051674 localization of cell (gain)
c-value
0.0
0.1
0.2
0.3
0.4
0.5
0.6
0.7
0.8
p-value
5e-05
2e-04
1e-03
5e-03
3e-02
Log-odds
0.5
1.0
1.5
2.0
2.5
3.0
3.5
4.0
Cellular_organisms - ps1
Archaea1 - ps2
Archaea2 - ps3
Archaea3 - ps4
Asgard/Eukaryota - ps5
Eukaryota - ps6
Amorpheae - ps7
Obazoa - ps8
Opisthokonta - ps9
Holomycota - ps10
Fungi - ps11
Fungi_1 - ps12
Fungi_2 - ps13
Fungi_3 - ps14
Dikarya - ps15
Ascomycota - ps16
Saccharo/Pezizomycotina - ps17
Saccharomycotina - ps18
Saccharomycotina_1 - ps19
Saccharomycotina_2 - ps20
Saccharomycotina_3 - ps21
Phaff./Saccharomycetaceae - ps22
Saccharomyco./mycetaceae - ps23
Saccharomycetaceae - ps24
Saccharomycetaceae_2 - ps25
Saccharomycetaceae_3 - ps26
Kaz./Nau./Saccharomyces - ps27
Saccharomyces - ps28
Arboricola_clade - ps29
Cerevisiae_clade - ps30
Saccharomyces_cerevisiae - ps31
Phylostratum

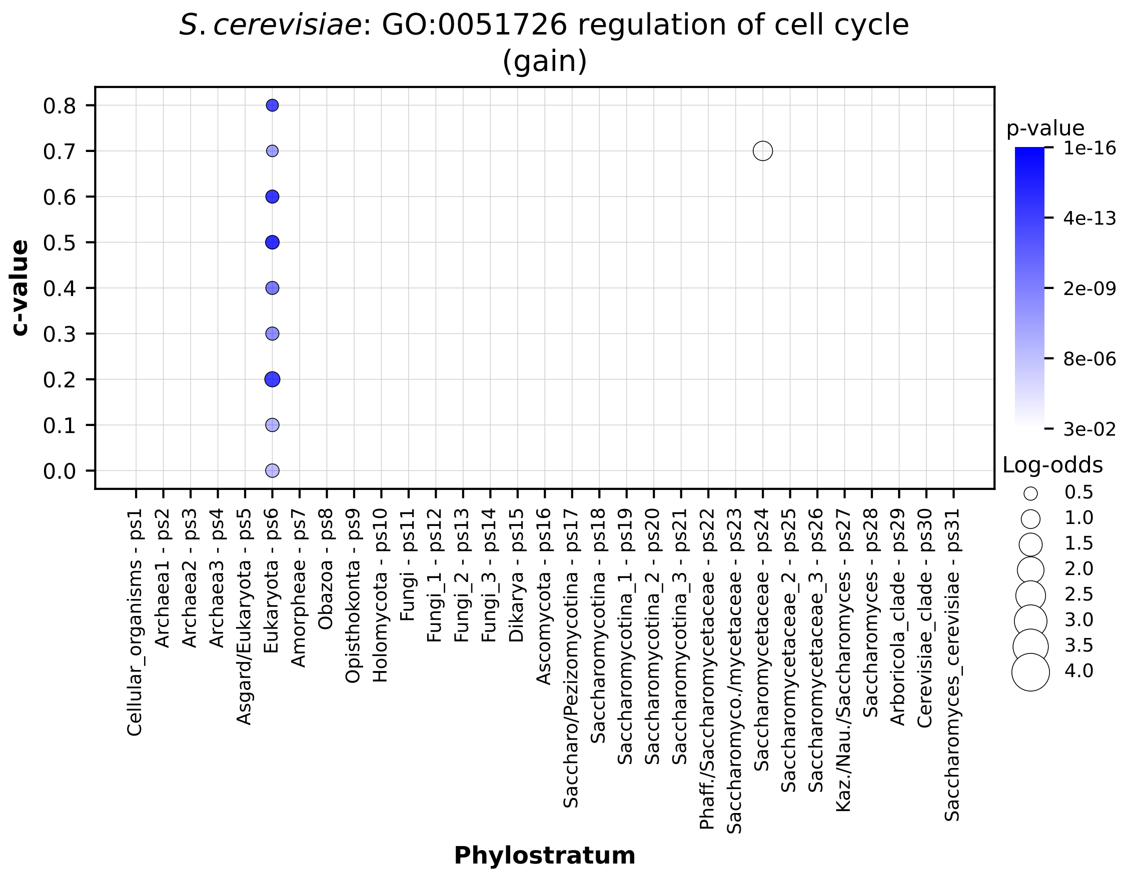

S. cerevisiae: GO:0051726 regulation of cell cycle
(gain)
c-value
0.8
0.7
0.6
0.5
0.4
0.3
0.2
0.1
0.0
p-value
1e-16
4e-13
2e-09
8e-06
3e-02
Log-odds
0.5
1.0
1.5
2.0
2.5
3.0
3.5
4.0
Cellular_organisms - ps1
Archaea1 - ps2
Archaea2 - ps3
Archaea3 - ps4
Asgard/Eukaryota - ps5
Eukaryota - ps6
Amorpheae - ps7
Obazoa - ps8
Opisthokonta - ps9
Holomycota - ps10
Fungi - ps11
Fungi_1 - ps12
Fungi_2 - ps13
Fungi_3 - ps14
Dikarya - ps15
Ascomycota - ps16
Saccharo/Pezizomycotina - ps17
Saccharomycotina - ps18
Saccharomycotina_1 - ps19
Saccharomycotina_2 - ps20
Saccharomycotina_3 - ps21
Phaff./Saccharomycetaceae - ps22
Saccharomyco./mycetaceae - ps23
Saccharomycetaceae - ps24
Saccharomycetaceae_2 - ps25
Saccharomycetaceae_3 - ps26
Kaz./Nau./Saccharomyces - ps27
Saccharomyces - ps28
Arboricola_clade - ps29
Cerevisiae_clade - ps30
Saccharomyces_cerevisiae - ps31
Phylostratum

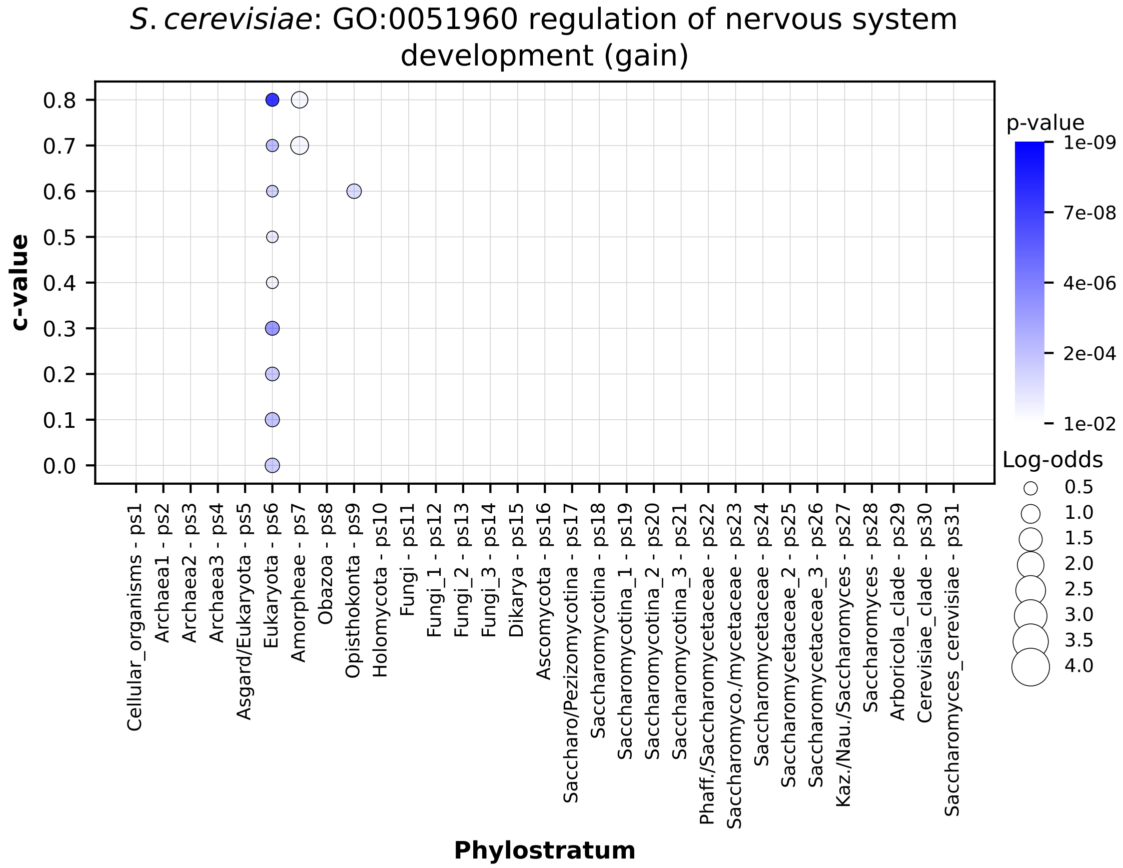

S. cerevisiae: GO:0051960 regulation of nervous system development (gain)
c-value
0.8
0.7
0.6
0.5
0.4
0.3
0.2
0.1
0.0
p-value
1e-09
7e-08
4e-06
2e-04
1e-02
Log-odds
0.5
1.0
1.5
2.0
2.5
3.0
3.5
4.0
Cellular_organisms - ps1
Archaea1 - ps2
Archaea2 - ps3
Archaea3 - ps4
Asgard/Eukaryota - ps5
Eukaryota - ps6
Amorpheae - ps7
Obazoa - ps8
Opisthokonta - ps9
Holomycota - ps10
Fungi - ps11
Fungi_1 - ps12
Fungi_2 - ps13
Fungi_3 - ps14
Dikarya - ps15
Ascomycota - ps16
Saccharo/Pezizomycotina - ps17
Saccharomycotina - ps18
Saccharomycotina_1 - ps19
Saccharomycotina_2 - ps20
Saccharomycotina_3 - ps21
Phaff./Saccharomycetaceae - ps22
Saccharomyco./mycetaceae - ps23
Saccharomycetaceae - ps24
Saccharomycetaceae_2 - ps25
Saccharomycetaceae_3 - ps26
Kaz./Nau./Saccharomyces - ps27
Saccharomyces - ps28
Arboricola_clade - ps29
Cerevisiae_clade - ps30
Saccharomyces_cerevisiae - ps31
Phylostratum

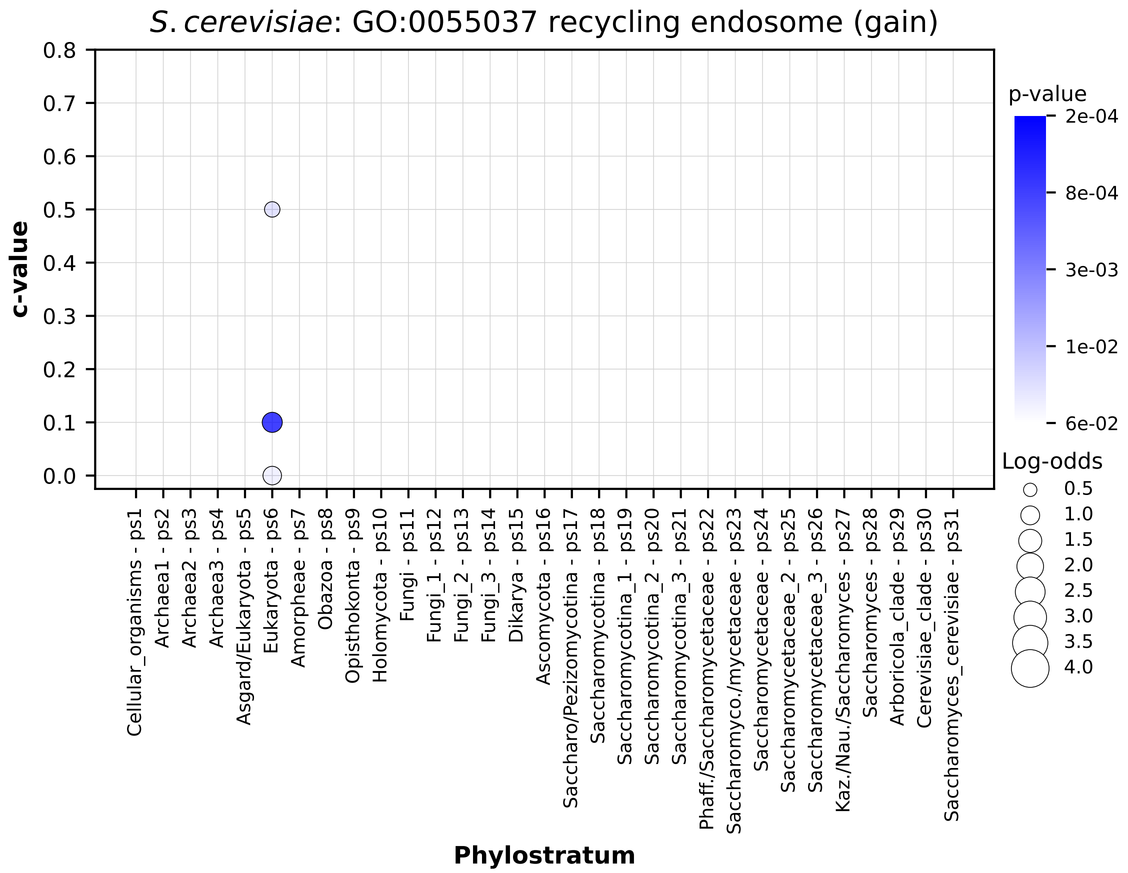

S. cerevisiae: GO:0055037 recycling endosome (gain)
c-value
0.8
0.7
0.6
0.5
0.4
0.3
0.2
0.1
0.0
p-value
2e-04
8e-04
3e-03
1e-02
6e-02
Log-odds
0.5
1.0
1.5
2.0
2.5
3.0
3.5
4.0
Cellular_organisms - ps1
Archaea1 - ps2
Archaea2 - ps3
Archaea3 - ps4
Asgard/Eukaryota - ps5
Eukaryota - ps6
Amorpheae - ps7
Obazoa - ps8
Opisthokonta - ps9
Holomycota - ps10
Fungi - ps11
Fungi_1 - ps12
Fungi_2 - ps13
Fungi_3 - ps14
Dikarya - ps15
Ascomycota - ps16
Saccharo/Pezizomycotina - ps17
Saccharomycotina - ps18
Saccharomycotina_1 - ps19
Saccharomycotina_2 - ps20
Saccharomycotina_3 - ps21
Phaff./Saccharomycetaceae - ps22
Saccharomyco./mycetaceae - ps23
Saccharomycetaceae - ps24
Saccharomycetaceae_2 - ps25
Saccharomycetaceae_3 - ps26
Kaz./Nau./Saccharomyces - ps27
Saccharomyces - ps28
Arboricola_clade - ps29
Cerevisiae_clade - ps30
Saccharomyces_cerevisiae - ps31
Phylostratum

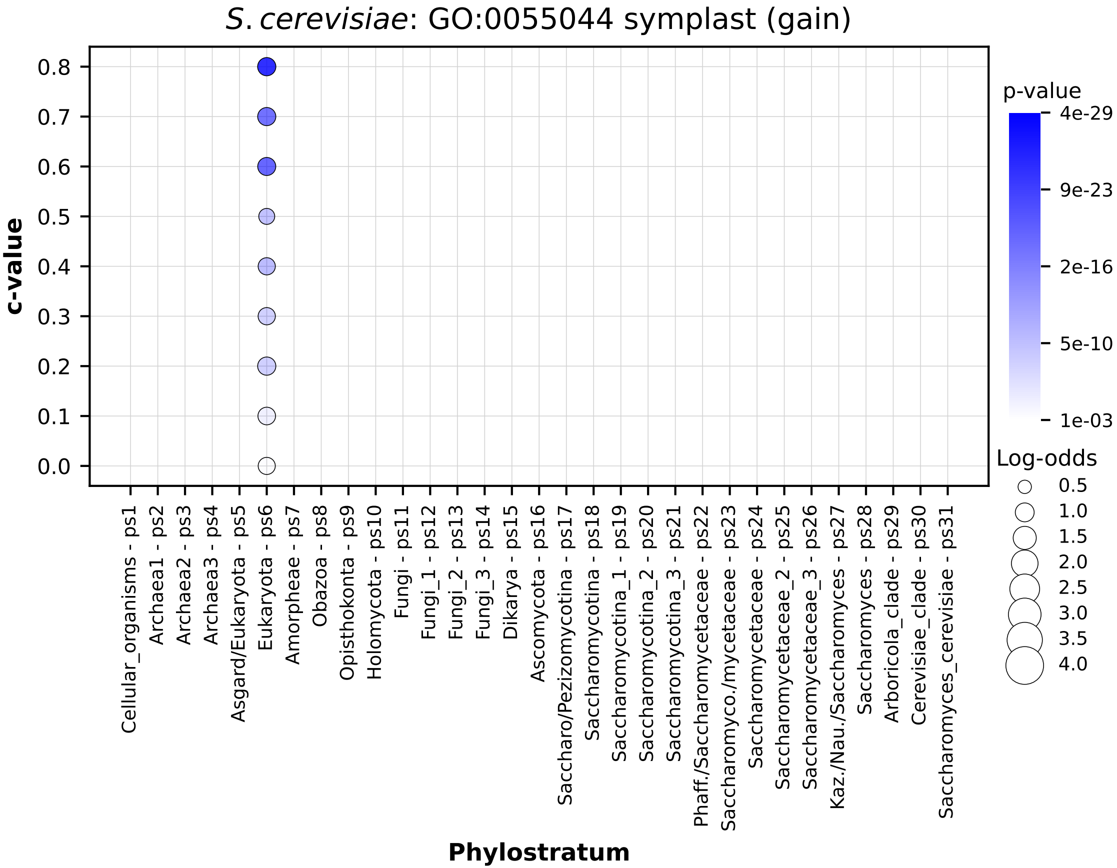

S. cerevisiae: GO:0055044 symplast (gain)
c-value
0.8
0.7
0.6
0.5
0.4
0.3
0.2
0.1
0.0
p-value
4e-29
9e-23
2e-16
5e-10
1e-03
Log-odds
0.5
1.0
1.5
2.0
2.5
3.0
3.5
4.0
Cellular_organisms - ps1
Archaea1 - ps2
Archaea2 - ps3
Archaea3 - ps4
Asgard/Eukaryota - ps5
Eukaryota - ps6
Amorpheae - ps7
Obazoa - ps8
Opisthokonta - ps9
Holomycota - ps10
Fungi - ps11
Fungi_1 - ps12
Fungi_2 - ps13
Fungi_3 - ps14
Dikarya - ps15
Ascomycota - ps16
Saccharo/Pezizomycotina - ps17
Saccharomycotina - ps18
Saccharomycotina_1 - ps19
Saccharomycotina_2 - ps20
Saccharomycotina_3 - ps21
Phaff./Saccharomycetaceae - ps22
Saccharomyco./mycetaceae - ps23
Saccharomycetaceae - ps24
Saccharomycetaceae_2 - ps25
Saccharomycetaceae_3 - ps26
Kaz./Nau./Saccharomyces - ps27
Saccharomyces - ps28
Arboricola_clade - ps29
Cerevisiae_clade - ps30
Saccharomyces_cerevisiae - ps31
Phylostratum

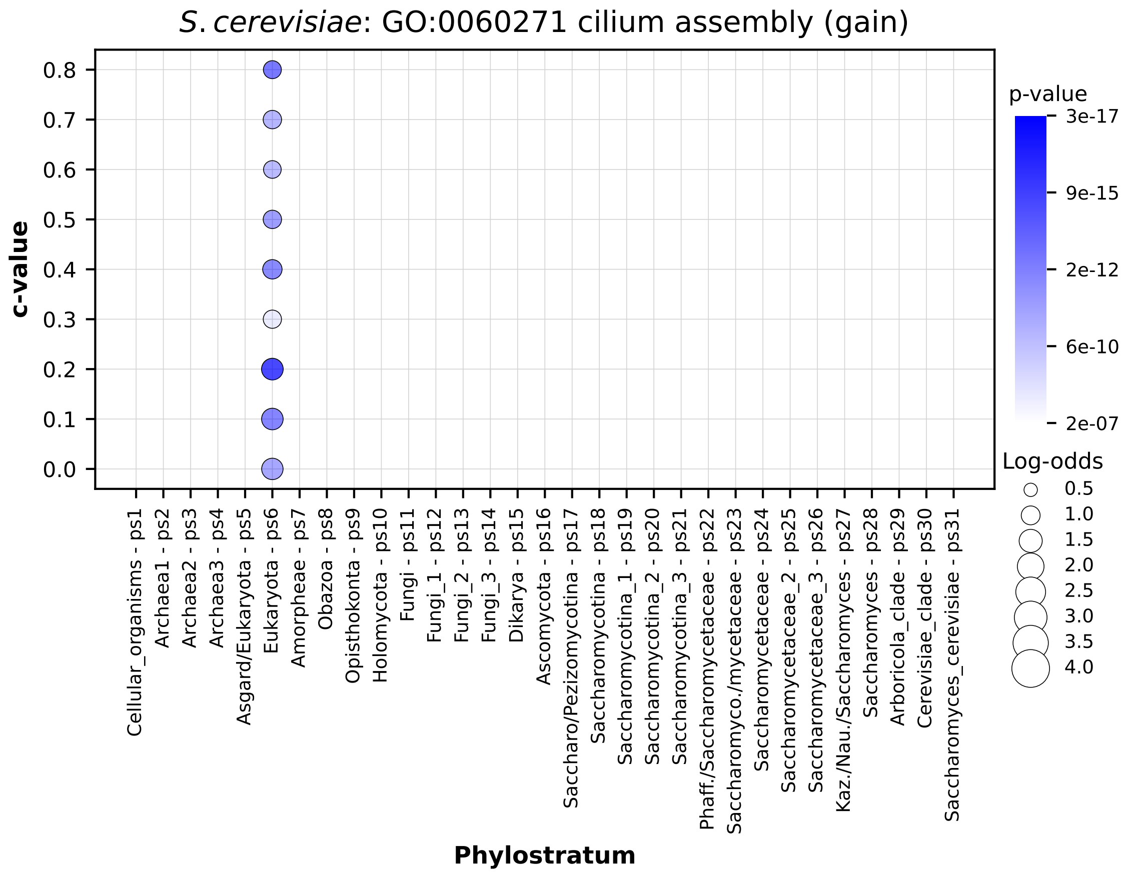

S. cerevisiae: GO:0060271 cilium assembly (gain)
c-value
0.8
0.7
0.6
0.5
0.4
0.3
0.2
0.1
0.0
p-value
3e-17
9e-15
2e-12
6e-10
2e-07
Log-odds
0.5
1.0
1.5
2.0
2.5
3.0
3.5
4.0
Cellular_organisms - ps1
Archaea1 - ps2
Archaea2 - ps3
Archaea3 - ps4
Asgard/Eukaryota - ps5
Eukaryota - ps6
Amorpheae - ps7
Obazoa - ps8
Opisthokonta - ps9
Holomycota - ps10
Fungi - ps11
Fungi_1 - ps12
Fungi_2 - ps13
Fungi_3 - ps14
Dikarya - ps15
Ascomycota - ps16
Saccharo/Pezizomycotina - ps17
Saccharomycotina - ps18
Saccharomycotina_1 - ps19
Saccharomycotina_2 - ps20
Saccharomycotina_3 - ps21
Phaff./Saccharomycetaceae - ps22
Saccharomyco./mycetaceae - ps23
Saccharomycetaceae - ps24
Saccharomycetaceae_2 - ps25
Saccharomycetaceae_3 - ps26
Kaz./Nau./Saccharomyces - ps27
Saccharomyces - ps28
Arboricola_clade - ps29
Cerevisiae_clade - ps30
Saccharomyces_cerevisiae - ps31
Phylostratum

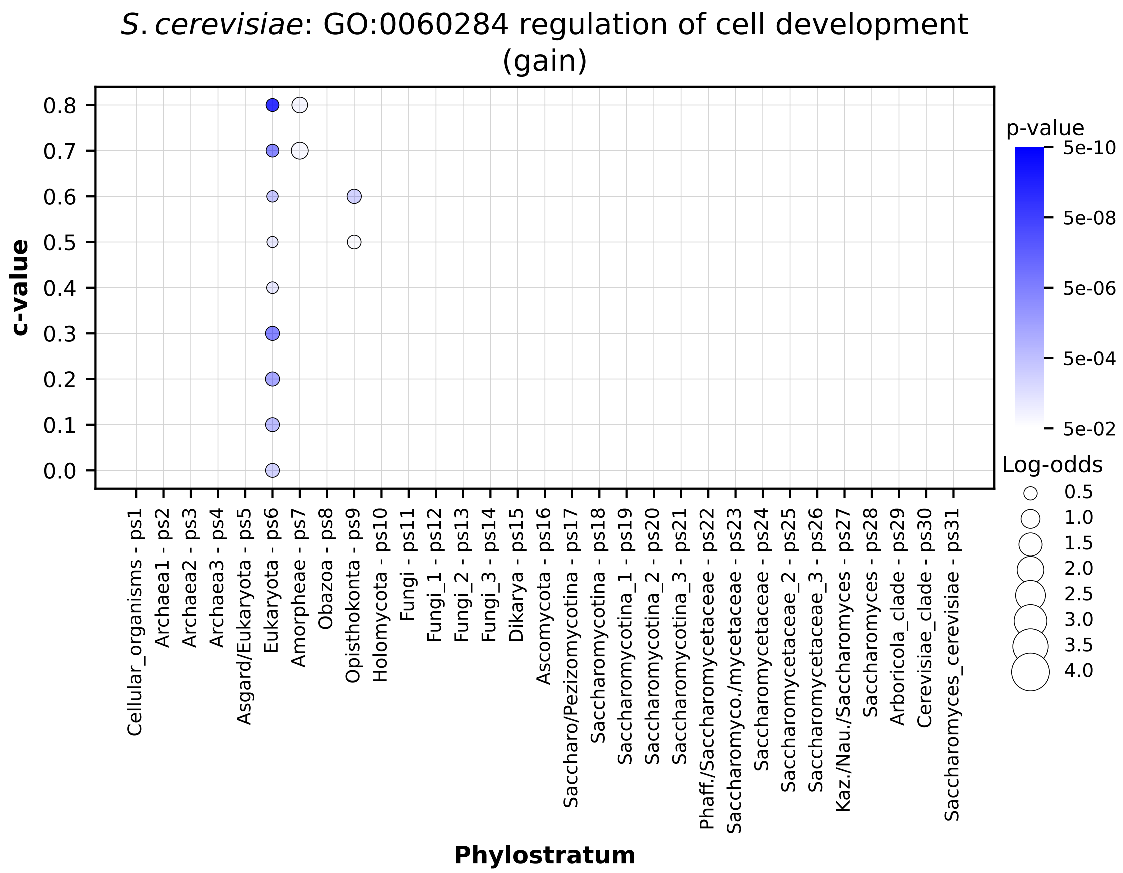

S. cerevisiae: GO:0060284 regulation of cell development (gain)
c-value
0.8
0.7
0.6
0.5
0.4
0.3
0.2
0.1
0.0
Phylostratum
Cellular_organisms - ps1
Archaea1 - ps2
Archaea2 - ps3
Archaea3 - ps4
Asgard/Eukaryota - ps5
Eukaryota - ps6
Amorpheae - ps7
Obazoa - ps8
Opisthokonta - ps9
Holomycota - ps10
Fungi - ps11
Fungi_1 - ps12
Fungi_2 - ps13
Fungi_3 - ps14
Dikarya - ps15
Ascomycota - ps16
Saccharo/Pezizomycotina - ps17
Saccharomycotina - ps18
Saccharomycotina_1 - ps19
Saccharomycotina_2 - ps20
Saccharomycotina_3 - ps21
Phaff./Saccharomycetaceae - ps22
Saccharomyco./mycetaceae - ps23
Saccharomycetaceae - ps24
Saccharomycetaceae_2 - ps25
Saccharomycetaceae_3 - ps26
Kaz./Nau./Saccharomyces - ps27
Saccharomyces - ps28
Arboricola_clade - ps29
Cerevisiae_clade - ps30
Saccharomyces_cerevisiae - ps31
p-value
5e-10
5e-08
5e-06
5e-04
5e-02
Log-odds
0.5
1.0
1.5
2.0
2.5
3.0
3.5
4.0

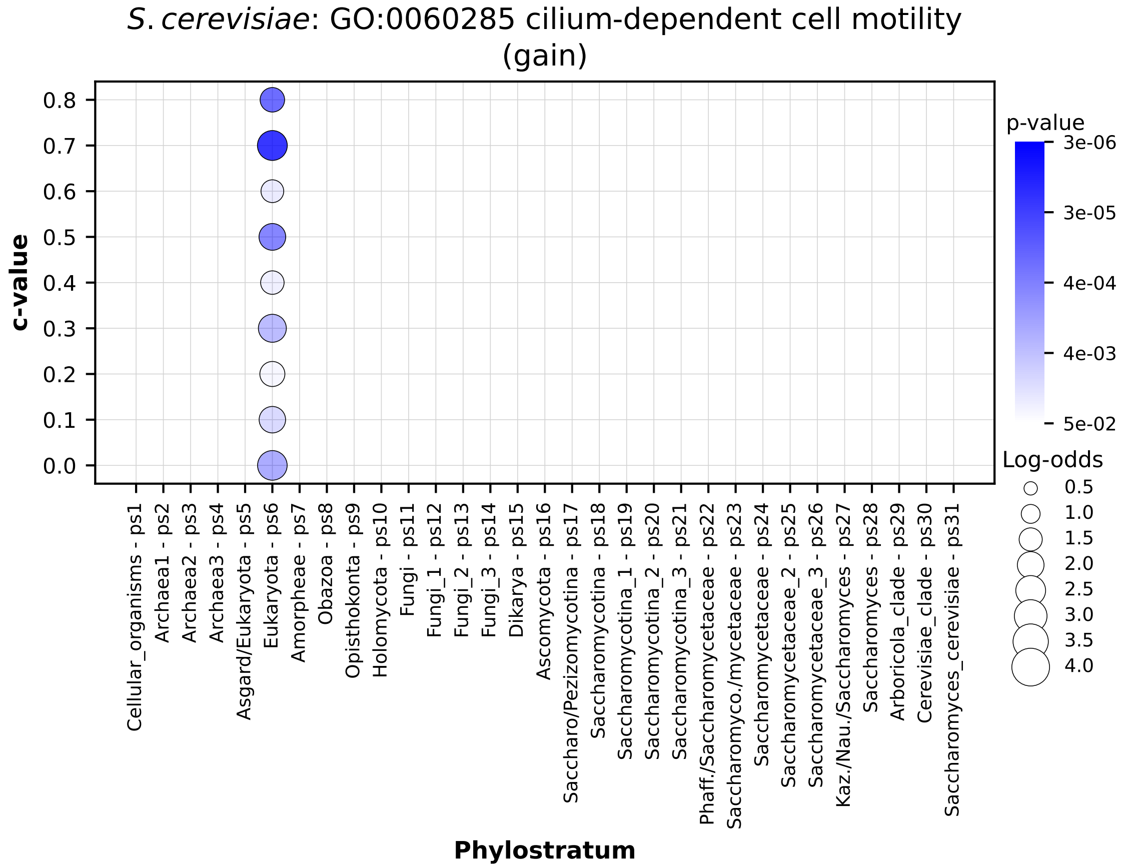

S. cerevisiae: GO:0060285 cilium-dependent cell motility
(gain)
c-value
0.8
0.7
0.6
0.5
0.4
0.3
0.2
0.1
0.0
p-value
3e-06
3e-05
4e-04
4e-03
5e-02
Log-odds
0.5
1.0
1.5
2.0
2.5
3.0
3.5
4.0
Cellular_organisms - ps1
Archaea1 - ps2
Archaea2 - ps3
Archaea3 - ps4
Asgard/Eukaryota - ps5
Eukaryota - ps6
Amorpheae - ps7
Obazoa - ps8
Opisthokonta - ps9
Holomycota - ps10
Fungi - ps11
Fungi_1 - ps12
Fungi_2 - ps13
Fungi_3 - ps14
Dikarya - ps15
Ascomycota - ps16
Saccharo/Pezizomycotina - ps17
Saccharomycotina - ps18
Saccharomycotina_1 - ps19
Saccharomycotina_2 - ps20
Saccharomycotina_3 - ps21
Phaff./Saccharomycetaceae - ps22
Saccharomyco./mycetaceae - ps23
Saccharomycetaceae - ps24
Saccharomycetaceae_2 - ps25
Saccharomycetaceae_3 - ps26
Kaz./Nau./Saccharomyces - ps27
Saccharomyces - ps28
Arboricola_clade - ps29
Cerevisiae_clade - ps30
Saccharomyces_cerevisiae - ps31
Phylostratum

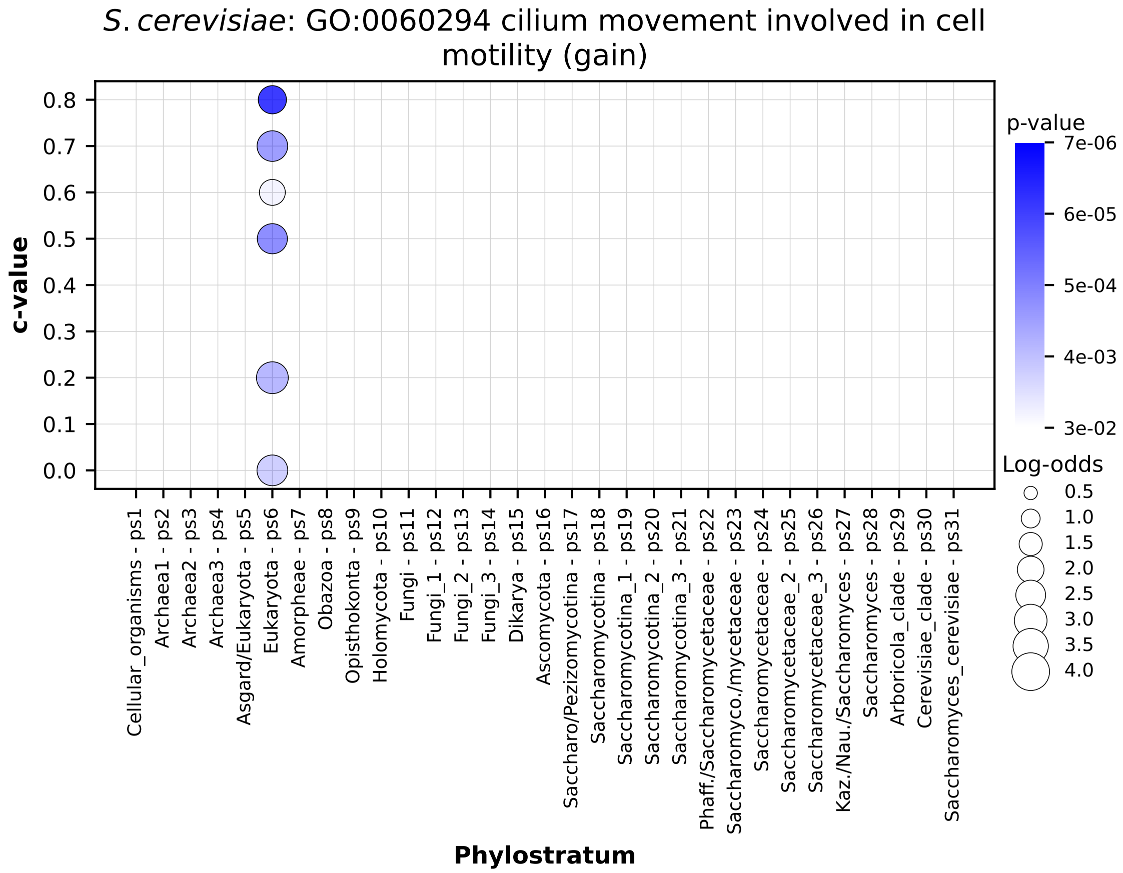

S. cerevisiae: GO:0060294 cilium movement involved in cell motility (gain)
c-value
0.8
0.7
0.6
0.5
0.4
0.3
0.2
0.1
0.0
p-value
7e-06
6e-05
5e-04
4e-03
3e-02
Log-odds
0.5
1.0
1.5
2.0
2.5
3.0
3.5
4.0
Cellular_organisms - ps1
Archaea1 - ps2
Archaea2 - ps3
Archaea3 - ps4
Asgard/Eukaryota - ps5
Eukaryota - ps6
Amorpheae - ps7
Obazoa - ps8
Opisthokonta - ps9
Holomycota - ps10
Fungi - ps11
Fungi_1 - ps12
Fungi_2 - ps13
Fungi_3 - ps14
Dikarya - ps15
Ascomycota - ps16
Saccharo/Pezizomycotina - ps17
Saccharomycotina - ps18
Saccharomycotina_1 - ps19
Saccharomycotina_2 - ps20
Saccharomycotina_3 - ps21
Phaff./Saccharomycetaceae - ps22
Saccharomyco./mycetaceae - ps23
Saccharomycetaceae - ps24
Saccharomycetaceae_2 - ps25
Saccharomycetaceae_3 - ps26
Kaz./Nau./Saccharomyces - ps27
Saccharomyces - ps28
Arboricola_clade - ps29
Cerevisiae_clade - ps30
Saccharomyces_cerevisiae - ps31
Phylostratum

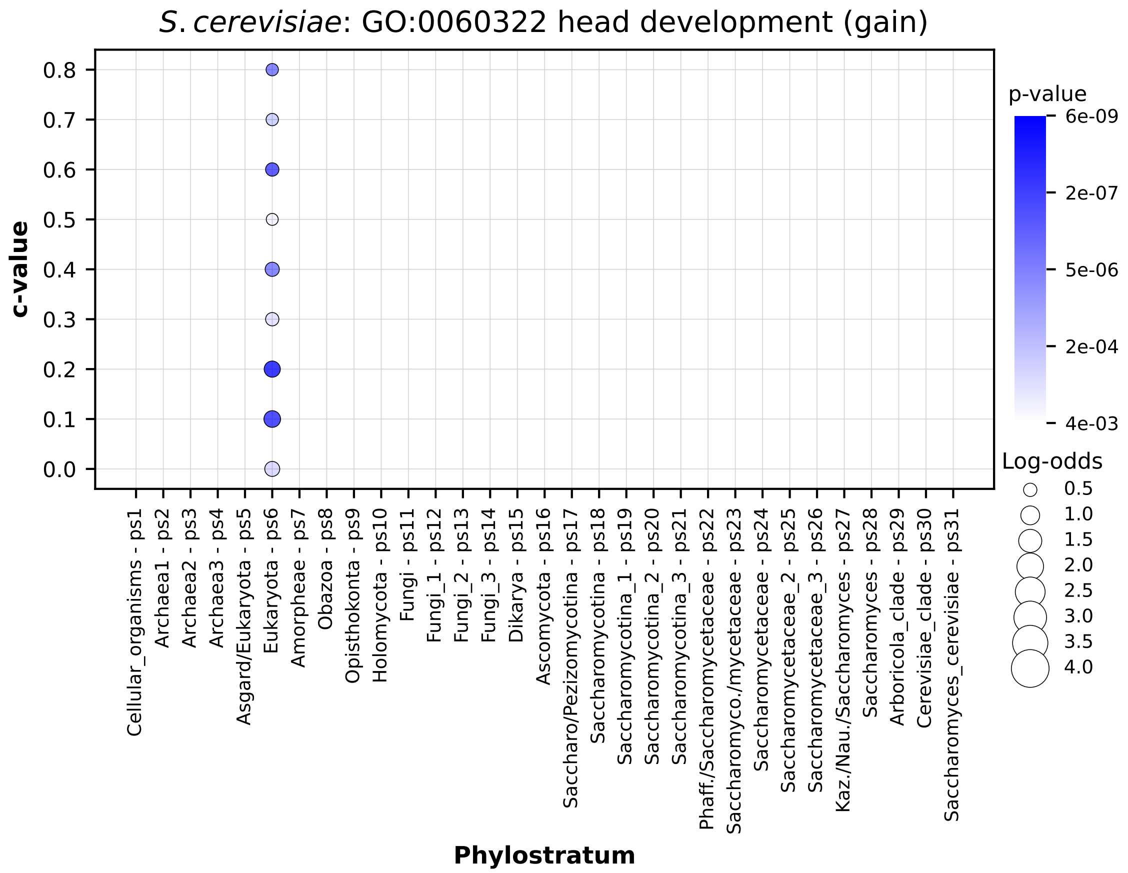

S. cerevisiae: GO:0060322 head development (gain)
c-value
0.0
0.1
0.2
0.3
0.4
0.5
0.6
0.7
0.8
p-value
6e-09
2e-07
5e-06
2e-04
4e-03
Log-odds
0.5
1.0
1.5
2.0
2.5
3.0
3.5
4.0
Cellular_organisms - ps1
Archaea1 - ps2
Archaea2 - ps3
Archaea3 - ps4
Asgard/Eukaryota - ps5
Eukaryota - ps6
Amorpheae - ps7
Obazoa - ps8
Opisthokonta - ps9
Holomycota - ps10
Fungi - ps11
Fungi_1 - ps12
Fungi_2 - ps13
Fungi_3 - ps14
Dikarya - ps15
Ascomycota - ps16
Saccharo/Pezizomycotina - ps17
Saccharomycotina - ps18
Saccharomycotina_1 - ps19
Saccharomycotina_2 - ps20
Saccharomycotina_3 - ps21
Phaff./Saccharomycetaceae - ps22
Saccharomyco./mycetaceae - ps23
Saccharomycetaceae - ps24
Saccharomycetaceae_2 - ps25
Saccharomycetaceae_3 - ps26
Kaz./Nau./Saccharomyces - ps27
Saccharomyces - ps28
Arboricola_clade - ps29
Cerevisiae_clade - ps30
Saccharomyces_cerevisiae - ps31
Phylostratum

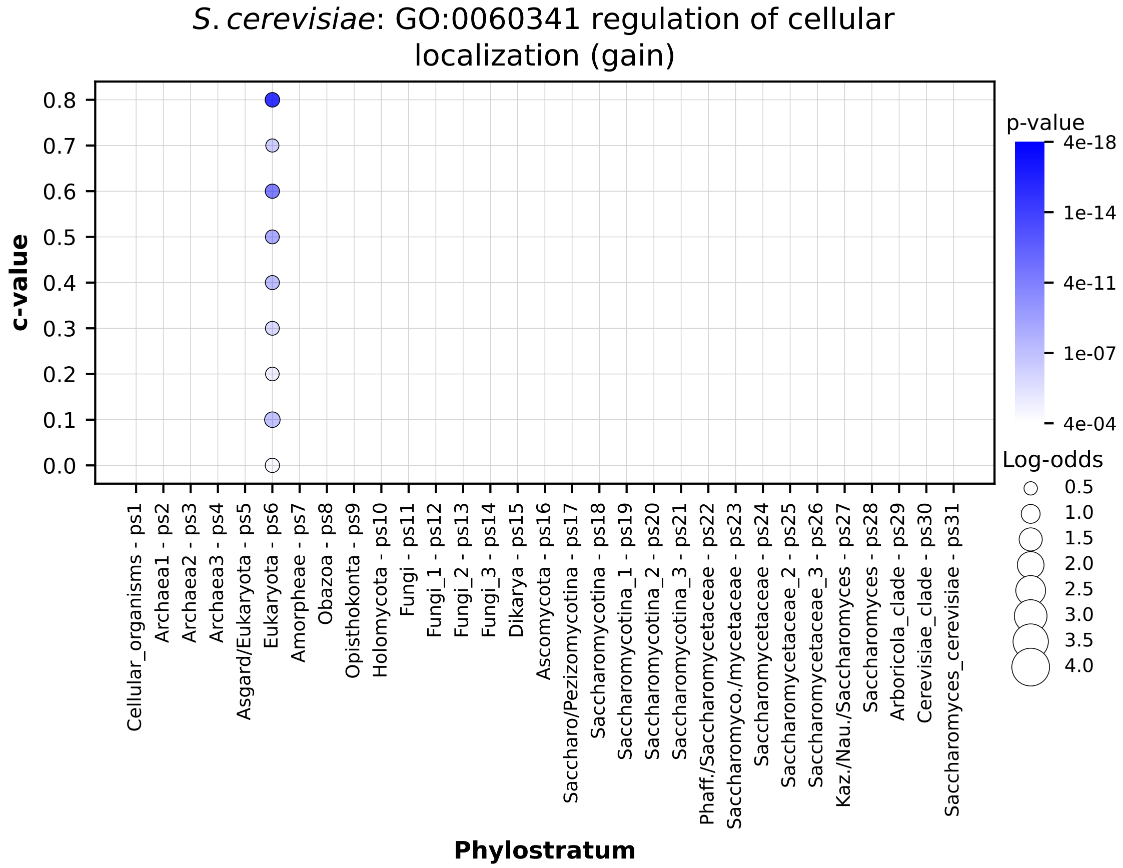

S. cerevisiae: GO:0060341 regulation of cellular localization (gain)
c-value
0.8
0.7
0.6
0.5
0.4
0.3
0.2
0.1
0.0
p-value
4e-18
1e-14
4e-11
1e-07
4e-04
Log-odds
0.5
1.0
1.5
2.0
2.5
3.0
3.5
4.0
Cellular_organisms - ps1
Archaea1 - ps2
Archaea2 - ps3
Archaea3 - ps4
Asgard/Eukaryota - ps5
Eukaryota - ps6
Amorpheae - ps7
Obazoa - ps8
Opisthokonta - ps9
Holomycota - ps10
Fungi - ps11
Fungi_1 - ps12
Fungi_2 - ps13
Fungi_3 - ps14
Dikarya - ps15
Ascomycota - ps16
Saccharo/Pezizomycotina - ps17
Saccharomycotina - ps18
Saccharomycotina_1 - ps19
Saccharomycotina_2 - ps20
Saccharomycotina_3 - ps21
Phaff./Saccharomycetaceae - ps22
Saccharomyco./mycetaceae - ps23
Saccharomycetaceae - ps24
Saccharomycetaceae_2 - ps25
Saccharomycetaceae_3 - ps26
Kaz./Nau./Saccharomyces - ps27
Saccharomyces - ps28
Arboricola_clade - ps29
Cerevisiae_clade - ps30
Saccharomyces_cerevisiae - ps31
Phylostratum

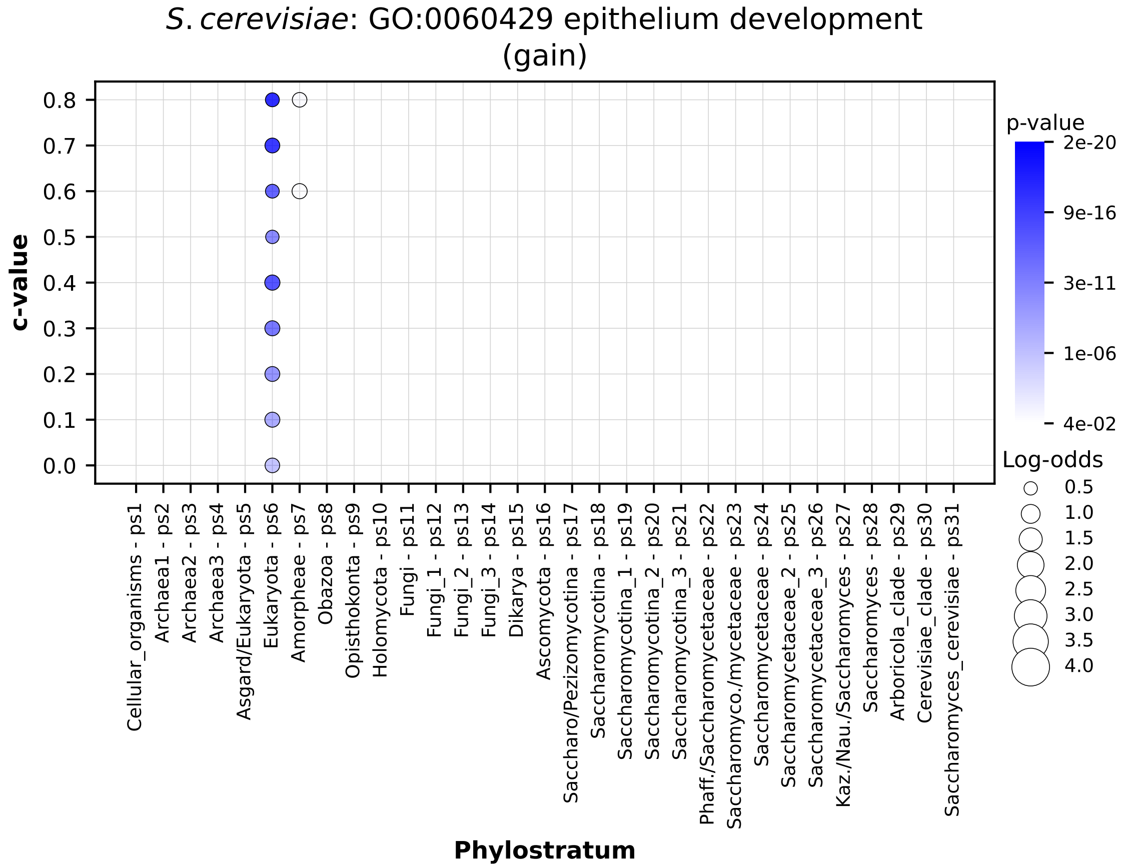

S. cerevisiae: GO:0060429 epithelium development
(gain)
c-value
0.8
0.7
0.6
0.5
0.4
0.3
0.2
0.1
0.0
p-value
2e-20
9e-16
3e-11
1e-06
4e-02
Log-odds
0.5
1.0
1.5
2.0
2.5
3.0
3.5
4.0
Cellular_organisms - ps1
Archaea1 - ps2
Archaea2 - ps3
Archaea3 - ps4
Asgard/Eukaryota - ps5
Eukaryota - ps6
Amorpheae - ps7
Obazoa - ps8
Opisthokonta - ps9
Holomycota - ps10
Fungi - ps11
Fungi_1 - ps12
Fungi_2 - ps13
Fungi_3 - ps14
Dikarya - ps15
Ascomycota - ps16
Saccharo/Pezizomycotina - ps17
Saccharomycotina - ps18
Saccharomycotina_1 - ps19
Saccharomycotina_2 - ps20
Saccharomycotina_3 - ps21
Phaff./Saccharomycetaceae - ps22
Saccharomyco./mycetaceae - ps23
Saccharomycetaceae - ps24
Saccharomycetaceae_2 - ps25
Saccharomycetaceae_3 - ps26
Kaz./Nau./Saccharomyces - ps27
Saccharomyces - ps28
Arboricola_clade - ps29
Cerevisiae_clade - ps30
Saccharomyces_cerevisiae - ps31
Phylostratum

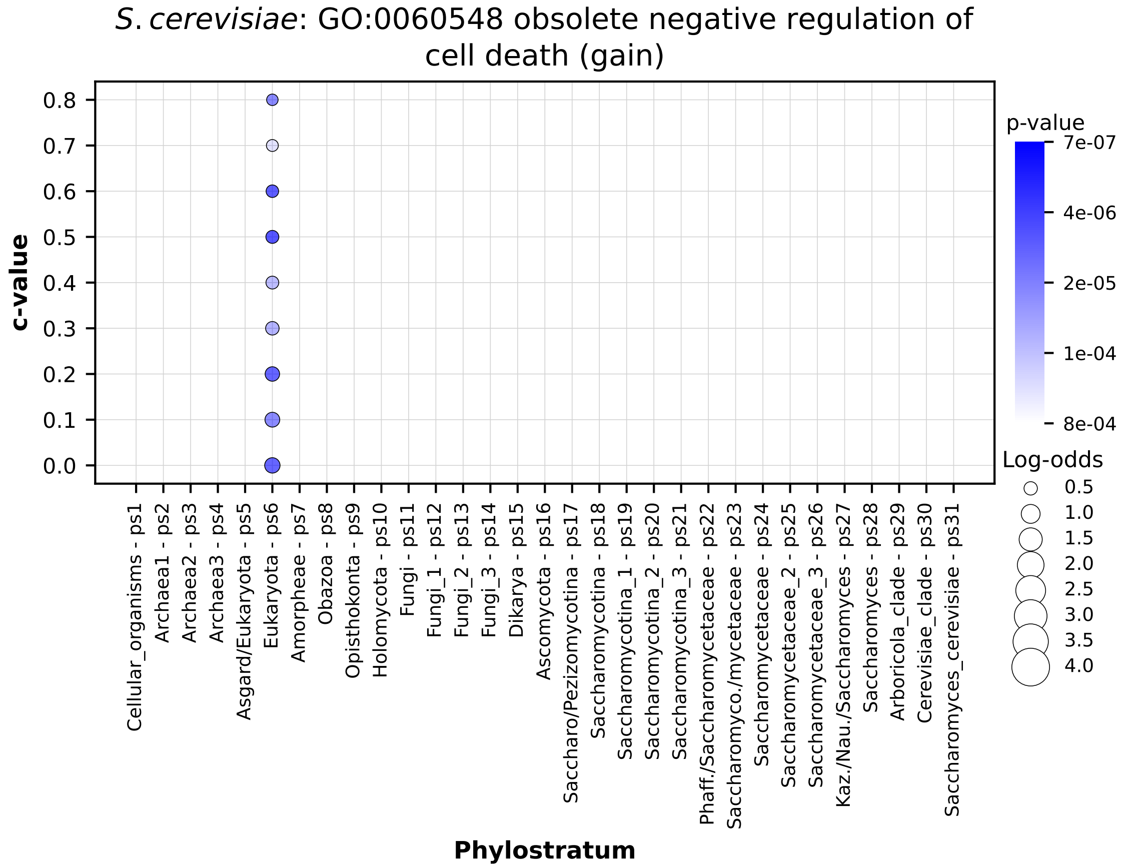

S. cerevisiae: GO:0060548 obsolete negative regulation of cell death (gain)
c-value
0.8
0.7
0.6
0.5
0.4
0.3
0.2
0.1
0.0
p-value
7e-07
4e-06
2e-05
1e-04
8e-04
Log-odds
0.5
1.0
1.5
2.0
2.5
3.0
3.5
4.0
Cellular_organisms - ps1
Archaea1 - ps2
Archaea2 - ps3
Archaea3 - ps4
Asgard/Eukaryota - ps5
Eukaryota - ps6
Amorpheae - ps7
Obazoa - ps8
Opisthokonta - ps9
Holomycota - ps10
Fungi - ps11
Fungi_1 - ps12
Fungi_2 - ps13
Fungi_3 - ps14
Dikarya - ps15
Ascomycota - ps16
Saccharo/Pezizomycotina - ps17
Saccharomycotina - ps18
Saccharomycotina_1 - ps19
Saccharomycotina_2 - ps20
Saccharomycotina_3 - ps21
Phaff./Saccharomycetaceae - ps22
Saccharomyco./mycetaceae - ps23
Saccharomycetaceae - ps24
Saccharomycetaceae_2 - ps25
Saccharomycetaceae_3 - ps26
Kaz./Nau./Saccharomyces - ps27
Saccharomyces - ps28
Arboricola_clade - ps29
Cerevisiae_clade - ps30
Saccharomyces_cerevisiae - ps31
Phylostratum

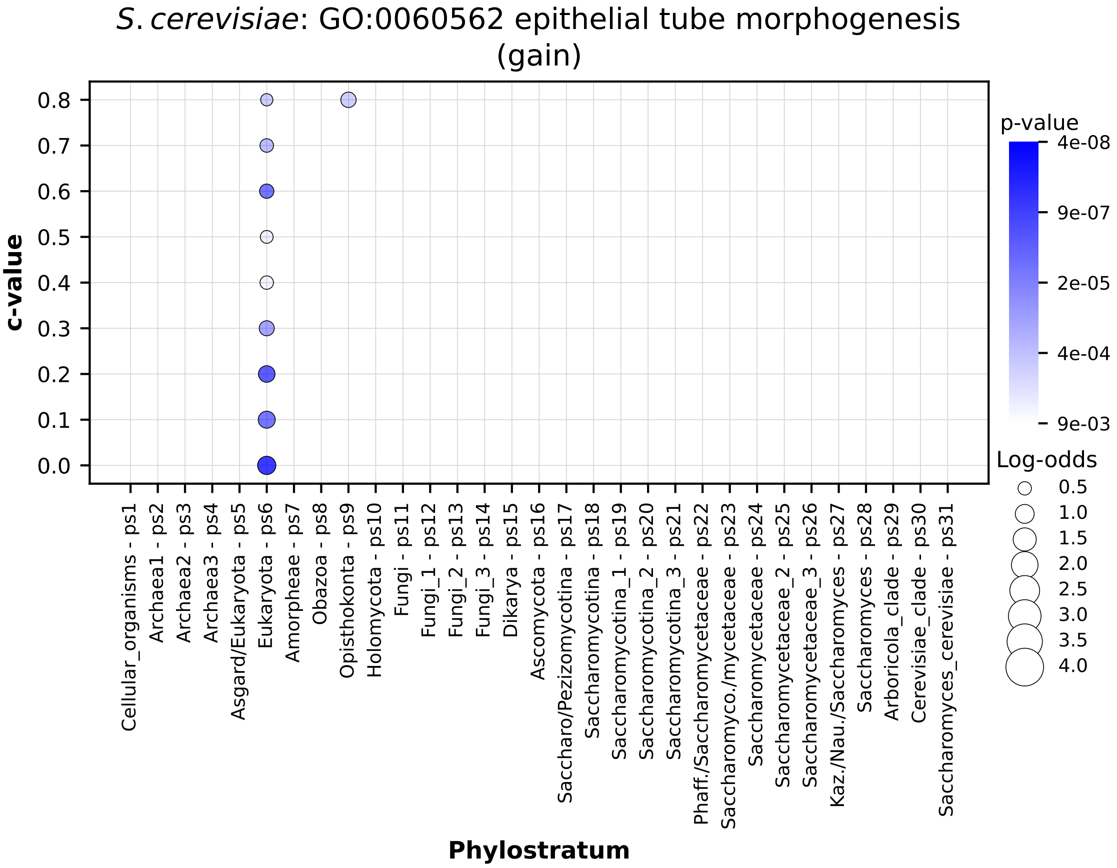

S. cerevisiae: GO:0060562 epithelial tube morphogenesis
(gain)
c-value
0.8
0.7
0.6
0.5
0.4
0.3
0.2
0.1
0.0
p-value
4e-08
9e-07
2e-05
4e-04
9e-03
Log-odds
0.5
1.0
1.5
2.0
2.5
3.0
3.5
4.0
Cellular_organisms - ps1
Archaea1 - ps2
Archaea2 - ps3
Archaea3 - ps4
Asgard/Eukaryota - ps5
Eukaryota - ps6
Amorpheae - ps7
Obazoa - ps8
Opisthokonta - ps9
Holomycota - ps10
Fungi - ps11
Fungi_1 - ps12
Fungi_2 - ps13
Fungi_3 - ps14
Dikarya - ps15
Ascomycota - ps16
Saccharo/Pezizomycotina - ps17
Saccharomycotina - ps18
Saccharomycotina_1 - ps19
Saccharomycotina_2 - ps20
Saccharomycotina_3 - ps21
Phaff./Saccharomycetaceae - ps22
Saccharomyco./mycetaceae - ps23
Saccharomycetaceae - ps24
Saccharomycetaceae_2 - ps25
Saccharomycetaceae_3 - ps26
Kaz./Nau./Saccharomyces - ps27
Saccharomyces - ps28
Arboricola_clade - ps29
Cerevisiae_clade - ps30
Saccharomyces_cerevisiae - ps31
Phylostratum

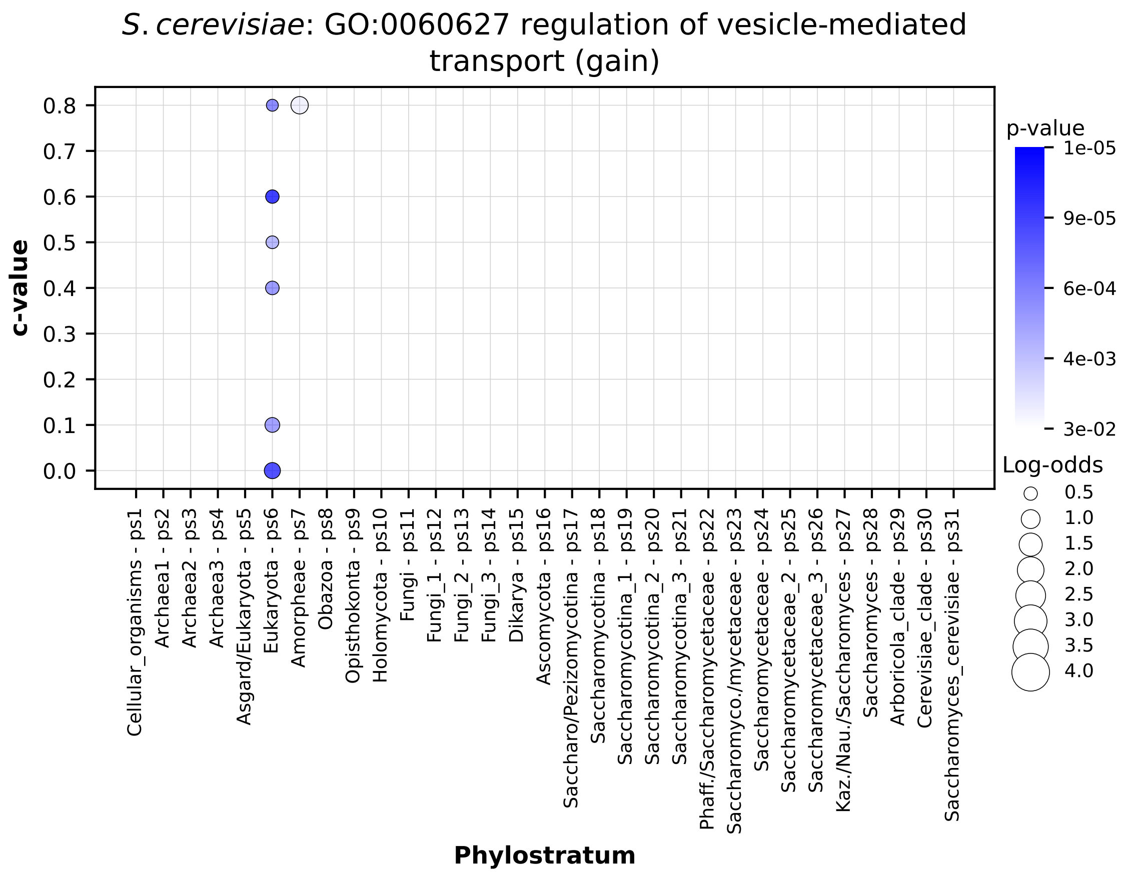

S. cerevisiae: GO:0060627 regulation of vesicle-mediated transport (gain)
c-value
0.8
0.7
0.6
0.5
0.4
0.3
0.2
0.1
0.0
p-value
1e-05
9e-05
6e-04
4e-03
3e-02
Log-odds
0.5
1.0
1.5
2.0
2.5
3.0
3.5
4.0
Cellular_organisms - ps1
Archaea1 - ps2
Archaea2 - ps3
Archaea3 - ps4
Asgard/Eukaryota - ps5
Eukaryota - ps6
Amorpheae - ps7
Obazoa - ps8
Opisthokonta - ps9
Holomycota - ps10
Fungi - ps11
Fungi_1 - ps12
Fungi_2 - ps13
Fungi_3 - ps14
Dikarya - ps15
Ascomycota - ps16
Saccharo/Pezizomycotina - ps17
Saccharomycotina - ps18
Saccharomycotina_1 - ps19
Saccharomycotina_2 - ps20
Saccharomycotina_3 - ps21
Phaff./Saccharomycetaceae - ps22
Saccharomyco./mycetaceae - ps23
Saccharomycetaceae - ps24
Saccharomycetaceae_2 - ps25
Saccharomycetaceae_3 - ps26
Kaz./Nau./Saccharomyces - ps27
Saccharomyces - ps28
Arboricola_clade - ps29
Cerevisiae_clade - ps30
Saccharomyces_cerevisiae - ps31
Phylostratum

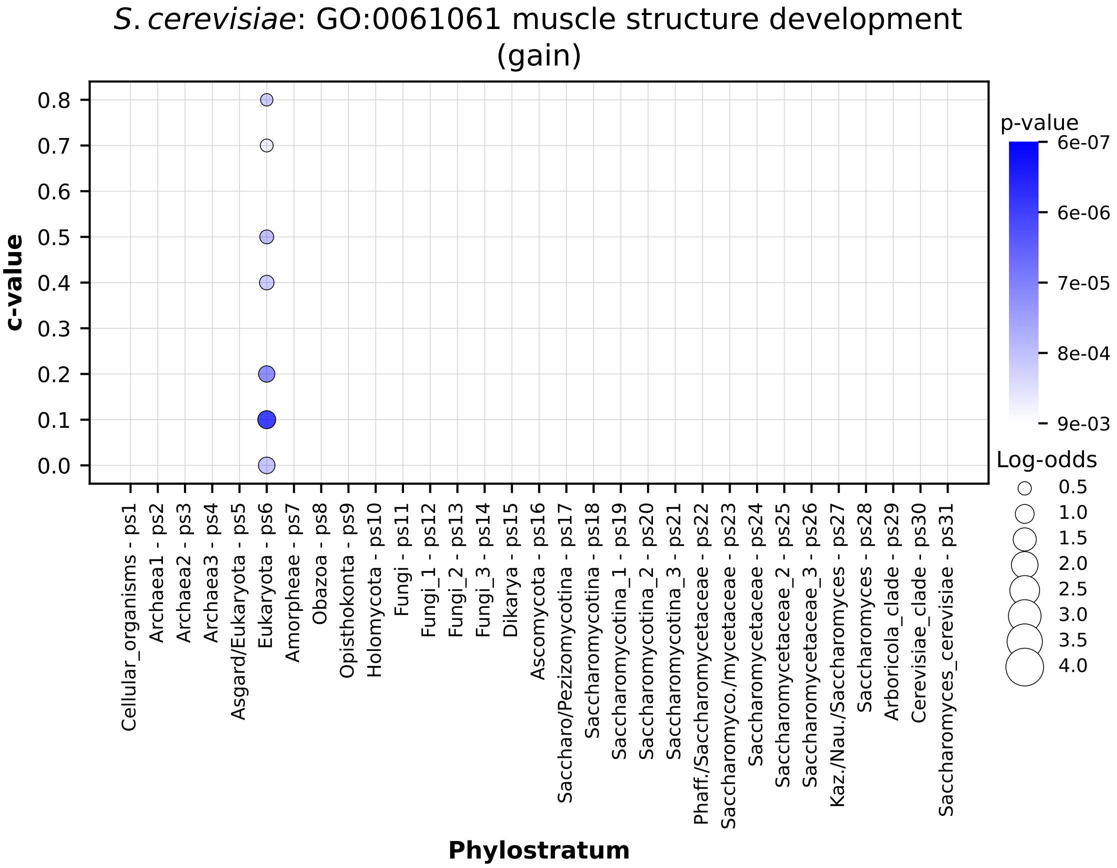

S. cerevisiae: GO:0061061 muscle structure development (gain)
c-value
0.8
0.7
0.6
0.5
0.4
0.3
0.2
0.1
0.0
p-value
6e-07
6e-06
7e-05
8e-04
9e-03
Log-odds
0.5
1.0
1.5
2.0
2.5
3.0
3.5
4.0
Cellular_organisms - ps1
Archaea1 - ps2
Archaea2 - ps3
Archaea3 - ps4
Asgard/Eukaryota - ps5
Eukaryota - ps6
Amorpheae - ps7
Obazoa - ps8
Opisthokonta - ps9
Holomycota - ps10
Fungi - ps11
Fungi_1 - ps12
Fungi_2 - ps13
Fungi_3 - ps14
Dikarya - ps15
Ascomycota - ps16
Saccharo/Pezizomycotina - ps17
Saccharomycotina - ps18
Saccharomycotina_1 - ps19
Saccharomycotina_2 - ps20
Saccharomycotina_3 - ps21
Phaff./Saccharomycetaceae - ps22
Saccharomyco./mycetaceae - ps23
Saccharomycetaceae - ps24
Saccharomycetaceae_2 - ps25
Saccharomycetaceae_3 - ps26
Kaz./Nau./Saccharomyces - ps27
Saccharomyces - ps28
Arboricola_clade - ps29
Cerevisiae_clade - ps30
Saccharomyces_cerevisiae - ps31
Phylostratum

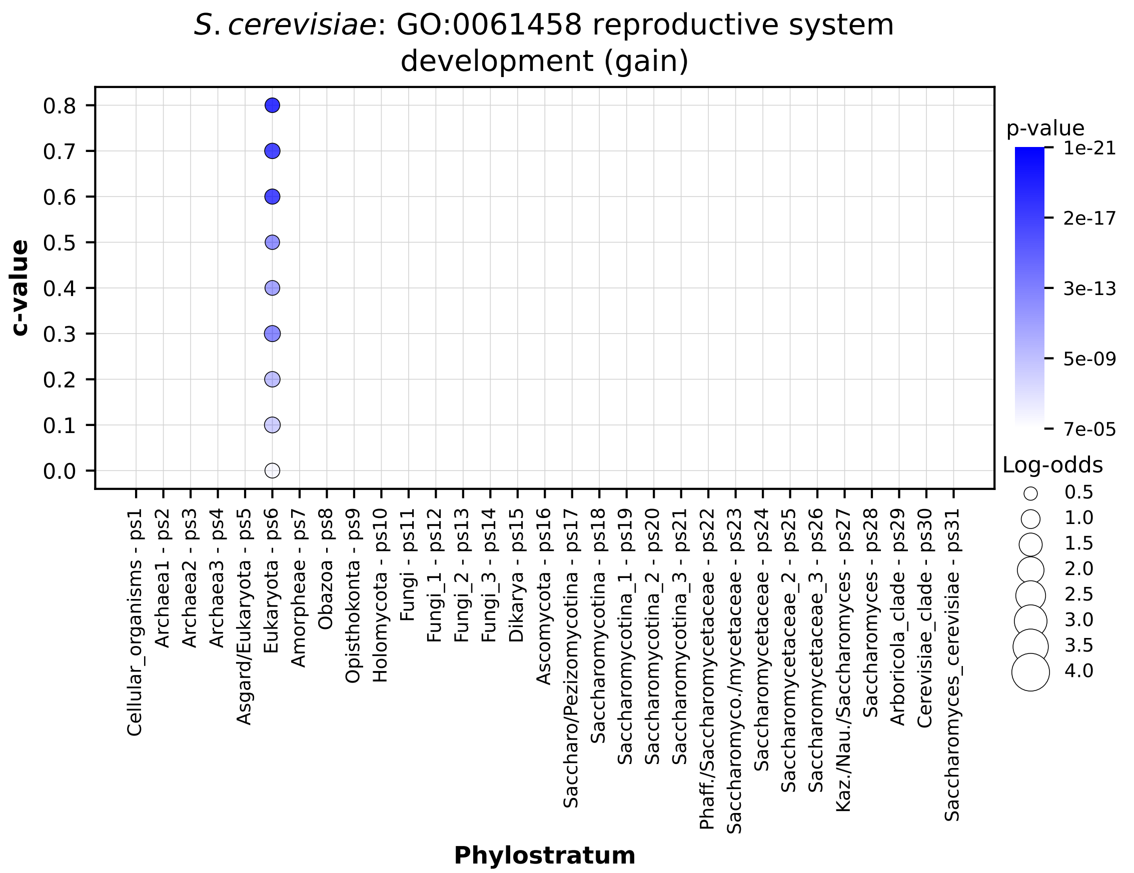

S. cerevisiae: GO:0061458 reproductive system development (gain)
c-value
0.8
0.7
0.6
0.5
0.4
0.3
0.2
0.1
0.0
p-value
1e-21
2e-17
3e-13
5e-09
7e-05
Log-odds
0.5
1.0
1.5
2.0
2.5
3.0
3.5
4.0
Cellular_organisms - ps1
Archaea1 - ps2
Archaea2 - ps3
Archaea3 - ps4
Asgard/Eukaryota - ps5
Eukaryota - ps6
Amorpheae - ps7
Obazoa - ps8
Opisthokonta - ps9
Holomycota - ps10
Fungi - ps11
Fungi_1 - ps12
Fungi_2 - ps13
Fungi_3 - ps14
Dikarya - ps15
Ascomycota - ps16
Saccharo/Pezizomycotina - ps17
Saccharomycotina - ps18
Saccharomycotina_1 - ps19
Saccharomycotina_2 - ps20
Saccharomycotina_3 - ps21
Phaff./Saccharomycetaceae - ps22
Saccharomyco./mycetaceae - ps23
Saccharomycetaceae - ps24
Saccharomycetaceae_2 - ps25
Saccharomycetaceae_3 - ps26
Kaz./Nau./Saccharomyces - ps27
Saccharomyces - ps28
Arboricola_clade - ps29
Cerevisiae_clade - ps30
Saccharomyces_cerevisiae - ps31
Phylostratum

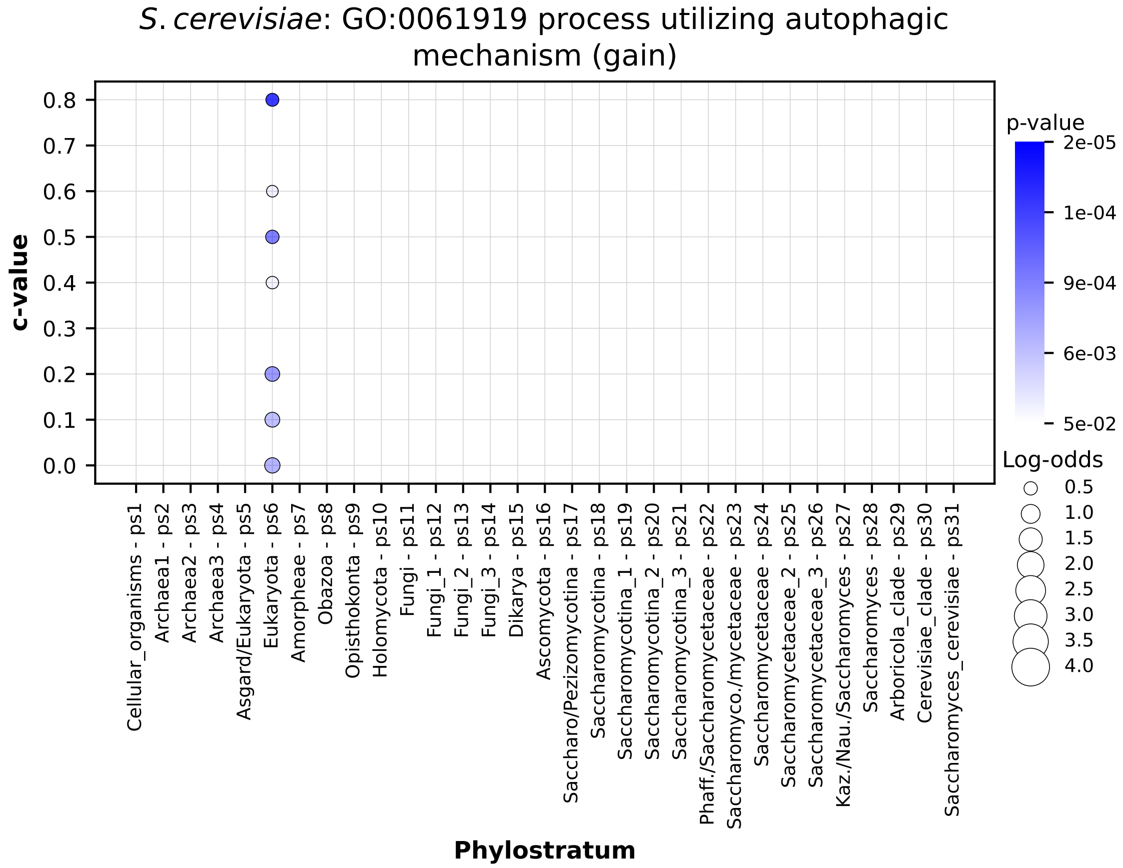

S. cerevisiae: GO:0061919 process utilizing autophagic mechanism (gain)
c-value
0.0
0.1
0.2
0.3
0.4
0.5
0.6
0.7
0.8
Phylostratum
Cellular_organisms - ps1
Archaea1 - ps2
Archaea2 - ps3
Archaea3 - ps4
Asgard/Eukaryota - ps5
Eukaryota - ps6
Amorpheae - ps7
Obazoa - ps8
Opisthokonta - ps9
Holomycota - ps10
Fungi - ps11
Fungi_1 - ps12
Fungi_2 - ps13
Fungi_3 - ps14
Dikarya - ps15
Ascomycota - ps16
Saccharo/Pezizomycotina - ps17
Saccharomycotina - ps18
Saccharomycotina_1 - ps19
Saccharomycotina_2 - ps20
Saccharomycotina_3 - ps21
Phaff./Saccharomycetaceae - ps22
Saccharomyco./mycetaceae - ps23
Saccharomycetaceae - ps24
Saccharomycetaceae_2 - ps25
Saccharomycetaceae_3 - ps26
Kaz./Nau./Saccharomyces - ps27
Saccharomyces - ps28
Arboricola_clade - ps29
Cerevisiae_clade - ps30
Saccharomyces_cerevisiae - ps31
p-value
2e-05
1e-04
9e-04
6e-03
5e-02
Log-odds
0.5
1.0
1.5
2.0
2.5
3.0
3.5
4.0

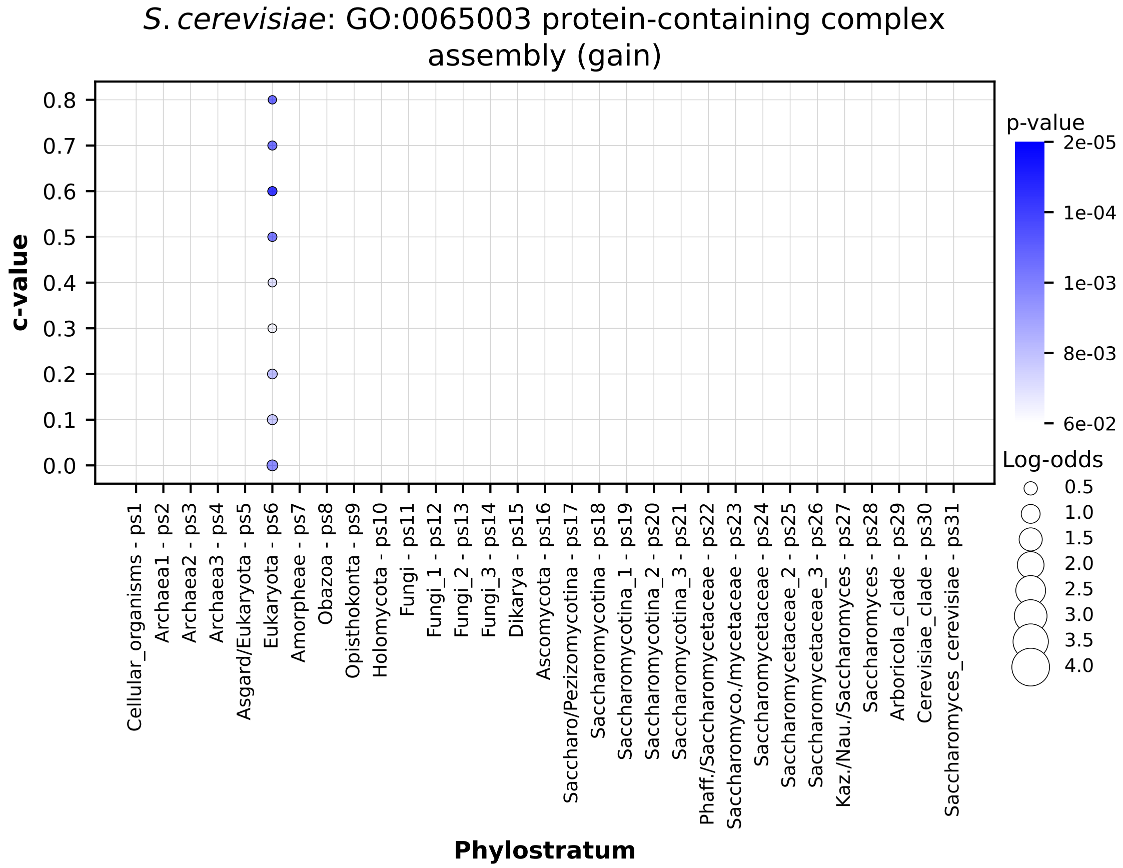

S. cerevisiae: GO:0065003 protein-containing complex assembly (gain)
c-value
0.8
0.7
0.6
0.5
0.4
0.3
0.2
0.1
0.0
p-value
2e-05
1e-04
1e-03
8e-03
6e-02
Log-odds
0.5
1.0
1.5
2.0
2.5
3.0
3.5
4.0
Cellular_organisms - ps1
Archaea1 - ps2
Archaea2 - ps3
Archaea3 - ps4
Asgard/Eukaryota - ps5
Eukaryota - ps6
Amorpheae - ps7
Obazoa - ps8
Opisthokonta - ps9
Holomycota - ps10
Fungi - ps11
Fungi_1 - ps12
Fungi_2 - ps13
Fungi_3 - ps14
Dikarya - ps15
Ascomycota - ps16
Saccharo/Pezizomycotina - ps17
Saccharomycotina - ps18
Saccharomycotina_1 - ps19
Saccharomycotina_2 - ps20
Saccharomycotina_3 - ps21
Phaff./Saccharomycetaceae - ps22
Saccharomyco./mycetaceae - ps23
Saccharomycetaceae - ps24
Saccharomycetaceae_2 - ps25
Saccharomycetaceae_3 - ps26
Kaz./Nau./Saccharomyces - ps27
Saccharomyces - ps28
Arboricola_clade - ps29
Cerevisiae_clade - ps30
Saccharomyces_cerevisiae - ps31
Phylostratum

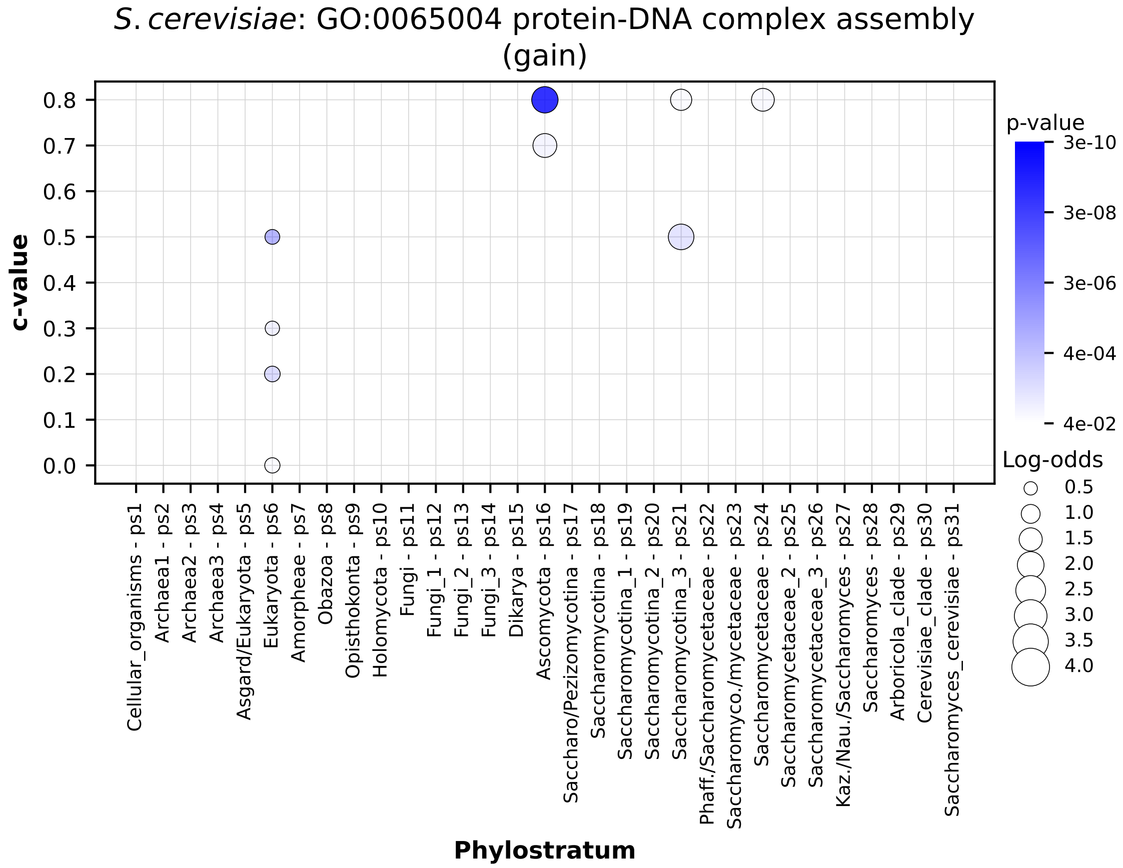

S. cerevisiae: GO:0065004 protein-DNA complex assembly
(gain)
c-value
0.8
0.7
0.6
0.5
0.4
0.3
0.2
0.1
0.0
p-value
3e-10
3e-08
3e-06
4e-04
4e-02
Log-odds
0.5
1.0
1.5
2.0
2.5
3.0
3.5
4.0
Cellular_organisms - ps1
Archaea1 - ps2
Archaea2 - ps3
Archaea3 - ps4
Asgard/Eukaryota - ps5
Eukaryota - ps6
Amorpheae - ps7
Obazoa - ps8
Opisthokonta - ps9
Holomycota - ps10
Fungi - ps11
Fungi_1 - ps12
Fungi_2 - ps13
Fungi_3 - ps14
Dikarya - ps15
Ascomycota - ps16
Saccharo/Pezizomycotina - ps17
Saccharomycotina - ps18
Saccharomycotina_1 - ps19
Saccharomycotina_2 - ps20
Saccharomycotina_3 - ps21
Phaff./Saccharomycetaceae - ps22
Saccharomyco./mycetaceae - ps23
Saccharomycetaceae - ps24
Saccharomycetaceae_2 - ps25
Saccharomycetaceae_3 - ps26
Kaz./Nau./Saccharomyces - ps27
Saccharomyces - ps28
Arboricola_clade - ps29
Cerevisiae_clade - ps30
Saccharomyces_cerevisiae - ps31
Phylostratum

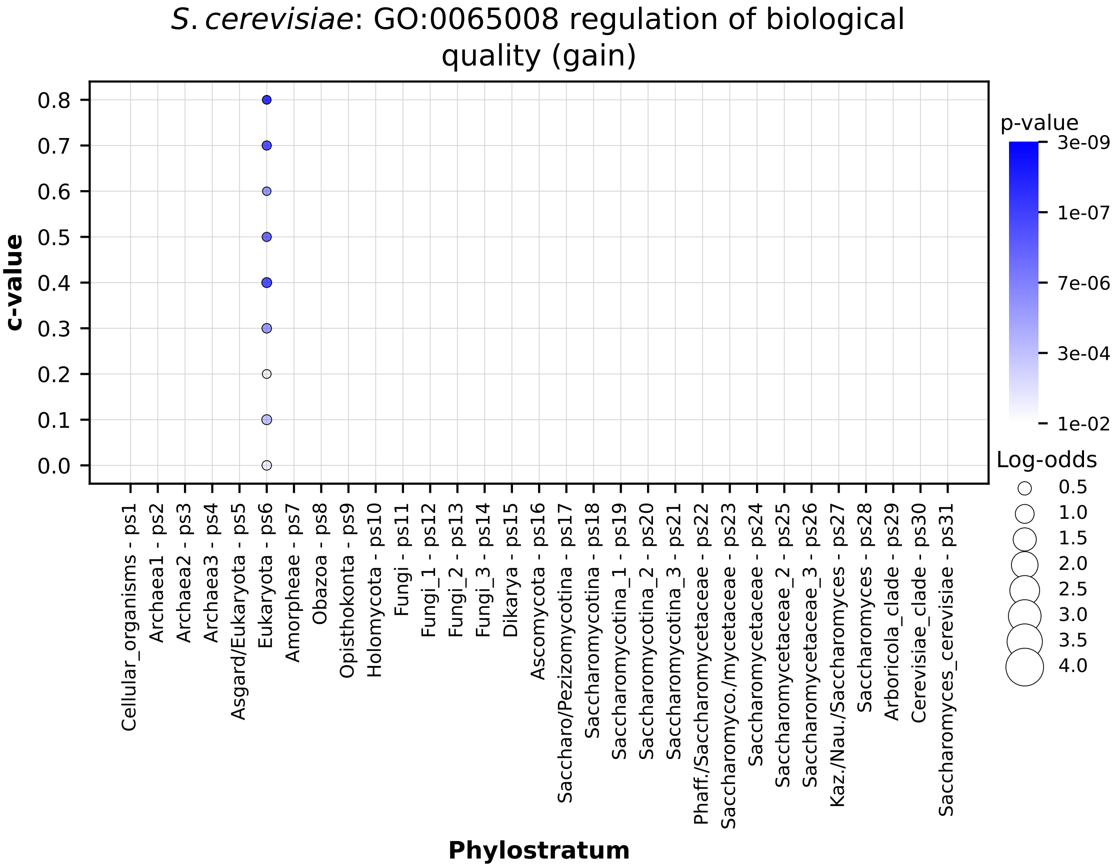
S. cerevisiae: GO:0065008 regulation of biological quality (gain)
c-value
0.0
0.1
0.2
0.3
0.4
0.5
0.6
0.7
0.8
p-value
3e-09
1e-07
7e-06
3e-04
1e-02
Log-odds
0.5
1.0
1.5
2.0
2.5
3.0
3.5
4.0
Cellular_organisms - ps1
Archaea1 - ps2
Archaea2 - ps3
Archaea3 - ps4
Asgard/Eukaryota - ps5
Eukaryota - ps6
Amorpheae - ps7
Obazoa - ps8
Opisthokonta - ps9
Holomycota - ps10
Fungi - ps11
Fungi_1 - ps12
Fungi_2 - ps13
Fungi_3 - ps14
Dikarya - ps15
Ascomycota - ps16
Saccharo/Pezizomycotina - ps17
Saccharomycotina - ps18
Saccharomycotina_1 - ps19
Saccharomycotina_2 - ps20
Saccharomycotina_3 - ps21
Phaff./Saccharomycetaceae - ps22
Saccharomyco./mycetaceae - ps23
Saccharomycetaceae - ps24
Saccharomycetaceae_2 - ps25
Saccharomycetaceae_3 - ps26
Kaz./Nau./Saccharomyces - ps27
Saccharomyces - ps28
Arboricola_clade - ps29
Cerevisiae_clade - ps30
Saccharomyces_cerevisiae - ps31
Phylostratum

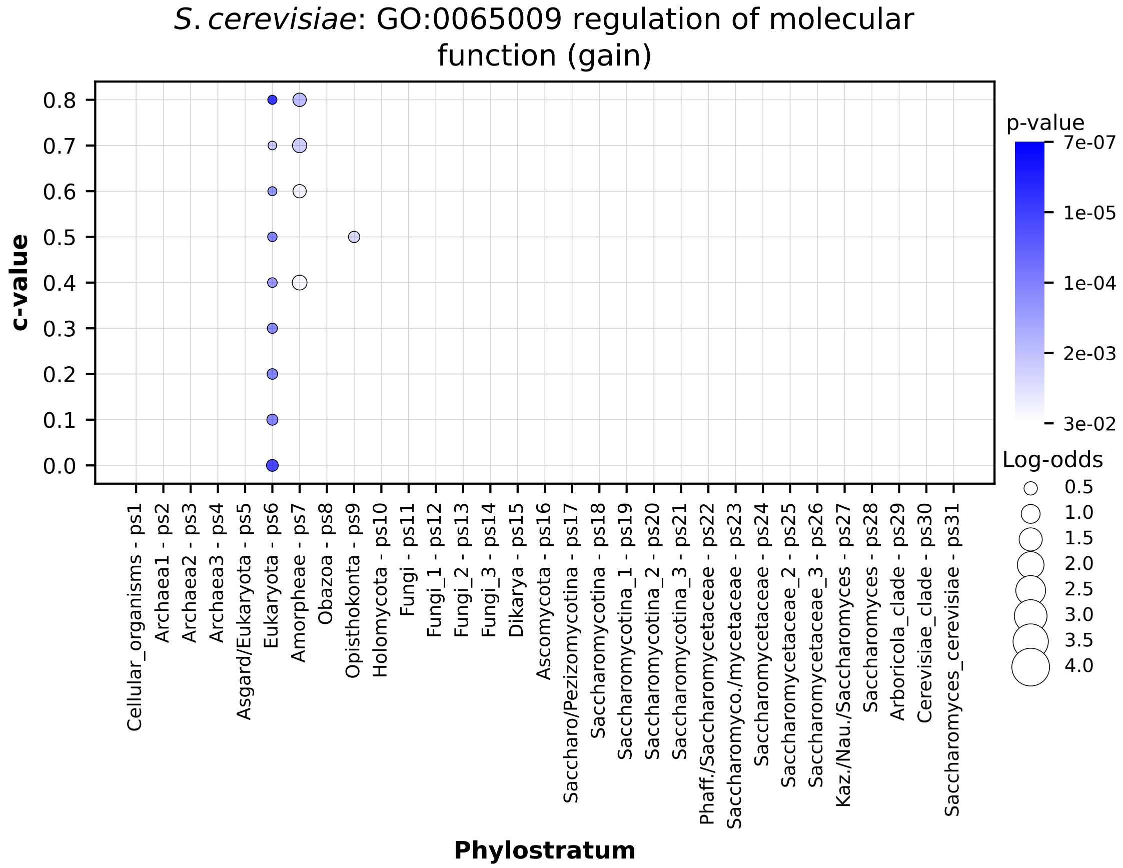
S. cerevisiae: GO:0065009 regulation of molecular function (gain)
c-value
0.8
0.7
0.6
0.5
0.4
0.3
0.2
0.1
0.0
Phylostratum
Cellular_organisms - ps1
Archaea1 - ps2
Archaea2 - ps3
Archaea3 - ps4
Asgard/Eukaryota - ps5
Eukaryota - ps6
Amorpheae - ps7
Obazoa - ps8
Opisthokonta - ps9
Holomycota - ps10
Fungi - ps11
Fungi_1 - ps12
Fungi_2 - ps13
Fungi_3 - ps14
Dikarya - ps15
Ascomycota - ps16
Saccharo/Pezizomycotina - ps17
Saccharomycotina - ps18
Saccharomycotina_1 - ps19
Saccharomycotina_2 - ps20
Saccharomycotina_3 - ps21
Phaff./Saccharomycetaceae - ps22
Saccharomyco./mycetaceae - ps23
Saccharomycetaceae - ps24
Saccharomycetaceae_2 - ps25
Saccharomycetaceae_3 - ps26
Kaz./Nau./Saccharomyces - ps27
Saccharomyces - ps28
Arboricola_clade - ps29
Cerevisiae_clade - ps30
Saccharomyces_cerevisiae - ps31
p-value
7e-07
1e-05
1e-04
2e-03
3e-02
Log-odds
0.5
1.0
1.5
2.0
2.5
3.0
3.5
4.0

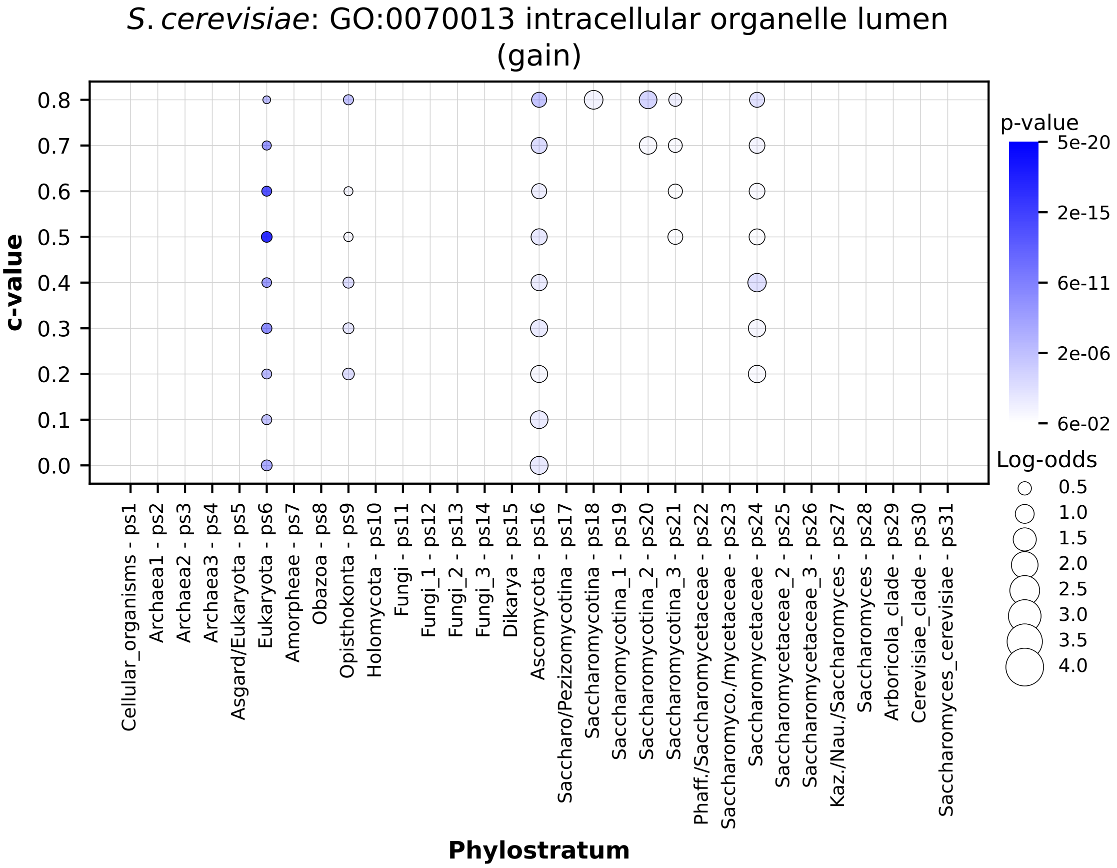

S. cerevisiae: GO:0070013 intracellular organelle lumen (gain)
c-value
0.0
0.1
0.2
0.3
0.4
0.5
0.6
0.7
0.8
Phylostratum
Cellular_organisms - ps1
Archaea1 - ps2
Archaea2 - ps3
Archaea3 - ps4
Asgard/Eukaryota - ps5
Eukaryota - ps6
Amorpheae - ps7
Obazoa - ps8
Opisthokonta - ps9
Holomycota - ps10
Fungi - ps11
Fungi_1 - ps12
Fungi_2 - ps13
Fungi_3 - ps14
Dikarya - ps15
Ascomycota - ps16
Saccharo/Pezizomycotina - ps17
Saccharomycotina - ps18
Saccharomycotina_1 - ps19
Saccharomycotina_2 - ps20
Saccharomycotina_3 - ps21
Phaff./Saccharomycetaceae - ps22
Saccharomyco./mycetaceae - ps23
Saccharomycetaceae - ps24
Saccharomycetaceae_2 - ps25
Saccharomycetaceae_3 - ps26
Kaz./Nau./Saccharomyces - ps27
Saccharomyces - ps28
Arboricola_clade - ps29
Cerevisiae_clade - ps30
Saccharomyces_cerevisiae - ps31
p-value
5e-20
2e-15
6e-11
2e-06
6e-02
Log-odds
0.5
1.0
1.5
2.0
2.5
3.0
3.5
4.0

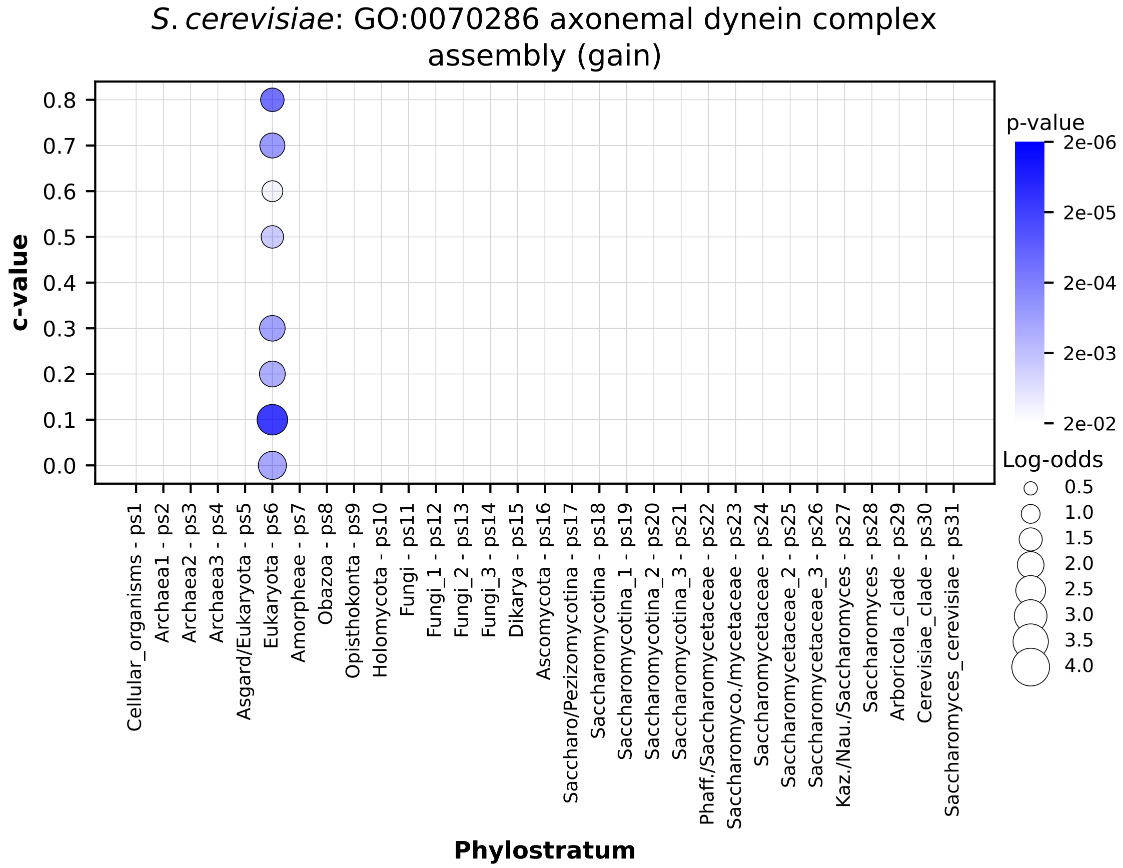

S. cerevisiae: GO:0070286 axonemal dynein complex assembly (gain)
c-value
0.8
0.7
0.6
0.5
0.4
0.3
0.2
0.1
0.0
p-value
2e-06
2e-05
2e-04
2e-03
2e-02
Log-odds
0.5
1.0
1.5
2.0
2.5
3.0
3.5
4.0
Cellular_organisms - ps1
Archaea1 - ps2
Archaea2 - ps3
Archaea3 - ps4
Asgard/Eukaryota - ps5
Eukaryota - ps6
Amorpheae - ps7
Obazoa - ps8
Opisthokonta - ps9
Holomycota - ps10
Fungi - ps11
Fungi_1 - ps12
Fungi_2 - ps13
Fungi_3 - ps14
Dikarya - ps15
Ascomycota - ps16
Saccharo/Pezizomycotina - ps17
Saccharomycotina - ps18
Saccharomycotina_1 - ps19
Saccharomycotina_2 - ps20
Saccharomycotina_3 - ps21
Phaff./Saccharomycetaceae - ps22
Saccharomyco./mycetaceae - ps23
Saccharomycetaceae - ps24
Saccharomycetaceae_2 - ps25
Saccharomycetaceae_3 - ps26
Kaz./Nau./Saccharomyces - ps27
Saccharomyces - ps28
Arboricola_clade - ps29
Cerevisiae_clade - ps30
Saccharomyces_cerevisiae - ps31
Phylostratum

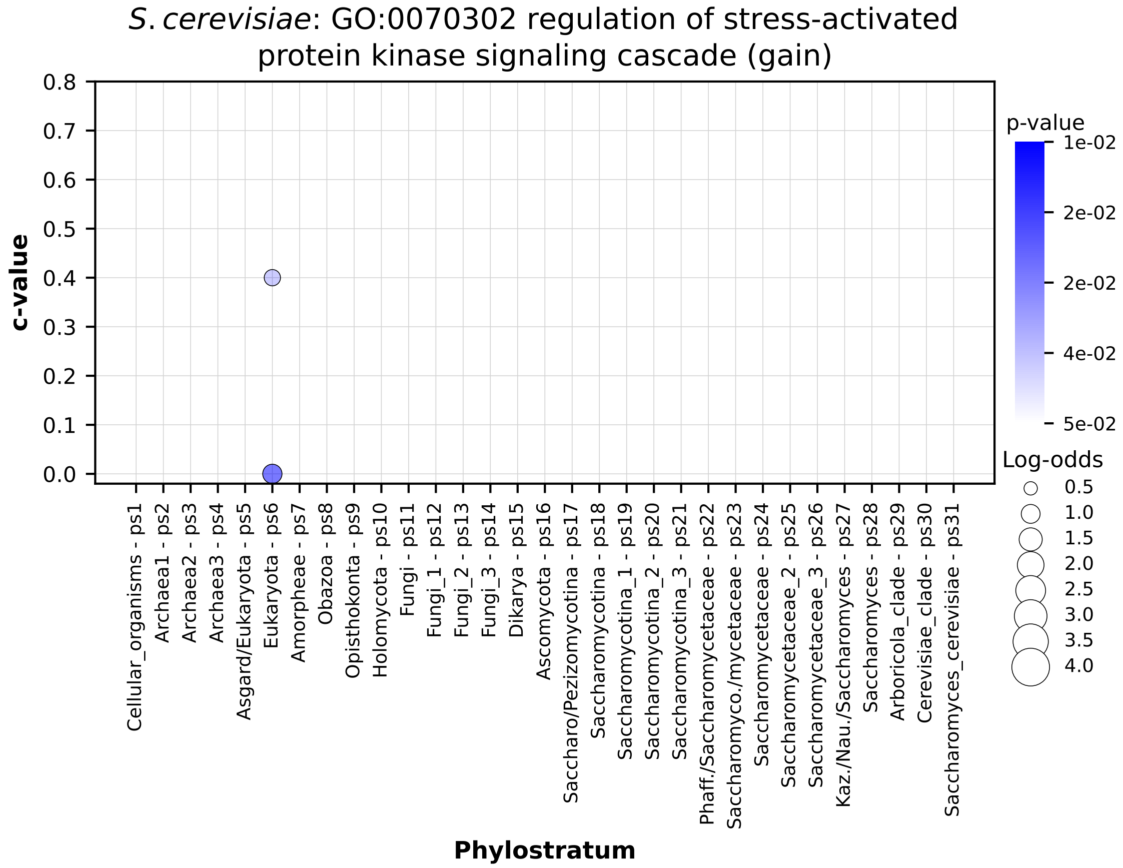

S. cerevisiae: GO:0070302 regulation of stress-activated protein kinase signaling cascade (gain)
c-value
0.8
0.7
0.6
0.5
0.4
0.3
0.2
0.1
0.0
Phylostratum
Cellular_organisms - ps1
Archaea1 - ps2
Archaea2 - ps3
Archaea3 - ps4
Asgard/Eukaryota - ps5
Eukaryota - ps6
Amorpheae - ps7
Obazoa - ps8
Opisthokonta - ps9
Holomycota - ps10
Fungi - ps11
Fungi_1 - ps12
Fungi_2 - ps13
Fungi_3 - ps14
Dikarya - ps15
Ascomycota - ps16
Saccharo/Pezizomycotina - ps17
Saccharomycotina - ps18
Saccharomycotina_1 - ps19
Saccharomycotina_2 - ps20
Saccharomycotina_3 - ps21
Phaff./Saccharomycetaceae - ps22
Saccharomyco./mycetaceae - ps23
Saccharomycetaceae - ps24
Saccharomycetaceae_2 - ps25
Saccharomycetaceae_3 - ps26
Kaz./Nau./Saccharomyces - ps27
Saccharomyces - ps28
Arboricola_clade - ps29
Cerevisiae_clade - ps30
Saccharomyces_cerevisiae - ps31
p-value
1e-02
2e-02
2e-02
4e-02
5e-02
Log-odds
0.5
1.0
1.5
2.0
2.5
3.0
3.5
4.0

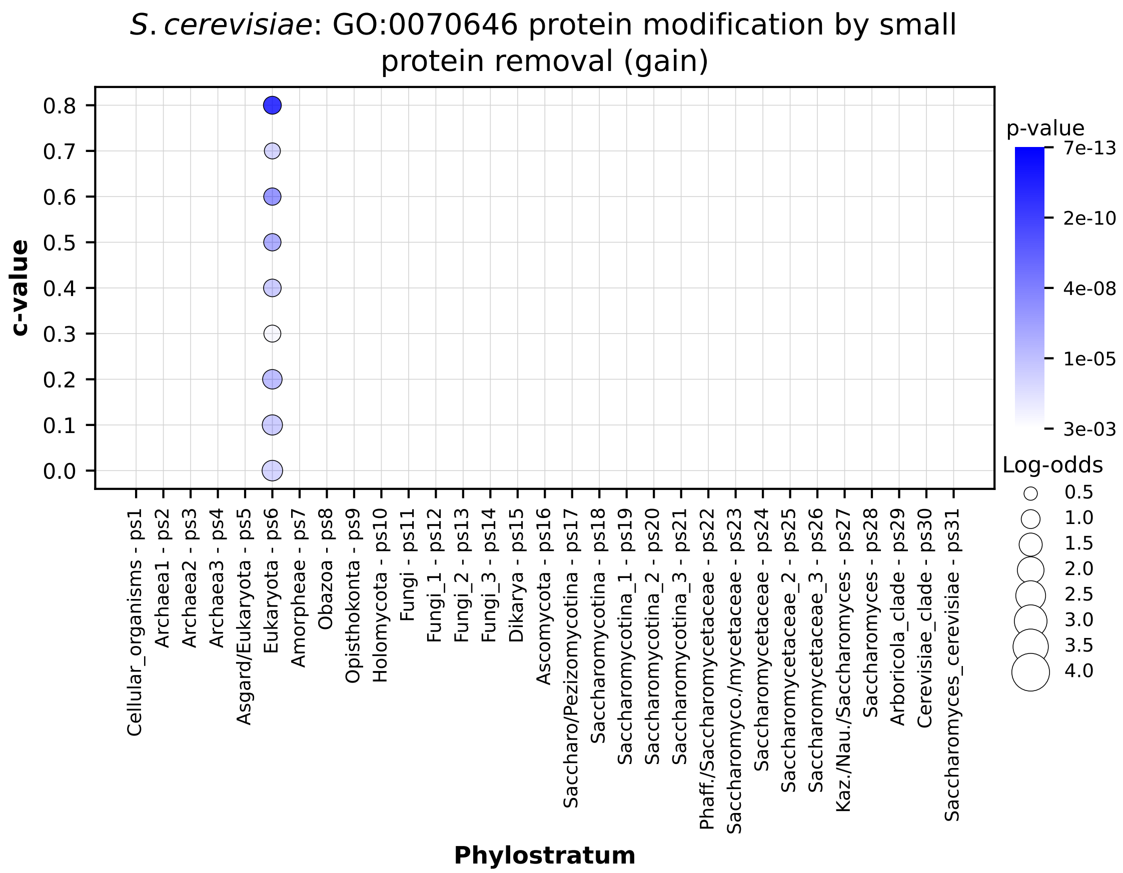
S. cerevisiae: GO:0070646 protein modification by small protein removal (gain)
c-value
0.8
0.7
0.6
0.5
0.4
0.3
0.2
0.1
0.0
p-value
7e-13
2e-10
4e-08
1e-05
3e-03
Log-odds
0.5
1.0
1.5
2.0
2.5
3.0
3.5
4.0
Cellular_organisms - ps1
Archaea1 - ps2
Archaea2 - ps3
Archaea3 - ps4
Asgard/Eukaryota - ps5
Eukaryota - ps6
Amorpheae - ps7
Obazoa - ps8
Opisthokonta - ps9
Holomycota - ps10
Fungi - ps11
Fungi_1 - ps12
Fungi_2 - ps13
Fungi_3 - ps14
Dikarya - ps15
Ascomycota - ps16
Saccharo/Pezizomycotina - ps17
Saccharomycotina - ps18
Saccharomycotina_1 - ps19
Saccharomycotina_2 - ps20
Saccharomycotina_3 - ps21
Phaff./Saccharomycetaceae - ps22
Saccharomyco./mycetaceae - ps23
Saccharomycetaceae - ps24
Saccharomycetaceae_2 - ps25
Saccharomycetaceae_3 - ps26
Kaz./Nau./Saccharomyces - ps27
Saccharomyces - ps28
Arboricola_clade - ps29
Cerevisiae_clade - ps30
Saccharomyces_cerevisiae - ps31
Phylostratum

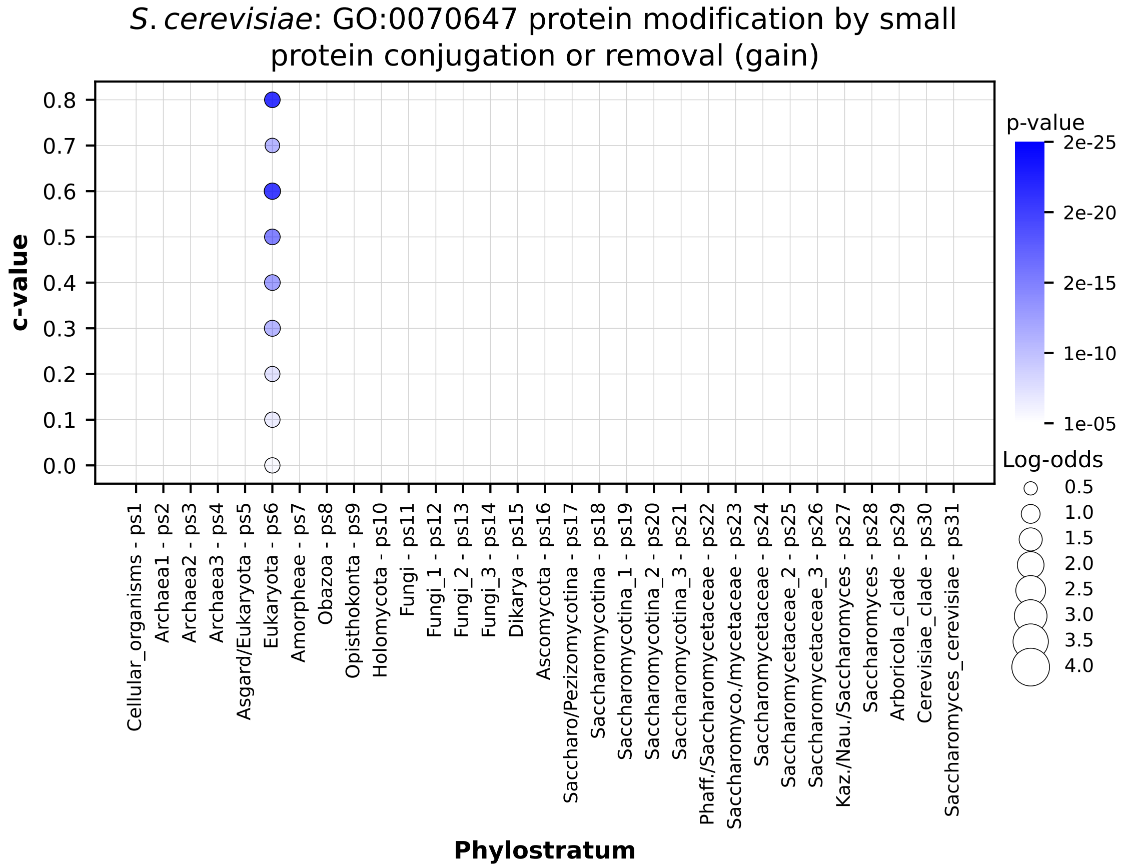

S. cerevisiae: GO:0070647 protein modification by small protein conjugation or removal (gain)
c-value
0.0
0.1
0.2
0.3
0.4
0.5
0.6
0.7
0.8
Phylostratum
Cellular_organisms - ps1
Archaea1 - ps2
Archaea2 - ps3
Archaea3 - ps4
Asgard/Eukaryota - ps5
Eukaryota - ps6
Amorpheae - ps7
Obazoa - ps8
Opisthokonta - ps9
Holomycota - ps10
Fungi - ps11
Fungi_1 - ps12
Fungi_2 - ps13
Fungi_3 - ps14
Dikarya - ps15
Ascomycota - ps16
Saccharo/Pezizomycotina - ps17
Saccharomycotina - ps18
Saccharomycotina_1 - ps19
Saccharomycotina_2 - ps20
Saccharomycotina_3 - ps21
Phaff./Saccharomycetaceae - ps22
Saccharomyco./mycetaceae - ps23
Saccharomycetaceae - ps24
Saccharomycetaceae_2 - ps25
Saccharomycetaceae_3 - ps26
Kaz./Nau./Saccharomyces - ps27
Saccharomyces - ps28
Arboricola_clade - ps29
Cerevisiae_clade - ps30
Saccharomyces_cerevisiae - ps31
p-value
2e-25
2e-20
2e-15
1e-10
1e-05
Log-odds
0.5
1.0
1.5
2.0
2.5
3.0
3.5
4.0

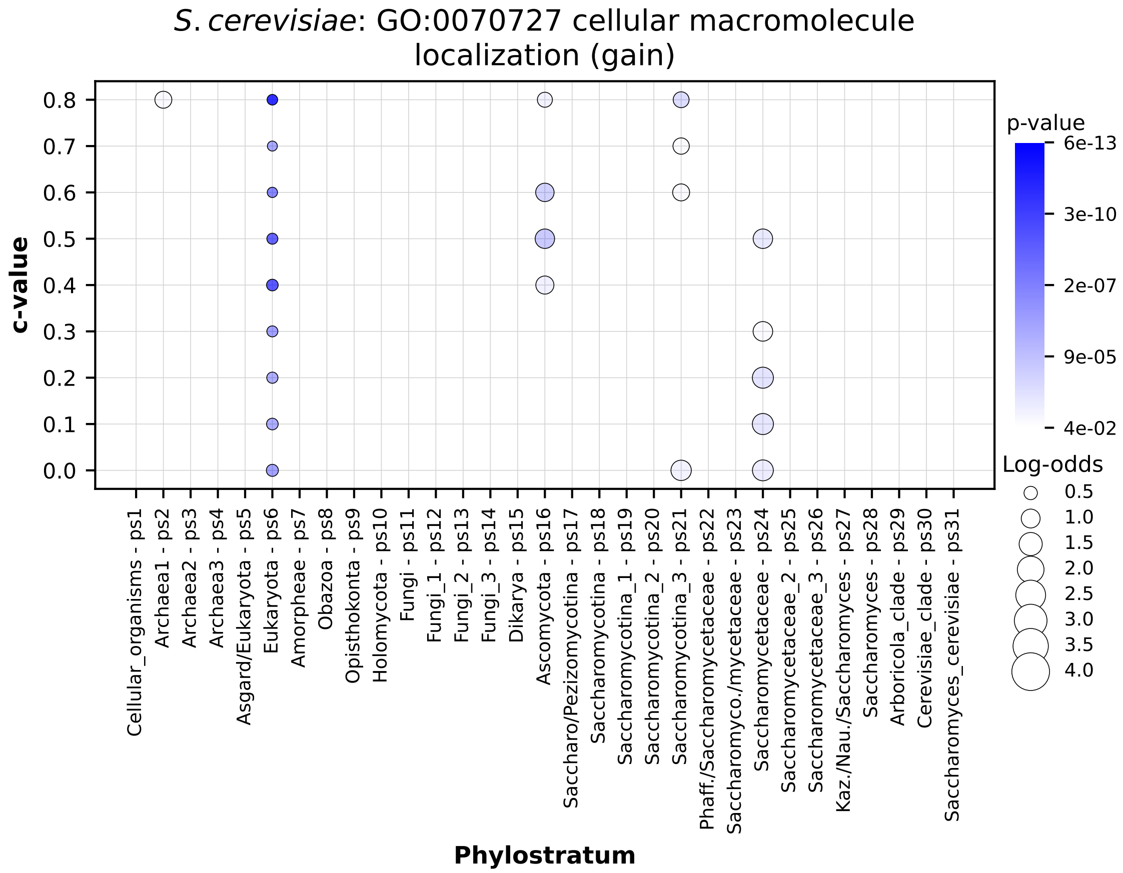

S. cerevisiae: GO:0070727 cellular macromolecule localization (gain)
c-value
0.8
0.7
0.6
0.5
0.4
0.3
0.2
0.1
0.0
p-value
6e-13
3e-10
2e-07
9e-05
4e-02
Log-odds
0.5
1.0
1.5
2.0
2.5
3.0
3.5
4.0
Cellular_organisms - ps1
Archaea1 - ps2
Archaea2 - ps3
Archaea3 - ps4
Asgard/Eukaryota - ps5
Eukaryota - ps6
Amorpheae - ps7
Obazoa - ps8
Opisthokonta - ps9
Holomycota - ps10
Fungi - ps11
Fungi_1 - ps12
Fungi_2 - ps13
Fungi_3 - ps14
Dikarya - ps15
Ascomycota - ps16
Saccharo/Pezizomycotina - ps17
Saccharomycotina - ps18
Saccharomycotina_1 - ps19
Saccharomycotina_2 - ps20
Saccharomycotina_3 - ps21
Phaff./Saccharomycetaceae - ps22
Saccharomyco./mycetaceae - ps23
Saccharomycetaceae - ps24
Saccharomycetaceae_2 - ps25
Saccharomycetaceae_3 - ps26
Kaz./Nau./Saccharomyces - ps27
Saccharomyces - ps28
Arboricola_clade - ps29
Cerevisiae_clade - ps30
Saccharomyces_cerevisiae - ps31
Phylostratum

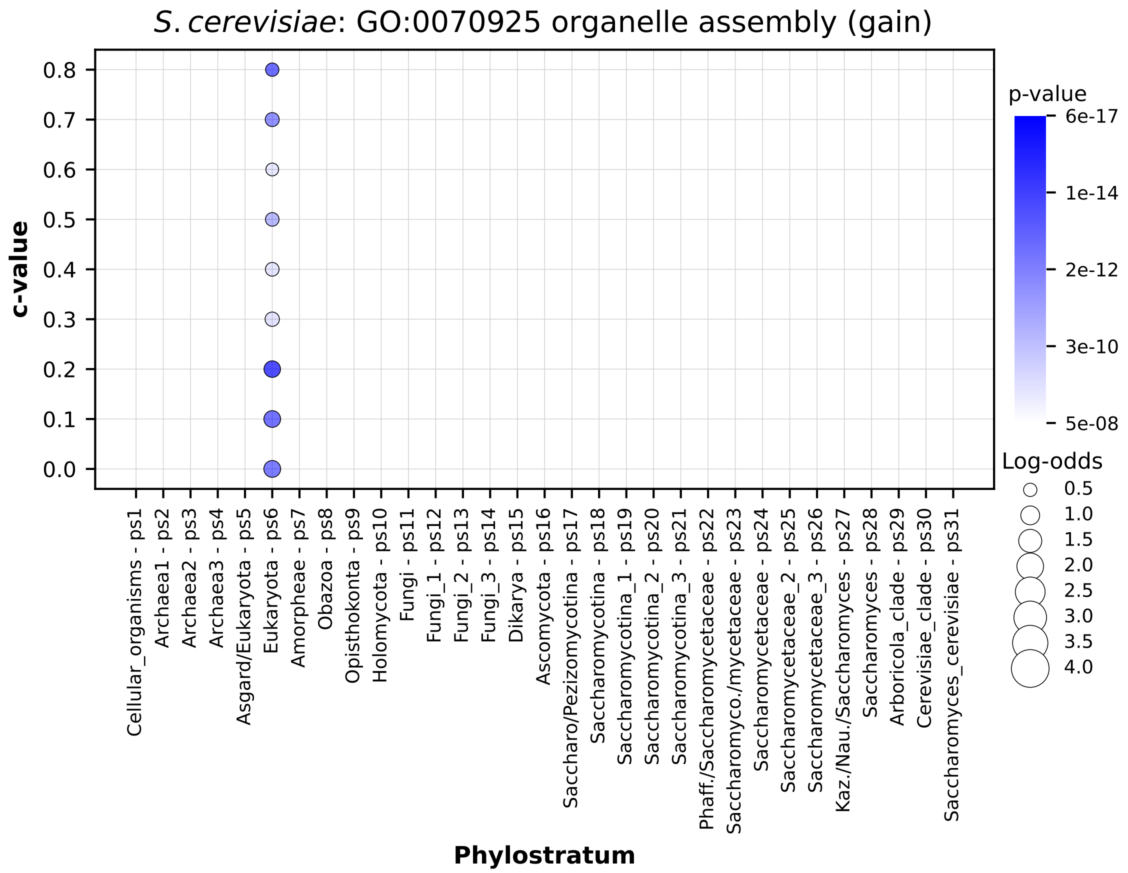

S. cerevisiae: GO:0070925 organelle assembly (gain)
c-value
0.8
0.7
0.6
0.5
0.4
0.3
0.2
0.1
0.0
p-value
6e-17
1e-14
2e-12
3e-10
5e-08
Log-odds
0.5
1.0
1.5
2.0
2.5
3.0
3.5
4.0
Cellular_organisms - ps1
Archaea1 - ps2
Archaea2 - ps3
Archaea3 - ps4
Asgard/Eukaryota - ps5
Eukaryota - ps6
Amorpheae - ps7
Obazoa - ps8
Opisthokonta - ps9
Holomycota - ps10
Fungi - ps11
Fungi_1 - ps12
Fungi_2 - ps13
Fungi_3 - ps14
Dikarya - ps15
Ascomycota - ps16
Saccharo/Pezizomycotina - ps17
Saccharomycotina - ps18
Saccharomycotina_1 - ps19
Saccharomycotina_2 - ps20
Saccharomycotina_3 - ps21
Phaff./Saccharomycetaceae - ps22
Saccharomyco./mycetaceae - ps23
Saccharomycetaceae - ps24
Saccharomycetaceae_2 - ps25
Saccharomycetaceae_3 - ps26
Kaz./Nau./Saccharomyces - ps27
Saccharomyces - ps28
Arboricola_clade - ps29
Cerevisiae_clade - ps30
Saccharomyces_cerevisiae - ps31
Phylostratum

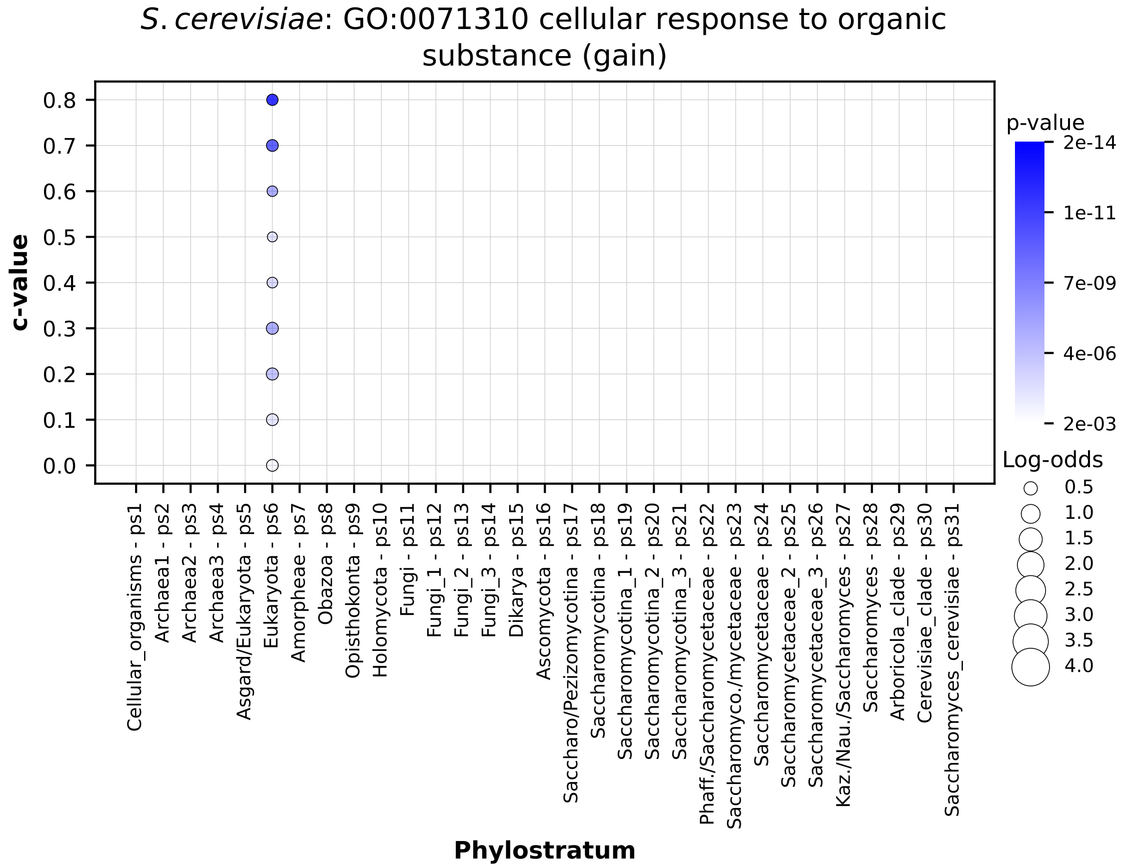
S. cerevisiae: GO:0071310 cellular response to organic substance (gain)
c-value
0.0
0.1
0.2
0.3
0.4
0.5
0.6
0.7
0.8
p-value
2e-14
1e-11
7e-09
4e-06
2e-03
Log-odds
0.5
1.0
1.5
2.0
2.5
3.0
3.5
4.0
Cellular_organisms - ps1
Archaea1 - ps2
Archaea2 - ps3
Archaea3 - ps4
Asgard/Eukaryota - ps5
Eukaryota - ps6
Amorpheae - ps7
Obazoa - ps8
Opisthokonta - ps9
Holomycota - ps10
Fungi - ps11
Fungi_1 - ps12
Fungi_2 - ps13
Fungi_3 - ps14
Dikarya - ps15
Ascomycota - ps16
Saccharo/Pezizomycotina - ps17
Saccharomycotina - ps18
Saccharomycotina_1 - ps19
Saccharomycotina_2 - ps20
Saccharomycotina_3 - ps21
Phaff./Saccharomycetaceae - ps22
Saccharomyco./mycetaceae - ps23
Saccharomycetaceae - ps24
Saccharomycetaceae_2 - ps25
Saccharomycetaceae_3 - ps26
Kaz./Nau./Saccharomyces - ps27
Saccharomyces - ps28
Arboricola_clade - ps29
Cerevisiae_clade - ps30
Saccharomyces_cerevisiae - ps31
Phylostratum

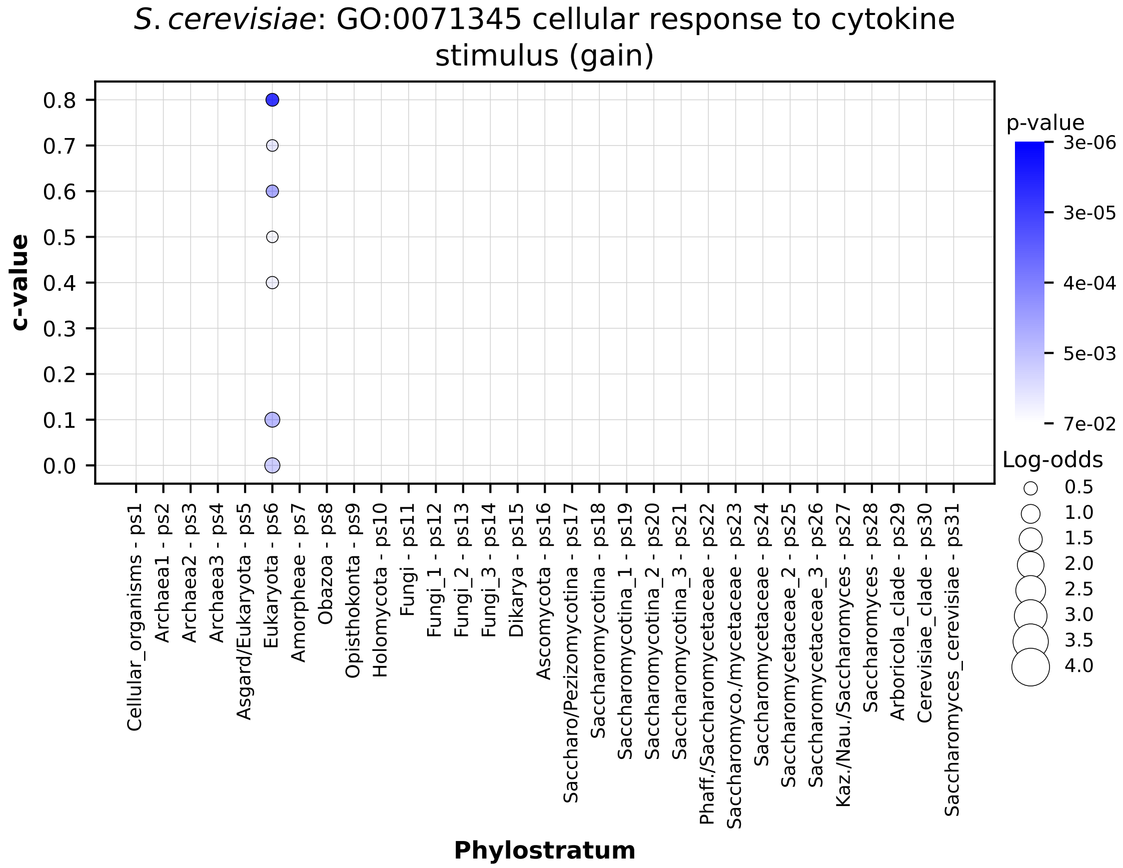
S. cerevisiae: GO:0071345 cellular response to cytokine stimulus (gain)
c-value
0.8
0.7
0.6
0.5
0.4
0.3
0.2
0.1
0.0
p-value
3e-06
3e-05
4e-04
5e-03
7e-02
Log-odds
0.5
1.0
1.5
2.0
2.5
3.0
3.5
4.0
Cellular_organisms - ps1
Archaea1 - ps2
Archaea2 - ps3
Archaea3 - ps4
Asgard/Eukaryota - ps5
Eukaryota - ps6
Amorpheae - ps7
Obazoa - ps8
Opisthokonta - ps9
Holomycota - ps10
Fungi - ps11
Fungi_1 - ps12
Fungi_2 - ps13
Fungi_3 - ps14
Dikarya - ps15
Ascomycota - ps16
Saccharo/Pezizomycotina - ps17
Saccharomycotina - ps18
Saccharomycotina_1 - ps19
Saccharomycotina_2 - ps20
Saccharomycotina_3 - ps21
Phaff./Saccharomycetaceae - ps22
Saccharomyco./mycetaceae - ps23
Saccharomycetaceae - ps24
Saccharomycetaceae_2 - ps25
Saccharomycetaceae_3 - ps26
Kaz./Nau./Saccharomyces - ps27
Saccharomyces - ps28
Arboricola_clade - ps29
Cerevisiae_clade - ps30
Saccharomyces_cerevisiae - ps31
Phylostratum

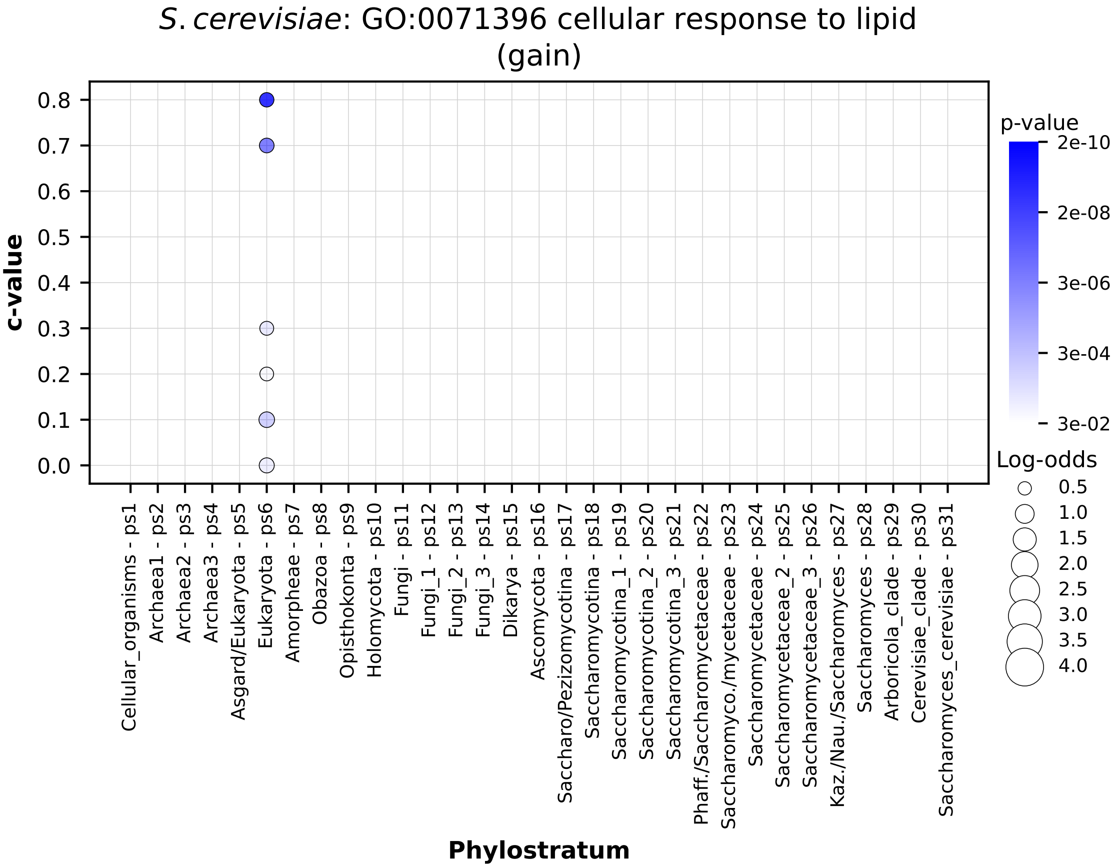

S. cerevisiae: GO:0071396 cellular response to lipid
(gain)
c-value
0.8
0.7
0.6
0.5
0.4
0.3
0.2
0.1
0.0
p-value
2e-10
2e-08
3e-06
3e-04
3e-02
Log-odds
0.5
1.0
1.5
2.0
2.5
3.0
3.5
4.0
Cellular_organisms - ps1
Archaea1 - ps2
Archaea2 - ps3
Archaea3 - ps4
Asgard/Eukaryota - ps5
Eukaryota - ps6
Amorpheae - ps7
Obazoa - ps8
Opisthokonta - ps9
Holomycota - ps10
Fungi - ps11
Fungi_1 - ps12
Fungi_2 - ps13
Fungi_3 - ps14
Dikarya - ps15
Ascomycota - ps16
Saccharo/Pezizomycotina - ps17
Saccharomycotina - ps18
Saccharomycotina_1 - ps19
Saccharomycotina_2 - ps20
Saccharomycotina_3 - ps21
Phaff./Saccharomycetaceae - ps22
Saccharomyco./mycetaceae - ps23
Saccharomycetaceae - ps24
Saccharomycetaceae_2 - ps25
Saccharomycetaceae_3 - ps26
Kaz./Nau./Saccharomyces - ps27
Saccharomyces - ps28
Arboricola_clade - ps29
Cerevisiae_clade - ps30
Saccharomyces_cerevisiae - ps31
Phylostratum

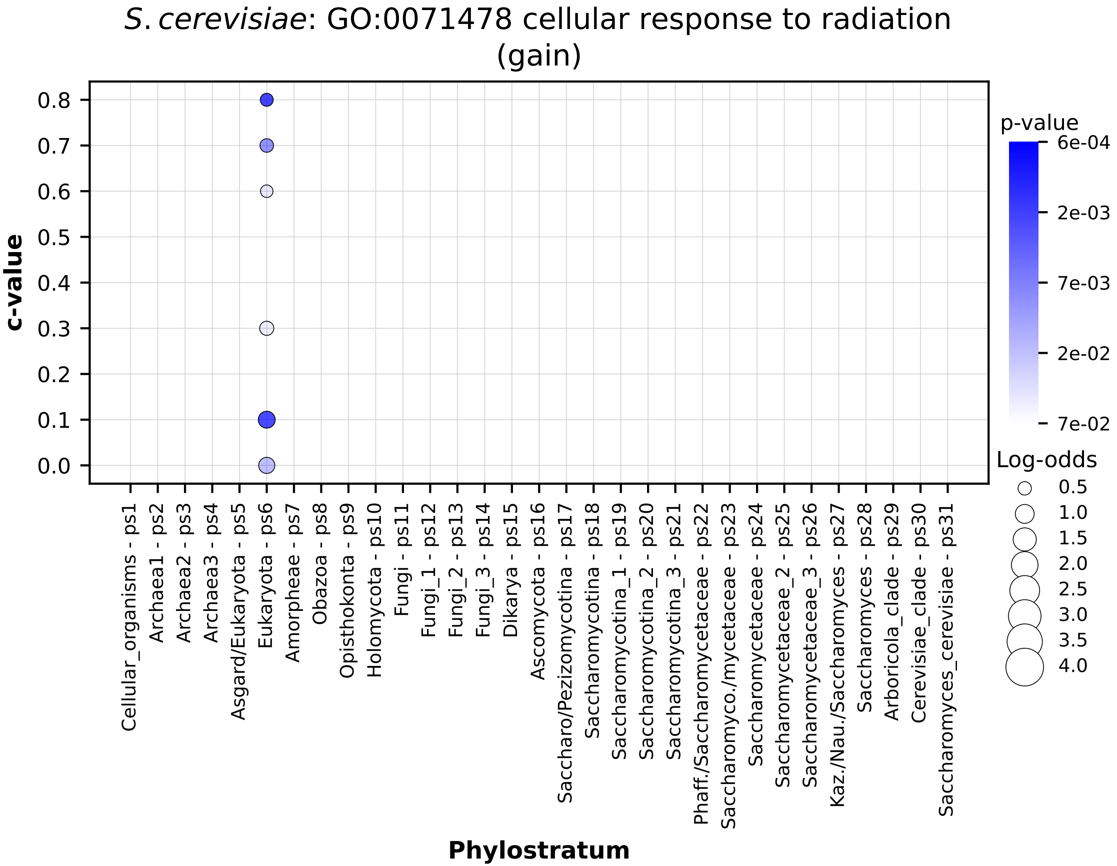

S. cerevisiae: GO:0071478 cellular response to radiation (gain)
c-value
0.0
0.1
0.2
0.3
0.4
0.5
0.6
0.7
0.8
p-value
6e-04
2e-03
7e-03
2e-02
7e-02
Log-odds
0.5
1.0
1.5
2.0
2.5
3.0
3.5
4.0
Cellular_organisms - ps1
Archaea1 - ps2
Archaea2 - ps3
Archaea3 - ps4
Asgard/Eukaryota - ps5
Eukaryota - ps6
Amorpheae - ps7
Obazoa - ps8
Opisthokonta - ps9
Holomycota - ps10
Fungi - ps11
Fungi_1 - ps12
Fungi_2 - ps13
Fungi_3 - ps14
Dikarya - ps15
Ascomycota - ps16
Saccharo/Pezizomycotina - ps17
Saccharomycotina - ps18
Saccharomycotina_1 - ps19
Saccharomycotina_2 - ps20
Saccharomycotina_3 - ps21
Phaff./Saccharomycetaceae - ps22
Saccharomyco./mycetaceae - ps23
Saccharomycetaceae - ps24
Saccharomycetaceae_2 - ps25
Saccharomycetaceae_3 - ps26
Kaz./Nau./Saccharomyces - ps27
Saccharomyces - ps28
Arboricola_clade - ps29
Cerevisiae_clade - ps30
Saccharomyces_cerevisiae - ps31
Phylostratum

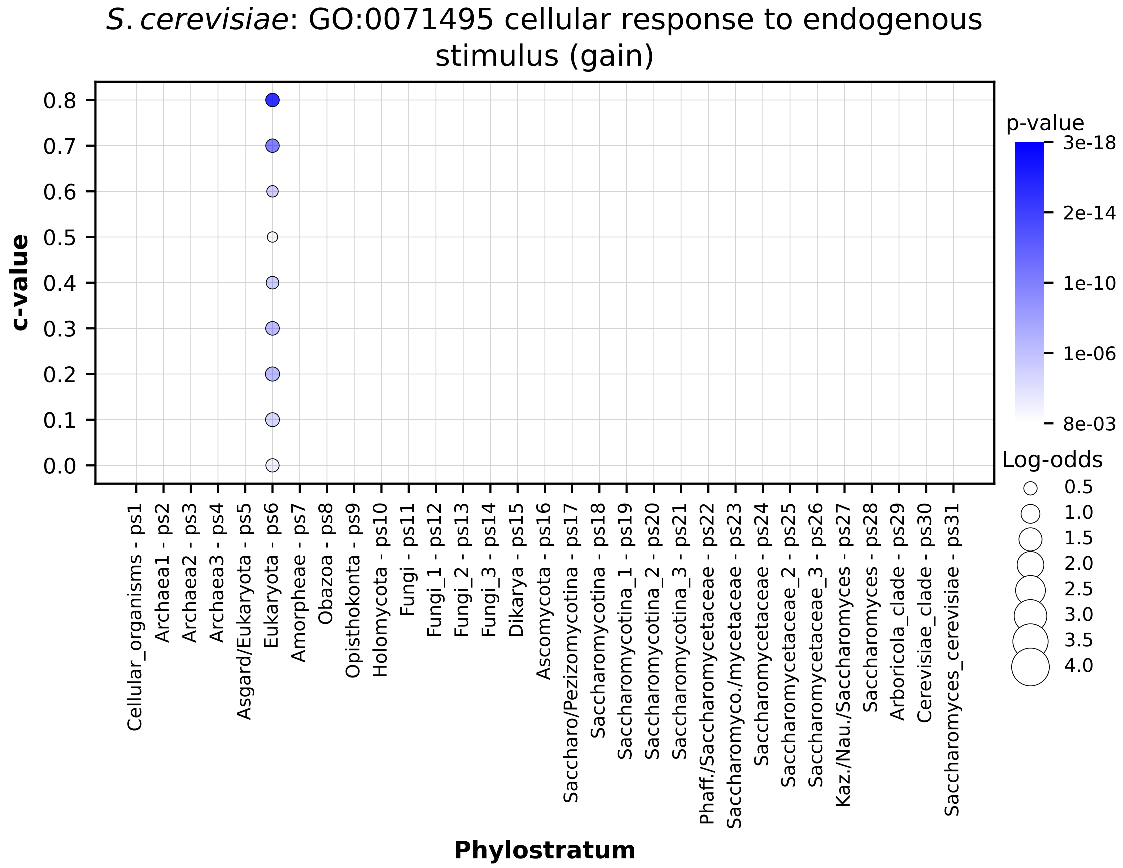

S. cerevisiae: GO:0071495 cellular response to endogenous stimulus (gain)
c-value
0.8
0.7
0.6
0.5
0.4
0.3
0.2
0.1
0.0
p-value
3e-18
2e-14
1e-10
1e-06
8e-03
Log-odds
0.5
1.0
1.5
2.0
2.5
3.0
3.5
4.0
Cellular_organisms - ps1
Archaea1 - ps2
Archaea2 - ps3
Archaea3 - ps4
Asgard/Eukaryota - ps5
Eukaryota - ps6
Amorpheae - ps7
Obazoa - ps8
Opisthokonta - ps9
Holomycota - ps10
Fungi - ps11
Fungi_1 - ps12
Fungi_2 - ps13
Fungi_3 - ps14
Dikarya - ps15
Ascomycota - ps16
Saccharo/Pezizomycotina - ps17
Saccharomycotina - ps18
Saccharomycotina_1 - ps19
Saccharomycotina_2 - ps20
Saccharomycotina_3 - ps21
Phaff./Saccharomycetaceae - ps22
Saccharomyco./mycetaceae - ps23
Saccharomycetaceae - ps24
Saccharomycetaceae_2 - ps25
Saccharomycetaceae_3 - ps26
Kaz./Nau./Saccharomyces - ps27
Saccharomyces - ps28
Arboricola_clade - ps29
Cerevisiae_clade - ps30
Saccharomyces_cerevisiae - ps31
Phylostratum

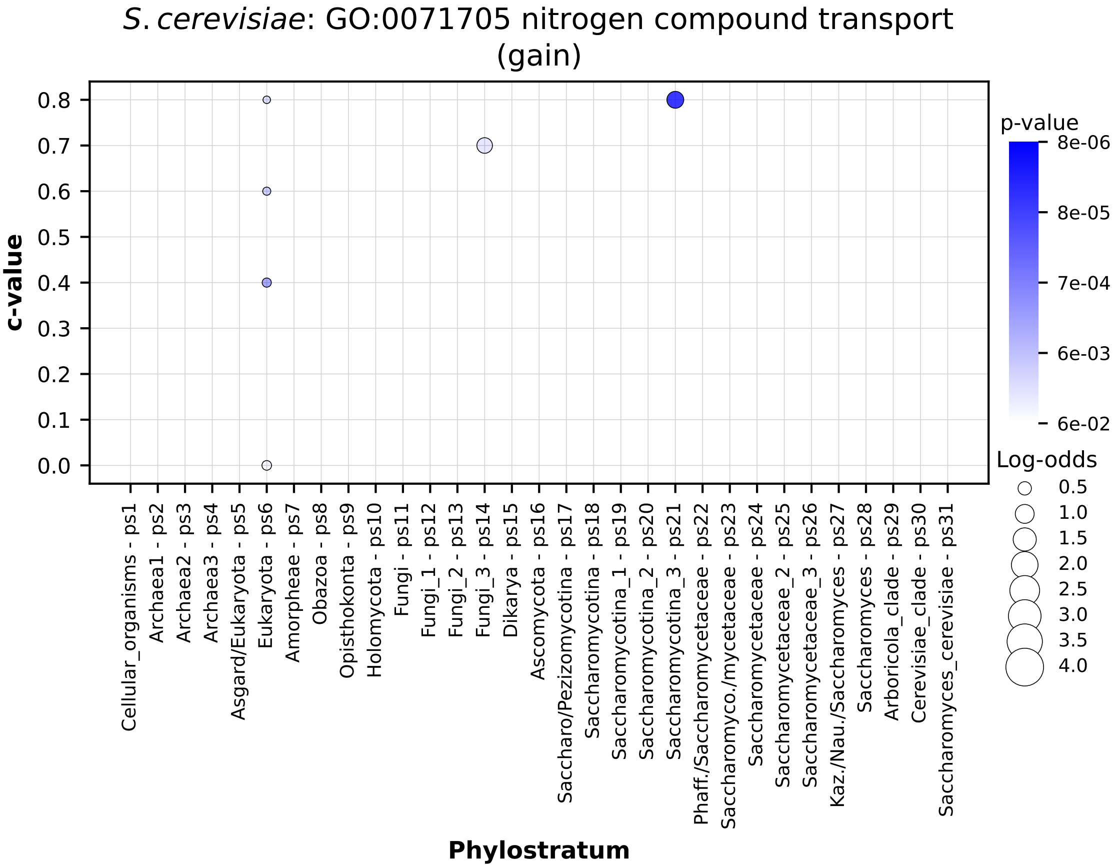

S. cerevisiae: GO:0071705 nitrogen compound transport
(gain)
c-value
0.0
0.1
0.2
0.3
0.4
0.5
0.6
0.7
0.8
p-value
8e-06
8e-05
7e-04
6e-03
6e-02
Log-odds
0.5
1.0
1.5
2.0
2.5
3.0
3.5
4.0
Cellular_organisms - ps1
Archaea1 - ps2
Archaea2 - ps3
Archaea3 - ps4
Asgard/Eukaryota - ps5
Eukaryota - ps6
Amorpheae - ps7
Obazoa - ps8
Opisthokonta - ps9
Holomycota - ps10
Fungi - ps11
Fungi_1 - ps12
Fungi_2 - ps13
Fungi_3 - ps14
Dikarya - ps15
Ascomycota - ps16
Saccharo/Pezizomycotina - ps17
Saccharomycotina - ps18
Saccharomycotina_1 - ps19
Saccharomycotina_2 - ps20
Saccharomycotina_3 - ps21
Phaff./Saccharomycetaceae - ps22
Saccharomyco./mycetaceae - ps23
Saccharomycetaceae - ps24
Saccharomycetaceae_2 - ps25
Saccharomycetaceae_3 - ps26
Kaz./Nau./Saccharomyces - ps27
Saccharomyces - ps28
Arboricola_clade - ps29
Cerevisiae_clade - ps30
Saccharomyces_cerevisiae - ps31
Phylostratum

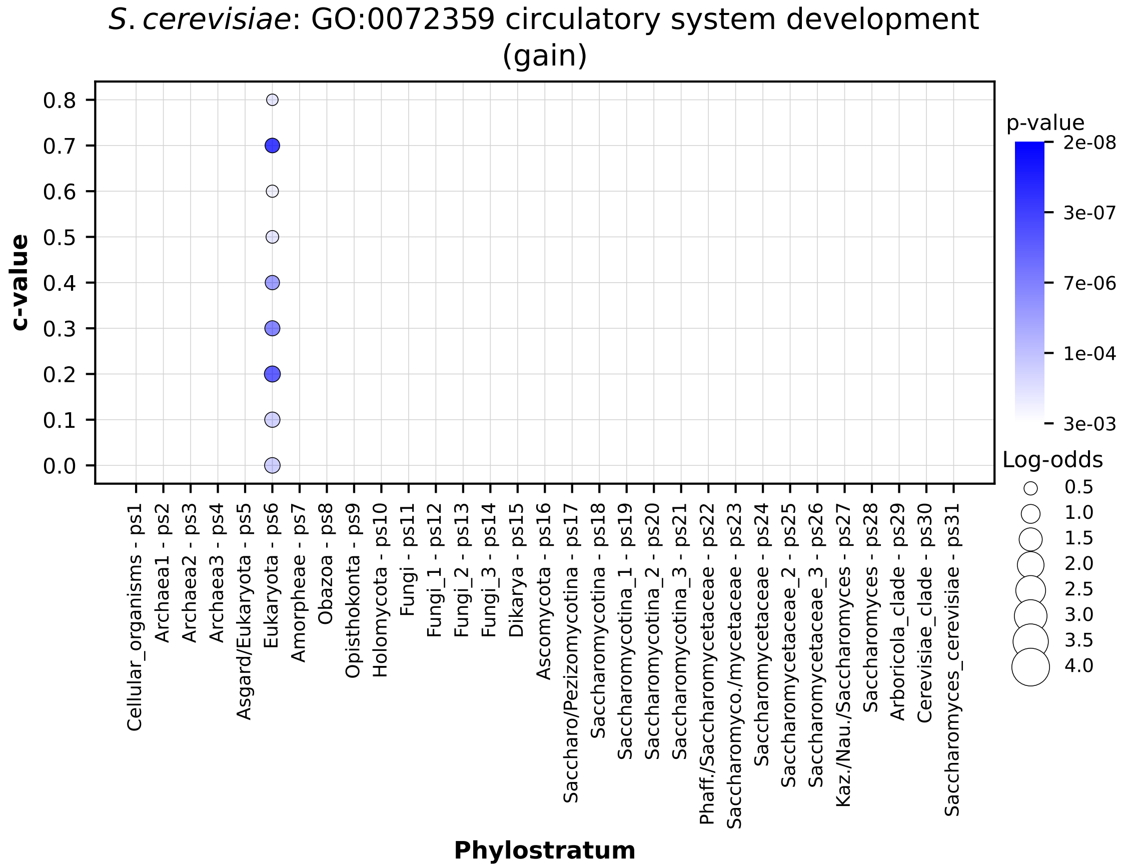

S. cerevisiae: GO:0072359 circulatory system development
(gain)
c-value
0.8
0.7
0.6
0.5
0.4
0.3
0.2
0.1
0.0
p-value
2e-08
3e-07
7e-06
1e-04
3e-03
Log-odds
0.5
1.0
1.5
2.0
2.5
3.0
3.5
4.0
Cellular_organisms - ps1
Archaea1 - ps2
Archaea2 - ps3
Archaea3 - ps4
Asgard/Eukaryota - ps5
Eukaryota - ps6
Amorpheae - ps7
Obazoa - ps8
Opisthokonta - ps9
Holomycota - ps10
Fungi - ps11
Fungi_1 - ps12
Fungi_2 - ps13
Fungi_3 - ps14
Dikarya - ps15
Ascomycota - ps16
Saccharo/Pezizomycotina - ps17
Saccharomycotina - ps18
Saccharomycotina_1 - ps19
Saccharomycotina_2 - ps20
Saccharomycotina_3 - ps21
Phaff./Saccharomycetaceae - ps22
Saccharomyco./mycetaceae - ps23
Saccharomycetaceae - ps24
Saccharomycetaceae_2 - ps25
Saccharomycetaceae_3 - ps26
Kaz./Nau./Saccharomyces - ps27
Saccharomyces - ps28
Arboricola_clade - ps29
Cerevisiae_clade - ps30
Saccharomyces_cerevisiae - ps31
Phylostratum

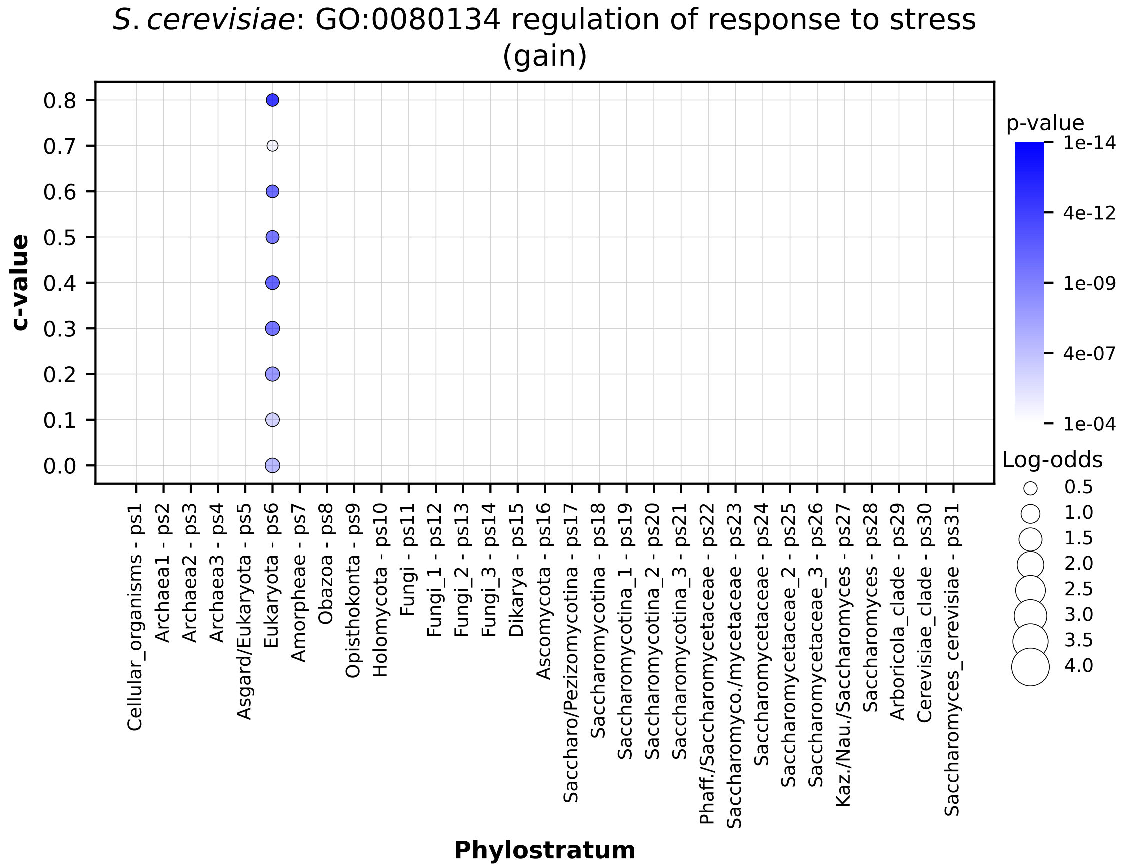

S. cerevisiae: GO:0080134 regulation of response to stress (gain)
c-value
0.0
0.1
0.2
0.3
0.4
0.5
0.6
0.7
0.8
Phylostratum
Cellular_organisms - ps1
Archaea1 - ps2
Archaea2 - ps3
Archaea3 - ps4
Asgard/Eukaryota - ps5
Eukaryota - ps6
Amorpheae - ps7
Obazoa - ps8
Opisthokonta - ps9
Holomycota - ps10
Fungi - ps11
Fungi_1 - ps12
Fungi_2 - ps13
Fungi_3 - ps14
Dikarya - ps15
Ascomycota - ps16
Saccharo/Pezizomycotina - ps17
Saccharomycotina - ps18
Saccharomycotina_1 - ps19
Saccharomycotina_2 - ps20
Saccharomycotina_3 - ps21
Phaff./Saccharomycetaceae - ps22
Saccharomyco./mycetaceae - ps23
Saccharomycetaceae - ps24
Saccharomycetaceae_2 - ps25
Saccharomycetaceae_3 - ps26
Kaz./Nau./Saccharomyces - ps27
Saccharomyces - ps28
Arboricola_clade - ps29
Cerevisiae_clade - ps30
Saccharomyces_cerevisiae - ps31
p-value
1e-14
4e-12
1e-09
4e-07
1e-04
Log-odds
0.5
1.0
1.5
2.0
2.5
3.0
3.5
4.0

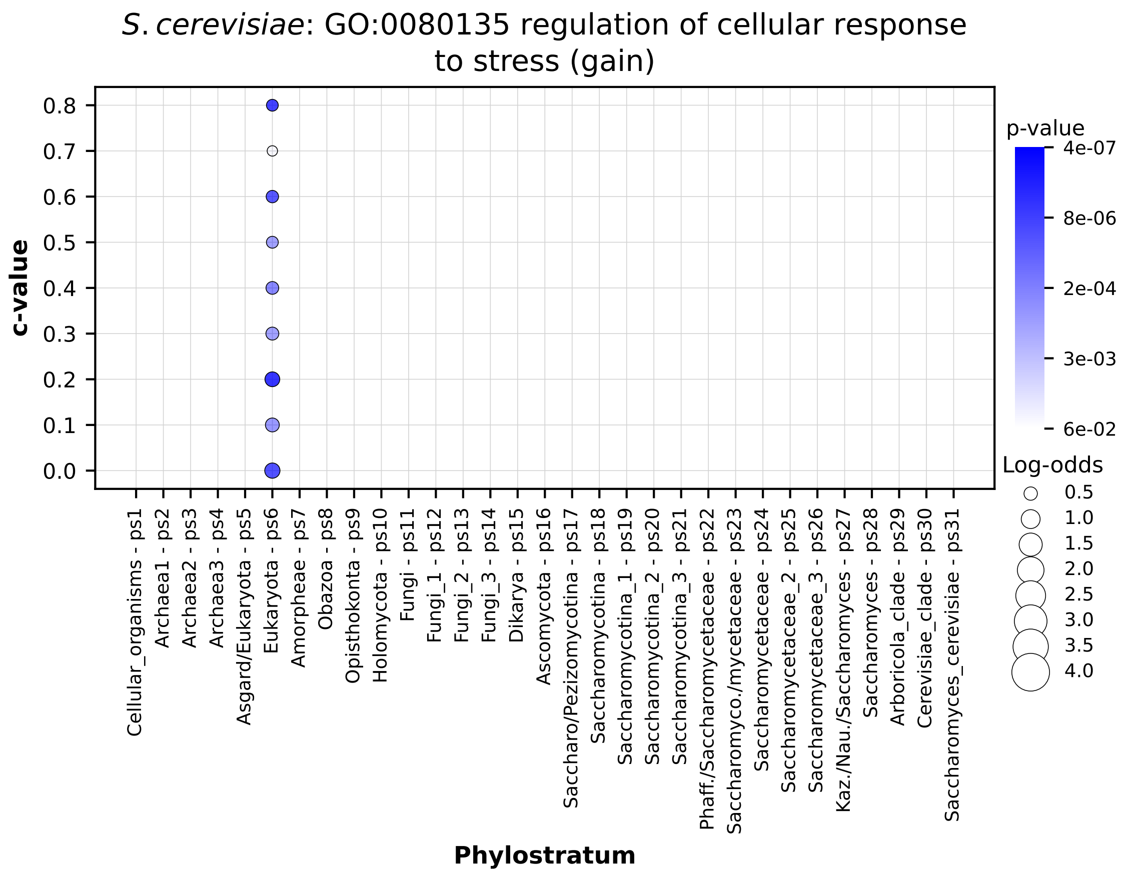

S. cerevisiae: GO:0080135 regulation of cellular response to stress (gain)
c-value
0.8
0.7
0.6
0.5
0.4
0.3
0.2
0.1
0.0
Phylostratum
Cellular_organisms - ps1
Archaea1 - ps2
Archaea2 - ps3
Archaea3 - ps4
Asgard/Eukaryota - ps5
Eukaryota - ps6
Amorpheae - ps7
Obazoa - ps8
Opisthokonta - ps9
Holomycota - ps10
Fungi - ps11
Fungi_1 - ps12
Fungi_2 - ps13
Fungi_3 - ps14
Dikarya - ps15
Ascomycota - ps16
Saccharo/Pezizomycotina - ps17
Saccharomycotina - ps18
Saccharomycotina_1 - ps19
Saccharomycotina_2 - ps20
Saccharomycotina_3 - ps21
Phaff./Saccharomycetaceae - ps22
Saccharomyco./mycetaceae - ps23
Saccharomycetaceae - ps24
Saccharomycetaceae_2 - ps25
Saccharomycetaceae_3 - ps26
Kaz./Nau./Saccharomyces - ps27
Saccharomyces - ps28
Arboricola_clade - ps29
Cerevisiae_clade - ps30
Saccharomyces_cerevisiae - ps31
p-value
4e-07
8e-06
2e-04
3e-03
6e-02
Log-odds
0.5
1.0
1.5
2.0
2.5
3.0
3.5
4.0

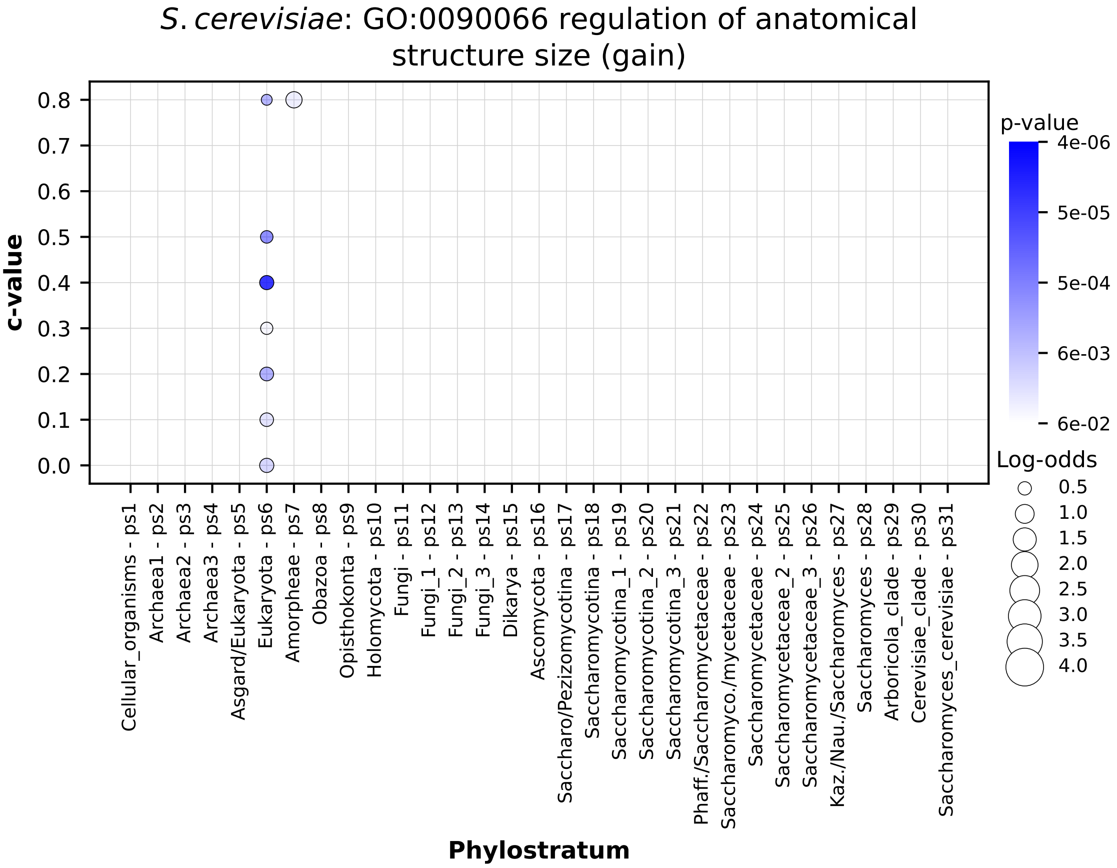

S. cerevisiae: GO:0090066 regulation of anatomical structure size (gain)
c-value
0.8
0.7
0.6
0.5
0.4
0.3
0.2
0.1
0.0
p-value
4e-06
5e-05
5e-04
6e-03
6e-02
Log-odds
0.5
1.0
1.5
2.0
2.5
3.0
3.5
4.0
Cellular_organisms - ps1
Archaea1 - ps2
Archaea2 - ps3
Archaea3 - ps4
Asgard/Eukaryota - ps5
Eukaryota - ps6
Amorpheae - ps7
Obazoa - ps8
Opisthokonta - ps9
Holomycota - ps10
Fungi - ps11
Fungi_1 - ps12
Fungi_2 - ps13
Fungi_3 - ps14
Dikarya - ps15
Ascomycota - ps16
Saccharo/Pezizomycotina - ps17
Saccharomycotina - ps18
Saccharomycotina_1 - ps19
Saccharomycotina_2 - ps20
Saccharomycotina_3 - ps21
Phaff./Saccharomycetaceae - ps22
Saccharomyco./mycetaceae - ps23
Saccharomycetaceae - ps24
Saccharomycetaceae_2 - ps25
Saccharomycetaceae_3 - ps26
Kaz./Nau./Saccharomyces - ps27
Saccharomyces - ps28
Arboricola_clade - ps29
Cerevisiae_clade - ps30
Saccharomyces_cerevisiae - ps31
Phylostratum

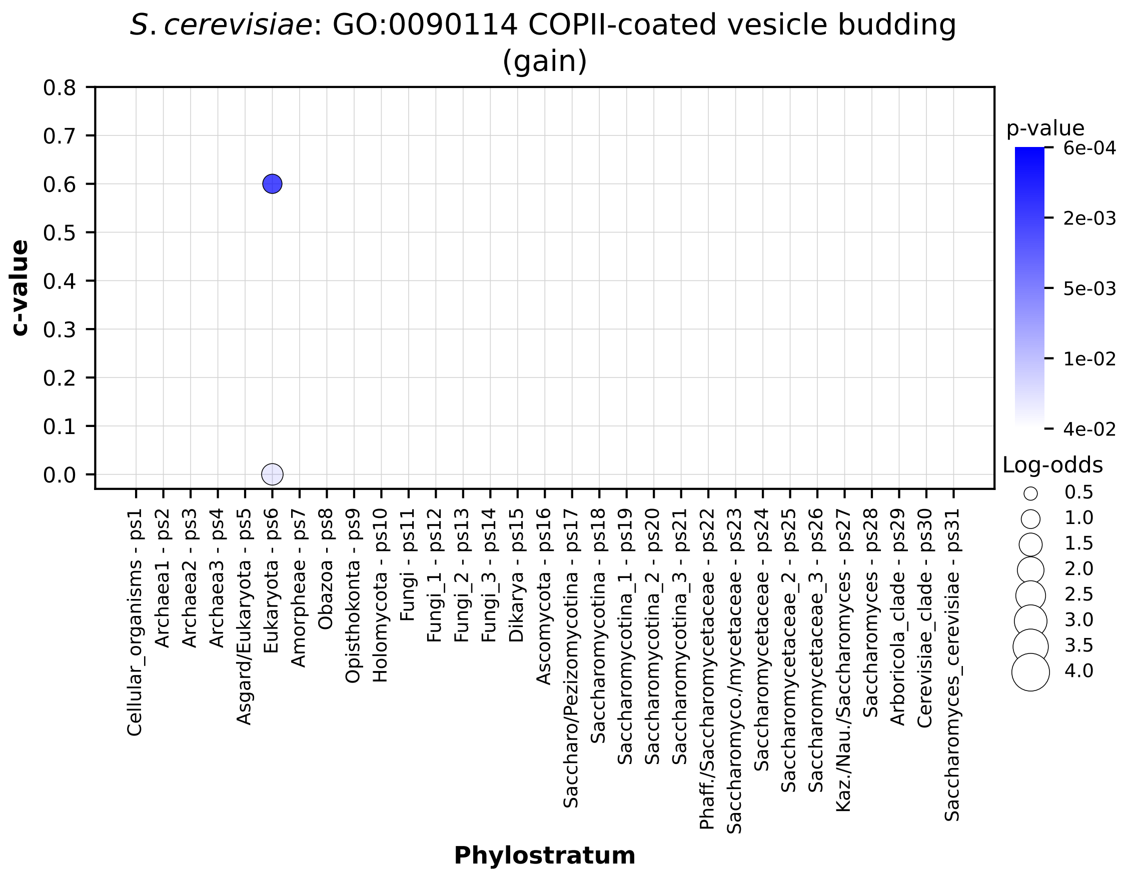

S. cerevisiae: GO:0090114 COPII-coated vesicle budding (gain)
c-value
0.8
0.7
0.6
0.5
0.4
0.3
0.2
0.1
0.0
Phylostratum
Cellular_organisms - ps1
Archaea1 - ps2
Archaea2 - ps3
Archaea3 - ps4
Asgard/Eukaryota - ps5
Eukaryota - ps6
Amorpheae - ps7
Obazoa - ps8
Opisthokonta - ps9
Holomycota - ps10
Fungi - ps11
Fungi_1 - ps12
Fungi_2 - ps13
Fungi_3 - ps14
Dikarya - ps15
Ascomycota - ps16
Saccharo/Pezizomycotina - ps17
Saccharomycotina - ps18
Saccharomycotina_1 - ps19
Saccharomycotina_2 - ps20
Saccharomycotina_3 - ps21
Phaff./Saccharomycetaceae - ps22
Saccharomyco./mycetaceae - ps23
Saccharomycetaceae - ps24
Saccharomycetaceae_2 - ps25
Saccharomycetaceae_3 - ps26
Kaz./Nau./Saccharomyces - ps27
Saccharomyces - ps28
Arboricola_clade - ps29
Cerevisiae_clade - ps30
Saccharomyces_cerevisiae - ps31
p-value
6e-04
2e-03
5e-03
1e-02
4e-02
Log-odds
0.5
1.0
1.5
2.0
2.5
3.0
3.5
4.0

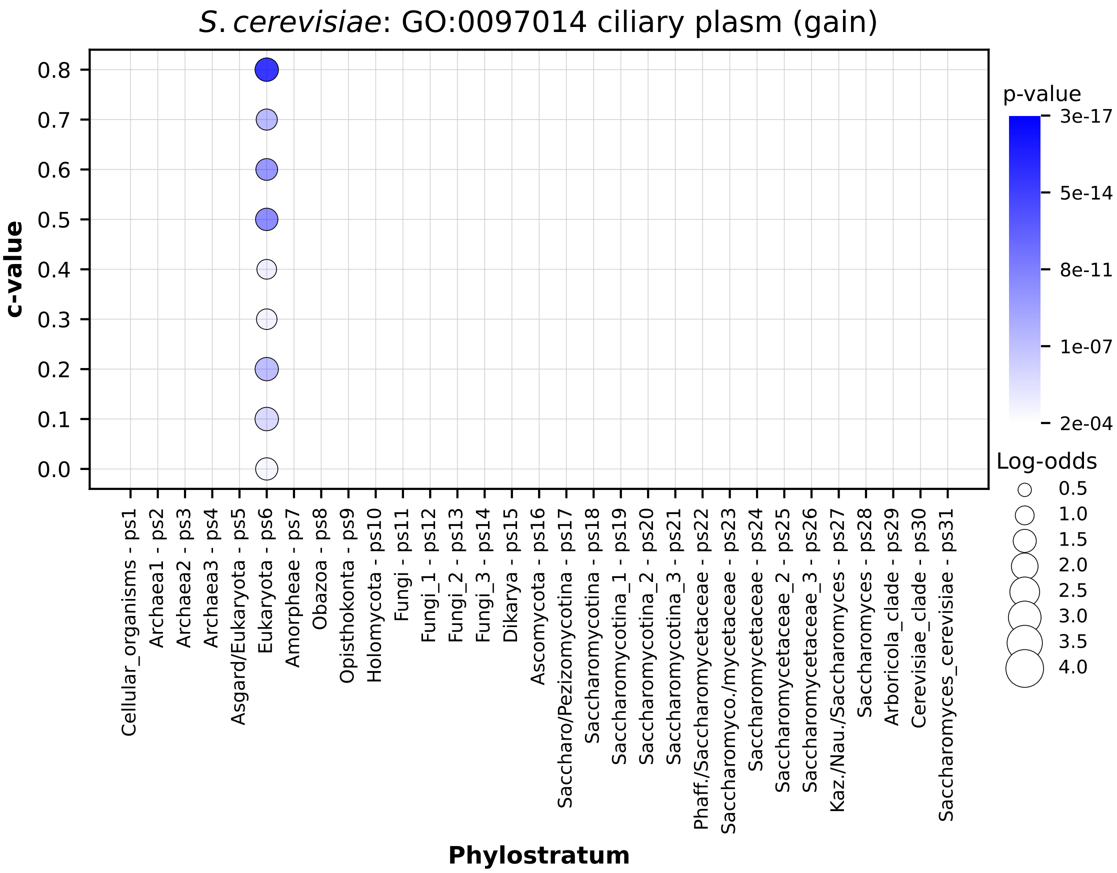
S. cerevisiae: GO:0097014 ciliary plasm (gain)
c-value
0.8
0.7
0.6
0.5
0.4
0.3
0.2
0.1
0.0
p-value
3e-17
5e-14
8e-11
1e-07
2e-04
Log-odds
0.5
1.0
1.5
2.0
2.5
3.0
3.5
4.0
Cellular_organisms - ps1
Archaea1 - ps2
Archaea2 - ps3
Archaea3 - ps4
Asgard/Eukaryota - ps5
Eukaryota - ps6
Amorpheae - ps7
Obazoa - ps8
Opisthokonta - ps9
Holomycota - ps10
Fungi - ps11
Fungi_1 - ps12
Fungi_2 - ps13
Fungi_3 - ps14
Dikarya - ps15
Ascomycota - ps16
Saccharo/Pezizomycotina - ps17
Saccharomycotina - ps18
Saccharomycotina_1 - ps19
Saccharomycotina_2 - ps20
Saccharomycotina_3 - ps21
Phaff./Saccharomycetaceae - ps22
Saccharomyco./mycetaceae - ps23
Saccharomycetaceae - ps24
Saccharomycetaceae_2 - ps25
Saccharomycetaceae_3 - ps26
Kaz./Nau./Saccharomyces - ps27
Saccharomyces - ps28
Arboricola_clade - ps29
Cerevisiae_clade - ps30
Saccharomyces_cerevisiae - ps31
Phylostratum

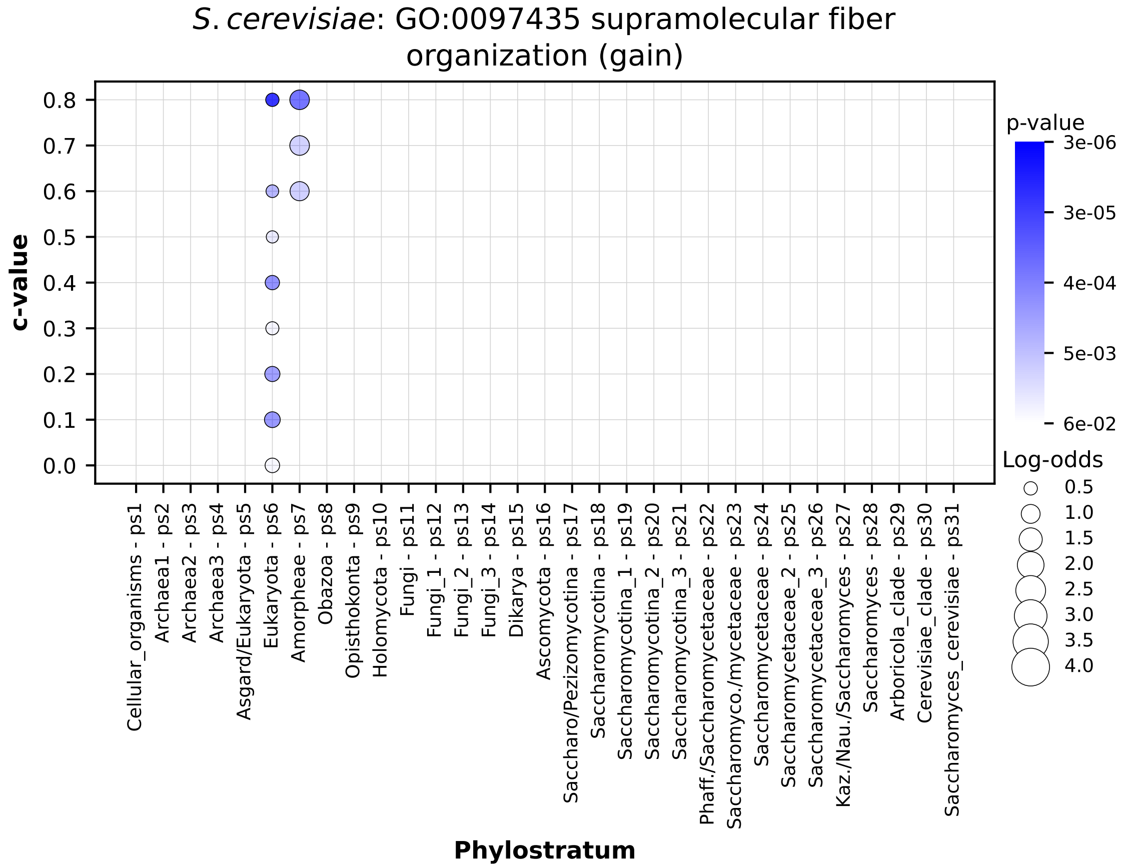

S. cerevisiae: GO:0097435 supramolecular fiber organization (gain)
c-value
0.0
0.1
0.2
0.3
0.4
0.5
0.6
0.7
0.8
p-value
3e-06
3e-05
4e-04
5e-03
6e-02
Log-odds
0.5
1.0
1.5
2.0
2.5
3.0
3.5
4.0
Cellular_organisms - ps1
Archaea1 - ps2
Archaea2 - ps3
Archaea3 - ps4
Asgard/Eukaryota - ps5
Eukaryota - ps6
Amorpheae - ps7
Obazoa - ps8
Opisthokonta - ps9
Holomycota - ps10
Fungi - ps11
Fungi_1 - ps12
Fungi_2 - ps13
Fungi_3 - ps14
Dikarya - ps15
Ascomycota - ps16
Saccharo/Pezizomycotina - ps17
Saccharomycotina - ps18
Saccharomycotina_1 - ps19
Saccharomycotina_2 - ps20
Saccharomycotina_3 - ps21
Phaff./Saccharomycetaceae - ps22
Saccharomyco./mycetaceae - ps23
Saccharomycetaceae - ps24
Saccharomycetaceae_2 - ps25
Saccharomycetaceae_3 - ps26
Kaz./Nau./Saccharomyces - ps27
Saccharomyces - ps28
Arboricola_clade - ps29
Cerevisiae_clade - ps30
Saccharomyces_cerevisiae - ps31
Phylostratum

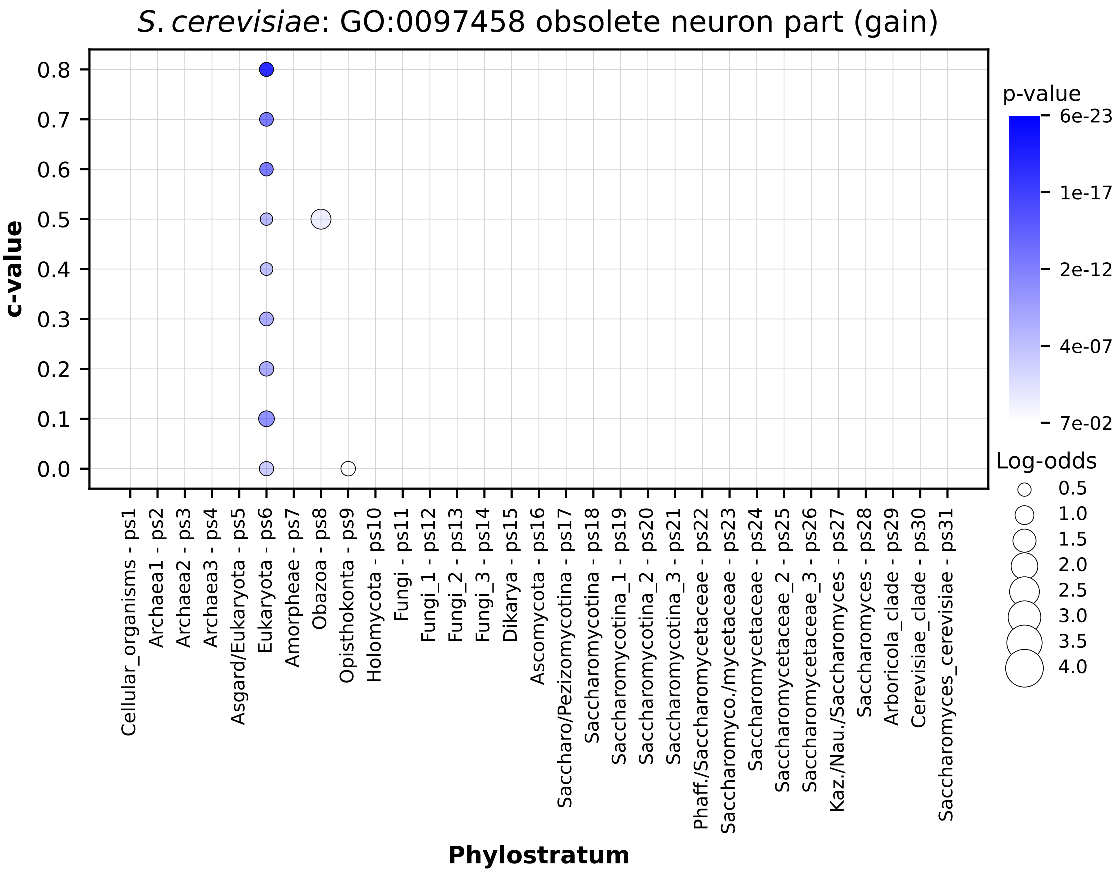

S. cerevisiae: GO:0097458 obsolete neuron part (gain)
c-value
0.8
0.7
0.6
0.5
0.4
0.3
0.2
0.1
0.0
Phylostratum
Cellular_organisms - ps1
Archaea1 - ps2
Archaea2 - ps3
Archaea3 - ps4
Asgard/Eukaryota - ps5
Eukaryota - ps6
Amorpheae - ps7
Obazoa - ps8
Opisthokonta - ps9
Holomycota - ps10
Fungi - ps11
Fungi_1 - ps12
Fungi_2 - ps13
Fungi_3 - ps14
Dikarya - ps15
Ascomycota - ps16
Saccharo/Pezizomycotina - ps17
Saccharomycotina - ps18
Saccharomycotina_1 - ps19
Saccharomycotina_2 - ps20
Saccharomycotina_3 - ps21
Phaff./Saccharomycetaceae - ps22
Saccharomyco./mycetaceae - ps23
Saccharomycetaceae - ps24
Saccharomycetaceae_2 - ps25
Saccharomycetaceae_3 - ps26
Kaz./Nau./Saccharomyces - ps27
Saccharomyces - ps28
Arboricola_clade - ps29
Cerevisiae_clade - ps30
Saccharomyces_cerevisiae - ps31
p-value
6e-23
1e-17
2e-12
4e-07
7e-02
Log-odds
0.5
1.0
1.5
2.0
2.5
3.0
3.5
4.0

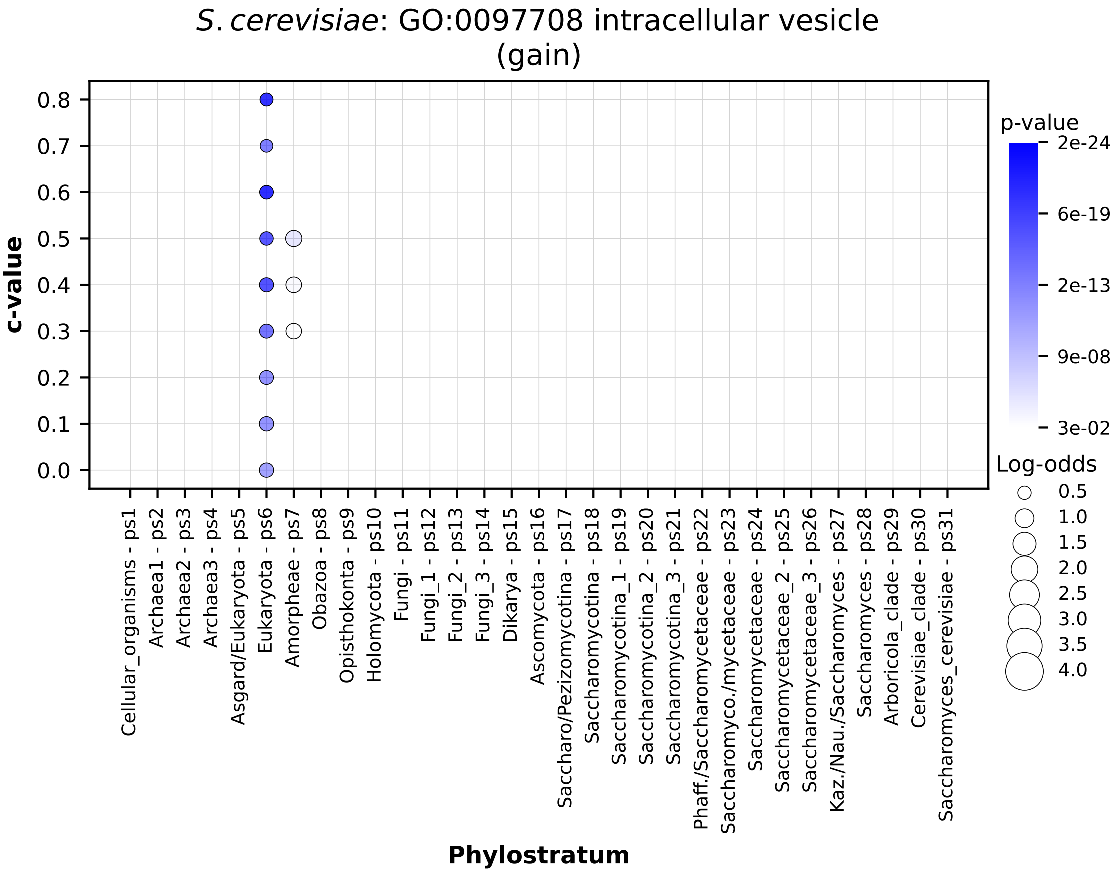

S. cerevisiae: GO:0097708 intracellular vesicle
(gain)
c-value
0.8
0.7
0.6
0.5
0.4
0.3
0.2
0.1
0.0
p-value
2e-24
6e-19
2e-13
9e-08
3e-02
Log-odds
0.5
1.0
1.5
2.0
2.5
3.0
3.5
4.0
Cellular_organisms - ps1
Archaea1 - ps2
Archaea2 - ps3
Archaea3 - ps4
Asgard/Eukaryota - ps5
Eukaryota - ps6
Amorpheae - ps7
Obazoa - ps8
Opisthokonta - ps9
Holomycota - ps10
Fungi - ps11
Fungi_1 - ps12
Fungi_2 - ps13
Fungi_3 - ps14
Dikarya - ps15
Ascomycota - ps16
Saccharo/Pezizomycotina - ps17
Saccharomycotina - ps18
Saccharomycotina_1 - ps19
Saccharomycotina_2 - ps20
Saccharomycotina_3 - ps21
Phaff./Saccharomycetaceae - ps22
Saccharomyco./mycetaceae - ps23
Saccharomycetaceae - ps24
Saccharomycetaceae_2 - ps25
Saccharomycetaceae_3 - ps26
Kaz./Nau./Saccharomyces - ps27
Saccharomyces - ps28
Arboricola_clade - ps29
Cerevisiae_clade - ps30
Saccharomyces_cerevisiae - ps31
Phylostratum

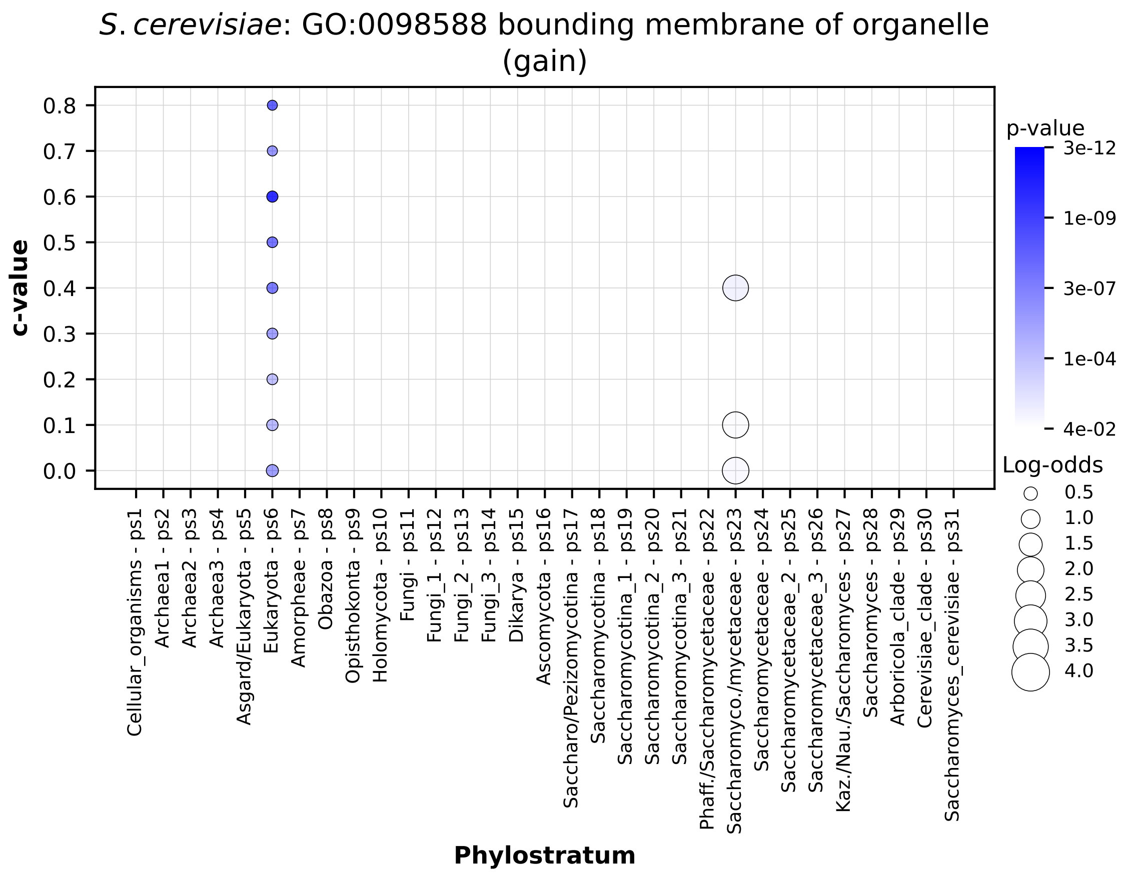

S. cerevisiae: GO:0098588 bounding membrane of organelle (gain)
c-value
0.0
0.1
0.2
0.3
0.4
0.5
0.6
0.7
0.8
p-value
3e-12
1e-09
3e-07
1e-04
4e-02
Log-odds
0.5
1.0
1.5
2.0
2.5
3.0
3.5
4.0
Cellular_organisms - ps1
Archaea1 - ps2
Archaea2 - ps3
Archaea3 - ps4
Asgard/Eukaryota - ps5
Eukaryota - ps6
Amorpheae - ps7
Obazoa - ps8
Opisthokonta - ps9
Holomycota - ps10
Fungi - ps11
Fungi_1 - ps12
Fungi_2 - ps13
Fungi_3 - ps14
Dikarya - ps15
Ascomycota - ps16
Saccharo/Pezizomycotina - ps17
Saccharomycotina - ps18
Saccharomycotina_1 - ps19
Saccharomycotina_2 - ps20
Saccharomycotina_3 - ps21
Phaff./Saccharomycetaceae - ps22
Saccharomyco./mycetaceae - ps23
Saccharomycetaceae - ps24
Saccharomycetaceae_2 - ps25
Saccharomycetaceae_3 - ps26
Kaz./Nau./Saccharomyces - ps27
Saccharomyces - ps28
Arboricola_clade - ps29
Cerevisiae_clade - ps30
Saccharomyces_cerevisiae - ps31
Phylostratum

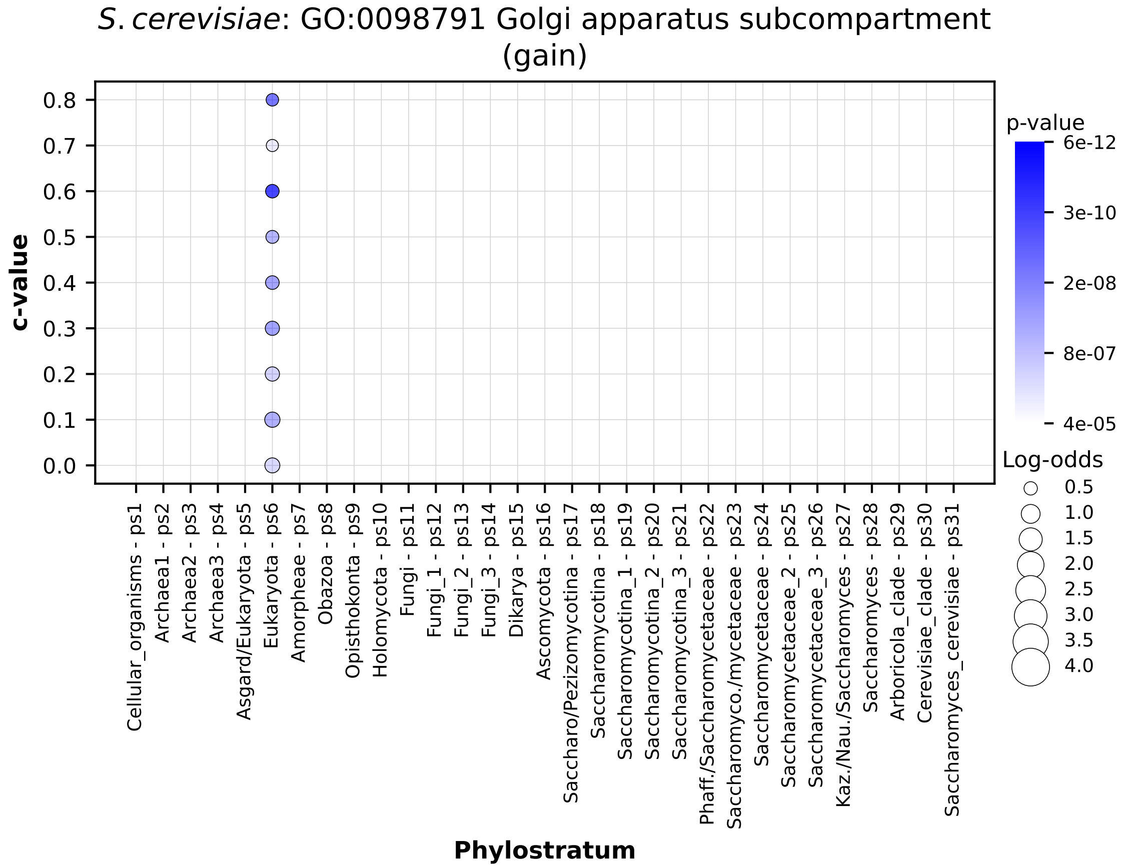

S. cerevisiae: GO:0098791 Golgi apparatus subcompartment (gain)
c-value
0.8
0.7
0.6
0.5
0.4
0.3
0.2
0.1
0.0
p-value
6e-12
3e-10
2e-08
8e-07
4e-05
Log-odds
0.5
1.0
1.5
2.0
2.5
3.0
3.5
4.0
Cellular_organisms - ps1
Archaea1 - ps2
Archaea2 - ps3
Archaea3 - ps4
Asgard/Eukaryota - ps5
Eukaryota - ps6
Amorpheae - ps7
Obazoa - ps8
Opisthokonta - ps9
Holomycota - ps10
Fungi - ps11
Fungi_1 - ps12
Fungi_2 - ps13
Fungi_3 - ps14
Dikarya - ps15
Ascomycota - ps16
Saccharo/Pezizomycotina - ps17
Saccharomycotina - ps18
Saccharomycotina_1 - ps19
Saccharomycotina_2 - ps20
Saccharomycotina_3 - ps21
Phaff./Saccharomycetaceae - ps22
Saccharomyco./mycetaceae - ps23
Saccharomycetaceae - ps24
Saccharomycetaceae_2 - ps25
Saccharomycetaceae_3 - ps26
Kaz./Nau./Saccharomyces - ps27
Saccharomyces - ps28
Arboricola_clade - ps29
Cerevisiae_clade - ps30
Saccharomyces_cerevisiae - ps31
Phylostratum

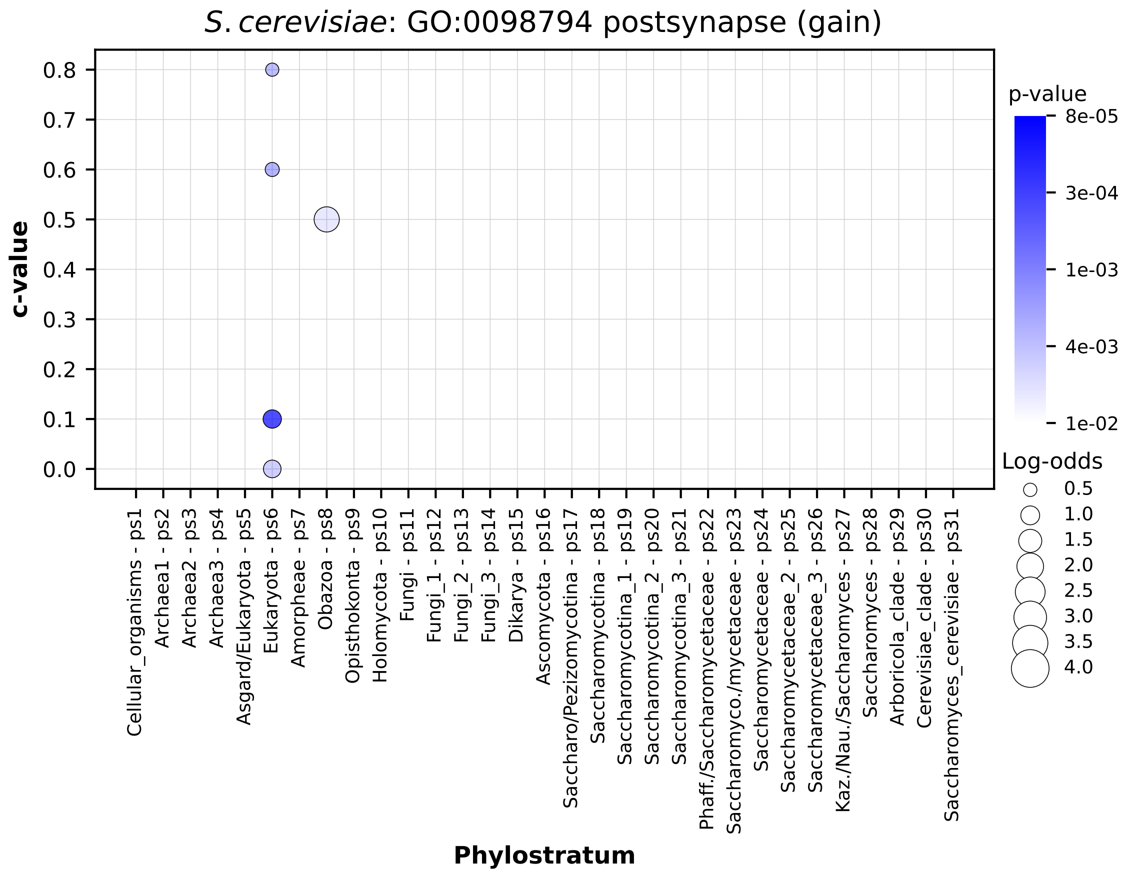

S. cerevisiae: GO:0098794 postsynapse (gain)
c-value
0.8
0.7
0.6
0.5
0.4
0.3
0.2
0.1
0.0
p-value
8e-05
3e-04
1e-03
4e-03
1e-02
Log-odds
0.5
1.0
1.5
2.0
2.5
3.0
3.5
4.0
Cellular_organisms - ps1
Archaea1 - ps2
Archaea2 - ps3
Archaea3 - ps4
Asgard/Eukaryota - ps5
Eukaryota - ps6
Amorpheae - ps7
Obazoa - ps8
Opisthokonta - ps9
Holomycota - ps10
Fungi - ps11
Fungi_1 - ps12
Fungi_2 - ps13
Fungi_3 - ps14
Dikarya - ps15
Ascomycota - ps16
Saccharo/Pezizomycotina - ps17
Saccharomycotina - ps18
Saccharomycotina_1 - ps19
Saccharomycotina_2 - ps20
Saccharomycotina_3 - ps21
Phaff./Saccharomycetaceae - ps22
Saccharomyco./mycetaceae - ps23
Saccharomycetaceae - ps24
Saccharomycetaceae_2 - ps25
Saccharomycetaceae_3 - ps26
Kaz./Nau./Saccharomyces - ps27
Saccharomyces - ps28
Arboricola_clade - ps29
Cerevisiae_clade - ps30
Saccharomyces_cerevisiae - ps31
Phylostratum

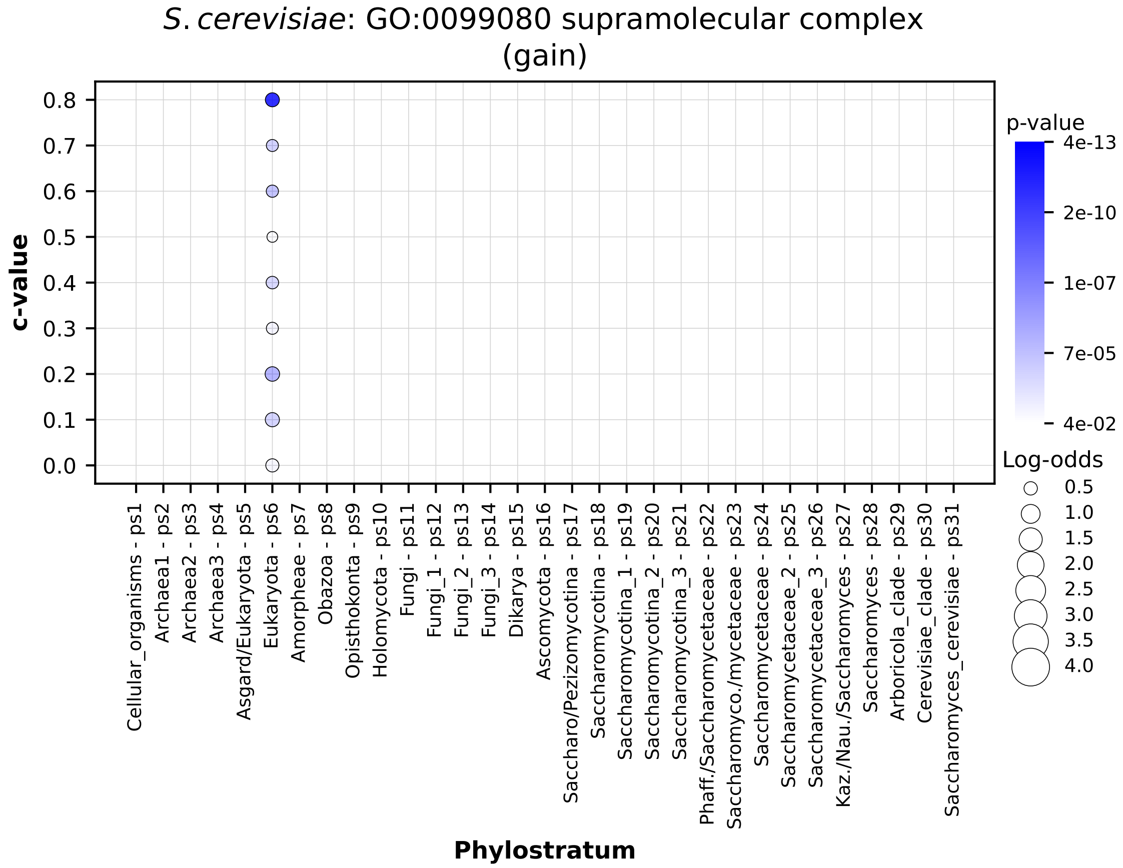

S. cerevisiae: GO:0099080 supramolecular complex
(gain)
c-value
0.8
0.7
0.6
0.5
0.4
0.3
0.2
0.1
0.0
p-value
4e-13
2e-10
1e-07
7e-05
4e-02
Log-odds
0.5
1.0
1.5
2.0
2.5
3.0
3.5
4.0
Cellular_organisms - ps1
Archaea1 - ps2
Archaea2 - ps3
Archaea3 - ps4
Asgard/Eukaryota - ps5
Eukaryota - ps6
Amorpheae - ps7
Obazoa - ps8
Opisthokonta - ps9
Holomycota - ps10
Fungi - ps11
Fungi_1 - ps12
Fungi_2 - ps13
Fungi_3 - ps14
Dikarya - ps15
Ascomycota - ps16
Saccharo/Pezizomycotina - ps17
Saccharomycotina - ps18
Saccharomycotina_1 - ps19
Saccharomycotina_2 - ps20
Saccharomycotina_3 - ps21
Phaff./Saccharomycetaceae - ps22
Saccharomyco./mycetaceae - ps23
Saccharomycetaceae - ps24
Saccharomycetaceae_2 - ps25
Saccharomycetaceae_3 - ps26
Kaz./Nau./Saccharomyces - ps27
Saccharomyces - ps28
Arboricola_clade - ps29
Cerevisiae_clade - ps30
Saccharomyces_cerevisiae - ps31
Phylostratum

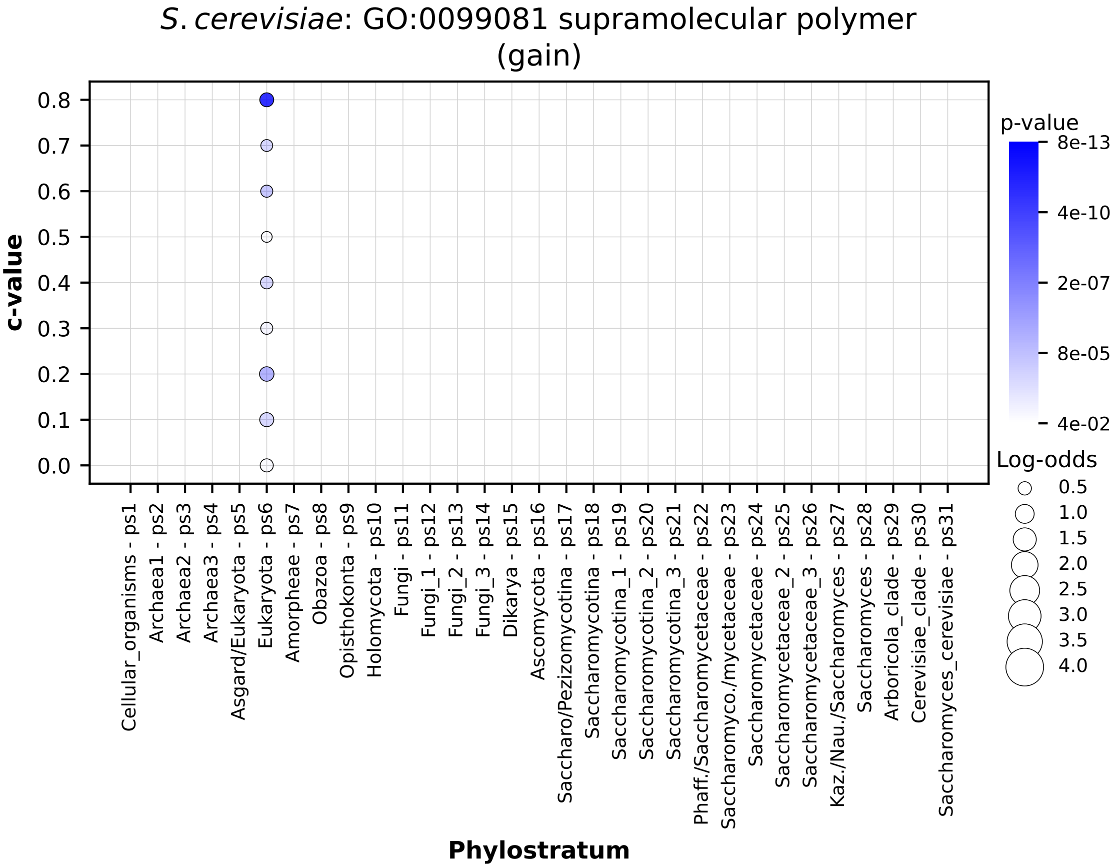

S. cerevisiae: GO:0099081 supramolecular polymer
(gain)
c-value
0.0
0.1
0.2
0.3
0.4
0.5
0.6
0.7
0.8
Phylostratum
Cellular_organisms - ps1
Archaea1 - ps2
Archaea2 - ps3
Archaea3 - ps4
Asgard/Eukaryota - ps5
Eukaryota - ps6
Amorpheae - ps7
Obazoa - ps8
Opisthokonta - ps9
Holomycota - ps10
Fungi - ps11
Fungi_1 - ps12
Fungi_2 - ps13
Fungi_3 - ps14
Dikarya - ps15
Ascomycota - ps16
Saccharo/Pezizomycotina - ps17
Saccharomycotina - ps18
Saccharomycotina_1 - ps19
Saccharomycotina_2 - ps20
Saccharomycotina_3 - ps21
Phaff./Saccharomycetaceae - ps22
Saccharomyco./mycetaceae - ps23
Saccharomycetaceae - ps24
Saccharomycetaceae_2 - ps25
Saccharomycetaceae_3 - ps26
Kaz./Nau./Saccharomyces - ps27
Saccharomyces - ps28
Arboricola_clade - ps29
Cerevisiae_clade - ps30
Saccharomyces_cerevisiae - ps31
p-value
8e-13
4e-10
2e-07
8e-05
4e-02
Log-odds
0.5
1.0
1.5
2.0
2.5
3.0
3.5
4.0

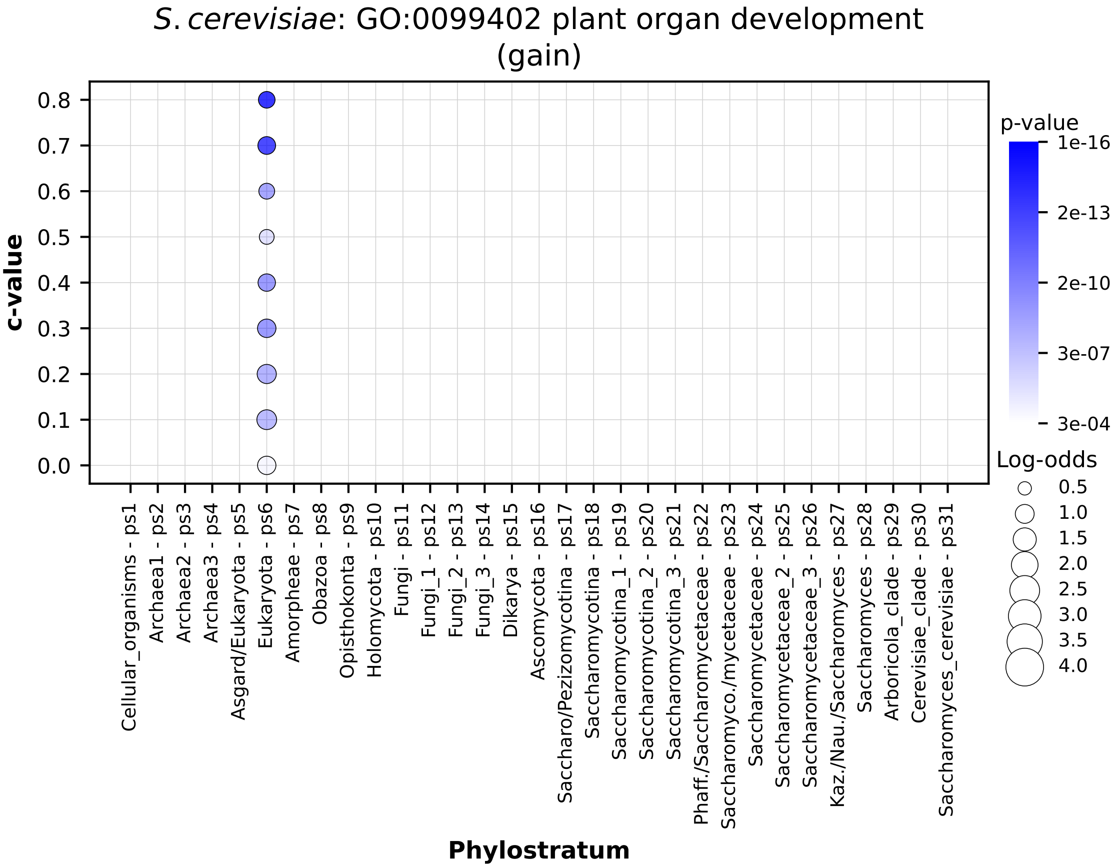

S. cerevisiae: GO:0099402 plant organ development
(gain)
c-value
0.8
0.7
0.6
0.5
0.4
0.3
0.2
0.1
0.0
p-value
1e-16
2e-13
2e-10
3e-07
3e-04
Log-odds
0.5
1.0
1.5
2.0
2.5
3.0
3.5
4.0
Cellular_organisms - ps1
Archaea1 - ps2
Archaea2 - ps3
Archaea3 - ps4
Asgard/Eukaryota - ps5
Eukaryota - ps6
Amorpheae - ps7
Obazoa - ps8
Opisthokonta - ps9
Holomycota - ps10
Fungi - ps11
Fungi_1 - ps12
Fungi_2 - ps13
Fungi_3 - ps14
Dikarya - ps15
Ascomycota - ps16
Saccharo/Pezizomycotina - ps17
Saccharomycotina - ps18
Saccharomycotina_1 - ps19
Saccharomycotina_2 - ps20
Saccharomycotina_3 - ps21
Phaff./Saccharomycetaceae - ps22
Saccharomyco./mycetaceae - ps23
Saccharomycetaceae - ps24
Saccharomycetaceae_2 - ps25
Saccharomycetaceae_3 - ps26
Kaz./Nau./Saccharomyces - ps27
Saccharomyces - ps28
Arboricola_clade - ps29
Cerevisiae_clade - ps30
Saccharomyces_cerevisiae - ps31
Phylostratum

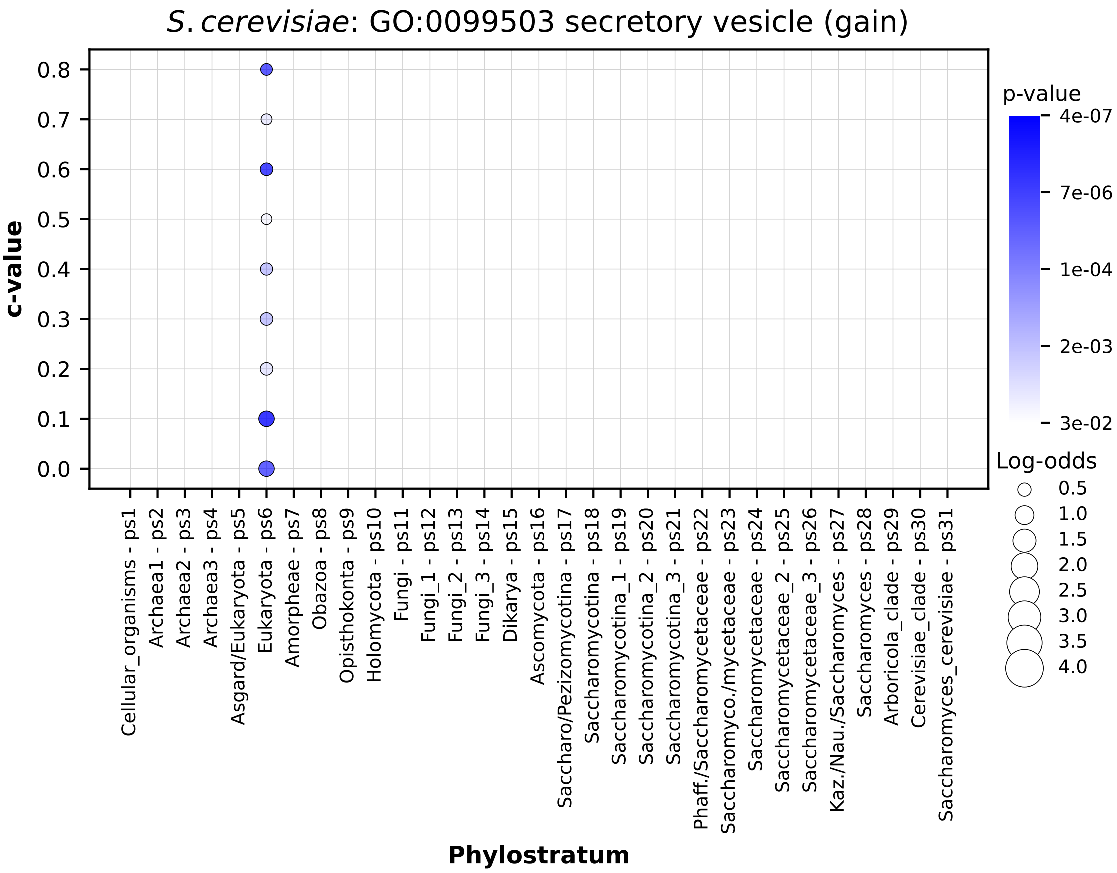

S. cerevisiae: GO:0099503 secretory vesicle (gain)
c-value
0.8
0.7
0.6
0.5
0.4
0.3
0.2
0.1
0.0
p-value
4e-07
7e-06
1e-04
2e-03
3e-02
Log-odds
0.5
1.0
1.5
2.0
2.5
3.0
3.5
4.0
Cellular_organisms - ps1
Archaea1 - ps2
Archaea2 - ps3
Archaea3 - ps4
Asgard/Eukaryota - ps5
Eukaryota - ps6
Amorpheae - ps7
Obazoa - ps8
Opisthokonta - ps9
Holomycota - ps10
Fungi - ps11
Fungi_1 - ps12
Fungi_2 - ps13
Fungi_3 - ps14
Dikarya - ps15
Ascomycota - ps16
Saccharo/Pezizomycotina - ps17
Saccharomycotina - ps18
Saccharomycotina_1 - ps19
Saccharomycotina_2 - ps20
Saccharomycotina_3 - ps21
Phaff./Saccharomycetaceae - ps22
Saccharomyco./mycetaceae - ps23
Saccharomycetaceae - ps24
Saccharomycetaceae_2 - ps25
Saccharomycetaceae_3 - ps26
Kaz./Nau./Saccharomyces - ps27
Saccharomyces - ps28
Arboricola_clade - ps29
Cerevisiae_clade - ps30
Saccharomyces_cerevisiae - ps31
Phylostratum

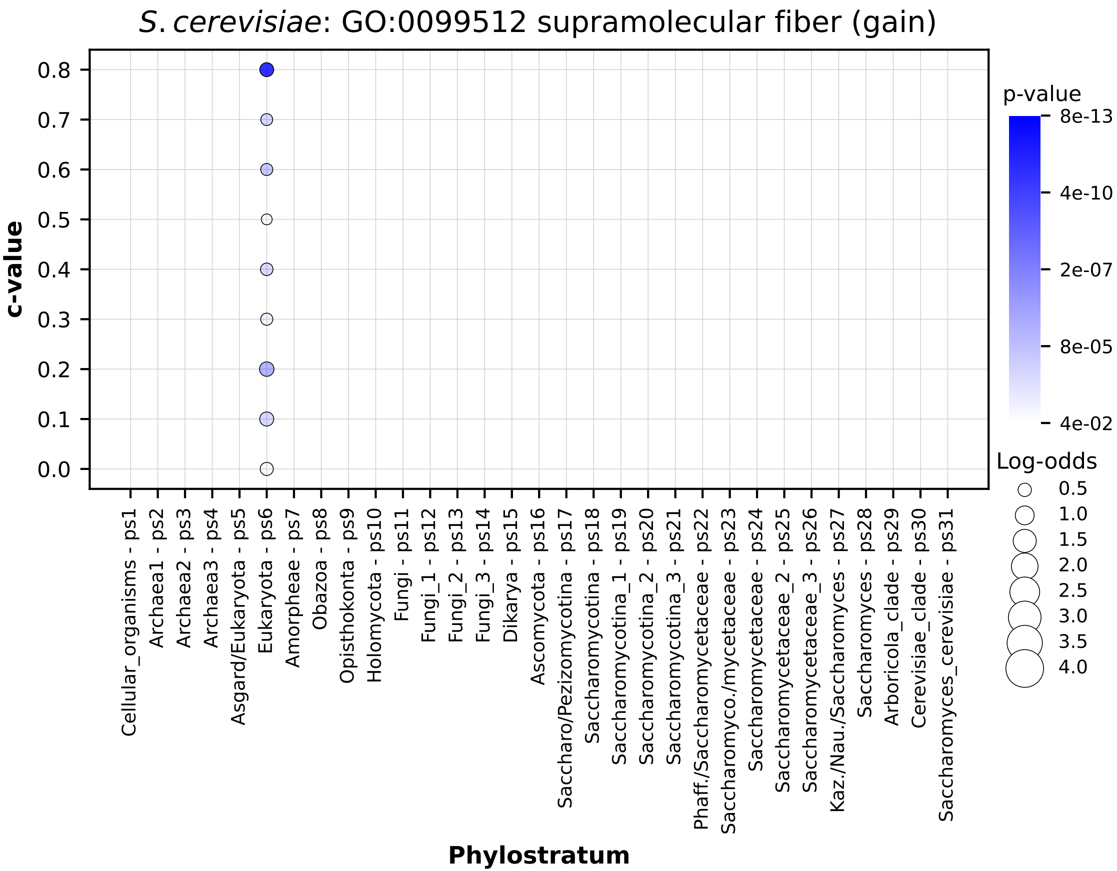

S. cerevisiae: GO:0099512 supramolecular fiber (gain)
c-value
0.8
0.7
0.6
0.5
0.4
0.3
0.2
0.1
0.0
p-value
8e-13
4e-10
2e-07
8e-05
4e-02
Log-odds
0.5
1.0
1.5
2.0
2.5
3.0
3.5
4.0
Cellular_organisms - ps1
Archaea1 - ps2
Archaea2 - ps3
Archaea3 - ps4
Asgard/Eukaryota - ps5
Eukaryota - ps6
Amorpheae - ps7
Obazoa - ps8
Opisthokonta - ps9
Holomycota - ps10
Fungi - ps11
Fungi_1 - ps12
Fungi_2 - ps13
Fungi_3 - ps14
Dikarya - ps15
Ascomycota - ps16
Saccharo/Pezizomycotina - ps17
Saccharomycotina - ps18
Saccharomycotina_1 - ps19
Saccharomycotina_2 - ps20
Saccharomycotina_3 - ps21
Phaff./Saccharomycetaceae - ps22
Saccharomyco./mycetaceae - ps23
Saccharomycetaceae - ps24
Saccharomycetaceae_2 - ps25
Saccharomycetaceae_3 - ps26
Kaz./Nau./Saccharomyces - ps27
Saccharomyces - ps28
Arboricola_clade - ps29
Cerevisiae_clade - ps30
Saccharomyces_cerevisiae - ps31
Phylostratum

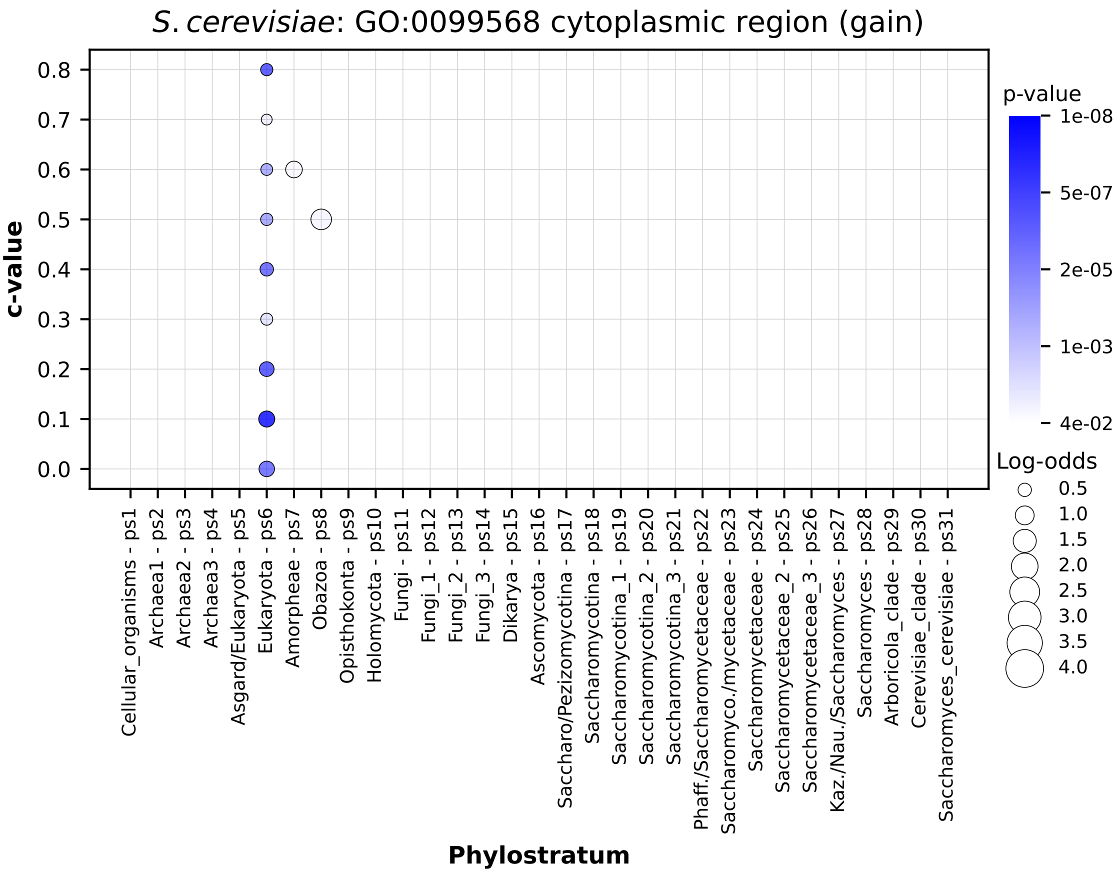

S. cerevisiae: GO:0099568 cytoplasmic region (gain)
c-value
0.0
0.1
0.2
0.3
0.4
0.5
0.6
0.7
0.8
p-value
1e-08
5e-07
2e-05
1e-03
4e-02
Log-odds
0.5
1.0
1.5
2.0
2.5
3.0
3.5
4.0
Cellular_organisms - ps1
Archaea1 - ps2
Archaea2 - ps3
Archaea3 - ps4
Asgard/Eukaryota - ps5
Eukaryota - ps6
Amorpheae - ps7
Obazoa - ps8
Opisthokonta - ps9
Holomycota - ps10
Fungi - ps11
Fungi_1 - ps12
Fungi_2 - ps13
Fungi_3 - ps14
Dikarya - ps15
Ascomycota - ps16
Saccharo/Pezizomycotina - ps17
Saccharomycotina - ps18
Saccharomycotina_1 - ps19
Saccharomycotina_2 - ps20
Saccharomycotina_3 - ps21
Phaff./Saccharomycetaceae - ps22
Saccharomyco./mycetaceae - ps23
Saccharomycetaceae - ps24
Saccharomycetaceae_2 - ps25
Saccharomycetaceae_3 - ps26
Kaz./Nau./Saccharomyces - ps27
Saccharomyces - ps28
Arboricola_clade - ps29
Cerevisiae_clade - ps30
Saccharomyces_cerevisiae - ps31
Phylostratum

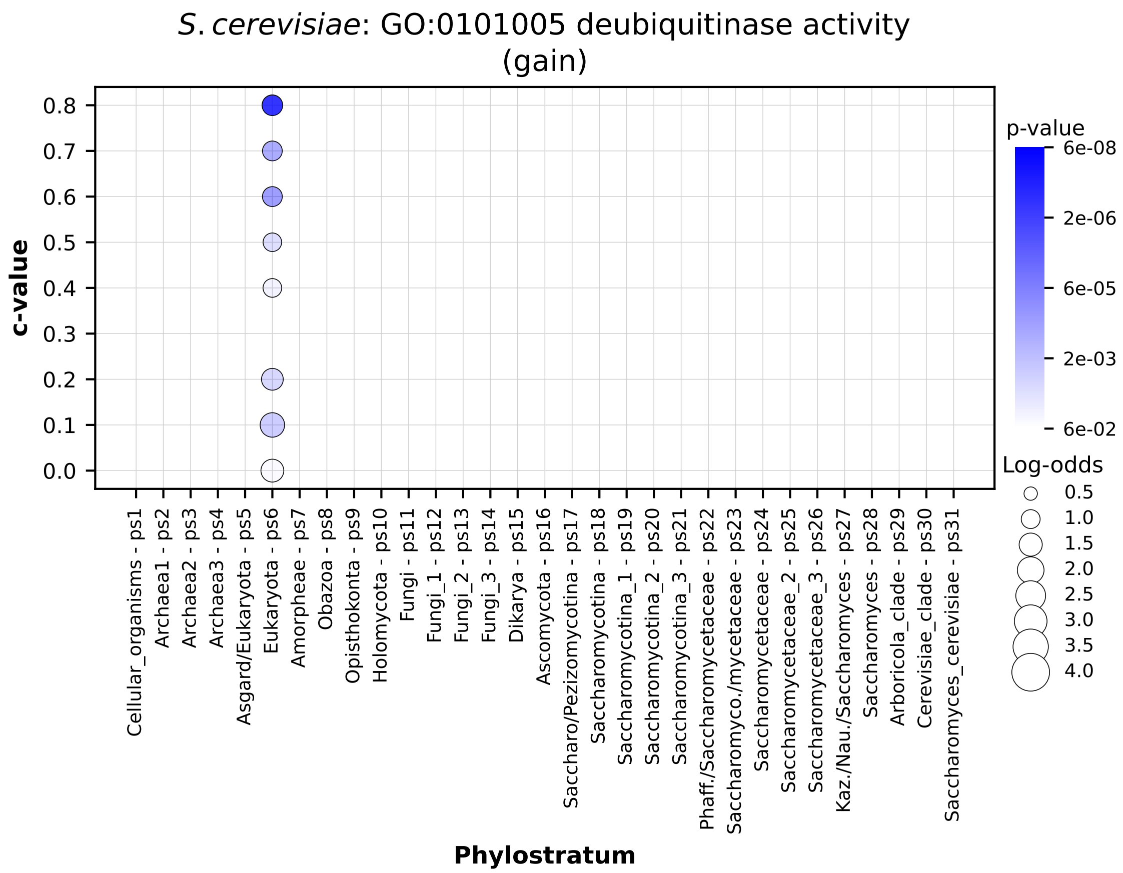

S. cerevisiae: GO:0101005 deubiquitinase activity
(gain)
c-value
0.8
0.7
0.6
0.5
0.4
0.3
0.2
0.1
0.0
p-value
6e-08
2e-06
6e-05
2e-03
6e-02
Log-odds
0.5
1.0
1.5
2.0
2.5
3.0
3.5
4.0
Cellular_organisms - ps1
Archaea1 - ps2
Archaea2 - ps3
Archaea3 - ps4
Asgard/Eukaryota - ps5
Eukaryota - ps6
Amorpheae - ps7
Obazoa - ps8
Opisthokonta - ps9
Holomycota - ps10
Fungi - ps11
Fungi_1 - ps12
Fungi_2 - ps13
Fungi_3 - ps14
Dikarya - ps15
Ascomycota - ps16
Saccharo/Pezizomycotina - ps17
Saccharomycotina - ps18
Saccharomycotina_1 - ps19
Saccharomycotina_2 - ps20
Saccharomycotina_3 - ps21
Phaff./Saccharomycetaceae - ps22
Saccharomyco./mycetaceae - ps23
Saccharomycetaceae - ps24
Saccharomycetaceae_2 - ps25
Saccharomycetaceae_3 - ps26
Kaz./Nau./Saccharomyces - ps27
Saccharomyces - ps28
Arboricola_clade - ps29
Cerevisiae_clade - ps30
Saccharomyces_cerevisiae - ps31
Phylostratum

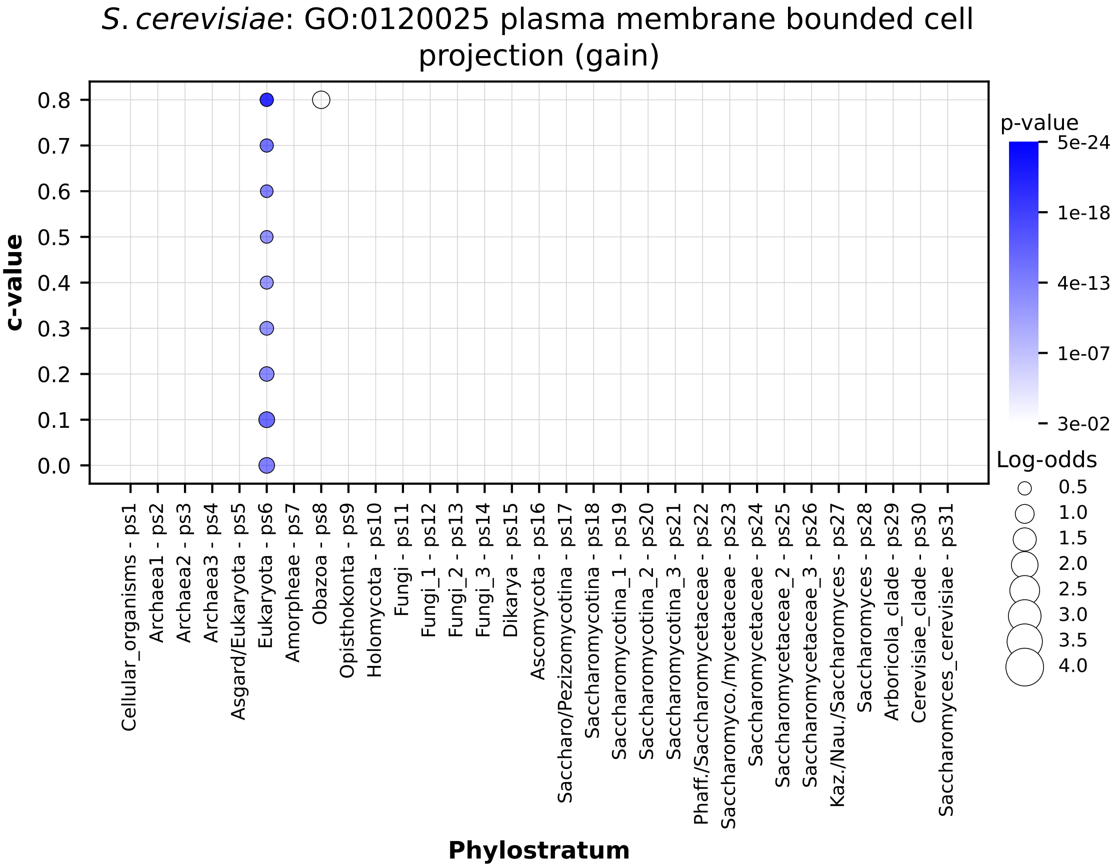

S. cerevisiae: GO:0120025 plasma membrane bounded cell projection (gain)
c-value
0.8
0.7
0.6
0.5
0.4
0.3
0.2
0.1
0.0
p-value
5e-24
1e-18
4e-13
1e-07
3e-02
Log-odds
0.5
1.0
1.5
2.0
2.5
3.0
3.5
4.0
Cellular_organisms - ps1
Archaea1 - ps2
Archaea2 - ps3
Archaea3 - ps4
Asgard/Eukaryota - ps5
Eukaryota - ps6
Amorpheae - ps7
Obazoa - ps8
Opisthokonta - ps9
Holomycota - ps10
Fungi - ps11
Fungi_1 - ps12
Fungi_2 - ps13
Fungi_3 - ps14
Dikarya - ps15
Ascomycota - ps16
Saccharo/Pezizomycotina - ps17
Saccharomycotina - ps18
Saccharomycotina_1 - ps19
Saccharomycotina_2 - ps20
Saccharomycotina_3 - ps21
Phaff./Saccharomycetaceae - ps22
Saccharomyco./mycetaceae - ps23
Saccharomycetaceae - ps24
Saccharomycetaceae_2 - ps25
Saccharomycetaceae_3 - ps26
Kaz./Nau./Saccharomyces - ps27
Saccharomyces - ps28
Arboricola_clade - ps29
Cerevisiae_clade - ps30
Saccharomyces_cerevisiae - ps31
Phylostratum

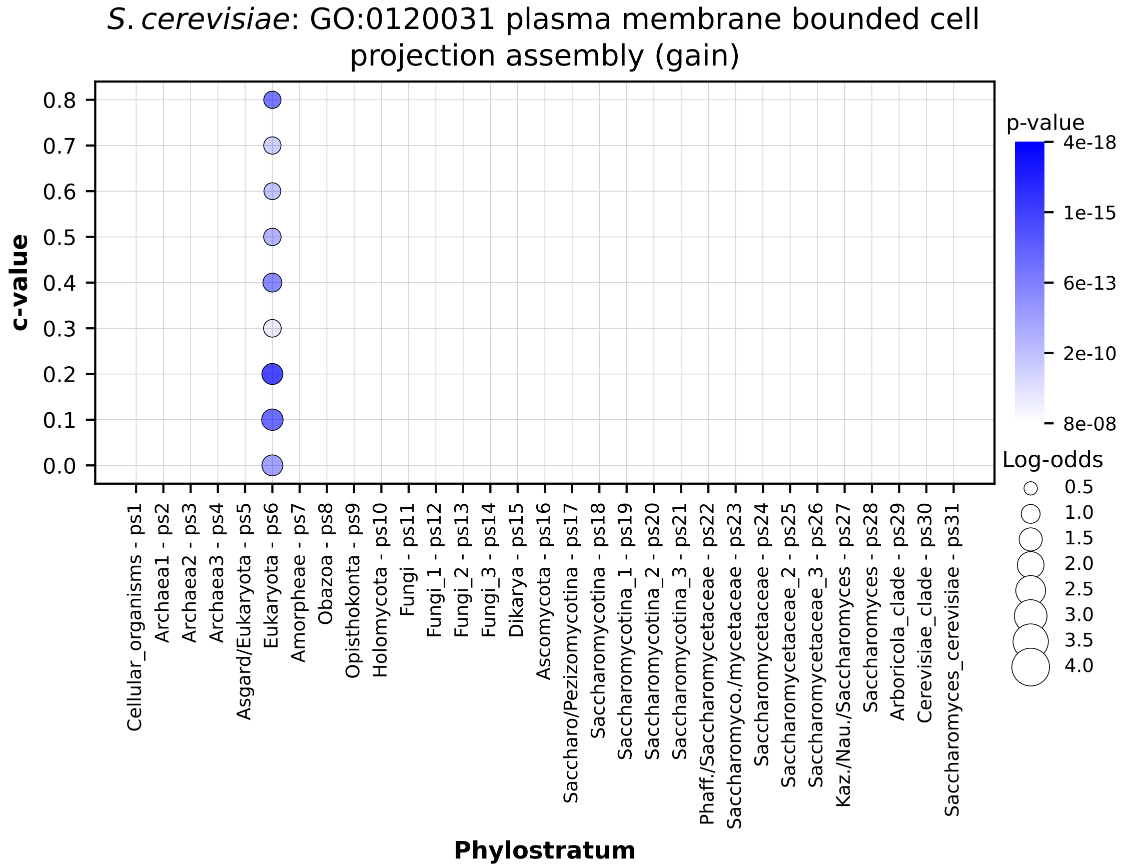

S. cerevisiae: GO:0120031 plasma membrane bounded cell projection assembly (gain)
c-value
0.0
0.1
0.2
0.3
0.4
0.5
0.6
0.7
0.8
p-value
4e-18
1e-15
6e-13
2e-10
8e-08
Log-odds
0.5
1.0
1.5
2.0
2.5
3.0
3.5
4.0
Cellular_organisms - ps1
Archaea1 - ps2
Archaea2 - ps3
Archaea3 - ps4
Asgard/Eukaryota - ps5
Eukaryota - ps6
Amorpheae - ps7
Obazoa - ps8
Opisthokonta - ps9
Holomycota - ps10
Fungi - ps11
Fungi_1 - ps12
Fungi_2 - ps13
Fungi_3 - ps14
Dikarya - ps15
Ascomycota - ps16
Saccharo/Pezizomycotina - ps17
Saccharomycotina - ps18
Saccharomycotina_1 - ps19
Saccharomycotina_2 - ps20
Saccharomycotina_3 - ps21
Phaff./Saccharomycetaceae - ps22
Saccharomyco./mycetaceae - ps23
Saccharomycetaceae - ps24
Saccharomycetaceae_2 - ps25
Saccharomycetaceae_3 - ps26
Kaz./Nau./Saccharomyces - ps27
Saccharomyces - ps28
Arboricola_clade - ps29
Cerevisiae_clade - ps30
Saccharomyces_cerevisiae - ps31
Phylostratum

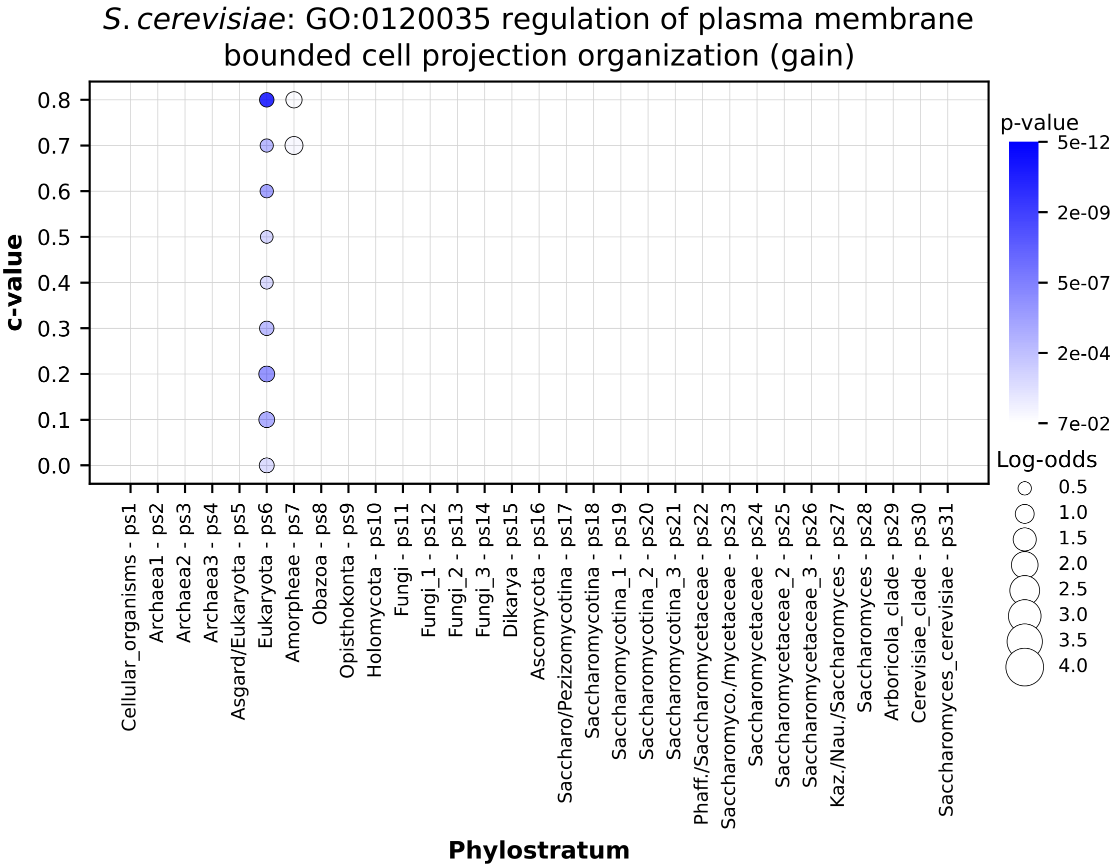

S. cerevisiae: GO:0120035 regulation of plasma membrane bounded cell projection organization (gain)
c-value
0.8
0.7
0.6
0.5
0.4
0.3
0.2
0.1
0.0
p-value
5e-12
2e-09
5e-07
2e-04
7e-02
Log-odds
0.5
1.0
1.5
2.0
2.5
3.0
3.5
4.0
Cellular_organisms - ps1
Archaea1 - ps2
Archaea2 - ps3
Archaea3 - ps4
Asgard/Eukaryota - ps5
Eukaryota - ps6
Amorpheae - ps7
Obazoa - ps8
Opisthokonta - ps9
Holomycota - ps10
Fungi - ps11
Fungi_1 - ps12
Fungi_2 - ps13
Fungi_3 - ps14
Dikarya - ps15
Ascomycota - ps16
Saccharo/Pezizomycotina - ps17
Saccharomycotina - ps18
Saccharomycotina_1 - ps19
Saccharomycotina_2 - ps20
Saccharomycotina_3 - ps21
Phaff./Saccharomycetaceae - ps22
Saccharomyco./mycetaceae - ps23
Saccharomycetaceae - ps24
Saccharomycetaceae_2 - ps25
Saccharomycetaceae_3 - ps26
Kaz./Nau./Saccharomyces - ps27
Saccharomyces - ps28
Arboricola_clade - ps29
Cerevisiae_clade - ps30
Saccharomyces_cerevisiae - ps31
Phylostratum

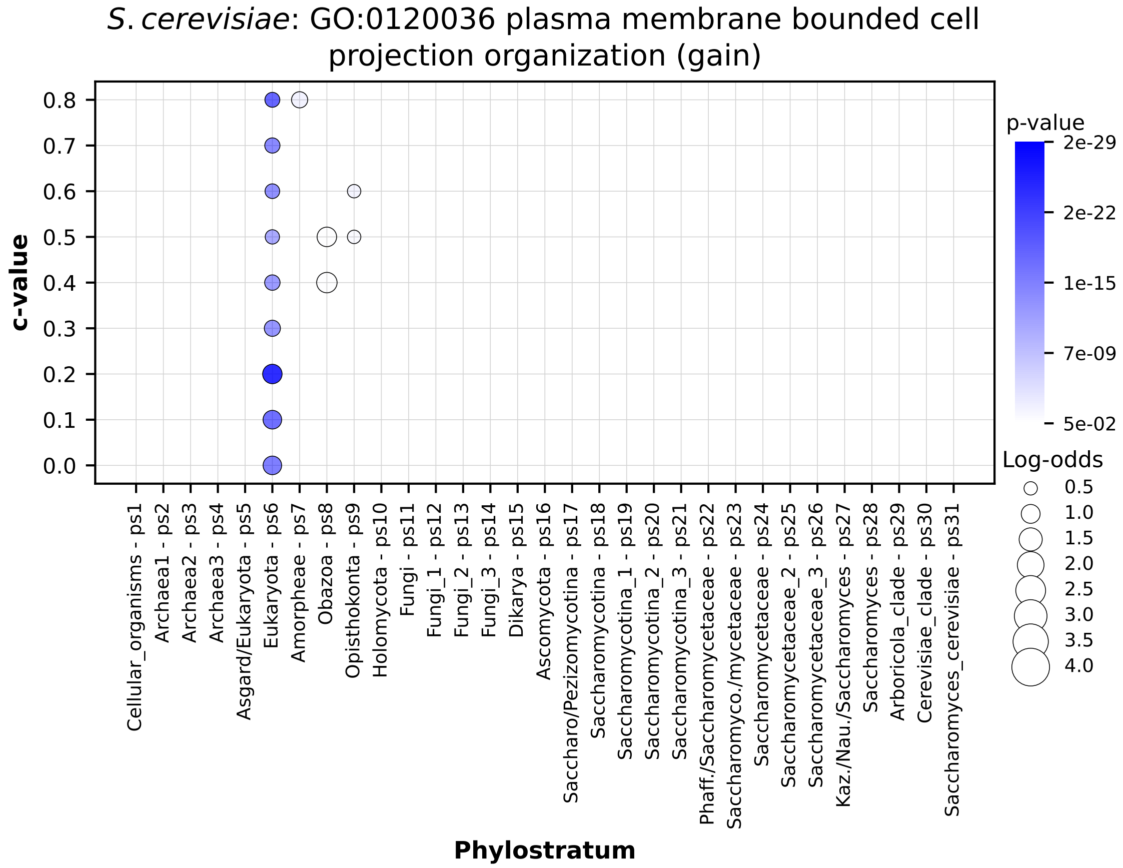

S. cerevisiae: GO:0120036 plasma membrane bounded cell projection organization (gain)
c-value
0.8
0.7
0.6
0.5
0.4
0.3
0.2
0.1
0.0
p-value
2e-29
2e-22
1e-15
7e-09
5e-02
Log-odds
0.5
1.0
1.5
2.0
2.5
3.0
3.5
4.0
Cellular_organisms - ps1
Archaea1 - ps2
Archaea2 - ps3
Archaea3 - ps4
Asgard/Eukaryota - ps5
Eukaryota - ps6
Amorpheae - ps7
Obazoa - ps8
Opisthokonta - ps9
Holomycota - ps10
Fungi - ps11
Fungi_1 - ps12
Fungi_2 - ps13
Fungi_3 - ps14
Dikarya - ps15
Ascomycota - ps16
Saccharo/Pezizomycotina - ps17
Saccharomycotina - ps18
Saccharomycotina_1 - ps19
Saccharomycotina_2 - ps20
Saccharomycotina_3 - ps21
Phaff./Saccharomycetaceae - ps22
Saccharomyco./mycetaceae - ps23
Saccharomycetaceae - ps24
Saccharomycetaceae_2 - ps25
Saccharomycetaceae_3 - ps26
Kaz./Nau./Saccharomyces - ps27
Saccharomyces - ps28
Arboricola_clade - ps29
Cerevisiae_clade - ps30
Saccharomyces_cerevisiae - ps31
Phylostratum

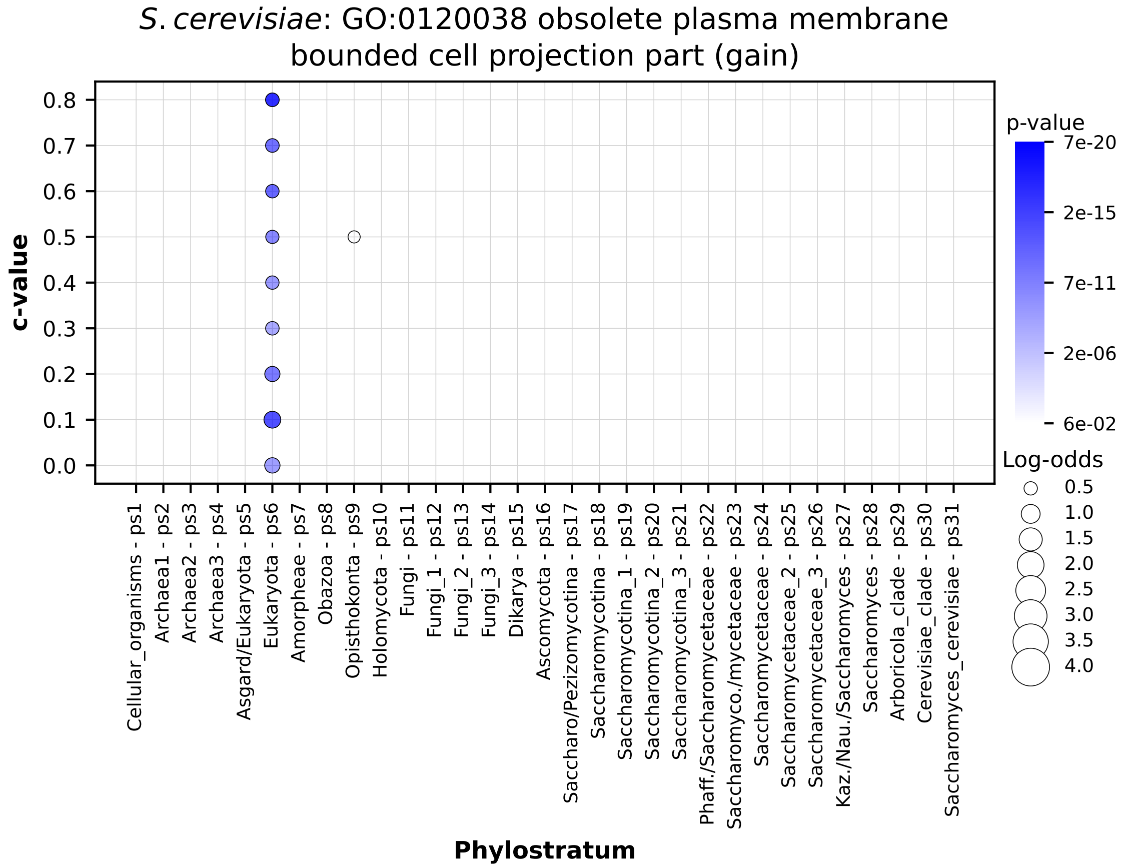

S. cerevisiae: GO:0120038 obsolete plasma membrane bounded cell projection part (gain)
c-value
0.8
0.7
0.6
0.5
0.4
0.3
0.2
0.1
0.0
p-value
7e-20
2e-15
7e-11
2e-06
6e-02
Log-odds
0.5
1.0
1.5
2.0
2.5
3.0
3.5
4.0
Cellular_organisms - ps1
Archaea1 - ps2
Archaea2 - ps3
Archaea3 - ps4
Asgard/Eukaryota - ps5
Eukaryota - ps6
Amorpheae - ps7
Obazoa - ps8
Opisthokonta - ps9
Holomycota - ps10
Fungi - ps11
Fungi_1 - ps12
Fungi_2 - ps13
Fungi_3 - ps14
Dikarya - ps15
Ascomycota - ps16
Saccharo/Pezizomycotina - ps17
Saccharomycotina - ps18
Saccharomycotina_1 - ps19
Saccharomycotina_2 - ps20
Saccharomycotina_3 - ps21
Phaff./Saccharomycetaceae - ps22
Saccharomyco./mycetaceae - ps23
Saccharomycetaceae - ps24
Saccharomycetaceae_2 - ps25
Saccharomycetaceae_3 - ps26
Kaz./Nau./Saccharomyces - ps27
Saccharomyces - ps28
Arboricola_clade - ps29
Cerevisiae_clade - ps30
Saccharomyces_cerevisiae - ps31
Phylostratum

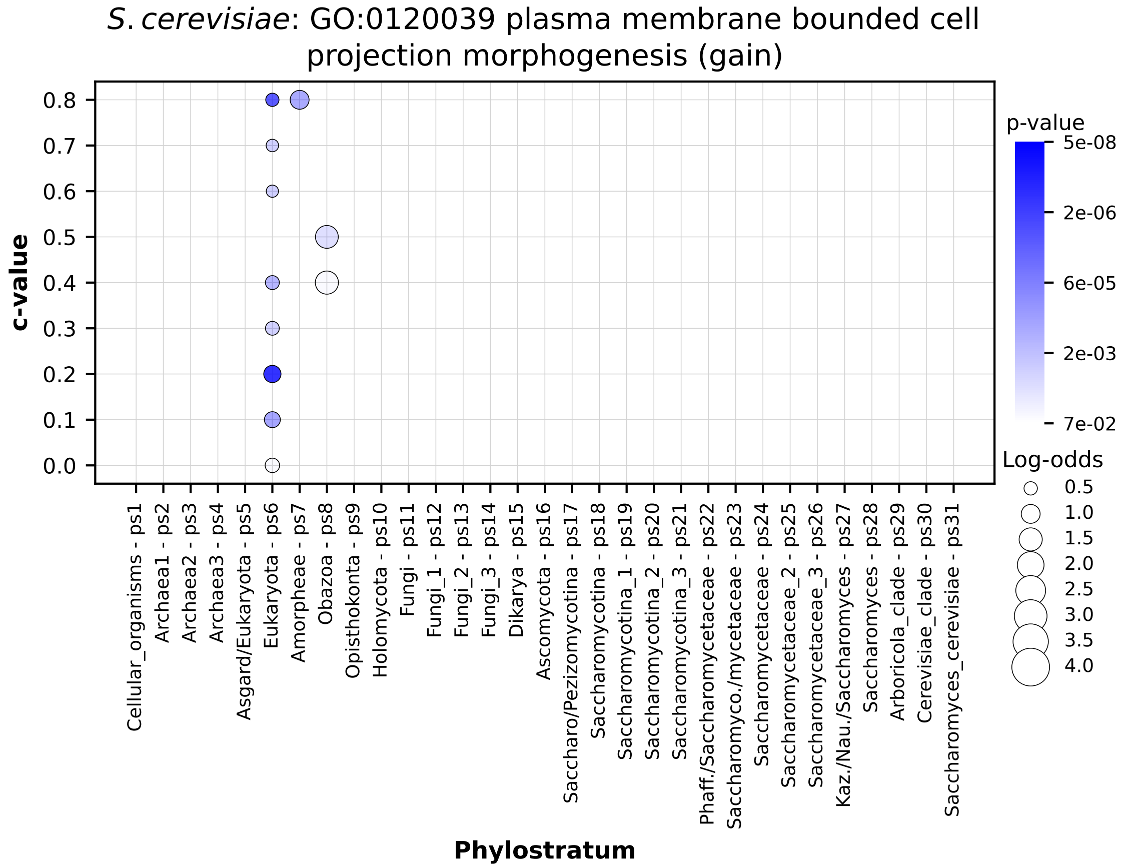

S. cerevisiae: GO:0120039 plasma membrane bounded cell projection morphogenesis (gain)
c-value
0.0
0.1
0.2
0.3
0.4
0.5
0.6
0.7
0.8
Phylostratum
Cellular_organisms - ps1
Archaea1 - ps2
Archaea2 - ps3
Archaea3 - ps4
Asgard/Eukaryota - ps5
Eukaryota - ps6
Amorpheae - ps7
Obazoa - ps8
Opisthokonta - ps9
Holomycota - ps10
Fungi - ps11
Fungi_1 - ps12
Fungi_2 - ps13
Fungi_3 - ps14
Dikarya - ps15
Ascomycota - ps16
Saccharo/Pezizomycotina - ps17
Saccharomycotina - ps18
Saccharomycotina_1 - ps19
Saccharomycotina_2 - ps20
Saccharomycotina_3 - ps21
Phaff./Saccharomycetaceae - ps22
Saccharomyco./mycetaceae - ps23
Saccharomycetaceae - ps24
Saccharomycetaceae_2 - ps25
Saccharomycetaceae_3 - ps26
Kaz./Nau./Saccharomyces - ps27
Saccharomyces - ps28
Arboricola_clade - ps29
Cerevisiae_clade - ps30
Saccharomyces_cerevisiae - ps31
p-value
5e-08
2e-06
6e-05
2e-03
7e-02
Log-odds
0.5
1.0
1.5
2.0
2.5
3.0
3.5
4.0

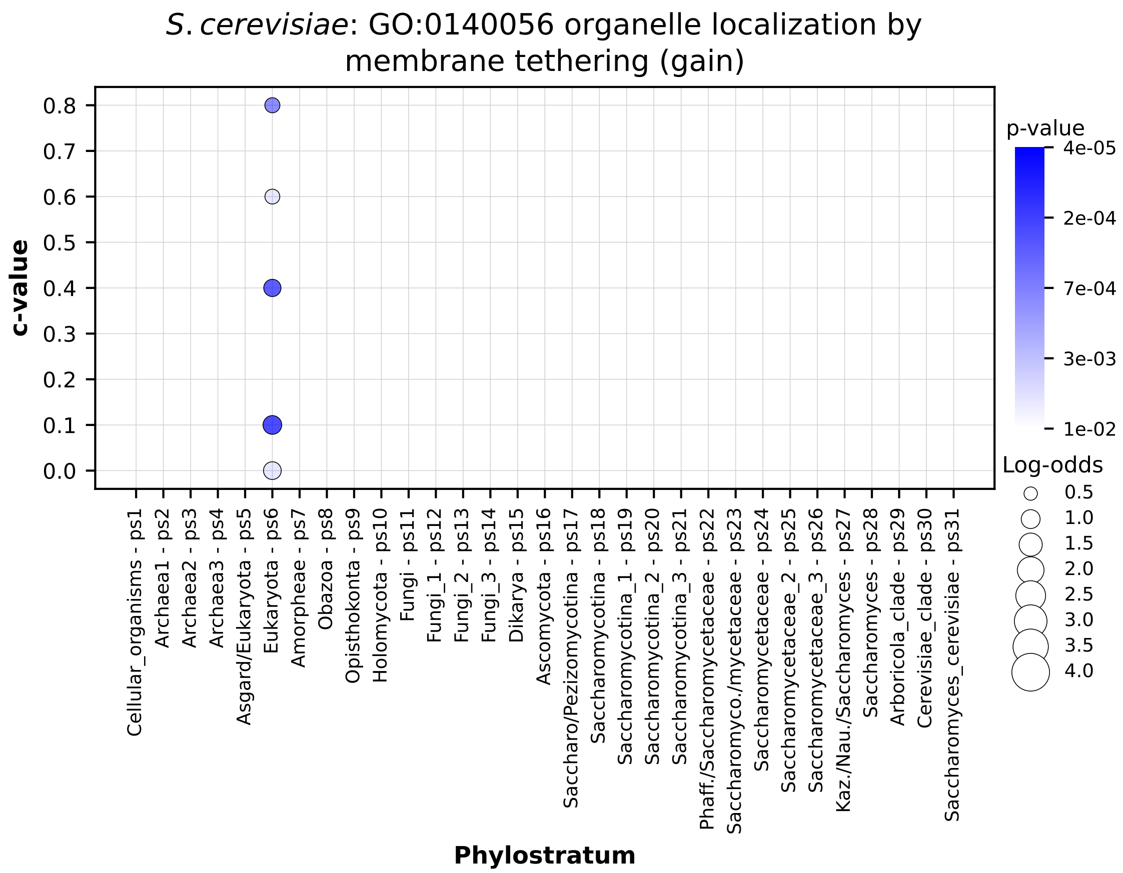

S. cerevisiae: GO:0140056 organelle localization by membrane tethering (gain)
c-value
0.8
0.7
0.6
0.5
0.4
0.3
0.2
0.1
0.0
p-value
4e-05
2e-04
7e-04
3e-03
1e-02
Log-odds
0.5
1.0
1.5
2.0
2.5
3.0
3.5
4.0
Cellular_organisms - ps1
Archaea1 - ps2
Archaea2 - ps3
Archaea3 - ps4
Asgard/Eukaryota - ps5
Eukaryota - ps6
Amorpheae - ps7
Obazoa - ps8
Opisthokonta - ps9
Holomycota - ps10
Fungi - ps11
Fungi_1 - ps12
Fungi_2 - ps13
Fungi_3 - ps14
Dikarya - ps15
Ascomycota - ps16
Saccharo/Pezizomycotina - ps17
Saccharomycotina - ps18
Saccharomycotina_1 - ps19
Saccharomycotina_2 - ps20
Saccharomycotina_3 - ps21
Phaff./Saccharomycetaceae - ps22
Saccharomyco./mycetaceae - ps23
Saccharomycetaceae - ps24
Saccharomycetaceae_2 - ps25
Saccharomycetaceae_3 - ps26
Kaz./Nau./Saccharomyces - ps27
Saccharomyces - ps28
Arboricola_clade - ps29
Cerevisiae_clade - ps30
Saccharomyces_cerevisiae - ps31
Phylostratum

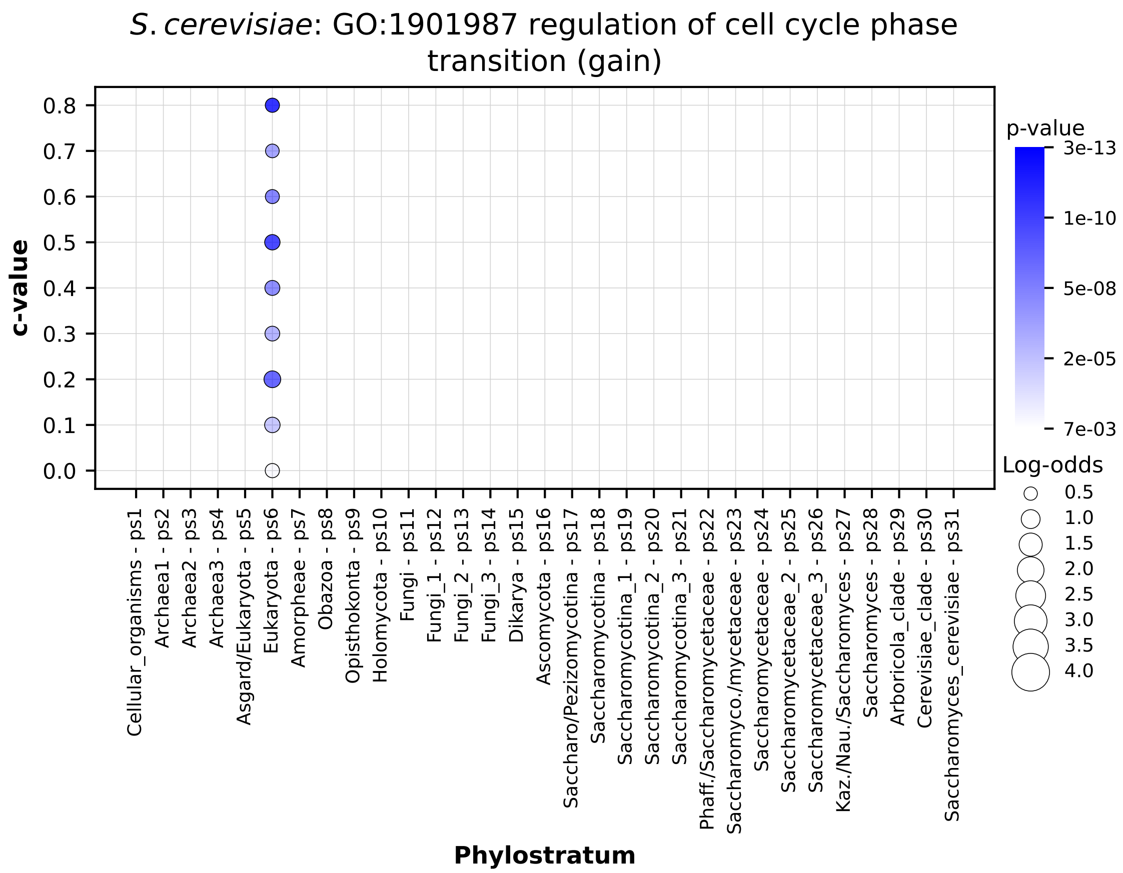

S. cerevisiae: GO:1901987 regulation of cell cycle phase transition (gain)
c-value
0.0
0.1
0.2
0.3
0.4
0.5
0.6
0.7
0.8
Phylostratum
Cellular_organisms - ps1
Archaea1 - ps2
Archaea2 - ps3
Archaea3 - ps4
Asgard/Eukaryota - ps5
Eukaryota - ps6
Amorpheae - ps7
Obazoa - ps8
Opisthokonta - ps9
Holomycota - ps10
Fungi - ps11
Fungi_1 - ps12
Fungi_2 - ps13
Fungi_3 - ps14
Dikarya - ps15
Ascomycota - ps16
Saccharo/Pezizomycotina - ps17
Saccharomycotina - ps18
Saccharomycotina_1 - ps19
Saccharomycotina_2 - ps20
Saccharomycotina_3 - ps21
Phaff./Saccharomycetaceae - ps22
Saccharomyco./mycetaceae - ps23
Saccharomycetaceae - ps24
Saccharomycetaceae_2 - ps25
Saccharomycetaceae_3 - ps26
Kaz./Nau./Saccharomyces - ps27
Saccharomyces - ps28
Arboricola_clade - ps29
Cerevisiae_clade - ps30
Saccharomyces_cerevisiae - ps31
p-value
3e-13
1e-10
5e-08
2e-05
7e-03
Log-odds
0.5
1.0
1.5
2.0
2.5
3.0
3.5
4.0

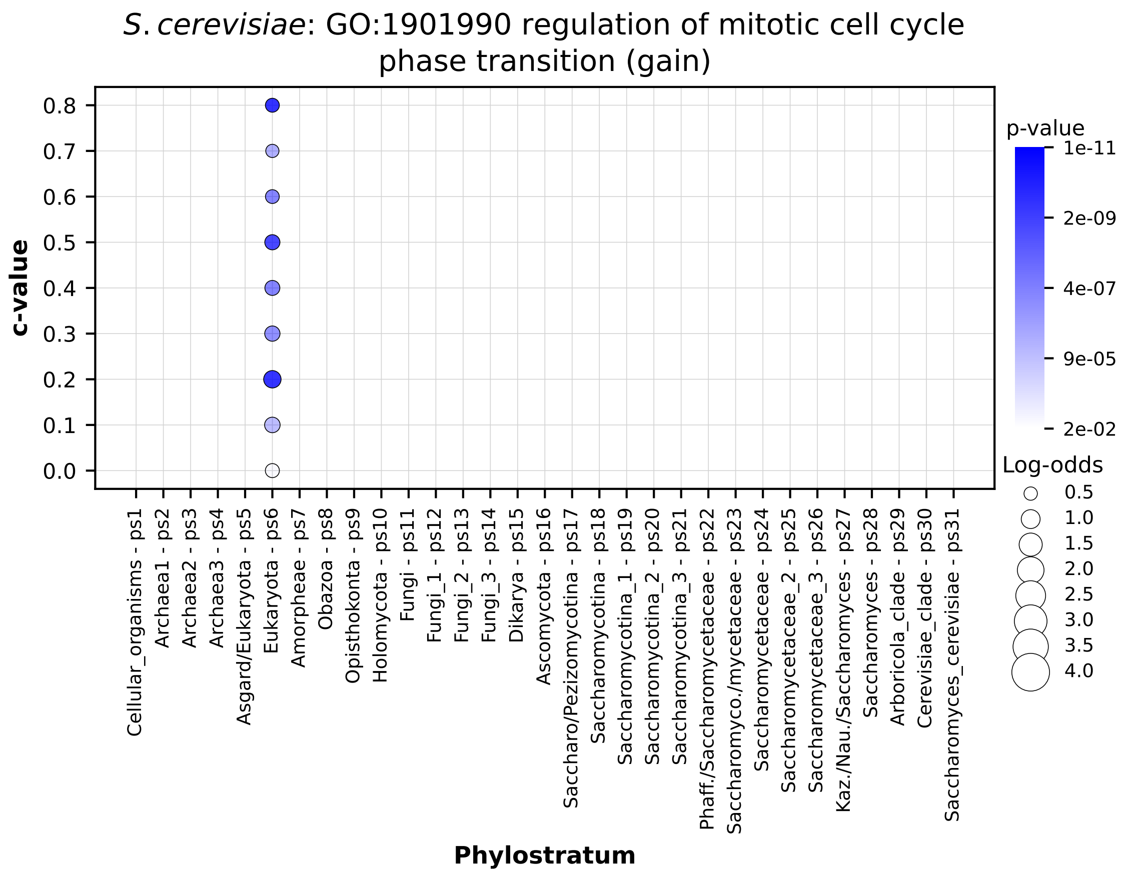

S. cerevisiae: GO:1901990 regulation of mitotic cell cycle phase transition (gain)
c-value
0.8
0.7
0.6
0.5
0.4
0.3
0.2
0.1
0.0
p-value
1e-11
2e-09
4e-07
9e-05
2e-02
Log-odds
0.5
1.0
1.5
2.0
2.5
3.0
3.5
4.0
Cellular_organisms - ps1
Archaea1 - ps2
Archaea2 - ps3
Archaea3 - ps4
Asgard/Eukaryota - ps5
Eukaryota - ps6
Amorpheae - ps7
Obazoa - ps8
Opisthokonta - ps9
Holomycota - ps10
Fungi - ps11
Fungi_1 - ps12
Fungi_2 - ps13
Fungi_3 - ps14
Dikarya - ps15
Ascomycota - ps16
Saccharo/Pezizomycotina - ps17
Saccharomycotina - ps18
Saccharomycotina_1 - ps19
Saccharomycotina_2 - ps20
Saccharomycotina_3 - ps21
Phaff./Saccharomycetaceae - ps22
Saccharomyco./mycetaceae - ps23
Saccharomycetaceae - ps24
Saccharomycetaceae_2 - ps25
Saccharomycetaceae_3 - ps26
Kaz./Nau./Saccharomyces - ps27
Saccharomyces - ps28
Arboricola_clade - ps29
Cerevisiae_clade - ps30
Saccharomyces_cerevisiae - ps31
Phylostratum

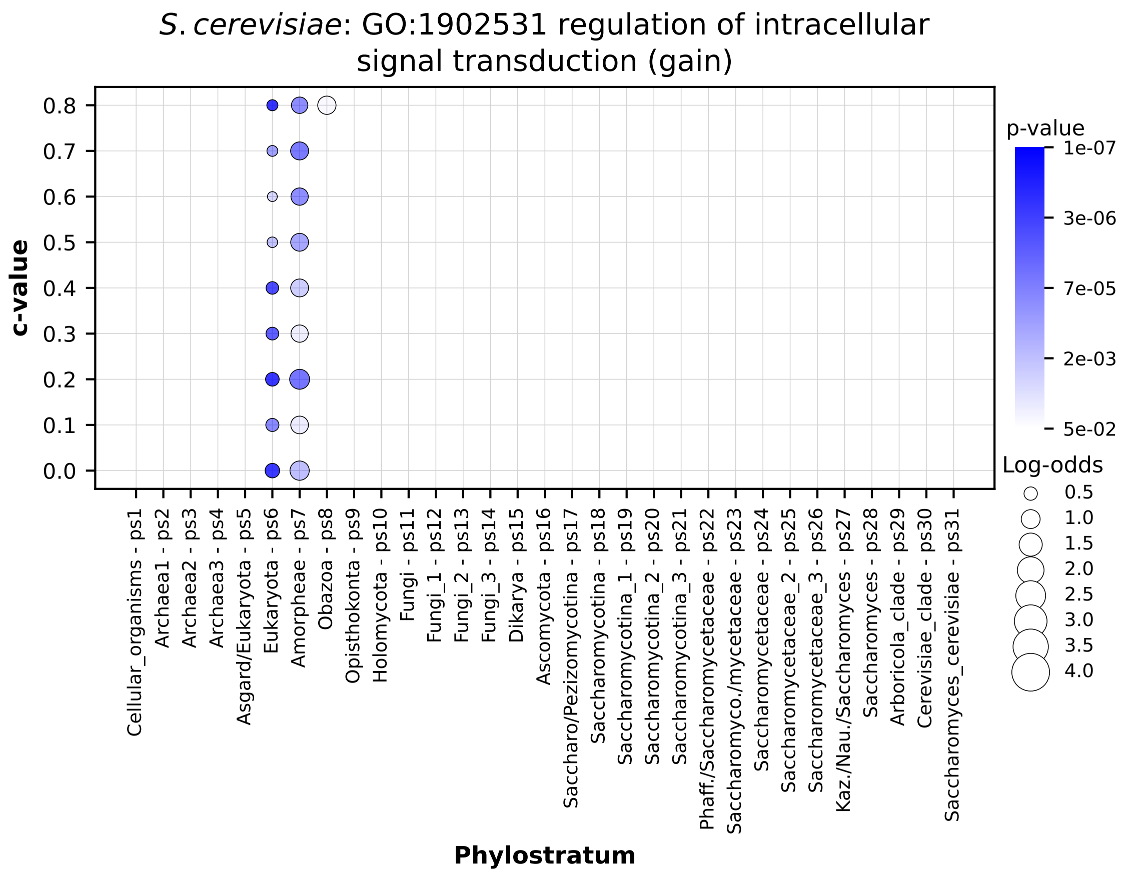

S. cerevisiae: GO:1902531 regulation of intracellular signal transduction (gain)
c-value
0.0
0.1
0.2
0.3
0.4
0.5
0.6
0.7
0.8
p-value
1e-07
3e-06
7e-05
2e-03
5e-02
Log-odds
0.5
1.0
1.5
2.0
2.5
3.0
3.5
4.0
Cellular_organisms - ps1
Archaea1 - ps2
Archaea2 - ps3
Archaea3 - ps4
Asgard/Eukaryota - ps5
Eukaryota - ps6
Amorpheae - ps7
Obazoa - ps8
Opisthokonta - ps9
Holomycota - ps10
Fungi - ps11
Fungi_1 - ps12
Fungi_2 - ps13
Fungi_3 - ps14
Dikarya - ps15
Ascomycota - ps16
Saccharo/Pezizomycotina - ps17
Saccharomycotina - ps18
Saccharomycotina_1 - ps19
Saccharomycotina_2 - ps20
Saccharomycotina_3 - ps21
Phaff./Saccharomycetaceae - ps22
Saccharomyco./mycetaceae - ps23
Saccharomycetaceae - ps24
Saccharomycetaceae_2 - ps25
Saccharomycetaceae_3 - ps26
Kaz./Nau./Saccharomyces - ps27
Saccharomyces - ps28
Arboricola_clade - ps29
Cerevisiae_clade - ps30
Saccharomyces_cerevisiae - ps31
Phylostratum

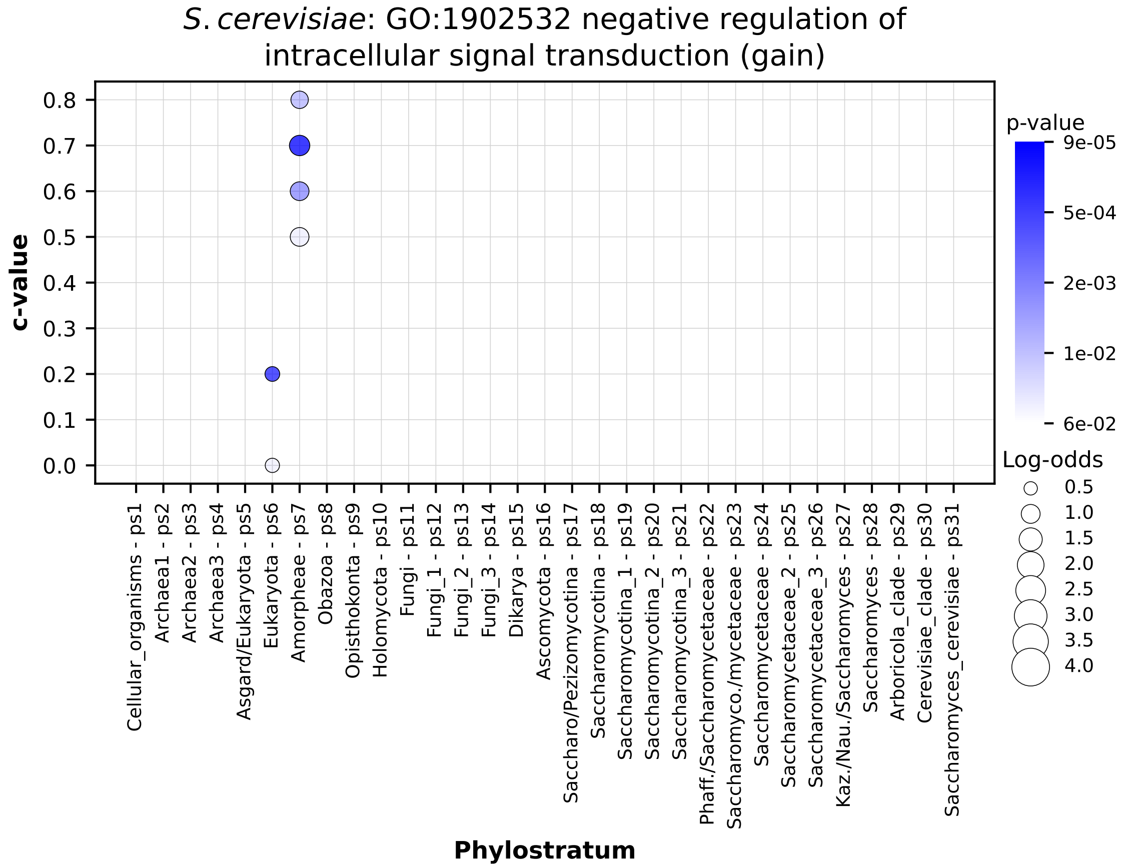

S. cerevisiae: GO:1902532 negative regulation of intracellular signal transduction (gain)
c-value
0.0
0.1
0.2
0.3
0.4
0.5
0.6
0.7
0.8
Phylostratum
Cellular_organisms - ps1
Archaea1 - ps2
Archaea2 - ps3
Archaea3 - ps4
Asgard/Eukaryota - ps5
Eukaryota - ps6
Amorpheae - ps7
Obazoa - ps8
Opisthokonta - ps9
Holomycota - ps10
Fungi - ps11
Fungi_1 - ps12
Fungi_2 - ps13
Fungi_3 - ps14
Dikarya - ps15
Ascomycota - ps16
Saccharo/Pezizomycotina - ps17
Saccharomycotina - ps18
Saccharomycotina_1 - ps19
Saccharomycotina_2 - ps20
Saccharomycotina_3 - ps21
Phaff./Saccharomycetaceae - ps22
Saccharomyco./mycetaceae - ps23
Saccharomycetaceae - ps24
Saccharomycetaceae_2 - ps25
Saccharomycetaceae_3 - ps26
Kaz./Nau./Saccharomyces - ps27
Saccharomyces - ps28
Arboricola_clade - ps29
Cerevisiae_clade - ps30
Saccharomyces_cerevisiae - ps31
p-value
9e-05
5e-04
2e-03
1e-02
6e-02
Log-odds
0.5
1.0
1.5
2.0
2.5
3.0
3.5
4.0

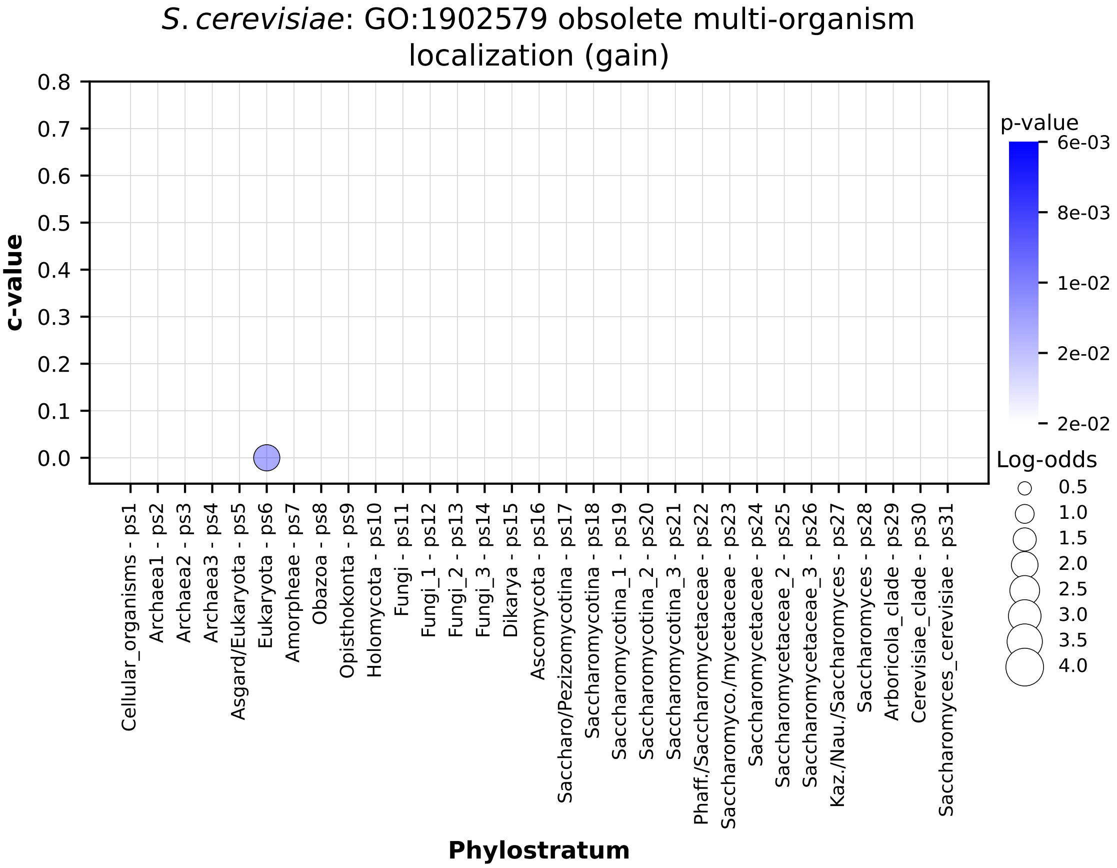

S. cerevisiae: GO:1902579 obsolete multi-organism localization (gain)
c-value
0.8
0.7
0.6
0.5
0.4
0.3
0.2
0.1
0.0
p-value
6e-03
8e-03
1e-02
2e-02
2e-02
Log-odds
0.5
1.0
1.5
2.0
2.5
3.0
3.5
4.0
Cellular_organisms - ps1
Archaea1 - ps2
Archaea2 - ps3
Archaea3 - ps4
Asgard/Eukaryota - ps5
Eukaryota - ps6
Amorpheae - ps7
Obazoa - ps8
Opisthokonta - ps9
Holomycota - ps10
Fungi - ps11
Fungi_1 - ps12
Fungi_2 - ps13
Fungi_3 - ps14
Dikarya - ps15
Ascomycota - ps16
Saccharo/Pezizomycotina - ps17
Saccharomycotina - ps18
Saccharomycotina_1 - ps19
Saccharomycotina_2 - ps20
Saccharomycotina_3 - ps21
Phaff./Saccharomycetaceae - ps22
Saccharomyco./mycetaceae - ps23
Saccharomycetaceae - ps24
Saccharomycetaceae_2 - ps25
Saccharomycetaceae_3 - ps26
Kaz./Nau./Saccharomyces - ps27
Saccharomyces - ps28
Arboricola_clade - ps29
Cerevisiae_clade - ps30
Saccharomyces_cerevisiae - ps31
Phylostratum

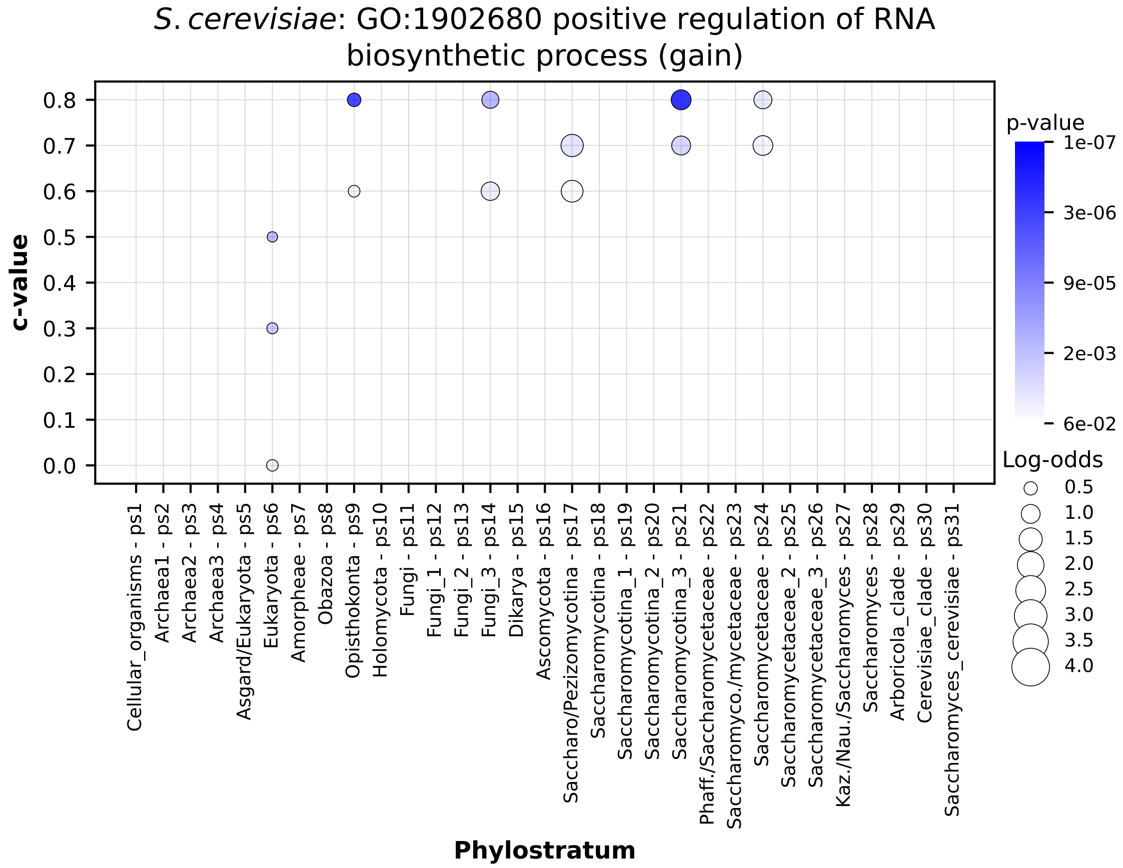

S. cerevisiae: GO:1902680 positive regulation of RNA biosynthetic process (gain)
c-value
0.8
0.7
0.6
0.5
0.4
0.3
0.2
0.1
0.0
p-value
1e-07
3e-06
9e-05
2e-03
6e-02
Log-odds
0.5
1.0
1.5
2.0
2.5
3.0
3.5
4.0
Cellular_organisms - ps1
Archaea1 - ps2
Archaea2 - ps3
Archaea3 - ps4
Asgard/Eukaryota - ps5
Eukaryota - ps6
Amorpheae - ps7
Obazoa - ps8
Opisthokonta - ps9
Holomycota - ps10
Fungi - ps11
Fungi_1 - ps12
Fungi_2 - ps13
Fungi_3 - ps14
Dikarya - ps15
Ascomycota - ps16
Saccharo/Pezizomycotina - ps17
Saccharomycotina - ps18
Saccharomycotina_1 - ps19
Saccharomycotina_2 - ps20
Saccharomycotina_3 - ps21
Phaff./Saccharomycetaceae - ps22
Saccharomyco./mycetaceae - ps23
Saccharomycetaceae - ps24
Saccharomycetaceae_2 - ps25
Saccharomycetaceae_3 - ps26
Kaz./Nau./Saccharomyces - ps27
Saccharomyces - ps28
Arboricola_clade - ps29
Cerevisiae_clade - ps30
Saccharomyces_cerevisiae - ps31
Phylostratum

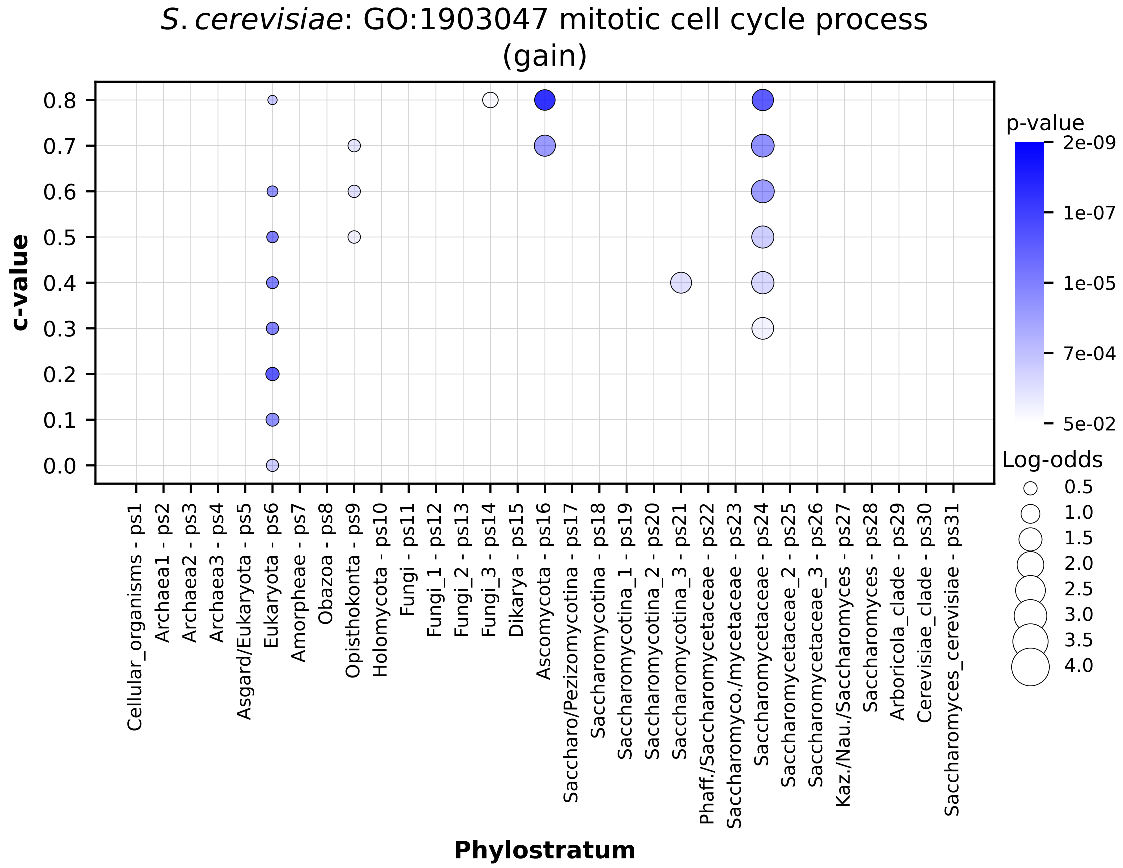

S. cerevisiae: GO:1903047 mitotic cell cycle process
(gain)
c-value
0.0
0.1
0.2
0.3
0.4
0.5
0.6
0.7
0.8
Phylostratum
Cellular_organisms - ps1
Archaea1 - ps2
Archaea2 - ps3
Archaea3 - ps4
Asgard/Eukaryota - ps5
Eukaryota - ps6
Amorpheae - ps7
Obazoa - ps8
Opisthokonta - ps9
Holomycota - ps10
Fungi - ps11
Fungi_1 - ps12
Fungi_2 - ps13
Fungi_3 - ps14
Dikarya - ps15
Ascomycota - ps16
Saccharo/Pezizomycotina - ps17
Saccharomycotina - ps18
Saccharomycotina_1 - ps19
Saccharomycotina_2 - ps20
Saccharomycotina_3 - ps21
Phaff./Saccharomycetaceae - ps22
Saccharomyco./mycetaceae - ps23
Saccharomycetaceae - ps24
Saccharomycetaceae_2 - ps25
Saccharomycetaceae_3 - ps26
Kaz./Nau./Saccharomyces - ps27
Saccharomyces - ps28
Arboricola_clade - ps29
Cerevisiae_clade - ps30
Saccharomyces_cerevisiae - ps31
p-value
2e-09
1e-07
1e-05
7e-04
5e-02
Log-odds
0.5
1.0
1.5
2.0
2.5
3.0
3.5
4.0

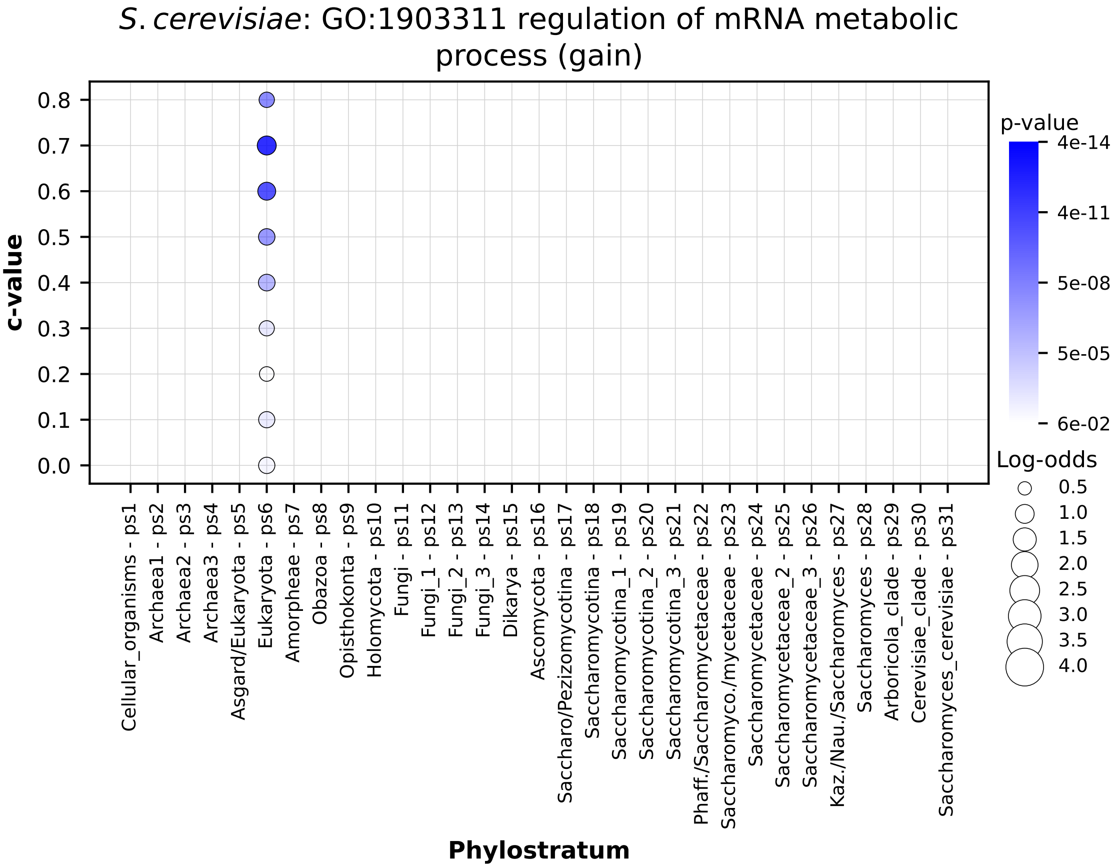

S. cerevisiae: GO:1903311 regulation of mRNA metabolic process (gain)
c-value
0.8
0.7
0.6
0.5
0.4
0.3
0.2
0.1
0.0
p-value
4e-14
4e-11
5e-08
5e-05
6e-02
Log-odds
0.5
1.0
1.5
2.0
2.5
3.0
3.5
4.0
Cellular_organisms - ps1
Archaea1 - ps2
Archaea2 - ps3
Archaea3 - ps4
Asgard/Eukaryota - ps5
Eukaryota - ps6
Amorpheae - ps7
Obazoa - ps8
Opisthokonta - ps9
Holomycota - ps10
Fungi - ps11
Fungi_1 - ps12
Fungi_2 - ps13
Fungi_3 - ps14
Dikarya - ps15
Ascomycota - ps16
Saccharo/Pezizomycotina - ps17
Saccharomycotina - ps18
Saccharomycotina_1 - ps19
Saccharomycotina_2 - ps20
Saccharomycotina_3 - ps21
Phaff./Saccharomycetaceae - ps22
Saccharomyco./mycetaceae - ps23
Saccharomycetaceae - ps24
Saccharomycetaceae_2 - ps25
Saccharomycetaceae_3 - ps26
Kaz./Nau./Saccharomyces - ps27
Saccharomyces - ps28
Arboricola_clade - ps29
Cerevisiae_clade - ps30
Saccharomyces_cerevisiae - ps31
Phylostratum

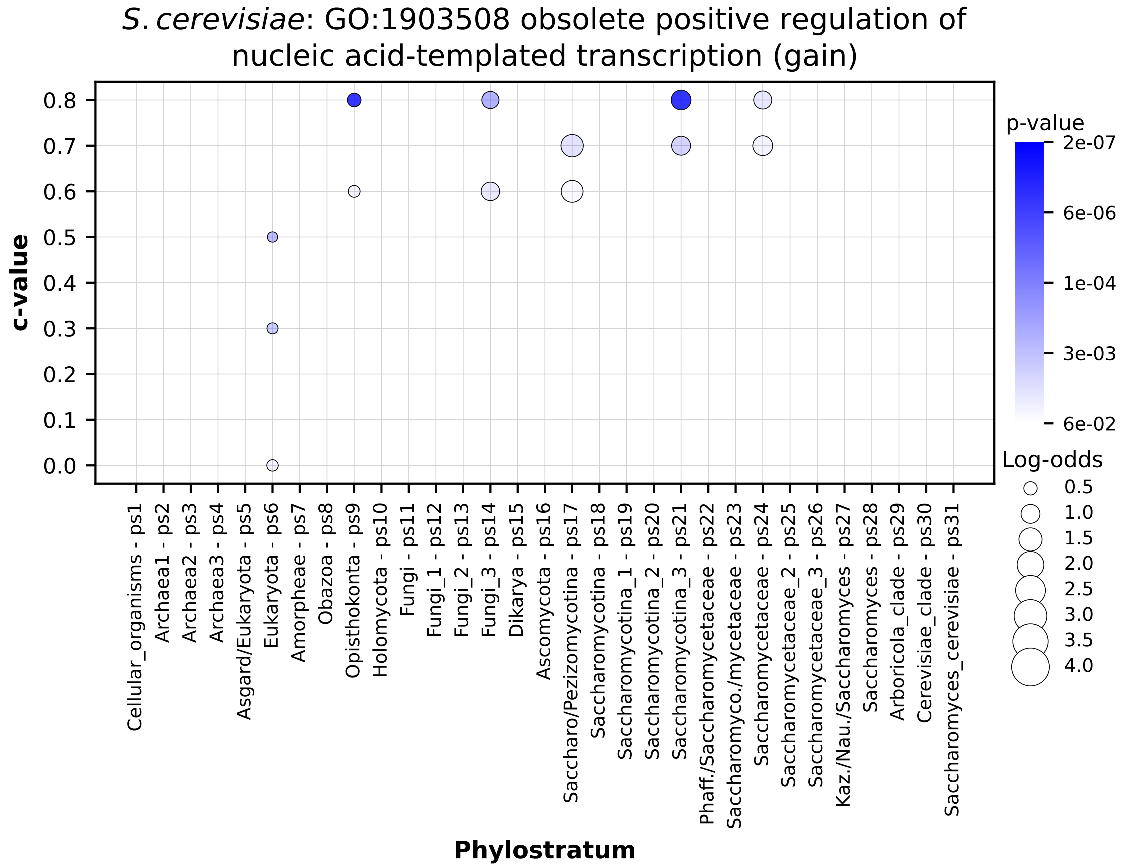

S. cerevisiae: GO:1903508 obsolete positive regulation of nucleic acid-templated transcription (gain)
c-value
0.0
0.1
0.2
0.3
0.4
0.5
0.6
0.7
0.8
p-value
2e-07
6e-06
1e-04
3e-03
6e-02
Log-odds
0.5
1.0
1.5
2.0
2.5
3.0
3.5
4.0
Cellular_organisms - ps1
Archaea1 - ps2
Archaea2 - ps3
Archaea3 - ps4
Asgard/Eukaryota - ps5
Eukaryota - ps6
Amorpheae - ps7
Obazoa - ps8
Opisthokonta - ps9
Holomycota - ps10
Fungi - ps11
Fungi_1 - ps12
Fungi_2 - ps13
Fungi_3 - ps14
Dikarya - ps15
Ascomycota - ps16
Saccharo/Pezizomycotina - ps17
Saccharomycotina - ps18
Saccharomycotina_1 - ps19
Saccharomycotina_2 - ps20
Saccharomycotina_3 - ps21
Phaff./Saccharomycetaceae - ps22
Saccharomyco./mycetaceae - ps23
Saccharomycetaceae - ps24
Saccharomycetaceae_2 - ps25
Saccharomycetaceae_3 - ps26
Kaz./Nau./Saccharomyces - ps27
Saccharomyces - ps28
Arboricola_clade - ps29
Cerevisiae_clade - ps30
Saccharomyces_cerevisiae - ps31
Phylostratum

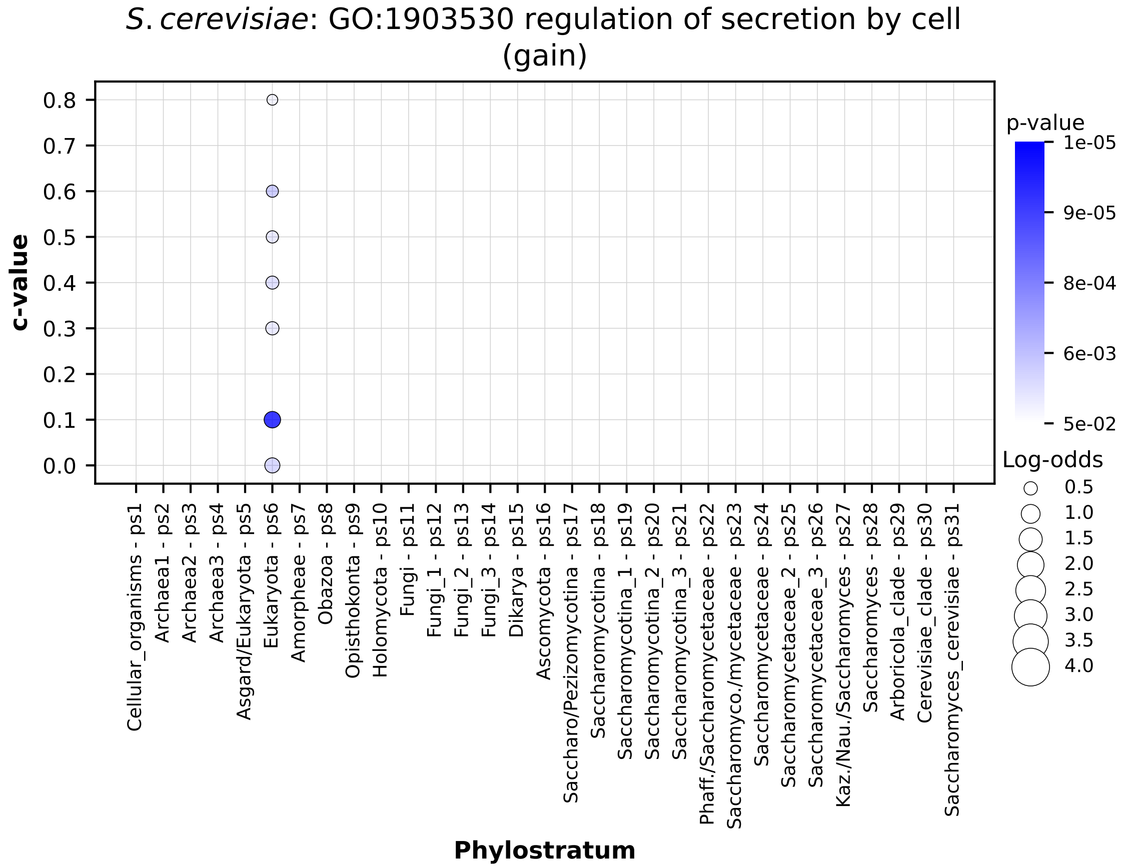

S. cerevisiae: GO:1903530 regulation of secretion by cell
(gain)
c-value
0.0
0.1
0.2
0.3
0.4
0.5
0.6
0.7
0.8
Phylostratum
Cellular_organisms - ps1
Archaea1 - ps2
Archaea2 - ps3
Archaea3 - ps4
Asgard/Eukaryota - ps5
Eukaryota - ps6
Amorpheae - ps7
Obazoa - ps8
Opisthokonta - ps9
Holomycota - ps10
Fungi - ps11
Fungi_1 - ps12
Fungi_2 - ps13
Fungi_3 - ps14
Dikarya - ps15
Ascomycota - ps16
Saccharo/Pezizomycotina - ps17
Saccharomycotina - ps18
Saccharomycotina_1 - ps19
Saccharomycotina_2 - ps20
Saccharomycotina_3 - ps21
Phaff./Saccharomycetaceae - ps22
Saccharomyco./mycetaceae - ps23
Saccharomycetaceae - ps24
Saccharomycetaceae_2 - ps25
Saccharomycetaceae_3 - ps26
Kaz./Nau./Saccharomyces - ps27
Saccharomyces - ps28
Arboricola_clade - ps29
Cerevisiae_clade - ps30
Saccharomyces_cerevisiae - ps31
p-value
1e-05
9e-05
8e-04
6e-03
5e-02
Log-odds
0.5
1.0
1.5
2.0
2.5
3.0
3.5
4.0

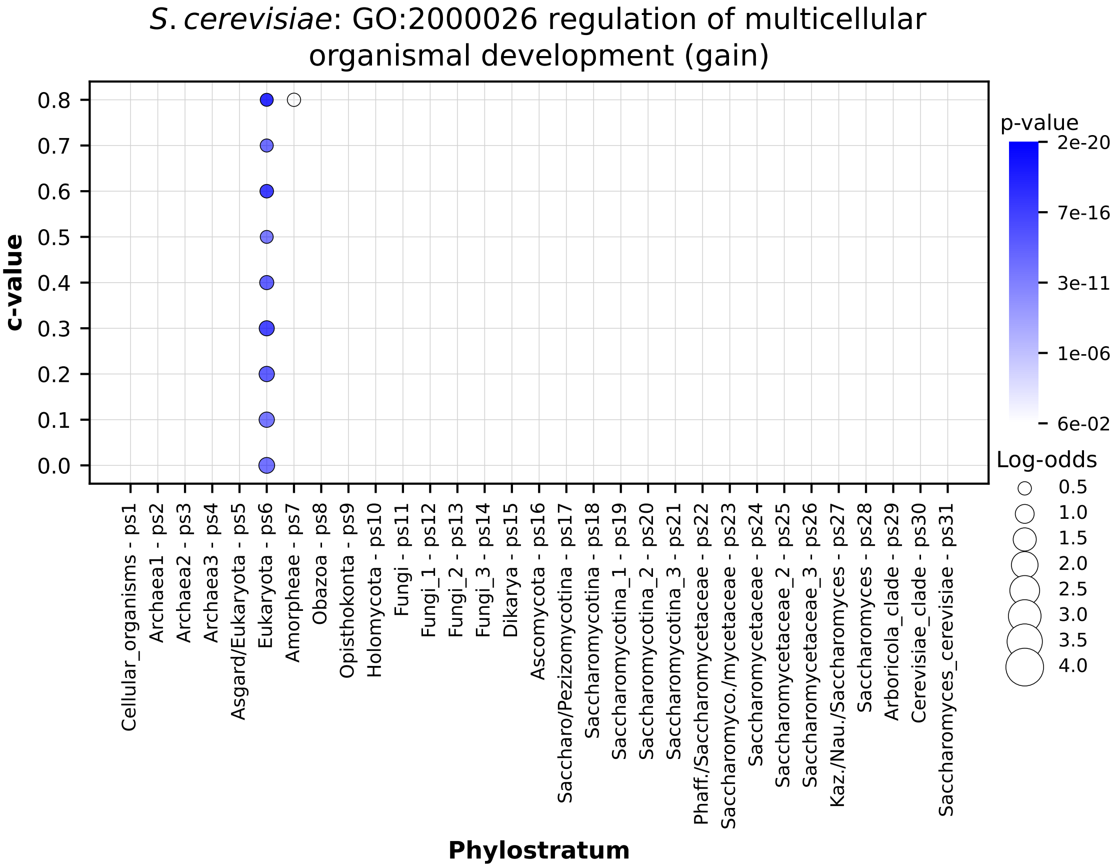

$S. cerevisiae$: GO:2000026 regulation of multicellular organismal development (gain)
c-value
0.8
0.7
0.6
0.5
0.4
0.3
0.2
0.1
0.0
Phylostratum
Cellular_organisms - ps1
Archaea1 - ps2
Archaea2 - ps3
Archaea3 - ps4
Asgard/Eukaryota - ps5
Eukaryota - ps6
Amorpheae - ps7
Obazoa - ps8
Opisthokonta - ps9
Holomycota - ps10
Fungi - ps11
Fungi_1 - ps12
Fungi_2 - ps13
Fungi_3 - ps14
Dikarya - ps15
Ascomycota - ps16
Saccharo/Pezizomycotina - ps17
Saccharomycotina - ps18
Saccharomycotina_1 - ps19
Saccharomycotina_2 - ps20
Saccharomycotina_3 - ps21
Phaff./Saccharomycetaceae - ps22
Saccharomyco./mycetaceae - ps23
Saccharomycetaceae - ps24
Saccharomycetaceae_2 - ps25
Saccharomycetaceae_3 - ps26
Kaz./Nau./Saccharomyces - ps27
Saccharomyces - ps28
Arboricola_clade - ps29
Cerevisiae_clade - ps30
Saccharomyces_cerevisiae - ps31
p-value
2e-20
7e-16
3e-11
1e-06
6e-02
Log-odds
0.5
1.0
1.5
2.0
2.5
3.0
3.5
4.0

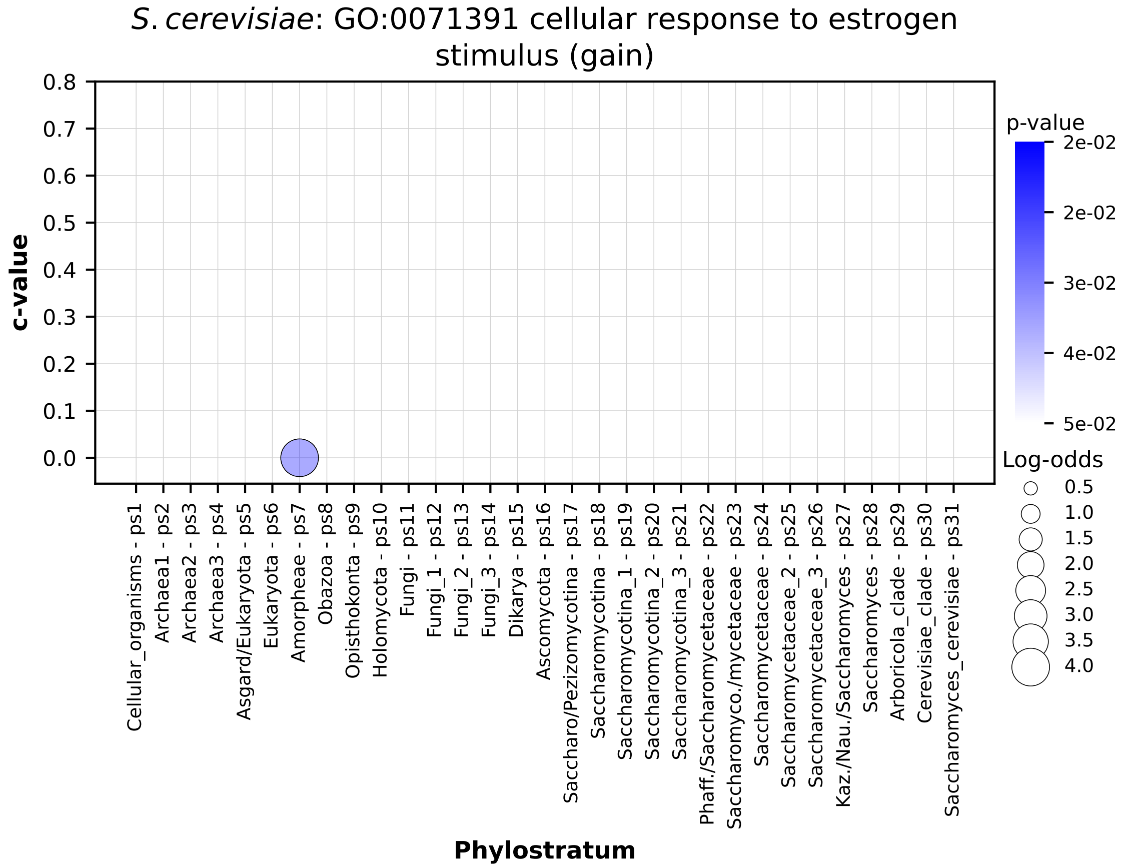
S. cerevisiae: GO:0071391 cellular response to estrogen stimulus (gain)
c-value
0.8
0.7
0.6
0.5
0.4
0.3
0.2
0.1
0.0
p-value
2e-02
2e-02
3e-02
4e-02
5e-02
Log-odds
0.5
1.0
1.5
2.0
2.5
3.0
3.5
4.0
Cellular_organisms - ps1
Archaea1 - ps2
Archaea2 - ps3
Archaea3 - ps4
Asgard/Eukaryota - ps5
Eukaryota - ps6
Amorpheae - ps7
Obazoa - ps8
Opisthokonta - ps9
Holomycota - ps10
Fungi - ps11
Fungi_1 - ps12
Fungi_2 - ps13
Fungi_3 - ps14
Dikarya - ps15
Ascomycota - ps16
Saccharo/Pezizomycotina - ps17
Saccharomycotina - ps18
Saccharomycotina_1 - ps19
Saccharomycotina_2 - ps20
Saccharomycotina_3 - ps21
Phaff./Saccharomycetaceae - ps22
Saccharomyco./mycetaceae - ps23
Saccharomycetaceae - ps24
Saccharomycetaceae_2 - ps25
Saccharomycetaceae_3 - ps26
Kaz./Nau./Saccharomyces - ps27
Saccharomyces - ps28
Arboricola_clade - ps29
Cerevisiae_clade - ps30
Saccharomyces_cerevisiae - ps31
Phylostratum

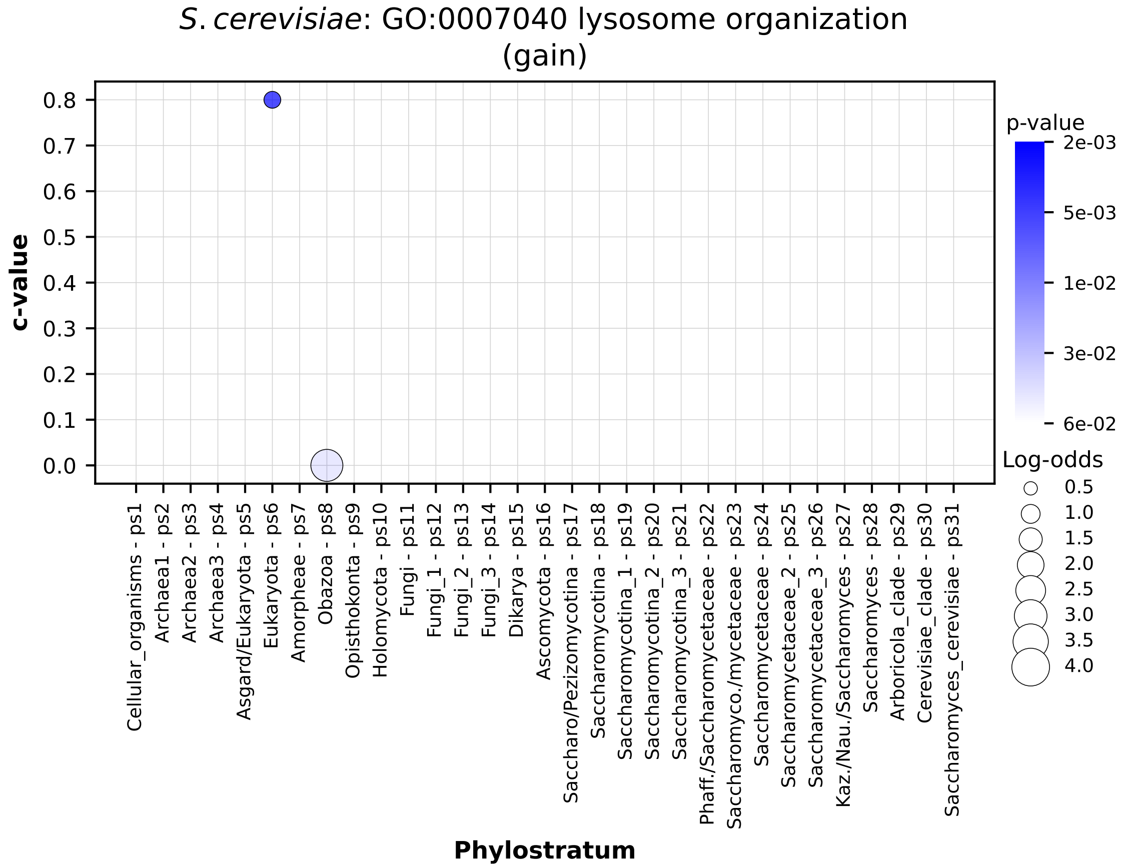

S. cerevisiae: GO:0007040 lysosome organization (gain)
c-value
0.8
0.7
0.6
0.5
0.4
0.3
0.2
0.1
0.0
p-value
2e-03
5e-03
1e-02
3e-02
6e-02
Log-odds
0.5
1.0
1.5
2.0
2.5
3.0
3.5
4.0
Cellular_organisms - ps1
Archaea1 - ps2
Archaea2 - ps3
Archaea3 - ps4
Asgard/Eukaryota - ps5
Eukaryota - ps6
Amorpheae - ps7
Obazoa - ps8
Opisthokonta - ps9
Holomycota - ps10
Fungi - ps11
Fungi_1 - ps12
Fungi_2 - ps13
Fungi_3 - ps14
Dikarya - ps15
Ascomycota - ps16
Saccharo/Pezizomycotina - ps17
Saccharomycotina - ps18
Saccharomycotina_1 - ps19
Saccharomycotina_2 - ps20
Saccharomycotina_3 - ps21
Phaff./Saccharomycetaceae - ps22
Saccharomyco./mycetaceae - ps23
Saccharomycetaceae - ps24
Saccharomycetaceae_2 - ps25
Saccharomycetaceae_3 - ps26
Kaz./Nau./Saccharomyces - ps27
Saccharomyces - ps28
Arboricola_clade - ps29
Cerevisiae_clade - ps30
Saccharomyces_cerevisiae - ps31
Phylostratum

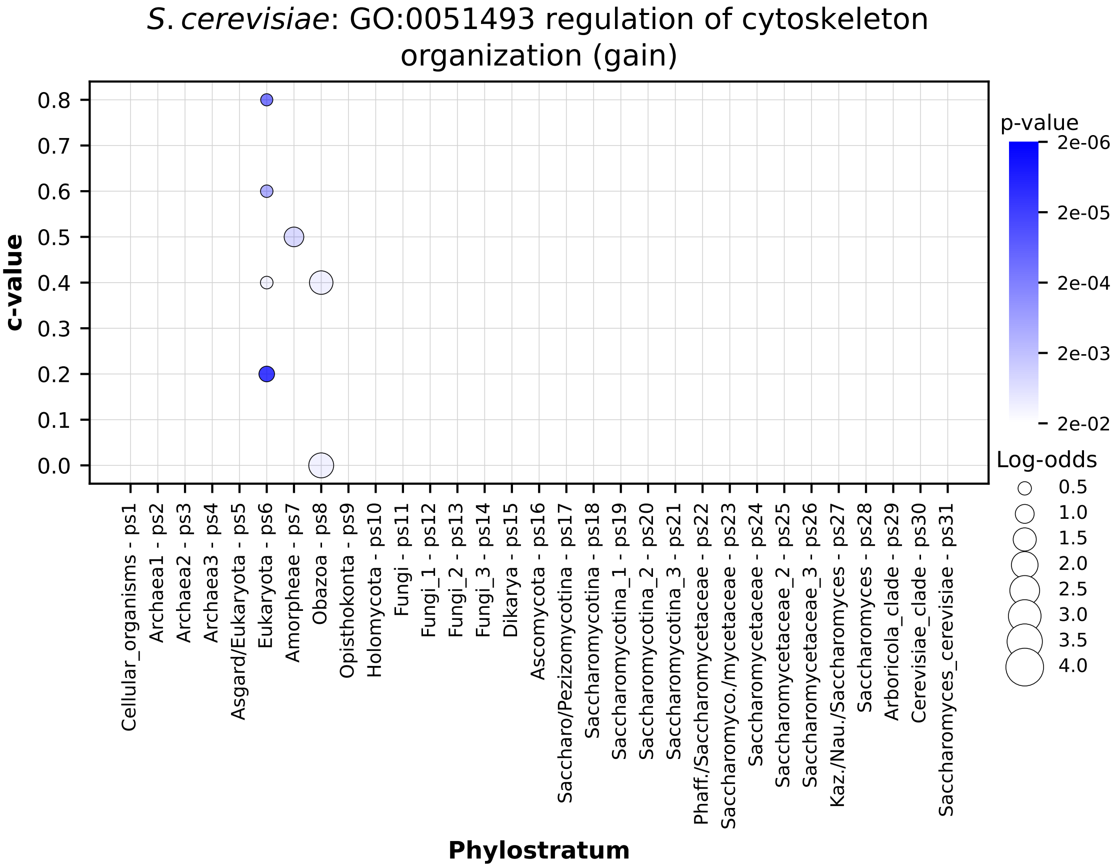

S. cerevisiae: GO:0051493 regulation of cytoskeleton organization (gain)
c-value
0.8
0.7
0.6
0.5
0.4
0.3
0.2
0.1
0.0
p-value
2e-06
2e-05
2e-04
2e-03
2e-02
Log-odds
0.5
1.0
1.5
2.0
2.5
3.0
3.5
4.0
Cellular_organisms - ps1
Archaea1 - ps2
Archaea2 - ps3
Archaea3 - ps4
Asgard/Eukaryota - ps5
Eukaryota - ps6
Amorpheae - ps7
Obazoa - ps8
Opisthokonta - ps9
Holomycota - ps10
Fungi - ps11
Fungi_1 - ps12
Fungi_2 - ps13
Fungi_3 - ps14
Dikarya - ps15
Ascomycota - ps16
Saccharo/Pezizomycotina - ps17
Saccharomycotina - ps18
Saccharomycotina_1 - ps19
Saccharomycotina_2 - ps20
Saccharomycotina_3 - ps21
Phaff./Saccharomycetaceae - ps22
Saccharomyco./mycetaceae - ps23
Saccharomycetaceae - ps24
Saccharomycetaceae_2 - ps25
Saccharomycetaceae_3 - ps26
Kaz./Nau./Saccharomyces - ps27
Saccharomyces - ps28
Arboricola_clade - ps29
Cerevisiae_clade - ps30
Saccharomyces_cerevisiae - ps31
Phylostratum

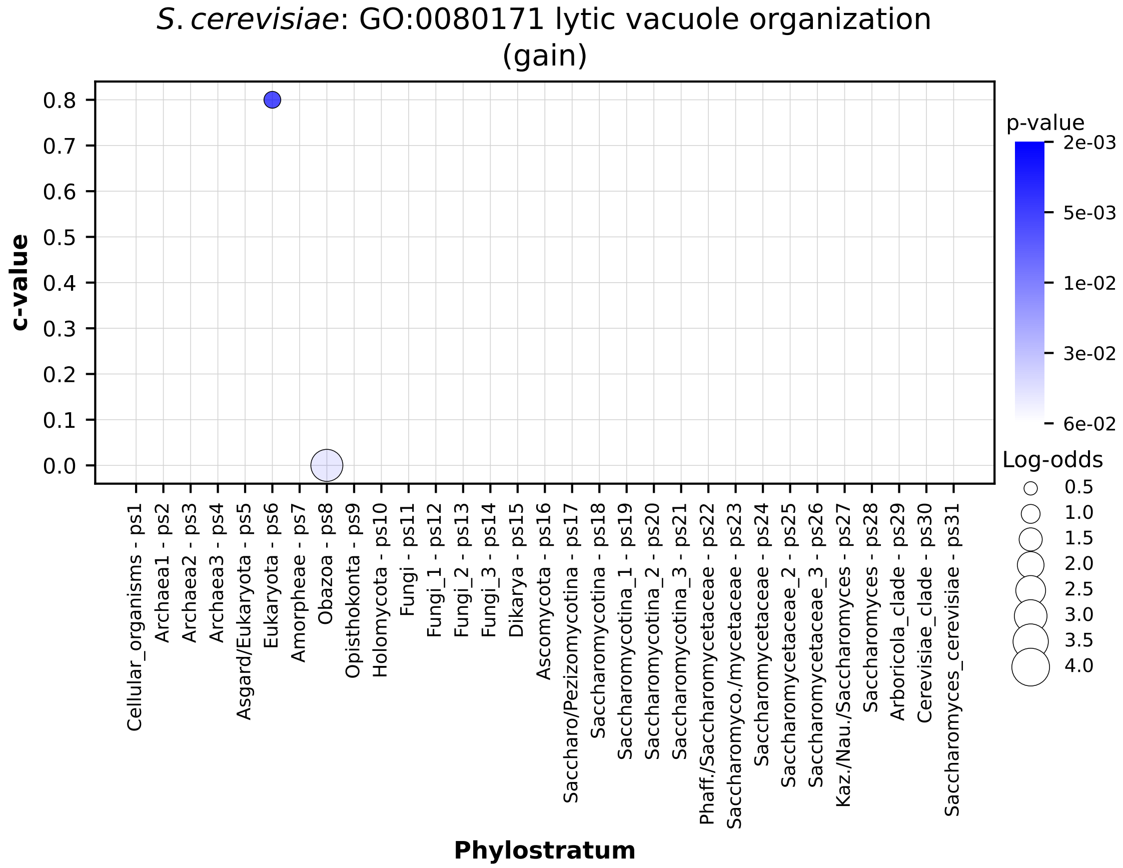

S. cerevisiae: GO:0080171 lytic vacuole organization
(gain)
c-value
0.8
0.7
0.6
0.5
0.4
0.3
0.2
0.1
0.0
p-value
2e-03
5e-03
1e-02
3e-02
6e-02
Log-odds
0.5
1.0
1.5
2.0
2.5
3.0
3.5
4.0
Cellular_organisms - ps1
Archaea1 - ps2
Archaea2 - ps3
Archaea3 - ps4
Asgard/Eukaryota - ps5
Eukaryota - ps6
Amorpheae - ps7
Obazoa - ps8
Opisthokonta - ps9
Holomycota - ps10
Fungi - ps11
Fungi_1 - ps12
Fungi_2 - ps13
Fungi_3 - ps14
Dikarya - ps15
Ascomycota - ps16
Saccharo/Pezizomycotina - ps17
Saccharomycotina - ps18
Saccharomycotina_1 - ps19
Saccharomycotina_2 - ps20
Saccharomycotina_3 - ps21
Phaff./Saccharomycetaceae - ps22
Saccharomyco./mycetaceae - ps23
Saccharomycetaceae - ps24
Saccharomycetaceae_2 - ps25
Saccharomycetaceae_3 - ps26
Kaz./Nau./Saccharomyces - ps27
Saccharomyces - ps28
Arboricola_clade - ps29
Cerevisiae_clade - ps30
Saccharomyces_cerevisiae - ps31
Phylostratum

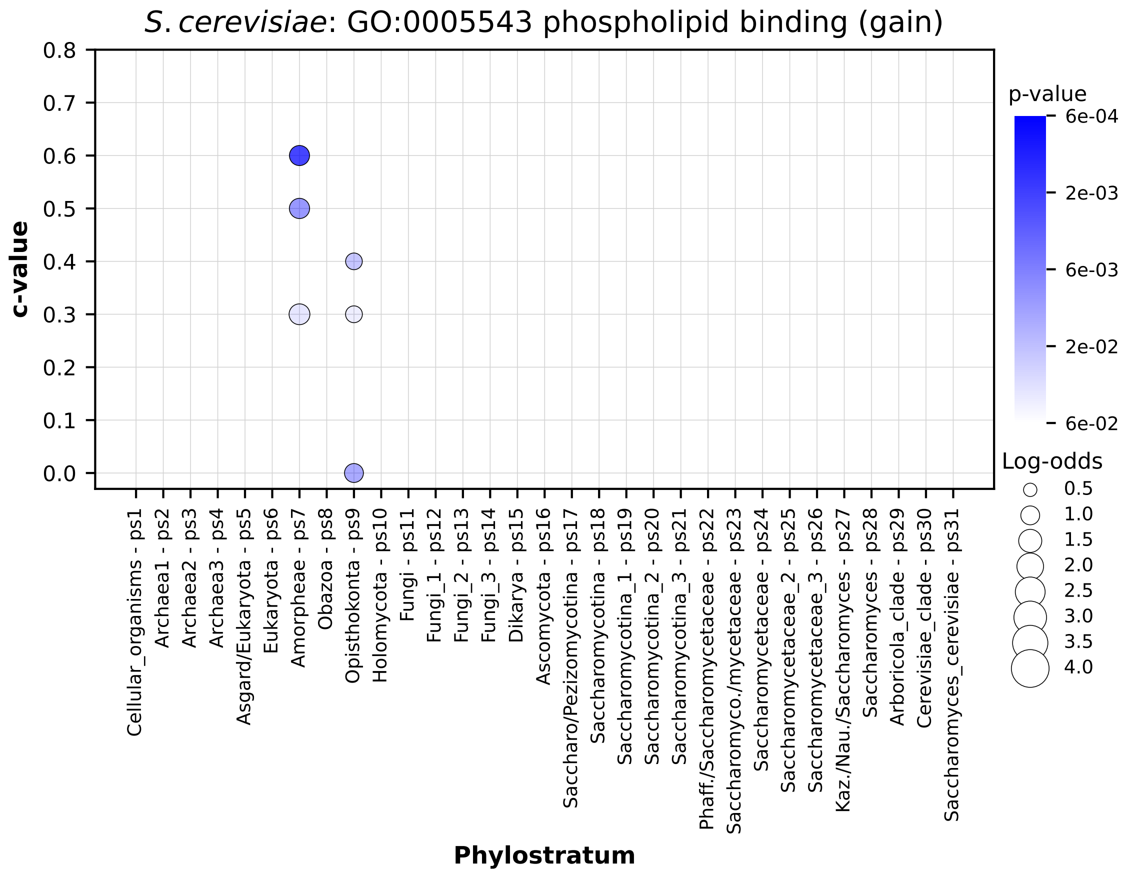
S. cerevisiae: GO:0005543 phospholipid binding (gain)
c-value
0.8
0.7
0.6
0.5
0.4
0.3
0.2
0.1
0.0
p-value
6e-04
2e-03
6e-03
2e-02
6e-02
Log-odds
0.5
1.0
1.5
2.0
2.5
3.0
3.5
4.0
Cellular_organisms - ps1
Archaea1 - ps2
Archaea2 - ps3
Archaea3 - ps4
Asgard/Eukaryota - ps5
Eukaryota - ps6
Amorpheae - ps7
Obazoa - ps8
Opisthokonta - ps9
Holomycota - ps10
Fungi - ps11
Fungi_1 - ps12
Fungi_2 - ps13
Fungi_3 - ps14
Dikarya - ps15
Ascomycota - ps16
Saccharo/Pezizomycotina - ps17
Saccharomycotina - ps18
Saccharomycotina_1 - ps19
Saccharomycotina_2 - ps20
Saccharomycotina_3 - ps21
Phaff./Saccharomycetaceae - ps22
Saccharomyco./mycetaceae - ps23
Saccharomycetaceae - ps24
Saccharomycetaceae_2 - ps25
Saccharomycetaceae_3 - ps26
Kaz./Nau./Saccharomyces - ps27
Saccharomyces - ps28
Arboricola_clade - ps29
Cerevisiae_clade - ps30
Saccharomyces_cerevisiae - ps31
Phylostratum

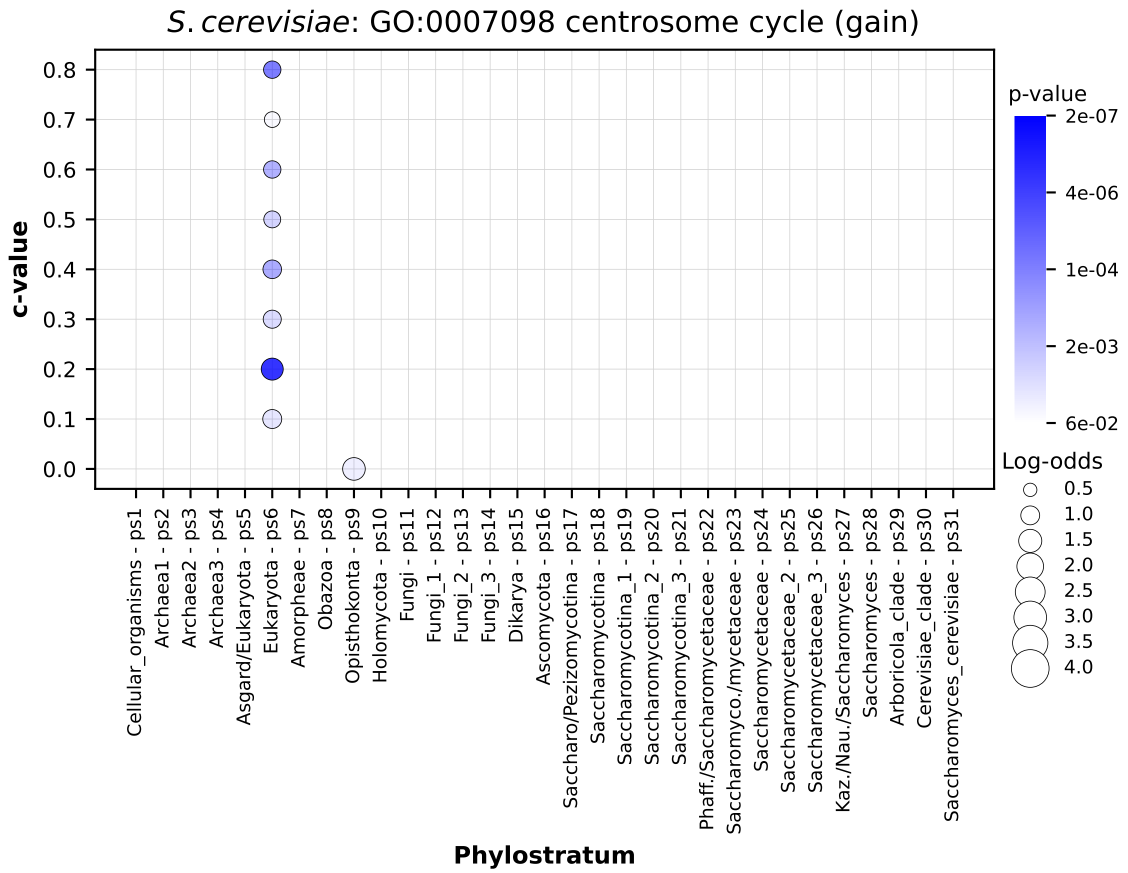

S. cerevisiae: GO:0007098 centrosome cycle (gain)
c-value
0.0
0.1
0.2
0.3
0.4
0.5
0.6
0.7
0.8
p-value
2e-07
4e-06
1e-04
2e-03
6e-02
Log-odds
0.5
1.0
1.5
2.0
2.5
3.0
3.5
4.0
Cellular_organisms - ps1
Archaea1 - ps2
Archaea2 - ps3
Archaea3 - ps4
Asgard/Eukaryota - ps5
Eukaryota - ps6
Amorpheae - ps7
Obazoa - ps8
Opisthokonta - ps9
Holomycota - ps10
Fungi - ps11
Fungi_1 - ps12
Fungi_2 - ps13
Fungi_3 - ps14
Dikarya - ps15
Ascomycota - ps16
Saccharo/Pezizomycotina - ps17
Saccharomycotina - ps18
Saccharomycotina_1 - ps19
Saccharomycotina_2 - ps20
Saccharomycotina_3 - ps21
Phaff./Saccharomycetaceae - ps22
Saccharomyco./mycetaceae - ps23
Saccharomycetaceae - ps24
Saccharomycetaceae_2 - ps25
Saccharomycetaceae_3 - ps26
Kaz./Nau./Saccharomyces - ps27
Saccharomyces - ps28
Arboricola_clade - ps29
Cerevisiae_clade - ps30
Saccharomyces_cerevisiae - ps31
Phylostratum

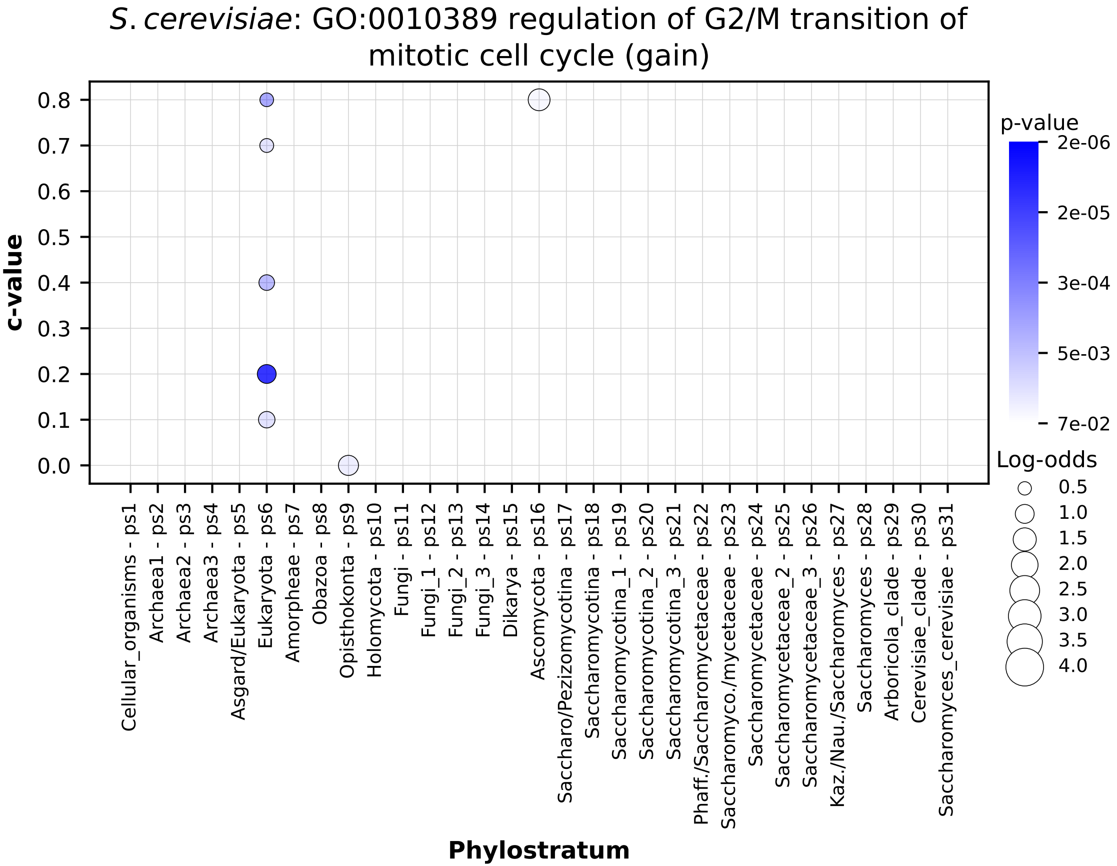

S. cerevisiae: GO:0010389 regulation of G2/M transition of mitotic cell cycle (gain)
c-value
0.8
0.7
0.6
0.5
0.4
0.3
0.2
0.1
0.0
p-value
2e-06
2e-05
3e-04
5e-03
7e-02
Log-odds
0.5
1.0
1.5
2.0
2.5
3.0
3.5
4.0
Cellular_organisms - ps1
Archaea1 - ps2
Archaea2 - ps3
Archaea3 - ps4
Asgard/Eukaryota - ps5
Eukaryota - ps6
Amorpheae - ps7
Obazoa - ps8
Opisthokonta - ps9
Holomycota - ps10
Fungi - ps11
Fungi_1 - ps12
Fungi_2 - ps13
Fungi_3 - ps14
Dikarya - ps15
Ascomycota - ps16
Saccharo/Pezizomycotina - ps17
Saccharomycotina - ps18
Saccharomycotina_1 - ps19
Saccharomycotina_2 - ps20
Saccharomycotina_3 - ps21
Phaff./Saccharomycetaceae - ps22
Saccharomyco./mycetaceae - ps23
Saccharomycetaceae - ps24
Saccharomycetaceae_2 - ps25
Saccharomycetaceae_3 - ps26
Kaz./Nau./Saccharomyces - ps27
Saccharomyces - ps28
Arboricola_clade - ps29
Cerevisiae_clade - ps30
Saccharomyces_cerevisiae - ps31
Phylostratum

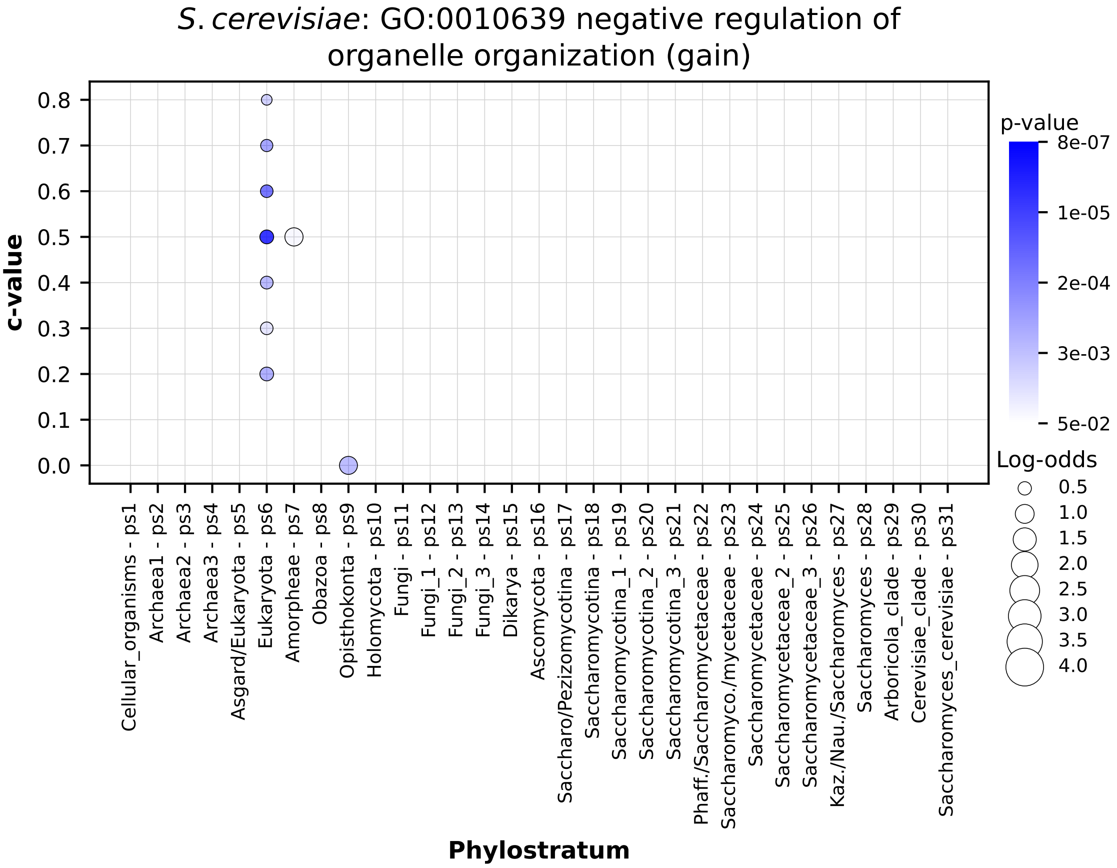

S. cerevisiae: GO:0010639 negative regulation of organelle organization (gain)
c-value
Phylostratum
p-value
8e-07
1e-05
2e-04
3e-03
5e-02
Log-odds
0.5
1.0
1.5
2.0
2.5
3.0
3.5
4.0
Cellular_organisms - ps1
Archaea1 - ps2
Archaea2 - ps3
Archaea3 - ps4
Asgard/Eukaryota - ps5
Eukaryota - ps6
Amorpheae - ps7
Obazoa - ps8
Opisthokonta - ps9
Holomycota - ps10
Fungi - ps11
Fungi_1 - ps12
Fungi_2 - ps13
Fungi_3 - ps14
Dikarya - ps15
Ascomycota - ps16
Saccharo/Pezizomycotina - ps17
Saccharomycotina - ps18
Saccharomycotina_1 - ps19
Saccharomycotina_2 - ps20
Saccharomycotina_3 - ps21
Phaff./Saccharomycetaceae - ps22
Saccharomyco./mycetaceae - ps23
Saccharomycetaceae - ps24
Saccharomycetaceae_2 - ps25
Saccharomycetaceae_3 - ps26
Kaz./Nau./Saccharomyces - ps27
Saccharomyces - ps28
Arboricola_clade - ps29
Cerevisiae_clade - ps30
Saccharomyces_cerevisiae - ps31

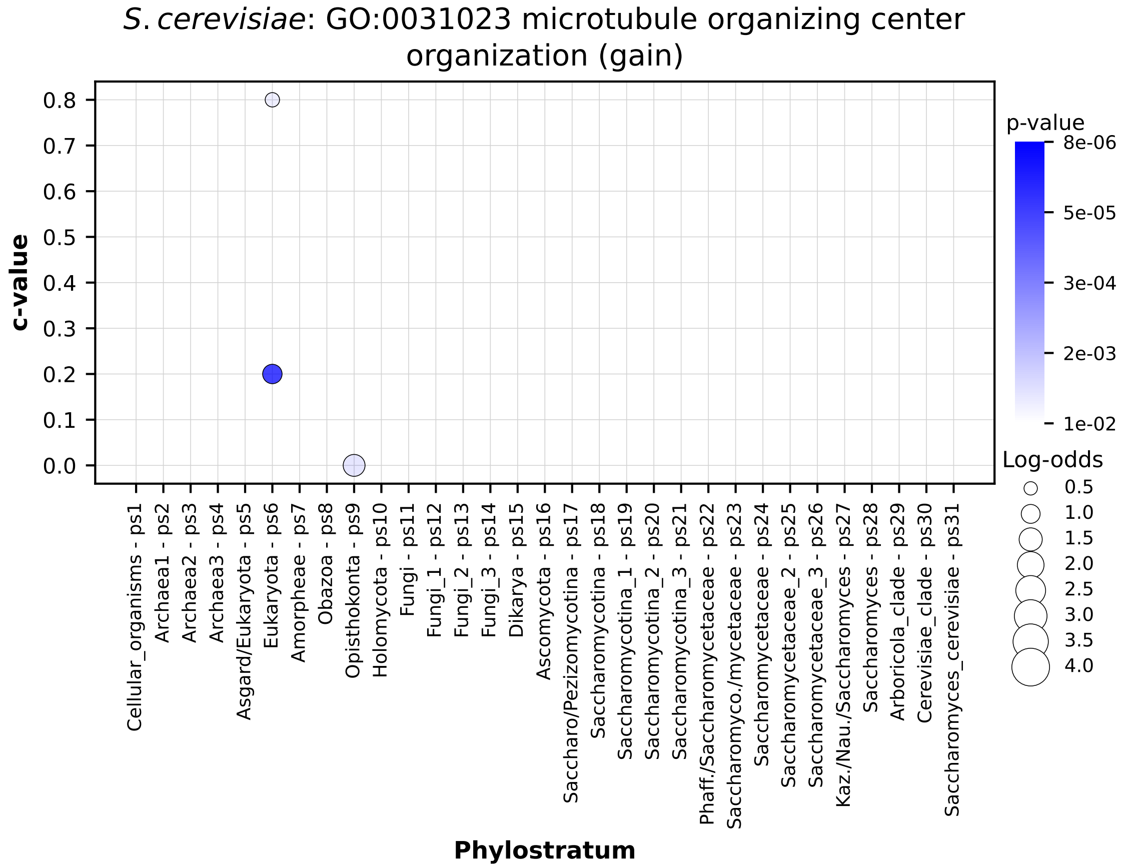

S. cerevisiae: GO:0031023 microtubule organizing center organization (gain)
c-value
0.0
0.1
0.2
0.3
0.4
0.5
0.6
0.7
0.8
p-value
8e-06
5e-05
3e-04
2e-03
1e-02
Log-odds
0.5
1.0
1.5
2.0
2.5
3.0
3.5
4.0
Cellular_organisms - ps1
Archaea1 - ps2
Archaea2 - ps3
Archaea3 - ps4
Asgard/Eukaryota - ps5
Eukaryota - ps6
Amorpheae - ps7
Obazoa - ps8
Opisthokonta - ps9
Holomycota - ps10
Fungi - ps11
Fungi_1 - ps12
Fungi_2 - ps13
Fungi_3 - ps14
Dikarya - ps15
Ascomycota - ps16
Saccharo/Pezizomycotina - ps17
Saccharomycotina - ps18
Saccharomycotina_1 - ps19
Saccharomycotina_2 - ps20
Saccharomycotina_3 - ps21
Phaff./Saccharomycetaceae - ps22
Saccharomyco./mycetaceae - ps23
Saccharomycetaceae - ps24
Saccharomycetaceae_2 - ps25
Saccharomycetaceae_3 - ps26
Kaz./Nau./Saccharomyces - ps27
Saccharomyces - ps28
Arboricola_clade - ps29
Cerevisiae_clade - ps30
Saccharomyces_cerevisiae - ps31
Phylostratum

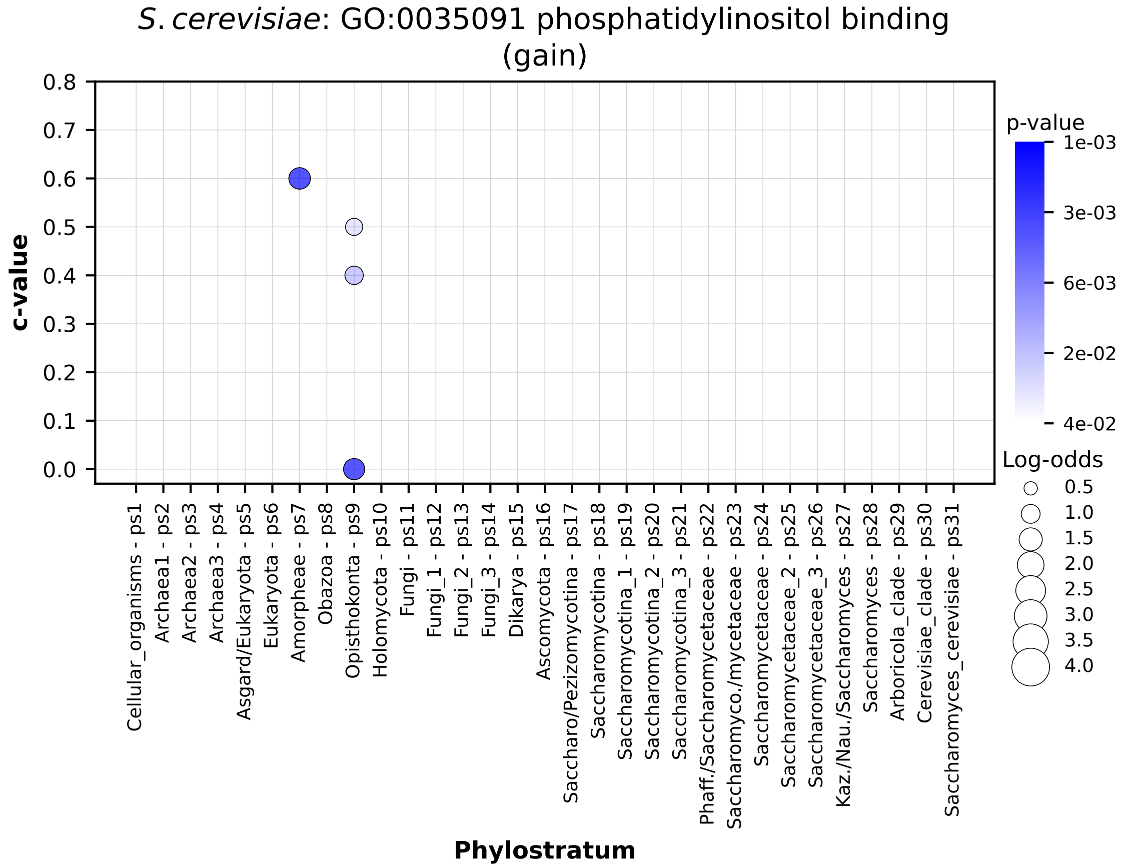

S. cerevisiae: GO:0035091 phosphatidylinositol binding
(gain)
c-value
0.8
0.7
0.6
0.5
0.4
0.3
0.2
0.1
0.0
Phylostratum
Cellular_organisms - ps1
Archaea1 - ps2
Archaea2 - ps3
Archaea3 - ps4
Asgard/Eukaryota - ps5
Eukaryota - ps6
Amorpheae - ps7
Obazoa - ps8
Opisthokonta - ps9
Holomycota - ps10
Fungi - ps11
Fungi_1 - ps12
Fungi_2 - ps13
Fungi_3 - ps14
Dikarya - ps15
Ascomycota - ps16
Saccharo/Pezizomycotina - ps17
Saccharomycotina - ps18
Saccharomycotina_1 - ps19
Saccharomycotina_2 - ps20
Saccharomycotina_3 - ps21
Phaff./Saccharomycetaceae - ps22
Saccharomyco./mycetaceae - ps23
Saccharomycetaceae - ps24
Saccharomycetaceae_2 - ps25
Saccharomycetaceae_3 - ps26
Kaz./Nau./Saccharomyces - ps27
Saccharomyces - ps28
Arboricola_clade - ps29
Cerevisiae_clade - ps30
Saccharomyces_cerevisiae - ps31
p-value
1e-03
3e-03
6e-03
2e-02
4e-02
Log-odds
0.5
1.0
1.5
2.0
2.5
3.0
3.5
4.0

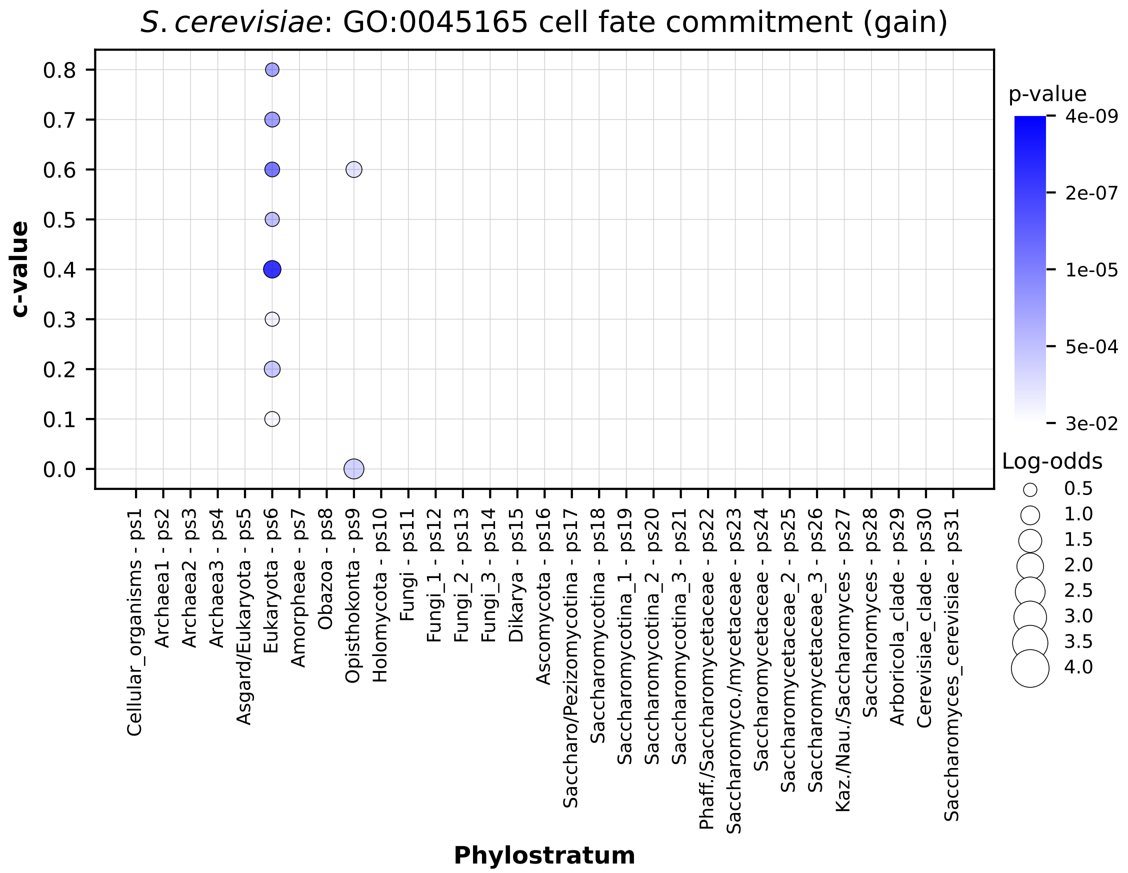
S. cerevisiae: GO:0045165 cell fate commitment (gain)
c-value
0.8
0.7
0.6
0.5
0.4
0.3
0.2
0.1
0.0
p-value
4e-09
2e-07
1e-05
5e-04
3e-02
Log-odds
0.5
1.0
1.5
2.0
2.5
3.0
3.5
4.0
Cellular_organisms - ps1
Archaea1 - ps2
Archaea2 - ps3
Archaea3 - ps4
Asgard/Eukaryota - ps5
Eukaryota - ps6
Amorpheae - ps7
Obazoa - ps8
Opisthokonta - ps9
Holomycota - ps10
Fungi - ps11
Fungi_1 - ps12
Fungi_2 - ps13
Fungi_3 - ps14
Dikarya - ps15
Ascomycota - ps16
Saccharo/Pezizomycotina - ps17
Saccharomycotina - ps18
Saccharomycotina_1 - ps19
Saccharomycotina_2 - ps20
Saccharomycotina_3 - ps21
Phaff./Saccharomycetaceae - ps22
Saccharomyco./mycetaceae - ps23
Saccharomycetaceae - ps24
Saccharomycetaceae_2 - ps25
Saccharomycetaceae_3 - ps26
Kaz./Nau./Saccharomyces - ps27
Saccharomyces - ps28
Arboricola_clade - ps29
Cerevisiae_clade - ps30
Saccharomyces_cerevisiae - ps31
Phylostratum

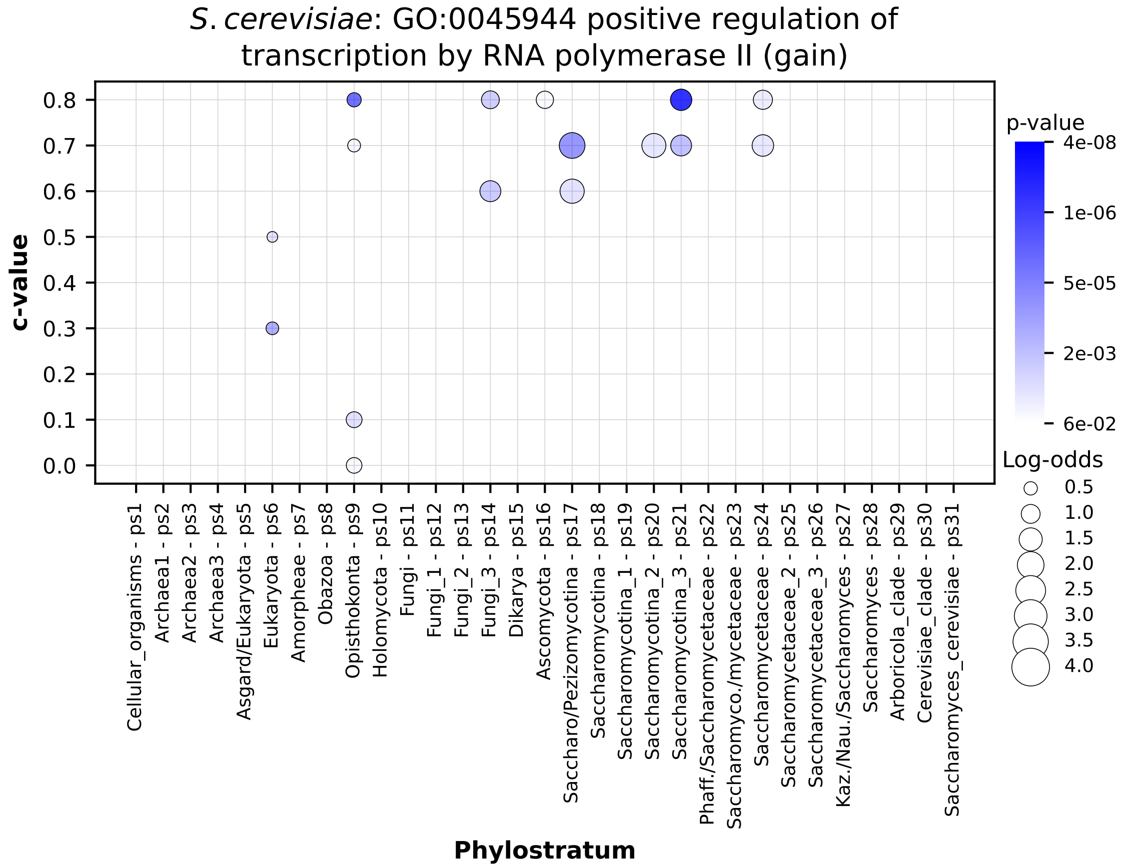

S. cerevisiae: GO:0045944 positive regulation of transcription by RNA polymerase II (gain)
c-value
0.8
0.7
0.6
0.5
0.4
0.3
0.2
0.1
0.0
p-value
4e-08
1e-06
5e-05
2e-03
6e-02
Log-odds
0.5
1.0
1.5
2.0
2.5
3.0
3.5
4.0
Cellular_organisms - ps1
Archaea1 - ps2
Archaea2 - ps3
Archaea3 - ps4
Asgard/Eukaryota - ps5
Eukaryota - ps6
Amorpheae - ps7
Obazoa - ps8
Opisthokonta - ps9
Holomycota - ps10
Fungi - ps11
Fungi_1 - ps12
Fungi_2 - ps13
Fungi_3 - ps14
Dikarya - ps15
Ascomycota - ps16
Saccharo/Pezizomycotina - ps17
Saccharomycotina - ps18
Saccharomycotina_1 - ps19
Saccharomycotina_2 - ps20
Saccharomycotina_3 - ps21
Phaff./Saccharomycetaceae - ps22
Saccharomyco./mycetaceae - ps23
Saccharomycetaceae - ps24
Saccharomycetaceae_2 - ps25
Saccharomycetaceae_3 - ps26
Kaz./Nau./Saccharomyces - ps27
Saccharomyces - ps28
Arboricola_clade - ps29
Cerevisiae_clade - ps30
Saccharomyces_cerevisiae - ps31
Phylostratum

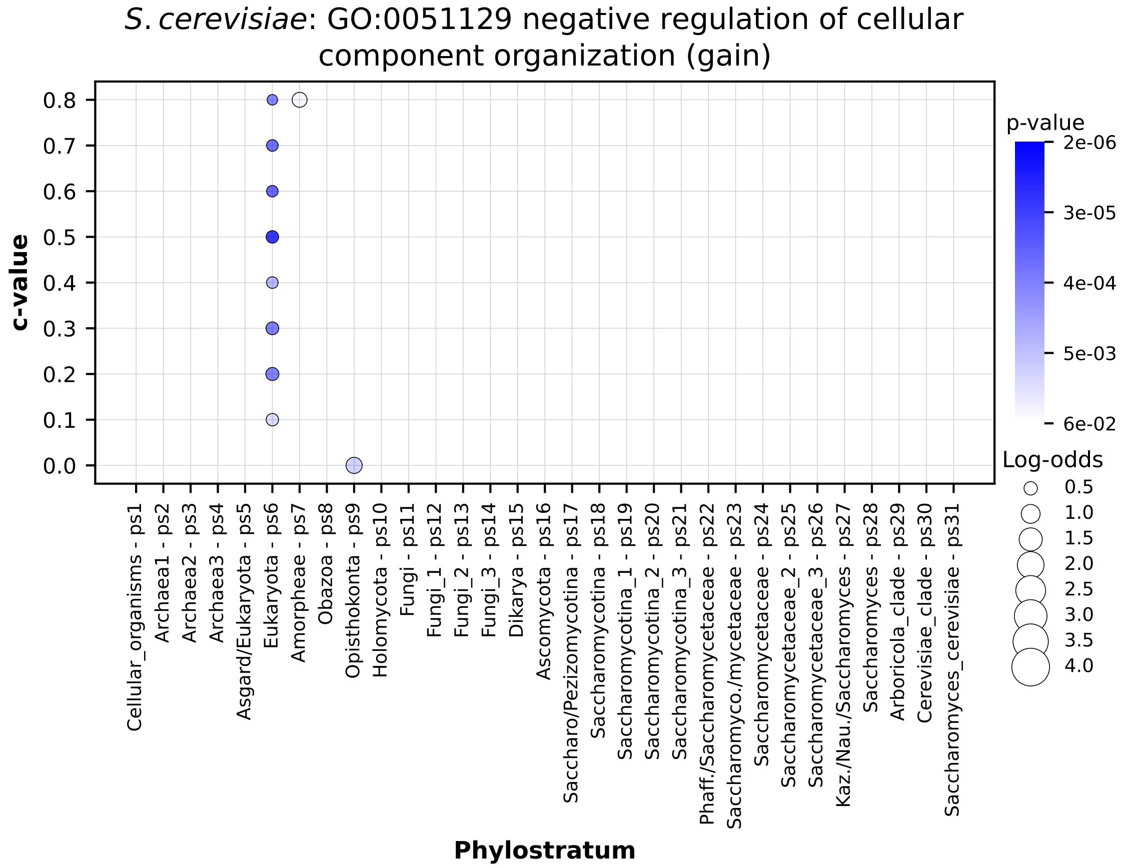

S. cerevisiae: GO:0051129 negative regulation of cellular component organization (gain)
c-value
0.8
0.7
0.6
0.5
0.4
0.3
0.2
0.1
0.0
p-value
2e-06
3e-05
4e-04
5e-03
6e-02
Log-odds
0.5
1.0
1.5
2.0
2.5
3.0
3.5
4.0
Cellular_organisms - ps1
Archaea1 - ps2
Archaea2 - ps3
Archaea3 - ps4
Asgard/Eukaryota - ps5
Eukaryota - ps6
Amorpheae - ps7
Obazoa - ps8
Opisthokonta - ps9
Holomycota - ps10
Fungi - ps11
Fungi_1 - ps12
Fungi_2 - ps13
Fungi_3 - ps14
Dikarya - ps15
Ascomycota - ps16
Saccharo/Pezizomycotina - ps17
Saccharomycotina - ps18
Saccharomycotina_1 - ps19
Saccharomycotina_2 - ps20
Saccharomycotina_3 - ps21
Phaff./Saccharomycetaceae - ps22
Saccharomyco./mycetaceae - ps23
Saccharomycetaceae - ps24
Saccharomycetaceae_2 - ps25
Saccharomycetaceae_3 - ps26
Kaz./Nau./Saccharomyces - ps27
Saccharomyces - ps28
Arboricola_clade - ps29
Cerevisiae_clade - ps30
Saccharomyces_cerevisiae - ps31
Phylostratum

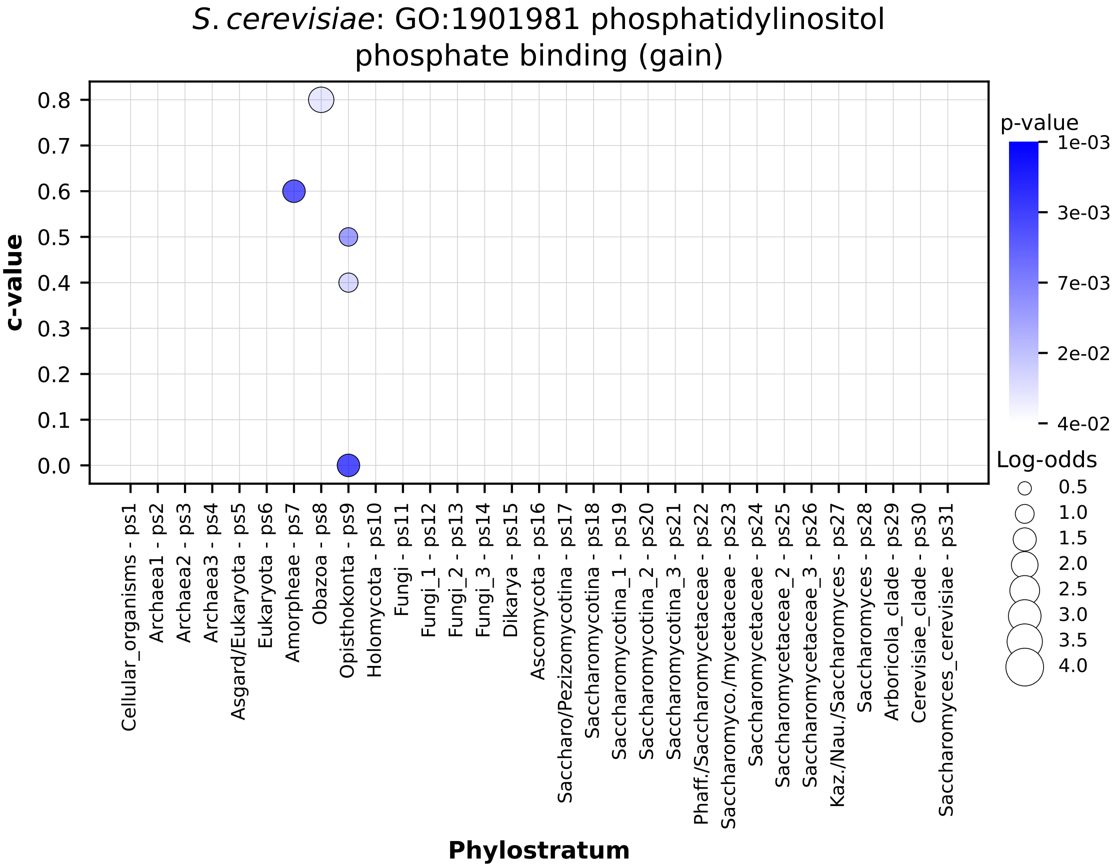

S. cerevisiae: GO:1901981 phosphatidylinositol phosphate binding (gain)
c-value
0.0
0.1
0.2
0.3
0.4
0.5
0.6
0.7
0.8
Phylostratum
Cellular_organisms - ps1
Archaea1 - ps2
Archaea2 - ps3
Archaea3 - ps4
Asgard/Eukaryota - ps5
Eukaryota - ps6
Amorpheae - ps7
Obazoa - ps8
Opisthokonta - ps9
Holomycota - ps10
Fungi - ps11
Fungi_1 - ps12
Fungi_2 - ps13
Fungi_3 - ps14
Dikarya - ps15
Ascomycota - ps16
Saccharo/Pezizomycotina - ps17
Saccharomycotina - ps18
Saccharomycotina_1 - ps19
Saccharomycotina_2 - ps20
Saccharomycotina_3 - ps21
Phaff./Saccharomycetaceae - ps22
Saccharomyco./mycetaceae - ps23
Saccharomycetaceae - ps24
Saccharomycetaceae_2 - ps25
Saccharomycetaceae_3 - ps26
Kaz./Nau./Saccharomyces - ps27
Saccharomyces - ps28
Arboricola_clade - ps29
Cerevisiae_clade - ps30
Saccharomyces_cerevisiae - ps31
p-value
1e-03
3e-03
7e-03
2e-02
4e-02
Log-odds
0.5
1.0
1.5
2.0
2.5
3.0
3.5
4.0

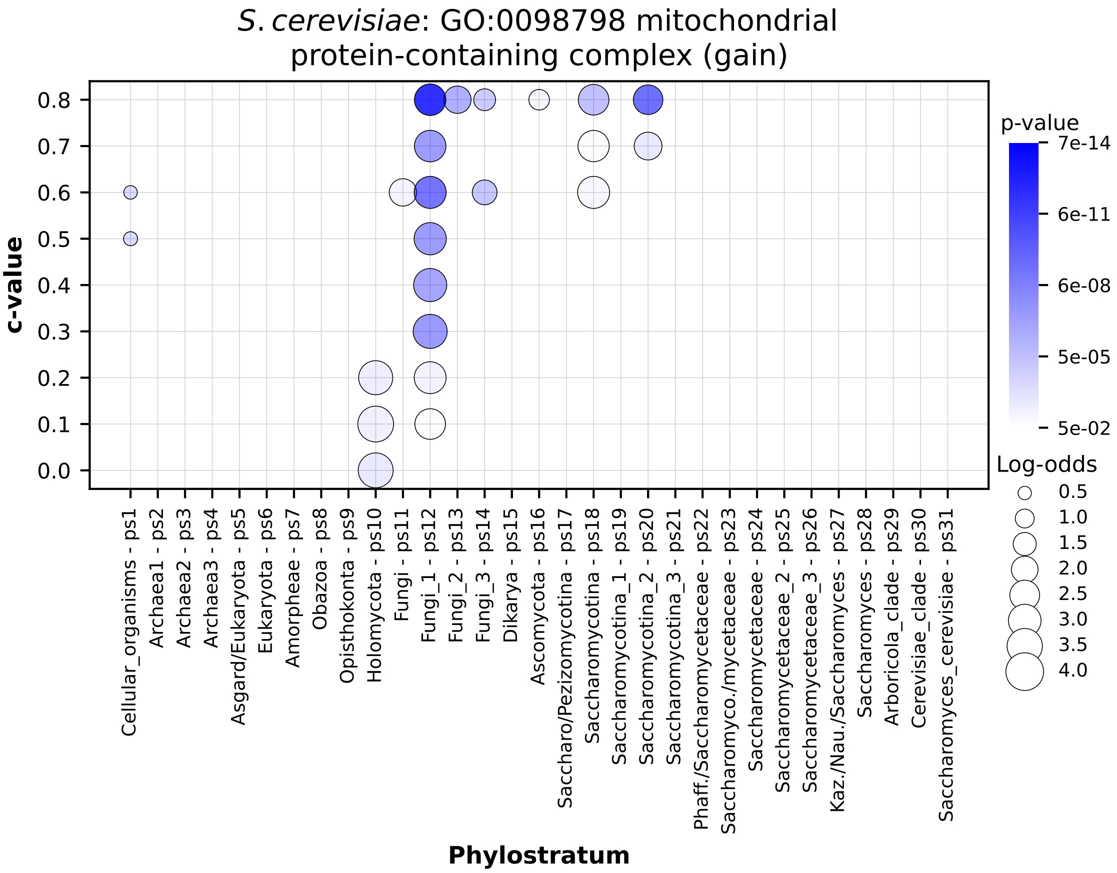

S. cerevisiae: GO:0098798 mitochondrial protein-containing complex (gain)
c-value
0.8
0.7
0.6
0.5
0.4
0.3
0.2
0.1
0.0
p-value
7e-14
6e-11
6e-08
5e-05
5e-02
Log-odds
0.5
1.0
1.5
2.0
2.5
3.0
3.5
4.0
Cellular_organisms - ps1
Archaea1 - ps2
Archaea2 - ps3
Archaea3 - ps4
Asgard/Eukaryota - ps5
Eukaryota - ps6
Amorpheae - ps7
Obazoa - ps8
Opisthokonta - ps9
Holomycota - ps10
Fungi - ps11
Fungi_1 - ps12
Fungi_2 - ps13
Fungi_3 - ps14
Dikarya - ps15
Ascomycota - ps16
Saccharo/Pezizomycotina - ps17
Saccharomycotina - ps18
Saccharomycotina_1 - ps19
Saccharomycotina_2 - ps20
Saccharomycotina_3 - ps21
Phaff./Saccharomycetaceae - ps22
Saccharomyco./mycetaceae - ps23
Saccharomycetaceae - ps24
Saccharomycetaceae_2 - ps25
Saccharomycetaceae_3 - ps26
Kaz./Nau./Saccharomyces - ps27
Saccharomyces - ps28
Arboricola_clade - ps29
Cerevisiae_clade - ps30
Saccharomyces_cerevisiae - ps31
Phylostratum

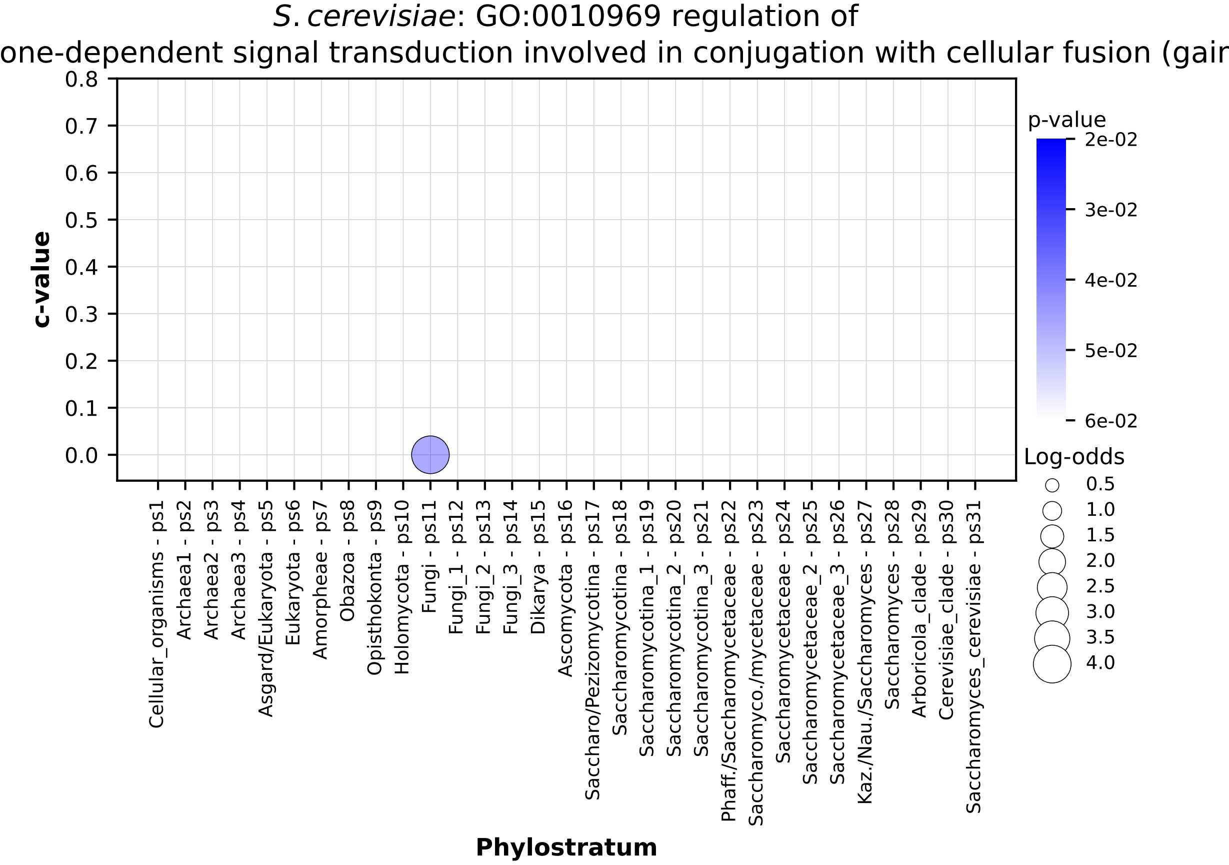

S. cerevisiae: GO:0010969 regulation of
one-dependent signal transduction involved in conjugation with cellular fusion (gair
c-value
0.8
0.7
0.6
0.5
0.4
0.3
0.2
0.1
0.0
p-value
2e-02
3e-02
4e-02
5e-02
6e-02
Log-odds
0.5
1.0
1.5
2.0
2.5
3.0
3.5
4.0
Cellular_organisms - ps1
Archaea1 - ps2
Archaea2 - ps3
Archaea3 - ps4
Asgard/Eukaryota - ps5
Eukaryota - ps6
Amorpheae - ps7
Obazoa - ps8
Opisthokonta - ps9
Holomycota - ps10
Fungi - ps11
Fungi_1 - ps12
Fungi_2 - ps13
Fungi_3 - ps14
Dikarya - ps15
Ascomycota - ps16
Saccharo/Pezizomycotina - ps17
Saccharomycotina - ps18
Saccharomycotina_1 - ps19
Saccharomycotina_2 - ps20
Saccharomycotina_3 - ps21
Phaff./Saccharomycetaceae - ps22
Saccharomyco./mycetaceae - ps23
Saccharomycetaceae - ps24
Saccharomycetaceae_2 - ps25
Saccharomycetaceae_3 - ps26
Kaz./Nau./Saccharomyces - ps27
Saccharomyces - ps28
Arboricola_clade - ps29
Cerevisiae_clade - ps30
Saccharomyces_cerevisiae - ps31
Phylostratum

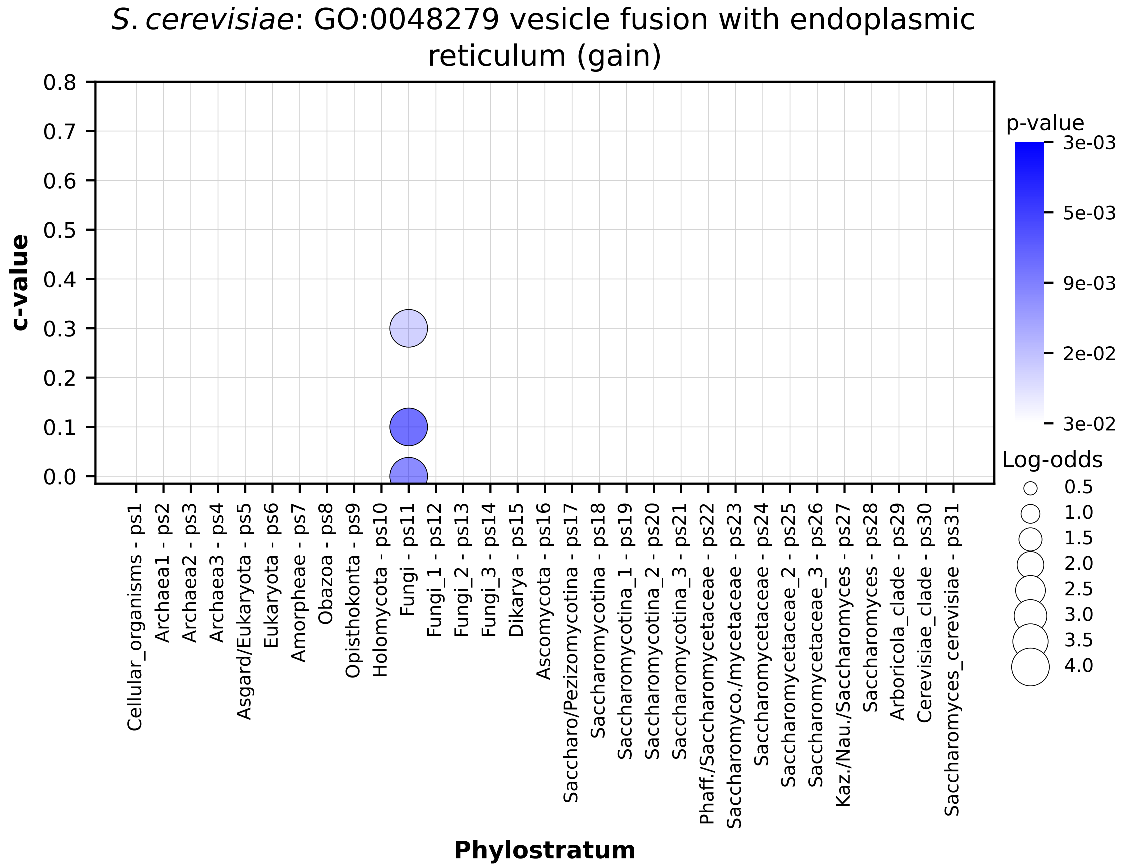

S. cerevisiae: GO:0048279 vesicle fusion with endoplasmic reticulum (gain)
c-value
0.8
0.7
0.6
0.5
0.4
0.3
0.2
0.1
0.0
p-value
3e-03
5e-03
9e-03
2e-02
3e-02
Log-odds
0.5
1.0
1.5
2.0
2.5
3.0
3.5
4.0
Cellular_organisms - ps1
Archaea1 - ps2
Archaea2 - ps3
Archaea3 - ps4
Asgard/Eukaryota - ps5
Eukaryota - ps6
Amorpheae - ps7
Obazoa - ps8
Opisthokonta - ps9
Holomycota - ps10
Fungi - ps11
Fungi_1 - ps12
Fungi_2 - ps13
Fungi_3 - ps14
Dikarya - ps15
Ascomycota - ps16
Saccharo/Pezizomycotina - ps17
Saccharomycotina - ps18
Saccharomycotina_1 - ps19
Saccharomycotina_2 - ps20
Saccharomycotina_3 - ps21
Phaff./Saccharomycetaceae - ps22
Saccharomyco./mycetaceae - ps23
Saccharomycetaceae - ps24
Saccharomycetaceae_2 - ps25
Saccharomycetaceae_3 - ps26
Kaz./Nau./Saccharomyces - ps27
Saccharomyces - ps28
Arboricola_clade - ps29
Cerevisiae_clade - ps30
Saccharomyces_cerevisiae - ps31
Phylostratum

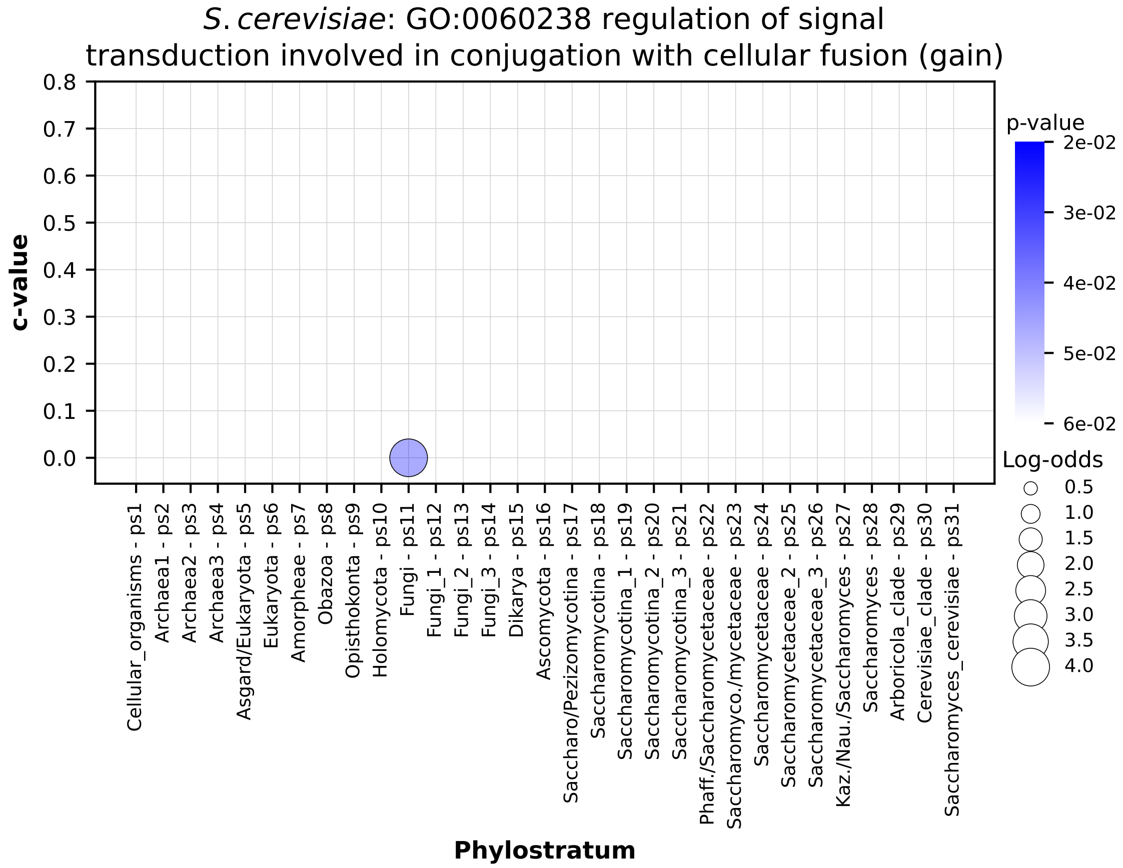

S. cerevisiae: GO:0060238 regulation of signal transduction involved in conjugation with cellular fusion (gain)
c-value
0.8
0.7
0.6
0.5
0.4
0.3
0.2
0.1
0.0
p-value
2e-02
3e-02
4e-02
5e-02
6e-02
Log-odds
0.5
1.0
1.5
2.0
2.5
3.0
3.5
4.0
Cellular_organisms - ps1
Archaea1 - ps2
Archaea2 - ps3
Archaea3 - ps4
Asgard/Eukaryota - ps5
Eukaryota - ps6
Amorpheae - ps7
Obazoa - ps8
Opisthokonta - ps9
Holomycota - ps10
Fungi - ps11
Fungi_1 - ps12
Fungi_2 - ps13
Fungi_3 - ps14
Dikarya - ps15
Ascomycota - ps16
Saccharo/Pezizomycotina - ps17
Saccharomycotina - ps18
Saccharomycotina_1 - ps19
Saccharomycotina_2 - ps20
Saccharomycotina_3 - ps21
Phaff./Saccharomycetaceae - ps22
Saccharomyco./mycetaceae - ps23
Saccharomycetaceae - ps24
Saccharomycetaceae_2 - ps25
Saccharomycetaceae_3 - ps26
Kaz./Nau./Saccharomyces - ps27
Saccharomyces - ps28
Arboricola_clade - ps29
Cerevisiae_clade - ps30
Saccharomyces_cerevisiae - ps31
Phylostratum

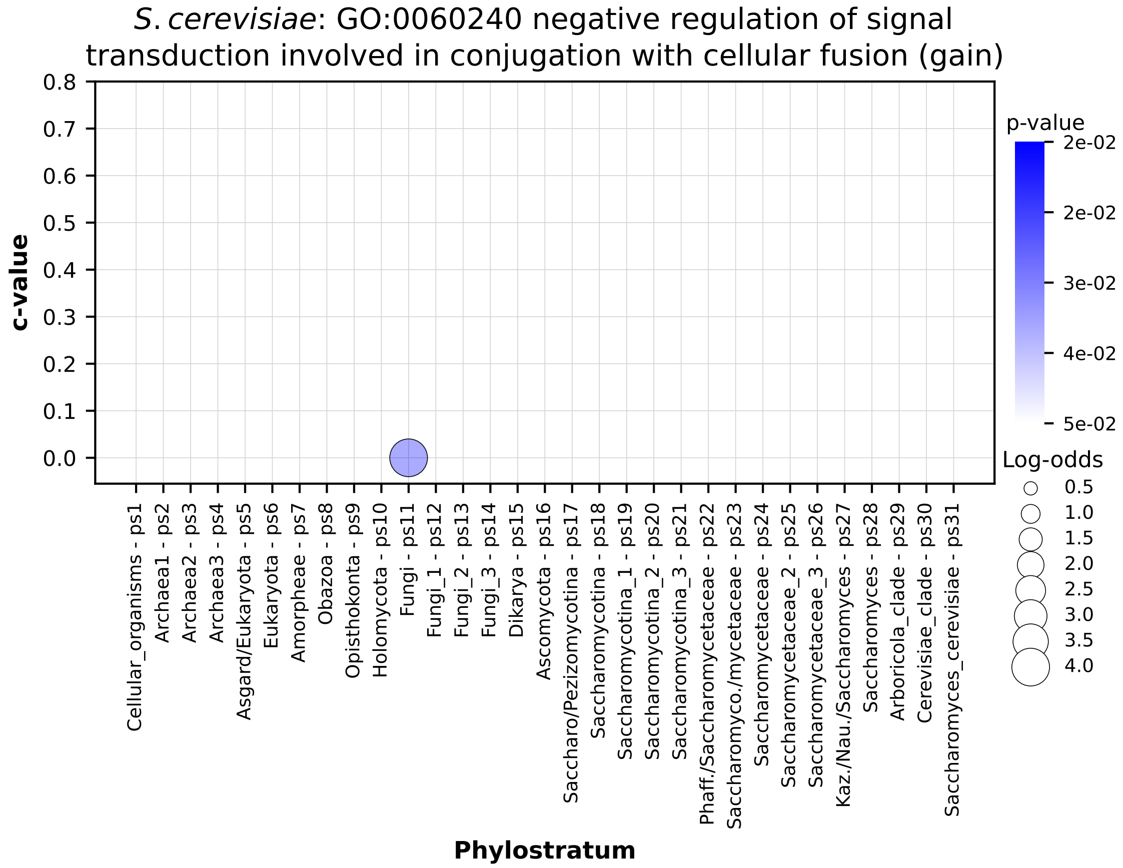
S. cerevisiae: GO:0060240 negative regulation of signal transduction involved in conjugation with cellular fusion (gain)
c-value
0.8
0.7
0.6
0.5
0.4
0.3
0.2
0.1
0.0
p-value
2e-02
2e-02
3e-02
4e-02
5e-02
Log-odds
0.5
1.0
1.5
2.0
2.5
3.0
3.5
4.0
Cellular_organisms - ps1
Archaea1 - ps2
Archaea2 - ps3
Archaea3 - ps4
Asgard/Eukaryota - ps5
Eukaryota - ps6
Amorpheae - ps7
Obazoa - ps8
Opisthokonta - ps9
Holomycota - ps10
Fungi - ps11
Fungi_1 - ps12
Fungi_2 - ps13
Fungi_3 - ps14
Dikarya - ps15
Ascomycota - ps16
Saccharo/Pezizomycotina - ps17
Saccharomycotina - ps18
Saccharomycotina_1 - ps19
Saccharomycotina_2 - ps20
Saccharomycotina_3 - ps21
Phaff./Saccharomycetaceae - ps22
Saccharomyco./mycetaceae - ps23
Saccharomycetaceae - ps24
Saccharomycetaceae_2 - ps25
Saccharomycetaceae_3 - ps26
Kaz./Nau./Saccharomyces - ps27
Saccharomyces - ps28
Arboricola_clade - ps29
Cerevisiae_clade - ps30
Saccharomyces_cerevisiae - ps31
Phylostratum

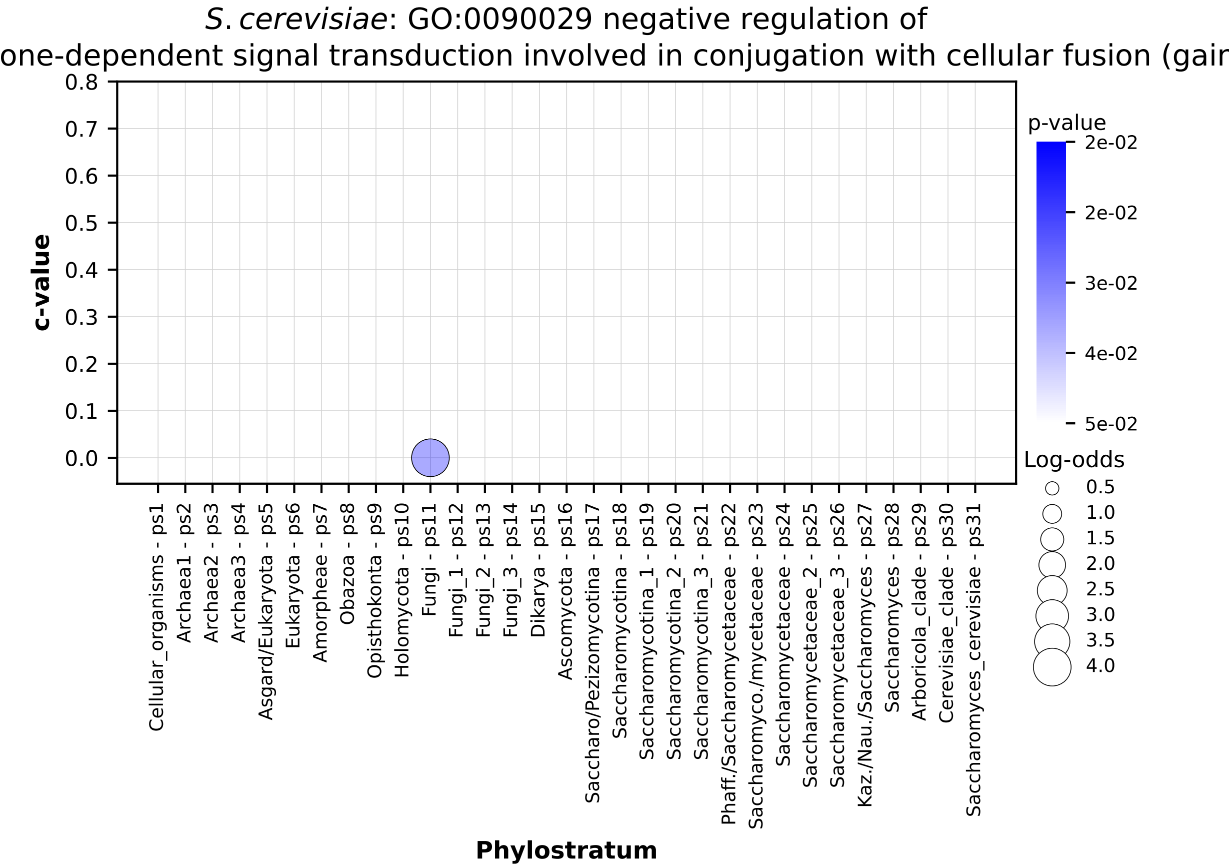

S. cerevisiae: GO:0090029 negative regulation of
one-dependent signal transduction involved in conjugation with cellular fusion (gai
c-value
0.8
0.7
0.6
0.5
0.4
0.3
0.2
0.1
0.0
p-value
2e-02
2e-02
3e-02
4e-02
5e-02
Log-odds
0.5
1.0
1.5
2.0
2.5
3.0
3.5
4.0
Cellular_organisms - ps1
Archaea1 - ps2
Archaea2 - ps3
Archaea3 - ps4
Asgard/Eukaryota - ps5
Eukaryota - ps6
Amorpheae - ps7
Obazoa - ps8
Opisthokonta - ps9
Holomycota - ps10
Fungi - ps11
Fungi_1 - ps12
Fungi_2 - ps13
Fungi_3 - ps14
Dikarya - ps15
Ascomycota - ps16
Saccharo/Pezizomycotina - ps17
Saccharomycotina - ps18
Saccharomycotina_1 - ps19
Saccharomycotina_2 - ps20
Saccharomycotina_3 - ps21
Phaff./Saccharomycetaceae - ps22
Saccharomyco./mycetaceae - ps23
Saccharomycetaceae - ps24
Saccharomycetaceae_2 - ps25
Saccharomycetaceae_3 - ps26
Kaz./Nau./Saccharomyces - ps27
Saccharomyces - ps28
Arboricola_clade - ps29
Cerevisiae_clade - ps30
Saccharomyces_cerevisiae - ps31
Phylostratum

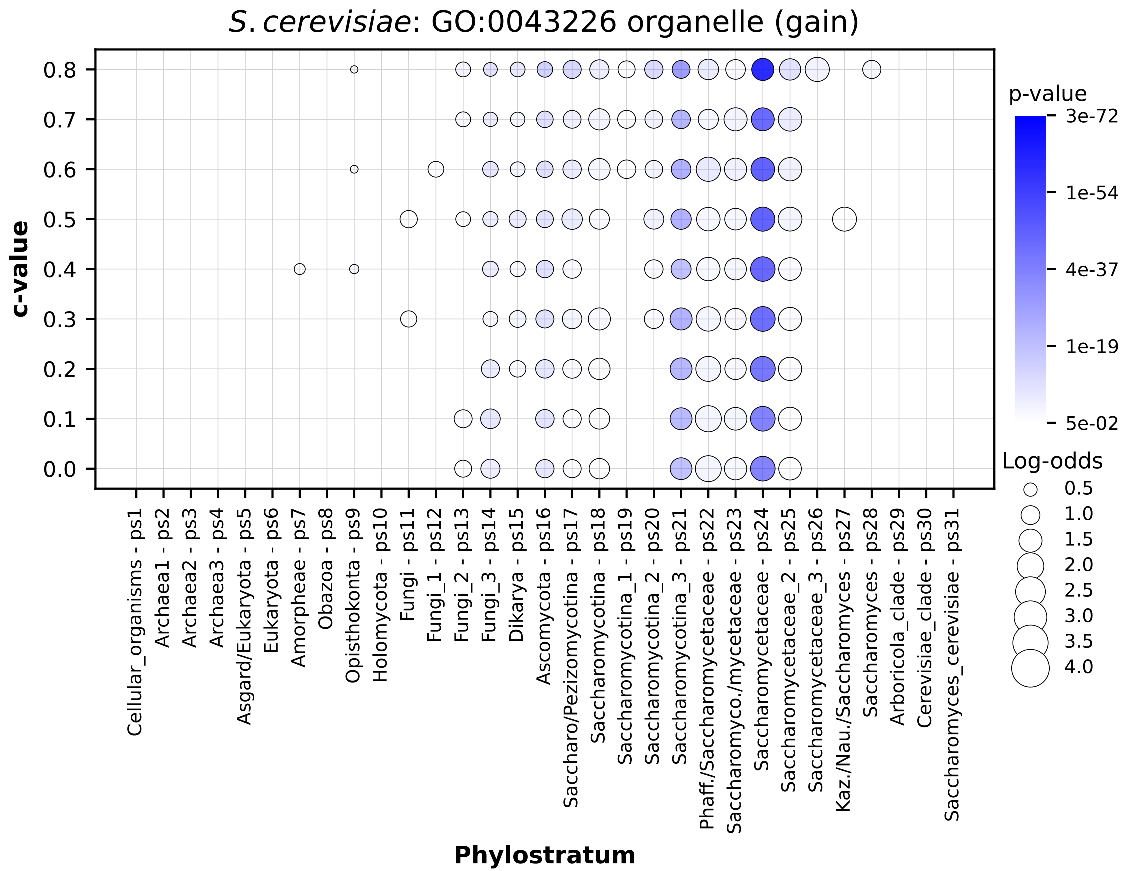

S. cerevisiae: GO:0043226 organelle (gain)
c-value
0.8
0.7
0.6
0.5
0.4
0.3
0.2
0.1
0.0
p-value
3e-72
1e-54
4e-37
1e-19
5e-02
Log-odds
0.5
1.0
1.5
2.0
2.5
3.0
3.5
4.0
Cellular_organisms - ps1
Archaea1 - ps2
Archaea2 - ps3
Archaea3 - ps4
Asgard/Eukaryota - ps5
Eukaryota - ps6
Amorpheae - ps7
Obazoa - ps8
Opisthokonta - ps9
Holomycota - ps10
Fungi - ps11
Fungi_1 - ps12
Fungi_2 - ps13
Fungi_3 - ps14
Dikarya - ps15
Ascomycota - ps16
Saccharo/Pezizomycotina - ps17
Saccharomycotina - ps18
Saccharomycotina_1 - ps19
Saccharomycotina_2 - ps20
Saccharomycotina_3 - ps21
Phaff./Saccharomycetaceae - ps22
Saccharomyco./mycetaceae - ps23
Saccharomycetaceae - ps24
Saccharomycetaceae_2 - ps25
Saccharomycetaceae_3 - ps26
Kaz./Nau./Saccharomyces - ps27
Saccharomyces - ps28
Arboricola_clade - ps29
Cerevisiae_clade - ps30
Saccharomyces_cerevisiae - ps31
Phylostratum

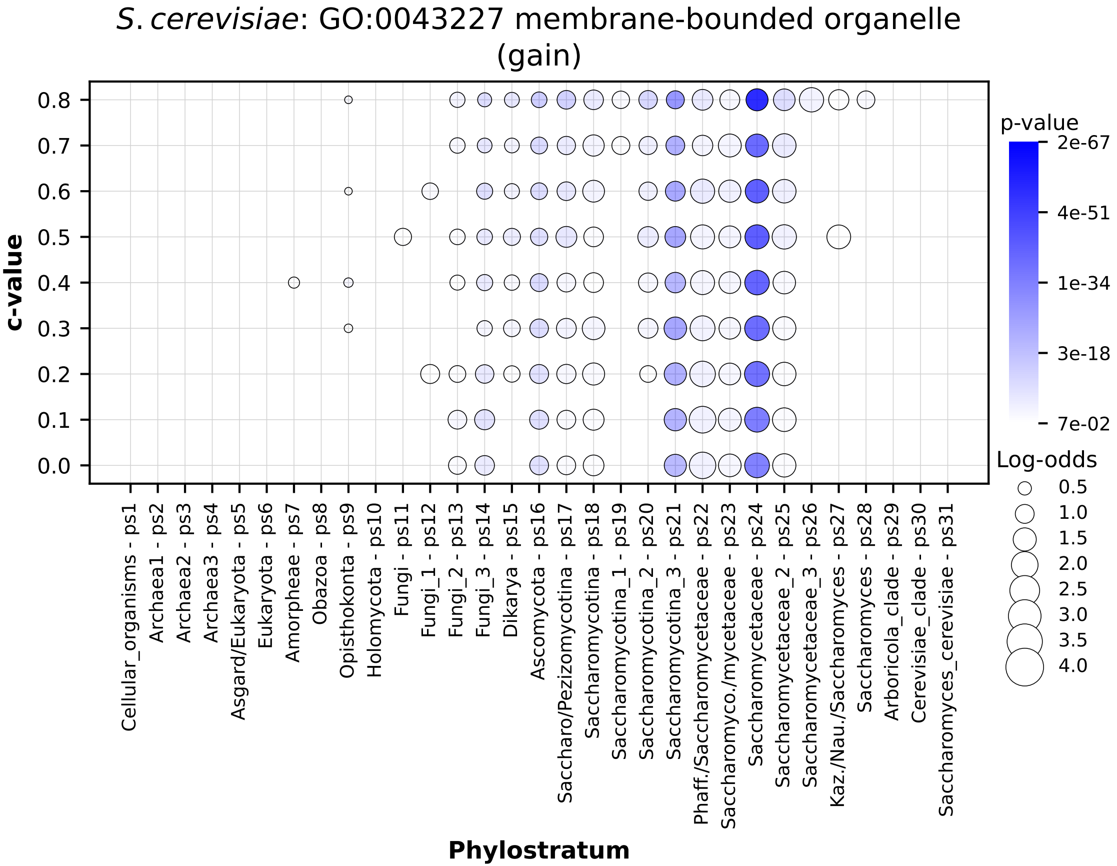
S. cerevisiae: GO:0043227 membrane-bounded organelle
(gain)
c-value
0.8
0.7
0.6
0.5
0.4
0.3
0.2
0.1
0.0
p-value
2e-67
4e-51
1e-34
3e-18
7e-02
Log-odds
0.5
1.0
1.5
2.0
2.5
3.0
3.5
4.0
Cellular_organisms - ps1
Archaea1 - ps2
Archaea2 - ps3
Archaea3 - ps4
Asgard/Eukaryota - ps5
Eukaryota - ps6
Amorpheae - ps7
Obazoa - ps8
Opisthokonta - ps9
Holomycota - ps10
Fungi - ps11
Fungi_1 - ps12
Fungi_2 - ps13
Fungi_3 - ps14
Dikarya - ps15
Ascomycota - ps16
Saccharo/Pezizomycotina - ps17
Saccharomycotina - ps18
Saccharomycotina_1 - ps19
Saccharomycotina_2 - ps20
Saccharomycotina_3 - ps21
Phaff./Saccharomycetaceae - ps22
Saccharomyco./mycetaceae - ps23
Saccharomycetaceae - ps24
Saccharomycetaceae_2 - ps25
Saccharomycetaceae_3 - ps26
Kaz./Nau./Saccharomyces - ps27
Saccharomyces - ps28
Arboricola_clade - ps29
Cerevisiae_clade - ps30
Saccharomyces_cerevisiae - ps31
Phylostratum

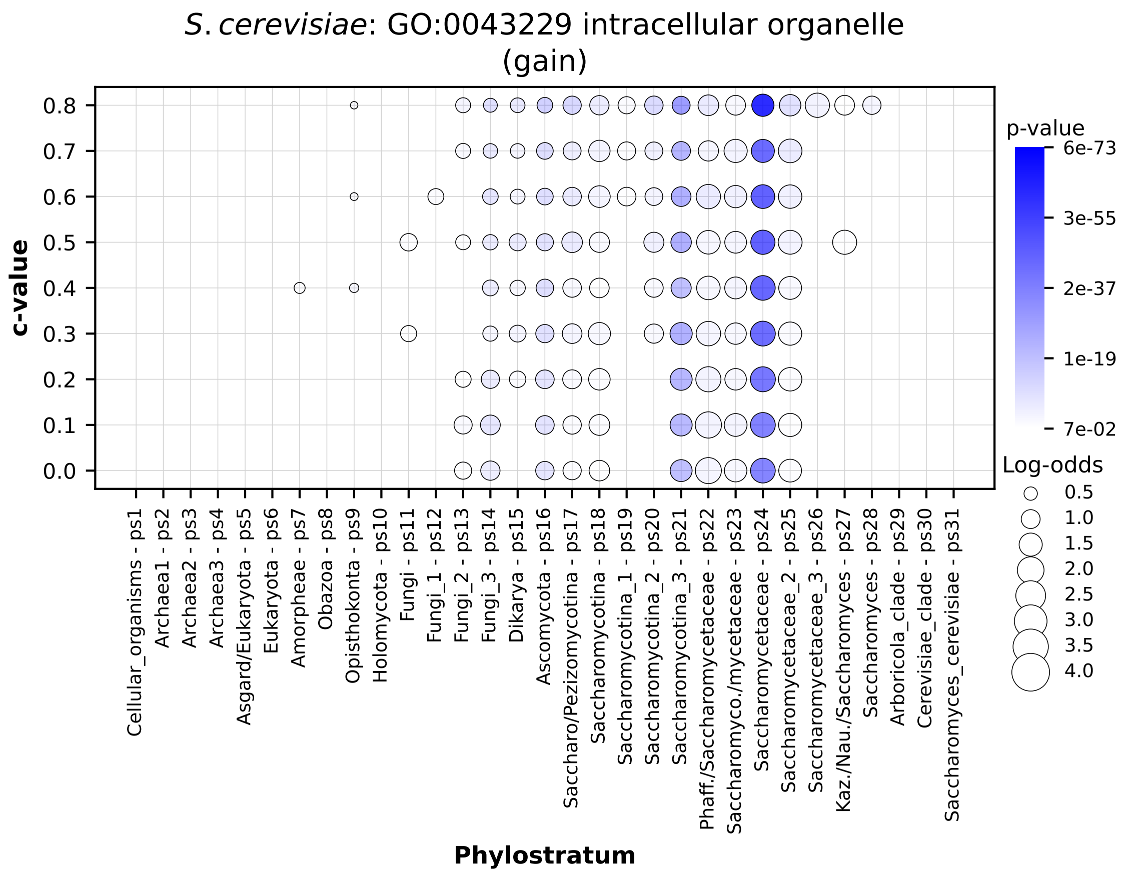
S. cerevisiae: GO:0043229 intracellular organelle (gain)
c-value
0.8
0.7
0.6
0.5
0.4
0.3
0.2
0.1
0.0
Phylostratum
Cellular_organisms - ps1
Archaea1 - ps2
Archaea2 - ps3
Archaea3 - ps4
Asgard/Eukaryota - ps5
Eukaryota - ps6
Amorpheae - ps7
Obazoa - ps8
Opisthokonta - ps9
Holomycota - ps10
Fungi - ps11
Fungi_1 - ps12
Fungi_2 - ps13
Fungi_3 - ps14
Dikarya - ps15
Ascomycota - ps16
Saccharo/Pezizomycotina - ps17
Saccharomycotina - ps18
Saccharomycotina_1 - ps19
Saccharomycotina_2 - ps20
Saccharomycotina_3 - ps21
Phaff./Saccharomycetaceae - ps22
Saccharomyco./mycetaceae - ps23
Saccharomycetaceae - ps24
Saccharomycetaceae_2 - ps25
Saccharomycetaceae_3 - ps26
Kaz./Nau./Saccharomyces - ps27
Saccharomyces - ps28
Arboricola_clade - ps29
Cerevisiae_clade - ps30
Saccharomyces_cerevisiae - ps31
p-value
6e-73
3e-55
2e-37
1e-19
7e-02
Log-odds
0.5
1.0
1.5
2.0
2.5
3.0
3.5
4.0

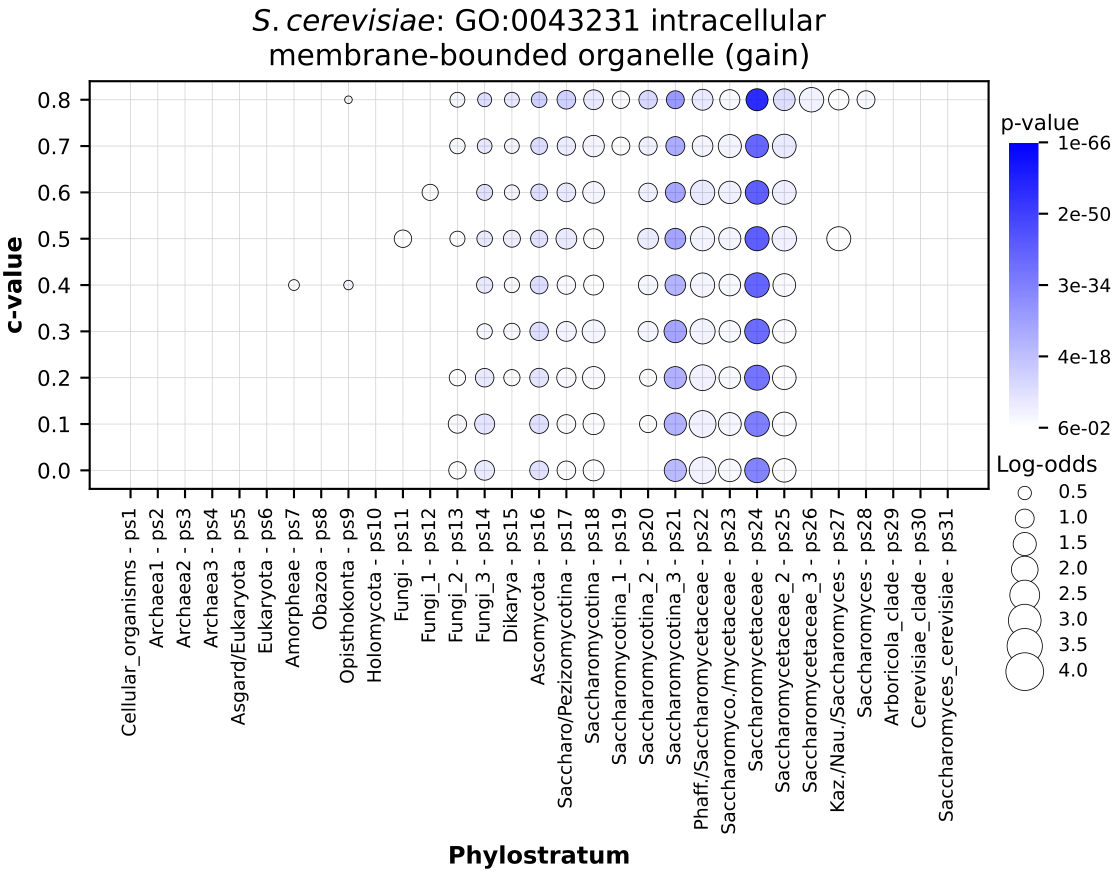

S. cerevisiae: GO:0043231 intracellular membrane-bounded organelle (gain)
c-value
0.8
0.7
0.6
0.5
0.4
0.3
0.2
0.1
0.0
p-value
1e-66
2e-50
3e-34
4e-18
6e-02
Log-odds
0.5
1.0
1.5
2.0
2.5
3.0
3.5
4.0
Cellular_organisms - ps1
Archaea1 - ps2
Archaea2 - ps3
Archaea3 - ps4
Asgard/Eukaryota - ps5
Eukaryota - ps6
Amorpheae - ps7
Obazoa - ps8
Opisthokonta - ps9
Holomycota - ps10
Fungi - ps11
Fungi_1 - ps12
Fungi_2 - ps13
Fungi_3 - ps14
Dikarya - ps15
Ascomycota - ps16
Saccharo/Pezizomycotina - ps17
Saccharomycotina - ps18
Saccharomycotina_1 - ps19
Saccharomycotina_2 - ps20
Saccharomycotina_3 - ps21
Phaff./Saccharomycetaceae - ps22
Saccharomyco./mycetaceae - ps23
Saccharomycetaceae - ps24
Saccharomycetaceae_2 - ps25
Saccharomycetaceae_3 - ps26
Kaz./Nau./Saccharomyces - ps27
Saccharomyces - ps28
Arboricola_clade - ps29
Cerevisiae_clade - ps30
Saccharomyces_cerevisiae - ps31
Phylostratum

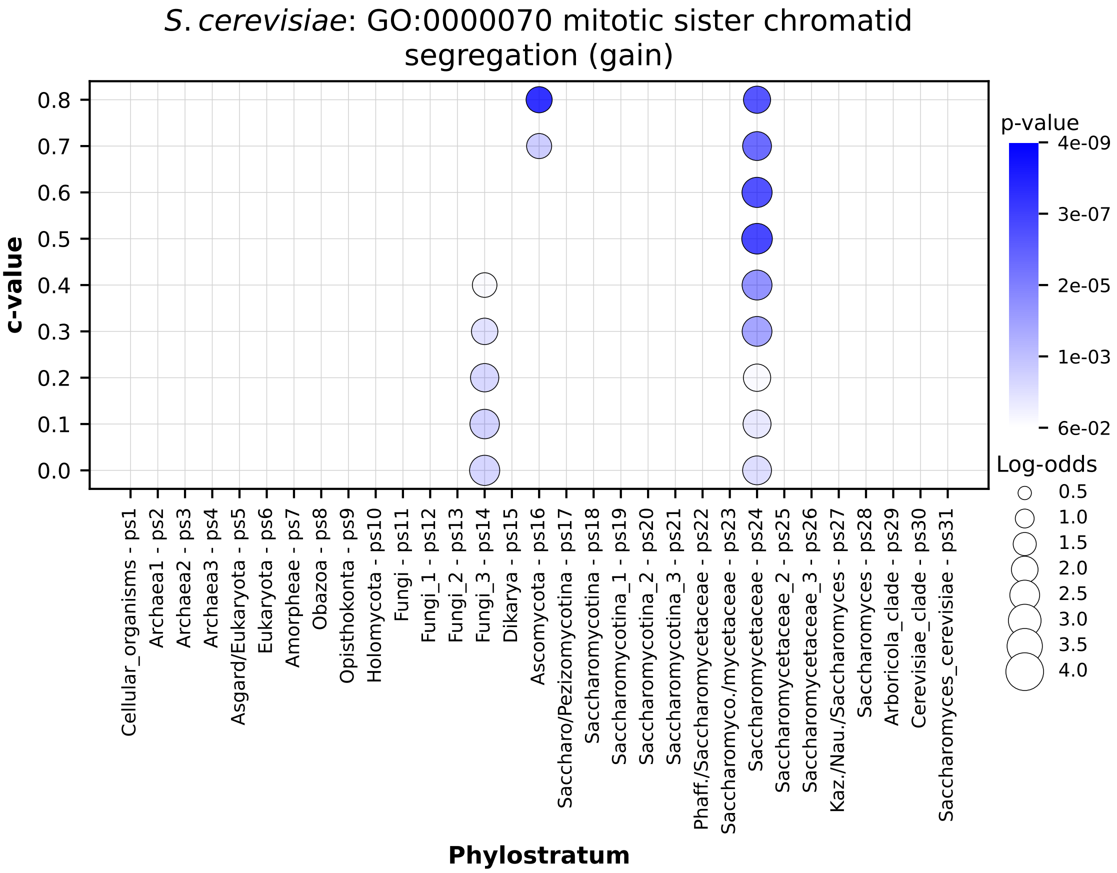

S. cerevisiae: GO:0000070 mitotic sister chromatid segregation (gain)
c-value
0.8
0.7
0.6
0.5
0.4
0.3
0.2
0.1
0.0
p-value
4e-09
3e-07
2e-05
1e-03
6e-02
Log-odds
0.5
1.0
1.5
2.0
2.5
3.0
3.5
4.0
Cellular_organisms - ps1
Archaea1 - ps2
Archaea2 - ps3
Archaea3 - ps4
Asgard/Eukaryota - ps5
Eukaryota - ps6
Amorpheae - ps7
Obazoa - ps8
Opisthokonta - ps9
Holomycota - ps10
Fungi - ps11
Fungi_1 - ps12
Fungi_2 - ps13
Fungi_3 - ps14
Dikarya - ps15
Ascomycota - ps16
Saccharo/Pezizomycotina - ps17
Saccharomycotina - ps18
Saccharomycotina_1 - ps19
Saccharomycotina_2 - ps20
Saccharomycotina_3 - ps21
Phaff./Saccharomycetaceae - ps22
Saccharomyco./mycetaceae - ps23
Saccharomycetaceae - ps24
Saccharomycetaceae_2 - ps25
Saccharomycetaceae_3 - ps26
Kaz./Nau./Saccharomyces - ps27
Saccharomyces - ps28
Arboricola_clade - ps29
Cerevisiae_clade - ps30
Saccharomyces_cerevisiae - ps31
Phylostratum

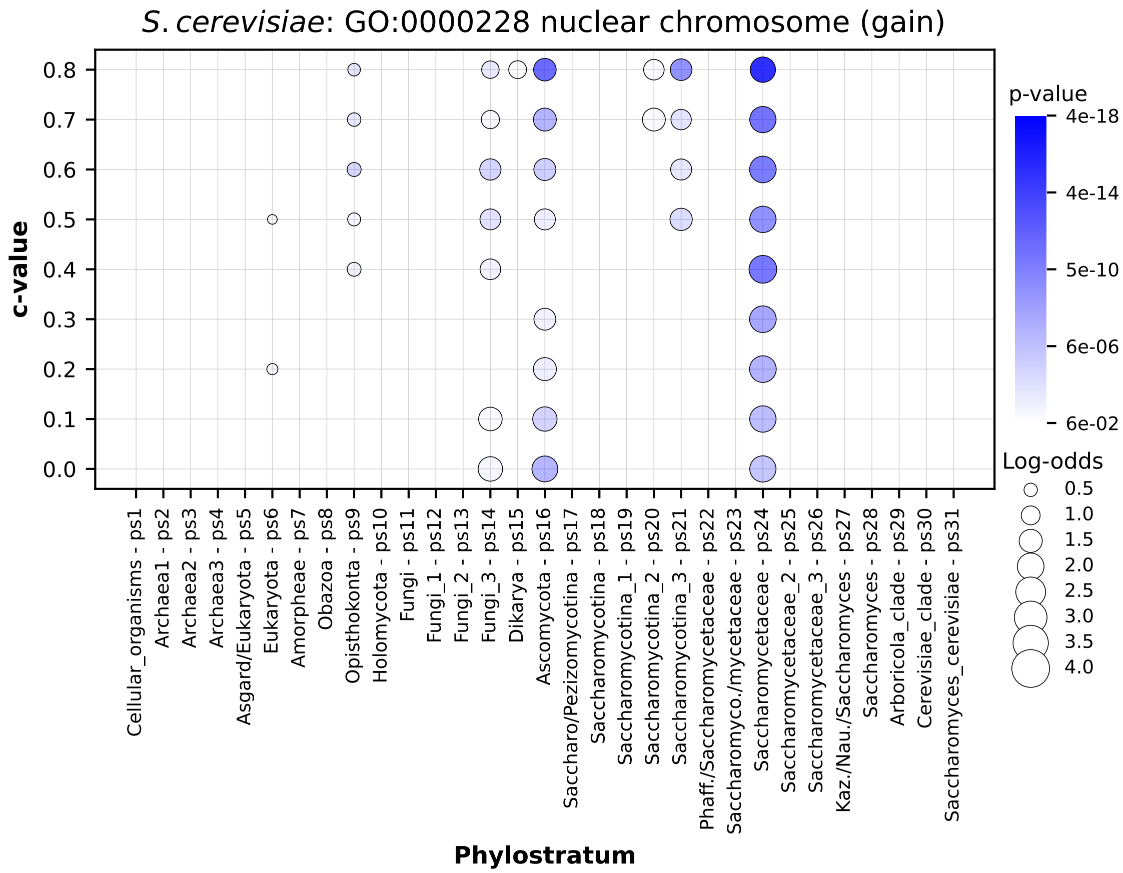
S. cerevisiae: GO:0000228 nuclear chromosome (gain)
c-value
0.8
0.7
0.6
0.5
0.4
0.3
0.2
0.1
0.0
p-value
4e-18
4e-14
5e-10
6e-06
6e-02
Log-odds
0.5
1.0
1.5
2.0
2.5
3.0
3.5
4.0
Cellular_organisms - ps1
Archaea1 - ps2
Archaea2 - ps3
Archaea3 - ps4
Asgard/Eukaryota - ps5
Eukaryota - ps6
Amorpheae - ps7
Obazoa - ps8
Opisthokonta - ps9
Holomycota - ps10
Fungi - ps11
Fungi_1 - ps12
Fungi_2 - ps13
Fungi_3 - ps14
Dikarya - ps15
Ascomycota - ps16
Saccharo/Pezizomycotina - ps17
Saccharomycotina - ps18
Saccharomycotina_1 - ps19
Saccharomycotina_2 - ps20
Saccharomycotina_3 - ps21
Phaff./Saccharomycetaceae - ps22
Saccharomyco./mycetaceae - ps23
Saccharomycetaceae - ps24
Saccharomycetaceae_2 - ps25
Saccharomycetaceae_3 - ps26
Kaz./Nau./Saccharomyces - ps27
Saccharomyces - ps28
Arboricola_clade - ps29
Cerevisiae_clade - ps30
Saccharomyces_cerevisiae - ps31
Phylostratum

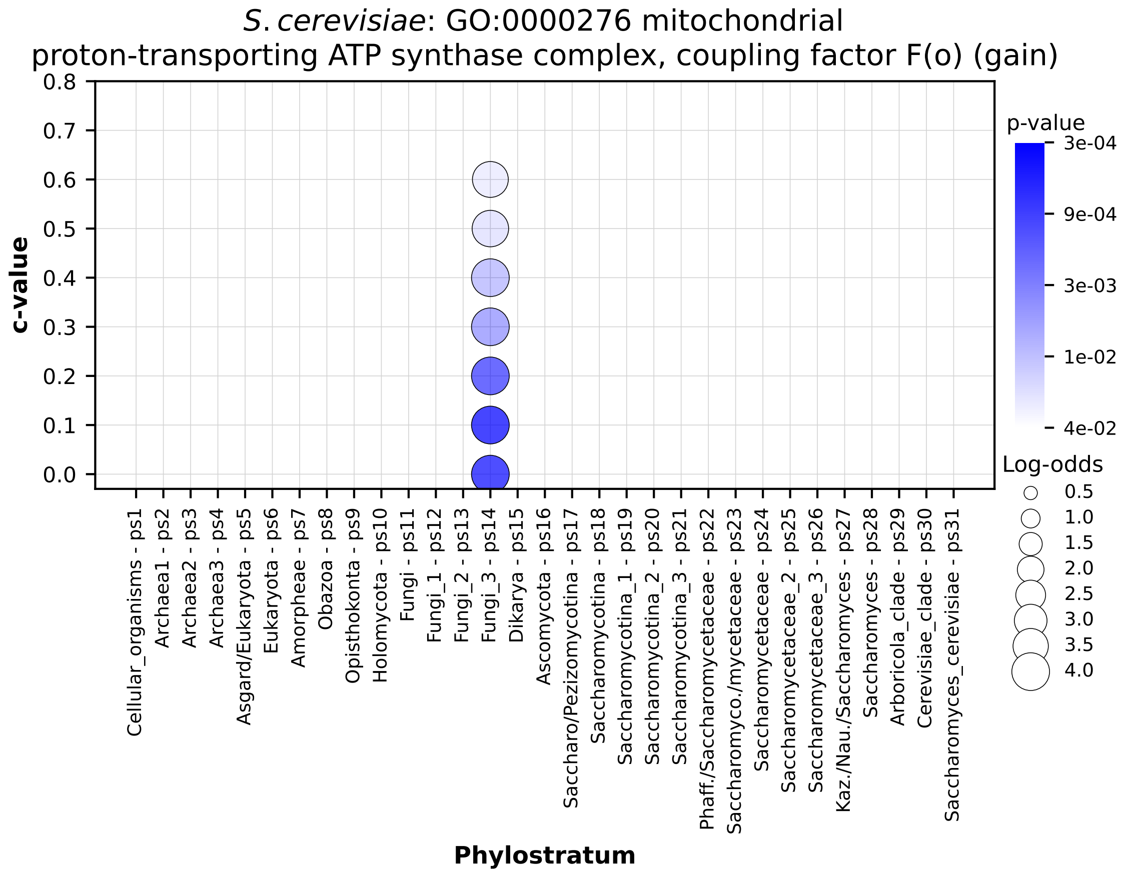

S. cerevisiae: GO:0000276 mitochondrial proton-transporting ATP synthase complex, coupling factor F(o) (gain)
c-value
0.8
0.7
0.6
0.5
0.4
0.3
0.2
0.1
0.0
p-value
3e-04
9e-04
3e-03
1e-02
4e-02
Log-odds
0.5
1.0
1.5
2.0
2.5
3.0
3.5
4.0
Cellular_organisms - ps1
Archaea1 - ps2
Archaea2 - ps3
Archaea3 - ps4
Asgard/Eukaryota - ps5
Eukaryota - ps6
Amorpheae - ps7
Obazoa - ps8
Opisthokonta - ps9
Holomycota - ps10
Fungi - ps11
Fungi_1 - ps12
Fungi_2 - ps13
Fungi_3 - ps14
Dikarya - ps15
Ascomycota - ps16
Saccharo/Pezizomycotina - ps17
Saccharomycotina - ps18
Saccharomycotina_1 - ps19
Saccharomycotina_2 - ps20
Saccharomycotina_3 - ps21
Phaff./Saccharomycetaceae - ps22
Saccharomyco./mycetaceae - ps23
Saccharomycetaceae - ps24
Saccharomycetaceae_2 - ps25
Saccharomycetaceae_3 - ps26
Kaz./Nau./Saccharomyces - ps27
Saccharomyces - ps28
Arboricola_clade - ps29
Cerevisiae_clade - ps30
Saccharomyces_cerevisiae - ps31
Phylostratum

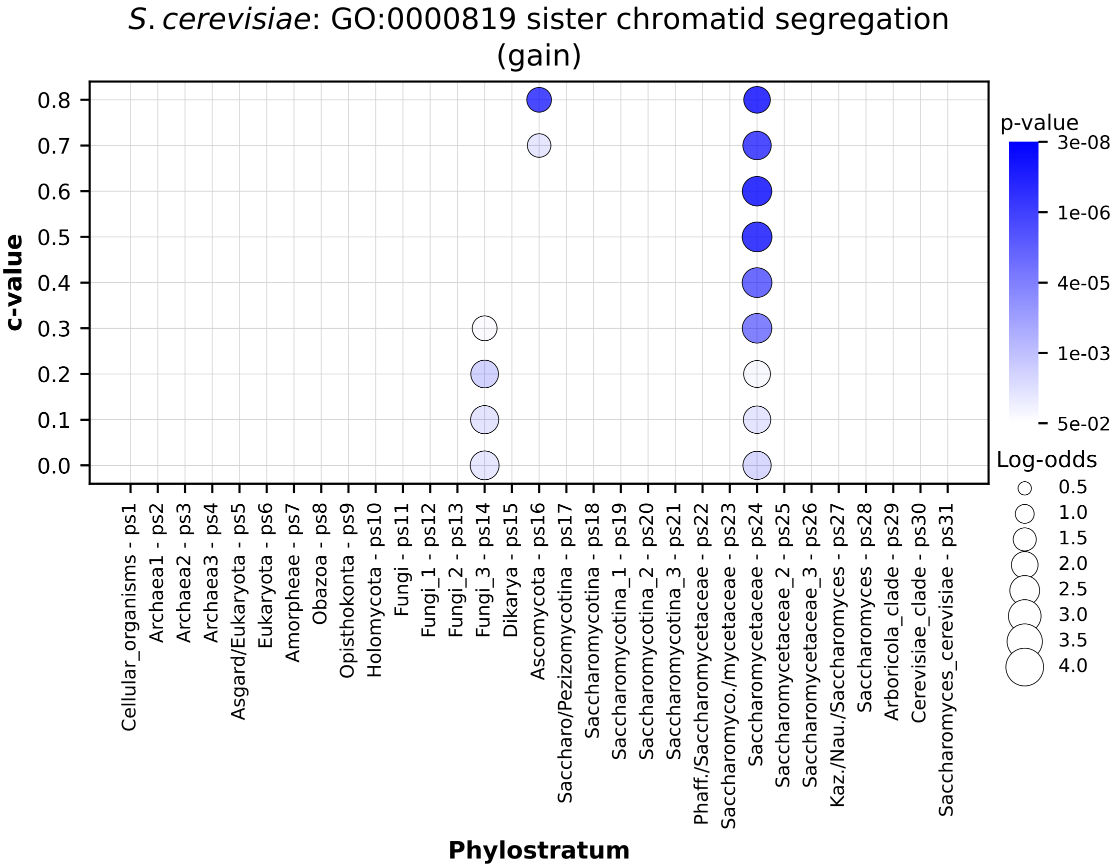

S. cerevisiae: GO:0000819 sister chromatid segregation
(gain)
c-value
0.8
0.7
0.6
0.5
0.4
0.3
0.2
0.1
0.0
Phylostratum
Cellular_organisms - ps1
Archaea1 - ps2
Archaea2 - ps3
Archaea3 - ps4
Asgard/Eukaryota - ps5
Eukaryota - ps6
Amorpheae - ps7
Obazoa - ps8
Opisthokonta - ps9
Holomycota - ps10
Fungi - ps11
Fungi_1 - ps12
Fungi_2 - ps13
Fungi_3 - ps14
Dikarya - ps15
Ascomycota - ps16
Saccharo/Pezizomycotina - ps17
Saccharomycotina - ps18
Saccharomycotina_1 - ps19
Saccharomycotina_2 - ps20
Saccharomycotina_3 - ps21
Phaff./Saccharomycetaceae - ps22
Saccharomyco./mycetaceae - ps23
Saccharomycetaceae - ps24
Saccharomycetaceae_2 - ps25
Saccharomycetaceae_3 - ps26
Kaz./Nau./Saccharomyces - ps27
Saccharomyces - ps28
Arboricola_clade - ps29
Cerevisiae_clade - ps30
Saccharomyces_cerevisiae - ps31
p-value
3e-08
1e-06
4e-05
1e-03
5e-02
Log-odds
0.5
1.0
1.5
2.0
2.5
3.0
3.5
4.0

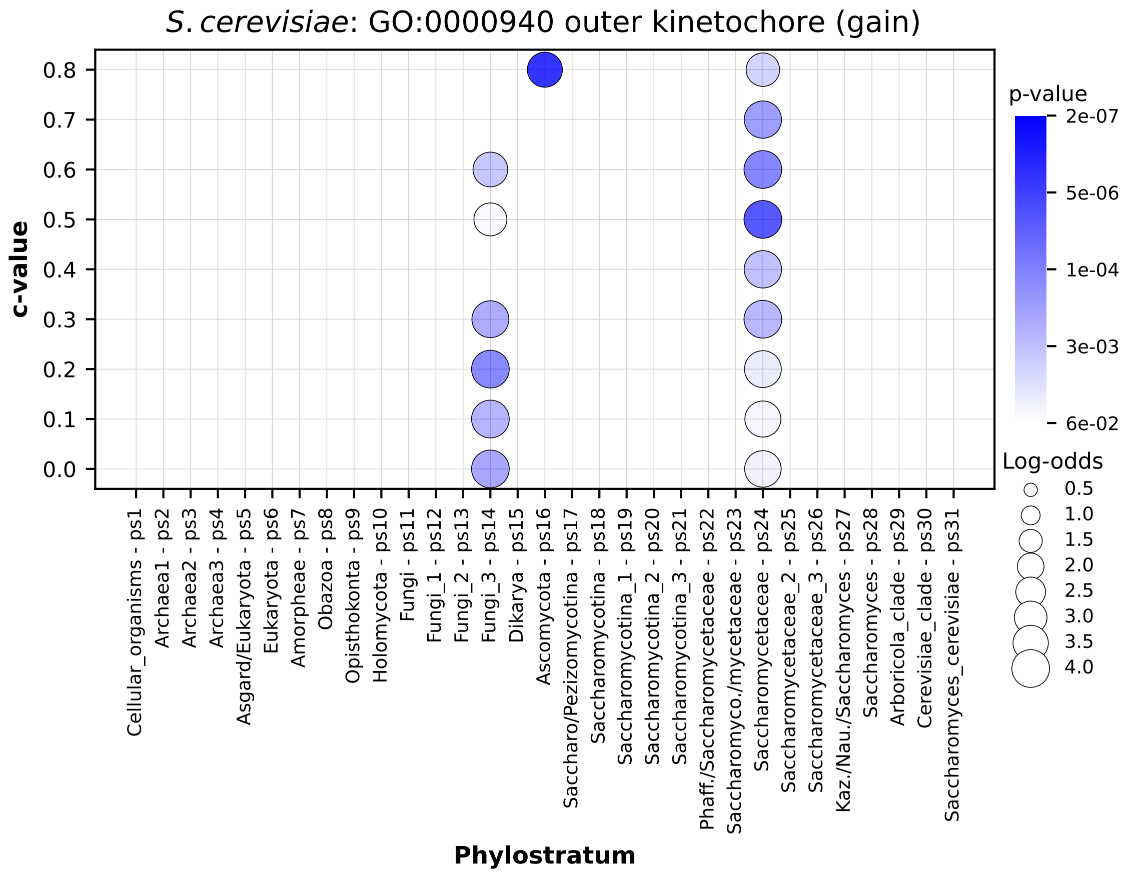

S. cerevisiae: GO:0000940 outer kinetochore (gain)
c-value
0.8
0.7
0.6
0.5
0.4
0.3
0.2
0.1
0.0
p-value
2e-07
5e-06
1e-04
3e-03
6e-02
Log-odds
0.5
1.0
1.5
2.0
2.5
3.0
3.5
4.0
Cellular_organisms - ps1
Archaea1 - ps2
Archaea2 - ps3
Archaea3 - ps4
Asgard/Eukaryota - ps5
Eukaryota - ps6
Amorpheae - ps7
Obazoa - ps8
Opisthokonta - ps9
Holomycota - ps10
Fungi - ps11
Fungi_1 - ps12
Fungi_2 - ps13
Fungi_3 - ps14
Dikarya - ps15
Ascomycota - ps16
Saccharo/Pezizomycotina - ps17
Saccharomycotina - ps18
Saccharomycotina_1 - ps19
Saccharomycotina_2 - ps20
Saccharomycotina_3 - ps21
Phaff./Saccharomycetaceae - ps22
Saccharomyco./mycetaceae - ps23
Saccharomycetaceae - ps24
Saccharomycetaceae_2 - ps25
Saccharomycetaceae_3 - ps26
Kaz./Nau./Saccharomyces - ps27
Saccharomyces - ps28
Arboricola_clade - ps29
Cerevisiae_clade - ps30
Saccharomyces_cerevisiae - ps31
Phylostratum

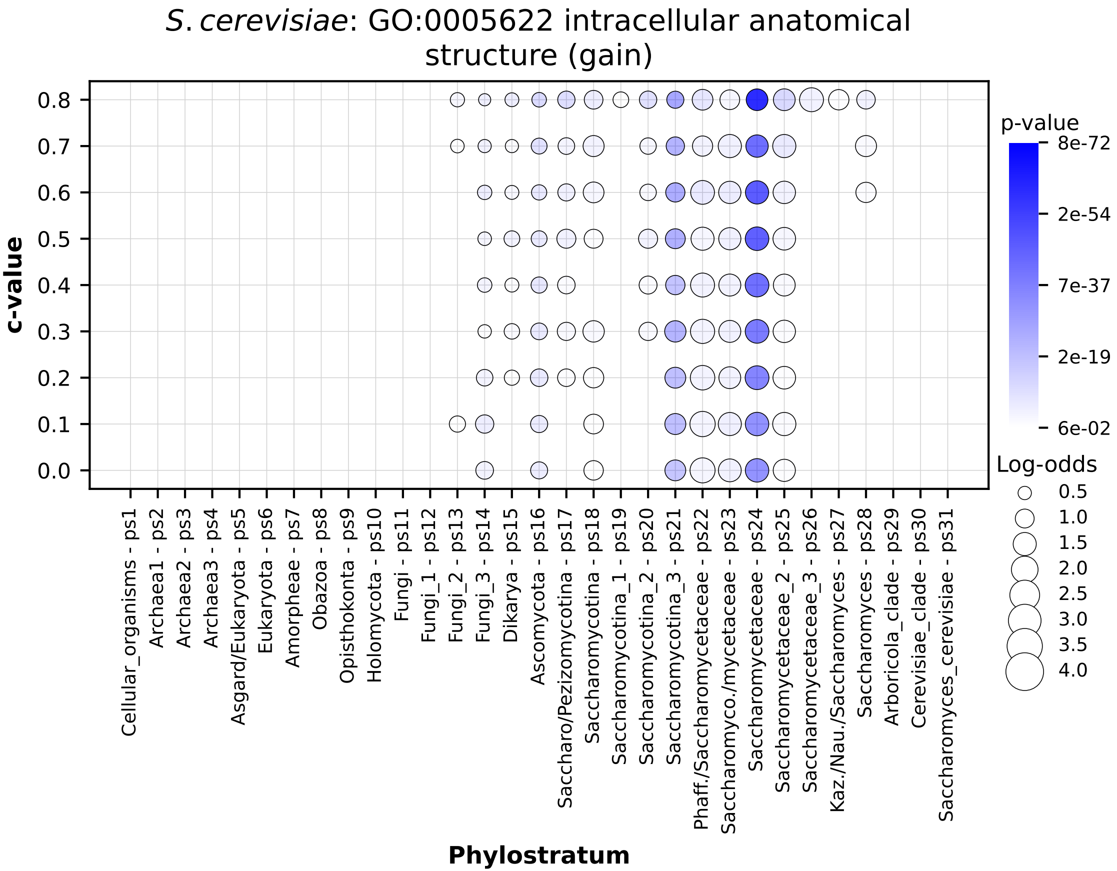
S. cerevisiae: GO:0005622 intracellular anatomical structure (gain)
c-value
0.8
0.7
0.6
0.5
0.4
0.3
0.2
0.1
0.0
p-value
8e-72
2e-54
7e-37
2e-19
6e-02
Log-odds
0.5
1.0
1.5
2.0
2.5
3.0
3.5
4.0
Cellular_organisms - ps1
Archaea1 - ps2
Archaea2 - ps3
Archaea3 - ps4
Asgard/Eukaryota - ps5
Eukaryota - ps6
Amorpheae - ps7
Obazoa - ps8
Opisthokonta - ps9
Holomycota - ps10
Fungi - ps11
Fungi_1 - ps12
Fungi_2 - ps13
Fungi_3 - ps14
Dikarya - ps15
Ascomycota - ps16
Saccharo/Pezizomycotina - ps17
Saccharomycotina - ps18
Saccharomycotina_1 - ps19
Saccharomycotina_2 - ps20
Saccharomycotina_3 - ps21
Phaff./Saccharomycetaceae - ps22
Saccharomyco./mycetaceae - ps23
Saccharomycetaceae - ps24
Saccharomycetaceae_2 - ps25
Saccharomycetaceae_3 - ps26
Kaz./Nau./Saccharomyces - ps27
Saccharomyces - ps28
Arboricola_clade - ps29
Cerevisiae_clade - ps30
Saccharomyces_cerevisiae - ps31
Phylostratum

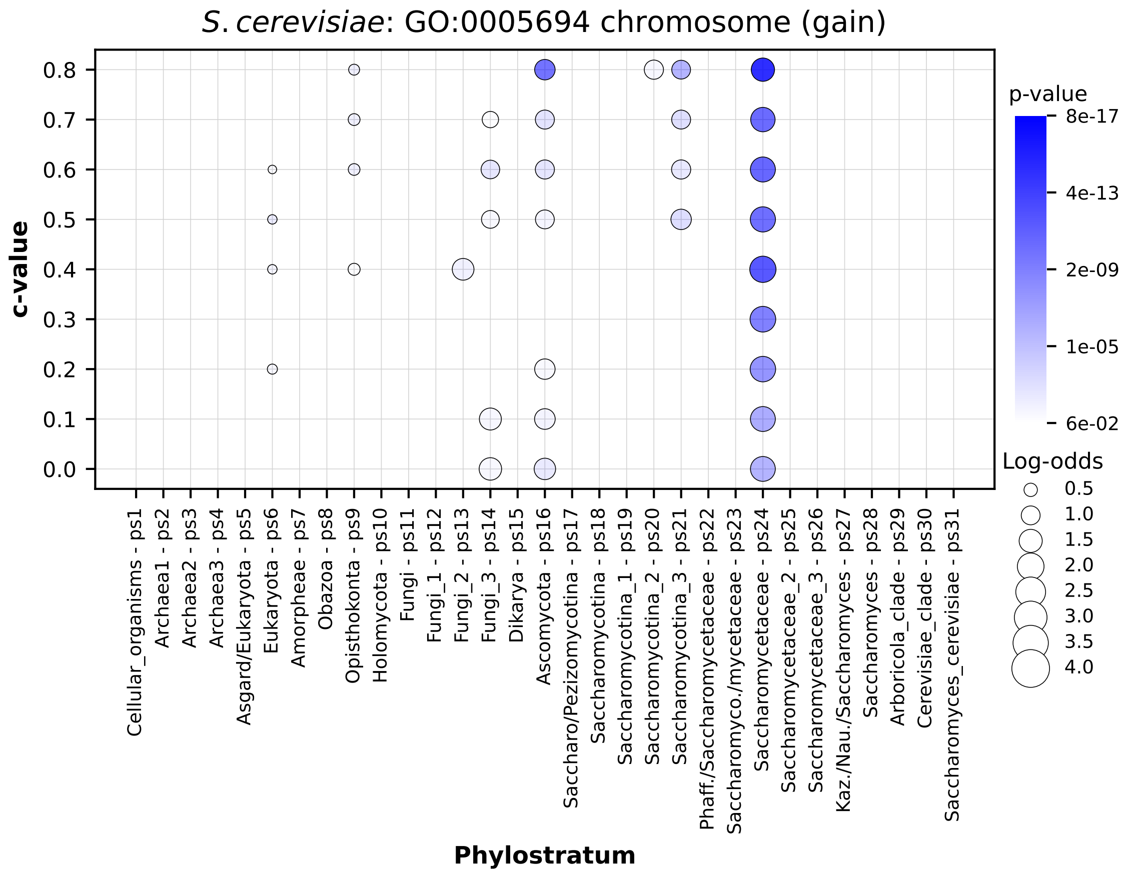

S. cerevisiae: GO:0005694 chromosome (gain)
c-value
0.8
0.7
0.6
0.5
0.4
0.3
0.2
0.1
0.0
p-value
8e-17
4e-13
2e-09
1e-05
6e-02
Log-odds
0.5
1.0
1.5
2.0
2.5
3.0
3.5
4.0
Cellular_organisms - ps1
Archaea1 - ps2
Archaea2 - ps3
Archaea3 - ps4
Asgard/Eukaryota - ps5
Eukaryota - ps6
Amorpheae - ps7
Obazoa - ps8
Opisthokonta - ps9
Holomycota - ps10
Fungi - ps11
Fungi_1 - ps12
Fungi_2 - ps13
Fungi_3 - ps14
Dikarya - ps15
Ascomycota - ps16
Saccharo/Pezizomycotina - ps17
Saccharomycotina - ps18
Saccharomycotina_1 - ps19
Saccharomycotina_2 - ps20
Saccharomycotina_3 - ps21
Phaff./Saccharomycetaceae - ps22
Saccharomyco./mycetaceae - ps23
Saccharomycetaceae - ps24
Saccharomycetaceae_2 - ps25
Saccharomycetaceae_3 - ps26
Kaz./Nau./Saccharomyces - ps27
Saccharomyces - ps28
Arboricola_clade - ps29
Cerevisiae_clade - ps30
Saccharomyces_cerevisiae - ps31
Phylostratum

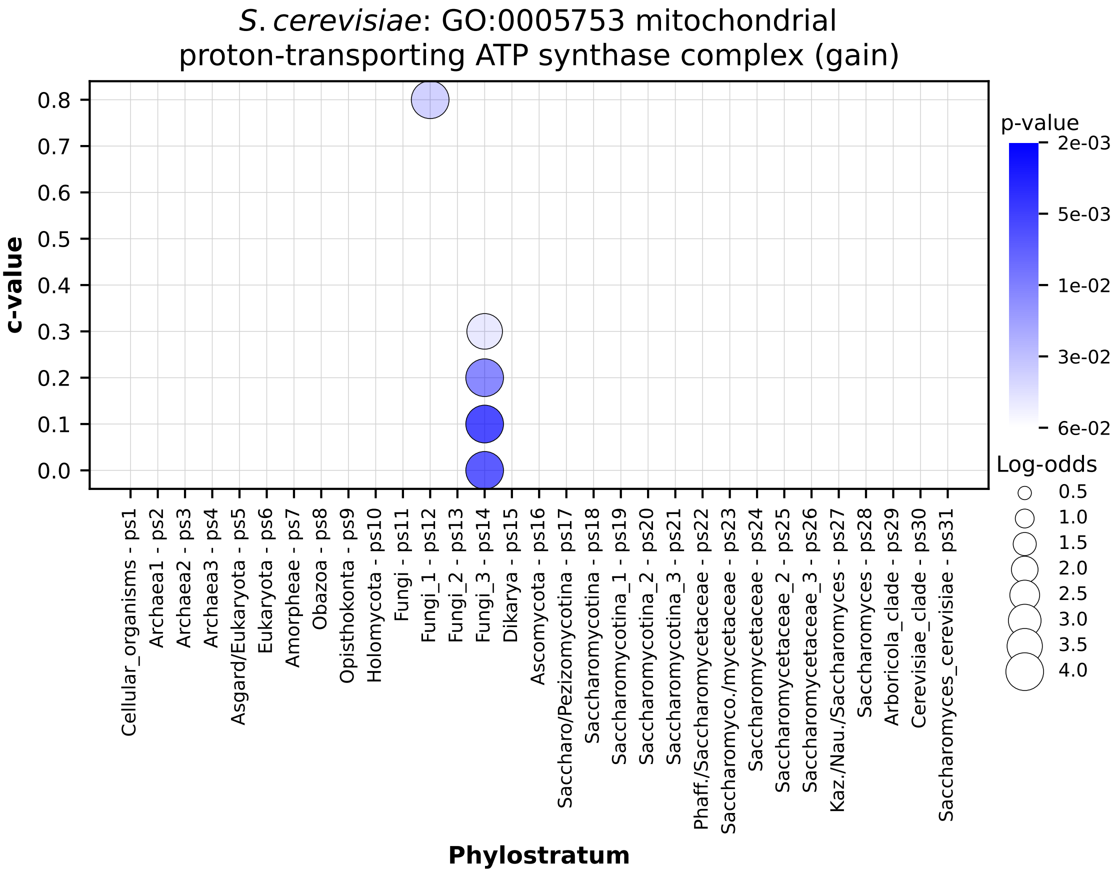

S. cerevisiae: GO:0005753 mitochondrial proton-transporting ATP synthase complex (gain)
c-value
0.8
0.7
0.6
0.5
0.4
0.3
0.2
0.1
0.0
p-value
2e-03
5e-03
1e-02
3e-02
6e-02
Log-odds
0.5
1.0
1.5
2.0
2.5
3.0
3.5
4.0
Cellular_organisms - ps1
Archaea1 - ps2
Archaea2 - ps3
Archaea3 - ps4
Asgard/Eukaryota - ps5
Eukaryota - ps6
Amorpheae - ps7
Obazoa - ps8
Opisthokonta - ps9
Holomycota - ps10
Fungi - ps11
Fungi_1 - ps12
Fungi_2 - ps13
Fungi_3 - ps14
Dikarya - ps15
Ascomycota - ps16
Saccharo/Pezizomycotina - ps17
Saccharomycotina - ps18
Saccharomycotina_1 - ps19
Saccharomycotina_2 - ps20
Saccharomycotina_3 - ps21
Phaff./Saccharomycetaceae - ps22
Saccharomyco./mycetaceae - ps23
Saccharomycetaceae - ps24
Saccharomycetaceae_2 - ps25
Saccharomycetaceae_3 - ps26
Kaz./Nau./Saccharomyces - ps27
Saccharomyces - ps28
Arboricola_clade - ps29
Cerevisiae_clade - ps30
Saccharomyces_cerevisiae - ps31
Phylostratum

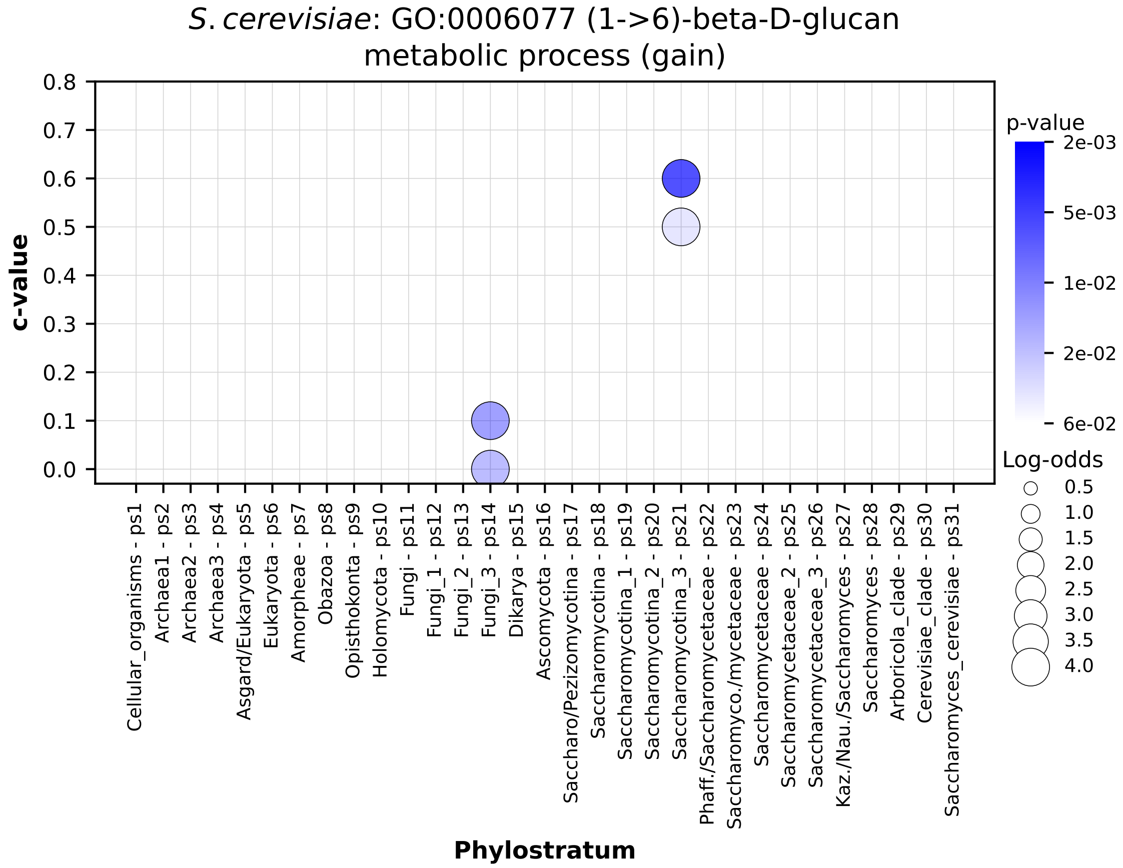

S. cerevisiae: GO:0006077 (1->6)-beta-D-glucan metabolic process (gain)
c-value
0.8
0.7
0.6
0.5
0.4
0.3
0.2
0.1
0.0
p-value
2e-03
5e-03
1e-02
2e-02
6e-02
Log-odds
0.5
1.0
1.5
2.0
2.5
3.0
3.5
4.0
Cellular_organisms - ps1
Archaea1 - ps2
Archaea2 - ps3
Archaea3 - ps4
Asgard/Eukaryota - ps5
Eukaryota - ps6
Amorpheae - ps7
Obazoa - ps8
Opisthokonta - ps9
Holomycota - ps10
Fungi - ps11
Fungi_1 - ps12
Fungi_2 - ps13
Fungi_3 - ps14
Dikarya - ps15
Ascomycota - ps16
Saccharo/Pezizomycotina - ps17
Saccharomycotina - ps18
Saccharomycotina_1 - ps19
Saccharomycotina_2 - ps20
Saccharomycotina_3 - ps21
Phaff./Saccharomycetaceae - ps22
Saccharomyco./mycetaceae - ps23
Saccharomycetaceae - ps24
Saccharomycetaceae_2 - ps25
Saccharomycetaceae_3 - ps26
Kaz./Nau./Saccharomyces - ps27
Saccharomyces - ps28
Arboricola_clade - ps29
Cerevisiae_clade - ps30
Saccharomyces_cerevisiae - ps31
Phylostratum

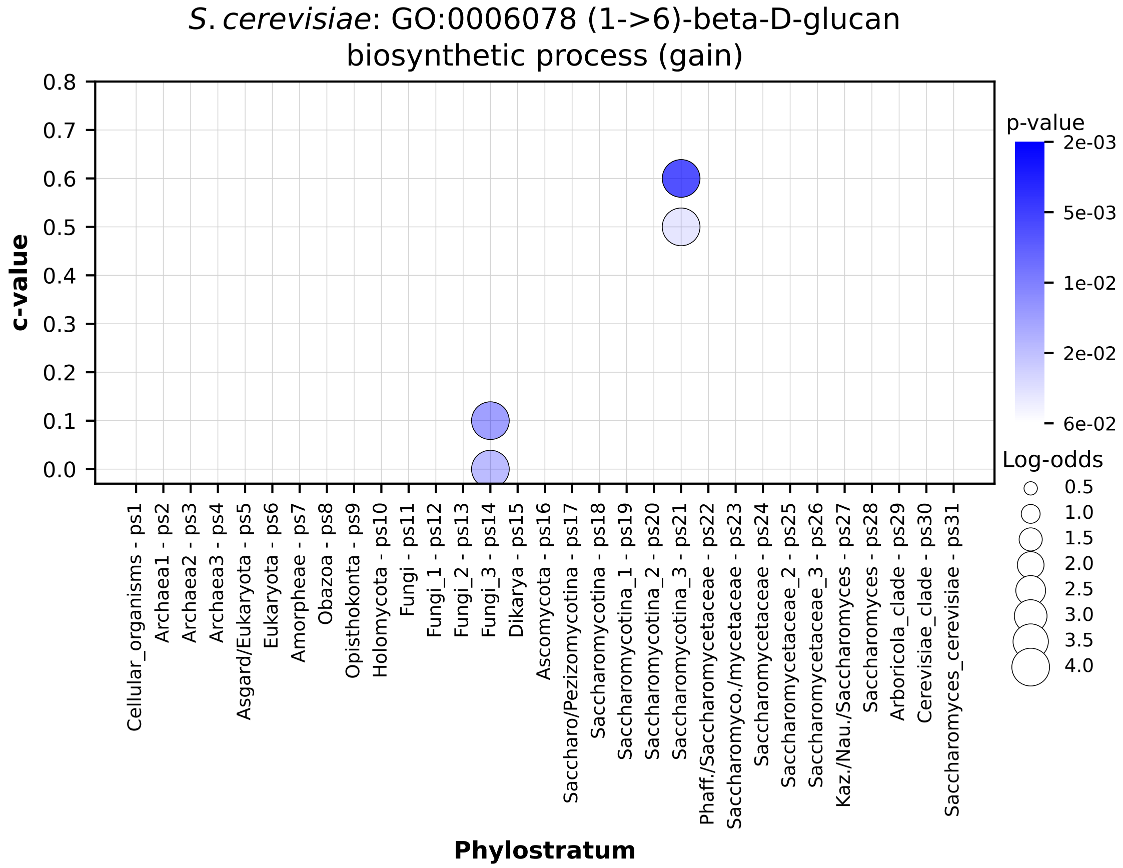

S. cerevisiae: GO:0006078 (1->6)-beta-D-glucan biosynthetic process (gain)
c-value
0.8
0.7
0.6
0.5
0.4
0.3
0.2
0.1
0.0
p-value
2e-03
5e-03
1e-02
2e-02
6e-02
Log-odds
0.5
1.0
1.5
2.0
2.5
3.0
3.5
4.0
Cellular_organisms - ps1
Archaea1 - ps2
Archaea2 - ps3
Archaea3 - ps4
Asgard/Eukaryota - ps5
Eukaryota - ps6
Amorpheae - ps7
Obazoa - ps8
Opisthokonta - ps9
Holomycota - ps10
Fungi - ps11
Fungi_1 - ps12
Fungi_2 - ps13
Fungi_3 - ps14
Dikarya - ps15
Ascomycota - ps16
Saccharo/Pezizomycotina - ps17
Saccharomycotina - ps18
Saccharomycotina_1 - ps19
Saccharomycotina_2 - ps20
Saccharomycotina_3 - ps21
Phaff./Saccharomycetaceae - ps22
Saccharomyco./mycetaceae - ps23
Saccharomycetaceae - ps24
Saccharomycetaceae_2 - ps25
Saccharomycetaceae_3 - ps26
Kaz./Nau./Saccharomyces - ps27
Saccharomyces - ps28
Arboricola_clade - ps29
Cerevisiae_clade - ps30
Saccharomyces_cerevisiae - ps31
Phylostratum

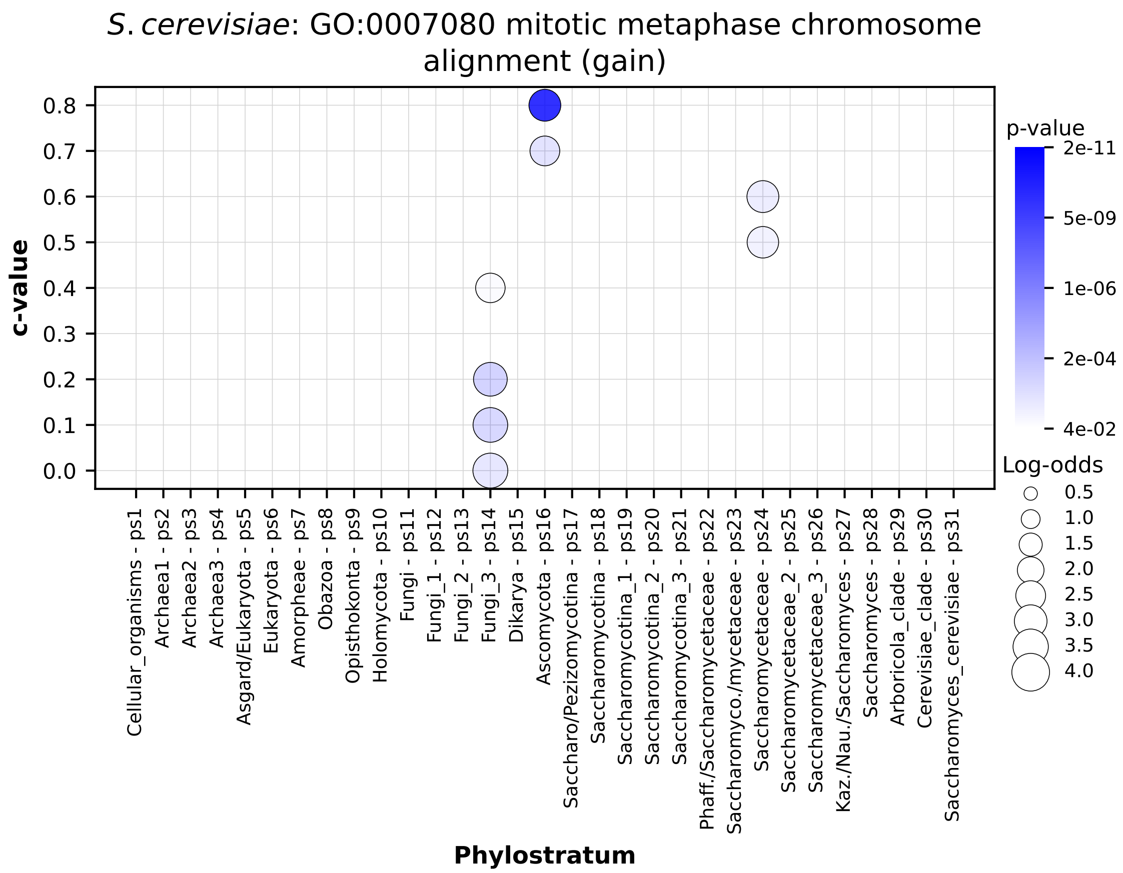

S. cerevisiae: GO:0007080 mitotic metaphase chromosome alignment (gain)
c-value
0.8
0.7
0.6
0.5
0.4
0.3
0.2
0.1
0.0
Phylostratum
Cellular_organisms - ps1
Archaea1 - ps2
Archaea2 - ps3
Archaea3 - ps4
Asgard/Eukaryota - ps5
Eukaryota - ps6
Amorpheae - ps7
Obazoa - ps8
Opisthokonta - ps9
Holomycota - ps10
Fungi - ps11
Fungi_1 - ps12
Fungi_2 - ps13
Fungi_3 - ps14
Dikarya - ps15
Ascomycota - ps16
Saccharo/Pezizomycotina - ps17
Saccharomycotina - ps18
Saccharomycotina_1 - ps19
Saccharomycotina_2 - ps20
Saccharomycotina_3 - ps21
Phaff./Saccharomycetaceae - ps22
Saccharomyco./mycetaceae - ps23
Saccharomycetaceae - ps24
Saccharomycetaceae_2 - ps25
Saccharomycetaceae_3 - ps26
Kaz./Nau./Saccharomyces - ps27
Saccharomyces - ps28
Arboricola_clade - ps29
Cerevisiae_clade - ps30
Saccharomyces_cerevisiae - ps31
p-value
2e-11
5e-09
1e-06
2e-04
4e-02
Log-odds
0.5
1.0
1.5
2.0
2.5
3.0
3.5
4.0

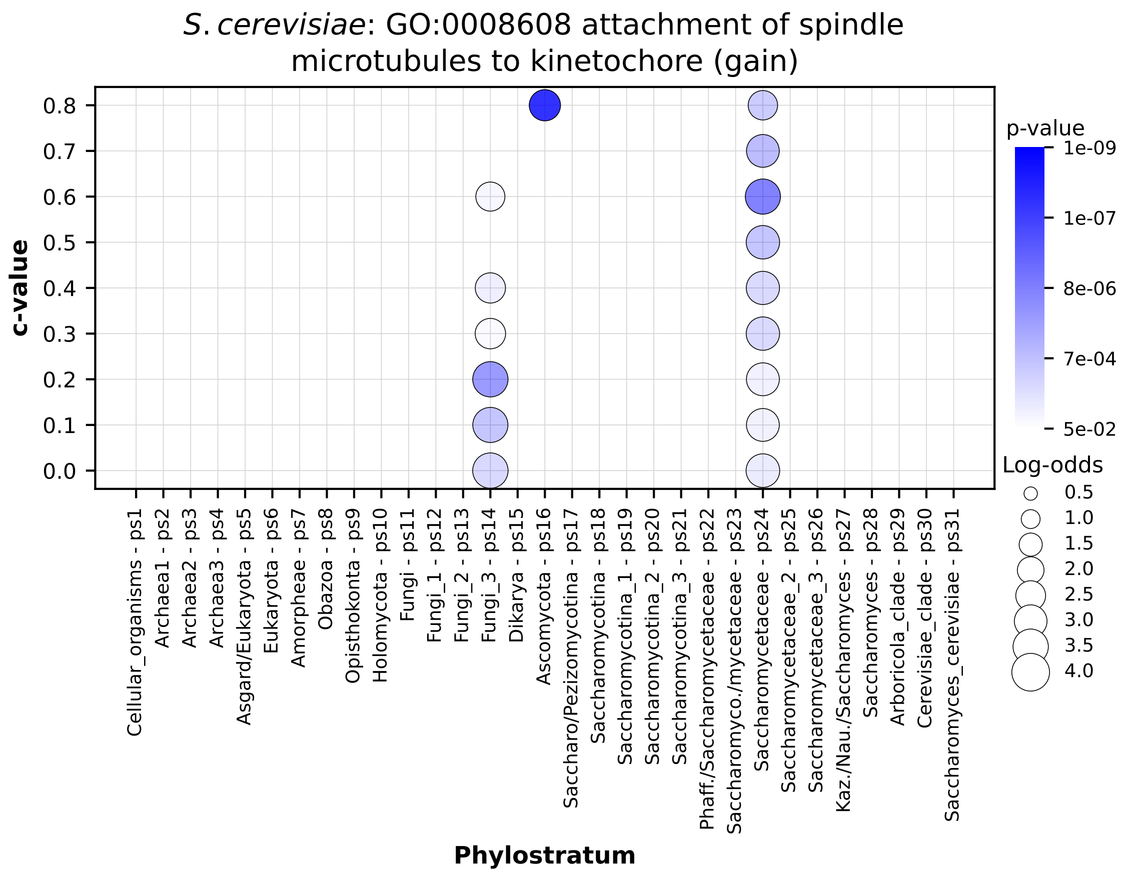

S. cerevisiae: GO:0008608 attachment of spindle microtubules to kinetochore (gain)
c-value
0.0
0.1
0.2
0.3
0.4
0.5
0.6
0.7
0.8
Phylostratum
Cellular_organisms - ps1
Archaea1 - ps2
Archaea2 - ps3
Archaea3 - ps4
Asgard/Eukaryota - ps5
Eukaryota - ps6
Amorpheae - ps7
Obazoa - ps8
Opisthokonta - ps9
Holomycota - ps10
Fungi - ps11
Fungi_1 - ps12
Fungi_2 - ps13
Fungi_3 - ps14
Dikarya - ps15
Ascomycota - ps16
Saccharo/Pezizomycotina - ps17
Saccharomycotina - ps18
Saccharomycotina_1 - ps19
Saccharomycotina_2 - ps20
Saccharomycotina_3 - ps21
Phaff./Saccharomycetaceae - ps22
Saccharomyco./mycetaceae - ps23
Saccharomycetaceae - ps24
Saccharomycetaceae_2 - ps25
Saccharomycetaceae_3 - ps26
Kaz./Nau./Saccharomyces - ps27
Saccharomyces - ps28
Arboricola_clade - ps29
Cerevisiae_clade - ps30
Saccharomyces_cerevisiae - ps31
p-value
1e-09
1e-07
8e-06
7e-04
5e-02
Log-odds
0.5
1.0
1.5
2.0
2.5
3.0
3.5
4.0

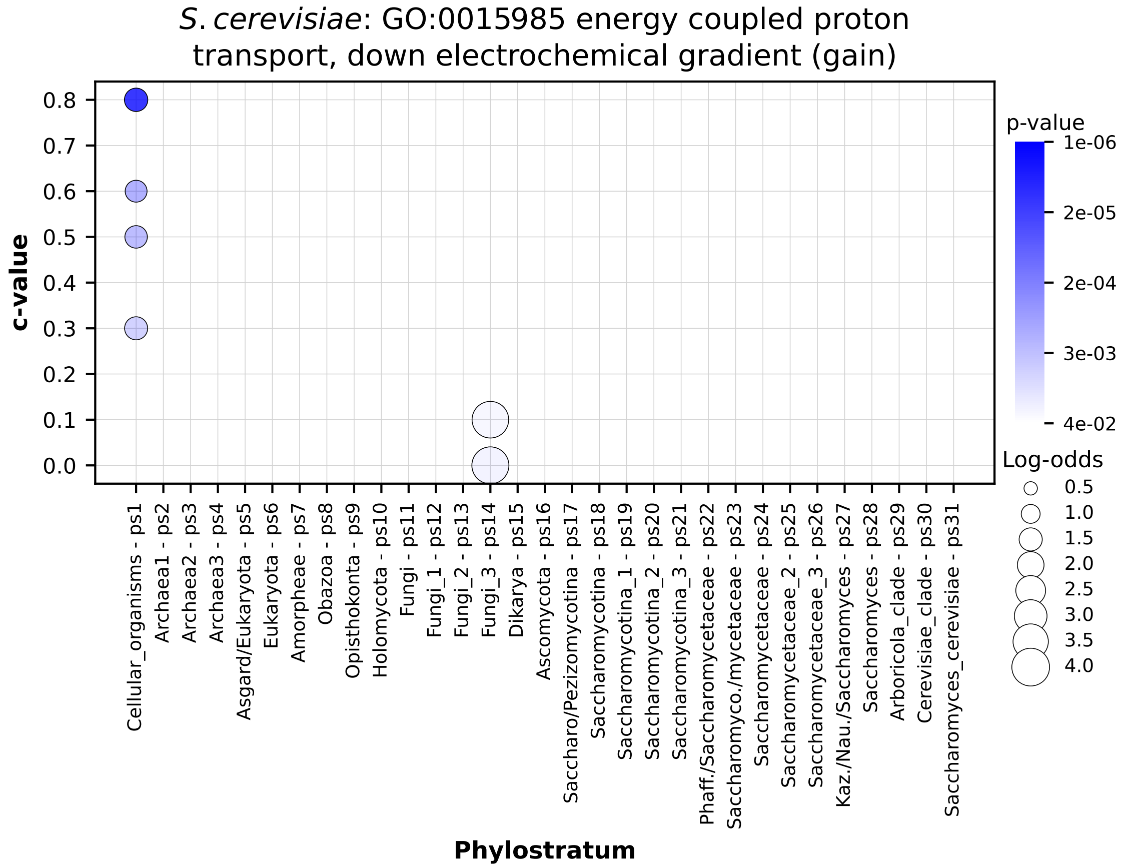

S. cerevisiae: GO:0015985 energy coupled proton transport, down electrochemical gradient (gain)
c-value
0.8
0.7
0.6
0.5
0.4
0.3
0.2
0.1
0.0
Phylostratum
Cellular_organisms - ps1
Archaea1 - ps2
Archaea2 - ps3
Archaea3 - ps4
Asgard/Eukaryota - ps5
Eukaryota - ps6
Amorpheae - ps7
Obazoa - ps8
Opisthokonta - ps9
Holomycota - ps10
Fungi - ps11
Fungi_1 - ps12
Fungi_2 - ps13
Fungi_3 - ps14
Dikarya - ps15
Ascomycota - ps16
Saccharo/Pezizomycotina - ps17
Saccharomycotina - ps18
Saccharomycotina_1 - ps19
Saccharomycotina_2 - ps20
Saccharomycotina_3 - ps21
Phaff./Saccharomycetaceae - ps22
Saccharomyco./mycetaceae - ps23
Saccharomycetaceae - ps24
Saccharomycetaceae_2 - ps25
Saccharomycetaceae_3 - ps26
Kaz./Nau./Saccharomyces - ps27
Saccharomyces - ps28
Arboricola_clade - ps29
Cerevisiae_clade - ps30
Saccharomyces_cerevisiae - ps31
p-value
1e-06
2e-05
2e-04
3e-03
4e-02
Log-odds
0.5
1.0
1.5
2.0
2.5
3.0
3.5
4.0

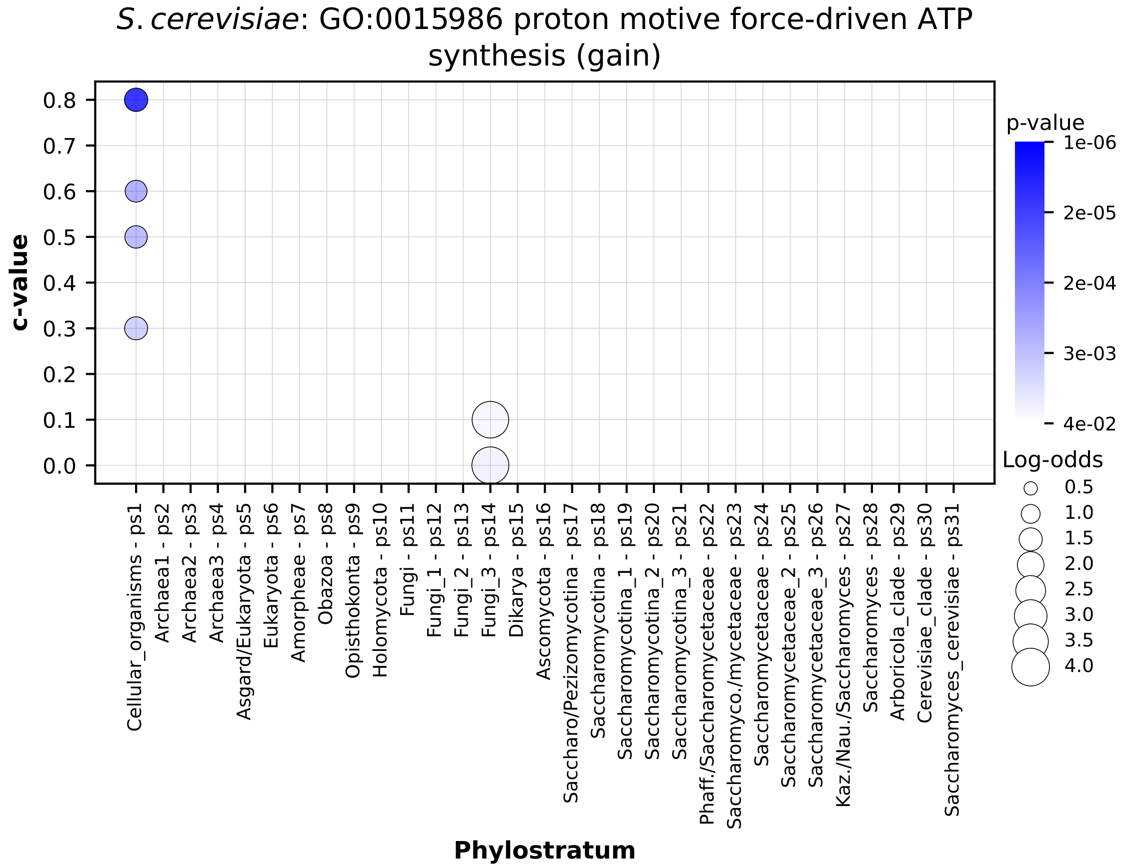

S. cerevisiae: GO:0015986 proton motive force-driven ATP synthesis (gain)
c-value
0.8
0.7
0.6
0.5
0.4
0.3
0.2
0.1
0.0
p-value
1e-06
2e-05
2e-04
3e-03
4e-02
Log-odds
0.5
1.0
1.5
2.0
2.5
3.0
3.5
4.0
Cellular_organisms - ps1
Archaea1 - ps2
Archaea2 - ps3
Archaea3 - ps4
Asgard/Eukaryota - ps5
Eukaryota - ps6
Amorpheae - ps7
Obazoa - ps8
Opisthokonta - ps9
Holomycota - ps10
Fungi - ps11
Fungi_1 - ps12
Fungi_2 - ps13
Fungi_3 - ps14
Dikarya - ps15
Ascomycota - ps16
Saccharo/Pezizomycotina - ps17
Saccharomycotina - ps18
Saccharomycotina_1 - ps19
Saccharomycotina_2 - ps20
Saccharomycotina_3 - ps21
Phaff./Saccharomycetaceae - ps22
Saccharomyco./mycetaceae - ps23
Saccharomycetaceae - ps24
Saccharomycetaceae_2 - ps25
Saccharomycetaceae_3 - ps26
Kaz./Nau./Saccharomyces - ps27
Saccharomyces - ps28
Arboricola_clade - ps29
Cerevisiae_clade - ps30
Saccharomyces_cerevisiae - ps31
Phylostratum

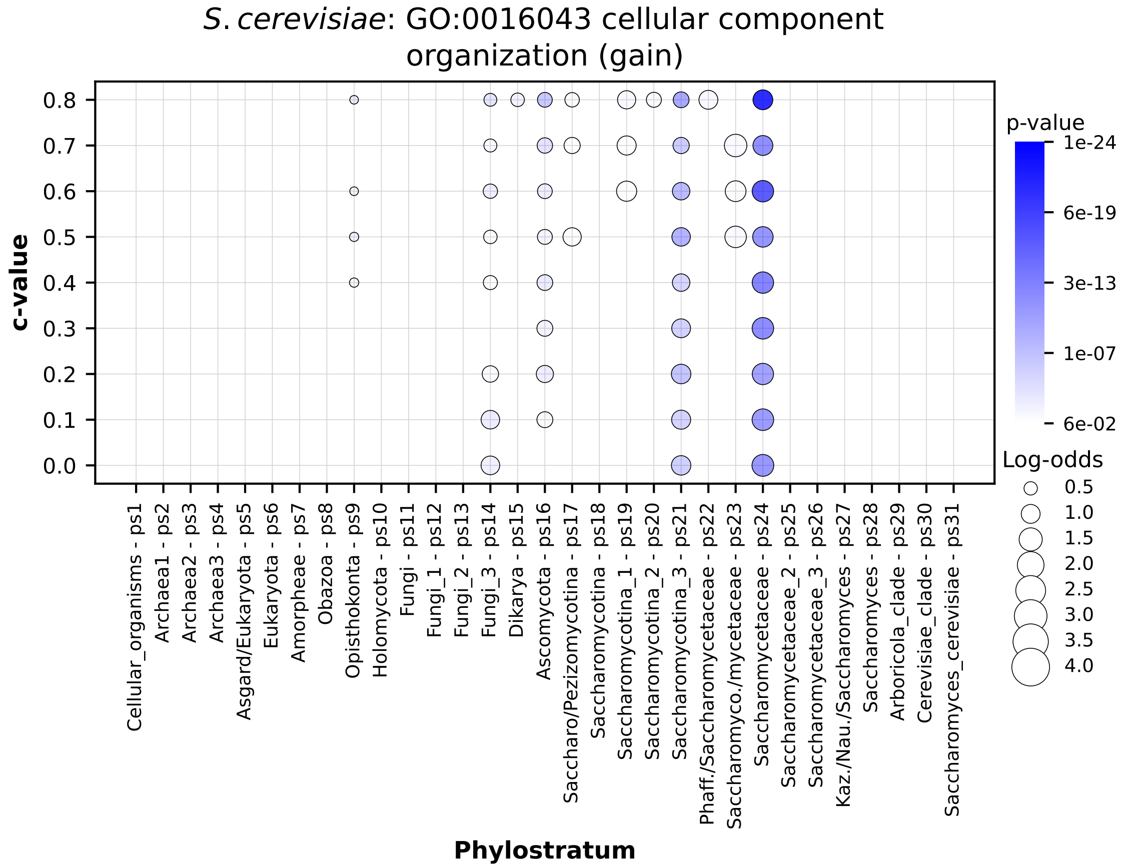

S. cerevisiae: GO:0016043 cellular component organization (gain)
c-value
0.8
0.7
0.6
0.5
0.4
0.3
0.2
0.1
0.0
p-value
1e-24
6e-19
3e-13
1e-07
6e-02
Log-odds
0.5
1.0
1.5
2.0
2.5
3.0
3.5
4.0
Cellular_organisms - ps1
Archaea1 - ps2
Archaea2 - ps3
Archaea3 - ps4
Asgard/Eukaryota - ps5
Eukaryota - ps6
Amorpheae - ps7
Obazoa - ps8
Opisthokonta - ps9
Holomycota - ps10
Fungi - ps11
Fungi_1 - ps12
Fungi_2 - ps13
Fungi_3 - ps14
Dikarya - ps15
Ascomycota - ps16
Saccharo/Pezizomycotina - ps17
Saccharomycotina - ps18
Saccharomycotina_1 - ps19
Saccharomycotina_2 - ps20
Saccharomycotina_3 - ps21
Phaff./Saccharomycetaceae - ps22
Saccharomyco./mycetaceae - ps23
Saccharomycetaceae - ps24
Saccharomycetaceae_2 - ps25
Saccharomycetaceae_3 - ps26
Kaz./Nau./Saccharomyces - ps27
Saccharomyces - ps28
Arboricola_clade - ps29
Cerevisiae_clade - ps30
Saccharomyces_cerevisiae - ps31
Phylostratum

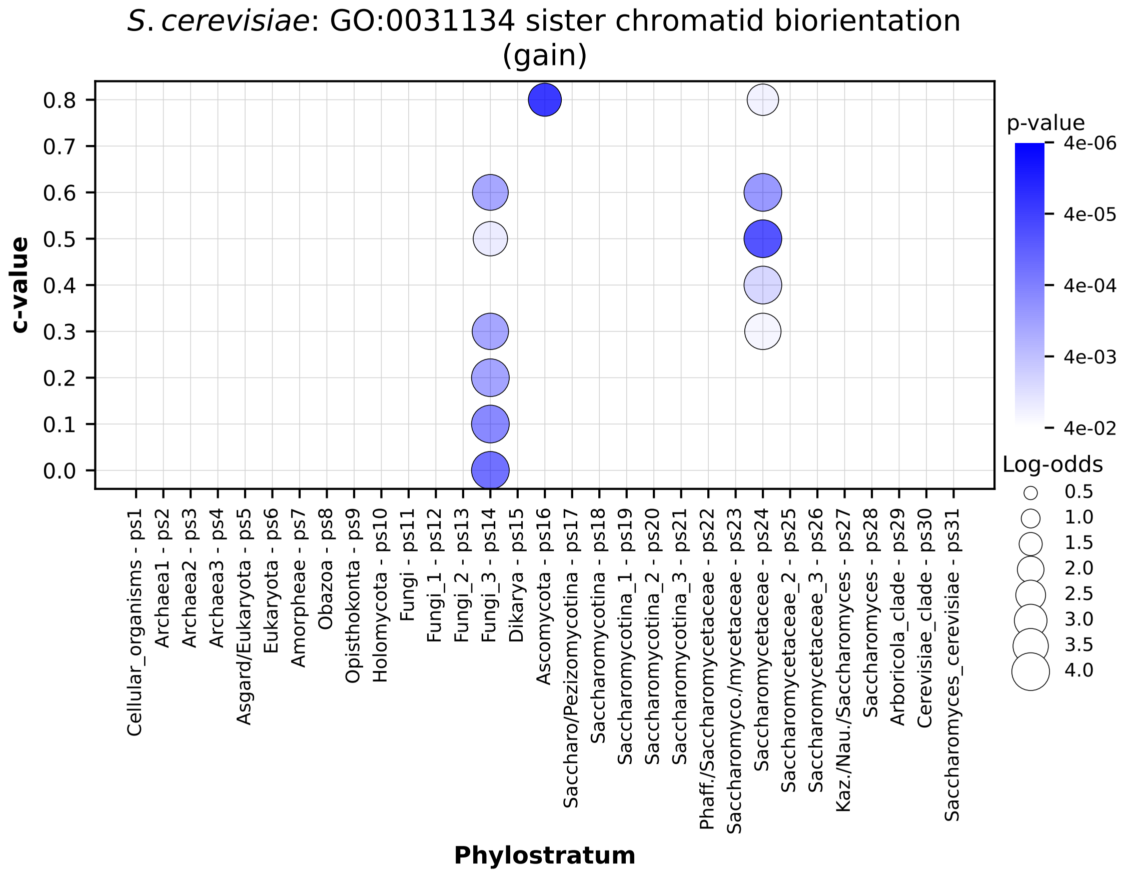
S. cerevisiae: GO:0031134 sister chromatid biorientation (gain)
c-value
0.0
0.1
0.2
0.3
0.4
0.5
0.6
0.7
0.8
p-value
4e-06
4e-05
4e-04
4e-03
4e-02
Log-odds
0.5
1.0
1.5
2.0
2.5
3.0
3.5
4.0
Cellular_organisms - ps1
Archaea1 - ps2
Archaea2 - ps3
Archaea3 - ps4
Asgard/Eukaryota - ps5
Eukaryota - ps6
Amorpheae - ps7
Obazoa - ps8
Opisthokonta - ps9
Holomycota - ps10
Fungi - ps11
Fungi_1 - ps12
Fungi_2 - ps13
Fungi_3 - ps14
Dikarya - ps15
Ascomycota - ps16
Saccharo/Pezizomycotina - ps17
Saccharomycotina - ps18
Saccharomycotina_1 - ps19
Saccharomycotina_2 - ps20
Saccharomycotina_3 - ps21
Phaff./Saccharomycetaceae - ps22
Saccharomyco./mycetaceae - ps23
Saccharomycetaceae - ps24
Saccharomycetaceae_2 - ps25
Saccharomycetaceae_3 - ps26
Kaz./Nau./Saccharomyces - ps27
Saccharomyces - ps28
Arboricola_clade - ps29
Cerevisiae_clade - ps30
Saccharomyces_cerevisiae - ps31
Phylostratum

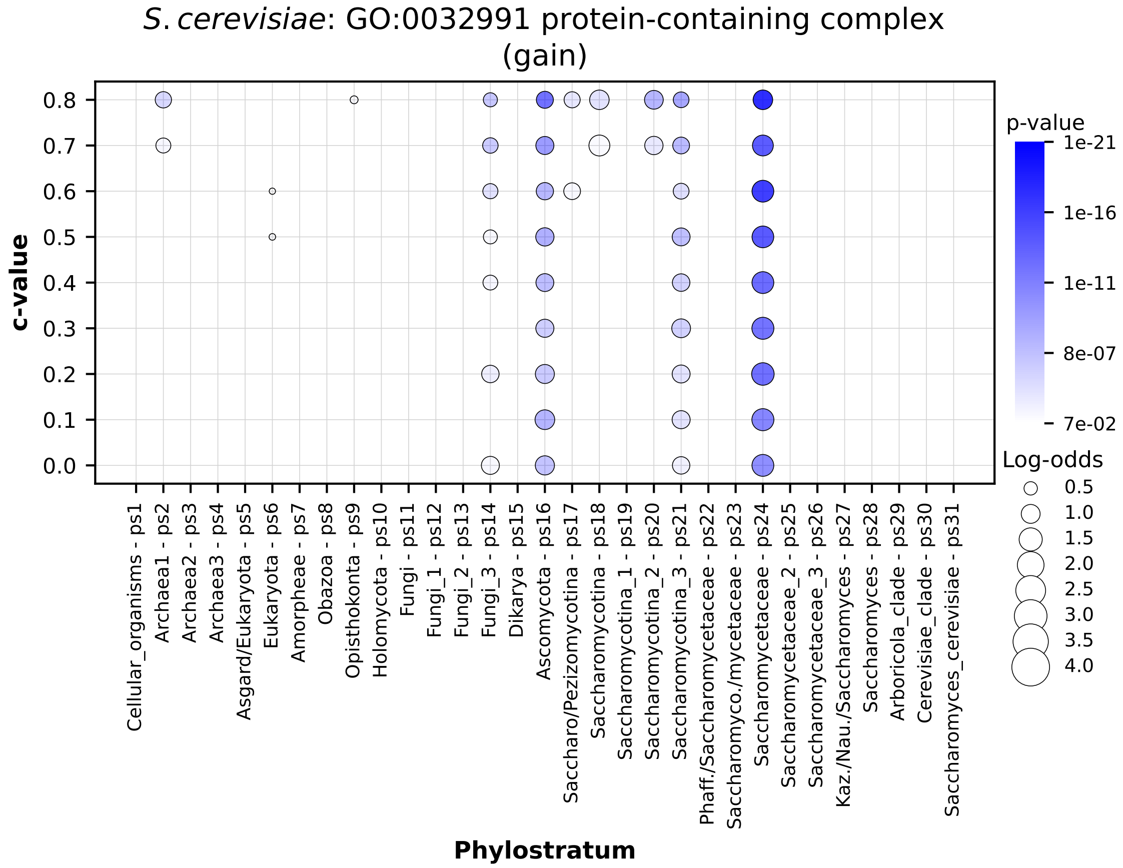
S. cerevisiae: GO:0032991 protein-containing complex (gain)
c-value
0.8
0.7
0.6
0.5
0.4
0.3
0.2
0.1
0.0
p-value
1e-21
1e-16
1e-11
8e-07
7e-02
Log-odds
0.5
1.0
1.5
2.0
2.5
3.0
3.5
4.0
Cellular_organisms - ps1
Archaea1 - ps2
Archaea2 - ps3
Archaea3 - ps4
Asgard/Eukaryota - ps5
Eukaryota - ps6
Amorpheae - ps7
Obazoa - ps8
Opisthokonta - ps9
Holomycota - ps10
Fungi - ps11
Fungi_1 - ps12
Fungi_2 - ps13
Fungi_3 - ps14
Dikarya - ps15
Ascomycota - ps16
Saccharo/Pezizomycotina - ps17
Saccharomycotina - ps18
Saccharomycotina_1 - ps19
Saccharomycotina_2 - ps20
Saccharomycotina_3 - ps21
Phaff./Saccharomycetaceae - ps22
Saccharomyco./mycetaceae - ps23
Saccharomycetaceae - ps24
Saccharomycetaceae_2 - ps25
Saccharomycetaceae_3 - ps26
Kaz./Nau./Saccharomyces - ps27
Saccharomyces - ps28
Arboricola_clade - ps29
Cerevisiae_clade - ps30
Saccharomyces_cerevisiae - ps31
Phylostratum

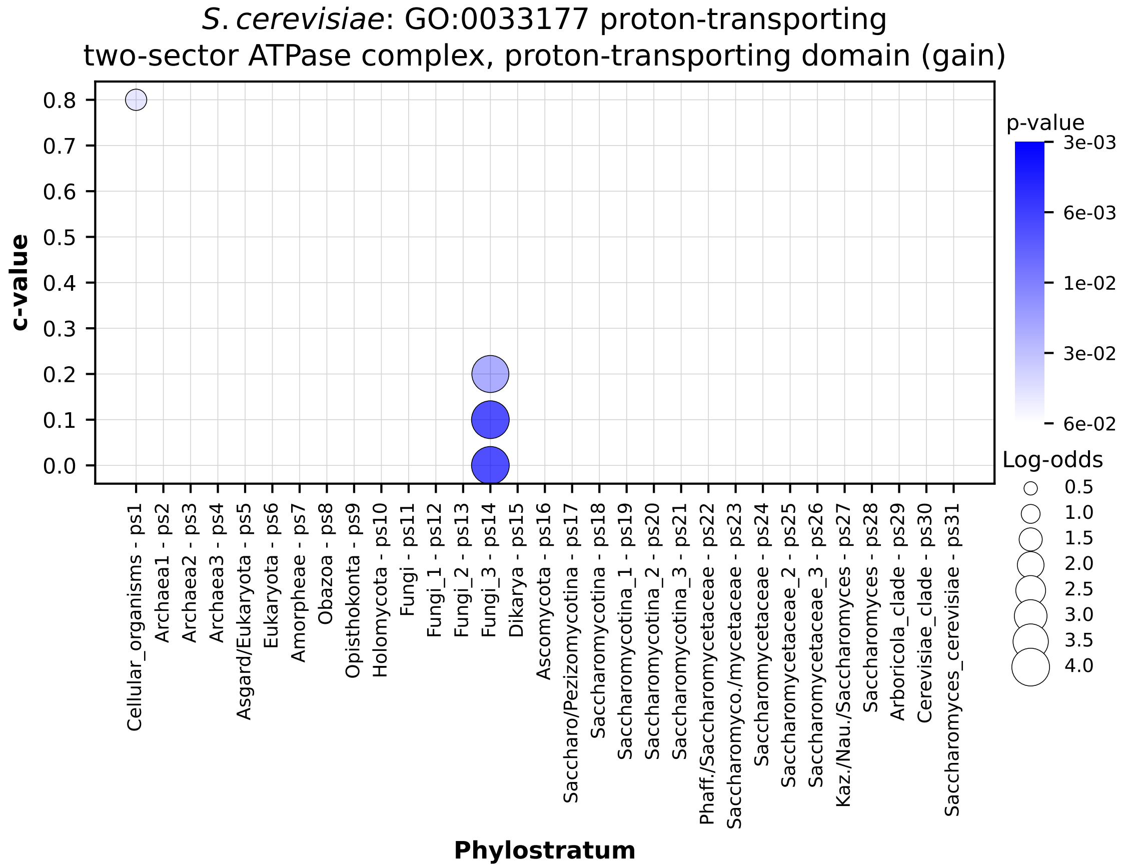

S. cerevisiae: GO:0033177 proton-transporting two-sector ATPase complex, proton-transporting domain (gain)
c-value
0.8
0.7
0.6
0.5
0.4
0.3
0.2
0.1
0.0
p-value
3e-03
6e-03
1e-02
3e-02
6e-02
Log-odds
0.5
1.0
1.5
2.0
2.5
3.0
3.5
4.0
Cellular_organisms - ps1
Archaea1 - ps2
Archaea2 - ps3
Archaea3 - ps4
Asgard/Eukaryota - ps5
Eukaryota - ps6
Amorpheae - ps7
Obazoa - ps8
Opisthokonta - ps9
Holomycota - ps10
Fungi - ps11
Fungi_1 - ps12
Fungi_2 - ps13
Fungi_3 - ps14
Dikarya - ps15
Ascomycota - ps16
Saccharo/Pezizomycotina - ps17
Saccharomycotina - ps18
Saccharomycotina_1 - ps19
Saccharomycotina_2 - ps20
Saccharomycotina_3 - ps21
Phaff./Saccharomycetaceae - ps22
Saccharomyco./mycetaceae - ps23
Saccharomycetaceae - ps24
Saccharomycetaceae_2 - ps25
Saccharomycetaceae_3 - ps26
Kaz./Nau./Saccharomyces - ps27
Saccharomyces - ps28
Arboricola_clade - ps29
Cerevisiae_clade - ps30
Saccharomyces_cerevisiae - ps31
Phylostratum

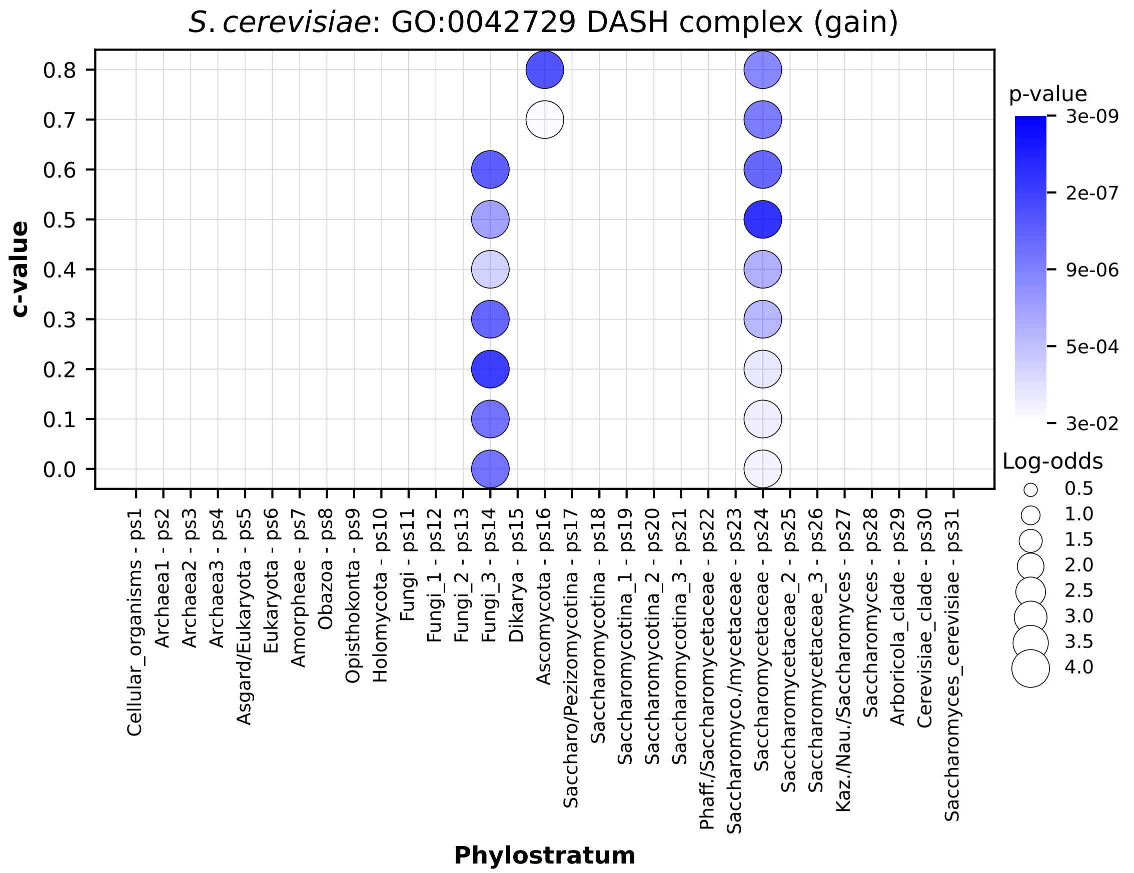

S. cerevisiae: GO:0042729 DASH complex (gain)
c-value
0.8
0.7
0.6
0.5
0.4
0.3
0.2
0.1
0.0
p-value
3e-09
2e-07
9e-06
5e-04
3e-02
Log-odds
0.5
1.0
1.5
2.0
2.5
3.0
3.5
4.0
Cellular_organisms - ps1
Archaea1 - ps2
Archaea2 - ps3
Archaea3 - ps4
Asgard/Eukaryota - ps5
Eukaryota - ps6
Amorpheae - ps7
Obazoa - ps8
Opisthokonta - ps9
Holomycota - ps10
Fungi - ps11
Fungi_1 - ps12
Fungi_2 - ps13
Fungi_3 - ps14
Dikarya - ps15
Ascomycota - ps16
Saccharo/Pezizomycotina - ps17
Saccharomycotina - ps18
Saccharomycotina_1 - ps19
Saccharomycotina_2 - ps20
Saccharomycotina_3 - ps21
Phaff./Saccharomycetaceae - ps22
Saccharomyco./mycetaceae - ps23
Saccharomycetaceae - ps24
Saccharomycetaceae_2 - ps25
Saccharomycetaceae_3 - ps26
Kaz./Nau./Saccharomyces - ps27
Saccharomyces - ps28
Arboricola_clade - ps29
Cerevisiae_clade - ps30
Saccharomyces_cerevisiae - ps31
Phylostratum

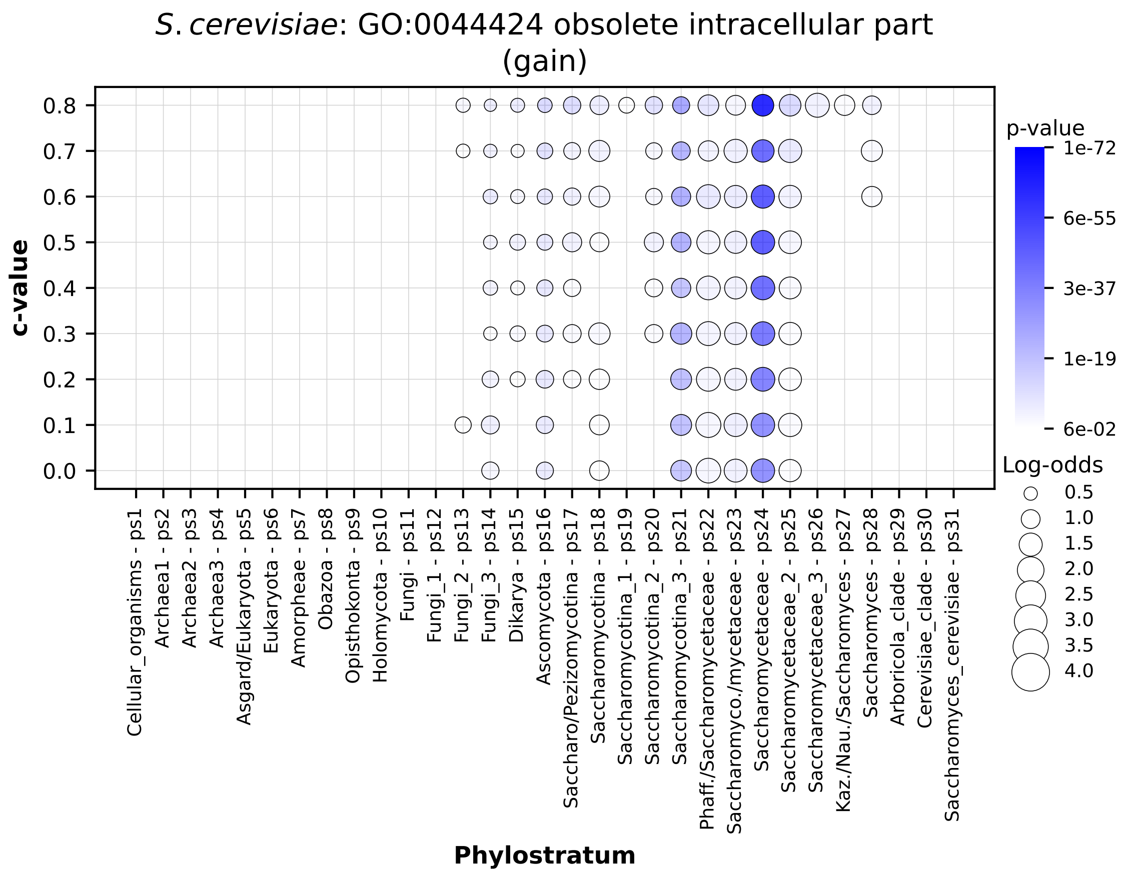

S. cerevisiae: GO:0044424 obsolete intracellular part
(gain)
c-value
0.8
0.7
0.6
0.5
0.4
0.3
0.2
0.1
0.0
p-value
1e-72
6e-55
3e-37
1e-19
6e-02
Log-odds
0.5
1.0
1.5
2.0
2.5
3.0
3.5
4.0
Cellular_organisms - ps1
Archaea1 - ps2
Archaea2 - ps3
Archaea3 - ps4
Asgard/Eukaryota - ps5
Eukaryota - ps6
Amorpheae - ps7
Obazoa - ps8
Opisthokonta - ps9
Holomycota - ps10
Fungi - ps11
Fungi_1 - ps12
Fungi_2 - ps13
Fungi_3 - ps14
Dikarya - ps15
Ascomycota - ps16
Saccharo/Pezizomycotina - ps17
Saccharomycotina - ps18
Saccharomycotina_1 - ps19
Saccharomycotina_2 - ps20
Saccharomycotina_3 - ps21
Phaff./Saccharomycetaceae - ps22
Saccharomyco./mycetaceae - ps23
Saccharomycetaceae - ps24
Saccharomycetaceae_2 - ps25
Saccharomycetaceae_3 - ps26
Kaz./Nau./Saccharomyces - ps27
Saccharomyces - ps28
Arboricola_clade - ps29
Cerevisiae_clade - ps30
Saccharomyces_cerevisiae - ps31
Phylostratum

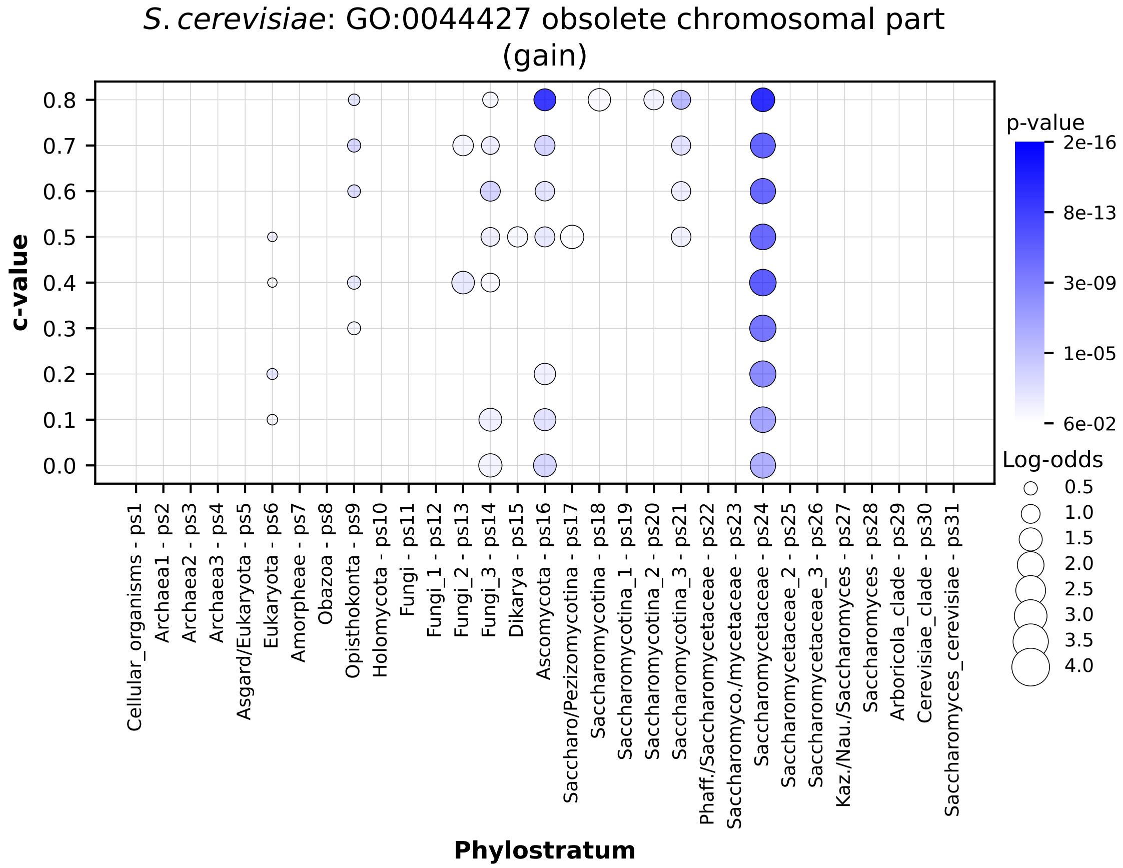

S. cerevisiae: GO:0044427 obsolete chromosomal part (gain)
c-value
0.8
0.7
0.6
0.5
0.4
0.3
0.2
0.1
0.0
p-value
2e-16
8e-13
3e-09
1e-05
6e-02
Log-odds
0.5
1.0
1.5
2.0
2.5
3.0
3.5
4.0
Cellular_organisms - ps1
Archaea1 - ps2
Archaea2 - ps3
Archaea3 - ps4
Asgard/Eukaryota - ps5
Eukaryota - ps6
Amorpheae - ps7
Obazoa - ps8
Opisthokonta - ps9
Holomycota - ps10
Fungi - ps11
Fungi_1 - ps12
Fungi_2 - ps13
Fungi_3 - ps14
Dikarya - ps15
Ascomycota - ps16
Saccharo/Pezizomycotina - ps17
Saccharomycotina - ps18
Saccharomycotina_1 - ps19
Saccharomycotina_2 - ps20
Saccharomycotina_3 - ps21
Phaff./Saccharomycetaceae - ps22
Saccharomyco./mycetaceae - ps23
Saccharomycetaceae - ps24
Saccharomycetaceae_2 - ps25
Saccharomycetaceae_3 - ps26
Kaz./Nau./Saccharomyces - ps27
Saccharomyces - ps28
Arboricola_clade - ps29
Cerevisiae_clade - ps30
Saccharomyces_cerevisiae - ps31
Phylostratum

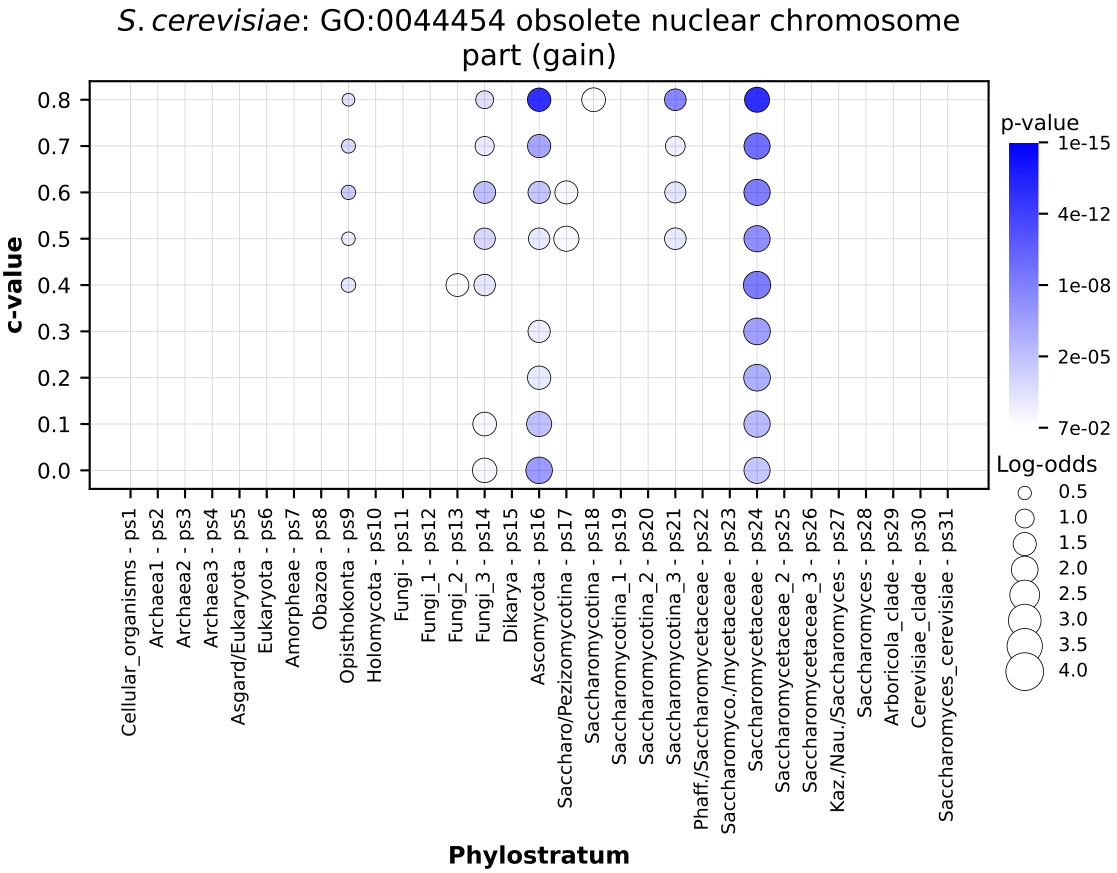

S. cerevisiae: GO:0044454 obsolete nuclear chromosome part (gain)
c-value
0.8
0.7
0.6
0.5
0.4
0.3
0.2
0.1
0.0
p-value
1e-15
4e-12
1e-08
2e-05
7e-02
Log-odds
0.5
1.0
1.5
2.0
2.5
3.0
3.5
4.0
Cellular_organisms - ps1
Archaea1 - ps2
Archaea2 - ps3
Archaea3 - ps4
Asgard/Eukaryota - ps5
Eukaryota - ps6
Amorpheae - ps7
Obazoa - ps8
Opisthokonta - ps9
Holomycota - ps10
Fungi - ps11
Fungi_1 - ps12
Fungi_2 - ps13
Fungi_3 - ps14
Dikarya - ps15
Ascomycota - ps16
Saccharo/Pezizomycotina - ps17
Saccharomycotina - ps18
Saccharomycotina_1 - ps19
Saccharomycotina_2 - ps20
Saccharomycotina_3 - ps21
Phaff./Saccharomycetaceae - ps22
Saccharomyco./mycetaceae - ps23
Saccharomycetaceae - ps24
Saccharomycetaceae_2 - ps25
Saccharomycetaceae_3 - ps26
Kaz./Nau./Saccharomyces - ps27
Saccharomyces - ps28
Arboricola_clade - ps29
Cerevisiae_clade - ps30
Saccharomyces_cerevisiae - ps31
Phylostratum

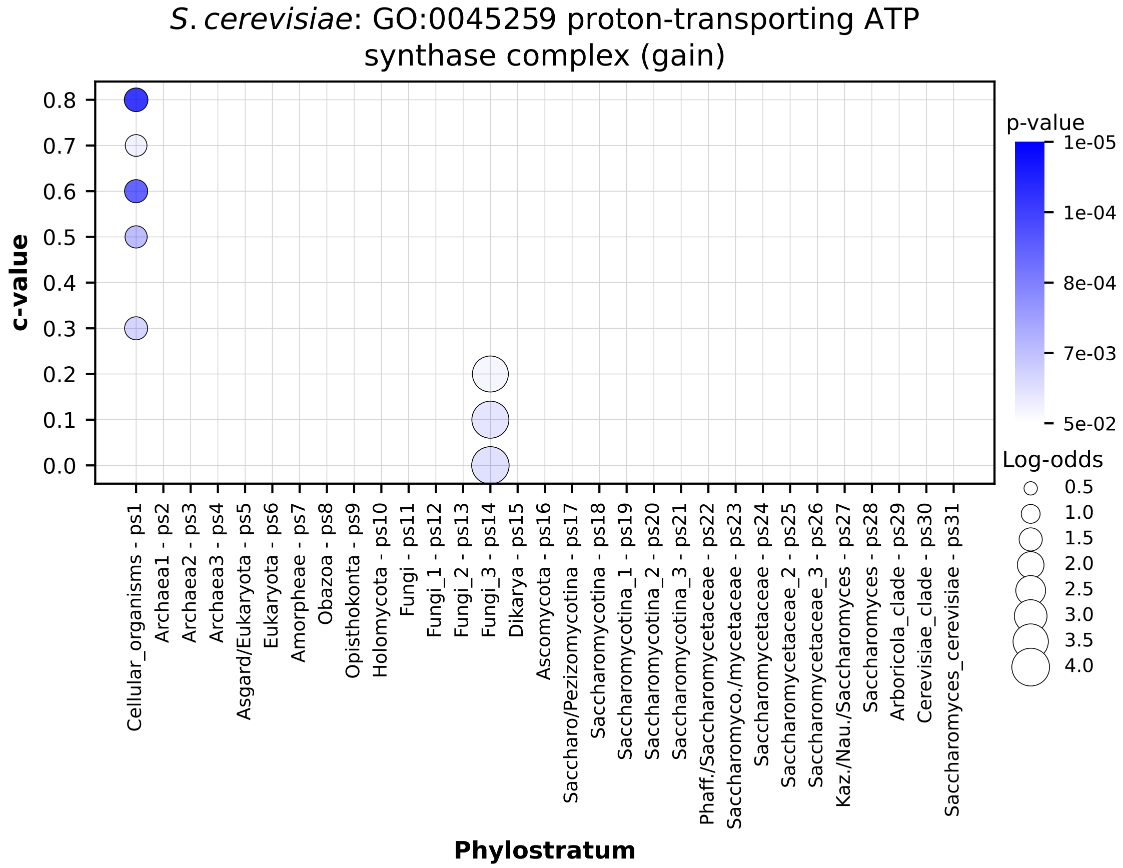

S. cerevisiae: GO:0045259 proton-transporting ATP synthase complex (gain)
c-value
0.8
0.7
0.6
0.5
0.4
0.3
0.2
0.1
0.0
p-value
1e-05
1e-04
8e-04
7e-03
5e-02
Log-odds
0.5
1.0
1.5
2.0
2.5
3.0
3.5
4.0
Cellular_organisms - ps1
Archaea1 - ps2
Archaea2 - ps3
Archaea3 - ps4
Asgard/Eukaryota - ps5
Eukaryota - ps6
Amorpheae - ps7
Obazoa - ps8
Opisthokonta - ps9
Holomycota - ps10
Fungi - ps11
Fungi_1 - ps12
Fungi_2 - ps13
Fungi_3 - ps14
Dikarya - ps15
Ascomycota - ps16
Saccharo/Pezizomycotina - ps17
Saccharomycotina - ps18
Saccharomycotina_1 - ps19
Saccharomycotina_2 - ps20
Saccharomycotina_3 - ps21
Phaff./Saccharomycetaceae - ps22
Saccharomyco./mycetaceae - ps23
Saccharomycetaceae - ps24
Saccharomycetaceae_2 - ps25
Saccharomycetaceae_3 - ps26
Kaz./Nau./Saccharomyces - ps27
Saccharomyces - ps28
Arboricola_clade - ps29
Cerevisiae_clade - ps30
Saccharomyces_cerevisiae - ps31
Phylostratum

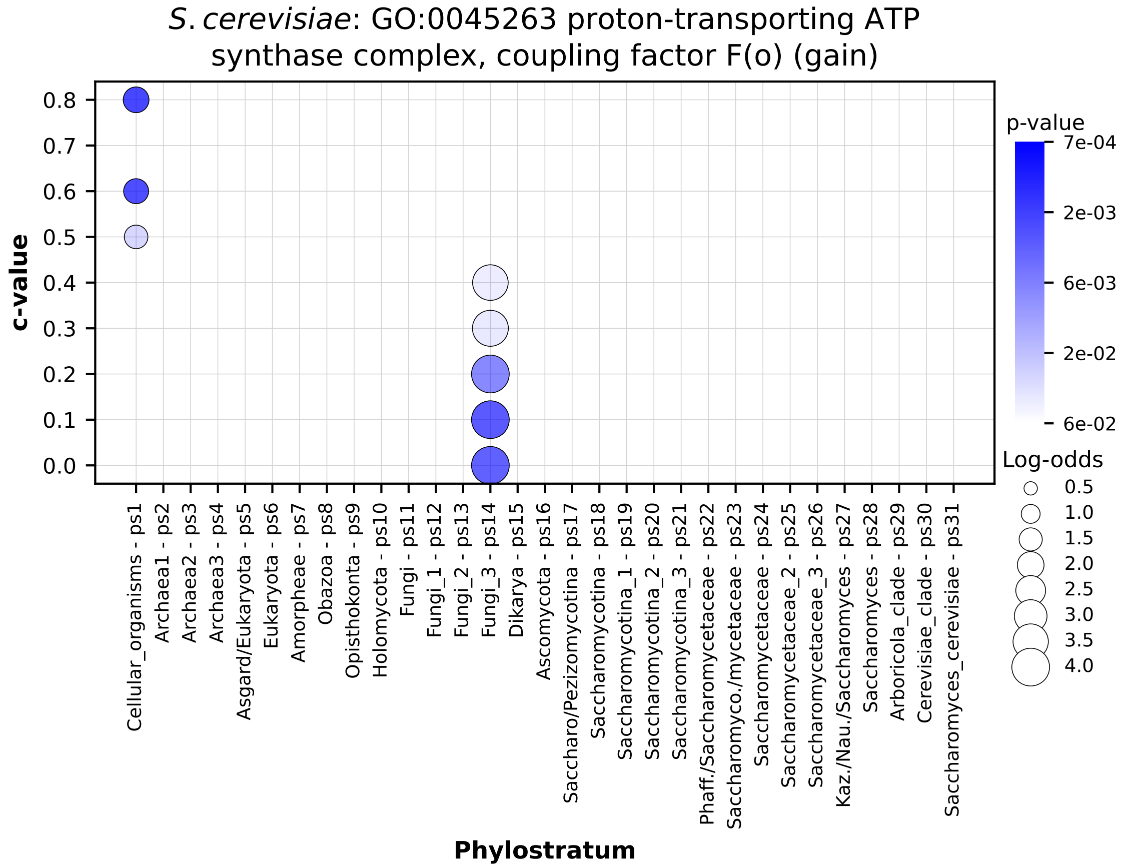

S. cerevisiae: GO:0045263 proton-transporting ATP synthase complex, coupling factor F(o) (gain)
c-value
0.8
0.7
0.6
0.5
0.4
0.3
0.2
0.1
0.0
p-value
7e-04
2e-03
6e-03
2e-02
6e-02
Log-odds
0.5
1.0
1.5
2.0
2.5
3.0
3.5
4.0
Cellular_organisms - ps1
Archaea1 - ps2
Archaea2 - ps3
Archaea3 - ps4
Asgard/Eukaryota - ps5
Eukaryota - ps6
Amorpheae - ps7
Obazoa - ps8
Opisthokonta - ps9
Holomycota - ps10
Fungi - ps11
Fungi_1 - ps12
Fungi_2 - ps13
Fungi_3 - ps14
Dikarya - ps15
Ascomycota - ps16
Saccharo/Pezizomycotina - ps17
Saccharomycotina - ps18
Saccharomycotina_1 - ps19
Saccharomycotina_2 - ps20
Saccharomycotina_3 - ps21
Phaff./Saccharomycetaceae - ps22
Saccharomyco./mycetaceae - ps23
Saccharomycetaceae - ps24
Saccharomycetaceae_2 - ps25
Saccharomycetaceae_3 - ps26
Kaz./Nau./Saccharomyces - ps27
Saccharomyces - ps28
Arboricola_clade - ps29
Cerevisiae_clade - ps30
Saccharomyces_cerevisiae - ps31
Phylostratum

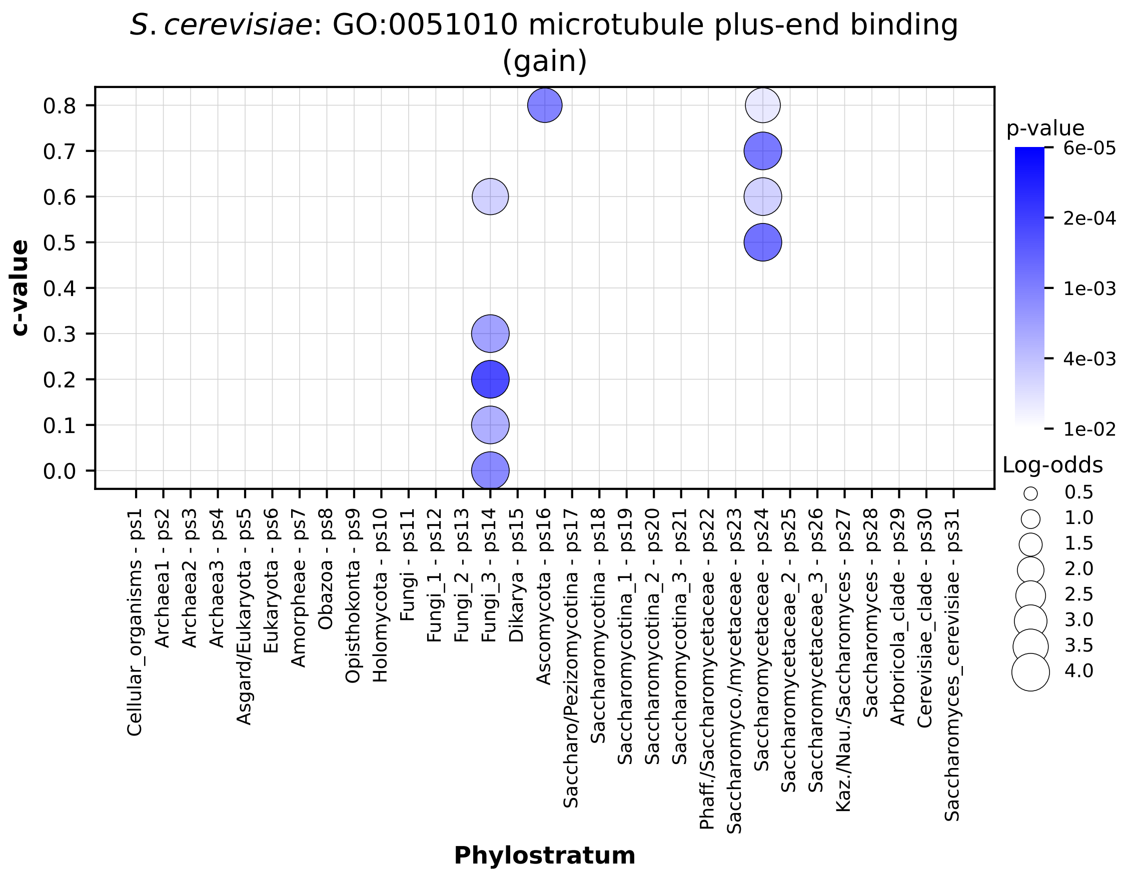

S. cerevisiae: GO:0051010 microtubule plus-end binding (gain)
c-value
0.8
0.7
0.6
0.5
0.4
0.3
0.2
0.1
0.0
p-value
6e-05
2e-04
1e-03
4e-03
1e-02
Log-odds
0.5
1.0
1.5
2.0
2.5
3.0
3.5
4.0
Cellular_organisms - ps1
Archaea1 - ps2
Archaea2 - ps3
Archaea3 - ps4
Asgard/Eukaryota - ps5
Eukaryota - ps6
Amorpheae - ps7
Obazoa - ps8
Opisthokonta - ps9
Holomycota - ps10
Fungi - ps11
Fungi_1 - ps12
Fungi_2 - ps13
Fungi_3 - ps14
Dikarya - ps15
Ascomycota - ps16
Saccharo/Pezizomycotina - ps17
Saccharomycotina - ps18
Saccharomycotina_1 - ps19
Saccharomycotina_2 - ps20
Saccharomycotina_3 - ps21
Phaff./Saccharomycetaceae - ps22
Saccharomyco./mycetaceae - ps23
Saccharomycetaceae - ps24
Saccharomycetaceae_2 - ps25
Saccharomycetaceae_3 - ps26
Kaz./Nau./Saccharomyces - ps27
Saccharomyces - ps28
Arboricola_clade - ps29
Cerevisiae_clade - ps30
Saccharomyces_cerevisiae - ps31
Phylostratum

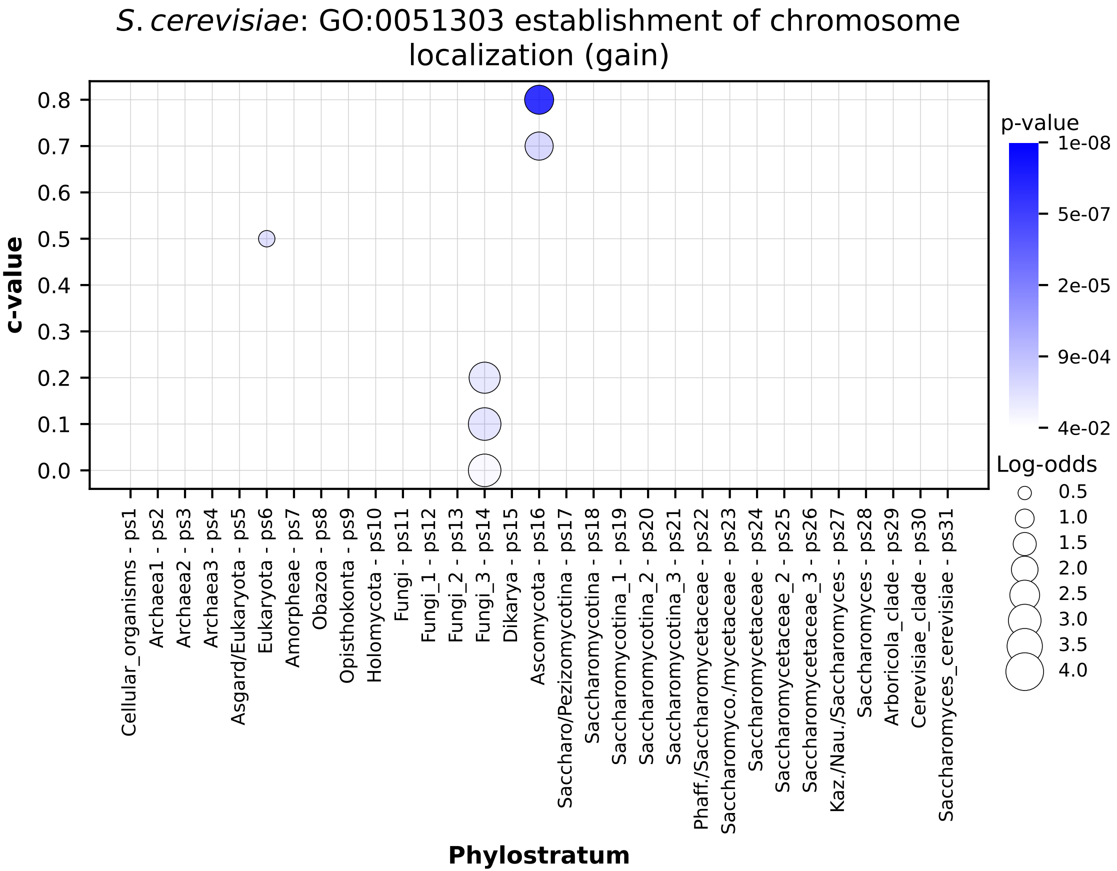

S. cerevisiae: GO:0051303 establishment of chromosome localization (gain)
c-value
0.0
0.1
0.2
0.3
0.4
0.5
0.6
0.7
0.8
Phylostratum
Cellular_organisms - ps1
Archaea1 - ps2
Archaea2 - ps3
Archaea3 - ps4
Asgard/Eukaryota - ps5
Eukaryota - ps6
Amorpheae - ps7
Obazoa - ps8
Opisthokonta - ps9
Holomycota - ps10
Fungi - ps11
Fungi_1 - ps12
Fungi_2 - ps13
Fungi_3 - ps14
Dikarya - ps15
Ascomycota - ps16
Saccharo/Pezizomycotina - ps17
Saccharomycotina - ps18
Saccharomycotina_1 - ps19
Saccharomycotina_2 - ps20
Saccharomycotina_3 - ps21
Phaff./Saccharomycetaceae - ps22
Saccharomyco./mycetaceae - ps23
Saccharomycetaceae - ps24
Saccharomycetaceae_2 - ps25
Saccharomycetaceae_3 - ps26
Kaz./Nau./Saccharomyces - ps27
Saccharomyces - ps28
Arboricola_clade - ps29
Cerevisiae_clade - ps30
Saccharomyces_cerevisiae - ps31
p-value
1e-08
5e-07
2e-05
9e-04
4e-02
Log-odds
0.5
1.0
1.5
2.0
2.5
3.0
3.5
4.0

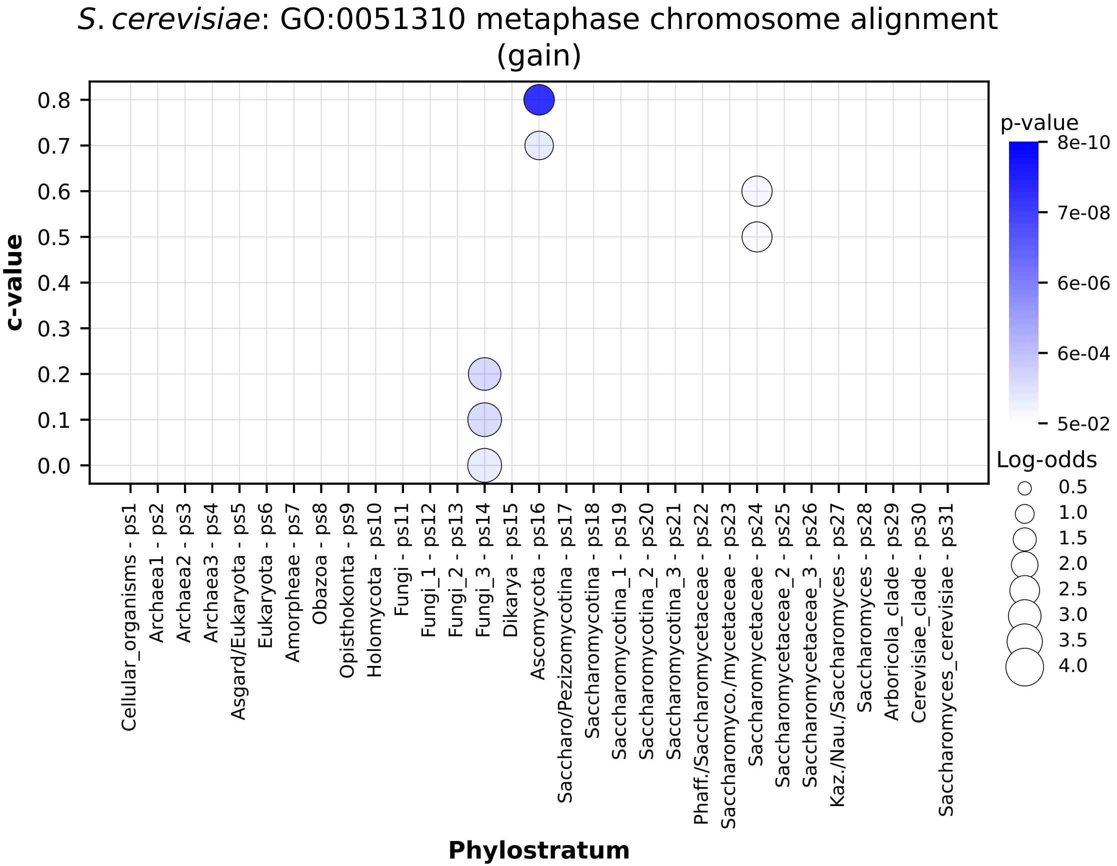

S. cerevisiae: GO:0051310 metaphase chromosome alignment (gain)
c-value
0.8
0.7
0.6
0.5
0.4
0.3
0.2
0.1
0.0
p-value
8e-10
7e-08
6e-06
6e-04
5e-02
Log-odds
0.5
1.0
1.5
2.0
2.5
3.0
3.5
4.0
Cellular_organisms - ps1
Archaea1 - ps2
Archaea2 - ps3
Archaea3 - ps4
Asgard/Eukaryota - ps5
Eukaryota - ps6
Amorpheae - ps7
Obazoa - ps8
Opisthokonta - ps9
Holomycota - ps10
Fungi - ps11
Fungi_1 - ps12
Fungi_2 - ps13
Fungi_3 - ps14
Dikarya - ps15
Ascomycota - ps16
Saccharo/Pezizomycotina - ps17
Saccharomycotina - ps18
Saccharomycotina_1 - ps19
Saccharomycotina_2 - ps20
Saccharomycotina_3 - ps21
Phaff./Saccharomycetaceae - ps22
Saccharomyco./mycetaceae - ps23
Saccharomycetaceae - ps24
Saccharomycetaceae_2 - ps25
Saccharomycetaceae_3 - ps26
Kaz./Nau./Saccharomyces - ps27
Saccharomyces - ps28
Arboricola_clade - ps29
Cerevisiae_clade - ps30
Saccharomyces_cerevisiae - ps31
Phylostratum

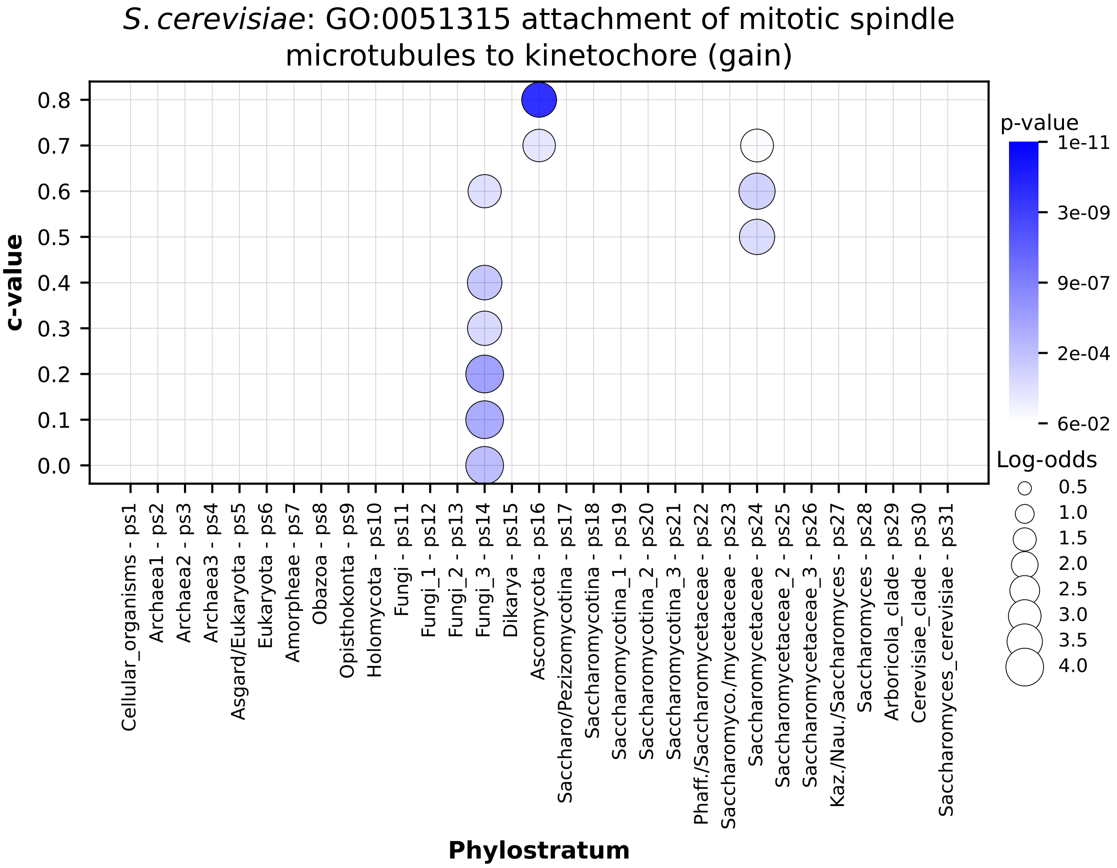

S. cerevisiae: GO:0051315 attachment of mitotic spindle microtubules to kinetochore (gain)
c-value
0.0
0.1
0.2
0.3
0.4
0.5
0.6
0.7
0.8
p-value
1e-11
3e-09
9e-07
2e-04
6e-02
Log-odds
0.5
1.0
1.5
2.0
2.5
3.0
3.5
4.0
Cellular_organisms - ps1
Archaea1 - ps2
Archaea2 - ps3
Archaea3 - ps4
Asgard/Eukaryota - ps5
Eukaryota - ps6
Amorpheae - ps7
Obazoa - ps8
Opisthokonta - ps9
Holomycota - ps10
Fungi - ps11
Fungi_1 - ps12
Fungi_2 - ps13
Fungi_3 - ps14
Dikarya - ps15
Ascomycota - ps16
Saccharo/Pezizomycotina - ps17
Saccharomycotina - ps18
Saccharomycotina_1 - ps19
Saccharomycotina_2 - ps20
Saccharomycotina_3 - ps21
Phaff./Saccharomycetaceae - ps22
Saccharomyco./mycetaceae - ps23
Saccharomycetaceae - ps24
Saccharomycetaceae_2 - ps25
Saccharomycetaceae_3 - ps26
Kaz./Nau./Saccharomyces - ps27
Saccharomyces - ps28
Arboricola_clade - ps29
Cerevisiae_clade - ps30
Saccharomyces_cerevisiae - ps31
Phylostratum

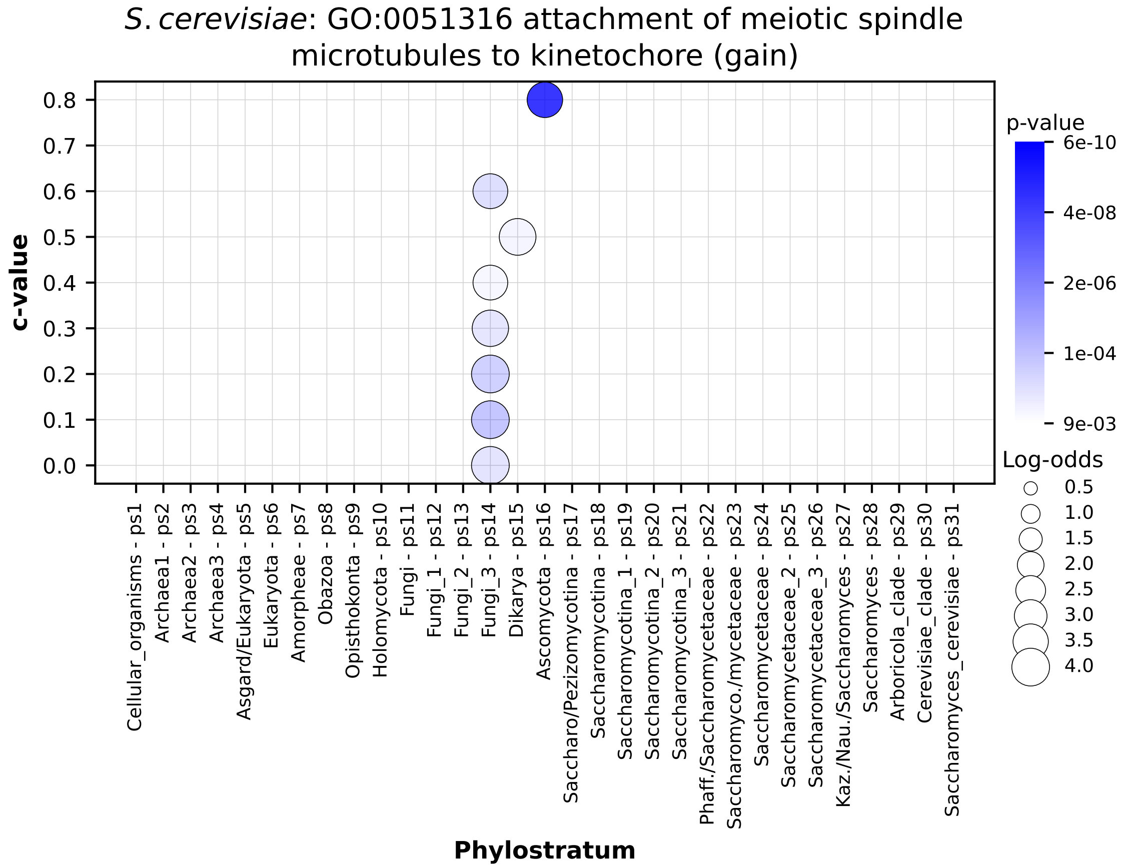

S. cerevisiae: GO:0051316 attachment of meiotic spindle microtubules to kinetochore (gain)
c-value
0.8
0.7
0.6
0.5
0.4
0.3
0.2
0.1
0.0
p-value
6e-10
4e-08
2e-06
1e-04
9e-03
Log-odds
0.5
1.0
1.5
2.0
2.5
3.0
3.5
4.0
Cellular_organisms - ps1
Archaea1 - ps2
Archaea2 - ps3
Archaea3 - ps4
Asgard/Eukaryota - ps5
Eukaryota - ps6
Amorpheae - ps7
Obazoa - ps8
Opisthokonta - ps9
Holomycota - ps10
Fungi - ps11
Fungi_1 - ps12
Fungi_2 - ps13
Fungi_3 - ps14
Dikarya - ps15
Ascomycota - ps16
Saccharo/Pezizomycotina - ps17
Saccharomycotina - ps18
Saccharomycotina_1 - ps19
Saccharomycotina_2 - ps20
Saccharomycotina_3 - ps21
Phaff./Saccharomycetaceae - ps22
Saccharomyco./mycetaceae - ps23
Saccharomycetaceae - ps24
Saccharomycetaceae_2 - ps25
Saccharomycetaceae_3 - ps26
Kaz./Nau./Saccharomyces - ps27
Saccharomyces - ps28
Arboricola_clade - ps29
Cerevisiae_clade - ps30
Saccharomyces_cerevisiae - ps31
Phylostratum

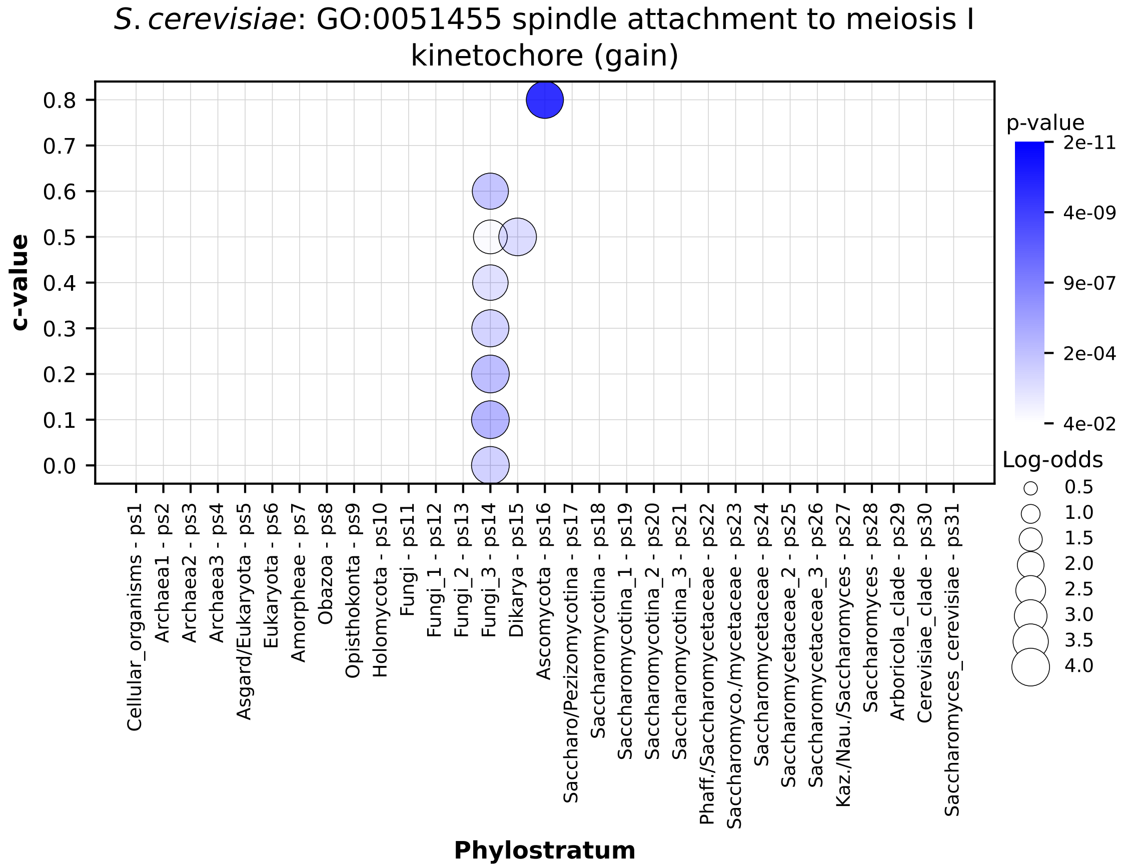

S. cerevisiae: GO:0051455 spindle attachment to meiosis I kinetochore (gain)
c-value
0.8
0.7
0.6
0.5
0.4
0.3
0.2
0.1
0.0
p-value
2e-11
4e-09
9e-07
2e-04
4e-02
Log-odds
0.5
1.0
1.5
2.0
2.5
3.0
3.5
4.0
Cellular_organisms - ps1
Archaea1 - ps2
Archaea2 - ps3
Archaea3 - ps4
Asgard/Eukaryota - ps5
Eukaryota - ps6
Amorpheae - ps7
Obazoa - ps8
Opisthokonta - ps9
Holomycota - ps10
Fungi - ps11
Fungi_1 - ps12
Fungi_2 - ps13
Fungi_3 - ps14
Dikarya - ps15
Ascomycota - ps16
Saccharo/Pezizomycotina - ps17
Saccharomycotina - ps18
Saccharomycotina_1 - ps19
Saccharomycotina_2 - ps20
Saccharomycotina_3 - ps21
Phaff./Saccharomycetaceae - ps22
Saccharomyco./mycetaceae - ps23
Saccharomycetaceae - ps24
Saccharomycetaceae_2 - ps25
Saccharomycetaceae_3 - ps26
Kaz./Nau./Saccharomyces - ps27
Saccharomyces - ps28
Arboricola_clade - ps29
Cerevisiae_clade - ps30
Saccharomyces_cerevisiae - ps31
Phylostratum

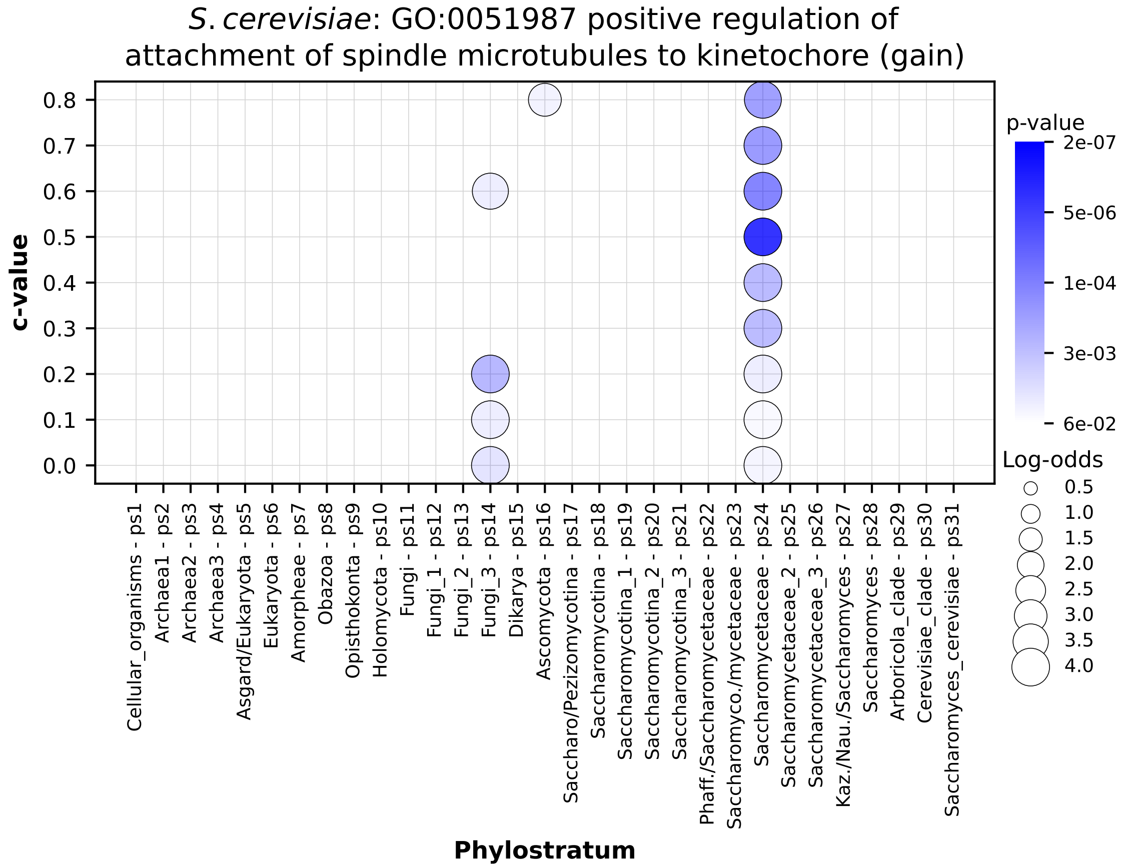

S. cerevisiae: GO:0051987 positive regulation of attachment of spindle microtubules to kinetochore (gain)
c-value
0.8
0.7
0.6
0.5
0.4
0.3
0.2
0.1
0.0
Phylostratum
Cellular_organisms - ps1
Archaea1 - ps2
Archaea2 - ps3
Archaea3 - ps4
Asgard/Eukaryota - ps5
Eukaryota - ps6
Amorpheae - ps7
Obazoa - ps8
Opisthokonta - ps9
Holomycota - ps10
Fungi - ps11
Fungi_1 - ps12
Fungi_2 - ps13
Fungi_3 - ps14
Dikarya - ps15
Ascomycota - ps16
Saccharo/Pezizomycotina - ps17
Saccharomycotina - ps18
Saccharomycotina_1 - ps19
Saccharomycotina_2 - ps20
Saccharomycotina_3 - ps21
Phaff./Saccharomycetaceae - ps22
Saccharomyco./mycetaceae - ps23
Saccharomycetaceae - ps24
Saccharomycetaceae_2 - ps25
Saccharomycetaceae_3 - ps26
Kaz./Nau./Saccharomyces - ps27
Saccharomyces - ps28
Arboricola_clade - ps29
Cerevisiae_clade - ps30
Saccharomyces_cerevisiae - ps31
p-value
2e-07
5e-06
1e-04
3e-03
6e-02
Log-odds
0.5
1.0
1.5
2.0
2.5
3.0
3.5
4.0

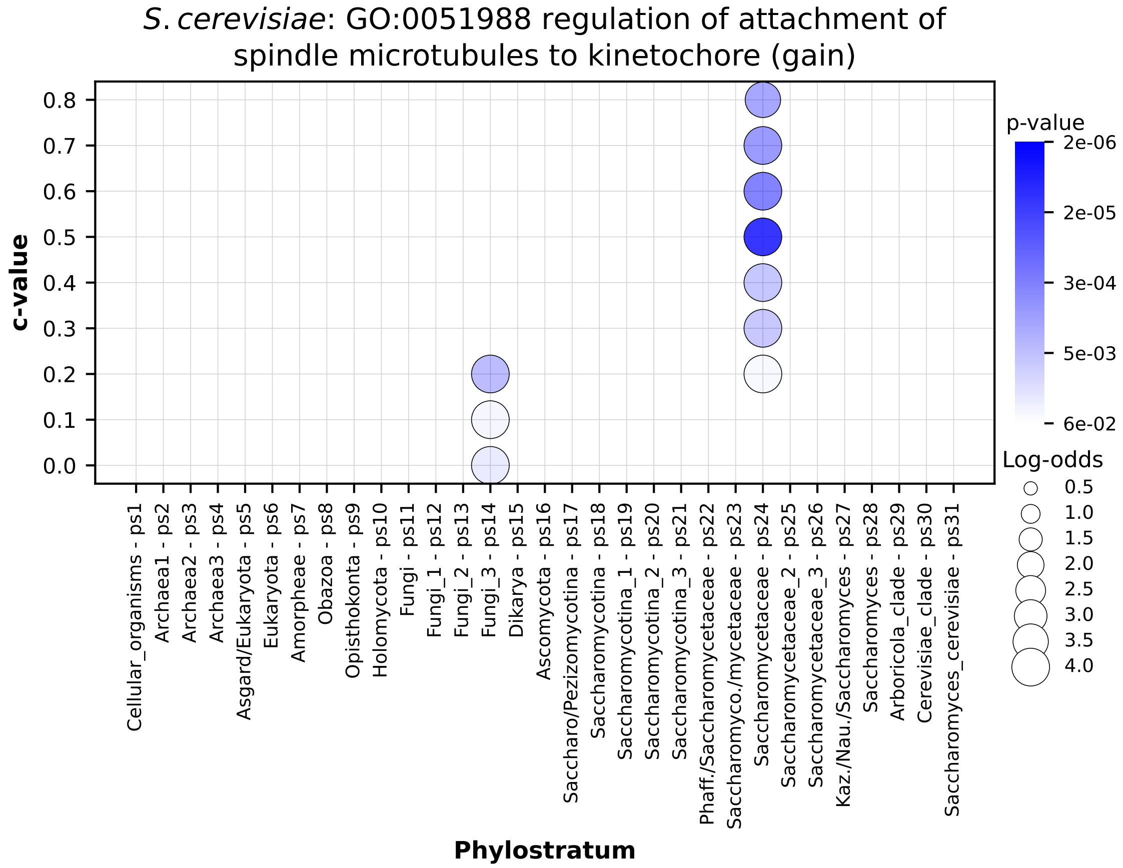

S. cerevisiae: GO:0051988 regulation of attachment of spindle microtubules to kinetochore (gain)
c-value
0.8
0.7
0.6
0.5
0.4
0.3
0.2
0.1
0.0
p-value
2e-06
2e-05
3e-04
5e-03
6e-02
Log-odds
0.5
1.0
1.5
2.0
2.5
3.0
3.5
4.0
Cellular_organisms - ps1
Archaea1 - ps2
Archaea2 - ps3
Archaea3 - ps4
Asgard/Eukaryota - ps5
Eukaryota - ps6
Amorpheae - ps7
Obazoa - ps8
Opisthokonta - ps9
Holomycota - ps10
Fungi - ps11
Fungi_1 - ps12
Fungi_2 - ps13
Fungi_3 - ps14
Dikarya - ps15
Ascomycota - ps16
Saccharo/Pezizomycotina - ps17
Saccharomycotina - ps18
Saccharomycotina_1 - ps19
Saccharomycotina_2 - ps20
Saccharomycotina_3 - ps21
Phaff./Saccharomycetaceae - ps22
Saccharomyco./mycetaceae - ps23
Saccharomycetaceae - ps24
Saccharomycetaceae_2 - ps25
Saccharomycetaceae_3 - ps26
Kaz./Nau./Saccharomyces - ps27
Saccharomyces - ps28
Arboricola_clade - ps29
Cerevisiae_clade - ps30
Saccharomyces_cerevisiae - ps31
Phylostratum

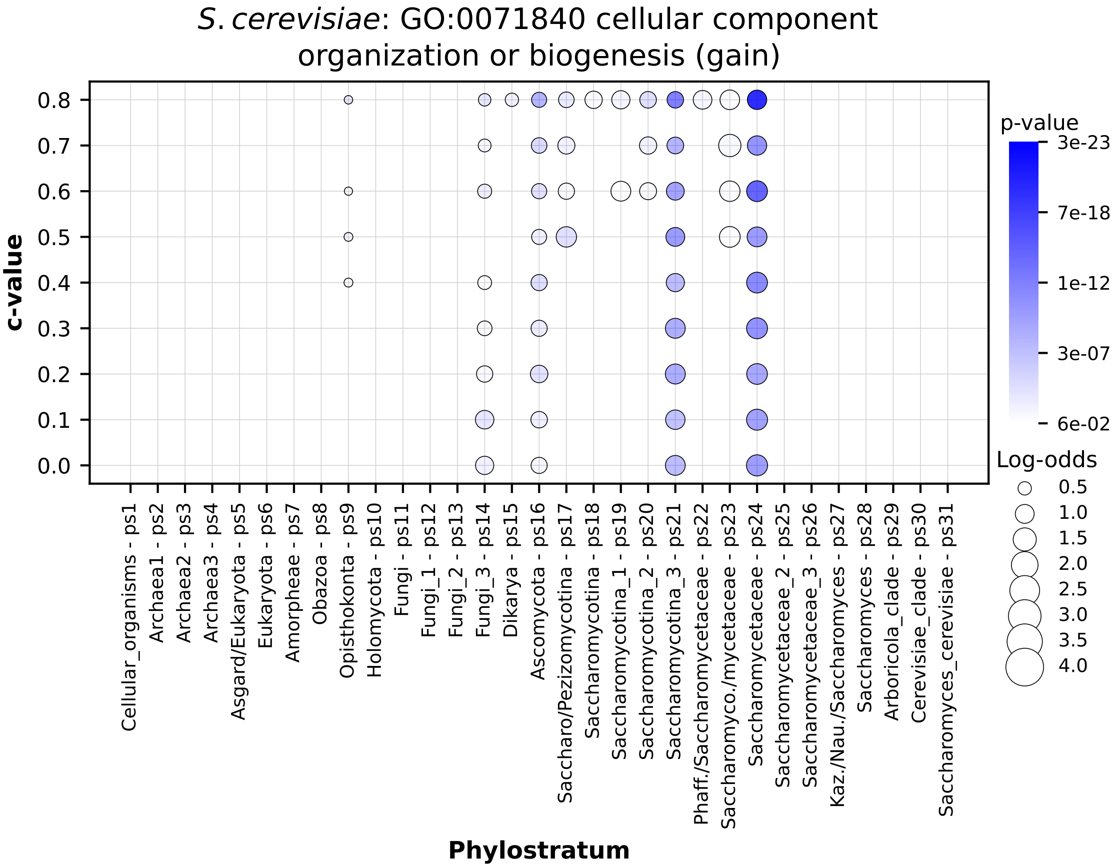
S. cerevisiae: GO:0071840 cellular component organization or biogenesis (gain)
c-value
0.8
0.7
0.6
0.5
0.4
0.3
0.2
0.1
0.0
Phylostratum
Cellular_organisms - ps1
Archaea1 - ps2
Archaea2 - ps3
Archaea3 - ps4
Asgard/Eukaryota - ps5
Eukaryota - ps6
Amorpheae - ps7
Obazoa - ps8
Opisthokonta - ps9
Holomycota - ps10
Fungi - ps11
Fungi_1 - ps12
Fungi_2 - ps13
Fungi_3 - ps14
Dikarya - ps15
Ascomycota - ps16
Saccharo/Pezizomycotina - ps17
Saccharomycotina - ps18
Saccharomycotina_1 - ps19
Saccharomycotina_2 - ps20
Saccharomycotina_3 - ps21
Phaff./Saccharomycetaceae - ps22
Saccharomyco./mycetaceae - ps23
Saccharomycetaceae - ps24
Saccharomycetaceae_2 - ps25
Saccharomycetaceae_3 - ps26
Kaz./Nau./Saccharomyces - ps27
Saccharomyces - ps28
Arboricola_clade - ps29
Cerevisiae_clade - ps30
Saccharomyces_cerevisiae - ps31
p-value
3e-23
7e-18
1e-12
3e-07
6e-02
Log-odds
0.5
1.0
1.5
2.0
2.5
3.0
3.5
4.0

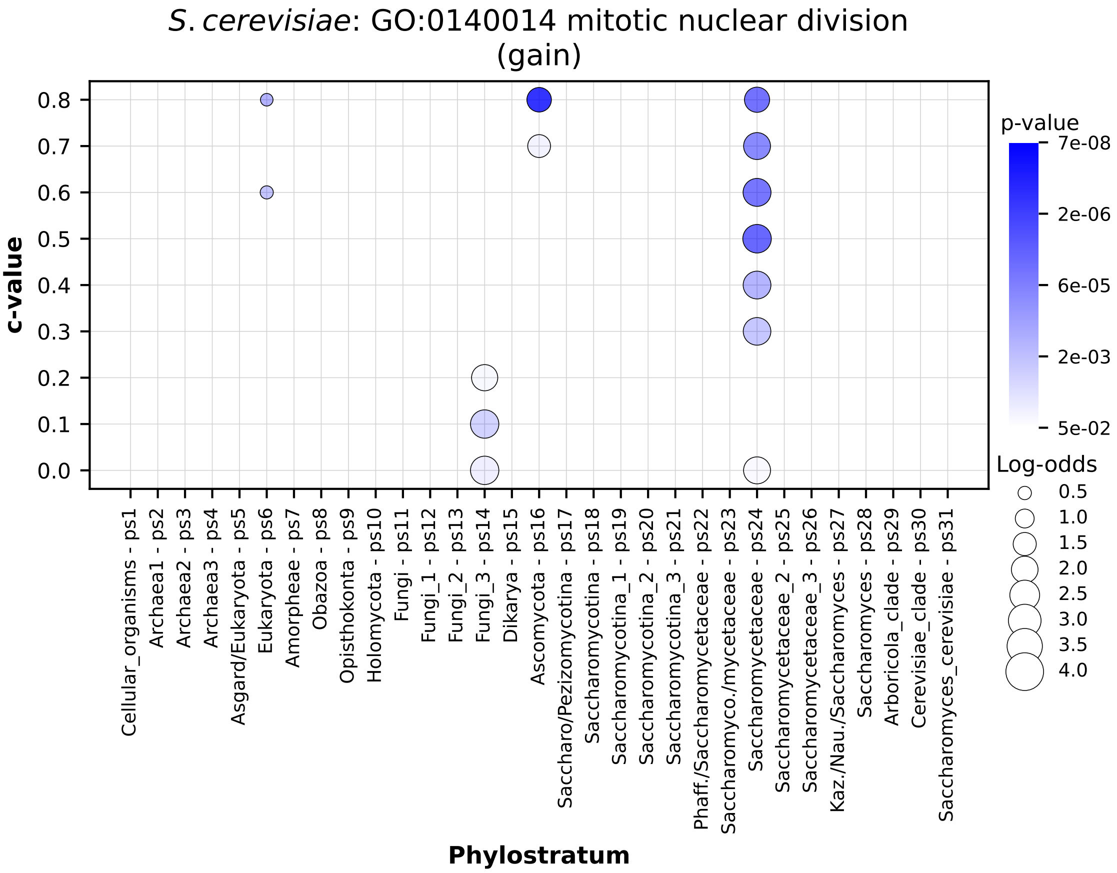

S. cerevisiae: GO:0140014 mitotic nuclear division (gain)
c-value
0.8
0.7
0.6
0.5
0.4
0.3
0.2
0.1
0.0
Phylostratum
Cellular_organisms - ps1
Archaea1 - ps2
Archaea2 - ps3
Archaea3 - ps4
Asgard/Eukaryota - ps5
Eukaryota - ps6
Amorpheae - ps7
Obazoa - ps8
Opisthokonta - ps9
Holomycota - ps10
Fungi - ps11
Fungi_1 - ps12
Fungi_2 - ps13
Fungi_3 - ps14
Dikarya - ps15
Ascomycota - ps16
Saccharo/Pezizomycotina - ps17
Saccharomycotina - ps18
Saccharomycotina_1 - ps19
Saccharomycotina_2 - ps20
Saccharomycotina_3 - ps21
Phaff./Saccharomycetaceae - ps22
Saccharomyco./mycetaceae - ps23
Saccharomycetaceae - ps24
Saccharomycetaceae_2 - ps25
Saccharomycetaceae_3 - ps26
Kaz./Nau./Saccharomyces - ps27
Saccharomyces - ps28
Arboricola_clade - ps29
Cerevisiae_clade - ps30
Saccharomyces_cerevisiae - ps31
p-value
7e-08
2e-06
6e-05
2e-03
5e-02
Log-odds
0.5
1.0
1.5
2.0
2.5
3.0
3.5
4.0

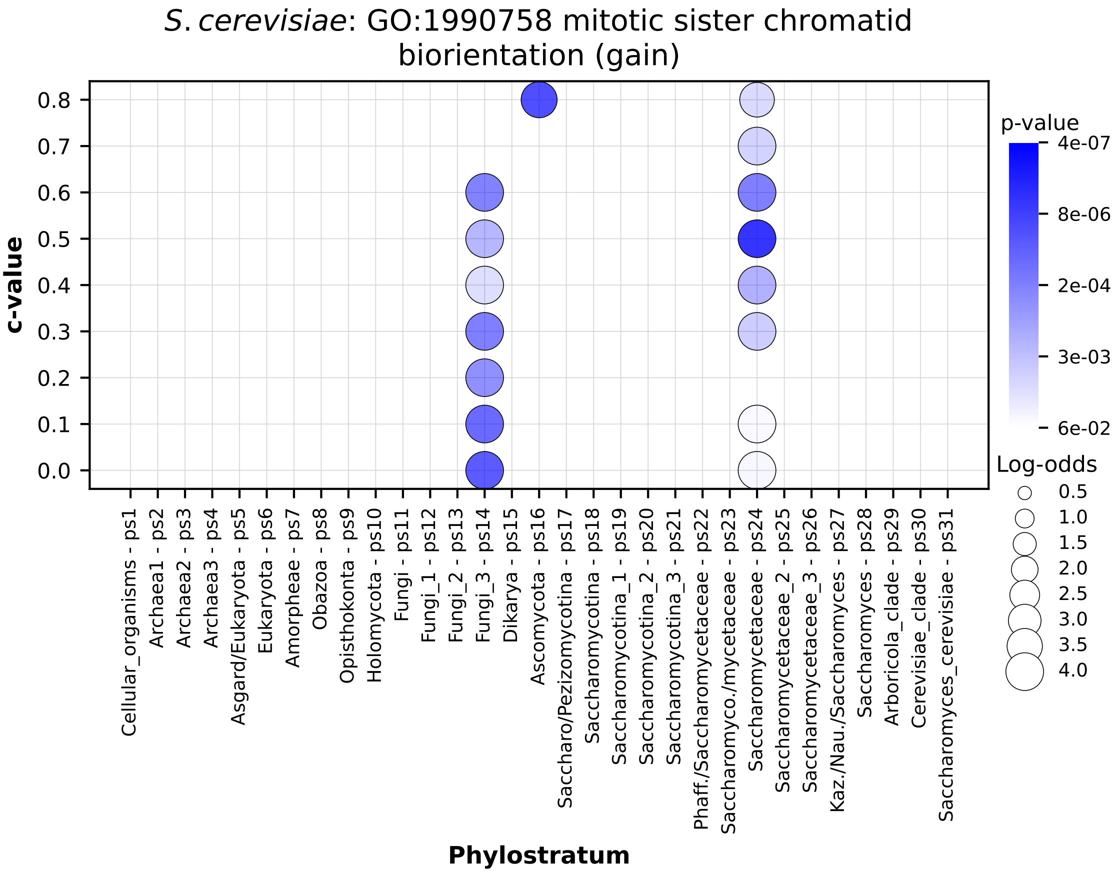

S. cerevisiae: GO:1990758 mitotic sister chromatid biorientation (gain)
c-value
0.0
0.1
0.2
0.3
0.4
0.5
0.6
0.7
0.8
p-value
4e-07
8e-06
2e-04
3e-03
6e-02
Log-odds
0.5
1.0
1.5
2.0
2.5
3.0
3.5
4.0
Cellular_organisms - ps1
Archaea1 - ps2
Archaea2 - ps3
Archaea3 - ps4
Asgard/Eukaryota - ps5
Eukaryota - ps6
Amorpheae - ps7
Obazoa - ps8
Opisthokonta - ps9
Holomycota - ps10
Fungi - ps11
Fungi_1 - ps12
Fungi_2 - ps13
Fungi_3 - ps14
Dikarya - ps15
Ascomycota - ps16
Saccharo/Pezizomycotina - ps17
Saccharomycotina - ps18
Saccharomycotina_1 - ps19
Saccharomycotina_2 - ps20
Saccharomycotina_3 - ps21
Phaff./Saccharomycetaceae - ps22
Saccharomyco./mycetaceae - ps23
Saccharomycetaceae - ps24
Saccharomycetaceae_2 - ps25
Saccharomycetaceae_3 - ps26
Kaz./Nau./Saccharomyces - ps27
Saccharomyces - ps28
Arboricola_clade - ps29
Cerevisiae_clade - ps30
Saccharomyces_cerevisiae - ps31
Phylostratum

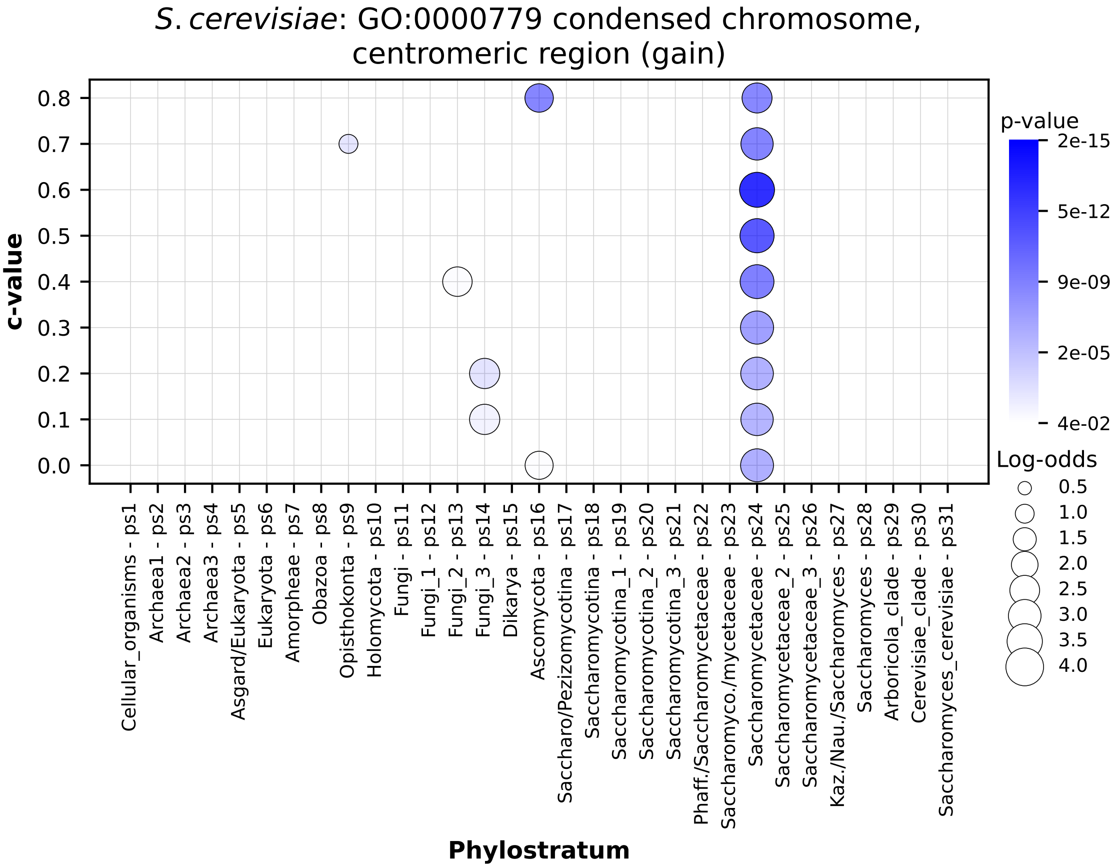
S. cerevisiae: GO:0000779 condensed chromosome, centromeric region (gain)
c-value
0.8
0.7
0.6
0.5
0.4
0.3
0.2
0.1
0.0
Phylostratum
Cellular_organisms - ps1
Archaea1 - ps2
Archaea2 - ps3
Archaea3 - ps4
Asgard/Eukaryota - ps5
Eukaryota - ps6
Amorpheae - ps7
Obazoa - ps8
Opisthokonta - ps9
Holomycota - ps10
Fungi - ps11
Fungi_1 - ps12
Fungi_2 - ps13
Fungi_3 - ps14
Dikarya - ps15
Ascomycota - ps16
Saccharo/Pezizomycotina - ps17
Saccharomycotina - ps18
Saccharomycotina_1 - ps19
Saccharomycotina_2 - ps20
Saccharomycotina_3 - ps21
Phaff./Saccharomycetaceae - ps22
Saccharomyco./mycetaceae - ps23
Saccharomycetaceae - ps24
Saccharomycetaceae_2 - ps25
Saccharomycetaceae_3 - ps26
Kaz./Nau./Saccharomyces - ps27
Saccharomyces - ps28
Arboricola_clade - ps29
Cerevisiae_clade - ps30
Saccharomyces_cerevisiae - ps31
p-value
2e-15
5e-12
9e-09
2e-05
4e-02
Log-odds
0.5
1.0
1.5
2.0
2.5
3.0
3.5
4.0

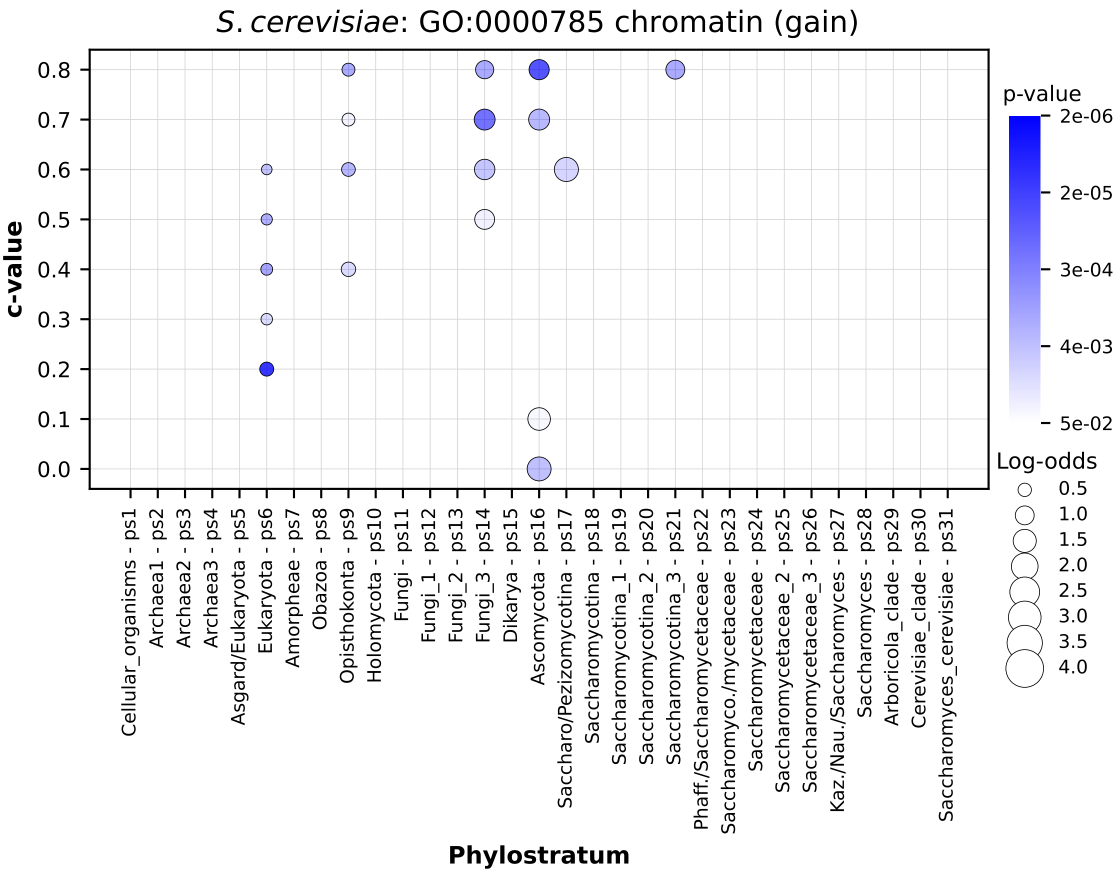

S. cerevisiae: GO:0000785 chromatin (gain)
c-value
0.0
0.1
0.2
0.3
0.4
0.5
0.6
0.7
0.8
Phylostratum
Cellular_organisms - ps1
Archaea1 - ps2
Archaea2 - ps3
Archaea3 - ps4
Asgard/Eukaryota - ps5
Eukaryota - ps6
Amorpheae - ps7
Obazoa - ps8
Opisthokonta - ps9
Holomycota - ps10
Fungi - ps11
Fungi_1 - ps12
Fungi_2 - ps13
Fungi_3 - ps14
Dikarya - ps15
Ascomycota - ps16
Saccharo/Pezizomycotina - ps17
Saccharomycotina - ps18
Saccharomycotina_1 - ps19
Saccharomycotina_2 - ps20
Saccharomycotina_3 - ps21
Phaff./Saccharomycetaceae - ps22
Saccharomyco./mycetaceae - ps23
Saccharomycetaceae - ps24
Saccharomycetaceae_2 - ps25
Saccharomycetaceae_3 - ps26
Kaz./Nau./Saccharomyces - ps27
Saccharomyces - ps28
Arboricola_clade - ps29
Cerevisiae_clade - ps30
Saccharomyces_cerevisiae - ps31
p-value
2e-06
2e-05
3e-04
4e-03
5e-02
Log-odds
0.5
1.0
1.5
2.0
2.5
3.0
3.5
4.0

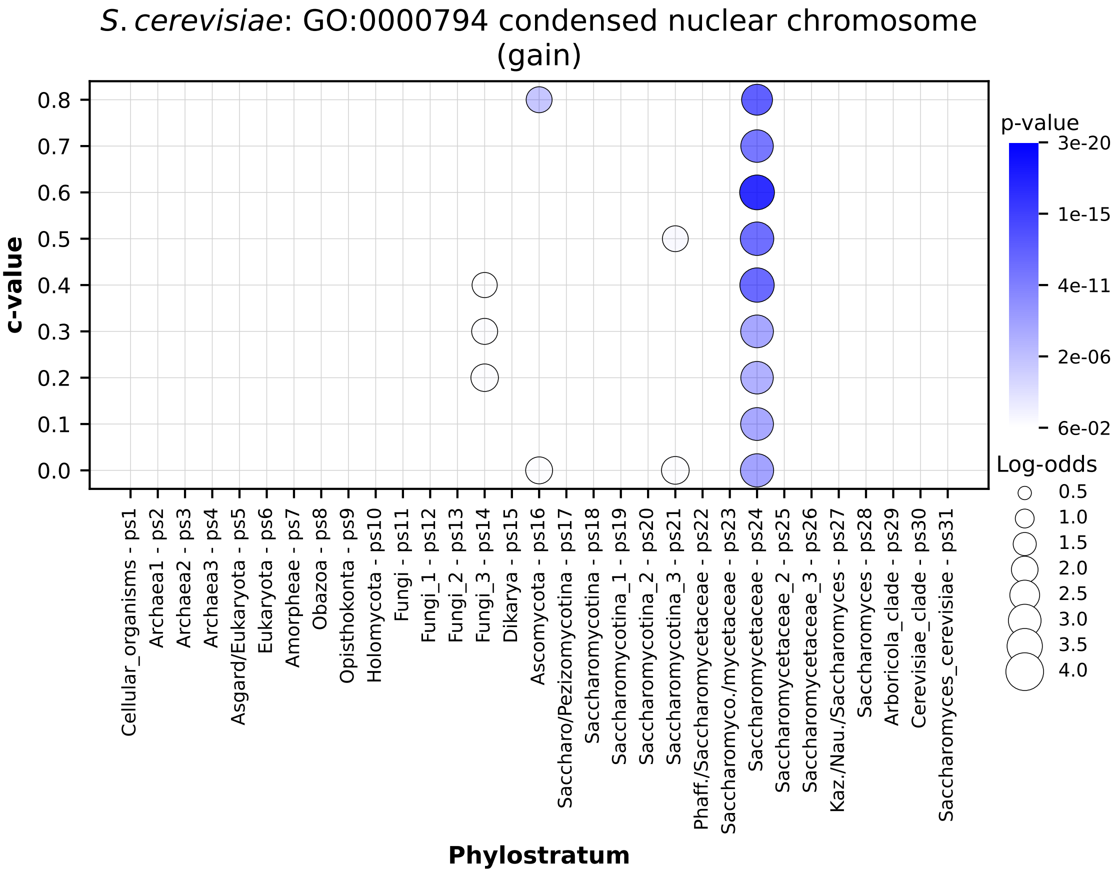

S. cerevisiae: GO:0000794 condensed nuclear chromosome
(gain)
c-value
0.8
0.7
0.6
0.5
0.4
0.3
0.2
0.1
0.0
p-value
3e-20
1e-15
4e-11
2e-06
6e-02
Log-odds
0.5
1.0
1.5
2.0
2.5
3.0
3.5
4.0
Cellular_organisms - ps1
Archaea1 - ps2
Archaea2 - ps3
Archaea3 - ps4
Asgard/Eukaryota - ps5
Eukaryota - ps6
Amorpheae - ps7
Obazoa - ps8
Opisthokonta - ps9
Holomycota - ps10
Fungi - ps11
Fungi_1 - ps12
Fungi_2 - ps13
Fungi_3 - ps14
Dikarya - ps15
Ascomycota - ps16
Saccharo/Pezizomycotina - ps17
Saccharomycotina - ps18
Saccharomycotina_1 - ps19
Saccharomycotina_2 - ps20
Saccharomycotina_3 - ps21
Phaff./Saccharomycetaceae - ps22
Saccharomyco./mycetaceae - ps23
Saccharomycetaceae - ps24
Saccharomycetaceae_2 - ps25
Saccharomycetaceae_3 - ps26
Kaz./Nau./Saccharomyces - ps27
Saccharomyces - ps28
Arboricola_clade - ps29
Cerevisiae_clade - ps30
Saccharomyces_cerevisiae - ps31
Phylostratum

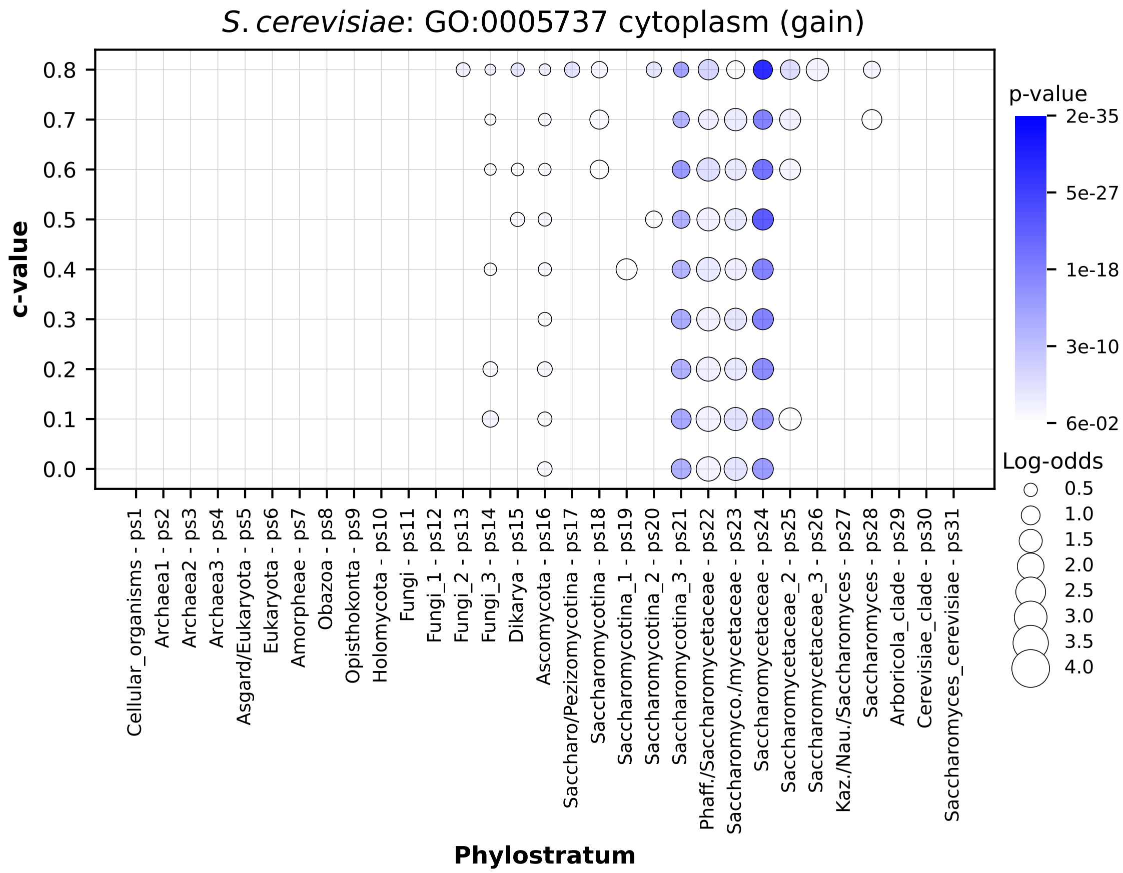

S. cerevisiae: GO:0005737 cytoplasm (gain)
c-value
0.8
0.7
0.6
0.5
0.4
0.3
0.2
0.1
0.0
Phylostratum
Cellular_organisms - ps1
Archaea1 - ps2
Archaea2 - ps3
Archaea3 - ps4
Asgard/Eukaryota - ps5
Eukaryota - ps6
Amorpheae - ps7
Obazoa - ps8
Opisthokonta - ps9
Holomycota - ps10
Fungi - ps11
Fungi_1 - ps12
Fungi_2 - ps13
Fungi_3 - ps14
Dikarya - ps15
Ascomycota - ps16
Saccharo/Pezizomycotina - ps17
Saccharomycotina - ps18
Saccharomycotina_1 - ps19
Saccharomycotina_2 - ps20
Saccharomycotina_3 - ps21
Phaff./Saccharomycetaceae - ps22
Saccharomyco./mycetaceae - ps23
Saccharomycetaceae - ps24
Saccharomycetaceae_2 - ps25
Saccharomycetaceae_3 - ps26
Kaz./Nau./Saccharomyces - ps27
Saccharomyces - ps28
Arboricola_clade - ps29
Cerevisiae_clade - ps30
Saccharomyces_cerevisiae - ps31
p-value
2e-35
5e-27
1e-18
3e-10
6e-02
Log-odds
0.5
1.0
1.5
2.0
2.5
3.0
3.5
4.0

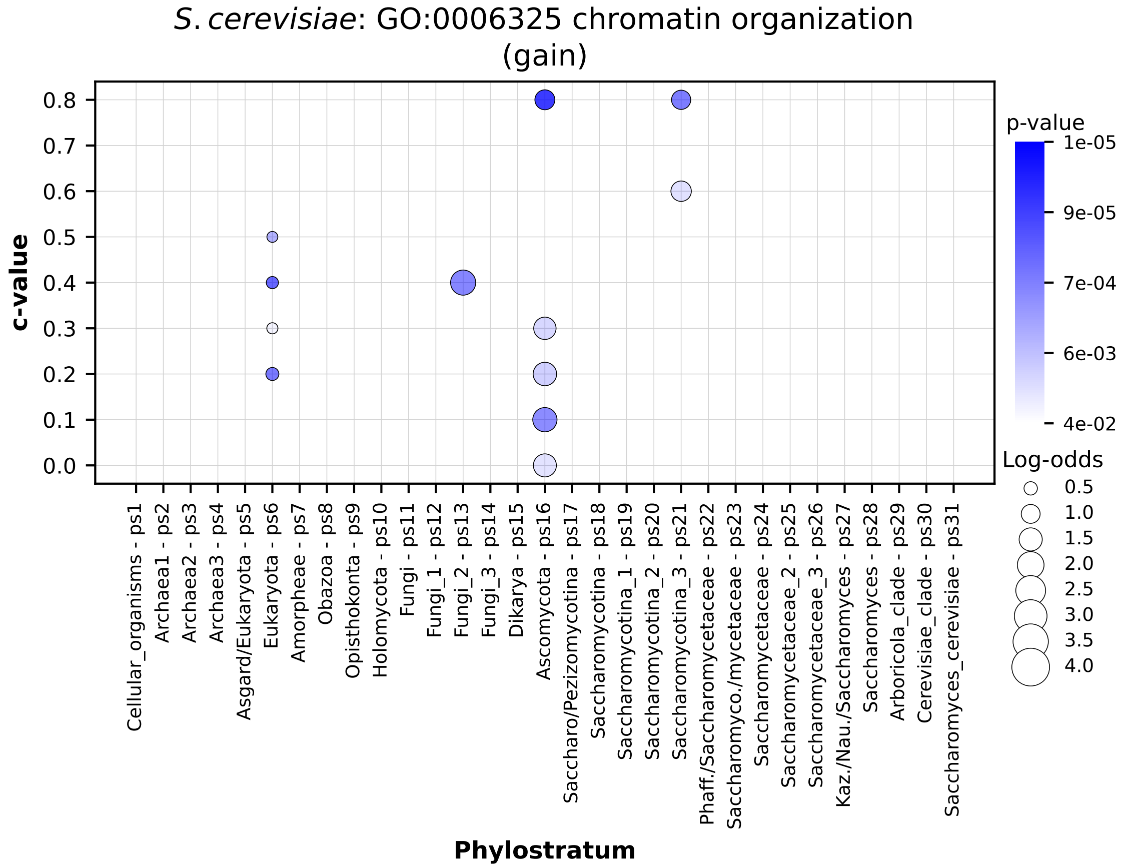

S. cerevisiae: GO:0006325 chromatin organization
(gain)
c-value
0.8
0.7
0.6
0.5
0.4
0.3
0.2
0.1
0.0
p-value
1e-05
9e-05
7e-04
6e-03
4e-02
Log-odds
0.5
1.0
1.5
2.0
2.5
3.0
3.5
4.0
Cellular_organisms - ps1
Archaea1 - ps2
Archaea2 - ps3
Archaea3 - ps4
Asgard/Eukaryota - ps5
Eukaryota - ps6
Amorpheae - ps7
Obazoa - ps8
Opisthokonta - ps9
Holomycota - ps10
Fungi - ps11
Fungi_1 - ps12
Fungi_2 - ps13
Fungi_3 - ps14
Dikarya - ps15
Ascomycota - ps16
Saccharo/Pezizomycotina - ps17
Saccharomycotina - ps18
Saccharomycotina_1 - ps19
Saccharomycotina_2 - ps20
Saccharomycotina_3 - ps21
Phaff./Saccharomycetaceae - ps22
Saccharomyco./mycetaceae - ps23
Saccharomycetaceae - ps24
Saccharomycetaceae_2 - ps25
Saccharomycetaceae_3 - ps26
Kaz./Nau./Saccharomyces - ps27
Saccharomyces - ps28
Arboricola_clade - ps29
Cerevisiae_clade - ps30
Saccharomyces_cerevisiae - ps31
Phylostratum

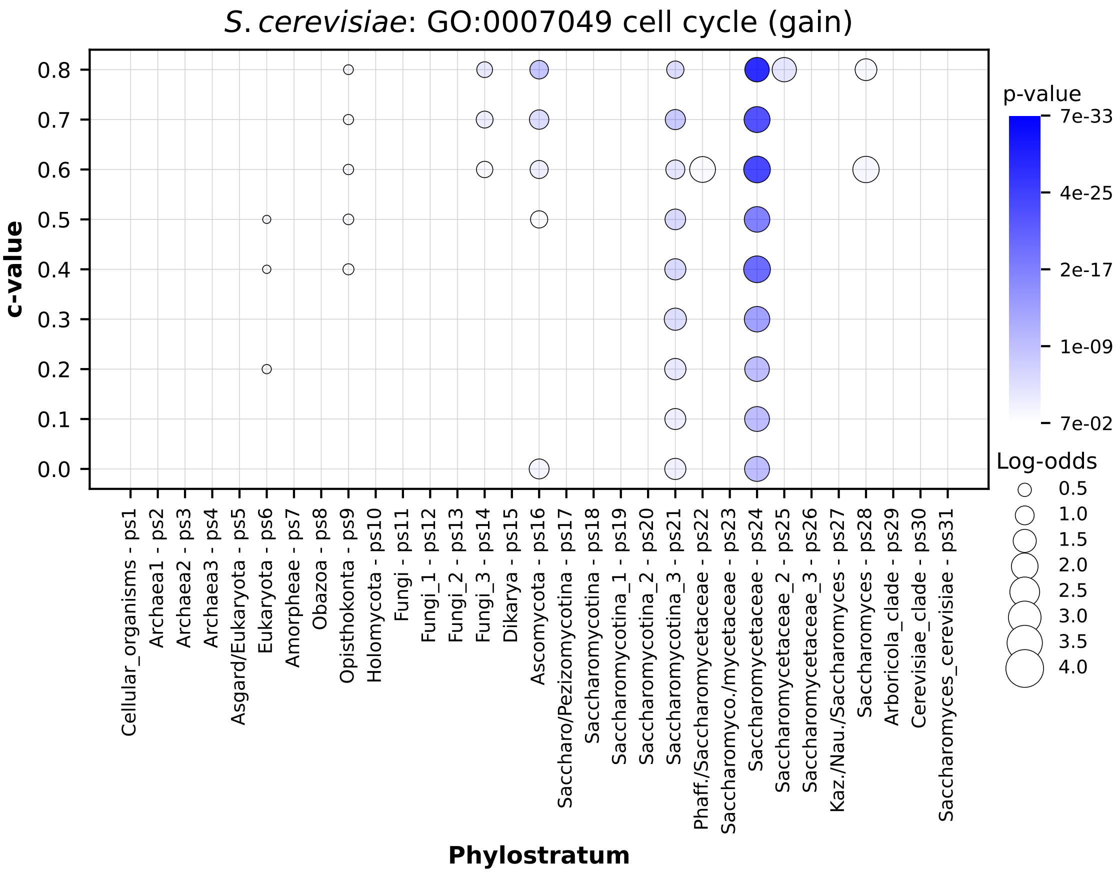

S. cerevisiae: GO:0007049 cell cycle (gain)
c-value
0.8
0.7
0.6
0.5
0.4
0.3
0.2
0.1
0.0
Phylostratum
Cellular_organisms - ps1
Archaea1 - ps2
Archaea2 - ps3
Archaea3 - ps4
Asgard/Eukaryota - ps5
Eukaryota - ps6
Amorpheae - ps7
Obazoa - ps8
Opisthokonta - ps9
Holomycota - ps10
Fungi - ps11
Fungi_1 - ps12
Fungi_2 - ps13
Fungi_3 - ps14
Dikarya - ps15
Ascomycota - ps16
Saccharo/Pezizomycotina - ps17
Saccharomycotina - ps18
Saccharomycotina_1 - ps19
Saccharomycotina_2 - ps20
Saccharomycotina_3 - ps21
Phaff./Saccharomycetaceae - ps22
Saccharomyco./mycetaceae - ps23
Saccharomycetaceae - ps24
Saccharomycetaceae_2 - ps25
Saccharomycetaceae_3 - ps26
Kaz./Nau./Saccharomyces - ps27
Saccharomyces - ps28
Arboricola_clade - ps29
Cerevisiae_clade - ps30
Saccharomyces_cerevisiae - ps31
p-value
7e-33
4e-25
2e-17
1e-09
7e-02
Log-odds
0.5
1.0
1.5
2.0
2.5
3.0
3.5
4.0

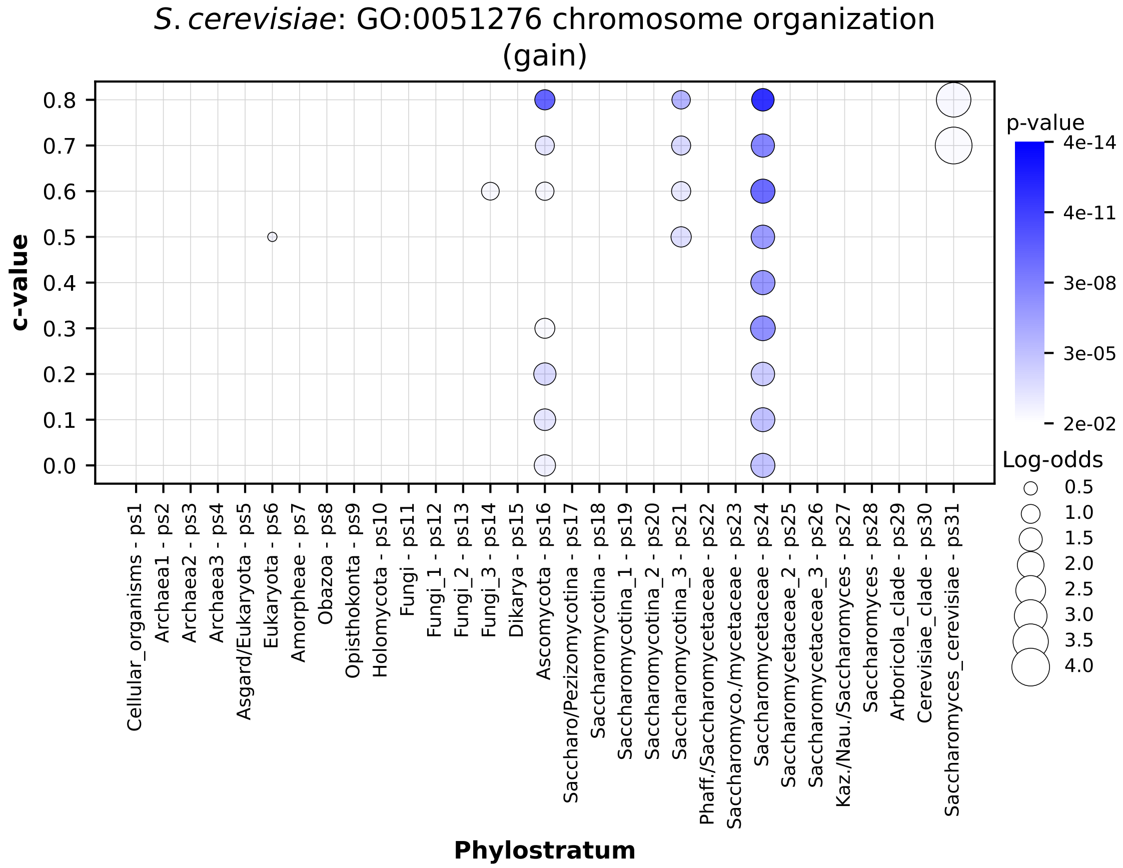
S. cerevisiae: GO:0051276 chromosome organization (gain)
c-value
0.0
0.1
0.2
0.3
0.4
0.5
0.6
0.7
0.8
Phylostratum
Cellular_organisms - ps1
Archaea1 - ps2
Archaea2 - ps3
Archaea3 - ps4
Asgard/Eukaryota - ps5
Eukaryota - ps6
Amorpheae - ps7
Obazoa - ps8
Opisthokonta - ps9
Holomycota - ps10
Fungi - ps11
Fungi_1 - ps12
Fungi_2 - ps13
Fungi_3 - ps14
Dikarya - ps15
Ascomycota - ps16
Saccharo/Pezizomycotina - ps17
Saccharomycotina - ps18
Saccharomycotina_1 - ps19
Saccharomycotina_2 - ps20
Saccharomycotina_3 - ps21
Phaff./Saccharomycetaceae - ps22
Saccharomyco./mycetaceae - ps23
Saccharomycetaceae - ps24
Saccharomycetaceae_2 - ps25
Saccharomycetaceae_3 - ps26
Kaz./Nau./Saccharomyces - ps27
Saccharomyces - ps28
Arboricola_clade - ps29
Cerevisiae_clade - ps30
Saccharomyces_cerevisiae - ps31
p-value
4e-14
4e-11
3e-08
3e-05
2e-02
Log-odds
0.5
1.0
1.5
2.0
2.5
3.0
3.5
4.0

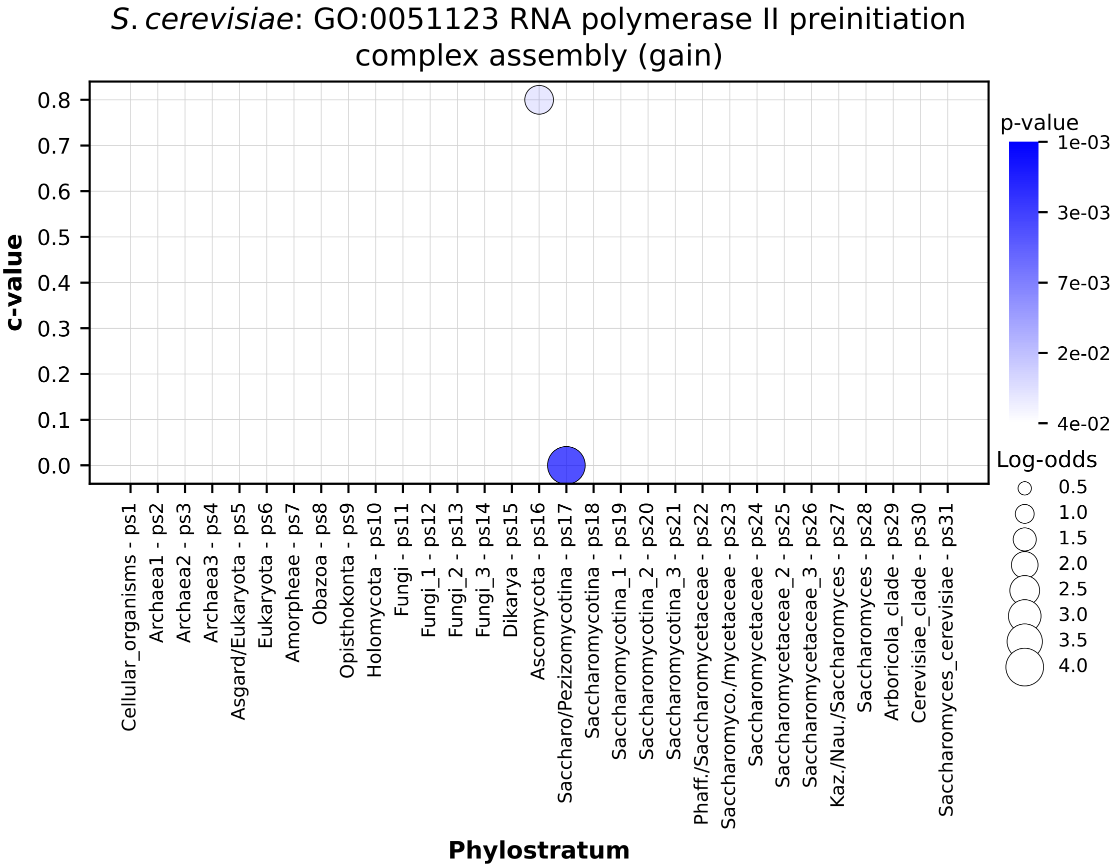

S. cerevisiae: GO:0051123 RNA polymerase II preinitiation complex assembly (gain)
c-value
0.8
0.7
0.6
0.5
0.4
0.3
0.2
0.1
0.0
p-value
1e-03
3e-03
7e-03
2e-02
4e-02
Log-odds
0.5
1.0
1.5
2.0
2.5
3.0
3.5
4.0
Cellular_organisms - ps1
Archaea1 - ps2
Archaea2 - ps3
Archaea3 - ps4
Asgard/Eukaryota - ps5
Eukaryota - ps6
Amorpheae - ps7
Obazoa - ps8
Opisthokonta - ps9
Holomycota - ps10
Fungi - ps11
Fungi_1 - ps12
Fungi_2 - ps13
Fungi_3 - ps14
Dikarya - ps15
Ascomycota - ps16
Saccharo/Pezizomycotina - ps17
Saccharomycotina - ps18
Saccharomycotina_1 - ps19
Saccharomycotina_2 - ps20
Saccharomycotina_3 - ps21
Phaff./Saccharomycetaceae - ps22
Saccharomyco./mycetaceae - ps23
Saccharomycetaceae - ps24
Saccharomycetaceae_2 - ps25
Saccharomycetaceae_3 - ps26
Kaz./Nau./Saccharomyces - ps27
Saccharomyces - ps28
Arboricola_clade - ps29
Cerevisiae_clade - ps30
Saccharomyces_cerevisiae - ps31
Phylostratum

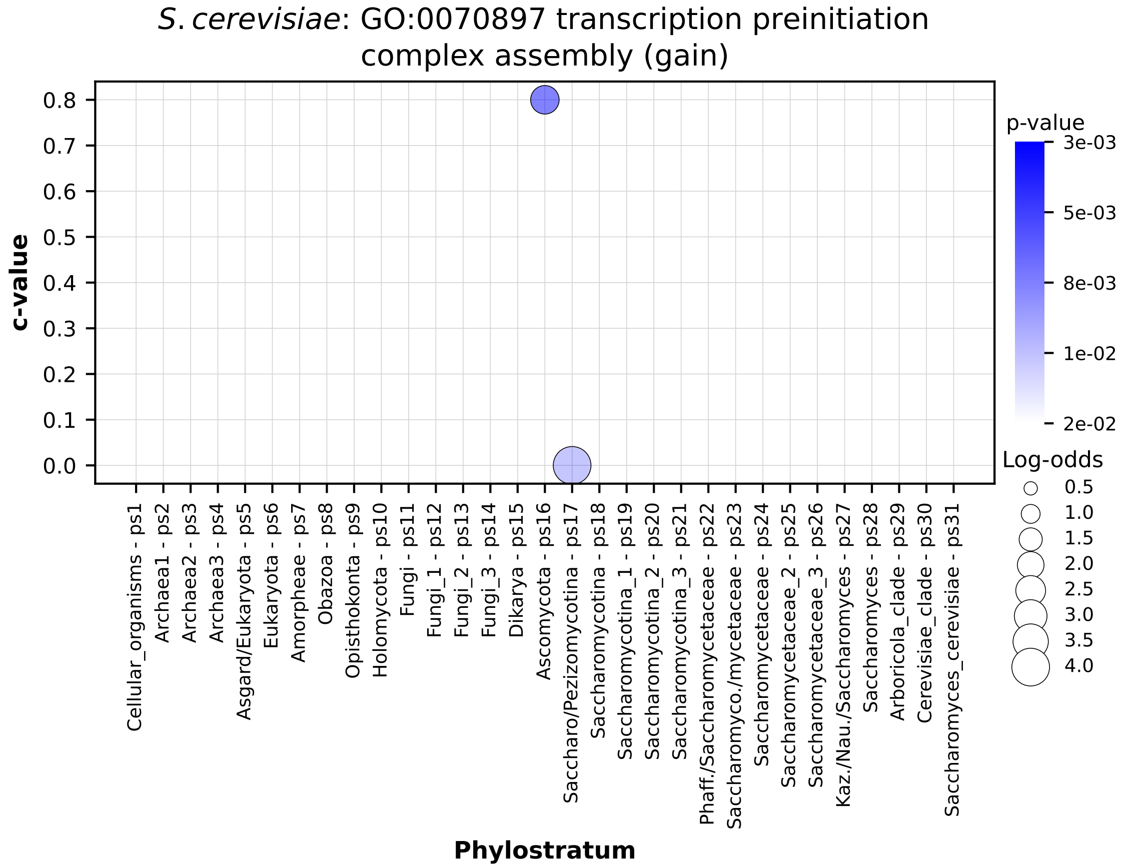
S. cerevisiae: GO:0070897 transcription preinitiation complex assembly (gain)
c-value
0.8
0.7
0.6
0.5
0.4
0.3
0.2
0.1
0.0
p-value
3e-03
5e-03
8e-03
1e-02
2e-02
Log-odds
0.5
1.0
1.5
2.0
2.5
3.0
3.5
4.0
Cellular_organisms - ps1
Archaea1 - ps2
Archaea2 - ps3
Archaea3 - ps4
Asgard/Eukaryota - ps5
Eukaryota - ps6
Amorpheae - ps7
Obazoa - ps8
Opisthokonta - ps9
Holomycota - ps10
Fungi - ps11
Fungi_1 - ps12
Fungi_2 - ps13
Fungi_3 - ps14
Dikarya - ps15
Ascomycota - ps16
Saccharo/Pezizomycotina - ps17
Saccharomycotina - ps18
Saccharomycotina_1 - ps19
Saccharomycotina_2 - ps20
Saccharomycotina_3 - ps21
Phaff./Saccharomycetaceae - ps22
Saccharomyco./mycetaceae - ps23
Saccharomycetaceae - ps24
Saccharomycetaceae_2 - ps25
Saccharomycetaceae_3 - ps26
Kaz./Nau./Saccharomyces - ps27
Saccharomyces - ps28
Arboricola_clade - ps29
Cerevisiae_clade - ps30
Saccharomyces_cerevisiae - ps31
Phylostratum

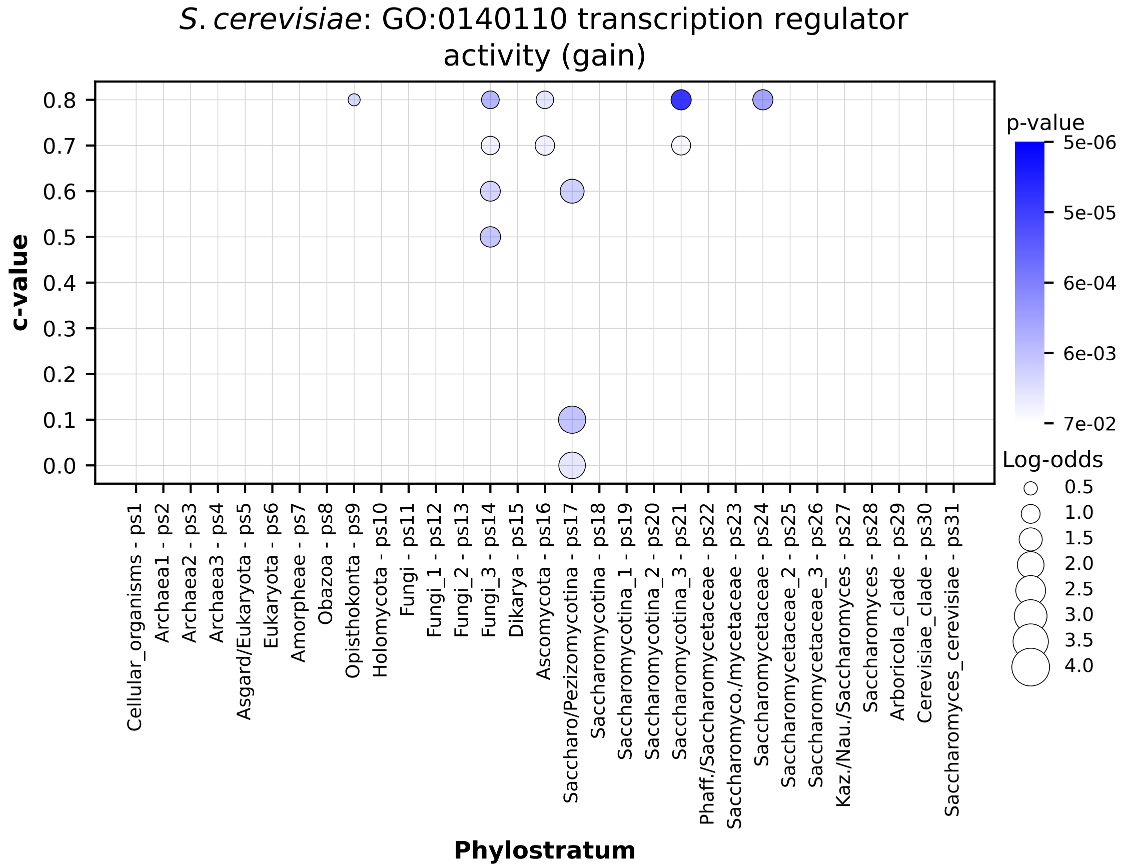
S. cerevisiae: GO:0140110 transcription regulator activity (gain)
c-value
0.8
0.7
0.6
0.5
0.4
0.3
0.2
0.1
0.0
p-value
5e-06
5e-05
6e-04
6e-03
7e-02
Log-odds
0.5
1.0
1.5
2.0
2.5
3.0
3.5
4.0
Cellular_organisms - ps1
Archaea1 - ps2
Archaea2 - ps3
Archaea3 - ps4
Asgard/Eukaryota - ps5
Eukaryota - ps6
Amorpheae - ps7
Obazoa - ps8
Opisthokonta - ps9
Holomycota - ps10
Fungi - ps11
Fungi_1 - ps12
Fungi_2 - ps13
Fungi_3 - ps14
Dikarya - ps15
Ascomycota - ps16
Saccharo/Pezizomycotina - ps17
Saccharomycotina - ps18
Saccharomycotina_1 - ps19
Saccharomycotina_2 - ps20
Saccharomycotina_3 - ps21
Phaff./Saccharomycetaceae - ps22
Saccharomyco./mycetaceae - ps23
Saccharomycetaceae - ps24
Saccharomycetaceae_2 - ps25
Saccharomycetaceae_3 - ps26
Kaz./Nau./Saccharomyces - ps27
Saccharomyces - ps28
Arboricola_clade - ps29
Cerevisiae_clade - ps30
Saccharomyces_cerevisiae - ps31
Phylostratum

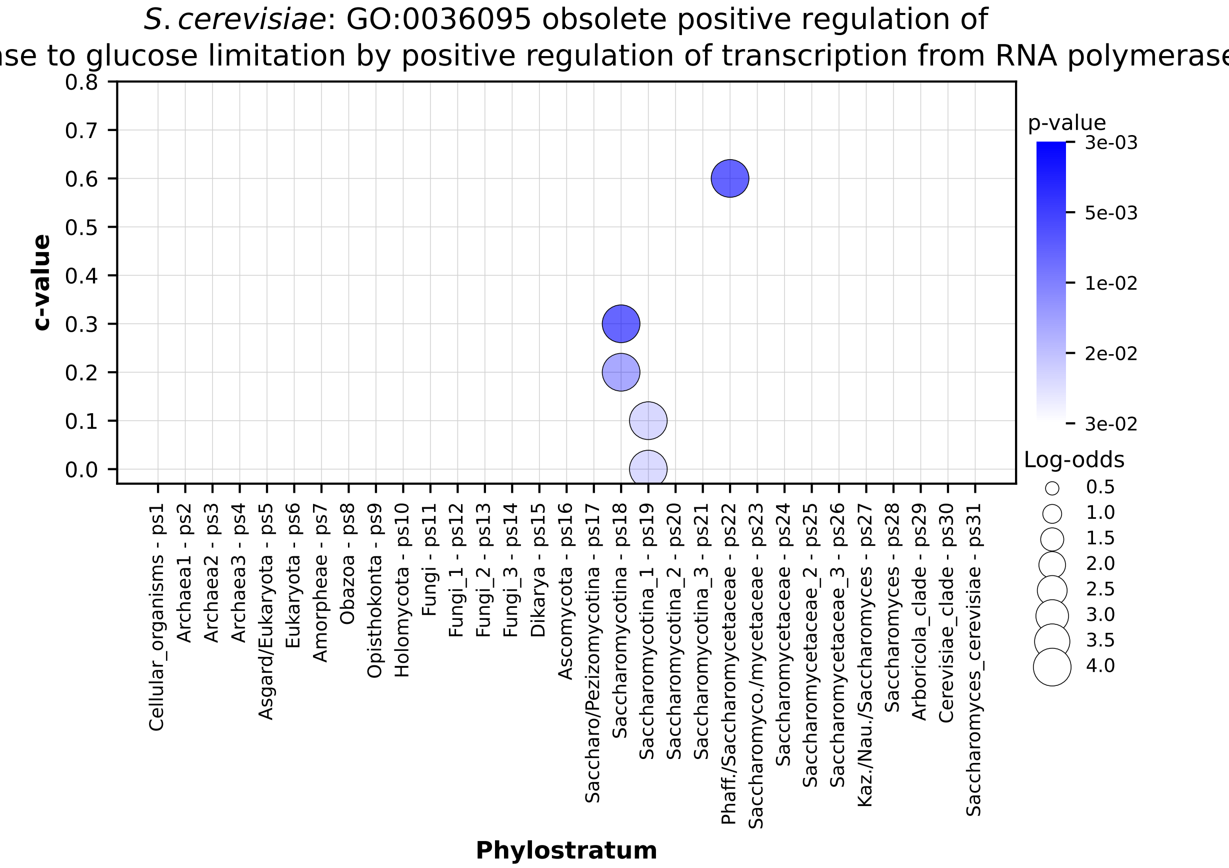

S. cerevisiae: GO:0036095 obsolete positive regulation of
nse to glucose limitation by positive regulation of transcription from RNA polymerase
c-value
0.8
0.7
0.6
0.5
0.4
0.3
0.2
0.1
0.0
p-value
3e-03
5e-03
1e-02
2e-02
3e-02
Log-odds
0.5
1.0
1.5
2.0
2.5
3.0
3.5
4.0
Cellular_organisms - ps1
Archaea1 - ps2
Archaea2 - ps3
Archaea3 - ps4
Asgard/Eukaryota - ps5
Eukaryota - ps6
Amorpheae - ps7
Obazoa - ps8
Opisthokonta - ps9
Holomycota - ps10
Fungi - ps11
Fungi_1 - ps12
Fungi_2 - ps13
Fungi_3 - ps14
Dikarya - ps15
Ascomycota - ps16
Saccharo/Pezizomycotina - ps17
Saccharomycotina - ps18
Saccharomycotina_1 - ps19
Saccharomycotina_2 - ps20
Saccharomycotina_3 - ps21
Phaff./Saccharomycetaceae - ps22
Saccharomyco./mycetaceae - ps23
Saccharomycetaceae - ps24
Saccharomycetaceae_2 - ps25
Saccharomycetaceae_3 - ps26
Kaz./Nau./Saccharomyces - ps27
Saccharomyces - ps28
Arboricola_clade - ps29
Cerevisiae_clade - ps30
Saccharomyces_cerevisiae - ps31
Phylostratum

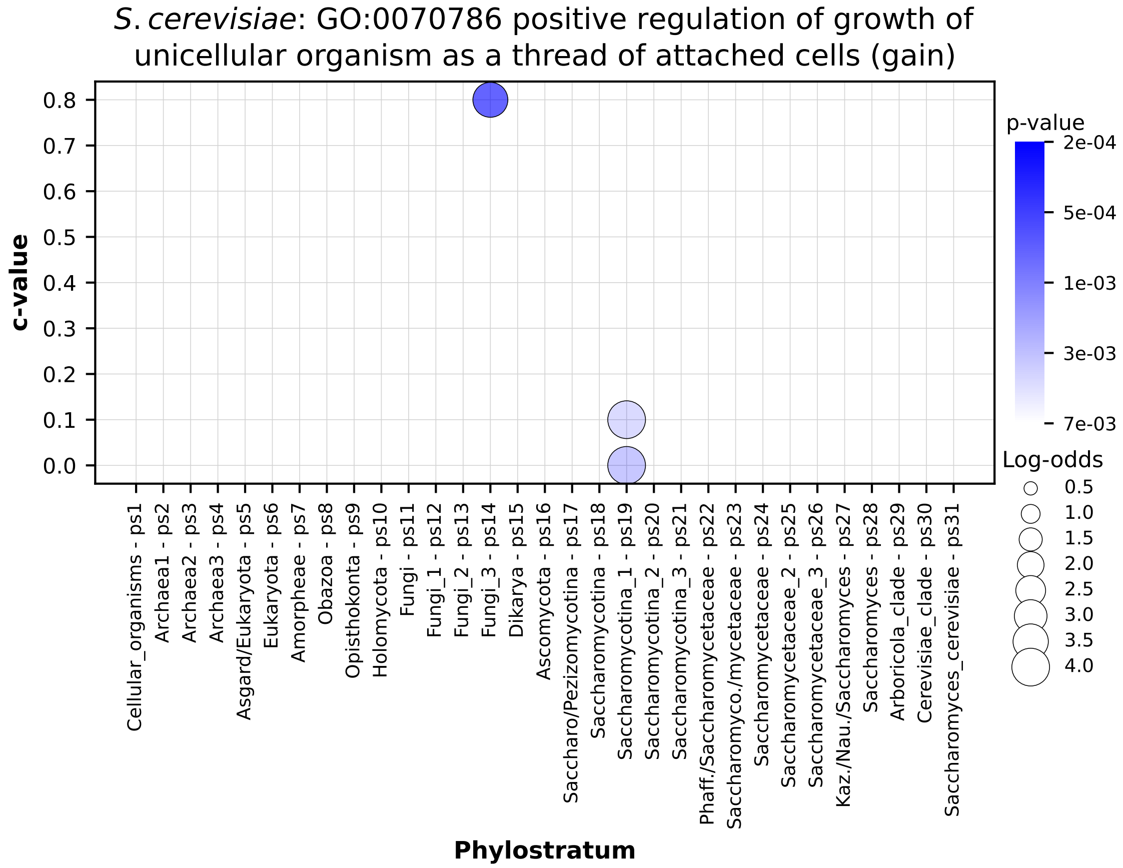
S. cerevisiae: GO:0070786 positive regulation of growth of unicellular organism as a thread of attached cells (gain)
c-value
0.8
0.7
0.6
0.5
0.4
0.3
0.2
0.1
0.0
p-value
2e-04
5e-04
1e-03
3e-03
7e-03
Log-odds
0.5
1.0
1.5
2.0
2.5
3.0
3.5
4.0
Cellular_organisms - ps1
Archaea1 - ps2
Archaea2 - ps3
Archaea3 - ps4
Asgard/Eukaryota - ps5
Eukaryota - ps6
Amorpheae - ps7
Obazoa - ps8
Opisthokonta - ps9
Holomycota - ps10
Fungi - ps11
Fungi_1 - ps12
Fungi_2 - ps13
Fungi_3 - ps14
Dikarya - ps15
Ascomycota - ps16
Saccharo/Pezizomycotina - ps17
Saccharomycotina - ps18
Saccharomycotina_1 - ps19
Saccharomycotina_2 - ps20
Saccharomycotina_3 - ps21
Phaff./Saccharomycetaceae - ps22
Saccharomyco./mycetaceae - ps23
Saccharomycetaceae - ps24
Saccharomycetaceae_2 - ps25
Saccharomycetaceae_3 - ps26
Kaz./Nau./Saccharomyces - ps27
Saccharomyces - ps28
Arboricola_clade - ps29
Cerevisiae_clade - ps30
Saccharomyces_cerevisiae - ps31
Phylostratum

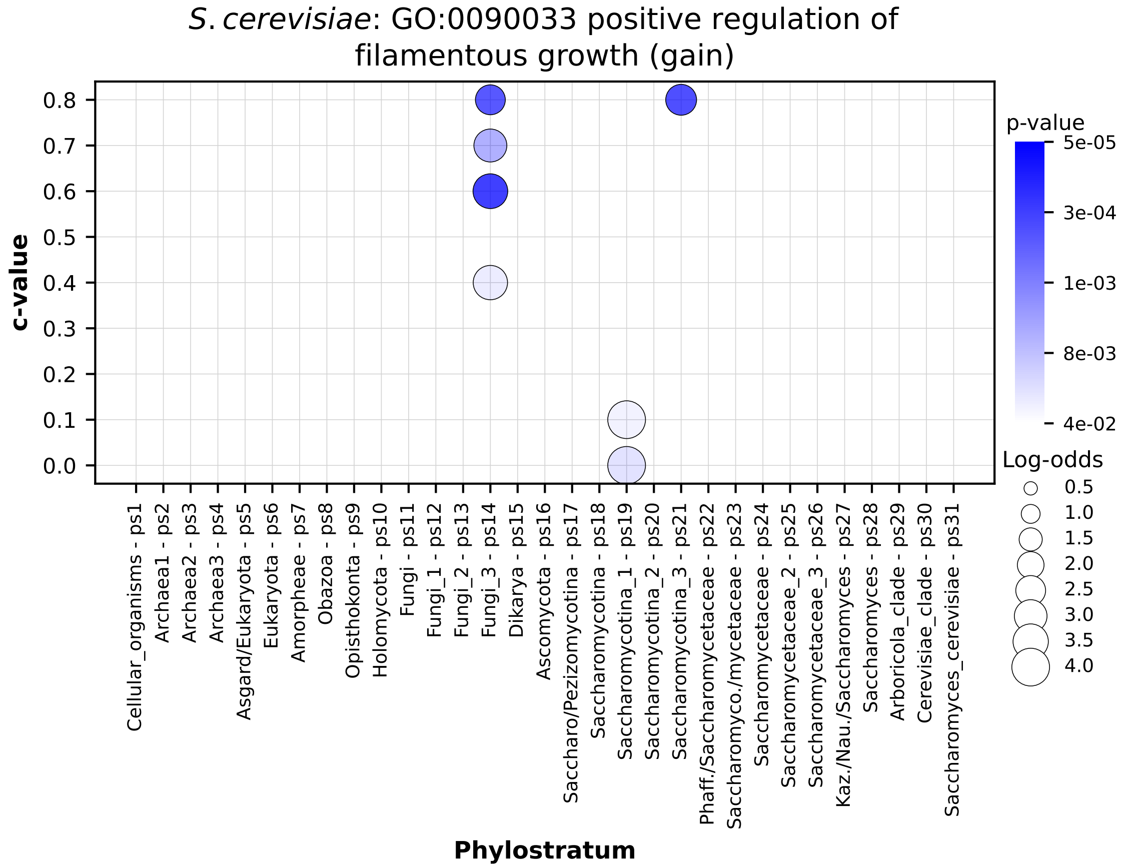

S. cerevisiae: GO:0090033 positive regulation of filamentous growth (gain)
c-value
0.0
0.1
0.2
0.3
0.4
0.5
0.6
0.7
0.8
Phylostratum
Cellular_organisms - ps1
Archaea1 - ps2
Archaea2 - ps3
Archaea3 - ps4
Asgard/Eukaryota - ps5
Eukaryota - ps6
Amorpheae - ps7
Obazoa - ps8
Opisthokonta - ps9
Holomycota - ps10
Fungi - ps11
Fungi_1 - ps12
Fungi_2 - ps13
Fungi_3 - ps14
Dikarya - ps15
Ascomycota - ps16
Saccharo/Pezizomycotina - ps17
Saccharomycotina - ps18
Saccharomycotina_1 - ps19
Saccharomycotina_2 - ps20
Saccharomycotina_3 - ps21
Phaff./Saccharomycetaceae - ps22
Saccharomyco./mycetaceae - ps23
Saccharomycetaceae - ps24
Saccharomycetaceae_2 - ps25
Saccharomycetaceae_3 - ps26
Kaz./Nau./Saccharomyces - ps27
Saccharomyces - ps28
Arboricola_clade - ps29
Cerevisiae_clade - ps30
Saccharomyces_cerevisiae - ps31
p-value
5e-05
3e-04
1e-03
8e-03
4e-02
Log-odds
0.5
1.0
1.5
2.0
2.5
3.0
3.5
4.0

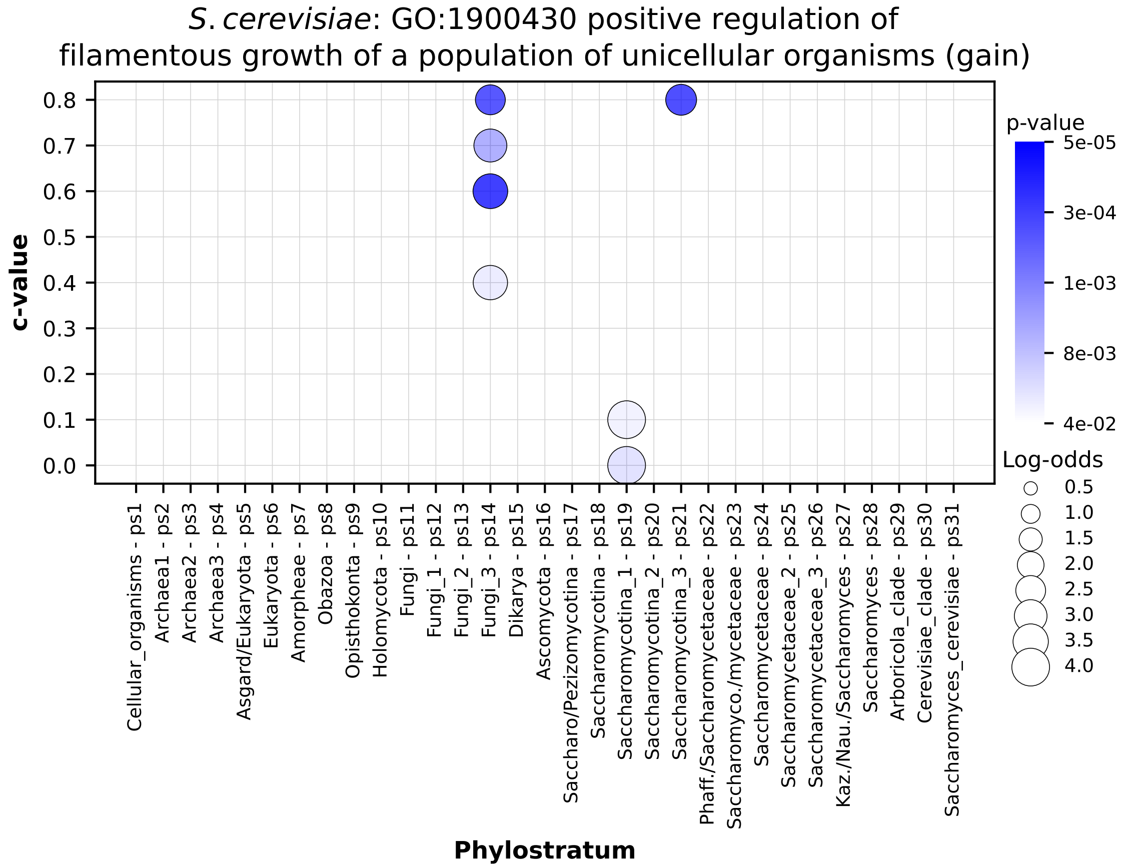

S. cerevisiae: GO:1900430 positive regulation of filamentous growth of a population of unicellular organisms (gain)
c-value
0.8
0.7
0.6
0.5
0.4
0.3
0.2
0.1
0.0
p-value
5e-05
3e-04
1e-03
8e-03
4e-02
Log-odds
0.5
1.0
1.5
2.0
2.5
3.0
3.5
4.0
Cellular_organisms - ps1
Archaea1 - ps2
Archaea2 - ps3
Archaea3 - ps4
Asgard/Eukaryota - ps5
Eukaryota - ps6
Amorpheae - ps7
Obazoa - ps8
Opisthokonta - ps9
Holomycota - ps10
Fungi - ps11
Fungi_1 - ps12
Fungi_2 - ps13
Fungi_3 - ps14
Dikarya - ps15
Ascomycota - ps16
Saccharo/Pezizomycotina - ps17
Saccharomycotina - ps18
Saccharomycotina_1 - ps19
Saccharomycotina_2 - ps20
Saccharomycotina_3 - ps21
Phaff./Saccharomycetaceae - ps22
Saccharomyco./mycetaceae - ps23
Saccharomycetaceae - ps24
Saccharomycetaceae_2 - ps25
Saccharomycetaceae_3 - ps26
Kaz./Nau./Saccharomyces - ps27
Saccharomyces - ps28
Arboricola_clade - ps29
Cerevisiae_clade - ps30
Saccharomyces_cerevisiae - ps31
Phylostratum

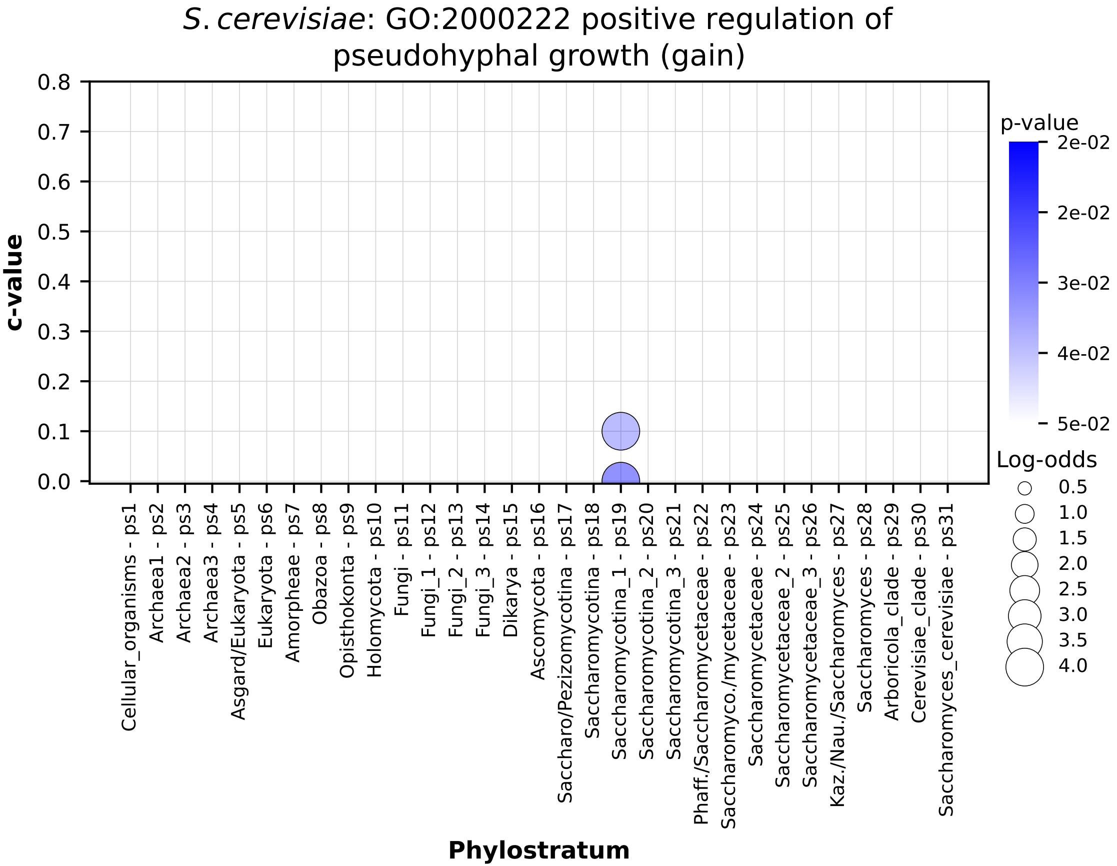
S. cerevisiae: GO:2000222 positive regulation of pseudohyphal growth (gain)
c-value
0.0
0.1
0.2
0.3
0.4
0.5
0.6
0.7
0.8
Phylostratum
Cellular_organisms - ps1
Archaea1 - ps2
Archaea2 - ps3
Archaea3 - ps4
Asgard/Eukaryota - ps5
Eukaryota - ps6
Amorpheae - ps7
Obazoa - ps8
Opisthokonta - ps9
Holomycota - ps10
Fungi - ps11
Fungi_1 - ps12
Fungi_2 - ps13
Fungi_3 - ps14
Dikarya - ps15
Ascomycota - ps16
Saccharo/Pezizomycotina - ps17
Saccharomycotina - ps18
Saccharomycotina_1 - ps19
Saccharomycotina_2 - ps20
Saccharomycotina_3 - ps21
Phaff./Saccharomycetaceae - ps22
Saccharomyco./mycetaceae - ps23
Saccharomycetaceae - ps24
Saccharomycetaceae_2 - ps25
Saccharomycetaceae_3 - ps26
Kaz./Nau./Saccharomyces - ps27
Saccharomyces - ps28
Arboricola_clade - ps29
Cerevisiae_clade - ps30
Saccharomyces_cerevisiae - ps31
p-value
2e-02
2e-02
3e-02
4e-02
5e-02
Log-odds
0.5
1.0
1.5
2.0
2.5
3.0
3.5
4.0

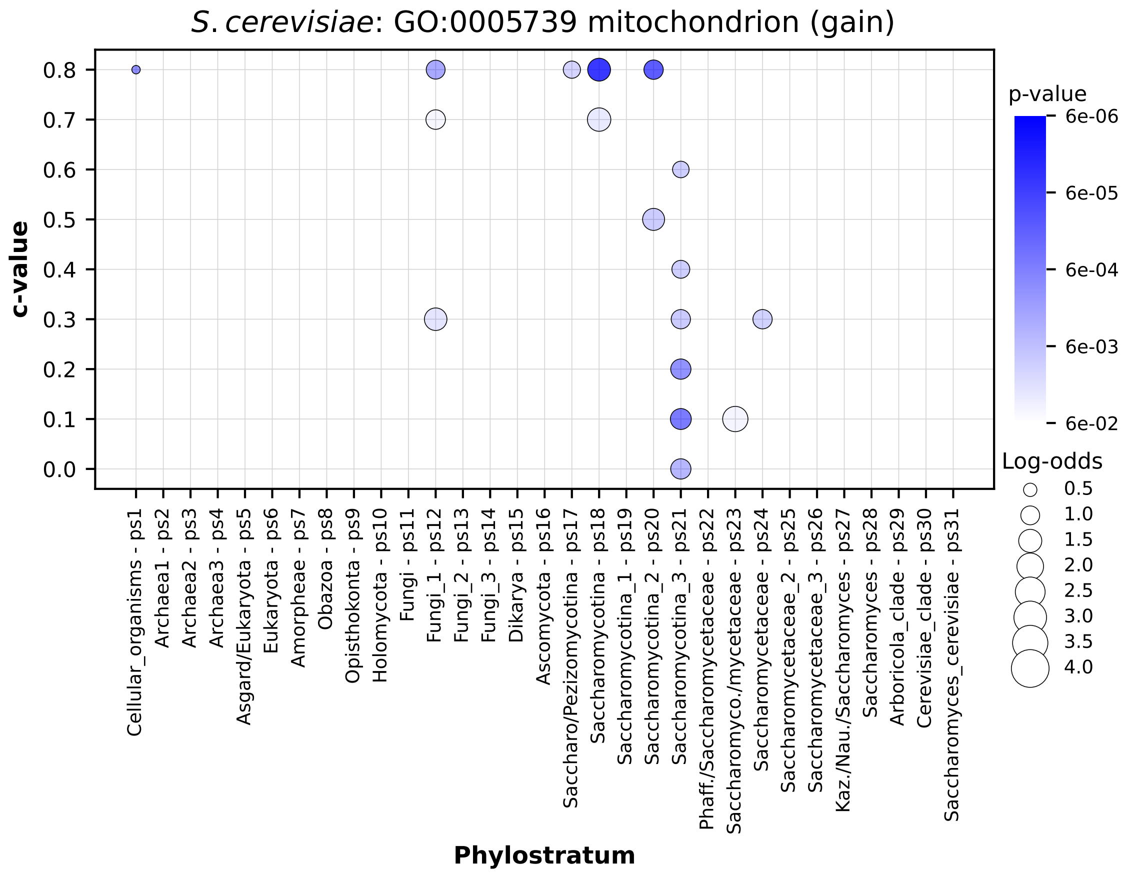

S. cerevisiae: GO:0005739 mitochondrion (gain)
c-value
0.8
0.7
0.6
0.5
0.4
0.3
0.2
0.1
0.0
Phylostratum
Cellular_organisms - ps1
Archaea1 - ps2
Archaea2 - ps3
Archaea3 - ps4
Asgard/Eukaryota - ps5
Eukaryota - ps6
Amorpheae - ps7
Obazoa - ps8
Opisthokonta - ps9
Holomycota - ps10
Fungi - ps11
Fungi_1 - ps12
Fungi_2 - ps13
Fungi_3 - ps14
Dikarya - ps15
Ascomycota - ps16
Saccharo/Pezizomycotina - ps17
Saccharomycotina - ps18
Saccharomycotina_1 - ps19
Saccharomycotina_2 - ps20
Saccharomycotina_3 - ps21
Phaff./Saccharomycetaceae - ps22
Saccharomyco./mycetaceae - ps23
Saccharomycetaceae - ps24
Saccharomycetaceae_2 - ps25
Saccharomycetaceae_3 - ps26
Kaz./Nau./Saccharomyces - ps27
Saccharomyces - ps28
Arboricola_clade - ps29
Cerevisiae_clade - ps30
Saccharomyces_cerevisiae - ps31
p-value
6e-06
6e-05
6e-04
6e-03
6e-02
Log-odds
0.5
1.0
1.5
2.0
2.5
3.0
3.5
4.0

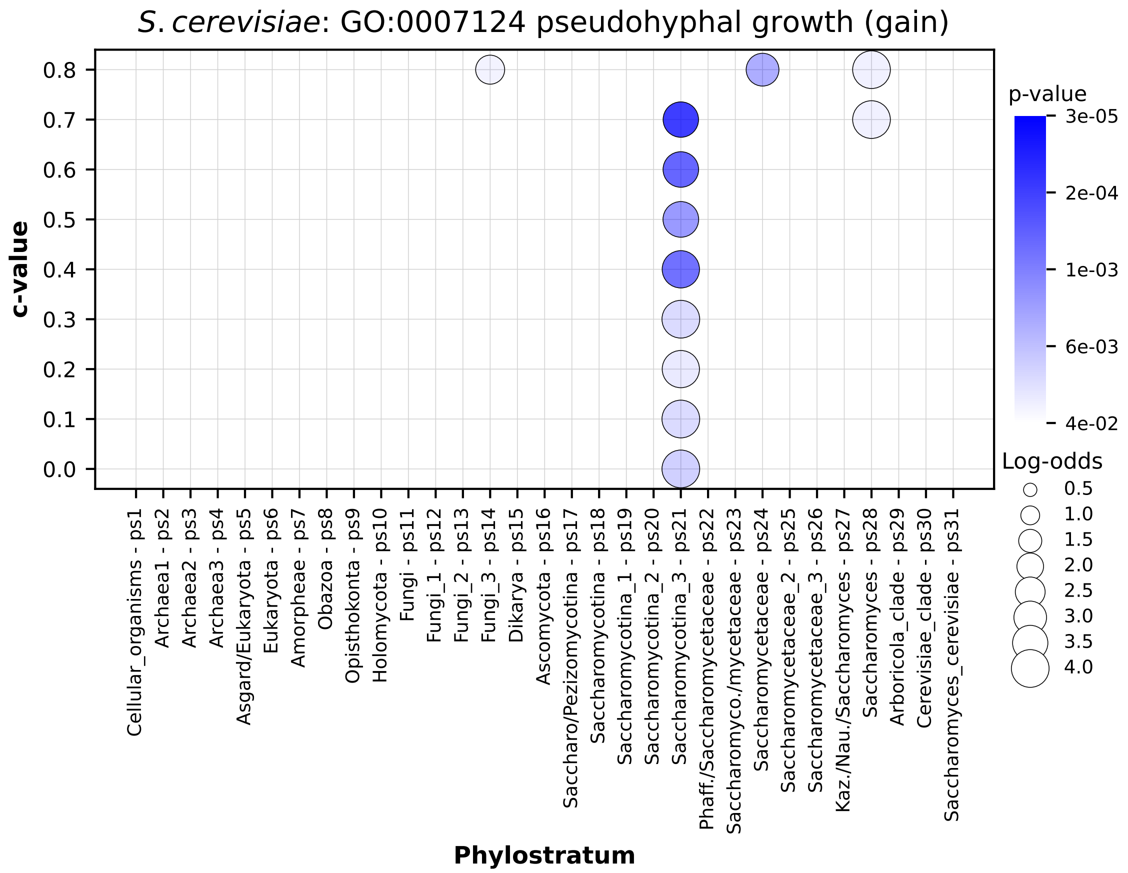

S. cerevisiae: GO:0007124 pseudohyphal growth (gain)
c-value
0.0
0.1
0.2
0.3
0.4
0.5
0.6
0.7
0.8
Phylostratum
Cellular_organisms - ps1
Archaea1 - ps2
Archaea2 - ps3
Archaea3 - ps4
Asgard/Eukaryota - ps5
Eukaryota - ps6
Amorpheae - ps7
Obazoa - ps8
Opisthokonta - ps9
Holomycota - ps10
Fungi - ps11
Fungi_1 - ps12
Fungi_2 - ps13
Fungi_3 - ps14
Dikarya - ps15
Ascomycota - ps16
Saccharo/Pezizomycotina - ps17
Saccharomycotina - ps18
Saccharomycotina_1 - ps19
Saccharomycotina_2 - ps20
Saccharomycotina_3 - ps21
Phaff./Saccharomycetaceae - ps22
Saccharomyco./mycetaceae - ps23
Saccharomycetaceae - ps24
Saccharomycetaceae_2 - ps25
Saccharomycetaceae_3 - ps26
Kaz./Nau./Saccharomyces - ps27
Saccharomyces - ps28
Arboricola_clade - ps29
Cerevisiae_clade - ps30
Saccharomyces_cerevisiae - ps31
p-value
3e-05
2e-04
1e-03
6e-03
4e-02
Log-odds
0.5
1.0
1.5
2.0
2.5
3.0
3.5
4.0

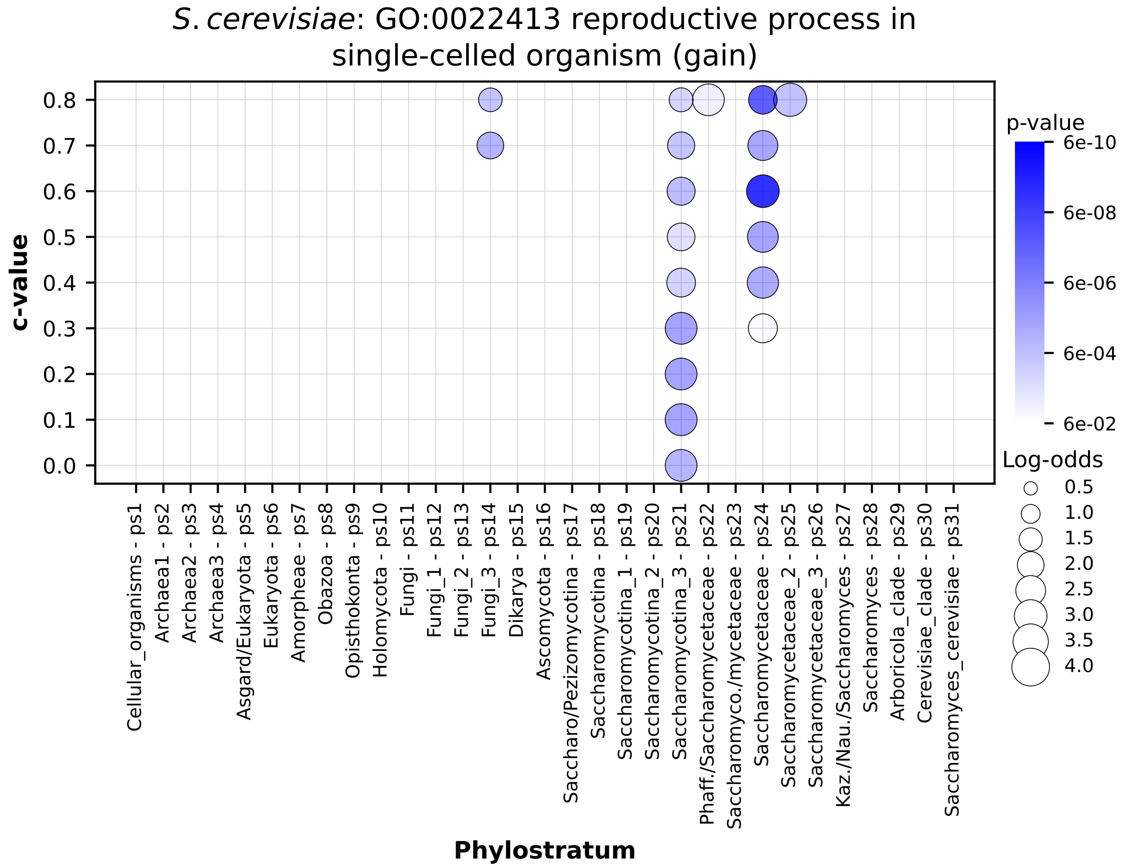

S. cerevisiae: GO:0022413 reproductive process in single-celled organism (gain)
c-value
0.8
0.7
0.6
0.5
0.4
0.3
0.2
0.1
0.0
p-value
6e-10
6e-08
6e-06
6e-04
6e-02
Log-odds
0.5
1.0
1.5
2.0
2.5
3.0
3.5
4.0
Cellular_organisms - ps1
Archaea1 - ps2
Archaea2 - ps3
Archaea3 - ps4
Asgard/Eukaryota - ps5
Eukaryota - ps6
Amorpheae - ps7
Obazoa - ps8
Opisthokonta - ps9
Holomycota - ps10
Fungi - ps11
Fungi_1 - ps12
Fungi_2 - ps13
Fungi_3 - ps14
Dikarya - ps15
Ascomycota - ps16
Saccharo/Pezizomycotina - ps17
Saccharomycotina - ps18
Saccharomycotina_1 - ps19
Saccharomycotina_2 - ps20
Saccharomycotina_3 - ps21
Phaff./Saccharomycetaceae - ps22
Saccharomyco./mycetaceae - ps23
Saccharomycetaceae - ps24
Saccharomycetaceae_2 - ps25
Saccharomycetaceae_3 - ps26
Kaz./Nau./Saccharomyces - ps27
Saccharomyces - ps28
Arboricola_clade - ps29
Cerevisiae_clade - ps30
Saccharomyces_cerevisiae - ps31
Phylostratum

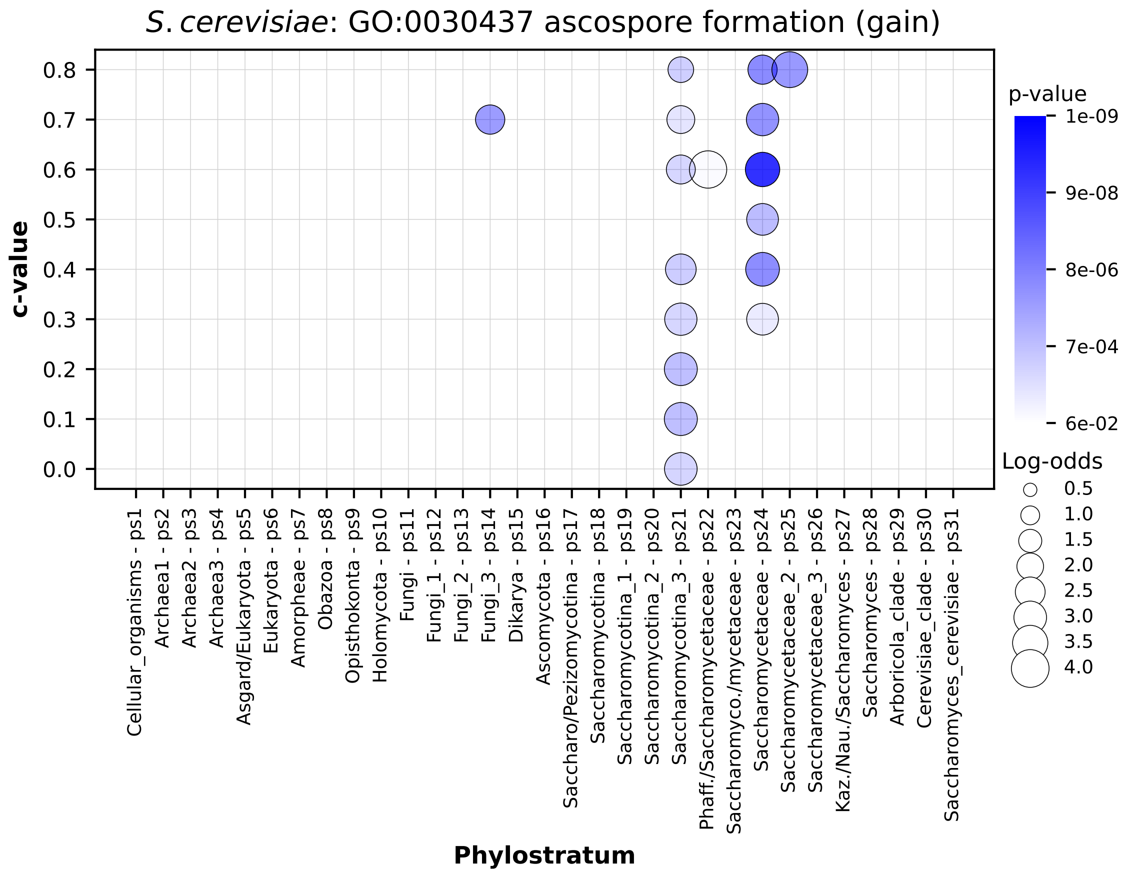

S. cerevisiae: GO:0030437 ascospore formation (gain)
c-value
0.8
0.7
0.6
0.5
0.4
0.3
0.2
0.1
0.0
p-value
1e-09
9e-08
8e-06
7e-04
6e-02
Log-odds
0.5
1.0
1.5
2.0
2.5
3.0
3.5
4.0
Cellular_organisms - ps1
Archaea1 - ps2
Archaea2 - ps3
Archaea3 - ps4
Asgard/Eukaryota - ps5
Eukaryota - ps6
Amorpheae - ps7
Obazoa - ps8
Opisthokonta - ps9
Holomycota - ps10
Fungi - ps11
Fungi_1 - ps12
Fungi_2 - ps13
Fungi_3 - ps14
Dikarya - ps15
Ascomycota - ps16
Saccharo/Pezizomycotina - ps17
Saccharomycotina - ps18
Saccharomycotina_1 - ps19
Saccharomycotina_2 - ps20
Saccharomycotina_3 - ps21
Phaff./Saccharomycetaceae - ps22
Saccharomyco./mycetaceae - ps23
Saccharomycetaceae - ps24
Saccharomycetaceae_2 - ps25
Saccharomycetaceae_3 - ps26
Kaz./Nau./Saccharomyces - ps27
Saccharomyces - ps28
Arboricola_clade - ps29
Cerevisiae_clade - ps30
Saccharomyces_cerevisiae - ps31
Phylostratum

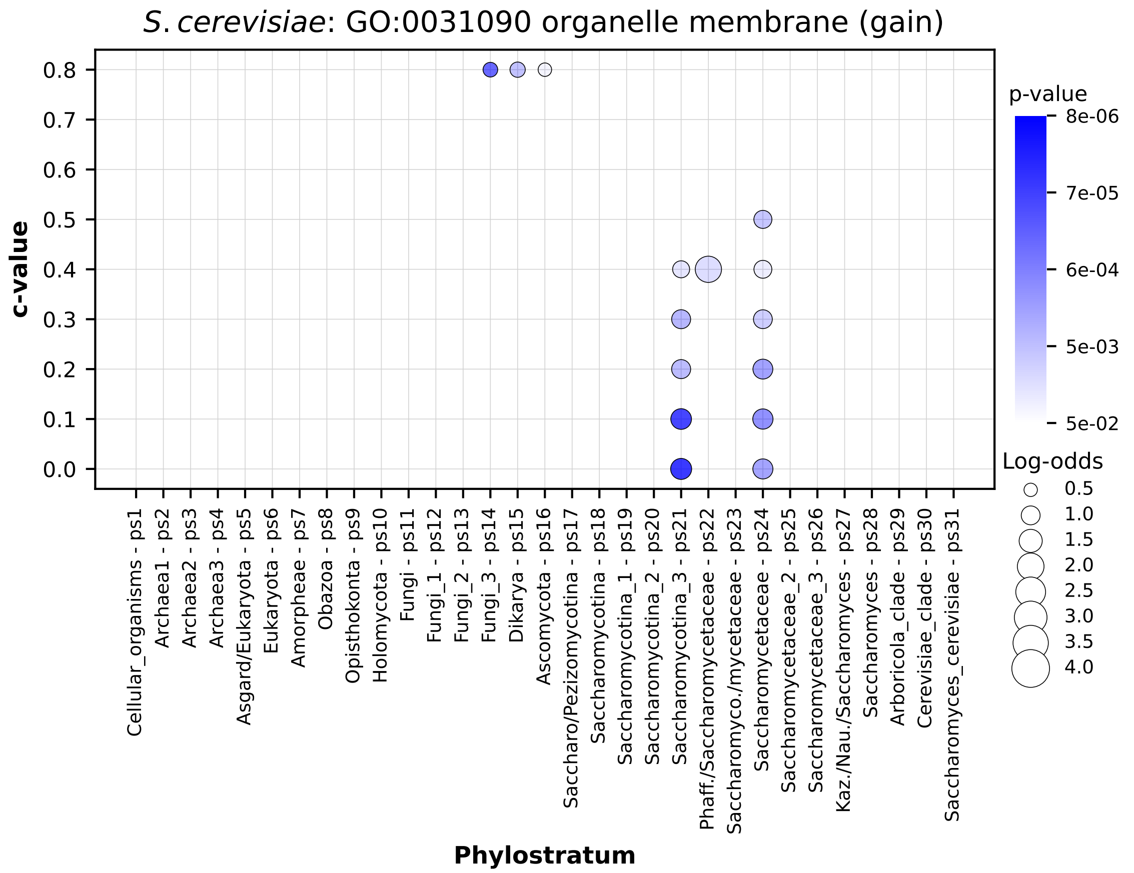

S. cerevisiae: GO:0031090 organelle membrane (gain)
c-value
0.8
0.7
0.6
0.5
0.4
0.3
0.2
0.1
0.0
p-value
8e-06
7e-05
6e-04
5e-03
5e-02
Log-odds
0.5
1.0
1.5
2.0
2.5
3.0
3.5
4.0
Cellular_organisms - ps1
Archaea1 - ps2
Archaea2 - ps3
Archaea3 - ps4
Asgard/Eukaryota - ps5
Eukaryota - ps6
Amorpheae - ps7
Obazoa - ps8
Opisthokonta - ps9
Holomycota - ps10
Fungi - ps11
Fungi_1 - ps12
Fungi_2 - ps13
Fungi_3 - ps14
Dikarya - ps15
Ascomycota - ps16
Saccharo/Pezizomycotina - ps17
Saccharomycotina - ps18
Saccharomycotina_1 - ps19
Saccharomycotina_2 - ps20
Saccharomycotina_3 - ps21
Phaff./Saccharomycetaceae - ps22
Saccharomyco./mycetaceae - ps23
Saccharomycetaceae - ps24
Saccharomycetaceae_2 - ps25
Saccharomycetaceae_3 - ps26
Kaz./Nau./Saccharomyces - ps27
Saccharomyces - ps28
Arboricola_clade - ps29
Cerevisiae_clade - ps30
Saccharomyces_cerevisiae - ps31
Phylostratum

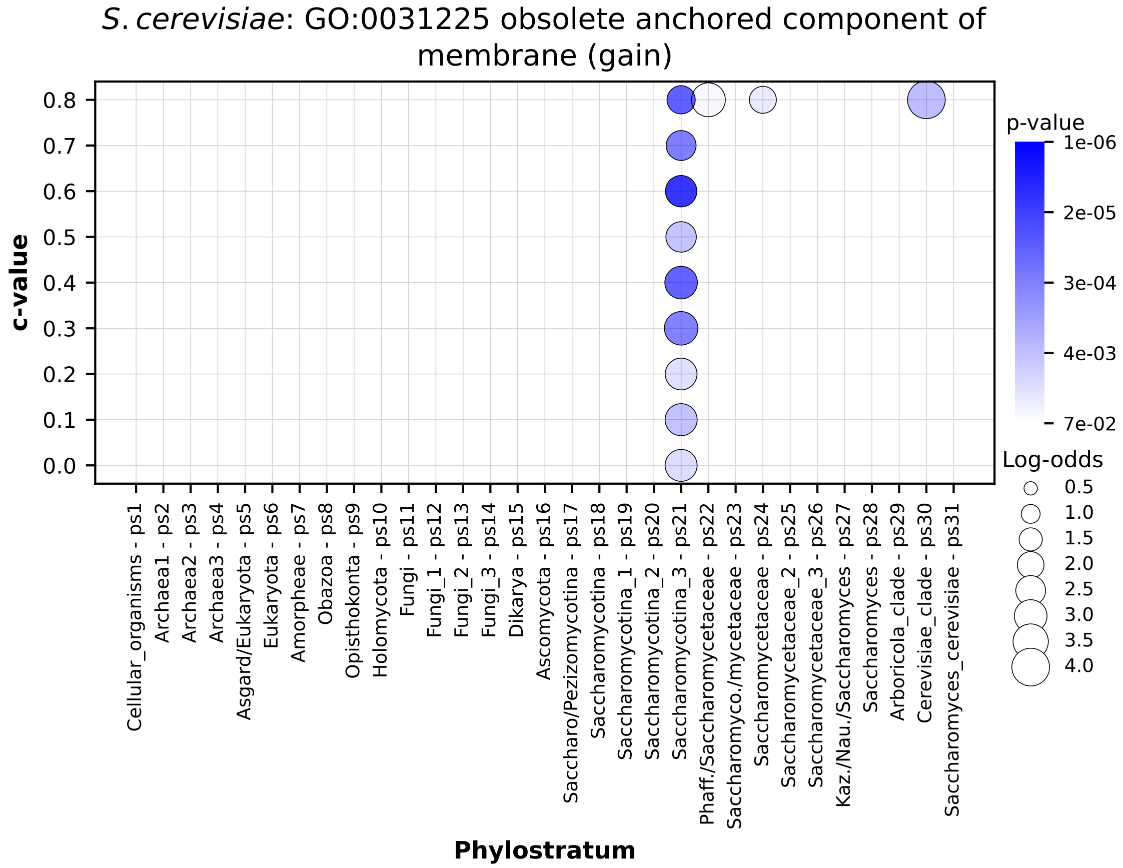

S. cerevisiae: GO:0031225 obsolete anchored component of membrane (gain)
c-value
0.0
0.1
0.2
0.3
0.4
0.5
0.6
0.7
0.8
p-value
1e-06
2e-05
3e-04
4e-03
7e-02
Log-odds
0.5
1.0
1.5
2.0
2.5
3.0
3.5
4.0
Cellular_organisms - ps1
Archaea1 - ps2
Archaea2 - ps3
Archaea3 - ps4
Asgard/Eukaryota - ps5
Eukaryota - ps6
Amorpheae - ps7
Obazoa - ps8
Opisthokonta - ps9
Holomycota - ps10
Fungi - ps11
Fungi_1 - ps12
Fungi_2 - ps13
Fungi_3 - ps14
Dikarya - ps15
Ascomycota - ps16
Saccharo/Pezizomycotina - ps17
Saccharomycotina - ps18
Saccharomycotina_1 - ps19
Saccharomycotina_2 - ps20
Saccharomycotina_3 - ps21
Phaff./Saccharomycetaceae - ps22
Saccharomyco./mycetaceae - ps23
Saccharomycetaceae - ps24
Saccharomycetaceae_2 - ps25
Saccharomycetaceae_3 - ps26
Kaz./Nau./Saccharomyces - ps27
Saccharomyces - ps28
Arboricola_clade - ps29
Cerevisiae_clade - ps30
Saccharomyces_cerevisiae - ps31
Phylostratum

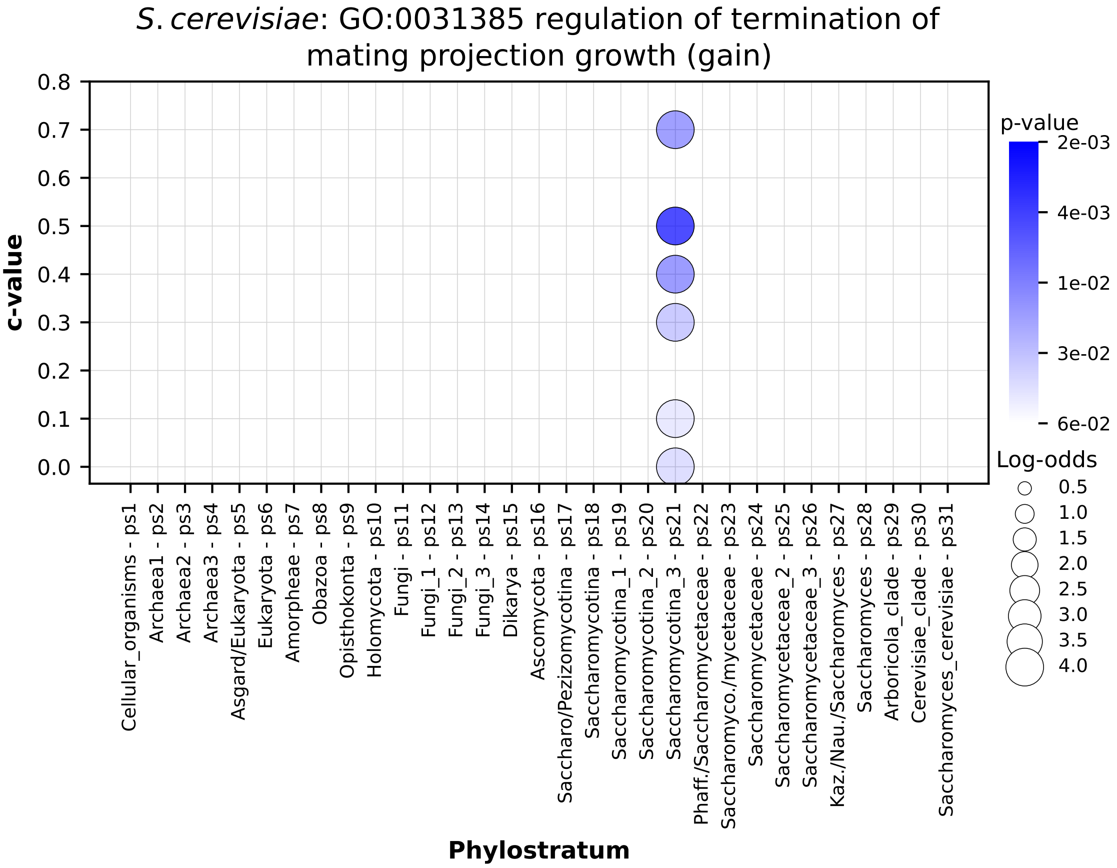

S. cerevisiae: GO:0031385 regulation of termination of mating projection growth (gain)
c-value
0.8
0.7
0.6
0.5
0.4
0.3
0.2
0.1
0.0
p-value
2e-03
4e-03
1e-02
3e-02
6e-02
Log-odds
0.5
1.0
1.5
2.0
2.5
3.0
3.5
4.0
Cellular_organisms - ps1
Archaea1 - ps2
Archaea2 - ps3
Archaea3 - ps4
Asgard/Eukaryota - ps5
Eukaryota - ps6
Amorpheae - ps7
Obazoa - ps8
Opisthokonta - ps9
Holomycota - ps10
Fungi - ps11
Fungi_1 - ps12
Fungi_2 - ps13
Fungi_3 - ps14
Dikarya - ps15
Ascomycota - ps16
Saccharo/Pezizomycotina - ps17
Saccharomycotina - ps18
Saccharomycotina_1 - ps19
Saccharomycotina_2 - ps20
Saccharomycotina_3 - ps21
Phaff./Saccharomycetaceae - ps22
Saccharomyco./mycetaceae - ps23
Saccharomycetaceae - ps24
Saccharomycetaceae_2 - ps25
Saccharomycetaceae_3 - ps26
Kaz./Nau./Saccharomyces - ps27
Saccharomyces - ps28
Arboricola_clade - ps29
Cerevisiae_clade - ps30
Saccharomyces_cerevisiae - ps31
Phylostratum

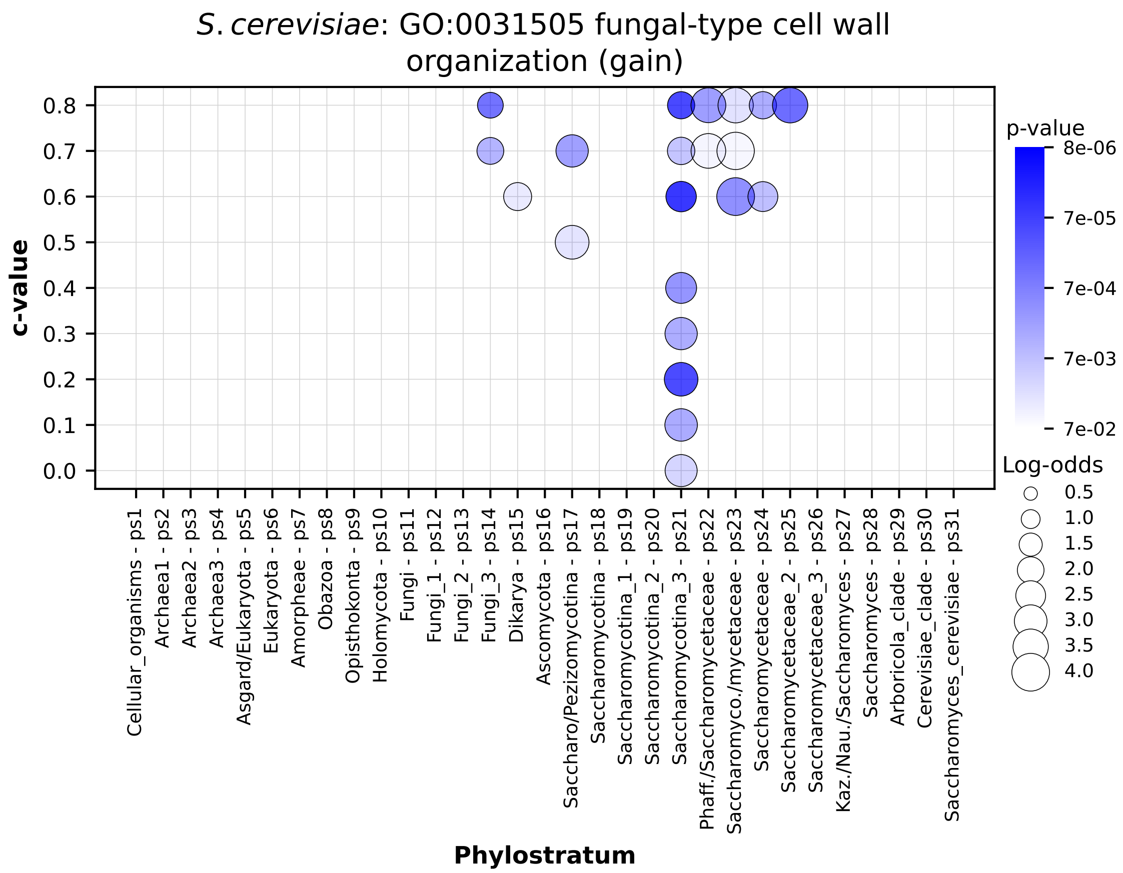

$S. cerevisiae$: GO:0031505 fungal-type cell wall organization (gain)
c-value
0.8
0.7
0.6
0.5
0.4
0.3
0.2
0.1
0.0
Phylostratum
Cellular_organisms - ps1
Archaea1 - ps2
Archaea2 - ps3
Archaea3 - ps4
Asgard/Eukaryota - ps5
Eukaryota - ps6
Amorpheae - ps7
Obazoa - ps8
Opisthokonta - ps9
Holomycota - ps10
Fungi - ps11
Fungi_1 - ps12
Fungi_2 - ps13
Fungi_3 - ps14
Dikarya - ps15
Ascomycota - ps16
Saccharo/Pezizomycotina - ps17
Saccharomycotina - ps18
Saccharomycotina_1 - ps19
Saccharomycotina_2 - ps20
Saccharomycotina_3 - ps21
Phaff./Saccharomycetaceae - ps22
Saccharomyco./mycetaceae - ps23
Saccharomycetaceae - ps24
Saccharomycetaceae_2 - ps25
Saccharomycetaceae_3 - ps26
Kaz./Nau./Saccharomyces - ps27
Saccharomyces - ps28
Arboricola_clade - ps29
Cerevisiae_clade - ps30
Saccharomyces_cerevisiae - ps31
p-value
8e-06
7e-05
7e-04
7e-03
7e-02
Log-odds
0.5
1.0
1.5
2.0
2.5
3.0
3.5
4.0

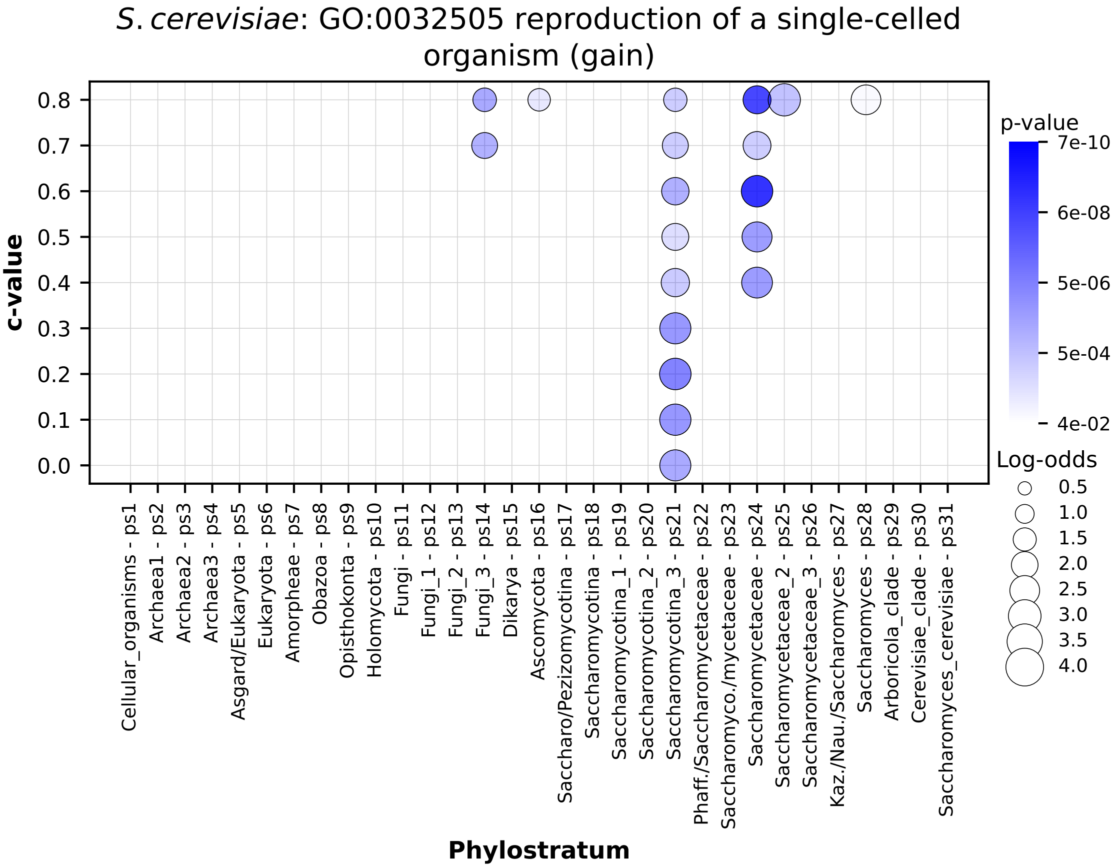

S. cerevisiae: GO:0032505 reproduction of a single-celled organism (gain)
c-value
0.8
0.7
0.6
0.5
0.4
0.3
0.2
0.1
0.0
p-value
7e-10
6e-08
5e-06
5e-04
4e-02
Log-odds
0.5
1.0
1.5
2.0
2.5
3.0
3.5
4.0
Cellular_organisms - ps1
Archaea1 - ps2
Archaea2 - ps3
Archaea3 - ps4
Asgard/Eukaryota - ps5
Eukaryota - ps6
Amorpheae - ps7
Obazoa - ps8
Opisthokonta - ps9
Holomycota - ps10
Fungi - ps11
Fungi_1 - ps12
Fungi_2 - ps13
Fungi_3 - ps14
Dikarya - ps15
Ascomycota - ps16
Saccharo/Pezizomycotina - ps17
Saccharomycotina - ps18
Saccharomycotina_1 - ps19
Saccharomycotina_2 - ps20
Saccharomycotina_3 - ps21
Phaff./Saccharomycetaceae - ps22
Saccharomyco./mycetaceae - ps23
Saccharomycetaceae - ps24
Saccharomycetaceae_2 - ps25
Saccharomycetaceae_3 - ps26
Kaz./Nau./Saccharomyces - ps27
Saccharomyces - ps28
Arboricola_clade - ps29
Cerevisiae_clade - ps30
Saccharomyces_cerevisiae - ps31
Phylostratum

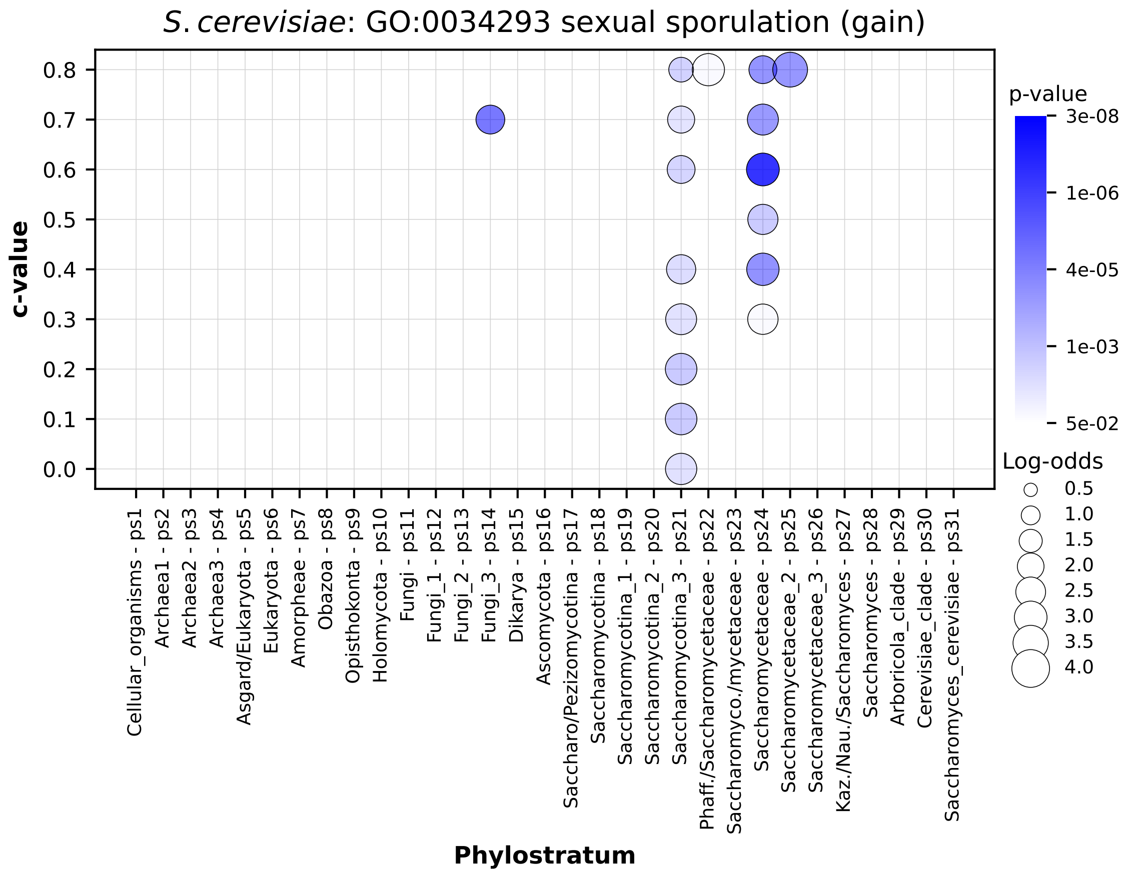

S. cerevisiae: GO:0034293 sexual sporulation (gain)
c-value
0.8
0.7
0.6
0.5
0.4
0.3
0.2
0.1
0.0
p-value
3e-08
1e-06
4e-05
1e-03
5e-02
Log-odds
0.5
1.0
1.5
2.0
2.5
3.0
3.5
4.0
Cellular_organisms - ps1
Archaea1 - ps2
Archaea2 - ps3
Archaea3 - ps4
Asgard/Eukaryota - ps5
Eukaryota - ps6
Amorpheae - ps7
Obazoa - ps8
Opisthokonta - ps9
Holomycota - ps10
Fungi - ps11
Fungi_1 - ps12
Fungi_2 - ps13
Fungi_3 - ps14
Dikarya - ps15
Ascomycota - ps16
Saccharo/Pezizomycotina - ps17
Saccharomycotina - ps18
Saccharomycotina_1 - ps19
Saccharomycotina_2 - ps20
Saccharomycotina_3 - ps21
Phaff./Saccharomycetaceae - ps22
Saccharomyco./mycetaceae - ps23
Saccharomycetaceae - ps24
Saccharomycetaceae_2 - ps25
Saccharomycetaceae_3 - ps26
Kaz./Nau./Saccharomyces - ps27
Saccharomyces - ps28
Arboricola_clade - ps29
Cerevisiae_clade - ps30
Saccharomyces_cerevisiae - ps31
Phylostratum

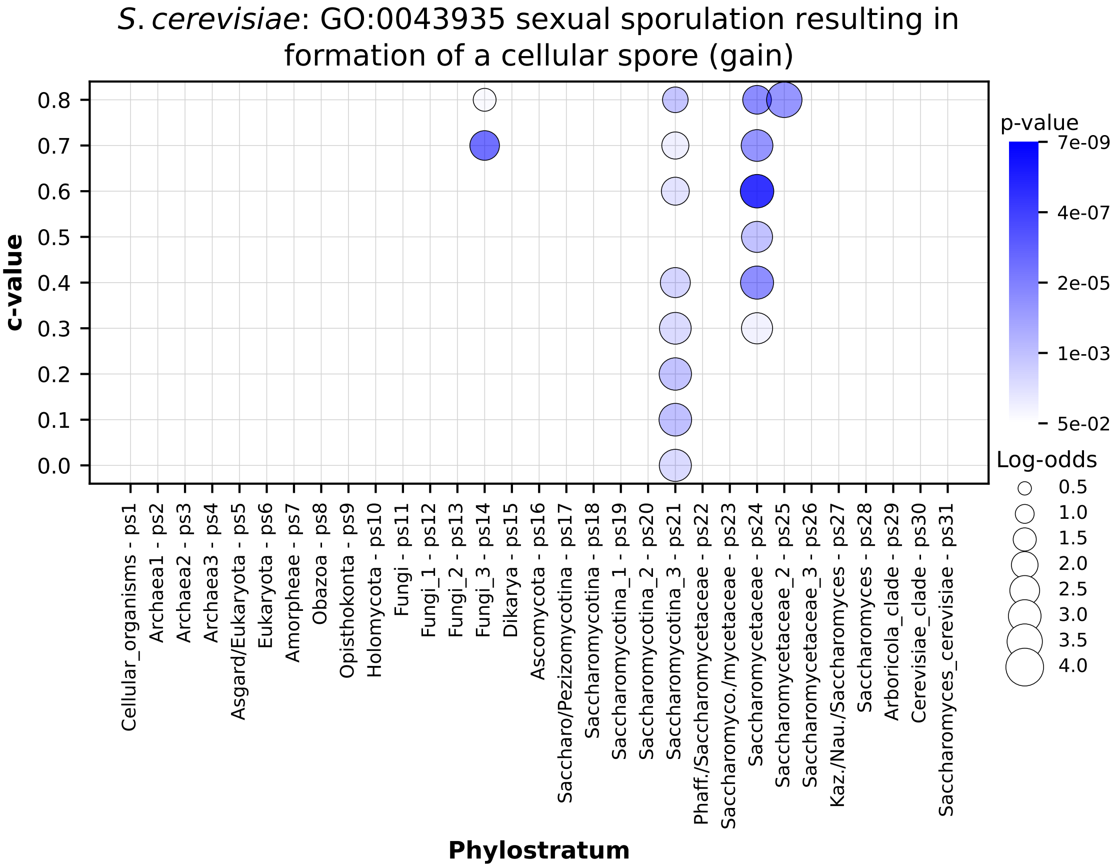

S. cerevisiae: GO:0043935 sexual sporulation resulting in formation of a cellular spore (gain)
c-value
0.8
0.7
0.6
0.5
0.4
0.3
0.2
0.1
0.0
p-value
7e-09
4e-07
2e-05
1e-03
5e-02
Log-odds
0.5
1.0
1.5
2.0
2.5
3.0
3.5
4.0
Cellular_organisms - ps1
Archaea1 - ps2
Archaea2 - ps3
Archaea3 - ps4
Asgard/Eukaryota - ps5
Eukaryota - ps6
Amorpheae - ps7
Obazoa - ps8
Opisthokonta - ps9
Holomycota - ps10
Fungi - ps11
Fungi_1 - ps12
Fungi_2 - ps13
Fungi_3 - ps14
Dikarya - ps15
Ascomycota - ps16
Saccharo/Pezizomycotina - ps17
Saccharomycotina - ps18
Saccharomycotina_1 - ps19
Saccharomycotina_2 - ps20
Saccharomycotina_3 - ps21
Phaff./Saccharomycetaceae - ps22
Saccharomyco./mycetaceae - ps23
Saccharomycetaceae - ps24
Saccharomycetaceae_2 - ps25
Saccharomycetaceae_3 - ps26
Kaz./Nau./Saccharomyces - ps27
Saccharomyces - ps28
Arboricola_clade - ps29
Cerevisiae_clade - ps30
Saccharomyces_cerevisiae - ps31
Phylostratum

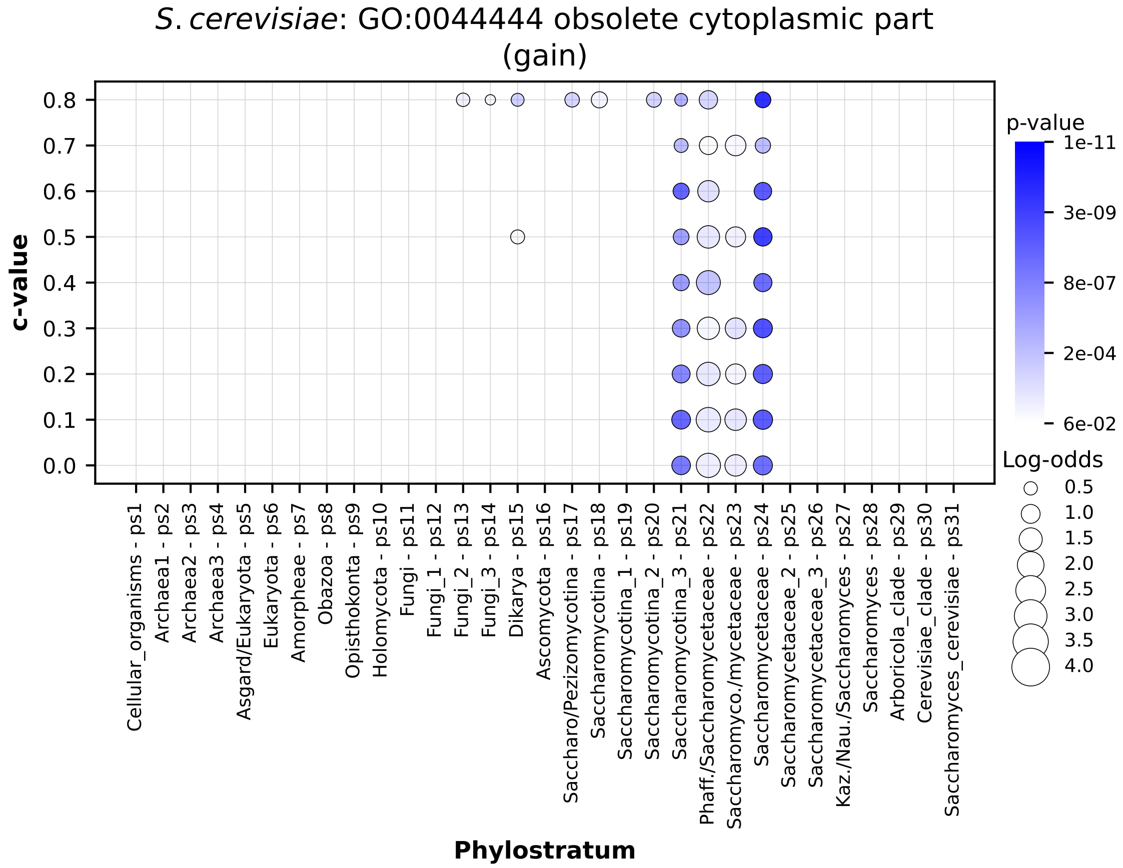
S. cerevisiae: GO:0044444 obsolete cytoplasmic part (gain)
c-value
0.8
0.7
0.6
0.5
0.4
0.3
0.2
0.1
0.0
p-value
1e-11
3e-09
8e-07
2e-04
6e-02
Log-odds
0.5
1.0
1.5
2.0
2.5
3.0
3.5
4.0
Cellular_organisms - ps1
Archaea1 - ps2
Archaea2 - ps3
Archaea3 - ps4
Asgard/Eukaryota - ps5
Eukaryota - ps6
Amorpheae - ps7
Obazoa - ps8
Opisthokonta - ps9
Holomycota - ps10
Fungi - ps11
Fungi_1 - ps12
Fungi_2 - ps13
Fungi_3 - ps14
Dikarya - ps15
Ascomycota - ps16
Saccharo/Pezizomycotina - ps17
Saccharomycotina - ps18
Saccharomycotina_1 - ps19
Saccharomycotina_2 - ps20
Saccharomycotina_3 - ps21
Phaff./Saccharomycetaceae - ps22
Saccharomyco./mycetaceae - ps23
Saccharomycetaceae - ps24
Saccharomycetaceae_2 - ps25
Saccharomycetaceae_3 - ps26
Kaz./Nau./Saccharomyces - ps27
Saccharomyces - ps28
Arboricola_clade - ps29
Cerevisiae_clade - ps30
Saccharomyces_cerevisiae - ps31
Phylostratum

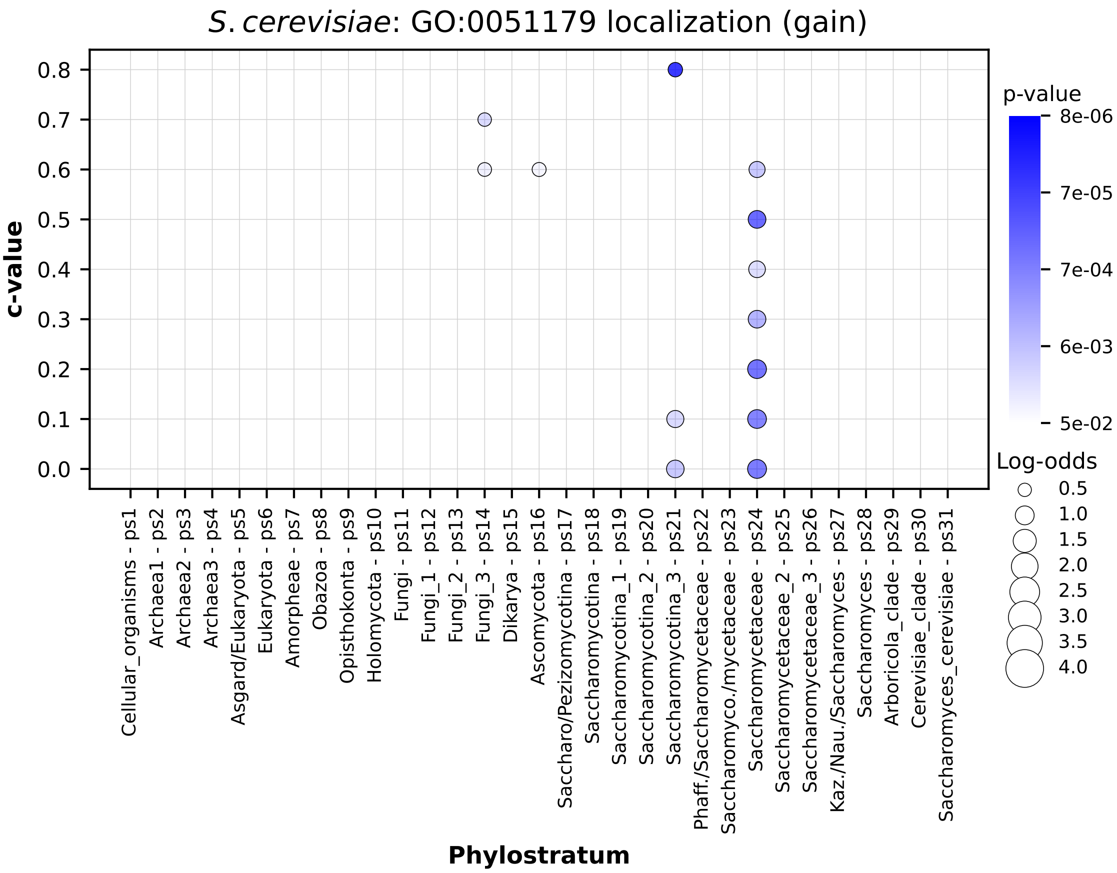

S. cerevisiae: GO:0051179 localization (gain)
c-value
0.8
0.7
0.6
0.5
0.4
0.3
0.2
0.1
0.0
p-value
8e-06
7e-05
7e-04
6e-03
5e-02
Log-odds
0.5
1.0
1.5
2.0
2.5
3.0
3.5
4.0
Cellular_organisms - ps1
Archaea1 - ps2
Archaea2 - ps3
Archaea3 - ps4
Asgard/Eukaryota - ps5
Eukaryota - ps6
Amorpheae - ps7
Obazoa - ps8
Opisthokonta - ps9
Holomycota - ps10
Fungi - ps11
Fungi_1 - ps12
Fungi_2 - ps13
Fungi_3 - ps14
Dikarya - ps15
Ascomycota - ps16
Saccharo/Pezizomycotina - ps17
Saccharomycotina - ps18
Saccharomycotina_1 - ps19
Saccharomycotina_2 - ps20
Saccharomycotina_3 - ps21
Phaff./Saccharomycetaceae - ps22
Saccharomyco./mycetaceae - ps23
Saccharomycetaceae - ps24
Saccharomycetaceae_2 - ps25
Saccharomycetaceae_3 - ps26
Kaz./Nau./Saccharomyces - ps27
Saccharomyces - ps28
Arboricola_clade - ps29
Cerevisiae_clade - ps30
Saccharomyces_cerevisiae - ps31
Phylostratum

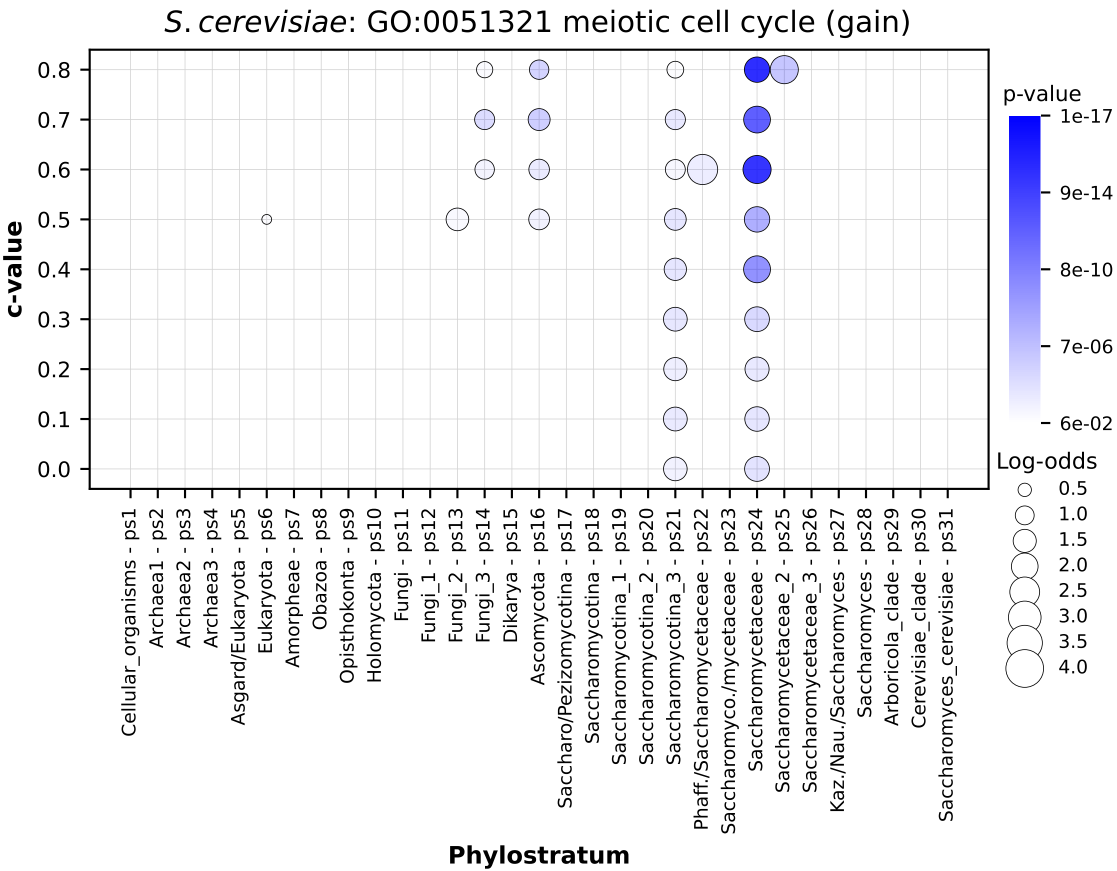

S. cerevisiae: GO:0051321 meiotic cell cycle (gain)
c-value
0.8
0.7
0.6
0.5
0.4
0.3
0.2
0.1
0.0
p-value
1e-17
9e-14
8e-10
7e-06
6e-02
Log-odds
0.5
1.0
1.5
2.0
2.5
3.0
3.5
4.0
Cellular_organisms - ps1
Archaea1 - ps2
Archaea2 - ps3
Archaea3 - ps4
Asgard/Eukaryota - ps5
Eukaryota - ps6
Amorpheae - ps7
Obazoa - ps8
Opisthokonta - ps9
Holomycota - ps10
Fungi - ps11
Fungi_1 - ps12
Fungi_2 - ps13
Fungi_3 - ps14
Dikarya - ps15
Ascomycota - ps16
Saccharo/Pezizomycotina - ps17
Saccharomycotina - ps18
Saccharomycotina_1 - ps19
Saccharomycotina_2 - ps20
Saccharomycotina_3 - ps21
Phaff./Saccharomycetaceae - ps22
Saccharomyco./mycetaceae - ps23
Saccharomycetaceae - ps24
Saccharomycetaceae_2 - ps25
Saccharomycetaceae_3 - ps26
Kaz./Nau./Saccharomyces - ps27
Saccharomyces - ps28
Arboricola_clade - ps29
Cerevisiae_clade - ps30
Saccharomyces_cerevisiae - ps31
Phylostratum

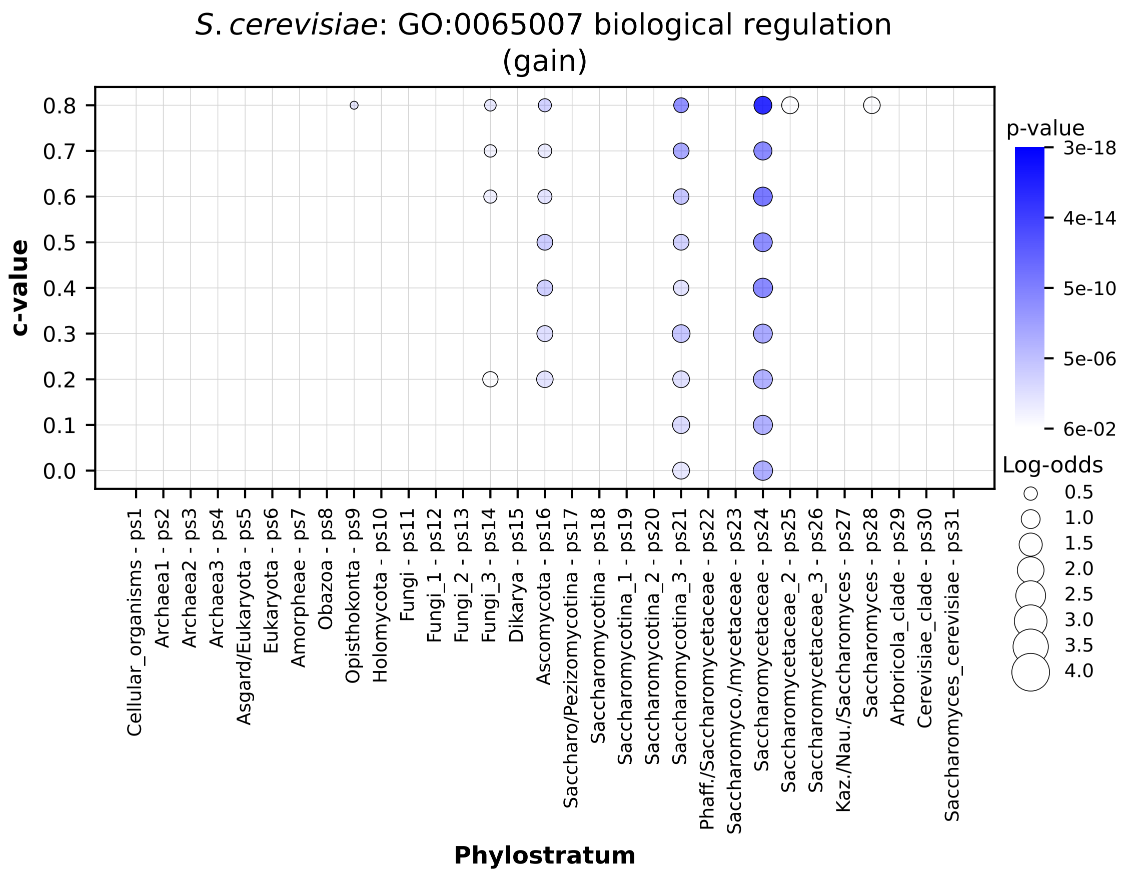

S. cerevisiae: GO:0065007 biological regulation
(gain)
c-value
0.8
0.7
0.6
0.5
0.4
0.3
0.2
0.1
0.0
p-value
3e-18
4e-14
5e-10
5e-06
6e-02
Log-odds
0.5
1.0
1.5
2.0
2.5
3.0
3.5
4.0
Cellular_organisms - ps1
Archaea1 - ps2
Archaea2 - ps3
Archaea3 - ps4
Asgard/Eukaryota - ps5
Eukaryota - ps6
Amorpheae - ps7
Obazoa - ps8
Opisthokonta - ps9
Holomycota - ps10
Fungi - ps11
Fungi_1 - ps12
Fungi_2 - ps13
Fungi_3 - ps14
Dikarya - ps15
Ascomycota - ps16
Saccharo/Pezizomycotina - ps17
Saccharomycotina - ps18
Saccharomycotina_1 - ps19
Saccharomycotina_2 - ps20
Saccharomycotina_3 - ps21
Phaff./Saccharomycetaceae - ps22
Saccharomyco./mycetaceae - ps23
Saccharomycetaceae - ps24
Saccharomycetaceae_2 - ps25
Saccharomycetaceae_3 - ps26
Kaz./Nau./Saccharomyces - ps27
Saccharomyces - ps28
Arboricola_clade - ps29
Cerevisiae_clade - ps30
Saccharomyces_cerevisiae - ps31
Phylostratum

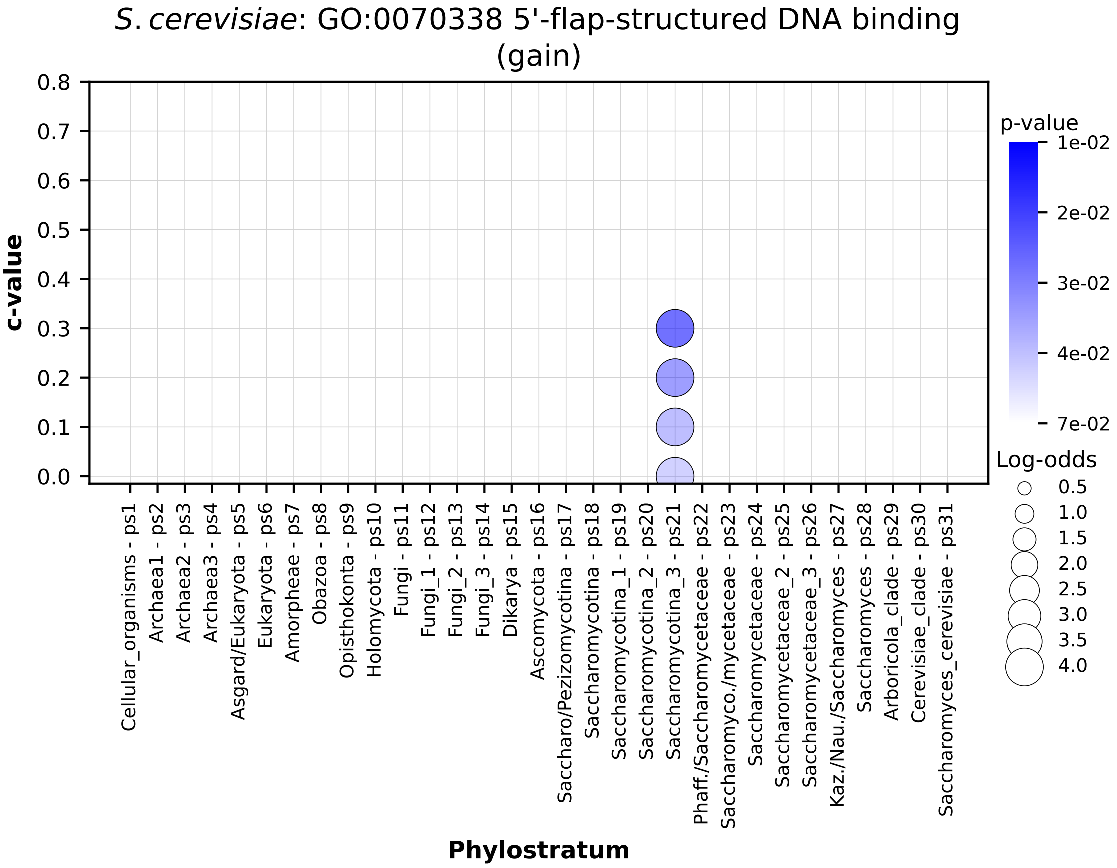

S. cerevisiae: GO:0070338 5'-flap-structured DNA binding (gain)
c-value
0.8
0.7
0.6
0.5
0.4
0.3
0.2
0.1
0.0
p-value
1e-02
2e-02
3e-02
4e-02
7e-02
Log-odds
0.5
1.0
1.5
2.0
2.5
3.0
3.5
4.0
Cellular_organisms - ps1
Archaea1 - ps2
Archaea2 - ps3
Archaea3 - ps4
Asgard/Eukaryota - ps5
Eukaryota - ps6
Amorpheae - ps7
Obazoa - ps8
Opisthokonta - ps9
Holomycota - ps10
Fungi - ps11
Fungi_1 - ps12
Fungi_2 - ps13
Fungi_3 - ps14
Dikarya - ps15
Ascomycota - ps16
Saccharo/Pezizomycotina - ps17
Saccharomycotina - ps18
Saccharomycotina_1 - ps19
Saccharomycotina_2 - ps20
Saccharomycotina_3 - ps21
Phaff./Saccharomycetaceae - ps22
Saccharomyco./mycetaceae - ps23
Saccharomycetaceae - ps24
Saccharomycetaceae_2 - ps25
Saccharomycetaceae_3 - ps26
Kaz./Nau./Saccharomyces - ps27
Saccharomyces - ps28
Arboricola_clade - ps29
Cerevisiae_clade - ps30
Saccharomyces_cerevisiae - ps31
Phylostratum

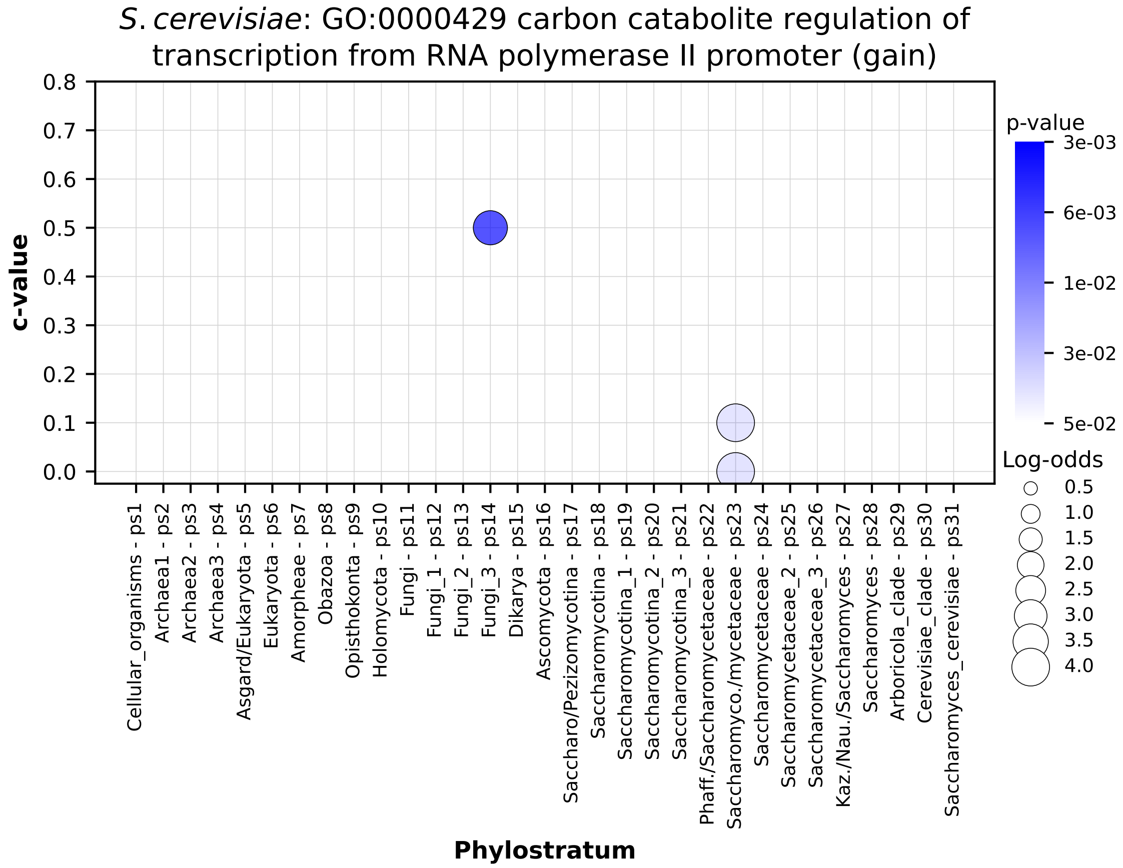
S. cerevisiae: GO:0000429 carbon catabolite regulation of transcription from RNA polymerase II promoter (gain)
c-value
0.8
0.7
0.6
0.5
0.4
0.3
0.2
0.1
0.0
Phylostratum
Cellular_organisms - ps1
Archaea1 - ps2
Archaea2 - ps3
Archaea3 - ps4
Asgard/Eukaryota - ps5
Eukaryota - ps6
Amorpheae - ps7
Obazoa - ps8
Opisthokonta - ps9
Holomycota - ps10
Fungi - ps11
Fungi_1 - ps12
Fungi_2 - ps13
Fungi_3 - ps14
Dikarya - ps15
Ascomycota - ps16
Saccharo/Pezizomycotina - ps17
Saccharomycotina - ps18
Saccharomycotina_1 - ps19
Saccharomycotina_2 - ps20
Saccharomycotina_3 - ps21
Phaff./Saccharomycetaceae - ps22
Saccharomyco./mycetaceae - ps23
Saccharomycetaceae - ps24
Saccharomycetaceae_2 - ps25
Saccharomycetaceae_3 - ps26
Kaz./Nau./Saccharomyces - ps27
Saccharomyces - ps28
Arboricola_clade - ps29
Cerevisiae_clade - ps30
Saccharomyces_cerevisiae - ps31
p-value
3e-03
6e-03
1e-02
3e-02
5e-02
Log-odds
0.5
1.0
1.5
2.0
2.5
3.0
3.5
4.0

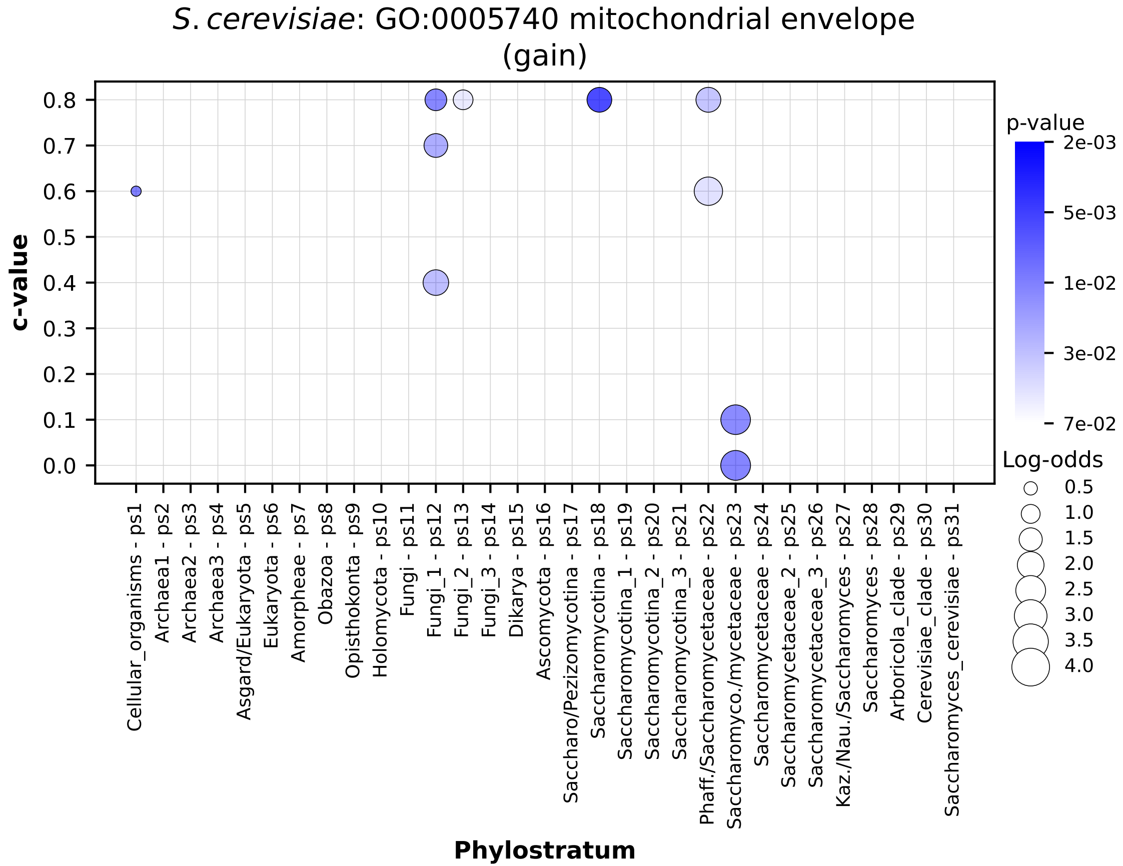

S. cerevisiae: GO:0005740 mitochondrial envelope
(gain)
c-value
0.8
0.7
0.6
0.5
0.4
0.3
0.2
0.1
0.0
p-value
2e-03
5e-03
1e-02
3e-02
7e-02
Log-odds
0.5
1.0
1.5
2.0
2.5
3.0
3.5
4.0
Cellular_organisms - ps1
Archaea1 - ps2
Archaea2 - ps3
Archaea3 - ps4
Asgard/Eukaryota - ps5
Eukaryota - ps6
Amorpheae - ps7
Obazoa - ps8
Opisthokonta - ps9
Holomycota - ps10
Fungi - ps11
Fungi_1 - ps12
Fungi_2 - ps13
Fungi_3 - ps14
Dikarya - ps15
Ascomycota - ps16
Saccharo/Pezizomycotina - ps17
Saccharomycotina - ps18
Saccharomycotina_1 - ps19
Saccharomycotina_2 - ps20
Saccharomycotina_3 - ps21
Phaff./Saccharomycetaceae - ps22
Saccharomyco./mycetaceae - ps23
Saccharomycetaceae - ps24
Saccharomycetaceae_2 - ps25
Saccharomycetaceae_3 - ps26
Kaz./Nau./Saccharomyces - ps27
Saccharomyces - ps28
Arboricola_clade - ps29
Cerevisiae_clade - ps30
Saccharomyces_cerevisiae - ps31
Phylostratum

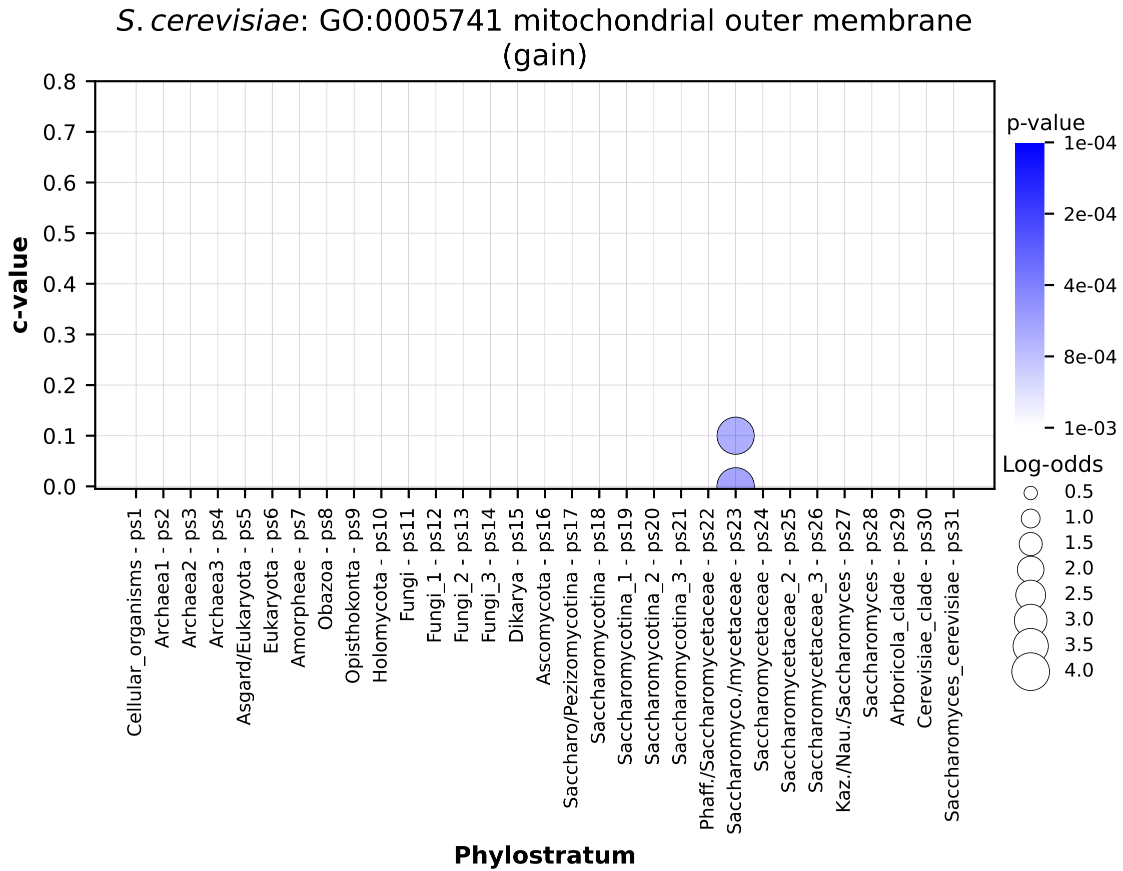

S. cerevisiae: GO:0005741 mitochondrial outer membrane (gain)
c-value
0.8
0.7
0.6
0.5
0.4
0.3
0.2
0.1
0.0
p-value
1e-04
2e-04
4e-04
8e-04
1e-03
Log-odds
0.5
1.0
1.5
2.0
2.5
3.0
3.5
4.0
Cellular_organisms - ps1
Archaea1 - ps2
Archaea2 - ps3
Archaea3 - ps4
Asgard/Eukaryota - ps5
Eukaryota - ps6
Amorpheae - ps7
Obazoa - ps8
Opisthokonta - ps9
Holomycota - ps10
Fungi - ps11
Fungi_1 - ps12
Fungi_2 - ps13
Fungi_3 - ps14
Dikarya - ps15
Ascomycota - ps16
Saccharo/Pezizomycotina - ps17
Saccharomycotina - ps18
Saccharomycotina_1 - ps19
Saccharomycotina_2 - ps20
Saccharomycotina_3 - ps21
Phaff./Saccharomycetaceae - ps22
Saccharomyco./mycetaceae - ps23
Saccharomycetaceae - ps24
Saccharomycetaceae_2 - ps25
Saccharomycetaceae_3 - ps26
Kaz./Nau./Saccharomyces - ps27
Saccharomyces - ps28
Arboricola_clade - ps29
Cerevisiae_clade - ps30
Saccharomyces_cerevisiae - ps31
Phylostratum

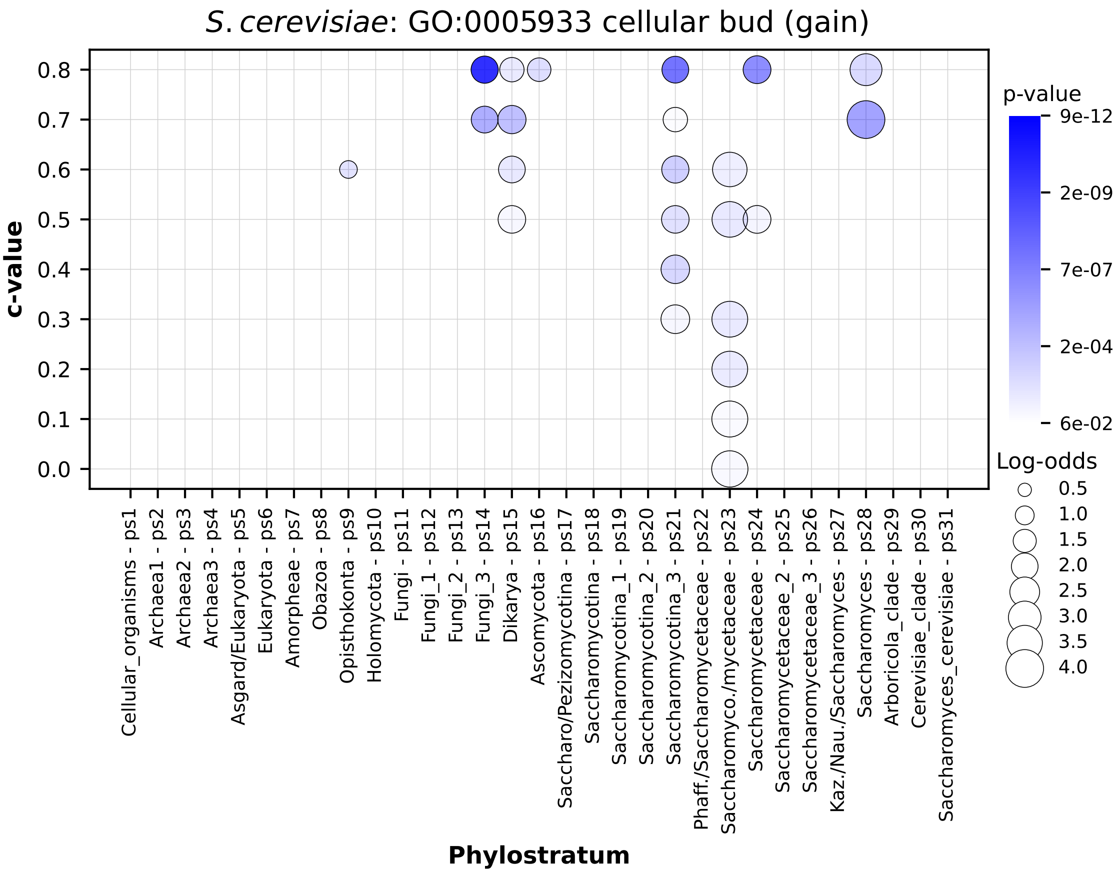

S. cerevisiae: GO:0005933 cellular bud (gain)
c-value
0.8
0.7
0.6
0.5
0.4
0.3
0.2
0.1
0.0
p-value
9e-12
2e-09
7e-07
2e-04
6e-02
Log-odds
0.5
1.0
1.5
2.0
2.5
3.0
3.5
4.0
Cellular_organisms - ps1
Archaea1 - ps2
Archaea2 - ps3
Archaea3 - ps4
Asgard/Eukaryota - ps5
Eukaryota - ps6
Amorpheae - ps7
Obazoa - ps8
Opisthokonta - ps9
Holomycota - ps10
Fungi - ps11
Fungi_1 - ps12
Fungi_2 - ps13
Fungi_3 - ps14
Dikarya - ps15
Ascomycota - ps16
Saccharo/Pezizomycotina - ps17
Saccharomycotina - ps18
Saccharomycotina_1 - ps19
Saccharomycotina_2 - ps20
Saccharomycotina_3 - ps21
Phaff./Saccharomycetaceae - ps22
Saccharomyco./mycetaceae - ps23
Saccharomycetaceae - ps24
Saccharomycetaceae_2 - ps25
Saccharomycetaceae_3 - ps26
Kaz./Nau./Saccharomyces - ps27
Saccharomyces - ps28
Arboricola_clade - ps29
Cerevisiae_clade - ps30
Saccharomyces_cerevisiae - ps31
Phylostratum

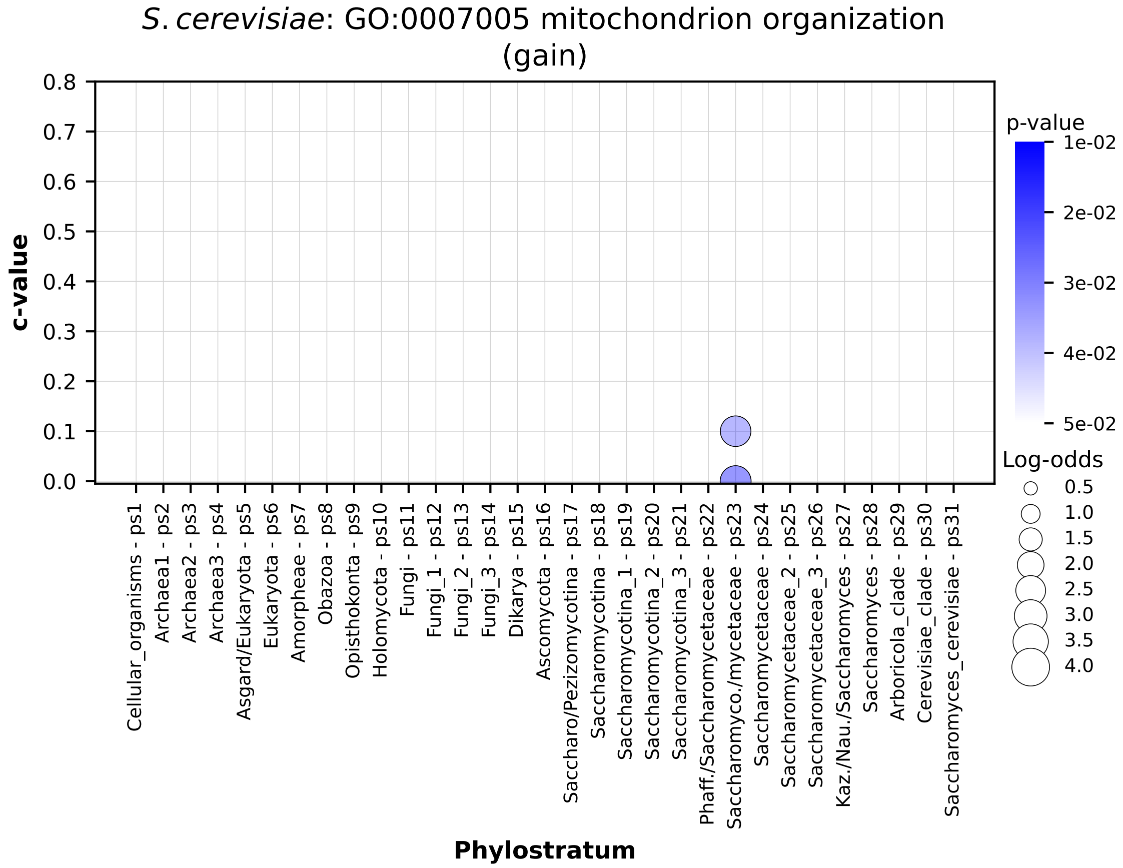
S. cerevisiae: GO:0007005 mitochondrion organization
(gain)
c-value
0.0
0.1
0.2
0.3
0.4
0.5
0.6
0.7
0.8
p-value
1e-02
2e-02
3e-02
4e-02
5e-02
Log-odds
0.5
1.0
1.5
2.0
2.5
3.0
3.5
4.0
Cellular_organisms - ps1
Archaea1 - ps2
Archaea2 - ps3
Archaea3 - ps4
Asgard/Eukaryota - ps5
Eukaryota - ps6
Amorpheae - ps7
Obazoa - ps8
Opisthokonta - ps9
Holomycota - ps10
Fungi - ps11
Fungi_1 - ps12
Fungi_2 - ps13
Fungi_3 - ps14
Dikarya - ps15
Ascomycota - ps16
Saccharo/Pezizomycotina - ps17
Saccharomycotina - ps18
Saccharomycotina_1 - ps19
Saccharomycotina_2 - ps20
Saccharomycotina_3 - ps21
Phaff./Saccharomycetaceae - ps22
Saccharomyco./mycetaceae - ps23
Saccharomycetaceae - ps24
Saccharomycetaceae_2 - ps25
Saccharomycetaceae_3 - ps26
Kaz./Nau./Saccharomyces - ps27
Saccharomyces - ps28
Arboricola_clade - ps29
Cerevisiae_clade - ps30
Saccharomyces_cerevisiae - ps31
Phylostratum

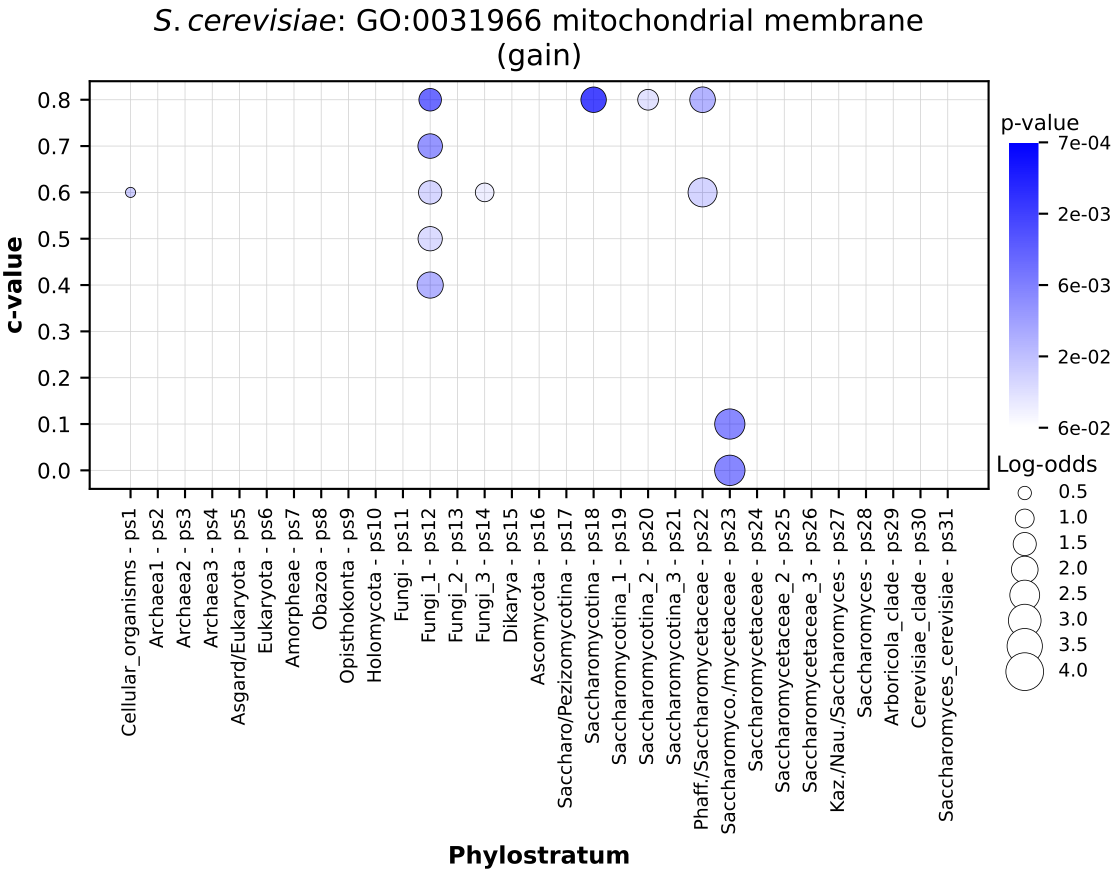

S. cerevisiae: GO:0031966 mitochondrial membrane
(gain)
c-value
0.8
0.7
0.6
0.5
0.4
0.3
0.2
0.1
0.0
Phylostratum
Cellular_organisms - ps1
Archaea1 - ps2
Archaea2 - ps3
Archaea3 - ps4
Asgard/Eukaryota - ps5
Eukaryota - ps6
Amorpheae - ps7
Obazoa - ps8
Opisthokonta - ps9
Holomycota - ps10
Fungi - ps11
Fungi_1 - ps12
Fungi_2 - ps13
Fungi_3 - ps14
Dikarya - ps15
Ascomycota - ps16
Saccharo/Pezizomycotina - ps17
Saccharomycotina - ps18
Saccharomycotina_1 - ps19
Saccharomycotina_2 - ps20
Saccharomycotina_3 - ps21
Phaff./Saccharomycetaceae - ps22
Saccharomyco./mycetaceae - ps23
Saccharomycetaceae - ps24
Saccharomycetaceae_2 - ps25
Saccharomycetaceae_3 - ps26
Kaz./Nau./Saccharomyces - ps27
Saccharomyces - ps28
Arboricola_clade - ps29
Cerevisiae_clade - ps30
Saccharomyces_cerevisiae - ps31
p-value
7e-04
2e-03
6e-03
2e-02
6e-02
Log-odds
0.5
1.0
1.5
2.0
2.5
3.0
3.5
4.0

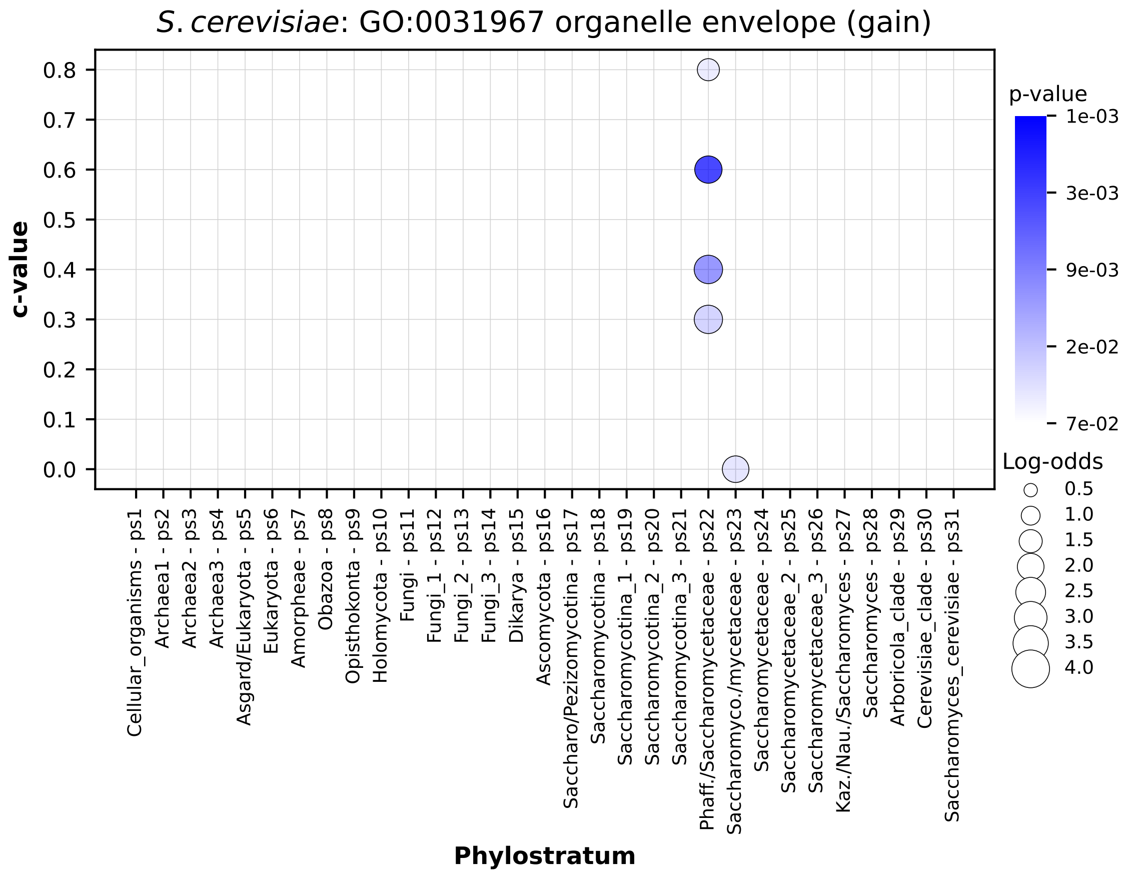

S. cerevisiae: GO:0031967 organelle envelope (gain)
c-value
0.8
0.7
0.6
0.5
0.4
0.3
0.2
0.1
0.0
p-value
1e-03
3e-03
9e-03
2e-02
7e-02
Log-odds
0.5
1.0
1.5
2.0
2.5
3.0
3.5
4.0
Cellular_organisms - ps1
Archaea1 - ps2
Archaea2 - ps3
Archaea3 - ps4
Asgard/Eukaryota - ps5
Eukaryota - ps6
Amorpheae - ps7
Obazoa - ps8
Opisthokonta - ps9
Holomycota - ps10
Fungi - ps11
Fungi_1 - ps12
Fungi_2 - ps13
Fungi_3 - ps14
Dikarya - ps15
Ascomycota - ps16
Saccharo/Pezizomycotina - ps17
Saccharomycotina - ps18
Saccharomycotina_1 - ps19
Saccharomycotina_2 - ps20
Saccharomycotina_3 - ps21
Phaff./Saccharomycetaceae - ps22
Saccharomyco./mycetaceae - ps23
Saccharomycetaceae - ps24
Saccharomycetaceae_2 - ps25
Saccharomycetaceae_3 - ps26
Kaz./Nau./Saccharomyces - ps27
Saccharomyces - ps28
Arboricola_clade - ps29
Cerevisiae_clade - ps30
Saccharomyces_cerevisiae - ps31
Phylostratum

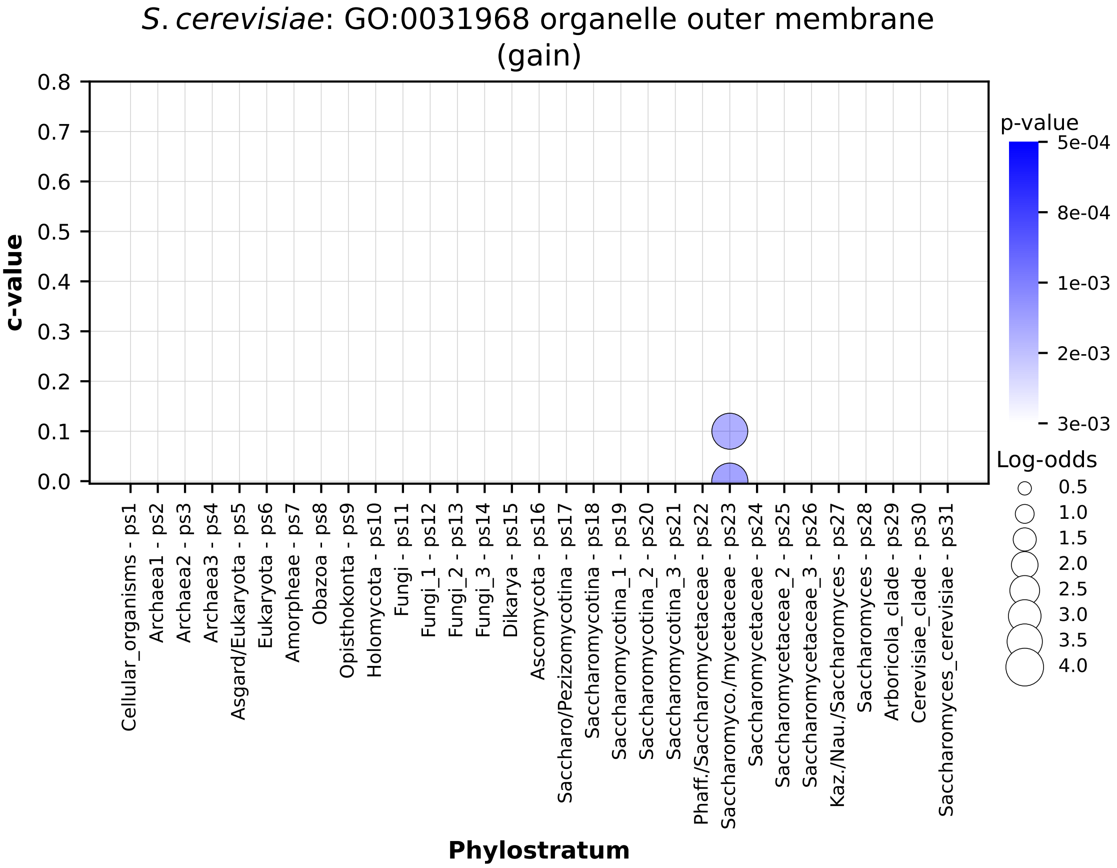

S. cerevisiae: GO:0031968 organelle outer membrane
(gain)
c-value
0.8
0.7
0.6
0.5
0.4
0.3
0.2
0.1
0.0
p-value
5e-04
8e-04
1e-03
2e-03
3e-03
Log-odds
0.5
1.0
1.5
2.0
2.5
3.0
3.5
4.0
Cellular_organisms - ps1
Archaea1 - ps2
Archaea2 - ps3
Archaea3 - ps4
Asgard/Eukaryota - ps5
Eukaryota - ps6
Amorpheae - ps7
Obazoa - ps8
Opisthokonta - ps9
Holomycota - ps10
Fungi - ps11
Fungi_1 - ps12
Fungi_2 - ps13
Fungi_3 - ps14
Dikarya - ps15
Ascomycota - ps16
Saccharo/Pezizomycotina - ps17
Saccharomycotina - ps18
Saccharomycotina_1 - ps19
Saccharomycotina_2 - ps20
Saccharomycotina_3 - ps21
Phaff./Saccharomycetaceae - ps22
Saccharomyco./mycetaceae - ps23
Saccharomycetaceae - ps24
Saccharomycetaceae_2 - ps25
Saccharomycetaceae_3 - ps26
Kaz./Nau./Saccharomyces - ps27
Saccharomyces - ps28
Arboricola_clade - ps29
Cerevisiae_clade - ps30
Saccharomyces_cerevisiae - ps31
Phylostratum

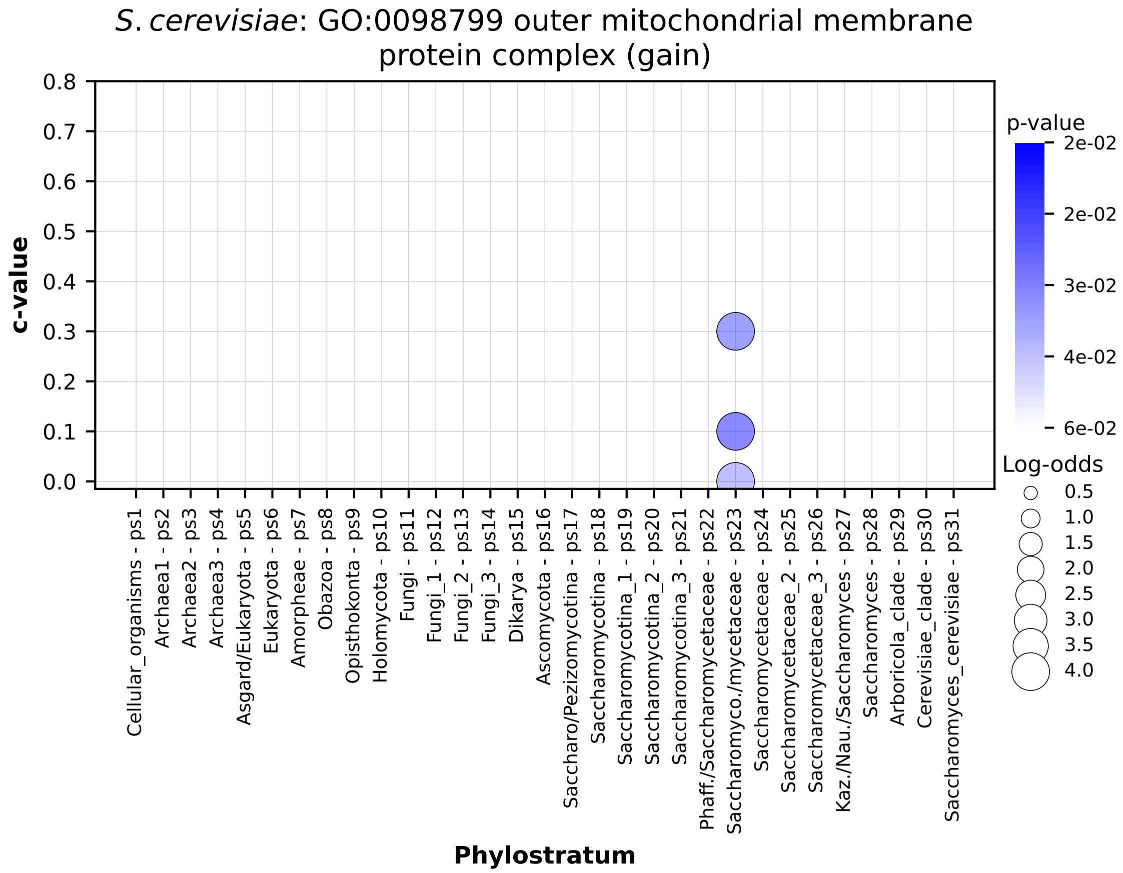
S. cerevisiae: GO:0098799 outer mitochondrial membrane protein complex (gain)
c-value
0.8
0.7
0.6
0.5
0.4
0.3
0.2
0.1
0.0
p-value
2e-02
2e-02
3e-02
4e-02
6e-02
Log-odds
0.5
1.0
1.5
2.0
2.5
3.0
3.5
4.0
Cellular_organisms - ps1
Archaea1 - ps2
Archaea2 - ps3
Archaea3 - ps4
Asgard/Eukaryota - ps5
Eukaryota - ps6
Amorpheae - ps7
Obazoa - ps8
Opisthokonta - ps9
Holomycota - ps10
Fungi - ps11
Fungi_1 - ps12
Fungi_2 - ps13
Fungi_3 - ps14
Dikarya - ps15
Ascomycota - ps16
Saccharo/Pezizomycotina - ps17
Saccharomycotina - ps18
Saccharomycotina_1 - ps19
Saccharomycotina_2 - ps20
Saccharomycotina_3 - ps21
Phaff./Saccharomycetaceae - ps22
Saccharomyco./mycetaceae - ps23
Saccharomycetaceae - ps24
Saccharomycetaceae_2 - ps25
Saccharomycetaceae_3 - ps26
Kaz./Nau./Saccharomyces - ps27
Saccharomyces - ps28
Arboricola_clade - ps29
Cerevisiae_clade - ps30
Saccharomyces_cerevisiae - ps31
Phylostratum

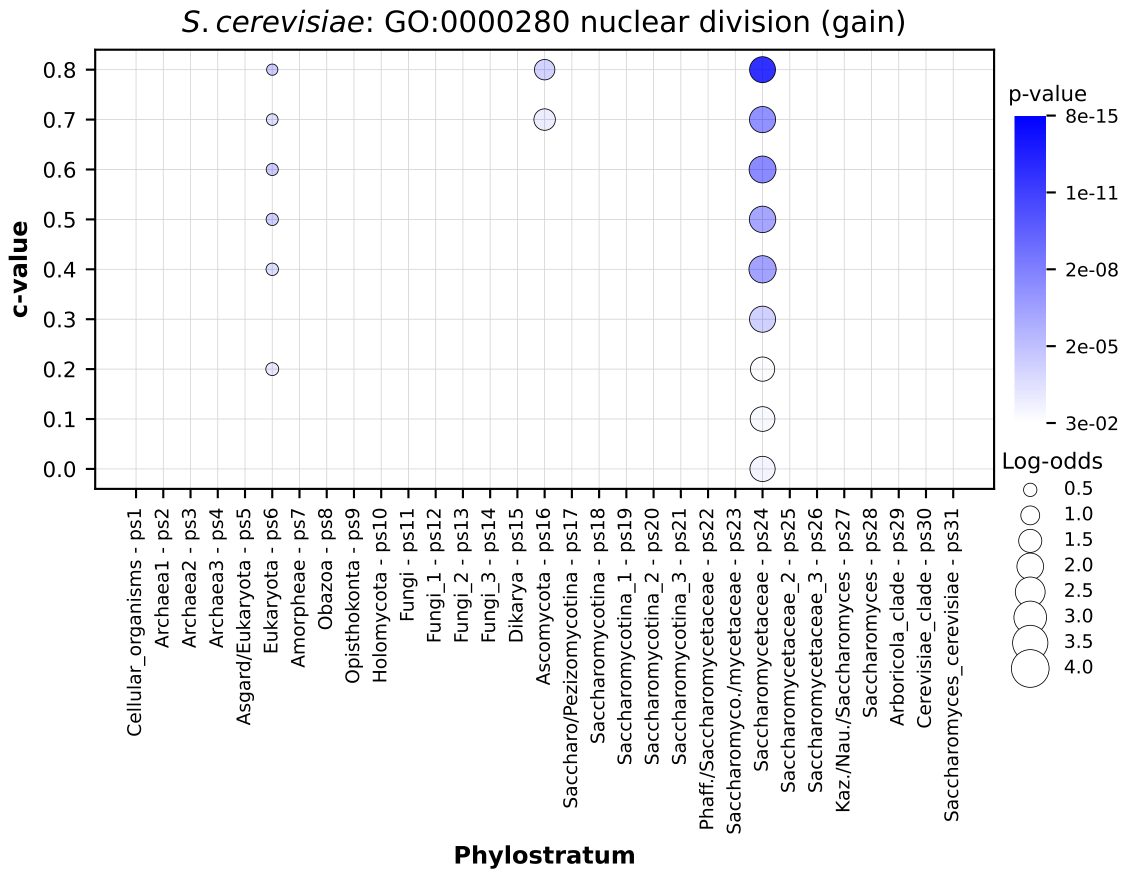

S. cerevisiae: GO:0000280 nuclear division (gain)
c-value
0.0
0.1
0.2
0.3
0.4
0.5
0.6
0.7
0.8
Phylostratum
Cellular_organisms - ps1
Archaea1 - ps2
Archaea2 - ps3
Archaea3 - ps4
Asgard/Eukaryota - ps5
Eukaryota - ps6
Amorpheae - ps7
Obazoa - ps8
Opisthokonta - ps9
Holomycota - ps10
Fungi - ps11
Fungi_1 - ps12
Fungi_2 - ps13
Fungi_3 - ps14
Dikarya - ps15
Ascomycota - ps16
Saccharo/Pezizomycotina - ps17
Saccharomycotina - ps18
Saccharomycotina_1 - ps19
Saccharomycotina_2 - ps20
Saccharomycotina_3 - ps21
Phaff./Saccharomycetaceae - ps22
Saccharomyco./mycetaceae - ps23
Saccharomycetaceae - ps24
Saccharomycetaceae_2 - ps25
Saccharomycetaceae_3 - ps26
Kaz./Nau./Saccharomyces - ps27
Saccharomyces - ps28
Arboricola_clade - ps29
Cerevisiae_clade - ps30
Saccharomyces_cerevisiae - ps31
p-value
8e-15
1e-11
2e-08
2e-05
3e-02
Log-odds
0.5
1.0
1.5
2.0
2.5
3.0
3.5
4.0

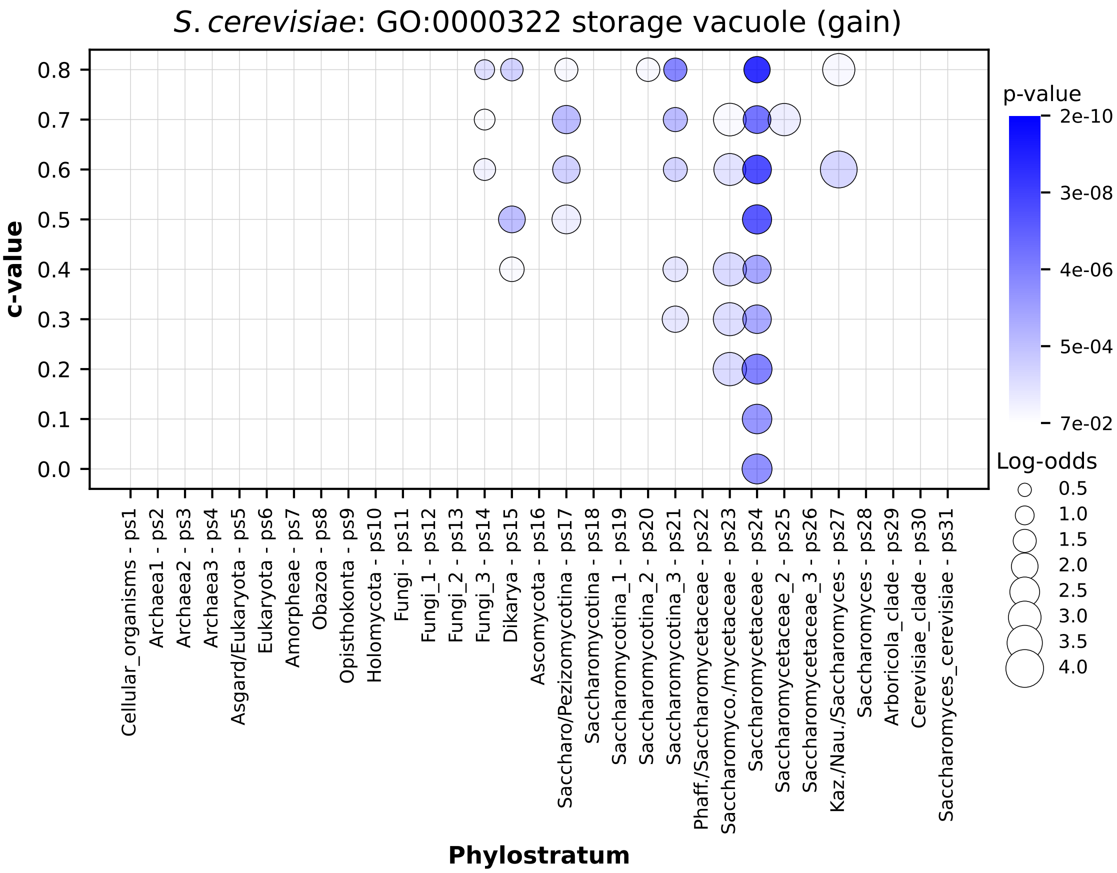

S. cerevisiae: GO:0000322 storage vacuole (gain)
c-value
0.8
0.7
0.6
0.5
0.4
0.3
0.2
0.1
0.0
Phylostratum
Cellular_organisms - ps1
Archaea1 - ps2
Archaea2 - ps3
Archaea3 - ps4
Asgard/Eukaryota - ps5
Eukaryota - ps6
Amorpheae - ps7
Obazoa - ps8
Opisthokonta - ps9
Holomycota - ps10
Fungi - ps11
Fungi_1 - ps12
Fungi_2 - ps13
Fungi_3 - ps14
Dikarya - ps15
Ascomycota - ps16
Saccharo/Pezizomycotina - ps17
Saccharomycotina - ps18
Saccharomycotina_1 - ps19
Saccharomycotina_2 - ps20
Saccharomycotina_3 - ps21
Phaff./Saccharomycetaceae - ps22
Saccharomyco./mycetaceae - ps23
Saccharomycetaceae - ps24
Saccharomycetaceae_2 - ps25
Saccharomycetaceae_3 - ps26
Kaz./Nau./Saccharomyces - ps27
Saccharomyces - ps28
Arboricola_clade - ps29
Cerevisiae_clade - ps30
Saccharomyces_cerevisiae - ps31
p-value
2e-10
3e-08
4e-06
5e-04
7e-02
Log-odds
0.5
1.0
1.5
2.0
2.5
3.0
3.5
4.0

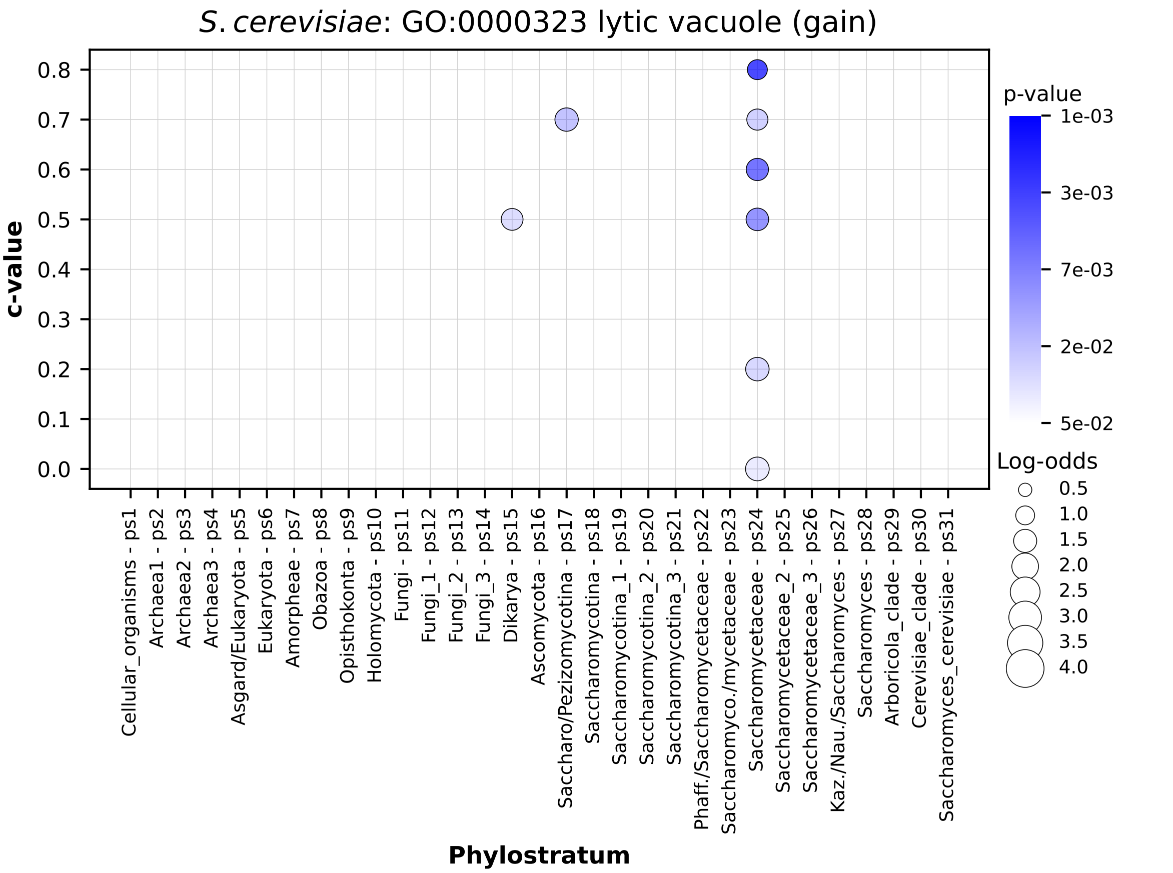
S. cerevisiae: GO:0000323 lytic vacuole (gain)
c-value
0.8
0.7
0.6
0.5
0.4
0.3
0.2
0.1
0.0
p-value
1e-03
3e-03
7e-03
2e-02
5e-02
Log-odds
0.5
1.0
1.5
2.0
2.5
3.0
3.5
4.0
Cellular_organisms - ps1
Archaea1 - ps2
Archaea2 - ps3
Archaea3 - ps4
Asgard/Eukaryota - ps5
Eukaryota - ps6
Amorpheae - ps7
Obazoa - ps8
Opisthokonta - ps9
Holomycota - ps10
Fungi - ps11
Fungi_1 - ps12
Fungi_2 - ps13
Fungi_3 - ps14
Dikarya - ps15
Ascomycota - ps16
Saccharo/Pezizomycotina - ps17
Saccharomycotina - ps18
Saccharomycotina_1 - ps19
Saccharomycotina_2 - ps20
Saccharomycotina_3 - ps21
Phaff./Saccharomycetaceae - ps22
Saccharomyco./mycetaceae - ps23
Saccharomycetaceae - ps24
Saccharomycetaceae_2 - ps25
Saccharomycetaceae_3 - ps26
Kaz./Nau./Saccharomyces - ps27
Saccharomyces - ps28
Arboricola_clade - ps29
Cerevisiae_clade - ps30
Saccharomyces_cerevisiae - ps31
Phylostratum

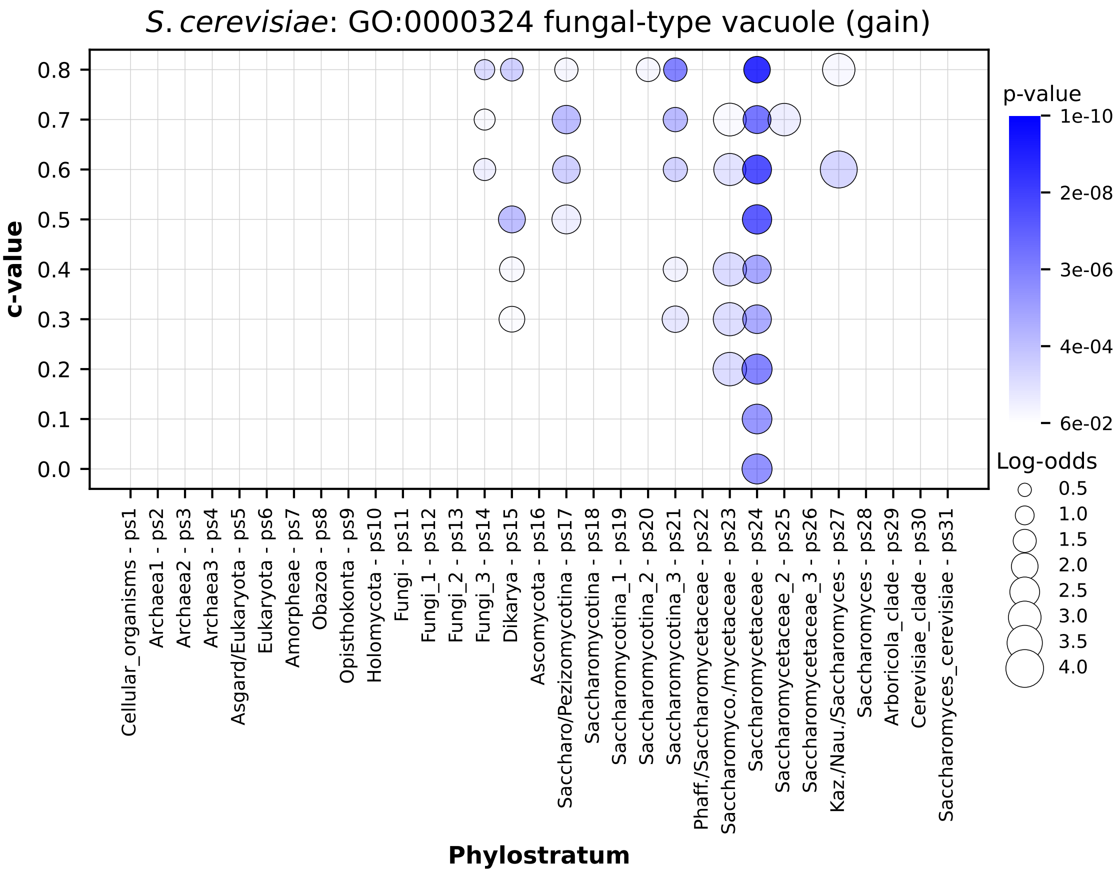

S. cerevisiae: GO:0000324 fungal-type vacuole (gain)
c-value
0.8
0.7
0.6
0.5
0.4
0.3
0.2
0.1
0.0
Phylostratum
Cellular_organisms - ps1
Archaea1 - ps2
Archaea2 - ps3
Archaea3 - ps4
Asgard/Eukaryota - ps5
Eukaryota - ps6
Amorpheae - ps7
Obazoa - ps8
Opisthokonta - ps9
Holomycota - ps10
Fungi - ps11
Fungi_1 - ps12
Fungi_2 - ps13
Fungi_3 - ps14
Dikarya - ps15
Ascomycota - ps16
Saccharo/Pezizomycotina - ps17
Saccharomycotina - ps18
Saccharomycotina_1 - ps19
Saccharomycotina_2 - ps20
Saccharomycotina_3 - ps21
Phaff./Saccharomycetaceae - ps22
Saccharomyco./mycetaceae - ps23
Saccharomycetaceae - ps24
Saccharomycetaceae_2 - ps25
Saccharomycetaceae_3 - ps26
Kaz./Nau./Saccharomyces - ps27
Saccharomyces - ps28
Arboricola_clade - ps29
Cerevisiae_clade - ps30
Saccharomyces_cerevisiae - ps31
p-value
1e-10
2e-08
3e-06
4e-04
6e-02
Log-odds
0.5
1.0
1.5
2.0
2.5
3.0
3.5
4.0

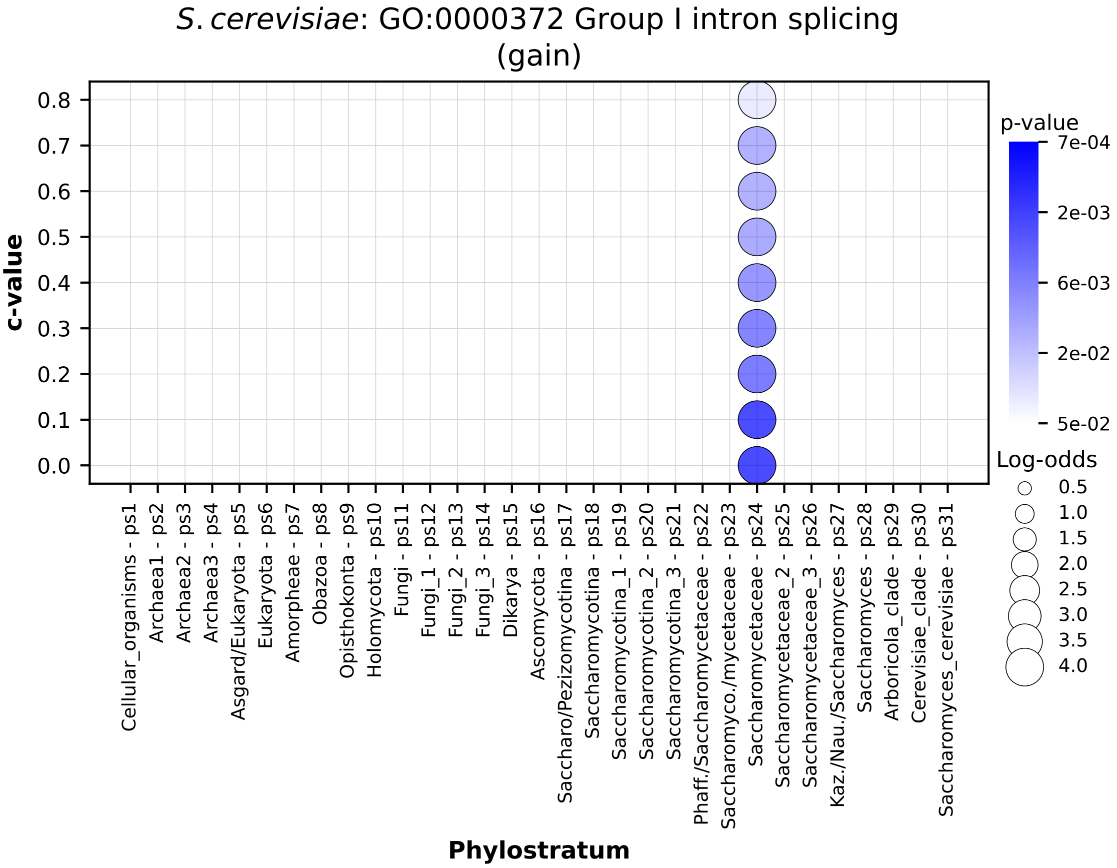

S. cerevisiae: GO:0000372 Group I intron splicing (gain)
c-value
0.8
0.7
0.6
0.5
0.4
0.3
0.2
0.1
0.0
p-value
7e-04
2e-03
6e-03
2e-02
5e-02
Log-odds
0.5
1.0
1.5
2.0
2.5
3.0
3.5
4.0
Cellular_organisms - ps1
Archaea1 - ps2
Archaea2 - ps3
Archaea3 - ps4
Asgard/Eukaryota - ps5
Eukaryota - ps6
Amorpheae - ps7
Obazoa - ps8
Opisthokonta - ps9
Holomycota - ps10
Fungi - ps11
Fungi_1 - ps12
Fungi_2 - ps13
Fungi_3 - ps14
Dikarya - ps15
Ascomycota - ps16
Saccharo/Pezizomycotina - ps17
Saccharomycotina - ps18
Saccharomycotina_1 - ps19
Saccharomycotina_2 - ps20
Saccharomycotina_3 - ps21
Phaff./Saccharomycetaceae - ps22
Saccharomyco./mycetaceae - ps23
Saccharomycetaceae - ps24
Saccharomycetaceae_2 - ps25
Saccharomycetaceae_3 - ps26
Kaz./Nau./Saccharomyces - ps27
Saccharomyces - ps28
Arboricola_clade - ps29
Cerevisiae_clade - ps30
Saccharomyces_cerevisiae - ps31
Phylostratum

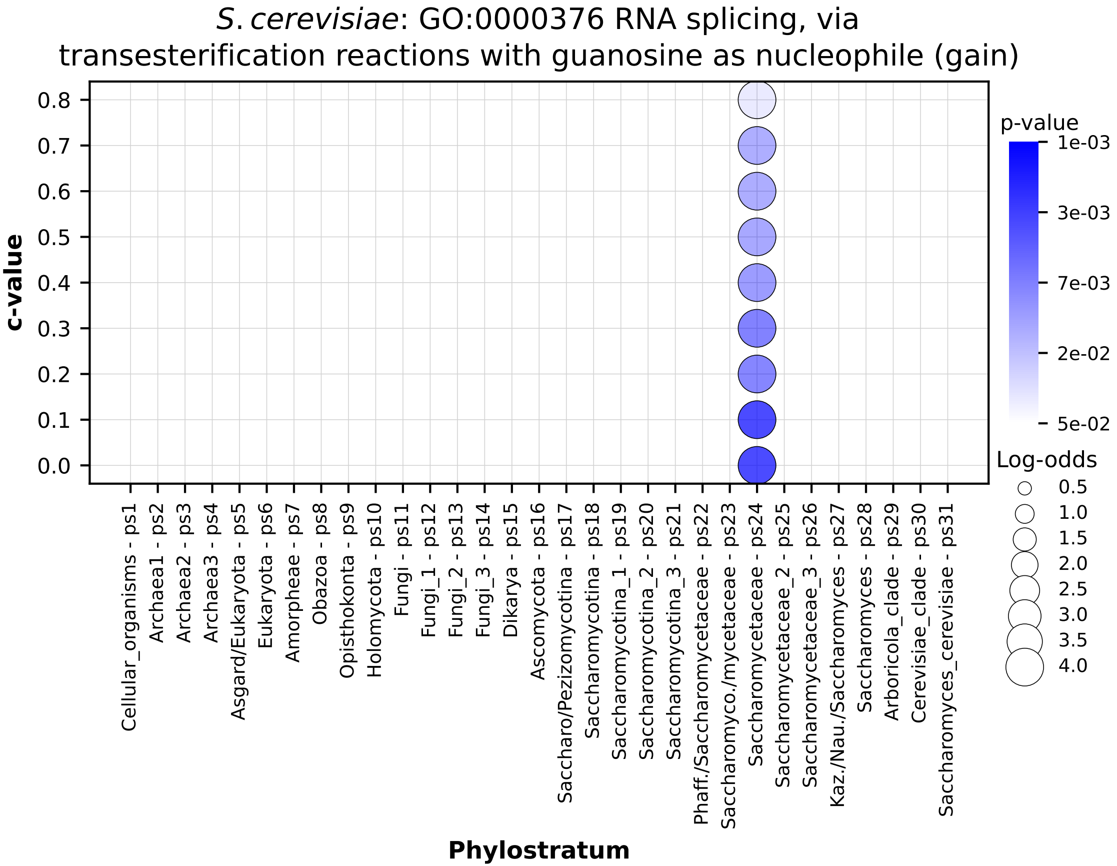

S. cerevisiae: GO:0000376 RNA splicing, via transesterification reactions with guanosine as nucleophile (gain)
c-value
0.0
0.1
0.2
0.3
0.4
0.5
0.6
0.7
0.8
Phylostratum
Cellular_organisms - ps1
Archaea1 - ps2
Archaea2 - ps3
Archaea3 - ps4
Asgard/Eukaryota - ps5
Eukaryota - ps6
Amorpheae - ps7
Obazoa - ps8
Opisthokonta - ps9
Holomycota - ps10
Fungi - ps11
Fungi_1 - ps12
Fungi_2 - ps13
Fungi_3 - ps14
Dikarya - ps15
Ascomycota - ps16
Saccharo/Pezizomycotina - ps17
Saccharomycotina - ps18
Saccharomycotina_1 - ps19
Saccharomycotina_2 - ps20
Saccharomycotina_3 - ps21
Phaff./Saccharomycetaceae - ps22
Saccharomyco./mycetaceae - ps23
Saccharomycetaceae - ps24
Saccharomycetaceae_2 - ps25
Saccharomycetaceae_3 - ps26
Kaz./Nau./Saccharomyces - ps27
Saccharomyces - ps28
Arboricola_clade - ps29
Cerevisiae_clade - ps30
Saccharomyces_cerevisiae - ps31
p-value
1e-03
3e-03
7e-03
2e-02
5e-02
Log-odds
0.5
1.0
1.5
2.0
2.5
3.0
3.5
4.0

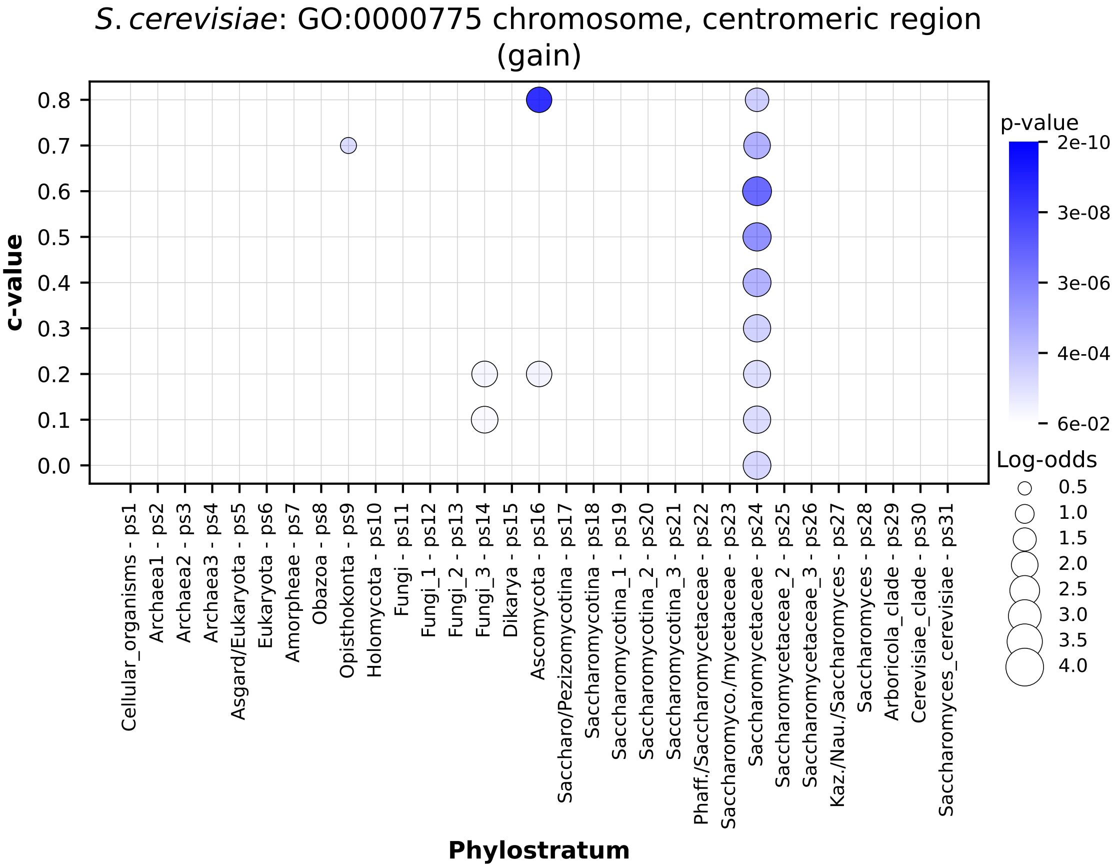

S. cerevisiae: GO:0000775 chromosome, centromeric region (gain)
c-value
0.8
0.7
0.6
0.5
0.4
0.3
0.2
0.1
0.0
p-value
2e-10
3e-08
3e-06
4e-04
6e-02
Log-odds
0.5
1.0
1.5
2.0
2.5
3.0
3.5
4.0
Cellular_organisms - ps1
Archaea1 - ps2
Archaea2 - ps3
Archaea3 - ps4
Asgard/Eukaryota - ps5
Eukaryota - ps6
Amorpheae - ps7
Obazoa - ps8
Opisthokonta - ps9
Holomycota - ps10
Fungi - ps11
Fungi_1 - ps12
Fungi_2 - ps13
Fungi_3 - ps14
Dikarya - ps15
Ascomycota - ps16
Saccharo/Pezizomycotina - ps17
Saccharomycotina - ps18
Saccharomycotina_1 - ps19
Saccharomycotina_2 - ps20
Saccharomycotina_3 - ps21
Phaff./Saccharomycetaceae - ps22
Saccharomyco./mycetaceae - ps23
Saccharomycetaceae - ps24
Saccharomycetaceae_2 - ps25
Saccharomycetaceae_3 - ps26
Kaz./Nau./Saccharomyces - ps27
Saccharomyces - ps28
Arboricola_clade - ps29
Cerevisiae_clade - ps30
Saccharomyces_cerevisiae - ps31
Phylostratum

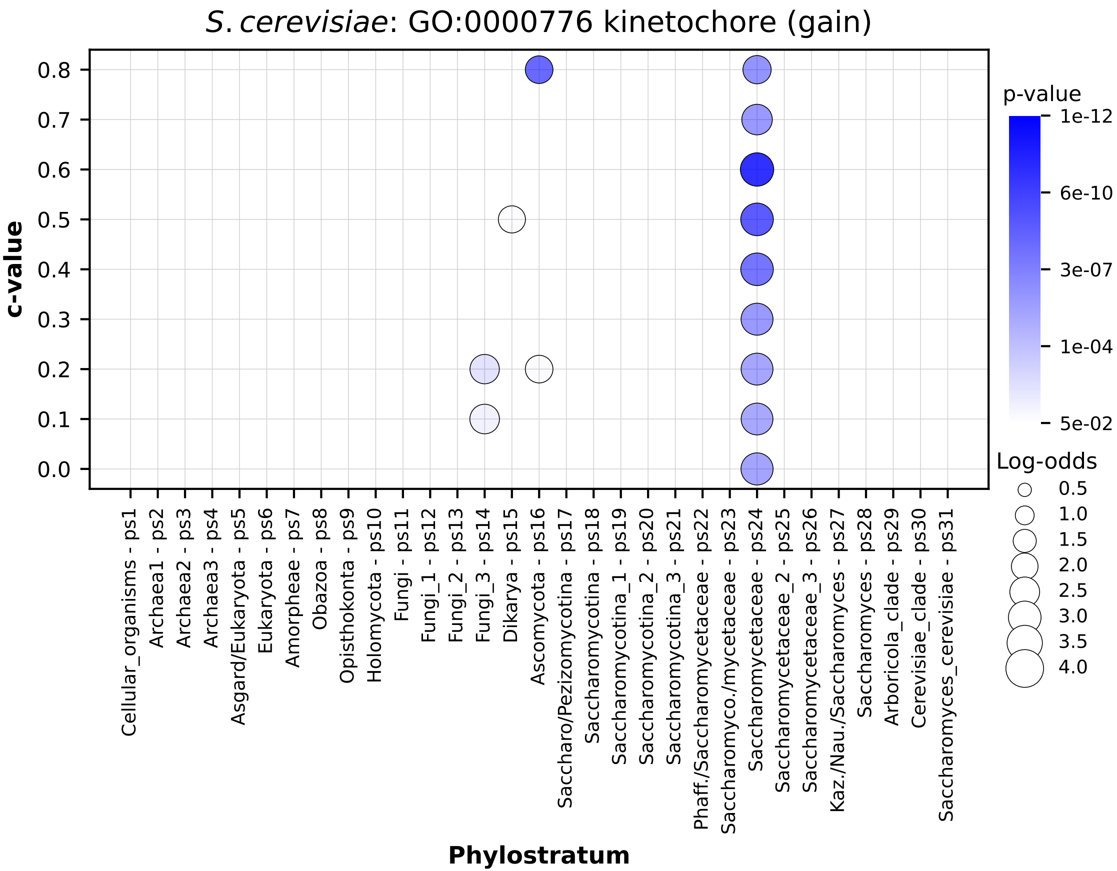

S. cerevisiae: GO:0000776 kinetochore (gain)
c-value
0.0
0.1
0.2
0.3
0.4
0.5
0.6
0.7
0.8
p-value
1e-12
6e-10
3e-07
1e-04
5e-02
Log-odds
0.5
1.0
1.5
2.0
2.5
3.0
3.5
4.0
Cellular_organisms - ps1
Archaea1 - ps2
Archaea2 - ps3
Archaea3 - ps4
Asgard/Eukaryota - ps5
Eukaryota - ps6
Amorpheae - ps7
Obazoa - ps8
Opisthokonta - ps9
Holomycota - ps10
Fungi - ps11
Fungi_1 - ps12
Fungi_2 - ps13
Fungi_3 - ps14
Dikarya - ps15
Ascomycota - ps16
Saccharo/Pezizomycotina - ps17
Saccharomycotina - ps18
Saccharomycotina_1 - ps19
Saccharomycotina_2 - ps20
Saccharomycotina_3 - ps21
Phaff./Saccharomycetaceae - ps22
Saccharomyco./mycetaceae - ps23
Saccharomycetaceae - ps24
Saccharomycetaceae_2 - ps25
Saccharomycetaceae_3 - ps26
Kaz./Nau./Saccharomyces - ps27
Saccharomyces - ps28
Arboricola_clade - ps29
Cerevisiae_clade - ps30
Saccharomyces_cerevisiae - ps31
Phylostratum

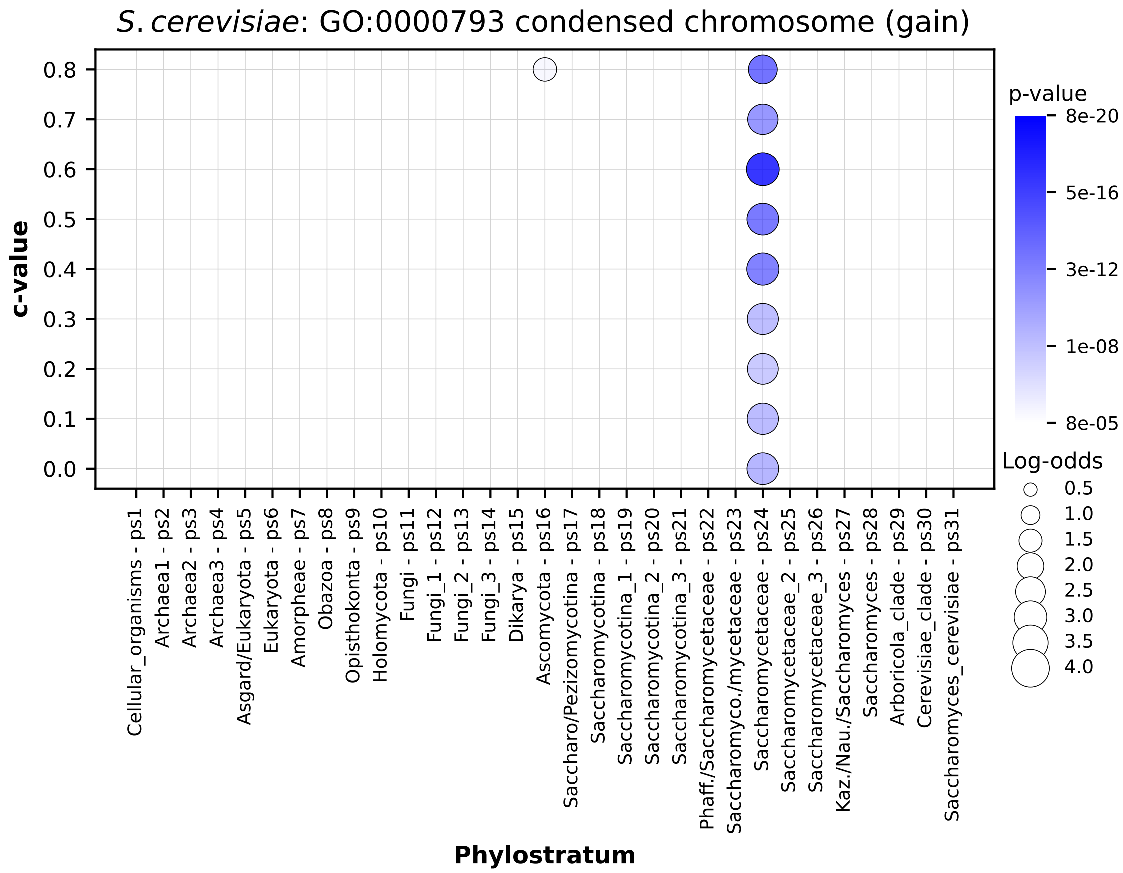

S. cerevisiae: GO:0000793 condensed chromosome (gain)
c-value
0.8
0.7
0.6
0.5
0.4
0.3
0.2
0.1
0.0
p-value
8e-20
5e-16
3e-12
1e-08
8e-05
Log-odds
0.5
1.0
1.5
2.0
2.5
3.0
3.5
4.0
Cellular_organisms - ps1
Archaea1 - ps2
Archaea2 - ps3
Archaea3 - ps4
Asgard/Eukaryota - ps5
Eukaryota - ps6
Amorpheae - ps7
Obazoa - ps8
Opisthokonta - ps9
Holomycota - ps10
Fungi - ps11
Fungi_1 - ps12
Fungi_2 - ps13
Fungi_3 - ps14
Dikarya - ps15
Ascomycota - ps16
Saccharo/Pezizomycotina - ps17
Saccharomycotina - ps18
Saccharomycotina_1 - ps19
Saccharomycotina_2 - ps20
Saccharomycotina_3 - ps21
Phaff./Saccharomycetaceae - ps22
Saccharomyco./mycetaceae - ps23
Saccharomycetaceae - ps24
Saccharomycetaceae_2 - ps25
Saccharomycetaceae_3 - ps26
Kaz./Nau./Saccharomyces - ps27
Saccharomyces - ps28
Arboricola_clade - ps29
Cerevisiae_clade - ps30
Saccharomyces_cerevisiae - ps31
Phylostratum

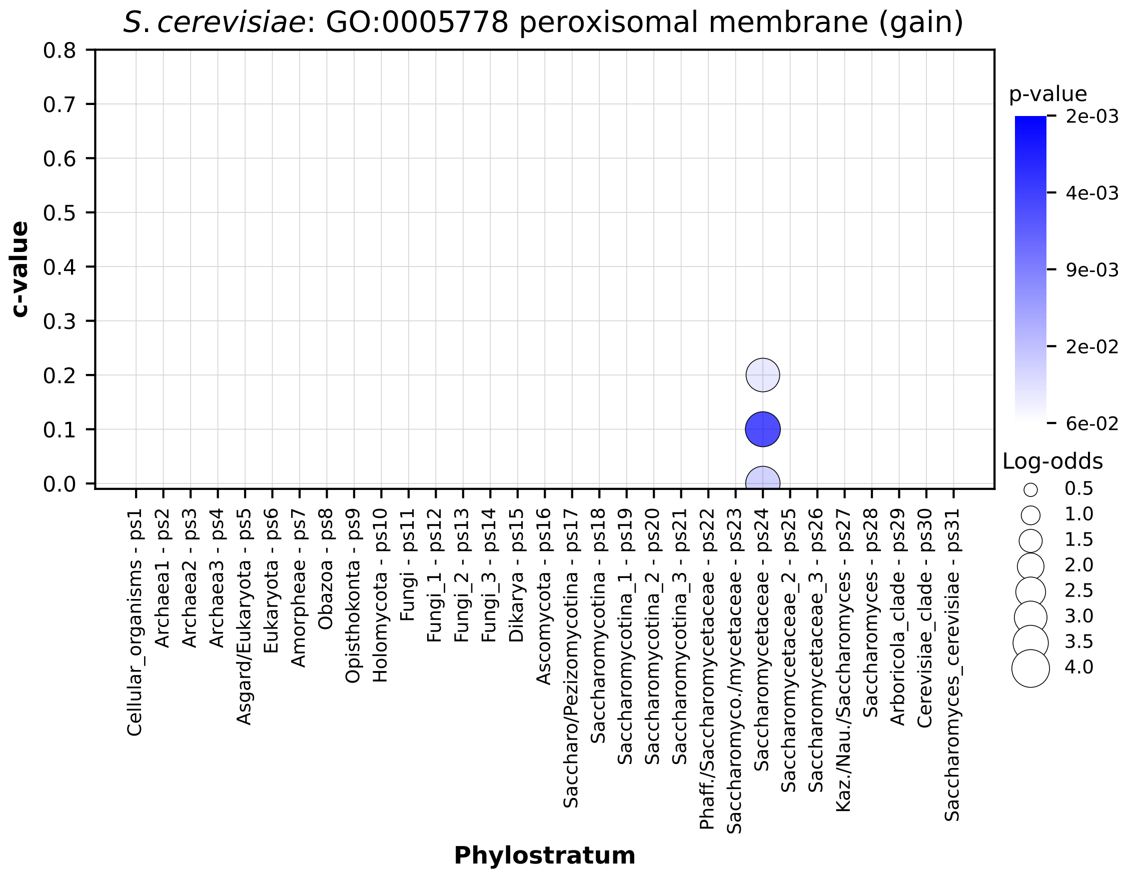
S. cerevisiae: GO:0005778 peroxisomal membrane (gain)
c-value
0.8
0.7
0.6
0.5
0.4
0.3
0.2
0.1
0.0
p-value
2e-03
4e-03
9e-03
2e-02
6e-02
Log-odds
0.5
1.0
1.5
2.0
2.5
3.0
3.5
4.0
Cellular_organisms - ps1
Archaea1 - ps2
Archaea2 - ps3
Archaea3 - ps4
Asgard/Eukaryota - ps5
Eukaryota - ps6
Amorpheae - ps7
Obazoa - ps8
Opisthokonta - ps9
Holomycota - ps10
Fungi - ps11
Fungi_1 - ps12
Fungi_2 - ps13
Fungi_3 - ps14
Dikarya - ps15
Ascomycota - ps16
Saccharo/Pezizomycotina - ps17
Saccharomycotina - ps18
Saccharomycotina_1 - ps19
Saccharomycotina_2 - ps20
Saccharomycotina_3 - ps21
Phaff./Saccharomycetaceae - ps22
Saccharomyco./mycetaceae - ps23
Saccharomycetaceae - ps24
Saccharomycetaceae_2 - ps25
Saccharomycetaceae_3 - ps26
Kaz./Nau./Saccharomyces - ps27
Saccharomyces - ps28
Arboricola_clade - ps29
Cerevisiae_clade - ps30
Saccharomyces_cerevisiae - ps31
Phylostratum

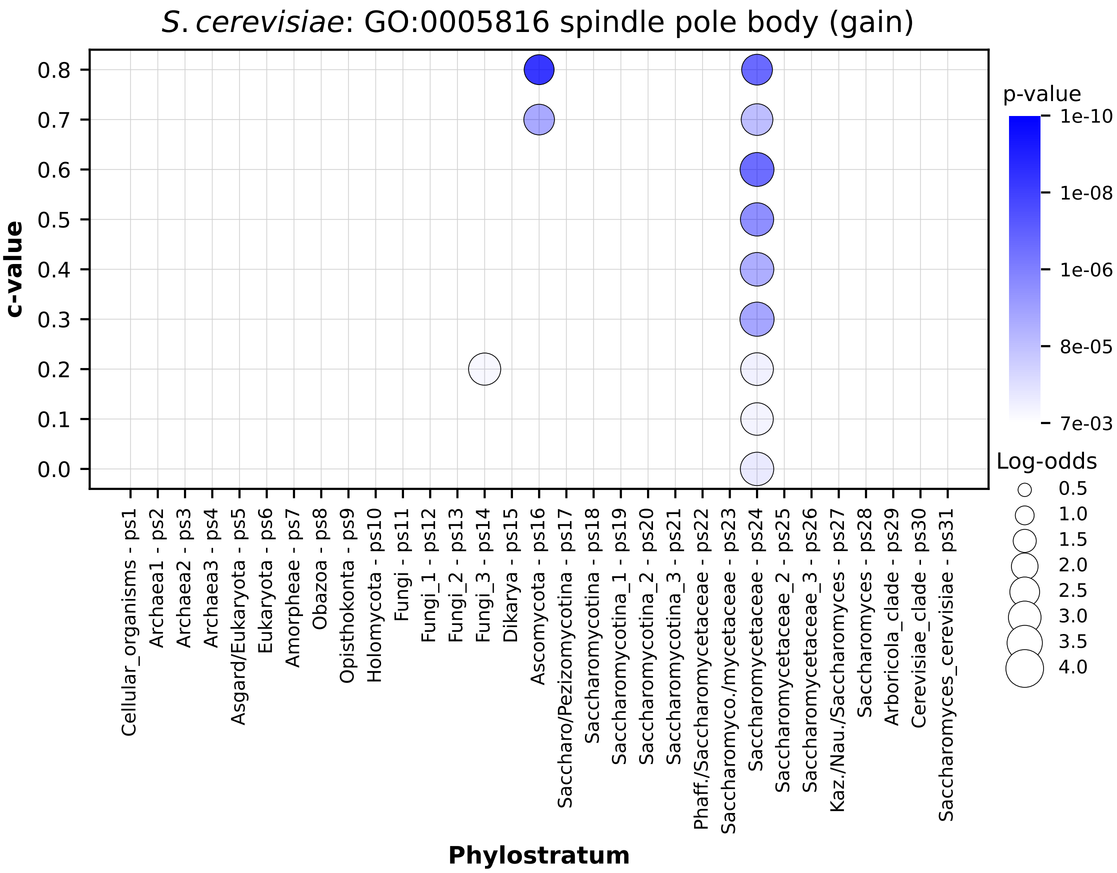

S. cerevisiae: GO:0005816 spindle pole body (gain)
c-value
0.8
0.7
0.6
0.5
0.4
0.3
0.2
0.1
0.0
p-value
1e-10
1e-08
1e-06
8e-05
7e-03
Log-odds
0.5
1.0
1.5
2.0
2.5
3.0
3.5
4.0
Cellular_organisms - ps1
Archaea1 - ps2
Archaea2 - ps3
Archaea3 - ps4
Asgard/Eukaryota - ps5
Eukaryota - ps6
Amorpheae - ps7
Obazoa - ps8
Opisthokonta - ps9
Holomycota - ps10
Fungi - ps11
Fungi_1 - ps12
Fungi_2 - ps13
Fungi_3 - ps14
Dikarya - ps15
Ascomycota - ps16
Saccharo/Pezizomycotina - ps17
Saccharomycotina - ps18
Saccharomycotina_1 - ps19
Saccharomycotina_2 - ps20
Saccharomycotina_3 - ps21
Phaff./Saccharomycetaceae - ps22
Saccharomyco./mycetaceae - ps23
Saccharomycetaceae - ps24
Saccharomycetaceae_2 - ps25
Saccharomycetaceae_3 - ps26
Kaz./Nau./Saccharomyces - ps27
Saccharomyces - ps28
Arboricola_clade - ps29
Cerevisiae_clade - ps30
Saccharomyces_cerevisiae - ps31
Phylostratum

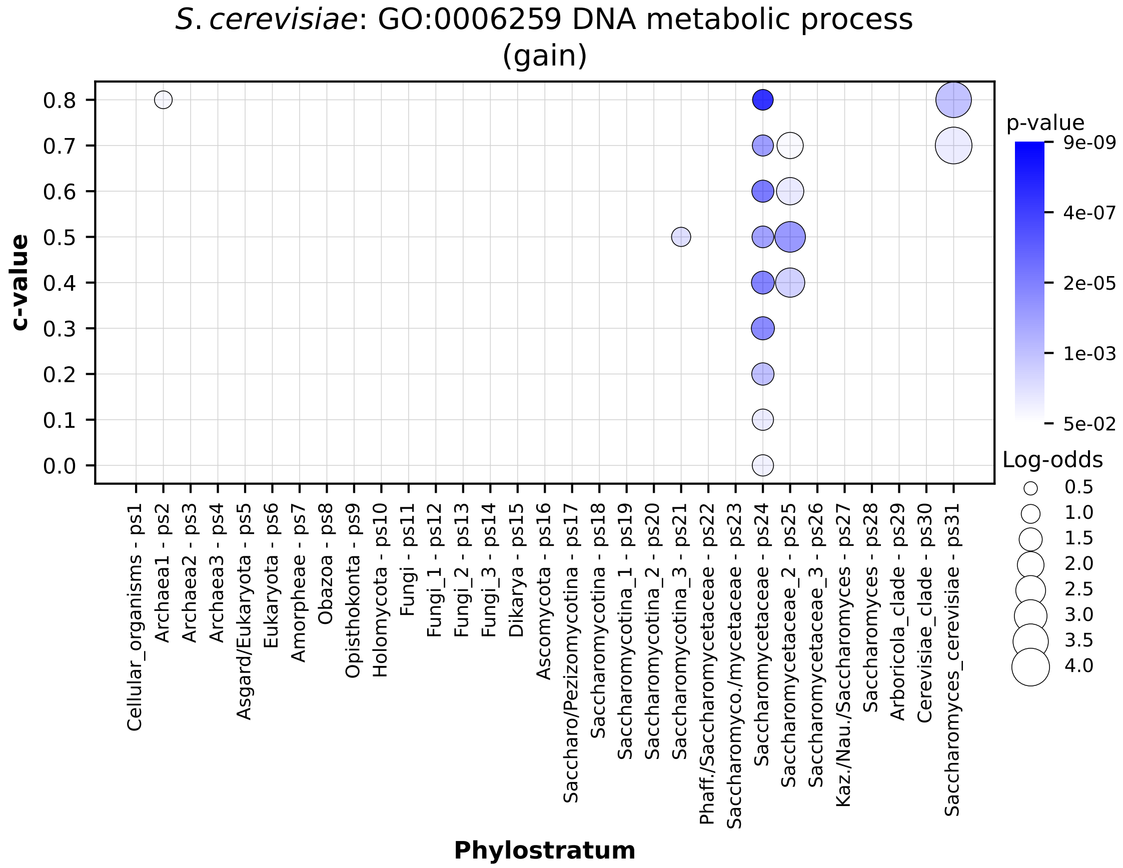

S. cerevisiae: GO:0006259 DNA metabolic process (gain)
c-value
0.8
0.7
0.6
0.5
0.4
0.3
0.2
0.1
0.0
p-value
9e-09
4e-07
2e-05
1e-03
5e-02
Log-odds
0.5
1.0
1.5
2.0
2.5
3.0
3.5
4.0
Cellular_organisms - ps1
Archaea1 - ps2
Archaea2 - ps3
Archaea3 - ps4
Asgard/Eukaryota - ps5
Eukaryota - ps6
Amorpheae - ps7
Obazoa - ps8
Opisthokonta - ps9
Holomycota - ps10
Fungi - ps11
Fungi_1 - ps12
Fungi_2 - ps13
Fungi_3 - ps14
Dikarya - ps15
Ascomycota - ps16
Saccharo/Pezizomycotina - ps17
Saccharomycotina - ps18
Saccharomycotina_1 - ps19
Saccharomycotina_2 - ps20
Saccharomycotina_3 - ps21
Phaff./Saccharomycetaceae - ps22
Saccharomyco./mycetaceae - ps23
Saccharomycetaceae - ps24
Saccharomycetaceae_2 - ps25
Saccharomycetaceae_3 - ps26
Kaz./Nau./Saccharomyces - ps27
Saccharomyces - ps28
Arboricola_clade - ps29
Cerevisiae_clade - ps30
Saccharomyces_cerevisiae - ps31
Phylostratum

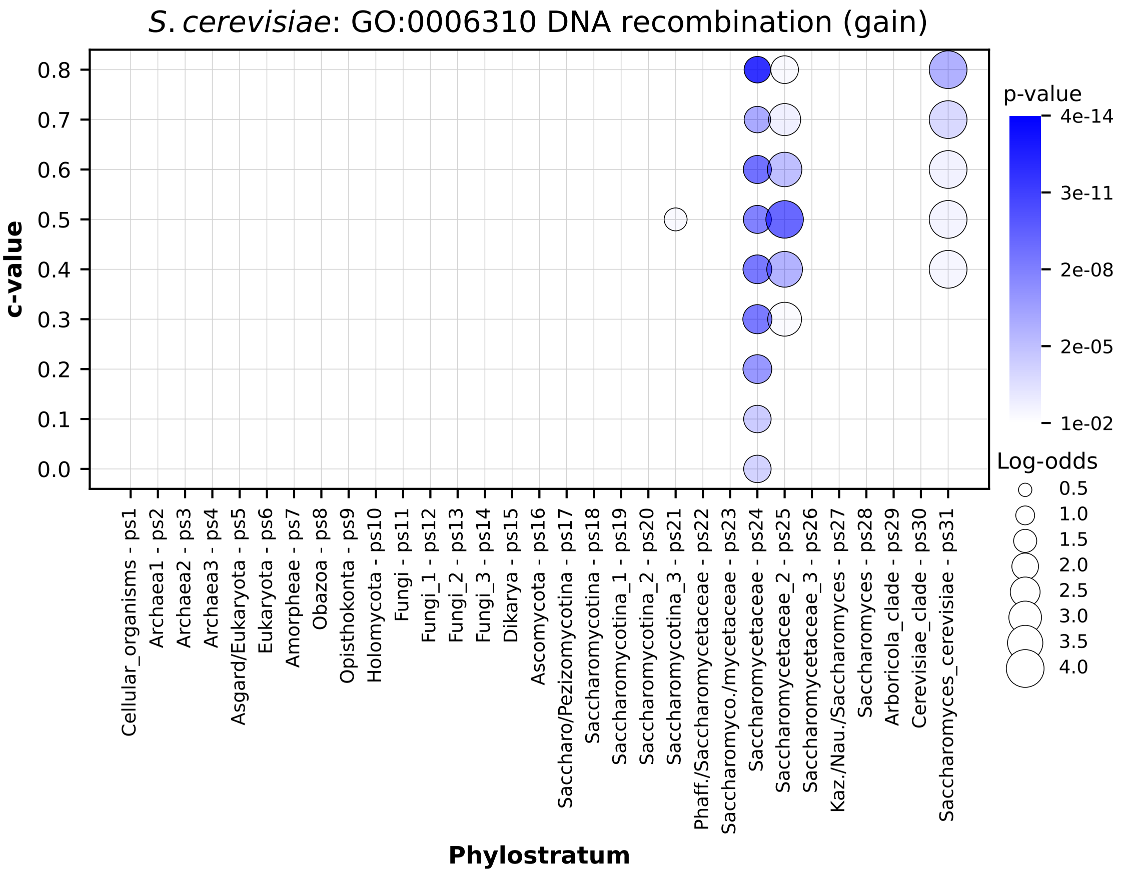

S. cerevisiae: GO:0006310 DNA recombination (gain)
c-value
0.0
0.1
0.2
0.3
0.4
0.5
0.6
0.7
0.8
Phylostratum
Cellular_organisms - ps1
Archaea1 - ps2
Archaea2 - ps3
Archaea3 - ps4
Asgard/Eukaryota - ps5
Eukaryota - ps6
Amorpheae - ps7
Obazoa - ps8
Opisthokonta - ps9
Holomycota - ps10
Fungi - ps11
Fungi_1 - ps12
Fungi_2 - ps13
Fungi_3 - ps14
Dikarya - ps15
Ascomycota - ps16
Saccharo/Pezizomycotina - ps17
Saccharomycotina - ps18
Saccharomycotina_1 - ps19
Saccharomycotina_2 - ps20
Saccharomycotina_3 - ps21
Phaff./Saccharomycetaceae - ps22
Saccharomyco./mycetaceae - ps23
Saccharomycetaceae - ps24
Saccharomycetaceae_2 - ps25
Saccharomycetaceae_3 - ps26
Kaz./Nau./Saccharomyces - ps27
Saccharomyces - ps28
Arboricola_clade - ps29
Cerevisiae_clade - ps30
Saccharomyces_cerevisiae - ps31
p-value
4e-14
3e-11
2e-08
2e-05
1e-02
Log-odds
0.5
1.0
1.5
2.0
2.5
3.0
3.5
4.0

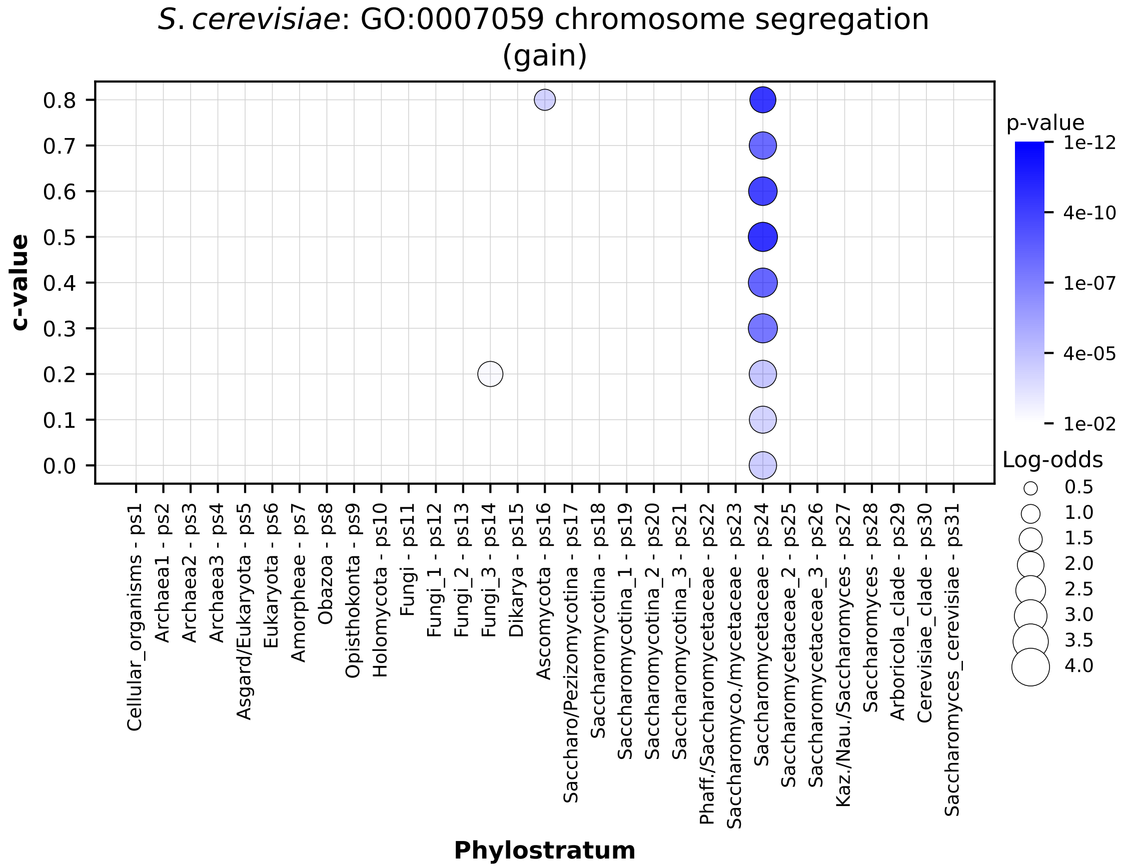

S. cerevisiae: GO:0007059 chromosome segregation
(gain)
c-value
0.8
0.7
0.6
0.5
0.4
0.3
0.2
0.1
0.0
p-value
1e-12
4e-10
1e-07
4e-05
1e-02
Log-odds
0.5
1.0
1.5
2.0
2.5
3.0
3.5
4.0
Cellular_organisms - ps1
Archaea1 - ps2
Archaea2 - ps3
Archaea3 - ps4
Asgard/Eukaryota - ps5
Eukaryota - ps6
Amorpheae - ps7
Obazoa - ps8
Opisthokonta - ps9
Holomycota - ps10
Fungi - ps11
Fungi_1 - ps12
Fungi_2 - ps13
Fungi_3 - ps14
Dikarya - ps15
Ascomycota - ps16
Saccharo/Pezizomycotina - ps17
Saccharomycotina - ps18
Saccharomycotina_1 - ps19
Saccharomycotina_2 - ps20
Saccharomycotina_3 - ps21
Phaff./Saccharomycetaceae - ps22
Saccharomyco./mycetaceae - ps23
Saccharomycetaceae - ps24
Saccharomycetaceae_2 - ps25
Saccharomycetaceae_3 - ps26
Kaz./Nau./Saccharomyces - ps27
Saccharomyces - ps28
Arboricola_clade - ps29
Cerevisiae_clade - ps30
Saccharomyces_cerevisiae - ps31
Phylostratum

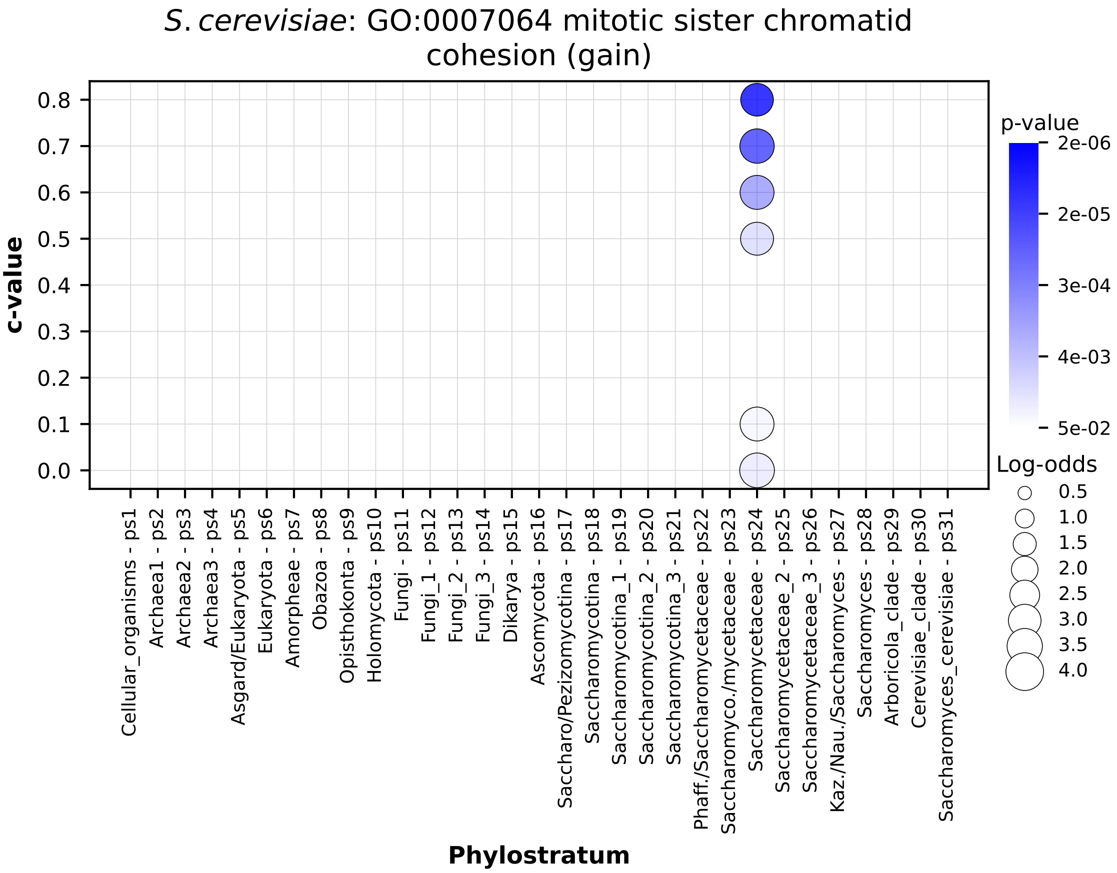

S. cerevisiae: GO:0007064 mitotic sister chromatid cohesion (gain)
c-value
0.8
0.7
0.6
0.5
0.4
0.3
0.2
0.1
0.0
p-value
2e-06
2e-05
3e-04
4e-03
5e-02
Log-odds
0.5
1.0
1.5
2.0
2.5
3.0
3.5
4.0
Cellular_organisms - ps1
Archaea1 - ps2
Archaea2 - ps3
Archaea3 - ps4
Asgard/Eukaryota - ps5
Eukaryota - ps6
Amorpheae - ps7
Obazoa - ps8
Opisthokonta - ps9
Holomycota - ps10
Fungi - ps11
Fungi_1 - ps12
Fungi_2 - ps13
Fungi_3 - ps14
Dikarya - ps15
Ascomycota - ps16
Saccharo/Pezizomycotina - ps17
Saccharomycotina - ps18
Saccharomycotina_1 - ps19
Saccharomycotina_2 - ps20
Saccharomycotina_3 - ps21
Phaff./Saccharomycetaceae - ps22
Saccharomyco./mycetaceae - ps23
Saccharomycetaceae - ps24
Saccharomycetaceae_2 - ps25
Saccharomycetaceae_3 - ps26
Kaz./Nau./Saccharomyces - ps27
Saccharomyces - ps28
Arboricola_clade - ps29
Cerevisiae_clade - ps30
Saccharomyces_cerevisiae - ps31
Phylostratum

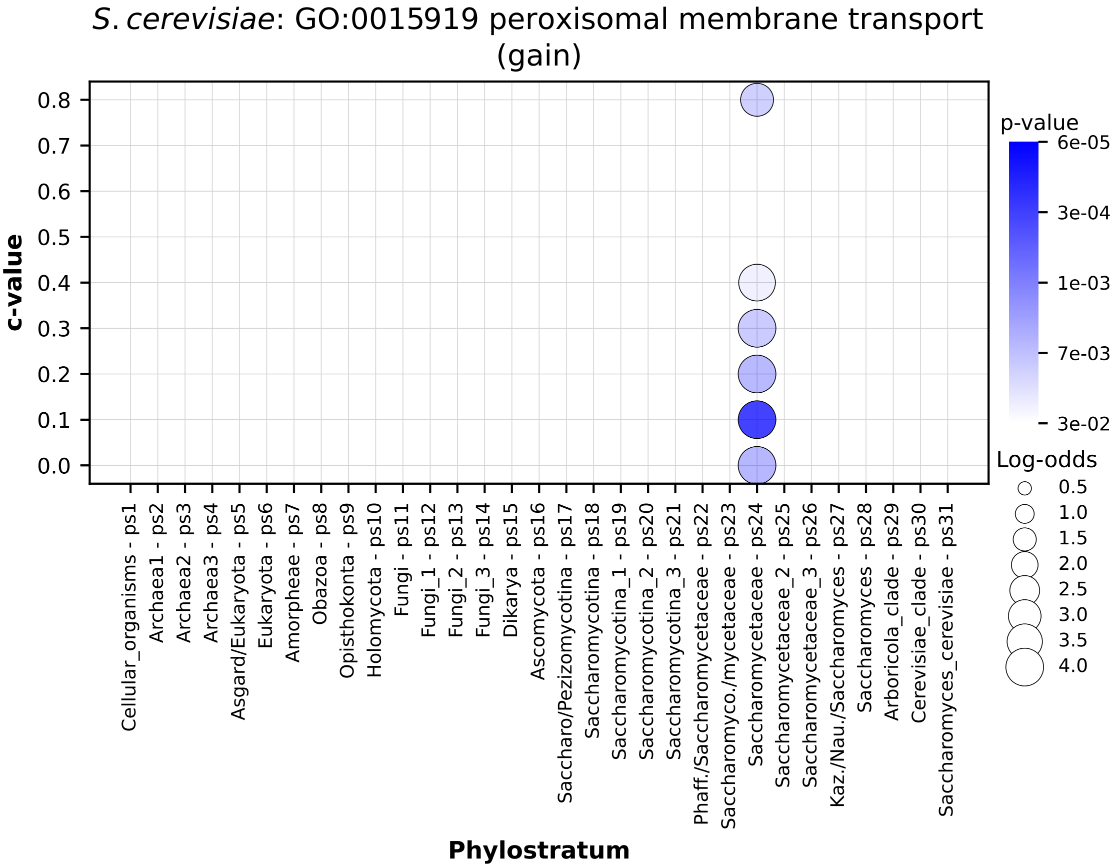

S. cerevisiae: GO:0015919 peroxisomal membrane transport (gain)
c-value
0.8
0.7
0.6
0.5
0.4
0.3
0.2
0.1
0.0
p-value
6e-05
3e-04
1e-03
7e-03
3e-02
Log-odds
0.5
1.0
1.5
2.0
2.5
3.0
3.5
4.0
Cellular_organisms - ps1
Archaea1 - ps2
Archaea2 - ps3
Archaea3 - ps4
Asgard/Eukaryota - ps5
Eukaryota - ps6
Amorpheae - ps7
Obazoa - ps8
Opisthokonta - ps9
Holomycota - ps10
Fungi - ps11
Fungi_1 - ps12
Fungi_2 - ps13
Fungi_3 - ps14
Dikarya - ps15
Ascomycota - ps16
Saccharo/Pezizomycotina - ps17
Saccharomycotina - ps18
Saccharomycotina_1 - ps19
Saccharomycotina_2 - ps20
Saccharomycotina_3 - ps21
Phaff./Saccharomycetaceae - ps22
Saccharomyco./mycetaceae - ps23
Saccharomycetaceae - ps24
Saccharomycetaceae_2 - ps25
Saccharomycetaceae_3 - ps26
Kaz./Nau./Saccharomyces - ps27
Saccharomyces - ps28
Arboricola_clade - ps29
Cerevisiae_clade - ps30
Saccharomyces_cerevisiae - ps31
Phylostratum

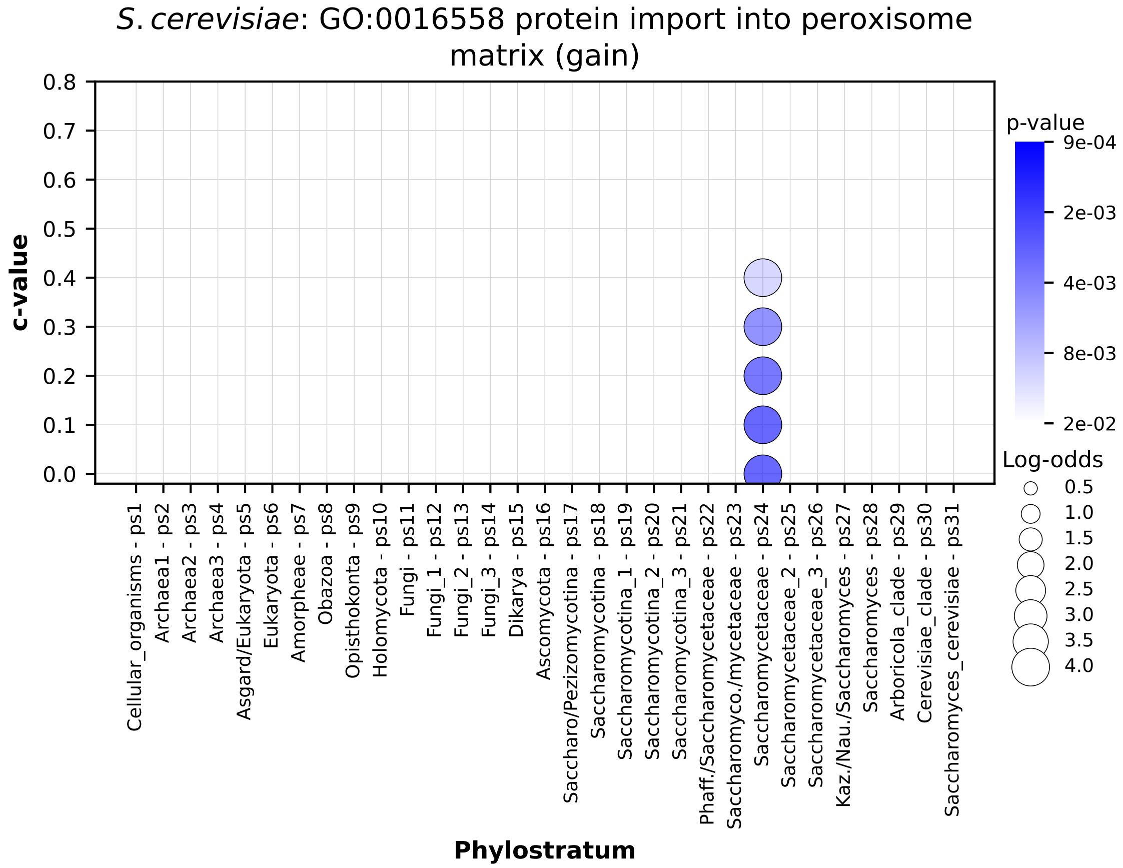

S. cerevisiae: GO:0016558 protein import into peroxisome matrix (gain)
c-value
0.8
0.7
0.6
0.5
0.4
0.3
0.2
0.1
0.0
p-value
9e-04
2e-03
4e-03
8e-03
2e-02
Log-odds
0.5
1.0
1.5
2.0
2.5
3.0
3.5
4.0
Cellular_organisms - ps1
Archaea1 - ps2
Archaea2 - ps3
Archaea3 - ps4
Asgard/Eukaryota - ps5
Eukaryota - ps6
Amorpheae - ps7
Obazoa - ps8
Opisthokonta - ps9
Holomycota - ps10
Fungi - ps11
Fungi_1 - ps12
Fungi_2 - ps13
Fungi_3 - ps14
Dikarya - ps15
Ascomycota - ps16
Saccharo/Pezizomycotina - ps17
Saccharomycotina - ps18
Saccharomycotina_1 - ps19
Saccharomycotina_2 - ps20
Saccharomycotina_3 - ps21
Phaff./Saccharomycetaceae - ps22
Saccharomyco./mycetaceae - ps23
Saccharomycetaceae - ps24
Saccharomycetaceae_2 - ps25
Saccharomycetaceae_3 - ps26
Kaz./Nau./Saccharomyces - ps27
Saccharomyces - ps28
Arboricola_clade - ps29
Cerevisiae_clade - ps30
Saccharomyces_cerevisiae - ps31
Phylostratum

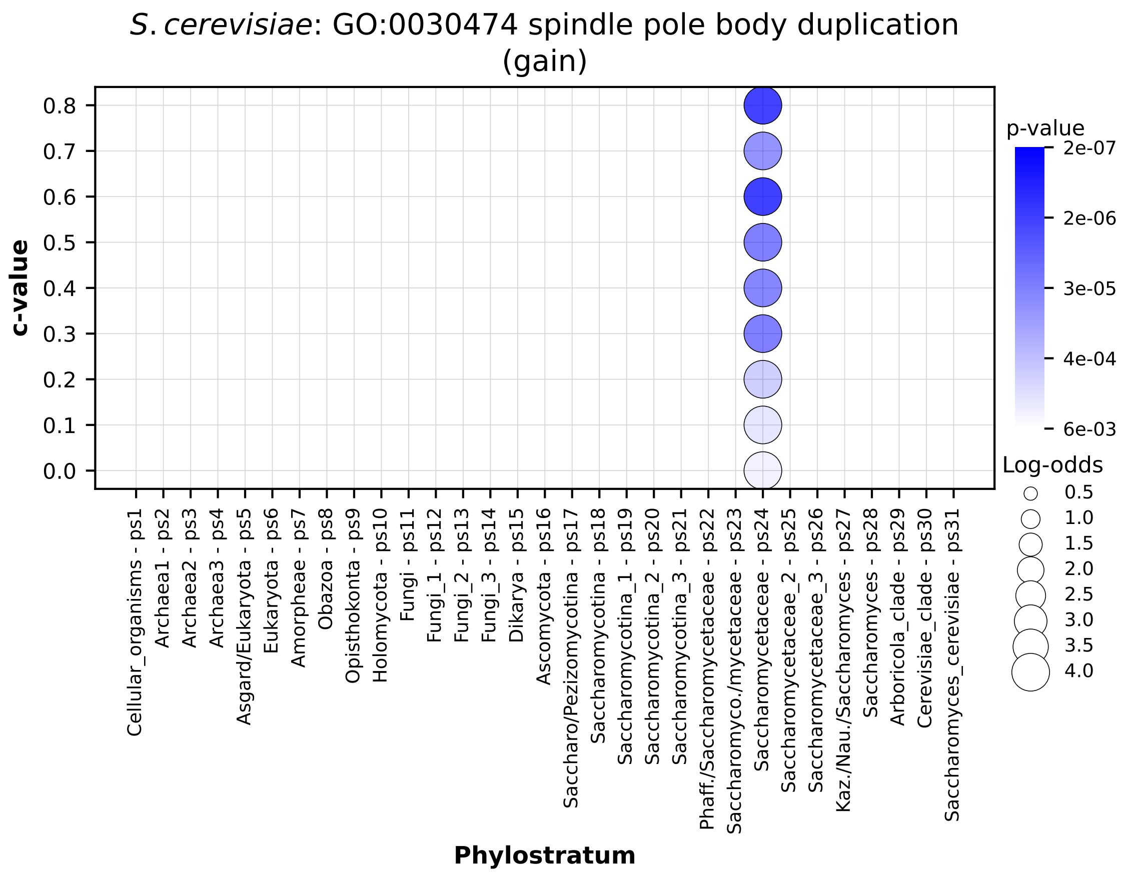

S. cerevisiae: GO:0030474 spindle pole body duplication
(gain)
c-value
0.8
0.7
0.6
0.5
0.4
0.3
0.2
0.1
0.0
p-value
2e-07
2e-06
3e-05
4e-04
6e-03
Log-odds
0.5
1.0
1.5
2.0
2.5
3.0
3.5
4.0
Cellular_organisms - ps1
Archaea1 - ps2
Archaea2 - ps3
Archaea3 - ps4
Asgard/Eukaryota - ps5
Eukaryota - ps6
Amorpheae - ps7
Obazoa - ps8
Opisthokonta - ps9
Holomycota - ps10
Fungi - ps11
Fungi_1 - ps12
Fungi_2 - ps13
Fungi_3 - ps14
Dikarya - ps15
Ascomycota - ps16
Saccharo/Pezizomycotina - ps17
Saccharomycotina - ps18
Saccharomycotina_1 - ps19
Saccharomycotina_2 - ps20
Saccharomycotina_3 - ps21
Phaff./Saccharomycetaceae - ps22
Saccharomyco./mycetaceae - ps23
Saccharomycetaceae - ps24
Saccharomycetaceae_2 - ps25
Saccharomycetaceae_3 - ps26
Kaz./Nau./Saccharomyces - ps27
Saccharomyces - ps28
Arboricola_clade - ps29
Cerevisiae_clade - ps30
Saccharomyces_cerevisiae - ps31
Phylostratum

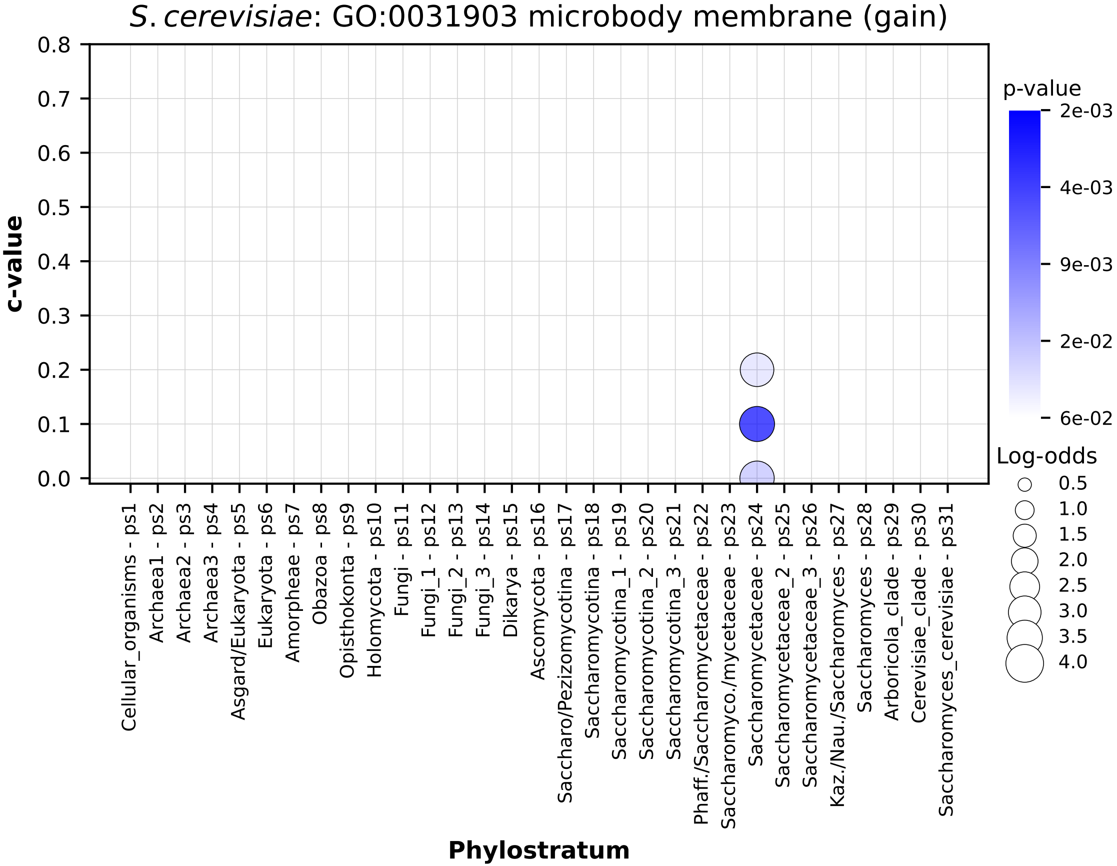

S. cerevisiae: GO:0031903 microbody membrane (gain)
c-value
0.8
0.7
0.6
0.5
0.4
0.3
0.2
0.1
0.0
p-value
2e-03
4e-03
9e-03
2e-02
6e-02
Log-odds
0.5
1.0
1.5
2.0
2.5
3.0
3.5
4.0
Cellular_organisms - ps1
Archaea1 - ps2
Archaea2 - ps3
Archaea3 - ps4
Asgard/Eukaryota - ps5
Eukaryota - ps6
Amorpheae - ps7
Obazoa - ps8
Opisthokonta - ps9
Holomycota - ps10
Fungi - ps11
Fungi_1 - ps12
Fungi_2 - ps13
Fungi_3 - ps14
Dikarya - ps15
Ascomycota - ps16
Saccharo/Pezizomycotina - ps17
Saccharomycotina - ps18
Saccharomycotina_1 - ps19
Saccharomycotina_2 - ps20
Saccharomycotina_3 - ps21
Phaff./Saccharomycetaceae - ps22
Saccharomyco./mycetaceae - ps23
Saccharomycetaceae - ps24
Saccharomycetaceae_2 - ps25
Saccharomycetaceae_3 - ps26
Kaz./Nau./Saccharomyces - ps27
Saccharomyces - ps28
Arboricola_clade - ps29
Cerevisiae_clade - ps30
Saccharomyces_cerevisiae - ps31
Phylostratum

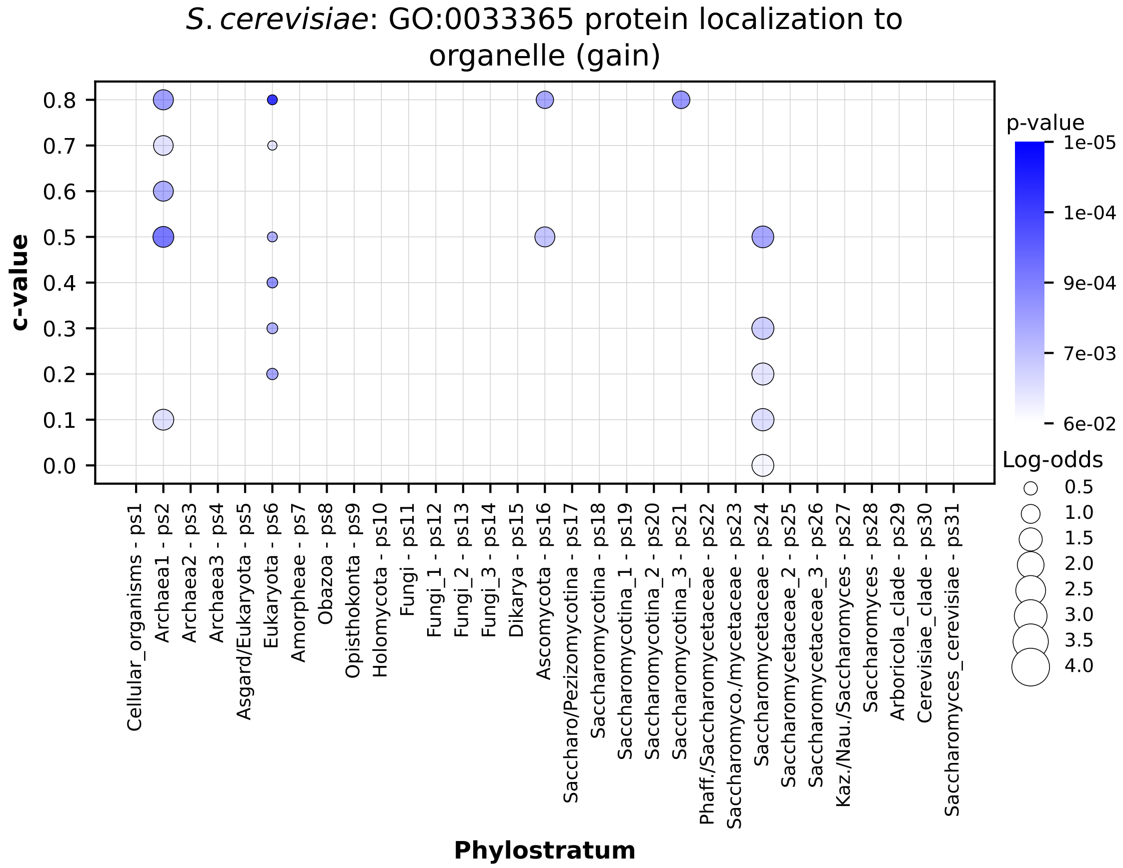

S. cerevisiae: GO:0033365 protein localization to organelle (gain)
c-value
0.8
0.7
0.6
0.5
0.4
0.3
0.2
0.1
0.0
p-value
1e-05
1e-04
9e-04
7e-03
6e-02
Log-odds
0.5
1.0
1.5
2.0
2.5
3.0
3.5
4.0
Cellular_organisms - ps1
Archaea1 - ps2
Archaea2 - ps3
Archaea3 - ps4
Asgard/Eukaryota - ps5
Eukaryota - ps6
Amorpheae - ps7
Obazoa - ps8
Opisthokonta - ps9
Holomycota - ps10
Fungi - ps11
Fungi_1 - ps12
Fungi_2 - ps13
Fungi_3 - ps14
Dikarya - ps15
Ascomycota - ps16
Saccharo/Pezizomycotina - ps17
Saccharomycotina - ps18
Saccharomycotina_1 - ps19
Saccharomycotina_2 - ps20
Saccharomycotina_3 - ps21
Phaff./Saccharomycetaceae - ps22
Saccharomyco./mycetaceae - ps23
Saccharomycetaceae - ps24
Saccharomycetaceae_2 - ps25
Saccharomycetaceae_3 - ps26
Kaz./Nau./Saccharomyces - ps27
Saccharomyces - ps28
Arboricola_clade - ps29
Cerevisiae_clade - ps30
Saccharomyces_cerevisiae - ps31
Phylostratum

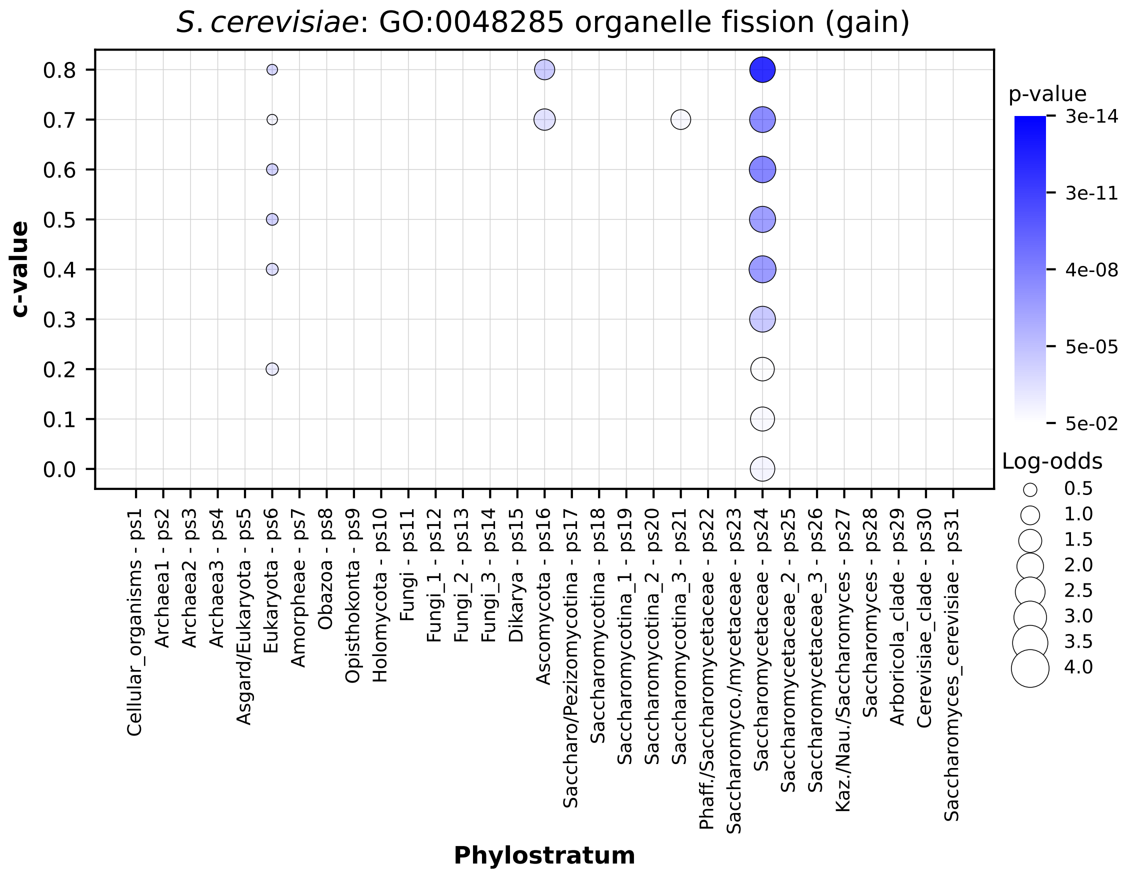

S. cerevisiae: GO:0048285 organelle fission (gain)
c-value
0.8
0.7
0.6
0.5
0.4
0.3
0.2
0.1
0.0
p-value
3e-14
3e-11
4e-08
5e-05
5e-02
Log-odds
0.5
1.0
1.5
2.0
2.5
3.0
3.5
4.0
Cellular_organisms - ps1
Archaea1 - ps2
Archaea2 - ps3
Archaea3 - ps4
Asgard/Eukaryota - ps5
Eukaryota - ps6
Amorpheae - ps7
Obazoa - ps8
Opisthokonta - ps9
Holomycota - ps10
Fungi - ps11
Fungi_1 - ps12
Fungi_2 - ps13
Fungi_3 - ps14
Dikarya - ps15
Ascomycota - ps16
Saccharo/Pezizomycotina - ps17
Saccharomycotina - ps18
Saccharomycotina_1 - ps19
Saccharomycotina_2 - ps20
Saccharomycotina_3 - ps21
Phaff./Saccharomycetaceae - ps22
Saccharomyco./mycetaceae - ps23
Saccharomycetaceae - ps24
Saccharomycetaceae_2 - ps25
Saccharomycetaceae_3 - ps26
Kaz./Nau./Saccharomyces - ps27
Saccharomyces - ps28
Arboricola_clade - ps29
Cerevisiae_clade - ps30
Saccharomyces_cerevisiae - ps31
Phylostratum

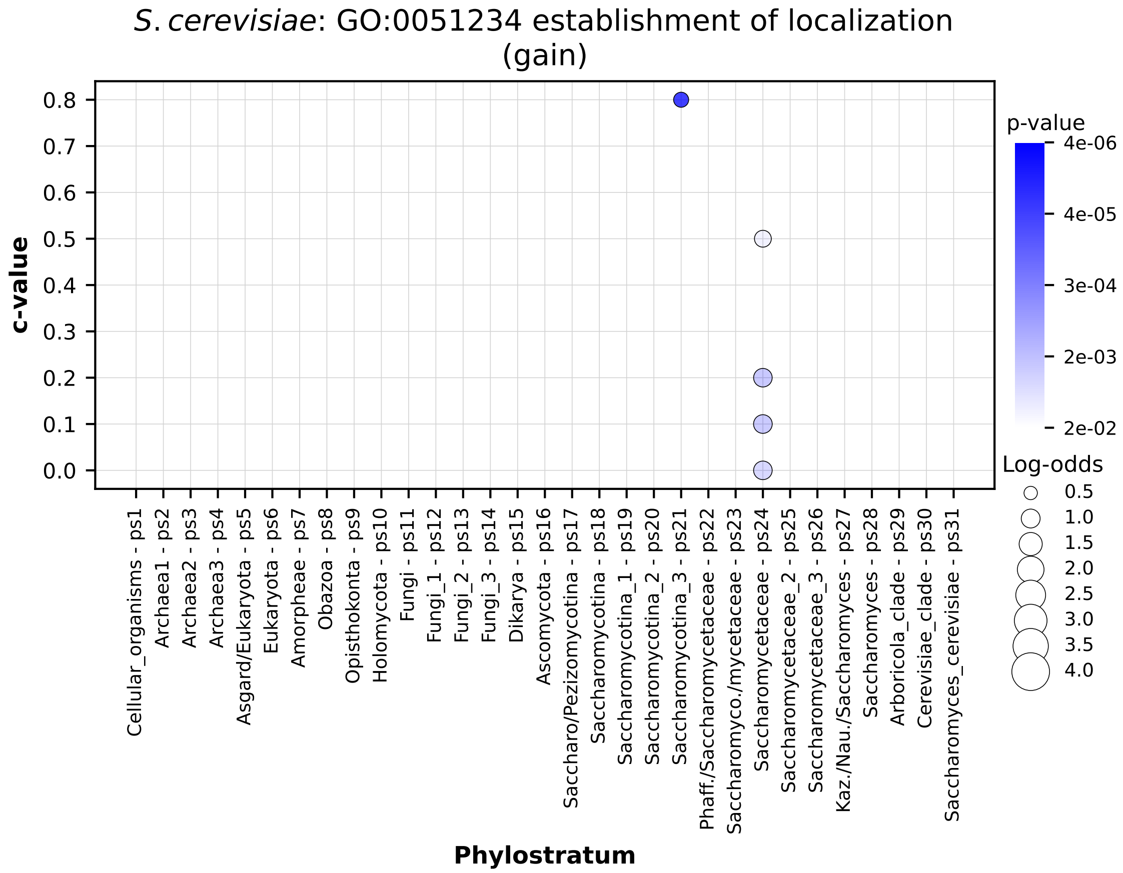

S. cerevisiae: GO:0051234 establishment of localization (gain)
c-value
0.8
0.7
0.6
0.5
0.4
0.3
0.2
0.1
0.0
p-value
4e-06
4e-05
3e-04
2e-03
2e-02
Log-odds
0.5
1.0
1.5
2.0
2.5
3.0
3.5
4.0
Cellular_organisms - ps1
Archaea1 - ps2
Archaea2 - ps3
Archaea3 - ps4
Asgard/Eukaryota - ps5
Eukaryota - ps6
Amorpheae - ps7
Obazoa - ps8
Opisthokonta - ps9
Holomycota - ps10
Fungi - ps11
Fungi_1 - ps12
Fungi_2 - ps13
Fungi_3 - ps14
Dikarya - ps15
Ascomycota - ps16
Saccharo/Pezizomycotina - ps17
Saccharomycotina - ps18
Saccharomycotina_1 - ps19
Saccharomycotina_2 - ps20
Saccharomycotina_3 - ps21
Phaff./Saccharomycetaceae - ps22
Saccharomyco./mycetaceae - ps23
Saccharomycetaceae - ps24
Saccharomycetaceae_2 - ps25
Saccharomycetaceae_3 - ps26
Kaz./Nau./Saccharomyces - ps27
Saccharomyces - ps28
Arboricola_clade - ps29
Cerevisiae_clade - ps30
Saccharomyces_cerevisiae - ps31
Phylostratum

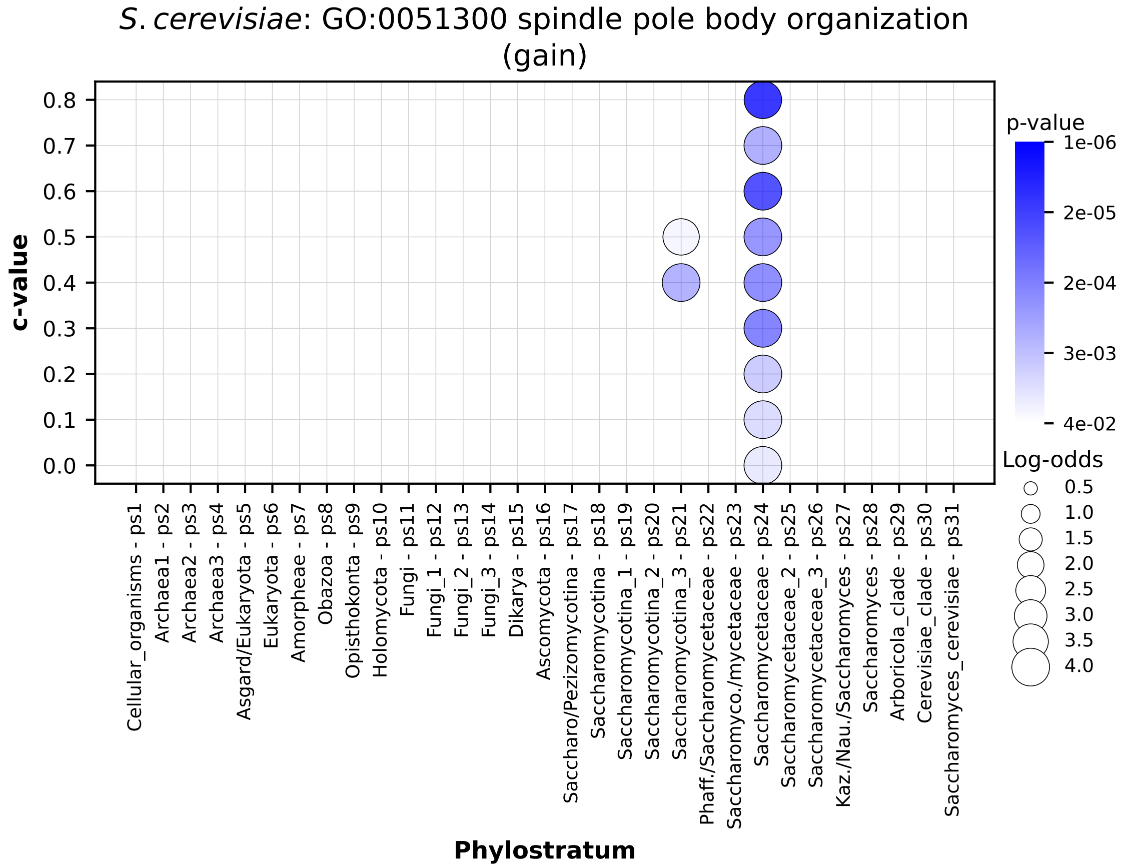

S. cerevisiae: GO:0051300 spindle pole body organization
(gain)
c-value
0.8
0.7
0.6
0.5
0.4
0.3
0.2
0.1
0.0
p-value
1e-06
2e-05
2e-04
3e-03
4e-02
Log-odds
0.5
1.0
1.5
2.0
2.5
3.0
3.5
4.0
Cellular_organisms - ps1
Archaea1 - ps2
Archaea2 - ps3
Archaea3 - ps4
Asgard/Eukaryota - ps5
Eukaryota - ps6
Amorpheae - ps7
Obazoa - ps8
Opisthokonta - ps9
Holomycota - ps10
Fungi - ps11
Fungi_1 - ps12
Fungi_2 - ps13
Fungi_3 - ps14
Dikarya - ps15
Ascomycota - ps16
Saccharo/Pezizomycotina - ps17
Saccharomycotina - ps18
Saccharomycotina_1 - ps19
Saccharomycotina_2 - ps20
Saccharomycotina_3 - ps21
Phaff./Saccharomycetaceae - ps22
Saccharomyco./mycetaceae - ps23
Saccharomycetaceae - ps24
Saccharomycetaceae_2 - ps25
Saccharomycetaceae_3 - ps26
Kaz./Nau./Saccharomyces - ps27
Saccharomyces - ps28
Arboricola_clade - ps29
Cerevisiae_clade - ps30
Saccharomyces_cerevisiae - ps31
Phylostratum

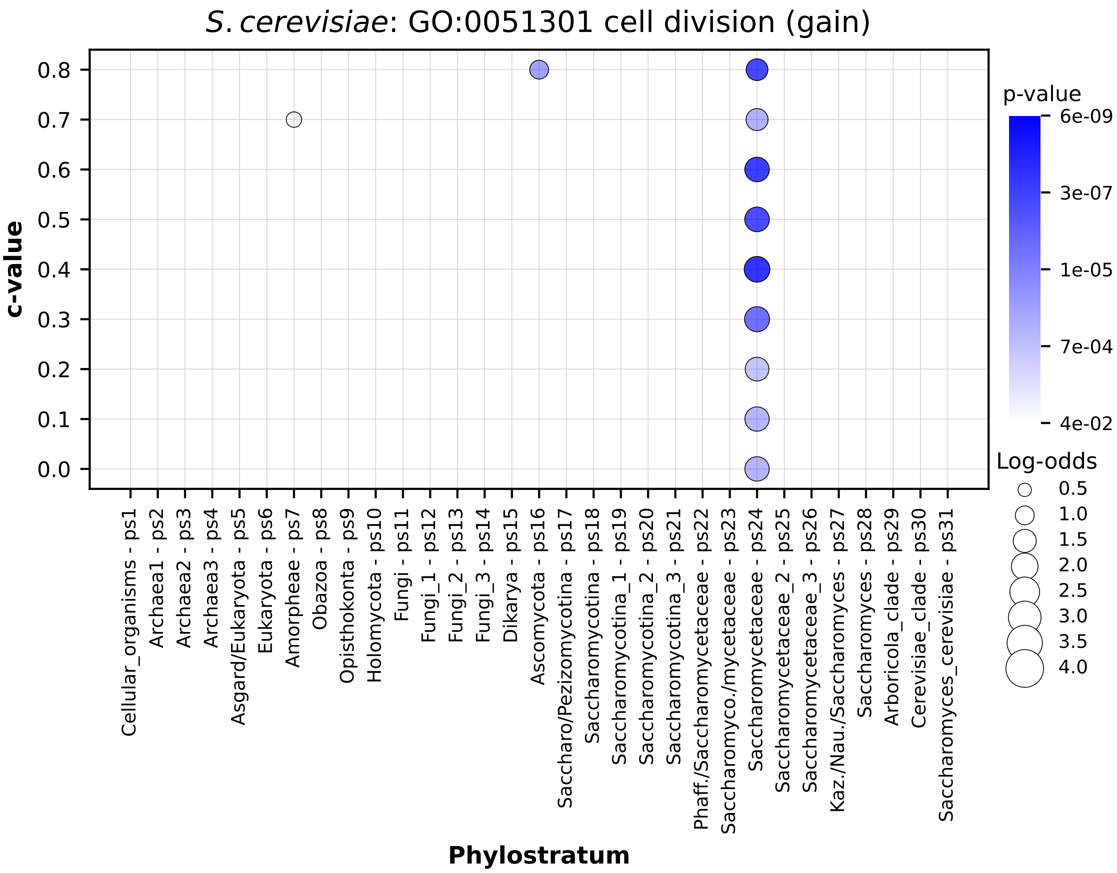

S. cerevisiae: GO:0051301 cell division (gain)
c-value
0.0
0.1
0.2
0.3
0.4
0.5
0.6
0.7
0.8
p-value
6e-09
3e-07
1e-05
7e-04
4e-02
Log-odds
0.5
1.0
1.5
2.0
2.5
3.0
3.5
4.0
Cellular_organisms - ps1
Archaea1 - ps2
Archaea2 - ps3
Archaea3 - ps4
Asgard/Eukaryota - ps5
Eukaryota - ps6
Amorpheae - ps7
Obazoa - ps8
Opisthokonta - ps9
Holomycota - ps10
Fungi - ps11
Fungi_1 - ps12
Fungi_2 - ps13
Fungi_3 - ps14
Dikarya - ps15
Ascomycota - ps16
Saccharo/Pezizomycotina - ps17
Saccharomycotina - ps18
Saccharomycotina_1 - ps19
Saccharomycotina_2 - ps20
Saccharomycotina_3 - ps21
Phaff./Saccharomycetaceae - ps22
Saccharomyco./mycetaceae - ps23
Saccharomycetaceae - ps24
Saccharomycetaceae_2 - ps25
Saccharomycetaceae_3 - ps26
Kaz./Nau./Saccharomyces - ps27
Saccharomyces - ps28
Arboricola_clade - ps29
Cerevisiae_clade - ps30
Saccharomyces_cerevisiae - ps31
Phylostratum

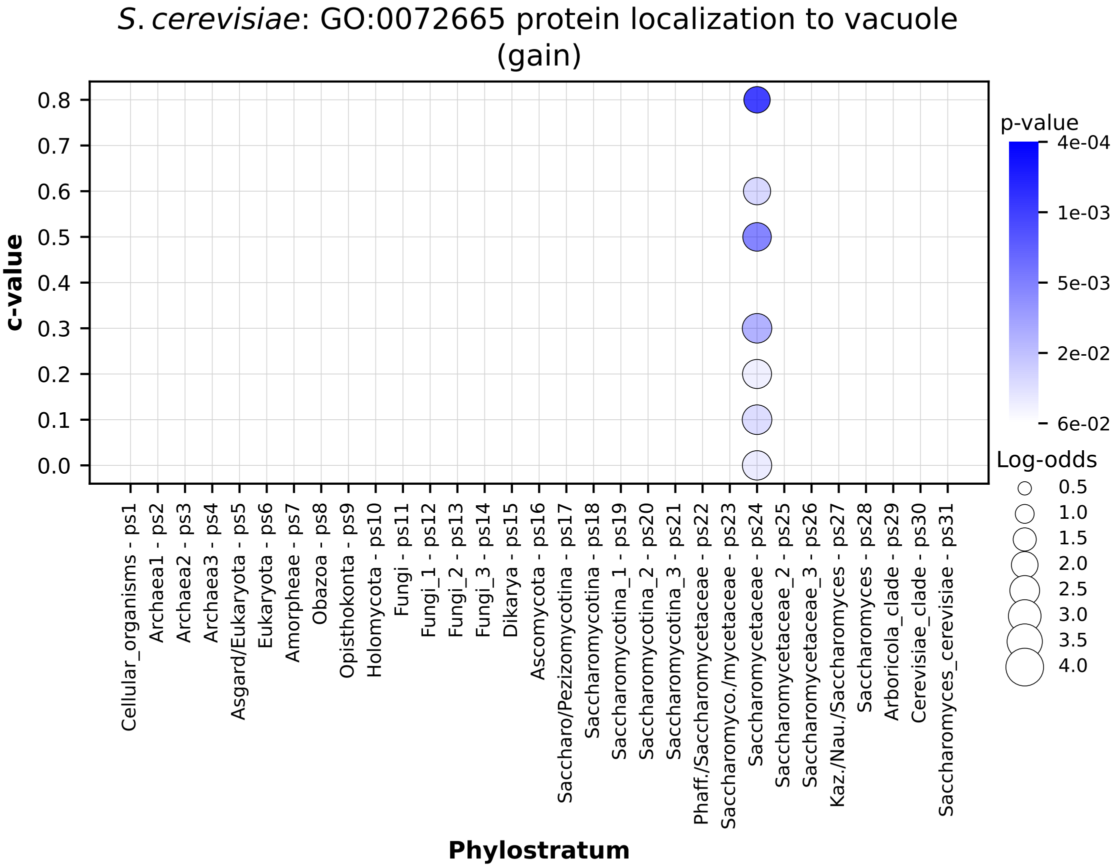

S. cerevisiae: GO:0072665 protein localization to vacuole
(gain)
c-value
0.0
0.1
0.2
0.3
0.4
0.5
0.6
0.7
0.8
p-value
4e-04
1e-03
5e-03
2e-02
6e-02
Log-odds
0.5
1.0
1.5
2.0
2.5
3.0
3.5
4.0
Cellular_organisms - ps1
Archaea1 - ps2
Archaea2 - ps3
Archaea3 - ps4
Asgard/Eukaryota - ps5
Eukaryota - ps6
Amorpheae - ps7
Obazoa - ps8
Opisthokonta - ps9
Holomycota - ps10
Fungi - ps11
Fungi_1 - ps12
Fungi_2 - ps13
Fungi_3 - ps14
Dikarya - ps15
Ascomycota - ps16
Saccharo/Pezizomycotina - ps17
Saccharomycotina - ps18
Saccharomycotina_1 - ps19
Saccharomycotina_2 - ps20
Saccharomycotina_3 - ps21
Phaff./Saccharomycetaceae - ps22
Saccharomyco./mycetaceae - ps23
Saccharomycetaceae - ps24
Saccharomycetaceae_2 - ps25
Saccharomycetaceae_3 - ps26
Kaz./Nau./Saccharomyces - ps27
Saccharomyces - ps28
Arboricola_clade - ps29
Cerevisiae_clade - ps30
Saccharomyces_cerevisiae - ps31
Phylostratum

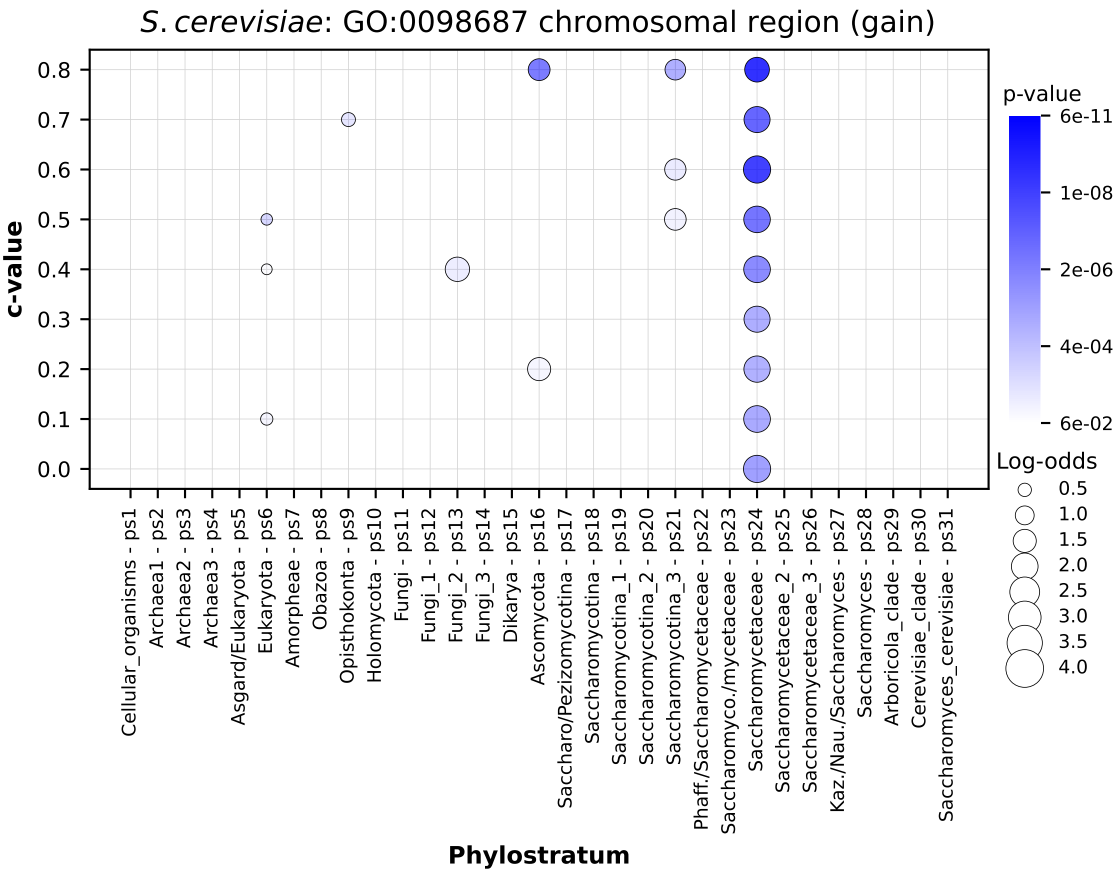

S. cerevisiae: GO:0098687 chromosomal region (gain)
c-value
0.8
0.7
0.6
0.5
0.4
0.3
0.2
0.1
0.0
p-value
6e-11
1e-08
2e-06
4e-04
6e-02
Log-odds
0.5
1.0
1.5
2.0
2.5
3.0
3.5
4.0
Cellular_organisms - ps1
Archaea1 - ps2
Archaea2 - ps3
Archaea3 - ps4
Asgard/Eukaryota - ps5
Eukaryota - ps6
Amorpheae - ps7
Obazoa - ps8
Opisthokonta - ps9
Holomycota - ps10
Fungi - ps11
Fungi_1 - ps12
Fungi_2 - ps13
Fungi_3 - ps14
Dikarya - ps15
Ascomycota - ps16
Saccharo/Pezizomycotina - ps17
Saccharomycotina - ps18
Saccharomycotina_1 - ps19
Saccharomycotina_2 - ps20
Saccharomycotina_3 - ps21
Phaff./Saccharomycetaceae - ps22
Saccharomyco./mycetaceae - ps23
Saccharomycetaceae - ps24
Saccharomycetaceae_2 - ps25
Saccharomycetaceae_3 - ps26
Kaz./Nau./Saccharomyces - ps27
Saccharomyces - ps28
Arboricola_clade - ps29
Cerevisiae_clade - ps30
Saccharomyces_cerevisiae - ps31
Phylostratum

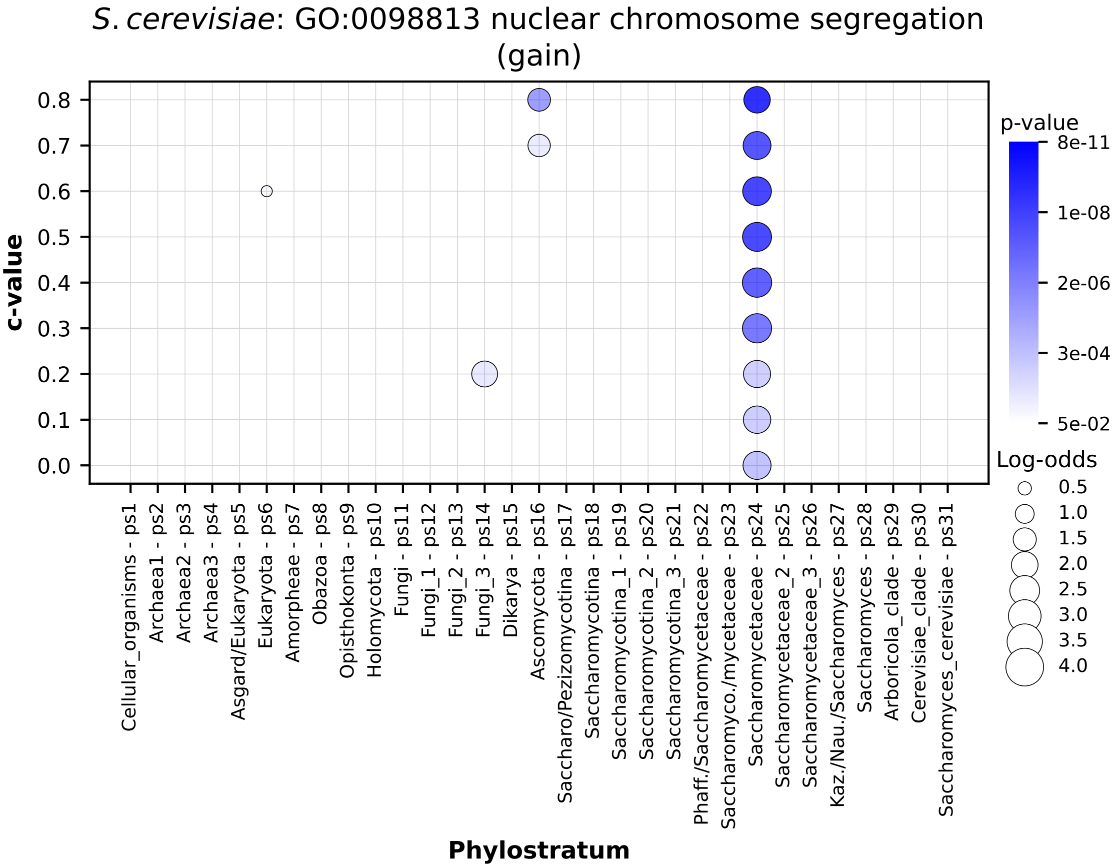
S. cerevisiae: GO:0098813 nuclear chromosome segregation (gain)
c-value
0.0
0.1
0.2
0.3
0.4
0.5
0.6
0.7
0.8
Phylostratum
Cellular_organisms - ps1
Archaea1 - ps2
Archaea2 - ps3
Archaea3 - ps4
Asgard/Eukaryota - ps5
Eukaryota - ps6
Amorpheae - ps7
Obazoa - ps8
Opisthokonta - ps9
Holomycota - ps10
Fungi - ps11
Fungi_1 - ps12
Fungi_2 - ps13
Fungi_3 - ps14
Dikarya - ps15
Ascomycota - ps16
Saccharo/Pezizomycotina - ps17
Saccharomycotina - ps18
Saccharomycotina_1 - ps19
Saccharomycotina_2 - ps20
Saccharomycotina_3 - ps21
Phaff./Saccharomycetaceae - ps22
Saccharomyco./mycetaceae - ps23
Saccharomycetaceae - ps24
Saccharomycetaceae_2 - ps25
Saccharomycetaceae_3 - ps26
Kaz./Nau./Saccharomyces - ps27
Saccharomyces - ps28
Arboricola_clade - ps29
Cerevisiae_clade - ps30
Saccharomyces_cerevisiae - ps31
p-value
8e-11
1e-08
2e-06
3e-04
5e-02
Log-odds
0.5
1.0
1.5
2.0
2.5
3.0
3.5
4.0

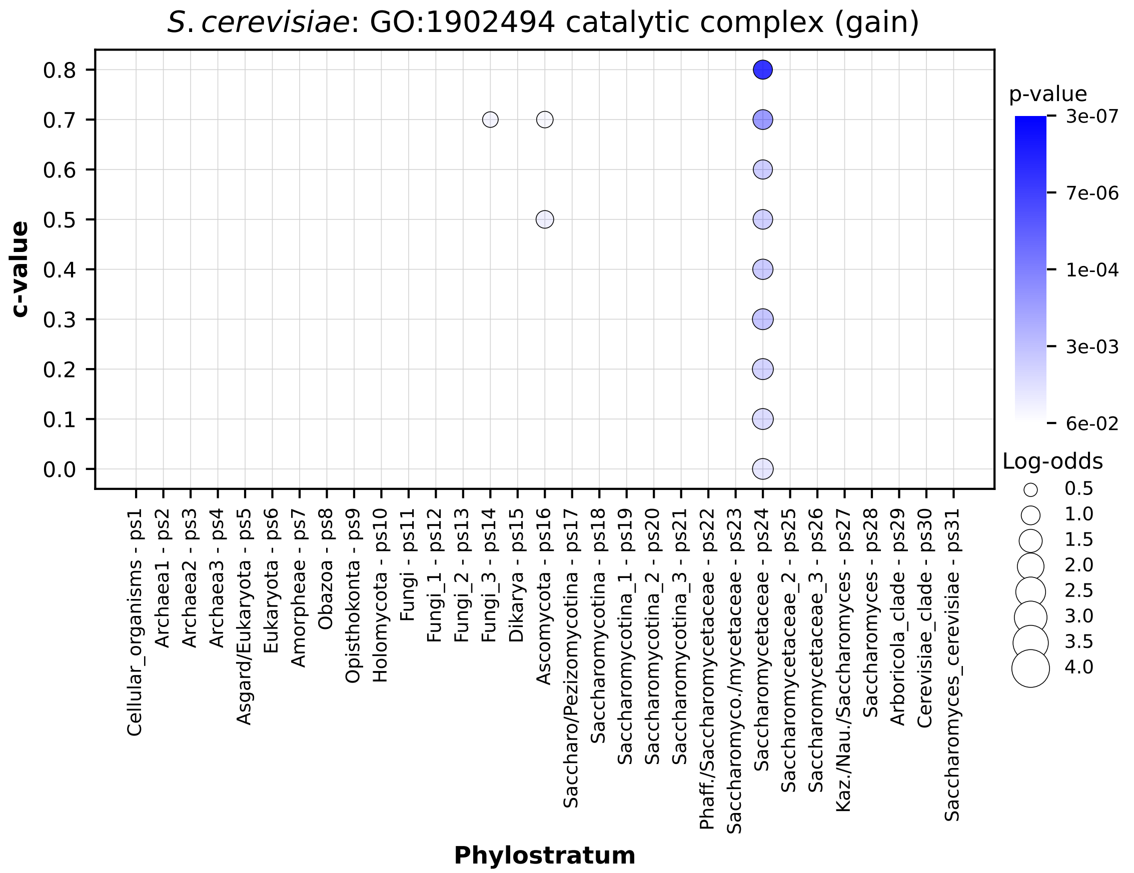

S. cerevisiae: GO:1902494 catalytic complex (gain)
c-value
0.8
0.7
0.6
0.5
0.4
0.3
0.2
0.1
0.0
p-value
3e-07
7e-06
1e-04
3e-03
6e-02
Log-odds
0.5
1.0
1.5
2.0
2.5
3.0
3.5
4.0
Cellular_organisms - ps1
Archaea1 - ps2
Archaea2 - ps3
Archaea3 - ps4
Asgard/Eukaryota - ps5
Eukaryota - ps6
Amorpheae - ps7
Obazoa - ps8
Opisthokonta - ps9
Holomycota - ps10
Fungi - ps11
Fungi_1 - ps12
Fungi_2 - ps13
Fungi_3 - ps14
Dikarya - ps15
Ascomycota - ps16
Saccharo/Pezizomycotina - ps17
Saccharomycotina - ps18
Saccharomycotina_1 - ps19
Saccharomycotina_2 - ps20
Saccharomycotina_3 - ps21
Phaff./Saccharomycetaceae - ps22
Saccharomyco./mycetaceae - ps23
Saccharomycetaceae - ps24
Saccharomycetaceae_2 - ps25
Saccharomycetaceae_3 - ps26
Kaz./Nau./Saccharomyces - ps27
Saccharomyces - ps28
Arboricola_clade - ps29
Cerevisiae_clade - ps30
Saccharomyces_cerevisiae - ps31
Phylostratum

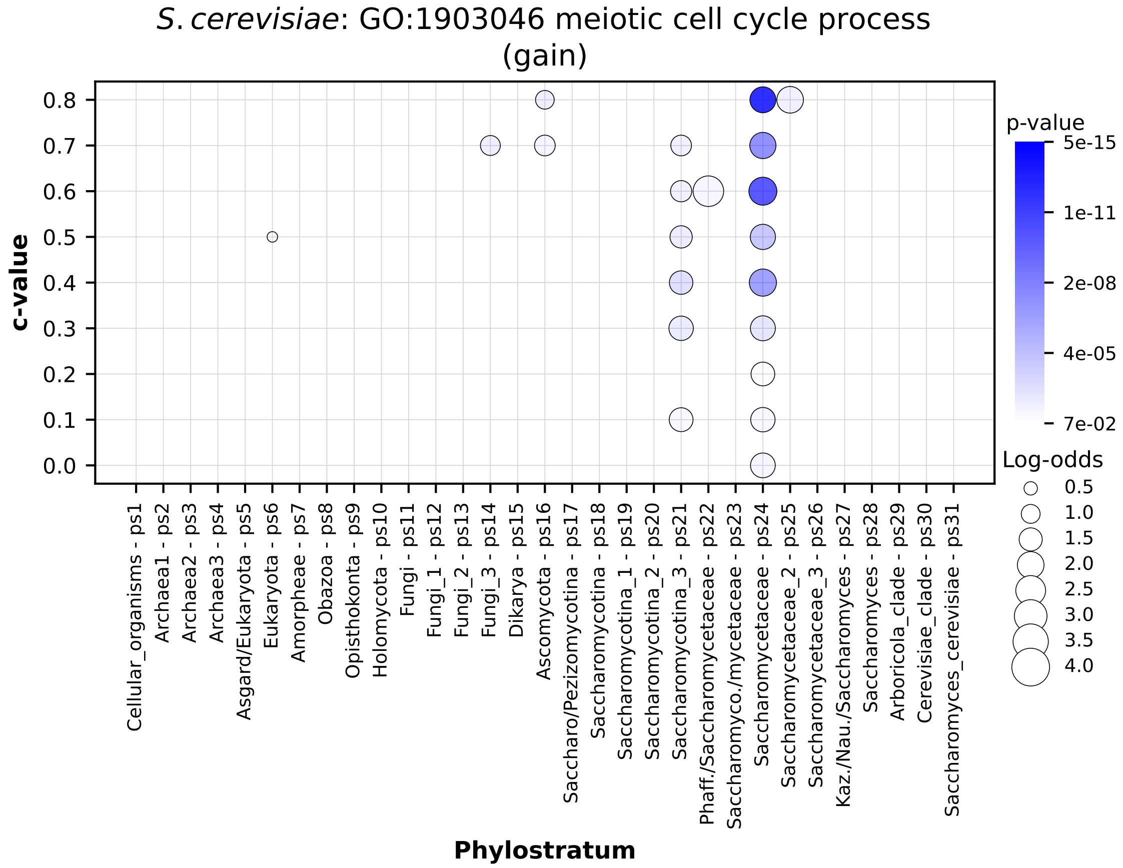
S. cerevisiae: GO:1903046 meiotic cell cycle process (gain)
c-value
0.8
0.7
0.6
0.5
0.4
0.3
0.2
0.1
0.0
p-value
5e-15
1e-11
2e-08
4e-05
7e-02
Log-odds
0.5
1.0
1.5
2.0
2.5
3.0
3.5
4.0
Cellular_organisms - ps1
Archaea1 - ps2
Archaea2 - ps3
Archaea3 - ps4
Asgard/Eukaryota - ps5
Eukaryota - ps6
Amorpheae - ps7
Obazoa - ps8
Opisthokonta - ps9
Holomycota - ps10
Fungi - ps11
Fungi_1 - ps12
Fungi_2 - ps13
Fungi_3 - ps14
Dikarya - ps15
Ascomycota - ps16
Saccharo/Pezizomycotina - ps17
Saccharomycotina - ps18
Saccharomycotina_1 - ps19
Saccharomycotina_2 - ps20
Saccharomycotina_3 - ps21
Phaff./Saccharomycetaceae - ps22
Saccharomyco./mycetaceae - ps23
Saccharomycetaceae - ps24
Saccharomycetaceae_2 - ps25
Saccharomycetaceae_3 - ps26
Kaz./Nau./Saccharomyces - ps27
Saccharomyces - ps28
Arboricola_clade - ps29
Cerevisiae_clade - ps30
Saccharomyces_cerevisiae - ps31
Phylostratum

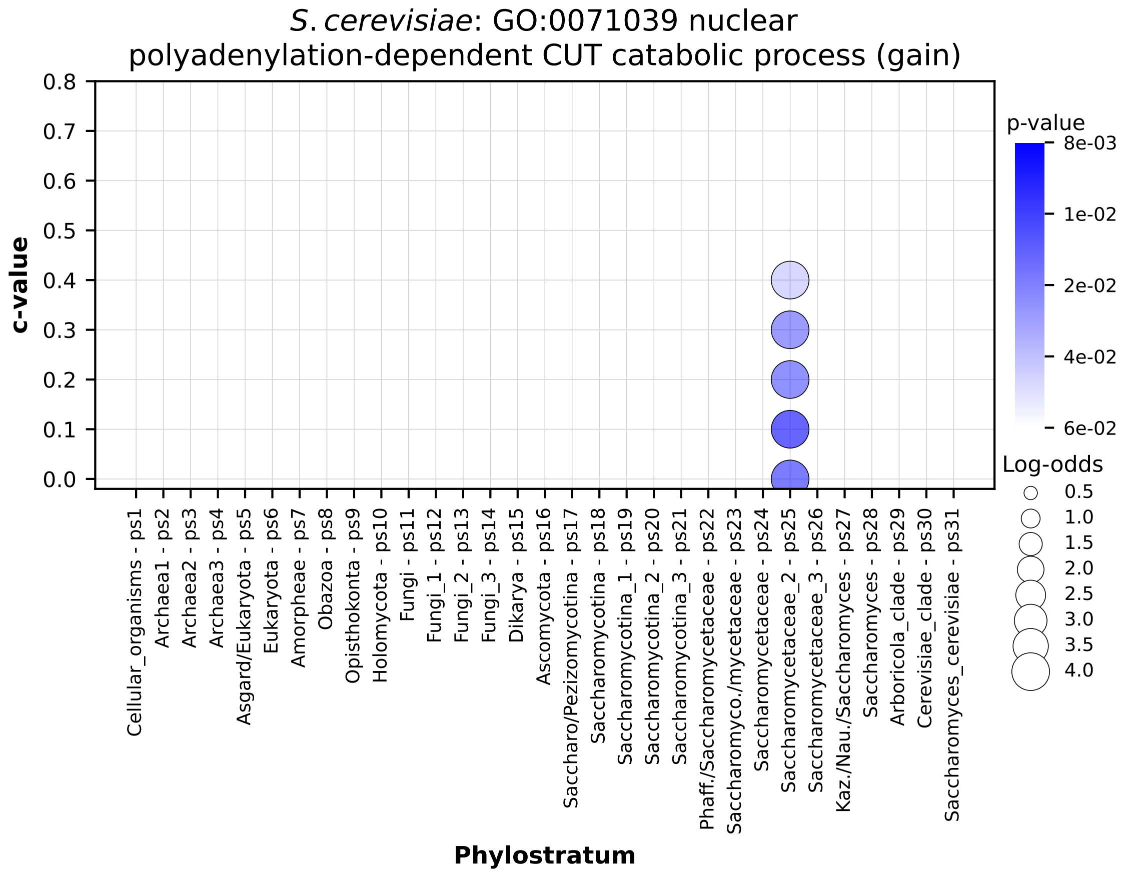

S. cerevisiae: GO:0071039 nuclear polyadenylation-dependent CUT catabolic process (gain)
c-value
0.8
0.7
0.6
0.5
0.4
0.3
0.2
0.1
0.0
p-value
8e-03
1e-02
2e-02
4e-02
6e-02
Log-odds
0.5
1.0
1.5
2.0
2.5
3.0
3.5
4.0
Cellular_organisms - ps1
Archaea1 - ps2
Archaea2 - ps3
Archaea3 - ps4
Asgard/Eukaryota - ps5
Eukaryota - ps6
Amorpheae - ps7
Obazoa - ps8
Opisthokonta - ps9
Holomycota - ps10
Fungi - ps11
Fungi_1 - ps12
Fungi_2 - ps13
Fungi_3 - ps14
Dikarya - ps15
Ascomycota - ps16
Saccharo/Pezizomycotina - ps17
Saccharomycotina - ps18
Saccharomycotina_1 - ps19
Saccharomycotina_2 - ps20
Saccharomycotina_3 - ps21
Phaff./Saccharomycetaceae - ps22
Saccharomyco./mycetaceae - ps23
Saccharomycetaceae - ps24
Saccharomycetaceae_2 - ps25
Saccharomycetaceae_3 - ps26
Kaz./Nau./Saccharomyces - ps27
Saccharomyces - ps28
Arboricola_clade - ps29
Cerevisiae_clade - ps30
Saccharomyces_cerevisiae - ps31
Phylostratum

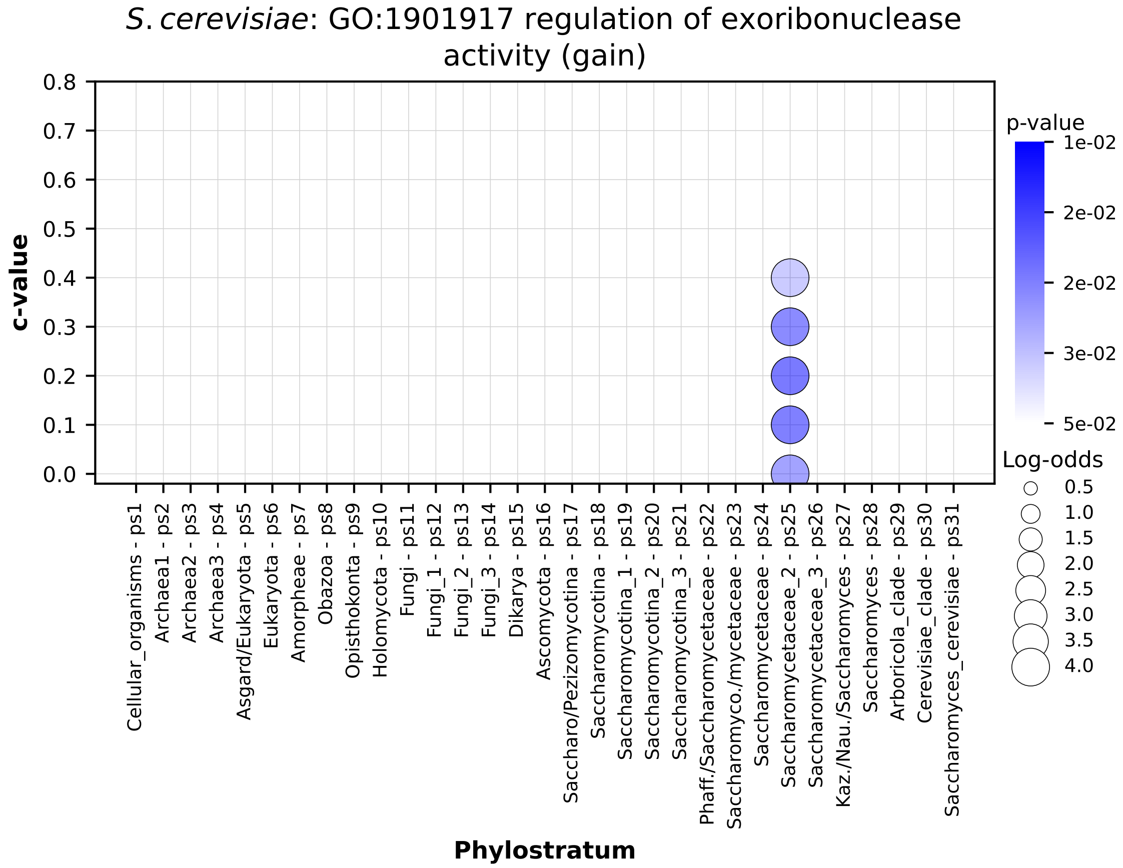
S. cerevisiae: GO:1901917 regulation of exoribonuclease activity (gain)
c-value
0.8
0.7
0.6
0.5
0.4
0.3
0.2
0.1
0.0
Phylostratum
Cellular_organisms - ps1
Archaea1 - ps2
Archaea2 - ps3
Archaea3 - ps4
Asgard/Eukaryota - ps5
Eukaryota - ps6
Amorpheae - ps7
Obazoa - ps8
Opisthokonta - ps9
Holomycota - ps10
Fungi - ps11
Fungi_1 - ps12
Fungi_2 - ps13
Fungi_3 - ps14
Dikarya - ps15
Ascomycota - ps16
Saccharo/Pezizomycotina - ps17
Saccharomycotina - ps18
Saccharomycotina_1 - ps19
Saccharomycotina_2 - ps20
Saccharomycotina_3 - ps21
Phaff./Saccharomycetaceae - ps22
Saccharomyco./mycetaceae - ps23
Saccharomycetaceae - ps24
Saccharomycetaceae_2 - ps25
Saccharomycetaceae_3 - ps26
Kaz./Nau./Saccharomyces - ps27
Saccharomyces - ps28
Arboricola_clade - ps29
Cerevisiae_clade - ps30
Saccharomyces_cerevisiae - ps31
p-value
1e-02
2e-02
2e-02
3e-02
5e-02
Log-odds
0.5
1.0
1.5
2.0
2.5
3.0
3.5
4.0

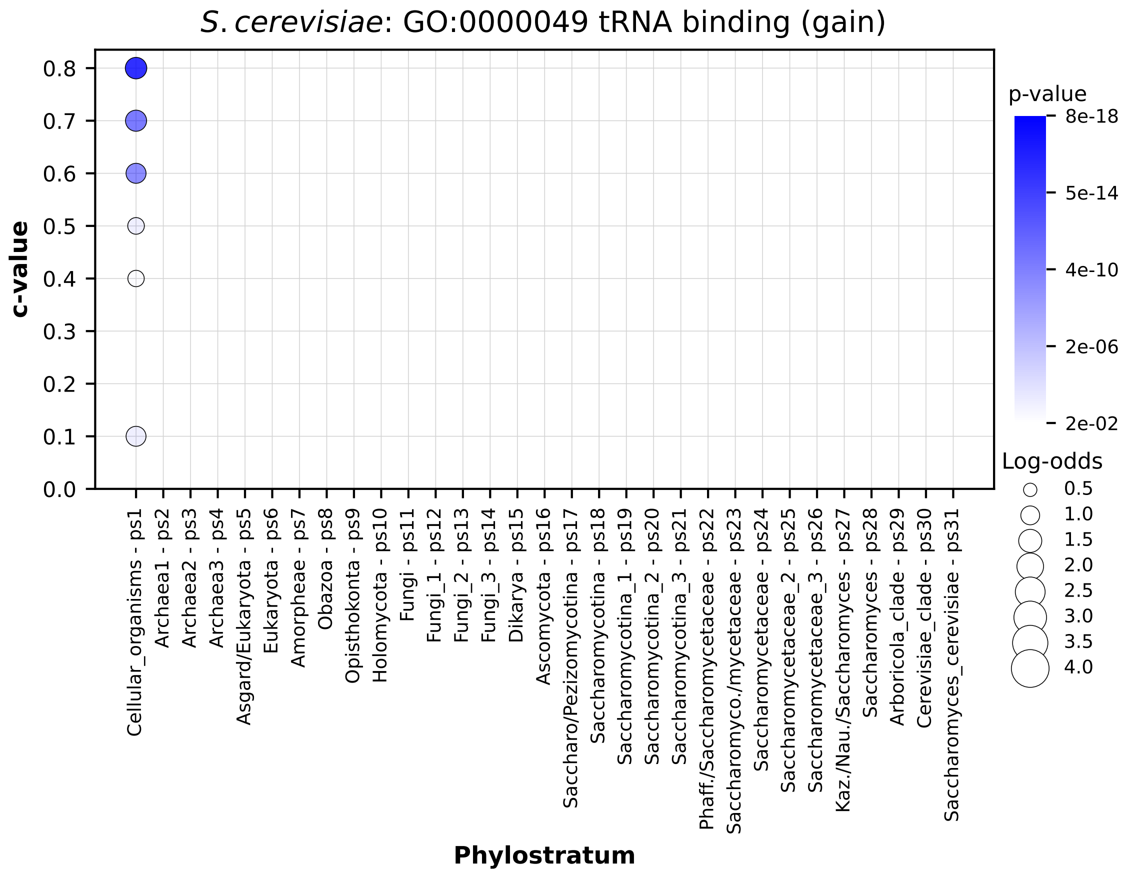

S. cerevisiae: GO:0000049 tRNA binding (gain)
c-value
0.0
0.1
0.2
0.3
0.4
0.5
0.6
0.7
0.8
p-value
8e-18
5e-14
4e-10
2e-06
2e-02
Log-odds
0.5
1.0
1.5
2.0
2.5
3.0
3.5
4.0
Cellular_organisms - ps1
Archaea1 - ps2
Archaea2 - ps3
Archaea3 - ps4
Asgard/Eukaryota - ps5
Eukaryota - ps6
Amorpheae - ps7
Obazoa - ps8
Opisthokonta - ps9
Holomycota - ps10
Fungi - ps11
Fungi_1 - ps12
Fungi_2 - ps13
Fungi_3 - ps14
Dikarya - ps15
Ascomycota - ps16
Saccharo/Pezizomycotina - ps17
Saccharomycotina - ps18
Saccharomycotina_1 - ps19
Saccharomycotina_2 - ps20
Saccharomycotina_3 - ps21
Phaff./Saccharomycetaceae - ps22
Saccharomyco./mycetaceae - ps23
Saccharomycetaceae - ps24
Saccharomycetaceae_2 - ps25
Saccharomycetaceae_3 - ps26
Kaz./Nau./Saccharomyces - ps27
Saccharomyces - ps28
Arboricola_clade - ps29
Cerevisiae_clade - ps30
Saccharomyces_cerevisiae - ps31
Phylostratum

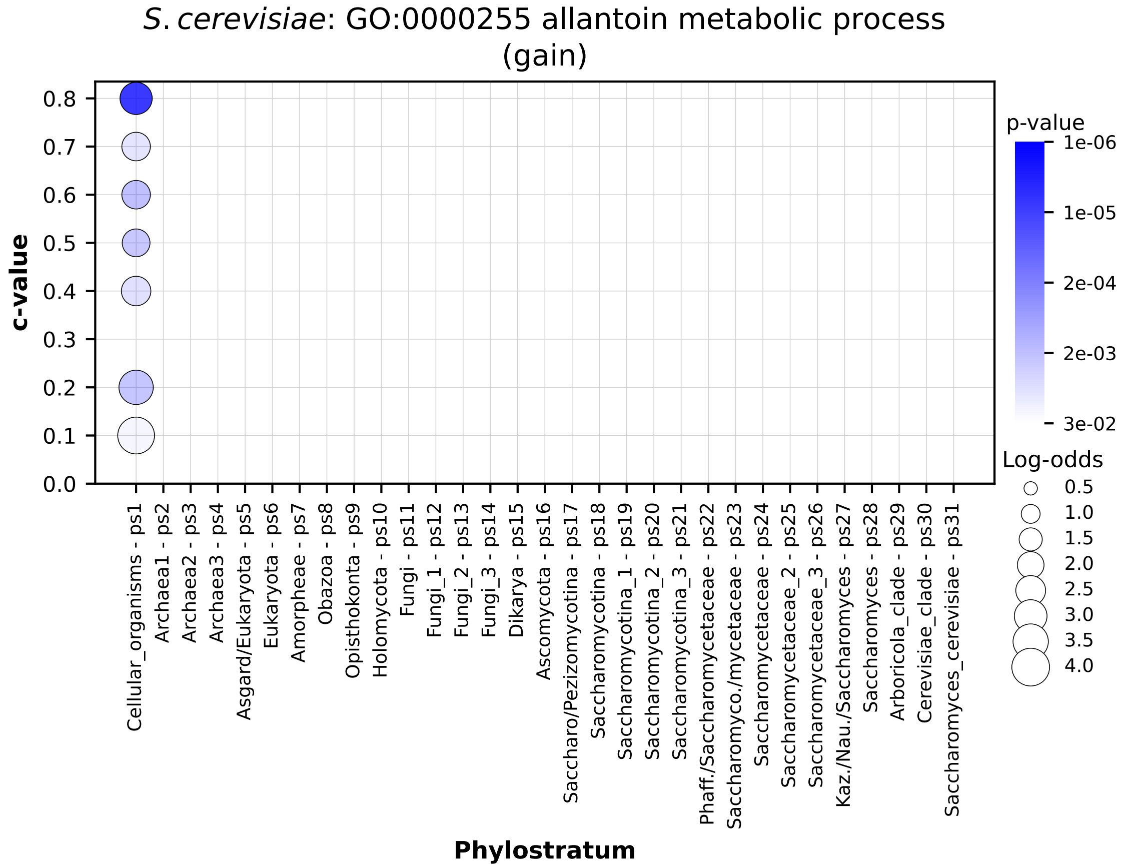
S. cerevisiae: GO:0000255 allantoin metabolic process
(gain)
c-value
0.0
0.1
0.2
0.3
0.4
0.5
0.6
0.7
0.8
p-value
1e-06
1e-05
2e-04
2e-03
3e-02
Log-odds
0.5
1.0
1.5
2.0
2.5
3.0
3.5
4.0
Cellular_organisms - ps1
Archaea1 - ps2
Archaea2 - ps3
Archaea3 - ps4
Asgard/Eukaryota - ps5
Eukaryota - ps6
Amorpheae - ps7
Obazoa - ps8
Opisthokonta - ps9
Holomycota - ps10
Fungi - ps11
Fungi_1 - ps12
Fungi_2 - ps13
Fungi_3 - ps14
Dikarya - ps15
Ascomycota - ps16
Saccharo/Pezizomycotina - ps17
Saccharomycotina - ps18
Saccharomycotina_1 - ps19
Saccharomycotina_2 - ps20
Saccharomycotina_3 - ps21
Phaff./Saccharomycetaceae - ps22
Saccharomyco./mycetaceae - ps23
Saccharomycetaceae - ps24
Saccharomycetaceae_2 - ps25
Saccharomycetaceae_3 - ps26
Kaz./Nau./Saccharomyces - ps27
Saccharomyces - ps28
Arboricola_clade - ps29
Cerevisiae_clade - ps30
Saccharomyces_cerevisiae - ps31
Phylostratum

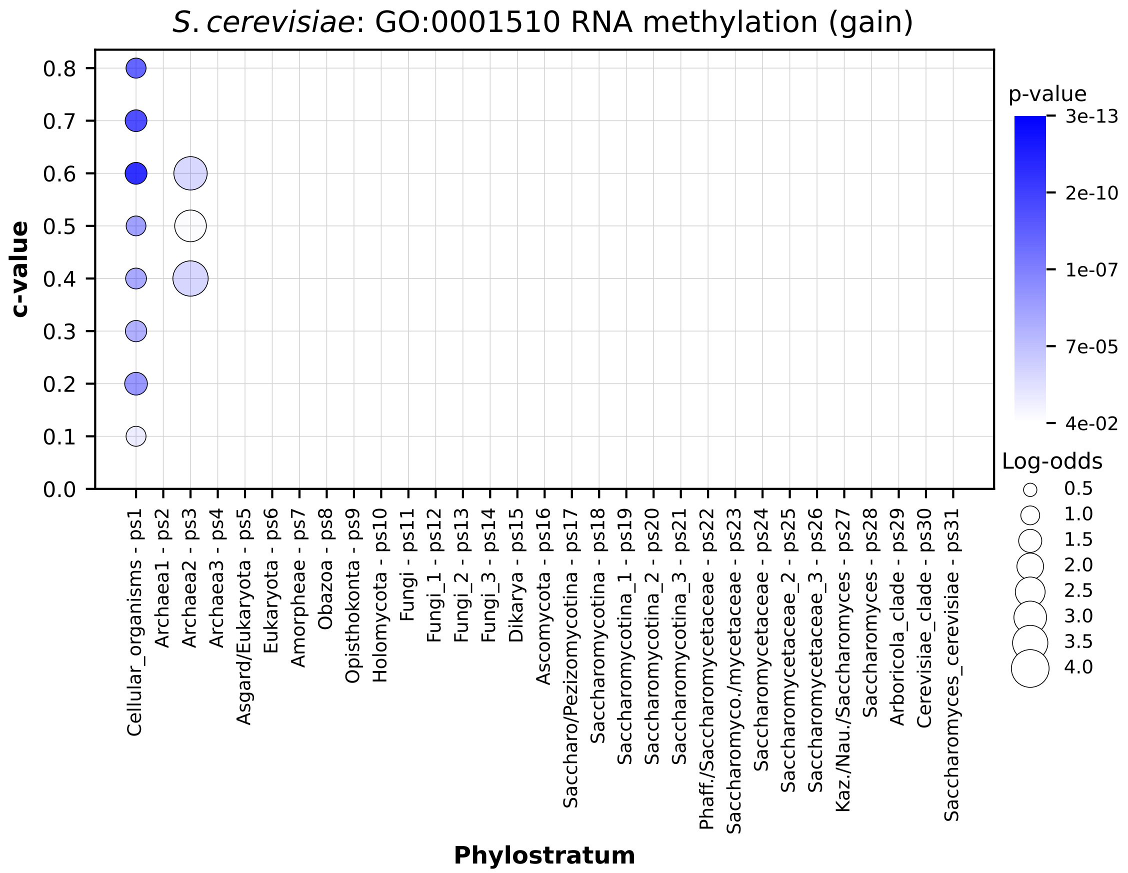

S. cerevisiae: GO:0001510 RNA methylation (gain)
c-value
0.8
0.7
0.6
0.5
0.4
0.3
0.2
0.1
0.0
p-value
3e-13
2e-10
1e-07
7e-05
4e-02
Log-odds
0.5
1.0
1.5
2.0
2.5
3.0
3.5
4.0
Cellular_organisms - ps1
Archaea1 - ps2
Archaea2 - ps3
Archaea3 - ps4
Asgard/Eukaryota - ps5
Eukaryota - ps6
Amorpheae - ps7
Obazoa - ps8
Opisthokonta - ps9
Holomycota - ps10
Fungi - ps11
Fungi_1 - ps12
Fungi_2 - ps13
Fungi_3 - ps14
Dikarya - ps15
Ascomycota - ps16
Saccharo/Pezizomycotina - ps17
Saccharomycotina - ps18
Saccharomycotina_1 - ps19
Saccharomycotina_2 - ps20
Saccharomycotina_3 - ps21
Phaff./Saccharomycetaceae - ps22
Saccharomyco./mycetaceae - ps23
Saccharomycetaceae - ps24
Saccharomycetaceae_2 - ps25
Saccharomycetaceae_3 - ps26
Kaz./Nau./Saccharomyces - ps27
Saccharomyces - ps28
Arboricola_clade - ps29
Cerevisiae_clade - ps30
Saccharomyces_cerevisiae - ps31
Phylostratum

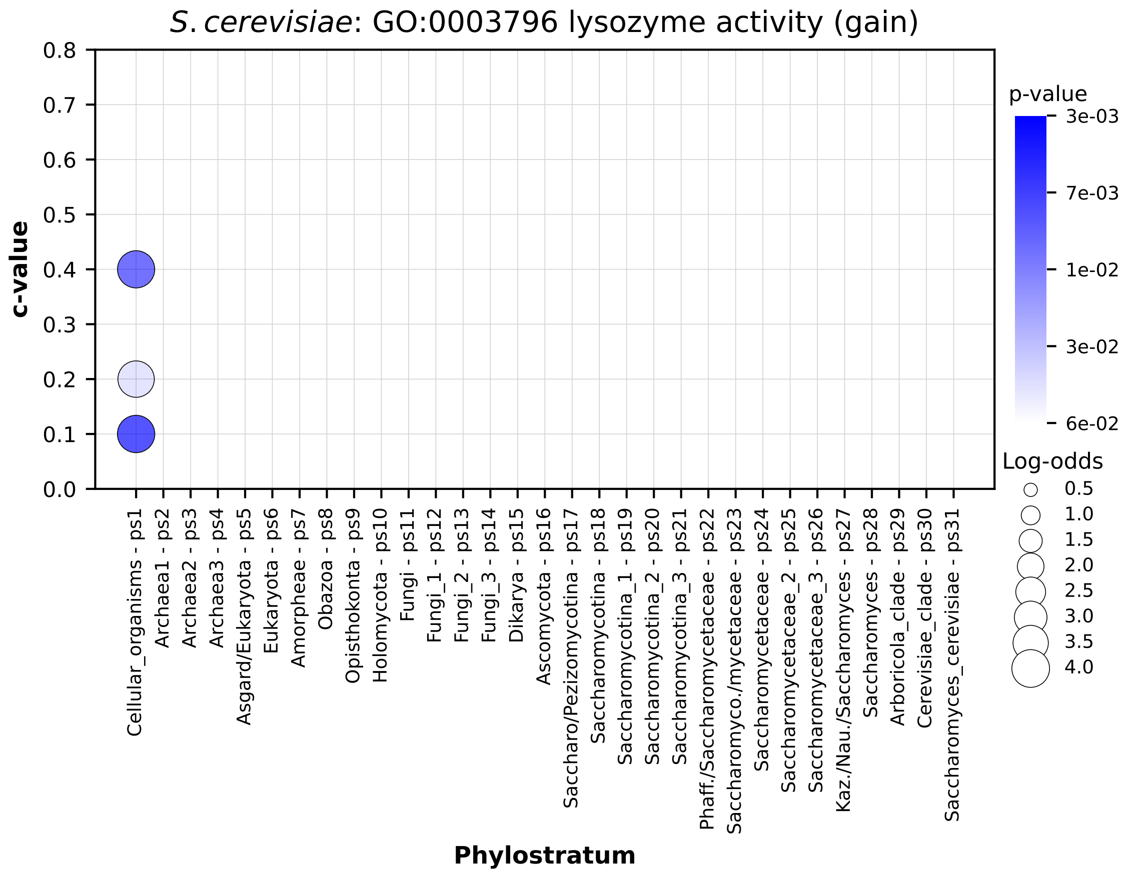

$S. cerevisiae$: GO:0003796 lysozyme activity (gain)
c-value
0.8
0.7
0.6
0.5
0.4
0.3
0.2
0.1
0.0
p-value
3e-03
7e-03
1e-02
3e-02
6e-02
Log-odds
0.5
1.0
1.5
2.0
2.5
3.0
3.5
4.0
Cellular_organisms - ps1
Archaea1 - ps2
Archaea2 - ps3
Archaea3 - ps4
Asgard/Eukaryota - ps5
Eukaryota - ps6
Amorpheae - ps7
Obazoa - ps8
Opisthokonta - ps9
Holomycota - ps10
Fungi - ps11
Fungi_1 - ps12
Fungi_2 - ps13
Fungi_3 - ps14
Dikarya - ps15
Ascomycota - ps16
Saccharo/Pezizomycotina - ps17
Saccharomycotina - ps18
Saccharomycotina_1 - ps19
Saccharomycotina_2 - ps20
Saccharomycotina_3 - ps21
Phaff./Saccharomycetaceae - ps22
Saccharomyco./mycetaceae - ps23
Saccharomycetaceae - ps24
Saccharomycetaceae_2 - ps25
Saccharomycetaceae_3 - ps26
Kaz./Nau./Saccharomyces - ps27
Saccharomyces - ps28
Arboricola_clade - ps29
Cerevisiae_clade - ps30
Saccharomyces_cerevisiae - ps31
Phylostratum

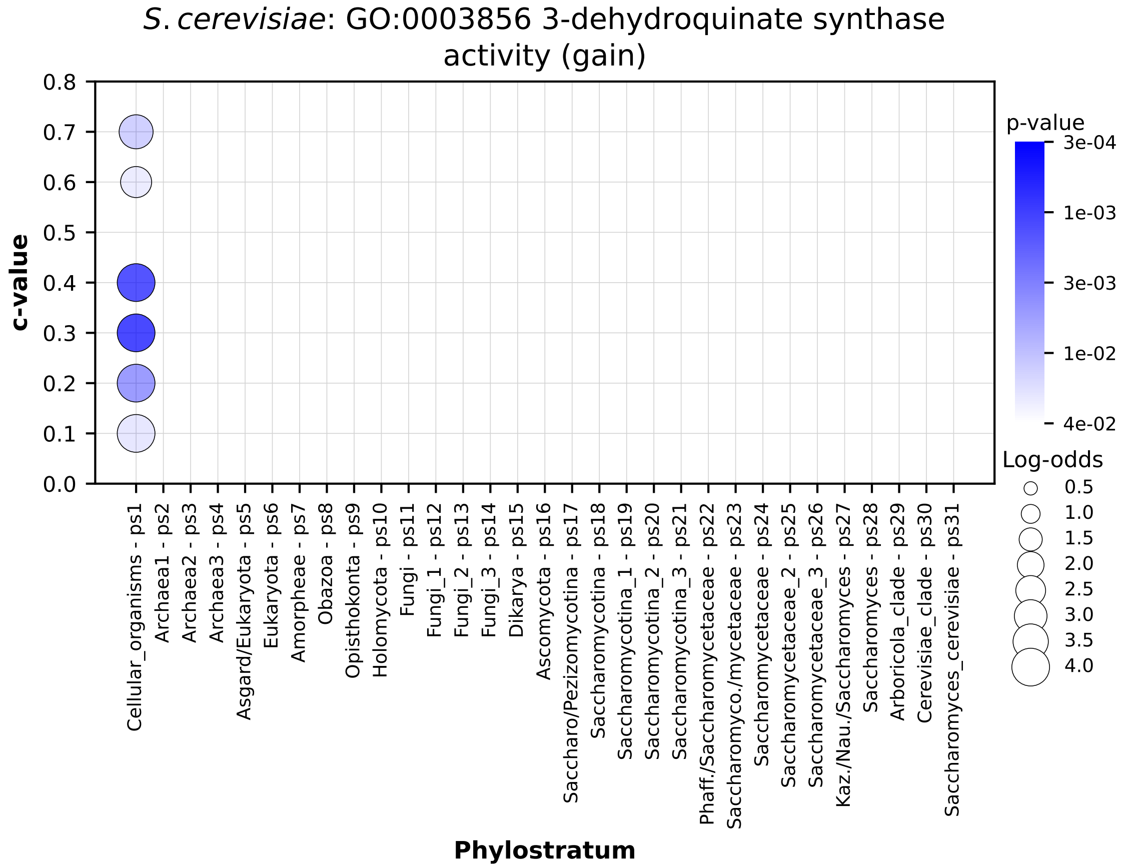

S. cerevisiae: GO:0003856 3-dehydroquinate synthase activity (gain)
c-value
0.0
0.1
0.2
0.3
0.4
0.5
0.6
0.7
0.8
p-value
3e-04
1e-03
3e-03
1e-02
4e-02
Log-odds
0.5
1.0
1.5
2.0
2.5
3.0
3.5
4.0
Cellular_organisms - ps1
Archaea1 - ps2
Archaea2 - ps3
Archaea3 - ps4
Asgard/Eukaryota - ps5
Eukaryota - ps6
Amorpheae - ps7
Obazoa - ps8
Opisthokonta - ps9
Holomycota - ps10
Fungi - ps11
Fungi_1 - ps12
Fungi_2 - ps13
Fungi_3 - ps14
Dikarya - ps15
Ascomycota - ps16
Saccharo/Pezizomycotina - ps17
Saccharomycotina - ps18
Saccharomycotina_1 - ps19
Saccharomycotina_2 - ps20
Saccharomycotina_3 - ps21
Phaff./Saccharomycetaceae - ps22
Saccharomyco./mycetaceae - ps23
Saccharomycetaceae - ps24
Saccharomycetaceae_2 - ps25
Saccharomycetaceae_3 - ps26
Kaz./Nau./Saccharomyces - ps27
Saccharomyces - ps28
Arboricola_clade - ps29
Cerevisiae_clade - ps30
Saccharomyces_cerevisiae - ps31
Phylostratum

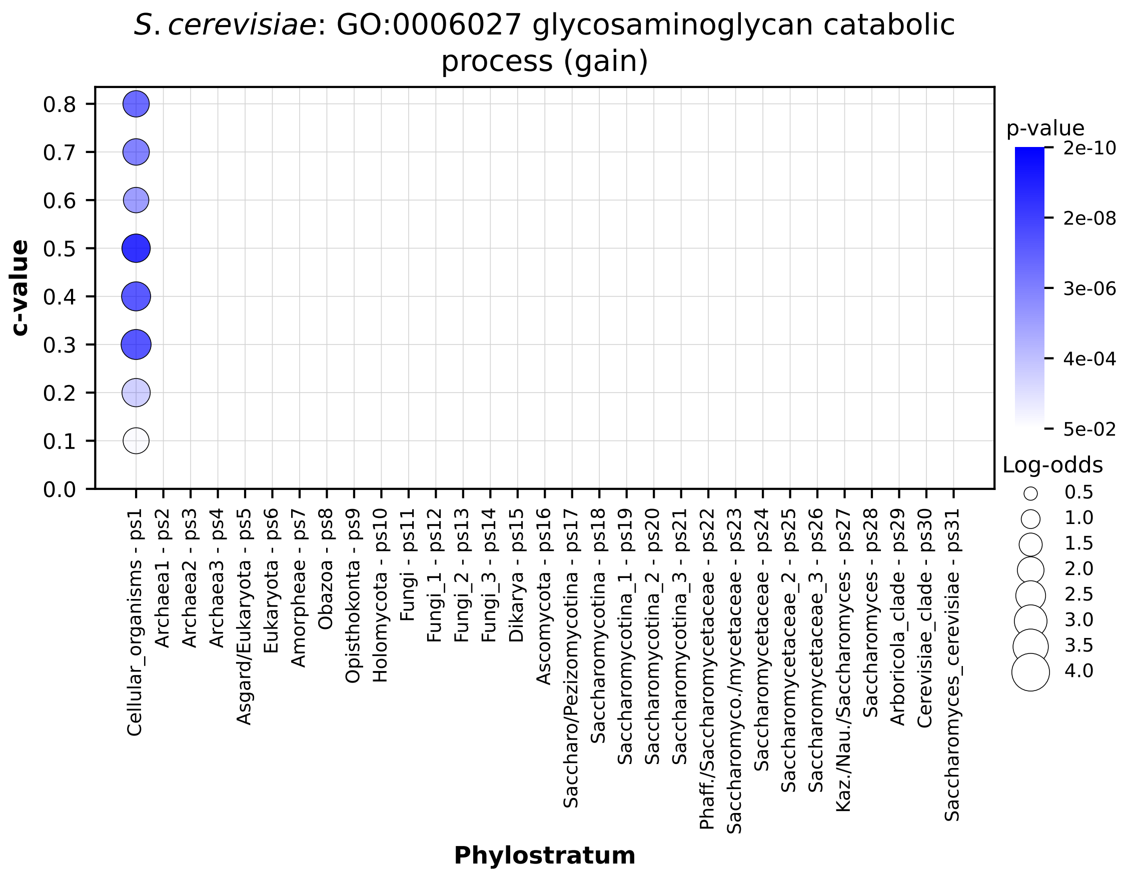

S. cerevisiae: GO:0006027 glycosaminoglycan catabolic process (gain)
c-value
0.0
0.1
0.2
0.3
0.4
0.5
0.6
0.7
0.8
Phylostratum
Cellular_organisms - ps1
Archaea1 - ps2
Archaea2 - ps3
Archaea3 - ps4
Asgard/Eukaryota - ps5
Eukaryota - ps6
Amorpheae - ps7
Obazoa - ps8
Opisthokonta - ps9
Holomycota - ps10
Fungi - ps11
Fungi_1 - ps12
Fungi_2 - ps13
Fungi_3 - ps14
Dikarya - ps15
Ascomycota - ps16
Saccharo/Pezizomycotina - ps17
Saccharomycotina - ps18
Saccharomycotina_1 - ps19
Saccharomycotina_2 - ps20
Saccharomycotina_3 - ps21
Phaff./Saccharomycetaceae - ps22
Saccharomyco./mycetaceae - ps23
Saccharomycetaceae - ps24
Saccharomycetaceae_2 - ps25
Saccharomycetaceae_3 - ps26
Kaz./Nau./Saccharomyces - ps27
Saccharomyces - ps28
Arboricola_clade - ps29
Cerevisiae_clade - ps30
Saccharomyces_cerevisiae - ps31
p-value
2e-10
2e-08
3e-06
4e-04
5e-02
Log-odds
0.5
1.0
1.5
2.0
2.5
3.0
3.5
4.0

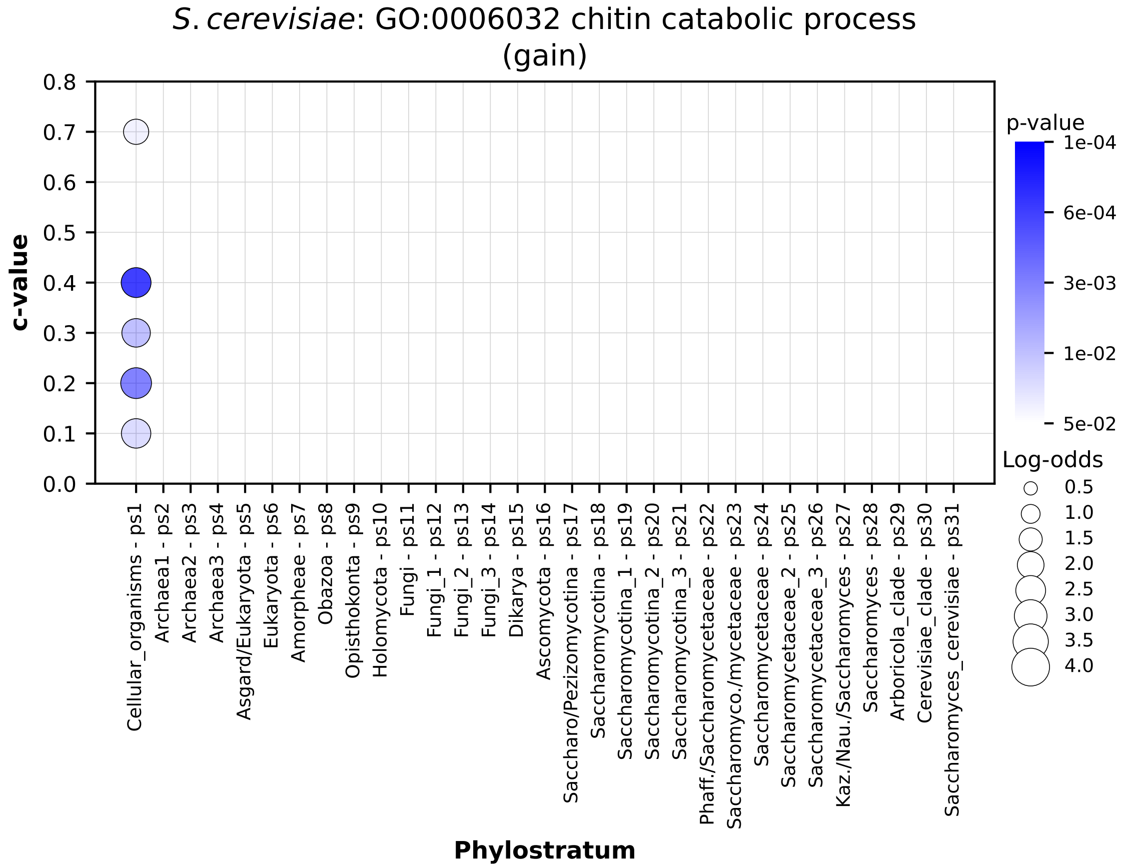

S. cerevisiae: GO:0006032 chitin catabolic process
(gain)
c-value
0.0
0.1
0.2
0.3
0.4
0.5
0.6
0.7
0.8
Phylostratum
Cellular_organisms - ps1
Archaea1 - ps2
Archaea2 - ps3
Archaea3 - ps4
Asgard/Eukaryota - ps5
Eukaryota - ps6
Amorpheae - ps7
Obazoa - ps8
Opisthokonta - ps9
Holomycota - ps10
Fungi - ps11
Fungi_1 - ps12
Fungi_2 - ps13
Fungi_3 - ps14
Dikarya - ps15
Ascomycota - ps16
Saccharo/Pezizomycotina - ps17
Saccharomycotina - ps18
Saccharomycotina_1 - ps19
Saccharomycotina_2 - ps20
Saccharomycotina_3 - ps21
Phaff./Saccharomycetaceae - ps22
Saccharomyco./mycetaceae - ps23
Saccharomycetaceae - ps24
Saccharomycetaceae_2 - ps25
Saccharomycetaceae_3 - ps26
Kaz./Nau./Saccharomyces - ps27
Saccharomyces - ps28
Arboricola_clade - ps29
Cerevisiae_clade - ps30
Saccharomyces_cerevisiae - ps31
p-value
1e-04
6e-04
3e-03
1e-02
5e-02
Log-odds
0.5
1.0
1.5
2.0
2.5
3.0
3.5
4.0

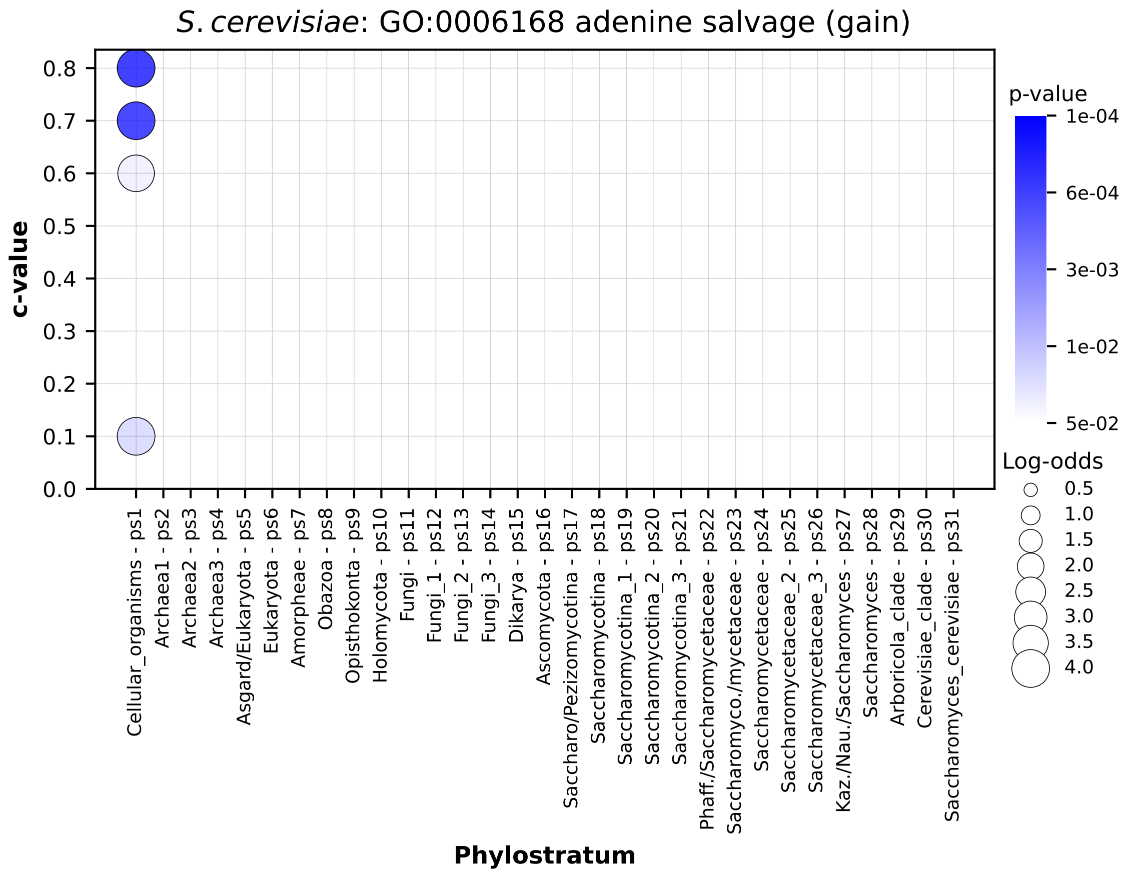

S. cerevisiae: GO:0006168 adenine salvage (gain)
c-value
0.0
0.1
0.2
0.3
0.4
0.5
0.6
0.7
0.8
p-value
1e-04
6e-04
3e-03
1e-02
5e-02
Log-odds
0.5
1.0
1.5
2.0
2.5
3.0
3.5
4.0
Cellular_organisms - ps1
Archaea1 - ps2
Archaea2 - ps3
Archaea3 - ps4
Asgard/Eukaryota - ps5
Eukaryota - ps6
Amorpheae - ps7
Obazoa - ps8
Opisthokonta - ps9
Holomycota - ps10
Fungi - ps11
Fungi_1 - ps12
Fungi_2 - ps13
Fungi_3 - ps14
Dikarya - ps15
Ascomycota - ps16
Saccharo/Pezizomycotina - ps17
Saccharomycotina - ps18
Saccharomycotina_1 - ps19
Saccharomycotina_2 - ps20
Saccharomycotina_3 - ps21
Phaff./Saccharomycetaceae - ps22
Saccharomyco./mycetaceae - ps23
Saccharomycetaceae - ps24
Saccharomycetaceae_2 - ps25
Saccharomycetaceae_3 - ps26
Kaz./Nau./Saccharomyces - ps27
Saccharomyces - ps28
Arboricola_clade - ps29
Cerevisiae_clade - ps30
Saccharomyces_cerevisiae - ps31
Phylostratum

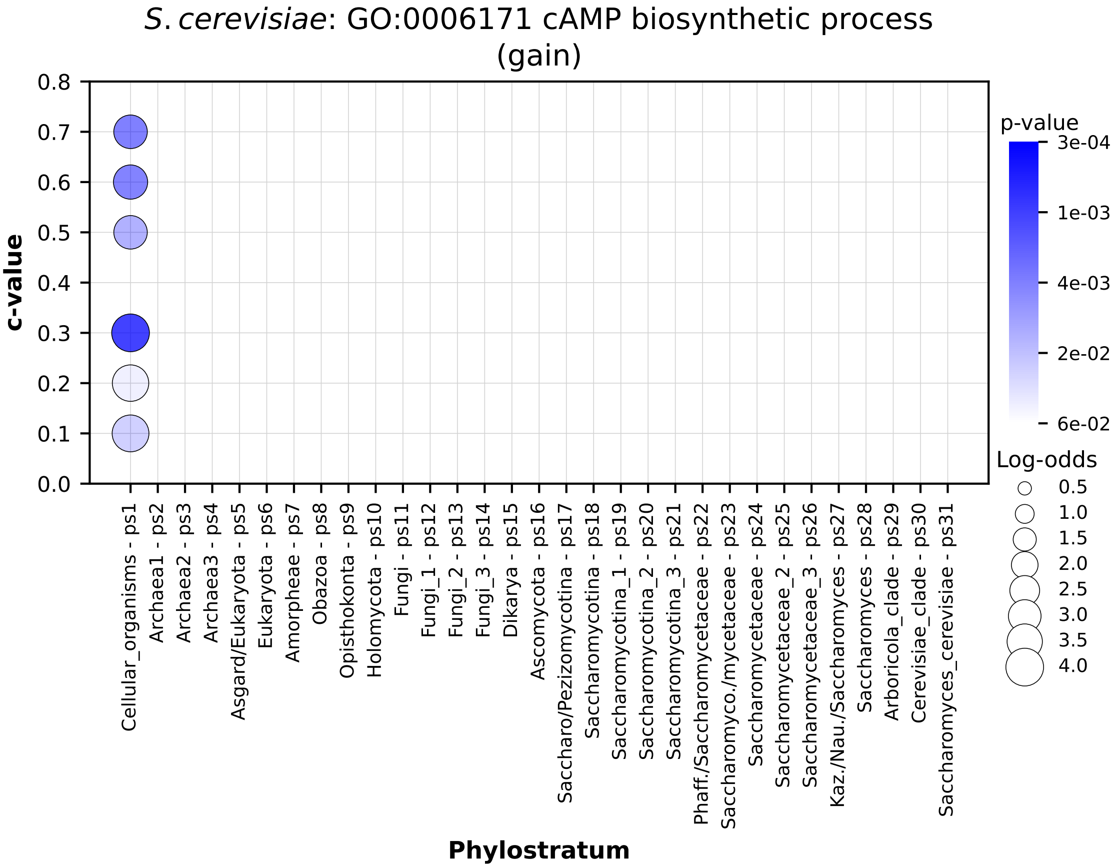

S. cerevisiae: GO:0006171 cAMP biosynthetic process
(gain)
c-value
0.0
0.1
0.2
0.3
0.4
0.5
0.6
0.7
0.8
p-value
3e-04
1e-03
4e-03
2e-02
6e-02
Log-odds
0.5
1.0
1.5
2.0
2.5
3.0
3.5
4.0
Cellular_organisms - ps1
Archaea1 - ps2
Archaea2 - ps3
Archaea3 - ps4
Asgard/Eukaryota - ps5
Eukaryota - ps6
Amorpheae - ps7
Obazoa - ps8
Opisthokonta - ps9
Holomycota - ps10
Fungi - ps11
Fungi_1 - ps12
Fungi_2 - ps13
Fungi_3 - ps14
Dikarya - ps15
Ascomycota - ps16
Saccharo/Pezizomycotina - ps17
Saccharomycotina - ps18
Saccharomycotina_1 - ps19
Saccharomycotina_2 - ps20
Saccharomycotina_3 - ps21
Phaff./Saccharomycetaceae - ps22
Saccharomyco./mycetaceae - ps23
Saccharomycetaceae - ps24
Saccharomycetaceae_2 - ps25
Saccharomycetaceae_3 - ps26
Kaz./Nau./Saccharomyces - ps27
Saccharomyces - ps28
Arboricola_clade - ps29
Cerevisiae_clade - ps30
Saccharomyces_cerevisiae - ps31
Phylostratum

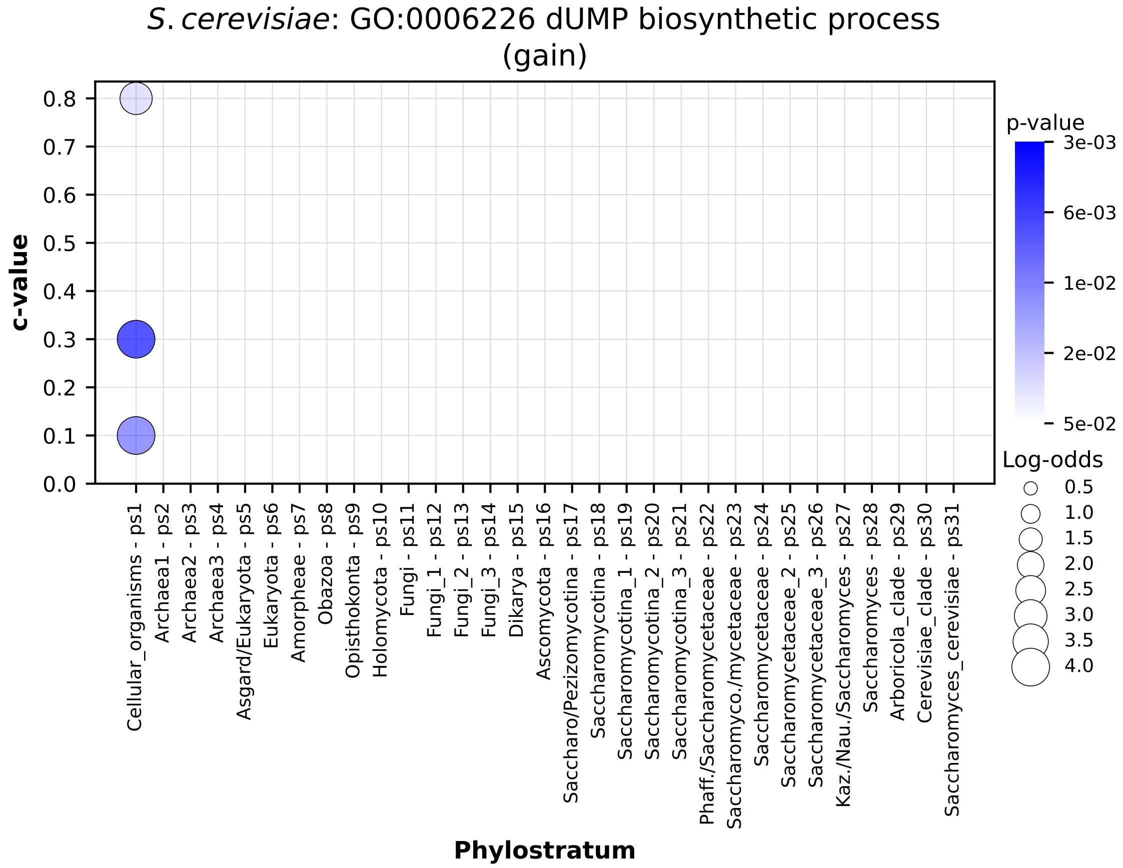

S. cerevisiae: GO:0006226 dUMP biosynthetic process
(gain)
c-value
0.0
0.1
0.2
0.3
0.4
0.5
0.6
0.7
0.8
p-value
3e-03
6e-03
1e-02
2e-02
5e-02
Log-odds
0.5
1.0
1.5
2.0
2.5
3.0
3.5
4.0
Cellular_organisms - ps1
Archaea1 - ps2
Archaea2 - ps3
Archaea3 - ps4
Asgard/Eukaryota - ps5
Eukaryota - ps6
Amorpheae - ps7
Obazoa - ps8
Opisthokonta - ps9
Holomycota - ps10
Fungi - ps11
Fungi_1 - ps12
Fungi_2 - ps13
Fungi_3 - ps14
Dikarya - ps15
Ascomycota - ps16
Saccharo/Pezizomycotina - ps17
Saccharomycotina - ps18
Saccharomycotina_1 - ps19
Saccharomycotina_2 - ps20
Saccharomycotina_3 - ps21
Phaff./Saccharomycetaceae - ps22
Saccharomyco./mycetaceae - ps23
Saccharomycetaceae - ps24
Saccharomycetaceae_2 - ps25
Saccharomycetaceae_3 - ps26
Kaz./Nau./Saccharomyces - ps27
Saccharomyces - ps28
Arboricola_clade - ps29
Cerevisiae_clade - ps30
Saccharomyces_cerevisiae - ps31
Phylostratum

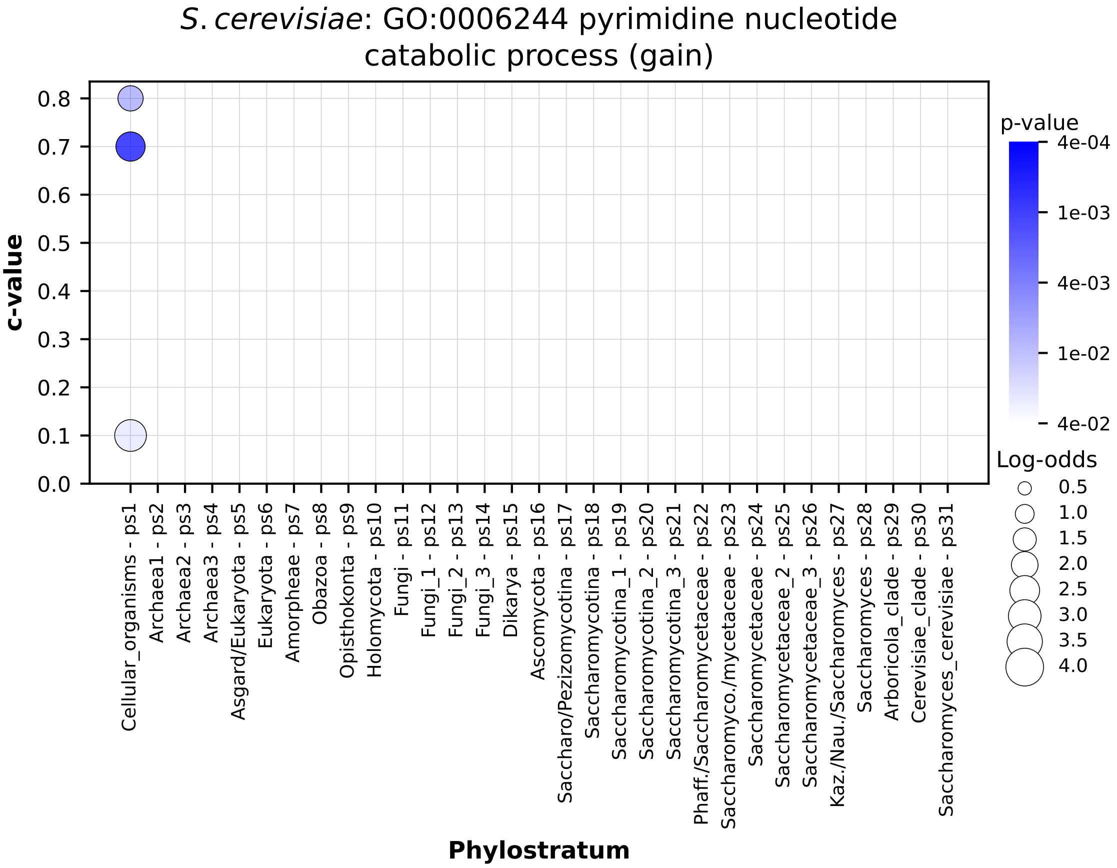

S. cerevisiae: GO:0006244 pyrimidine nucleotide catabolic process (gain)
c-value
0.0
0.1
0.2
0.3
0.4
0.5
0.6
0.7
0.8
Phylostratum
Cellular_organisms - ps1
Archaea1 - ps2
Archaea2 - ps3
Archaea3 - ps4
Asgard/Eukaryota - ps5
Eukaryota - ps6
Amorpheae - ps7
Obazoa - ps8
Opisthokonta - ps9
Holomycota - ps10
Fungi - ps11
Fungi_1 - ps12
Fungi_2 - ps13
Fungi_3 - ps14
Dikarya - ps15
Ascomycota - ps16
Saccharo/Pezizomycotina - ps17
Saccharomycotina - ps18
Saccharomycotina_1 - ps19
Saccharomycotina_2 - ps20
Saccharomycotina_3 - ps21
Phaff./Saccharomycetaceae - ps22
Saccharomyco./mycetaceae - ps23
Saccharomycetaceae - ps24
Saccharomycetaceae_2 - ps25
Saccharomycetaceae_3 - ps26
Kaz./Nau./Saccharomyces - ps27
Saccharomyces - ps28
Arboricola_clade - ps29
Cerevisiae_clade - ps30
Saccharomyces_cerevisiae - ps31
p-value
4e-04
1e-03
4e-03
1e-02
4e-02
Log-odds
0.5
1.0
1.5
2.0
2.5
3.0
3.5
4.0

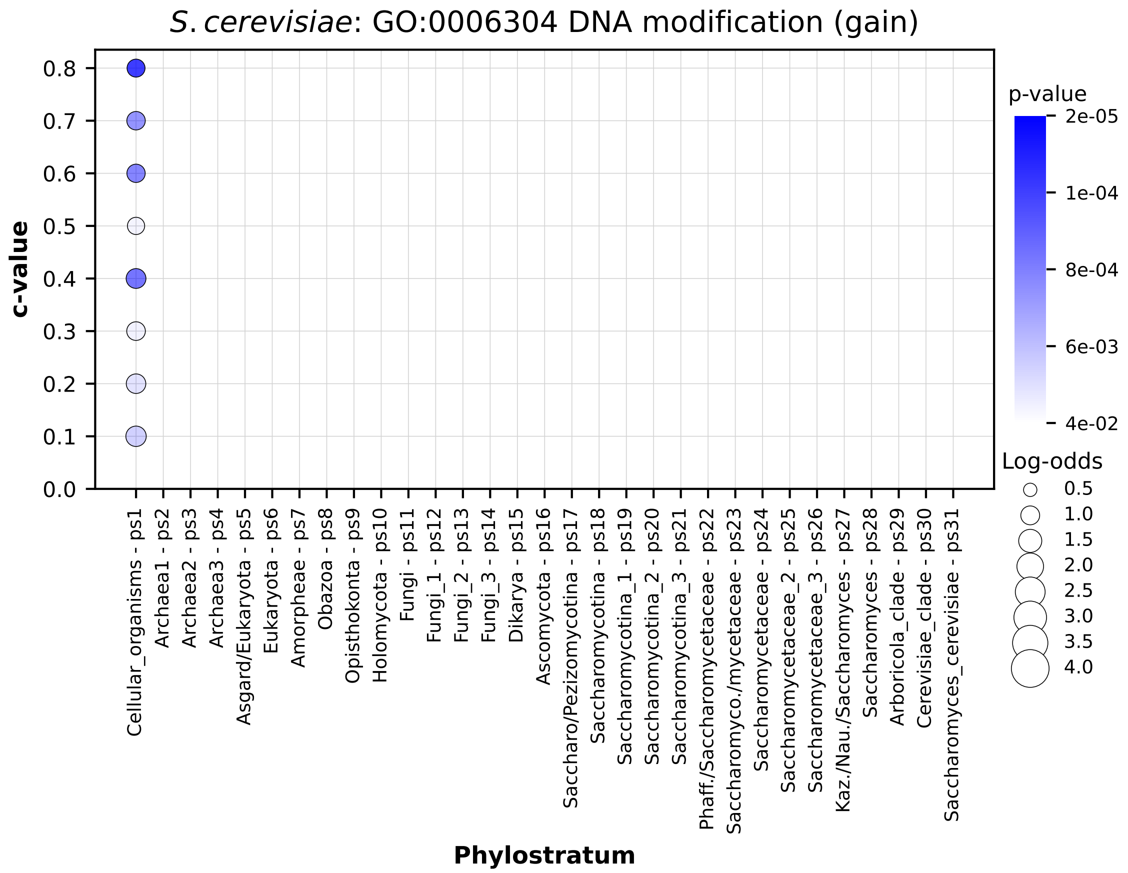

S. cerevisiae: GO:0006304 DNA modification (gain)
c-value
0.0
0.1
0.2
0.3
0.4
0.5
0.6
0.7
0.8
p-value
2e-05
1e-04
8e-04
6e-03
4e-02
Log-odds
0.5
1.0
1.5
2.0
2.5
3.0
3.5
4.0
Cellular_organisms - ps1
Archaea1 - ps2
Archaea2 - ps3
Archaea3 - ps4
Asgard/Eukaryota - ps5
Eukaryota - ps6
Amorpheae - ps7
Obazoa - ps8
Opisthokonta - ps9
Holomycota - ps10
Fungi - ps11
Fungi_1 - ps12
Fungi_2 - ps13
Fungi_3 - ps14
Dikarya - ps15
Ascomycota - ps16
Saccharo/Pezizomycotina - ps17
Saccharomycotina - ps18
Saccharomycotina_1 - ps19
Saccharomycotina_2 - ps20
Saccharomycotina_3 - ps21
Phaff./Saccharomycetaceae - ps22
Saccharomyco./mycetaceae - ps23
Saccharomycetaceae - ps24
Saccharomycetaceae_2 - ps25
Saccharomycetaceae_3 - ps26
Kaz./Nau./Saccharomyces - ps27
Saccharomyces - ps28
Arboricola_clade - ps29
Cerevisiae_clade - ps30
Saccharomyces_cerevisiae - ps31
Phylostratum

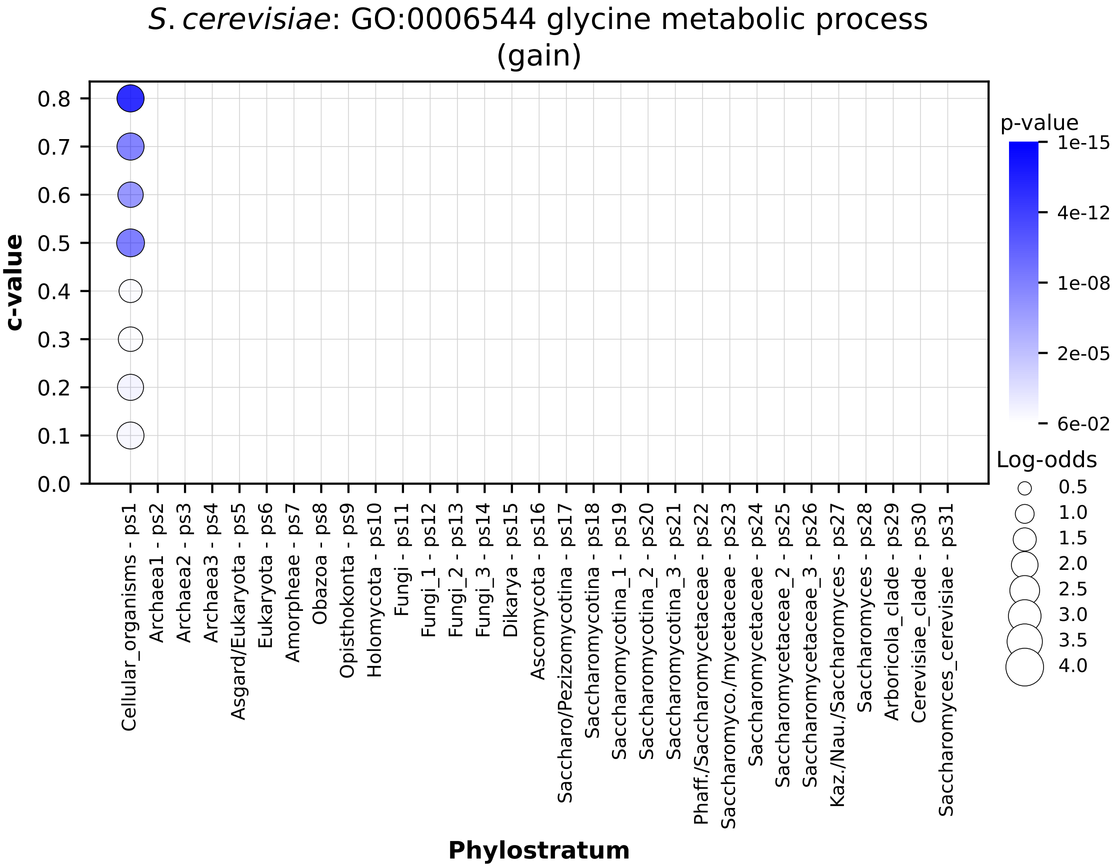

S. cerevisiae: GO:0006544 glycine metabolic process
(gain)
c-value
0.8
0.7
0.6
0.5
0.4
0.3
0.2
0.1
0.0
Phylostratum
Cellular_organisms - ps1
Archaea1 - ps2
Archaea2 - ps3
Archaea3 - ps4
Asgard/Eukaryota - ps5
Eukaryota - ps6
Amorpheae - ps7
Obazoa - ps8
Opisthokonta - ps9
Holomycota - ps10
Fungi - ps11
Fungi_1 - ps12
Fungi_2 - ps13
Fungi_3 - ps14
Dikarya - ps15
Ascomycota - ps16
Saccharo/Pezizomycotina - ps17
Saccharomycotina - ps18
Saccharomycotina_1 - ps19
Saccharomycotina_2 - ps20
Saccharomycotina_3 - ps21
Phaff./Saccharomycetaceae - ps22
Saccharomyco./mycetaceae - ps23
Saccharomycetaceae - ps24
Saccharomycetaceae_2 - ps25
Saccharomycetaceae_3 - ps26
Kaz./Nau./Saccharomyces - ps27
Saccharomyces - ps28
Arboricola_clade - ps29
Cerevisiae_clade - ps30
Saccharomyces_cerevisiae - ps31
p-value
1e-15
4e-12
1e-08
2e-05
6e-02
Log-odds
0.5
1.0
1.5
2.0
2.5
3.0
3.5
4.0

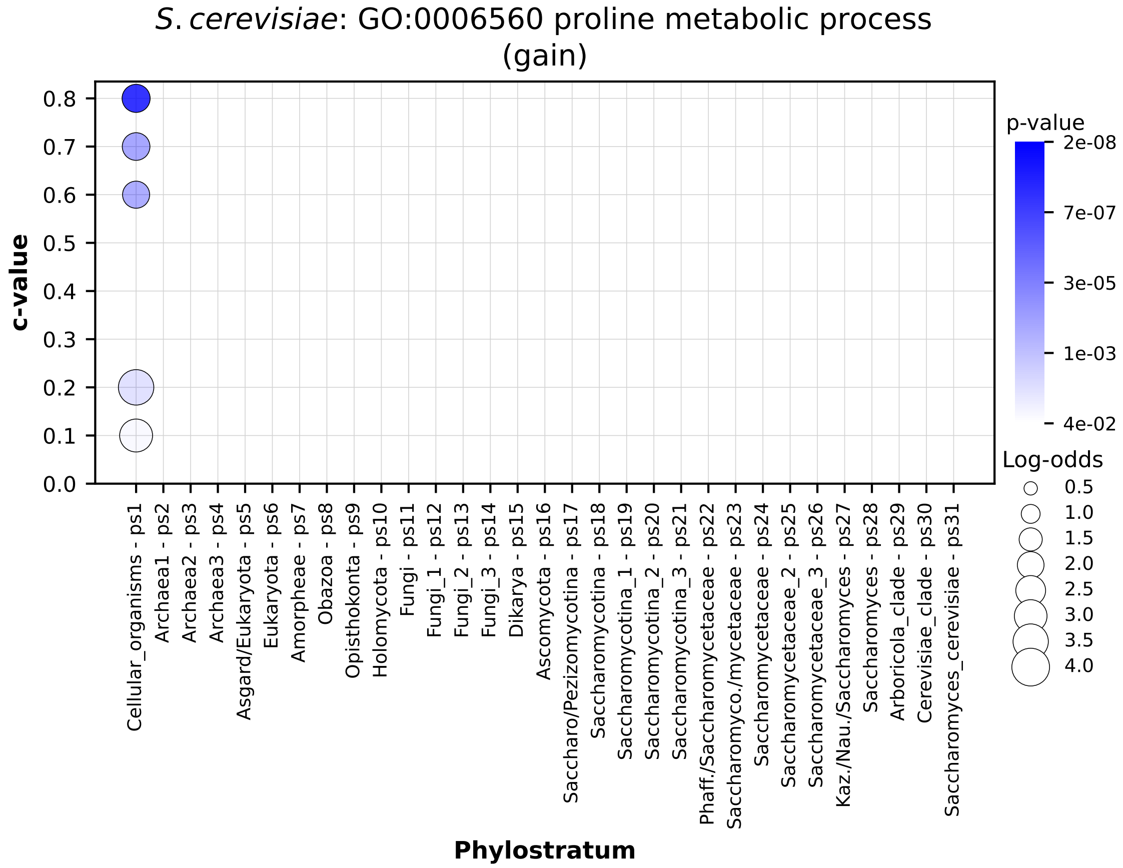
S. cerevisiae: GO:0006560 proline metabolic process
(gain)
c-value
0.0
0.1
0.2
0.3
0.4
0.5
0.6
0.7
0.8
p-value
2e-08
7e-07
3e-05
1e-03
4e-02
Log-odds
0.5
1.0
1.5
2.0
2.5
3.0
3.5
4.0
Cellular_organisms - ps1
Archaea1 - ps2
Archaea2 - ps3
Archaea3 - ps4
Asgard/Eukaryota - ps5
Eukaryota - ps6
Amorpheae - ps7
Obazoa - ps8
Opisthokonta - ps9
Holomycota - ps10
Fungi - ps11
Fungi_1 - ps12
Fungi_2 - ps13
Fungi_3 - ps14
Dikarya - ps15
Ascomycota - ps16
Saccharo/Pezizomycotina - ps17
Saccharomycotina - ps18
Saccharomycotina_1 - ps19
Saccharomycotina_2 - ps20
Saccharomycotina_3 - ps21
Phaff./Saccharomycetaceae - ps22
Saccharomyco./mycetaceae - ps23
Saccharomycetaceae - ps24
Saccharomycetaceae_2 - ps25
Saccharomycetaceae_3 - ps26
Kaz./Nau./Saccharomyces - ps27
Saccharomyces - ps28
Arboricola_clade - ps29
Cerevisiae_clade - ps30
Saccharomyces_cerevisiae - ps31
Phylostratum

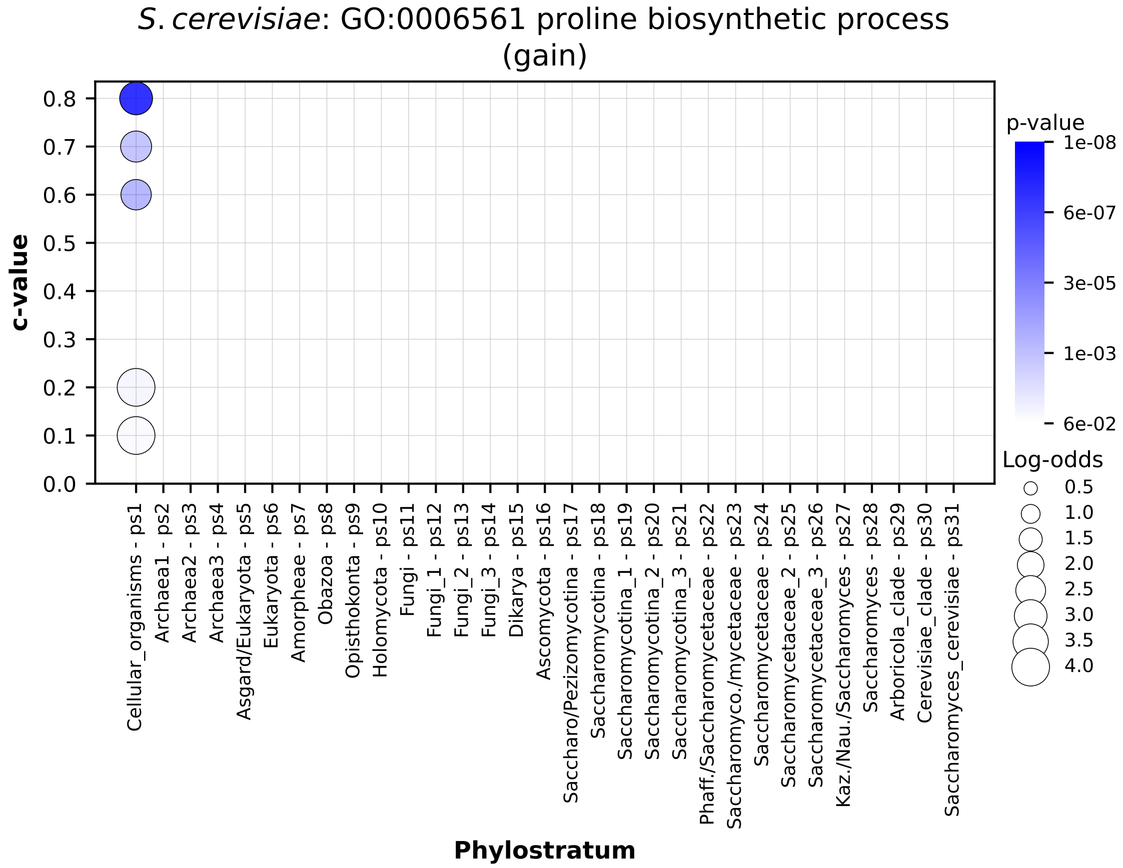

S. cerevisiae: GO:0006561 proline biosynthetic process
(gain)
c-value
0.0
0.1
0.2
0.3
0.4
0.5
0.6
0.7
0.8
Phylostratum
Cellular_organisms - ps1
Archaea1 - ps2
Archaea2 - ps3
Archaea3 - ps4
Asgard/Eukaryota - ps5
Eukaryota - ps6
Amorpheae - ps7
Obazoa - ps8
Opisthokonta - ps9
Holomycota - ps10
Fungi - ps11
Fungi_1 - ps12
Fungi_2 - ps13
Fungi_3 - ps14
Dikarya - ps15
Ascomycota - ps16
Saccharo/Pezizomycotina - ps17
Saccharomycotina - ps18
Saccharomycotina_1 - ps19
Saccharomycotina_2 - ps20
Saccharomycotina_3 - ps21
Phaff./Saccharomycetaceae - ps22
Saccharomyco./mycetaceae - ps23
Saccharomycetaceae - ps24
Saccharomycetaceae_2 - ps25
Saccharomycetaceae_3 - ps26
Kaz./Nau./Saccharomyces - ps27
Saccharomyces - ps28
Arboricola_clade - ps29
Cerevisiae_clade - ps30
Saccharomyces_cerevisiae - ps31
p-value
1e-08
6e-07
3e-05
1e-03
6e-02
Log-odds
0.5
1.0
1.5
2.0
2.5
3.0
3.5
4.0

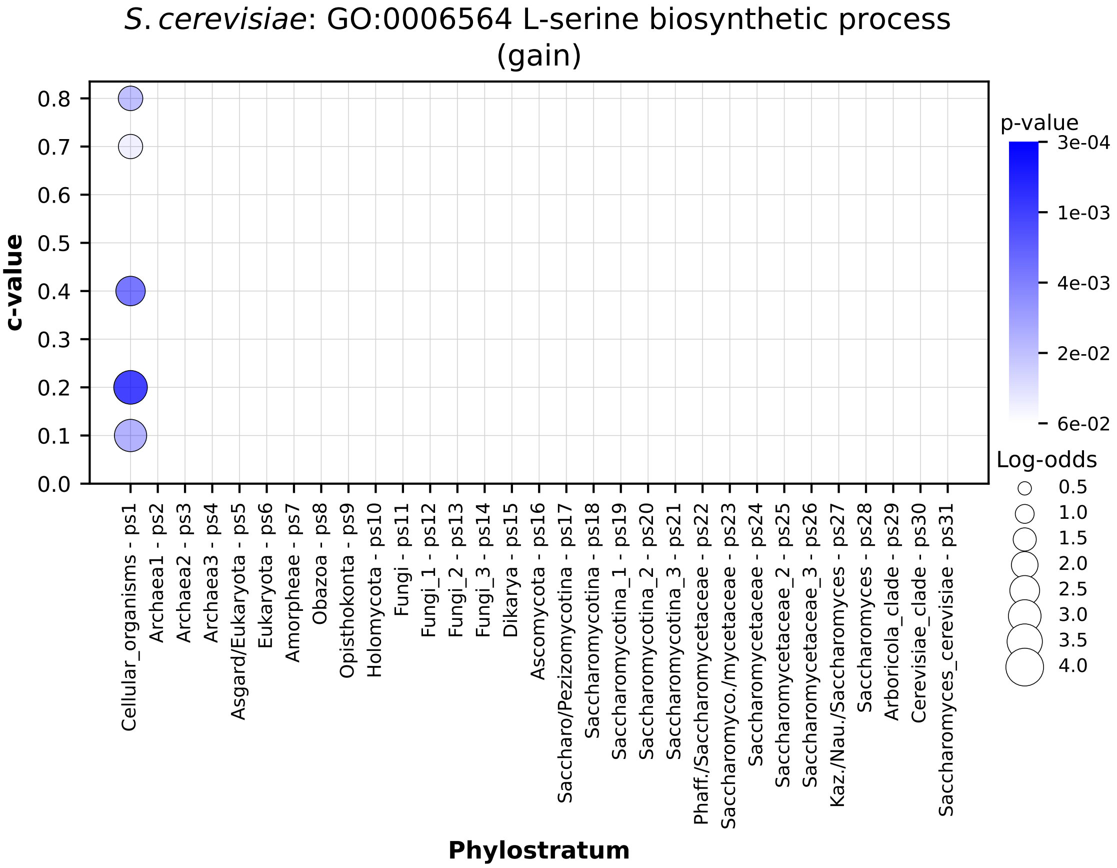

S. cerevisiae: GO:0006564 L-serine biosynthetic process (gain)
c-value
0.0
0.1
0.2
0.3
0.4
0.5
0.6
0.7
0.8
p-value
3e-04
1e-03
4e-03
2e-02
6e-02
Log-odds
0.5
1.0
1.5
2.0
2.5
3.0
3.5
4.0
Cellular_organisms - ps1
Archaea1 - ps2
Archaea2 - ps3
Archaea3 - ps4
Asgard/Eukaryota - ps5
Eukaryota - ps6
Amorpheae - ps7
Obazoa - ps8
Opisthokonta - ps9
Holomycota - ps10
Fungi - ps11
Fungi_1 - ps12
Fungi_2 - ps13
Fungi_3 - ps14
Dikarya - ps15
Ascomycota - ps16
Saccharo/Pezizomycotina - ps17
Saccharomycotina - ps18
Saccharomycotina_1 - ps19
Saccharomycotina_2 - ps20
Saccharomycotina_3 - ps21
Phaff./Saccharomycetaceae - ps22
Saccharomyco./mycetaceae - ps23
Saccharomycetaceae - ps24
Saccharomycetaceae_2 - ps25
Saccharomycetaceae_3 - ps26
Kaz./Nau./Saccharomyces - ps27
Saccharomyces - ps28
Arboricola_clade - ps29
Cerevisiae_clade - ps30
Saccharomyces_cerevisiae - ps31
Phylostratum

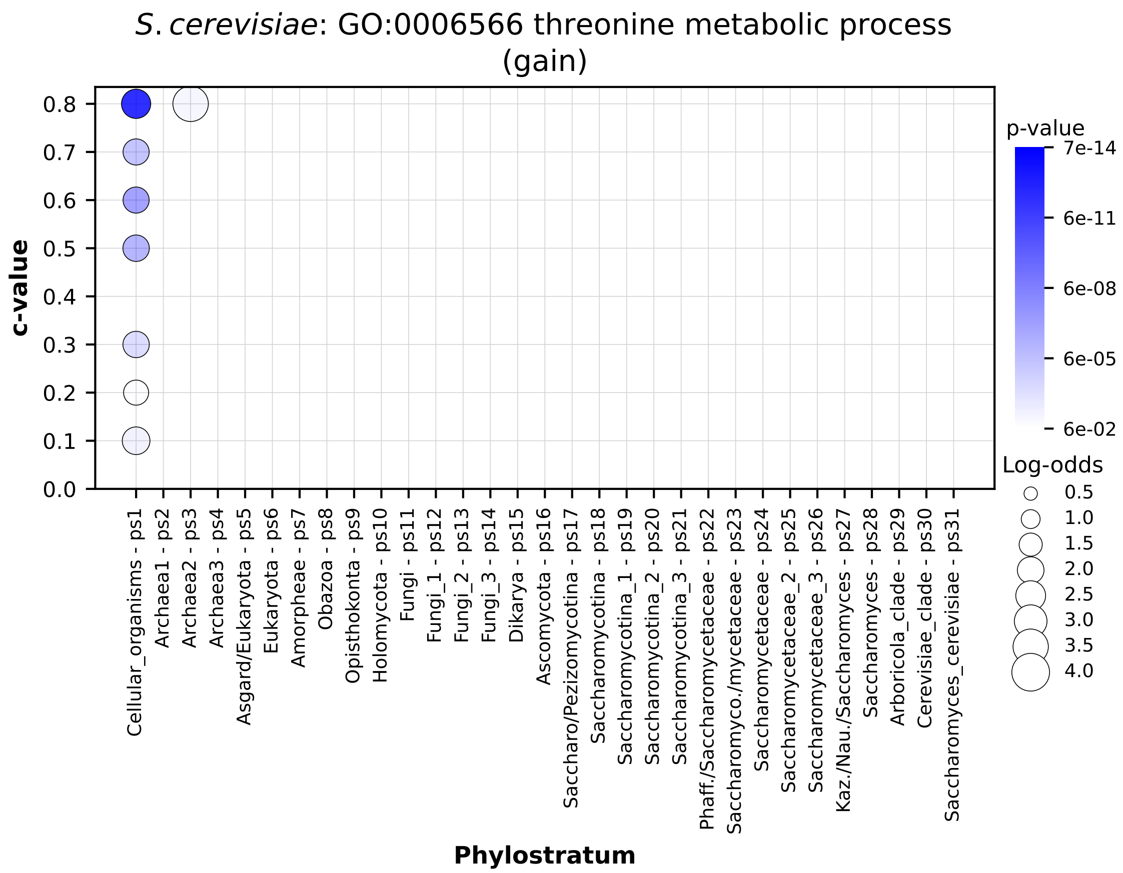

S. cerevisiae: GO:0006566 threonine metabolic process
(gain)
c-value
0.0
0.1
0.2
0.3
0.4
0.5
0.6
0.7
0.8
Phylostratum
Cellular_organisms - ps1
Archaea1 - ps2
Archaea2 - ps3
Archaea3 - ps4
Asgard/Eukaryota - ps5
Eukaryota - ps6
Amorpheae - ps7
Obazoa - ps8
Opisthokonta - ps9
Holomycota - ps10
Fungi - ps11
Fungi_1 - ps12
Fungi_2 - ps13
Fungi_3 - ps14
Dikarya - ps15
Ascomycota - ps16
Saccharo/Pezizomycotina - ps17
Saccharomycotina - ps18
Saccharomycotina_1 - ps19
Saccharomycotina_2 - ps20
Saccharomycotina_3 - ps21
Phaff./Saccharomycetaceae - ps22
Saccharomyco./mycetaceae - ps23
Saccharomycetaceae - ps24
Saccharomycetaceae_2 - ps25
Saccharomycetaceae_3 - ps26
Kaz./Nau./Saccharomyces - ps27
Saccharomyces - ps28
Arboricola_clade - ps29
Cerevisiae_clade - ps30
Saccharomyces_cerevisiae - ps31
p-value
7e-14
6e-11
6e-08
6e-05
6e-02
Log-odds
0.5
1.0
1.5
2.0
2.5
3.0
3.5
4.0

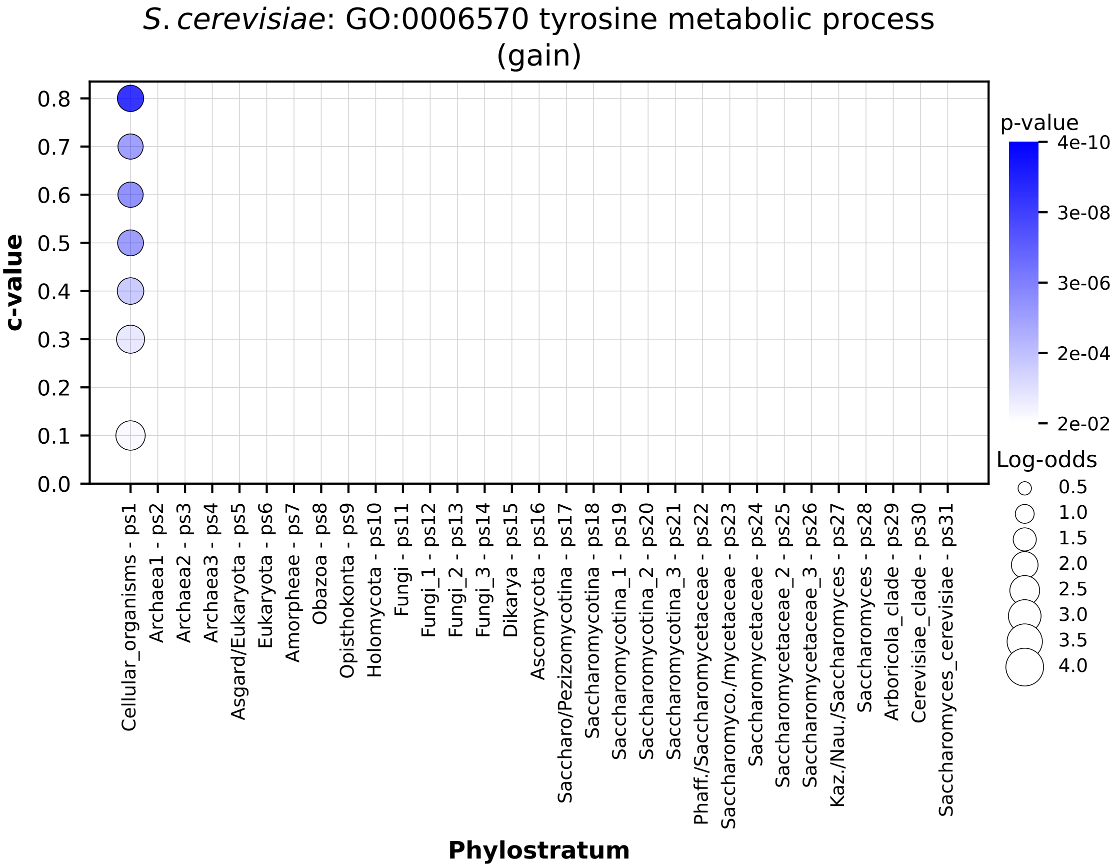

S. cerevisiae: GO:0006570 tyrosine metabolic process
(gain)
c-value
0.0
0.1
0.2
0.3
0.4
0.5
0.6
0.7
0.8
p-value
4e-10
3e-08
3e-06
2e-04
2e-02
Log-odds
0.5
1.0
1.5
2.0
2.5
3.0
3.5
4.0
Cellular_organisms - ps1
Archaea1 - ps2
Archaea2 - ps3
Archaea3 - ps4
Asgard/Eukaryota - ps5
Eukaryota - ps6
Amorpheae - ps7
Obazoa - ps8
Opisthokonta - ps9
Holomycota - ps10
Fungi - ps11
Fungi_1 - ps12
Fungi_2 - ps13
Fungi_3 - ps14
Dikarya - ps15
Ascomycota - ps16
Saccharo/Pezizomycotina - ps17
Saccharomycotina - ps18
Saccharomycotina_1 - ps19
Saccharomycotina_2 - ps20
Saccharomycotina_3 - ps21
Phaff./Saccharomycetaceae - ps22
Saccharomyco./mycetaceae - ps23
Saccharomycetaceae - ps24
Saccharomycetaceae_2 - ps25
Saccharomycetaceae_3 - ps26
Kaz./Nau./Saccharomyces - ps27
Saccharomyces - ps28
Arboricola_clade - ps29
Cerevisiae_clade - ps30
Saccharomyces_cerevisiae - ps31
Phylostratum

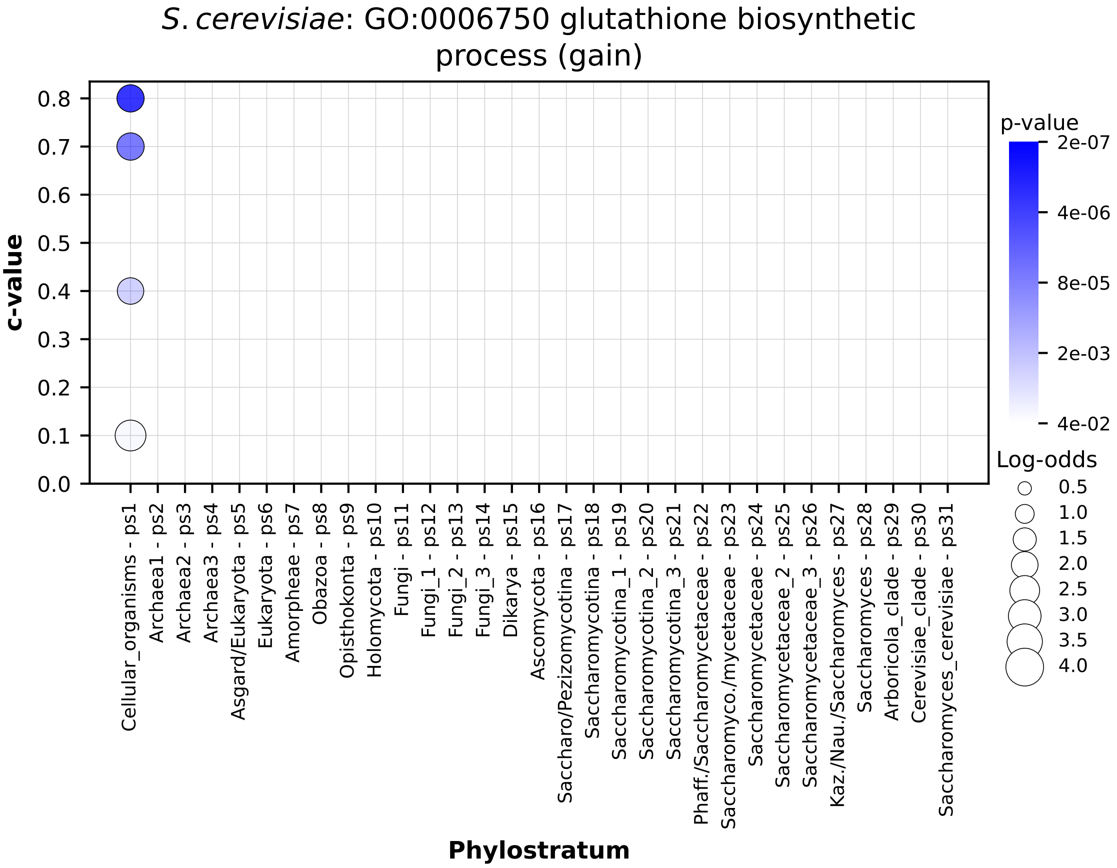
S. cerevisiae: GO:0006750 glutathione biosynthetic process (gain)
c-value
0.8
0.7
0.6
0.5
0.4
0.3
0.2
0.1
0.0
p-value
2e-07
4e-06
8e-05
2e-03
4e-02
Log-odds
0.5
1.0
1.5
2.0
2.5
3.0
3.5
4.0
Cellular_organisms - ps1
Archaea1 - ps2
Archaea2 - ps3
Archaea3 - ps4
Asgard/Eukaryota - ps5
Eukaryota - ps6
Amorpheae - ps7
Obazoa - ps8
Opisthokonta - ps9
Holomycota - ps10
Fungi - ps11
Fungi_1 - ps12
Fungi_2 - ps13
Fungi_3 - ps14
Dikarya - ps15
Ascomycota - ps16
Saccharo/Pezizomycotina - ps17
Saccharomycotina - ps18
Saccharomycotina_1 - ps19
Saccharomycotina_2 - ps20
Saccharomycotina_3 - ps21
Phaff./Saccharomycetaceae - ps22
Saccharomyco./mycetaceae - ps23
Saccharomycetaceae - ps24
Saccharomycetaceae_2 - ps25
Saccharomycetaceae_3 - ps26
Kaz./Nau./Saccharomyces - ps27
Saccharomyces - ps28
Arboricola_clade - ps29
Cerevisiae_clade - ps30
Saccharomyces_cerevisiae - ps31
Phylostratum

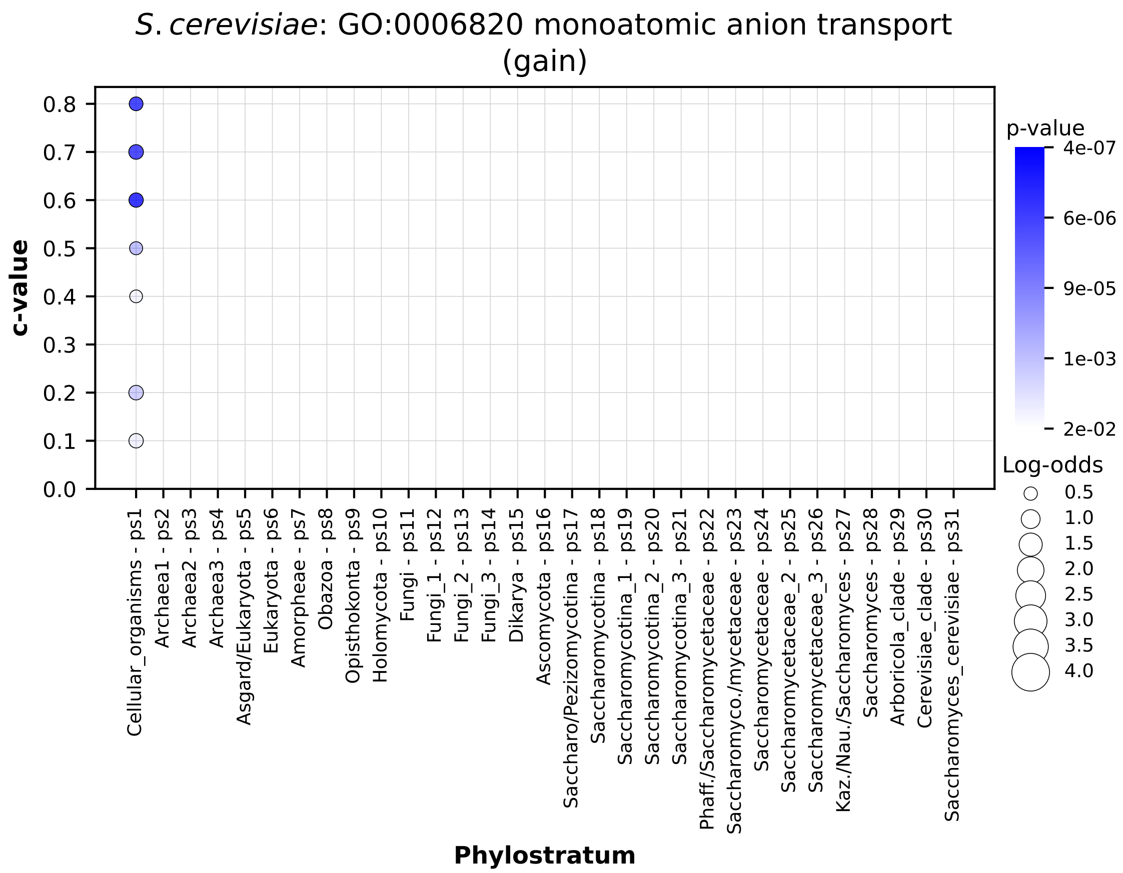

S. cerevisiae: GO:0006820 monoatomic anion transport (gain)
c-value
0.0
0.1
0.2
0.3
0.4
0.5
0.6
0.7
0.8
p-value
4e-07
6e-06
9e-05
1e-03
2e-02
Log-odds
0.5
1.0
1.5
2.0
2.5
3.0
3.5
4.0
Cellular_organisms - ps1
Archaea1 - ps2
Archaea2 - ps3
Archaea3 - ps4
Asgard/Eukaryota - ps5
Eukaryota - ps6
Amorpheae - ps7
Obazoa - ps8
Opisthokonta - ps9
Holomycota - ps10
Fungi - ps11
Fungi_1 - ps12
Fungi_2 - ps13
Fungi_3 - ps14
Dikarya - ps15
Ascomycota - ps16
Saccharo/Pezizomycotina - ps17
Saccharomycotina - ps18
Saccharomycotina_1 - ps19
Saccharomycotina_2 - ps20
Saccharomycotina_3 - ps21
Phaff./Saccharomycetaceae - ps22
Saccharomyco./mycetaceae - ps23
Saccharomycetaceae - ps24
Saccharomycetaceae_2 - ps25
Saccharomycetaceae_3 - ps26
Kaz./Nau./Saccharomyces - ps27
Saccharomyces - ps28
Arboricola_clade - ps29
Cerevisiae_clade - ps30
Saccharomyces_cerevisiae - ps31
Phylostratum

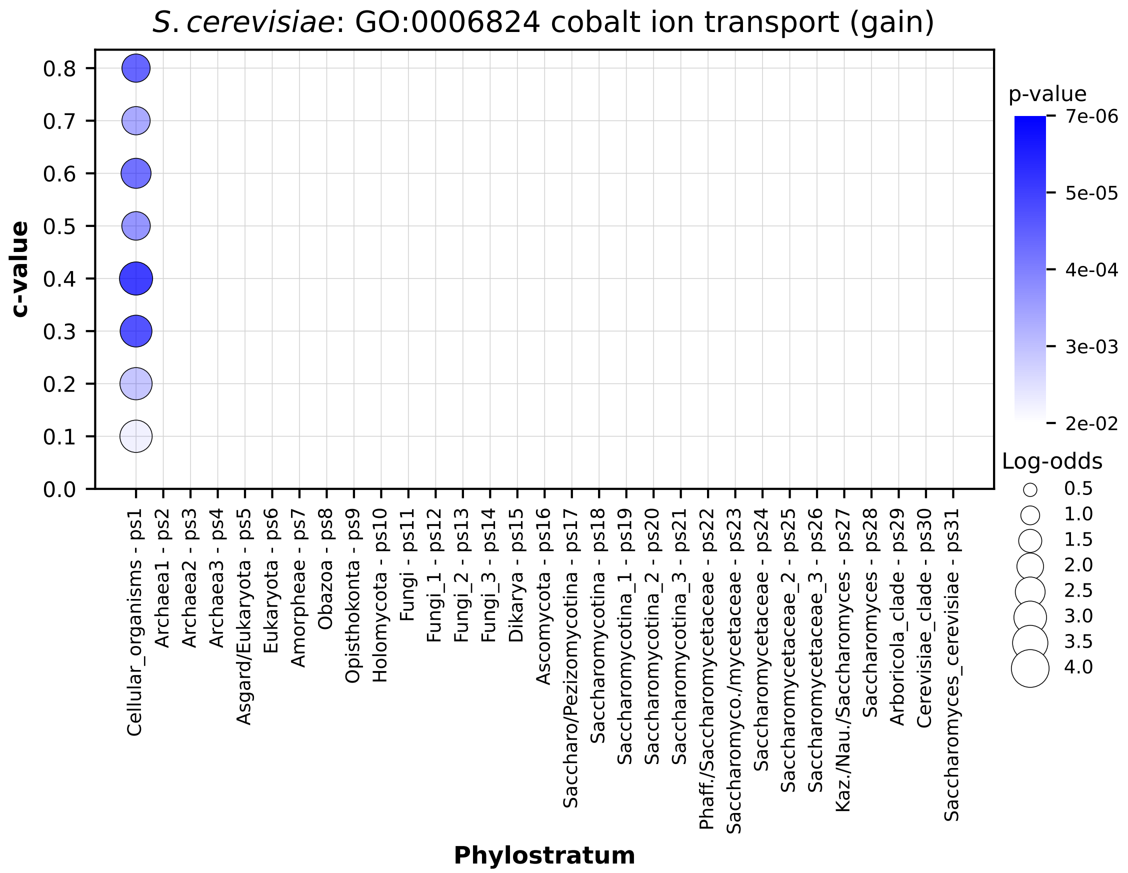

S. cerevisiae: GO:0006824 cobalt ion transport (gain)
c-value
Phylostratum
p-value
7e-06
5e-05
4e-04
3e-03
2e-02
Log-odds
0.5
1.0
1.5
2.0
2.5
3.0
3.5
4.0
Cellular_organisms - ps1
Archaea1 - ps2
Archaea2 - ps3
Archaea3 - ps4
Asgard/Eukaryota - ps5
Eukaryota - ps6
Amorpheae - ps7
Obazoa - ps8
Opisthokonta - ps9
Holomycota - ps10
Fungi - ps11
Fungi_1 - ps12
Fungi_2 - ps13
Fungi_3 - ps14
Dikarya - ps15
Ascomycota - ps16
Saccharo/Pezizomycotina - ps17
Saccharomycotina - ps18
Saccharomycotina_1 - ps19
Saccharomycotina_2 - ps20
Saccharomycotina_3 - ps21
Phaff./Saccharomycetaceae - ps22
Saccharomyco./mycetaceae - ps23
Saccharomycetaceae - ps24
Saccharomycetaceae_2 - ps25
Saccharomycetaceae_3 - ps26
Kaz./Nau./Saccharomyces - ps27
Saccharomyces - ps28
Arboricola_clade - ps29
Cerevisiae_clade - ps30
Saccharomyces_cerevisiae - ps31

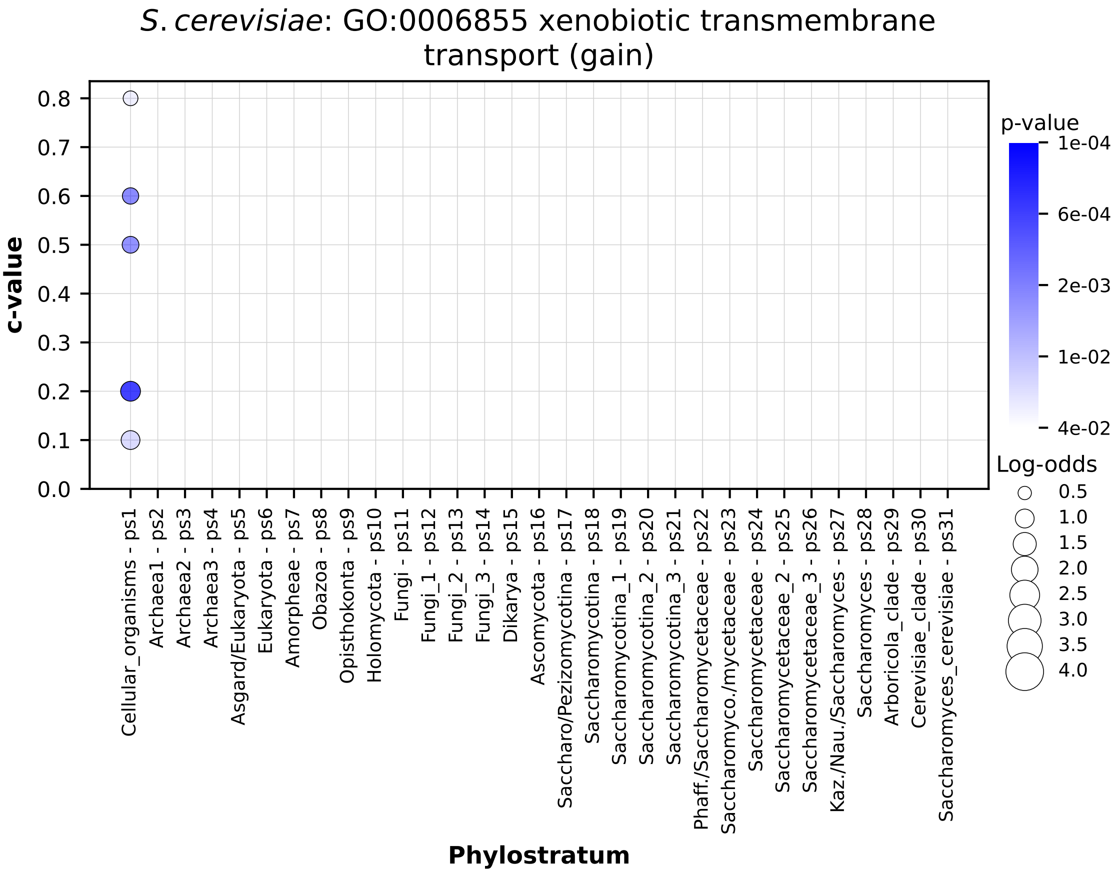

S. cerevisiae: GO:0006855 xenobiotic transmembrane transport (gain)
c-value
0.8
0.7
0.6
0.5
0.4
0.3
0.2
0.1
0.0
p-value
1e-04
6e-04
2e-03
1e-02
4e-02
Log-odds
0.5
1.0
1.5
2.0
2.5
3.0
3.5
4.0
Cellular_organisms - ps1
Archaea1 - ps2
Archaea2 - ps3
Archaea3 - ps4
Asgard/Eukaryota - ps5
Eukaryota - ps6
Amorpheae - ps7
Obazoa - ps8
Opisthokonta - ps9
Holomycota - ps10
Fungi - ps11
Fungi_1 - ps12
Fungi_2 - ps13
Fungi_3 - ps14
Dikarya - ps15
Ascomycota - ps16
Saccharo/Pezizomycotina - ps17
Saccharomycotina - ps18
Saccharomycotina_1 - ps19
Saccharomycotina_2 - ps20
Saccharomycotina_3 - ps21
Phaff./Saccharomycetaceae - ps22
Saccharomyco./mycetaceae - ps23
Saccharomycetaceae - ps24
Saccharomycetaceae_2 - ps25
Saccharomycetaceae_3 - ps26
Kaz./Nau./Saccharomyces - ps27
Saccharomyces - ps28
Arboricola_clade - ps29
Cerevisiae_clade - ps30
Saccharomyces_cerevisiae - ps31
Phylostratum

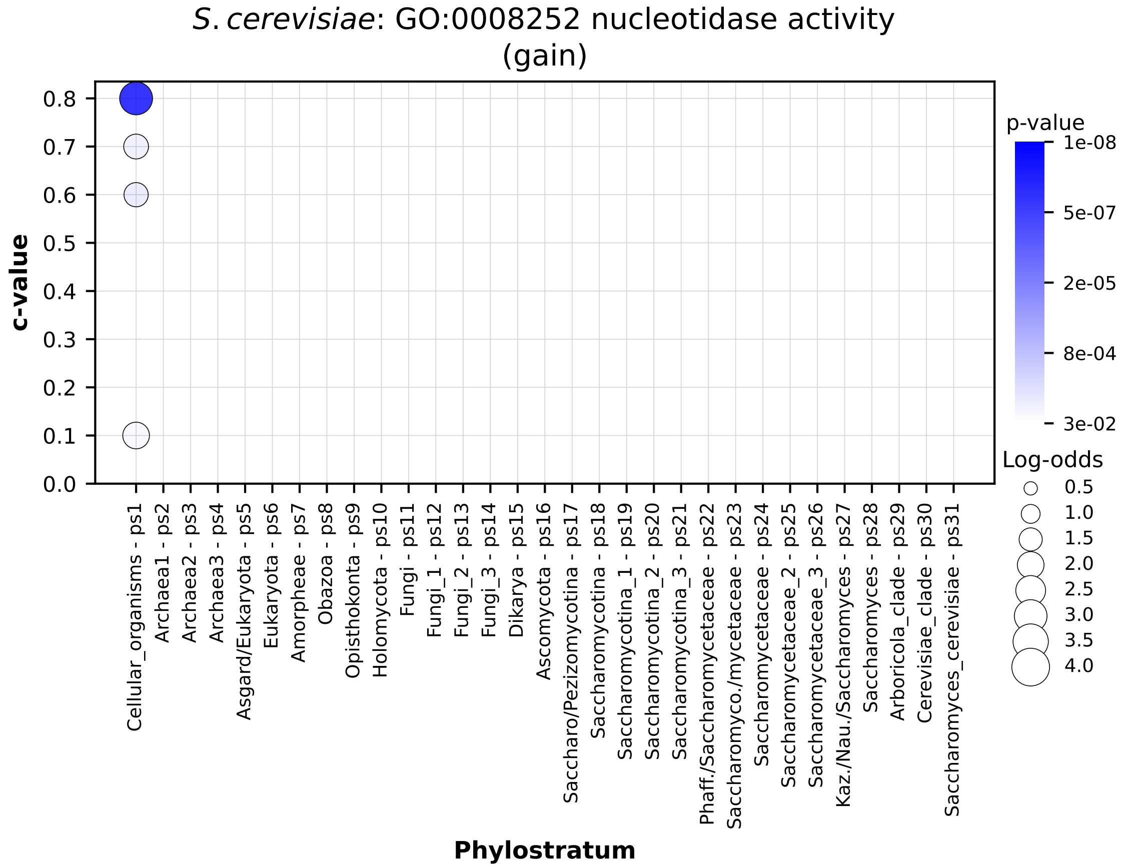

S. cerevisiae: GO:0008252 nucleotidase activity
(gain)
c-value
0.0
0.1
0.2
0.3
0.4
0.5
0.6
0.7
0.8
p-value
1e-08
5e-07
2e-05
8e-04
3e-02
Log-odds
0.5
1.0
1.5
2.0
2.5
3.0
3.5
4.0
Cellular_organisms - ps1
Archaea1 - ps2
Archaea2 - ps3
Archaea3 - ps4
Asgard/Eukaryota - ps5
Eukaryota - ps6
Amorpheae - ps7
Obazoa - ps8
Opisthokonta - ps9
Holomycota - ps10
Fungi - ps11
Fungi_1 - ps12
Fungi_2 - ps13
Fungi_3 - ps14
Dikarya - ps15
Ascomycota - ps16
Saccharo/Pezizomycotina - ps17
Saccharomycotina - ps18
Saccharomycotina_1 - ps19
Saccharomycotina_2 - ps20
Saccharomycotina_3 - ps21
Phaff./Saccharomycetaceae - ps22
Saccharomyco./mycetaceae - ps23
Saccharomycetaceae - ps24
Saccharomycetaceae_2 - ps25
Saccharomycetaceae_3 - ps26
Kaz./Nau./Saccharomyces - ps27
Saccharomyces - ps28
Arboricola_clade - ps29
Cerevisiae_clade - ps30
Saccharomyces_cerevisiae - ps31
Phylostratum

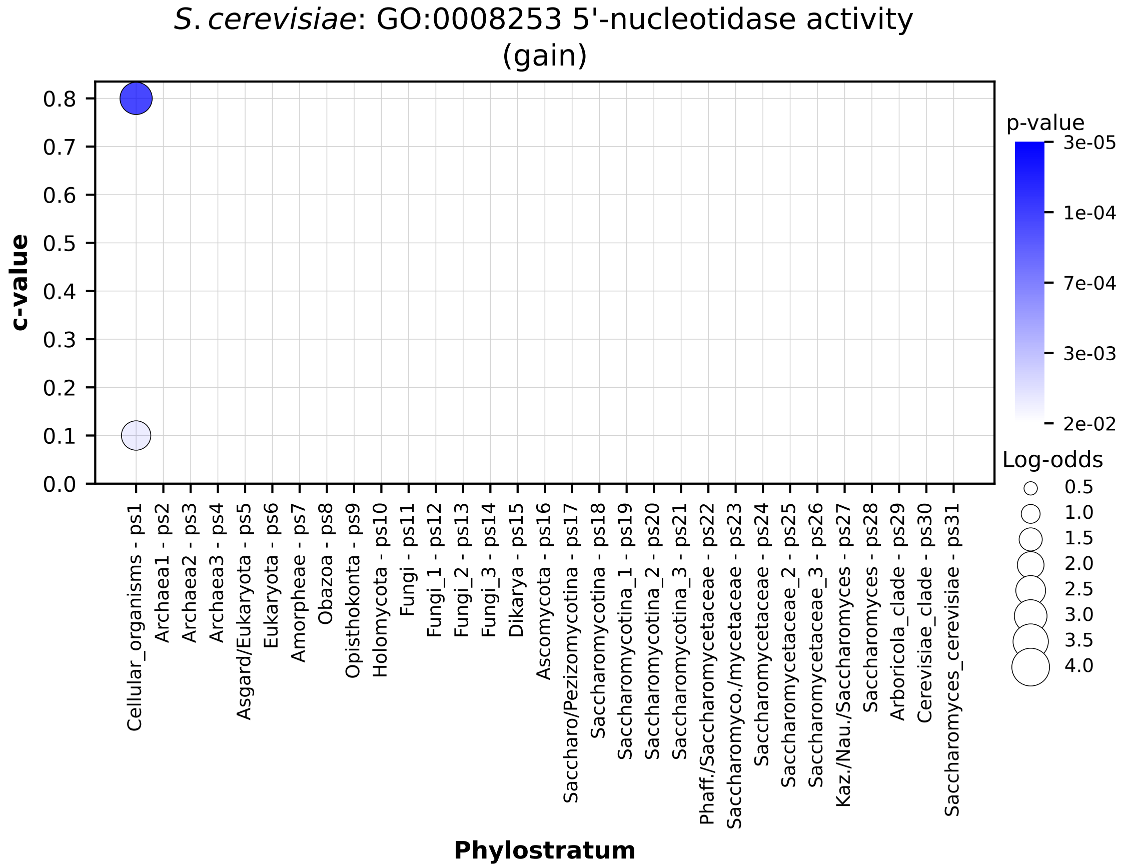

S. cerevisiae: GO:0008253 5'-nucleotidase activity (gain)
c-value
0.0
0.1
0.2
0.3
0.4
0.5
0.6
0.7
0.8
Phylostratum
Cellular_organisms - ps1
Archaea1 - ps2
Archaea2 - ps3
Archaea3 - ps4
Asgard/Eukaryota - ps5
Eukaryota - ps6
Amorpheae - ps7
Obazoa - ps8
Opisthokonta - ps9
Holomycota - ps10
Fungi - ps11
Fungi_1 - ps12
Fungi_2 - ps13
Fungi_3 - ps14
Dikarya - ps15
Ascomycota - ps16
Saccharo/Pezizomycotina - ps17
Saccharomycotina - ps18
Saccharomycotina_1 - ps19
Saccharomycotina_2 - ps20
Saccharomycotina_3 - ps21
Phaff./Saccharomycetaceae - ps22
Saccharomyco./mycetaceae - ps23
Saccharomycetaceae - ps24
Saccharomycetaceae_2 - ps25
Saccharomycetaceae_3 - ps26
Kaz./Nau./Saccharomyces - ps27
Saccharomyces - ps28
Arboricola_clade - ps29
Cerevisiae_clade - ps30
Saccharomyces_cerevisiae - ps31
p-value
3e-05
1e-04
7e-04
3e-03
2e-02
Log-odds
0.5
1.0
1.5
2.0
2.5
3.0
3.5
4.0

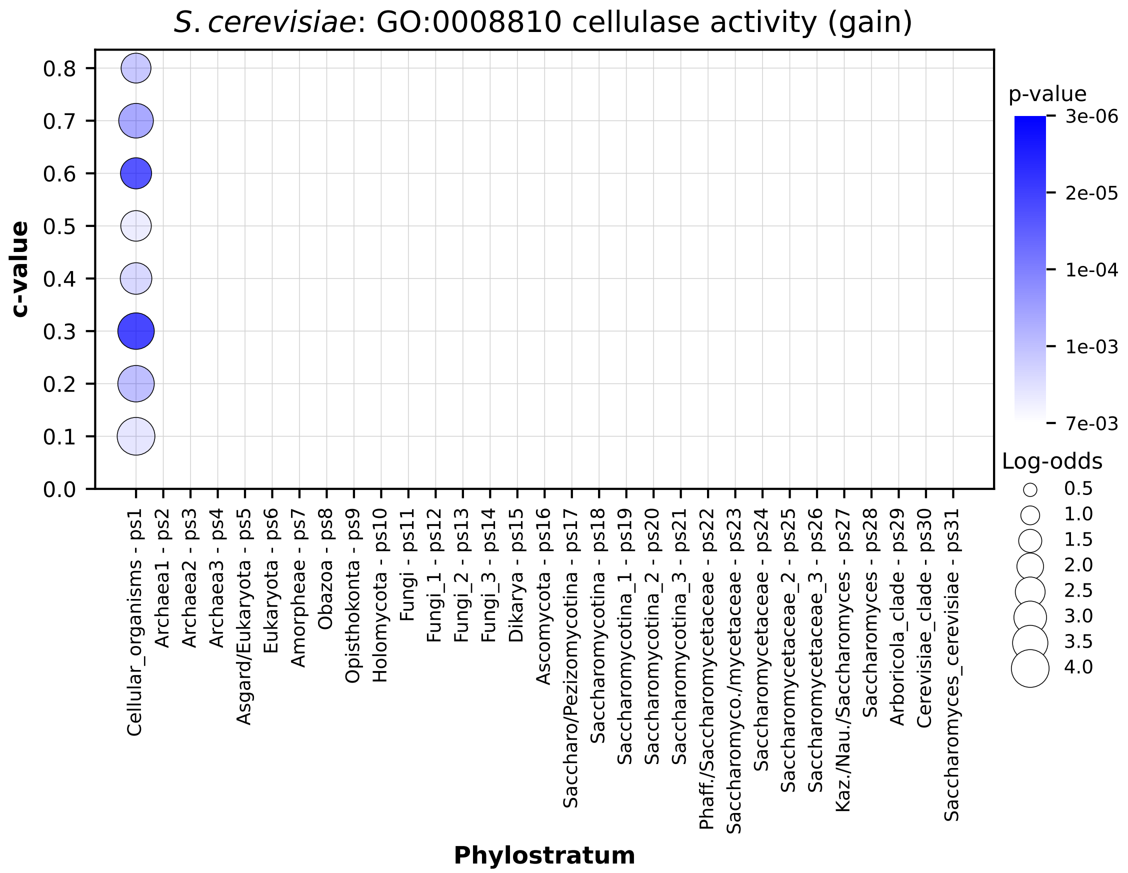
S. cerevisiae: GO:0008810 cellulase activity (gain)
c-value
0.0
0.1
0.2
0.3
0.4
0.5
0.6
0.7
0.8
p-value
3e-06
2e-05
1e-04
1e-03
7e-03
Log-odds
0.5
1.0
1.5
2.0
2.5
3.0
3.5
4.0
Cellular_organisms - ps1
Archaea1 - ps2
Archaea2 - ps3
Archaea3 - ps4
Asgard/Eukaryota - ps5
Eukaryota - ps6
Amorpheae - ps7
Obazoa - ps8
Opisthokonta - ps9
Holomycota - ps10
Fungi - ps11
Fungi_1 - ps12
Fungi_2 - ps13
Fungi_3 - ps14
Dikarya - ps15
Ascomycota - ps16
Saccharo/Pezizomycotina - ps17
Saccharomycotina - ps18
Saccharomycotina_1 - ps19
Saccharomycotina_2 - ps20
Saccharomycotina_3 - ps21
Phaff./Saccharomycetaceae - ps22
Saccharomyco./mycetaceae - ps23
Saccharomycetaceae - ps24
Saccharomycetaceae_2 - ps25
Saccharomycetaceae_3 - ps26
Kaz./Nau./Saccharomyces - ps27
Saccharomyces - ps28
Arboricola_clade - ps29
Cerevisiae_clade - ps30
Saccharomyces_cerevisiae - ps31
Phylostratum

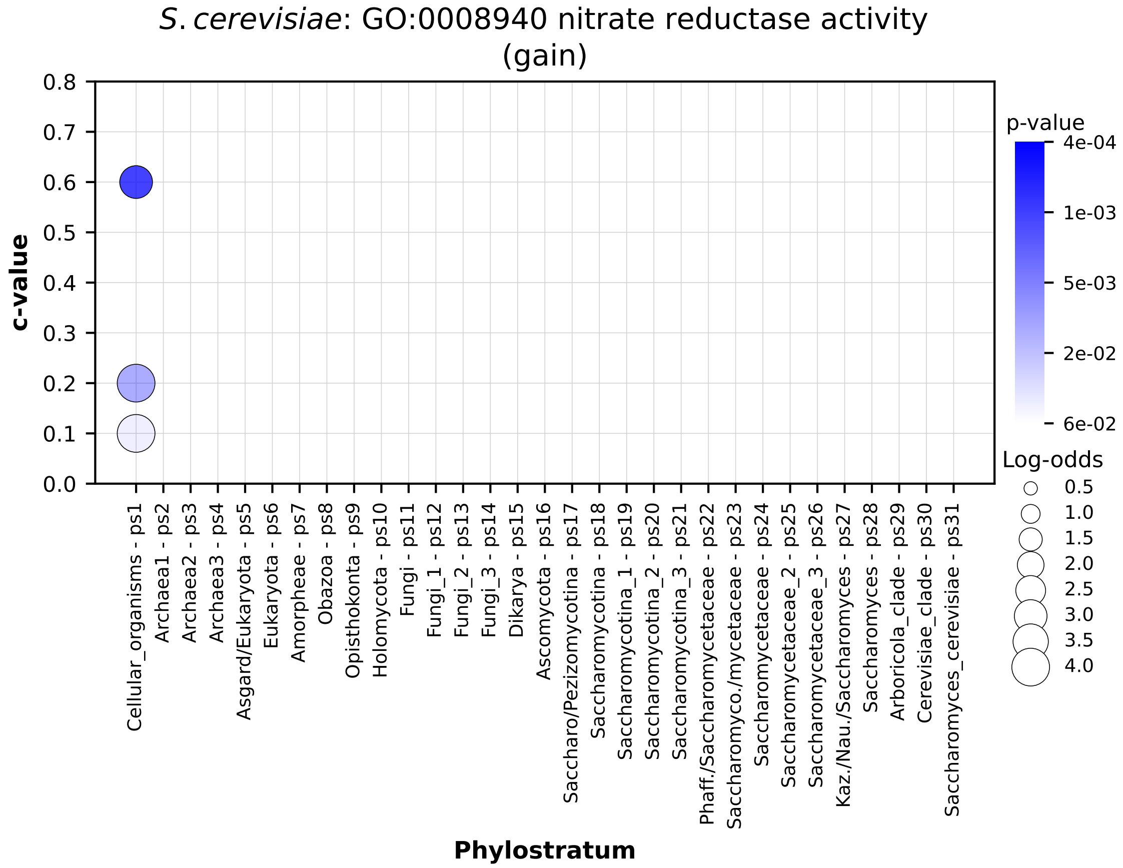

S. cerevisiae: GO:0008940 nitrate reductase activity (gain)
c-value
0.0
0.1
0.2
0.3
0.4
0.5
0.6
0.7
0.8
Phylostratum
Cellular_organisms - ps1
Archaea1 - ps2
Archaea2 - ps3
Archaea3 - ps4
Asgard/Eukaryota - ps5
Eukaryota - ps6
Amorpheae - ps7
Obazoa - ps8
Opisthokonta - ps9
Holomycota - ps10
Fungi - ps11
Fungi_1 - ps12
Fungi_2 - ps13
Fungi_3 - ps14
Dikarya - ps15
Ascomycota - ps16
Saccharo/Pezizomycotina - ps17
Saccharomycotina - ps18
Saccharomycotina_1 - ps19
Saccharomycotina_2 - ps20
Saccharomycotina_3 - ps21
Phaff./Saccharomycetaceae - ps22
Saccharomyco./mycetaceae - ps23
Saccharomycetaceae - ps24
Saccharomycetaceae_2 - ps25
Saccharomycetaceae_3 - ps26
Kaz./Nau./Saccharomyces - ps27
Saccharomyces - ps28
Arboricola_clade - ps29
Cerevisiae_clade - ps30
Saccharomyces_cerevisiae - ps31
p-value
4e-04
1e-03
5e-03
2e-02
6e-02
Log-odds
0.5
1.0
1.5
2.0
2.5
3.0
3.5
4.0

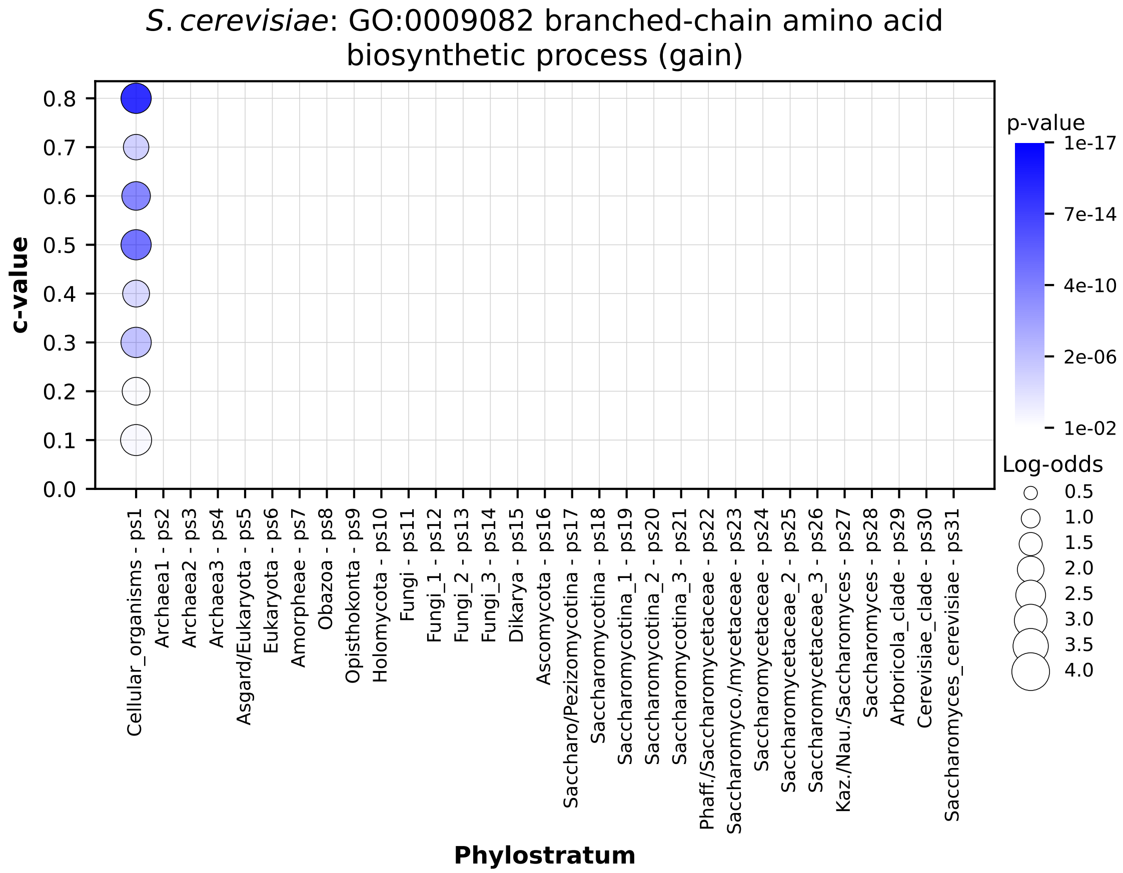
S. cerevisiae: GO:0009082 branched-chain amino acid biosynthetic process (gain)
c-value
0.0
0.1
0.2
0.3
0.4
0.5
0.6
0.7
0.8
Phylostratum
Cellular_organisms - ps1
Archaea1 - ps2
Archaea2 - ps3
Archaea3 - ps4
Asgard/Eukaryota - ps5
Eukaryota - ps6
Amorpheae - ps7
Obazoa - ps8
Opisthokonta - ps9
Holomycota - ps10
Fungi - ps11
Fungi_1 - ps12
Fungi_2 - ps13
Fungi_3 - ps14
Dikarya - ps15
Ascomycota - ps16
Saccharo/Pezizomycotina - ps17
Saccharomycotina - ps18
Saccharomycotina_1 - ps19
Saccharomycotina_2 - ps20
Saccharomycotina_3 - ps21
Phaff./Saccharomycetaceae - ps22
Saccharomyco./mycetaceae - ps23
Saccharomycetaceae - ps24
Saccharomycetaceae_2 - ps25
Saccharomycetaceae_3 - ps26
Kaz./Nau./Saccharomyces - ps27
Saccharomyces - ps28
Arboricola_clade - ps29
Cerevisiae_clade - ps30
Saccharomyces_cerevisiae - ps31
p-value
1e-17
7e-14
4e-10
2e-06
1e-02
Log-odds
0.5
1.0
1.5
2.0
2.5
3.0
3.5
4.0

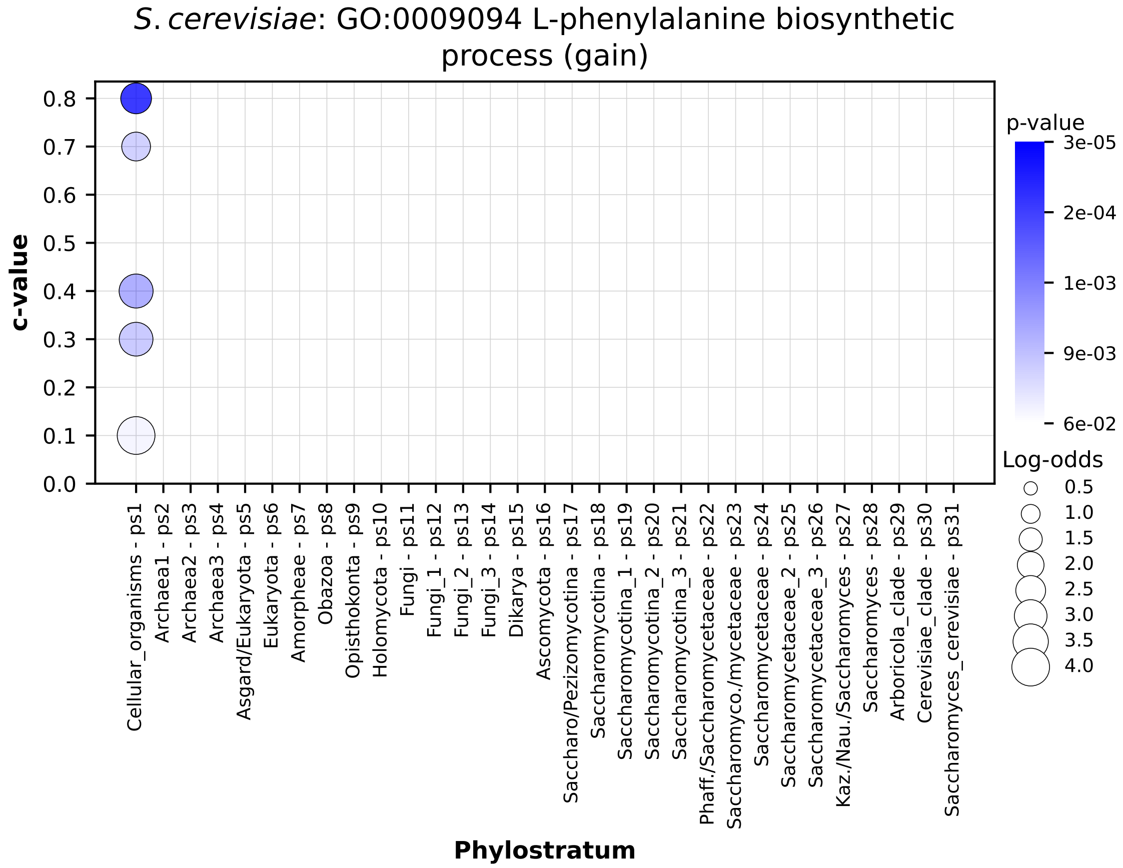

S. cerevisiae: GO:0009094 L-phenylalanine biosynthetic process (gain)
c-value
0.0
0.1
0.2
0.3
0.4
0.5
0.6
0.7
0.8
p-value
3e-05
2e-04
1e-03
9e-03
6e-02
Log-odds
0.5
1.0
1.5
2.0
2.5
3.0
3.5
4.0
Cellular_organisms - ps1
Archaea1 - ps2
Archaea2 - ps3
Archaea3 - ps4
Asgard/Eukaryota - ps5
Eukaryota - ps6
Amorpheae - ps7
Obazoa - ps8
Opisthokonta - ps9
Holomycota - ps10
Fungi - ps11
Fungi_1 - ps12
Fungi_2 - ps13
Fungi_3 - ps14
Dikarya - ps15
Ascomycota - ps16
Saccharo/Pezizomycotina - ps17
Saccharomycotina - ps18
Saccharomycotina_1 - ps19
Saccharomycotina_2 - ps20
Saccharomycotina_3 - ps21
Phaff./Saccharomycetaceae - ps22
Saccharomyco./mycetaceae - ps23
Saccharomycetaceae - ps24
Saccharomycetaceae_2 - ps25
Saccharomycetaceae_3 - ps26
Kaz./Nau./Saccharomyces - ps27
Saccharomyces - ps28
Arboricola_clade - ps29
Cerevisiae_clade - ps30
Saccharomyces_cerevisiae - ps31
Phylostratum

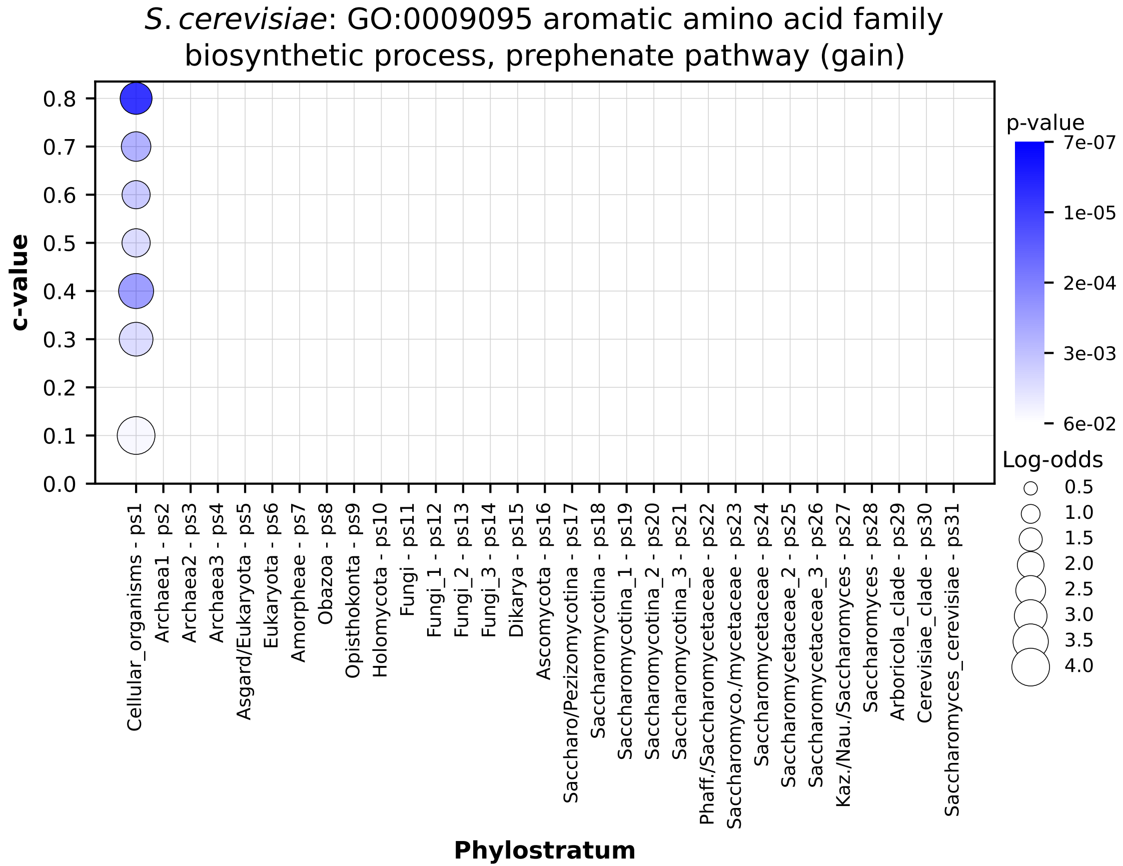

S. cerevisiae: GO:0009095 aromatic amino acid family biosynthetic process, prephenate pathway (gain)
c-value
0.0
0.1
0.2
0.3
0.4
0.5
0.6
0.7
0.8
Phylostratum
Cellular_organisms - ps1
Archaea1 - ps2
Archaea2 - ps3
Archaea3 - ps4
Asgard/Eukaryota - ps5
Eukaryota - ps6
Amorpheae - ps7
Obazoa - ps8
Opisthokonta - ps9
Holomycota - ps10
Fungi - ps11
Fungi_1 - ps12
Fungi_2 - ps13
Fungi_3 - ps14
Dikarya - ps15
Ascomycota - ps16
Saccharo/Pezizomycotina - ps17
Saccharomycotina - ps18
Saccharomycotina_1 - ps19
Saccharomycotina_2 - ps20
Saccharomycotina_3 - ps21
Phaff./Saccharomycetaceae - ps22
Saccharomyco./mycetaceae - ps23
Saccharomycetaceae - ps24
Saccharomycetaceae_2 - ps25
Saccharomycetaceae_3 - ps26
Kaz./Nau./Saccharomyces - ps27
Saccharomyces - ps28
Arboricola_clade - ps29
Cerevisiae_clade - ps30
Saccharomyces_cerevisiae - ps31
p-value
7e-07
1e-05
2e-04
3e-03
6e-02
Log-odds
0.5
1.0
1.5
2.0
2.5
3.0
3.5
4.0

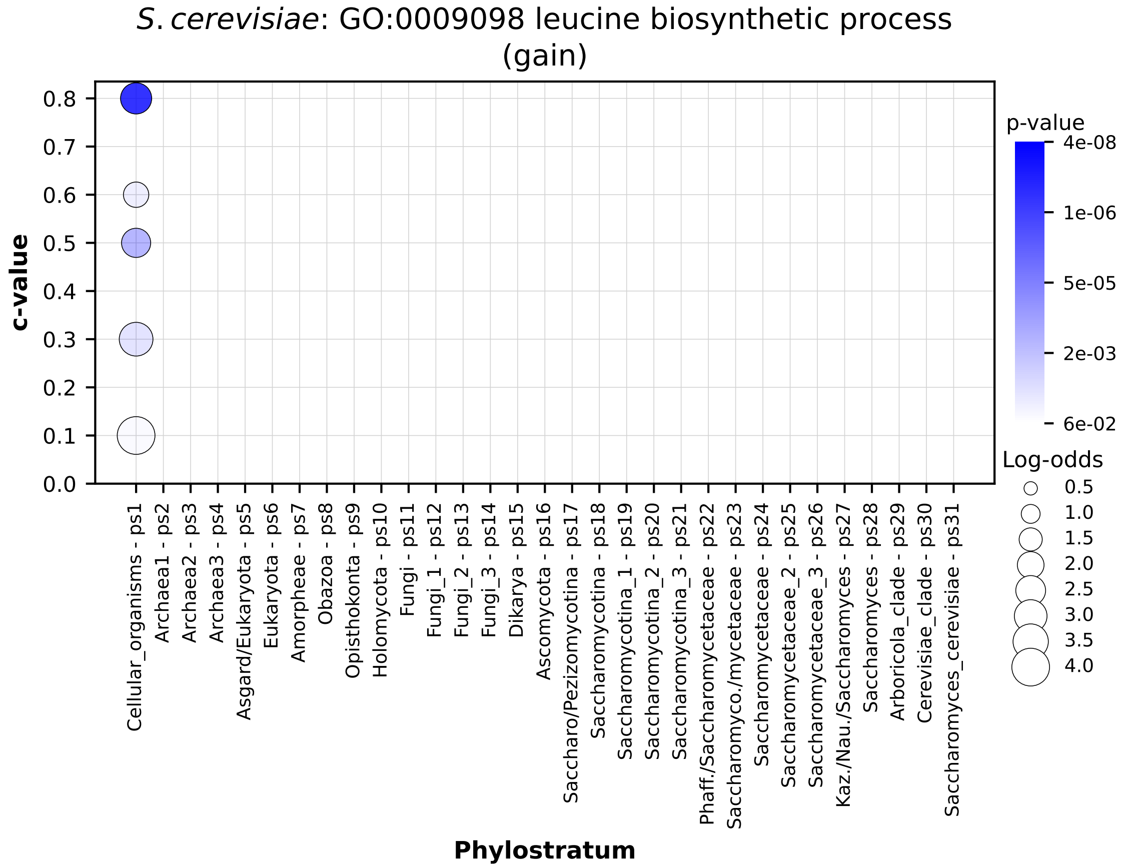

S. cerevisiae: GO:0009098 leucine biosynthetic process (gain)
c-value
0.0
0.1
0.2
0.3
0.4
0.5
0.6
0.7
0.8
p-value
4e-08
1e-06
5e-05
2e-03
6e-02
Log-odds
0.5
1.0
1.5
2.0
2.5
3.0
3.5
4.0
Cellular_organisms - ps1
Archaea1 - ps2
Archaea2 - ps3
Archaea3 - ps4
Asgard/Eukaryota - ps5
Eukaryota - ps6
Amorpheae - ps7
Obazoa - ps8
Opisthokonta - ps9
Holomycota - ps10
Fungi - ps11
Fungi_1 - ps12
Fungi_2 - ps13
Fungi_3 - ps14
Dikarya - ps15
Ascomycota - ps16
Saccharo/Pezizomycotina - ps17
Saccharomycotina - ps18
Saccharomycotina_1 - ps19
Saccharomycotina_2 - ps20
Saccharomycotina_3 - ps21
Phaff./Saccharomycetaceae - ps22
Saccharomyco./mycetaceae - ps23
Saccharomycetaceae - ps24
Saccharomycetaceae_2 - ps25
Saccharomycetaceae_3 - ps26
Kaz./Nau./Saccharomyces - ps27
Saccharomyces - ps28
Arboricola_clade - ps29
Cerevisiae_clade - ps30
Saccharomyces_cerevisiae - ps31
Phylostratum

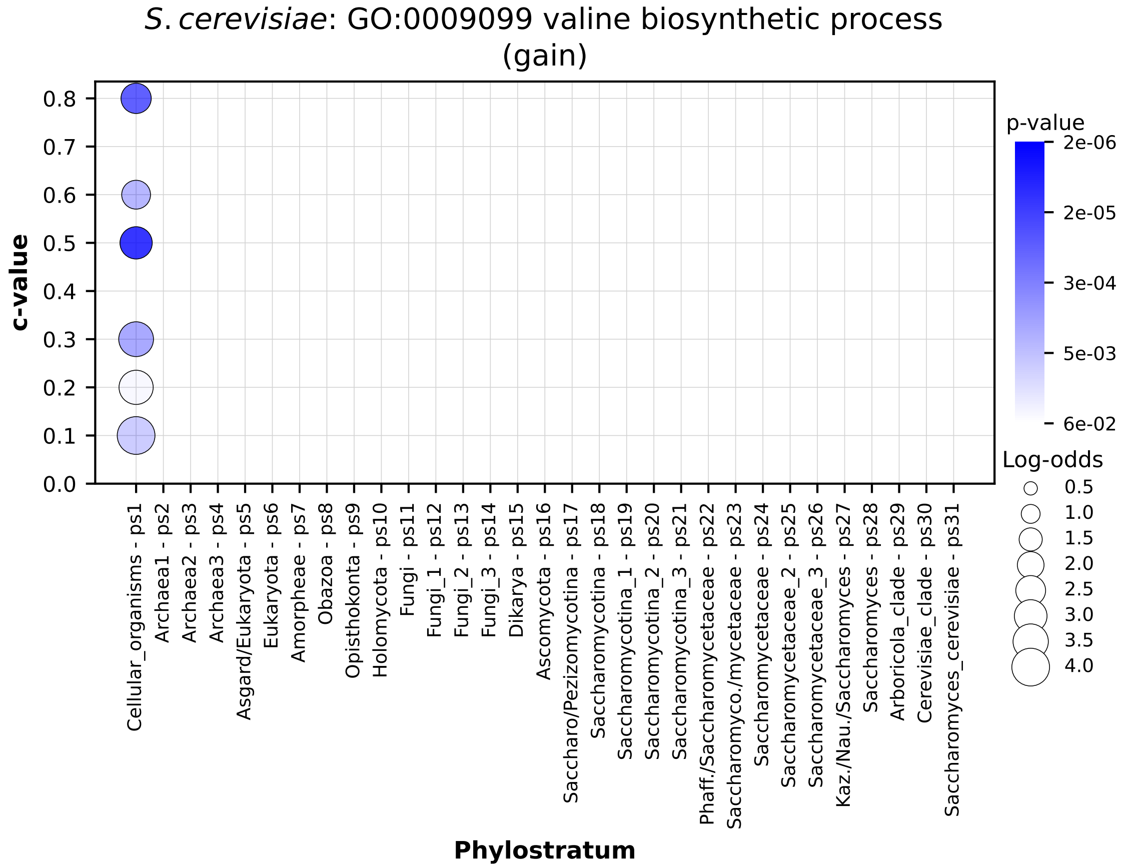
S. cerevisiae: GO:0009099 valine biosynthetic process
(gain)
c-value
0.8
0.7
0.6
0.5
0.4
0.3
0.2
0.1
0.0
p-value
2e-06
2e-05
3e-04
5e-03
6e-02
Log-odds
0.5
1.0
1.5
2.0
2.5
3.0
3.5
4.0
Cellular_organisms - ps1
Archaea1 - ps2
Archaea2 - ps3
Archaea3 - ps4
Asgard/Eukaryota - ps5
Eukaryota - ps6
Amorpheae - ps7
Obazoa - ps8
Opisthokonta - ps9
Holomycota - ps10
Fungi - ps11
Fungi_1 - ps12
Fungi_2 - ps13
Fungi_3 - ps14
Dikarya - ps15
Ascomycota - ps16
Saccharo/Pezizomycotina - ps17
Saccharomycotina - ps18
Saccharomycotina_1 - ps19
Saccharomycotina_2 - ps20
Saccharomycotina_3 - ps21
Phaff./Saccharomycetaceae - ps22
Saccharomyco./mycetaceae - ps23
Saccharomycetaceae - ps24
Saccharomycetaceae_2 - ps25
Saccharomycetaceae_3 - ps26
Kaz./Nau./Saccharomyces - ps27
Saccharomyces - ps28
Arboricola_clade - ps29
Cerevisiae_clade - ps30
Saccharomyces_cerevisiae - ps31
Phylostratum

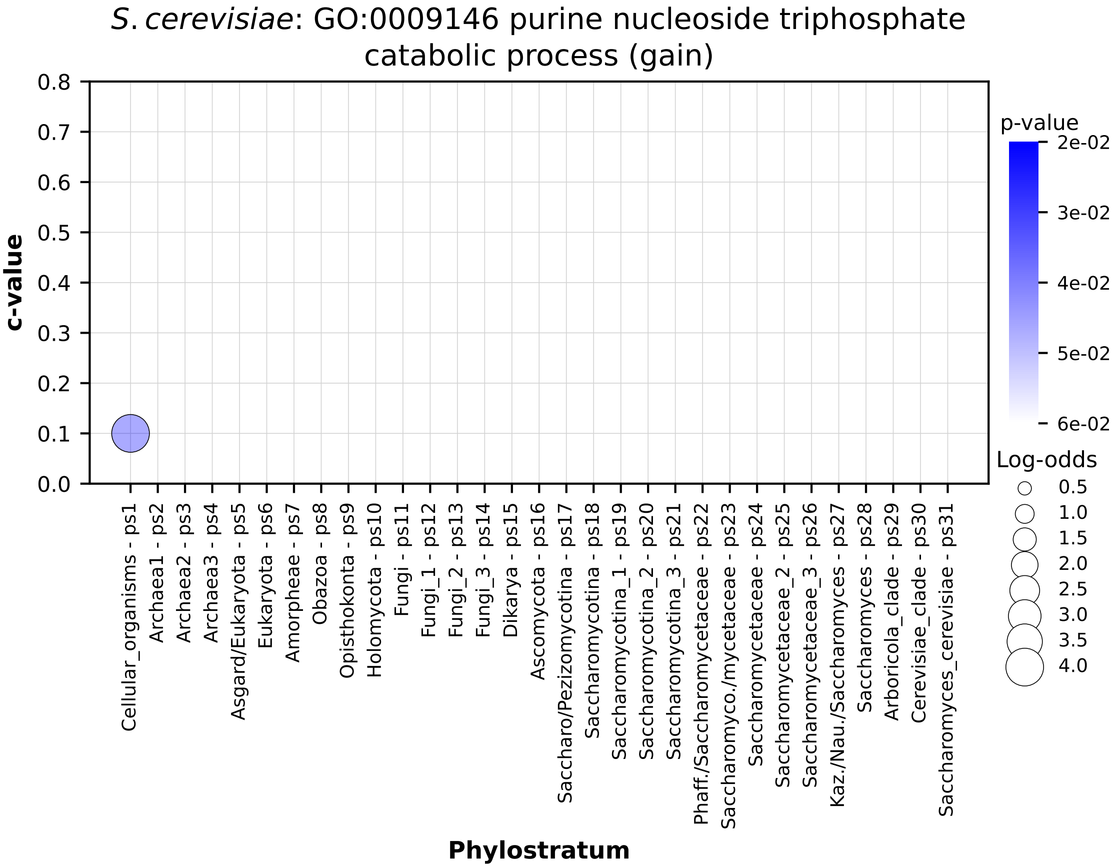

S. cerevisiae: GO:0009146 purine nucleoside triphosphate catabolic process (gain)
c-value
0.8
0.7
0.6
0.5
0.4
0.3
0.2
0.1
0.0
p-value
2e-02
3e-02
4e-02
5e-02
6e-02
Log-odds
0.5
1.0
1.5
2.0
2.5
3.0
3.5
4.0
Cellular_organisms - ps1
Archaea1 - ps2
Archaea2 - ps3
Archaea3 - ps4
Asgard/Eukaryota - ps5
Eukaryota - ps6
Amorpheae - ps7
Obazoa - ps8
Opisthokonta - ps9
Holomycota - ps10
Fungi - ps11
Fungi_1 - ps12
Fungi_2 - ps13
Fungi_3 - ps14
Dikarya - ps15
Ascomycota - ps16
Saccharo/Pezizomycotina - ps17
Saccharomycotina - ps18
Saccharomycotina_1 - ps19
Saccharomycotina_2 - ps20
Saccharomycotina_3 - ps21
Phaff./Saccharomycetaceae - ps22
Saccharomyco./mycetaceae - ps23
Saccharomycetaceae - ps24
Saccharomycetaceae_2 - ps25
Saccharomycetaceae_3 - ps26
Kaz./Nau./Saccharomyces - ps27
Saccharomyces - ps28
Arboricola_clade - ps29
Cerevisiae_clade - ps30
Saccharomyces_cerevisiae - ps31
Phylostratum

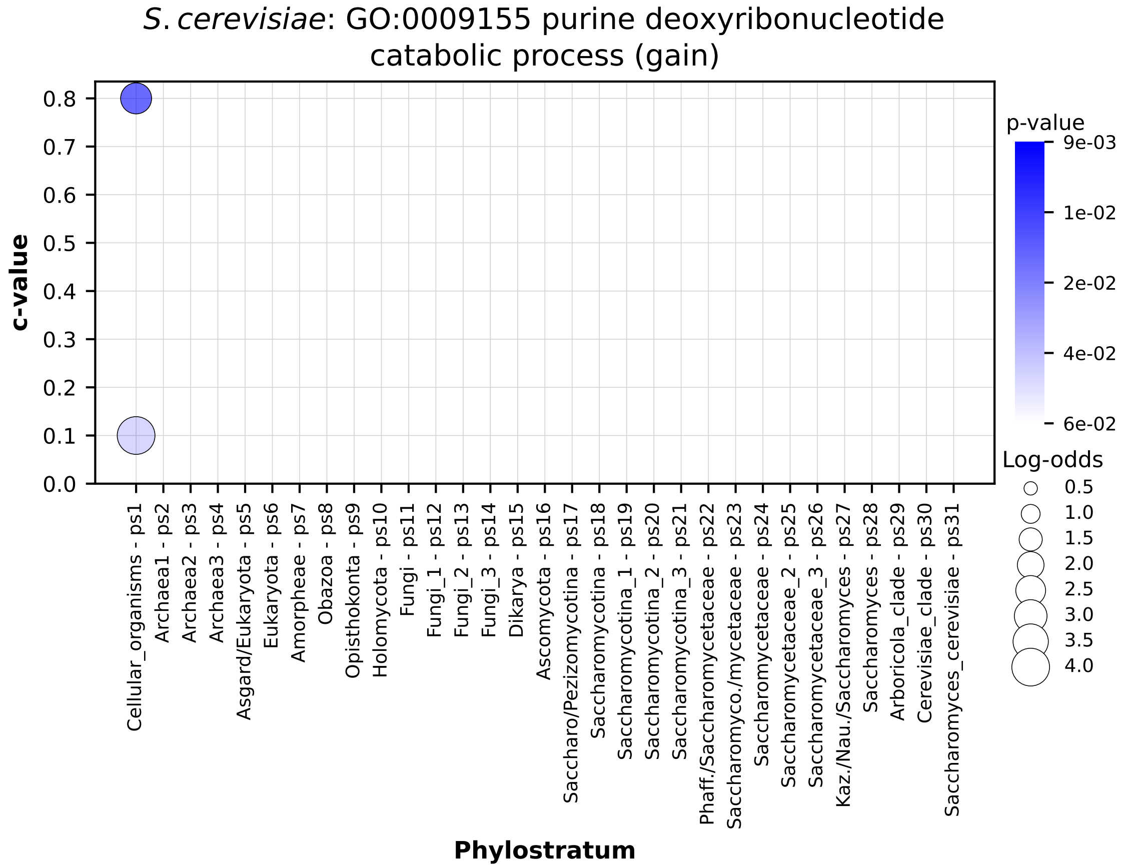
S. cerevisiae: GO:0009155 purine deoxyribonucleotide catabolic process (gain)
c-value
0.0
0.1
0.2
0.3
0.4
0.5
0.6
0.7
0.8
p-value
9e-03
1e-02
2e-02
4e-02
6e-02
Log-odds
0.5
1.0
1.5
2.0
2.5
3.0
3.5
4.0
Cellular_organisms - ps1
Archaea1 - ps2
Archaea2 - ps3
Archaea3 - ps4
Asgard/Eukaryota - ps5
Eukaryota - ps6
Amorpheae - ps7
Obazoa - ps8
Opisthokonta - ps9
Holomycota - ps10
Fungi - ps11
Fungi_1 - ps12
Fungi_2 - ps13
Fungi_3 - ps14
Dikarya - ps15
Ascomycota - ps16
Saccharo/Pezizomycotina - ps17
Saccharomycotina - ps18
Saccharomycotina_1 - ps19
Saccharomycotina_2 - ps20
Saccharomycotina_3 - ps21
Phaff./Saccharomycetaceae - ps22
Saccharomyco./mycetaceae - ps23
Saccharomycetaceae - ps24
Saccharomycetaceae_2 - ps25
Saccharomycetaceae_3 - ps26
Kaz./Nau./Saccharomyces - ps27
Saccharomyces - ps28
Arboricola_clade - ps29
Cerevisiae_clade - ps30
Saccharomyces_cerevisiae - ps31
Phylostratum

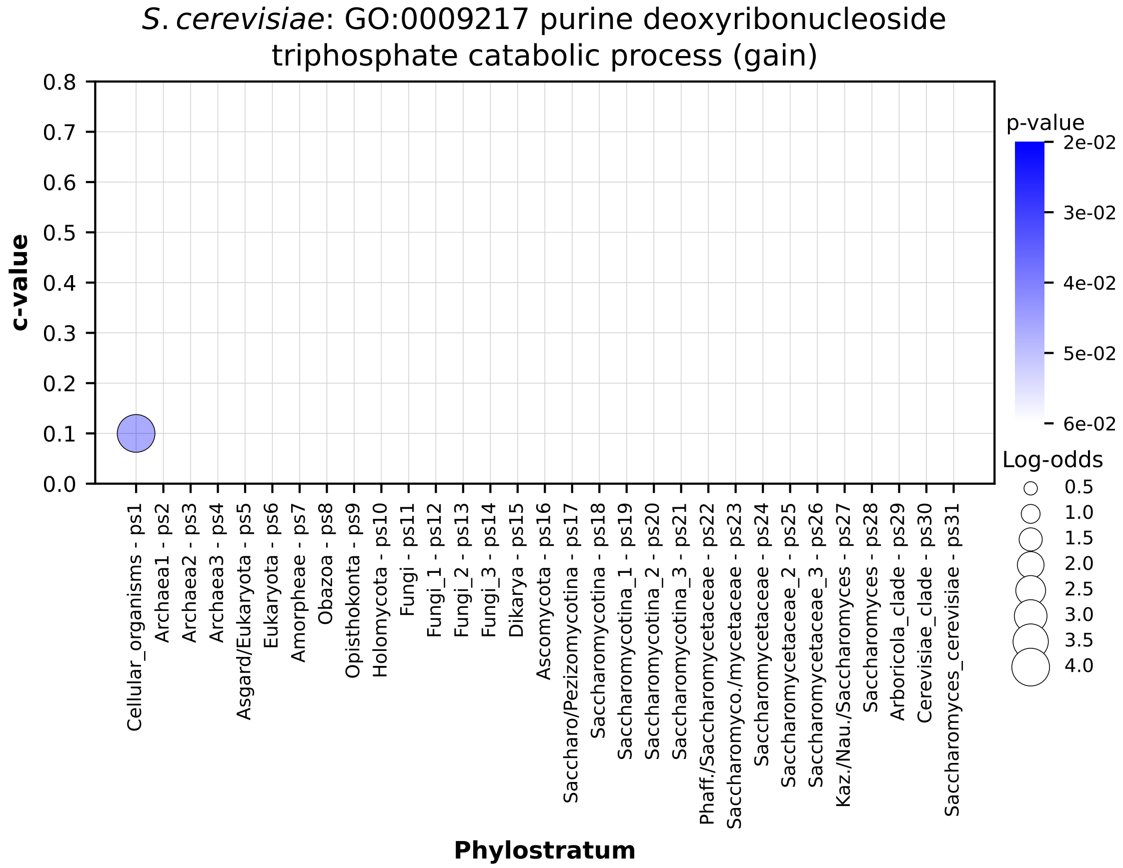
S. cerevisiae: GO:0009217 purine deoxyribonucleoside triphosphate catabolic process (gain)
c-value
0.0
0.1
0.2
0.3
0.4
0.5
0.6
0.7
0.8
p-value
2e-02
3e-02
4e-02
5e-02
6e-02
Log-odds
0.5
1.0
1.5
2.0
2.5
3.0
3.5
4.0
Cellular_organisms - ps1
Archaea1 - ps2
Archaea2 - ps3
Archaea3 - ps4
Asgard/Eukaryota - ps5
Eukaryota - ps6
Amorpheae - ps7
Obazoa - ps8
Opisthokonta - ps9
Holomycota - ps10
Fungi - ps11
Fungi_1 - ps12
Fungi_2 - ps13
Fungi_3 - ps14
Dikarya - ps15
Ascomycota - ps16
Saccharo/Pezizomycotina - ps17
Saccharomycotina - ps18
Saccharomycotina_1 - ps19
Saccharomycotina_2 - ps20
Saccharomycotina_3 - ps21
Phaff./Saccharomycetaceae - ps22
Saccharomyco./mycetaceae - ps23
Saccharomycetaceae - ps24
Saccharomycetaceae_2 - ps25
Saccharomycetaceae_3 - ps26
Kaz./Nau./Saccharomyces - ps27
Saccharomyces - ps28
Arboricola_clade - ps29
Cerevisiae_clade - ps30
Saccharomyces_cerevisiae - ps31
Phylostratum

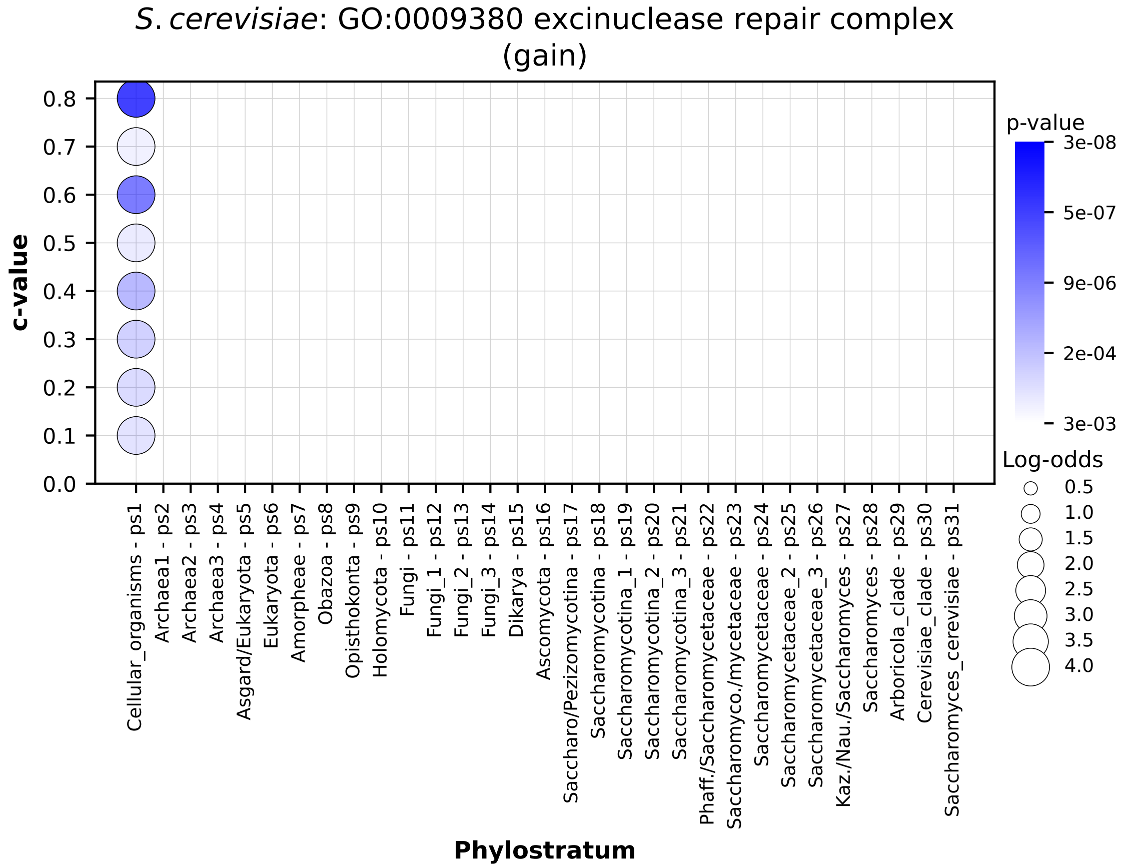

S. cerevisiae: GO:0009380 excinuclease repair complex (gain)
c-value
0.0
0.1
0.2
0.3
0.4
0.5
0.6
0.7
0.8
Phylostratum
Cellular_organisms - ps1
Archaea1 - ps2
Archaea2 - ps3
Archaea3 - ps4
Asgard/Eukaryota - ps5
Eukaryota - ps6
Amorpheae - ps7
Obazoa - ps8
Opisthokonta - ps9
Holomycota - ps10
Fungi - ps11
Fungi_1 - ps12
Fungi_2 - ps13
Fungi_3 - ps14
Dikarya - ps15
Ascomycota - ps16
Saccharo/Pezizomycotina - ps17
Saccharomycotina - ps18
Saccharomycotina_1 - ps19
Saccharomycotina_2 - ps20
Saccharomycotina_3 - ps21
Phaff./Saccharomycetaceae - ps22
Saccharomyco./mycetaceae - ps23
Saccharomycetaceae - ps24
Saccharomycetaceae_2 - ps25
Saccharomycetaceae_3 - ps26
Kaz./Nau./Saccharomyces - ps27
Saccharomyces - ps28
Arboricola_clade - ps29
Cerevisiae_clade - ps30
Saccharomyces_cerevisiae - ps31
p-value
3e-08
5e-07
9e-06
2e-04
3e-03
Log-odds
0.5
1.0
1.5
2.0
2.5
3.0
3.5
4.0

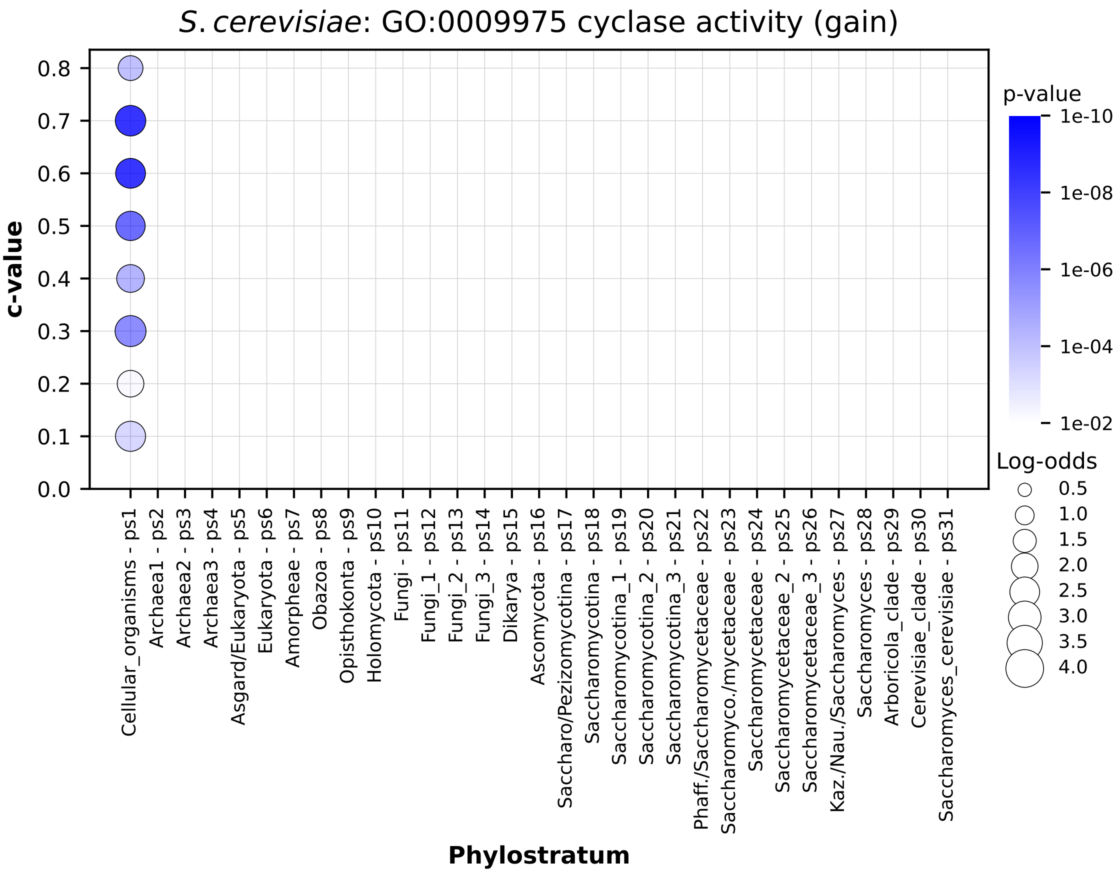

S. cerevisiae: GO:0009975 cyclase activity (gain)
c-value
0.0
0.1
0.2
0.3
0.4
0.5
0.6
0.7
0.8
p-value
1e-10
1e-08
1e-06
1e-04
1e-02
Log-odds
0.5
1.0
1.5
2.0
2.5
3.0
3.5
4.0
Cellular_organisms - ps1
Archaea1 - ps2
Archaea2 - ps3
Archaea3 - ps4
Asgard/Eukaryota - ps5
Eukaryota - ps6
Amorpheae - ps7
Obazoa - ps8
Opisthokonta - ps9
Holomycota - ps10
Fungi - ps11
Fungi_1 - ps12
Fungi_2 - ps13
Fungi_3 - ps14
Dikarya - ps15
Ascomycota - ps16
Saccharo/Pezizomycotina - ps17
Saccharomycotina - ps18
Saccharomycotina_1 - ps19
Saccharomycotina_2 - ps20
Saccharomycotina_3 - ps21
Phaff./Saccharomycetaceae - ps22
Saccharomyco./mycetaceae - ps23
Saccharomycetaceae - ps24
Saccharomycetaceae_2 - ps25
Saccharomycetaceae_3 - ps26
Kaz./Nau./Saccharomyces - ps27
Saccharomyces - ps28
Arboricola_clade - ps29
Cerevisiae_clade - ps30
Saccharomyces_cerevisiae - ps31
Phylostratum

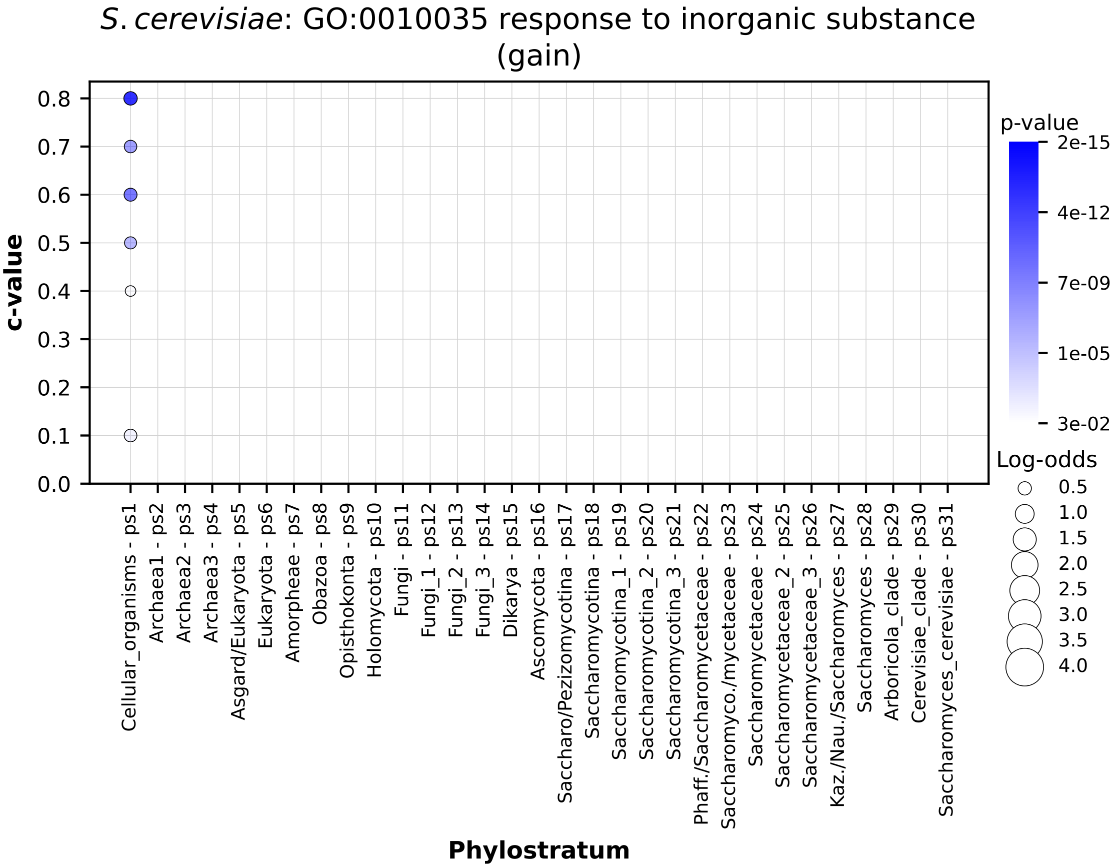

S. cerevisiae: GO:0010035 response to inorganic substance (gain)
c-value
0.0
0.1
0.2
0.3
0.4
0.5
0.6
0.7
0.8
Phylostratum
Cellular_organisms - ps1
Archaea1 - ps2
Archaea2 - ps3
Archaea3 - ps4
Asgard/Eukaryota - ps5
Eukaryota - ps6
Amorpheae - ps7
Obazoa - ps8
Opisthokonta - ps9
Holomycota - ps10
Fungi - ps11
Fungi_1 - ps12
Fungi_2 - ps13
Fungi_3 - ps14
Dikarya - ps15
Ascomycota - ps16
Saccharo/Pezizomycotina - ps17
Saccharomycotina - ps18
Saccharomycotina_1 - ps19
Saccharomycotina_2 - ps20
Saccharomycotina_3 - ps21
Phaff./Saccharomycetaceae - ps22
Saccharomyco./mycetaceae - ps23
Saccharomycetaceae - ps24
Saccharomycetaceae_2 - ps25
Saccharomycetaceae_3 - ps26
Kaz./Nau./Saccharomyces - ps27
Saccharomyces - ps28
Arboricola_clade - ps29
Cerevisiae_clade - ps30
Saccharomyces_cerevisiae - ps31
p-value
2e-15
4e-12
7e-09
1e-05
3e-02
Log-odds
0.5
1.0
1.5
2.0
2.5
3.0
3.5
4.0

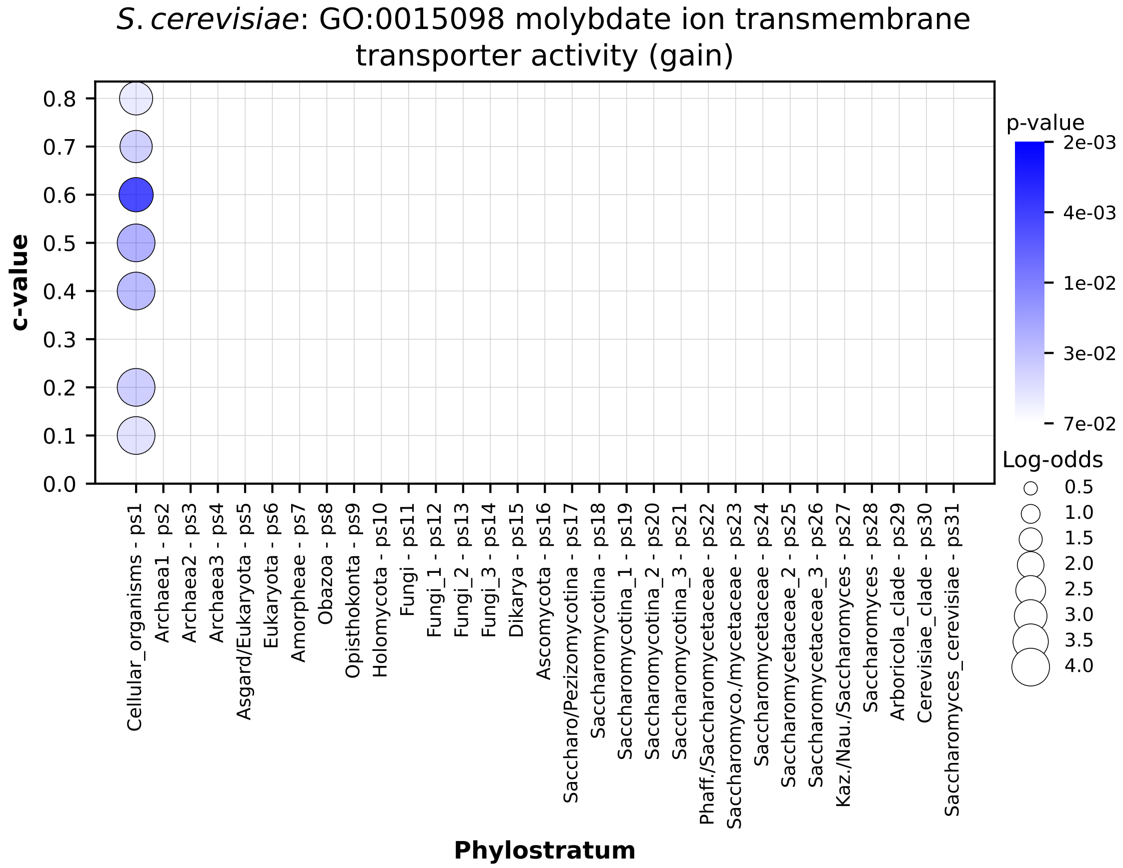
S. cerevisiae: GO:0015098 molybdate ion transmembrane transporter activity (gain)
c-value
0.0
0.1
0.2
0.3
0.4
0.5
0.6
0.7
0.8
Phylostratum
Cellular_organisms - ps1
Archaea1 - ps2
Archaea2 - ps3
Archaea3 - ps4
Asgard/Eukaryota - ps5
Eukaryota - ps6
Amorpheae - ps7
Obazoa - ps8
Opisthokonta - ps9
Holomycota - ps10
Fungi - ps11
Fungi_1 - ps12
Fungi_2 - ps13
Fungi_3 - ps14
Dikarya - ps15
Ascomycota - ps16
Saccharo/Pezizomycotina - ps17
Saccharomycotina - ps18
Saccharomycotina_1 - ps19
Saccharomycotina_2 - ps20
Saccharomycotina_3 - ps21
Phaff./Saccharomycetaceae - ps22
Saccharomyco./mycetaceae - ps23
Saccharomycetaceae - ps24
Saccharomycetaceae_2 - ps25
Saccharomycetaceae_3 - ps26
Kaz./Nau./Saccharomyces - ps27
Saccharomyces - ps28
Arboricola_clade - ps29
Cerevisiae_clade - ps30
Saccharomyces_cerevisiae - ps31
p-value
2e-03
4e-03
1e-02
3e-02
7e-02
Log-odds
0.5
1.0
1.5
2.0
2.5
3.0
3.5
4.0

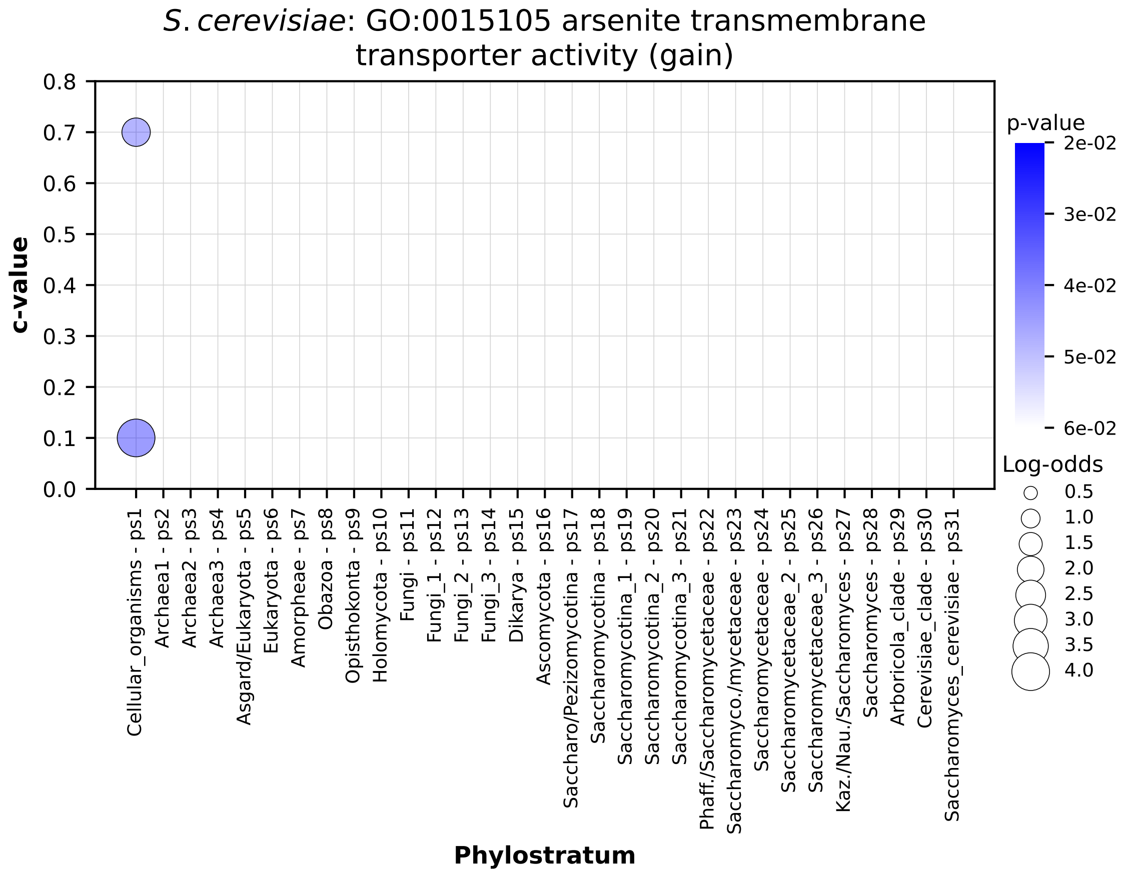
S. cerevisiae: GO:0015105 arsenite transmembrane transporter activity (gain)
c-value
0.0
0.1
0.2
0.3
0.4
0.5
0.6
0.7
0.8
Phylostratum
Cellular_organisms - ps1
Archaea1 - ps2
Archaea2 - ps3
Archaea3 - ps4
Asgard/Eukaryota - ps5
Eukaryota - ps6
Amorpheae - ps7
Obazoa - ps8
Opisthokonta - ps9
Holomycota - ps10
Fungi - ps11
Fungi_1 - ps12
Fungi_2 - ps13
Fungi_3 - ps14
Dikarya - ps15
Ascomycota - ps16
Saccharo/Pezizomycotina - ps17
Saccharomycotina - ps18
Saccharomycotina_1 - ps19
Saccharomycotina_2 - ps20
Saccharomycotina_3 - ps21
Phaff./Saccharomycetaceae - ps22
Saccharomyco./mycetaceae - ps23
Saccharomycetaceae - ps24
Saccharomycetaceae_2 - ps25
Saccharomycetaceae_3 - ps26
Kaz./Nau./Saccharomyces - ps27
Saccharomyces - ps28
Arboricola_clade - ps29
Cerevisiae_clade - ps30
Saccharomyces_cerevisiae - ps31
p-value
2e-02
3e-02
4e-02
5e-02
6e-02
Log-odds
0.5
1.0
1.5
2.0
2.5
3.0
3.5
4.0

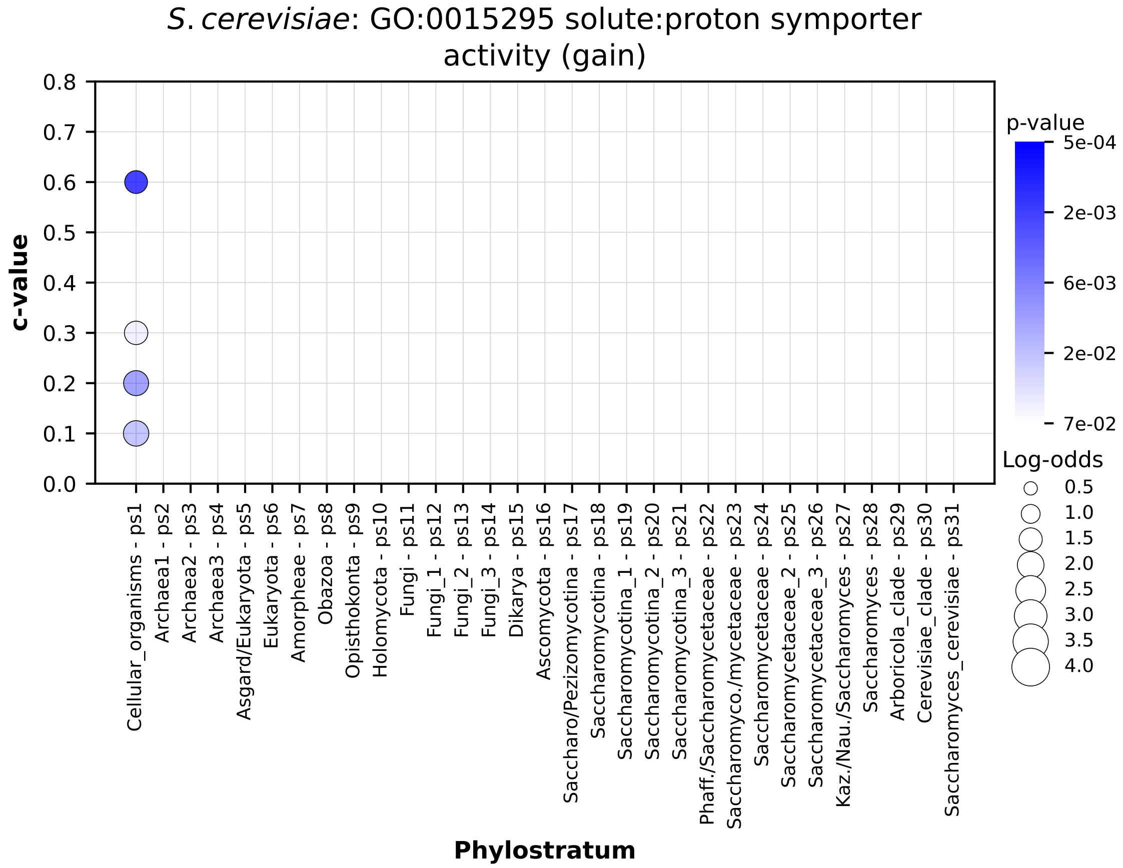

S. cerevisiae: GO:0015295 solute:proton symporter activity (gain)
c-value
0.0
0.1
0.2
0.3
0.4
0.5
0.6
0.7
0.8
p-value
5e-04
2e-03
6e-03
2e-02
7e-02
Log-odds
0.5
1.0
1.5
2.0
2.5
3.0
3.5
4.0
Cellular_organisms - ps1
Archaea1 - ps2
Archaea2 - ps3
Archaea3 - ps4
Asgard/Eukaryota - ps5
Eukaryota - ps6
Amorpheae - ps7
Obazoa - ps8
Opisthokonta - ps9
Holomycota - ps10
Fungi - ps11
Fungi_1 - ps12
Fungi_2 - ps13
Fungi_3 - ps14
Dikarya - ps15
Ascomycota - ps16
Saccharo/Pezizomycotina - ps17
Saccharomycotina - ps18
Saccharomycotina_1 - ps19
Saccharomycotina_2 - ps20
Saccharomycotina_3 - ps21
Phaff./Saccharomycetaceae - ps22
Saccharomyco./mycetaceae - ps23
Saccharomycetaceae - ps24
Saccharomycetaceae_2 - ps25
Saccharomycetaceae_3 - ps26
Kaz./Nau./Saccharomyces - ps27
Saccharomyces - ps28
Arboricola_clade - ps29
Cerevisiae_clade - ps30
Saccharomyces_cerevisiae - ps31
Phylostratum

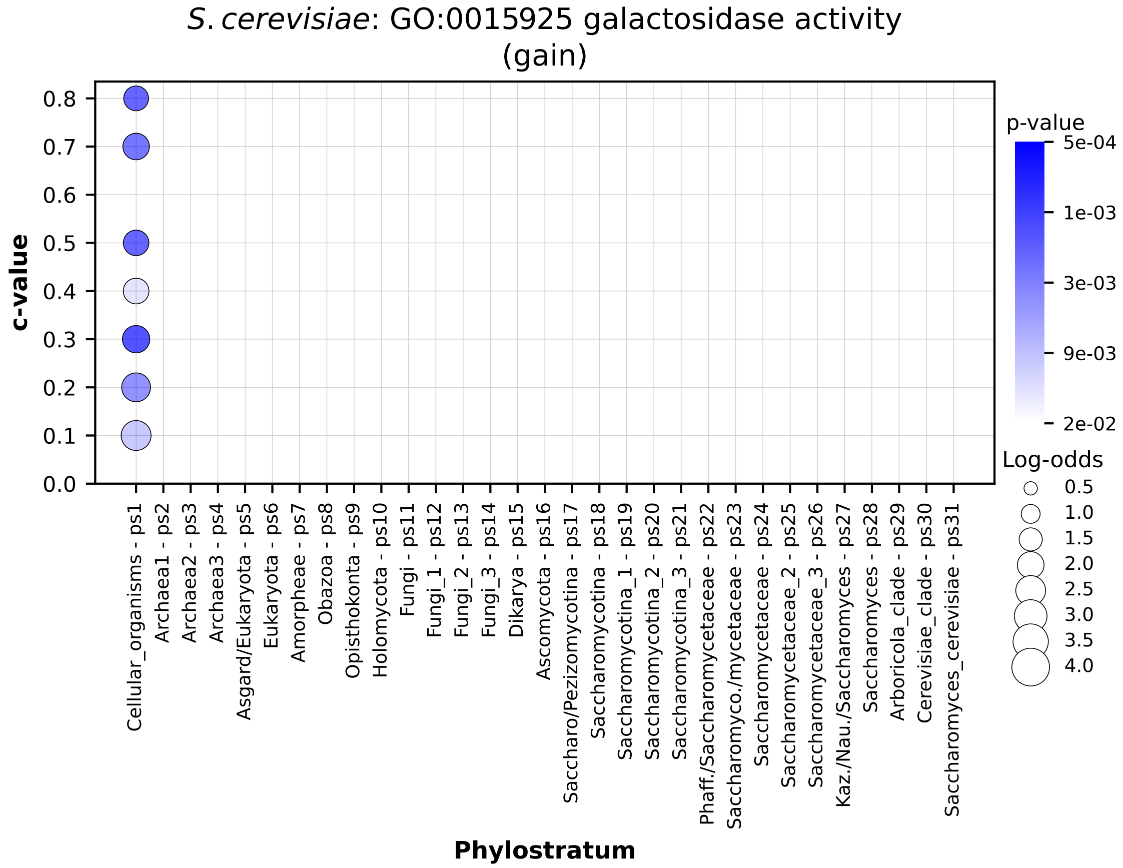

S. cerevisiae: GO:0015925 galactosidase activity
(gain)
c-value
0.0
0.1
0.2
0.3
0.4
0.5
0.6
0.7
0.8
p-value
5e-04
1e-03
3e-03
9e-03
2e-02
Log-odds
0.5
1.0
1.5
2.0
2.5
3.0
3.5
4.0
Cellular_organisms - ps1
Archaea1 - ps2
Archaea2 - ps3
Archaea3 - ps4
Asgard/Eukaryota - ps5
Eukaryota - ps6
Amorpheae - ps7
Obazoa - ps8
Opisthokonta - ps9
Holomycota - ps10
Fungi - ps11
Fungi_1 - ps12
Fungi_2 - ps13
Fungi_3 - ps14
Dikarya - ps15
Ascomycota - ps16
Saccharo/Pezizomycotina - ps17
Saccharomycotina - ps18
Saccharomycotina_1 - ps19
Saccharomycotina_2 - ps20
Saccharomycotina_3 - ps21
Phaff./Saccharomycetaceae - ps22
Saccharomyco./mycetaceae - ps23
Saccharomycetaceae - ps24
Saccharomycetaceae_2 - ps25
Saccharomycetaceae_3 - ps26
Kaz./Nau./Saccharomyces - ps27
Saccharomyces - ps28
Arboricola_clade - ps29
Cerevisiae_clade - ps30
Saccharomyces_cerevisiae - ps31
Phylostratum

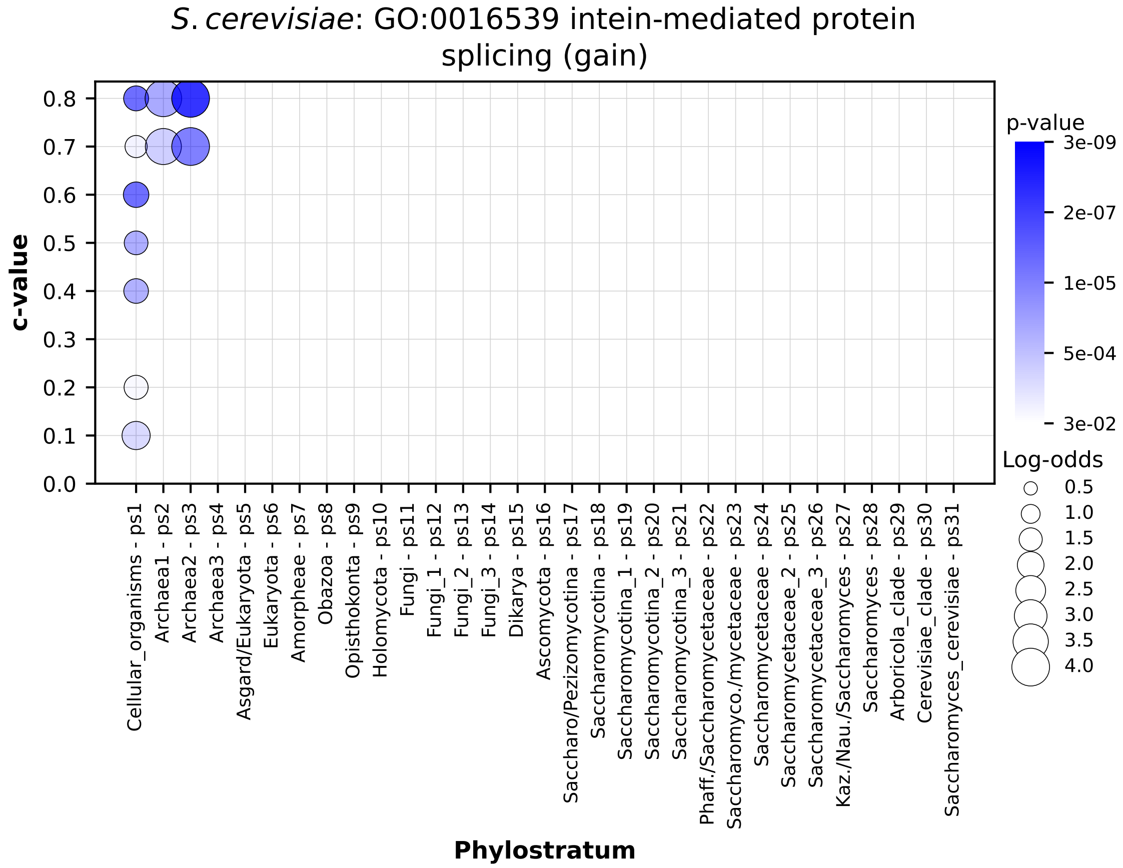

S. cerevisiae: GO:0016539 intein-mediated protein splicing (gain)
c-value
0.8
0.7
0.6
0.5
0.4
0.3
0.2
0.1
0.0
p-value
3e-09
2e-07
1e-05
5e-04
3e-02
Log-odds
0.5
1.0
1.5
2.0
2.5
3.0
3.5
4.0
Cellular_organisms - ps1
Archaea1 - ps2
Archaea2 - ps3
Archaea3 - ps4
Asgard/Eukaryota - ps5
Eukaryota - ps6
Amorpheae - ps7
Obazoa - ps8
Opisthokonta - ps9
Holomycota - ps10
Fungi - ps11
Fungi_1 - ps12
Fungi_2 - ps13
Fungi_3 - ps14
Dikarya - ps15
Ascomycota - ps16
Saccharo/Pezizomycotina - ps17
Saccharomycotina - ps18
Saccharomycotina_1 - ps19
Saccharomycotina_2 - ps20
Saccharomycotina_3 - ps21
Phaff./Saccharomycetaceae - ps22
Saccharomyco./mycetaceae - ps23
Saccharomycetaceae - ps24
Saccharomycetaceae_2 - ps25
Saccharomycetaceae_3 - ps26
Kaz./Nau./Saccharomyces - ps27
Saccharomyces - ps28
Arboricola_clade - ps29
Cerevisiae_clade - ps30
Saccharomyces_cerevisiae - ps31
Phylostratum

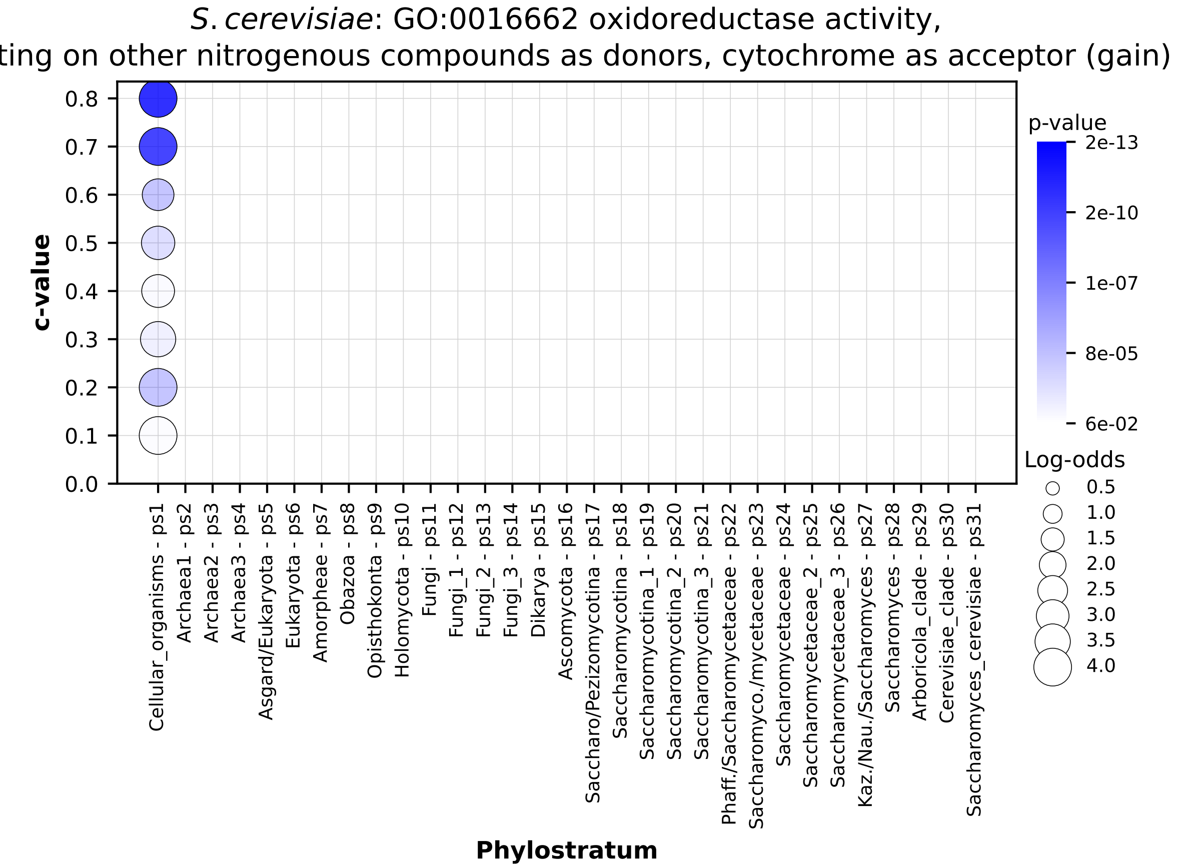

S. cerevisiae: GO:0016662 oxidoreductase activity,
ting on other nitrogenous compounds as donors, cytochrome as acceptor (gain)
c-value
0.8
0.7
0.6
0.5
0.4
0.3
0.2
0.1
0.0
p-value
2e-13
2e-10
1e-07
8e-05
6e-02
Log-odds
0.5
1.0
1.5
2.0
2.5
3.0
3.5
4.0
Cellular_organisms - ps1
Archaea1 - ps2
Archaea2 - ps3
Archaea3 - ps4
Asgard/Eukaryota - ps5
Eukaryota - ps6
Amorpheae - ps7
Obazoa - ps8
Opisthokonta - ps9
Holomycota - ps10
Fungi - ps11
Fungi_1 - ps12
Fungi_2 - ps13
Fungi_3 - ps14
Dikarya - ps15
Ascomycota - ps16
Saccharo/Pezizomycotina - ps17
Saccharomycotina - ps18
Saccharomycotina_1 - ps19
Saccharomycotina_2 - ps20
Saccharomycotina_3 - ps21
Phaff./Saccharomycetaceae - ps22
Saccharomyco./mycetaceae - ps23
Saccharomycetaceae - ps24
Saccharomycetaceae_2 - ps25
Saccharomycetaceae_3 - ps26
Kaz./Nau./Saccharomyces - ps27
Saccharomyces - ps28
Arboricola_clade - ps29
Cerevisiae_clade - ps30
Saccharomyces_cerevisiae - ps31
Phylostratum

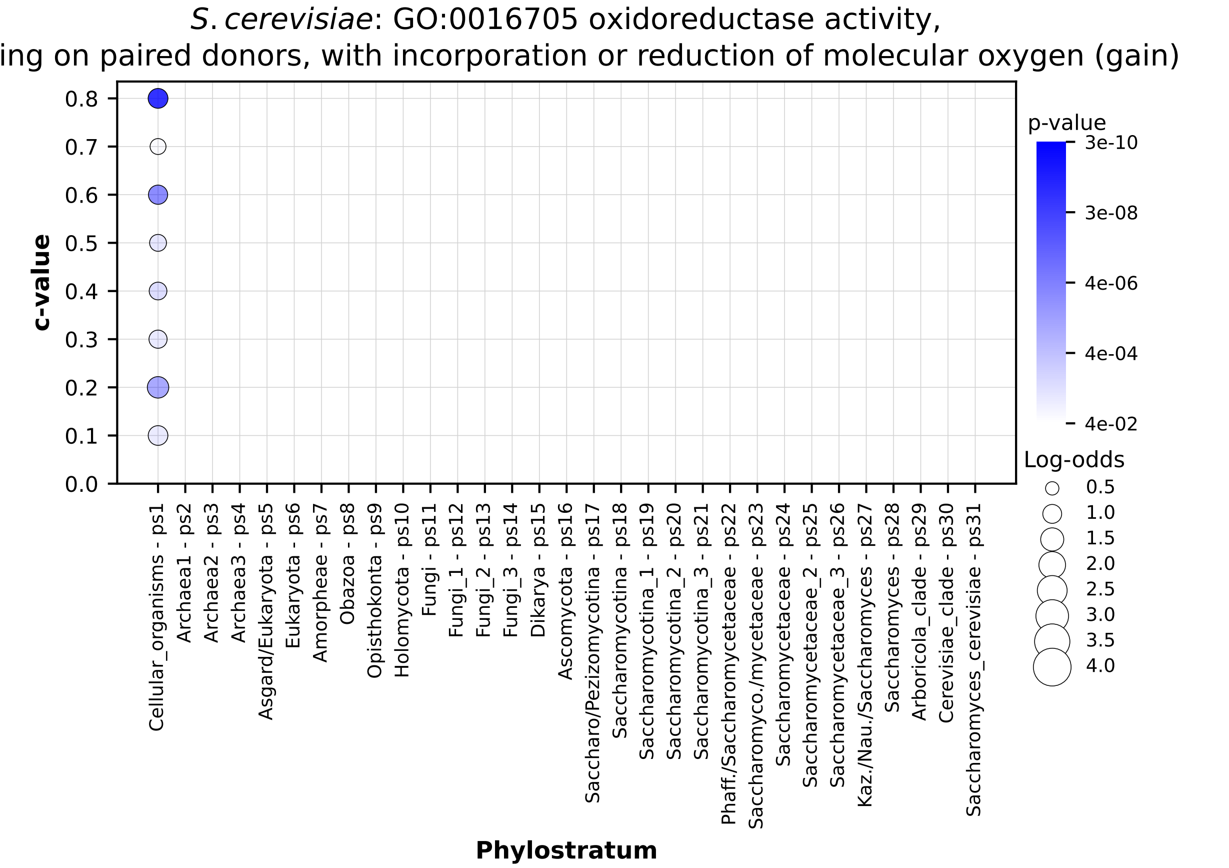

S. cerevisiae: GO:0016705 oxidoreductase activity,
ing on paired donors, with incorporation or reduction of molecular oxygen (gain)
c-value
0.0
0.1
0.2
0.3
0.4
0.5
0.6
0.7
0.8
Phylostratum
Cellular_organisms - ps1
Archaea1 - ps2
Archaea2 - ps3
Archaea3 - ps4
Asgard/Eukaryota - ps5
Eukaryota - ps6
Amorpheae - ps7
Obazoa - ps8
Opisthokonta - ps9
Holomycota - ps10
Fungi - ps11
Fungi_1 - ps12
Fungi_2 - ps13
Fungi_3 - ps14
Dikarya - ps15
Ascomycota - ps16
Saccharo/Pezizomycotina - ps17
Saccharomycotina - ps18
Saccharomycotina_1 - ps19
Saccharomycotina_2 - ps20
Saccharomycotina_3 - ps21
Phaff./Saccharomycetaceae - ps22
Saccharomyco./mycetaceae - ps23
Saccharomycetaceae - ps24
Saccharomycetaceae_2 - ps25
Saccharomycetaceae_3 - ps26
Kaz./Nau./Saccharomyces - ps27
Saccharomyces - ps28
Arboricola_clade - ps29
Cerevisiae_clade - ps30
Saccharomyces_cerevisiae - ps31
p-value
3e-10
3e-08
4e-06
4e-04
4e-02
Log-odds
0.5
1.0
1.5
2.0
2.5
3.0
3.5
4.0

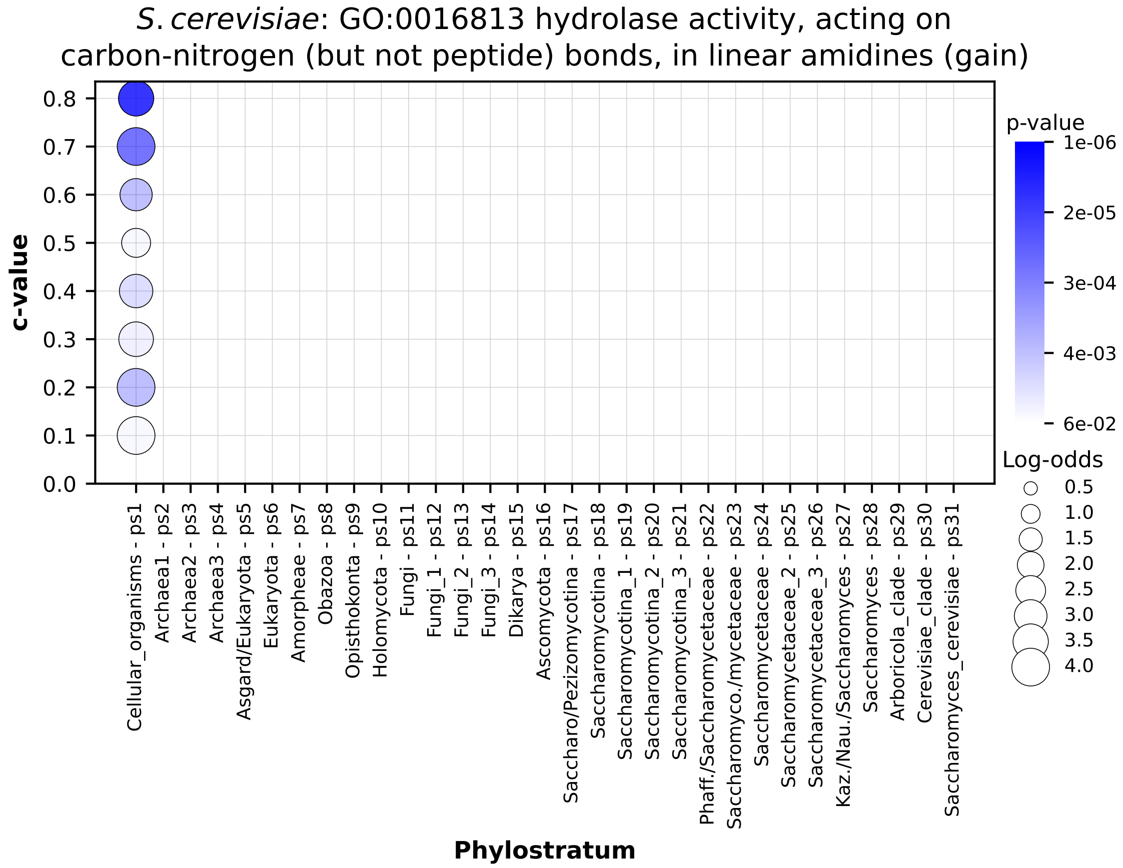

S. cerevisiae: GO:0016813 hydrolase activity, acting on carbon-nitrogen (but not peptide) bonds, in linear amidines (gain)
c-value
0.0
0.1
0.2
0.3
0.4
0.5
0.6
0.7
0.8
Phylostratum
Cellular_organisms - ps1
Archaea1 - ps2
Archaea2 - ps3
Archaea3 - ps4
Asgard/Eukaryota - ps5
Eukaryota - ps6
Amorpheae - ps7
Obazoa - ps8
Opisthokonta - ps9
Holomycota - ps10
Fungi - ps11
Fungi_1 - ps12
Fungi_2 - ps13
Fungi_3 - ps14
Dikarya - ps15
Ascomycota - ps16
Saccharo/Pezizomycotina - ps17
Saccharomycotina - ps18
Saccharomycotina_1 - ps19
Saccharomycotina_2 - ps20
Saccharomycotina_3 - ps21
Phaff./Saccharomycetaceae - ps22
Saccharomyco./mycetaceae - ps23
Saccharomycetaceae - ps24
Saccharomycetaceae_2 - ps25
Saccharomycetaceae_3 - ps26
Kaz./Nau./Saccharomyces - ps27
Saccharomyces - ps28
Arboricola_clade - ps29
Cerevisiae_clade - ps30
Saccharomyces_cerevisiae - ps31
p-value
1e-06
2e-05
3e-04
4e-03
6e-02
Log-odds
0.5
1.0
1.5
2.0
2.5
3.0
3.5
4.0

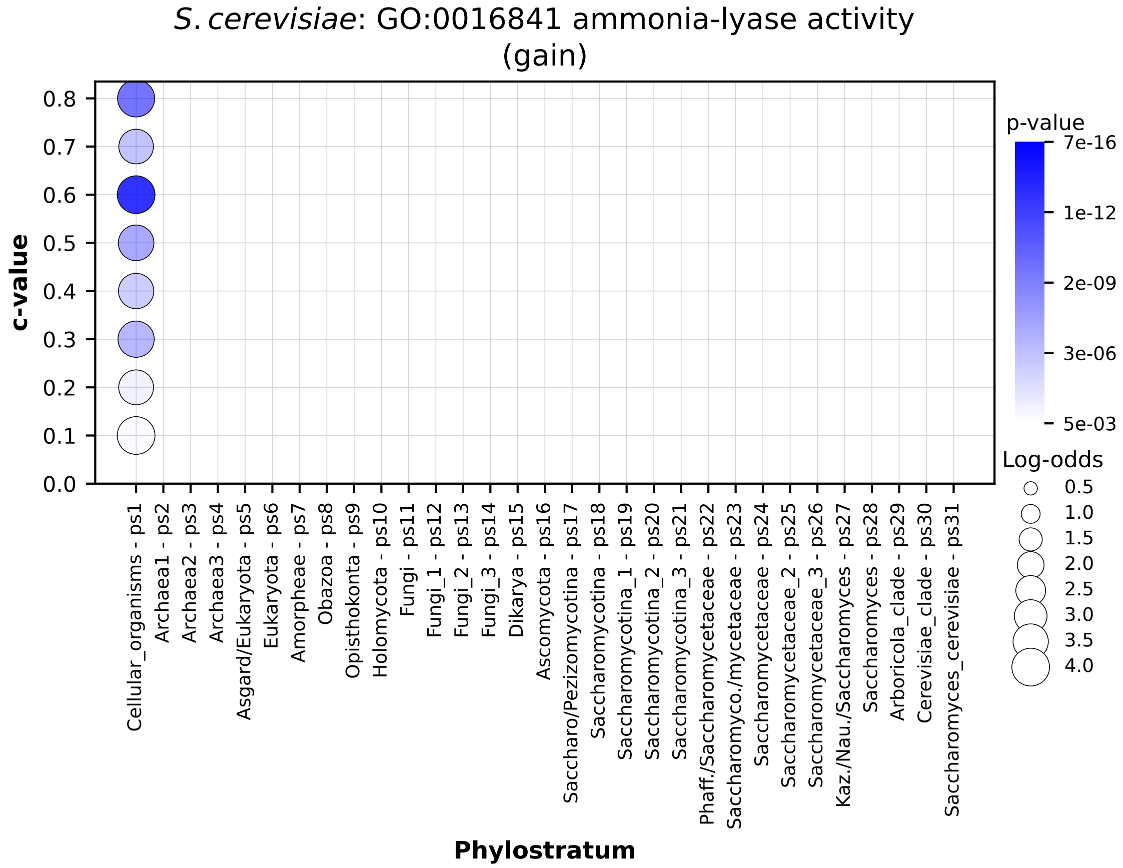

S. cerevisiae: GO:0016841 ammonia-lyase activity
(gain)
c-value
0.8
0.7
0.6
0.5
0.4
0.3
0.2
0.1
0.0
Phylostratum
Cellular_organisms - ps1
Archaea1 - ps2
Archaea2 - ps3
Archaea3 - ps4
Asgard/Eukaryota - ps5
Eukaryota - ps6
Amorpheae - ps7
Obazoa - ps8
Opisthokonta - ps9
Holomycota - ps10
Fungi - ps11
Fungi_1 - ps12
Fungi_2 - ps13
Fungi_3 - ps14
Dikarya - ps15
Ascomycota - ps16
Saccharo/Pezizomycotina - ps17
Saccharomycotina - ps18
Saccharomycotina_1 - ps19
Saccharomycotina_2 - ps20
Saccharomycotina_3 - ps21
Phaff./Saccharomycetaceae - ps22
Saccharomyco./mycetaceae - ps23
Saccharomycetaceae - ps24
Saccharomycetaceae_2 - ps25
Saccharomycetaceae_3 - ps26
Kaz./Nau./Saccharomyces - ps27
Saccharomyces - ps28
Arboricola_clade - ps29
Cerevisiae_clade - ps30
Saccharomyces_cerevisiae - ps31
p-value
7e-16
1e-12
2e-09
3e-06
5e-03
Log-odds
0.5
1.0
1.5
2.0
2.5
3.0
3.5
4.0

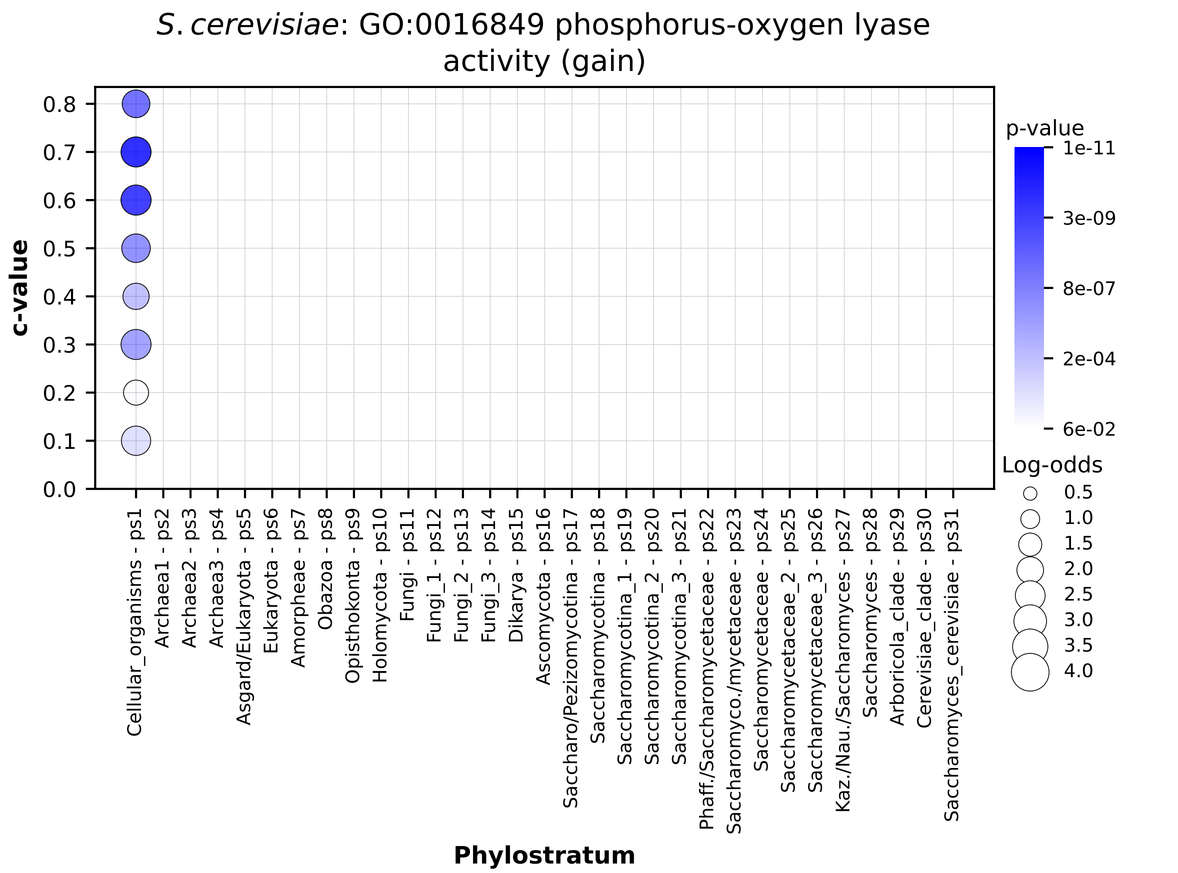
S. cerevisiae: GO:0016849 phosphorus-oxygen lyase activity (gain)
c-value
0.0
0.1
0.2
0.3
0.4
0.5
0.6
0.7
0.8
p-value
1e-11
3e-09
8e-07
2e-04
6e-02
Log-odds
0.5
1.0
1.5
2.0
2.5
3.0
3.5
4.0
Cellular_organisms - ps1
Archaea1 - ps2
Archaea2 - ps3
Archaea3 - ps4
Asgard/Eukaryota - ps5
Eukaryota - ps6
Amorpheae - ps7
Obazoa - ps8
Opisthokonta - ps9
Holomycota - ps10
Fungi - ps11
Fungi_1 - ps12
Fungi_2 - ps13
Fungi_3 - ps14
Dikarya - ps15
Ascomycota - ps16
Saccharo/Pezizomycotina - ps17
Saccharomycotina - ps18
Saccharomycotina_1 - ps19
Saccharomycotina_2 - ps20
Saccharomycotina_3 - ps21
Phaff./Saccharomycetaceae - ps22
Saccharomyco./mycetaceae - ps23
Saccharomycetaceae - ps24
Saccharomycetaceae_2 - ps25
Saccharomycetaceae_3 - ps26
Kaz./Nau./Saccharomyces - ps27
Saccharomyces - ps28
Arboricola_clade - ps29
Cerevisiae_clade - ps30
Saccharomyces_cerevisiae - ps31
Phylostratum

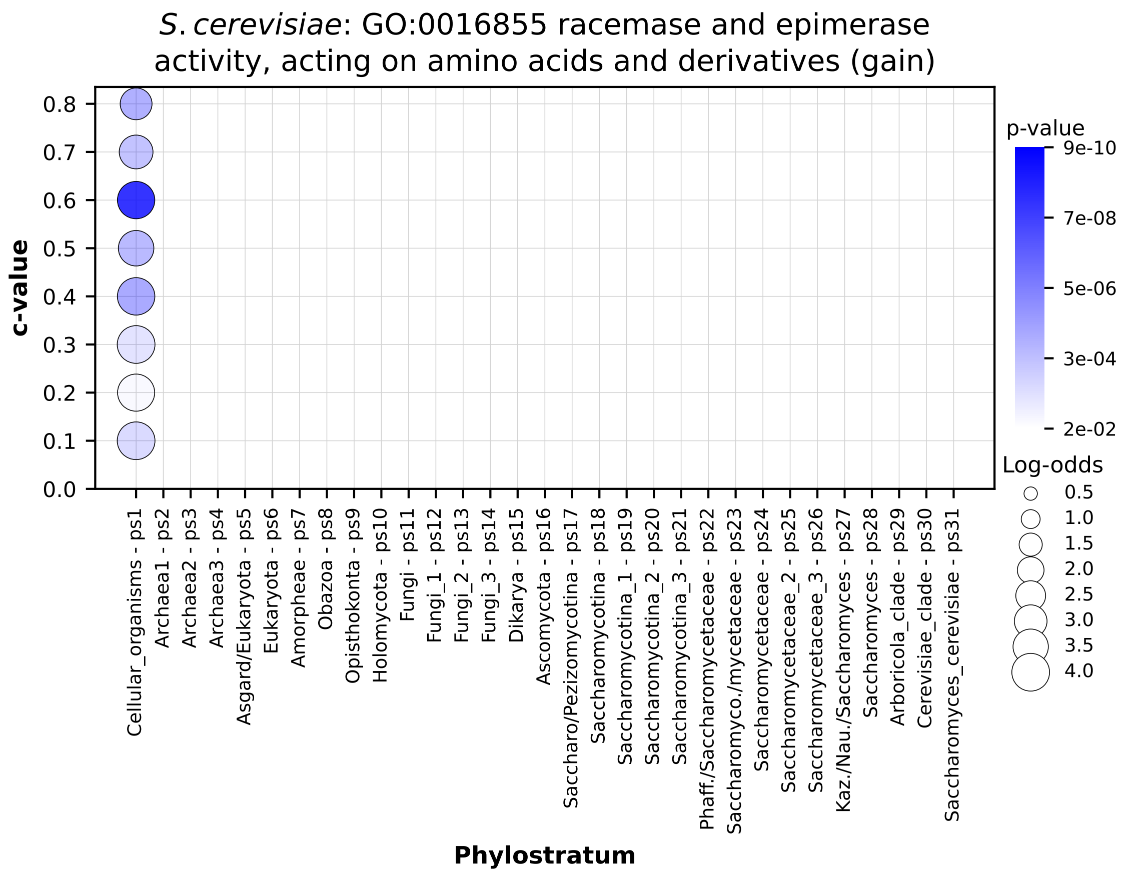
S. cerevisiae: GO:0016855 racemase and epimerase activity, acting on amino acids and derivatives (gain)
c-value
0.0
0.1
0.2
0.3
0.4
0.5
0.6
0.7
0.8
p-value
9e-10
7e-08
5e-06
3e-04
2e-02
Log-odds
0.5
1.0
1.5
2.0
2.5
3.0
3.5
4.0
Cellular_organisms - ps1
Archaea1 - ps2
Archaea2 - ps3
Archaea3 - ps4
Asgard/Eukaryota - ps5
Eukaryota - ps6
Amorpheae - ps7
Obazoa - ps8
Opisthokonta - ps9
Holomycota - ps10
Fungi - ps11
Fungi_1 - ps12
Fungi_2 - ps13
Fungi_3 - ps14
Dikarya - ps15
Ascomycota - ps16
Saccharo/Pezizomycotina - ps17
Saccharomycotina - ps18
Saccharomycotina_1 - ps19
Saccharomycotina_2 - ps20
Saccharomycotina_3 - ps21
Phaff./Saccharomycetaceae - ps22
Saccharomyco./mycetaceae - ps23
Saccharomycetaceae - ps24
Saccharomycetaceae_2 - ps25
Saccharomycetaceae_3 - ps26
Kaz./Nau./Saccharomyces - ps27
Saccharomyces - ps28
Arboricola_clade - ps29
Cerevisiae_clade - ps30
Saccharomyces_cerevisiae - ps31
Phylostratum

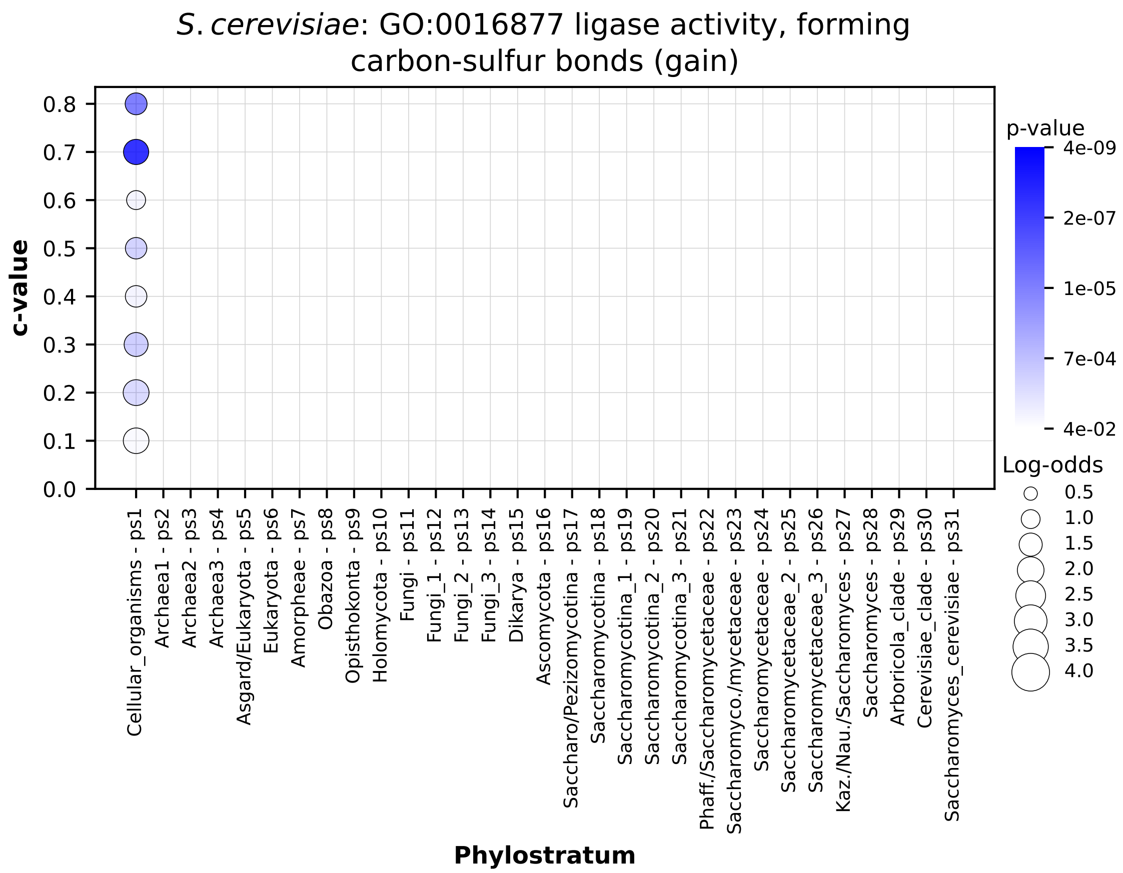

S. cerevisiae: GO:0016877 ligase activity, forming carbon-sulfur bonds (gain)
c-value
0.0
0.1
0.2
0.3
0.4
0.5
0.6
0.7
0.8
Phylostratum
Cellular_organisms - ps1
Archaea1 - ps2
Archaea2 - ps3
Archaea3 - ps4
Asgard/Eukaryota - ps5
Eukaryota - ps6
Amorpheae - ps7
Obazoa - ps8
Opisthokonta - ps9
Holomycota - ps10
Fungi - ps11
Fungi_1 - ps12
Fungi_2 - ps13
Fungi_3 - ps14
Dikarya - ps15
Ascomycota - ps16
Saccharo/Pezizomycotina - ps17
Saccharomycotina - ps18
Saccharomycotina_1 - ps19
Saccharomycotina_2 - ps20
Saccharomycotina_3 - ps21
Phaff./Saccharomycetaceae - ps22
Saccharomyco./mycetaceae - ps23
Saccharomycetaceae - ps24
Saccharomycetaceae_2 - ps25
Saccharomycetaceae_3 - ps26
Kaz./Nau./Saccharomyces - ps27
Saccharomyces - ps28
Arboricola_clade - ps29
Cerevisiae_clade - ps30
Saccharomyces_cerevisiae - ps31
p-value
4e-09
2e-07
1e-05
7e-04
4e-02
Log-odds
0.5
1.0
1.5
2.0
2.5
3.0
3.5
4.0

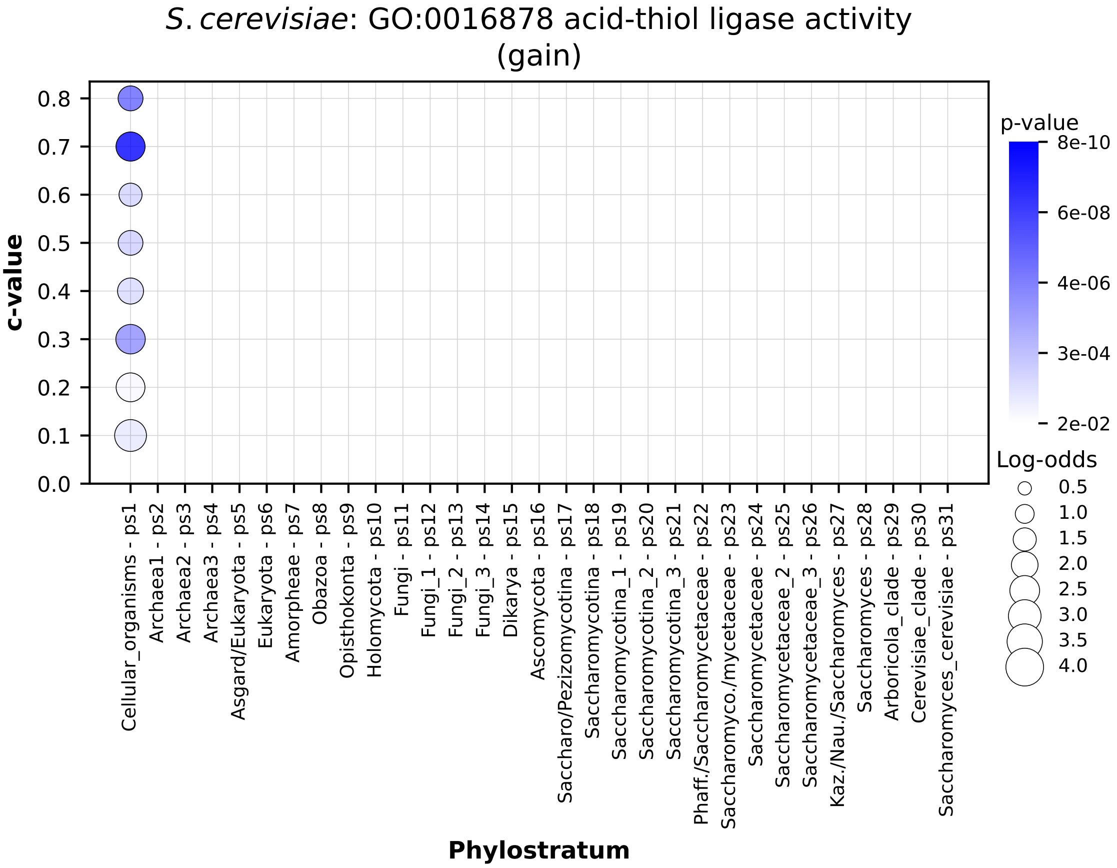

S. cerevisiae: GO:0016878 acid-thiol ligase activity
(gain)
c-value
0.0
0.1
0.2
0.3
0.4
0.5
0.6
0.7
0.8
p-value
8e-10
6e-08
4e-06
3e-04
2e-02
Log-odds
0.5
1.0
1.5
2.0
2.5
3.0
3.5
4.0
Cellular_organisms - ps1
Archaea1 - ps2
Archaea2 - ps3
Archaea3 - ps4
Asgard/Eukaryota - ps5
Eukaryota - ps6
Amorpheae - ps7
Obazoa - ps8
Opisthokonta - ps9
Holomycota - ps10
Fungi - ps11
Fungi_1 - ps12
Fungi_2 - ps13
Fungi_3 - ps14
Dikarya - ps15
Ascomycota - ps16
Saccharo/Pezizomycotina - ps17
Saccharomycotina - ps18
Saccharomycotina_1 - ps19
Saccharomycotina_2 - ps20
Saccharomycotina_3 - ps21
Phaff./Saccharomycetaceae - ps22
Saccharomyco./mycetaceae - ps23
Saccharomycetaceae - ps24
Saccharomycetaceae_2 - ps25
Saccharomycetaceae_3 - ps26
Kaz./Nau./Saccharomyces - ps27
Saccharomyces - ps28
Arboricola_clade - ps29
Cerevisiae_clade - ps30
Saccharomyces_cerevisiae - ps31
Phylostratum

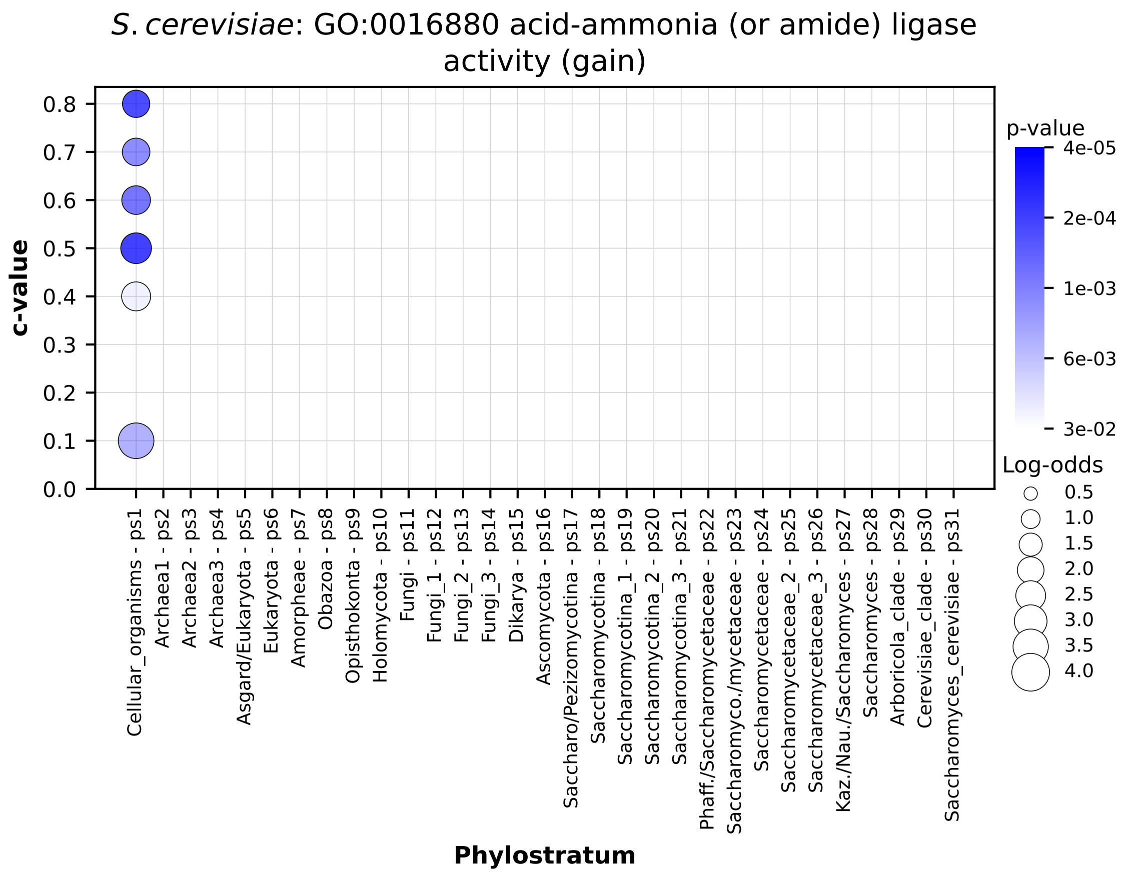

S. cerevisiae: GO:0016880 acid-ammonia (or amide) ligase activity (gain)
c-value
0.0
0.1
0.2
0.3
0.4
0.5
0.6
0.7
0.8
p-value
4e-05
2e-04
1e-03
6e-03
3e-02
Log-odds
0.5
1.0
1.5
2.0
2.5
3.0
3.5
4.0
Cellular_organisms - ps1
Archaea1 - ps2
Archaea2 - ps3
Archaea3 - ps4
Asgard/Eukaryota - ps5
Eukaryota - ps6
Amorpheae - ps7
Obazoa - ps8
Opisthokonta - ps9
Holomycota - ps10
Fungi - ps11
Fungi_1 - ps12
Fungi_2 - ps13
Fungi_3 - ps14
Dikarya - ps15
Ascomycota - ps16
Saccharo/Pezizomycotina - ps17
Saccharomycotina - ps18
Saccharomycotina_1 - ps19
Saccharomycotina_2 - ps20
Saccharomycotina_3 - ps21
Phaff./Saccharomycetaceae - ps22
Saccharomyco./mycetaceae - ps23
Saccharomycetaceae - ps24
Saccharomycetaceae_2 - ps25
Saccharomycetaceae_3 - ps26
Kaz./Nau./Saccharomyces - ps27
Saccharomyces - ps28
Arboricola_clade - ps29
Cerevisiae_clade - ps30
Saccharomyces_cerevisiae - ps31
Phylostratum

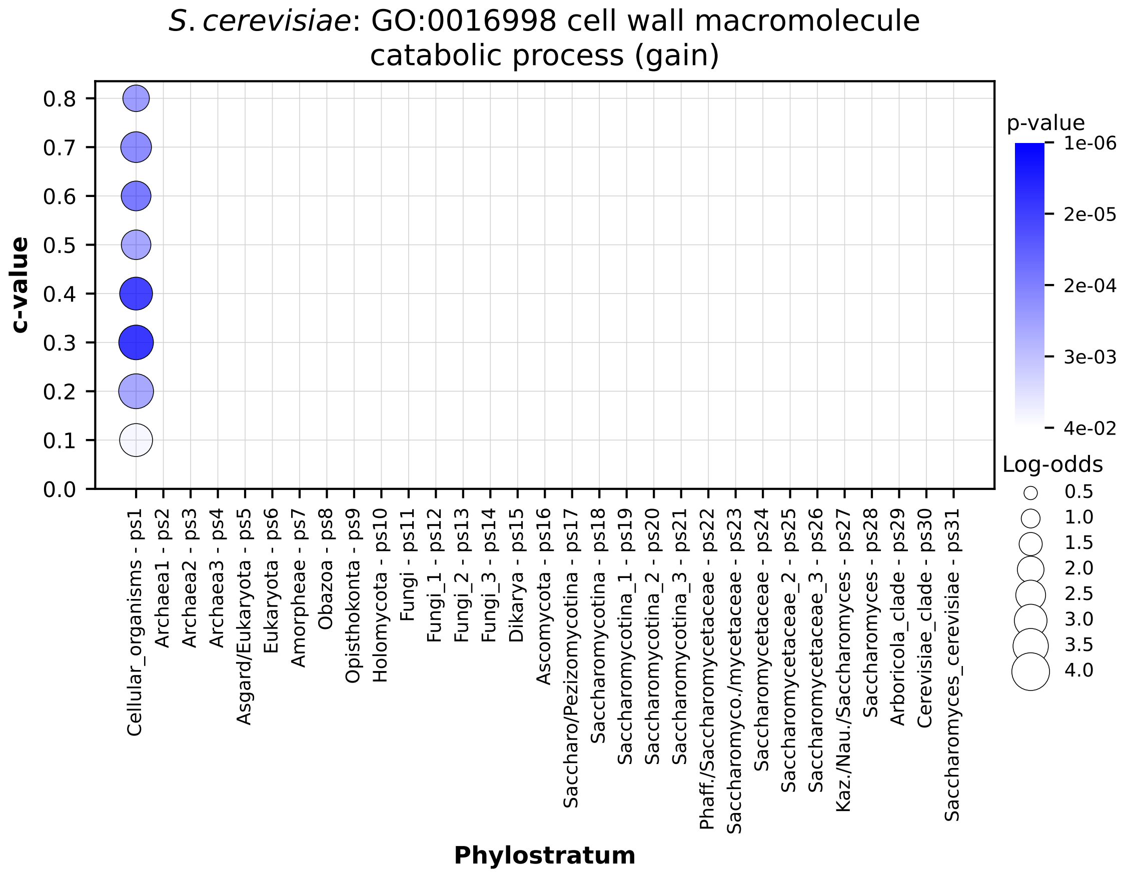

S. cerevisiae: GO:0016998 cell wall macromolecule catabolic process (gain)
c-value
0.0
0.1
0.2
0.3
0.4
0.5
0.6
0.7
0.8
p-value
1e-06
2e-05
2e-04
3e-03
4e-02
Log-odds
0.5
1.0
1.5
2.0
2.5
3.0
3.5
4.0
Cellular_organisms - ps1
Archaea1 - ps2
Archaea2 - ps3
Archaea3 - ps4
Asgard/Eukaryota - ps5
Eukaryota - ps6
Amorpheae - ps7
Obazoa - ps8
Opisthokonta - ps9
Holomycota - ps10
Fungi - ps11
Fungi_1 - ps12
Fungi_2 - ps13
Fungi_3 - ps14
Dikarya - ps15
Ascomycota - ps16
Saccharo/Pezizomycotina - ps17
Saccharomycotina - ps18
Saccharomycotina_1 - ps19
Saccharomycotina_2 - ps20
Saccharomycotina_3 - ps21
Phaff./Saccharomycetaceae - ps22
Saccharomyco./mycetaceae - ps23
Saccharomycetaceae - ps24
Saccharomycetaceae_2 - ps25
Saccharomycetaceae_3 - ps26
Kaz./Nau./Saccharomyces - ps27
Saccharomyces - ps28
Arboricola_clade - ps29
Cerevisiae_clade - ps30
Saccharomyces_cerevisiae - ps31
Phylostratum

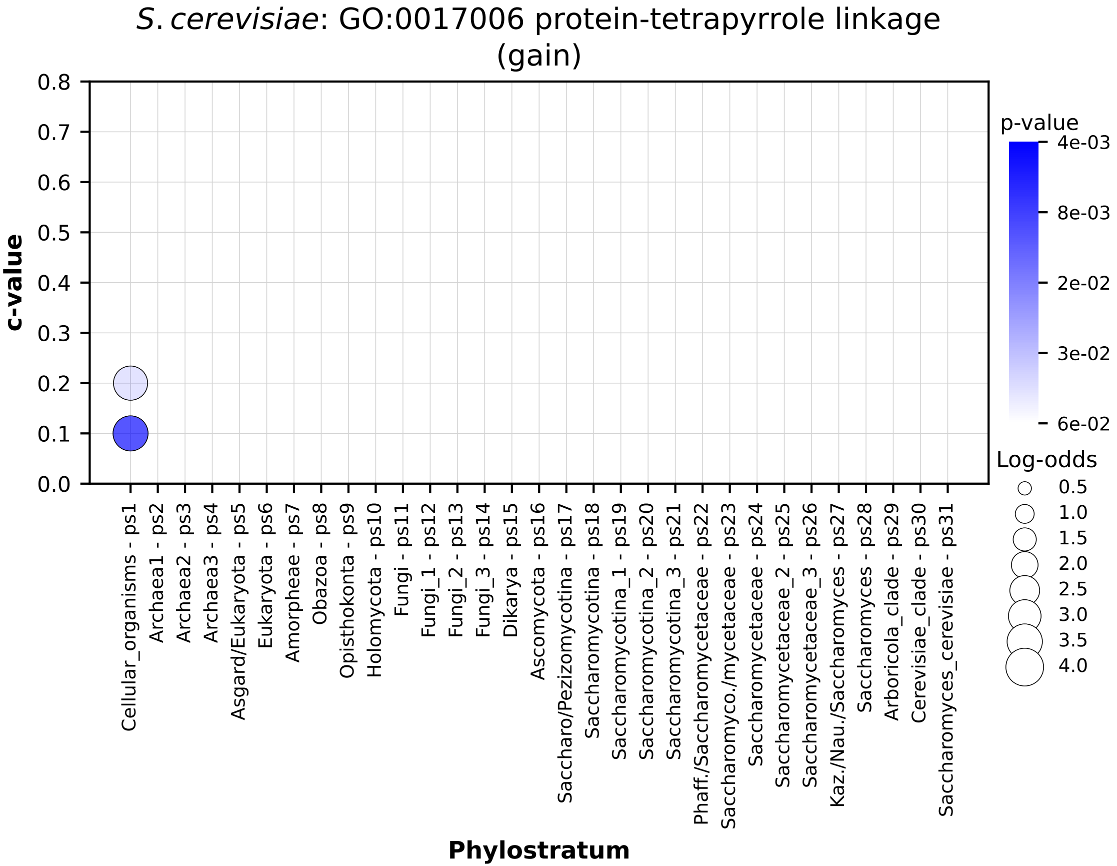

S. cerevisiae: GO:0017006 protein-tetrapyrrole linkage
(gain)
c-value
0.0
0.1
0.2
0.3
0.4
0.5
0.6
0.7
0.8
Phylostratum
Cellular_organisms - ps1
Archaea1 - ps2
Archaea2 - ps3
Archaea3 - ps4
Asgard/Eukaryota - ps5
Eukaryota - ps6
Amorpheae - ps7
Obazoa - ps8
Opisthokonta - ps9
Holomycota - ps10
Fungi - ps11
Fungi_1 - ps12
Fungi_2 - ps13
Fungi_3 - ps14
Dikarya - ps15
Ascomycota - ps16
Saccharo/Pezizomycotina - ps17
Saccharomycotina - ps18
Saccharomycotina_1 - ps19
Saccharomycotina_2 - ps20
Saccharomycotina_3 - ps21
Phaff./Saccharomycetaceae - ps22
Saccharomyco./mycetaceae - ps23
Saccharomycetaceae - ps24
Saccharomycetaceae_2 - ps25
Saccharomycetaceae_3 - ps26
Kaz./Nau./Saccharomyces - ps27
Saccharomyces - ps28
Arboricola_clade - ps29
Cerevisiae_clade - ps30
Saccharomyces_cerevisiae - ps31
p-value
4e-03
8e-03
2e-02
3e-02
6e-02
Log-odds
0.5
1.0
1.5
2.0
2.5
3.0
3.5
4.0

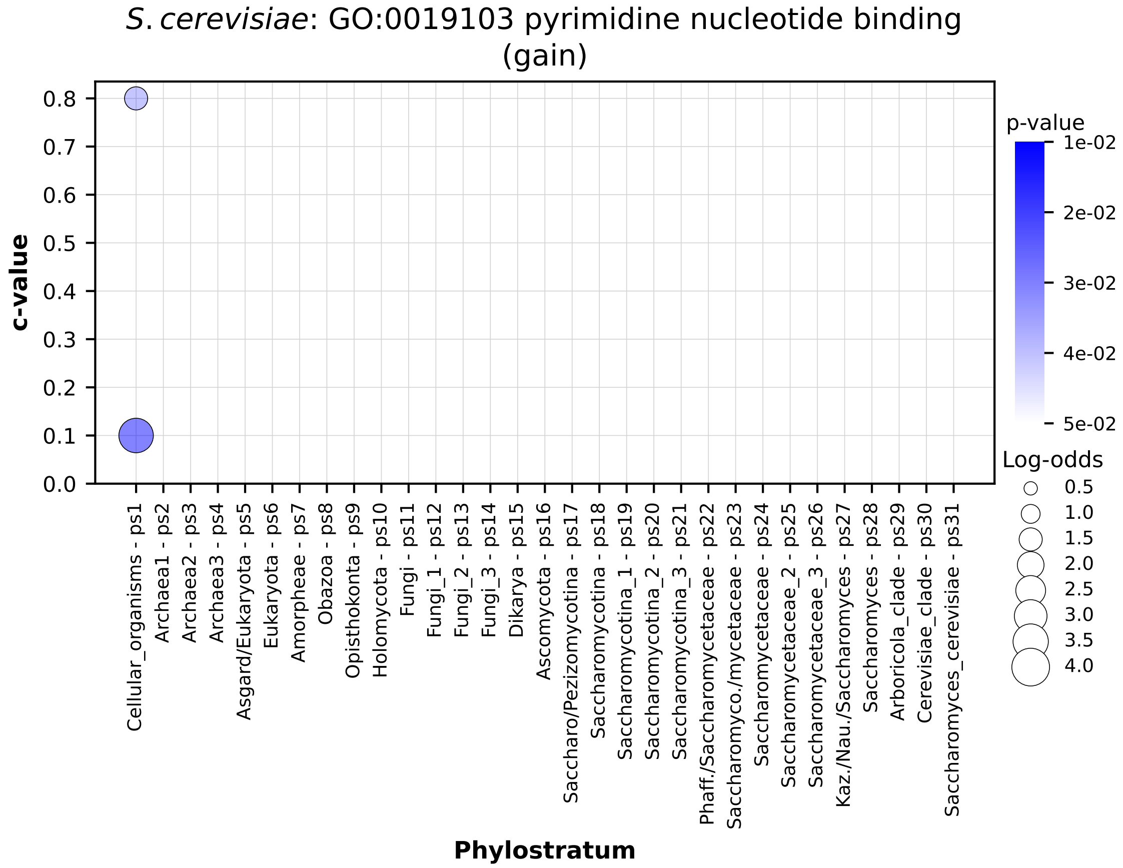

S. cerevisiae: GO:0019103 pyrimidine nucleotide binding (gain)
c-value
0.0
0.1
0.2
0.3
0.4
0.5
0.6
0.7
0.8
p-value
1e-02
2e-02
3e-02
4e-02
5e-02
Log-odds
0.5
1.0
1.5
2.0
2.5
3.0
3.5
4.0
Cellular_organisms - ps1
Archaea1 - ps2
Archaea2 - ps3
Archaea3 - ps4
Asgard/Eukaryota - ps5
Eukaryota - ps6
Amorpheae - ps7
Obazoa - ps8
Opisthokonta - ps9
Holomycota - ps10
Fungi - ps11
Fungi_1 - ps12
Fungi_2 - ps13
Fungi_3 - ps14
Dikarya - ps15
Ascomycota - ps16
Saccharo/Pezizomycotina - ps17
Saccharomycotina - ps18
Saccharomycotina_1 - ps19
Saccharomycotina_2 - ps20
Saccharomycotina_3 - ps21
Phaff./Saccharomycetaceae - ps22
Saccharomyco./mycetaceae - ps23
Saccharomycetaceae - ps24
Saccharomycetaceae_2 - ps25
Saccharomycetaceae_3 - ps26
Kaz./Nau./Saccharomyces - ps27
Saccharomyces - ps28
Arboricola_clade - ps29
Cerevisiae_clade - ps30
Saccharomyces_cerevisiae - ps31
Phylostratum

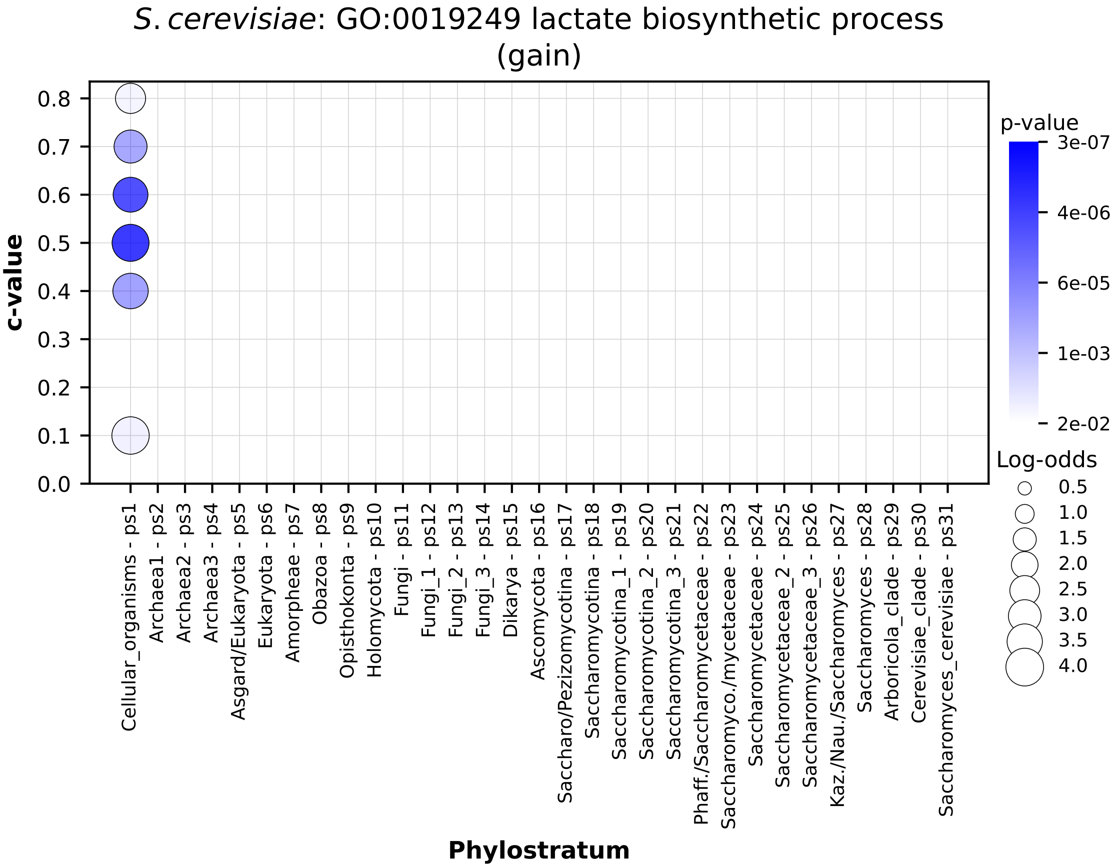

S. cerevisiae: GO:0019249 lactate biosynthetic process
(gain)
c-value
0.0
0.1
0.2
0.3
0.4
0.5
0.6
0.7
0.8
p-value
3e-07
4e-06
6e-05
1e-03
2e-02
Log-odds
0.5
1.0
1.5
2.0
2.5
3.0
3.5
4.0
Cellular_organisms - ps1
Archaea1 - ps2
Archaea2 - ps3
Archaea3 - ps4
Asgard/Eukaryota - ps5
Eukaryota - ps6
Amorpheae - ps7
Obazoa - ps8
Opisthokonta - ps9
Holomycota - ps10
Fungi - ps11
Fungi_1 - ps12
Fungi_2 - ps13
Fungi_3 - ps14
Dikarya - ps15
Ascomycota - ps16
Saccharo/Pezizomycotina - ps17
Saccharomycotina - ps18
Saccharomycotina_1 - ps19
Saccharomycotina_2 - ps20
Saccharomycotina_3 - ps21
Phaff./Saccharomycetaceae - ps22
Saccharomyco./mycetaceae - ps23
Saccharomycetaceae - ps24
Saccharomycetaceae_2 - ps25
Saccharomycetaceae_3 - ps26
Kaz./Nau./Saccharomyces - ps27
Saccharomyces - ps28
Arboricola_clade - ps29
Cerevisiae_clade - ps30
Saccharomyces_cerevisiae - ps31
Phylostratum

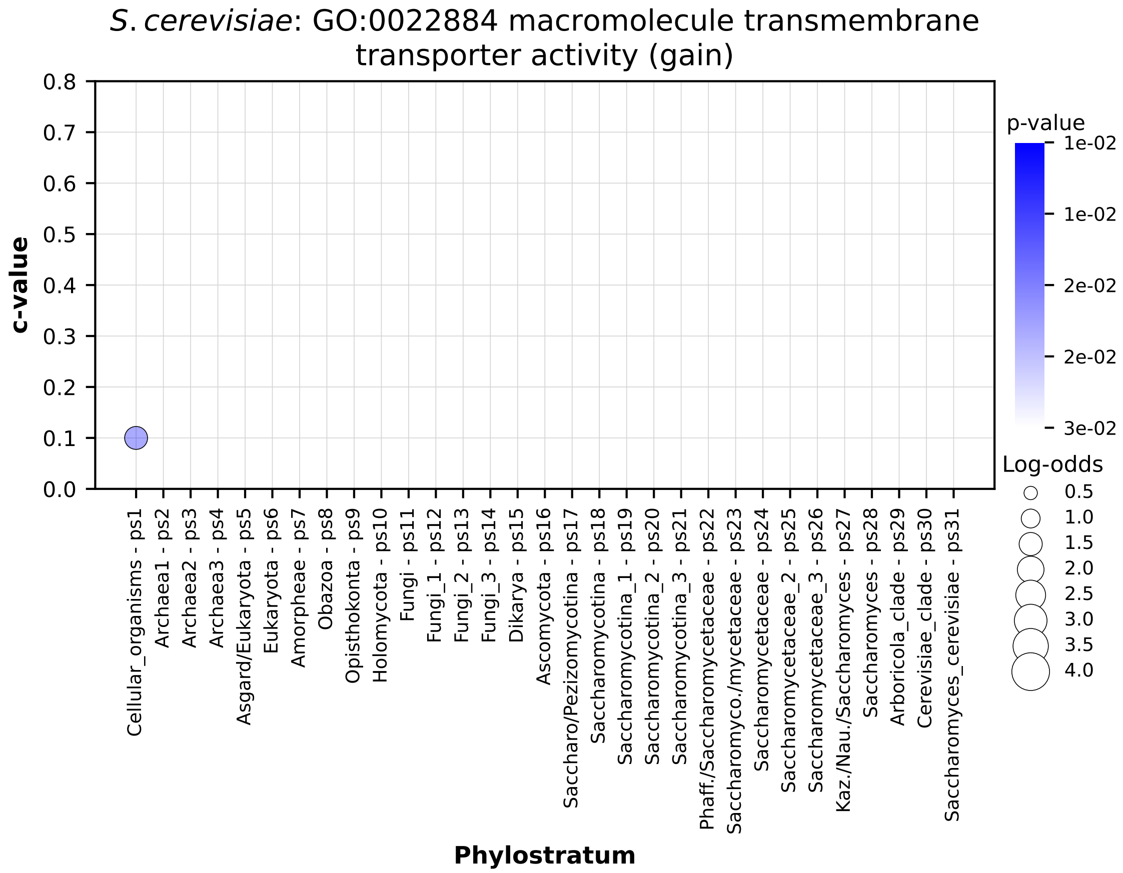

S. cerevisiae: GO:0022884 macromolecule transmembrane transporter activity (gain)
c-value
0.8
0.7
0.6
0.5
0.4
0.3
0.2
0.1
0.0
Phylostratum
Cellular_organisms - ps1
Archaea1 - ps2
Archaea2 - ps3
Archaea3 - ps4
Asgard/Eukaryota - ps5
Eukaryota - ps6
Amorpheae - ps7
Obazoa - ps8
Opisthokonta - ps9
Holomycota - ps10
Fungi - ps11
Fungi_1 - ps12
Fungi_2 - ps13
Fungi_3 - ps14
Dikarya - ps15
Ascomycota - ps16
Saccharo/Pezizomycotina - ps17
Saccharomycotina - ps18
Saccharomycotina_1 - ps19
Saccharomycotina_2 - ps20
Saccharomycotina_3 - ps21
Phaff./Saccharomycetaceae - ps22
Saccharomyco./mycetaceae - ps23
Saccharomycetaceae - ps24
Saccharomycetaceae_2 - ps25
Saccharomycetaceae_3 - ps26
Kaz./Nau./Saccharomyces - ps27
Saccharomyces - ps28
Arboricola_clade - ps29
Cerevisiae_clade - ps30
Saccharomyces_cerevisiae - ps31
p-value
1e-02
1e-02
2e-02
2e-02
3e-02
Log-odds
0.5
1.0
1.5
2.0
2.5
3.0
3.5
4.0

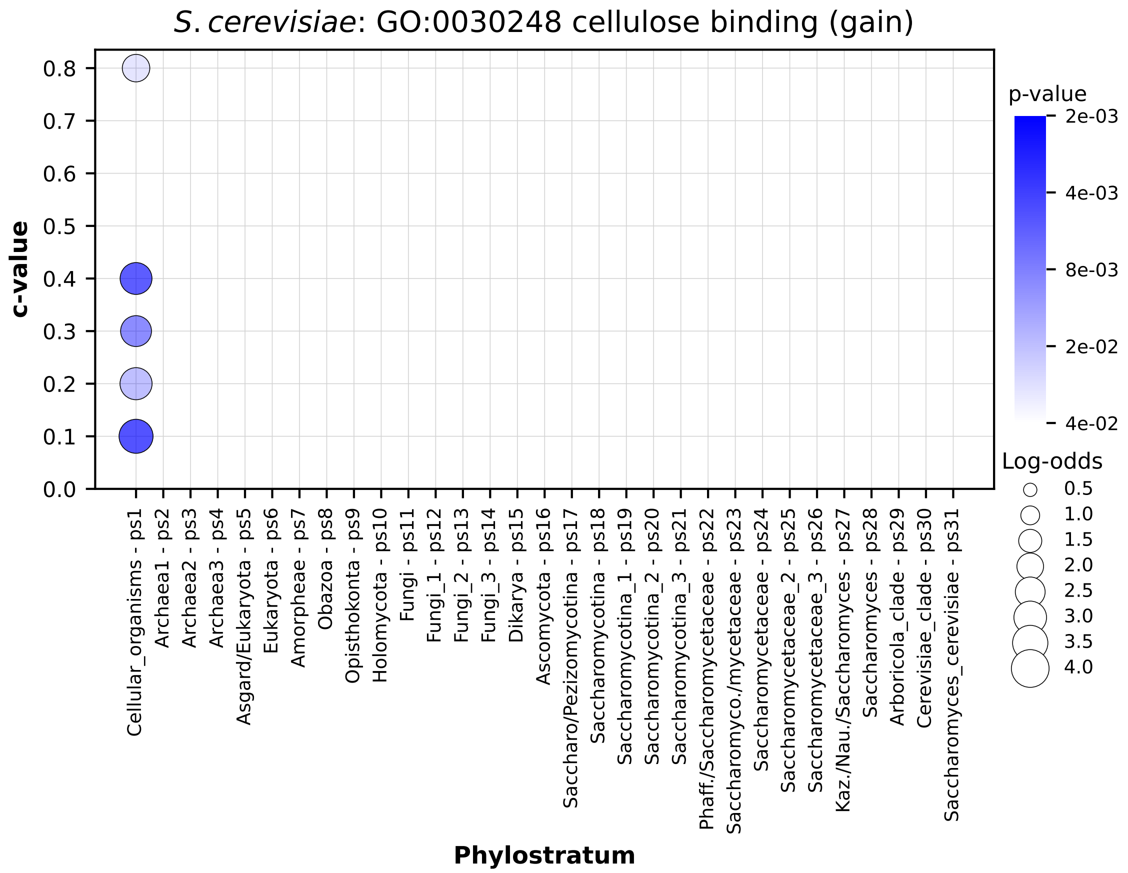

S. cerevisiae: GO:0030248 cellulose binding (gain)
c-value
0.0
0.1
0.2
0.3
0.4
0.5
0.6
0.7
0.8
p-value
2e-03
4e-03
8e-03
2e-02
4e-02
Log-odds
0.5
1.0
1.5
2.0
2.5
3.0
3.5
4.0
Cellular_organisms - ps1
Archaea1 - ps2
Archaea2 - ps3
Archaea3 - ps4
Asgard/Eukaryota - ps5
Eukaryota - ps6
Amorpheae - ps7
Obazoa - ps8
Opisthokonta - ps9
Holomycota - ps10
Fungi - ps11
Fungi_1 - ps12
Fungi_2 - ps13
Fungi_3 - ps14
Dikarya - ps15
Ascomycota - ps16
Saccharo/Pezizomycotina - ps17
Saccharomycotina - ps18
Saccharomycotina_1 - ps19
Saccharomycotina_2 - ps20
Saccharomycotina_3 - ps21
Phaff./Saccharomycetaceae - ps22
Saccharomyco./mycetaceae - ps23
Saccharomycetaceae - ps24
Saccharomycetaceae_2 - ps25
Saccharomycetaceae_3 - ps26
Kaz./Nau./Saccharomyces - ps27
Saccharomyces - ps28
Arboricola_clade - ps29
Cerevisiae_clade - ps30
Saccharomyces_cerevisiae - ps31
Phylostratum

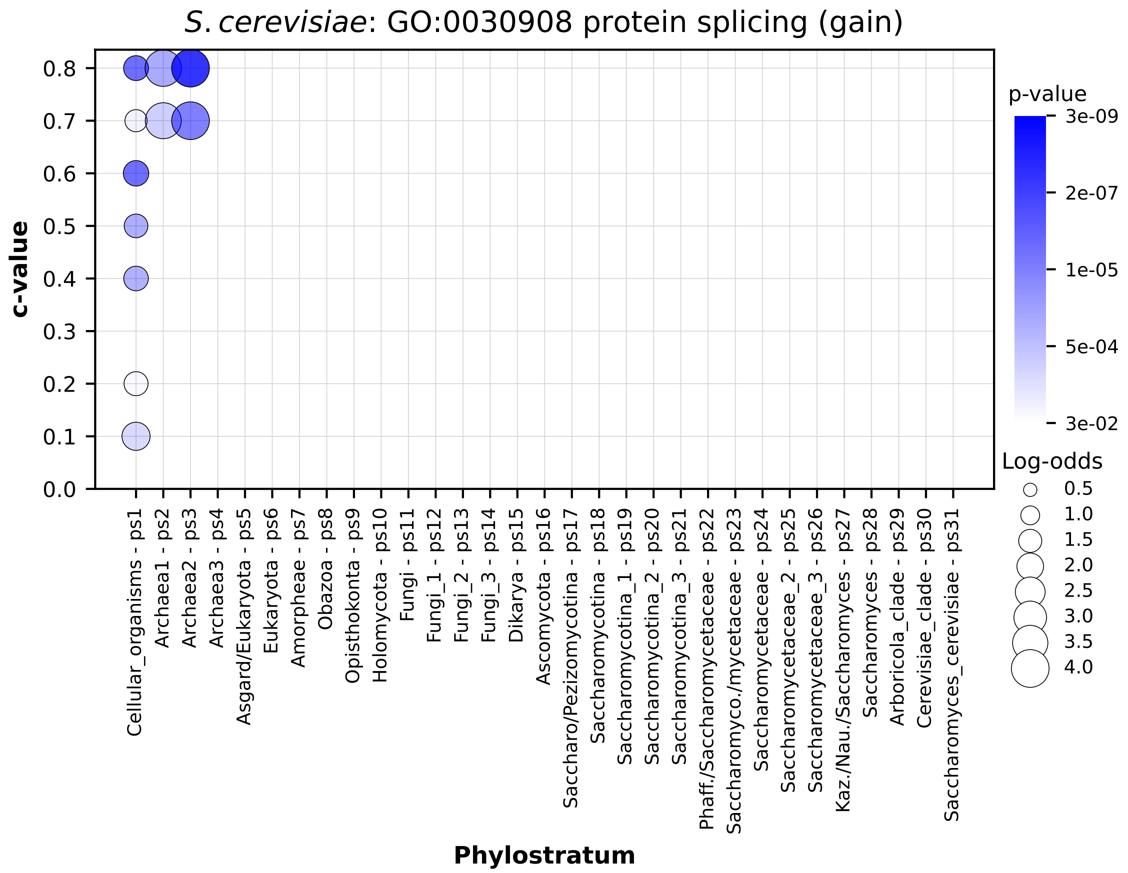

S. cerevisiae: GO:0030908 protein splicing (gain)
c-value
0.0
0.1
0.2
0.3
0.4
0.5
0.6
0.7
0.8
Phylostratum
Cellular_organisms - ps1
Archaea1 - ps2
Archaea2 - ps3
Archaea3 - ps4
Asgard/Eukaryota - ps5
Eukaryota - ps6
Amorpheae - ps7
Obazoa - ps8
Opisthokonta - ps9
Holomycota - ps10
Fungi - ps11
Fungi_1 - ps12
Fungi_2 - ps13
Fungi_3 - ps14
Dikarya - ps15
Ascomycota - ps16
Saccharo/Pezizomycotina - ps17
Saccharomycotina - ps18
Saccharomycotina_1 - ps19
Saccharomycotina_2 - ps20
Saccharomycotina_3 - ps21
Phaff./Saccharomycetaceae - ps22
Saccharomyco./mycetaceae - ps23
Saccharomycetaceae - ps24
Saccharomycetaceae_2 - ps25
Saccharomycetaceae_3 - ps26
Kaz./Nau./Saccharomyces - ps27
Saccharomyces - ps28
Arboricola_clade - ps29
Cerevisiae_clade - ps30
Saccharomyces_cerevisiae - ps31
p-value
3e-09
2e-07
1e-05
5e-04
3e-02
Log-odds
0.5
1.0
1.5
2.0
2.5
3.0
3.5
4.0

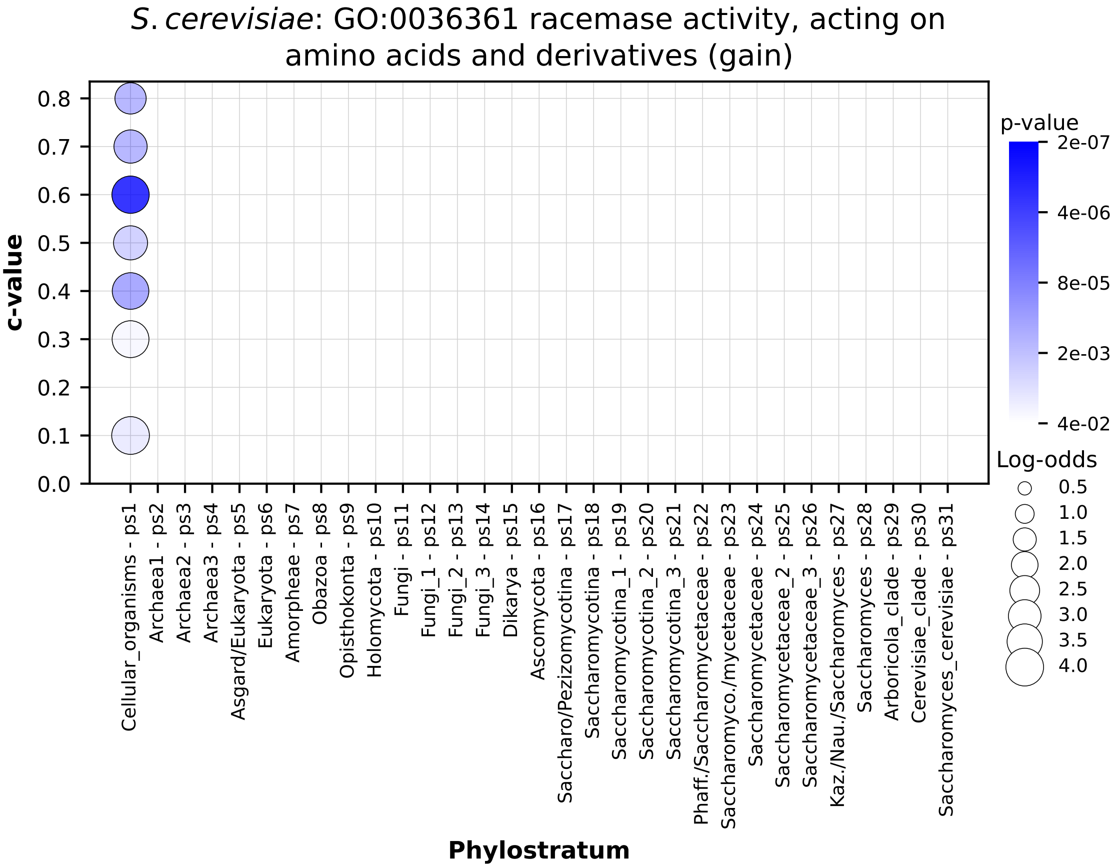

S. cerevisiae: GO:0036361 racemase activity, acting on amino acids and derivatives (gain)
c-value
0.0
0.1
0.2
0.3
0.4
0.5
0.6
0.7
0.8
p-value
2e-07
4e-06
8e-05
2e-03
4e-02
Log-odds
0.5
1.0
1.5
2.0
2.5
3.0
3.5
4.0
Cellular_organisms - ps1
Archaea1 - ps2
Archaea2 - ps3
Archaea3 - ps4
Asgard/Eukaryota - ps5
Eukaryota - ps6
Amorpheae - ps7
Obazoa - ps8
Opisthokonta - ps9
Holomycota - ps10
Fungi - ps11
Fungi_1 - ps12
Fungi_2 - ps13
Fungi_3 - ps14
Dikarya - ps15
Ascomycota - ps16
Saccharo/Pezizomycotina - ps17
Saccharomycotina - ps18
Saccharomycotina_1 - ps19
Saccharomycotina_2 - ps20
Saccharomycotina_3 - ps21
Phaff./Saccharomycetaceae - ps22
Saccharomyco./mycetaceae - ps23
Saccharomycetaceae - ps24
Saccharomycetaceae_2 - ps25
Saccharomycetaceae_3 - ps26
Kaz./Nau./Saccharomyces - ps27
Saccharomyces - ps28
Arboricola_clade - ps29
Cerevisiae_clade - ps30
Saccharomyces_cerevisiae - ps31
Phylostratum

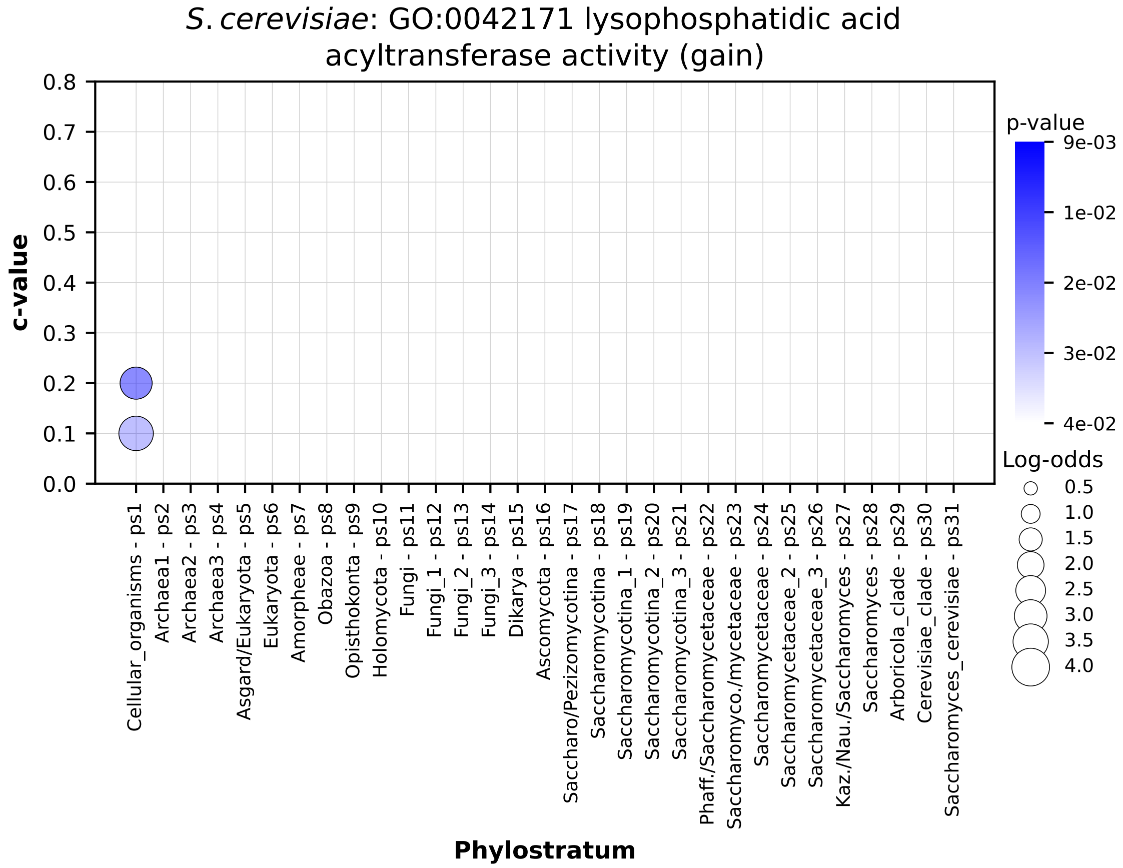

S. cerevisiae: GO:0042171 lysophosphatidic acid acyltransferase activity (gain)
c-value
0.8
0.7
0.6
0.5
0.4
0.3
0.2
0.1
0.0
p-value
9e-03
1e-02
2e-02
3e-02
4e-02
Log-odds
0.5
1.0
1.5
2.0
2.5
3.0
3.5
4.0
Cellular_organisms - ps1
Archaea1 - ps2
Archaea2 - ps3
Archaea3 - ps4
Asgard/Eukaryota - ps5
Eukaryota - ps6
Amorpheae - ps7
Obazoa - ps8
Opisthokonta - ps9
Holomycota - ps10
Fungi - ps11
Fungi_1 - ps12
Fungi_2 - ps13
Fungi_3 - ps14
Dikarya - ps15
Ascomycota - ps16
Saccharo/Pezizomycotina - ps17
Saccharomycotina - ps18
Saccharomycotina_1 - ps19
Saccharomycotina_2 - ps20
Saccharomycotina_3 - ps21
Phaff./Saccharomycetaceae - ps22
Saccharomyco./mycetaceae - ps23
Saccharomycetaceae - ps24
Saccharomycetaceae_2 - ps25
Saccharomycetaceae_3 - ps26
Kaz./Nau./Saccharomyces - ps27
Saccharomyces - ps28
Arboricola_clade - ps29
Cerevisiae_clade - ps30
Saccharomyces_cerevisiae - ps31
Phylostratum

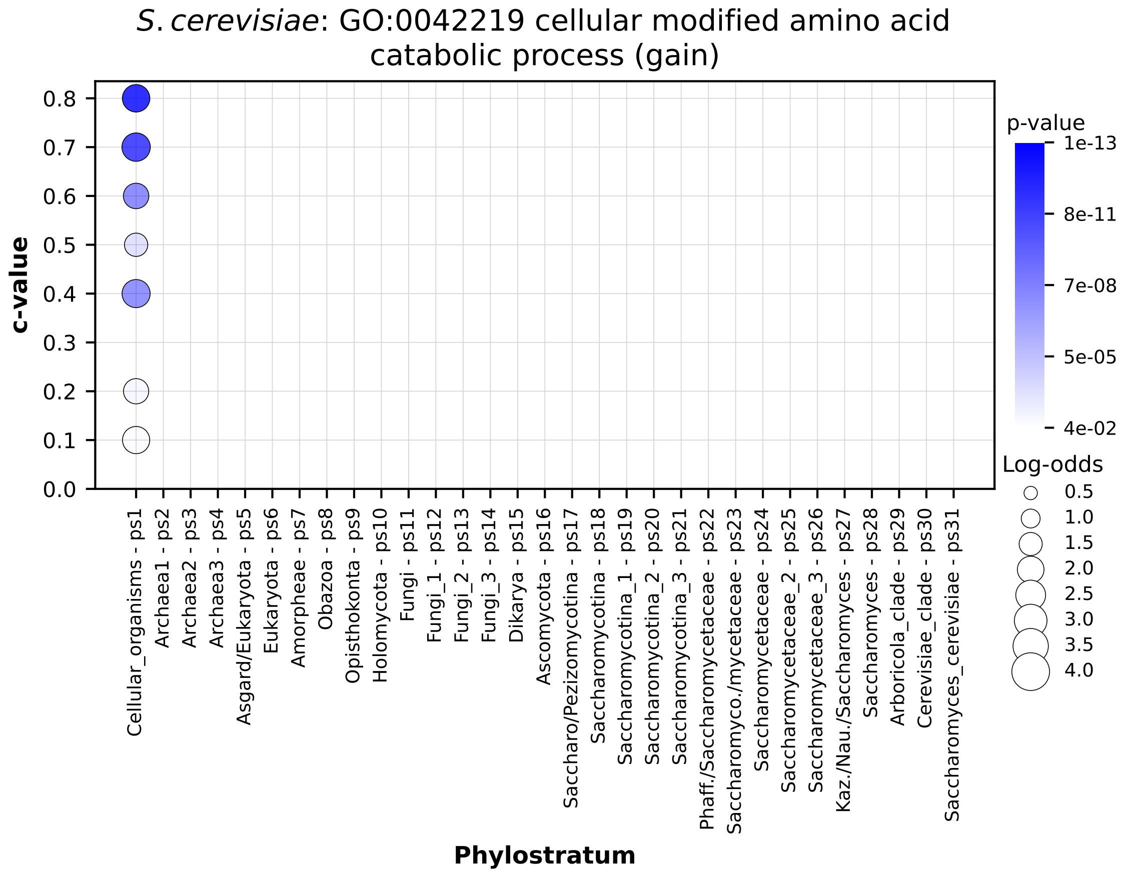
S. cerevisiae: GO:0042219 cellular modified amino acid catabolic process (gain)
c-value
0.0
0.1
0.2
0.3
0.4
0.5
0.6
0.7
0.8
Phylostratum
Cellular_organisms - ps1
Archaea1 - ps2
Archaea2 - ps3
Archaea3 - ps4
Asgard/Eukaryota - ps5
Eukaryota - ps6
Amorpheae - ps7
Obazoa - ps8
Opisthokonta - ps9
Holomycota - ps10
Fungi - ps11
Fungi_1 - ps12
Fungi_2 - ps13
Fungi_3 - ps14
Dikarya - ps15
Ascomycota - ps16
Saccharo/Pezizomycotina - ps17
Saccharomycotina - ps18
Saccharomycotina_1 - ps19
Saccharomycotina_2 - ps20
Saccharomycotina_3 - ps21
Phaff./Saccharomycetaceae - ps22
Saccharomyco./mycetaceae - ps23
Saccharomycetaceae - ps24
Saccharomycetaceae_2 - ps25
Saccharomycetaceae_3 - ps26
Kaz./Nau./Saccharomyces - ps27
Saccharomyces - ps28
Arboricola_clade - ps29
Cerevisiae_clade - ps30
Saccharomyces_cerevisiae - ps31
p-value
1e-13
8e-11
7e-08
5e-05
4e-02
Log-odds
0.5
1.0
1.5
2.0
2.5
3.0
3.5
4.0

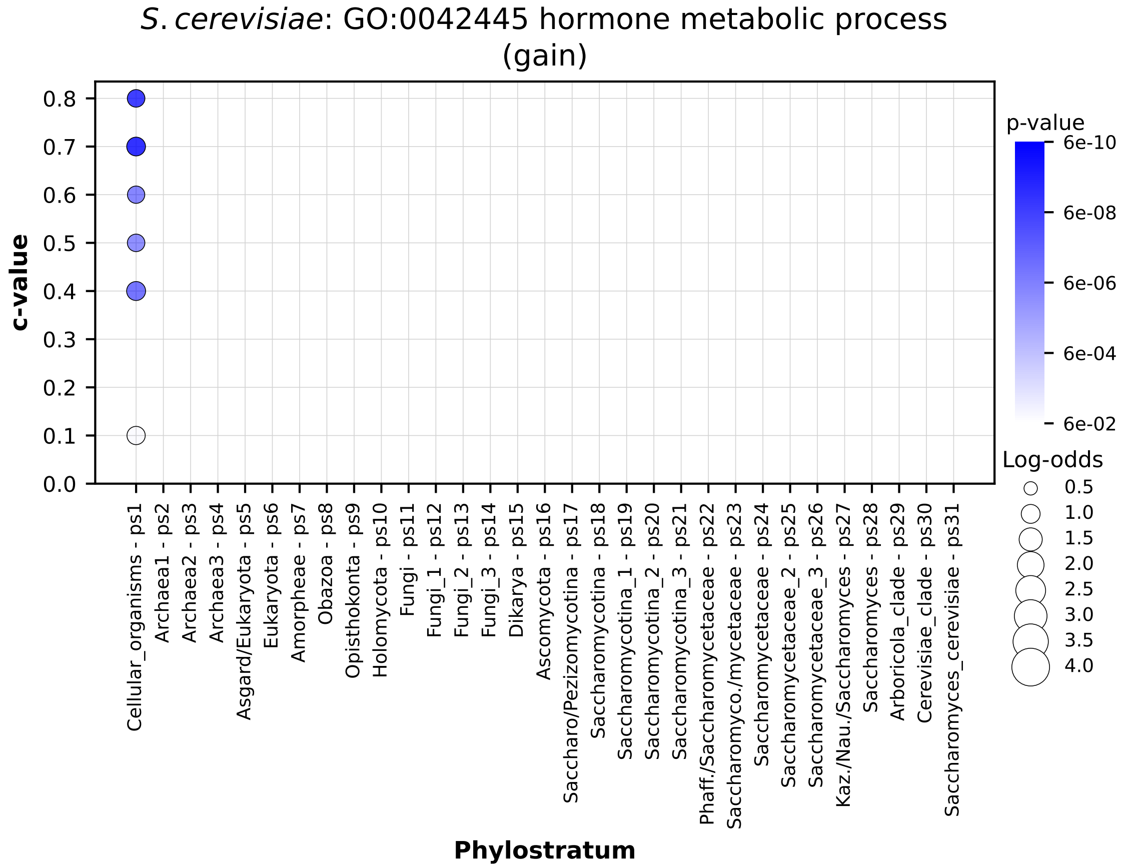

S. cerevisiae: GO:0042445 hormone metabolic process
(gain)
c-value
0.0
0.1
0.2
0.3
0.4
0.5
0.6
0.7
0.8
Phylostratum
Cellular_organisms - ps1
Archaea1 - ps2
Archaea2 - ps3
Archaea3 - ps4
Asgard/Eukaryota - ps5
Eukaryota - ps6
Amorpheae - ps7
Obazoa - ps8
Opisthokonta - ps9
Holomycota - ps10
Fungi - ps11
Fungi_1 - ps12
Fungi_2 - ps13
Fungi_3 - ps14
Dikarya - ps15
Ascomycota - ps16
Saccharo/Pezizomycotina - ps17
Saccharomycotina - ps18
Saccharomycotina_1 - ps19
Saccharomycotina_2 - ps20
Saccharomycotina_3 - ps21
Phaff./Saccharomycetaceae - ps22
Saccharomyco./mycetaceae - ps23
Saccharomycetaceae - ps24
Saccharomycetaceae_2 - ps25
Saccharomycetaceae_3 - ps26
Kaz./Nau./Saccharomyces - ps27
Saccharomyces - ps28
Arboricola_clade - ps29
Cerevisiae_clade - ps30
Saccharomyces_cerevisiae - ps31
p-value
6e-10
6e-08
6e-06
6e-04
6e-02
Log-odds
0.5
1.0
1.5
2.0
2.5
3.0
3.5
4.0

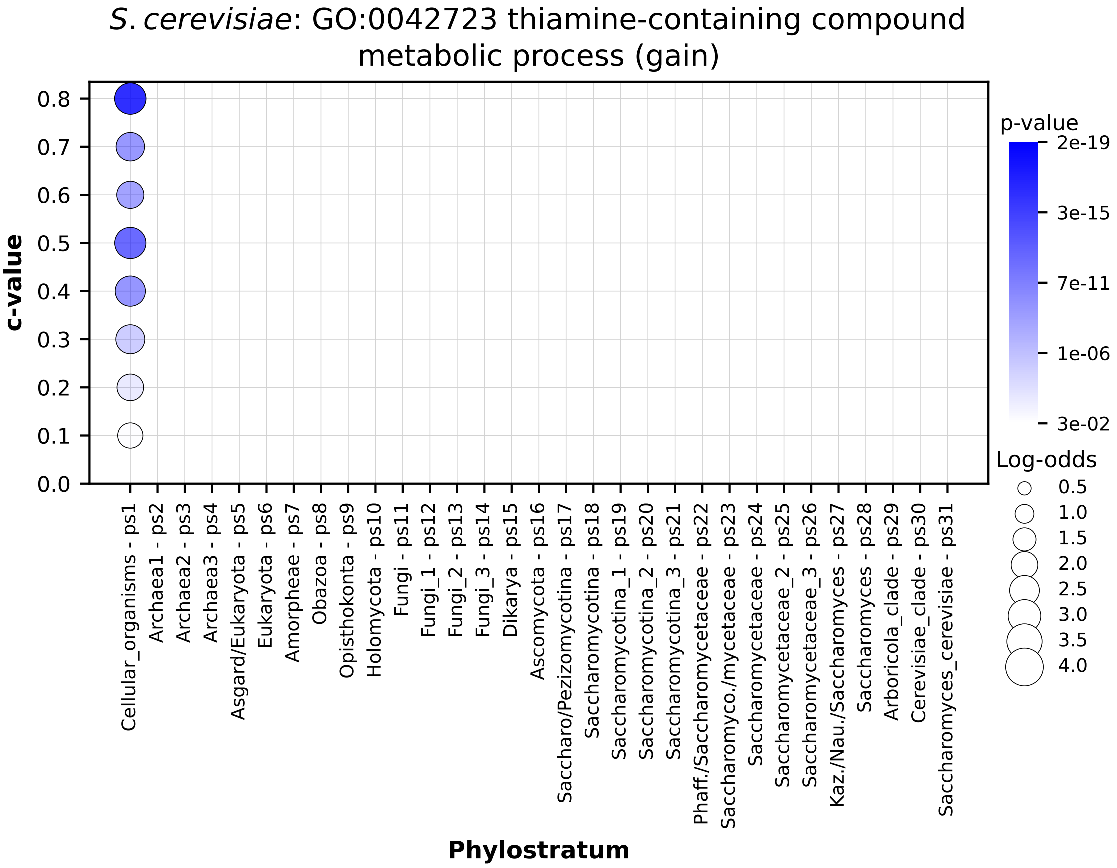

S. cerevisiae: GO:0042723 thiamine-containing compound metabolic process (gain)
c-value
0.0
0.1
0.2
0.3
0.4
0.5
0.6
0.7
0.8
p-value
2e-19
3e-15
7e-11
1e-06
3e-02
Log-odds
0.5
1.0
1.5
2.0
2.5
3.0
3.5
4.0
Cellular_organisms - ps1
Archaea1 - ps2
Archaea2 - ps3
Archaea3 - ps4
Asgard/Eukaryota - ps5
Eukaryota - ps6
Amorpheae - ps7
Obazoa - ps8
Opisthokonta - ps9
Holomycota - ps10
Fungi - ps11
Fungi_1 - ps12
Fungi_2 - ps13
Fungi_3 - ps14
Dikarya - ps15
Ascomycota - ps16
Saccharo/Pezizomycotina - ps17
Saccharomycotina - ps18
Saccharomycotina_1 - ps19
Saccharomycotina_2 - ps20
Saccharomycotina_3 - ps21
Phaff./Saccharomycetaceae - ps22
Saccharomyco./mycetaceae - ps23
Saccharomycetaceae - ps24
Saccharomycetaceae_2 - ps25
Saccharomycetaceae_3 - ps26
Kaz./Nau./Saccharomyces - ps27
Saccharomyces - ps28
Arboricola_clade - ps29
Cerevisiae_clade - ps30
Saccharomyces_cerevisiae - ps31
Phylostratum

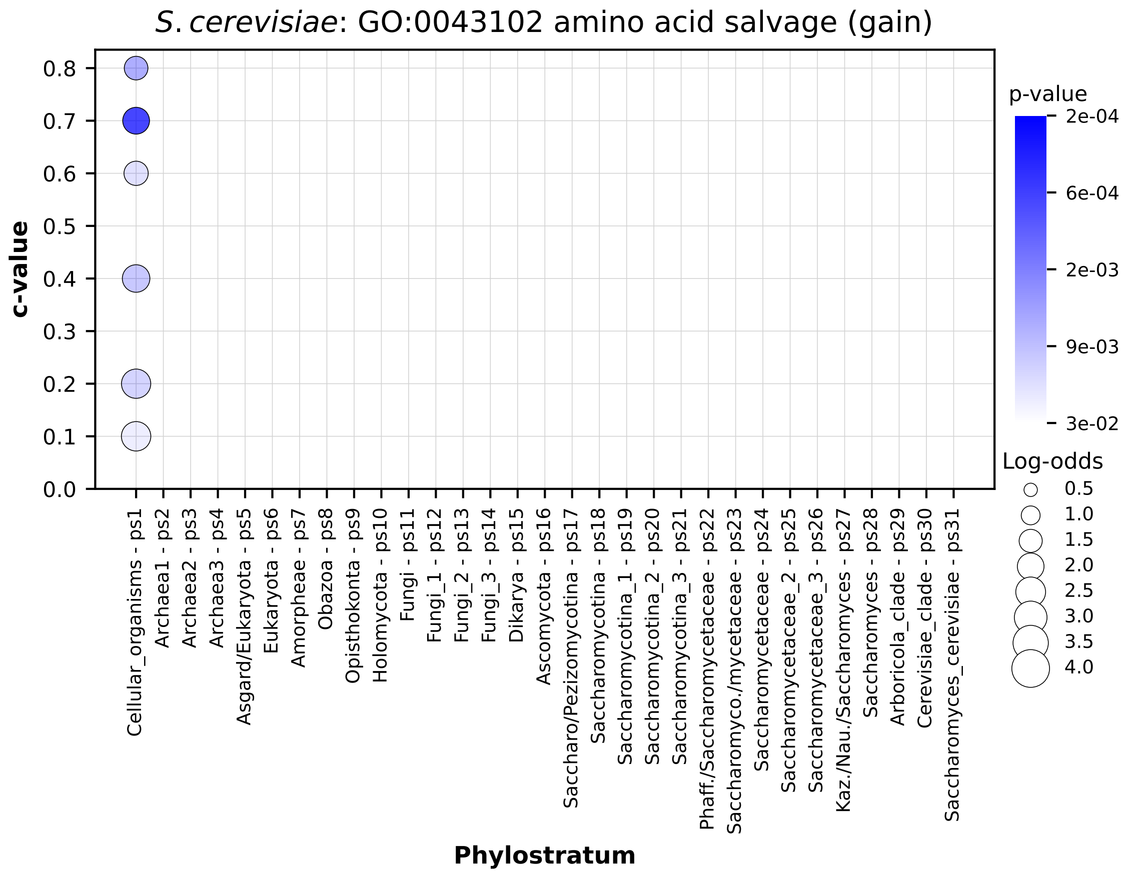

S. cerevisiae: GO:0043102 amino acid salvage (gain)
c-value
0.0
0.1
0.2
0.3
0.4
0.5
0.6
0.7
0.8
Phylostratum
Cellular_organisms - ps1
Archaea1 - ps2
Archaea2 - ps3
Archaea3 - ps4
Asgard/Eukaryota - ps5
Eukaryota - ps6
Amorpheae - ps7
Obazoa - ps8
Opisthokonta - ps9
Holomycota - ps10
Fungi - ps11
Fungi_1 - ps12
Fungi_2 - ps13
Fungi_3 - ps14
Dikarya - ps15
Ascomycota - ps16
Saccharo/Pezizomycotina - ps17
Saccharomycotina - ps18
Saccharomycotina_1 - ps19
Saccharomycotina_2 - ps20
Saccharomycotina_3 - ps21
Phaff./Saccharomycetaceae - ps22
Saccharomyco./mycetaceae - ps23
Saccharomycetaceae - ps24
Saccharomycetaceae_2 - ps25
Saccharomycetaceae_3 - ps26
Kaz./Nau./Saccharomyces - ps27
Saccharomyces - ps28
Arboricola_clade - ps29
Cerevisiae_clade - ps30
Saccharomyces_cerevisiae - ps31
p-value
2e-04
6e-04
2e-03
9e-03
3e-02
Log-odds
0.5
1.0
1.5
2.0
2.5
3.0
3.5
4.0

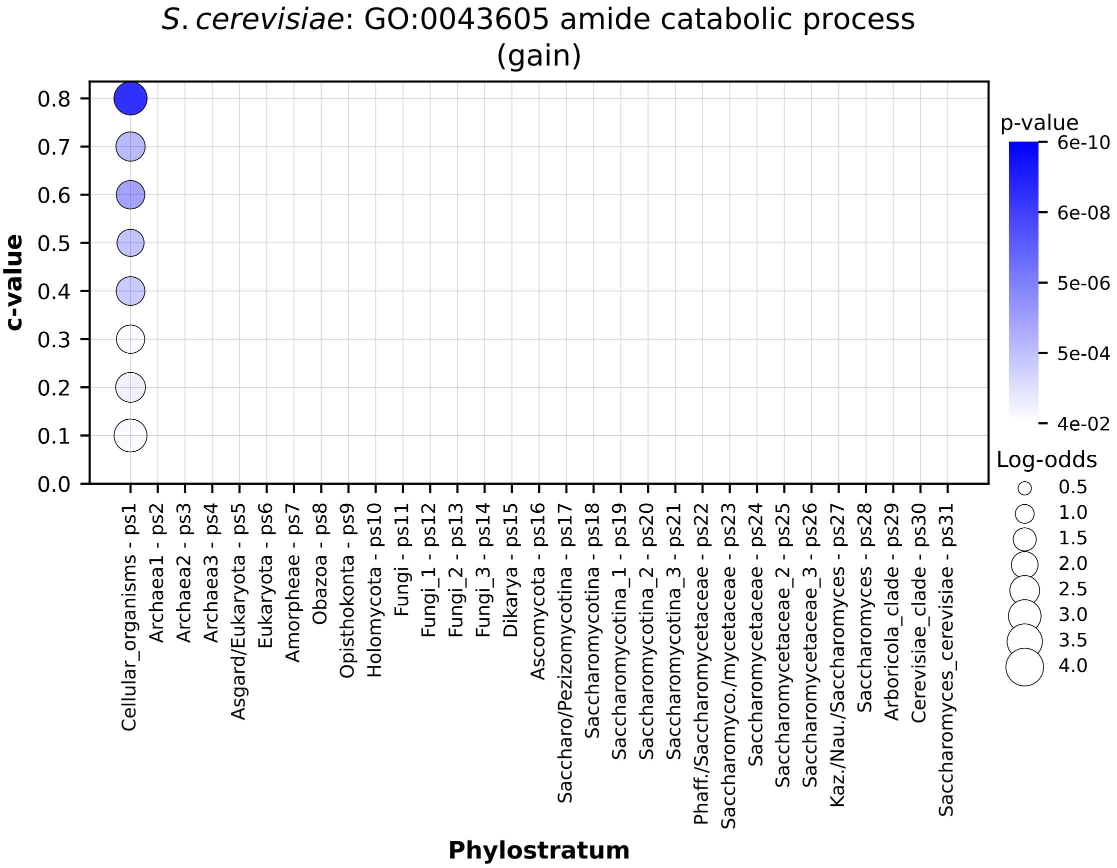

S. cerevisiae: GO:0043605 amide catabolic process
(gain)
c-value
0.8
0.7
0.6
0.5
0.4
0.3
0.2
0.1
0.0
p-value
6e-10
6e-08
5e-06
5e-04
4e-02
Log-odds
0.5
1.0
1.5
2.0
2.5
3.0
3.5
4.0
Cellular_organisms - ps1
Archaea1 - ps2
Archaea2 - ps3
Archaea3 - ps4
Asgard/Eukaryota - ps5
Eukaryota - ps6
Amorpheae - ps7
Obazoa - ps8
Opisthokonta - ps9
Holomycota - ps10
Fungi - ps11
Fungi_1 - ps12
Fungi_2 - ps13
Fungi_3 - ps14
Dikarya - ps15
Ascomycota - ps16
Saccharo/Pezizomycotina - ps17
Saccharomycotina - ps18
Saccharomycotina_1 - ps19
Saccharomycotina_2 - ps20
Saccharomycotina_3 - ps21
Phaff./Saccharomycetaceae - ps22
Saccharomyco./mycetaceae - ps23
Saccharomycetaceae - ps24
Saccharomycetaceae_2 - ps25
Saccharomycetaceae_3 - ps26
Kaz./Nau./Saccharomyces - ps27
Saccharomyces - ps28
Arboricola_clade - ps29
Cerevisiae_clade - ps30
Saccharomyces_cerevisiae - ps31
Phylostratum

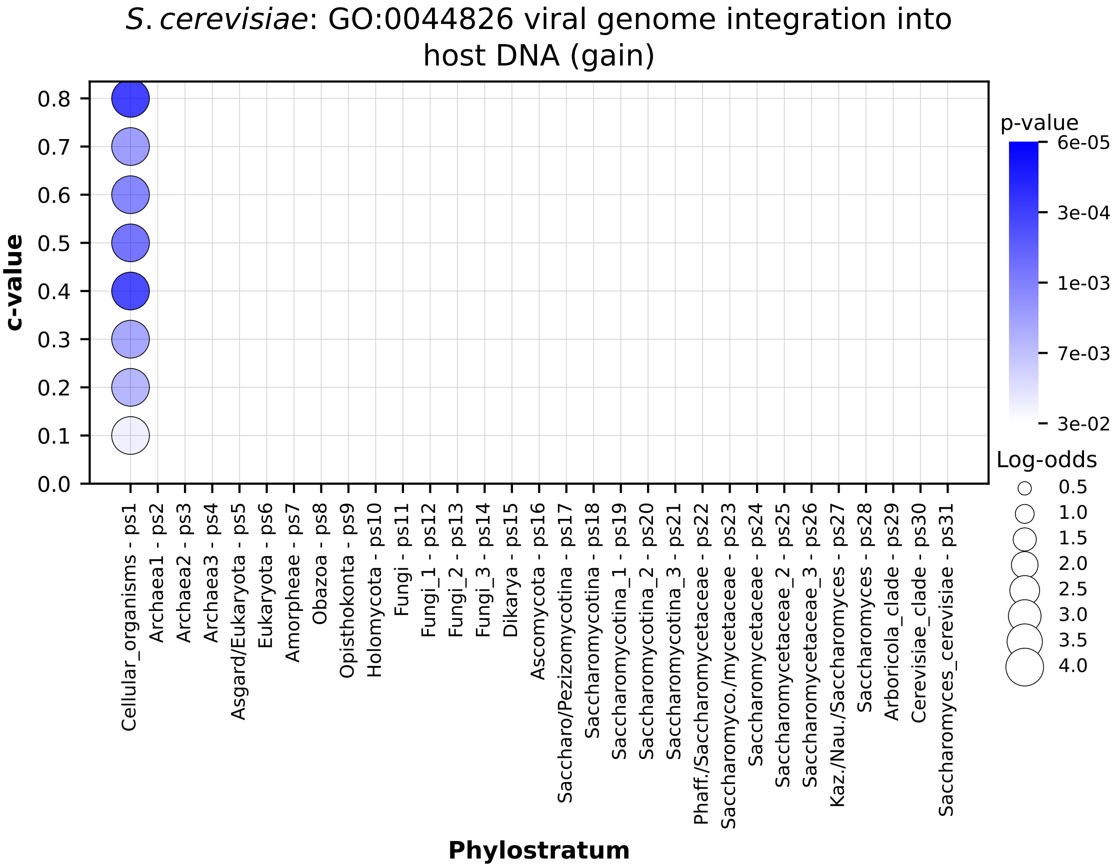

S. cerevisiae: GO:0044826 viral genome integration into host DNA (gain)
c-value
0.8
0.7
0.6
0.5
0.4
0.3
0.2
0.1
0.0
Phylostratum
Cellular_organisms - ps1
Archaea1 - ps2
Archaea2 - ps3
Archaea3 - ps4
Asgard/Eukaryota - ps5
Eukaryota - ps6
Amorpheae - ps7
Obazoa - ps8
Opisthokonta - ps9
Holomycota - ps10
Fungi - ps11
Fungi_1 - ps12
Fungi_2 - ps13
Fungi_3 - ps14
Dikarya - ps15
Ascomycota - ps16
Saccharo/Pezizomycotina - ps17
Saccharomycotina - ps18
Saccharomycotina_1 - ps19
Saccharomycotina_2 - ps20
Saccharomycotina_3 - ps21
Phaff./Saccharomycetaceae - ps22
Saccharomyco./mycetaceae - ps23
Saccharomycetaceae - ps24
Saccharomycetaceae_2 - ps25
Saccharomycetaceae_3 - ps26
Kaz./Nau./Saccharomyces - ps27
Saccharomyces - ps28
Arboricola_clade - ps29
Cerevisiae_clade - ps30
Saccharomyces_cerevisiae - ps31
p-value
6e-05
3e-04
1e-03
7e-03
3e-02
Log-odds
0.5
1.0
1.5
2.0
2.5
3.0
3.5
4.0

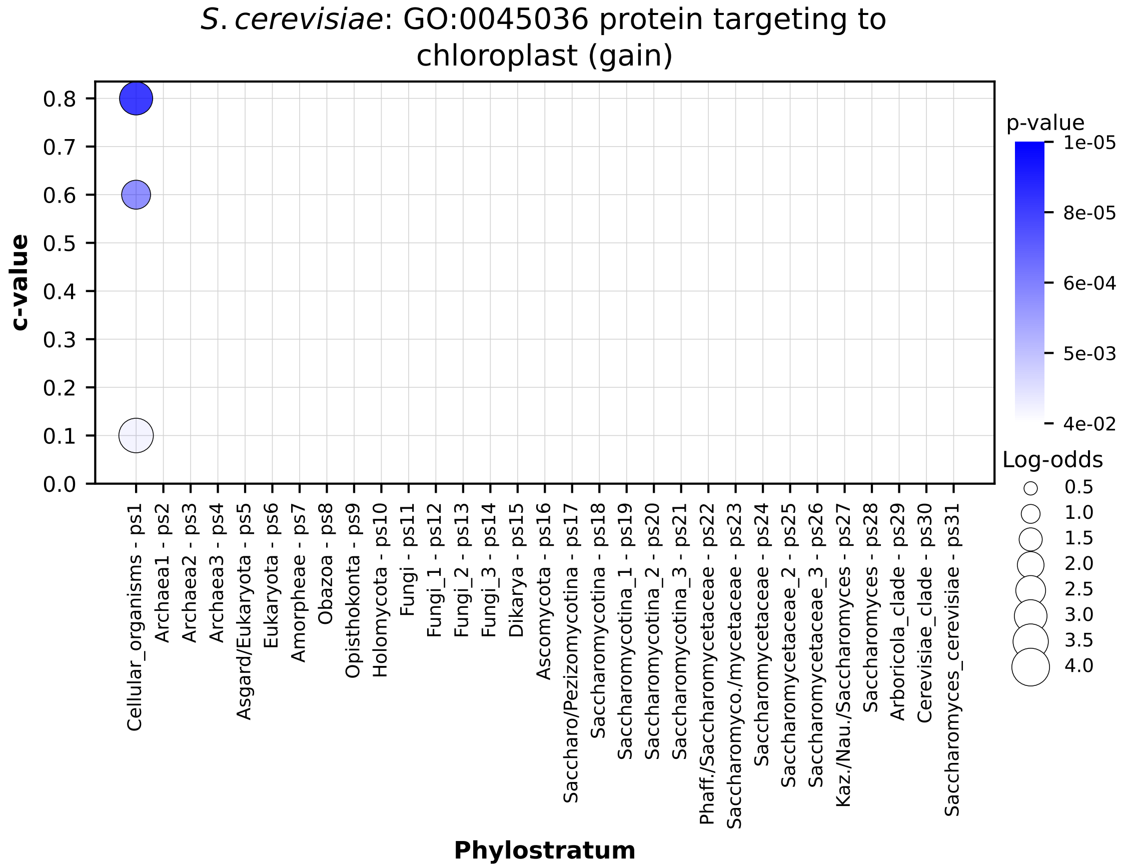

S. cerevisiae: GO:0045036 protein targeting to chloroplast (gain)
c-value
0.0
0.1
0.2
0.3
0.4
0.5
0.6
0.7
0.8
Phylostratum
Cellular_organisms - ps1
Archaea1 - ps2
Archaea2 - ps3
Archaea3 - ps4
Asgard/Eukaryota - ps5
Eukaryota - ps6
Amorpheae - ps7
Obazoa - ps8
Opisthokonta - ps9
Holomycota - ps10
Fungi - ps11
Fungi_1 - ps12
Fungi_2 - ps13
Fungi_3 - ps14
Dikarya - ps15
Ascomycota - ps16
Saccharo/Pezizomycotina - ps17
Saccharomycotina - ps18
Saccharomycotina_1 - ps19
Saccharomycotina_2 - ps20
Saccharomycotina_3 - ps21
Phaff./Saccharomycetaceae - ps22
Saccharomyco./mycetaceae - ps23
Saccharomycetaceae - ps24
Saccharomycetaceae_2 - ps25
Saccharomycetaceae_3 - ps26
Kaz./Nau./Saccharomyces - ps27
Saccharomyces - ps28
Arboricola_clade - ps29
Cerevisiae_clade - ps30
Saccharomyces_cerevisiae - ps31
p-value
1e-05
8e-05
6e-04
5e-03
4e-02
Log-odds
0.5
1.0
1.5
2.0
2.5
3.0
3.5
4.0

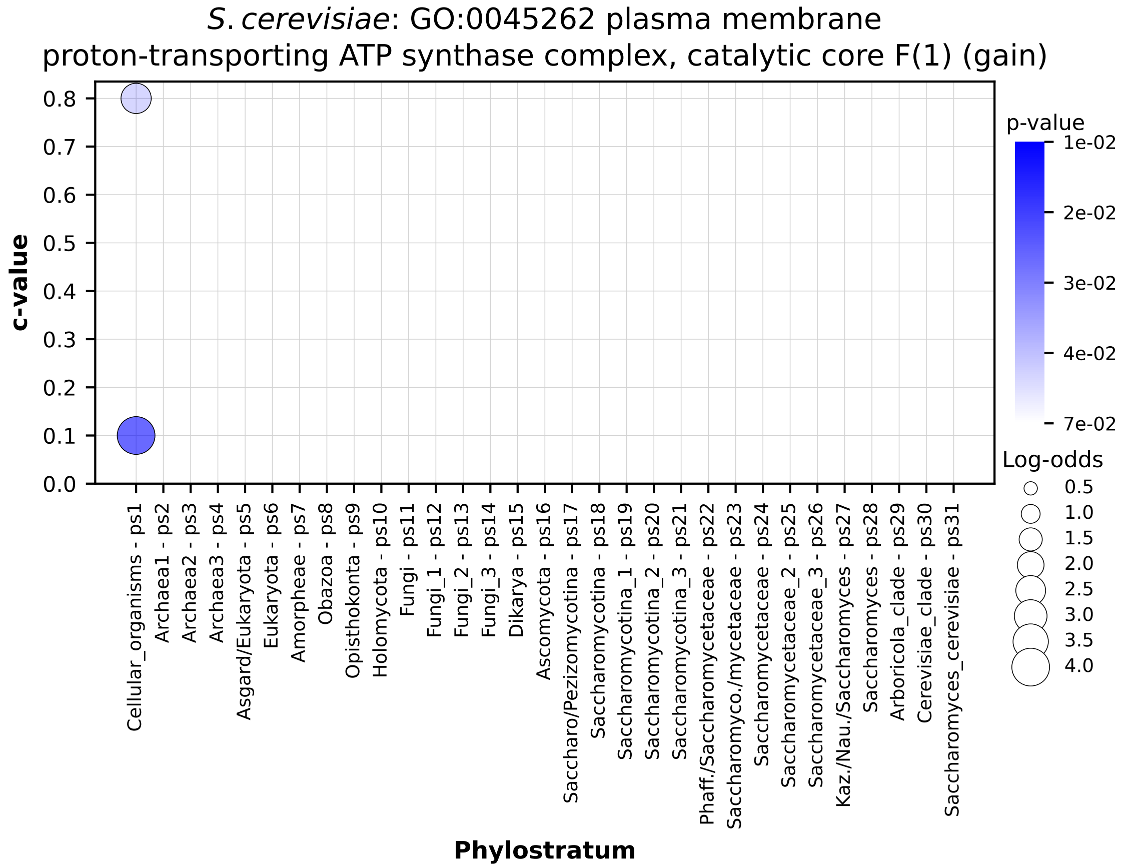

S. cerevisiae: GO:0045262 plasma membrane
proton-transporting ATP synthase complex, catalytic core F(1) (gain)
c-value
0.0
0.1
0.2
0.3
0.4
0.5
0.6
0.7
0.8
Phylostratum
Cellular_organisms - ps1
Archaea1 - ps2
Archaea2 - ps3
Archaea3 - ps4
Asgard/Eukaryota - ps5
Eukaryota - ps6
Amorpheae - ps7
Obazoa - ps8
Opisthokonta - ps9
Holomycota - ps10
Fungi - ps11
Fungi_1 - ps12
Fungi_2 - ps13
Fungi_3 - ps14
Dikarya - ps15
Ascomycota - ps16
Saccharo/Pezizomycotina - ps17
Saccharomycotina - ps18
Saccharomycotina_1 - ps19
Saccharomycotina_2 - ps20
Saccharomycotina_3 - ps21
Phaff./Saccharomycetaceae - ps22
Saccharomyco./mycetaceae - ps23
Saccharomycetaceae - ps24
Saccharomycetaceae_2 - ps25
Saccharomycetaceae_3 - ps26
Kaz./Nau./Saccharomyces - ps27
Saccharomyces - ps28
Arboricola_clade - ps29
Cerevisiae_clade - ps30
Saccharomyces_cerevisiae - ps31
p-value
1e-02
2e-02
3e-02
4e-02
7e-02
Log-odds
0.5
1.0
1.5
2.0
2.5
3.0
3.5
4.0

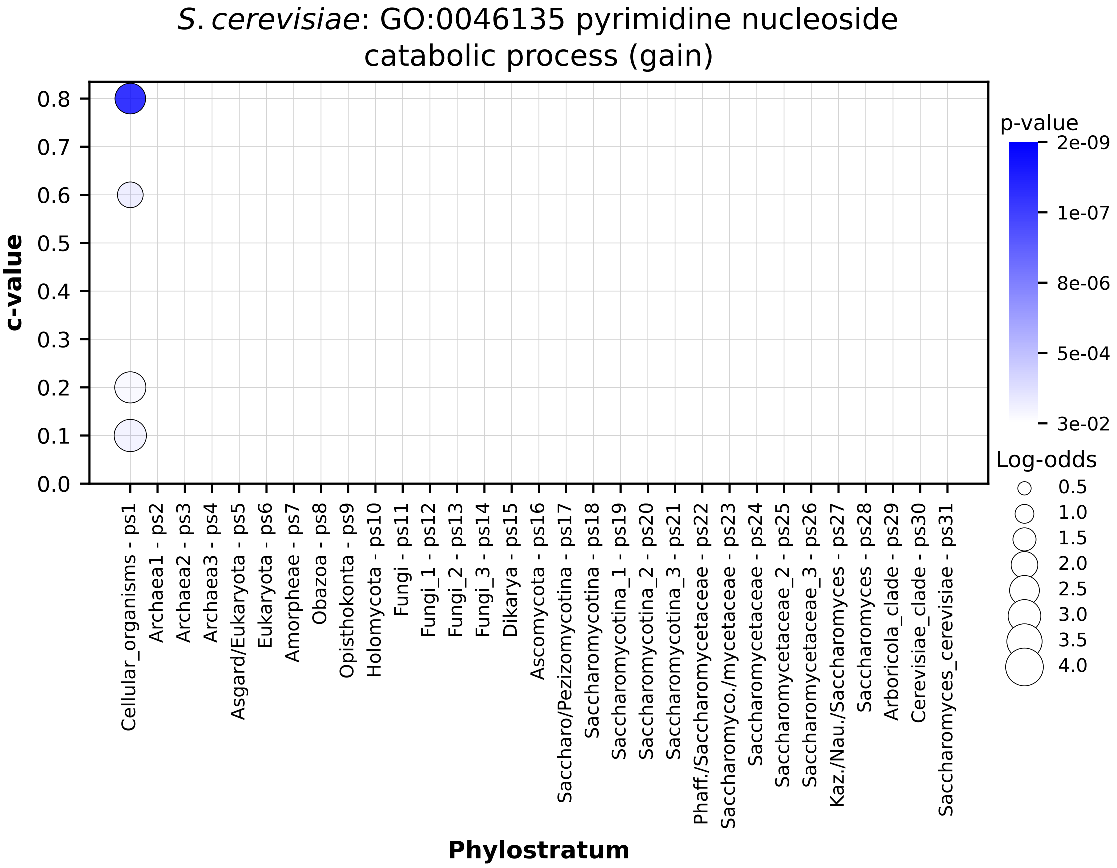

S. cerevisiae: GO:0046135 pyrimidine nucleoside catabolic process (gain)
c-value
0.0
0.1
0.2
0.3
0.4
0.5
0.6
0.7
0.8
p-value
2e-09
1e-07
8e-06
5e-04
3e-02
Log-odds
0.5
1.0
1.5
2.0
2.5
3.0
3.5
4.0
Cellular_organisms - ps1
Archaea1 - ps2
Archaea2 - ps3
Archaea3 - ps4
Asgard/Eukaryota - ps5
Eukaryota - ps6
Amorpheae - ps7
Obazoa - ps8
Opisthokonta - ps9
Holomycota - ps10
Fungi - ps11
Fungi_1 - ps12
Fungi_2 - ps13
Fungi_3 - ps14
Dikarya - ps15
Ascomycota - ps16
Saccharo/Pezizomycotina - ps17
Saccharomycotina - ps18
Saccharomycotina_1 - ps19
Saccharomycotina_2 - ps20
Saccharomycotina_3 - ps21
Phaff./Saccharomycetaceae - ps22
Saccharomyco./mycetaceae - ps23
Saccharomycetaceae - ps24
Saccharomycetaceae_2 - ps25
Saccharomycetaceae_3 - ps26
Kaz./Nau./Saccharomyces - ps27
Saccharomyces - ps28
Arboricola_clade - ps29
Cerevisiae_clade - ps30
Saccharomyces_cerevisiae - ps31
Phylostratum

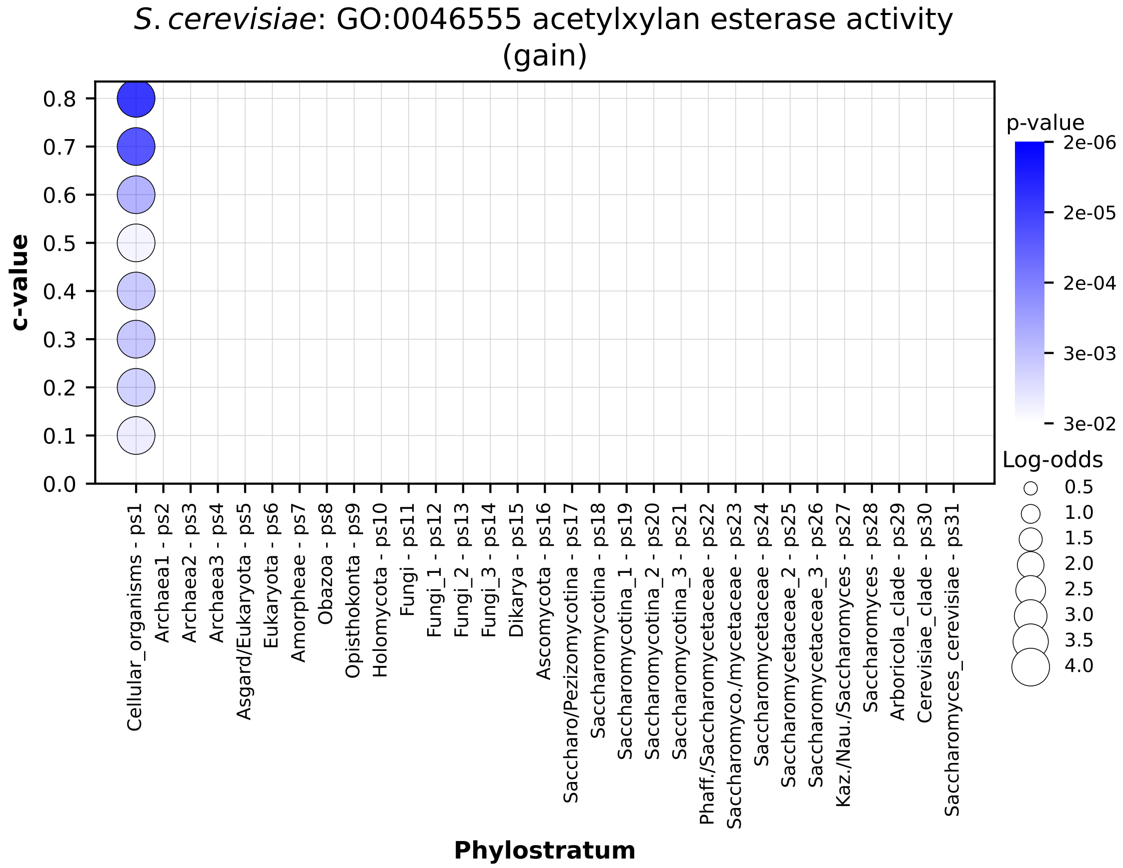

S. cerevisiae: GO:0046555 acetylxylan esterase activity (gain)
c-value
0.0
0.1
0.2
0.3
0.4
0.5
0.6
0.7
0.8
p-value
2e-06
2e-05
2e-04
3e-03
3e-02
Log-odds
0.5
1.0
1.5
2.0
2.5
3.0
3.5
4.0
Cellular_organisms - ps1
Archaea1 - ps2
Archaea2 - ps3
Archaea3 - ps4
Asgard/Eukaryota - ps5
Eukaryota - ps6
Amorpheae - ps7
Obazoa - ps8
Opisthokonta - ps9
Holomycota - ps10
Fungi - ps11
Fungi_1 - ps12
Fungi_2 - ps13
Fungi_3 - ps14
Dikarya - ps15
Ascomycota - ps16
Saccharo/Pezizomycotina - ps17
Saccharomycotina - ps18
Saccharomycotina_1 - ps19
Saccharomycotina_2 - ps20
Saccharomycotina_3 - ps21
Phaff./Saccharomycetaceae - ps22
Saccharomyco./mycetaceae - ps23
Saccharomycetaceae - ps24
Saccharomycetaceae_2 - ps25
Saccharomycetaceae_3 - ps26
Kaz./Nau./Saccharomyces - ps27
Saccharomyces - ps28
Arboricola_clade - ps29
Cerevisiae_clade - ps30
Saccharomyces_cerevisiae - ps31
Phylostratum

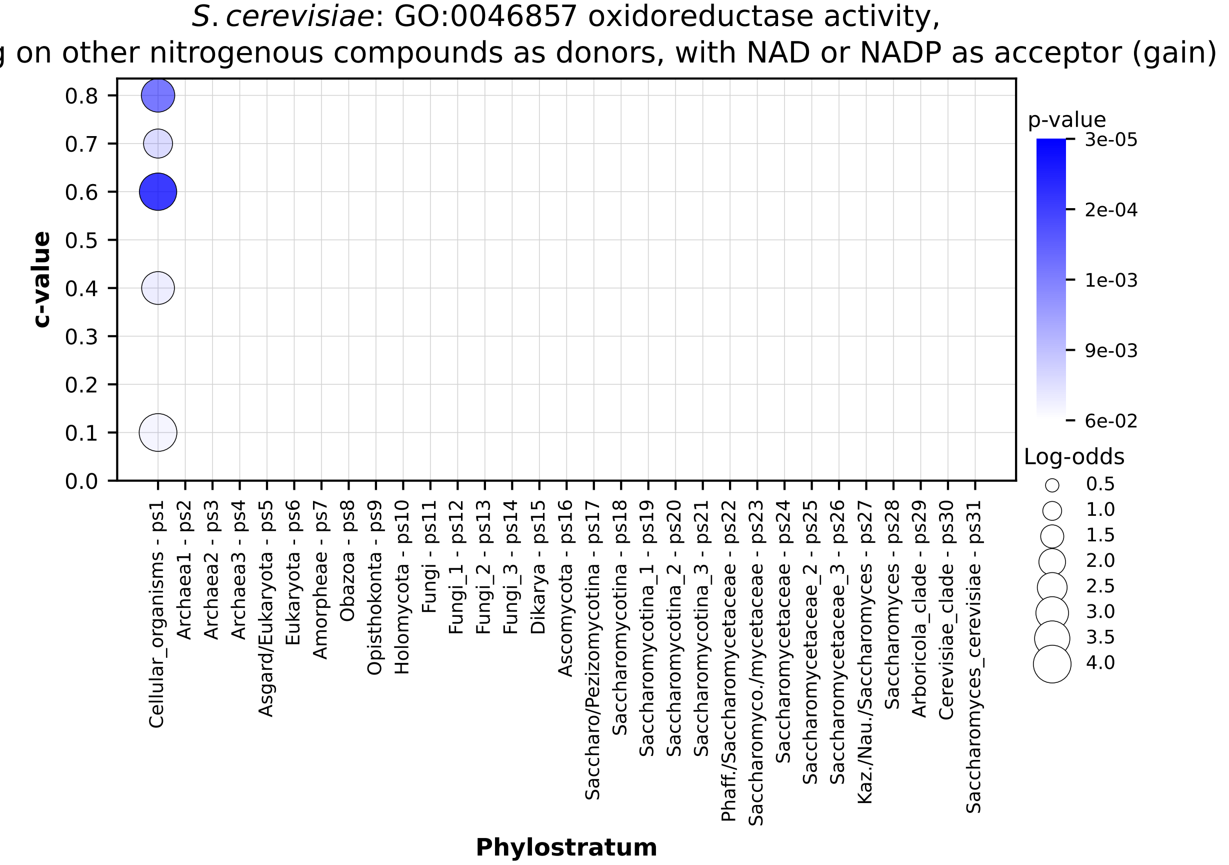

S. cerevisiae: GO:0046857 oxidoreductase activity,
g on other nitrogenous compounds as donors, with NAD or NADP as acceptor (gain)
c-value
0.0
0.1
0.2
0.3
0.4
0.5
0.6
0.7
0.8
p-value
3e-05
2e-04
1e-03
9e-03
6e-02
Log-odds
0.5
1.0
1.5
2.0
2.5
3.0
3.5
4.0
Cellular_organisms - ps1
Archaea1 - ps2
Archaea2 - ps3
Archaea3 - ps4
Asgard/Eukaryota - ps5
Eukaryota - ps6
Amorpheae - ps7
Obazoa - ps8
Opisthokonta - ps9
Holomycota - ps10
Fungi - ps11
Fungi_1 - ps12
Fungi_2 - ps13
Fungi_3 - ps14
Dikarya - ps15
Ascomycota - ps16
Saccharo/Pezizomycotina - ps17
Saccharomycotina - ps18
Saccharomycotina_1 - ps19
Saccharomycotina_2 - ps20
Saccharomycotina_3 - ps21
Phaff./Saccharomycetaceae - ps22
Saccharomyco./mycetaceae - ps23
Saccharomycetaceae - ps24
Saccharomycetaceae_2 - ps25
Saccharomycetaceae_3 - ps26
Kaz./Nau./Saccharomyces - ps27
Saccharomyces - ps28
Arboricola_clade - ps29
Cerevisiae_clade - ps30
Saccharomyces_cerevisiae - ps31
Phylostratum

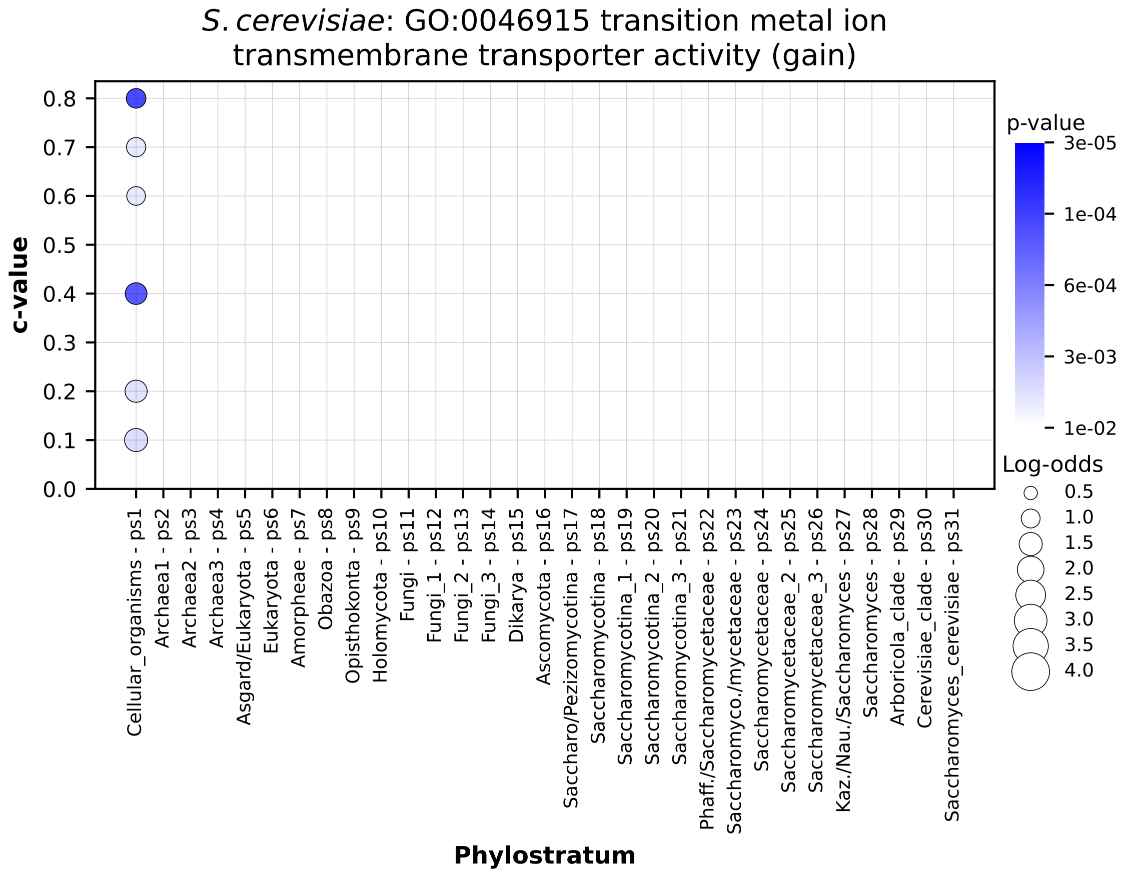
S. cerevisiae: GO:0046915 transition metal ion transmembrane transporter activity (gain)
c-value
0.0
0.1
0.2
0.3
0.4
0.5
0.6
0.7
0.8
p-value
3e-05
1e-04
6e-04
3e-03
1e-02
Log-odds
0.5
1.0
1.5
2.0
2.5
3.0
3.5
4.0
Cellular_organisms - ps1
Archaea1 - ps2
Archaea2 - ps3
Archaea3 - ps4
Asgard/Eukaryota - ps5
Eukaryota - ps6
Amorpheae - ps7
Obazoa - ps8
Opisthokonta - ps9
Holomycota - ps10
Fungi - ps11
Fungi_1 - ps12
Fungi_2 - ps13
Fungi_3 - ps14
Dikarya - ps15
Ascomycota - ps16
Saccharo/Pezizomycotina - ps17
Saccharomycotina - ps18
Saccharomycotina_1 - ps19
Saccharomycotina_2 - ps20
Saccharomycotina_3 - ps21
Phaff./Saccharomycetaceae - ps22
Saccharomyco./mycetaceae - ps23
Saccharomycetaceae - ps24
Saccharomycetaceae_2 - ps25
Saccharomycetaceae_3 - ps26
Kaz./Nau./Saccharomyces - ps27
Saccharomyces - ps28
Arboricola_clade - ps29
Cerevisiae_clade - ps30
Saccharomyces_cerevisiae - ps31
Phylostratum

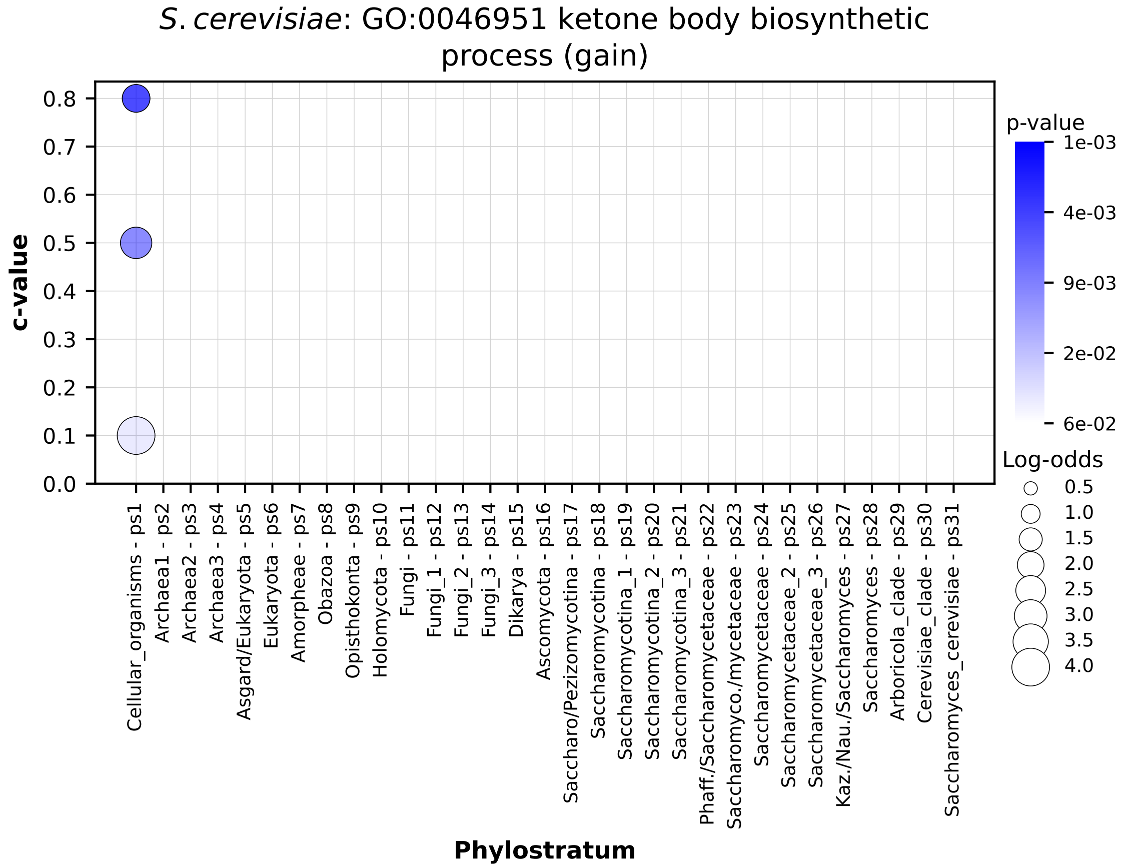

S. cerevisiae: GO:0046951 ketone body biosynthetic process (gain)
c-value
0.0
0.1
0.2
0.3
0.4
0.5
0.6
0.7
0.8
Phylostratum
Cellular_organisms - ps1
Archaea1 - ps2
Archaea2 - ps3
Archaea3 - ps4
Asgard/Eukaryota - ps5
Eukaryota - ps6
Amorpheae - ps7
Obazoa - ps8
Opisthokonta - ps9
Holomycota - ps10
Fungi - ps11
Fungi_1 - ps12
Fungi_2 - ps13
Fungi_3 - ps14
Dikarya - ps15
Ascomycota - ps16
Saccharo/Pezizomycotina - ps17
Saccharomycotina - ps18
Saccharomycotina_1 - ps19
Saccharomycotina_2 - ps20
Saccharomycotina_3 - ps21
Phaff./Saccharomycetaceae - ps22
Saccharomyco./mycetaceae - ps23
Saccharomycetaceae - ps24
Saccharomycetaceae_2 - ps25
Saccharomycetaceae_3 - ps26
Kaz./Nau./Saccharomyces - ps27
Saccharomyces - ps28
Arboricola_clade - ps29
Cerevisiae_clade - ps30
Saccharomyces_cerevisiae - ps31
p-value
1e-03
4e-03
9e-03
2e-02
6e-02
Log-odds
0.5
1.0
1.5
2.0
2.5
3.0
3.5
4.0

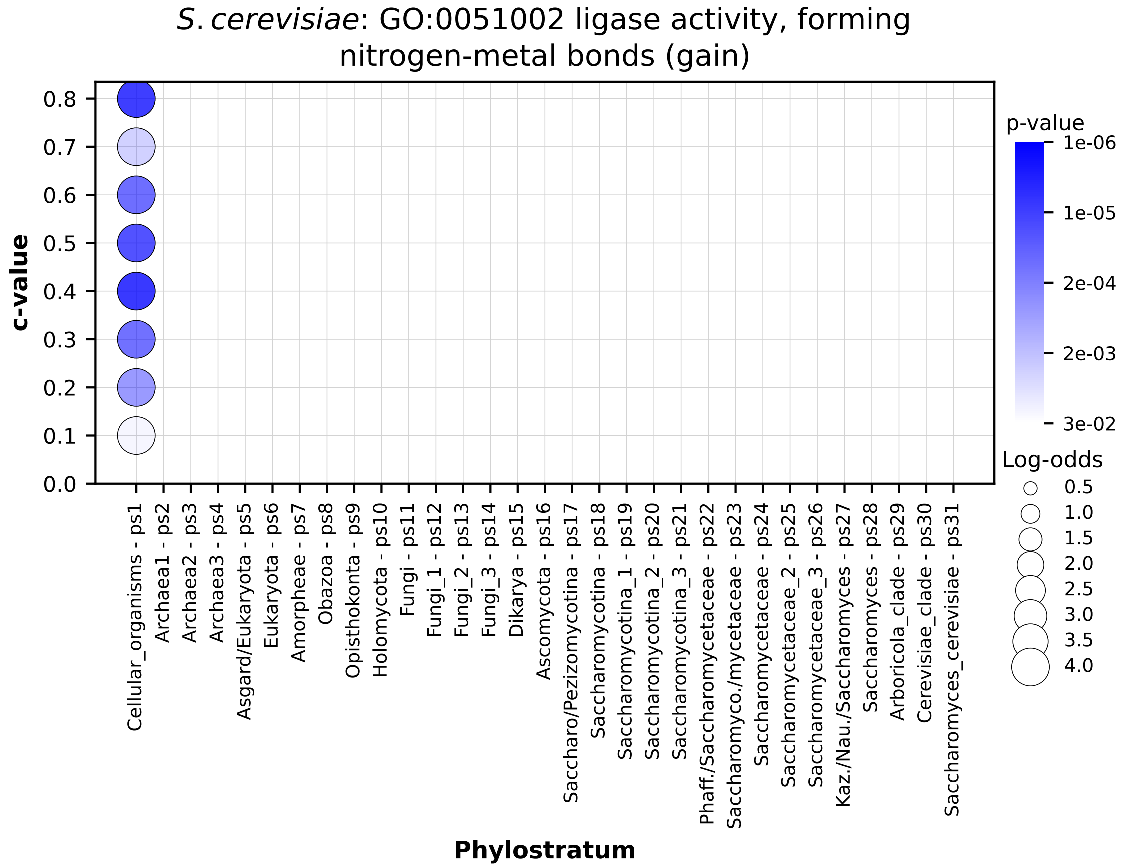

S. cerevisiae: GO:0051002 ligase activity, forming nitrogen-metal bonds (gain)
c-value
0.0
0.1
0.2
0.3
0.4
0.5
0.6
0.7
0.8
Phylostratum
Cellular_organisms - ps1
Archaea1 - ps2
Archaea2 - ps3
Archaea3 - ps4
Asgard/Eukaryota - ps5
Eukaryota - ps6
Amorpheae - ps7
Obazoa - ps8
Opisthokonta - ps9
Holomycota - ps10
Fungi - ps11
Fungi_1 - ps12
Fungi_2 - ps13
Fungi_3 - ps14
Dikarya - ps15
Ascomycota - ps16
Saccharo/Pezizomycotina - ps17
Saccharomycotina - ps18
Saccharomycotina_1 - ps19
Saccharomycotina_2 - ps20
Saccharomycotina_3 - ps21
Phaff./Saccharomycetaceae - ps22
Saccharomyco./mycetaceae - ps23
Saccharomycetaceae - ps24
Saccharomycetaceae_2 - ps25
Saccharomycetaceae_3 - ps26
Kaz./Nau./Saccharomyces - ps27
Saccharomyces - ps28
Arboricola_clade - ps29
Cerevisiae_clade - ps30
Saccharomyces_cerevisiae - ps31
p-value
1e-06
1e-05
2e-04
2e-03
3e-02
Log-odds
0.5
1.0
1.5
2.0
2.5
3.0
3.5
4.0

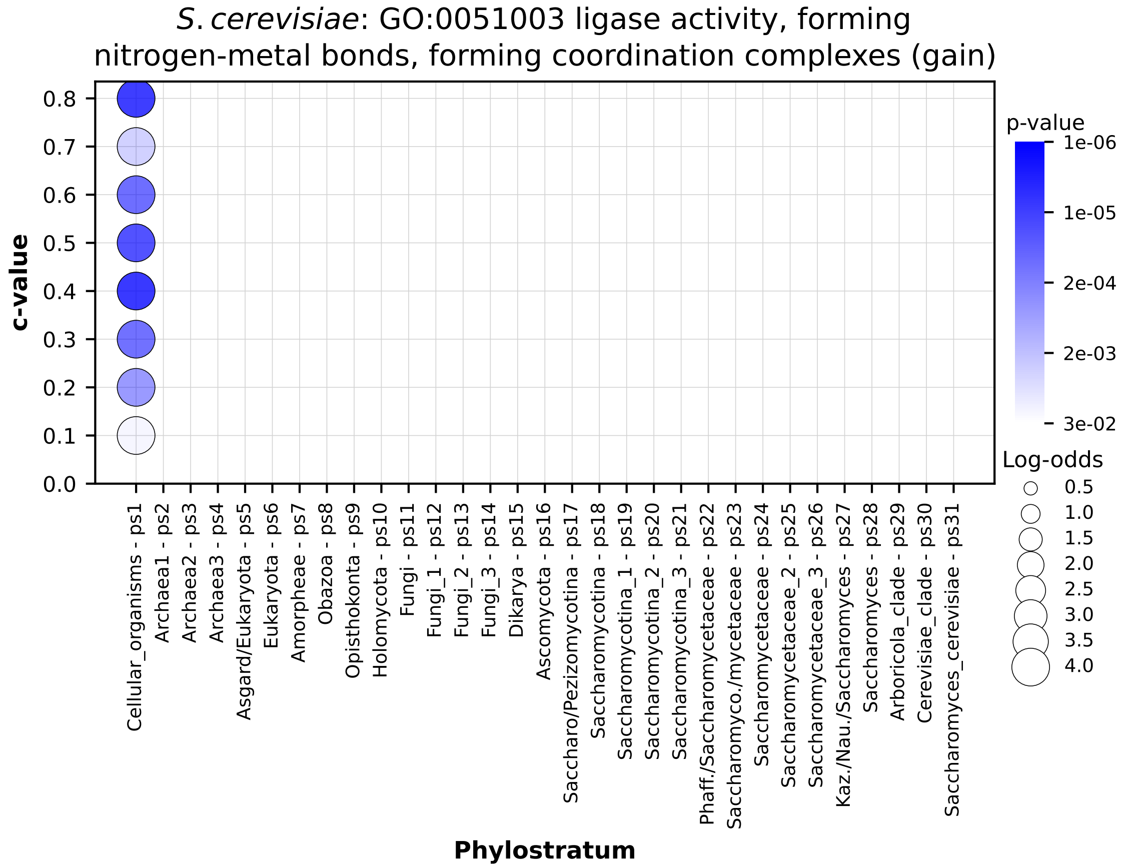
S. cerevisiae: GO:0051003 ligase activity, forming nitrogen-metal bonds, forming coordination complexes (gain)
c-value
0.0
0.1
0.2
0.3
0.4
0.5
0.6
0.7
0.8
p-value
1e-06
1e-05
2e-04
2e-03
3e-02
Log-odds
0.5
1.0
1.5
2.0
2.5
3.0
3.5
4.0
Cellular_organisms - ps1
Archaea1 - ps2
Archaea2 - ps3
Archaea3 - ps4
Asgard/Eukaryota - ps5
Eukaryota - ps6
Amorpheae - ps7
Obazoa - ps8
Opisthokonta - ps9
Holomycota - ps10
Fungi - ps11
Fungi_1 - ps12
Fungi_2 - ps13
Fungi_3 - ps14
Dikarya - ps15
Ascomycota - ps16
Saccharo/Pezizomycotina - ps17
Saccharomycotina - ps18
Saccharomycotina_1 - ps19
Saccharomycotina_2 - ps20
Saccharomycotina_3 - ps21
Phaff./Saccharomycetaceae - ps22
Saccharomyco./mycetaceae - ps23
Saccharomycetaceae - ps24
Saccharomycetaceae_2 - ps25
Saccharomycetaceae_3 - ps26
Kaz./Nau./Saccharomyces - ps27
Saccharomyces - ps28
Arboricola_clade - ps29
Cerevisiae_clade - ps30
Saccharomyces_cerevisiae - ps31
Phylostratum

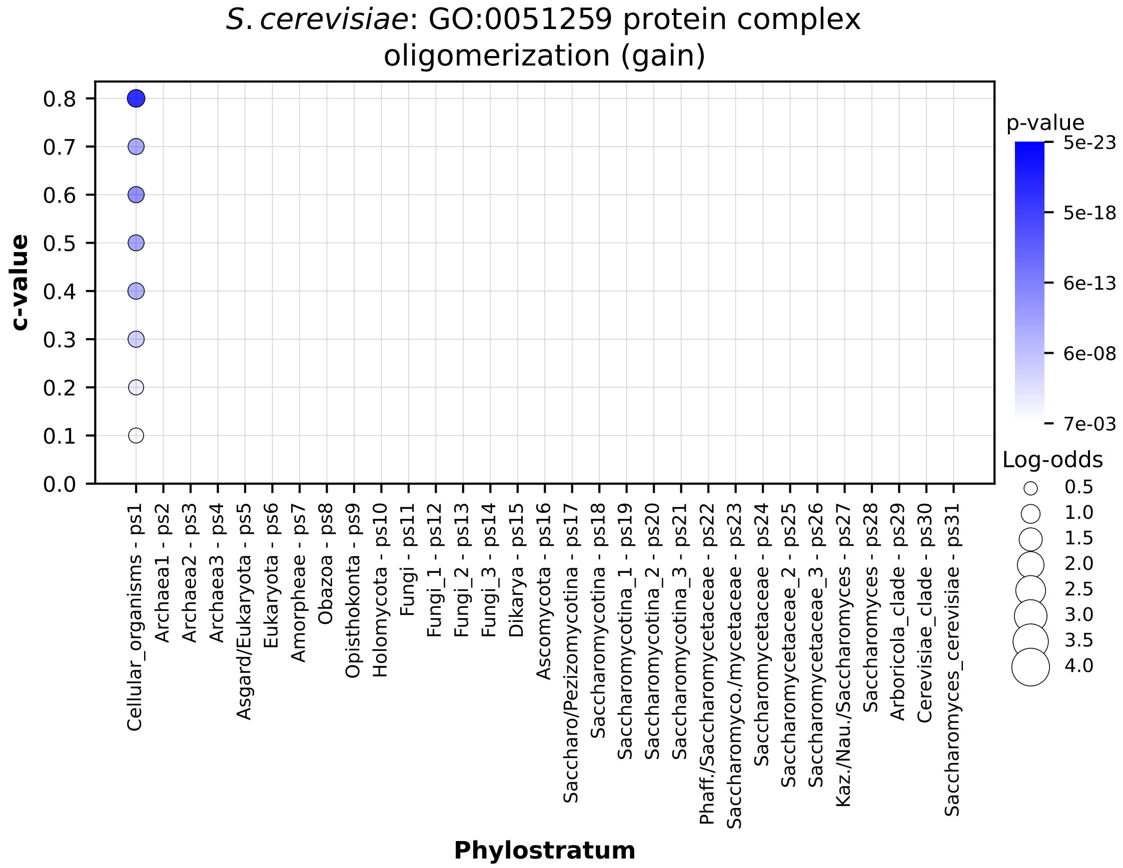

$S. cerevisiae$: GO:0051259 protein complex oligomerization (gain)
c-value
0.0
0.1
0.2
0.3
0.4
0.5
0.6
0.7
0.8
p-value
5e-23
5e-18
6e-13
6e-08
7e-03
Log-odds
0.5
1.0
1.5
2.0
2.5
3.0
3.5
4.0
Cellular_organisms - ps1
Archaea1 - ps2
Archaea2 - ps3
Archaea3 - ps4
Asgard/Eukaryota - ps5
Eukaryota - ps6
Amorpheae - ps7
Obazoa - ps8
Opisthokonta - ps9
Holomycota - ps10
Fungi - ps11
Fungi_1 - ps12
Fungi_2 - ps13
Fungi_3 - ps14
Dikarya - ps15
Ascomycota - ps16
Saccharo/Pezizomycotina - ps17
Saccharomycotina - ps18
Saccharomycotina_1 - ps19
Saccharomycotina_2 - ps20
Saccharomycotina_3 - ps21
Phaff./Saccharomycetaceae - ps22
Saccharomyco./mycetaceae - ps23
Saccharomycetaceae - ps24
Saccharomycetaceae_2 - ps25
Saccharomycetaceae_3 - ps26
Kaz./Nau./Saccharomyces - ps27
Saccharomyces - ps28
Arboricola_clade - ps29
Cerevisiae_clade - ps30
Saccharomyces_cerevisiae - ps31
Phylostratum

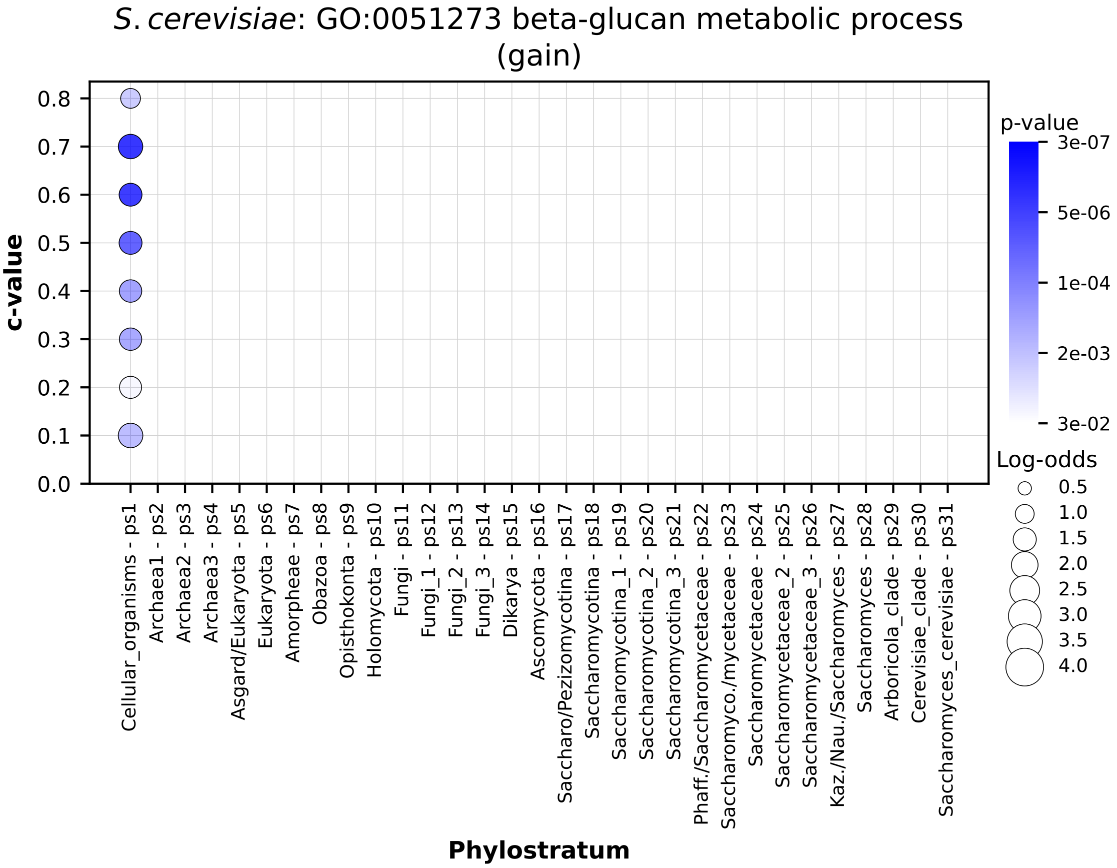

S. cerevisiae: GO:0051273 beta-glucan metabolic process
(gain)
c-value
0.0
0.1
0.2
0.3
0.4
0.5
0.6
0.7
0.8
p-value
3e-07
5e-06
1e-04
2e-03
3e-02
Log-odds
0.5
1.0
1.5
2.0
2.5
3.0
3.5
4.0
Cellular_organisms - ps1
Archaea1 - ps2
Archaea2 - ps3
Archaea3 - ps4
Asgard/Eukaryota - ps5
Eukaryota - ps6
Amorpheae - ps7
Obazoa - ps8
Opisthokonta - ps9
Holomycota - ps10
Fungi - ps11
Fungi_1 - ps12
Fungi_2 - ps13
Fungi_3 - ps14
Dikarya - ps15
Ascomycota - ps16
Saccharo/Pezizomycotina - ps17
Saccharomycotina - ps18
Saccharomycotina_1 - ps19
Saccharomycotina_2 - ps20
Saccharomycotina_3 - ps21
Phaff./Saccharomycetaceae - ps22
Saccharomyco./mycetaceae - ps23
Saccharomycetaceae - ps24
Saccharomycetaceae_2 - ps25
Saccharomycetaceae_3 - ps26
Kaz./Nau./Saccharomyces - ps27
Saccharomyces - ps28
Arboricola_clade - ps29
Cerevisiae_clade - ps30
Saccharomyces_cerevisiae - ps31
Phylostratum

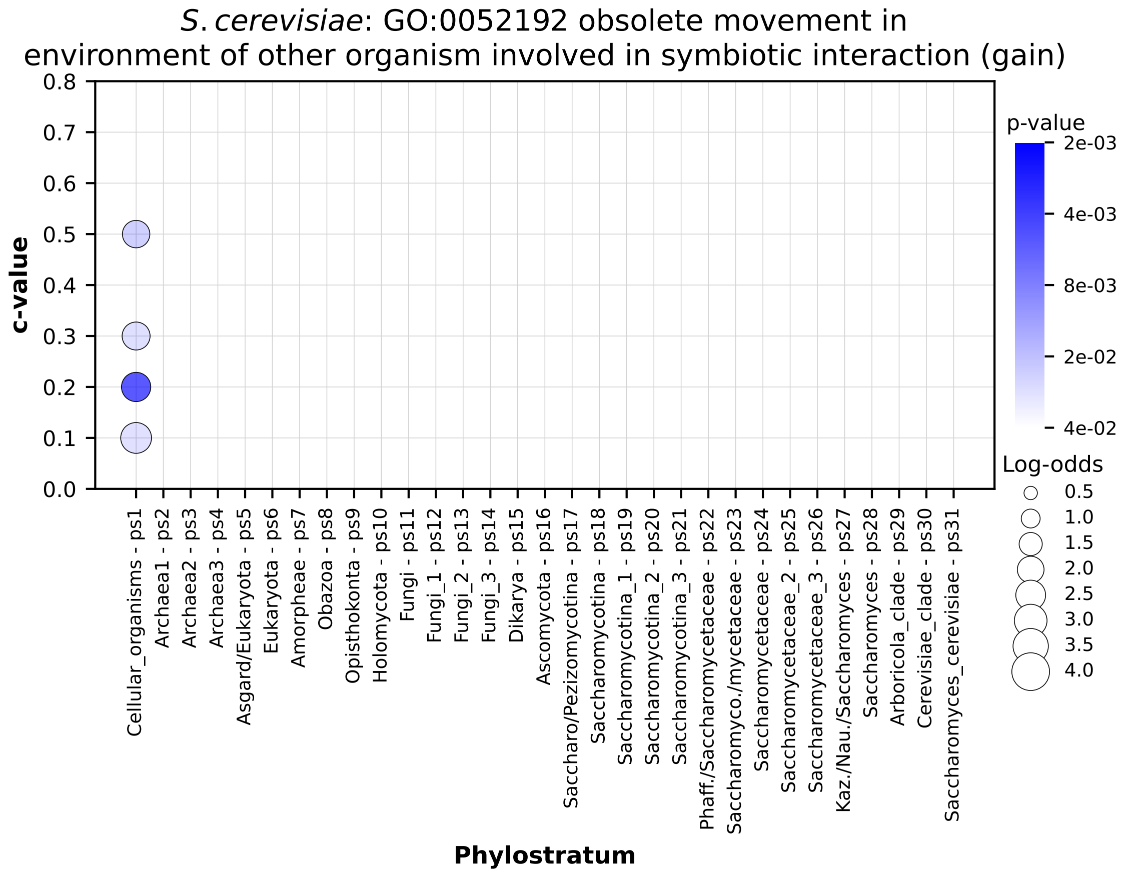

S. cerevisiae: GO:0052192 obsolete movement in environment of other organism involved in symbiotic interaction (gain)
c-value
0.0
0.1
0.2
0.3
0.4
0.5
0.6
0.7
0.8
p-value
2e-03
4e-03
8e-03
2e-02
4e-02
Log-odds
0.5
1.0
1.5
2.0
2.5
3.0
3.5
4.0
Cellular_organisms - ps1
Archaea1 - ps2
Archaea2 - ps3
Archaea3 - ps4
Asgard/Eukaryota - ps5
Eukaryota - ps6
Amorpheae - ps7
Obazoa - ps8
Opisthokonta - ps9
Holomycota - ps10
Fungi - ps11
Fungi_1 - ps12
Fungi_2 - ps13
Fungi_3 - ps14
Dikarya - ps15
Ascomycota - ps16
Saccharo/Pezizomycotina - ps17
Saccharomycotina - ps18
Saccharomycotina_1 - ps19
Saccharomycotina_2 - ps20
Saccharomycotina_3 - ps21
Phaff./Saccharomycetaceae - ps22
Saccharomyco./mycetaceae - ps23
Saccharomycetaceae - ps24
Saccharomycetaceae_2 - ps25
Saccharomycetaceae_3 - ps26
Kaz./Nau./Saccharomyces - ps27
Saccharomyces - ps28
Arboricola_clade - ps29
Cerevisiae_clade - ps30
Saccharomyces_cerevisiae - ps31
Phylostratum

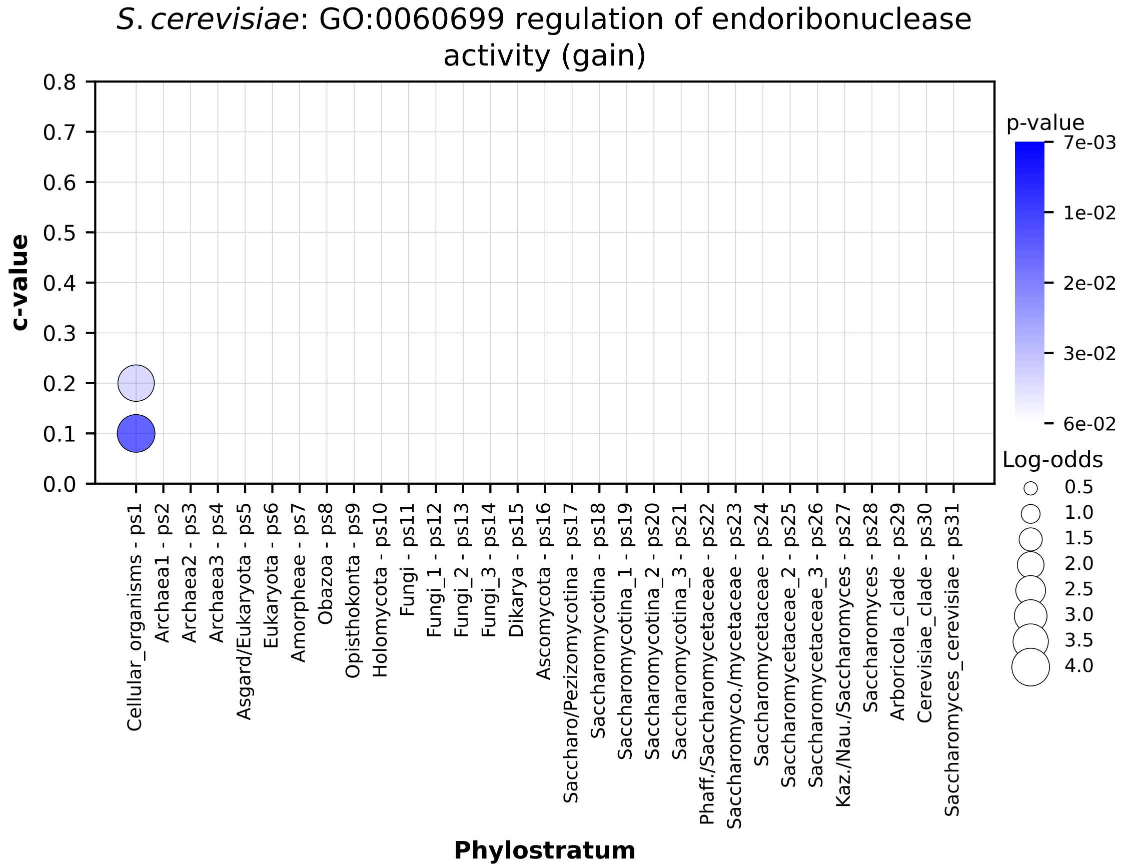

S. cerevisiae: GO:0060699 regulation of endoribonuclease activity (gain)
c-value
0.0
0.1
0.2
0.3
0.4
0.5
0.6
0.7
0.8
Phylostratum
Cellular_organisms - ps1
Archaea1 - ps2
Archaea2 - ps3
Archaea3 - ps4
Asgard/Eukaryota - ps5
Eukaryota - ps6
Amorpheae - ps7
Obazoa - ps8
Opisthokonta - ps9
Holomycota - ps10
Fungi - ps11
Fungi_1 - ps12
Fungi_2 - ps13
Fungi_3 - ps14
Dikarya - ps15
Ascomycota - ps16
Saccharo/Pezizomycotina - ps17
Saccharomycotina - ps18
Saccharomycotina_1 - ps19
Saccharomycotina_2 - ps20
Saccharomycotina_3 - ps21
Phaff./Saccharomycetaceae - ps22
Saccharomyco./mycetaceae - ps23
Saccharomycetaceae - ps24
Saccharomycetaceae_2 - ps25
Saccharomycetaceae_3 - ps26
Kaz./Nau./Saccharomyces - ps27
Saccharomyces - ps28
Arboricola_clade - ps29
Cerevisiae_clade - ps30
Saccharomyces_cerevisiae - ps31
p-value
7e-03
1e-02
2e-02
3e-02
6e-02
Log-odds
0.5
1.0
1.5
2.0
2.5
3.0
3.5
4.0

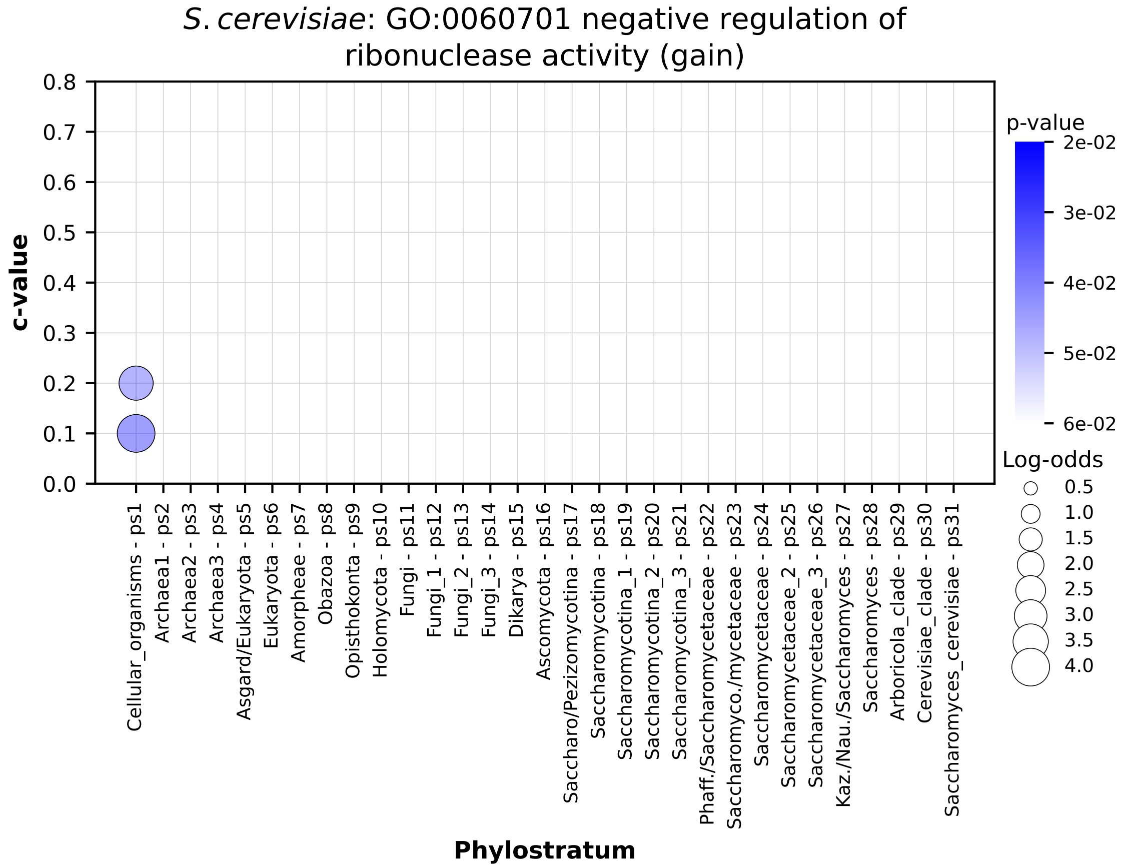

S. cerevisiae: GO:0060701 negative regulation of ribonuclease activity (gain)
c-value
0.0
0.1
0.2
0.3
0.4
0.5
0.6
0.7
0.8
p-value
2e-02
3e-02
4e-02
5e-02
6e-02
Log-odds
0.5
1.0
1.5
2.0
2.5
3.0
3.5
4.0
Cellular_organisms - ps1
Archaea1 - ps2
Archaea2 - ps3
Archaea3 - ps4
Asgard/Eukaryota - ps5
Eukaryota - ps6
Amorpheae - ps7
Obazoa - ps8
Opisthokonta - ps9
Holomycota - ps10
Fungi - ps11
Fungi_1 - ps12
Fungi_2 - ps13
Fungi_3 - ps14
Dikarya - ps15
Ascomycota - ps16
Saccharo/Pezizomycotina - ps17
Saccharomycotina - ps18
Saccharomycotina_1 - ps19
Saccharomycotina_2 - ps20
Saccharomycotina_3 - ps21
Phaff./Saccharomycetaceae - ps22
Saccharomyco./mycetaceae - ps23
Saccharomycetaceae - ps24
Saccharomycetaceae_2 - ps25
Saccharomycetaceae_3 - ps26
Kaz./Nau./Saccharomyces - ps27
Saccharomyces - ps28
Arboricola_clade - ps29
Cerevisiae_clade - ps30
Saccharomyces_cerevisiae - ps31
Phylostratum

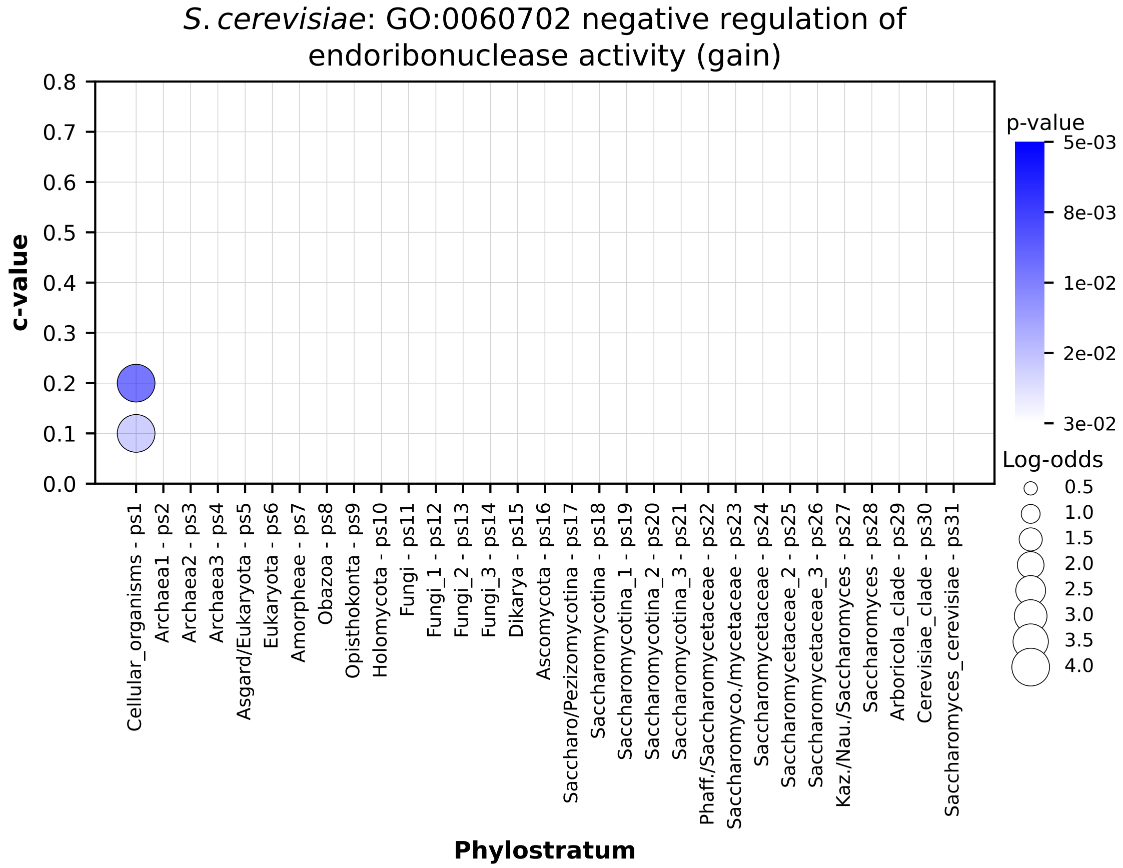

S. cerevisiae: GO:0060702 negative regulation of endoribonuclease activity (gain)
c-value
0.8
0.7
0.6
0.5
0.4
0.3
0.2
0.1
0.0
p-value
5e-03
8e-03
1e-02
2e-02
3e-02
Log-odds
0.5
1.0
1.5
2.0
2.5
3.0
3.5
4.0
Cellular_organisms - ps1
Archaea1 - ps2
Archaea2 - ps3
Archaea3 - ps4
Asgard/Eukaryota - ps5
Eukaryota - ps6
Amorpheae - ps7
Obazoa - ps8
Opisthokonta - ps9
Holomycota - ps10
Fungi - ps11
Fungi_1 - ps12
Fungi_2 - ps13
Fungi_3 - ps14
Dikarya - ps15
Ascomycota - ps16
Saccharo/Pezizomycotina - ps17
Saccharomycotina - ps18
Saccharomycotina_1 - ps19
Saccharomycotina_2 - ps20
Saccharomycotina_3 - ps21
Phaff./Saccharomycetaceae - ps22
Saccharomyco./mycetaceae - ps23
Saccharomycetaceae - ps24
Saccharomycetaceae_2 - ps25
Saccharomycetaceae_3 - ps26
Kaz./Nau./Saccharomyces - ps27
Saccharomyces - ps28
Arboricola_clade - ps29
Cerevisiae_clade - ps30
Saccharomyces_cerevisiae - ps31
Phylostratum

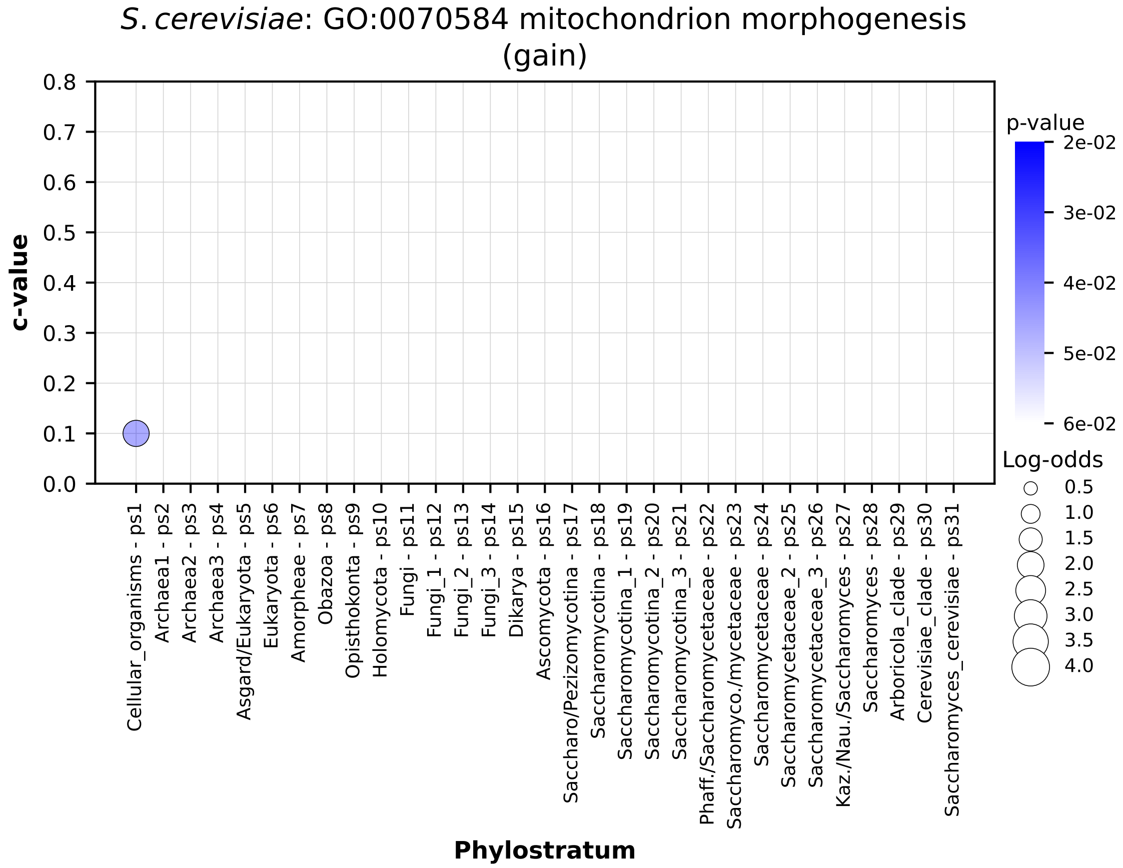

S. cerevisiae: GO:0070584 mitochondrion morphogenesis (gain)
c-value
0.0
0.1
0.2
0.3
0.4
0.5
0.6
0.7
0.8
Phylostratum
Cellular_organisms - ps1
Archaea1 - ps2
Archaea2 - ps3
Archaea3 - ps4
Asgard/Eukaryota - ps5
Eukaryota - ps6
Amorpheae - ps7
Obazoa - ps8
Opisthokonta - ps9
Holomycota - ps10
Fungi - ps11
Fungi_1 - ps12
Fungi_2 - ps13
Fungi_3 - ps14
Dikarya - ps15
Ascomycota - ps16
Saccharo/Pezizomycotina - ps17
Saccharomycotina - ps18
Saccharomycotina_1 - ps19
Saccharomycotina_2 - ps20
Saccharomycotina_3 - ps21
Phaff./Saccharomycetaceae - ps22
Saccharomyco./mycetaceae - ps23
Saccharomycetaceae - ps24
Saccharomycetaceae_2 - ps25
Saccharomycetaceae_3 - ps26
Kaz./Nau./Saccharomyces - ps27
Saccharomyces - ps28
Arboricola_clade - ps29
Cerevisiae_clade - ps30
Saccharomyces_cerevisiae - ps31
p-value
2e-02
3e-02
4e-02
5e-02
6e-02
Log-odds
0.5
1.0
1.5
2.0
2.5
3.0
3.5
4.0

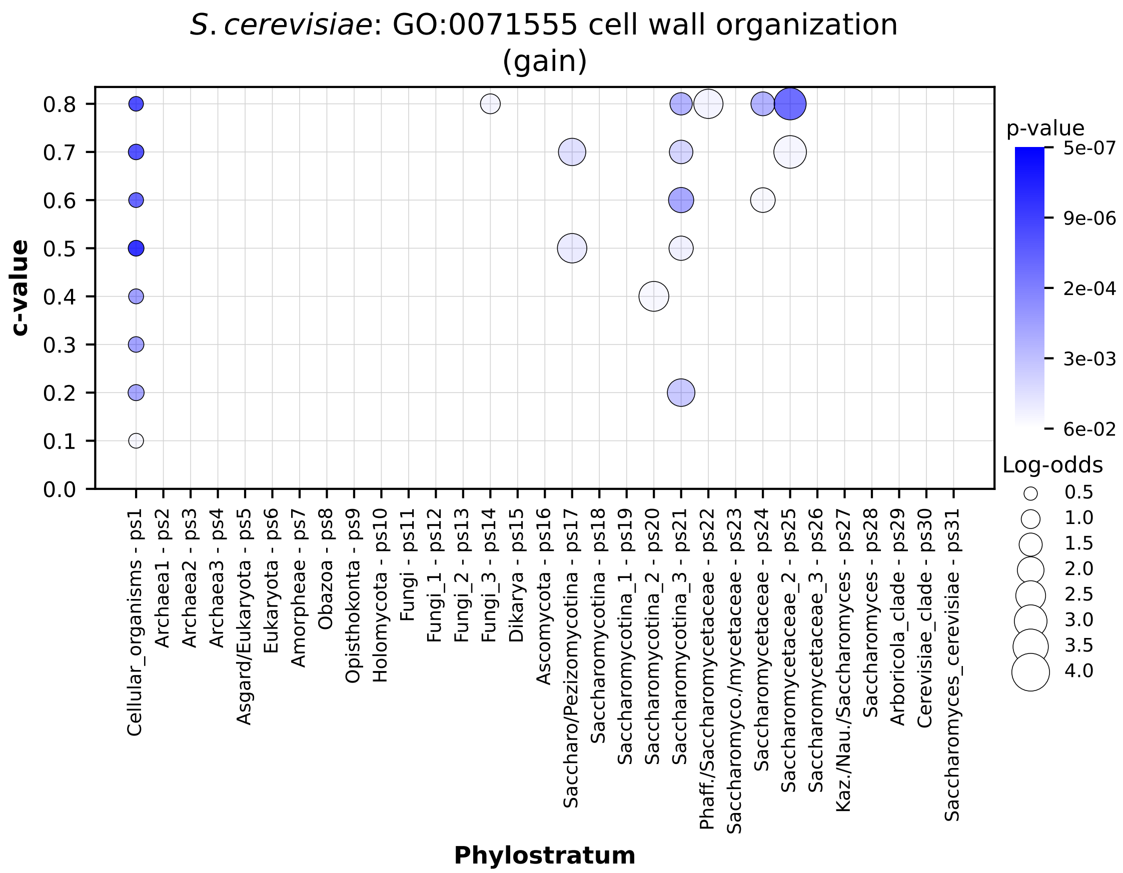

S. cerevisiae: GO:0071555 cell wall organization
(gain)
c-value
0.0
0.1
0.2
0.3
0.4
0.5
0.6
0.7
0.8
Phylostratum
Cellular_organisms - ps1
Archaea1 - ps2
Archaea2 - ps3
Archaea3 - ps4
Asgard/Eukaryota - ps5
Eukaryota - ps6
Amorpheae - ps7
Obazoa - ps8
Opisthokonta - ps9
Holomycota - ps10
Fungi - ps11
Fungi_1 - ps12
Fungi_2 - ps13
Fungi_3 - ps14
Dikarya - ps15
Ascomycota - ps16
Saccharo/Pezizomycotina - ps17
Saccharomycotina - ps18
Saccharomycotina_1 - ps19
Saccharomycotina_2 - ps20
Saccharomycotina_3 - ps21
Phaff./Saccharomycetaceae - ps22
Saccharomyco./mycetaceae - ps23
Saccharomycetaceae - ps24
Saccharomycetaceae_2 - ps25
Saccharomycetaceae_3 - ps26
Kaz./Nau./Saccharomyces - ps27
Saccharomyces - ps28
Arboricola_clade - ps29
Cerevisiae_clade - ps30
Saccharomyces_cerevisiae - ps31
p-value
5e-07
9e-06
2e-04
3e-03
6e-02
Log-odds
0.5
1.0
1.5
2.0
2.5
3.0
3.5
4.0

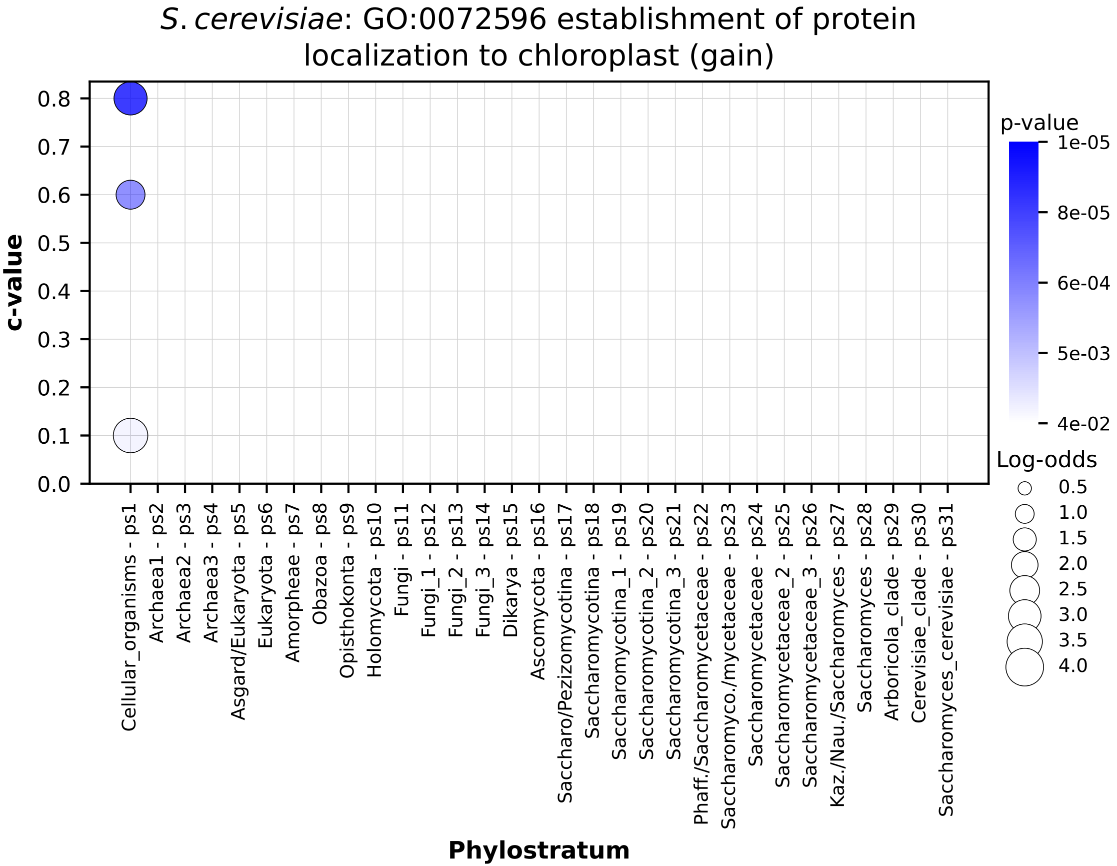

S. cerevisiae: GO:0072596 establishment of protein localization to chloroplast (gain)
c-value
0.8
0.7
0.6
0.5
0.4
0.3
0.2
0.1
0.0
p-value
1e-05
8e-05
6e-04
5e-03
4e-02
Log-odds
0.5
1.0
1.5
2.0
2.5
3.0
3.5
4.0
Cellular_organisms - ps1
Archaea1 - ps2
Archaea2 - ps3
Archaea3 - ps4
Asgard/Eukaryota - ps5
Eukaryota - ps6
Amorpheae - ps7
Obazoa - ps8
Opisthokonta - ps9
Holomycota - ps10
Fungi - ps11
Fungi_1 - ps12
Fungi_2 - ps13
Fungi_3 - ps14
Dikarya - ps15
Ascomycota - ps16
Saccharo/Pezizomycotina - ps17
Saccharomycotina - ps18
Saccharomycotina_1 - ps19
Saccharomycotina_2 - ps20
Saccharomycotina_3 - ps21
Phaff./Saccharomycetaceae - ps22
Saccharomyco./mycetaceae - ps23
Saccharomycetaceae - ps24
Saccharomycetaceae_2 - ps25
Saccharomycetaceae_3 - ps26
Kaz./Nau./Saccharomyces - ps27
Saccharomyces - ps28
Arboricola_clade - ps29
Cerevisiae_clade - ps30
Saccharomyces_cerevisiae - ps31
Phylostratum

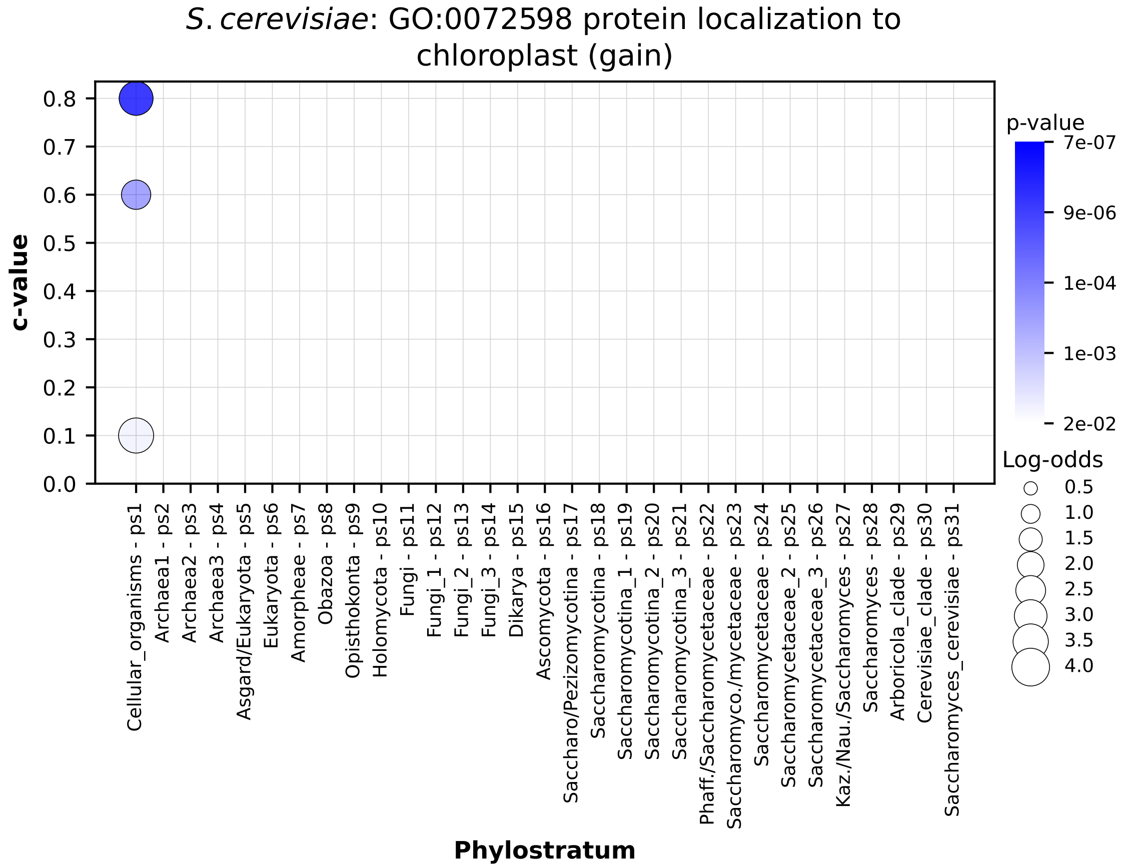

S. cerevisiae: GO:0072598 protein localization to chloroplast (gain)
c-value
0.0
0.1
0.2
0.3
0.4
0.5
0.6
0.7
0.8
Phylostratum
Cellular_organisms - ps1
Archaea1 - ps2
Archaea2 - ps3
Archaea3 - ps4
Asgard/Eukaryota - ps5
Eukaryota - ps6
Amorpheae - ps7
Obazoa - ps8
Opisthokonta - ps9
Holomycota - ps10
Fungi - ps11
Fungi_1 - ps12
Fungi_2 - ps13
Fungi_3 - ps14
Dikarya - ps15
Ascomycota - ps16
Saccharo/Pezizomycotina - ps17
Saccharomycotina - ps18
Saccharomycotina_1 - ps19
Saccharomycotina_2 - ps20
Saccharomycotina_3 - ps21
Phaff./Saccharomycetaceae - ps22
Saccharomyco./mycetaceae - ps23
Saccharomycetaceae - ps24
Saccharomycetaceae_2 - ps25
Saccharomycetaceae_3 - ps26
Kaz./Nau./Saccharomyces - ps27
Saccharomyces - ps28
Arboricola_clade - ps29
Cerevisiae_clade - ps30
Saccharomyces_cerevisiae - ps31
p-value
7e-07
9e-06
1e-04
1e-03
2e-02
Log-odds
0.5
1.0
1.5
2.0
2.5
3.0
3.5
4.0

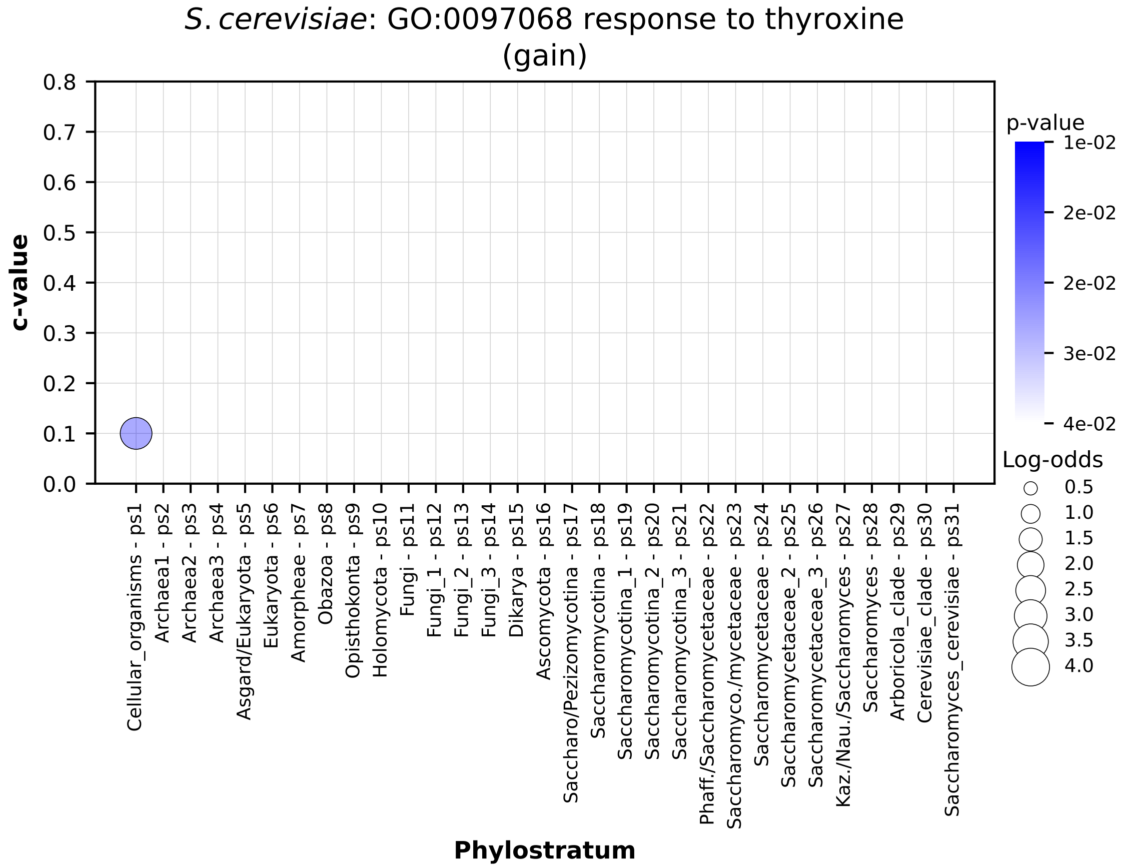

S. cerevisiae: GO:0097068 response to thyroxine
(gain)
c-value
0.8
0.7
0.6
0.5
0.4
0.3
0.2
0.1
0.0
Phylostratum
Cellular_organisms - ps1
Archaea1 - ps2
Archaea2 - ps3
Archaea3 - ps4
Asgard/Eukaryota - ps5
Eukaryota - ps6
Amorpheae - ps7
Obazoa - ps8
Opisthokonta - ps9
Holomycota - ps10
Fungi - ps11
Fungi_1 - ps12
Fungi_2 - ps13
Fungi_3 - ps14
Dikarya - ps15
Ascomycota - ps16
Saccharo/Pezizomycotina - ps17
Saccharomycotina - ps18
Saccharomycotina_1 - ps19
Saccharomycotina_2 - ps20
Saccharomycotina_3 - ps21
Phaff./Saccharomycetaceae - ps22
Saccharomyco./mycetaceae - ps23
Saccharomycetaceae - ps24
Saccharomycetaceae_2 - ps25
Saccharomycetaceae_3 - ps26
Kaz./Nau./Saccharomyces - ps27
Saccharomyces - ps28
Arboricola_clade - ps29
Cerevisiae_clade - ps30
Saccharomyces_cerevisiae - ps31
p-value
1e-02
2e-02
2e-02
3e-02
4e-02
Log-odds
0.5
1.0
1.5
2.0
2.5
3.0
3.5
4.0

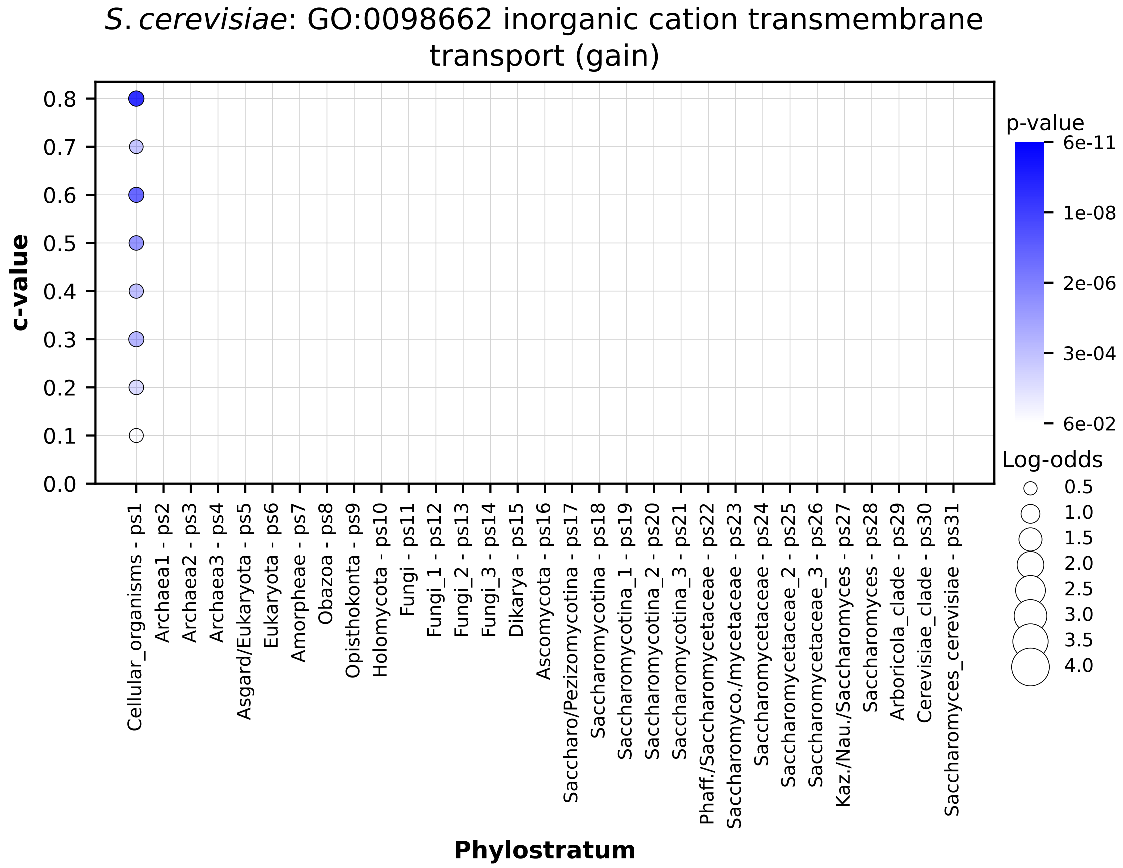

S. cerevisiae: GO:0098662 inorganic cation transmembrane transport (gain)
c-value
0.8
0.7
0.6
0.5
0.4
0.3
0.2
0.1
0.0
Phylostratum
Cellular_organisms - ps1
Archaea1 - ps2
Archaea2 - ps3
Archaea3 - ps4
Asgard/Eukaryota - ps5
Eukaryota - ps6
Amorpheae - ps7
Obazoa - ps8
Opisthokonta - ps9
Holomycota - ps10
Fungi - ps11
Fungi_1 - ps12
Fungi_2 - ps13
Fungi_3 - ps14
Dikarya - ps15
Ascomycota - ps16
Saccharo/Pezizomycotina - ps17
Saccharomycotina - ps18
Saccharomycotina_1 - ps19
Saccharomycotina_2 - ps20
Saccharomycotina_3 - ps21
Phaff./Saccharomycetaceae - ps22
Saccharomyco./mycetaceae - ps23
Saccharomycetaceae - ps24
Saccharomycetaceae_2 - ps25
Saccharomycetaceae_3 - ps26
Kaz./Nau./Saccharomyces - ps27
Saccharomyces - ps28
Arboricola_clade - ps29
Cerevisiae_clade - ps30
Saccharomyces_cerevisiae - ps31
p-value
6e-11
1e-08
2e-06
3e-04
6e-02
Log-odds
0.5
1.0
1.5
2.0
2.5
3.0
3.5
4.0

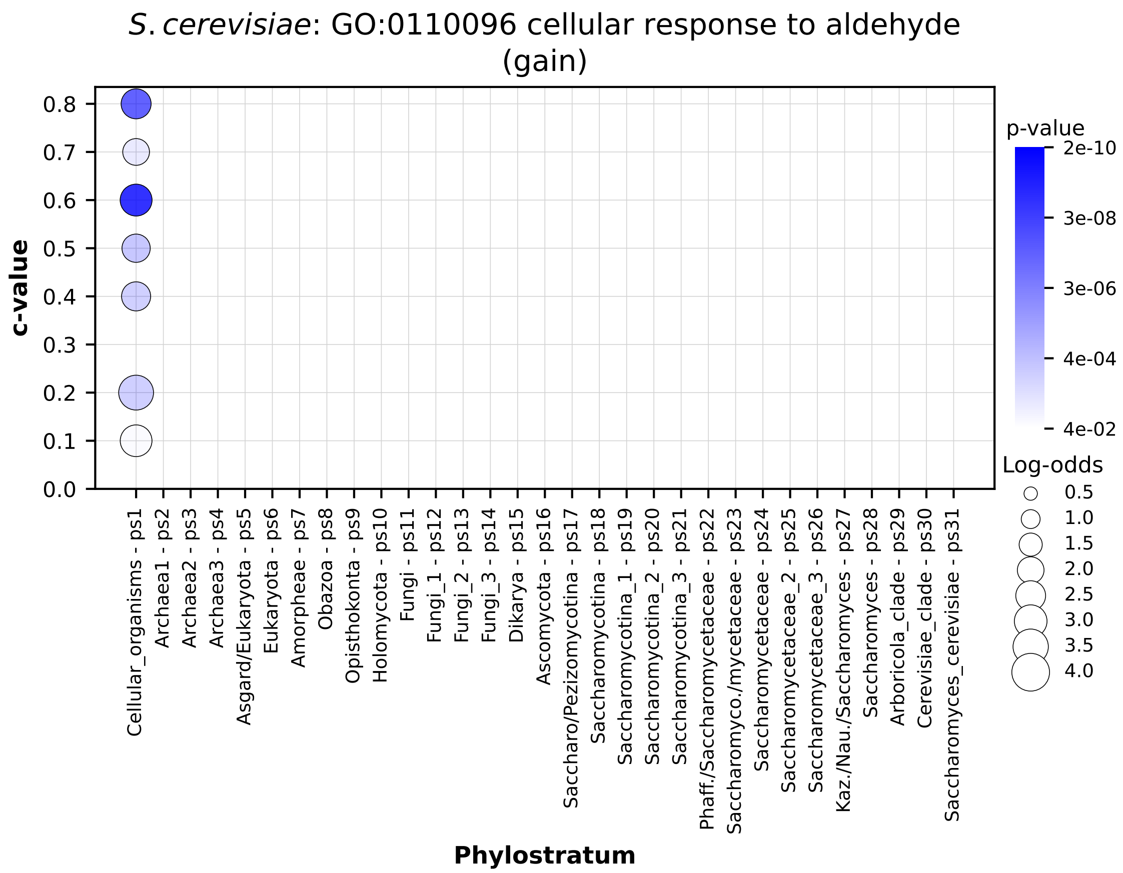

S. cerevisiae: GO:0110096 cellular response to aldehyde (gain)
c-value
0.0
0.1
0.2
0.3
0.4
0.5
0.6
0.7
0.8
Phylostratum
Cellular_organisms - ps1
Archaea1 - ps2
Archaea2 - ps3
Archaea3 - ps4
Asgard/Eukaryota - ps5
Eukaryota - ps6
Amorpheae - ps7
Obazoa - ps8
Opisthokonta - ps9
Holomycota - ps10
Fungi - ps11
Fungi_1 - ps12
Fungi_2 - ps13
Fungi_3 - ps14
Dikarya - ps15
Ascomycota - ps16
Saccharo/Pezizomycotina - ps17
Saccharomycotina - ps18
Saccharomycotina_1 - ps19
Saccharomycotina_2 - ps20
Saccharomycotina_3 - ps21
Phaff./Saccharomycetaceae - ps22
Saccharomyco./mycetaceae - ps23
Saccharomycetaceae - ps24
Saccharomycetaceae_2 - ps25
Saccharomycetaceae_3 - ps26
Kaz./Nau./Saccharomyces - ps27
Saccharomyces - ps28
Arboricola_clade - ps29
Cerevisiae_clade - ps30
Saccharomyces_cerevisiae - ps31
p-value
2e-10
3e-08
3e-06
4e-04
4e-02
Log-odds
0.5
1.0
1.5
2.0
2.5
3.0
3.5
4.0

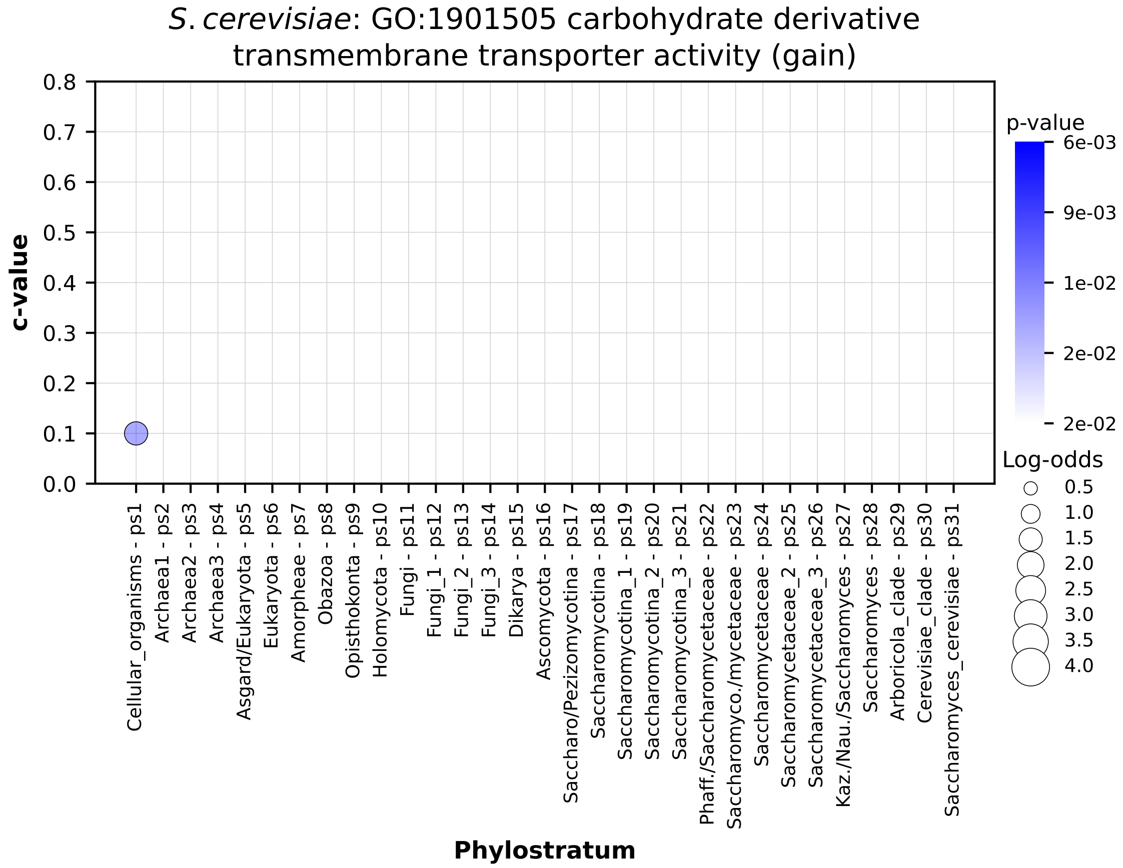

S. cerevisiae: GO:1901505 carbohydrate derivative transmembrane transporter activity (gain)
c-value
0.8
0.7
0.6
0.5
0.4
0.3
0.2
0.1
0.0
p-value
6e-03
9e-03
1e-02
2e-02
2e-02
Log-odds
0.5
1.0
1.5
2.0
2.5
3.0
3.5
4.0
Cellular_organisms - ps1
Archaea1 - ps2
Archaea2 - ps3
Archaea3 - ps4
Asgard/Eukaryota - ps5
Eukaryota - ps6
Amorpheae - ps7
Obazoa - ps8
Opisthokonta - ps9
Holomycota - ps10
Fungi - ps11
Fungi_1 - ps12
Fungi_2 - ps13
Fungi_3 - ps14
Dikarya - ps15
Ascomycota - ps16
Saccharo/Pezizomycotina - ps17
Saccharomycotina - ps18
Saccharomycotina_1 - ps19
Saccharomycotina_2 - ps20
Saccharomycotina_3 - ps21
Phaff./Saccharomycetaceae - ps22
Saccharomyco./mycetaceae - ps23
Saccharomycetaceae - ps24
Saccharomycetaceae_2 - ps25
Saccharomycetaceae_3 - ps26
Kaz./Nau./Saccharomyces - ps27
Saccharomyces - ps28
Arboricola_clade - ps29
Cerevisiae_clade - ps30
Saccharomyces_cerevisiae - ps31
Phylostratum

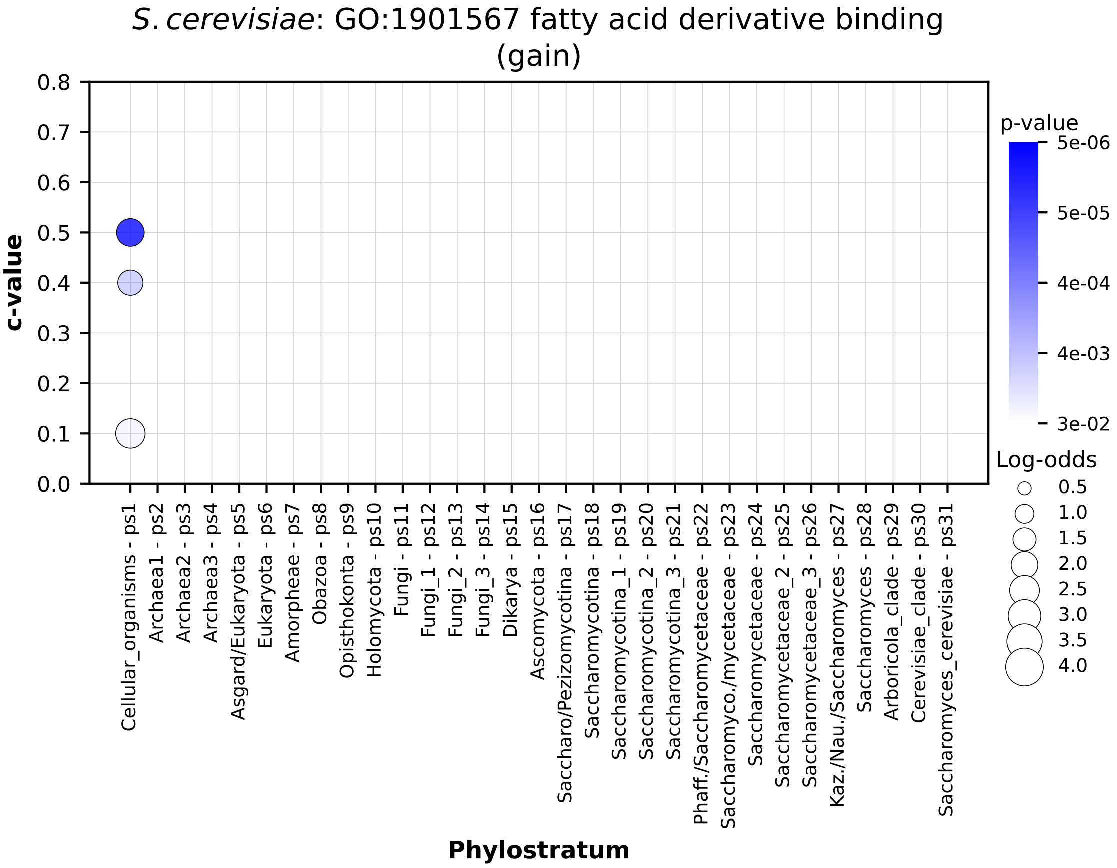

S. cerevisiae: GO:1901567 fatty acid derivative binding (gain)
c-value
0.8
0.7
0.6
0.5
0.4
0.3
0.2
0.1
0.0
Phylostratum
Cellular_organisms - ps1
Archaea1 - ps2
Archaea2 - ps3
Archaea3 - ps4
Asgard/Eukaryota - ps5
Eukaryota - ps6
Amorpheae - ps7
Obazoa - ps8
Opisthokonta - ps9
Holomycota - ps10
Fungi - ps11
Fungi_1 - ps12
Fungi_2 - ps13
Fungi_3 - ps14
Dikarya - ps15
Ascomycota - ps16
Saccharo/Pezizomycotina - ps17
Saccharomycotina - ps18
Saccharomycotina_1 - ps19
Saccharomycotina_2 - ps20
Saccharomycotina_3 - ps21
Phaff./Saccharomycetaceae - ps22
Saccharomyco./mycetaceae - ps23
Saccharomycetaceae - ps24
Saccharomycetaceae_2 - ps25
Saccharomycetaceae_3 - ps26
Kaz./Nau./Saccharomyces - ps27
Saccharomyces - ps28
Arboricola_clade - ps29
Cerevisiae_clade - ps30
Saccharomyces_cerevisiae - ps31
p-value
5e-06
5e-05
4e-04
4e-03
3e-02
Log-odds
0.5
1.0
1.5
2.0
2.5
3.0
3.5
4.0

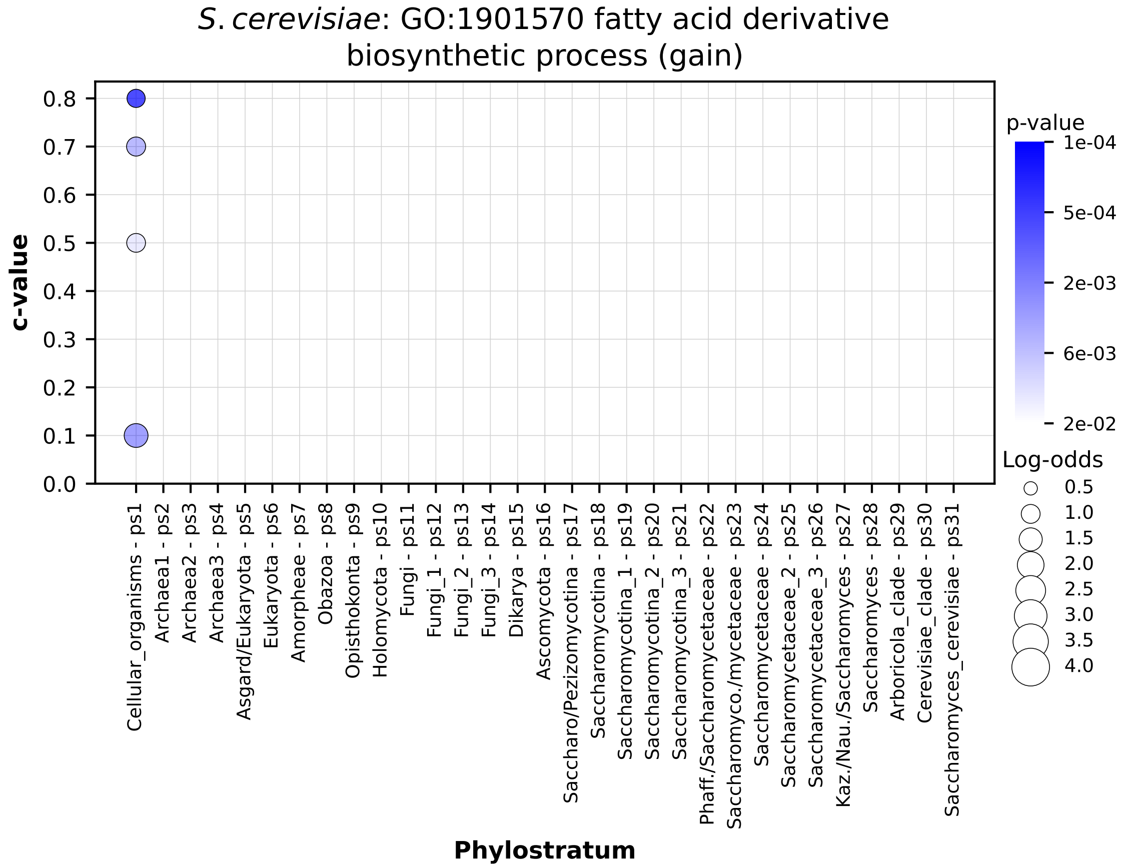
S. cerevisiae: GO:1901570 fatty acid derivative biosynthetic process (gain)
c-value
0.0
0.1
0.2
0.3
0.4
0.5
0.6
0.7
0.8
p-value
1e-04
5e-04
2e-03
6e-03
2e-02
Log-odds
0.5
1.0
1.5
2.0
2.5
3.0
3.5
4.0
Cellular_organisms - ps1
Archaea1 - ps2
Archaea2 - ps3
Archaea3 - ps4
Asgard/Eukaryota - ps5
Eukaryota - ps6
Amorpheae - ps7
Obazoa - ps8
Opisthokonta - ps9
Holomycota - ps10
Fungi - ps11
Fungi_1 - ps12
Fungi_2 - ps13
Fungi_3 - ps14
Dikarya - ps15
Ascomycota - ps16
Saccharo/Pezizomycotina - ps17
Saccharomycotina - ps18
Saccharomycotina_1 - ps19
Saccharomycotina_2 - ps20
Saccharomycotina_3 - ps21
Phaff./Saccharomycetaceae - ps22
Saccharomyco./mycetaceae - ps23
Saccharomycetaceae - ps24
Saccharomycetaceae_2 - ps25
Saccharomycetaceae_3 - ps26
Kaz./Nau./Saccharomyces - ps27
Saccharomyces - ps28
Arboricola_clade - ps29
Cerevisiae_clade - ps30
Saccharomyces_cerevisiae - ps31
Phylostratum

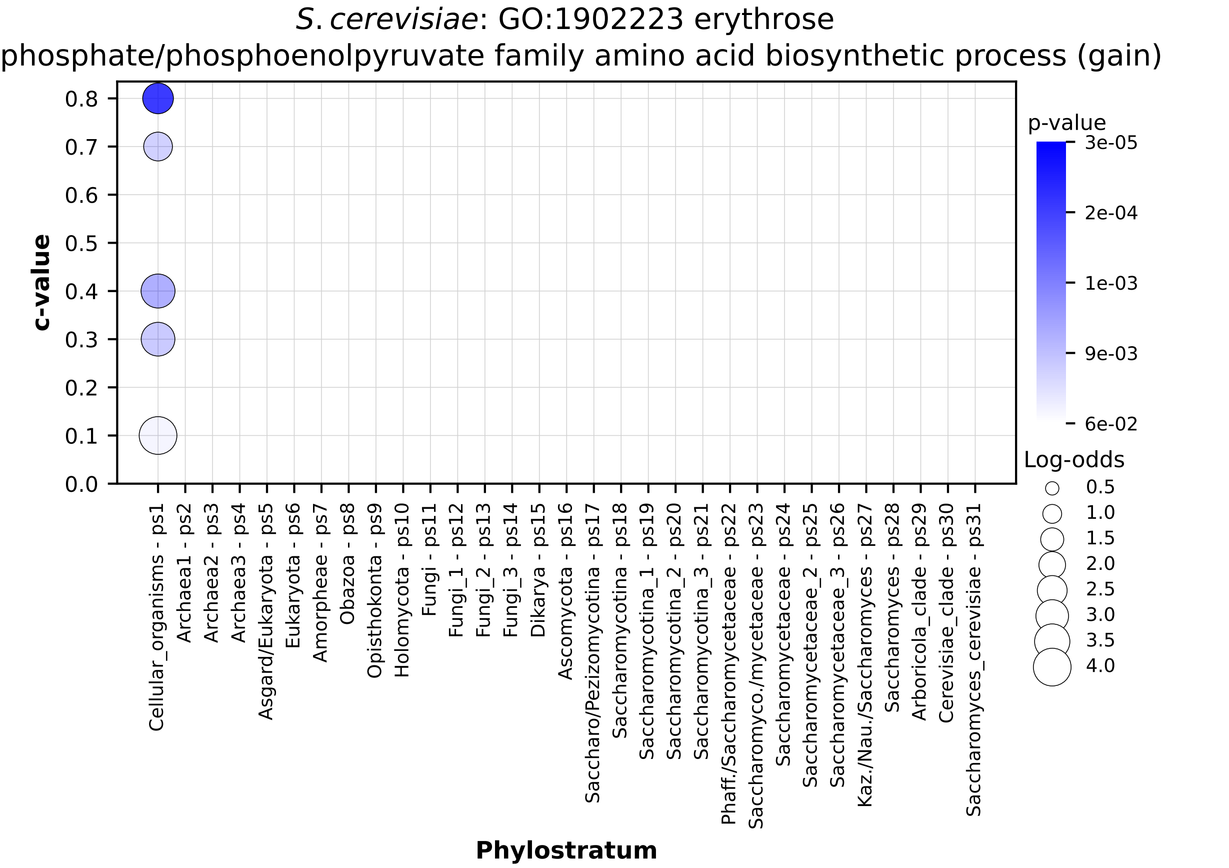

S. cerevisiae: GO:1902223 erythrose phosphate/phosphoenolpyruvate family amino acid biosynthetic process (gain)
c-value
0.0
0.1
0.2
0.3
0.4
0.5
0.6
0.7
0.8
p-value
3e-05
2e-04
1e-03
9e-03
6e-02
Log-odds
0.5
1.0
1.5
2.0
2.5
3.0
3.5
4.0
Cellular_organisms - ps1
Archaea1 - ps2
Archaea2 - ps3
Archaea3 - ps4
Asgard/Eukaryota - ps5
Eukaryota - ps6
Amorpheae - ps7
Obazoa - ps8
Opisthokonta - ps9
Holomycota - ps10
Fungi - ps11
Fungi_1 - ps12
Fungi_2 - ps13
Fungi_3 - ps14
Dikarya - ps15
Ascomycota - ps16
Saccharo/Pezizomycotina - ps17
Saccharomycotina - ps18
Saccharomycotina_1 - ps19
Saccharomycotina_2 - ps20
Saccharomycotina_3 - ps21
Phaff./Saccharomycetaceae - ps22
Saccharomyco./mycetaceae - ps23
Saccharomycetaceae - ps24
Saccharomycetaceae_2 - ps25
Saccharomycetaceae_3 - ps26
Kaz./Nau./Saccharomyces - ps27
Saccharomyces - ps28
Arboricola_clade - ps29
Cerevisiae_clade - ps30
Saccharomyces_cerevisiae - ps31
Phylostratum

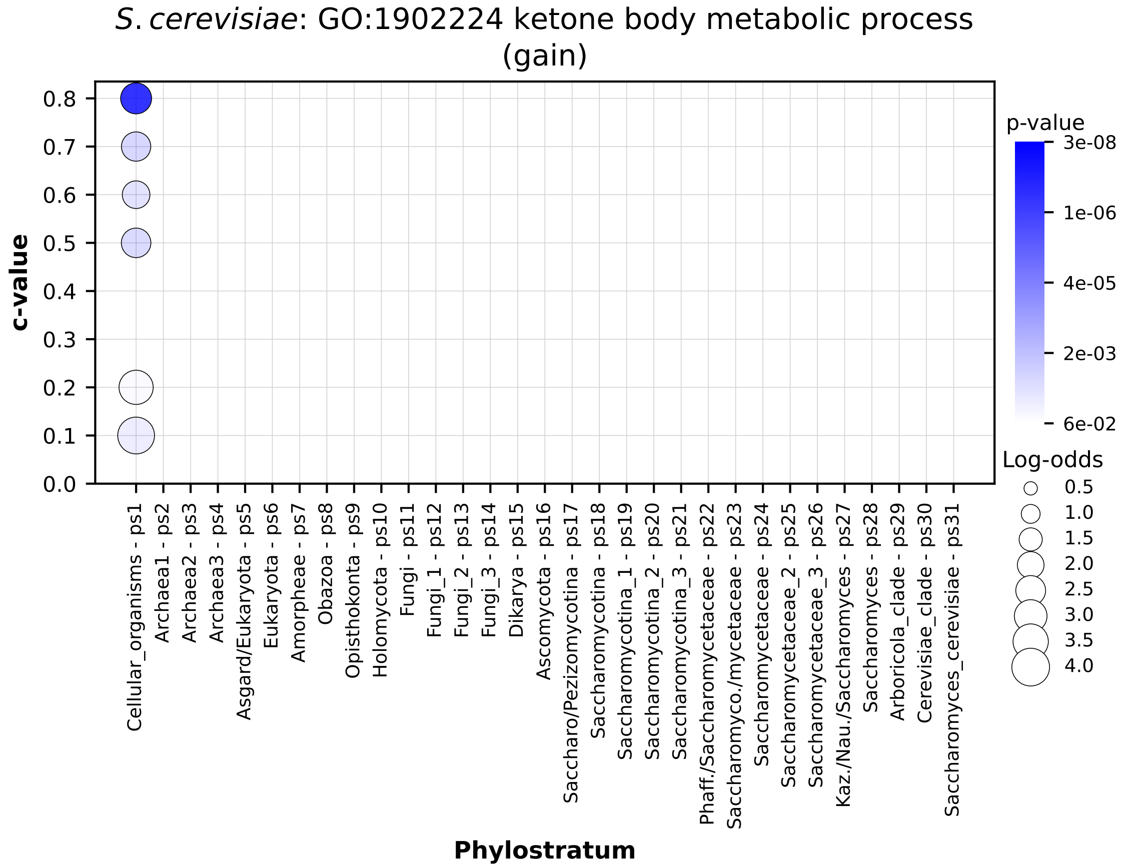

S. cerevisiae: GO:1902224 ketone body metabolic process
(gain)
c-value
0.0
0.1
0.2
0.3
0.4
0.5
0.6
0.7
0.8
p-value
3e-08
1e-06
4e-05
2e-03
6e-02
Log-odds
0.5
1.0
1.5
2.0
2.5
3.0
3.5
4.0
Cellular_organisms - ps1
Archaea1 - ps2
Archaea2 - ps3
Archaea3 - ps4
Asgard/Eukaryota - ps5
Eukaryota - ps6
Amorpheae - ps7
Obazoa - ps8
Opisthokonta - ps9
Holomycota - ps10
Fungi - ps11
Fungi_1 - ps12
Fungi_2 - ps13
Fungi_3 - ps14
Dikarya - ps15
Ascomycota - ps16
Saccharo/Pezizomycotina - ps17
Saccharomycotina - ps18
Saccharomycotina_1 - ps19
Saccharomycotina_2 - ps20
Saccharomycotina_3 - ps21
Phaff./Saccharomycetaceae - ps22
Saccharomyco./mycetaceae - ps23
Saccharomycetaceae - ps24
Saccharomycetaceae_2 - ps25
Saccharomycetaceae_3 - ps26
Kaz./Nau./Saccharomyces - ps27
Saccharomyces - ps28
Arboricola_clade - ps29
Cerevisiae_clade - ps30
Saccharomyces_cerevisiae - ps31
Phylostratum

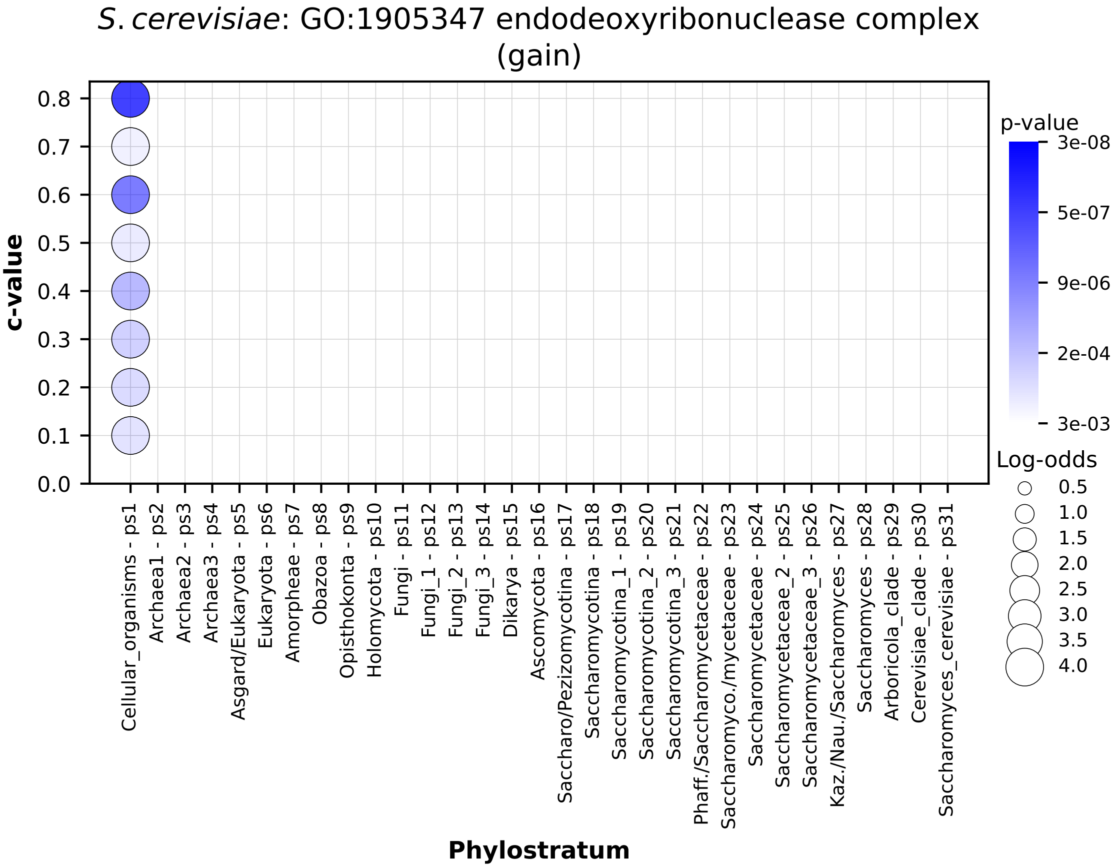
S. cerevisiae: GO:1905347 endodeoxyribonuclease complex
(gain)
c-value
0.0
0.1
0.2
0.3
0.4
0.5
0.6
0.7
0.8
Phylostratum
Cellular_organisms - ps1
Archaea1 - ps2
Archaea2 - ps3
Archaea3 - ps4
Asgard/Eukaryota - ps5
Eukaryota - ps6
Amorpheae - ps7
Obazoa - ps8
Opisthokonta - ps9
Holomycota - ps10
Fungi - ps11
Fungi_1 - ps12
Fungi_2 - ps13
Fungi_3 - ps14
Dikarya - ps15
Ascomycota - ps16
Saccharo/Pezizomycotina - ps17
Saccharomycotina - ps18
Saccharomycotina_1 - ps19
Saccharomycotina_2 - ps20
Saccharomycotina_3 - ps21
Phaff./Saccharomycetaceae - ps22
Saccharomyco./mycetaceae - ps23
Saccharomycetaceae - ps24
Saccharomycetaceae_2 - ps25
Saccharomycetaceae_3 - ps26
Kaz./Nau./Saccharomyces - ps27
Saccharomyces - ps28
Arboricola_clade - ps29
Cerevisiae_clade - ps30
Saccharomyces_cerevisiae - ps31
p-value
3e-08
5e-07
9e-06
2e-04
3e-03
Log-odds
0.5
1.0
1.5
2.0
2.5
3.0
3.5
4.0

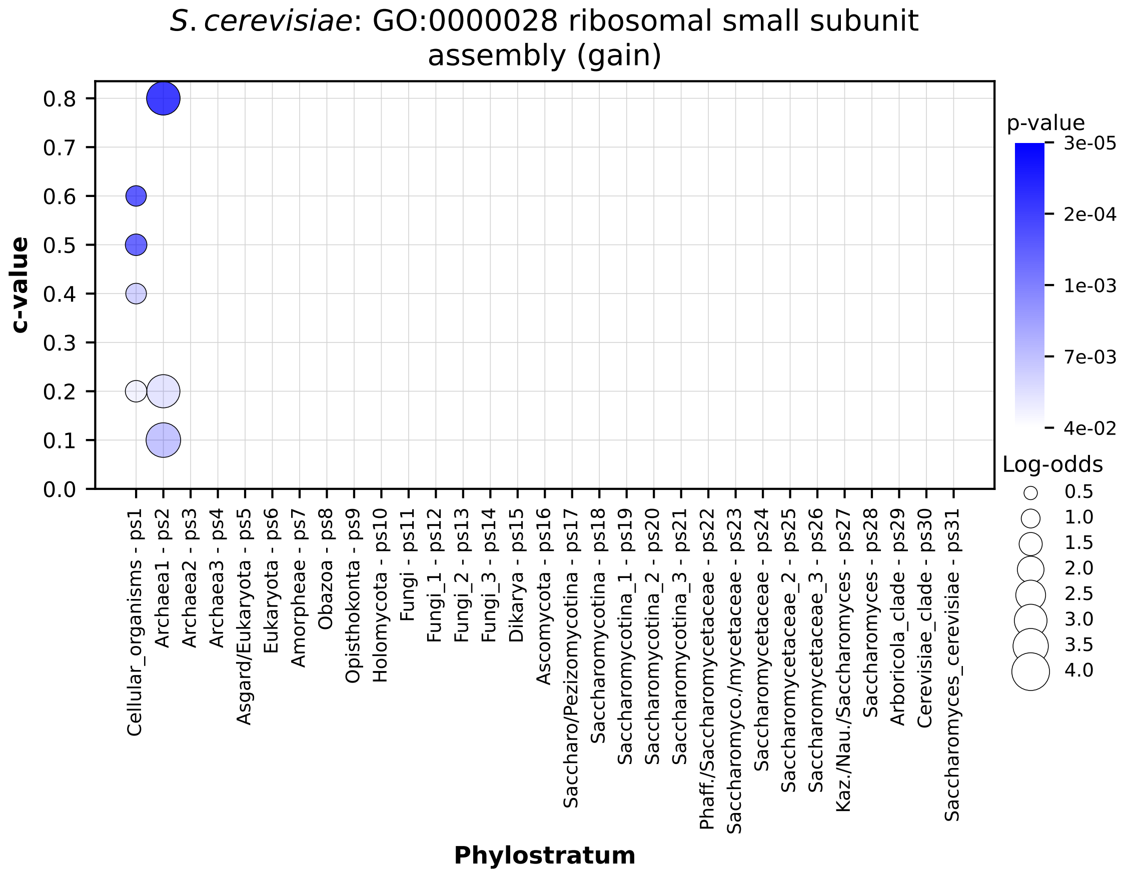
S. cerevisiae: GO:0000028 ribosomal small subunit assembly (gain)
c-value
0.0
0.1
0.2
0.3
0.4
0.5
0.6
0.7
0.8
Phylostratum
Cellular_organisms - ps1
Archaea1 - ps2
Archaea2 - ps3
Archaea3 - ps4
Asgard/Eukaryota - ps5
Eukaryota - ps6
Amorpheae - ps7
Obazoa - ps8
Opisthokonta - ps9
Holomycota - ps10
Fungi - ps11
Fungi_1 - ps12
Fungi_2 - ps13
Fungi_3 - ps14
Dikarya - ps15
Ascomycota - ps16
Saccharo/Pezizomycotina - ps17
Saccharomycotina - ps18
Saccharomycotina_1 - ps19
Saccharomycotina_2 - ps20
Saccharomycotina_3 - ps21
Phaff./Saccharomycetaceae - ps22
Saccharomyco./mycetaceae - ps23
Saccharomycetaceae - ps24
Saccharomycetaceae_2 - ps25
Saccharomycetaceae_3 - ps26
Kaz./Nau./Saccharomyces - ps27
Saccharomyces - ps28
Arboricola_clade - ps29
Cerevisiae_clade - ps30
Saccharomyces_cerevisiae - ps31
p-value
3e-05
2e-04
1e-03
7e-03
4e-02
Log-odds
0.5
1.0
1.5
2.0
2.5
3.0
3.5
4.0

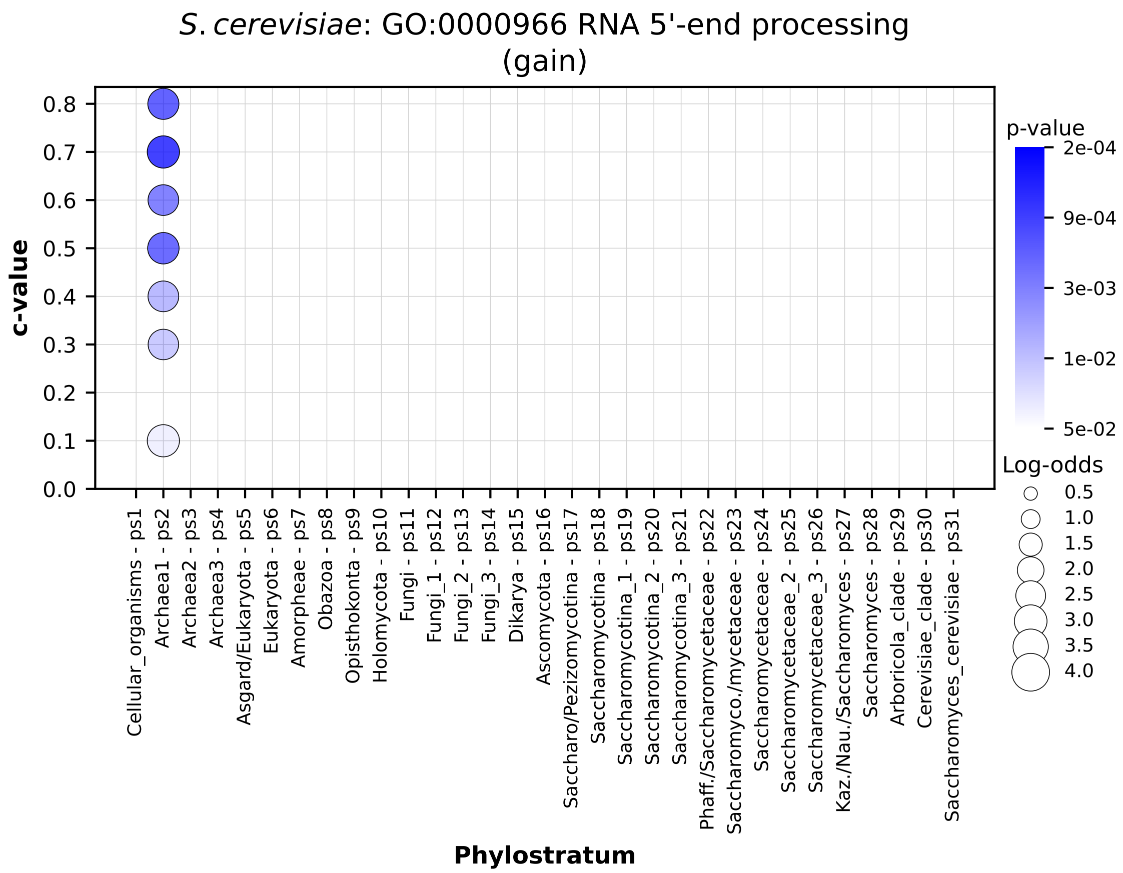

S. cerevisiae: GO:0000966 RNA 5'-end processing
(gain)
c-value
0.0
0.1
0.2
0.3
0.4
0.5
0.6
0.7
0.8
p-value
2e-04
9e-04
3e-03
1e-02
5e-02
Log-odds
0.5
1.0
1.5
2.0
2.5
3.0
3.5
4.0
Cellular_organisms - ps1
Archaea1 - ps2
Archaea2 - ps3
Archaea3 - ps4
Asgard/Eukaryota - ps5
Eukaryota - ps6
Amorpheae - ps7
Obazoa - ps8
Opisthokonta - ps9
Holomycota - ps10
Fungi - ps11
Fungi_1 - ps12
Fungi_2 - ps13
Fungi_3 - ps14
Dikarya - ps15
Ascomycota - ps16
Saccharo/Pezizomycotina - ps17
Saccharomycotina - ps18
Saccharomycotina_1 - ps19
Saccharomycotina_2 - ps20
Saccharomycotina_3 - ps21
Phaff./Saccharomycetaceae - ps22
Saccharomyco./mycetaceae - ps23
Saccharomycetaceae - ps24
Saccharomycetaceae_2 - ps25
Saccharomycetaceae_3 - ps26
Kaz./Nau./Saccharomyces - ps27
Saccharomyces - ps28
Arboricola_clade - ps29
Cerevisiae_clade - ps30
Saccharomyces_cerevisiae - ps31
Phylostratum

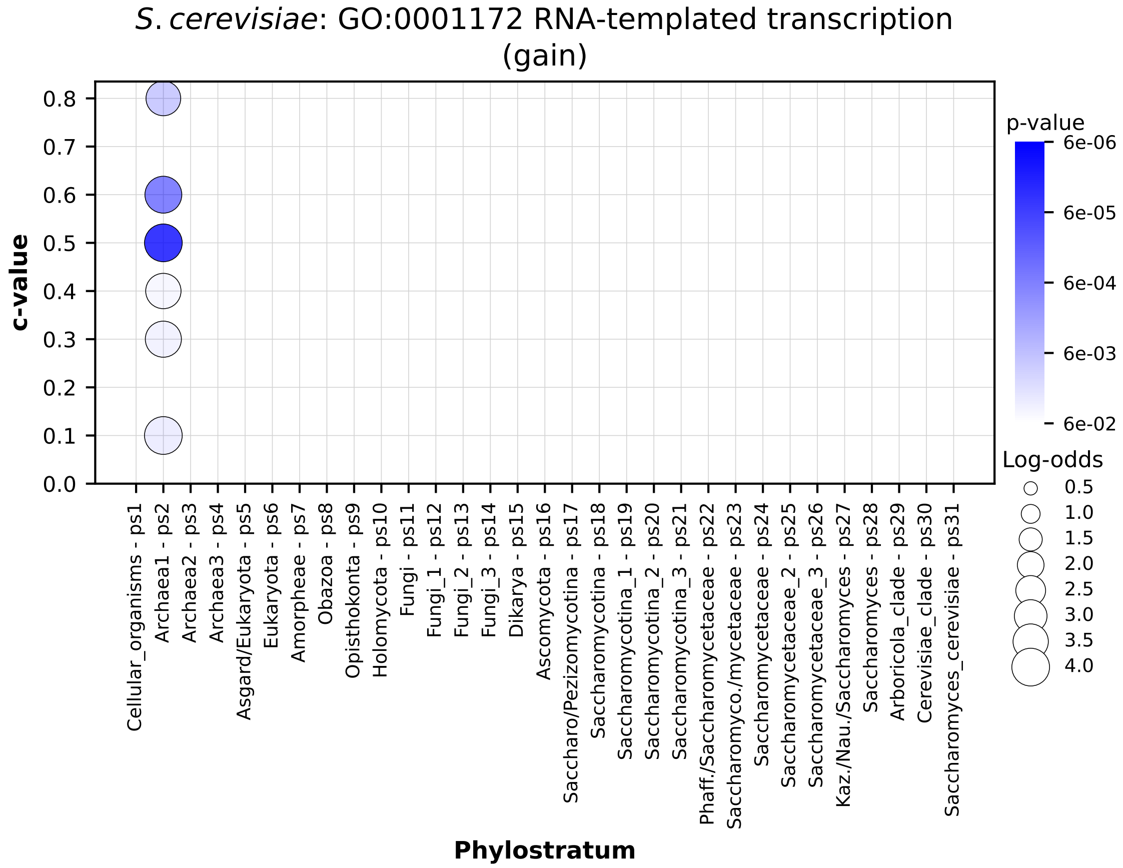

S. cerevisiae: GO:0001172 RNA-templated transcription (gain)
c-value
0.0
0.1
0.2
0.3
0.4
0.5
0.6
0.7
0.8
Phylostratum
Cellular_organisms - ps1
Archaea1 - ps2
Archaea2 - ps3
Archaea3 - ps4
Asgard/Eukaryota - ps5
Eukaryota - ps6
Amorpheae - ps7
Obazoa - ps8
Opisthokonta - ps9
Holomycota - ps10
Fungi - ps11
Fungi_1 - ps12
Fungi_2 - ps13
Fungi_3 - ps14
Dikarya - ps15
Ascomycota - ps16
Saccharo/Pezizomycotina - ps17
Saccharomycotina - ps18
Saccharomycotina_1 - ps19
Saccharomycotina_2 - ps20
Saccharomycotina_3 - ps21
Phaff./Saccharomycetaceae - ps22
Saccharomyco./mycetaceae - ps23
Saccharomycetaceae - ps24
Saccharomycetaceae_2 - ps25
Saccharomycetaceae_3 - ps26
Kaz./Nau./Saccharomyces - ps27
Saccharomyces - ps28
Arboricola_clade - ps29
Cerevisiae_clade - ps30
Saccharomyces_cerevisiae - ps31
p-value
6e-06
6e-05
6e-04
6e-03
6e-02
Log-odds
0.5
1.0
1.5
2.0
2.5
3.0
3.5
4.0

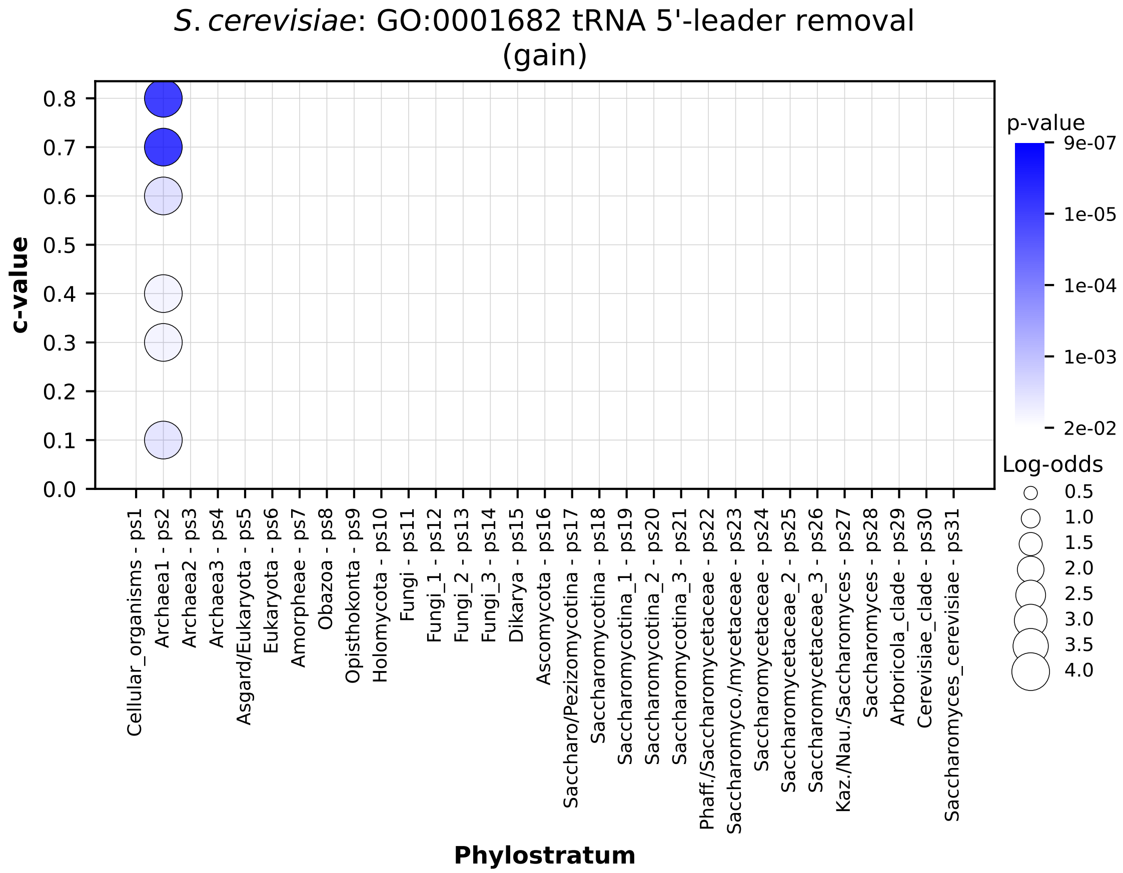

S. cerevisiae: GO:0001682 tRNA 5'-leader removal
(gain)
c-value
0.0
0.1
0.2
0.3
0.4
0.5
0.6
0.7
0.8
p-value
9e-07
1e-05
1e-04
1e-03
2e-02
Log-odds
0.5
1.0
1.5
2.0
2.5
3.0
3.5
4.0
Cellular_organisms - ps1
Archaea1 - ps2
Archaea2 - ps3
Archaea3 - ps4
Asgard/Eukaryota - ps5
Eukaryota - ps6
Amorpheae - ps7
Obazoa - ps8
Opisthokonta - ps9
Holomycota - ps10
Fungi - ps11
Fungi_1 - ps12
Fungi_2 - ps13
Fungi_3 - ps14
Dikarya - ps15
Ascomycota - ps16
Saccharo/Pezizomycotina - ps17
Saccharomycotina - ps18
Saccharomycotina_1 - ps19
Saccharomycotina_2 - ps20
Saccharomycotina_3 - ps21
Phaff./Saccharomycetaceae - ps22
Saccharomyco./mycetaceae - ps23
Saccharomycetaceae - ps24
Saccharomycetaceae_2 - ps25
Saccharomycetaceae_3 - ps26
Kaz./Nau./Saccharomyces - ps27
Saccharomyces - ps28
Arboricola_clade - ps29
Cerevisiae_clade - ps30
Saccharomyces_cerevisiae - ps31
Phylostratum

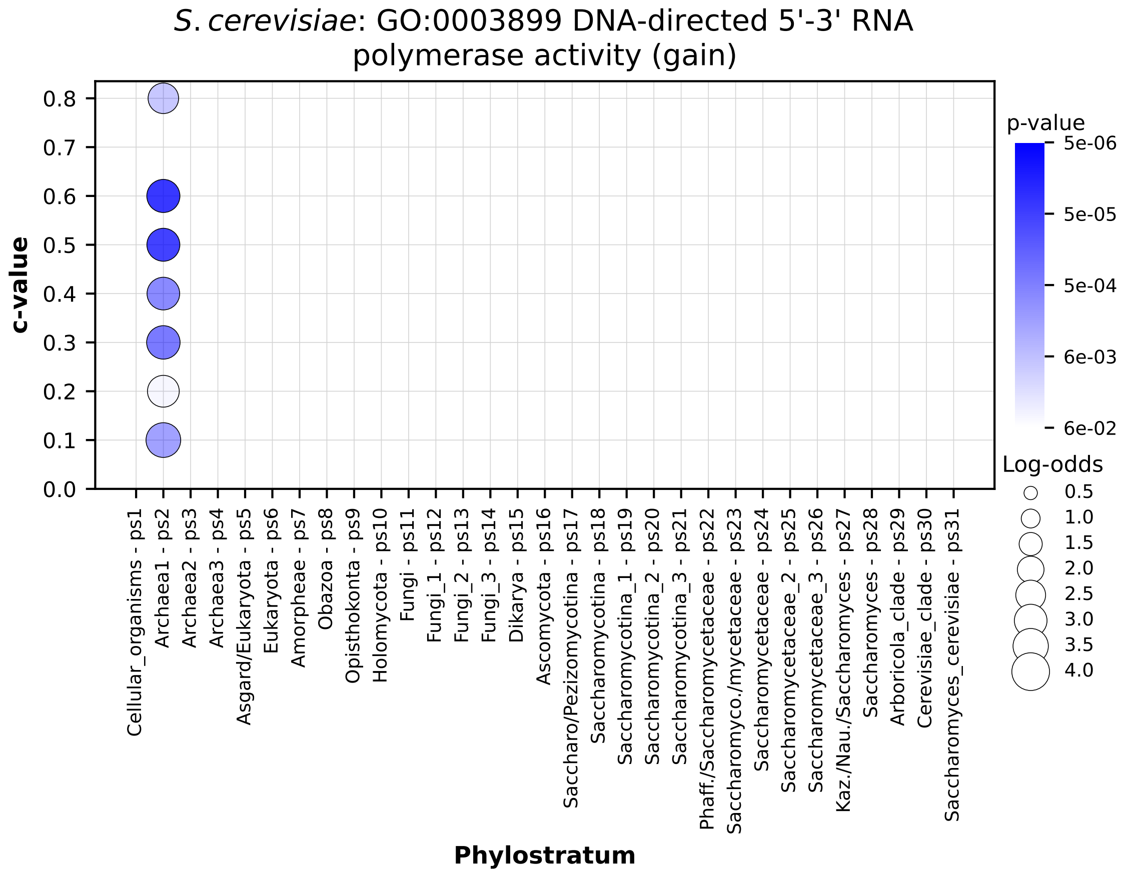
S. cerevisiae: GO:0003899 DNA-directed 5'-3' RNA polymerase activity (gain)
c-value
0.0
0.1
0.2
0.3
0.4
0.5
0.6
0.7
0.8
p-value
5e-06
5e-05
5e-04
6e-03
6e-02
Log-odds
0.5
1.0
1.5
2.0
2.5
3.0
3.5
4.0
Cellular_organisms - ps1
Archaea1 - ps2
Archaea2 - ps3
Archaea3 - ps4
Asgard/Eukaryota - ps5
Eukaryota - ps6
Amorpheae - ps7
Obazoa - ps8
Opisthokonta - ps9
Holomycota - ps10
Fungi - ps11
Fungi_1 - ps12
Fungi_2 - ps13
Fungi_3 - ps14
Dikarya - ps15
Ascomycota - ps16
Saccharo/Pezizomycotina - ps17
Saccharomycotina - ps18
Saccharomycotina_1 - ps19
Saccharomycotina_2 - ps20
Saccharomycotina_3 - ps21
Phaff./Saccharomycetaceae - ps22
Saccharomyco./mycetaceae - ps23
Saccharomycetaceae - ps24
Saccharomycetaceae_2 - ps25
Saccharomycetaceae_3 - ps26
Kaz./Nau./Saccharomyces - ps27
Saccharomyces - ps28
Arboricola_clade - ps29
Cerevisiae_clade - ps30
Saccharomyces_cerevisiae - ps31
Phylostratum

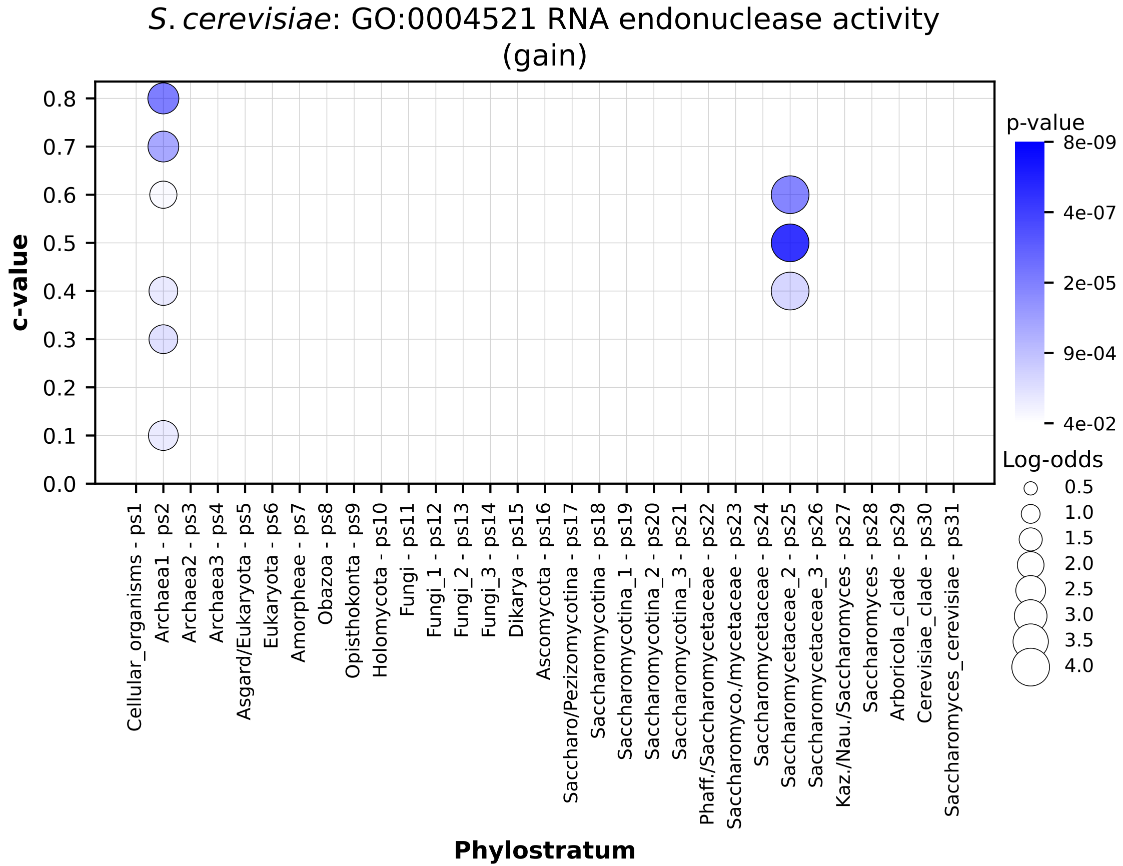

S. cerevisiae: GO:0004521 RNA endonuclease activity
(gain)
c-value
0.0
0.1
0.2
0.3
0.4
0.5
0.6
0.7
0.8
p-value
8e-09
4e-07
2e-05
9e-04
4e-02
Log-odds
0.5
1.0
1.5
2.0
2.5
3.0
3.5
4.0
Cellular_organisms - ps1
Archaea1 - ps2
Archaea2 - ps3
Archaea3 - ps4
Asgard/Eukaryota - ps5
Eukaryota - ps6
Amorpheae - ps7
Obazoa - ps8
Opisthokonta - ps9
Holomycota - ps10
Fungi - ps11
Fungi_1 - ps12
Fungi_2 - ps13
Fungi_3 - ps14
Dikarya - ps15
Ascomycota - ps16
Saccharo/Pezizomycotina - ps17
Saccharomycotina - ps18
Saccharomycotina_1 - ps19
Saccharomycotina_2 - ps20
Saccharomycotina_3 - ps21
Phaff./Saccharomycetaceae - ps22
Saccharomyco./mycetaceae - ps23
Saccharomycetaceae - ps24
Saccharomycetaceae_2 - ps25
Saccharomycetaceae_3 - ps26
Kaz./Nau./Saccharomyces - ps27
Saccharomyces - ps28
Arboricola_clade - ps29
Cerevisiae_clade - ps30
Saccharomyces_cerevisiae - ps31
Phylostratum

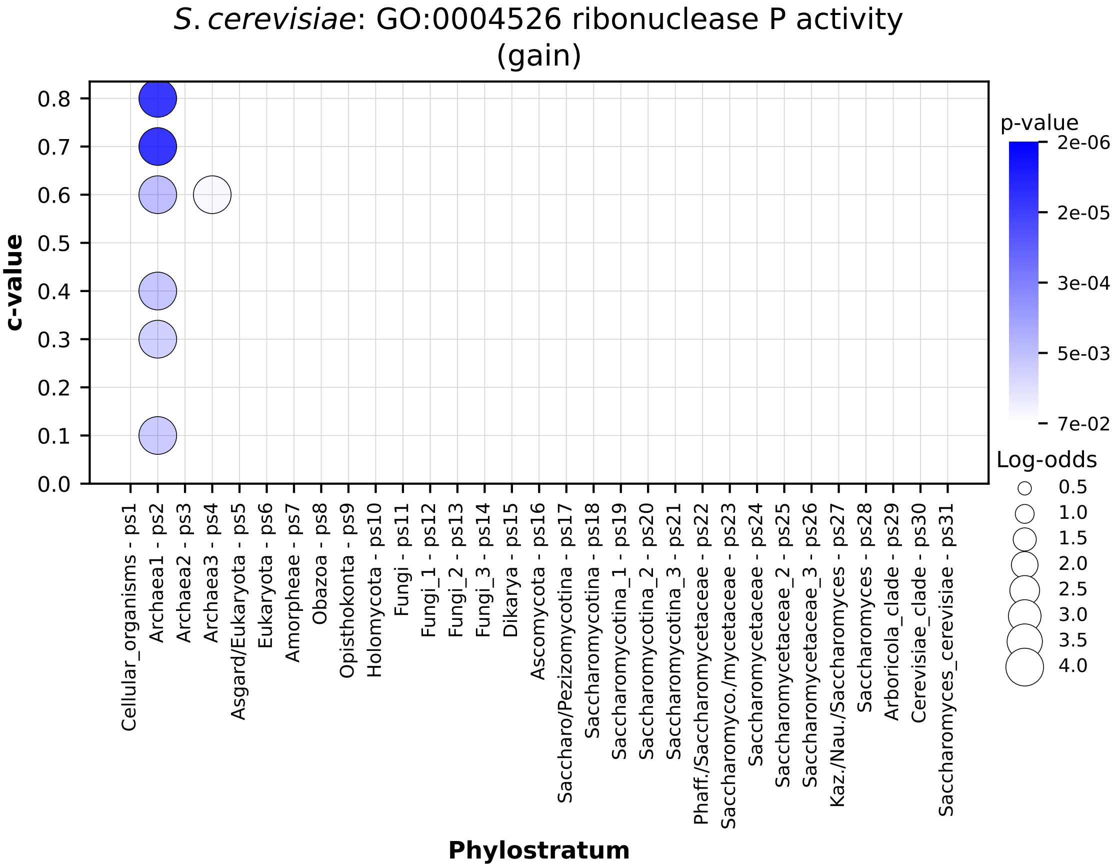

S. cerevisiae: GO:0004526 ribonuclease P activity
(gain)
c-value
0.8
0.7
0.6
0.5
0.4
0.3
0.2
0.1
0.0
p-value
2e-06
2e-05
3e-04
5e-03
7e-02
Log-odds
0.5
1.0
1.5
2.0
2.5
3.0
3.5
4.0
Cellular_organisms - ps1
Archaea1 - ps2
Archaea2 - ps3
Archaea3 - ps4
Asgard/Eukaryota - ps5
Eukaryota - ps6
Amorpheae - ps7
Obazoa - ps8
Opisthokonta - ps9
Holomycota - ps10
Fungi - ps11
Fungi_1 - ps12
Fungi_2 - ps13
Fungi_3 - ps14
Dikarya - ps15
Ascomycota - ps16
Saccharo/Pezizomycotina - ps17
Saccharomycotina - ps18
Saccharomycotina_1 - ps19
Saccharomycotina_2 - ps20
Saccharomycotina_3 - ps21
Phaff./Saccharomycetaceae - ps22
Saccharomyco./mycetaceae - ps23
Saccharomycetaceae - ps24
Saccharomycetaceae_2 - ps25
Saccharomycetaceae_3 - ps26
Kaz./Nau./Saccharomyces - ps27
Saccharomyces - ps28
Arboricola_clade - ps29
Cerevisiae_clade - ps30
Saccharomyces_cerevisiae - ps31
Phylostratum

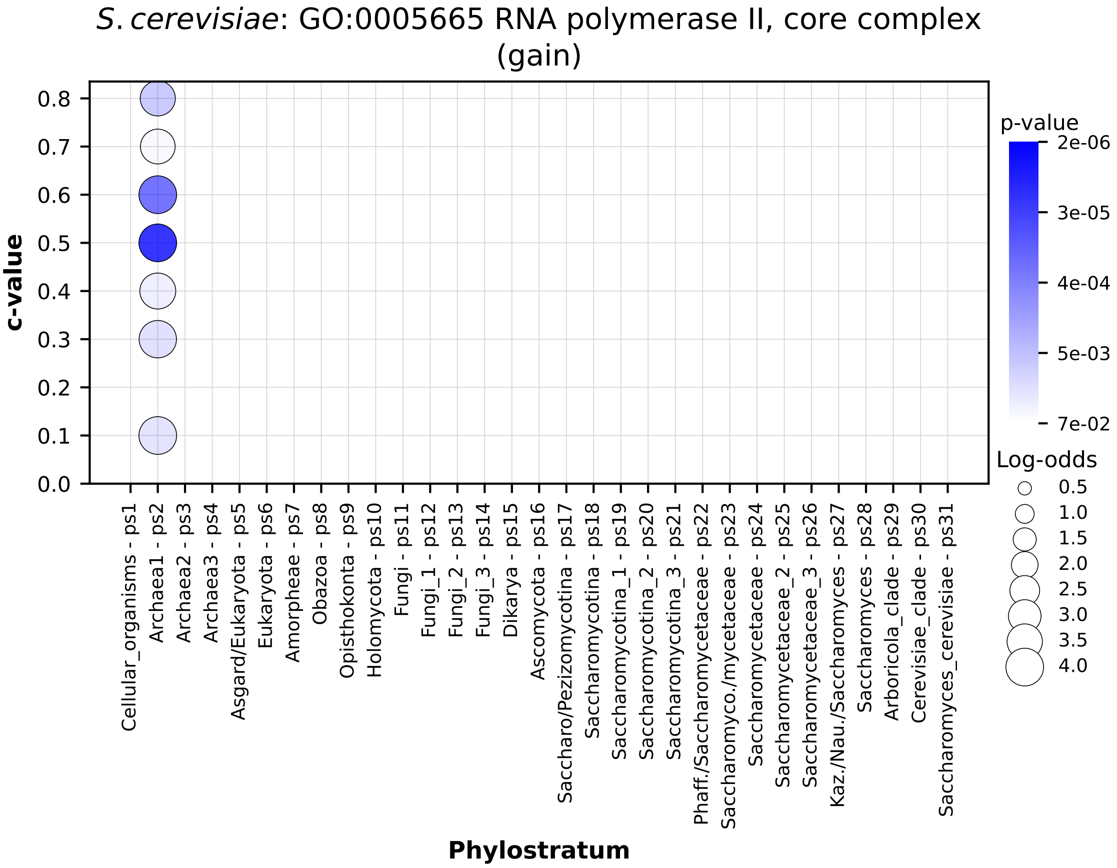

S. cerevisiae: GO:0005665 RNA polymerase II, core complex (gain)
c-value
0.0
0.1
0.2
0.3
0.4
0.5
0.6
0.7
0.8
p-value
2e-06
3e-05
4e-04
5e-03
7e-02
Log-odds
0.5
1.0
1.5
2.0
2.5
3.0
3.5
4.0
Cellular_organisms - ps1
Archaea1 - ps2
Archaea2 - ps3
Archaea3 - ps4
Asgard/Eukaryota - ps5
Eukaryota - ps6
Amorpheae - ps7
Obazoa - ps8
Opisthokonta - ps9
Holomycota - ps10
Fungi - ps11
Fungi_1 - ps12
Fungi_2 - ps13
Fungi_3 - ps14
Dikarya - ps15
Ascomycota - ps16
Saccharo/Pezizomycotina - ps17
Saccharomycotina - ps18
Saccharomycotina_1 - ps19
Saccharomycotina_2 - ps20
Saccharomycotina_3 - ps21
Phaff./Saccharomycetaceae - ps22
Saccharomyco./mycetaceae - ps23
Saccharomycetaceae - ps24
Saccharomycetaceae_2 - ps25
Saccharomycetaceae_3 - ps26
Kaz./Nau./Saccharomyces - ps27
Saccharomyces - ps28
Arboricola_clade - ps29
Cerevisiae_clade - ps30
Saccharomyces_cerevisiae - ps31
Phylostratum

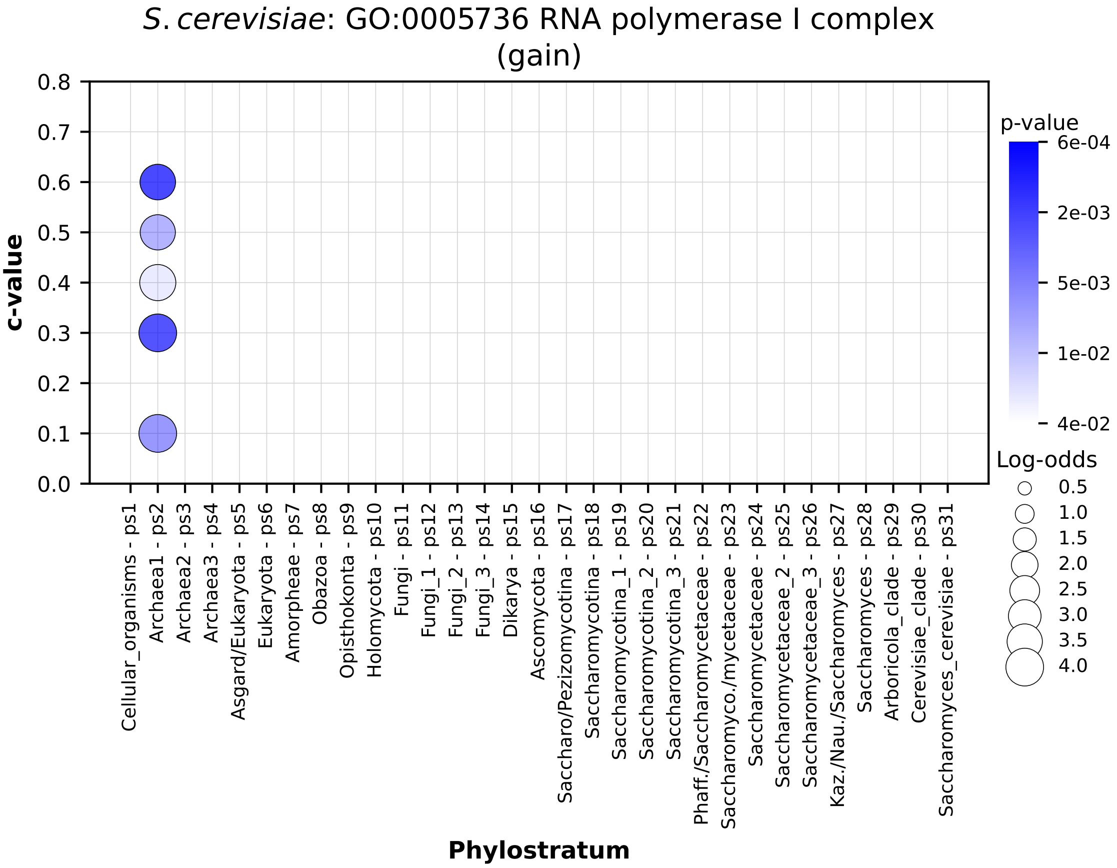
S. cerevisiae: GO:0005736 RNA polymerase I complex
(gain)
c-value
0.0
0.1
0.2
0.3
0.4
0.5
0.6
0.7
0.8
p-value
6e-04
2e-03
5e-03
1e-02
4e-02
Log-odds
0.5
1.0
1.5
2.0
2.5
3.0
3.5
4.0
Cellular_organisms - ps1
Archaea1 - ps2
Archaea2 - ps3
Archaea3 - ps4
Asgard/Eukaryota - ps5
Eukaryota - ps6
Amorpheae - ps7
Obazoa - ps8
Opisthokonta - ps9
Holomycota - ps10
Fungi - ps11
Fungi_1 - ps12
Fungi_2 - ps13
Fungi_3 - ps14
Dikarya - ps15
Ascomycota - ps16
Saccharo/Pezizomycotina - ps17
Saccharomycotina - ps18
Saccharomycotina_1 - ps19
Saccharomycotina_2 - ps20
Saccharomycotina_3 - ps21
Phaff./Saccharomycetaceae - ps22
Saccharomyco./mycetaceae - ps23
Saccharomycetaceae - ps24
Saccharomycetaceae_2 - ps25
Saccharomycetaceae_3 - ps26
Kaz./Nau./Saccharomyces - ps27
Saccharomyces - ps28
Arboricola_clade - ps29
Cerevisiae_clade - ps30
Saccharomyces_cerevisiae - ps31
Phylostratum

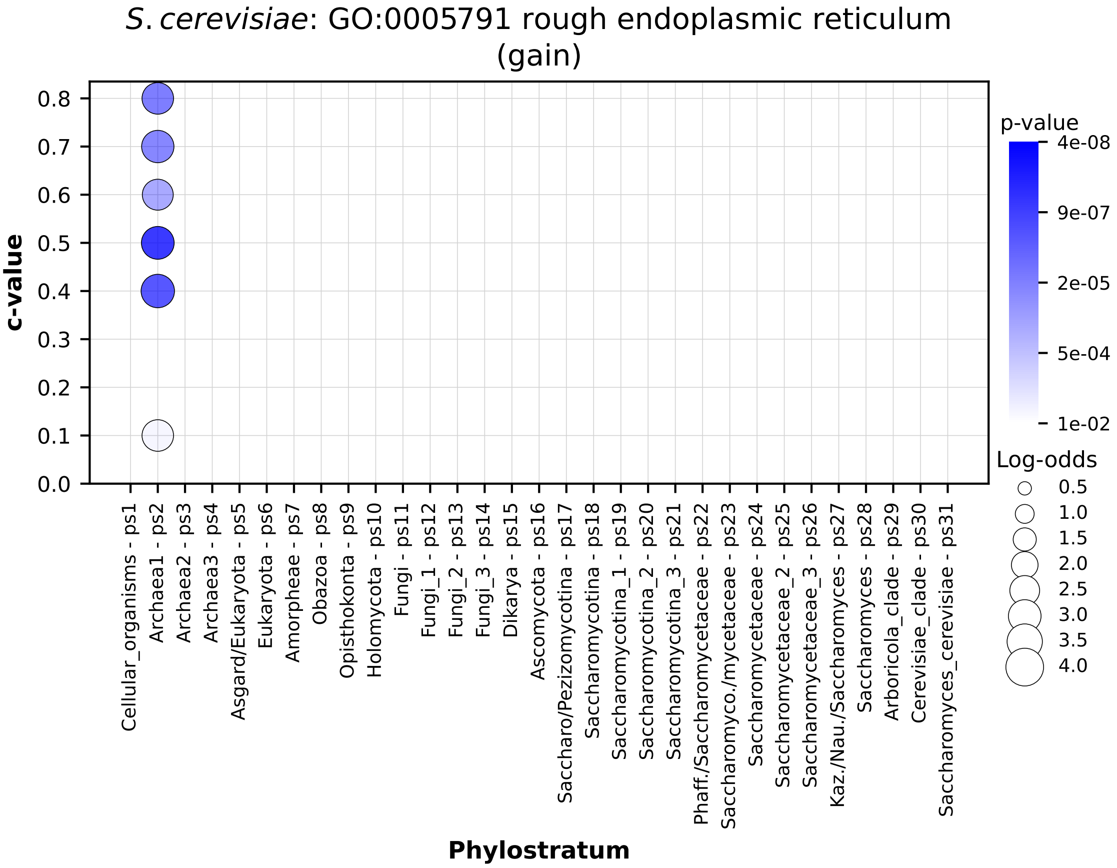

S. cerevisiae: GO:0005791 rough endoplasmic reticulum
(gain)
c-value
0.8
0.7
0.6
0.5
0.4
0.3
0.2
0.1
0.0
p-value
4e-08
9e-07
2e-05
5e-04
1e-02
Log-odds
0.5
1.0
1.5
2.0
2.5
3.0
3.5
4.0
Cellular_organisms - ps1
Archaea1 - ps2
Archaea2 - ps3
Archaea3 - ps4
Asgard/Eukaryota - ps5
Eukaryota - ps6
Amorpheae - ps7
Obazoa - ps8
Opisthokonta - ps9
Holomycota - ps10
Fungi - ps11
Fungi_1 - ps12
Fungi_2 - ps13
Fungi_3 - ps14
Dikarya - ps15
Ascomycota - ps16
Saccharo/Pezizomycotina - ps17
Saccharomycotina - ps18
Saccharomycotina_1 - ps19
Saccharomycotina_2 - ps20
Saccharomycotina_3 - ps21
Phaff./Saccharomycetaceae - ps22
Saccharomyco./mycetaceae - ps23
Saccharomycetaceae - ps24
Saccharomycetaceae_2 - ps25
Saccharomycetaceae_3 - ps26
Kaz./Nau./Saccharomyces - ps27
Saccharomyces - ps28
Arboricola_clade - ps29
Cerevisiae_clade - ps30
Saccharomyces_cerevisiae - ps31
Phylostratum

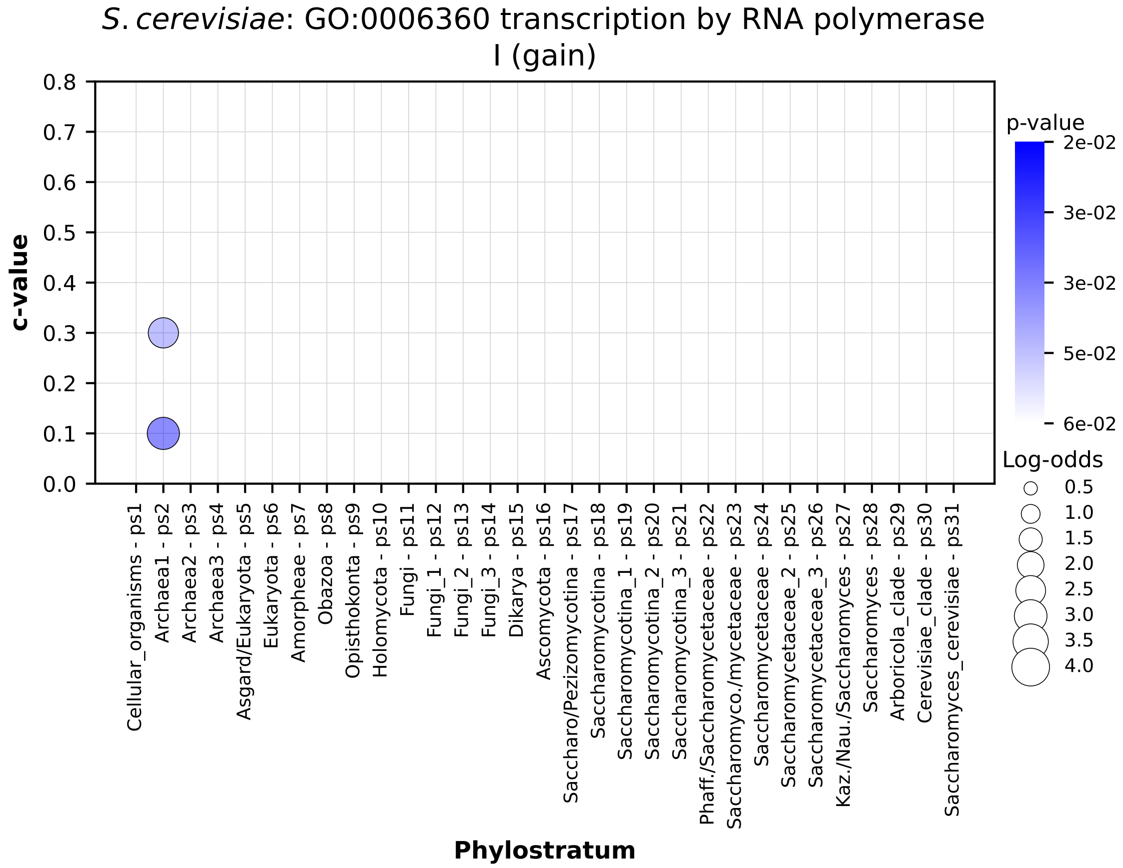

S. cerevisiae: GO:0006360 transcription by RNA polymerase I (gain)
c-value
0.0
0.1
0.2
0.3
0.4
0.5
0.6
0.7
0.8
Phylostratum
Cellular_organisms - ps1
Archaea1 - ps2
Archaea2 - ps3
Archaea3 - ps4
Asgard/Eukaryota - ps5
Eukaryota - ps6
Amorpheae - ps7
Obazoa - ps8
Opisthokonta - ps9
Holomycota - ps10
Fungi - ps11
Fungi_1 - ps12
Fungi_2 - ps13
Fungi_3 - ps14
Dikarya - ps15
Ascomycota - ps16
Saccharo/Pezizomycotina - ps17
Saccharomycotina - ps18
Saccharomycotina_1 - ps19
Saccharomycotina_2 - ps20
Saccharomycotina_3 - ps21
Phaff./Saccharomycetaceae - ps22
Saccharomyco./mycetaceae - ps23
Saccharomycetaceae - ps24
Saccharomycetaceae_2 - ps25
Saccharomycetaceae_3 - ps26
Kaz./Nau./Saccharomyces - ps27
Saccharomyces - ps28
Arboricola_clade - ps29
Cerevisiae_clade - ps30
Saccharomyces_cerevisiae - ps31
p-value
2e-02
3e-02
3e-02
5e-02
6e-02
Log-odds
0.5
1.0
1.5
2.0
2.5
3.0
3.5
4.0

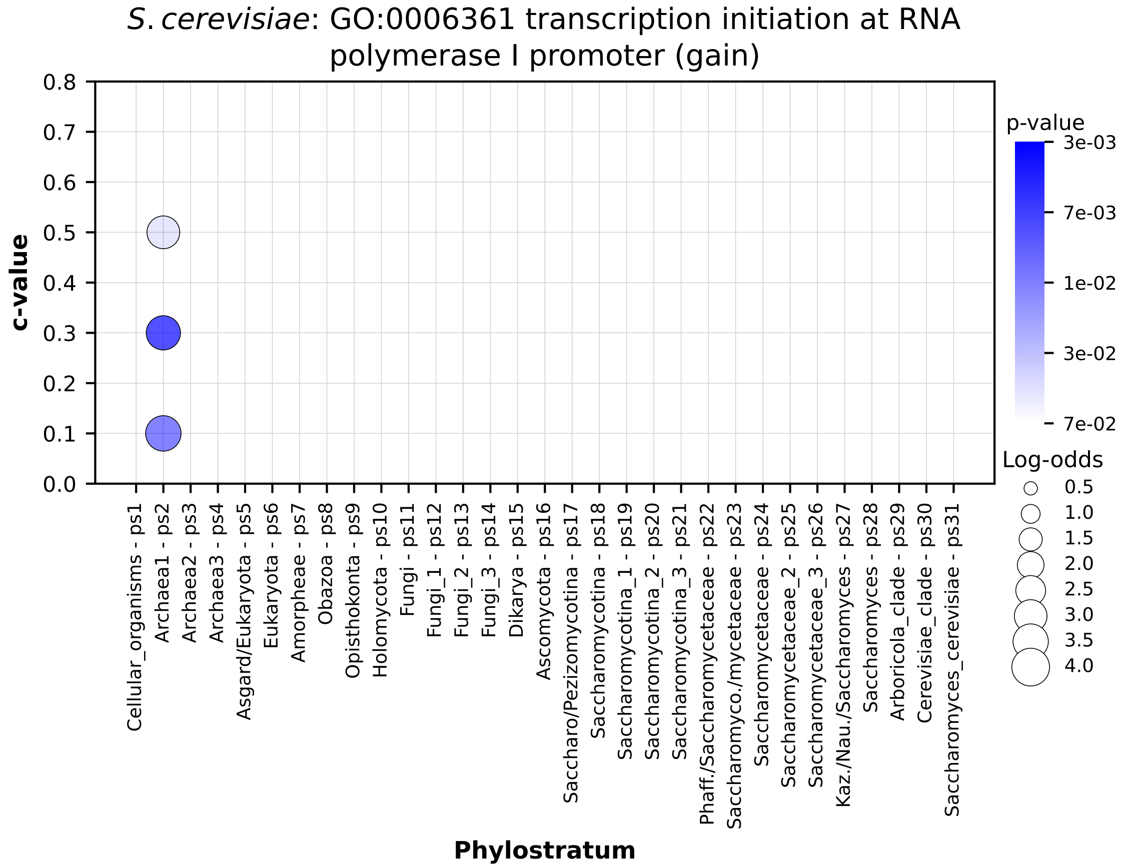

S. cerevisiae: GO:0006361 transcription initiation at RNA polymerase I promoter (gain)
c-value
0.0
0.1
0.2
0.3
0.4
0.5
0.6
0.7
0.8
p-value
3e-03
7e-03
1e-02
3e-02
7e-02
Log-odds
0.5
1.0
1.5
2.0
2.5
3.0
3.5
4.0
Cellular_organisms - ps1
Archaea1 - ps2
Archaea2 - ps3
Archaea3 - ps4
Asgard/Eukaryota - ps5
Eukaryota - ps6
Amorpheae - ps7
Obazoa - ps8
Opisthokonta - ps9
Holomycota - ps10
Fungi - ps11
Fungi_1 - ps12
Fungi_2 - ps13
Fungi_3 - ps14
Dikarya - ps15
Ascomycota - ps16
Saccharo/Pezizomycotina - ps17
Saccharomycotina - ps18
Saccharomycotina_1 - ps19
Saccharomycotina_2 - ps20
Saccharomycotina_3 - ps21
Phaff./Saccharomycetaceae - ps22
Saccharomyco./mycetaceae - ps23
Saccharomycetaceae - ps24
Saccharomycetaceae_2 - ps25
Saccharomycetaceae_3 - ps26
Kaz./Nau./Saccharomyces - ps27
Saccharomyces - ps28
Arboricola_clade - ps29
Cerevisiae_clade - ps30
Saccharomyces_cerevisiae - ps31
Phylostratum

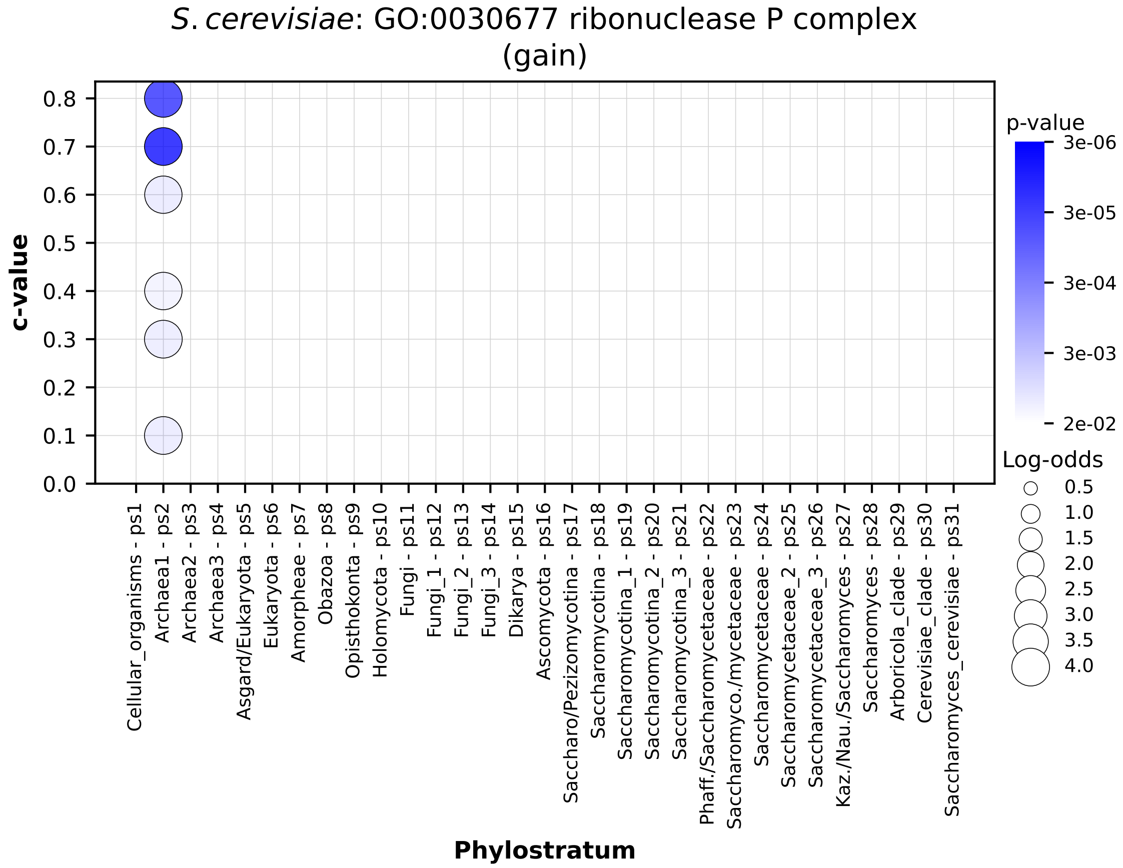

S. cerevisiae: GO:0030677 ribonuclease P complex
(gain)
c-value
0.0
0.1
0.2
0.3
0.4
0.5
0.6
0.7
0.8
p-value
3e-06
3e-05
3e-04
3e-03
2e-02
Log-odds
0.5
1.0
1.5
2.0
2.5
3.0
3.5
4.0
Cellular_organisms - ps1
Archaea1 - ps2
Archaea2 - ps3
Archaea3 - ps4
Asgard/Eukaryota - ps5
Eukaryota - ps6
Amorpheae - ps7
Obazoa - ps8
Opisthokonta - ps9
Holomycota - ps10
Fungi - ps11
Fungi_1 - ps12
Fungi_2 - ps13
Fungi_3 - ps14
Dikarya - ps15
Ascomycota - ps16
Saccharo/Pezizomycotina - ps17
Saccharomycotina - ps18
Saccharomycotina_1 - ps19
Saccharomycotina_2 - ps20
Saccharomycotina_3 - ps21
Phaff./Saccharomycetaceae - ps22
Saccharomyco./mycetaceae - ps23
Saccharomycetaceae - ps24
Saccharomycetaceae_2 - ps25
Saccharomycetaceae_3 - ps26
Kaz./Nau./Saccharomyces - ps27
Saccharomyces - ps28
Arboricola_clade - ps29
Cerevisiae_clade - ps30
Saccharomyces_cerevisiae - ps31
Phylostratum

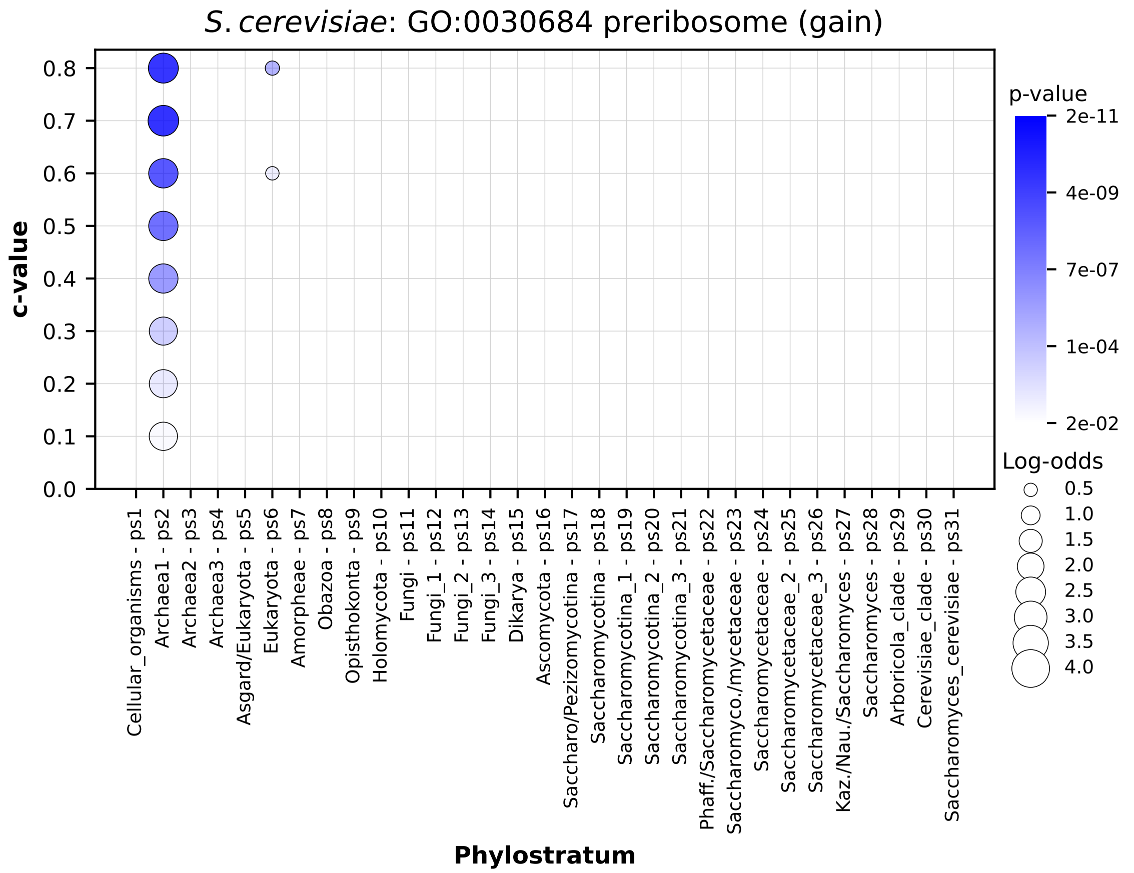

S. cerevisiae: GO:0030684 preribosome (gain)
c-value
0.0
0.1
0.2
0.3
0.4
0.5
0.6
0.7
0.8
p-value
2e-11
4e-09
7e-07
1e-04
2e-02
Log-odds
0.5
1.0
1.5
2.0
2.5
3.0
3.5
4.0
Cellular_organisms - ps1
Archaea1 - ps2
Archaea2 - ps3
Archaea3 - ps4
Asgard/Eukaryota - ps5
Eukaryota - ps6
Amorpheae - ps7
Obazoa - ps8
Opisthokonta - ps9
Holomycota - ps10
Fungi - ps11
Fungi_1 - ps12
Fungi_2 - ps13
Fungi_3 - ps14
Dikarya - ps15
Ascomycota - ps16
Saccharo/Pezizomycotina - ps17
Saccharomycotina - ps18
Saccharomycotina_1 - ps19
Saccharomycotina_2 - ps20
Saccharomycotina_3 - ps21
Phaff./Saccharomycetaceae - ps22
Saccharomyco./mycetaceae - ps23
Saccharomycetaceae - ps24
Saccharomycetaceae_2 - ps25
Saccharomycetaceae_3 - ps26
Kaz./Nau./Saccharomyces - ps27
Saccharomyces - ps28
Arboricola_clade - ps29
Cerevisiae_clade - ps30
Saccharomyces_cerevisiae - ps31
Phylostratum

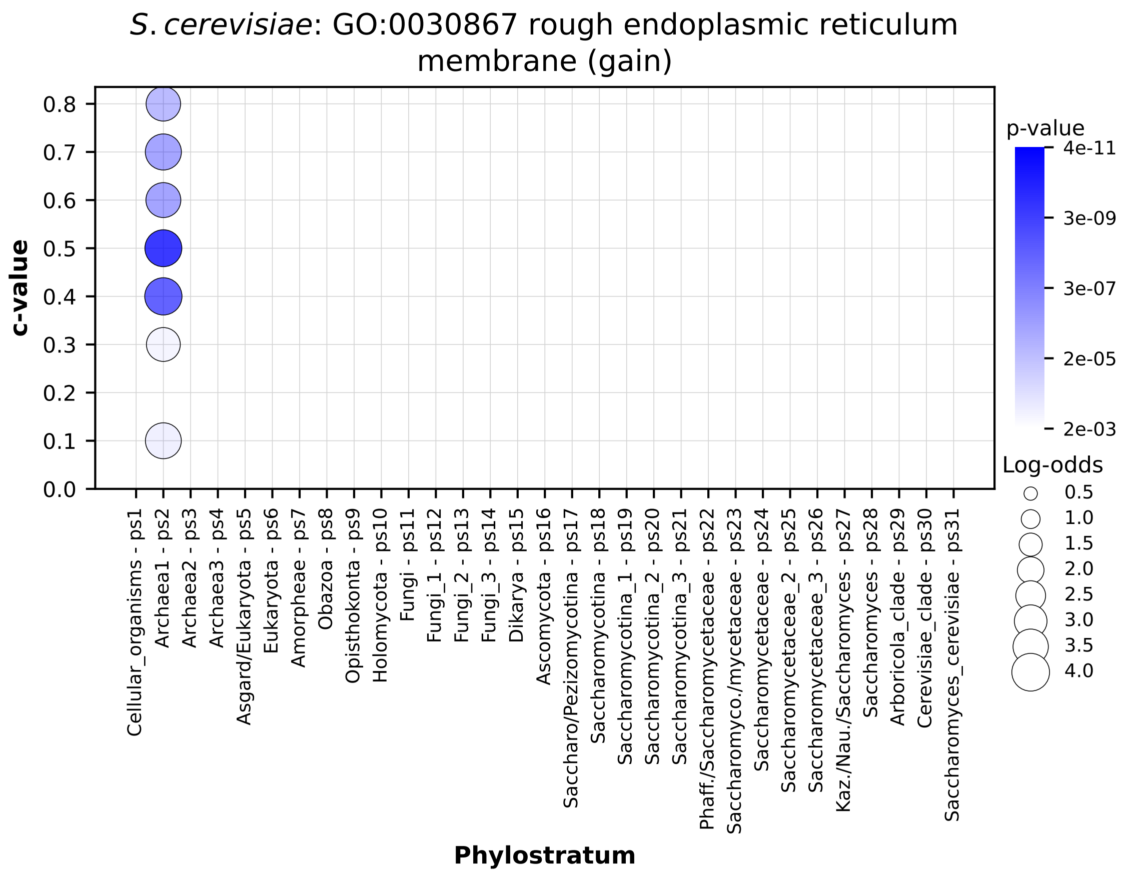

S. cerevisiae: GO:0030867 rough endoplasmic reticulum membrane (gain)
c-value
0.0
0.1
0.2
0.3
0.4
0.5
0.6
0.7
0.8
Phylostratum
Cellular_organisms - ps1
Archaea1 - ps2
Archaea2 - ps3
Archaea3 - ps4
Asgard/Eukaryota - ps5
Eukaryota - ps6
Amorpheae - ps7
Obazoa - ps8
Opisthokonta - ps9
Holomycota - ps10
Fungi - ps11
Fungi_1 - ps12
Fungi_2 - ps13
Fungi_3 - ps14
Dikarya - ps15
Ascomycota - ps16
Saccharo/Pezizomycotina - ps17
Saccharomycotina - ps18
Saccharomycotina_1 - ps19
Saccharomycotina_2 - ps20
Saccharomycotina_3 - ps21
Phaff./Saccharomycetaceae - ps22
Saccharomyco./mycetaceae - ps23
Saccharomycetaceae - ps24
Saccharomycetaceae_2 - ps25
Saccharomycetaceae_3 - ps26
Kaz./Nau./Saccharomyces - ps27
Saccharomyces - ps28
Arboricola_clade - ps29
Cerevisiae_clade - ps30
Saccharomyces_cerevisiae - ps31
p-value
4e-11
3e-09
3e-07
2e-05
2e-03
Log-odds
0.5
1.0
1.5
2.0
2.5
3.0
3.5
4.0

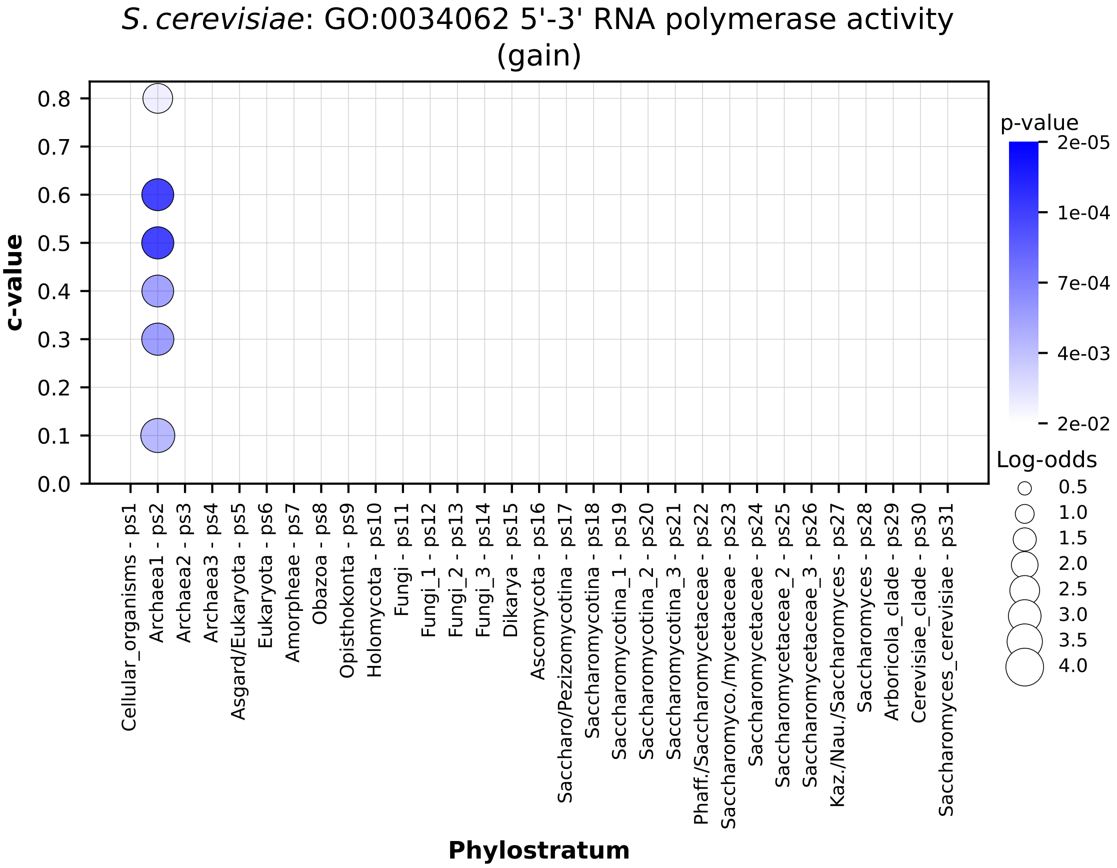

S. cerevisiae: GO:0034062 5'-3' RNA polymerase activity (gain)
c-value
0.8
0.7
0.6
0.5
0.4
0.3
0.2
0.1
0.0
Phylostratum
Cellular_organisms - ps1
Archaea1 - ps2
Archaea2 - ps3
Archaea3 - ps4
Asgard/Eukaryota - ps5
Eukaryota - ps6
Amorpheae - ps7
Obazoa - ps8
Opisthokonta - ps9
Holomycota - ps10
Fungi - ps11
Fungi_1 - ps12
Fungi_2 - ps13
Fungi_3 - ps14
Dikarya - ps15
Ascomycota - ps16
Saccharo/Pezizomycotina - ps17
Saccharomycotina - ps18
Saccharomycotina_1 - ps19
Saccharomycotina_2 - ps20
Saccharomycotina_3 - ps21
Phaff./Saccharomycetaceae - ps22
Saccharomyco./mycetaceae - ps23
Saccharomycetaceae - ps24
Saccharomycetaceae_2 - ps25
Saccharomycetaceae_3 - ps26
Kaz./Nau./Saccharomyces - ps27
Saccharomyces - ps28
Arboricola_clade - ps29
Cerevisiae_clade - ps30
Saccharomyces_cerevisiae - ps31
p-value
2e-05
1e-04
7e-04
4e-03
2e-02
Log-odds
0.5
1.0
1.5
2.0
2.5
3.0
3.5
4.0

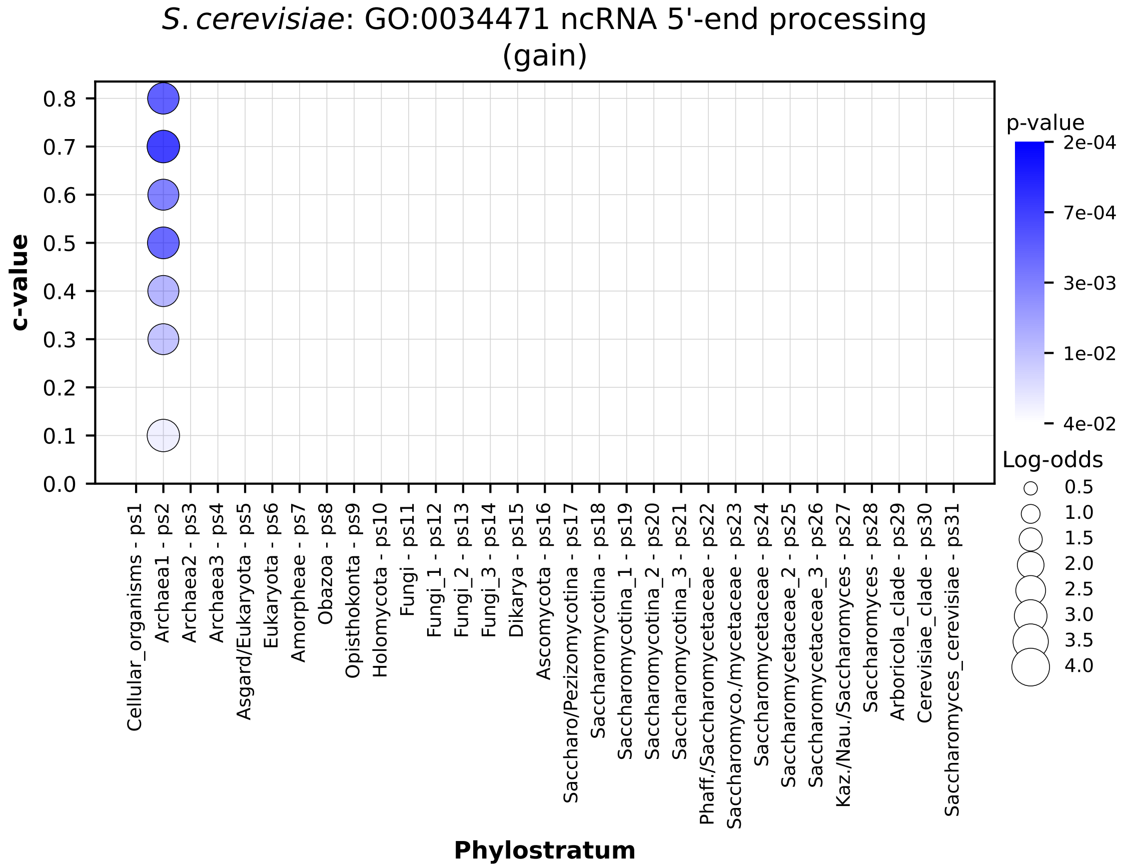

S. cerevisiae: GO:0034471 ncRNA 5'-end processing
(gain)
c-value
0.0
0.1
0.2
0.3
0.4
0.5
0.6
0.7
0.8
Phylostratum
Cellular_organisms - ps1
Archaea1 - ps2
Archaea2 - ps3
Archaea3 - ps4
Asgard/Eukaryota - ps5
Eukaryota - ps6
Amorpheae - ps7
Obazoa - ps8
Opisthokonta - ps9
Holomycota - ps10
Fungi - ps11
Fungi_1 - ps12
Fungi_2 - ps13
Fungi_3 - ps14
Dikarya - ps15
Ascomycota - ps16
Saccharo/Pezizomycotina - ps17
Saccharomycotina - ps18
Saccharomycotina_1 - ps19
Saccharomycotina_2 - ps20
Saccharomycotina_3 - ps21
Phaff./Saccharomycetaceae - ps22
Saccharomyco./mycetaceae - ps23
Saccharomycetaceae - ps24
Saccharomycetaceae_2 - ps25
Saccharomycetaceae_3 - ps26
Kaz./Nau./Saccharomyces - ps27
Saccharomyces - ps28
Arboricola_clade - ps29
Cerevisiae_clade - ps30
Saccharomyces_cerevisiae - ps31
p-value
2e-04
7e-04
3e-03
1e-02
4e-02
Log-odds
0.5
1.0
1.5
2.0
2.5
3.0
3.5
4.0

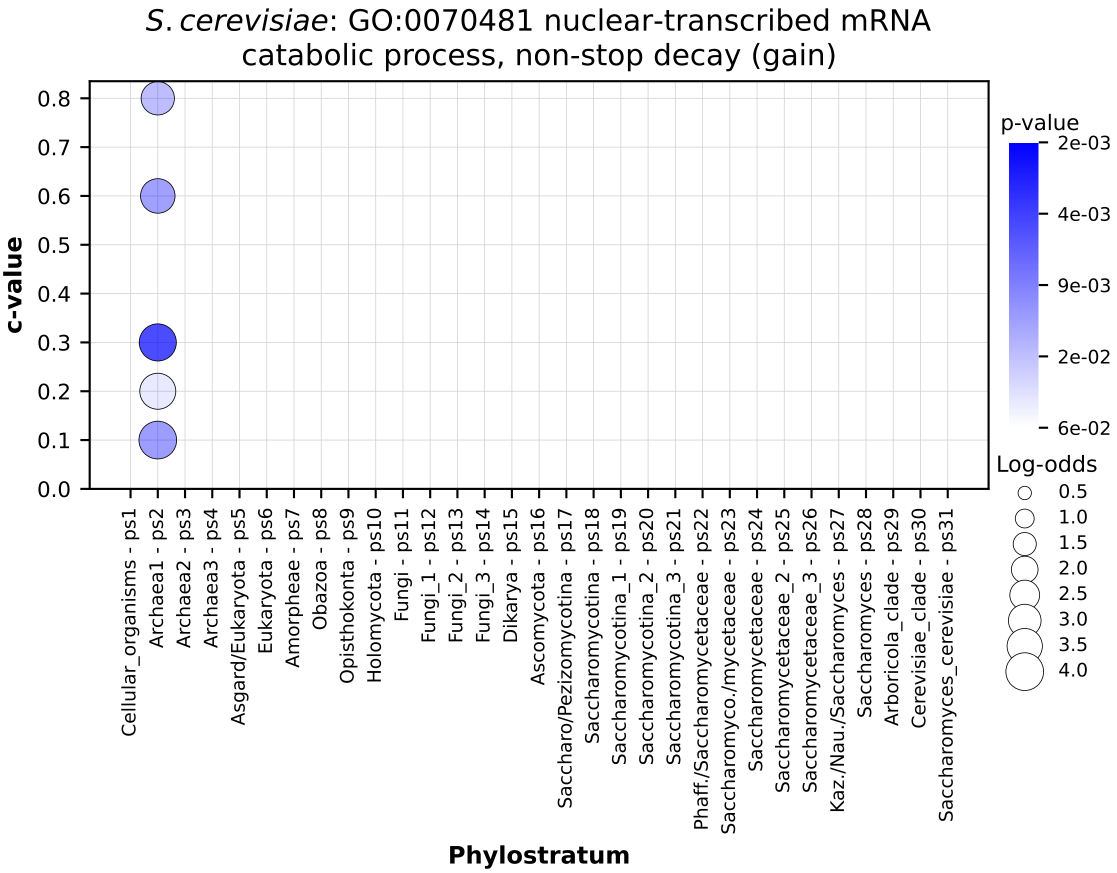

S. cerevisiae: GO:0070481 nuclear-transcribed mRNA catabolic process, non-stop decay (gain)
c-value
0.0
0.1
0.2
0.3
0.4
0.5
0.6
0.7
0.8
p-value
2e-03
4e-03
9e-03
2e-02
6e-02
Log-odds
0.5
1.0
1.5
2.0
2.5
3.0
3.5
4.0
Cellular_organisms - ps1
Archaea1 - ps2
Archaea2 - ps3
Archaea3 - ps4
Asgard/Eukaryota - ps5
Eukaryota - ps6
Amorpheae - ps7
Obazoa - ps8
Opisthokonta - ps9
Holomycota - ps10
Fungi - ps11
Fungi_1 - ps12
Fungi_2 - ps13
Fungi_3 - ps14
Dikarya - ps15
Ascomycota - ps16
Saccharo/Pezizomycotina - ps17
Saccharomycotina - ps18
Saccharomycotina_1 - ps19
Saccharomycotina_2 - ps20
Saccharomycotina_3 - ps21
Phaff./Saccharomycetaceae - ps22
Saccharomyco./mycetaceae - ps23
Saccharomycetaceae - ps24
Saccharomycetaceae_2 - ps25
Saccharomycetaceae_3 - ps26
Kaz./Nau./Saccharomyces - ps27
Saccharomyces - ps28
Arboricola_clade - ps29
Cerevisiae_clade - ps30
Saccharomyces_cerevisiae - ps31
Phylostratum

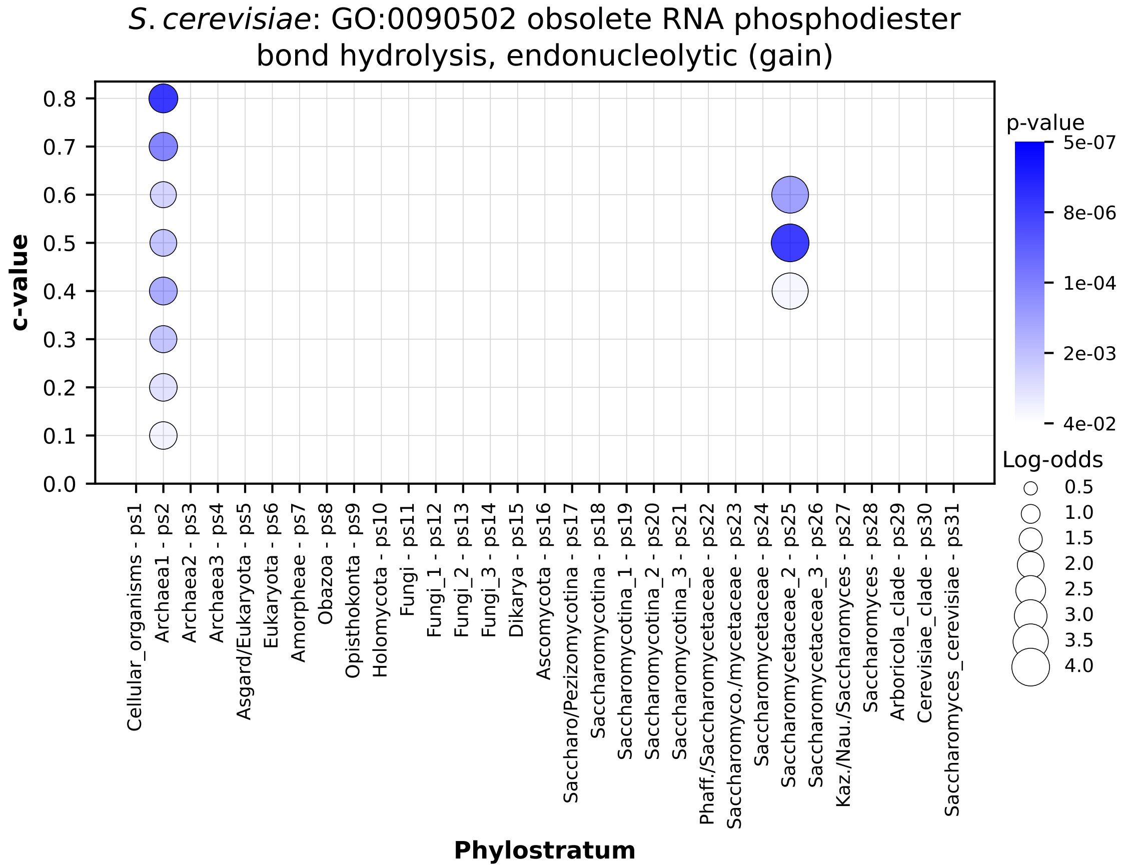

S. cerevisiae: GO:0090502 obsolete RNA phosphodiester bond hydrolysis, endonucleolytic (gain)
c-value
0.0
0.1
0.2
0.3
0.4
0.5
0.6
0.7
0.8
p-value
5e-07
8e-06
1e-04
2e-03
4e-02
Log-odds
0.5
1.0
1.5
2.0
2.5
3.0
3.5
4.0
Cellular_organisms - ps1
Archaea1 - ps2
Archaea2 - ps3
Archaea3 - ps4
Asgard/Eukaryota - ps5
Eukaryota - ps6
Amorpheae - ps7
Obazoa - ps8
Opisthokonta - ps9
Holomycota - ps10
Fungi - ps11
Fungi_1 - ps12
Fungi_2 - ps13
Fungi_3 - ps14
Dikarya - ps15
Ascomycota - ps16
Saccharo/Pezizomycotina - ps17
Saccharomycotina - ps18
Saccharomycotina_1 - ps19
Saccharomycotina_2 - ps20
Saccharomycotina_3 - ps21
Phaff./Saccharomycetaceae - ps22
Saccharomyco./mycetaceae - ps23
Saccharomycetaceae - ps24
Saccharomycetaceae_2 - ps25
Saccharomycetaceae_3 - ps26
Kaz./Nau./Saccharomyces - ps27
Saccharomyces - ps28
Arboricola_clade - ps29
Cerevisiae_clade - ps30
Saccharomyces_cerevisiae - ps31
Phylostratum

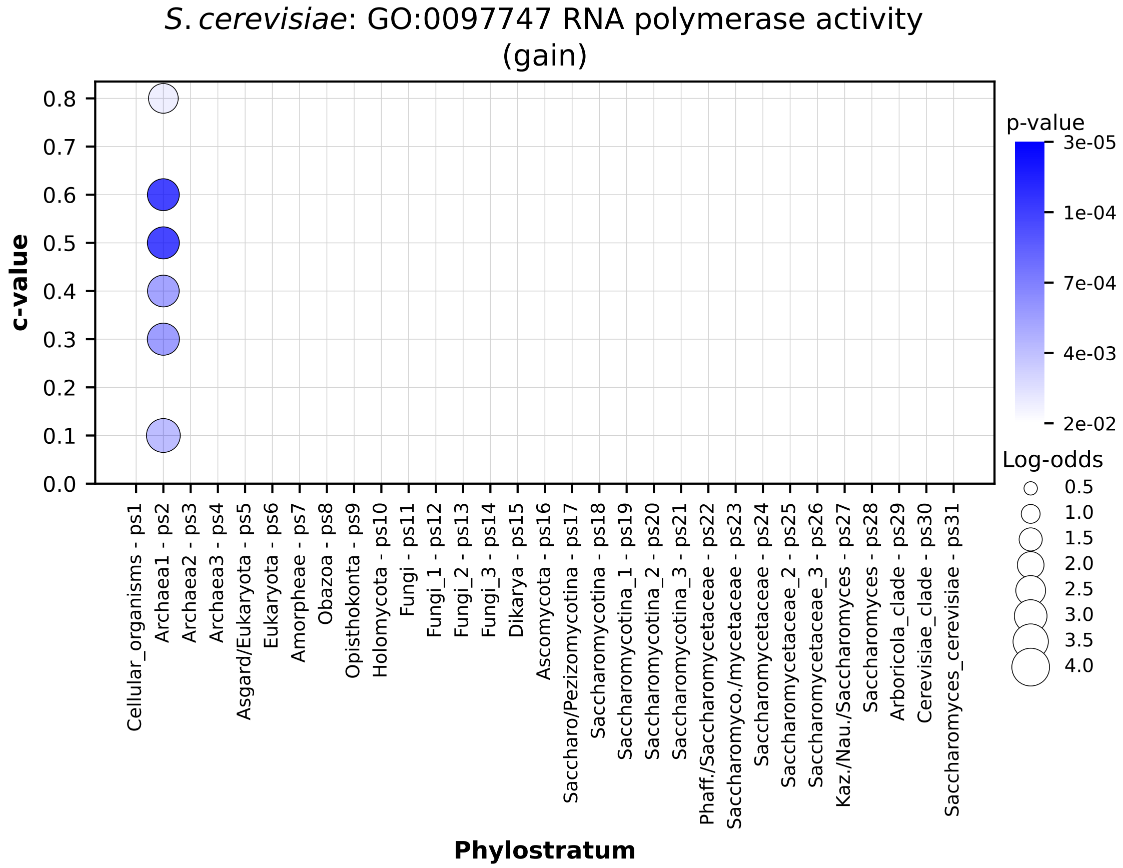

S. cerevisiae: GO:0097747 RNA polymerase activity
(gain)
c-value
0.0
0.1
0.2
0.3
0.4
0.5
0.6
0.7
0.8
Phylostratum
Cellular_organisms - ps1
Archaea1 - ps2
Archaea2 - ps3
Archaea3 - ps4
Asgard/Eukaryota - ps5
Eukaryota - ps6
Amorpheae - ps7
Obazoa - ps8
Opisthokonta - ps9
Holomycota - ps10
Fungi - ps11
Fungi_1 - ps12
Fungi_2 - ps13
Fungi_3 - ps14
Dikarya - ps15
Ascomycota - ps16
Saccharo/Pezizomycotina - ps17
Saccharomycotina - ps18
Saccharomycotina_1 - ps19
Saccharomycotina_2 - ps20
Saccharomycotina_3 - ps21
Phaff./Saccharomycetaceae - ps22
Saccharomyco./mycetaceae - ps23
Saccharomycetaceae - ps24
Saccharomycetaceae_2 - ps25
Saccharomycetaceae_3 - ps26
Kaz./Nau./Saccharomyces - ps27
Saccharomyces - ps28
Arboricola_clade - ps29
Cerevisiae_clade - ps30
Saccharomyces_cerevisiae - ps31
p-value
3e-05
1e-04
7e-04
4e-03
2e-02
Log-odds
0.5
1.0
1.5
2.0
2.5
3.0
3.5
4.0

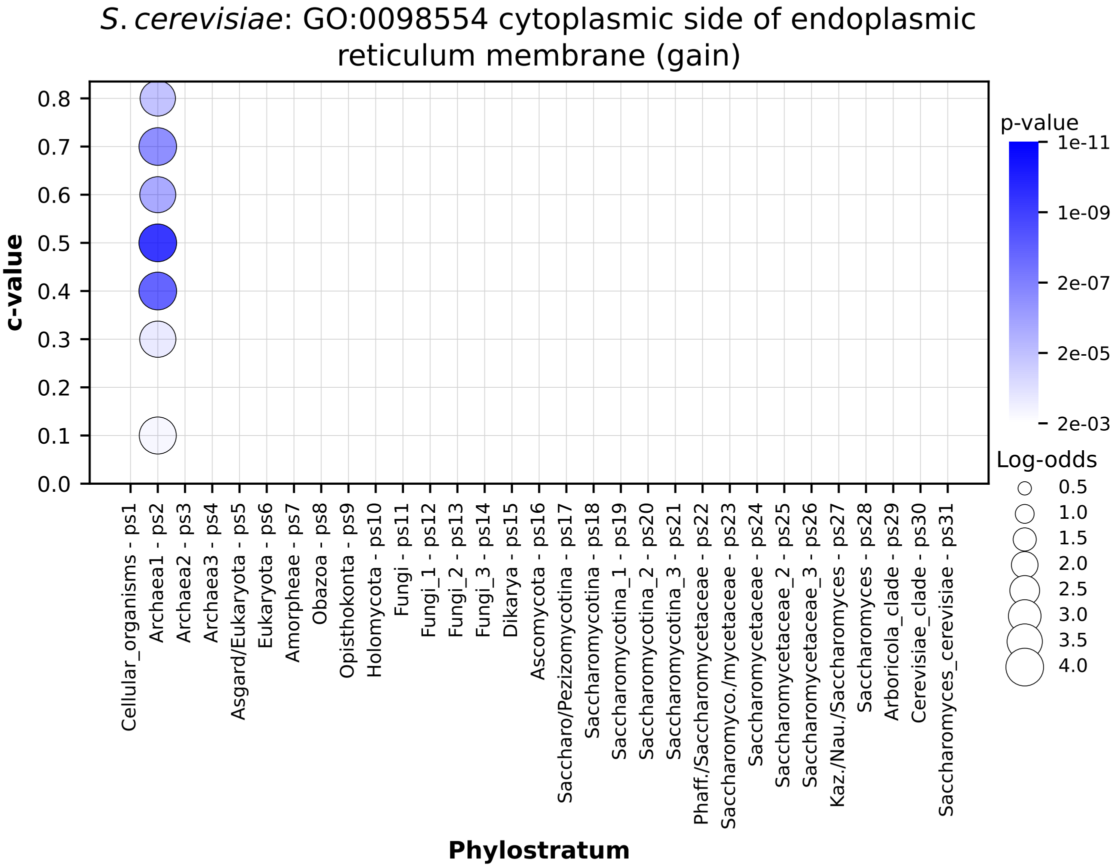

S. cerevisiae: GO:0098554 cytoplasmic side of endoplasmic reticulum membrane (gain)
c-value
0.0
0.1
0.2
0.3
0.4
0.5
0.6
0.7
0.8
p-value
1e-11
1e-09
2e-07
2e-05
2e-03
Log-odds
0.5
1.0
1.5
2.0
2.5
3.0
3.5
4.0
Cellular_organisms - ps1
Archaea1 - ps2
Archaea2 - ps3
Archaea3 - ps4
Asgard/Eukaryota - ps5
Eukaryota - ps6
Amorpheae - ps7
Obazoa - ps8
Opisthokonta - ps9
Holomycota - ps10
Fungi - ps11
Fungi_1 - ps12
Fungi_2 - ps13
Fungi_3 - ps14
Dikarya - ps15
Ascomycota - ps16
Saccharo/Pezizomycotina - ps17
Saccharomycotina - ps18
Saccharomycotina_1 - ps19
Saccharomycotina_2 - ps20
Saccharomycotina_3 - ps21
Phaff./Saccharomycetaceae - ps22
Saccharomyco./mycetaceae - ps23
Saccharomycetaceae - ps24
Saccharomycetaceae_2 - ps25
Saccharomycetaceae_3 - ps26
Kaz./Nau./Saccharomyces - ps27
Saccharomyces - ps28
Arboricola_clade - ps29
Cerevisiae_clade - ps30
Saccharomyces_cerevisiae - ps31
Phylostratum

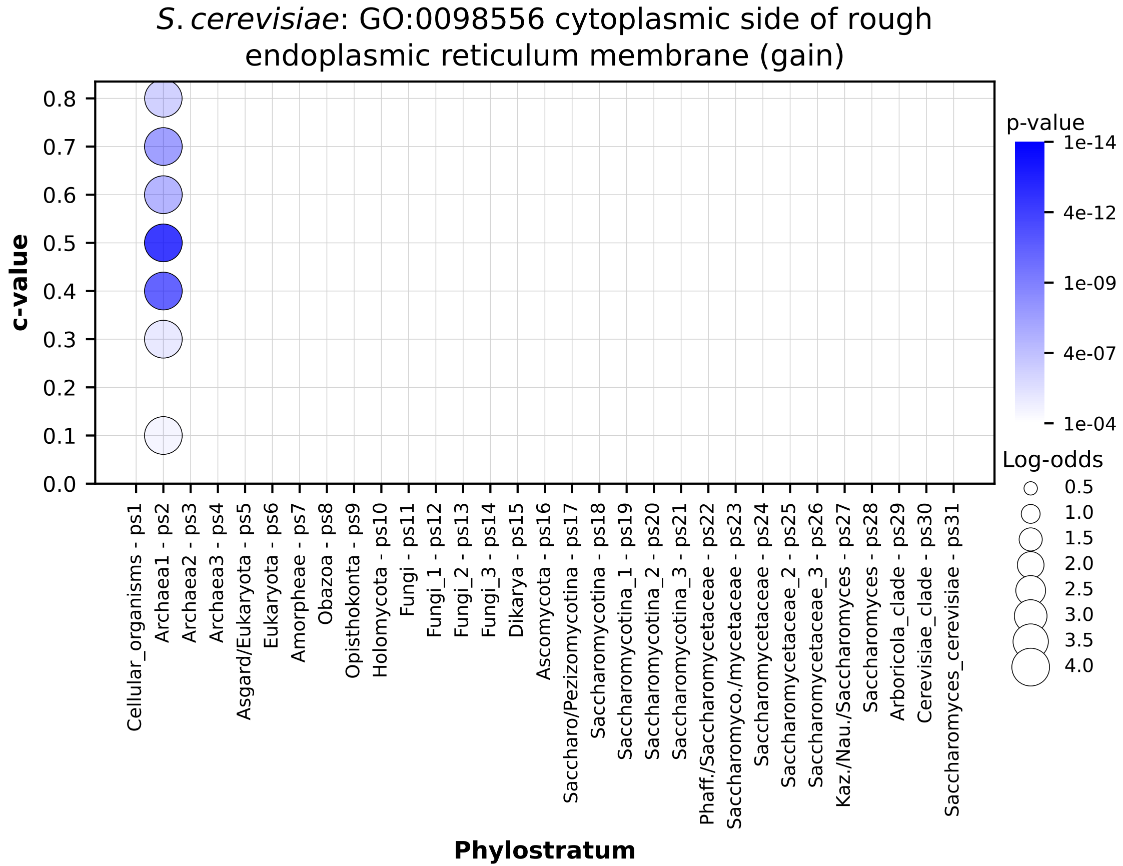

S. cerevisiae: GO:0098556 cytoplasmic side of rough endoplasmic reticulum membrane (gain)
c-value
0.0
0.1
0.2
0.3
0.4
0.5
0.6
0.7
0.8
Phylostratum
Cellular_organisms - ps1
Archaea1 - ps2
Archaea2 - ps3
Archaea3 - ps4
Asgard/Eukaryota - ps5
Eukaryota - ps6
Amorpheae - ps7
Obazoa - ps8
Opisthokonta - ps9
Holomycota - ps10
Fungi - ps11
Fungi_1 - ps12
Fungi_2 - ps13
Fungi_3 - ps14
Dikarya - ps15
Ascomycota - ps16
Saccharo/Pezizomycotina - ps17
Saccharomycotina - ps18
Saccharomycotina_1 - ps19
Saccharomycotina_2 - ps20
Saccharomycotina_3 - ps21
Phaff./Saccharomycetaceae - ps22
Saccharomyco./mycetaceae - ps23
Saccharomycetaceae - ps24
Saccharomycetaceae_2 - ps25
Saccharomycetaceae_3 - ps26
Kaz./Nau./Saccharomyces - ps27
Saccharomyces - ps28
Arboricola_clade - ps29
Cerevisiae_clade - ps30
Saccharomyces_cerevisiae - ps31
p-value
1e-14
4e-12
1e-09
4e-07
1e-04
Log-odds
0.5
1.0
1.5
2.0
2.5
3.0
3.5
4.0

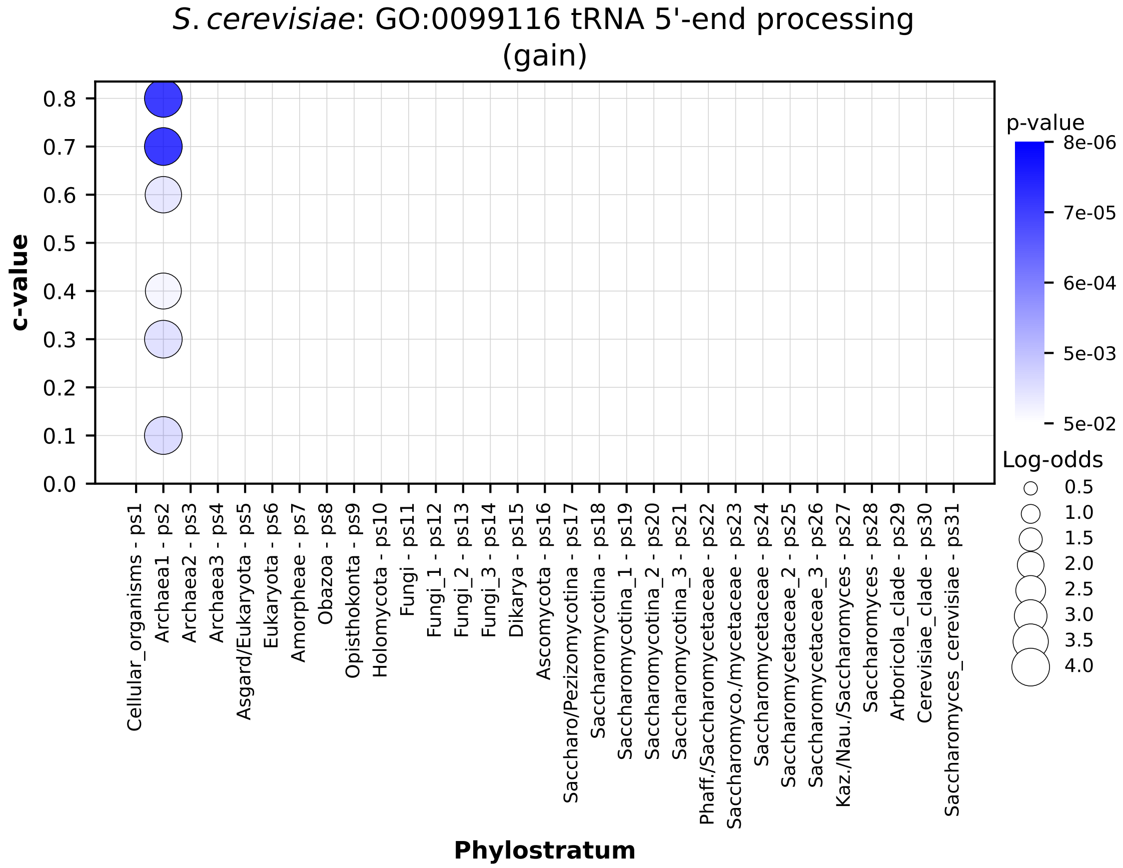

S. cerevisiae: GO:0099116 tRNA 5'-end processing (gain)
c-value
0.0
0.1
0.2
0.3
0.4
0.5
0.6
0.7
0.8
Phylostratum
Cellular_organisms - ps1
Archaea1 - ps2
Archaea2 - ps3
Archaea3 - ps4
Asgard/Eukaryota - ps5
Eukaryota - ps6
Amorpheae - ps7
Obazoa - ps8
Opisthokonta - ps9
Holomycota - ps10
Fungi - ps11
Fungi_1 - ps12
Fungi_2 - ps13
Fungi_3 - ps14
Dikarya - ps15
Ascomycota - ps16
Saccharo/Pezizomycotina - ps17
Saccharomycotina - ps18
Saccharomycotina_1 - ps19
Saccharomycotina_2 - ps20
Saccharomycotina_3 - ps21
Phaff./Saccharomycetaceae - ps22
Saccharomyco./mycetaceae - ps23
Saccharomycetaceae - ps24
Saccharomycetaceae_2 - ps25
Saccharomycetaceae_3 - ps26
Kaz./Nau./Saccharomyces - ps27
Saccharomyces - ps28
Arboricola_clade - ps29
Cerevisiae_clade - ps30
Saccharomyces_cerevisiae - ps31
p-value
8e-06
7e-05
6e-04
5e-03
5e-02
Log-odds
0.5
1.0
1.5
2.0
2.5
3.0
3.5
4.0

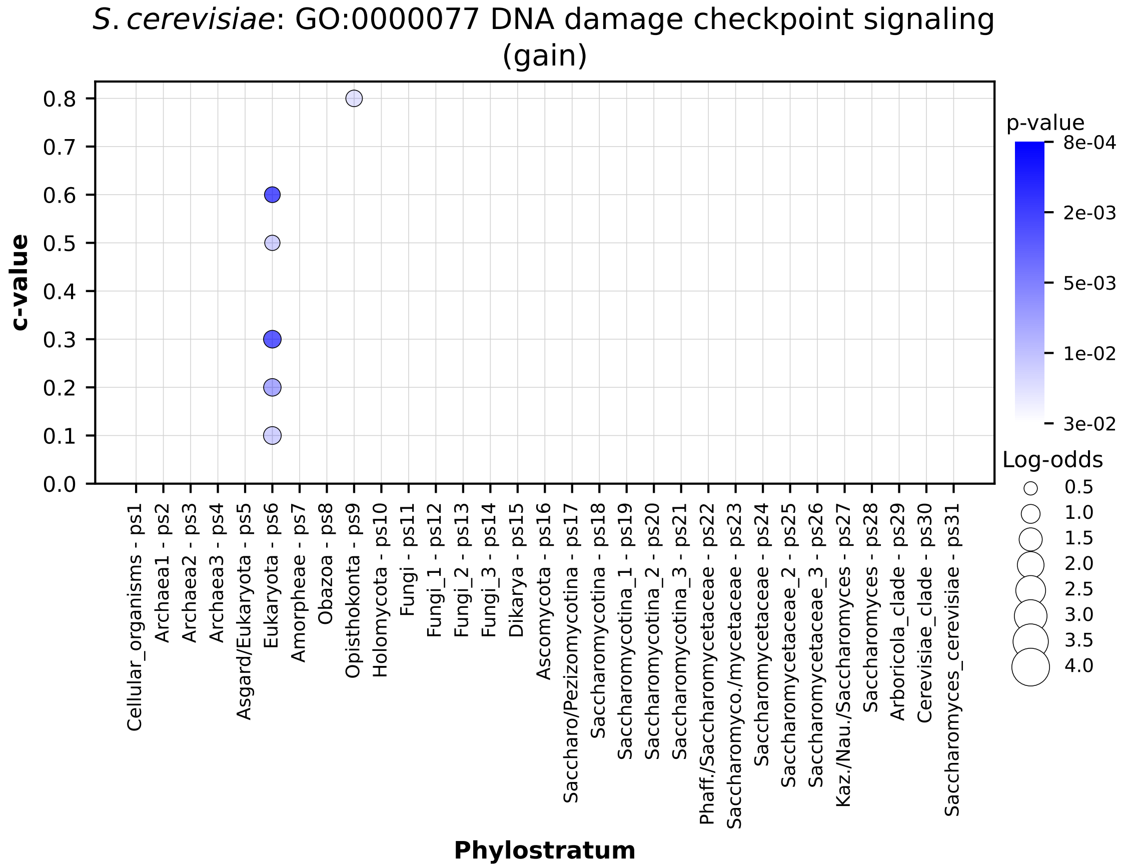

S. cerevisiae: GO:0000077 DNA damage checkpoint signaling (gain)
c-value
0.8
0.7
0.6
0.5
0.4
0.3
0.2
0.1
0.0
p-value
8e-04
2e-03
5e-03
1e-02
3e-02
Log-odds
0.5
1.0
1.5
2.0
2.5
3.0
3.5
4.0
Cellular_organisms - ps1
Archaea1 - ps2
Archaea2 - ps3
Archaea3 - ps4
Asgard/Eukaryota - ps5
Eukaryota - ps6
Amorpheae - ps7
Obazoa - ps8
Opisthokonta - ps9
Holomycota - ps10
Fungi - ps11
Fungi_1 - ps12
Fungi_2 - ps13
Fungi_3 - ps14
Dikarya - ps15
Ascomycota - ps16
Saccharo/Pezizomycotina - ps17
Saccharomycotina - ps18
Saccharomycotina_1 - ps19
Saccharomycotina_2 - ps20
Saccharomycotina_3 - ps21
Phaff./Saccharomycetaceae - ps22
Saccharomyco./mycetaceae - ps23
Saccharomycetaceae - ps24
Saccharomycetaceae_2 - ps25
Saccharomycetaceae_3 - ps26
Kaz./Nau./Saccharomyces - ps27
Saccharomyces - ps28
Arboricola_clade - ps29
Cerevisiae_clade - ps30
Saccharomyces_cerevisiae - ps31
Phylostratum

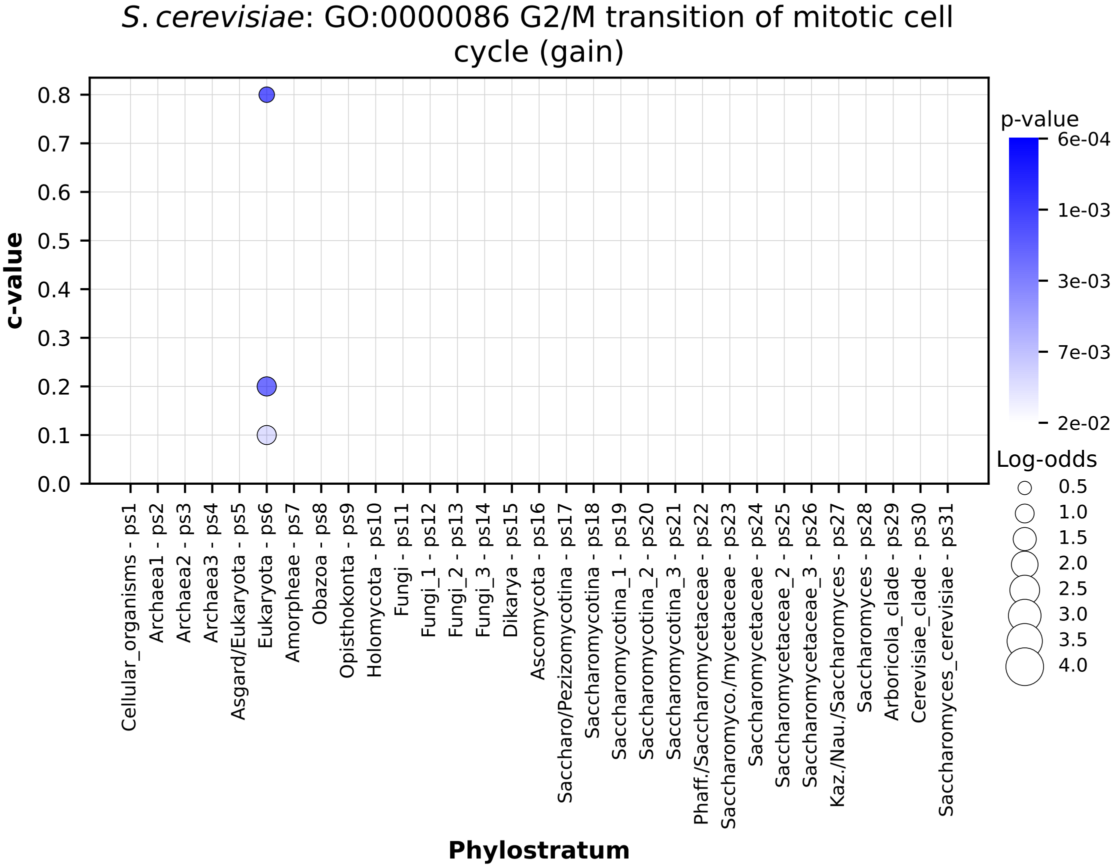

S. cerevisiae: GO:0000086 G2/M transition of mitotic cell cycle (gain)
c-value
0.0
0.1
0.2
0.3
0.4
0.5
0.6
0.7
0.8
p-value
6e-04
1e-03
3e-03
7e-03
2e-02
Log-odds
0.5
1.0
1.5
2.0
2.5
3.0
3.5
4.0
Cellular_organisms - ps1
Archaea1 - ps2
Archaea2 - ps3
Archaea3 - ps4
Asgard/Eukaryota - ps5
Eukaryota - ps6
Amorpheae - ps7
Obazoa - ps8
Opisthokonta - ps9
Holomycota - ps10
Fungi - ps11
Fungi_1 - ps12
Fungi_2 - ps13
Fungi_3 - ps14
Dikarya - ps15
Ascomycota - ps16
Saccharo/Pezizomycotina - ps17
Saccharomycotina - ps18
Saccharomycotina_1 - ps19
Saccharomycotina_2 - ps20
Saccharomycotina_3 - ps21
Phaff./Saccharomycetaceae - ps22
Saccharomyco./mycetaceae - ps23
Saccharomycetaceae - ps24
Saccharomycetaceae_2 - ps25
Saccharomycetaceae_3 - ps26
Kaz./Nau./Saccharomyces - ps27
Saccharomyces - ps28
Arboricola_clade - ps29
Cerevisiae_clade - ps30
Saccharomyces_cerevisiae - ps31
Phylostratum

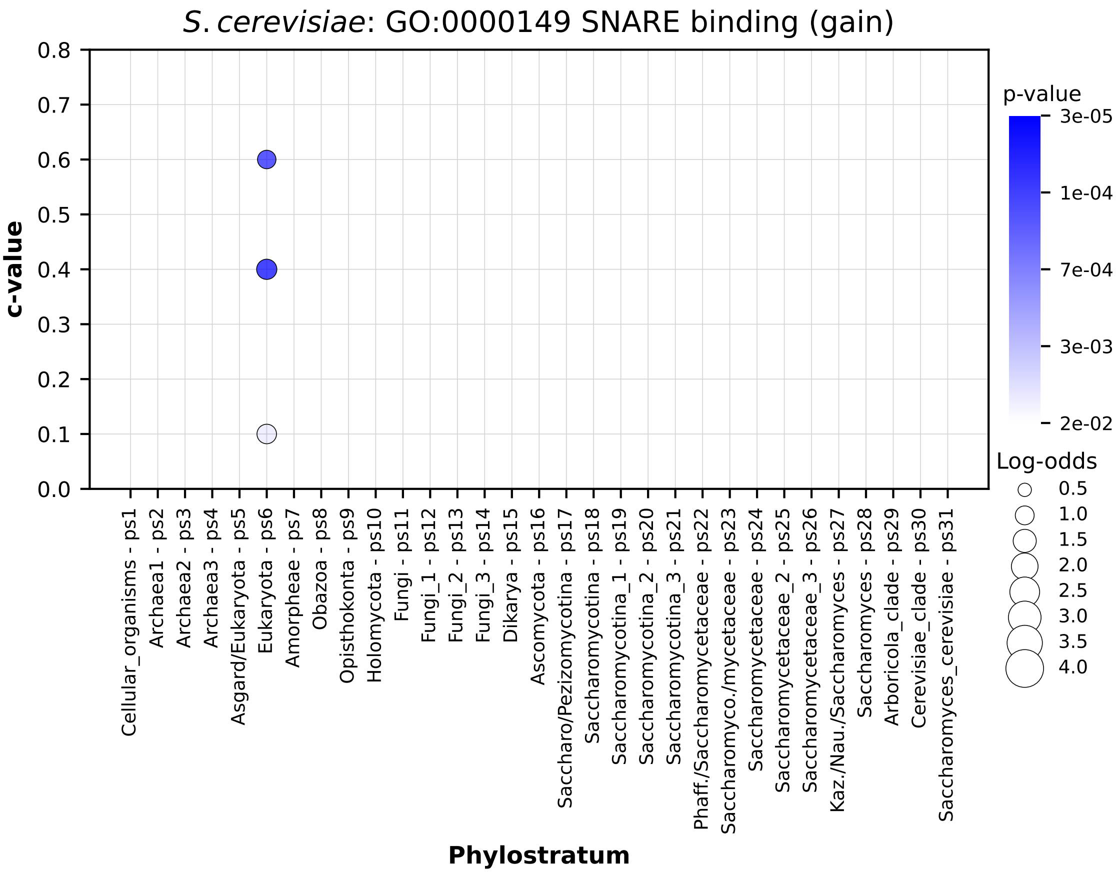

S. cerevisiae: GO:0000149 SNARE binding (gain)
c-value
0.0
0.1
0.2
0.3
0.4
0.5
0.6
0.7
0.8
Phylostratum
Cellular_organisms - ps1
Archaea1 - ps2
Archaea2 - ps3
Archaea3 - ps4
Asgard/Eukaryota - ps5
Eukaryota - ps6
Amorpheae - ps7
Obazoa - ps8
Opisthokonta - ps9
Holomycota - ps10
Fungi - ps11
Fungi_1 - ps12
Fungi_2 - ps13
Fungi_3 - ps14
Dikarya - ps15
Ascomycota - ps16
Saccharo/Pezizomycotina - ps17
Saccharomycotina - ps18
Saccharomycotina_1 - ps19
Saccharomycotina_2 - ps20
Saccharomycotina_3 - ps21
Phaff./Saccharomycetaceae - ps22
Saccharomyco./mycetaceae - ps23
Saccharomycetaceae - ps24
Saccharomycetaceae_2 - ps25
Saccharomycetaceae_3 - ps26
Kaz./Nau./Saccharomyces - ps27
Saccharomyces - ps28
Arboricola_clade - ps29
Cerevisiae_clade - ps30
Saccharomyces_cerevisiae - ps31
p-value
3e-05
1e-04
7e-04
3e-03
2e-02
Log-odds
0.5
1.0
1.5
2.0
2.5
3.0
3.5
4.0

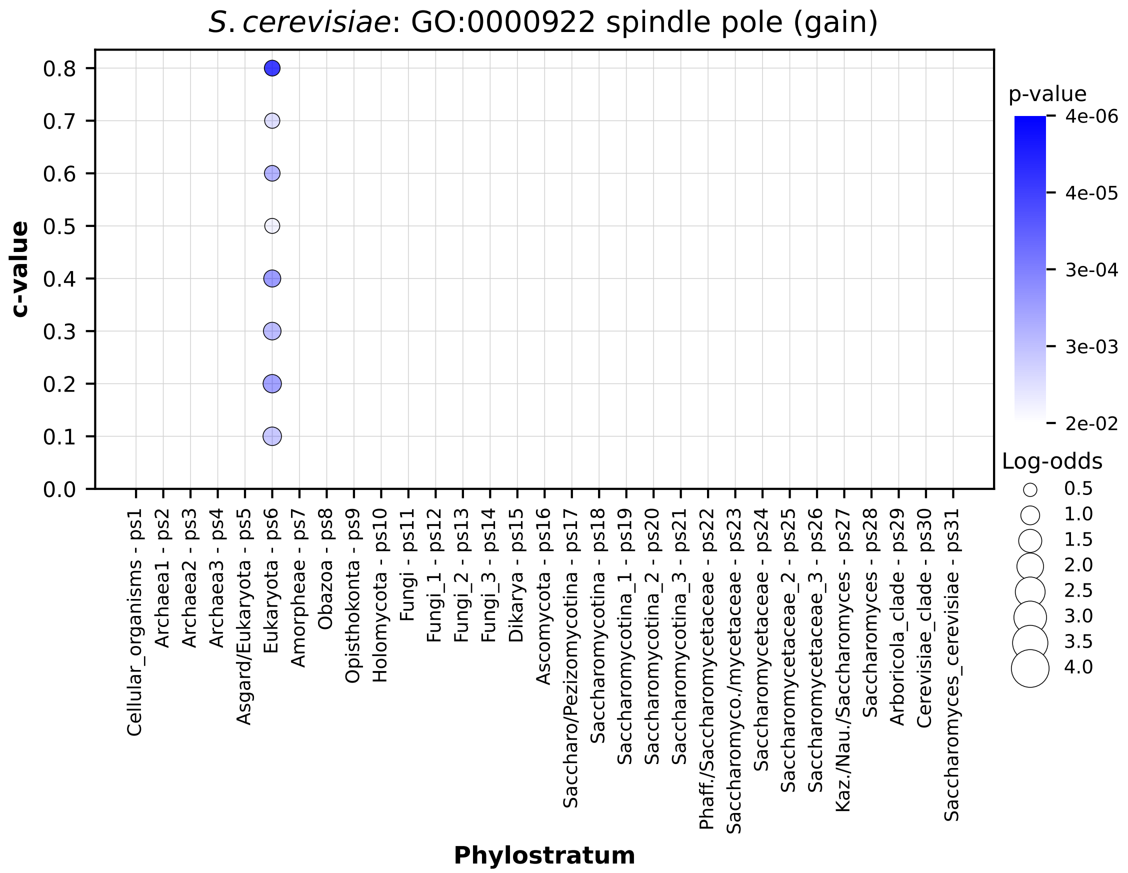

S. cerevisiae: GO:0000922 spindle pole (gain)
c-value
0.8
0.7
0.6
0.5
0.4
0.3
0.2
0.1
0.0
p-value
4e-06
4e-05
3e-04
3e-03
2e-02
Log-odds
0.5
1.0
1.5
2.0
2.5
3.0
3.5
4.0
Cellular_organisms - ps1
Archaea1 - ps2
Archaea2 - ps3
Archaea3 - ps4
Asgard/Eukaryota - ps5
Eukaryota - ps6
Amorpheae - ps7
Obazoa - ps8
Opisthokonta - ps9
Holomycota - ps10
Fungi - ps11
Fungi_1 - ps12
Fungi_2 - ps13
Fungi_3 - ps14
Dikarya - ps15
Ascomycota - ps16
Saccharo/Pezizomycotina - ps17
Saccharomycotina - ps18
Saccharomycotina_1 - ps19
Saccharomycotina_2 - ps20
Saccharomycotina_3 - ps21
Phaff./Saccharomycetaceae - ps22
Saccharomyco./mycetaceae - ps23
Saccharomycetaceae - ps24
Saccharomycetaceae_2 - ps25
Saccharomycetaceae_3 - ps26
Kaz./Nau./Saccharomyces - ps27
Saccharomyces - ps28
Arboricola_clade - ps29
Cerevisiae_clade - ps30
Saccharomyces_cerevisiae - ps31
Phylostratum

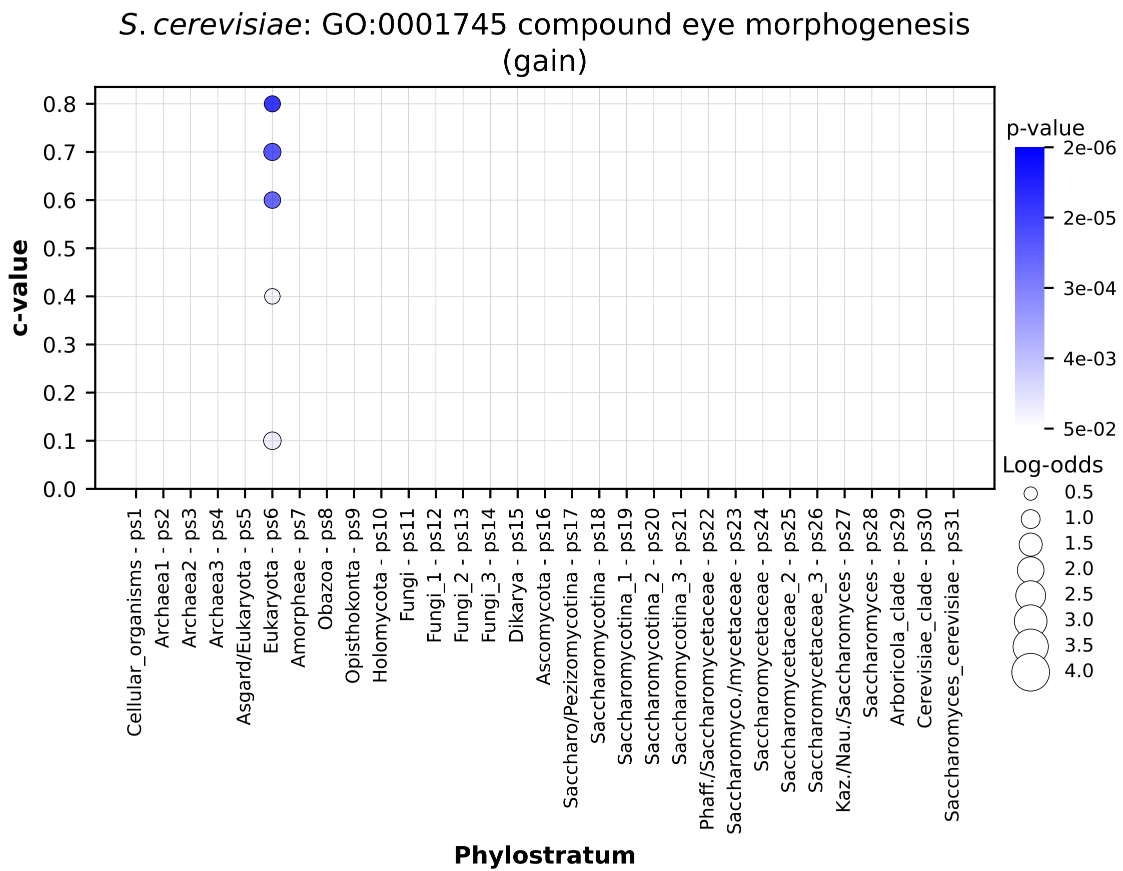

S. cerevisiae: GO:0001745 compound eye morphogenesis
(gain)
c-value
0.0
0.1
0.2
0.3
0.4
0.5
0.6
0.7
0.8
p-value
2e-06
2e-05
3e-04
4e-03
5e-02
Log-odds
0.5
1.0
1.5
2.0
2.5
3.0
3.5
4.0
Cellular_organisms - ps1
Archaea1 - ps2
Archaea2 - ps3
Archaea3 - ps4
Asgard/Eukaryota - ps5
Eukaryota - ps6
Amorpheae - ps7
Obazoa - ps8
Opisthokonta - ps9
Holomycota - ps10
Fungi - ps11
Fungi_1 - ps12
Fungi_2 - ps13
Fungi_3 - ps14
Dikarya - ps15
Ascomycota - ps16
Saccharo/Pezizomycotina - ps17
Saccharomycotina - ps18
Saccharomycotina_1 - ps19
Saccharomycotina_2 - ps20
Saccharomycotina_3 - ps21
Phaff./Saccharomycetaceae - ps22
Saccharomyco./mycetaceae - ps23
Saccharomycetaceae - ps24
Saccharomycetaceae_2 - ps25
Saccharomycetaceae_3 - ps26
Kaz./Nau./Saccharomyces - ps27
Saccharomyces - ps28
Arboricola_clade - ps29
Cerevisiae_clade - ps30
Saccharomyces_cerevisiae - ps31
Phylostratum

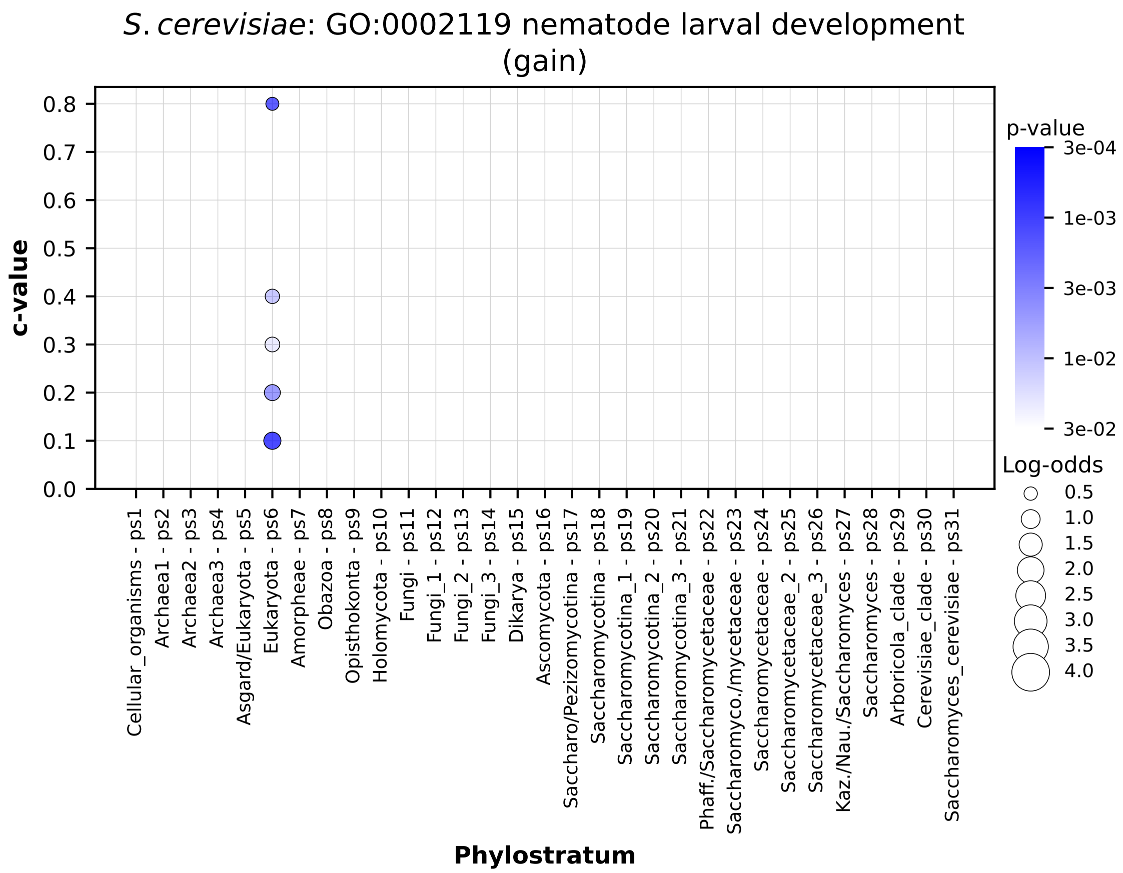

S. cerevisiae: GO:0002119 nematode larval development
(gain)
c-value
0.0
0.1
0.2
0.3
0.4
0.5
0.6
0.7
0.8
Phylostratum
Cellular_organisms - ps1
Archaea1 - ps2
Archaea2 - ps3
Archaea3 - ps4
Asgard/Eukaryota - ps5
Eukaryota - ps6
Amorpheae - ps7
Obazoa - ps8
Opisthokonta - ps9
Holomycota - ps10
Fungi - ps11
Fungi_1 - ps12
Fungi_2 - ps13
Fungi_3 - ps14
Dikarya - ps15
Ascomycota - ps16
Saccharo/Pezizomycotina - ps17
Saccharomycotina - ps18
Saccharomycotina_1 - ps19
Saccharomycotina_2 - ps20
Saccharomycotina_3 - ps21
Phaff./Saccharomycetaceae - ps22
Saccharomyco./mycetaceae - ps23
Saccharomycetaceae - ps24
Saccharomycetaceae_2 - ps25
Saccharomycetaceae_3 - ps26
Kaz./Nau./Saccharomyces - ps27
Saccharomyces - ps28
Arboricola_clade - ps29
Cerevisiae_clade - ps30
Saccharomyces_cerevisiae - ps31
p-value
3e-04
1e-03
3e-03
1e-02
3e-02
Log-odds
0.5
1.0
1.5
2.0
2.5
3.0
3.5
4.0

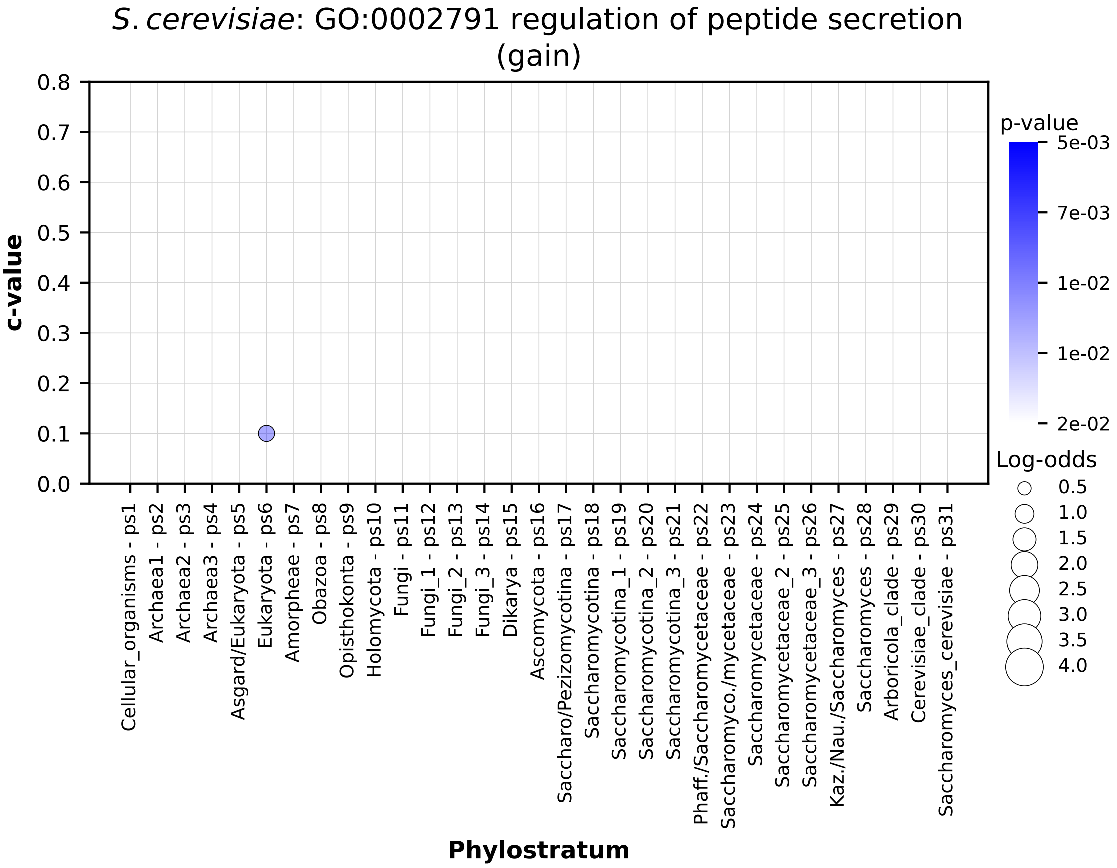

S. cerevisiae: GO:0002791 regulation of peptide secretion
(gain)
c-value
0.8
0.7
0.6
0.5
0.4
0.3
0.2
0.1
0.0
p-value
5e-03
7e-03
1e-02
1e-02
2e-02
Log-odds
0.5
1.0
1.5
2.0
2.5
3.0
3.5
4.0
Cellular_organisms - ps1
Archaea1 - ps2
Archaea2 - ps3
Archaea3 - ps4
Asgard/Eukaryota - ps5
Eukaryota - ps6
Amorpheae - ps7
Obazoa - ps8
Opisthokonta - ps9
Holomycota - ps10
Fungi - ps11
Fungi_1 - ps12
Fungi_2 - ps13
Fungi_3 - ps14
Dikarya - ps15
Ascomycota - ps16
Saccharo/Pezizomycotina - ps17
Saccharomycotina - ps18
Saccharomycotina_1 - ps19
Saccharomycotina_2 - ps20
Saccharomycotina_3 - ps21
Phaff./Saccharomycetaceae - ps22
Saccharomyco./mycetaceae - ps23
Saccharomycetaceae - ps24
Saccharomycetaceae_2 - ps25
Saccharomycetaceae_3 - ps26
Kaz./Nau./Saccharomyces - ps27
Saccharomyces - ps28
Arboricola_clade - ps29
Cerevisiae_clade - ps30
Saccharomyces_cerevisiae - ps31
Phylostratum

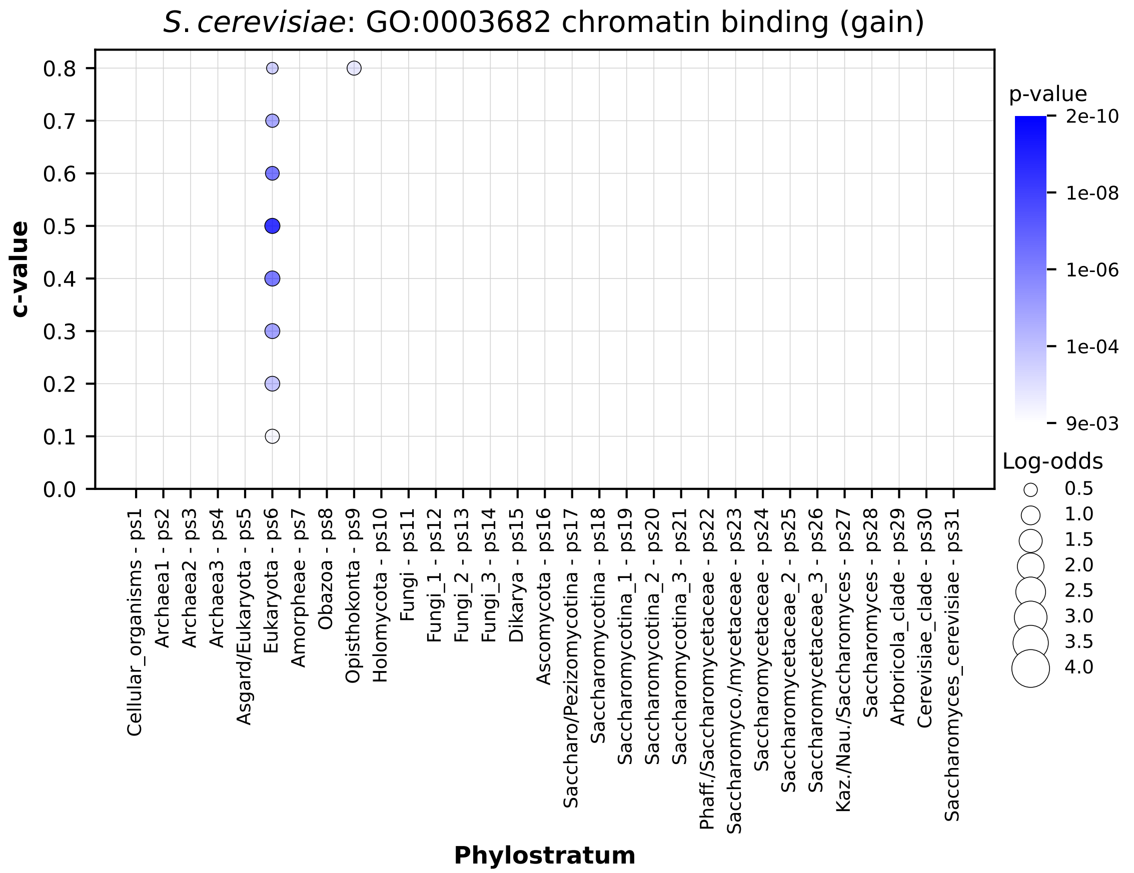

S. cerevisiae: GO:0003682 chromatin binding (gain)
c-value
0.0
0.1
0.2
0.3
0.4
0.5
0.6
0.7
0.8
Phylostratum
Cellular_organisms - ps1
Archaea1 - ps2
Archaea2 - ps3
Archaea3 - ps4
Asgard/Eukaryota - ps5
Eukaryota - ps6
Amorpheae - ps7
Obazoa - ps8
Opisthokonta - ps9
Holomycota - ps10
Fungi - ps11
Fungi_1 - ps12
Fungi_2 - ps13
Fungi_3 - ps14
Dikarya - ps15
Ascomycota - ps16
Saccharo/Pezizomycotina - ps17
Saccharomycotina - ps18
Saccharomycotina_1 - ps19
Saccharomycotina_2 - ps20
Saccharomycotina_3 - ps21
Phaff./Saccharomycetaceae - ps22
Saccharomyco./mycetaceae - ps23
Saccharomycetaceae - ps24
Saccharomycetaceae_2 - ps25
Saccharomycetaceae_3 - ps26
Kaz./Nau./Saccharomyces - ps27
Saccharomyces - ps28
Arboricola_clade - ps29
Cerevisiae_clade - ps30
Saccharomyces_cerevisiae - ps31
p-value
2e-10
1e-08
1e-06
1e-04
9e-03
Log-odds
0.5
1.0
1.5
2.0
2.5
3.0
3.5
4.0

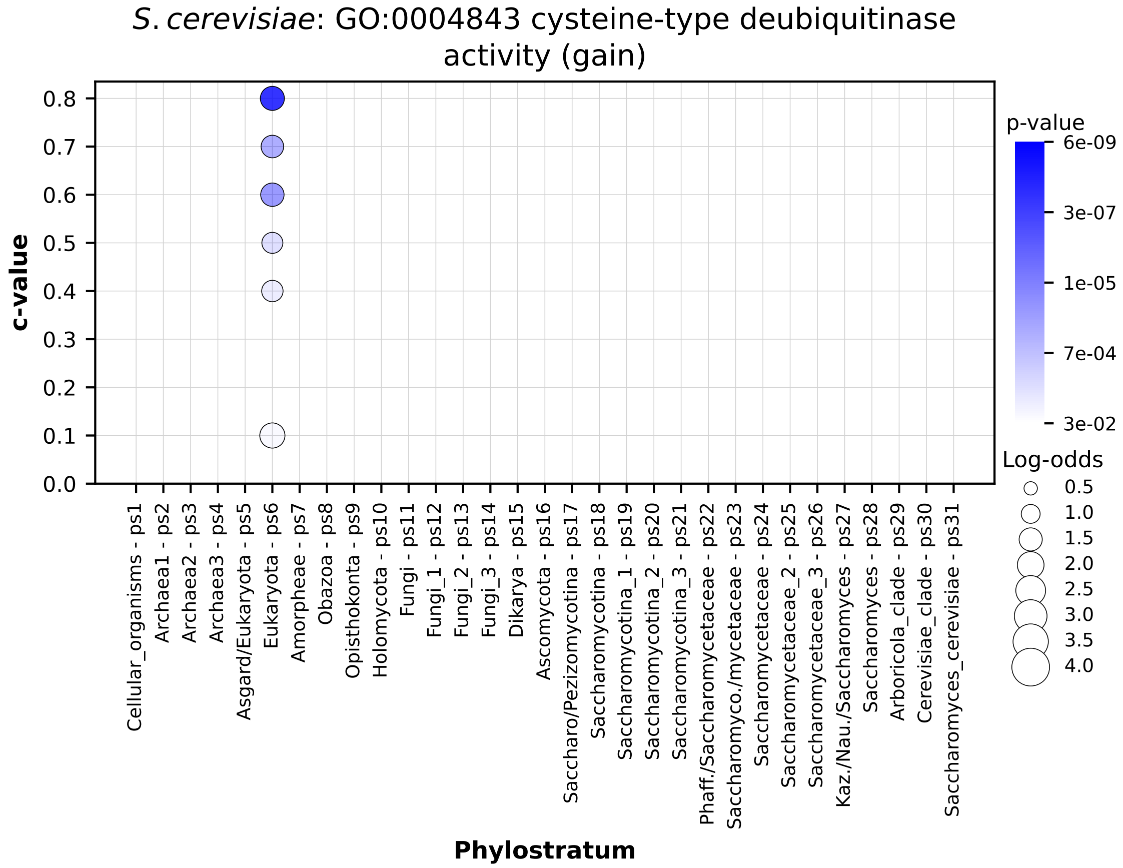

S. cerevisiae: GO:0004843 cysteine-type deubiquitinase activity (gain)
c-value
0.0
0.1
0.2
0.3
0.4
0.5
0.6
0.7
0.8
Phylostratum
Cellular_organisms - ps1
Archaea1 - ps2
Archaea2 - ps3
Archaea3 - ps4
Asgard/Eukaryota - ps5
Eukaryota - ps6
Amorpheae - ps7
Obazoa - ps8
Opisthokonta - ps9
Holomycota - ps10
Fungi - ps11
Fungi_1 - ps12
Fungi_2 - ps13
Fungi_3 - ps14
Dikarya - ps15
Ascomycota - ps16
Saccharo/Pezizomycotina - ps17
Saccharomycotina - ps18
Saccharomycotina_1 - ps19
Saccharomycotina_2 - ps20
Saccharomycotina_3 - ps21
Phaff./Saccharomycetaceae - ps22
Saccharomyco./mycetaceae - ps23
Saccharomycetaceae - ps24
Saccharomycetaceae_2 - ps25
Saccharomycetaceae_3 - ps26
Kaz./Nau./Saccharomyces - ps27
Saccharomyces - ps28
Arboricola_clade - ps29
Cerevisiae_clade - ps30
Saccharomyces_cerevisiae - ps31
p-value
6e-09
3e-07
1e-05
7e-04
3e-02
Log-odds
0.5
1.0
1.5
2.0
2.5
3.0
3.5
4.0

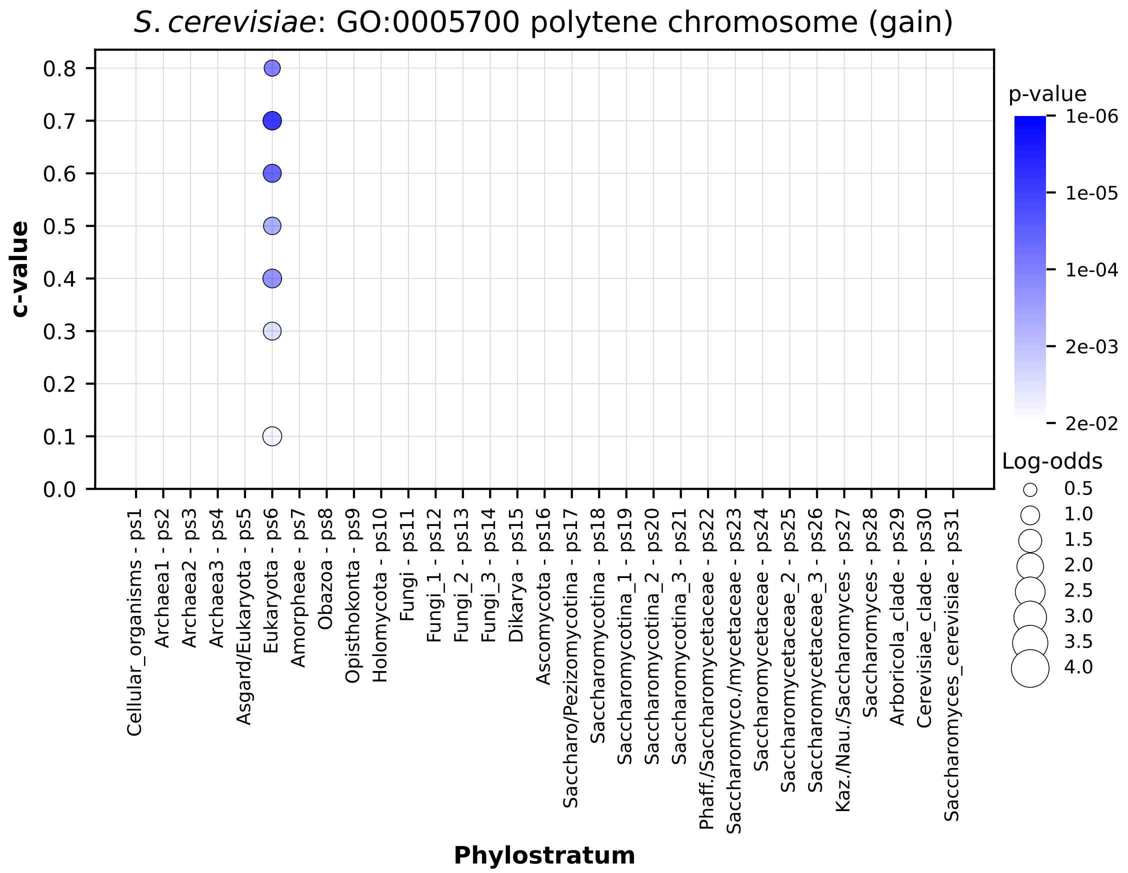

S. cerevisiae: GO:0005700 polytene chromosome (gain)
c-value
0.0
0.1
0.2
0.3
0.4
0.5
0.6
0.7
0.8
Phylostratum
Cellular_organisms - ps1
Archaea1 - ps2
Archaea2 - ps3
Archaea3 - ps4
Asgard/Eukaryota - ps5
Eukaryota - ps6
Amorpheae - ps7
Obazoa - ps8
Opisthokonta - ps9
Holomycota - ps10
Fungi - ps11
Fungi_1 - ps12
Fungi_2 - ps13
Fungi_3 - ps14
Dikarya - ps15
Ascomycota - ps16
Saccharo/Pezizomycotina - ps17
Saccharomycotina - ps18
Saccharomycotina_1 - ps19
Saccharomycotina_2 - ps20
Saccharomycotina_3 - ps21
Phaff./Saccharomycetaceae - ps22
Saccharomyco./mycetaceae - ps23
Saccharomycetaceae - ps24
Saccharomycetaceae_2 - ps25
Saccharomycetaceae_3 - ps26
Kaz./Nau./Saccharomyces - ps27
Saccharomyces - ps28
Arboricola_clade - ps29
Cerevisiae_clade - ps30
Saccharomyces_cerevisiae - ps31
p-value
1e-06
1e-05
1e-04
2e-03
2e-02
Log-odds
0.5
1.0
1.5
2.0
2.5
3.0
3.5
4.0

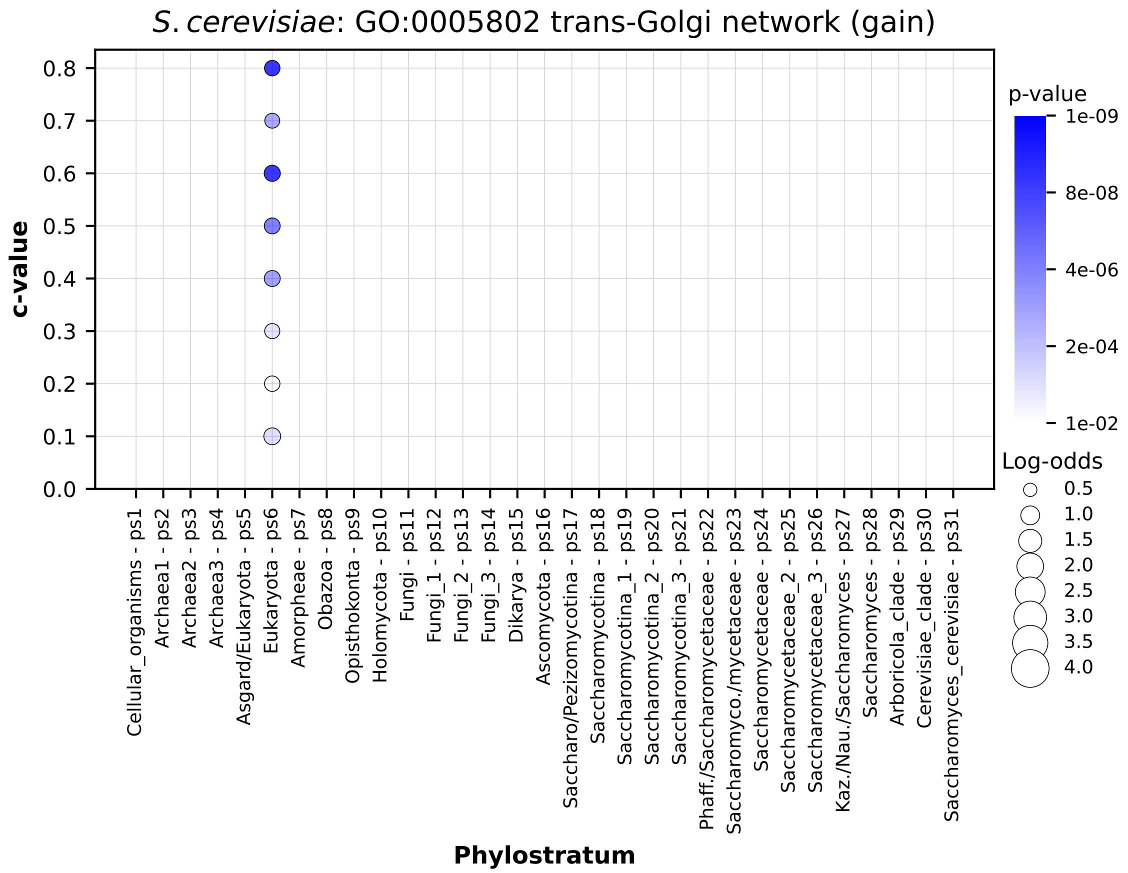

S. cerevisiae: GO:0005802 trans-Golgi network (gain)
c-value
0.0
0.1
0.2
0.3
0.4
0.5
0.6
0.7
0.8
Phylostratum
Cellular_organisms - ps1
Archaea1 - ps2
Archaea2 - ps3
Archaea3 - ps4
Asgard/Eukaryota - ps5
Eukaryota - ps6
Amorpheae - ps7
Obazoa - ps8
Opisthokonta - ps9
Holomycota - ps10
Fungi - ps11
Fungi_1 - ps12
Fungi_2 - ps13
Fungi_3 - ps14
Dikarya - ps15
Ascomycota - ps16
Saccharo/Pezizomycotina - ps17
Saccharomycotina - ps18
Saccharomycotina_1 - ps19
Saccharomycotina_2 - ps20
Saccharomycotina_3 - ps21
Phaff./Saccharomycetaceae - ps22
Saccharomyco./mycetaceae - ps23
Saccharomycetaceae - ps24
Saccharomycetaceae_2 - ps25
Saccharomycetaceae_3 - ps26
Kaz./Nau./Saccharomyces - ps27
Saccharomyces - ps28
Arboricola_clade - ps29
Cerevisiae_clade - ps30
Saccharomyces_cerevisiae - ps31
p-value
1e-09
8e-08
4e-06
2e-04
1e-02
Log-odds
0.5
1.0
1.5
2.0
2.5
3.0
3.5
4.0

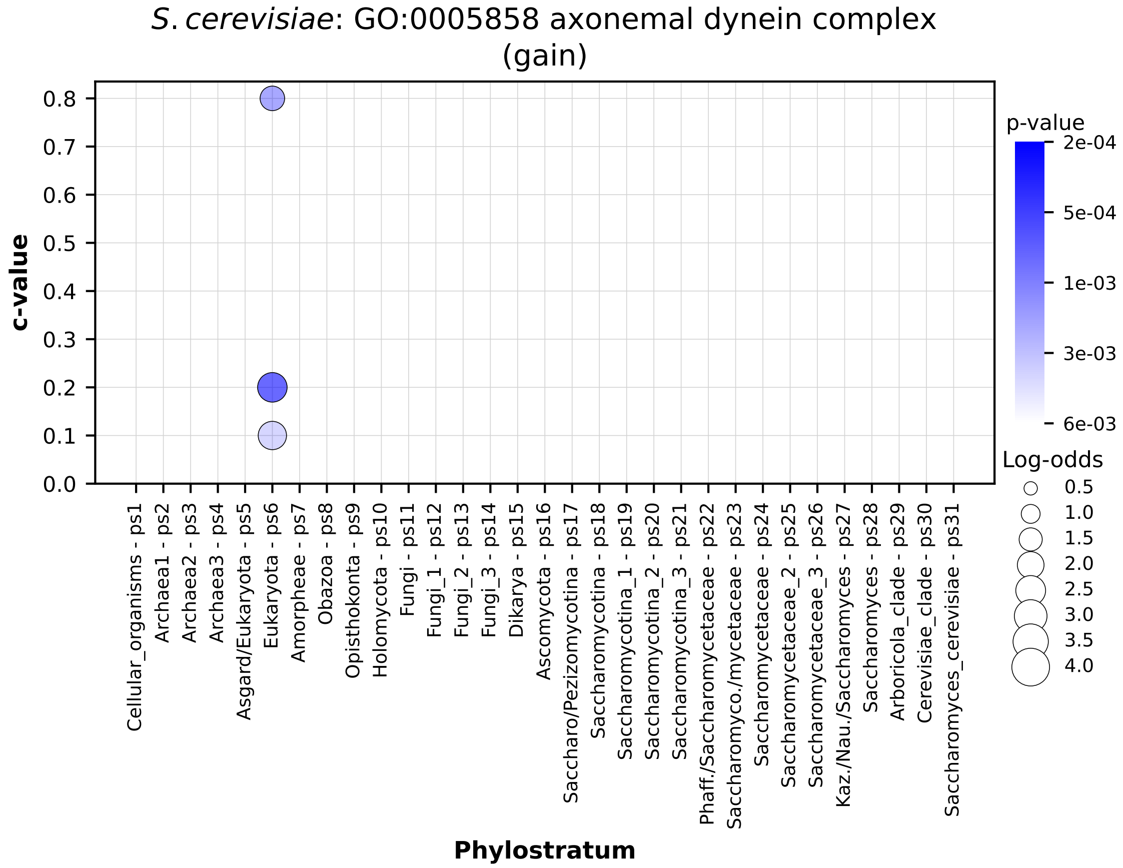
S. cerevisiae: GO:0005858 axonemal dynein complex
(gain)
c-value
0.0
0.1
0.2
0.3
0.4
0.5
0.6
0.7
0.8
p-value
2e-04
5e-04
1e-03
3e-03
6e-03
Log-odds
0.5
1.0
1.5
2.0
2.5
3.0
3.5
4.0
Cellular_organisms - ps1
Archaea1 - ps2
Archaea2 - ps3
Archaea3 - ps4
Asgard/Eukaryota - ps5
Eukaryota - ps6
Amorpheae - ps7
Obazoa - ps8
Opisthokonta - ps9
Holomycota - ps10
Fungi - ps11
Fungi_1 - ps12
Fungi_2 - ps13
Fungi_3 - ps14
Dikarya - ps15
Ascomycota - ps16
Saccharo/Pezizomycotina - ps17
Saccharomycotina - ps18
Saccharomycotina_1 - ps19
Saccharomycotina_2 - ps20
Saccharomycotina_3 - ps21
Phaff./Saccharomycetaceae - ps22
Saccharomyco./mycetaceae - ps23
Saccharomycetaceae - ps24
Saccharomycetaceae_2 - ps25
Saccharomycetaceae_3 - ps26
Kaz./Nau./Saccharomyces - ps27
Saccharomyces - ps28
Arboricola_clade - ps29
Cerevisiae_clade - ps30
Saccharomyces_cerevisiae - ps31
Phylostratum

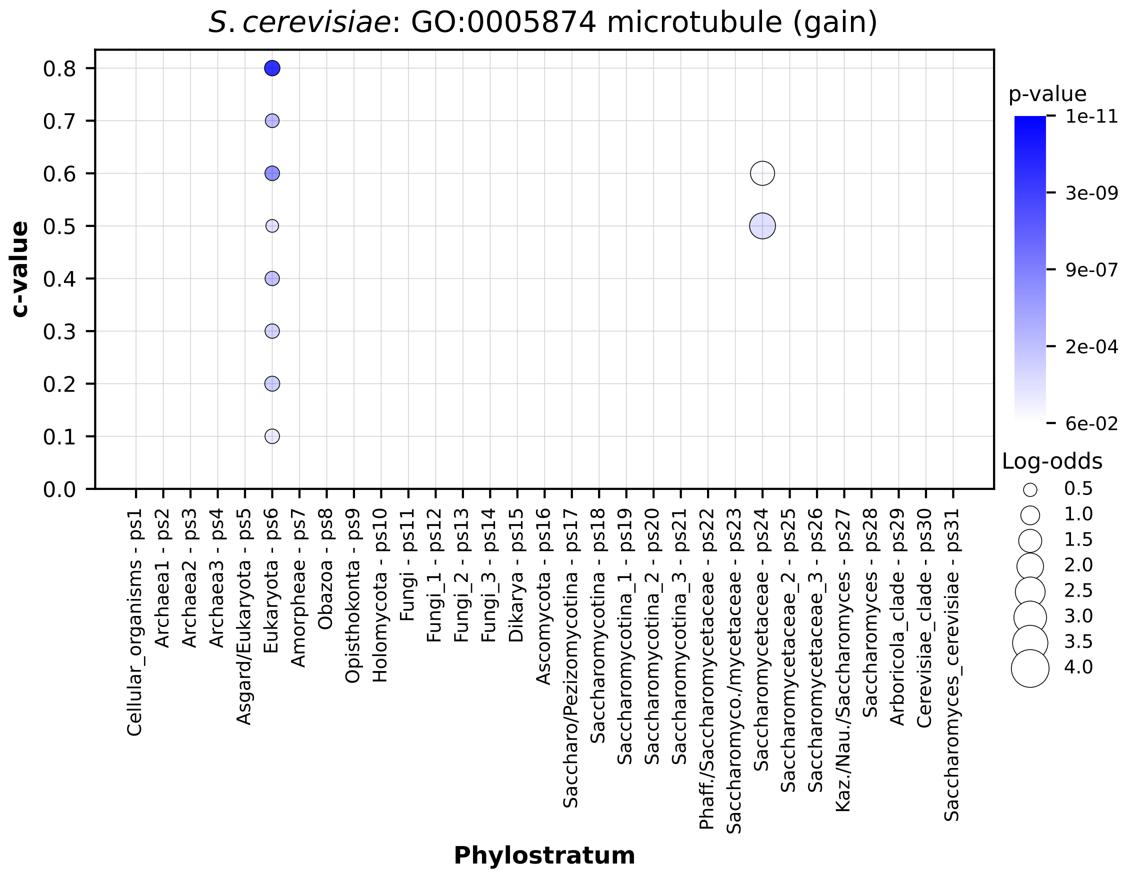

S. cerevisiae: GO:0005874 microtubule (gain)
c-value
0.0
0.1
0.2
0.3
0.4
0.5
0.6
0.7
0.8
p-value
1e-11
3e-09
9e-07
2e-04
6e-02
Log-odds
0.5
1.0
1.5
2.0
2.5
3.0
3.5
4.0
Cellular_organisms - ps1
Archaea1 - ps2
Archaea2 - ps3
Archaea3 - ps4
Asgard/Eukaryota - ps5
Eukaryota - ps6
Amorpheae - ps7
Obazoa - ps8
Opisthokonta - ps9
Holomycota - ps10
Fungi - ps11
Fungi_1 - ps12
Fungi_2 - ps13
Fungi_3 - ps14
Dikarya - ps15
Ascomycota - ps16
Saccharo/Pezizomycotina - ps17
Saccharomycotina - ps18
Saccharomycotina_1 - ps19
Saccharomycotina_2 - ps20
Saccharomycotina_3 - ps21
Phaff./Saccharomycetaceae - ps22
Saccharomyco./mycetaceae - ps23
Saccharomycetaceae - ps24
Saccharomycetaceae_2 - ps25
Saccharomycetaceae_3 - ps26
Kaz./Nau./Saccharomyces - ps27
Saccharomyces - ps28
Arboricola_clade - ps29
Cerevisiae_clade - ps30
Saccharomyces_cerevisiae - ps31
Phylostratum

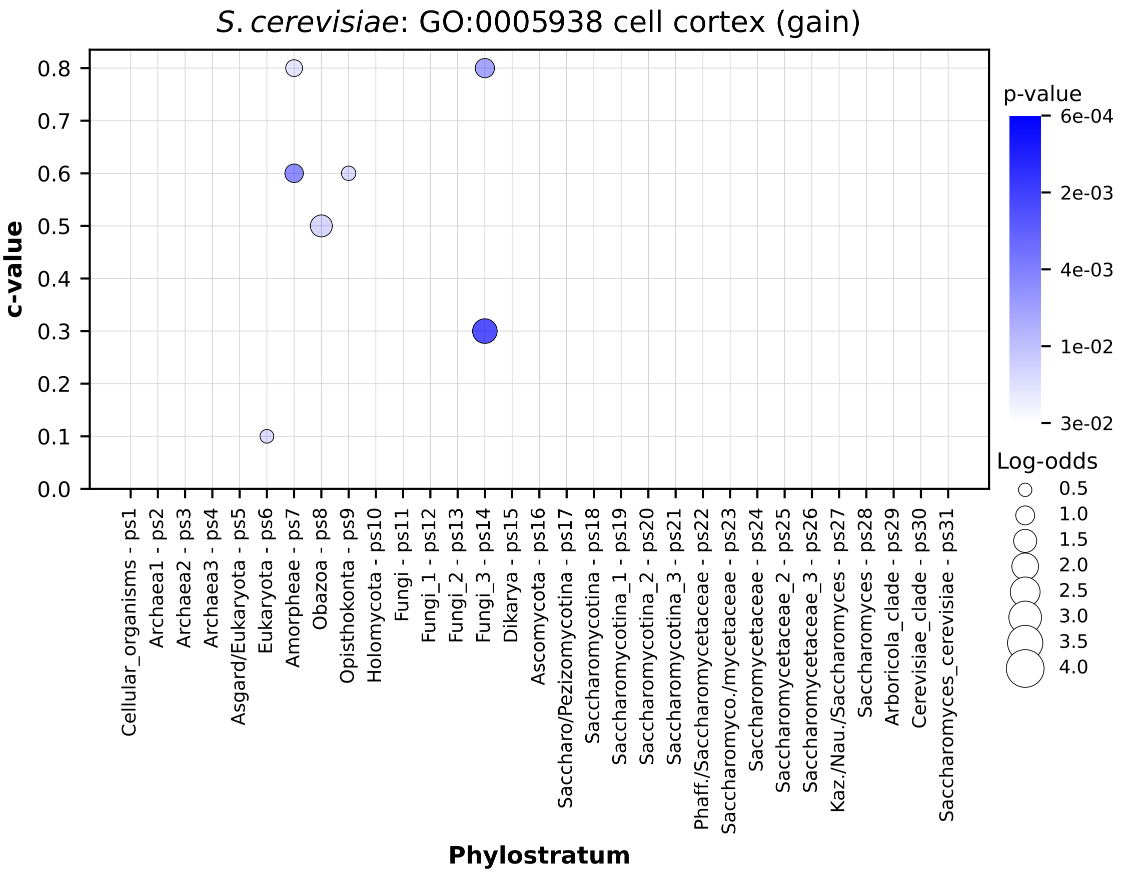

S. cerevisiae: GO:0005938 cell cortex (gain)
c-value
0.0
0.1
0.2
0.3
0.4
0.5
0.6
0.7
0.8
p-value
6e-04
2e-03
4e-03
1e-02
3e-02
Log-odds
0.5
1.0
1.5
2.0
2.5
3.0
3.5
4.0
Cellular_organisms - ps1
Archaea1 - ps2
Archaea2 - ps3
Archaea3 - ps4
Asgard/Eukaryota - ps5
Eukaryota - ps6
Amorpheae - ps7
Obazoa - ps8
Opisthokonta - ps9
Holomycota - ps10
Fungi - ps11
Fungi_1 - ps12
Fungi_2 - ps13
Fungi_3 - ps14
Dikarya - ps15
Ascomycota - ps16
Saccharo/Pezizomycotina - ps17
Saccharomycotina - ps18
Saccharomycotina_1 - ps19
Saccharomycotina_2 - ps20
Saccharomycotina_3 - ps21
Phaff./Saccharomycetaceae - ps22
Saccharomyco./mycetaceae - ps23
Saccharomycetaceae - ps24
Saccharomycetaceae_2 - ps25
Saccharomycetaceae_3 - ps26
Kaz./Nau./Saccharomyces - ps27
Saccharomyces - ps28
Arboricola_clade - ps29
Cerevisiae_clade - ps30
Saccharomyces_cerevisiae - ps31
Phylostratum

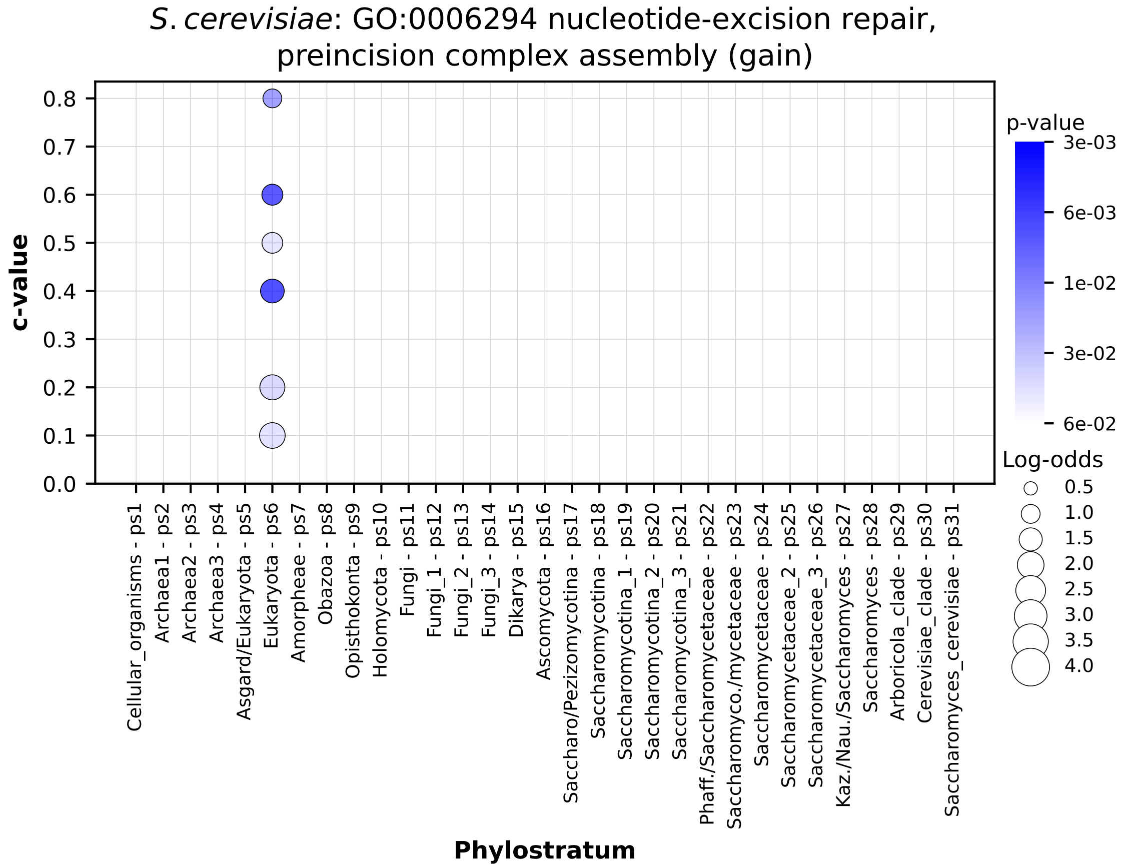

S. cerevisiae: GO:0006294 nucleotide-excision repair, preincision complex assembly (gain)
c-value
0.0
0.1
0.2
0.3
0.4
0.5
0.6
0.7
0.8
Phylostratum
Cellular_organisms - ps1
Archaea1 - ps2
Archaea2 - ps3
Archaea3 - ps4
Asgard/Eukaryota - ps5
Eukaryota - ps6
Amorpheae - ps7
Obazoa - ps8
Opisthokonta - ps9
Holomycota - ps10
Fungi - ps11
Fungi_1 - ps12
Fungi_2 - ps13
Fungi_3 - ps14
Dikarya - ps15
Ascomycota - ps16
Saccharo/Pezizomycotina - ps17
Saccharomycotina - ps18
Saccharomycotina_1 - ps19
Saccharomycotina_2 - ps20
Saccharomycotina_3 - ps21
Phaff./Saccharomycetaceae - ps22
Saccharomyco./mycetaceae - ps23
Saccharomycetaceae - ps24
Saccharomycetaceae_2 - ps25
Saccharomycetaceae_3 - ps26
Kaz./Nau./Saccharomyces - ps27
Saccharomyces - ps28
Arboricola_clade - ps29
Cerevisiae_clade - ps30
Saccharomyces_cerevisiae - ps31
p-value
3e-03
6e-03
1e-02
3e-02
6e-02
Log-odds
0.5
1.0
1.5
2.0
2.5
3.0
3.5
4.0

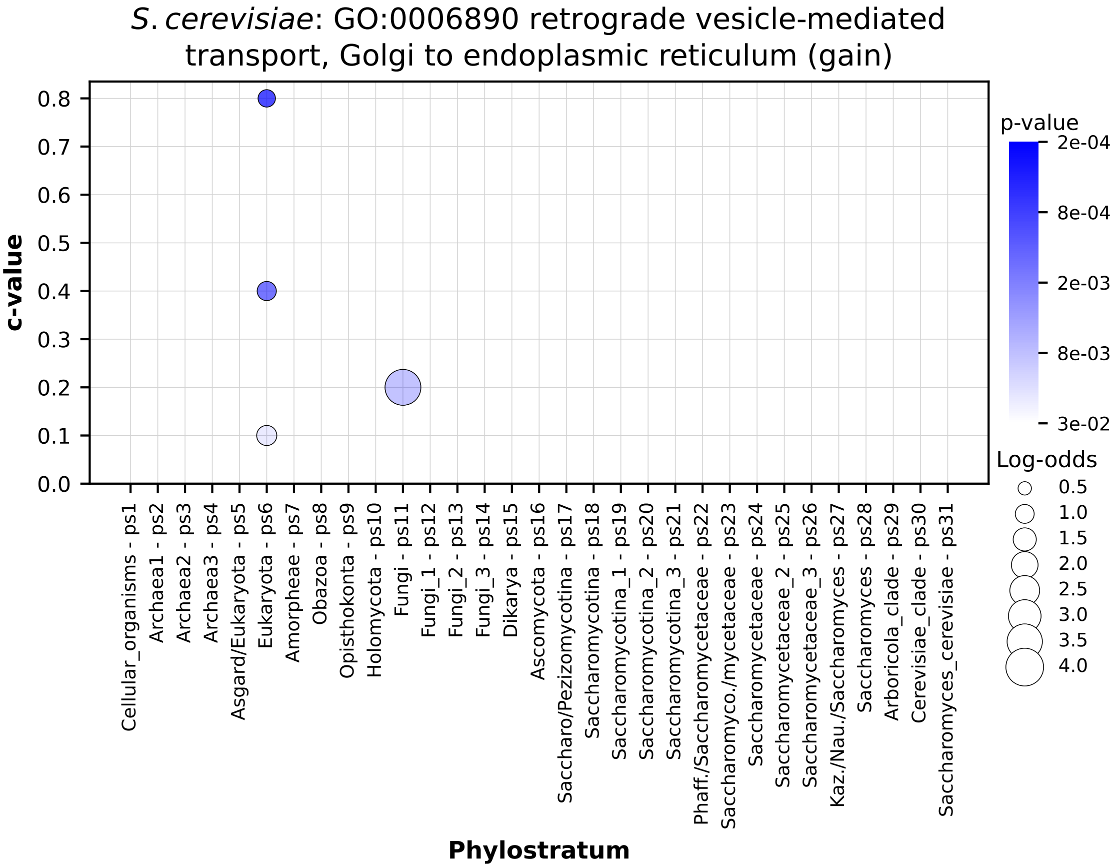

S. cerevisiae: GO:0006890 retrograde vesicle-mediated transport, Golgi to endoplasmic reticulum (gain)
c-value
0.0
0.1
0.2
0.3
0.4
0.5
0.6
0.7
0.8
Phylostratum
Cellular_organisms - ps1
Archaea1 - ps2
Archaea2 - ps3
Archaea3 - ps4
Asgard/Eukaryota - ps5
Eukaryota - ps6
Amorpheae - ps7
Obazoa - ps8
Opisthokonta - ps9
Holomycota - ps10
Fungi - ps11
Fungi_1 - ps12
Fungi_2 - ps13
Fungi_3 - ps14
Dikarya - ps15
Ascomycota - ps16
Saccharo/Pezizomycotina - ps17
Saccharomycotina - ps18
Saccharomycotina_1 - ps19
Saccharomycotina_2 - ps20
Saccharomycotina_3 - ps21
Phaff./Saccharomycetaceae - ps22
Saccharomyco./mycetaceae - ps23
Saccharomycetaceae - ps24
Saccharomycetaceae_2 - ps25
Saccharomycetaceae_3 - ps26
Kaz./Nau./Saccharomyces - ps27
Saccharomyces - ps28
Arboricola_clade - ps29
Cerevisiae_clade - ps30
Saccharomyces_cerevisiae - ps31
p-value
2e-04
8e-04
2e-03
8e-03
3e-02
Log-odds
0.5
1.0
1.5
2.0
2.5
3.0
3.5
4.0

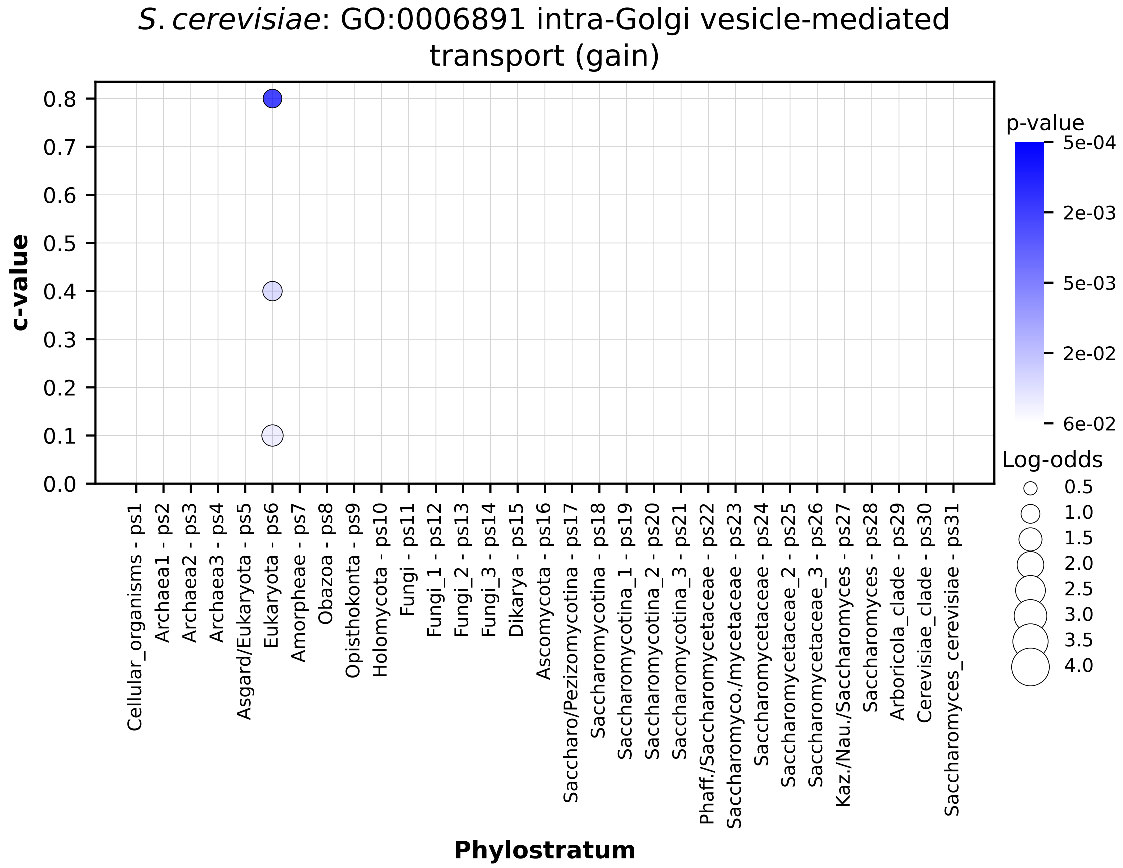

S. cerevisiae: GO:0006891 intra-Golgi vesicle-mediated transport (gain)
c-value
0.0
0.1
0.2
0.3
0.4
0.5
0.6
0.7
0.8
Phylostratum
Cellular_organisms - ps1
Archaea1 - ps2
Archaea2 - ps3
Archaea3 - ps4
Asgard/Eukaryota - ps5
Eukaryota - ps6
Amorpheae - ps7
Obazoa - ps8
Opisthokonta - ps9
Holomycota - ps10
Fungi - ps11
Fungi_1 - ps12
Fungi_2 - ps13
Fungi_3 - ps14
Dikarya - ps15
Ascomycota - ps16
Saccharo/Pezizomycotina - ps17
Saccharomycotina - ps18
Saccharomycotina_1 - ps19
Saccharomycotina_2 - ps20
Saccharomycotina_3 - ps21
Phaff./Saccharomycetaceae - ps22
Saccharomyco./mycetaceae - ps23
Saccharomycetaceae - ps24
Saccharomycetaceae_2 - ps25
Saccharomycetaceae_3 - ps26
Kaz./Nau./Saccharomyces - ps27
Saccharomyces - ps28
Arboricola_clade - ps29
Cerevisiae_clade - ps30
Saccharomyces_cerevisiae - ps31
p-value
5e-04
2e-03
5e-03
2e-02
6e-02
Log-odds
0.5
1.0
1.5
2.0
2.5
3.0
3.5
4.0

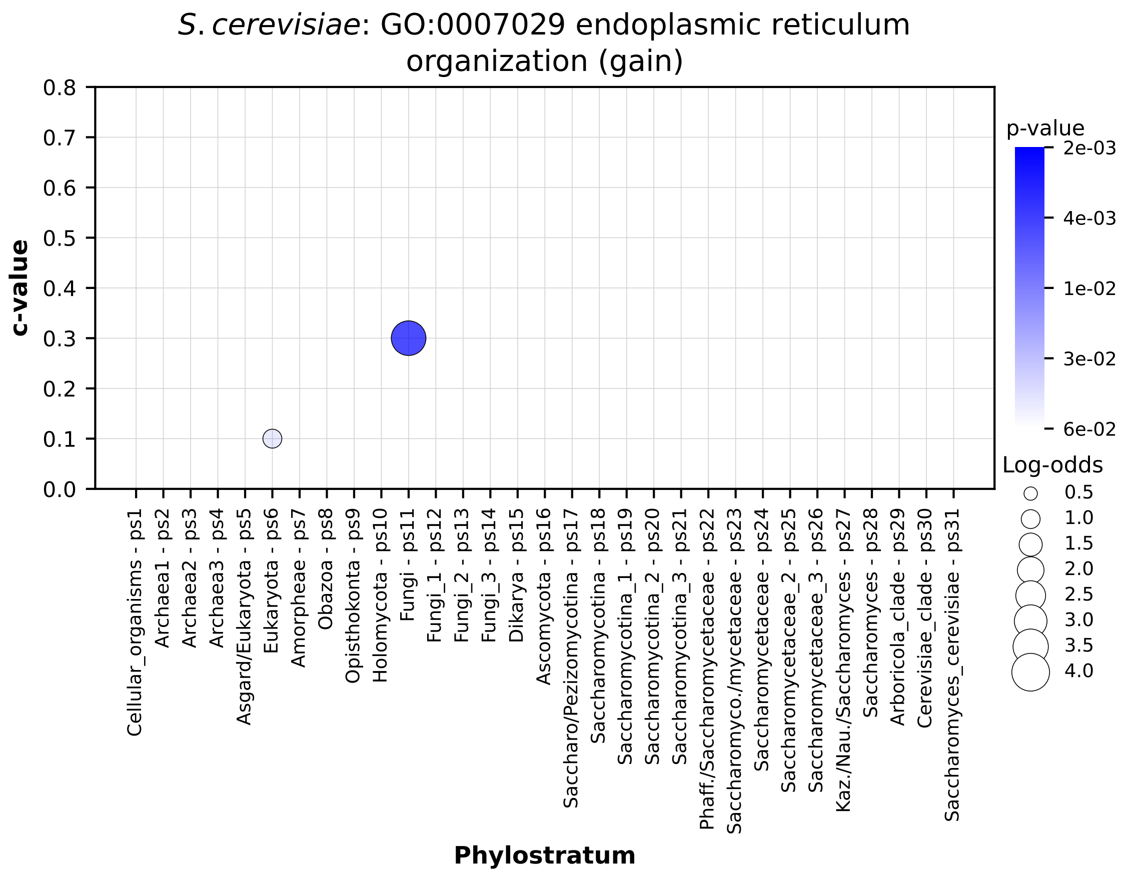

S. cerevisiae: GO:0007029 endoplasmic reticulum organization (gain)
c-value
0.8
0.7
0.6
0.5
0.4
0.3
0.2
0.1
0.0
Phylostratum
Cellular_organisms - ps1
Archaea1 - ps2
Archaea2 - ps3
Archaea3 - ps4
Asgard/Eukaryota - ps5
Eukaryota - ps6
Amorpheae - ps7
Obazoa - ps8
Opisthokonta - ps9
Holomycota - ps10
Fungi - ps11
Fungi_1 - ps12
Fungi_2 - ps13
Fungi_3 - ps14
Dikarya - ps15
Ascomycota - ps16
Saccharo/Pezizomycotina - ps17
Saccharomycotina - ps18
Saccharomycotina_1 - ps19
Saccharomycotina_2 - ps20
Saccharomycotina_3 - ps21
Phaff./Saccharomycetaceae - ps22
Saccharomyco./mycetaceae - ps23
Saccharomycetaceae - ps24
Saccharomycetaceae_2 - ps25
Saccharomycetaceae_3 - ps26
Kaz./Nau./Saccharomyces - ps27
Saccharomyces - ps28
Arboricola_clade - ps29
Cerevisiae_clade - ps30
Saccharomyces_cerevisiae - ps31
p-value
2e-03
4e-03
1e-02
3e-02
6e-02
Log-odds
0.5
1.0
1.5
2.0
2.5
3.0
3.5
4.0

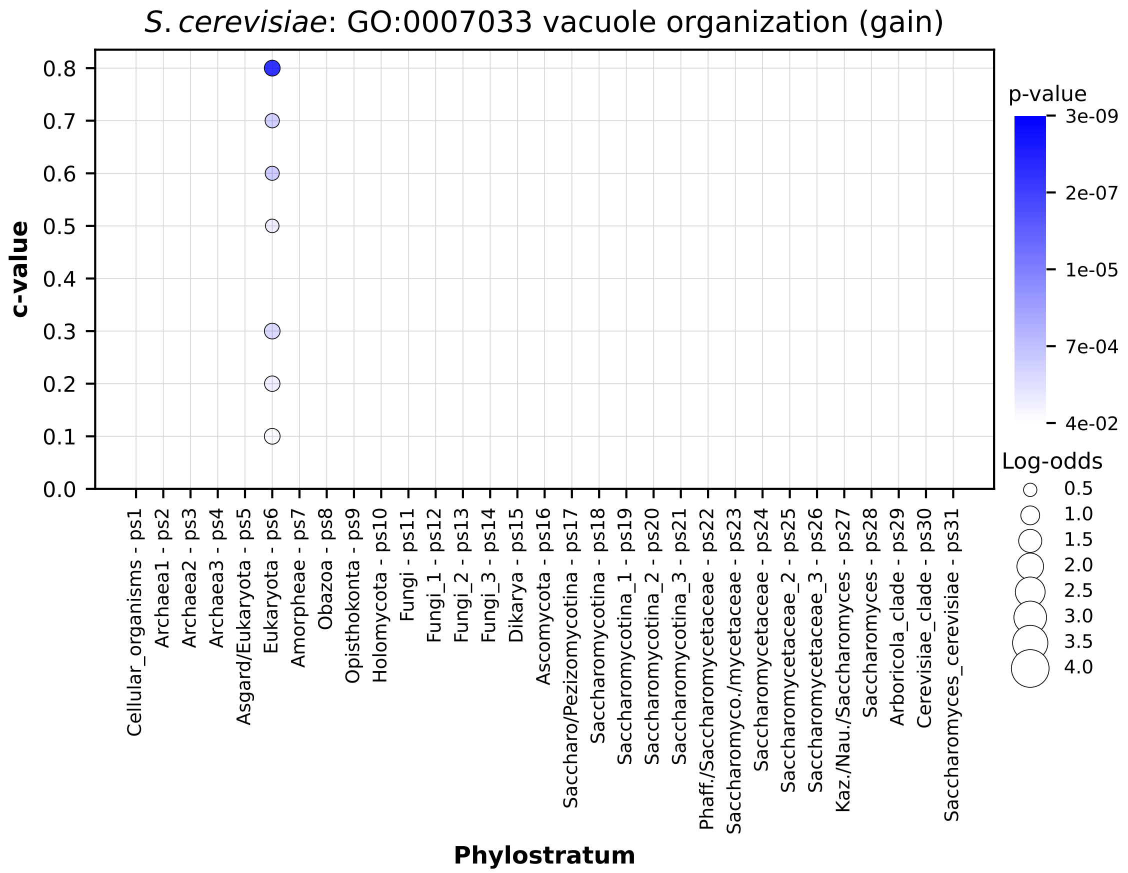

S. cerevisiae: GO:0007033 vacuole organization (gain)
c-value
0.0
0.1
0.2
0.3
0.4
0.5
0.6
0.7
0.8
p-value
3e-09
2e-07
1e-05
7e-04
4e-02
Log-odds
0.5
1.0
1.5
2.0
2.5
3.0
3.5
4.0
Cellular_organisms - ps1
Archaea1 - ps2
Archaea2 - ps3
Archaea3 - ps4
Asgard/Eukaryota - ps5
Eukaryota - ps6
Amorpheae - ps7
Obazoa - ps8
Opisthokonta - ps9
Holomycota - ps10
Fungi - ps11
Fungi_1 - ps12
Fungi_2 - ps13
Fungi_3 - ps14
Dikarya - ps15
Ascomycota - ps16
Saccharo/Pezizomycotina - ps17
Saccharomycotina - ps18
Saccharomycotina_1 - ps19
Saccharomycotina_2 - ps20
Saccharomycotina_3 - ps21
Phaff./Saccharomycetaceae - ps22
Saccharomyco./mycetaceae - ps23
Saccharomycetaceae - ps24
Saccharomycetaceae_2 - ps25
Saccharomycetaceae_3 - ps26
Kaz./Nau./Saccharomyces - ps27
Saccharomyces - ps28
Arboricola_clade - ps29
Cerevisiae_clade - ps30
Saccharomyces_cerevisiae - ps31
Phylostratum

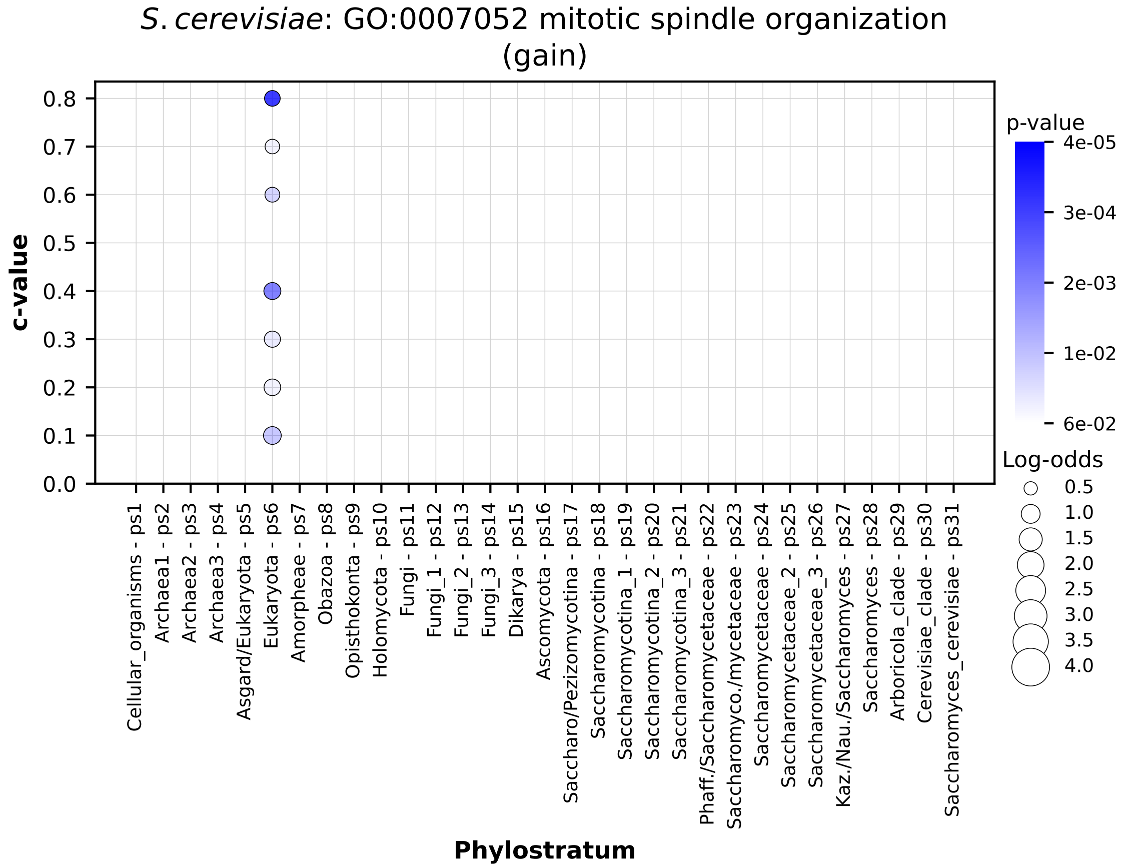

S. cerevisiae: GO:0007052 mitotic spindle organization
(gain)
c-value
0.0
0.1
0.2
0.3
0.4
0.5
0.6
0.7
0.8
p-value
4e-05
3e-04
2e-03
1e-02
6e-02
Log-odds
0.5
1.0
1.5
2.0
2.5
3.0
3.5
4.0
Cellular_organisms - ps1
Archaea1 - ps2
Archaea2 - ps3
Archaea3 - ps4
Asgard/Eukaryota - ps5
Eukaryota - ps6
Amorpheae - ps7
Obazoa - ps8
Opisthokonta - ps9
Holomycota - ps10
Fungi - ps11
Fungi_1 - ps12
Fungi_2 - ps13
Fungi_3 - ps14
Dikarya - ps15
Ascomycota - ps16
Saccharo/Pezizomycotina - ps17
Saccharomycotina - ps18
Saccharomycotina_1 - ps19
Saccharomycotina_2 - ps20
Saccharomycotina_3 - ps21
Phaff./Saccharomycetaceae - ps22
Saccharomyco./mycetaceae - ps23
Saccharomycetaceae - ps24
Saccharomycetaceae_2 - ps25
Saccharomycetaceae_3 - ps26
Kaz./Nau./Saccharomyces - ps27
Saccharomyces - ps28
Arboricola_clade - ps29
Cerevisiae_clade - ps30
Saccharomyces_cerevisiae - ps31
Phylostratum

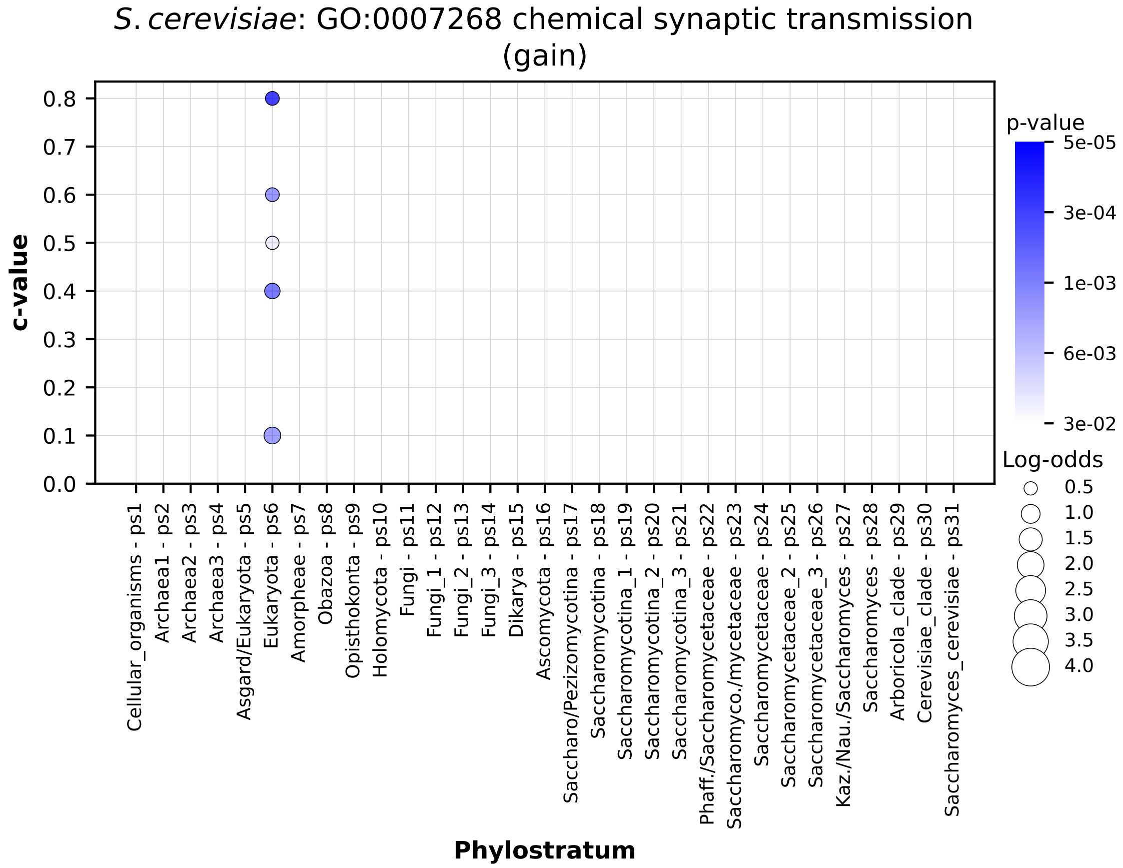

S. cerevisiae: GO:0007268 chemical synaptic transmission (gain)
c-value
0.0
0.1
0.2
0.3
0.4
0.5
0.6
0.7
0.8
Phylostratum
Cellular_organisms - ps1
Archaea1 - ps2
Archaea2 - ps3
Archaea3 - ps4
Asgard/Eukaryota - ps5
Eukaryota - ps6
Amorpheae - ps7
Obazoa - ps8
Opisthokonta - ps9
Holomycota - ps10
Fungi - ps11
Fungi_1 - ps12
Fungi_2 - ps13
Fungi_3 - ps14
Dikarya - ps15
Ascomycota - ps16
Saccharo/Pezizomycotina - ps17
Saccharomycotina - ps18
Saccharomycotina_1 - ps19
Saccharomycotina_2 - ps20
Saccharomycotina_3 - ps21
Phaff./Saccharomycetaceae - ps22
Saccharomyco./mycetaceae - ps23
Saccharomycetaceae - ps24
Saccharomycetaceae_2 - ps25
Saccharomycetaceae_3 - ps26
Kaz./Nau./Saccharomyces - ps27
Saccharomyces - ps28
Arboricola_clade - ps29
Cerevisiae_clade - ps30
Saccharomyces_cerevisiae - ps31
p-value
5e-05
3e-04
1e-03
6e-03
3e-02
Log-odds
0.5
1.0
1.5
2.0
2.5
3.0
3.5
4.0

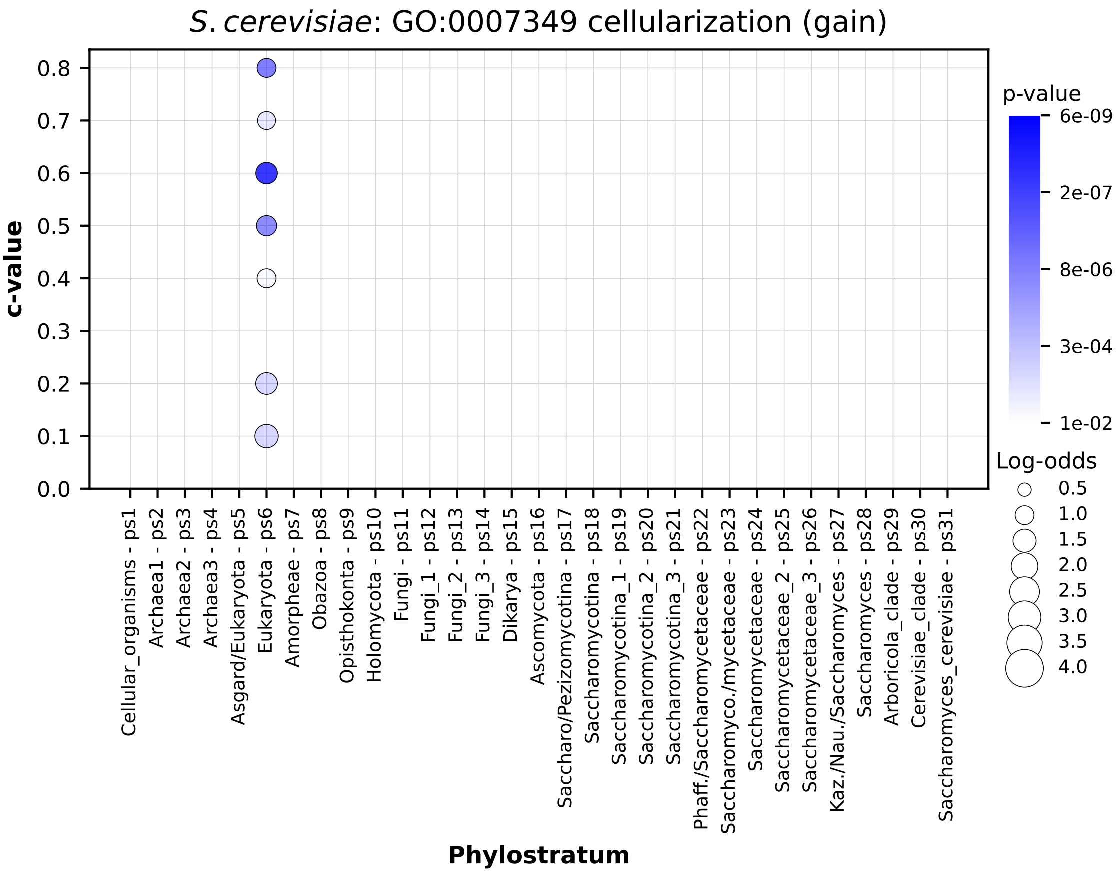

S. cerevisiae: GO:0007349 cellularization (gain)
c-value
0.0
0.1
0.2
0.3
0.4
0.5
0.6
0.7
0.8
Phylostratum
Cellular_organisms - ps1
Archaea1 - ps2
Archaea2 - ps3
Archaea3 - ps4
Asgard/Eukaryota - ps5
Eukaryota - ps6
Amorpheae - ps7
Obazoa - ps8
Opisthokonta - ps9
Holomycota - ps10
Fungi - ps11
Fungi_1 - ps12
Fungi_2 - ps13
Fungi_3 - ps14
Dikarya - ps15
Ascomycota - ps16
Saccharo/Pezizomycotina - ps17
Saccharomycotina - ps18
Saccharomycotina_1 - ps19
Saccharomycotina_2 - ps20
Saccharomycotina_3 - ps21
Phaff./Saccharomycetaceae - ps22
Saccharomyco./mycetaceae - ps23
Saccharomycetaceae - ps24
Saccharomycetaceae_2 - ps25
Saccharomycetaceae_3 - ps26
Kaz./Nau./Saccharomyces - ps27
Saccharomyces - ps28
Arboricola_clade - ps29
Cerevisiae_clade - ps30
Saccharomyces_cerevisiae - ps31
p-value
6e-09
2e-07
8e-06
3e-04
1e-02
Log-odds
0.5
1.0
1.5
2.0
2.5
3.0
3.5
4.0

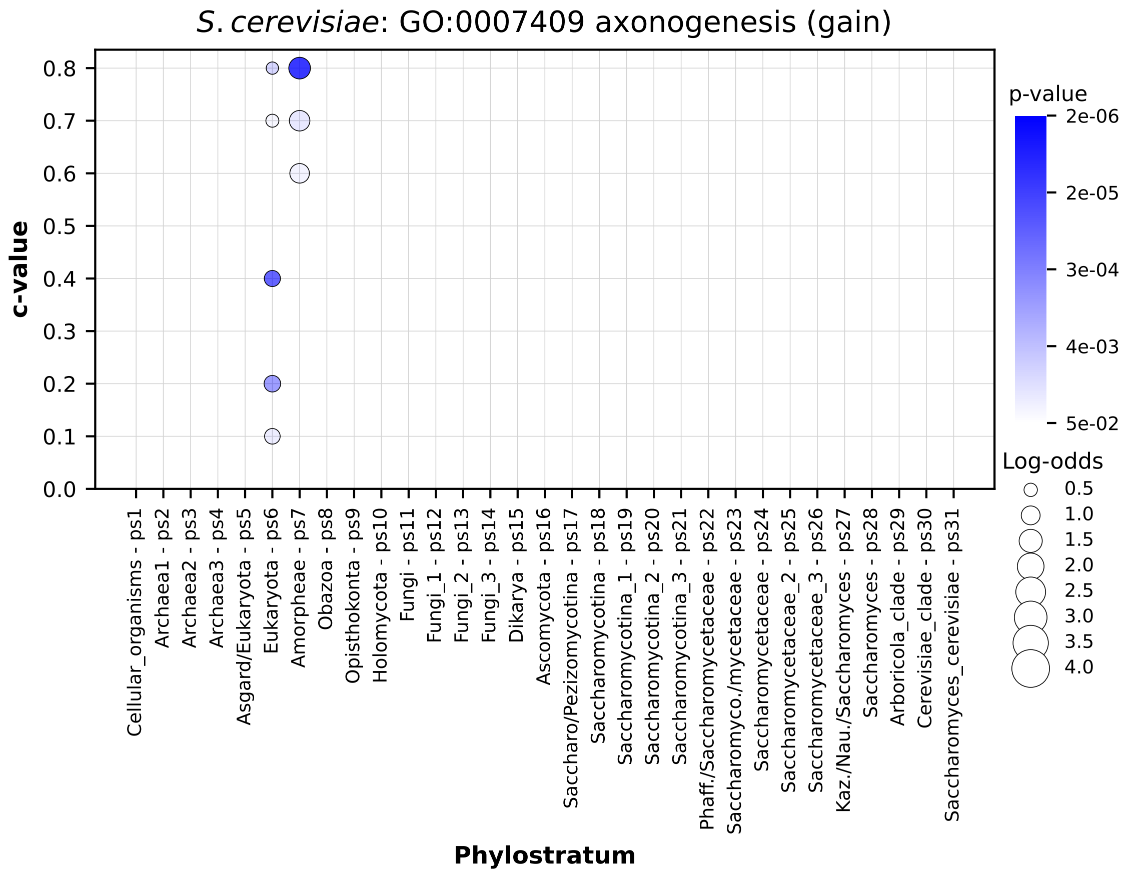
S. cerevisiae: GO:0007409 axonogenesis (gain)
c-value
0.0
0.1
0.2
0.3
0.4
0.5
0.6
0.7
0.8
p-value
2e-06
2e-05
3e-04
4e-03
5e-02
Log-odds
0.5
1.0
1.5
2.0
2.5
3.0
3.5
4.0
Cellular_organisms - ps1
Archaea1 - ps2
Archaea2 - ps3
Archaea3 - ps4
Asgard/Eukaryota - ps5
Eukaryota - ps6
Amorpheae - ps7
Obazoa - ps8
Opisthokonta - ps9
Holomycota - ps10
Fungi - ps11
Fungi_1 - ps12
Fungi_2 - ps13
Fungi_3 - ps14
Dikarya - ps15
Ascomycota - ps16
Saccharo/Pezizomycotina - ps17
Saccharomycotina - ps18
Saccharomycotina_1 - ps19
Saccharomycotina_2 - ps20
Saccharomycotina_3 - ps21
Phaff./Saccharomycetaceae - ps22
Saccharomyco./mycetaceae - ps23
Saccharomycetaceae - ps24
Saccharomycetaceae_2 - ps25
Saccharomycetaceae_3 - ps26
Kaz./Nau./Saccharomyces - ps27
Saccharomyces - ps28
Arboricola_clade - ps29
Cerevisiae_clade - ps30
Saccharomyces_cerevisiae - ps31
Phylostratum

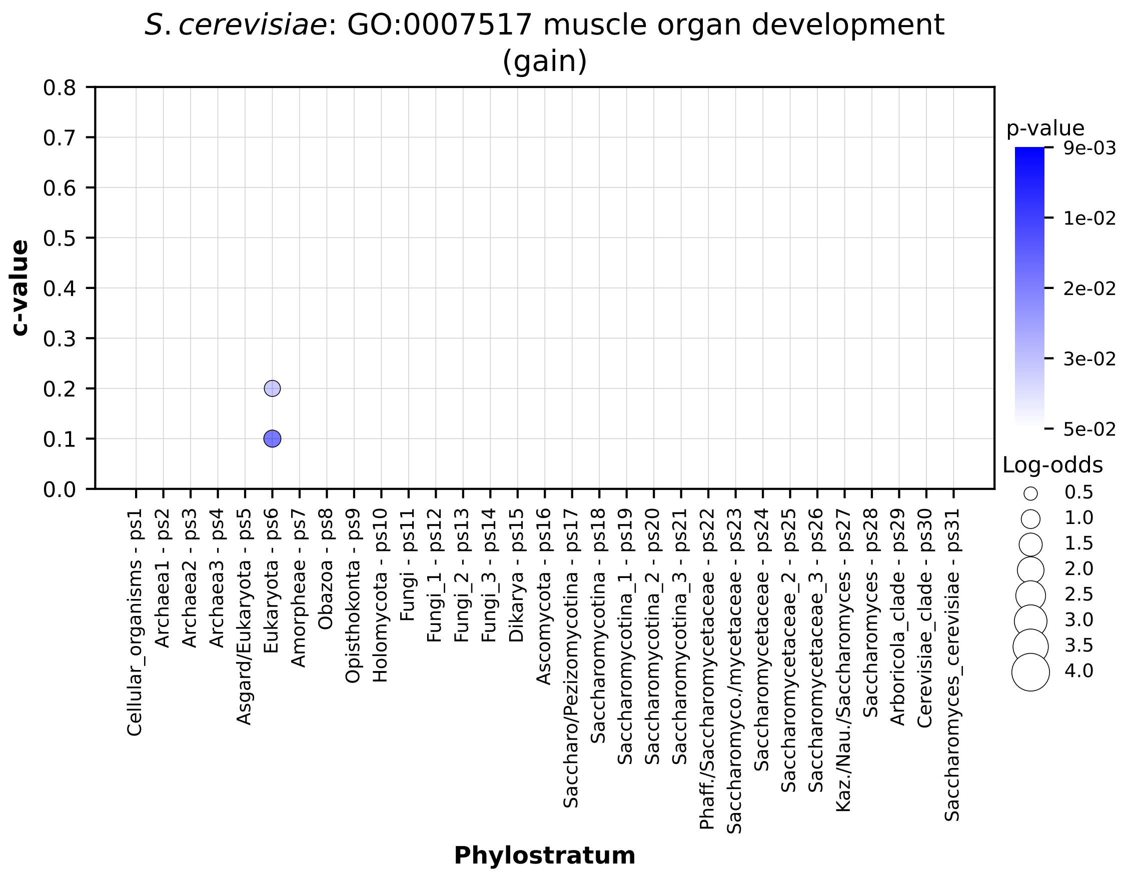

S. cerevisiae: GO:0007517 muscle organ development (gain)
c-value
0.0
0.1
0.2
0.3
0.4
0.5
0.6
0.7
0.8
Phylostratum
Cellular_organisms - ps1
Archaea1 - ps2
Archaea2 - ps3
Archaea3 - ps4
Asgard/Eukaryota - ps5
Eukaryota - ps6
Amorpheae - ps7
Obazoa - ps8
Opisthokonta - ps9
Holomycota - ps10
Fungi - ps11
Fungi_1 - ps12
Fungi_2 - ps13
Fungi_3 - ps14
Dikarya - ps15
Ascomycota - ps16
Saccharo/Pezizomycotina - ps17
Saccharomycotina - ps18
Saccharomycotina_1 - ps19
Saccharomycotina_2 - ps20
Saccharomycotina_3 - ps21
Phaff./Saccharomycetaceae - ps22
Saccharomyco./mycetaceae - ps23
Saccharomycetaceae - ps24
Saccharomycetaceae_2 - ps25
Saccharomycetaceae_3 - ps26
Kaz./Nau./Saccharomyces - ps27
Saccharomyces - ps28
Arboricola_clade - ps29
Cerevisiae_clade - ps30
Saccharomyces_cerevisiae - ps31
p-value
9e-03
1e-02
2e-02
3e-02
5e-02
Log-odds
0.5
1.0
1.5
2.0
2.5
3.0
3.5
4.0

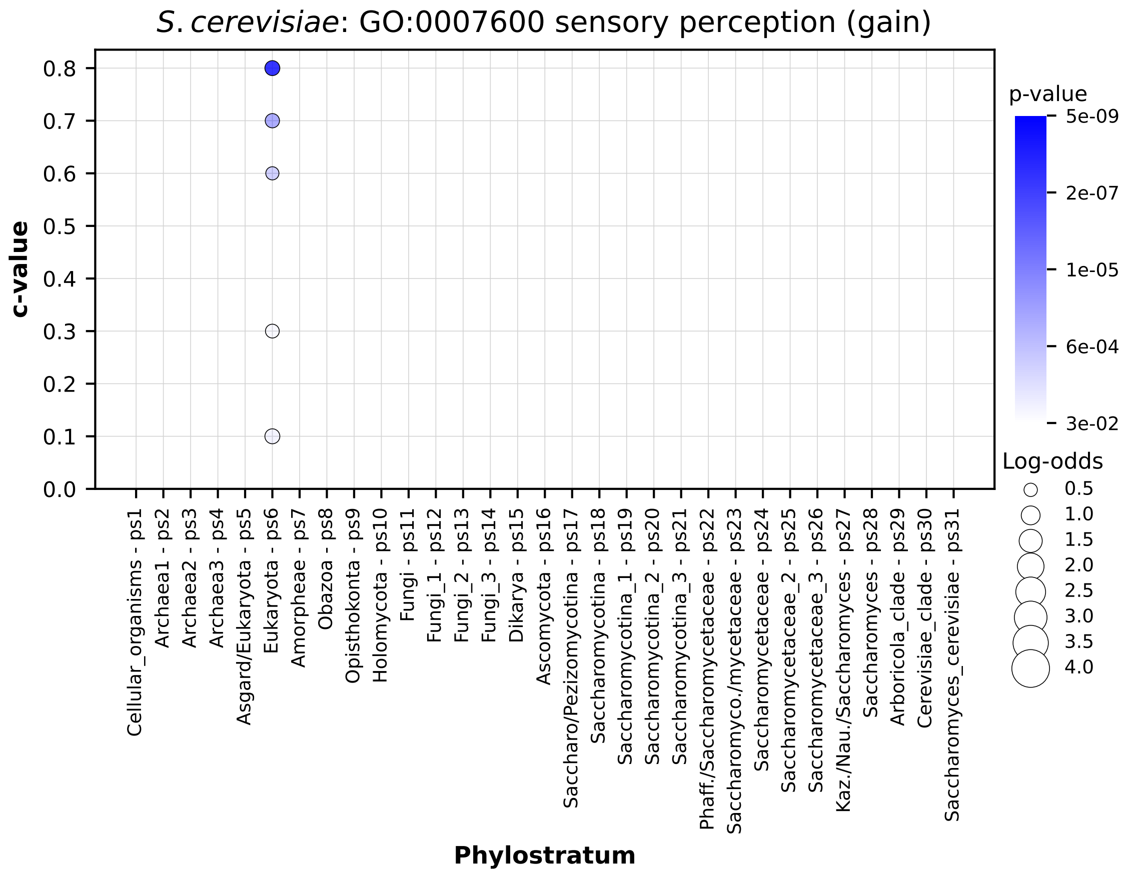

S. cerevisiae: GO:0007600 sensory perception (gain)
c-value
0.0
0.1
0.2
0.3
0.4
0.5
0.6
0.7
0.8
p-value
5e-09
2e-07
1e-05
6e-04
3e-02
Log-odds
0.5
1.0
1.5
2.0
2.5
3.0
3.5
4.0
Cellular_organisms - ps1
Archaea1 - ps2
Archaea2 - ps3
Archaea3 - ps4
Asgard/Eukaryota - ps5
Eukaryota - ps6
Amorpheae - ps7
Obazoa - ps8
Opisthokonta - ps9
Holomycota - ps10
Fungi - ps11
Fungi_1 - ps12
Fungi_2 - ps13
Fungi_3 - ps14
Dikarya - ps15
Ascomycota - ps16
Saccharo/Pezizomycotina - ps17
Saccharomycotina - ps18
Saccharomycotina_1 - ps19
Saccharomycotina_2 - ps20
Saccharomycotina_3 - ps21
Phaff./Saccharomycetaceae - ps22
Saccharomyco./mycetaceae - ps23
Saccharomycetaceae - ps24
Saccharomycetaceae_2 - ps25
Saccharomycetaceae_3 - ps26
Kaz./Nau./Saccharomyces - ps27
Saccharomyces - ps28
Arboricola_clade - ps29
Cerevisiae_clade - ps30
Saccharomyces_cerevisiae - ps31
Phylostratum

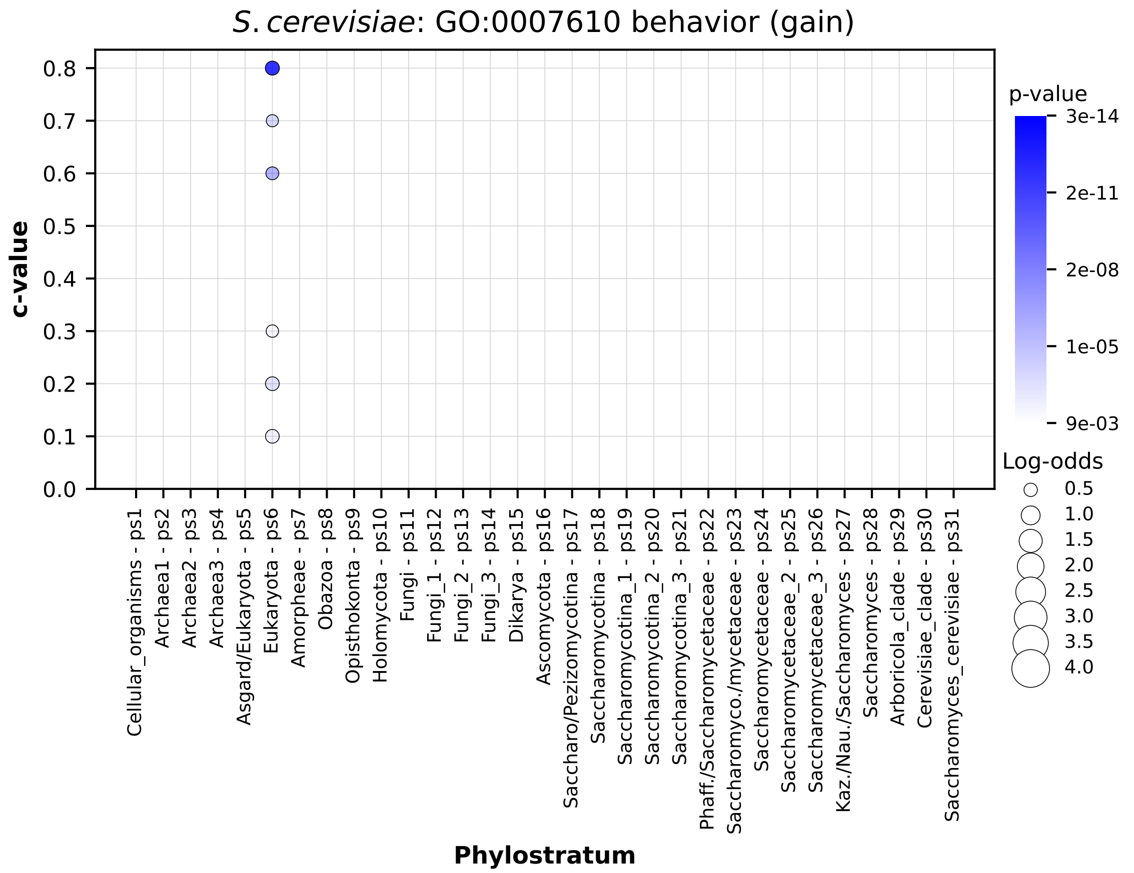
S. cerevisiae: GO:0007610 behavior (gain)
c-value
0.0
0.1
0.2
0.3
0.4
0.5
0.6
0.7
0.8
p-value
3e-14
2e-11
2e-08
1e-05
9e-03
Log-odds
0.5
1.0
1.5
2.0
2.5
3.0
3.5
4.0
Cellular_organisms - ps1
Archaea1 - ps2
Archaea2 - ps3
Archaea3 - ps4
Asgard/Eukaryota - ps5
Eukaryota - ps6
Amorpheae - ps7
Obazoa - ps8
Opisthokonta - ps9
Holomycota - ps10
Fungi - ps11
Fungi_1 - ps12
Fungi_2 - ps13
Fungi_3 - ps14
Dikarya - ps15
Ascomycota - ps16
Saccharo/Pezizomycotina - ps17
Saccharomycotina - ps18
Saccharomycotina_1 - ps19
Saccharomycotina_2 - ps20
Saccharomycotina_3 - ps21
Phaff./Saccharomycetaceae - ps22
Saccharomyco./mycetaceae - ps23
Saccharomycetaceae - ps24
Saccharomycetaceae_2 - ps25
Saccharomycetaceae_3 - ps26
Kaz./Nau./Saccharomyces - ps27
Saccharomyces - ps28
Arboricola_clade - ps29
Cerevisiae_clade - ps30
Saccharomyces_cerevisiae - ps31
Phylostratum

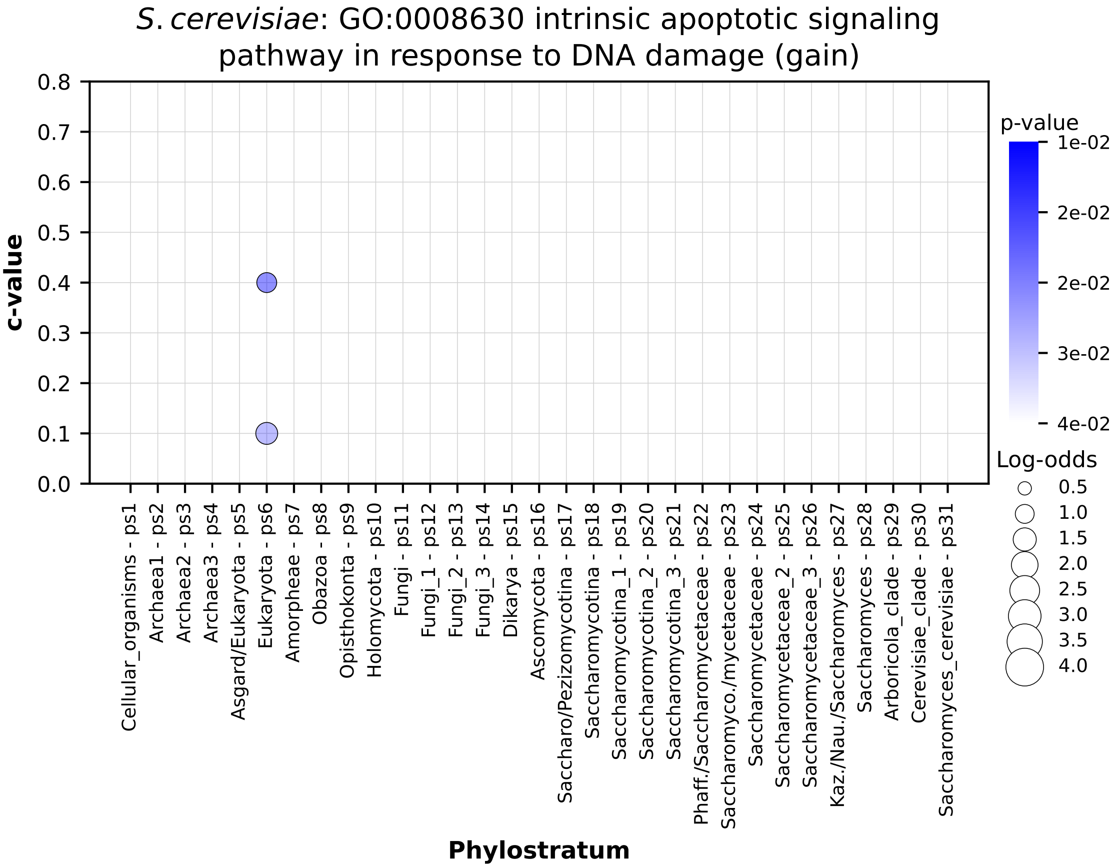

S. cerevisiae: GO:0008630 intrinsic apoptotic signaling pathway in response to DNA damage (gain)
c-value
0.0
0.1
0.2
0.3
0.4
0.5
0.6
0.7
0.8
Phylostratum
Cellular_organisms - ps1
Archaea1 - ps2
Archaea2 - ps3
Archaea3 - ps4
Asgard/Eukaryota - ps5
Eukaryota - ps6
Amorpheae - ps7
Obazoa - ps8
Opisthokonta - ps9
Holomycota - ps10
Fungi - ps11
Fungi_1 - ps12
Fungi_2 - ps13
Fungi_3 - ps14
Dikarya - ps15
Ascomycota - ps16
Saccharo/Pezizomycotina - ps17
Saccharomycotina - ps18
Saccharomycotina_1 - ps19
Saccharomycotina_2 - ps20
Saccharomycotina_3 - ps21
Phaff./Saccharomycetaceae - ps22
Saccharomyco./mycetaceae - ps23
Saccharomycetaceae - ps24
Saccharomycetaceae_2 - ps25
Saccharomycetaceae_3 - ps26
Kaz./Nau./Saccharomyces - ps27
Saccharomyces - ps28
Arboricola_clade - ps29
Cerevisiae_clade - ps30
Saccharomyces_cerevisiae - ps31
p-value
1e-02
2e-02
2e-02
3e-02
4e-02
Log-odds
0.5
1.0
1.5
2.0
2.5
3.0
3.5
4.0

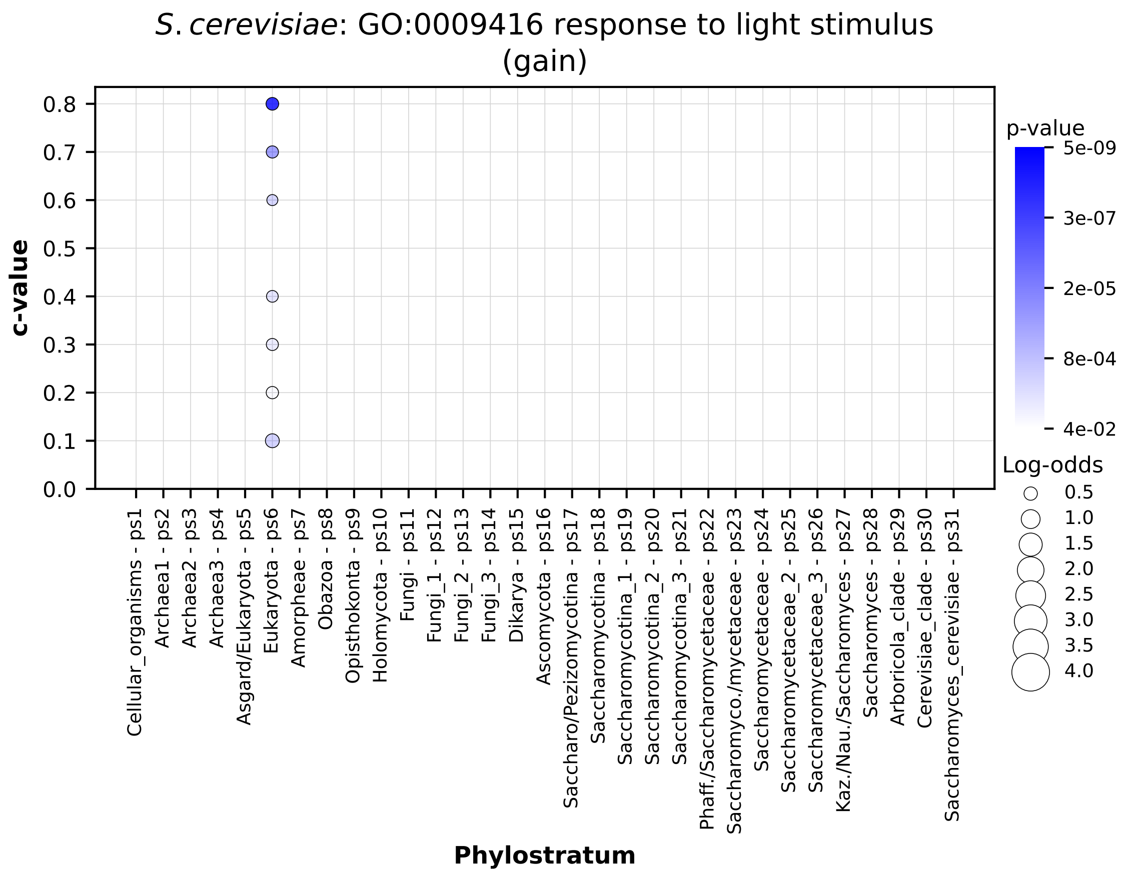

S. cerevisiae: GO:0009416 response to light stimulus (gain)
c-value
0.0
0.1
0.2
0.3
0.4
0.5
0.6
0.7
0.8
p-value
5e-09
3e-07
2e-05
8e-04
4e-02
Log-odds
0.5
1.0
1.5
2.0
2.5
3.0
3.5
4.0
Cellular_organisms - ps1
Archaea1 - ps2
Archaea2 - ps3
Archaea3 - ps4
Asgard/Eukaryota - ps5
Eukaryota - ps6
Amorpheae - ps7
Obazoa - ps8
Opisthokonta - ps9
Holomycota - ps10
Fungi - ps11
Fungi_1 - ps12
Fungi_2 - ps13
Fungi_3 - ps14
Dikarya - ps15
Ascomycota - ps16
Saccharo/Pezizomycotina - ps17
Saccharomycotina - ps18
Saccharomycotina_1 - ps19
Saccharomycotina_2 - ps20
Saccharomycotina_3 - ps21
Phaff./Saccharomycetaceae - ps22
Saccharomyco./mycetaceae - ps23
Saccharomycetaceae - ps24
Saccharomycetaceae_2 - ps25
Saccharomycetaceae_3 - ps26
Kaz./Nau./Saccharomyces - ps27
Saccharomyces - ps28
Arboricola_clade - ps29
Cerevisiae_clade - ps30
Saccharomyces_cerevisiae - ps31
Phylostratum

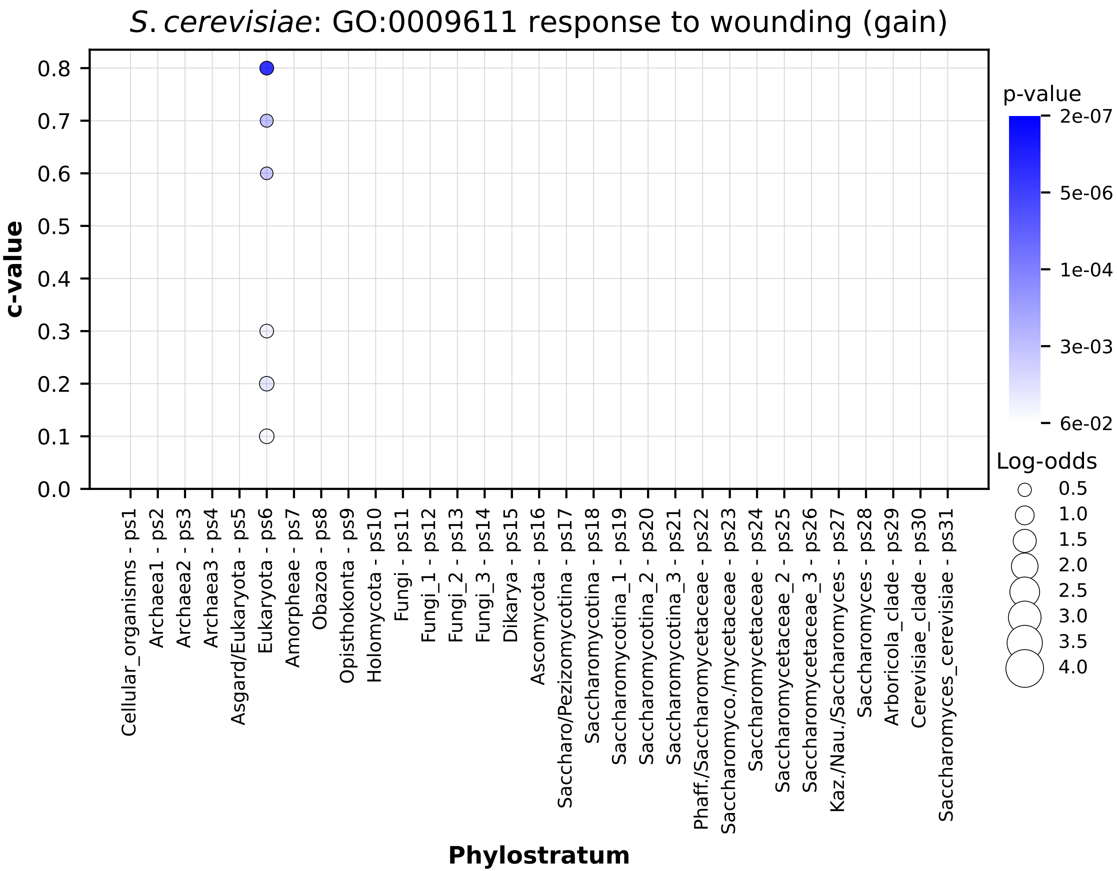

S. cerevisiae: GO:0009611 response to wounding (gain)
c-value
0.0
0.1
0.2
0.3
0.4
0.5
0.6
0.7
0.8
p-value
2e-07
5e-06
1e-04
3e-03
6e-02
Log-odds
0.5
1.0
1.5
2.0
2.5
3.0
3.5
4.0
Cellular_organisms - ps1
Archaea1 - ps2
Archaea2 - ps3
Archaea3 - ps4
Asgard/Eukaryota - ps5
Eukaryota - ps6
Amorpheae - ps7
Obazoa - ps8
Opisthokonta - ps9
Holomycota - ps10
Fungi - ps11
Fungi_1 - ps12
Fungi_2 - ps13
Fungi_3 - ps14
Dikarya - ps15
Ascomycota - ps16
Saccharo/Pezizomycotina - ps17
Saccharomycotina - ps18
Saccharomycotina_1 - ps19
Saccharomycotina_2 - ps20
Saccharomycotina_3 - ps21
Phaff./Saccharomycetaceae - ps22
Saccharomyco./mycetaceae - ps23
Saccharomycetaceae - ps24
Saccharomycetaceae_2 - ps25
Saccharomycetaceae_3 - ps26
Kaz./Nau./Saccharomyces - ps27
Saccharomyces - ps28
Arboricola_clade - ps29
Cerevisiae_clade - ps30
Saccharomyces_cerevisiae - ps31
Phylostratum

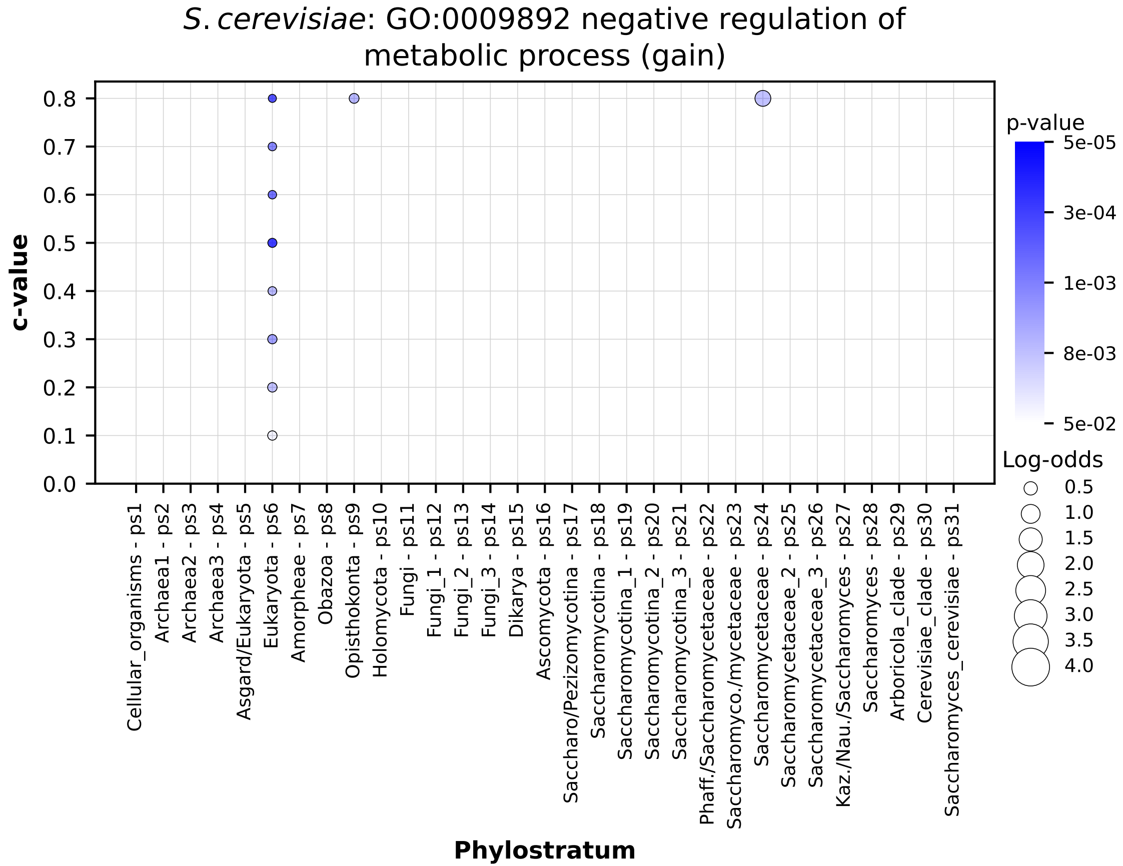

S. cerevisiae: GO:0009892 negative regulation of metabolic process (gain)
c-value
0.0
0.1
0.2
0.3
0.4
0.5
0.6
0.7
0.8
Phylostratum
Cellular_organisms - ps1
Archaea1 - ps2
Archaea2 - ps3
Archaea3 - ps4
Asgard/Eukaryota - ps5
Eukaryota - ps6
Amorpheae - ps7
Obazoa - ps8
Opisthokonta - ps9
Holomycota - ps10
Fungi - ps11
Fungi_1 - ps12
Fungi_2 - ps13
Fungi_3 - ps14
Dikarya - ps15
Ascomycota - ps16
Saccharo/Pezizomycotina - ps17
Saccharomycotina - ps18
Saccharomycotina_1 - ps19
Saccharomycotina_2 - ps20
Saccharomycotina_3 - ps21
Phaff./Saccharomycetaceae - ps22
Saccharomyco./mycetaceae - ps23
Saccharomycetaceae - ps24
Saccharomycetaceae_2 - ps25
Saccharomycetaceae_3 - ps26
Kaz./Nau./Saccharomyces - ps27
Saccharomyces - ps28
Arboricola_clade - ps29
Cerevisiae_clade - ps30
Saccharomyces_cerevisiae - ps31
p-value
5e-05
3e-04
1e-03
8e-03
5e-02
Log-odds
0.5
1.0
1.5
2.0
2.5
3.0
3.5
4.0

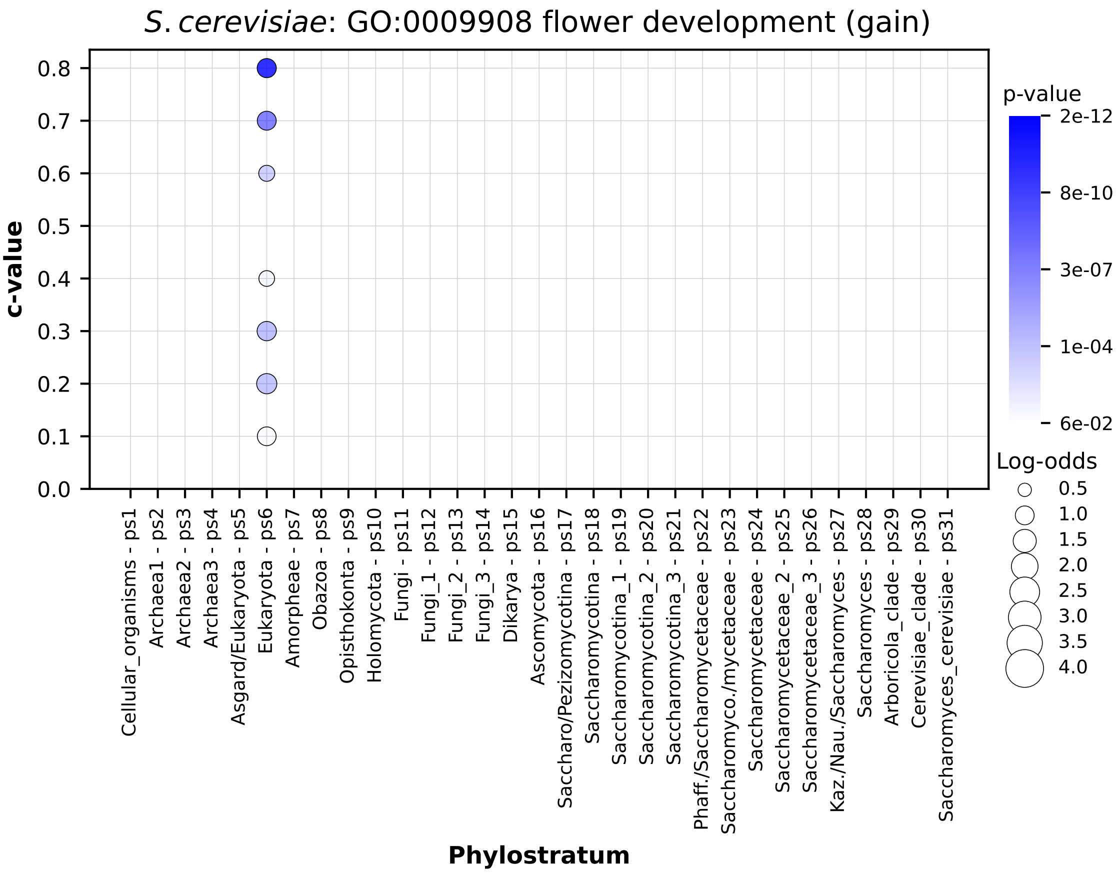

S. cerevisiae: GO:0009908 flower development (gain)
c-value
0.0
0.1
0.2
0.3
0.4
0.5
0.6
0.7
0.8
p-value
2e-12
8e-10
3e-07
1e-04
6e-02
Log-odds
0.5
1.0
1.5
2.0
2.5
3.0
3.5
4.0
Cellular_organisms - ps1
Archaea1 - ps2
Archaea2 - ps3
Archaea3 - ps4
Asgard/Eukaryota - ps5
Eukaryota - ps6
Amorpheae - ps7
Obazoa - ps8
Opisthokonta - ps9
Holomycota - ps10
Fungi - ps11
Fungi_1 - ps12
Fungi_2 - ps13
Fungi_3 - ps14
Dikarya - ps15
Ascomycota - ps16
Saccharo/Pezizomycotina - ps17
Saccharomycotina - ps18
Saccharomycotina_1 - ps19
Saccharomycotina_2 - ps20
Saccharomycotina_3 - ps21
Phaff./Saccharomycetaceae - ps22
Saccharomyco./mycetaceae - ps23
Saccharomycetaceae - ps24
Saccharomycetaceae_2 - ps25
Saccharomycetaceae_3 - ps26
Kaz./Nau./Saccharomyces - ps27
Saccharomyces - ps28
Arboricola_clade - ps29
Cerevisiae_clade - ps30
Saccharomyces_cerevisiae - ps31
Phylostratum

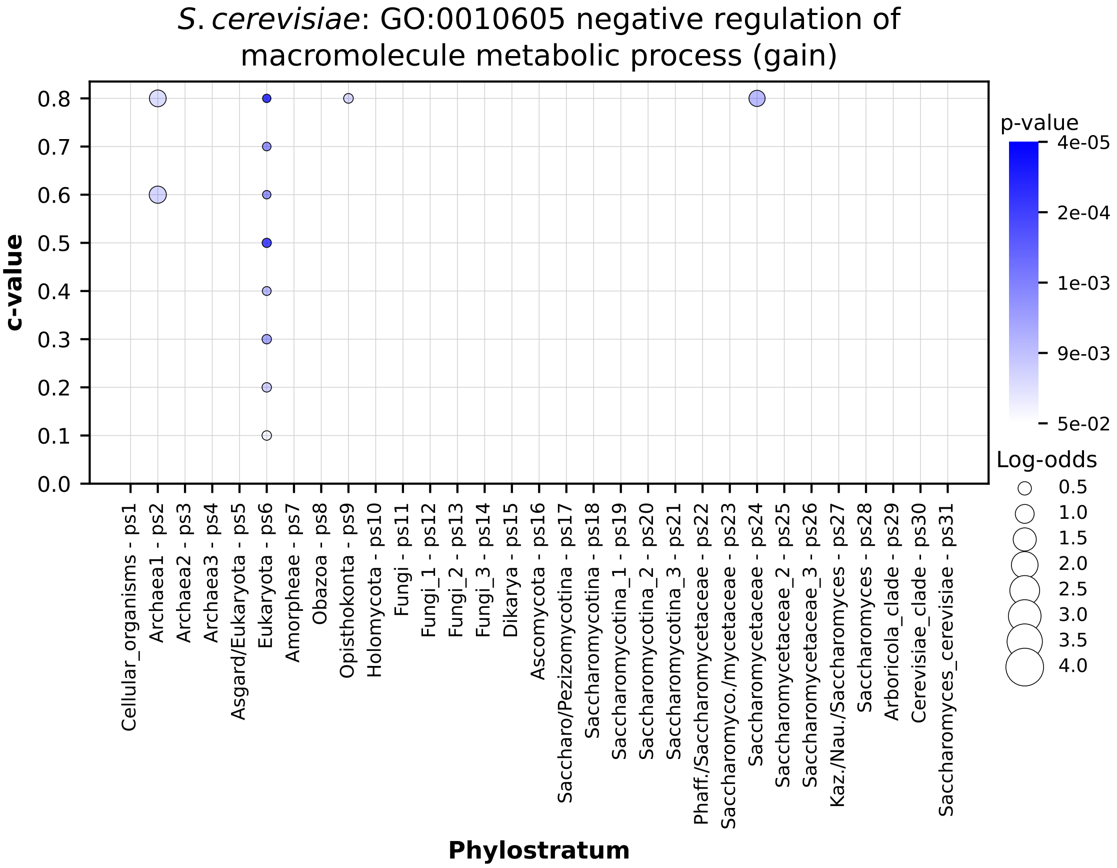

S. cerevisiae: GO:0010605 negative regulation of macromolecule metabolic process (gain)
c-value
0.8
0.7
0.6
0.5
0.4
0.3
0.2
0.1
0.0
p-value
4e-05
2e-04
1e-03
9e-03
5e-02
Log-odds
0.5
1.0
1.5
2.0
2.5
3.0
3.5
4.0
Cellular_organisms - ps1
Archaea1 - ps2
Archaea2 - ps3
Archaea3 - ps4
Asgard/Eukaryota - ps5
Eukaryota - ps6
Amorpheae - ps7
Obazoa - ps8
Opisthokonta - ps9
Holomycota - ps10
Fungi - ps11
Fungi_1 - ps12
Fungi_2 - ps13
Fungi_3 - ps14
Dikarya - ps15
Ascomycota - ps16
Saccharo/Pezizomycotina - ps17
Saccharomycotina - ps18
Saccharomycotina_1 - ps19
Saccharomycotina_2 - ps20
Saccharomycotina_3 - ps21
Phaff./Saccharomycetaceae - ps22
Saccharomyco./mycetaceae - ps23
Saccharomycetaceae - ps24
Saccharomycetaceae_2 - ps25
Saccharomycetaceae_3 - ps26
Kaz./Nau./Saccharomyces - ps27
Saccharomyces - ps28
Arboricola_clade - ps29
Cerevisiae_clade - ps30
Saccharomyces_cerevisiae - ps31
Phylostratum

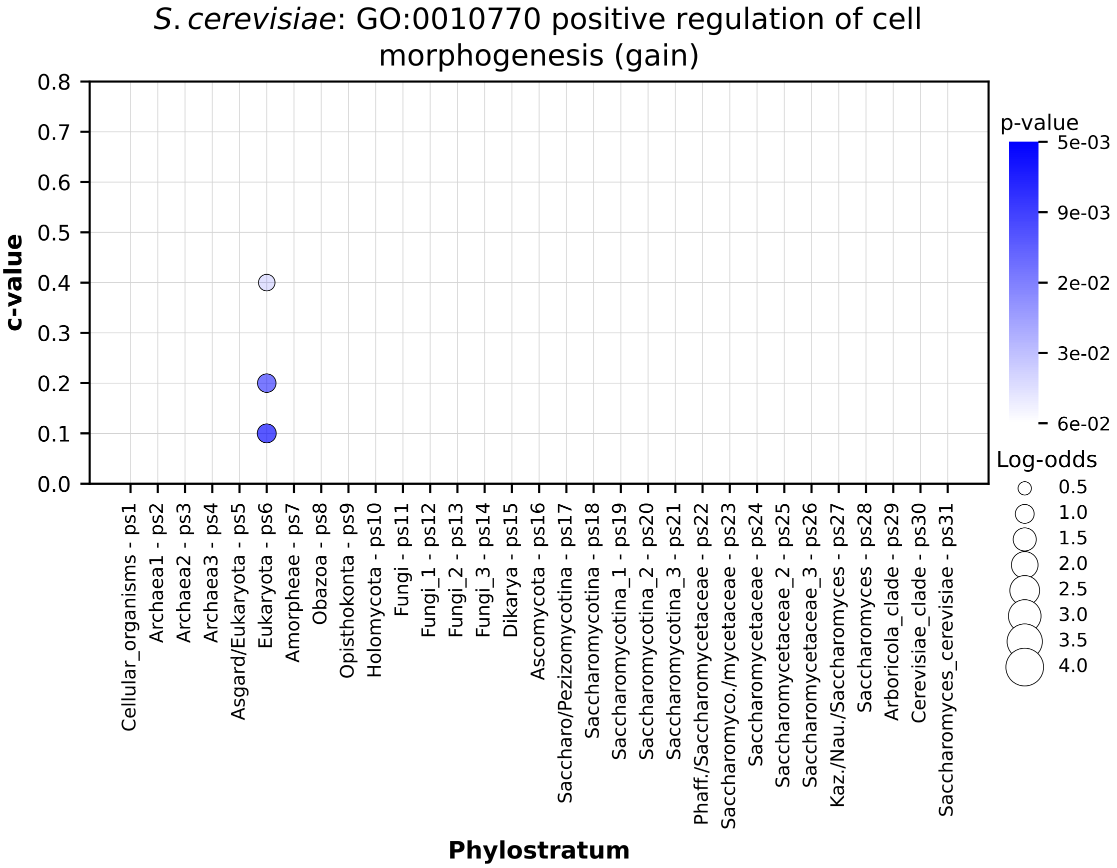
S. cerevisiae: GO:0010770 positive regulation of cell morphogenesis (gain)
c-value
0.0
0.1
0.2
0.3
0.4
0.5
0.6
0.7
0.8
Phylostratum
Cellular_organisms - ps1
Archaea1 - ps2
Archaea2 - ps3
Archaea3 - ps4
Asgard/Eukaryota - ps5
Eukaryota - ps6
Amorpheae - ps7
Obazoa - ps8
Opisthokonta - ps9
Holomycota - ps10
Fungi - ps11
Fungi_1 - ps12
Fungi_2 - ps13
Fungi_3 - ps14
Dikarya - ps15
Ascomycota - ps16
Saccharo/Pezizomycotina - ps17
Saccharomycotina - ps18
Saccharomycotina_1 - ps19
Saccharomycotina_2 - ps20
Saccharomycotina_3 - ps21
Phaff./Saccharomycetaceae - ps22
Saccharomyco./mycetaceae - ps23
Saccharomycetaceae - ps24
Saccharomycetaceae_2 - ps25
Saccharomycetaceae_3 - ps26
Kaz./Nau./Saccharomyces - ps27
Saccharomyces - ps28
Arboricola_clade - ps29
Cerevisiae_clade - ps30
Saccharomyces_cerevisiae - ps31
p-value
5e-03
9e-03
2e-02
3e-02
6e-02
Log-odds
0.5
1.0
1.5
2.0
2.5
3.0
3.5
4.0

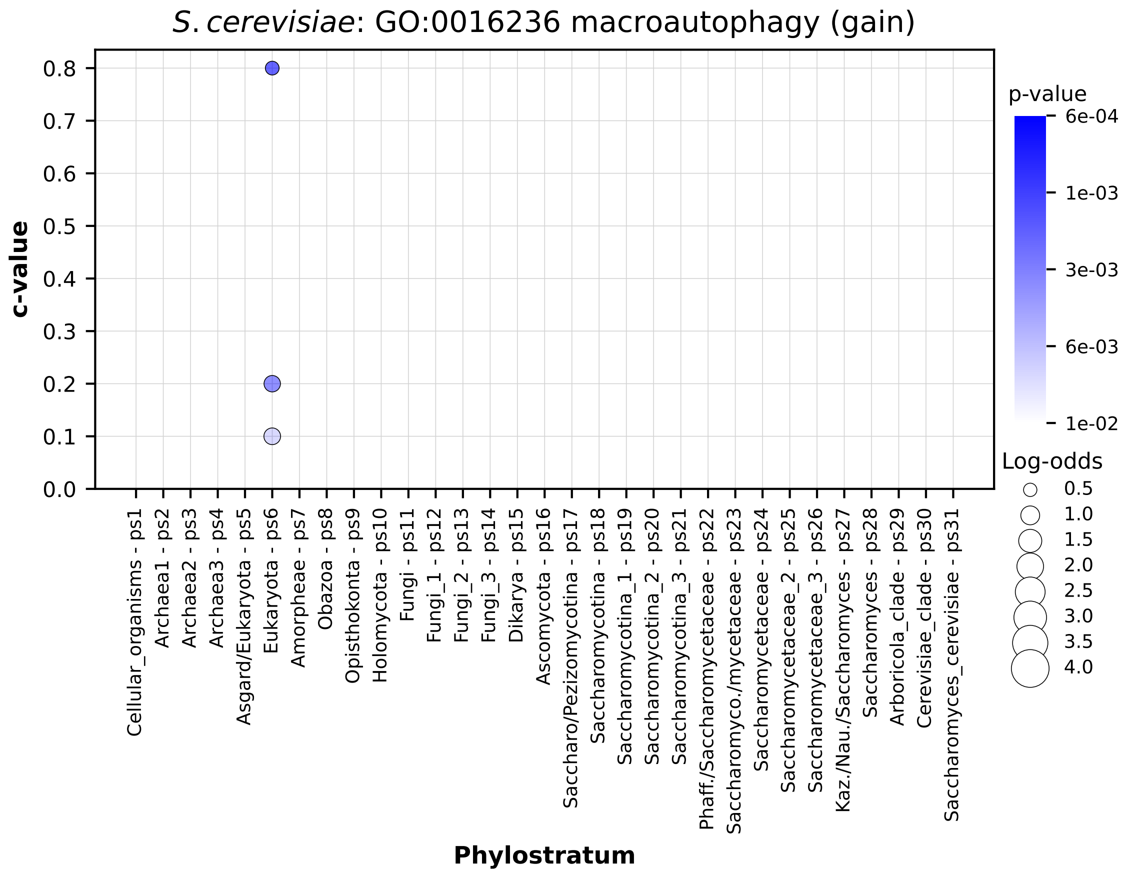

S. cerevisiae: GO:0016236 macroautophagy (gain)
c-value
0.0
0.1
0.2
0.3
0.4
0.5
0.6
0.7
0.8
Phylostratum
Cellular_organisms - ps1
Archaea1 - ps2
Archaea2 - ps3
Archaea3 - ps4
Asgard/Eukaryota - ps5
Eukaryota - ps6
Amorpheae - ps7
Obazoa - ps8
Opisthokonta - ps9
Holomycota - ps10
Fungi - ps11
Fungi_1 - ps12
Fungi_2 - ps13
Fungi_3 - ps14
Dikarya - ps15
Ascomycota - ps16
Saccharo/Pezizomycotina - ps17
Saccharomycotina - ps18
Saccharomycotina_1 - ps19
Saccharomycotina_2 - ps20
Saccharomycotina_3 - ps21
Phaff./Saccharomycetaceae - ps22
Saccharomyco./mycetaceae - ps23
Saccharomycetaceae - ps24
Saccharomycetaceae_2 - ps25
Saccharomycetaceae_3 - ps26
Kaz./Nau./Saccharomyces - ps27
Saccharomyces - ps28
Arboricola_clade - ps29
Cerevisiae_clade - ps30
Saccharomyces_cerevisiae - ps31
p-value
6e-04
1e-03
3e-03
6e-03
1e-02
Log-odds
0.5
1.0
1.5
2.0
2.5
3.0
3.5
4.0

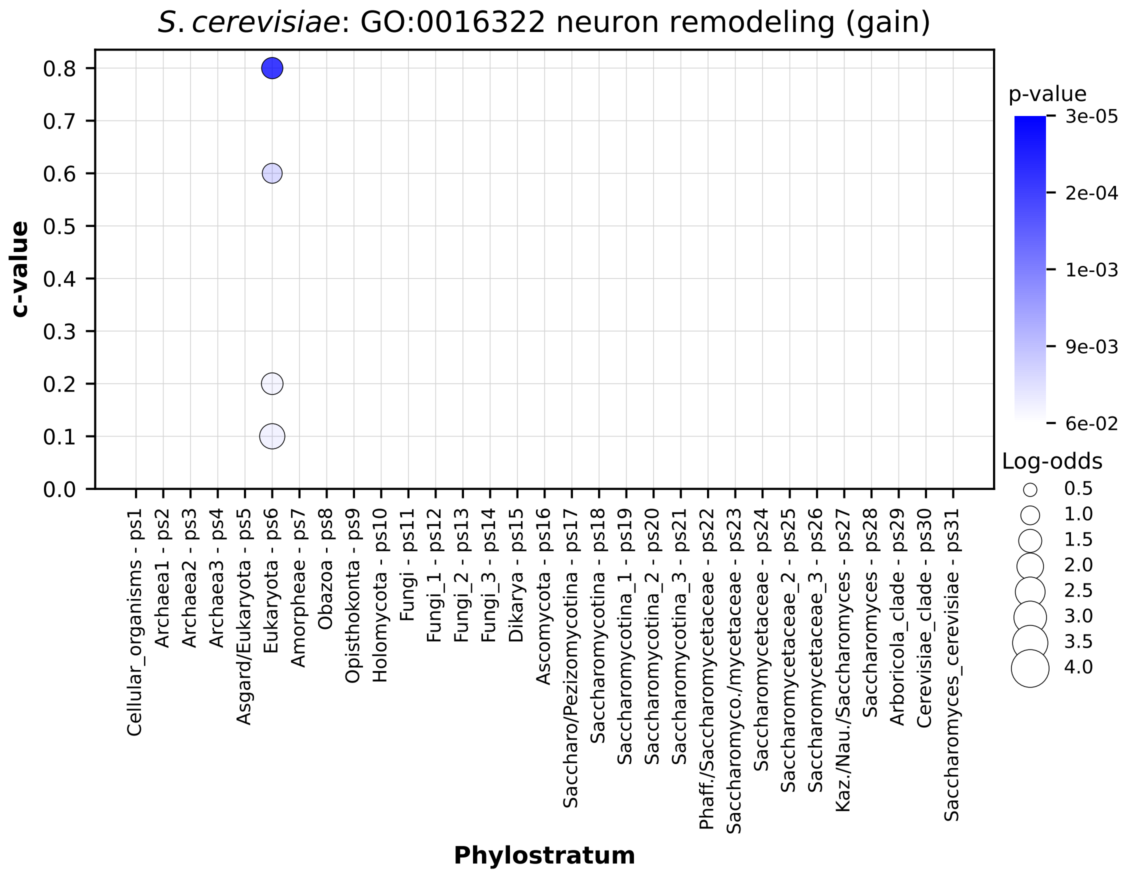

S. cerevisiae: GO:0016322 neuron remodeling (gain)
c-value
0.0
0.1
0.2
0.3
0.4
0.5
0.6
0.7
0.8
Phylostratum
Cellular_organisms - ps1
Archaea1 - ps2
Archaea2 - ps3
Archaea3 - ps4
Asgard/Eukaryota - ps5
Eukaryota - ps6
Amorpheae - ps7
Obazoa - ps8
Opisthokonta - ps9
Holomycota - ps10
Fungi - ps11
Fungi_1 - ps12
Fungi_2 - ps13
Fungi_3 - ps14
Dikarya - ps15
Ascomycota - ps16
Saccharo/Pezizomycotina - ps17
Saccharomycotina - ps18
Saccharomycotina_1 - ps19
Saccharomycotina_2 - ps20
Saccharomycotina_3 - ps21
Phaff./Saccharomycetaceae - ps22
Saccharomyco./mycetaceae - ps23
Saccharomycetaceae - ps24
Saccharomycetaceae_2 - ps25
Saccharomycetaceae_3 - ps26
Kaz./Nau./Saccharomyces - ps27
Saccharomyces - ps28
Arboricola_clade - ps29
Cerevisiae_clade - ps30
Saccharomyces_cerevisiae - ps31
p-value
3e-05
2e-04
1e-03
9e-03
6e-02
Log-odds
0.5
1.0
1.5
2.0
2.5
3.0
3.5
4.0

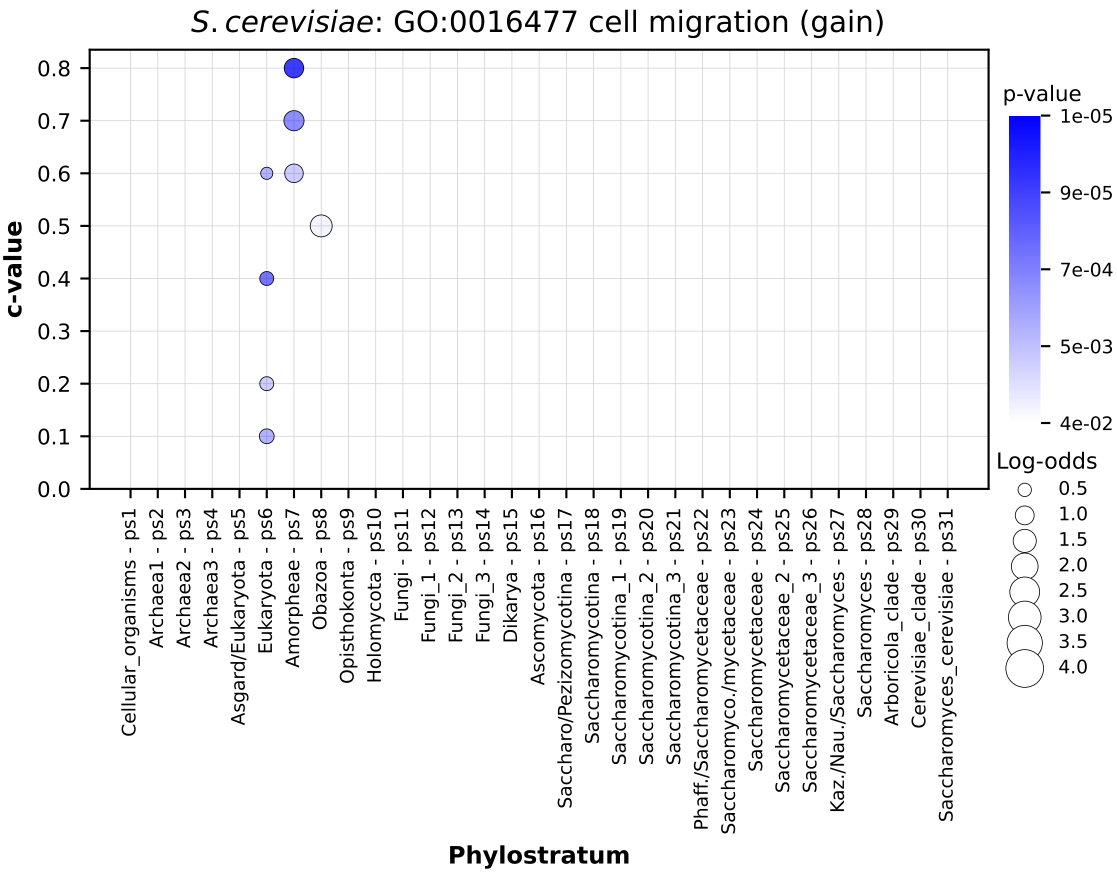

S. cerevisiae: GO:0016477 cell migration (gain)
c-value
0.0
0.1
0.2
0.3
0.4
0.5
0.6
0.7
0.8
Phylostratum
Cellular_organisms - ps1
Archaea1 - ps2
Archaea2 - ps3
Archaea3 - ps4
Asgard/Eukaryota - ps5
Eukaryota - ps6
Amorpheae - ps7
Obazoa - ps8
Opisthokonta - ps9
Holomycota - ps10
Fungi - ps11
Fungi_1 - ps12
Fungi_2 - ps13
Fungi_3 - ps14
Dikarya - ps15
Ascomycota - ps16
Saccharo/Pezizomycotina - ps17
Saccharomycotina - ps18
Saccharomycotina_1 - ps19
Saccharomycotina_2 - ps20
Saccharomycotina_3 - ps21
Phaff./Saccharomycetaceae - ps22
Saccharomyco./mycetaceae - ps23
Saccharomycetaceae - ps24
Saccharomycetaceae_2 - ps25
Saccharomycetaceae_3 - ps26
Kaz./Nau./Saccharomyces - ps27
Saccharomyces - ps28
Arboricola_clade - ps29
Cerevisiae_clade - ps30
Saccharomyces_cerevisiae - ps31
p-value
1e-05
9e-05
7e-04
5e-03
4e-02
Log-odds
0.5
1.0
1.5
2.0
2.5
3.0
3.5
4.0

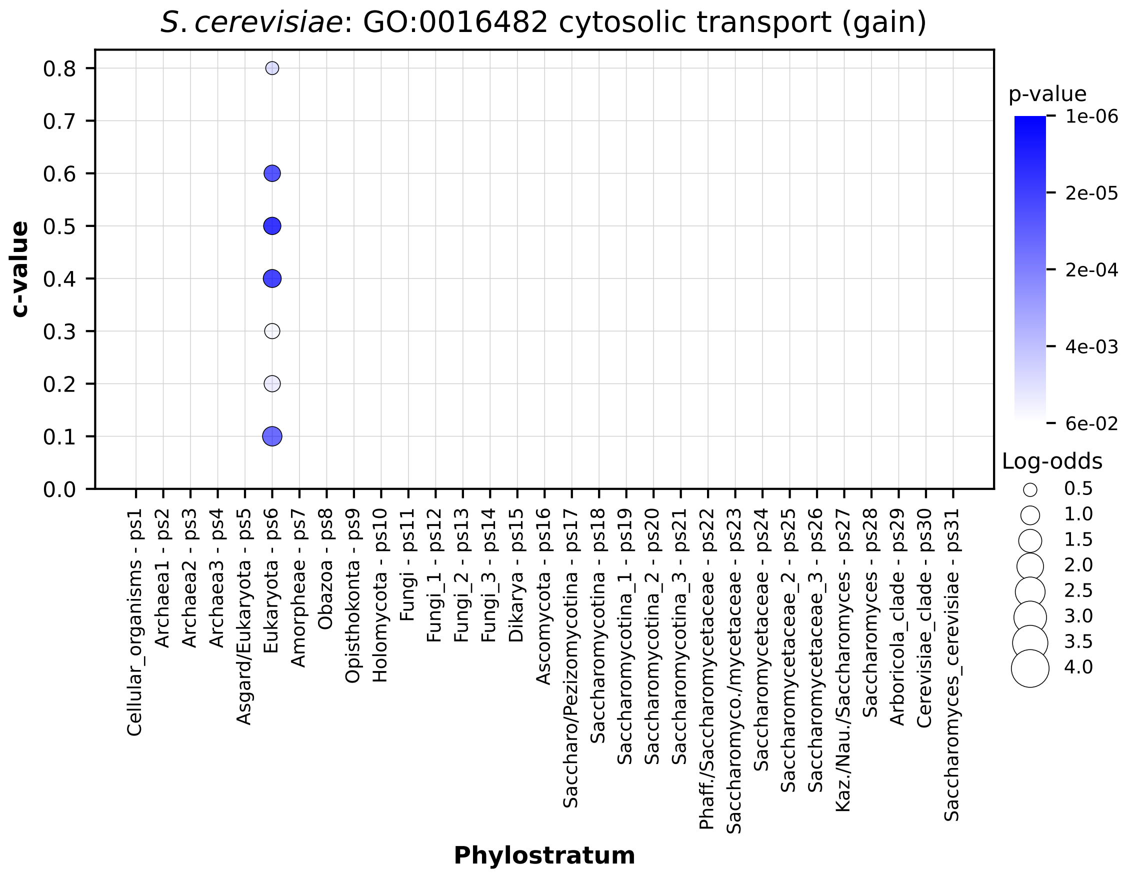

S. cerevisiae: GO:0016482 cytosolic transport (gain)
c-value
0.0
0.1
0.2
0.3
0.4
0.5
0.6
0.7
0.8
p-value
1e-06
2e-05
2e-04
4e-03
6e-02
Log-odds
0.5
1.0
1.5
2.0
2.5
3.0
3.5
4.0
Cellular_organisms - ps1
Archaea1 - ps2
Archaea2 - ps3
Archaea3 - ps4
Asgard/Eukaryota - ps5
Eukaryota - ps6
Amorpheae - ps7
Obazoa - ps8
Opisthokonta - ps9
Holomycota - ps10
Fungi - ps11
Fungi_1 - ps12
Fungi_2 - ps13
Fungi_3 - ps14
Dikarya - ps15
Ascomycota - ps16
Saccharo/Pezizomycotina - ps17
Saccharomycotina - ps18
Saccharomycotina_1 - ps19
Saccharomycotina_2 - ps20
Saccharomycotina_3 - ps21
Phaff./Saccharomycetaceae - ps22
Saccharomyco./mycetaceae - ps23
Saccharomycetaceae - ps24
Saccharomycetaceae_2 - ps25
Saccharomycetaceae_3 - ps26
Kaz./Nau./Saccharomyces - ps27
Saccharomyces - ps28
Arboricola_clade - ps29
Cerevisiae_clade - ps30
Saccharomyces_cerevisiae - ps31
Phylostratum

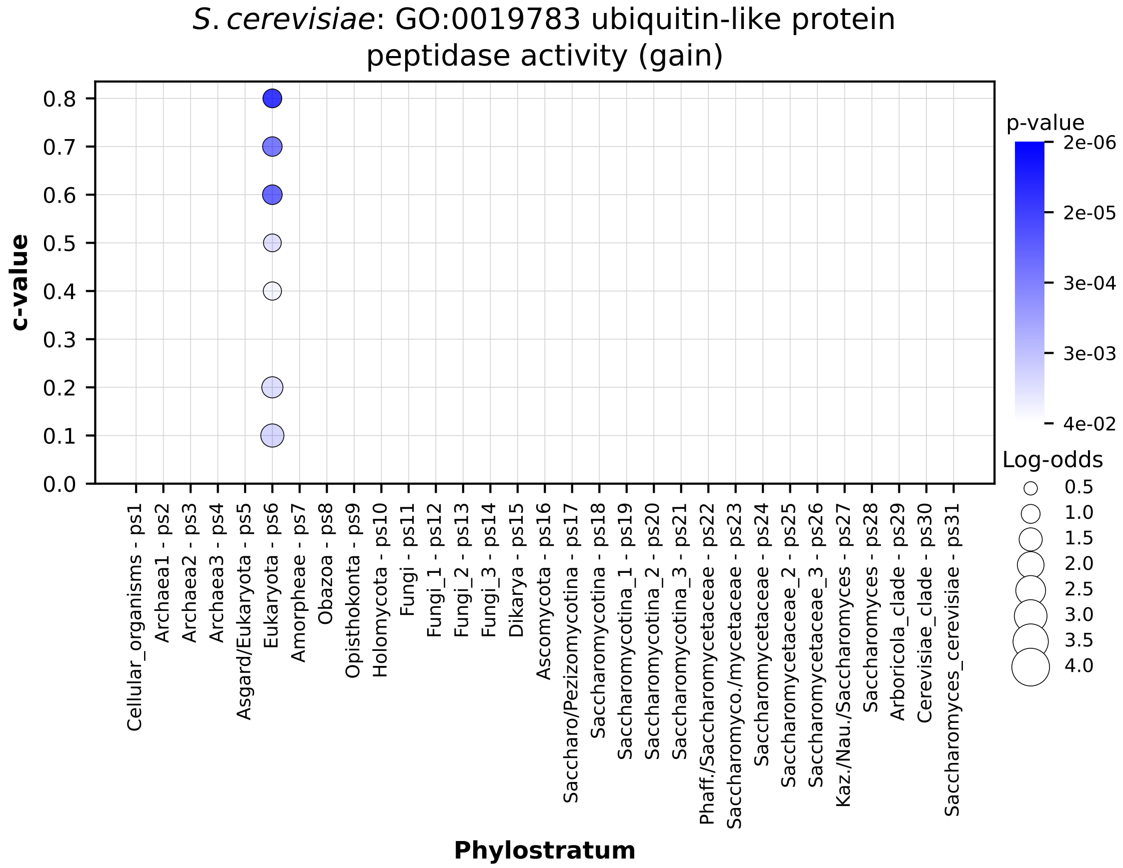

S. cerevisiae: GO:0019783 ubiquitin-like protein peptidase activity (gain)
c-value
0.8
0.7
0.6
0.5
0.4
0.3
0.2
0.1
0.0
p-value
2e-06
2e-05
3e-04
3e-03
4e-02
Log-odds
0.5
1.0
1.5
2.0
2.5
3.0
3.5
4.0
Cellular_organisms - ps1
Archaea1 - ps2
Archaea2 - ps3
Archaea3 - ps4
Asgard/Eukaryota - ps5
Eukaryota - ps6
Amorpheae - ps7
Obazoa - ps8
Opisthokonta - ps9
Holomycota - ps10
Fungi - ps11
Fungi_1 - ps12
Fungi_2 - ps13
Fungi_3 - ps14
Dikarya - ps15
Ascomycota - ps16
Saccharo/Pezizomycotina - ps17
Saccharomycotina - ps18
Saccharomycotina_1 - ps19
Saccharomycotina_2 - ps20
Saccharomycotina_3 - ps21
Phaff./Saccharomycetaceae - ps22
Saccharomyco./mycetaceae - ps23
Saccharomycetaceae - ps24
Saccharomycetaceae_2 - ps25
Saccharomycetaceae_3 - ps26
Kaz./Nau./Saccharomyces - ps27
Saccharomyces - ps28
Arboricola_clade - ps29
Cerevisiae_clade - ps30
Saccharomyces_cerevisiae - ps31
Phylostratum

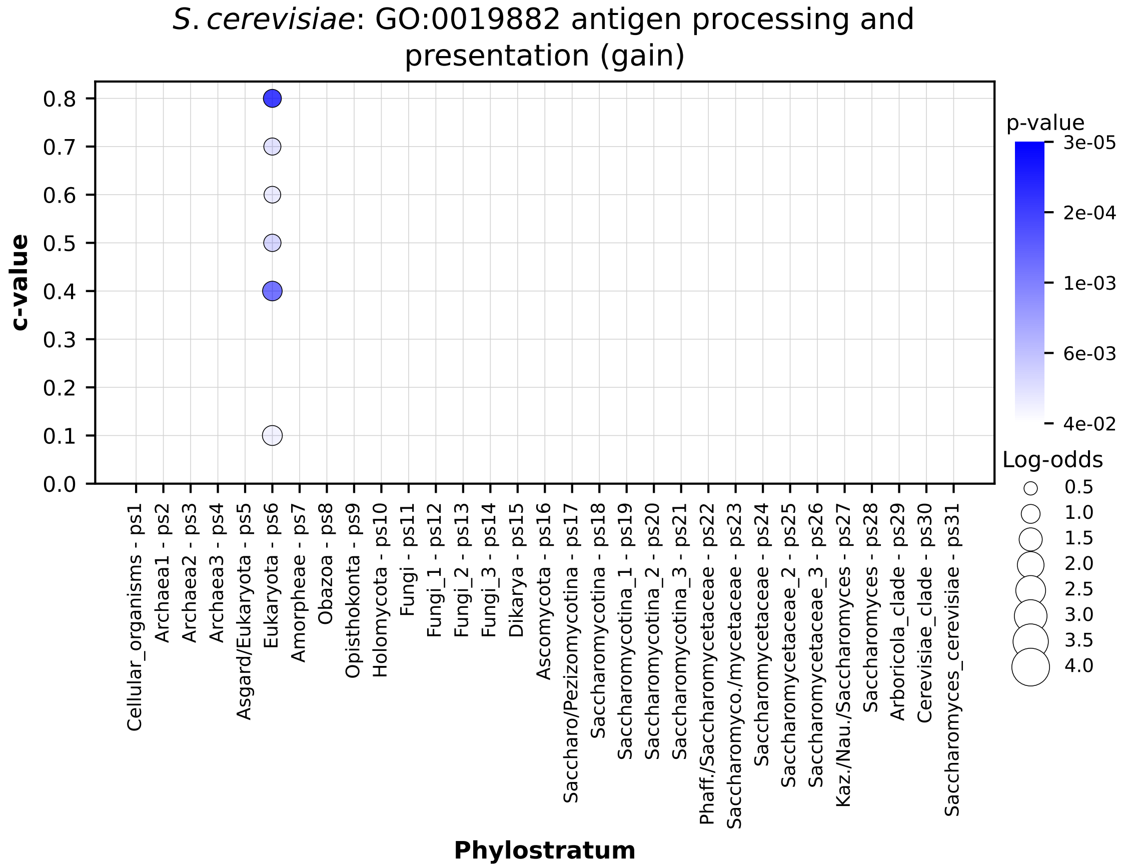

S. cerevisiae: GO:0019882 antigen processing and presentation (gain)
c-value
0.8
0.7
0.6
0.5
0.4
0.3
0.2
0.1
0.0
p-value
3e-05
2e-04
1e-03
6e-03
4e-02
Log-odds
0.5
1.0
1.5
2.0
2.5
3.0
3.5
4.0
Cellular_organisms - ps1
Archaea1 - ps2
Archaea2 - ps3
Archaea3 - ps4
Asgard/Eukaryota - ps5
Eukaryota - ps6
Amorpheae - ps7
Obazoa - ps8
Opisthokonta - ps9
Holomycota - ps10
Fungi - ps11
Fungi_1 - ps12
Fungi_2 - ps13
Fungi_3 - ps14
Dikarya - ps15
Ascomycota - ps16
Saccharo/Pezizomycotina - ps17
Saccharomycotina - ps18
Saccharomycotina_1 - ps19
Saccharomycotina_2 - ps20
Saccharomycotina_3 - ps21
Phaff./Saccharomycetaceae - ps22
Saccharomyco./mycetaceae - ps23
Saccharomycetaceae - ps24
Saccharomycetaceae_2 - ps25
Saccharomycetaceae_3 - ps26
Kaz./Nau./Saccharomyces - ps27
Saccharomyces - ps28
Arboricola_clade - ps29
Cerevisiae_clade - ps30
Saccharomyces_cerevisiae - ps31
Phylostratum

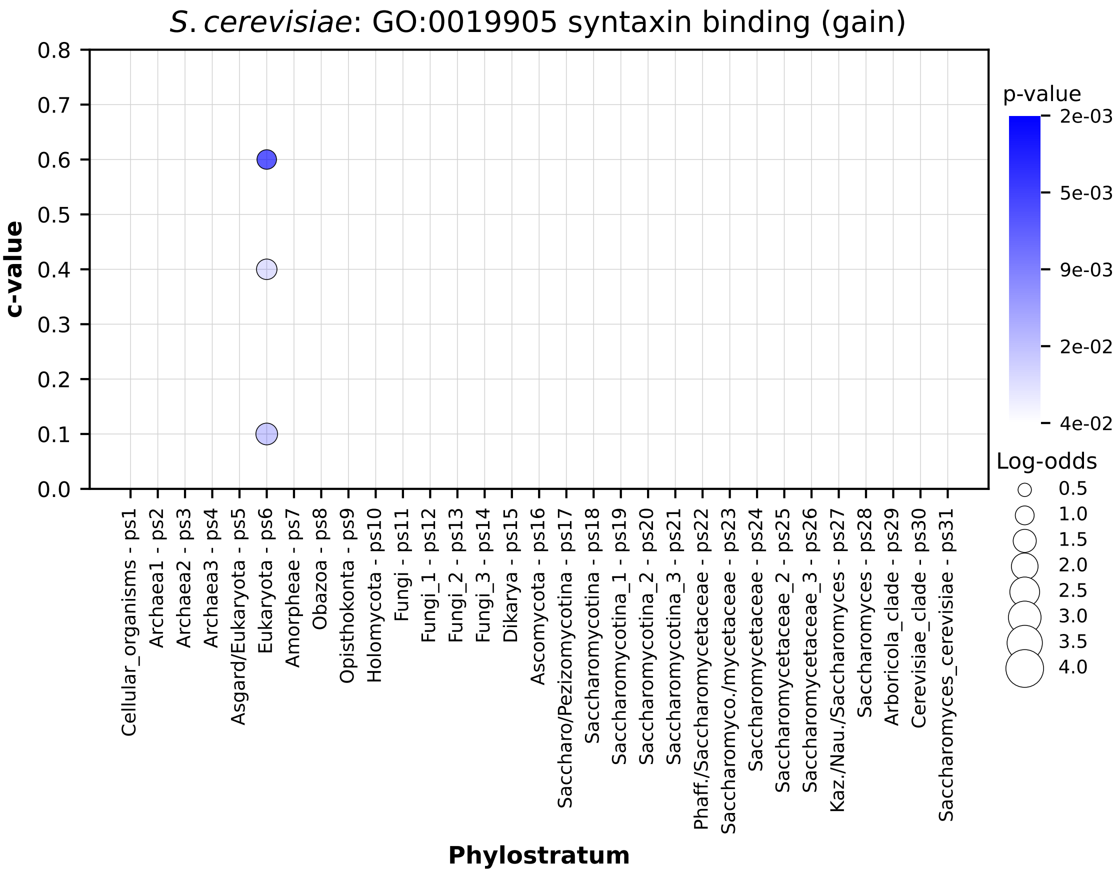

S. cerevisiae: GO:0019905 syntaxin binding (gain)
c-value
0.0
0.1
0.2
0.3
0.4
0.5
0.6
0.7
0.8
Phylostratum
Cellular_organisms - ps1
Archaea1 - ps2
Archaea2 - ps3
Archaea3 - ps4
Asgard/Eukaryota - ps5
Eukaryota - ps6
Amorpheae - ps7
Obazoa - ps8
Opisthokonta - ps9
Holomycota - ps10
Fungi - ps11
Fungi_1 - ps12
Fungi_2 - ps13
Fungi_3 - ps14
Dikarya - ps15
Ascomycota - ps16
Saccharo/Pezizomycotina - ps17
Saccharomycotina - ps18
Saccharomycotina_1 - ps19
Saccharomycotina_2 - ps20
Saccharomycotina_3 - ps21
Phaff./Saccharomycetaceae - ps22
Saccharomyco./mycetaceae - ps23
Saccharomycetaceae - ps24
Saccharomycetaceae_2 - ps25
Saccharomycetaceae_3 - ps26
Kaz./Nau./Saccharomyces - ps27
Saccharomyces - ps28
Arboricola_clade - ps29
Cerevisiae_clade - ps30
Saccharomyces_cerevisiae - ps31
p-value
2e-03
5e-03
9e-03
2e-02
4e-02
Log-odds
0.5
1.0
1.5
2.0
2.5
3.0
3.5
4.0

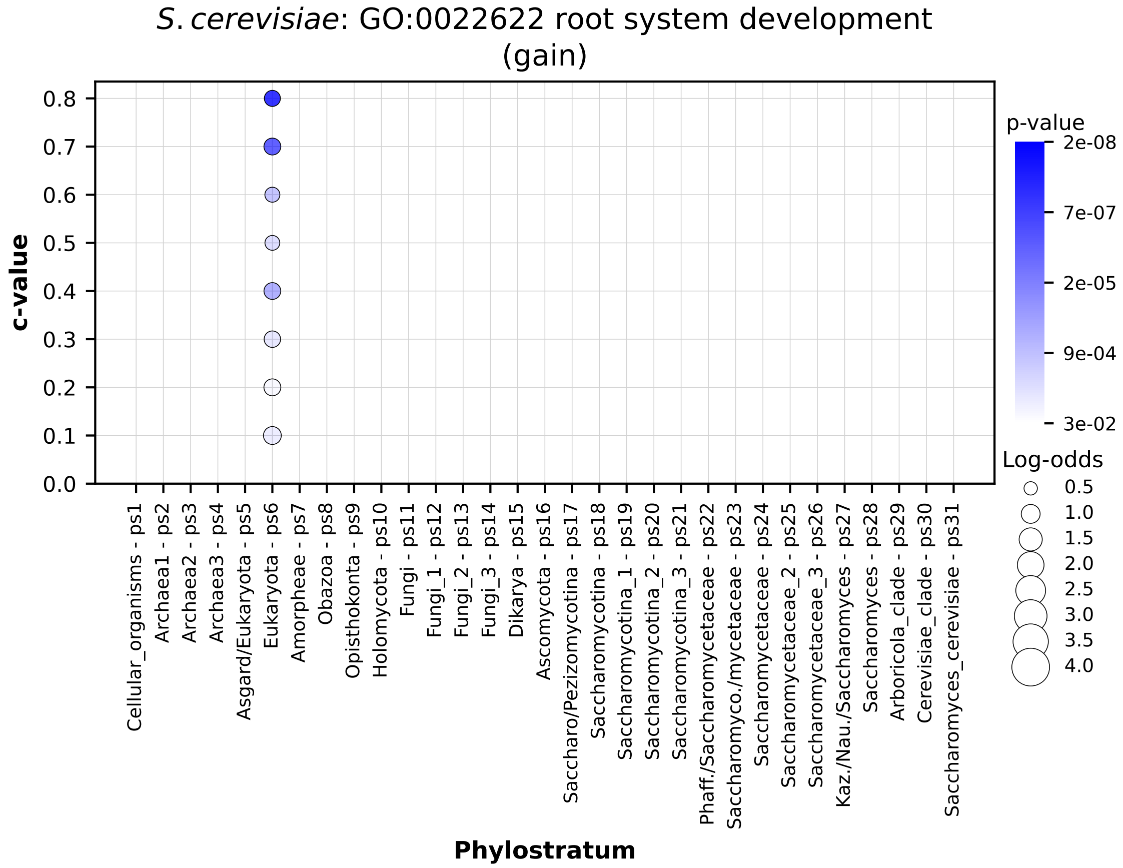

S. cerevisiae: GO:0022622 root system development
(gain)
c-value
0.0
0.1
0.2
0.3
0.4
0.5
0.6
0.7
0.8
p-value
2e-08
7e-07
2e-05
9e-04
3e-02
Log-odds
0.5
1.0
1.5
2.0
2.5
3.0
3.5
4.0
Cellular_organisms - ps1
Archaea1 - ps2
Archaea2 - ps3
Archaea3 - ps4
Asgard/Eukaryota - ps5
Eukaryota - ps6
Amorpheae - ps7
Obazoa - ps8
Opisthokonta - ps9
Holomycota - ps10
Fungi - ps11
Fungi_1 - ps12
Fungi_2 - ps13
Fungi_3 - ps14
Dikarya - ps15
Ascomycota - ps16
Saccharo/Pezizomycotina - ps17
Saccharomycotina - ps18
Saccharomycotina_1 - ps19
Saccharomycotina_2 - ps20
Saccharomycotina_3 - ps21
Phaff./Saccharomycetaceae - ps22
Saccharomyco./mycetaceae - ps23
Saccharomycetaceae - ps24
Saccharomycetaceae_2 - ps25
Saccharomycetaceae_3 - ps26
Kaz./Nau./Saccharomyces - ps27
Saccharomyces - ps28
Arboricola_clade - ps29
Cerevisiae_clade - ps30
Saccharomyces_cerevisiae - ps31
Phylostratum

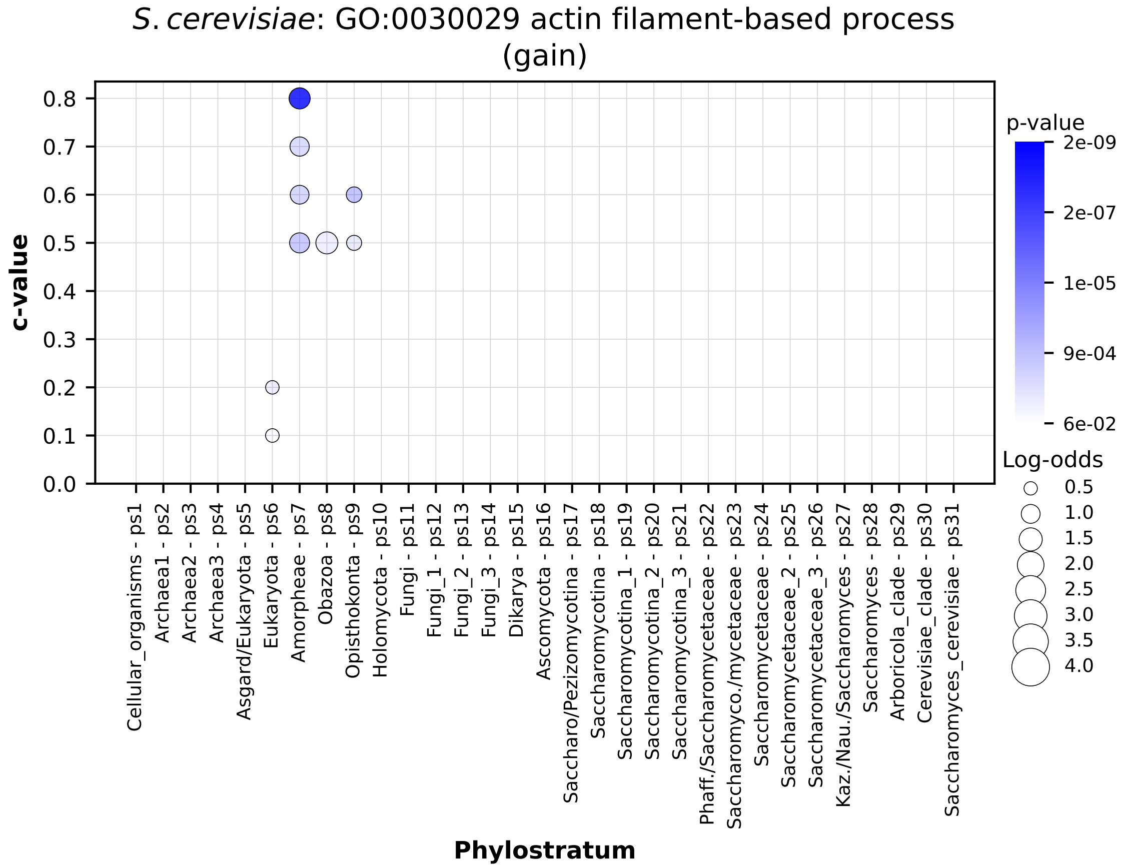
S. cerevisiae: GO:0030029 actin filament-based process (gain)
c-value
0.0
0.1
0.2
0.3
0.4
0.5
0.6
0.7
0.8
Phylostratum
Cellular_organisms - ps1
Archaea1 - ps2
Archaea2 - ps3
Archaea3 - ps4
Asgard/Eukaryota - ps5
Eukaryota - ps6
Amorpheae - ps7
Obazoa - ps8
Opisthokonta - ps9
Holomycota - ps10
Fungi - ps11
Fungi_1 - ps12
Fungi_2 - ps13
Fungi_3 - ps14
Dikarya - ps15
Ascomycota - ps16
Saccharo/Pezizomycotina - ps17
Saccharomycotina - ps18
Saccharomycotina_1 - ps19
Saccharomycotina_2 - ps20
Saccharomycotina_3 - ps21
Phaff./Saccharomycetaceae - ps22
Saccharomyco./mycetaceae - ps23
Saccharomycetaceae - ps24
Saccharomycetaceae_2 - ps25
Saccharomycetaceae_3 - ps26
Kaz./Nau./Saccharomyces - ps27
Saccharomyces - ps28
Arboricola_clade - ps29
Cerevisiae_clade - ps30
Saccharomyces_cerevisiae - ps31
p-value
2e-09
2e-07
1e-05
9e-04
6e-02
Log-odds
0.5
1.0
1.5
2.0
2.5
3.0
3.5
4.0

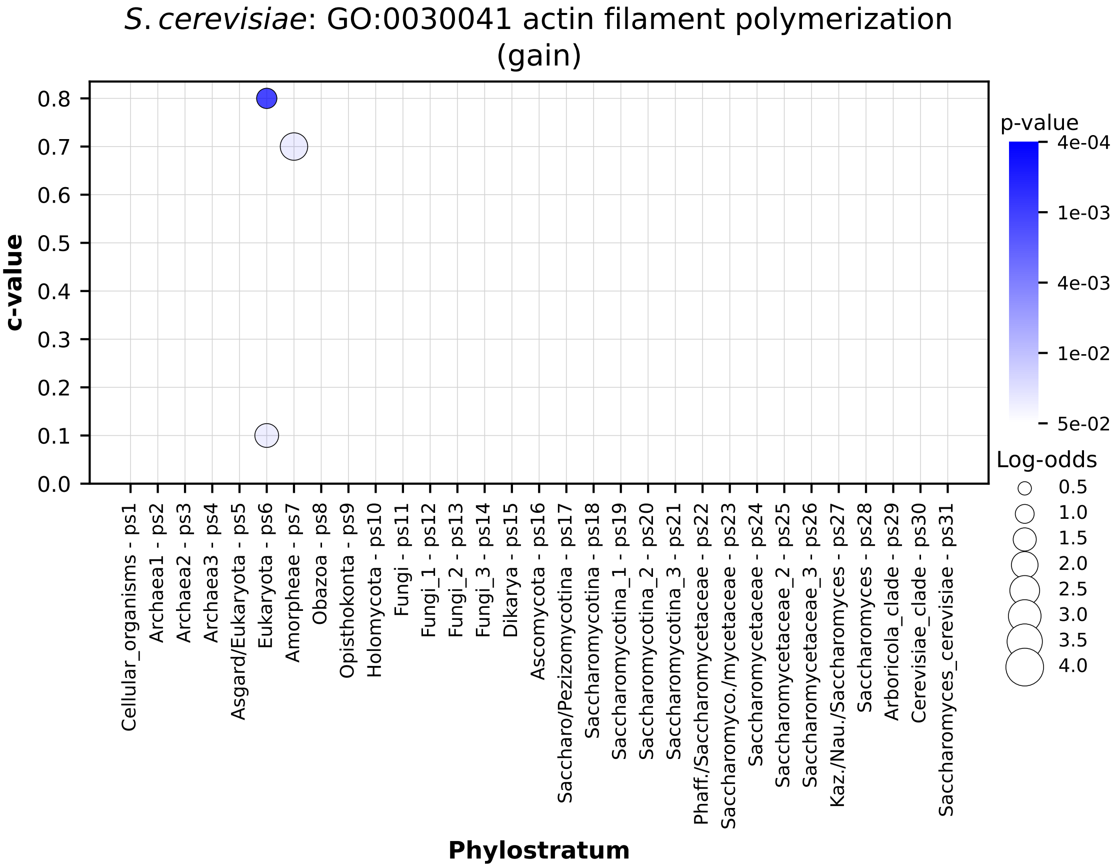

S. cerevisiae: GO:0030041 actin filament polymerization
(gain)
c-value
0.0
0.1
0.2
0.3
0.4
0.5
0.6
0.7
0.8
Phylostratum
Cellular_organisms - ps1
Archaea1 - ps2
Archaea2 - ps3
Archaea3 - ps4
Asgard/Eukaryota - ps5
Eukaryota - ps6
Amorpheae - ps7
Obazoa - ps8
Opisthokonta - ps9
Holomycota - ps10
Fungi - ps11
Fungi_1 - ps12
Fungi_2 - ps13
Fungi_3 - ps14
Dikarya - ps15
Ascomycota - ps16
Saccharo/Pezizomycotina - ps17
Saccharomycotina - ps18
Saccharomycotina_1 - ps19
Saccharomycotina_2 - ps20
Saccharomycotina_3 - ps21
Phaff./Saccharomycetaceae - ps22
Saccharomyco./mycetaceae - ps23
Saccharomycetaceae - ps24
Saccharomycetaceae_2 - ps25
Saccharomycetaceae_3 - ps26
Kaz./Nau./Saccharomyces - ps27
Saccharomyces - ps28
Arboricola_clade - ps29
Cerevisiae_clade - ps30
Saccharomyces_cerevisiae - ps31
p-value
4e-04
1e-03
4e-03
1e-02
5e-02
Log-odds
0.5
1.0
1.5
2.0
2.5
3.0
3.5
4.0

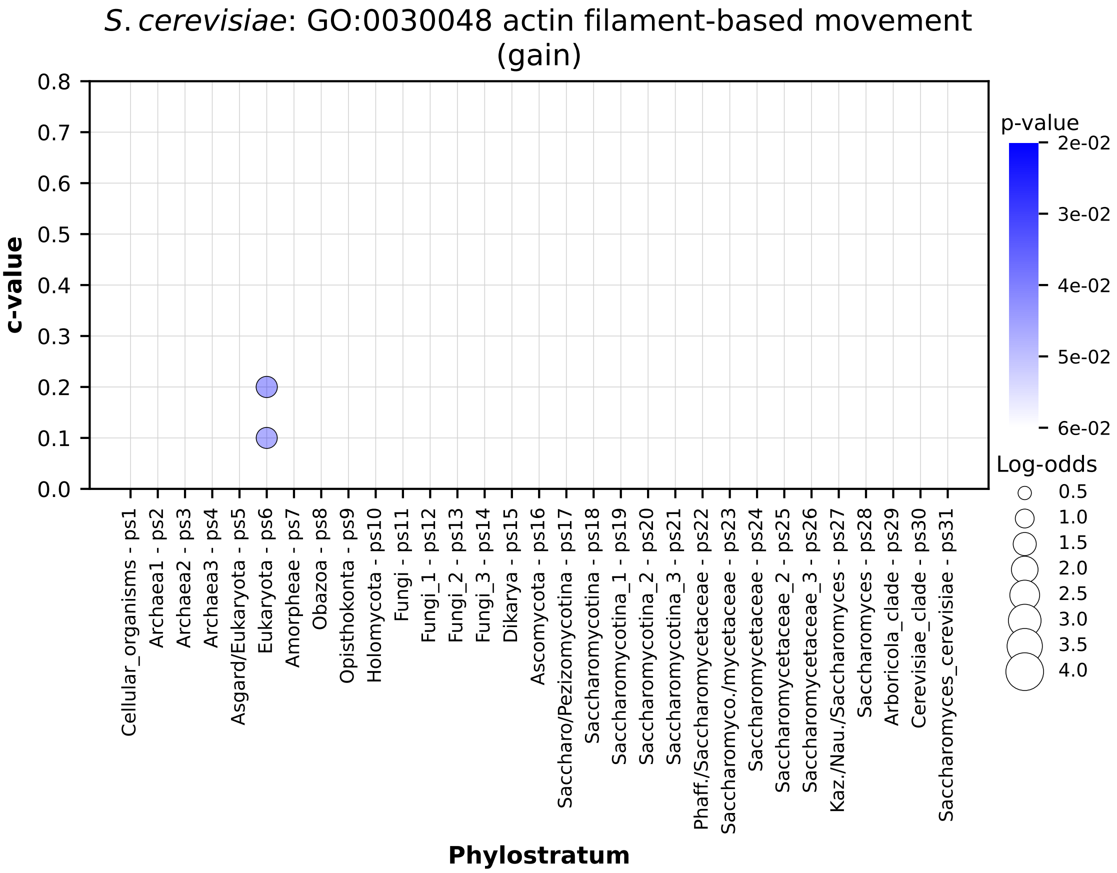

S. cerevisiae: GO:0030048 actin filament-based movement (gain)
c-value
0.8
0.7
0.6
0.5
0.4
0.3
0.2
0.1
0.0
p-value
2e-02
3e-02
4e-02
5e-02
6e-02
Log-odds
0.5
1.0
1.5
2.0
2.5
3.0
3.5
4.0
Cellular_organisms - ps1
Archaea1 - ps2
Archaea2 - ps3
Archaea3 - ps4
Asgard/Eukaryota - ps5
Eukaryota - ps6
Amorpheae - ps7
Obazoa - ps8
Opisthokonta - ps9
Holomycota - ps10
Fungi - ps11
Fungi_1 - ps12
Fungi_2 - ps13
Fungi_3 - ps14
Dikarya - ps15
Ascomycota - ps16
Saccharo/Pezizomycotina - ps17
Saccharomycotina - ps18
Saccharomycotina_1 - ps19
Saccharomycotina_2 - ps20
Saccharomycotina_3 - ps21
Phaff./Saccharomycetaceae - ps22
Saccharomyco./mycetaceae - ps23
Saccharomycetaceae - ps24
Saccharomycetaceae_2 - ps25
Saccharomycetaceae_3 - ps26
Kaz./Nau./Saccharomyces - ps27
Saccharomyces - ps28
Arboricola_clade - ps29
Cerevisiae_clade - ps30
Saccharomyces_cerevisiae - ps31
Phylostratum

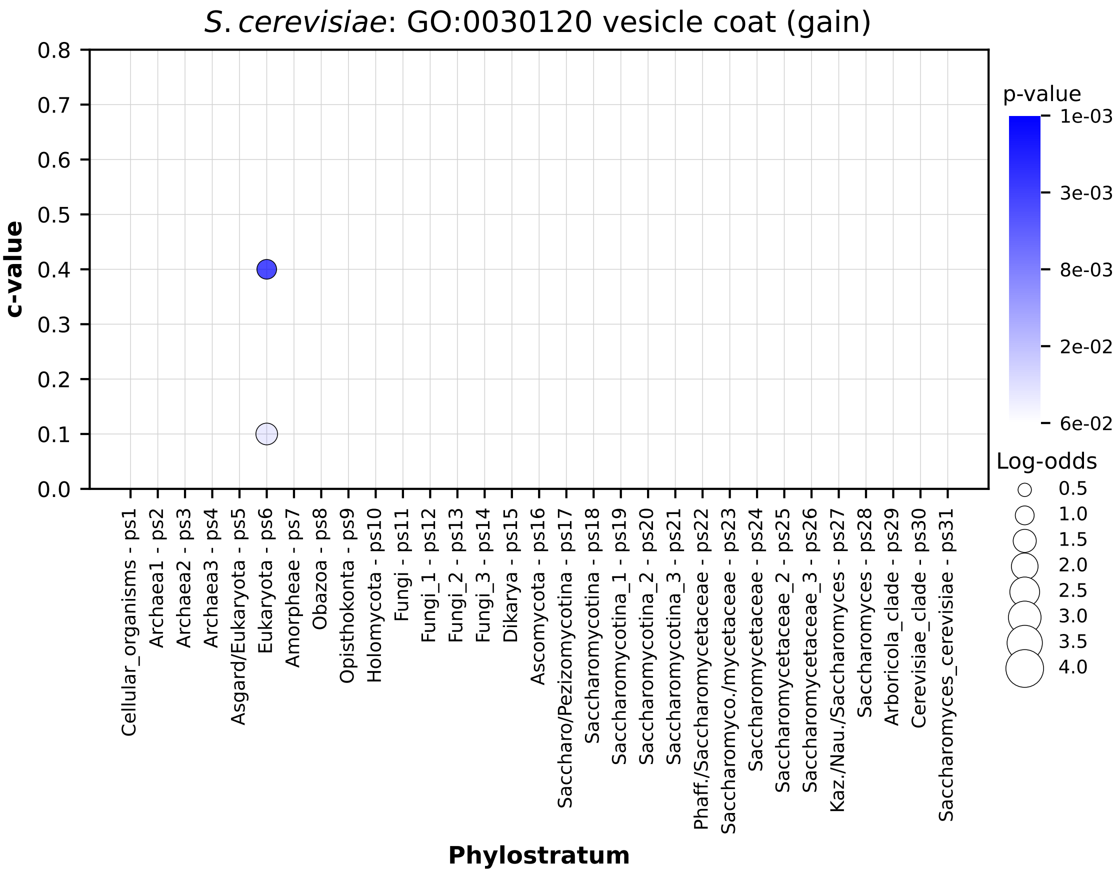

S. cerevisiae: GO:0030120 vesicle coat (gain)
c-value
0.0
0.1
0.2
0.3
0.4
0.5
0.6
0.7
0.8
Phylostratum
Cellular_organisms - ps1
Archaea1 - ps2
Archaea2 - ps3
Archaea3 - ps4
Asgard/Eukaryota - ps5
Eukaryota - ps6
Amorpheae - ps7
Obazoa - ps8
Opisthokonta - ps9
Holomycota - ps10
Fungi - ps11
Fungi_1 - ps12
Fungi_2 - ps13
Fungi_3 - ps14
Dikarya - ps15
Ascomycota - ps16
Saccharo/Pezizomycotina - ps17
Saccharomycotina - ps18
Saccharomycotina_1 - ps19
Saccharomycotina_2 - ps20
Saccharomycotina_3 - ps21
Phaff./Saccharomycetaceae - ps22
Saccharomyco./mycetaceae - ps23
Saccharomycetaceae - ps24
Saccharomycetaceae_2 - ps25
Saccharomycetaceae_3 - ps26
Kaz./Nau./Saccharomyces - ps27
Saccharomyces - ps28
Arboricola_clade - ps29
Cerevisiae_clade - ps30
Saccharomyces_cerevisiae - ps31
p-value
1e-03
3e-03
8e-03
2e-02
6e-02
Log-odds
0.5
1.0
1.5
2.0
2.5
3.0
3.5
4.0

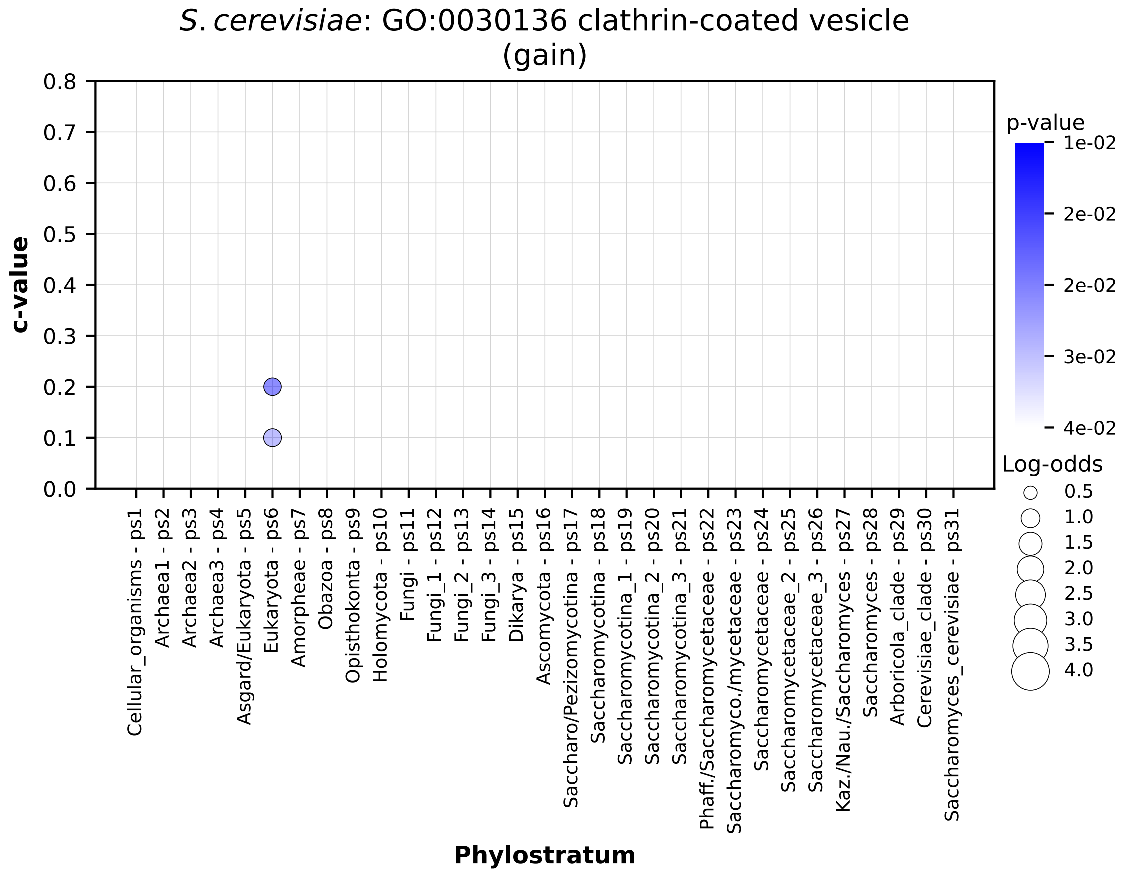

S. cerevisiae: GO:0030136 clathrin-coated vesicle
(gain)
c-value
0.0
0.1
0.2
0.3
0.4
0.5
0.6
0.7
0.8
p-value
1e-02
2e-02
2e-02
3e-02
4e-02
Log-odds
0.5
1.0
1.5
2.0
2.5
3.0
3.5
4.0
Cellular_organisms - ps1
Archaea1 - ps2
Archaea2 - ps3
Archaea3 - ps4
Asgard/Eukaryota - ps5
Eukaryota - ps6
Amorpheae - ps7
Obazoa - ps8
Opisthokonta - ps9
Holomycota - ps10
Fungi - ps11
Fungi_1 - ps12
Fungi_2 - ps13
Fungi_3 - ps14
Dikarya - ps15
Ascomycota - ps16
Saccharo/Pezizomycotina - ps17
Saccharomycotina - ps18
Saccharomycotina_1 - ps19
Saccharomycotina_2 - ps20
Saccharomycotina_3 - ps21
Phaff./Saccharomycetaceae - ps22
Saccharomyco./mycetaceae - ps23
Saccharomycetaceae - ps24
Saccharomycetaceae_2 - ps25
Saccharomycetaceae_3 - ps26
Kaz./Nau./Saccharomyces - ps27
Saccharomyces - ps28
Arboricola_clade - ps29
Cerevisiae_clade - ps30
Saccharomyces_cerevisiae - ps31
Phylostratum

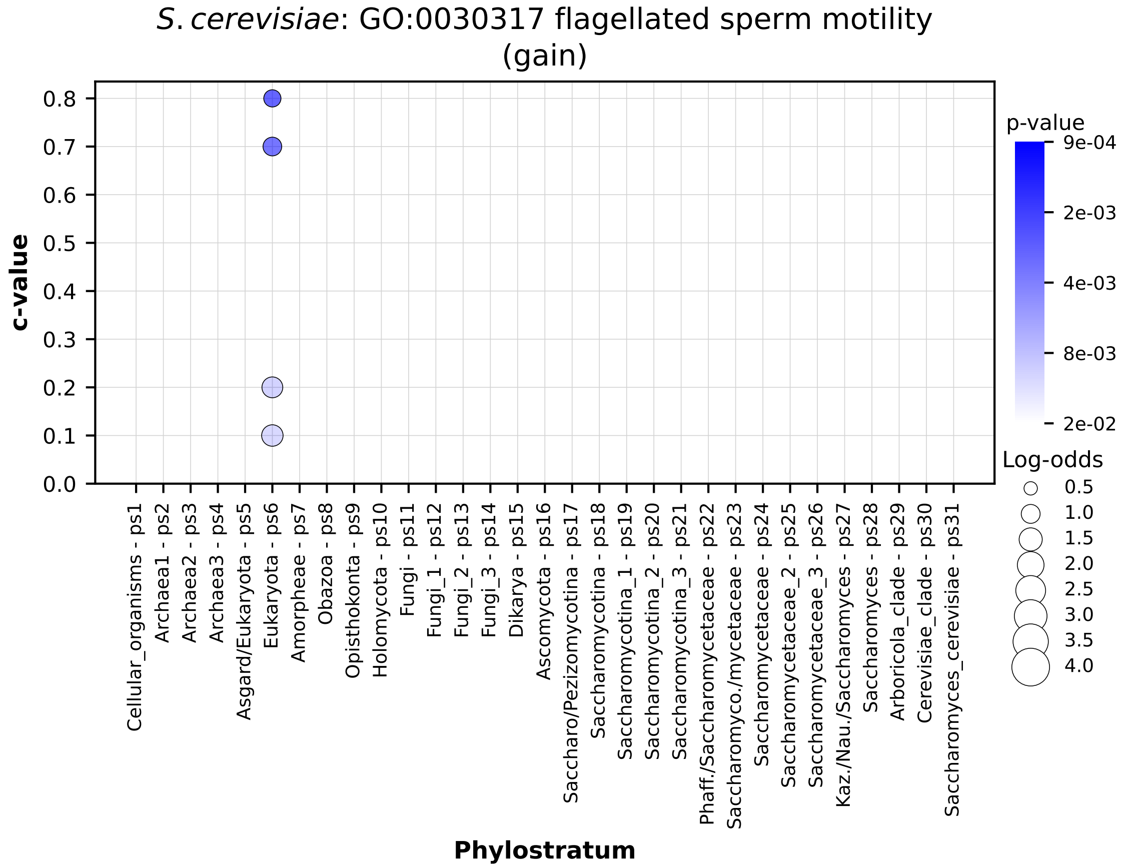

S. cerevisiae: GO:0030317 flagellated sperm motility
(gain)
c-value
0.8
0.7
0.6
0.5
0.4
0.3
0.2
0.1
0.0
p-value
9e-04
2e-03
4e-03
8e-03
2e-02
Log-odds
0.5
1.0
1.5
2.0
2.5
3.0
3.5
4.0
Cellular_organisms - ps1
Archaea1 - ps2
Archaea2 - ps3
Archaea3 - ps4
Asgard/Eukaryota - ps5
Eukaryota - ps6
Amorpheae - ps7
Obazoa - ps8
Opisthokonta - ps9
Holomycota - ps10
Fungi - ps11
Fungi_1 - ps12
Fungi_2 - ps13
Fungi_3 - ps14
Dikarya - ps15
Ascomycota - ps16
Saccharo/Pezizomycotina - ps17
Saccharomycotina - ps18
Saccharomycotina_1 - ps19
Saccharomycotina_2 - ps20
Saccharomycotina_3 - ps21
Phaff./Saccharomycetaceae - ps22
Saccharomyco./mycetaceae - ps23
Saccharomycetaceae - ps24
Saccharomycetaceae_2 - ps25
Saccharomycetaceae_3 - ps26
Kaz./Nau./Saccharomyces - ps27
Saccharomyces - ps28
Arboricola_clade - ps29
Cerevisiae_clade - ps30
Saccharomyces_cerevisiae - ps31
Phylostratum

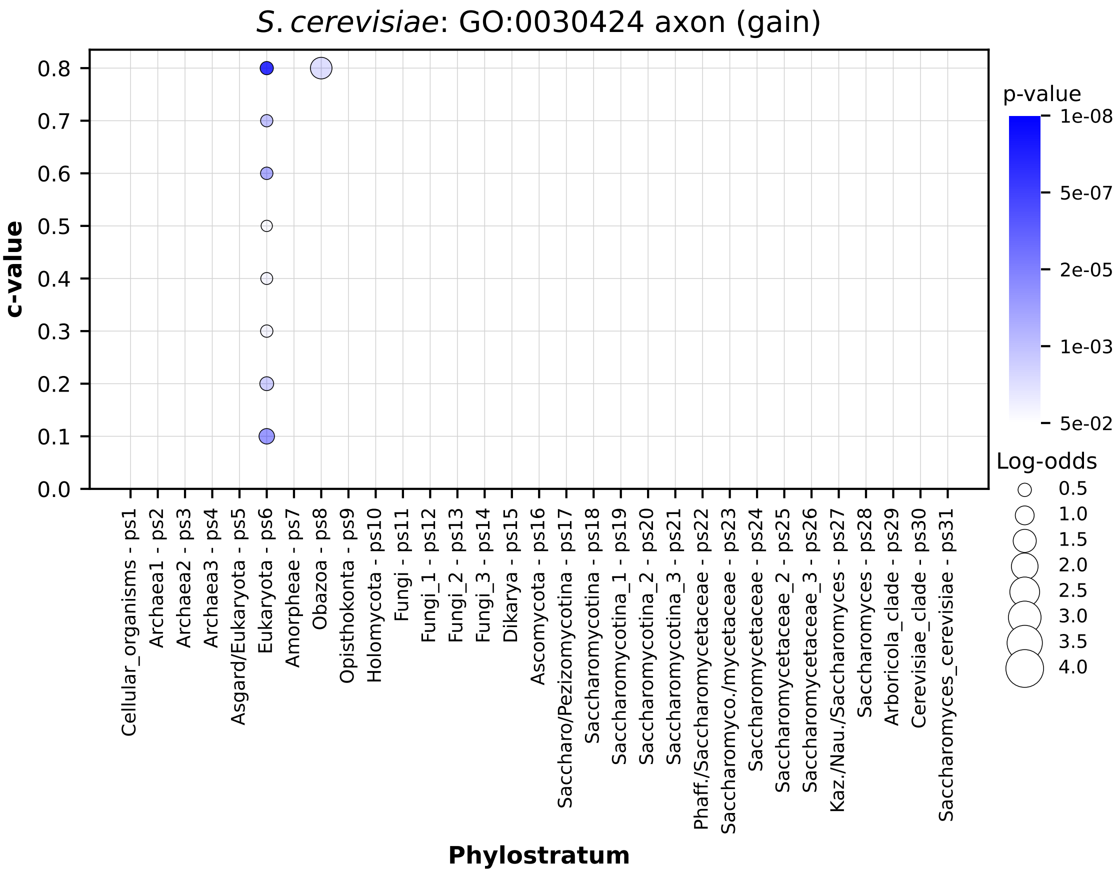

S. cerevisiae: GO:0030424 axon (gain)
c-value
0.0
0.1
0.2
0.3
0.4
0.5
0.6
0.7
0.8
Phylostratum
Cellular_organisms - ps1
Archaea1 - ps2
Archaea2 - ps3
Archaea3 - ps4
Asgard/Eukaryota - ps5
Eukaryota - ps6
Amorpheae - ps7
Obazoa - ps8
Opisthokonta - ps9
Holomycota - ps10
Fungi - ps11
Fungi_1 - ps12
Fungi_2 - ps13
Fungi_3 - ps14
Dikarya - ps15
Ascomycota - ps16
Saccharo/Pezizomycotina - ps17
Saccharomycotina - ps18
Saccharomycotina_1 - ps19
Saccharomycotina_2 - ps20
Saccharomycotina_3 - ps21
Phaff./Saccharomycetaceae - ps22
Saccharomyco./mycetaceae - ps23
Saccharomycetaceae - ps24
Saccharomycetaceae_2 - ps25
Saccharomycetaceae_3 - ps26
Kaz./Nau./Saccharomyces - ps27
Saccharomyces - ps28
Arboricola_clade - ps29
Cerevisiae_clade - ps30
Saccharomyces_cerevisiae - ps31
p-value
1e-08
5e-07
2e-05
1e-03
5e-02
Log-odds
0.5
1.0
1.5
2.0
2.5
3.0
3.5
4.0

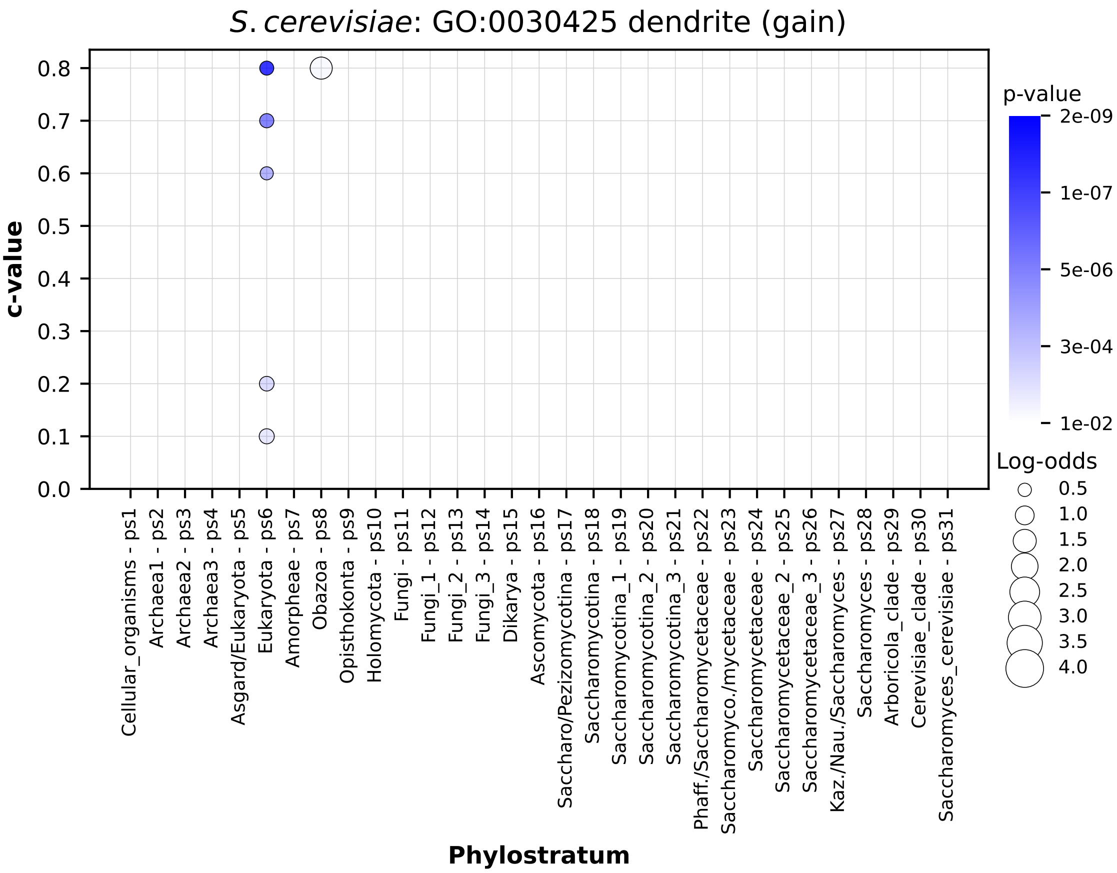

S. cerevisiae: GO:0030425 dendrite (gain)
c-value
0.0
0.1
0.2
0.3
0.4
0.5
0.6
0.7
0.8
p-value
2e-09
1e-07
5e-06
3e-04
1e-02
Log-odds
0.5
1.0
1.5
2.0
2.5
3.0
3.5
4.0
Cellular_organisms - ps1
Archaea1 - ps2
Archaea2 - ps3
Archaea3 - ps4
Asgard/Eukaryota - ps5
Eukaryota - ps6
Amorpheae - ps7
Obazoa - ps8
Opisthokonta - ps9
Holomycota - ps10
Fungi - ps11
Fungi_1 - ps12
Fungi_2 - ps13
Fungi_3 - ps14
Dikarya - ps15
Ascomycota - ps16
Saccharo/Pezizomycotina - ps17
Saccharomycotina - ps18
Saccharomycotina_1 - ps19
Saccharomycotina_2 - ps20
Saccharomycotina_3 - ps21
Phaff./Saccharomycetaceae - ps22
Saccharomyco./mycetaceae - ps23
Saccharomycetaceae - ps24
Saccharomycetaceae_2 - ps25
Saccharomycetaceae_3 - ps26
Kaz./Nau./Saccharomyces - ps27
Saccharomyces - ps28
Arboricola_clade - ps29
Cerevisiae_clade - ps30
Saccharomyces_cerevisiae - ps31
Phylostratum

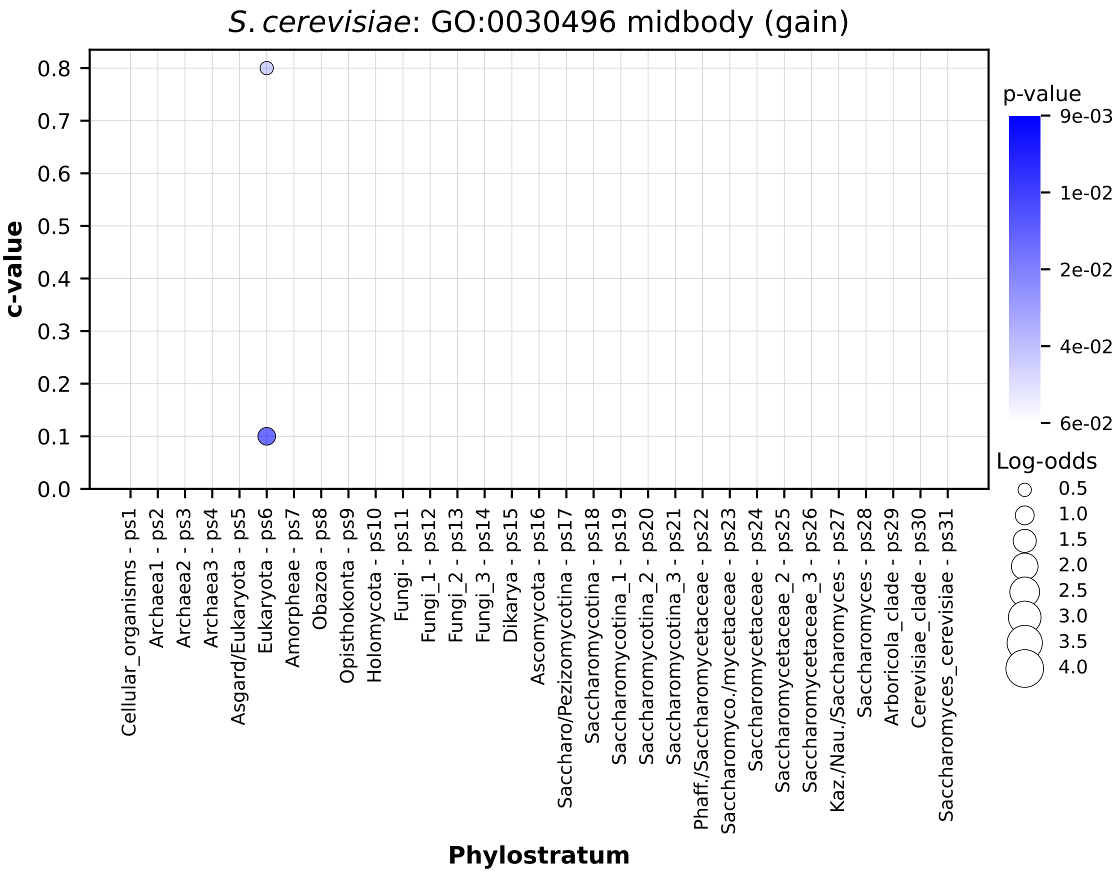
S. cerevisiae: GO:0030496 midbody (gain)
c-value
0.0
0.1
0.2
0.3
0.4
0.5
0.6
0.7
0.8
Phylostratum
Cellular_organisms - ps1
Archaea1 - ps2
Archaea2 - ps3
Archaea3 - ps4
Asgard/Eukaryota - ps5
Eukaryota - ps6
Amorpheae - ps7
Obazoa - ps8
Opisthokonta - ps9
Holomycota - ps10
Fungi - ps11
Fungi_1 - ps12
Fungi_2 - ps13
Fungi_3 - ps14
Dikarya - ps15
Ascomycota - ps16
Saccharo/Pezizomycotina - ps17
Saccharomycotina - ps18
Saccharomycotina_1 - ps19
Saccharomycotina_2 - ps20
Saccharomycotina_3 - ps21
Phaff./Saccharomycetaceae - ps22
Saccharomyco./mycetaceae - ps23
Saccharomycetaceae - ps24
Saccharomycetaceae_2 - ps25
Saccharomycetaceae_3 - ps26
Kaz./Nau./Saccharomyces - ps27
Saccharomyces - ps28
Arboricola_clade - ps29
Cerevisiae_clade - ps30
Saccharomyces_cerevisiae - ps31
p-value
9e-03
1e-02
2e-02
4e-02
6e-02
Log-odds
0.5
1.0
1.5
2.0
2.5
3.0
3.5
4.0

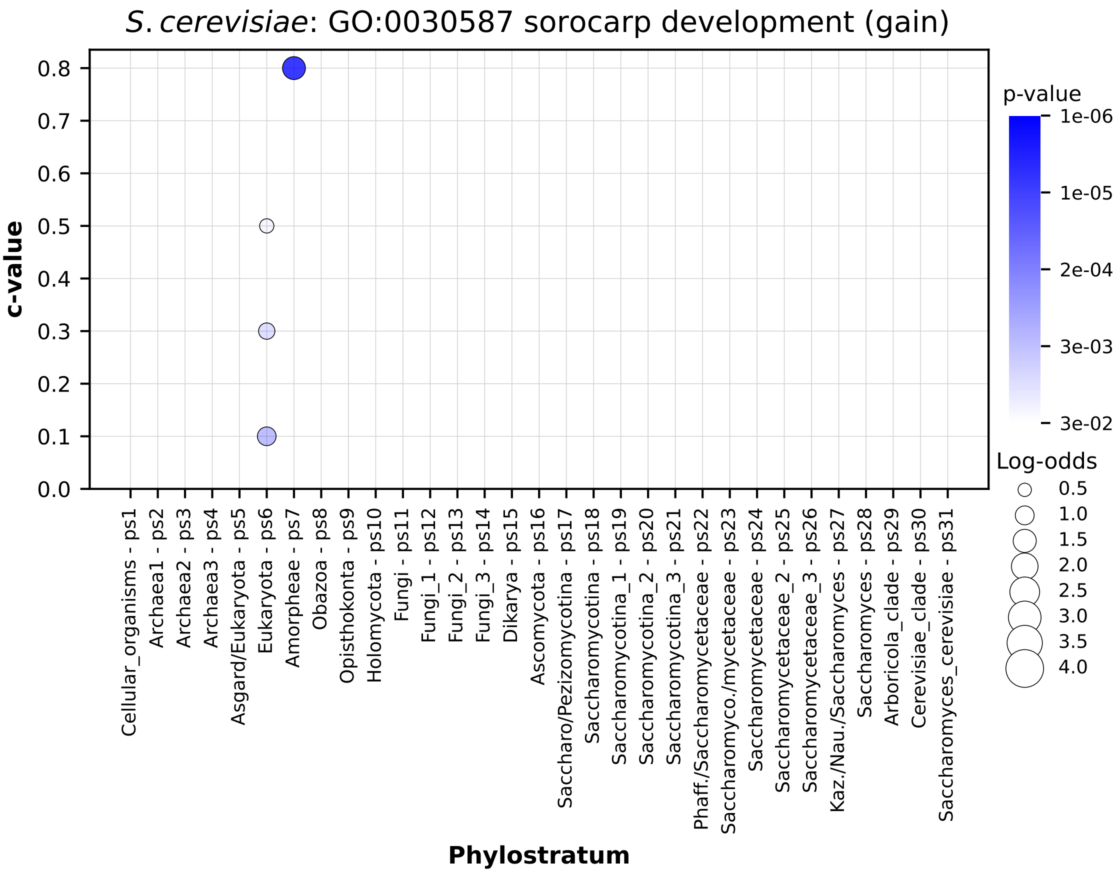

S. cerevisiae: GO:0030587 sorocarp development (gain)
c-value
0.0
0.1
0.2
0.3
0.4
0.5
0.6
0.7
0.8
p-value
1e-06
1e-05
2e-04
3e-03
3e-02
Log-odds
0.5
1.0
1.5
2.0
2.5
3.0
3.5
4.0
Cellular_organisms - ps1
Archaea1 - ps2
Archaea2 - ps3
Archaea3 - ps4
Asgard/Eukaryota - ps5
Eukaryota - ps6
Amorpheae - ps7
Obazoa - ps8
Opisthokonta - ps9
Holomycota - ps10
Fungi - ps11
Fungi_1 - ps12
Fungi_2 - ps13
Fungi_3 - ps14
Dikarya - ps15
Ascomycota - ps16
Saccharo/Pezizomycotina - ps17
Saccharomycotina - ps18
Saccharomycotina_1 - ps19
Saccharomycotina_2 - ps20
Saccharomycotina_3 - ps21
Phaff./Saccharomycetaceae - ps22
Saccharomyco./mycetaceae - ps23
Saccharomycetaceae - ps24
Saccharomycetaceae_2 - ps25
Saccharomycetaceae_3 - ps26
Kaz./Nau./Saccharomyces - ps27
Saccharomyces - ps28
Arboricola_clade - ps29
Cerevisiae_clade - ps30
Saccharomyces_cerevisiae - ps31
Phylostratum

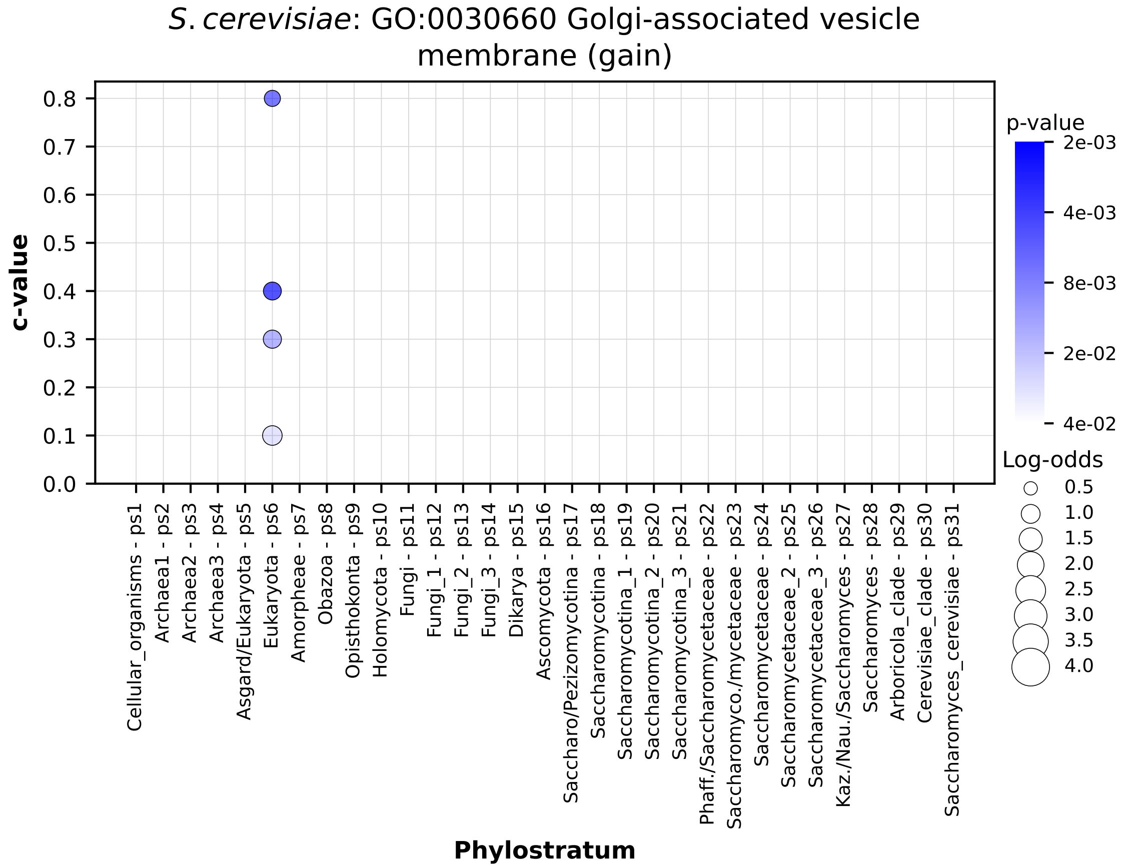

S. cerevisiae: GO:0030660 Golgi-associated vesicle membrane (gain)
c-value
0.0
0.1
0.2
0.3
0.4
0.5
0.6
0.7
0.8
Phylostratum
Cellular_organisms - ps1
Archaea1 - ps2
Archaea2 - ps3
Archaea3 - ps4
Asgard/Eukaryota - ps5
Eukaryota - ps6
Amorpheae - ps7
Obazoa - ps8
Opisthokonta - ps9
Holomycota - ps10
Fungi - ps11
Fungi_1 - ps12
Fungi_2 - ps13
Fungi_3 - ps14
Dikarya - ps15
Ascomycota - ps16
Saccharo/Pezizomycotina - ps17
Saccharomycotina - ps18
Saccharomycotina_1 - ps19
Saccharomycotina_2 - ps20
Saccharomycotina_3 - ps21
Phaff./Saccharomycetaceae - ps22
Saccharomyco./mycetaceae - ps23
Saccharomycetaceae - ps24
Saccharomycetaceae_2 - ps25
Saccharomycetaceae_3 - ps26
Kaz./Nau./Saccharomyces - ps27
Saccharomyces - ps28
Arboricola_clade - ps29
Cerevisiae_clade - ps30
Saccharomyces_cerevisiae - ps31
p-value
2e-03
4e-03
8e-03
2e-02
4e-02
Log-odds
0.5
1.0
1.5
2.0
2.5
3.0
3.5
4.0

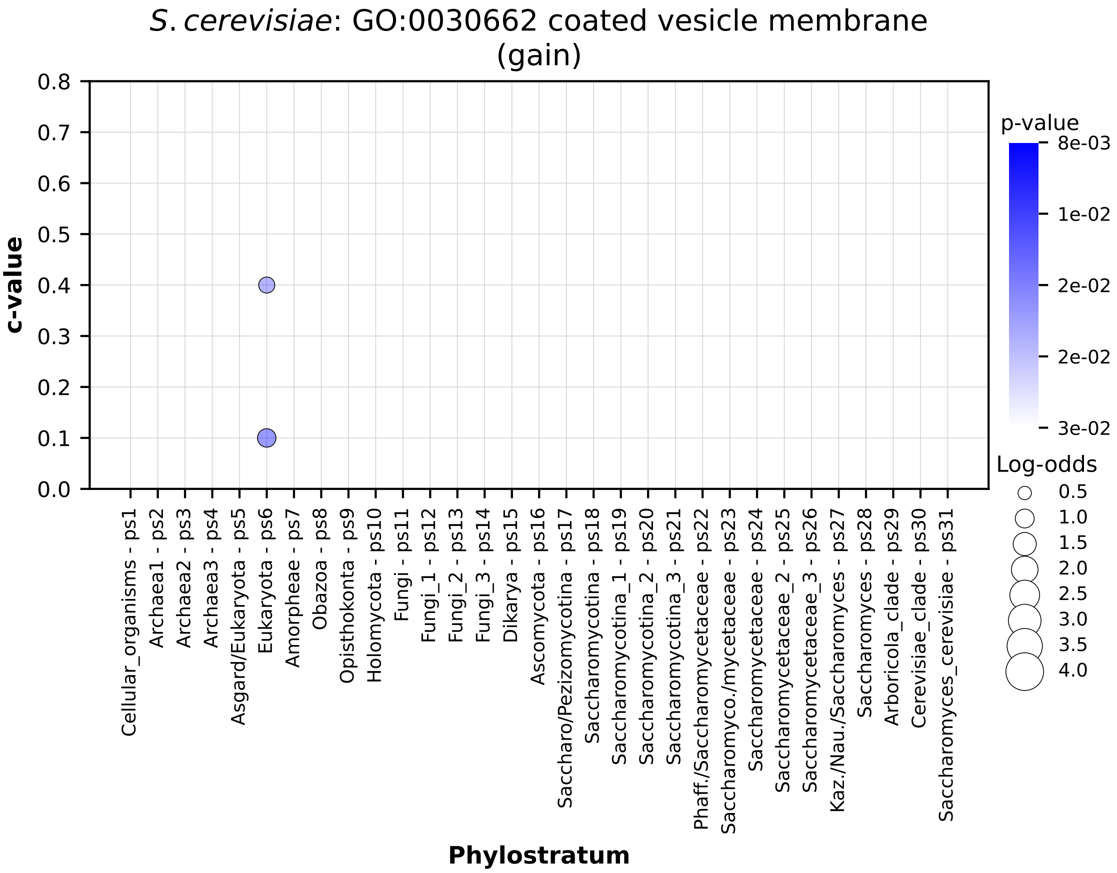

S. cerevisiae: GO:0030662 coated vesicle membrane
(gain)
c-value
0.0
0.1
0.2
0.3
0.4
0.5
0.6
0.7
0.8
p-value
8e-03
1e-02
2e-02
2e-02
3e-02
Log-odds
0.5
1.0
1.5
2.0
2.5
3.0
3.5
4.0
Cellular_organisms - ps1
Archaea1 - ps2
Archaea2 - ps3
Archaea3 - ps4
Asgard/Eukaryota - ps5
Eukaryota - ps6
Amorpheae - ps7
Obazoa - ps8
Opisthokonta - ps9
Holomycota - ps10
Fungi - ps11
Fungi_1 - ps12
Fungi_2 - ps13
Fungi_3 - ps14
Dikarya - ps15
Ascomycota - ps16
Saccharo/Pezizomycotina - ps17
Saccharomycotina - ps18
Saccharomycotina_1 - ps19
Saccharomycotina_2 - ps20
Saccharomycotina_3 - ps21
Phaff./Saccharomycetaceae - ps22
Saccharomyco./mycetaceae - ps23
Saccharomycetaceae - ps24
Saccharomycetaceae_2 - ps25
Saccharomycetaceae_3 - ps26
Kaz./Nau./Saccharomyces - ps27
Saccharomyces - ps28
Arboricola_clade - ps29
Cerevisiae_clade - ps30
Saccharomyces_cerevisiae - ps31
Phylostratum

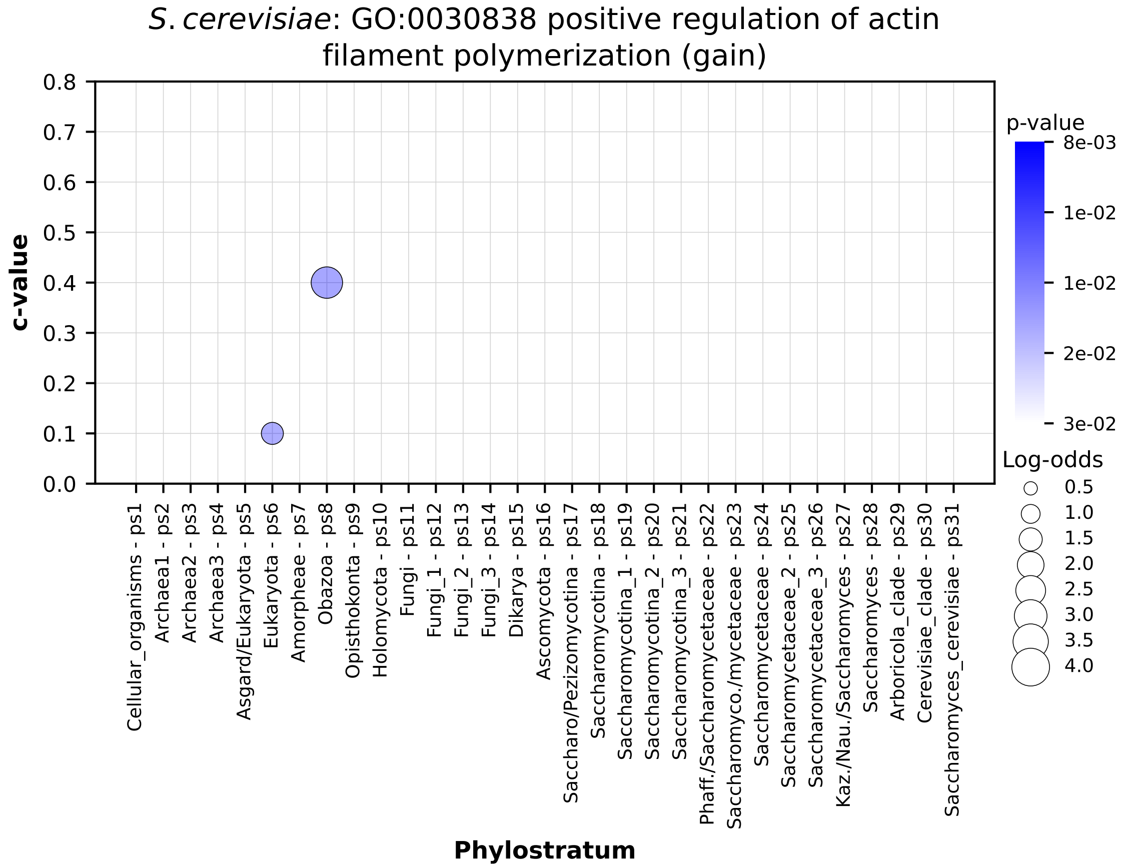

S. cerevisiae: GO:0030838 positive regulation of actin filament polymerization (gain)
c-value
0.8
0.7
0.6
0.5
0.4
0.3
0.2
0.1
0.0
Phylostratum
Cellular_organisms - ps1
Archaea1 - ps2
Archaea2 - ps3
Archaea3 - ps4
Asgard/Eukaryota - ps5
Eukaryota - ps6
Amorpheae - ps7
Obazoa - ps8
Opisthokonta - ps9
Holomycota - ps10
Fungi - ps11
Fungi_1 - ps12
Fungi_2 - ps13
Fungi_3 - ps14
Dikarya - ps15
Ascomycota - ps16
Saccharo/Pezizomycotina - ps17
Saccharomycotina - ps18
Saccharomycotina_1 - ps19
Saccharomycotina_2 - ps20
Saccharomycotina_3 - ps21
Phaff./Saccharomycetaceae - ps22
Saccharomyco./mycetaceae - ps23
Saccharomycetaceae - ps24
Saccharomycetaceae_2 - ps25
Saccharomycetaceae_3 - ps26
Kaz./Nau./Saccharomyces - ps27
Saccharomyces - ps28
Arboricola_clade - ps29
Cerevisiae_clade - ps30
Saccharomyces_cerevisiae - ps31
p-value
8e-03
1e-02
1e-02
2e-02
3e-02
Log-odds
0.5
1.0
1.5
2.0
2.5
3.0
3.5
4.0

S. cerevisiae: GO:0030855 epithelial cell differentiation (gain)

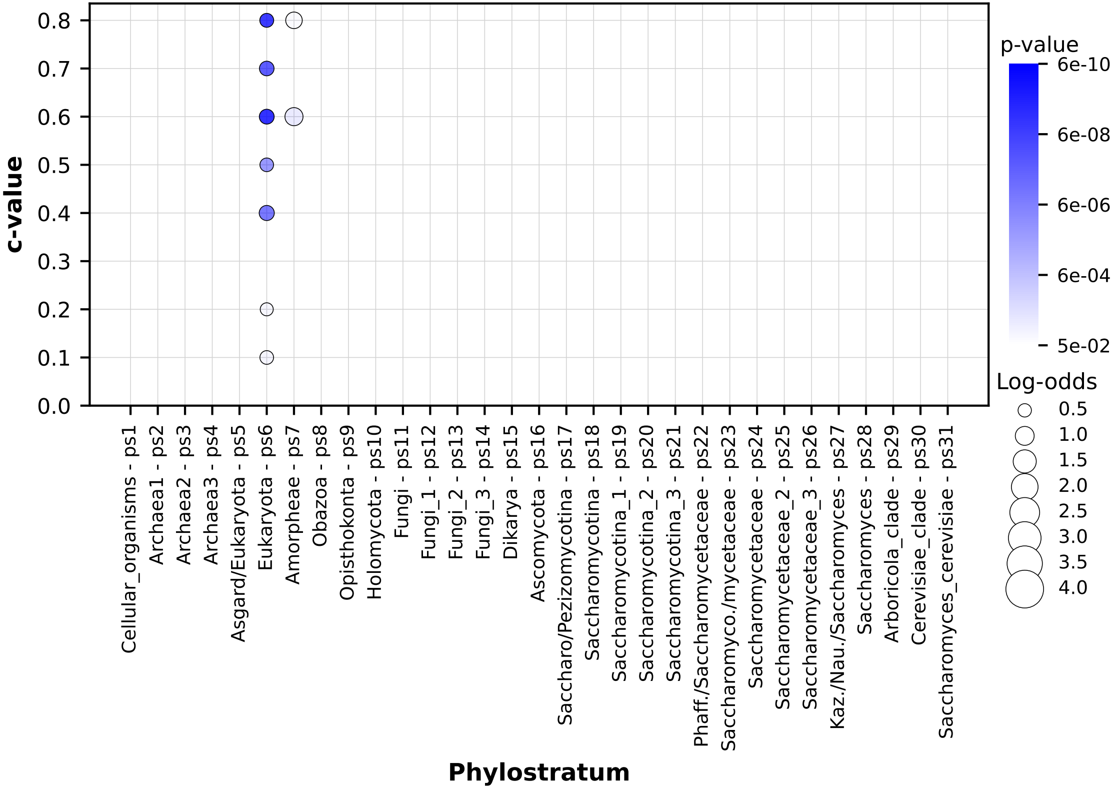

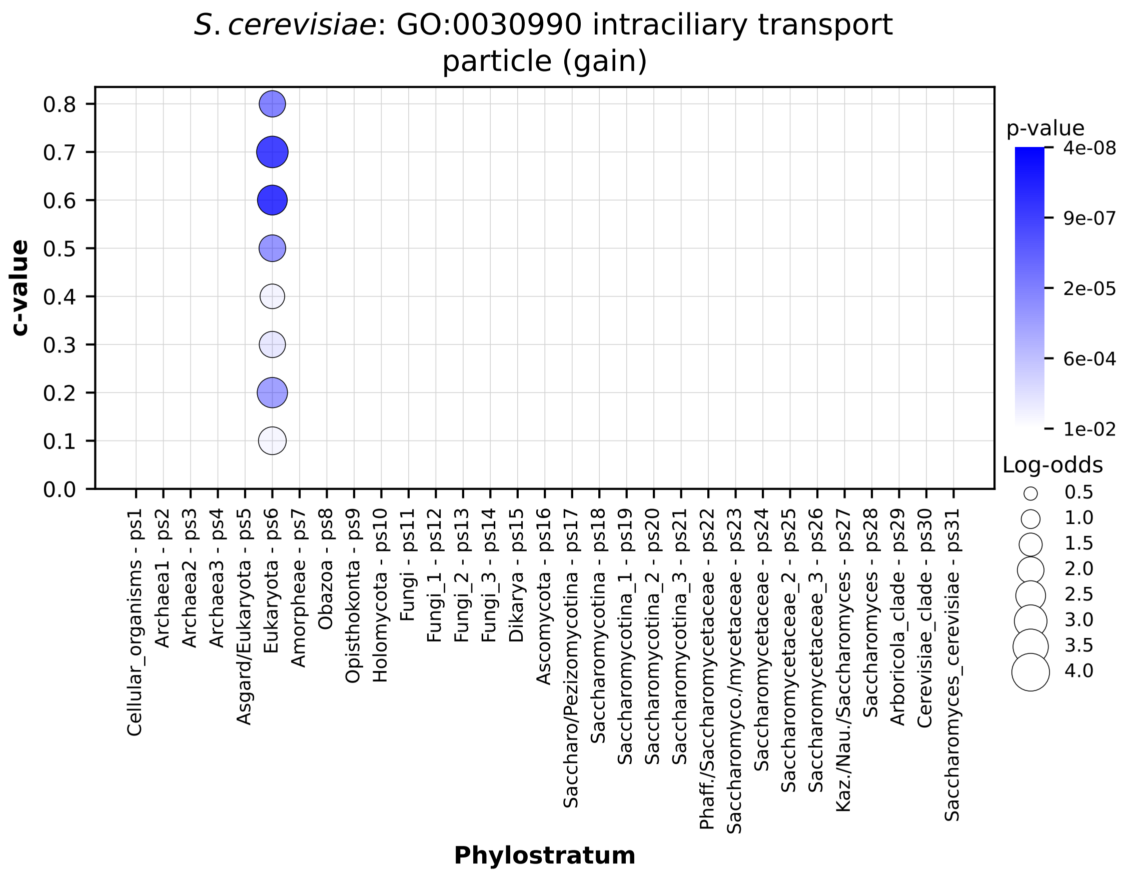

S. cerevisiae: GO:0030990 intraciliary transport particle (gain)
c-value
0.0
0.1
0.2
0.3
0.4
0.5
0.6
0.7
0.8
p-value
4e-08
9e-07
2e-05
6e-04
1e-02
Log-odds
0.5
1.0
1.5
2.0
2.5
3.0
3.5
4.0
Cellular_organisms - ps1
Archaea1 - ps2
Archaea2 - ps3
Archaea3 - ps4
Asgard/Eukaryota - ps5
Eukaryota - ps6
Amorpheae - ps7
Obazoa - ps8
Opisthokonta - ps9
Holomycota - ps10
Fungi - ps11
Fungi_1 - ps12
Fungi_2 - ps13
Fungi_3 - ps14
Dikarya - ps15
Ascomycota - ps16
Saccharo/Pezizomycotina - ps17
Saccharomycotina - ps18
Saccharomycotina_1 - ps19
Saccharomycotina_2 - ps20
Saccharomycotina_3 - ps21
Phaff./Saccharomycetaceae - ps22
Saccharomyco./mycetaceae - ps23
Saccharomycetaceae - ps24
Saccharomycetaceae_2 - ps25
Saccharomycetaceae_3 - ps26
Kaz./Nau./Saccharomyces - ps27
Saccharomyces - ps28
Arboricola_clade - ps29
Cerevisiae_clade - ps30
Saccharomyces_cerevisiae - ps31
Phylostratum

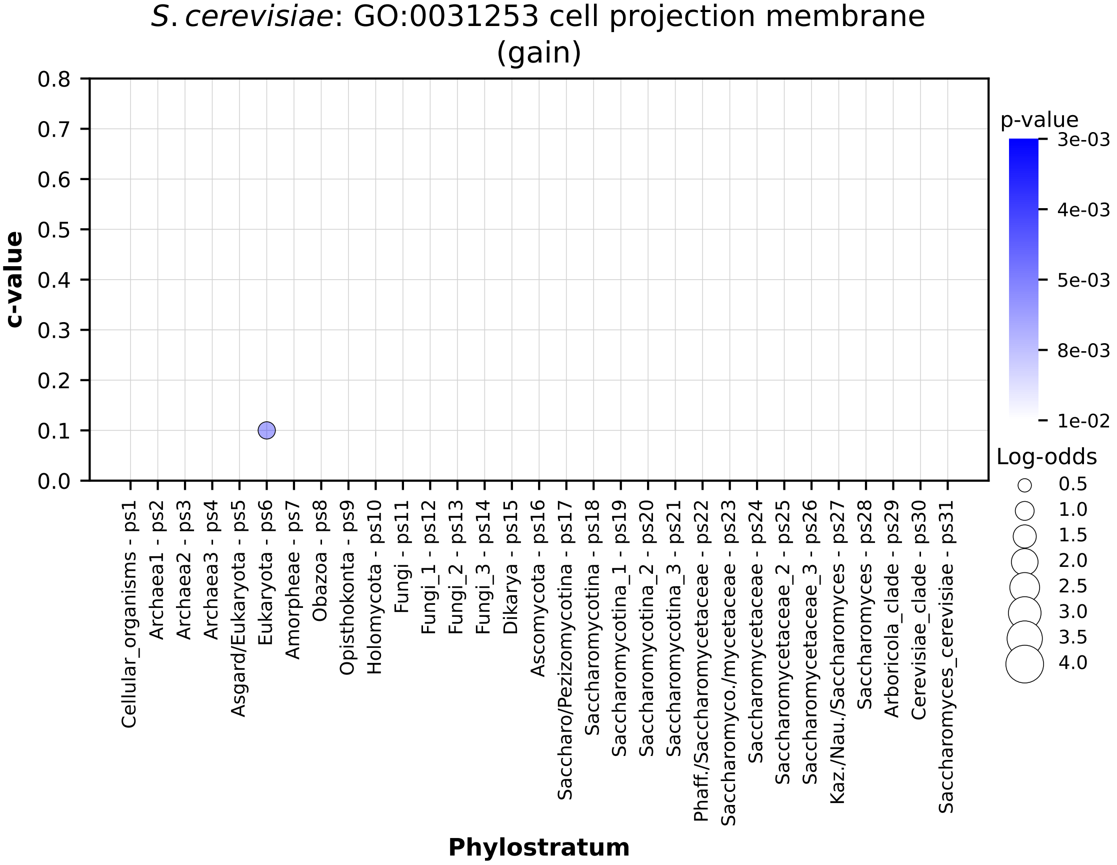

S. cerevisiae: GO:0031253 cell projection membrane
(gain)
c-value
0.0
0.1
0.2
0.3
0.4
0.5
0.6
0.7
0.8
p-value
3e-03
4e-03
5e-03
8e-03
1e-02
Log-odds
0.5
1.0
1.5
2.0
2.5
3.0
3.5
4.0
Cellular_organisms - ps1
Archaea1 - ps2
Archaea2 - ps3
Archaea3 - ps4
Asgard/Eukaryota - ps5
Eukaryota - ps6
Amorpheae - ps7
Obazoa - ps8
Opisthokonta - ps9
Holomycota - ps10
Fungi - ps11
Fungi_1 - ps12
Fungi_2 - ps13
Fungi_3 - ps14
Dikarya - ps15
Ascomycota - ps16
Saccharo/Pezizomycotina - ps17
Saccharomycotina - ps18
Saccharomycotina_1 - ps19
Saccharomycotina_2 - ps20
Saccharomycotina_3 - ps21
Phaff./Saccharomycetaceae - ps22
Saccharomyco./mycetaceae - ps23
Saccharomycetaceae - ps24
Saccharomycetaceae_2 - ps25
Saccharomycetaceae_3 - ps26
Kaz./Nau./Saccharomyces - ps27
Saccharomyces - ps28
Arboricola_clade - ps29
Cerevisiae_clade - ps30
Saccharomyces_cerevisiae - ps31
Phylostratum

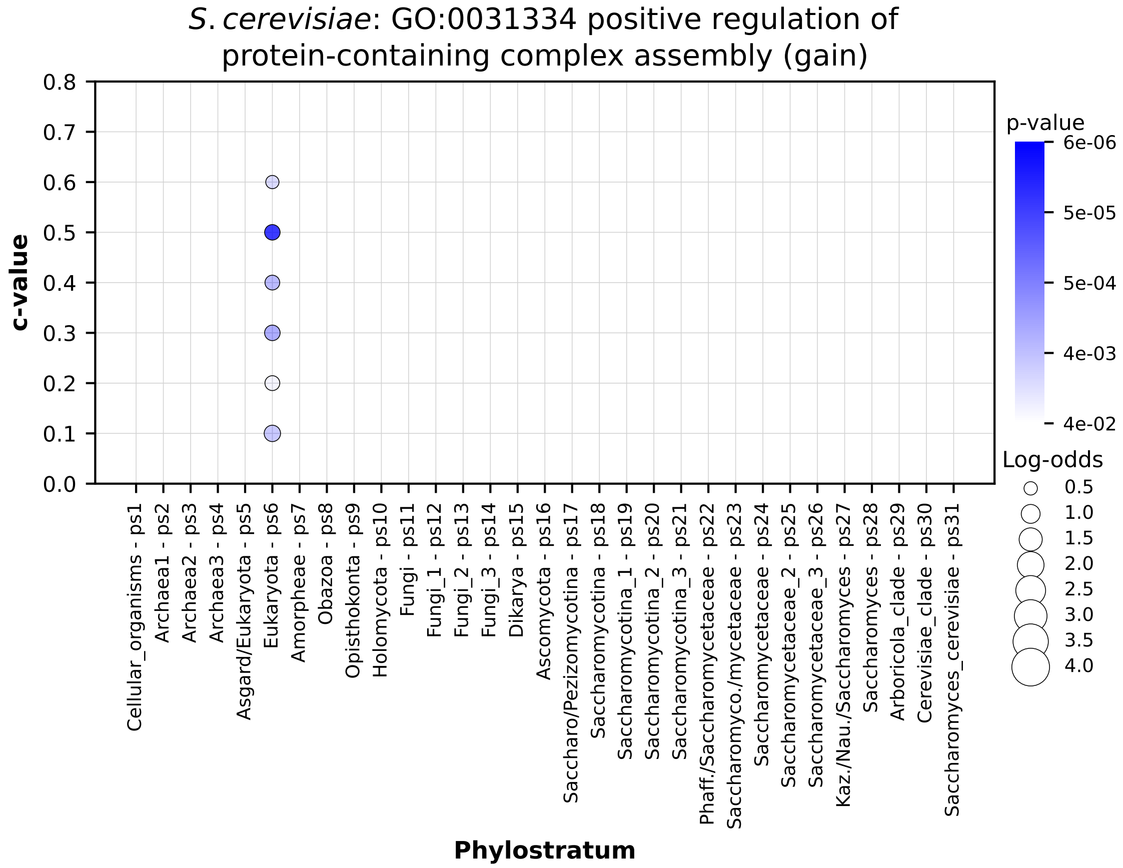

S. cerevisiae: GO:0031334 positive regulation of protein-containing complex assembly (gain)
c-value
0.0
0.1
0.2
0.3
0.4
0.5
0.6
0.7
0.8
Phylostratum
Cellular_organisms - ps1
Archaea1 - ps2
Archaea2 - ps3
Archaea3 - ps4
Asgard/Eukaryota - ps5
Eukaryota - ps6
Amorpheae - ps7
Obazoa - ps8
Opisthokonta - ps9
Holomycota - ps10
Fungi - ps11
Fungi_1 - ps12
Fungi_2 - ps13
Fungi_3 - ps14
Dikarya - ps15
Ascomycota - ps16
Saccharo/Pezizomycotina - ps17
Saccharomycotina - ps18
Saccharomycotina_1 - ps19
Saccharomycotina_2 - ps20
Saccharomycotina_3 - ps21
Phaff./Saccharomycetaceae - ps22
Saccharomyco./mycetaceae - ps23
Saccharomycetaceae - ps24
Saccharomycetaceae_2 - ps25
Saccharomycetaceae_3 - ps26
Kaz./Nau./Saccharomyces - ps27
Saccharomyces - ps28
Arboricola_clade - ps29
Cerevisiae_clade - ps30
Saccharomyces_cerevisiae - ps31
p-value
6e-06
5e-05
5e-04
4e-03
4e-02
Log-odds
0.5
1.0
1.5
2.0
2.5
3.0
3.5
4.0

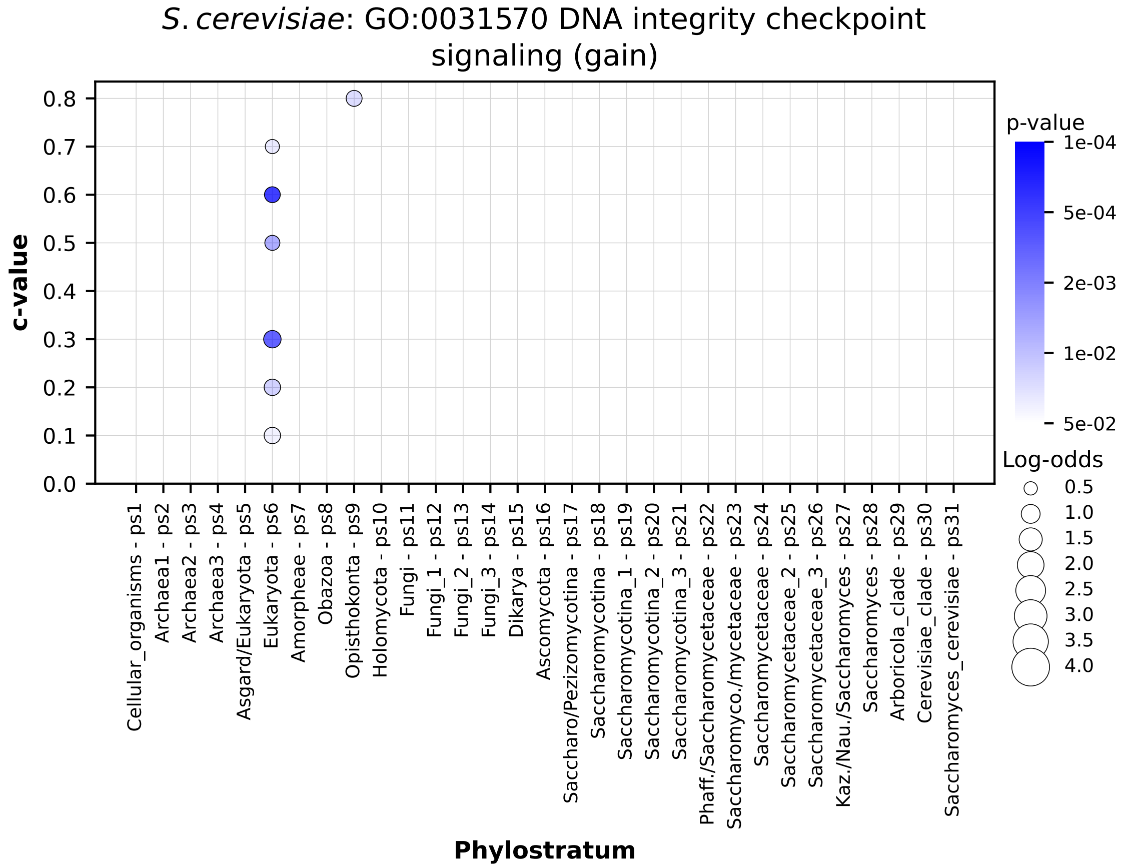

S. cerevisiae: GO:0031570 DNA integrity checkpoint signaling (gain)
c-value
0.0
0.1
0.2
0.3
0.4
0.5
0.6
0.7
0.8
p-value
1e-04
5e-04
2e-03
1e-02
5e-02
Log-odds
0.5
1.0
1.5
2.0
2.5
3.0
3.5
4.0
Cellular_organisms - ps1
Archaea1 - ps2
Archaea2 - ps3
Archaea3 - ps4
Asgard/Eukaryota - ps5
Eukaryota - ps6
Amorpheae - ps7
Obazoa - ps8
Opisthokonta - ps9
Holomycota - ps10
Fungi - ps11
Fungi_1 - ps12
Fungi_2 - ps13
Fungi_3 - ps14
Dikarya - ps15
Ascomycota - ps16
Saccharo/Pezizomycotina - ps17
Saccharomycotina - ps18
Saccharomycotina_1 - ps19
Saccharomycotina_2 - ps20
Saccharomycotina_3 - ps21
Phaff./Saccharomycetaceae - ps22
Saccharomyco./mycetaceae - ps23
Saccharomycetaceae - ps24
Saccharomycetaceae_2 - ps25
Saccharomycetaceae_3 - ps26
Kaz./Nau./Saccharomyces - ps27
Saccharomyces - ps28
Arboricola_clade - ps29
Cerevisiae_clade - ps30
Saccharomyces_cerevisiae - ps31
Phylostratum

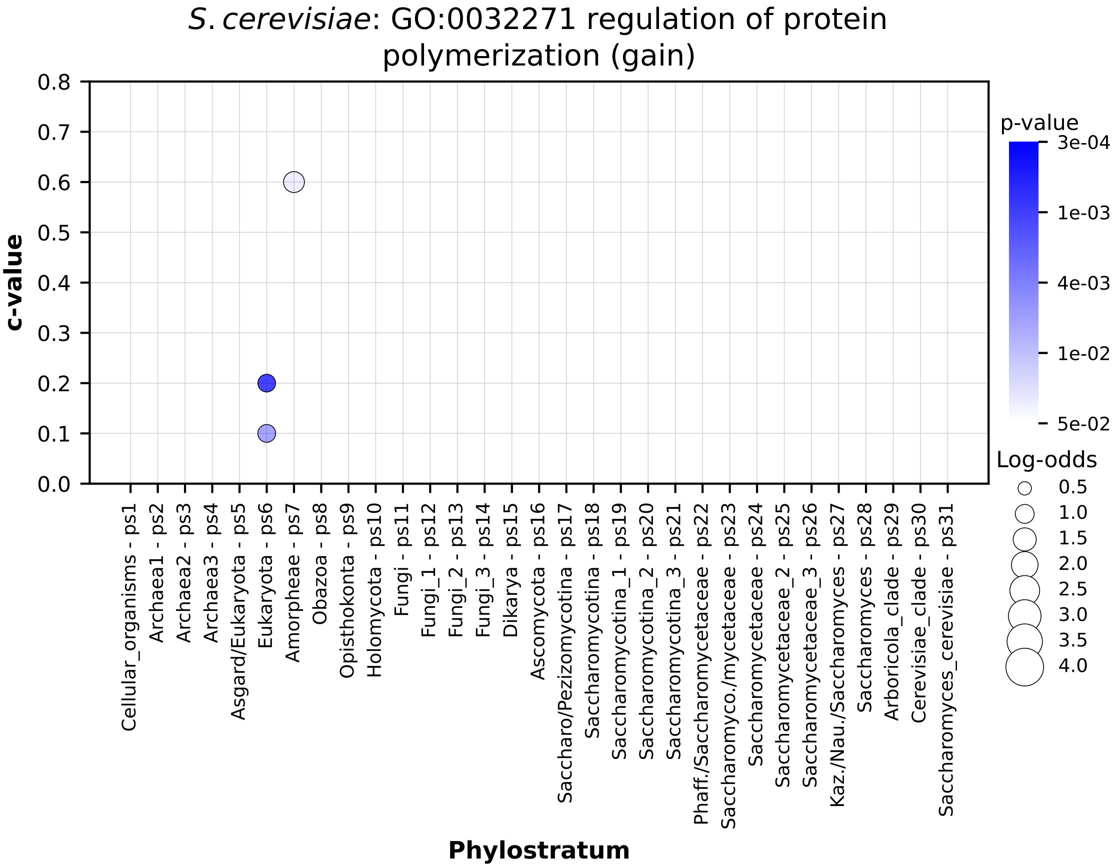

S. cerevisiae: GO:0032271 regulation of protein polymerization (gain)
c-value
0.0
0.1
0.2
0.3
0.4
0.5
0.6
0.7
0.8
p-value
3e-04
1e-03
4e-03
1e-02
5e-02
Log-odds
0.5
1.0
1.5
2.0
2.5
3.0
3.5
4.0
Cellular_organisms - ps1
Archaea1 - ps2
Archaea2 - ps3
Archaea3 - ps4
Asgard/Eukaryota - ps5
Eukaryota - ps6
Amorpheae - ps7
Obazoa - ps8
Opisthokonta - ps9
Holomycota - ps10
Fungi - ps11
Fungi_1 - ps12
Fungi_2 - ps13
Fungi_3 - ps14
Dikarya - ps15
Ascomycota - ps16
Saccharo/Pezizomycotina - ps17
Saccharomycotina - ps18
Saccharomycotina_1 - ps19
Saccharomycotina_2 - ps20
Saccharomycotina_3 - ps21
Phaff./Saccharomycetaceae - ps22
Saccharomyco./mycetaceae - ps23
Saccharomycetaceae - ps24
Saccharomycetaceae_2 - ps25
Saccharomycetaceae_3 - ps26
Kaz./Nau./Saccharomyces - ps27
Saccharomyces - ps28
Arboricola_clade - ps29
Cerevisiae_clade - ps30
Saccharomyces_cerevisiae - ps31
Phylostratum

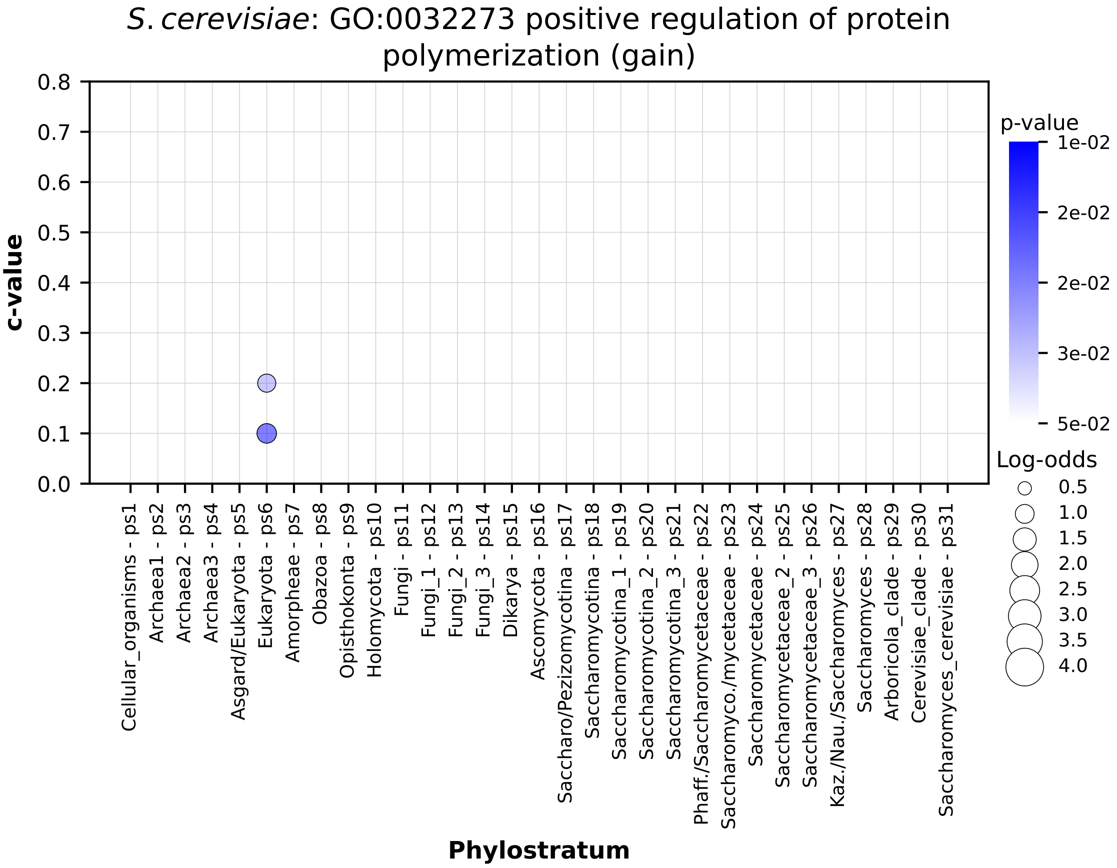

S. cerevisiae: GO:0032273 positive regulation of protein polymerization (gain)
c-value
0.8
0.7
0.6
0.5
0.4
0.3
0.2
0.1
0.0
p-value
1e-02
2e-02
2e-02
3e-02
5e-02
Log-odds
0.5
1.0
1.5
2.0
2.5
3.0
3.5
4.0
Cellular_organisms - ps1
Archaea1 - ps2
Archaea2 - ps3
Archaea3 - ps4
Asgard/Eukaryota - ps5
Eukaryota - ps6
Amorpheae - ps7
Obazoa - ps8
Opisthokonta - ps9
Holomycota - ps10
Fungi - ps11
Fungi_1 - ps12
Fungi_2 - ps13
Fungi_3 - ps14
Dikarya - ps15
Ascomycota - ps16
Saccharo/Pezizomycotina - ps17
Saccharomycotina - ps18
Saccharomycotina_1 - ps19
Saccharomycotina_2 - ps20
Saccharomycotina_3 - ps21
Phaff./Saccharomycetaceae - ps22
Saccharomyco./mycetaceae - ps23
Saccharomycetaceae - ps24
Saccharomycetaceae_2 - ps25
Saccharomycetaceae_3 - ps26
Kaz./Nau./Saccharomyces - ps27
Saccharomyces - ps28
Arboricola_clade - ps29
Cerevisiae_clade - ps30
Saccharomyces_cerevisiae - ps31
Phylostratum

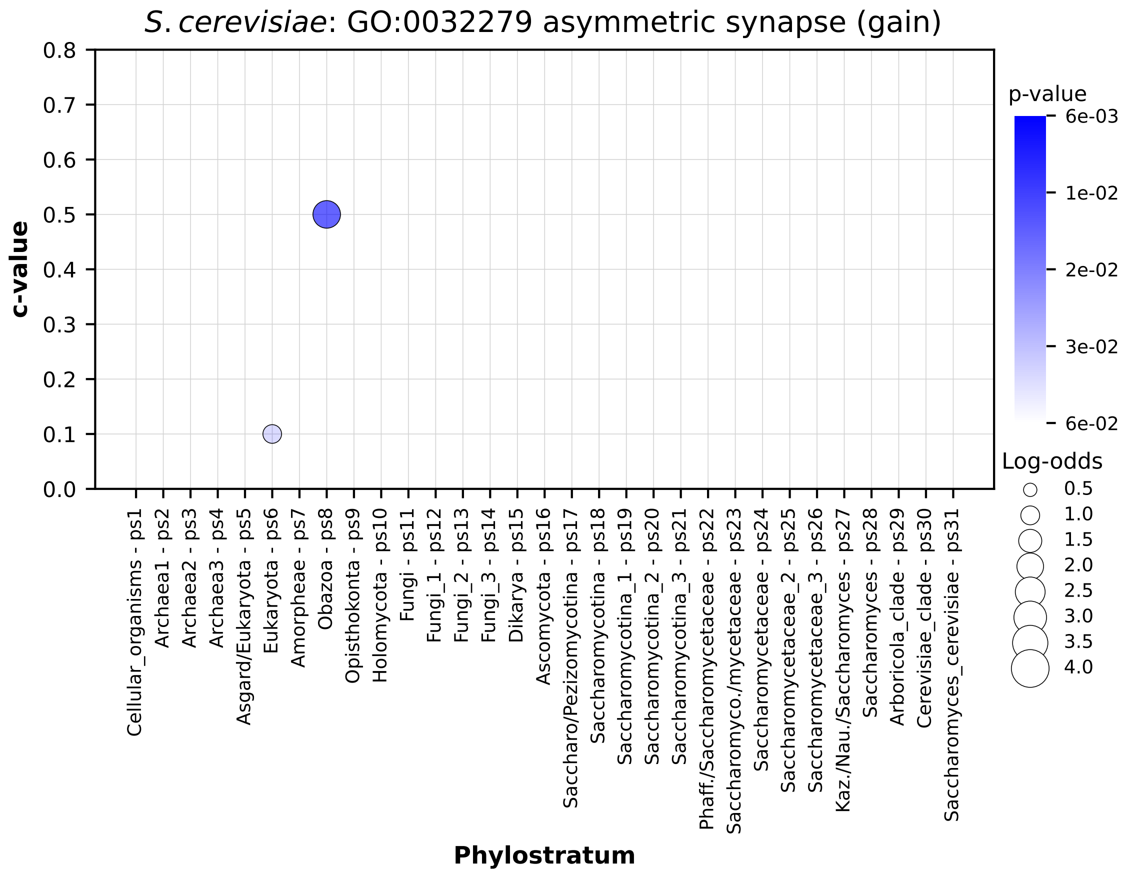

S. cerevisiae: GO:0032279 asymmetric synapse (gain)
c-value
0.0
0.1
0.2
0.3
0.4
0.5
0.6
0.7
0.8
Phylostratum
Cellular_organisms - ps1
Archaea1 - ps2
Archaea2 - ps3
Archaea3 - ps4
Asgard/Eukaryota - ps5
Eukaryota - ps6
Amorpheae - ps7
Obazoa - ps8
Opisthokonta - ps9
Holomycota - ps10
Fungi - ps11
Fungi_1 - ps12
Fungi_2 - ps13
Fungi_3 - ps14
Dikarya - ps15
Ascomycota - ps16
Saccharo/Pezizomycotina - ps17
Saccharomycotina - ps18
Saccharomycotina_1 - ps19
Saccharomycotina_2 - ps20
Saccharomycotina_3 - ps21
Phaff./Saccharomycetaceae - ps22
Saccharomyco./mycetaceae - ps23
Saccharomycetaceae - ps24
Saccharomycetaceae_2 - ps25
Saccharomycetaceae_3 - ps26
Kaz./Nau./Saccharomyces - ps27
Saccharomyces - ps28
Arboricola_clade - ps29
Cerevisiae_clade - ps30
Saccharomyces_cerevisiae - ps31
p-value
6e-03
1e-02
2e-02
3e-02
6e-02
Log-odds
0.5
1.0
1.5
2.0
2.5
3.0
3.5
4.0

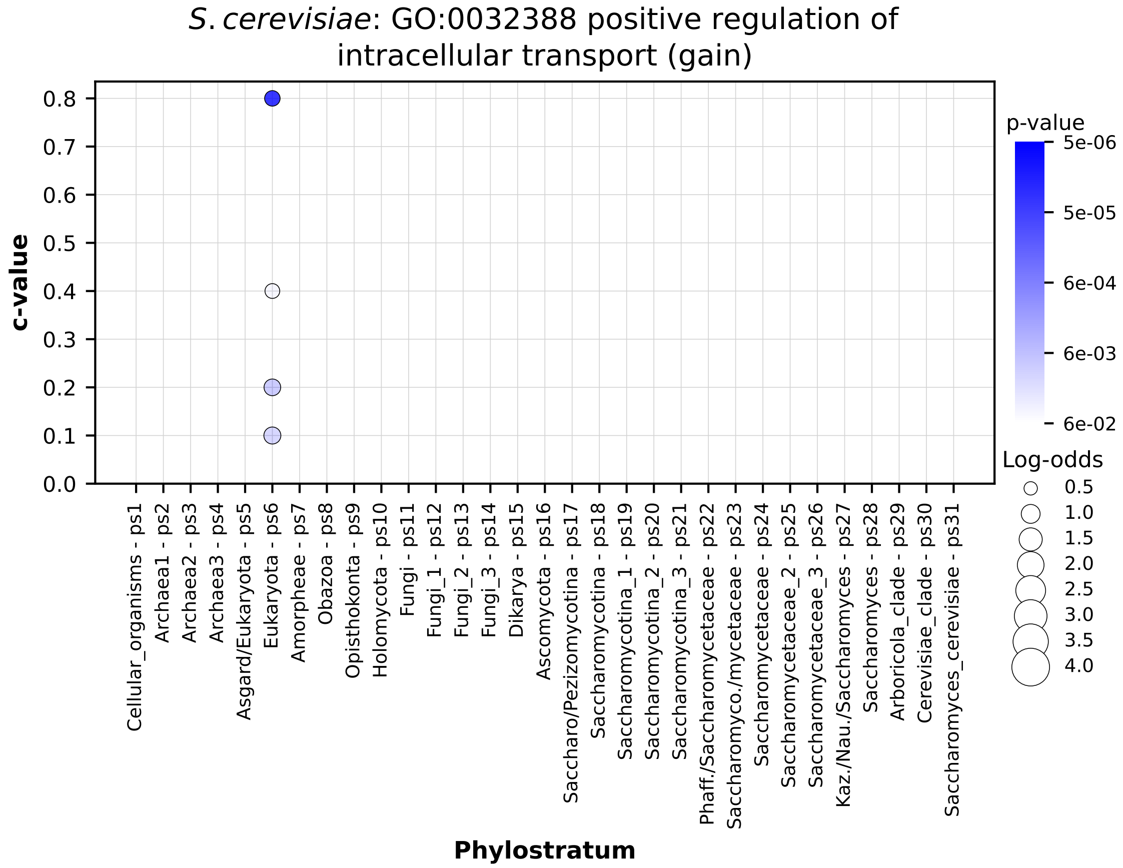

S. cerevisiae: GO:0032388 positive regulation of intracellular transport (gain)
c-value
0.0
0.1
0.2
0.3
0.4
0.5
0.6
0.7
0.8
p-value
5e-06
5e-05
6e-04
6e-03
6e-02
Log-odds
0.5
1.0
1.5
2.0
2.5
3.0
3.5
4.0
Cellular_organisms - ps1
Archaea1 - ps2
Archaea2 - ps3
Archaea3 - ps4
Asgard/Eukaryota - ps5
Eukaryota - ps6
Amorpheae - ps7
Obazoa - ps8
Opisthokonta - ps9
Holomycota - ps10
Fungi - ps11
Fungi_1 - ps12
Fungi_2 - ps13
Fungi_3 - ps14
Dikarya - ps15
Ascomycota - ps16
Saccharo/Pezizomycotina - ps17
Saccharomycotina - ps18
Saccharomycotina_1 - ps19
Saccharomycotina_2 - ps20
Saccharomycotina_3 - ps21
Phaff./Saccharomycetaceae - ps22
Saccharomyco./mycetaceae - ps23
Saccharomycetaceae - ps24
Saccharomycetaceae_2 - ps25
Saccharomycetaceae_3 - ps26
Kaz./Nau./Saccharomyces - ps27
Saccharomyces - ps28
Arboricola_clade - ps29
Cerevisiae_clade - ps30
Saccharomyces_cerevisiae - ps31
Phylostratum

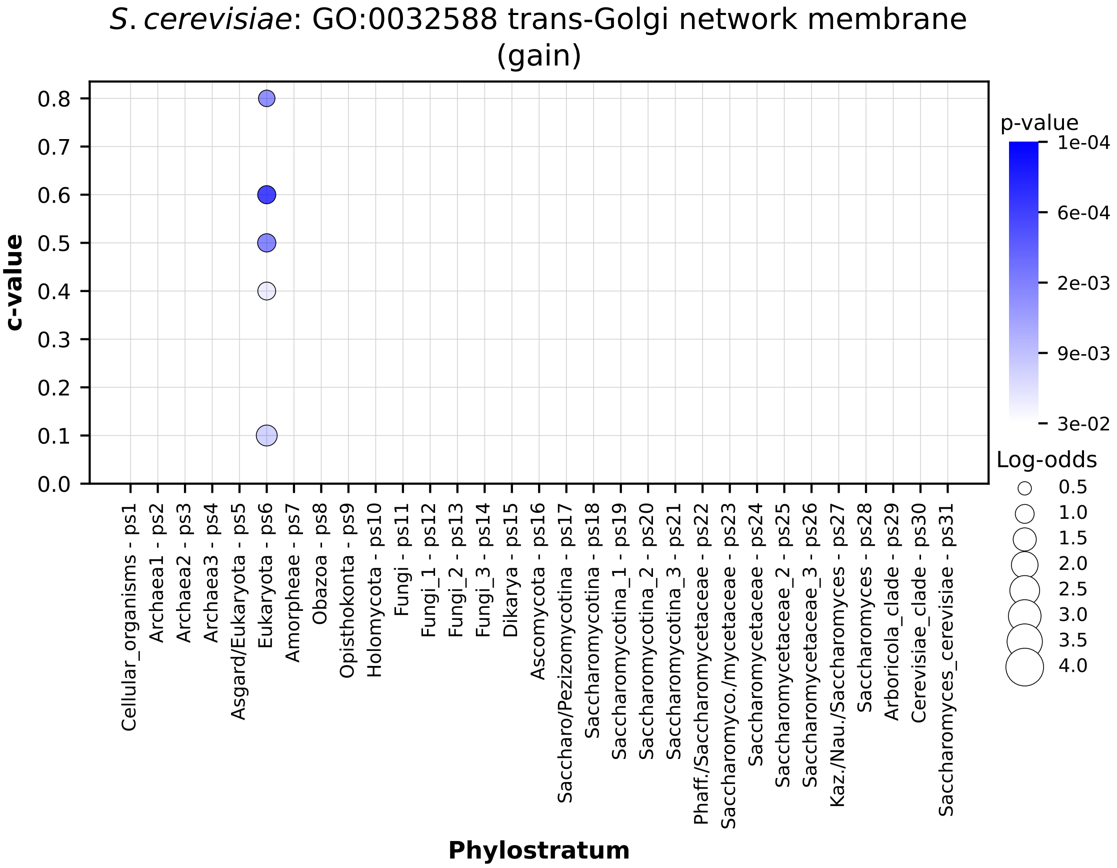
S. cerevisiae: GO:0032588 trans-Golgi network membrane (gain)
c-value
0.8
0.7
0.6
0.5
0.4
0.3
0.2
0.1
0.0
p-value
1e-04
6e-04
2e-03
9e-03
3e-02
Log-odds
0.5
1.0
1.5
2.0
2.5
3.0
3.5
4.0
Cellular_organisms - ps1
Archaea1 - ps2
Archaea2 - ps3
Archaea3 - ps4
Asgard/Eukaryota - ps5
Eukaryota - ps6
Amorpheae - ps7
Obazoa - ps8
Opisthokonta - ps9
Holomycota - ps10
Fungi - ps11
Fungi_1 - ps12
Fungi_2 - ps13
Fungi_3 - ps14
Dikarya - ps15
Ascomycota - ps16
Saccharo/Pezizomycotina - ps17
Saccharomycotina - ps18
Saccharomycotina_1 - ps19
Saccharomycotina_2 - ps20
Saccharomycotina_3 - ps21
Phaff./Saccharomycetaceae - ps22
Saccharomyco./mycetaceae - ps23
Saccharomycetaceae - ps24
Saccharomycetaceae_2 - ps25
Saccharomycetaceae_3 - ps26
Kaz./Nau./Saccharomyces - ps27
Saccharomyces - ps28
Arboricola_clade - ps29
Cerevisiae_clade - ps30
Saccharomyces_cerevisiae - ps31
Phylostratum

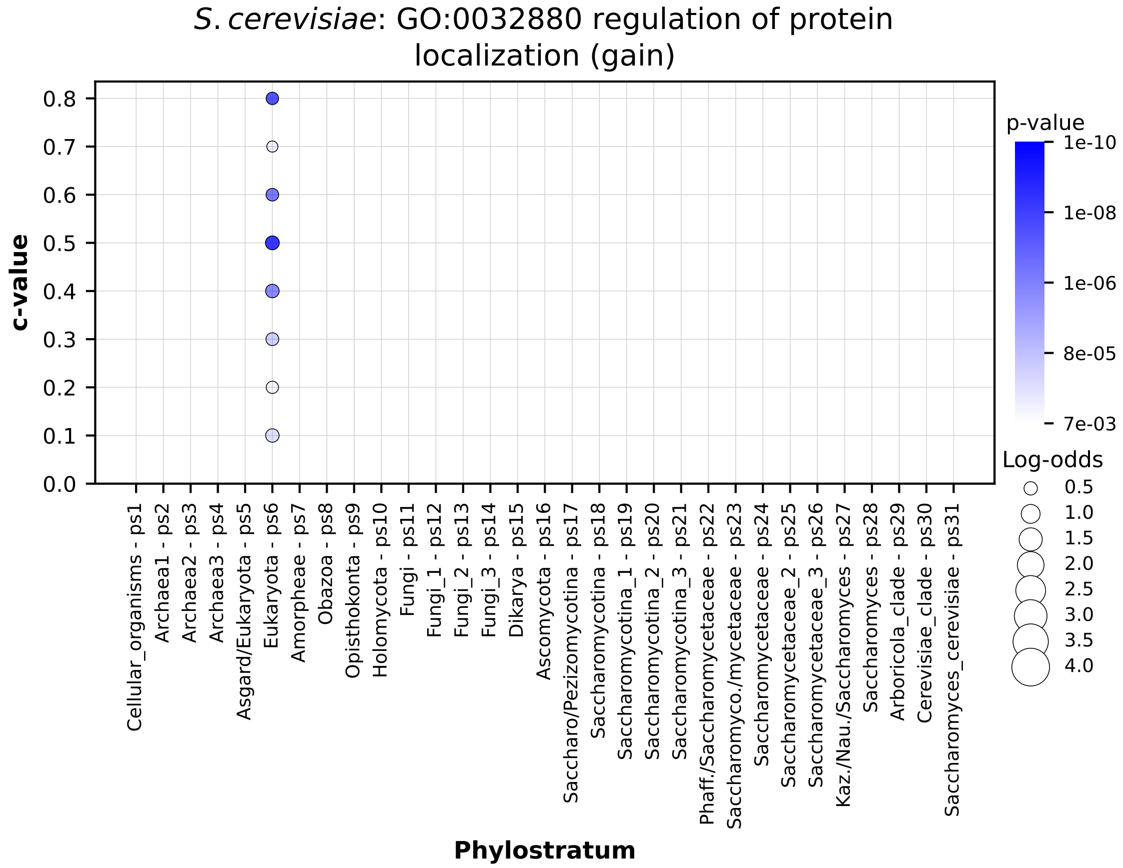

S. cerevisiae: GO:0032880 regulation of protein localization (gain)
c-value
0.0
0.1
0.2
0.3
0.4
0.5
0.6
0.7
0.8
p-value
1e-10
1e-08
1e-06
8e-05
7e-03
Log-odds
0.5
1.0
1.5
2.0
2.5
3.0
3.5
4.0
Cellular_organisms - ps1
Archaea1 - ps2
Archaea2 - ps3
Archaea3 - ps4
Asgard/Eukaryota - ps5
Eukaryota - ps6
Amorpheae - ps7
Obazoa - ps8
Opisthokonta - ps9
Holomycota - ps10
Fungi - ps11
Fungi_1 - ps12
Fungi_2 - ps13
Fungi_3 - ps14
Dikarya - ps15
Ascomycota - ps16
Saccharo/Pezizomycotina - ps17
Saccharomycotina - ps18
Saccharomycotina_1 - ps19
Saccharomycotina_2 - ps20
Saccharomycotina_3 - ps21
Phaff./Saccharomycetaceae - ps22
Saccharomyco./mycetaceae - ps23
Saccharomycetaceae - ps24
Saccharomycetaceae_2 - ps25
Saccharomycetaceae_3 - ps26
Kaz./Nau./Saccharomyces - ps27
Saccharomyces - ps28
Arboricola_clade - ps29
Cerevisiae_clade - ps30
Saccharomyces_cerevisiae - ps31
Phylostratum

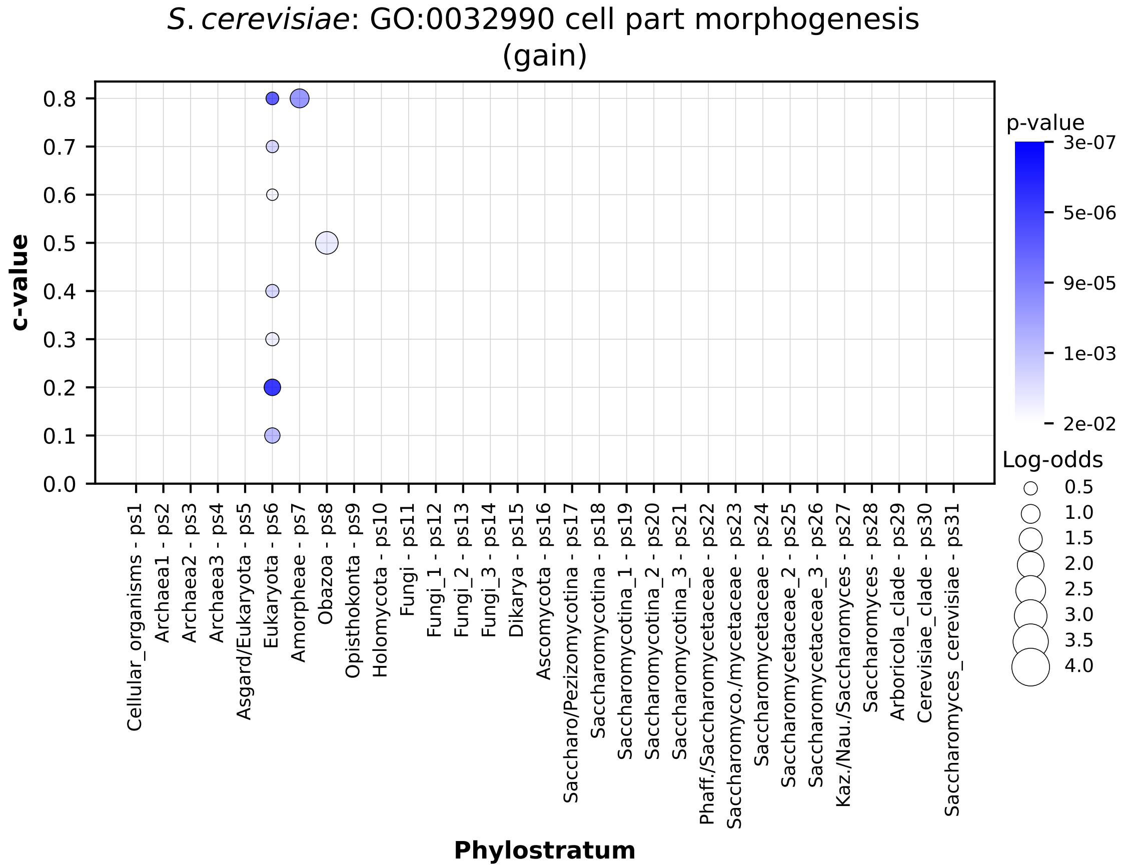

S. cerevisiae: GO:0032990 cell part morphogenesis
(gain)
c-value
0.8
0.7
0.6
0.5
0.4
0.3
0.2
0.1
0.0
p-value
3e-07
5e-06
9e-05
1e-03
2e-02
Log-odds
0.5
1.0
1.5
2.0
2.5
3.0
3.5
4.0
Cellular_organisms - ps1
Archaea1 - ps2
Archaea2 - ps3
Archaea3 - ps4
Asgard/Eukaryota - ps5
Eukaryota - ps6
Amorpheae - ps7
Obazoa - ps8
Opisthokonta - ps9
Holomycota - ps10
Fungi - ps11
Fungi_1 - ps12
Fungi_2 - ps13
Fungi_3 - ps14
Dikarya - ps15
Ascomycota - ps16
Saccharo/Pezizomycotina - ps17
Saccharomycotina - ps18
Saccharomycotina_1 - ps19
Saccharomycotina_2 - ps20
Saccharomycotina_3 - ps21
Phaff./Saccharomycetaceae - ps22
Saccharomyco./mycetaceae - ps23
Saccharomycetaceae - ps24
Saccharomycetaceae_2 - ps25
Saccharomycetaceae_3 - ps26
Kaz./Nau./Saccharomyces - ps27
Saccharomyces - ps28
Arboricola_clade - ps29
Cerevisiae_clade - ps30
Saccharomyces_cerevisiae - ps31
Phylostratum

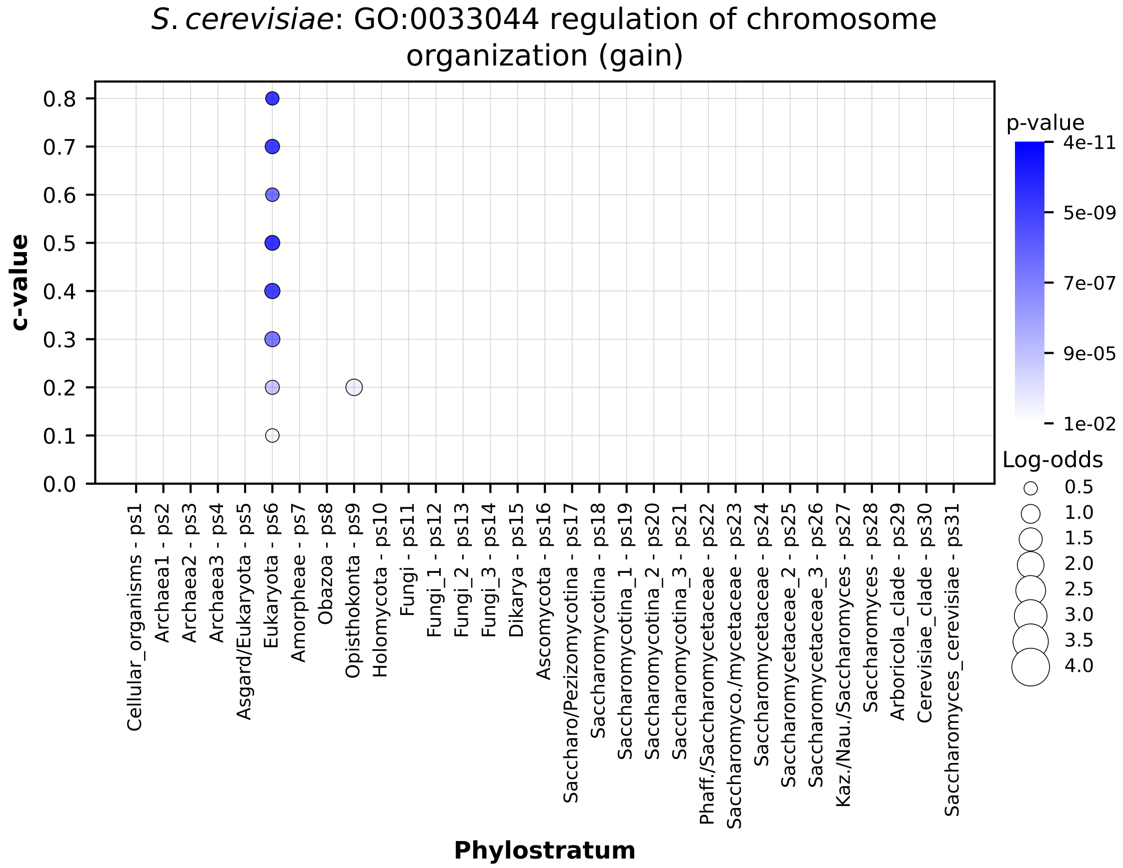

S. cerevisiae: GO:0033044 regulation of chromosome organization (gain)
c-value
0.0
0.1
0.2
0.3
0.4
0.5
0.6
0.7
0.8
Phylostratum
Cellular_organisms - ps1
Archaea1 - ps2
Archaea2 - ps3
Archaea3 - ps4
Asgard/Eukaryota - ps5
Eukaryota - ps6
Amorpheae - ps7
Obazoa - ps8
Opisthokonta - ps9
Holomycota - ps10
Fungi - ps11
Fungi_1 - ps12
Fungi_2 - ps13
Fungi_3 - ps14
Dikarya - ps15
Ascomycota - ps16
Saccharo/Pezizomycotina - ps17
Saccharomycotina - ps18
Saccharomycotina_1 - ps19
Saccharomycotina_2 - ps20
Saccharomycotina_3 - ps21
Phaff./Saccharomycetaceae - ps22
Saccharomyco./mycetaceae - ps23
Saccharomycetaceae - ps24
Saccharomycetaceae_2 - ps25
Saccharomycetaceae_3 - ps26
Kaz./Nau./Saccharomyces - ps27
Saccharomyces - ps28
Arboricola_clade - ps29
Cerevisiae_clade - ps30
Saccharomyces_cerevisiae - ps31
p-value
4e-11
5e-09
7e-07
9e-05
1e-02
Log-odds
0.5
1.0
1.5
2.0
2.5
3.0
3.5
4.0

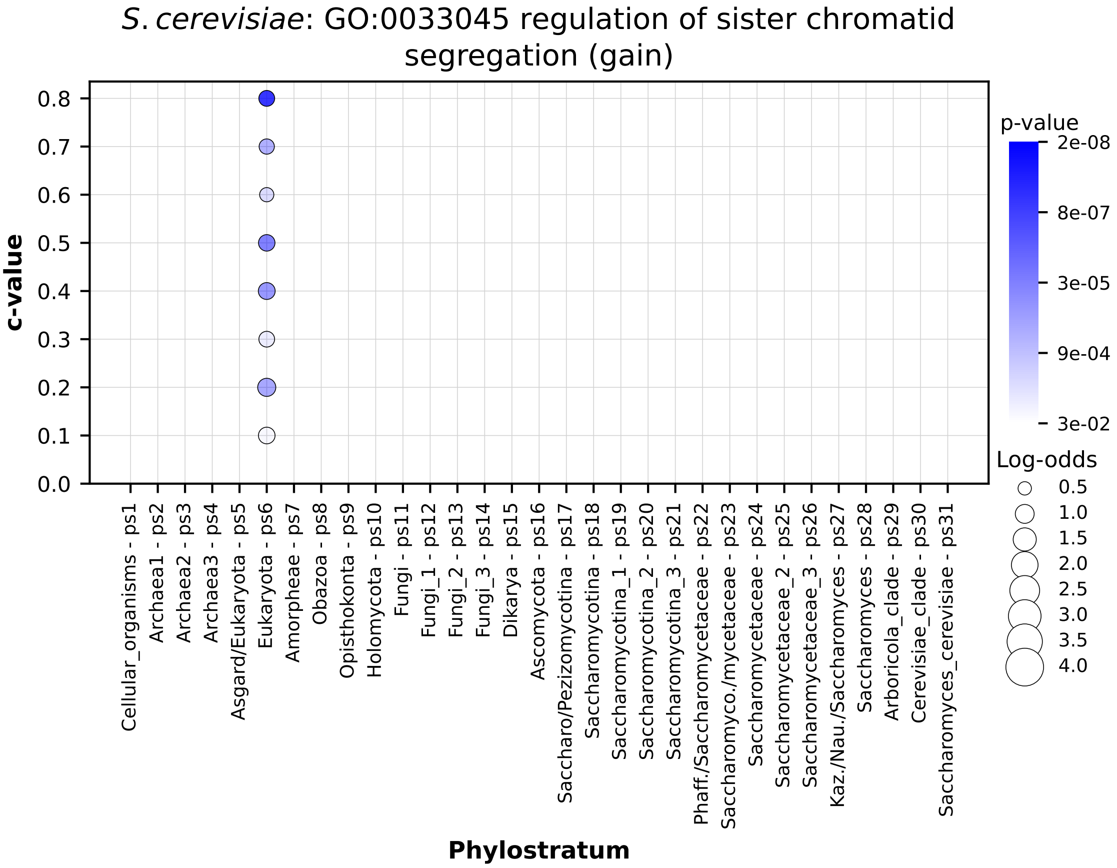

S. cerevisiae: GO:0033045 regulation of sister chromatid segregation (gain)
c-value
0.0
0.1
0.2
0.3
0.4
0.5
0.6
0.7
0.8
Phylostratum
Cellular_organisms - ps1
Archaea1 - ps2
Archaea2 - ps3
Archaea3 - ps4
Asgard/Eukaryota - ps5
Eukaryota - ps6
Amorpheae - ps7
Obazoa - ps8
Opisthokonta - ps9
Holomycota - ps10
Fungi - ps11
Fungi_1 - ps12
Fungi_2 - ps13
Fungi_3 - ps14
Dikarya - ps15
Ascomycota - ps16
Saccharo/Pezizomycotina - ps17
Saccharomycotina - ps18
Saccharomycotina_1 - ps19
Saccharomycotina_2 - ps20
Saccharomycotina_3 - ps21
Phaff./Saccharomycetaceae - ps22
Saccharomyco./mycetaceae - ps23
Saccharomycetaceae - ps24
Saccharomycetaceae_2 - ps25
Saccharomycetaceae_3 - ps26
Kaz./Nau./Saccharomyces - ps27
Saccharomyces - ps28
Arboricola_clade - ps29
Cerevisiae_clade - ps30
Saccharomyces_cerevisiae - ps31
p-value
2e-08
8e-07
3e-05
9e-04
3e-02
Log-odds
0.5
1.0
1.5
2.0
2.5
3.0
3.5
4.0

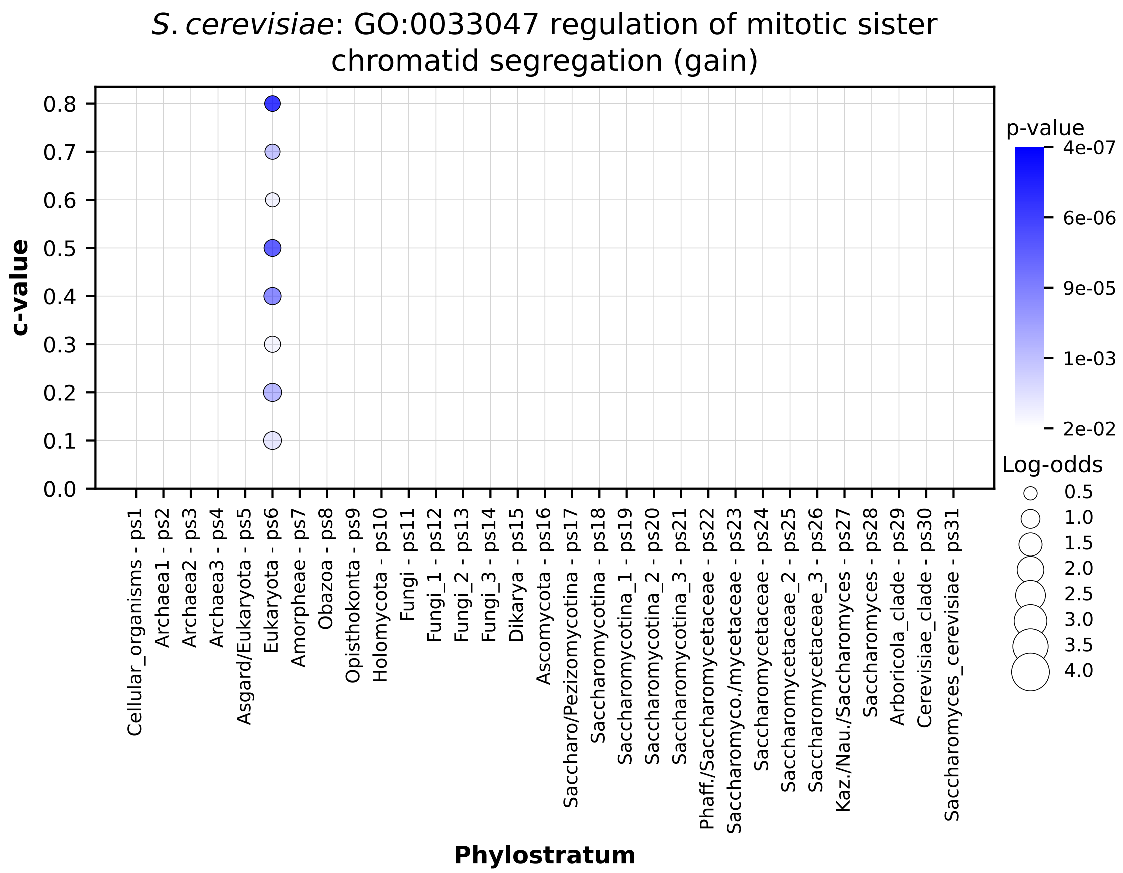

S. cerevisiae: GO:0033047 regulation of mitotic sister chromatid segregation (gain)
c-value
0.0
0.1
0.2
0.3
0.4
0.5
0.6
0.7
0.8
p-value
4e-07
6e-06
9e-05
1e-03
2e-02
Log-odds
0.5
1.0
1.5
2.0
2.5
3.0
3.5
4.0
Cellular_organisms - ps1
Archaea1 - ps2
Archaea2 - ps3
Archaea3 - ps4
Asgard/Eukaryota - ps5
Eukaryota - ps6
Amorpheae - ps7
Obazoa - ps8
Opisthokonta - ps9
Holomycota - ps10
Fungi - ps11
Fungi_1 - ps12
Fungi_2 - ps13
Fungi_3 - ps14
Dikarya - ps15
Ascomycota - ps16
Saccharo/Pezizomycotina - ps17
Saccharomycotina - ps18
Saccharomycotina_1 - ps19
Saccharomycotina_2 - ps20
Saccharomycotina_3 - ps21
Phaff./Saccharomycetaceae - ps22
Saccharomyco./mycetaceae - ps23
Saccharomycetaceae - ps24
Saccharomycetaceae_2 - ps25
Saccharomycetaceae_3 - ps26
Kaz./Nau./Saccharomyces - ps27
Saccharomyces - ps28
Arboricola_clade - ps29
Cerevisiae_clade - ps30
Saccharomyces_cerevisiae - ps31
Phylostratum

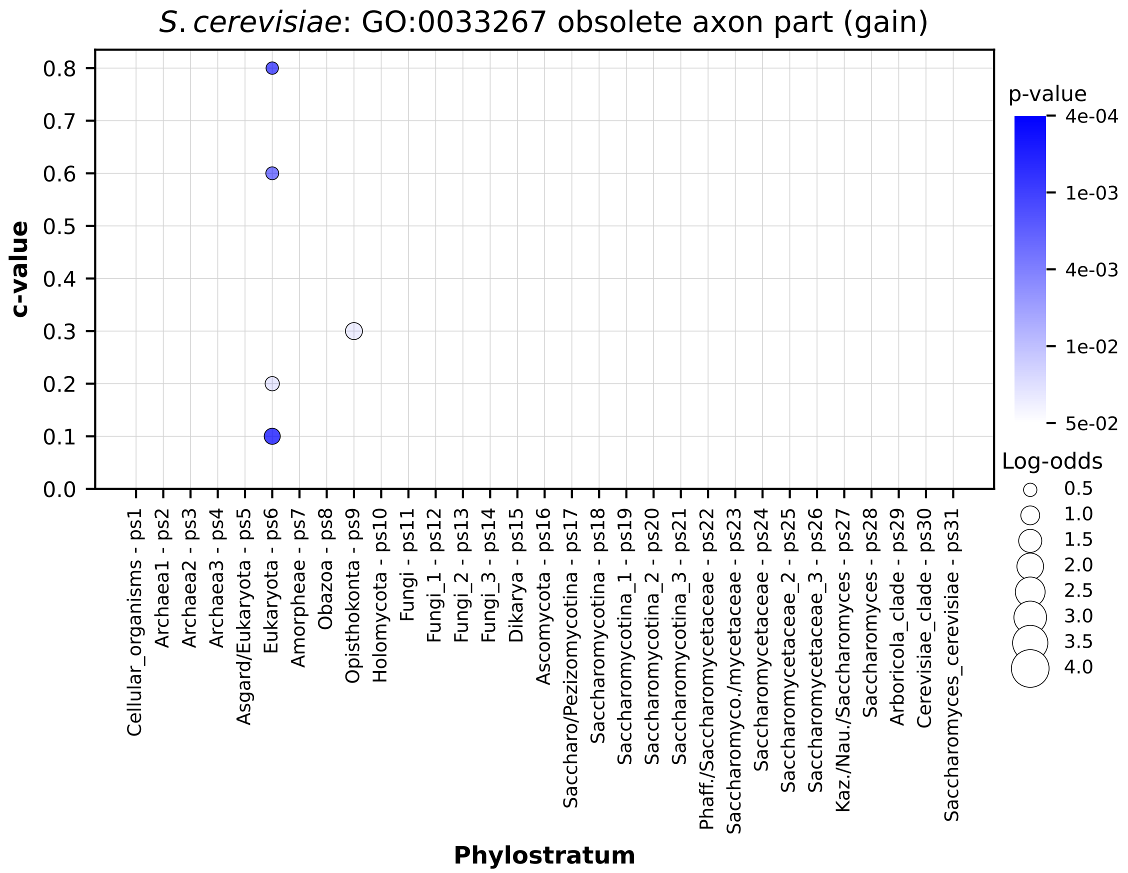

S. cerevisiae: GO:0033267 obsolete axon part (gain)
c-value
0.0
0.1
0.2
0.3
0.4
0.5
0.6
0.7
0.8
Phylostratum
Cellular_organisms - ps1
Archaea1 - ps2
Archaea2 - ps3
Archaea3 - ps4
Asgard/Eukaryota - ps5
Eukaryota - ps6
Amorpheae - ps7
Obazoa - ps8
Opisthokonta - ps9
Holomycota - ps10
Fungi - ps11
Fungi_1 - ps12
Fungi_2 - ps13
Fungi_3 - ps14
Dikarya - ps15
Ascomycota - ps16
Saccharo/Pezizomycotina - ps17
Saccharomycotina - ps18
Saccharomycotina_1 - ps19
Saccharomycotina_2 - ps20
Saccharomycotina_3 - ps21
Phaff./Saccharomycetaceae - ps22
Saccharomyco./mycetaceae - ps23
Saccharomycetaceae - ps24
Saccharomycetaceae_2 - ps25
Saccharomycetaceae_3 - ps26
Kaz./Nau./Saccharomyces - ps27
Saccharomyces - ps28
Arboricola_clade - ps29
Cerevisiae_clade - ps30
Saccharomyces_cerevisiae - ps31
p-value
4e-04
1e-03
4e-03
1e-02
5e-02
Log-odds
0.5
1.0
1.5
2.0
2.5
3.0
3.5
4.0

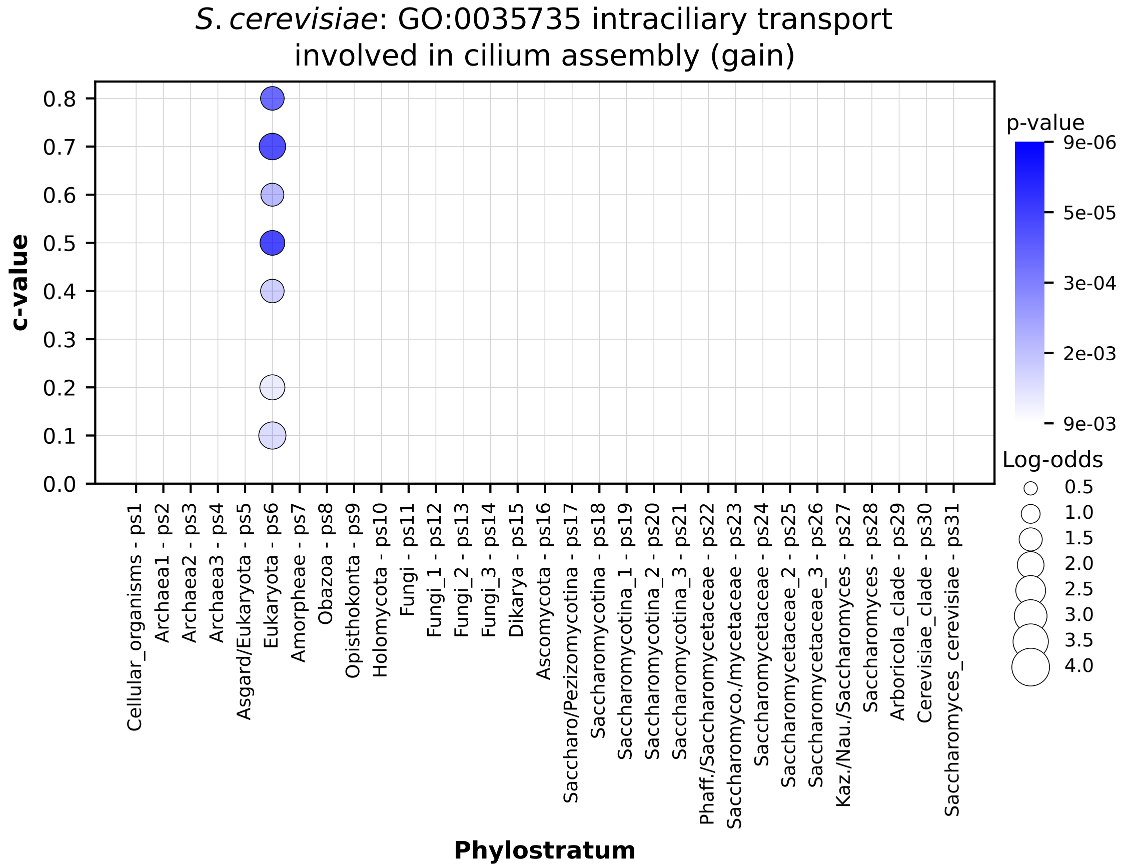

S. cerevisiae: GO:0035735 intraciliary transport involved in cilium assembly (gain)
c-value
0.0
0.1
0.2
0.3
0.4
0.5
0.6
0.7
0.8
p-value
9e-06
5e-05
3e-04
2e-03
9e-03
Log-odds
0.5
1.0
1.5
2.0
2.5
3.0
3.5
4.0
Cellular_organisms - ps1
Archaea1 - ps2
Archaea2 - ps3
Archaea3 - ps4
Asgard/Eukaryota - ps5
Eukaryota - ps6
Amorpheae - ps7
Obazoa - ps8
Opisthokonta - ps9
Holomycota - ps10
Fungi - ps11
Fungi_1 - ps12
Fungi_2 - ps13
Fungi_3 - ps14
Dikarya - ps15
Ascomycota - ps16
Saccharo/Pezizomycotina - ps17
Saccharomycotina - ps18
Saccharomycotina_1 - ps19
Saccharomycotina_2 - ps20
Saccharomycotina_3 - ps21
Phaff./Saccharomycetaceae - ps22
Saccharomyco./mycetaceae - ps23
Saccharomycetaceae - ps24
Saccharomycetaceae_2 - ps25
Saccharomycetaceae_3 - ps26
Kaz./Nau./Saccharomyces - ps27
Saccharomyces - ps28
Arboricola_clade - ps29
Cerevisiae_clade - ps30
Saccharomyces_cerevisiae - ps31
Phylostratum

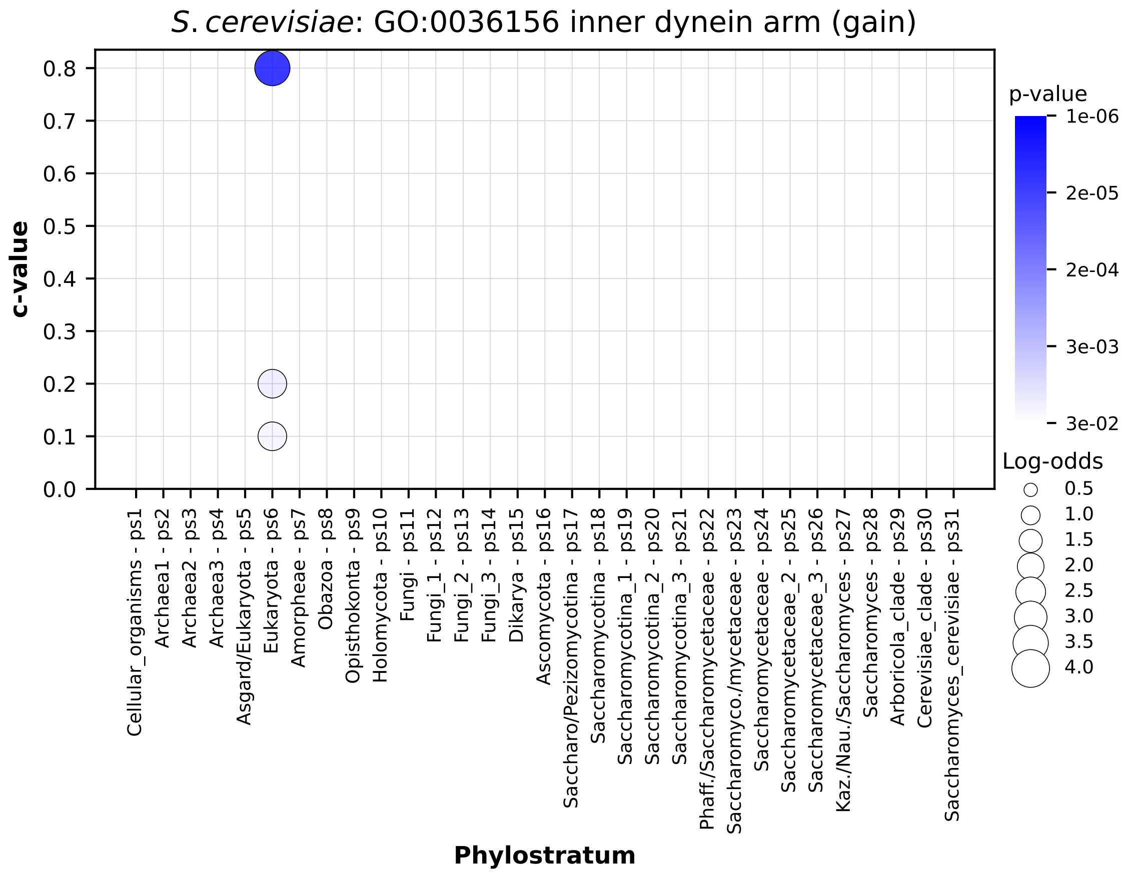
S. cerevisiae: GO:0036156 inner dynein arm (gain)
c-value
0.0
0.1
0.2
0.3
0.4
0.5
0.6
0.7
0.8
Phylostratum
Cellular_organisms - ps1
Archaea1 - ps2
Archaea2 - ps3
Archaea3 - ps4
Asgard/Eukaryota - ps5
Eukaryota - ps6
Amorpheae - ps7
Obazoa - ps8
Opisthokonta - ps9
Holomycota - ps10
Fungi - ps11
Fungi_1 - ps12
Fungi_2 - ps13
Fungi_3 - ps14
Dikarya - ps15
Ascomycota - ps16
Saccharo/Pezizomycotina - ps17
Saccharomycotina - ps18
Saccharomycotina_1 - ps19
Saccharomycotina_2 - ps20
Saccharomycotina_3 - ps21
Phaff./Saccharomycetaceae - ps22
Saccharomyco./mycetaceae - ps23
Saccharomycetaceae - ps24
Saccharomycetaceae_2 - ps25
Saccharomycetaceae_3 - ps26
Kaz./Nau./Saccharomyces - ps27
Saccharomyces - ps28
Arboricola_clade - ps29
Cerevisiae_clade - ps30
Saccharomyces_cerevisiae - ps31
p-value
1e-06
2e-05
2e-04
3e-03
3e-02
Log-odds
0.5
1.0
1.5
2.0
2.5
3.0
3.5
4.0

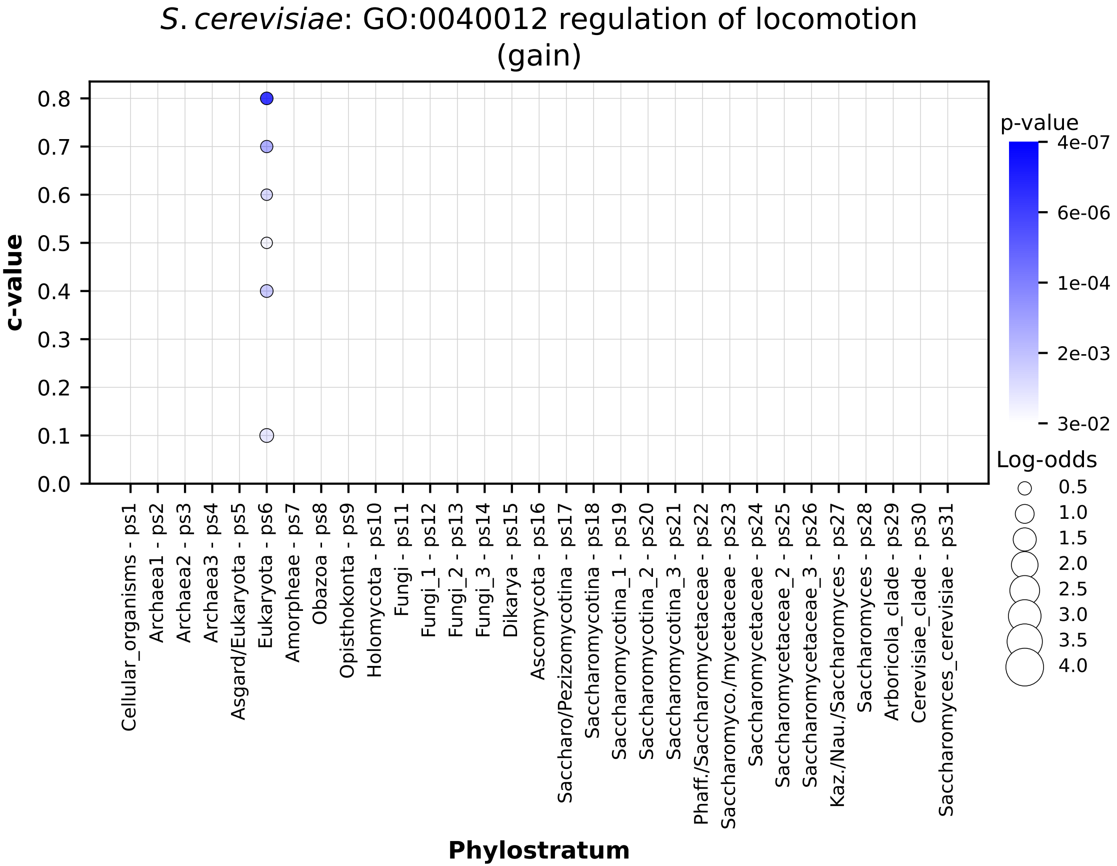

S. cerevisiae: GO:0040012 regulation of locomotion (gain)
c-value
0.0
0.1
0.2
0.3
0.4
0.5
0.6
0.7
0.8
Phylostratum
Cellular_organisms - ps1
Archaea1 - ps2
Archaea2 - ps3
Archaea3 - ps4
Asgard/Eukaryota - ps5
Eukaryota - ps6
Amorpheae - ps7
Obazoa - ps8
Opisthokonta - ps9
Holomycota - ps10
Fungi - ps11
Fungi_1 - ps12
Fungi_2 - ps13
Fungi_3 - ps14
Dikarya - ps15
Ascomycota - ps16
Saccharo/Pezizomycotina - ps17
Saccharomycotina - ps18
Saccharomycotina_1 - ps19
Saccharomycotina_2 - ps20
Saccharomycotina_3 - ps21
Phaff./Saccharomycetaceae - ps22
Saccharomyco./mycetaceae - ps23
Saccharomycetaceae - ps24
Saccharomycetaceae_2 - ps25
Saccharomycetaceae_3 - ps26
Kaz./Nau./Saccharomyces - ps27
Saccharomyces - ps28
Arboricola_clade - ps29
Cerevisiae_clade - ps30
Saccharomyces_cerevisiae - ps31
p-value
4e-07
6e-06
1e-04
2e-03
3e-02
Log-odds
0.5
1.0
1.5
2.0
2.5
3.0
3.5
4.0

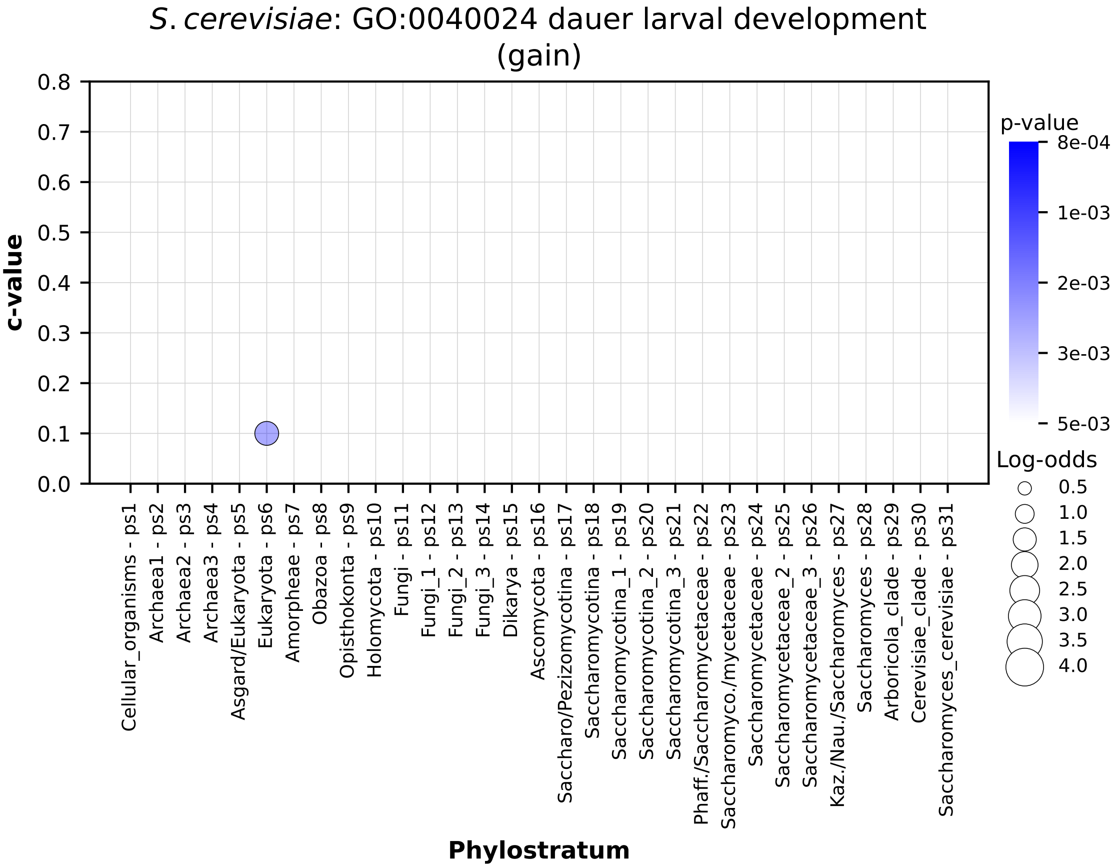
S. cerevisiae: GO:0040024 dauer larval development
(gain)
c-value
0.0
0.1
0.2
0.3
0.4
0.5
0.6
0.7
0.8
p-value
8e-04
1e-03
2e-03
3e-03
5e-03
Log-odds
0.5
1.0
1.5
2.0
2.5
3.0
3.5
4.0
Cellular_organisms - ps1
Archaea1 - ps2
Archaea2 - ps3
Archaea3 - ps4
Asgard/Eukaryota - ps5
Eukaryota - ps6
Amorpheae - ps7
Obazoa - ps8
Opisthokonta - ps9
Holomycota - ps10
Fungi - ps11
Fungi_1 - ps12
Fungi_2 - ps13
Fungi_3 - ps14
Dikarya - ps15
Ascomycota - ps16
Saccharo/Pezizomycotina - ps17
Saccharomycotina - ps18
Saccharomycotina_1 - ps19
Saccharomycotina_2 - ps20
Saccharomycotina_3 - ps21
Phaff./Saccharomycetaceae - ps22
Saccharomyco./mycetaceae - ps23
Saccharomycetaceae - ps24
Saccharomycetaceae_2 - ps25
Saccharomycetaceae_3 - ps26
Kaz./Nau./Saccharomyces - ps27
Saccharomyces - ps28
Arboricola_clade - ps29
Cerevisiae_clade - ps30
Saccharomyces_cerevisiae - ps31
Phylostratum

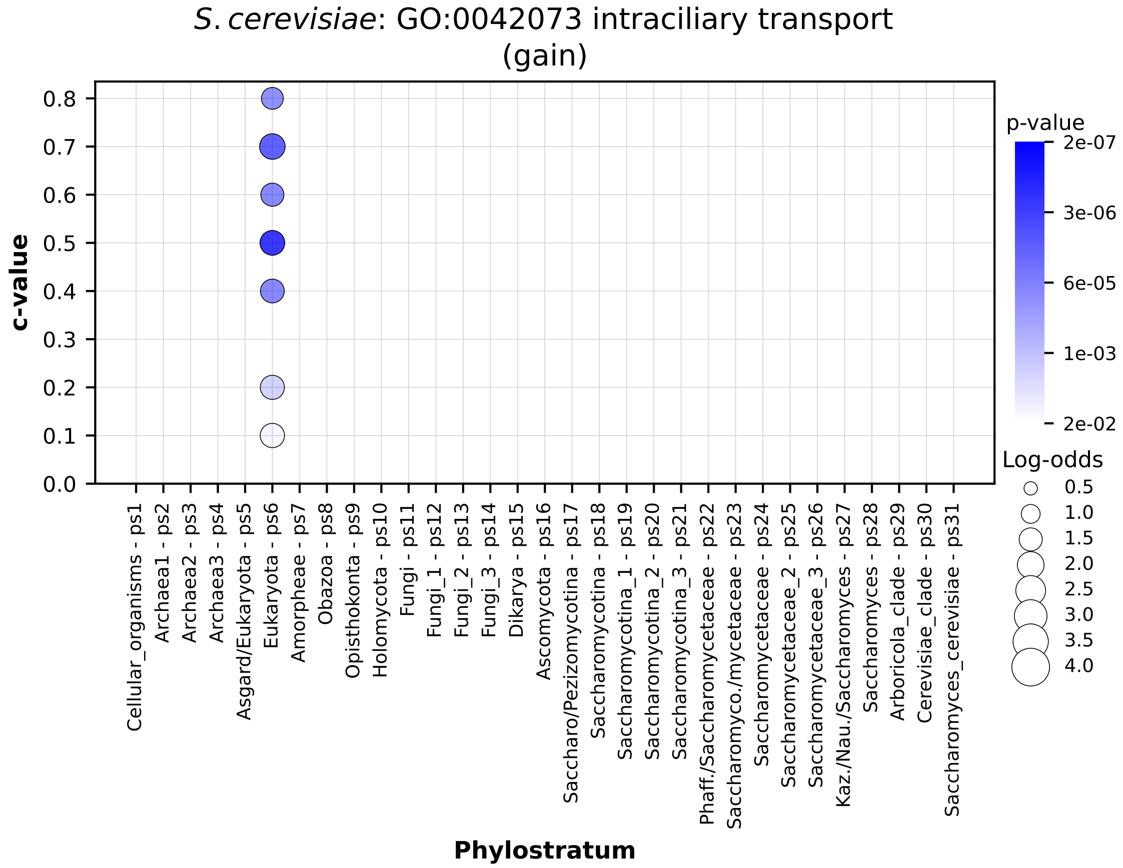

S. cerevisiae: GO:0042073 intraciliary transport
(gain)
c-value
0.8
0.7
0.6
0.5
0.4
0.3
0.2
0.1
0.0
p-value
2e-07
3e-06
6e-05
1e-03
2e-02
Log-odds
0.5
1.0
1.5
2.0
2.5
3.0
3.5
4.0
Cellular_organisms - ps1
Archaea1 - ps2
Archaea2 - ps3
Archaea3 - ps4
Asgard/Eukaryota - ps5
Eukaryota - ps6
Amorpheae - ps7
Obazoa - ps8
Opisthokonta - ps9
Holomycota - ps10
Fungi - ps11
Fungi_1 - ps12
Fungi_2 - ps13
Fungi_3 - ps14
Dikarya - ps15
Ascomycota - ps16
Saccharo/Pezizomycotina - ps17
Saccharomycotina - ps18
Saccharomycotina_1 - ps19
Saccharomycotina_2 - ps20
Saccharomycotina_3 - ps21
Phaff./Saccharomycetaceae - ps22
Saccharomyco./mycetaceae - ps23
Saccharomycetaceae - ps24
Saccharomycetaceae_2 - ps25
Saccharomycetaceae_3 - ps26
Kaz./Nau./Saccharomyces - ps27
Saccharomyces - ps28
Arboricola_clade - ps29
Cerevisiae_clade - ps30
Saccharomyces_cerevisiae - ps31
Phylostratum

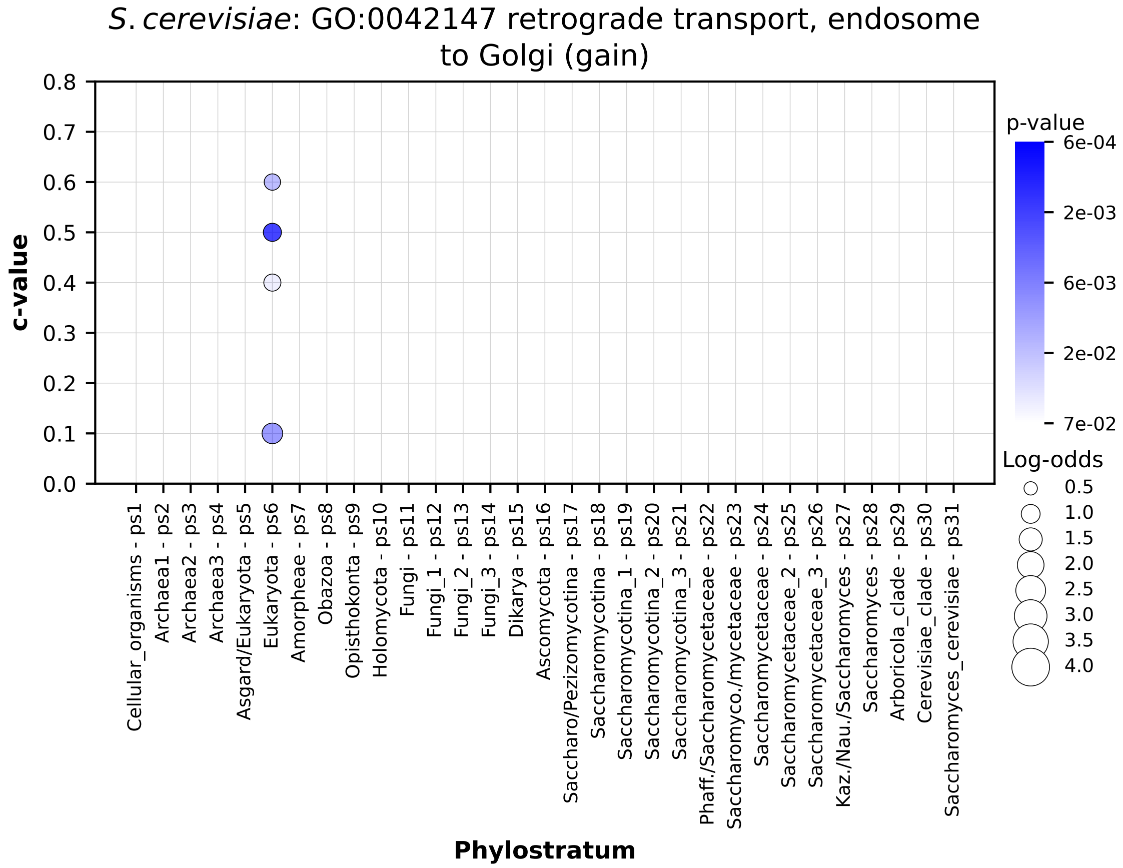

S. cerevisiae: GO:0042147 retrograde transport, endosome to Golgi (gain)
c-value
0.0
0.1
0.2
0.3
0.4
0.5
0.6
0.7
0.8
Phylostratum
Cellular_organisms - ps1
Archaea1 - ps2
Archaea2 - ps3
Archaea3 - ps4
Asgard/Eukaryota - ps5
Eukaryota - ps6
Amorpheae - ps7
Obazoa - ps8
Opisthokonta - ps9
Holomycota - ps10
Fungi - ps11
Fungi_1 - ps12
Fungi_2 - ps13
Fungi_3 - ps14
Dikarya - ps15
Ascomycota - ps16
Saccharo/Pezizomycotina - ps17
Saccharomycotina - ps18
Saccharomycotina_1 - ps19
Saccharomycotina_2 - ps20
Saccharomycotina_3 - ps21
Phaff./Saccharomycetaceae - ps22
Saccharomyco./mycetaceae - ps23
Saccharomycetaceae - ps24
Saccharomycetaceae_2 - ps25
Saccharomycetaceae_3 - ps26
Kaz./Nau./Saccharomyces - ps27
Saccharomyces - ps28
Arboricola_clade - ps29
Cerevisiae_clade - ps30
Saccharomyces_cerevisiae - ps31
p-value
6e-04
2e-03
6e-03
2e-02
7e-02
Log-odds
0.5
1.0
1.5
2.0
2.5
3.0
3.5
4.0

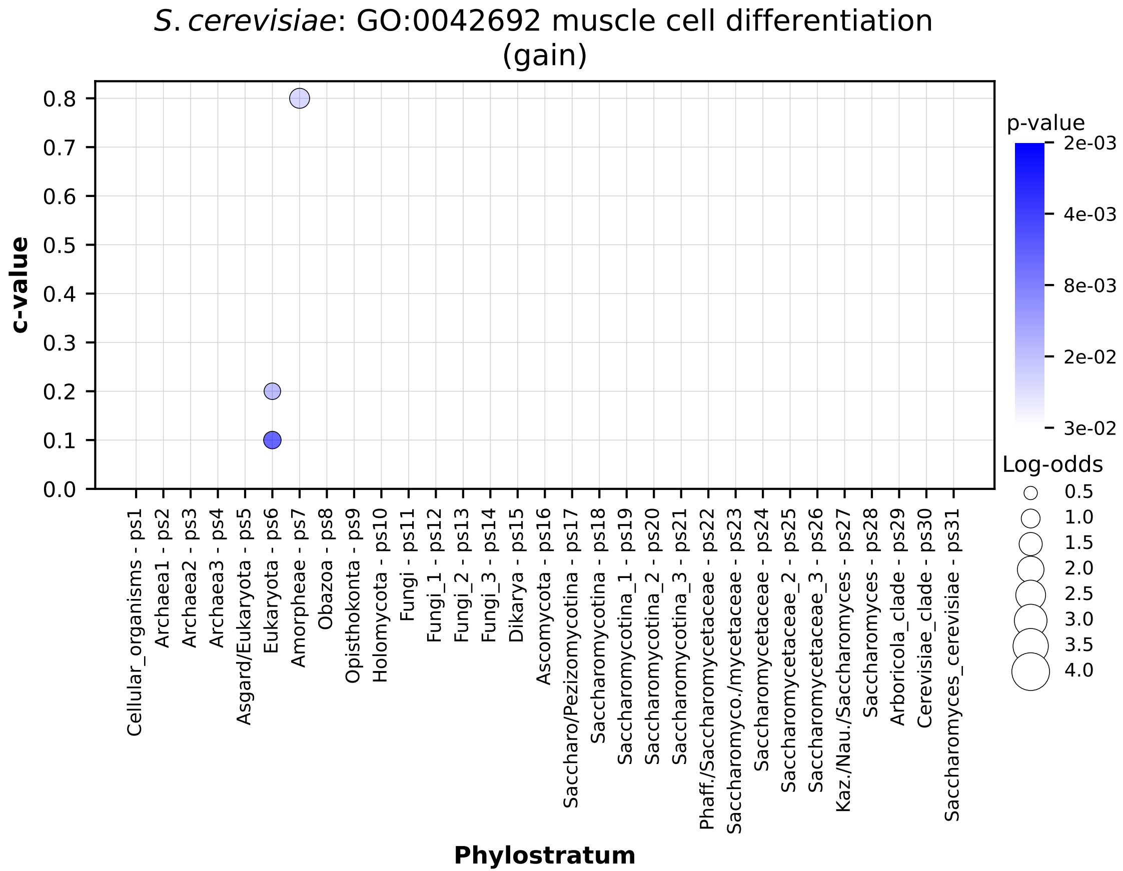

S. cerevisiae: GO:0042692 muscle cell differentiation
(gain)
c-value
0.0
0.1
0.2
0.3
0.4
0.5
0.6
0.7
0.8
p-value
2e-03
4e-03
8e-03
2e-02
3e-02
Log-odds
0.5
1.0
1.5
2.0
2.5
3.0
3.5
4.0
Cellular_organisms - ps1
Archaea1 - ps2
Archaea2 - ps3
Archaea3 - ps4
Asgard/Eukaryota - ps5
Eukaryota - ps6
Amorpheae - ps7
Obazoa - ps8
Opisthokonta - ps9
Holomycota - ps10
Fungi - ps11
Fungi_1 - ps12
Fungi_2 - ps13
Fungi_3 - ps14
Dikarya - ps15
Ascomycota - ps16
Saccharo/Pezizomycotina - ps17
Saccharomycotina - ps18
Saccharomycotina_1 - ps19
Saccharomycotina_2 - ps20
Saccharomycotina_3 - ps21
Phaff./Saccharomycetaceae - ps22
Saccharomyco./mycetaceae - ps23
Saccharomycetaceae - ps24
Saccharomycetaceae_2 - ps25
Saccharomycetaceae_3 - ps26
Kaz./Nau./Saccharomyces - ps27
Saccharomyces - ps28
Arboricola_clade - ps29
Cerevisiae_clade - ps30
Saccharomyces_cerevisiae - ps31
Phylostratum

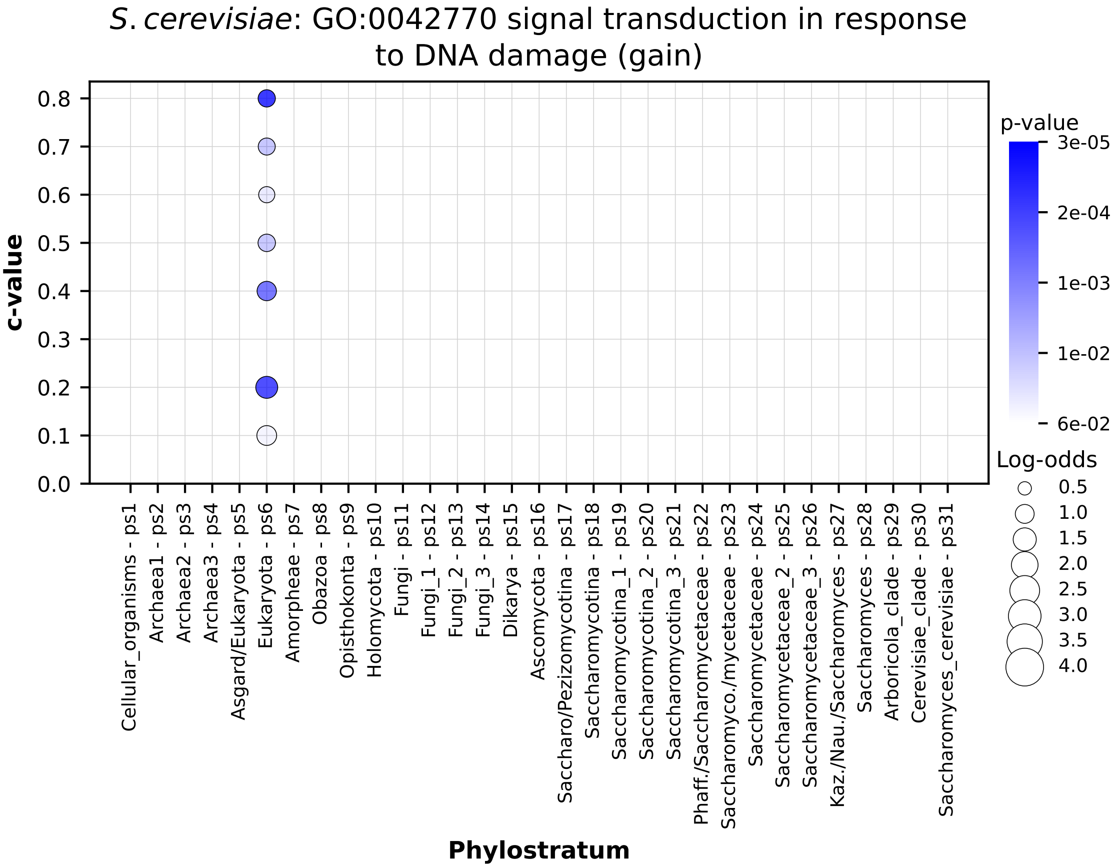

S. cerevisiae: GO:0042770 signal transduction in response to DNA damage (gain)
c-value
0.0
0.1
0.2
0.3
0.4
0.5
0.6
0.7
0.8
Phylostratum
Cellular_organisms - ps1
Archaea1 - ps2
Archaea2 - ps3
Archaea3 - ps4
Asgard/Eukaryota - ps5
Eukaryota - ps6
Amorpheae - ps7
Obazoa - ps8
Opisthokonta - ps9
Holomycota - ps10
Fungi - ps11
Fungi_1 - ps12
Fungi_2 - ps13
Fungi_3 - ps14
Dikarya - ps15
Ascomycota - ps16
Saccharo/Pezizomycotina - ps17
Saccharomycotina - ps18
Saccharomycotina_1 - ps19
Saccharomycotina_2 - ps20
Saccharomycotina_3 - ps21
Phaff./Saccharomycetaceae - ps22
Saccharomyco./mycetaceae - ps23
Saccharomycetaceae - ps24
Saccharomycetaceae_2 - ps25
Saccharomycetaceae_3 - ps26
Kaz./Nau./Saccharomyces - ps27
Saccharomyces - ps28
Arboricola_clade - ps29
Cerevisiae_clade - ps30
Saccharomyces_cerevisiae - ps31
p-value
3e-05
2e-04
1e-03
1e-02
6e-02
Log-odds
0.5
1.0
1.5
2.0
2.5
3.0
3.5
4.0

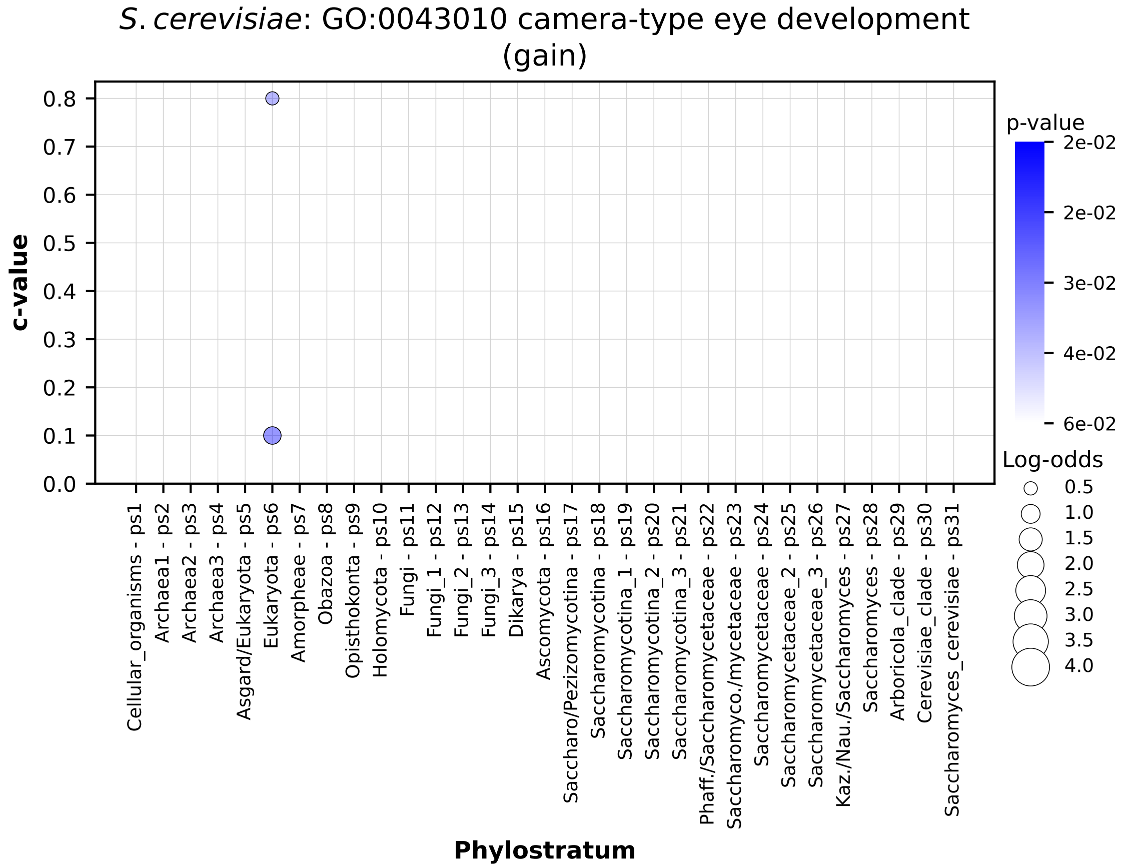
S. cerevisiae: GO:0043010 camera-type eye development (gain)
c-value
0.0
0.1
0.2
0.3
0.4
0.5
0.6
0.7
0.8
p-value
2e-02
2e-02
3e-02
4e-02
6e-02
Log-odds
0.5
1.0
1.5
2.0
2.5
3.0
3.5
4.0
Cellular_organisms - ps1
Archaea1 - ps2
Archaea2 - ps3
Archaea3 - ps4
Asgard/Eukaryota - ps5
Eukaryota - ps6
Amorpheae - ps7
Obazoa - ps8
Opisthokonta - ps9
Holomycota - ps10
Fungi - ps11
Fungi_1 - ps12
Fungi_2 - ps13
Fungi_3 - ps14
Dikarya - ps15
Ascomycota - ps16
Saccharo/Pezizomycotina - ps17
Saccharomycotina - ps18
Saccharomycotina_1 - ps19
Saccharomycotina_2 - ps20
Saccharomycotina_3 - ps21
Phaff./Saccharomycetaceae - ps22
Saccharomyco./mycetaceae - ps23
Saccharomycetaceae - ps24
Saccharomycetaceae_2 - ps25
Saccharomycetaceae_3 - ps26
Kaz./Nau./Saccharomyces - ps27
Saccharomyces - ps28
Arboricola_clade - ps29
Cerevisiae_clade - ps30
Saccharomyces_cerevisiae - ps31
Phylostratum

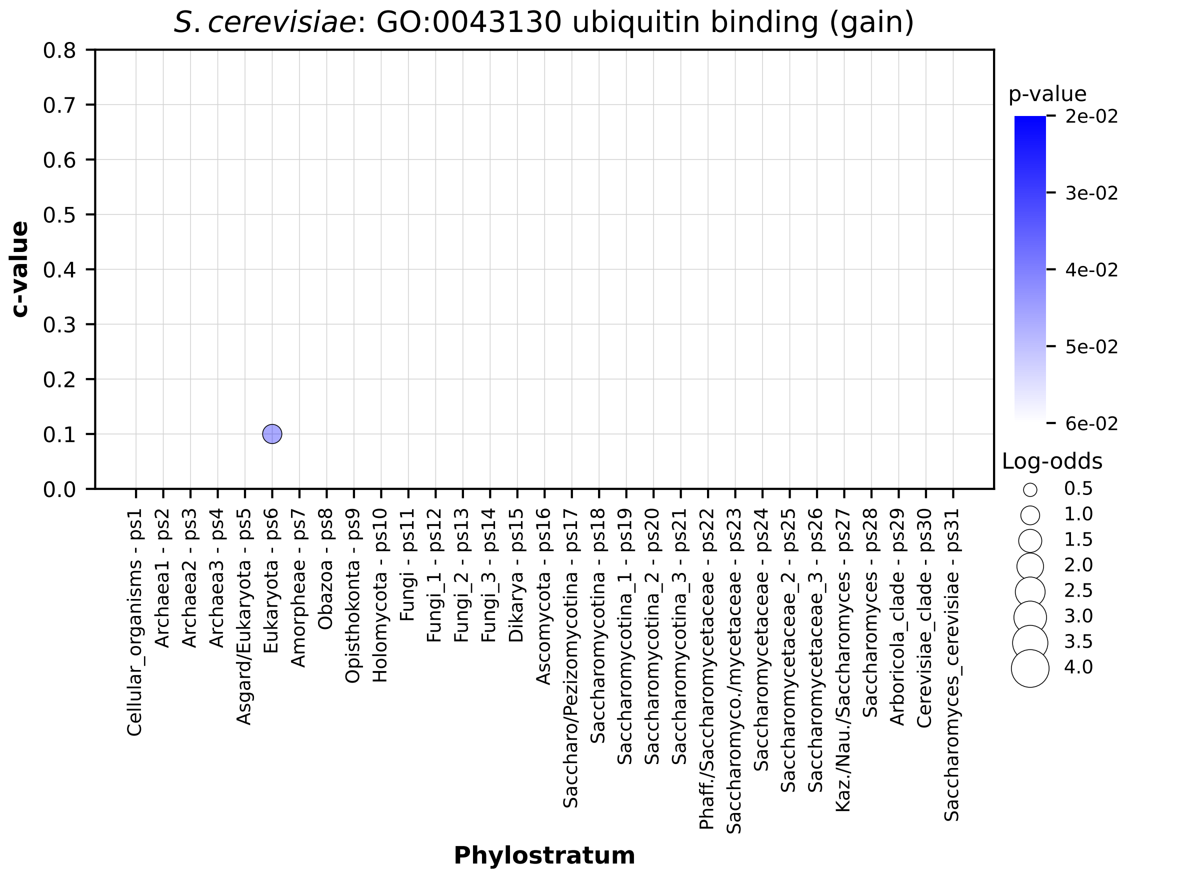
S. cerevisiae: GO:0043130 ubiquitin binding (gain)
c-value
0.0
0.1
0.2
0.3
0.4
0.5
0.6
0.7
0.8
Phylostratum
Cellular_organisms - ps1
Archaea1 - ps2
Archaea2 - ps3
Archaea3 - ps4
Asgard/Eukaryota - ps5
Eukaryota - ps6
Amorpheae - ps7
Obazoa - ps8
Opisthokonta - ps9
Holomycota - ps10
Fungi - ps11
Fungi_1 - ps12
Fungi_2 - ps13
Fungi_3 - ps14
Dikarya - ps15
Ascomycota - ps16
Saccharo/Pezizomycotina - ps17
Saccharomycotina - ps18
Saccharomycotina_1 - ps19
Saccharomycotina_2 - ps20
Saccharomycotina_3 - ps21
Phaff./Saccharomycetaceae - ps22
Saccharomyco./mycetaceae - ps23
Saccharomycetaceae - ps24
Saccharomycetaceae_2 - ps25
Saccharomycetaceae_3 - ps26
Kaz./Nau./Saccharomyces - ps27
Saccharomyces - ps28
Arboricola_clade - ps29
Cerevisiae_clade - ps30
Saccharomyces_cerevisiae - ps31
p-value
2e-02
3e-02
4e-02
5e-02
6e-02
Log-odds
0.5
1.0
1.5
2.0
2.5
3.0
3.5
4.0

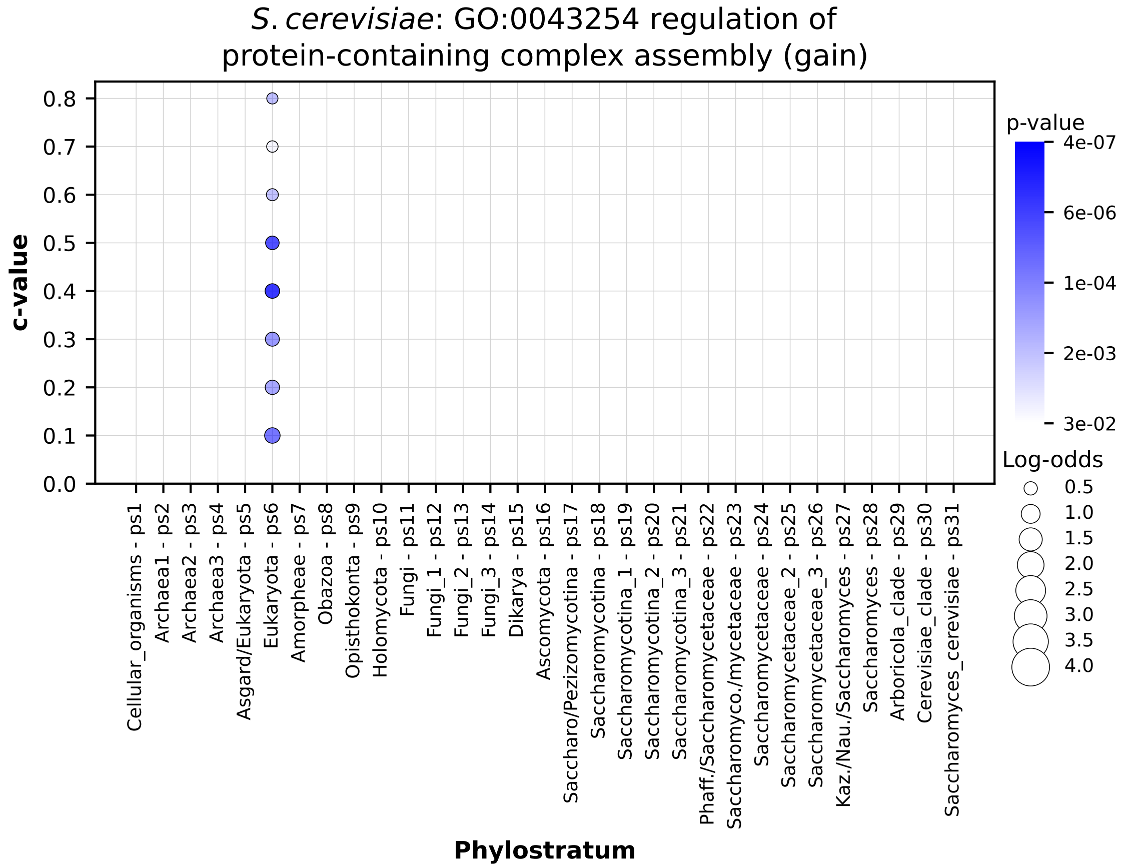

S. cerevisiae: GO:0043254 regulation of protein-containing complex assembly (gain)
c-value
0.0
0.1
0.2
0.3
0.4
0.5
0.6
0.7
0.8
p-value
4e-07
6e-06
1e-04
2e-03
3e-02
Log-odds
0.5
1.0
1.5
2.0
2.5
3.0
3.5
4.0
Cellular_organisms - ps1
Archaea1 - ps2
Archaea2 - ps3
Archaea3 - ps4
Asgard/Eukaryota - ps5
Eukaryota - ps6
Amorpheae - ps7
Obazoa - ps8
Opisthokonta - ps9
Holomycota - ps10
Fungi - ps11
Fungi_1 - ps12
Fungi_2 - ps13
Fungi_3 - ps14
Dikarya - ps15
Ascomycota - ps16
Saccharo/Pezizomycotina - ps17
Saccharomycotina - ps18
Saccharomycotina_1 - ps19
Saccharomycotina_2 - ps20
Saccharomycotina_3 - ps21
Phaff./Saccharomycetaceae - ps22
Saccharomyco./mycetaceae - ps23
Saccharomycetaceae - ps24
Saccharomycetaceae_2 - ps25
Saccharomycetaceae_3 - ps26
Kaz./Nau./Saccharomyces - ps27
Saccharomyces - ps28
Arboricola_clade - ps29
Cerevisiae_clade - ps30
Saccharomyces_cerevisiae - ps31
Phylostratum

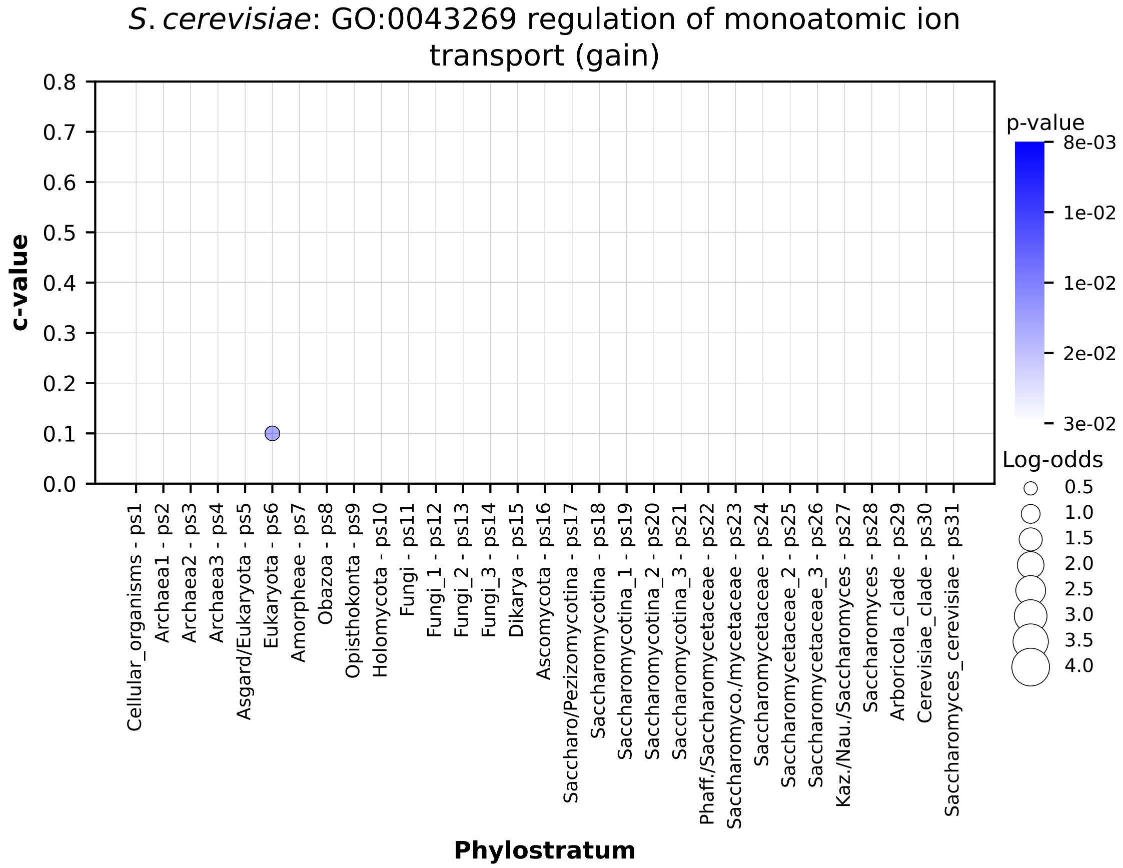
S. cerevisiae: GO:0043269 regulation of monoatomic ion transport (gain)
c-value
0.0
0.1
0.2
0.3
0.4
0.5
0.6
0.7
0.8
Phylostratum
Cellular_organisms - ps1
Archaea1 - ps2
Archaea2 - ps3
Archaea3 - ps4
Asgard/Eukaryota - ps5
Eukaryota - ps6
Amorpheae - ps7
Obazoa - ps8
Opisthokonta - ps9
Holomycota - ps10
Fungi - ps11
Fungi_1 - ps12
Fungi_2 - ps13
Fungi_3 - ps14
Dikarya - ps15
Ascomycota - ps16
Saccharo/Pezizomycotina - ps17
Saccharomycotina - ps18
Saccharomycotina_1 - ps19
Saccharomycotina_2 - ps20
Saccharomycotina_3 - ps21
Phaff./Saccharomycetaceae - ps22
Saccharomyco./mycetaceae - ps23
Saccharomycetaceae - ps24
Saccharomycetaceae_2 - ps25
Saccharomycetaceae_3 - ps26
Kaz./Nau./Saccharomyces - ps27
Saccharomyces - ps28
Arboricola_clade - ps29
Cerevisiae_clade - ps30
Saccharomyces_cerevisiae - ps31
p-value
8e-03
1e-02
1e-02
2e-02
3e-02
Log-odds
0.5
1.0
1.5
2.0
2.5
3.0
3.5
4.0

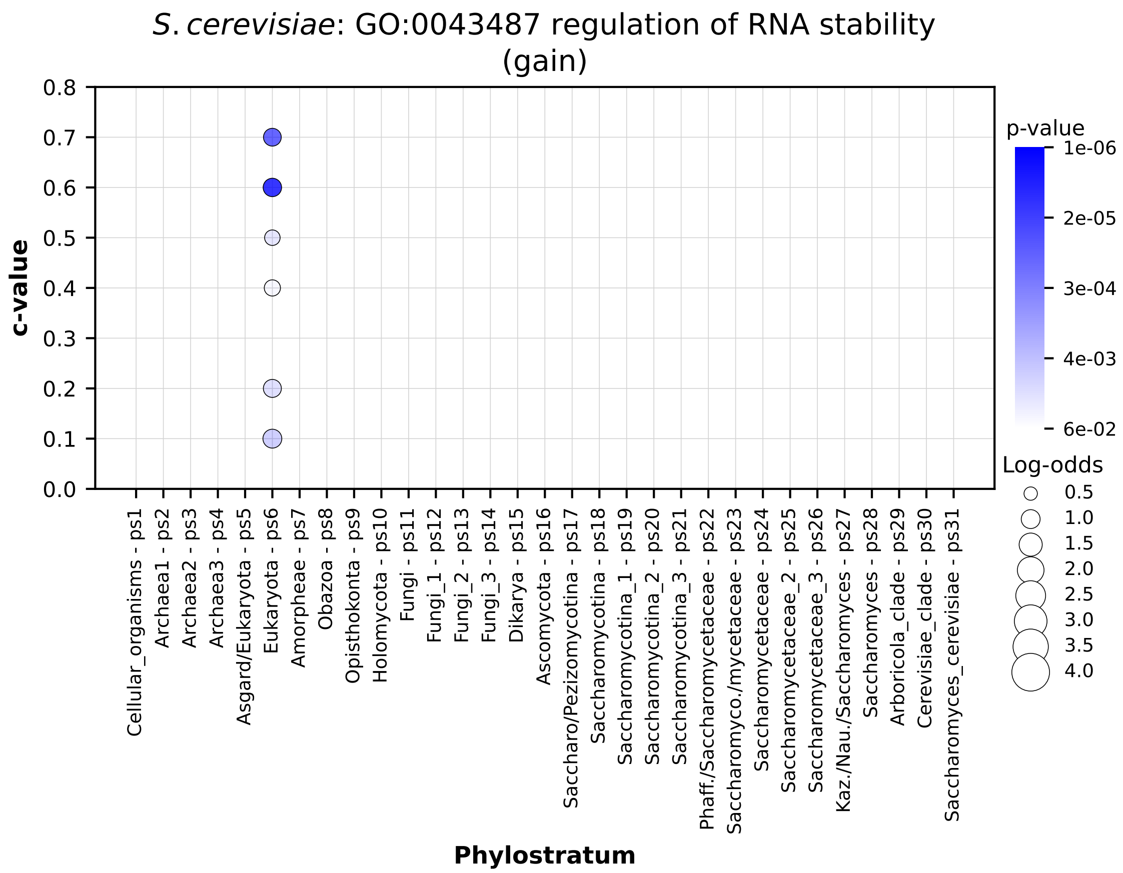

S. cerevisiae: GO:0043487 regulation of RNA stability (gain)
c-value
0.0
0.1
0.2
0.3
0.4
0.5
0.6
0.7
0.8
Phylostratum
Cellular_organisms - ps1
Archaea1 - ps2
Archaea2 - ps3
Archaea3 - ps4
Asgard/Eukaryota - ps5
Eukaryota - ps6
Amorpheae - ps7
Obazoa - ps8
Opisthokonta - ps9
Holomycota - ps10
Fungi - ps11
Fungi_1 - ps12
Fungi_2 - ps13
Fungi_3 - ps14
Dikarya - ps15
Ascomycota - ps16
Saccharo/Pezizomycotina - ps17
Saccharomycotina - ps18
Saccharomycotina_1 - ps19
Saccharomycotina_2 - ps20
Saccharomycotina_3 - ps21
Phaff./Saccharomycetaceae - ps22
Saccharomyco./mycetaceae - ps23
Saccharomycetaceae - ps24
Saccharomycetaceae_2 - ps25
Saccharomycetaceae_3 - ps26
Kaz./Nau./Saccharomyces - ps27
Saccharomyces - ps28
Arboricola_clade - ps29
Cerevisiae_clade - ps30
Saccharomyces_cerevisiae - ps31
p-value
1e-06
2e-05
3e-04
4e-03
6e-02
Log-odds
0.5
1.0
1.5
2.0
2.5
3.0
3.5
4.0

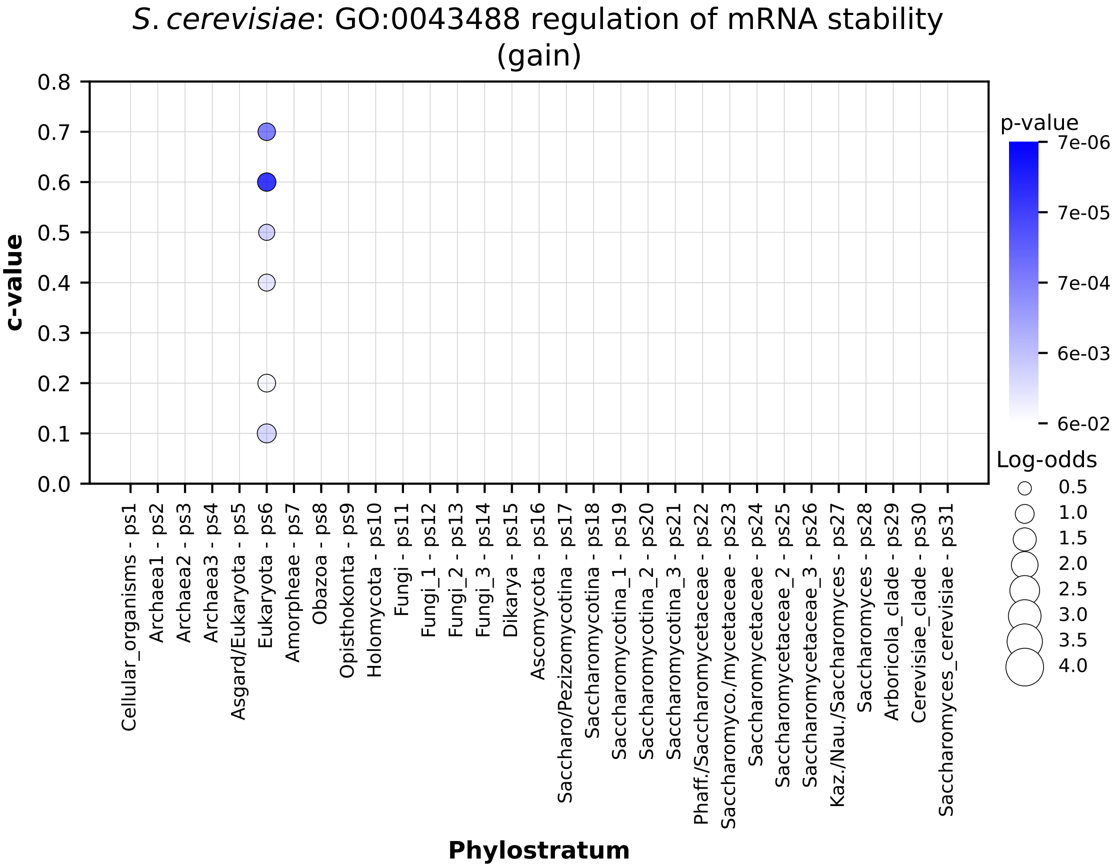
S. cerevisiae: GO:0043488 regulation of mRNA stability (gain)
c-value
0.0
0.1
0.2
0.3
0.4
0.5
0.6
0.7
0.8
p-value
7e-06
7e-05
7e-04
6e-03
6e-02
Log-odds
0.5
1.0
1.5
2.0
2.5
3.0
3.5
4.0
Cellular_organisms - ps1
Archaea1 - ps2
Archaea2 - ps3
Archaea3 - ps4
Asgard/Eukaryota - ps5
Eukaryota - ps6
Amorpheae - ps7
Obazoa - ps8
Opisthokonta - ps9
Holomycota - ps10
Fungi - ps11
Fungi_1 - ps12
Fungi_2 - ps13
Fungi_3 - ps14
Dikarya - ps15
Ascomycota - ps16
Saccharo/Pezizomycotina - ps17
Saccharomycotina - ps18
Saccharomycotina_1 - ps19
Saccharomycotina_2 - ps20
Saccharomycotina_3 - ps21
Phaff./Saccharomycetaceae - ps22
Saccharomyco./mycetaceae - ps23
Saccharomycetaceae - ps24
Saccharomycetaceae_2 - ps25
Saccharomycetaceae_3 - ps26
Kaz./Nau./Saccharomyces - ps27
Saccharomyces - ps28
Arboricola_clade - ps29
Cerevisiae_clade - ps30
Saccharomyces_cerevisiae - ps31
Phylostratum

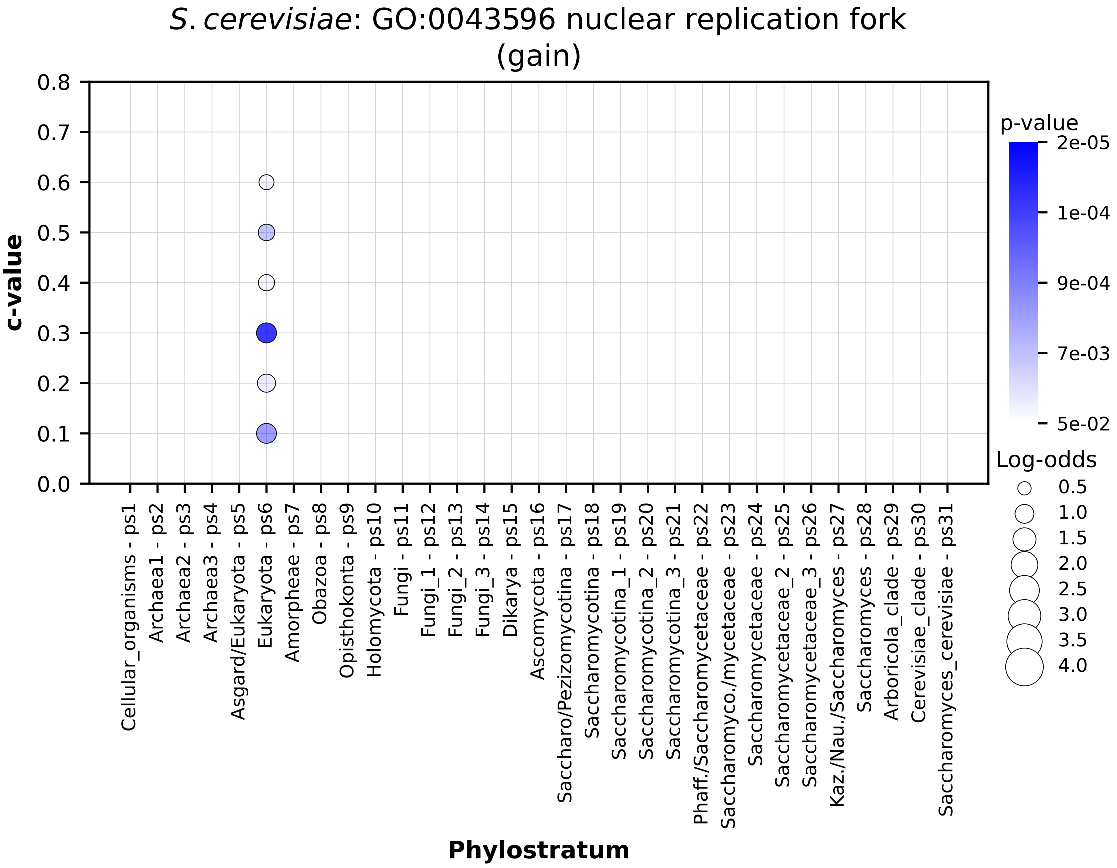

S. cerevisiae: GO:0043596 nuclear replication fork
(gain)
c-value
0.8
0.7
0.6
0.5
0.4
0.3
0.2
0.1
0.0
p-value
2e-05
1e-04
9e-04
7e-03
5e-02
Log-odds
0.5
1.0
1.5
2.0
2.5
3.0
3.5
4.0
Cellular_organisms - ps1
Archaea1 - ps2
Archaea2 - ps3
Archaea3 - ps4
Asgard/Eukaryota - ps5
Eukaryota - ps6
Amorpheae - ps7
Obazoa - ps8
Opisthokonta - ps9
Holomycota - ps10
Fungi - ps11
Fungi_1 - ps12
Fungi_2 - ps13
Fungi_3 - ps14
Dikarya - ps15
Ascomycota - ps16
Saccharo/Pezizomycotina - ps17
Saccharomycotina - ps18
Saccharomycotina_1 - ps19
Saccharomycotina_2 - ps20
Saccharomycotina_3 - ps21
Phaff./Saccharomycetaceae - ps22
Saccharomyco./mycetaceae - ps23
Saccharomycetaceae - ps24
Saccharomycetaceae_2 - ps25
Saccharomycetaceae_3 - ps26
Kaz./Nau./Saccharomyces - ps27
Saccharomyces - ps28
Arboricola_clade - ps29
Cerevisiae_clade - ps30
Saccharomyces_cerevisiae - ps31
Phylostratum

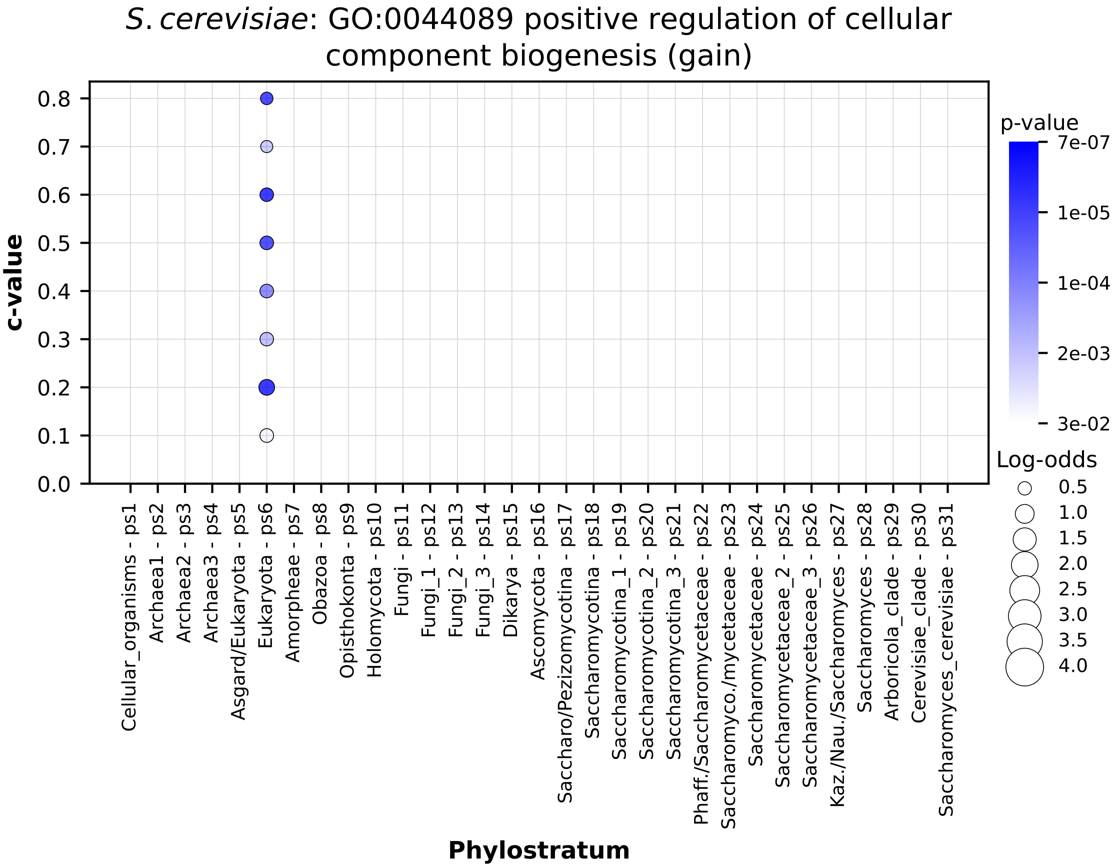

S. cerevisiae: GO:0044089 positive regulation of cellular component biogenesis (gain)
c-value
0.0
0.1
0.2
0.3
0.4
0.5
0.6
0.7
0.8
Phylostratum
Cellular_organisms - ps1
Archaea1 - ps2
Archaea2 - ps3
Archaea3 - ps4
Asgard/Eukaryota - ps5
Eukaryota - ps6
Amorpheae - ps7
Obazoa - ps8
Opisthokonta - ps9
Holomycota - ps10
Fungi - ps11
Fungi_1 - ps12
Fungi_2 - ps13
Fungi_3 - ps14
Dikarya - ps15
Ascomycota - ps16
Saccharo/Pezizomycotina - ps17
Saccharomycotina - ps18
Saccharomycotina_1 - ps19
Saccharomycotina_2 - ps20
Saccharomycotina_3 - ps21
Phaff./Saccharomycetaceae - ps22
Saccharomyco./mycetaceae - ps23
Saccharomycetaceae - ps24
Saccharomycetaceae_2 - ps25
Saccharomycetaceae_3 - ps26
Kaz./Nau./Saccharomyces - ps27
Saccharomyces - ps28
Arboricola_clade - ps29
Cerevisiae_clade - ps30
Saccharomyces_cerevisiae - ps31
p-value
7e-07
1e-05
1e-04
2e-03
3e-02
Log-odds
0.5
1.0
1.5
2.0
2.5
3.0
3.5
4.0

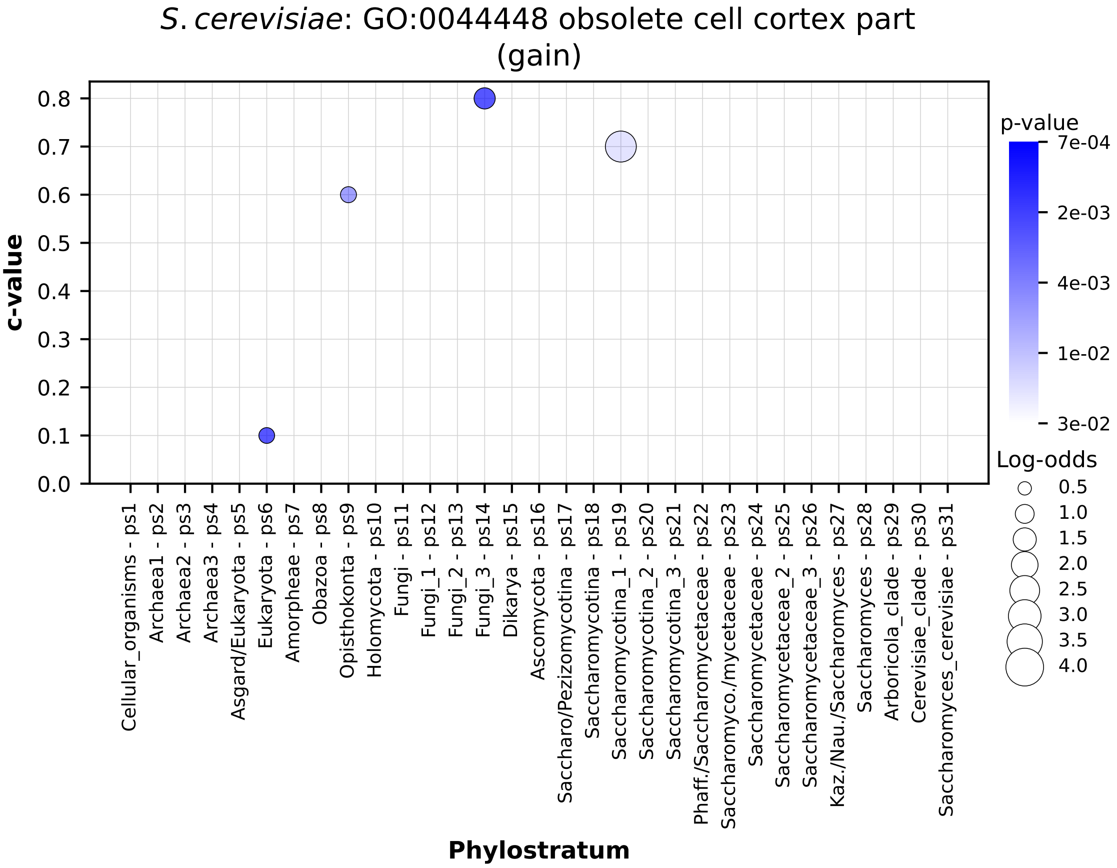

S. cerevisiae: GO:0044448 obsolete cell cortex part
(gain)
c-value
0.0
0.1
0.2
0.3
0.4
0.5
0.6
0.7
0.8
p-value
7e-04
2e-03
4e-03
1e-02
3e-02
Log-odds
0.5
1.0
1.5
2.0
2.5
3.0
3.5
4.0
Cellular_organisms - ps1
Archaea1 - ps2
Archaea2 - ps3
Archaea3 - ps4
Asgard/Eukaryota - ps5
Eukaryota - ps6
Amorpheae - ps7
Obazoa - ps8
Opisthokonta - ps9
Holomycota - ps10
Fungi - ps11
Fungi_1 - ps12
Fungi_2 - ps13
Fungi_3 - ps14
Dikarya - ps15
Ascomycota - ps16
Saccharo/Pezizomycotina - ps17
Saccharomycotina - ps18
Saccharomycotina_1 - ps19
Saccharomycotina_2 - ps20
Saccharomycotina_3 - ps21
Phaff./Saccharomycetaceae - ps22
Saccharomyco./mycetaceae - ps23
Saccharomycetaceae - ps24
Saccharomycetaceae_2 - ps25
Saccharomycetaceae_3 - ps26
Kaz./Nau./Saccharomyces - ps27
Saccharomyces - ps28
Arboricola_clade - ps29
Cerevisiae_clade - ps30
Saccharomyces_cerevisiae - ps31
Phylostratum

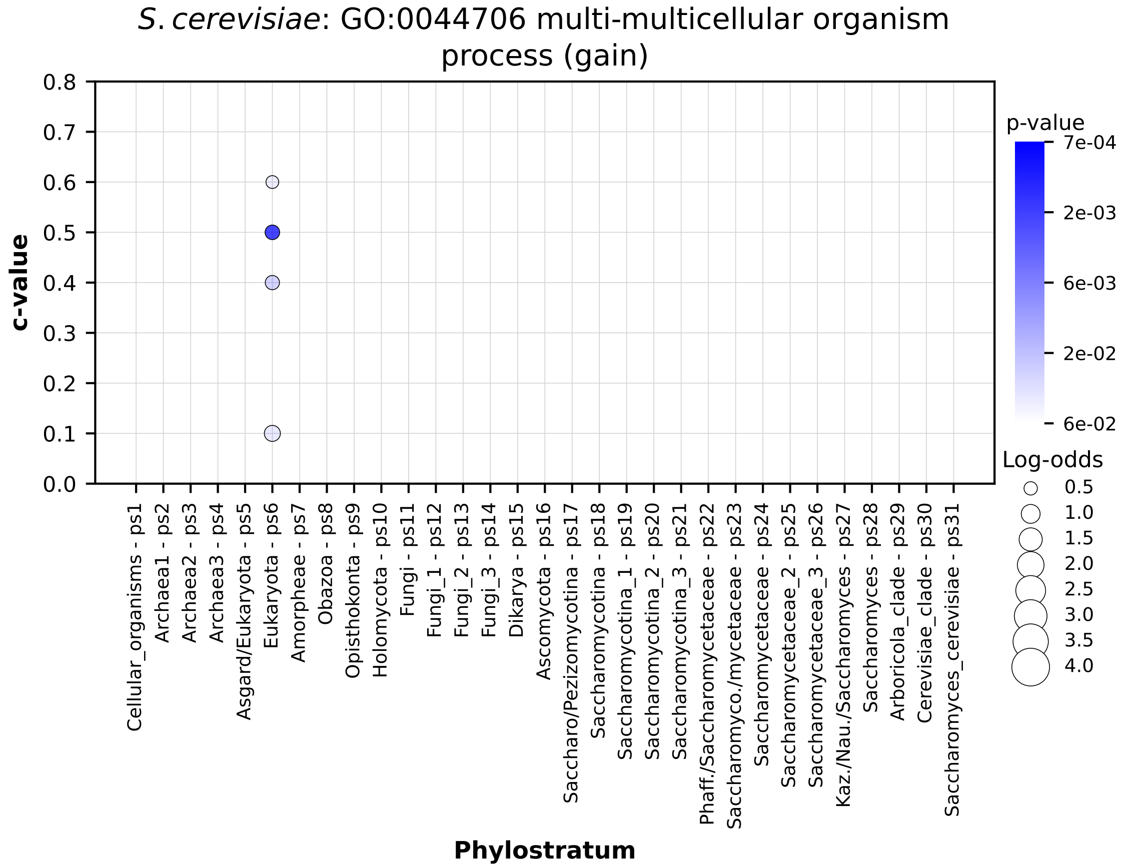

S. cerevisiae: GO:0044706 multi-multicellular organism process (gain)
c-value
0.8
0.7
0.6
0.5
0.4
0.3
0.2
0.1
0.0
p-value
7e-04
2e-03
6e-03
2e-02
6e-02
Log-odds
0.5
1.0
1.5
2.0
2.5
3.0
3.5
4.0
Cellular_organisms - ps1
Archaea1 - ps2
Archaea2 - ps3
Archaea3 - ps4
Asgard/Eukaryota - ps5
Eukaryota - ps6
Amorpheae - ps7
Obazoa - ps8
Opisthokonta - ps9
Holomycota - ps10
Fungi - ps11
Fungi_1 - ps12
Fungi_2 - ps13
Fungi_3 - ps14
Dikarya - ps15
Ascomycota - ps16
Saccharo/Pezizomycotina - ps17
Saccharomycotina - ps18
Saccharomycotina_1 - ps19
Saccharomycotina_2 - ps20
Saccharomycotina_3 - ps21
Phaff./Saccharomycetaceae - ps22
Saccharomyco./mycetaceae - ps23
Saccharomycetaceae - ps24
Saccharomycetaceae_2 - ps25
Saccharomycetaceae_3 - ps26
Kaz./Nau./Saccharomyces - ps27
Saccharomyces - ps28
Arboricola_clade - ps29
Cerevisiae_clade - ps30
Saccharomyces_cerevisiae - ps31
Phylostratum

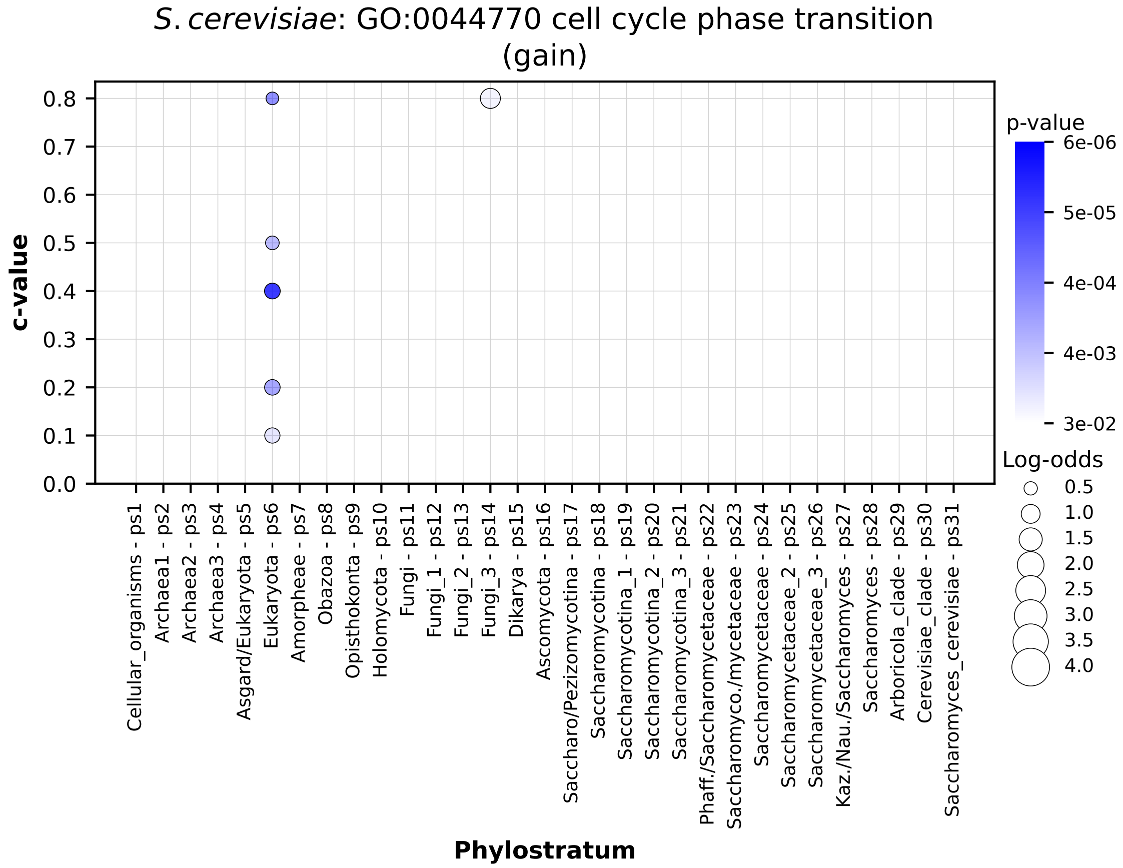
S. cerevisiae: GO:0044770 cell cycle phase transition (gain)
c-value
0.0
0.1
0.2
0.3
0.4
0.5
0.6
0.7
0.8
p-value
6e-06
5e-05
4e-04
4e-03
3e-02
Log-odds
0.5
1.0
1.5
2.0
2.5
3.0
3.5
4.0
Cellular_organisms - ps1
Archaea1 - ps2
Archaea2 - ps3
Archaea3 - ps4
Asgard/Eukaryota - ps5
Eukaryota - ps6
Amorpheae - ps7
Obazoa - ps8
Opisthokonta - ps9
Holomycota - ps10
Fungi - ps11
Fungi_1 - ps12
Fungi_2 - ps13
Fungi_3 - ps14
Dikarya - ps15
Ascomycota - ps16
Saccharo/Pezizomycotina - ps17
Saccharomycotina - ps18
Saccharomycotina_1 - ps19
Saccharomycotina_2 - ps20
Saccharomycotina_3 - ps21
Phaff./Saccharomycetaceae - ps22
Saccharomyco./mycetaceae - ps23
Saccharomycetaceae - ps24
Saccharomycetaceae_2 - ps25
Saccharomycetaceae_3 - ps26
Kaz./Nau./Saccharomyces - ps27
Saccharomyces - ps28
Arboricola_clade - ps29
Cerevisiae_clade - ps30
Saccharomyces_cerevisiae - ps31
Phylostratum

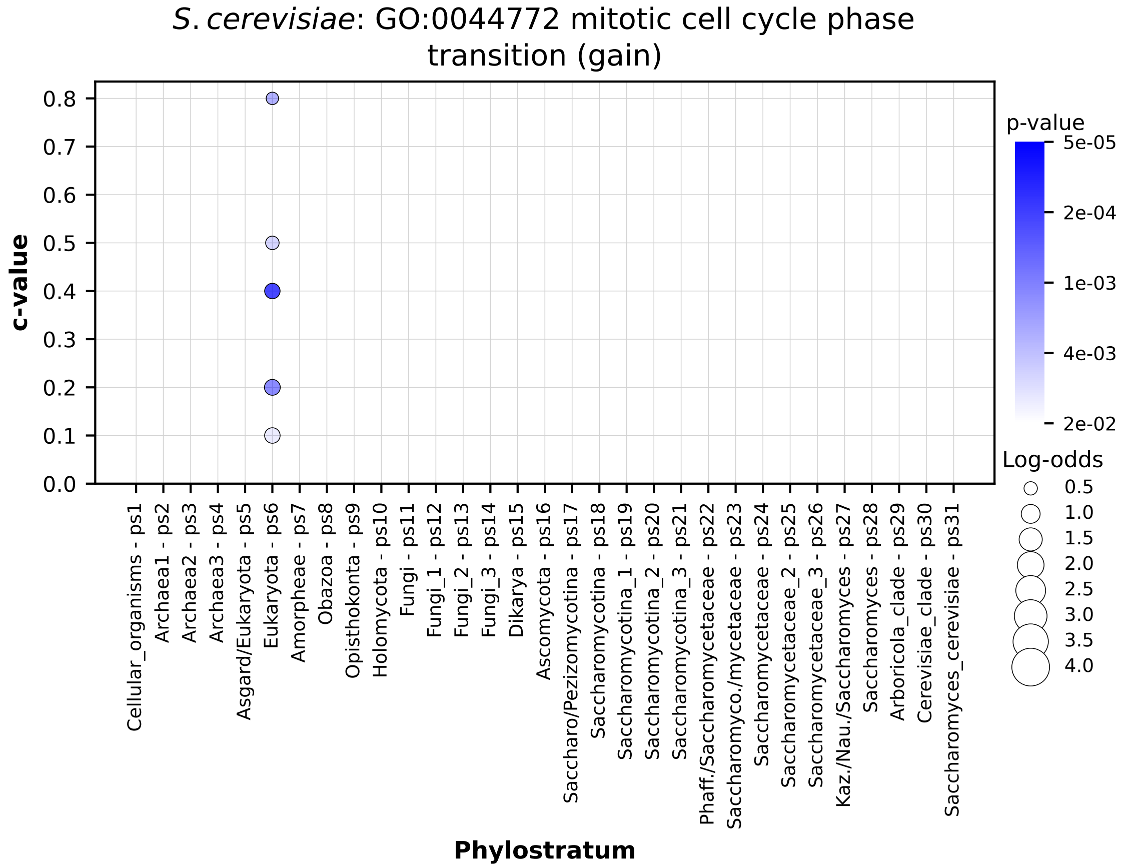

S. cerevisiae: GO:0044772 mitotic cell cycle phase transition (gain)
c-value
0.0
0.1
0.2
0.3
0.4
0.5
0.6
0.7
0.8
Phylostratum
Cellular_organisms - ps1
Archaea1 - ps2
Archaea2 - ps3
Archaea3 - ps4
Asgard/Eukaryota - ps5
Eukaryota - ps6
Amorpheae - ps7
Obazoa - ps8
Opisthokonta - ps9
Holomycota - ps10
Fungi - ps11
Fungi_1 - ps12
Fungi_2 - ps13
Fungi_3 - ps14
Dikarya - ps15
Ascomycota - ps16
Saccharo/Pezizomycotina - ps17
Saccharomycotina - ps18
Saccharomycotina_1 - ps19
Saccharomycotina_2 - ps20
Saccharomycotina_3 - ps21
Phaff./Saccharomycetaceae - ps22
Saccharomyco./mycetaceae - ps23
Saccharomycetaceae - ps24
Saccharomycetaceae_2 - ps25
Saccharomycetaceae_3 - ps26
Kaz./Nau./Saccharomyces - ps27
Saccharomyces - ps28
Arboricola_clade - ps29
Cerevisiae_clade - ps30
Saccharomyces_cerevisiae - ps31
p-value
5e-05
2e-04
1e-03
4e-03
2e-02
Log-odds
0.5
1.0
1.5
2.0
2.5
3.0
3.5
4.0

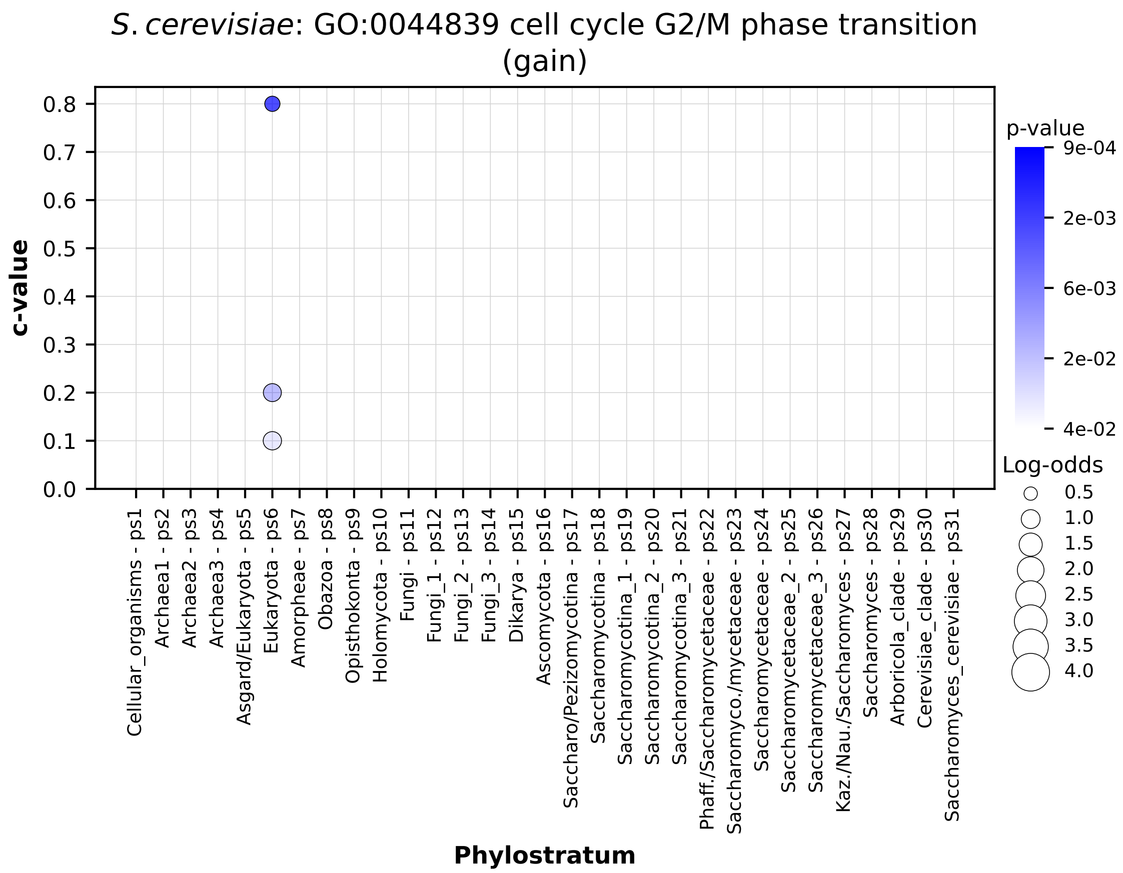

S. cerevisiae: GO:0044839 cell cycle G2/M phase transition (gain)
c-value
0.0
0.1
0.2
0.3
0.4
0.5
0.6
0.7
0.8
p-value
9e-04
2e-03
6e-03
2e-02
4e-02
Log-odds
0.5
1.0
1.5
2.0
2.5
3.0
3.5
4.0
Cellular_organisms - ps1
Archaea1 - ps2
Archaea2 - ps3
Archaea3 - ps4
Asgard/Eukaryota - ps5
Eukaryota - ps6
Amorpheae - ps7
Obazoa - ps8
Opisthokonta - ps9
Holomycota - ps10
Fungi - ps11
Fungi_1 - ps12
Fungi_2 - ps13
Fungi_3 - ps14
Dikarya - ps15
Ascomycota - ps16
Saccharo/Pezizomycotina - ps17
Saccharomycotina - ps18
Saccharomycotina_1 - ps19
Saccharomycotina_2 - ps20
Saccharomycotina_3 - ps21
Phaff./Saccharomycetaceae - ps22
Saccharomyco./mycetaceae - ps23
Saccharomycetaceae - ps24
Saccharomycetaceae_2 - ps25
Saccharomycetaceae_3 - ps26
Kaz./Nau./Saccharomyces - ps27
Saccharomyces - ps28
Arboricola_clade - ps29
Cerevisiae_clade - ps30
Saccharomyces_cerevisiae - ps31
Phylostratum

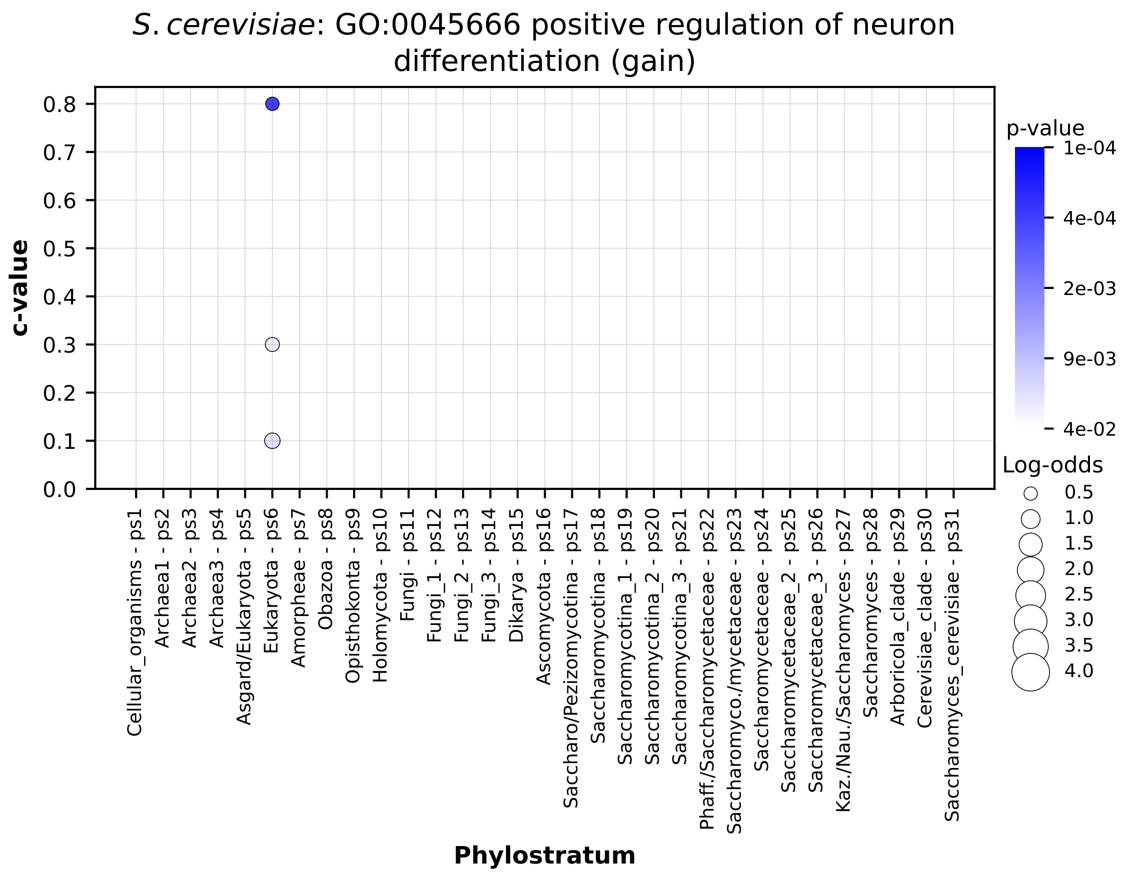

S. cerevisiae: GO:0045666 positive regulation of neuron differentiation (gain)
c-value
0.8
0.7
0.6
0.5
0.4
0.3
0.2
0.1
0.0
p-value
1e-04
4e-04
2e-03
9e-03
4e-02
Log-odds
0.5
1.0
1.5
2.0
2.5
3.0
3.5
4.0
Cellular_organisms - ps1
Archaea1 - ps2
Archaea2 - ps3
Archaea3 - ps4
Asgard/Eukaryota - ps5
Eukaryota - ps6
Amorpheae - ps7
Obazoa - ps8
Opisthokonta - ps9
Holomycota - ps10
Fungi - ps11
Fungi_1 - ps12
Fungi_2 - ps13
Fungi_3 - ps14
Dikarya - ps15
Ascomycota - ps16
Saccharo/Pezizomycotina - ps17
Saccharomycotina - ps18
Saccharomycotina_1 - ps19
Saccharomycotina_2 - ps20
Saccharomycotina_3 - ps21
Phaff./Saccharomycetaceae - ps22
Saccharomyco./mycetaceae - ps23
Saccharomycetaceae - ps24
Saccharomycetaceae_2 - ps25
Saccharomycetaceae_3 - ps26
Kaz./Nau./Saccharomyces - ps27
Saccharomyces - ps28
Arboricola_clade - ps29
Cerevisiae_clade - ps30
Saccharomyces_cerevisiae - ps31
Phylostratum

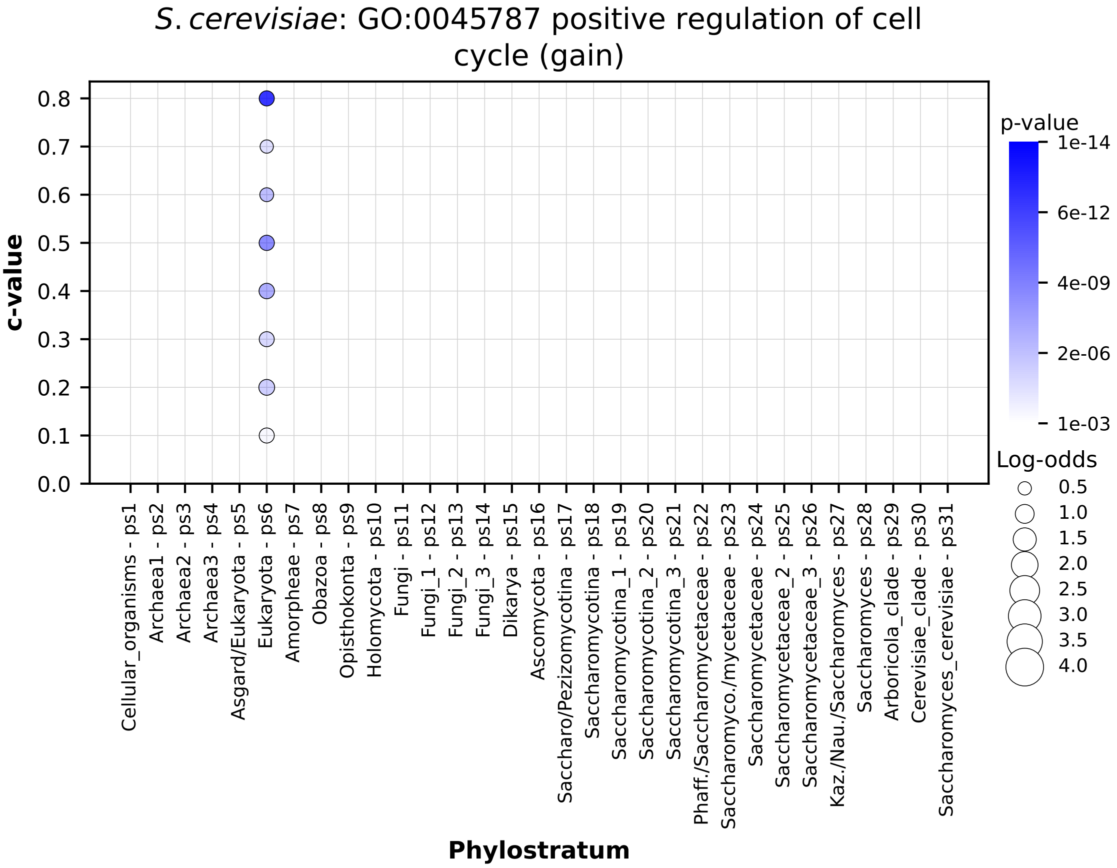

S. cerevisiae: GO:0045787 positive regulation of cell cycle (gain)
c-value
0.0
0.1
0.2
0.3
0.4
0.5
0.6
0.7
0.8
p-value
1e-14
6e-12
4e-09
2e-06
1e-03
Log-odds
0.5
1.0
1.5
2.0
2.5
3.0
3.5
4.0
Cellular_organisms - ps1
Archaea1 - ps2
Archaea2 - ps3
Archaea3 - ps4
Asgard/Eukaryota - ps5
Eukaryota - ps6
Amorpheae - ps7
Obazoa - ps8
Opisthokonta - ps9
Holomycota - ps10
Fungi - ps11
Fungi_1 - ps12
Fungi_2 - ps13
Fungi_3 - ps14
Dikarya - ps15
Ascomycota - ps16
Saccharo/Pezizomycotina - ps17
Saccharomycotina - ps18
Saccharomycotina_1 - ps19
Saccharomycotina_2 - ps20
Saccharomycotina_3 - ps21
Phaff./Saccharomycetaceae - ps22
Saccharomyco./mycetaceae - ps23
Saccharomycetaceae - ps24
Saccharomycetaceae_2 - ps25
Saccharomycetaceae_3 - ps26
Kaz./Nau./Saccharomyces - ps27
Saccharomyces - ps28
Arboricola_clade - ps29
Cerevisiae_clade - ps30
Saccharomyces_cerevisiae - ps31
Phylostratum

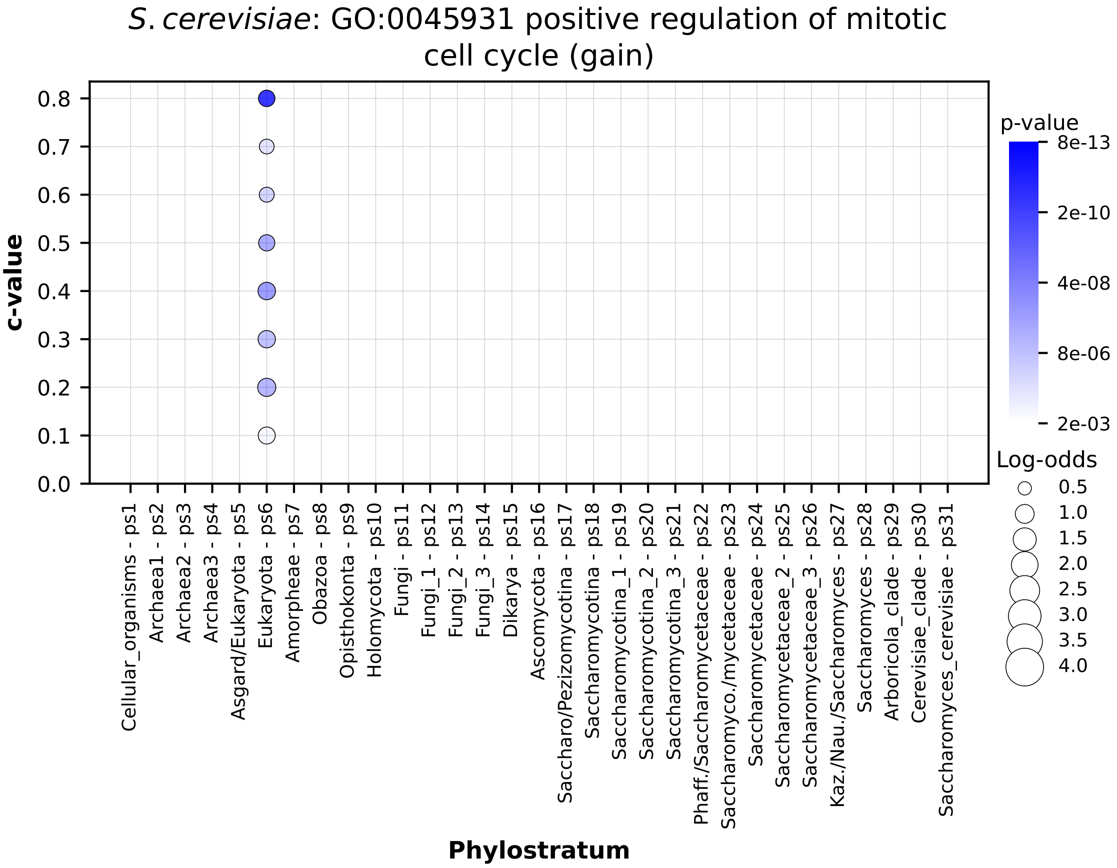
S. cerevisiae: GO:0045931 positive regulation of mitotic cell cycle (gain)
c-value
0.0
0.1
0.2
0.3
0.4
0.5
0.6
0.7
0.8
Phylostratum
Cellular_organisms - ps1
Archaea1 - ps2
Archaea2 - ps3
Archaea3 - ps4
Asgard/Eukaryota - ps5
Eukaryota - ps6
Amorpheae - ps7
Obazoa - ps8
Opisthokonta - ps9
Holomycota - ps10
Fungi - ps11
Fungi_1 - ps12
Fungi_2 - ps13
Fungi_3 - ps14
Dikarya - ps15
Ascomycota - ps16
Saccharo/Pezizomycotina - ps17
Saccharomycotina - ps18
Saccharomycotina_1 - ps19
Saccharomycotina_2 - ps20
Saccharomycotina_3 - ps21
Phaff./Saccharomycetaceae - ps22
Saccharomyco./mycetaceae - ps23
Saccharomycetaceae - ps24
Saccharomycetaceae_2 - ps25
Saccharomycetaceae_3 - ps26
Kaz./Nau./Saccharomyces - ps27
Saccharomyces - ps28
Arboricola_clade - ps29
Cerevisiae_clade - ps30
Saccharomyces_cerevisiae - ps31
p-value
8e-13
2e-10
4e-08
8e-06
2e-03
Log-odds
0.5
1.0
1.5
2.0
2.5
3.0
3.5
4.0

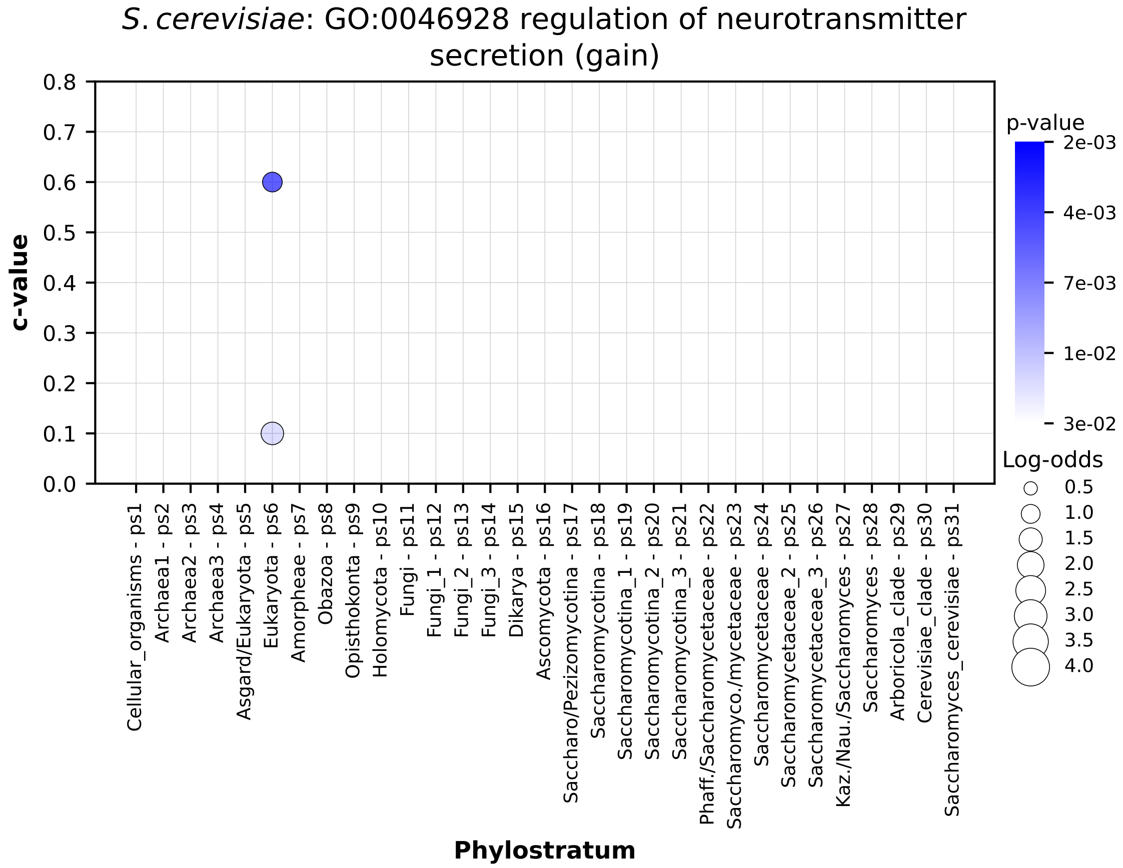

S. cerevisiae: GO:0046928 regulation of neurotransmitter secretion (gain)
c-value
0.8
0.7
0.6
0.5
0.4
0.3
0.2
0.1
0.0
p-value
2e-03
4e-03
7e-03
1e-02
3e-02
Log-odds
0.5
1.0
1.5
2.0
2.5
3.0
3.5
4.0
Cellular_organisms - ps1
Archaea1 - ps2
Archaea2 - ps3
Archaea3 - ps4
Asgard/Eukaryota - ps5
Eukaryota - ps6
Amorpheae - ps7
Obazoa - ps8
Opisthokonta - ps9
Holomycota - ps10
Fungi - ps11
Fungi_1 - ps12
Fungi_2 - ps13
Fungi_3 - ps14
Dikarya - ps15
Ascomycota - ps16
Saccharo/Pezizomycotina - ps17
Saccharomycotina - ps18
Saccharomycotina_1 - ps19
Saccharomycotina_2 - ps20
Saccharomycotina_3 - ps21
Phaff./Saccharomycetaceae - ps22
Saccharomyco./mycetaceae - ps23
Saccharomycetaceae - ps24
Saccharomycetaceae_2 - ps25
Saccharomycetaceae_3 - ps26
Kaz./Nau./Saccharomyces - ps27
Saccharomyces - ps28
Arboricola_clade - ps29
Cerevisiae_clade - ps30
Saccharomyces_cerevisiae - ps31
Phylostratum

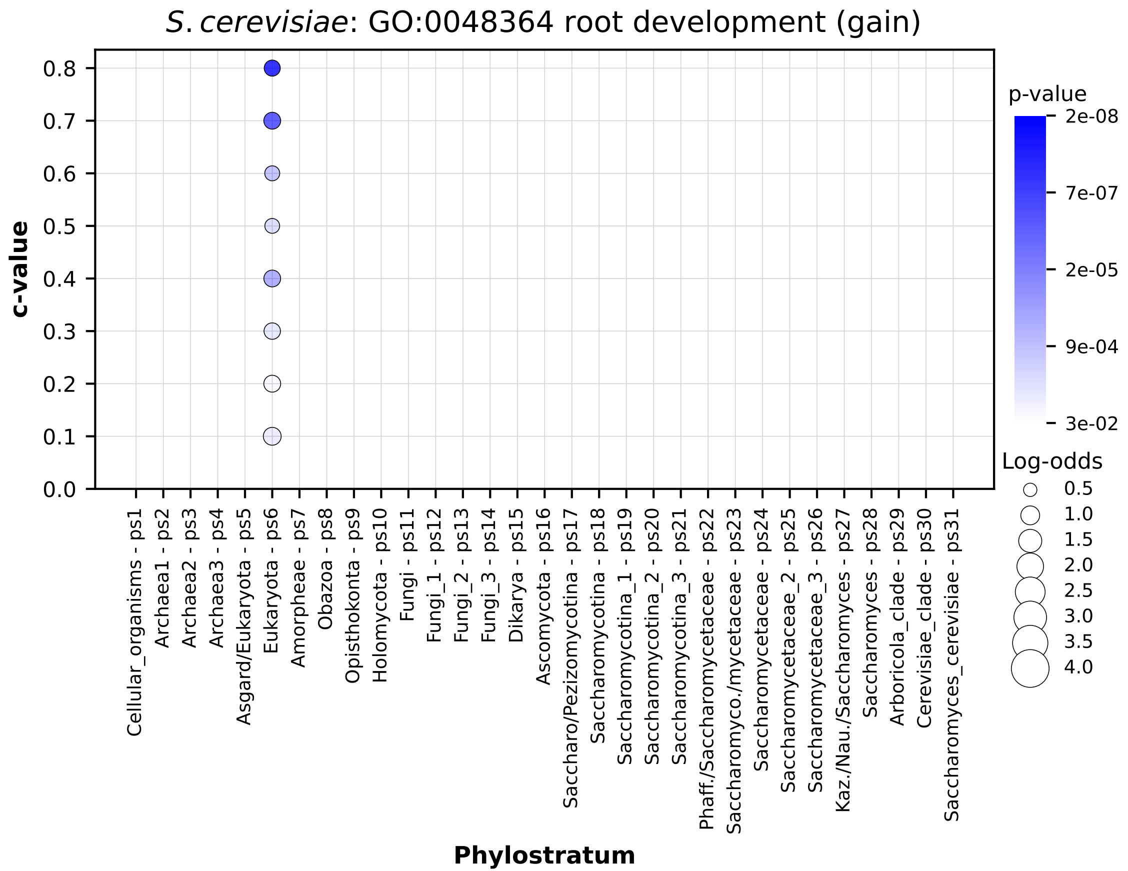

S. cerevisiae: GO:0048364 root development (gain)
c-value
0.0
0.1
0.2
0.3
0.4
0.5
0.6
0.7
0.8
p-value
2e-08
7e-07
2e-05
9e-04
3e-02
Log-odds
0.5
1.0
1.5
2.0
2.5
3.0
3.5
4.0
Cellular_organisms - ps1
Archaea1 - ps2
Archaea2 - ps3
Archaea3 - ps4
Asgard/Eukaryota - ps5
Eukaryota - ps6
Amorpheae - ps7
Obazoa - ps8
Opisthokonta - ps9
Holomycota - ps10
Fungi - ps11
Fungi_1 - ps12
Fungi_2 - ps13
Fungi_3 - ps14
Dikarya - ps15
Ascomycota - ps16
Saccharo/Pezizomycotina - ps17
Saccharomycotina - ps18
Saccharomycotina_1 - ps19
Saccharomycotina_2 - ps20
Saccharomycotina_3 - ps21
Phaff./Saccharomycetaceae - ps22
Saccharomyco./mycetaceae - ps23
Saccharomycetaceae - ps24
Saccharomycetaceae_2 - ps25
Saccharomycetaceae_3 - ps26
Kaz./Nau./Saccharomyces - ps27
Saccharomyces - ps28
Arboricola_clade - ps29
Cerevisiae_clade - ps30
Saccharomyces_cerevisiae - ps31
Phylostratum

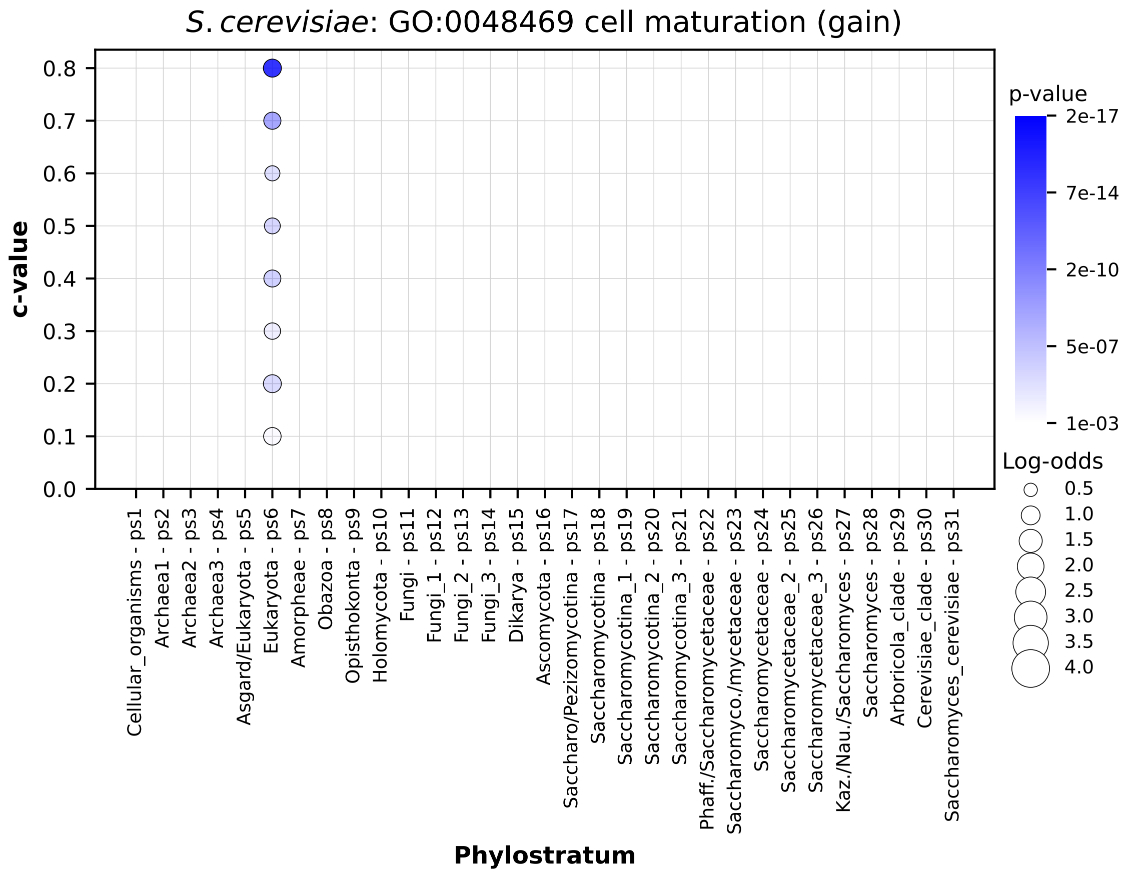

S. cerevisiae: GO:0048469 cell maturation (gain)
c-value
0.0
0.1
0.2
0.3
0.4
0.5
0.6
0.7
0.8
p-value
2e-17
7e-14
2e-10
5e-07
1e-03
Log-odds
0.5
1.0
1.5
2.0
2.5
3.0
3.5
4.0
Cellular_organisms - ps1
Archaea1 - ps2
Archaea2 - ps3
Archaea3 - ps4
Asgard/Eukaryota - ps5
Eukaryota - ps6
Amorpheae - ps7
Obazoa - ps8
Opisthokonta - ps9
Holomycota - ps10
Fungi - ps11
Fungi_1 - ps12
Fungi_2 - ps13
Fungi_3 - ps14
Dikarya - ps15
Ascomycota - ps16
Saccharo/Pezizomycotina - ps17
Saccharomycotina - ps18
Saccharomycotina_1 - ps19
Saccharomycotina_2 - ps20
Saccharomycotina_3 - ps21
Phaff./Saccharomycetaceae - ps22
Saccharomyco./mycetaceae - ps23
Saccharomycetaceae - ps24
Saccharomycetaceae_2 - ps25
Saccharomycetaceae_3 - ps26
Kaz./Nau./Saccharomyces - ps27
Saccharomyces - ps28
Arboricola_clade - ps29
Cerevisiae_clade - ps30
Saccharomyces_cerevisiae - ps31
Phylostratum

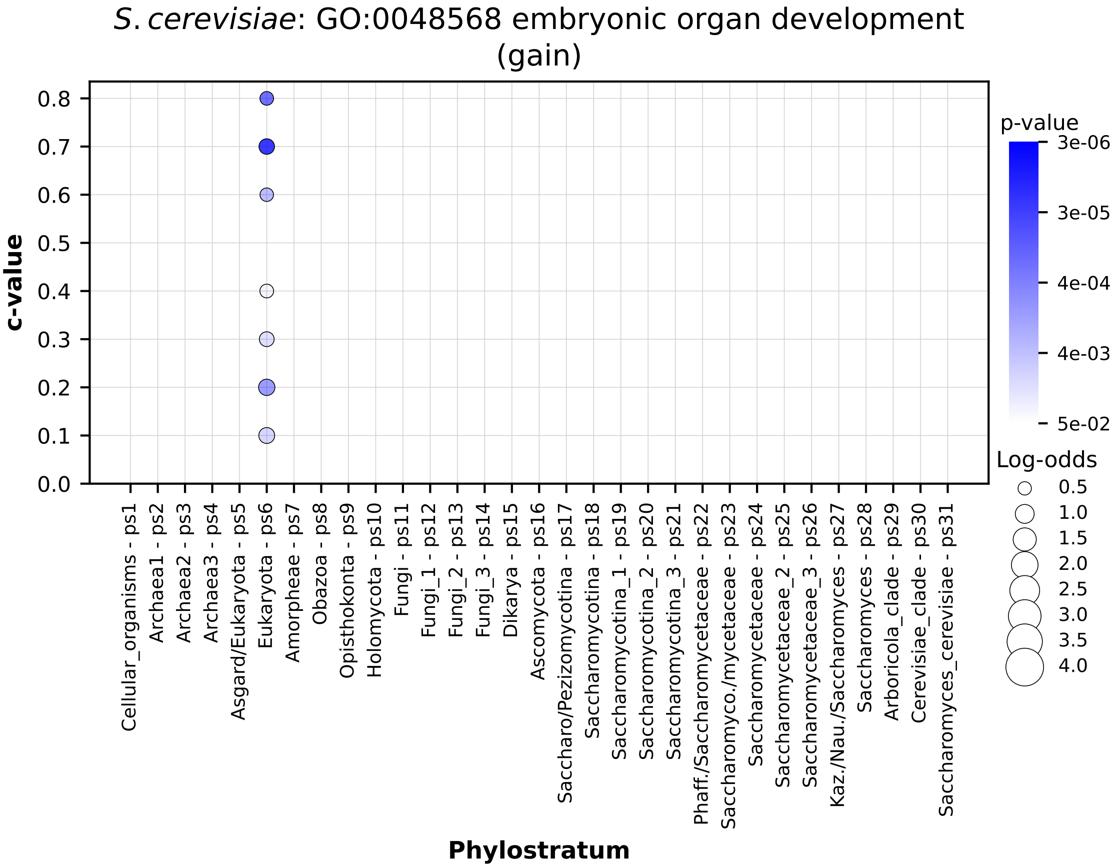

S. cerevisiae: GO:0048568 embryonic organ development
(gain)
c-value
0.0
0.1
0.2
0.3
0.4
0.5
0.6
0.7
0.8
p-value
3e-06
3e-05
4e-04
4e-03
5e-02
Log-odds
0.5
1.0
1.5
2.0
2.5
3.0
3.5
4.0
Cellular_organisms - ps1
Archaea1 - ps2
Archaea2 - ps3
Archaea3 - ps4
Asgard/Eukaryota - ps5
Eukaryota - ps6
Amorpheae - ps7
Obazoa - ps8
Opisthokonta - ps9
Holomycota - ps10
Fungi - ps11
Fungi_1 - ps12
Fungi_2 - ps13
Fungi_3 - ps14
Dikarya - ps15
Ascomycota - ps16
Saccharo/Pezizomycotina - ps17
Saccharomycotina - ps18
Saccharomycotina_1 - ps19
Saccharomycotina_2 - ps20
Saccharomycotina_3 - ps21
Phaff./Saccharomycetaceae - ps22
Saccharomyco./mycetaceae - ps23
Saccharomycetaceae - ps24
Saccharomycetaceae_2 - ps25
Saccharomycetaceae_3 - ps26
Kaz./Nau./Saccharomyces - ps27
Saccharomyces - ps28
Arboricola_clade - ps29
Cerevisiae_clade - ps30
Saccharomyces_cerevisiae - ps31
Phylostratum

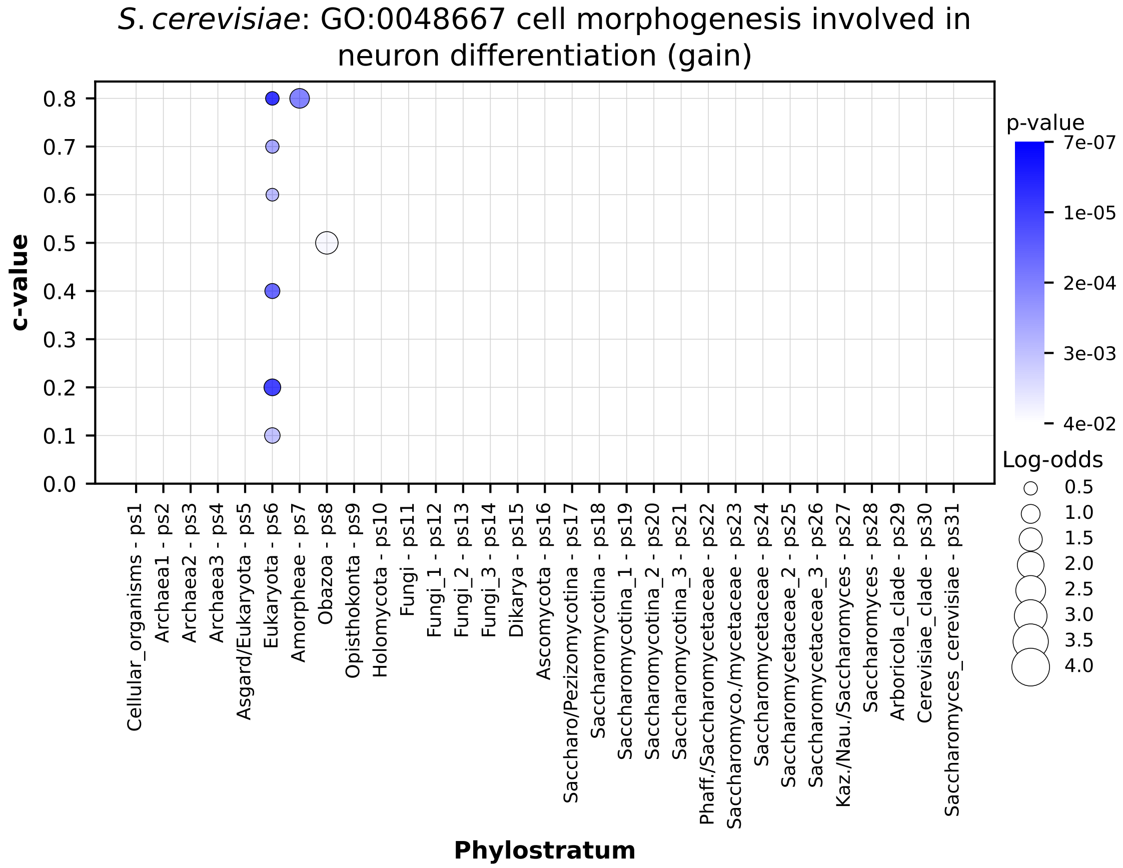
S. cerevisiae: GO:0048667 cell morphogenesis involved in neuron differentiation (gain)
c-value
0.0
0.1
0.2
0.3
0.4
0.5
0.6
0.7
0.8
Phylostratum
Cellular_organisms - ps1
Archaea1 - ps2
Archaea2 - ps3
Archaea3 - ps4
Asgard/Eukaryota - ps5
Eukaryota - ps6
Amorpheae - ps7
Obazoa - ps8
Opisthokonta - ps9
Holomycota - ps10
Fungi - ps11
Fungi_1 - ps12
Fungi_2 - ps13
Fungi_3 - ps14
Dikarya - ps15
Ascomycota - ps16
Saccharo/Pezizomycotina - ps17
Saccharomycotina - ps18
Saccharomycotina_1 - ps19
Saccharomycotina_2 - ps20
Saccharomycotina_3 - ps21
Phaff./Saccharomycetaceae - ps22
Saccharomyco./mycetaceae - ps23
Saccharomycetaceae - ps24
Saccharomycetaceae_2 - ps25
Saccharomycetaceae_3 - ps26
Kaz./Nau./Saccharomyces - ps27
Saccharomyces - ps28
Arboricola_clade - ps29
Cerevisiae_clade - ps30
Saccharomyces_cerevisiae - ps31
p-value
7e-07
1e-05
2e-04
3e-03
4e-02
Log-odds
0.5
1.0
1.5
2.0
2.5
3.0
3.5
4.0

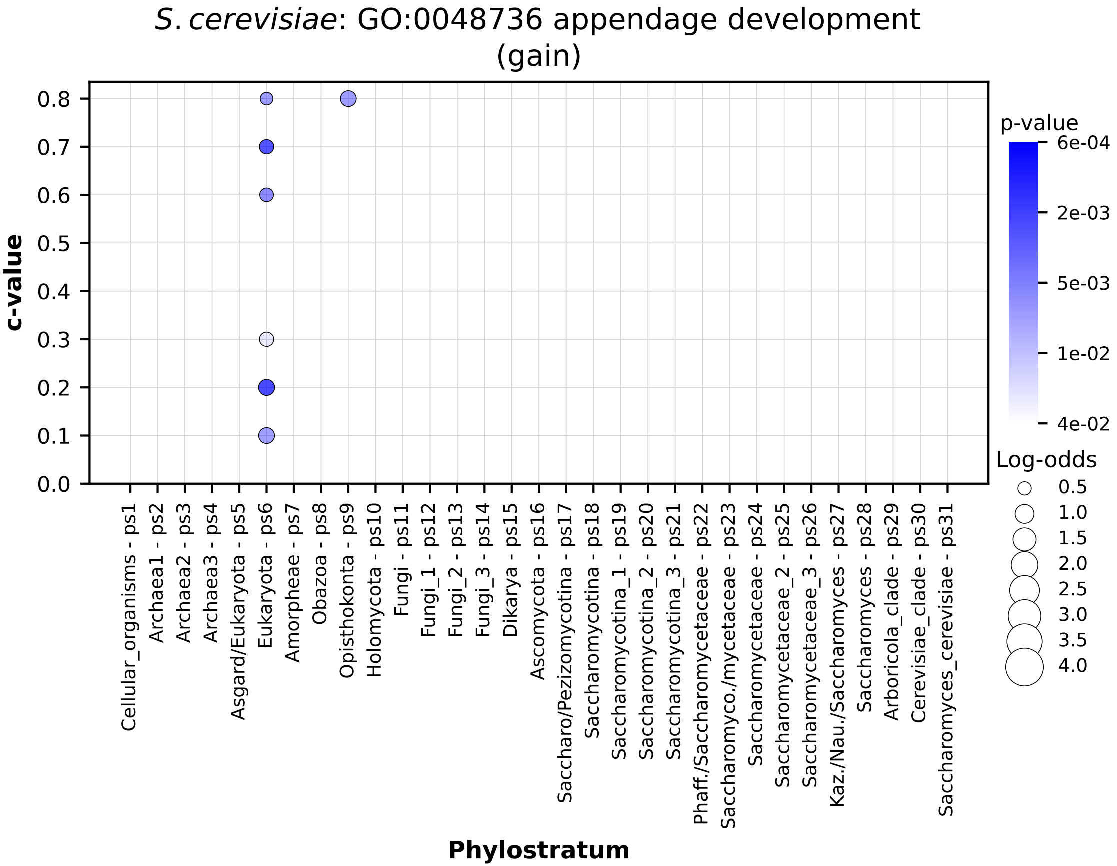

S. cerevisiae: GO:0048736 appendage development (gain)
c-value
0.0
0.1
0.2
0.3
0.4
0.5
0.6
0.7
0.8
Phylostratum
Cellular_organisms - ps1
Archaea1 - ps2
Archaea2 - ps3
Archaea3 - ps4
Asgard/Eukaryota - ps5
Eukaryota - ps6
Amorpheae - ps7
Obazoa - ps8
Opisthokonta - ps9
Holomycota - ps10
Fungi - ps11
Fungi_1 - ps12
Fungi_2 - ps13
Fungi_3 - ps14
Dikarya - ps15
Ascomycota - ps16
Saccharo/Pezizomycotina - ps17
Saccharomycotina - ps18
Saccharomycotina_1 - ps19
Saccharomycotina_2 - ps20
Saccharomycotina_3 - ps21
Phaff./Saccharomycetaceae - ps22
Saccharomyco./mycetaceae - ps23
Saccharomycetaceae - ps24
Saccharomycetaceae_2 - ps25
Saccharomycetaceae_3 - ps26
Kaz./Nau./Saccharomyces - ps27
Saccharomyces - ps28
Arboricola_clade - ps29
Cerevisiae_clade - ps30
Saccharomyces_cerevisiae - ps31
p-value
6e-04
2e-03
5e-03
1e-02
4e-02
Log-odds
0.5
1.0
1.5
2.0
2.5
3.0
3.5
4.0

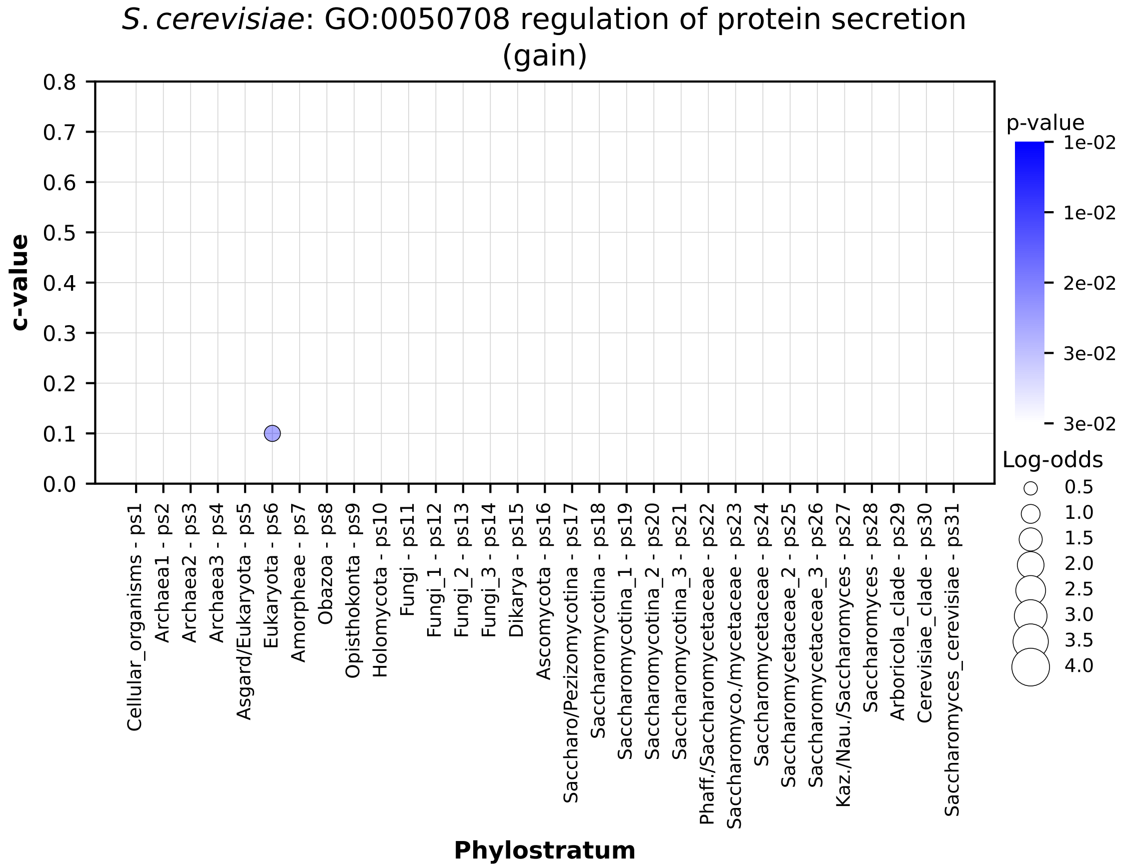

S. cerevisiae: GO:0050708 regulation of protein secretion
(gain)
c-value
0.0
0.1
0.2
0.3
0.4
0.5
0.6
0.7
0.8
Phylostratum
Cellular_organisms - ps1
Archaea1 - ps2
Archaea2 - ps3
Archaea3 - ps4
Asgard/Eukaryota - ps5
Eukaryota - ps6
Amorpheae - ps7
Obazoa - ps8
Opisthokonta - ps9
Holomycota - ps10
Fungi - ps11
Fungi_1 - ps12
Fungi_2 - ps13
Fungi_3 - ps14
Dikarya - ps15
Ascomycota - ps16
Saccharo/Pezizomycotina - ps17
Saccharomycotina - ps18
Saccharomycotina_1 - ps19
Saccharomycotina_2 - ps20
Saccharomycotina_3 - ps21
Phaff./Saccharomycetaceae - ps22
Saccharomyco./mycetaceae - ps23
Saccharomycetaceae - ps24
Saccharomycetaceae_2 - ps25
Saccharomycetaceae_3 - ps26
Kaz./Nau./Saccharomyces - ps27
Saccharomyces - ps28
Arboricola_clade - ps29
Cerevisiae_clade - ps30
Saccharomyces_cerevisiae - ps31
p-value
1e-02
1e-02
2e-02
3e-02
3e-02
Log-odds
0.5
1.0
1.5
2.0
2.5
3.0
3.5
4.0

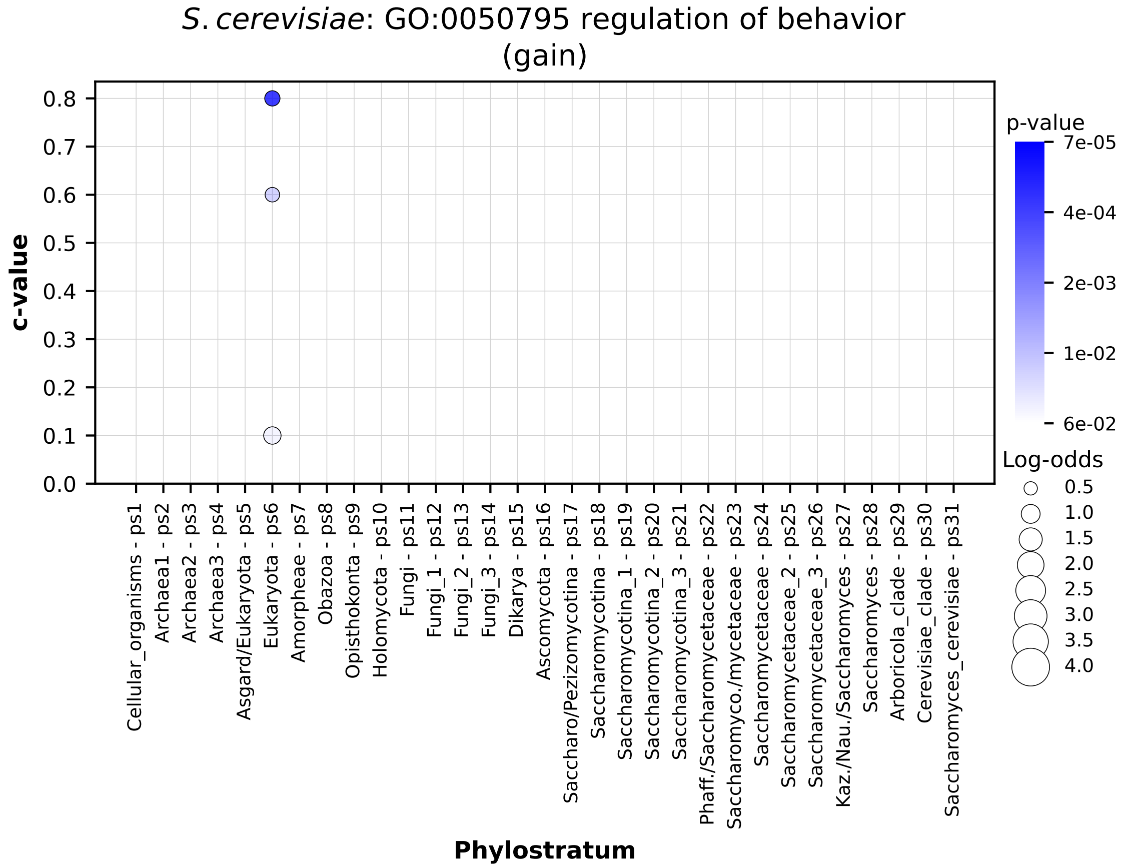
S. cerevisiae: GO:0050795 regulation of behavior
(gain)
c-value
0.0
0.1
0.2
0.3
0.4
0.5
0.6
0.7
0.8
Phylostratum
Cellular_organisms - ps1
Archaea1 - ps2
Archaea2 - ps3
Archaea3 - ps4
Asgard/Eukaryota - ps5
Eukaryota - ps6
Amorpheae - ps7
Obazoa - ps8
Opisthokonta - ps9
Holomycota - ps10
Fungi - ps11
Fungi_1 - ps12
Fungi_2 - ps13
Fungi_3 - ps14
Dikarya - ps15
Ascomycota - ps16
Saccharo/Pezizomycotina - ps17
Saccharomycotina - ps18
Saccharomycotina_1 - ps19
Saccharomycotina_2 - ps20
Saccharomycotina_3 - ps21
Phaff./Saccharomycetaceae - ps22
Saccharomyco./mycetaceae - ps23
Saccharomycetaceae - ps24
Saccharomycetaceae_2 - ps25
Saccharomycetaceae_3 - ps26
Kaz./Nau./Saccharomyces - ps27
Saccharomyces - ps28
Arboricola_clade - ps29
Cerevisiae_clade - ps30
Saccharomyces_cerevisiae - ps31
p-value
7e-05
4e-04
2e-03
1e-02
6e-02
Log-odds
0.5
1.0
1.5
2.0
2.5
3.0
3.5
4.0

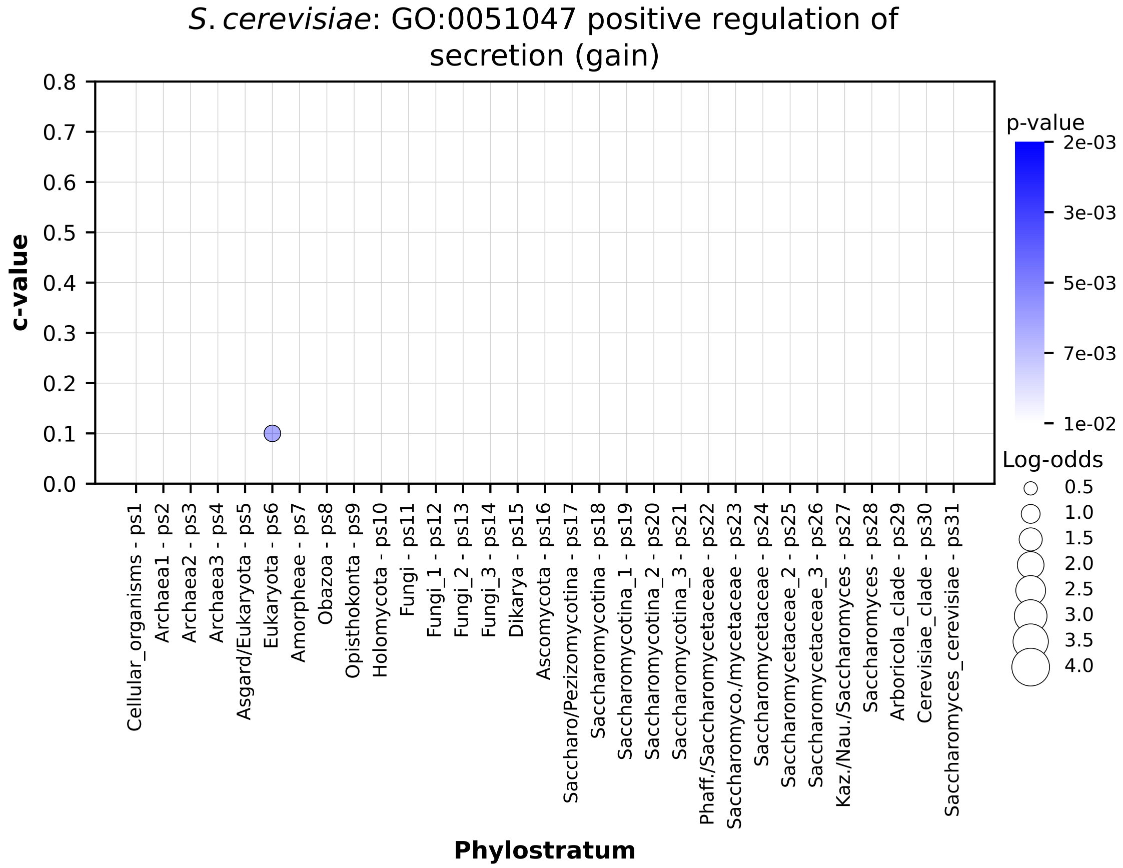

S. cerevisiae: GO:0051047 positive regulation of secretion (gain)
c-value
0.8
0.7
0.6
0.5
0.4
0.3
0.2
0.1
0.0
p-value
2e-03
3e-03
5e-03
7e-03
1e-02
Log-odds
0.5
1.0
1.5
2.0
2.5
3.0
3.5
4.0
Cellular_organisms - ps1
Archaea1 - ps2
Archaea2 - ps3
Archaea3 - ps4
Asgard/Eukaryota - ps5
Eukaryota - ps6
Amorpheae - ps7
Obazoa - ps8
Opisthokonta - ps9
Holomycota - ps10
Fungi - ps11
Fungi_1 - ps12
Fungi_2 - ps13
Fungi_3 - ps14
Dikarya - ps15
Ascomycota - ps16
Saccharo/Pezizomycotina - ps17
Saccharomycotina - ps18
Saccharomycotina_1 - ps19
Saccharomycotina_2 - ps20
Saccharomycotina_3 - ps21
Phaff./Saccharomycetaceae - ps22
Saccharomyco./mycetaceae - ps23
Saccharomycetaceae - ps24
Saccharomycetaceae_2 - ps25
Saccharomycetaceae_3 - ps26
Kaz./Nau./Saccharomyces - ps27
Saccharomyces - ps28
Arboricola_clade - ps29
Cerevisiae_clade - ps30
Saccharomyces_cerevisiae - ps31
Phylostratum

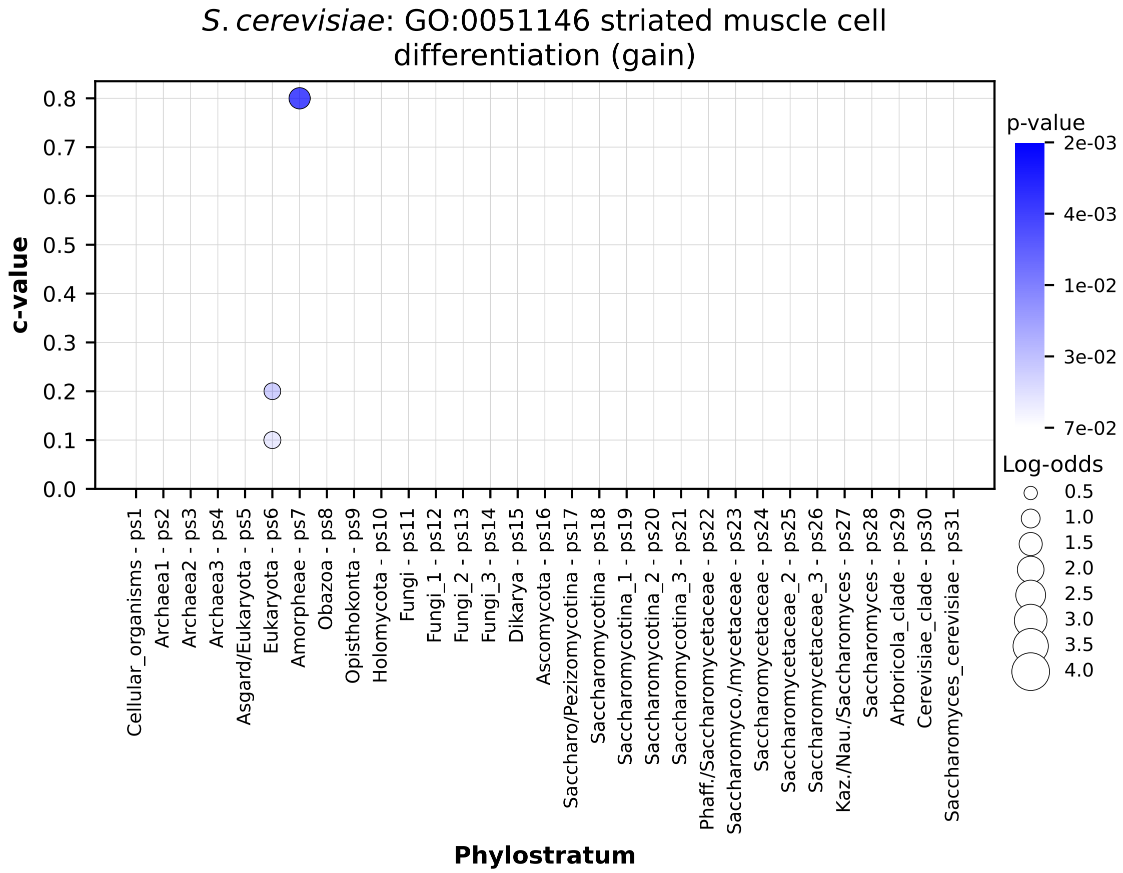

S. cerevisiae: GO:0051146 striated muscle cell differentiation (gain)
c-value
0.0
0.1
0.2
0.3
0.4
0.5
0.6
0.7
0.8
p-value
2e-03
4e-03
1e-02
3e-02
7e-02
Log-odds
0.5
1.0
1.5
2.0
2.5
3.0
3.5
4.0
Cellular_organisms - ps1
Archaea1 - ps2
Archaea2 - ps3
Archaea3 - ps4
Asgard/Eukaryota - ps5
Eukaryota - ps6
Amorpheae - ps7
Obazoa - ps8
Opisthokonta - ps9
Holomycota - ps10
Fungi - ps11
Fungi_1 - ps12
Fungi_2 - ps13
Fungi_3 - ps14
Dikarya - ps15
Ascomycota - ps16
Saccharo/Pezizomycotina - ps17
Saccharomycotina - ps18
Saccharomycotina_1 - ps19
Saccharomycotina_2 - ps20
Saccharomycotina_3 - ps21
Phaff./Saccharomycetaceae - ps22
Saccharomyco./mycetaceae - ps23
Saccharomycetaceae - ps24
Saccharomycetaceae_2 - ps25
Saccharomycetaceae_3 - ps26
Kaz./Nau./Saccharomyces - ps27
Saccharomyces - ps28
Arboricola_clade - ps29
Cerevisiae_clade - ps30
Saccharomyces_cerevisiae - ps31
Phylostratum

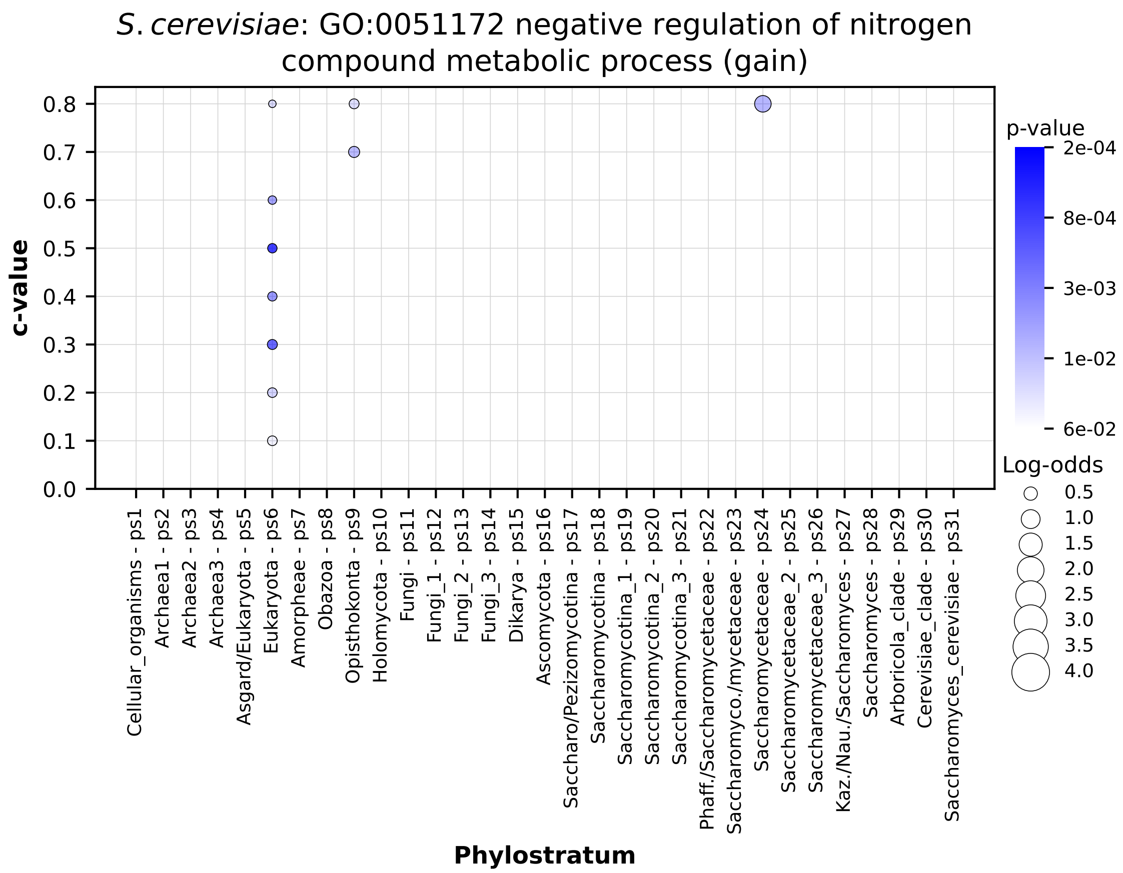

S. cerevisiae: GO:0051172 negative regulation of nitrogen compound metabolic process (gain)
c-value
0.0
0.1
0.2
0.3
0.4
0.5
0.6
0.7
0.8
Phylostratum
Cellular_organisms - ps1
Archaea1 - ps2
Archaea2 - ps3
Archaea3 - ps4
Asgard/Eukaryota - ps5
Eukaryota - ps6
Amorpheae - ps7
Obazoa - ps8
Opisthokonta - ps9
Holomycota - ps10
Fungi - ps11
Fungi_1 - ps12
Fungi_2 - ps13
Fungi_3 - ps14
Dikarya - ps15
Ascomycota - ps16
Saccharo/Pezizomycotina - ps17
Saccharomycotina - ps18
Saccharomycotina_1 - ps19
Saccharomycotina_2 - ps20
Saccharomycotina_3 - ps21
Phaff./Saccharomycetaceae - ps22
Saccharomyco./mycetaceae - ps23
Saccharomycetaceae - ps24
Saccharomycetaceae_2 - ps25
Saccharomycetaceae_3 - ps26
Kaz./Nau./Saccharomyces - ps27
Saccharomyces - ps28
Arboricola_clade - ps29
Cerevisiae_clade - ps30
Saccharomyces_cerevisiae - ps31
p-value
2e-04
8e-04
3e-03
1e-02
6e-02
Log-odds
0.5
1.0
1.5
2.0
2.5
3.0
3.5
4.0

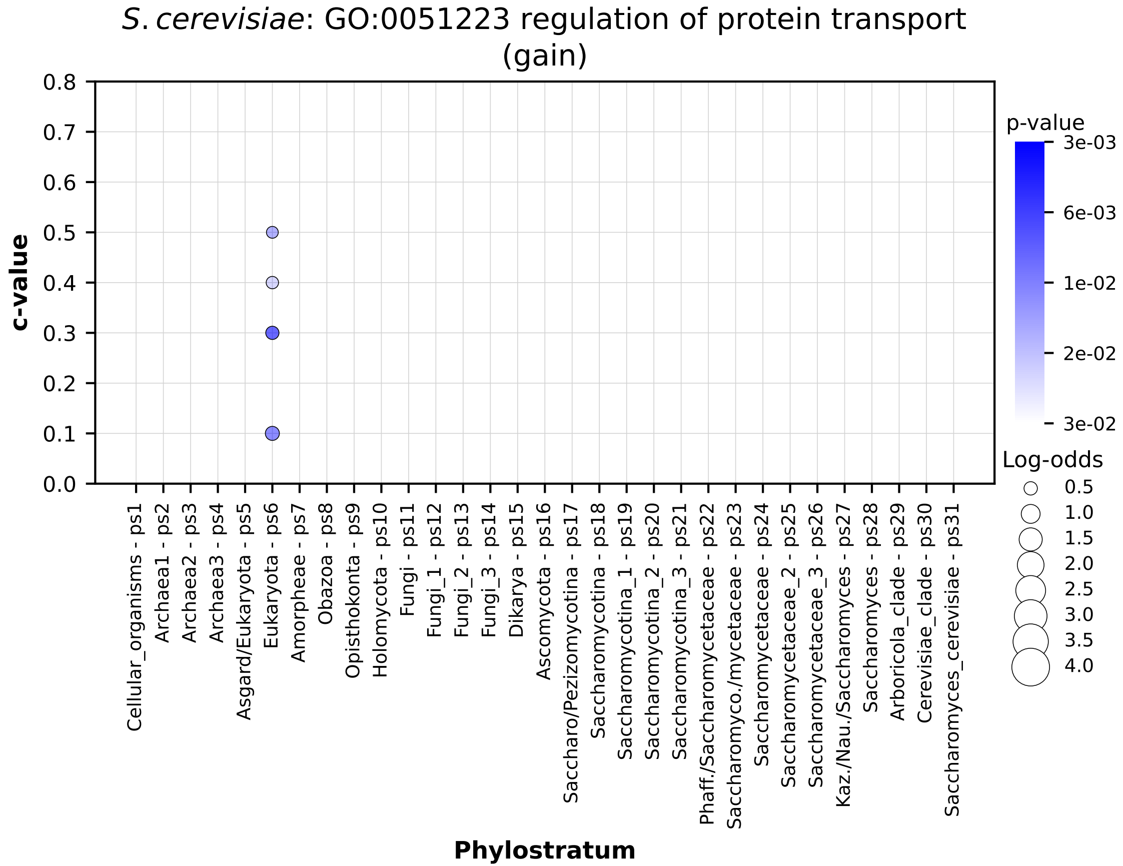

S. cerevisiae: GO:0051223 regulation of protein transport (gain)
c-value
0.8
0.7
0.6
0.5
0.4
0.3
0.2
0.1
0.0
Phylostratum
Cellular_organisms - ps1
Archaea1 - ps2
Archaea2 - ps3
Archaea3 - ps4
Asgard/Eukaryota - ps5
Eukaryota - ps6
Amorpheae - ps7
Obazoa - ps8
Opisthokonta - ps9
Holomycota - ps10
Fungi - ps11
Fungi_1 - ps12
Fungi_2 - ps13
Fungi_3 - ps14
Dikarya - ps15
Ascomycota - ps16
Saccharo/Pezizomycotina - ps17
Saccharomycotina - ps18
Saccharomycotina_1 - ps19
Saccharomycotina_2 - ps20
Saccharomycotina_3 - ps21
Phaff./Saccharomycetaceae - ps22
Saccharomyco./mycetaceae - ps23
Saccharomycetaceae - ps24
Saccharomycetaceae_2 - ps25
Saccharomycetaceae_3 - ps26
Kaz./Nau./Saccharomyces - ps27
Saccharomyces - ps28
Arboricola_clade - ps29
Cerevisiae_clade - ps30
Saccharomyces_cerevisiae - ps31
p-value
3e-03
6e-03
1e-02
2e-02
3e-02
Log-odds
0.5
1.0
1.5
2.0
2.5
3.0
3.5
4.0

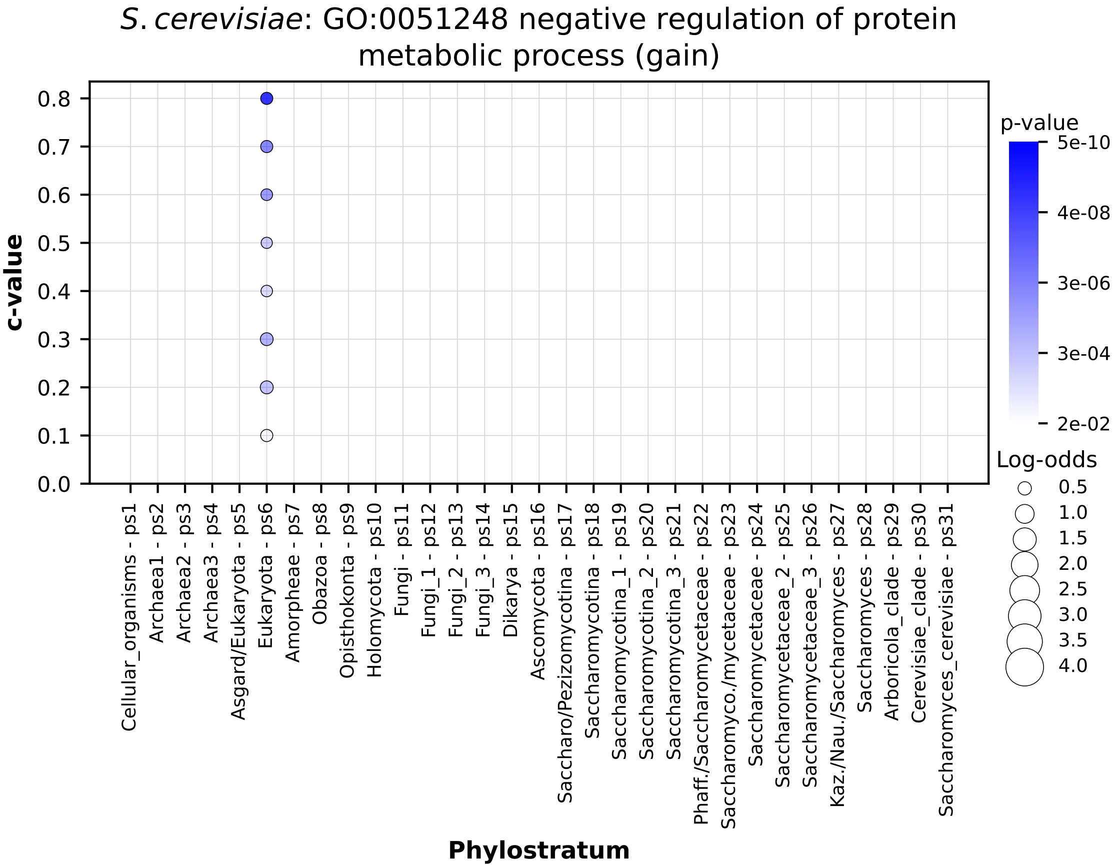

S. cerevisiae: GO:0051248 negative regulation of protein metabolic process (gain)
c-value
0.8
0.7
0.6
0.5
0.4
0.3
0.2
0.1
0.0
Phylostratum
Cellular_organisms - ps1
Archaea1 - ps2
Archaea2 - ps3
Archaea3 - ps4
Asgard/Eukaryota - ps5
Eukaryota - ps6
Amorpheae - ps7
Obazoa - ps8
Opisthokonta - ps9
Holomycota - ps10
Fungi - ps11
Fungi_1 - ps12
Fungi_2 - ps13
Fungi_3 - ps14
Dikarya - ps15
Ascomycota - ps16
Saccharo/Pezizomycotina - ps17
Saccharomycotina - ps18
Saccharomycotina_1 - ps19
Saccharomycotina_2 - ps20
Saccharomycotina_3 - ps21
Phaff./Saccharomycetaceae - ps22
Saccharomyco./mycetaceae - ps23
Saccharomycetaceae - ps24
Saccharomycetaceae_2 - ps25
Saccharomycetaceae_3 - ps26
Kaz./Nau./Saccharomyces - ps27
Saccharomyces - ps28
Arboricola_clade - ps29
Cerevisiae_clade - ps30
Saccharomyces_cerevisiae - ps31
p-value
5e-10
4e-08
3e-06
3e-04
2e-02
Log-odds
0.5
1.0
1.5
2.0
2.5
3.0
3.5
4.0

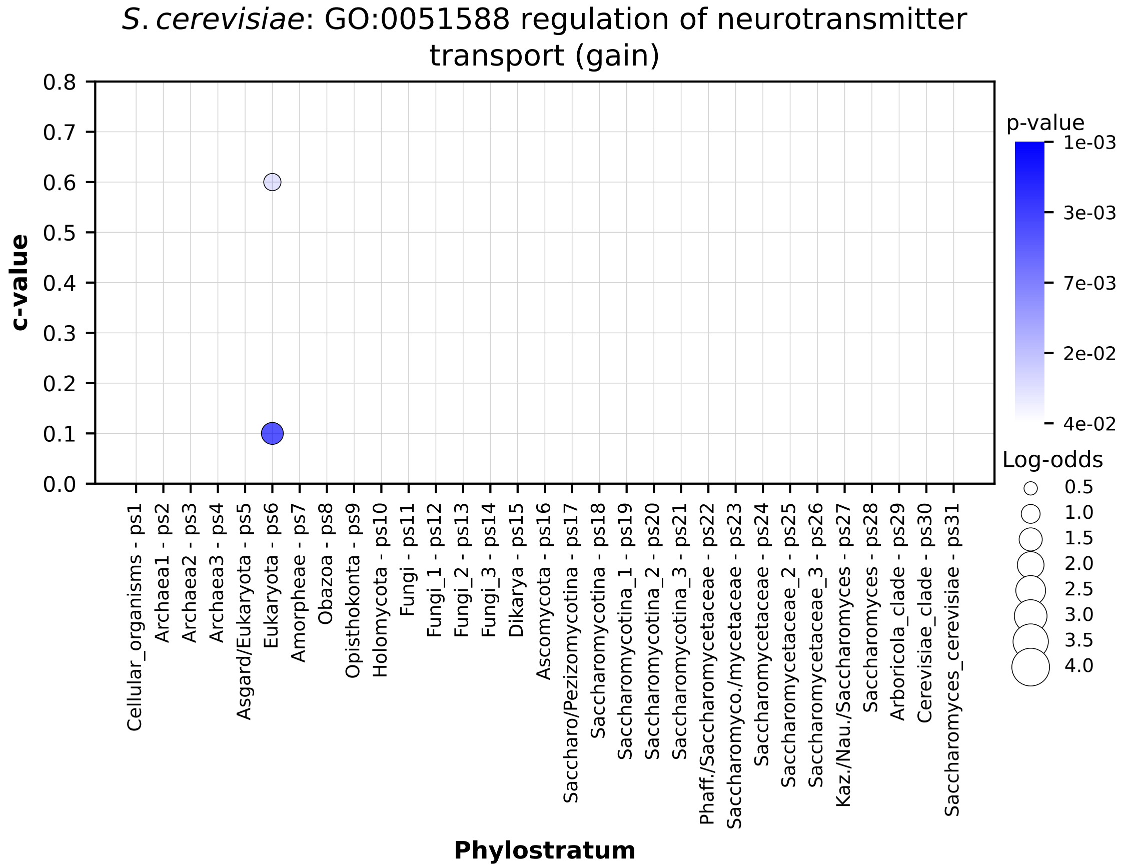

S. cerevisiae: GO:0051588 regulation of neurotransmitter transport (gain)
c-value
0.0
0.1
0.2
0.3
0.4
0.5
0.6
0.7
0.8
Phylostratum
Cellular_organisms - ps1
Archaea1 - ps2
Archaea2 - ps3
Archaea3 - ps4
Asgard/Eukaryota - ps5
Eukaryota - ps6
Amorpheae - ps7
Obazoa - ps8
Opisthokonta - ps9
Holomycota - ps10
Fungi - ps11
Fungi_1 - ps12
Fungi_2 - ps13
Fungi_3 - ps14
Dikarya - ps15
Ascomycota - ps16
Saccharo/Pezizomycotina - ps17
Saccharomycotina - ps18
Saccharomycotina_1 - ps19
Saccharomycotina_2 - ps20
Saccharomycotina_3 - ps21
Phaff./Saccharomycetaceae - ps22
Saccharomyco./mycetaceae - ps23
Saccharomycetaceae - ps24
Saccharomycetaceae_2 - ps25
Saccharomycetaceae_3 - ps26
Kaz./Nau./Saccharomyces - ps27
Saccharomyces - ps28
Arboricola_clade - ps29
Cerevisiae_clade - ps30
Saccharomyces_cerevisiae - ps31
p-value
1e-03
3e-03
7e-03
2e-02
4e-02
Log-odds
0.5
1.0
1.5
2.0
2.5
3.0
3.5
4.0

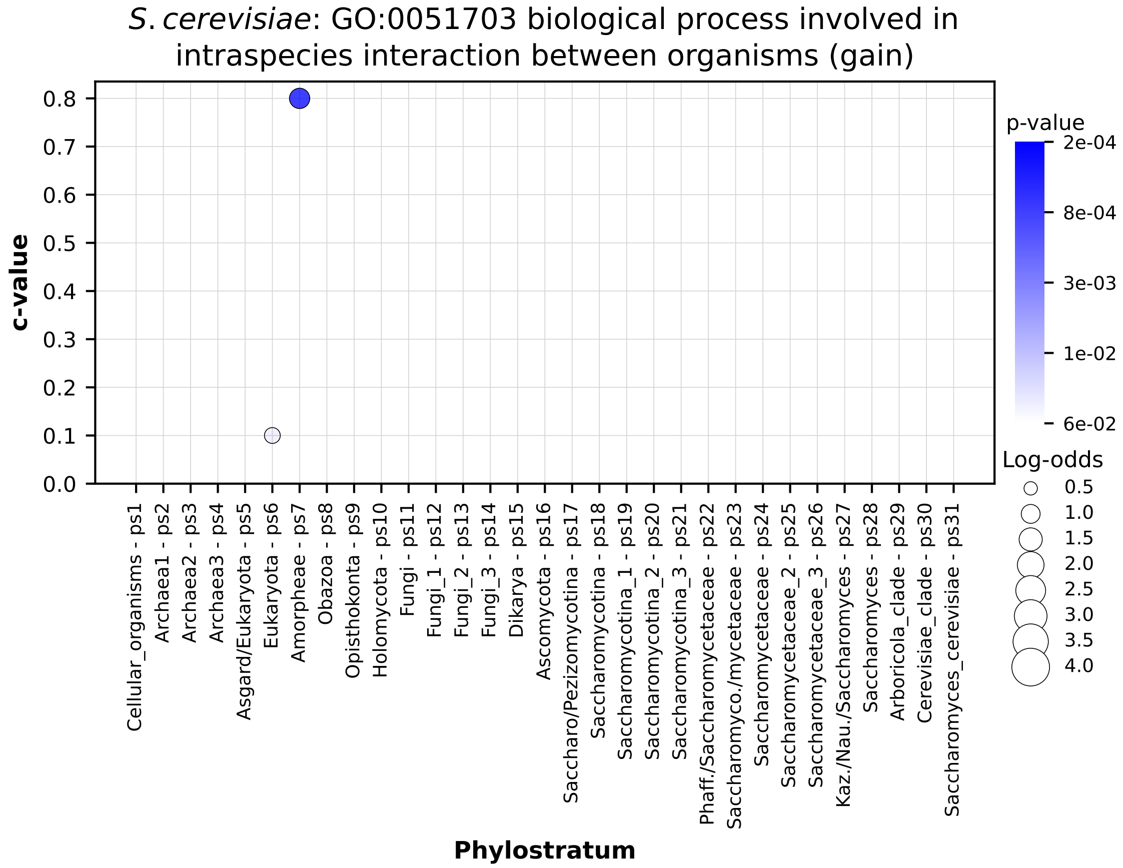

S. cerevisiae: GO:0051703 biological process involved in intraspecies interaction between organisms (gain)
c-value
0.0
0.1
0.2
0.3
0.4
0.5
0.6
0.7
0.8
p-value
2e-04
8e-04
3e-03
1e-02
6e-02
Log-odds
0.5
1.0
1.5
2.0
2.5
3.0
3.5
4.0
Cellular_organisms - ps1
Archaea1 - ps2
Archaea2 - ps3
Archaea3 - ps4
Asgard/Eukaryota - ps5
Eukaryota - ps6
Amorpheae - ps7
Obazoa - ps8
Opisthokonta - ps9
Holomycota - ps10
Fungi - ps11
Fungi_1 - ps12
Fungi_2 - ps13
Fungi_3 - ps14
Dikarya - ps15
Ascomycota - ps16
Saccharo/Pezizomycotina - ps17
Saccharomycotina - ps18
Saccharomycotina_1 - ps19
Saccharomycotina_2 - ps20
Saccharomycotina_3 - ps21
Phaff./Saccharomycetaceae - ps22
Saccharomyco./mycetaceae - ps23
Saccharomycetaceae - ps24
Saccharomycetaceae_2 - ps25
Saccharomycetaceae_3 - ps26
Kaz./Nau./Saccharomyces - ps27
Saccharomyces - ps28
Arboricola_clade - ps29
Cerevisiae_clade - ps30
Saccharomyces_cerevisiae - ps31
Phylostratum

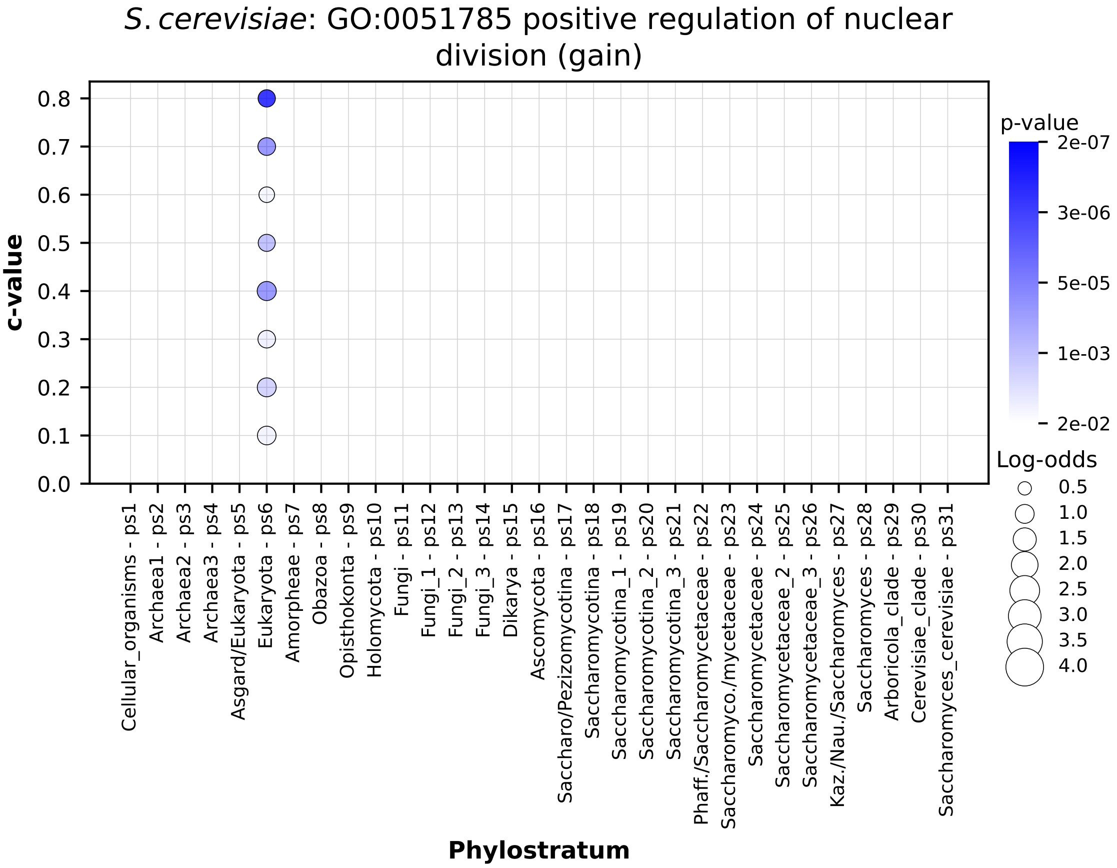

S. cerevisiae: GO:0051785 positive regulation of nuclear division (gain)
c-value
0.0
0.1
0.2
0.3
0.4
0.5
0.6
0.7
0.8
p-value
2e-07
3e-06
5e-05
1e-03
2e-02
Log-odds
0.5
1.0
1.5
2.0
2.5
3.0
3.5
4.0
Cellular_organisms - ps1
Archaea1 - ps2
Archaea2 - ps3
Archaea3 - ps4
Asgard/Eukaryota - ps5
Eukaryota - ps6
Amorpheae - ps7
Obazoa - ps8
Opisthokonta - ps9
Holomycota - ps10
Fungi - ps11
Fungi_1 - ps12
Fungi_2 - ps13
Fungi_3 - ps14
Dikarya - ps15
Ascomycota - ps16
Saccharo/Pezizomycotina - ps17
Saccharomycotina - ps18
Saccharomycotina_1 - ps19
Saccharomycotina_2 - ps20
Saccharomycotina_3 - ps21
Phaff./Saccharomycetaceae - ps22
Saccharomyco./mycetaceae - ps23
Saccharomycetaceae - ps24
Saccharomycetaceae_2 - ps25
Saccharomycetaceae_3 - ps26
Kaz./Nau./Saccharomyces - ps27
Saccharomyces - ps28
Arboricola_clade - ps29
Cerevisiae_clade - ps30
Saccharomyces_cerevisiae - ps31
Phylostratum

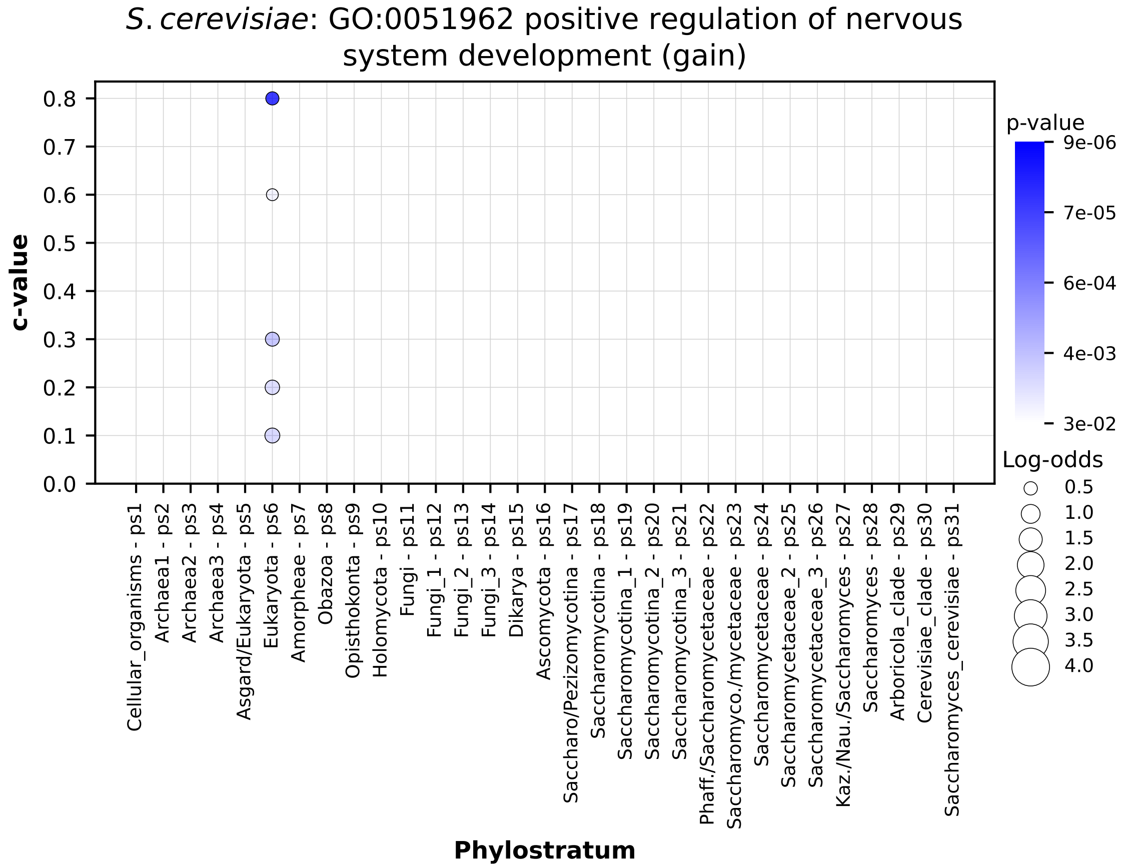

S. cerevisiae: GO:0051962 positive regulation of nervous system development (gain)
c-value
0.0
0.1
0.2
0.3
0.4
0.5
0.6
0.7
0.8
p-value
9e-06
7e-05
6e-04
4e-03
3e-02
Log-odds
0.5
1.0
1.5
2.0
2.5
3.0
3.5
4.0
Cellular_organisms - ps1
Archaea1 - ps2
Archaea2 - ps3
Archaea3 - ps4
Asgard/Eukaryota - ps5
Eukaryota - ps6
Amorpheae - ps7
Obazoa - ps8
Opisthokonta - ps9
Holomycota - ps10
Fungi - ps11
Fungi_1 - ps12
Fungi_2 - ps13
Fungi_3 - ps14
Dikarya - ps15
Ascomycota - ps16
Saccharo/Pezizomycotina - ps17
Saccharomycotina - ps18
Saccharomycotina_1 - ps19
Saccharomycotina_2 - ps20
Saccharomycotina_3 - ps21
Phaff./Saccharomycetaceae - ps22
Saccharomyco./mycetaceae - ps23
Saccharomycetaceae - ps24
Saccharomycetaceae_2 - ps25
Saccharomycetaceae_3 - ps26
Kaz./Nau./Saccharomyces - ps27
Saccharomyces - ps28
Arboricola_clade - ps29
Cerevisiae_clade - ps30
Saccharomyces_cerevisiae - ps31
Phylostratum

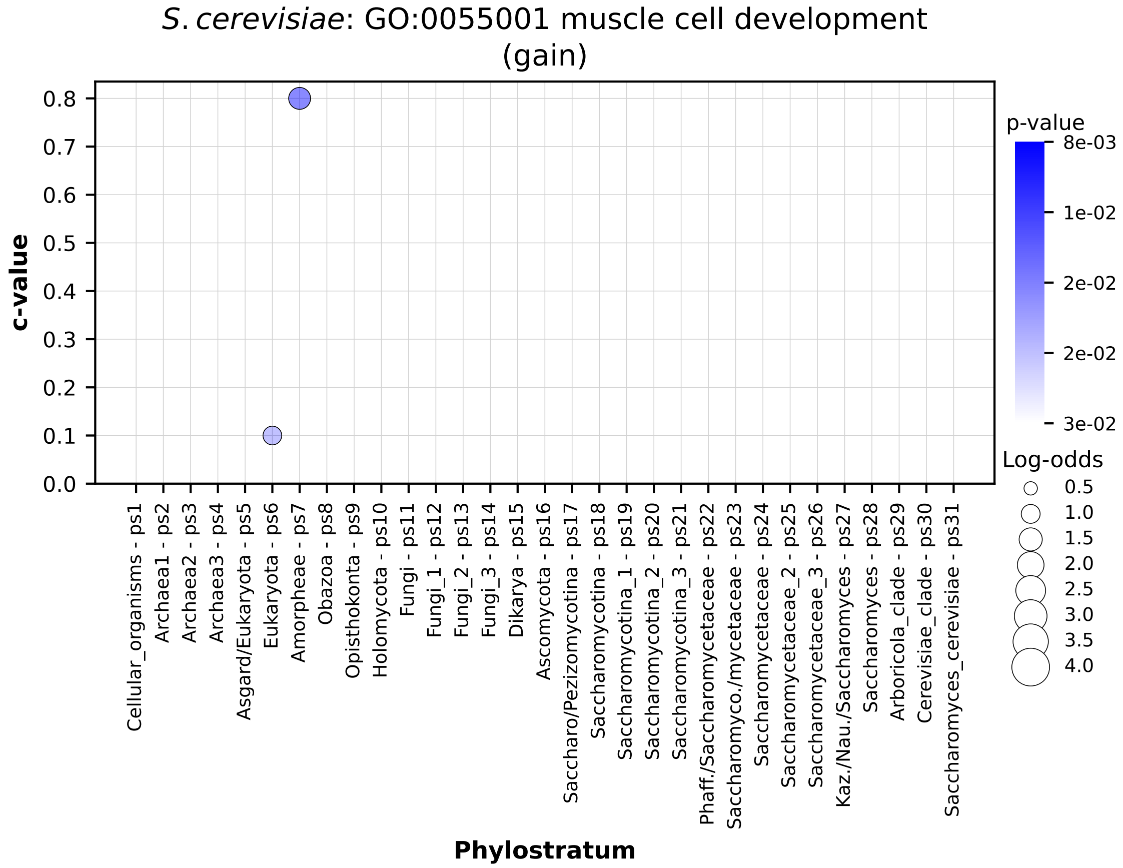
S. cerevisiae: GO:0055001 muscle cell development
(gain)
c-value
0.0
0.1
0.2
0.3
0.4
0.5
0.6
0.7
0.8
p-value
8e-03
1e-02
2e-02
2e-02
3e-02
Log-odds
0.5
1.0
1.5
2.0
2.5
3.0
3.5
4.0
Cellular_organisms - ps1
Archaea1 - ps2
Archaea2 - ps3
Archaea3 - ps4
Asgard/Eukaryota - ps5
Eukaryota - ps6
Amorpheae - ps7
Obazoa - ps8
Opisthokonta - ps9
Holomycota - ps10
Fungi - ps11
Fungi_1 - ps12
Fungi_2 - ps13
Fungi_3 - ps14
Dikarya - ps15
Ascomycota - ps16
Saccharo/Pezizomycotina - ps17
Saccharomycotina - ps18
Saccharomycotina_1 - ps19
Saccharomycotina_2 - ps20
Saccharomycotina_3 - ps21
Phaff./Saccharomycetaceae - ps22
Saccharomyco./mycetaceae - ps23
Saccharomycetaceae - ps24
Saccharomycetaceae_2 - ps25
Saccharomycetaceae_3 - ps26
Kaz./Nau./Saccharomyces - ps27
Saccharomyces - ps28
Arboricola_clade - ps29
Cerevisiae_clade - ps30
Saccharomyces_cerevisiae - ps31
Phylostratum

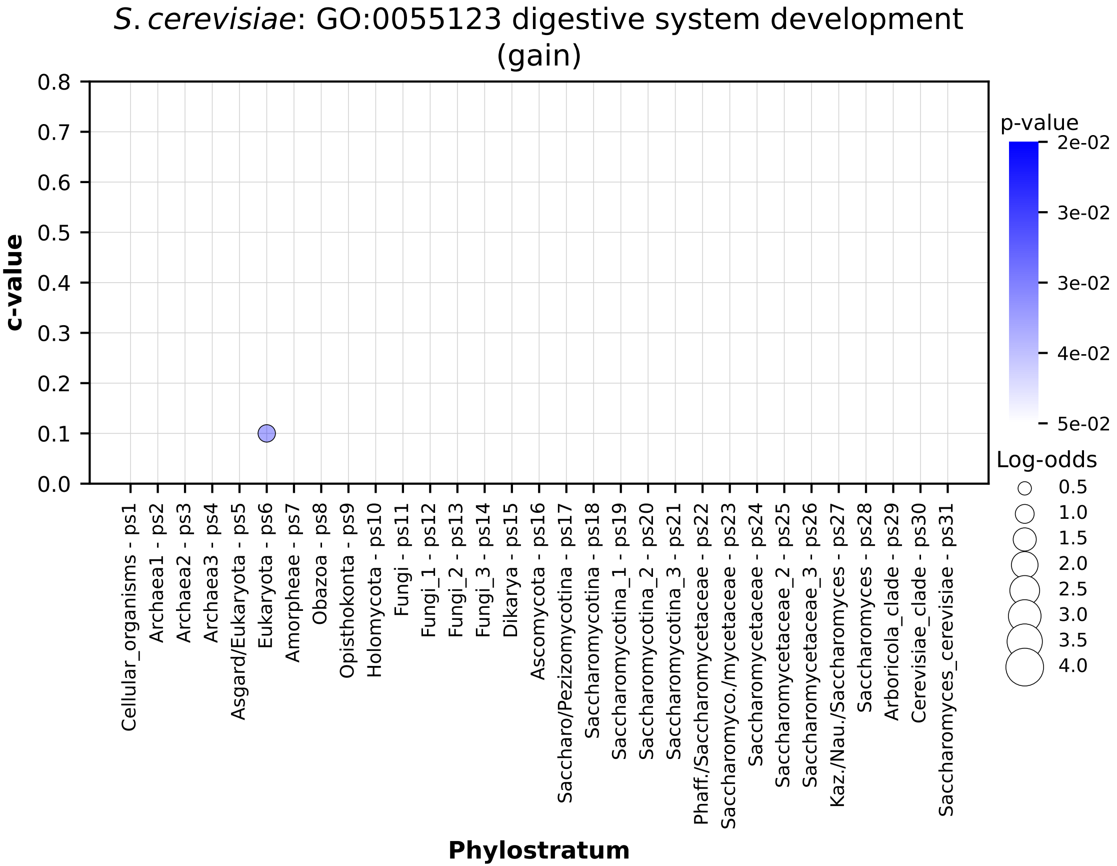

S. cerevisiae: GO:0055123 digestive system development (gain)
c-value
0.8
0.7
0.6
0.5
0.4
0.3
0.2
0.1
0.0
p-value
2e-02
3e-02
3e-02
4e-02
5e-02
Log-odds
0.5
1.0
1.5
2.0
2.5
3.0
3.5
4.0
Cellular_organisms - ps1
Archaea1 - ps2
Archaea2 - ps3
Archaea3 - ps4
Asgard/Eukaryota - ps5
Eukaryota - ps6
Amorpheae - ps7
Obazoa - ps8
Opisthokonta - ps9
Holomycota - ps10
Fungi - ps11
Fungi_1 - ps12
Fungi_2 - ps13
Fungi_3 - ps14
Dikarya - ps15
Ascomycota - ps16
Saccharo/Pezizomycotina - ps17
Saccharomycotina - ps18
Saccharomycotina_1 - ps19
Saccharomycotina_2 - ps20
Saccharomycotina_3 - ps21
Phaff./Saccharomycetaceae - ps22
Saccharomyco./mycetaceae - ps23
Saccharomycetaceae - ps24
Saccharomycetaceae_2 - ps25
Saccharomycetaceae_3 - ps26
Kaz./Nau./Saccharomyces - ps27
Saccharomyces - ps28
Arboricola_clade - ps29
Cerevisiae_clade - ps30
Saccharomyces_cerevisiae - ps31
Phylostratum

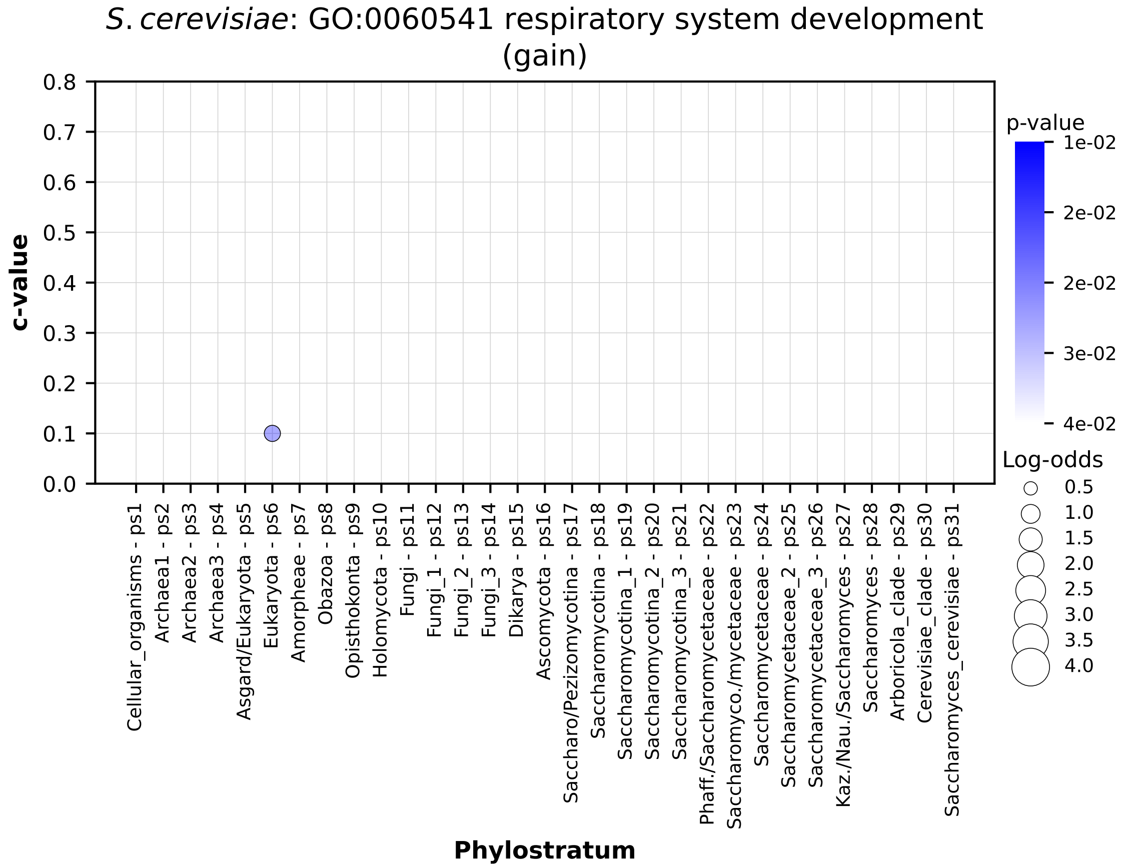

S. cerevisiae: GO:0060541 respiratory system development
(gain)
c-value
0.0
0.1
0.2
0.3
0.4
0.5
0.6
0.7
0.8
Phylostratum
Cellular_organisms - ps1
Archaea1 - ps2
Archaea2 - ps3
Archaea3 - ps4
Asgard/Eukaryota - ps5
Eukaryota - ps6
Amorpheae - ps7
Obazoa - ps8
Opisthokonta - ps9
Holomycota - ps10
Fungi - ps11
Fungi_1 - ps12
Fungi_2 - ps13
Fungi_3 - ps14
Dikarya - ps15
Ascomycota - ps16
Saccharo/Pezizomycotina - ps17
Saccharomycotina - ps18
Saccharomycotina_1 - ps19
Saccharomycotina_2 - ps20
Saccharomycotina_3 - ps21
Phaff./Saccharomycetaceae - ps22
Saccharomyco./mycetaceae - ps23
Saccharomycetaceae - ps24
Saccharomycetaceae_2 - ps25
Saccharomycetaceae_3 - ps26
Kaz./Nau./Saccharomyces - ps27
Saccharomyces - ps28
Arboricola_clade - ps29
Cerevisiae_clade - ps30
Saccharomyces_cerevisiae - ps31
p-value
1e-02
2e-02
2e-02
3e-02
4e-02
Log-odds
0.5
1.0
1.5
2.0
2.5
3.0
3.5
4.0

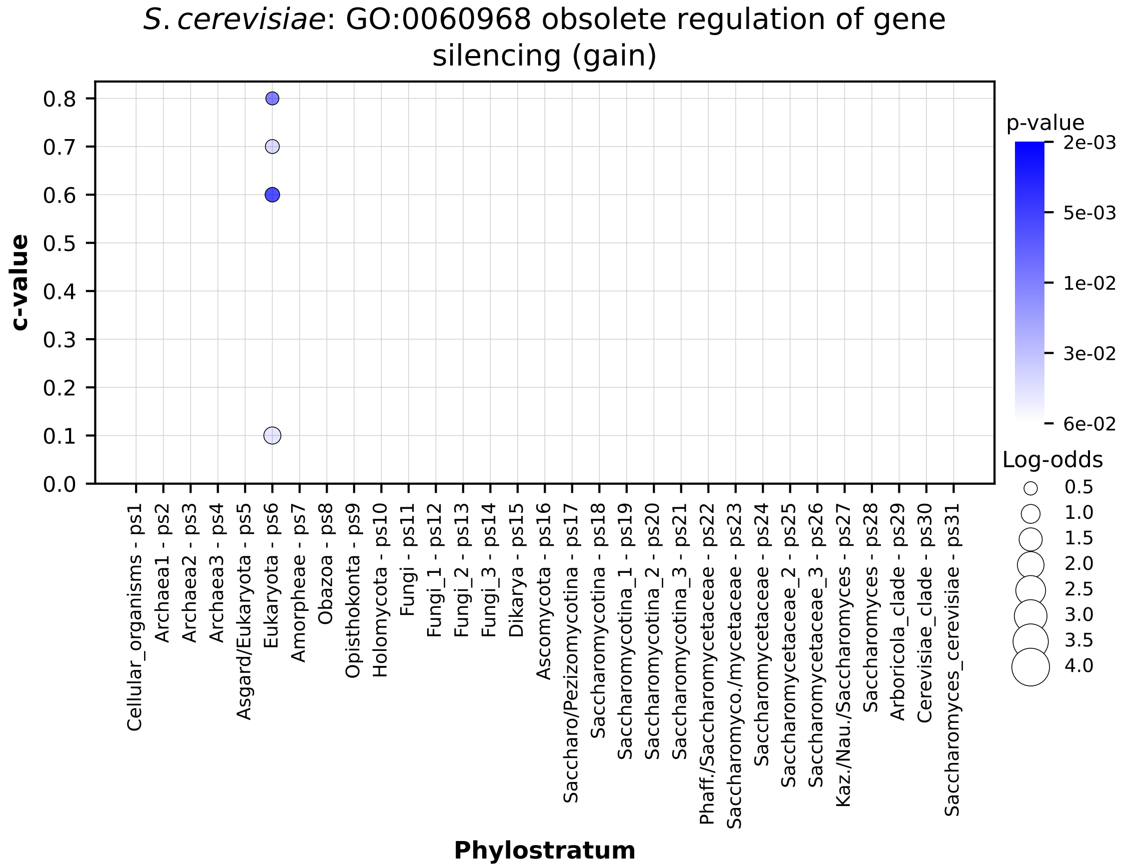

S. cerevisiae: GO:0060968 obsolete regulation of gene silencing (gain)
c-value
0.0
0.1
0.2
0.3
0.4
0.5
0.6
0.7
0.8
Phylostratum
Cellular_organisms - ps1
Archaea1 - ps2
Archaea2 - ps3
Archaea3 - ps4
Asgard/Eukaryota - ps5
Eukaryota - ps6
Amorpheae - ps7
Obazoa - ps8
Opisthokonta - ps9
Holomycota - ps10
Fungi - ps11
Fungi_1 - ps12
Fungi_2 - ps13
Fungi_3 - ps14
Dikarya - ps15
Ascomycota - ps16
Saccharo/Pezizomycotina - ps17
Saccharomycotina - ps18
Saccharomycotina_1 - ps19
Saccharomycotina_2 - ps20
Saccharomycotina_3 - ps21
Phaff./Saccharomycetaceae - ps22
Saccharomyco./mycetaceae - ps23
Saccharomycetaceae - ps24
Saccharomycetaceae_2 - ps25
Saccharomycetaceae_3 - ps26
Kaz./Nau./Saccharomyces - ps27
Saccharomyces - ps28
Arboricola_clade - ps29
Cerevisiae_clade - ps30
Saccharomyces_cerevisiae - ps31
p-value
2e-03
5e-03
1e-02
3e-02
6e-02
Log-odds
0.5
1.0
1.5
2.0
2.5
3.0
3.5
4.0

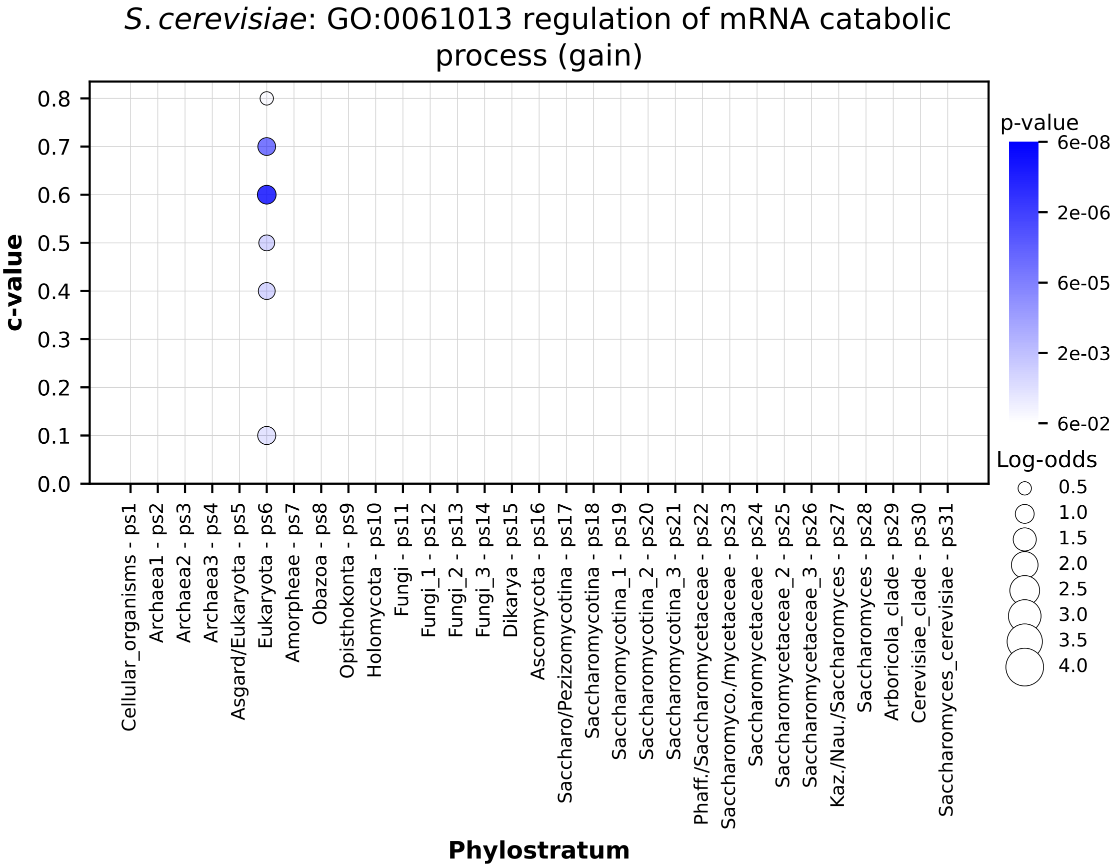

S. cerevisiae: GO:0061013 regulation of mRNA catabolic process (gain)
c-value
0.0
0.1
0.2
0.3
0.4
0.5
0.6
0.7
0.8
Phylostratum
Cellular_organisms - ps1
Archaea1 - ps2
Archaea2 - ps3
Archaea3 - ps4
Asgard/Eukaryota - ps5
Eukaryota - ps6
Amorpheae - ps7
Obazoa - ps8
Opisthokonta - ps9
Holomycota - ps10
Fungi - ps11
Fungi_1 - ps12
Fungi_2 - ps13
Fungi_3 - ps14
Dikarya - ps15
Ascomycota - ps16
Saccharo/Pezizomycotina - ps17
Saccharomycotina - ps18
Saccharomycotina_1 - ps19
Saccharomycotina_2 - ps20
Saccharomycotina_3 - ps21
Phaff./Saccharomycetaceae - ps22
Saccharomyco./mycetaceae - ps23
Saccharomycetaceae - ps24
Saccharomycetaceae_2 - ps25
Saccharomycetaceae_3 - ps26
Kaz./Nau./Saccharomyces - ps27
Saccharomyces - ps28
Arboricola_clade - ps29
Cerevisiae_clade - ps30
Saccharomyces_cerevisiae - ps31
p-value
6e-08
2e-06
6e-05
2e-03
6e-02
Log-odds
0.5
1.0
1.5
2.0
2.5
3.0
3.5
4.0

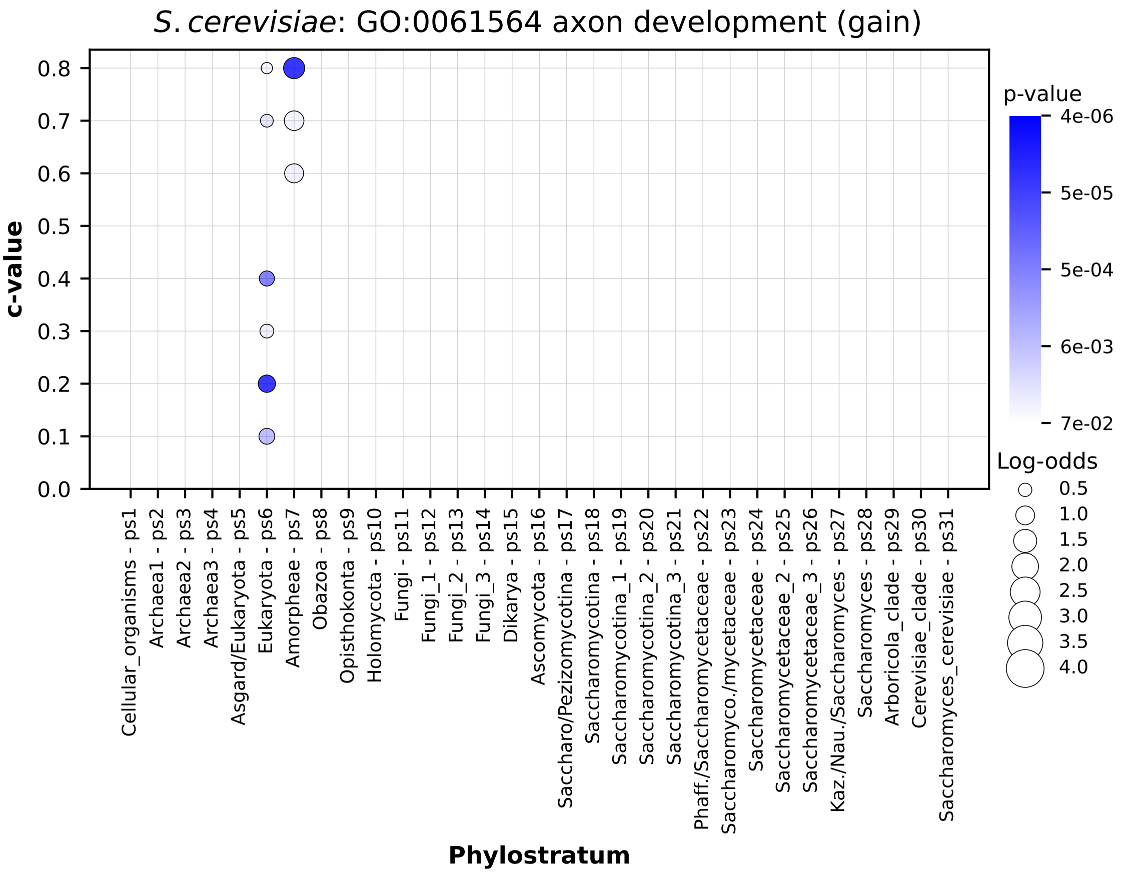
S. cerevisiae: GO:0061564 axon development (gain)
c-value
0.0
0.1
0.2
0.3
0.4
0.5
0.6
0.7
0.8
Phylostratum
Cellular_organisms - ps1
Archaea1 - ps2
Archaea2 - ps3
Archaea3 - ps4
Asgard/Eukaryota - ps5
Eukaryota - ps6
Amorpheae - ps7
Obazoa - ps8
Opisthokonta - ps9
Holomycota - ps10
Fungi - ps11
Fungi_1 - ps12
Fungi_2 - ps13
Fungi_3 - ps14
Dikarya - ps15
Ascomycota - ps16
Saccharo/Pezizomycotina - ps17
Saccharomycotina - ps18
Saccharomycotina_1 - ps19
Saccharomycotina_2 - ps20
Saccharomycotina_3 - ps21
Phaff./Saccharomycetaceae - ps22
Saccharomyco./mycetaceae - ps23
Saccharomycetaceae - ps24
Saccharomycetaceae_2 - ps25
Saccharomycetaceae_3 - ps26
Kaz./Nau./Saccharomyces - ps27
Saccharomyces - ps28
Arboricola_clade - ps29
Cerevisiae_clade - ps30
Saccharomyces_cerevisiae - ps31
p-value
4e-06
5e-05
5e-04
6e-03
7e-02
Log-odds
0.5
1.0
1.5
2.0
2.5
3.0
3.5
4.0

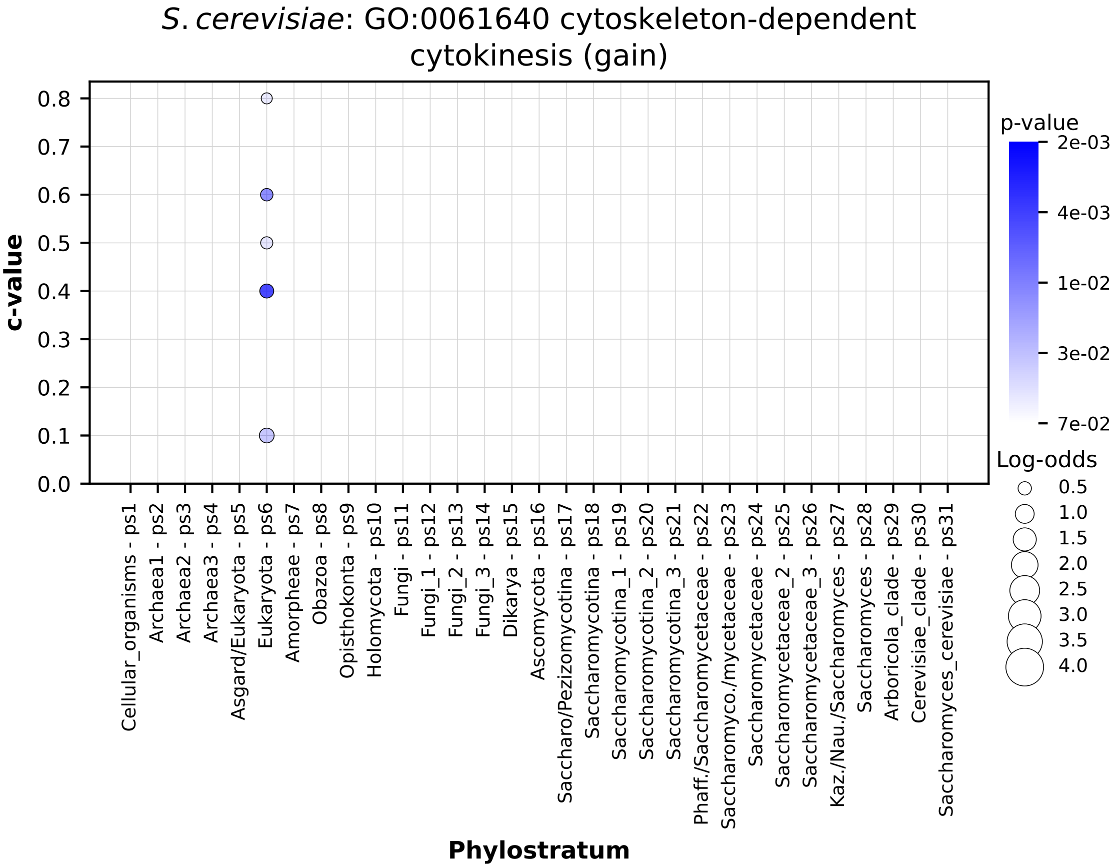

S. cerevisiae: GO:0061640 cytoskeleton-dependent cytokinesis (gain)
c-value
0.0
0.1
0.2
0.3
0.4
0.5
0.6
0.7
0.8
p-value
2e-03
4e-03
1e-02
3e-02
7e-02
Log-odds
0.5
1.0
1.5
2.0
2.5
3.0
3.5
4.0
Cellular_organisms - ps1
Archaea1 - ps2
Archaea2 - ps3
Archaea3 - ps4
Asgard/Eukaryota - ps5
Eukaryota - ps6
Amorpheae - ps7
Obazoa - ps8
Opisthokonta - ps9
Holomycota - ps10
Fungi - ps11
Fungi_1 - ps12
Fungi_2 - ps13
Fungi_3 - ps14
Dikarya - ps15
Ascomycota - ps16
Saccharo/Pezizomycotina - ps17
Saccharomycotina - ps18
Saccharomycotina_1 - ps19
Saccharomycotina_2 - ps20
Saccharomycotina_3 - ps21
Phaff./Saccharomycetaceae - ps22
Saccharomyco./mycetaceae - ps23
Saccharomycetaceae - ps24
Saccharomycetaceae_2 - ps25
Saccharomycetaceae_3 - ps26
Kaz./Nau./Saccharomyces - ps27
Saccharomyces - ps28
Arboricola_clade - ps29
Cerevisiae_clade - ps30
Saccharomyces_cerevisiae - ps31
Phylostratum

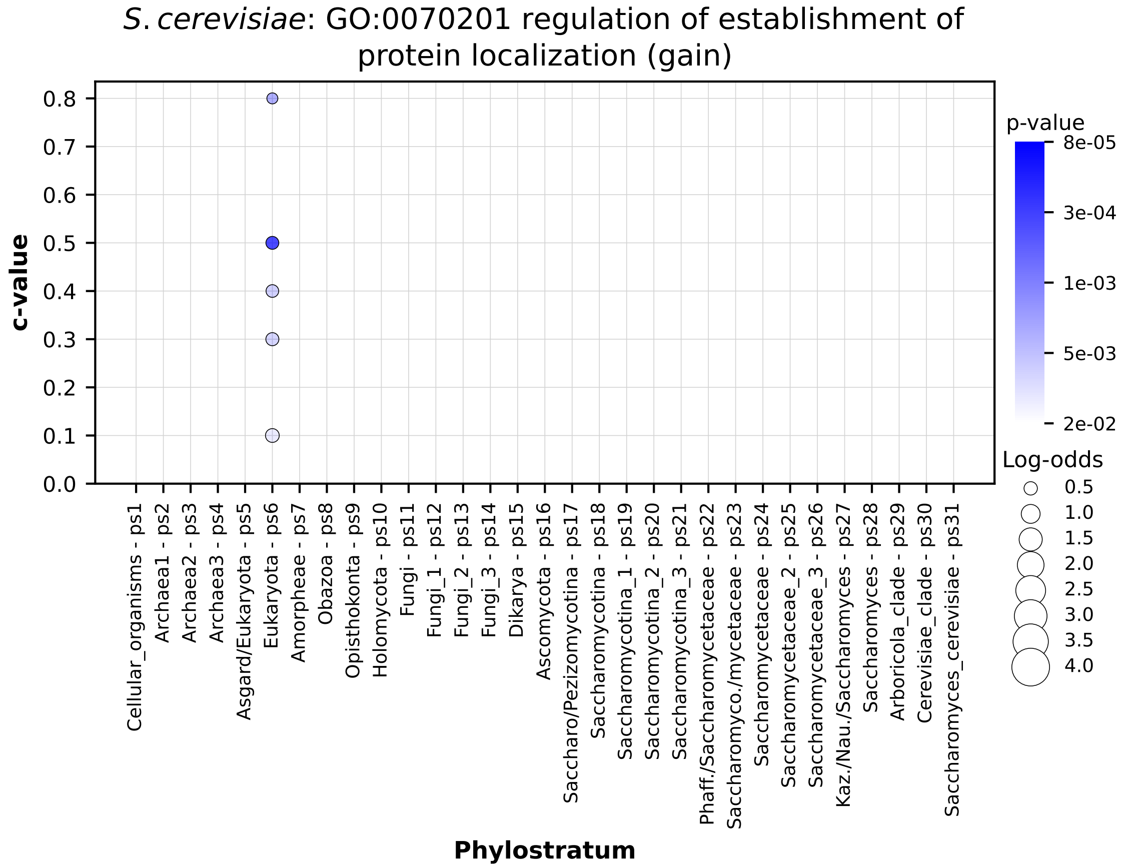

S. cerevisiae: GO:0070201 regulation of establishment of protein localization (gain)
c-value
0.0
0.1
0.2
0.3
0.4
0.5
0.6
0.7
0.8
Phylostratum
Cellular_organisms - ps1
Archaea1 - ps2
Archaea2 - ps3
Archaea3 - ps4
Asgard/Eukaryota - ps5
Eukaryota - ps6
Amorpheae - ps7
Obazoa - ps8
Opisthokonta - ps9
Holomycota - ps10
Fungi - ps11
Fungi_1 - ps12
Fungi_2 - ps13
Fungi_3 - ps14
Dikarya - ps15
Ascomycota - ps16
Saccharo/Pezizomycotina - ps17
Saccharomycotina - ps18
Saccharomycotina_1 - ps19
Saccharomycotina_2 - ps20
Saccharomycotina_3 - ps21
Phaff./Saccharomycetaceae - ps22
Saccharomyco./mycetaceae - ps23
Saccharomycetaceae - ps24
Saccharomycetaceae_2 - ps25
Saccharomycetaceae_3 - ps26
Kaz./Nau./Saccharomyces - ps27
Saccharomyces - ps28
Arboricola_clade - ps29
Cerevisiae_clade - ps30
Saccharomyces_cerevisiae - ps31
p-value
8e-05
3e-04
1e-03
5e-03
2e-02
Log-odds
0.5
1.0
1.5
2.0
2.5
3.0
3.5
4.0

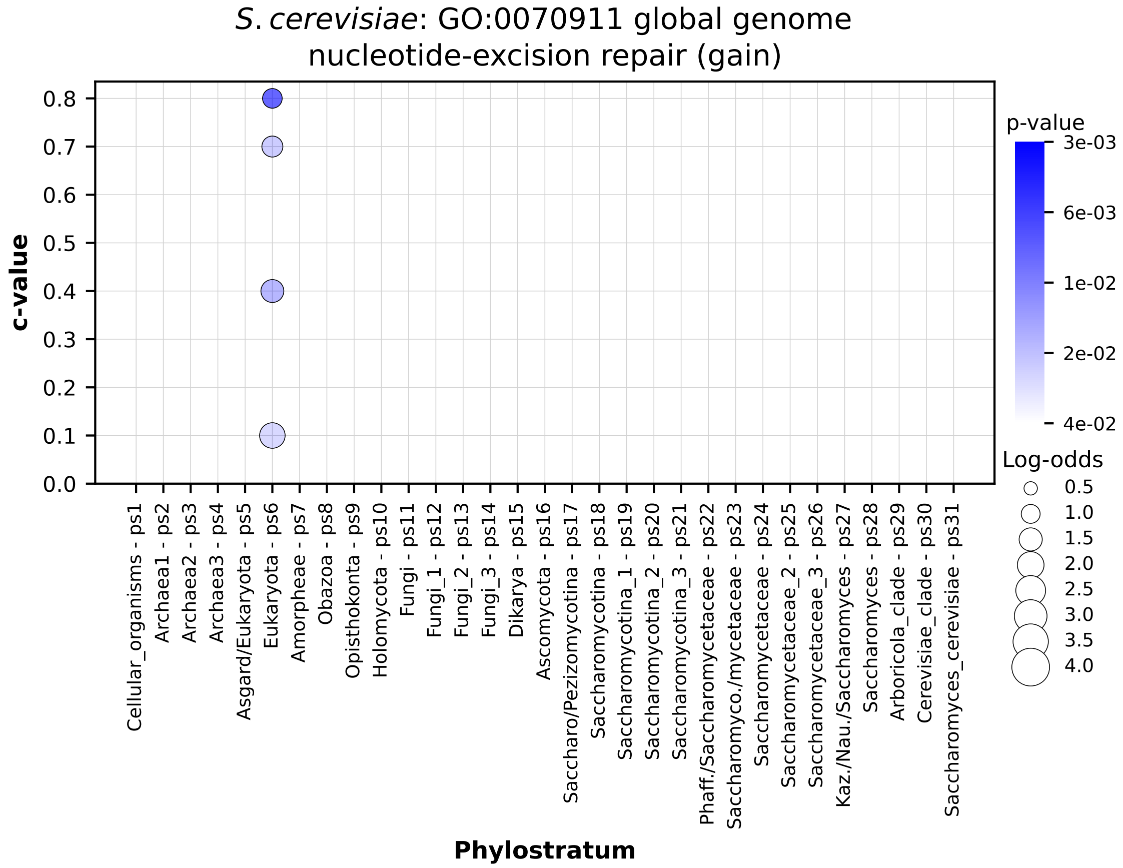

S. cerevisiae: GO:0070911 global genome nucleotide-excision repair (gain)
c-value
0.0
0.1
0.2
0.3
0.4
0.5
0.6
0.7
0.8
p-value
3e-03
6e-03
1e-02
2e-02
4e-02
Log-odds
0.5
1.0
1.5
2.0
2.5
3.0
3.5
4.0
Cellular_organisms - ps1
Archaea1 - ps2
Archaea2 - ps3
Archaea3 - ps4
Asgard/Eukaryota - ps5
Eukaryota - ps6
Amorpheae - ps7
Obazoa - ps8
Opisthokonta - ps9
Holomycota - ps10
Fungi - ps11
Fungi_1 - ps12
Fungi_2 - ps13
Fungi_3 - ps14
Dikarya - ps15
Ascomycota - ps16
Saccharo/Pezizomycotina - ps17
Saccharomycotina - ps18
Saccharomycotina_1 - ps19
Saccharomycotina_2 - ps20
Saccharomycotina_3 - ps21
Phaff./Saccharomycetaceae - ps22
Saccharomyco./mycetaceae - ps23
Saccharomycetaceae - ps24
Saccharomycetaceae_2 - ps25
Saccharomycetaceae_3 - ps26
Kaz./Nau./Saccharomyces - ps27
Saccharomyces - ps28
Arboricola_clade - ps29
Cerevisiae_clade - ps30
Saccharomyces_cerevisiae - ps31
Phylostratum

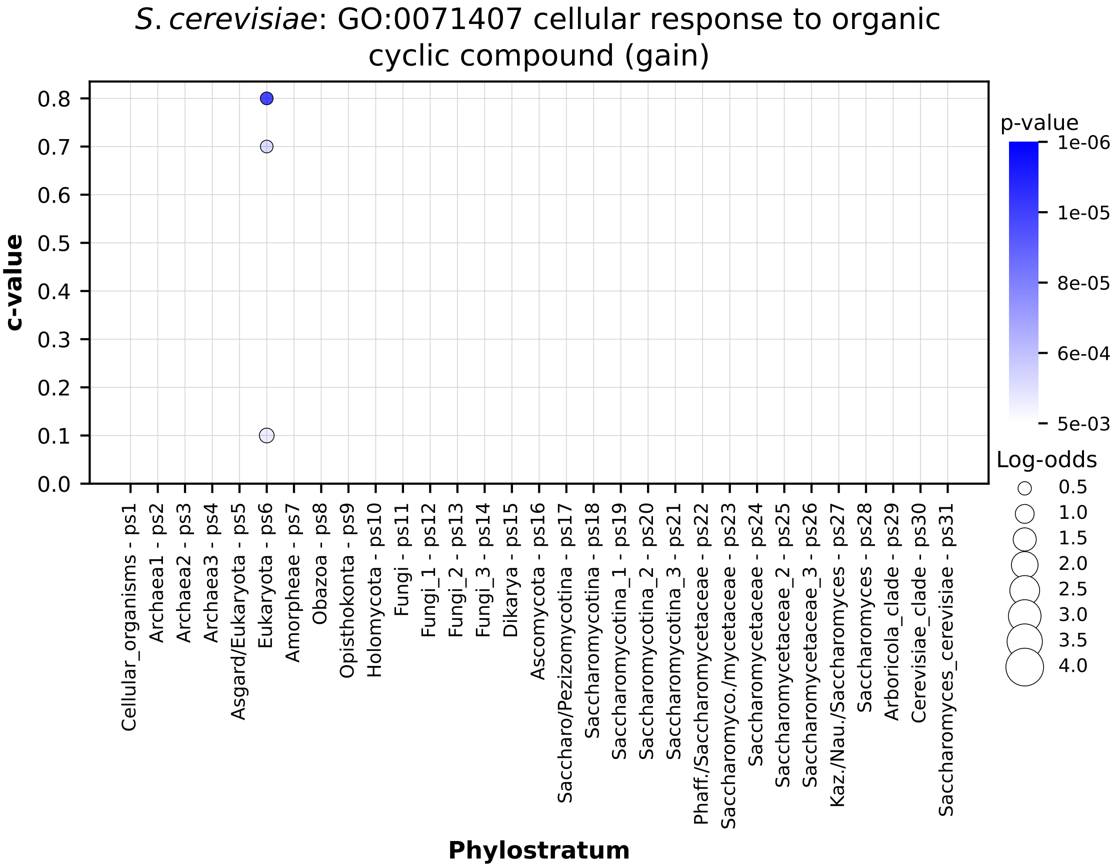

S. cerevisiae: GO:0071407 cellular response to organic cyclic compound (gain)
c-value
0.0
0.1
0.2
0.3
0.4
0.5
0.6
0.7
0.8
Phylostratum
Cellular_organisms - ps1
Archaea1 - ps2
Archaea2 - ps3
Archaea3 - ps4
Asgard/Eukaryota - ps5
Eukaryota - ps6
Amorpheae - ps7
Obazoa - ps8
Opisthokonta - ps9
Holomycota - ps10
Fungi - ps11
Fungi_1 - ps12
Fungi_2 - ps13
Fungi_3 - ps14
Dikarya - ps15
Ascomycota - ps16
Saccharo/Pezizomycotina - ps17
Saccharomycotina - ps18
Saccharomycotina_1 - ps19
Saccharomycotina_2 - ps20
Saccharomycotina_3 - ps21
Phaff./Saccharomycetaceae - ps22
Saccharomyco./mycetaceae - ps23
Saccharomycetaceae - ps24
Saccharomycetaceae_2 - ps25
Saccharomycetaceae_3 - ps26
Kaz./Nau./Saccharomyces - ps27
Saccharomyces - ps28
Arboricola_clade - ps29
Cerevisiae_clade - ps30
Saccharomyces_cerevisiae - ps31
p-value
1e-06
1e-05
8e-05
6e-04
5e-03
Log-odds
0.5
1.0
1.5
2.0
2.5
3.0
3.5
4.0

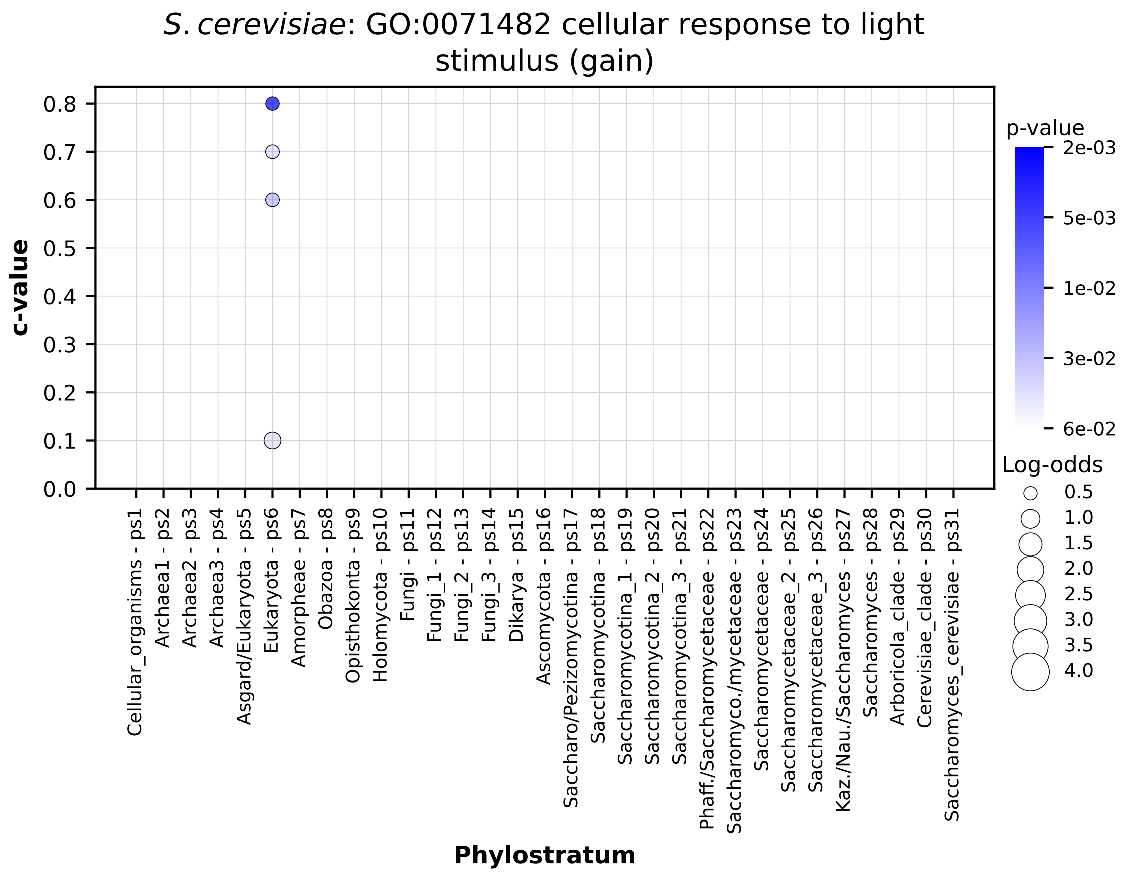
S. cerevisiae: GO:0071482 cellular response to light stimulus (gain)
c-value
0.0
0.1
0.2
0.3
0.4
0.5
0.6
0.7
0.8
p-value
2e-03
5e-03
1e-02
3e-02
6e-02
Log-odds
0.5
1.0
1.5
2.0
2.5
3.0
3.5
4.0
Cellular_organisms - ps1
Archaea1 - ps2
Archaea2 - ps3
Archaea3 - ps4
Asgard/Eukaryota - ps5
Eukaryota - ps6
Amorpheae - ps7
Obazoa - ps8
Opisthokonta - ps9
Holomycota - ps10
Fungi - ps11
Fungi_1 - ps12
Fungi_2 - ps13
Fungi_3 - ps14
Dikarya - ps15
Ascomycota - ps16
Saccharo/Pezizomycotina - ps17
Saccharomycotina - ps18
Saccharomycotina_1 - ps19
Saccharomycotina_2 - ps20
Saccharomycotina_3 - ps21
Phaff./Saccharomycetaceae - ps22
Saccharomyco./mycetaceae - ps23
Saccharomycetaceae - ps24
Saccharomycetaceae_2 - ps25
Saccharomycetaceae_3 - ps26
Kaz./Nau./Saccharomyces - ps27
Saccharomyces - ps28
Arboricola_clade - ps29
Cerevisiae_clade - ps30
Saccharomyces_cerevisiae - ps31
Phylostratum

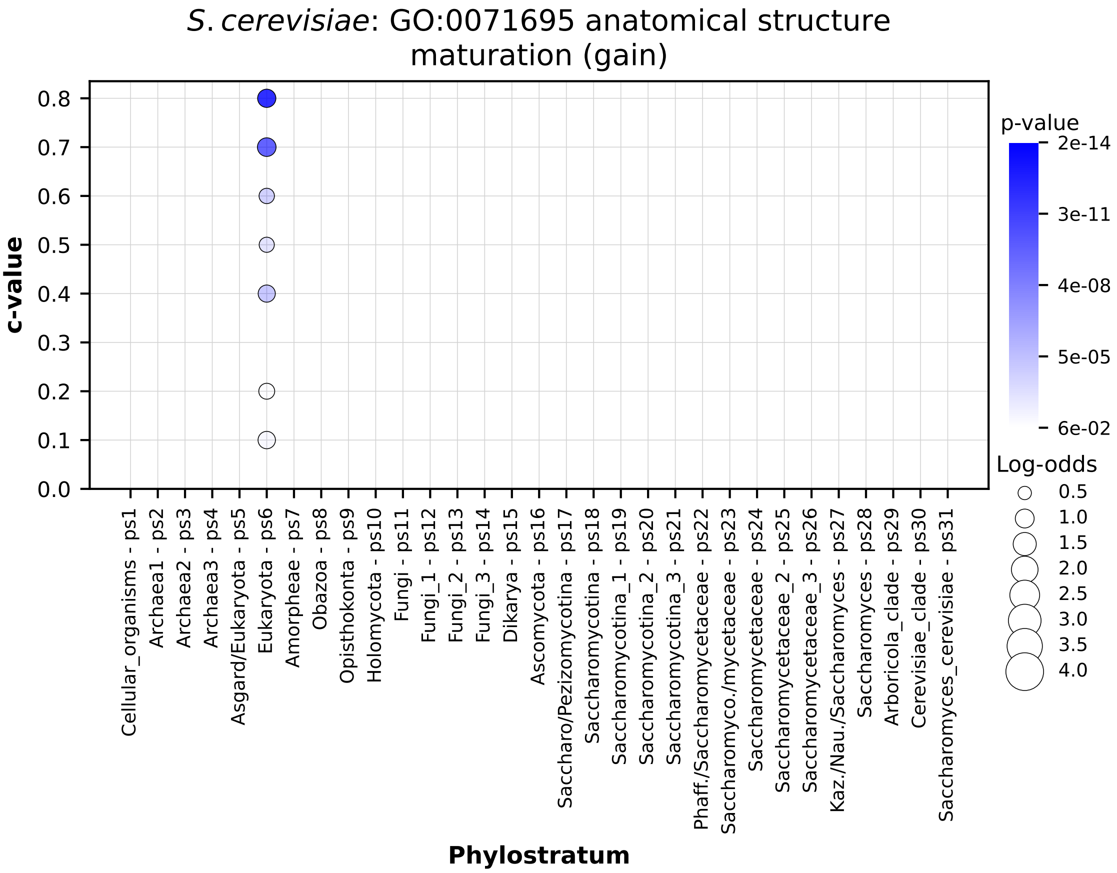
S. cerevisiae: GO:0071695 anatomical structure maturation (gain)
c-value
0.0
0.1
0.2
0.3
0.4
0.5
0.6
0.7
0.8
Phylostratum
Cellular_organisms - ps1
Archaea1 - ps2
Archaea2 - ps3
Archaea3 - ps4
Asgard/Eukaryota - ps5
Eukaryota - ps6
Amorpheae - ps7
Obazoa - ps8
Opisthokonta - ps9
Holomycota - ps10
Fungi - ps11
Fungi_1 - ps12
Fungi_2 - ps13
Fungi_3 - ps14
Dikarya - ps15
Ascomycota - ps16
Saccharo/Pezizomycotina - ps17
Saccharomycotina - ps18
Saccharomycotina_1 - ps19
Saccharomycotina_2 - ps20
Saccharomycotina_3 - ps21
Phaff./Saccharomycetaceae - ps22
Saccharomyco./mycetaceae - ps23
Saccharomycetaceae - ps24
Saccharomycetaceae_2 - ps25
Saccharomycetaceae_3 - ps26
Kaz./Nau./Saccharomyces - ps27
Saccharomyces - ps28
Arboricola_clade - ps29
Cerevisiae_clade - ps30
Saccharomyces_cerevisiae - ps31
p-value
2e-14
3e-11
4e-08
5e-05
6e-02
Log-odds
0.5
1.0
1.5
2.0
2.5
3.0
3.5
4.0

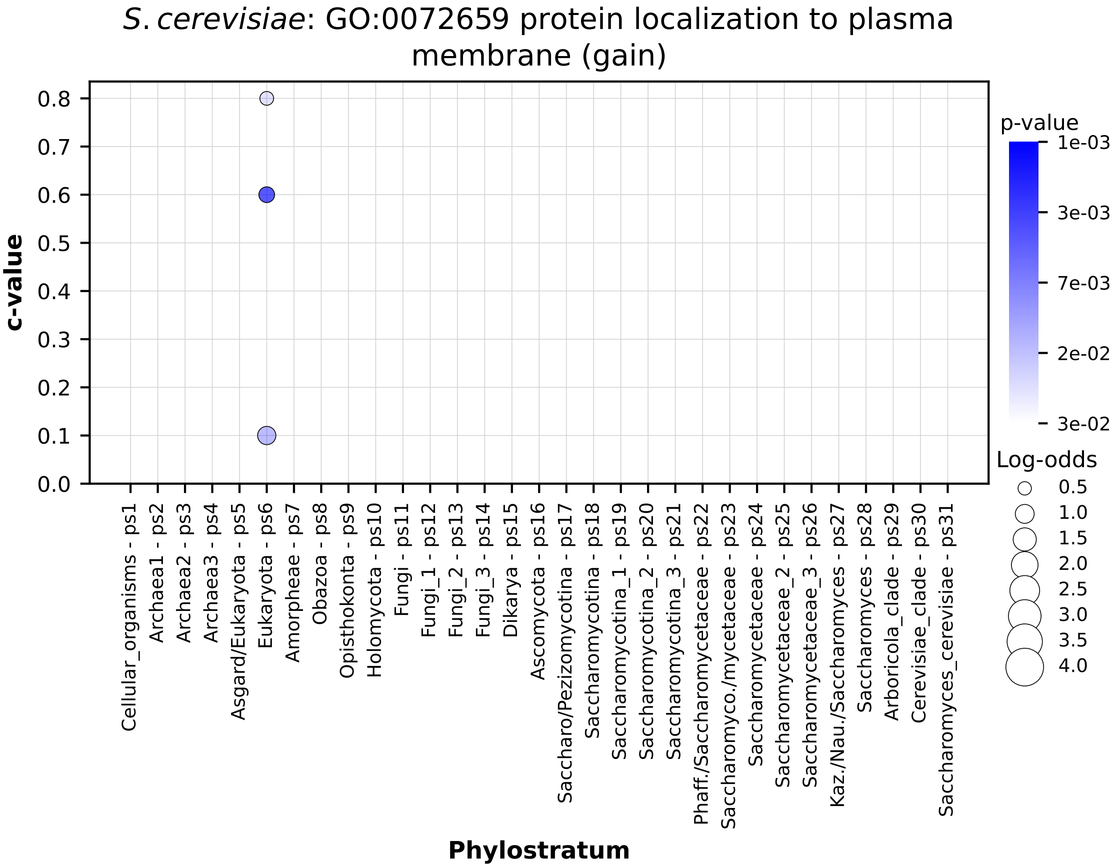

S. cerevisiae: GO:0072659 protein localization to plasma membrane (gain)
c-value
0.0
0.1
0.2
0.3
0.4
0.5
0.6
0.7
0.8
p-value
1e-03
3e-03
7e-03
2e-02
3e-02
Log-odds
0.5
1.0
1.5
2.0
2.5
3.0
3.5
4.0
Cellular_organisms - ps1
Archaea1 - ps2
Archaea2 - ps3
Archaea3 - ps4
Asgard/Eukaryota - ps5
Eukaryota - ps6
Amorpheae - ps7
Obazoa - ps8
Opisthokonta - ps9
Holomycota - ps10
Fungi - ps11
Fungi_1 - ps12
Fungi_2 - ps13
Fungi_3 - ps14
Dikarya - ps15
Ascomycota - ps16
Saccharo/Pezizomycotina - ps17
Saccharomycotina - ps18
Saccharomycotina_1 - ps19
Saccharomycotina_2 - ps20
Saccharomycotina_3 - ps21
Phaff./Saccharomycetaceae - ps22
Saccharomyco./mycetaceae - ps23
Saccharomycetaceae - ps24
Saccharomycetaceae_2 - ps25
Saccharomycetaceae_3 - ps26
Kaz./Nau./Saccharomyces - ps27
Saccharomyces - ps28
Arboricola_clade - ps29
Cerevisiae_clade - ps30
Saccharomyces_cerevisiae - ps31
Phylostratum

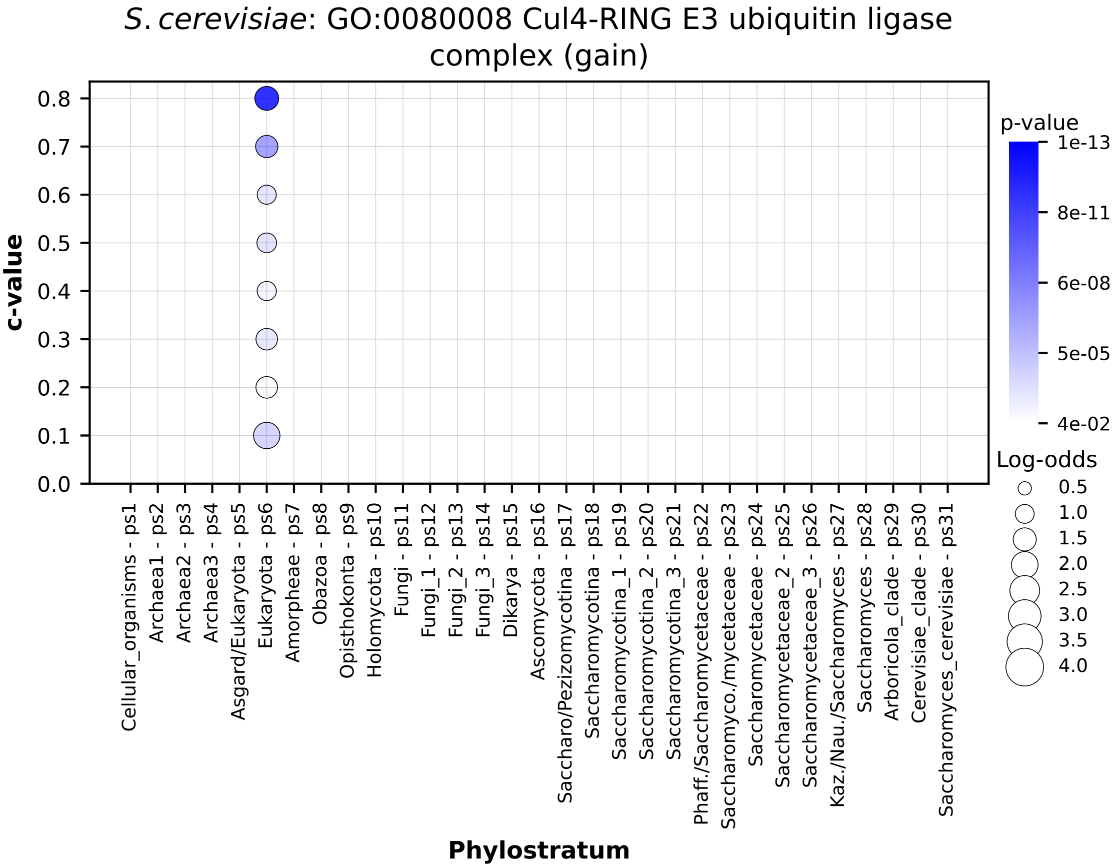

S. cerevisiae: GO:0080008 Cul4-RING E3 ubiquitin ligase complex (gain)
c-value
0.0
0.1
0.2
0.3
0.4
0.5
0.6
0.7
0.8
p-value
1e-13
8e-11
6e-08
5e-05
4e-02
Log-odds
0.5
1.0
1.5
2.0
2.5
3.0
3.5
4.0
Cellular_organisms - ps1
Archaea1 - ps2
Archaea2 - ps3
Archaea3 - ps4
Asgard/Eukaryota - ps5
Eukaryota - ps6
Amorpheae - ps7
Obazoa - ps8
Opisthokonta - ps9
Holomycota - ps10
Fungi - ps11
Fungi_1 - ps12
Fungi_2 - ps13
Fungi_3 - ps14
Dikarya - ps15
Ascomycota - ps16
Saccharo/Pezizomycotina - ps17
Saccharomycotina - ps18
Saccharomycotina_1 - ps19
Saccharomycotina_2 - ps20
Saccharomycotina_3 - ps21
Phaff./Saccharomycetaceae - ps22
Saccharomyco./mycetaceae - ps23
Saccharomycetaceae - ps24
Saccharomycetaceae_2 - ps25
Saccharomycetaceae_3 - ps26
Kaz./Nau./Saccharomyces - ps27
Saccharomyces - ps28
Arboricola_clade - ps29
Cerevisiae_clade - ps30
Saccharomyces_cerevisiae - ps31
Phylostratum

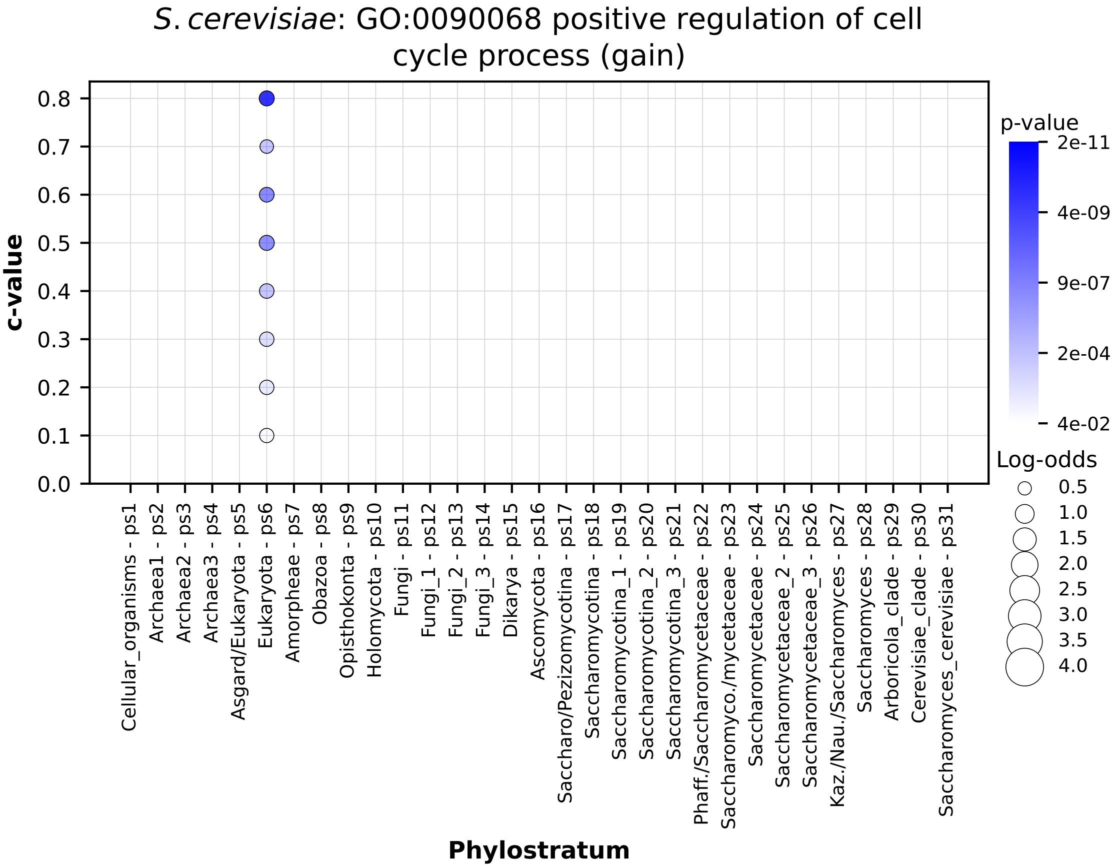

S. cerevisiae: GO:0090068 positive regulation of cell cycle process (gain)
c-value
0.0
0.1
0.2
0.3
0.4
0.5
0.6
0.7
0.8
p-value
2e-11
4e-09
9e-07
2e-04
4e-02
Log-odds
0.5
1.0
1.5
2.0
2.5
3.0
3.5
4.0
Cellular_organisms - ps1
Archaea1 - ps2
Archaea2 - ps3
Archaea3 - ps4
Asgard/Eukaryota - ps5
Eukaryota - ps6
Amorpheae - ps7
Obazoa - ps8
Opisthokonta - ps9
Holomycota - ps10
Fungi - ps11
Fungi_1 - ps12
Fungi_2 - ps13
Fungi_3 - ps14
Dikarya - ps15
Ascomycota - ps16
Saccharo/Pezizomycotina - ps17
Saccharomycotina - ps18
Saccharomycotina_1 - ps19
Saccharomycotina_2 - ps20
Saccharomycotina_3 - ps21
Phaff./Saccharomycetaceae - ps22
Saccharomyco./mycetaceae - ps23
Saccharomycetaceae - ps24
Saccharomycetaceae_2 - ps25
Saccharomycetaceae_3 - ps26
Kaz./Nau./Saccharomyces - ps27
Saccharomyces - ps28
Arboricola_clade - ps29
Cerevisiae_clade - ps30
Saccharomyces_cerevisiae - ps31
Phylostratum

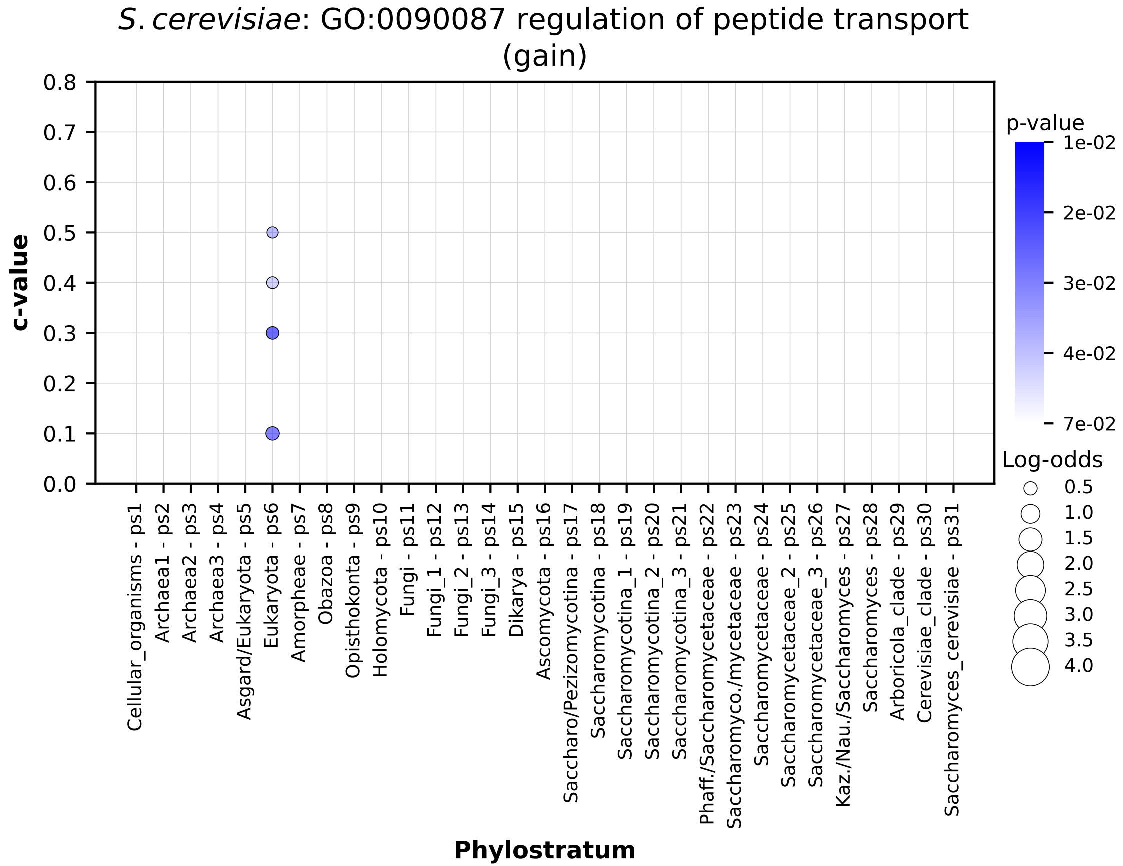

S. cerevisiae: GO:0090087 regulation of peptide transport (gain)
c-value
0.8
0.7
0.6
0.5
0.4
0.3
0.2
0.1
0.0
Phylostratum
Cellular_organisms - ps1
Archaea1 - ps2
Archaea2 - ps3
Archaea3 - ps4
Asgard/Eukaryota - ps5
Eukaryota - ps6
Amorpheae - ps7
Obazoa - ps8
Opisthokonta - ps9
Holomycota - ps10
Fungi - ps11
Fungi_1 - ps12
Fungi_2 - ps13
Fungi_3 - ps14
Dikarya - ps15
Ascomycota - ps16
Saccharo/Pezizomycotina - ps17
Saccharomycotina - ps18
Saccharomycotina_1 - ps19
Saccharomycotina_2 - ps20
Saccharomycotina_3 - ps21
Phaff./Saccharomycetaceae - ps22
Saccharomyco./mycetaceae - ps23
Saccharomycetaceae - ps24
Saccharomycetaceae_2 - ps25
Saccharomycetaceae_3 - ps26
Kaz./Nau./Saccharomyces - ps27
Saccharomyces - ps28
Arboricola_clade - ps29
Cerevisiae_clade - ps30
Saccharomyces_cerevisiae - ps31
p-value
1e-02
2e-02
3e-02
4e-02
7e-02
Log-odds
0.5
1.0
1.5
2.0
2.5
3.0
3.5
4.0

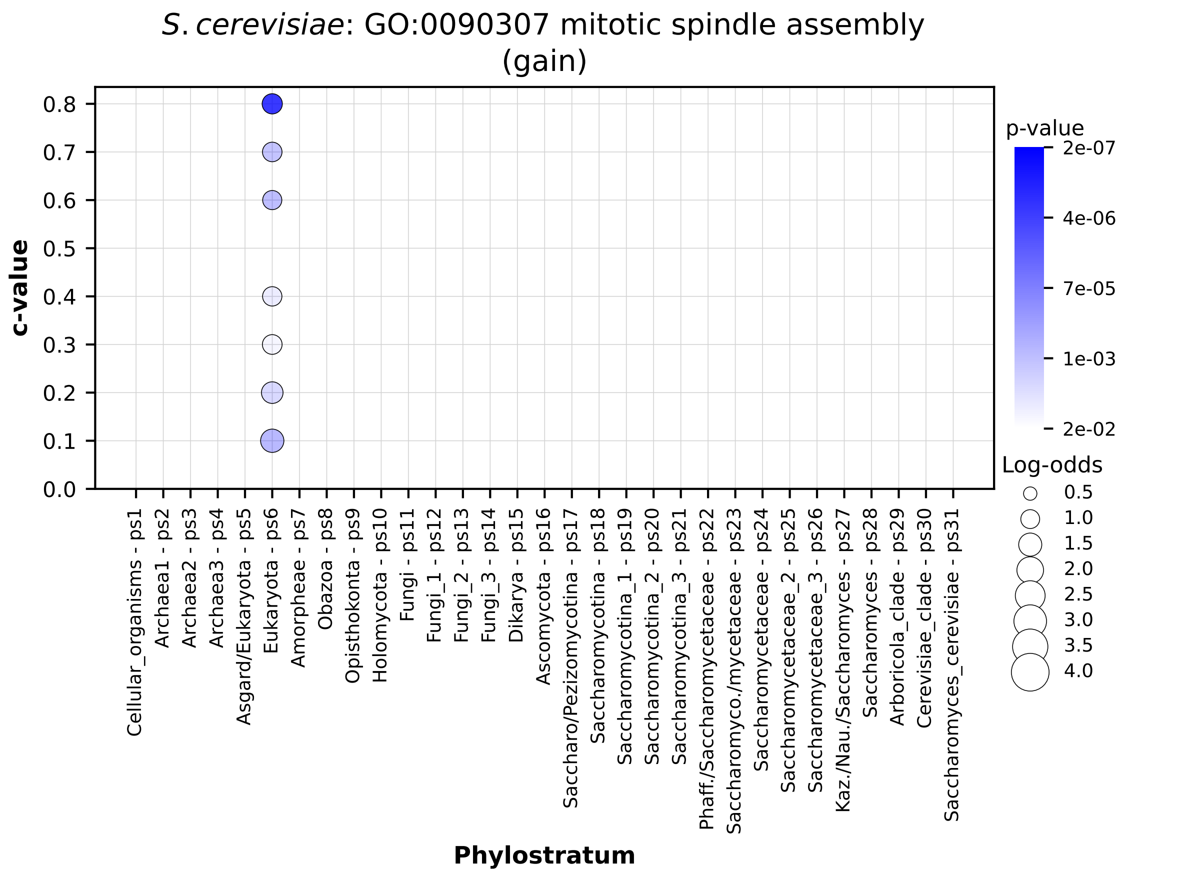

S. cerevisiae: GO:0090307 mitotic spindle assembly
(gain)
c-value
0.8
0.7
0.6
0.5
0.4
0.3
0.2
0.1
0.0
p-value
2e-07
4e-06
7e-05
1e-03
2e-02
Log-odds
0.5
1.0
1.5
2.0
2.5
3.0
3.5
4.0
Cellular_organisms - ps1
Archaea1 - ps2
Archaea2 - ps3
Archaea3 - ps4
Asgard/Eukaryota - ps5
Eukaryota - ps6
Amorpheae - ps7
Obazoa - ps8
Opisthokonta - ps9
Holomycota - ps10
Fungi - ps11
Fungi_1 - ps12
Fungi_2 - ps13
Fungi_3 - ps14
Dikarya - ps15
Ascomycota - ps16
Saccharo/Pezizomycotina - ps17
Saccharomycotina - ps18
Saccharomycotina_1 - ps19
Saccharomycotina_2 - ps20
Saccharomycotina_3 - ps21
Phaff./Saccharomycetaceae - ps22
Saccharomyco./mycetaceae - ps23
Saccharomycetaceae - ps24
Saccharomycetaceae_2 - ps25
Saccharomycetaceae_3 - ps26
Kaz./Nau./Saccharomyces - ps27
Saccharomyces - ps28
Arboricola_clade - ps29
Cerevisiae_clade - ps30
Saccharomyces_cerevisiae - ps31
Phylostratum

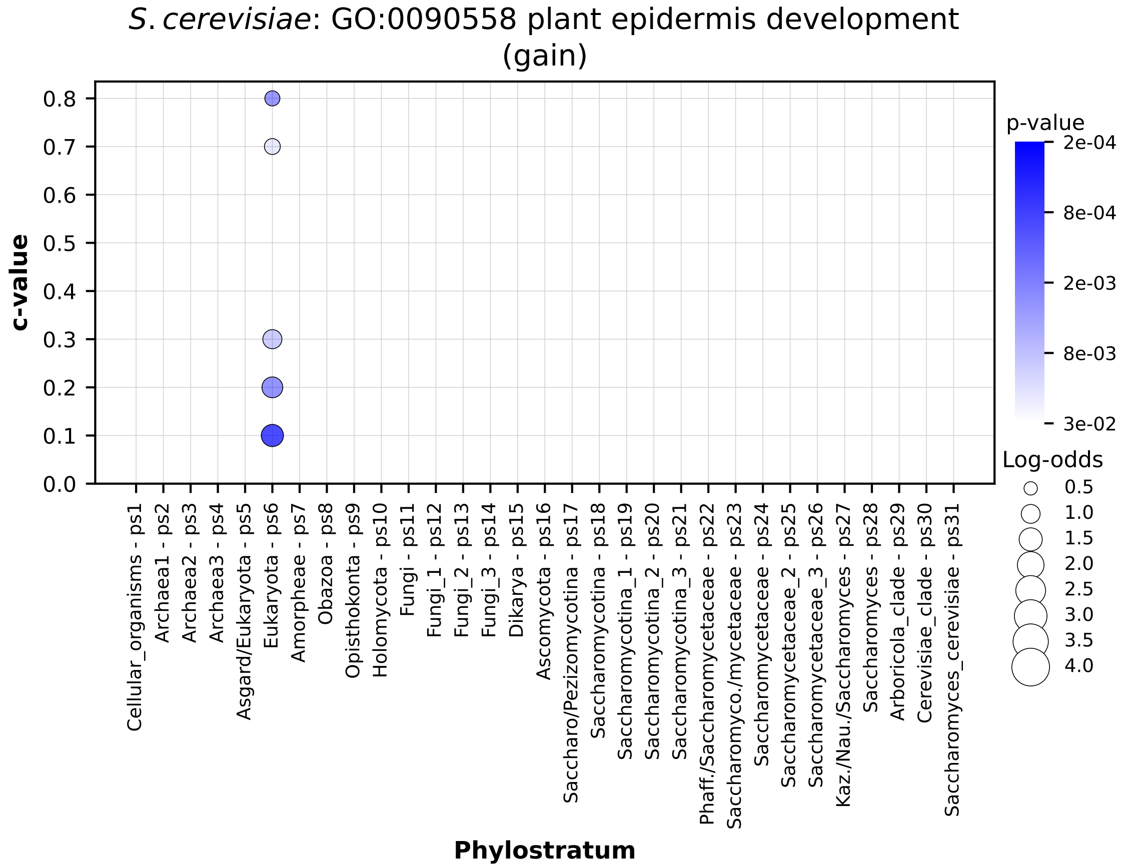

S. cerevisiae: GO:0090558 plant epidermis development (gain)
c-value
0.0
0.1
0.2
0.3
0.4
0.5
0.6
0.7
0.8
p-value
2e-04
8e-04
2e-03
8e-03
3e-02
Log-odds
0.5
1.0
1.5
2.0
2.5
3.0
3.5
4.0
Cellular_organisms - ps1
Archaea1 - ps2
Archaea2 - ps3
Archaea3 - ps4
Asgard/Eukaryota - ps5
Eukaryota - ps6
Amorpheae - ps7
Obazoa - ps8
Opisthokonta - ps9
Holomycota - ps10
Fungi - ps11
Fungi_1 - ps12
Fungi_2 - ps13
Fungi_3 - ps14
Dikarya - ps15
Ascomycota - ps16
Saccharo/Pezizomycotina - ps17
Saccharomycotina - ps18
Saccharomycotina_1 - ps19
Saccharomycotina_2 - ps20
Saccharomycotina_3 - ps21
Phaff./Saccharomycetaceae - ps22
Saccharomyco./mycetaceae - ps23
Saccharomycetaceae - ps24
Saccharomycetaceae_2 - ps25
Saccharomycetaceae_3 - ps26
Kaz./Nau./Saccharomyces - ps27
Saccharomyces - ps28
Arboricola_clade - ps29
Cerevisiae_clade - ps30
Saccharomyces_cerevisiae - ps31
Phylostratum

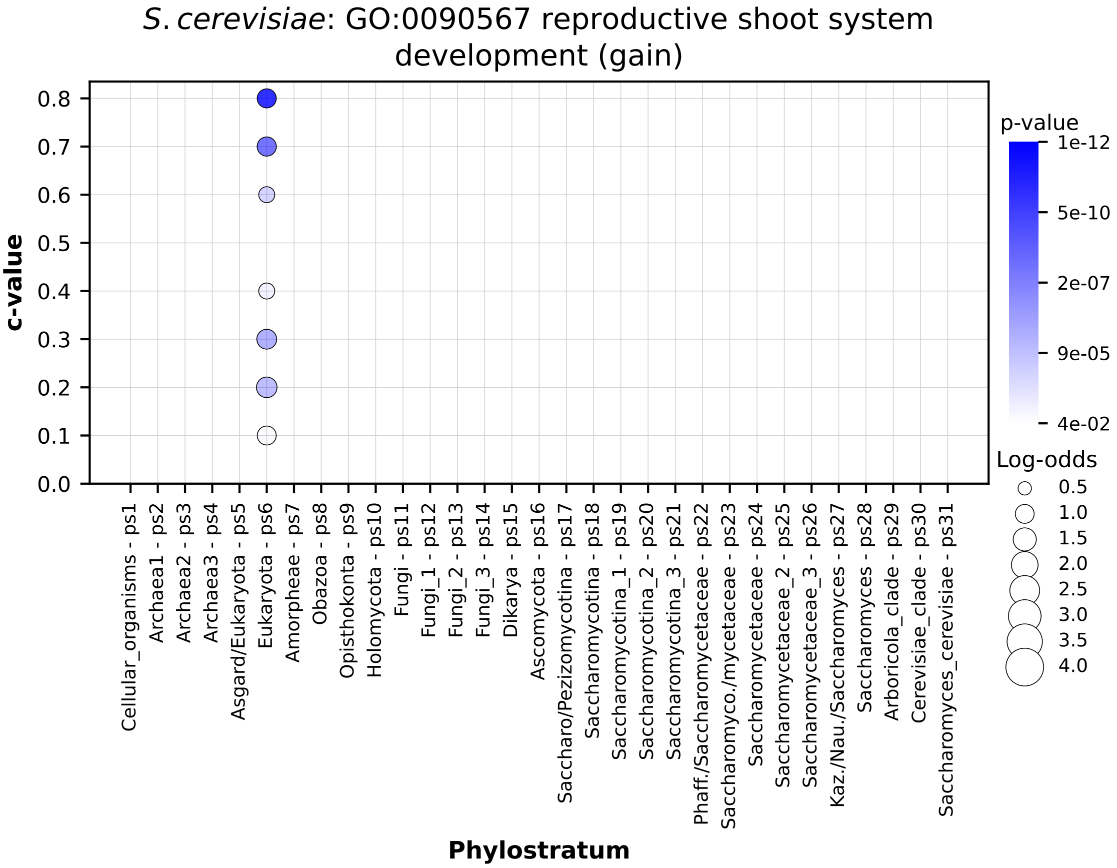

S. cerevisiae: GO:0090567 reproductive shoot system development (gain)
c-value
0.0
0.1
0.2
0.3
0.4
0.5
0.6
0.7
0.8
Phylostratum
Cellular_organisms - ps1
Archaea1 - ps2
Archaea2 - ps3
Archaea3 - ps4
Asgard/Eukaryota - ps5
Eukaryota - ps6
Amorpheae - ps7
Obazoa - ps8
Opisthokonta - ps9
Holomycota - ps10
Fungi - ps11
Fungi_1 - ps12
Fungi_2 - ps13
Fungi_3 - ps14
Dikarya - ps15
Ascomycota - ps16
Saccharo/Pezizomycotina - ps17
Saccharomycotina - ps18
Saccharomycotina_1 - ps19
Saccharomycotina_2 - ps20
Saccharomycotina_3 - ps21
Phaff./Saccharomycetaceae - ps22
Saccharomyco./mycetaceae - ps23
Saccharomycetaceae - ps24
Saccharomycetaceae_2 - ps25
Saccharomycetaceae_3 - ps26
Kaz./Nau./Saccharomyces - ps27
Saccharomyces - ps28
Arboricola_clade - ps29
Cerevisiae_clade - ps30
Saccharomyces_cerevisiae - ps31
p-value
1e-12
5e-10
2e-07
9e-05
4e-02
Log-odds
0.5
1.0
1.5
2.0
2.5
3.0
3.5
4.0

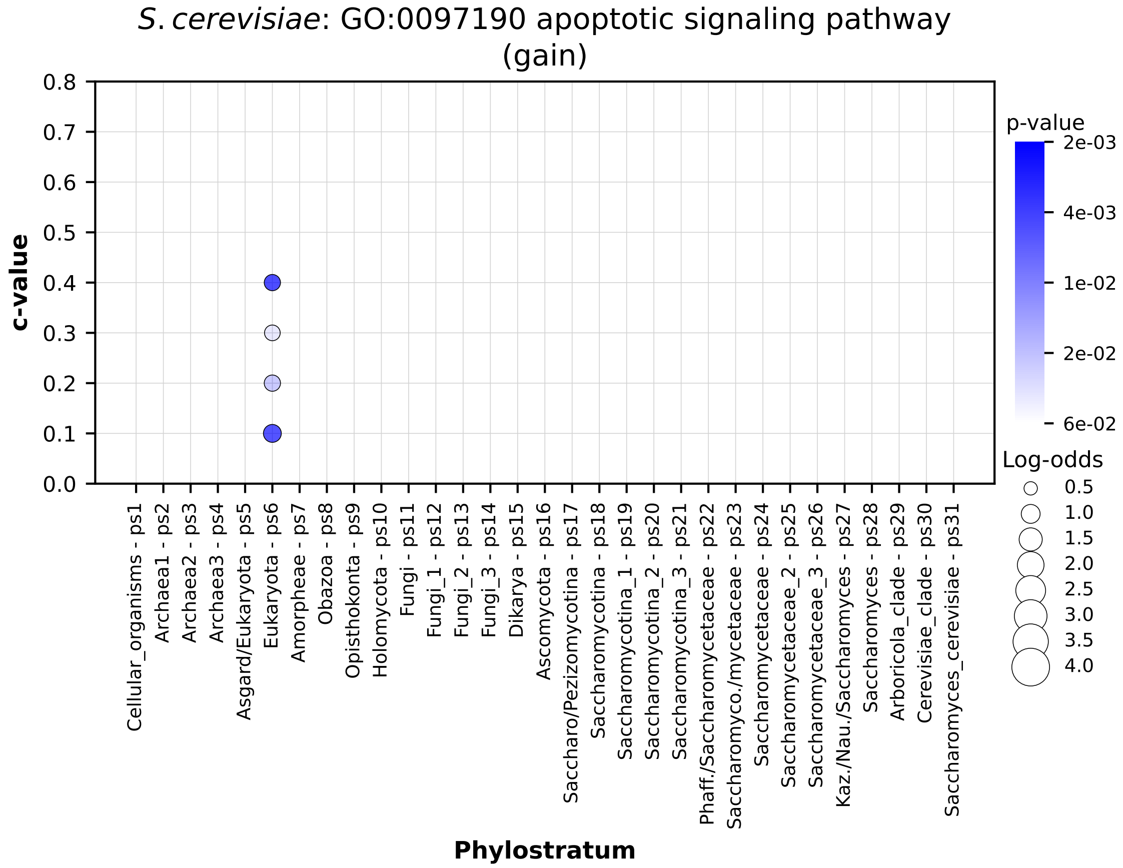

S. cerevisiae: GO:0097190 apoptotic signaling pathway (gain)
c-value
0.0
0.1
0.2
0.3
0.4
0.5
0.6
0.7
0.8
p-value
2e-03
4e-03
1e-02
2e-02
6e-02
Log-odds
0.5
1.0
1.5
2.0
2.5
3.0
3.5
4.0
Cellular_organisms - ps1
Archaea1 - ps2
Archaea2 - ps3
Archaea3 - ps4
Asgard/Eukaryota - ps5
Eukaryota - ps6
Amorpheae - ps7
Obazoa - ps8
Opisthokonta - ps9
Holomycota - ps10
Fungi - ps11
Fungi_1 - ps12
Fungi_2 - ps13
Fungi_3 - ps14
Dikarya - ps15
Ascomycota - ps16
Saccharo/Pezizomycotina - ps17
Saccharomycotina - ps18
Saccharomycotina_1 - ps19
Saccharomycotina_2 - ps20
Saccharomycotina_3 - ps21
Phaff./Saccharomycetaceae - ps22
Saccharomyco./mycetaceae - ps23
Saccharomycetaceae - ps24
Saccharomycetaceae_2 - ps25
Saccharomycetaceae_3 - ps26
Kaz./Nau./Saccharomyces - ps27
Saccharomyces - ps28
Arboricola_clade - ps29
Cerevisiae_clade - ps30
Saccharomyces_cerevisiae - ps31
Phylostratum

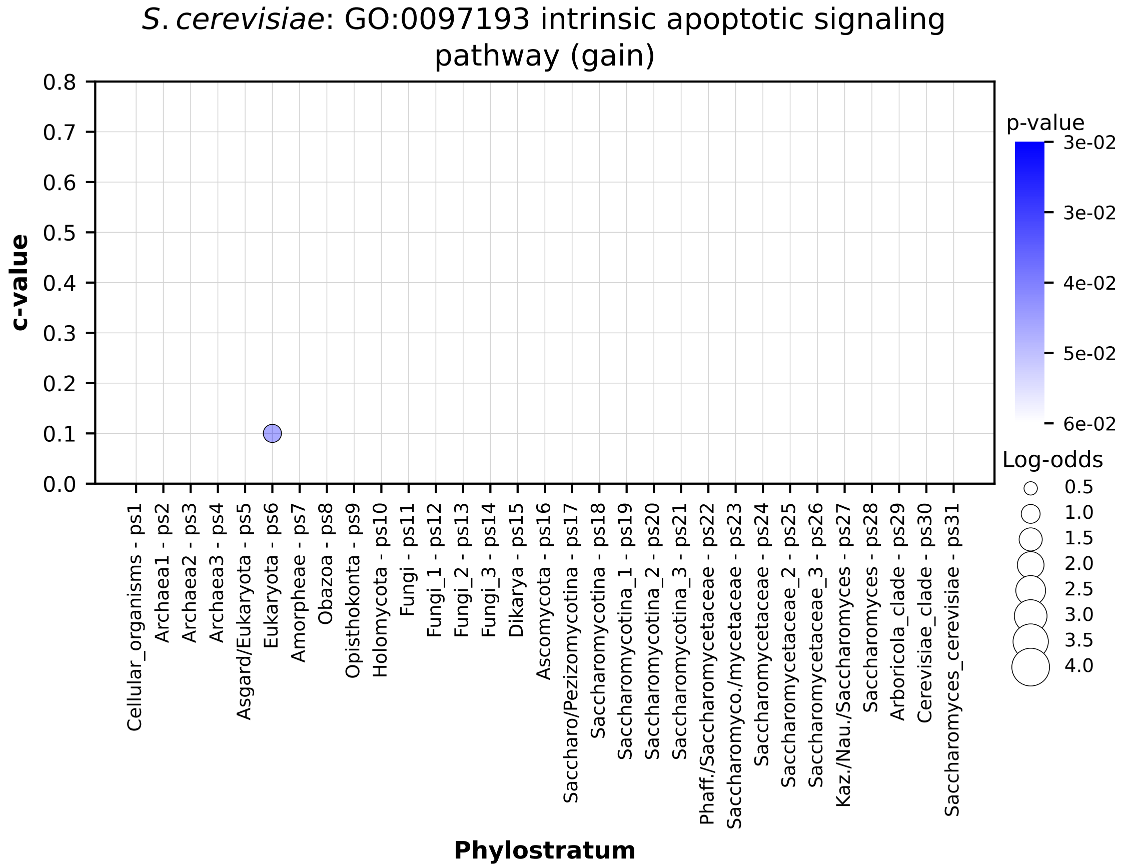

S. cerevisiae: GO:0097193 intrinsic apoptotic signaling pathway (gain)
c-value
0.0
0.1
0.2
0.3
0.4
0.5
0.6
0.7
0.8
Phylostratum
Cellular_organisms - ps1
Archaea1 - ps2
Archaea2 - ps3
Archaea3 - ps4
Asgard/Eukaryota - ps5
Eukaryota - ps6
Amorpheae - ps7
Obazoa - ps8
Opisthokonta - ps9
Holomycota - ps10
Fungi - ps11
Fungi_1 - ps12
Fungi_2 - ps13
Fungi_3 - ps14
Dikarya - ps15
Ascomycota - ps16
Saccharo/Pezizomycotina - ps17
Saccharomycotina - ps18
Saccharomycotina_1 - ps19
Saccharomycotina_2 - ps20
Saccharomycotina_3 - ps21
Phaff./Saccharomycetaceae - ps22
Saccharomyco./mycetaceae - ps23
Saccharomycetaceae - ps24
Saccharomycetaceae_2 - ps25
Saccharomycetaceae_3 - ps26
Kaz./Nau./Saccharomyces - ps27
Saccharomyces - ps28
Arboricola_clade - ps29
Cerevisiae_clade - ps30
Saccharomyces_cerevisiae - ps31
p-value
3e-02
3e-02
4e-02
5e-02
6e-02
Log-odds
0.5
1.0
1.5
2.0
2.5
3.0
3.5
4.0

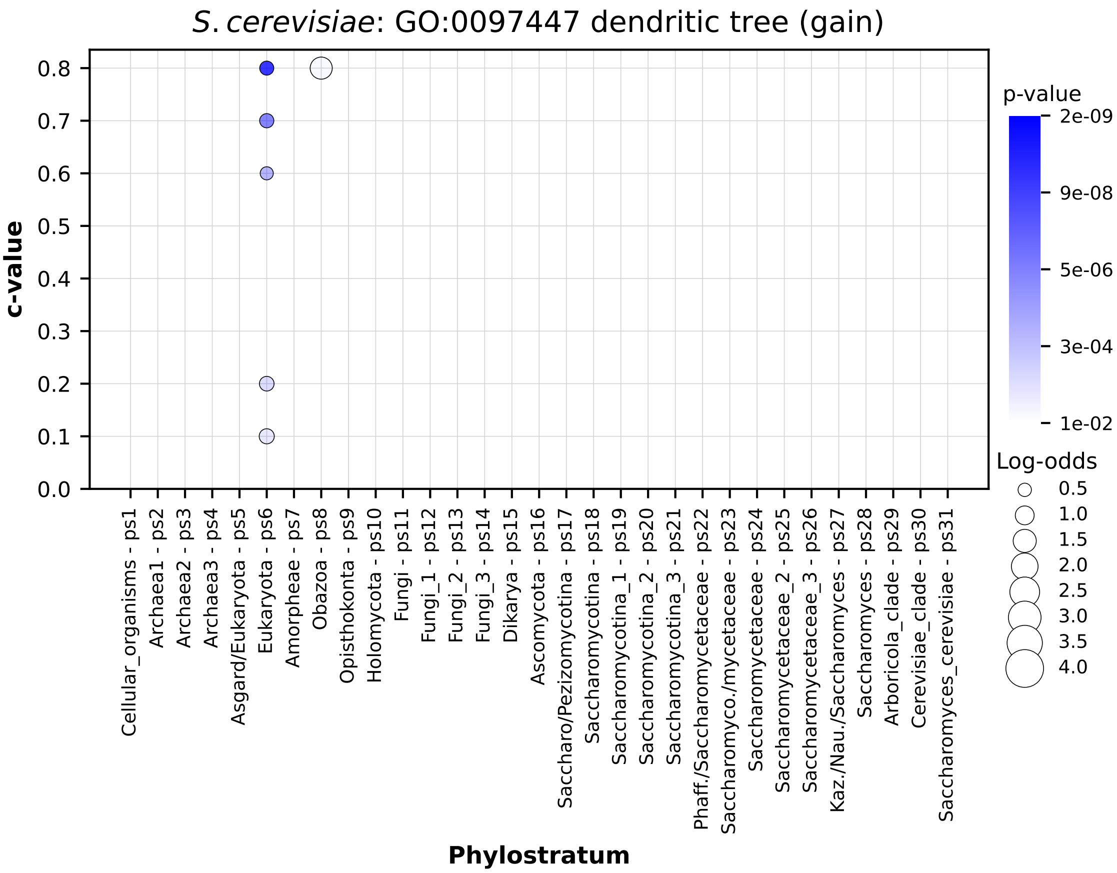

S. cerevisiae: GO:0097447 dendritic tree (gain)
c-value
0.0
0.1
0.2
0.3
0.4
0.5
0.6
0.7
0.8
p-value
2e-09
9e-08
5e-06
3e-04
1e-02
Log-odds
0.5
1.0
1.5
2.0
2.5
3.0
3.5
4.0
Cellular_organisms - ps1
Archaea1 - ps2
Archaea2 - ps3
Archaea3 - ps4
Asgard/Eukaryota - ps5
Eukaryota - ps6
Amorpheae - ps7
Obazoa - ps8
Opisthokonta - ps9
Holomycota - ps10
Fungi - ps11
Fungi_1 - ps12
Fungi_2 - ps13
Fungi_3 - ps14
Dikarya - ps15
Ascomycota - ps16
Saccharo/Pezizomycotina - ps17
Saccharomycotina - ps18
Saccharomycotina_1 - ps19
Saccharomycotina_2 - ps20
Saccharomycotina_3 - ps21
Phaff./Saccharomycetaceae - ps22
Saccharomyco./mycetaceae - ps23
Saccharomycetaceae - ps24
Saccharomycetaceae_2 - ps25
Saccharomycetaceae_3 - ps26
Kaz./Nau./Saccharomyces - ps27
Saccharomyces - ps28
Arboricola_clade - ps29
Cerevisiae_clade - ps30
Saccharomyces_cerevisiae - ps31
Phylostratum

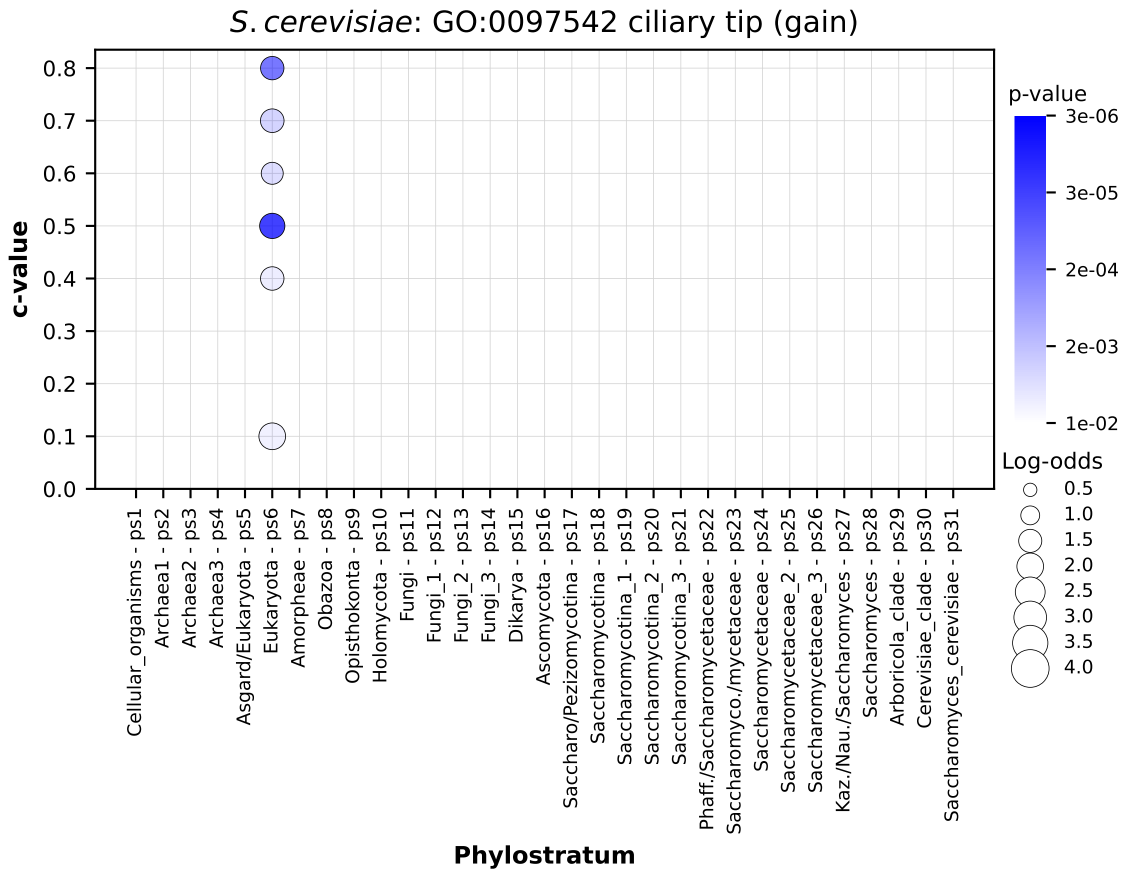
S. cerevisiae: GO:0097542 ciliary tip (gain)
c-value
0.0
0.1
0.2
0.3
0.4
0.5
0.6
0.7
0.8
p-value
3e-06
3e-05
2e-04
2e-03
1e-02
Log-odds
0.5
1.0
1.5
2.0
2.5
3.0
3.5
4.0
Cellular_organisms - ps1
Archaea1 - ps2
Archaea2 - ps3
Archaea3 - ps4
Asgard/Eukaryota - ps5
Eukaryota - ps6
Amorpheae - ps7
Obazoa - ps8
Opisthokonta - ps9
Holomycota - ps10
Fungi - ps11
Fungi_1 - ps12
Fungi_2 - ps13
Fungi_3 - ps14
Dikarya - ps15
Ascomycota - ps16
Saccharo/Pezizomycotina - ps17
Saccharomycotina - ps18
Saccharomycotina_1 - ps19
Saccharomycotina_2 - ps20
Saccharomycotina_3 - ps21
Phaff./Saccharomycetaceae - ps22
Saccharomyco./mycetaceae - ps23
Saccharomycetaceae - ps24
Saccharomycetaceae_2 - ps25
Saccharomycetaceae_3 - ps26
Kaz./Nau./Saccharomyces - ps27
Saccharomyces - ps28
Arboricola_clade - ps29
Cerevisiae_clade - ps30
Saccharomyces_cerevisiae - ps31
Phylostratum

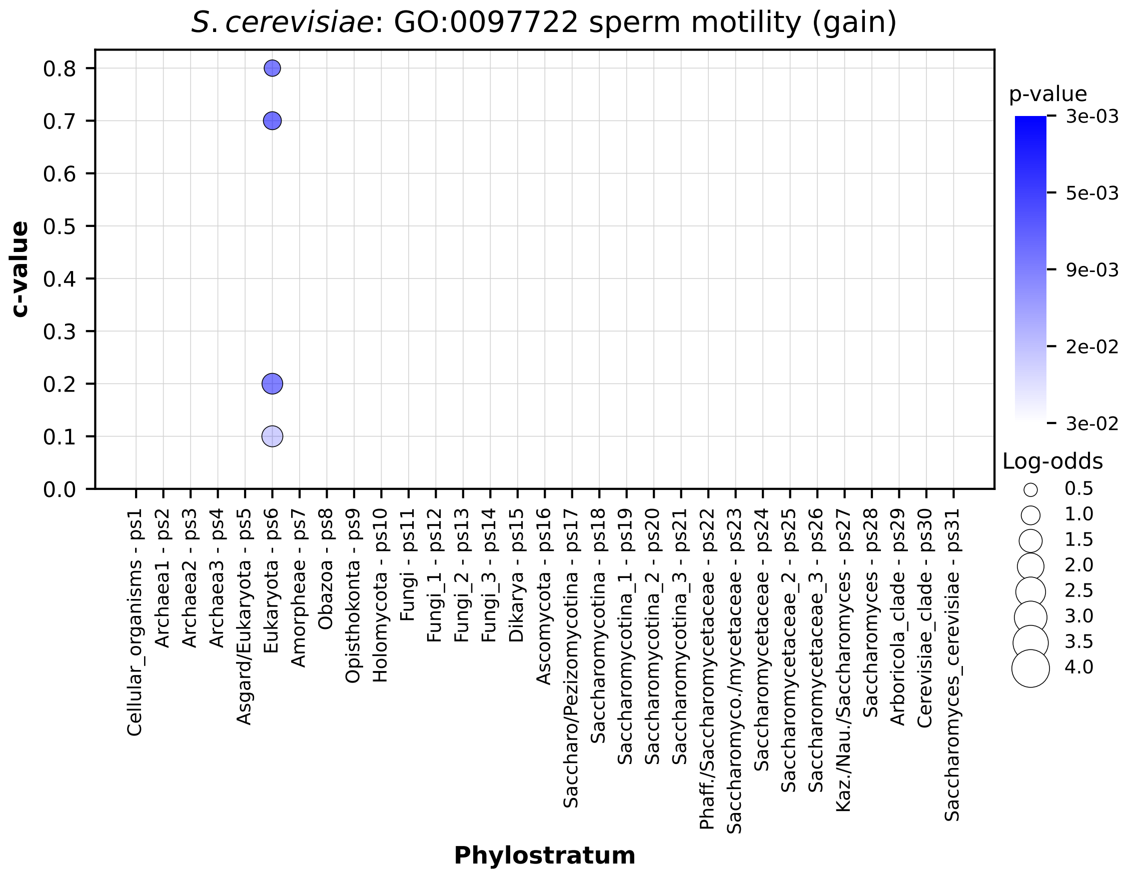

S. cerevisiae: GO:0097722 sperm motility (gain)
c-value
0.0
0.1
0.2
0.3
0.4
0.5
0.6
0.7
0.8
p-value
3e-03
5e-03
9e-03
2e-02
3e-02
Log-odds
0.5
1.0
1.5
2.0
2.5
3.0
3.5
4.0
Cellular_organisms - ps1
Archaea1 - ps2
Archaea2 - ps3
Archaea3 - ps4
Asgard/Eukaryota - ps5
Eukaryota - ps6
Amorpheae - ps7
Obazoa - ps8
Opisthokonta - ps9
Holomycota - ps10
Fungi - ps11
Fungi_1 - ps12
Fungi_2 - ps13
Fungi_3 - ps14
Dikarya - ps15
Ascomycota - ps16
Saccharo/Pezizomycotina - ps17
Saccharomycotina - ps18
Saccharomycotina_1 - ps19
Saccharomycotina_2 - ps20
Saccharomycotina_3 - ps21
Phaff./Saccharomycetaceae - ps22
Saccharomyco./mycetaceae - ps23
Saccharomycetaceae - ps24
Saccharomycetaceae_2 - ps25
Saccharomycetaceae_3 - ps26
Kaz./Nau./Saccharomyces - ps27
Saccharomyces - ps28
Arboricola_clade - ps29
Cerevisiae_clade - ps30
Saccharomyces_cerevisiae - ps31
Phylostratum

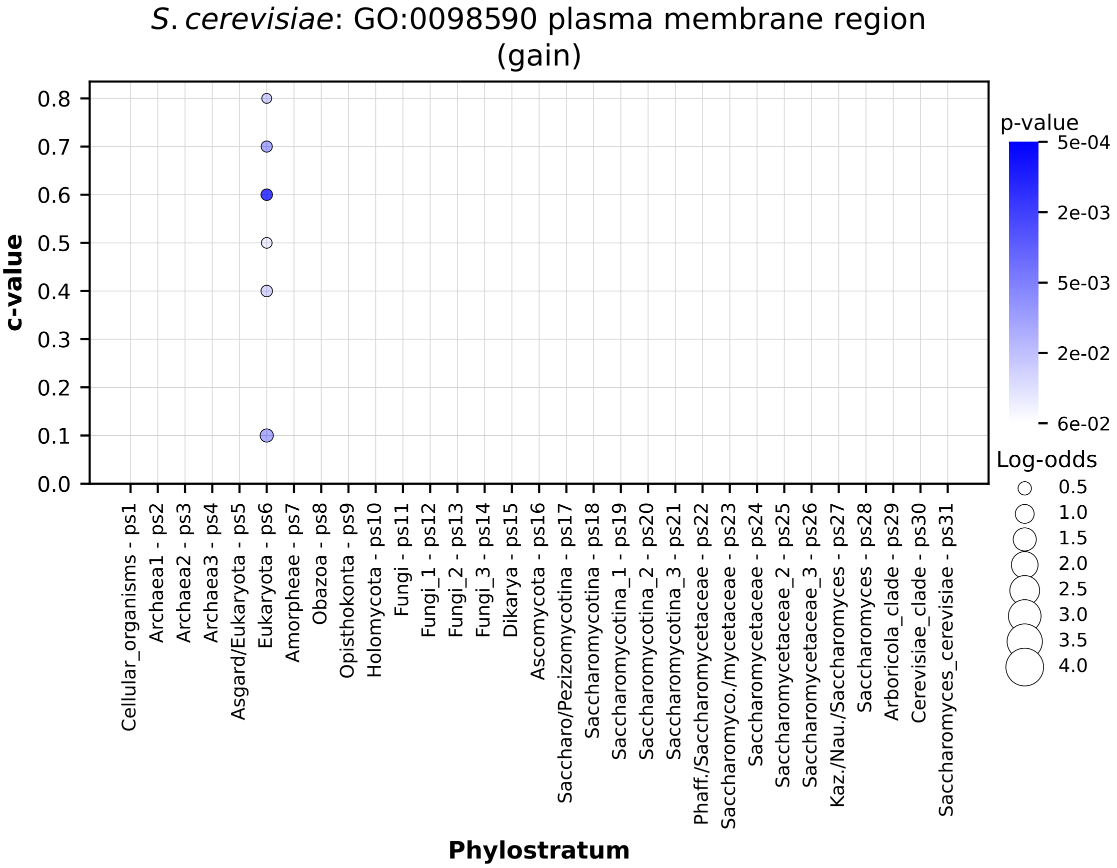

S. cerevisiae: GO:0098590 plasma membrane region
(gain)
c-value
0.0
0.1
0.2
0.3
0.4
0.5
0.6
0.7
0.8
p-value
5e-04
2e-03
5e-03
2e-02
6e-02
Log-odds
0.5
1.0
1.5
2.0
2.5
3.0
3.5
4.0
Cellular_organisms - ps1
Archaea1 - ps2
Archaea2 - ps3
Archaea3 - ps4
Asgard/Eukaryota - ps5
Eukaryota - ps6
Amorpheae - ps7
Obazoa - ps8
Opisthokonta - ps9
Holomycota - ps10
Fungi - ps11
Fungi_1 - ps12
Fungi_2 - ps13
Fungi_3 - ps14
Dikarya - ps15
Ascomycota - ps16
Saccharo/Pezizomycotina - ps17
Saccharomycotina - ps18
Saccharomycotina_1 - ps19
Saccharomycotina_2 - ps20
Saccharomycotina_3 - ps21
Phaff./Saccharomycetaceae - ps22
Saccharomyco./mycetaceae - ps23
Saccharomycetaceae - ps24
Saccharomycetaceae_2 - ps25
Saccharomycetaceae_3 - ps26
Kaz./Nau./Saccharomyces - ps27
Saccharomyces - ps28
Arboricola_clade - ps29
Cerevisiae_clade - ps30
Saccharomyces_cerevisiae - ps31
Phylostratum

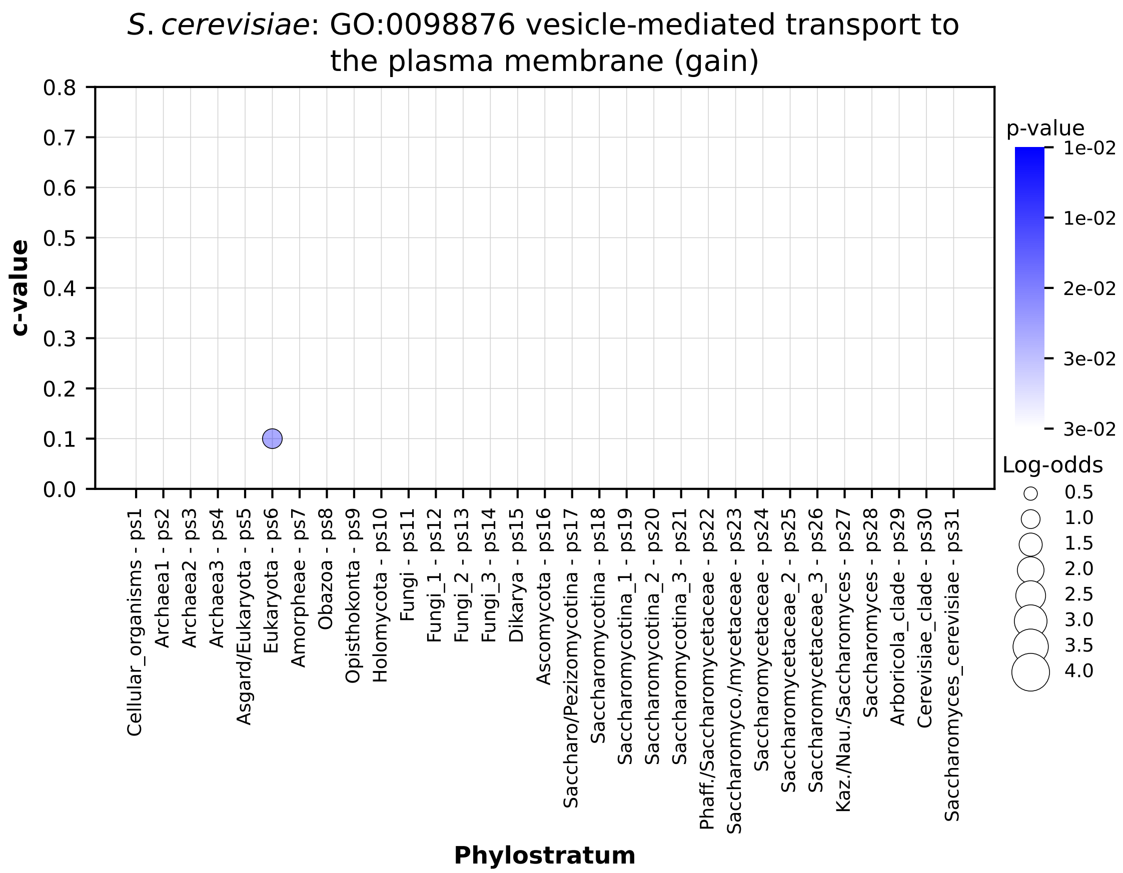
S. cerevisiae: GO:0098876 vesicle-mediated transport to the plasma membrane (gain)
c-value
0.8
0.7
0.6
0.5
0.4
0.3
0.2
0.1
0.0
Phylostratum
Cellular_organisms - ps1
Archaea1 - ps2
Archaea2 - ps3
Archaea3 - ps4
Asgard/Eukaryota - ps5
Eukaryota - ps6
Amorpheae - ps7
Obazoa - ps8
Opisthokonta - ps9
Holomycota - ps10
Fungi - ps11
Fungi_1 - ps12
Fungi_2 - ps13
Fungi_3 - ps14
Dikarya - ps15
Ascomycota - ps16
Saccharo/Pezizomycotina - ps17
Saccharomycotina - ps18
Saccharomycotina_1 - ps19
Saccharomycotina_2 - ps20
Saccharomycotina_3 - ps21
Phaff./Saccharomycetaceae - ps22
Saccharomyco./mycetaceae - ps23
Saccharomycetaceae - ps24
Saccharomycetaceae_2 - ps25
Saccharomycetaceae_3 - ps26
Kaz./Nau./Saccharomyces - ps27
Saccharomyces - ps28
Arboricola_clade - ps29
Cerevisiae_clade - ps30
Saccharomyces_cerevisiae - ps31
p-value
1e-02
1e-02
2e-02
3e-02
3e-02
Log-odds
0.5
1.0
1.5
2.0
2.5
3.0
3.5
4.0

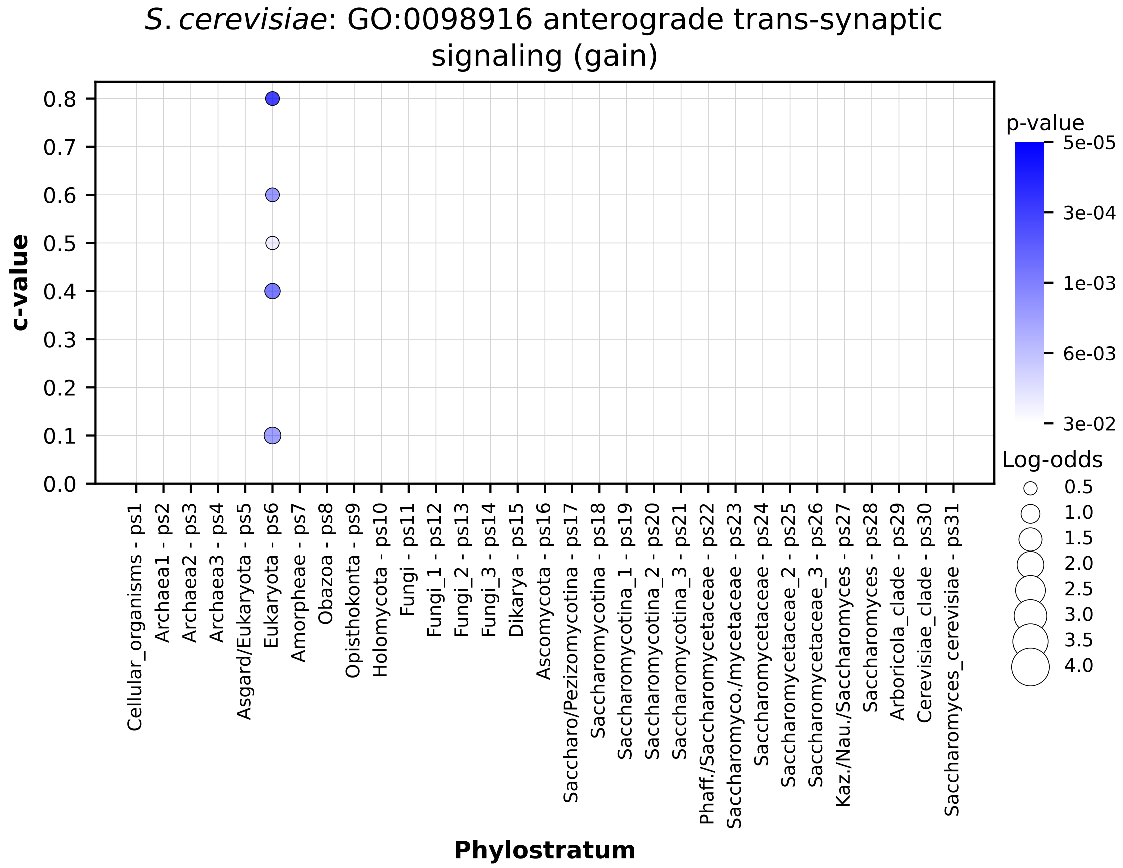

S. cerevisiae: GO:0098916 anterograde trans-synaptic signaling (gain)
c-value
0.0
0.1
0.2
0.3
0.4
0.5
0.6
0.7
0.8
p-value
5e-05
3e-04
1e-03
6e-03
3e-02
Log-odds
0.5
1.0
1.5
2.0
2.5
3.0
3.5
4.0
Cellular_organisms - ps1
Archaea1 - ps2
Archaea2 - ps3
Archaea3 - ps4
Asgard/Eukaryota - ps5
Eukaryota - ps6
Amorpheae - ps7
Obazoa - ps8
Opisthokonta - ps9
Holomycota - ps10
Fungi - ps11
Fungi_1 - ps12
Fungi_2 - ps13
Fungi_3 - ps14
Dikarya - ps15
Ascomycota - ps16
Saccharo/Pezizomycotina - ps17
Saccharomycotina - ps18
Saccharomycotina_1 - ps19
Saccharomycotina_2 - ps20
Saccharomycotina_3 - ps21
Phaff./Saccharomycetaceae - ps22
Saccharomyco./mycetaceae - ps23
Saccharomycetaceae - ps24
Saccharomycetaceae_2 - ps25
Saccharomycetaceae_3 - ps26
Kaz./Nau./Saccharomyces - ps27
Saccharomyces - ps28
Arboricola_clade - ps29
Cerevisiae_clade - ps30
Saccharomyces_cerevisiae - ps31
Phylostratum

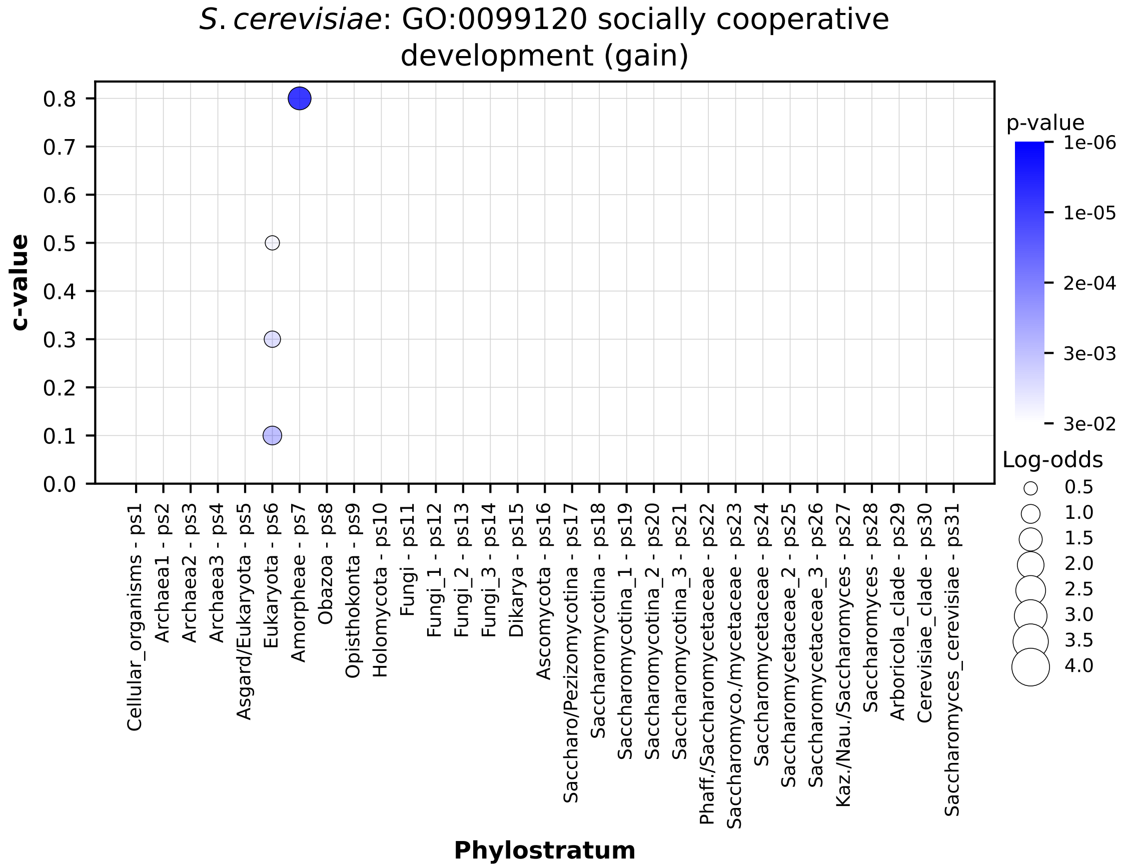

S. cerevisiae: GO:0099120 socially cooperative development (gain)
c-value
0.0
0.1
0.2
0.3
0.4
0.5
0.6
0.7
0.8
p-value
1e-06
1e-05
2e-04
3e-03
3e-02
Log-odds
0.5
1.0
1.5
2.0
2.5
3.0
3.5
4.0
Cellular_organisms - ps1
Archaea1 - ps2
Archaea2 - ps3
Archaea3 - ps4
Asgard/Eukaryota - ps5
Eukaryota - ps6
Amorpheae - ps7
Obazoa - ps8
Opisthokonta - ps9
Holomycota - ps10
Fungi - ps11
Fungi_1 - ps12
Fungi_2 - ps13
Fungi_3 - ps14
Dikarya - ps15
Ascomycota - ps16
Saccharo/Pezizomycotina - ps17
Saccharomycotina - ps18
Saccharomycotina_1 - ps19
Saccharomycotina_2 - ps20
Saccharomycotina_3 - ps21
Phaff./Saccharomycetaceae - ps22
Saccharomyco./mycetaceae - ps23
Saccharomycetaceae - ps24
Saccharomycetaceae_2 - ps25
Saccharomycetaceae_3 - ps26
Kaz./Nau./Saccharomyces - ps27
Saccharomyces - ps28
Arboricola_clade - ps29
Cerevisiae_clade - ps30
Saccharomyces_cerevisiae - ps31
Phylostratum

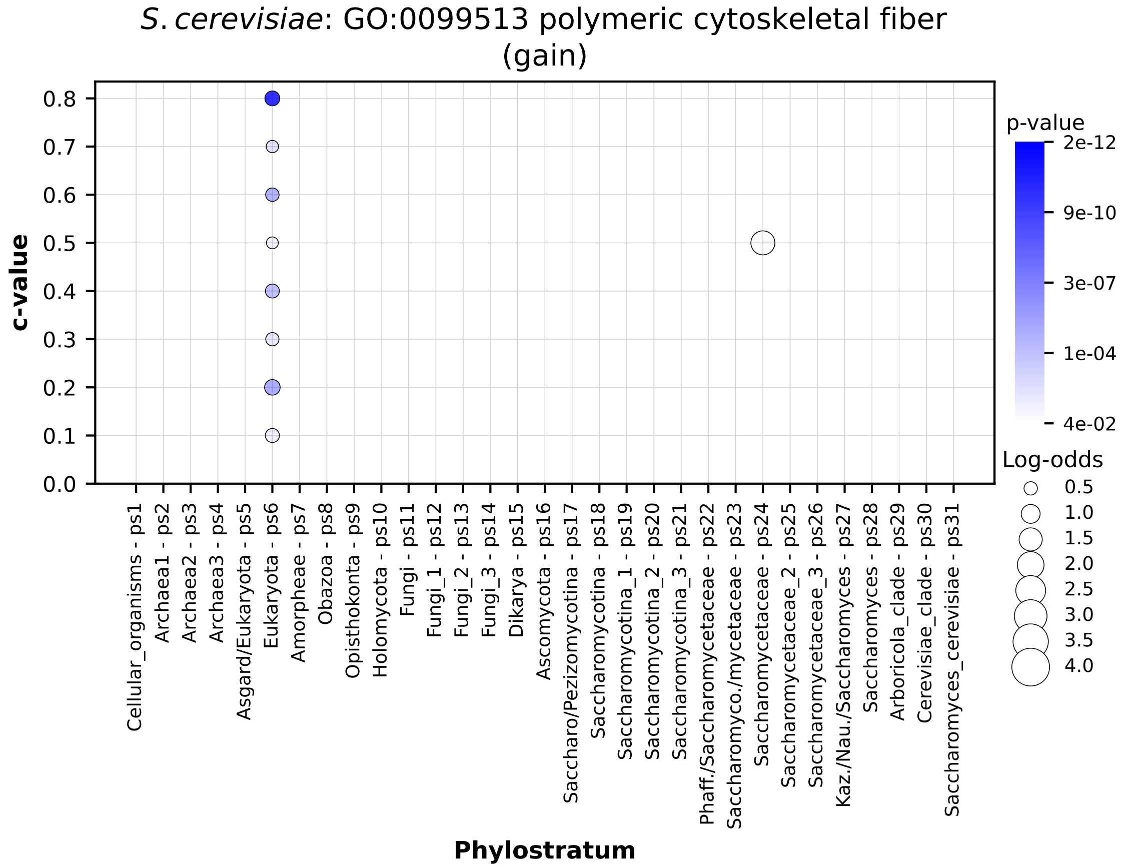
S. cerevisiae: GO:0099513 polymeric cytoskeletal fiber (gain)
c-value
0.0
0.1
0.2
0.3
0.4
0.5
0.6
0.7
0.8
Phylostratum
Cellular_organisms - ps1
Archaea1 - ps2
Archaea2 - ps3
Archaea3 - ps4
Asgard/Eukaryota - ps5
Eukaryota - ps6
Amorpheae - ps7
Obazoa - ps8
Opisthokonta - ps9
Holomycota - ps10
Fungi - ps11
Fungi_1 - ps12
Fungi_2 - ps13
Fungi_3 - ps14
Dikarya - ps15
Ascomycota - ps16
Saccharo/Pezizomycotina - ps17
Saccharomycotina - ps18
Saccharomycotina_1 - ps19
Saccharomycotina_2 - ps20
Saccharomycotina_3 - ps21
Phaff./Saccharomycetaceae - ps22
Saccharomyco./mycetaceae - ps23
Saccharomycetaceae - ps24
Saccharomycetaceae_2 - ps25
Saccharomycetaceae_3 - ps26
Kaz./Nau./Saccharomyces - ps27
Saccharomyces - ps28
Arboricola_clade - ps29
Cerevisiae_clade - ps30
Saccharomyces_cerevisiae - ps31
p-value
2e-12
9e-10
3e-07
1e-04
4e-02
Log-odds
0.5
1.0
1.5
2.0
2.5
3.0
3.5
4.0

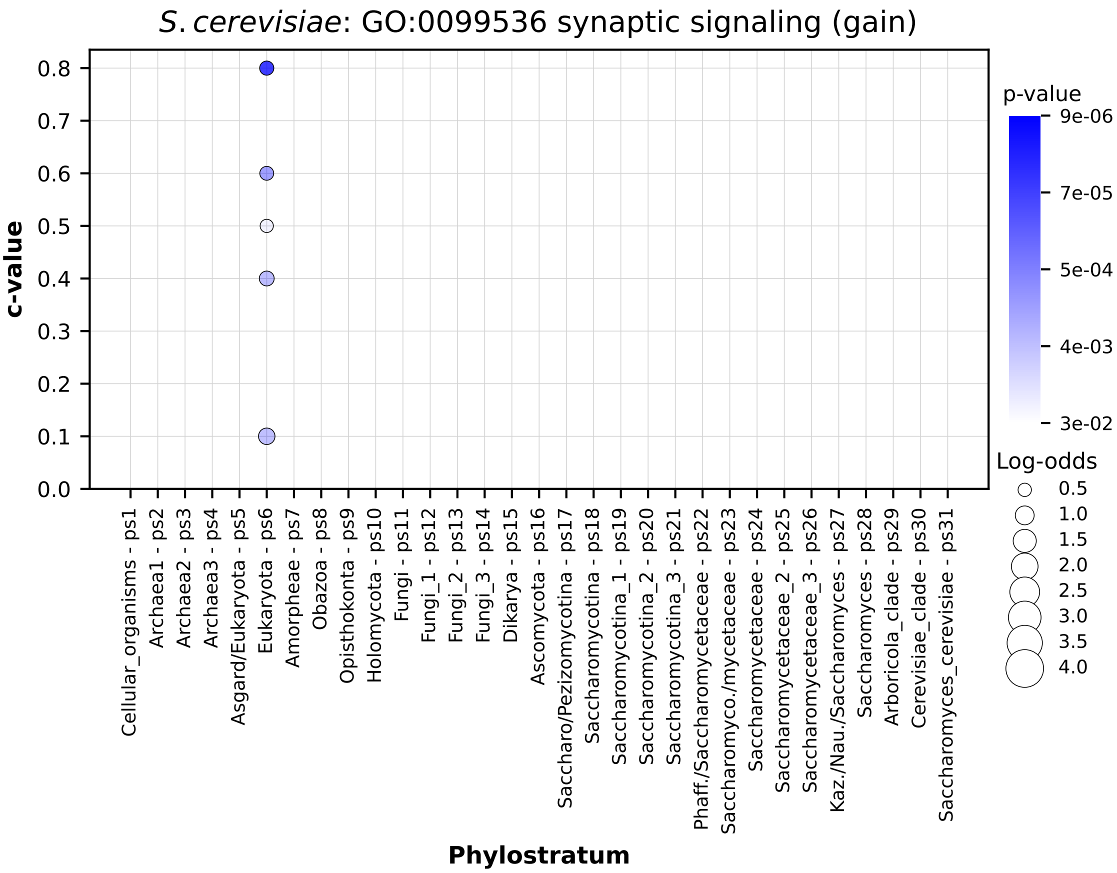
S. cerevisiae: GO:0099536 synaptic signaling (gain)
c-value
0.0
0.1
0.2
0.3
0.4
0.5
0.6
0.7
0.8
p-value
9e-06
7e-05
5e-04
4e-03
3e-02
Log-odds
0.5
1.0
1.5
2.0
2.5
3.0
3.5
4.0
Cellular_organisms - ps1
Archaea1 - ps2
Archaea2 - ps3
Archaea3 - ps4
Asgard/Eukaryota - ps5
Eukaryota - ps6
Amorpheae - ps7
Obazoa - ps8
Opisthokonta - ps9
Holomycota - ps10
Fungi - ps11
Fungi_1 - ps12
Fungi_2 - ps13
Fungi_3 - ps14
Dikarya - ps15
Ascomycota - ps16
Saccharo/Pezizomycotina - ps17
Saccharomycotina - ps18
Saccharomycotina_1 - ps19
Saccharomycotina_2 - ps20
Saccharomycotina_3 - ps21
Phaff./Saccharomycetaceae - ps22
Saccharomyco./mycetaceae - ps23
Saccharomycetaceae - ps24
Saccharomycetaceae_2 - ps25
Saccharomycetaceae_3 - ps26
Kaz./Nau./Saccharomyces - ps27
Saccharomyces - ps28
Arboricola_clade - ps29
Cerevisiae_clade - ps30
Saccharomyces_cerevisiae - ps31
Phylostratum

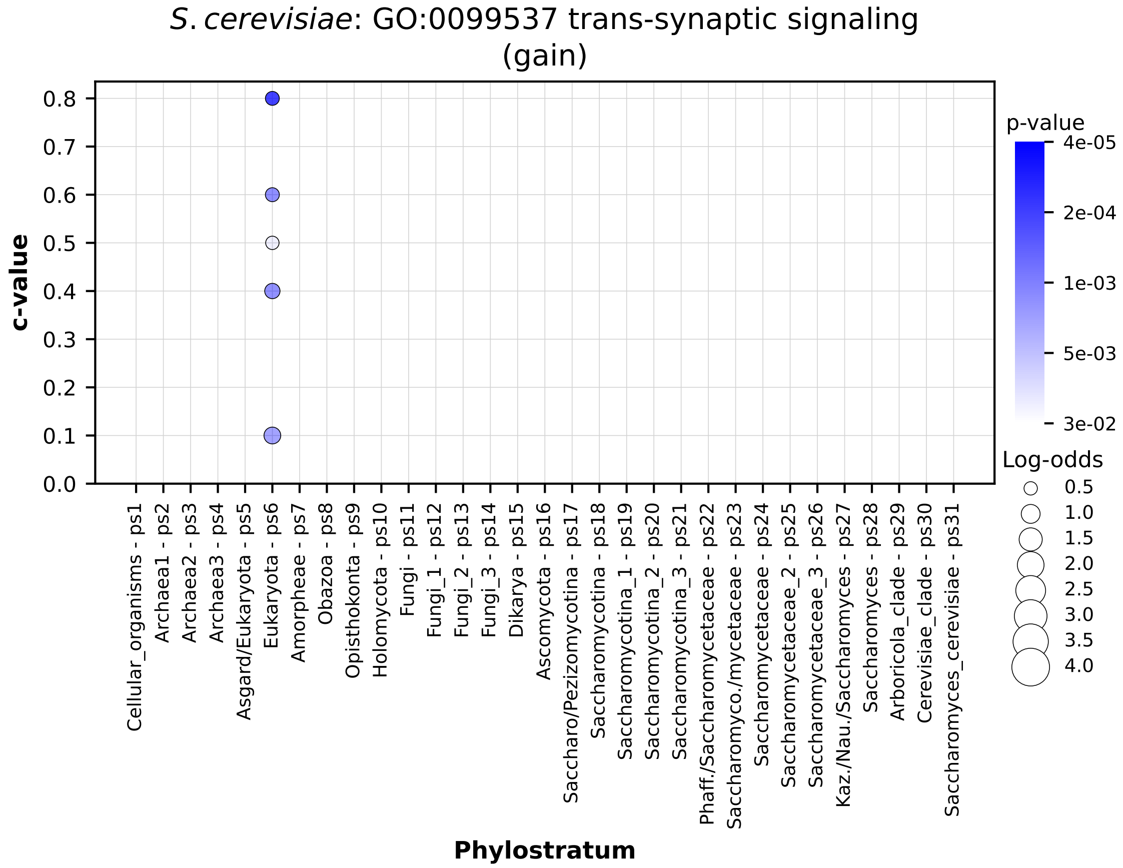
S. cerevisiae: GO:0099537 trans-synaptic signaling
(gain)
c-value
0.0
0.1
0.2
0.3
0.4
0.5
0.6
0.7
0.8
p-value
4e-05
2e-04
1e-03
5e-03
3e-02
Log-odds
0.5
1.0
1.5
2.0
2.5
3.0
3.5
4.0
Cellular_organisms - ps1
Archaea1 - ps2
Archaea2 - ps3
Archaea3 - ps4
Asgard/Eukaryota - ps5
Eukaryota - ps6
Amorpheae - ps7
Obazoa - ps8
Opisthokonta - ps9
Holomycota - ps10
Fungi - ps11
Fungi_1 - ps12
Fungi_2 - ps13
Fungi_3 - ps14
Dikarya - ps15
Ascomycota - ps16
Saccharo/Pezizomycotina - ps17
Saccharomycotina - ps18
Saccharomycotina_1 - ps19
Saccharomycotina_2 - ps20
Saccharomycotina_3 - ps21
Phaff./Saccharomycetaceae - ps22
Saccharomyco./mycetaceae - ps23
Saccharomycetaceae - ps24
Saccharomycetaceae_2 - ps25
Saccharomycetaceae_3 - ps26
Kaz./Nau./Saccharomyces - ps27
Saccharomyces - ps28
Arboricola_clade - ps29
Cerevisiae_clade - ps30
Saccharomyces_cerevisiae - ps31
Phylostratum

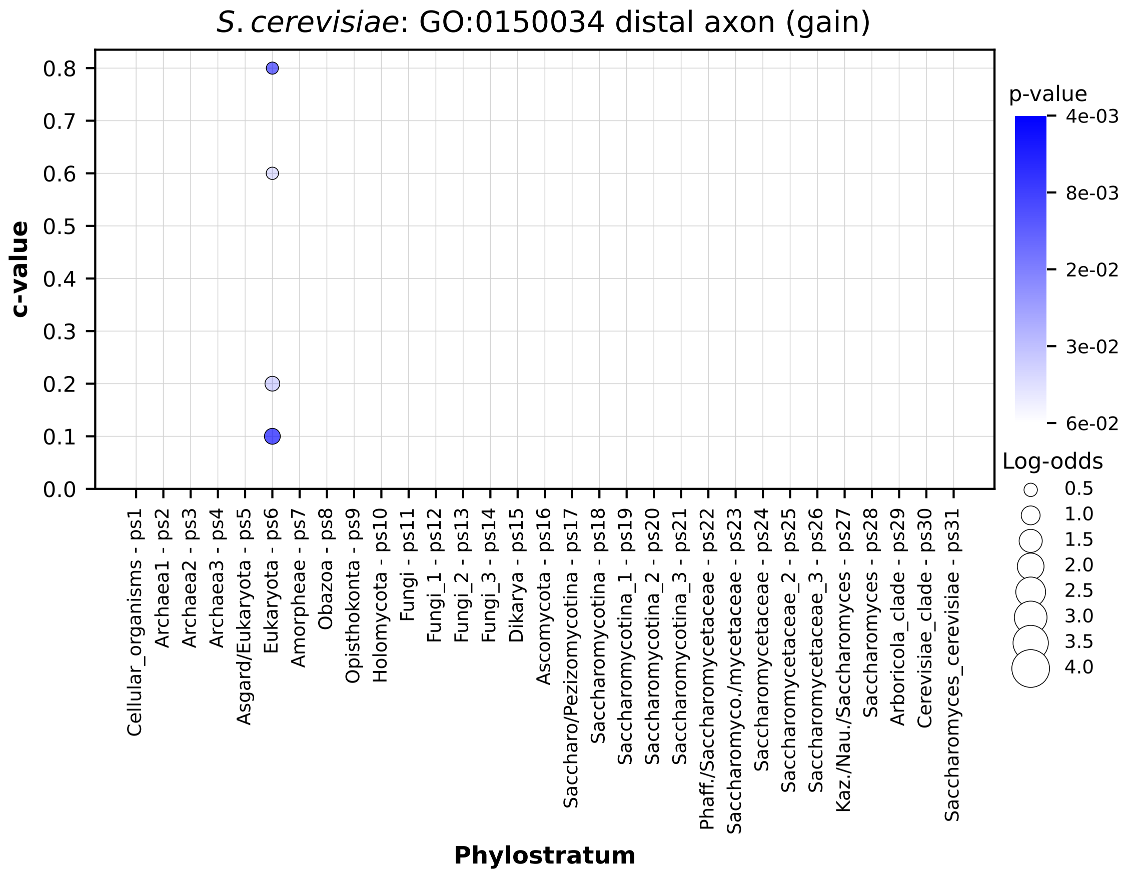

S. cerevisiae: GO:0150034 distal axon (gain)
c-value
0.0
0.1
0.2
0.3
0.4
0.5
0.6
0.7
0.8
p-value
4e-03
8e-03
2e-02
3e-02
6e-02
Log-odds
0.5
1.0
1.5
2.0
2.5
3.0
3.5
4.0
Cellular_organisms - ps1
Archaea1 - ps2
Archaea2 - ps3
Archaea3 - ps4
Asgard/Eukaryota - ps5
Eukaryota - ps6
Amorpheae - ps7
Obazoa - ps8
Opisthokonta - ps9
Holomycota - ps10
Fungi - ps11
Fungi_1 - ps12
Fungi_2 - ps13
Fungi_3 - ps14
Dikarya - ps15
Ascomycota - ps16
Saccharo/Pezizomycotina - ps17
Saccharomycotina - ps18
Saccharomycotina_1 - ps19
Saccharomycotina_2 - ps20
Saccharomycotina_3 - ps21
Phaff./Saccharomycetaceae - ps22
Saccharomyco./mycetaceae - ps23
Saccharomycetaceae - ps24
Saccharomycetaceae_2 - ps25
Saccharomycetaceae_3 - ps26
Kaz./Nau./Saccharomyces - ps27
Saccharomyces - ps28
Arboricola_clade - ps29
Cerevisiae_clade - ps30
Saccharomyces_cerevisiae - ps31
Phylostratum

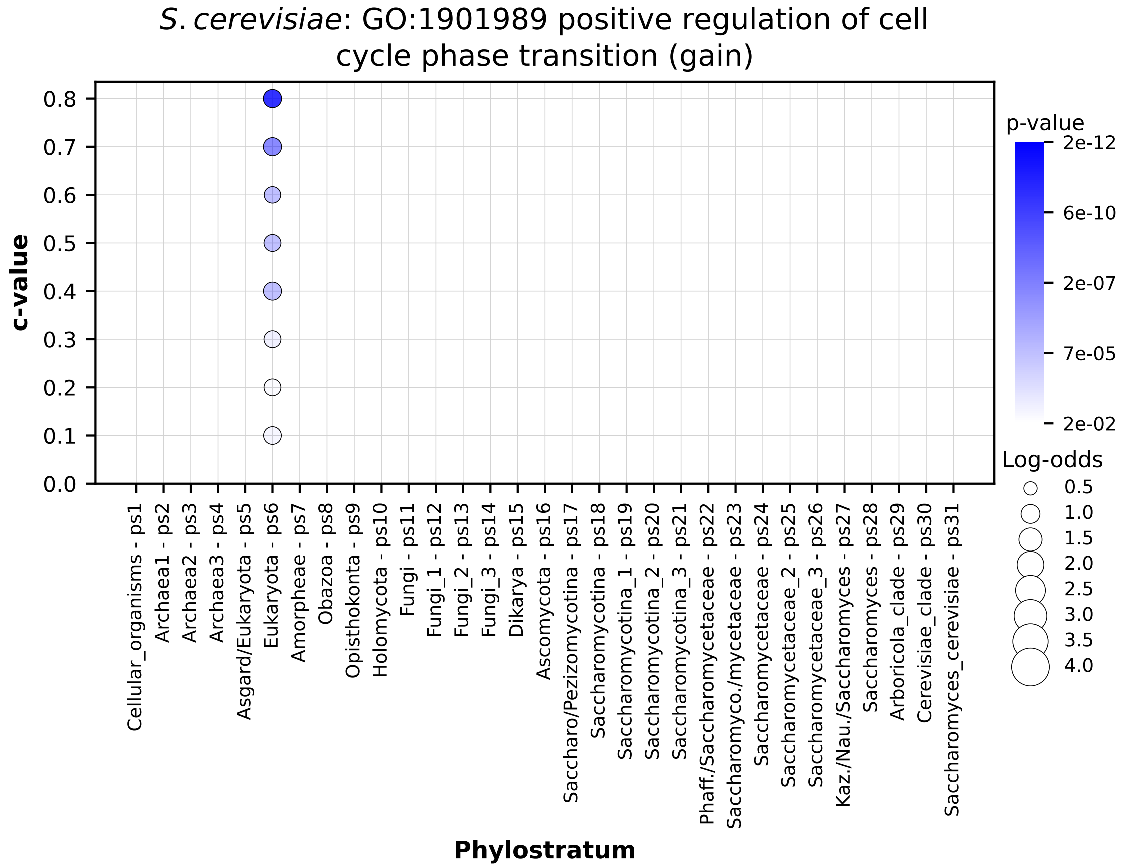
S. cerevisiae: GO:1901989 positive regulation of cell cycle phase transition (gain)
c-value
0.0
0.1
0.2
0.3
0.4
0.5
0.6
0.7
0.8
p-value
2e-12
6e-10
2e-07
7e-05
2e-02
Log-odds
0.5
1.0
1.5
2.0
2.5
3.0
3.5
4.0
Cellular_organisms - ps1
Archaea1 - ps2
Archaea2 - ps3
Archaea3 - ps4
Asgard/Eukaryota - ps5
Eukaryota - ps6
Amorpheae - ps7
Obazoa - ps8
Opisthokonta - ps9
Holomycota - ps10
Fungi - ps11
Fungi_1 - ps12
Fungi_2 - ps13
Fungi_3 - ps14
Dikarya - ps15
Ascomycota - ps16
Saccharo/Pezizomycotina - ps17
Saccharomycotina - ps18
Saccharomycotina_1 - ps19
Saccharomycotina_2 - ps20
Saccharomycotina_3 - ps21
Phaff./Saccharomycetaceae - ps22
Saccharomyco./mycetaceae - ps23
Saccharomycetaceae - ps24
Saccharomycetaceae_2 - ps25
Saccharomycetaceae_3 - ps26
Kaz./Nau./Saccharomyces - ps27
Saccharomyces - ps28
Arboricola_clade - ps29
Cerevisiae_clade - ps30
Saccharomyces_cerevisiae - ps31
Phylostratum

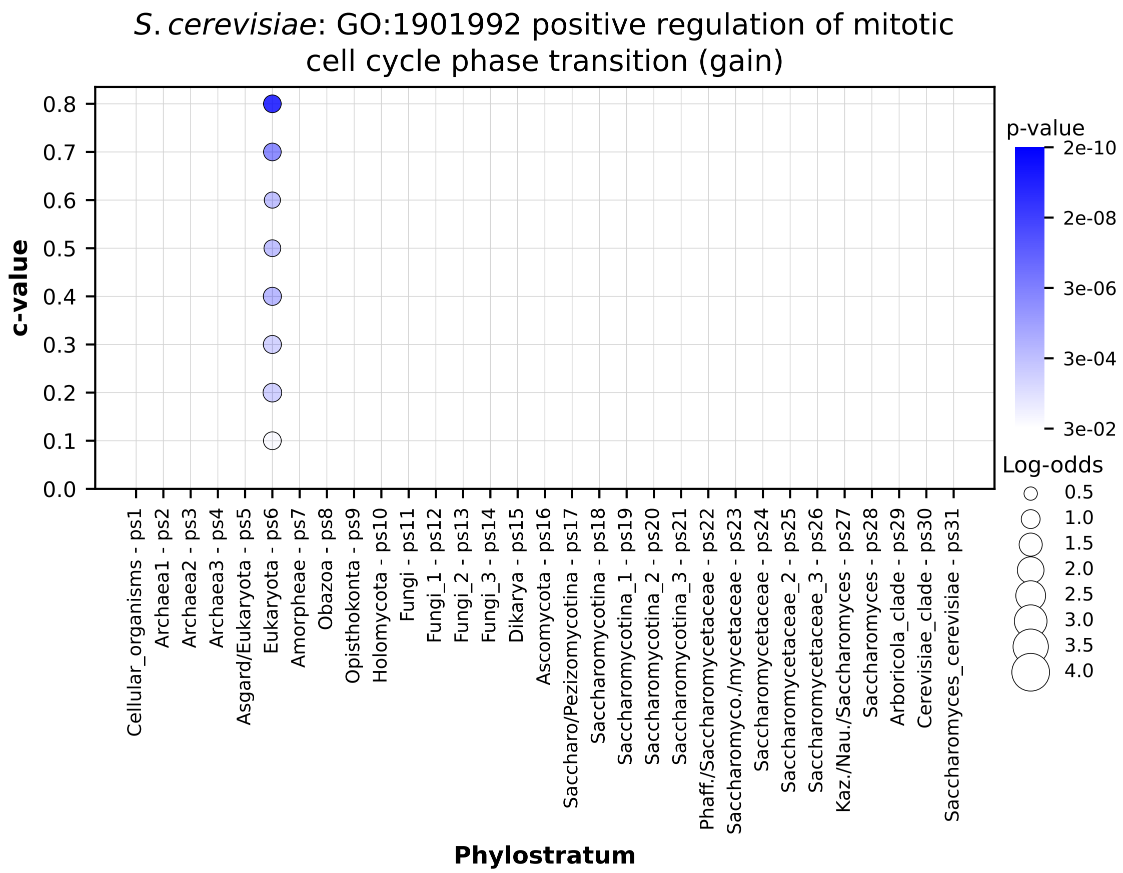

S. cerevisiae: GO:1901992 positive regulation of mitotic cell cycle phase transition (gain)
c-value
0.0
0.1
0.2
0.3
0.4
0.5
0.6
0.7
0.8
Phylostratum
Cellular_organisms - ps1
Archaea1 - ps2
Archaea2 - ps3
Archaea3 - ps4
Asgard/Eukaryota - ps5
Eukaryota - ps6
Amorpheae - ps7
Obazoa - ps8
Opisthokonta - ps9
Holomycota - ps10
Fungi - ps11
Fungi_1 - ps12
Fungi_2 - ps13
Fungi_3 - ps14
Dikarya - ps15
Ascomycota - ps16
Saccharo/Pezizomycotina - ps17
Saccharomycotina - ps18
Saccharomycotina_1 - ps19
Saccharomycotina_2 - ps20
Saccharomycotina_3 - ps21
Phaff./Saccharomycetaceae - ps22
Saccharomyco./mycetaceae - ps23
Saccharomycetaceae - ps24
Saccharomycetaceae_2 - ps25
Saccharomycetaceae_3 - ps26
Kaz./Nau./Saccharomyces - ps27
Saccharomyces - ps28
Arboricola_clade - ps29
Cerevisiae_clade - ps30
Saccharomyces_cerevisiae - ps31
p-value
2e-10
2e-08
3e-06
3e-04
3e-02
Log-odds
0.5
1.0
1.5
2.0
2.5
3.0
3.5
4.0

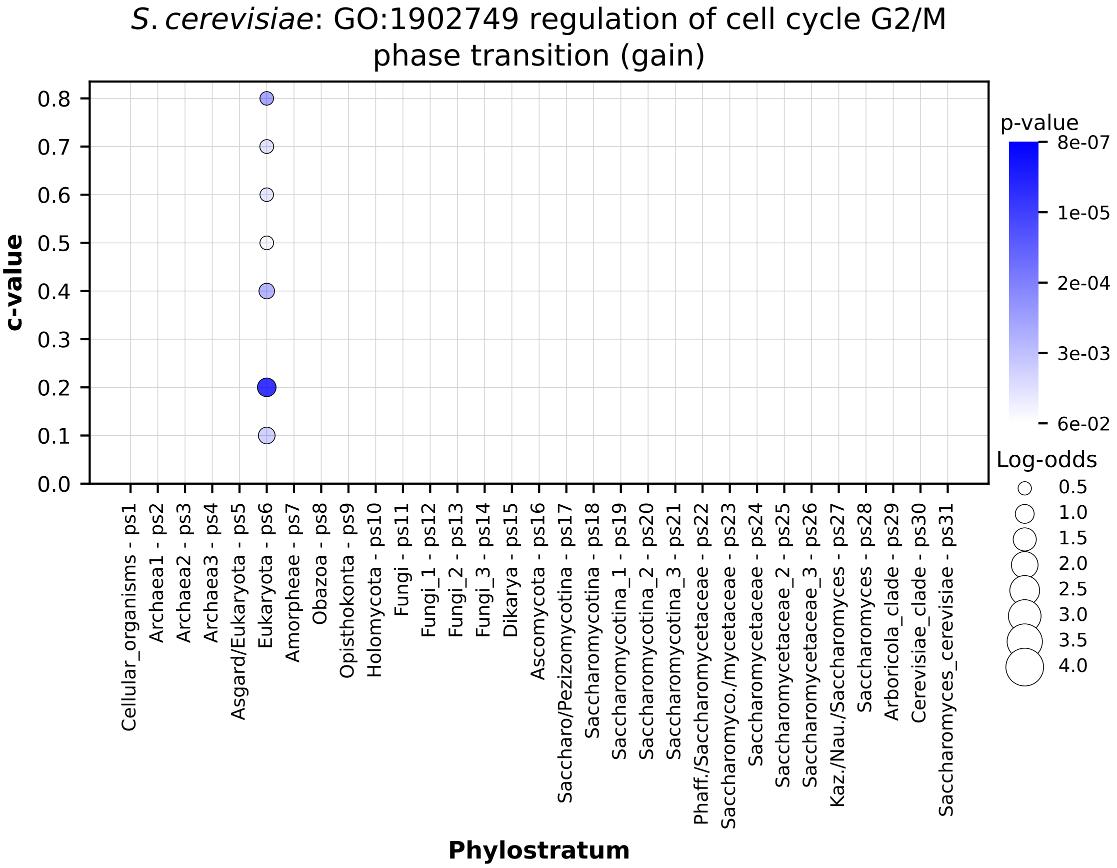
S. cerevisiae: GO:1902749 regulation of cell cycle G2/M phase transition (gain)
c-value
0.0
0.1
0.2
0.3
0.4
0.5
0.6
0.7
0.8
p-value
8e-07
1e-05
2e-04
3e-03
6e-02
Log-odds
0.5
1.0
1.5
2.0
2.5
3.0
3.5
4.0
Cellular_organisms - ps1
Archaea1 - ps2
Archaea2 - ps3
Archaea3 - ps4
Asgard/Eukaryota - ps5
Eukaryota - ps6
Amorpheae - ps7
Obazoa - ps8
Opisthokonta - ps9
Holomycota - ps10
Fungi - ps11
Fungi_1 - ps12
Fungi_2 - ps13
Fungi_3 - ps14
Dikarya - ps15
Ascomycota - ps16
Saccharo/Pezizomycotina - ps17
Saccharomycotina - ps18
Saccharomycotina_1 - ps19
Saccharomycotina_2 - ps20
Saccharomycotina_3 - ps21
Phaff./Saccharomycetaceae - ps22
Saccharomyco./mycetaceae - ps23
Saccharomycetaceae - ps24
Saccharomycetaceae_2 - ps25
Saccharomycetaceae_3 - ps26
Kaz./Nau./Saccharomyces - ps27
Saccharomyces - ps28
Arboricola_clade - ps29
Cerevisiae_clade - ps30
Saccharomyces_cerevisiae - ps31
Phylostratum

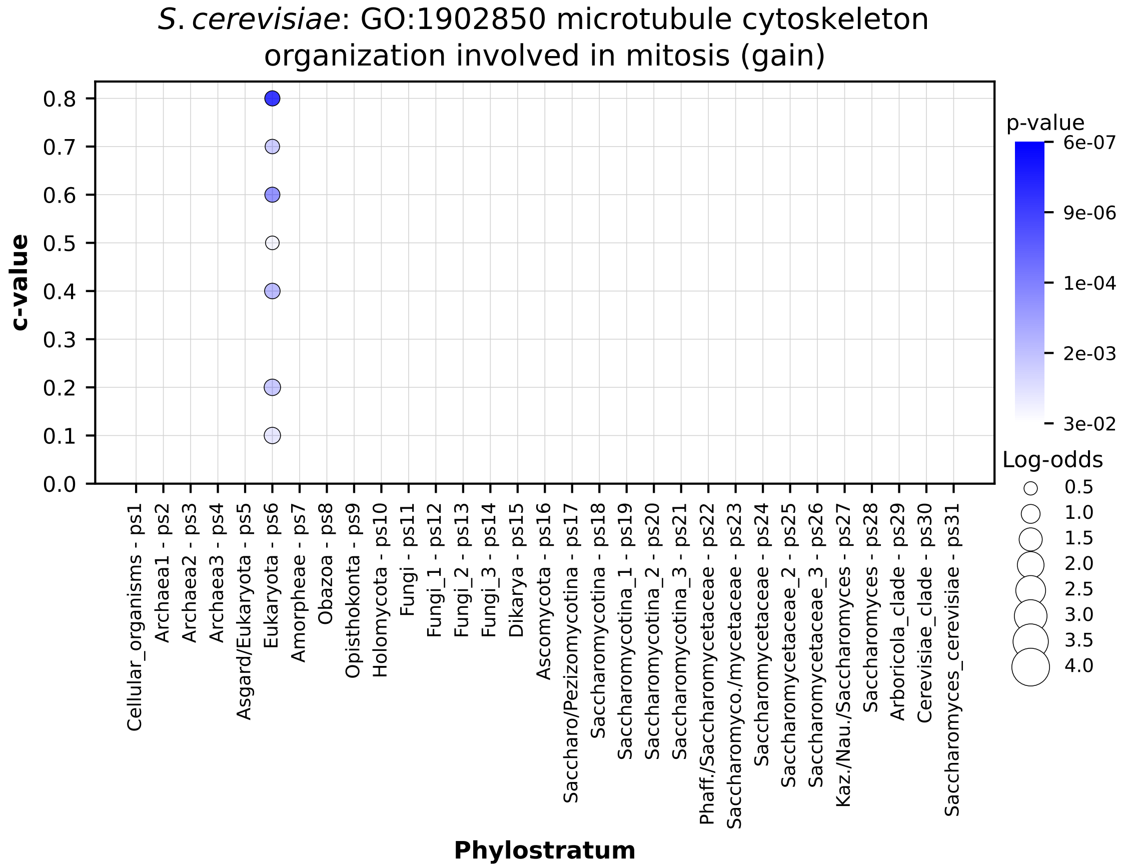

S. cerevisiae: GO:1902850 microtubule cytoskeleton organization involved in mitosis (gain)
c-value
0.8
0.7
0.6
0.5
0.4
0.3
0.2
0.1
0.0
p-value
6e-07
9e-06
1e-04
2e-03
3e-02
Log-odds
0.5
1.0
1.5
2.0
2.5
3.0
3.5
4.0
Cellular_organisms - ps1
Archaea1 - ps2
Archaea2 - ps3
Archaea3 - ps4
Asgard/Eukaryota - ps5
Eukaryota - ps6
Amorpheae - ps7
Obazoa - ps8
Opisthokonta - ps9
Holomycota - ps10
Fungi - ps11
Fungi_1 - ps12
Fungi_2 - ps13
Fungi_3 - ps14
Dikarya - ps15
Ascomycota - ps16
Saccharo/Pezizomycotina - ps17
Saccharomycotina - ps18
Saccharomycotina_1 - ps19
Saccharomycotina_2 - ps20
Saccharomycotina_3 - ps21
Phaff./Saccharomycetaceae - ps22
Saccharomyco./mycetaceae - ps23
Saccharomycetaceae - ps24
Saccharomycetaceae_2 - ps25
Saccharomycetaceae_3 - ps26
Kaz./Nau./Saccharomyces - ps27
Saccharomyces - ps28
Arboricola_clade - ps29
Cerevisiae_clade - ps30
Saccharomyces_cerevisiae - ps31
Phylostratum

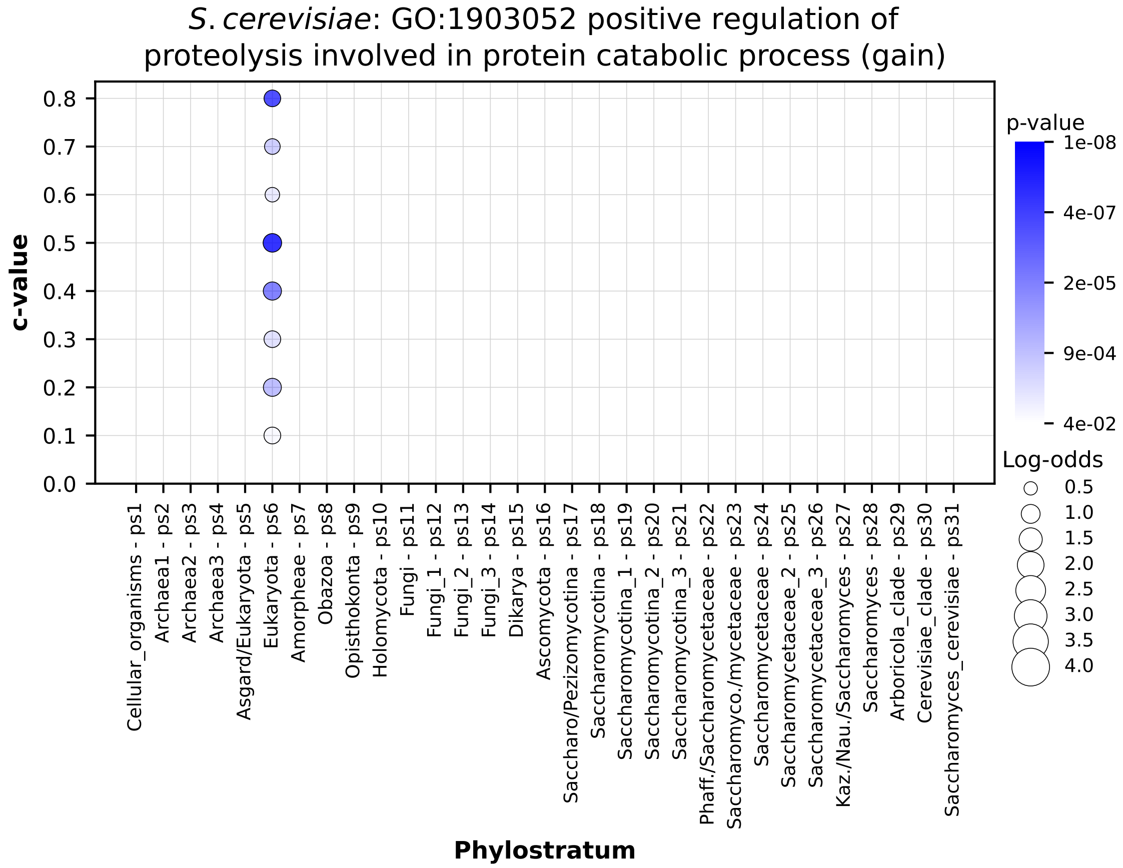

S. cerevisiae: GO:1903052 positive regulation of proteolysis involved in protein catabolic process (gain)
c-value
0.0
0.1
0.2
0.3
0.4
0.5
0.6
0.7
0.8
p-value
1e-08
4e-07
2e-05
9e-04
4e-02
Log-odds
0.5
1.0
1.5
2.0
2.5
3.0
3.5
4.0
Cellular_organisms - ps1
Archaea1 - ps2
Archaea2 - ps3
Archaea3 - ps4
Asgard/Eukaryota - ps5
Eukaryota - ps6
Amorpheae - ps7
Obazoa - ps8
Opisthokonta - ps9
Holomycota - ps10
Fungi - ps11
Fungi_1 - ps12
Fungi_2 - ps13
Fungi_3 - ps14
Dikarya - ps15
Ascomycota - ps16
Saccharo/Pezizomycotina - ps17
Saccharomycotina - ps18
Saccharomycotina_1 - ps19
Saccharomycotina_2 - ps20
Saccharomycotina_3 - ps21
Phaff./Saccharomycetaceae - ps22
Saccharomyco./mycetaceae - ps23
Saccharomycetaceae - ps24
Saccharomycetaceae_2 - ps25
Saccharomycetaceae_3 - ps26
Kaz./Nau./Saccharomyces - ps27
Saccharomyces - ps28
Arboricola_clade - ps29
Cerevisiae_clade - ps30
Saccharomyces_cerevisiae - ps31
Phylostratum

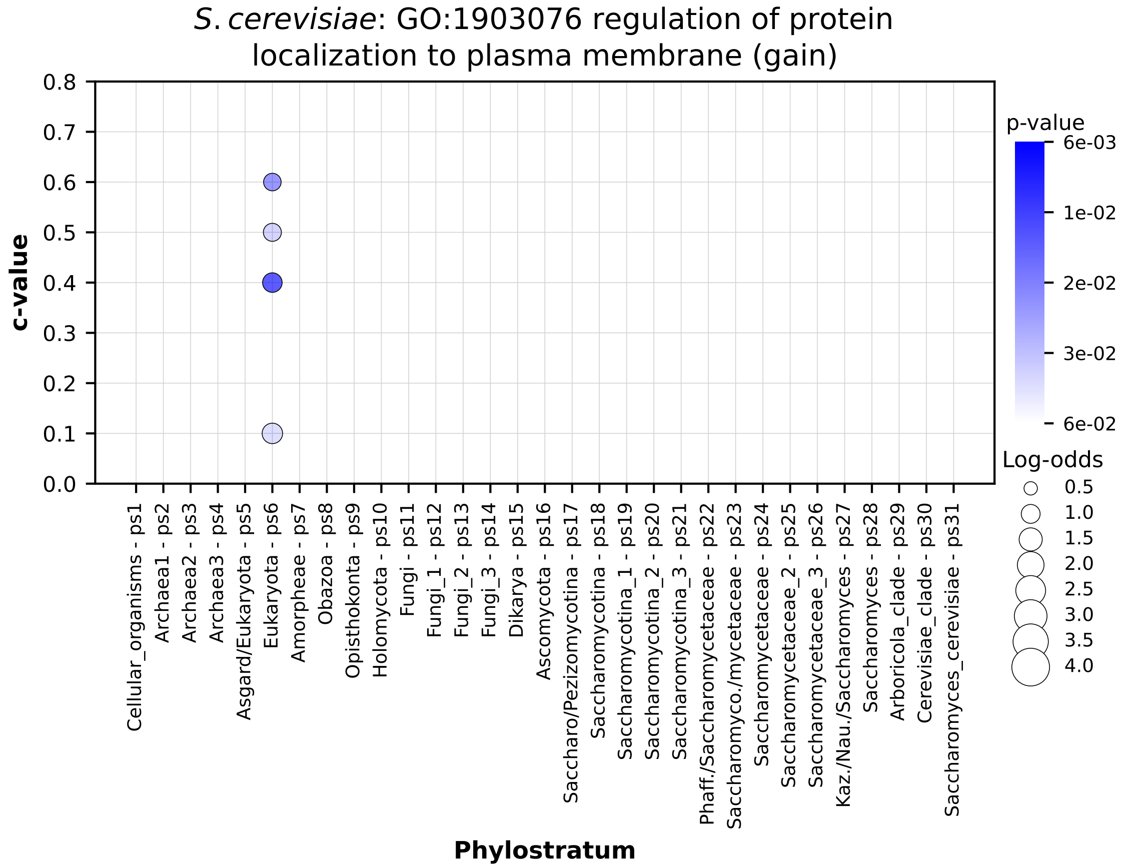

S. cerevisiae: GO:1903076 regulation of protein localization to plasma membrane (gain)
c-value
0.8
0.7
0.6
0.5
0.4
0.3
0.2
0.1
0.0
Phylostratum
Cellular_organisms - ps1
Archaea1 - ps2
Archaea2 - ps3
Archaea3 - ps4
Asgard/Eukaryota - ps5
Eukaryota - ps6
Amorpheae - ps7
Obazoa - ps8
Opisthokonta - ps9
Holomycota - ps10
Fungi - ps11
Fungi_1 - ps12
Fungi_2 - ps13
Fungi_3 - ps14
Dikarya - ps15
Ascomycota - ps16
Saccharo/Pezizomycotina - ps17
Saccharomycotina - ps18
Saccharomycotina_1 - ps19
Saccharomycotina_2 - ps20
Saccharomycotina_3 - ps21
Phaff./Saccharomycetaceae - ps22
Saccharomyco./mycetaceae - ps23
Saccharomycetaceae - ps24
Saccharomycetaceae_2 - ps25
Saccharomycetaceae_3 - ps26
Kaz./Nau./Saccharomyces - ps27
Saccharomyces - ps28
Arboricola_clade - ps29
Cerevisiae_clade - ps30
Saccharomyces_cerevisiae - ps31
p-value
6e-03
1e-02
2e-02
3e-02
6e-02
Log-odds
0.5
1.0
1.5
2.0
2.5
3.0
3.5
4.0

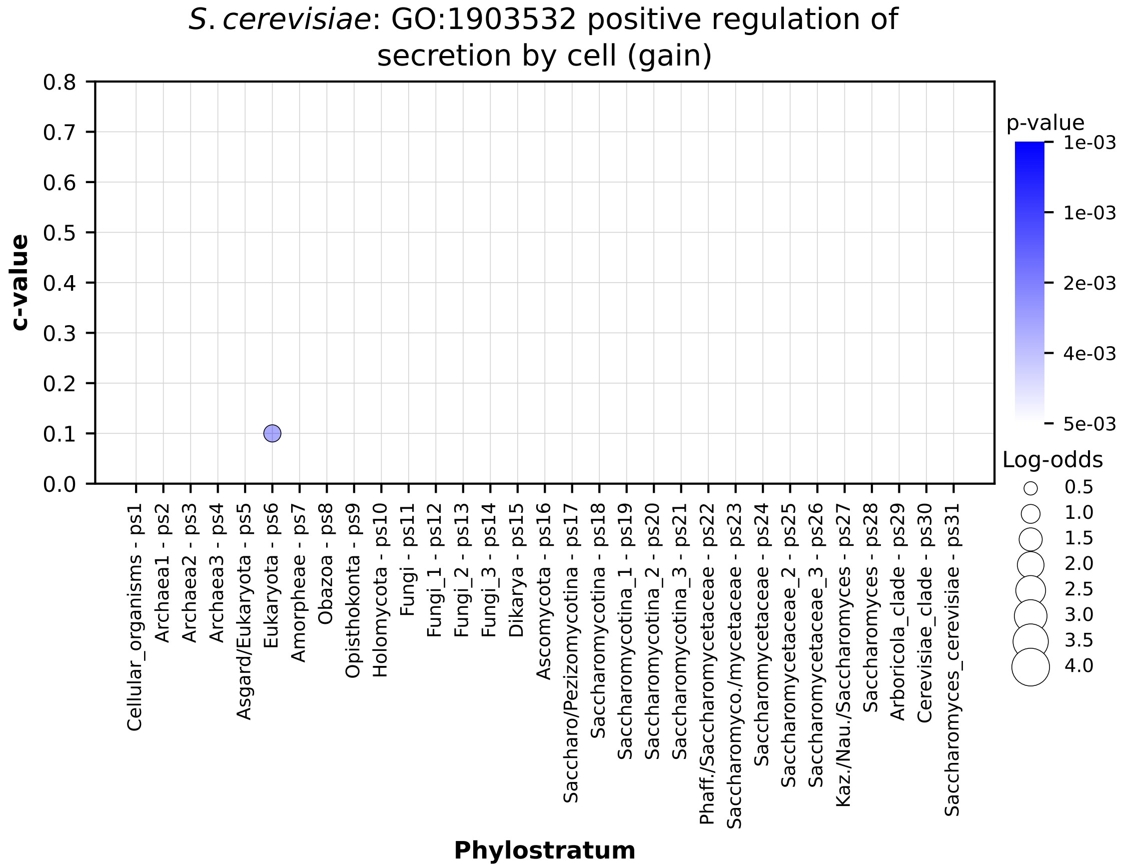

S. cerevisiae: GO:1903532 positive regulation of secretion by cell (gain)
c-value
0.8
0.7
0.6
0.5
0.4
0.3
0.2
0.1
0.0
p-value
1e-03
1e-03
2e-03
4e-03
5e-03
Log-odds
0.5
1.0
1.5
2.0
2.5
3.0
3.5
4.0
Cellular_organisms - ps1
Archaea1 - ps2
Archaea2 - ps3
Archaea3 - ps4
Asgard/Eukaryota - ps5
Eukaryota - ps6
Amorpheae - ps7
Obazoa - ps8
Opisthokonta - ps9
Holomycota - ps10
Fungi - ps11
Fungi_1 - ps12
Fungi_2 - ps13
Fungi_3 - ps14
Dikarya - ps15
Ascomycota - ps16
Saccharo/Pezizomycotina - ps17
Saccharomycotina - ps18
Saccharomycotina_1 - ps19
Saccharomycotina_2 - ps20
Saccharomycotina_3 - ps21
Phaff./Saccharomycetaceae - ps22
Saccharomyco./mycetaceae - ps23
Saccharomycetaceae - ps24
Saccharomycetaceae_2 - ps25
Saccharomycetaceae_3 - ps26
Kaz./Nau./Saccharomyces - ps27
Saccharomyces - ps28
Arboricola_clade - ps29
Cerevisiae_clade - ps30
Saccharomyces_cerevisiae - ps31
Phylostratum

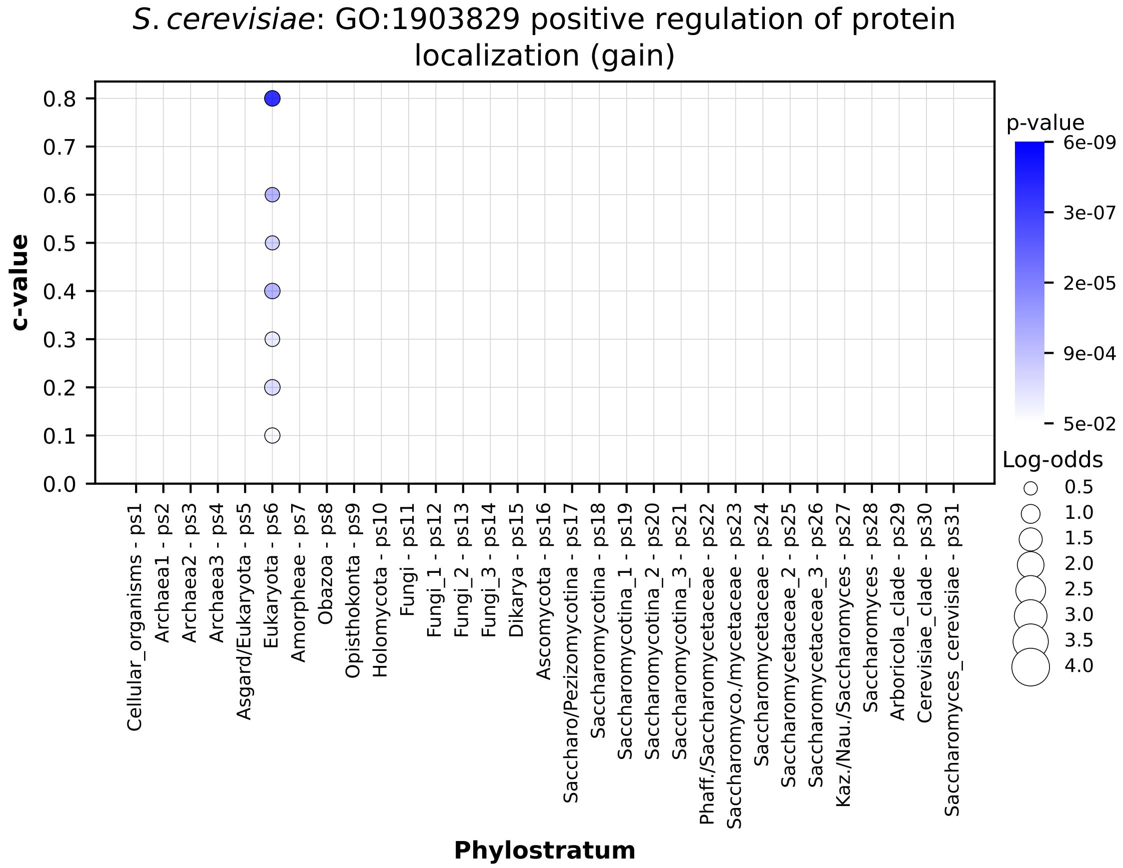

S. cerevisiae: GO:1903829 positive regulation of protein localization (gain)
c-value
0.0
0.1
0.2
0.3
0.4
0.5
0.6
0.7
0.8
Phylostratum
Cellular_organisms - ps1
Archaea1 - ps2
Archaea2 - ps3
Archaea3 - ps4
Asgard/Eukaryota - ps5
Eukaryota - ps6
Amorpheae - ps7
Obazoa - ps8
Opisthokonta - ps9
Holomycota - ps10
Fungi - ps11
Fungi_1 - ps12
Fungi_2 - ps13
Fungi_3 - ps14
Dikarya - ps15
Ascomycota - ps16
Saccharo/Pezizomycotina - ps17
Saccharomycotina - ps18
Saccharomycotina_1 - ps19
Saccharomycotina_2 - ps20
Saccharomycotina_3 - ps21
Phaff./Saccharomycetaceae - ps22
Saccharomyco./mycetaceae - ps23
Saccharomycetaceae - ps24
Saccharomycetaceae_2 - ps25
Saccharomycetaceae_3 - ps26
Kaz./Nau./Saccharomyces - ps27
Saccharomyces - ps28
Arboricola_clade - ps29
Cerevisiae_clade - ps30
Saccharomyces_cerevisiae - ps31
p-value
6e-09
3e-07
2e-05
9e-04
5e-02
Log-odds
0.5
1.0
1.5
2.0
2.5
3.0
3.5
4.0

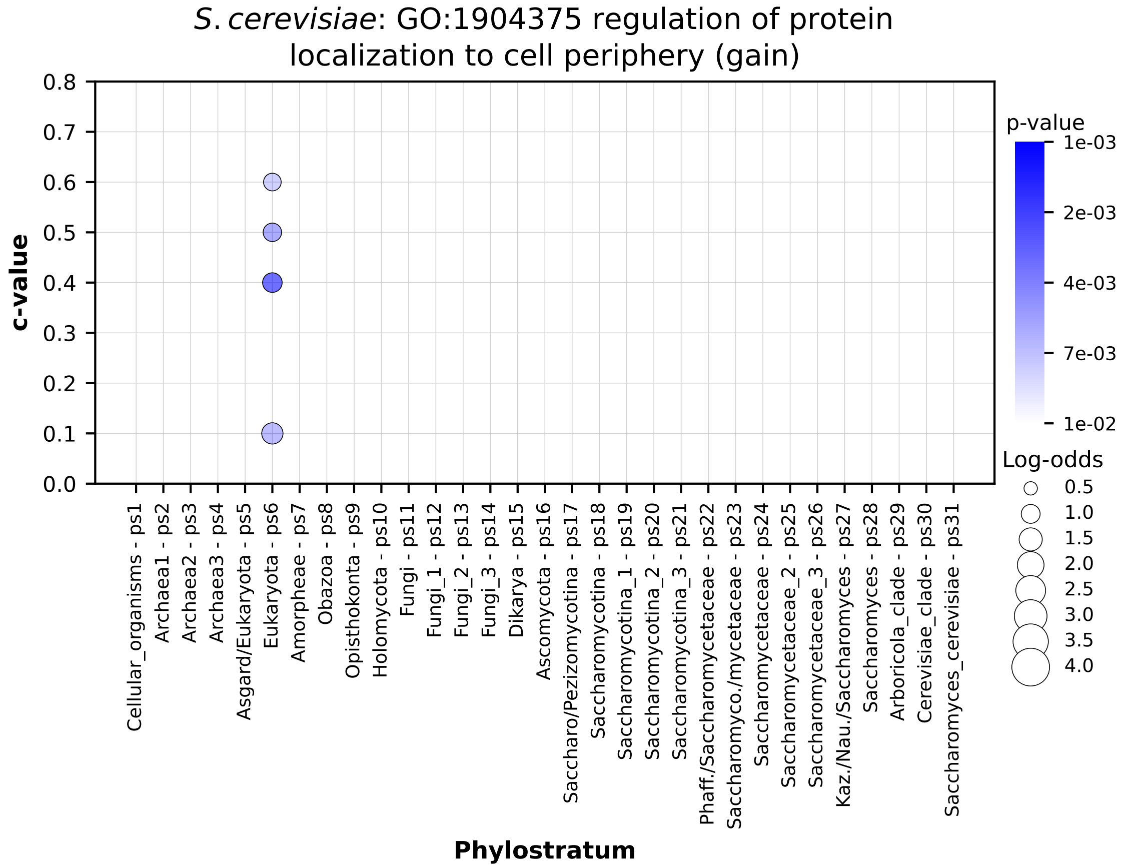

S. cerevisiae: GO:1904375 regulation of protein localization to cell periphery (gain)
c-value
0.8
0.7
0.6
0.5
0.4
0.3
0.2
0.1
0.0
Phylostratum
Cellular_organisms - ps1
Archaea1 - ps2
Archaea2 - ps3
Archaea3 - ps4
Asgard/Eukaryota - ps5
Eukaryota - ps6
Amorpheae - ps7
Obazoa - ps8
Opisthokonta - ps9
Holomycota - ps10
Fungi - ps11
Fungi_1 - ps12
Fungi_2 - ps13
Fungi_3 - ps14
Dikarya - ps15
Ascomycota - ps16
Saccharo/Pezizomycotina - ps17
Saccharomycotina - ps18
Saccharomycotina_1 - ps19
Saccharomycotina_2 - ps20
Saccharomycotina_3 - ps21
Phaff./Saccharomycetaceae - ps22
Saccharomyco./mycetaceae - ps23
Saccharomycetaceae - ps24
Saccharomycetaceae_2 - ps25
Saccharomycetaceae_3 - ps26
Kaz./Nau./Saccharomyces - ps27
Saccharomyces - ps28
Arboricola_clade - ps29
Cerevisiae_clade - ps30
Saccharomyces_cerevisiae - ps31
p-value
1e-03
2e-03
4e-03
7e-03
1e-02
Log-odds
0.5
1.0
1.5
2.0
2.5
3.0
3.5
4.0

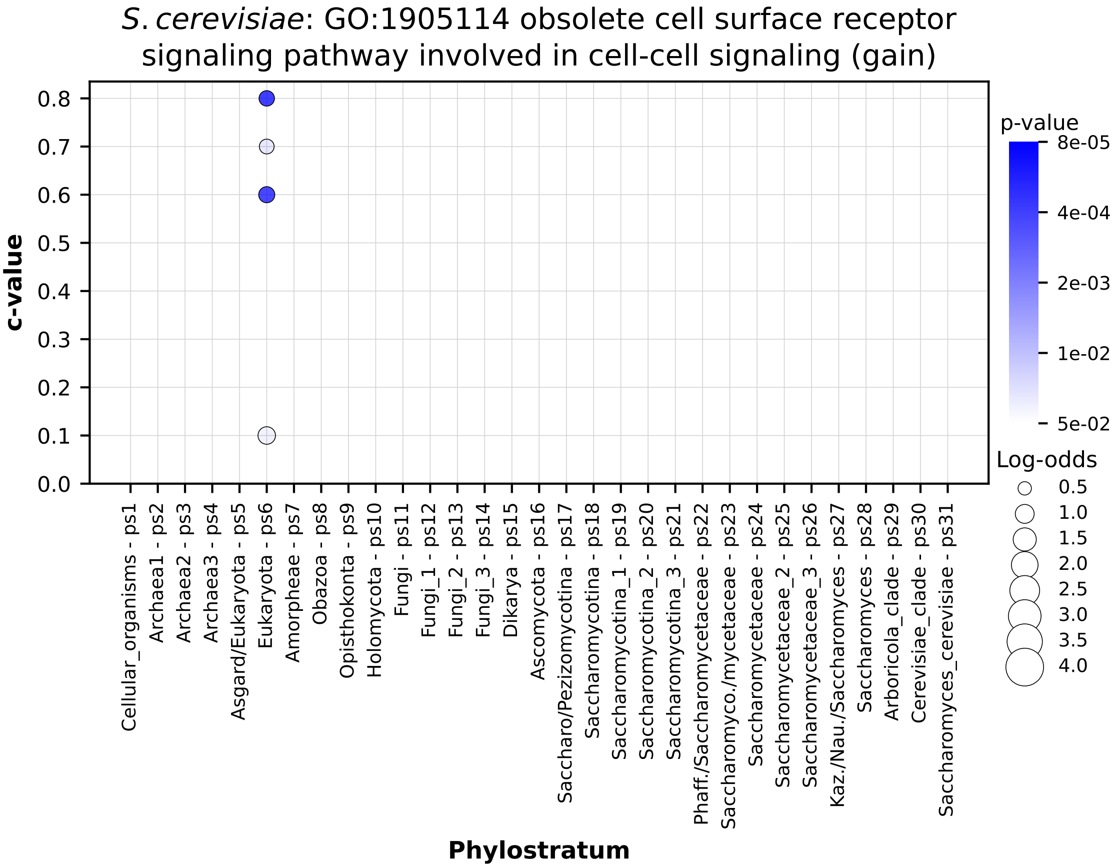

S. cerevisiae: GO:1905114 obsolete cell surface receptor signaling pathway involved in cell-cell signaling (gain)
c-value
0.8
0.7
0.6
0.5
0.4
0.3
0.2
0.1
0.0
p-value
8e-05
4e-04
2e-03
1e-02
5e-02
Log-odds
0.5
1.0
1.5
2.0
2.5
3.0
3.5
4.0
Cellular_organisms - ps1
Archaea1 - ps2
Archaea2 - ps3
Archaea3 - ps4
Asgard/Eukaryota - ps5
Eukaryota - ps6
Amorpheae - ps7
Obazoa - ps8
Opisthokonta - ps9
Holomycota - ps10
Fungi - ps11
Fungi_1 - ps12
Fungi_2 - ps13
Fungi_3 - ps14
Dikarya - ps15
Ascomycota - ps16
Saccharo/Pezizomycotina - ps17
Saccharomycotina - ps18
Saccharomycotina_1 - ps19
Saccharomycotina_2 - ps20
Saccharomycotina_3 - ps21
Phaff./Saccharomycetaceae - ps22
Saccharomyco./mycetaceae - ps23
Saccharomycetaceae - ps24
Saccharomycetaceae_2 - ps25
Saccharomycetaceae_3 - ps26
Kaz./Nau./Saccharomyces - ps27
Saccharomyces - ps28
Arboricola_clade - ps29
Cerevisiae_clade - ps30
Saccharomyces_cerevisiae - ps31
Phylostratum

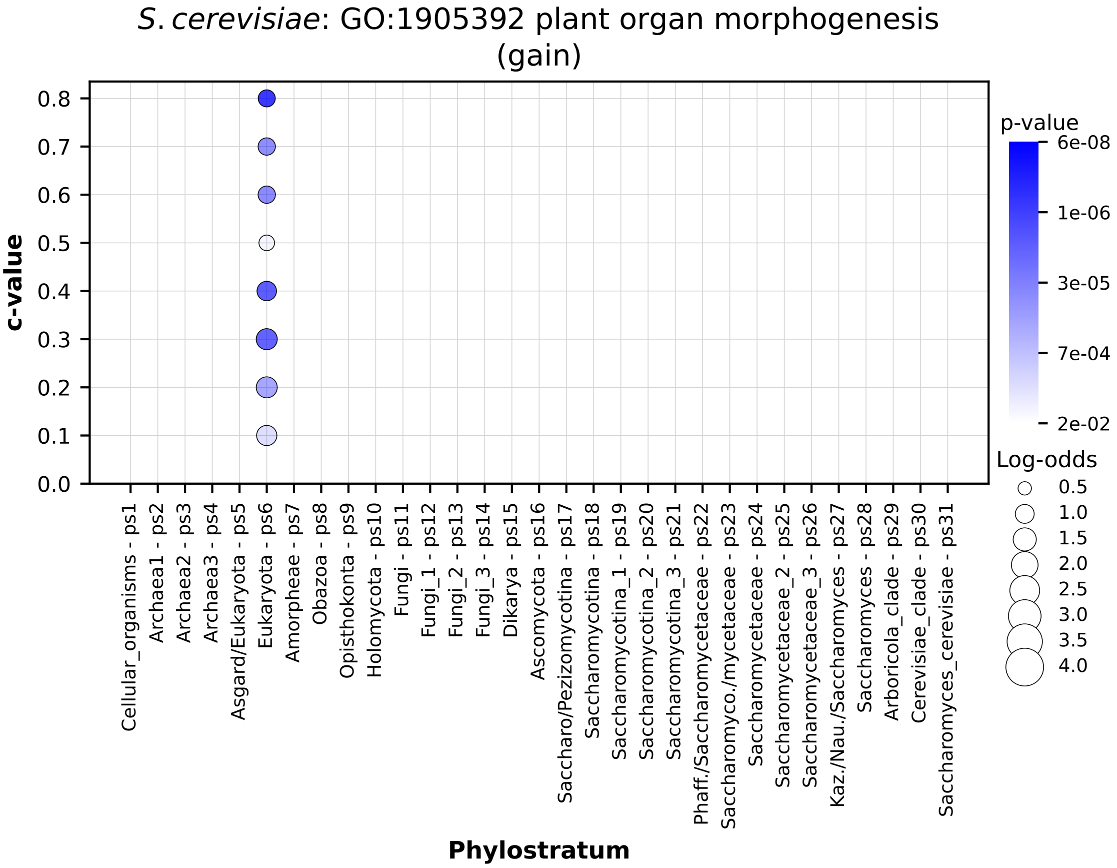

S. cerevisiae: GO:1905392 plant organ morphogenesis
(gain)
c-value
0.0
0.1
0.2
0.3
0.4
0.5
0.6
0.7
0.8
p-value
6e-08
1e-06
3e-05
7e-04
2e-02
Log-odds
0.5
1.0
1.5
2.0
2.5
3.0
3.5
4.0
Cellular_organisms - ps1
Archaea1 - ps2
Archaea2 - ps3
Archaea3 - ps4
Asgard/Eukaryota - ps5
Eukaryota - ps6
Amorpheae - ps7
Obazoa - ps8
Opisthokonta - ps9
Holomycota - ps10
Fungi - ps11
Fungi_1 - ps12
Fungi_2 - ps13
Fungi_3 - ps14
Dikarya - ps15
Ascomycota - ps16
Saccharo/Pezizomycotina - ps17
Saccharomycotina - ps18
Saccharomycotina_1 - ps19
Saccharomycotina_2 - ps20
Saccharomycotina_3 - ps21
Phaff./Saccharomycetaceae - ps22
Saccharomyco./mycetaceae - ps23
Saccharomycetaceae - ps24
Saccharomycetaceae_2 - ps25
Saccharomycetaceae_3 - ps26
Kaz./Nau./Saccharomyces - ps27
Saccharomyces - ps28
Arboricola_clade - ps29
Cerevisiae_clade - ps30
Saccharomyces_cerevisiae - ps31
Phylostratum

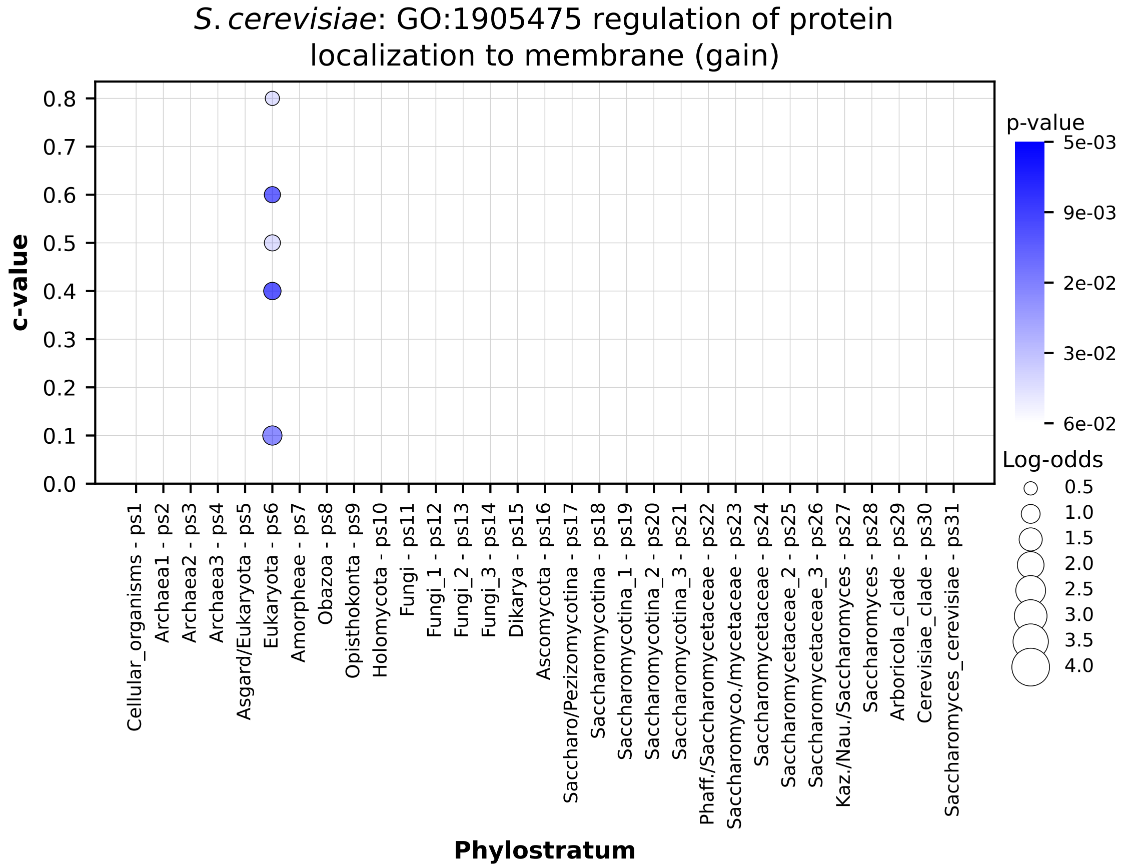

S. cerevisiae: GO:1905475 regulation of protein localization to membrane (gain)
c-value
0.0
0.1
0.2
0.3
0.4
0.5
0.6
0.7
0.8
Phylostratum
Cellular_organisms - ps1
Archaea1 - ps2
Archaea2 - ps3
Archaea3 - ps4
Asgard/Eukaryota - ps5
Eukaryota - ps6
Amorpheae - ps7
Obazoa - ps8
Opisthokonta - ps9
Holomycota - ps10
Fungi - ps11
Fungi_1 - ps12
Fungi_2 - ps13
Fungi_3 - ps14
Dikarya - ps15
Ascomycota - ps16
Saccharo/Pezizomycotina - ps17
Saccharomycotina - ps18
Saccharomycotina_1 - ps19
Saccharomycotina_2 - ps20
Saccharomycotina_3 - ps21
Phaff./Saccharomycetaceae - ps22
Saccharomyco./mycetaceae - ps23
Saccharomycetaceae - ps24
Saccharomycetaceae_2 - ps25
Saccharomycetaceae_3 - ps26
Kaz./Nau./Saccharomyces - ps27
Saccharomyces - ps28
Arboricola_clade - ps29
Cerevisiae_clade - ps30
Saccharomyces_cerevisiae - ps31
p-value
5e-03
9e-03
2e-02
3e-02
6e-02
Log-odds
0.5
1.0
1.5
2.0
2.5
3.0
3.5
4.0

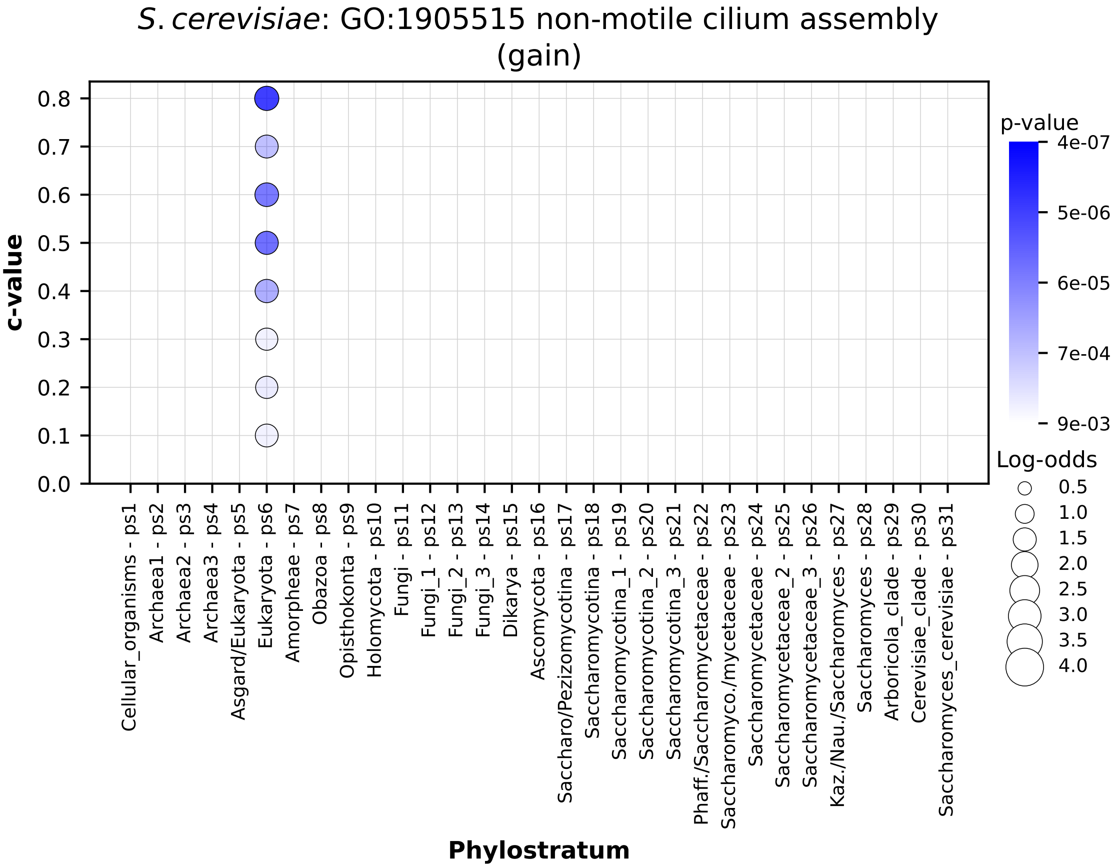

S. cerevisiae: GO:1905515 non-motile cilium assembly
(gain)
c-value
0.0
0.1
0.2
0.3
0.4
0.5
0.6
0.7
0.8
p-value
4e-07
5e-06
6e-05
7e-04
9e-03
Log-odds
0.5
1.0
1.5
2.0
2.5
3.0
3.5
4.0
Cellular_organisms - ps1
Archaea1 - ps2
Archaea2 - ps3
Archaea3 - ps4
Asgard/Eukaryota - ps5
Eukaryota - ps6
Amorpheae - ps7
Obazoa - ps8
Opisthokonta - ps9
Holomycota - ps10
Fungi - ps11
Fungi_1 - ps12
Fungi_2 - ps13
Fungi_3 - ps14
Dikarya - ps15
Ascomycota - ps16
Saccharo/Pezizomycotina - ps17
Saccharomycotina - ps18
Saccharomycotina_1 - ps19
Saccharomycotina_2 - ps20
Saccharomycotina_3 - ps21
Phaff./Saccharomycetaceae - ps22
Saccharomyco./mycetaceae - ps23
Saccharomycetaceae - ps24
Saccharomycetaceae_2 - ps25
Saccharomycetaceae_3 - ps26
Kaz./Nau./Saccharomyces - ps27
Saccharomyces - ps28
Arboricola_clade - ps29
Cerevisiae_clade - ps30
Saccharomyces_cerevisiae - ps31
Phylostratum

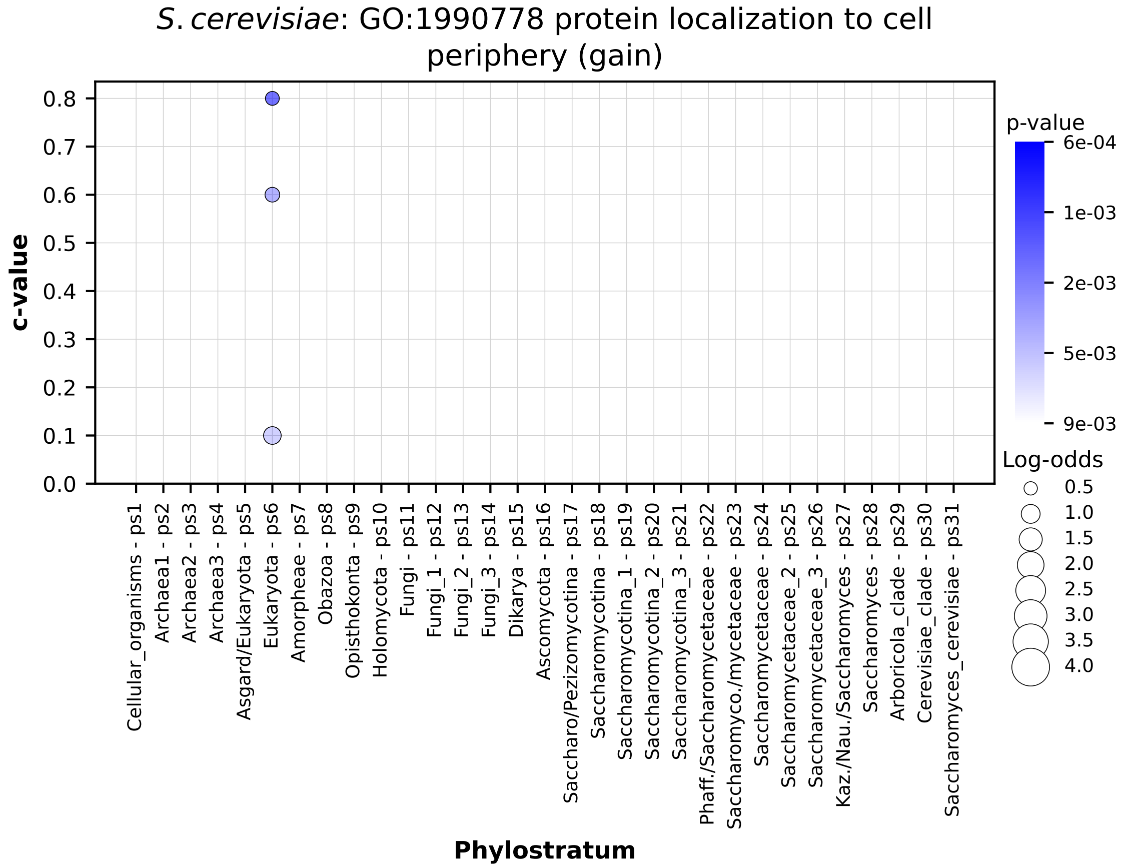

S. cerevisiae: GO:1990778 protein localization to cell periphery (gain)
c-value
0.0
0.1
0.2
0.3
0.4
0.5
0.6
0.7
0.8
p-value
6e-04
1e-03
2e-03
5e-03
9e-03
Log-odds
0.5
1.0
1.5
2.0
2.5
3.0
3.5
4.0
Cellular_organisms - ps1
Archaea1 - ps2
Archaea2 - ps3
Archaea3 - ps4
Asgard/Eukaryota - ps5
Eukaryota - ps6
Amorpheae - ps7
Obazoa - ps8
Opisthokonta - ps9
Holomycota - ps10
Fungi - ps11
Fungi_1 - ps12
Fungi_2 - ps13
Fungi_3 - ps14
Dikarya - ps15
Ascomycota - ps16
Saccharo/Pezizomycotina - ps17
Saccharomycotina - ps18
Saccharomycotina_1 - ps19
Saccharomycotina_2 - ps20
Saccharomycotina_3 - ps21
Phaff./Saccharomycetaceae - ps22
Saccharomyco./mycetaceae - ps23
Saccharomycetaceae - ps24
Saccharomycetaceae_2 - ps25
Saccharomycetaceae_3 - ps26
Kaz./Nau./Saccharomyces - ps27
Saccharomyces - ps28
Arboricola_clade - ps29
Cerevisiae_clade - ps30
Saccharomyces_cerevisiae - ps31
Phylostratum

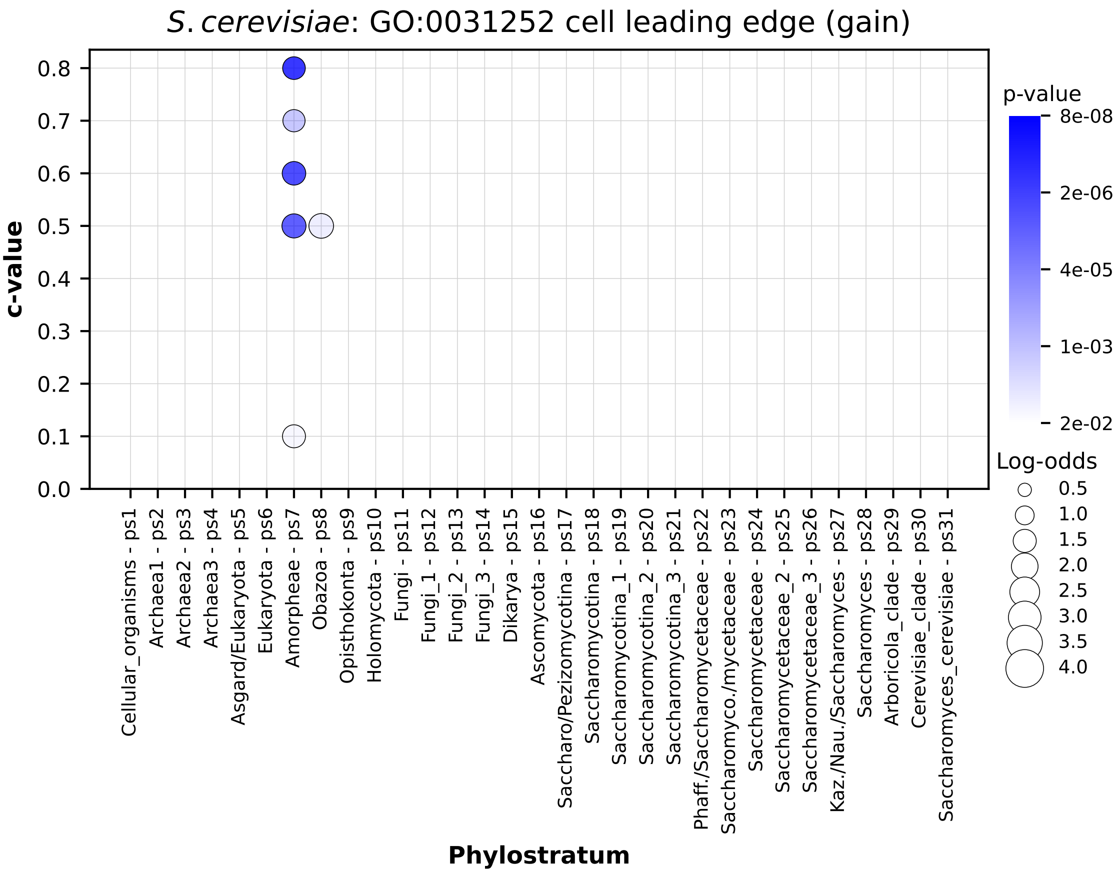

S. cerevisiae: GO:0031252 cell leading edge (gain)
c-value
0.8
0.7
0.6
0.5
0.4
0.3
0.2
0.1
0.0
p-value
8e-08
2e-06
4e-05
1e-03
2e-02
Log-odds
0.5
1.0
1.5
2.0
2.5
3.0
3.5
4.0
Cellular_organisms - ps1
Archaea1 - ps2
Archaea2 - ps3
Archaea3 - ps4
Asgard/Eukaryota - ps5
Eukaryota - ps6
Amorpheae - ps7
Obazoa - ps8
Opisthokonta - ps9
Holomycota - ps10
Fungi - ps11
Fungi_1 - ps12
Fungi_2 - ps13
Fungi_3 - ps14
Dikarya - ps15
Ascomycota - ps16
Saccharo/Pezizomycotina - ps17
Saccharomycotina - ps18
Saccharomycotina_1 - ps19
Saccharomycotina_2 - ps20
Saccharomycotina_3 - ps21
Phaff./Saccharomycetaceae - ps22
Saccharomyco./mycetaceae - ps23
Saccharomycetaceae - ps24
Saccharomycetaceae_2 - ps25
Saccharomycetaceae_3 - ps26
Kaz./Nau./Saccharomyces - ps27
Saccharomyces - ps28
Arboricola_clade - ps29
Cerevisiae_clade - ps30
Saccharomyces_cerevisiae - ps31
Phylostratum

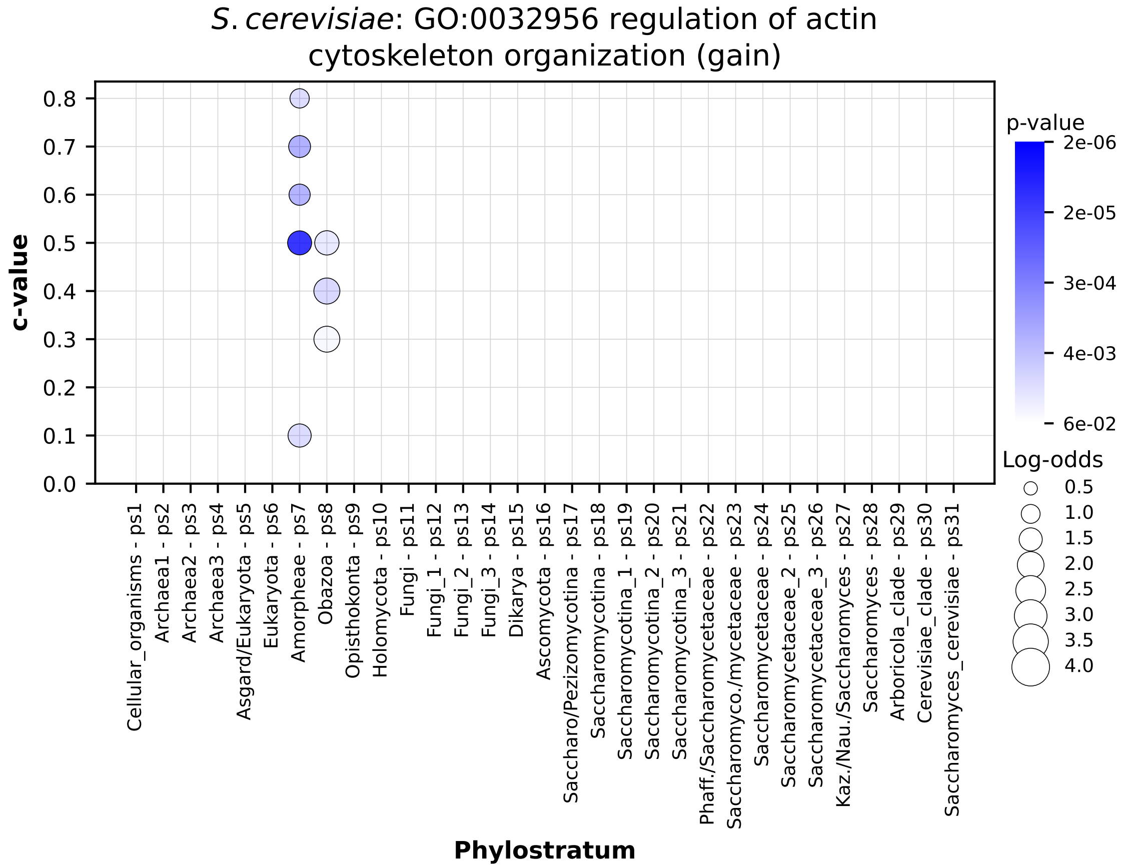

S. cerevisiae: GO:0032956 regulation of actin cytoskeleton organization (gain)
c-value
0.0
0.1
0.2
0.3
0.4
0.5
0.6
0.7
0.8
Phylostratum
Cellular_organisms - ps1
Archaea1 - ps2
Archaea2 - ps3
Archaea3 - ps4
Asgard/Eukaryota - ps5
Eukaryota - ps6
Amorpheae - ps7
Obazoa - ps8
Opisthokonta - ps9
Holomycota - ps10
Fungi - ps11
Fungi_1 - ps12
Fungi_2 - ps13
Fungi_3 - ps14
Dikarya - ps15
Ascomycota - ps16
Saccharo/Pezizomycotina - ps17
Saccharomycotina - ps18
Saccharomycotina_1 - ps19
Saccharomycotina_2 - ps20
Saccharomycotina_3 - ps21
Phaff./Saccharomycetaceae - ps22
Saccharomyco./mycetaceae - ps23
Saccharomycetaceae - ps24
Saccharomycetaceae_2 - ps25
Saccharomycetaceae_3 - ps26
Kaz./Nau./Saccharomyces - ps27
Saccharomyces - ps28
Arboricola_clade - ps29
Cerevisiae_clade - ps30
Saccharomyces_cerevisiae - ps31
p-value
2e-06
2e-05
3e-04
4e-03
6e-02
Log-odds
0.5
1.0
1.5
2.0
2.5
3.0
3.5
4.0

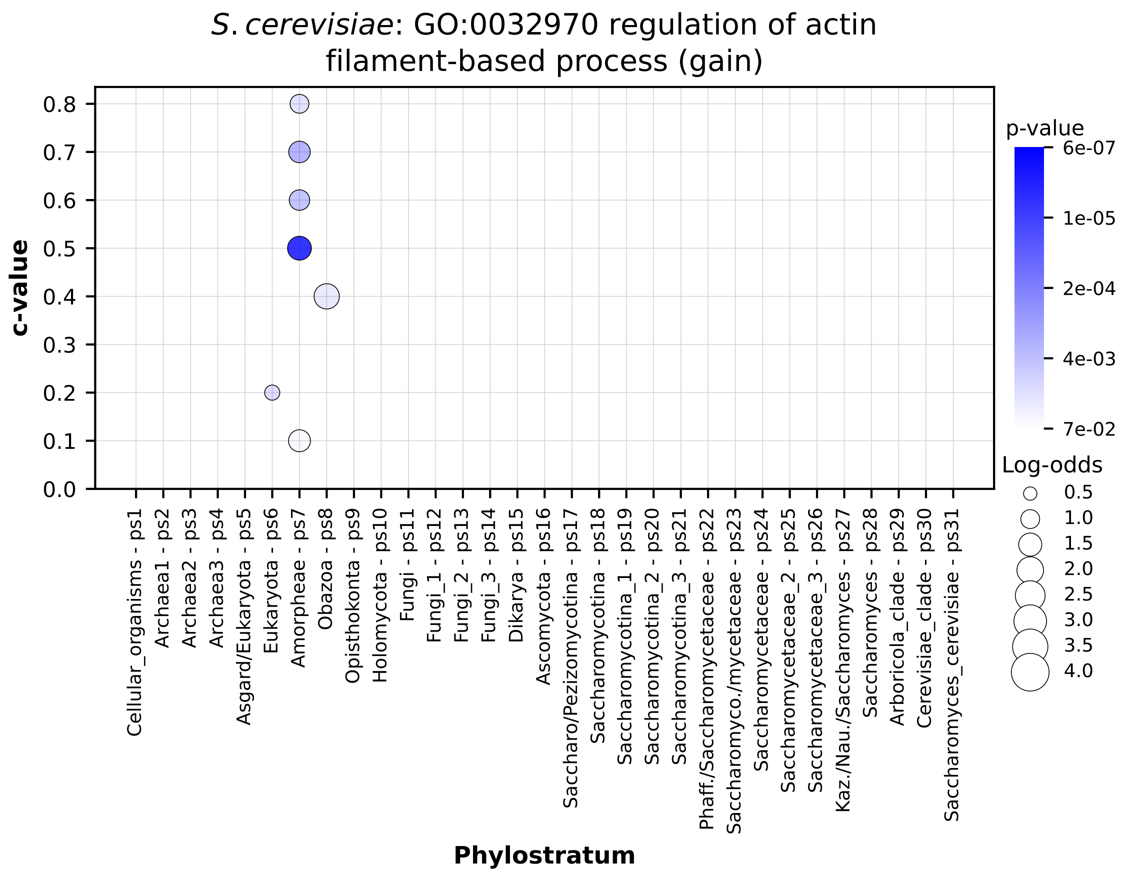

S. cerevisiae: GO:0032970 regulation of actin filament-based process (gain)
c-value
0.0
0.1
0.2
0.3
0.4
0.5
0.6
0.7
0.8
Phylostratum
Cellular_organisms - ps1
Archaea1 - ps2
Archaea2 - ps3
Archaea3 - ps4
Asgard/Eukaryota - ps5
Eukaryota - ps6
Amorpheae - ps7
Obazoa - ps8
Opisthokonta - ps9
Holomycota - ps10
Fungi - ps11
Fungi_1 - ps12
Fungi_2 - ps13
Fungi_3 - ps14
Dikarya - ps15
Ascomycota - ps16
Saccharo/Pezizomycotina - ps17
Saccharomycotina - ps18
Saccharomycotina_1 - ps19
Saccharomycotina_2 - ps20
Saccharomycotina_3 - ps21
Phaff./Saccharomycetaceae - ps22
Saccharomyco./mycetaceae - ps23
Saccharomycetaceae - ps24
Saccharomycetaceae_2 - ps25
Saccharomycetaceae_3 - ps26
Kaz./Nau./Saccharomyces - ps27
Saccharomyces - ps28
Arboricola_clade - ps29
Cerevisiae_clade - ps30
Saccharomyces_cerevisiae - ps31
p-value
6e-07
1e-05
2e-04
4e-03
7e-02
Log-odds
0.5
1.0
1.5
2.0
2.5
3.0
3.5
4.0

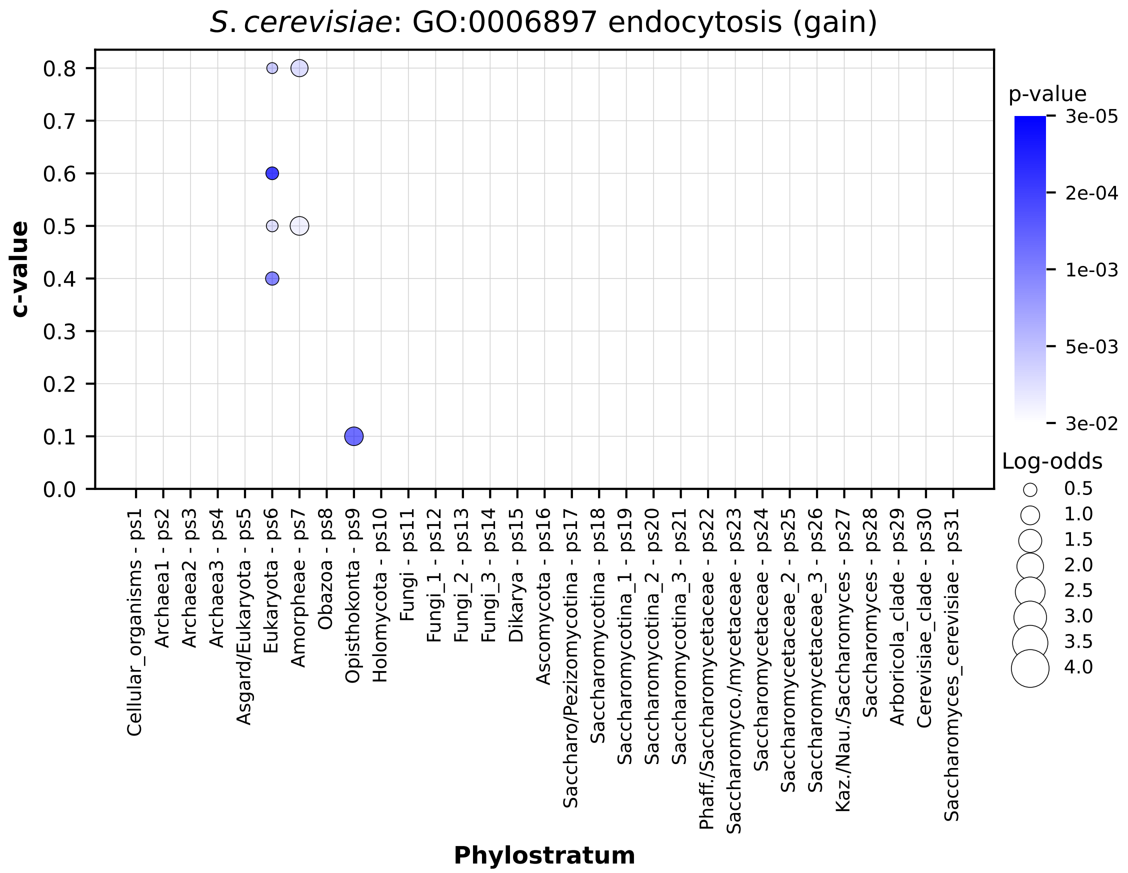

S. cerevisiae: GO:0006897 endocytosis (gain)
c-value
0.0
0.1
0.2
0.3
0.4
0.5
0.6
0.7
0.8
Phylostratum
Cellular_organisms - ps1
Archaea1 - ps2
Archaea2 - ps3
Archaea3 - ps4
Asgard/Eukaryota - ps5
Eukaryota - ps6
Amorpheae - ps7
Obazoa - ps8
Opisthokonta - ps9
Holomycota - ps10
Fungi - ps11
Fungi_1 - ps12
Fungi_2 - ps13
Fungi_3 - ps14
Dikarya - ps15
Ascomycota - ps16
Saccharo/Pezizomycotina - ps17
Saccharomycotina - ps18
Saccharomycotina_1 - ps19
Saccharomycotina_2 - ps20
Saccharomycotina_3 - ps21
Phaff./Saccharomycetaceae - ps22
Saccharomyco./mycetaceae - ps23
Saccharomycetaceae - ps24
Saccharomycetaceae_2 - ps25
Saccharomycetaceae_3 - ps26
Kaz./Nau./Saccharomyces - ps27
Saccharomyces - ps28
Arboricola_clade - ps29
Cerevisiae_clade - ps30
Saccharomyces_cerevisiae - ps31
p-value
3e-05
2e-04
1e-03
5e-03
3e-02
Log-odds
0.5
1.0
1.5
2.0
2.5
3.0
3.5
4.0

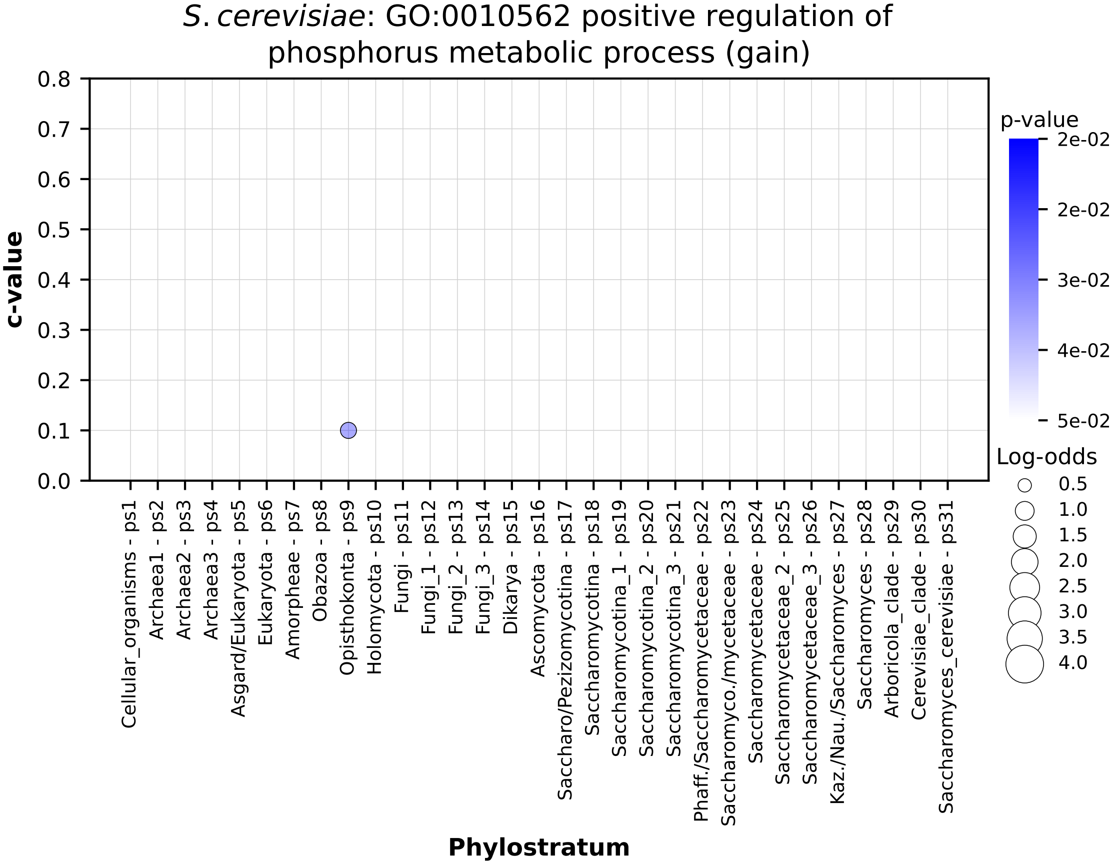

S. cerevisiae: GO:0010562 positive regulation of phosphorus metabolic process (gain)
c-value
0.8
0.7
0.6
0.5
0.4
0.3
0.2
0.1
0.0
p-value
2e-02
2e-02
3e-02
4e-02
5e-02
Log-odds
0.5
1.0
1.5
2.0
2.5
3.0
3.5
4.0
Cellular_organisms - ps1
Archaea1 - ps2
Archaea2 - ps3
Archaea3 - ps4
Asgard/Eukaryota - ps5
Eukaryota - ps6
Amorpheae - ps7
Obazoa - ps8
Opisthokonta - ps9
Holomycota - ps10
Fungi - ps11
Fungi_1 - ps12
Fungi_2 - ps13
Fungi_3 - ps14
Dikarya - ps15
Ascomycota - ps16
Saccharo/Pezizomycotina - ps17
Saccharomycotina - ps18
Saccharomycotina_1 - ps19
Saccharomycotina_2 - ps20
Saccharomycotina_3 - ps21
Phaff./Saccharomycetaceae - ps22
Saccharomyco./mycetaceae - ps23
Saccharomycetaceae - ps24
Saccharomycetaceae_2 - ps25
Saccharomycetaceae_3 - ps26
Kaz./Nau./Saccharomyces - ps27
Saccharomyces - ps28
Arboricola_clade - ps29
Cerevisiae_clade - ps30
Saccharomyces_cerevisiae - ps31
Phylostratum

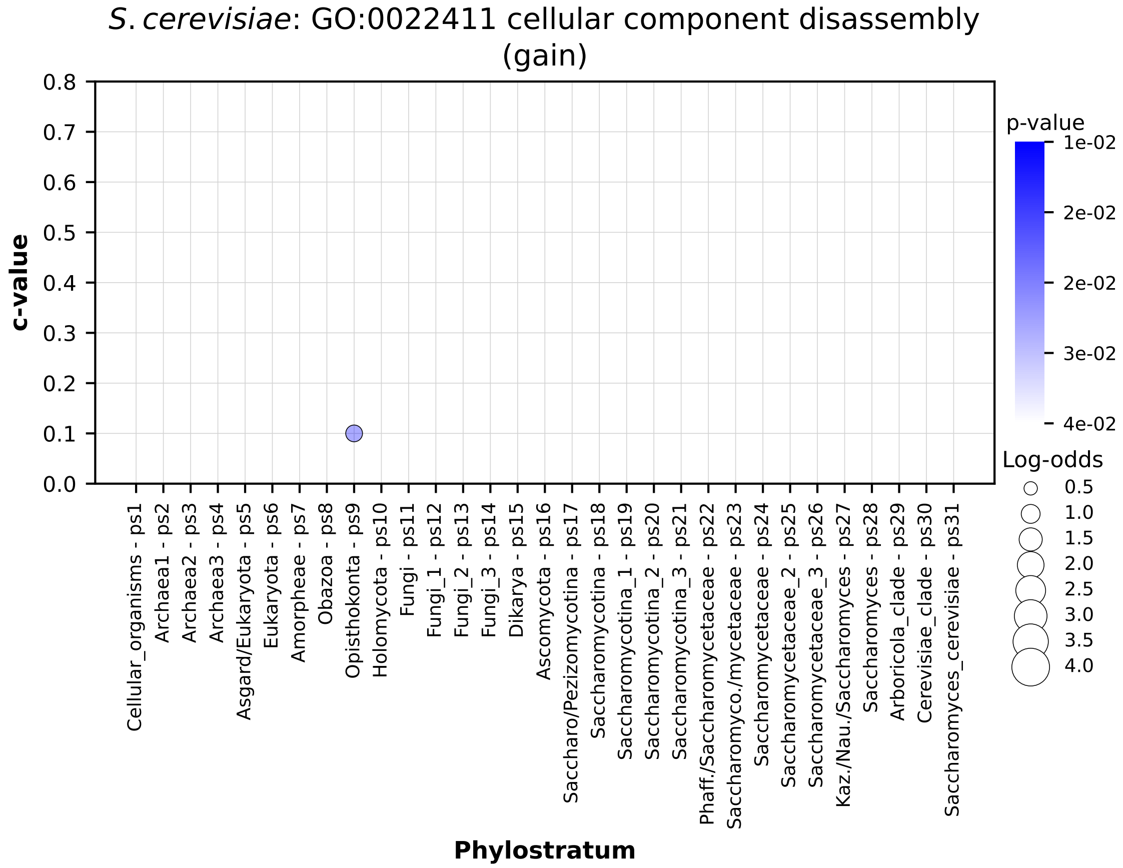

S. cerevisiae: GO:0022411 cellular component disassembly (gain)
c-value
0.8
0.7
0.6
0.5
0.4
0.3
0.2
0.1
0.0
p-value
1e-02
2e-02
2e-02
3e-02
4e-02
Log-odds
0.5
1.0
1.5
2.0
2.5
3.0
3.5
4.0
Cellular_organisms - ps1
Archaea1 - ps2
Archaea2 - ps3
Archaea3 - ps4
Asgard/Eukaryota - ps5
Eukaryota - ps6
Amorpheae - ps7
Obazoa - ps8
Opisthokonta - ps9
Holomycota - ps10
Fungi - ps11
Fungi_1 - ps12
Fungi_2 - ps13
Fungi_3 - ps14
Dikarya - ps15
Ascomycota - ps16
Saccharo/Pezizomycotina - ps17
Saccharomycotina - ps18
Saccharomycotina_1 - ps19
Saccharomycotina_2 - ps20
Saccharomycotina_3 - ps21
Phaff./Saccharomycetaceae - ps22
Saccharomyco./mycetaceae - ps23
Saccharomycetaceae - ps24
Saccharomycetaceae_2 - ps25
Saccharomycetaceae_3 - ps26
Kaz./Nau./Saccharomyces - ps27
Saccharomyces - ps28
Arboricola_clade - ps29
Cerevisiae_clade - ps30
Saccharomyces_cerevisiae - ps31
Phylostratum

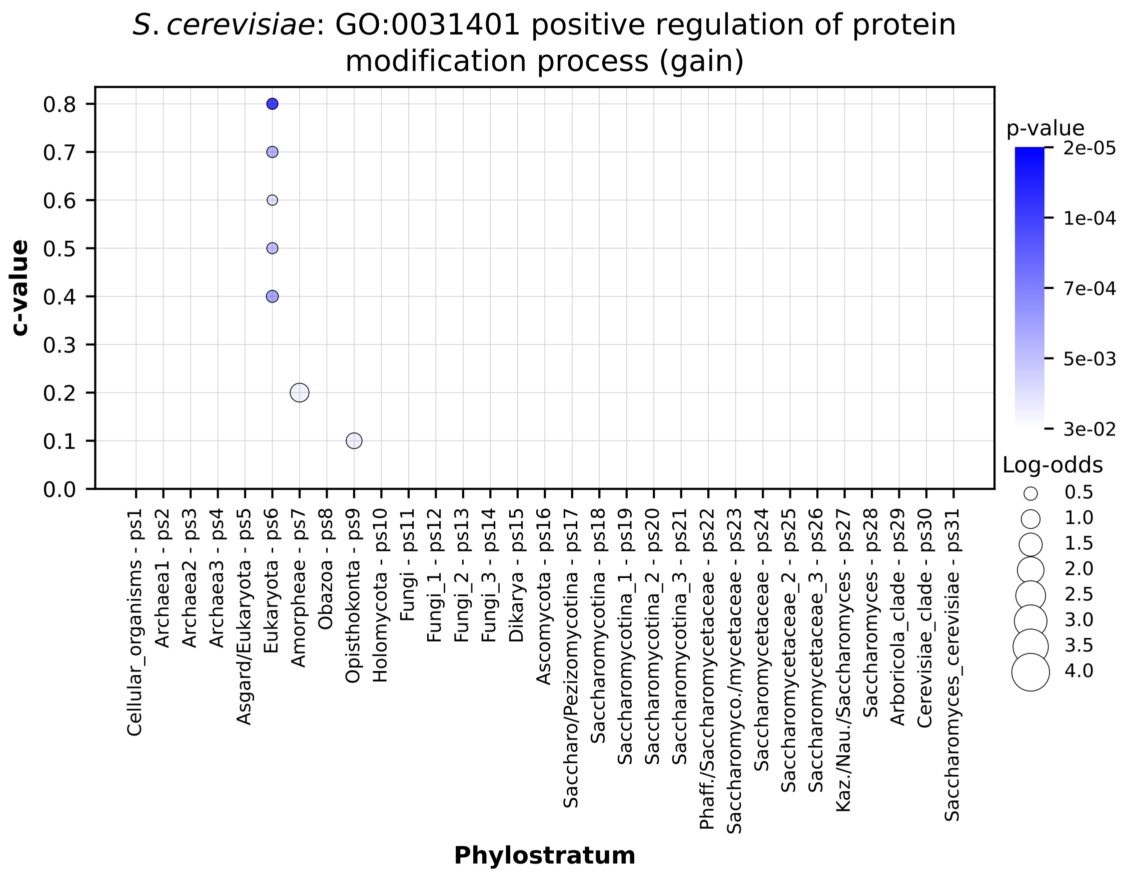

S. cerevisiae: GO:0031401 positive regulation of protein modification process (gain)
c-value
0.0
0.1
0.2
0.3
0.4
0.5
0.6
0.7
0.8
Phylostratum
Cellular_organisms - ps1
Archaea1 - ps2
Archaea2 - ps3
Archaea3 - ps4
Asgard/Eukaryota - ps5
Eukaryota - ps6
Amorpheae - ps7
Obazoa - ps8
Opisthokonta - ps9
Holomycota - ps10
Fungi - ps11
Fungi_1 - ps12
Fungi_2 - ps13
Fungi_3 - ps14
Dikarya - ps15
Ascomycota - ps16
Saccharo/Pezizomycotina - ps17
Saccharomycotina - ps18
Saccharomycotina_1 - ps19
Saccharomycotina_2 - ps20
Saccharomycotina_3 - ps21
Phaff./Saccharomycetaceae - ps22
Saccharomyco./mycetaceae - ps23
Saccharomycetaceae - ps24
Saccharomycetaceae_2 - ps25
Saccharomycetaceae_3 - ps26
Kaz./Nau./Saccharomyces - ps27
Saccharomyces - ps28
Arboricola_clade - ps29
Cerevisiae_clade - ps30
Saccharomyces_cerevisiae - ps31
p-value
2e-05
1e-04
7e-04
5e-03
3e-02
Log-odds
0.5
1.0
1.5
2.0
2.5
3.0
3.5
4.0

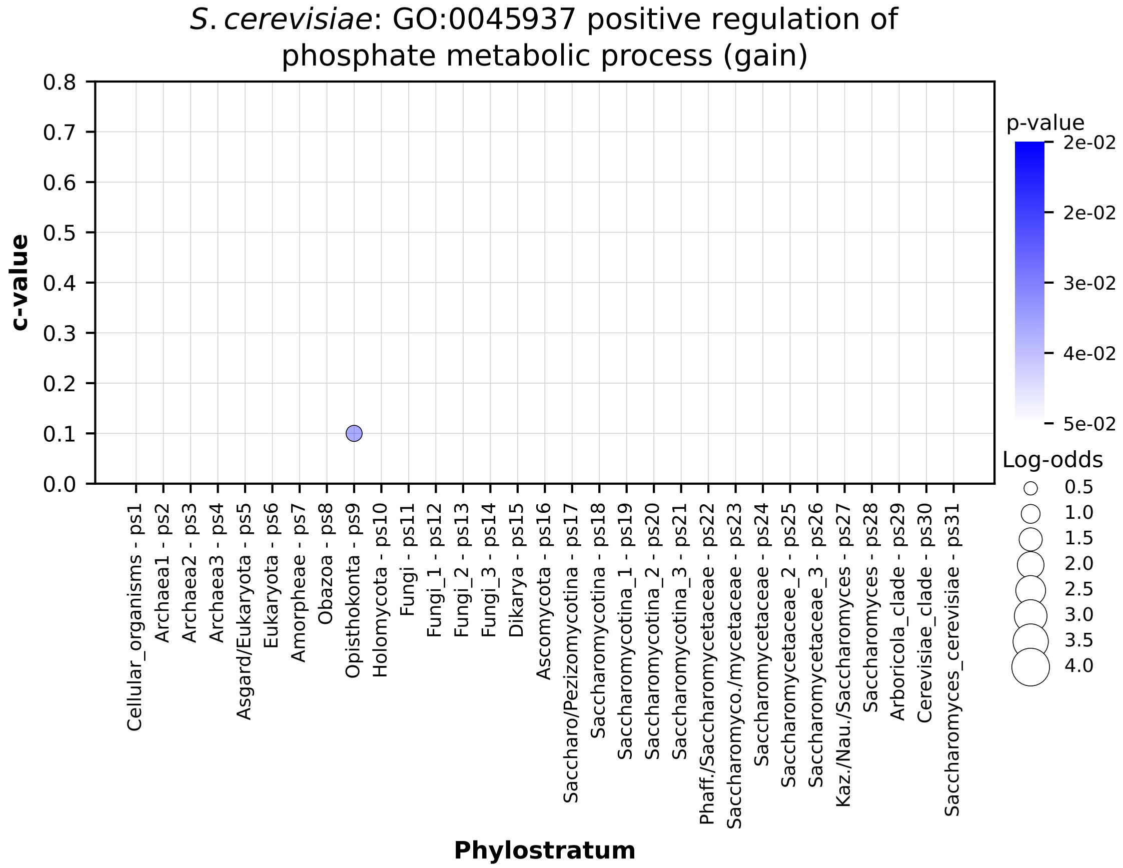

S. cerevisiae: GO:0045937 positive regulation of phosphate metabolic process (gain)
c-value
0.8
0.7
0.6
0.5
0.4
0.3
0.2
0.1
0.0
p-value
2e-02
2e-02
3e-02
4e-02
5e-02
Log-odds
0.5
1.0
1.5
2.0
2.5
3.0
3.5
4.0
Cellular_organisms - ps1
Archaea1 - ps2
Archaea2 - ps3
Archaea3 - ps4
Asgard/Eukaryota - ps5
Eukaryota - ps6
Amorpheae - ps7
Obazoa - ps8
Opisthokonta - ps9
Holomycota - ps10
Fungi - ps11
Fungi_1 - ps12
Fungi_2 - ps13
Fungi_3 - ps14
Dikarya - ps15
Ascomycota - ps16
Saccharo/Pezizomycotina - ps17
Saccharomycotina - ps18
Saccharomycotina_1 - ps19
Saccharomycotina_2 - ps20
Saccharomycotina_3 - ps21
Phaff./Saccharomycetaceae - ps22
Saccharomyco./mycetaceae - ps23
Saccharomycetaceae - ps24
Saccharomycetaceae_2 - ps25
Saccharomycetaceae_3 - ps26
Kaz./Nau./Saccharomyces - ps27
Saccharomyces - ps28
Arboricola_clade - ps29
Cerevisiae_clade - ps30
Saccharomyces_cerevisiae - ps31
Phylostratum

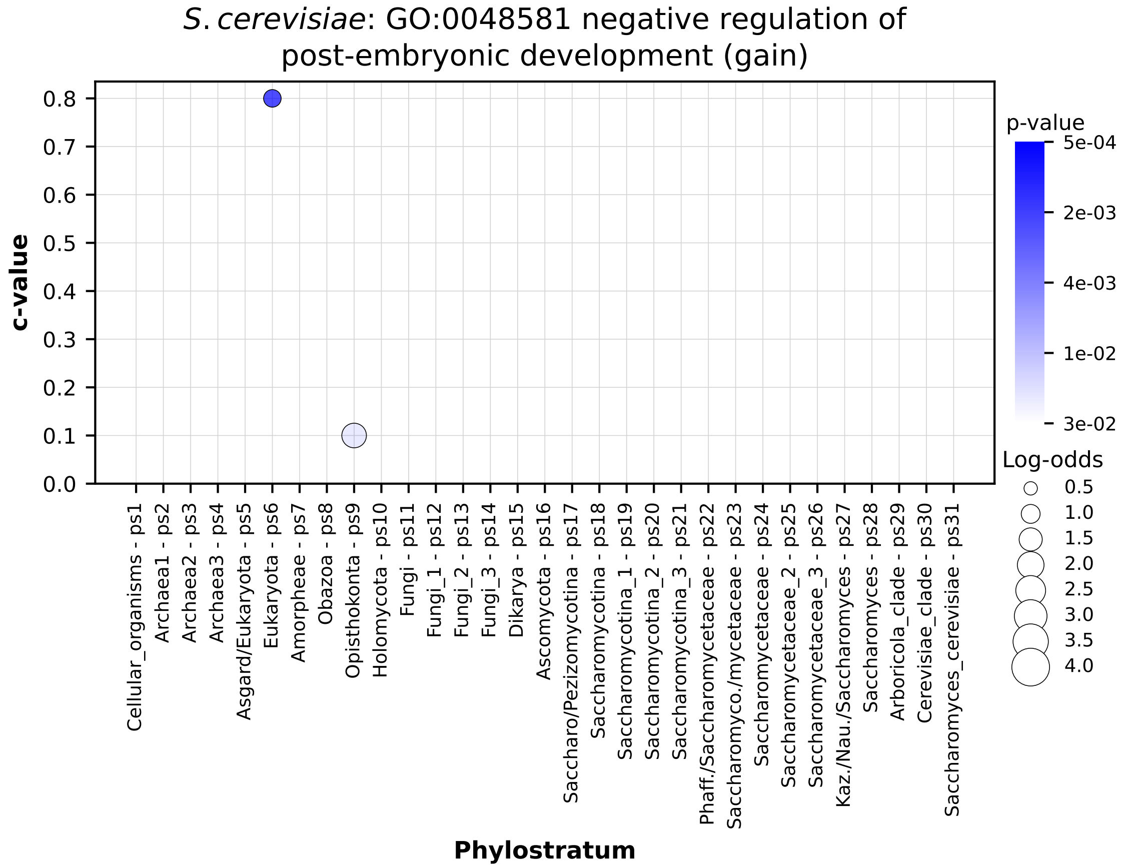

S. cerevisiae: GO:0048581 negative regulation of post-embryonic development (gain)
c-value
0.0
0.1
0.2
0.3
0.4
0.5
0.6
0.7
0.8
Phylostratum
Cellular_organisms - ps1
Archaea1 - ps2
Archaea2 - ps3
Archaea3 - ps4
Asgard/Eukaryota - ps5
Eukaryota - ps6
Amorpheae - ps7
Obazoa - ps8
Opisthokonta - ps9
Holomycota - ps10
Fungi - ps11
Fungi_1 - ps12
Fungi_2 - ps13
Fungi_3 - ps14
Dikarya - ps15
Ascomycota - ps16
Saccharo/Pezizomycotina - ps17
Saccharomycotina - ps18
Saccharomycotina_1 - ps19
Saccharomycotina_2 - ps20
Saccharomycotina_3 - ps21
Phaff./Saccharomycetaceae - ps22
Saccharomyco./mycetaceae - ps23
Saccharomycetaceae - ps24
Saccharomycetaceae_2 - ps25
Saccharomycetaceae_3 - ps26
Kaz./Nau./Saccharomyces - ps27
Saccharomyces - ps28
Arboricola_clade - ps29
Cerevisiae_clade - ps30
Saccharomyces_cerevisiae - ps31
p-value
5e-04
2e-03
4e-03
1e-02
3e-02
Log-odds
0.5
1.0
1.5
2.0
2.5
3.0
3.5
4.0

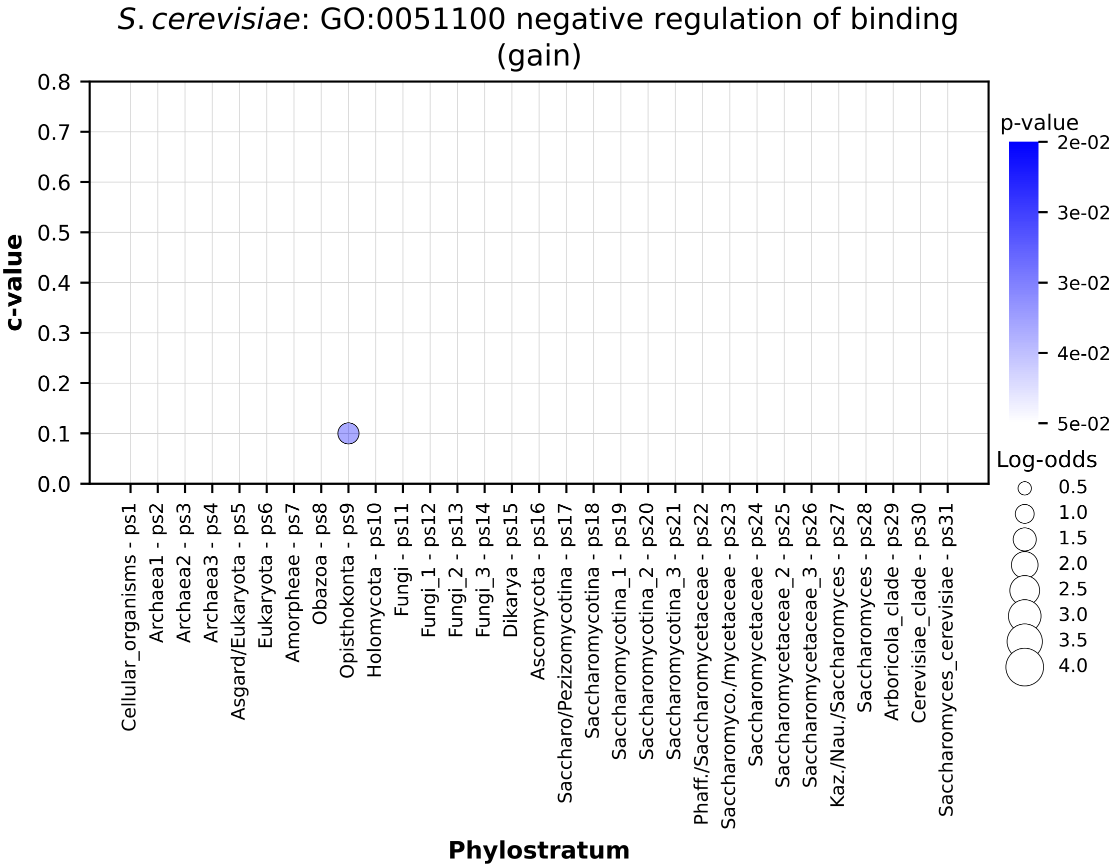

S. cerevisiae: GO:0051100 negative regulation of binding (gain)
c-value
0.8
0.7
0.6
0.5
0.4
0.3
0.2
0.1
0.0
Phylostratum
Cellular_organisms - ps1
Archaea1 - ps2
Archaea2 - ps3
Archaea3 - ps4
Asgard/Eukaryota - ps5
Eukaryota - ps6
Amorpheae - ps7
Obazoa - ps8
Opisthokonta - ps9
Holomycota - ps10
Fungi - ps11
Fungi_1 - ps12
Fungi_2 - ps13
Fungi_3 - ps14
Dikarya - ps15
Ascomycota - ps16
Saccharo/Pezizomycotina - ps17
Saccharomycotina - ps18
Saccharomycotina_1 - ps19
Saccharomycotina_2 - ps20
Saccharomycotina_3 - ps21
Phaff./Saccharomycetaceae - ps22
Saccharomyco./mycetaceae - ps23
Saccharomycetaceae - ps24
Saccharomycetaceae_2 - ps25
Saccharomycetaceae_3 - ps26
Kaz./Nau./Saccharomyces - ps27
Saccharomyces - ps28
Arboricola_clade - ps29
Cerevisiae_clade - ps30
Saccharomyces_cerevisiae - ps31
p-value
2e-02
3e-02
3e-02
4e-02
5e-02
Log-odds
0.5
1.0
1.5
2.0
2.5
3.0
3.5
4.0

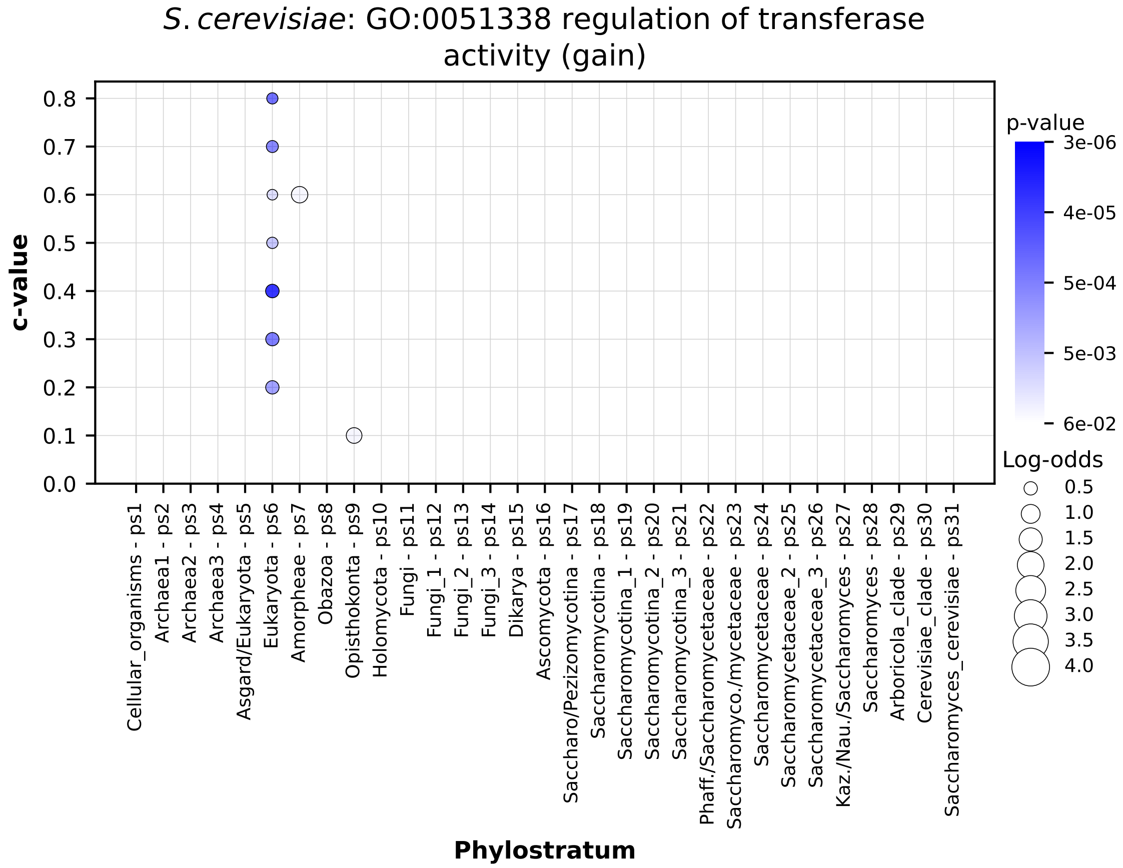

S. cerevisiae: GO:0051338 regulation of transferase activity (gain)
c-value
0.0
0.1
0.2
0.3
0.4
0.5
0.6
0.7
0.8
p-value
3e-06
4e-05
5e-04
5e-03
6e-02
Log-odds
0.5
1.0
1.5
2.0
2.5
3.0
3.5
4.0
Cellular_organisms - ps1
Archaea1 - ps2
Archaea2 - ps3
Archaea3 - ps4
Asgard/Eukaryota - ps5
Eukaryota - ps6
Amorpheae - ps7
Obazoa - ps8
Opisthokonta - ps9
Holomycota - ps10
Fungi - ps11
Fungi_1 - ps12
Fungi_2 - ps13
Fungi_3 - ps14
Dikarya - ps15
Ascomycota - ps16
Saccharo/Pezizomycotina - ps17
Saccharomycotina - ps18
Saccharomycotina_1 - ps19
Saccharomycotina_2 - ps20
Saccharomycotina_3 - ps21
Phaff./Saccharomycetaceae - ps22
Saccharomyco./mycetaceae - ps23
Saccharomycetaceae - ps24
Saccharomycetaceae_2 - ps25
Saccharomycetaceae_3 - ps26
Kaz./Nau./Saccharomyces - ps27
Saccharomyces - ps28
Arboricola_clade - ps29
Cerevisiae_clade - ps30
Saccharomyces_cerevisiae - ps31
Phylostratum

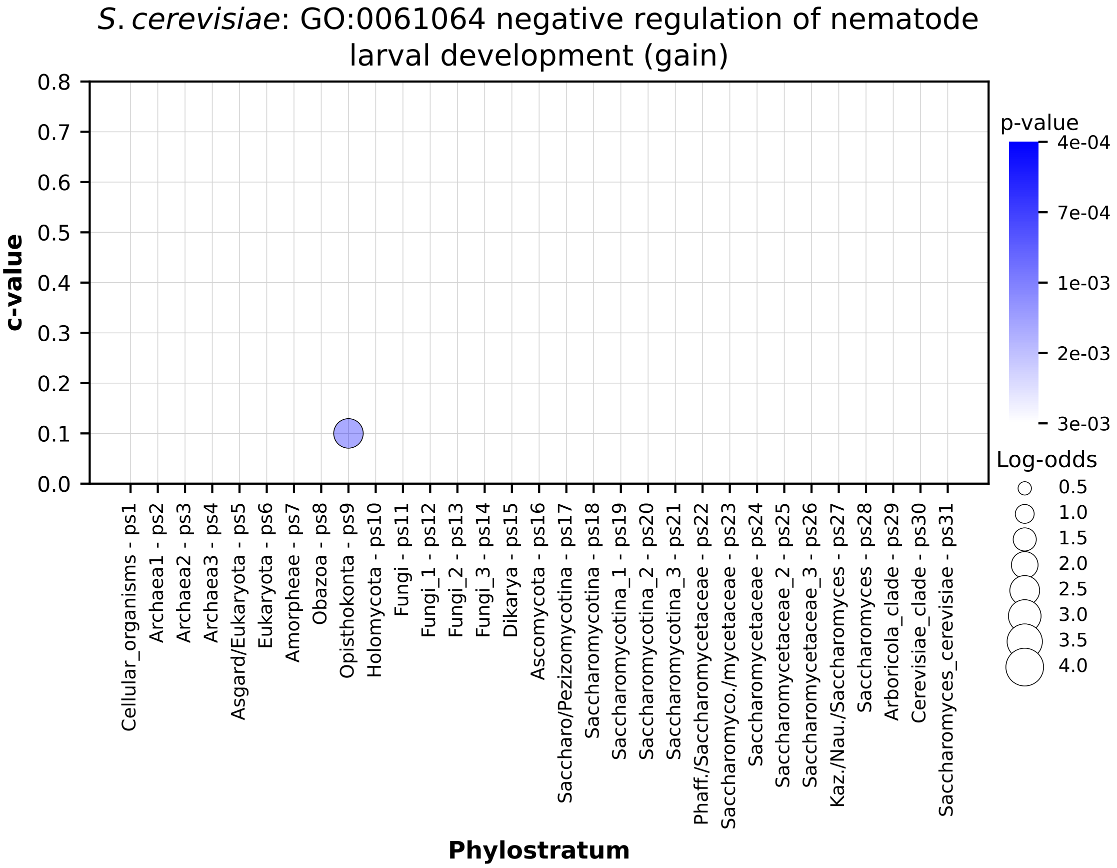

S. cerevisiae: GO:0061064 negative regulation of nematode larval development (gain)
c-value
0.0
0.1
0.2
0.3
0.4
0.5
0.6
0.7
0.8
Phylostratum
Cellular_organisms - ps1
Archaea1 - ps2
Archaea2 - ps3
Archaea3 - ps4
Asgard/Eukaryota - ps5
Eukaryota - ps6
Amorpheae - ps7
Obazoa - ps8
Opisthokonta - ps9
Holomycota - ps10
Fungi - ps11
Fungi_1 - ps12
Fungi_2 - ps13
Fungi_3 - ps14
Dikarya - ps15
Ascomycota - ps16
Saccharo/Pezizomycotina - ps17
Saccharomycotina - ps18
Saccharomycotina_1 - ps19
Saccharomycotina_2 - ps20
Saccharomycotina_3 - ps21
Phaff./Saccharomycetaceae - ps22
Saccharomyco./mycetaceae - ps23
Saccharomycetaceae - ps24
Saccharomycetaceae_2 - ps25
Saccharomycetaceae_3 - ps26
Kaz./Nau./Saccharomyces - ps27
Saccharomyces - ps28
Arboricola_clade - ps29
Cerevisiae_clade - ps30
Saccharomyces_cerevisiae - ps31
p-value
4e-04
7e-04
1e-03
2e-03
3e-03
Log-odds
0.5
1.0
1.5
2.0
2.5
3.0
3.5
4.0

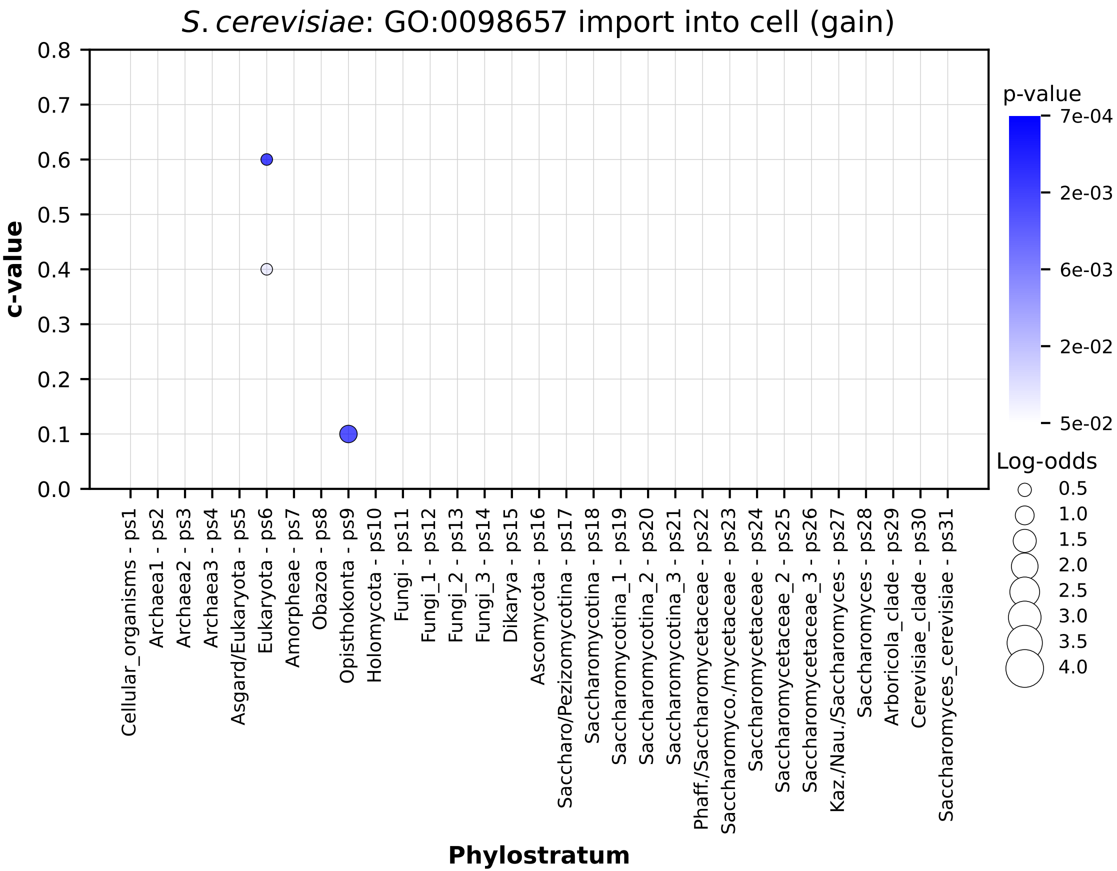

S. cerevisiae: GO:0098657 import into cell (gain)
c-value
0.0
0.1
0.2
0.3
0.4
0.5
0.6
0.7
0.8
p-value
7e-04
2e-03
6e-03
2e-02
5e-02
Log-odds
0.5
1.0
1.5
2.0
2.5
3.0
3.5
4.0
Cellular_organisms - ps1
Archaea1 - ps2
Archaea2 - ps3
Archaea3 - ps4
Asgard/Eukaryota - ps5
Eukaryota - ps6
Amorpheae - ps7
Obazoa - ps8
Opisthokonta - ps9
Holomycota - ps10
Fungi - ps11
Fungi_1 - ps12
Fungi_2 - ps13
Fungi_3 - ps14
Dikarya - ps15
Ascomycota - ps16
Saccharo/Pezizomycotina - ps17
Saccharomycotina - ps18
Saccharomycotina_1 - ps19
Saccharomycotina_2 - ps20
Saccharomycotina_3 - ps21
Phaff./Saccharomycetaceae - ps22
Saccharomyco./mycetaceae - ps23
Saccharomycetaceae - ps24
Saccharomycetaceae_2 - ps25
Saccharomycetaceae_3 - ps26
Kaz./Nau./Saccharomyces - ps27
Saccharomyces - ps28
Arboricola_clade - ps29
Cerevisiae_clade - ps30
Saccharomyces_cerevisiae - ps31
Phylostratum

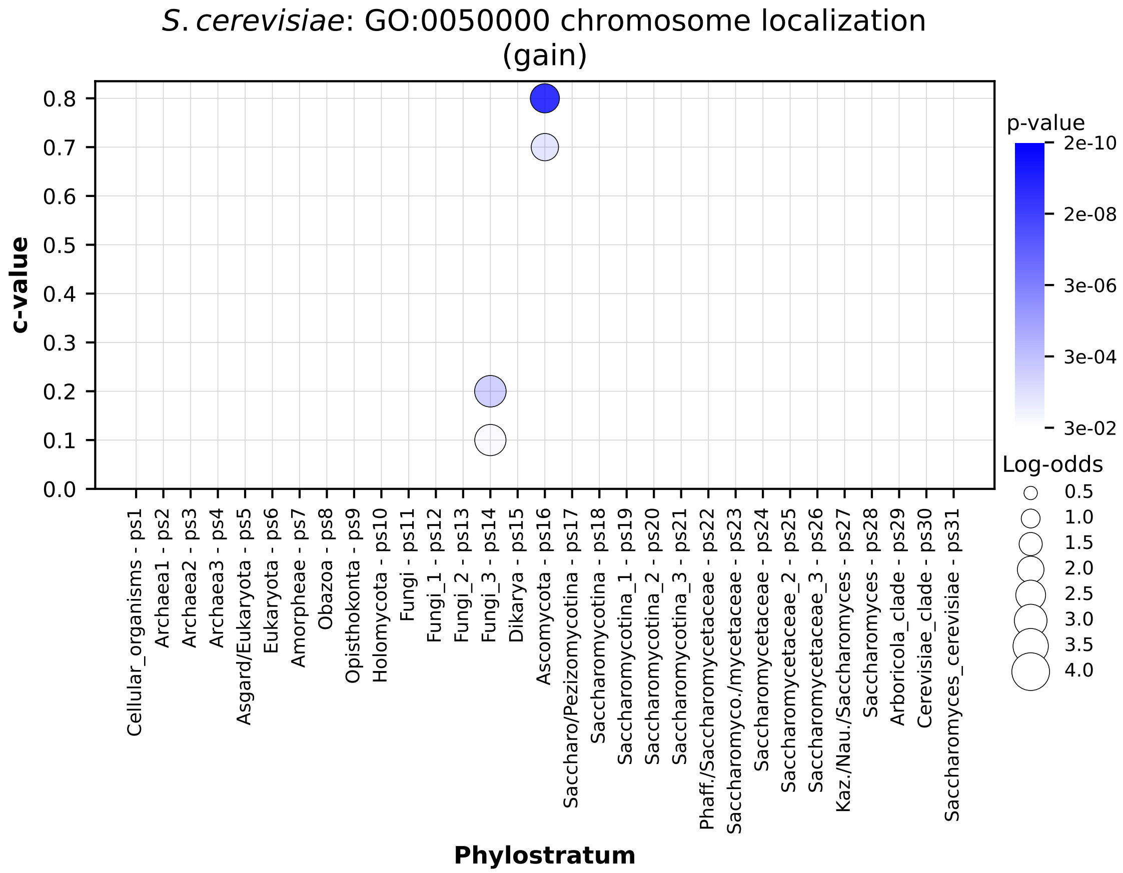

S. cerevisiae: GO:0050000 chromosome localization
(gain)
c-value
0.0
0.1
0.2
0.3
0.4
0.5
0.6
0.7
0.8
p-value
2e-10
2e-08
3e-06
3e-04
3e-02
Log-odds
0.5
1.0
1.5
2.0
2.5
3.0
3.5
4.0
Cellular_organisms - ps1
Archaea1 - ps2
Archaea2 - ps3
Archaea3 - ps4
Asgard/Eukaryota - ps5
Eukaryota - ps6
Amorpheae - ps7
Obazoa - ps8
Opisthokonta - ps9
Holomycota - ps10
Fungi - ps11
Fungi_1 - ps12
Fungi_2 - ps13
Fungi_3 - ps14
Dikarya - ps15
Ascomycota - ps16
Saccharo/Pezizomycotina - ps17
Saccharomycotina - ps18
Saccharomycotina_1 - ps19
Saccharomycotina_2 - ps20
Saccharomycotina_3 - ps21
Phaff./Saccharomycetaceae - ps22
Saccharomyco./mycetaceae - ps23
Saccharomycetaceae - ps24
Saccharomycetaceae_2 - ps25
Saccharomycetaceae_3 - ps26
Kaz./Nau./Saccharomyces - ps27
Saccharomyces - ps28
Arboricola_clade - ps29
Cerevisiae_clade - ps30
Saccharomyces_cerevisiae - ps31
Phylostratum

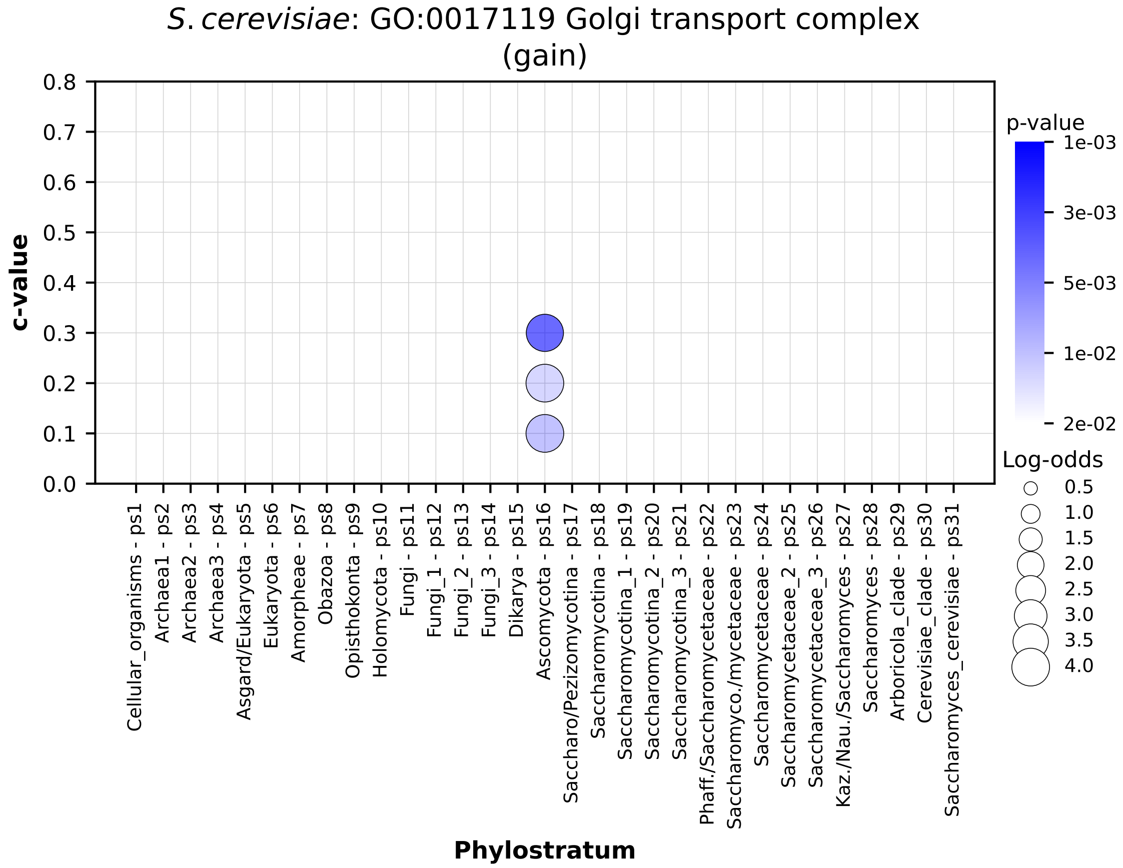

S. cerevisiae: GO:0017119 Golgi transport complex
(gain)
c-value
0.0
0.1
0.2
0.3
0.4
0.5
0.6
0.7
0.8
p-value
1e-03
3e-03
5e-03
1e-02
2e-02
Log-odds
0.5
1.0
1.5
2.0
2.5
3.0
3.5
4.0
Cellular_organisms - ps1
Archaea1 - ps2
Archaea2 - ps3
Archaea3 - ps4
Asgard/Eukaryota - ps5
Eukaryota - ps6
Amorpheae - ps7
Obazoa - ps8
Opisthokonta - ps9
Holomycota - ps10
Fungi - ps11
Fungi_1 - ps12
Fungi_2 - ps13
Fungi_3 - ps14
Dikarya - ps15
Ascomycota - ps16
Saccharo/Pezizomycotina - ps17
Saccharomycotina - ps18
Saccharomycotina_1 - ps19
Saccharomycotina_2 - ps20
Saccharomycotina_3 - ps21
Phaff./Saccharomycetaceae - ps22
Saccharomyco./mycetaceae - ps23
Saccharomycetaceae - ps24
Saccharomycetaceae_2 - ps25
Saccharomycetaceae_3 - ps26
Kaz./Nau./Saccharomyces - ps27
Saccharomyces - ps28
Arboricola_clade - ps29
Cerevisiae_clade - ps30
Saccharomyces_cerevisiae - ps31
Phylostratum

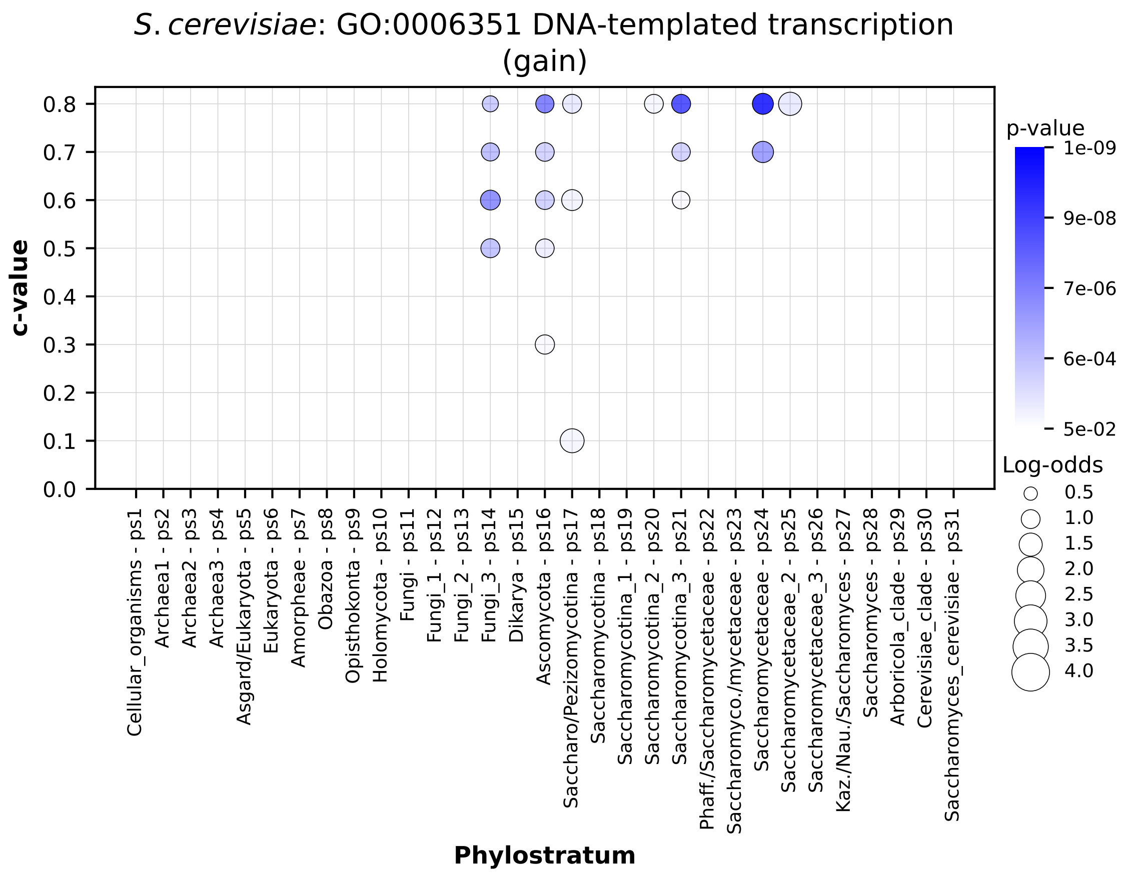

S. cerevisiae: GO:0006351 DNA-templated transcription (gain)
c-value
0.0
0.1
0.2
0.3
0.4
0.5
0.6
0.7
0.8
p-value
1e-09
9e-08
7e-06
6e-04
5e-02
Log-odds
0.5
1.0
1.5
2.0
2.5
3.0
3.5
4.0
Cellular_organisms - ps1
Archaea1 - ps2
Archaea2 - ps3
Archaea3 - ps4
Asgard/Eukaryota - ps5
Eukaryota - ps6
Amorpheae - ps7
Obazoa - ps8
Opisthokonta - ps9
Holomycota - ps10
Fungi - ps11
Fungi_1 - ps12
Fungi_2 - ps13
Fungi_3 - ps14
Dikarya - ps15
Ascomycota - ps16
Saccharo/Pezizomycotina - ps17
Saccharomycotina - ps18
Saccharomycotina_1 - ps19
Saccharomycotina_2 - ps20
Saccharomycotina_3 - ps21
Phaff./Saccharomycetaceae - ps22
Saccharomyco./mycetaceae - ps23
Saccharomycetaceae - ps24
Saccharomycetaceae_2 - ps25
Saccharomycetaceae_3 - ps26
Kaz./Nau./Saccharomyces - ps27
Saccharomyces - ps28
Arboricola_clade - ps29
Cerevisiae_clade - ps30
Saccharomyces_cerevisiae - ps31
Phylostratum

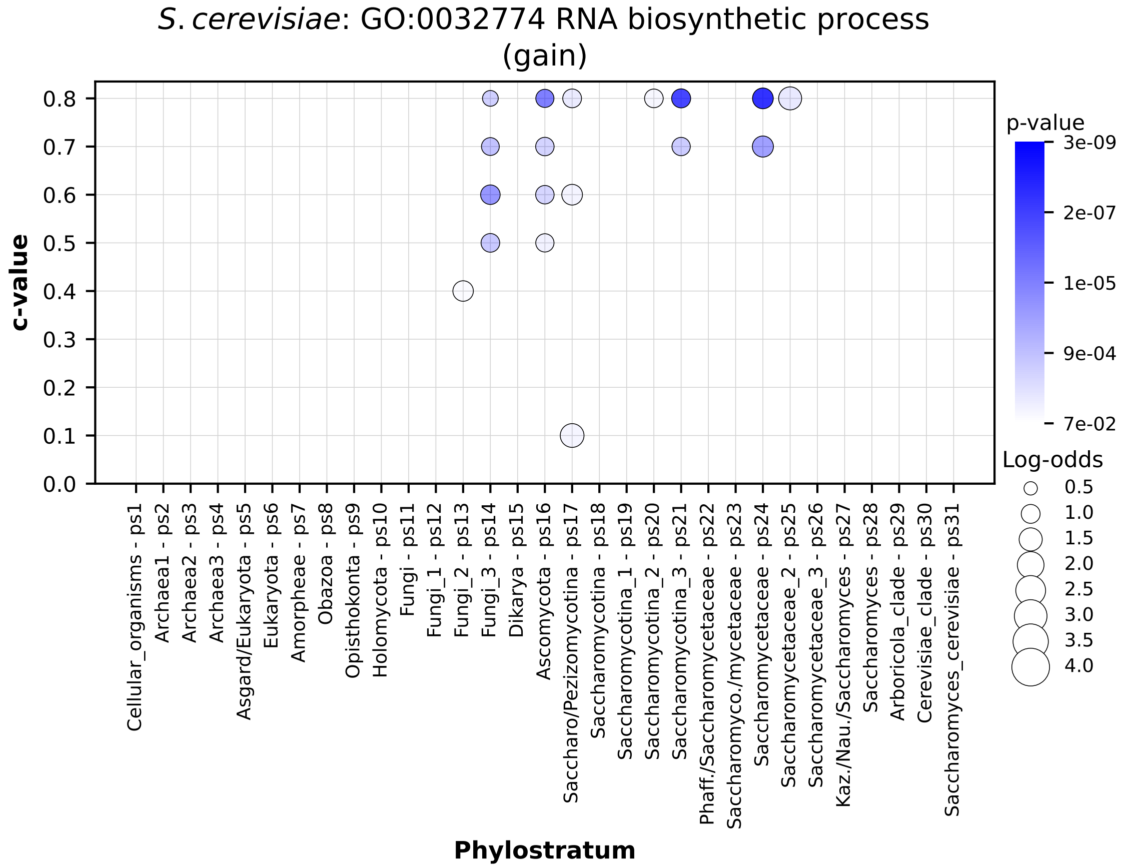

S. cerevisiae: GO:0032774 RNA biosynthetic process
(gain)
c-value
0.8
0.7
0.6
0.5
0.4
0.3
0.2
0.1
0.0
Phylostratum
Cellular_organisms - ps1
Archaea1 - ps2
Archaea2 - ps3
Archaea3 - ps4
Asgard/Eukaryota - ps5
Eukaryota - ps6
Amorpheae - ps7
Obazoa - ps8
Opisthokonta - ps9
Holomycota - ps10
Fungi - ps11
Fungi_1 - ps12
Fungi_2 - ps13
Fungi_3 - ps14
Dikarya - ps15
Ascomycota - ps16
Saccharo/Pezizomycotina - ps17
Saccharomycotina - ps18
Saccharomycotina_1 - ps19
Saccharomycotina_2 - ps20
Saccharomycotina_3 - ps21
Phaff./Saccharomycetaceae - ps22
Saccharomyco./mycetaceae - ps23
Saccharomycetaceae - ps24
Saccharomycetaceae_2 - ps25
Saccharomycetaceae_3 - ps26
Kaz./Nau./Saccharomyces - ps27
Saccharomyces - ps28
Arboricola_clade - ps29
Cerevisiae_clade - ps30
Saccharomyces_cerevisiae - ps31
p-value
3e-09
2e-07
1e-05
9e-04
7e-02
Log-odds
0.5
1.0
1.5
2.0
2.5
3.0
3.5
4.0

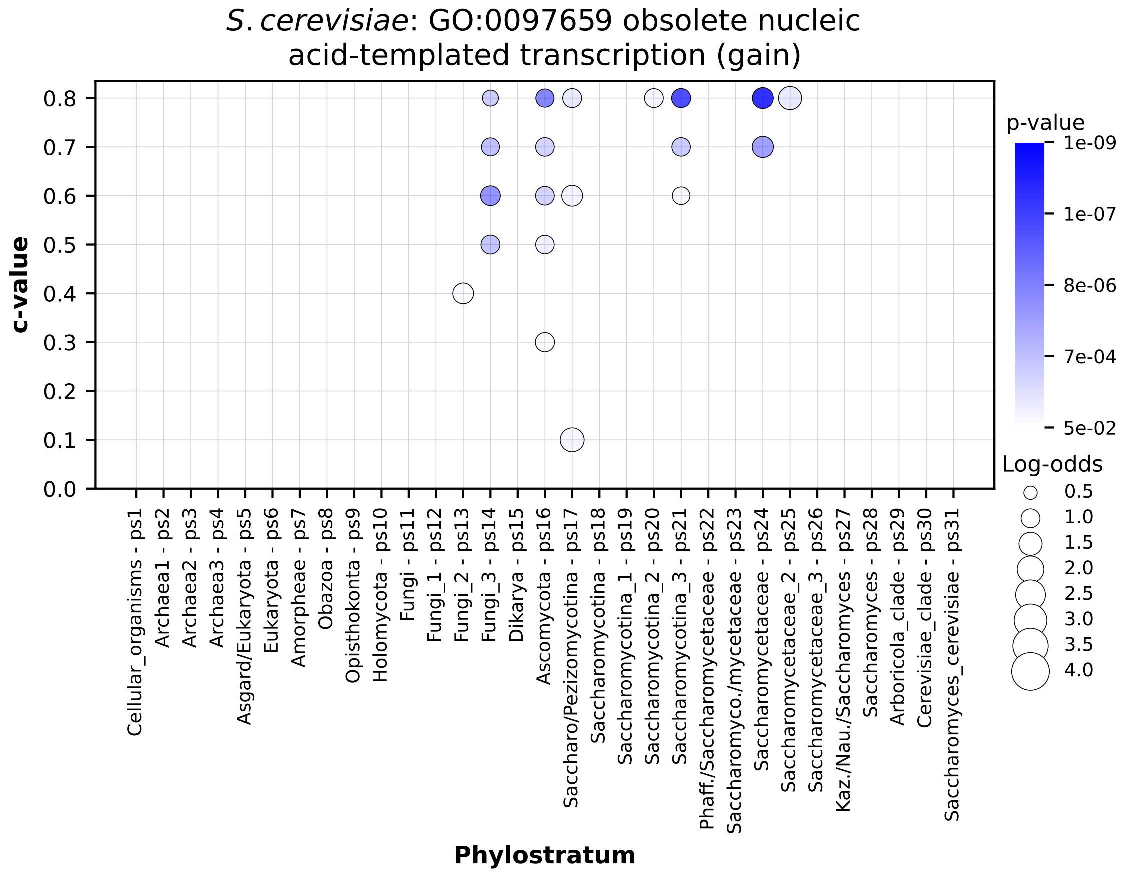

S. cerevisiae: GO:0097659 obsolete nucleic acid-templated transcription (gain)
c-value
Phylostratum
p-value
1e-09
1e-07
8e-06
7e-04
5e-02
Log-odds
0.5
1.0
1.5
2.0
2.5
3.0
3.5
4.0
Cellular_organisms - ps1
Archaea1 - ps2
Archaea2 - ps3
Archaea3 - ps4
Asgard/Eukaryota - ps5
Eukaryota - ps6
Amorpheae - ps7
Obazoa - ps8
Opisthokonta - ps9
Holomycota - ps10
Fungi - ps11
Fungi_1 - ps12
Fungi_2 - ps13
Fungi_3 - ps14
Dikarya - ps15
Ascomycota - ps16
Saccharo/Pezizomycotina - ps17
Saccharomycotina - ps18
Saccharomycotina_1 - ps19
Saccharomycotina_2 - ps20
Saccharomycotina_3 - ps21
Phaff./Saccharomycetaceae - ps22
Saccharomyco./mycetaceae - ps23
Saccharomycetaceae - ps24
Saccharomycetaceae_2 - ps25
Saccharomycetaceae_3 - ps26
Kaz./Nau./Saccharomyces - ps27
Saccharomyces - ps28
Arboricola_clade - ps29
Cerevisiae_clade - ps30
Saccharomyces_cerevisiae - ps31

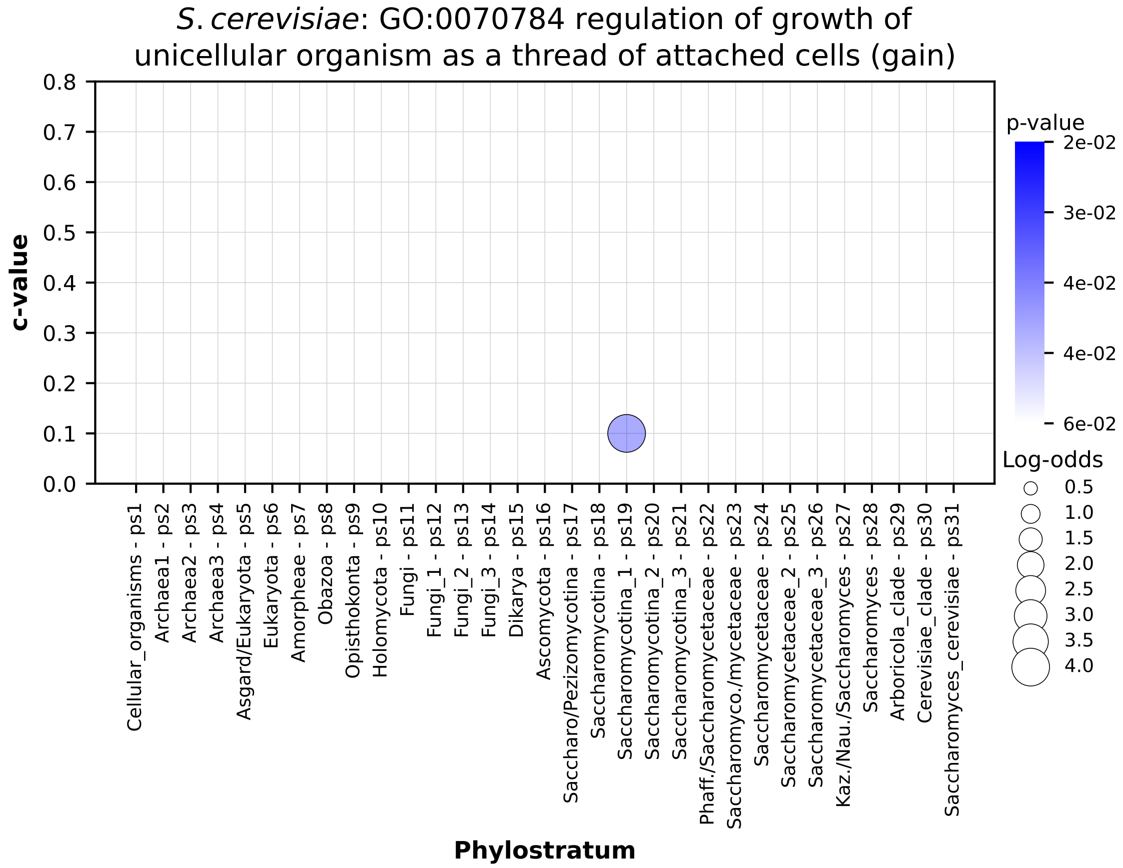
S. cerevisiae: GO:0070784 regulation of growth of unicellular organism as a thread of attached cells (gain)
c-value
0.0
0.1
0.2
0.3
0.4
0.5
0.6
0.7
0.8
Phylostratum
Cellular_organisms - ps1
Archaea1 - ps2
Archaea2 - ps3
Archaea3 - ps4
Asgard/Eukaryota - ps5
Eukaryota - ps6
Amorpheae - ps7
Obazoa - ps8
Opisthokonta - ps9
Holomycota - ps10
Fungi - ps11
Fungi_1 - ps12
Fungi_2 - ps13
Fungi_3 - ps14
Dikarya - ps15
Ascomycota - ps16
Saccharo/Pezizomycotina - ps17
Saccharomycotina - ps18
Saccharomycotina_1 - ps19
Saccharomycotina_2 - ps20
Saccharomycotina_3 - ps21
Phaff./Saccharomycetaceae - ps22
Saccharomyco./mycetaceae - ps23
Saccharomycetaceae - ps24
Saccharomycetaceae_2 - ps25
Saccharomycetaceae_3 - ps26
Kaz./Nau./Saccharomyces - ps27
Saccharomyces - ps28
Arboricola_clade - ps29
Cerevisiae_clade - ps30
Saccharomyces_cerevisiae - ps31
p-value
2e-02
3e-02
4e-02
4e-02
6e-02
Log-odds
0.5
1.0
1.5
2.0
2.5
3.0
3.5
4.0

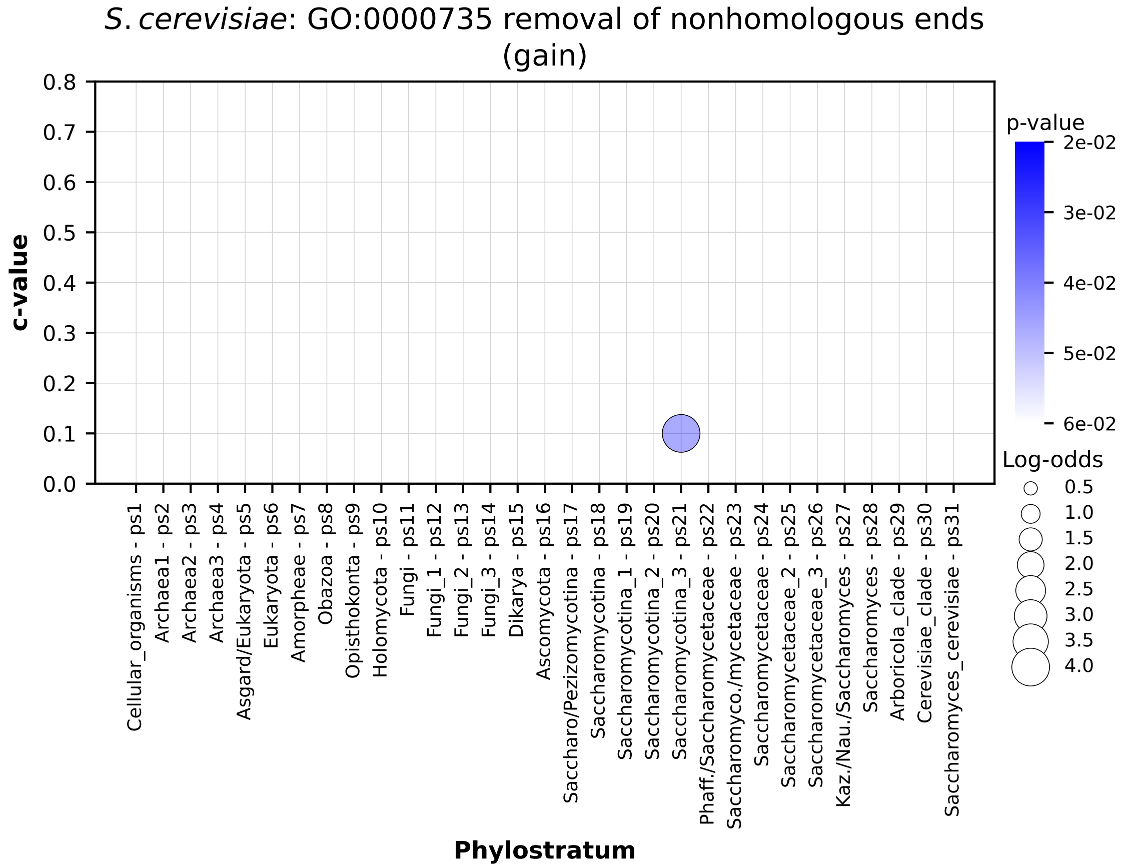
S. cerevisiae: GO:0000735 removal of nonhomologous ends
(gain)
c-value
0.8
0.7
0.6
0.5
0.4
0.3
0.2
0.1
0.0
p-value
2e-02
3e-02
4e-02
5e-02
6e-02
Log-odds
0.5
1.0
1.5
2.0
2.5
3.0
3.5
4.0
Cellular_organisms - ps1
Archaea1 - ps2
Archaea2 - ps3
Archaea3 - ps4
Asgard/Eukaryota - ps5
Eukaryota - ps6
Amorpheae - ps7
Obazoa - ps8
Opisthokonta - ps9
Holomycota - ps10
Fungi - ps11
Fungi_1 - ps12
Fungi_2 - ps13
Fungi_3 - ps14
Dikarya - ps15
Ascomycota - ps16
Saccharo/Pezizomycotina - ps17
Saccharomycotina - ps18
Saccharomycotina_1 - ps19
Saccharomycotina_2 - ps20
Saccharomycotina_3 - ps21
Phaff./Saccharomycetaceae - ps22
Saccharomyco./mycetaceae - ps23
Saccharomycetaceae - ps24
Saccharomycetaceae_2 - ps25
Saccharomycetaceae_3 - ps26
Kaz./Nau./Saccharomyces - ps27
Saccharomyces - ps28
Arboricola_clade - ps29
Cerevisiae_clade - ps30
Saccharomyces_cerevisiae - ps31
Phylostratum

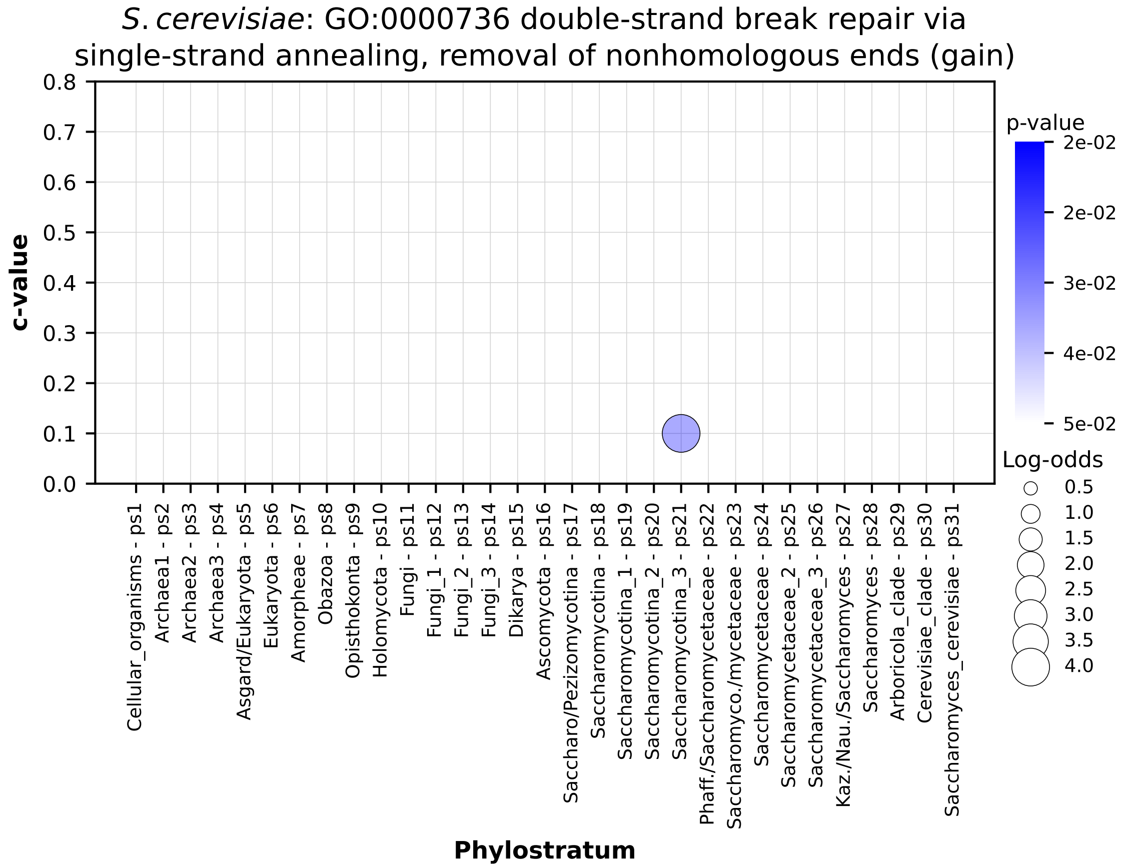

S. cerevisiae: GO:0000736 double-strand break repair via single-strand annealing, removal of nonhomologous ends (gain)
c-value
0.8
0.7
0.6
0.5
0.4
0.3
0.2
0.1
0.0
Phylostratum
Cellular_organisms - ps1
Archaea1 - ps2
Archaea2 - ps3
Archaea3 - ps4
Asgard/Eukaryota - ps5
Eukaryota - ps6
Amorpheae - ps7
Obazoa - ps8
Opisthokonta - ps9
Holomycota - ps10
Fungi - ps11
Fungi_1 - ps12
Fungi_2 - ps13
Fungi_3 - ps14
Dikarya - ps15
Ascomycota - ps16
Saccharo/Pezizomycotina - ps17
Saccharomycotina - ps18
Saccharomycotina_1 - ps19
Saccharomycotina_2 - ps20
Saccharomycotina_3 - ps21
Phaff./Saccharomycetaceae - ps22
Saccharomyco./mycetaceae - ps23
Saccharomycetaceae - ps24
Saccharomycetaceae_2 - ps25
Saccharomycetaceae_3 - ps26
Kaz./Nau./Saccharomyces - ps27
Saccharomyces - ps28
Arboricola_clade - ps29
Cerevisiae_clade - ps30
Saccharomyces_cerevisiae - ps31
p-value
2e-02
2e-02
3e-02
4e-02
5e-02
Log-odds
0.5
1.0
1.5
2.0
2.5
3.0
3.5
4.0

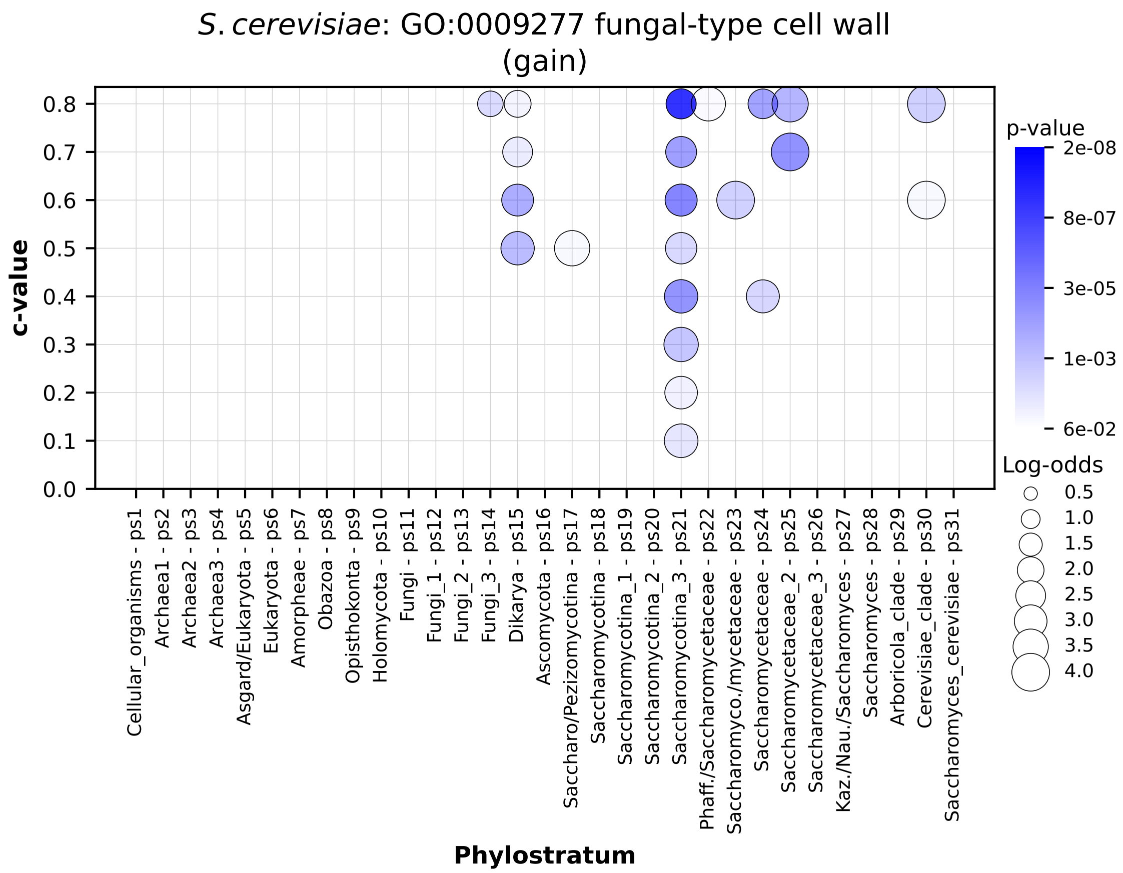

S. cerevisiae: GO:0009277 fungal-type cell wall
(gain)
c-value
0.0
0.1
0.2
0.3
0.4
0.5
0.6
0.7
0.8
Phylostratum
Cellular_organisms - ps1
Archaea1 - ps2
Archaea2 - ps3
Archaea3 - ps4
Asgard/Eukaryota - ps5
Eukaryota - ps6
Amorpheae - ps7
Obazoa - ps8
Opisthokonta - ps9
Holomycota - ps10
Fungi - ps11
Fungi_1 - ps12
Fungi_2 - ps13
Fungi_3 - ps14
Dikarya - ps15
Ascomycota - ps16
Saccharo/Pezizomycotina - ps17
Saccharomycotina - ps18
Saccharomycotina_1 - ps19
Saccharomycotina_2 - ps20
Saccharomycotina_3 - ps21
Phaff./Saccharomycetaceae - ps22
Saccharomyco./mycetaceae - ps23
Saccharomycetaceae - ps24
Saccharomycetaceae_2 - ps25
Saccharomycetaceae_3 - ps26
Kaz./Nau./Saccharomyces - ps27
Saccharomyces - ps28
Arboricola_clade - ps29
Cerevisiae_clade - ps30
Saccharomyces_cerevisiae - ps31
p-value
2e-08
8e-07
3e-05
1e-03
6e-02
Log-odds
0.5
1.0
1.5
2.0
2.5
3.0
3.5
4.0

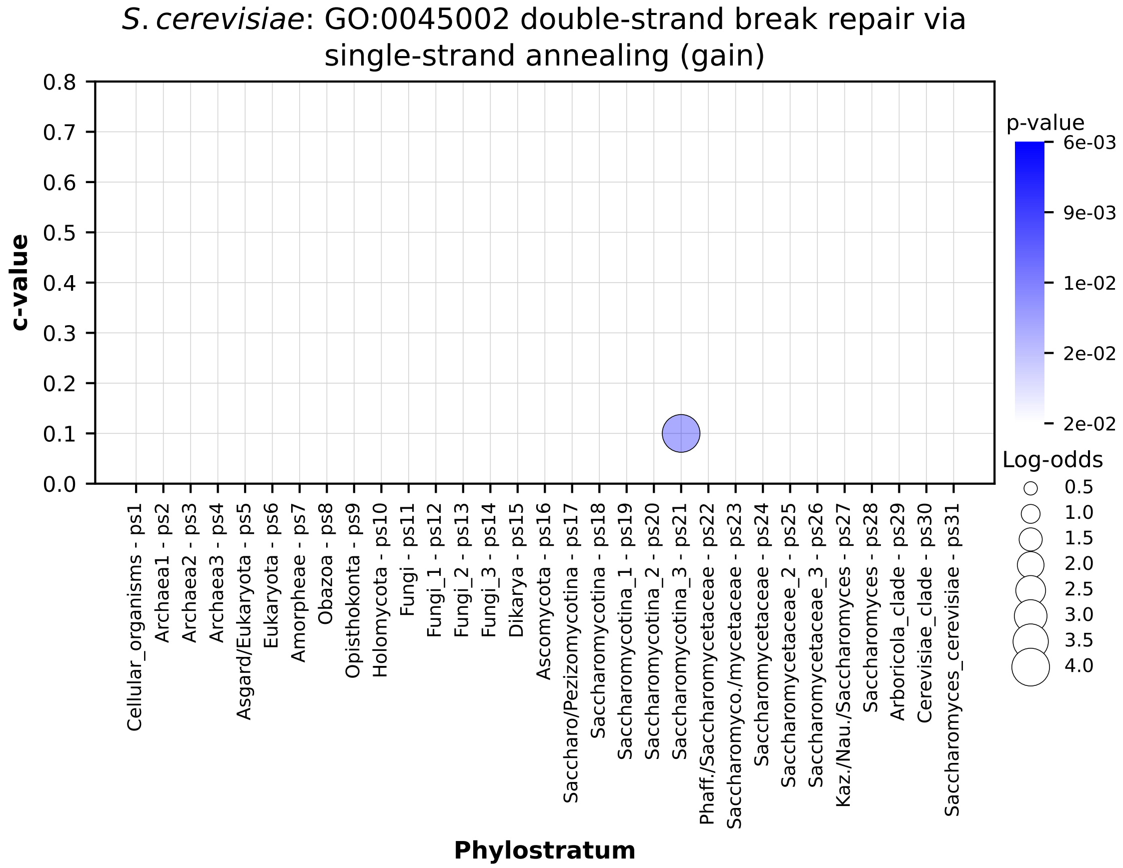

S. cerevisiae: GO:0045002 double-strand break repair via single-strand annealing (gain)
c-value
0.8
0.7
0.6
0.5
0.4
0.3
0.2
0.1
0.0
p-value
6e-03
9e-03
1e-02
2e-02
2e-02
Log-odds
0.5
1.0
1.5
2.0
2.5
3.0
3.5
4.0
Cellular_organisms - ps1
Archaea1 - ps2
Archaea2 - ps3
Archaea3 - ps4
Asgard/Eukaryota - ps5
Eukaryota - ps6
Amorpheae - ps7
Obazoa - ps8
Opisthokonta - ps9
Holomycota - ps10
Fungi - ps11
Fungi_1 - ps12
Fungi_2 - ps13
Fungi_3 - ps14
Dikarya - ps15
Ascomycota - ps16
Saccharo/Pezizomycotina - ps17
Saccharomycotina - ps18
Saccharomycotina_1 - ps19
Saccharomycotina_2 - ps20
Saccharomycotina_3 - ps21
Phaff./Saccharomycetaceae - ps22
Saccharomyco./mycetaceae - ps23
Saccharomycetaceae - ps24
Saccharomycetaceae_2 - ps25
Saccharomycetaceae_3 - ps26
Kaz./Nau./Saccharomyces - ps27
Saccharomyces - ps28
Arboricola_clade - ps29
Cerevisiae_clade - ps30
Saccharomyces_cerevisiae - ps31
Phylostratum

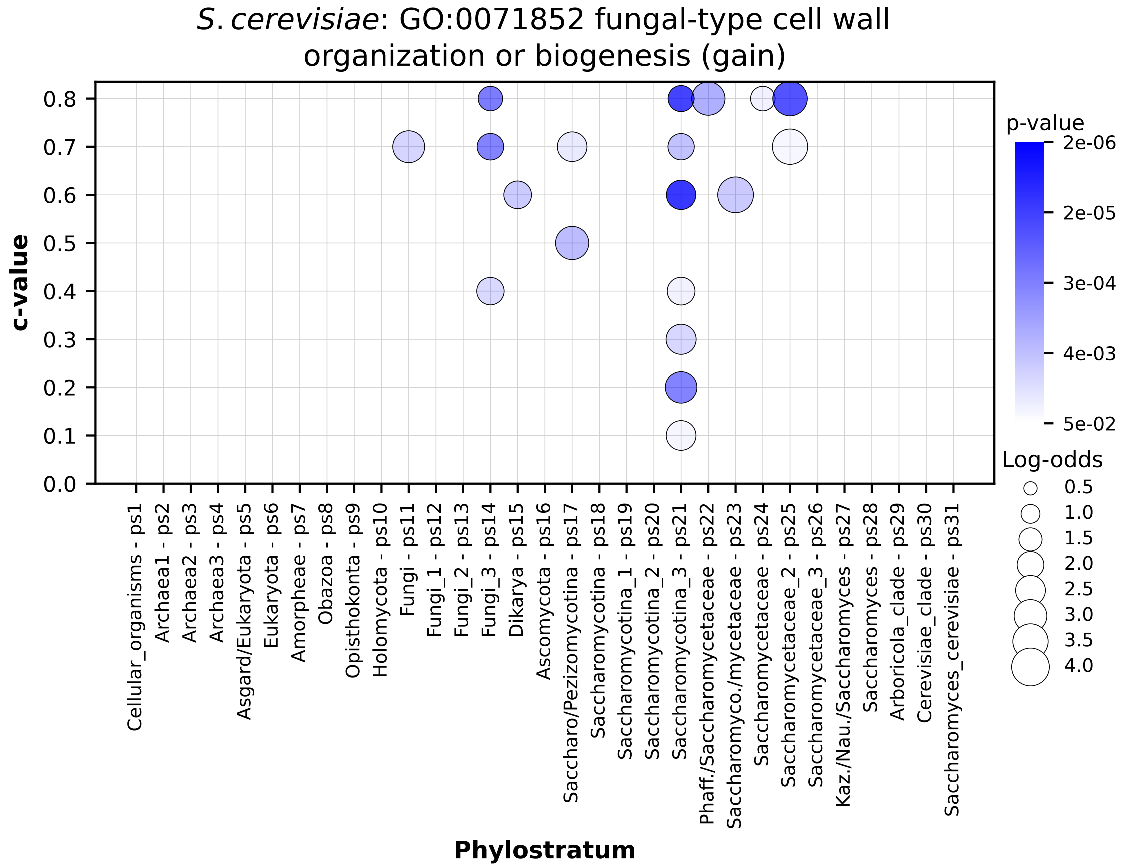
S. cerevisiae: GO:0071852 fungal-type cell wall organization or biogenesis (gain)
c-value
Phylostratum
p-value
2e-06
2e-05
3e-04
4e-03
5e-02
Log-odds
0.5
1.0
1.5
2.0
2.5
3.0
3.5
4.0
Cellular_organisms - ps1
Archaea1 - ps2
Archaea2 - ps3
Archaea3 - ps4
Asgard/Eukaryota - ps5
Eukaryota - ps6
Amorpheae - ps7
Obazoa - ps8
Opisthokonta - ps9
Holomycota - ps10
Fungi - ps11
Fungi_1 - ps12
Fungi_2 - ps13
Fungi_3 - ps14
Dikarya - ps15
Ascomycota - ps16
Saccharo/Pezizomycotina - ps17
Saccharomycotina - ps18
Saccharomycotina_1 - ps19
Saccharomycotina_2 - ps20
Saccharomycotina_3 - ps21
Phaff./Saccharomycetaceae - ps22
Saccharomyco./mycetaceae - ps23
Saccharomycetaceae - ps24
Saccharomycetaceae_2 - ps25
Saccharomycetaceae_3 - ps26
Kaz./Nau./Saccharomyces - ps27
Saccharomyces - ps28
Arboricola_clade - ps29
Cerevisiae_clade - ps30
Saccharomyces_cerevisiae - ps31

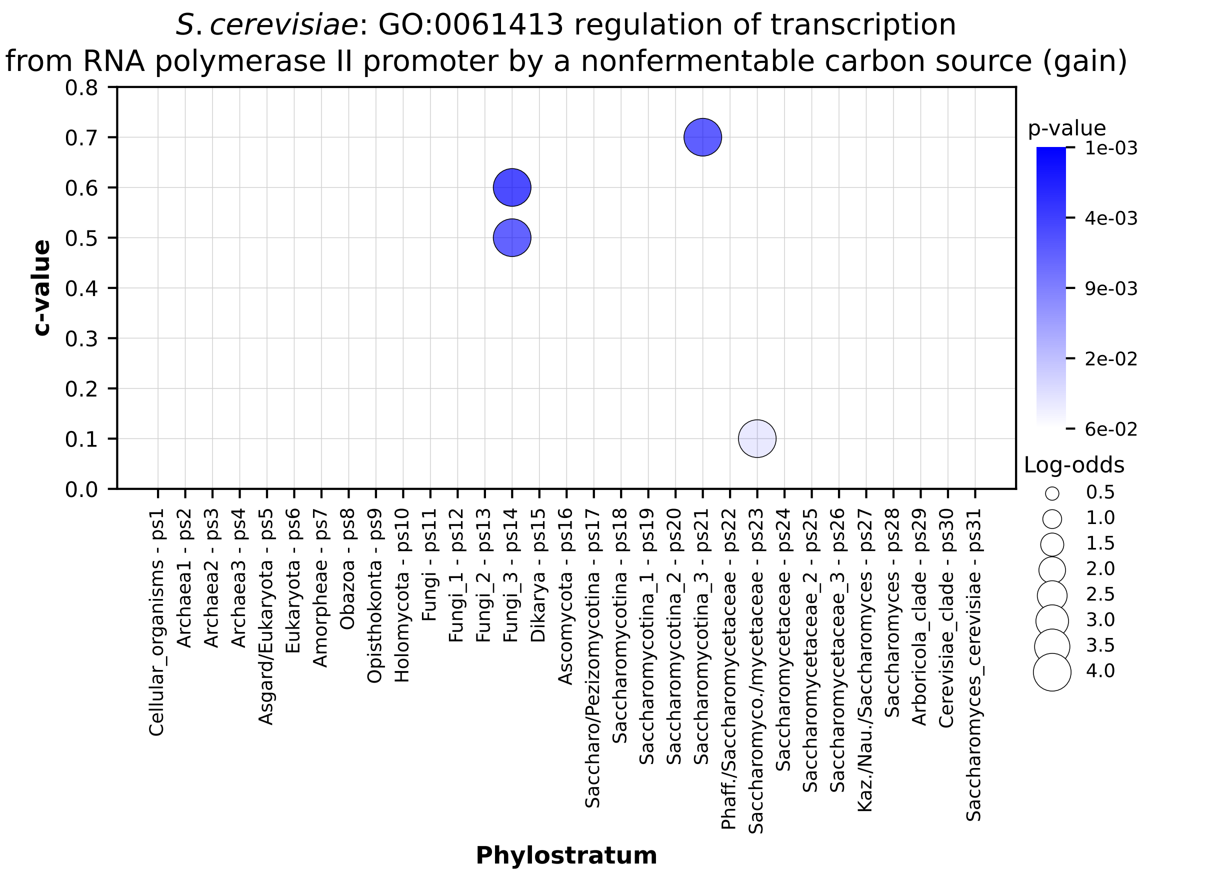

S. cerevisiae: GO:0061413 regulation of transcription
from RNA polymerase II promoter by a nonfermentable carbon source (gain)
c-value
0.0
0.1
0.2
0.3
0.4
0.5
0.6
0.7
0.8
p-value
1e-03
4e-03
9e-03
2e-02
6e-02
Log-odds
0.5
1.0
1.5
2.0
2.5
3.0
3.5
4.0
Cellular_organisms - ps1
Archaea1 - ps2
Archaea2 - ps3
Archaea3 - ps4
Asgard/Eukaryota - ps5
Eukaryota - ps6
Amorpheae - ps7
Obazoa - ps8
Opisthokonta - ps9
Holomycota - ps10
Fungi - ps11
Fungi_1 - ps12
Fungi_2 - ps13
Fungi_3 - ps14
Dikarya - ps15
Ascomycota - ps16
Saccharo/Pezizomycotina - ps17
Saccharomycotina - ps18
Saccharomycotina_1 - ps19
Saccharomycotina_2 - ps20
Saccharomycotina_3 - ps21
Phaff./Saccharomycetaceae - ps22
Saccharomyco./mycetaceae - ps23
Saccharomycetaceae - ps24
Saccharomycetaceae_2 - ps25
Saccharomycetaceae_3 - ps26
Kaz./Nau./Saccharomyces - ps27
Saccharomyces - ps28
Arboricola_clade - ps29
Cerevisiae_clade - ps30
Saccharomyces_cerevisiae - ps31
Phylostratum

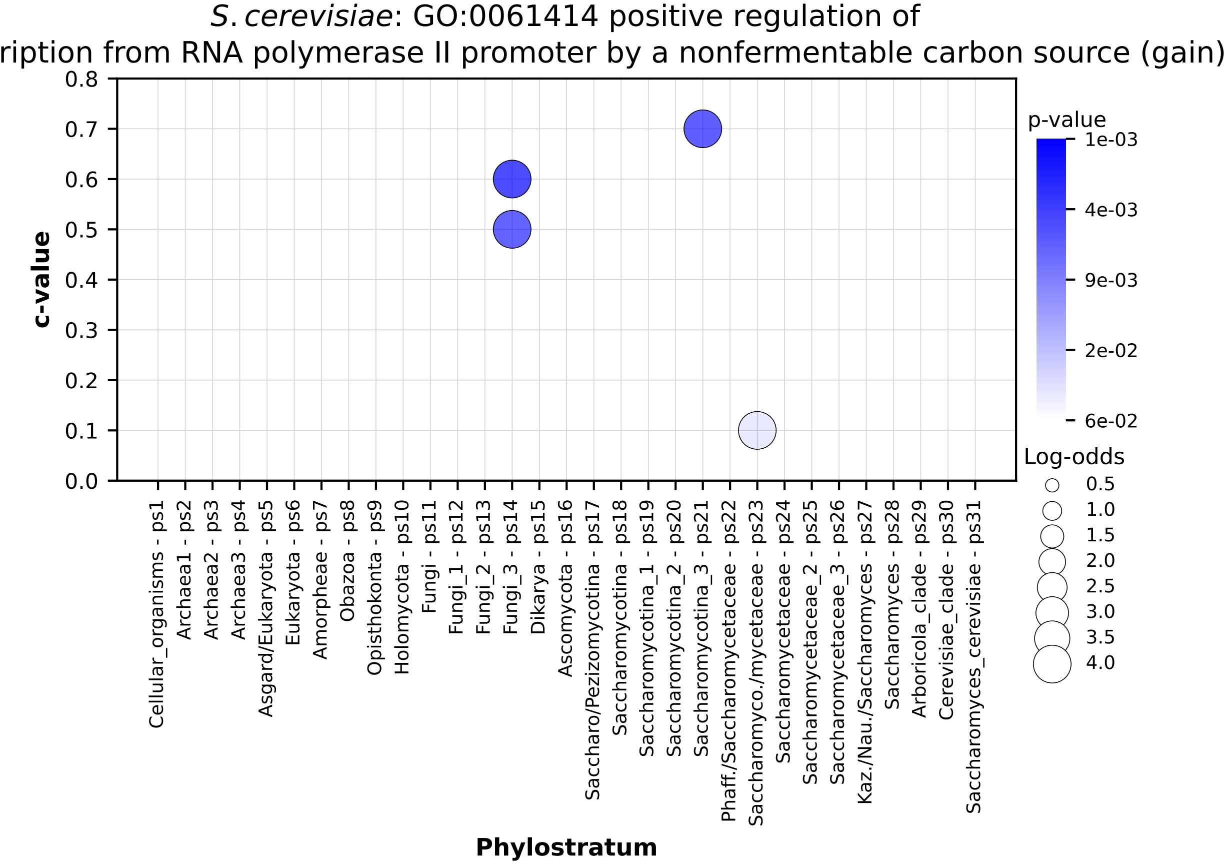
S. cerevisiae: GO:0061414 positive regulation of
ription from RNA polymerase II promoter by a nonfermentable carbon source (gain)
c-value
0.8
0.7
0.6
0.5
0.4
0.3
0.2
0.1
0.0
p-value
1e-03
4e-03
9e-03
2e-02
6e-02
Log-odds
0.5
1.0
1.5
2.0
2.5
3.0
3.5
4.0
Cellular_organisms - ps1
Archaea1 - ps2
Archaea2 - ps3
Archaea3 - ps4
Asgard/Eukaryota - ps5
Eukaryota - ps6
Amorpheae - ps7
Obazoa - ps8
Opisthokonta - ps9
Holomycota - ps10
Fungi - ps11
Fungi_1 - ps12
Fungi_2 - ps13
Fungi_3 - ps14
Dikarya - ps15
Ascomycota - ps16
Saccharo/Pezizomycotina - ps17
Saccharomycotina - ps18
Saccharomycotina_1 - ps19
Saccharomycotina_2 - ps20
Saccharomycotina_3 - ps21
Phaff./Saccharomycetaceae - ps22
Saccharomyco./mycetaceae - ps23
Saccharomycetaceae - ps24
Saccharomycetaceae_2 - ps25
Saccharomycetaceae_3 - ps26
Kaz./Nau./Saccharomyces - ps27
Saccharomyces - ps28
Arboricola_clade - ps29
Cerevisiae_clade - ps30
Saccharomyces_cerevisiae - ps31
Phylostratum

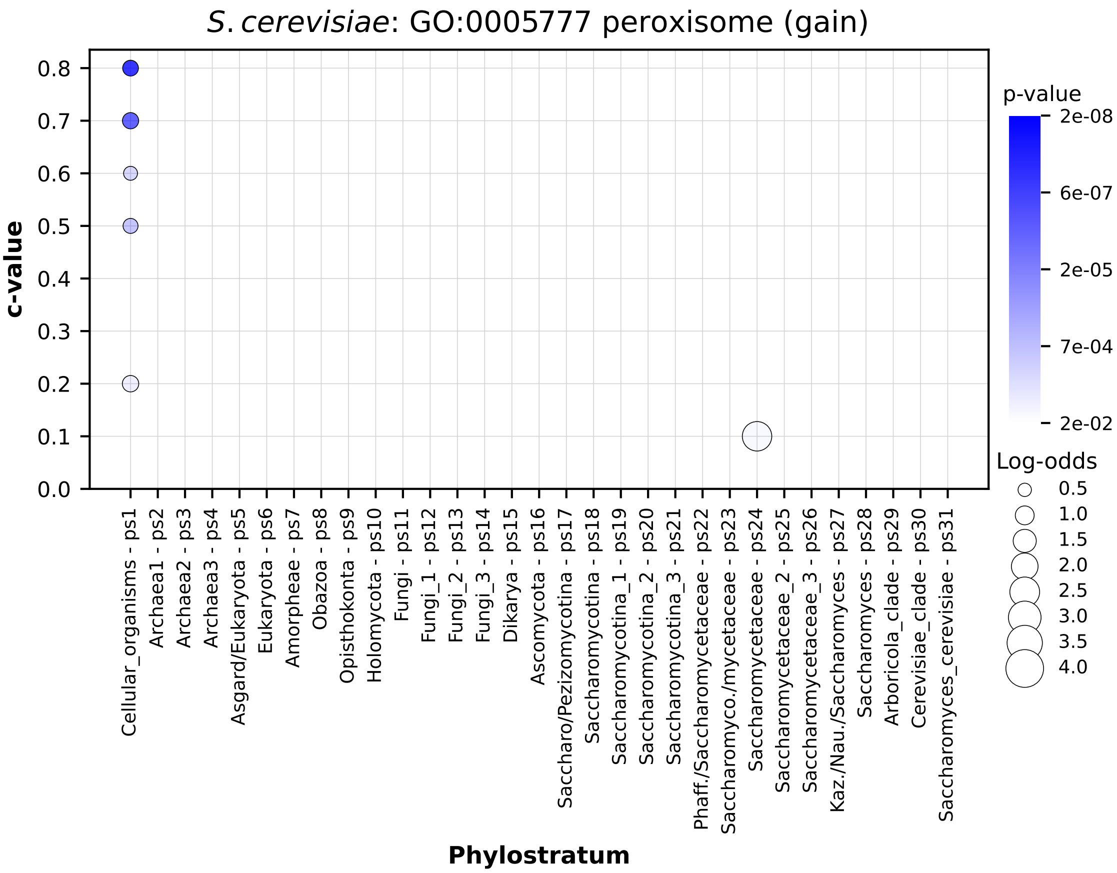

S. cerevisiae: GO:0005777 peroxisome (gain)
c-value
0.0
0.1
0.2
0.3
0.4
0.5
0.6
0.7
0.8
Phylostratum
Cellular_organisms - ps1
Archaea1 - ps2
Archaea2 - ps3
Archaea3 - ps4
Asgard/Eukaryota - ps5
Eukaryota - ps6
Amorpheae - ps7
Obazoa - ps8
Opisthokonta - ps9
Holomycota - ps10
Fungi - ps11
Fungi_1 - ps12
Fungi_2 - ps13
Fungi_3 - ps14
Dikarya - ps15
Ascomycota - ps16
Saccharo/Pezizomycotina - ps17
Saccharomycotina - ps18
Saccharomycotina_1 - ps19
Saccharomycotina_2 - ps20
Saccharomycotina_3 - ps21
Phaff./Saccharomycetaceae - ps22
Saccharomyco./mycetaceae - ps23
Saccharomycetaceae - ps24
Saccharomycetaceae_2 - ps25
Saccharomycetaceae_3 - ps26
Kaz./Nau./Saccharomyces - ps27
Saccharomyces - ps28
Arboricola_clade - ps29
Cerevisiae_clade - ps30
Saccharomyces_cerevisiae - ps31
p-value
2e-08
6e-07
2e-05
7e-04
2e-02
Log-odds
0.5
1.0
1.5
2.0
2.5
3.0
3.5
4.0

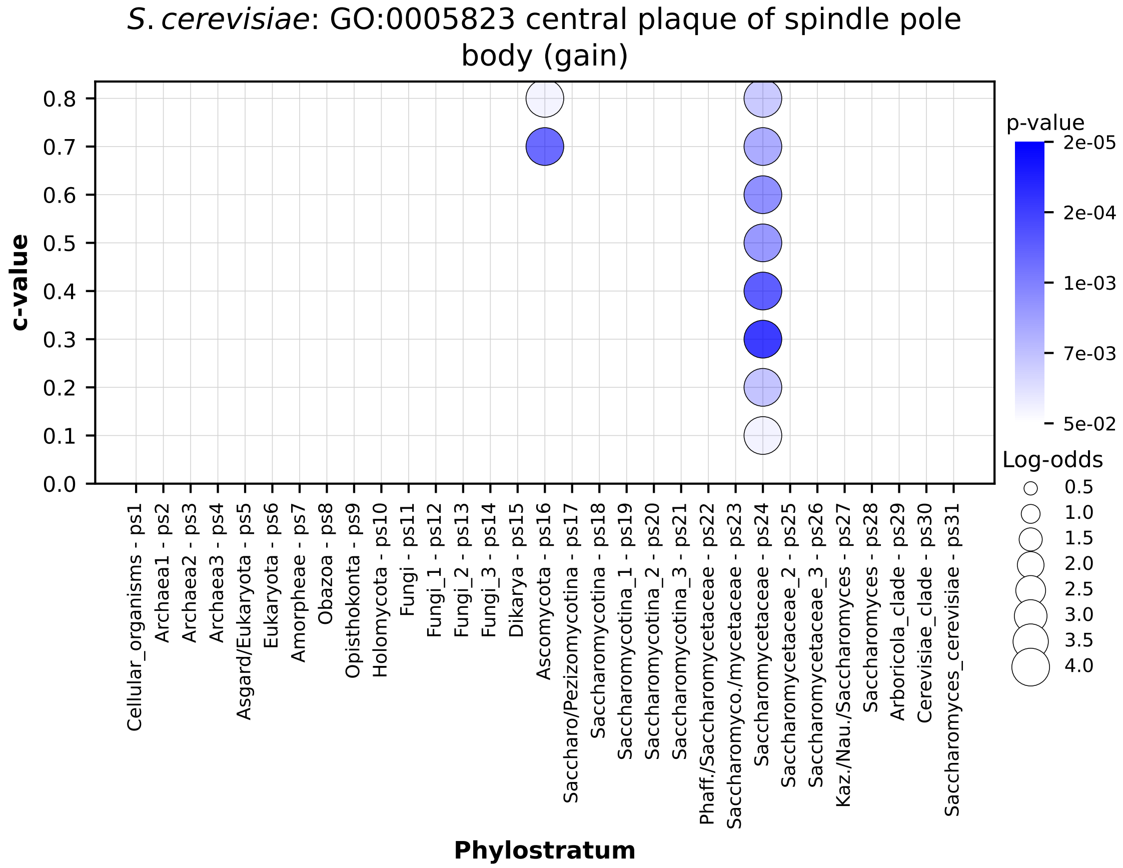

S. cerevisiae: GO:0005823 central plaque of spindle pole body (gain)
c-value
0.8
0.7
0.6
0.5
0.4
0.3
0.2
0.1
0.0
p-value
2e-05
2e-04
1e-03
7e-03
5e-02
Log-odds
0.5
1.0
1.5
2.0
2.5
3.0
3.5
4.0
Cellular_organisms - ps1
Archaea1 - ps2
Archaea2 - ps3
Archaea3 - ps4
Asgard/Eukaryota - ps5
Eukaryota - ps6
Amorpheae - ps7
Obazoa - ps8
Opisthokonta - ps9
Holomycota - ps10
Fungi - ps11
Fungi_1 - ps12
Fungi_2 - ps13
Fungi_3 - ps14
Dikarya - ps15
Ascomycota - ps16
Saccharo/Pezizomycotina - ps17
Saccharomycotina - ps18
Saccharomycotina_1 - ps19
Saccharomycotina_2 - ps20
Saccharomycotina_3 - ps21
Phaff./Saccharomycetaceae - ps22
Saccharomyco./mycetaceae - ps23
Saccharomycetaceae - ps24
Saccharomycetaceae_2 - ps25
Saccharomycetaceae_3 - ps26
Kaz./Nau./Saccharomyces - ps27
Saccharomyces - ps28
Arboricola_clade - ps29
Cerevisiae_clade - ps30
Saccharomyces_cerevisiae - ps31
Phylostratum

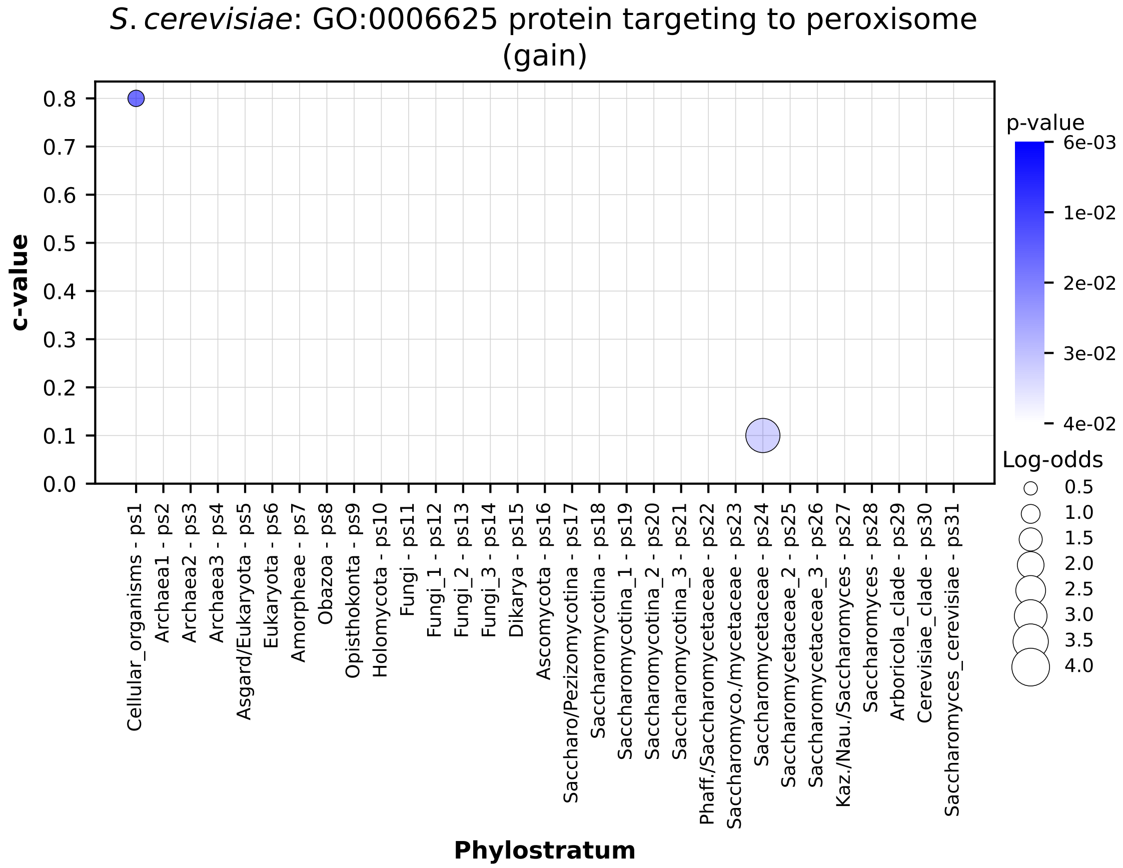

S. cerevisiae: GO:0006625 protein targeting to peroxisome (gain)
c-value
0.0
0.1
0.2
0.3
0.4
0.5
0.6
0.7
0.8
p-value
6e-03
1e-02
2e-02
3e-02
4e-02
Log-odds
0.5
1.0
1.5
2.0
2.5
3.0
3.5
4.0
Cellular_organisms - ps1
Archaea1 - ps2
Archaea2 - ps3
Archaea3 - ps4
Asgard/Eukaryota - ps5
Eukaryota - ps6
Amorpheae - ps7
Obazoa - ps8
Opisthokonta - ps9
Holomycota - ps10
Fungi - ps11
Fungi_1 - ps12
Fungi_2 - ps13
Fungi_3 - ps14
Dikarya - ps15
Ascomycota - ps16
Saccharo/Pezizomycotina - ps17
Saccharomycotina - ps18
Saccharomycotina_1 - ps19
Saccharomycotina_2 - ps20
Saccharomycotina_3 - ps21
Phaff./Saccharomycetaceae - ps22
Saccharomyco./mycetaceae - ps23
Saccharomycetaceae - ps24
Saccharomycetaceae_2 - ps25
Saccharomycetaceae_3 - ps26
Kaz./Nau./Saccharomyces - ps27
Saccharomyces - ps28
Arboricola_clade - ps29
Cerevisiae_clade - ps30
Saccharomyces_cerevisiae - ps31
Phylostratum

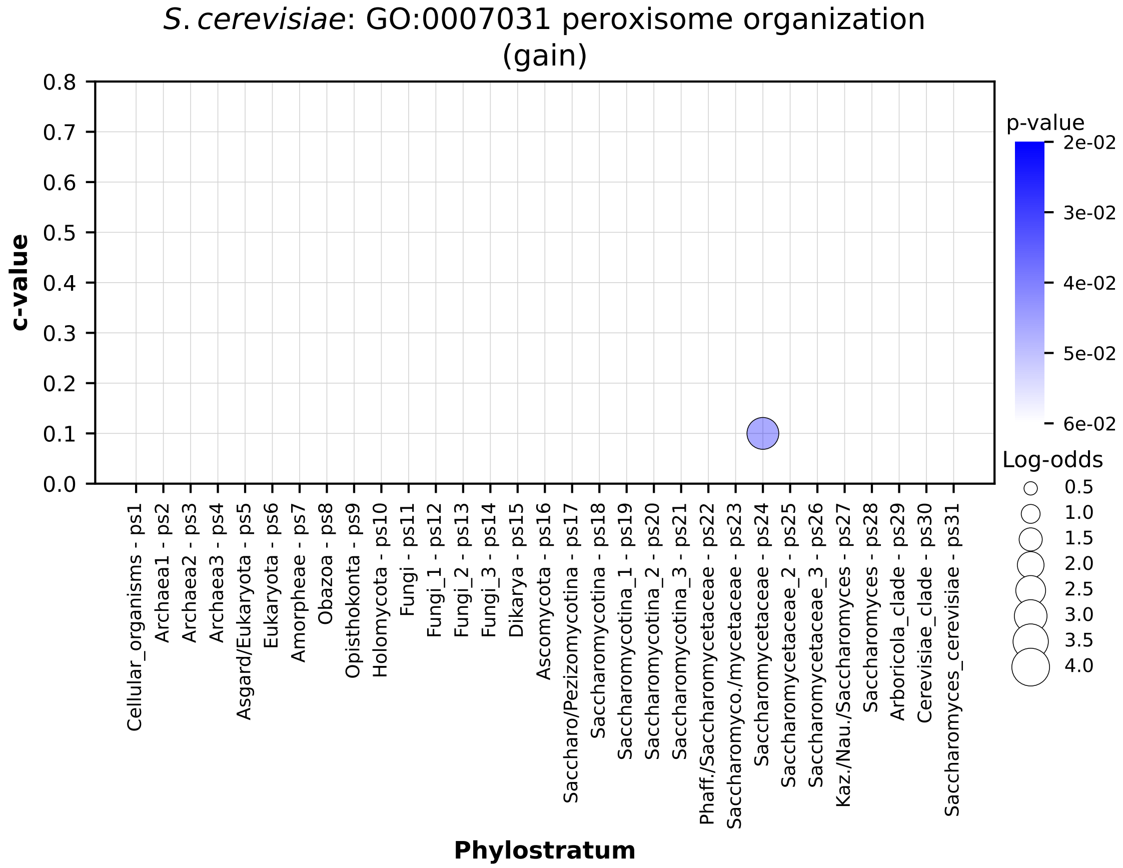

S. cerevisiae: GO:0007031 peroxisome organization (gain)
c-value
0.0
0.1
0.2
0.3
0.4
0.5
0.6
0.7
0.8
p-value
2e-02
3e-02
4e-02
5e-02
6e-02
Log-odds
0.5
1.0
1.5
2.0
2.5
3.0
3.5
4.0
Cellular_organisms - ps1
Archaea1 - ps2
Archaea2 - ps3
Archaea3 - ps4
Asgard/Eukaryota - ps5
Eukaryota - ps6
Amorpheae - ps7
Obazoa - ps8
Opisthokonta - ps9
Holomycota - ps10
Fungi - ps11
Fungi_1 - ps12
Fungi_2 - ps13
Fungi_3 - ps14
Dikarya - ps15
Ascomycota - ps16
Saccharo/Pezizomycotina - ps17
Saccharomycotina - ps18
Saccharomycotina_1 - ps19
Saccharomycotina_2 - ps20
Saccharomycotina_3 - ps21
Phaff./Saccharomycetaceae - ps22
Saccharomyco./mycetaceae - ps23
Saccharomycetaceae - ps24
Saccharomycetaceae_2 - ps25
Saccharomycetaceae_3 - ps26
Kaz./Nau./Saccharomyces - ps27
Saccharomyces - ps28
Arboricola_clade - ps29
Cerevisiae_clade - ps30
Saccharomyces_cerevisiae - ps31
Phylostratum

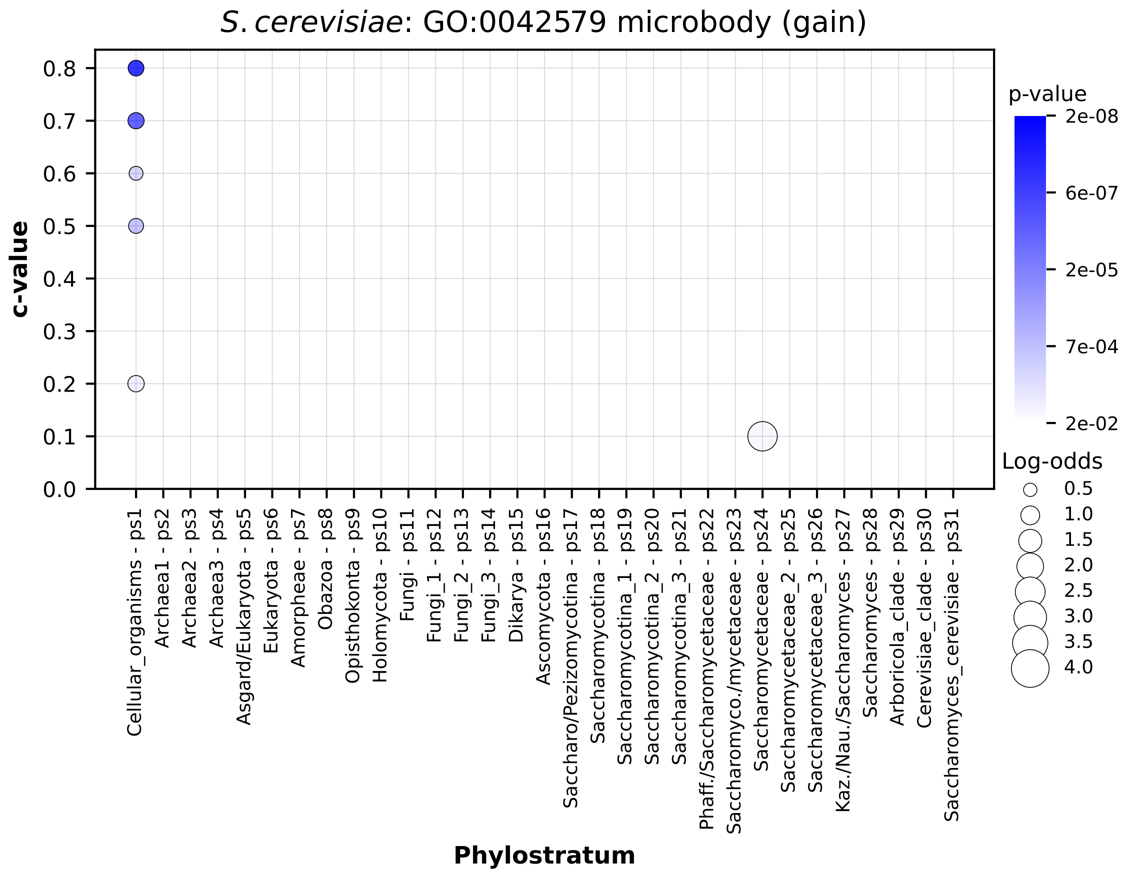

S. cerevisiae: GO:0042579 microbody (gain)
c-value
0.8
0.7
0.6
0.5
0.4
0.3
0.2
0.1
0.0
Phylostratum
Cellular_organisms - ps1
Archaea1 - ps2
Archaea2 - ps3
Archaea3 - ps4
Asgard/Eukaryota - ps5
Eukaryota - ps6
Amorpheae - ps7
Obazoa - ps8
Opisthokonta - ps9
Holomycota - ps10
Fungi - ps11
Fungi_1 - ps12
Fungi_2 - ps13
Fungi_3 - ps14
Dikarya - ps15
Ascomycota - ps16
Saccharo/Pezizomycotina - ps17
Saccharomycotina - ps18
Saccharomycotina_1 - ps19
Saccharomycotina_2 - ps20
Saccharomycotina_3 - ps21
Phaff./Saccharomycetaceae - ps22
Saccharomyco./mycetaceae - ps23
Saccharomycetaceae - ps24
Saccharomycetaceae_2 - ps25
Saccharomycetaceae_3 - ps26
Kaz./Nau./Saccharomyces - ps27
Saccharomyces - ps28
Arboricola_clade - ps29
Cerevisiae_clade - ps30
Saccharomyces_cerevisiae - ps31
p-value
2e-08
6e-07
2e-05
7e-04
2e-02
Log-odds
0.5
1.0
1.5
2.0
2.5
3.0
3.5
4.0

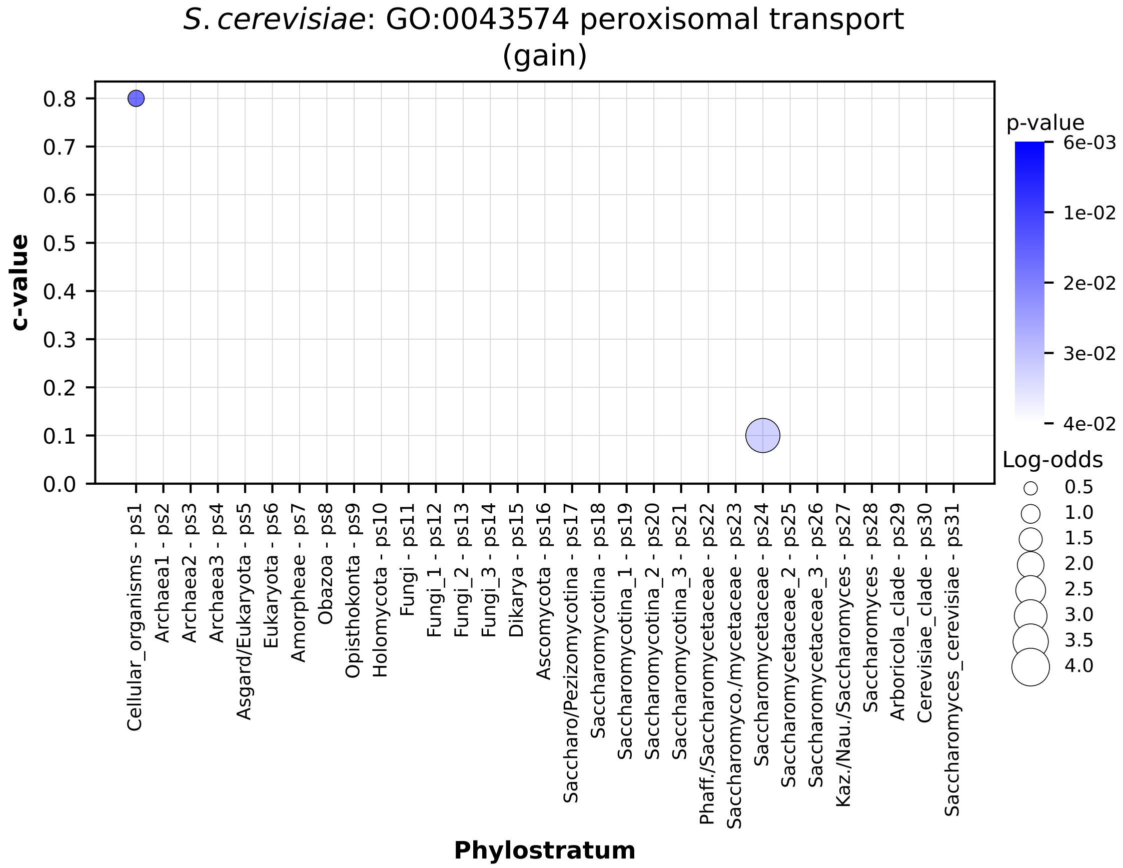

S. cerevisiae: GO:0043574 peroxisomal transport
(gain)
c-value
0.0
0.1
0.2
0.3
0.4
0.5
0.6
0.7
0.8
p-value
6e-03
1e-02
2e-02
3e-02
4e-02
Log-odds
0.5
1.0
1.5
2.0
2.5
3.0
3.5
4.0
Cellular_organisms - ps1
Archaea1 - ps2
Archaea2 - ps3
Archaea3 - ps4
Asgard/Eukaryota - ps5
Eukaryota - ps6
Amorpheae - ps7
Obazoa - ps8
Opisthokonta - ps9
Holomycota - ps10
Fungi - ps11
Fungi_1 - ps12
Fungi_2 - ps13
Fungi_3 - ps14
Dikarya - ps15
Ascomycota - ps16
Saccharo/Pezizomycotina - ps17
Saccharomycotina - ps18
Saccharomycotina_1 - ps19
Saccharomycotina_2 - ps20
Saccharomycotina_3 - ps21
Phaff./Saccharomycetaceae - ps22
Saccharomyco./mycetaceae - ps23
Saccharomycetaceae - ps24
Saccharomycetaceae_2 - ps25
Saccharomycetaceae_3 - ps26
Kaz./Nau./Saccharomyces - ps27
Saccharomyces - ps28
Arboricola_clade - ps29
Cerevisiae_clade - ps30
Saccharomyces_cerevisiae - ps31
Phylostratum

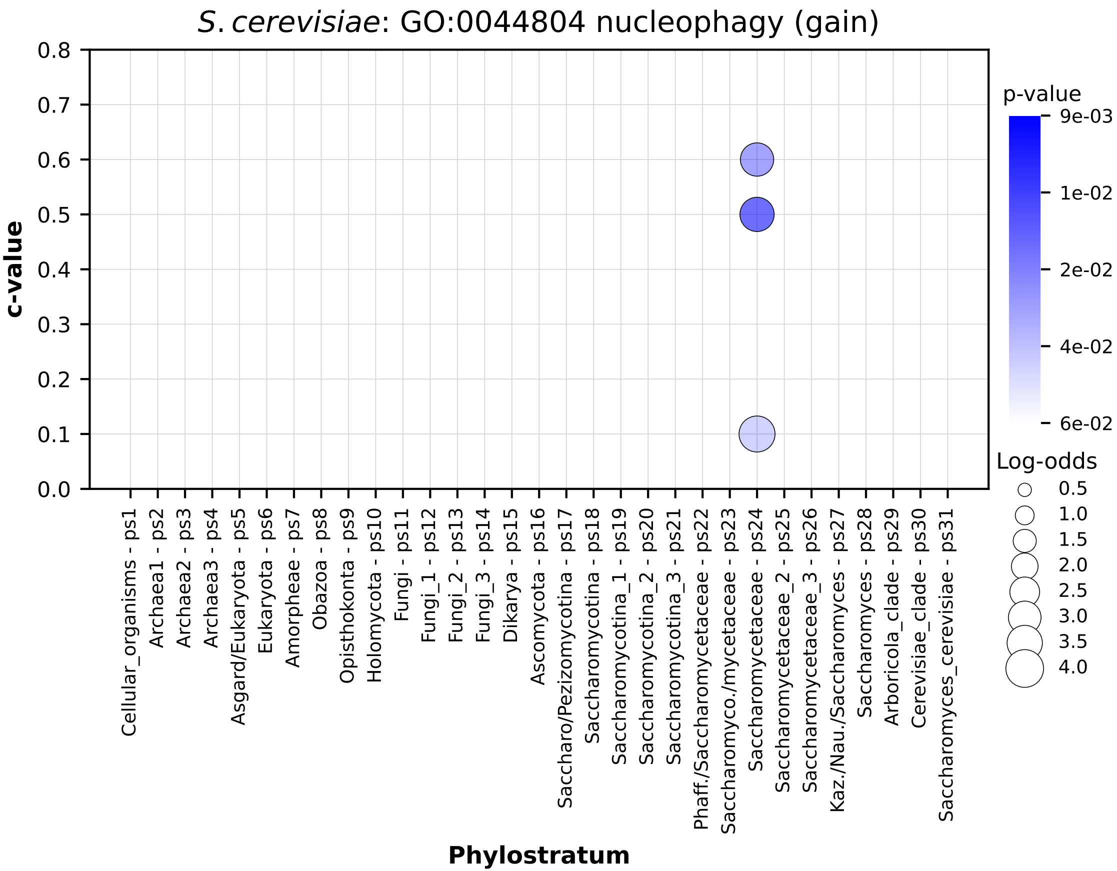
S. cerevisiae: GO:0044804 nucleophagy (gain)
c-value
0.0
0.1
0.2
0.3
0.4
0.5
0.6
0.7
0.8
p-value
9e-03
1e-02
2e-02
4e-02
6e-02
Log-odds
0.5
1.0
1.5
2.0
2.5
3.0
3.5
4.0
Cellular_organisms - ps1
Archaea1 - ps2
Archaea2 - ps3
Archaea3 - ps4
Asgard/Eukaryota - ps5
Eukaryota - ps6
Amorpheae - ps7
Obazoa - ps8
Opisthokonta - ps9
Holomycota - ps10
Fungi - ps11
Fungi_1 - ps12
Fungi_2 - ps13
Fungi_3 - ps14
Dikarya - ps15
Ascomycota - ps16
Saccharo/Pezizomycotina - ps17
Saccharomycotina - ps18
Saccharomycotina_1 - ps19
Saccharomycotina_2 - ps20
Saccharomycotina_3 - ps21
Phaff./Saccharomycetaceae - ps22
Saccharomyco./mycetaceae - ps23
Saccharomycetaceae - ps24
Saccharomycetaceae_2 - ps25
Saccharomycetaceae_3 - ps26
Kaz./Nau./Saccharomyces - ps27
Saccharomyces - ps28
Arboricola_clade - ps29
Cerevisiae_clade - ps30
Saccharomyces_cerevisiae - ps31
Phylostratum

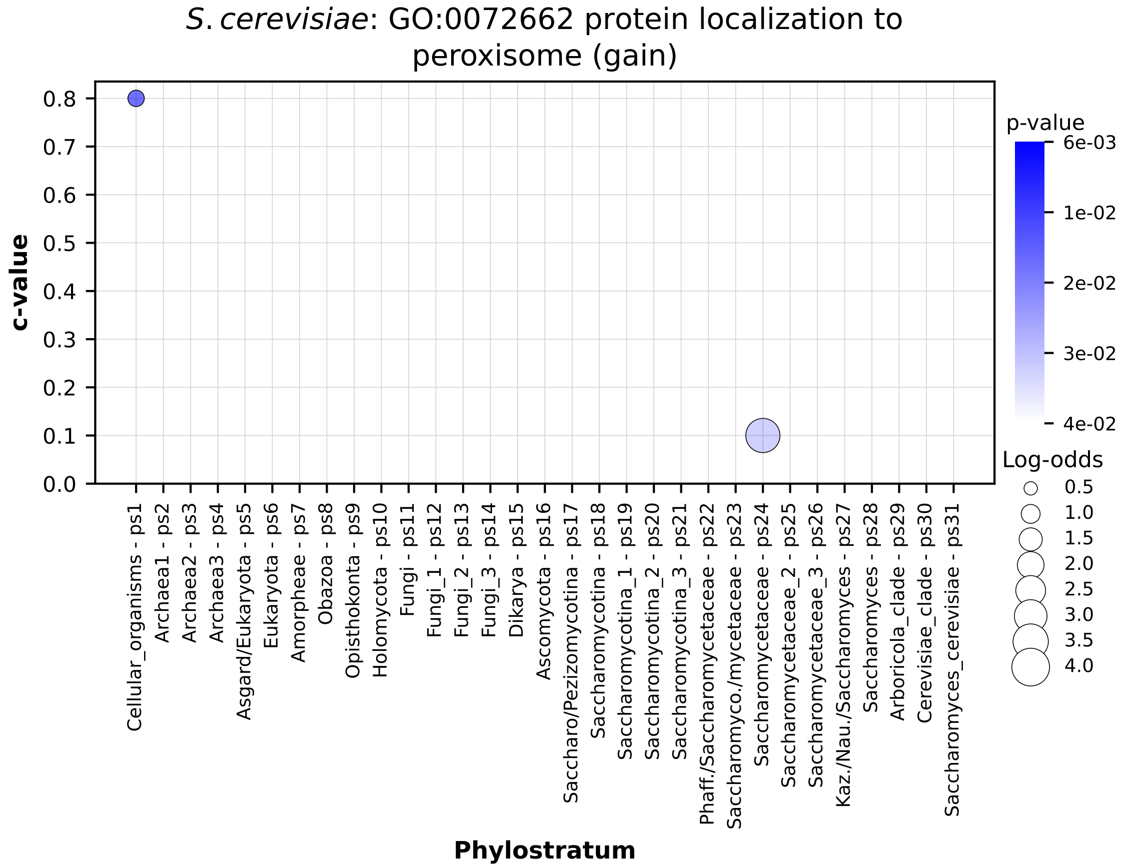

S. cerevisiae: GO:0072662 protein localization to peroxisome (gain)
c-value
0.0
0.1
0.2
0.3
0.4
0.5
0.6
0.7
0.8
p-value
6e-03
1e-02
2e-02
3e-02
4e-02
Log-odds
0.5
1.0
1.5
2.0
2.5
3.0
3.5
4.0
Cellular_organisms - ps1
Archaea1 - ps2
Archaea2 - ps3
Archaea3 - ps4
Asgard/Eukaryota - ps5
Eukaryota - ps6
Amorpheae - ps7
Obazoa - ps8
Opisthokonta - ps9
Holomycota - ps10
Fungi - ps11
Fungi_1 - ps12
Fungi_2 - ps13
Fungi_3 - ps14
Dikarya - ps15
Ascomycota - ps16
Saccharo/Pezizomycotina - ps17
Saccharomycotina - ps18
Saccharomycotina_1 - ps19
Saccharomycotina_2 - ps20
Saccharomycotina_3 - ps21
Phaff./Saccharomycetaceae - ps22
Saccharomyco./mycetaceae - ps23
Saccharomycetaceae - ps24
Saccharomycetaceae_2 - ps25
Saccharomycetaceae_3 - ps26
Kaz./Nau./Saccharomyces - ps27
Saccharomyces - ps28
Arboricola_clade - ps29
Cerevisiae_clade - ps30
Saccharomyces_cerevisiae - ps31
Phylostratum

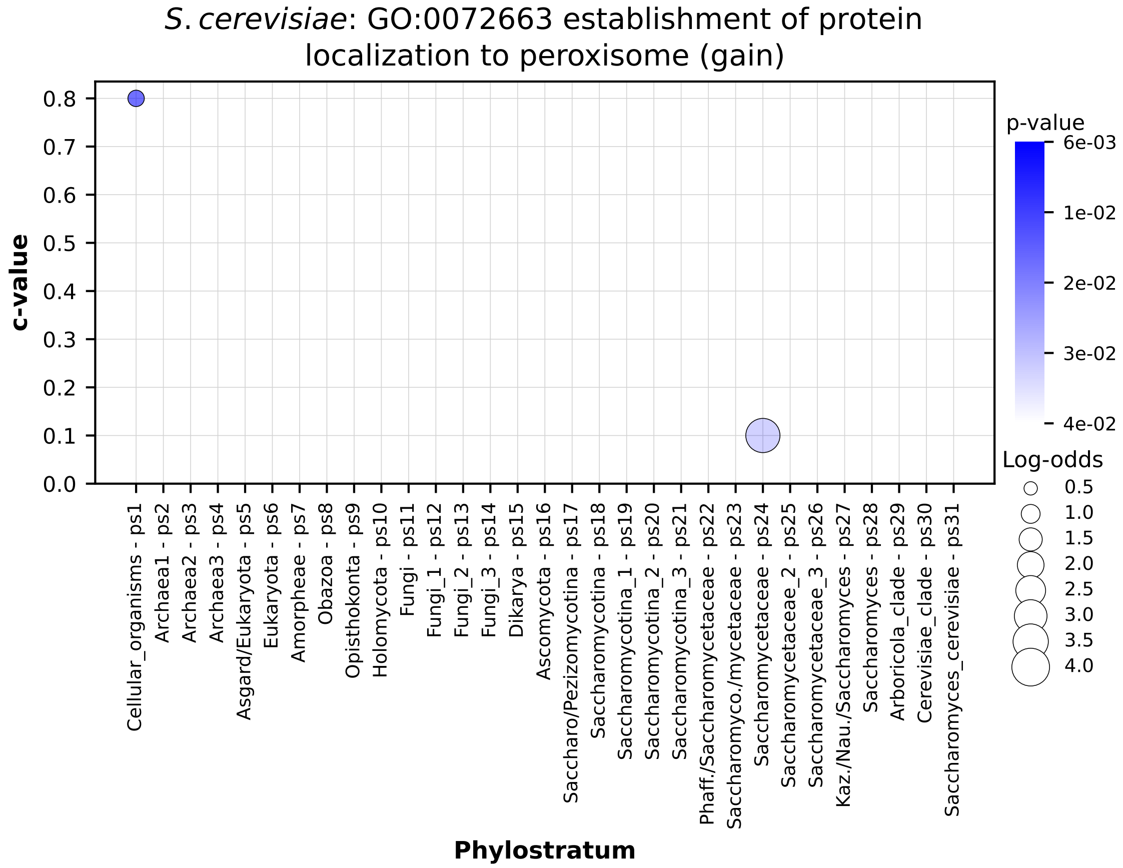

S. cerevisiae: GO:0072663 establishment of protein localization to peroxisome (gain)
c-value
0.0
0.1
0.2
0.3
0.4
0.5
0.6
0.7
0.8
p-value
6e-03
1e-02
2e-02
3e-02
4e-02
Log-odds
0.5
1.0
1.5
2.0
2.5
3.0
3.5
4.0
Cellular_organisms - ps1
Archaea1 - ps2
Archaea2 - ps3
Archaea3 - ps4
Asgard/Eukaryota - ps5
Eukaryota - ps6
Amorpheae - ps7
Obazoa - ps8
Opisthokonta - ps9
Holomycota - ps10
Fungi - ps11
Fungi_1 - ps12
Fungi_2 - ps13
Fungi_3 - ps14
Dikarya - ps15
Ascomycota - ps16
Saccharo/Pezizomycotina - ps17
Saccharomycotina - ps18
Saccharomycotina_1 - ps19
Saccharomycotina_2 - ps20
Saccharomycotina_3 - ps21
Phaff./Saccharomycetaceae - ps22
Saccharomyco./mycetaceae - ps23
Saccharomycetaceae - ps24
Saccharomycetaceae_2 - ps25
Saccharomycetaceae_3 - ps26
Kaz./Nau./Saccharomyces - ps27
Saccharomyces - ps28
Arboricola_clade - ps29
Cerevisiae_clade - ps30
Saccharomyces_cerevisiae - ps31
Phylostratum

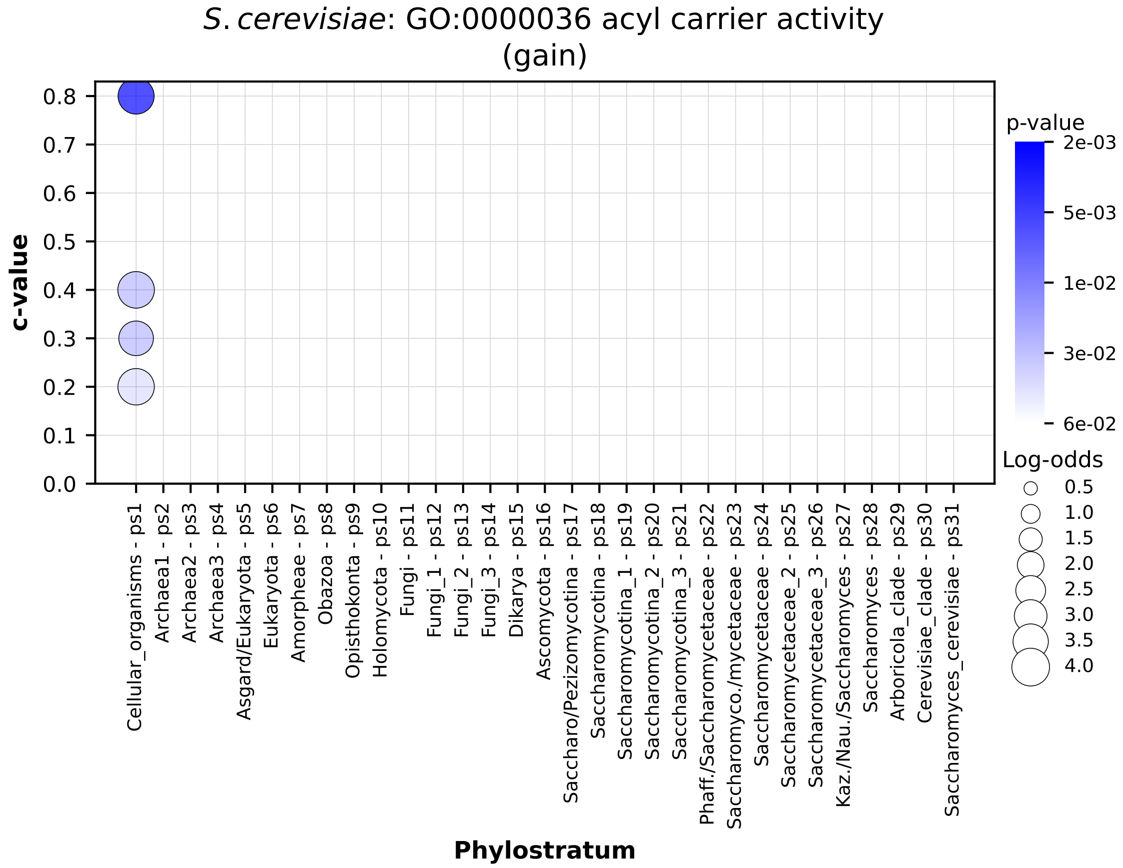

S. cerevisiae: GO:0000036 acyl carrier activity (gain)
c-value
0.0
0.1
0.2
0.3
0.4
0.5
0.6
0.7
0.8
Phylostratum
Cellular_organisms - ps1
Archaea1 - ps2
Archaea2 - ps3
Archaea3 - ps4
Asgard/Eukaryota - ps5
Eukaryota - ps6
Amorpheae - ps7
Obazoa - ps8
Opisthokonta - ps9
Holomycota - ps10
Fungi - ps11
Fungi_1 - ps12
Fungi_2 - ps13
Fungi_3 - ps14
Dikarya - ps15
Ascomycota - ps16
Saccharo/Pezizomycotina - ps17
Saccharomycotina - ps18
Saccharomycotina_1 - ps19
Saccharomycotina_2 - ps20
Saccharomycotina_3 - ps21
Phaff./Saccharomycetaceae - ps22
Saccharomyco./mycetaceae - ps23
Saccharomycetaceae - ps24
Saccharomycetaceae_2 - ps25
Saccharomycetaceae_3 - ps26
Kaz./Nau./Saccharomyces - ps27
Saccharomyces - ps28
Arboricola_clade - ps29
Cerevisiae_clade - ps30
Saccharomyces_cerevisiae - ps31
p-value
2e-03
5e-03
1e-02
3e-02
6e-02
Log-odds
0.5
1.0
1.5
2.0
2.5
3.0
3.5
4.0

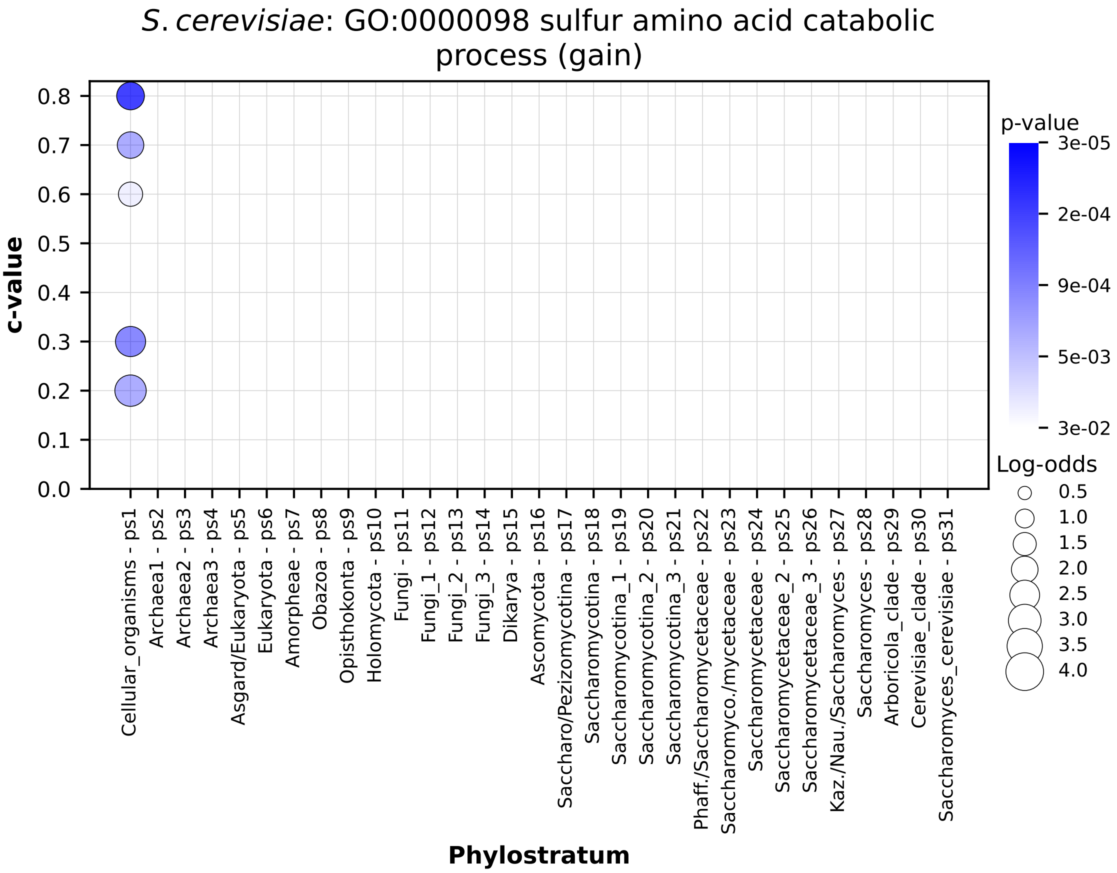

S. cerevisiae: GO:0000098 sulfur amino acid catabolic process (gain)
c-value
0.0
0.1
0.2
0.3
0.4
0.5
0.6
0.7
0.8
Phylostratum
Cellular_organisms - ps1
Archaea1 - ps2
Archaea2 - ps3
Archaea3 - ps4
Asgard/Eukaryota - ps5
Eukaryota - ps6
Amorpheae - ps7
Obazoa - ps8
Opisthokonta - ps9
Holomycota - ps10
Fungi - ps11
Fungi_1 - ps12
Fungi_2 - ps13
Fungi_3 - ps14
Dikarya - ps15
Ascomycota - ps16
Saccharo/Pezizomycotina - ps17
Saccharomycotina - ps18
Saccharomycotina_1 - ps19
Saccharomycotina_2 - ps20
Saccharomycotina_3 - ps21
Phaff./Saccharomycetaceae - ps22
Saccharomyco./mycetaceae - ps23
Saccharomycetaceae - ps24
Saccharomycetaceae_2 - ps25
Saccharomycetaceae_3 - ps26
Kaz./Nau./Saccharomyces - ps27
Saccharomyces - ps28
Arboricola_clade - ps29
Cerevisiae_clade - ps30
Saccharomyces_cerevisiae - ps31
p-value
3e-05
2e-04
9e-04
5e-03
3e-02
Log-odds
0.5
1.0
1.5
2.0
2.5
3.0
3.5
4.0

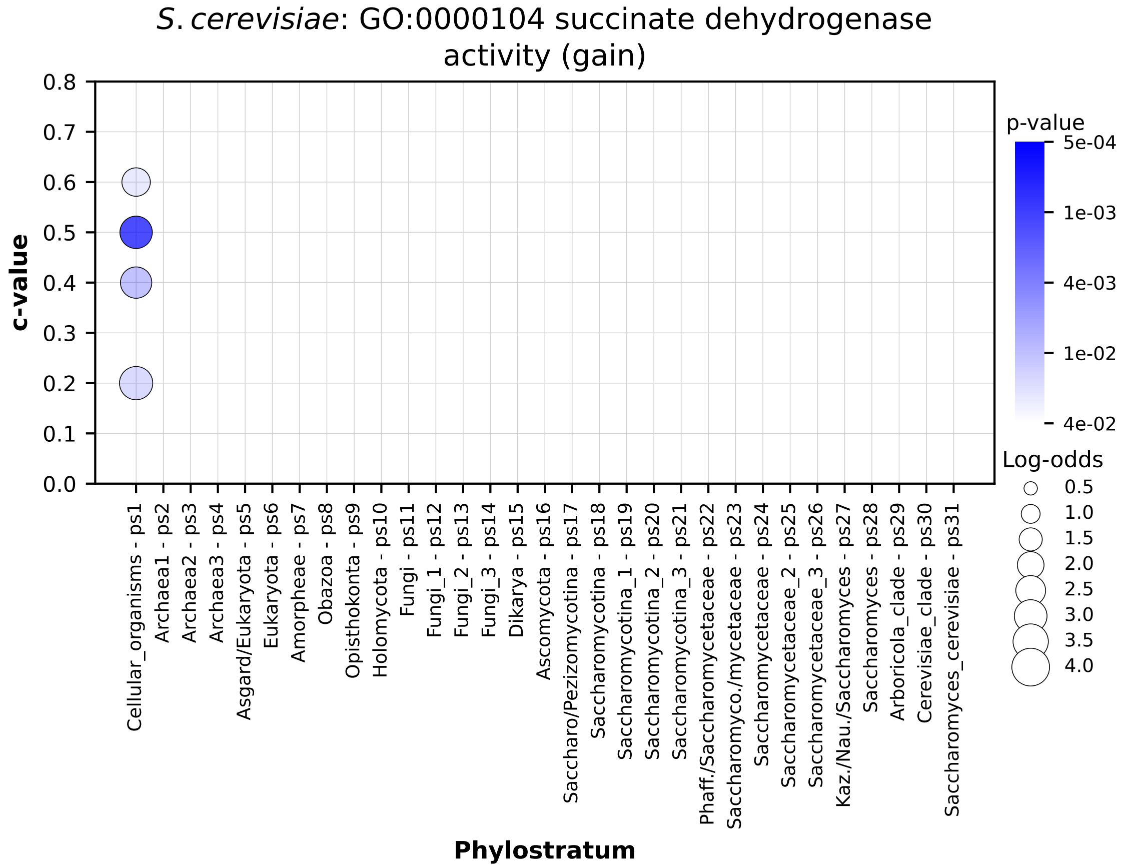

S. cerevisiae: GO:0000104 succinate dehydrogenase activity (gain)
c-value
0.8
0.7
0.6
0.5
0.4
0.3
0.2
0.1
0.0
Phylostratum
Cellular_organisms - ps1
Archaea1 - ps2
Archaea2 - ps3
Archaea3 - ps4
Asgard/Eukaryota - ps5
Eukaryota - ps6
Amorpheae - ps7
Obazoa - ps8
Opisthokonta - ps9
Holomycota - ps10
Fungi - ps11
Fungi_1 - ps12
Fungi_2 - ps13
Fungi_3 - ps14
Dikarya - ps15
Ascomycota - ps16
Saccharo/Pezizomycotina - ps17
Saccharomycotina - ps18
Saccharomycotina_1 - ps19
Saccharomycotina_2 - ps20
Saccharomycotina_3 - ps21
Phaff./Saccharomycetaceae - ps22
Saccharomyco./mycetaceae - ps23
Saccharomycetaceae - ps24
Saccharomycetaceae_2 - ps25
Saccharomycetaceae_3 - ps26
Kaz./Nau./Saccharomyces - ps27
Saccharomyces - ps28
Arboricola_clade - ps29
Cerevisiae_clade - ps30
Saccharomyces_cerevisiae - ps31
p-value
5e-04
1e-03
4e-03
1e-02
4e-02
Log-odds
0.5
1.0
1.5
2.0
2.5
3.0
3.5
4.0

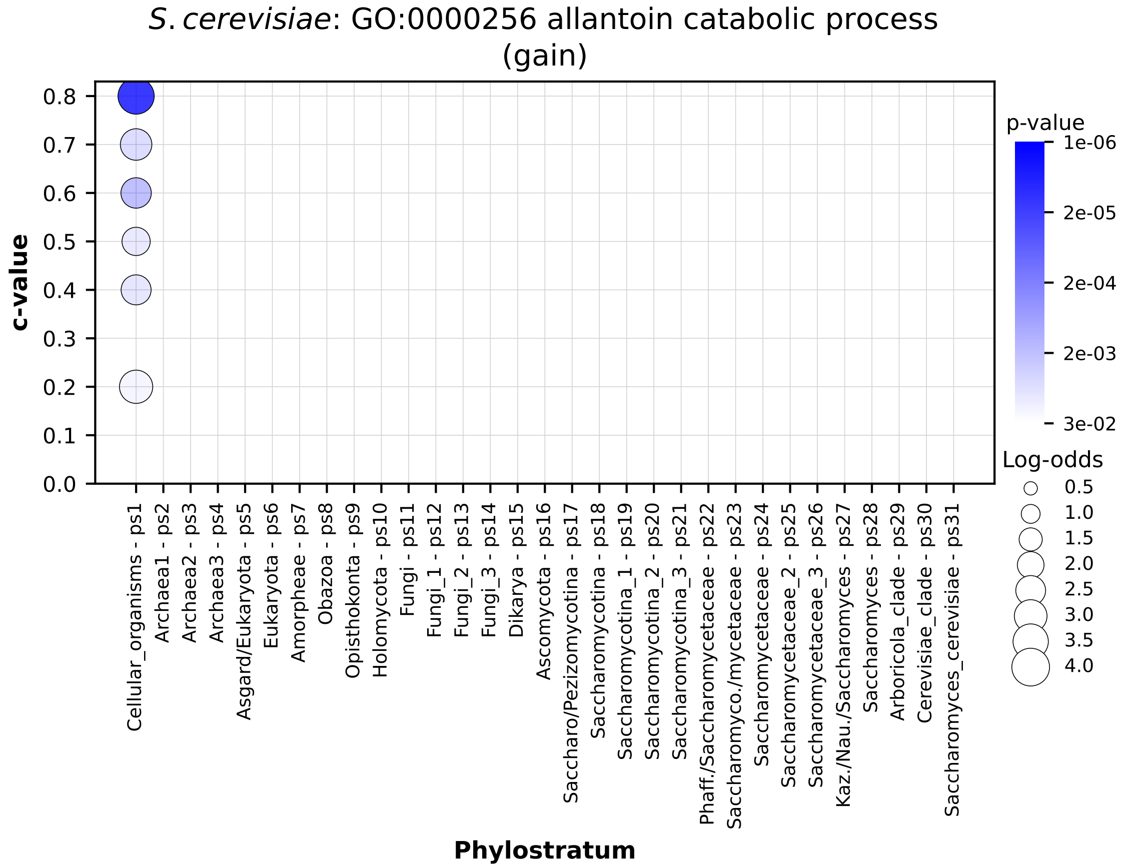

S. cerevisiae: GO:0000256 allantoin catabolic process (gain)
c-value
0.0
0.1
0.2
0.3
0.4
0.5
0.6
0.7
0.8
p-value
1e-06
2e-05
2e-04
2e-03
3e-02
Log-odds
0.5
1.0
1.5
2.0
2.5
3.0
3.5
4.0
Cellular_organisms - ps1
Archaea1 - ps2
Archaea2 - ps3
Archaea3 - ps4
Asgard/Eukaryota - ps5
Eukaryota - ps6
Amorpheae - ps7
Obazoa - ps8
Opisthokonta - ps9
Holomycota - ps10
Fungi - ps11
Fungi_1 - ps12
Fungi_2 - ps13
Fungi_3 - ps14
Dikarya - ps15
Ascomycota - ps16
Saccharo/Pezizomycotina - ps17
Saccharomycotina - ps18
Saccharomycotina_1 - ps19
Saccharomycotina_2 - ps20
Saccharomycotina_3 - ps21
Phaff./Saccharomycetaceae - ps22
Saccharomyco./mycetaceae - ps23
Saccharomycetaceae - ps24
Saccharomycetaceae_2 - ps25
Saccharomycetaceae_3 - ps26
Kaz./Nau./Saccharomyces - ps27
Saccharomyces - ps28
Arboricola_clade - ps29
Cerevisiae_clade - ps30
Saccharomyces_cerevisiae - ps31
Phylostratum

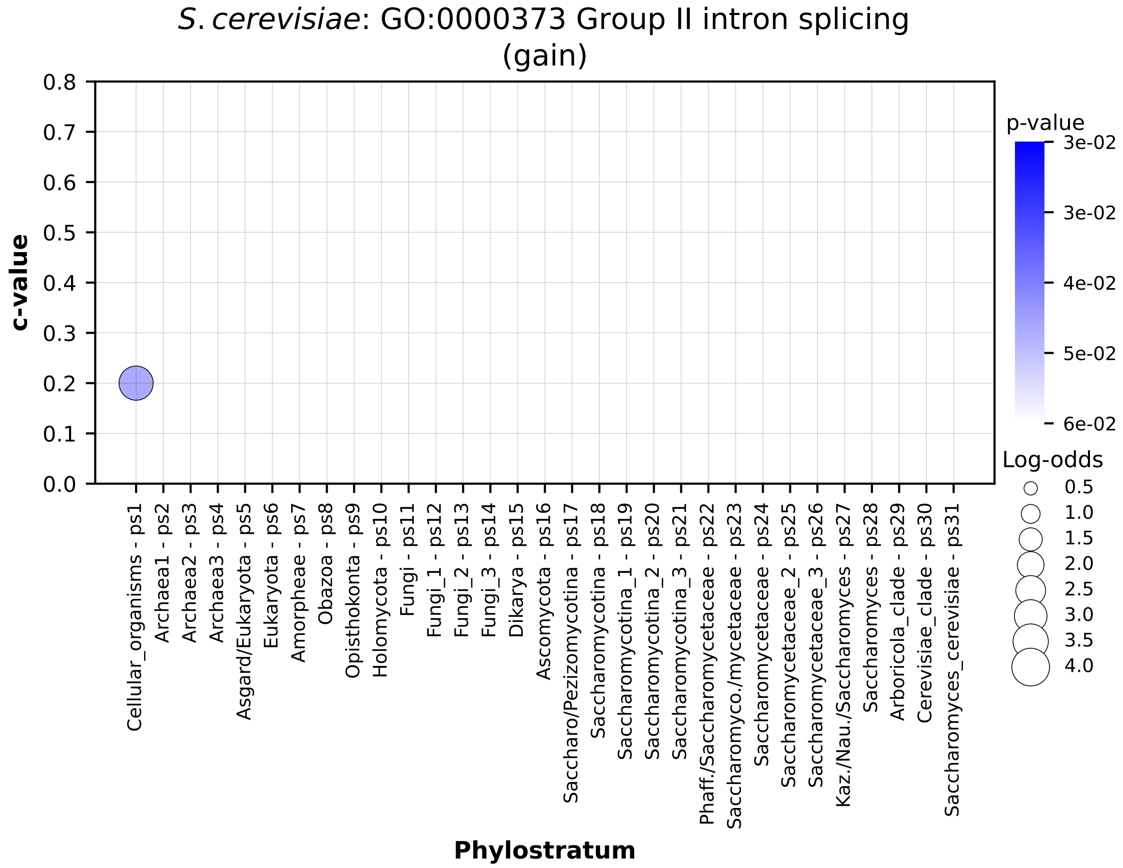
S. cerevisiae: GO:0000373 Group II intron splicing
(gain)
c-value
0.0
0.1
0.2
0.3
0.4
0.5
0.6
0.7
0.8
p-value
3e-02
3e-02
4e-02
5e-02
6e-02
Log-odds
0.5
1.0
1.5
2.0
2.5
3.0
3.5
4.0
Cellular_organisms - ps1
Archaea1 - ps2
Archaea2 - ps3
Archaea3 - ps4
Asgard/Eukaryota - ps5
Eukaryota - ps6
Amorpheae - ps7
Obazoa - ps8
Opisthokonta - ps9
Holomycota - ps10
Fungi - ps11
Fungi_1 - ps12
Fungi_2 - ps13
Fungi_3 - ps14
Dikarya - ps15
Ascomycota - ps16
Saccharo/Pezizomycotina - ps17
Saccharomycotina - ps18
Saccharomycotina_1 - ps19
Saccharomycotina_2 - ps20
Saccharomycotina_3 - ps21
Phaff./Saccharomycetaceae - ps22
Saccharomyco./mycetaceae - ps23
Saccharomycetaceae - ps24
Saccharomycetaceae_2 - ps25
Saccharomycetaceae_3 - ps26
Kaz./Nau./Saccharomyces - ps27
Saccharomyces - ps28
Arboricola_clade - ps29
Cerevisiae_clade - ps30
Saccharomyces_cerevisiae - ps31
Phylostratum

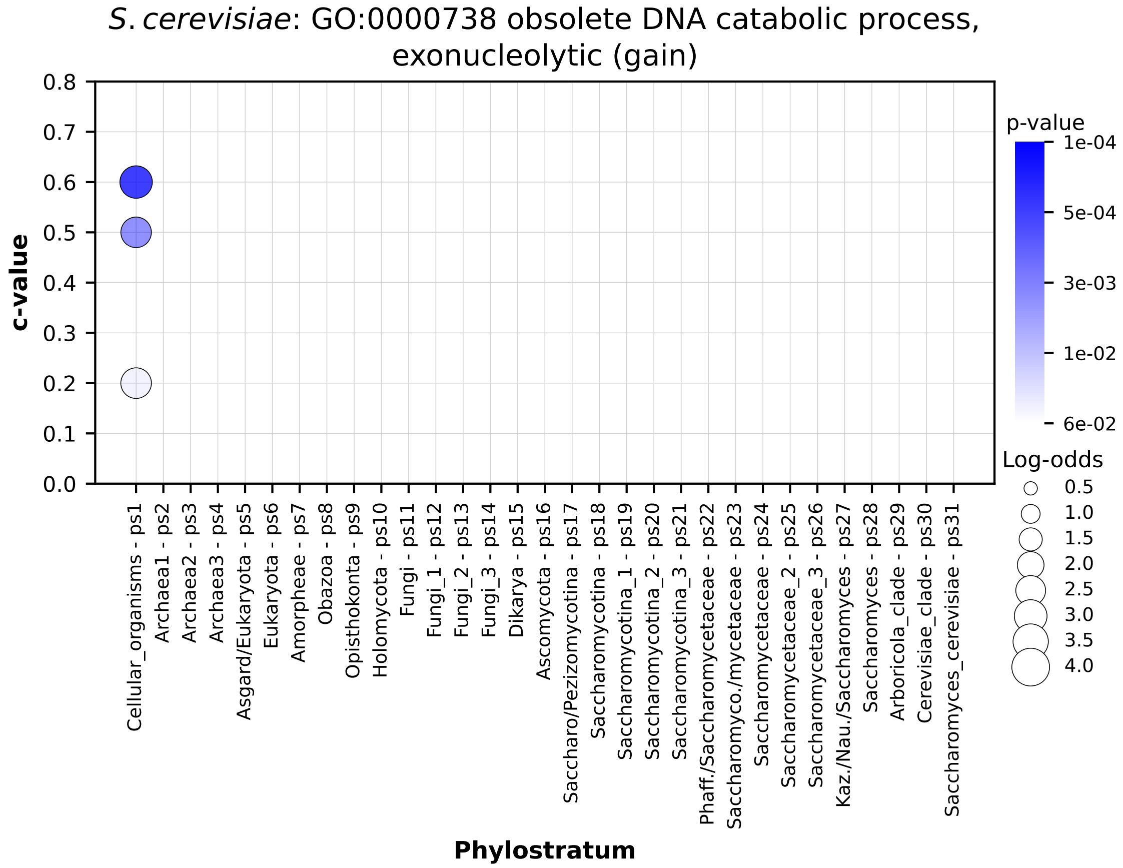

S. cerevisiae: GO:0000738 obsolete DNA catabolic process, exonucleolytic (gain)
c-value
0.8
0.7
0.6
0.5
0.4
0.3
0.2
0.1
0.0
p-value
1e-04
5e-04
3e-03
1e-02
6e-02
Log-odds
0.5
1.0
1.5
2.0
2.5
3.0
3.5
4.0
Cellular_organisms - ps1
Archaea1 - ps2
Archaea2 - ps3
Archaea3 - ps4
Asgard/Eukaryota - ps5
Eukaryota - ps6
Amorpheae - ps7
Obazoa - ps8
Opisthokonta - ps9
Holomycota - ps10
Fungi - ps11
Fungi_1 - ps12
Fungi_2 - ps13
Fungi_3 - ps14
Dikarya - ps15
Ascomycota - ps16
Saccharo/Pezizomycotina - ps17
Saccharomycotina - ps18
Saccharomycotina_1 - ps19
Saccharomycotina_2 - ps20
Saccharomycotina_3 - ps21
Phaff./Saccharomycetaceae - ps22
Saccharomyco./mycetaceae - ps23
Saccharomycetaceae - ps24
Saccharomycetaceae_2 - ps25
Saccharomycetaceae_3 - ps26
Kaz./Nau./Saccharomyces - ps27
Saccharomyces - ps28
Arboricola_clade - ps29
Cerevisiae_clade - ps30
Saccharomyces_cerevisiae - ps31
Phylostratum

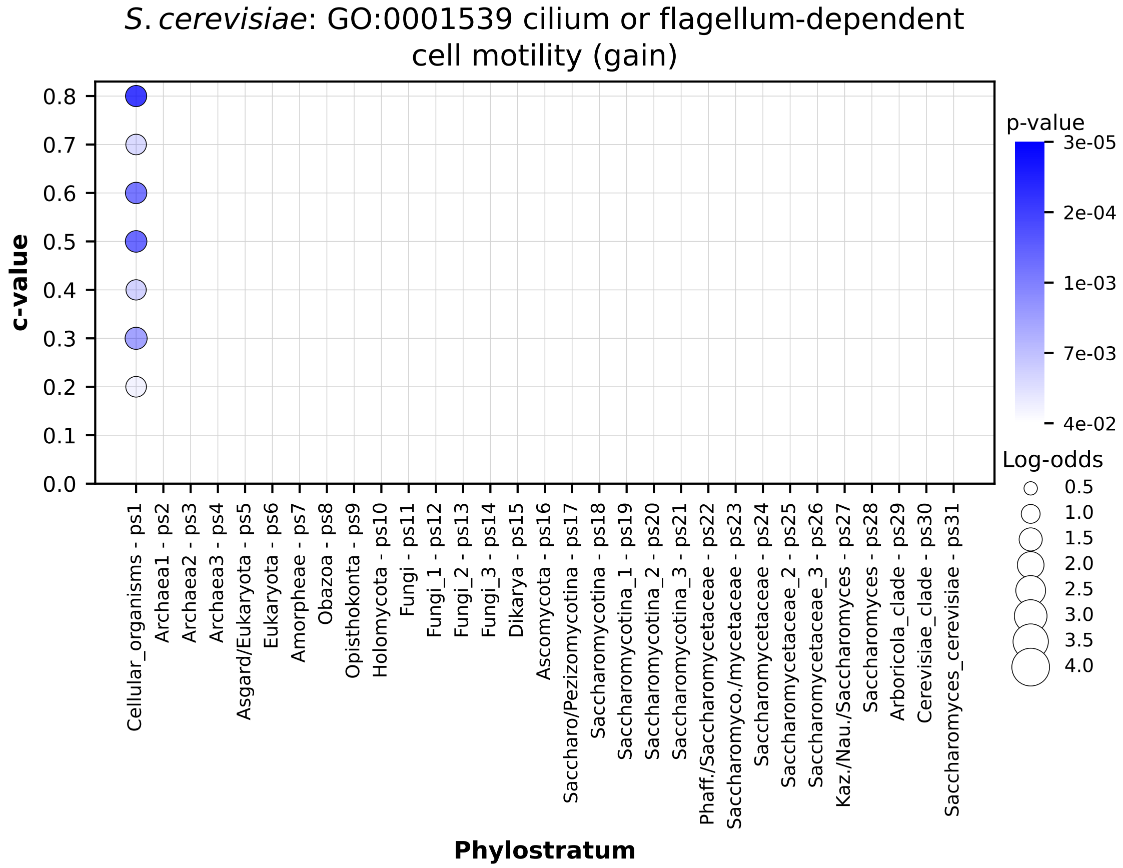

S. cerevisiae: GO:0001539 cilium or flagellum-dependent cell motility (gain)
c-value
0.8
0.7
0.6
0.5
0.4
0.3
0.2
0.1
0.0
p-value
3e-05
2e-04
1e-03
7e-03
4e-02
Log-odds
0.5
1.0
1.5
2.0
2.5
3.0
3.5
4.0
Cellular_organisms - ps1
Archaea1 - ps2
Archaea2 - ps3
Archaea3 - ps4
Asgard/Eukaryota - ps5
Eukaryota - ps6
Amorpheae - ps7
Obazoa - ps8
Opisthokonta - ps9
Holomycota - ps10
Fungi - ps11
Fungi_1 - ps12
Fungi_2 - ps13
Fungi_3 - ps14
Dikarya - ps15
Ascomycota - ps16
Saccharo/Pezizomycotina - ps17
Saccharomycotina - ps18
Saccharomycotina_1 - ps19
Saccharomycotina_2 - ps20
Saccharomycotina_3 - ps21
Phaff./Saccharomycetaceae - ps22
Saccharomyco./mycetaceae - ps23
Saccharomycetaceae - ps24
Saccharomycetaceae_2 - ps25
Saccharomycetaceae_3 - ps26
Kaz./Nau./Saccharomyces - ps27
Saccharomyces - ps28
Arboricola_clade - ps29
Cerevisiae_clade - ps30
Saccharomyces_cerevisiae - ps31
Phylostratum

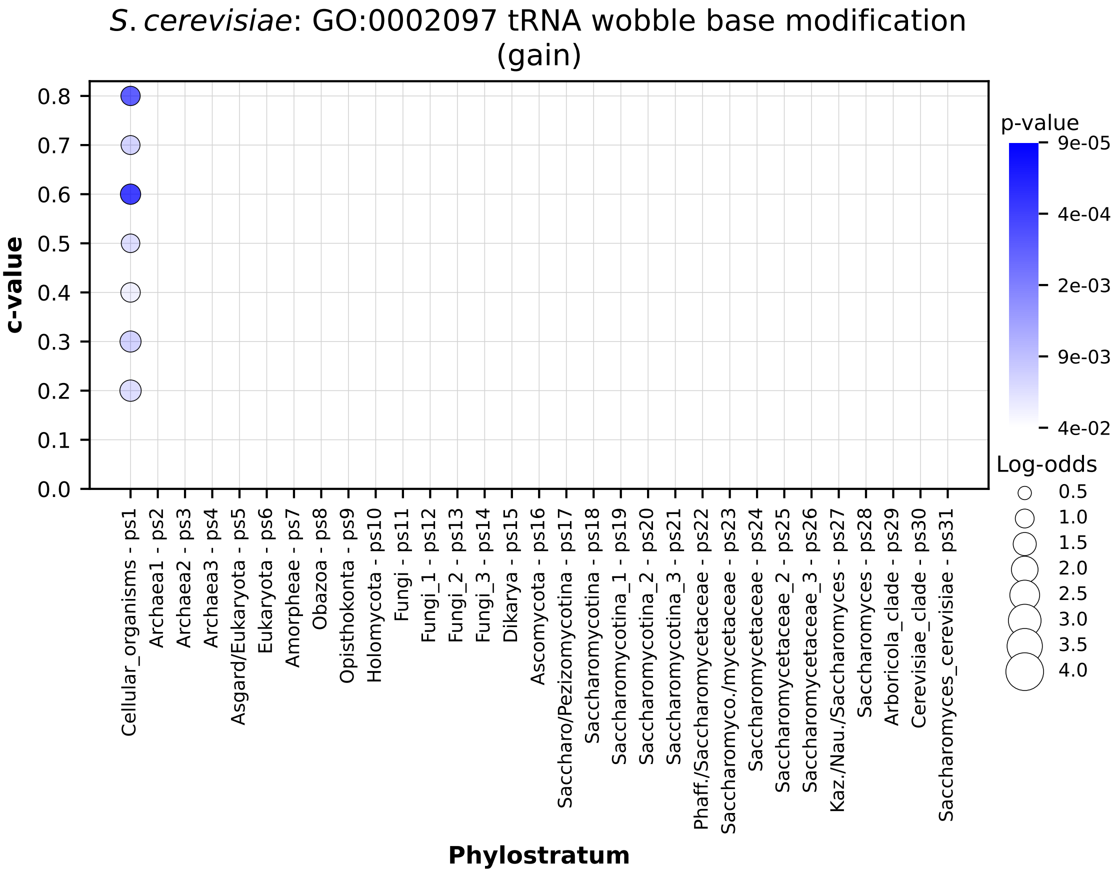

S. cerevisiae: GO:0002097 tRNA wobble base modification (gain)
c-value
0.0
0.1
0.2
0.3
0.4
0.5
0.6
0.7
0.8
Phylostratum
Cellular_organisms - ps1
Archaea1 - ps2
Archaea2 - ps3
Archaea3 - ps4
Asgard/Eukaryota - ps5
Eukaryota - ps6
Amorpheae - ps7
Obazoa - ps8
Opisthokonta - ps9
Holomycota - ps10
Fungi - ps11
Fungi_1 - ps12
Fungi_2 - ps13
Fungi_3 - ps14
Dikarya - ps15
Ascomycota - ps16
Saccharo/Pezizomycotina - ps17
Saccharomycotina - ps18
Saccharomycotina_1 - ps19
Saccharomycotina_2 - ps20
Saccharomycotina_3 - ps21
Phaff./Saccharomycetaceae - ps22
Saccharomyco./mycetaceae - ps23
Saccharomycetaceae - ps24
Saccharomycetaceae_2 - ps25
Saccharomycetaceae_3 - ps26
Kaz./Nau./Saccharomyces - ps27
Saccharomyces - ps28
Arboricola_clade - ps29
Cerevisiae_clade - ps30
Saccharomyces_cerevisiae - ps31
p-value
9e-05
4e-04
2e-03
9e-03
4e-02
Log-odds
0.5
1.0
1.5
2.0
2.5
3.0
3.5
4.0

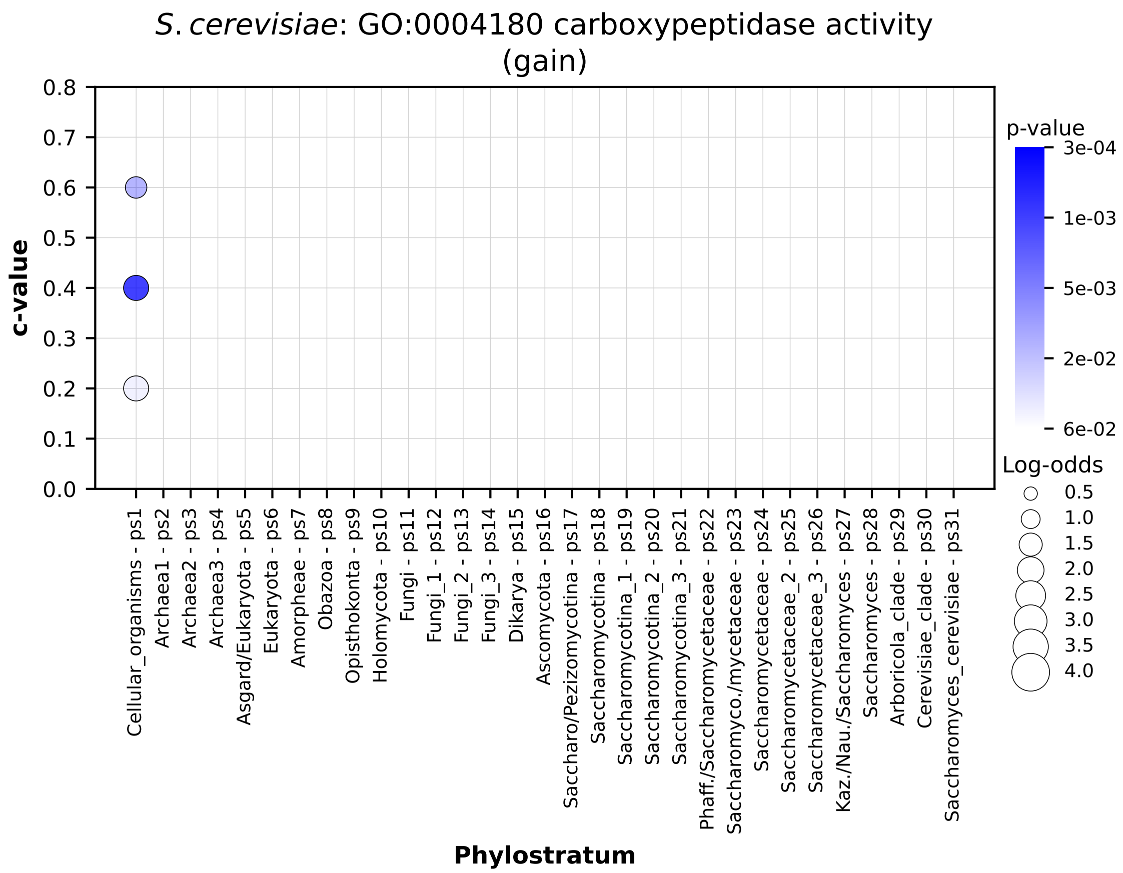

S. cerevisiae: GO:0004180 carboxypeptidase activity (gain)
c-value
0.0
0.1
0.2
0.3
0.4
0.5
0.6
0.7
0.8
Phylostratum
Cellular_organisms - ps1
Archaea1 - ps2
Archaea2 - ps3
Archaea3 - ps4
Asgard/Eukaryota - ps5
Eukaryota - ps6
Amorpheae - ps7
Obazoa - ps8
Opisthokonta - ps9
Holomycota - ps10
Fungi - ps11
Fungi_1 - ps12
Fungi_2 - ps13
Fungi_3 - ps14
Dikarya - ps15
Ascomycota - ps16
Saccharo/Pezizomycotina - ps17
Saccharomycotina - ps18
Saccharomycotina_1 - ps19
Saccharomycotina_2 - ps20
Saccharomycotina_3 - ps21
Phaff./Saccharomycetaceae - ps22
Saccharomyco./mycetaceae - ps23
Saccharomycetaceae - ps24
Saccharomycetaceae_2 - ps25
Saccharomycetaceae_3 - ps26
Kaz./Nau./Saccharomyces - ps27
Saccharomyces - ps28
Arboricola_clade - ps29
Cerevisiae_clade - ps30
Saccharomyces_cerevisiae - ps31
p-value
3e-04
1e-03
5e-03
2e-02
6e-02
Log-odds
0.5
1.0
1.5
2.0
2.5
3.0
3.5
4.0

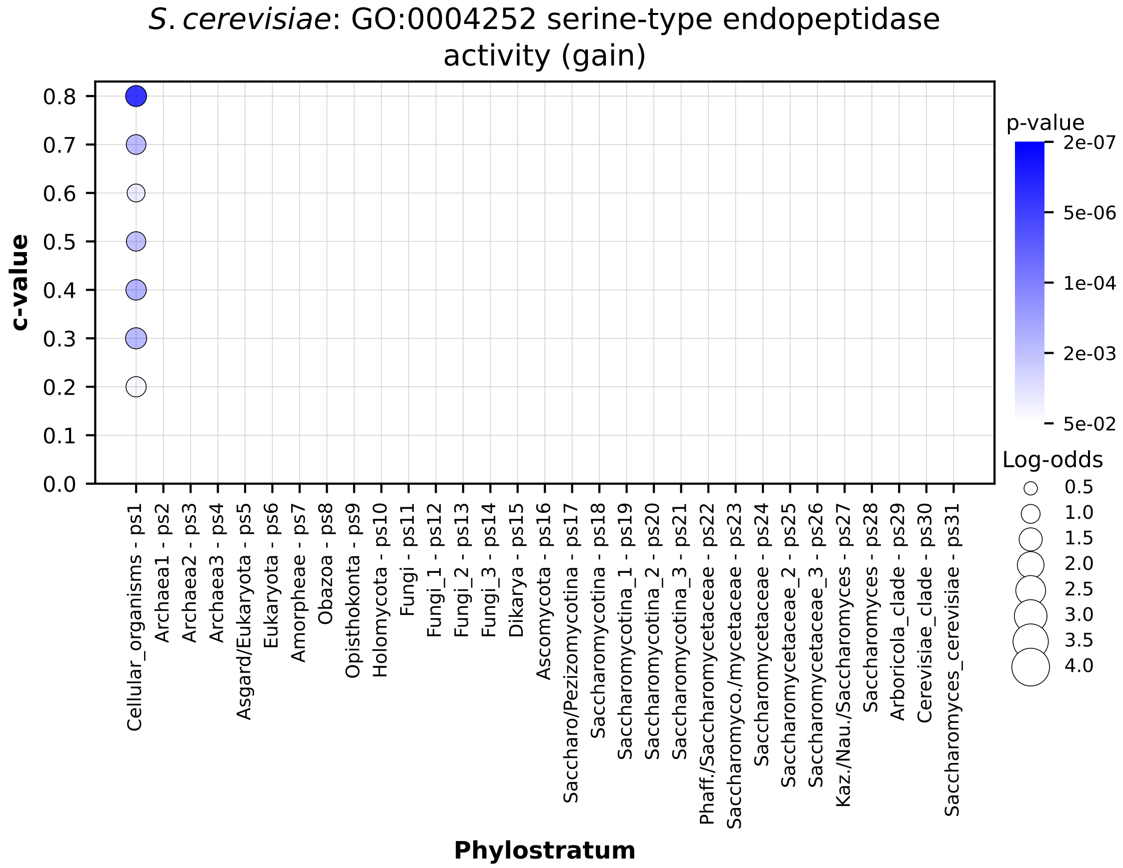

S. cerevisiae: GO:0004252 serine-type endopeptidase activity (gain)
c-value
0.0
0.1
0.2
0.3
0.4
0.5
0.6
0.7
0.8
Phylostratum
Cellular_organisms - ps1
Archaea1 - ps2
Archaea2 - ps3
Archaea3 - ps4
Asgard/Eukaryota - ps5
Eukaryota - ps6
Amorpheae - ps7
Obazoa - ps8
Opisthokonta - ps9
Holomycota - ps10
Fungi - ps11
Fungi_1 - ps12
Fungi_2 - ps13
Fungi_3 - ps14
Dikarya - ps15
Ascomycota - ps16
Saccharo/Pezizomycotina - ps17
Saccharomycotina - ps18
Saccharomycotina_1 - ps19
Saccharomycotina_2 - ps20
Saccharomycotina_3 - ps21
Phaff./Saccharomycetaceae - ps22
Saccharomyco./mycetaceae - ps23
Saccharomycetaceae - ps24
Saccharomycetaceae_2 - ps25
Saccharomycetaceae_3 - ps26
Kaz./Nau./Saccharomyces - ps27
Saccharomyces - ps28
Arboricola_clade - ps29
Cerevisiae_clade - ps30
Saccharomyces_cerevisiae - ps31
p-value
2e-07
5e-06
1e-04
2e-03
5e-02
Log-odds
0.5
1.0
1.5
2.0
2.5
3.0
3.5
4.0

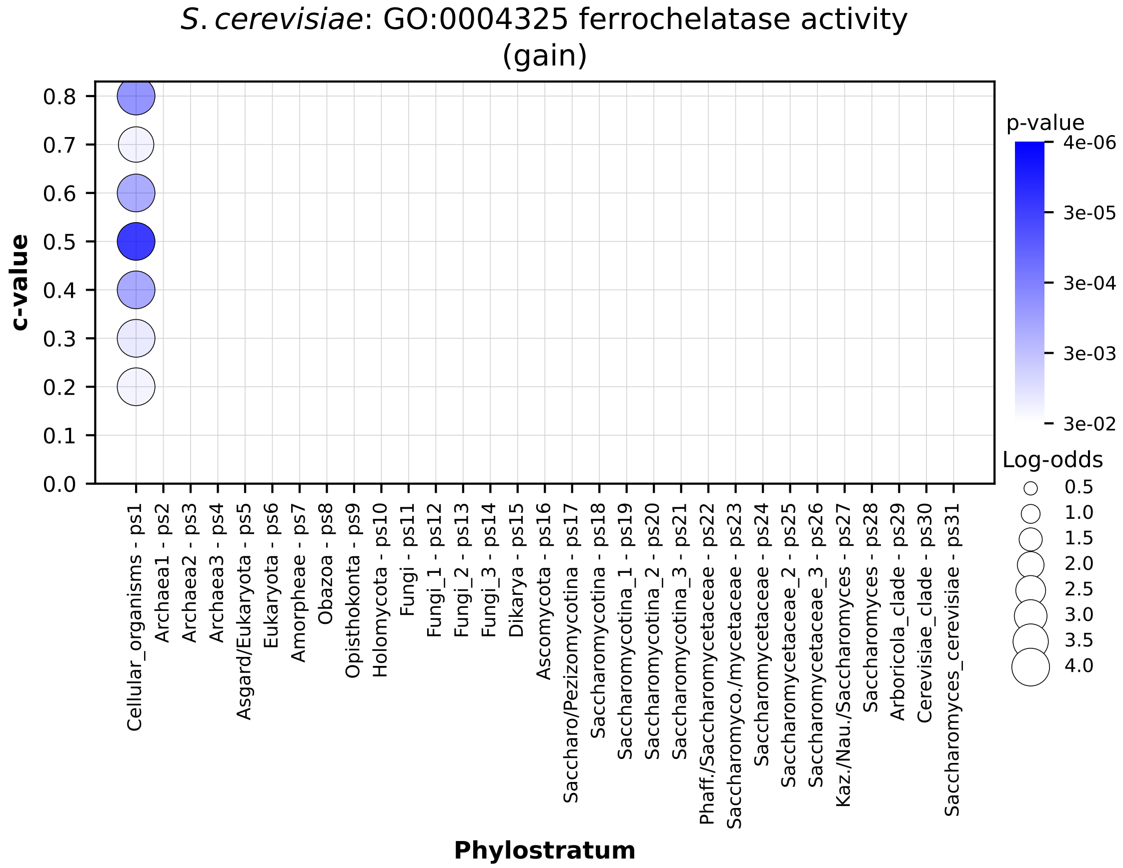

S. cerevisiae: GO:0004325 ferrochelatase activity
(gain)
c-value
0.8
0.7
0.6
0.5
0.4
0.3
0.2
0.1
0.0
Phylostratum
Cellular_organisms - ps1
Archaea1 - ps2
Archaea2 - ps3
Archaea3 - ps4
Asgard/Eukaryota - ps5
Eukaryota - ps6
Amorpheae - ps7
Obazoa - ps8
Opisthokonta - ps9
Holomycota - ps10
Fungi - ps11
Fungi_1 - ps12
Fungi_2 - ps13
Fungi_3 - ps14
Dikarya - ps15
Ascomycota - ps16
Saccharo/Pezizomycotina - ps17
Saccharomycotina - ps18
Saccharomycotina_1 - ps19
Saccharomycotina_2 - ps20
Saccharomycotina_3 - ps21
Phaff./Saccharomycetaceae - ps22
Saccharomyco./mycetaceae - ps23
Saccharomycetaceae - ps24
Saccharomycetaceae_2 - ps25
Saccharomycetaceae_3 - ps26
Kaz./Nau./Saccharomyces - ps27
Saccharomyces - ps28
Arboricola_clade - ps29
Cerevisiae_clade - ps30
Saccharomyces_cerevisiae - ps31
p-value
4e-06
3e-05
3e-04
3e-03
3e-02
Log-odds
0.5
1.0
1.5
2.0
2.5
3.0
3.5
4.0

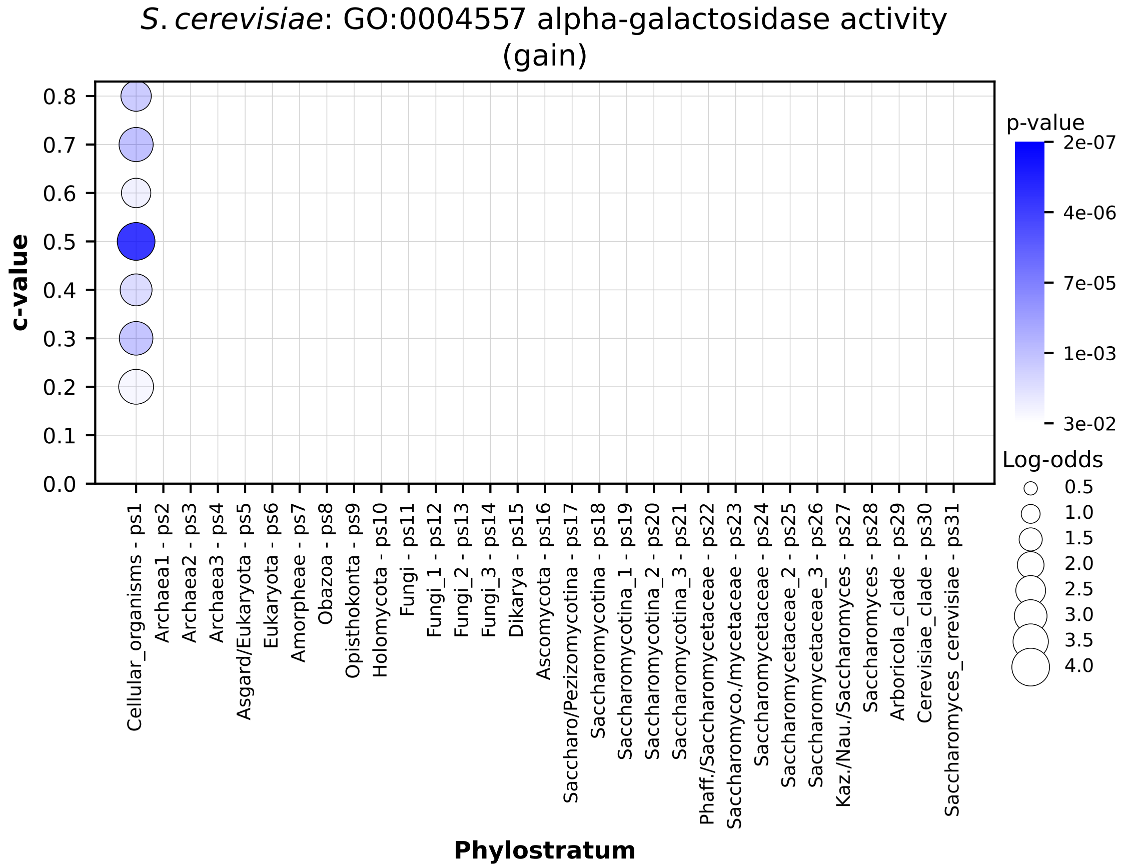

S. cerevisiae: GO:0004557 alpha-galactosidase activity (gain)
c-value
0.8
0.7
0.6
0.5
0.4
0.3
0.2
0.1
0.0
Phylostratum
Cellular_organisms - ps1
Archaea1 - ps2
Archaea2 - ps3
Archaea3 - ps4
Asgard/Eukaryota - ps5
Eukaryota - ps6
Amorpheae - ps7
Obazoa - ps8
Opisthokonta - ps9
Holomycota - ps10
Fungi - ps11
Fungi_1 - ps12
Fungi_2 - ps13
Fungi_3 - ps14
Dikarya - ps15
Ascomycota - ps16
Saccharo/Pezizomycotina - ps17
Saccharomycotina - ps18
Saccharomycotina_1 - ps19
Saccharomycotina_2 - ps20
Saccharomycotina_3 - ps21
Phaff./Saccharomycetaceae - ps22
Saccharomyco./mycetaceae - ps23
Saccharomycetaceae - ps24
Saccharomycetaceae_2 - ps25
Saccharomycetaceae_3 - ps26
Kaz./Nau./Saccharomyces - ps27
Saccharomyces - ps28
Arboricola_clade - ps29
Cerevisiae_clade - ps30
Saccharomyces_cerevisiae - ps31
p-value
2e-07
4e-06
7e-05
1e-03
3e-02
Log-odds
0.5
1.0
1.5
2.0
2.5
3.0
3.5
4.0

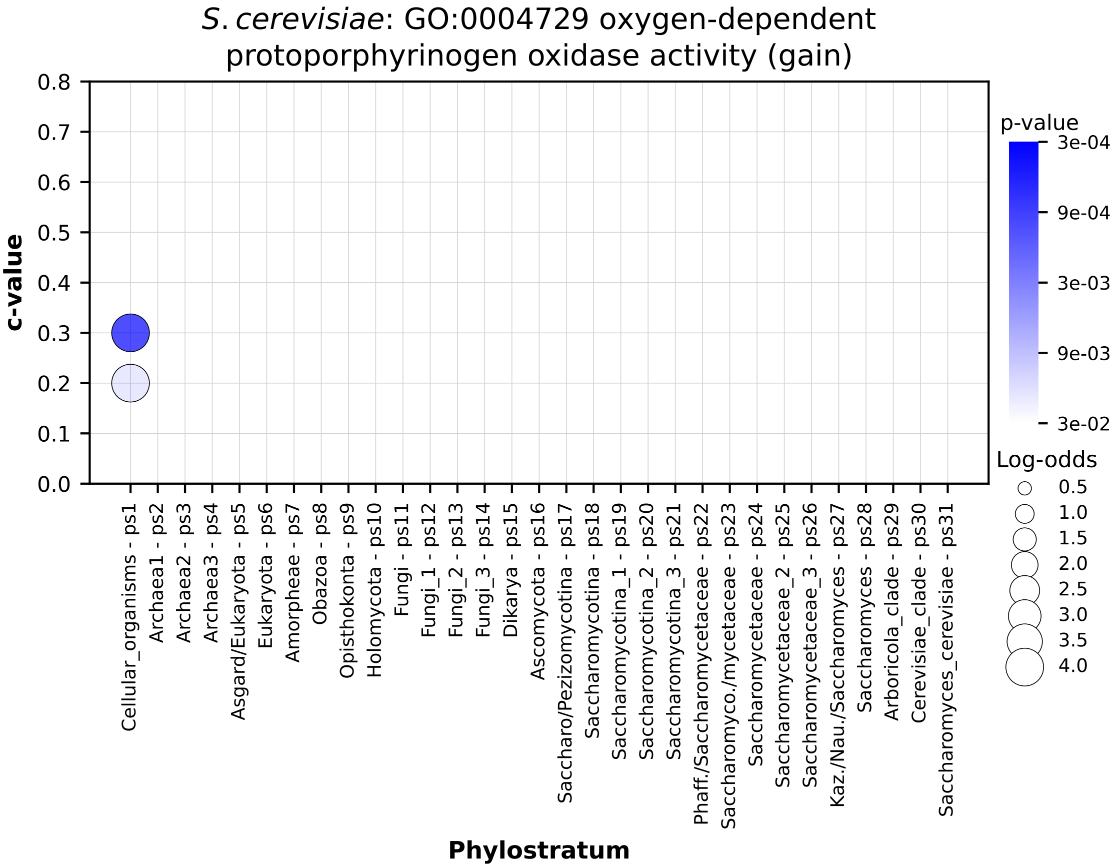

S. cerevisiae: GO:0004729 oxygen-dependent protoporphyrinogen oxidase activity (gain)
c-value
0.8
0.7
0.6
0.5
0.4
0.3
0.2
0.1
0.0
p-value
3e-04
9e-04
3e-03
9e-03
3e-02
Log-odds
0.5
1.0
1.5
2.0
2.5
3.0
3.5
4.0
Cellular_organisms - ps1
Archaea1 - ps2
Archaea2 - ps3
Archaea3 - ps4
Asgard/Eukaryota - ps5
Eukaryota - ps6
Amorpheae - ps7
Obazoa - ps8
Opisthokonta - ps9
Holomycota - ps10
Fungi - ps11
Fungi_1 - ps12
Fungi_2 - ps13
Fungi_3 - ps14
Dikarya - ps15
Ascomycota - ps16
Saccharo/Pezizomycotina - ps17
Saccharomycotina - ps18
Saccharomycotina_1 - ps19
Saccharomycotina_2 - ps20
Saccharomycotina_3 - ps21
Phaff./Saccharomycetaceae - ps22
Saccharomyco./mycetaceae - ps23
Saccharomycetaceae - ps24
Saccharomycetaceae_2 - ps25
Saccharomycetaceae_3 - ps26
Kaz./Nau./Saccharomyces - ps27
Saccharomyces - ps28
Arboricola_clade - ps29
Cerevisiae_clade - ps30
Saccharomyces_cerevisiae - ps31
Phylostratum

S. cerevisiae: GO:0004764 shikimate 3-dehydrogenase (NADP+) activity (gain)

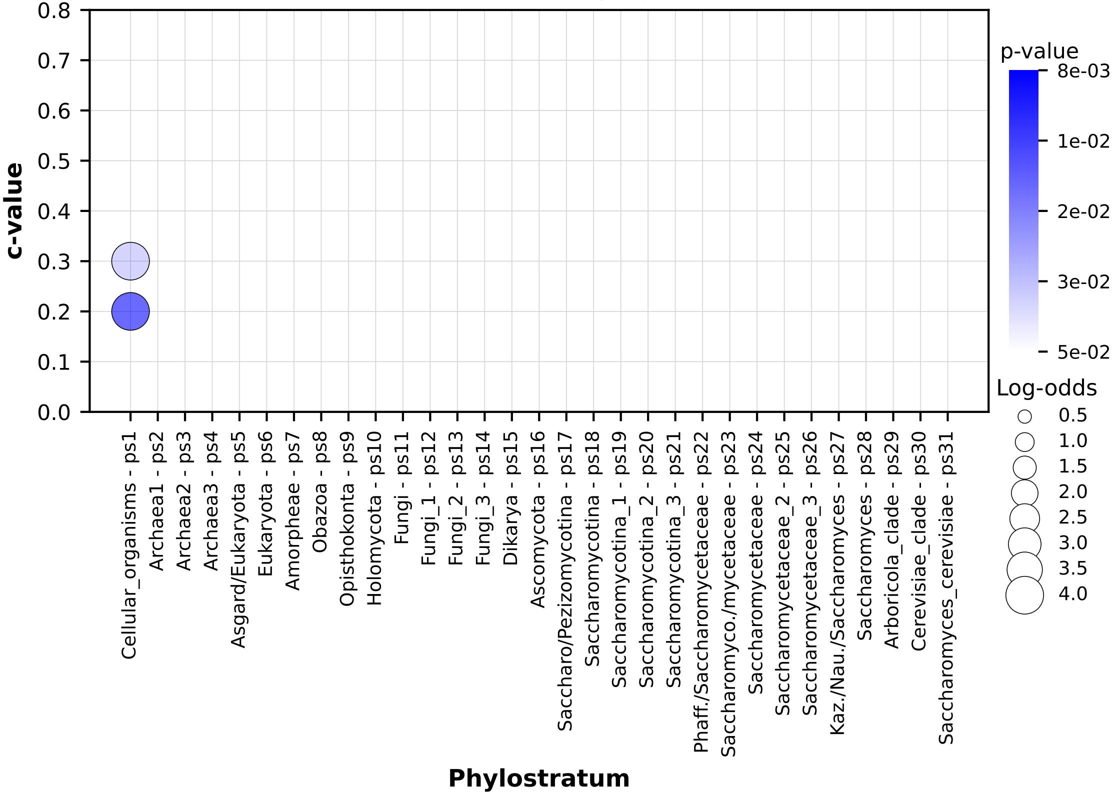

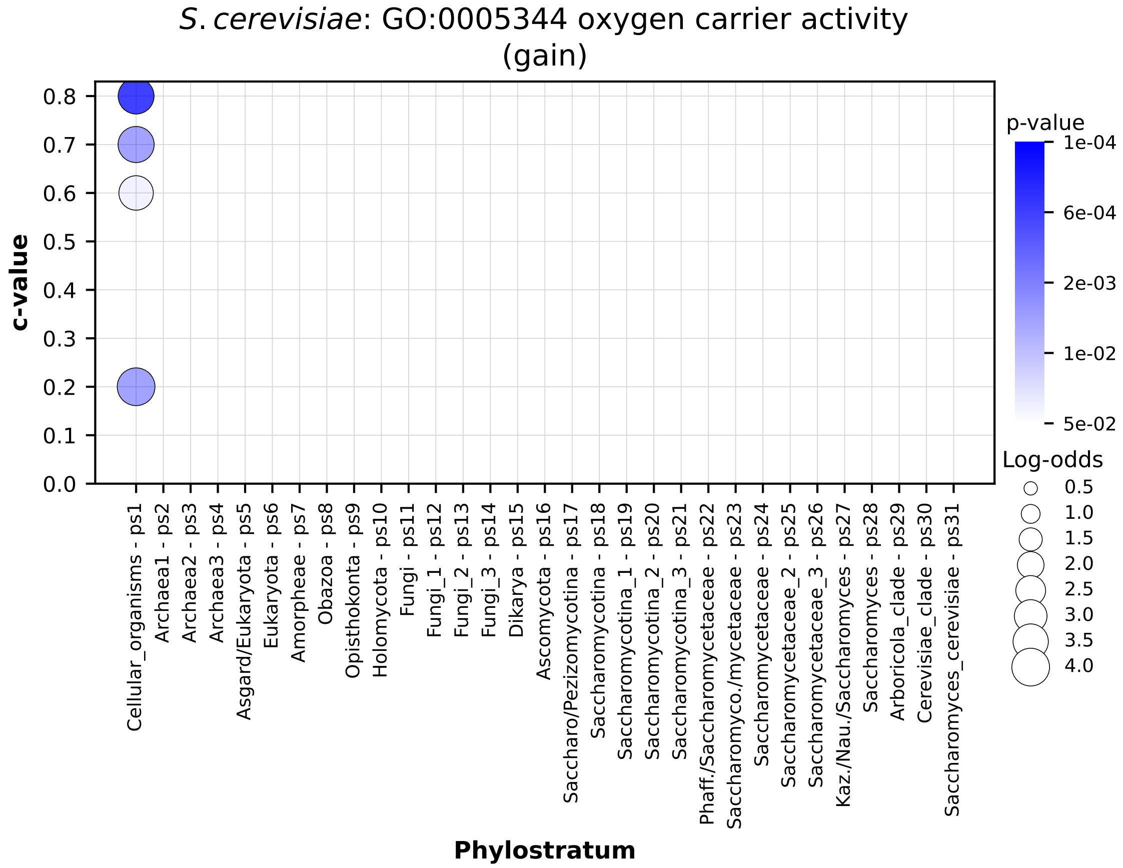

S. cerevisiae: GO:0005344 oxygen carrier activity
(gain)
c-value
0.0
0.1
0.2
0.3
0.4
0.5
0.6
0.7
0.8
p-value
1e-04
6e-04
2e-03
1e-02
5e-02
Log-odds
0.5
1.0
1.5
2.0
2.5
3.0
3.5
4.0
Cellular_organisms - ps1
Archaea1 - ps2
Archaea2 - ps3
Archaea3 - ps4
Asgard/Eukaryota - ps5
Eukaryota - ps6
Amorpheae - ps7
Obazoa - ps8
Opisthokonta - ps9
Holomycota - ps10
Fungi - ps11
Fungi_1 - ps12
Fungi_2 - ps13
Fungi_3 - ps14
Dikarya - ps15
Ascomycota - ps16
Saccharo/Pezizomycotina - ps17
Saccharomycotina - ps18
Saccharomycotina_1 - ps19
Saccharomycotina_2 - ps20
Saccharomycotina_3 - ps21
Phaff./Saccharomycetaceae - ps22
Saccharomyco./mycetaceae - ps23
Saccharomycetaceae - ps24
Saccharomycetaceae_2 - ps25
Saccharomycetaceae_3 - ps26
Kaz./Nau./Saccharomyces - ps27
Saccharomyces - ps28
Arboricola_clade - ps29
Cerevisiae_clade - ps30
Saccharomyces_cerevisiae - ps31
Phylostratum

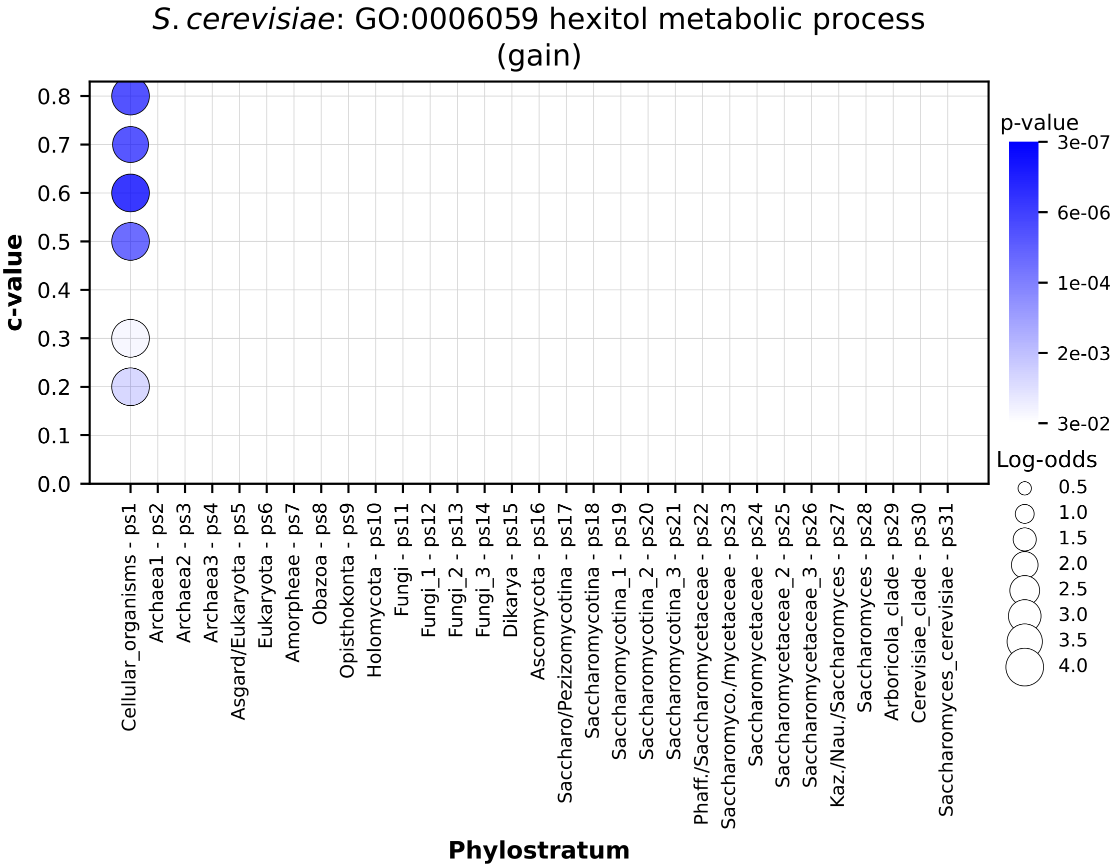

S. cerevisiae: GO:0006059 hexitol metabolic process
(gain)
c-value
0.8
0.7
0.6
0.5
0.4
0.3
0.2
0.1
0.0
p-value
3e-07
6e-06
1e-04
2e-03
3e-02
Log-odds
0.5
1.0
1.5
2.0
2.5
3.0
3.5
4.0
Cellular_organisms - ps1
Archaea1 - ps2
Archaea2 - ps3
Archaea3 - ps4
Asgard/Eukaryota - ps5
Eukaryota - ps6
Amorpheae - ps7
Obazoa - ps8
Opisthokonta - ps9
Holomycota - ps10
Fungi - ps11
Fungi_1 - ps12
Fungi_2 - ps13
Fungi_3 - ps14
Dikarya - ps15
Ascomycota - ps16
Saccharo/Pezizomycotina - ps17
Saccharomycotina - ps18
Saccharomycotina_1 - ps19
Saccharomycotina_2 - ps20
Saccharomycotina_3 - ps21
Phaff./Saccharomycetaceae - ps22
Saccharomyco./mycetaceae - ps23
Saccharomycetaceae - ps24
Saccharomycetaceae_2 - ps25
Saccharomycetaceae_3 - ps26
Kaz./Nau./Saccharomyces - ps27
Saccharomyces - ps28
Arboricola_clade - ps29
Cerevisiae_clade - ps30
Saccharomyces_cerevisiae - ps31
Phylostratum

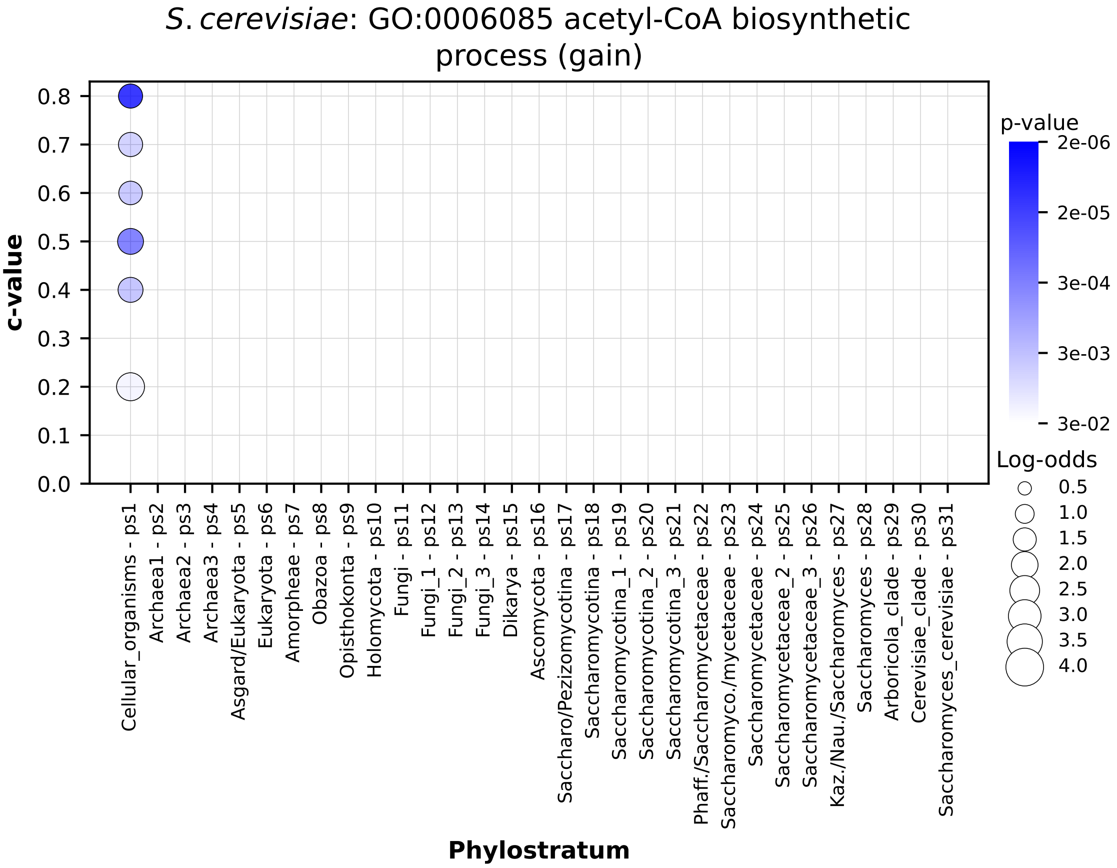

S. cerevisiae: GO:0006085 acetyl-CoA biosynthetic process (gain)
c-value
0.8
0.7
0.6
0.5
0.4
0.3
0.2
0.1
0.0
p-value
2e-06
2e-05
3e-04
3e-03
3e-02
Log-odds
0.5
1.0
1.5
2.0
2.5
3.0
3.5
4.0
Cellular_organisms - ps1
Archaea1 - ps2
Archaea2 - ps3
Archaea3 - ps4
Asgard/Eukaryota - ps5
Eukaryota - ps6
Amorpheae - ps7
Obazoa - ps8
Opisthokonta - ps9
Holomycota - ps10
Fungi - ps11
Fungi_1 - ps12
Fungi_2 - ps13
Fungi_3 - ps14
Dikarya - ps15
Ascomycota - ps16
Saccharo/Pezizomycotina - ps17
Saccharomycotina - ps18
Saccharomycotina_1 - ps19
Saccharomycotina_2 - ps20
Saccharomycotina_3 - ps21
Phaff./Saccharomycetaceae - ps22
Saccharomyco./mycetaceae - ps23
Saccharomycetaceae - ps24
Saccharomycetaceae_2 - ps25
Saccharomycetaceae_3 - ps26
Kaz./Nau./Saccharomyces - ps27
Saccharomyces - ps28
Arboricola_clade - ps29
Cerevisiae_clade - ps30
Saccharomyces_cerevisiae - ps31
Phylostratum

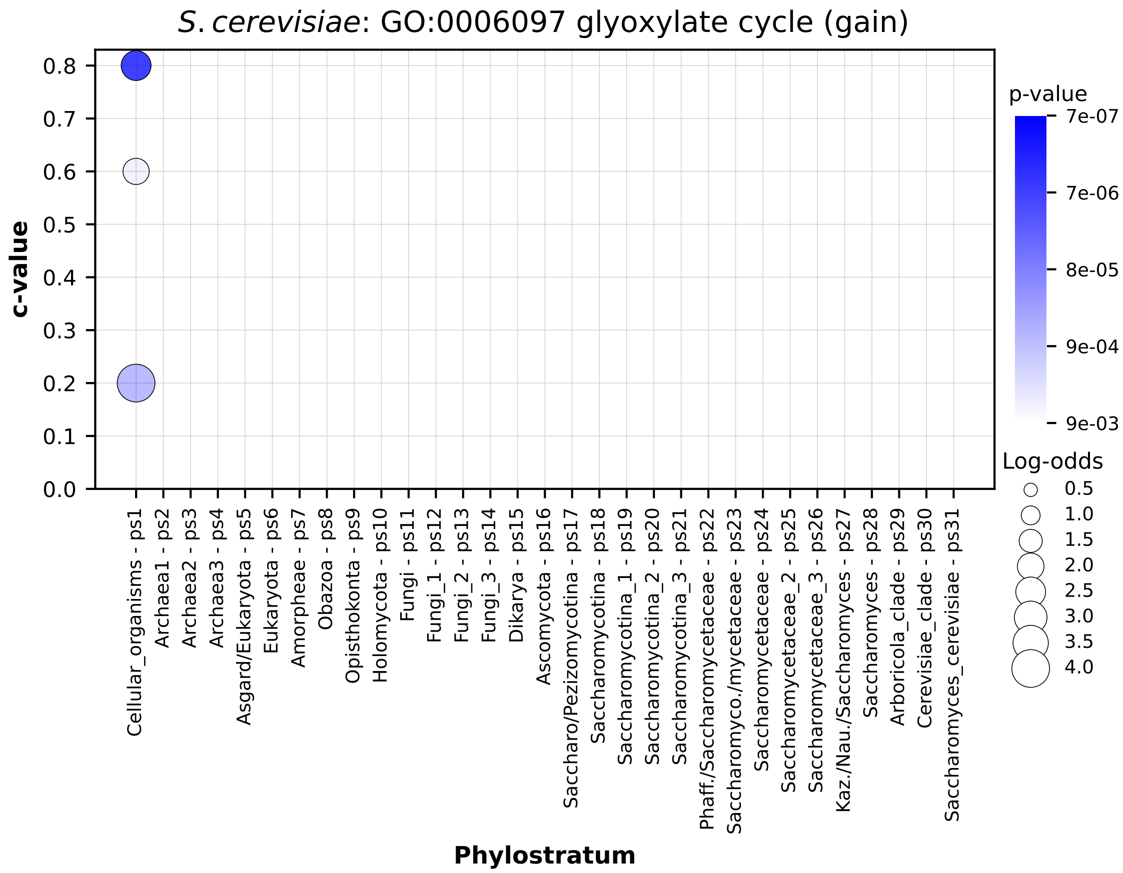

S. cerevisiae: GO:0006097 glyoxylate cycle (gain)
c-value
0.0
0.1
0.2
0.3
0.4
0.5
0.6
0.7
0.8
p-value
7e-07
7e-06
8e-05
9e-04
9e-03
Log-odds
0.5
1.0
1.5
2.0
2.5
3.0
3.5
4.0
Cellular_organisms - ps1
Archaea1 - ps2
Archaea2 - ps3
Archaea3 - ps4
Asgard/Eukaryota - ps5
Eukaryota - ps6
Amorpheae - ps7
Obazoa - ps8
Opisthokonta - ps9
Holomycota - ps10
Fungi - ps11
Fungi_1 - ps12
Fungi_2 - ps13
Fungi_3 - ps14
Dikarya - ps15
Ascomycota - ps16
Saccharo/Pezizomycotina - ps17
Saccharomycotina - ps18
Saccharomycotina_1 - ps19
Saccharomycotina_2 - ps20
Saccharomycotina_3 - ps21
Phaff./Saccharomycetaceae - ps22
Saccharomyco./mycetaceae - ps23
Saccharomycetaceae - ps24
Saccharomycetaceae_2 - ps25
Saccharomycetaceae_3 - ps26
Kaz./Nau./Saccharomyces - ps27
Saccharomyces - ps28
Arboricola_clade - ps29
Cerevisiae_clade - ps30
Saccharomyces_cerevisiae - ps31
Phylostratum

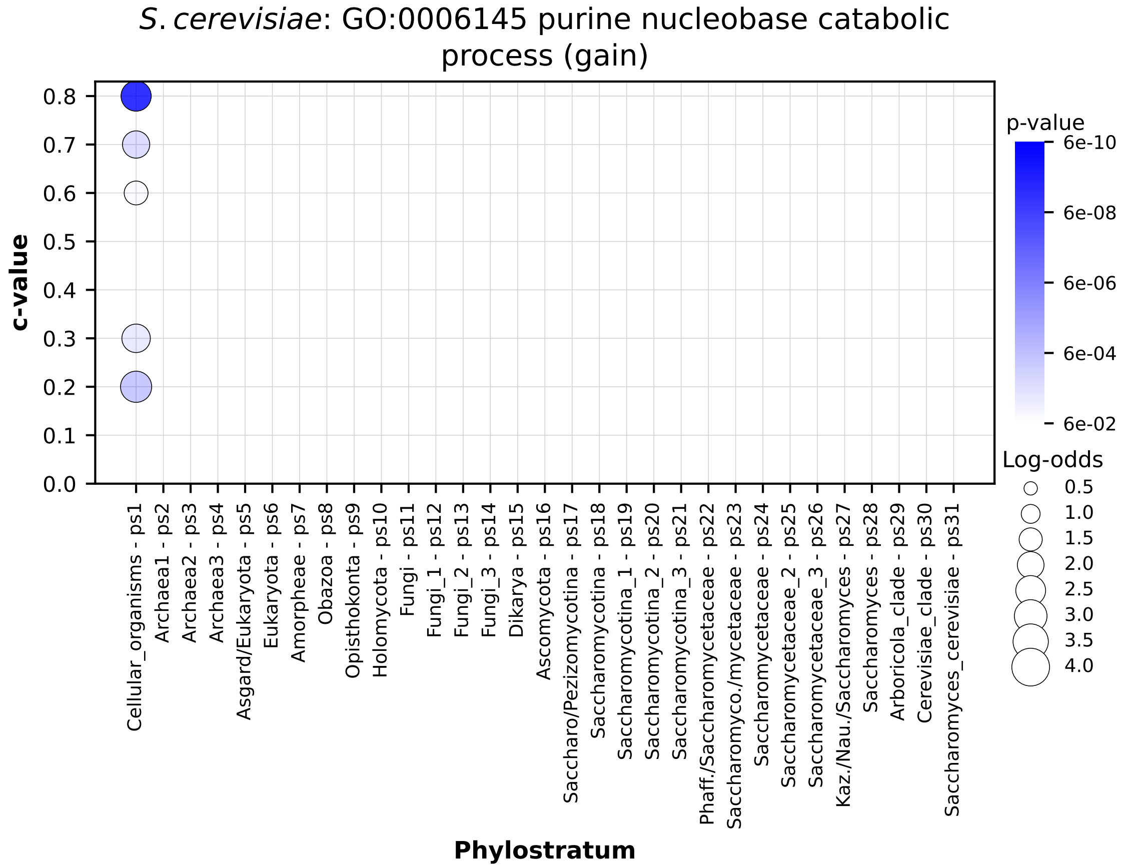

S. cerevisiae: GO:0006145 purine nucleobase catabolic process (gain)
c-value
0.8
0.7
0.6
0.5
0.4
0.3
0.2
0.1
0.0
Phylostratum
Cellular_organisms - ps1
Archaea1 - ps2
Archaea2 - ps3
Archaea3 - ps4
Asgard/Eukaryota - ps5
Eukaryota - ps6
Amorpheae - ps7
Obazoa - ps8
Opisthokonta - ps9
Holomycota - ps10
Fungi - ps11
Fungi_1 - ps12
Fungi_2 - ps13
Fungi_3 - ps14
Dikarya - ps15
Ascomycota - ps16
Saccharo/Pezizomycotina - ps17
Saccharomycotina - ps18
Saccharomycotina_1 - ps19
Saccharomycotina_2 - ps20
Saccharomycotina_3 - ps21
Phaff./Saccharomycetaceae - ps22
Saccharomyco./mycetaceae - ps23
Saccharomycetaceae - ps24
Saccharomycetaceae_2 - ps25
Saccharomycetaceae_3 - ps26
Kaz./Nau./Saccharomyces - ps27
Saccharomyces - ps28
Arboricola_clade - ps29
Cerevisiae_clade - ps30
Saccharomyces_cerevisiae - ps31
p-value
6e-10
6e-08
6e-06
6e-04
6e-02
Log-odds
0.5
1.0
1.5
2.0
2.5
3.0
3.5
4.0

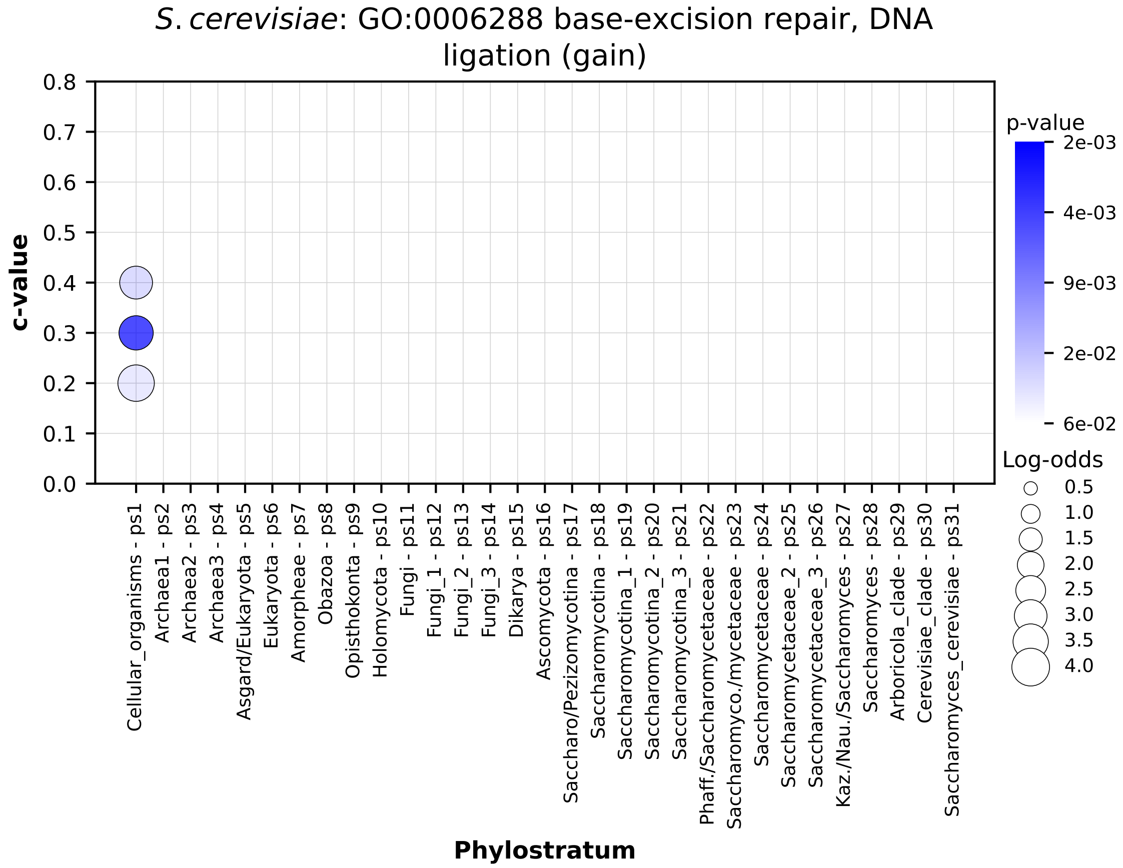

S. cerevisiae: GO:0006288 base-excision repair, DNA ligation (gain)
c-value
0.8
0.7
0.6
0.5
0.4
0.3
0.2
0.1
0.0
Phylostratum
Cellular_organisms - ps1
Archaea1 - ps2
Archaea2 - ps3
Archaea3 - ps4
Asgard/Eukaryota - ps5
Eukaryota - ps6
Amorpheae - ps7
Obazoa - ps8
Opisthokonta - ps9
Holomycota - ps10
Fungi - ps11
Fungi_1 - ps12
Fungi_2 - ps13
Fungi_3 - ps14
Dikarya - ps15
Ascomycota - ps16
Saccharo/Pezizomycotina - ps17
Saccharomycotina - ps18
Saccharomycotina_1 - ps19
Saccharomycotina_2 - ps20
Saccharomycotina_3 - ps21
Phaff./Saccharomycetaceae - ps22
Saccharomyco./mycetaceae - ps23
Saccharomycetaceae - ps24
Saccharomycetaceae_2 - ps25
Saccharomycetaceae_3 - ps26
Kaz./Nau./Saccharomyces - ps27
Saccharomyces - ps28
Arboricola_clade - ps29
Cerevisiae_clade - ps30
Saccharomyces_cerevisiae - ps31
p-value
2e-03
4e-03
9e-03
2e-02
6e-02
Log-odds
0.5
1.0
1.5
2.0
2.5
3.0
3.5
4.0

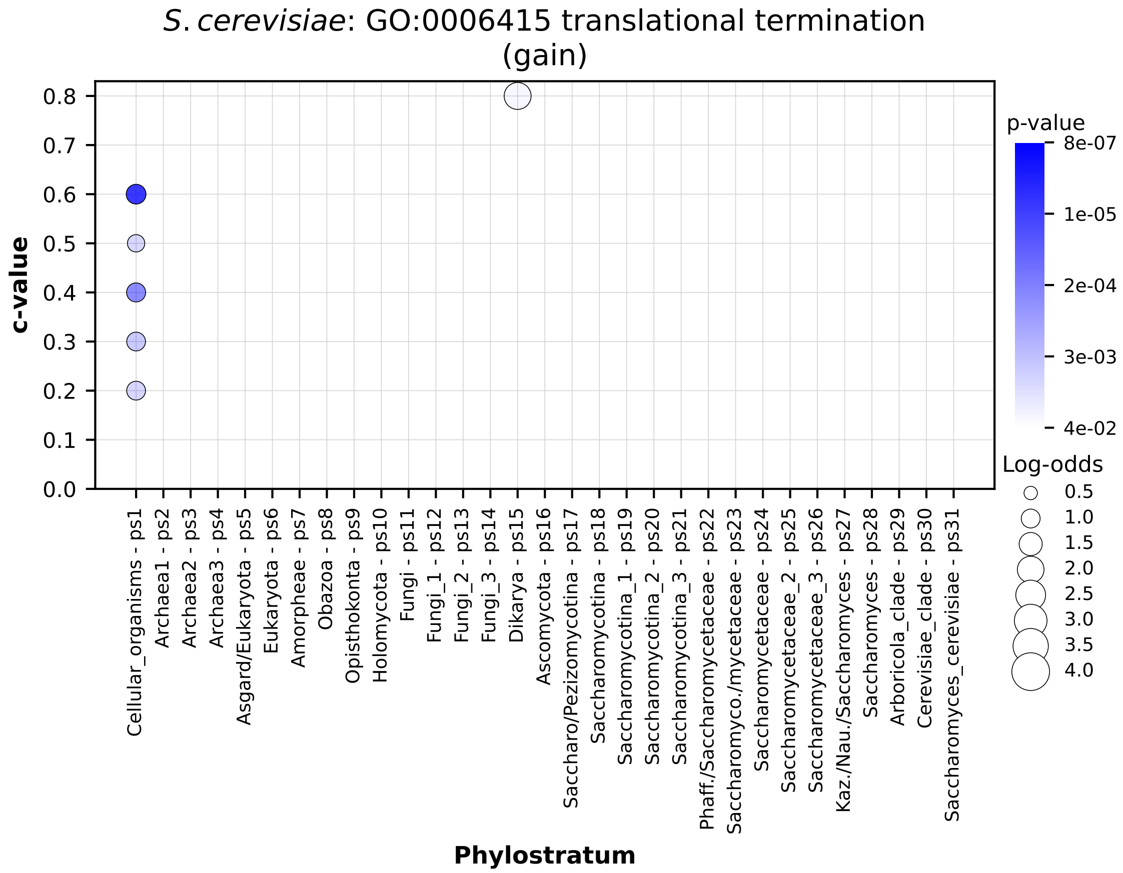

S. cerevisiae: GO:0006415 translational termination
(gain)
c-value
0.0
0.1
0.2
0.3
0.4
0.5
0.6
0.7
0.8
p-value
8e-07
1e-05
2e-04
3e-03
4e-02
Log-odds
0.5
1.0
1.5
2.0
2.5
3.0
3.5
4.0
Cellular_organisms - ps1
Archaea1 - ps2
Archaea2 - ps3
Archaea3 - ps4
Asgard/Eukaryota - ps5
Eukaryota - ps6
Amorpheae - ps7
Obazoa - ps8
Opisthokonta - ps9
Holomycota - ps10
Fungi - ps11
Fungi_1 - ps12
Fungi_2 - ps13
Fungi_3 - ps14
Dikarya - ps15
Ascomycota - ps16
Saccharo/Pezizomycotina - ps17
Saccharomycotina - ps18
Saccharomycotina_1 - ps19
Saccharomycotina_2 - ps20
Saccharomycotina_3 - ps21
Phaff./Saccharomycetaceae - ps22
Saccharomyco./mycetaceae - ps23
Saccharomycetaceae - ps24
Saccharomycetaceae_2 - ps25
Saccharomycetaceae_3 - ps26
Kaz./Nau./Saccharomyces - ps27
Saccharomyces - ps28
Arboricola_clade - ps29
Cerevisiae_clade - ps30
Saccharomyces_cerevisiae - ps31
Phylostratum

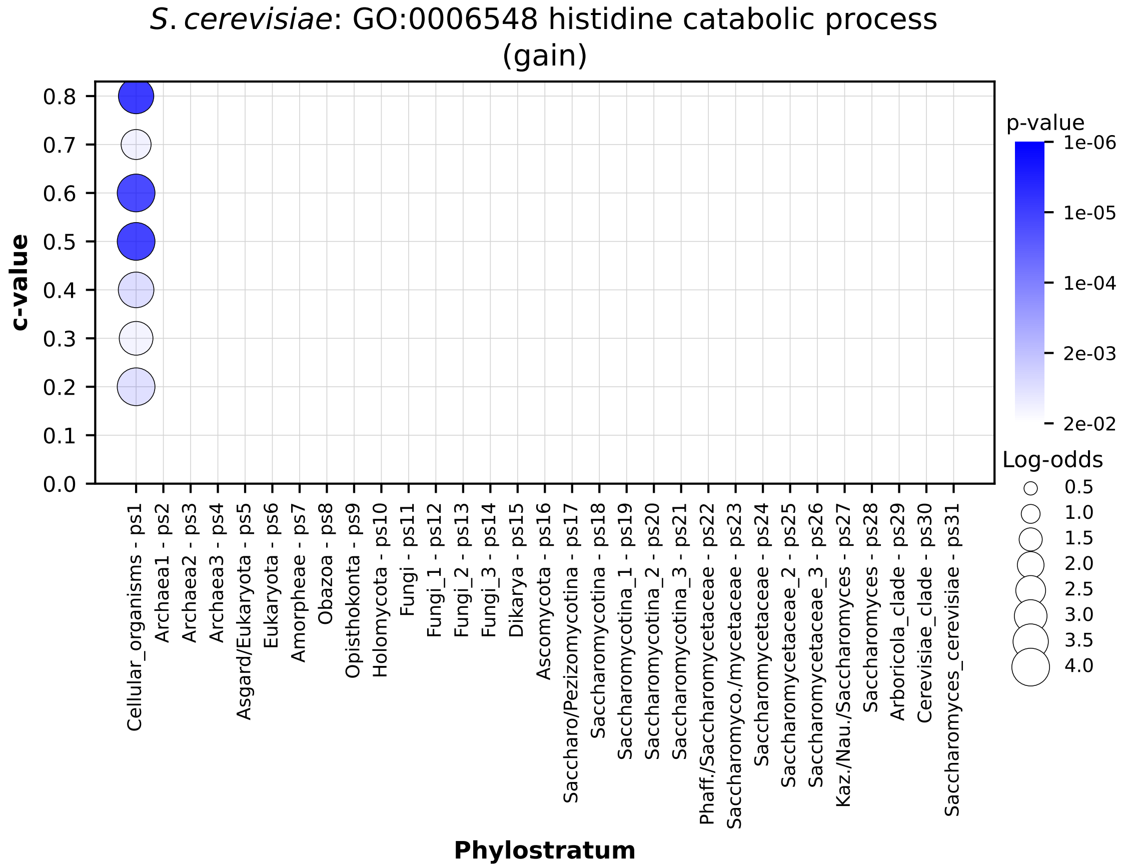

S. cerevisiae: GO:0006548 histidine catabolic process
(gain)
c-value
0.0
0.1
0.2
0.3
0.4
0.5
0.6
0.7
0.8
Phylostratum
Cellular_organisms - ps1
Archaea1 - ps2
Archaea2 - ps3
Archaea3 - ps4
Asgard/Eukaryota - ps5
Eukaryota - ps6
Amorpheae - ps7
Obazoa - ps8
Opisthokonta - ps9
Holomycota - ps10
Fungi - ps11
Fungi_1 - ps12
Fungi_2 - ps13
Fungi_3 - ps14
Dikarya - ps15
Ascomycota - ps16
Saccharo/Pezizomycotina - ps17
Saccharomycotina - ps18
Saccharomycotina_1 - ps19
Saccharomycotina_2 - ps20
Saccharomycotina_3 - ps21
Phaff./Saccharomycetaceae - ps22
Saccharomyco./mycetaceae - ps23
Saccharomycetaceae - ps24
Saccharomycetaceae_2 - ps25
Saccharomycetaceae_3 - ps26
Kaz./Nau./Saccharomyces - ps27
Saccharomyces - ps28
Arboricola_clade - ps29
Cerevisiae_clade - ps30
Saccharomyces_cerevisiae - ps31
p-value
1e-06
1e-05
1e-04
2e-03
2e-02
Log-odds
0.5
1.0
1.5
2.0
2.5
3.0
3.5
4.0

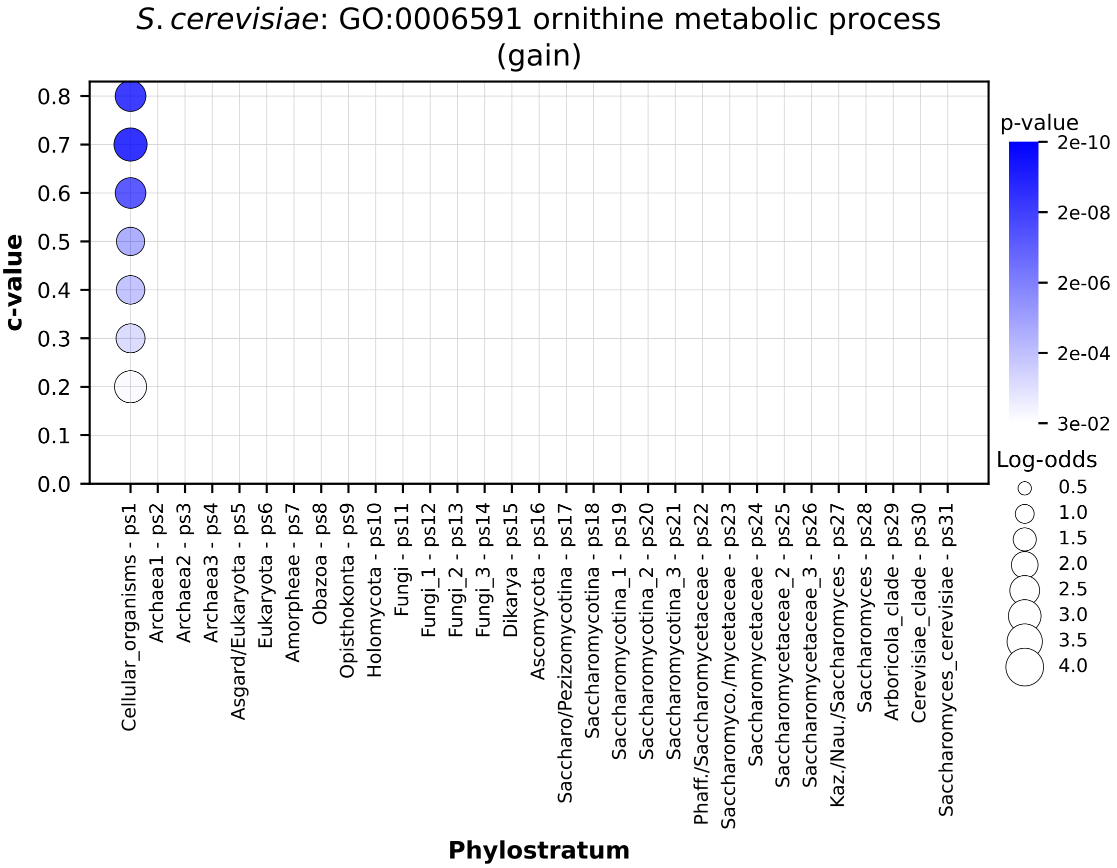

S. cerevisiae: GO:0006591 ornithine metabolic process (gain)
c-value
0.8
0.7
0.6
0.5
0.4
0.3
0.2
0.1
0.0
Phylostratum
Cellular_organisms - ps1
Archaea1 - ps2
Archaea2 - ps3
Archaea3 - ps4
Asgard/Eukaryota - ps5
Eukaryota - ps6
Amorpheae - ps7
Obazoa - ps8
Opisthokonta - ps9
Holomycota - ps10
Fungi - ps11
Fungi_1 - ps12
Fungi_2 - ps13
Fungi_3 - ps14
Dikarya - ps15
Ascomycota - ps16
Saccharo/Pezizomycotina - ps17
Saccharomycotina - ps18
Saccharomycotina_1 - ps19
Saccharomycotina_2 - ps20
Saccharomycotina_3 - ps21
Phaff./Saccharomycetaceae - ps22
Saccharomyco./mycetaceae - ps23
Saccharomycetaceae - ps24
Saccharomycetaceae_2 - ps25
Saccharomycetaceae_3 - ps26
Kaz./Nau./Saccharomyces - ps27
Saccharomyces - ps28
Arboricola_clade - ps29
Cerevisiae_clade - ps30
Saccharomyces_cerevisiae - ps31
p-value
2e-10
2e-08
2e-06
2e-04
3e-02
Log-odds
0.5
1.0
1.5
2.0
2.5
3.0
3.5
4.0

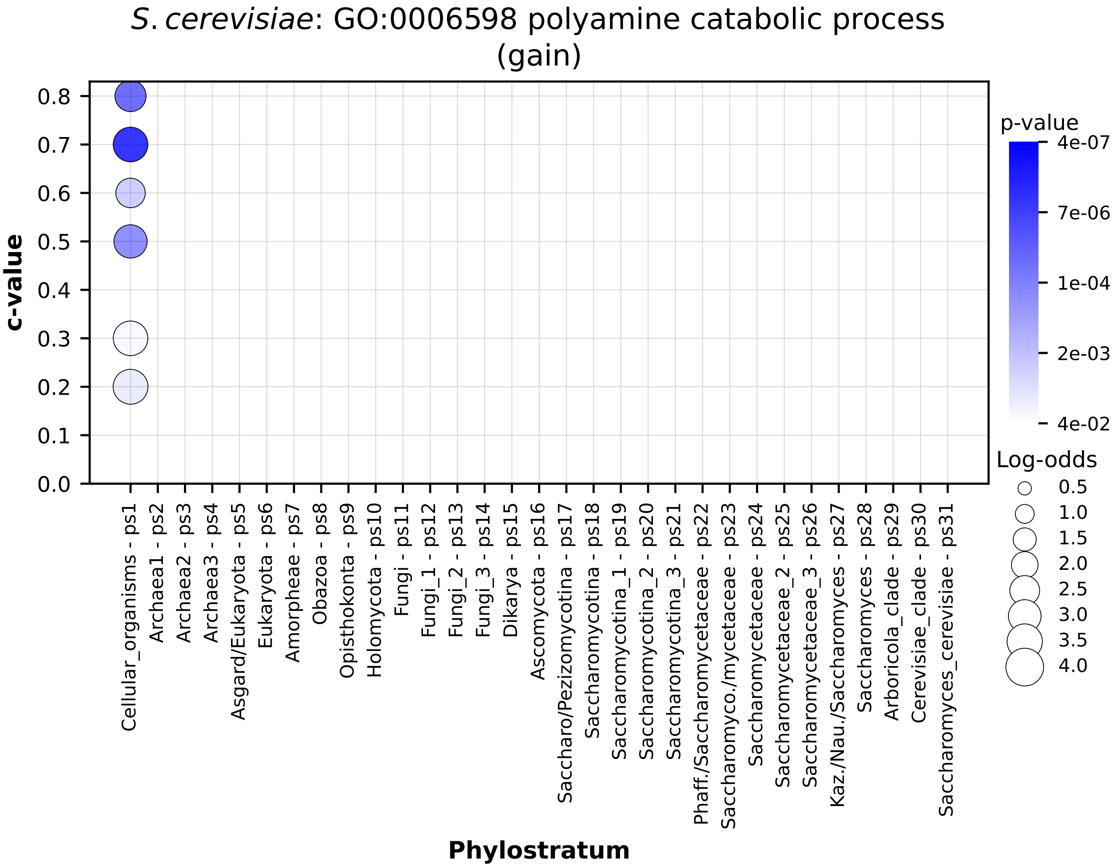

S. cerevisiae: GO:0006598 polyamine catabolic process
(gain)
c-value
0.0
0.1
0.2
0.3
0.4
0.5
0.6
0.7
0.8
Phylostratum
Cellular_organisms - ps1
Archaea1 - ps2
Archaea2 - ps3
Archaea3 - ps4
Asgard/Eukaryota - ps5
Eukaryota - ps6
Amorpheae - ps7
Obazoa - ps8
Opisthokonta - ps9
Holomycota - ps10
Fungi - ps11
Fungi_1 - ps12
Fungi_2 - ps13
Fungi_3 - ps14
Dikarya - ps15
Ascomycota - ps16
Saccharo/Pezizomycotina - ps17
Saccharomycotina - ps18
Saccharomycotina_1 - ps19
Saccharomycotina_2 - ps20
Saccharomycotina_3 - ps21
Phaff./Saccharomycetaceae - ps22
Saccharomyco./mycetaceae - ps23
Saccharomycetaceae - ps24
Saccharomycetaceae_2 - ps25
Saccharomycetaceae_3 - ps26
Kaz./Nau./Saccharomyces - ps27
Saccharomyces - ps28
Arboricola_clade - ps29
Cerevisiae_clade - ps30
Saccharomyces_cerevisiae - ps31
p-value
4e-07
7e-06
1e-04
2e-03
4e-02
Log-odds
0.5
1.0
1.5
2.0
2.5
3.0
3.5
4.0

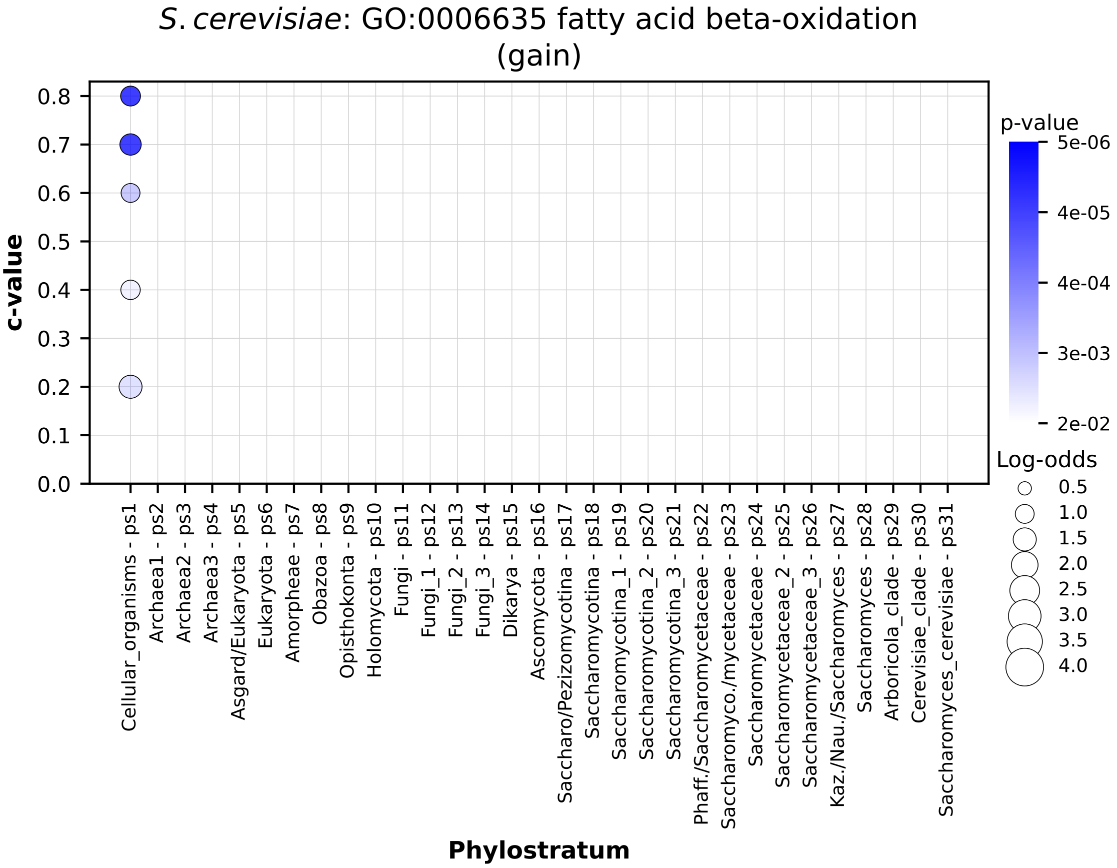

S. cerevisiae: GO:0006635 fatty acid beta-oxidation (gain)
c-value
0.0
0.1
0.2
0.3
0.4
0.5
0.6
0.7
0.8
Phylostratum
Cellular_organisms - ps1
Archaea1 - ps2
Archaea2 - ps3
Archaea3 - ps4
Asgard/Eukaryota - ps5
Eukaryota - ps6
Amorpheae - ps7
Obazoa - ps8
Opisthokonta - ps9
Holomycota - ps10
Fungi - ps11
Fungi_1 - ps12
Fungi_2 - ps13
Fungi_3 - ps14
Dikarya - ps15
Ascomycota - ps16
Saccharo/Pezizomycotina - ps17
Saccharomycotina - ps18
Saccharomycotina_1 - ps19
Saccharomycotina_2 - ps20
Saccharomycotina_3 - ps21
Phaff./Saccharomycetaceae - ps22
Saccharomyco./mycetaceae - ps23
Saccharomycetaceae - ps24
Saccharomycetaceae_2 - ps25
Saccharomycetaceae_3 - ps26
Kaz./Nau./Saccharomyces - ps27
Saccharomyces - ps28
Arboricola_clade - ps29
Cerevisiae_clade - ps30
Saccharomyces_cerevisiae - ps31
p-value
5e-06
4e-05
4e-04
3e-03
2e-02
Log-odds
0.5
1.0
1.5
2.0
2.5
3.0
3.5
4.0

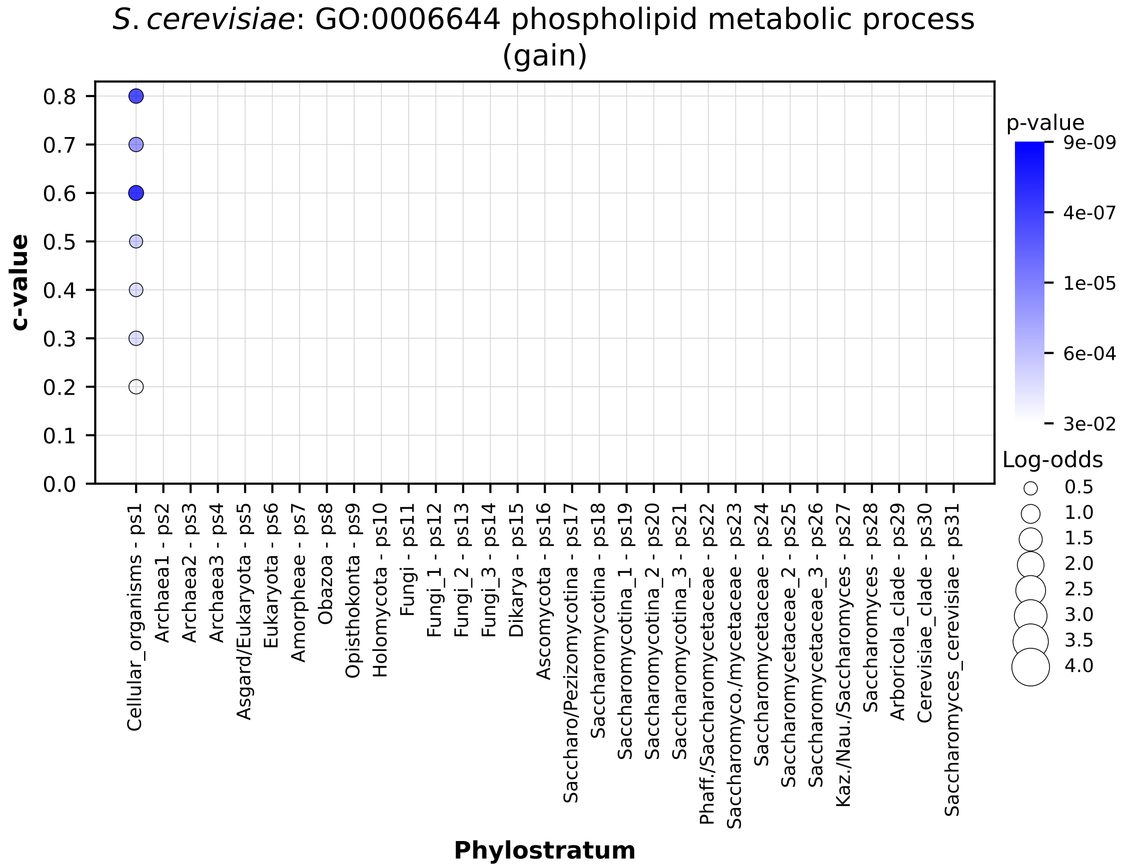

S. cerevisiae: GO:0006644 phospholipid metabolic process (gain)
c-value
0.0
0.1
0.2
0.3
0.4
0.5
0.6
0.7
0.8
p-value
9e-09
4e-07
1e-05
6e-04
3e-02
Log-odds
0.5
1.0
1.5
2.0
2.5
3.0
3.5
4.0
Cellular_organisms - ps1
Archaea1 - ps2
Archaea2 - ps3
Archaea3 - ps4
Asgard/Eukaryota - ps5
Eukaryota - ps6
Amorpheae - ps7
Obazoa - ps8
Opisthokonta - ps9
Holomycota - ps10
Fungi - ps11
Fungi_1 - ps12
Fungi_2 - ps13
Fungi_3 - ps14
Dikarya - ps15
Ascomycota - ps16
Saccharo/Pezizomycotina - ps17
Saccharomycotina - ps18
Saccharomycotina_1 - ps19
Saccharomycotina_2 - ps20
Saccharomycotina_3 - ps21
Phaff./Saccharomycetaceae - ps22
Saccharomyco./mycetaceae - ps23
Saccharomycetaceae - ps24
Saccharomycetaceae_2 - ps25
Saccharomycetaceae_3 - ps26
Kaz./Nau./Saccharomyces - ps27
Saccharomyces - ps28
Arboricola_clade - ps29
Cerevisiae_clade - ps30
Saccharomyces_cerevisiae - ps31
Phylostratum

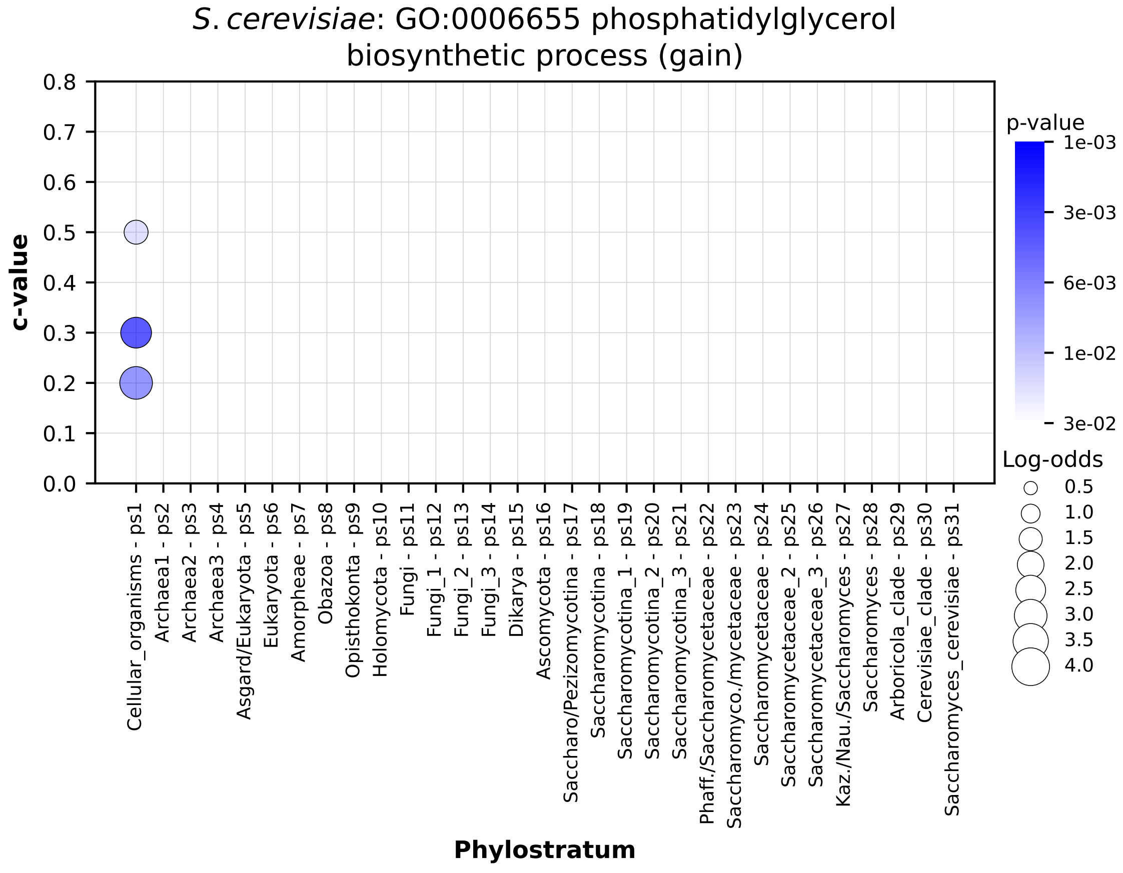

S. cerevisiae: GO:0006655 phosphatidylglycerol biosynthetic process (gain)
c-value
0.0
0.1
0.2
0.3
0.4
0.5
0.6
0.7
0.8
Phylostratum
Cellular_organisms - ps1
Archaea1 - ps2
Archaea2 - ps3
Archaea3 - ps4
Asgard/Eukaryota - ps5
Eukaryota - ps6
Amorpheae - ps7
Obazoa - ps8
Opisthokonta - ps9
Holomycota - ps10
Fungi - ps11
Fungi_1 - ps12
Fungi_2 - ps13
Fungi_3 - ps14
Dikarya - ps15
Ascomycota - ps16
Saccharo/Pezizomycotina - ps17
Saccharomycotina - ps18
Saccharomycotina_1 - ps19
Saccharomycotina_2 - ps20
Saccharomycotina_3 - ps21
Phaff./Saccharomycetaceae - ps22
Saccharomyco./mycetaceae - ps23
Saccharomycetaceae - ps24
Saccharomycetaceae_2 - ps25
Saccharomycetaceae_3 - ps26
Kaz./Nau./Saccharomyces - ps27
Saccharomyces - ps28
Arboricola_clade - ps29
Cerevisiae_clade - ps30
Saccharomyces_cerevisiae - ps31
p-value
1e-03
3e-03
6e-03
1e-02
3e-02
Log-odds
0.5
1.0
1.5
2.0
2.5
3.0
3.5
4.0

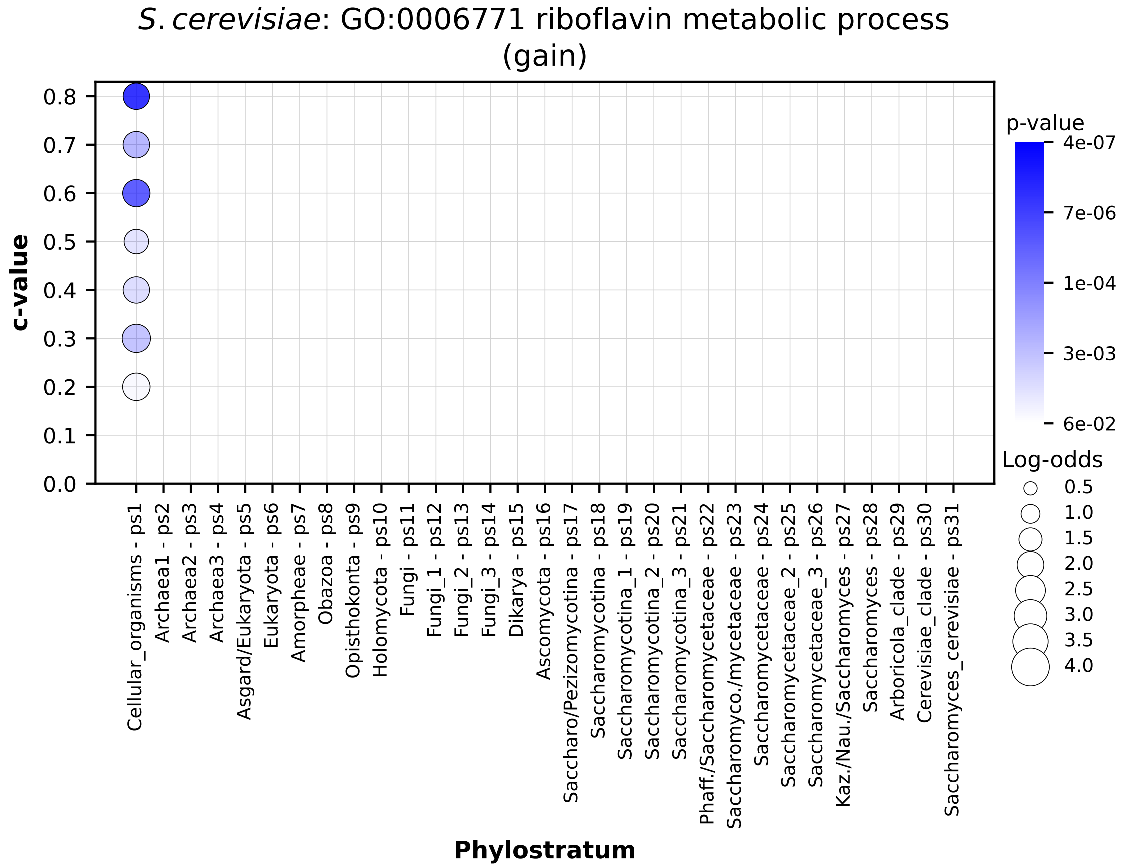

S. cerevisiae: GO:0006771 riboflavin metabolic process (gain)
c-value
0.8
0.7
0.6
0.5
0.4
0.3
0.2
0.1
0.0
p-value
4e-07
7e-06
1e-04
3e-03
6e-02
Log-odds
0.5
1.0
1.5
2.0
2.5
3.0
3.5
4.0
Cellular_organisms - ps1
Archaea1 - ps2
Archaea2 - ps3
Archaea3 - ps4
Asgard/Eukaryota - ps5
Eukaryota - ps6
Amorpheae - ps7
Obazoa - ps8
Opisthokonta - ps9
Holomycota - ps10
Fungi - ps11
Fungi_1 - ps12
Fungi_2 - ps13
Fungi_3 - ps14
Dikarya - ps15
Ascomycota - ps16
Saccharo/Pezizomycotina - ps17
Saccharomycotina - ps18
Saccharomycotina_1 - ps19
Saccharomycotina_2 - ps20
Saccharomycotina_3 - ps21
Phaff./Saccharomycetaceae - ps22
Saccharomyco./mycetaceae - ps23
Saccharomycetaceae - ps24
Saccharomycetaceae_2 - ps25
Saccharomycetaceae_3 - ps26
Kaz./Nau./Saccharomyces - ps27
Saccharomyces - ps28
Arboricola_clade - ps29
Cerevisiae_clade - ps30
Saccharomyces_cerevisiae - ps31
Phylostratum

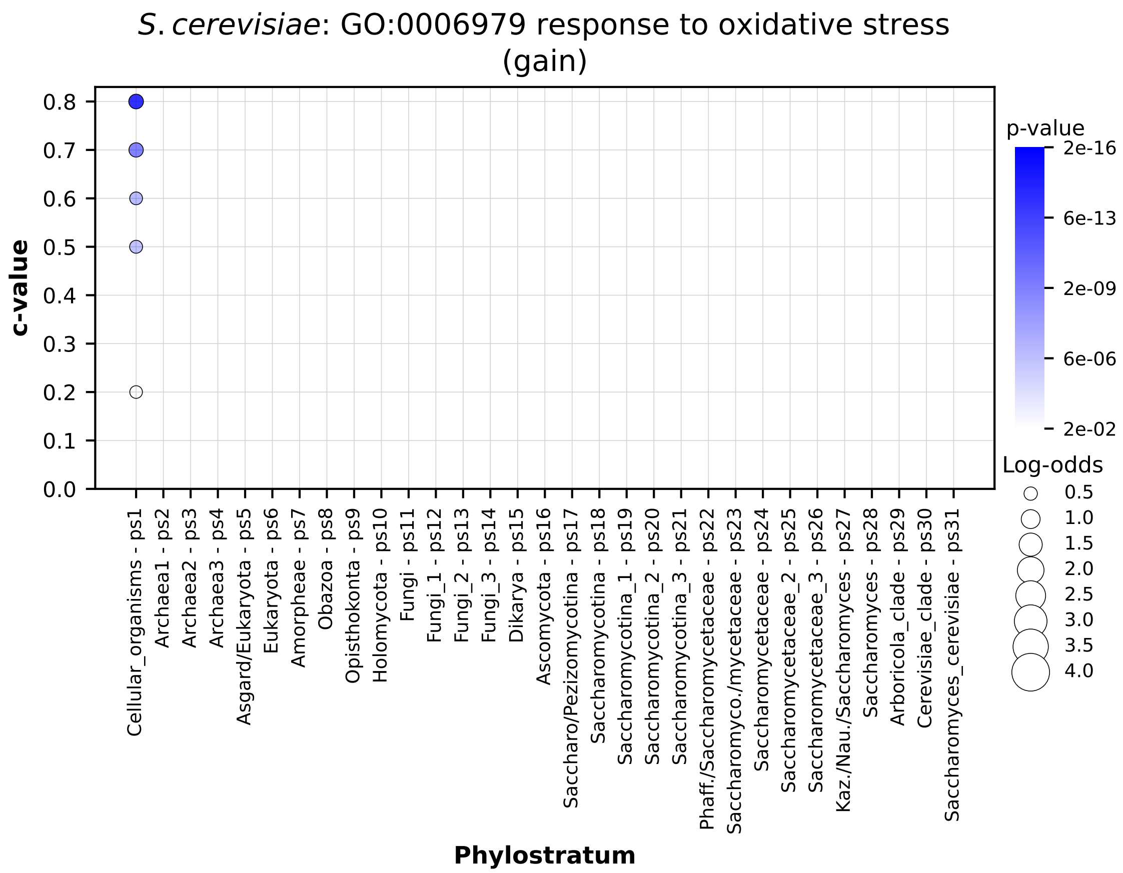

S. cerevisiae: GO:0006979 response to oxidative stress
(gain)
c-value
0.0
0.1
0.2
0.3
0.4
0.5
0.6
0.7
0.8
p-value
2e-16
6e-13
2e-09
6e-06
2e-02
Log-odds
0.5
1.0
1.5
2.0
2.5
3.0
3.5
4.0
Cellular_organisms - ps1
Archaea1 - ps2
Archaea2 - ps3
Archaea3 - ps4
Asgard/Eukaryota - ps5
Eukaryota - ps6
Amorpheae - ps7
Obazoa - ps8
Opisthokonta - ps9
Holomycota - ps10
Fungi - ps11
Fungi_1 - ps12
Fungi_2 - ps13
Fungi_3 - ps14
Dikarya - ps15
Ascomycota - ps16
Saccharo/Pezizomycotina - ps17
Saccharomycotina - ps18
Saccharomycotina_1 - ps19
Saccharomycotina_2 - ps20
Saccharomycotina_3 - ps21
Phaff./Saccharomycetaceae - ps22
Saccharomyco./mycetaceae - ps23
Saccharomycetaceae - ps24
Saccharomycetaceae_2 - ps25
Saccharomycetaceae_3 - ps26
Kaz./Nau./Saccharomyces - ps27
Saccharomyces - ps28
Arboricola_clade - ps29
Cerevisiae_clade - ps30
Saccharomyces_cerevisiae - ps31
Phylostratum

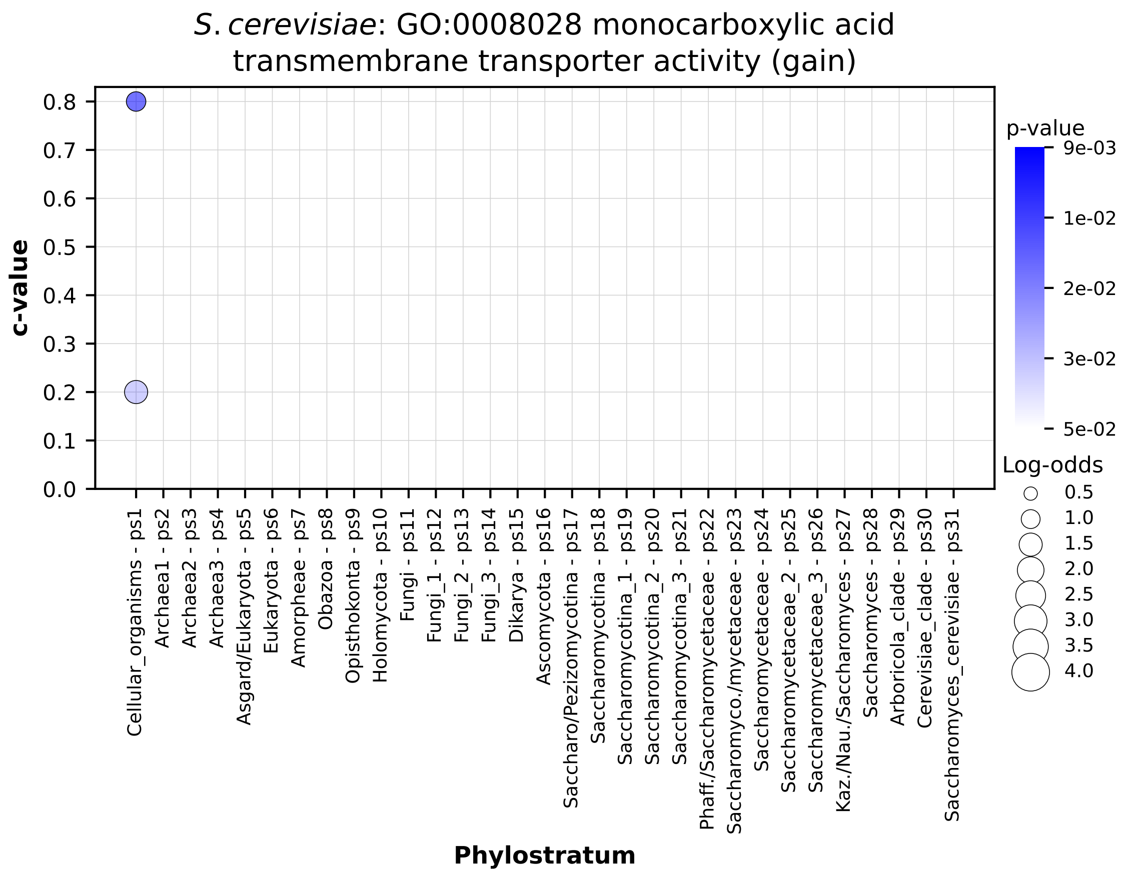

S. cerevisiae: GO:0008028 monocarboxylic acid transmembrane transporter activity (gain)
c-value
0.0
0.1
0.2
0.3
0.4
0.5
0.6
0.7
0.8
Phylostratum
Cellular_organisms - ps1
Archaea1 - ps2
Archaea2 - ps3
Archaea3 - ps4
Asgard/Eukaryota - ps5
Eukaryota - ps6
Amorpheae - ps7
Obazoa - ps8
Opisthokonta - ps9
Holomycota - ps10
Fungi - ps11
Fungi_1 - ps12
Fungi_2 - ps13
Fungi_3 - ps14
Dikarya - ps15
Ascomycota - ps16
Saccharo/Pezizomycotina - ps17
Saccharomycotina - ps18
Saccharomycotina_1 - ps19
Saccharomycotina_2 - ps20
Saccharomycotina_3 - ps21
Phaff./Saccharomycetaceae - ps22
Saccharomyco./mycetaceae - ps23
Saccharomycetaceae - ps24
Saccharomycetaceae_2 - ps25
Saccharomycetaceae_3 - ps26
Kaz./Nau./Saccharomyces - ps27
Saccharomyces - ps28
Arboricola_clade - ps29
Cerevisiae_clade - ps30
Saccharomyces_cerevisiae - ps31
p-value
9e-03
1e-02
2e-02
3e-02
5e-02
Log-odds
0.5
1.0
1.5
2.0
2.5
3.0
3.5
4.0

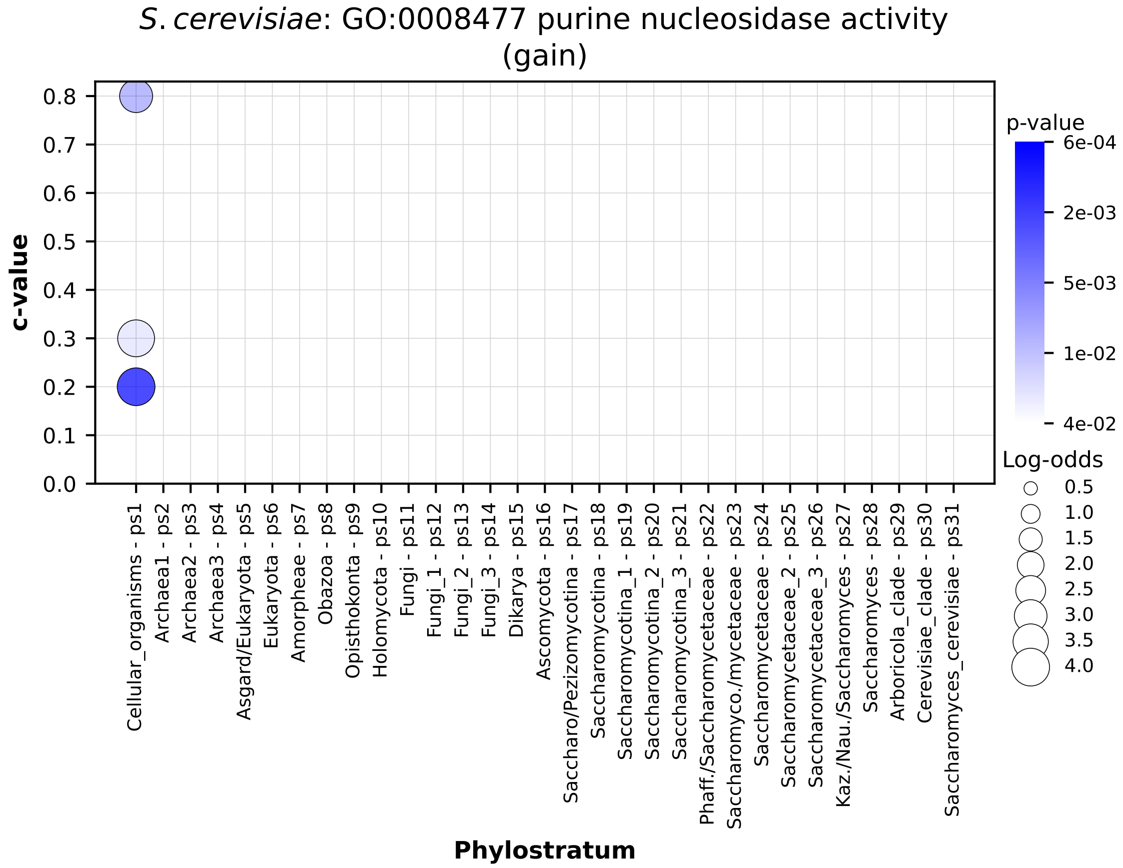
S. cerevisiae: GO:0008477 purine nucleosidase activity
(gain)
c-value
0.0
0.1
0.2
0.3
0.4
0.5
0.6
0.7
0.8
p-value
6e-04
2e-03
5e-03
1e-02
4e-02
Log-odds
0.5
1.0
1.5
2.0
2.5
3.0
3.5
4.0
Cellular_organisms - ps1
Archaea1 - ps2
Archaea2 - ps3
Archaea3 - ps4
Asgard/Eukaryota - ps5
Eukaryota - ps6
Amorpheae - ps7
Obazoa - ps8
Opisthokonta - ps9
Holomycota - ps10
Fungi - ps11
Fungi_1 - ps12
Fungi_2 - ps13
Fungi_3 - ps14
Dikarya - ps15
Ascomycota - ps16
Saccharo/Pezizomycotina - ps17
Saccharomycotina - ps18
Saccharomycotina_1 - ps19
Saccharomycotina_2 - ps20
Saccharomycotina_3 - ps21
Phaff./Saccharomycetaceae - ps22
Saccharomyco./mycetaceae - ps23
Saccharomycetaceae - ps24
Saccharomycetaceae_2 - ps25
Saccharomycetaceae_3 - ps26
Kaz./Nau./Saccharomyces - ps27
Saccharomyces - ps28
Arboricola_clade - ps29
Cerevisiae_clade - ps30
Saccharomyces_cerevisiae - ps31
Phylostratum

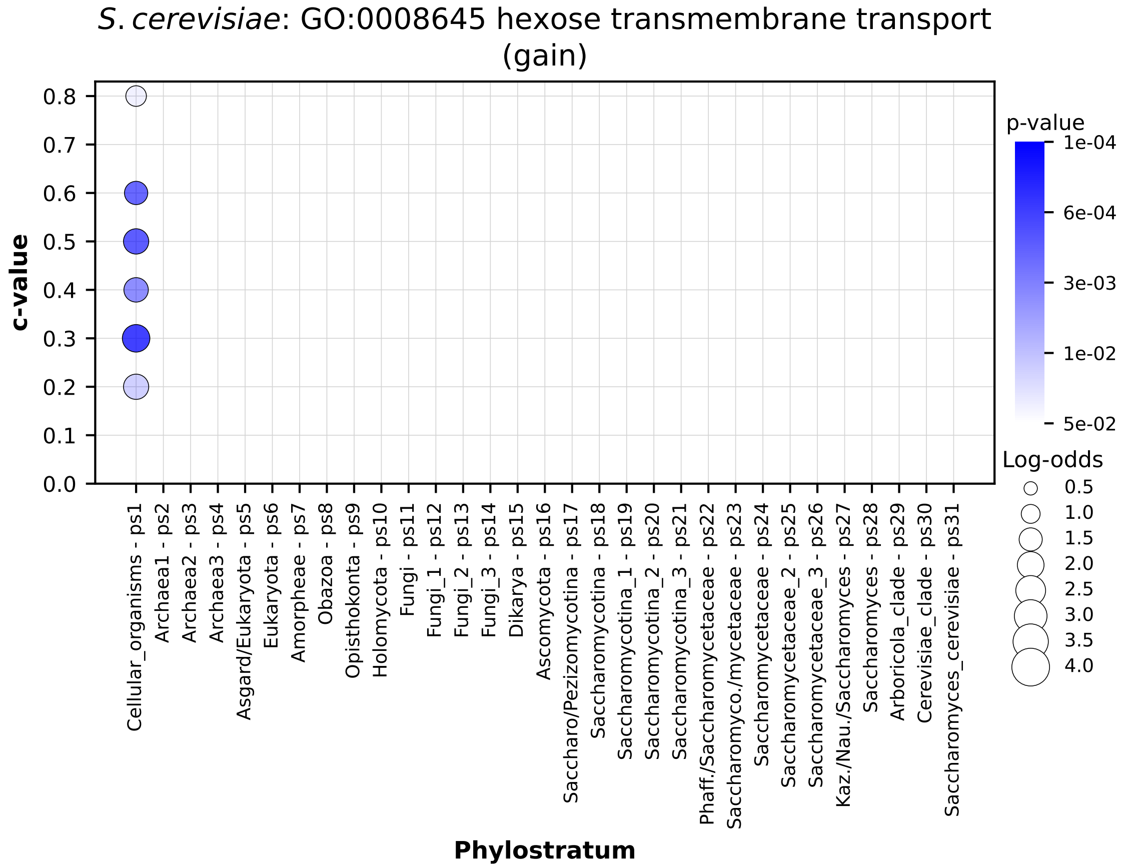
S. cerevisiae: GO:0008645 hexose transmembrane transport
(gain)
c-value
0.0
0.1
0.2
0.3
0.4
0.5
0.6
0.7
0.8
p-value
1e-04
6e-04
3e-03
1e-02
5e-02
Log-odds
0.5
1.0
1.5
2.0
2.5
3.0
3.5
4.0
Cellular_organisms - ps1
Archaea1 - ps2
Archaea2 - ps3
Archaea3 - ps4
Asgard/Eukaryota - ps5
Eukaryota - ps6
Amorpheae - ps7
Obazoa - ps8
Opisthokonta - ps9
Holomycota - ps10
Fungi - ps11
Fungi_1 - ps12
Fungi_2 - ps13
Fungi_3 - ps14
Dikarya - ps15
Ascomycota - ps16
Saccharo/Pezizomycotina - ps17
Saccharomycotina - ps18
Saccharomycotina_1 - ps19
Saccharomycotina_2 - ps20
Saccharomycotina_3 - ps21
Phaff./Saccharomycetaceae - ps22
Saccharomyco./mycetaceae - ps23
Saccharomycetaceae - ps24
Saccharomycetaceae_2 - ps25
Saccharomycetaceae_3 - ps26
Kaz./Nau./Saccharomyces - ps27
Saccharomyces - ps28
Arboricola_clade - ps29
Cerevisiae_clade - ps30
Saccharomyces_cerevisiae - ps31
Phylostratum

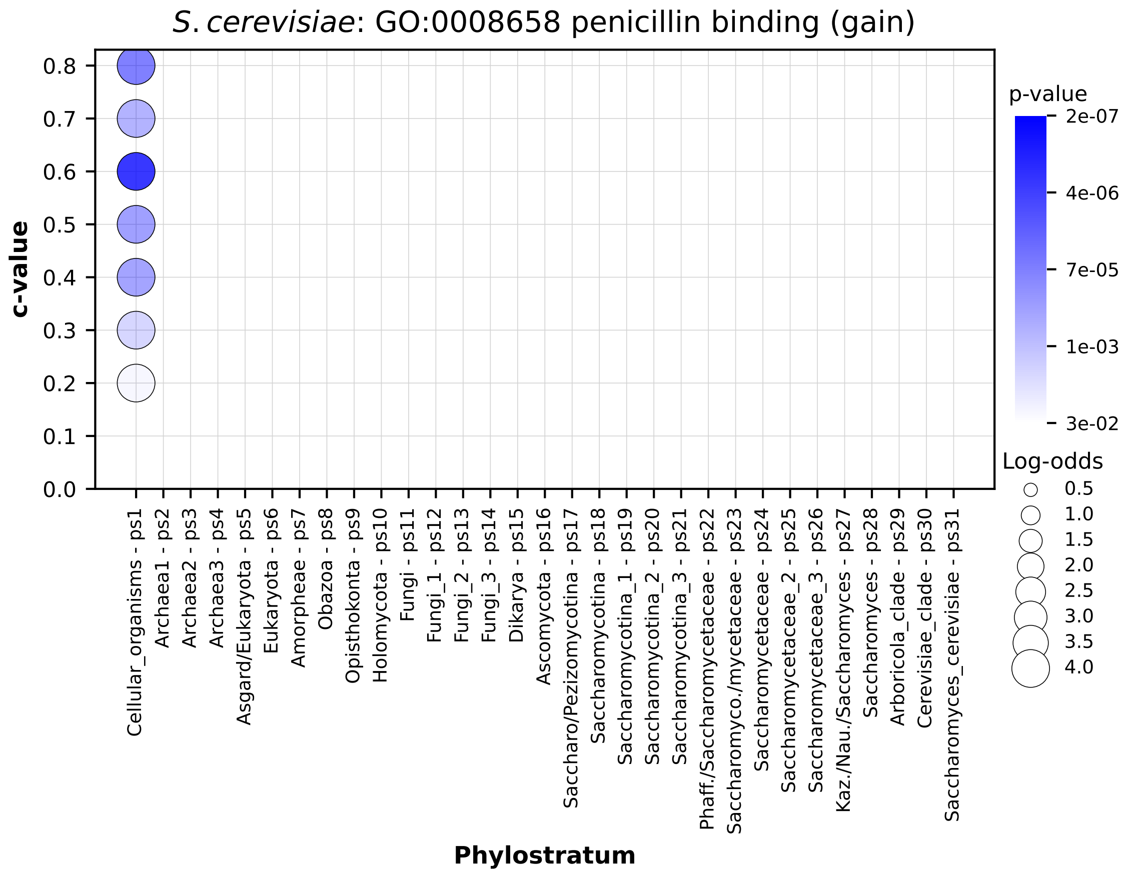

S. cerevisiae: GO:0008658 penicillin binding (gain)
c-value
0.8
0.7
0.6
0.5
0.4
0.3
0.2
0.1
0.0
p-value
2e-07
4e-06
7e-05
1e-03
3e-02
Log-odds
0.5
1.0
1.5
2.0
2.5
3.0
3.5
4.0
Cellular_organisms - ps1
Archaea1 - ps2
Archaea2 - ps3
Archaea3 - ps4
Asgard/Eukaryota - ps5
Eukaryota - ps6
Amorpheae - ps7
Obazoa - ps8
Opisthokonta - ps9
Holomycota - ps10
Fungi - ps11
Fungi_1 - ps12
Fungi_2 - ps13
Fungi_3 - ps14
Dikarya - ps15
Ascomycota - ps16
Saccharo/Pezizomycotina - ps17
Saccharomycotina - ps18
Saccharomycotina_1 - ps19
Saccharomycotina_2 - ps20
Saccharomycotina_3 - ps21
Phaff./Saccharomycetaceae - ps22
Saccharomyco./mycetaceae - ps23
Saccharomycetaceae - ps24
Saccharomycetaceae_2 - ps25
Saccharomycetaceae_3 - ps26
Kaz./Nau./Saccharomyces - ps27
Saccharomyces - ps28
Arboricola_clade - ps29
Cerevisiae_clade - ps30
Saccharomyces_cerevisiae - ps31
Phylostratum

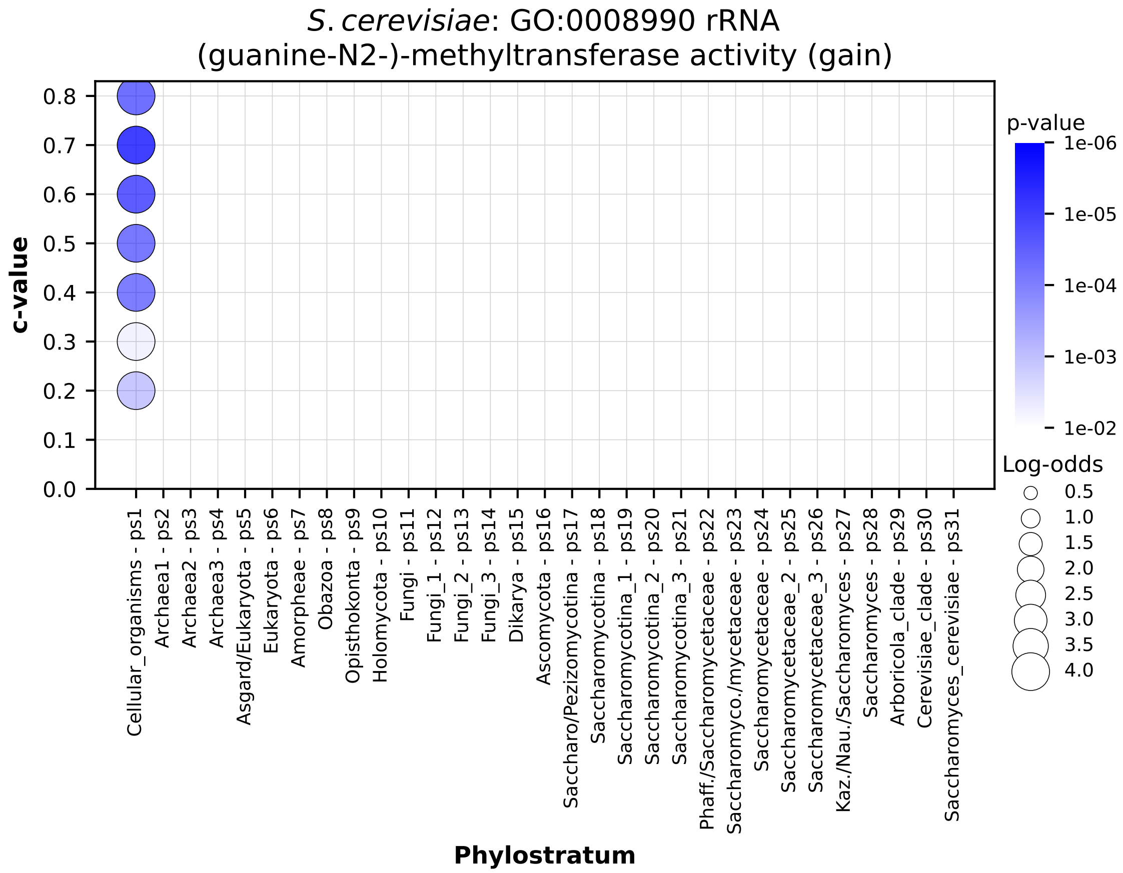
S. cerevisiae: GO:0008990 rRNA
(guanine-N2-)-methyltransferase activity (gain)
c-value
p-value
1e-06
1e-05
1e-04
1e-03
1e-02
Log-odds
0.5
1.0
1.5
2.0
2.5
3.0
3.5
4.0
0.0
0.1
0.2
0.3
0.4
0.5
0.6
0.7
0.8
Cellular_organisms - ps1
Archaea1 - ps2
Archaea2 - ps3
Archaea3 - ps4
Asgard/Eukaryota - ps5
Eukaryota - ps6
Amorpheae - ps7
Obazoa - ps8
Opisthokonta - ps9
Holomycota - ps10
Fungi - ps11
Fungi_1 - ps12
Fungi_2 - ps13
Fungi_3 - ps14
Dikarya - ps15
Ascomycota - ps16
Saccharo/Pezizomycotina - ps17
Saccharomycotina - ps18
Saccharomycotina_1 - ps19
Saccharomycotina_2 - ps20
Saccharomycotina_3 - ps21
Phaff./Saccharomycetaceae - ps22
Saccharomyco./mycetaceae - ps23
Saccharomycetaceae - ps24
Saccharomycetaceae_2 - ps25
Saccharomycetaceae_3 - ps26
Kaz./Nau./Saccharomyces - ps27
Saccharomyces - ps28
Arboricola_clade - ps29
Cerevisiae_clade - ps30
Saccharomyces_cerevisiae - ps31
Phylostratum

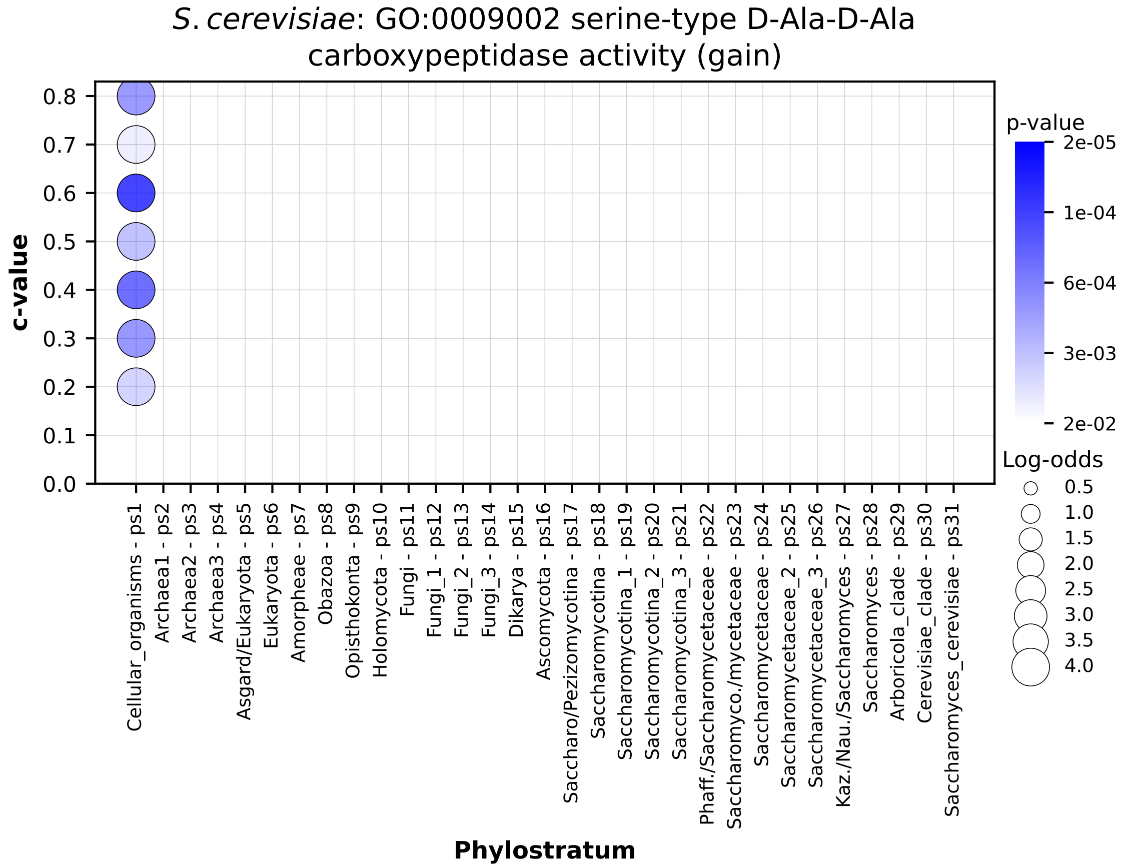

S. cerevisiae: GO:0009002 serine-type D-Ala-D-Ala carboxypeptidase activity (gain)
c-value
0.0
0.1
0.2
0.3
0.4
0.5
0.6
0.7
0.8
p-value
2e-05
1e-04
6e-04
3e-03
2e-02
Log-odds
0.5
1.0
1.5
2.0
2.5
3.0
3.5
4.0
Cellular_organisms - ps1
Archaea1 - ps2
Archaea2 - ps3
Archaea3 - ps4
Asgard/Eukaryota - ps5
Eukaryota - ps6
Amorpheae - ps7
Obazoa - ps8
Opisthokonta - ps9
Holomycota - ps10
Fungi - ps11
Fungi_1 - ps12
Fungi_2 - ps13
Fungi_3 - ps14
Dikarya - ps15
Ascomycota - ps16
Saccharo/Pezizomycotina - ps17
Saccharomycotina - ps18
Saccharomycotina_1 - ps19
Saccharomycotina_2 - ps20
Saccharomycotina_3 - ps21
Phaff./Saccharomycetaceae - ps22
Saccharomyco./mycetaceae - ps23
Saccharomycetaceae - ps24
Saccharomycetaceae_2 - ps25
Saccharomycetaceae_3 - ps26
Kaz./Nau./Saccharomyces - ps27
Saccharomyces - ps28
Arboricola_clade - ps29
Cerevisiae_clade - ps30
Saccharomyces_cerevisiae - ps31
Phylostratum

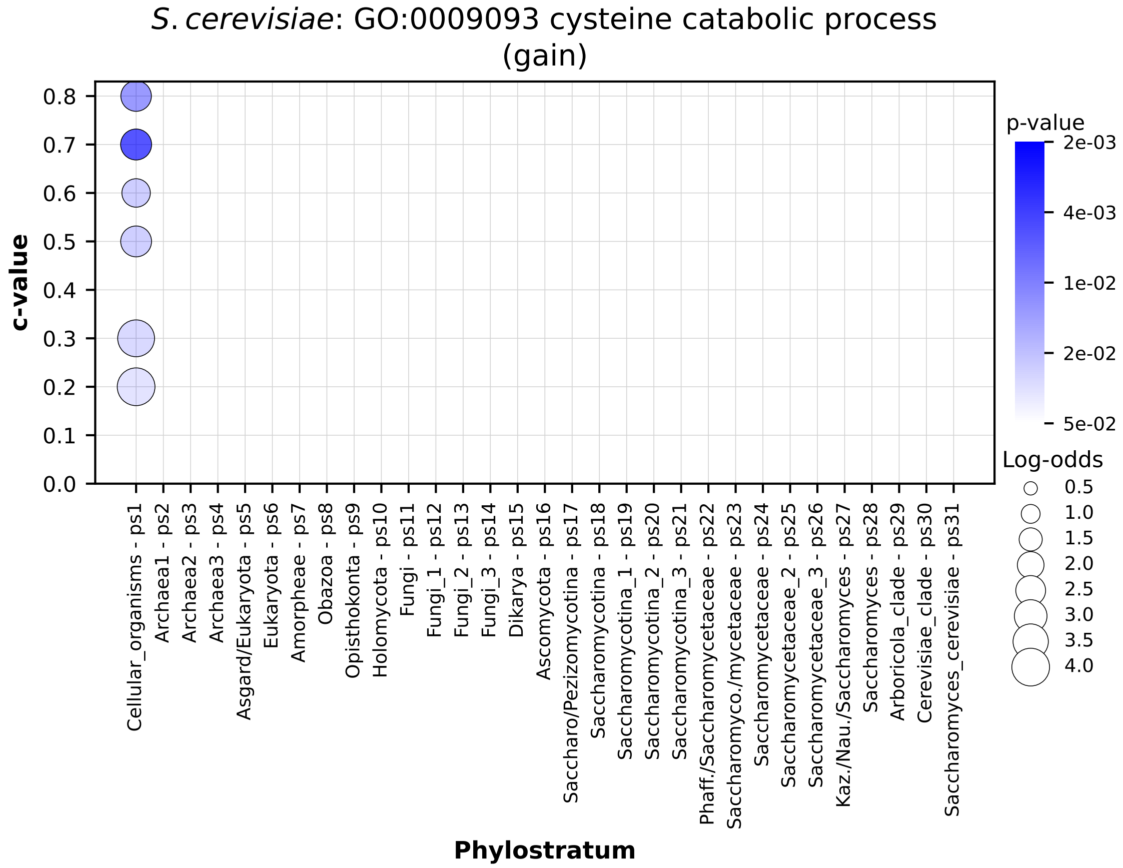

S. cerevisiae: GO:0009093 cysteine catabolic process
(gain)
c-value
0.0
0.1
0.2
0.3
0.4
0.5
0.6
0.7
0.8
p-value
2e-03
4e-03
1e-02
2e-02
5e-02
Log-odds
0.5
1.0
1.5
2.0
2.5
3.0
3.5
4.0
Cellular_organisms - ps1
Archaea1 - ps2
Archaea2 - ps3
Archaea3 - ps4
Asgard/Eukaryota - ps5
Eukaryota - ps6
Amorpheae - ps7
Obazoa - ps8
Opisthokonta - ps9
Holomycota - ps10
Fungi - ps11
Fungi_1 - ps12
Fungi_2 - ps13
Fungi_3 - ps14
Dikarya - ps15
Ascomycota - ps16
Saccharo/Pezizomycotina - ps17
Saccharomycotina - ps18
Saccharomycotina_1 - ps19
Saccharomycotina_2 - ps20
Saccharomycotina_3 - ps21
Phaff./Saccharomycetaceae - ps22
Saccharomyco./mycetaceae - ps23
Saccharomycetaceae - ps24
Saccharomycetaceae_2 - ps25
Saccharomycetaceae_3 - ps26
Kaz./Nau./Saccharomyces - ps27
Saccharomyces - ps28
Arboricola_clade - ps29
Cerevisiae_clade - ps30
Saccharomyces_cerevisiae - ps31
Phylostratum

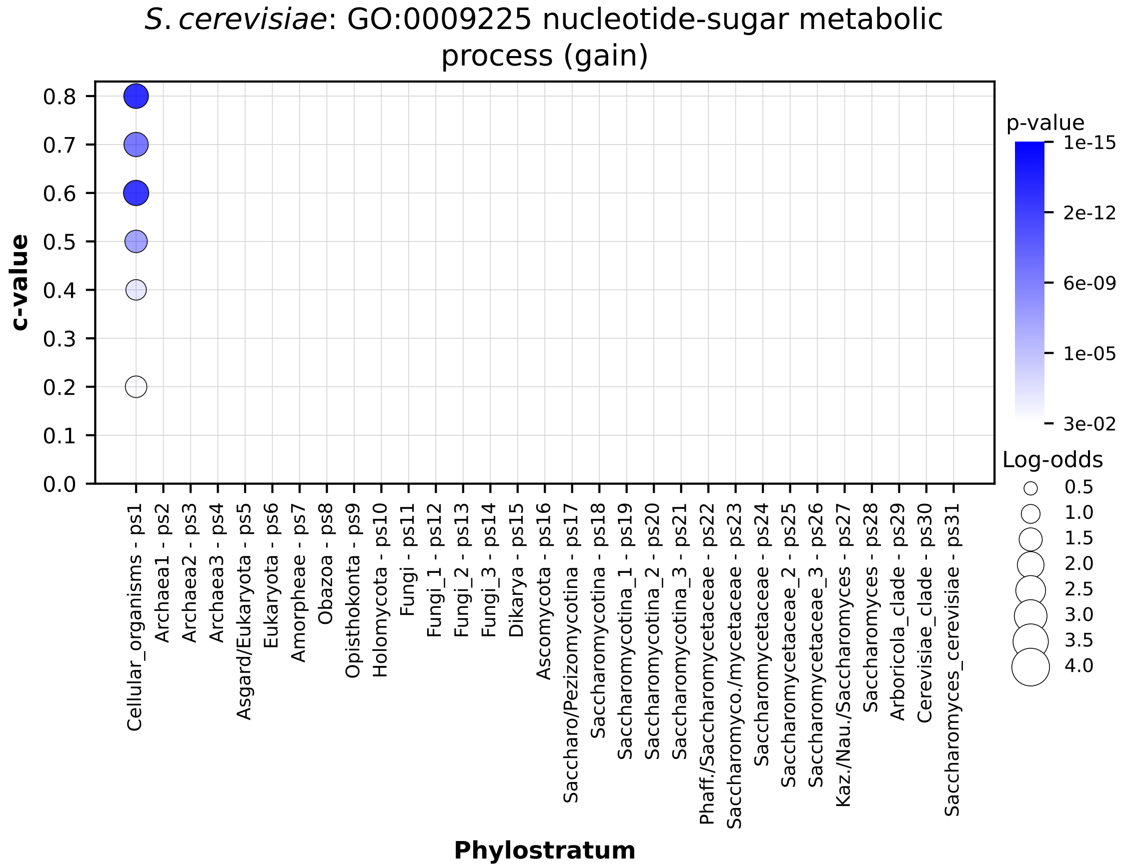

S. cerevisiae: GO:0009225 nucleotide-sugar metabolic process (gain)
c-value
0.0
0.1
0.2
0.3
0.4
0.5
0.6
0.7
0.8
Phylostratum
Cellular_organisms - ps1
Archaea1 - ps2
Archaea2 - ps3
Archaea3 - ps4
Asgard/Eukaryota - ps5
Eukaryota - ps6
Amorpheae - ps7
Obazoa - ps8
Opisthokonta - ps9
Holomycota - ps10
Fungi - ps11
Fungi_1 - ps12
Fungi_2 - ps13
Fungi_3 - ps14
Dikarya - ps15
Ascomycota - ps16
Saccharo/Pezizomycotina - ps17
Saccharomycotina - ps18
Saccharomycotina_1 - ps19
Saccharomycotina_2 - ps20
Saccharomycotina_3 - ps21
Phaff./Saccharomycetaceae - ps22
Saccharomyco./mycetaceae - ps23
Saccharomycetaceae - ps24
Saccharomycetaceae_2 - ps25
Saccharomycetaceae_3 - ps26
Kaz./Nau./Saccharomyces - ps27
Saccharomyces - ps28
Arboricola_clade - ps29
Cerevisiae_clade - ps30
Saccharomyces_cerevisiae - ps31
p-value
1e-15
2e-12
6e-09
1e-05
3e-02
Log-odds
0.5
1.0
1.5
2.0
2.5
3.0
3.5
4.0

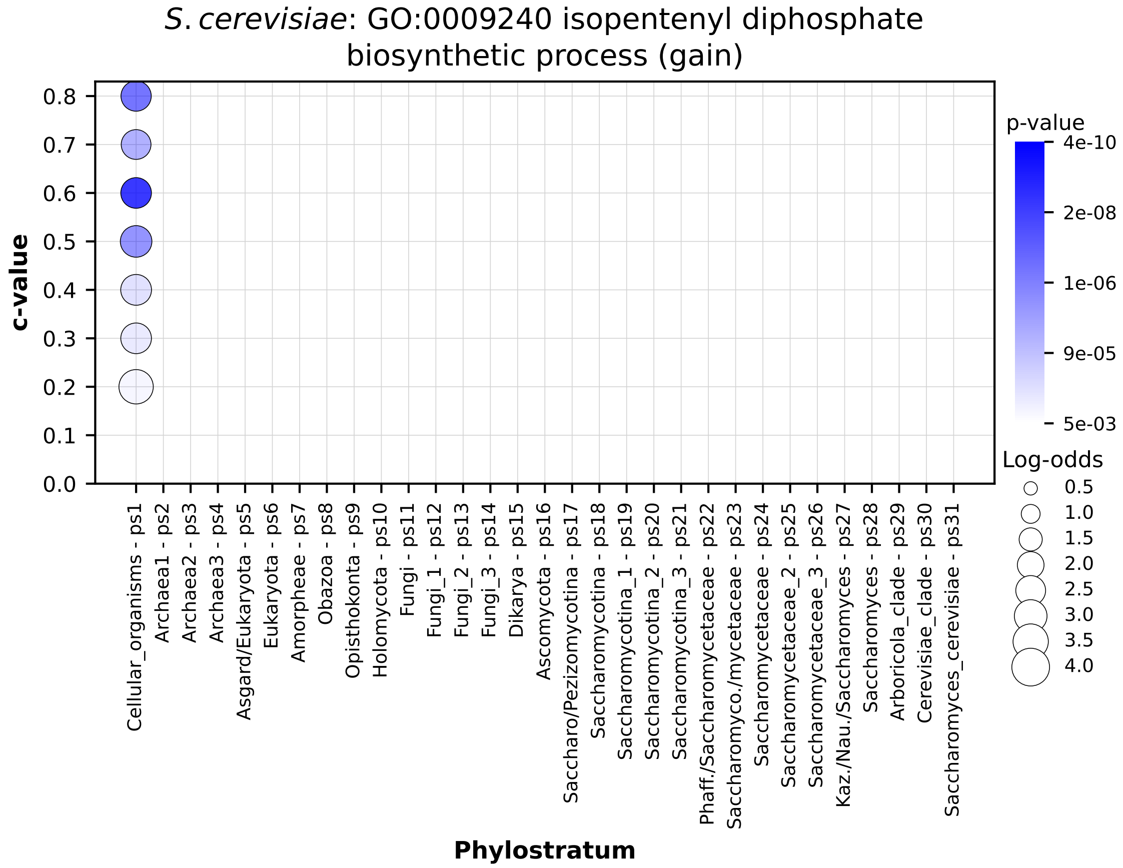

S. cerevisiae: GO:0009240 isopentenyl diphosphate biosynthetic process (gain)
c-value
0.0
0.1
0.2
0.3
0.4
0.5
0.6
0.7
0.8
Phylostratum
Cellular_organisms - ps1
Archaea1 - ps2
Archaea2 - ps3
Archaea3 - ps4
Asgard/Eukaryota - ps5
Eukaryota - ps6
Amorpheae - ps7
Obazoa - ps8
Opisthokonta - ps9
Holomycota - ps10
Fungi - ps11
Fungi_1 - ps12
Fungi_2 - ps13
Fungi_3 - ps14
Dikarya - ps15
Ascomycota - ps16
Saccharo/Pezizomycotina - ps17
Saccharomycotina - ps18
Saccharomycotina_1 - ps19
Saccharomycotina_2 - ps20
Saccharomycotina_3 - ps21
Phaff./Saccharomycetaceae - ps22
Saccharomyco./mycetaceae - ps23
Saccharomycetaceae - ps24
Saccharomycetaceae_2 - ps25
Saccharomycetaceae_3 - ps26
Kaz./Nau./Saccharomyces - ps27
Saccharomyces - ps28
Arboricola_clade - ps29
Cerevisiae_clade - ps30
Saccharomyces_cerevisiae - ps31
p-value
4e-10
2e-08
1e-06
9e-05
5e-03
Log-odds
0.5
1.0
1.5
2.0
2.5
3.0
3.5
4.0

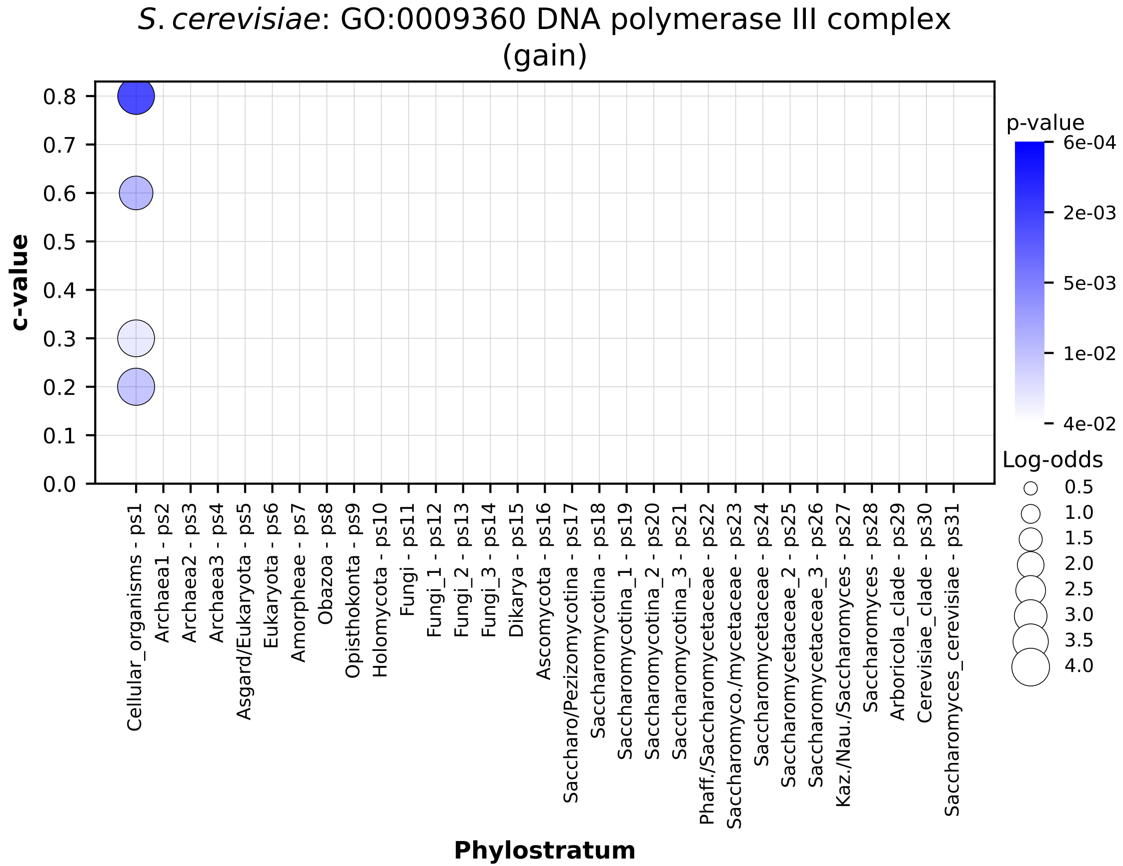

S. cerevisiae: GO:0009360 DNA polymerase III complex
(gain)
c-value
0.0
0.1
0.2
0.3
0.4
0.5
0.6
0.7
0.8
Phylostratum
Cellular_organisms - ps1
Archaea1 - ps2
Archaea2 - ps3
Archaea3 - ps4
Asgard/Eukaryota - ps5
Eukaryota - ps6
Amorpheae - ps7
Obazoa - ps8
Opisthokonta - ps9
Holomycota - ps10
Fungi - ps11
Fungi_1 - ps12
Fungi_2 - ps13
Fungi_3 - ps14
Dikarya - ps15
Ascomycota - ps16
Saccharo/Pezizomycotina - ps17
Saccharomycotina - ps18
Saccharomycotina_1 - ps19
Saccharomycotina_2 - ps20
Saccharomycotina_3 - ps21
Phaff./Saccharomycetaceae - ps22
Saccharomyco./mycetaceae - ps23
Saccharomycetaceae - ps24
Saccharomycetaceae_2 - ps25
Saccharomycetaceae_3 - ps26
Kaz./Nau./Saccharomyces - ps27
Saccharomyces - ps28
Arboricola_clade - ps29
Cerevisiae_clade - ps30
Saccharomyces_cerevisiae - ps31
p-value
6e-04
2e-03
5e-03
1e-02
4e-02
Log-odds
0.5
1.0
1.5
2.0
2.5
3.0
3.5
4.0

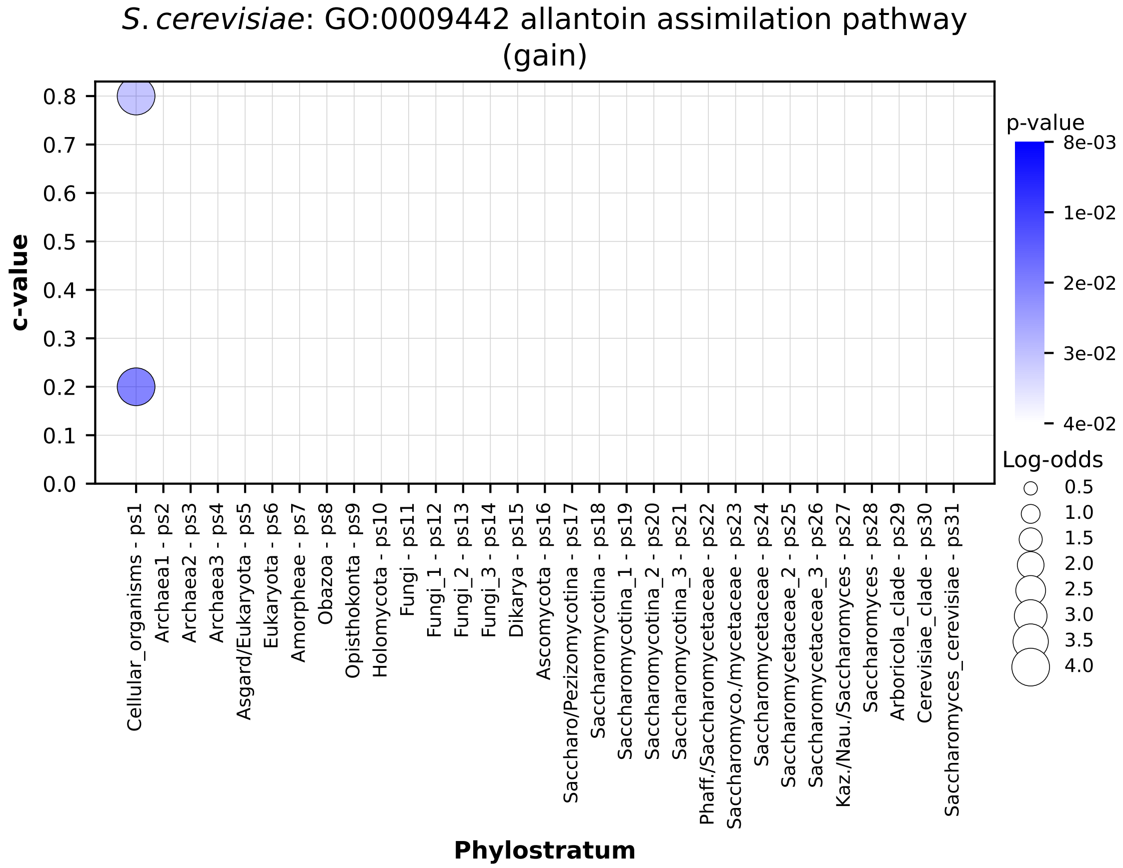

S. cerevisiae: GO:0009442 allantoin assimilation pathway
(gain)
c-value
0.0
0.1
0.2
0.3
0.4
0.5
0.6
0.7
0.8
Phylostratum
Cellular_organisms - ps1
Archaea1 - ps2
Archaea2 - ps3
Archaea3 - ps4
Asgard/Eukaryota - ps5
Eukaryota - ps6
Amorpheae - ps7
Obazoa - ps8
Opisthokonta - ps9
Holomycota - ps10
Fungi - ps11
Fungi_1 - ps12
Fungi_2 - ps13
Fungi_3 - ps14
Dikarya - ps15
Ascomycota - ps16
Saccharo/Pezizomycotina - ps17
Saccharomycotina - ps18
Saccharomycotina_1 - ps19
Saccharomycotina_2 - ps20
Saccharomycotina_3 - ps21
Phaff./Saccharomycetaceae - ps22
Saccharomyco./mycetaceae - ps23
Saccharomycetaceae - ps24
Saccharomycetaceae_2 - ps25
Saccharomycetaceae_3 - ps26
Kaz./Nau./Saccharomyces - ps27
Saccharomyces - ps28
Arboricola_clade - ps29
Cerevisiae_clade - ps30
Saccharomyces_cerevisiae - ps31
p-value
8e-03
1e-02
2e-02
3e-02
4e-02
Log-odds
0.5
1.0
1.5
2.0
2.5
3.0
3.5
4.0

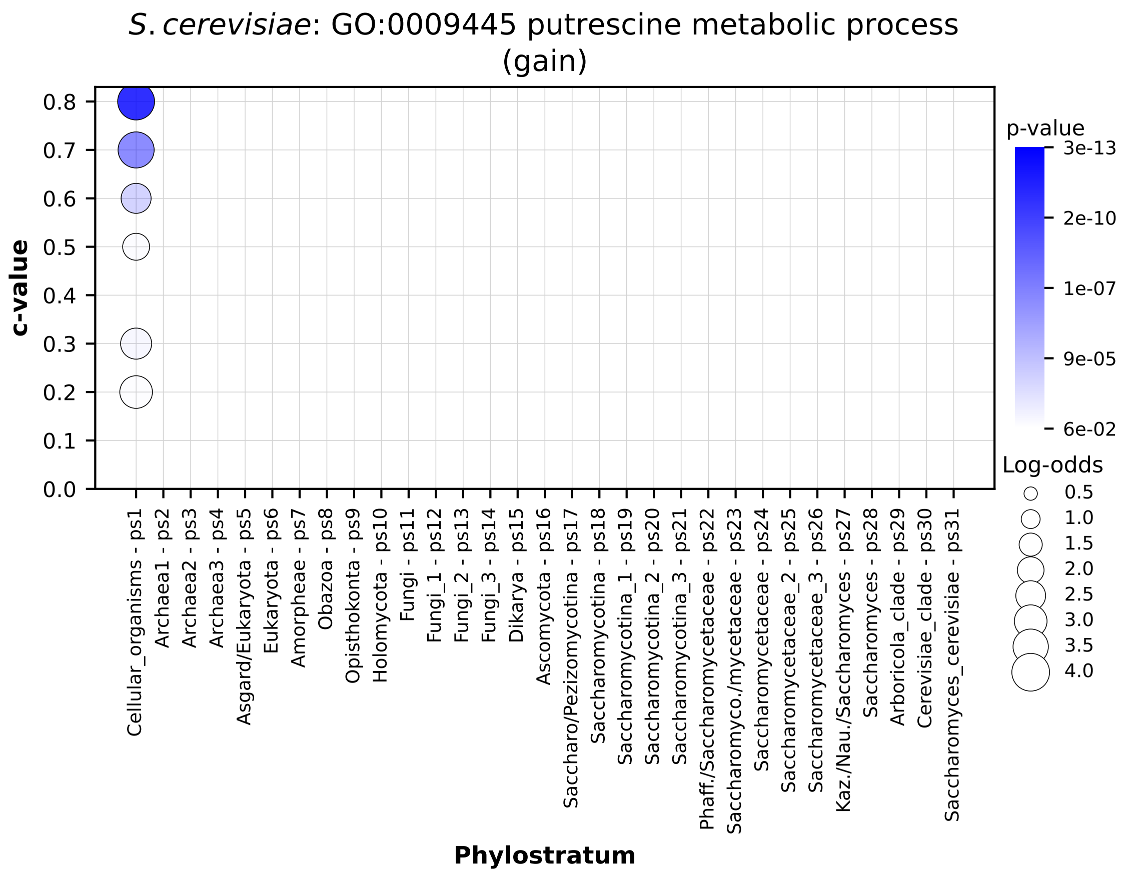

S. cerevisiae: GO:0009445 putrescine metabolic process (gain)
c-value
0.0
0.1
0.2
0.3
0.4
0.5
0.6
0.7
0.8
Phylostratum
Cellular_organisms - ps1
Archaea1 - ps2
Archaea2 - ps3
Archaea3 - ps4
Asgard/Eukaryota - ps5
Eukaryota - ps6
Amorpheae - ps7
Obazoa - ps8
Opisthokonta - ps9
Holomycota - ps10
Fungi - ps11
Fungi_1 - ps12
Fungi_2 - ps13
Fungi_3 - ps14
Dikarya - ps15
Ascomycota - ps16
Saccharo/Pezizomycotina - ps17
Saccharomycotina - ps18
Saccharomycotina_1 - ps19
Saccharomycotina_2 - ps20
Saccharomycotina_3 - ps21
Phaff./Saccharomycetaceae - ps22
Saccharomyco./mycetaceae - ps23
Saccharomycetaceae - ps24
Saccharomycetaceae_2 - ps25
Saccharomycetaceae_3 - ps26
Kaz./Nau./Saccharomyces - ps27
Saccharomyces - ps28
Arboricola_clade - ps29
Cerevisiae_clade - ps30
Saccharomyces_cerevisiae - ps31
p-value
3e-13
2e-10
1e-07
9e-05
6e-02
Log-odds
0.5
1.0
1.5
2.0
2.5
3.0
3.5
4.0

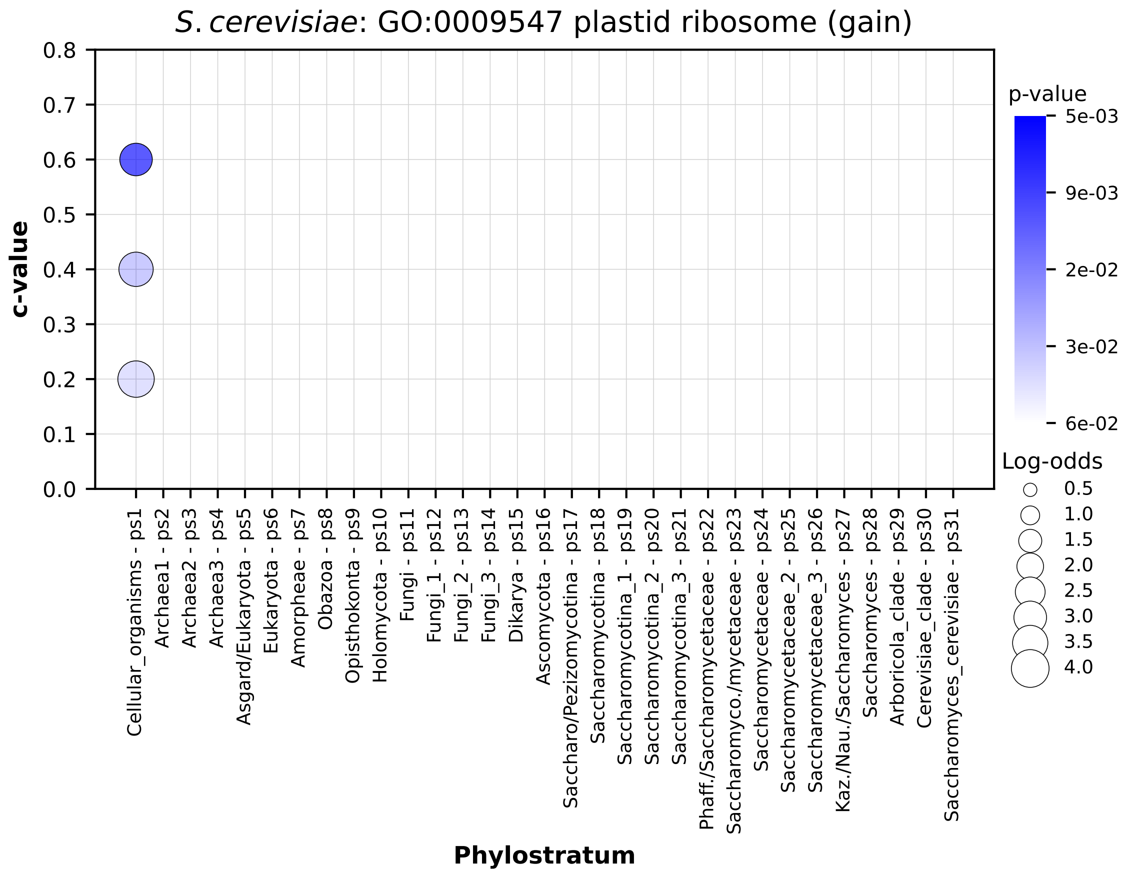

S. cerevisiae: GO:0009547 plastid ribosome (gain)
c-value
0.8
0.7
0.6
0.5
0.4
0.3
0.2
0.1
0.0
Phylostratum
Cellular_organisms - ps1
Archaea1 - ps2
Archaea2 - ps3
Archaea3 - ps4
Asgard/Eukaryota - ps5
Eukaryota - ps6
Amorpheae - ps7
Obazoa - ps8
Opisthokonta - ps9
Holomycota - ps10
Fungi - ps11
Fungi_1 - ps12
Fungi_2 - ps13
Fungi_3 - ps14
Dikarya - ps15
Ascomycota - ps16
Saccharo/Pezizomycotina - ps17
Saccharomycotina - ps18
Saccharomycotina_1 - ps19
Saccharomycotina_2 - ps20
Saccharomycotina_3 - ps21
Phaff./Saccharomycetaceae - ps22
Saccharomyco./mycetaceae - ps23
Saccharomycetaceae - ps24
Saccharomycetaceae_2 - ps25
Saccharomycetaceae_3 - ps26
Kaz./Nau./Saccharomyces - ps27
Saccharomyces - ps28
Arboricola_clade - ps29
Cerevisiae_clade - ps30
Saccharomyces_cerevisiae - ps31
p-value
5e-03
9e-03
2e-02
3e-02
6e-02
Log-odds
0.5
1.0
1.5
2.0
2.5
3.0
3.5
4.0

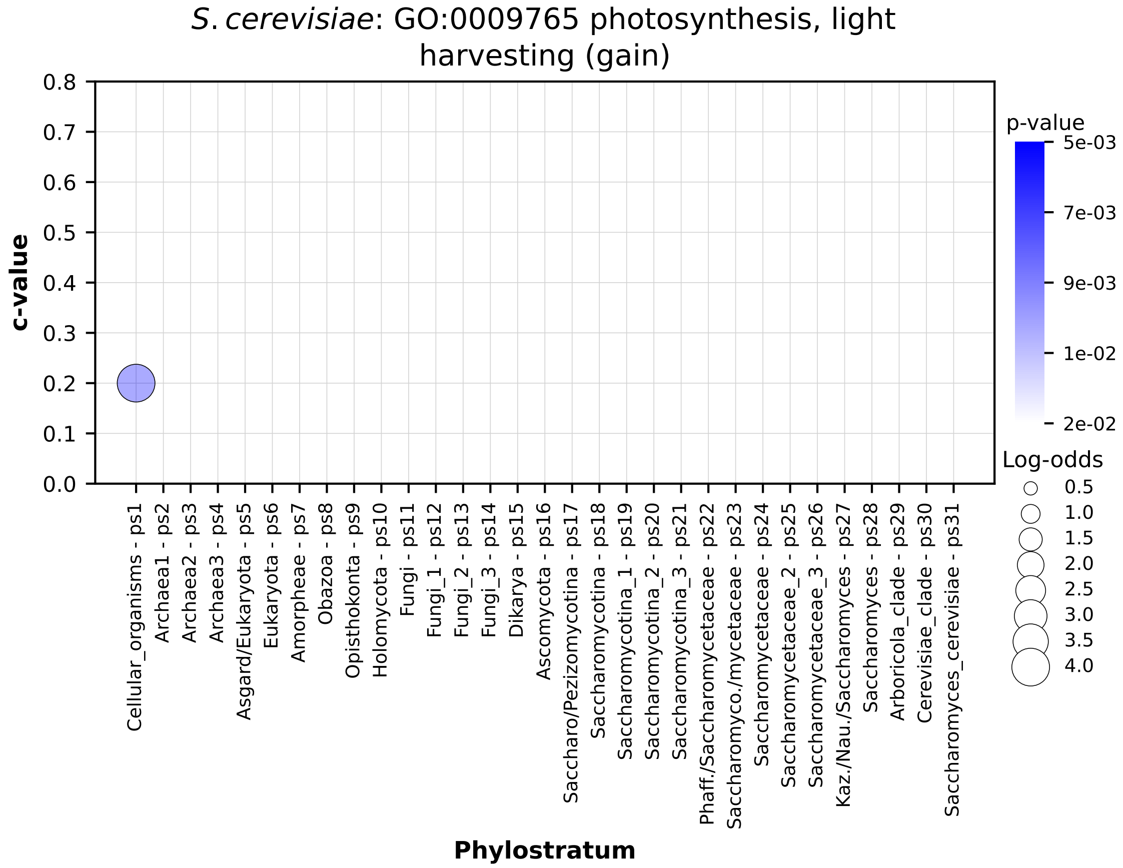

S. cerevisiae: GO:0009765 photosynthesis, light harvesting (gain)
c-value
0.8
0.7
0.6
0.5
0.4
0.3
0.2
0.1
0.0
p-value
5e-03
7e-03
9e-03
1e-02
2e-02
Log-odds
0.5
1.0
1.5
2.0
2.5
3.0
3.5
4.0
Cellular_organisms - ps1
Archaea1 - ps2
Archaea2 - ps3
Archaea3 - ps4
Asgard/Eukaryota - ps5
Eukaryota - ps6
Amorpheae - ps7
Obazoa - ps8
Opisthokonta - ps9
Holomycota - ps10
Fungi - ps11
Fungi_1 - ps12
Fungi_2 - ps13
Fungi_3 - ps14
Dikarya - ps15
Ascomycota - ps16
Saccharo/Pezizomycotina - ps17
Saccharomycotina - ps18
Saccharomycotina_1 - ps19
Saccharomycotina_2 - ps20
Saccharomycotina_3 - ps21
Phaff./Saccharomycetaceae - ps22
Saccharomyco./mycetaceae - ps23
Saccharomycetaceae - ps24
Saccharomycetaceae_2 - ps25
Saccharomycetaceae_3 - ps26
Kaz./Nau./Saccharomyces - ps27
Saccharomyces - ps28
Arboricola_clade - ps29
Cerevisiae_clade - ps30
Saccharomyces_cerevisiae - ps31
Phylostratum

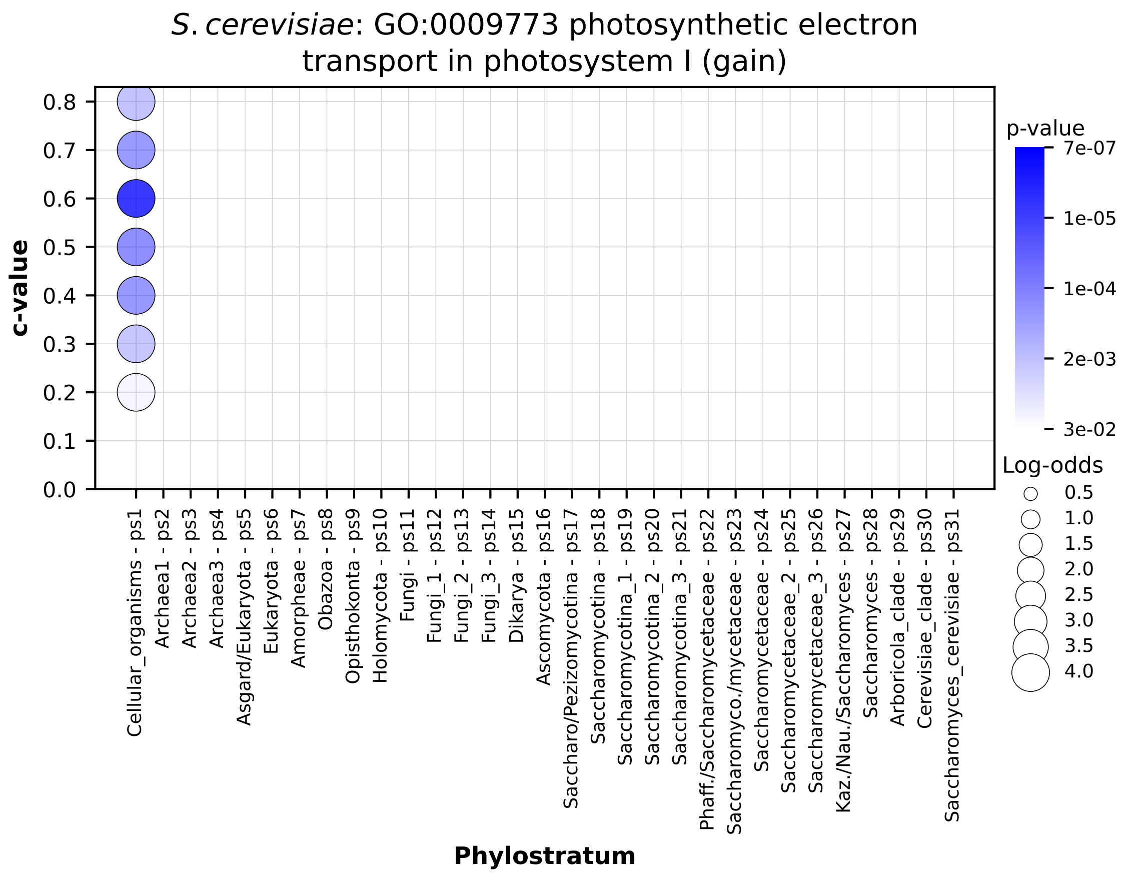

S. cerevisiae: GO:0009773 photosynthetic electron transport in photosystem I (gain)
c-value
0.0
0.1
0.2
0.3
0.4
0.5
0.6
0.7
0.8
p-value
7e-07
1e-05
1e-04
2e-03
3e-02
Log-odds
0.5
1.0
1.5
2.0
2.5
3.0
3.5
4.0
Cellular_organisms - ps1
Archaea1 - ps2
Archaea2 - ps3
Archaea3 - ps4
Asgard/Eukaryota - ps5
Eukaryota - ps6
Amorpheae - ps7
Obazoa - ps8
Opisthokonta - ps9
Holomycota - ps10
Fungi - ps11
Fungi_1 - ps12
Fungi_2 - ps13
Fungi_3 - ps14
Dikarya - ps15
Ascomycota - ps16
Saccharo/Pezizomycotina - ps17
Saccharomycotina - ps18
Saccharomycotina_1 - ps19
Saccharomycotina_2 - ps20
Saccharomycotina_3 - ps21
Phaff./Saccharomycetaceae - ps22
Saccharomyco./mycetaceae - ps23
Saccharomycetaceae - ps24
Saccharomycetaceae_2 - ps25
Saccharomycetaceae_3 - ps26
Kaz./Nau./Saccharomyces - ps27
Saccharomyces - ps28
Arboricola_clade - ps29
Cerevisiae_clade - ps30
Saccharomyces_cerevisiae - ps31
Phylostratum

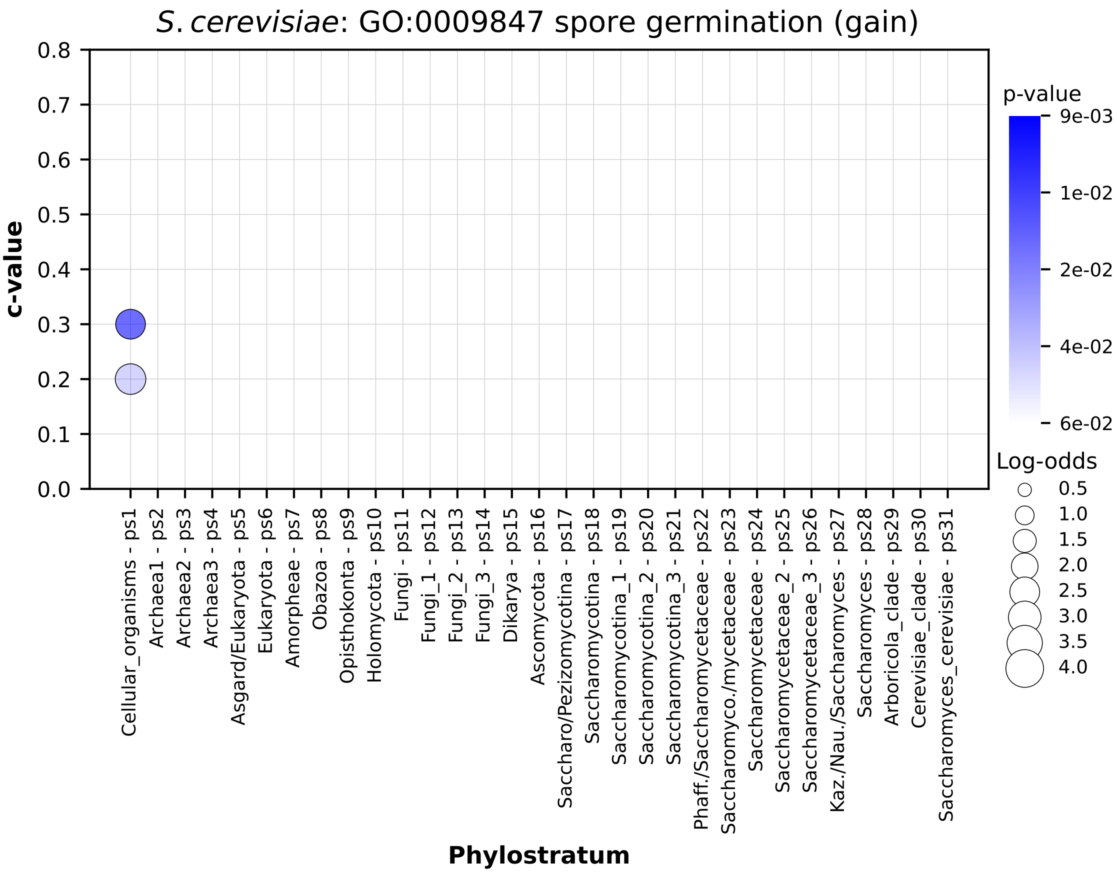

S. cerevisiae: GO:0009847 spore germination (gain)
c-value
0.8
0.7
0.6
0.5
0.4
0.3
0.2
0.1
0.0
p-value
9e-03
1e-02
2e-02
4e-02
6e-02
Log-odds
0.5
1.0
1.5
2.0
2.5
3.0
3.5
4.0
Cellular_organisms - ps1
Archaea1 - ps2
Archaea2 - ps3
Archaea3 - ps4
Asgard/Eukaryota - ps5
Eukaryota - ps6
Amorpheae - ps7
Obazoa - ps8
Opisthokonta - ps9
Holomycota - ps10
Fungi - ps11
Fungi_1 - ps12
Fungi_2 - ps13
Fungi_3 - ps14
Dikarya - ps15
Ascomycota - ps16
Saccharo/Pezizomycotina - ps17
Saccharomycotina - ps18
Saccharomycotina_1 - ps19
Saccharomycotina_2 - ps20
Saccharomycotina_3 - ps21
Phaff./Saccharomycetaceae - ps22
Saccharomyco./mycetaceae - ps23
Saccharomycetaceae - ps24
Saccharomycetaceae_2 - ps25
Saccharomycetaceae_3 - ps26
Kaz./Nau./Saccharomyces - ps27
Saccharomyces - ps28
Arboricola_clade - ps29
Cerevisiae_clade - ps30
Saccharomyces_cerevisiae - ps31
Phylostratum

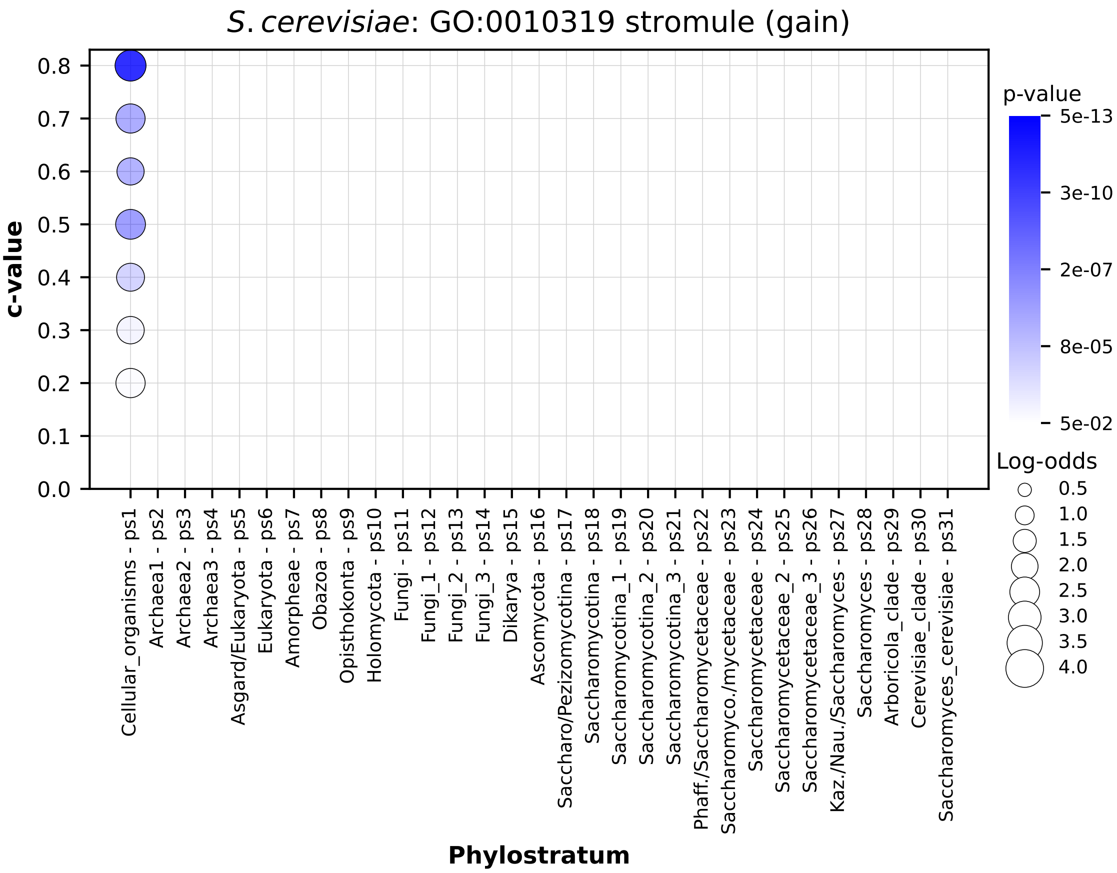

S. cerevisiae: GO:0010319 stromule (gain)
c-value
0.0
0.1
0.2
0.3
0.4
0.5
0.6
0.7
0.8
p-value
5e-13
3e-10
2e-07
8e-05
5e-02
Log-odds
0.5
1.0
1.5
2.0
2.5
3.0
3.5
4.0
Cellular_organisms - ps1
Archaea1 - ps2
Archaea2 - ps3
Archaea3 - ps4
Asgard/Eukaryota - ps5
Eukaryota - ps6
Amorpheae - ps7
Obazoa - ps8
Opisthokonta - ps9
Holomycota - ps10
Fungi - ps11
Fungi_1 - ps12
Fungi_2 - ps13
Fungi_3 - ps14
Dikarya - ps15
Ascomycota - ps16
Saccharo/Pezizomycotina - ps17
Saccharomycotina - ps18
Saccharomycotina_1 - ps19
Saccharomycotina_2 - ps20
Saccharomycotina_3 - ps21
Phaff./Saccharomycetaceae - ps22
Saccharomyco./mycetaceae - ps23
Saccharomycetaceae - ps24
Saccharomycetaceae_2 - ps25
Saccharomycetaceae_3 - ps26
Kaz./Nau./Saccharomyces - ps27
Saccharomyces - ps28
Arboricola_clade - ps29
Cerevisiae_clade - ps30
Saccharomyces_cerevisiae - ps31
Phylostratum

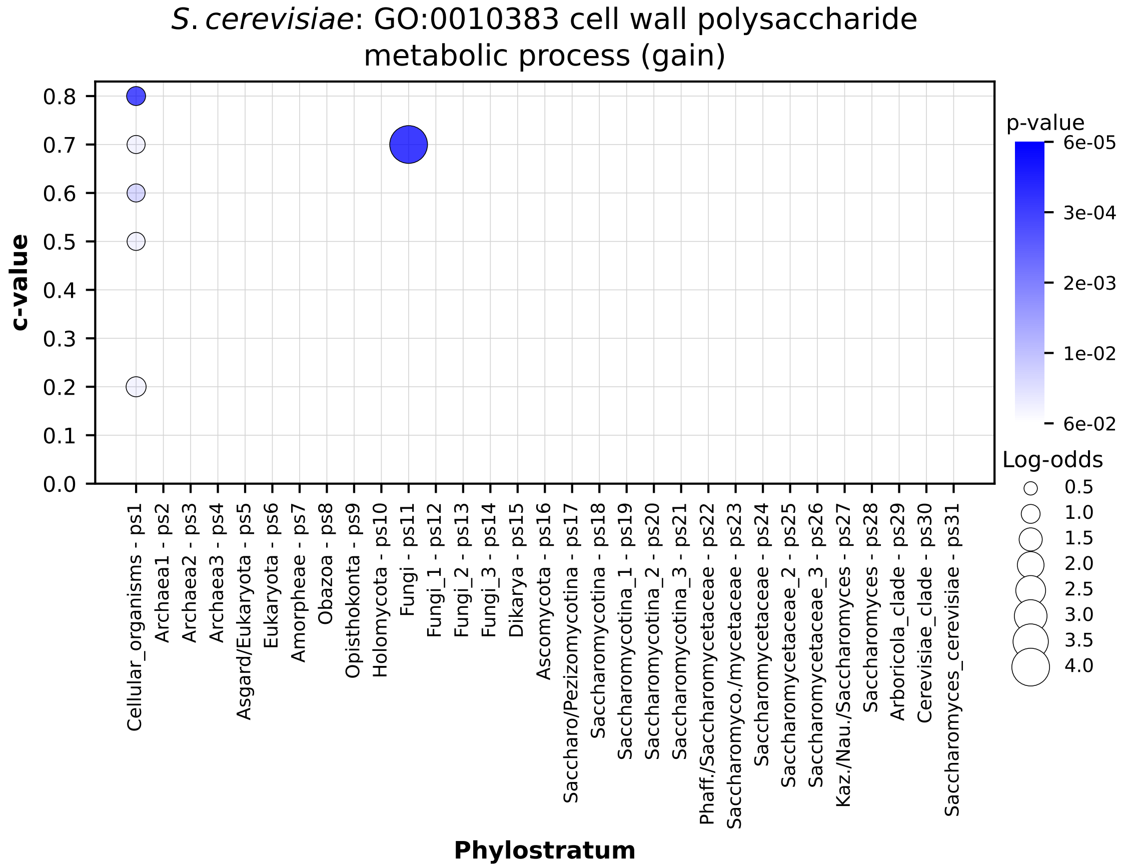

S. cerevisiae: GO:0010383 cell wall polysaccharide metabolic process (gain)
c-value
0.8
0.7
0.6
0.5
0.4
0.3
0.2
0.1
0.0
p-value
6e-05
3e-04
2e-03
1e-02
6e-02
Log-odds
0.5
1.0
1.5
2.0
2.5
3.0
3.5
4.0
Cellular_organisms - ps1
Archaea1 - ps2
Archaea2 - ps3
Archaea3 - ps4
Asgard/Eukaryota - ps5
Eukaryota - ps6
Amorpheae - ps7
Obazoa - ps8
Opisthokonta - ps9
Holomycota - ps10
Fungi - ps11
Fungi_1 - ps12
Fungi_2 - ps13
Fungi_3 - ps14
Dikarya - ps15
Ascomycota - ps16
Saccharo/Pezizomycotina - ps17
Saccharomycotina - ps18
Saccharomycotina_1 - ps19
Saccharomycotina_2 - ps20
Saccharomycotina_3 - ps21
Phaff./Saccharomycetaceae - ps22
Saccharomyco./mycetaceae - ps23
Saccharomycetaceae - ps24
Saccharomycetaceae_2 - ps25
Saccharomycetaceae_3 - ps26
Kaz./Nau./Saccharomyces - ps27
Saccharomyces - ps28
Arboricola_clade - ps29
Cerevisiae_clade - ps30
Saccharomyces_cerevisiae - ps31
Phylostratum

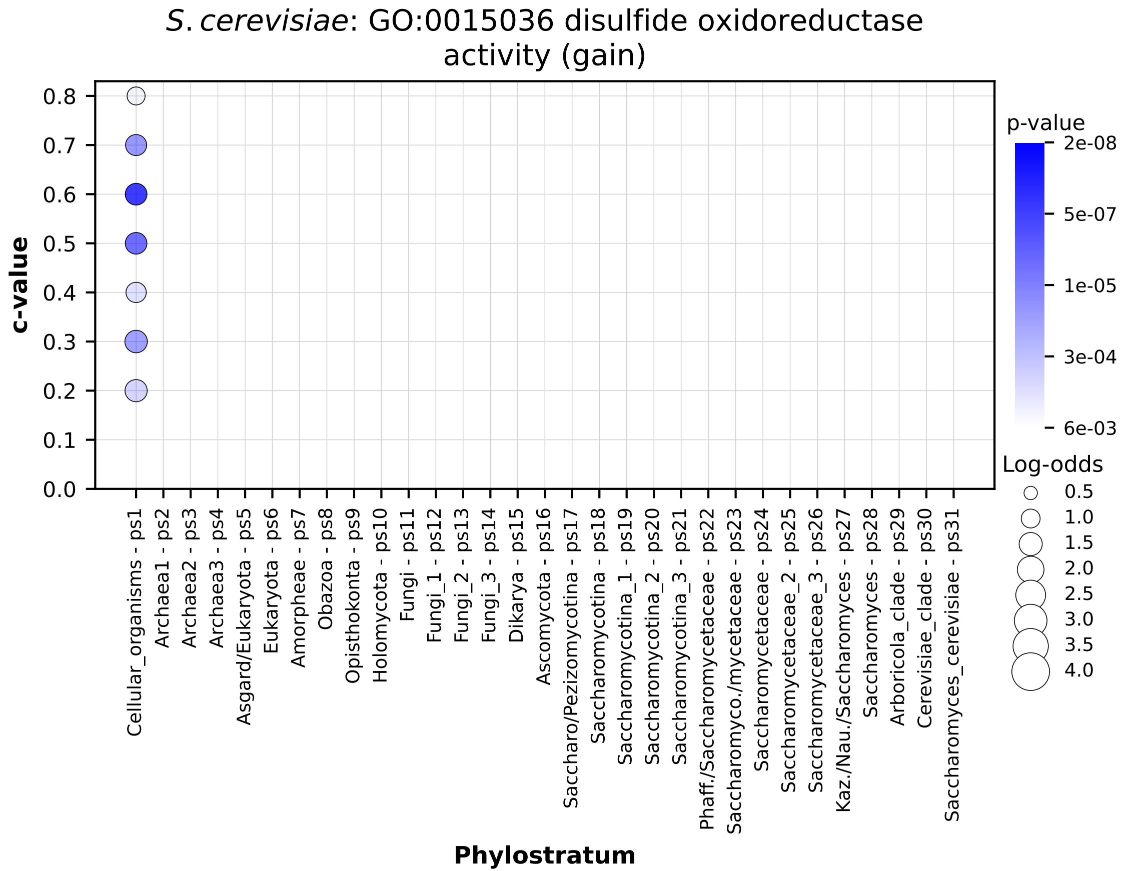

S. cerevisiae: GO:0015036 disulfide oxidoreductase activity (gain)
c-value
0.0
0.1
0.2
0.3
0.4
0.5
0.6
0.7
0.8
Phylostratum
Cellular_organisms - ps1
Archaea1 - ps2
Archaea2 - ps3
Archaea3 - ps4
Asgard/Eukaryota - ps5
Eukaryota - ps6
Amorpheae - ps7
Obazoa - ps8
Opisthokonta - ps9
Holomycota - ps10
Fungi - ps11
Fungi_1 - ps12
Fungi_2 - ps13
Fungi_3 - ps14
Dikarya - ps15
Ascomycota - ps16
Saccharo/Pezizomycotina - ps17
Saccharomycotina - ps18
Saccharomycotina_1 - ps19
Saccharomycotina_2 - ps20
Saccharomycotina_3 - ps21
Phaff./Saccharomycetaceae - ps22
Saccharomyco./mycetaceae - ps23
Saccharomycetaceae - ps24
Saccharomycetaceae_2 - ps25
Saccharomycetaceae_3 - ps26
Kaz./Nau./Saccharomyces - ps27
Saccharomyces - ps28
Arboricola_clade - ps29
Cerevisiae_clade - ps30
Saccharomyces_cerevisiae - ps31
p-value
2e-08
5e-07
1e-05
3e-04
6e-03
Log-odds
0.5
1.0
1.5
2.0
2.5
3.0
3.5
4.0

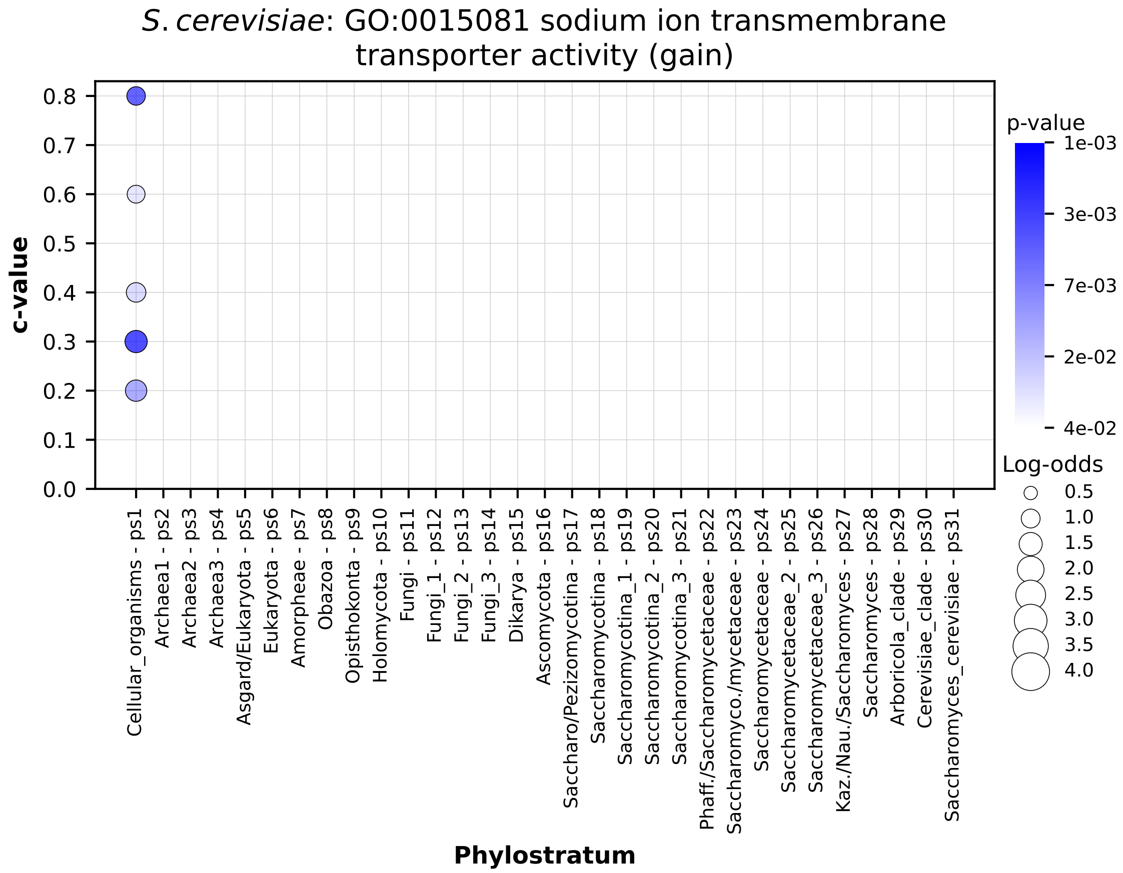

S. cerevisiae: GO:0015081 sodium ion transmembrane transporter activity (gain)
c-value
0.0
0.1
0.2
0.3
0.4
0.5
0.6
0.7
0.8
Phylostratum
Cellular_organisms - ps1
Archaea1 - ps2
Archaea2 - ps3
Archaea3 - ps4
Asgard/Eukaryota - ps5
Eukaryota - ps6
Amorpheae - ps7
Obazoa - ps8
Opisthokonta - ps9
Holomycota - ps10
Fungi - ps11
Fungi_1 - ps12
Fungi_2 - ps13
Fungi_3 - ps14
Dikarya - ps15
Ascomycota - ps16
Saccharo/Pezizomycotina - ps17
Saccharomycotina - ps18
Saccharomycotina_1 - ps19
Saccharomycotina_2 - ps20
Saccharomycotina_3 - ps21
Phaff./Saccharomycetaceae - ps22
Saccharomyco./mycetaceae - ps23
Saccharomycetaceae - ps24
Saccharomycetaceae_2 - ps25
Saccharomycetaceae_3 - ps26
Kaz./Nau./Saccharomyces - ps27
Saccharomyces - ps28
Arboricola_clade - ps29
Cerevisiae_clade - ps30
Saccharomyces_cerevisiae - ps31
p-value
1e-03
3e-03
7e-03
2e-02
4e-02
Log-odds
0.5
1.0
1.5
2.0
2.5
3.0
3.5
4.0

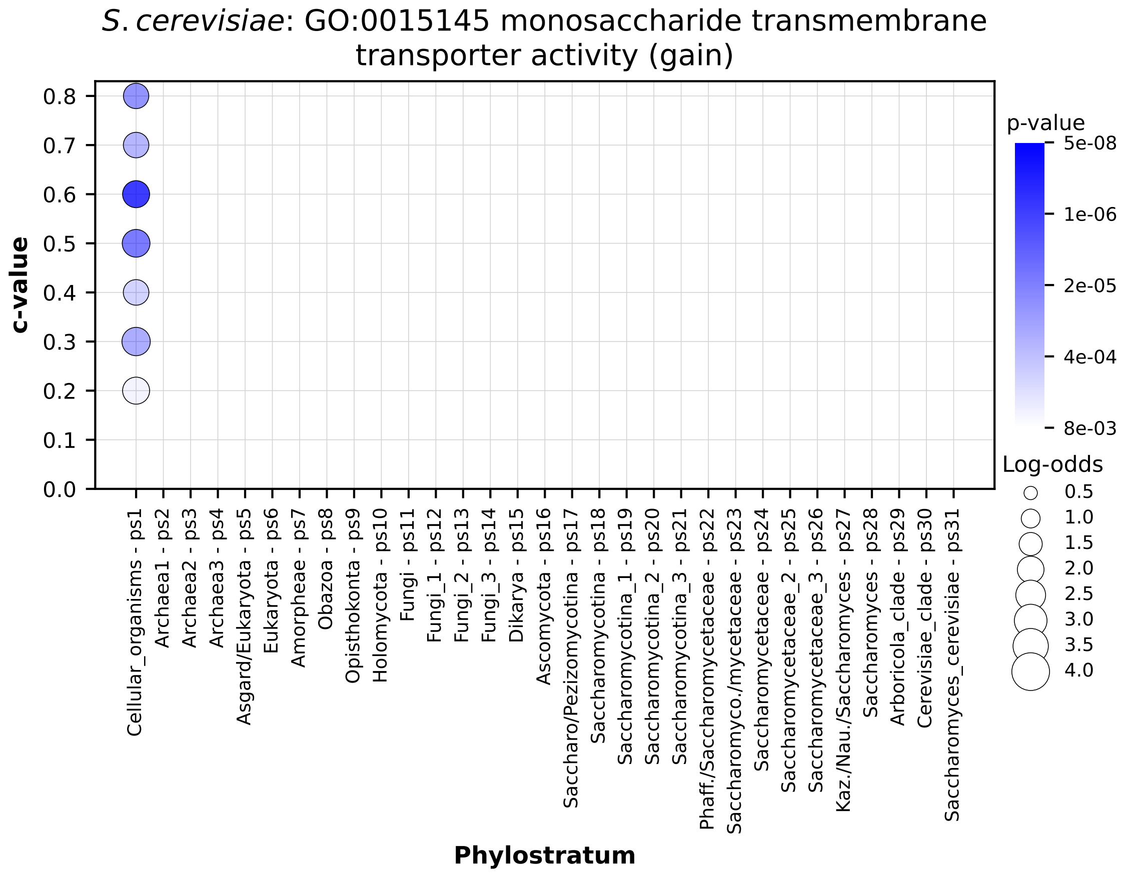

S. cerevisiae: GO:0015145 monosaccharide transmembrane transporter activity (gain)
c-value
0.0
0.1
0.2
0.3
0.4
0.5
0.6
0.7
0.8
p-value
5e-08
1e-06
2e-05
4e-04
8e-03
Log-odds
0.5
1.0
1.5
2.0
2.5
3.0
3.5
4.0
Cellular_organisms - ps1
Archaea1 - ps2
Archaea2 - ps3
Archaea3 - ps4
Asgard/Eukaryota - ps5
Eukaryota - ps6
Amorpheae - ps7
Obazoa - ps8
Opisthokonta - ps9
Holomycota - ps10
Fungi - ps11
Fungi_1 - ps12
Fungi_2 - ps13
Fungi_3 - ps14
Dikarya - ps15
Ascomycota - ps16
Saccharo/Pezizomycotina - ps17
Saccharomycotina - ps18
Saccharomycotina_1 - ps19
Saccharomycotina_2 - ps20
Saccharomycotina_3 - ps21
Phaff./Saccharomycetaceae - ps22
Saccharomyco./mycetaceae - ps23
Saccharomycetaceae - ps24
Saccharomycetaceae_2 - ps25
Saccharomycetaceae_3 - ps26
Kaz./Nau./Saccharomyces - ps27
Saccharomyces - ps28
Arboricola_clade - ps29
Cerevisiae_clade - ps30
Saccharomyces_cerevisiae - ps31
Phylostratum

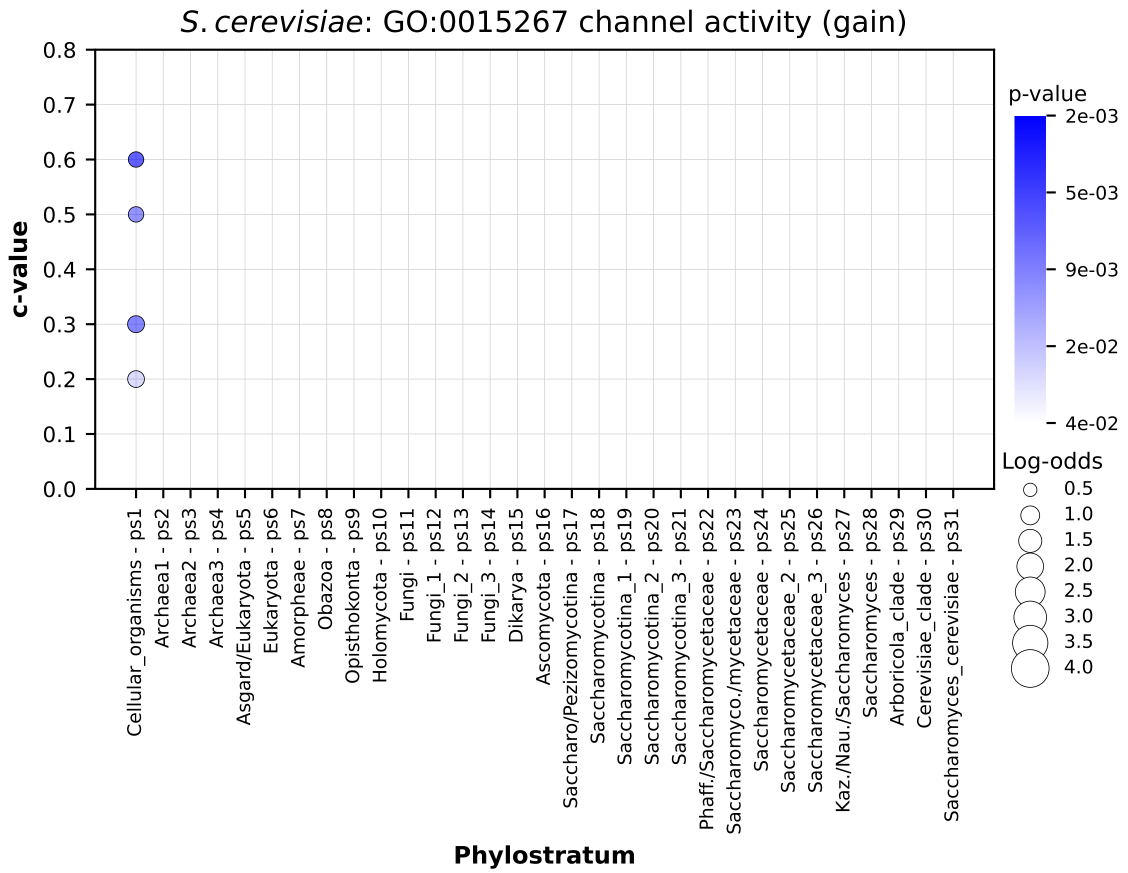

S. cerevisiae: GO:0015267 channel activity (gain)
c-value
0.0
0.1
0.2
0.3
0.4
0.5
0.6
0.7
0.8
Phylostratum
Cellular_organisms - ps1
Archaea1 - ps2
Archaea2 - ps3
Archaea3 - ps4
Asgard/Eukaryota - ps5
Eukaryota - ps6
Amorpheae - ps7
Obazoa - ps8
Opisthokonta - ps9
Holomycota - ps10
Fungi - ps11
Fungi_1 - ps12
Fungi_2 - ps13
Fungi_3 - ps14
Dikarya - ps15
Ascomycota - ps16
Saccharo/Pezizomycotina - ps17
Saccharomycotina - ps18
Saccharomycotina_1 - ps19
Saccharomycotina_2 - ps20
Saccharomycotina_3 - ps21
Phaff./Saccharomycetaceae - ps22
Saccharomyco./mycetaceae - ps23
Saccharomycetaceae - ps24
Saccharomycetaceae_2 - ps25
Saccharomycetaceae_3 - ps26
Kaz./Nau./Saccharomyces - ps27
Saccharomyces - ps28
Arboricola_clade - ps29
Cerevisiae_clade - ps30
Saccharomyces_cerevisiae - ps31
p-value
2e-03
5e-03
9e-03
2e-02
4e-02
Log-odds
0.5
1.0
1.5
2.0
2.5
3.0
3.5
4.0

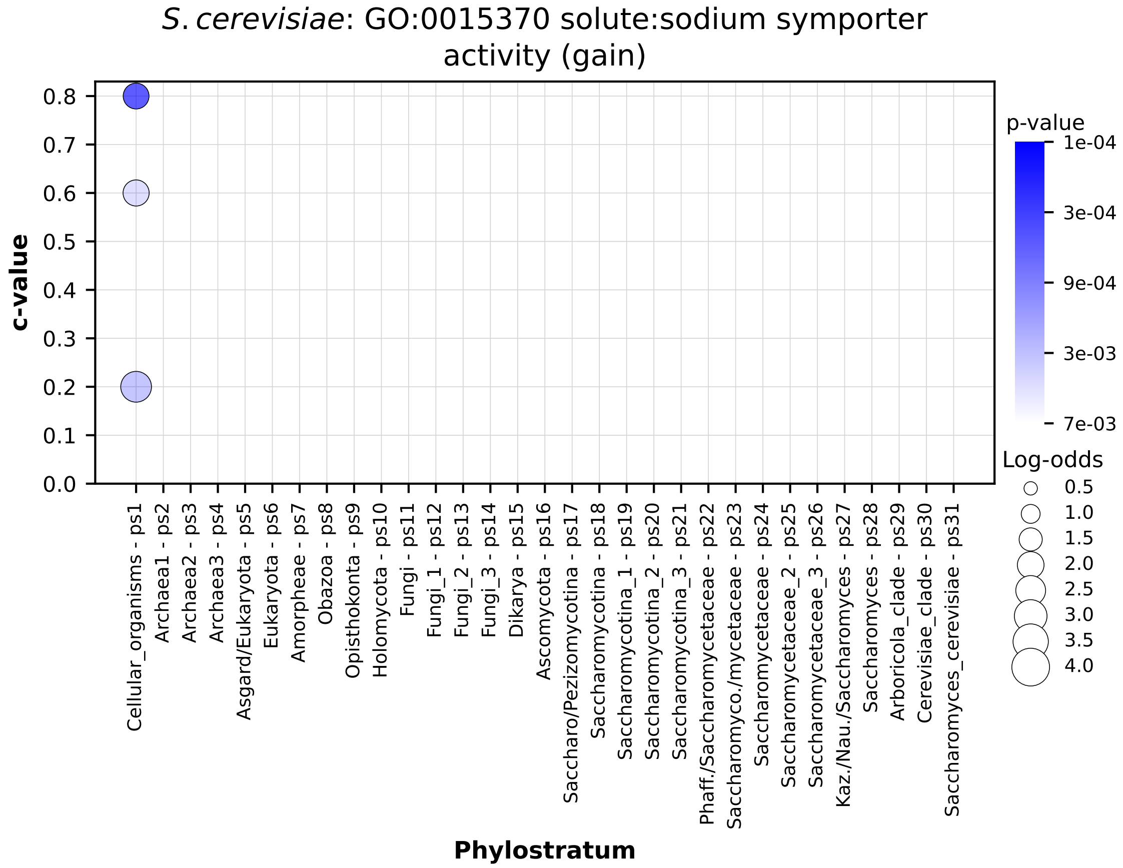

S. cerevisiae: GO:0015370 solute:sodium symporter activity (gain)
c-value
0.0
0.1
0.2
0.3
0.4
0.5
0.6
0.7
0.8
p-value
1e-04
3e-04
9e-04
3e-03
7e-03
Log-odds
0.5
1.0
1.5
2.0
2.5
3.0
3.5
4.0
Cellular_organisms - ps1
Archaea1 - ps2
Archaea2 - ps3
Archaea3 - ps4
Asgard/Eukaryota - ps5
Eukaryota - ps6
Amorpheae - ps7
Obazoa - ps8
Opisthokonta - ps9
Holomycota - ps10
Fungi - ps11
Fungi_1 - ps12
Fungi_2 - ps13
Fungi_3 - ps14
Dikarya - ps15
Ascomycota - ps16
Saccharo/Pezizomycotina - ps17
Saccharomycotina - ps18
Saccharomycotina_1 - ps19
Saccharomycotina_2 - ps20
Saccharomycotina_3 - ps21
Phaff./Saccharomycetaceae - ps22
Saccharomyco./mycetaceae - ps23
Saccharomycetaceae - ps24
Saccharomycetaceae_2 - ps25
Saccharomycetaceae_3 - ps26
Kaz./Nau./Saccharomyces - ps27
Saccharomyces - ps28
Arboricola_clade - ps29
Cerevisiae_clade - ps30
Saccharomyces_cerevisiae - ps31
Phylostratum

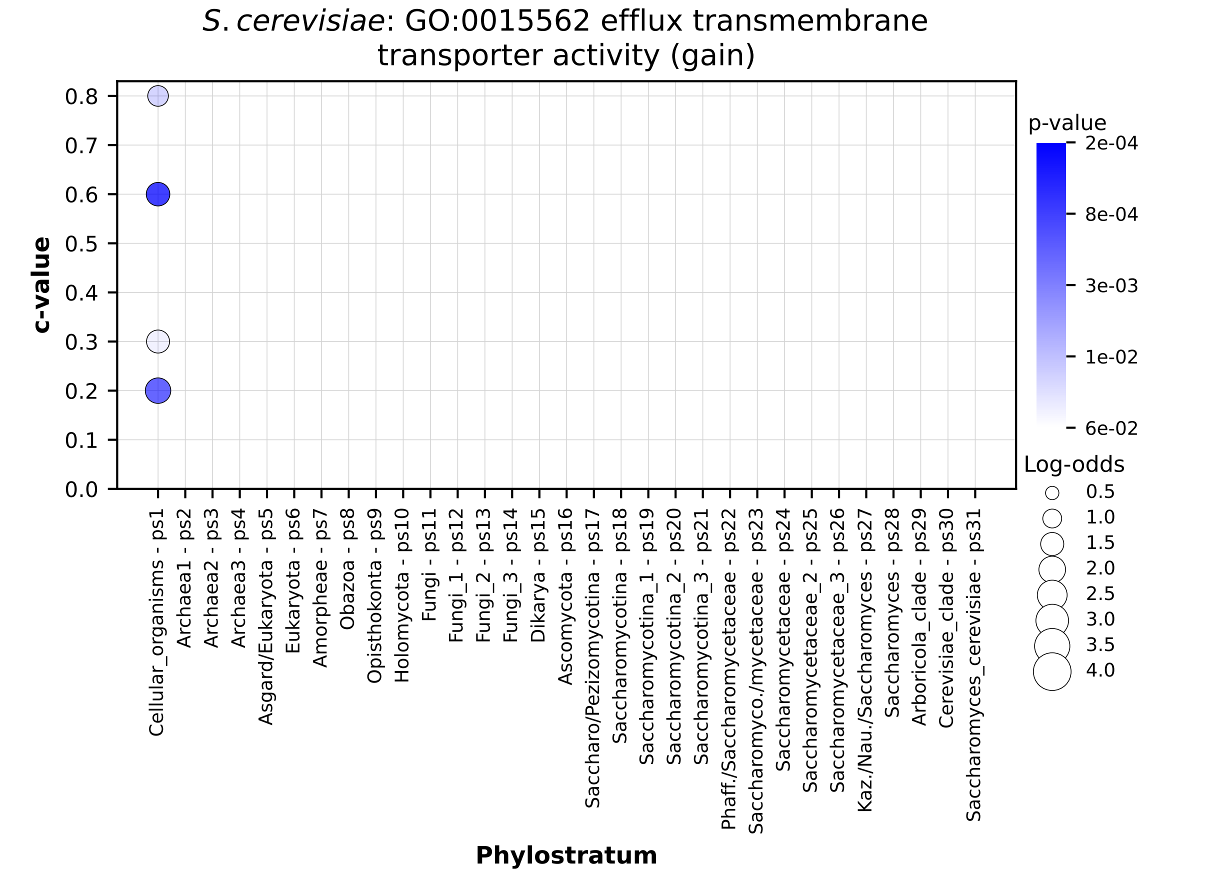
S. cerevisiae: GO:0015562 efflux transmembrane transporter activity (gain)
c-value
0.0
0.1
0.2
0.3
0.4
0.5
0.6
0.7
0.8
Phylostratum
Cellular_organisms - ps1
Archaea1 - ps2
Archaea2 - ps3
Archaea3 - ps4
Asgard/Eukaryota - ps5
Eukaryota - ps6
Amorpheae - ps7
Obazoa - ps8
Opisthokonta - ps9
Holomycota - ps10
Fungi - ps11
Fungi_1 - ps12
Fungi_2 - ps13
Fungi_3 - ps14
Dikarya - ps15
Ascomycota - ps16
Saccharo/Pezizomycotina - ps17
Saccharomycotina - ps18
Saccharomycotina_1 - ps19
Saccharomycotina_2 - ps20
Saccharomycotina_3 - ps21
Phaff./Saccharomycetaceae - ps22
Saccharomyco./mycetaceae - ps23
Saccharomycetaceae - ps24
Saccharomycetaceae_2 - ps25
Saccharomycetaceae_3 - ps26
Kaz./Nau./Saccharomyces - ps27
Saccharomyces - ps28
Arboricola_clade - ps29
Cerevisiae_clade - ps30
Saccharomyces_cerevisiae - ps31
p-value
2e-04
8e-04
3e-03
1e-02
6e-02
Log-odds
0.5
1.0
1.5
2.0
2.5
3.0
3.5
4.0

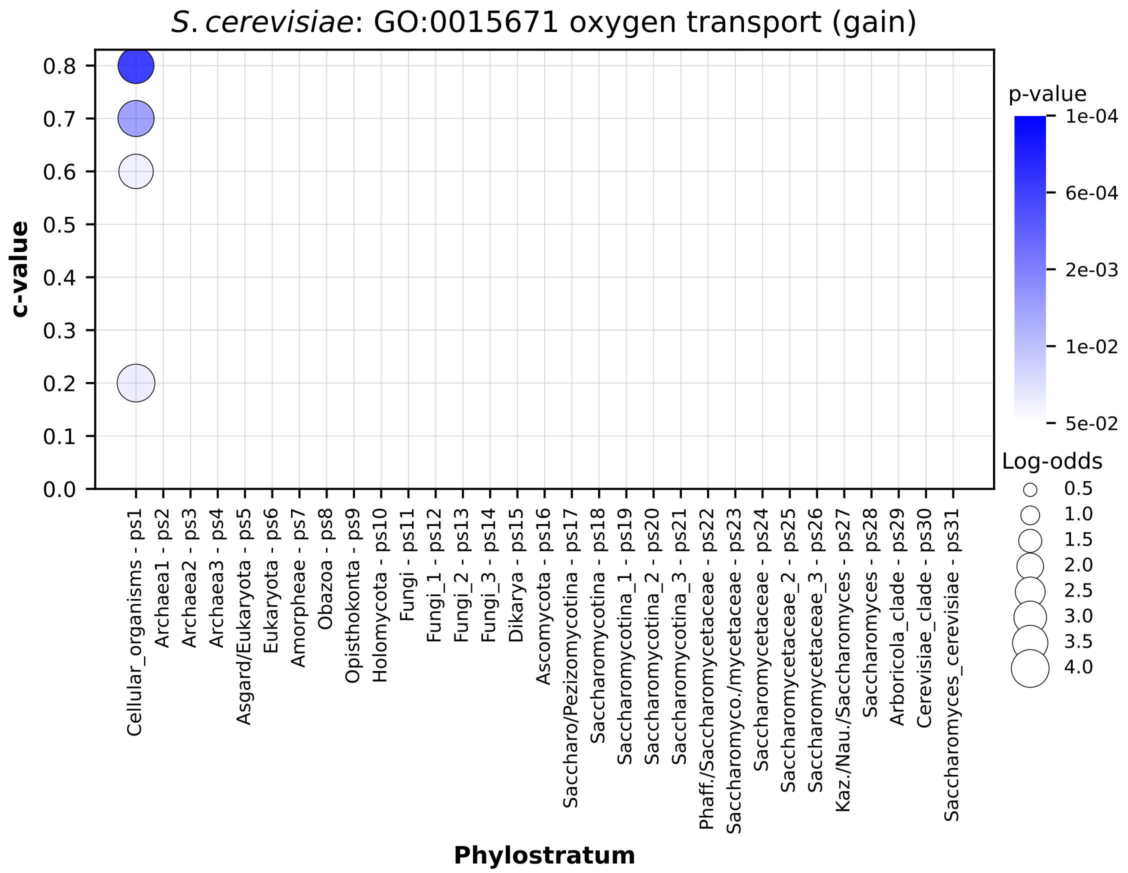

S. cerevisiae: GO:0015671 oxygen transport (gain)
c-value
0.0
0.1
0.2
0.3
0.4
0.5
0.6
0.7
0.8
p-value
1e-04
6e-04
2e-03
1e-02
5e-02
Log-odds
0.5
1.0
1.5
2.0
2.5
3.0
3.5
4.0
Cellular_organisms - ps1
Archaea1 - ps2
Archaea2 - ps3
Archaea3 - ps4
Asgard/Eukaryota - ps5
Eukaryota - ps6
Amorpheae - ps7
Obazoa - ps8
Opisthokonta - ps9
Holomycota - ps10
Fungi - ps11
Fungi_1 - ps12
Fungi_2 - ps13
Fungi_3 - ps14
Dikarya - ps15
Ascomycota - ps16
Saccharo/Pezizomycotina - ps17
Saccharomycotina - ps18
Saccharomycotina_1 - ps19
Saccharomycotina_2 - ps20
Saccharomycotina_3 - ps21
Phaff./Saccharomycetaceae - ps22
Saccharomyco./mycetaceae - ps23
Saccharomycetaceae - ps24
Saccharomycetaceae_2 - ps25
Saccharomycetaceae_3 - ps26
Kaz./Nau./Saccharomyces - ps27
Saccharomyces - ps28
Arboricola_clade - ps29
Cerevisiae_clade - ps30
Saccharomyces_cerevisiae - ps31
Phylostratum

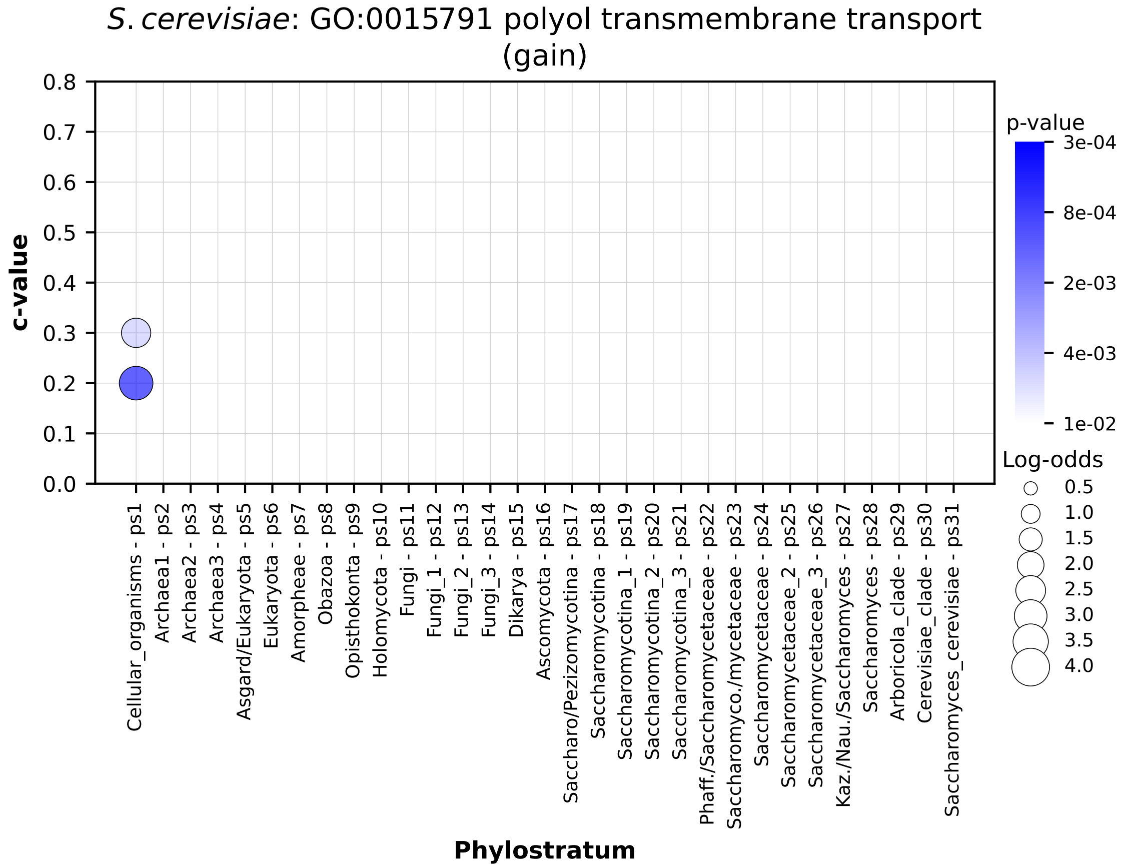

S. cerevisiae: GO:0015791 polyol transmembrane transport (gain)
c-value
0.8
0.7
0.6
0.5
0.4
0.3
0.2
0.1
0.0
Phylostratum
Cellular_organisms - ps1
Archaea1 - ps2
Archaea2 - ps3
Archaea3 - ps4
Asgard/Eukaryota - ps5
Eukaryota - ps6
Amorpheae - ps7
Obazoa - ps8
Opisthokonta - ps9
Holomycota - ps10
Fungi - ps11
Fungi_1 - ps12
Fungi_2 - ps13
Fungi_3 - ps14
Dikarya - ps15
Ascomycota - ps16
Saccharo/Pezizomycotina - ps17
Saccharomycotina - ps18
Saccharomycotina_1 - ps19
Saccharomycotina_2 - ps20
Saccharomycotina_3 - ps21
Phaff./Saccharomycetaceae - ps22
Saccharomyco./mycetaceae - ps23
Saccharomycetaceae - ps24
Saccharomycetaceae_2 - ps25
Saccharomycetaceae_3 - ps26
Kaz./Nau./Saccharomyces - ps27
Saccharomyces - ps28
Arboricola_clade - ps29
Cerevisiae_clade - ps30
Saccharomyces_cerevisiae - ps31
p-value
3e-04
8e-04
2e-03
4e-03
1e-02
Log-odds
0.5
1.0
1.5
2.0
2.5
3.0
3.5
4.0

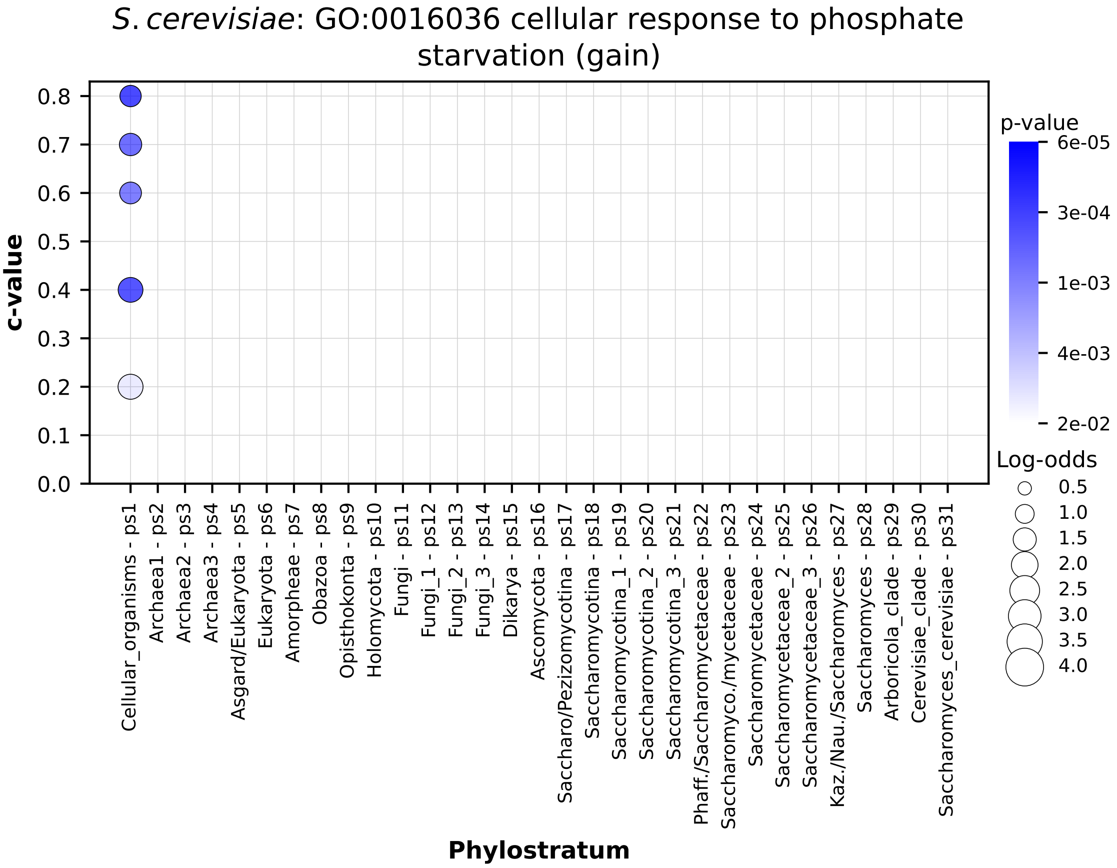

$S. cerevisiae$: GO:0016036 cellular response to phosphate starvation (gain)
c-value
0.0
0.1
0.2
0.3
0.4
0.5
0.6
0.7
0.8
Phylostratum
Cellular_organisms - ps1
Archaea1 - ps2
Archaea2 - ps3
Archaea3 - ps4
Asgard/Eukaryota - ps5
Eukaryota - ps6
Amorpheae - ps7
Obazoa - ps8
Opisthokonta - ps9
Holomycota - ps10
Fungi - ps11
Fungi_1 - ps12
Fungi_2 - ps13
Fungi_3 - ps14
Dikarya - ps15
Ascomycota - ps16
Saccharo/Pezizomycotina - ps17
Saccharomycotina - ps18
Saccharomycotina_1 - ps19
Saccharomycotina_2 - ps20
Saccharomycotina_3 - ps21
Phaff./Saccharomycetaceae - ps22
Saccharomyco./mycetaceae - ps23
Saccharomycetaceae - ps24
Saccharomycetaceae_2 - ps25
Saccharomycetaceae_3 - ps26
Kaz./Nau./Saccharomyces - ps27
Saccharomyces - ps28
Arboricola_clade - ps29
Cerevisiae_clade - ps30
Saccharomyces_cerevisiae - ps31
p-value
6e-05
3e-04
1e-03
4e-03
2e-02
Log-odds
0.5
1.0
1.5
2.0
2.5
3.0
3.5
4.0

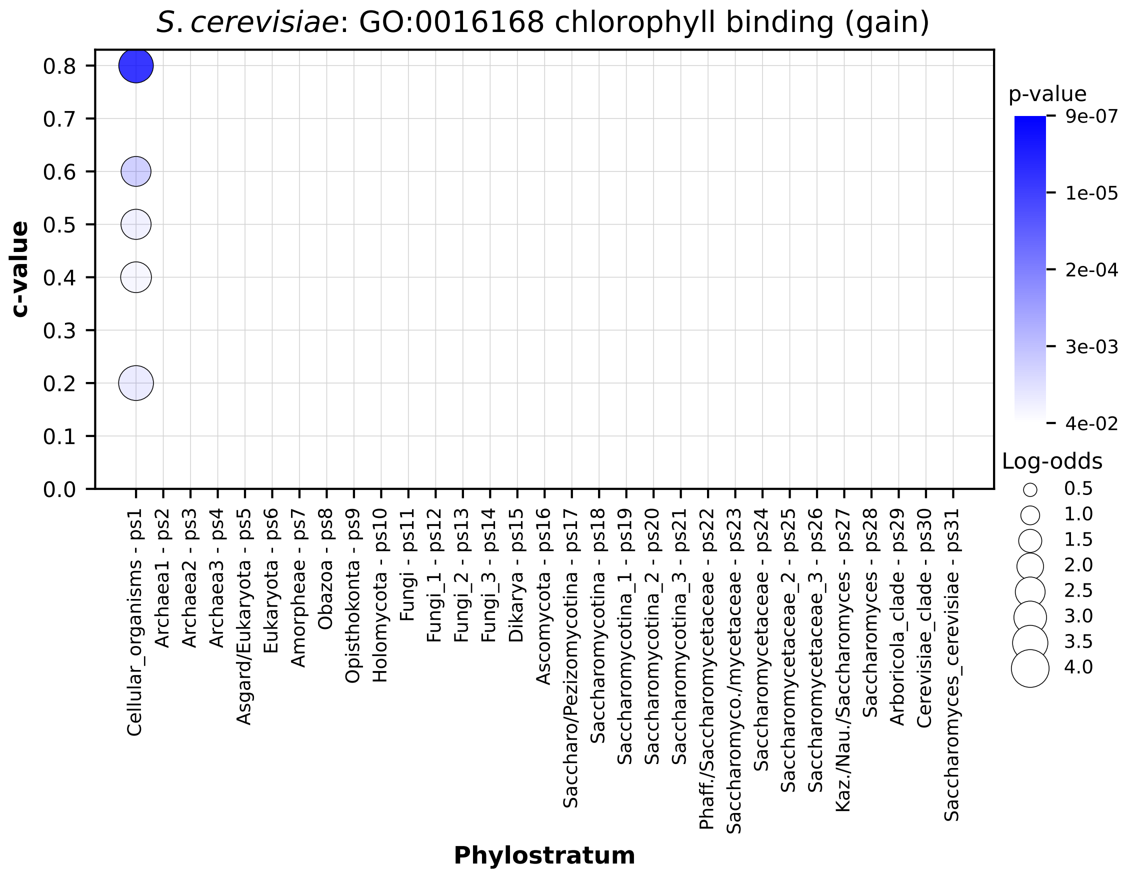

S. cerevisiae: GO:0016168 chlorophyll binding (gain)
c-value
0.0
0.1
0.2
0.3
0.4
0.5
0.6
0.7
0.8
Phylostratum
Cellular_organisms - ps1
Archaea1 - ps2
Archaea2 - ps3
Archaea3 - ps4
Asgard/Eukaryota - ps5
Eukaryota - ps6
Amorpheae - ps7
Obazoa - ps8
Opisthokonta - ps9
Holomycota - ps10
Fungi - ps11
Fungi_1 - ps12
Fungi_2 - ps13
Fungi_3 - ps14
Dikarya - ps15
Ascomycota - ps16
Saccharo/Pezizomycotina - ps17
Saccharomycotina - ps18
Saccharomycotina_1 - ps19
Saccharomycotina_2 - ps20
Saccharomycotina_3 - ps21
Phaff./Saccharomycetaceae - ps22
Saccharomyco./mycetaceae - ps23
Saccharomycetaceae - ps24
Saccharomycetaceae_2 - ps25
Saccharomycetaceae_3 - ps26
Kaz./Nau./Saccharomyces - ps27
Saccharomyces - ps28
Arboricola_clade - ps29
Cerevisiae_clade - ps30
Saccharomyces_cerevisiae - ps31
p-value
9e-07
1e-05
2e-04
3e-03
4e-02
Log-odds
0.5
1.0
1.5
2.0
2.5
3.0
3.5
4.0

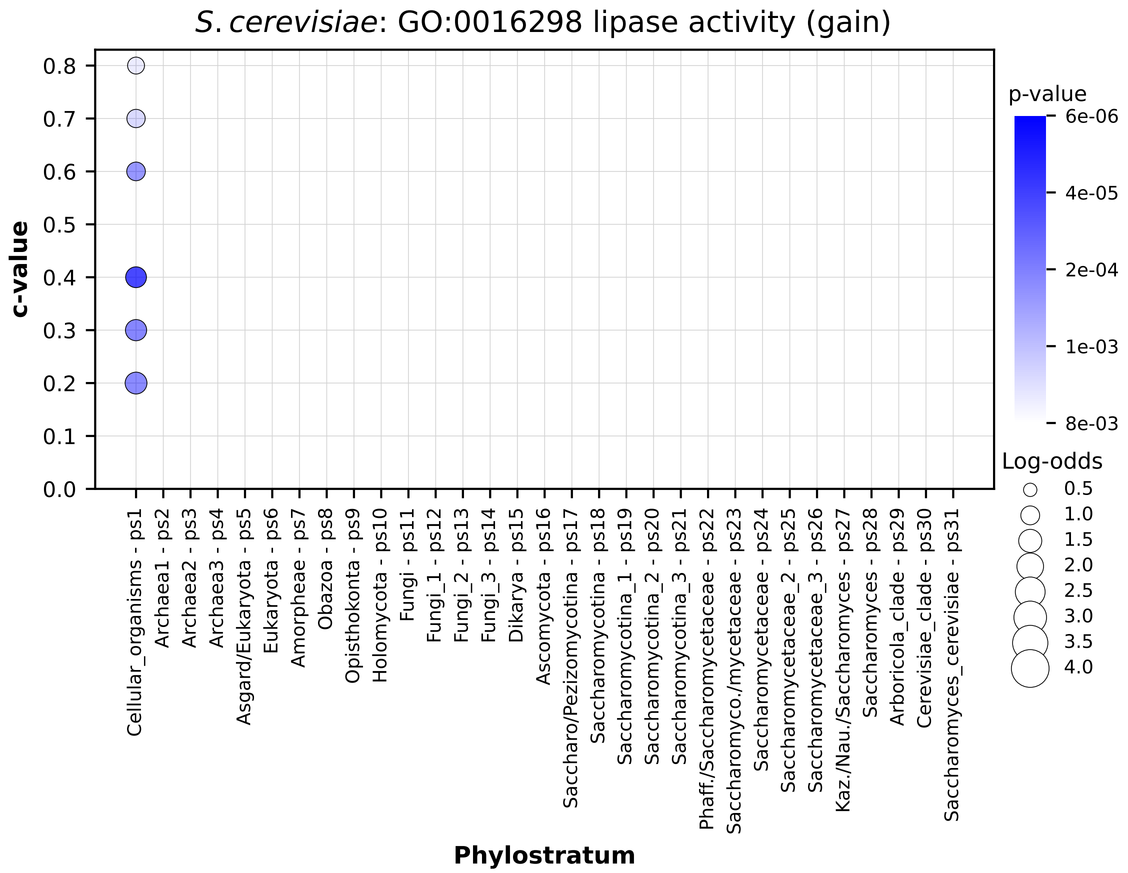
S. cerevisiae: GO:0016298 lipase activity (gain)
c-value
0.0
0.1
0.2
0.3
0.4
0.5
0.6
0.7
0.8
Phylostratum
Cellular_organisms - ps1
Archaea1 - ps2
Archaea2 - ps3
Archaea3 - ps4
Asgard/Eukaryota - ps5
Eukaryota - ps6
Amorpheae - ps7
Obazoa - ps8
Opisthokonta - ps9
Holomycota - ps10
Fungi - ps11
Fungi_1 - ps12
Fungi_2 - ps13
Fungi_3 - ps14
Dikarya - ps15
Ascomycota - ps16
Saccharo/Pezizomycotina - ps17
Saccharomycotina - ps18
Saccharomycotina_1 - ps19
Saccharomycotina_2 - ps20
Saccharomycotina_3 - ps21
Phaff./Saccharomycetaceae - ps22
Saccharomyco./mycetaceae - ps23
Saccharomycetaceae - ps24
Saccharomycetaceae_2 - ps25
Saccharomycetaceae_3 - ps26
Kaz./Nau./Saccharomyces - ps27
Saccharomyces - ps28
Arboricola_clade - ps29
Cerevisiae_clade - ps30
Saccharomyces_cerevisiae - ps31
p-value
6e-06
4e-05
2e-04
1e-03
8e-03
Log-odds
0.5
1.0
1.5
2.0
2.5
3.0
3.5
4.0

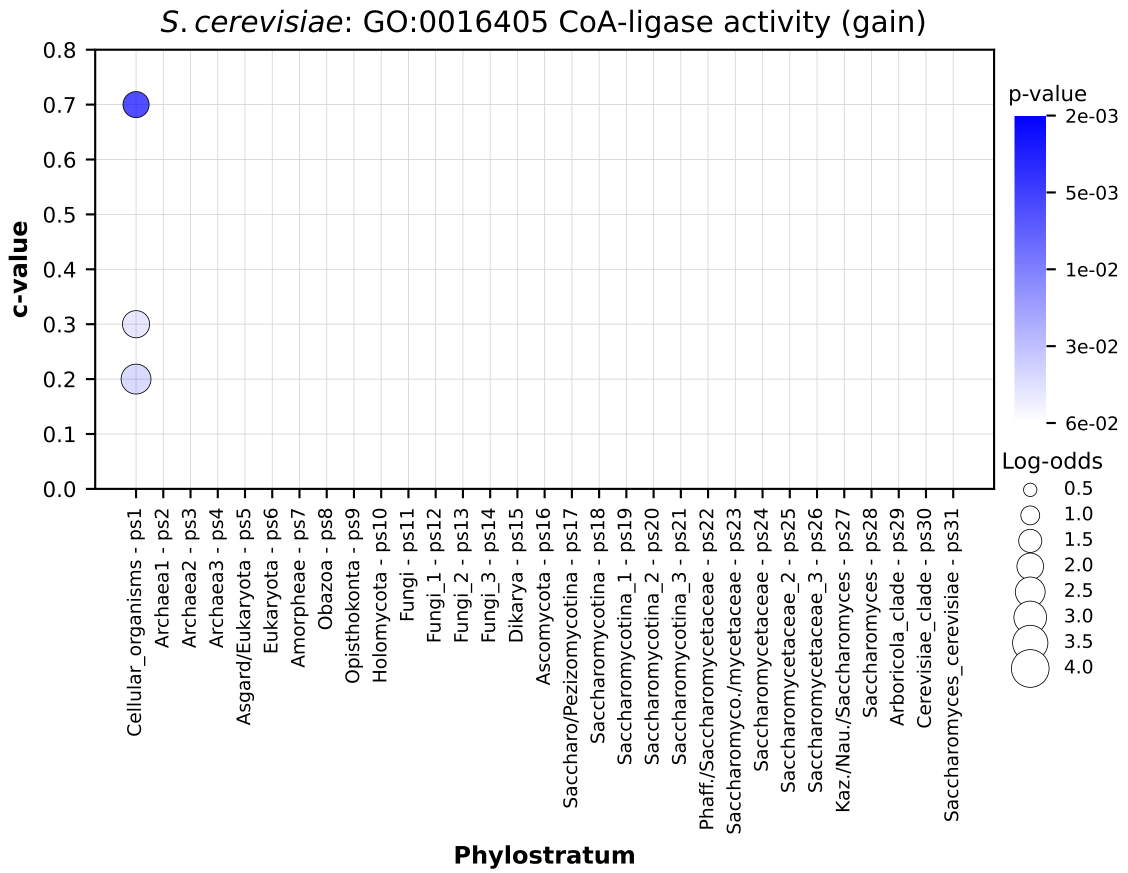

S. cerevisiae: GO:0016405 CoA-ligase activity (gain)
c-value
0.0
0.1
0.2
0.3
0.4
0.5
0.6
0.7
0.8
p-value
2e-03
5e-03
1e-02
3e-02
6e-02
Log-odds
0.5
1.0
1.5
2.0
2.5
3.0
3.5
4.0
Cellular_organisms - ps1
Archaea1 - ps2
Archaea2 - ps3
Archaea3 - ps4
Asgard/Eukaryota - ps5
Eukaryota - ps6
Amorpheae - ps7
Obazoa - ps8
Opisthokonta - ps9
Holomycota - ps10
Fungi - ps11
Fungi_1 - ps12
Fungi_2 - ps13
Fungi_3 - ps14
Dikarya - ps15
Ascomycota - ps16
Saccharo/Pezizomycotina - ps17
Saccharomycotina - ps18
Saccharomycotina_1 - ps19
Saccharomycotina_2 - ps20
Saccharomycotina_3 - ps21
Phaff./Saccharomycetaceae - ps22
Saccharomyco./mycetaceae - ps23
Saccharomycetaceae - ps24
Saccharomycetaceae_2 - ps25
Saccharomycetaceae_3 - ps26
Kaz./Nau./Saccharomyces - ps27
Saccharomyces - ps28
Arboricola_clade - ps29
Cerevisiae_clade - ps30
Saccharomyces_cerevisiae - ps31
Phylostratum

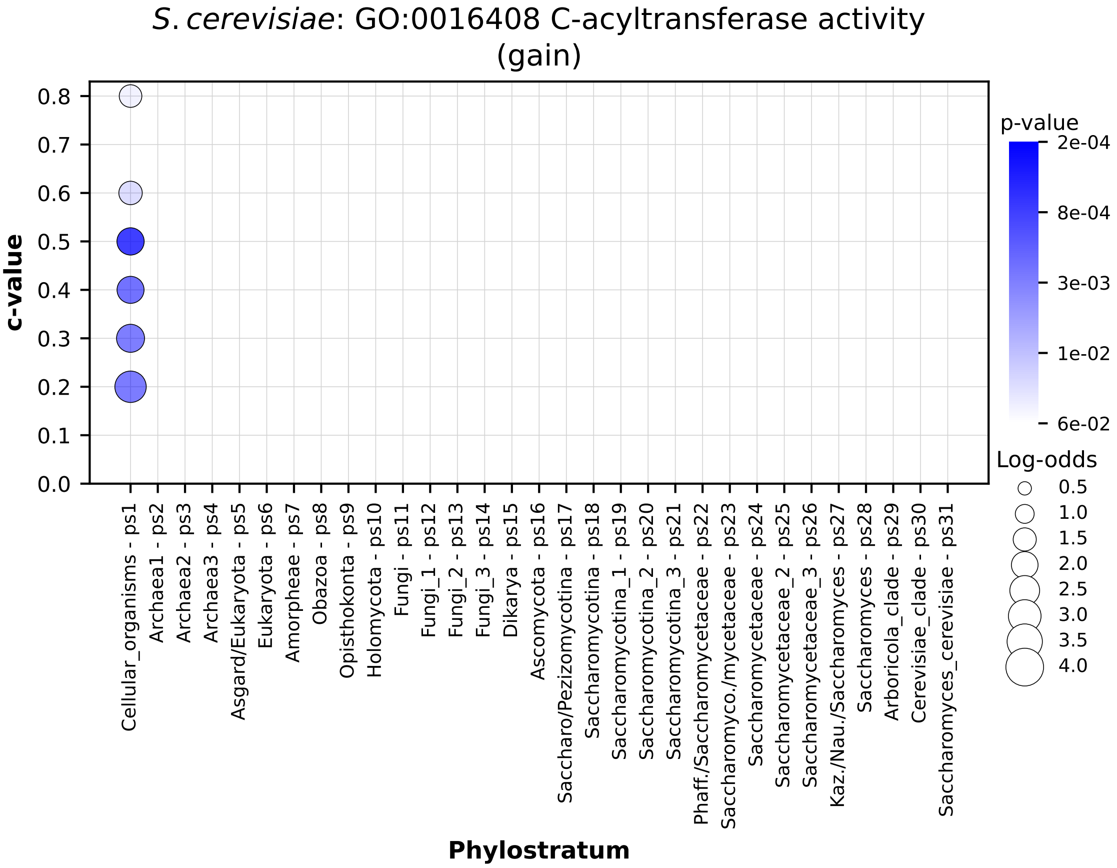

S. cerevisiae: GO:0016408 C-acyltransferase activity (gain)
c-value
0.8
0.7
0.6
0.5
0.4
0.3
0.2
0.1
0.0
p-value
2e-04
8e-04
3e-03
1e-02
6e-02
Log-odds
0.5
1.0
1.5
2.0
2.5
3.0
3.5
4.0
Cellular_organisms - ps1
Archaea1 - ps2
Archaea2 - ps3
Archaea3 - ps4
Asgard/Eukaryota - ps5
Eukaryota - ps6
Amorpheae - ps7
Obazoa - ps8
Opisthokonta - ps9
Holomycota - ps10
Fungi - ps11
Fungi_1 - ps12
Fungi_2 - ps13
Fungi_3 - ps14
Dikarya - ps15
Ascomycota - ps16
Saccharo/Pezizomycotina - ps17
Saccharomycotina - ps18
Saccharomycotina_1 - ps19
Saccharomycotina_2 - ps20
Saccharomycotina_3 - ps21
Phaff./Saccharomycetaceae - ps22
Saccharomyco./mycetaceae - ps23
Saccharomycetaceae - ps24
Saccharomycetaceae_2 - ps25
Saccharomycetaceae_3 - ps26
Kaz./Nau./Saccharomyces - ps27
Saccharomyces - ps28
Arboricola_clade - ps29
Cerevisiae_clade - ps30
Saccharomyces_cerevisiae - ps31
Phylostratum

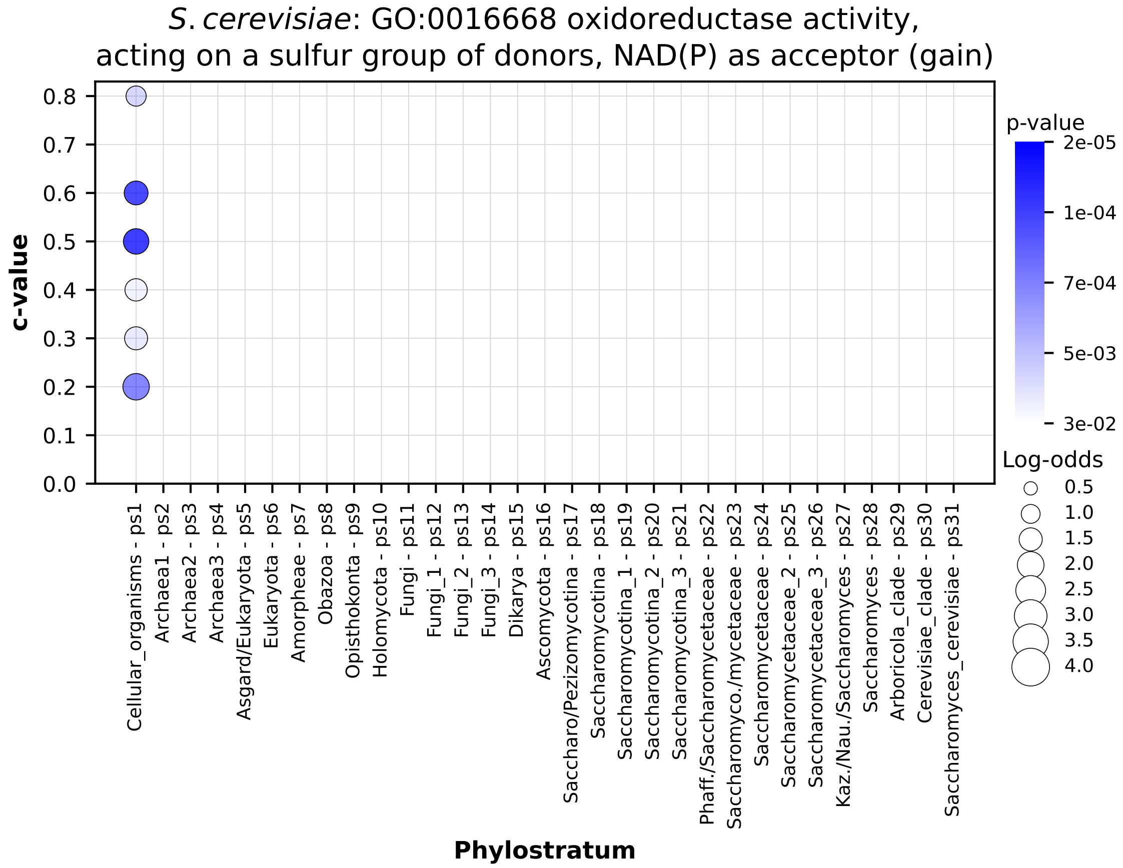

S. cerevisiae: GO:0016668 oxidoreductase activity,
acting on a sulfur group of donors, NAD(P) as acceptor (gain)
c-value
0.0
0.1
0.2
0.3
0.4
0.5
0.6
0.7
0.8
p-value
2e-05
1e-04
7e-04
5e-03
3e-02
Log-odds
0.5
1.0
1.5
2.0
2.5
3.0
3.5
4.0
Cellular_organisms - ps1
Archaea1 - ps2
Archaea2 - ps3
Archaea3 - ps4
Asgard/Eukaryota - ps5
Eukaryota - ps6
Amorpheae - ps7
Obazoa - ps8
Opisthokonta - ps9
Holomycota - ps10
Fungi - ps11
Fungi_1 - ps12
Fungi_2 - ps13
Fungi_3 - ps14
Dikarya - ps15
Ascomycota - ps16
Saccharo/Pezizomycotina - ps17
Saccharomycotina - ps18
Saccharomycotina_1 - ps19
Saccharomycotina_2 - ps20
Saccharomycotina_3 - ps21
Phaff./Saccharomycetaceae - ps22
Saccharomyco./mycetaceae - ps23
Saccharomycetaceae - ps24
Saccharomycetaceae_2 - ps25
Saccharomycetaceae_3 - ps26
Kaz./Nau./Saccharomyces - ps27
Saccharomyces - ps28
Arboricola_clade - ps29
Cerevisiae_clade - ps30
Saccharomyces_cerevisiae - ps31
Phylostratum

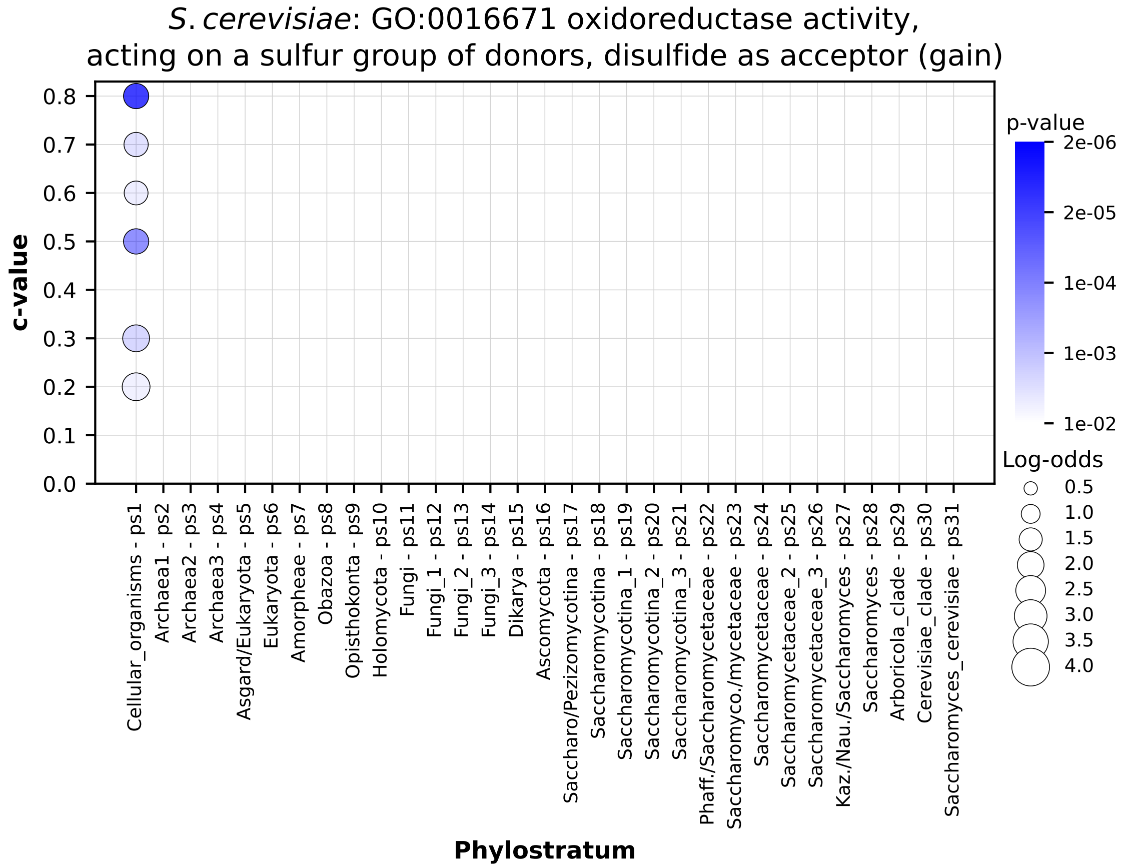

S. cerevisiae: GO:0016671 oxidoreductase activity, acting on a sulfur group of donors, disulfide as acceptor (gain)
c-value
0.0
0.1
0.2
0.3
0.4
0.5
0.6
0.7
0.8
Phylostratum
Cellular_organisms - ps1
Archaea1 - ps2
Archaea2 - ps3
Archaea3 - ps4
Asgard/Eukaryota - ps5
Eukaryota - ps6
Amorpheae - ps7
Obazoa - ps8
Opisthokonta - ps9
Holomycota - ps10
Fungi - ps11
Fungi_1 - ps12
Fungi_2 - ps13
Fungi_3 - ps14
Dikarya - ps15
Ascomycota - ps16
Saccharo/Pezizomycotina - ps17
Saccharomycotina - ps18
Saccharomycotina_1 - ps19
Saccharomycotina_2 - ps20
Saccharomycotina_3 - ps21
Phaff./Saccharomycetaceae - ps22
Saccharomyco./mycetaceae - ps23
Saccharomycetaceae - ps24
Saccharomycetaceae_2 - ps25
Saccharomycetaceae_3 - ps26
Kaz./Nau./Saccharomyces - ps27
Saccharomyces - ps28
Arboricola_clade - ps29
Cerevisiae_clade - ps30
Saccharomyces_cerevisiae - ps31
p-value
2e-06
2e-05
1e-04
1e-03
1e-02
Log-odds
0.5
1.0
1.5
2.0
2.5
3.0
3.5
4.0

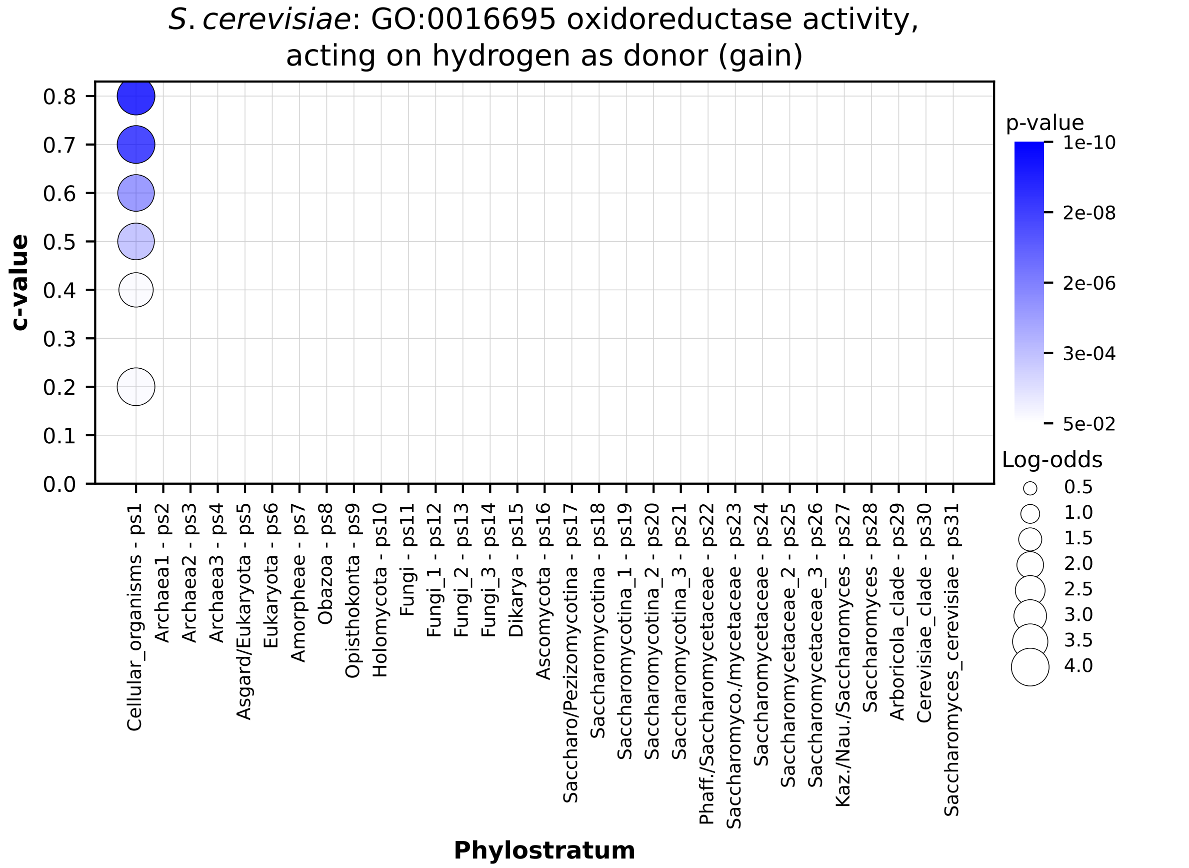

S. cerevisiae: GO:0016695 oxidoreductase activity, acting on hydrogen as donor (gain)
c-value
0.0
0.1
0.2
0.3
0.4
0.5
0.6
0.7
0.8
Phylostratum
Cellular_organisms - ps1
Archaea1 - ps2
Archaea2 - ps3
Archaea3 - ps4
Asgard/Eukaryota - ps5
Eukaryota - ps6
Amorpheae - ps7
Obazoa - ps8
Opisthokonta - ps9
Holomycota - ps10
Fungi - ps11
Fungi_1 - ps12
Fungi_2 - ps13
Fungi_3 - ps14
Dikarya - ps15
Ascomycota - ps16
Saccharo/Pezizomycotina - ps17
Saccharomycotina - ps18
Saccharomycotina_1 - ps19
Saccharomycotina_2 - ps20
Saccharomycotina_3 - ps21
Phaff./Saccharomycetaceae - ps22
Saccharomyco./mycetaceae - ps23
Saccharomycetaceae - ps24
Saccharomycetaceae_2 - ps25
Saccharomycetaceae_3 - ps26
Kaz./Nau./Saccharomyces - ps27
Saccharomyces - ps28
Arboricola_clade - ps29
Cerevisiae_clade - ps30
Saccharomyces_cerevisiae - ps31
p-value
1e-10
2e-08
2e-06
3e-04
5e-02
Log-odds
0.5
1.0
1.5
2.0
2.5
3.0
3.5
4.0

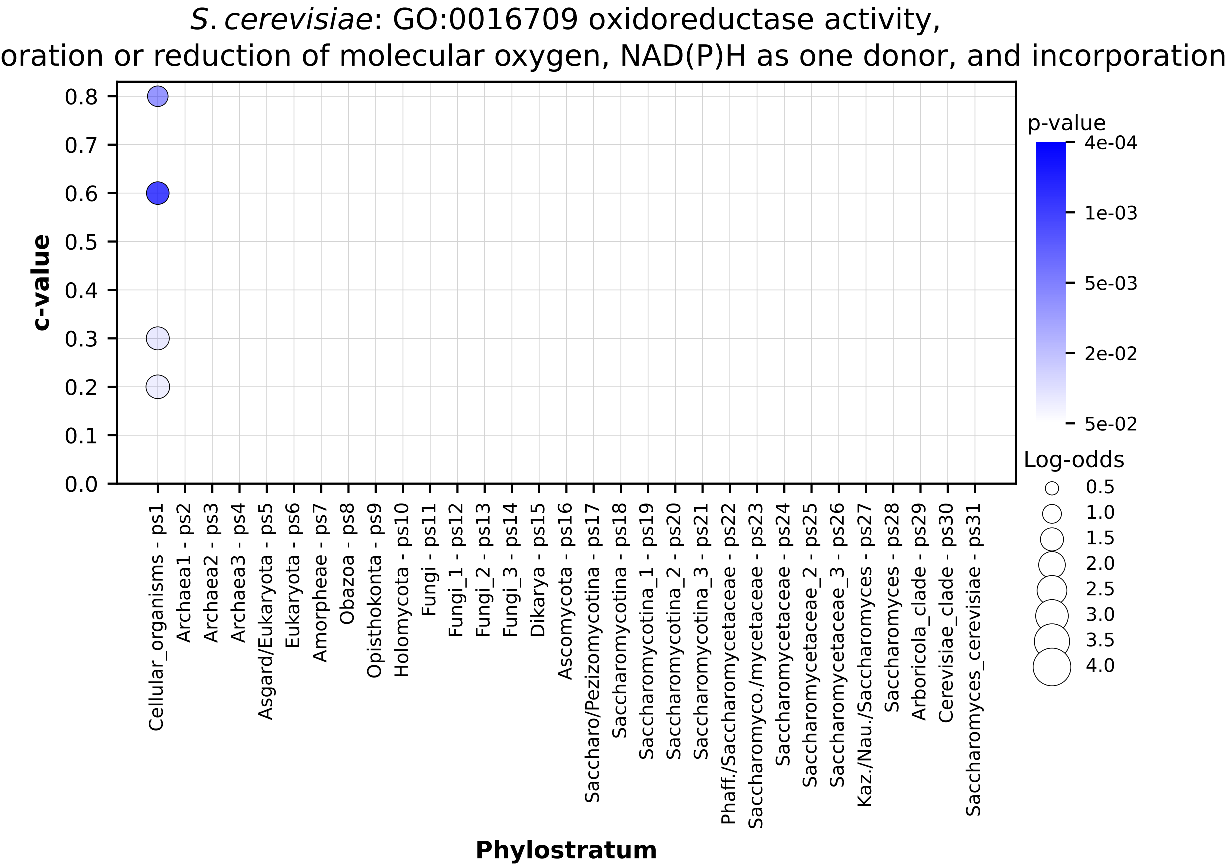

S. cerevisiae: GO:0016709 oxidoreductase activity,
oration or reduction of molecular oxygen, NAD(P)H as one donor, and incorporation
c-value
0.0
0.1
0.2
0.3
0.4
0.5
0.6
0.7
0.8
Phylostratum
Cellular_organisms - ps1
Archaea1 - ps2
Archaea2 - ps3
Archaea3 - ps4
Asgard/Eukaryota - ps5
Eukaryota - ps6
Amorpheae - ps7
Obazoa - ps8
Opisthokonta - ps9
Holomycota - ps10
Fungi - ps11
Fungi_1 - ps12
Fungi_2 - ps13
Fungi_3 - ps14
Dikarya - ps15
Ascomycota - ps16
Saccharo/Pezizomycotina - ps17
Saccharomycotina - ps18
Saccharomycotina_1 - ps19
Saccharomycotina_2 - ps20
Saccharomycotina_3 - ps21
Phaff./Saccharomycetaceae - ps22
Saccharomyco./mycetaceae - ps23
Saccharomycetaceae - ps24
Saccharomycetaceae_2 - ps25
Saccharomycetaceae_3 - ps26
Kaz./Nau./Saccharomyces - ps27
Saccharomyces - ps28
Arboricola_clade - ps29
Cerevisiae_clade - ps30
Saccharomyces_cerevisiae - ps31
p-value
4e-04
1e-03
5e-03
2e-02
5e-02
Log-odds
0.5
1.0
1.5
2.0
2.5
3.0
3.5
4.0

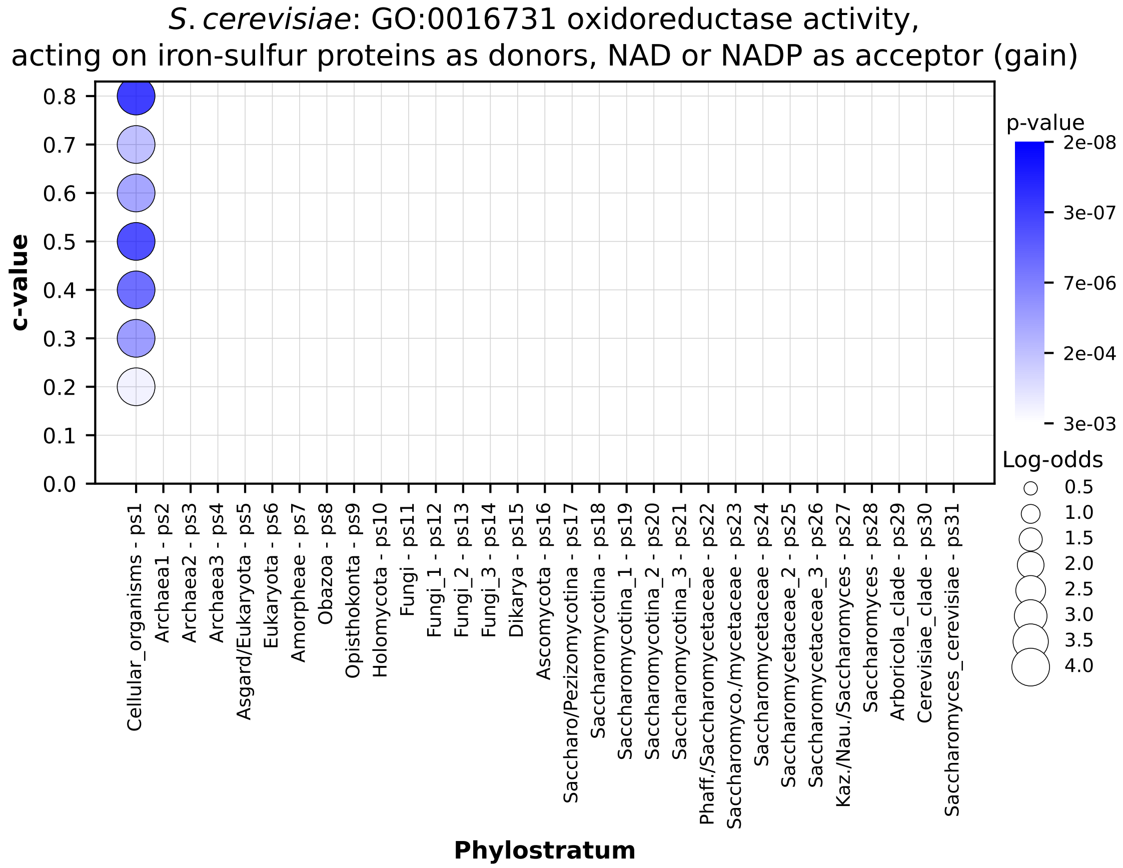

S. cerevisiae: GO:0016731 oxidoreductase activity,
acting on iron-sulfur proteins as donors, NAD or NADP as acceptor (gain)
c-value
0.0
0.1
0.2
0.3
0.4
0.5
0.6
0.7
0.8
p-value
2e-08
3e-07
7e-06
2e-04
3e-03
Log-odds
0.5
1.0
1.5
2.0
2.5
3.0
3.5
4.0
Cellular_organisms - ps1
Archaea1 - ps2
Archaea2 - ps3
Archaea3 - ps4
Asgard/Eukaryota - ps5
Eukaryota - ps6
Amorpheae - ps7
Obazoa - ps8
Opisthokonta - ps9
Holomycota - ps10
Fungi - ps11
Fungi_1 - ps12
Fungi_2 - ps13
Fungi_3 - ps14
Dikarya - ps15
Ascomycota - ps16
Saccharo/Pezizomycotina - ps17
Saccharomycotina - ps18
Saccharomycotina_1 - ps19
Saccharomycotina_2 - ps20
Saccharomycotina_3 - ps21
Phaff./Saccharomycetaceae - ps22
Saccharomyco./mycetaceae - ps23
Saccharomycetaceae - ps24
Saccharomycetaceae_2 - ps25
Saccharomycetaceae_3 - ps26
Kaz./Nau./Saccharomyces - ps27
Saccharomyces - ps28
Arboricola_clade - ps29
Cerevisiae_clade - ps30
Saccharomyces_cerevisiae - ps31
Phylostratum

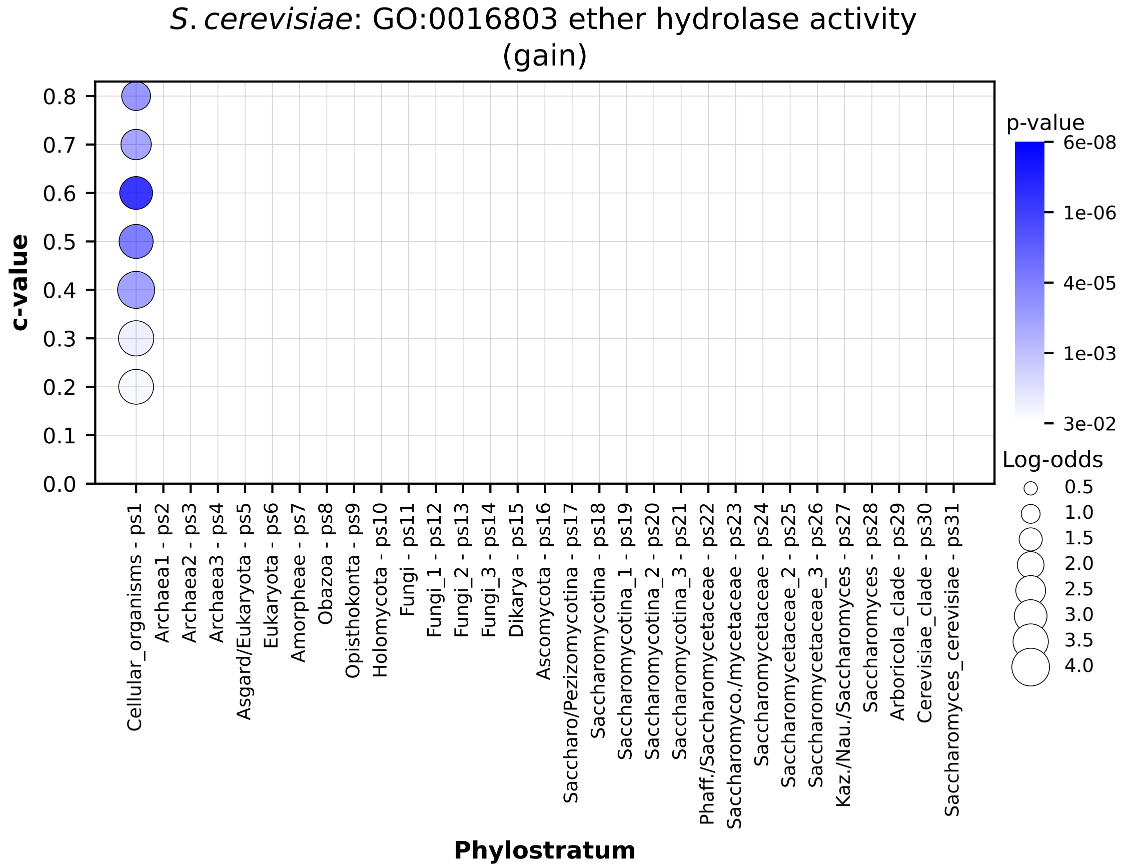

S. cerevisiae: GO:0016803 ether hydrolase activity (gain)
c-value
0.0
0.1
0.2
0.3
0.4
0.5
0.6
0.7
0.8
Phylostratum
Cellular_organisms - ps1
Archaea1 - ps2
Archaea2 - ps3
Archaea3 - ps4
Asgard/Eukaryota - ps5
Eukaryota - ps6
Amorpheae - ps7
Obazoa - ps8
Opisthokonta - ps9
Holomycota - ps10
Fungi - ps11
Fungi_1 - ps12
Fungi_2 - ps13
Fungi_3 - ps14
Dikarya - ps15
Ascomycota - ps16
Saccharo/Pezizomycotina - ps17
Saccharomycotina - ps18
Saccharomycotina_1 - ps19
Saccharomycotina_2 - ps20
Saccharomycotina_3 - ps21
Phaff./Saccharomycetaceae - ps22
Saccharomyco./mycetaceae - ps23
Saccharomycetaceae - ps24
Saccharomycetaceae_2 - ps25
Saccharomycetaceae_3 - ps26
Kaz./Nau./Saccharomyces - ps27
Saccharomyces - ps28
Arboricola_clade - ps29
Cerevisiae_clade - ps30
Saccharomyces_cerevisiae - ps31
p-value
6e-08
1e-06
4e-05
1e-03
3e-02
Log-odds
0.5
1.0
1.5
2.0
2.5
3.0
3.5
4.0

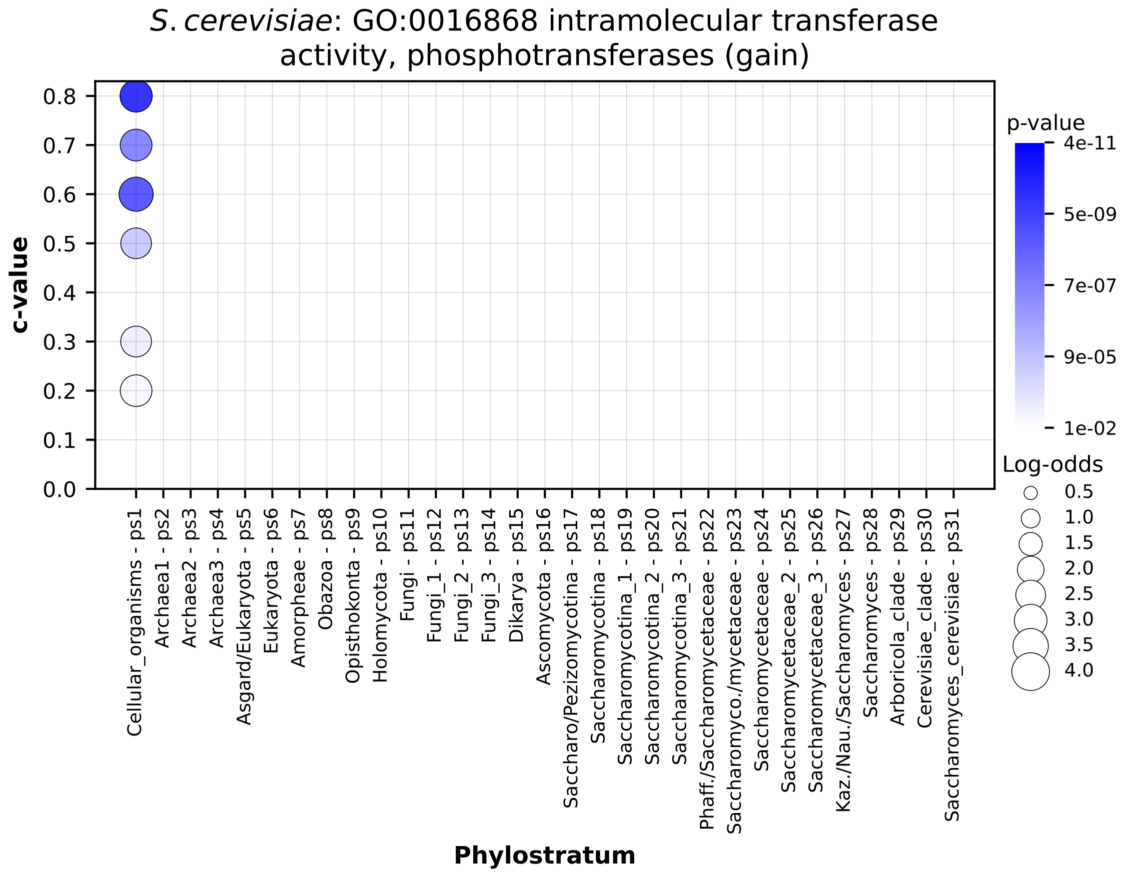

S. cerevisiae: GO:0016868 intramolecular transferase activity, phosphotransferases (gain)
c-value
0.0
0.1
0.2
0.3
0.4
0.5
0.6
0.7
0.8
p-value
4e-11
5e-09
7e-07
9e-05
1e-02
Log-odds
0.5
1.0
1.5
2.0
2.5
3.0
3.5
4.0
Cellular_organisms - ps1
Archaea1 - ps2
Archaea2 - ps3
Archaea3 - ps4
Asgard/Eukaryota - ps5
Eukaryota - ps6
Amorpheae - ps7
Obazoa - ps8
Opisthokonta - ps9
Holomycota - ps10
Fungi - ps11
Fungi_1 - ps12
Fungi_2 - ps13
Fungi_3 - ps14
Dikarya - ps15
Ascomycota - ps16
Saccharo/Pezizomycotina - ps17
Saccharomycotina - ps18
Saccharomycotina_1 - ps19
Saccharomycotina_2 - ps20
Saccharomycotina_3 - ps21
Phaff./Saccharomycetaceae - ps22
Saccharomyco./mycetaceae - ps23
Saccharomycetaceae - ps24
Saccharomycetaceae_2 - ps25
Saccharomycetaceae_3 - ps26
Kaz./Nau./Saccharomyces - ps27
Saccharomyces - ps28
Arboricola_clade - ps29
Cerevisiae_clade - ps30
Saccharomyces_cerevisiae - ps31
Phylostratum

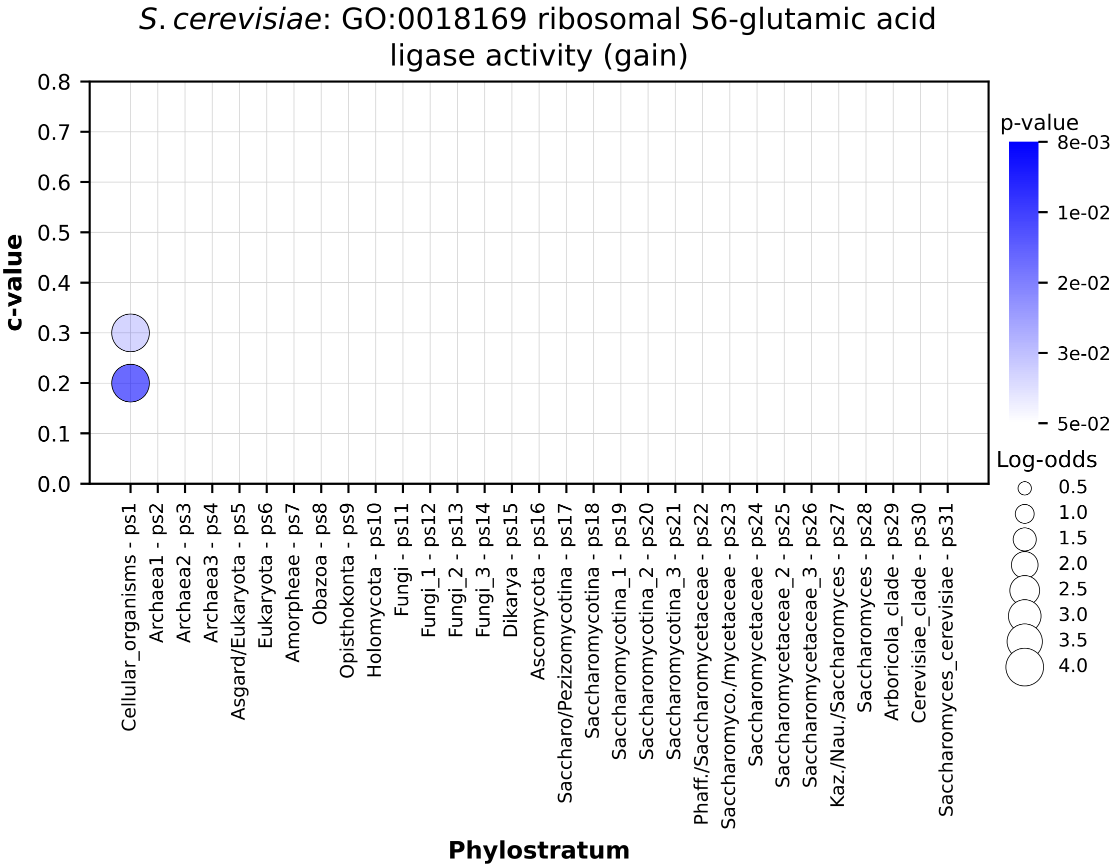

S. cerevisiae: GO:0018169 ribosomal S6-glutamic acid ligase activity (gain)
c-value
0.8
0.7
0.6
0.5
0.4
0.3
0.2
0.1
0.0
p-value
8e-03
1e-02
2e-02
3e-02
5e-02
Log-odds
0.5
1.0
1.5
2.0
2.5
3.0
3.5
4.0
Cellular_organisms - ps1
Archaea1 - ps2
Archaea2 - ps3
Archaea3 - ps4
Asgard/Eukaryota - ps5
Eukaryota - ps6
Amorpheae - ps7
Obazoa - ps8
Opisthokonta - ps9
Holomycota - ps10
Fungi - ps11
Fungi_1 - ps12
Fungi_2 - ps13
Fungi_3 - ps14
Dikarya - ps15
Ascomycota - ps16
Saccharo/Pezizomycotina - ps17
Saccharomycotina - ps18
Saccharomycotina_1 - ps19
Saccharomycotina_2 - ps20
Saccharomycotina_3 - ps21
Phaff./Saccharomycetaceae - ps22
Saccharomyco./mycetaceae - ps23
Saccharomycetaceae - ps24
Saccharomycetaceae_2 - ps25
Saccharomycetaceae_3 - ps26
Kaz./Nau./Saccharomyces - ps27
Saccharomyces - ps28
Arboricola_clade - ps29
Cerevisiae_clade - ps30
Saccharomyces_cerevisiae - ps31
Phylostratum

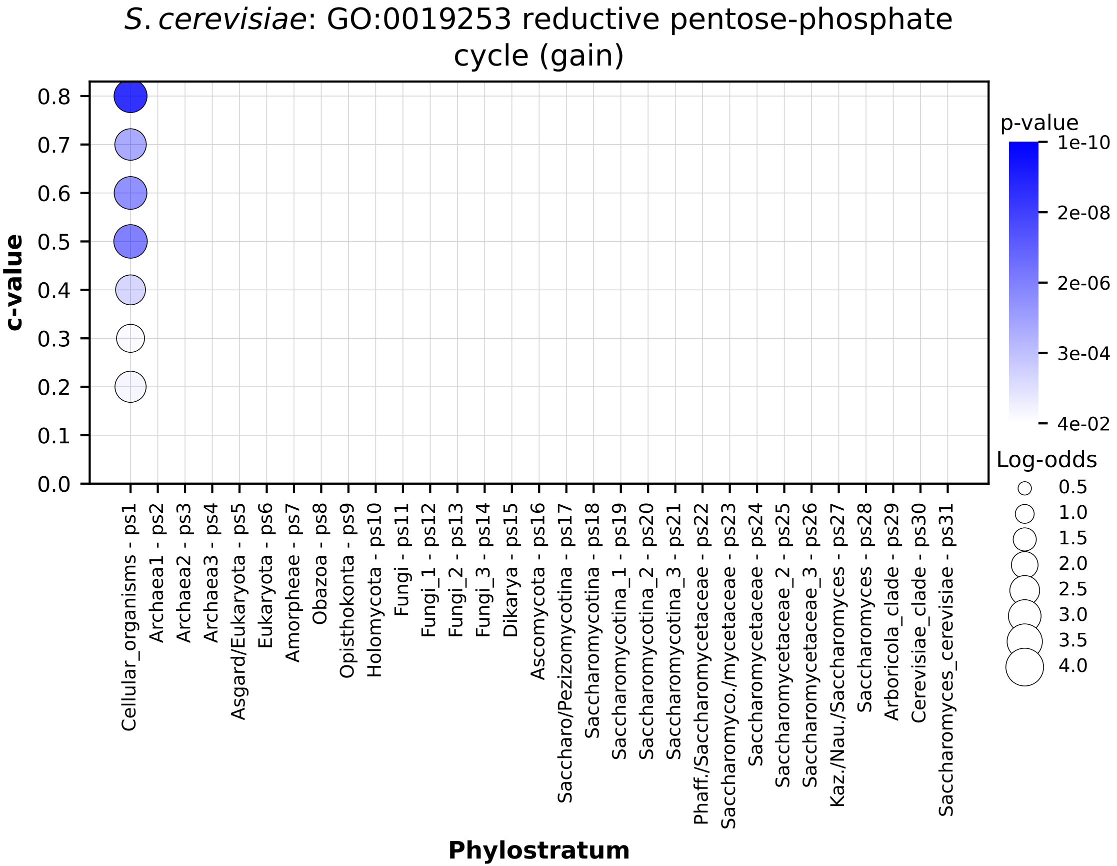

S. cerevisiae: GO:0019253 reductive pentose-phosphate cycle (gain)
c-value
0.8
0.7
0.6
0.5
0.4
0.3
0.2
0.1
0.0
p-value
1e-10
2e-08
2e-06
3e-04
4e-02
Log-odds
0.5
1.0
1.5
2.0
2.5
3.0
3.5
4.0
Cellular_organisms - ps1
Archaea1 - ps2
Archaea2 - ps3
Archaea3 - ps4
Asgard/Eukaryota - ps5
Eukaryota - ps6
Amorpheae - ps7
Obazoa - ps8
Opisthokonta - ps9
Holomycota - ps10
Fungi - ps11
Fungi_1 - ps12
Fungi_2 - ps13
Fungi_3 - ps14
Dikarya - ps15
Ascomycota - ps16
Saccharo/Pezizomycotina - ps17
Saccharomycotina - ps18
Saccharomycotina_1 - ps19
Saccharomycotina_2 - ps20
Saccharomycotina_3 - ps21
Phaff./Saccharomycetaceae - ps22
Saccharomyco./mycetaceae - ps23
Saccharomycetaceae - ps24
Saccharomycetaceae_2 - ps25
Saccharomycetaceae_3 - ps26
Kaz./Nau./Saccharomyces - ps27
Saccharomyces - ps28
Arboricola_clade - ps29
Cerevisiae_clade - ps30
Saccharomyces_cerevisiae - ps31
Phylostratum

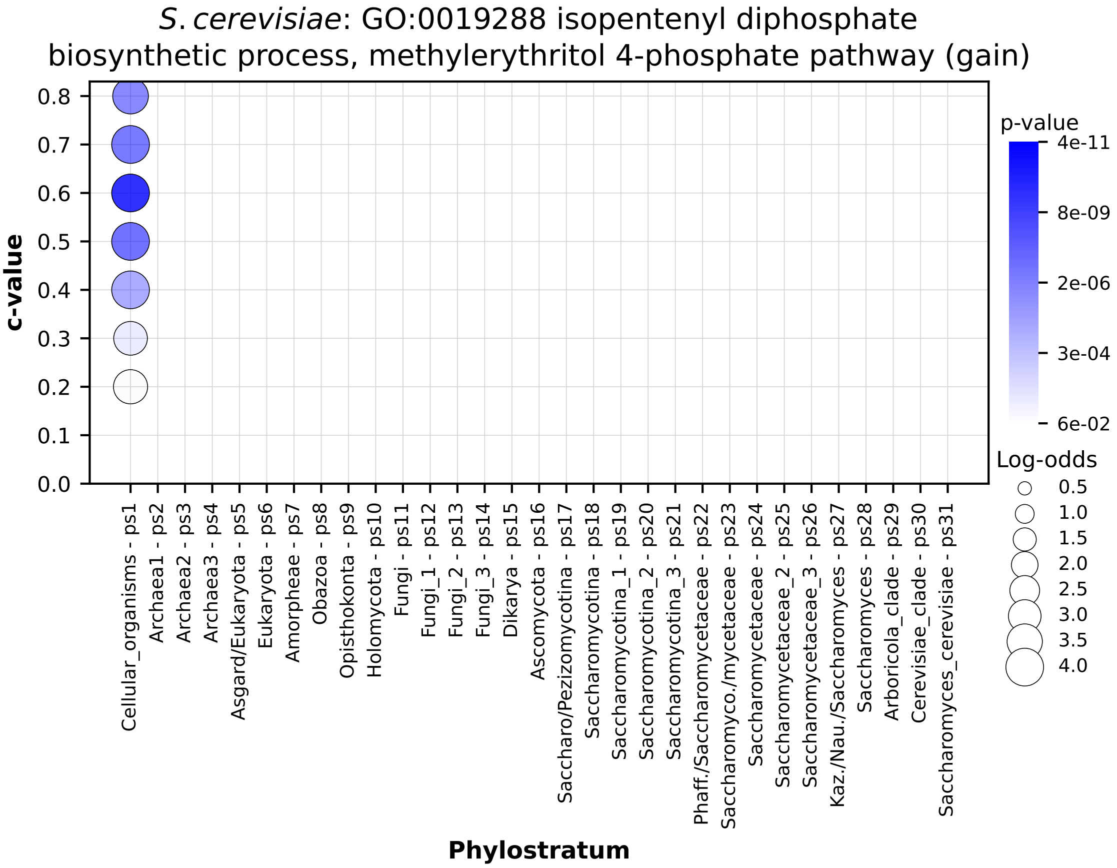

S. cerevisiae: GO:0019288 isopentenyl diphosphate biosynthetic process, methylerythritol 4-phosphate pathway (gain)
c-value
0.8
0.7
0.6
0.5
0.4
0.3
0.2
0.1
0.0
p-value
4e-11
8e-09
2e-06
3e-04
6e-02
Log-odds
0.5
1.0
1.5
2.0
2.5
3.0
3.5
4.0
Cellular_organisms - ps1
Archaea1 - ps2
Archaea2 - ps3
Archaea3 - ps4
Asgard/Eukaryota - ps5
Eukaryota - ps6
Amorpheae - ps7
Obazoa - ps8
Opisthokonta - ps9
Holomycota - ps10
Fungi - ps11
Fungi_1 - ps12
Fungi_2 - ps13
Fungi_3 - ps14
Dikarya - ps15
Ascomycota - ps16
Saccharo/Pezizomycotina - ps17
Saccharomycotina - ps18
Saccharomycotina_1 - ps19
Saccharomycotina_2 - ps20
Saccharomycotina_3 - ps21
Phaff./Saccharomycetaceae - ps22
Saccharomyco./mycetaceae - ps23
Saccharomycetaceae - ps24
Saccharomycetaceae_2 - ps25
Saccharomycetaceae_3 - ps26
Kaz./Nau./Saccharomyces - ps27
Saccharomyces - ps28
Arboricola_clade - ps29
Cerevisiae_clade - ps30
Saccharomyces_cerevisiae - ps31
Phylostratum

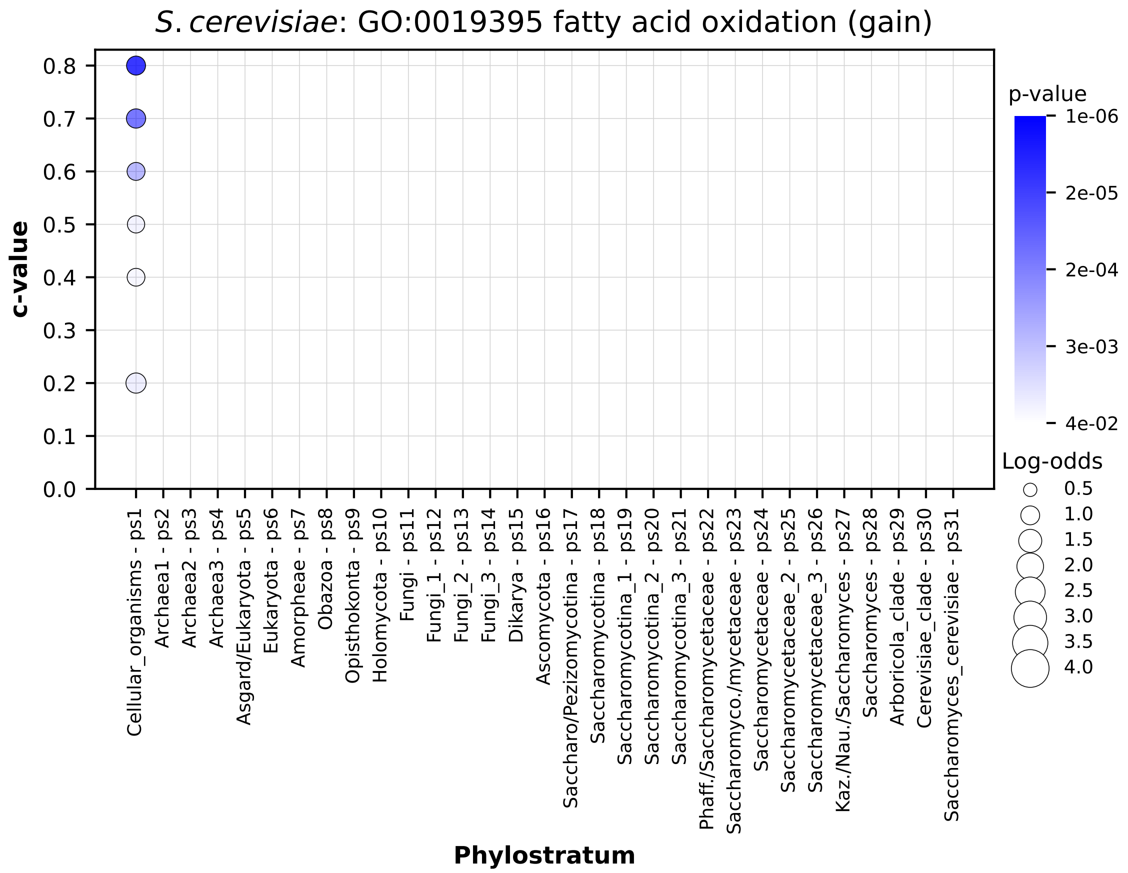

S. cerevisiae: GO:0019395 fatty acid oxidation (gain)
c-value
0.8
0.7
0.6
0.5
0.4
0.3
0.2
0.1
0.0
p-value
1e-06
2e-05
2e-04
3e-03
4e-02
Log-odds
0.5
1.0
1.5
2.0
2.5
3.0
3.5
4.0
Cellular_organisms - ps1
Archaea1 - ps2
Archaea2 - ps3
Archaea3 - ps4
Asgard/Eukaryota - ps5
Eukaryota - ps6
Amorpheae - ps7
Obazoa - ps8
Opisthokonta - ps9
Holomycota - ps10
Fungi - ps11
Fungi_1 - ps12
Fungi_2 - ps13
Fungi_3 - ps14
Dikarya - ps15
Ascomycota - ps16
Saccharo/Pezizomycotina - ps17
Saccharomycotina - ps18
Saccharomycotina_1 - ps19
Saccharomycotina_2 - ps20
Saccharomycotina_3 - ps21
Phaff./Saccharomycetaceae - ps22
Saccharomyco./mycetaceae - ps23
Saccharomycetaceae - ps24
Saccharomycetaceae_2 - ps25
Saccharomycetaceae_3 - ps26
Kaz./Nau./Saccharomyces - ps27
Saccharomyces - ps28
Arboricola_clade - ps29
Cerevisiae_clade - ps30
Saccharomyces_cerevisiae - ps31
Phylostratum

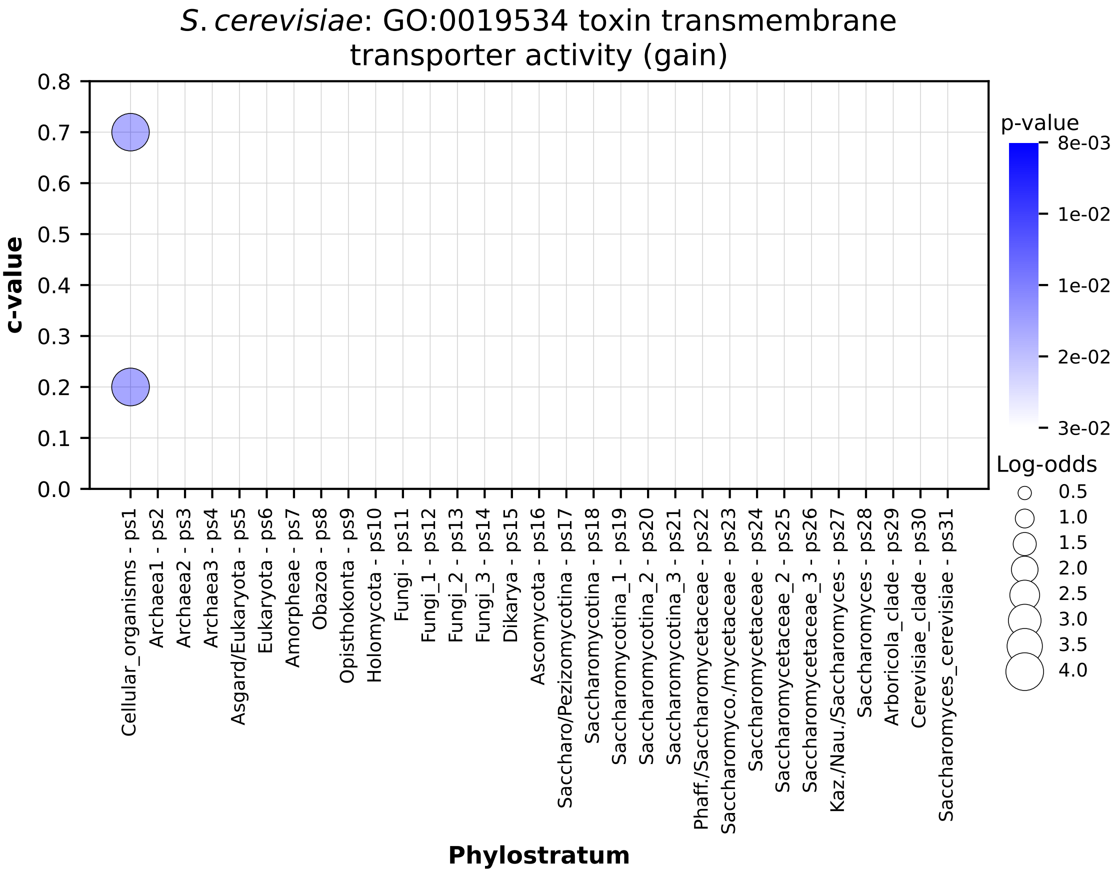

S. cerevisiae: GO:0019534 toxin transmembrane transporter activity (gain)
c-value
0.8
0.7
0.6
0.5
0.4
0.3
0.2
0.1
0.0
p-value
8e-03
1e-02
1e-02
2e-02
3e-02
Log-odds
0.5
1.0
1.5
2.0
2.5
3.0
3.5
4.0
Cellular_organisms - ps1
Archaea1 - ps2
Archaea2 - ps3
Archaea3 - ps4
Asgard/Eukaryota - ps5
Eukaryota - ps6
Amorpheae - ps7
Obazoa - ps8
Opisthokonta - ps9
Holomycota - ps10
Fungi - ps11
Fungi_1 - ps12
Fungi_2 - ps13
Fungi_3 - ps14
Dikarya - ps15
Ascomycota - ps16
Saccharo/Pezizomycotina - ps17
Saccharomycotina - ps18
Saccharomycotina_1 - ps19
Saccharomycotina_2 - ps20
Saccharomycotina_3 - ps21
Phaff./Saccharomycetaceae - ps22
Saccharomyco./mycetaceae - ps23
Saccharomycetaceae - ps24
Saccharomycetaceae_2 - ps25
Saccharomycetaceae_3 - ps26
Kaz./Nau./Saccharomyces - ps27
Saccharomyces - ps28
Arboricola_clade - ps29
Cerevisiae_clade - ps30
Saccharomyces_cerevisiae - ps31
Phylostratum

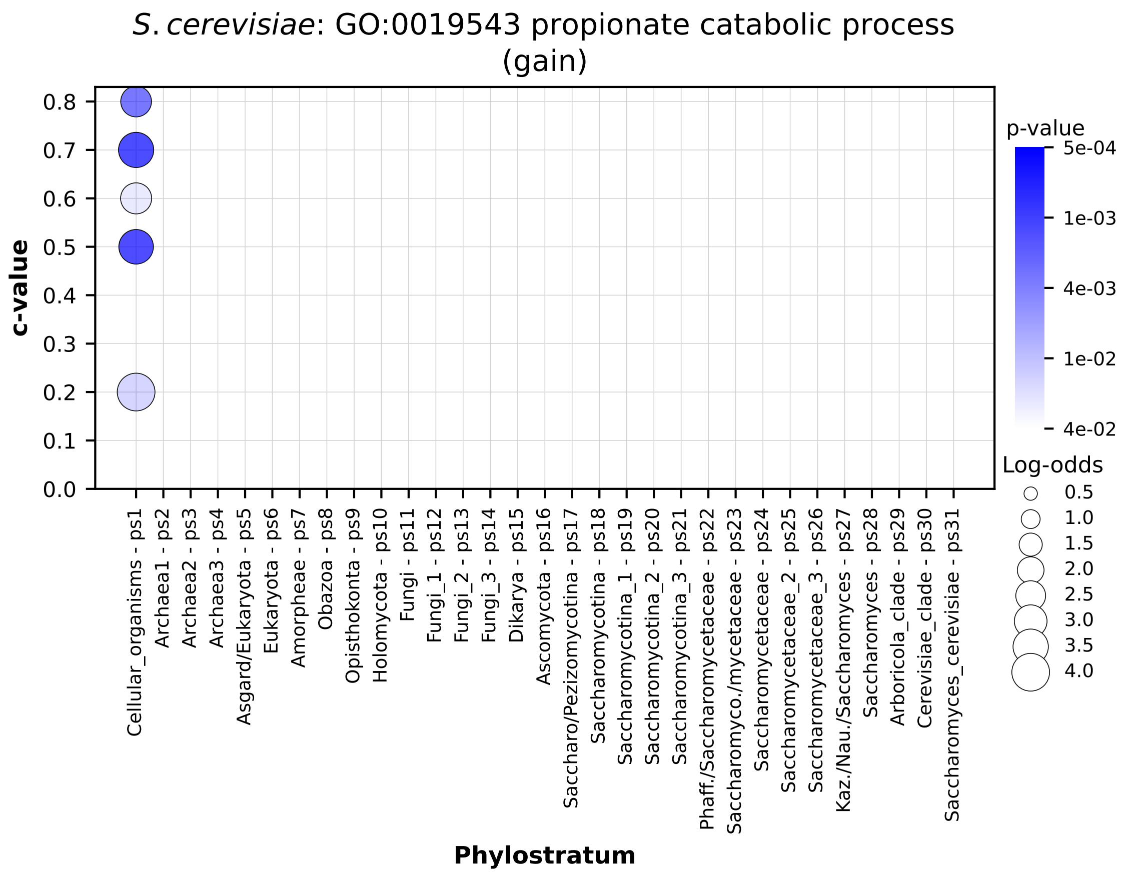

S. cerevisiae: GO:0019543 propionate catabolic process (gain)
c-value
0.0
0.1
0.2
0.3
0.4
0.5
0.6
0.7
0.8
p-value
5e-04
1e-03
4e-03
1e-02
4e-02
Log-odds
0.5
1.0
1.5
2.0
2.5
3.0
3.5
4.0
Cellular_organisms - ps1
Archaea1 - ps2
Archaea2 - ps3
Archaea3 - ps4
Asgard/Eukaryota - ps5
Eukaryota - ps6
Amorpheae - ps7
Obazoa - ps8
Opisthokonta - ps9
Holomycota - ps10
Fungi - ps11
Fungi_1 - ps12
Fungi_2 - ps13
Fungi_3 - ps14
Dikarya - ps15
Ascomycota - ps16
Saccharo/Pezizomycotina - ps17
Saccharomycotina - ps18
Saccharomycotina_1 - ps19
Saccharomycotina_2 - ps20
Saccharomycotina_3 - ps21
Phaff./Saccharomycetaceae - ps22
Saccharomyco./mycetaceae - ps23
Saccharomycetaceae - ps24
Saccharomycetaceae_2 - ps25
Saccharomycetaceae_3 - ps26
Kaz./Nau./Saccharomyces - ps27
Saccharomyces - ps28
Arboricola_clade - ps29
Cerevisiae_clade - ps30
Saccharomyces_cerevisiae - ps31
Phylostratum

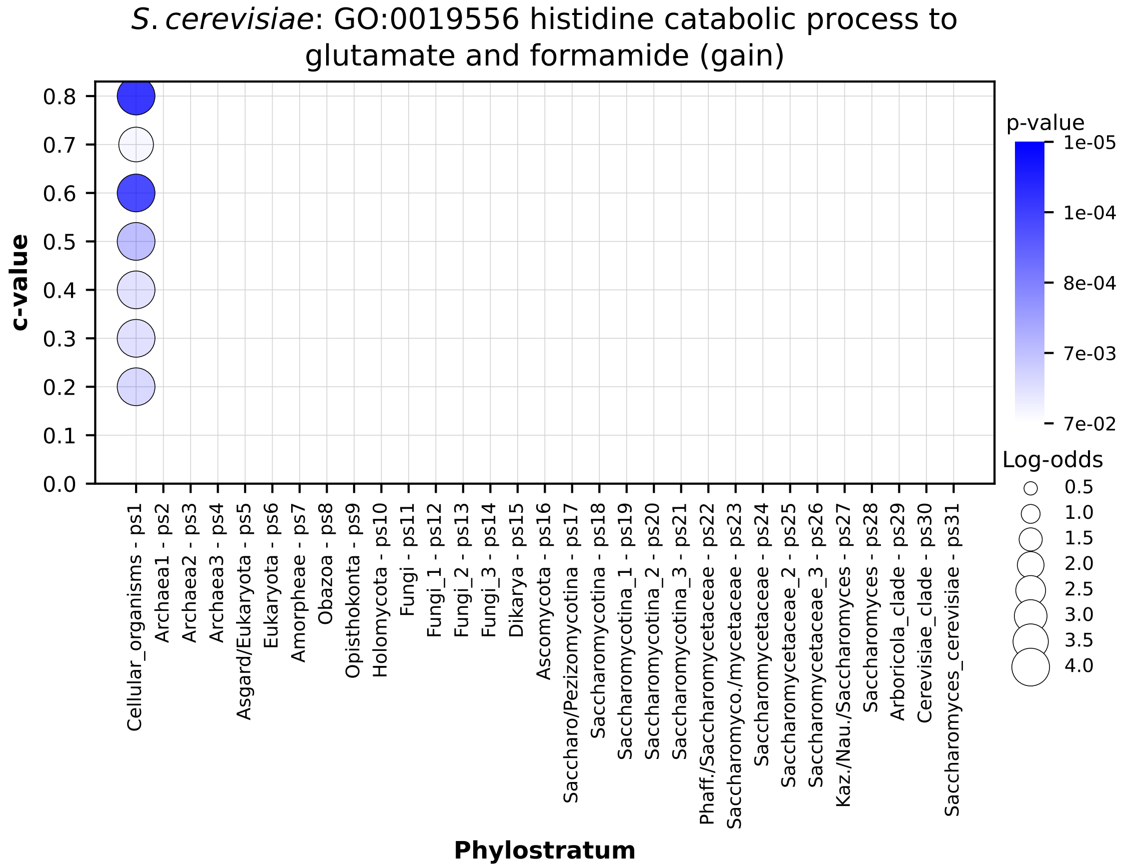

S. cerevisiae: GO:0019556 histidine catabolic process to glutamate and formamide (gain)
c-value
0.8
0.7
0.6
0.5
0.4
0.3
0.2
0.1
0.0
p-value
1e-05
1e-04
8e-04
7e-03
7e-02
Log-odds
0.5
1.0
1.5
2.0
2.5
3.0
3.5
4.0
Cellular_organisms - ps1
Archaea1 - ps2
Archaea2 - ps3
Archaea3 - ps4
Asgard/Eukaryota - ps5
Eukaryota - ps6
Amorpheae - ps7
Obazoa - ps8
Opisthokonta - ps9
Holomycota - ps10
Fungi - ps11
Fungi_1 - ps12
Fungi_2 - ps13
Fungi_3 - ps14
Dikarya - ps15
Ascomycota - ps16
Saccharo/Pezizomycotina - ps17
Saccharomycotina - ps18
Saccharomycotina_1 - ps19
Saccharomycotina_2 - ps20
Saccharomycotina_3 - ps21
Phaff./Saccharomycetaceae - ps22
Saccharomyco./mycetaceae - ps23
Saccharomycetaceae - ps24
Saccharomycetaceae_2 - ps25
Saccharomycetaceae_3 - ps26
Kaz./Nau./Saccharomyces - ps27
Saccharomyces - ps28
Arboricola_clade - ps29
Cerevisiae_clade - ps30
Saccharomyces_cerevisiae - ps31
Phylostratum

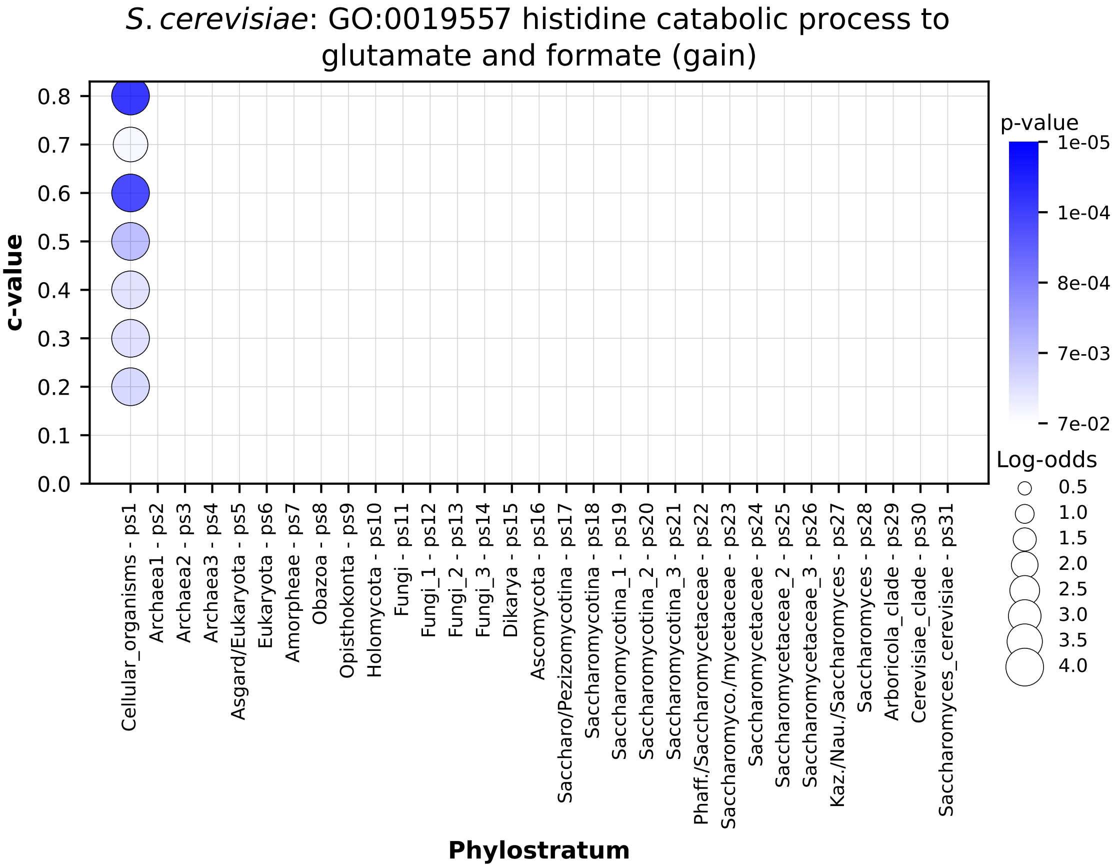

S. cerevisiae: GO:0019557 histidine catabolic process to glutamate and formate (gain)
c-value
0.0
0.1
0.2
0.3
0.4
0.5
0.6
0.7
0.8
p-value
1e-05
1e-04
8e-04
7e-03
7e-02
Log-odds
0.5
1.0
1.5
2.0
2.5
3.0
3.5
4.0
Cellular_organisms - ps1
Archaea1 - ps2
Archaea2 - ps3
Archaea3 - ps4
Asgard/Eukaryota - ps5
Eukaryota - ps6
Amorpheae - ps7
Obazoa - ps8
Opisthokonta - ps9
Holomycota - ps10
Fungi - ps11
Fungi_1 - ps12
Fungi_2 - ps13
Fungi_3 - ps14
Dikarya - ps15
Ascomycota - ps16
Saccharo/Pezizomycotina - ps17
Saccharomycotina - ps18
Saccharomycotina_1 - ps19
Saccharomycotina_2 - ps20
Saccharomycotina_3 - ps21
Phaff./Saccharomycetaceae - ps22
Saccharomyco./mycetaceae - ps23
Saccharomycetaceae - ps24
Saccharomycetaceae_2 - ps25
Saccharomycetaceae_3 - ps26
Kaz./Nau./Saccharomyces - ps27
Saccharomyces - ps28
Arboricola_clade - ps29
Cerevisiae_clade - ps30
Saccharomyces_cerevisiae - ps31
Phylostratum

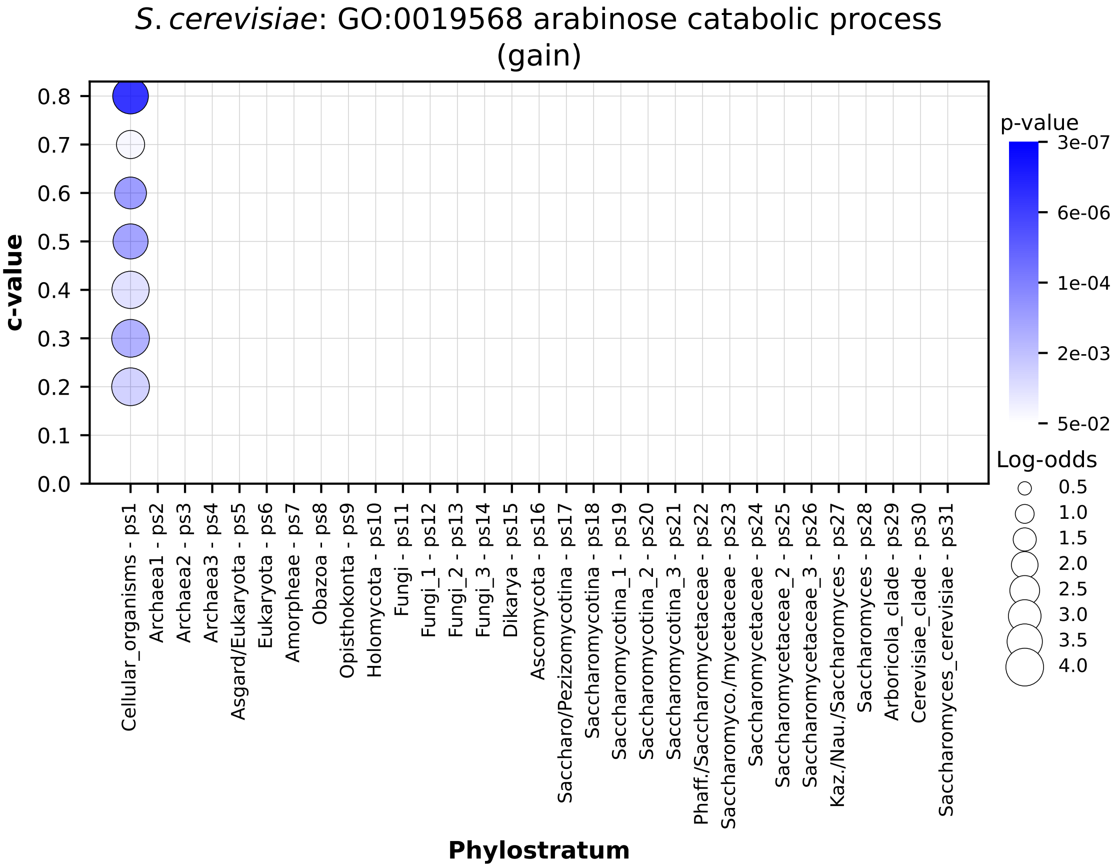

S. cerevisiae: GO:0019568 arabinose catabolic process
(gain)
c-value
0.0
0.1
0.2
0.3
0.4
0.5
0.6
0.7
0.8
Phylostratum
Cellular_organisms - ps1
Archaea1 - ps2
Archaea2 - ps3
Archaea3 - ps4
Asgard/Eukaryota - ps5
Eukaryota - ps6
Amorpheae - ps7
Obazoa - ps8
Opisthokonta - ps9
Holomycota - ps10
Fungi - ps11
Fungi_1 - ps12
Fungi_2 - ps13
Fungi_3 - ps14
Dikarya - ps15
Ascomycota - ps16
Saccharo/Pezizomycotina - ps17
Saccharomycotina - ps18
Saccharomycotina_1 - ps19
Saccharomycotina_2 - ps20
Saccharomycotina_3 - ps21
Phaff./Saccharomycetaceae - ps22
Saccharomyco./mycetaceae - ps23
Saccharomycetaceae - ps24
Saccharomycetaceae_2 - ps25
Saccharomycetaceae_3 - ps26
Kaz./Nau./Saccharomyces - ps27
Saccharomyces - ps28
Arboricola_clade - ps29
Cerevisiae_clade - ps30
Saccharomyces_cerevisiae - ps31
p-value
3e-07
6e-06
1e-04
2e-03
5e-02
Log-odds
0.5
1.0
1.5
2.0
2.5
3.0
3.5
4.0

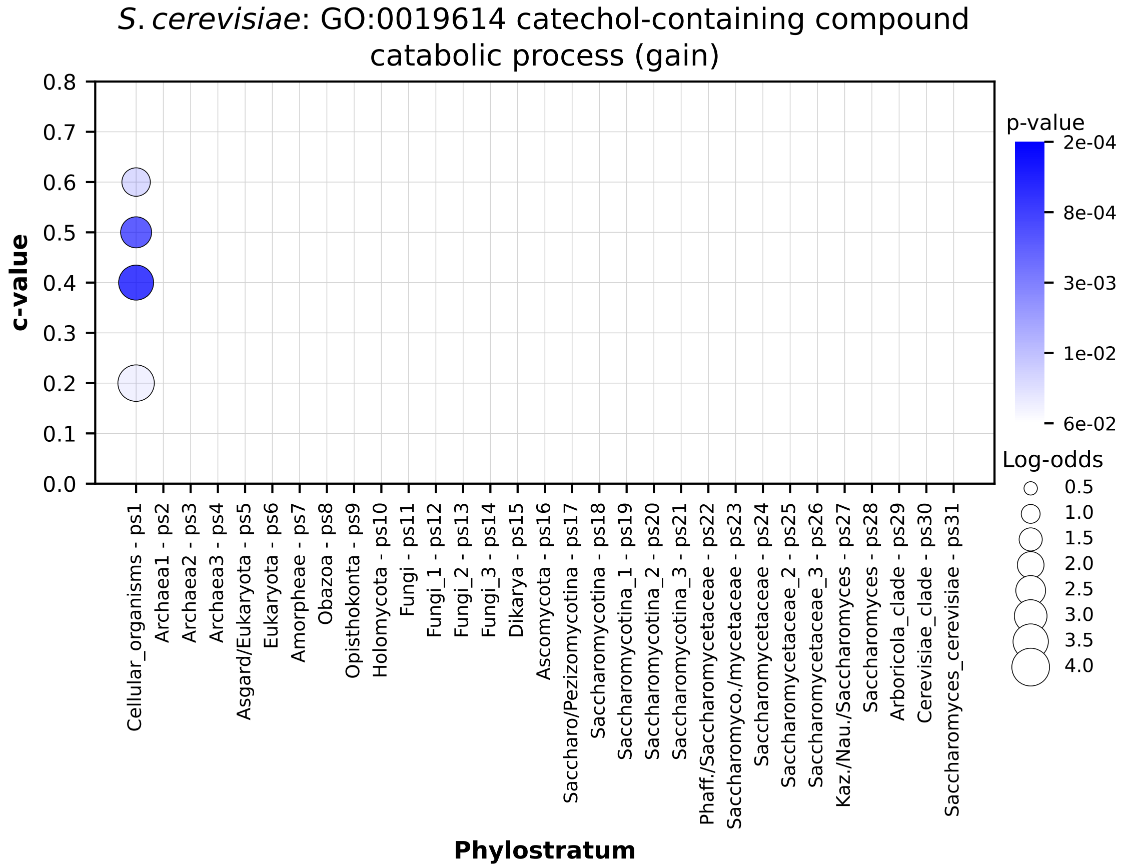

S. cerevisiae: GO:0019614 catechol-containing compound catabolic process (gain)
c-value
0.0
0.1
0.2
0.3
0.4
0.5
0.6
0.7
0.8
Phylostratum
Cellular_organisms - ps1
Archaea1 - ps2
Archaea2 - ps3
Archaea3 - ps4
Asgard/Eukaryota - ps5
Eukaryota - ps6
Amorpheae - ps7
Obazoa - ps8
Opisthokonta - ps9
Holomycota - ps10
Fungi - ps11
Fungi_1 - ps12
Fungi_2 - ps13
Fungi_3 - ps14
Dikarya - ps15
Ascomycota - ps16
Saccharo/Pezizomycotina - ps17
Saccharomycotina - ps18
Saccharomycotina_1 - ps19
Saccharomycotina_2 - ps20
Saccharomycotina_3 - ps21
Phaff./Saccharomycetaceae - ps22
Saccharomyco./mycetaceae - ps23
Saccharomycetaceae - ps24
Saccharomycetaceae_2 - ps25
Saccharomycetaceae_3 - ps26
Kaz./Nau./Saccharomyces - ps27
Saccharomyces - ps28
Arboricola_clade - ps29
Cerevisiae_clade - ps30
Saccharomyces_cerevisiae - ps31
p-value
2e-04
8e-04
3e-03
1e-02
6e-02
Log-odds
0.5
1.0
1.5
2.0
2.5
3.0
3.5
4.0

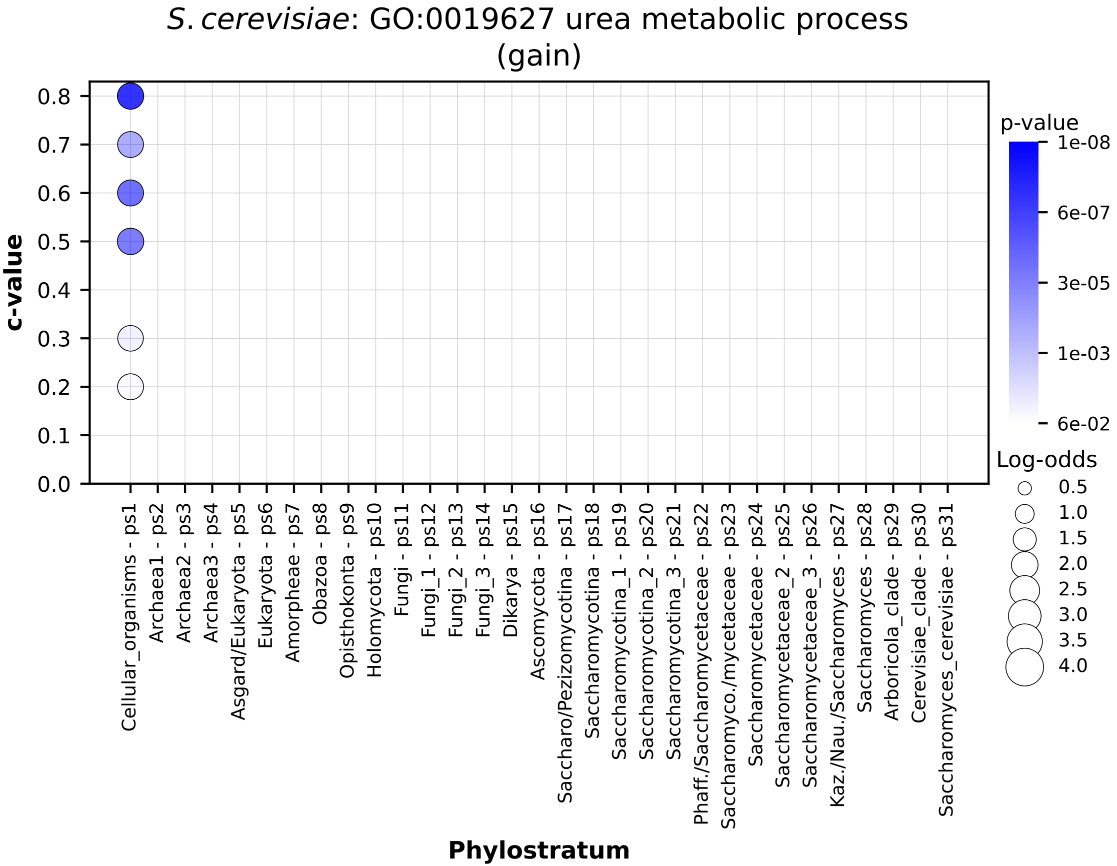

S. cerevisiae: GO:0019627 urea metabolic process
(gain)
c-value
0.8
0.7
0.6
0.5
0.4
0.3
0.2
0.1
0.0
p-value
1e-08
6e-07
3e-05
1e-03
6e-02
Log-odds
0.5
1.0
1.5
2.0
2.5
3.0
3.5
4.0
Cellular_organisms - ps1
Archaea1 - ps2
Archaea2 - ps3
Archaea3 - ps4
Asgard/Eukaryota - ps5
Eukaryota - ps6
Amorpheae - ps7
Obazoa - ps8
Opisthokonta - ps9
Holomycota - ps10
Fungi - ps11
Fungi_1 - ps12
Fungi_2 - ps13
Fungi_3 - ps14
Dikarya - ps15
Ascomycota - ps16
Saccharo/Pezizomycotina - ps17
Saccharomycotina - ps18
Saccharomycotina_1 - ps19
Saccharomycotina_2 - ps20
Saccharomycotina_3 - ps21
Phaff./Saccharomycetaceae - ps22
Saccharomyco./mycetaceae - ps23
Saccharomycetaceae - ps24
Saccharomycetaceae_2 - ps25
Saccharomycetaceae_3 - ps26
Kaz./Nau./Saccharomyces - ps27
Saccharomyces - ps28
Arboricola_clade - ps29
Cerevisiae_clade - ps30
Saccharomyces_cerevisiae - ps31
Phylostratum

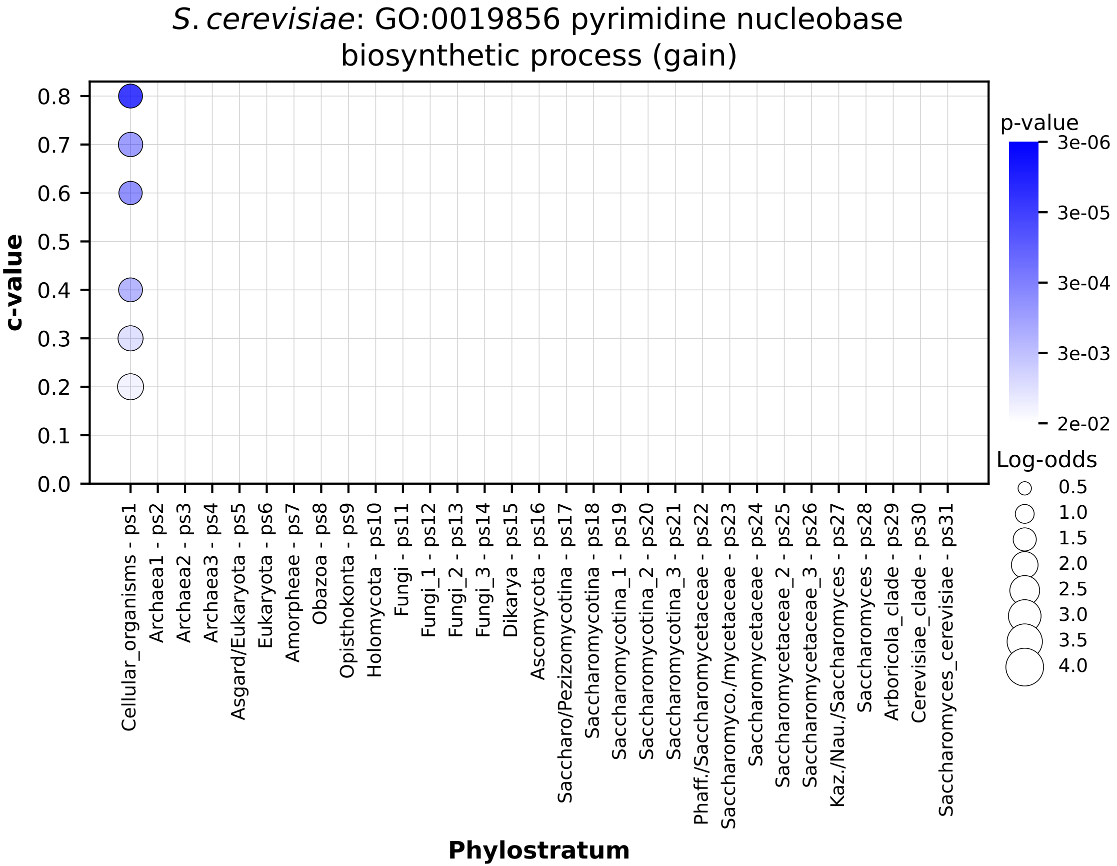

S. cerevisiae: GO:0019856 pyrimidine nucleobase biosynthetic process (gain)
c-value
0.0
0.1
0.2
0.3
0.4
0.5
0.6
0.7
0.8
p-value
3e-06
3e-05
3e-04
3e-03
2e-02
Log-odds
0.5
1.0
1.5
2.0
2.5
3.0
3.5
4.0
Cellular_organisms - ps1
Archaea1 - ps2
Archaea2 - ps3
Archaea3 - ps4
Asgard/Eukaryota - ps5
Eukaryota - ps6
Amorpheae - ps7
Obazoa - ps8
Opisthokonta - ps9
Holomycota - ps10
Fungi - ps11
Fungi_1 - ps12
Fungi_2 - ps13
Fungi_3 - ps14
Dikarya - ps15
Ascomycota - ps16
Saccharo/Pezizomycotina - ps17
Saccharomycotina - ps18
Saccharomycotina_1 - ps19
Saccharomycotina_2 - ps20
Saccharomycotina_3 - ps21
Phaff./Saccharomycetaceae - ps22
Saccharomyco./mycetaceae - ps23
Saccharomycetaceae - ps24
Saccharomycetaceae_2 - ps25
Saccharomycetaceae_3 - ps26
Kaz./Nau./Saccharomyces - ps27
Saccharomyces - ps28
Arboricola_clade - ps29
Cerevisiae_clade - ps30
Saccharomyces_cerevisiae - ps31
Phylostratum

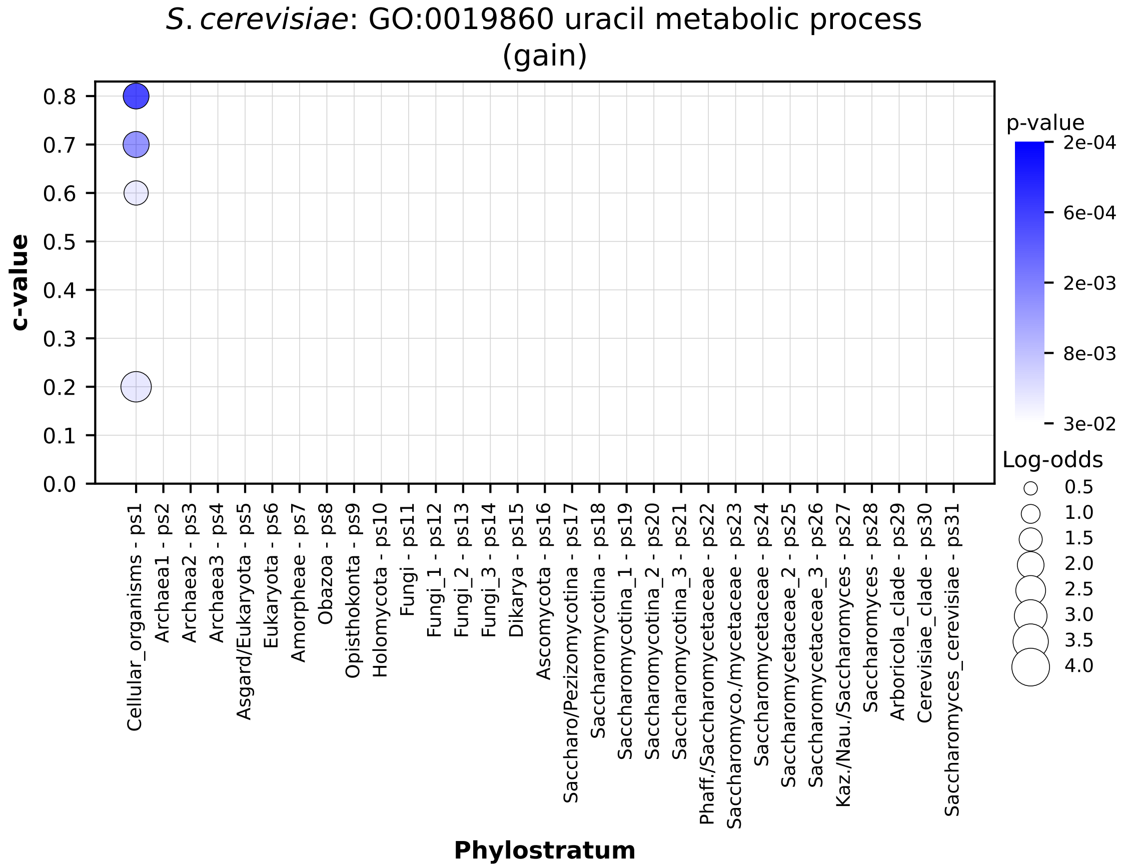

S. cerevisiae: GO:0019860 uracil metabolic process (gain)
c-value
0.0
0.1
0.2
0.3
0.4
0.5
0.6
0.7
0.8
p-value
2e-04
6e-04
2e-03
8e-03
3e-02
Log-odds
0.5
1.0
1.5
2.0
2.5
3.0
3.5
4.0
Cellular_organisms - ps1
Archaea1 - ps2
Archaea2 - ps3
Archaea3 - ps4
Asgard/Eukaryota - ps5
Eukaryota - ps6
Amorpheae - ps7
Obazoa - ps8
Opisthokonta - ps9
Holomycota - ps10
Fungi - ps11
Fungi_1 - ps12
Fungi_2 - ps13
Fungi_3 - ps14
Dikarya - ps15
Ascomycota - ps16
Saccharo/Pezizomycotina - ps17
Saccharomycotina - ps18
Saccharomycotina_1 - ps19
Saccharomycotina_2 - ps20
Saccharomycotina_3 - ps21
Phaff./Saccharomycetaceae - ps22
Saccharomyco./mycetaceae - ps23
Saccharomycetaceae - ps24
Saccharomycetaceae_2 - ps25
Saccharomycetaceae_3 - ps26
Kaz./Nau./Saccharomyces - ps27
Saccharomyces - ps28
Arboricola_clade - ps29
Cerevisiae_clade - ps30
Saccharomyces_cerevisiae - ps31
Phylostratum

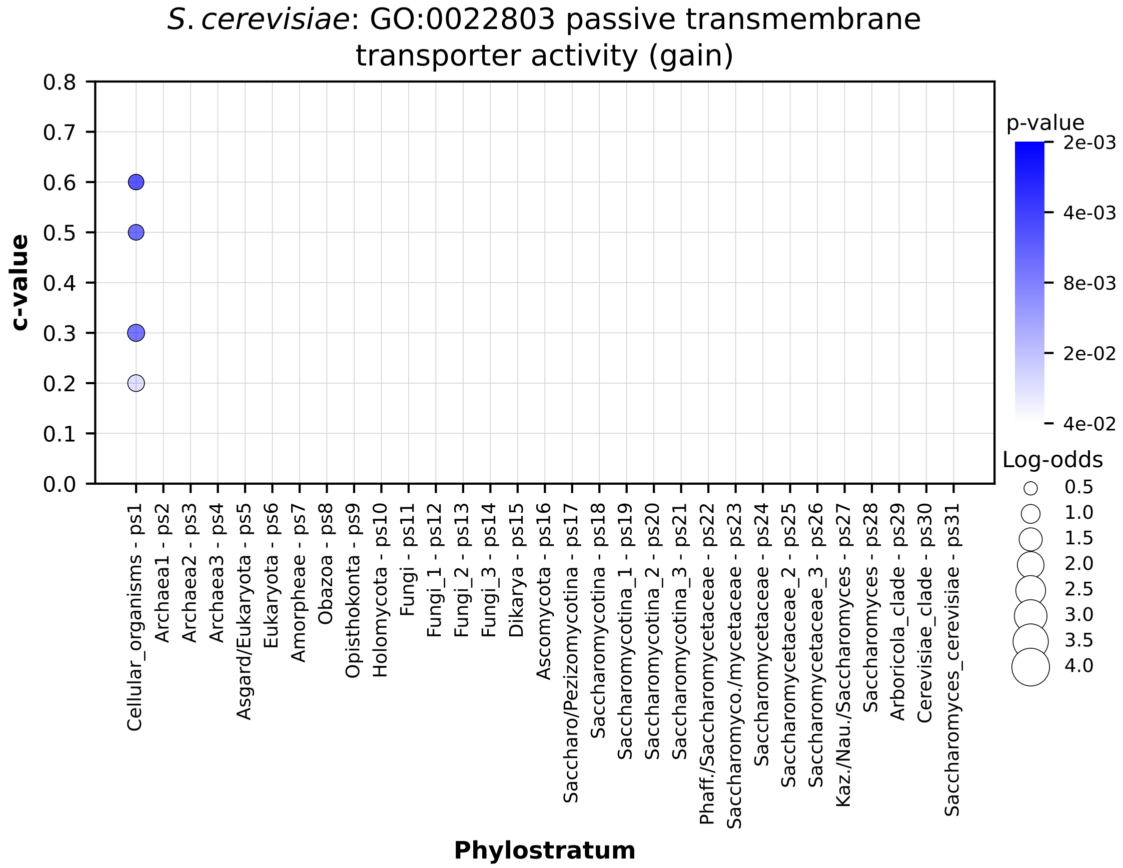

S. cerevisiae: GO:0022803 passive transmembrane transporter activity (gain)
c-value
0.8
0.7
0.6
0.5
0.4
0.3
0.2
0.1
0.0
p-value
2e-03
4e-03
8e-03
2e-02
4e-02
Log-odds
0.5
1.0
1.5
2.0
2.5
3.0
3.5
4.0
Cellular_organisms - ps1
Archaea1 - ps2
Archaea2 - ps3
Archaea3 - ps4
Asgard/Eukaryota - ps5
Eukaryota - ps6
Amorpheae - ps7
Obazoa - ps8
Opisthokonta - ps9
Holomycota - ps10
Fungi - ps11
Fungi_1 - ps12
Fungi_2 - ps13
Fungi_3 - ps14
Dikarya - ps15
Ascomycota - ps16
Saccharo/Pezizomycotina - ps17
Saccharomycotina - ps18
Saccharomycotina_1 - ps19
Saccharomycotina_2 - ps20
Saccharomycotina_3 - ps21
Phaff./Saccharomycetaceae - ps22
Saccharomyco./mycetaceae - ps23
Saccharomycetaceae - ps24
Saccharomycetaceae_2 - ps25
Saccharomycetaceae_3 - ps26
Kaz./Nau./Saccharomyces - ps27
Saccharomyces - ps28
Arboricola_clade - ps29
Cerevisiae_clade - ps30
Saccharomyces_cerevisiae - ps31
Phylostratum

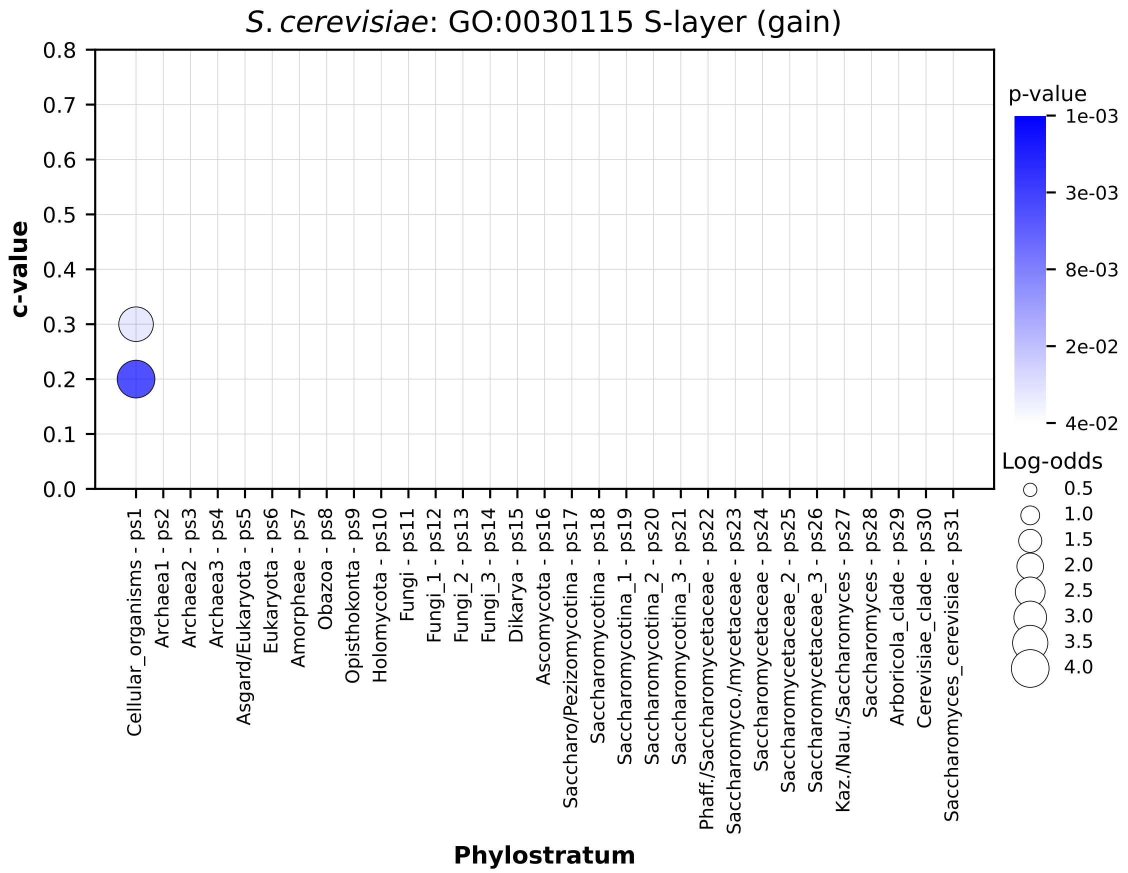

S. cerevisiae: GO:0030115 S-layer (gain)
c-value
0.0
0.1
0.2
0.3
0.4
0.5
0.6
0.7
0.8
p-value
1e-03
3e-03
8e-03
2e-02
4e-02
Log-odds
0.5
1.0
1.5
2.0
2.5
3.0
3.5
4.0
Cellular_organisms - ps1
Archaea1 - ps2
Archaea2 - ps3
Archaea3 - ps4
Asgard/Eukaryota - ps5
Eukaryota - ps6
Amorpheae - ps7
Obazoa - ps8
Opisthokonta - ps9
Holomycota - ps10
Fungi - ps11
Fungi_1 - ps12
Fungi_2 - ps13
Fungi_3 - ps14
Dikarya - ps15
Ascomycota - ps16
Saccharo/Pezizomycotina - ps17
Saccharomycotina - ps18
Saccharomycotina_1 - ps19
Saccharomycotina_2 - ps20
Saccharomycotina_3 - ps21
Phaff./Saccharomycetaceae - ps22
Saccharomyco./mycetaceae - ps23
Saccharomycetaceae - ps24
Saccharomycetaceae_2 - ps25
Saccharomycetaceae_3 - ps26
Kaz./Nau./Saccharomyces - ps27
Saccharomyces - ps28
Arboricola_clade - ps29
Cerevisiae_clade - ps30
Saccharomyces_cerevisiae - ps31
Phylostratum

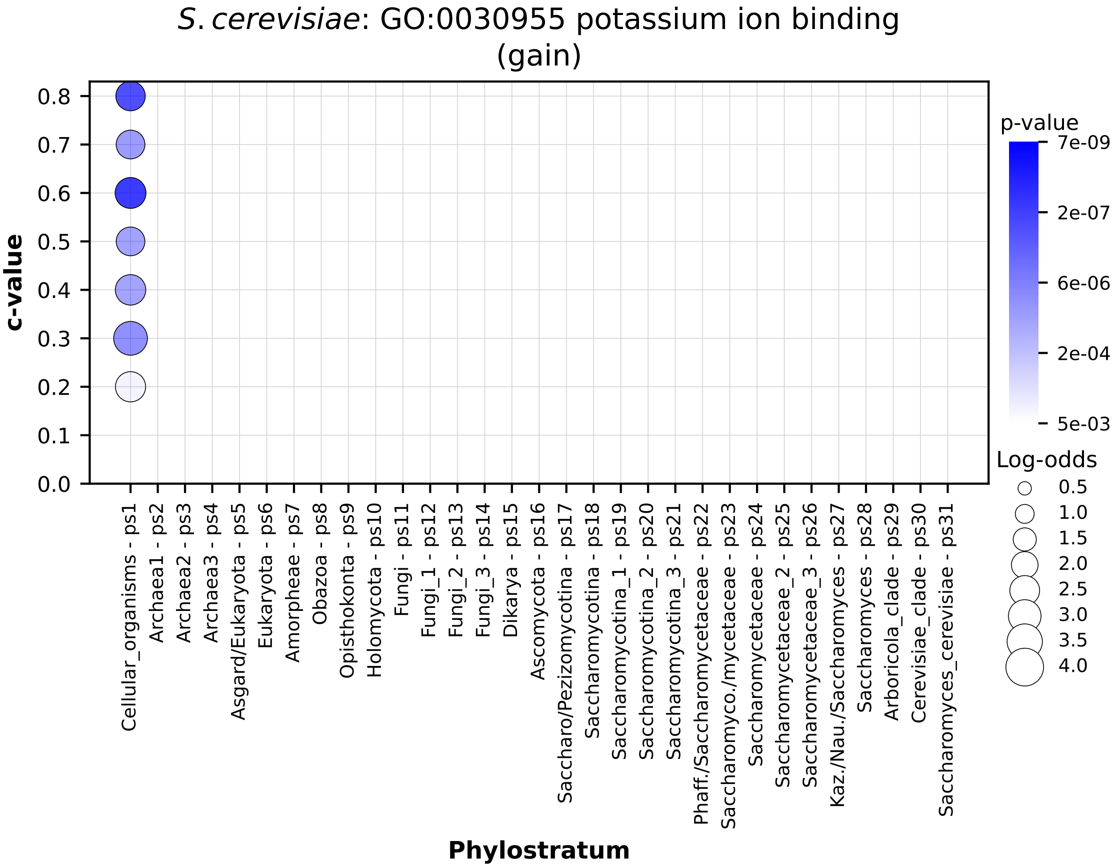

S. cerevisiae: GO:0030955 potassium ion binding
(gain)
c-value
0.0
0.1
0.2
0.3
0.4
0.5
0.6
0.7
0.8
p-value
7e-09
2e-07
6e-06
2e-04
5e-03
Log-odds
0.5
1.0
1.5
2.0
2.5
3.0
3.5
4.0
Cellular_organisms - ps1
Archaea1 - ps2
Archaea2 - ps3
Archaea3 - ps4
Asgard/Eukaryota - ps5
Eukaryota - ps6
Amorpheae - ps7
Obazoa - ps8
Opisthokonta - ps9
Holomycota - ps10
Fungi - ps11
Fungi_1 - ps12
Fungi_2 - ps13
Fungi_3 - ps14
Dikarya - ps15
Ascomycota - ps16
Saccharo/Pezizomycotina - ps17
Saccharomycotina - ps18
Saccharomycotina_1 - ps19
Saccharomycotina_2 - ps20
Saccharomycotina_3 - ps21
Phaff./Saccharomycetaceae - ps22
Saccharomyco./mycetaceae - ps23
Saccharomycetaceae - ps24
Saccharomycetaceae_2 - ps25
Saccharomycetaceae_3 - ps26
Kaz./Nau./Saccharomyces - ps27
Saccharomyces - ps28
Arboricola_clade - ps29
Cerevisiae_clade - ps30
Saccharomyces_cerevisiae - ps31
Phylostratum

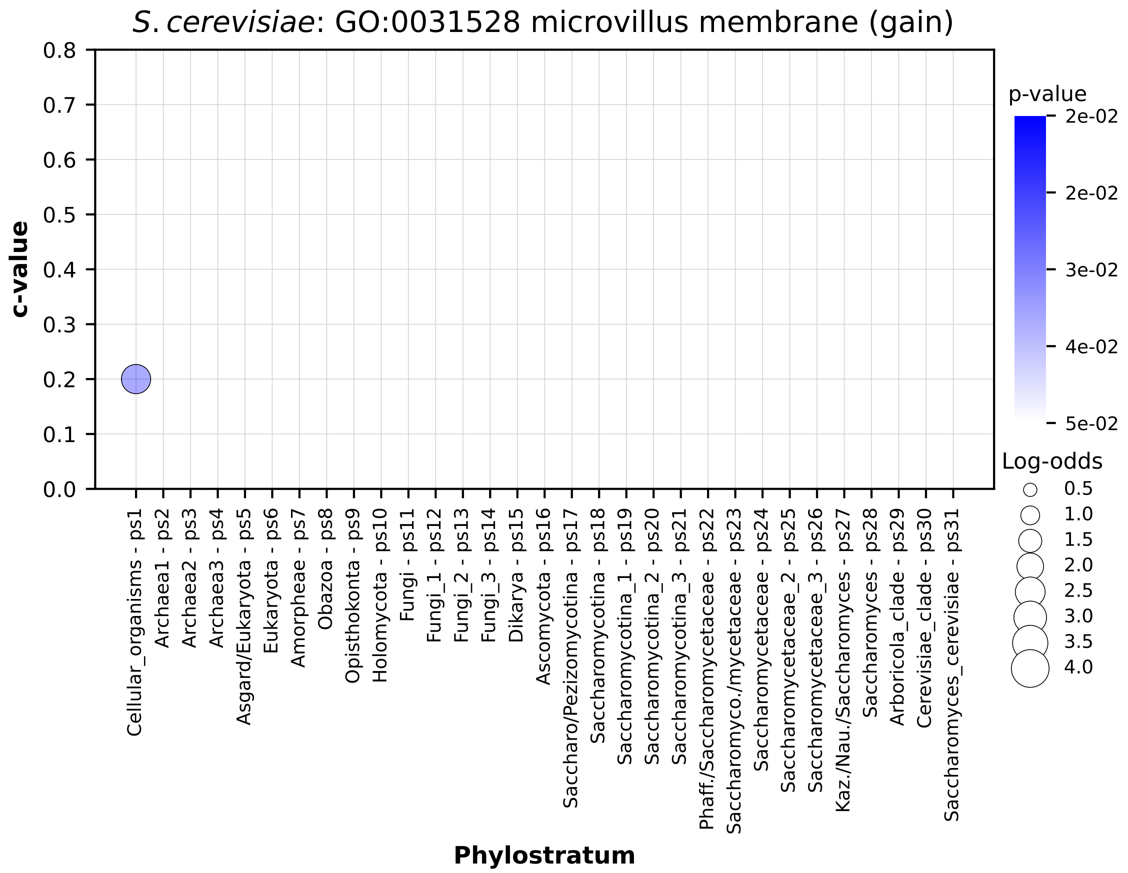

S. cerevisiae: GO:0031528 microvillus membrane (gain)
c-value
0.0
0.1
0.2
0.3
0.4
0.5
0.6
0.7
0.8
p-value
2e-02
2e-02
3e-02
4e-02
5e-02
Log-odds
0.5
1.0
1.5
2.0
2.5
3.0
3.5
4.0
Cellular_organisms - ps1
Archaea1 - ps2
Archaea2 - ps3
Archaea3 - ps4
Asgard/Eukaryota - ps5
Eukaryota - ps6
Amorpheae - ps7
Obazoa - ps8
Opisthokonta - ps9
Holomycota - ps10
Fungi - ps11
Fungi_1 - ps12
Fungi_2 - ps13
Fungi_3 - ps14
Dikarya - ps15
Ascomycota - ps16
Saccharo/Pezizomycotina - ps17
Saccharomycotina - ps18
Saccharomycotina_1 - ps19
Saccharomycotina_2 - ps20
Saccharomycotina_3 - ps21
Phaff./Saccharomycetaceae - ps22
Saccharomyco./mycetaceae - ps23
Saccharomycetaceae - ps24
Saccharomycetaceae_2 - ps25
Saccharomycetaceae_3 - ps26
Kaz./Nau./Saccharomyces - ps27
Saccharomyces - ps28
Arboricola_clade - ps29
Cerevisiae_clade - ps30
Saccharomyces_cerevisiae - ps31
Phylostratum

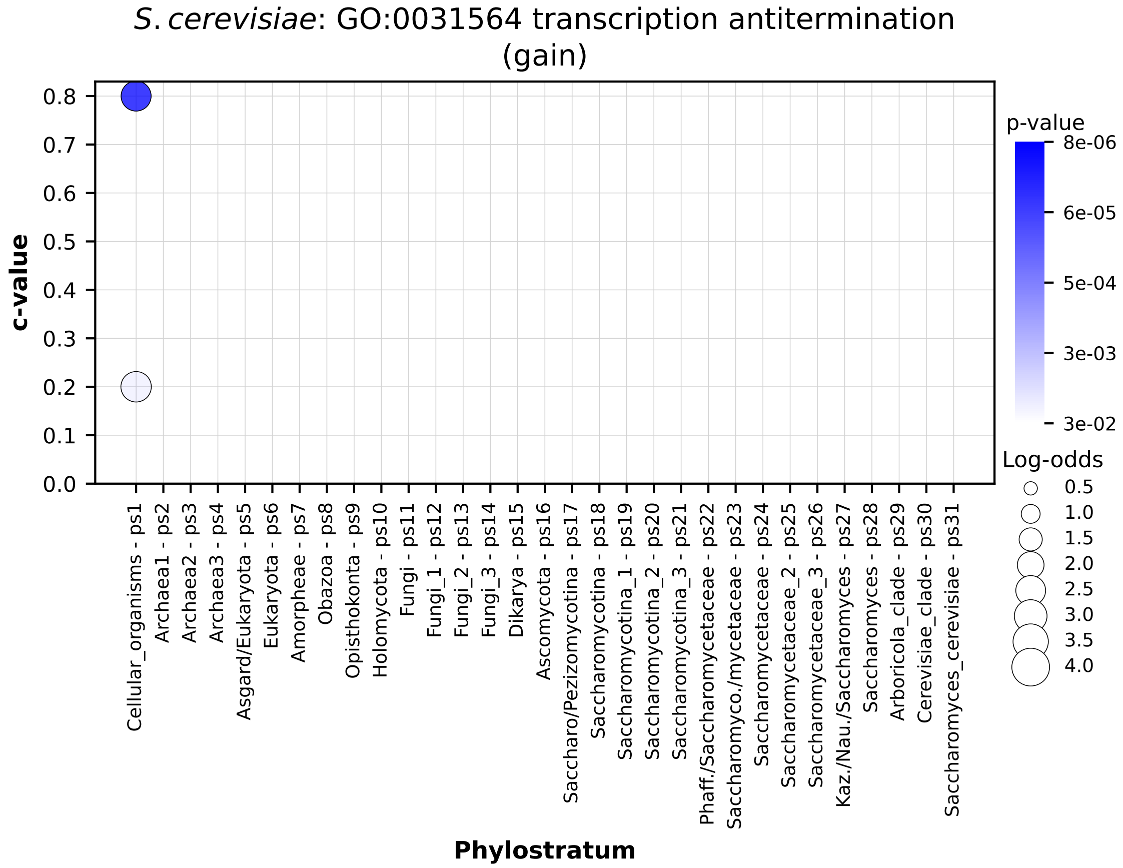

S. cerevisiae: GO:0031564 transcription antitermination (gain)
c-value
0.8
0.7
0.6
0.5
0.4
0.3
0.2
0.1
0.0
p-value
8e-06
6e-05
5e-04
3e-03
3e-02
Log-odds
0.5
1.0
1.5
2.0
2.5
3.0
3.5
4.0
Cellular_organisms - ps1
Archaea1 - ps2
Archaea2 - ps3
Archaea3 - ps4
Asgard/Eukaryota - ps5
Eukaryota - ps6
Amorpheae - ps7
Obazoa - ps8
Opisthokonta - ps9
Holomycota - ps10
Fungi - ps11
Fungi_1 - ps12
Fungi_2 - ps13
Fungi_3 - ps14
Dikarya - ps15
Ascomycota - ps16
Saccharo/Pezizomycotina - ps17
Saccharomycotina - ps18
Saccharomycotina_1 - ps19
Saccharomycotina_2 - ps20
Saccharomycotina_3 - ps21
Phaff./Saccharomycetaceae - ps22
Saccharomyco./mycetaceae - ps23
Saccharomycetaceae - ps24
Saccharomycetaceae_2 - ps25
Saccharomycetaceae_3 - ps26
Kaz./Nau./Saccharomyces - ps27
Saccharomyces - ps28
Arboricola_clade - ps29
Cerevisiae_clade - ps30
Saccharomyces_cerevisiae - ps31
Phylostratum

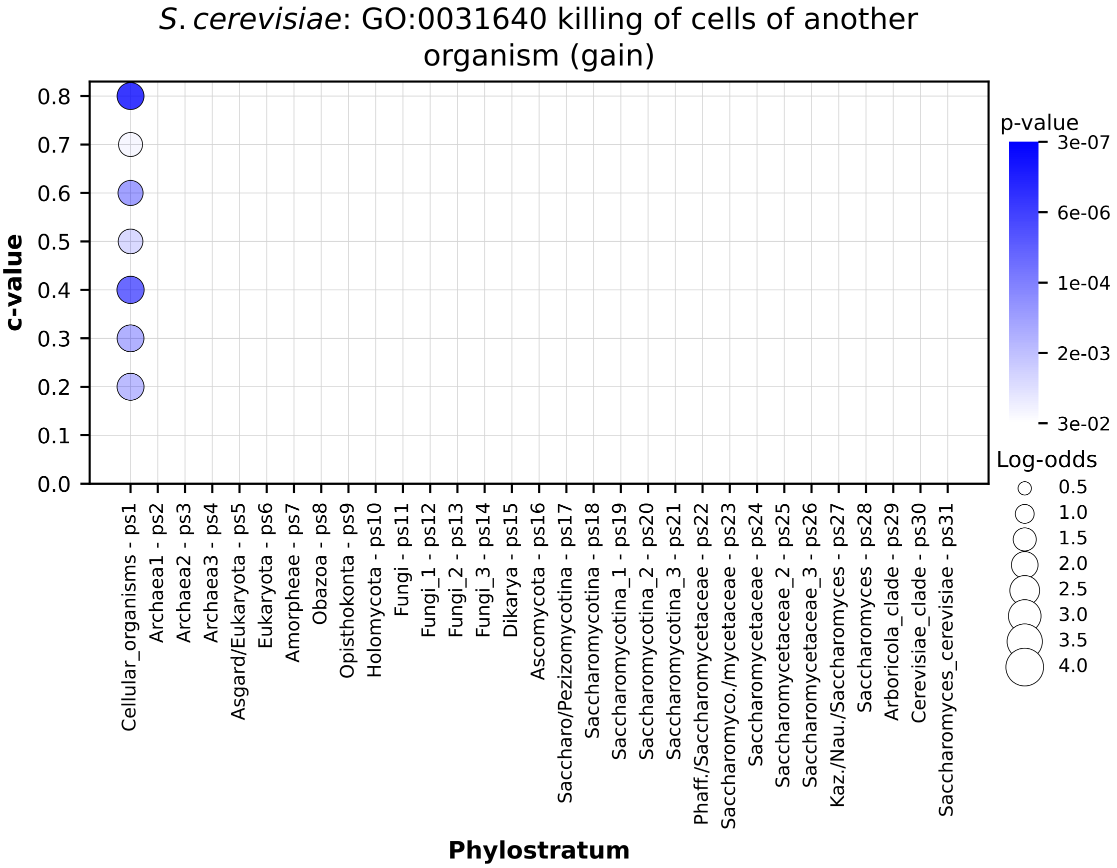
S. cerevisiae: GO:0031640 killing of cells of another organism (gain)
c-value
0.0
0.1
0.2
0.3
0.4
0.5
0.6
0.7
0.8
Phylostratum
Cellular_organisms - ps1
Archaea1 - ps2
Archaea2 - ps3
Archaea3 - ps4
Asgard/Eukaryota - ps5
Eukaryota - ps6
Amorpheae - ps7
Obazoa - ps8
Opisthokonta - ps9
Holomycota - ps10
Fungi - ps11
Fungi_1 - ps12
Fungi_2 - ps13
Fungi_3 - ps14
Dikarya - ps15
Ascomycota - ps16
Saccharo/Pezizomycotina - ps17
Saccharomycotina - ps18
Saccharomycotina_1 - ps19
Saccharomycotina_2 - ps20
Saccharomycotina_3 - ps21
Phaff./Saccharomycetaceae - ps22
Saccharomyco./mycetaceae - ps23
Saccharomycetaceae - ps24
Saccharomycetaceae_2 - ps25
Saccharomycetaceae_3 - ps26
Kaz./Nau./Saccharomyces - ps27
Saccharomyces - ps28
Arboricola_clade - ps29
Cerevisiae_clade - ps30
Saccharomyces_cerevisiae - ps31
p-value
3e-07
6e-06
1e-04
2e-03
3e-02
Log-odds
0.5
1.0
1.5
2.0
2.5
3.0
3.5
4.0

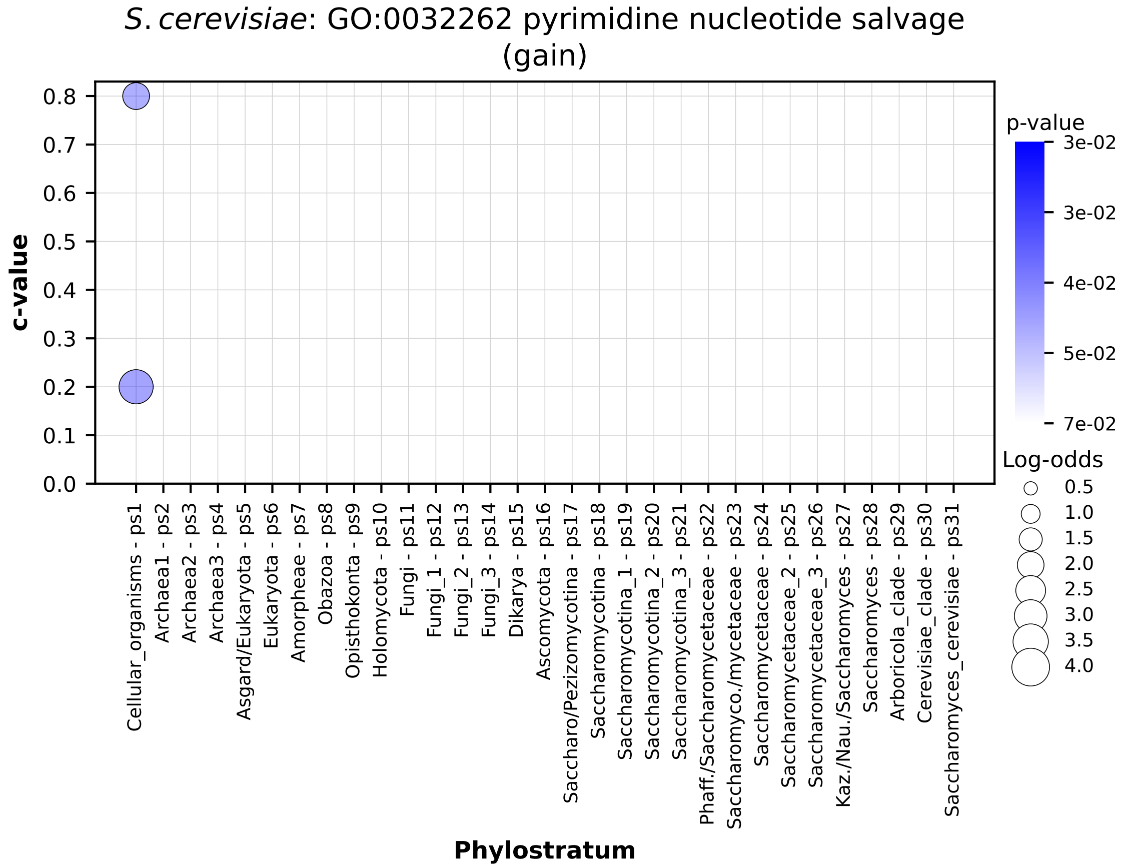

S. cerevisiae: GO:0032262 pyrimidine nucleotide salvage
(gain)
c-value
0.0
0.1
0.2
0.3
0.4
0.5
0.6
0.7
0.8
Phylostratum
Cellular_organisms - ps1
Archaea1 - ps2
Archaea2 - ps3
Archaea3 - ps4
Asgard/Eukaryota - ps5
Eukaryota - ps6
Amorpheae - ps7
Obazoa - ps8
Opisthokonta - ps9
Holomycota - ps10
Fungi - ps11
Fungi_1 - ps12
Fungi_2 - ps13
Fungi_3 - ps14
Dikarya - ps15
Ascomycota - ps16
Saccharo/Pezizomycotina - ps17
Saccharomycotina - ps18
Saccharomycotina_1 - ps19
Saccharomycotina_2 - ps20
Saccharomycotina_3 - ps21
Phaff./Saccharomycetaceae - ps22
Saccharomyco./mycetaceae - ps23
Saccharomycetaceae - ps24
Saccharomycetaceae_2 - ps25
Saccharomycetaceae_3 - ps26
Kaz./Nau./Saccharomyces - ps27
Saccharomyces - ps28
Arboricola_clade - ps29
Cerevisiae_clade - ps30
Saccharomyces_cerevisiae - ps31
p-value
3e-02
3e-02
4e-02
5e-02
7e-02
Log-odds
0.5
1.0
1.5
2.0
2.5
3.0
3.5
4.0

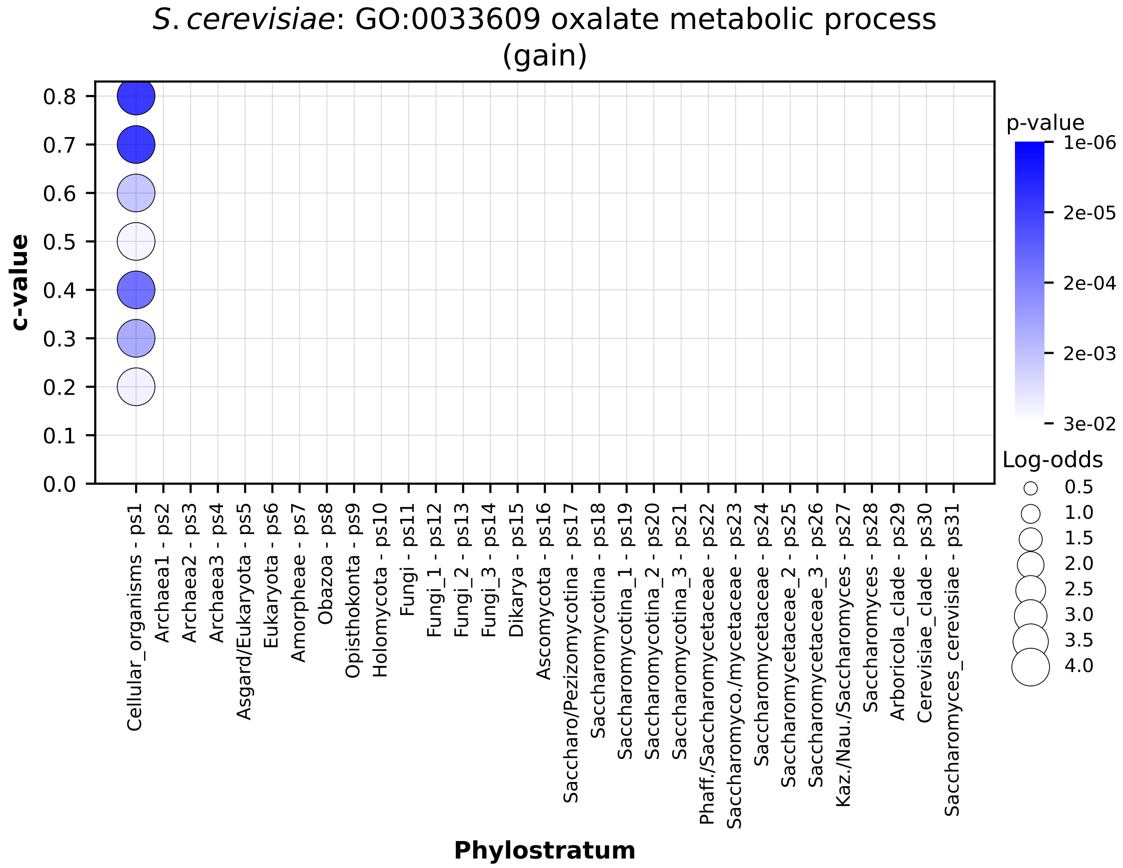

S. cerevisiae: GO:0033609 oxalate metabolic process
(gain)
c-value
0.0
0.1
0.2
0.3
0.4
0.5
0.6
0.7
0.8
p-value
1e-06
2e-05
2e-04
2e-03
3e-02
Log-odds
0.5
1.0
1.5
2.0
2.5
3.0
3.5
4.0
Cellular_organisms - ps1
Archaea1 - ps2
Archaea2 - ps3
Archaea3 - ps4
Asgard/Eukaryota - ps5
Eukaryota - ps6
Amorpheae - ps7
Obazoa - ps8
Opisthokonta - ps9
Holomycota - ps10
Fungi - ps11
Fungi_1 - ps12
Fungi_2 - ps13
Fungi_3 - ps14
Dikarya - ps15
Ascomycota - ps16
Saccharo/Pezizomycotina - ps17
Saccharomycotina - ps18
Saccharomycotina_1 - ps19
Saccharomycotina_2 - ps20
Saccharomycotina_3 - ps21
Phaff./Saccharomycetaceae - ps22
Saccharomyco./mycetaceae - ps23
Saccharomycetaceae - ps24
Saccharomycetaceae_2 - ps25
Saccharomycetaceae_3 - ps26
Kaz./Nau./Saccharomyces - ps27
Saccharomyces - ps28
Arboricola_clade - ps29
Cerevisiae_clade - ps30
Saccharomyces_cerevisiae - ps31
Phylostratum

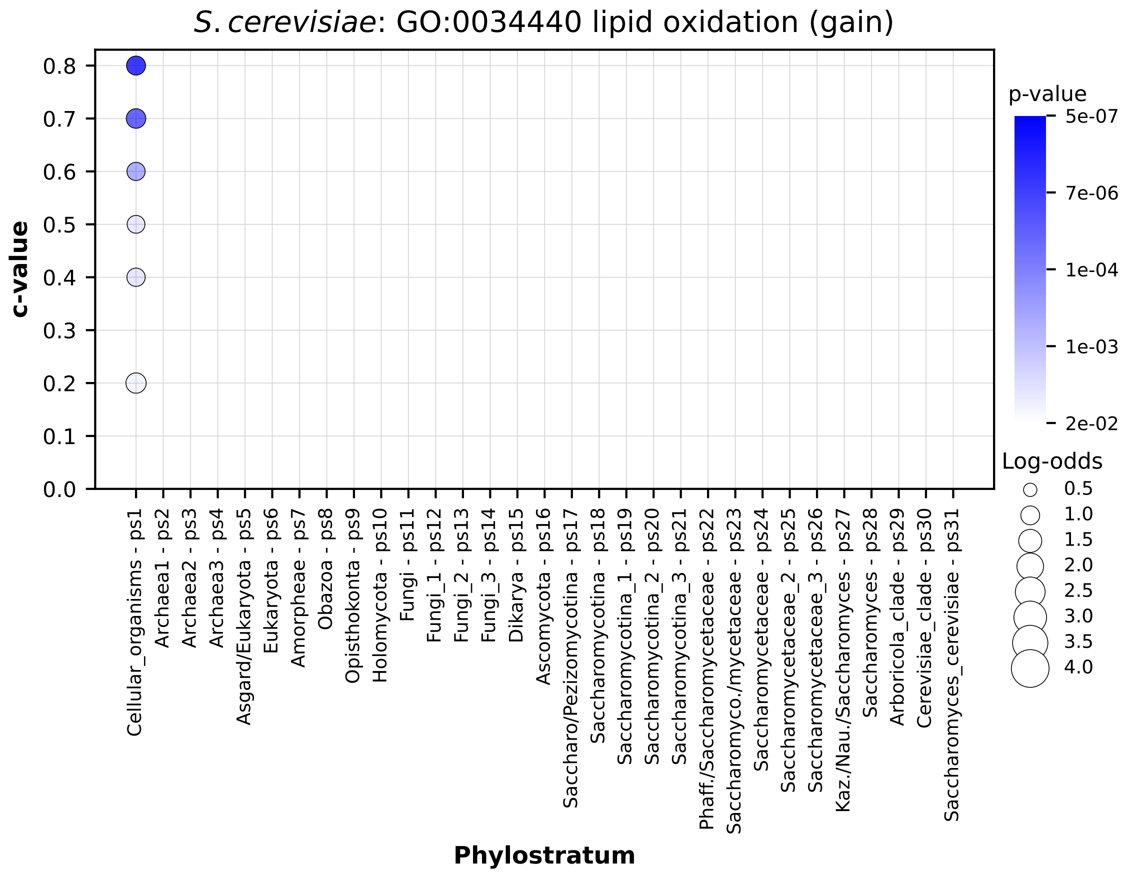

S. cerevisiae: GO:0034440 lipid oxidation (gain)
c-value
0.0
0.1
0.2
0.3
0.4
0.5
0.6
0.7
0.8
Phylostratum
Cellular_organisms - ps1
Archaea1 - ps2
Archaea2 - ps3
Archaea3 - ps4
Asgard/Eukaryota - ps5
Eukaryota - ps6
Amorpheae - ps7
Obazoa - ps8
Opisthokonta - ps9
Holomycota - ps10
Fungi - ps11
Fungi_1 - ps12
Fungi_2 - ps13
Fungi_3 - ps14
Dikarya - ps15
Ascomycota - ps16
Saccharo/Pezizomycotina - ps17
Saccharomycotina - ps18
Saccharomycotina_1 - ps19
Saccharomycotina_2 - ps20
Saccharomycotina_3 - ps21
Phaff./Saccharomycetaceae - ps22
Saccharomyco./mycetaceae - ps23
Saccharomycetaceae - ps24
Saccharomycetaceae_2 - ps25
Saccharomycetaceae_3 - ps26
Kaz./Nau./Saccharomyces - ps27
Saccharomyces - ps28
Arboricola_clade - ps29
Cerevisiae_clade - ps30
Saccharomyces_cerevisiae - ps31
p-value
5e-07
7e-06
1e-04
1e-03
2e-02
Log-odds
0.5
1.0
1.5
2.0
2.5
3.0
3.5
4.0

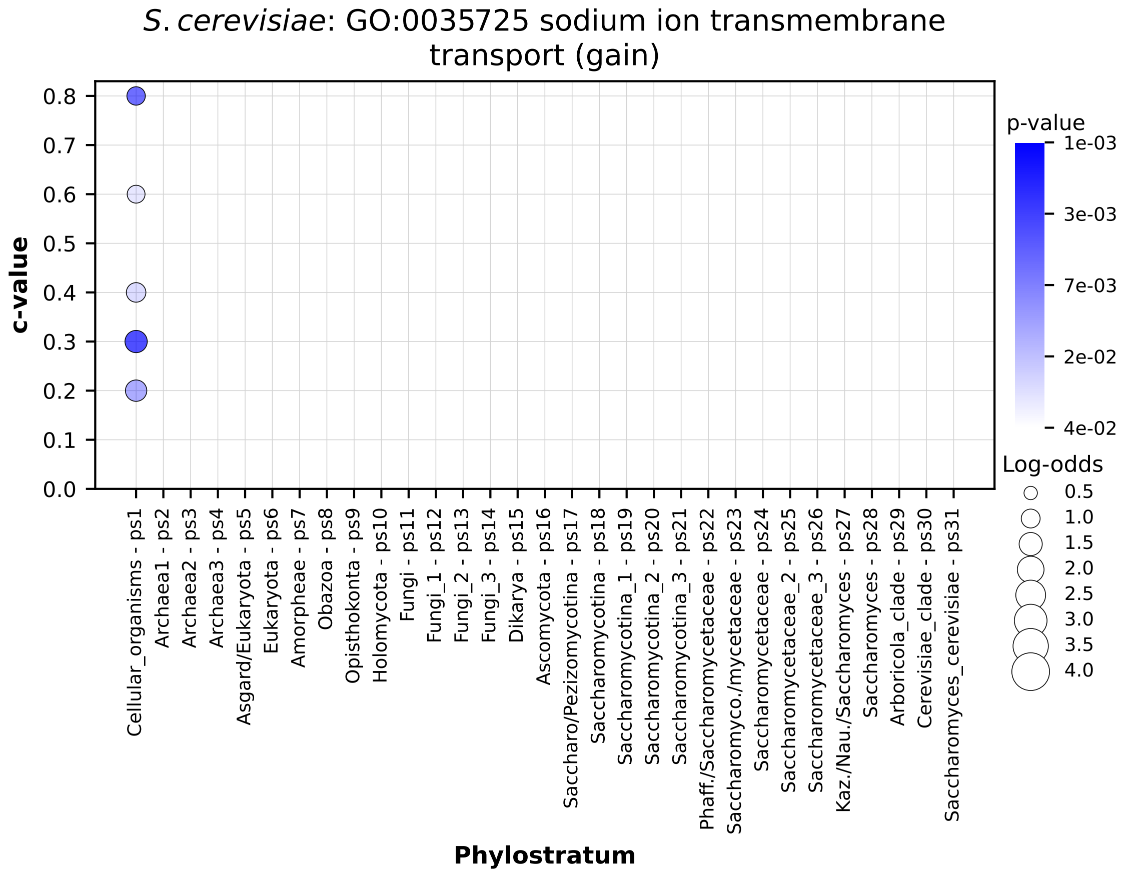

S. cerevisiae: GO:0035725 sodium ion transmembrane transport (gain)
c-value
0.0
0.1
0.2
0.3
0.4
0.5
0.6
0.7
0.8
p-value
1e-03
3e-03
7e-03
2e-02
4e-02
Log-odds
0.5
1.0
1.5
2.0
2.5
3.0
3.5
4.0
Cellular_organisms - ps1
Archaea1 - ps2
Archaea2 - ps3
Archaea3 - ps4
Asgard/Eukaryota - ps5
Eukaryota - ps6
Amorpheae - ps7
Obazoa - ps8
Opisthokonta - ps9
Holomycota - ps10
Fungi - ps11
Fungi_1 - ps12
Fungi_2 - ps13
Fungi_3 - ps14
Dikarya - ps15
Ascomycota - ps16
Saccharo/Pezizomycotina - ps17
Saccharomycotina - ps18
Saccharomycotina_1 - ps19
Saccharomycotina_2 - ps20
Saccharomycotina_3 - ps21
Phaff./Saccharomycetaceae - ps22
Saccharomyco./mycetaceae - ps23
Saccharomycetaceae - ps24
Saccharomycetaceae_2 - ps25
Saccharomycetaceae_3 - ps26
Kaz./Nau./Saccharomyces - ps27
Saccharomyces - ps28
Arboricola_clade - ps29
Cerevisiae_clade - ps30
Saccharomyces_cerevisiae - ps31
Phylostratum

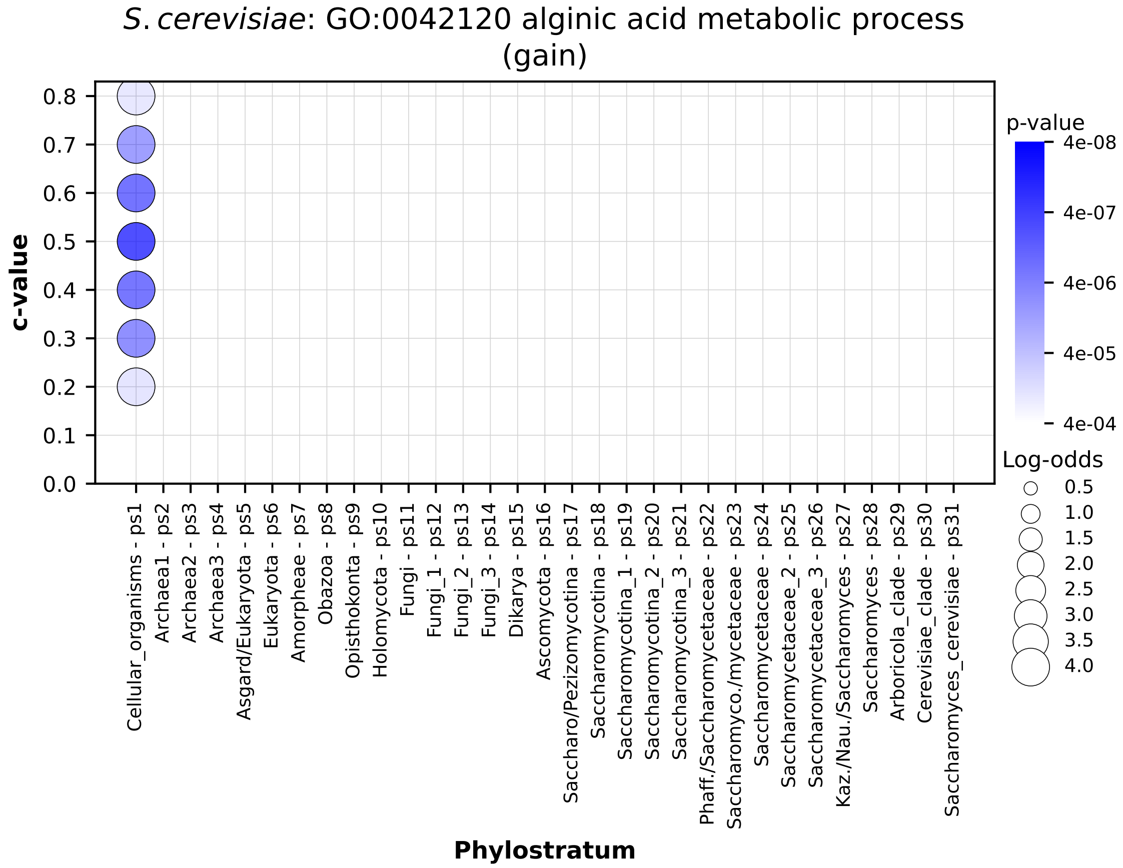

S. cerevisiae: GO:0042120 alginic acid metabolic process
(gain)
c-value
0.0
0.1
0.2
0.3
0.4
0.5
0.6
0.7
0.8
p-value
4e-08
4e-07
4e-06
4e-05
4e-04
Log-odds
0.5
1.0
1.5
2.0
2.5
3.0
3.5
4.0
Cellular_organisms - ps1
Archaea1 - ps2
Archaea2 - ps3
Archaea3 - ps4
Asgard/Eukaryota - ps5
Eukaryota - ps6
Amorpheae - ps7
Obazoa - ps8
Opisthokonta - ps9
Holomycota - ps10
Fungi - ps11
Fungi_1 - ps12
Fungi_2 - ps13
Fungi_3 - ps14
Dikarya - ps15
Ascomycota - ps16
Saccharo/Pezizomycotina - ps17
Saccharomycotina - ps18
Saccharomycotina_1 - ps19
Saccharomycotina_2 - ps20
Saccharomycotina_3 - ps21
Phaff./Saccharomycetaceae - ps22
Saccharomyco./mycetaceae - ps23
Saccharomycetaceae - ps24
Saccharomycetaceae_2 - ps25
Saccharomycetaceae_3 - ps26
Kaz./Nau./Saccharomyces - ps27
Saccharomyces - ps28
Arboricola_clade - ps29
Cerevisiae_clade - ps30
Saccharomyces_cerevisiae - ps31
Phylostratum

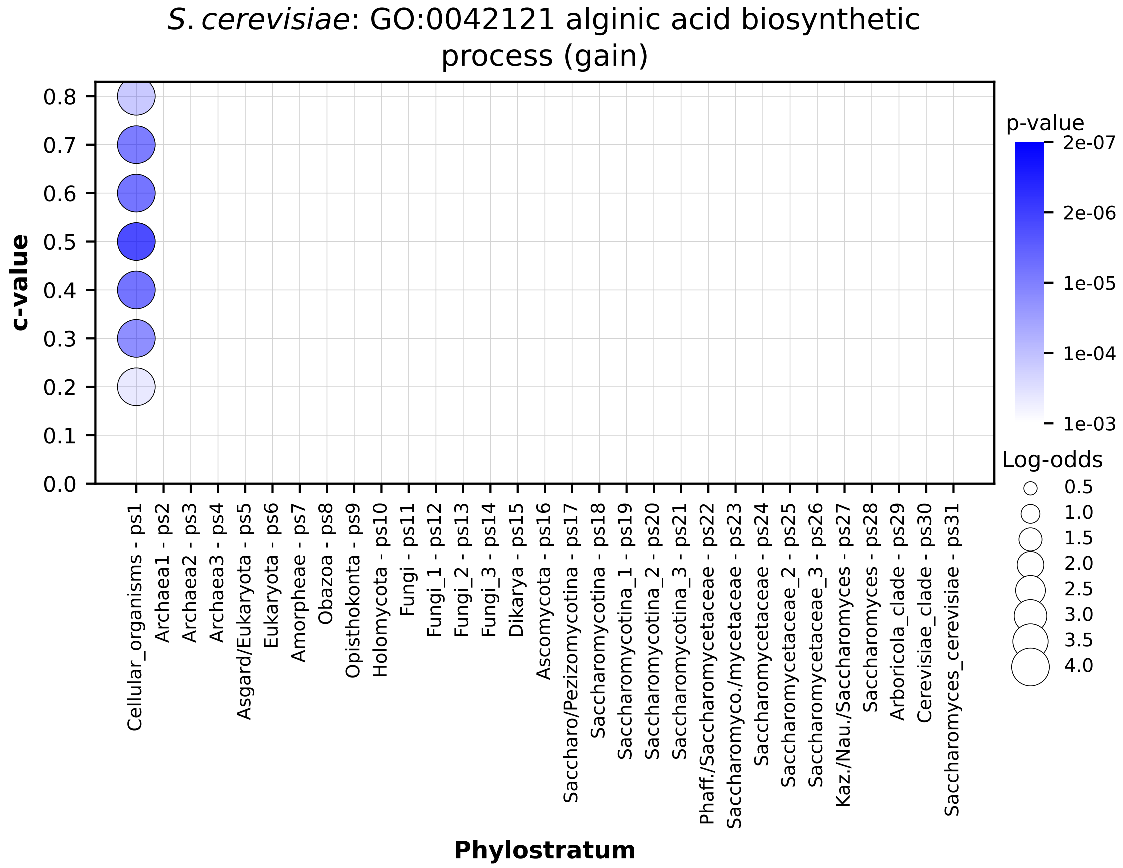

S. cerevisiae: GO:0042121 alginic acid biosynthetic process (gain)
c-value
0.8
0.7
0.6
0.5
0.4
0.3
0.2
0.1
0.0
p-value
2e-07
2e-06
1e-05
1e-04
1e-03
Log-odds
0.5
1.0
1.5
2.0
2.5
3.0
3.5
4.0
Cellular_organisms - ps1
Archaea1 - ps2
Archaea2 - ps3
Archaea3 - ps4
Asgard/Eukaryota - ps5
Eukaryota - ps6
Amorpheae - ps7
Obazoa - ps8
Opisthokonta - ps9
Holomycota - ps10
Fungi - ps11
Fungi_1 - ps12
Fungi_2 - ps13
Fungi_3 - ps14
Dikarya - ps15
Ascomycota - ps16
Saccharo/Pezizomycotina - ps17
Saccharomycotina - ps18
Saccharomycotina_1 - ps19
Saccharomycotina_2 - ps20
Saccharomycotina_3 - ps21
Phaff./Saccharomycetaceae - ps22
Saccharomyco./mycetaceae - ps23
Saccharomycetaceae - ps24
Saccharomycetaceae_2 - ps25
Saccharomycetaceae_3 - ps26
Kaz./Nau./Saccharomyces - ps27
Saccharomyces - ps28
Arboricola_clade - ps29
Cerevisiae_clade - ps30
Saccharomyces_cerevisiae - ps31
Phylostratum

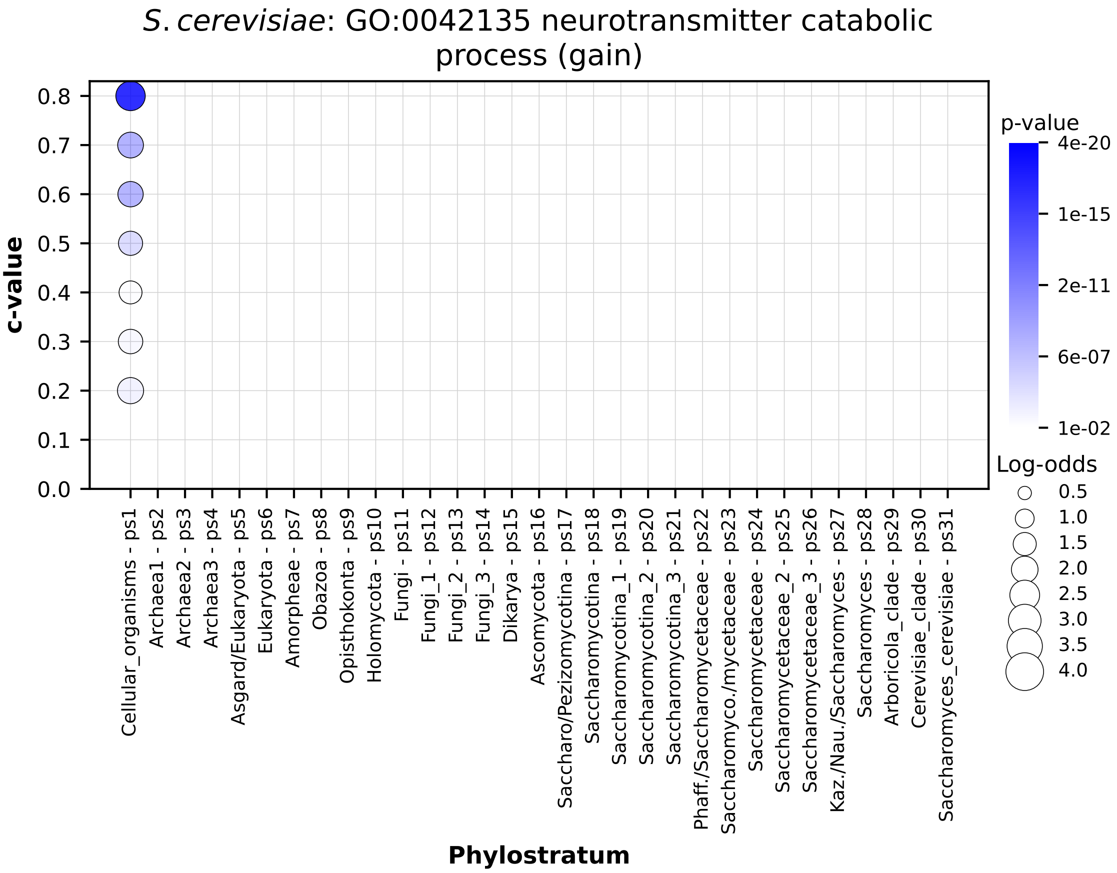

S. cerevisiae: GO:0042135 neurotransmitter catabolic process (gain)
c-value
0.0
0.1
0.2
0.3
0.4
0.5
0.6
0.7
0.8
Phylostratum
Cellular_organisms - ps1
Archaea1 - ps2
Archaea2 - ps3
Archaea3 - ps4
Asgard/Eukaryota - ps5
Eukaryota - ps6
Amorpheae - ps7
Obazoa - ps8
Opisthokonta - ps9
Holomycota - ps10
Fungi - ps11
Fungi_1 - ps12
Fungi_2 - ps13
Fungi_3 - ps14
Dikarya - ps15
Ascomycota - ps16
Saccharo/Pezizomycotina - ps17
Saccharomycotina - ps18
Saccharomycotina_1 - ps19
Saccharomycotina_2 - ps20
Saccharomycotina_3 - ps21
Phaff./Saccharomycetaceae - ps22
Saccharomyco./mycetaceae - ps23
Saccharomycetaceae - ps24
Saccharomycetaceae_2 - ps25
Saccharomycetaceae_3 - ps26
Kaz./Nau./Saccharomyces - ps27
Saccharomyces - ps28
Arboricola_clade - ps29
Cerevisiae_clade - ps30
Saccharomyces_cerevisiae - ps31
p-value
4e-20
1e-15
2e-11
6e-07
1e-02
Log-odds
0.5
1.0
1.5
2.0
2.5
3.0
3.5
4.0

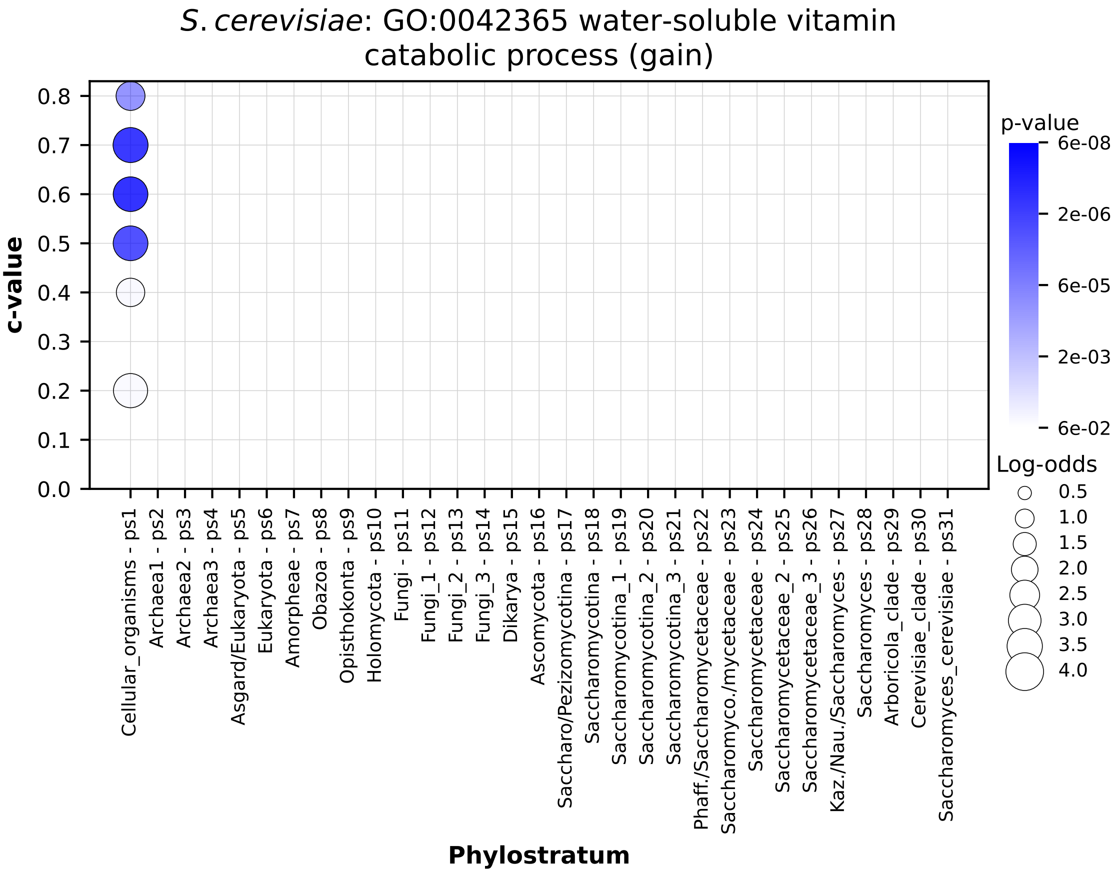

S. cerevisiae: GO:0042365 water-soluble vitamin catabolic process (gain)
c-value
0.0
0.1
0.2
0.3
0.4
0.5
0.6
0.7
0.8
p-value
6e-08
2e-06
6e-05
2e-03
6e-02
Log-odds
0.5
1.0
1.5
2.0
2.5
3.0
3.5
4.0
Cellular_organisms - ps1
Archaea1 - ps2
Archaea2 - ps3
Archaea3 - ps4
Asgard/Eukaryota - ps5
Eukaryota - ps6
Amorpheae - ps7
Obazoa - ps8
Opisthokonta - ps9
Holomycota - ps10
Fungi - ps11
Fungi_1 - ps12
Fungi_2 - ps13
Fungi_3 - ps14
Dikarya - ps15
Ascomycota - ps16
Saccharo/Pezizomycotina - ps17
Saccharomycotina - ps18
Saccharomycotina_1 - ps19
Saccharomycotina_2 - ps20
Saccharomycotina_3 - ps21
Phaff./Saccharomycetaceae - ps22
Saccharomyco./mycetaceae - ps23
Saccharomycetaceae - ps24
Saccharomycetaceae_2 - ps25
Saccharomycetaceae_3 - ps26
Kaz./Nau./Saccharomyces - ps27
Saccharomyces - ps28
Arboricola_clade - ps29
Cerevisiae_clade - ps30
Saccharomyces_cerevisiae - ps31
Phylostratum

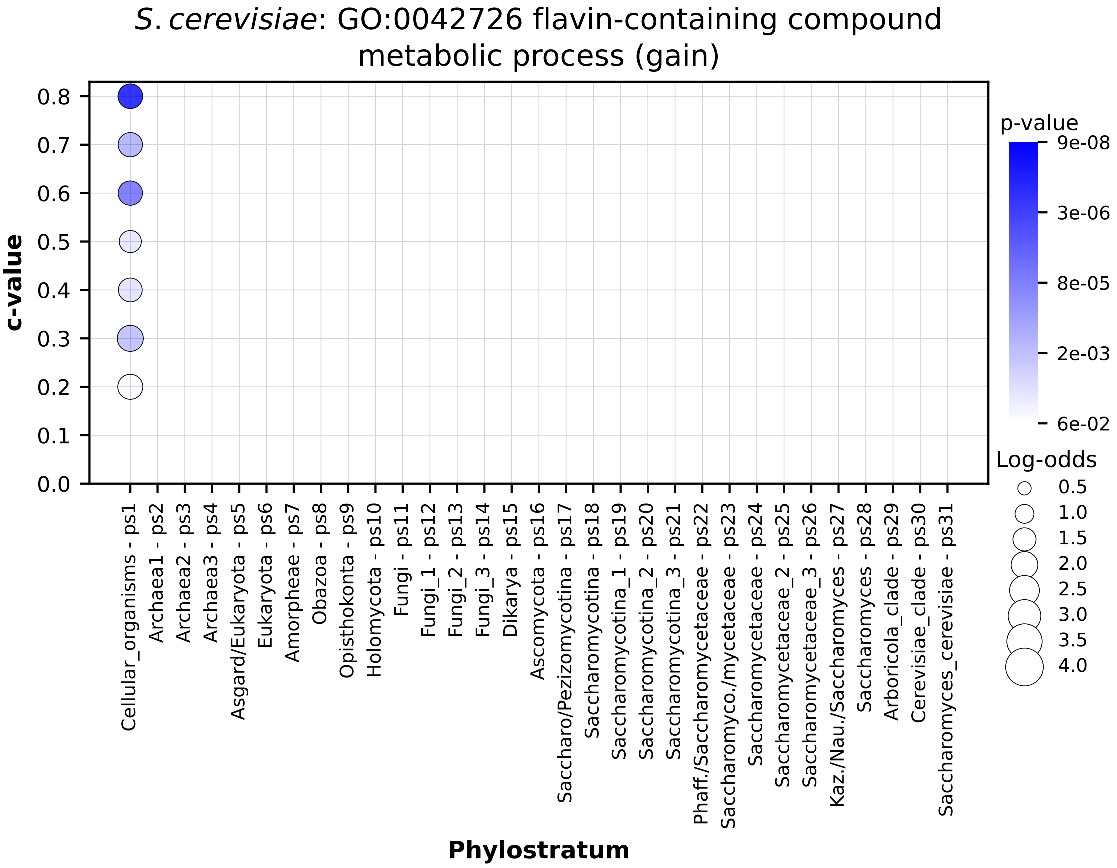

S. cerevisiae: GO:0042726 flavin-containing compound metabolic process (gain)
c-value
0.0
0.1
0.2
0.3
0.4
0.5
0.6
0.7
0.8
p-value
9e-08
3e-06
8e-05
2e-03
6e-02
Log-odds
0.5
1.0
1.5
2.0
2.5
3.0
3.5
4.0
Cellular_organisms - ps1
Archaea1 - ps2
Archaea2 - ps3
Archaea3 - ps4
Asgard/Eukaryota - ps5
Eukaryota - ps6
Amorpheae - ps7
Obazoa - ps8
Opisthokonta - ps9
Holomycota - ps10
Fungi - ps11
Fungi_1 - ps12
Fungi_2 - ps13
Fungi_3 - ps14
Dikarya - ps15
Ascomycota - ps16
Saccharo/Pezizomycotina - ps17
Saccharomycotina - ps18
Saccharomycotina_1 - ps19
Saccharomycotina_2 - ps20
Saccharomycotina_3 - ps21
Phaff./Saccharomycetaceae - ps22
Saccharomyco./mycetaceae - ps23
Saccharomycetaceae - ps24
Saccharomycetaceae_2 - ps25
Saccharomycetaceae_3 - ps26
Kaz./Nau./Saccharomyces - ps27
Saccharomyces - ps28
Arboricola_clade - ps29
Cerevisiae_clade - ps30
Saccharomyces_cerevisiae - ps31
Phylostratum

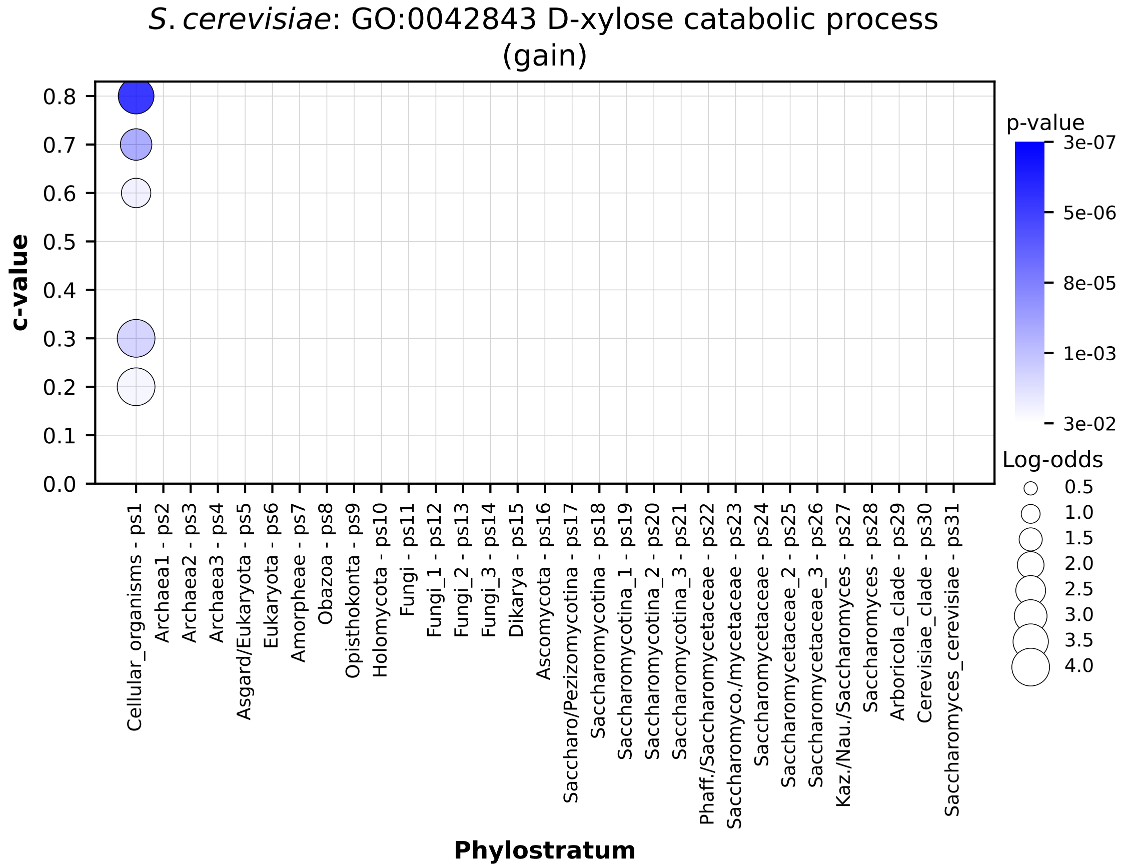

S. cerevisiae: GO:0042843 D-xylose catabolic process (gain)
c-value
0.8
0.7
0.6
0.5
0.4
0.3
0.2
0.1
0.0
p-value
3e-07
5e-06
8e-05
1e-03
3e-02
Log-odds
0.5
1.0
1.5
2.0
2.5
3.0
3.5
4.0
Cellular_organisms - ps1
Archaea1 - ps2
Archaea2 - ps3
Archaea3 - ps4
Asgard/Eukaryota - ps5
Eukaryota - ps6
Amorpheae - ps7
Obazoa - ps8
Opisthokonta - ps9
Holomycota - ps10
Fungi - ps11
Fungi_1 - ps12
Fungi_2 - ps13
Fungi_3 - ps14
Dikarya - ps15
Ascomycota - ps16
Saccharo/Pezizomycotina - ps17
Saccharomycotina - ps18
Saccharomycotina_1 - ps19
Saccharomycotina_2 - ps20
Saccharomycotina_3 - ps21
Phaff./Saccharomycetaceae - ps22
Saccharomyco./mycetaceae - ps23
Saccharomycetaceae - ps24
Saccharomycetaceae_2 - ps25
Saccharomycetaceae_3 - ps26
Kaz./Nau./Saccharomyces - ps27
Saccharomyces - ps28
Arboricola_clade - ps29
Cerevisiae_clade - ps30
Saccharomyces_cerevisiae - ps31
Phylostratum

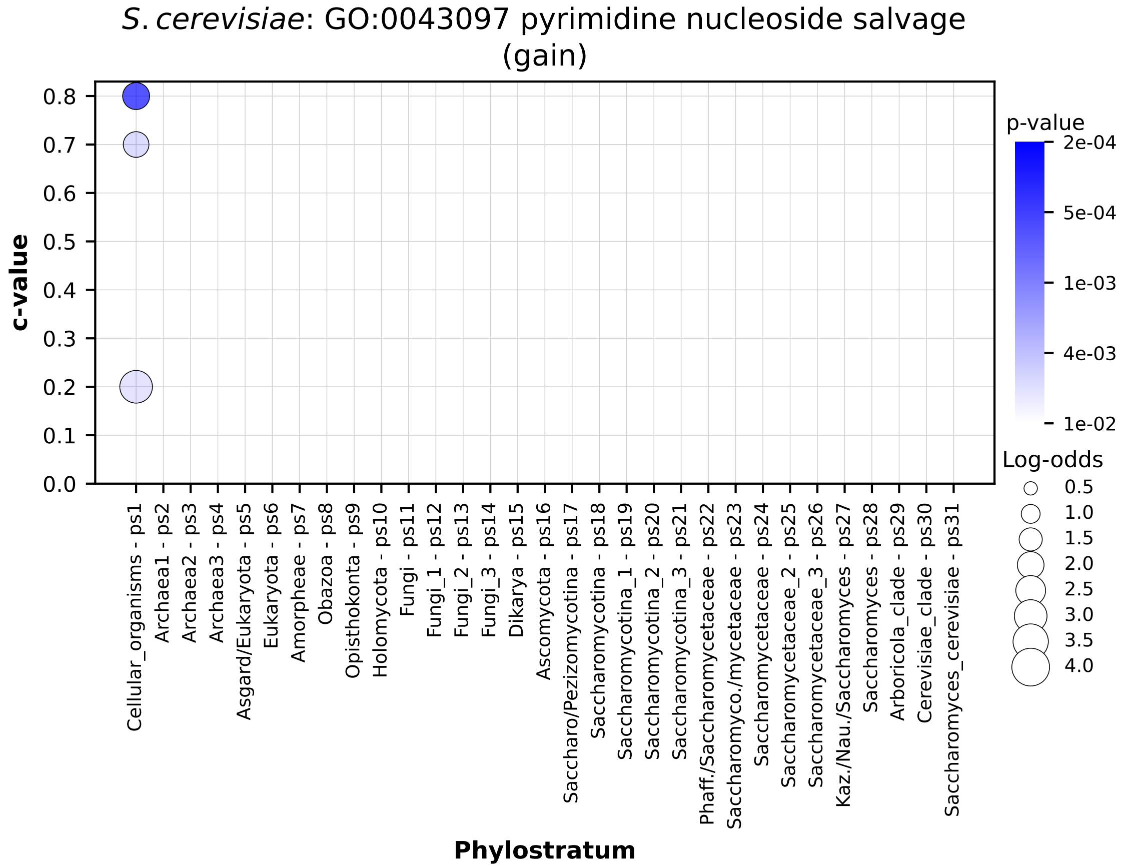

S. cerevisiae: GO:0043097 pyrimidine nucleoside salvage (gain)
c-value
0.0
0.1
0.2
0.3
0.4
0.5
0.6
0.7
0.8
Phylostratum
Cellular_organisms - ps1
Archaea1 - ps2
Archaea2 - ps3
Archaea3 - ps4
Asgard/Eukaryota - ps5
Eukaryota - ps6
Amorpheae - ps7
Obazoa - ps8
Opisthokonta - ps9
Holomycota - ps10
Fungi - ps11
Fungi_1 - ps12
Fungi_2 - ps13
Fungi_3 - ps14
Dikarya - ps15
Ascomycota - ps16
Saccharo/Pezizomycotina - ps17
Saccharomycotina - ps18
Saccharomycotina_1 - ps19
Saccharomycotina_2 - ps20
Saccharomycotina_3 - ps21
Phaff./Saccharomycetaceae - ps22
Saccharomyco./mycetaceae - ps23
Saccharomycetaceae - ps24
Saccharomycetaceae_2 - ps25
Saccharomycetaceae_3 - ps26
Kaz./Nau./Saccharomyces - ps27
Saccharomyces - ps28
Arboricola_clade - ps29
Cerevisiae_clade - ps30
Saccharomyces_cerevisiae - ps31
p-value
2e-04
5e-04
1e-03
4e-03
1e-02
Log-odds
0.5
1.0
1.5
2.0
2.5
3.0
3.5
4.0

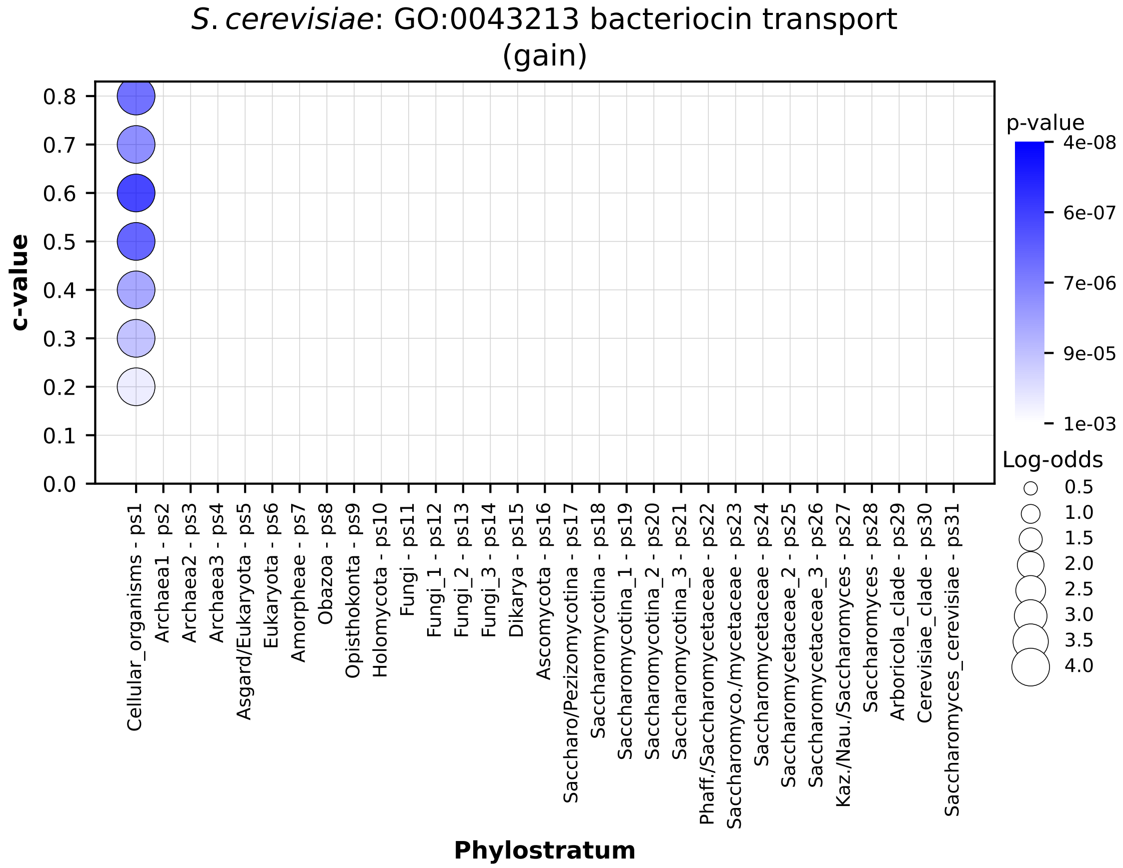
S. cerevisiae: GO:0043213 bacteriocin transport
(gain)
c-value
0.0
0.1
0.2
0.3
0.4
0.5
0.6
0.7
0.8
Phylostratum
Cellular_organisms - ps1
Archaea1 - ps2
Archaea2 - ps3
Archaea3 - ps4
Asgard/Eukaryota - ps5
Eukaryota - ps6
Amorpheae - ps7
Obazoa - ps8
Opisthokonta - ps9
Holomycota - ps10
Fungi - ps11
Fungi_1 - ps12
Fungi_2 - ps13
Fungi_3 - ps14
Dikarya - ps15
Ascomycota - ps16
Saccharo/Pezizomycotina - ps17
Saccharomycotina - ps18
Saccharomycotina_1 - ps19
Saccharomycotina_2 - ps20
Saccharomycotina_3 - ps21
Phaff./Saccharomycetaceae - ps22
Saccharomyco./mycetaceae - ps23
Saccharomycetaceae - ps24
Saccharomycetaceae_2 - ps25
Saccharomycetaceae_3 - ps26
Kaz./Nau./Saccharomyces - ps27
Saccharomyces - ps28
Arboricola_clade - ps29
Cerevisiae_clade - ps30
Saccharomyces_cerevisiae - ps31
p-value
4e-08
6e-07
7e-06
9e-05
1e-03
Log-odds
0.5
1.0
1.5
2.0
2.5
3.0
3.5
4.0

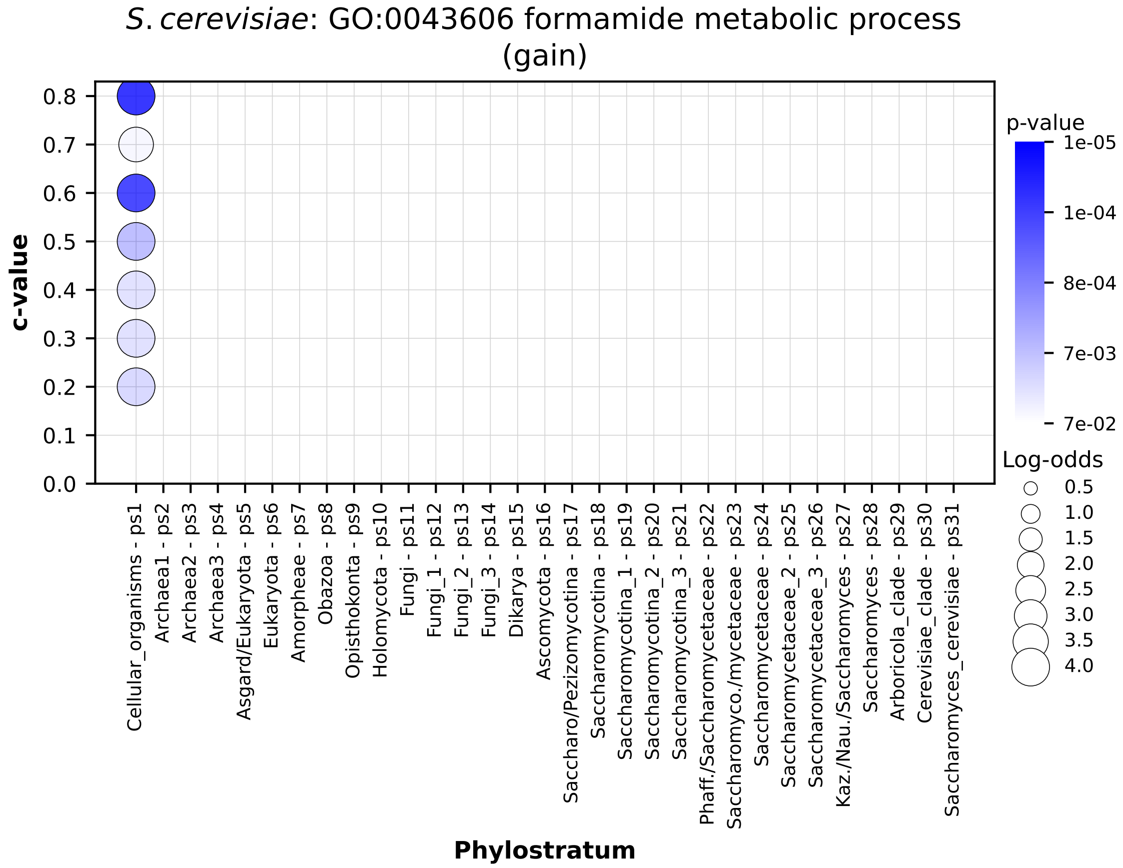

S. cerevisiae: GO:0043606 formamide metabolic process
(gain)
c-value
0.8
0.7
0.6
0.5
0.4
0.3
0.2
0.1
0.0
p-value
1e-05
1e-04
8e-04
7e-03
7e-02
Log-odds
0.5
1.0
1.5
2.0
2.5
3.0
3.5
4.0
Cellular_organisms - ps1
Archaea1 - ps2
Archaea2 - ps3
Archaea3 - ps4
Asgard/Eukaryota - ps5
Eukaryota - ps6
Amorpheae - ps7
Obazoa - ps8
Opisthokonta - ps9
Holomycota - ps10
Fungi - ps11
Fungi_1 - ps12
Fungi_2 - ps13
Fungi_3 - ps14
Dikarya - ps15
Ascomycota - ps16
Saccharo/Pezizomycotina - ps17
Saccharomycotina - ps18
Saccharomycotina_1 - ps19
Saccharomycotina_2 - ps20
Saccharomycotina_3 - ps21
Phaff./Saccharomycetaceae - ps22
Saccharomyco./mycetaceae - ps23
Saccharomycetaceae - ps24
Saccharomycetaceae_2 - ps25
Saccharomycetaceae_3 - ps26
Kaz./Nau./Saccharomyces - ps27
Saccharomyces - ps28
Arboricola_clade - ps29
Cerevisiae_clade - ps30
Saccharomyces_cerevisiae - ps31
Phylostratum

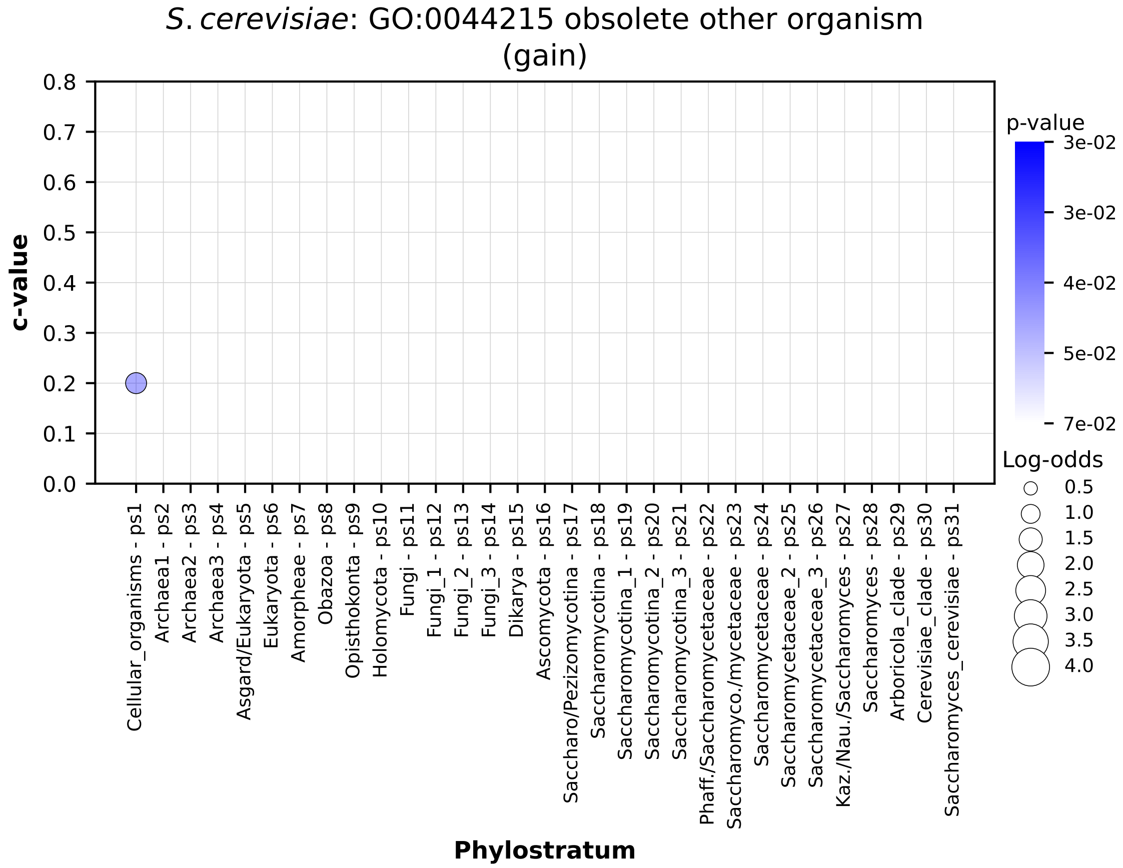

S. cerevisiae: GO:0044215 obsolete other organism (gain)
c-value
0.8
0.7
0.6
0.5
0.4
0.3
0.2
0.1
0.0
p-value
3e-02
3e-02
4e-02
5e-02
7e-02
Log-odds
0.5
1.0
1.5
2.0
2.5
3.0
3.5
4.0
Cellular_organisms - ps1
Archaea1 - ps2
Archaea2 - ps3
Archaea3 - ps4
Asgard/Eukaryota - ps5
Eukaryota - ps6
Amorpheae - ps7
Obazoa - ps8
Opisthokonta - ps9
Holomycota - ps10
Fungi - ps11
Fungi_1 - ps12
Fungi_2 - ps13
Fungi_3 - ps14
Dikarya - ps15
Ascomycota - ps16
Saccharo/Pezizomycotina - ps17
Saccharomycotina - ps18
Saccharomycotina_1 - ps19
Saccharomycotina_2 - ps20
Saccharomycotina_3 - ps21
Phaff./Saccharomycetaceae - ps22
Saccharomyco./mycetaceae - ps23
Saccharomycetaceae - ps24
Saccharomycetaceae_2 - ps25
Saccharomycetaceae_3 - ps26
Kaz./Nau./Saccharomyces - ps27
Saccharomyces - ps28
Arboricola_clade - ps29
Cerevisiae_clade - ps30
Saccharomyces_cerevisiae - ps31
Phylostratum

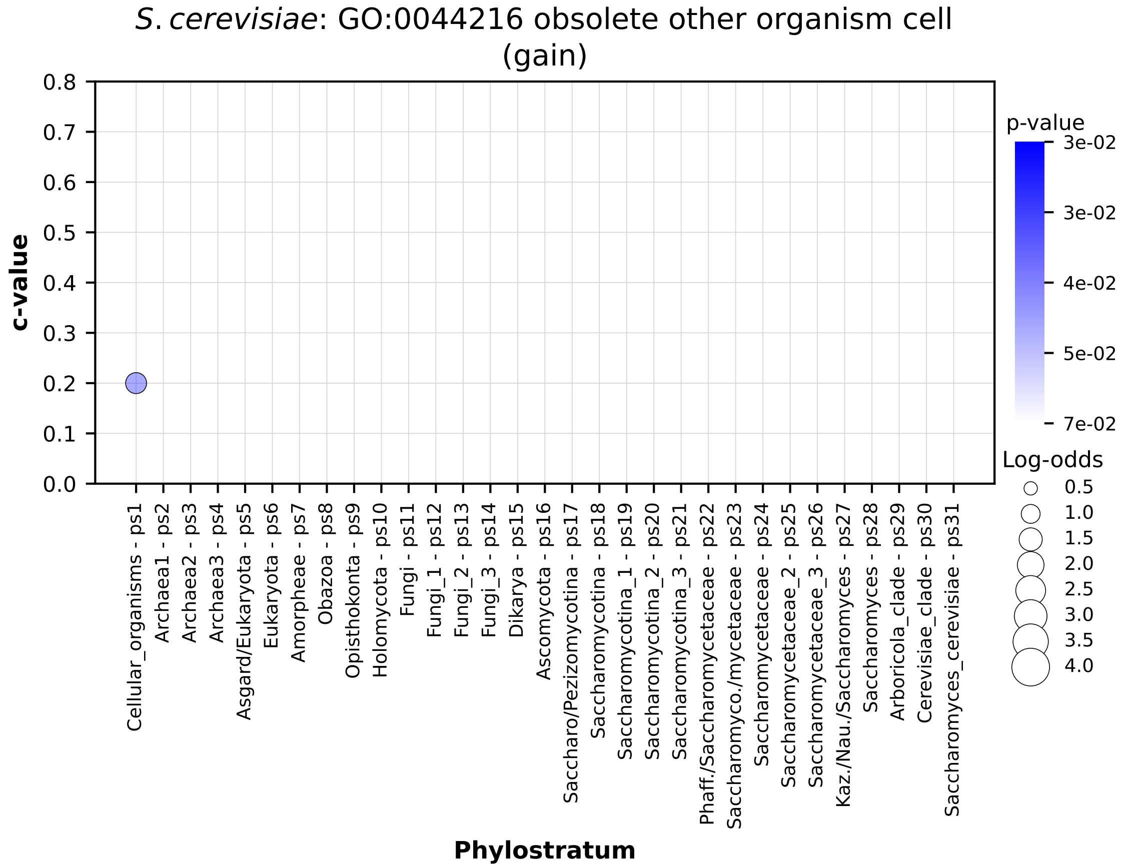

S. cerevisiae: GO:0044216 obsolete other organism cell (gain)
c-value
0.8
0.7
0.6
0.5
0.4
0.3
0.2
0.1
0.0
p-value
3e-02
3e-02
4e-02
5e-02
7e-02
Log-odds
0.5
1.0
1.5
2.0
2.5
3.0
3.5
4.0
Cellular_organisms - ps1
Archaea1 - ps2
Archaea2 - ps3
Archaea3 - ps4
Asgard/Eukaryota - ps5
Eukaryota - ps6
Amorpheae - ps7
Obazoa - ps8
Opisthokonta - ps9
Holomycota - ps10
Fungi - ps11
Fungi_1 - ps12
Fungi_2 - ps13
Fungi_3 - ps14
Dikarya - ps15
Ascomycota - ps16
Saccharo/Pezizomycotina - ps17
Saccharomycotina - ps18
Saccharomycotina_1 - ps19
Saccharomycotina_2 - ps20
Saccharomycotina_3 - ps21
Phaff./Saccharomycetaceae - ps22
Saccharomyco./mycetaceae - ps23
Saccharomycetaceae - ps24
Saccharomycetaceae_2 - ps25
Saccharomycetaceae_3 - ps26
Kaz./Nau./Saccharomyces - ps27
Saccharomyces - ps28
Arboricola_clade - ps29
Cerevisiae_clade - ps30
Saccharomyces_cerevisiae - ps31
Phylostratum

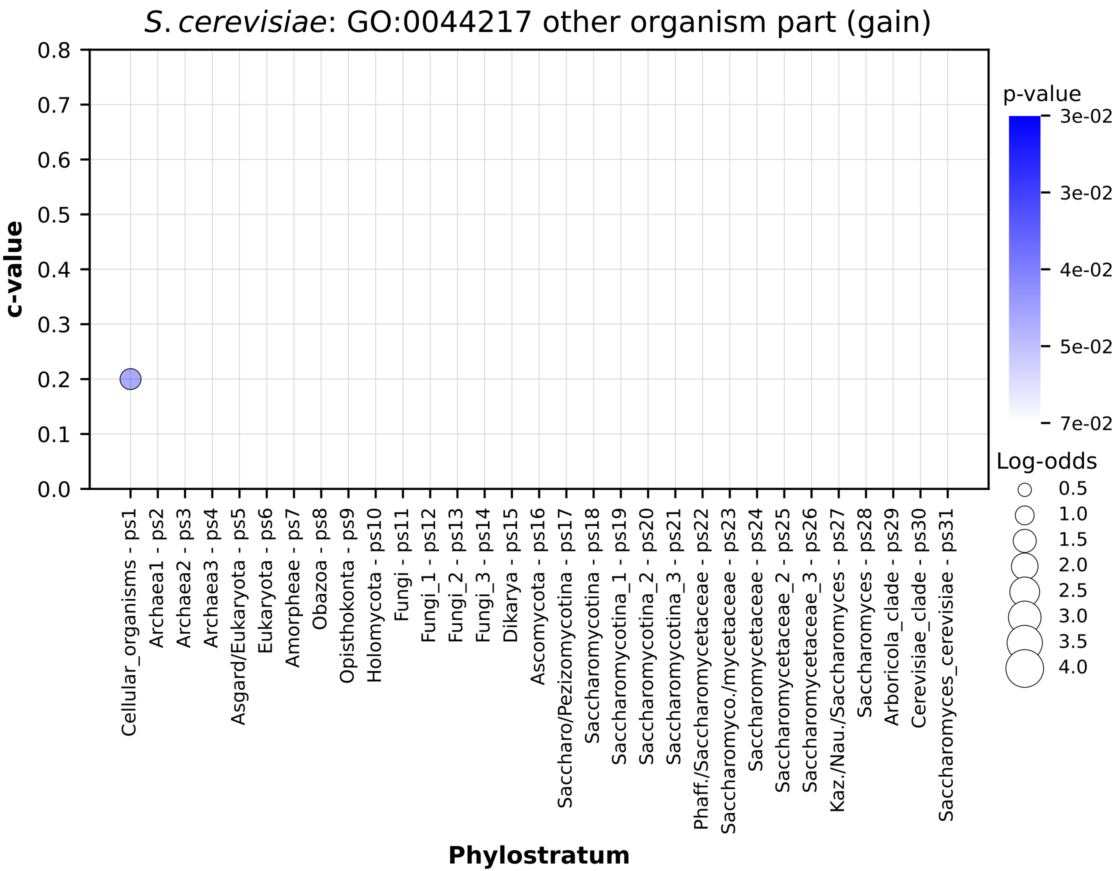

S. cerevisiae: GO:0044217 other organism part (gain)
c-value
0.0
0.1
0.2
0.3
0.4
0.5
0.6
0.7
0.8
Phylostratum
Cellular_organisms - ps1
Archaea1 - ps2
Archaea2 - ps3
Archaea3 - ps4
Asgard/Eukaryota - ps5
Eukaryota - ps6
Amorpheae - ps7
Obazoa - ps8
Opisthokonta - ps9
Holomycota - ps10
Fungi - ps11
Fungi_1 - ps12
Fungi_2 - ps13
Fungi_3 - ps14
Dikarya - ps15
Ascomycota - ps16
Saccharo/Pezizomycotina - ps17
Saccharomycotina - ps18
Saccharomycotina_1 - ps19
Saccharomycotina_2 - ps20
Saccharomycotina_3 - ps21
Phaff./Saccharomycetaceae - ps22
Saccharomyco./mycetaceae - ps23
Saccharomycetaceae - ps24
Saccharomycetaceae_2 - ps25
Saccharomycetaceae_3 - ps26
Kaz./Nau./Saccharomyces - ps27
Saccharomyces - ps28
Arboricola_clade - ps29
Cerevisiae_clade - ps30
Saccharomyces_cerevisiae - ps31
p-value
3e-02
3e-02
4e-02
5e-02
7e-02
Log-odds
0.5
1.0
1.5
2.0
2.5
3.0
3.5
4.0

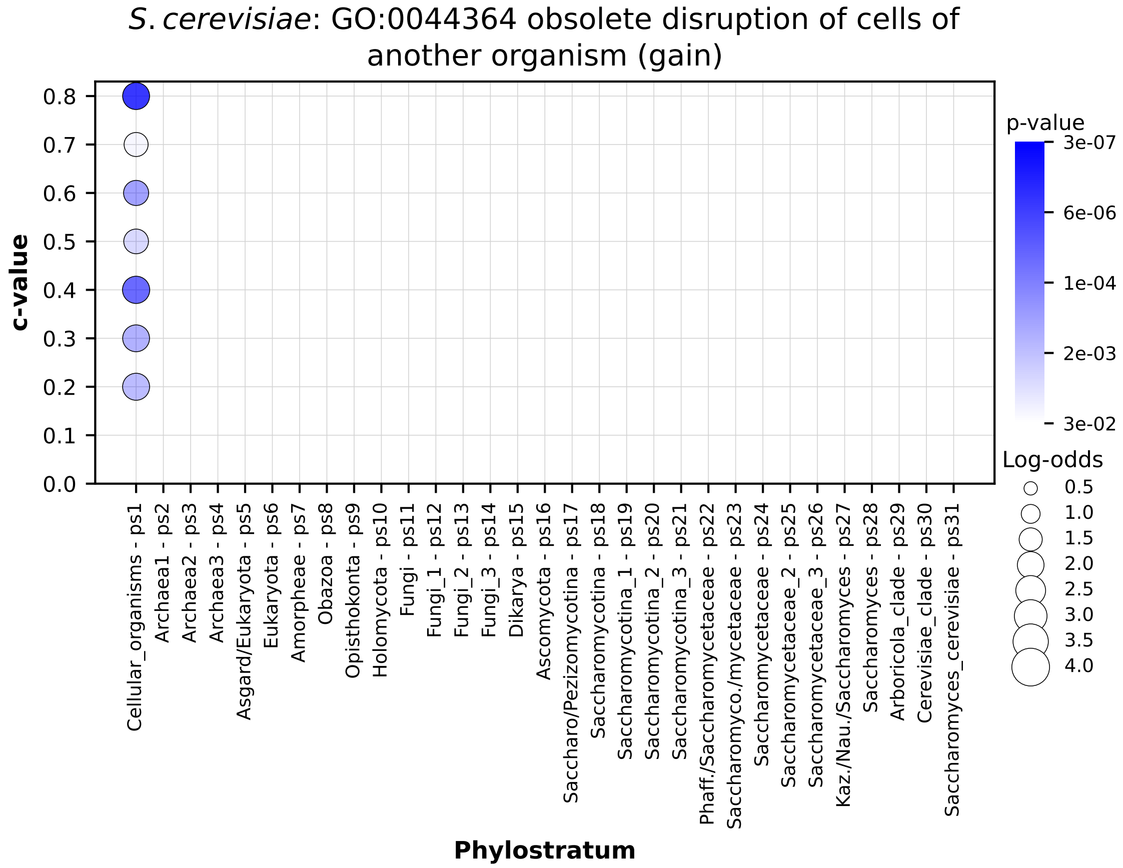

S. cerevisiae: GO:0044364 obsolete disruption of cells of another organism (gain)
c-value
0.0
0.1
0.2
0.3
0.4
0.5
0.6
0.7
0.8
Phylostratum
Cellular_organisms - ps1
Archaea1 - ps2
Archaea2 - ps3
Archaea3 - ps4
Asgard/Eukaryota - ps5
Eukaryota - ps6
Amorpheae - ps7
Obazoa - ps8
Opisthokonta - ps9
Holomycota - ps10
Fungi - ps11
Fungi_1 - ps12
Fungi_2 - ps13
Fungi_3 - ps14
Dikarya - ps15
Ascomycota - ps16
Saccharo/Pezizomycotina - ps17
Saccharomycotina - ps18
Saccharomycotina_1 - ps19
Saccharomycotina_2 - ps20
Saccharomycotina_3 - ps21
Phaff./Saccharomycetaceae - ps22
Saccharomyco./mycetaceae - ps23
Saccharomycetaceae - ps24
Saccharomycetaceae_2 - ps25
Saccharomycetaceae_3 - ps26
Kaz./Nau./Saccharomyces - ps27
Saccharomyces - ps28
Arboricola_clade - ps29
Cerevisiae_clade - ps30
Saccharomyces_cerevisiae - ps31
p-value
3e-07
6e-06
1e-04
2e-03
3e-02
Log-odds
0.5
1.0
1.5
2.0
2.5
3.0
3.5
4.0

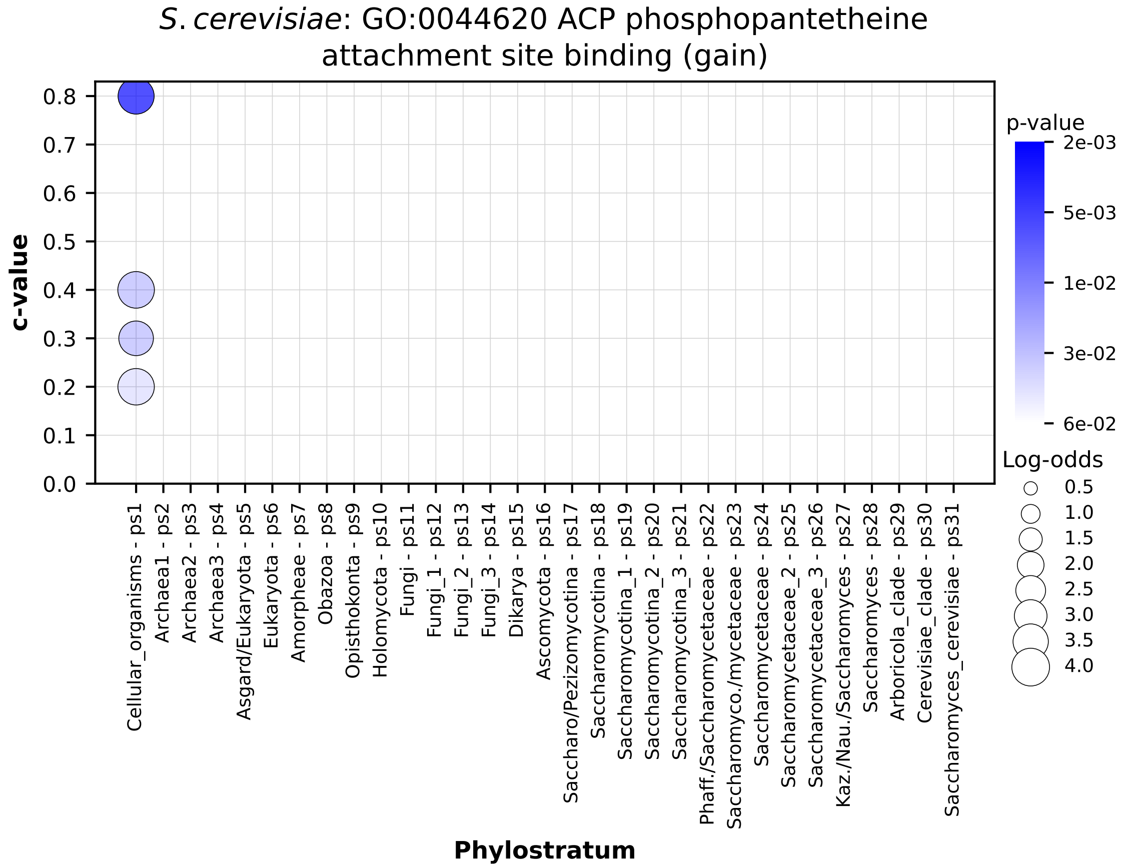

S. cerevisiae: GO:0044620 ACP phosphopantetheine attachment site binding (gain)
c-value
0.8
0.7
0.6
0.5
0.4
0.3
0.2
0.1
0.0
p-value
2e-03
5e-03
1e-02
3e-02
6e-02
Log-odds
0.5
1.0
1.5
2.0
2.5
3.0
3.5
4.0
Cellular_organisms - ps1
Archaea1 - ps2
Archaea2 - ps3
Archaea3 - ps4
Asgard/Eukaryota - ps5
Eukaryota - ps6
Amorpheae - ps7
Obazoa - ps8
Opisthokonta - ps9
Holomycota - ps10
Fungi - ps11
Fungi_1 - ps12
Fungi_2 - ps13
Fungi_3 - ps14
Dikarya - ps15
Ascomycota - ps16
Saccharo/Pezizomycotina - ps17
Saccharomycotina - ps18
Saccharomycotina_1 - ps19
Saccharomycotina_2 - ps20
Saccharomycotina_3 - ps21
Phaff./Saccharomycetaceae - ps22
Saccharomyco./mycetaceae - ps23
Saccharomycetaceae - ps24
Saccharomycetaceae_2 - ps25
Saccharomycetaceae_3 - ps26
Kaz./Nau./Saccharomyces - ps27
Saccharomyces - ps28
Arboricola_clade - ps29
Cerevisiae_clade - ps30
Saccharomyces_cerevisiae - ps31
Phylostratum

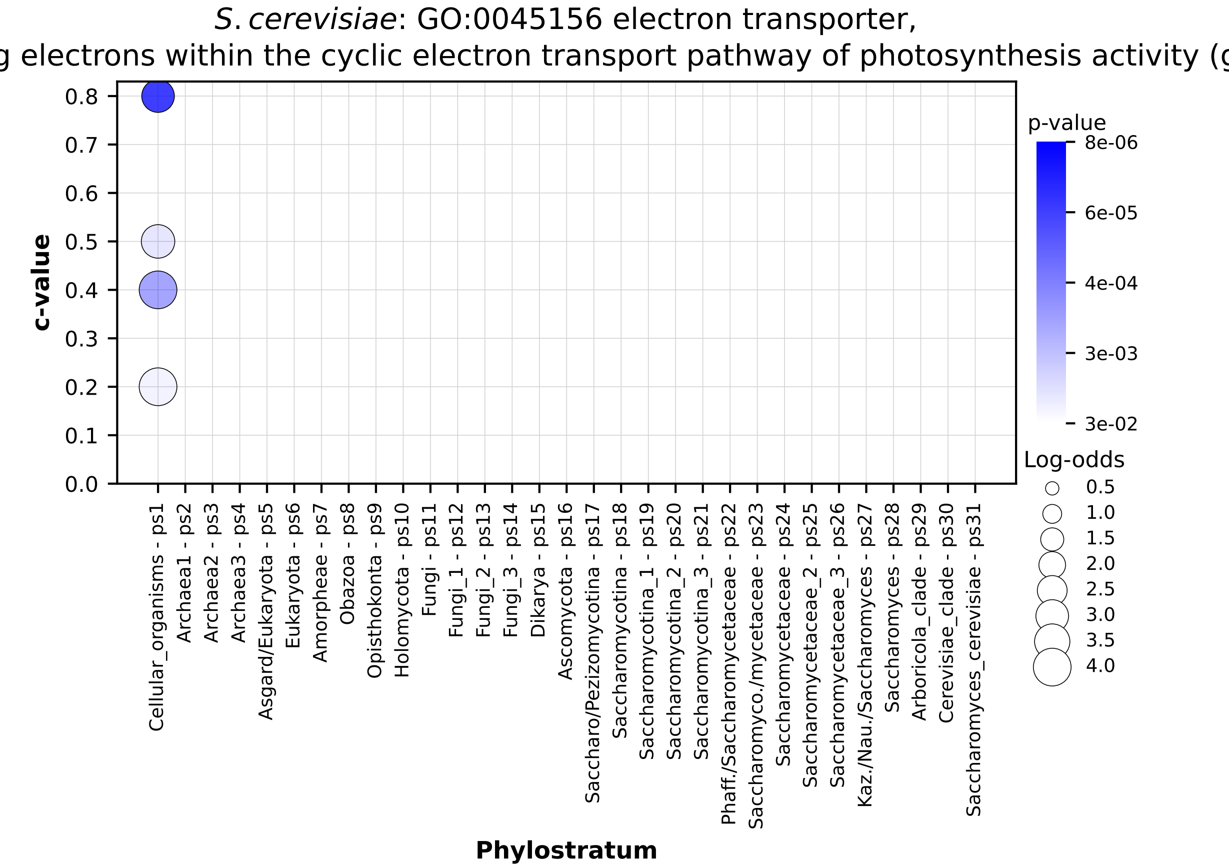

S. cerevisiae: GO:0045156 electron transporter,
g electrons within the cyclic electron transport pathway of photosynthesis activity (g
c-value
0.8
0.7
0.6
0.5
0.4
0.3
0.2
0.1
0.0
p-value
8e-06
6e-05
4e-04
3e-03
3e-02
Log-odds
0.5
1.0
1.5
2.0
2.5
3.0
3.5
4.0
Cellular_organisms - ps1
Archaea1 - ps2
Archaea2 - ps3
Archaea3 - ps4
Asgard/Eukaryota - ps5
Eukaryota - ps6
Amorpheae - ps7
Obazoa - ps8
Opisthokonta - ps9
Holomycota - ps10
Fungi - ps11
Fungi_1 - ps12
Fungi_2 - ps13
Fungi_3 - ps14
Dikarya - ps15
Ascomycota - ps16
Saccharo/Pezizomycotina - ps17
Saccharomycotina - ps18
Saccharomycotina_1 - ps19
Saccharomycotina_2 - ps20
Saccharomycotina_3 - ps21
Phaff./Saccharomycetaceae - ps22
Saccharomyco./mycetaceae - ps23
Saccharomycetaceae - ps24
Saccharomycetaceae_2 - ps25
Saccharomycetaceae_3 - ps26
Kaz./Nau./Saccharomyces - ps27
Saccharomyces - ps28
Arboricola_clade - ps29
Cerevisiae_clade - ps30
Saccharomyces_cerevisiae - ps31
Phylostratum

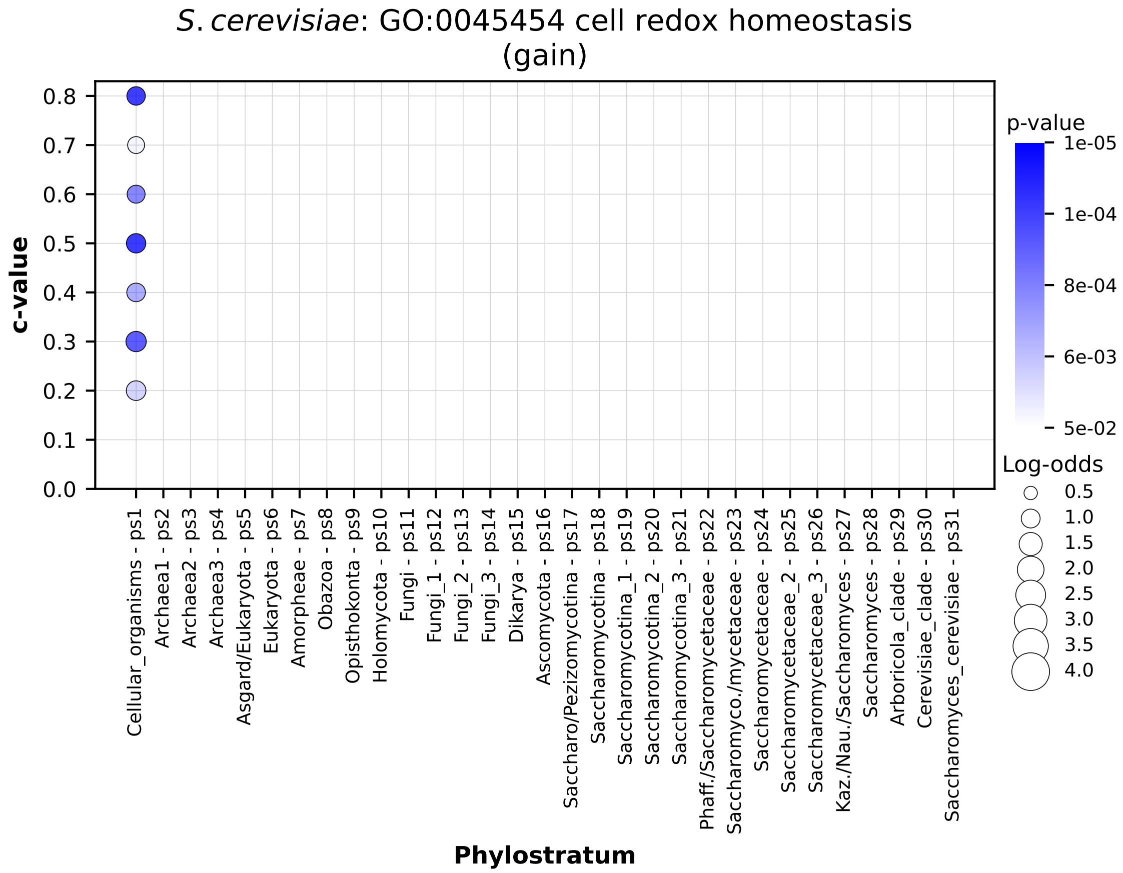
S. cerevisiae: GO:0045454 cell redox homeostasis
(gain)
c-value
0.0
0.1
0.2
0.3
0.4
0.5
0.6
0.7
0.8
Phylostratum
Cellular_organisms - ps1
Archaea1 - ps2
Archaea2 - ps3
Archaea3 - ps4
Asgard/Eukaryota - ps5
Eukaryota - ps6
Amorpheae - ps7
Obazoa - ps8
Opisthokonta - ps9
Holomycota - ps10
Fungi - ps11
Fungi_1 - ps12
Fungi_2 - ps13
Fungi_3 - ps14
Dikarya - ps15
Ascomycota - ps16
Saccharo/Pezizomycotina - ps17
Saccharomycotina - ps18
Saccharomycotina_1 - ps19
Saccharomycotina_2 - ps20
Saccharomycotina_3 - ps21
Phaff./Saccharomycetaceae - ps22
Saccharomyco./mycetaceae - ps23
Saccharomycetaceae - ps24
Saccharomycetaceae_2 - ps25
Saccharomycetaceae_3 - ps26
Kaz./Nau./Saccharomyces - ps27
Saccharomyces - ps28
Arboricola_clade - ps29
Cerevisiae_clade - ps30
Saccharomyces_cerevisiae - ps31
p-value
1e-05
1e-04
8e-04
6e-03
5e-02
Log-odds
0.5
1.0
1.5
2.0
2.5
3.0
3.5
4.0

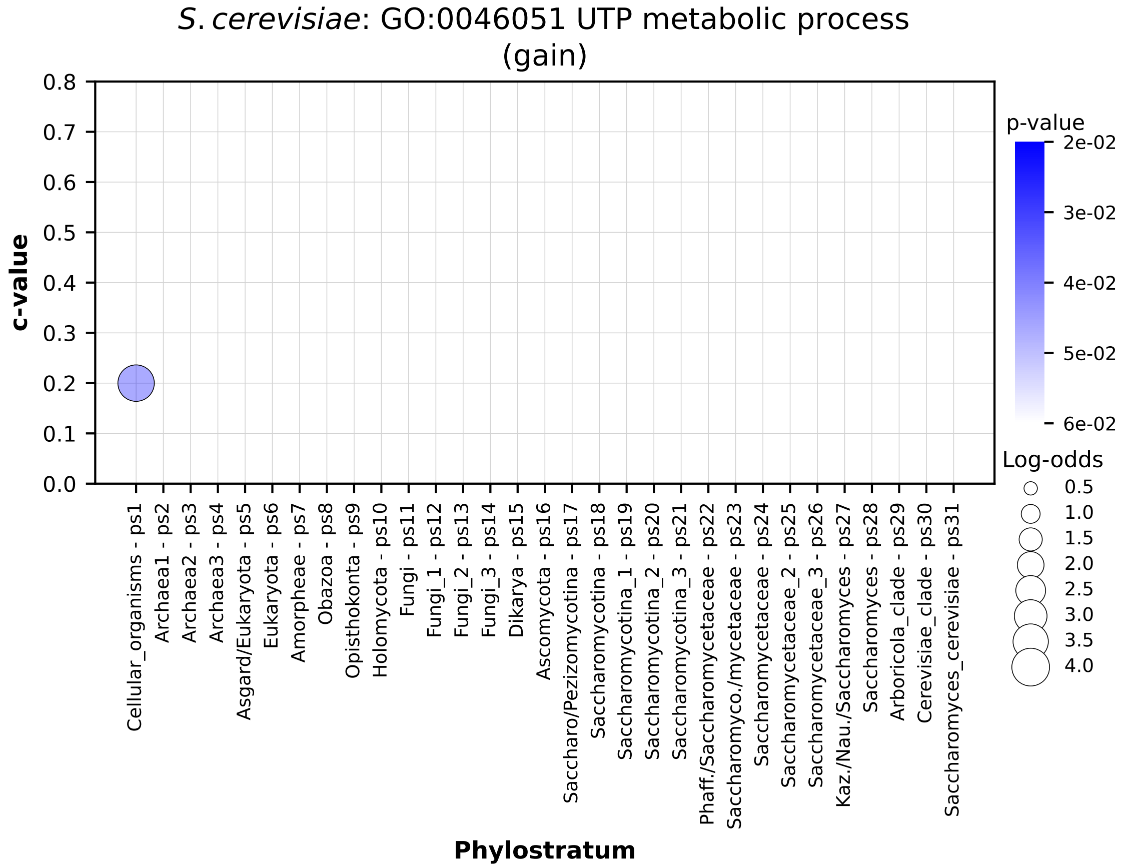
S. cerevisiae: GO:0046051 UTP metabolic process
(gain)
c-value
0.0
0.1
0.2
0.3
0.4
0.5
0.6
0.7
0.8
Phylostratum
Cellular_organisms - ps1
Archaea1 - ps2
Archaea2 - ps3
Archaea3 - ps4
Asgard/Eukaryota - ps5
Eukaryota - ps6
Amorpheae - ps7
Obazoa - ps8
Opisthokonta - ps9
Holomycota - ps10
Fungi - ps11
Fungi_1 - ps12
Fungi_2 - ps13
Fungi_3 - ps14
Dikarya - ps15
Ascomycota - ps16
Saccharo/Pezizomycotina - ps17
Saccharomycotina - ps18
Saccharomycotina_1 - ps19
Saccharomycotina_2 - ps20
Saccharomycotina_3 - ps21
Phaff./Saccharomycetaceae - ps22
Saccharomyco./mycetaceae - ps23
Saccharomycetaceae - ps24
Saccharomycetaceae_2 - ps25
Saccharomycetaceae_3 - ps26
Kaz./Nau./Saccharomyces - ps27
Saccharomyces - ps28
Arboricola_clade - ps29
Cerevisiae_clade - ps30
Saccharomyces_cerevisiae - ps31
p-value
2e-02
3e-02
4e-02
5e-02
6e-02
Log-odds
0.5
1.0
1.5
2.0
2.5
3.0
3.5
4.0

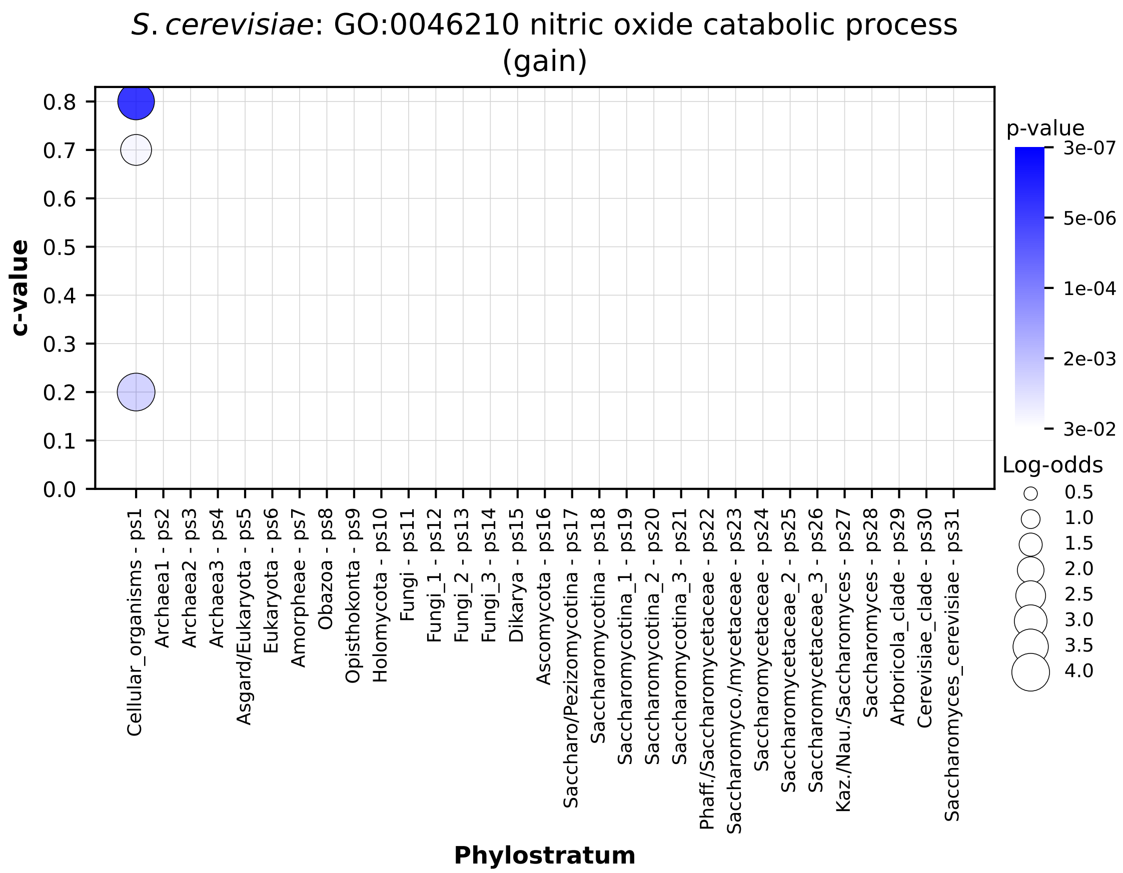

S. cerevisiae: GO:0046210 nitric oxide catabolic process (gain)
c-value
0.0
0.1
0.2
0.3
0.4
0.5
0.6
0.7
0.8
p-value
3e-07
5e-06
1e-04
2e-03
3e-02
Log-odds
0.5
1.0
1.5
2.0
2.5
3.0
3.5
4.0
Cellular_organisms - ps1
Archaea1 - ps2
Archaea2 - ps3
Archaea3 - ps4
Asgard/Eukaryota - ps5
Eukaryota - ps6
Amorpheae - ps7
Obazoa - ps8
Opisthokonta - ps9
Holomycota - ps10
Fungi - ps11
Fungi_1 - ps12
Fungi_2 - ps13
Fungi_3 - ps14
Dikarya - ps15
Ascomycota - ps16
Saccharo/Pezizomycotina - ps17
Saccharomycotina - ps18
Saccharomycotina_1 - ps19
Saccharomycotina_2 - ps20
Saccharomycotina_3 - ps21
Phaff./Saccharomycetaceae - ps22
Saccharomyco./mycetaceae - ps23
Saccharomycetaceae - ps24
Saccharomycetaceae_2 - ps25
Saccharomycetaceae_3 - ps26
Kaz./Nau./Saccharomyces - ps27
Saccharomyces - ps28
Arboricola_clade - ps29
Cerevisiae_clade - ps30
Saccharomyces_cerevisiae - ps31
Phylostratum

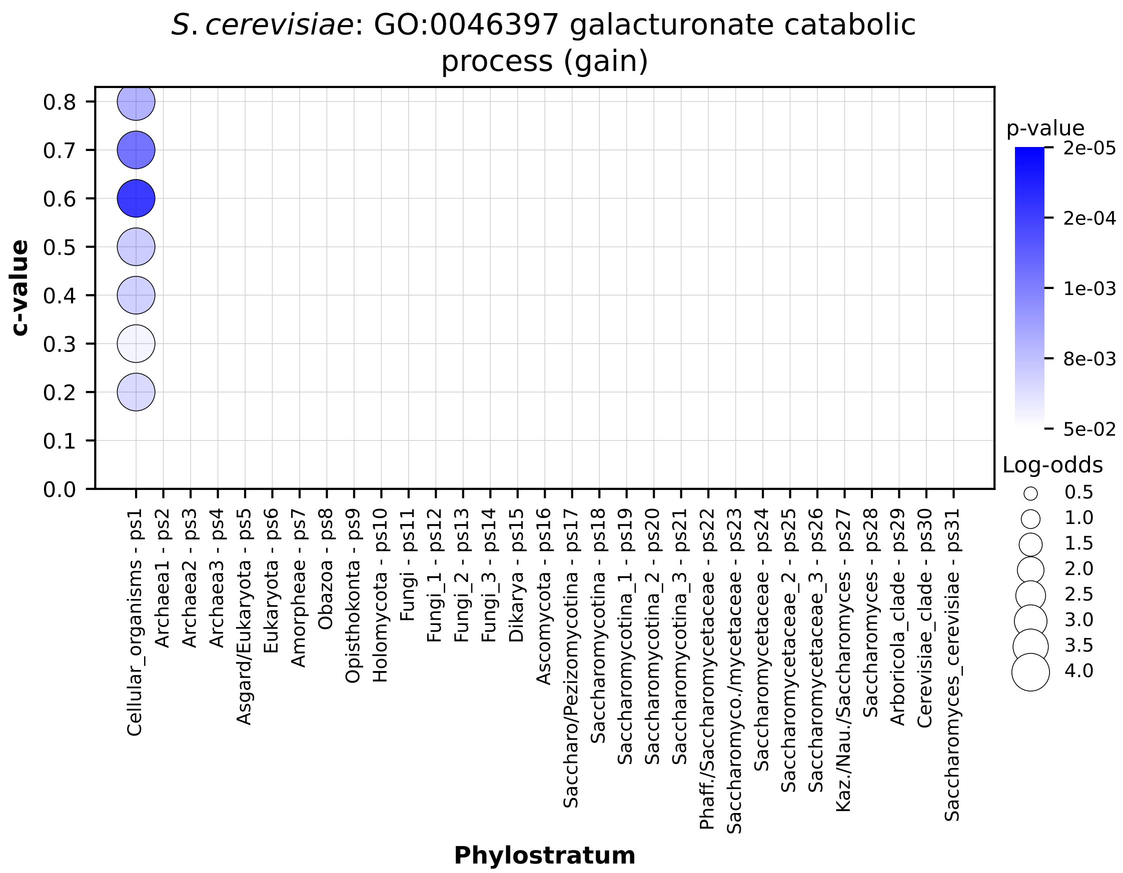
S. cerevisiae: GO:0046397 galacturonate catabolic process (gain)
c-value
0.0
0.1
0.2
0.3
0.4
0.5
0.6
0.7
0.8
p-value
2e-05
2e-04
1e-03
8e-03
5e-02
Log-odds
0.5
1.0
1.5
2.0
2.5
3.0
3.5
4.0
Cellular_organisms - ps1
Archaea1 - ps2
Archaea2 - ps3
Archaea3 - ps4
Asgard/Eukaryota - ps5
Eukaryota - ps6
Amorpheae - ps7
Obazoa - ps8
Opisthokonta - ps9
Holomycota - ps10
Fungi - ps11
Fungi_1 - ps12
Fungi_2 - ps13
Fungi_3 - ps14
Dikarya - ps15
Ascomycota - ps16
Saccharo/Pezizomycotina - ps17
Saccharomycotina - ps18
Saccharomycotina_1 - ps19
Saccharomycotina_2 - ps20
Saccharomycotina_3 - ps21
Phaff./Saccharomycetaceae - ps22
Saccharomyco./mycetaceae - ps23
Saccharomycetaceae - ps24
Saccharomycetaceae_2 - ps25
Saccharomycetaceae_3 - ps26
Kaz./Nau./Saccharomyces - ps27
Saccharomyces - ps28
Arboricola_clade - ps29
Cerevisiae_clade - ps30
Saccharomyces_cerevisiae - ps31
Phylostratum

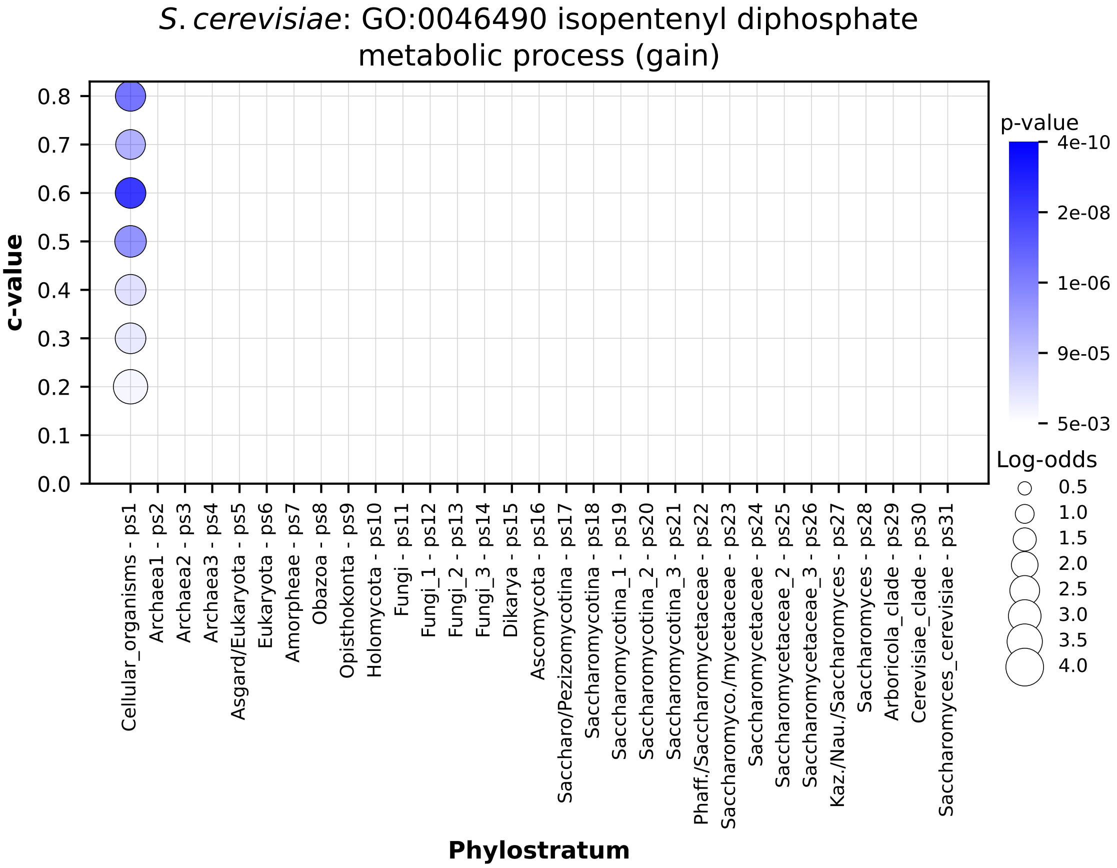

S. cerevisiae: GO:0046490 isopentenyl diphosphate metabolic process (gain)
c-value
0.0
0.1
0.2
0.3
0.4
0.5
0.6
0.7
0.8
Phylostratum
Cellular_organisms - ps1
Archaea1 - ps2
Archaea2 - ps3
Archaea3 - ps4
Asgard/Eukaryota - ps5
Eukaryota - ps6
Amorpheae - ps7
Obazoa - ps8
Opisthokonta - ps9
Holomycota - ps10
Fungi - ps11
Fungi_1 - ps12
Fungi_2 - ps13
Fungi_3 - ps14
Dikarya - ps15
Ascomycota - ps16
Saccharo/Pezizomycotina - ps17
Saccharomycotina - ps18
Saccharomycotina_1 - ps19
Saccharomycotina_2 - ps20
Saccharomycotina_3 - ps21
Phaff./Saccharomycetaceae - ps22
Saccharomyco./mycetaceae - ps23
Saccharomycetaceae - ps24
Saccharomycetaceae_2 - ps25
Saccharomycetaceae_3 - ps26
Kaz./Nau./Saccharomyces - ps27
Saccharomyces - ps28
Arboricola_clade - ps29
Cerevisiae_clade - ps30
Saccharomyces_cerevisiae - ps31
p-value
4e-10
2e-08
1e-06
9e-05
5e-03
Log-odds
0.5
1.0
1.5
2.0
2.5
3.0
3.5
4.0

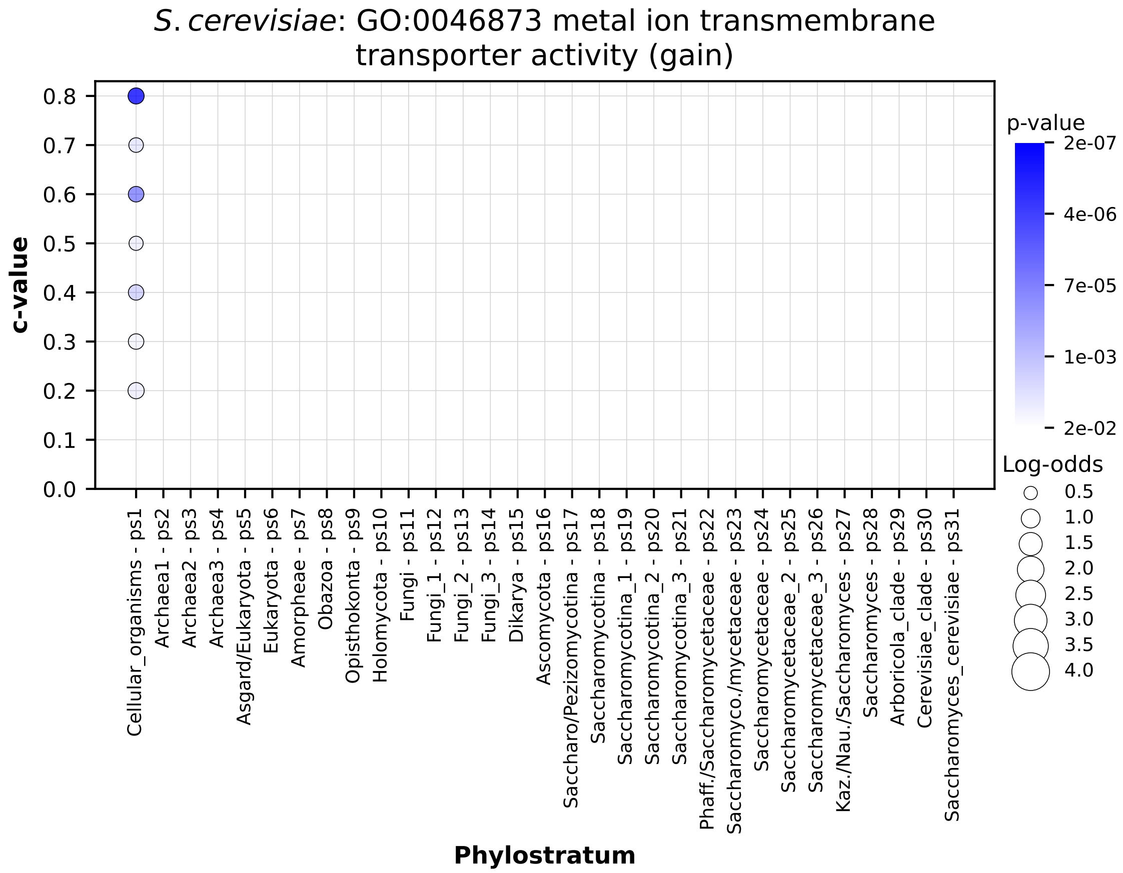

S. cerevisiae: GO:0046873 metal ion transmembrane transporter activity (gain)
c-value
0.0
0.1
0.2
0.3
0.4
0.5
0.6
0.7
0.8
Phylostratum
Cellular_organisms - ps1
Archaea1 - ps2
Archaea2 - ps3
Archaea3 - ps4
Asgard/Eukaryota - ps5
Eukaryota - ps6
Amorpheae - ps7
Obazoa - ps8
Opisthokonta - ps9
Holomycota - ps10
Fungi - ps11
Fungi_1 - ps12
Fungi_2 - ps13
Fungi_3 - ps14
Dikarya - ps15
Ascomycota - ps16
Saccharo/Pezizomycotina - ps17
Saccharomycotina - ps18
Saccharomycotina_1 - ps19
Saccharomycotina_2 - ps20
Saccharomycotina_3 - ps21
Phaff./Saccharomycetaceae - ps22
Saccharomyco./mycetaceae - ps23
Saccharomycetaceae - ps24
Saccharomycetaceae_2 - ps25
Saccharomycetaceae_3 - ps26
Kaz./Nau./Saccharomyces - ps27
Saccharomyces - ps28
Arboricola_clade - ps29
Cerevisiae_clade - ps30
Saccharomyces_cerevisiae - ps31
p-value
2e-07
4e-06
7e-05
1e-03
2e-02
Log-odds
0.5
1.0
1.5
2.0
2.5
3.0
3.5
4.0

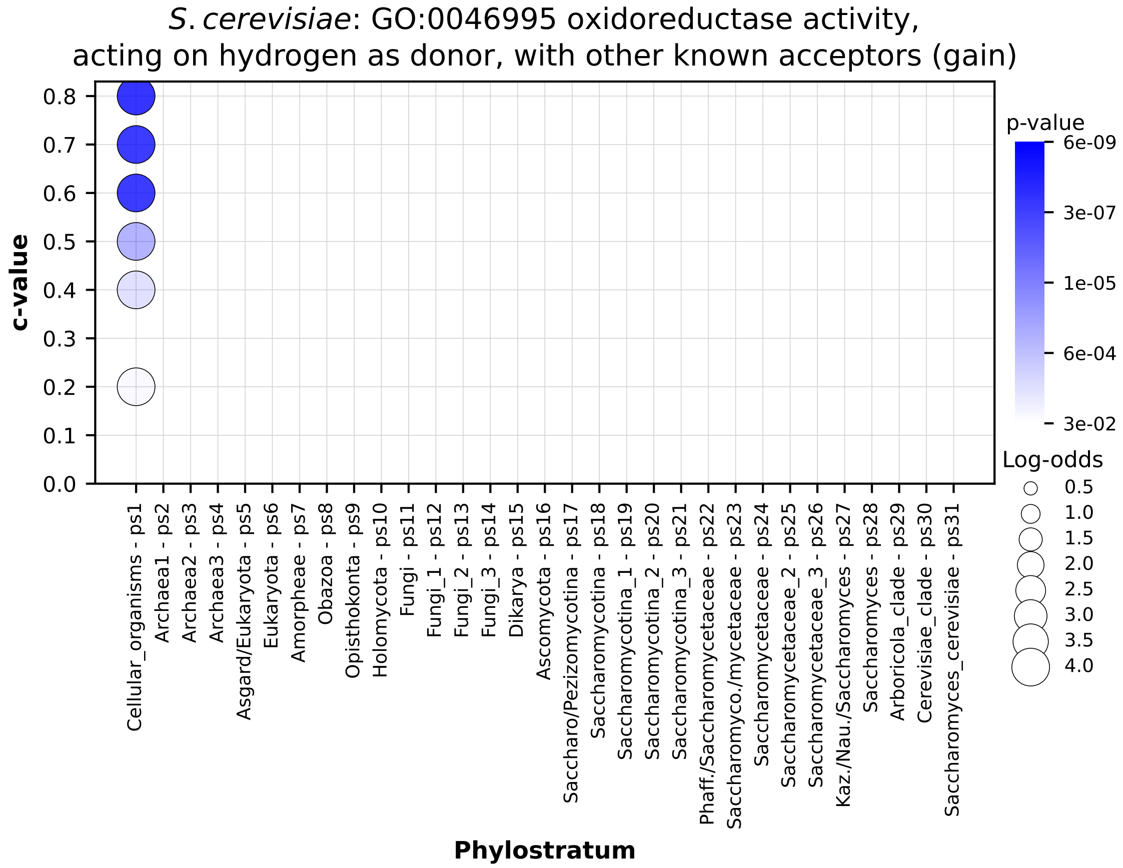

S. cerevisiae: GO:0046995 oxidoreductase activity,
acting on hydrogen as donor, with other known acceptors (gain)
c-value
0.8
0.7
0.6
0.5
0.4
0.3
0.2
0.1
0.0
p-value
6e-09
3e-07
1e-05
6e-04
3e-02
Log-odds
0.5
1.0
1.5
2.0
2.5
3.0
3.5
4.0
Cellular_organisms - ps1
Archaea1 - ps2
Archaea2 - ps3
Archaea3 - ps4
Asgard/Eukaryota - ps5
Eukaryota - ps6
Amorpheae - ps7
Obazoa - ps8
Opisthokonta - ps9
Holomycota - ps10
Fungi - ps11
Fungi_1 - ps12
Fungi_2 - ps13
Fungi_3 - ps14
Dikarya - ps15
Ascomycota - ps16
Saccharo/Pezizomycotina - ps17
Saccharomycotina - ps18
Saccharomycotina_1 - ps19
Saccharomycotina_2 - ps20
Saccharomycotina_3 - ps21
Phaff./Saccharomycetaceae - ps22
Saccharomyco./mycetaceae - ps23
Saccharomycetaceae - ps24
Saccharomycetaceae_2 - ps25
Saccharomycetaceae_3 - ps26
Kaz./Nau./Saccharomyces - ps27
Saccharomyces - ps28
Arboricola_clade - ps29
Cerevisiae_clade - ps30
Saccharomyces_cerevisiae - ps31
Phylostratum

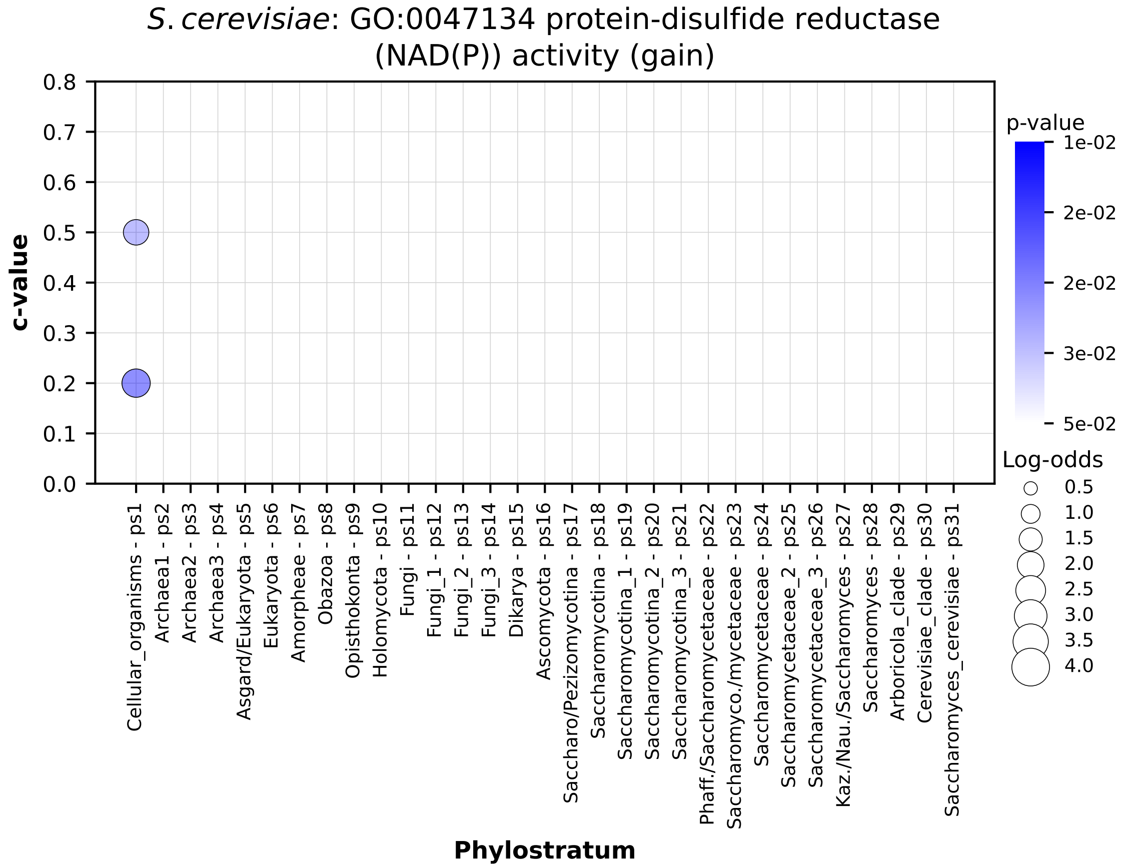

S. cerevisiae: GO:0047134 protein-disulfide reductase (NAD(P)) activity (gain)
c-value
0.0
0.1
0.2
0.3
0.4
0.5
0.6
0.7
0.8
p-value
1e-02
2e-02
2e-02
3e-02
5e-02
Log-odds
0.5
1.0
1.5
2.0
2.5
3.0
3.5
4.0
Cellular_organisms - ps1
Archaea1 - ps2
Archaea2 - ps3
Archaea3 - ps4
Asgard/Eukaryota - ps5
Eukaryota - ps6
Amorpheae - ps7
Obazoa - ps8
Opisthokonta - ps9
Holomycota - ps10
Fungi - ps11
Fungi_1 - ps12
Fungi_2 - ps13
Fungi_3 - ps14
Dikarya - ps15
Ascomycota - ps16
Saccharo/Pezizomycotina - ps17
Saccharomycotina - ps18
Saccharomycotina_1 - ps19
Saccharomycotina_2 - ps20
Saccharomycotina_3 - ps21
Phaff./Saccharomycetaceae - ps22
Saccharomyco./mycetaceae - ps23
Saccharomycetaceae - ps24
Saccharomycetaceae_2 - ps25
Saccharomycetaceae_3 - ps26
Kaz./Nau./Saccharomyces - ps27
Saccharomyces - ps28
Arboricola_clade - ps29
Cerevisiae_clade - ps30
Saccharomyces_cerevisiae - ps31
Phylostratum

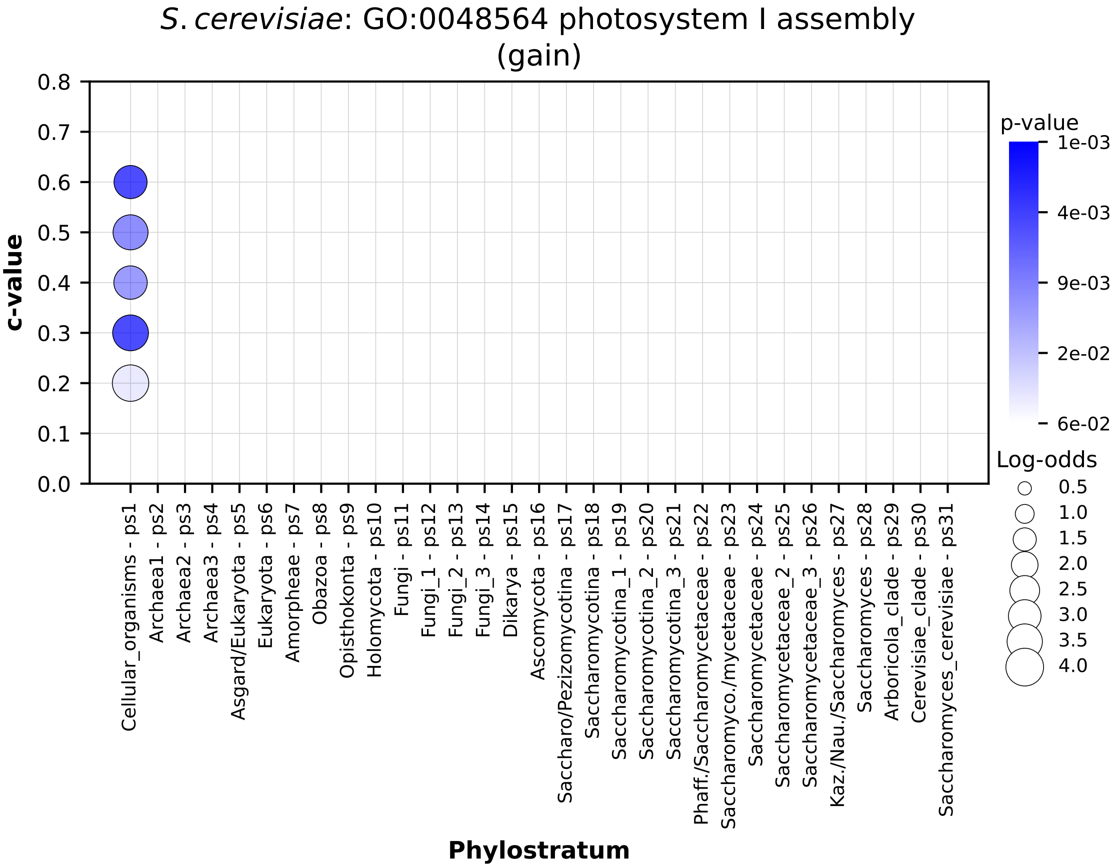

S. cerevisiae: GO:0048564 photosystem I assembly
(gain)
c-value
0.0
0.1
0.2
0.3
0.4
0.5
0.6
0.7
0.8
Phylostratum
Cellular_organisms - ps1
Archaea1 - ps2
Archaea2 - ps3
Archaea3 - ps4
Asgard/Eukaryota - ps5
Eukaryota - ps6
Amorpheae - ps7
Obazoa - ps8
Opisthokonta - ps9
Holomycota - ps10
Fungi - ps11
Fungi_1 - ps12
Fungi_2 - ps13
Fungi_3 - ps14
Dikarya - ps15
Ascomycota - ps16
Saccharo/Pezizomycotina - ps17
Saccharomycotina - ps18
Saccharomycotina_1 - ps19
Saccharomycotina_2 - ps20
Saccharomycotina_3 - ps21
Phaff./Saccharomycetaceae - ps22
Saccharomyco./mycetaceae - ps23
Saccharomycetaceae - ps24
Saccharomycetaceae_2 - ps25
Saccharomycetaceae_3 - ps26
Kaz./Nau./Saccharomyces - ps27
Saccharomyces - ps28
Arboricola_clade - ps29
Cerevisiae_clade - ps30
Saccharomyces_cerevisiae - ps31
p-value
1e-03
4e-03
9e-03
2e-02
6e-02
Log-odds
0.5
1.0
1.5
2.0
2.5
3.0
3.5
4.0

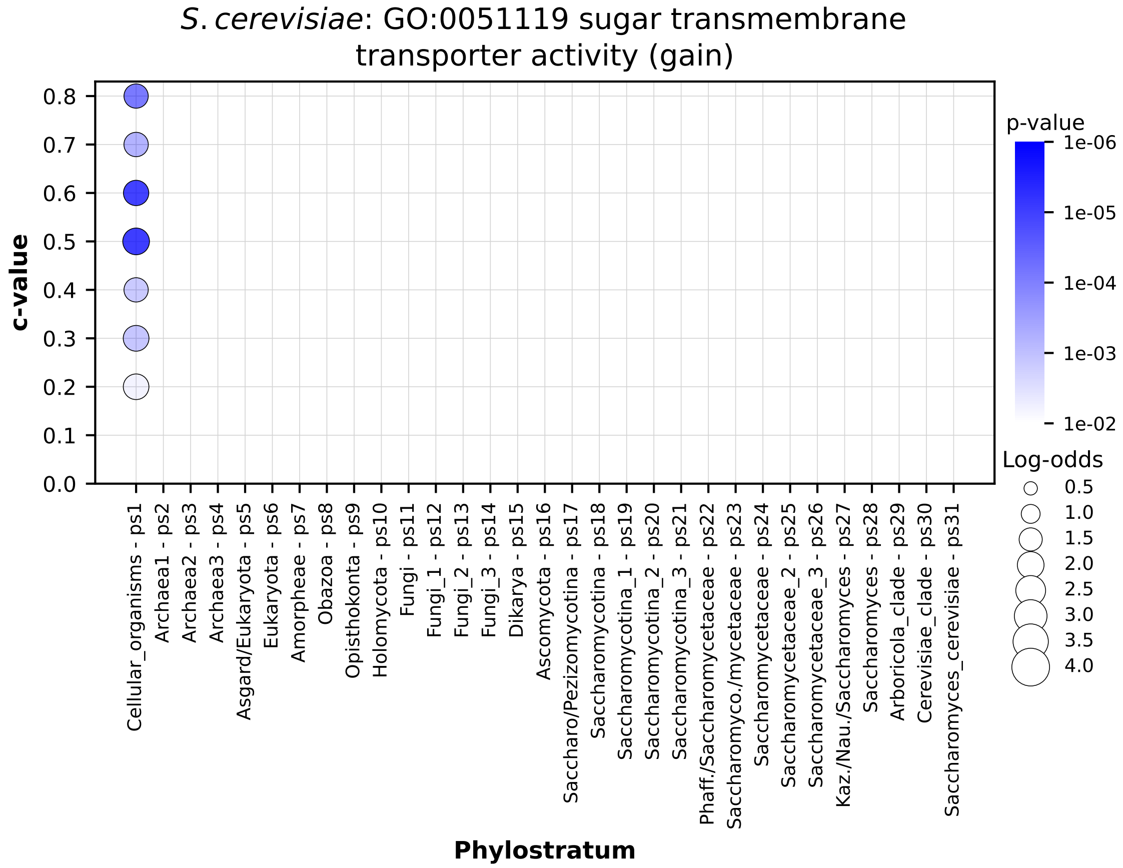

S. cerevisiae: GO:0051119 sugar transmembrane transporter activity (gain)
c-value
0.0
0.1
0.2
0.3
0.4
0.5
0.6
0.7
0.8
p-value
1e-06
1e-05
1e-04
1e-03
1e-02
Log-odds
0.5
1.0
1.5
2.0
2.5
3.0
3.5
4.0
Cellular_organisms - ps1
Archaea1 - ps2
Archaea2 - ps3
Archaea3 - ps4
Asgard/Eukaryota - ps5
Eukaryota - ps6
Amorpheae - ps7
Obazoa - ps8
Opisthokonta - ps9
Holomycota - ps10
Fungi - ps11
Fungi_1 - ps12
Fungi_2 - ps13
Fungi_3 - ps14
Dikarya - ps15
Ascomycota - ps16
Saccharo/Pezizomycotina - ps17
Saccharomycotina - ps18
Saccharomycotina_1 - ps19
Saccharomycotina_2 - ps20
Saccharomycotina_3 - ps21
Phaff./Saccharomycetaceae - ps22
Saccharomyco./mycetaceae - ps23
Saccharomycetaceae - ps24
Saccharomycetaceae_2 - ps25
Saccharomycetaceae_3 - ps26
Kaz./Nau./Saccharomyces - ps27
Saccharomyces - ps28
Arboricola_clade - ps29
Cerevisiae_clade - ps30
Saccharomyces_cerevisiae - ps31
Phylostratum

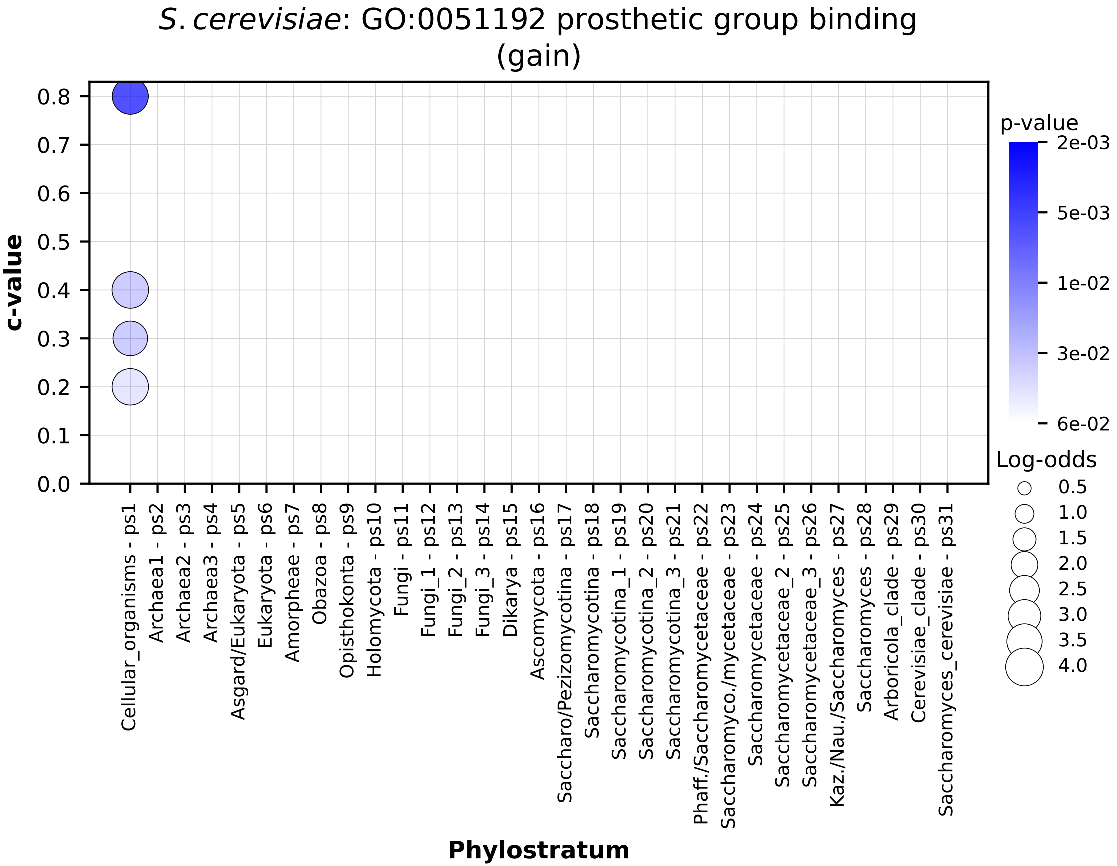

S. cerevisiae: GO:0051192 prosthetic group binding
(gain)
c-value
0.0
0.1
0.2
0.3
0.4
0.5
0.6
0.7
0.8
p-value
2e-03
5e-03
1e-02
3e-02
6e-02
Log-odds
0.5
1.0
1.5
2.0
2.5
3.0
3.5
4.0
Cellular_organisms - ps1
Archaea1 - ps2
Archaea2 - ps3
Archaea3 - ps4
Asgard/Eukaryota - ps5
Eukaryota - ps6
Amorpheae - ps7
Obazoa - ps8
Opisthokonta - ps9
Holomycota - ps10
Fungi - ps11
Fungi_1 - ps12
Fungi_2 - ps13
Fungi_3 - ps14
Dikarya - ps15
Ascomycota - ps16
Saccharo/Pezizomycotina - ps17
Saccharomycotina - ps18
Saccharomycotina_1 - ps19
Saccharomycotina_2 - ps20
Saccharomycotina_3 - ps21
Phaff./Saccharomycetaceae - ps22
Saccharomyco./mycetaceae - ps23
Saccharomycetaceae - ps24
Saccharomycetaceae_2 - ps25
Saccharomycetaceae_3 - ps26
Kaz./Nau./Saccharomyces - ps27
Saccharomyces - ps28
Arboricola_clade - ps29
Cerevisiae_clade - ps30
Saccharomyces_cerevisiae - ps31
Phylostratum

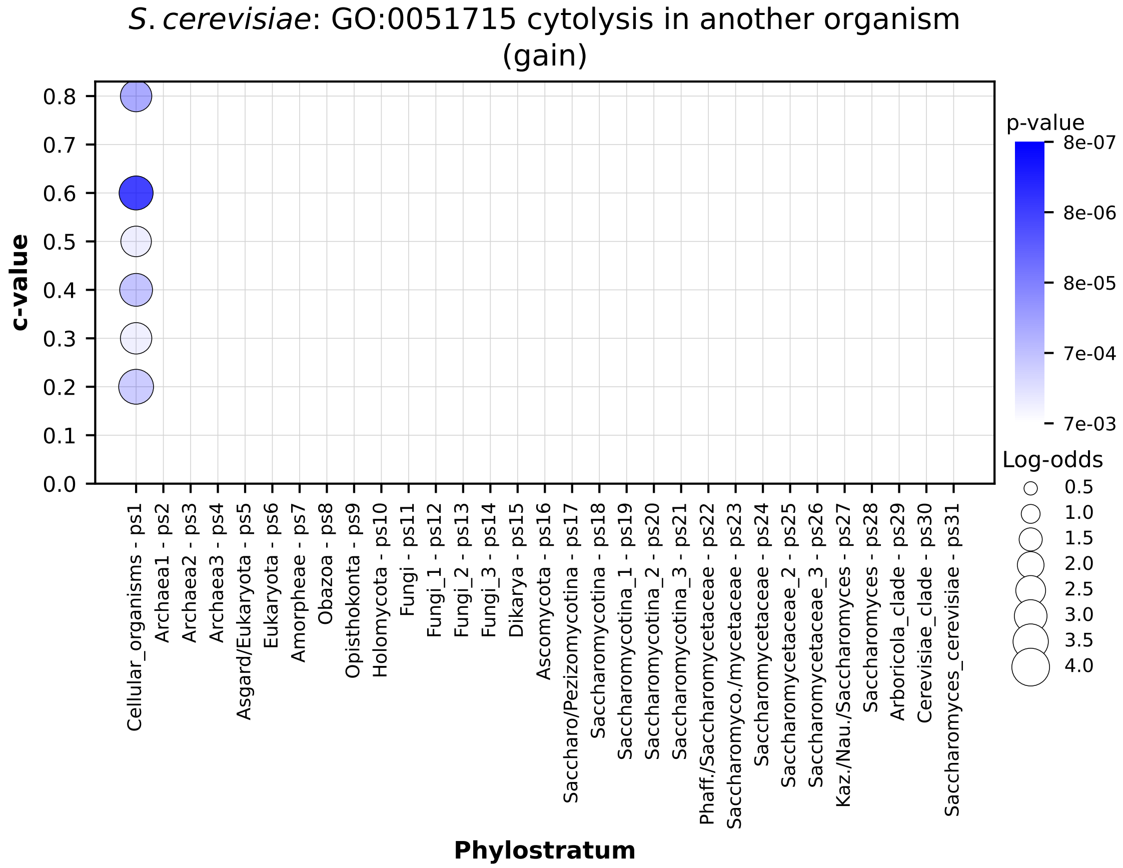

S. cerevisiae: GO:0051715 cytolysis in another organism
(gain)
c-value
0.0
0.1
0.2
0.3
0.4
0.5
0.6
0.7
0.8
p-value
8e-07
8e-06
8e-05
7e-04
7e-03
Log-odds
0.5
1.0
1.5
2.0
2.5
3.0
3.5
4.0
Cellular_organisms - ps1
Archaea1 - ps2
Archaea2 - ps3
Archaea3 - ps4
Asgard/Eukaryota - ps5
Eukaryota - ps6
Amorpheae - ps7
Obazoa - ps8
Opisthokonta - ps9
Holomycota - ps10
Fungi - ps11
Fungi_1 - ps12
Fungi_2 - ps13
Fungi_3 - ps14
Dikarya - ps15
Ascomycota - ps16
Saccharo/Pezizomycotina - ps17
Saccharomycotina - ps18
Saccharomycotina_1 - ps19
Saccharomycotina_2 - ps20
Saccharomycotina_3 - ps21
Phaff./Saccharomycetaceae - ps22
Saccharomyco./mycetaceae - ps23
Saccharomycetaceae - ps24
Saccharomycetaceae_2 - ps25
Saccharomycetaceae_3 - ps26
Kaz./Nau./Saccharomyces - ps27
Saccharomyces - ps28
Arboricola_clade - ps29
Cerevisiae_clade - ps30
Saccharomyces_cerevisiae - ps31
Phylostratum

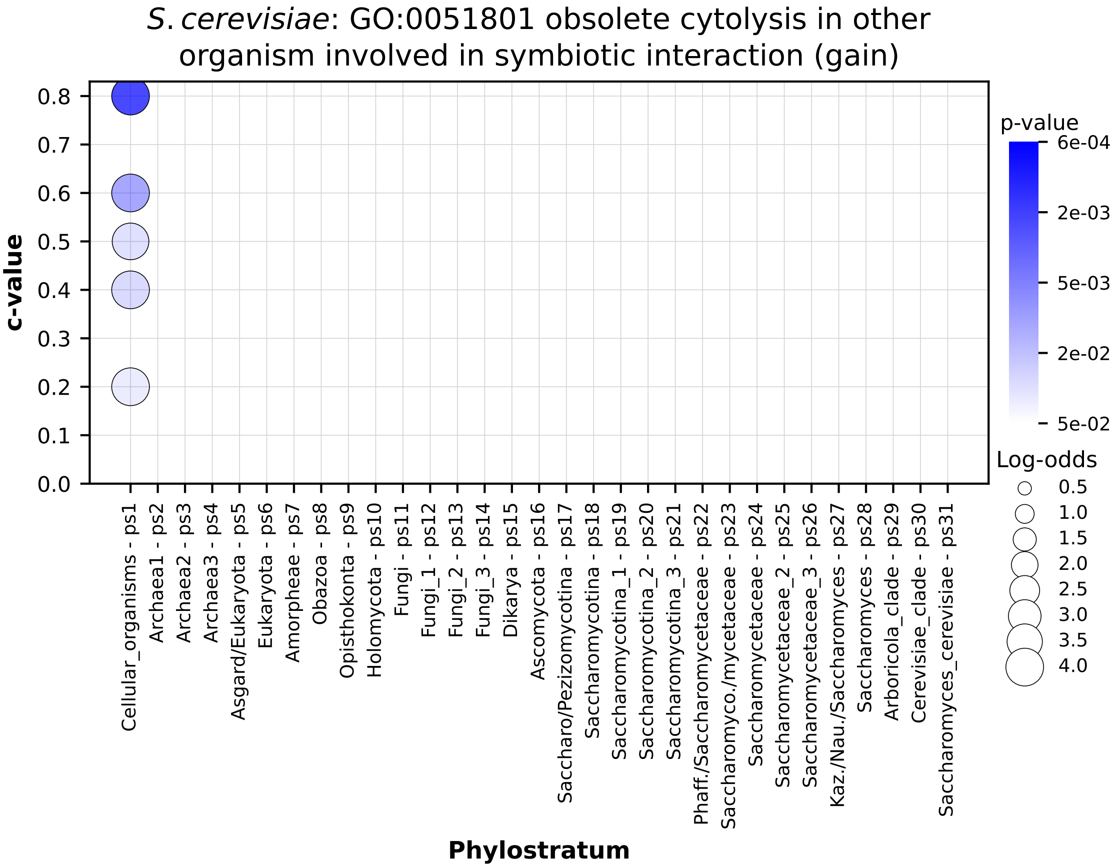

S. cerevisiae: GO:0051801 obsolete cytolysis in other organism involved in symbiotic interaction (gain)
c-value
0.0
0.1
0.2
0.3
0.4
0.5
0.6
0.7
0.8
p-value
6e-04
2e-03
5e-03
2e-02
5e-02
Log-odds
0.5
1.0
1.5
2.0
2.5
3.0
3.5
4.0
Cellular_organisms - ps1
Archaea1 - ps2
Archaea2 - ps3
Archaea3 - ps4
Asgard/Eukaryota - ps5
Eukaryota - ps6
Amorpheae - ps7
Obazoa - ps8
Opisthokonta - ps9
Holomycota - ps10
Fungi - ps11
Fungi_1 - ps12
Fungi_2 - ps13
Fungi_3 - ps14
Dikarya - ps15
Ascomycota - ps16
Saccharo/Pezizomycotina - ps17
Saccharomycotina - ps18
Saccharomycotina_1 - ps19
Saccharomycotina_2 - ps20
Saccharomycotina_3 - ps21
Phaff./Saccharomycetaceae - ps22
Saccharomyco./mycetaceae - ps23
Saccharomycetaceae - ps24
Saccharomycetaceae_2 - ps25
Saccharomycetaceae_3 - ps26
Kaz./Nau./Saccharomyces - ps27
Saccharomyces - ps28
Arboricola_clade - ps29
Cerevisiae_clade - ps30
Saccharomyces_cerevisiae - ps31
Phylostratum

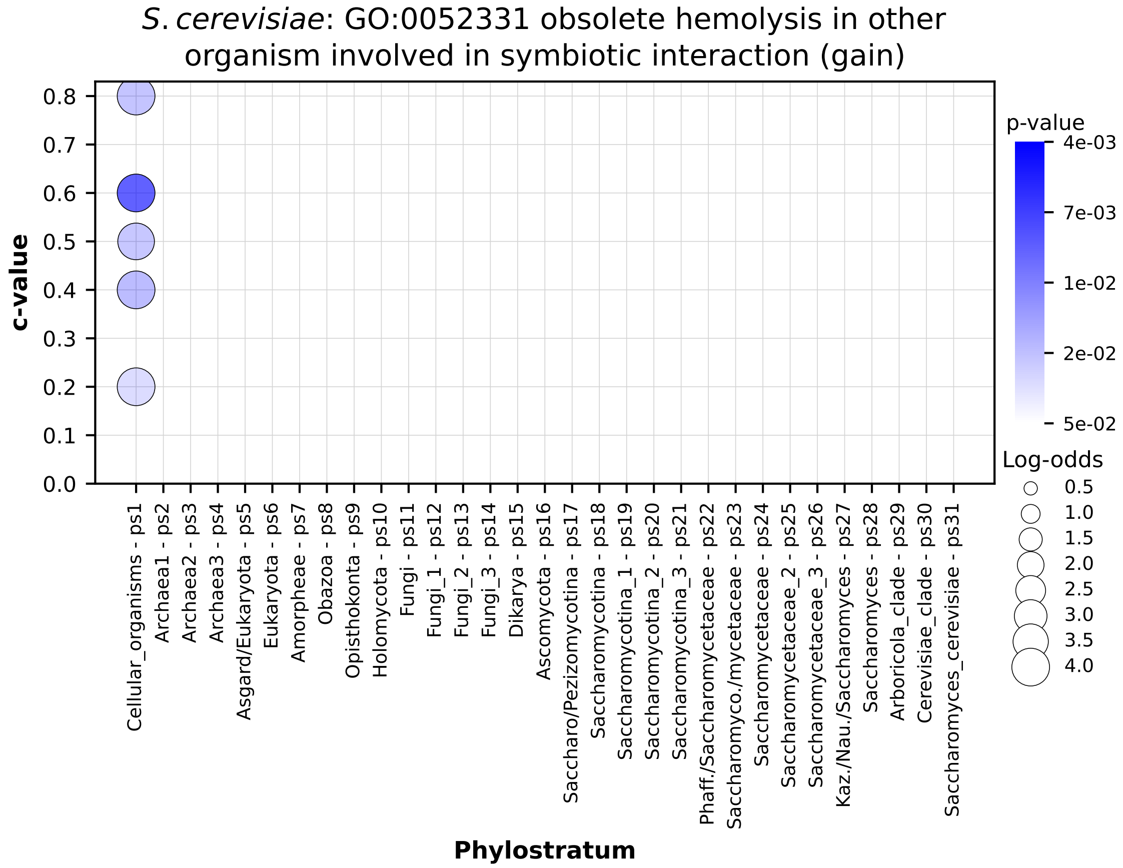

S. cerevisiae: GO:0052331 obsolete hemolysis in other organism involved in symbiotic interaction (gain)
c-value
0.0
0.1
0.2
0.3
0.4
0.5
0.6
0.7
0.8
Phylostratum
Cellular_organisms - ps1
Archaea1 - ps2
Archaea2 - ps3
Archaea3 - ps4
Asgard/Eukaryota - ps5
Eukaryota - ps6
Amorpheae - ps7
Obazoa - ps8
Opisthokonta - ps9
Holomycota - ps10
Fungi - ps11
Fungi_1 - ps12
Fungi_2 - ps13
Fungi_3 - ps14
Dikarya - ps15
Ascomycota - ps16
Saccharo/Pezizomycotina - ps17
Saccharomycotina - ps18
Saccharomycotina_1 - ps19
Saccharomycotina_2 - ps20
Saccharomycotina_3 - ps21
Phaff./Saccharomycetaceae - ps22
Saccharomyco./mycetaceae - ps23
Saccharomycetaceae - ps24
Saccharomycetaceae_2 - ps25
Saccharomycetaceae_3 - ps26
Kaz./Nau./Saccharomyces - ps27
Saccharomyces - ps28
Arboricola_clade - ps29
Cerevisiae_clade - ps30
Saccharomyces_cerevisiae - ps31
p-value
4e-03
7e-03
1e-02
2e-02
5e-02
Log-odds
0.5
1.0
1.5
2.0
2.5
3.0
3.5
4.0

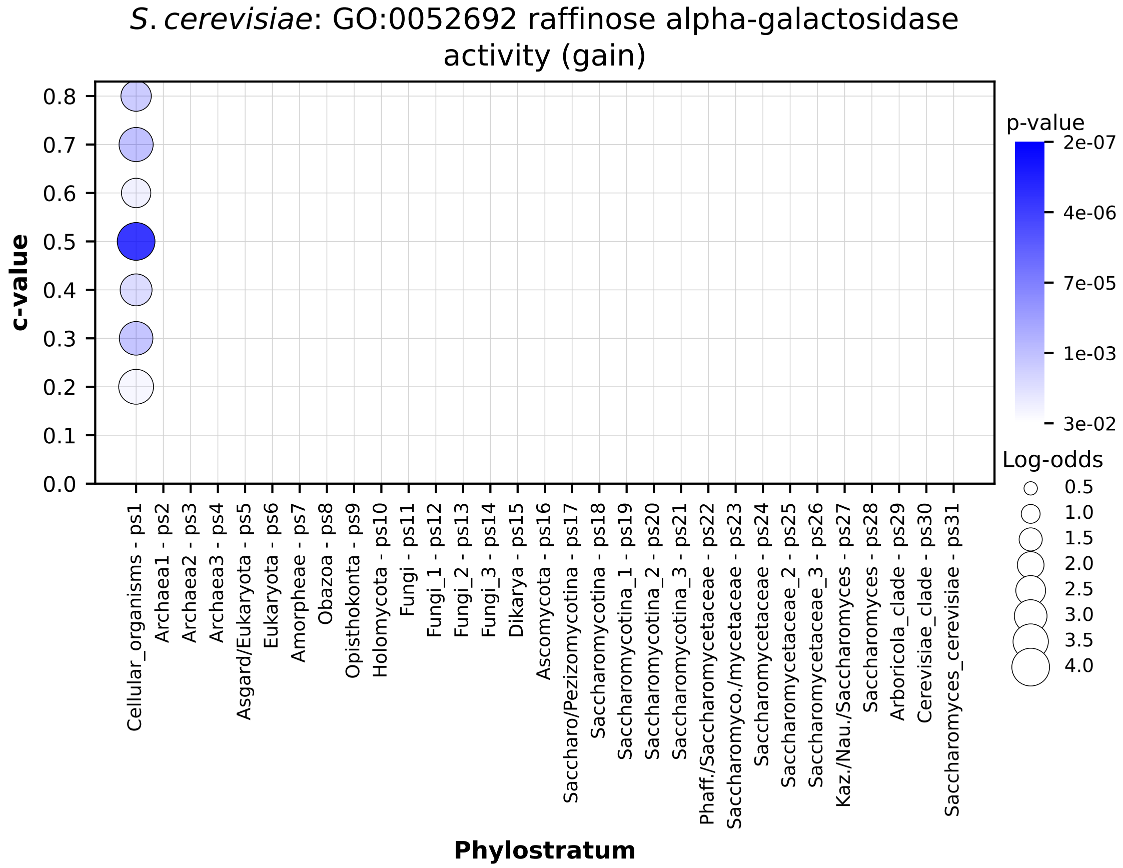

S. cerevisiae: GO:0052692 raffinose alpha-galactosidase activity (gain)
c-value
0.0
0.1
0.2
0.3
0.4
0.5
0.6
0.7
0.8
Phylostratum
Cellular_organisms - ps1
Archaea1 - ps2
Archaea2 - ps3
Archaea3 - ps4
Asgard/Eukaryota - ps5
Eukaryota - ps6
Amorpheae - ps7
Obazoa - ps8
Opisthokonta - ps9
Holomycota - ps10
Fungi - ps11
Fungi_1 - ps12
Fungi_2 - ps13
Fungi_3 - ps14
Dikarya - ps15
Ascomycota - ps16
Saccharo/Pezizomycotina - ps17
Saccharomycotina - ps18
Saccharomycotina_1 - ps19
Saccharomycotina_2 - ps20
Saccharomycotina_3 - ps21
Phaff./Saccharomycetaceae - ps22
Saccharomyco./mycetaceae - ps23
Saccharomycetaceae - ps24
Saccharomycetaceae_2 - ps25
Saccharomycetaceae_3 - ps26
Kaz./Nau./Saccharomyces - ps27
Saccharomyces - ps28
Arboricola_clade - ps29
Cerevisiae_clade - ps30
Saccharomyces_cerevisiae - ps31
p-value
2e-07
4e-06
7e-05
1e-03
3e-02
Log-odds
0.5
1.0
1.5
2.0
2.5
3.0
3.5
4.0

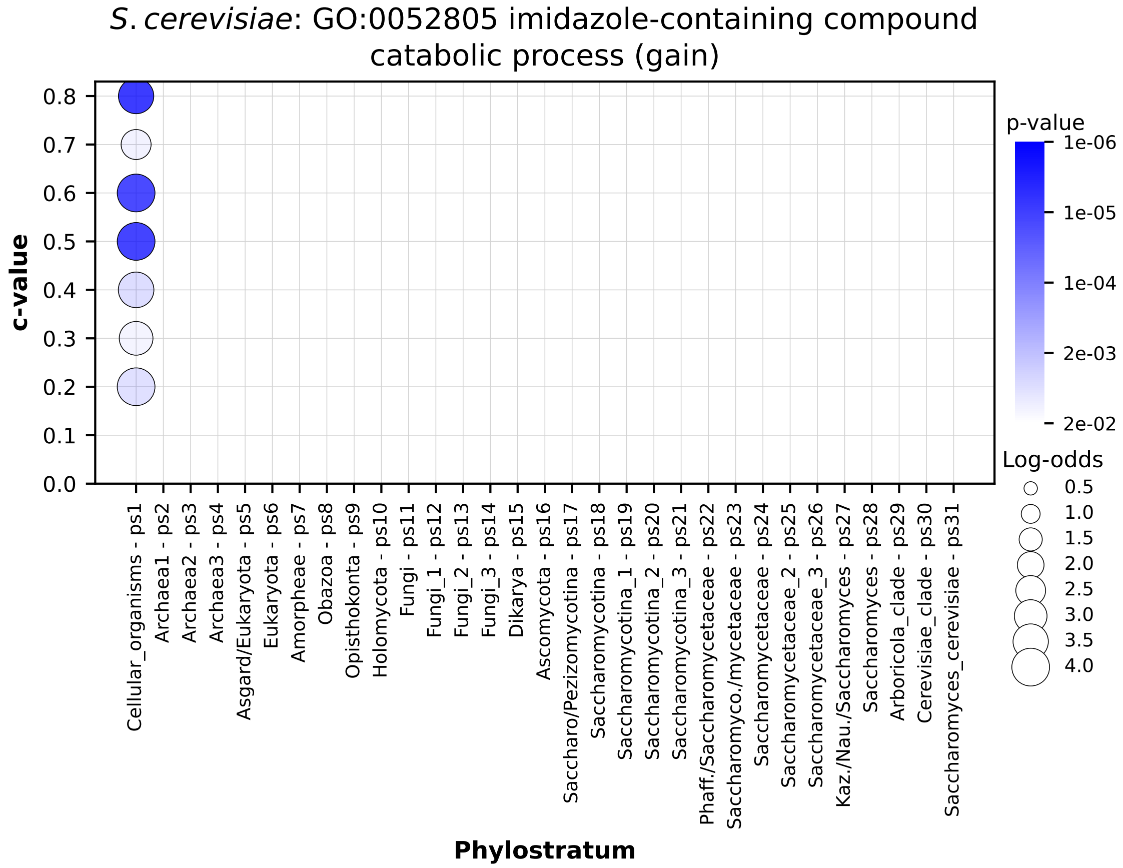

S. cerevisiae: GO:0052805 imidazole-containing compound catabolic process (gain)
c-value
0.0
0.1
0.2
0.3
0.4
0.5
0.6
0.7
0.8
p-value
1e-06
1e-05
1e-04
2e-03
2e-02
Log-odds
0.5
1.0
1.5
2.0
2.5
3.0
3.5
4.0
Cellular_organisms - ps1
Archaea1 - ps2
Archaea2 - ps3
Archaea3 - ps4
Asgard/Eukaryota - ps5
Eukaryota - ps6
Amorpheae - ps7
Obazoa - ps8
Opisthokonta - ps9
Holomycota - ps10
Fungi - ps11
Fungi_1 - ps12
Fungi_2 - ps13
Fungi_3 - ps14
Dikarya - ps15
Ascomycota - ps16
Saccharo/Pezizomycotina - ps17
Saccharomycotina - ps18
Saccharomycotina_1 - ps19
Saccharomycotina_2 - ps20
Saccharomycotina_3 - ps21
Phaff./Saccharomycetaceae - ps22
Saccharomyco./mycetaceae - ps23
Saccharomycetaceae - ps24
Saccharomycetaceae_2 - ps25
Saccharomycetaceae_3 - ps26
Kaz./Nau./Saccharomyces - ps27
Saccharomyces - ps28
Arboricola_clade - ps29
Cerevisiae_clade - ps30
Saccharomyces_cerevisiae - ps31
Phylostratum

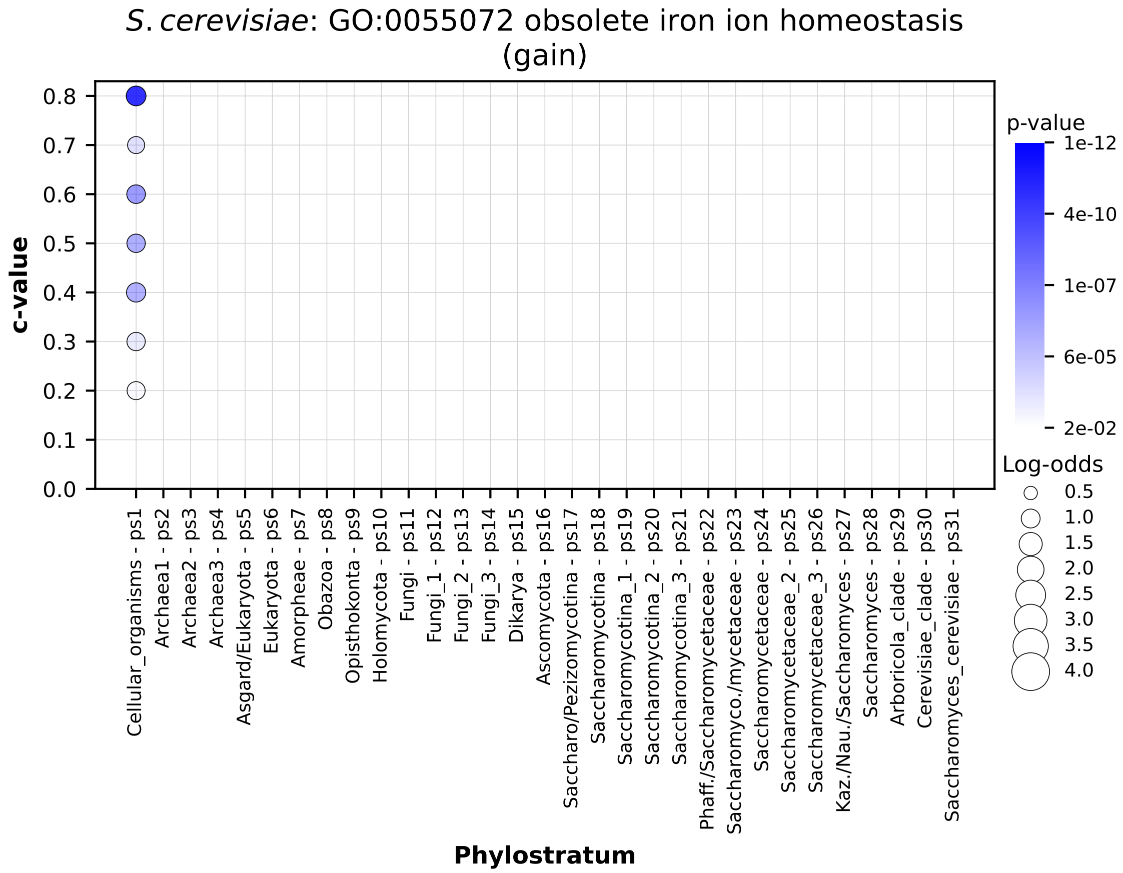

S. cerevisiae: GO:0055072 obsolete iron ion homeostasis (gain)
c-value
0.0
0.1
0.2
0.3
0.4
0.5
0.6
0.7
0.8
Phylostratum
Cellular_organisms - ps1
Archaea1 - ps2
Archaea2 - ps3
Archaea3 - ps4
Asgard/Eukaryota - ps5
Eukaryota - ps6
Amorpheae - ps7
Obazoa - ps8
Opisthokonta - ps9
Holomycota - ps10
Fungi - ps11
Fungi_1 - ps12
Fungi_2 - ps13
Fungi_3 - ps14
Dikarya - ps15
Ascomycota - ps16
Saccharo/Pezizomycotina - ps17
Saccharomycotina - ps18
Saccharomycotina_1 - ps19
Saccharomycotina_2 - ps20
Saccharomycotina_3 - ps21
Phaff./Saccharomycetaceae - ps22
Saccharomyco./mycetaceae - ps23
Saccharomycetaceae - ps24
Saccharomycetaceae_2 - ps25
Saccharomycetaceae_3 - ps26
Kaz./Nau./Saccharomyces - ps27
Saccharomyces - ps28
Arboricola_clade - ps29
Cerevisiae_clade - ps30
Saccharomyces_cerevisiae - ps31
p-value
1e-12
4e-10
1e-07
6e-05
2e-02
Log-odds
0.5
1.0
1.5
2.0
2.5
3.0
3.5
4.0

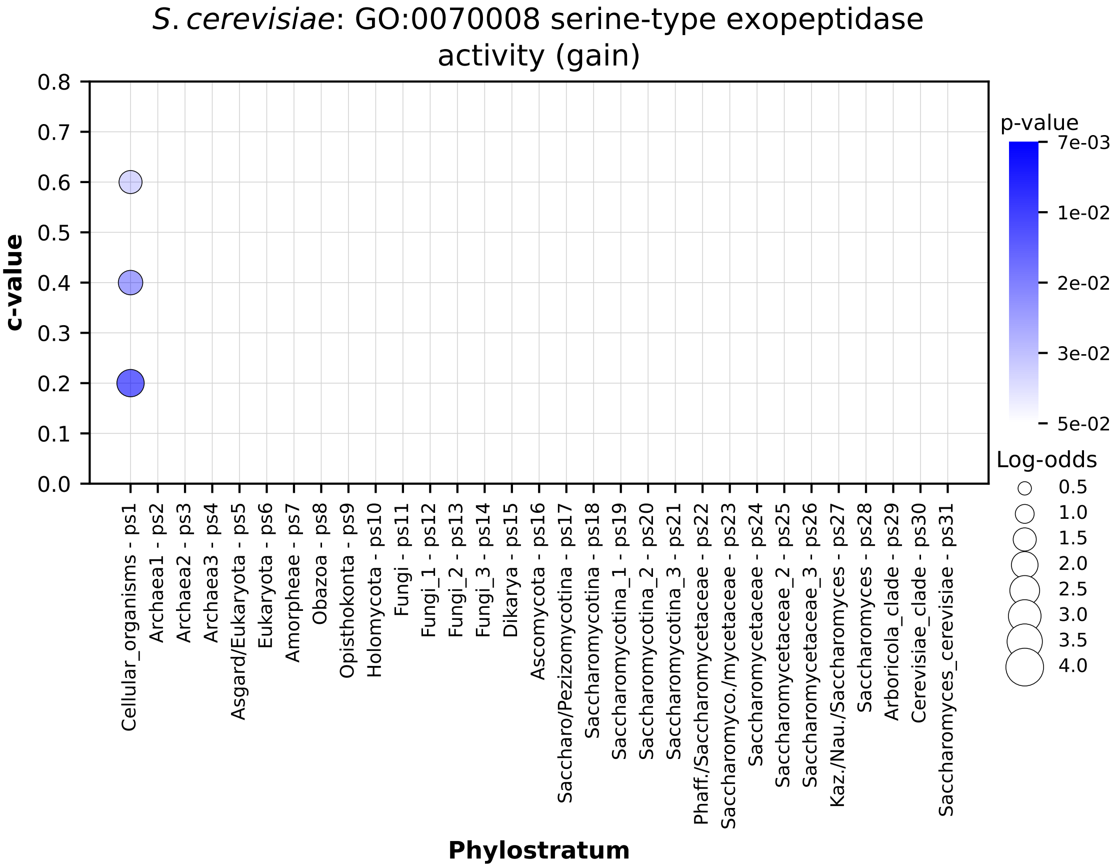

S. cerevisiae: GO:0070008 serine-type exopeptidase activity (gain)
c-value
0.8
0.7
0.6
0.5
0.4
0.3
0.2
0.1
0.0
p-value
7e-03
1e-02
2e-02
3e-02
5e-02
Log-odds
0.5
1.0
1.5
2.0
2.5
3.0
3.5
4.0
Cellular_organisms - ps1
Archaea1 - ps2
Archaea2 - ps3
Archaea3 - ps4
Asgard/Eukaryota - ps5
Eukaryota - ps6
Amorpheae - ps7
Obazoa - ps8
Opisthokonta - ps9
Holomycota - ps10
Fungi - ps11
Fungi_1 - ps12
Fungi_2 - ps13
Fungi_3 - ps14
Dikarya - ps15
Ascomycota - ps16
Saccharo/Pezizomycotina - ps17
Saccharomycotina - ps18
Saccharomycotina_1 - ps19
Saccharomycotina_2 - ps20
Saccharomycotina_3 - ps21
Phaff./Saccharomycetaceae - ps22
Saccharomyco./mycetaceae - ps23
Saccharomycetaceae - ps24
Saccharomycetaceae_2 - ps25
Saccharomycetaceae_3 - ps26
Kaz./Nau./Saccharomyces - ps27
Saccharomyces - ps28
Arboricola_clade - ps29
Cerevisiae_clade - ps30
Saccharomyces_cerevisiae - ps31
Phylostratum

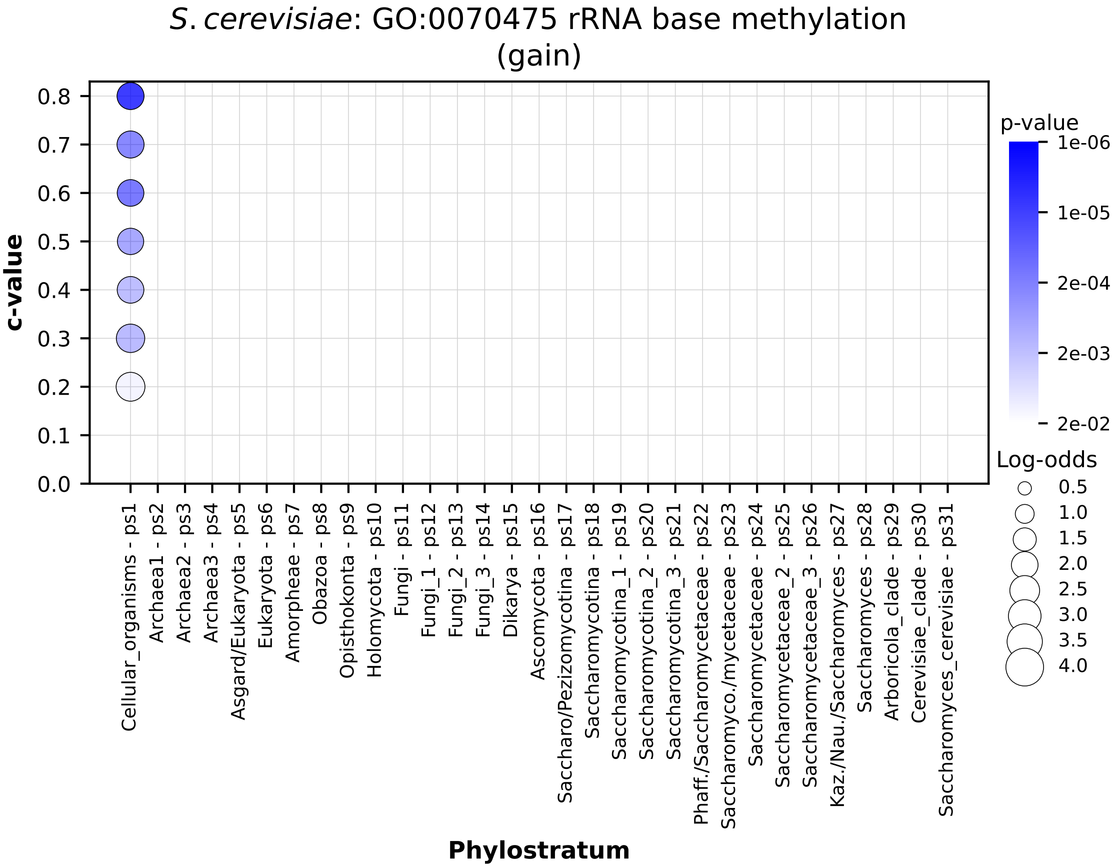

S. cerevisiae: GO:0070475 rRNA base methylation
(gain)
c-value
0.8
0.7
0.6
0.5
0.4
0.3
0.2
0.1
0.0
p-value
1e-06
1e-05
2e-04
2e-03
2e-02
Log-odds
0.5
1.0
1.5
2.0
2.5
3.0
3.5
4.0
Cellular_organisms - ps1
Archaea1 - ps2
Archaea2 - ps3
Archaea3 - ps4
Asgard/Eukaryota - ps5
Eukaryota - ps6
Amorpheae - ps7
Obazoa - ps8
Opisthokonta - ps9
Holomycota - ps10
Fungi - ps11
Fungi_1 - ps12
Fungi_2 - ps13
Fungi_3 - ps14
Dikarya - ps15
Ascomycota - ps16
Saccharo/Pezizomycotina - ps17
Saccharomycotina - ps18
Saccharomycotina_1 - ps19
Saccharomycotina_2 - ps20
Saccharomycotina_3 - ps21
Phaff./Saccharomycetaceae - ps22
Saccharomyco./mycetaceae - ps23
Saccharomycetaceae - ps24
Saccharomycetaceae_2 - ps25
Saccharomycetaceae_3 - ps26
Kaz./Nau./Saccharomyces - ps27
Saccharomyces - ps28
Arboricola_clade - ps29
Cerevisiae_clade - ps30
Saccharomyces_cerevisiae - ps31
Phylostratum

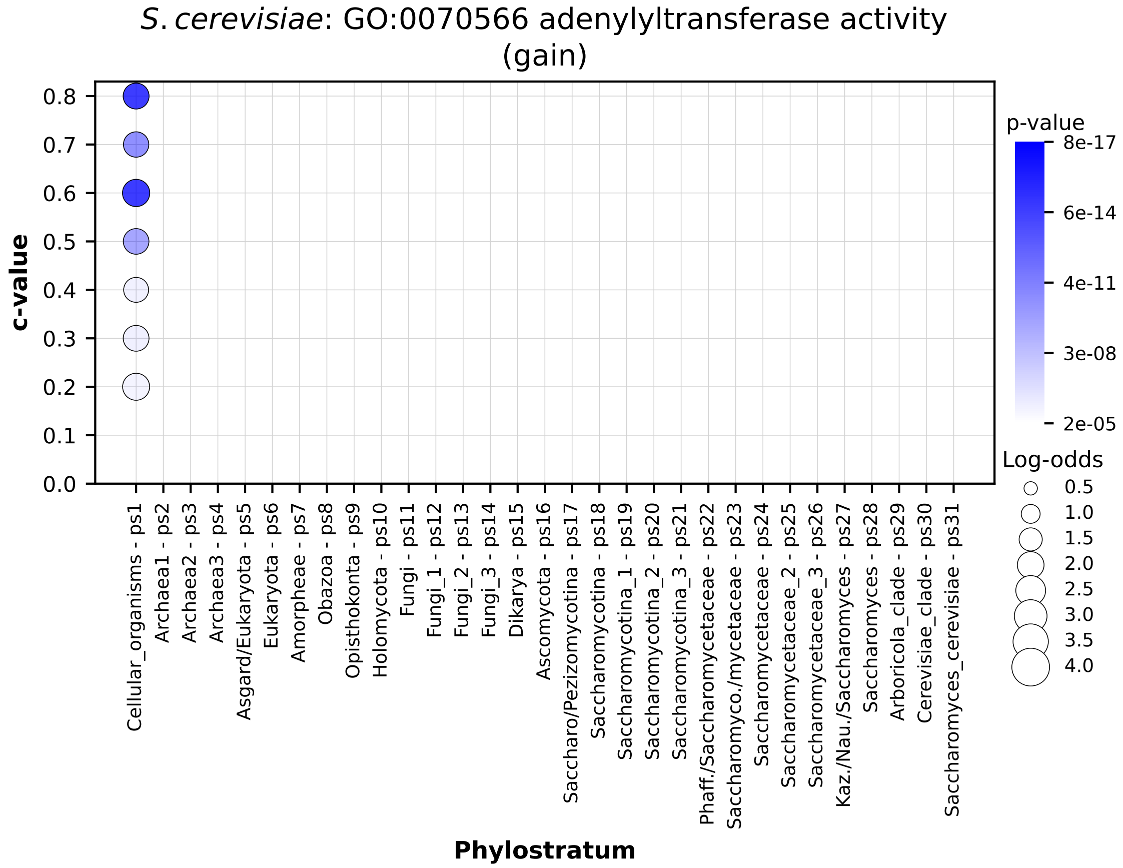

S. cerevisiae: GO:0070566 adenylyltransferase activity (gain)
c-value
0.0
0.1
0.2
0.3
0.4
0.5
0.6
0.7
0.8
p-value
8e-17
6e-14
4e-11
3e-08
2e-05
Log-odds
0.5
1.0
1.5
2.0
2.5
3.0
3.5
4.0
Cellular_organisms - ps1
Archaea1 - ps2
Archaea2 - ps3
Archaea3 - ps4
Asgard/Eukaryota - ps5
Eukaryota - ps6
Amorpheae - ps7
Obazoa - ps8
Opisthokonta - ps9
Holomycota - ps10
Fungi - ps11
Fungi_1 - ps12
Fungi_2 - ps13
Fungi_3 - ps14
Dikarya - ps15
Ascomycota - ps16
Saccharo/Pezizomycotina - ps17
Saccharomycotina - ps18
Saccharomycotina_1 - ps19
Saccharomycotina_2 - ps20
Saccharomycotina_3 - ps21
Phaff./Saccharomycetaceae - ps22
Saccharomyco./mycetaceae - ps23
Saccharomycetaceae - ps24
Saccharomycetaceae_2 - ps25
Saccharomycetaceae_3 - ps26
Kaz./Nau./Saccharomyces - ps27
Saccharomyces - ps28
Arboricola_clade - ps29
Cerevisiae_clade - ps30
Saccharomyces_cerevisiae - ps31
Phylostratum

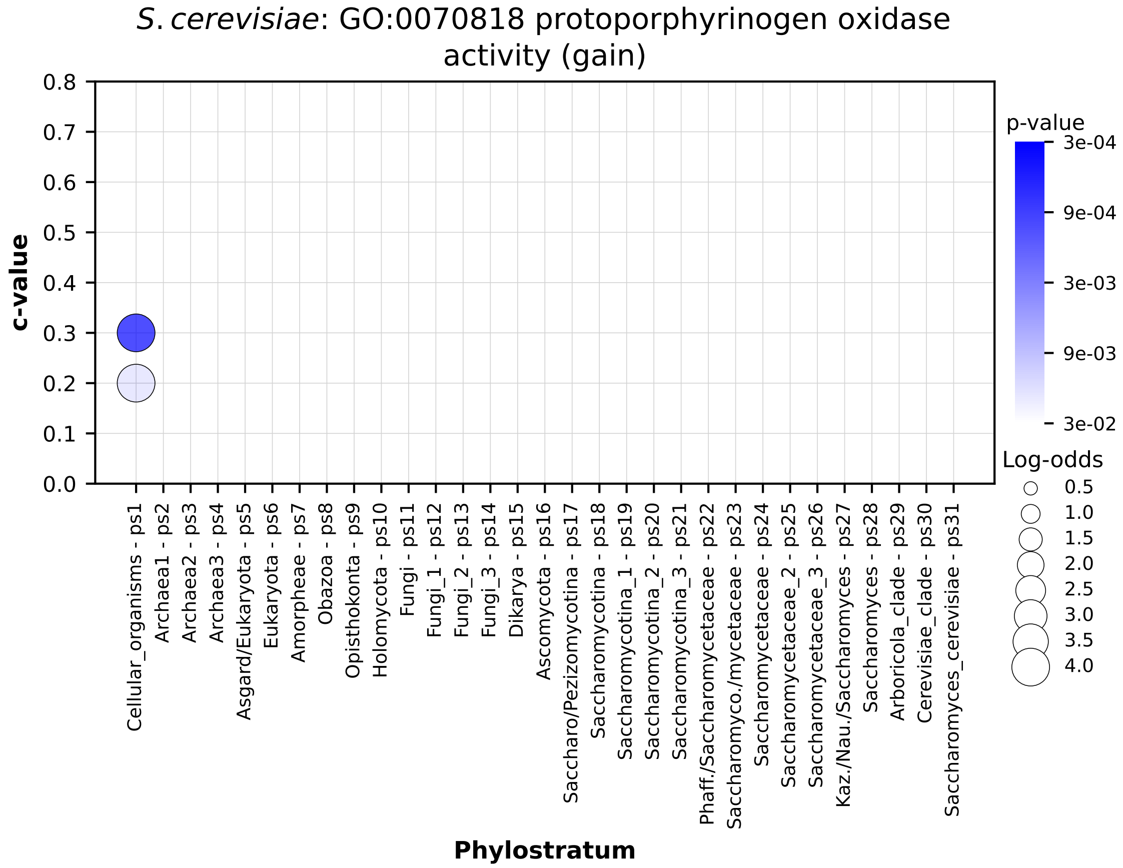

S. cerevisiae: GO:0070818 protoporphyrinogen oxidase activity (gain)
c-value
0.0
0.1
0.2
0.3
0.4
0.5
0.6
0.7
0.8
Phylostratum
Cellular_organisms - ps1
Archaea1 - ps2
Archaea2 - ps3
Archaea3 - ps4
Asgard/Eukaryota - ps5
Eukaryota - ps6
Amorpheae - ps7
Obazoa - ps8
Opisthokonta - ps9
Holomycota - ps10
Fungi - ps11
Fungi_1 - ps12
Fungi_2 - ps13
Fungi_3 - ps14
Dikarya - ps15
Ascomycota - ps16
Saccharo/Pezizomycotina - ps17
Saccharomycotina - ps18
Saccharomycotina_1 - ps19
Saccharomycotina_2 - ps20
Saccharomycotina_3 - ps21
Phaff./Saccharomycetaceae - ps22
Saccharomyco./mycetaceae - ps23
Saccharomycetaceae - ps24
Saccharomycetaceae_2 - ps25
Saccharomycetaceae_3 - ps26
Kaz./Nau./Saccharomyces - ps27
Saccharomyces - ps28
Arboricola_clade - ps29
Cerevisiae_clade - ps30
Saccharomyces_cerevisiae - ps31
p-value
3e-04
9e-04
3e-03
9e-03
3e-02
Log-odds
0.5
1.0
1.5
2.0
2.5
3.0
3.5
4.0

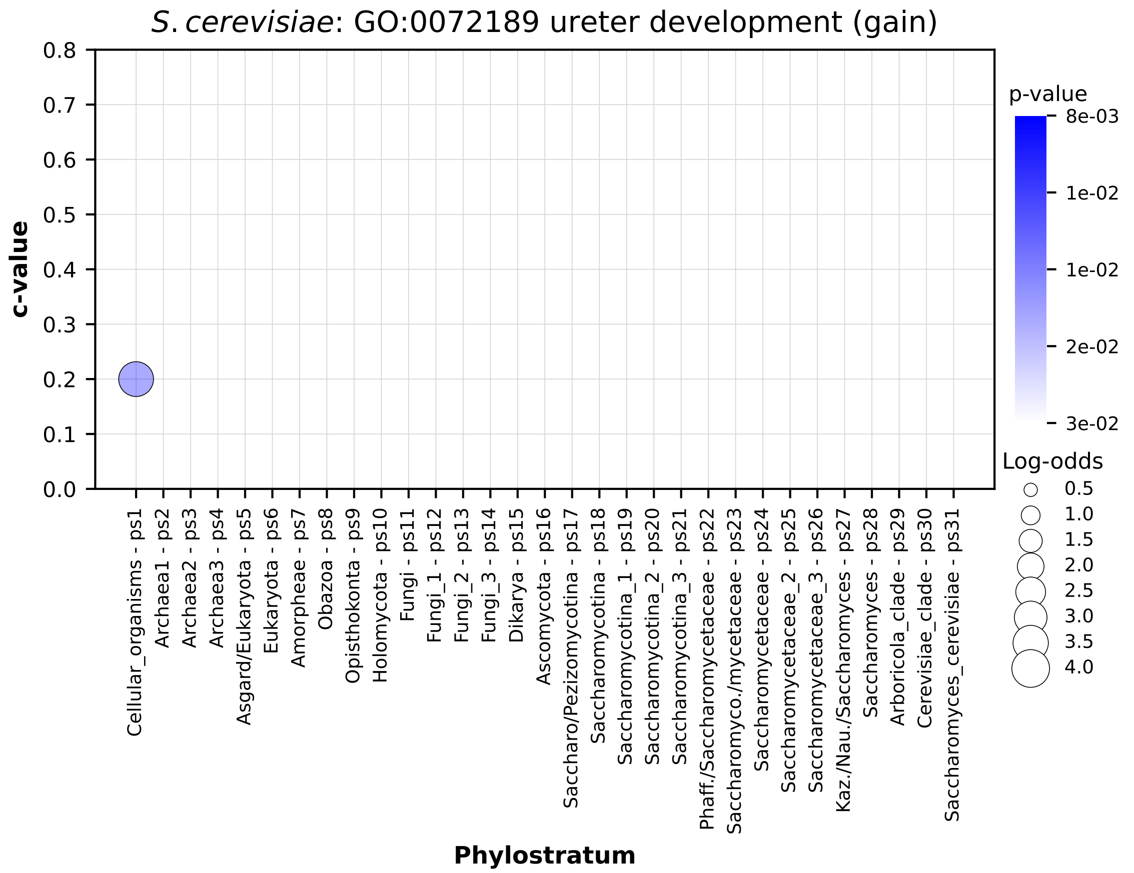

S. cerevisiae: GO:0072189 ureter development (gain)
c-value
0.0
0.1
0.2
0.3
0.4
0.5
0.6
0.7
0.8
Phylostratum
Cellular_organisms - ps1
Archaea1 - ps2
Archaea2 - ps3
Archaea3 - ps4
Asgard/Eukaryota - ps5
Eukaryota - ps6
Amorpheae - ps7
Obazoa - ps8
Opisthokonta - ps9
Holomycota - ps10
Fungi - ps11
Fungi_1 - ps12
Fungi_2 - ps13
Fungi_3 - ps14
Dikarya - ps15
Ascomycota - ps16
Saccharo/Pezizomycotina - ps17
Saccharomycotina - ps18
Saccharomycotina_1 - ps19
Saccharomycotina_2 - ps20
Saccharomycotina_3 - ps21
Phaff./Saccharomycetaceae - ps22
Saccharomyco./mycetaceae - ps23
Saccharomycetaceae - ps24
Saccharomycetaceae_2 - ps25
Saccharomycetaceae_3 - ps26
Kaz./Nau./Saccharomyces - ps27
Saccharomyces - ps28
Arboricola_clade - ps29
Cerevisiae_clade - ps30
Saccharomyces_cerevisiae - ps31
p-value
8e-03
1e-02
1e-02
2e-02
3e-02
Log-odds
0.5
1.0
1.5
2.0
2.5
3.0
3.5
4.0

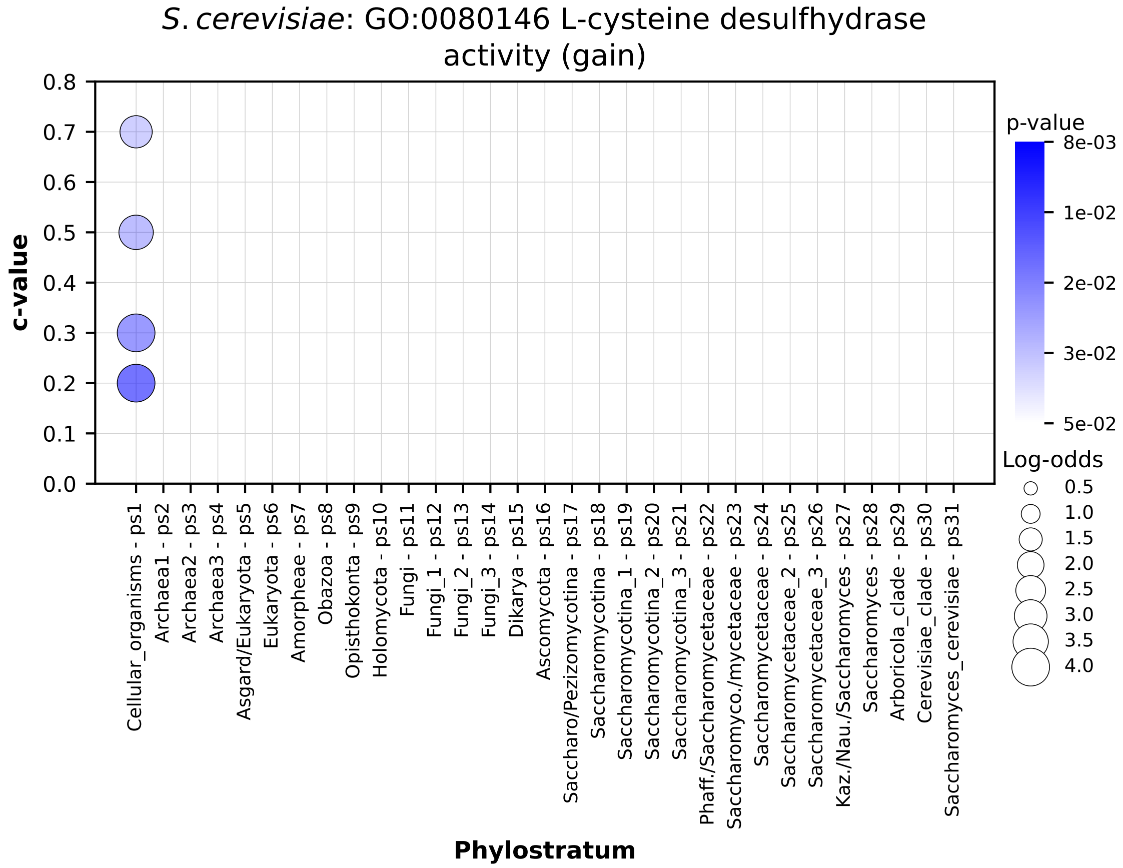

S. cerevisiae: GO:0080146 L-cysteine desulfhydrase activity (gain)
c-value
0.0
0.1
0.2
0.3
0.4
0.5
0.6
0.7
0.8
Phylostratum
Cellular_organisms - ps1
Archaea1 - ps2
Archaea2 - ps3
Archaea3 - ps4
Asgard/Eukaryota - ps5
Eukaryota - ps6
Amorpheae - ps7
Obazoa - ps8
Opisthokonta - ps9
Holomycota - ps10
Fungi - ps11
Fungi_1 - ps12
Fungi_2 - ps13
Fungi_3 - ps14
Dikarya - ps15
Ascomycota - ps16
Saccharo/Pezizomycotina - ps17
Saccharomycotina - ps18
Saccharomycotina_1 - ps19
Saccharomycotina_2 - ps20
Saccharomycotina_3 - ps21
Phaff./Saccharomycetaceae - ps22
Saccharomyco./mycetaceae - ps23
Saccharomycetaceae - ps24
Saccharomycetaceae_2 - ps25
Saccharomycetaceae_3 - ps26
Kaz./Nau./Saccharomyces - ps27
Saccharomyces - ps28
Arboricola_clade - ps29
Cerevisiae_clade - ps30
Saccharomyces_cerevisiae - ps31
p-value
8e-03
1e-02
2e-02
3e-02
5e-02
Log-odds
0.5
1.0
1.5
2.0
2.5
3.0
3.5
4.0

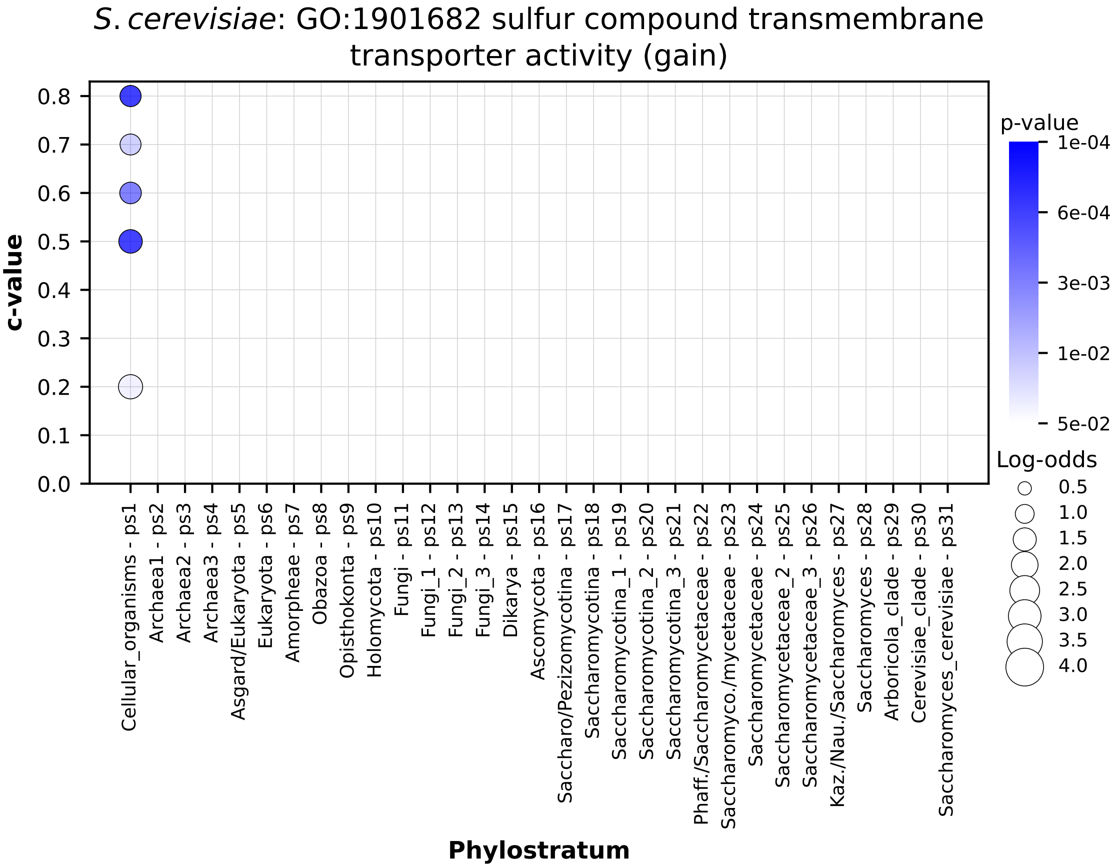

S. cerevisiae: GO:1901682 sulfur compound transmembrane transporter activity (gain)
c-value
0.0
0.1
0.2
0.3
0.4
0.5
0.6
0.7
0.8
p-value
1e-04
6e-04
3e-03
1e-02
5e-02
Log-odds
0.5
1.0
1.5
2.0
2.5
3.0
3.5
4.0
Cellular_organisms - ps1
Archaea1 - ps2
Archaea2 - ps3
Archaea3 - ps4
Asgard/Eukaryota - ps5
Eukaryota - ps6
Amorpheae - ps7
Obazoa - ps8
Opisthokonta - ps9
Holomycota - ps10
Fungi - ps11
Fungi_1 - ps12
Fungi_2 - ps13
Fungi_3 - ps14
Dikarya - ps15
Ascomycota - ps16
Saccharo/Pezizomycotina - ps17
Saccharomycotina - ps18
Saccharomycotina_1 - ps19
Saccharomycotina_2 - ps20
Saccharomycotina_3 - ps21
Phaff./Saccharomycetaceae - ps22
Saccharomyco./mycetaceae - ps23
Saccharomycetaceae - ps24
Saccharomycetaceae_2 - ps25
Saccharomycetaceae_3 - ps26
Kaz./Nau./Saccharomyces - ps27
Saccharomyces - ps28
Arboricola_clade - ps29
Cerevisiae_clade - ps30
Saccharomyces_cerevisiae - ps31
Phylostratum

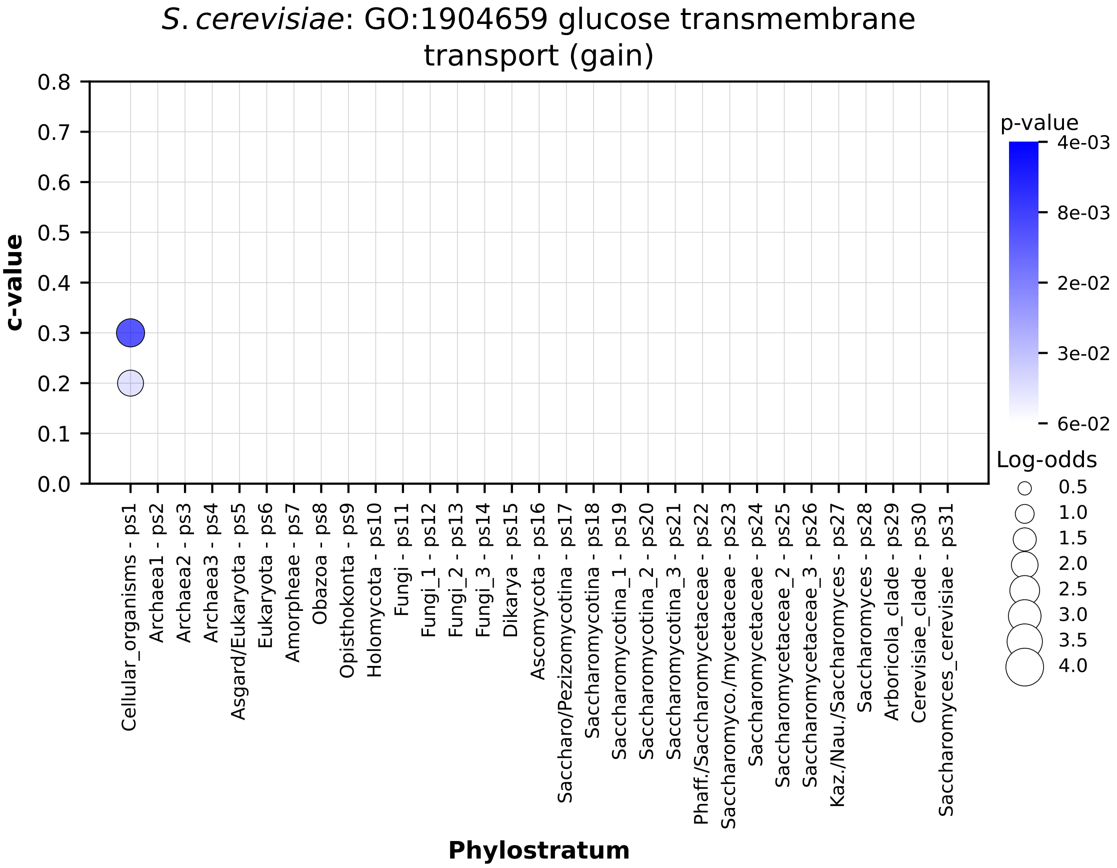

S. cerevisiae: GO:1904659 glucose transmembrane transport (gain)
c-value
0.0
0.1
0.2
0.3
0.4
0.5
0.6
0.7
0.8
p-value
4e-03
8e-03
2e-02
3e-02
6e-02
Log-odds
0.5
1.0
1.5
2.0
2.5
3.0
3.5
4.0
Cellular_organisms - ps1
Archaea1 - ps2
Archaea2 - ps3
Archaea3 - ps4
Asgard/Eukaryota - ps5
Eukaryota - ps6
Amorpheae - ps7
Obazoa - ps8
Opisthokonta - ps9
Holomycota - ps10
Fungi - ps11
Fungi_1 - ps12
Fungi_2 - ps13
Fungi_3 - ps14
Dikarya - ps15
Ascomycota - ps16
Saccharo/Pezizomycotina - ps17
Saccharomycotina - ps18
Saccharomycotina_1 - ps19
Saccharomycotina_2 - ps20
Saccharomycotina_3 - ps21
Phaff./Saccharomycetaceae - ps22
Saccharomyco./mycetaceae - ps23
Saccharomycetaceae - ps24
Saccharomycetaceae_2 - ps25
Saccharomycetaceae_3 - ps26
Kaz./Nau./Saccharomyces - ps27
Saccharomyces - ps28
Arboricola_clade - ps29
Cerevisiae_clade - ps30
Saccharomyces_cerevisiae - ps31
Phylostratum

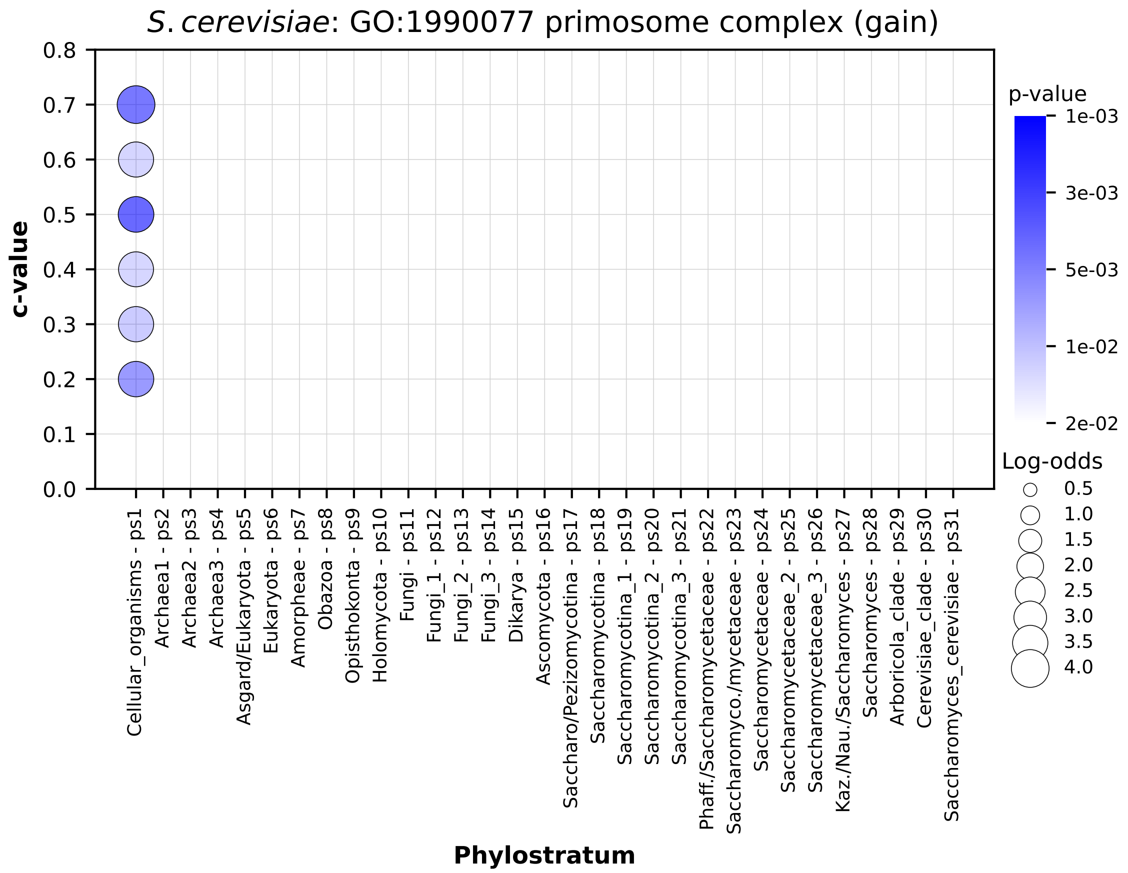
S. cerevisiae: GO:1990077 primosome complex (gain)
c-value
0.0
0.1
0.2
0.3
0.4
0.5
0.6
0.7
0.8
Phylostratum
Cellular_organisms - ps1
Archaea1 - ps2
Archaea2 - ps3
Archaea3 - ps4
Asgard/Eukaryota - ps5
Eukaryota - ps6
Amorpheae - ps7
Obazoa - ps8
Opisthokonta - ps9
Holomycota - ps10
Fungi - ps11
Fungi_1 - ps12
Fungi_2 - ps13
Fungi_3 - ps14
Dikarya - ps15
Ascomycota - ps16
Saccharo/Pezizomycotina - ps17
Saccharomycotina - ps18
Saccharomycotina_1 - ps19
Saccharomycotina_2 - ps20
Saccharomycotina_3 - ps21
Phaff./Saccharomycetaceae - ps22
Saccharomyco./mycetaceae - ps23
Saccharomycetaceae - ps24
Saccharomycetaceae_2 - ps25
Saccharomycetaceae_3 - ps26
Kaz./Nau./Saccharomyces - ps27
Saccharomyces - ps28
Arboricola_clade - ps29
Cerevisiae_clade - ps30
Saccharomyces_cerevisiae - ps31
p-value
1e-03
3e-03
5e-03
1e-02
2e-02
Log-odds
0.5
1.0
1.5
2.0
2.5
3.0
3.5
4.0

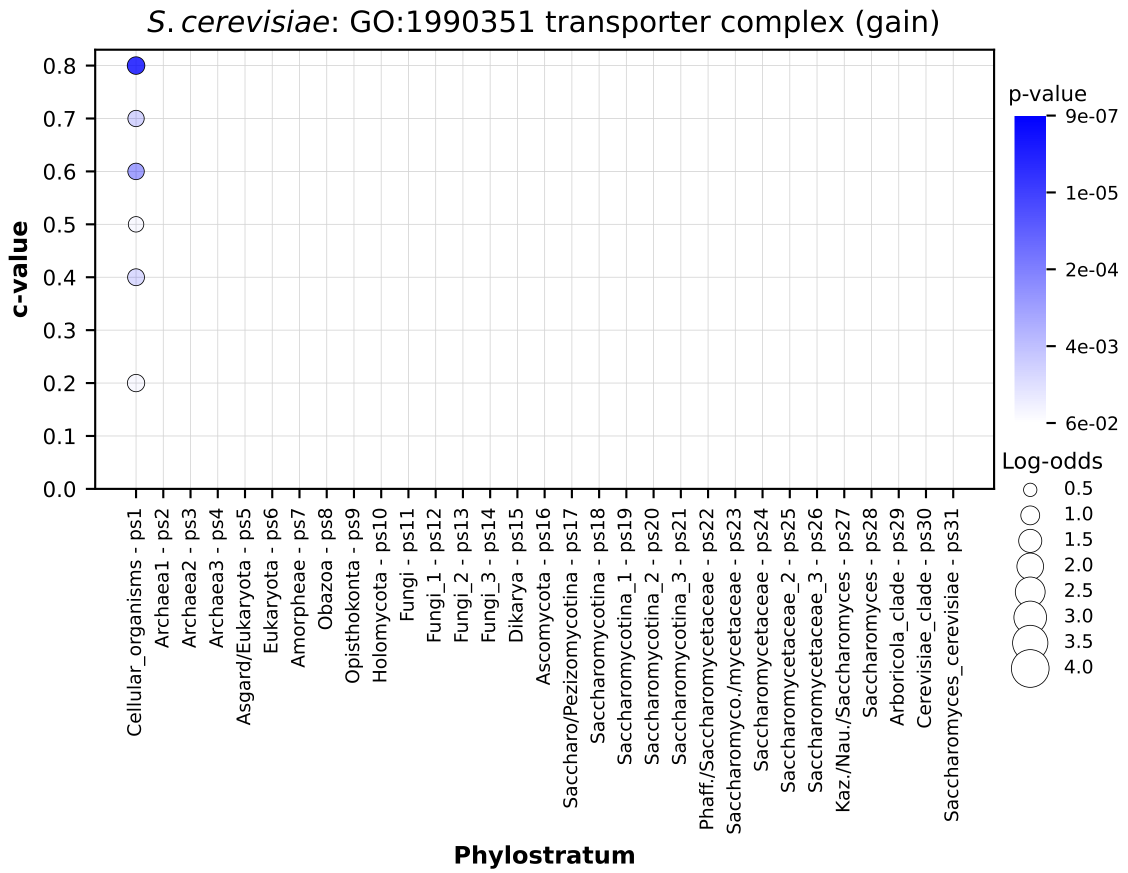

S. cerevisiae: GO:1990351 transporter complex (gain)
c-value
0.0
0.1
0.2
0.3
0.4
0.5
0.6
0.7
0.8
Phylostratum
Cellular_organisms - ps1
Archaea1 - ps2
Archaea2 - ps3
Archaea3 - ps4
Asgard/Eukaryota - ps5
Eukaryota - ps6
Amorpheae - ps7
Obazoa - ps8
Opisthokonta - ps9
Holomycota - ps10
Fungi - ps11
Fungi_1 - ps12
Fungi_2 - ps13
Fungi_3 - ps14
Dikarya - ps15
Ascomycota - ps16
Saccharo/Pezizomycotina - ps17
Saccharomycotina - ps18
Saccharomycotina_1 - ps19
Saccharomycotina_2 - ps20
Saccharomycotina_3 - ps21
Phaff./Saccharomycetaceae - ps22
Saccharomyco./mycetaceae - ps23
Saccharomycetaceae - ps24
Saccharomycetaceae_2 - ps25
Saccharomycetaceae_3 - ps26
Kaz./Nau./Saccharomyces - ps27
Saccharomyces - ps28
Arboricola_clade - ps29
Cerevisiae_clade - ps30
Saccharomyces_cerevisiae - ps31
p-value
9e-07
1e-05
2e-04
4e-03
6e-02
Log-odds
0.5
1.0
1.5
2.0
2.5
3.0
3.5
4.0

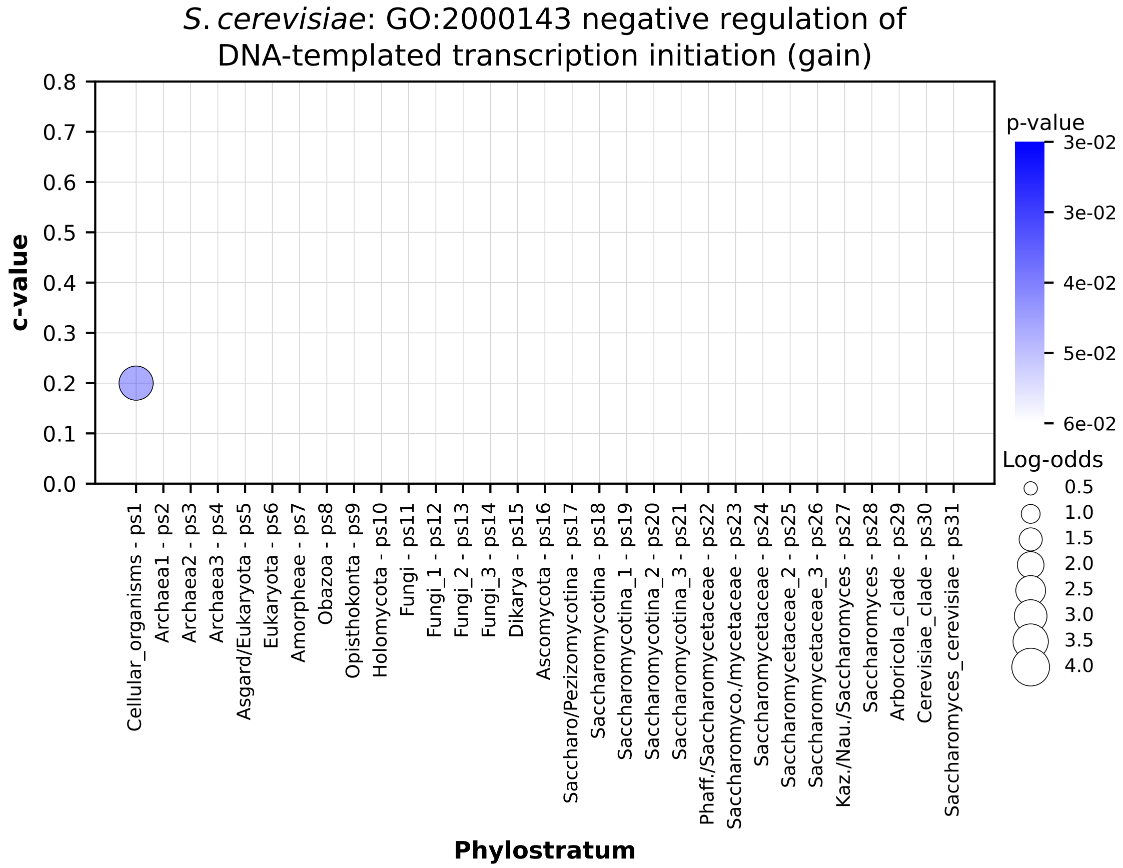
S. cerevisiae: GO:2000143 negative regulation of DNA-templated transcription initiation (gain)
c-value
0.8
0.7
0.6
0.5
0.4
0.3
0.2
0.1
0.0
p-value
3e-02
3e-02
4e-02
5e-02
6e-02
Log-odds
0.5
1.0
1.5
2.0
2.5
3.0
3.5
4.0
Cellular_organisms - ps1
Archaea1 - ps2
Archaea2 - ps3
Archaea3 - ps4
Asgard/Eukaryota - ps5
Eukaryota - ps6
Amorpheae - ps7
Obazoa - ps8
Opisthokonta - ps9
Holomycota - ps10
Fungi - ps11
Fungi_1 - ps12
Fungi_2 - ps13
Fungi_3 - ps14
Dikarya - ps15
Ascomycota - ps16
Saccharo/Pezizomycotina - ps17
Saccharomycotina - ps18
Saccharomycotina_1 - ps19
Saccharomycotina_2 - ps20
Saccharomycotina_3 - ps21
Phaff./Saccharomycetaceae - ps22
Saccharomyco./mycetaceae - ps23
Saccharomycetaceae - ps24
Saccharomycetaceae_2 - ps25
Saccharomycetaceae_3 - ps26
Kaz./Nau./Saccharomyces - ps27
Saccharomyces - ps28
Arboricola_clade - ps29
Cerevisiae_clade - ps30
Saccharomyces_cerevisiae - ps31
Phylostratum

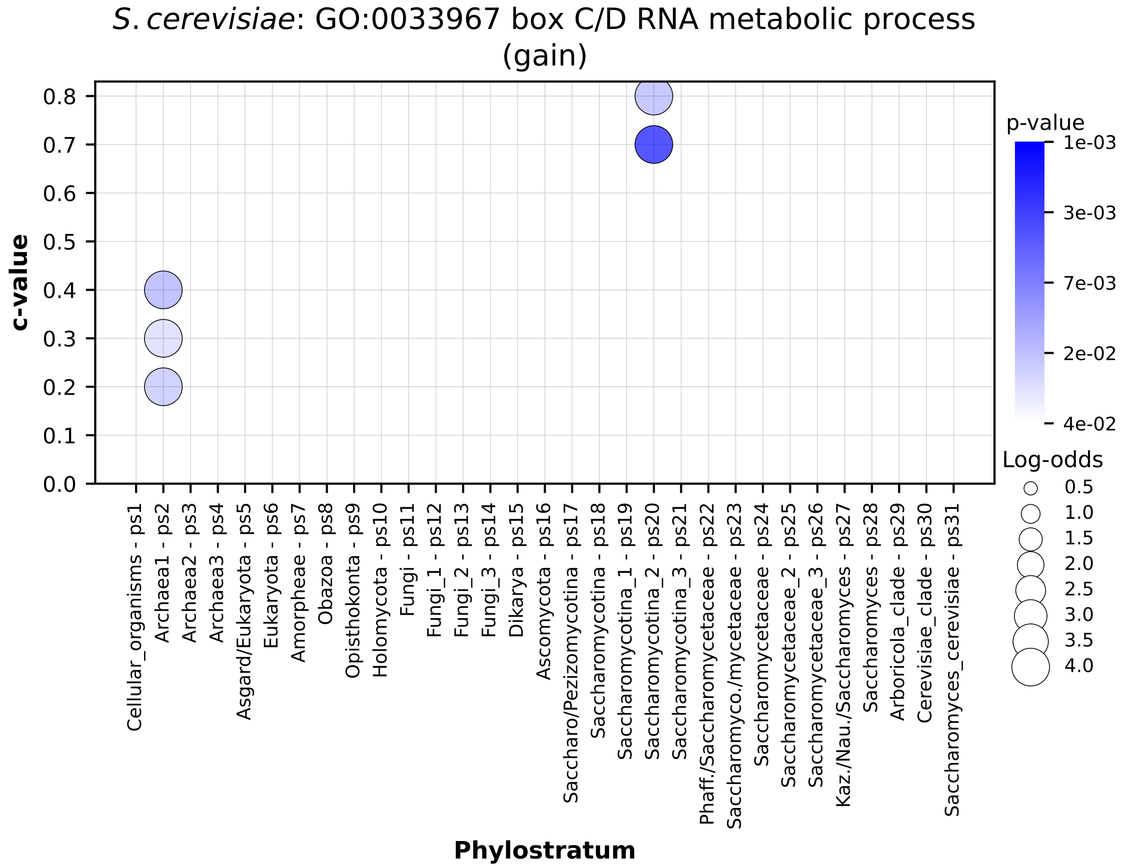

S. cerevisiae: GO:0033967 box C/D RNA metabolic process (gain)
c-value
0.0
0.1
0.2
0.3
0.4
0.5
0.6
0.7
0.8
Phylostratum
Cellular_organisms - ps1
Archaea1 - ps2
Archaea2 - ps3
Archaea3 - ps4
Asgard/Eukaryota - ps5
Eukaryota - ps6
Amorpheae - ps7
Obazoa - ps8
Opisthokonta - ps9
Holomycota - ps10
Fungi - ps11
Fungi_1 - ps12
Fungi_2 - ps13
Fungi_3 - ps14
Dikarya - ps15
Ascomycota - ps16
Saccharo/Pezizomycotina - ps17
Saccharomycotina - ps18
Saccharomycotina_1 - ps19
Saccharomycotina_2 - ps20
Saccharomycotina_3 - ps21
Phaff./Saccharomycetaceae - ps22
Saccharomyco./mycetaceae - ps23
Saccharomycetaceae - ps24
Saccharomycetaceae_2 - ps25
Saccharomycetaceae_3 - ps26
Kaz./Nau./Saccharomyces - ps27
Saccharomyces - ps28
Arboricola_clade - ps29
Cerevisiae_clade - ps30
Saccharomyces_cerevisiae - ps31
p-value
1e-03
3e-03
7e-03
2e-02
4e-02
Log-odds
0.5
1.0
1.5
2.0
2.5
3.0
3.5
4.0

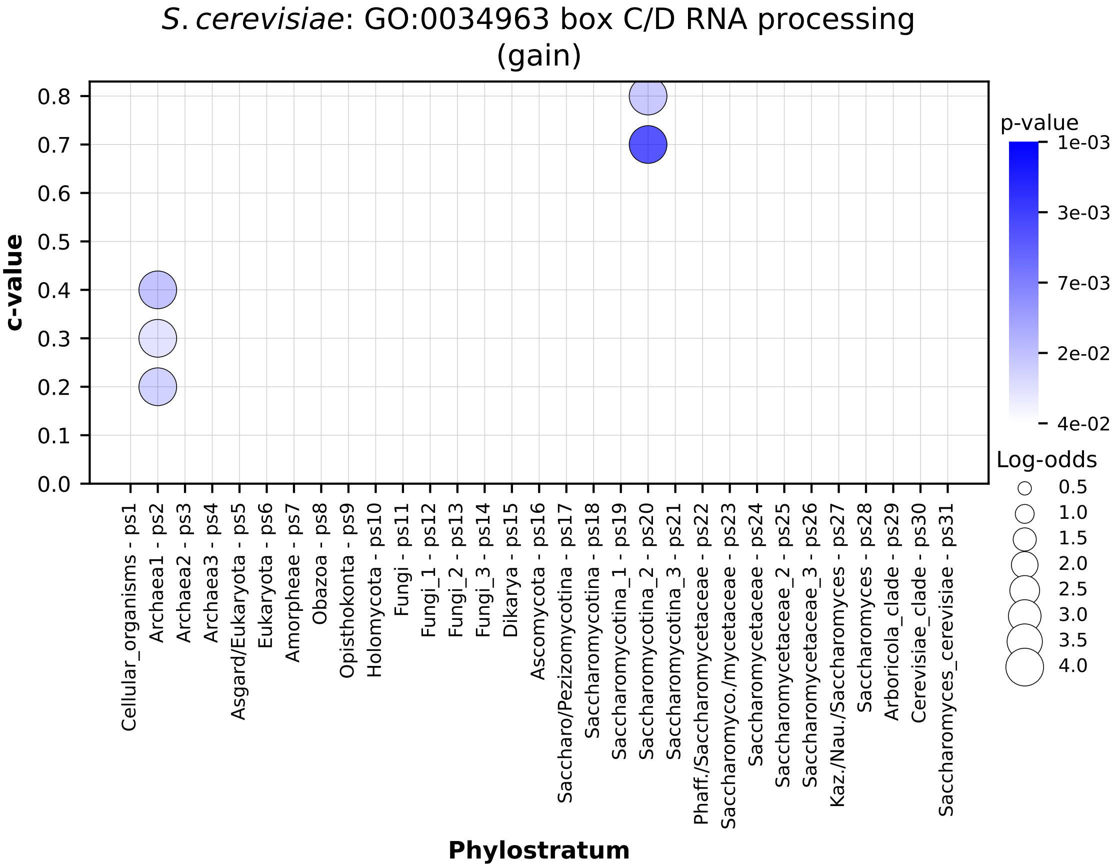
S. cerevisiae: GO:0034963 box C/D RNA processing (gain)
c-value
0.0
0.1
0.2
0.3
0.4
0.5
0.6
0.7
0.8
p-value
1e-03
3e-03
7e-03
2e-02
4e-02
Log-odds
0.5
1.0
1.5
2.0
2.5
3.0
3.5
4.0
Cellular_organisms - ps1
Archaea1 - ps2
Archaea2 - ps3
Archaea3 - ps4
Asgard/Eukaryota - ps5
Eukaryota - ps6
Amorpheae - ps7
Obazoa - ps8
Opisthokonta - ps9
Holomycota - ps10
Fungi - ps11
Fungi_1 - ps12
Fungi_2 - ps13
Fungi_3 - ps14
Dikarya - ps15
Ascomycota - ps16
Saccharo/Pezizomycotina - ps17
Saccharomycotina - ps18
Saccharomycotina_1 - ps19
Saccharomycotina_2 - ps20
Saccharomycotina_3 - ps21
Phaff./Saccharomycetaceae - ps22
Saccharomyco./mycetaceae - ps23
Saccharomycetaceae - ps24
Saccharomycetaceae_2 - ps25
Saccharomycetaceae_3 - ps26
Kaz./Nau./Saccharomyces - ps27
Saccharomyces - ps28
Arboricola_clade - ps29
Cerevisiae_clade - ps30
Saccharomyces_cerevisiae - ps31
Phylostratum

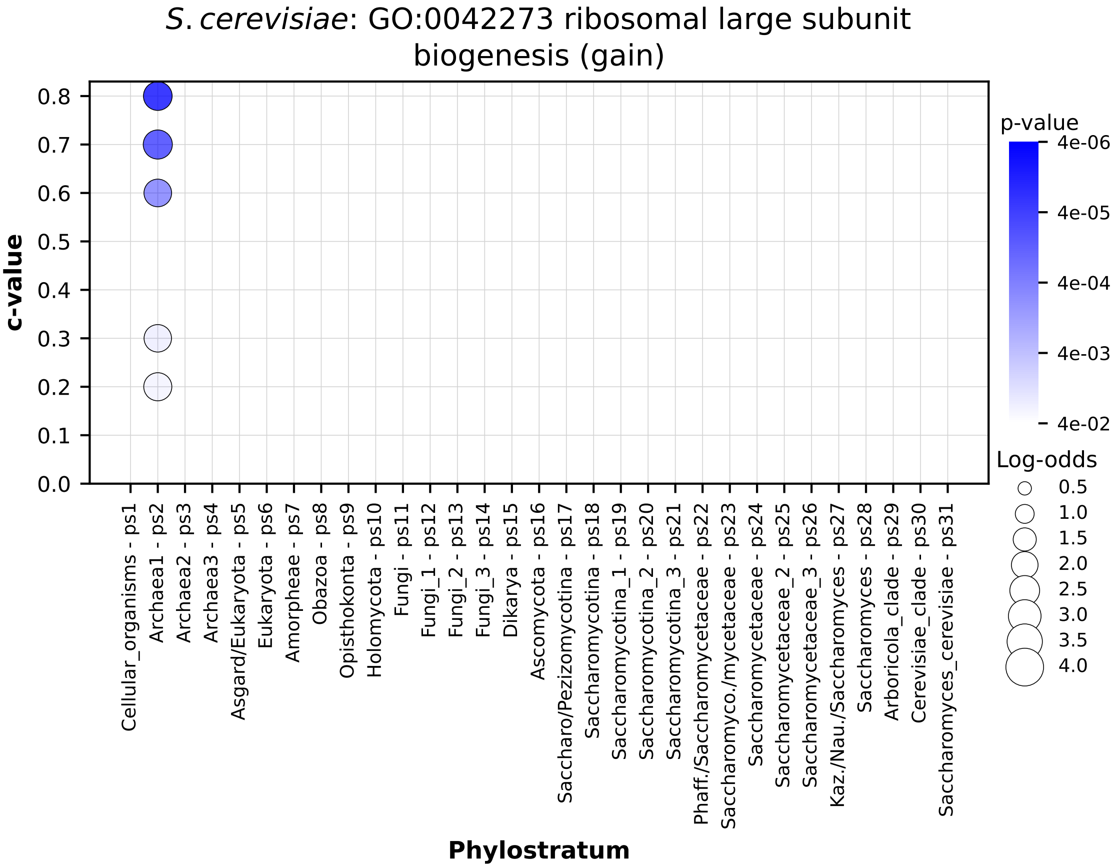

S. cerevisiae: GO:0042273 ribosomal large subunit biogenesis (gain)
c-value
0.8
0.7
0.6
0.5
0.4
0.3
0.2
0.1
0.0
Phylostratum
Cellular_organisms - ps1
Archaea1 - ps2
Archaea2 - ps3
Archaea3 - ps4
Asgard/Eukaryota - ps5
Eukaryota - ps6
Amorpheae - ps7
Obazoa - ps8
Opisthokonta - ps9
Holomycota - ps10
Fungi - ps11
Fungi_1 - ps12
Fungi_2 - ps13
Fungi_3 - ps14
Dikarya - ps15
Ascomycota - ps16
Saccharo/Pezizomycotina - ps17
Saccharomycotina - ps18
Saccharomycotina_1 - ps19
Saccharomycotina_2 - ps20
Saccharomycotina_3 - ps21
Phaff./Saccharomycetaceae - ps22
Saccharomyco./mycetaceae - ps23
Saccharomycetaceae - ps24
Saccharomycetaceae_2 - ps25
Saccharomycetaceae_3 - ps26
Kaz./Nau./Saccharomyces - ps27
Saccharomyces - ps28
Arboricola_clade - ps29
Cerevisiae_clade - ps30
Saccharomyces_cerevisiae - ps31
p-value
4e-06
4e-05
4e-04
4e-03
4e-02
Log-odds
0.5
1.0
1.5
2.0
2.5
3.0
3.5
4.0

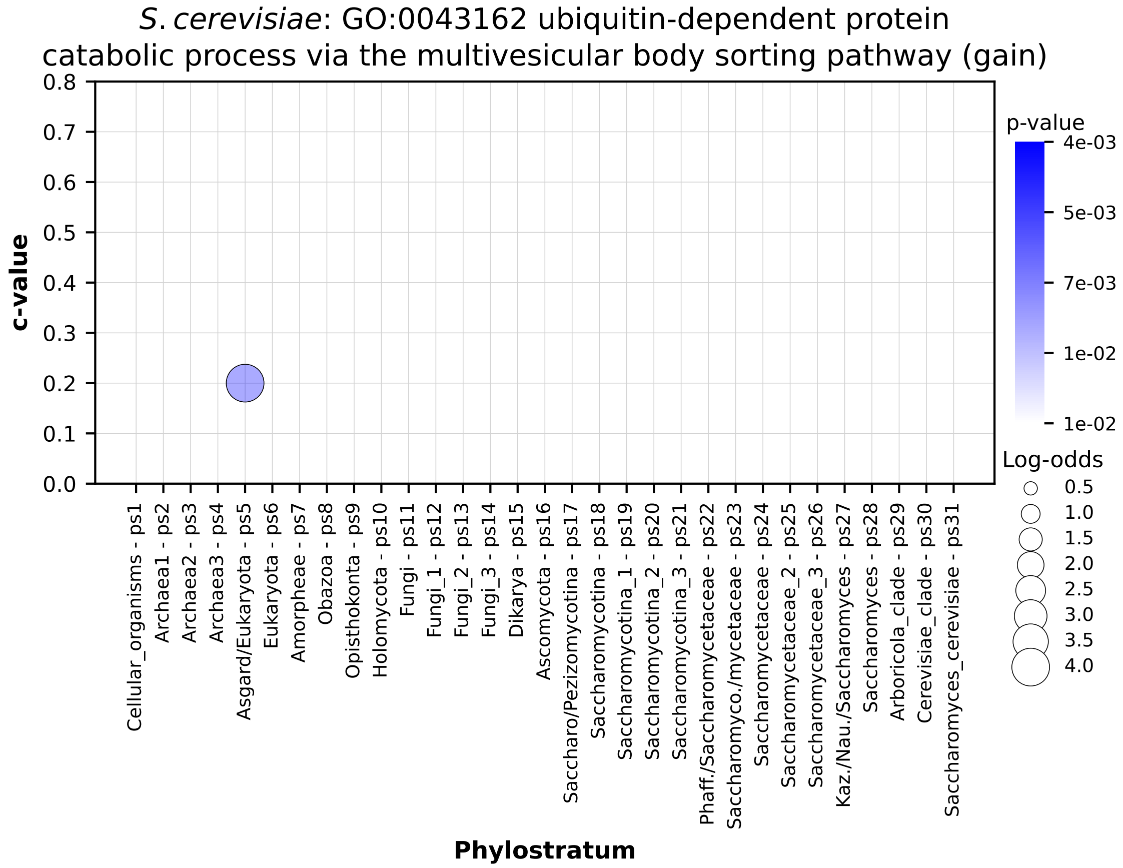
S. cerevisiae: GO:0043162 ubiquitin-dependent protein catabolic process via the multivesicular body sorting pathway (gain)
c-value
0.0
0.1
0.2
0.3
0.4
0.5
0.6
0.7
0.8
Phylostratum
Cellular_organisms - ps1
Archaea1 - ps2
Archaea2 - ps3
Archaea3 - ps4
Asgard/Eukaryota - ps5
Eukaryota - ps6
Amorpheae - ps7
Obazoa - ps8
Opisthokonta - ps9
Holomycota - ps10
Fungi - ps11
Fungi_1 - ps12
Fungi_2 - ps13
Fungi_3 - ps14
Dikarya - ps15
Ascomycota - ps16
Saccharo/Pezizomycotina - ps17
Saccharomycotina - ps18
Saccharomycotina_1 - ps19
Saccharomycotina_2 - ps20
Saccharomycotina_3 - ps21
Phaff./Saccharomycetaceae - ps22
Saccharomyco./mycetaceae - ps23
Saccharomycetaceae - ps24
Saccharomycetaceae_2 - ps25
Saccharomycetaceae_3 - ps26
Kaz./Nau./Saccharomyces - ps27
Saccharomyces - ps28
Arboricola_clade - ps29
Cerevisiae_clade - ps30
Saccharomyces_cerevisiae - ps31
p-value
4e-03
5e-03
7e-03
1e-02
1e-02
Log-odds
0.5
1.0
1.5
2.0
2.5
3.0
3.5
4.0

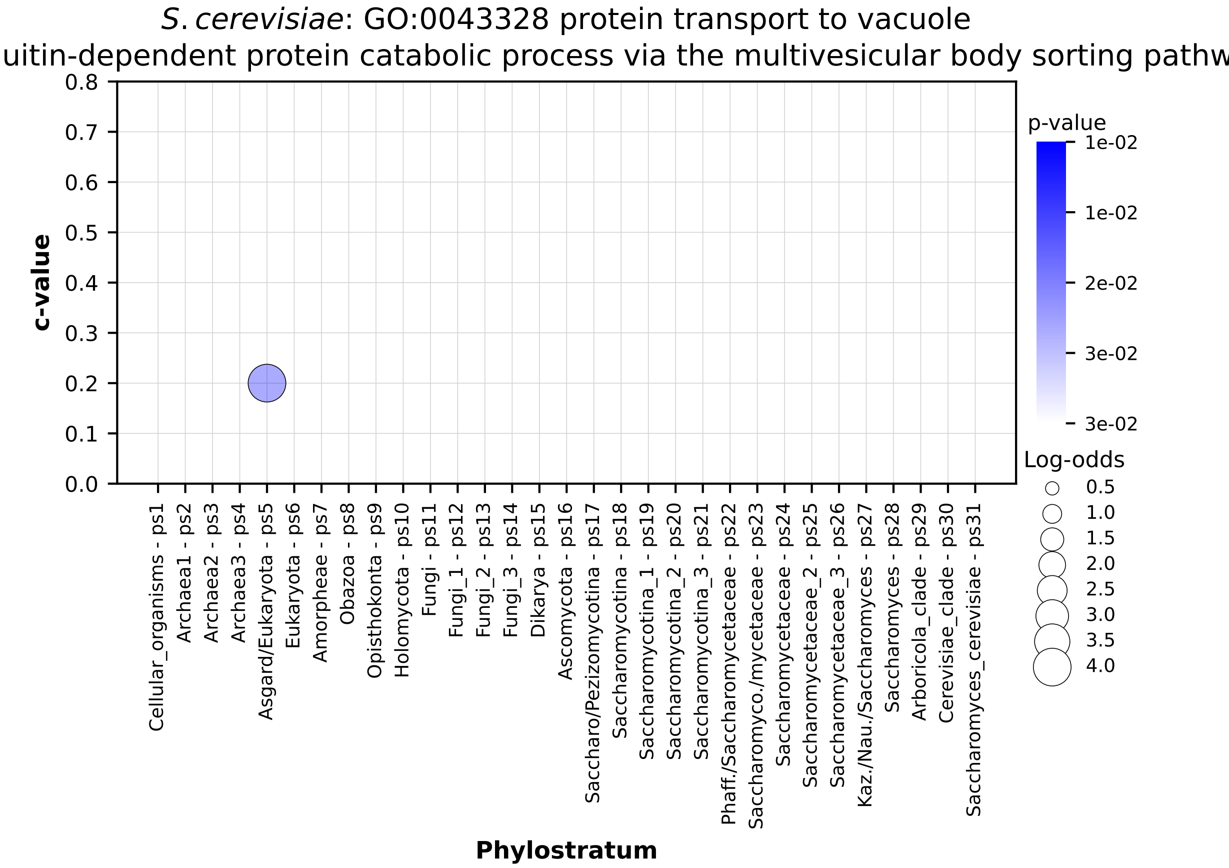

S. cerevisiae: GO:0043328 protein transport to vacuole
uitin-dependent protein catabolic process via the multivesicular body sorting pathw
c-value
0.8
0.7
0.6
0.5
0.4
0.3
0.2
0.1
0.0
p-value
1e-02
1e-02
2e-02
3e-02
3e-02
Log-odds
0.5
1.0
1.5
2.0
2.5
3.0
3.5
4.0
Cellular_organisms - ps1
Archaea1 - ps2
Archaea2 - ps3
Archaea3 - ps4
Asgard/Eukaryota - ps5
Eukaryota - ps6
Amorpheae - ps7
Obazoa - ps8
Opisthokonta - ps9
Holomycota - ps10
Fungi - ps11
Fungi_1 - ps12
Fungi_2 - ps13
Fungi_3 - ps14
Dikarya - ps15
Ascomycota - ps16
Saccharo/Pezizomycotina - ps17
Saccharomycotina - ps18
Saccharomycotina_1 - ps19
Saccharomycotina_2 - ps20
Saccharomycotina_3 - ps21
Phaff./Saccharomycetaceae - ps22
Saccharomyco./mycetaceae - ps23
Saccharomycetaceae - ps24
Saccharomycetaceae_2 - ps25
Saccharomycetaceae_3 - ps26
Kaz./Nau./Saccharomyces - ps27
Saccharomyces - ps28
Arboricola_clade - ps29
Cerevisiae_clade - ps30
Saccharomyces_cerevisiae - ps31
Phylostratum

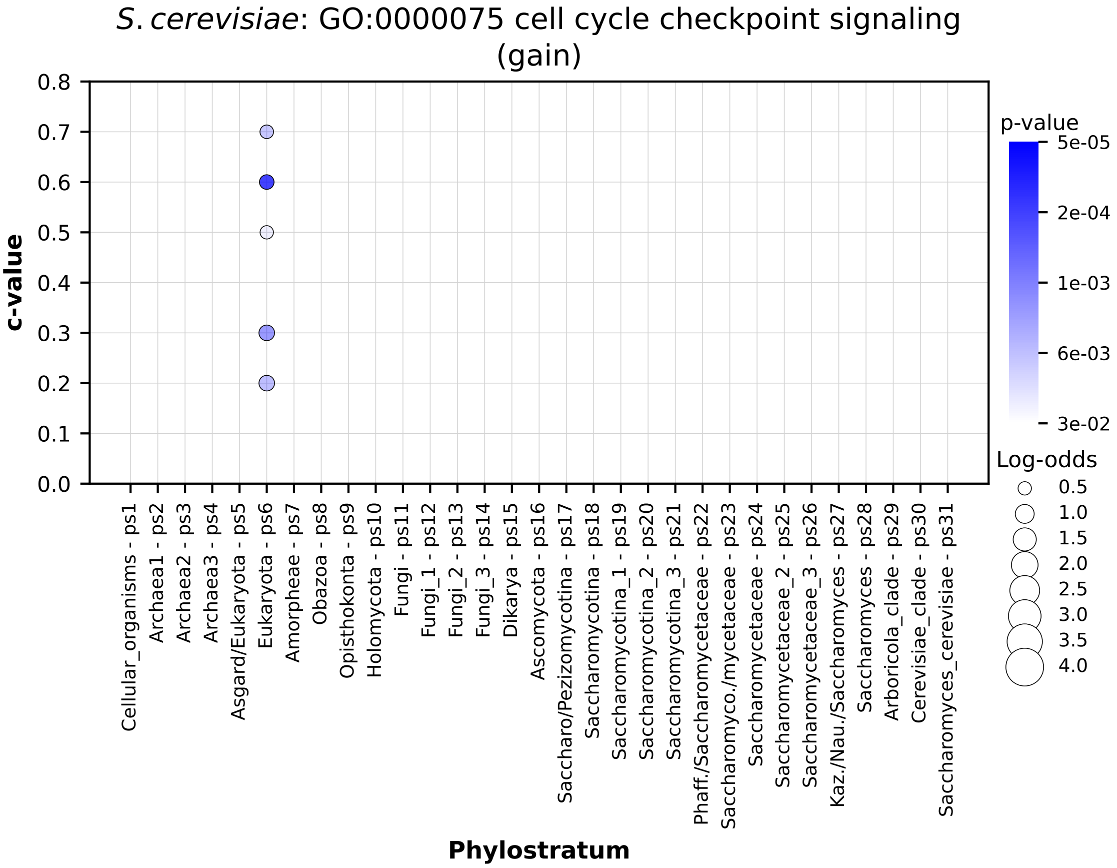

S. cerevisiae: GO:0000075 cell cycle checkpoint signaling (gain)
c-value
0.8
0.7
0.6
0.5
0.4
0.3
0.2
0.1
0.0
p-value
5e-05
2e-04
1e-03
6e-03
3e-02
Log-odds
0.5
1.0
1.5
2.0
2.5
3.0
3.5
4.0
Cellular_organisms - ps1
Archaea1 - ps2
Archaea2 - ps3
Archaea3 - ps4
Asgard/Eukaryota - ps5
Eukaryota - ps6
Amorpheae - ps7
Obazoa - ps8
Opisthokonta - ps9
Holomycota - ps10
Fungi - ps11
Fungi_1 - ps12
Fungi_2 - ps13
Fungi_3 - ps14
Dikarya - ps15
Ascomycota - ps16
Saccharo/Pezizomycotina - ps17
Saccharomycotina - ps18
Saccharomycotina_1 - ps19
Saccharomycotina_2 - ps20
Saccharomycotina_3 - ps21
Phaff./Saccharomycetaceae - ps22
Saccharomyco./mycetaceae - ps23
Saccharomycetaceae - ps24
Saccharomycetaceae_2 - ps25
Saccharomycetaceae_3 - ps26
Kaz./Nau./Saccharomyces - ps27
Saccharomyces - ps28
Arboricola_clade - ps29
Cerevisiae_clade - ps30
Saccharomyces_cerevisiae - ps31
Phylostratum

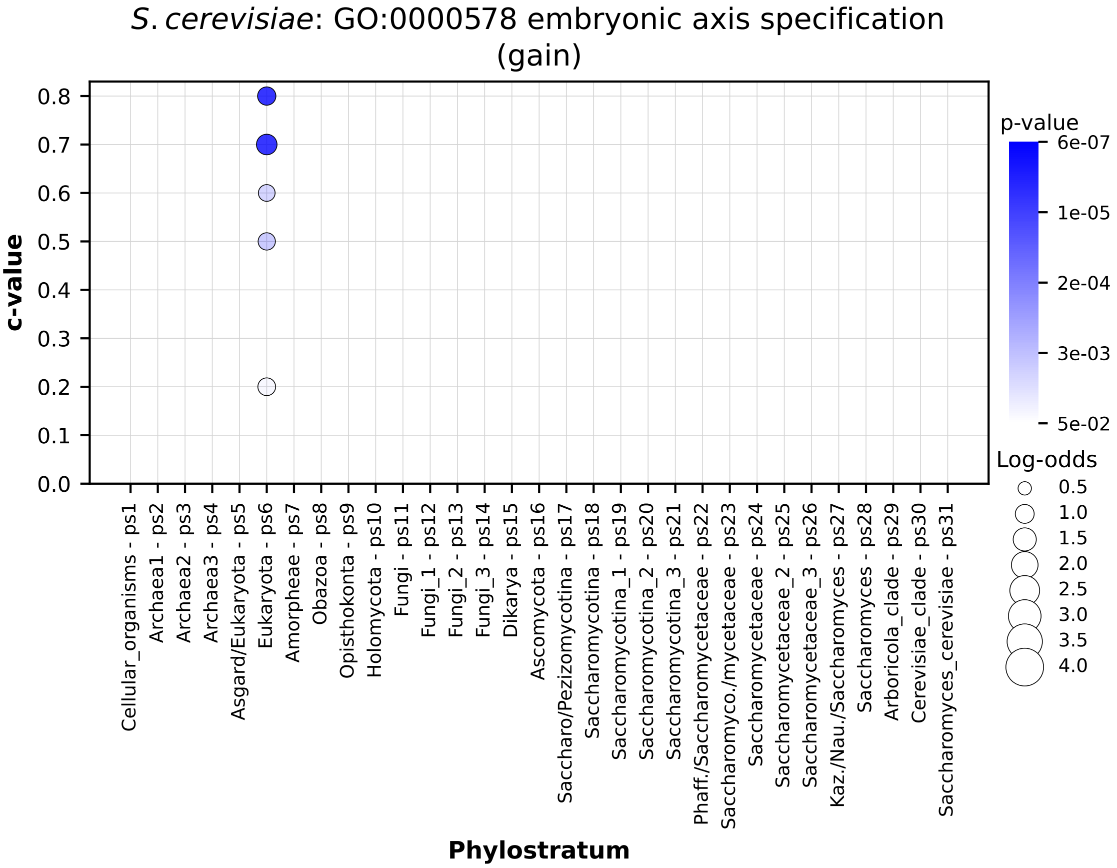

S. cerevisiae: GO:0000578 embryonic axis specification
(gain)
c-value
0.0
0.1
0.2
0.3
0.4
0.5
0.6
0.7
0.8
p-value
6e-07
1e-05
2e-04
3e-03
5e-02
Log-odds
0.5
1.0
1.5
2.0
2.5
3.0
3.5
4.0
Cellular_organisms - ps1
Archaea1 - ps2
Archaea2 - ps3
Archaea3 - ps4
Asgard/Eukaryota - ps5
Eukaryota - ps6
Amorpheae - ps7
Obazoa - ps8
Opisthokonta - ps9
Holomycota - ps10
Fungi - ps11
Fungi_1 - ps12
Fungi_2 - ps13
Fungi_3 - ps14
Dikarya - ps15
Ascomycota - ps16
Saccharo/Pezizomycotina - ps17
Saccharomycotina - ps18
Saccharomycotina_1 - ps19
Saccharomycotina_2 - ps20
Saccharomycotina_3 - ps21
Phaff./Saccharomycetaceae - ps22
Saccharomyco./mycetaceae - ps23
Saccharomycetaceae - ps24
Saccharomycetaceae_2 - ps25
Saccharomycetaceae_3 - ps26
Kaz./Nau./Saccharomyces - ps27
Saccharomyces - ps28
Arboricola_clade - ps29
Cerevisiae_clade - ps30
Saccharomyces_cerevisiae - ps31
Phylostratum

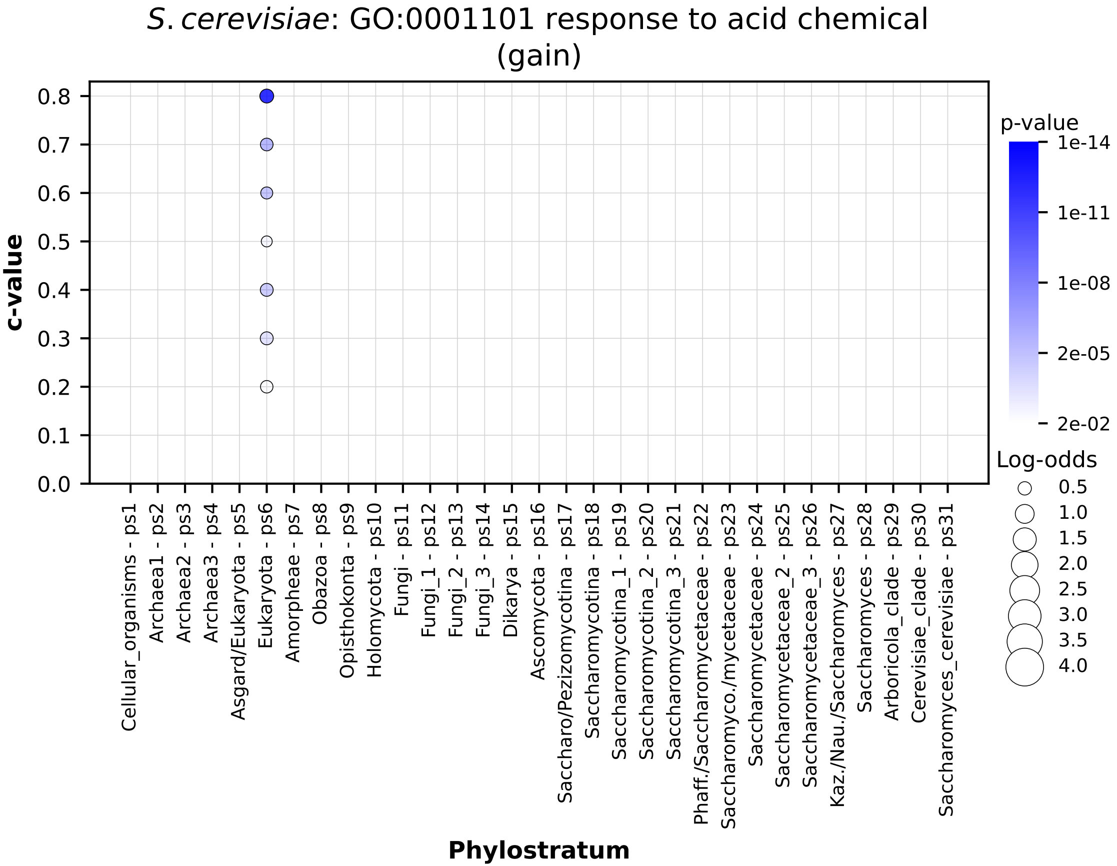

S. cerevisiae: GO:0001101 response to acid chemical (gain)
c-value
0.0
0.1
0.2
0.3
0.4
0.5
0.6
0.7
0.8
p-value
1e-14
1e-11
1e-08
2e-05
2e-02
Log-odds
0.5
1.0
1.5
2.0
2.5
3.0
3.5
4.0
Cellular_organisms - ps1
Archaea1 - ps2
Archaea2 - ps3
Archaea3 - ps4
Asgard/Eukaryota - ps5
Eukaryota - ps6
Amorpheae - ps7
Obazoa - ps8
Opisthokonta - ps9
Holomycota - ps10
Fungi - ps11
Fungi_1 - ps12
Fungi_2 - ps13
Fungi_3 - ps14
Dikarya - ps15
Ascomycota - ps16
Saccharo/Pezizomycotina - ps17
Saccharomycotina - ps18
Saccharomycotina_1 - ps19
Saccharomycotina_2 - ps20
Saccharomycotina_3 - ps21
Phaff./Saccharomycetaceae - ps22
Saccharomyco./mycetaceae - ps23
Saccharomycetaceae - ps24
Saccharomycetaceae_2 - ps25
Saccharomycetaceae_3 - ps26
Kaz./Nau./Saccharomyces - ps27
Saccharomyces - ps28
Arboricola_clade - ps29
Cerevisiae_clade - ps30
Saccharomyces_cerevisiae - ps31
Phylostratum

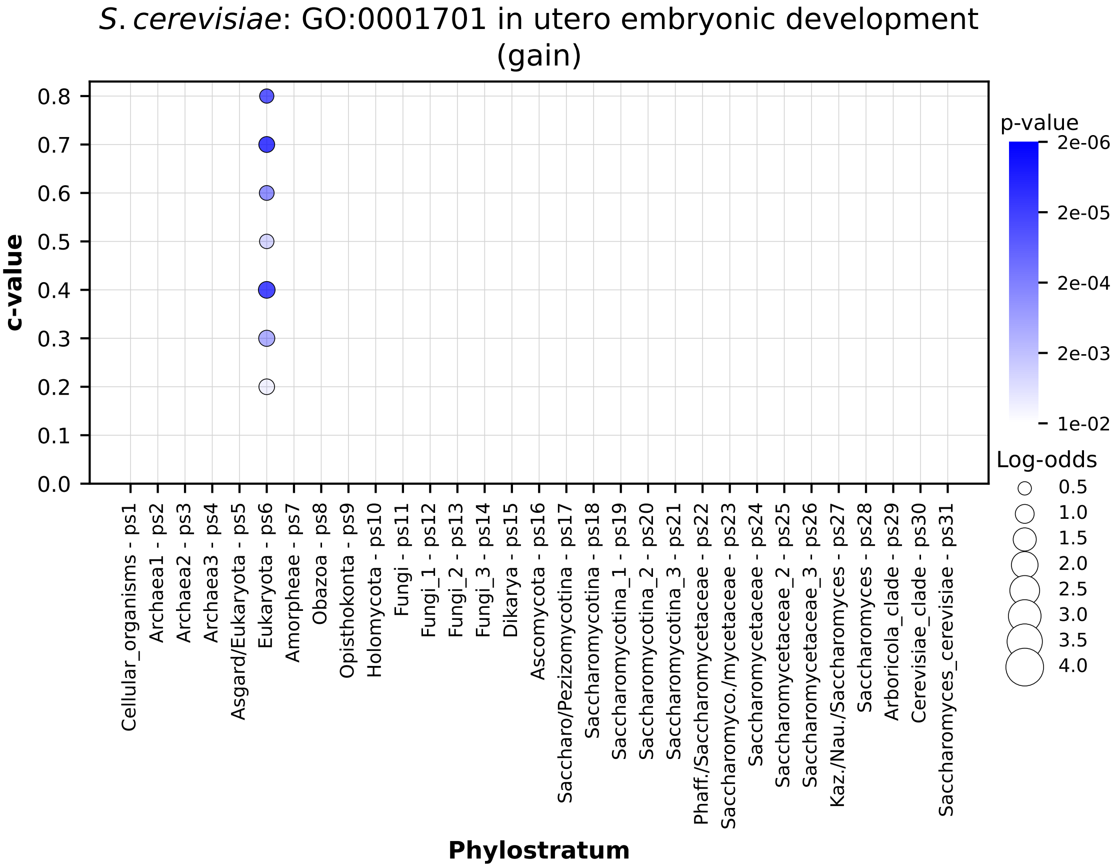

S. cerevisiae: GO:0001701 in utero embryonic development
(gain)
c-value
0.0
0.1
0.2
0.3
0.4
0.5
0.6
0.7
0.8
p-value
2e-06
2e-05
2e-04
2e-03
1e-02
Log-odds
0.5
1.0
1.5
2.0
2.5
3.0
3.5
4.0
Cellular_organisms - ps1
Archaea1 - ps2
Archaea2 - ps3
Archaea3 - ps4
Asgard/Eukaryota - ps5
Eukaryota - ps6
Amorpheae - ps7
Obazoa - ps8
Opisthokonta - ps9
Holomycota - ps10
Fungi - ps11
Fungi_1 - ps12
Fungi_2 - ps13
Fungi_3 - ps14
Dikarya - ps15
Ascomycota - ps16
Saccharo/Pezizomycotina - ps17
Saccharomycotina - ps18
Saccharomycotina_1 - ps19
Saccharomycotina_2 - ps20
Saccharomycotina_3 - ps21
Phaff./Saccharomycetaceae - ps22
Saccharomyco./mycetaceae - ps23
Saccharomycetaceae - ps24
Saccharomycetaceae_2 - ps25
Saccharomycetaceae_3 - ps26
Kaz./Nau./Saccharomyces - ps27
Saccharomyces - ps28
Arboricola_clade - ps29
Cerevisiae_clade - ps30
Saccharomyces_cerevisiae - ps31
Phylostratum

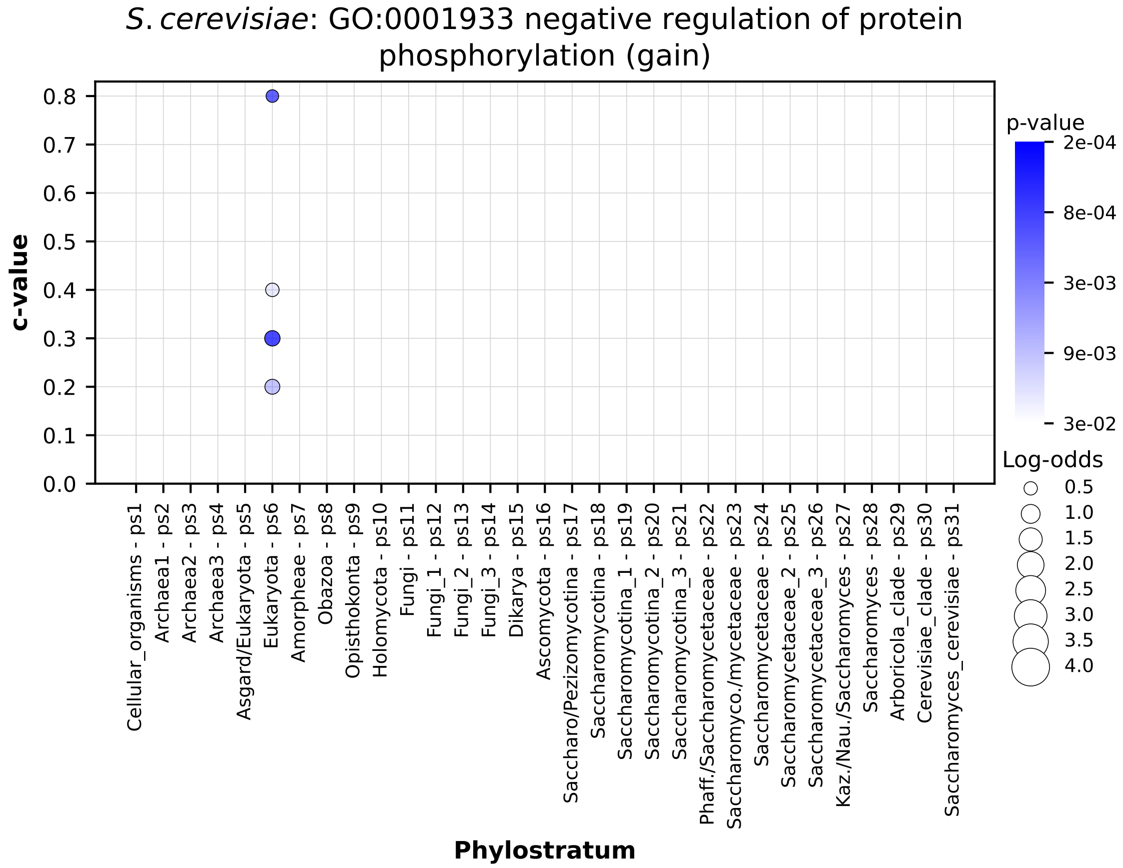

S. cerevisiae: GO:0001933 negative regulation of protein phosphorylation (gain)
c-value
0.0
0.1
0.2
0.3
0.4
0.5
0.6
0.7
0.8
Phylostratum
Cellular_organisms - ps1
Archaea1 - ps2
Archaea2 - ps3
Archaea3 - ps4
Asgard/Eukaryota - ps5
Eukaryota - ps6
Amorpheae - ps7
Obazoa - ps8
Opisthokonta - ps9
Holomycota - ps10
Fungi - ps11
Fungi_1 - ps12
Fungi_2 - ps13
Fungi_3 - ps14
Dikarya - ps15
Ascomycota - ps16
Saccharo/Pezizomycotina - ps17
Saccharomycotina - ps18
Saccharomycotina_1 - ps19
Saccharomycotina_2 - ps20
Saccharomycotina_3 - ps21
Phaff./Saccharomycetaceae - ps22
Saccharomyco./mycetaceae - ps23
Saccharomycetaceae - ps24
Saccharomycetaceae_2 - ps25
Saccharomycetaceae_3 - ps26
Kaz./Nau./Saccharomyces - ps27
Saccharomyces - ps28
Arboricola_clade - ps29
Cerevisiae_clade - ps30
Saccharomyces_cerevisiae - ps31
p-value
2e-04
8e-04
3e-03
9e-03
3e-02
Log-odds
0.5
1.0
1.5
2.0
2.5
3.0
3.5
4.0

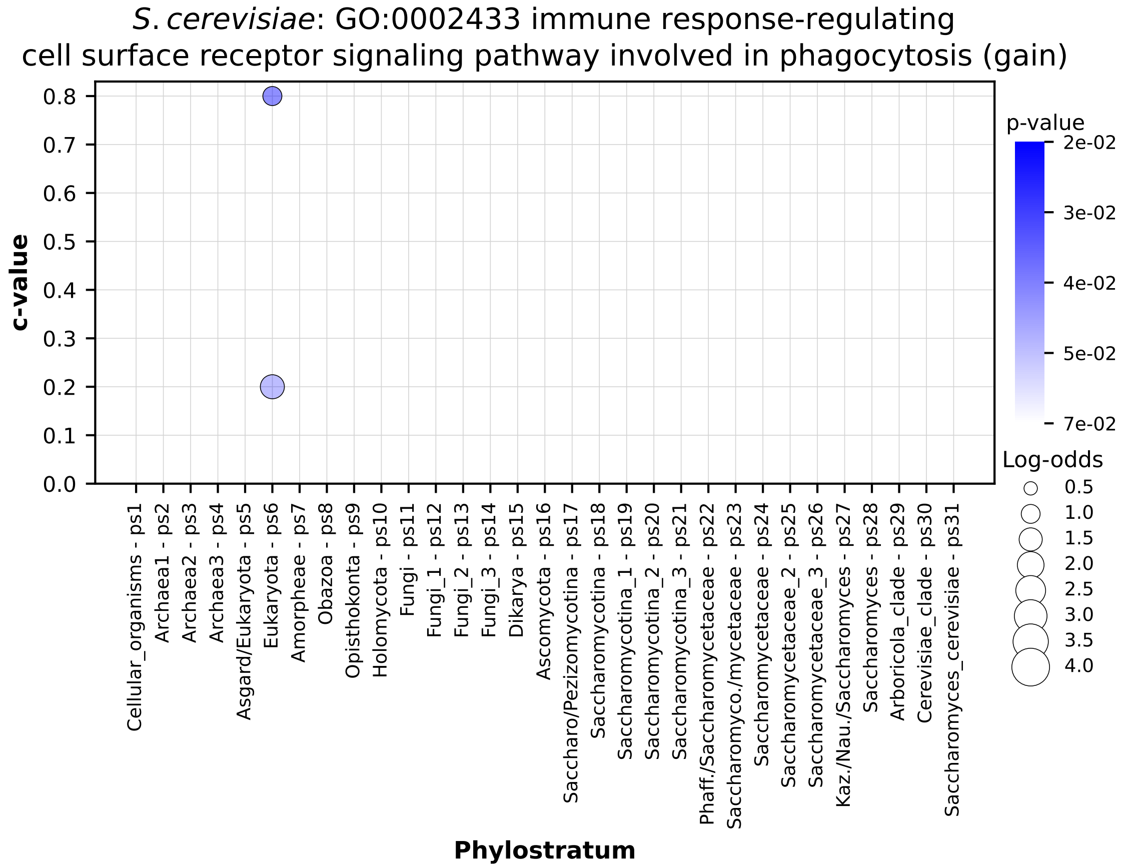

S. cerevisiae: GO:0002433 immune response-regulating
cell surface receptor signaling pathway involved in phagocytosis (gain)
c-value
0.0
0.1
0.2
0.3
0.4
0.5
0.6
0.7
0.8
p-value
2e-02
3e-02
4e-02
5e-02
7e-02
Log-odds
0.5
1.0
1.5
2.0
2.5
3.0
3.5
4.0
Cellular_organisms - ps1
Archaea1 - ps2
Archaea2 - ps3
Archaea3 - ps4
Asgard/Eukaryota - ps5
Eukaryota - ps6
Amorpheae - ps7
Obazoa - ps8
Opisthokonta - ps9
Holomycota - ps10
Fungi - ps11
Fungi_1 - ps12
Fungi_2 - ps13
Fungi_3 - ps14
Dikarya - ps15
Ascomycota - ps16
Saccharo/Pezizomycotina - ps17
Saccharomycotina - ps18
Saccharomycotina_1 - ps19
Saccharomycotina_2 - ps20
Saccharomycotina_3 - ps21
Phaff./Saccharomycetaceae - ps22
Saccharomyco./mycetaceae - ps23
Saccharomycetaceae - ps24
Saccharomycetaceae_2 - ps25
Saccharomycetaceae_3 - ps26
Kaz./Nau./Saccharomyces - ps27
Saccharomyces - ps28
Arboricola_clade - ps29
Cerevisiae_clade - ps30
Saccharomyces_cerevisiae - ps31
Phylostratum

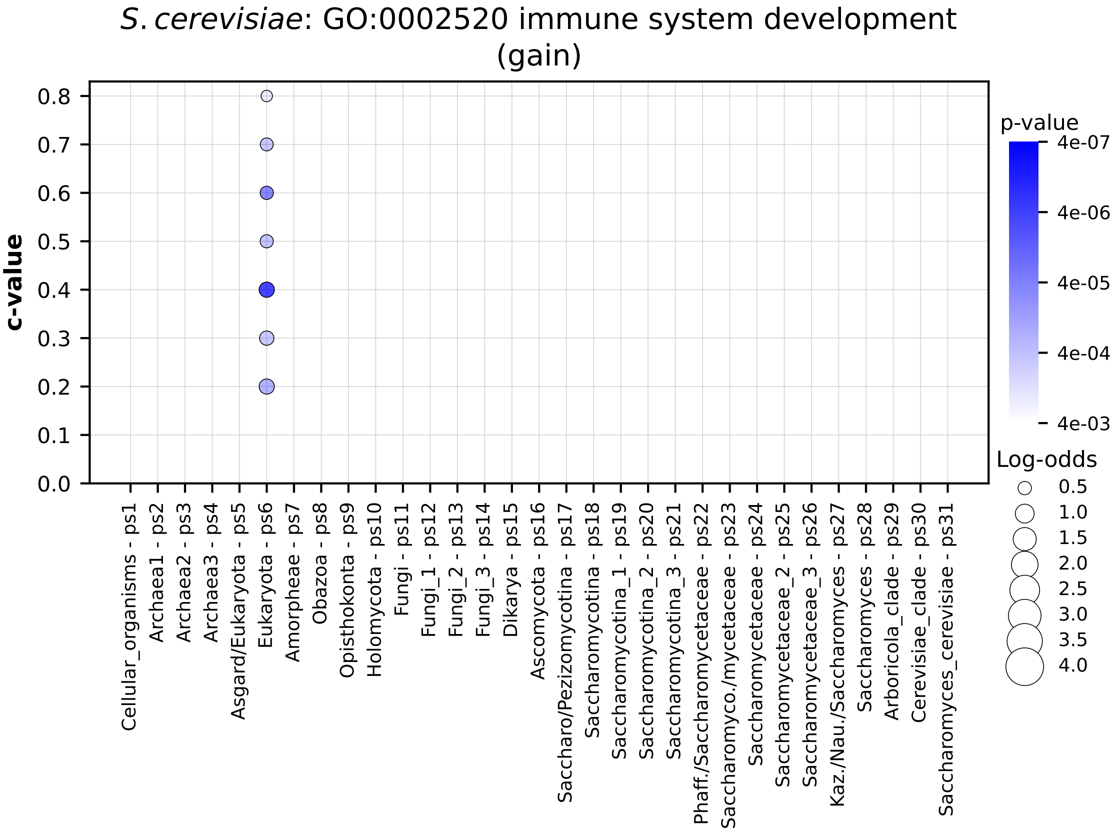

S. cerevisiae: GO:0002520 immune system development
(gain)
c-value
0.8
0.7
0.6
0.5
0.4
0.3
0.2
0.1
0.0
p-value
4e-07
4e-06
4e-05
4e-04
4e-03
Log-odds
0.5
1.0
1.5
2.0
2.5
3.0
3.5
4.0
Cellular_organisms - ps1
Archaea1 - ps2
Archaea2 - ps3
Archaea3 - ps4
Asgard/Eukaryota - ps5
Eukaryota - ps6
Amorpheae - ps7
Obazoa - ps8
Opisthokonta - ps9
Holomycota - ps10
Fungi - ps11
Fungi_1 - ps12
Fungi_2 - ps13
Fungi_3 - ps14
Dikarya - ps15
Ascomycota - ps16
Saccharo/Pezizomycotina - ps17
Saccharomycotina - ps18
Saccharomycotina_1 - ps19
Saccharomycotina_2 - ps20
Saccharomycotina_3 - ps21
Phaff./Saccharomycetaceae - ps22
Saccharomyco./mycetaceae - ps23
Saccharomycetaceae - ps24
Saccharomycetaceae_2 - ps25
Saccharomycetaceae_3 - ps26
Kaz./Nau./Saccharomyces - ps27
Saccharomyces - ps28
Arboricola_clade - ps29
Cerevisiae_clade - ps30
Saccharomyces_cerevisiae - ps31
Phylostratum

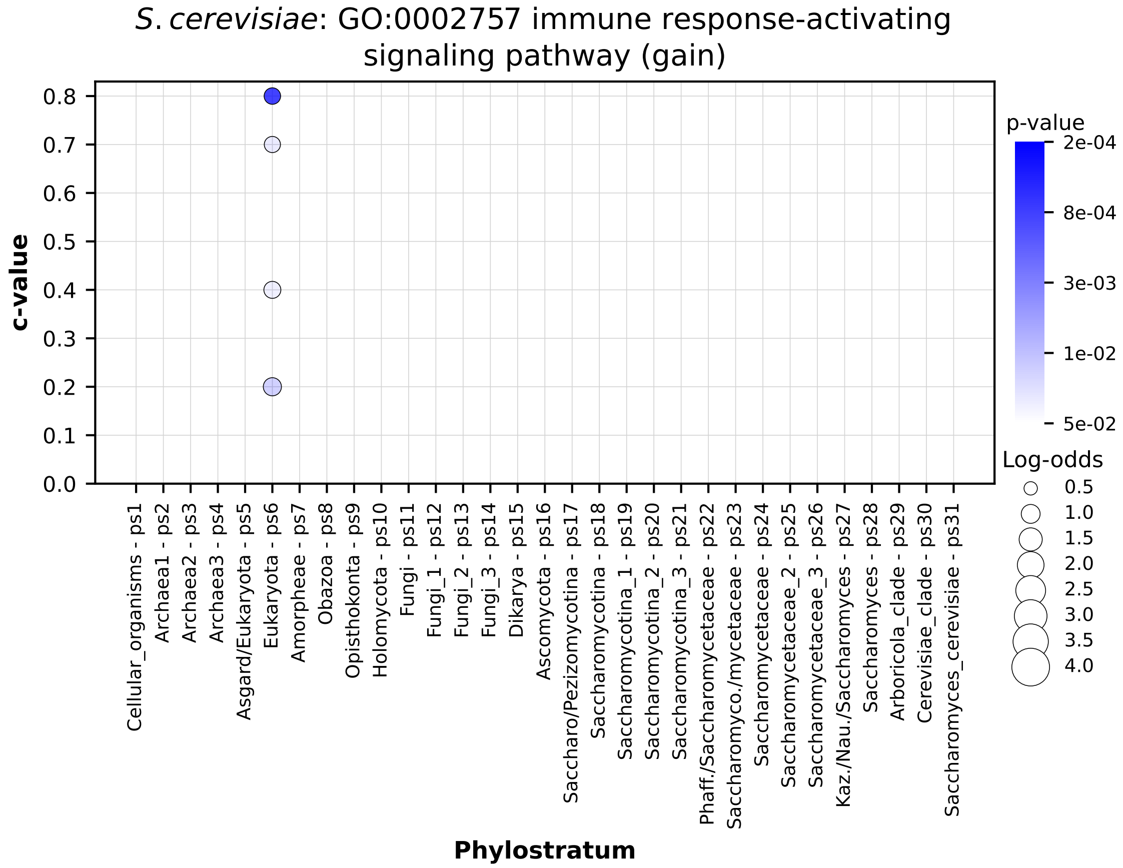
S. cerevisiae: GO:0002757 immune response-activating signaling pathway (gain)
c-value
0.0
0.1
0.2
0.3
0.4
0.5
0.6
0.7
0.8
p-value
2e-04
8e-04
3e-03
1e-02
5e-02
Log-odds
0.5
1.0
1.5
2.0
2.5
3.0
3.5
4.0
Cellular_organisms - ps1
Archaea1 - ps2
Archaea2 - ps3
Archaea3 - ps4
Asgard/Eukaryota - ps5
Eukaryota - ps6
Amorpheae - ps7
Obazoa - ps8
Opisthokonta - ps9
Holomycota - ps10
Fungi - ps11
Fungi_1 - ps12
Fungi_2 - ps13
Fungi_3 - ps14
Dikarya - ps15
Ascomycota - ps16
Saccharo/Pezizomycotina - ps17
Saccharomycotina - ps18
Saccharomycotina_1 - ps19
Saccharomycotina_2 - ps20
Saccharomycotina_3 - ps21
Phaff./Saccharomycetaceae - ps22
Saccharomyco./mycetaceae - ps23
Saccharomycetaceae - ps24
Saccharomycetaceae_2 - ps25
Saccharomycetaceae_3 - ps26
Kaz./Nau./Saccharomyces - ps27
Saccharomyces - ps28
Arboricola_clade - ps29
Cerevisiae_clade - ps30
Saccharomyces_cerevisiae - ps31
Phylostratum

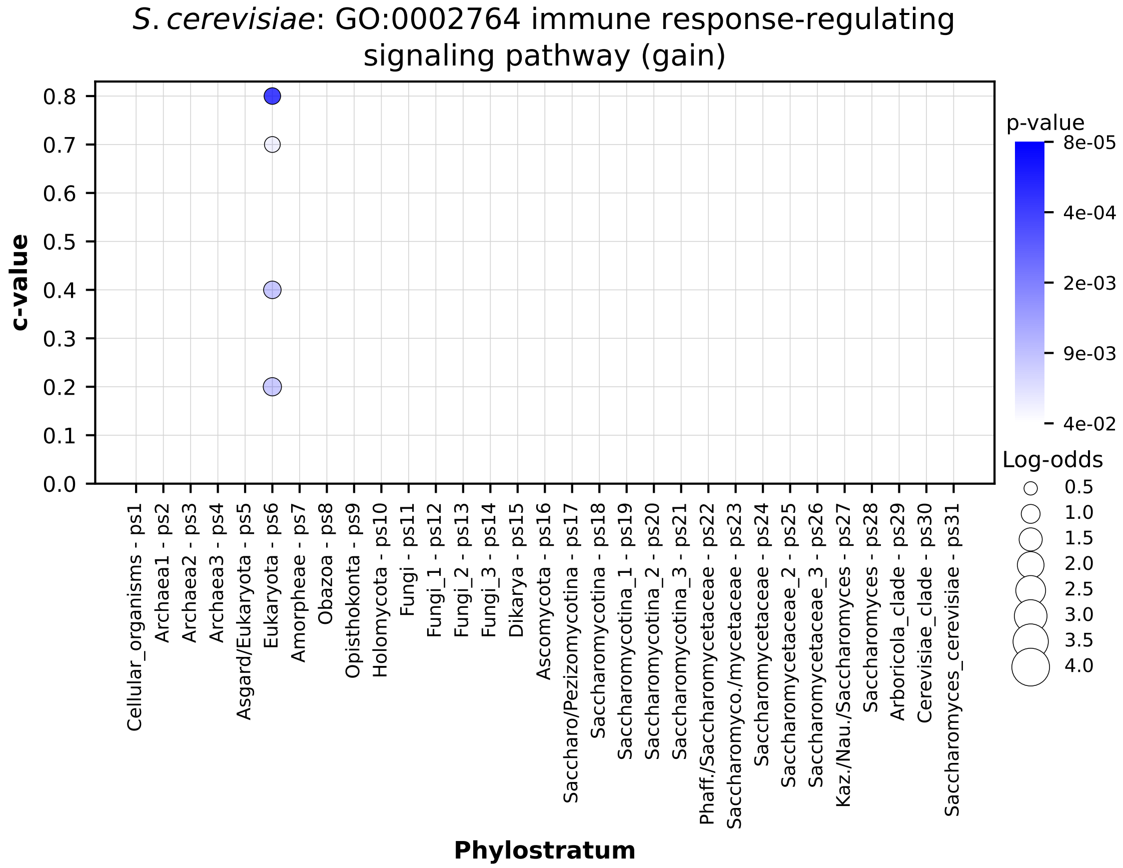
S. cerevisiae: GO:0002764 immune response-regulating signaling pathway (gain)
c-value
0.0
0.1
0.2
0.3
0.4
0.5
0.6
0.7
0.8
p-value
8e-05
4e-04
2e-03
9e-03
4e-02
Log-odds
0.5
1.0
1.5
2.0
2.5
3.0
3.5
4.0
Cellular_organisms - ps1
Archaea1 - ps2
Archaea2 - ps3
Archaea3 - ps4
Asgard/Eukaryota - ps5
Eukaryota - ps6
Amorpheae - ps7
Obazoa - ps8
Opisthokonta - ps9
Holomycota - ps10
Fungi - ps11
Fungi_1 - ps12
Fungi_2 - ps13
Fungi_3 - ps14
Dikarya - ps15
Ascomycota - ps16
Saccharo/Pezizomycotina - ps17
Saccharomycotina - ps18
Saccharomycotina_1 - ps19
Saccharomycotina_2 - ps20
Saccharomycotina_3 - ps21
Phaff./Saccharomycetaceae - ps22
Saccharomyco./mycetaceae - ps23
Saccharomycetaceae - ps24
Saccharomycetaceae_2 - ps25
Saccharomycetaceae_3 - ps26
Kaz./Nau./Saccharomyces - ps27
Saccharomyces - ps28
Arboricola_clade - ps29
Cerevisiae_clade - ps30
Saccharomyces_cerevisiae - ps31
Phylostratum

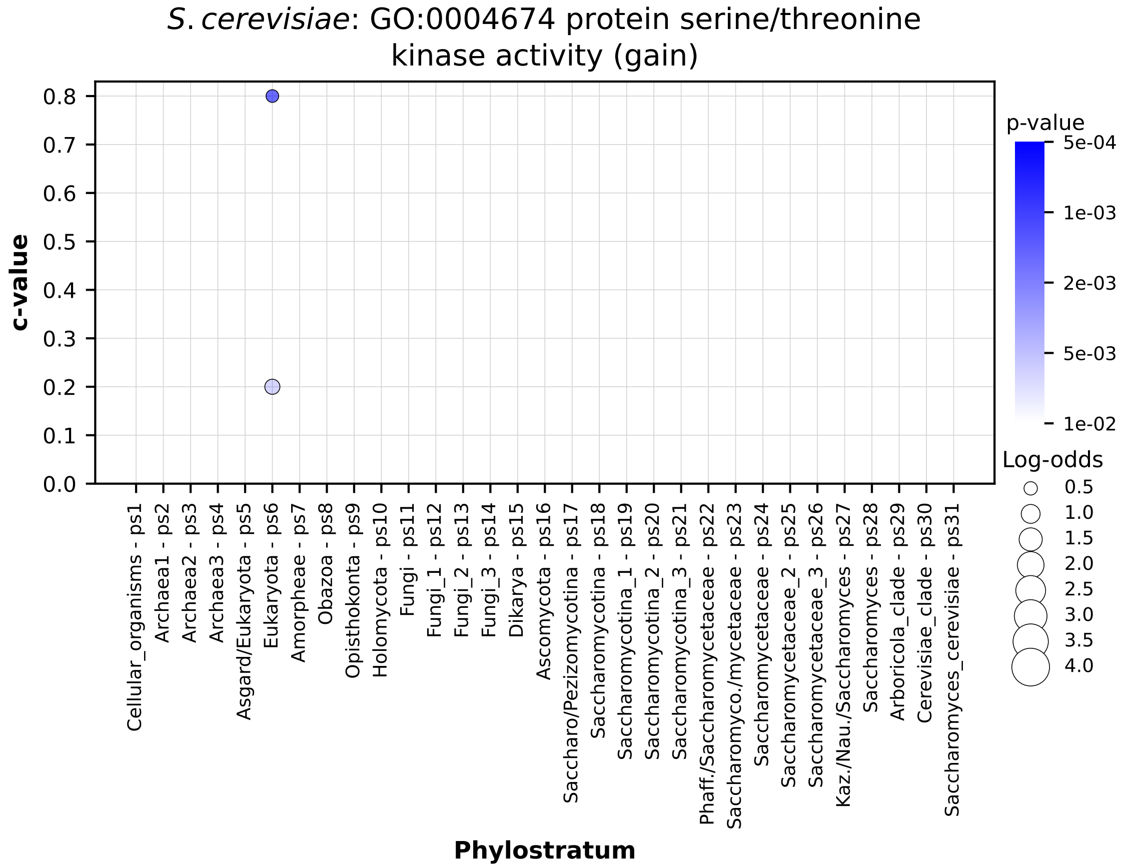
S. cerevisiae: GO:0004674 protein serine/threonine kinase activity (gain)
c-value
0.0
0.1
0.2
0.3
0.4
0.5
0.6
0.7
0.8
Phylostratum
Cellular_organisms - ps1
Archaea1 - ps2
Archaea2 - ps3
Archaea3 - ps4
Asgard/Eukaryota - ps5
Eukaryota - ps6
Amorpheae - ps7
Obazoa - ps8
Opisthokonta - ps9
Holomycota - ps10
Fungi - ps11
Fungi_1 - ps12
Fungi_2 - ps13
Fungi_3 - ps14
Dikarya - ps15
Ascomycota - ps16
Saccharo/Pezizomycotina - ps17
Saccharomycotina - ps18
Saccharomycotina_1 - ps19
Saccharomycotina_2 - ps20
Saccharomycotina_3 - ps21
Phaff./Saccharomycetaceae - ps22
Saccharomyco./mycetaceae - ps23
Saccharomycetaceae - ps24
Saccharomycetaceae_2 - ps25
Saccharomycetaceae_3 - ps26
Kaz./Nau./Saccharomyces - ps27
Saccharomyces - ps28
Arboricola_clade - ps29
Cerevisiae_clade - ps30
Saccharomyces_cerevisiae - ps31
p-value
5e-04
1e-03
2e-03
5e-03
1e-02
Log-odds
0.5
1.0
1.5
2.0
2.5
3.0
3.5
4.0

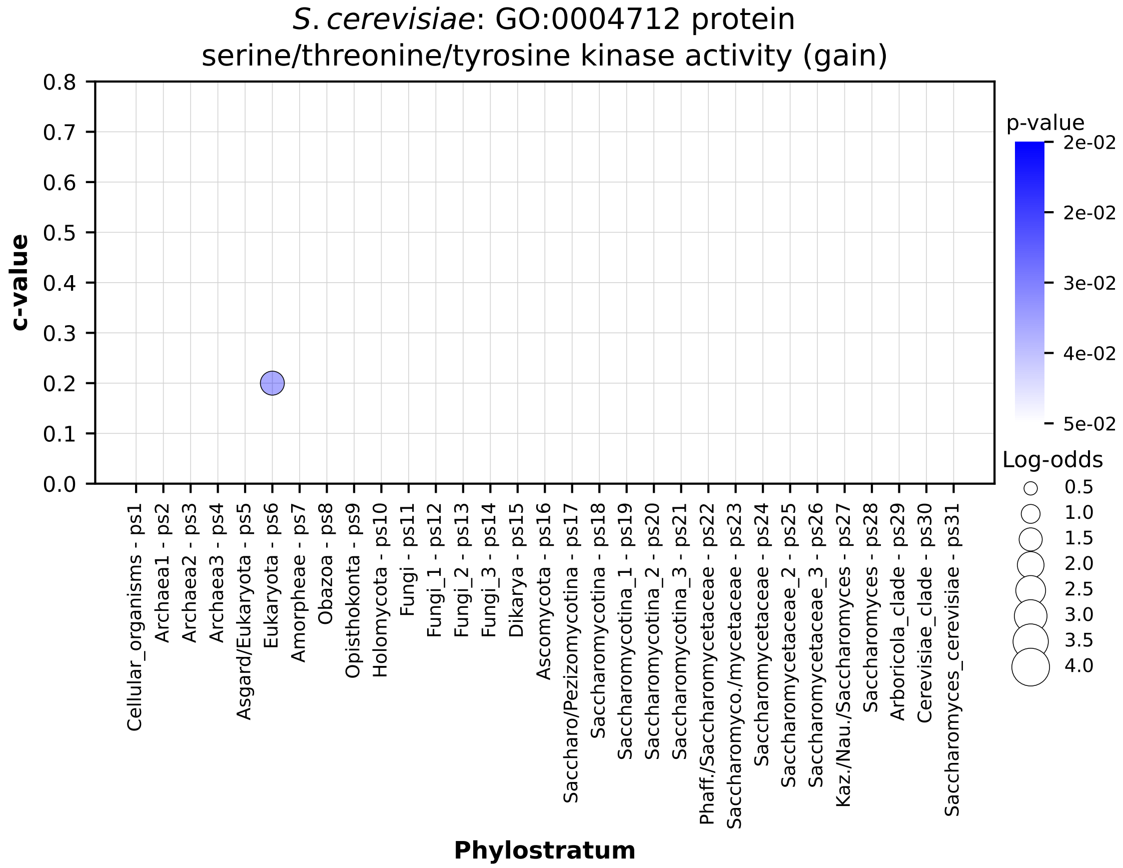
S. cerevisiae: GO:0004712 protein serine/threonine/tyrosine kinase activity (gain)
c-value
0.8
0.7
0.6
0.5
0.4
0.3
0.2
0.1
0.0
p-value
2e-02
2e-02
3e-02
4e-02
5e-02
Log-odds
0.5
1.0
1.5
2.0
2.5
3.0
3.5
4.0
Cellular_organisms - ps1
Archaea1 - ps2
Archaea2 - ps3
Archaea3 - ps4
Asgard/Eukaryota - ps5
Eukaryota - ps6
Amorpheae - ps7
Obazoa - ps8
Opisthokonta - ps9
Holomycota - ps10
Fungi - ps11
Fungi_1 - ps12
Fungi_2 - ps13
Fungi_3 - ps14
Dikarya - ps15
Ascomycota - ps16
Saccharo/Pezizomycotina - ps17
Saccharomycotina - ps18
Saccharomycotina_1 - ps19
Saccharomycotina_2 - ps20
Saccharomycotina_3 - ps21
Phaff./Saccharomycetaceae - ps22
Saccharomyco./mycetaceae - ps23
Saccharomycetaceae - ps24
Saccharomycetaceae_2 - ps25
Saccharomycetaceae_3 - ps26
Kaz./Nau./Saccharomyces - ps27
Saccharomyces - ps28
Arboricola_clade - ps29
Cerevisiae_clade - ps30
Saccharomyces_cerevisiae - ps31
Phylostratum

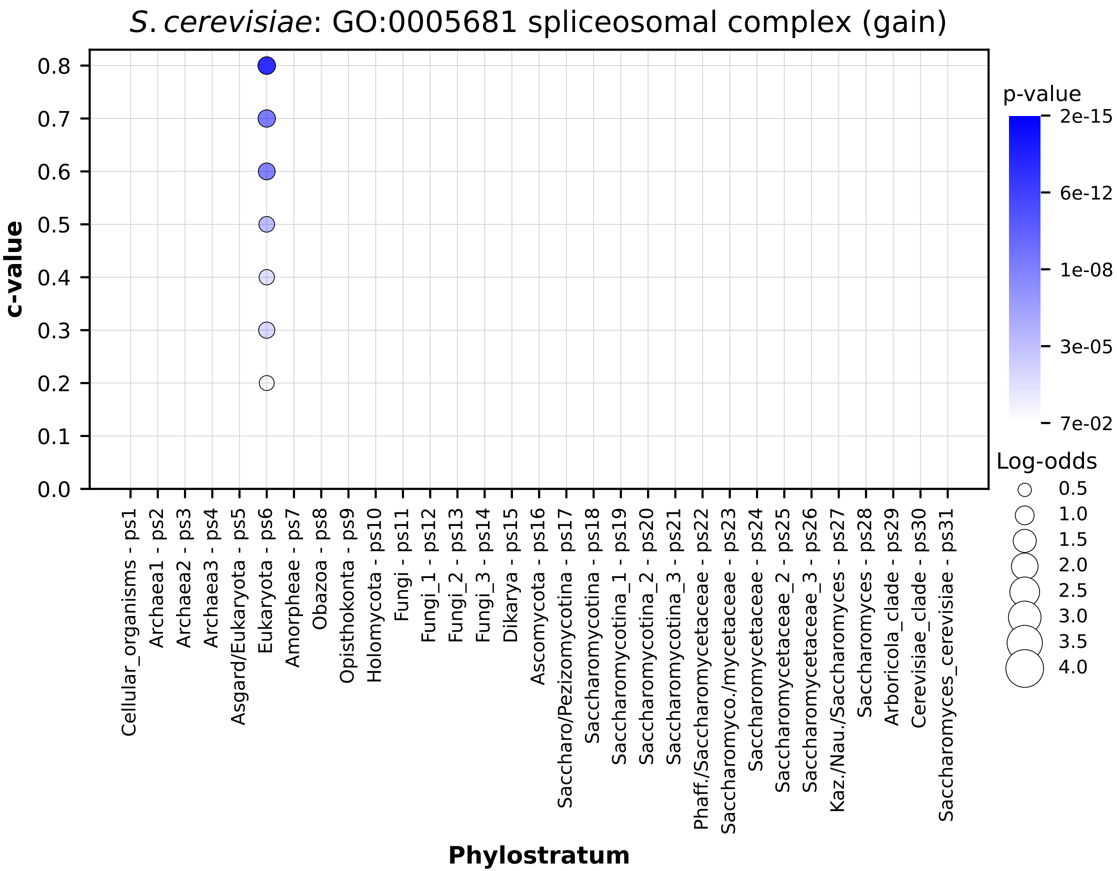

S. cerevisiae: GO:0005681 spliceosomal complex (gain)
c-value
0.0
0.1
0.2
0.3
0.4
0.5
0.6
0.7
0.8
p-value
2e-15
6e-12
1e-08
3e-05
7e-02
Log-odds
0.5
1.0
1.5
2.0
2.5
3.0
3.5
4.0
Cellular_organisms - ps1
Archaea1 - ps2
Archaea2 - ps3
Archaea3 - ps4
Asgard/Eukaryota - ps5
Eukaryota - ps6
Amorpheae - ps7
Obazoa - ps8
Opisthokonta - ps9
Holomycota - ps10
Fungi - ps11
Fungi_1 - ps12
Fungi_2 - ps13
Fungi_3 - ps14
Dikarya - ps15
Ascomycota - ps16
Saccharo/Pezizomycotina - ps17
Saccharomycotina - ps18
Saccharomycotina_1 - ps19
Saccharomycotina_2 - ps20
Saccharomycotina_3 - ps21
Phaff./Saccharomycetaceae - ps22
Saccharomyco./mycetaceae - ps23
Saccharomycetaceae - ps24
Saccharomycetaceae_2 - ps25
Saccharomycetaceae_3 - ps26
Kaz./Nau./Saccharomyces - ps27
Saccharomyces - ps28
Arboricola_clade - ps29
Cerevisiae_clade - ps30
Saccharomyces_cerevisiae - ps31
Phylostratum

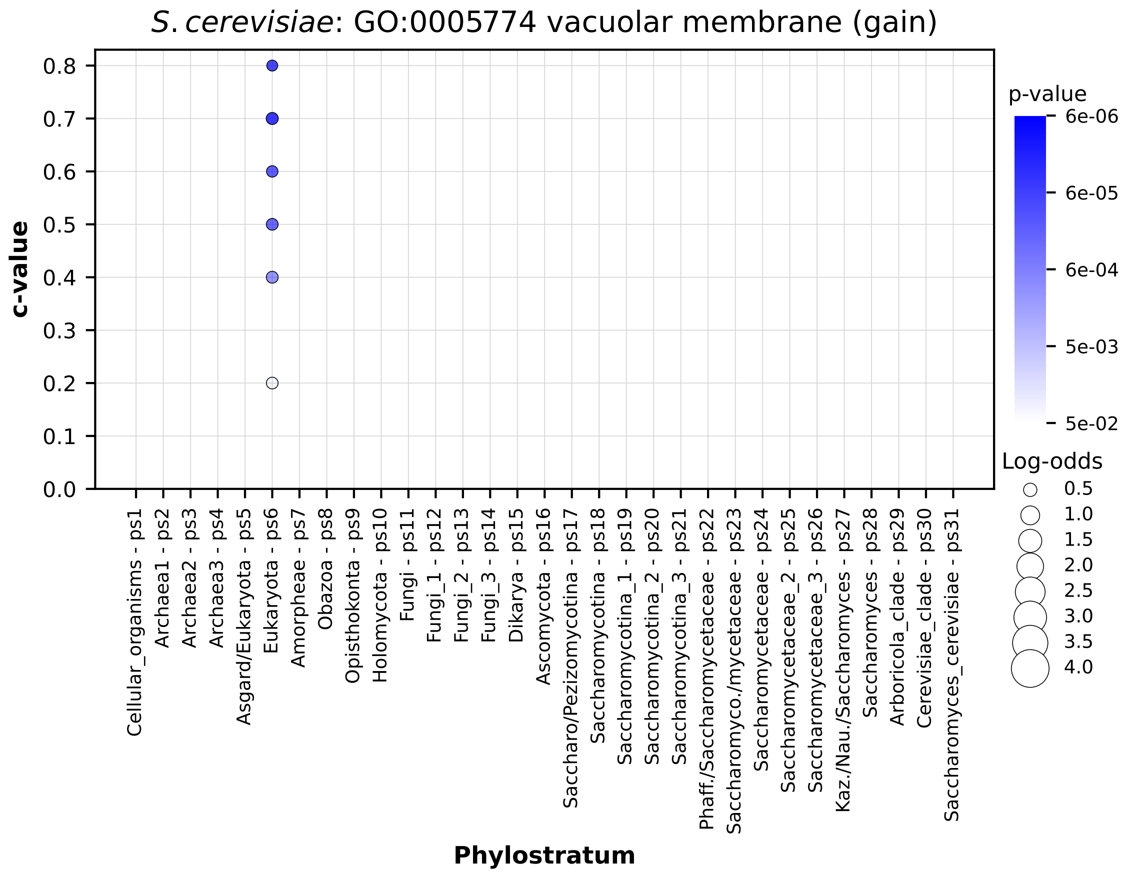

S. cerevisiae: GO:0005774 vacuolar membrane (gain)
c-value
0.0
0.1
0.2
0.3
0.4
0.5
0.6
0.7
0.8
p-value
6e-06
6e-05
6e-04
5e-03
5e-02
Log-odds
0.5
1.0
1.5
2.0
2.5
3.0
3.5
4.0
Cellular_organisms - ps1
Archaea1 - ps2
Archaea2 - ps3
Archaea3 - ps4
Asgard/Eukaryota - ps5
Eukaryota - ps6
Amorpheae - ps7
Obazoa - ps8
Opisthokonta - ps9
Holomycota - ps10
Fungi - ps11
Fungi_1 - ps12
Fungi_2 - ps13
Fungi_3 - ps14
Dikarya - ps15
Ascomycota - ps16
Saccharo/Pezizomycotina - ps17
Saccharomycotina - ps18
Saccharomycotina_1 - ps19
Saccharomycotina_2 - ps20
Saccharomycotina_3 - ps21
Phaff./Saccharomycetaceae - ps22
Saccharomyco./mycetaceae - ps23
Saccharomycetaceae - ps24
Saccharomycetaceae_2 - ps25
Saccharomycetaceae_3 - ps26
Kaz./Nau./Saccharomyces - ps27
Saccharomyces - ps28
Arboricola_clade - ps29
Cerevisiae_clade - ps30
Saccharomyces_cerevisiae - ps31
Phylostratum

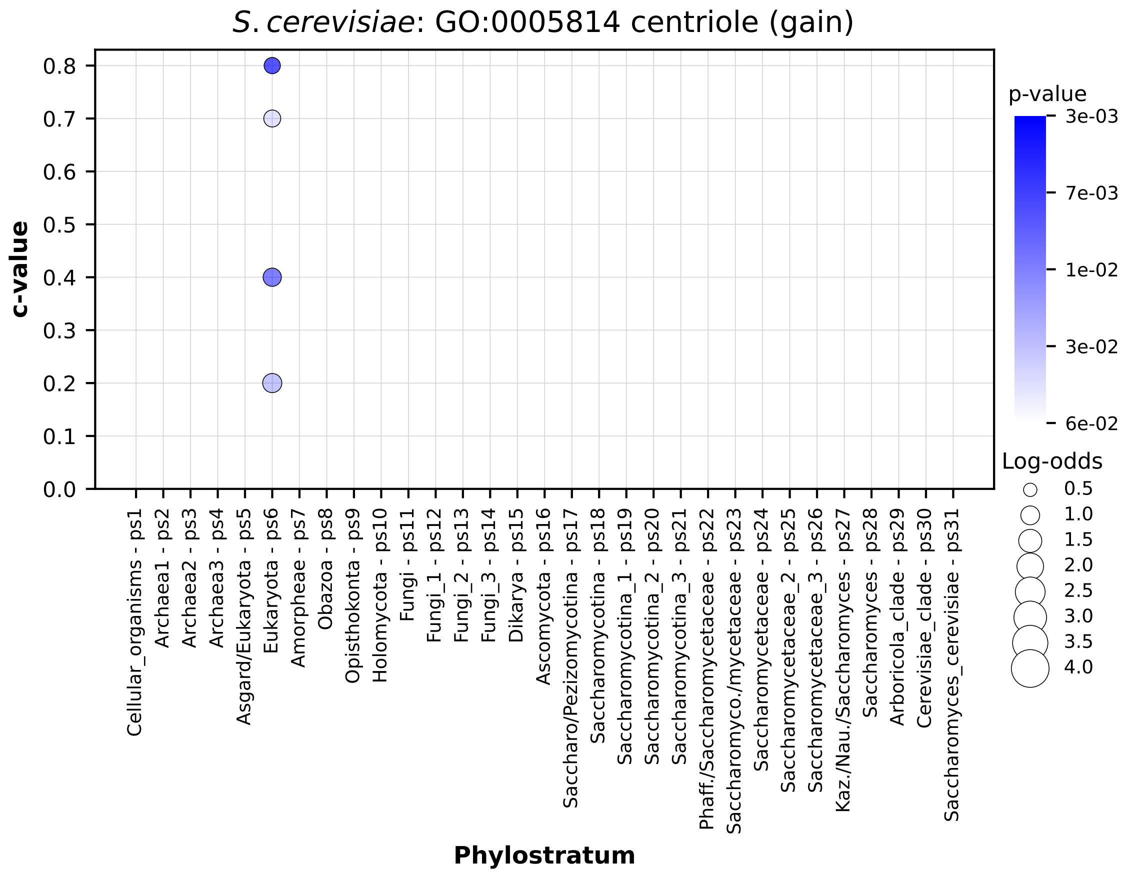

S. cerevisiae: GO:0005814 centriole (gain)
c-value
0.0
0.1
0.2
0.3
0.4
0.5
0.6
0.7
0.8
p-value
3e-03
7e-03
1e-02
3e-02
6e-02
Log-odds
0.5
1.0
1.5
2.0
2.5
3.0
3.5
4.0
Cellular_organisms - ps1
Archaea1 - ps2
Archaea2 - ps3
Archaea3 - ps4
Asgard/Eukaryota - ps5
Eukaryota - ps6
Amorpheae - ps7
Obazoa - ps8
Opisthokonta - ps9
Holomycota - ps10
Fungi - ps11
Fungi_1 - ps12
Fungi_2 - ps13
Fungi_3 - ps14
Dikarya - ps15
Ascomycota - ps16
Saccharo/Pezizomycotina - ps17
Saccharomycotina - ps18
Saccharomycotina_1 - ps19
Saccharomycotina_2 - ps20
Saccharomycotina_3 - ps21
Phaff./Saccharomycetaceae - ps22
Saccharomyco./mycetaceae - ps23
Saccharomycetaceae - ps24
Saccharomycetaceae_2 - ps25
Saccharomycetaceae_3 - ps26
Kaz./Nau./Saccharomyces - ps27
Saccharomyces - ps28
Arboricola_clade - ps29
Cerevisiae_clade - ps30
Saccharomyces_cerevisiae - ps31
Phylostratum

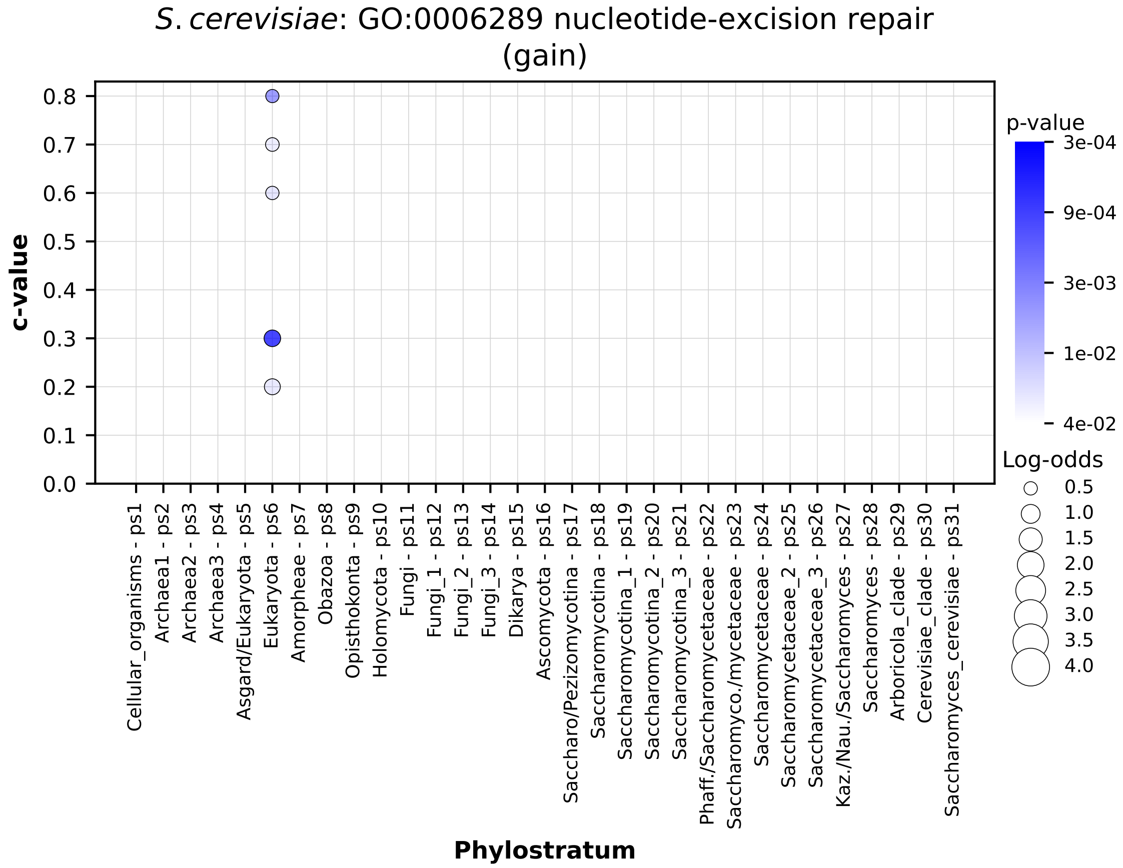

S. cerevisiae: GO:0006289 nucleotide-excision repair (gain)
c-value
0.0
0.1
0.2
0.3
0.4
0.5
0.6
0.7
0.8
p-value
3e-04
9e-04
3e-03
1e-02
4e-02
Log-odds
0.5
1.0
1.5
2.0
2.5
3.0
3.5
4.0
Cellular_organisms - ps1
Archaea1 - ps2
Archaea2 - ps3
Archaea3 - ps4
Asgard/Eukaryota - ps5
Eukaryota - ps6
Amorpheae - ps7
Obazoa - ps8
Opisthokonta - ps9
Holomycota - ps10
Fungi - ps11
Fungi_1 - ps12
Fungi_2 - ps13
Fungi_3 - ps14
Dikarya - ps15
Ascomycota - ps16
Saccharo/Pezizomycotina - ps17
Saccharomycotina - ps18
Saccharomycotina_1 - ps19
Saccharomycotina_2 - ps20
Saccharomycotina_3 - ps21
Phaff./Saccharomycetaceae - ps22
Saccharomyco./mycetaceae - ps23
Saccharomycetaceae - ps24
Saccharomycetaceae_2 - ps25
Saccharomycetaceae_3 - ps26
Kaz./Nau./Saccharomyces - ps27
Saccharomyces - ps28
Arboricola_clade - ps29
Cerevisiae_clade - ps30
Saccharomyces_cerevisiae - ps31
Phylostratum

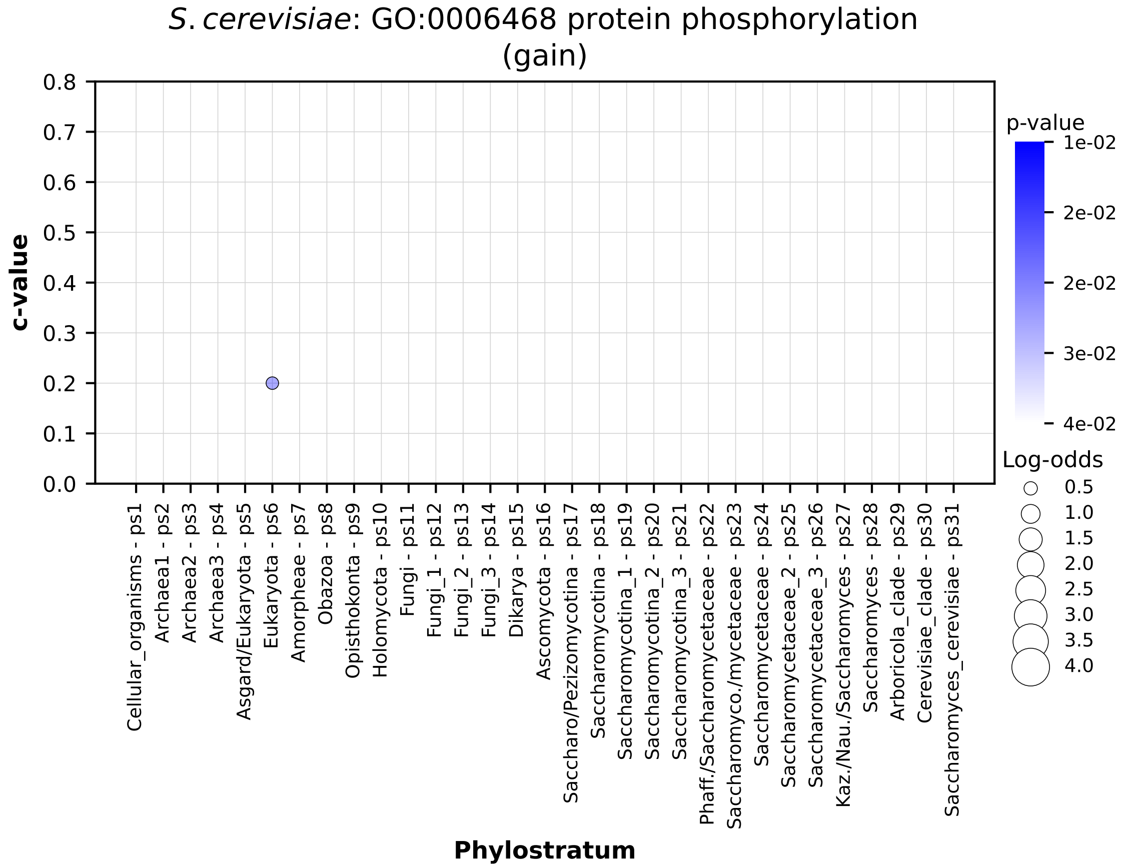
S. cerevisiae: GO:0006468 protein phosphorylation
(gain)
c-value
0.8
0.7
0.6
0.5
0.4
0.3
0.2
0.1
0.0
p-value
1e-02
2e-02
2e-02
3e-02
4e-02
Log-odds
0.5
1.0
1.5
2.0
2.5
3.0
3.5
4.0
Cellular_organisms - ps1
Archaea1 - ps2
Archaea2 - ps3
Archaea3 - ps4
Asgard/Eukaryota - ps5
Eukaryota - ps6
Amorpheae - ps7
Obazoa - ps8
Opisthokonta - ps9
Holomycota - ps10
Fungi - ps11
Fungi_1 - ps12
Fungi_2 - ps13
Fungi_3 - ps14
Dikarya - ps15
Ascomycota - ps16
Saccharo/Pezizomycotina - ps17
Saccharomycotina - ps18
Saccharomycotina_1 - ps19
Saccharomycotina_2 - ps20
Saccharomycotina_3 - ps21
Phaff./Saccharomycetaceae - ps22
Saccharomyco./mycetaceae - ps23
Saccharomycetaceae - ps24
Saccharomycetaceae_2 - ps25
Saccharomycetaceae_3 - ps26
Kaz./Nau./Saccharomyces - ps27
Saccharomyces - ps28
Arboricola_clade - ps29
Cerevisiae_clade - ps30
Saccharomyces_cerevisiae - ps31
Phylostratum

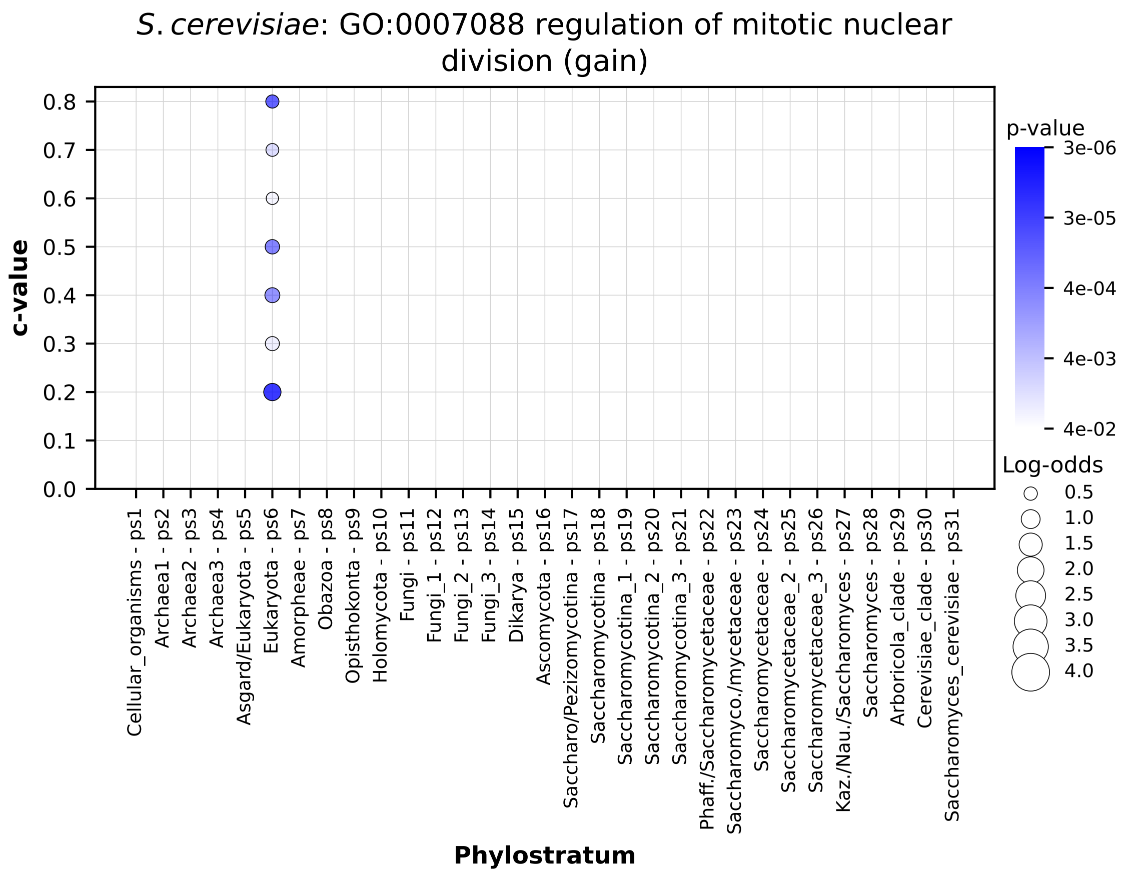

S. cerevisiae: GO:0007088 regulation of mitotic nuclear division (gain)
c-value
0.0
0.1
0.2
0.3
0.4
0.5
0.6
0.7
0.8
p-value
3e-06
3e-05
4e-04
4e-03
4e-02
Log-odds
0.5
1.0
1.5
2.0
2.5
3.0
3.5
4.0
Cellular_organisms - ps1
Archaea1 - ps2
Archaea2 - ps3
Archaea3 - ps4
Asgard/Eukaryota - ps5
Eukaryota - ps6
Amorpheae - ps7
Obazoa - ps8
Opisthokonta - ps9
Holomycota - ps10
Fungi - ps11
Fungi_1 - ps12
Fungi_2 - ps13
Fungi_3 - ps14
Dikarya - ps15
Ascomycota - ps16
Saccharo/Pezizomycotina - ps17
Saccharomycotina - ps18
Saccharomycotina_1 - ps19
Saccharomycotina_2 - ps20
Saccharomycotina_3 - ps21
Phaff./Saccharomycetaceae - ps22
Saccharomyco./mycetaceae - ps23
Saccharomycetaceae - ps24
Saccharomycetaceae_2 - ps25
Saccharomycetaceae_3 - ps26
Kaz./Nau./Saccharomyces - ps27
Saccharomyces - ps28
Arboricola_clade - ps29
Cerevisiae_clade - ps30
Saccharomyces_cerevisiae - ps31
Phylostratum

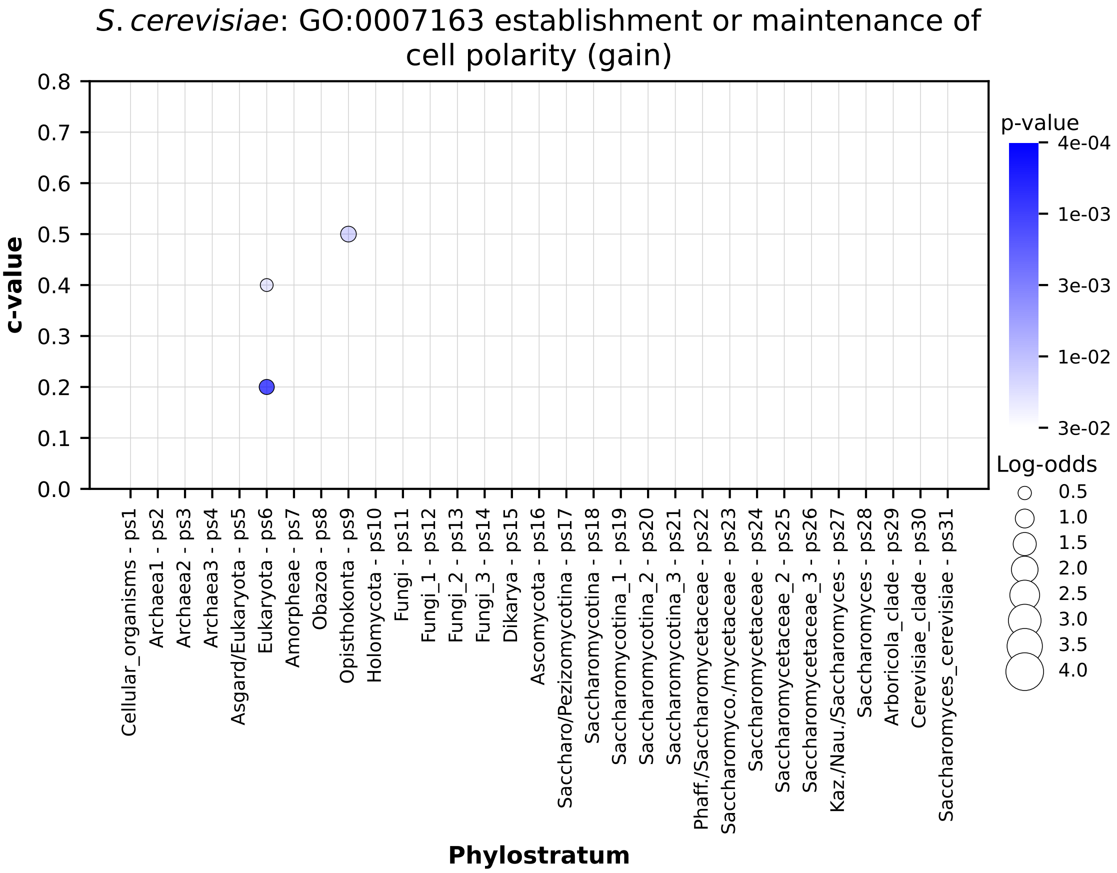

S. cerevisiae: GO:0007163 establishment or maintenance of cell polarity (gain)
c-value
0.8
0.7
0.6
0.5
0.4
0.3
0.2
0.1
0.0
p-value
4e-04
1e-03
3e-03
1e-02
3e-02
Log-odds
0.5
1.0
1.5
2.0
2.5
3.0
3.5
4.0
Cellular_organisms - ps1
Archaea1 - ps2
Archaea2 - ps3
Archaea3 - ps4
Asgard/Eukaryota - ps5
Eukaryota - ps6
Amorpheae - ps7
Obazoa - ps8
Opisthokonta - ps9
Holomycota - ps10
Fungi - ps11
Fungi_1 - ps12
Fungi_2 - ps13
Fungi_3 - ps14
Dikarya - ps15
Ascomycota - ps16
Saccharo/Pezizomycotina - ps17
Saccharomycotina - ps18
Saccharomycotina_1 - ps19
Saccharomycotina_2 - ps20
Saccharomycotina_3 - ps21
Phaff./Saccharomycetaceae - ps22
Saccharomyco./mycetaceae - ps23
Saccharomycetaceae - ps24
Saccharomycetaceae_2 - ps25
Saccharomycetaceae_3 - ps26
Kaz./Nau./Saccharomyces - ps27
Saccharomyces - ps28
Arboricola_clade - ps29
Cerevisiae_clade - ps30
Saccharomyces_cerevisiae - ps31
Phylostratum

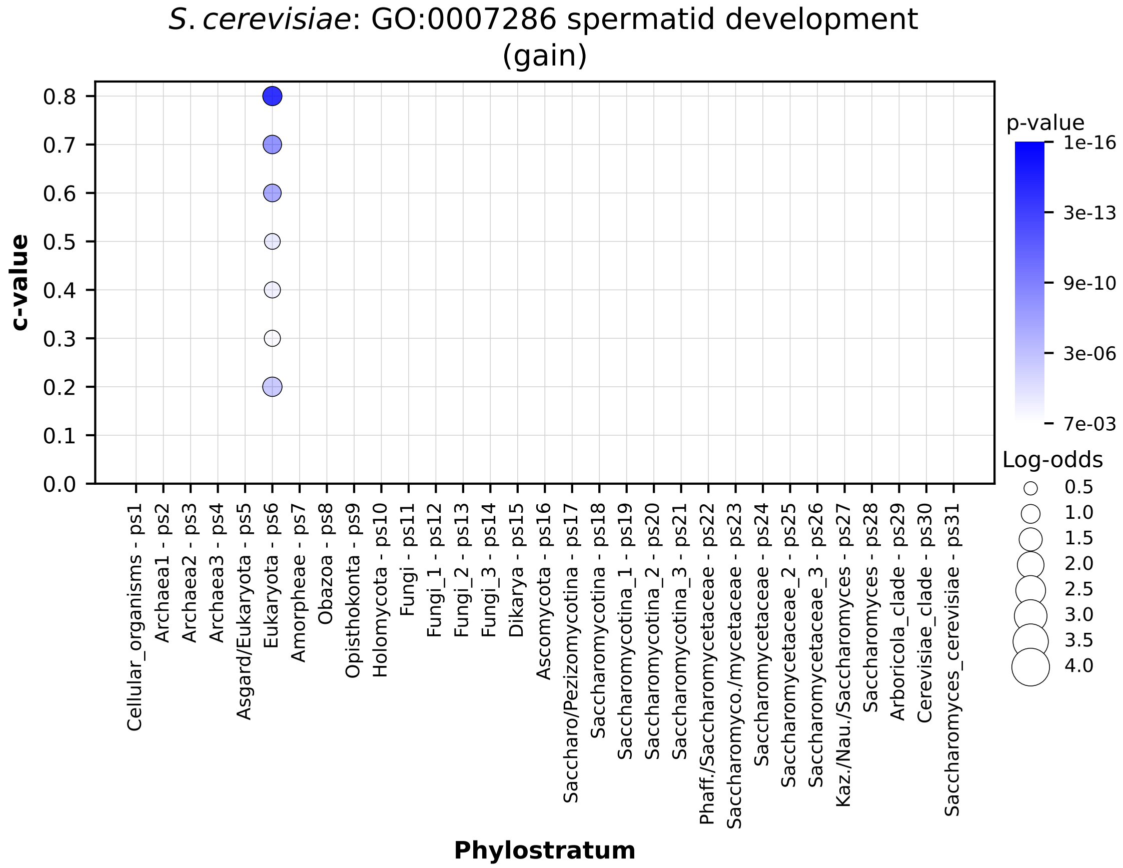
S. cerevisiae: GO:0007286 spermatid development
(gain)
c-value
0.0
0.1
0.2
0.3
0.4
0.5
0.6
0.7
0.8
p-value
1e-16
3e-13
9e-10
3e-06
7e-03
Log-odds
0.5
1.0
1.5
2.0
2.5
3.0
3.5
4.0
Cellular_organisms - ps1
Archaea1 - ps2
Archaea2 - ps3
Archaea3 - ps4
Asgard/Eukaryota - ps5
Eukaryota - ps6
Amorpheae - ps7
Obazoa - ps8
Opisthokonta - ps9
Holomycota - ps10
Fungi - ps11
Fungi_1 - ps12
Fungi_2 - ps13
Fungi_3 - ps14
Dikarya - ps15
Ascomycota - ps16
Saccharo/Pezizomycotina - ps17
Saccharomycotina - ps18
Saccharomycotina_1 - ps19
Saccharomycotina_2 - ps20
Saccharomycotina_3 - ps21
Phaff./Saccharomycetaceae - ps22
Saccharomyco./mycetaceae - ps23
Saccharomycetaceae - ps24
Saccharomycetaceae_2 - ps25
Saccharomycetaceae_3 - ps26
Kaz./Nau./Saccharomyces - ps27
Saccharomyces - ps28
Arboricola_clade - ps29
Cerevisiae_clade - ps30
Saccharomyces_cerevisiae - ps31
Phylostratum

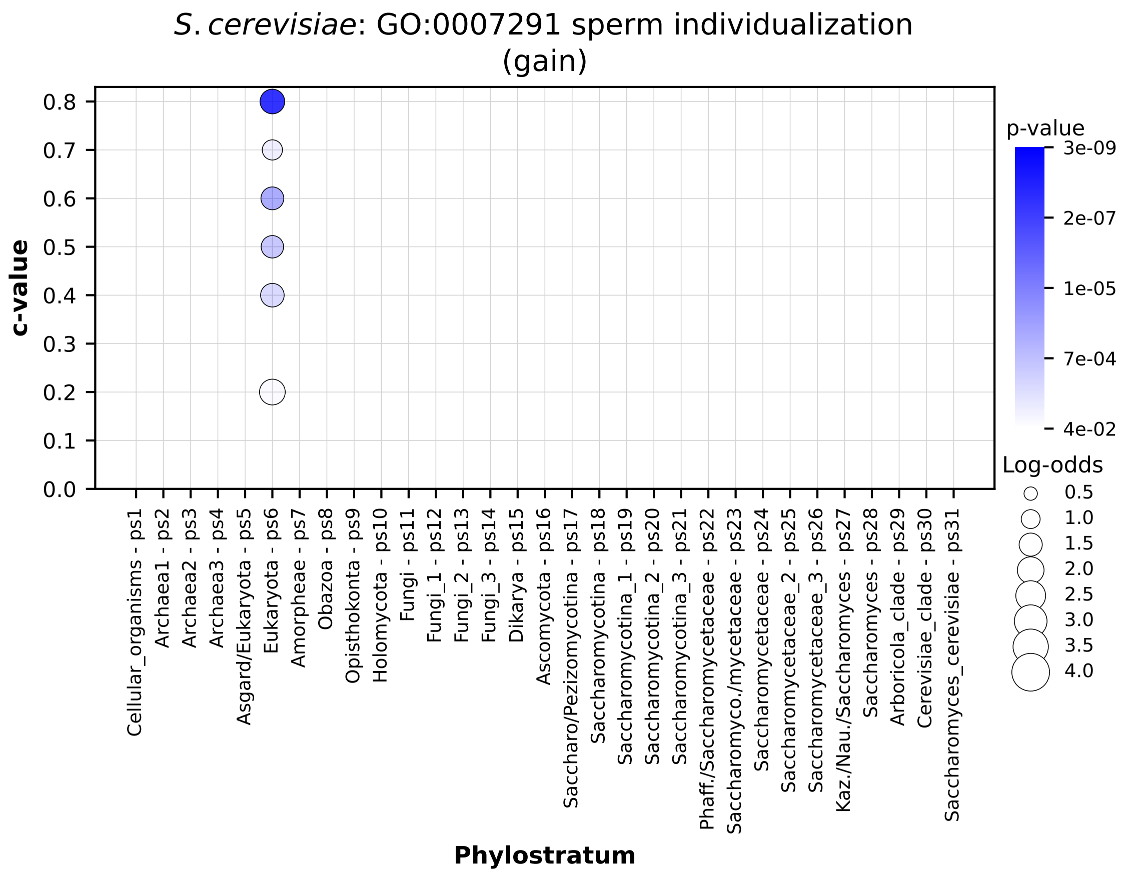

S. cerevisiae: GO:0007291 sperm individualization
(gain)
c-value
0.0
0.1
0.2
0.3
0.4
0.5
0.6
0.7
0.8
p-value
3e-09
2e-07
1e-05
7e-04
4e-02
Log-odds
0.5
1.0
1.5
2.0
2.5
3.0
3.5
4.0
Cellular_organisms - ps1
Archaea1 - ps2
Archaea2 - ps3
Archaea3 - ps4
Asgard/Eukaryota - ps5
Eukaryota - ps6
Amorpheae - ps7
Obazoa - ps8
Opisthokonta - ps9
Holomycota - ps10
Fungi - ps11
Fungi_1 - ps12
Fungi_2 - ps13
Fungi_3 - ps14
Dikarya - ps15
Ascomycota - ps16
Saccharo/Pezizomycotina - ps17
Saccharomycotina - ps18
Saccharomycotina_1 - ps19
Saccharomycotina_2 - ps20
Saccharomycotina_3 - ps21
Phaff./Saccharomycetaceae - ps22
Saccharomyco./mycetaceae - ps23
Saccharomycetaceae - ps24
Saccharomycetaceae_2 - ps25
Saccharomycetaceae_3 - ps26
Kaz./Nau./Saccharomyces - ps27
Saccharomyces - ps28
Arboricola_clade - ps29
Cerevisiae_clade - ps30
Saccharomyces_cerevisiae - ps31
Phylostratum

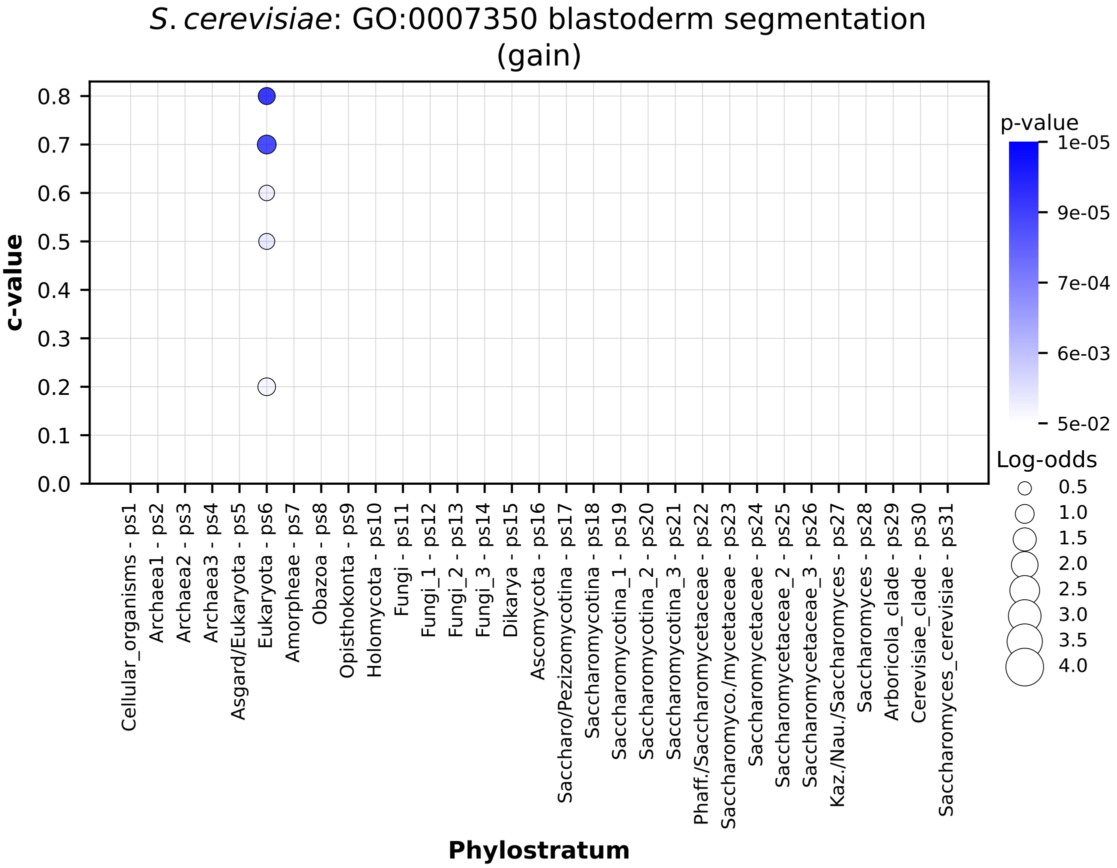

S. cerevisiae: GO:0007350 blastoderm segmentation
(gain)
c-value
0.8
0.7
0.6
0.5
0.4
0.3
0.2
0.1
0.0
p-value
1e-05
9e-05
7e-04
6e-03
5e-02
Log-odds
0.5
1.0
1.5
2.0
2.5
3.0
3.5
4.0
Cellular_organisms - ps1
Archaea1 - ps2
Archaea2 - ps3
Archaea3 - ps4
Asgard/Eukaryota - ps5
Eukaryota - ps6
Amorpheae - ps7
Obazoa - ps8
Opisthokonta - ps9
Holomycota - ps10
Fungi - ps11
Fungi_1 - ps12
Fungi_2 - ps13
Fungi_3 - ps14
Dikarya - ps15
Ascomycota - ps16
Saccharo/Pezizomycotina - ps17
Saccharomycotina - ps18
Saccharomycotina_1 - ps19
Saccharomycotina_2 - ps20
Saccharomycotina_3 - ps21
Phaff./Saccharomycetaceae - ps22
Saccharomyco./mycetaceae - ps23
Saccharomycetaceae - ps24
Saccharomycetaceae_2 - ps25
Saccharomycetaceae_3 - ps26
Kaz./Nau./Saccharomyces - ps27
Saccharomyces - ps28
Arboricola_clade - ps29
Cerevisiae_clade - ps30
Saccharomyces_cerevisiae - ps31
Phylostratum

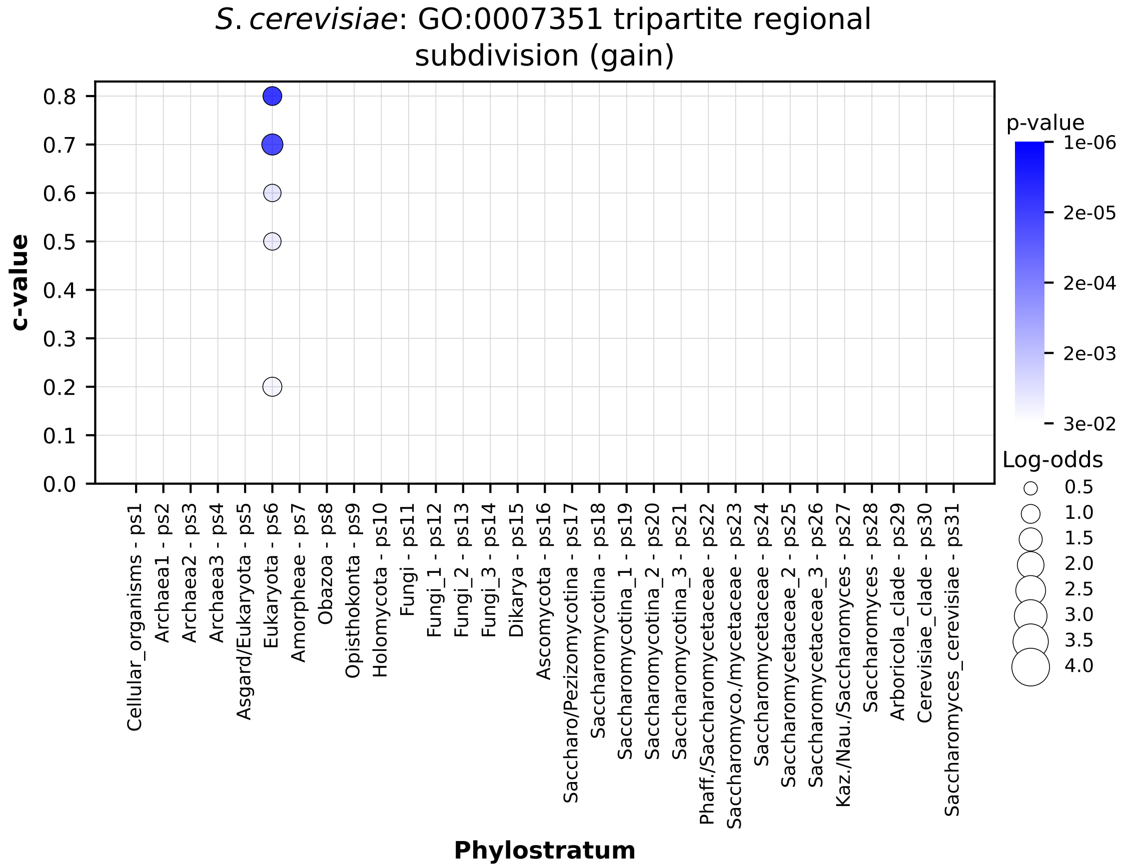

S. cerevisiae: GO:0007351 tripartite regional subdivision (gain)
c-value
0.0
0.1
0.2
0.3
0.4
0.5
0.6
0.7
0.8
p-value
1e-06
2e-05
2e-04
2e-03
3e-02
Log-odds
0.5
1.0
1.5
2.0
2.5
3.0
3.5
4.0
Cellular_organisms - ps1
Archaea1 - ps2
Archaea2 - ps3
Archaea3 - ps4
Asgard/Eukaryota - ps5
Eukaryota - ps6
Amorpheae - ps7
Obazoa - ps8
Opisthokonta - ps9
Holomycota - ps10
Fungi - ps11
Fungi_1 - ps12
Fungi_2 - ps13
Fungi_3 - ps14
Dikarya - ps15
Ascomycota - ps16
Saccharo/Pezizomycotina - ps17
Saccharomycotina - ps18
Saccharomycotina_1 - ps19
Saccharomycotina_2 - ps20
Saccharomycotina_3 - ps21
Phaff./Saccharomycetaceae - ps22
Saccharomyco./mycetaceae - ps23
Saccharomycetaceae - ps24
Saccharomycetaceae_2 - ps25
Saccharomycetaceae_3 - ps26
Kaz./Nau./Saccharomyces - ps27
Saccharomyces - ps28
Arboricola_clade - ps29
Cerevisiae_clade - ps30
Saccharomyces_cerevisiae - ps31
Phylostratum

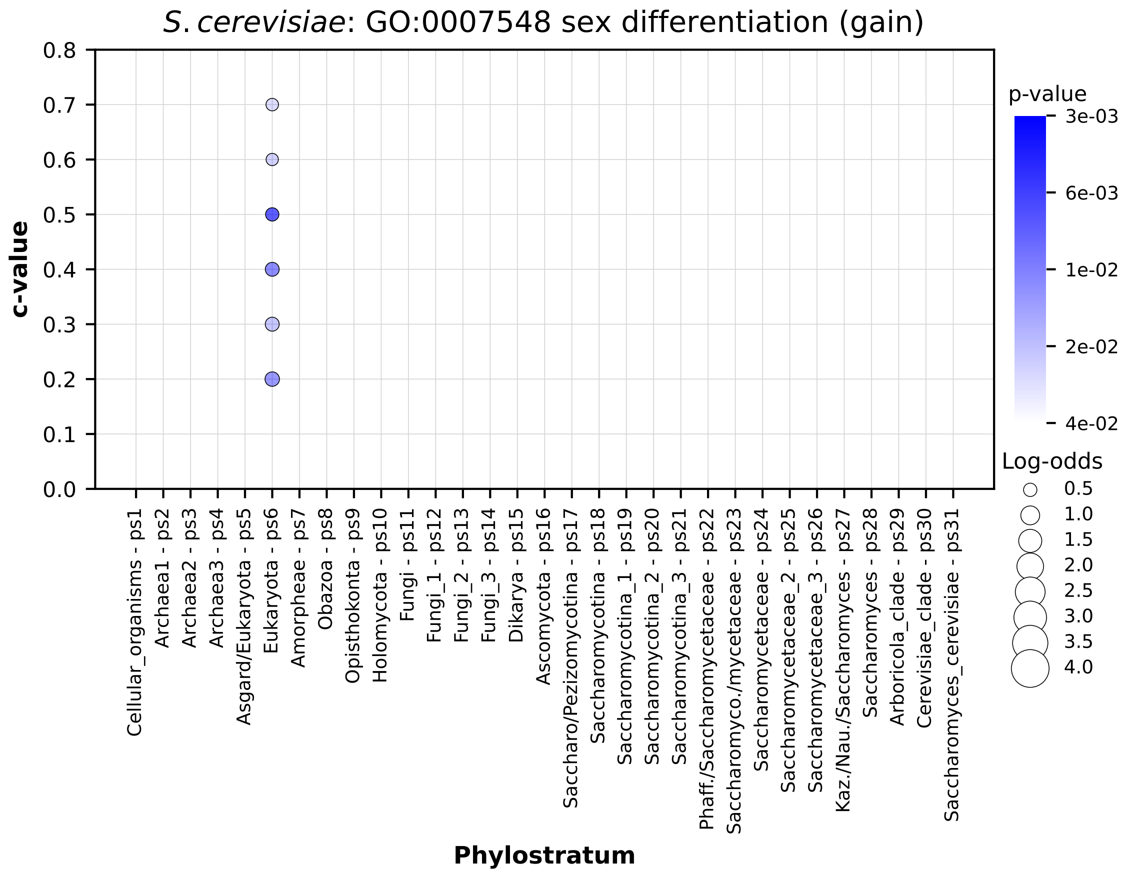

S. cerevisiae: GO:0007548 sex differentiation (gain)
c-value
0.0
0.1
0.2
0.3
0.4
0.5
0.6
0.7
0.8
p-value
3e-03
6e-03
1e-02
2e-02
4e-02
Log-odds
0.5
1.0
1.5
2.0
2.5
3.0
3.5
4.0
Cellular_organisms - ps1
Archaea1 - ps2
Archaea2 - ps3
Archaea3 - ps4
Asgard/Eukaryota - ps5
Eukaryota - ps6
Amorpheae - ps7
Obazoa - ps8
Opisthokonta - ps9
Holomycota - ps10
Fungi - ps11
Fungi_1 - ps12
Fungi_2 - ps13
Fungi_3 - ps14
Dikarya - ps15
Ascomycota - ps16
Saccharo/Pezizomycotina - ps17
Saccharomycotina - ps18
Saccharomycotina_1 - ps19
Saccharomycotina_2 - ps20
Saccharomycotina_3 - ps21
Phaff./Saccharomycetaceae - ps22
Saccharomyco./mycetaceae - ps23
Saccharomycetaceae - ps24
Saccharomycetaceae_2 - ps25
Saccharomycetaceae_3 - ps26
Kaz./Nau./Saccharomyces - ps27
Saccharomyces - ps28
Arboricola_clade - ps29
Cerevisiae_clade - ps30
Saccharomyces_cerevisiae - ps31
Phylostratum

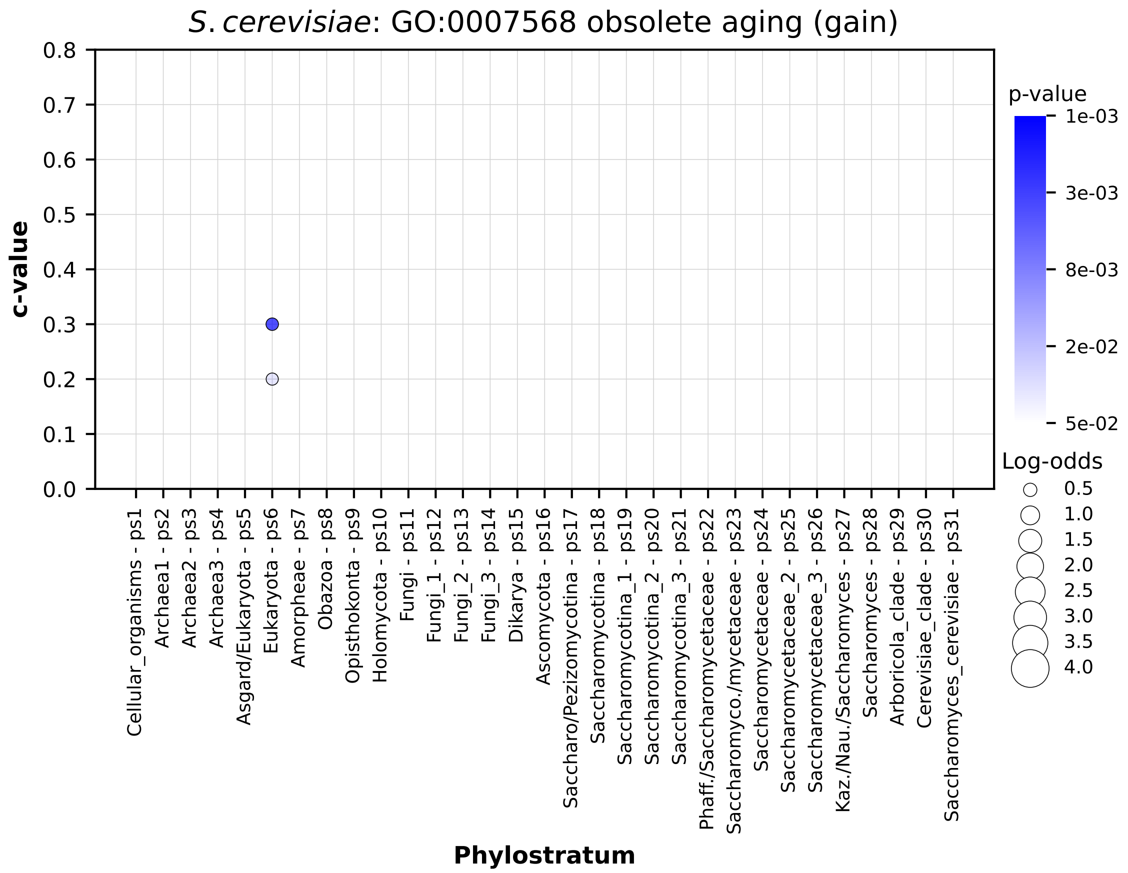

S. cerevisiae: GO:0007568 obsolete aging (gain)
c-value
0.0
0.1
0.2
0.3
0.4
0.5
0.6
0.7
0.8
p-value
1e-03
3e-03
8e-03
2e-02
5e-02
Log-odds
0.5
1.0
1.5
2.0
2.5
3.0
3.5
4.0
Cellular_organisms - ps1
Archaea1 - ps2
Archaea2 - ps3
Archaea3 - ps4
Asgard/Eukaryota - ps5
Eukaryota - ps6
Amorpheae - ps7
Obazoa - ps8
Opisthokonta - ps9
Holomycota - ps10
Fungi - ps11
Fungi_1 - ps12
Fungi_2 - ps13
Fungi_3 - ps14
Dikarya - ps15
Ascomycota - ps16
Saccharo/Pezizomycotina - ps17
Saccharomycotina - ps18
Saccharomycotina_1 - ps19
Saccharomycotina_2 - ps20
Saccharomycotina_3 - ps21
Phaff./Saccharomycetaceae - ps22
Saccharomyco./mycetaceae - ps23
Saccharomycetaceae - ps24
Saccharomycetaceae_2 - ps25
Saccharomycetaceae_3 - ps26
Kaz./Nau./Saccharomyces - ps27
Saccharomyces - ps28
Arboricola_clade - ps29
Cerevisiae_clade - ps30
Saccharomyces_cerevisiae - ps31
Phylostratum

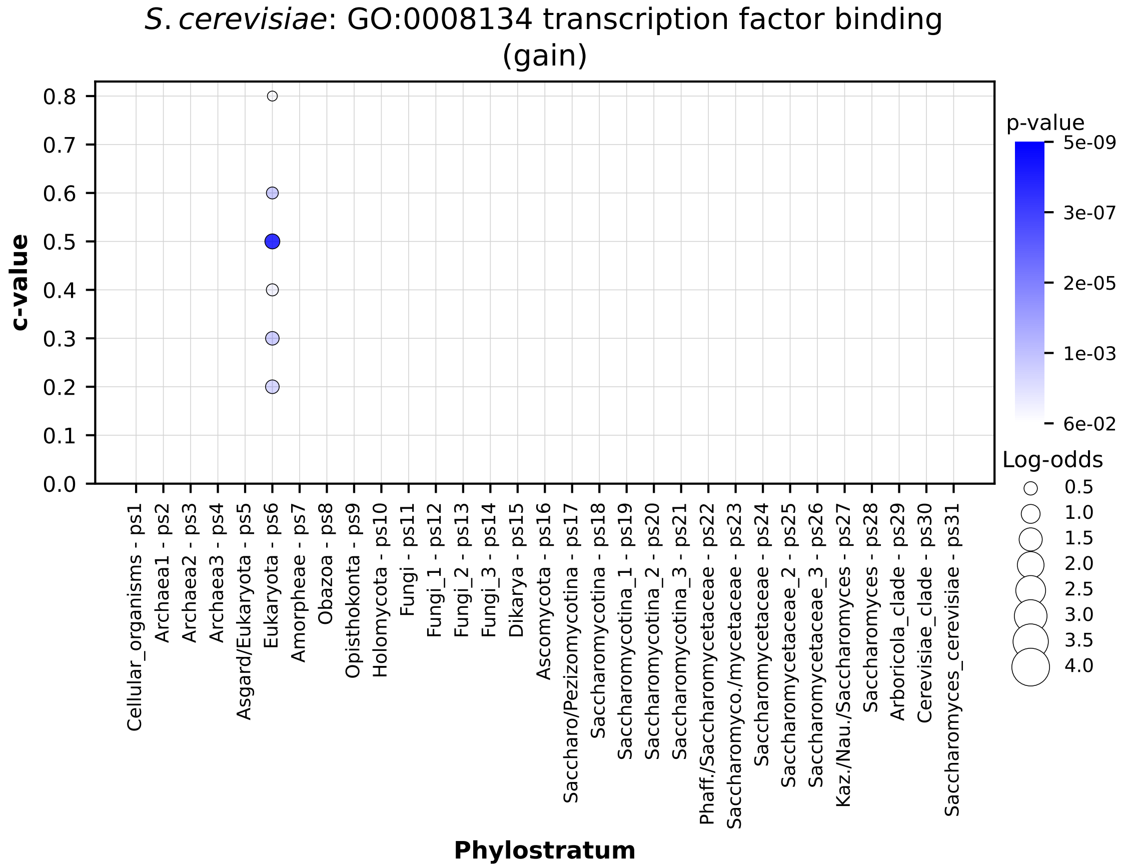

S. cerevisiae: GO:0008134 transcription factor binding
(gain)
c-value
0.8
0.7
0.6
0.5
0.4
0.3
0.2
0.1
0.0
p-value
5e-09
3e-07
2e-05
1e-03
6e-02
Log-odds
0.5
1.0
1.5
2.0
2.5
3.0
3.5
4.0
Cellular_organisms - ps1
Archaea1 - ps2
Archaea2 - ps3
Archaea3 - ps4
Asgard/Eukaryota - ps5
Eukaryota - ps6
Amorpheae - ps7
Obazoa - ps8
Opisthokonta - ps9
Holomycota - ps10
Fungi - ps11
Fungi_1 - ps12
Fungi_2 - ps13
Fungi_3 - ps14
Dikarya - ps15
Ascomycota - ps16
Saccharo/Pezizomycotina - ps17
Saccharomycotina - ps18
Saccharomycotina_1 - ps19
Saccharomycotina_2 - ps20
Saccharomycotina_3 - ps21
Phaff./Saccharomycetaceae - ps22
Saccharomyco./mycetaceae - ps23
Saccharomycetaceae - ps24
Saccharomycetaceae_2 - ps25
Saccharomycetaceae_3 - ps26
Kaz./Nau./Saccharomyces - ps27
Saccharomyces - ps28
Arboricola_clade - ps29
Cerevisiae_clade - ps30
Saccharomyces_cerevisiae - ps31
Phylostratum

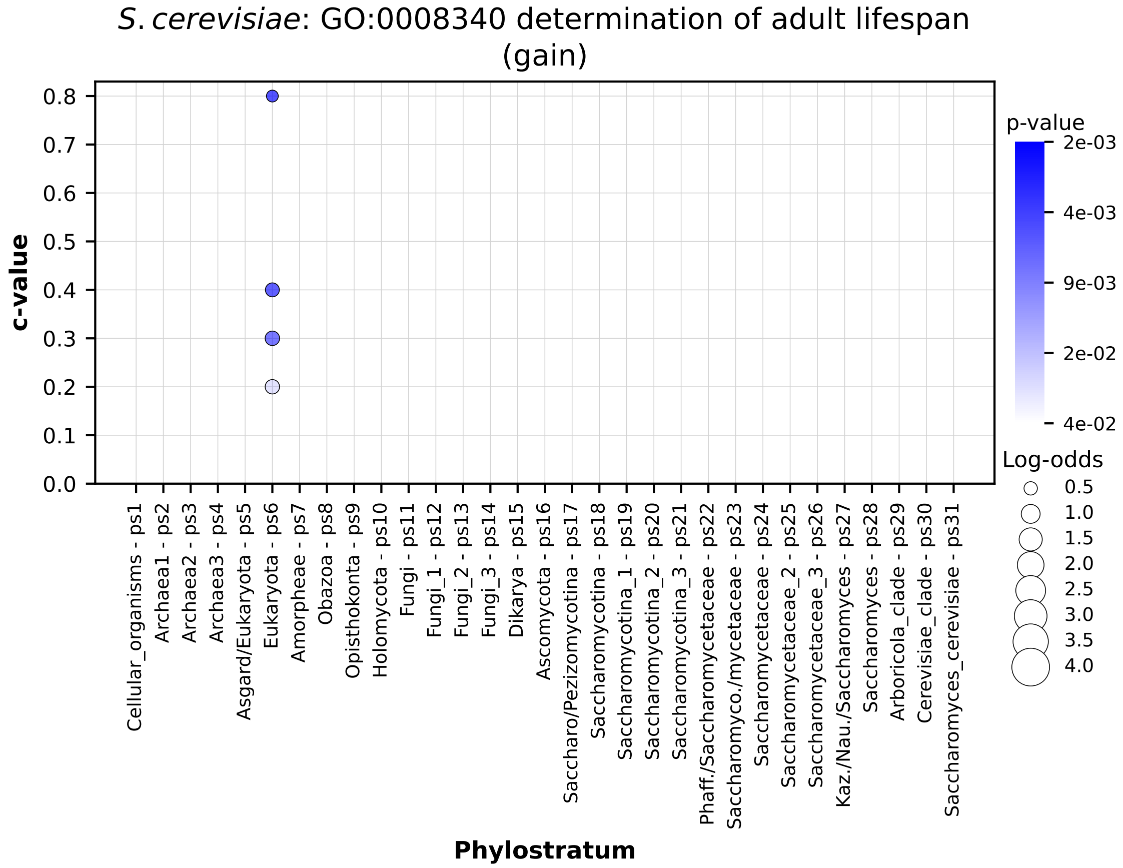

S. cerevisiae: GO:0008340 determination of adult lifespan (gain)
c-value
0.0
0.1
0.2
0.3
0.4
0.5
0.6
0.7
0.8
p-value
2e-03
4e-03
9e-03
2e-02
4e-02
Log-odds
0.5
1.0
1.5
2.0
2.5
3.0
3.5
4.0
Cellular_organisms - ps1
Archaea1 - ps2
Archaea2 - ps3
Archaea3 - ps4
Asgard/Eukaryota - ps5
Eukaryota - ps6
Amorpheae - ps7
Obazoa - ps8
Opisthokonta - ps9
Holomycota - ps10
Fungi - ps11
Fungi_1 - ps12
Fungi_2 - ps13
Fungi_3 - ps14
Dikarya - ps15
Ascomycota - ps16
Saccharo/Pezizomycotina - ps17
Saccharomycotina - ps18
Saccharomycotina_1 - ps19
Saccharomycotina_2 - ps20
Saccharomycotina_3 - ps21
Phaff./Saccharomycetaceae - ps22
Saccharomyco./mycetaceae - ps23
Saccharomycetaceae - ps24
Saccharomycetaceae_2 - ps25
Saccharomycetaceae_3 - ps26
Kaz./Nau./Saccharomyces - ps27
Saccharomyces - ps28
Arboricola_clade - ps29
Cerevisiae_clade - ps30
Saccharomyces_cerevisiae - ps31
Phylostratum

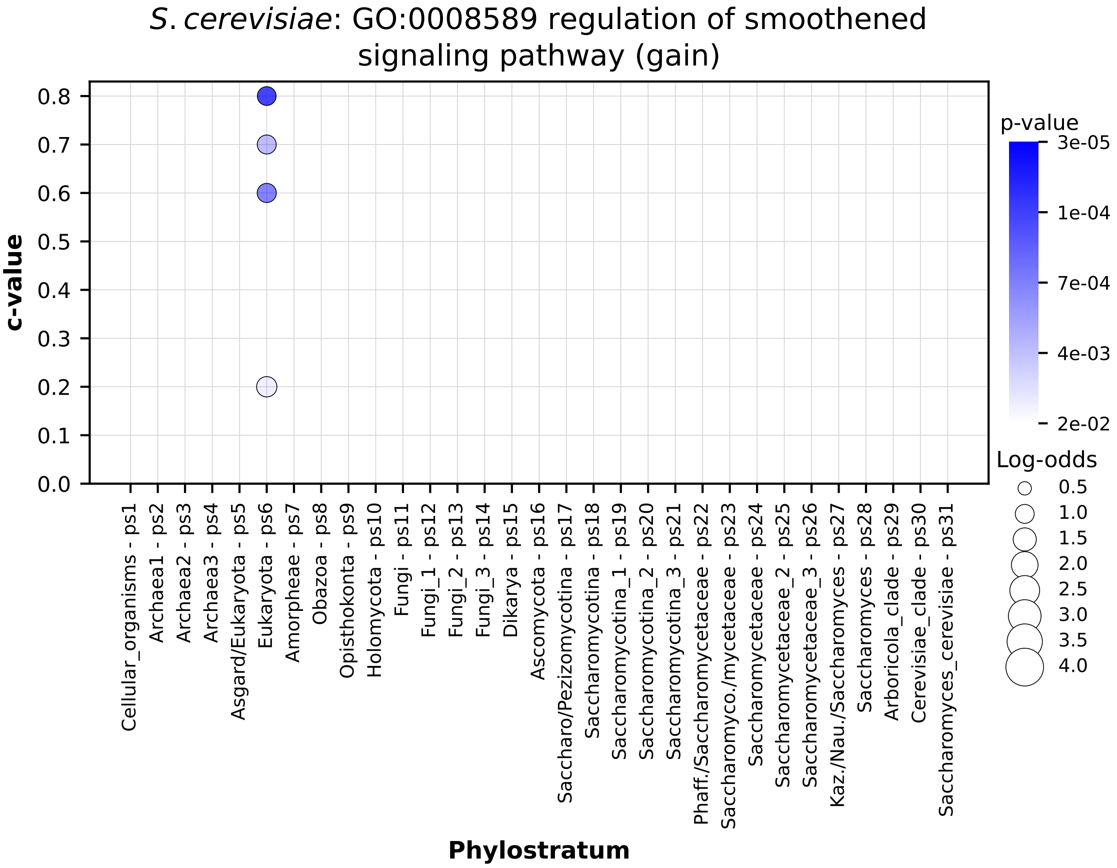

S. cerevisiae: GO:0008589 regulation of smoothened signaling pathway (gain)
c-value
0.0
0.1
0.2
0.3
0.4
0.5
0.6
0.7
0.8
Phylostratum
Cellular_organisms - ps1
Archaea1 - ps2
Archaea2 - ps3
Archaea3 - ps4
Asgard/Eukaryota - ps5
Eukaryota - ps6
Amorpheae - ps7
Obazoa - ps8
Opisthokonta - ps9
Holomycota - ps10
Fungi - ps11
Fungi_1 - ps12
Fungi_2 - ps13
Fungi_3 - ps14
Dikarya - ps15
Ascomycota - ps16
Saccharo/Pezizomycotina - ps17
Saccharomycotina - ps18
Saccharomycotina_1 - ps19
Saccharomycotina_2 - ps20
Saccharomycotina_3 - ps21
Phaff./Saccharomycetaceae - ps22
Saccharomyco./mycetaceae - ps23
Saccharomycetaceae - ps24
Saccharomycetaceae_2 - ps25
Saccharomycetaceae_3 - ps26
Kaz./Nau./Saccharomyces - ps27
Saccharomyces - ps28
Arboricola_clade - ps29
Cerevisiae_clade - ps30
Saccharomyces_cerevisiae - ps31
p-value
3e-05
1e-04
7e-04
4e-03
2e-02
Log-odds
0.5
1.0
1.5
2.0
2.5
3.0
3.5
4.0

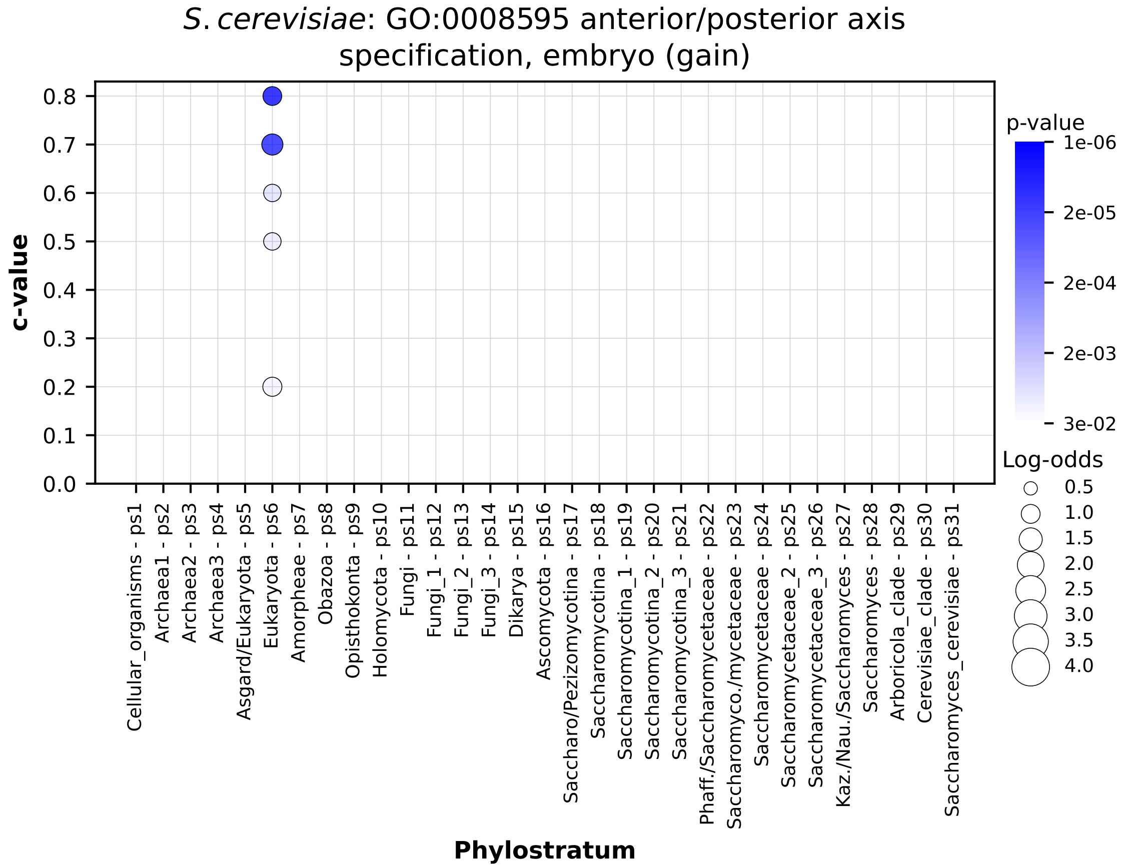

S. cerevisiae: GO:0008595 anterior/posterior axis specification, embryo (gain)
c-value
0.0
0.1
0.2
0.3
0.4
0.5
0.6
0.7
0.8
p-value
1e-06
2e-05
2e-04
2e-03
3e-02
Log-odds
0.5
1.0
1.5
2.0
2.5
3.0
3.5
4.0
Cellular_organisms - ps1
Archaea1 - ps2
Archaea2 - ps3
Archaea3 - ps4
Asgard/Eukaryota - ps5
Eukaryota - ps6
Amorpheae - ps7
Obazoa - ps8
Opisthokonta - ps9
Holomycota - ps10
Fungi - ps11
Fungi_1 - ps12
Fungi_2 - ps13
Fungi_3 - ps14
Dikarya - ps15
Ascomycota - ps16
Saccharo/Pezizomycotina - ps17
Saccharomycotina - ps18
Saccharomycotina_1 - ps19
Saccharomycotina_2 - ps20
Saccharomycotina_3 - ps21
Phaff./Saccharomycetaceae - ps22
Saccharomyco./mycetaceae - ps23
Saccharomycetaceae - ps24
Saccharomycetaceae_2 - ps25
Saccharomycetaceae_3 - ps26
Kaz./Nau./Saccharomyces - ps27
Saccharomyces - ps28
Arboricola_clade - ps29
Cerevisiae_clade - ps30
Saccharomyces_cerevisiae - ps31
Phylostratum

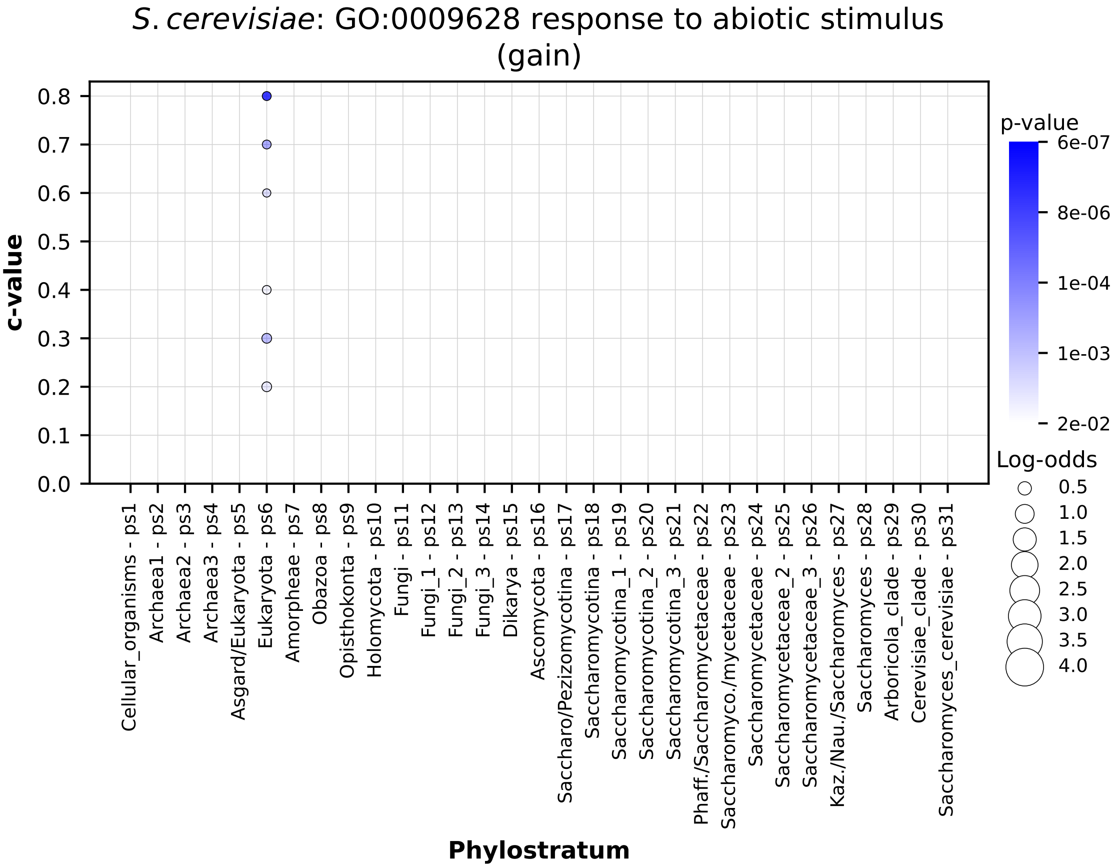
S. cerevisiae: GO:0009628 response to abiotic stimulus (gain)
c-value
0.0
0.1
0.2
0.3
0.4
0.5
0.6
0.7
0.8
Phylostratum
Cellular_organisms - ps1
Archaea1 - ps2
Archaea2 - ps3
Archaea3 - ps4
Asgard/Eukaryota - ps5
Eukaryota - ps6
Amorpheae - ps7
Obazoa - ps8
Opisthokonta - ps9
Holomycota - ps10
Fungi - ps11
Fungi_1 - ps12
Fungi_2 - ps13
Fungi_3 - ps14
Dikarya - ps15
Ascomycota - ps16
Saccharo/Pezizomycotina - ps17
Saccharomycotina - ps18
Saccharomycotina_1 - ps19
Saccharomycotina_2 - ps20
Saccharomycotina_3 - ps21
Phaff./Saccharomycetaceae - ps22
Saccharomyco./mycetaceae - ps23
Saccharomycetaceae - ps24
Saccharomycetaceae_2 - ps25
Saccharomycetaceae_3 - ps26
Kaz./Nau./Saccharomyces - ps27
Saccharomyces - ps28
Arboricola_clade - ps29
Cerevisiae_clade - ps30
Saccharomyces_cerevisiae - ps31
p-value
6e-07
8e-06
1e-04
1e-03
2e-02
Log-odds
0.5
1.0
1.5
2.0
2.5
3.0
3.5
4.0

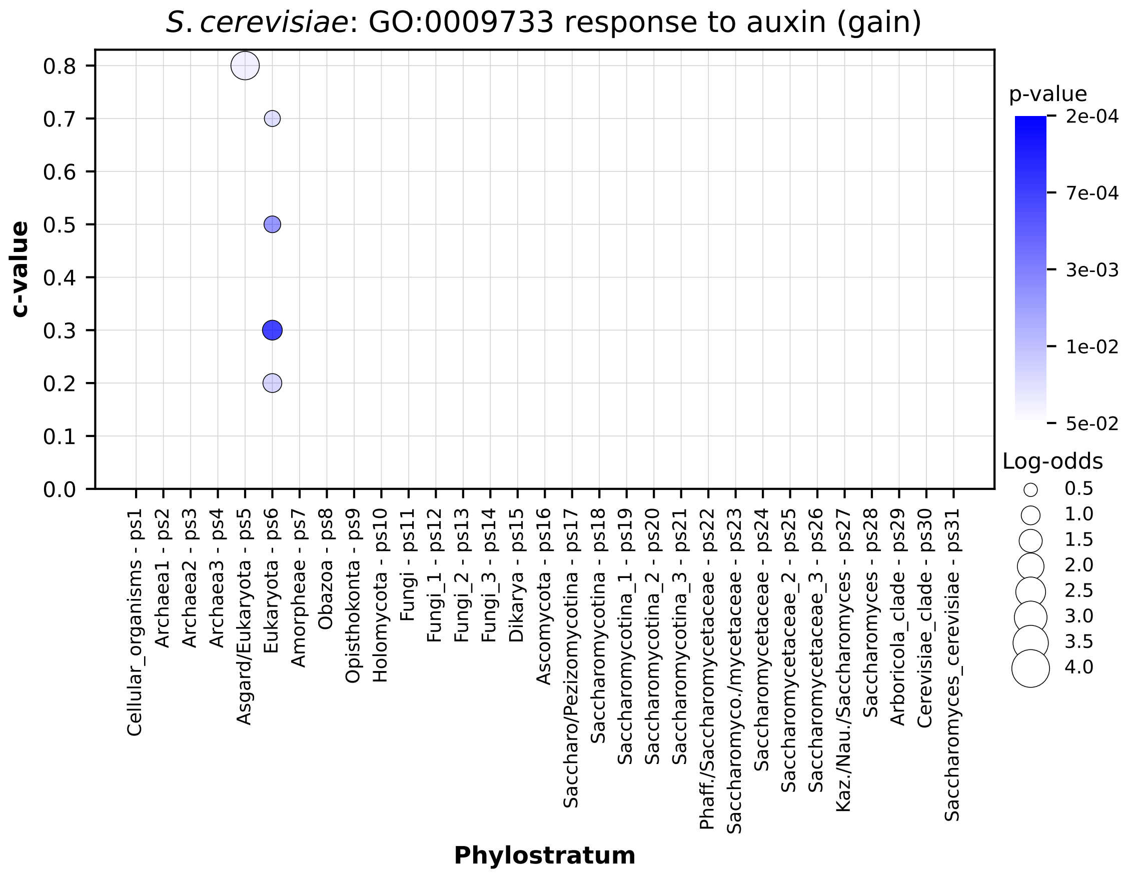

S. cerevisiae: GO:0009733 response to auxin (gain)
c-value
0.0
0.1
0.2
0.3
0.4
0.5
0.6
0.7
0.8
p-value
2e-04
7e-04
3e-03
1e-02
5e-02
Log-odds
0.5
1.0
1.5
2.0
2.5
3.0
3.5
4.0
Cellular_organisms - ps1
Archaea1 - ps2
Archaea2 - ps3
Archaea3 - ps4
Asgard/Eukaryota - ps5
Eukaryota - ps6
Amorpheae - ps7
Obazoa - ps8
Opisthokonta - ps9
Holomycota - ps10
Fungi - ps11
Fungi_1 - ps12
Fungi_2 - ps13
Fungi_3 - ps14
Dikarya - ps15
Ascomycota - ps16
Saccharo/Pezizomycotina - ps17
Saccharomycotina - ps18
Saccharomycotina_1 - ps19
Saccharomycotina_2 - ps20
Saccharomycotina_3 - ps21
Phaff./Saccharomycetaceae - ps22
Saccharomyco./mycetaceae - ps23
Saccharomycetaceae - ps24
Saccharomycetaceae_2 - ps25
Saccharomycetaceae_3 - ps26
Kaz./Nau./Saccharomyces - ps27
Saccharomyces - ps28
Arboricola_clade - ps29
Cerevisiae_clade - ps30
Saccharomyces_cerevisiae - ps31
Phylostratum

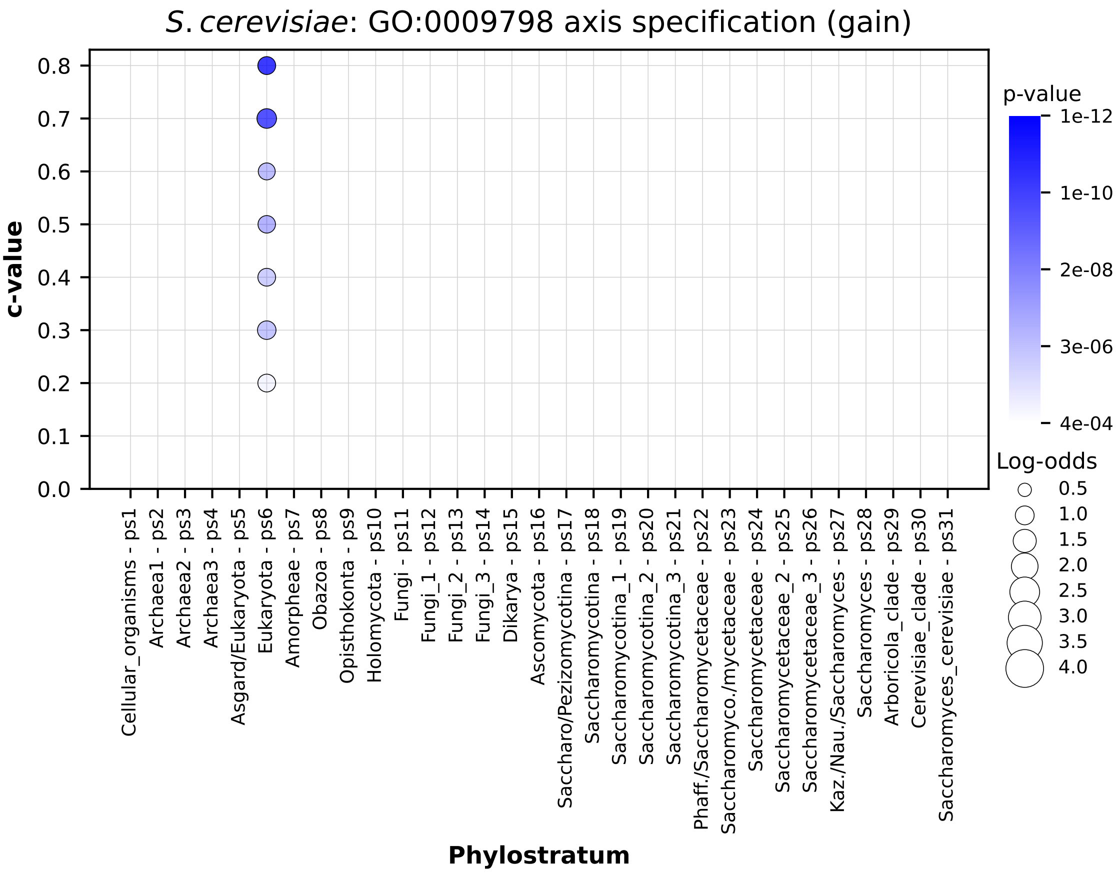

S. cerevisiae: GO:0009798 axis specification (gain)
c-value
0.0
0.1
0.2
0.3
0.4
0.5
0.6
0.7
0.8
p-value
1e-12
1e-10
2e-08
3e-06
4e-04
Log-odds
0.5
1.0
1.5
2.0
2.5
3.0
3.5
4.0
Cellular_organisms - ps1
Archaea1 - ps2
Archaea2 - ps3
Archaea3 - ps4
Asgard/Eukaryota - ps5
Eukaryota - ps6
Amorpheae - ps7
Obazoa - ps8
Opisthokonta - ps9
Holomycota - ps10
Fungi - ps11
Fungi_1 - ps12
Fungi_2 - ps13
Fungi_3 - ps14
Dikarya - ps15
Ascomycota - ps16
Saccharo/Pezizomycotina - ps17
Saccharomycotina - ps18
Saccharomycotina_1 - ps19
Saccharomycotina_2 - ps20
Saccharomycotina_3 - ps21
Phaff./Saccharomycetaceae - ps22
Saccharomyco./mycetaceae - ps23
Saccharomycetaceae - ps24
Saccharomycetaceae_2 - ps25
Saccharomycetaceae_3 - ps26
Kaz./Nau./Saccharomyces - ps27
Saccharomyces - ps28
Arboricola_clade - ps29
Cerevisiae_clade - ps30
Saccharomyces_cerevisiae - ps31
Phylostratum

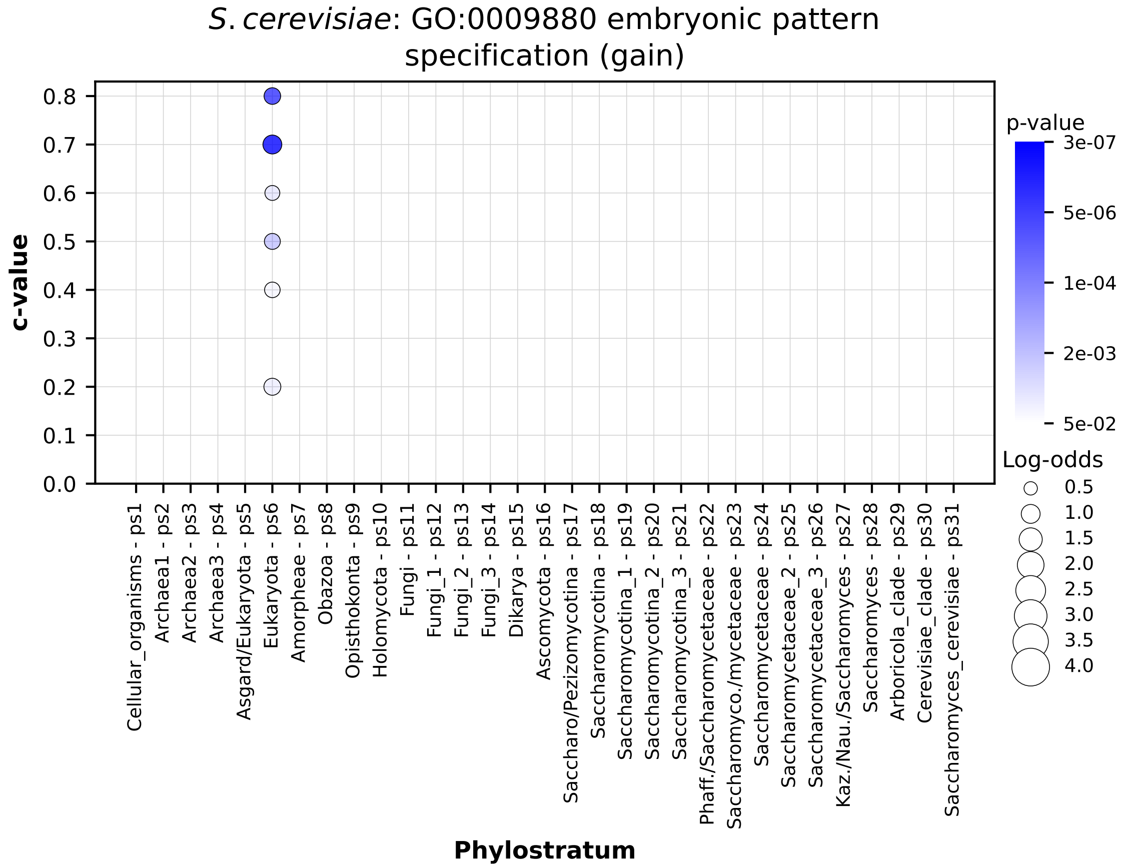

S. cerevisiae: GO:0009880 embryonic pattern specification (gain)
c-value
0.8
0.7
0.6
0.5
0.4
0.3
0.2
0.1
0.0
p-value
3e-07
5e-06
1e-04
2e-03
5e-02
Log-odds
0.5
1.0
1.5
2.0
2.5
3.0
3.5
4.0
Cellular_organisms - ps1
Archaea1 - ps2
Archaea2 - ps3
Archaea3 - ps4
Asgard/Eukaryota - ps5
Eukaryota - ps6
Amorpheae - ps7
Obazoa - ps8
Opisthokonta - ps9
Holomycota - ps10
Fungi - ps11
Fungi_1 - ps12
Fungi_2 - ps13
Fungi_3 - ps14
Dikarya - ps15
Ascomycota - ps16
Saccharo/Pezizomycotina - ps17
Saccharomycotina - ps18
Saccharomycotina_1 - ps19
Saccharomycotina_2 - ps20
Saccharomycotina_3 - ps21
Phaff./Saccharomycetaceae - ps22
Saccharomyco./mycetaceae - ps23
Saccharomycetaceae - ps24
Saccharomycetaceae_2 - ps25
Saccharomycetaceae_3 - ps26
Kaz./Nau./Saccharomyces - ps27
Saccharomyces - ps28
Arboricola_clade - ps29
Cerevisiae_clade - ps30
Saccharomyces_cerevisiae - ps31
Phylostratum

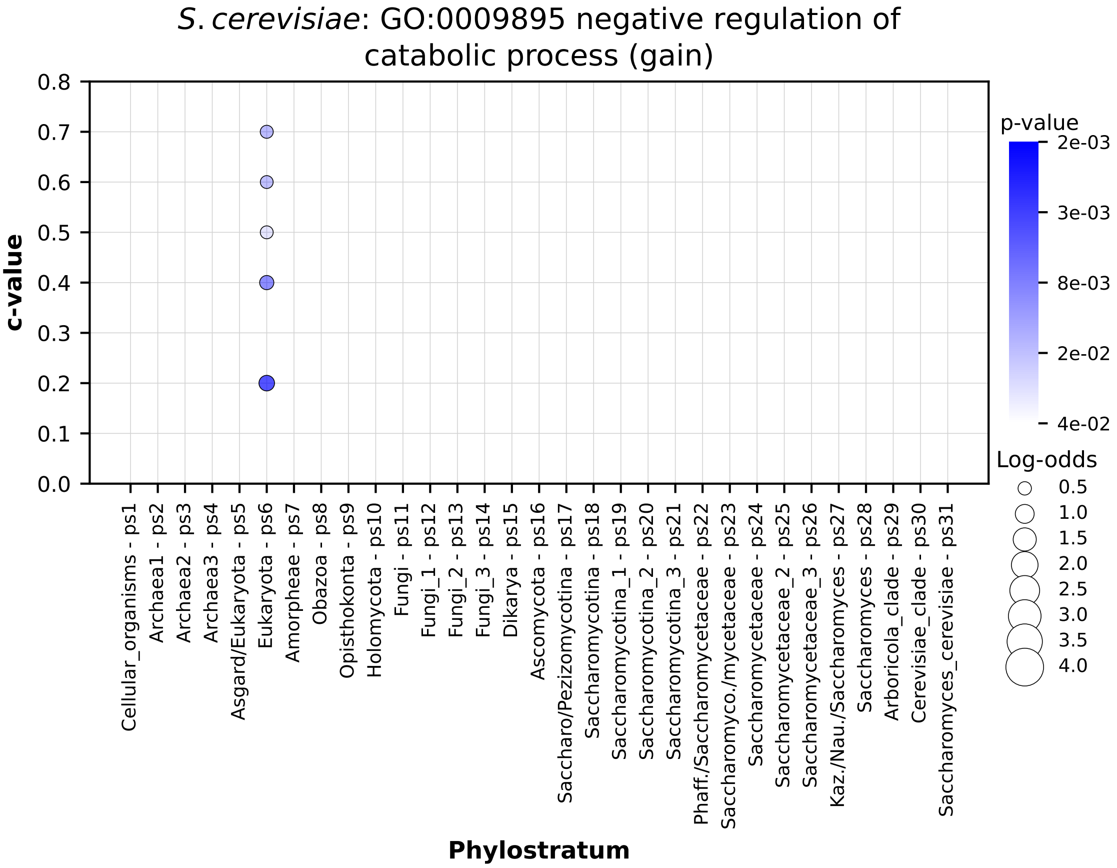

S. cerevisiae: GO:0009895 negative regulation of catabolic process (gain)
c-value
0.8
0.7
0.6
0.5
0.4
0.3
0.2
0.1
0.0
p-value
2e-03
3e-03
8e-03
2e-02
4e-02
Log-odds
0.5
1.0
1.5
2.0
2.5
3.0
3.5
4.0
Cellular_organisms - ps1
Archaea1 - ps2
Archaea2 - ps3
Archaea3 - ps4
Asgard/Eukaryota - ps5
Eukaryota - ps6
Amorpheae - ps7
Obazoa - ps8
Opisthokonta - ps9
Holomycota - ps10
Fungi - ps11
Fungi_1 - ps12
Fungi_2 - ps13
Fungi_3 - ps14
Dikarya - ps15
Ascomycota - ps16
Saccharo/Pezizomycotina - ps17
Saccharomycotina - ps18
Saccharomycotina_1 - ps19
Saccharomycotina_2 - ps20
Saccharomycotina_3 - ps21
Phaff./Saccharomycetaceae - ps22
Saccharomyco./mycetaceae - ps23
Saccharomycetaceae - ps24
Saccharomycetaceae_2 - ps25
Saccharomycetaceae_3 - ps26
Kaz./Nau./Saccharomyces - ps27
Saccharomyces - ps28
Arboricola_clade - ps29
Cerevisiae_clade - ps30
Saccharomyces_cerevisiae - ps31
Phylostratum

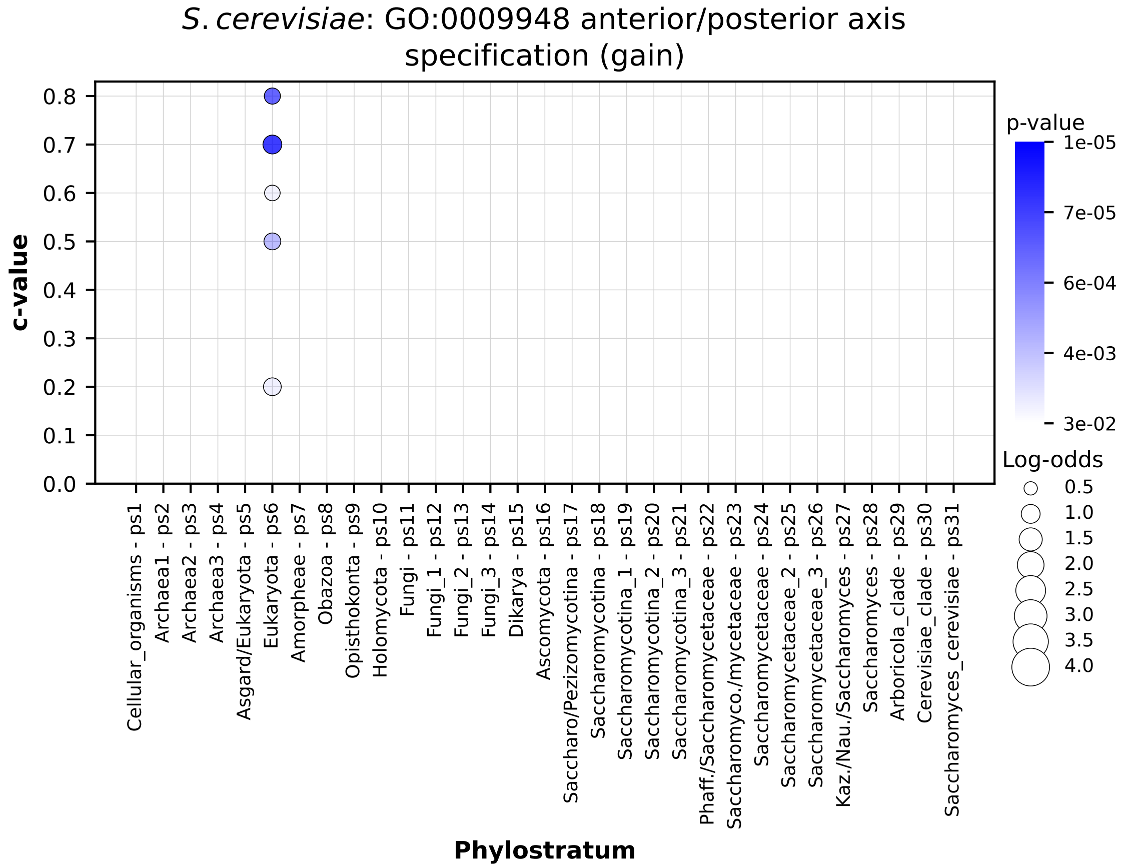
S. cerevisiae: GO:0009948 anterior/posterior axis specification (gain)
c-value
0.0
0.1
0.2
0.3
0.4
0.5
0.6
0.7
0.8
p-value
1e-05
7e-05
6e-04
4e-03
3e-02
Log-odds
0.5
1.0
1.5
2.0
2.5
3.0
3.5
4.0
Cellular_organisms - ps1
Archaea1 - ps2
Archaea2 - ps3
Archaea3 - ps4
Asgard/Eukaryota - ps5
Eukaryota - ps6
Amorpheae - ps7
Obazoa - ps8
Opisthokonta - ps9
Holomycota - ps10
Fungi - ps11
Fungi_1 - ps12
Fungi_2 - ps13
Fungi_3 - ps14
Dikarya - ps15
Ascomycota - ps16
Saccharo/Pezizomycotina - ps17
Saccharomycotina - ps18
Saccharomycotina_1 - ps19
Saccharomycotina_2 - ps20
Saccharomycotina_3 - ps21
Phaff./Saccharomycetaceae - ps22
Saccharomyco./mycetaceae - ps23
Saccharomycetaceae - ps24
Saccharomycetaceae_2 - ps25
Saccharomycetaceae_3 - ps26
Kaz./Nau./Saccharomyces - ps27
Saccharomyces - ps28
Arboricola_clade - ps29
Cerevisiae_clade - ps30
Saccharomyces_cerevisiae - ps31
Phylostratum

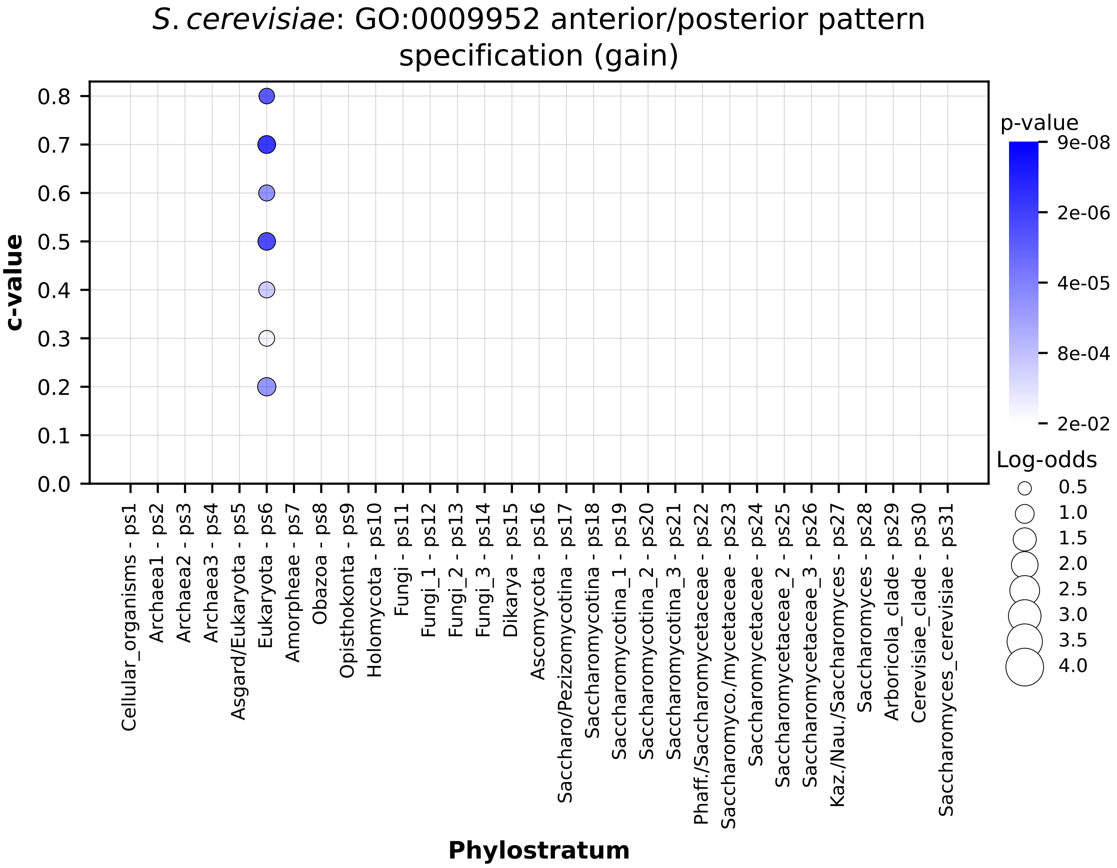

S. cerevisiae: GO:0009952 anterior/posterior pattern specification (gain)
c-value
0.0
0.1
0.2
0.3
0.4
0.5
0.6
0.7
0.8
p-value
9e-08
2e-06
4e-05
8e-04
2e-02
Log-odds
0.5
1.0
1.5
2.0
2.5
3.0
3.5
4.0
Cellular_organisms - ps1
Archaea1 - ps2
Archaea2 - ps3
Archaea3 - ps4
Asgard/Eukaryota - ps5
Eukaryota - ps6
Amorpheae - ps7
Obazoa - ps8
Opisthokonta - ps9
Holomycota - ps10
Fungi - ps11
Fungi_1 - ps12
Fungi_2 - ps13
Fungi_3 - ps14
Dikarya - ps15
Ascomycota - ps16
Saccharo/Pezizomycotina - ps17
Saccharomycotina - ps18
Saccharomycotina_1 - ps19
Saccharomycotina_2 - ps20
Saccharomycotina_3 - ps21
Phaff./Saccharomycetaceae - ps22
Saccharomyco./mycetaceae - ps23
Saccharomycetaceae - ps24
Saccharomycetaceae_2 - ps25
Saccharomycetaceae_3 - ps26
Kaz./Nau./Saccharomyces - ps27
Saccharomyces - ps28
Arboricola_clade - ps29
Cerevisiae_clade - ps30
Saccharomyces_cerevisiae - ps31
Phylostratum

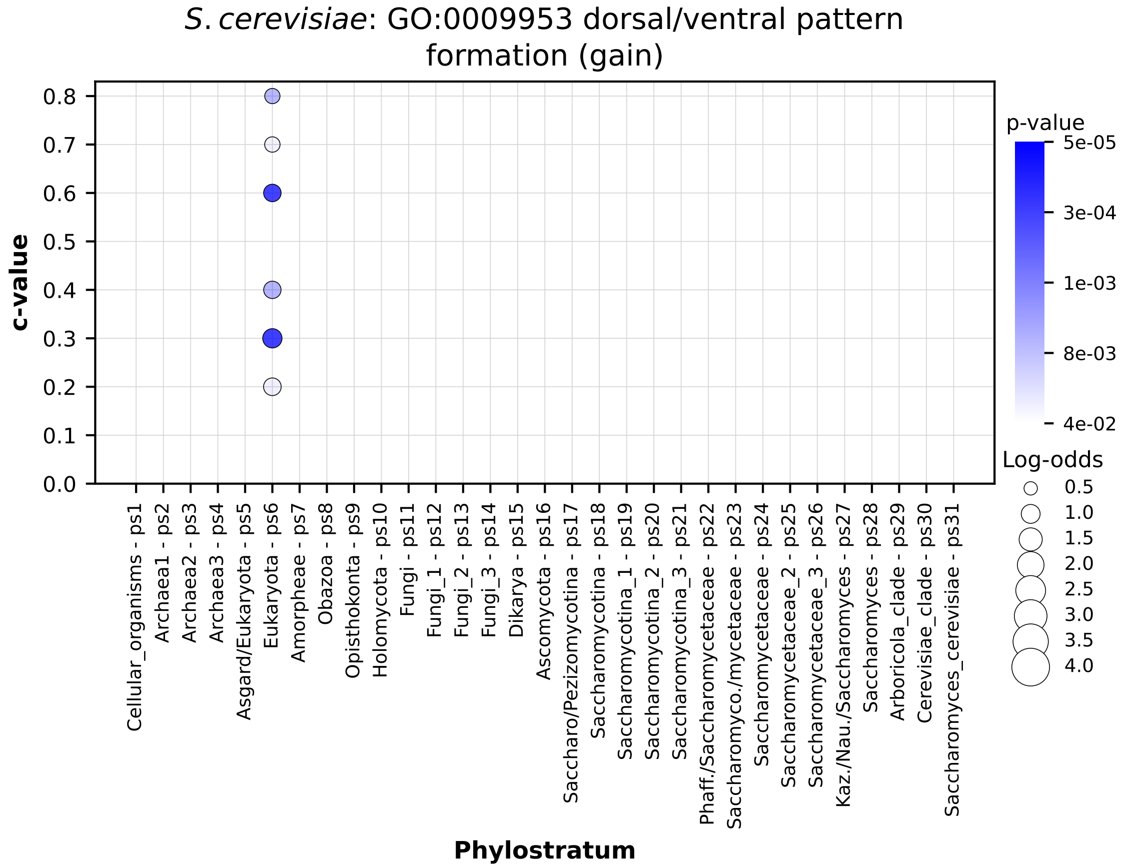

S. cerevisiae: GO:0009953 dorsal/ventral pattern formation (gain)
c-value
0.0
0.1
0.2
0.3
0.4
0.5
0.6
0.7
0.8
Phylostratum
Cellular_organisms - ps1
Archaea1 - ps2
Archaea2 - ps3
Archaea3 - ps4
Asgard/Eukaryota - ps5
Eukaryota - ps6
Amorpheae - ps7
Obazoa - ps8
Opisthokonta - ps9
Holomycota - ps10
Fungi - ps11
Fungi_1 - ps12
Fungi_2 - ps13
Fungi_3 - ps14
Dikarya - ps15
Ascomycota - ps16
Saccharo/Pezizomycotina - ps17
Saccharomycotina - ps18
Saccharomycotina_1 - ps19
Saccharomycotina_2 - ps20
Saccharomycotina_3 - ps21
Phaff./Saccharomycetaceae - ps22
Saccharomyco./mycetaceae - ps23
Saccharomycetaceae - ps24
Saccharomycetaceae_2 - ps25
Saccharomycetaceae_3 - ps26
Kaz./Nau./Saccharomyces - ps27
Saccharomyces - ps28
Arboricola_clade - ps29
Cerevisiae_clade - ps30
Saccharomyces_cerevisiae - ps31
p-value
5e-05
3e-04
1e-03
8e-03
4e-02
Log-odds
0.5
1.0
1.5
2.0
2.5
3.0
3.5
4.0

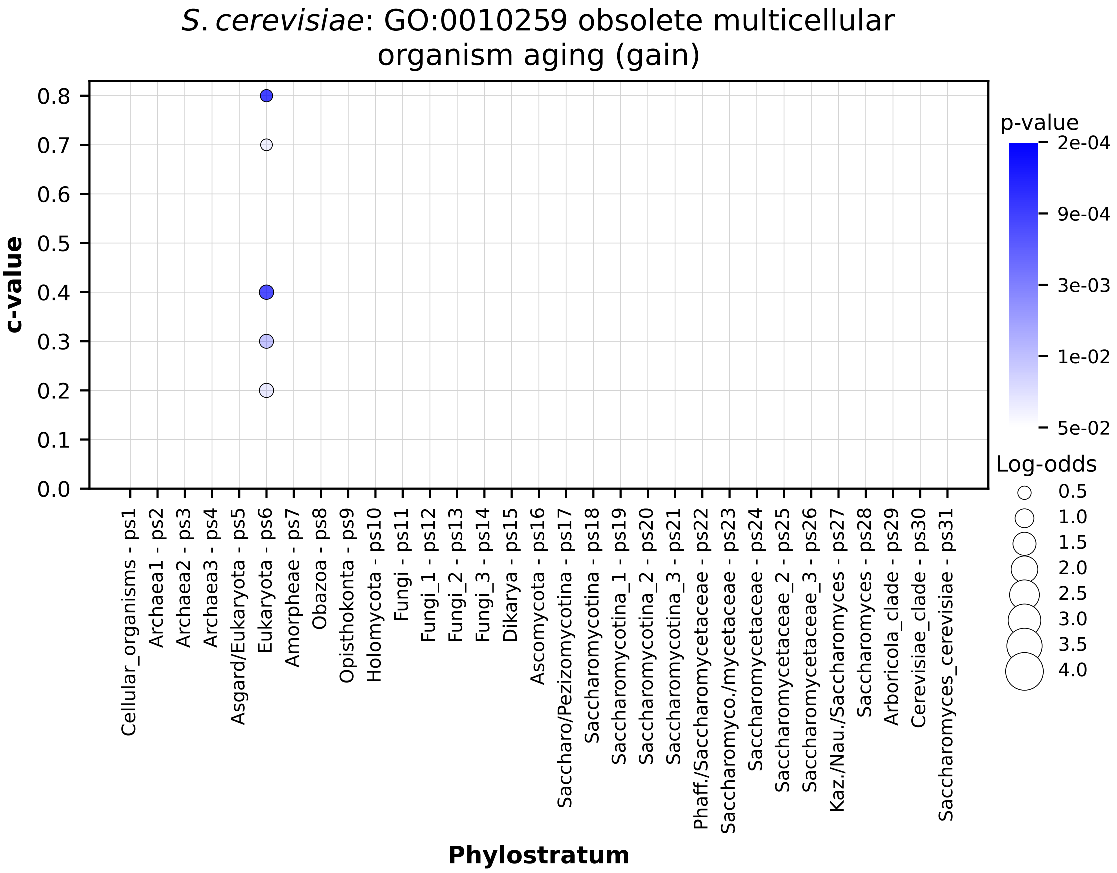

S. cerevisiae: GO:0010259 obsolete multicellular organism aging (gain)
c-value
0.0
0.1
0.2
0.3
0.4
0.5
0.6
0.7
0.8
Phylostratum
Cellular_organisms - ps1
Archaea1 - ps2
Archaea2 - ps3
Archaea3 - ps4
Asgard/Eukaryota - ps5
Eukaryota - ps6
Amorpheae - ps7
Obazoa - ps8
Opisthokonta - ps9
Holomycota - ps10
Fungi - ps11
Fungi_1 - ps12
Fungi_2 - ps13
Fungi_3 - ps14
Dikarya - ps15
Ascomycota - ps16
Saccharo/Pezizomycotina - ps17
Saccharomycotina - ps18
Saccharomycotina_1 - ps19
Saccharomycotina_2 - ps20
Saccharomycotina_3 - ps21
Phaff./Saccharomycetaceae - ps22
Saccharomyco./mycetaceae - ps23
Saccharomycetaceae - ps24
Saccharomycetaceae_2 - ps25
Saccharomycetaceae_3 - ps26
Kaz./Nau./Saccharomyces - ps27
Saccharomyces - ps28
Arboricola_clade - ps29
Cerevisiae_clade - ps30
Saccharomyces_cerevisiae - ps31
p-value
2e-04
9e-04
3e-03
1e-02
5e-02
Log-odds
0.5
1.0
1.5
2.0
2.5
3.0
3.5
4.0

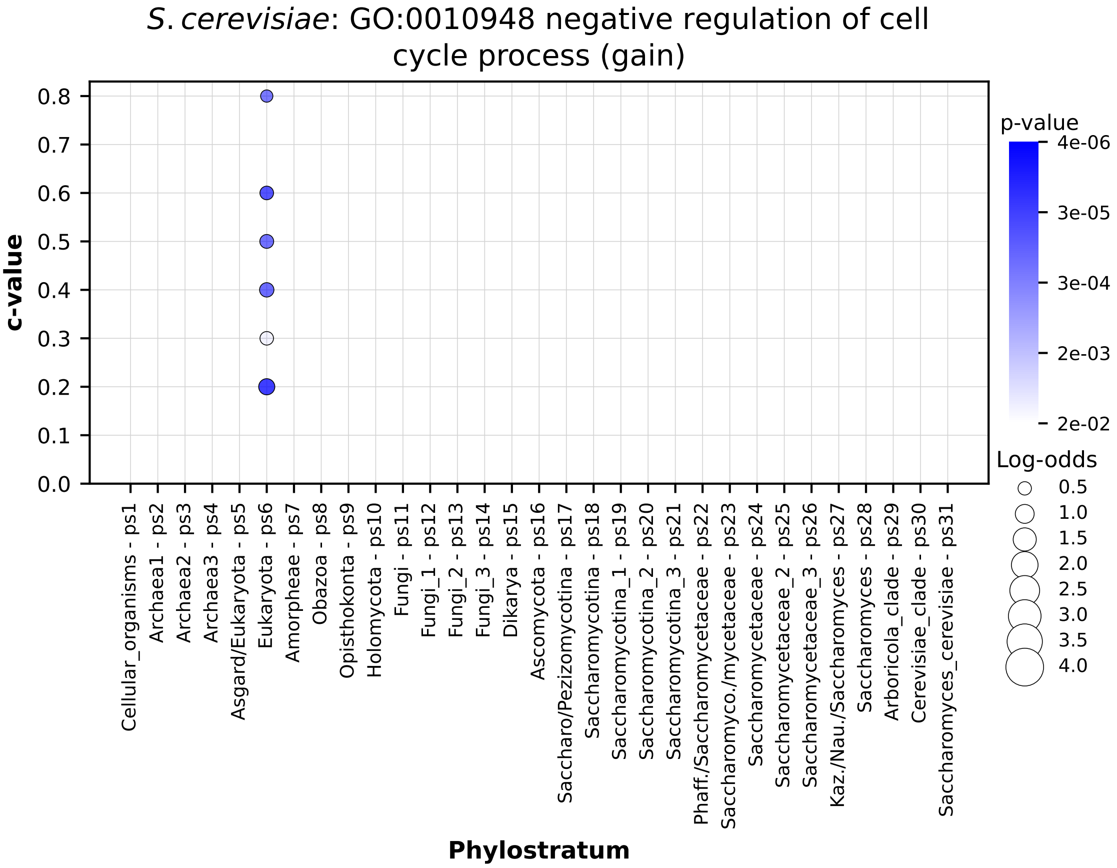
S. cerevisiae: GO:0010948 negative regulation of cell cycle process (gain)
c-value
0.0
0.1
0.2
0.3
0.4
0.5
0.6
0.7
0.8
p-value
4e-06
3e-05
3e-04
2e-03
2e-02
Log-odds
0.5
1.0
1.5
2.0
2.5
3.0
3.5
4.0
Cellular_organisms - ps1
Archaea1 - ps2
Archaea2 - ps3
Archaea3 - ps4
Asgard/Eukaryota - ps5
Eukaryota - ps6
Amorpheae - ps7
Obazoa - ps8
Opisthokonta - ps9
Holomycota - ps10
Fungi - ps11
Fungi_1 - ps12
Fungi_2 - ps13
Fungi_3 - ps14
Dikarya - ps15
Ascomycota - ps16
Saccharo/Pezizomycotina - ps17
Saccharomycotina - ps18
Saccharomycotina_1 - ps19
Saccharomycotina_2 - ps20
Saccharomycotina_3 - ps21
Phaff./Saccharomycetaceae - ps22
Saccharomyco./mycetaceae - ps23
Saccharomycetaceae - ps24
Saccharomycetaceae_2 - ps25
Saccharomycetaceae_3 - ps26
Kaz./Nau./Saccharomyces - ps27
Saccharomyces - ps28
Arboricola_clade - ps29
Cerevisiae_clade - ps30
Saccharomyces_cerevisiae - ps31
Phylostratum

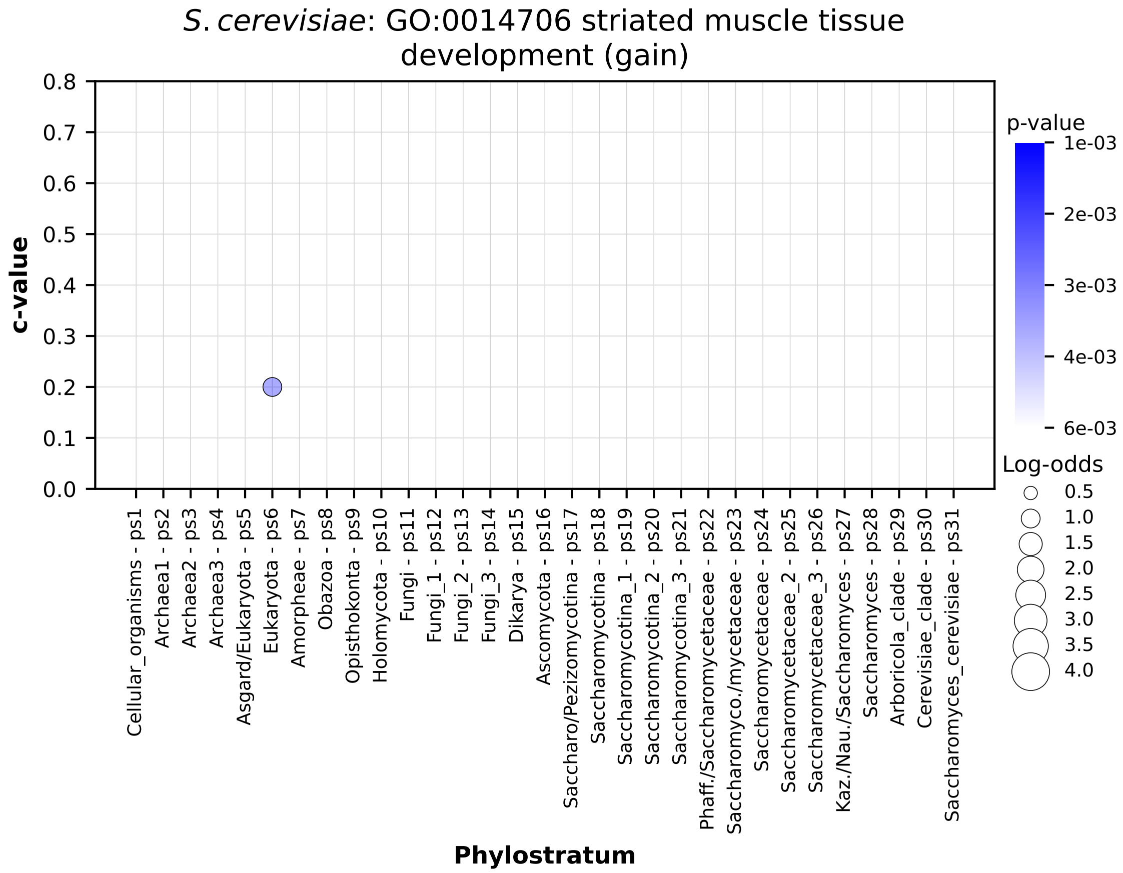

S. cerevisiae: GO:0014706 striated muscle tissue development (gain)
c-value
0.0
0.1
0.2
0.3
0.4
0.5
0.6
0.7
0.8
Phylostratum
Cellular_organisms - ps1
Archaea1 - ps2
Archaea2 - ps3
Archaea3 - ps4
Asgard/Eukaryota - ps5
Eukaryota - ps6
Amorpheae - ps7
Obazoa - ps8
Opisthokonta - ps9
Holomycota - ps10
Fungi - ps11
Fungi_1 - ps12
Fungi_2 - ps13
Fungi_3 - ps14
Dikarya - ps15
Ascomycota - ps16
Saccharo/Pezizomycotina - ps17
Saccharomycotina - ps18
Saccharomycotina_1 - ps19
Saccharomycotina_2 - ps20
Saccharomycotina_3 - ps21
Phaff./Saccharomycetaceae - ps22
Saccharomyco./mycetaceae - ps23
Saccharomycetaceae - ps24
Saccharomycetaceae_2 - ps25
Saccharomycetaceae_3 - ps26
Kaz./Nau./Saccharomyces - ps27
Saccharomyces - ps28
Arboricola_clade - ps29
Cerevisiae_clade - ps30
Saccharomyces_cerevisiae - ps31
p-value
1e-03
2e-03
3e-03
4e-03
6e-03
Log-odds
0.5
1.0
1.5
2.0
2.5
3.0
3.5
4.0

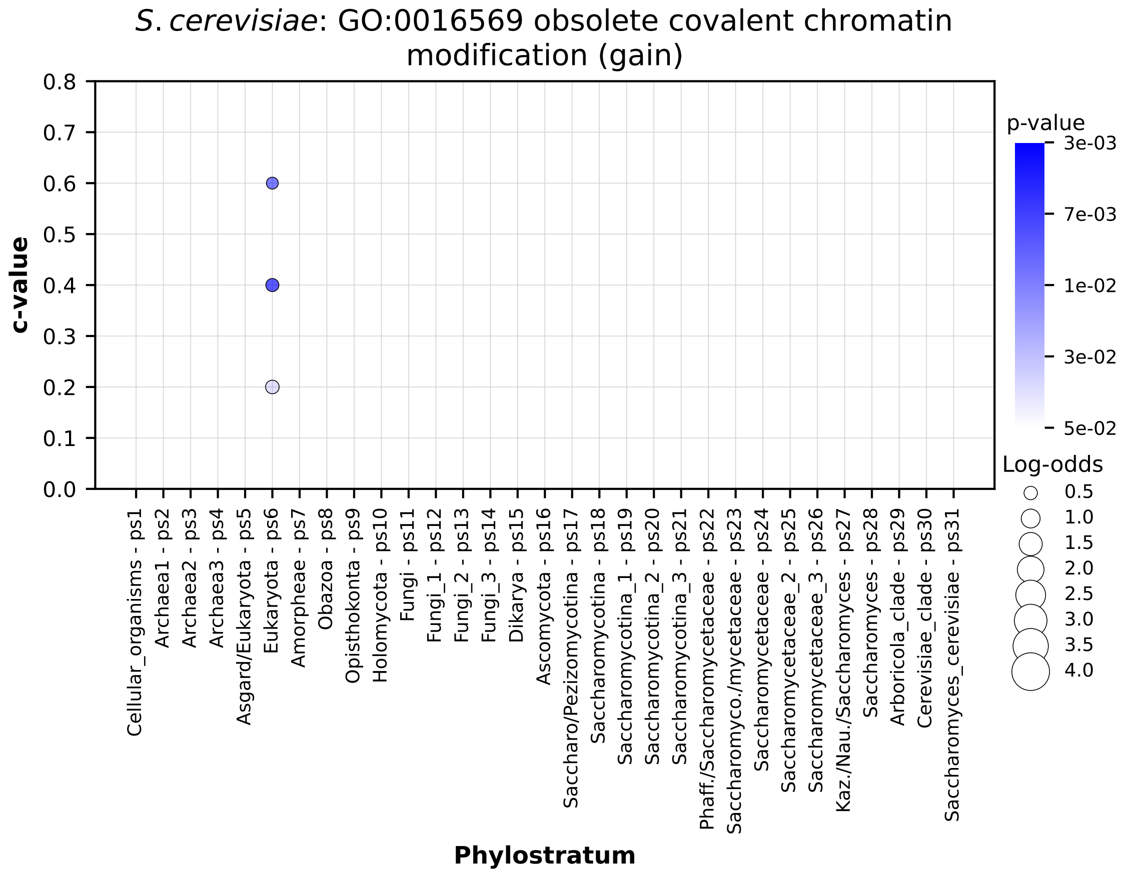

S. cerevisiae: GO:0016569 obsolete covalent chromatin modification (gain)
c-value
0.0
0.1
0.2
0.3
0.4
0.5
0.6
0.7
0.8
p-value
3e-03
7e-03
1e-02
3e-02
5e-02
Log-odds
0.5
1.0
1.5
2.0
2.5
3.0
3.5
4.0
Cellular_organisms - ps1
Archaea1 - ps2
Archaea2 - ps3
Archaea3 - ps4
Asgard/Eukaryota - ps5
Eukaryota - ps6
Amorpheae - ps7
Obazoa - ps8
Opisthokonta - ps9
Holomycota - ps10
Fungi - ps11
Fungi_1 - ps12
Fungi_2 - ps13
Fungi_3 - ps14
Dikarya - ps15
Ascomycota - ps16
Saccharo/Pezizomycotina - ps17
Saccharomycotina - ps18
Saccharomycotina_1 - ps19
Saccharomycotina_2 - ps20
Saccharomycotina_3 - ps21
Phaff./Saccharomycetaceae - ps22
Saccharomyco./mycetaceae - ps23
Saccharomycetaceae - ps24
Saccharomycetaceae_2 - ps25
Saccharomycetaceae_3 - ps26
Kaz./Nau./Saccharomyces - ps27
Saccharomyces - ps28
Arboricola_clade - ps29
Cerevisiae_clade - ps30
Saccharomyces_cerevisiae - ps31
Phylostratum

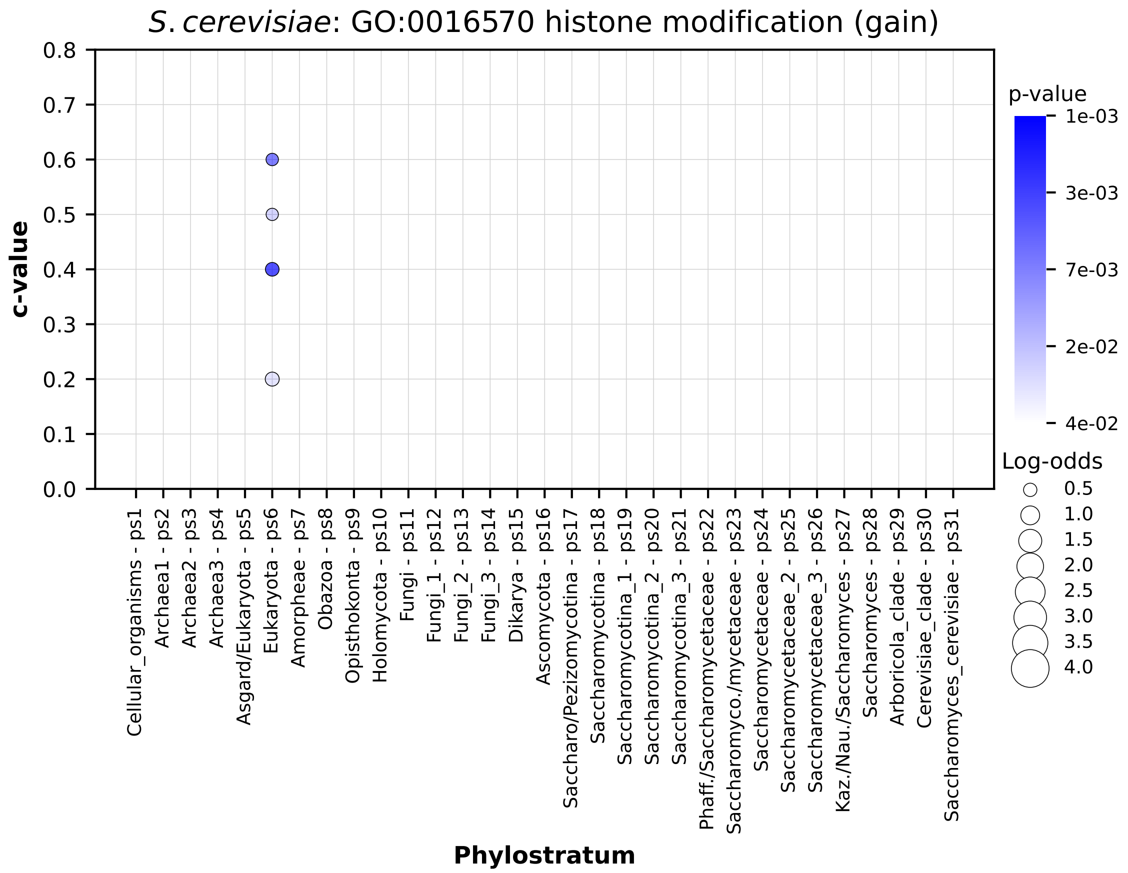
S. cerevisiae: GO:0016570 histone modification (gain)
c-value
0.8
0.7
0.6
0.5
0.4
0.3
0.2
0.1
0.0
Phylostratum
Cellular_organisms - ps1
Archaea1 - ps2
Archaea2 - ps3
Archaea3 - ps4
Asgard/Eukaryota - ps5
Eukaryota - ps6
Amorpheae - ps7
Obazoa - ps8
Opisthokonta - ps9
Holomycota - ps10
Fungi - ps11
Fungi_1 - ps12
Fungi_2 - ps13
Fungi_3 - ps14
Dikarya - ps15
Ascomycota - ps16
Saccharo/Pezizomycotina - ps17
Saccharomycotina - ps18
Saccharomycotina_1 - ps19
Saccharomycotina_2 - ps20
Saccharomycotina_3 - ps21
Phaff./Saccharomycetaceae - ps22
Saccharomyco./mycetaceae - ps23
Saccharomycetaceae - ps24
Saccharomycetaceae_2 - ps25
Saccharomycetaceae_3 - ps26
Kaz./Nau./Saccharomyces - ps27
Saccharomyces - ps28
Arboricola_clade - ps29
Cerevisiae_clade - ps30
Saccharomyces_cerevisiae - ps31
p-value
1e-03
3e-03
7e-03
2e-02
4e-02
Log-odds
0.5
1.0
1.5
2.0
2.5
3.0
3.5
4.0

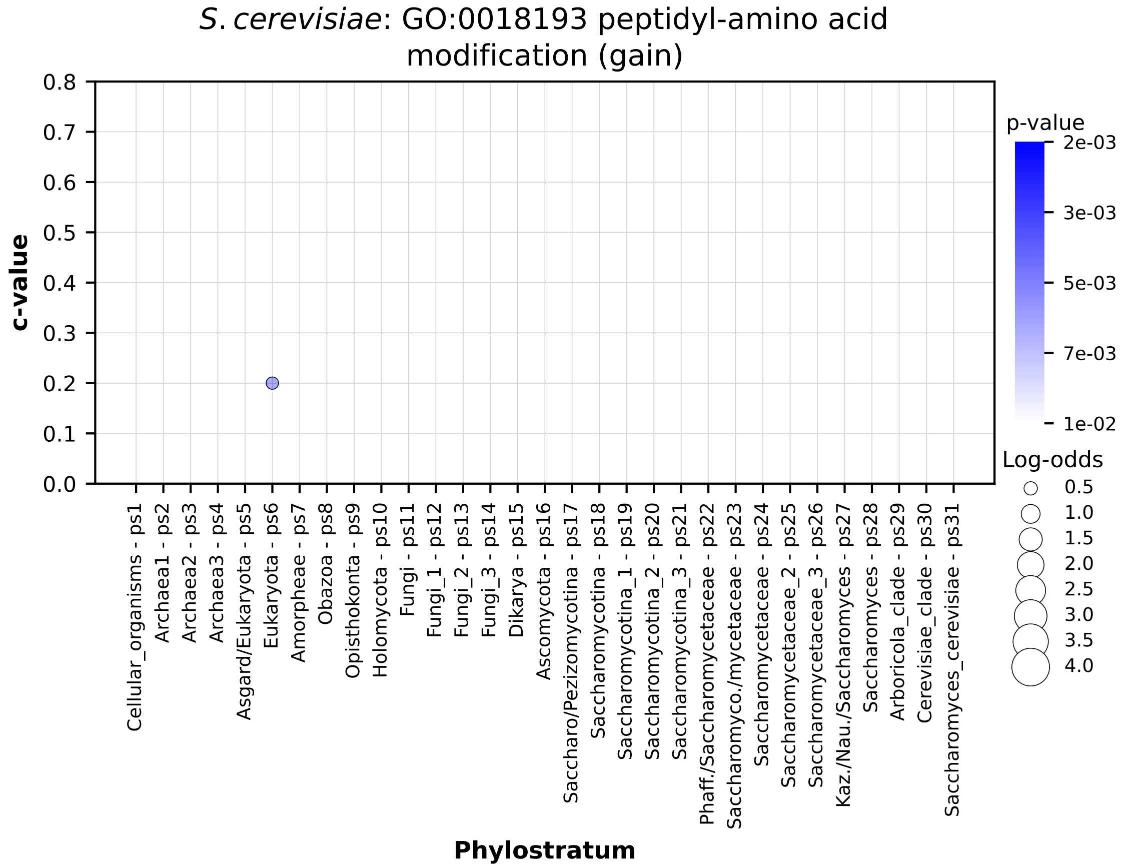

S. cerevisiae: GO:0018193 peptidyl-amino acid modification (gain)
c-value
0.8
0.7
0.6
0.5
0.4
0.3
0.2
0.1
0.0
Phylostratum
Cellular_organisms - ps1
Archaea1 - ps2
Archaea2 - ps3
Archaea3 - ps4
Asgard/Eukaryota - ps5
Eukaryota - ps6
Amorpheae - ps7
Obazoa - ps8
Opisthokonta - ps9
Holomycota - ps10
Fungi - ps11
Fungi_1 - ps12
Fungi_2 - ps13
Fungi_3 - ps14
Dikarya - ps15
Ascomycota - ps16
Saccharo/Pezizomycotina - ps17
Saccharomycotina - ps18
Saccharomycotina_1 - ps19
Saccharomycotina_2 - ps20
Saccharomycotina_3 - ps21
Phaff./Saccharomycetaceae - ps22
Saccharomyco./mycetaceae - ps23
Saccharomycetaceae - ps24
Saccharomycetaceae_2 - ps25
Saccharomycetaceae_3 - ps26
Kaz./Nau./Saccharomyces - ps27
Saccharomyces - ps28
Arboricola_clade - ps29
Cerevisiae_clade - ps30
Saccharomyces_cerevisiae - ps31
p-value
2e-03
3e-03
5e-03
7e-03
1e-02
Log-odds
0.5
1.0
1.5
2.0
2.5
3.0
3.5
4.0

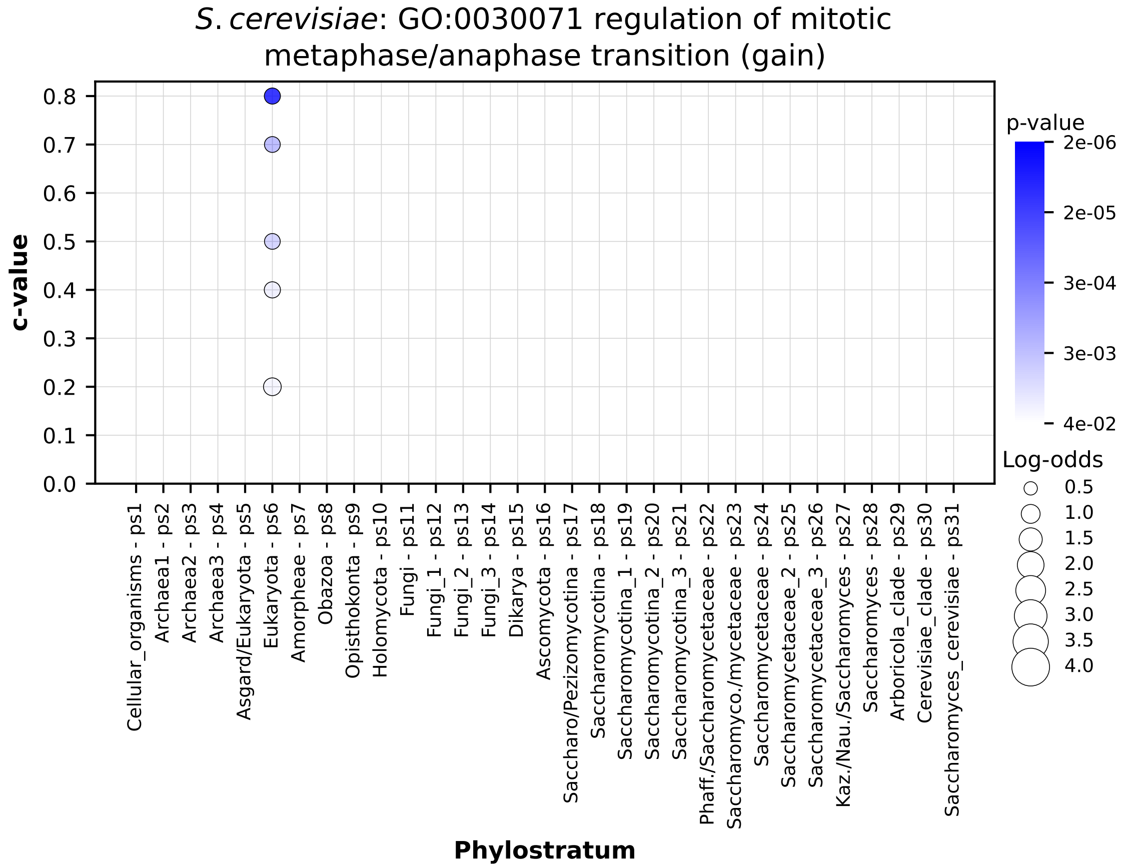
S. cerevisiae: GO:0030071 regulation of mitotic metaphase/anaphase transition (gain)
c-value
0.0
0.1
0.2
0.3
0.4
0.5
0.6
0.7
0.8
p-value
2e-06
2e-05
3e-04
3e-03
4e-02
Log-odds
0.5
1.0
1.5
2.0
2.5
3.0
3.5
4.0
Cellular_organisms - ps1
Archaea1 - ps2
Archaea2 - ps3
Archaea3 - ps4
Asgard/Eukaryota - ps5
Eukaryota - ps6
Amorpheae - ps7
Obazoa - ps8
Opisthokonta - ps9
Holomycota - ps10
Fungi - ps11
Fungi_1 - ps12
Fungi_2 - ps13
Fungi_3 - ps14
Dikarya - ps15
Ascomycota - ps16
Saccharo/Pezizomycotina - ps17
Saccharomycotina - ps18
Saccharomycotina_1 - ps19
Saccharomycotina_2 - ps20
Saccharomycotina_3 - ps21
Phaff./Saccharomycetaceae - ps22
Saccharomyco./mycetaceae - ps23
Saccharomycetaceae - ps24
Saccharomycetaceae_2 - ps25
Saccharomycetaceae_3 - ps26
Kaz./Nau./Saccharomyces - ps27
Saccharomyces - ps28
Arboricola_clade - ps29
Cerevisiae_clade - ps30
Saccharomyces_cerevisiae - ps31
Phylostratum

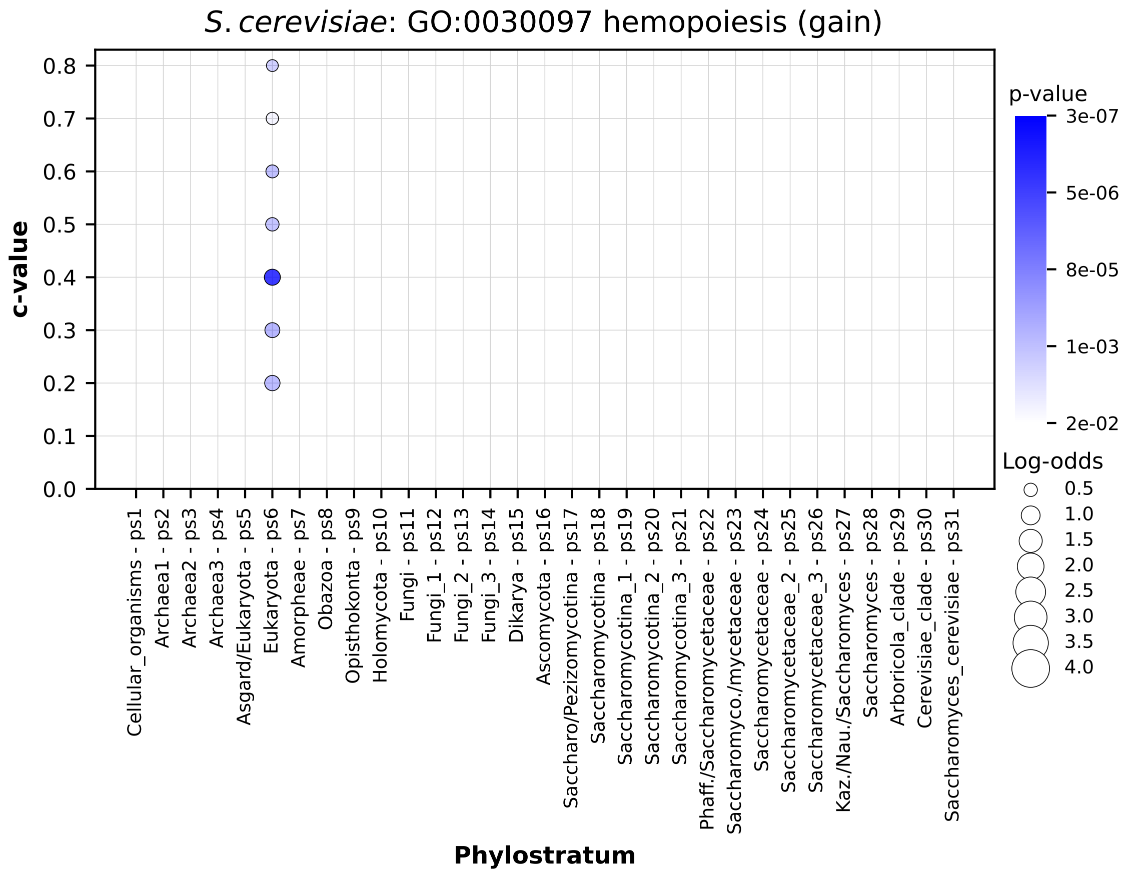
S. cerevisiae: GO:0030097 hemopoiesis (gain)
c-value
0.0
0.1
0.2
0.3
0.4
0.5
0.6
0.7
0.8
Phylostratum
Cellular_organisms - ps1
Archaea1 - ps2
Archaea2 - ps3
Archaea3 - ps4
Asgard/Eukaryota - ps5
Eukaryota - ps6
Amorpheae - ps7
Obazoa - ps8
Opisthokonta - ps9
Holomycota - ps10
Fungi - ps11
Fungi_1 - ps12
Fungi_2 - ps13
Fungi_3 - ps14
Dikarya - ps15
Ascomycota - ps16
Saccharo/Pezizomycotina - ps17
Saccharomycotina - ps18
Saccharomycotina_1 - ps19
Saccharomycotina_2 - ps20
Saccharomycotina_3 - ps21
Phaff./Saccharomycetaceae - ps22
Saccharomyco./mycetaceae - ps23
Saccharomycetaceae - ps24
Saccharomycetaceae_2 - ps25
Saccharomycetaceae_3 - ps26
Kaz./Nau./Saccharomyces - ps27
Saccharomyces - ps28
Arboricola_clade - ps29
Cerevisiae_clade - ps30
Saccharomyces_cerevisiae - ps31
p-value
3e-07
5e-06
8e-05
1e-03
2e-02
Log-odds
0.5
1.0
1.5
2.0
2.5
3.0
3.5
4.0

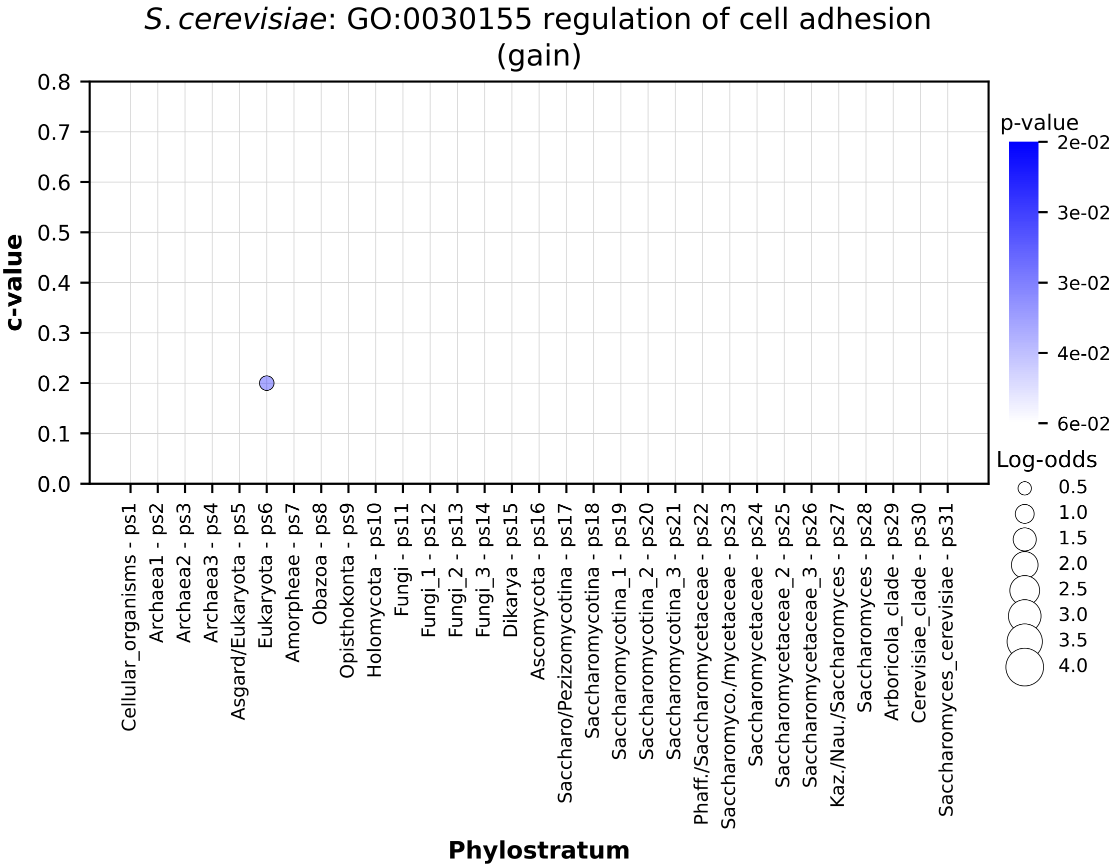

S. cerevisiae: GO:0030155 regulation of cell adhesion (gain)
c-value
0.8
0.7
0.6
0.5
0.4
0.3
0.2
0.1
0.0
Phylostratum
Cellular_organisms - ps1
Archaea1 - ps2
Archaea2 - ps3
Archaea3 - ps4
Asgard/Eukaryota - ps5
Eukaryota - ps6
Amorpheae - ps7
Obazoa - ps8
Opisthokonta - ps9
Holomycota - ps10
Fungi - ps11
Fungi_1 - ps12
Fungi_2 - ps13
Fungi_3 - ps14
Dikarya - ps15
Ascomycota - ps16
Saccharo/Pezizomycotina - ps17
Saccharomycotina - ps18
Saccharomycotina_1 - ps19
Saccharomycotina_2 - ps20
Saccharomycotina_3 - ps21
Phaff./Saccharomycetaceae - ps22
Saccharomyco./mycetaceae - ps23
Saccharomycetaceae - ps24
Saccharomycetaceae_2 - ps25
Saccharomycetaceae_3 - ps26
Kaz./Nau./Saccharomyces - ps27
Saccharomyces - ps28
Arboricola_clade - ps29
Cerevisiae_clade - ps30
Saccharomyces_cerevisiae - ps31
p-value
2e-02
3e-02
3e-02
4e-02
6e-02
Log-odds
0.5
1.0
1.5
2.0
2.5
3.0
3.5
4.0

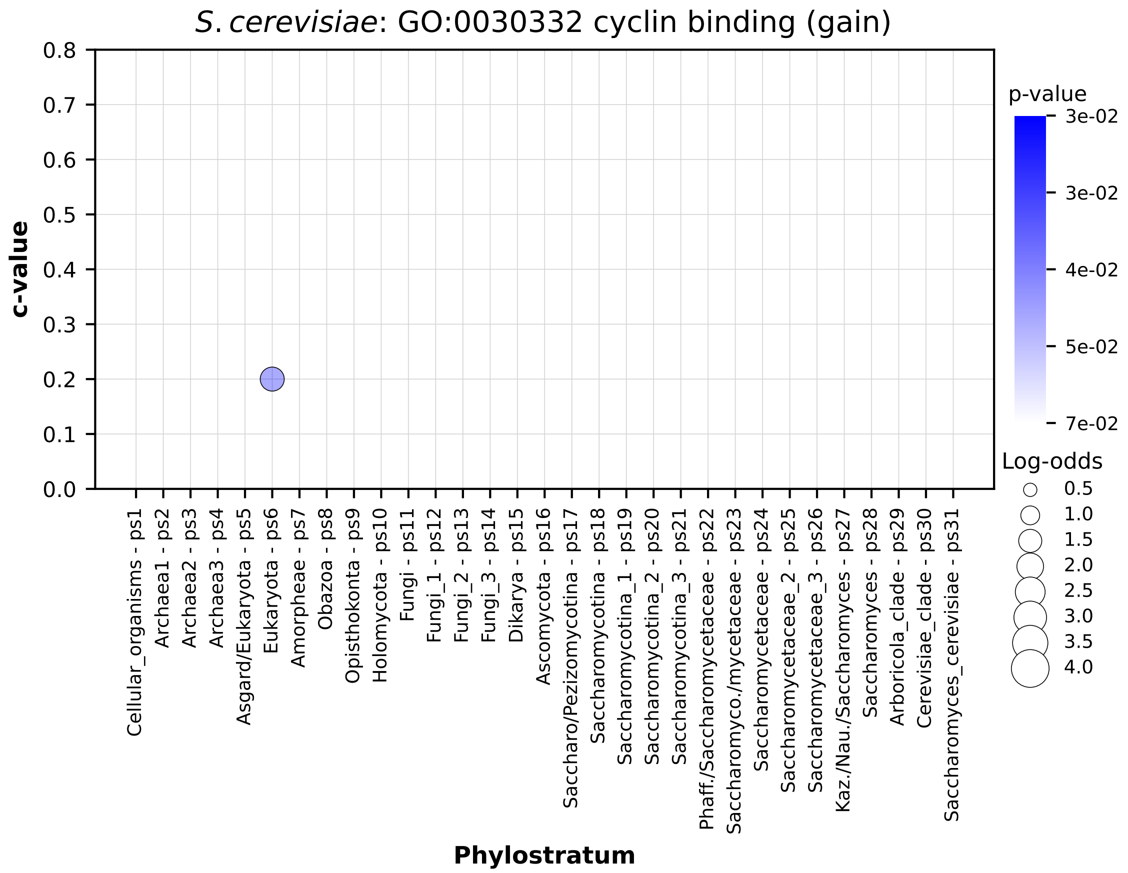

S. cerevisiae: GO:0030332 cyclin binding (gain)
c-value
0.0
0.1
0.2
0.3
0.4
0.5
0.6
0.7
0.8
Phylostratum
Cellular_organisms - ps1
Archaea1 - ps2
Archaea2 - ps3
Archaea3 - ps4
Asgard/Eukaryota - ps5
Eukaryota - ps6
Amorpheae - ps7
Obazoa - ps8
Opisthokonta - ps9
Holomycota - ps10
Fungi - ps11
Fungi_1 - ps12
Fungi_2 - ps13
Fungi_3 - ps14
Dikarya - ps15
Ascomycota - ps16
Saccharo/Pezizomycotina - ps17
Saccharomycotina - ps18
Saccharomycotina_1 - ps19
Saccharomycotina_2 - ps20
Saccharomycotina_3 - ps21
Phaff./Saccharomycetaceae - ps22
Saccharomyco./mycetaceae - ps23
Saccharomycetaceae - ps24
Saccharomycetaceae_2 - ps25
Saccharomycetaceae_3 - ps26
Kaz./Nau./Saccharomyces - ps27
Saccharomyces - ps28
Arboricola_clade - ps29
Cerevisiae_clade - ps30
Saccharomyces_cerevisiae - ps31
p-value
3e-02
3e-02
4e-02
5e-02
7e-02
Log-odds
0.5
1.0
1.5
2.0
2.5
3.0
3.5
4.0

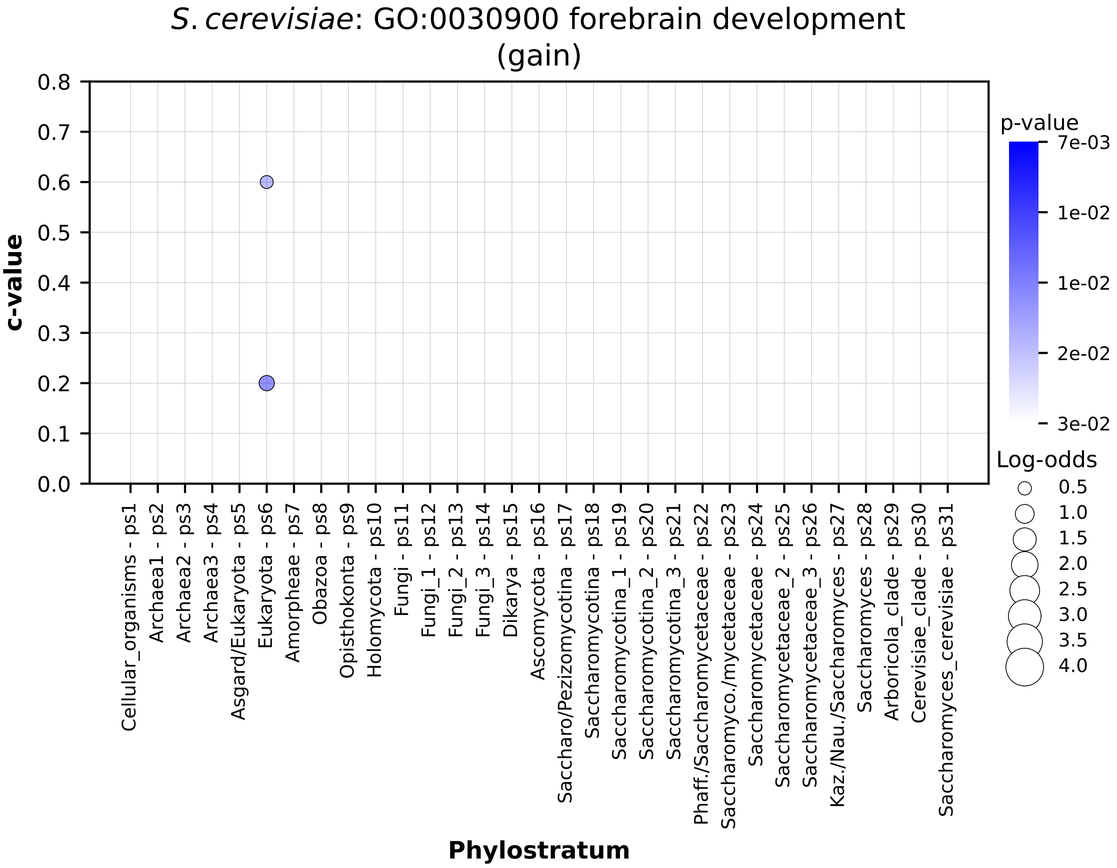

S. cerevisiae: GO:0030900 forebrain development
(gain)
c-value
0.8
0.7
0.6
0.5
0.4
0.3
0.2
0.1
0.0
p-value
7e-03
1e-02
1e-02
2e-02
3e-02
Log-odds
0.5
1.0
1.5
2.0
2.5
3.0
3.5
4.0
Cellular_organisms - ps1
Archaea1 - ps2
Archaea2 - ps3
Archaea3 - ps4
Asgard/Eukaryota - ps5
Eukaryota - ps6
Amorpheae - ps7
Obazoa - ps8
Opisthokonta - ps9
Holomycota - ps10
Fungi - ps11
Fungi_1 - ps12
Fungi_2 - ps13
Fungi_3 - ps14
Dikarya - ps15
Ascomycota - ps16
Saccharo/Pezizomycotina - ps17
Saccharomycotina - ps18
Saccharomycotina_1 - ps19
Saccharomycotina_2 - ps20
Saccharomycotina_3 - ps21
Phaff./Saccharomycetaceae - ps22
Saccharomyco./mycetaceae - ps23
Saccharomycetaceae - ps24
Saccharomycetaceae_2 - ps25
Saccharomycetaceae_3 - ps26
Kaz./Nau./Saccharomyces - ps27
Saccharomyces - ps28
Arboricola_clade - ps29
Cerevisiae_clade - ps30
Saccharomyces_cerevisiae - ps31
Phylostratum

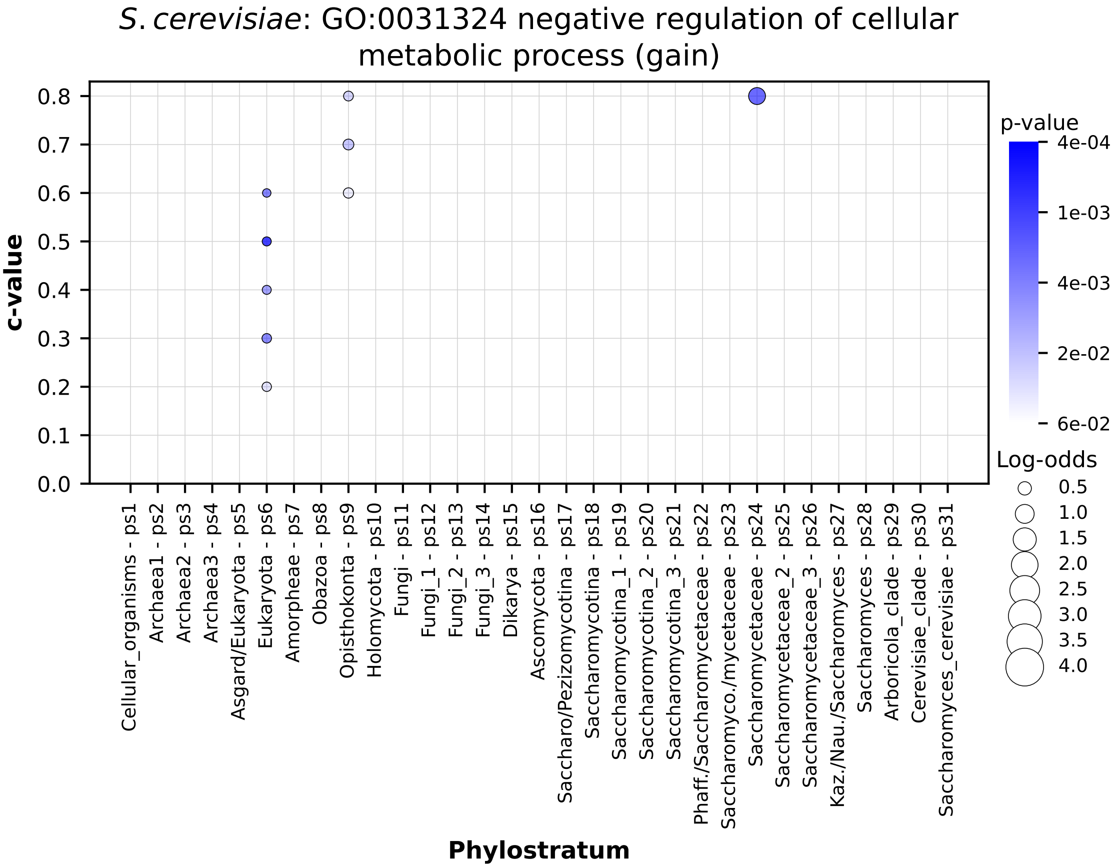

S. cerevisiae: GO:0031324 negative regulation of cellular metabolic process (gain)
c-value
0.0
0.1
0.2
0.3
0.4
0.5
0.6
0.7
0.8
Phylostratum
Cellular_organisms - ps1
Archaea1 - ps2
Archaea2 - ps3
Archaea3 - ps4
Asgard/Eukaryota - ps5
Eukaryota - ps6
Amorpheae - ps7
Obazoa - ps8
Opisthokonta - ps9
Holomycota - ps10
Fungi - ps11
Fungi_1 - ps12
Fungi_2 - ps13
Fungi_3 - ps14
Dikarya - ps15
Ascomycota - ps16
Saccharo/Pezizomycotina - ps17
Saccharomycotina - ps18
Saccharomycotina_1 - ps19
Saccharomycotina_2 - ps20
Saccharomycotina_3 - ps21
Phaff./Saccharomycetaceae - ps22
Saccharomyco./mycetaceae - ps23
Saccharomycetaceae - ps24
Saccharomycetaceae_2 - ps25
Saccharomycetaceae_3 - ps26
Kaz./Nau./Saccharomyces - ps27
Saccharomyces - ps28
Arboricola_clade - ps29
Cerevisiae_clade - ps30
Saccharomyces_cerevisiae - ps31
p-value
4e-04
1e-03
4e-03
2e-02
6e-02
Log-odds
0.5
1.0
1.5
2.0
2.5
3.0
3.5
4.0

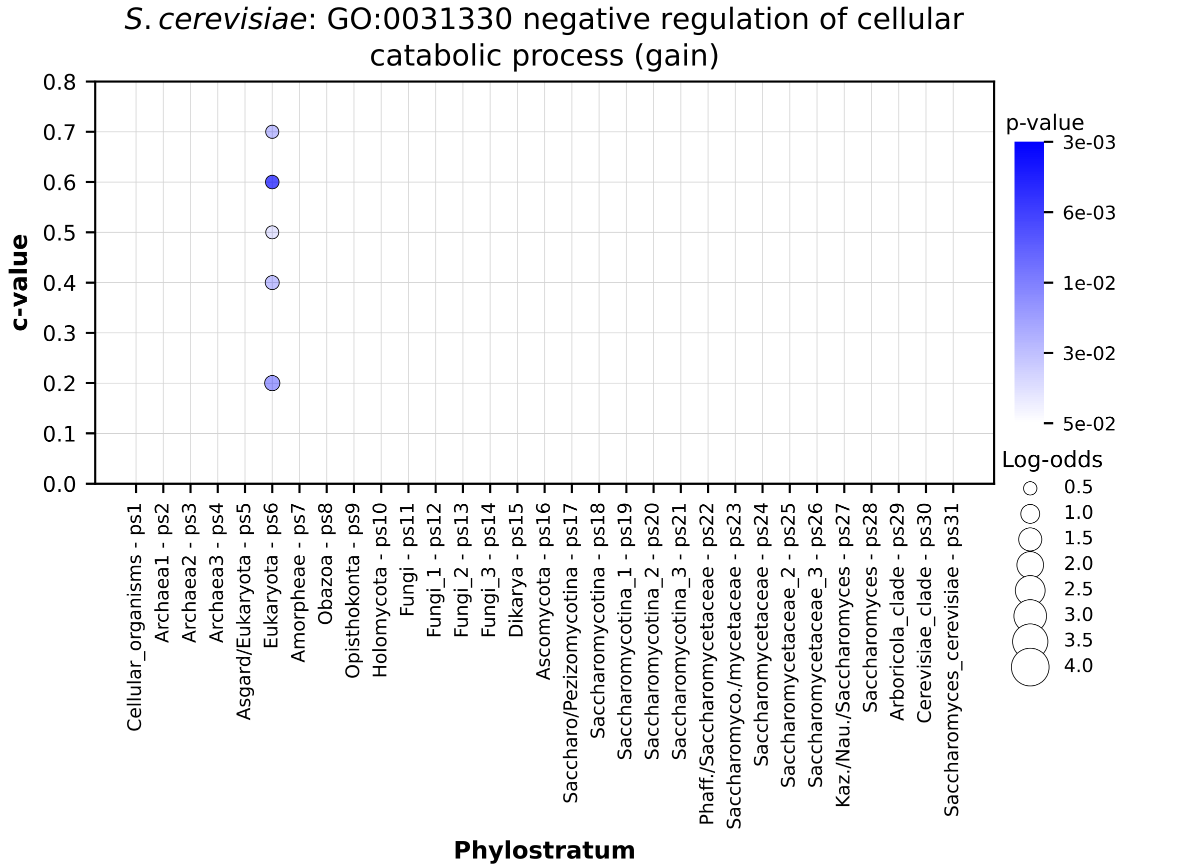

S. cerevisiae: GO:0031330 negative regulation of cellular catabolic process (gain)
c-value
0.0
0.1
0.2
0.3
0.4
0.5
0.6
0.7
0.8
Phylostratum
Cellular_organisms - ps1
Archaea1 - ps2
Archaea2 - ps3
Archaea3 - ps4
Asgard/Eukaryota - ps5
Eukaryota - ps6
Amorpheae - ps7
Obazoa - ps8
Opisthokonta - ps9
Holomycota - ps10
Fungi - ps11
Fungi_1 - ps12
Fungi_2 - ps13
Fungi_3 - ps14
Dikarya - ps15
Ascomycota - ps16
Saccharo/Pezizomycotina - ps17
Saccharomycotina - ps18
Saccharomycotina_1 - ps19
Saccharomycotina_2 - ps20
Saccharomycotina_3 - ps21
Phaff./Saccharomycetaceae - ps22
Saccharomyco./mycetaceae - ps23
Saccharomycetaceae - ps24
Saccharomycetaceae_2 - ps25
Saccharomycetaceae_3 - ps26
Kaz./Nau./Saccharomyces - ps27
Saccharomyces - ps28
Arboricola_clade - ps29
Cerevisiae_clade - ps30
Saccharomyces_cerevisiae - ps31
p-value
3e-03
6e-03
1e-02
3e-02
5e-02
Log-odds
0.5
1.0
1.5
2.0
2.5
3.0
3.5
4.0

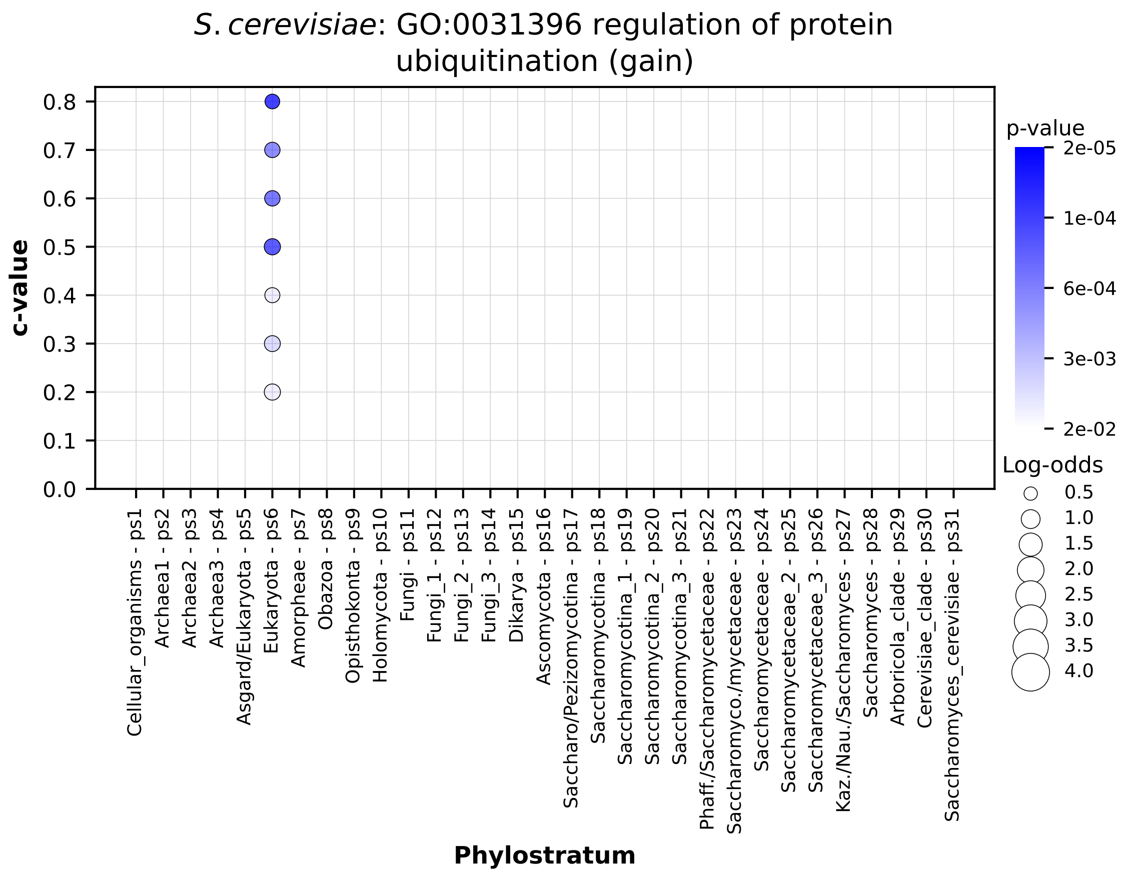

S. cerevisiae: GO:0031396 regulation of protein ubiquitination (gain)
c-value
0.0
0.1
0.2
0.3
0.4
0.5
0.6
0.7
0.8
Phylostratum
Cellular_organisms - ps1
Archaea1 - ps2
Archaea2 - ps3
Archaea3 - ps4
Asgard/Eukaryota - ps5
Eukaryota - ps6
Amorpheae - ps7
Obazoa - ps8
Opisthokonta - ps9
Holomycota - ps10
Fungi - ps11
Fungi_1 - ps12
Fungi_2 - ps13
Fungi_3 - ps14
Dikarya - ps15
Ascomycota - ps16
Saccharo/Pezizomycotina - ps17
Saccharomycotina - ps18
Saccharomycotina_1 - ps19
Saccharomycotina_2 - ps20
Saccharomycotina_3 - ps21
Phaff./Saccharomycetaceae - ps22
Saccharomyco./mycetaceae - ps23
Saccharomycetaceae - ps24
Saccharomycetaceae_2 - ps25
Saccharomycetaceae_3 - ps26
Kaz./Nau./Saccharomyces - ps27
Saccharomyces - ps28
Arboricola_clade - ps29
Cerevisiae_clade - ps30
Saccharomyces_cerevisiae - ps31
p-value
2e-05
1e-04
6e-04
3e-03
2e-02
Log-odds
0.5
1.0
1.5
2.0
2.5
3.0
3.5
4.0

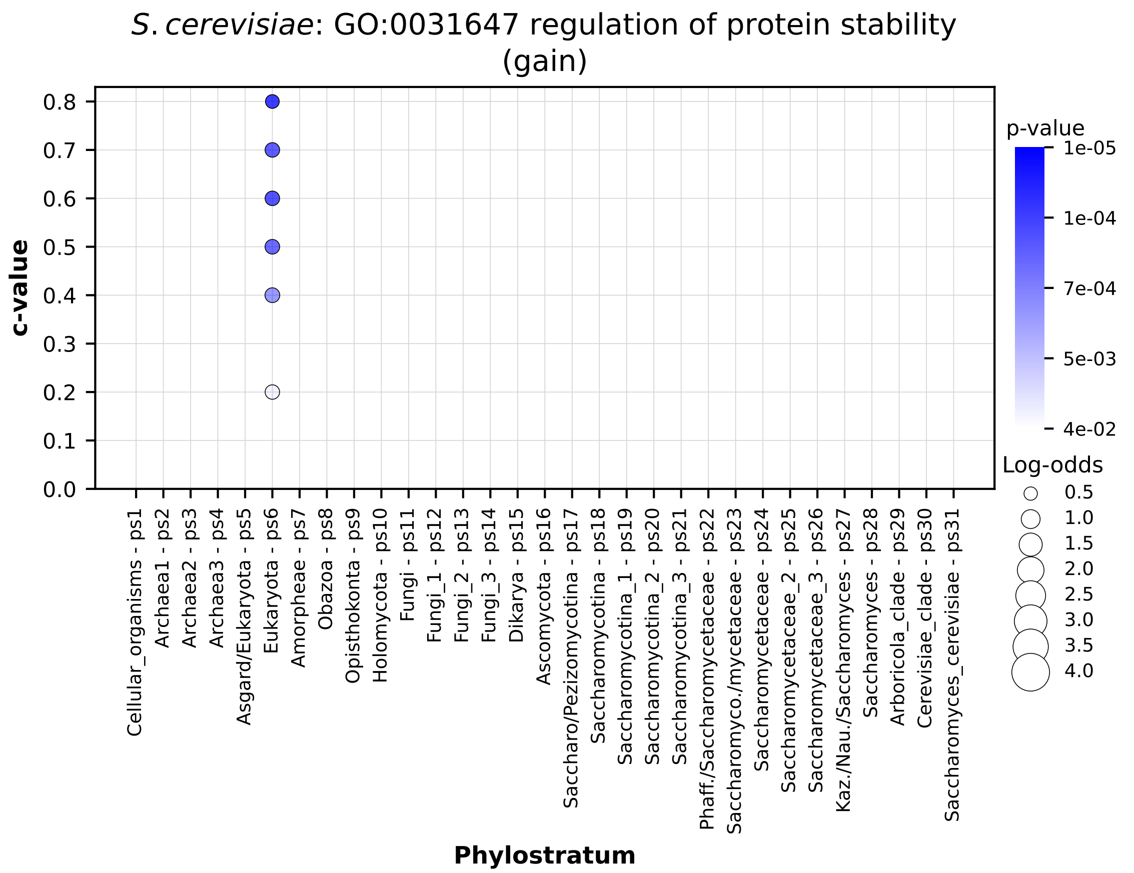

S. cerevisiae: GO:0031647 regulation of protein stability
(gain)
c-value
0.0
0.1
0.2
0.3
0.4
0.5
0.6
0.7
0.8
Phylostratum
Cellular_organisms - ps1
Archaea1 - ps2
Archaea2 - ps3
Archaea3 - ps4
Asgard/Eukaryota - ps5
Eukaryota - ps6
Amorpheae - ps7
Obazoa - ps8
Opisthokonta - ps9
Holomycota - ps10
Fungi - ps11
Fungi_1 - ps12
Fungi_2 - ps13
Fungi_3 - ps14
Dikarya - ps15
Ascomycota - ps16
Saccharo/Pezizomycotina - ps17
Saccharomycotina - ps18
Saccharomycotina_1 - ps19
Saccharomycotina_2 - ps20
Saccharomycotina_3 - ps21
Phaff./Saccharomycetaceae - ps22
Saccharomyco./mycetaceae - ps23
Saccharomycetaceae - ps24
Saccharomycetaceae_2 - ps25
Saccharomycetaceae_3 - ps26
Kaz./Nau./Saccharomyces - ps27
Saccharomyces - ps28
Arboricola_clade - ps29
Cerevisiae_clade - ps30
Saccharomyces_cerevisiae - ps31
p-value
1e-05
1e-04
7e-04
5e-03
4e-02
Log-odds
0.5
1.0
1.5
2.0
2.5
3.0
3.5
4.0

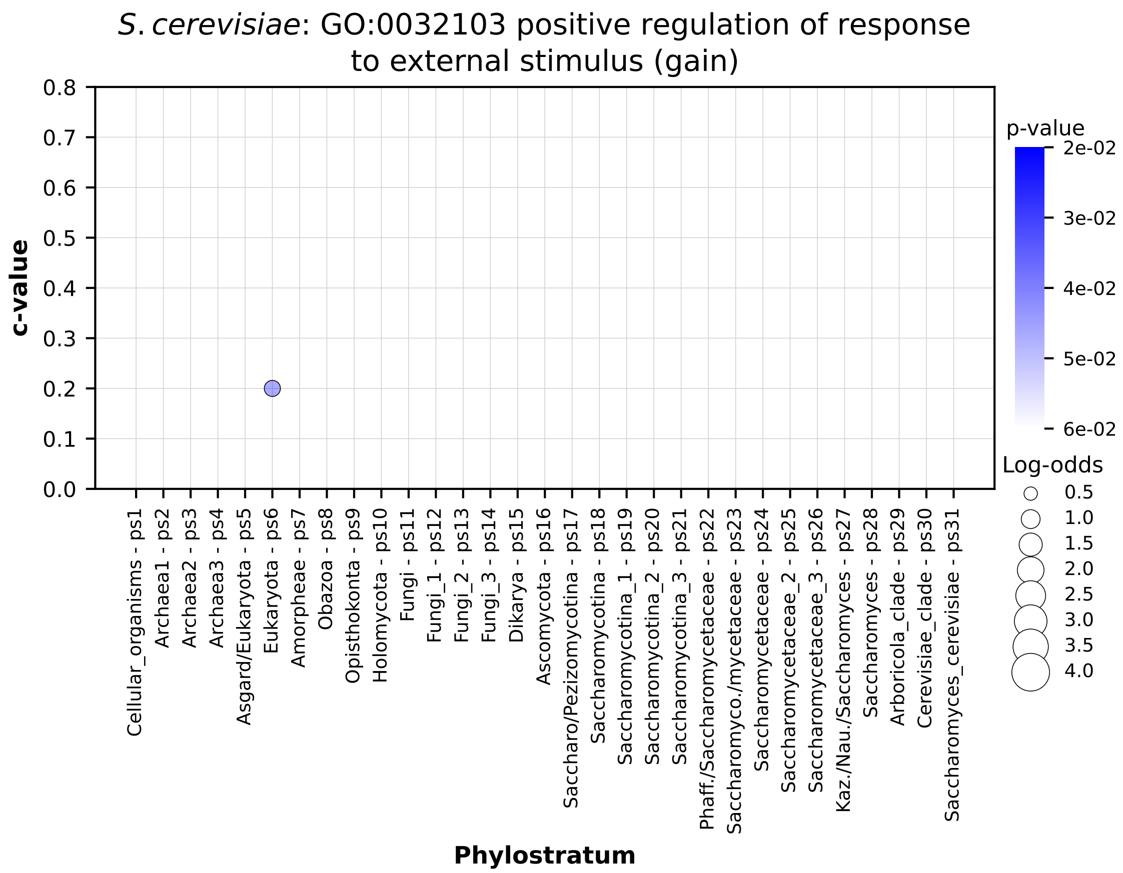

S. cerevisiae: GO:0032103 positive regulation of response to external stimulus (gain)
c-value
0.8
0.7
0.6
0.5
0.4
0.3
0.2
0.1
0.0
Phylostratum
Cellular_organisms - ps1
Archaea1 - ps2
Archaea2 - ps3
Archaea3 - ps4
Asgard/Eukaryota - ps5
Eukaryota - ps6
Amorpheae - ps7
Obazoa - ps8
Opisthokonta - ps9
Holomycota - ps10
Fungi - ps11
Fungi_1 - ps12
Fungi_2 - ps13
Fungi_3 - ps14
Dikarya - ps15
Ascomycota - ps16
Saccharo/Pezizomycotina - ps17
Saccharomycotina - ps18
Saccharomycotina_1 - ps19
Saccharomycotina_2 - ps20
Saccharomycotina_3 - ps21
Phaff./Saccharomycetaceae - ps22
Saccharomyco./mycetaceae - ps23
Saccharomycetaceae - ps24
Saccharomycetaceae_2 - ps25
Saccharomycetaceae_3 - ps26
Kaz./Nau./Saccharomyces - ps27
Saccharomyces - ps28
Arboricola_clade - ps29
Cerevisiae_clade - ps30
Saccharomyces_cerevisiae - ps31
p-value
2e-02
3e-02
4e-02
5e-02
6e-02
Log-odds
0.5
1.0
1.5
2.0
2.5
3.0
3.5
4.0

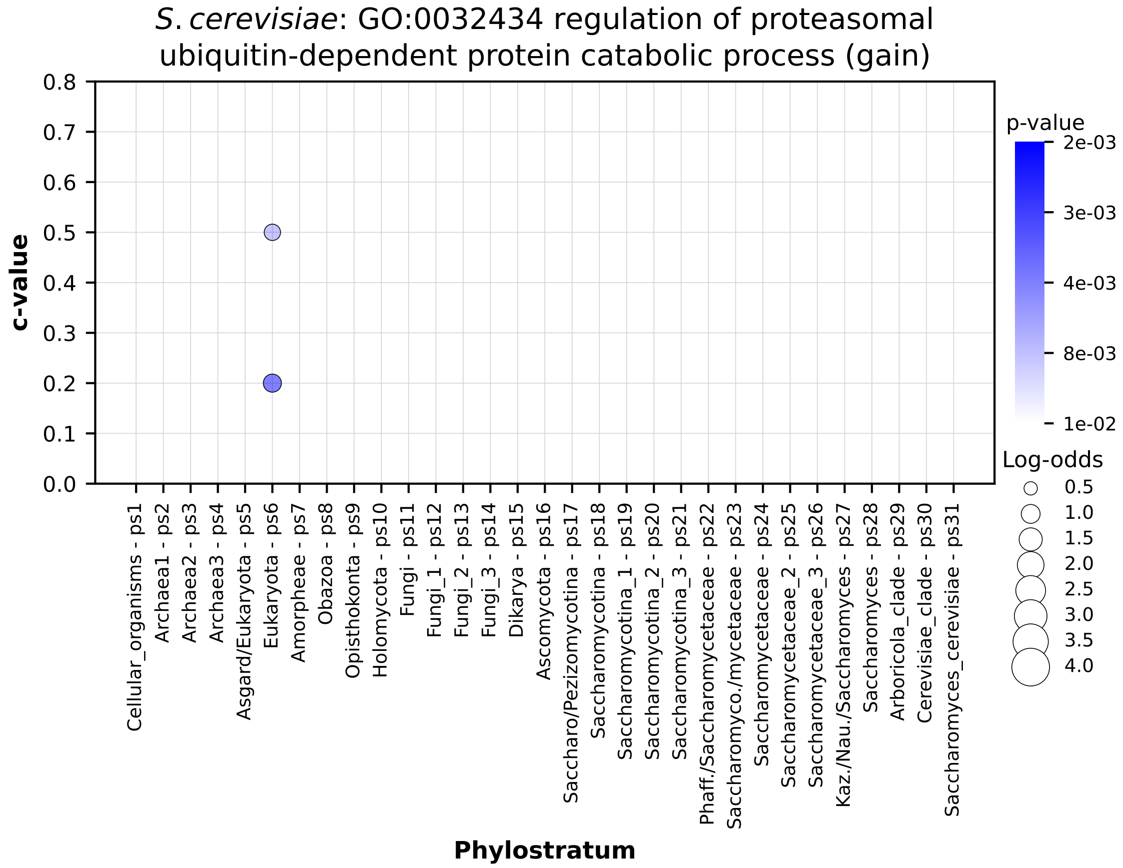

S. cerevisiae: GO:0032434 regulation of proteasomal ubiquitin-dependent protein catabolic process (gain)
c-value
0.0
0.1
0.2
0.3
0.4
0.5
0.6
0.7
0.8
p-value
2e-03
3e-03
4e-03
8e-03
1e-02
Log-odds
0.5
1.0
1.5
2.0
2.5
3.0
3.5
4.0
Cellular_organisms - ps1
Archaea1 - ps2
Archaea2 - ps3
Archaea3 - ps4
Asgard/Eukaryota - ps5
Eukaryota - ps6
Amorpheae - ps7
Obazoa - ps8
Opisthokonta - ps9
Holomycota - ps10
Fungi - ps11
Fungi_1 - ps12
Fungi_2 - ps13
Fungi_3 - ps14
Dikarya - ps15
Ascomycota - ps16
Saccharo/Pezizomycotina - ps17
Saccharomycotina - ps18
Saccharomycotina_1 - ps19
Saccharomycotina_2 - ps20
Saccharomycotina_3 - ps21
Phaff./Saccharomycetaceae - ps22
Saccharomyco./mycetaceae - ps23
Saccharomycetaceae - ps24
Saccharomycetaceae_2 - ps25
Saccharomycetaceae_3 - ps26
Kaz./Nau./Saccharomyces - ps27
Saccharomyces - ps28
Arboricola_clade - ps29
Cerevisiae_clade - ps30
Saccharomyces_cerevisiae - ps31
Phylostratum

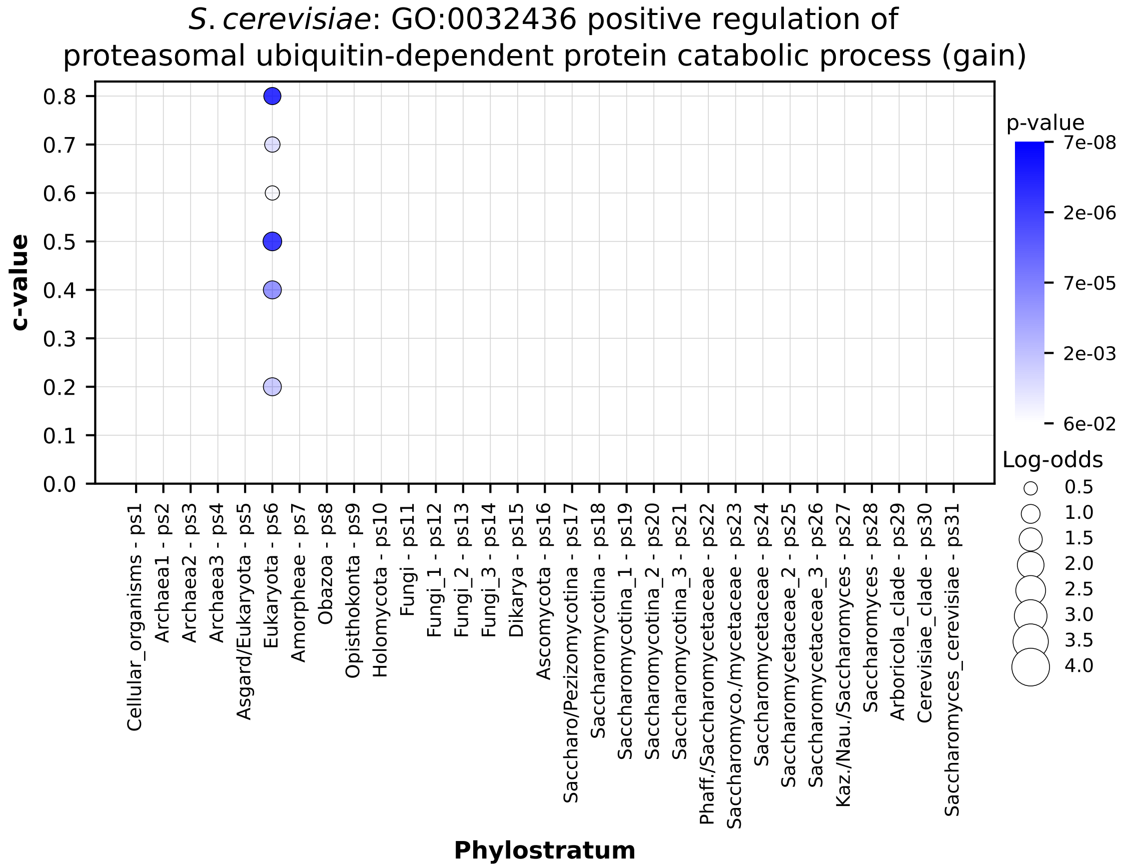

S. cerevisiae: GO:0032436 positive regulation of
proteasomal ubiquitin-dependent protein catabolic process (gain)
c-value
0.8
0.7
0.6
0.5
0.4
0.3
0.2
0.1
0.0
p-value
7e-08
2e-06
7e-05
2e-03
6e-02
Log-odds
0.5
1.0
1.5
2.0
2.5
3.0
3.5
4.0
Cellular_organisms - ps1
Archaea1 - ps2
Archaea2 - ps3
Archaea3 - ps4
Asgard/Eukaryota - ps5
Eukaryota - ps6
Amorpheae - ps7
Obazoa - ps8
Opisthokonta - ps9
Holomycota - ps10
Fungi - ps11
Fungi_1 - ps12
Fungi_2 - ps13
Fungi_3 - ps14
Dikarya - ps15
Ascomycota - ps16
Saccharo/Pezizomycotina - ps17
Saccharomycotina - ps18
Saccharomycotina_1 - ps19
Saccharomycotina_2 - ps20
Saccharomycotina_3 - ps21
Phaff./Saccharomycetaceae - ps22
Saccharomyco./mycetaceae - ps23
Saccharomycetaceae - ps24
Saccharomycetaceae_2 - ps25
Saccharomycetaceae_3 - ps26
Kaz./Nau./Saccharomyces - ps27
Saccharomyces - ps28
Arboricola_clade - ps29
Cerevisiae_clade - ps30
Saccharomyces_cerevisiae - ps31
Phylostratum

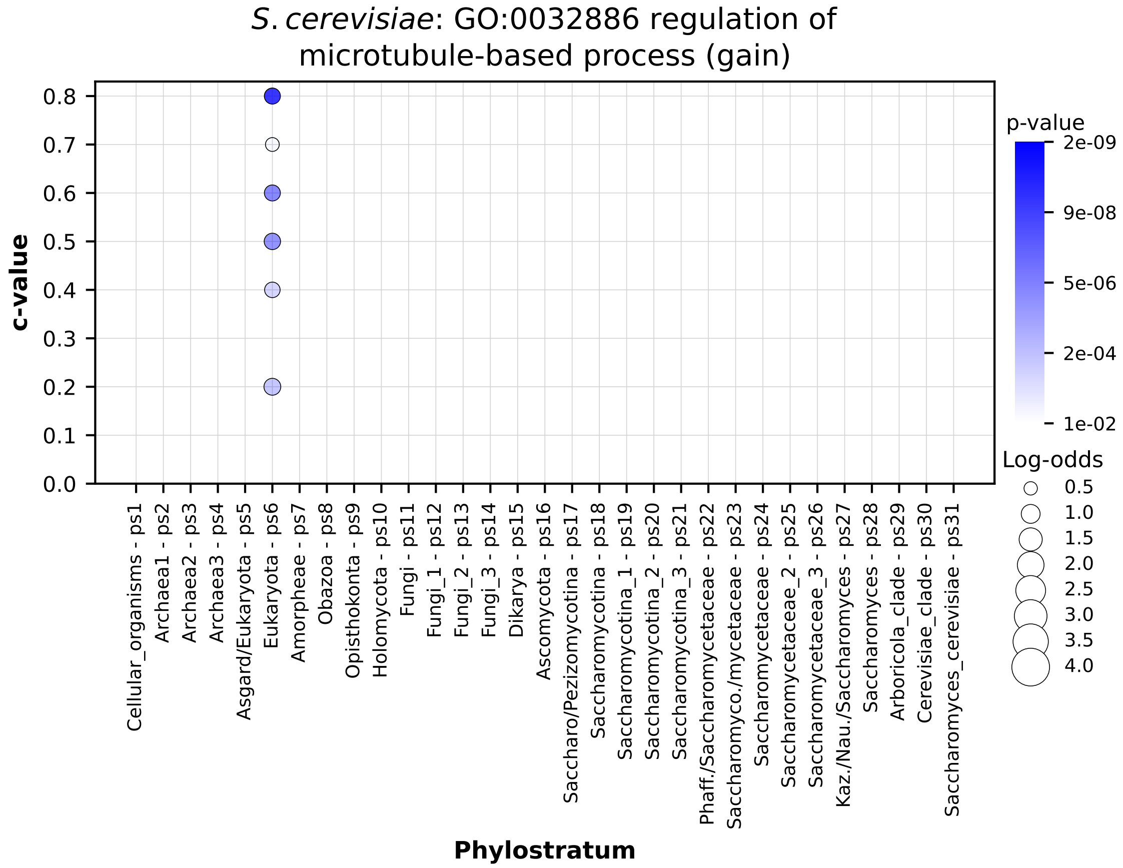
S. cerevisiae: GO:0032886 regulation of microtubule-based process (gain)
c-value
0.0
0.1
0.2
0.3
0.4
0.5
0.6
0.7
0.8
p-value
2e-09
9e-08
5e-06
2e-04
1e-02
Log-odds
0.5
1.0
1.5
2.0
2.5
3.0
3.5
4.0
Cellular_organisms - ps1
Archaea1 - ps2
Archaea2 - ps3
Archaea3 - ps4
Asgard/Eukaryota - ps5
Eukaryota - ps6
Amorpheae - ps7
Obazoa - ps8
Opisthokonta - ps9
Holomycota - ps10
Fungi - ps11
Fungi_1 - ps12
Fungi_2 - ps13
Fungi_3 - ps14
Dikarya - ps15
Ascomycota - ps16
Saccharo/Pezizomycotina - ps17
Saccharomycotina - ps18
Saccharomycotina_1 - ps19
Saccharomycotina_2 - ps20
Saccharomycotina_3 - ps21
Phaff./Saccharomycetaceae - ps22
Saccharomyco./mycetaceae - ps23
Saccharomycetaceae - ps24
Saccharomycetaceae_2 - ps25
Saccharomycetaceae_3 - ps26
Kaz./Nau./Saccharomyces - ps27
Saccharomyces - ps28
Arboricola_clade - ps29
Cerevisiae_clade - ps30
Saccharomyces_cerevisiae - ps31
Phylostratum

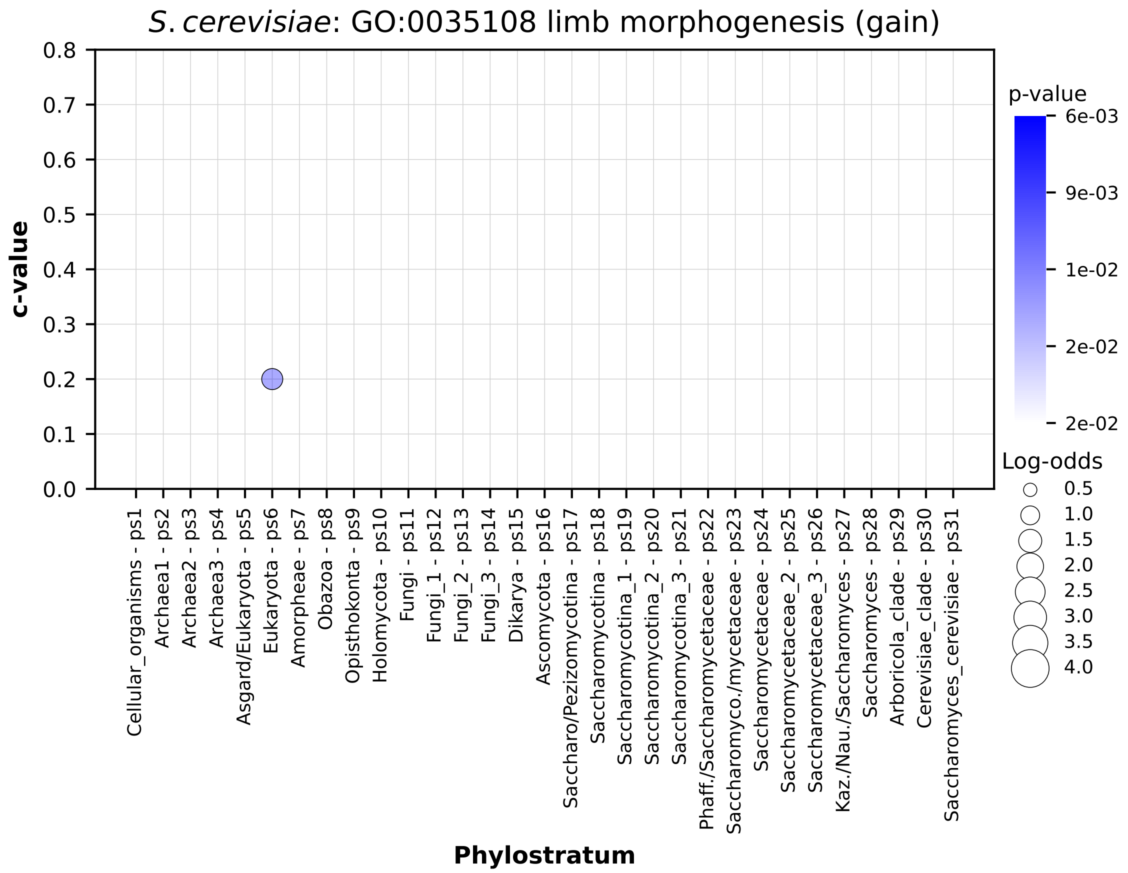

S. cerevisiae: GO:0035108 limb morphogenesis (gain)
c-value
0.0
0.1
0.2
0.3
0.4
0.5
0.6
0.7
0.8
Phylostratum
Cellular_organisms - ps1
Archaea1 - ps2
Archaea2 - ps3
Archaea3 - ps4
Asgard/Eukaryota - ps5
Eukaryota - ps6
Amorpheae - ps7
Obazoa - ps8
Opisthokonta - ps9
Holomycota - ps10
Fungi - ps11
Fungi_1 - ps12
Fungi_2 - ps13
Fungi_3 - ps14
Dikarya - ps15
Ascomycota - ps16
Saccharo/Pezizomycotina - ps17
Saccharomycotina - ps18
Saccharomycotina_1 - ps19
Saccharomycotina_2 - ps20
Saccharomycotina_3 - ps21
Phaff./Saccharomycetaceae - ps22
Saccharomyco./mycetaceae - ps23
Saccharomycetaceae - ps24
Saccharomycetaceae_2 - ps25
Saccharomycetaceae_3 - ps26
Kaz./Nau./Saccharomyces - ps27
Saccharomyces - ps28
Arboricola_clade - ps29
Cerevisiae_clade - ps30
Saccharomyces_cerevisiae - ps31
p-value
6e-03
9e-03
1e-02
2e-02
2e-02
Log-odds
0.5
1.0
1.5
2.0
2.5
3.0
3.5
4.0

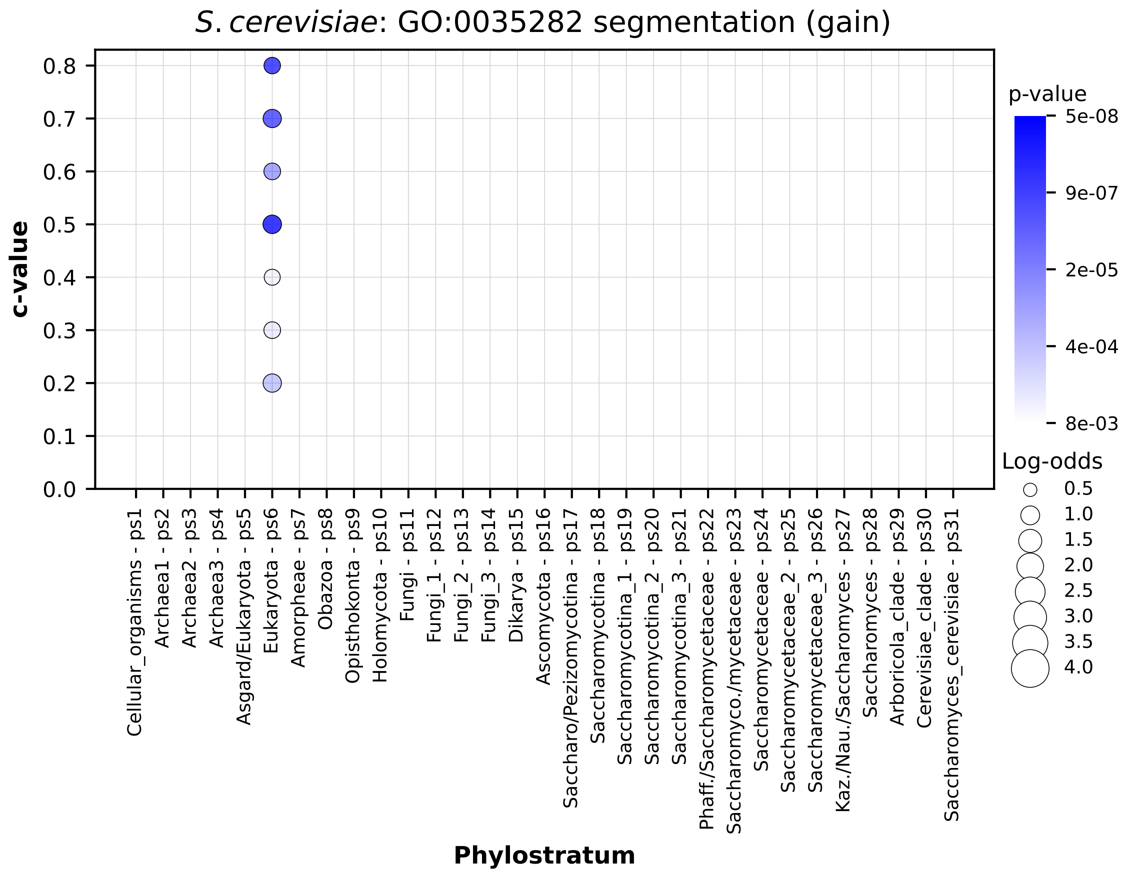

S. cerevisiae: GO:0035282 segmentation (gain)
c-value
0.8
0.7
0.6
0.5
0.4
0.3
0.2
0.1
0.0
p-value
5e-08
9e-07
2e-05
4e-04
8e-03
Log-odds
0.5
1.0
1.5
2.0
2.5
3.0
3.5
4.0
Cellular_organisms - ps1
Archaea1 - ps2
Archaea2 - ps3
Archaea3 - ps4
Asgard/Eukaryota - ps5
Eukaryota - ps6
Amorpheae - ps7
Obazoa - ps8
Opisthokonta - ps9
Holomycota - ps10
Fungi - ps11
Fungi_1 - ps12
Fungi_2 - ps13
Fungi_3 - ps14
Dikarya - ps15
Ascomycota - ps16
Saccharo/Pezizomycotina - ps17
Saccharomycotina - ps18
Saccharomycotina_1 - ps19
Saccharomycotina_2 - ps20
Saccharomycotina_3 - ps21
Phaff./Saccharomycetaceae - ps22
Saccharomyco./mycetaceae - ps23
Saccharomycetaceae - ps24
Saccharomycetaceae_2 - ps25
Saccharomycetaceae_3 - ps26
Kaz./Nau./Saccharomyces - ps27
Saccharomyces - ps28
Arboricola_clade - ps29
Cerevisiae_clade - ps30
Saccharomyces_cerevisiae - ps31
Phylostratum

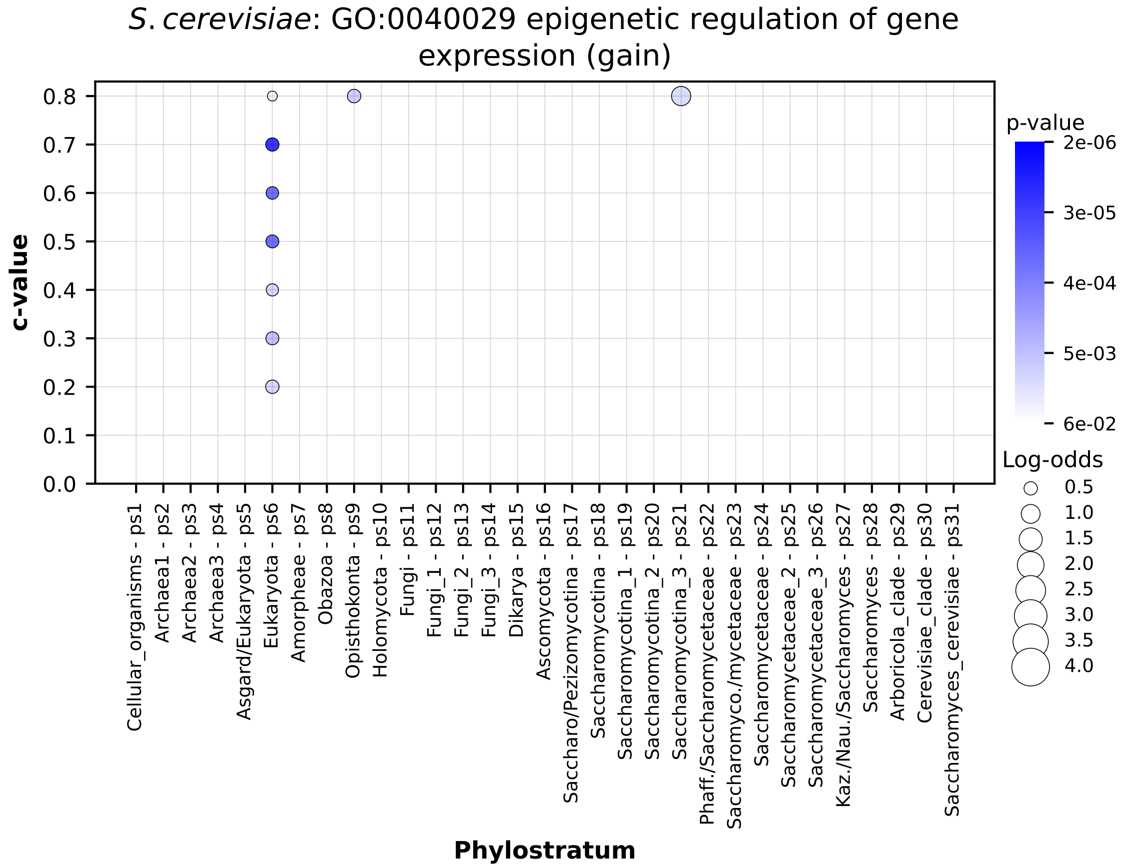

S. cerevisiae: GO:0040029 epigenetic regulation of gene expression (gain)
c-value
0.8
0.7
0.6
0.5
0.4
0.3
0.2
0.1
0.0
Phylostratum
Cellular_organisms - ps1
Archaea1 - ps2
Archaea2 - ps3
Archaea3 - ps4
Asgard/Eukaryota - ps5
Eukaryota - ps6
Amorpheae - ps7
Obazoa - ps8
Opisthokonta - ps9
Holomycota - ps10
Fungi - ps11
Fungi_1 - ps12
Fungi_2 - ps13
Fungi_3 - ps14
Dikarya - ps15
Ascomycota - ps16
Saccharo/Pezizomycotina - ps17
Saccharomycotina - ps18
Saccharomycotina_1 - ps19
Saccharomycotina_2 - ps20
Saccharomycotina_3 - ps21
Phaff./Saccharomycetaceae - ps22
Saccharomyco./mycetaceae - ps23
Saccharomycetaceae - ps24
Saccharomycetaceae_2 - ps25
Saccharomycetaceae_3 - ps26
Kaz./Nau./Saccharomyces - ps27
Saccharomyces - ps28
Arboricola_clade - ps29
Cerevisiae_clade - ps30
Saccharomyces_cerevisiae - ps31
p-value
2e-06
3e-05
4e-04
5e-03
6e-02
Log-odds
0.5
1.0
1.5
2.0
2.5
3.0
3.5
4.0

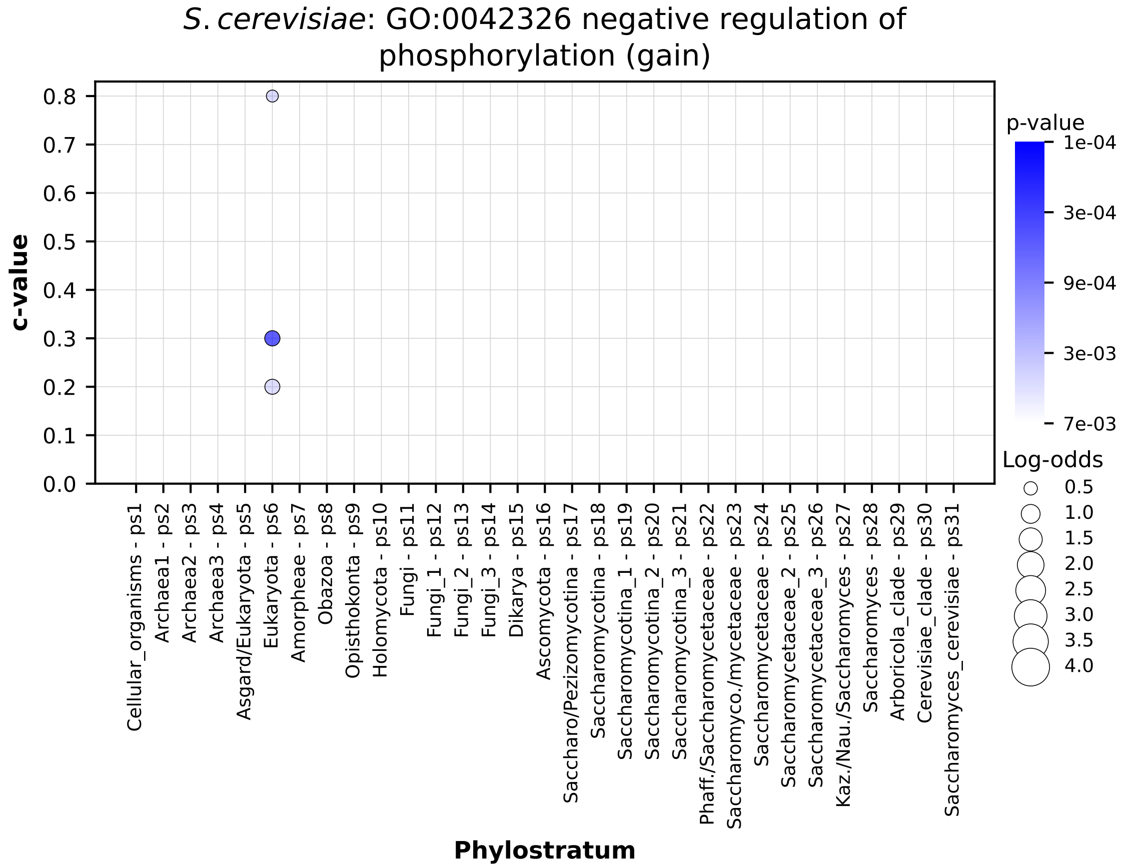

S. cerevisiae: GO:0042326 negative regulation of phosphorylation (gain)
c-value
0.0
0.1
0.2
0.3
0.4
0.5
0.6
0.7
0.8
Phylostratum
Cellular_organisms - ps1
Archaea1 - ps2
Archaea2 - ps3
Archaea3 - ps4
Asgard/Eukaryota - ps5
Eukaryota - ps6
Amorpheae - ps7
Obazoa - ps8
Opisthokonta - ps9
Holomycota - ps10
Fungi - ps11
Fungi_1 - ps12
Fungi_2 - ps13
Fungi_3 - ps14
Dikarya - ps15
Ascomycota - ps16
Saccharo/Pezizomycotina - ps17
Saccharomycotina - ps18
Saccharomycotina_1 - ps19
Saccharomycotina_2 - ps20
Saccharomycotina_3 - ps21
Phaff./Saccharomycetaceae - ps22
Saccharomyco./mycetaceae - ps23
Saccharomycetaceae - ps24
Saccharomycetaceae_2 - ps25
Saccharomycetaceae_3 - ps26
Kaz./Nau./Saccharomyces - ps27
Saccharomyces - ps28
Arboricola_clade - ps29
Cerevisiae_clade - ps30
Saccharomyces_cerevisiae - ps31
p-value
1e-04
3e-04
9e-04
3e-03
7e-03
Log-odds
0.5
1.0
1.5
2.0
2.5
3.0
3.5
4.0

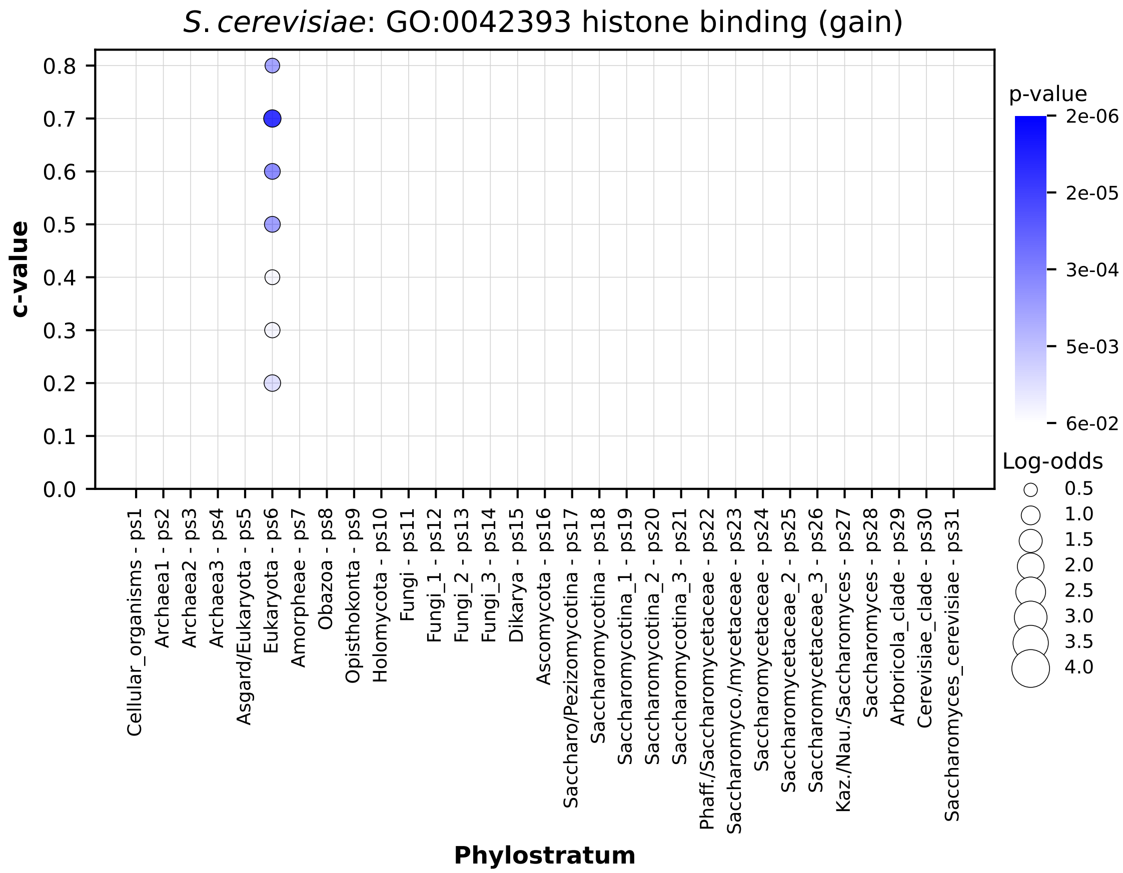

S. cerevisiae: GO:0042393 histone binding (gain)
c-value
0.0
0.1
0.2
0.3
0.4
0.5
0.6
0.7
0.8
Phylostratum
Cellular_organisms - ps1
Archaea1 - ps2
Archaea2 - ps3
Archaea3 - ps4
Asgard/Eukaryota - ps5
Eukaryota - ps6
Amorpheae - ps7
Obazoa - ps8
Opisthokonta - ps9
Holomycota - ps10
Fungi - ps11
Fungi_1 - ps12
Fungi_2 - ps13
Fungi_3 - ps14
Dikarya - ps15
Ascomycota - ps16
Saccharo/Pezizomycotina - ps17
Saccharomycotina - ps18
Saccharomycotina_1 - ps19
Saccharomycotina_2 - ps20
Saccharomycotina_3 - ps21
Phaff./Saccharomycetaceae - ps22
Saccharomyco./mycetaceae - ps23
Saccharomycetaceae - ps24
Saccharomycetaceae_2 - ps25
Saccharomycetaceae_3 - ps26
Kaz./Nau./Saccharomyces - ps27
Saccharomyces - ps28
Arboricola_clade - ps29
Cerevisiae_clade - ps30
Saccharomyces_cerevisiae - ps31
p-value
2e-06
2e-05
3e-04
5e-03
6e-02
Log-odds
0.5
1.0
1.5
2.0
2.5
3.0
3.5
4.0

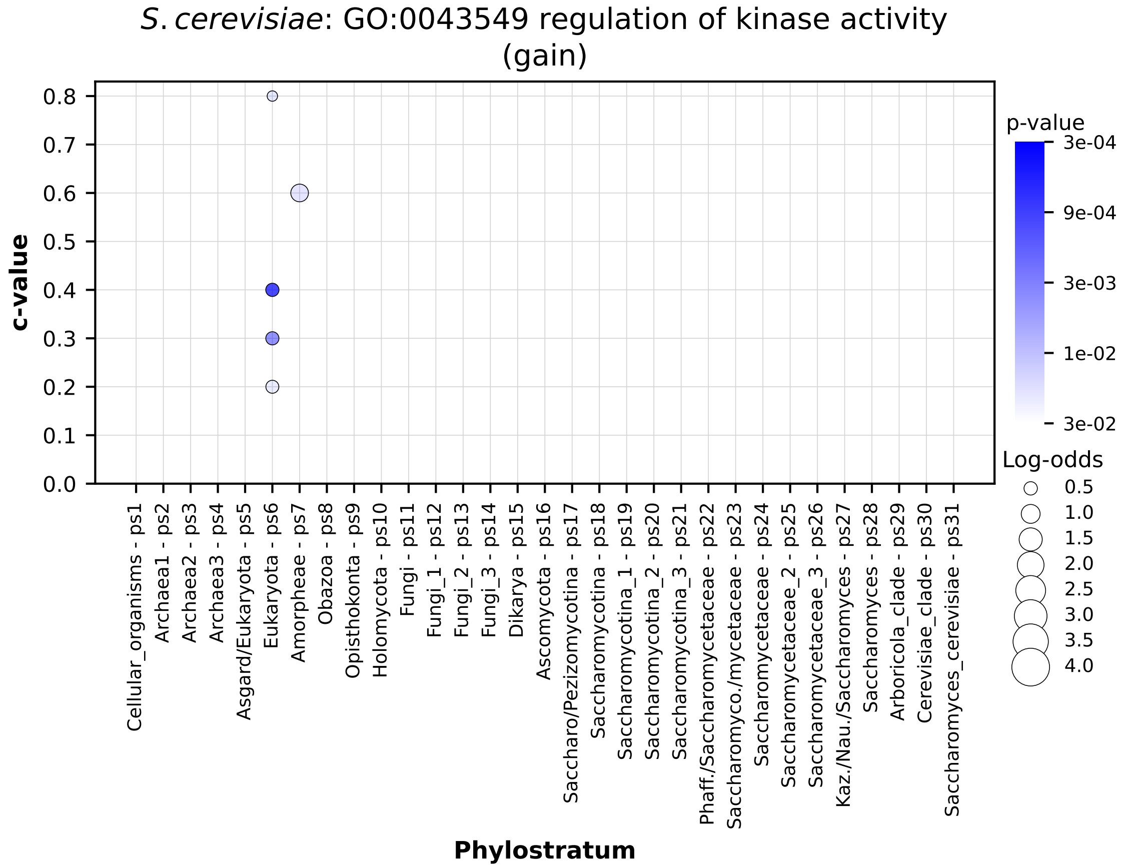

S. cerevisiae: GO:0043549 regulation of kinase activity
(gain)
c-value
0.0
0.1
0.2
0.3
0.4
0.5
0.6
0.7
0.8
Phylostratum
Cellular_organisms - ps1
Archaea1 - ps2
Archaea2 - ps3
Archaea3 - ps4
Asgard/Eukaryota - ps5
Eukaryota - ps6
Amorpheae - ps7
Obazoa - ps8
Opisthokonta - ps9
Holomycota - ps10
Fungi - ps11
Fungi_1 - ps12
Fungi_2 - ps13
Fungi_3 - ps14
Dikarya - ps15
Ascomycota - ps16
Saccharo/Pezizomycotina - ps17
Saccharomycotina - ps18
Saccharomycotina_1 - ps19
Saccharomycotina_2 - ps20
Saccharomycotina_3 - ps21
Phaff./Saccharomycetaceae - ps22
Saccharomyco./mycetaceae - ps23
Saccharomycetaceae - ps24
Saccharomycetaceae_2 - ps25
Saccharomycetaceae_3 - ps26
Kaz./Nau./Saccharomyces - ps27
Saccharomyces - ps28
Arboricola_clade - ps29
Cerevisiae_clade - ps30
Saccharomyces_cerevisiae - ps31
p-value
3e-04
9e-04
3e-03
1e-02
3e-02
Log-odds
0.5
1.0
1.5
2.0
2.5
3.0
3.5
4.0

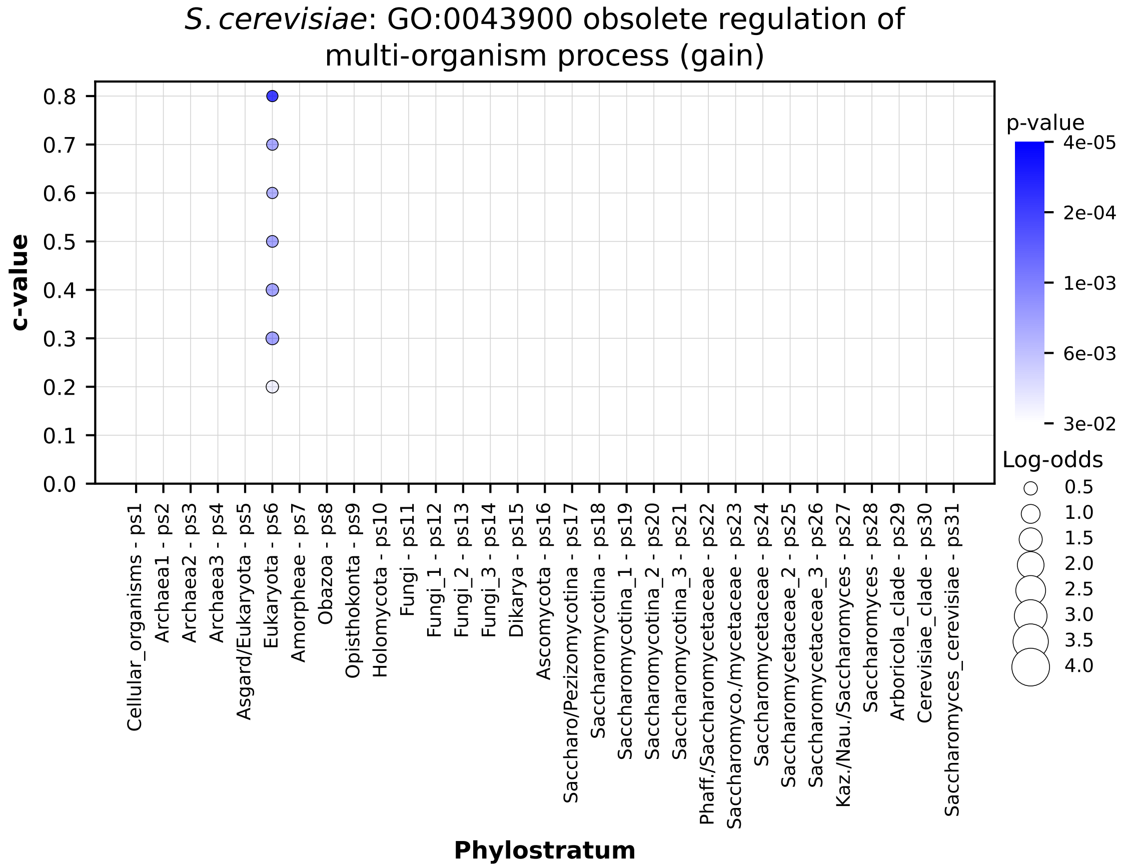
S. cerevisiae: GO:0043900 obsolete regulation of multi-organism process (gain)
c-value
0.0
0.1
0.2
0.3
0.4
0.5
0.6
0.7
0.8
p-value
4e-05
2e-04
1e-03
6e-03
3e-02
Log-odds
0.5
1.0
1.5
2.0
2.5
3.0
3.5
4.0
Cellular_organisms - ps1
Archaea1 - ps2
Archaea2 - ps3
Archaea3 - ps4
Asgard/Eukaryota - ps5
Eukaryota - ps6
Amorpheae - ps7
Obazoa - ps8
Opisthokonta - ps9
Holomycota - ps10
Fungi - ps11
Fungi_1 - ps12
Fungi_2 - ps13
Fungi_3 - ps14
Dikarya - ps15
Ascomycota - ps16
Saccharo/Pezizomycotina - ps17
Saccharomycotina - ps18
Saccharomycotina_1 - ps19
Saccharomycotina_2 - ps20
Saccharomycotina_3 - ps21
Phaff./Saccharomycetaceae - ps22
Saccharomyco./mycetaceae - ps23
Saccharomycetaceae - ps24
Saccharomycetaceae_2 - ps25
Saccharomycetaceae_3 - ps26
Kaz./Nau./Saccharomyces - ps27
Saccharomyces - ps28
Arboricola_clade - ps29
Cerevisiae_clade - ps30
Saccharomyces_cerevisiae - ps31
Phylostratum

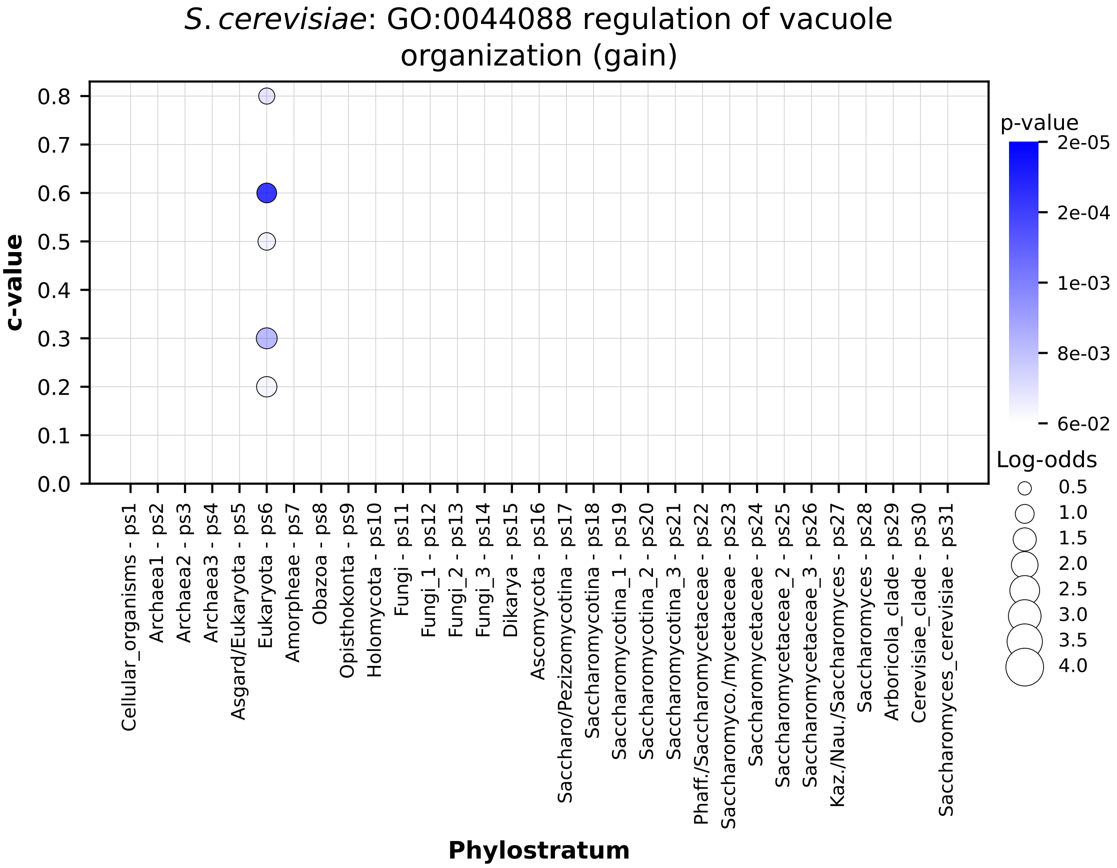

S. cerevisiae: GO:0044088 regulation of vacuole organization (gain)
c-value
0.0
0.1
0.2
0.3
0.4
0.5
0.6
0.7
0.8
p-value
2e-05
2e-04
1e-03
8e-03
6e-02
Log-odds
0.5
1.0
1.5
2.0
2.5
3.0
3.5
4.0
Cellular_organisms - ps1
Archaea1 - ps2
Archaea2 - ps3
Archaea3 - ps4
Asgard/Eukaryota - ps5
Eukaryota - ps6
Amorpheae - ps7
Obazoa - ps8
Opisthokonta - ps9
Holomycota - ps10
Fungi - ps11
Fungi_1 - ps12
Fungi_2 - ps13
Fungi_3 - ps14
Dikarya - ps15
Ascomycota - ps16
Saccharo/Pezizomycotina - ps17
Saccharomycotina - ps18
Saccharomycotina_1 - ps19
Saccharomycotina_2 - ps20
Saccharomycotina_3 - ps21
Phaff./Saccharomycetaceae - ps22
Saccharomyco./mycetaceae - ps23
Saccharomycetaceae - ps24
Saccharomycetaceae_2 - ps25
Saccharomycetaceae_3 - ps26
Kaz./Nau./Saccharomyces - ps27
Saccharomyces - ps28
Arboricola_clade - ps29
Cerevisiae_clade - ps30
Saccharomyces_cerevisiae - ps31
Phylostratum

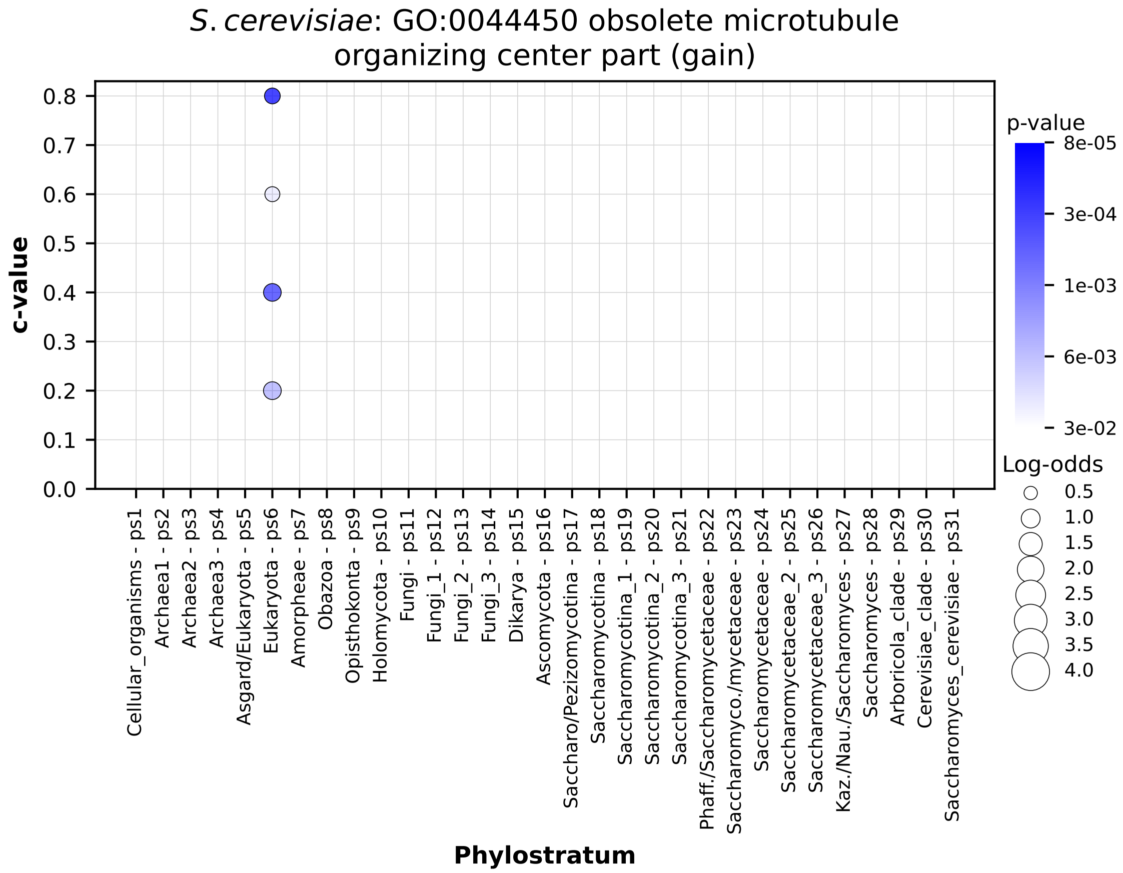

S. cerevisiae: GO:0044450 obsolete microtubule organizing center part (gain)
c-value
0.8
0.7
0.6
0.5
0.4
0.3
0.2
0.1
0.0
Phylostratum
Cellular_organisms - ps1
Archaea1 - ps2
Archaea2 - ps3
Archaea3 - ps4
Asgard/Eukaryota - ps5
Eukaryota - ps6
Amorpheae - ps7
Obazoa - ps8
Opisthokonta - ps9
Holomycota - ps10
Fungi - ps11
Fungi_1 - ps12
Fungi_2 - ps13
Fungi_3 - ps14
Dikarya - ps15
Ascomycota - ps16
Saccharo/Pezizomycotina - ps17
Saccharomycotina - ps18
Saccharomycotina_1 - ps19
Saccharomycotina_2 - ps20
Saccharomycotina_3 - ps21
Phaff./Saccharomycetaceae - ps22
Saccharomyco./mycetaceae - ps23
Saccharomycetaceae - ps24
Saccharomycetaceae_2 - ps25
Saccharomycetaceae_3 - ps26
Kaz./Nau./Saccharomyces - ps27
Saccharomyces - ps28
Arboricola_clade - ps29
Cerevisiae_clade - ps30
Saccharomyces_cerevisiae - ps31
p-value
8e-05
3e-04
1e-03
6e-03
3e-02
Log-odds
0.5
1.0
1.5
2.0
2.5
3.0
3.5
4.0

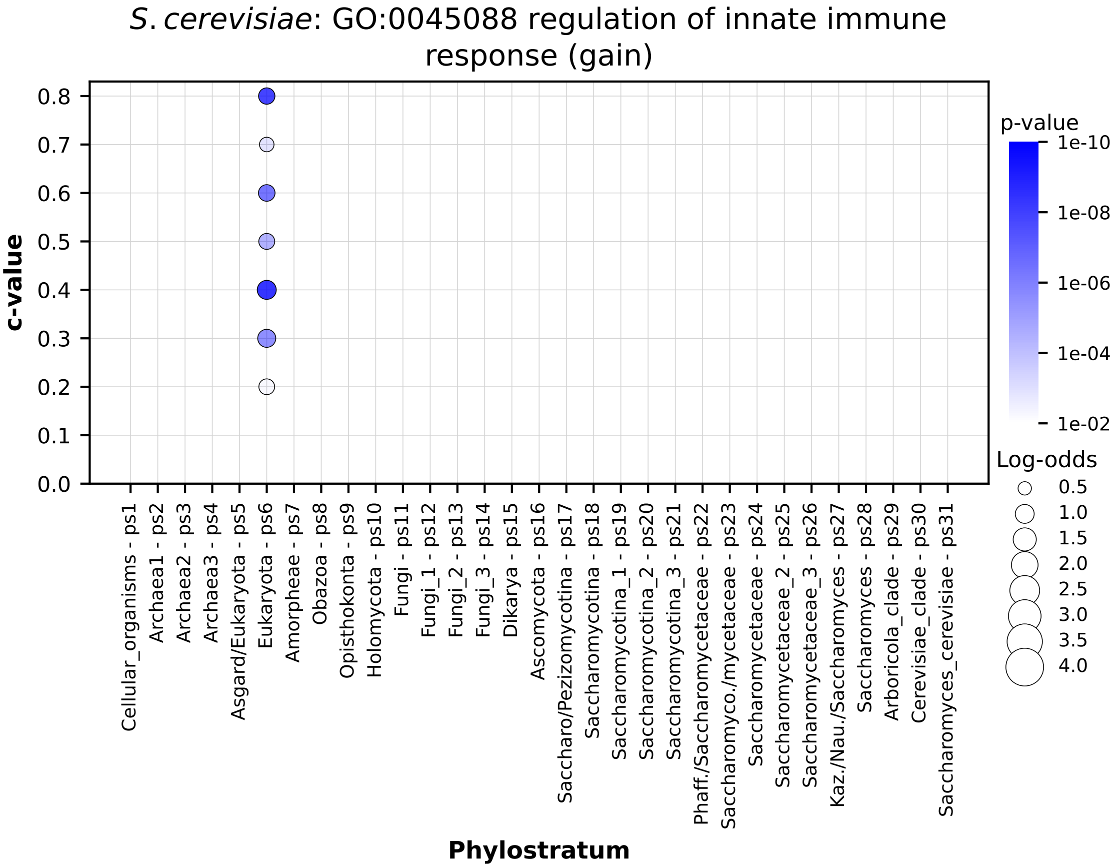

S. cerevisiae: GO:0045088 regulation of innate immune response (gain)
c-value
0.0
0.1
0.2
0.3
0.4
0.5
0.6
0.7
0.8
p-value
1e-10
1e-08
1e-06
1e-04
1e-02
Log-odds
0.5
1.0
1.5
2.0
2.5
3.0
3.5
4.0
Cellular_organisms - ps1
Archaea1 - ps2
Archaea2 - ps3
Archaea3 - ps4
Asgard/Eukaryota - ps5
Eukaryota - ps6
Amorpheae - ps7
Obazoa - ps8
Opisthokonta - ps9
Holomycota - ps10
Fungi - ps11
Fungi_1 - ps12
Fungi_2 - ps13
Fungi_3 - ps14
Dikarya - ps15
Ascomycota - ps16
Saccharo/Pezizomycotina - ps17
Saccharomycotina - ps18
Saccharomycotina_1 - ps19
Saccharomycotina_2 - ps20
Saccharomycotina_3 - ps21
Phaff./Saccharomycetaceae - ps22
Saccharomyco./mycetaceae - ps23
Saccharomycetaceae - ps24
Saccharomycetaceae_2 - ps25
Saccharomycetaceae_3 - ps26
Kaz./Nau./Saccharomyces - ps27
Saccharomyces - ps28
Arboricola_clade - ps29
Cerevisiae_clade - ps30
Saccharomyces_cerevisiae - ps31
Phylostratum

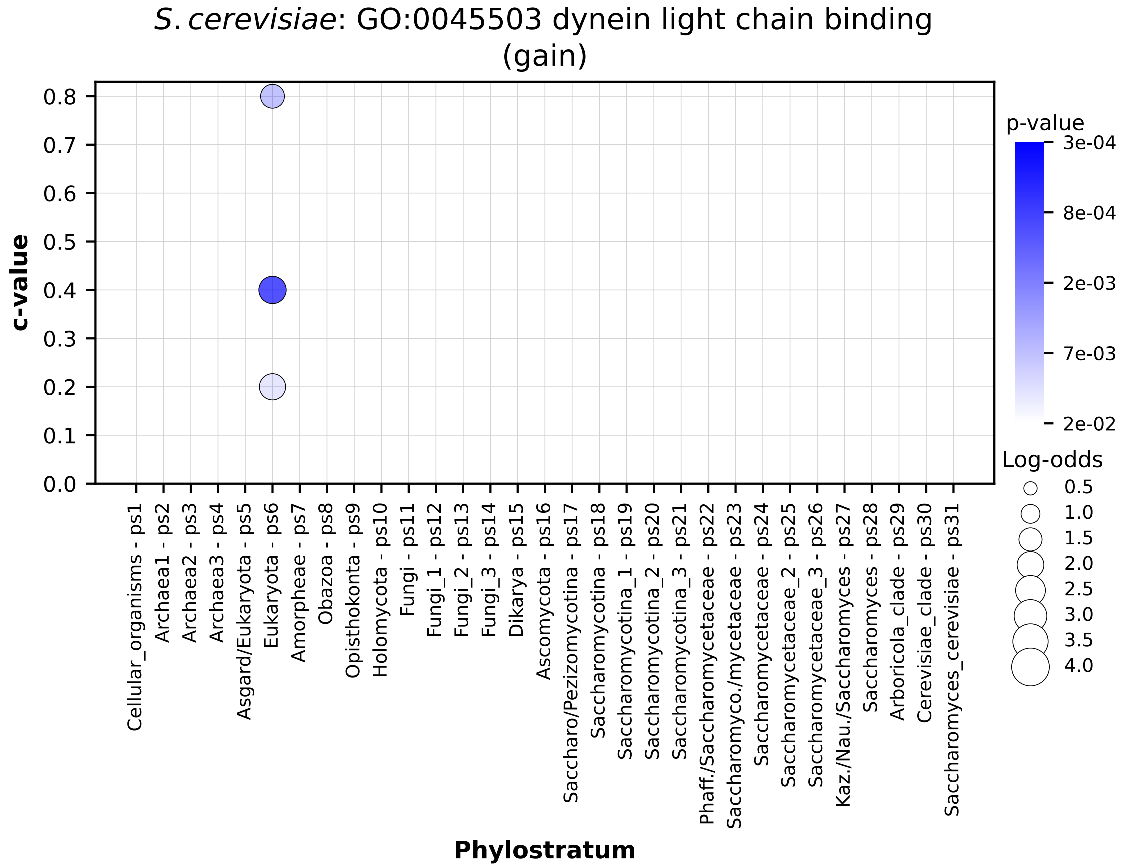

S. cerevisiae: GO:0045503 dynein light chain binding
(gain)
c-value
0.8
0.7
0.6
0.5
0.4
0.3
0.2
0.1
0.0
p-value
3e-04
8e-04
2e-03
7e-03
2e-02
Log-odds
0.5
1.0
1.5
2.0
2.5
3.0
3.5
4.0
Cellular_organisms - ps1
Archaea1 - ps2
Archaea2 - ps3
Archaea3 - ps4
Asgard/Eukaryota - ps5
Eukaryota - ps6
Amorpheae - ps7
Obazoa - ps8
Opisthokonta - ps9
Holomycota - ps10
Fungi - ps11
Fungi_1 - ps12
Fungi_2 - ps13
Fungi_3 - ps14
Dikarya - ps15
Ascomycota - ps16
Saccharo/Pezizomycotina - ps17
Saccharomycotina - ps18
Saccharomycotina_1 - ps19
Saccharomycotina_2 - ps20
Saccharomycotina_3 - ps21
Phaff./Saccharomycetaceae - ps22
Saccharomyco./mycetaceae - ps23
Saccharomycetaceae - ps24
Saccharomycetaceae_2 - ps25
Saccharomycetaceae_3 - ps26
Kaz./Nau./Saccharomyces - ps27
Saccharomyces - ps28
Arboricola_clade - ps29
Cerevisiae_clade - ps30
Saccharomyces_cerevisiae - ps31
Phylostratum

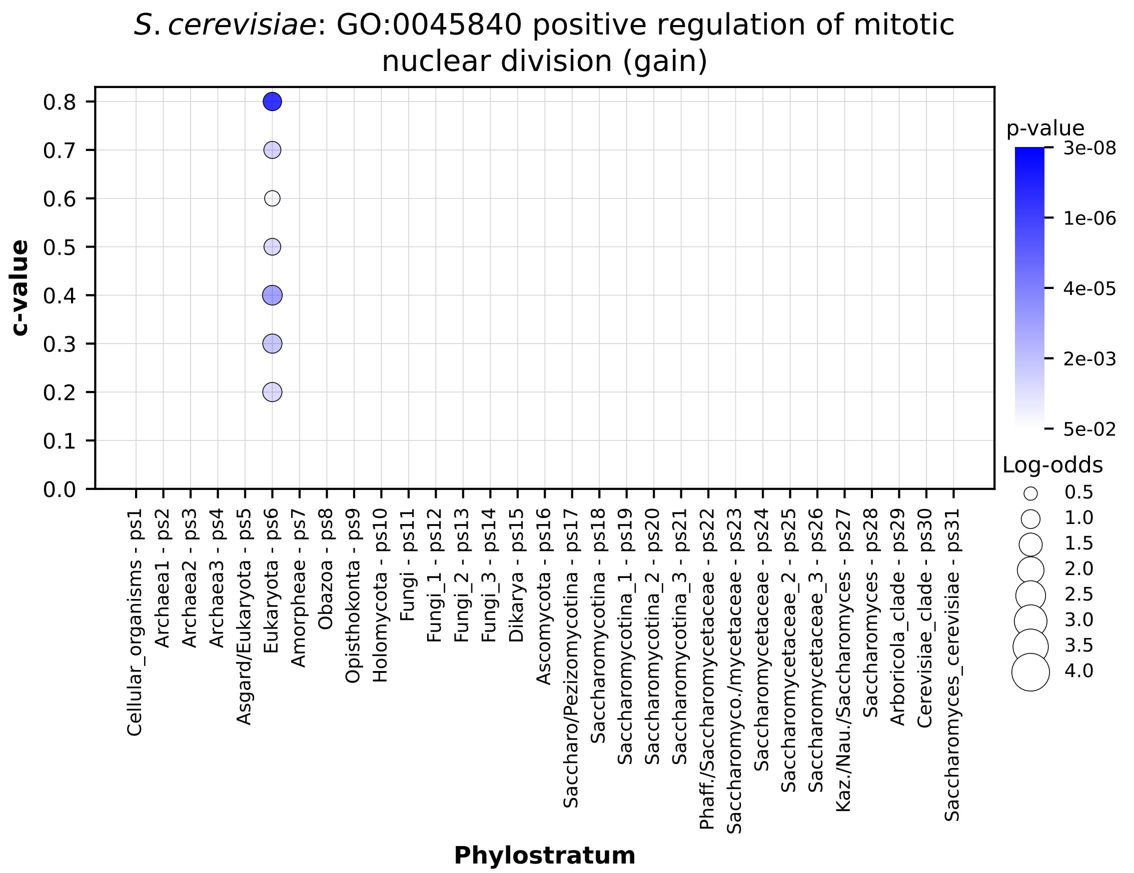

S. cerevisiae: GO:0045840 positive regulation of mitotic nuclear division (gain)
c-value
0.0
0.1
0.2
0.3
0.4
0.5
0.6
0.7
0.8
Phylostratum
Cellular_organisms - ps1
Archaea1 - ps2
Archaea2 - ps3
Archaea3 - ps4
Asgard/Eukaryota - ps5
Eukaryota - ps6
Amorpheae - ps7
Obazoa - ps8
Opisthokonta - ps9
Holomycota - ps10
Fungi - ps11
Fungi_1 - ps12
Fungi_2 - ps13
Fungi_3 - ps14
Dikarya - ps15
Ascomycota - ps16
Saccharo/Pezizomycotina - ps17
Saccharomycotina - ps18
Saccharomycotina_1 - ps19
Saccharomycotina_2 - ps20
Saccharomycotina_3 - ps21
Phaff./Saccharomycetaceae - ps22
Saccharomyco./mycetaceae - ps23
Saccharomycetaceae - ps24
Saccharomycetaceae_2 - ps25
Saccharomycetaceae_3 - ps26
Kaz./Nau./Saccharomyces - ps27
Saccharomyces - ps28
Arboricola_clade - ps29
Cerevisiae_clade - ps30
Saccharomyces_cerevisiae - ps31
p-value
3e-08
1e-06
4e-05
2e-03
5e-02
Log-odds
0.5
1.0
1.5
2.0
2.5
3.0
3.5
4.0

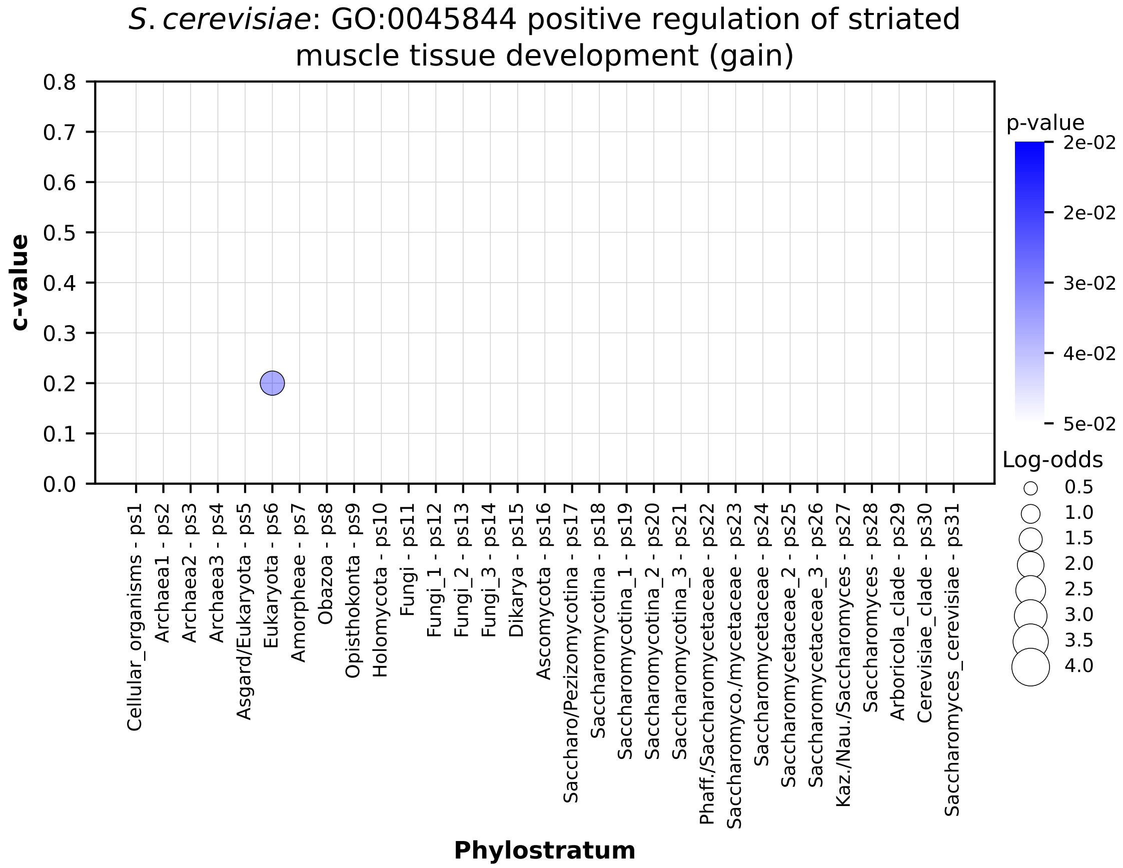
S. cerevisiae: GO:0045844 positive regulation of striated muscle tissue development (gain)
c-value
0.0
0.1
0.2
0.3
0.4
0.5
0.6
0.7
0.8
Phylostratum
Cellular_organisms - ps1
Archaea1 - ps2
Archaea2 - ps3
Archaea3 - ps4
Asgard/Eukaryota - ps5
Eukaryota - ps6
Amorpheae - ps7
Obazoa - ps8
Opisthokonta - ps9
Holomycota - ps10
Fungi - ps11
Fungi_1 - ps12
Fungi_2 - ps13
Fungi_3 - ps14
Dikarya - ps15
Ascomycota - ps16
Saccharo/Pezizomycotina - ps17
Saccharomycotina - ps18
Saccharomycotina_1 - ps19
Saccharomycotina_2 - ps20
Saccharomycotina_3 - ps21
Phaff./Saccharomycetaceae - ps22
Saccharomyco./mycetaceae - ps23
Saccharomycetaceae - ps24
Saccharomycetaceae_2 - ps25
Saccharomycetaceae_3 - ps26
Kaz./Nau./Saccharomyces - ps27
Saccharomyces - ps28
Arboricola_clade - ps29
Cerevisiae_clade - ps30
Saccharomyces_cerevisiae - ps31
p-value
2e-02
2e-02
3e-02
4e-02
5e-02
Log-odds
0.5
1.0
1.5
2.0
2.5
3.0
3.5
4.0

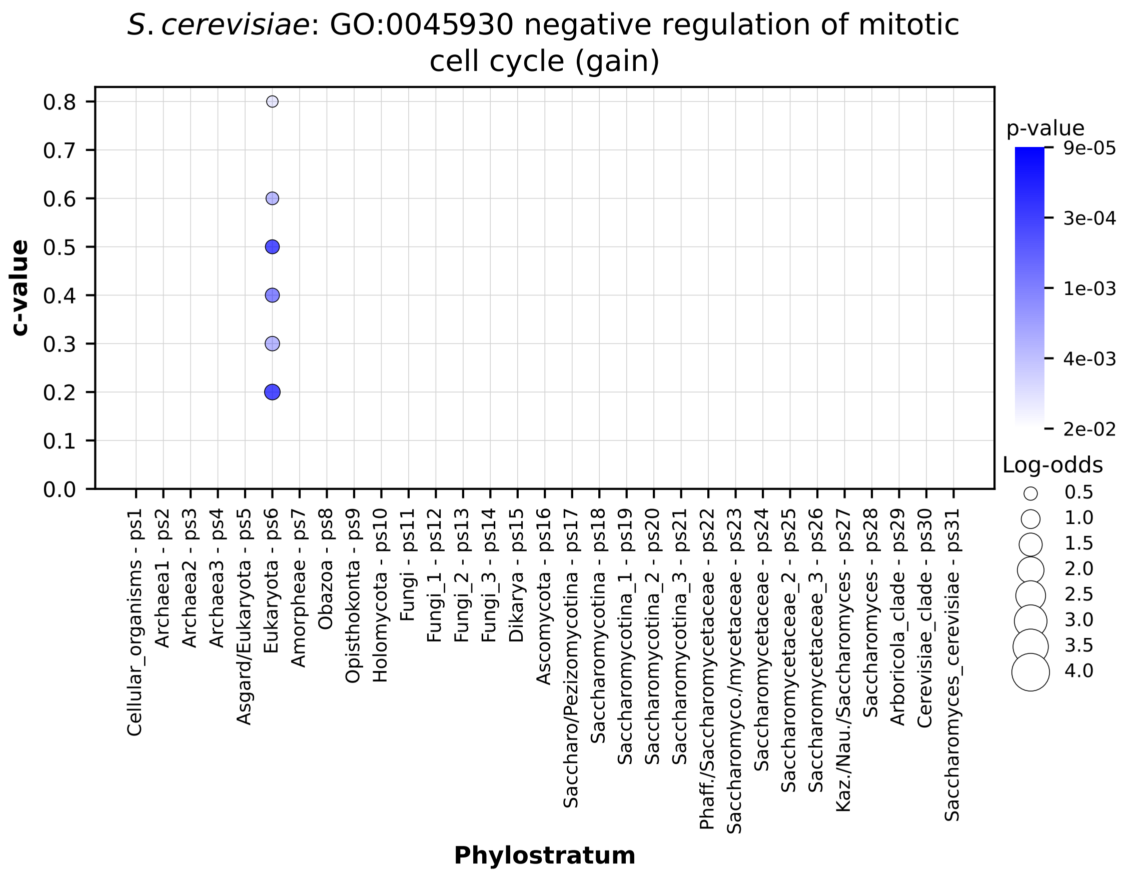

S. cerevisiae: GO:0045930 negative regulation of mitotic cell cycle (gain)
c-value
0.0
0.1
0.2
0.3
0.4
0.5
0.6
0.7
0.8
Phylostratum
Cellular_organisms - ps1
Archaea1 - ps2
Archaea2 - ps3
Archaea3 - ps4
Asgard/Eukaryota - ps5
Eukaryota - ps6
Amorpheae - ps7
Obazoa - ps8
Opisthokonta - ps9
Holomycota - ps10
Fungi - ps11
Fungi_1 - ps12
Fungi_2 - ps13
Fungi_3 - ps14
Dikarya - ps15
Ascomycota - ps16
Saccharo/Pezizomycotina - ps17
Saccharomycotina - ps18
Saccharomycotina_1 - ps19
Saccharomycotina_2 - ps20
Saccharomycotina_3 - ps21
Phaff./Saccharomycetaceae - ps22
Saccharomyco./mycetaceae - ps23
Saccharomycetaceae - ps24
Saccharomycetaceae_2 - ps25
Saccharomycetaceae_3 - ps26
Kaz./Nau./Saccharomyces - ps27
Saccharomyces - ps28
Arboricola_clade - ps29
Cerevisiae_clade - ps30
Saccharomyces_cerevisiae - ps31
p-value
9e-05
3e-04
1e-03
4e-03
2e-02
Log-odds
0.5
1.0
1.5
2.0
2.5
3.0
3.5
4.0

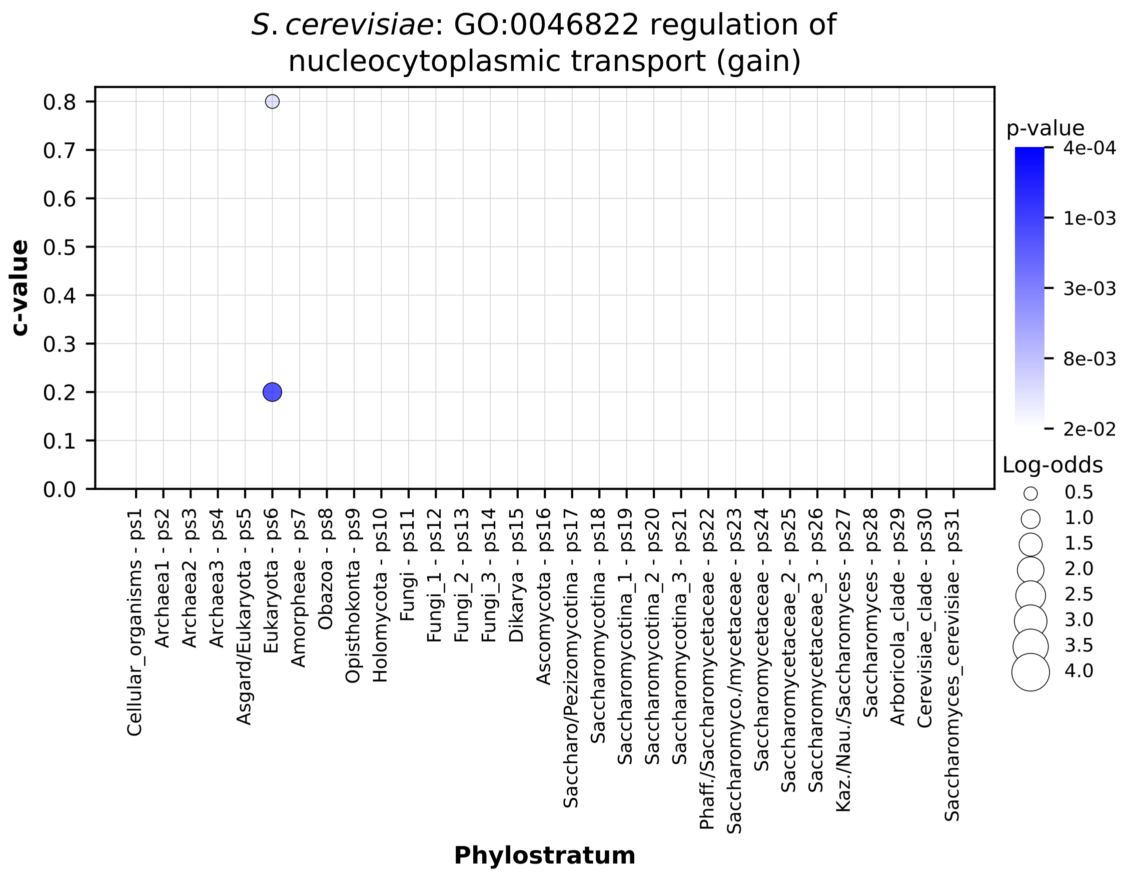

S. cerevisiae: GO:0046822 regulation of nucleocytoplasmic transport (gain)
c-value
0.0
0.1
0.2
0.3
0.4
0.5
0.6
0.7
0.8
p-value
4e-04
1e-03
3e-03
8e-03
2e-02
Log-odds
0.5
1.0
1.5
2.0
2.5
3.0
3.5
4.0
Cellular_organisms - ps1
Archaea1 - ps2
Archaea2 - ps3
Archaea3 - ps4
Asgard/Eukaryota - ps5
Eukaryota - ps6
Amorpheae - ps7
Obazoa - ps8
Opisthokonta - ps9
Holomycota - ps10
Fungi - ps11
Fungi_1 - ps12
Fungi_2 - ps13
Fungi_3 - ps14
Dikarya - ps15
Ascomycota - ps16
Saccharo/Pezizomycotina - ps17
Saccharomycotina - ps18
Saccharomycotina_1 - ps19
Saccharomycotina_2 - ps20
Saccharomycotina_3 - ps21
Phaff./Saccharomycetaceae - ps22
Saccharomyco./mycetaceae - ps23
Saccharomycetaceae - ps24
Saccharomycetaceae_2 - ps25
Saccharomycetaceae_3 - ps26
Kaz./Nau./Saccharomyces - ps27
Saccharomyces - ps28
Arboricola_clade - ps29
Cerevisiae_clade - ps30
Saccharomyces_cerevisiae - ps31
Phylostratum

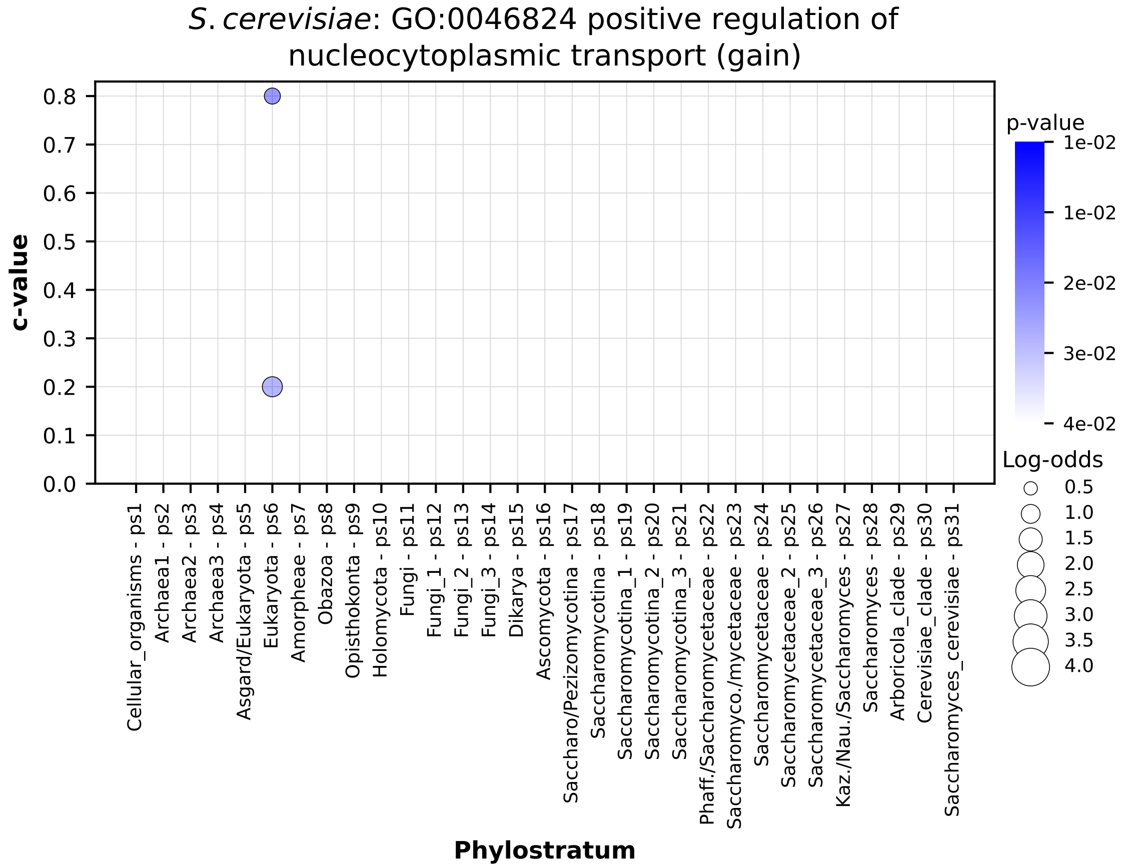

S. cerevisiae: GO:0046824 positive regulation of nucleocytoplasmic transport (gain)
c-value
0.8
0.7
0.6
0.5
0.4
0.3
0.2
0.1
0.0
p-value
1e-02
1e-02
2e-02
3e-02
4e-02
Log-odds
0.5
1.0
1.5
2.0
2.5
3.0
3.5
4.0
Cellular_organisms - ps1
Archaea1 - ps2
Archaea2 - ps3
Archaea3 - ps4
Asgard/Eukaryota - ps5
Eukaryota - ps6
Amorpheae - ps7
Obazoa - ps8
Opisthokonta - ps9
Holomycota - ps10
Fungi - ps11
Fungi_1 - ps12
Fungi_2 - ps13
Fungi_3 - ps14
Dikarya - ps15
Ascomycota - ps16
Saccharo/Pezizomycotina - ps17
Saccharomycotina - ps18
Saccharomycotina_1 - ps19
Saccharomycotina_2 - ps20
Saccharomycotina_3 - ps21
Phaff./Saccharomycetaceae - ps22
Saccharomyco./mycetaceae - ps23
Saccharomycetaceae - ps24
Saccharomycetaceae_2 - ps25
Saccharomycetaceae_3 - ps26
Kaz./Nau./Saccharomyces - ps27
Saccharomyces - ps28
Arboricola_clade - ps29
Cerevisiae_clade - ps30
Saccharomyces_cerevisiae - ps31
Phylostratum

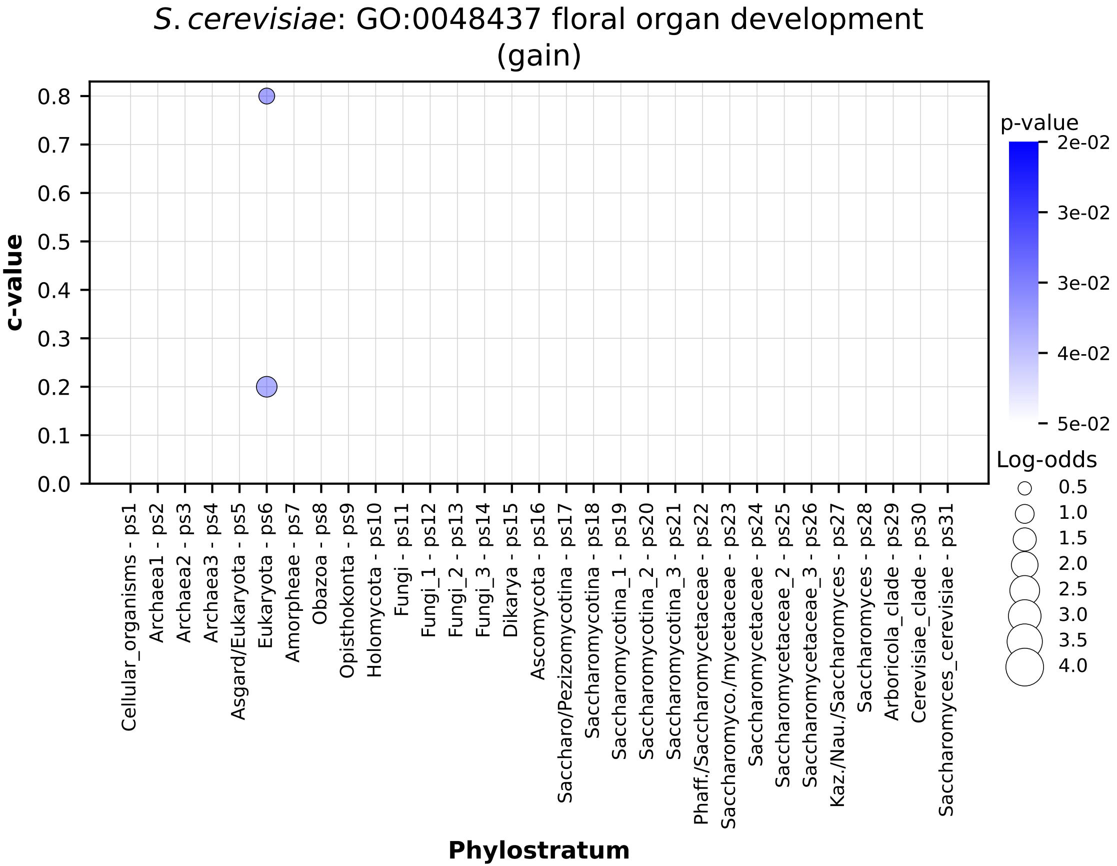
S. cerevisiae: GO:0048437 floral organ development
(gain)
c-value
0.0
0.1
0.2
0.3
0.4
0.5
0.6
0.7
0.8
p-value
2e-02
3e-02
3e-02
4e-02
5e-02
Log-odds
0.5
1.0
1.5
2.0
2.5
3.0
3.5
4.0
Cellular_organisms - ps1
Archaea1 - ps2
Archaea2 - ps3
Archaea3 - ps4
Asgard/Eukaryota - ps5
Eukaryota - ps6
Amorpheae - ps7
Obazoa - ps8
Opisthokonta - ps9
Holomycota - ps10
Fungi - ps11
Fungi_1 - ps12
Fungi_2 - ps13
Fungi_3 - ps14
Dikarya - ps15
Ascomycota - ps16
Saccharo/Pezizomycotina - ps17
Saccharomycotina - ps18
Saccharomycotina_1 - ps19
Saccharomycotina_2 - ps20
Saccharomycotina_3 - ps21
Phaff./Saccharomycetaceae - ps22
Saccharomyco./mycetaceae - ps23
Saccharomycetaceae - ps24
Saccharomycetaceae_2 - ps25
Saccharomycetaceae_3 - ps26
Kaz./Nau./Saccharomyces - ps27
Saccharomyces - ps28
Arboricola_clade - ps29
Cerevisiae_clade - ps30
Saccharomyces_cerevisiae - ps31
Phylostratum

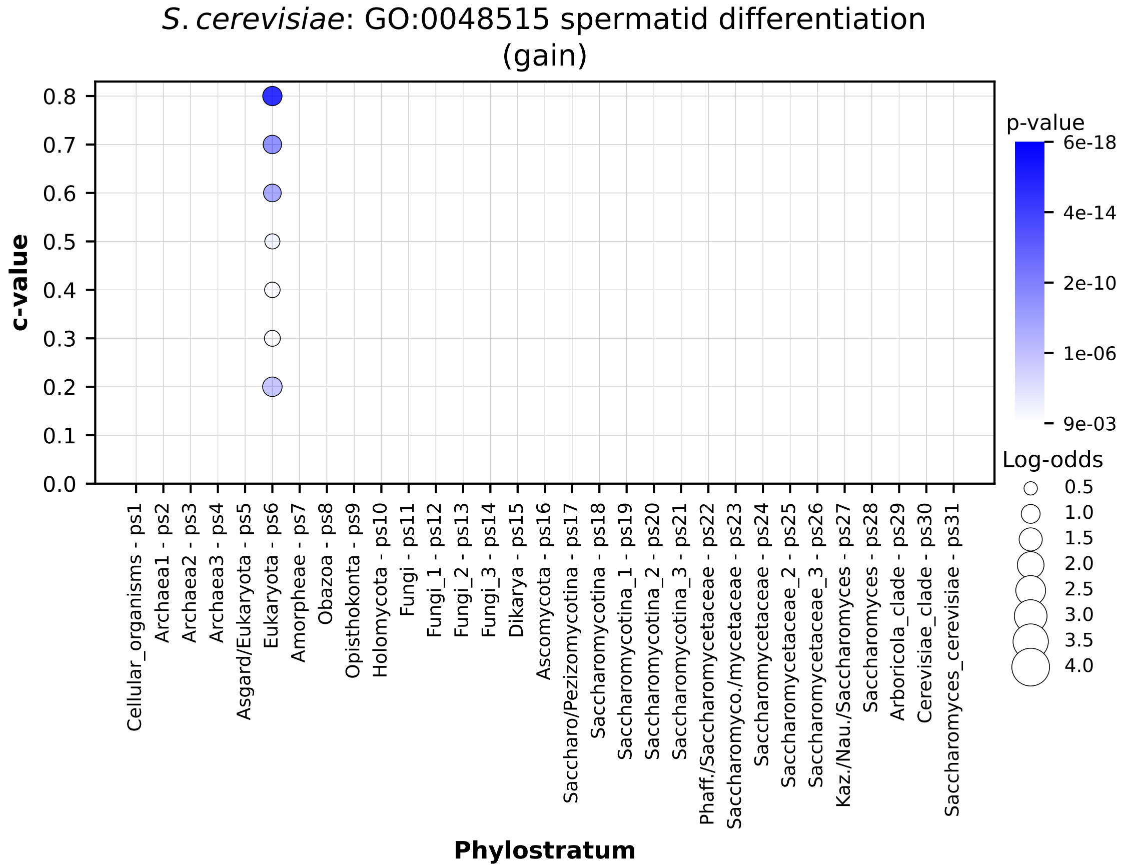

S. cerevisiae: GO:0048515 spermatid differentiation
(gain)
c-value
0.8
0.7
0.6
0.5
0.4
0.3
0.2
0.1
0.0
p-value
6e-18
4e-14
2e-10
1e-06
9e-03
Log-odds
0.5
1.0
1.5
2.0
2.5
3.0
3.5
4.0
Cellular_organisms - ps1
Archaea1 - ps2
Archaea2 - ps3
Archaea3 - ps4
Asgard/Eukaryota - ps5
Eukaryota - ps6
Amorpheae - ps7
Obazoa - ps8
Opisthokonta - ps9
Holomycota - ps10
Fungi - ps11
Fungi_1 - ps12
Fungi_2 - ps13
Fungi_3 - ps14
Dikarya - ps15
Ascomycota - ps16
Saccharo/Pezizomycotina - ps17
Saccharomycotina - ps18
Saccharomycotina_1 - ps19
Saccharomycotina_2 - ps20
Saccharomycotina_3 - ps21
Phaff./Saccharomycetaceae - ps22
Saccharomyco./mycetaceae - ps23
Saccharomycetaceae - ps24
Saccharomycetaceae_2 - ps25
Saccharomycetaceae_3 - ps26
Kaz./Nau./Saccharomyces - ps27
Saccharomyces - ps28
Arboricola_clade - ps29
Cerevisiae_clade - ps30
Saccharomyces_cerevisiae - ps31
Phylostratum

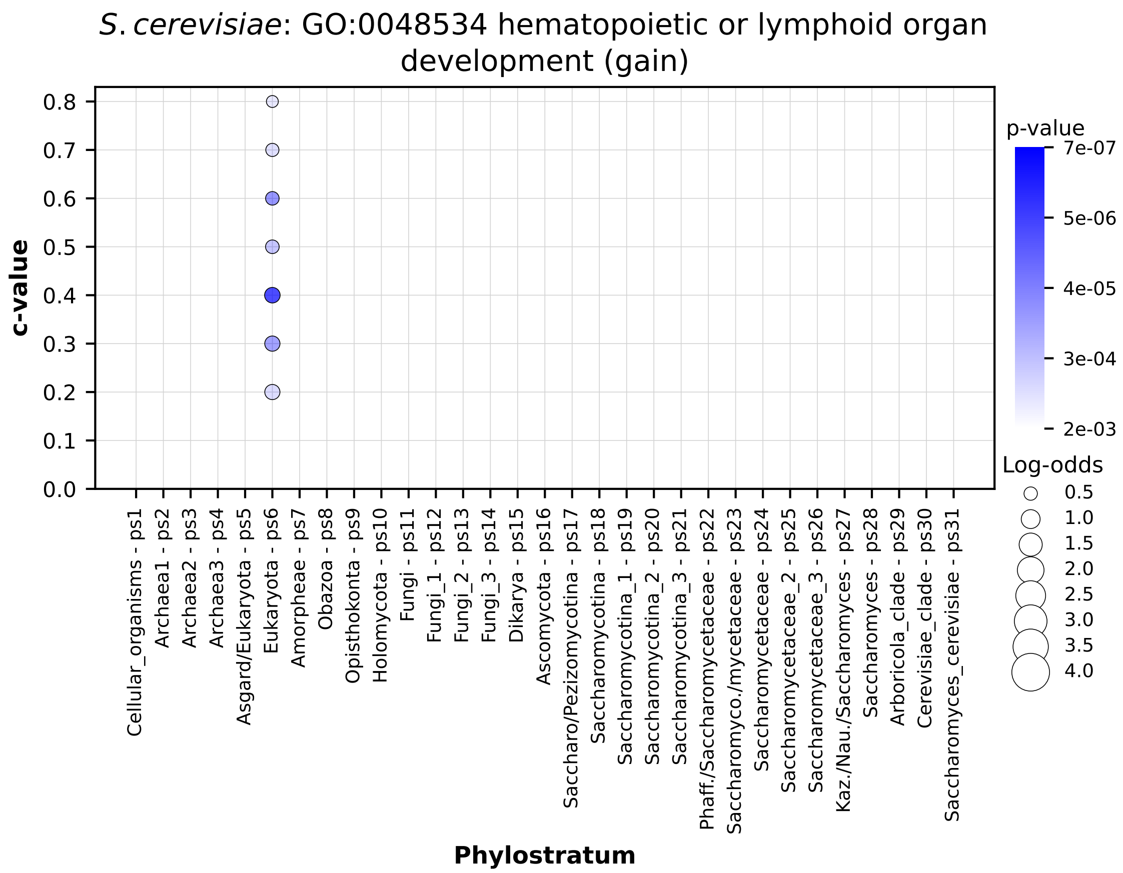

S. cerevisiae: GO:0048534 hematopoietic or lymphoid organ development (gain)
c-value
0.0
0.1
0.2
0.3
0.4
0.5
0.6
0.7
0.8
Phylostratum
Cellular_organisms - ps1
Archaea1 - ps2
Archaea2 - ps3
Archaea3 - ps4
Asgard/Eukaryota - ps5
Eukaryota - ps6
Amorpheae - ps7
Obazoa - ps8
Opisthokonta - ps9
Holomycota - ps10
Fungi - ps11
Fungi_1 - ps12
Fungi_2 - ps13
Fungi_3 - ps14
Dikarya - ps15
Ascomycota - ps16
Saccharo/Pezizomycotina - ps17
Saccharomycotina - ps18
Saccharomycotina_1 - ps19
Saccharomycotina_2 - ps20
Saccharomycotina_3 - ps21
Phaff./Saccharomycetaceae - ps22
Saccharomyco./mycetaceae - ps23
Saccharomycetaceae - ps24
Saccharomycetaceae_2 - ps25
Saccharomycetaceae_3 - ps26
Kaz./Nau./Saccharomyces - ps27
Saccharomyces - ps28
Arboricola_clade - ps29
Cerevisiae_clade - ps30
Saccharomyces_cerevisiae - ps31
p-value
7e-07
5e-06
4e-05
3e-04
2e-03
Log-odds
0.5
1.0
1.5
2.0
2.5
3.0
3.5
4.0

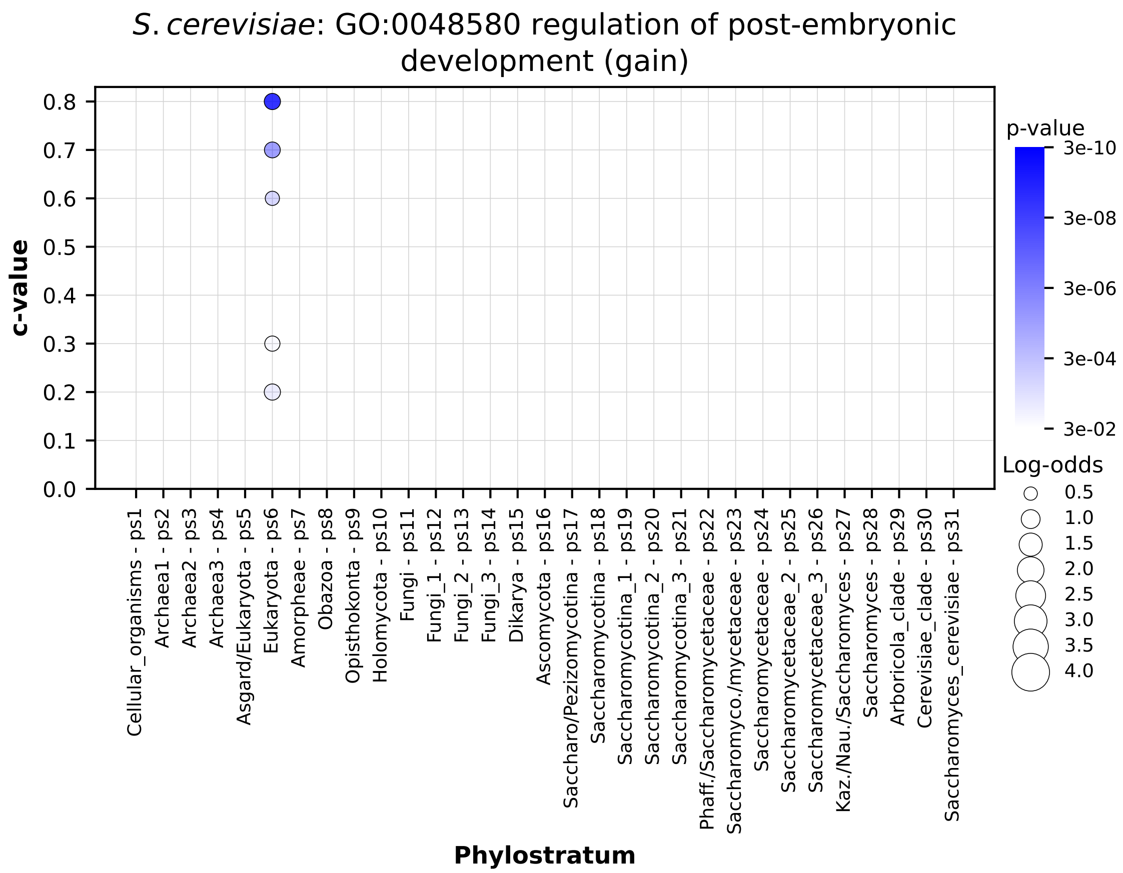

S. cerevisiae: GO:0048580 regulation of post-embryonic development (gain)
c-value
0.0
0.1
0.2
0.3
0.4
0.5
0.6
0.7
0.8
p-value
3e-10
3e-08
3e-06
3e-04
3e-02
Log-odds
0.5
1.0
1.5
2.0
2.5
3.0
3.5
4.0
Cellular_organisms - ps1
Archaea1 - ps2
Archaea2 - ps3
Archaea3 - ps4
Asgard/Eukaryota - ps5
Eukaryota - ps6
Amorpheae - ps7
Obazoa - ps8
Opisthokonta - ps9
Holomycota - ps10
Fungi - ps11
Fungi_1 - ps12
Fungi_2 - ps13
Fungi_3 - ps14
Dikarya - ps15
Ascomycota - ps16
Saccharo/Pezizomycotina - ps17
Saccharomycotina - ps18
Saccharomycotina_1 - ps19
Saccharomycotina_2 - ps20
Saccharomycotina_3 - ps21
Phaff./Saccharomycetaceae - ps22
Saccharomyco./mycetaceae - ps23
Saccharomycetaceae - ps24
Saccharomycetaceae_2 - ps25
Saccharomycetaceae_3 - ps26
Kaz./Nau./Saccharomyces - ps27
Saccharomyces - ps28
Arboricola_clade - ps29
Cerevisiae_clade - ps30
Saccharomyces_cerevisiae - ps31
Phylostratum

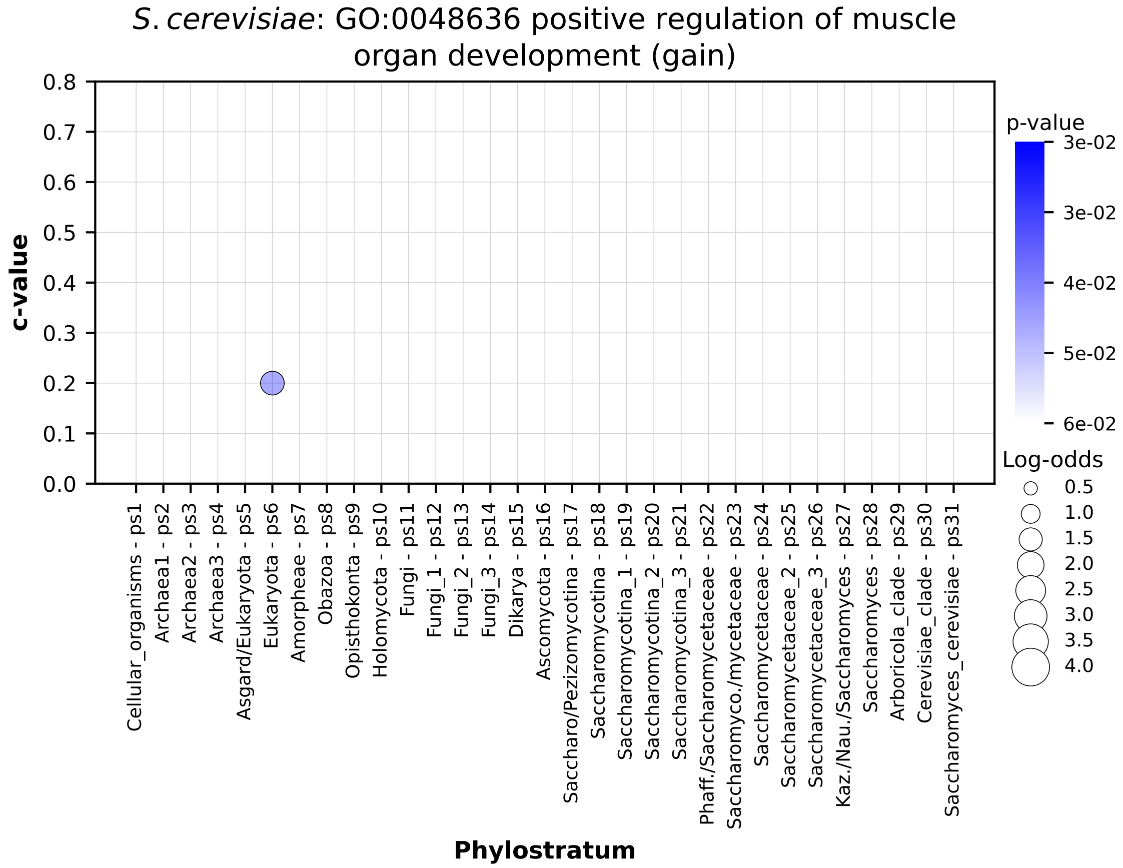

S. cerevisiae: GO:0048636 positive regulation of muscle organ development (gain)
c-value
0.8
0.7
0.6
0.5
0.4
0.3
0.2
0.1
0.0
Phylostratum
Cellular_organisms - ps1
Archaea1 - ps2
Archaea2 - ps3
Archaea3 - ps4
Asgard/Eukaryota - ps5
Eukaryota - ps6
Amorpheae - ps7
Obazoa - ps8
Opisthokonta - ps9
Holomycota - ps10
Fungi - ps11
Fungi_1 - ps12
Fungi_2 - ps13
Fungi_3 - ps14
Dikarya - ps15
Ascomycota - ps16
Saccharo/Pezizomycotina - ps17
Saccharomycotina - ps18
Saccharomycotina_1 - ps19
Saccharomycotina_2 - ps20
Saccharomycotina_3 - ps21
Phaff./Saccharomycetaceae - ps22
Saccharomyco./mycetaceae - ps23
Saccharomycetaceae - ps24
Saccharomycetaceae_2 - ps25
Saccharomycetaceae_3 - ps26
Kaz./Nau./Saccharomyces - ps27
Saccharomyces - ps28
Arboricola_clade - ps29
Cerevisiae_clade - ps30
Saccharomyces_cerevisiae - ps31
p-value
3e-02
3e-02
4e-02
5e-02
6e-02
Log-odds
0.5
1.0
1.5
2.0
2.5
3.0
3.5
4.0

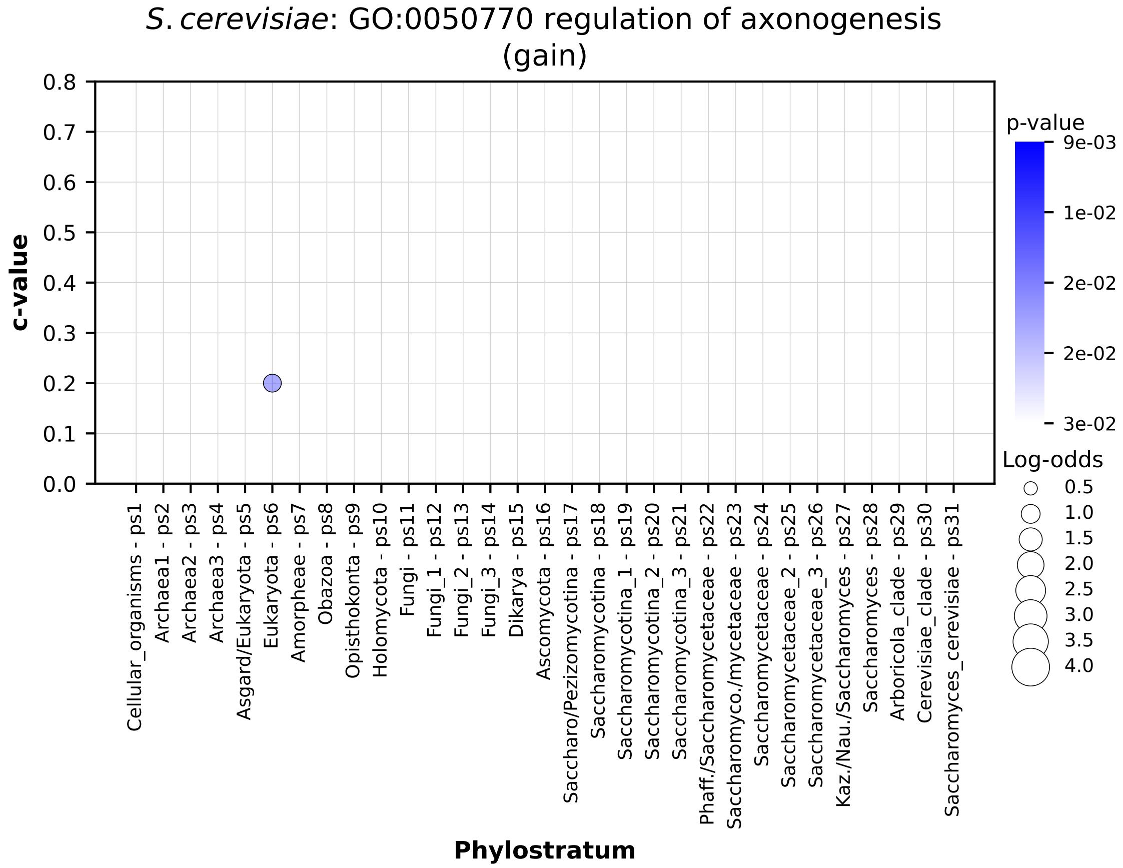

S. cerevisiae: GO:0050770 regulation of axonogenesis (gain)
c-value
0.8
0.7
0.6
0.5
0.4
0.3
0.2
0.1
0.0
p-value
9e-03
1e-02
2e-02
2e-02
3e-02
Log-odds
0.5
1.0
1.5
2.0
2.5
3.0
3.5
4.0
Cellular_organisms - ps1
Archaea1 - ps2
Archaea2 - ps3
Archaea3 - ps4
Asgard/Eukaryota - ps5
Eukaryota - ps6
Amorpheae - ps7
Obazoa - ps8
Opisthokonta - ps9
Holomycota - ps10
Fungi - ps11
Fungi_1 - ps12
Fungi_2 - ps13
Fungi_3 - ps14
Dikarya - ps15
Ascomycota - ps16
Saccharo/Pezizomycotina - ps17
Saccharomycotina - ps18
Saccharomycotina_1 - ps19
Saccharomycotina_2 - ps20
Saccharomycotina_3 - ps21
Phaff./Saccharomycetaceae - ps22
Saccharomyco./mycetaceae - ps23
Saccharomycetaceae - ps24
Saccharomycetaceae_2 - ps25
Saccharomycetaceae_3 - ps26
Kaz./Nau./Saccharomyces - ps27
Saccharomyces - ps28
Arboricola_clade - ps29
Cerevisiae_clade - ps30
Saccharomyces_cerevisiae - ps31
Phylostratum

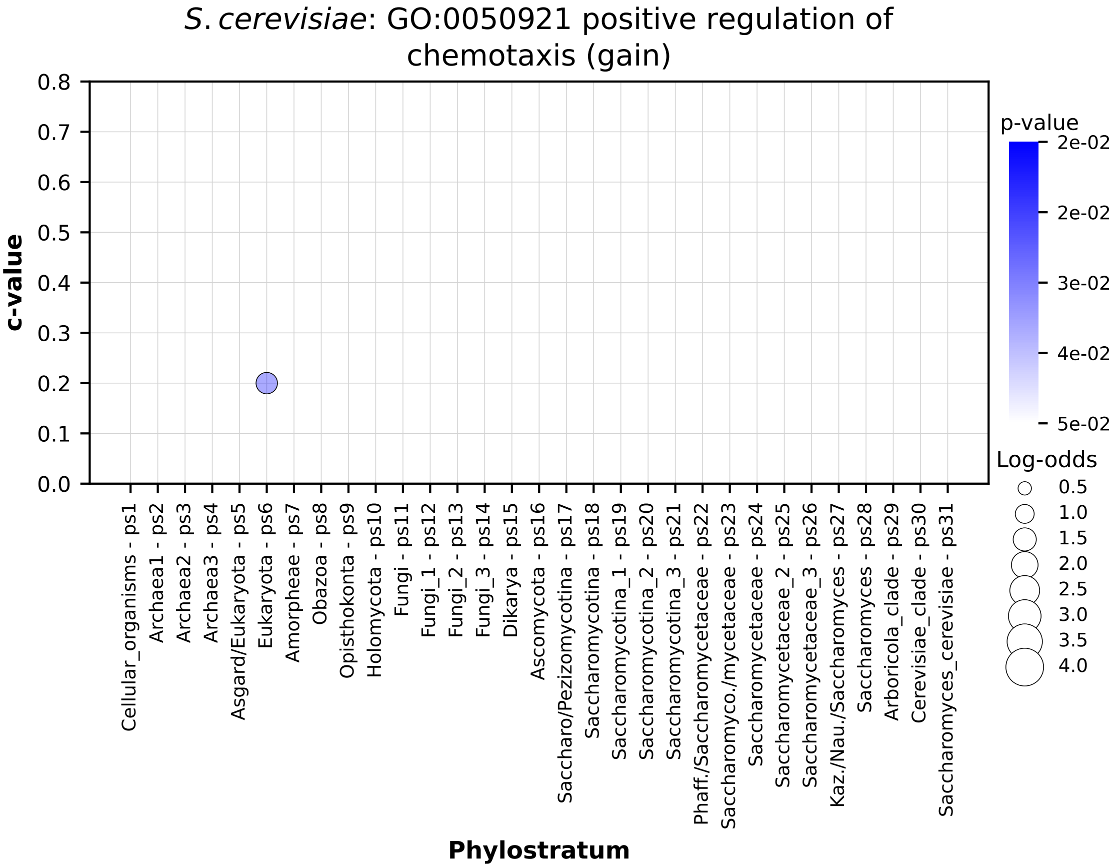
S. cerevisiae: GO:0050921 positive regulation of chemotaxis (gain)
c-value
0.8
0.7
0.6
0.5
0.4
0.3
0.2
0.1
0.0
p-value
2e-02
2e-02
3e-02
4e-02
5e-02
Log-odds
0.5
1.0
1.5
2.0
2.5
3.0
3.5
4.0
Cellular_organisms - ps1
Archaea1 - ps2
Archaea2 - ps3
Archaea3 - ps4
Asgard/Eukaryota - ps5
Eukaryota - ps6
Amorpheae - ps7
Obazoa - ps8
Opisthokonta - ps9
Holomycota - ps10
Fungi - ps11
Fungi_1 - ps12
Fungi_2 - ps13
Fungi_3 - ps14
Dikarya - ps15
Ascomycota - ps16
Saccharo/Pezizomycotina - ps17
Saccharomycotina - ps18
Saccharomycotina_1 - ps19
Saccharomycotina_2 - ps20
Saccharomycotina_3 - ps21
Phaff./Saccharomycetaceae - ps22
Saccharomyco./mycetaceae - ps23
Saccharomycetaceae - ps24
Saccharomycetaceae_2 - ps25
Saccharomycetaceae_3 - ps26
Kaz./Nau./Saccharomyces - ps27
Saccharomyces - ps28
Arboricola_clade - ps29
Cerevisiae_clade - ps30
Saccharomyces_cerevisiae - ps31
Phylostratum

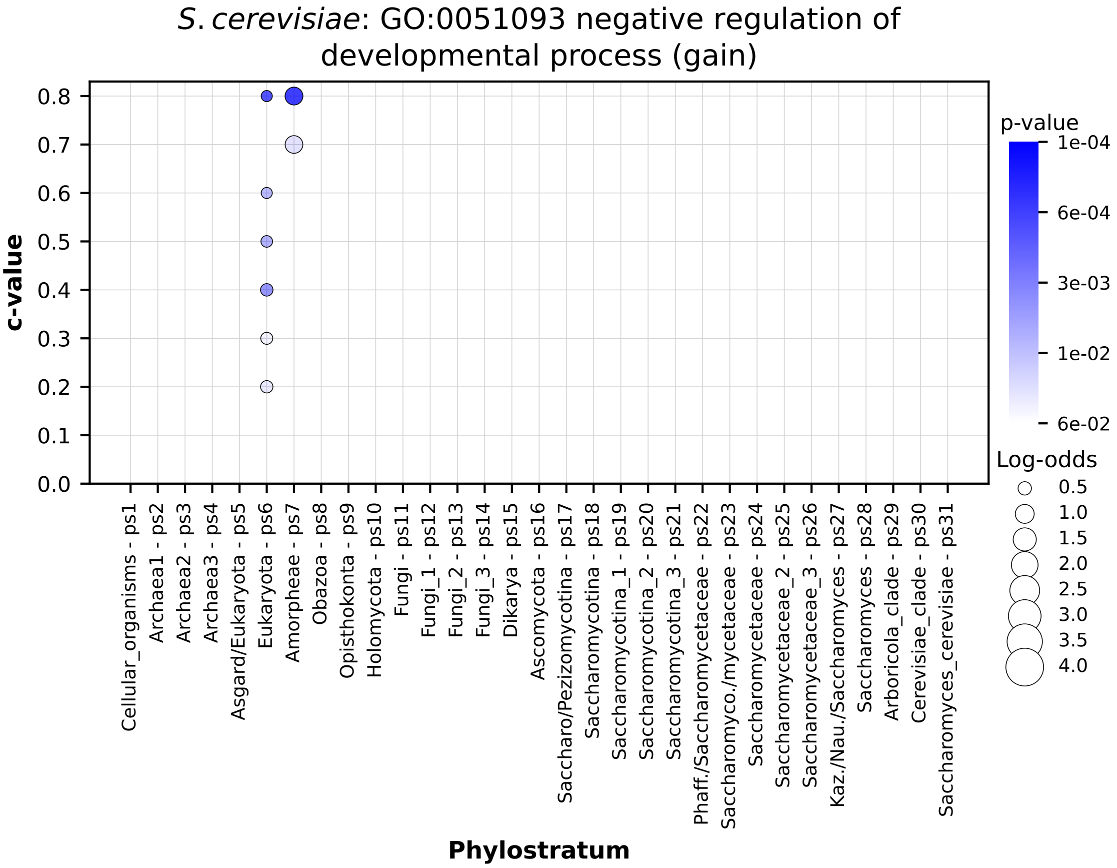

S. cerevisiae: GO:0051093 negative regulation of developmental process (gain)
c-value
0.0
0.1
0.2
0.3
0.4
0.5
0.6
0.7
0.8
p-value
1e-04
6e-04
3e-03
1e-02
6e-02
Log-odds
0.5
1.0
1.5
2.0
2.5
3.0
3.5
4.0
Cellular_organisms - ps1
Archaea1 - ps2
Archaea2 - ps3
Archaea3 - ps4
Asgard/Eukaryota - ps5
Eukaryota - ps6
Amorpheae - ps7
Obazoa - ps8
Opisthokonta - ps9
Holomycota - ps10
Fungi - ps11
Fungi_1 - ps12
Fungi_2 - ps13
Fungi_3 - ps14
Dikarya - ps15
Ascomycota - ps16
Saccharo/Pezizomycotina - ps17
Saccharomycotina - ps18
Saccharomycotina_1 - ps19
Saccharomycotina_2 - ps20
Saccharomycotina_3 - ps21
Phaff./Saccharomycetaceae - ps22
Saccharomyco./mycetaceae - ps23
Saccharomycetaceae - ps24
Saccharomycetaceae_2 - ps25
Saccharomycetaceae_3 - ps26
Kaz./Nau./Saccharomyces - ps27
Saccharomyces - ps28
Arboricola_clade - ps29
Cerevisiae_clade - ps30
Saccharomyces_cerevisiae - ps31
Phylostratum

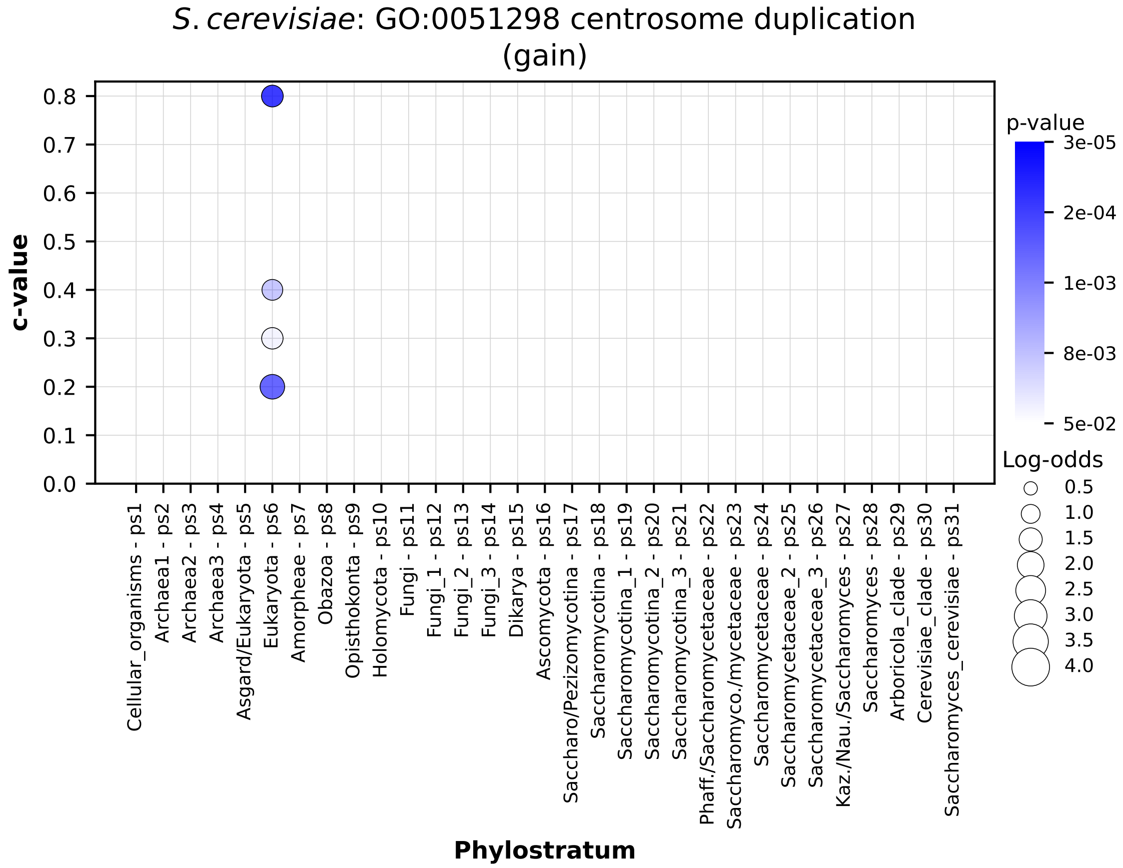

S. cerevisiae: GO:0051298 centrosome duplication
(gain)
c-value
0.8
0.7
0.6
0.5
0.4
0.3
0.2
0.1
0.0
p-value
3e-05
2e-04
1e-03
8e-03
5e-02
Log-odds
0.5
1.0
1.5
2.0
2.5
3.0
3.5
4.0
Cellular_organisms - ps1
Archaea1 - ps2
Archaea2 - ps3
Archaea3 - ps4
Asgard/Eukaryota - ps5
Eukaryota - ps6
Amorpheae - ps7
Obazoa - ps8
Opisthokonta - ps9
Holomycota - ps10
Fungi - ps11
Fungi_1 - ps12
Fungi_2 - ps13
Fungi_3 - ps14
Dikarya - ps15
Ascomycota - ps16
Saccharo/Pezizomycotina - ps17
Saccharomycotina - ps18
Saccharomycotina_1 - ps19
Saccharomycotina_2 - ps20
Saccharomycotina_3 - ps21
Phaff./Saccharomycetaceae - ps22
Saccharomyco./mycetaceae - ps23
Saccharomycetaceae - ps24
Saccharomycetaceae_2 - ps25
Saccharomycetaceae_3 - ps26
Kaz./Nau./Saccharomyces - ps27
Saccharomyces - ps28
Arboricola_clade - ps29
Cerevisiae_clade - ps30
Saccharomyces_cerevisiae - ps31
Phylostratum

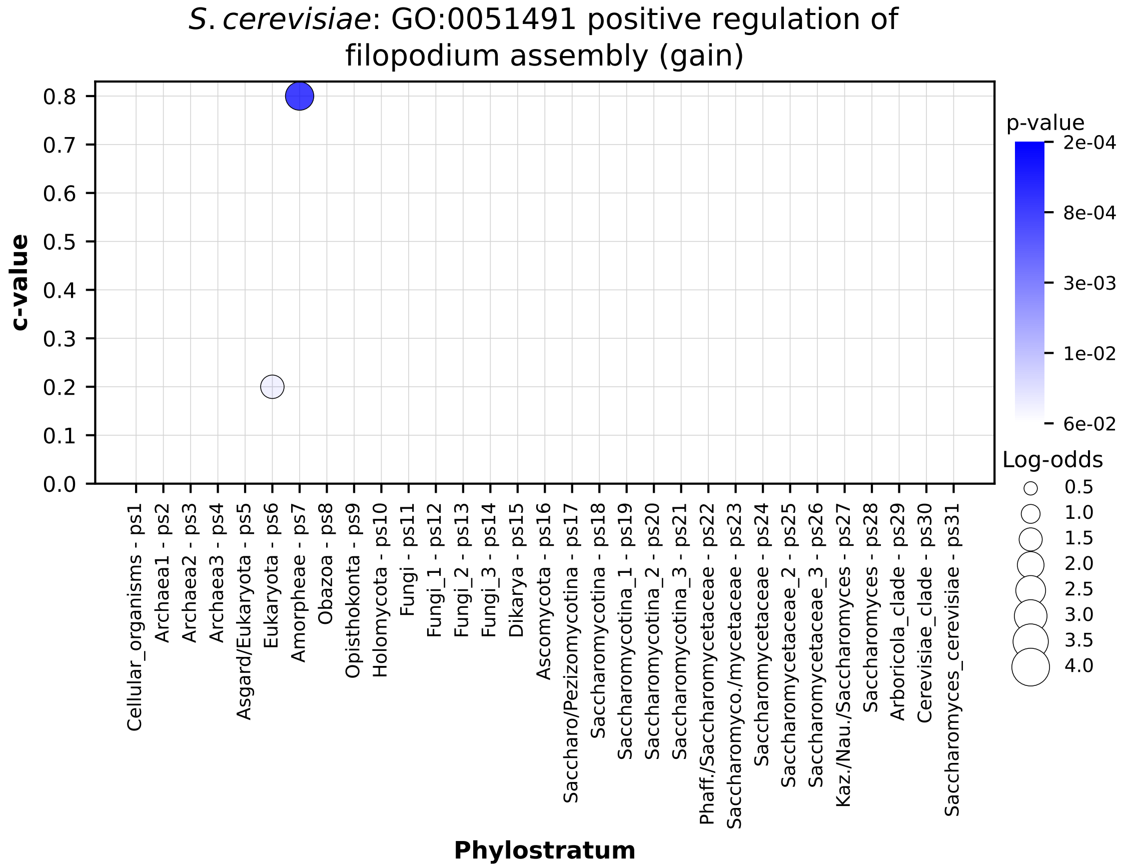
S. cerevisiae: GO:0051491 positive regulation of filopodium assembly (gain)
c-value
Phylostratum
p-value
2e-04
8e-04
3e-03
1e-02
6e-02
Log-odds
0.5
1.0
1.5
2.0
2.5
3.0
3.5
4.0
Cellular_organisms - ps1
Archaea1 - ps2
Archaea2 - ps3
Archaea3 - ps4
Asgard/Eukaryota - ps5
Eukaryota - ps6
Amorpheae - ps7
Obazoa - ps8
Opisthokonta - ps9
Holomycota - ps10
Fungi - ps11
Fungi_1 - ps12
Fungi_2 - ps13
Fungi_3 - ps14
Dikarya - ps15
Ascomycota - ps16
Saccharo/Pezizomycotina - ps17
Saccharomycotina - ps18
Saccharomycotina_1 - ps19
Saccharomycotina_2 - ps20
Saccharomycotina_3 - ps21
Phaff./Saccharomycetaceae - ps22
Saccharomyco./mycetaceae - ps23
Saccharomycetaceae - ps24
Saccharomycetaceae_2 - ps25
Saccharomycetaceae_3 - ps26
Kaz./Nau./Saccharomyces - ps27
Saccharomyces - ps28
Arboricola_clade - ps29
Cerevisiae_clade - ps30
Saccharomyces_cerevisiae - ps31

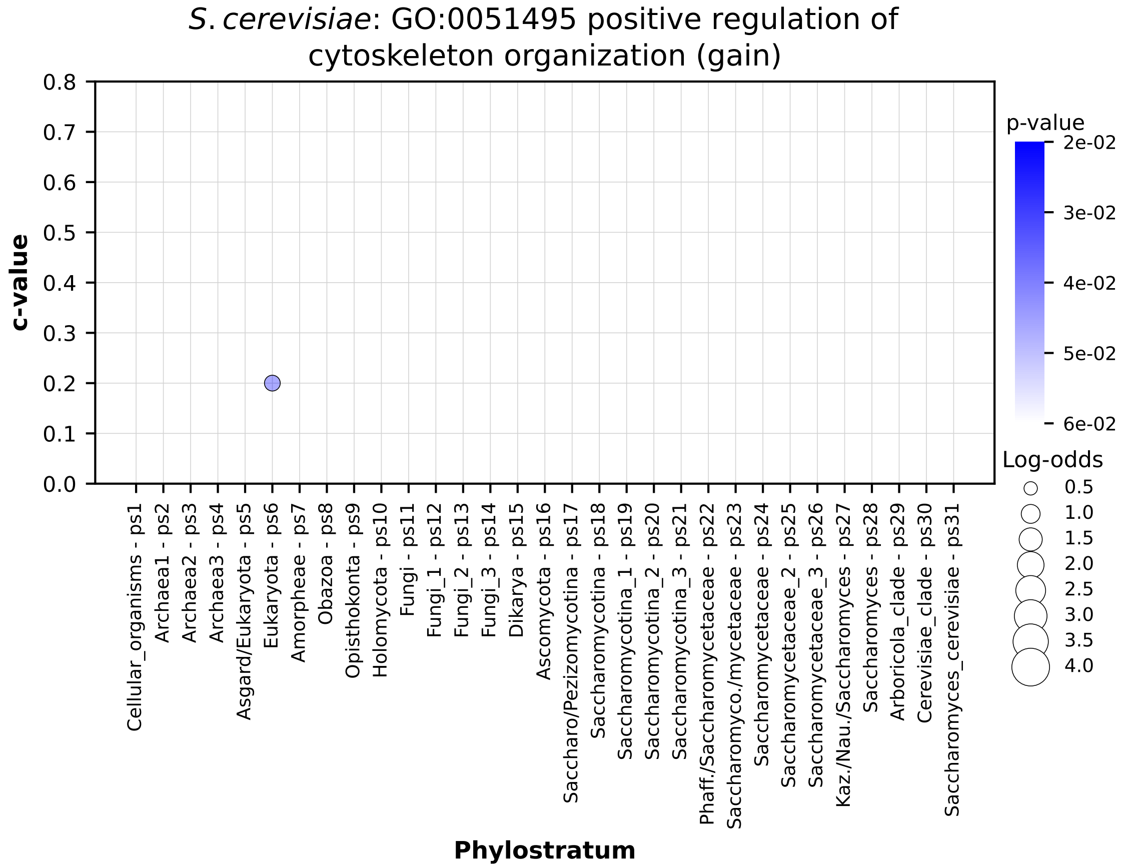

S. cerevisiae: GO:0051495 positive regulation of cytoskeleton organization (gain)
c-value
0.8
0.7
0.6
0.5
0.4
0.3
0.2
0.1
0.0
Phylostratum
Cellular_organisms - ps1
Archaea1 - ps2
Archaea2 - ps3
Archaea3 - ps4
Asgard/Eukaryota - ps5
Eukaryota - ps6
Amorpheae - ps7
Obazoa - ps8
Opisthokonta - ps9
Holomycota - ps10
Fungi - ps11
Fungi_1 - ps12
Fungi_2 - ps13
Fungi_3 - ps14
Dikarya - ps15
Ascomycota - ps16
Saccharo/Pezizomycotina - ps17
Saccharomycotina - ps18
Saccharomycotina_1 - ps19
Saccharomycotina_2 - ps20
Saccharomycotina_3 - ps21
Phaff./Saccharomycetaceae - ps22
Saccharomyco./mycetaceae - ps23
Saccharomycetaceae - ps24
Saccharomycetaceae_2 - ps25
Saccharomycetaceae_3 - ps26
Kaz./Nau./Saccharomyces - ps27
Saccharomyces - ps28
Arboricola_clade - ps29
Cerevisiae_clade - ps30
Saccharomyces_cerevisiae - ps31
p-value
2e-02
3e-02
4e-02
5e-02
6e-02
Log-odds
0.5
1.0
1.5
2.0
2.5
3.0
3.5
4.0

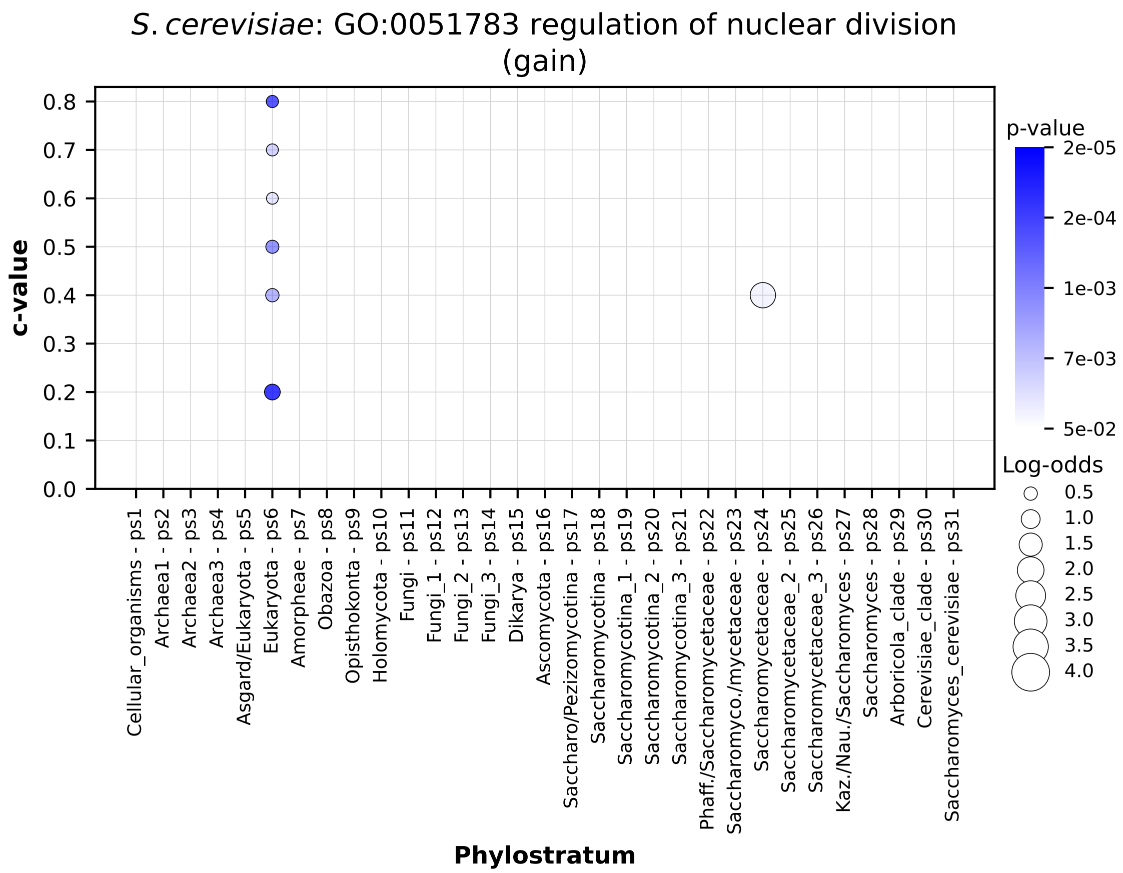

S. cerevisiae: GO:0051783 regulation of nuclear division
(gain)
c-value
0.0
0.1
0.2
0.3
0.4
0.5
0.6
0.7
0.8
p-value
2e-05
2e-04
1e-03
7e-03
5e-02
Log-odds
0.5
1.0
1.5
2.0
2.5
3.0
3.5
4.0
Cellular_organisms - ps1
Archaea1 - ps2
Archaea2 - ps3
Archaea3 - ps4
Asgard/Eukaryota - ps5
Eukaryota - ps6
Amorpheae - ps7
Obazoa - ps8
Opisthokonta - ps9
Holomycota - ps10
Fungi - ps11
Fungi_1 - ps12
Fungi_2 - ps13
Fungi_3 - ps14
Dikarya - ps15
Ascomycota - ps16
Saccharo/Pezizomycotina - ps17
Saccharomycotina - ps18
Saccharomycotina_1 - ps19
Saccharomycotina_2 - ps20
Saccharomycotina_3 - ps21
Phaff./Saccharomycetaceae - ps22
Saccharomyco./mycetaceae - ps23
Saccharomycetaceae - ps24
Saccharomycetaceae_2 - ps25
Saccharomycetaceae_3 - ps26
Kaz./Nau./Saccharomyces - ps27
Saccharomyces - ps28
Arboricola_clade - ps29
Cerevisiae_clade - ps30
Saccharomyces_cerevisiae - ps31
Phylostratum

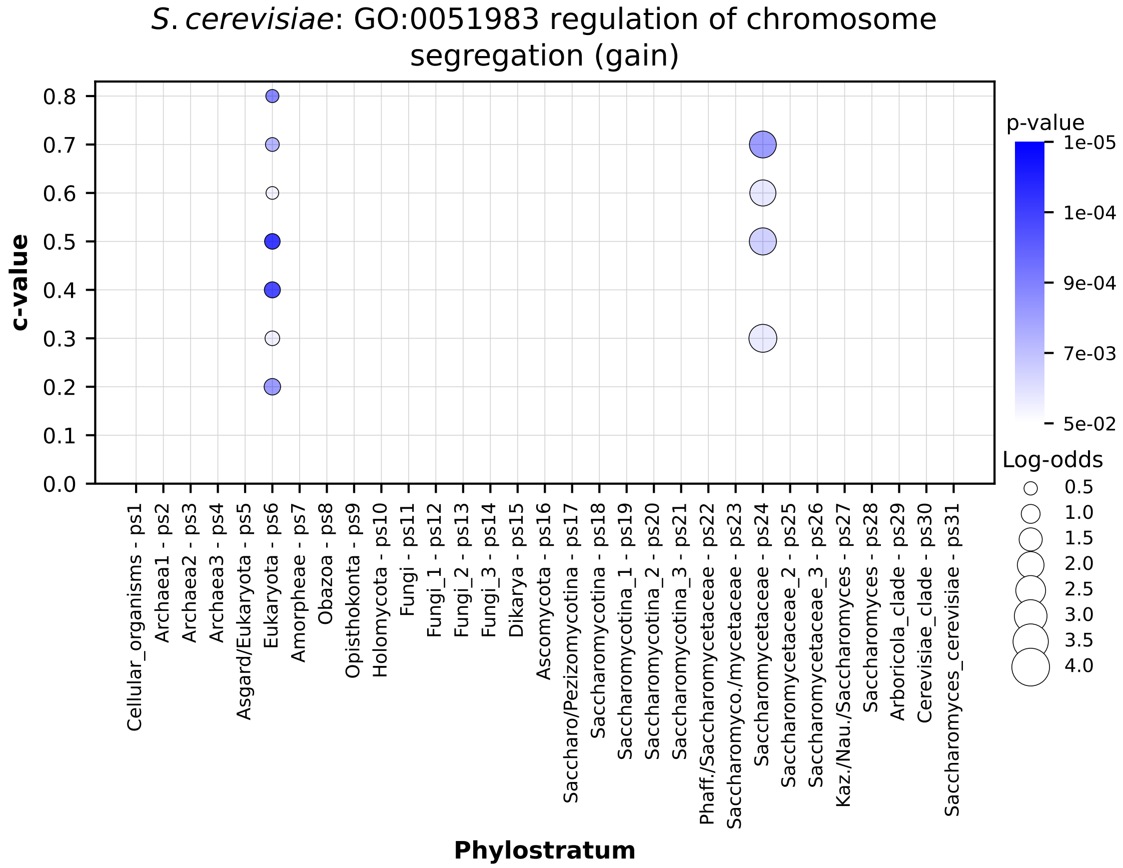

S. cerevisiae: GO:0051983 regulation of chromosome segregation (gain)
c-value
0.8
0.7
0.6
0.5
0.4
0.3
0.2
0.1
0.0
p-value
1e-05
1e-04
9e-04
7e-03
5e-02
Log-odds
0.5
1.0
1.5
2.0
2.5
3.0
3.5
4.0
Cellular_organisms - ps1
Archaea1 - ps2
Archaea2 - ps3
Archaea3 - ps4
Asgard/Eukaryota - ps5
Eukaryota - ps6
Amorpheae - ps7
Obazoa - ps8
Opisthokonta - ps9
Holomycota - ps10
Fungi - ps11
Fungi_1 - ps12
Fungi_2 - ps13
Fungi_3 - ps14
Dikarya - ps15
Ascomycota - ps16
Saccharo/Pezizomycotina - ps17
Saccharomycotina - ps18
Saccharomycotina_1 - ps19
Saccharomycotina_2 - ps20
Saccharomycotina_3 - ps21
Phaff./Saccharomycetaceae - ps22
Saccharomyco./mycetaceae - ps23
Saccharomycetaceae - ps24
Saccharomycetaceae_2 - ps25
Saccharomycetaceae_3 - ps26
Kaz./Nau./Saccharomyces - ps27
Saccharomyces - ps28
Arboricola_clade - ps29
Cerevisiae_clade - ps30
Saccharomyces_cerevisiae - ps31
Phylostratum

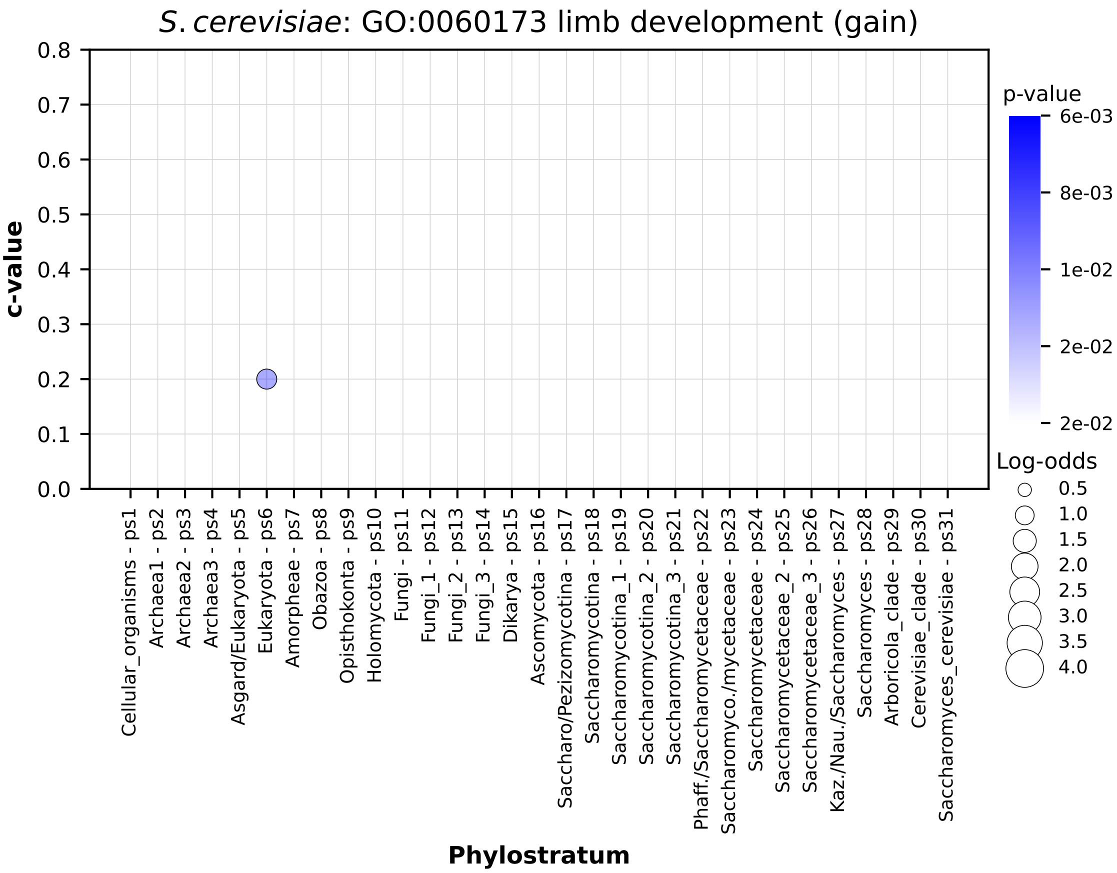

S. cerevisiae: GO:0060173 limb development (gain)
c-value
0.8
0.7
0.6
0.5
0.4
0.3
0.2
0.1
0.0
p-value
6e-03
8e-03
1e-02
2e-02
2e-02
Log-odds
0.5
1.0
1.5
2.0
2.5
3.0
3.5
4.0
Cellular_organisms - ps1
Archaea1 - ps2
Archaea2 - ps3
Archaea3 - ps4
Asgard/Eukaryota - ps5
Eukaryota - ps6
Amorpheae - ps7
Obazoa - ps8
Opisthokonta - ps9
Holomycota - ps10
Fungi - ps11
Fungi_1 - ps12
Fungi_2 - ps13
Fungi_3 - ps14
Dikarya - ps15
Ascomycota - ps16
Saccharo/Pezizomycotina - ps17
Saccharomycotina - ps18
Saccharomycotina_1 - ps19
Saccharomycotina_2 - ps20
Saccharomycotina_3 - ps21
Phaff./Saccharomycetaceae - ps22
Saccharomyco./mycetaceae - ps23
Saccharomycetaceae - ps24
Saccharomycetaceae_2 - ps25
Saccharomycetaceae_3 - ps26
Kaz./Nau./Saccharomyces - ps27
Saccharomyces - ps28
Arboricola_clade - ps29
Cerevisiae_clade - ps30
Saccharomyces_cerevisiae - ps31
Phylostratum

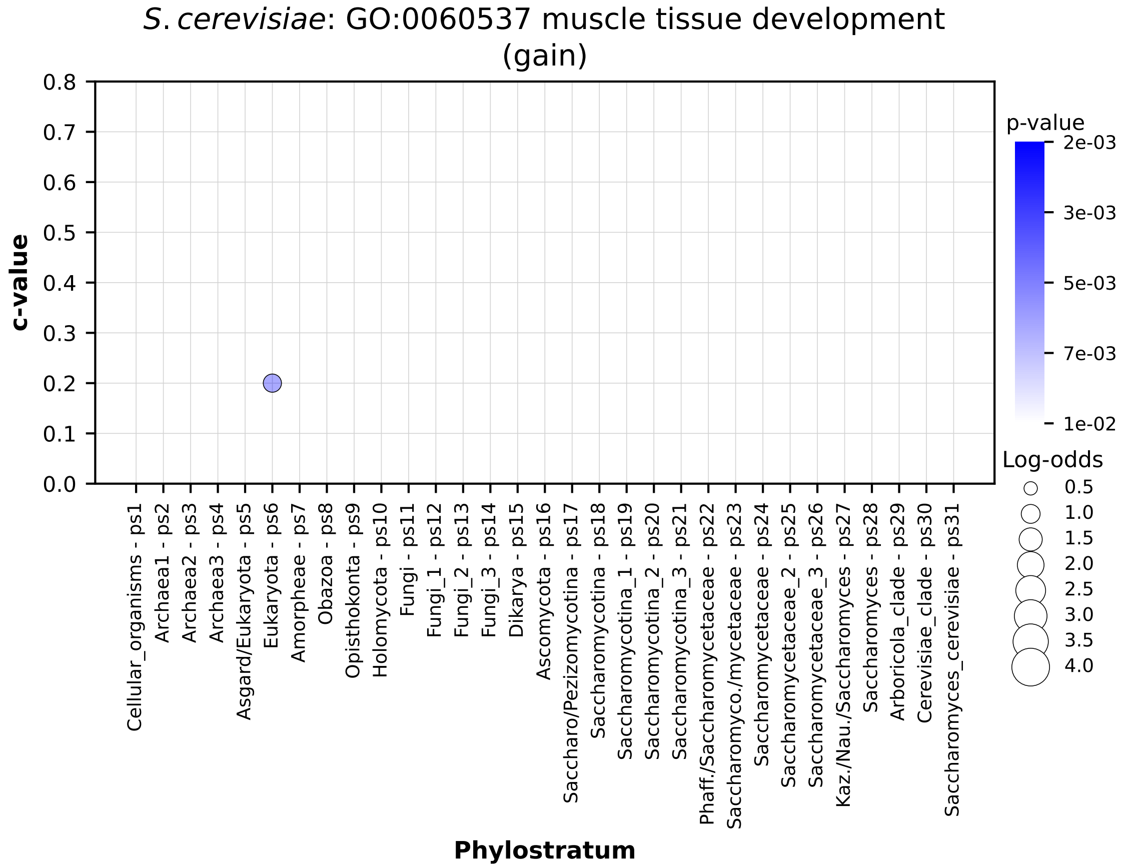

S. cerevisiae: GO:0060537 muscle tissue development
(gain)
c-value
0.8
0.7
0.6
0.5
0.4
0.3
0.2
0.1
0.0
p-value
2e-03
3e-03
5e-03
7e-03
1e-02
Log-odds
0.5
1.0
1.5
2.0
2.5
3.0
3.5
4.0
Cellular_organisms - ps1
Archaea1 - ps2
Archaea2 - ps3
Archaea3 - ps4
Asgard/Eukaryota - ps5
Eukaryota - ps6
Amorpheae - ps7
Obazoa - ps8
Opisthokonta - ps9
Holomycota - ps10
Fungi - ps11
Fungi_1 - ps12
Fungi_2 - ps13
Fungi_3 - ps14
Dikarya - ps15
Ascomycota - ps16
Saccharo/Pezizomycotina - ps17
Saccharomycotina - ps18
Saccharomycotina_1 - ps19
Saccharomycotina_2 - ps20
Saccharomycotina_3 - ps21
Phaff./Saccharomycetaceae - ps22
Saccharomyco./mycetaceae - ps23
Saccharomycetaceae - ps24
Saccharomycetaceae_2 - ps25
Saccharomycetaceae_3 - ps26
Kaz./Nau./Saccharomyces - ps27
Saccharomyces - ps28
Arboricola_clade - ps29
Cerevisiae_clade - ps30
Saccharomyces_cerevisiae - ps31
Phylostratum

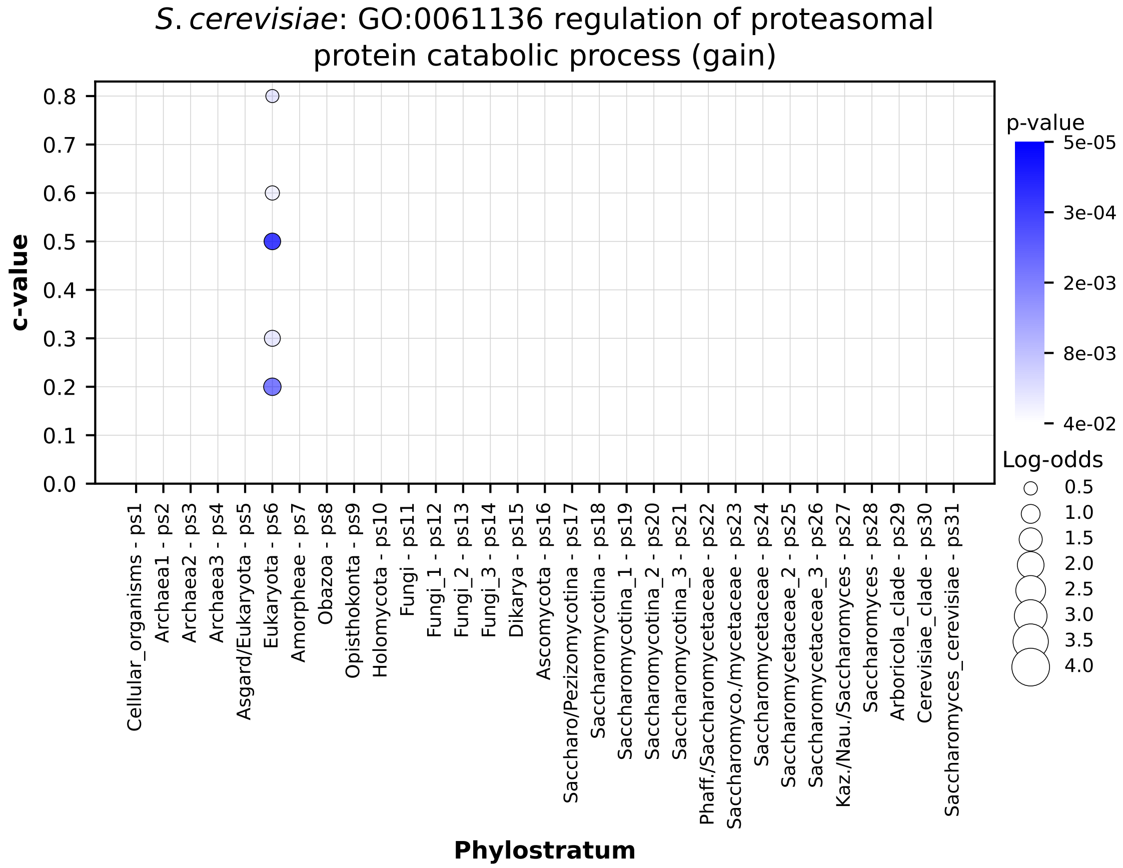

S. cerevisiae: GO:0061136 regulation of proteasomal protein catabolic process (gain)
c-value
0.0
0.1
0.2
0.3
0.4
0.5
0.6
0.7
0.8
Phylostratum
Cellular_organisms - ps1
Archaea1 - ps2
Archaea2 - ps3
Archaea3 - ps4
Asgard/Eukaryota - ps5
Eukaryota - ps6
Amorpheae - ps7
Obazoa - ps8
Opisthokonta - ps9
Holomycota - ps10
Fungi - ps11
Fungi_1 - ps12
Fungi_2 - ps13
Fungi_3 - ps14
Dikarya - ps15
Ascomycota - ps16
Saccharo/Pezizomycotina - ps17
Saccharomycotina - ps18
Saccharomycotina_1 - ps19
Saccharomycotina_2 - ps20
Saccharomycotina_3 - ps21
Phaff./Saccharomycetaceae - ps22
Saccharomyco./mycetaceae - ps23
Saccharomycetaceae - ps24
Saccharomycetaceae_2 - ps25
Saccharomycetaceae_3 - ps26
Kaz./Nau./Saccharomyces - ps27
Saccharomyces - ps28
Arboricola_clade - ps29
Cerevisiae_clade - ps30
Saccharomyces_cerevisiae - ps31
p-value
5e-05
3e-04
2e-03
8e-03
4e-02
Log-odds
0.5
1.0
1.5
2.0
2.5
3.0
3.5
4.0

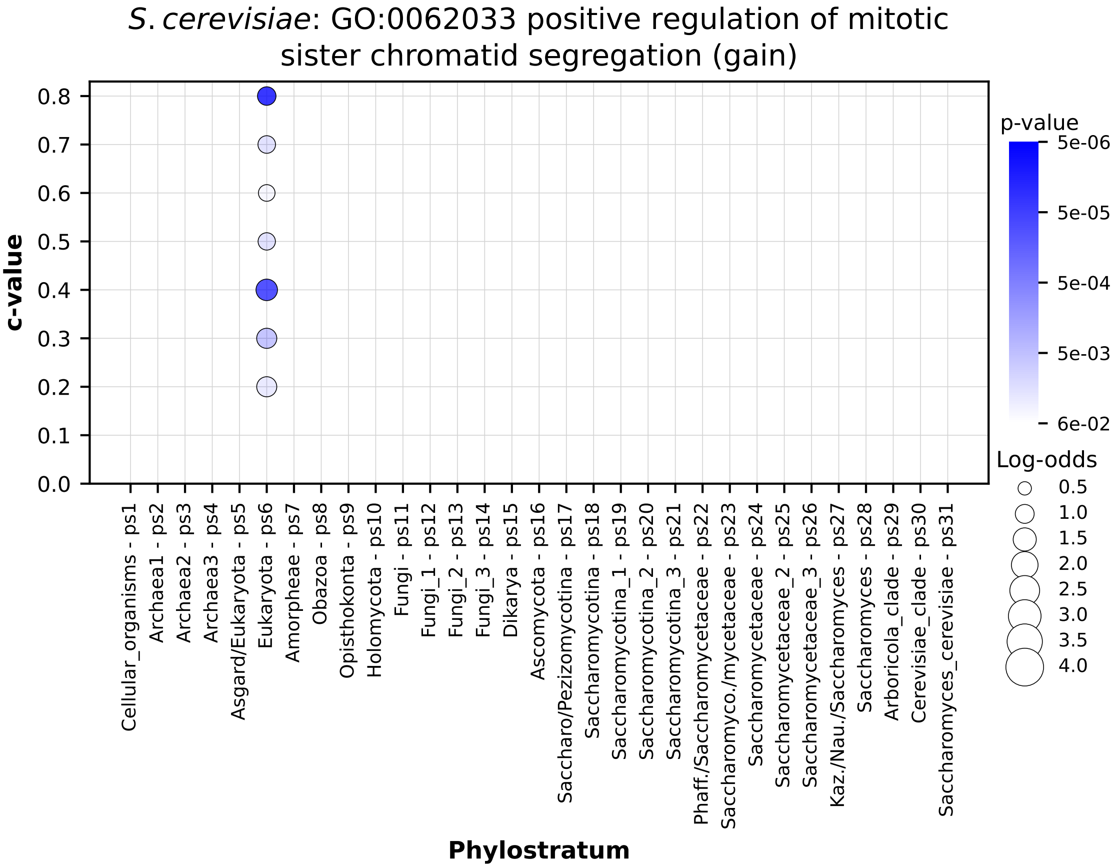

S. cerevisiae: GO:0062033 positive regulation of mitotic sister chromatid segregation (gain)
c-value
0.0
0.1
0.2
0.3
0.4
0.5
0.6
0.7
0.8
p-value
5e-06
5e-05
5e-04
5e-03
6e-02
Log-odds
0.5
1.0
1.5
2.0
2.5
3.0
3.5
4.0
Cellular_organisms - ps1
Archaea1 - ps2
Archaea2 - ps3
Archaea3 - ps4
Asgard/Eukaryota - ps5
Eukaryota - ps6
Amorpheae - ps7
Obazoa - ps8
Opisthokonta - ps9
Holomycota - ps10
Fungi - ps11
Fungi_1 - ps12
Fungi_2 - ps13
Fungi_3 - ps14
Dikarya - ps15
Ascomycota - ps16
Saccharo/Pezizomycotina - ps17
Saccharomycotina - ps18
Saccharomycotina_1 - ps19
Saccharomycotina_2 - ps20
Saccharomycotina_3 - ps21
Phaff./Saccharomycetaceae - ps22
Saccharomyco./mycetaceae - ps23
Saccharomycetaceae - ps24
Saccharomycetaceae_2 - ps25
Saccharomycetaceae_3 - ps26
Kaz./Nau./Saccharomyces - ps27
Saccharomyces - ps28
Arboricola_clade - ps29
Cerevisiae_clade - ps30
Saccharomyces_cerevisiae - ps31
Phylostratum

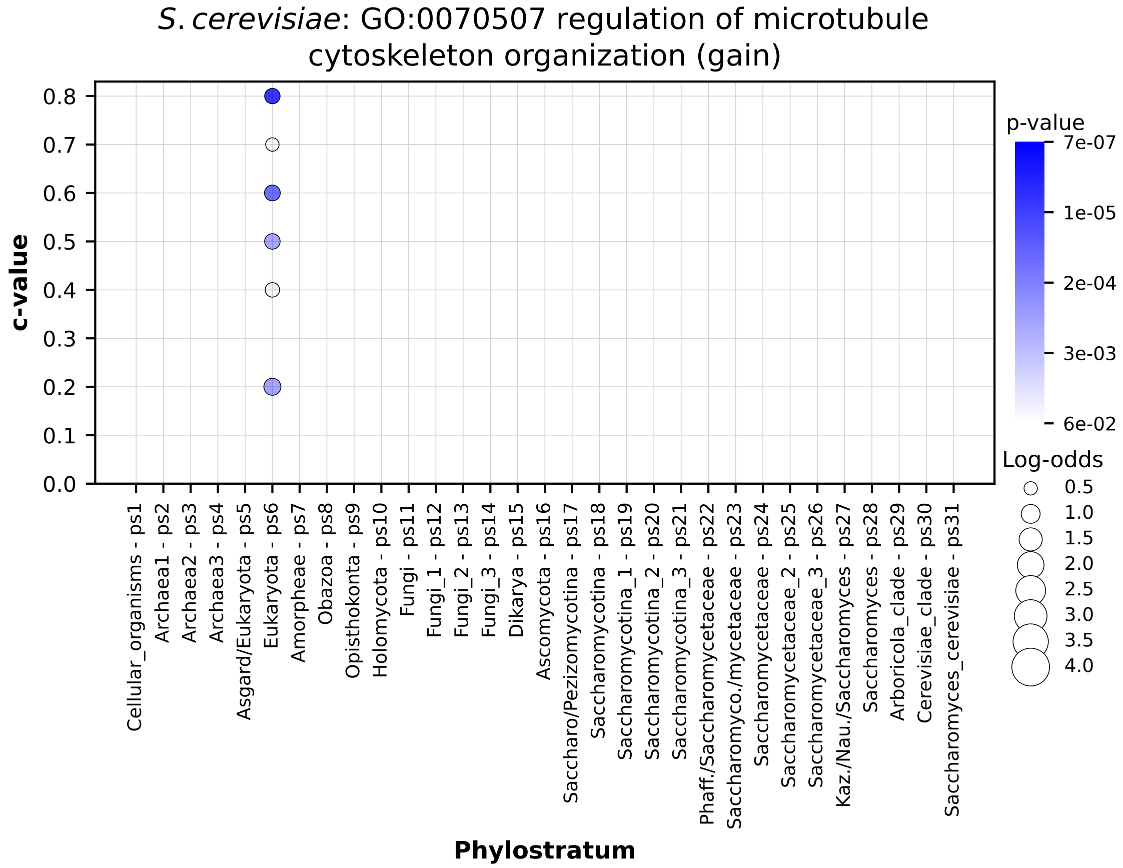

S. cerevisiae: GO:0070507 regulation of microtubule cytoskeleton organization (gain)
c-value
0.0
0.1
0.2
0.3
0.4
0.5
0.6
0.7
0.8
Phylostratum
Cellular_organisms - ps1
Archaea1 - ps2
Archaea2 - ps3
Archaea3 - ps4
Asgard/Eukaryota - ps5
Eukaryota - ps6
Amorpheae - ps7
Obazoa - ps8
Opisthokonta - ps9
Holomycota - ps10
Fungi - ps11
Fungi_1 - ps12
Fungi_2 - ps13
Fungi_3 - ps14
Dikarya - ps15
Ascomycota - ps16
Saccharo/Pezizomycotina - ps17
Saccharomycotina - ps18
Saccharomycotina_1 - ps19
Saccharomycotina_2 - ps20
Saccharomycotina_3 - ps21
Phaff./Saccharomycetaceae - ps22
Saccharomyco./mycetaceae - ps23
Saccharomycetaceae - ps24
Saccharomycetaceae_2 - ps25
Saccharomycetaceae_3 - ps26
Kaz./Nau./Saccharomyces - ps27
Saccharomyces - ps28
Arboricola_clade - ps29
Cerevisiae_clade - ps30
Saccharomyces_cerevisiae - ps31
p-value
7e-07
1e-05
2e-04
3e-03
6e-02
Log-odds
0.5
1.0
1.5
2.0
2.5
3.0
3.5
4.0

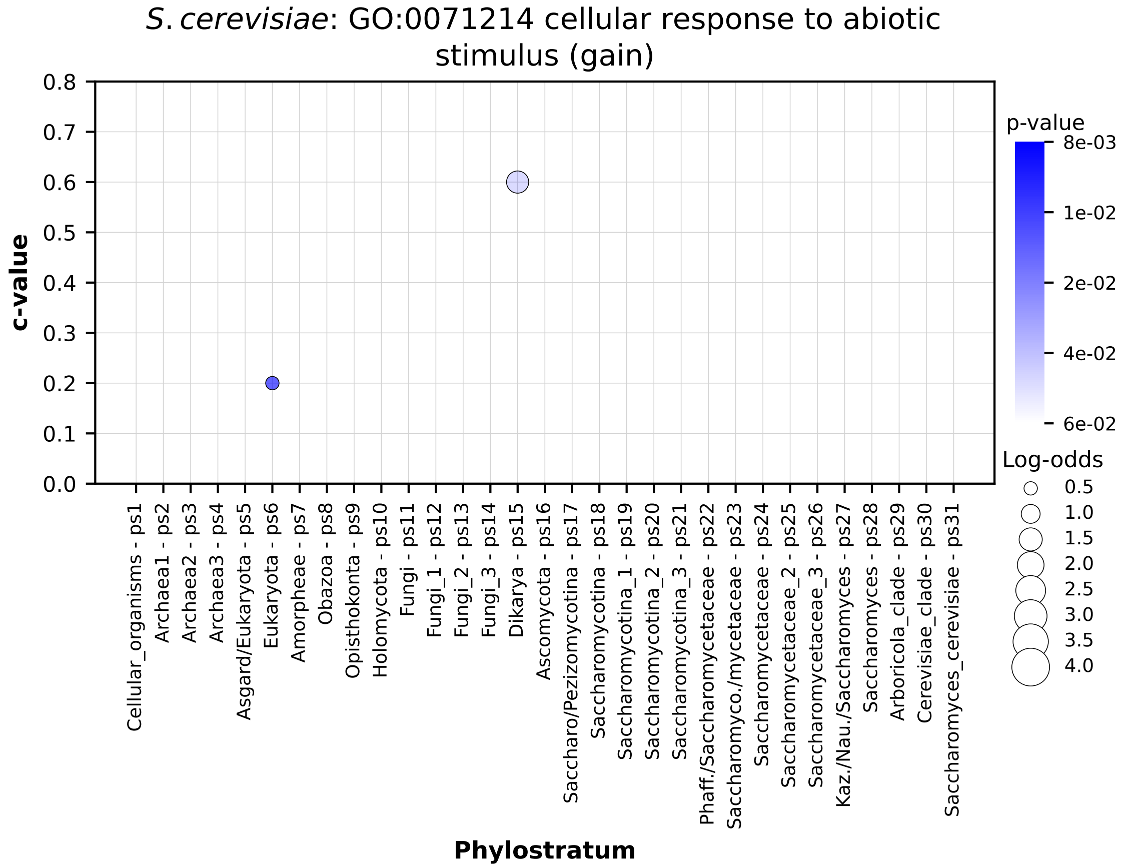

S. cerevisiae: GO:0071214 cellular response to abiotic stimulus (gain)
c-value
0.0
0.1
0.2
0.3
0.4
0.5
0.6
0.7
0.8
Phylostratum
Cellular_organisms - ps1
Archaea1 - ps2
Archaea2 - ps3
Archaea3 - ps4
Asgard/Eukaryota - ps5
Eukaryota - ps6
Amorpheae - ps7
Obazoa - ps8
Opisthokonta - ps9
Holomycota - ps10
Fungi - ps11
Fungi_1 - ps12
Fungi_2 - ps13
Fungi_3 - ps14
Dikarya - ps15
Ascomycota - ps16
Saccharo/Pezizomycotina - ps17
Saccharomycotina - ps18
Saccharomycotina_1 - ps19
Saccharomycotina_2 - ps20
Saccharomycotina_3 - ps21
Phaff./Saccharomycetaceae - ps22
Saccharomyco./mycetaceae - ps23
Saccharomycetaceae - ps24
Saccharomycetaceae_2 - ps25
Saccharomycetaceae_3 - ps26
Kaz./Nau./Saccharomyces - ps27
Saccharomyces - ps28
Arboricola_clade - ps29
Cerevisiae_clade - ps30
Saccharomyces_cerevisiae - ps31
p-value
8e-03
1e-02
2e-02
4e-02
6e-02
Log-odds
0.5
1.0
1.5
2.0
2.5
3.0
3.5
4.0

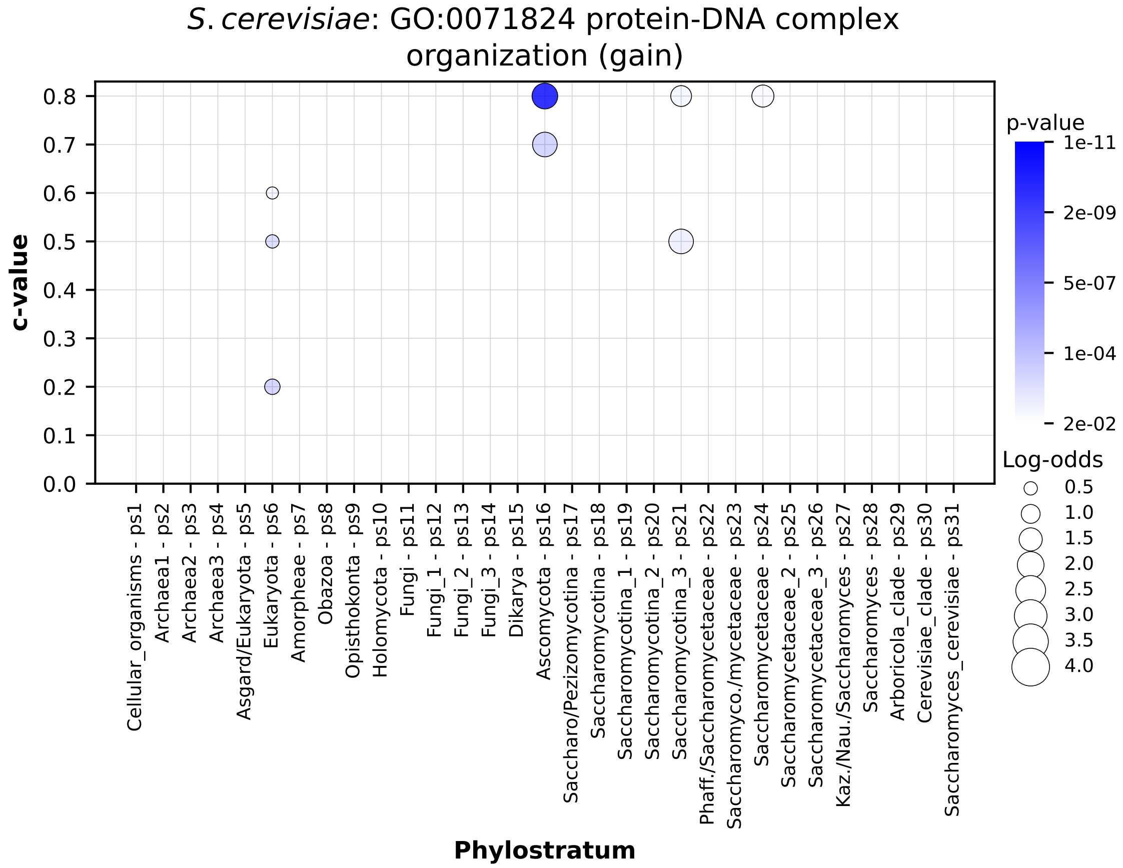

S. cerevisiae: GO:0071824 protein-DNA complex organization (gain)
c-value
0.0
0.1
0.2
0.3
0.4
0.5
0.6
0.7
0.8
p-value
1e-11
2e-09
5e-07
1e-04
2e-02
Log-odds
0.5
1.0
1.5
2.0
2.5
3.0
3.5
4.0
Cellular_organisms - ps1
Archaea1 - ps2
Archaea2 - ps3
Archaea3 - ps4
Asgard/Eukaryota - ps5
Eukaryota - ps6
Amorpheae - ps7
Obazoa - ps8
Opisthokonta - ps9
Holomycota - ps10
Fungi - ps11
Fungi_1 - ps12
Fungi_2 - ps13
Fungi_3 - ps14
Dikarya - ps15
Ascomycota - ps16
Saccharo/Pezizomycotina - ps17
Saccharomycotina - ps18
Saccharomycotina_1 - ps19
Saccharomycotina_2 - ps20
Saccharomycotina_3 - ps21
Phaff./Saccharomycetaceae - ps22
Saccharomyco./mycetaceae - ps23
Saccharomycetaceae - ps24
Saccharomycetaceae_2 - ps25
Saccharomycetaceae_3 - ps26
Kaz./Nau./Saccharomyces - ps27
Saccharomyces - ps28
Arboricola_clade - ps29
Cerevisiae_clade - ps30
Saccharomyces_cerevisiae - ps31
Phylostratum

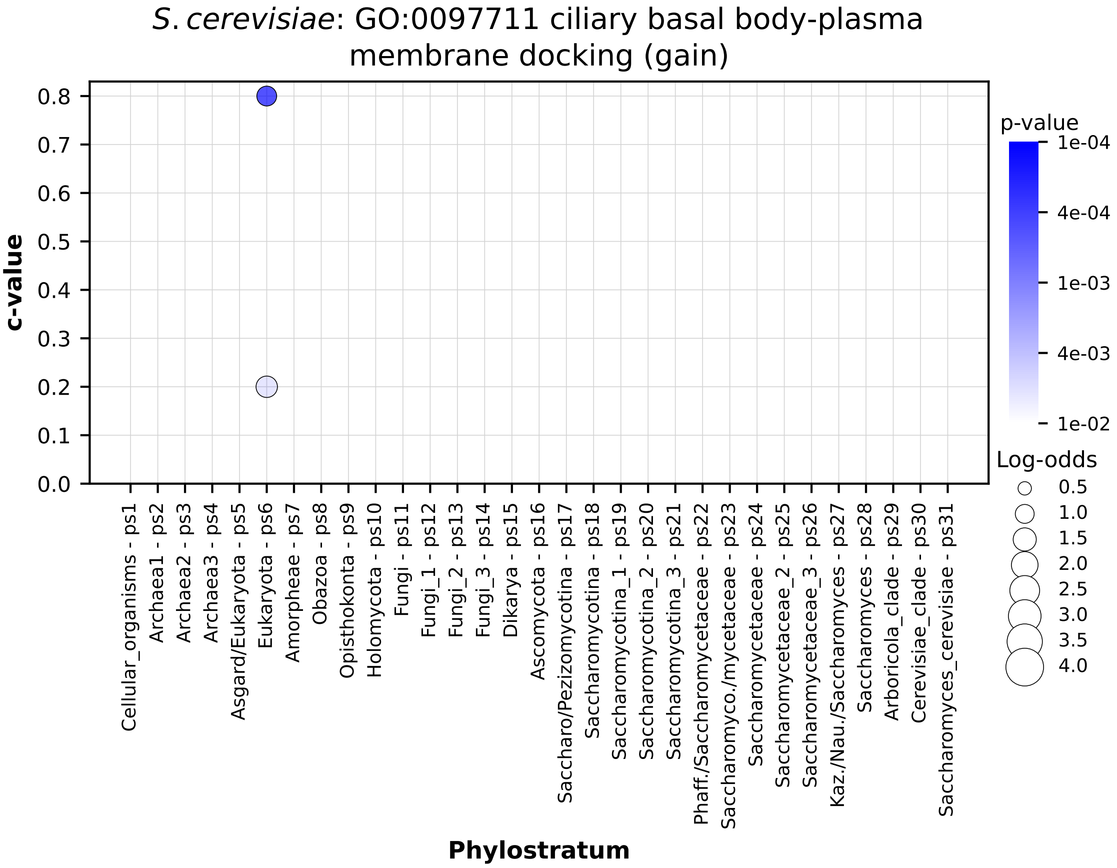

S. cerevisiae: GO:0097711 ciliary basal body-plasma membrane docking (gain)
c-value
0.0
0.1
0.2
0.3
0.4
0.5
0.6
0.7
0.8
Phylostratum
Cellular_organisms - ps1
Archaea1 - ps2
Archaea2 - ps3
Archaea3 - ps4
Asgard/Eukaryota - ps5
Eukaryota - ps6
Amorpheae - ps7
Obazoa - ps8
Opisthokonta - ps9
Holomycota - ps10
Fungi - ps11
Fungi_1 - ps12
Fungi_2 - ps13
Fungi_3 - ps14
Dikarya - ps15
Ascomycota - ps16
Saccharo/Pezizomycotina - ps17
Saccharomycotina - ps18
Saccharomycotina_1 - ps19
Saccharomycotina_2 - ps20
Saccharomycotina_3 - ps21
Phaff./Saccharomycetaceae - ps22
Saccharomyco./mycetaceae - ps23
Saccharomycetaceae - ps24
Saccharomycetaceae_2 - ps25
Saccharomycetaceae_3 - ps26
Kaz./Nau./Saccharomyces - ps27
Saccharomyces - ps28
Arboricola_clade - ps29
Cerevisiae_clade - ps30
Saccharomyces_cerevisiae - ps31
p-value
1e-04
4e-04
1e-03
4e-03
1e-02
Log-odds
0.5
1.0
1.5
2.0
2.5
3.0
3.5
4.0

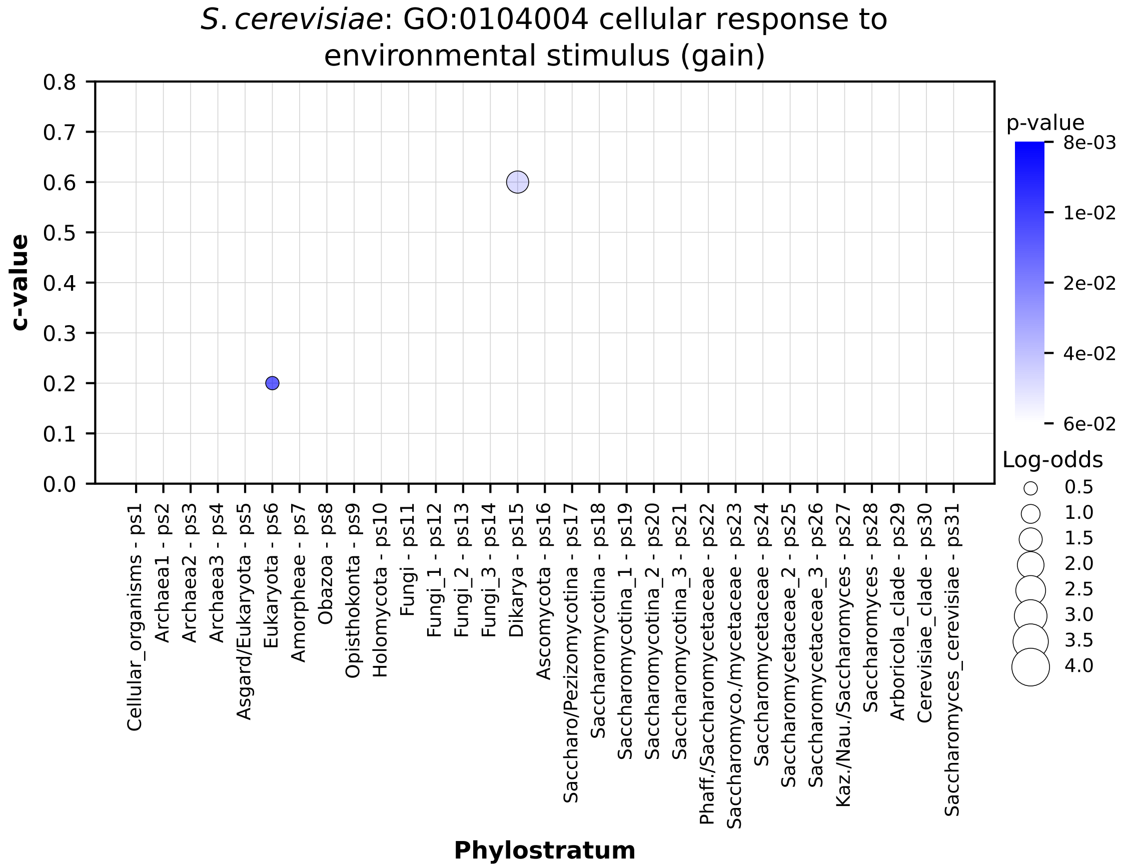

S. cerevisiae: GO:0104004 cellular response to environmental stimulus (gain)
c-value
0.8
0.7
0.6
0.5
0.4
0.3
0.2
0.1
0.0
Phylostratum
Cellular_organisms - ps1
Archaea1 - ps2
Archaea2 - ps3
Archaea3 - ps4
Asgard/Eukaryota - ps5
Eukaryota - ps6
Amorpheae - ps7
Obazoa - ps8
Opisthokonta - ps9
Holomycota - ps10
Fungi - ps11
Fungi_1 - ps12
Fungi_2 - ps13
Fungi_3 - ps14
Dikarya - ps15
Ascomycota - ps16
Saccharo/Pezizomycotina - ps17
Saccharomycotina - ps18
Saccharomycotina_1 - ps19
Saccharomycotina_2 - ps20
Saccharomycotina_3 - ps21
Phaff./Saccharomycetaceae - ps22
Saccharomyco./mycetaceae - ps23
Saccharomycetaceae - ps24
Saccharomycetaceae_2 - ps25
Saccharomycetaceae_3 - ps26
Kaz./Nau./Saccharomyces - ps27
Saccharomyces - ps28
Arboricola_clade - ps29
Cerevisiae_clade - ps30
Saccharomyces_cerevisiae - ps31
p-value
8e-03
1e-02
2e-02
4e-02
6e-02
Log-odds
0.5
1.0
1.5
2.0
2.5
3.0
3.5
4.0

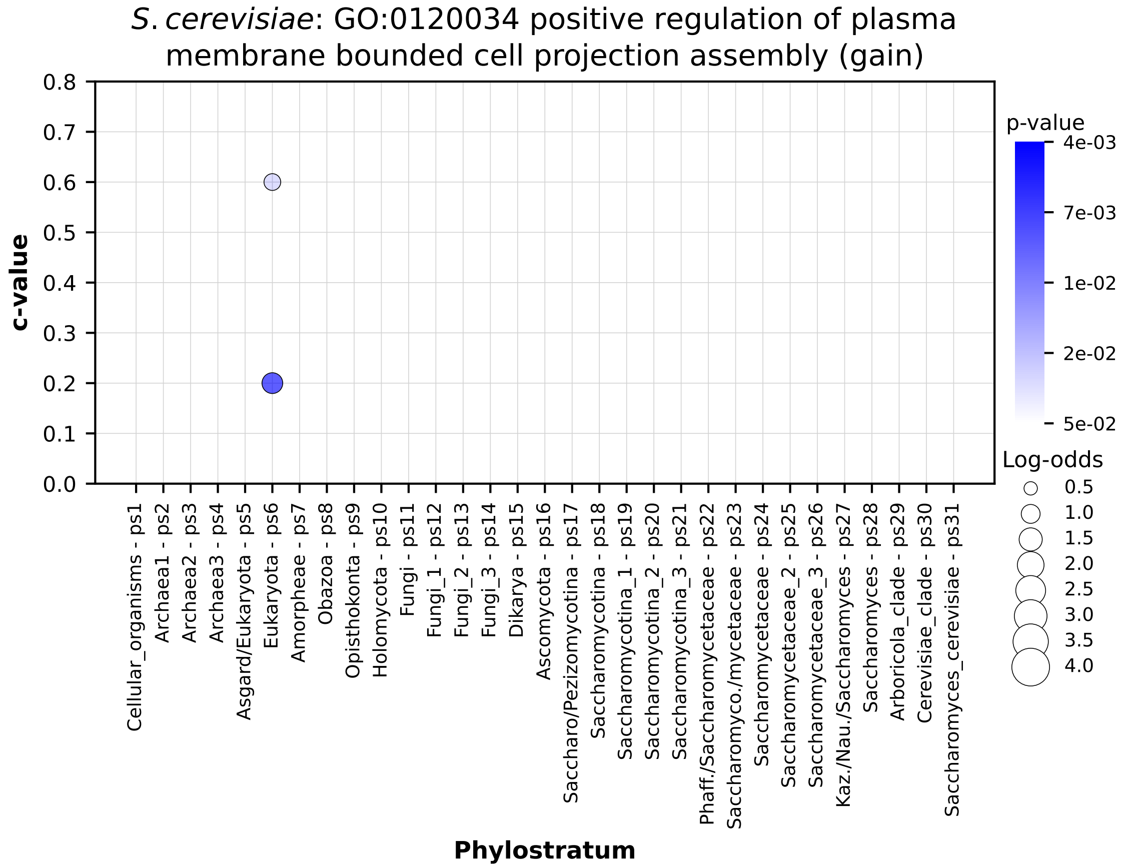

S. cerevisiae: GO:0120034 positive regulation of plasma membrane bounded cell projection assembly (gain)
c-value
0.8
0.7
0.6
0.5
0.4
0.3
0.2
0.1
0.0
p-value
4e-03
7e-03
1e-02
2e-02
5e-02
Log-odds
0.5
1.0
1.5
2.0
2.5
3.0
3.5
4.0
Cellular_organisms - ps1
Archaea1 - ps2
Archaea2 - ps3
Archaea3 - ps4
Asgard/Eukaryota - ps5
Eukaryota - ps6
Amorpheae - ps7
Obazoa - ps8
Opisthokonta - ps9
Holomycota - ps10
Fungi - ps11
Fungi_1 - ps12
Fungi_2 - ps13
Fungi_3 - ps14
Dikarya - ps15
Ascomycota - ps16
Saccharo/Pezizomycotina - ps17
Saccharomycotina - ps18
Saccharomycotina_1 - ps19
Saccharomycotina_2 - ps20
Saccharomycotina_3 - ps21
Phaff./Saccharomycetaceae - ps22
Saccharomyco./mycetaceae - ps23
Saccharomycetaceae - ps24
Saccharomycetaceae_2 - ps25
Saccharomycetaceae_3 - ps26
Kaz./Nau./Saccharomyces - ps27
Saccharomyces - ps28
Arboricola_clade - ps29
Cerevisiae_clade - ps30
Saccharomyces_cerevisiae - ps31
Phylostratum

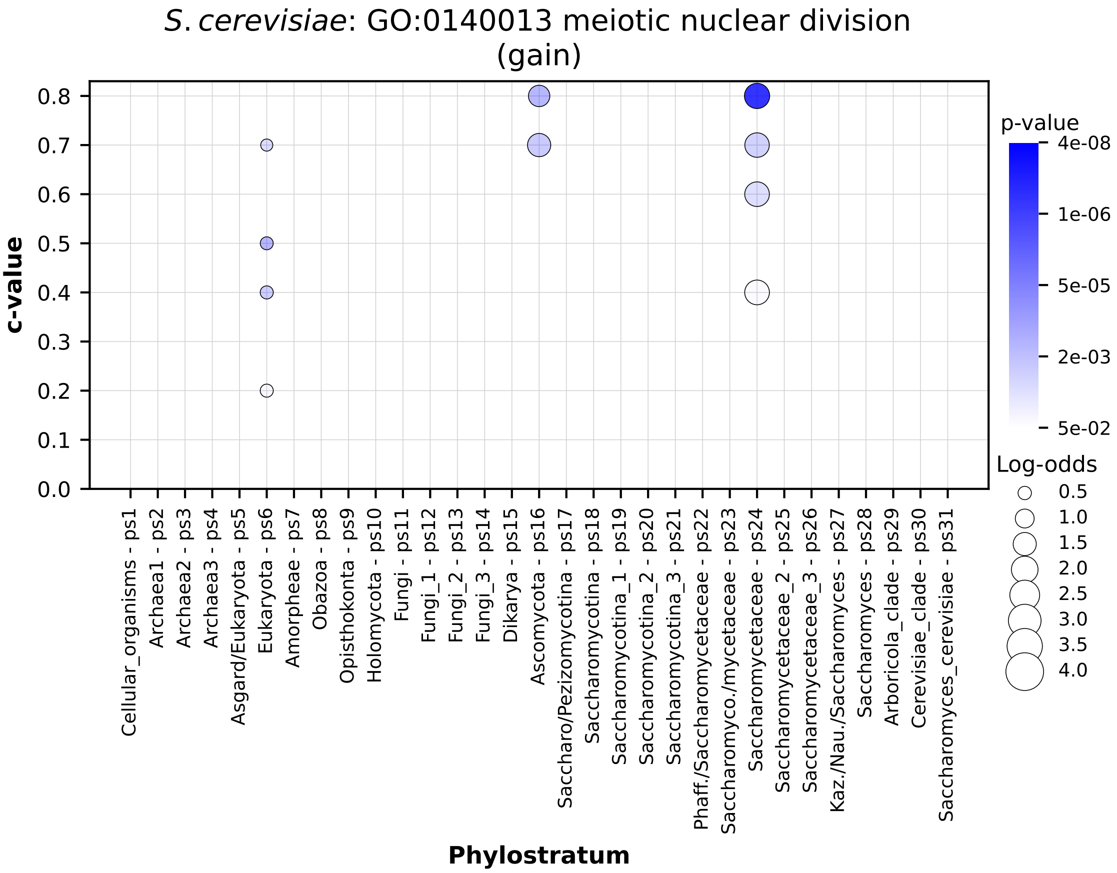

S. cerevisiae: GO:0140013 meiotic nuclear division (gain)
c-value
0.0
0.1
0.2
0.3
0.4
0.5
0.6
0.7
0.8
Phylostratum
Cellular_organisms - ps1
Archaea1 - ps2
Archaea2 - ps3
Archaea3 - ps4
Asgard/Eukaryota - ps5
Eukaryota - ps6
Amorpheae - ps7
Obazoa - ps8
Opisthokonta - ps9
Holomycota - ps10
Fungi - ps11
Fungi_1 - ps12
Fungi_2 - ps13
Fungi_3 - ps14
Dikarya - ps15
Ascomycota - ps16
Saccharo/Pezizomycotina - ps17
Saccharomycotina - ps18
Saccharomycotina_1 - ps19
Saccharomycotina_2 - ps20
Saccharomycotina_3 - ps21
Phaff./Saccharomycetaceae - ps22
Saccharomyco./mycetaceae - ps23
Saccharomycetaceae - ps24
Saccharomycetaceae_2 - ps25
Saccharomycetaceae_3 - ps26
Kaz./Nau./Saccharomyces - ps27
Saccharomyces - ps28
Arboricola_clade - ps29
Cerevisiae_clade - ps30
Saccharomyces_cerevisiae - ps31
p-value
4e-08
1e-06
5e-05
2e-03
5e-02
Log-odds
0.5
1.0
1.5
2.0
2.5
3.0
3.5
4.0

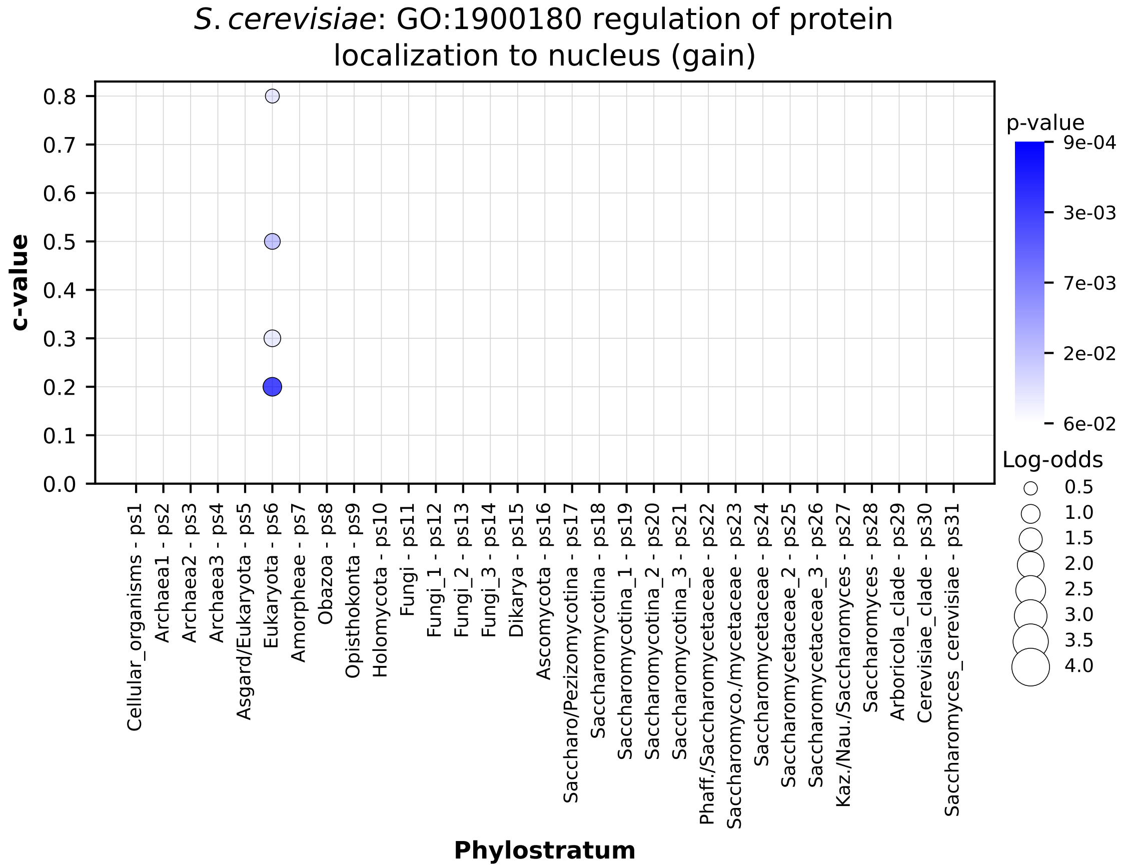

$S. cerevisiae$: GO:1900180 regulation of protein localization to nucleus (gain)
c-value
0.0
0.1
0.2
0.3
0.4
0.5
0.6
0.7
0.8
Phylostratum
Cellular_organisms - ps1
Archaea1 - ps2
Archaea2 - ps3
Archaea3 - ps4
Asgard/Eukaryota - ps5
Eukaryota - ps6
Amorpheae - ps7
Obazoa - ps8
Opisthokonta - ps9
Holomycota - ps10
Fungi - ps11
Fungi_1 - ps12
Fungi_2 - ps13
Fungi_3 - ps14
Dikarya - ps15
Ascomycota - ps16
Saccharo/Pezizomycotina - ps17
Saccharomycotina - ps18
Saccharomycotina_1 - ps19
Saccharomycotina_2 - ps20
Saccharomycotina_3 - ps21
Phaff./Saccharomycetaceae - ps22
Saccharomyco./mycetaceae - ps23
Saccharomycetaceae - ps24
Saccharomycetaceae_2 - ps25
Saccharomycetaceae_3 - ps26
Kaz./Nau./Saccharomyces - ps27
Saccharomyces - ps28
Arboricola_clade - ps29
Cerevisiae_clade - ps30
Saccharomyces_cerevisiae - ps31
p-value
9e-04
3e-03
7e-03
2e-02
6e-02
Log-odds
0.5
1.0
1.5
2.0
2.5
3.0
3.5
4.0

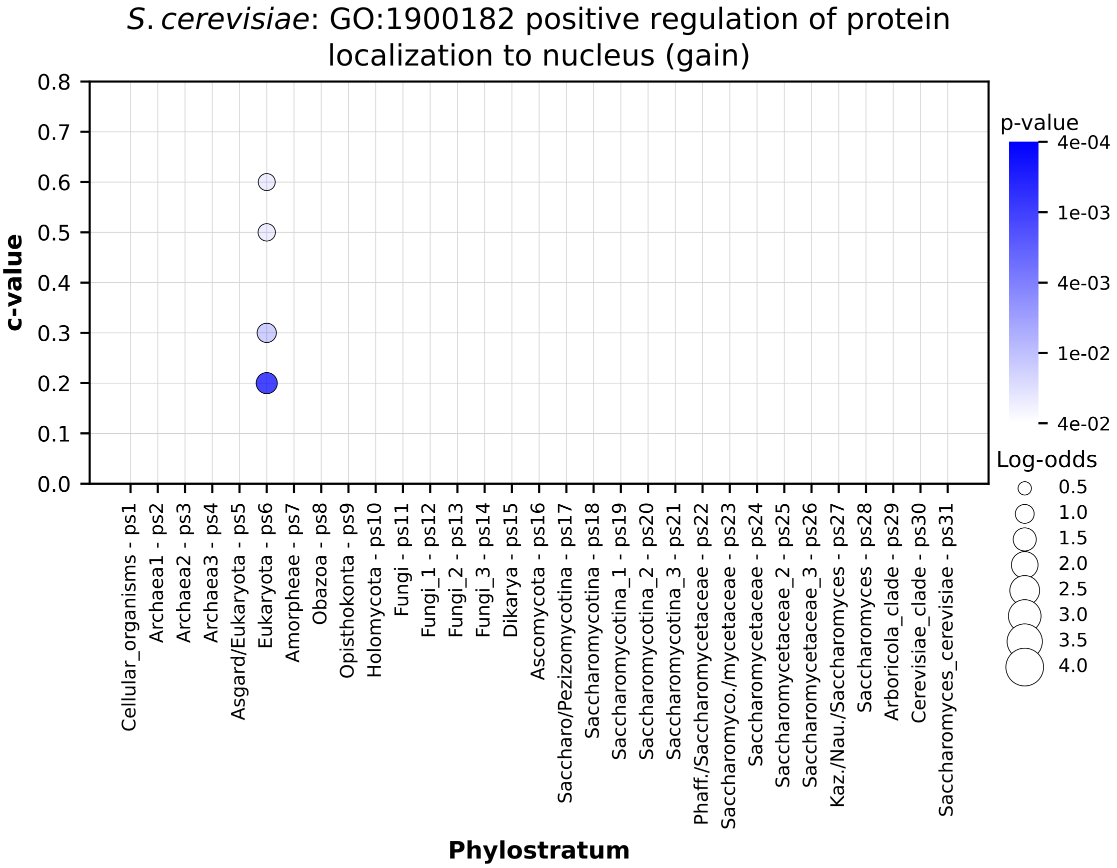

S. cerevisiae: GO:1900182 positive regulation of protein localization to nucleus (gain)
c-value
0.8
0.7
0.6
0.5
0.4
0.3
0.2
0.1
0.0
p-value
4e-04
1e-03
4e-03
1e-02
4e-02
Log-odds
0.5
1.0
1.5
2.0
2.5
3.0
3.5
4.0
Cellular_organisms - ps1
Archaea1 - ps2
Archaea2 - ps3
Archaea3 - ps4
Asgard/Eukaryota - ps5
Eukaryota - ps6
Amorpheae - ps7
Obazoa - ps8
Opisthokonta - ps9
Holomycota - ps10
Fungi - ps11
Fungi_1 - ps12
Fungi_2 - ps13
Fungi_3 - ps14
Dikarya - ps15
Ascomycota - ps16
Saccharo/Pezizomycotina - ps17
Saccharomycotina - ps18
Saccharomycotina_1 - ps19
Saccharomycotina_2 - ps20
Saccharomycotina_3 - ps21
Phaff./Saccharomycetaceae - ps22
Saccharomyco./mycetaceae - ps23
Saccharomycetaceae - ps24
Saccharomycetaceae_2 - ps25
Saccharomycetaceae_3 - ps26
Kaz./Nau./Saccharomyces - ps27
Saccharomyces - ps28
Arboricola_clade - ps29
Cerevisiae_clade - ps30
Saccharomyces_cerevisiae - ps31
Phylostratum

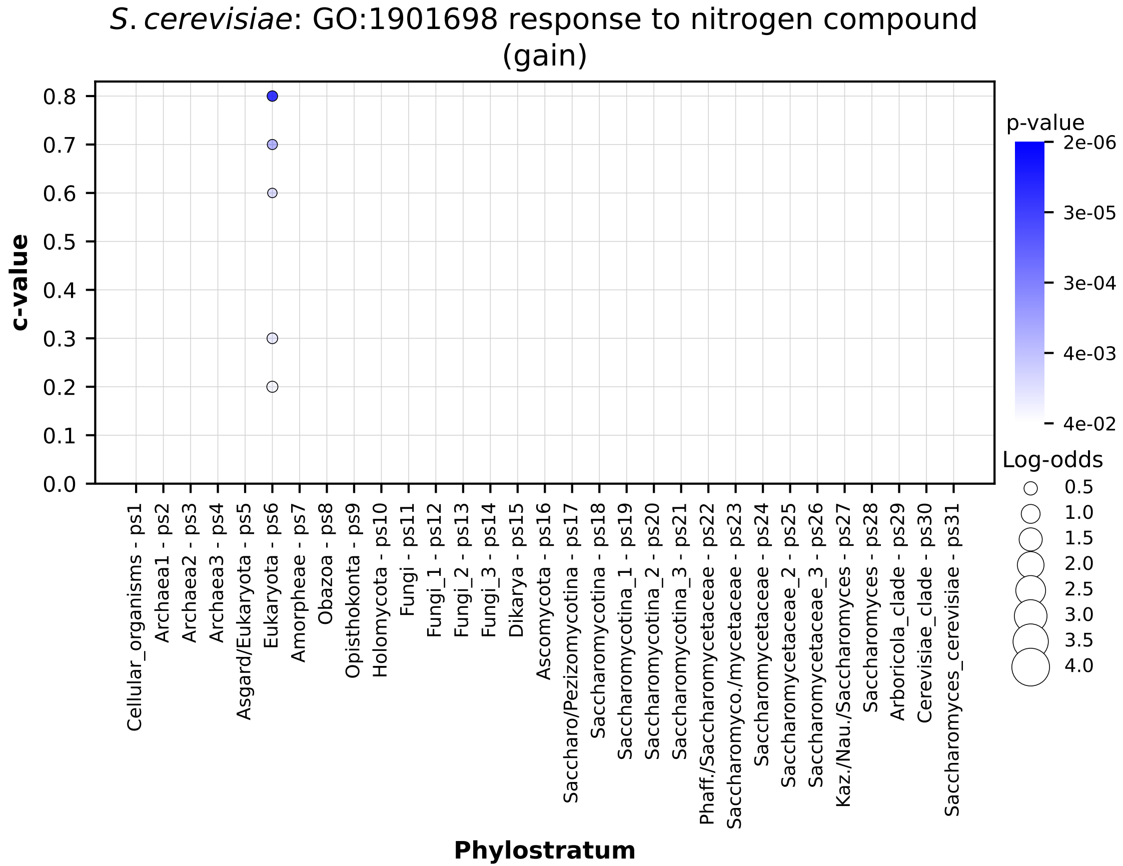

S. cerevisiae: GO:1901698 response to nitrogen compound (gain)
c-value
0.8
0.7
0.6
0.5
0.4
0.3
0.2
0.1
0.0
Phylostratum
Cellular_organisms - ps1
Archaea1 - ps2
Archaea2 - ps3
Archaea3 - ps4
Asgard/Eukaryota - ps5
Eukaryota - ps6
Amorpheae - ps7
Obazoa - ps8
Opisthokonta - ps9
Holomycota - ps10
Fungi - ps11
Fungi_1 - ps12
Fungi_2 - ps13
Fungi_3 - ps14
Dikarya - ps15
Ascomycota - ps16
Saccharo/Pezizomycotina - ps17
Saccharomycotina - ps18
Saccharomycotina_1 - ps19
Saccharomycotina_2 - ps20
Saccharomycotina_3 - ps21
Phaff./Saccharomycetaceae - ps22
Saccharomyco./mycetaceae - ps23
Saccharomycetaceae - ps24
Saccharomycetaceae_2 - ps25
Saccharomycetaceae_3 - ps26
Kaz./Nau./Saccharomyces - ps27
Saccharomyces - ps28
Arboricola_clade - ps29
Cerevisiae_clade - ps30
Saccharomyces_cerevisiae - ps31
p-value
2e-06
3e-05
3e-04
4e-03
4e-02
Log-odds
0.5
1.0
1.5
2.0
2.5
3.0
3.5
4.0

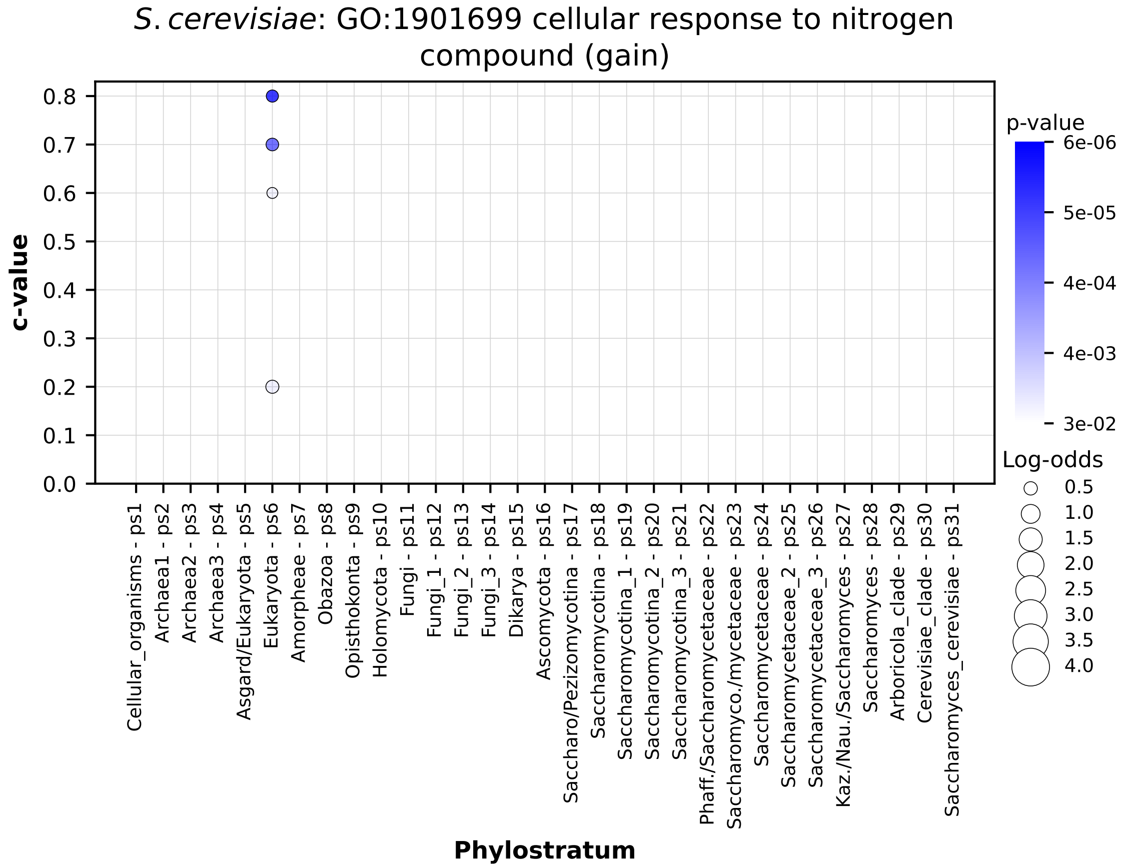

S. cerevisiae: GO:1901699 cellular response to nitrogen compound (gain)
c-value
0.8
0.7
0.6
0.5
0.4
0.3
0.2
0.1
0.0
p-value
6e-06
5e-05
4e-04
4e-03
3e-02
Log-odds
0.5
1.0
1.5
2.0
2.5
3.0
3.5
4.0
Cellular_organisms - ps1
Archaea1 - ps2
Archaea2 - ps3
Archaea3 - ps4
Asgard/Eukaryota - ps5
Eukaryota - ps6
Amorpheae - ps7
Obazoa - ps8
Opisthokonta - ps9
Holomycota - ps10
Fungi - ps11
Fungi_1 - ps12
Fungi_2 - ps13
Fungi_3 - ps14
Dikarya - ps15
Ascomycota - ps16
Saccharo/Pezizomycotina - ps17
Saccharomycotina - ps18
Saccharomycotina_1 - ps19
Saccharomycotina_2 - ps20
Saccharomycotina_3 - ps21
Phaff./Saccharomycetaceae - ps22
Saccharomyco./mycetaceae - ps23
Saccharomycetaceae - ps24
Saccharomycetaceae_2 - ps25
Saccharomycetaceae_3 - ps26
Kaz./Nau./Saccharomyces - ps27
Saccharomyces - ps28
Arboricola_clade - ps29
Cerevisiae_clade - ps30
Saccharomyces_cerevisiae - ps31
Phylostratum

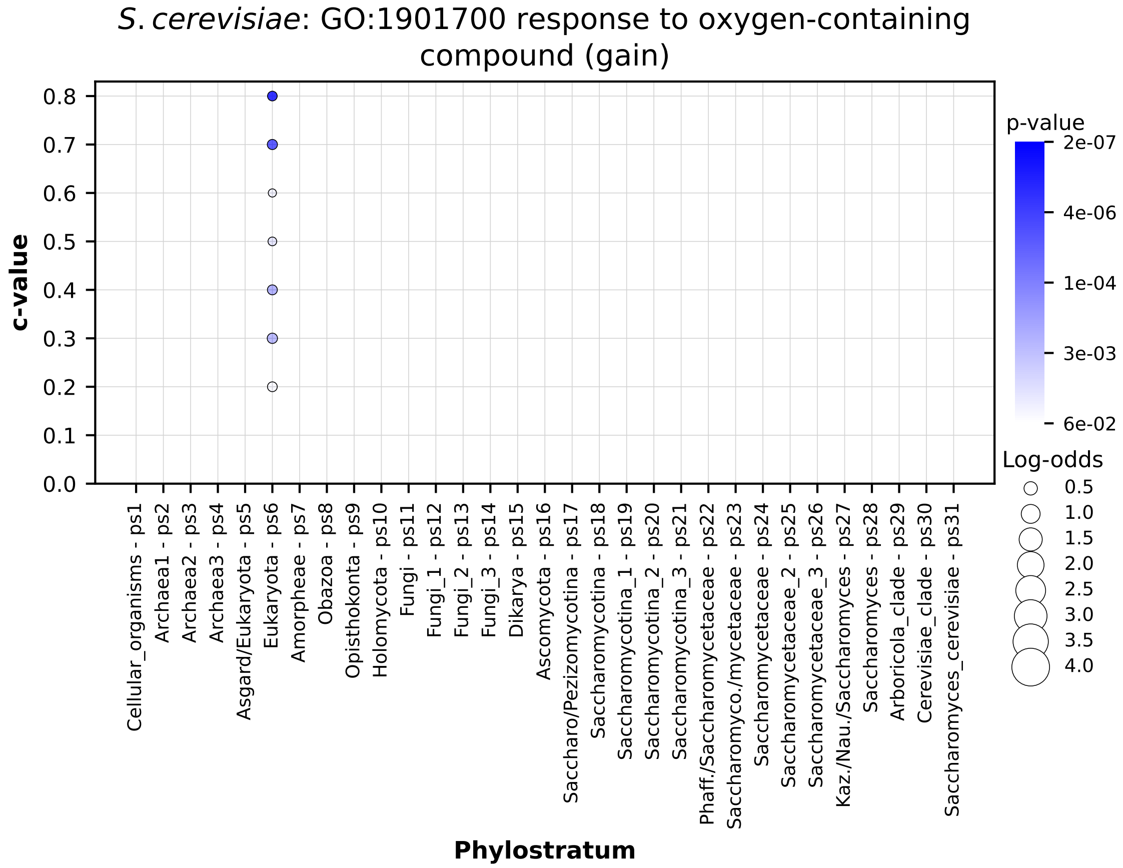

S. cerevisiae: GO:1901700 response to oxygen-containing compound (gain)
c-value
0.8
0.7
0.6
0.5
0.4
0.3
0.2
0.1
0.0
p-value
2e-07
4e-06
1e-04
3e-03
6e-02
Log-odds
0.5
1.0
1.5
2.0
2.5
3.0
3.5
4.0
Cellular_organisms - ps1
Archaea1 - ps2
Archaea2 - ps3
Archaea3 - ps4
Asgard/Eukaryota - ps5
Eukaryota - ps6
Amorpheae - ps7
Obazoa - ps8
Opisthokonta - ps9
Holomycota - ps10
Fungi - ps11
Fungi_1 - ps12
Fungi_2 - ps13
Fungi_3 - ps14
Dikarya - ps15
Ascomycota - ps16
Saccharo/Pezizomycotina - ps17
Saccharomycotina - ps18
Saccharomycotina_1 - ps19
Saccharomycotina_2 - ps20
Saccharomycotina_3 - ps21
Phaff./Saccharomycetaceae - ps22
Saccharomyco./mycetaceae - ps23
Saccharomycetaceae - ps24
Saccharomycetaceae_2 - ps25
Saccharomycetaceae_3 - ps26
Kaz./Nau./Saccharomyces - ps27
Saccharomyces - ps28
Arboricola_clade - ps29
Cerevisiae_clade - ps30
Saccharomyces_cerevisiae - ps31
Phylostratum

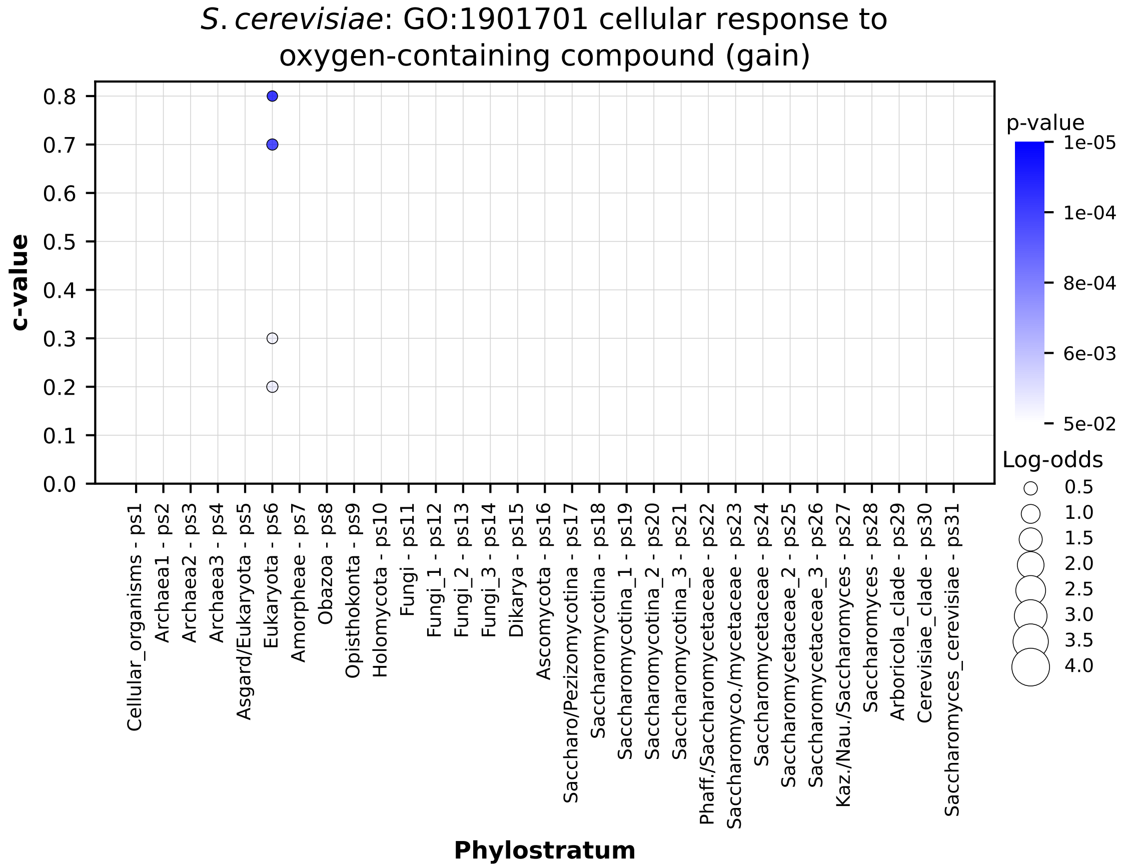
S. cerevisiae: GO:1901701 cellular response to oxygen-containing compound (gain)
c-value
0.0
0.1
0.2
0.3
0.4
0.5
0.6
0.7
0.8
Phylostratum
Cellular_organisms - ps1
Archaea1 - ps2
Archaea2 - ps3
Archaea3 - ps4
Asgard/Eukaryota - ps5
Eukaryota - ps6
Amorpheae - ps7
Obazoa - ps8
Opisthokonta - ps9
Holomycota - ps10
Fungi - ps11
Fungi_1 - ps12
Fungi_2 - ps13
Fungi_3 - ps14
Dikarya - ps15
Ascomycota - ps16
Saccharo/Pezizomycotina - ps17
Saccharomycotina - ps18
Saccharomycotina_1 - ps19
Saccharomycotina_2 - ps20
Saccharomycotina_3 - ps21
Phaff./Saccharomycetaceae - ps22
Saccharomyco./mycetaceae - ps23
Saccharomycetaceae - ps24
Saccharomycetaceae_2 - ps25
Saccharomycetaceae_3 - ps26
Kaz./Nau./Saccharomyces - ps27
Saccharomyces - ps28
Arboricola_clade - ps29
Cerevisiae_clade - ps30
Saccharomyces_cerevisiae - ps31
p-value
1e-05
1e-04
8e-04
6e-03
5e-02
Log-odds
0.5
1.0
1.5
2.0
2.5
3.0
3.5
4.0

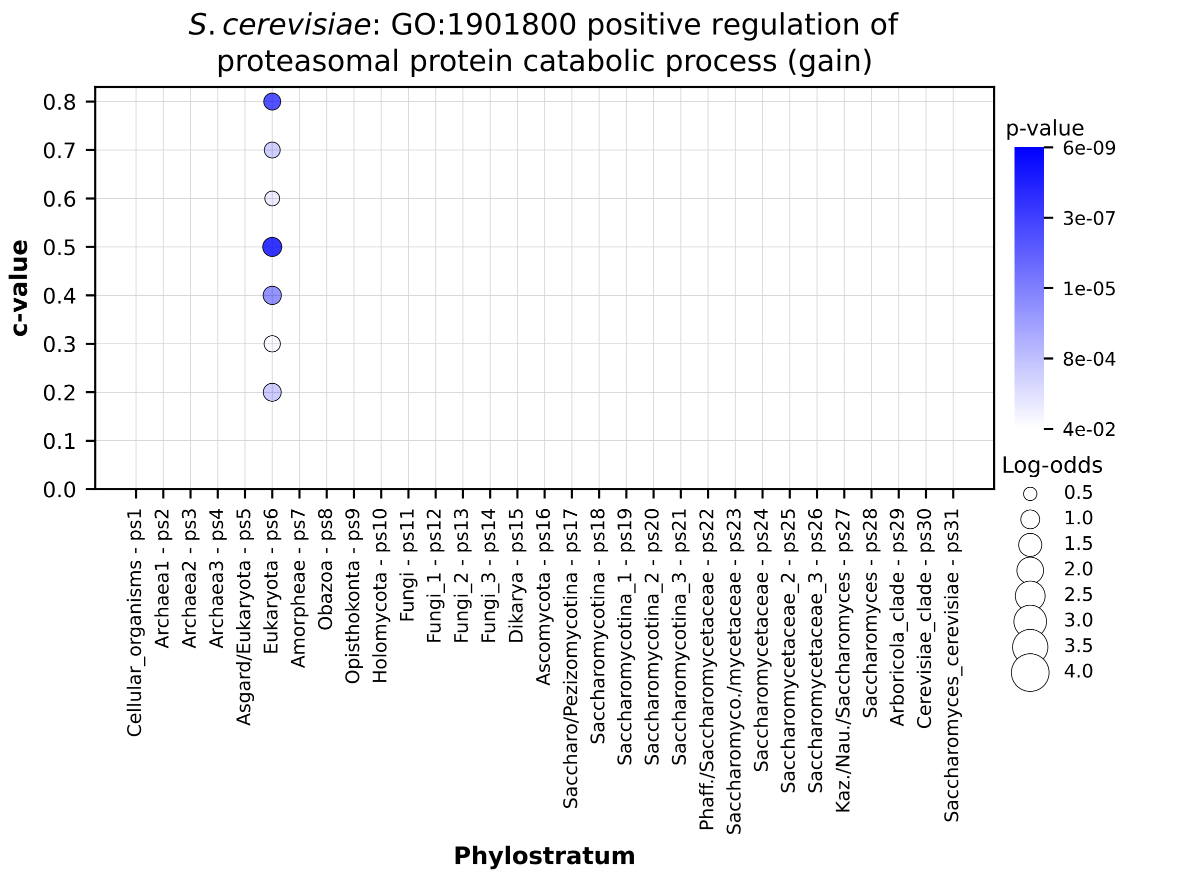

S. cerevisiae: GO:1901800 positive regulation of proteasomal protein catabolic process (gain)
c-value
0.0
0.1
0.2
0.3
0.4
0.5
0.6
0.7
0.8
p-value
6e-09
3e-07
1e-05
8e-04
4e-02
Log-odds
0.5
1.0
1.5
2.0
2.5
3.0
3.5
4.0
Cellular_organisms - ps1
Archaea1 - ps2
Archaea2 - ps3
Archaea3 - ps4
Asgard/Eukaryota - ps5
Eukaryota - ps6
Amorpheae - ps7
Obazoa - ps8
Opisthokonta - ps9
Holomycota - ps10
Fungi - ps11
Fungi_1 - ps12
Fungi_2 - ps13
Fungi_3 - ps14
Dikarya - ps15
Ascomycota - ps16
Saccharo/Pezizomycotina - ps17
Saccharomycotina - ps18
Saccharomycotina_1 - ps19
Saccharomycotina_2 - ps20
Saccharomycotina_3 - ps21
Phaff./Saccharomycetaceae - ps22
Saccharomyco./mycetaceae - ps23
Saccharomycetaceae - ps24
Saccharomycetaceae_2 - ps25
Saccharomycetaceae_3 - ps26
Kaz./Nau./Saccharomyces - ps27
Saccharomyces - ps28
Arboricola_clade - ps29
Cerevisiae_clade - ps30
Saccharomyces_cerevisiae - ps31
Phylostratum

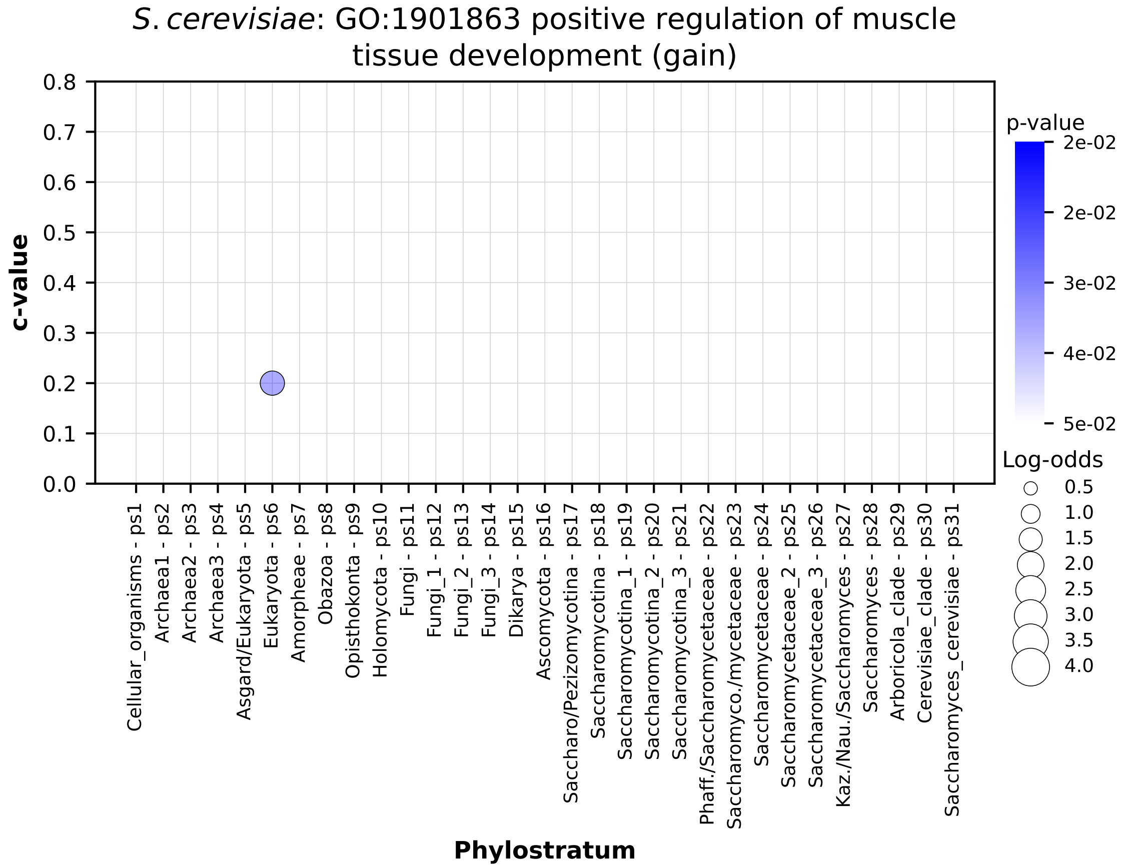

S. cerevisiae: GO:1901863 positive regulation of muscle tissue development (gain)
c-value
0.0
0.1
0.2
0.3
0.4
0.5
0.6
0.7
0.8
Phylostratum
Cellular_organisms - ps1
Archaea1 - ps2
Archaea2 - ps3
Archaea3 - ps4
Asgard/Eukaryota - ps5
Eukaryota - ps6
Amorpheae - ps7
Obazoa - ps8
Opisthokonta - ps9
Holomycota - ps10
Fungi - ps11
Fungi_1 - ps12
Fungi_2 - ps13
Fungi_3 - ps14
Dikarya - ps15
Ascomycota - ps16
Saccharo/Pezizomycotina - ps17
Saccharomycotina - ps18
Saccharomycotina_1 - ps19
Saccharomycotina_2 - ps20
Saccharomycotina_3 - ps21
Phaff./Saccharomycetaceae - ps22
Saccharomyco./mycetaceae - ps23
Saccharomycetaceae - ps24
Saccharomycetaceae_2 - ps25
Saccharomycetaceae_3 - ps26
Kaz./Nau./Saccharomyces - ps27
Saccharomyces - ps28
Arboricola_clade - ps29
Cerevisiae_clade - ps30
Saccharomyces_cerevisiae - ps31
p-value
2e-02
2e-02
3e-02
4e-02
5e-02
Log-odds
0.5
1.0
1.5
2.0
2.5
3.0
3.5
4.0

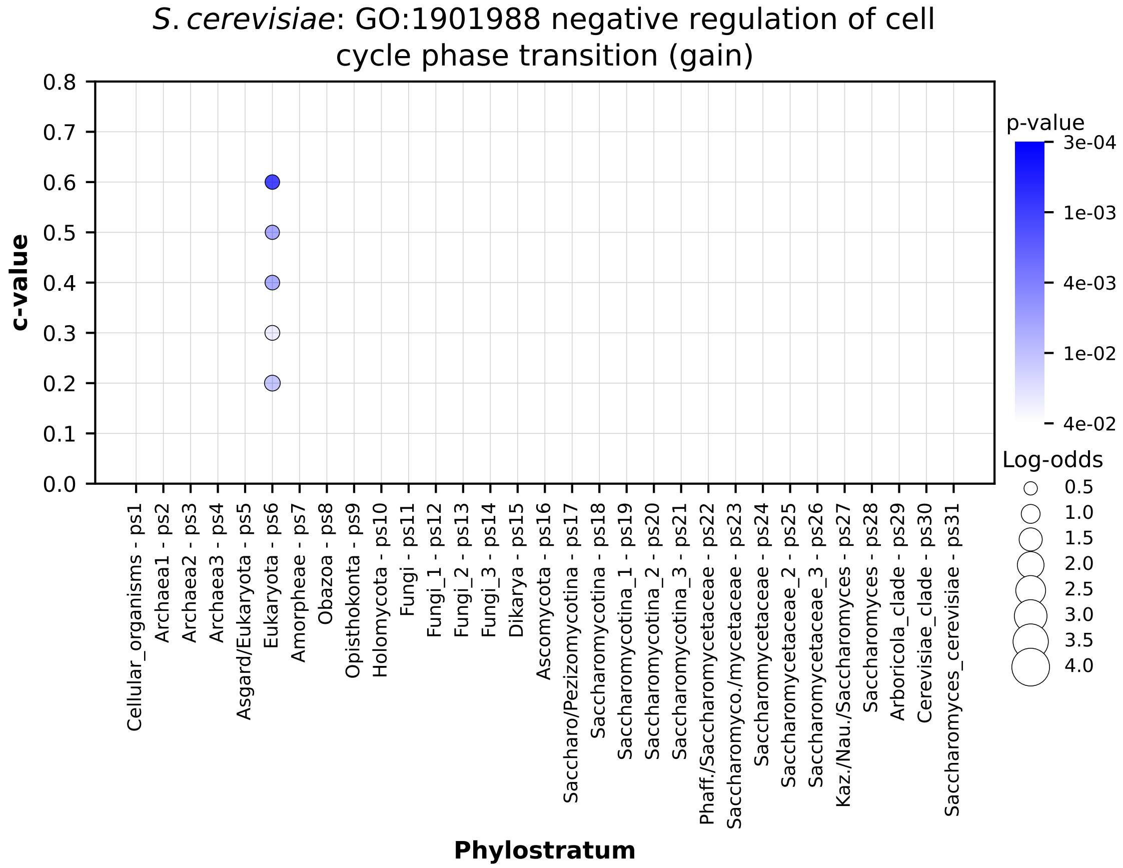

S. cerevisiae: GO:1901988 negative regulation of cell cycle phase transition (gain)
c-value
0.0
0.1
0.2
0.3
0.4
0.5
0.6
0.7
0.8
p-value
3e-04
1e-03
4e-03
1e-02
4e-02
Log-odds
0.5
1.0
1.5
2.0
2.5
3.0
3.5
4.0
Cellular_organisms - ps1
Archaea1 - ps2
Archaea2 - ps3
Archaea3 - ps4
Asgard/Eukaryota - ps5
Eukaryota - ps6
Amorpheae - ps7
Obazoa - ps8
Opisthokonta - ps9
Holomycota - ps10
Fungi - ps11
Fungi_1 - ps12
Fungi_2 - ps13
Fungi_3 - ps14
Dikarya - ps15
Ascomycota - ps16
Saccharo/Pezizomycotina - ps17
Saccharomycotina - ps18
Saccharomycotina_1 - ps19
Saccharomycotina_2 - ps20
Saccharomycotina_3 - ps21
Phaff./Saccharomycetaceae - ps22
Saccharomyco./mycetaceae - ps23
Saccharomycetaceae - ps24
Saccharomycetaceae_2 - ps25
Saccharomycetaceae_3 - ps26
Kaz./Nau./Saccharomyces - ps27
Saccharomyces - ps28
Arboricola_clade - ps29
Cerevisiae_clade - ps30
Saccharomyces_cerevisiae - ps31
Phylostratum

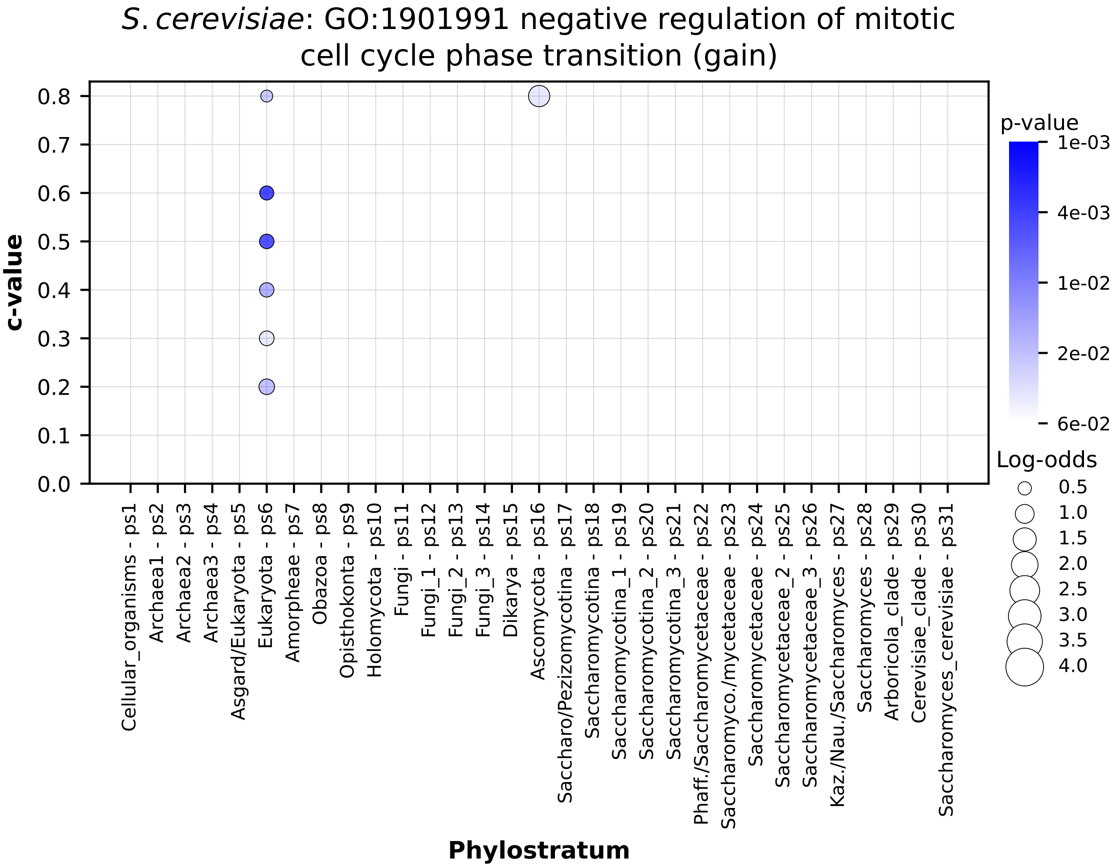

S. cerevisiae: GO:1901991 negative regulation of mitotic cell cycle phase transition (gain)
c-value
0.0
0.1
0.2
0.3
0.4
0.5
0.6
0.7
0.8
Phylostratum
Cellular_organisms - ps1
Archaea1 - ps2
Archaea2 - ps3
Archaea3 - ps4
Asgard/Eukaryota - ps5
Eukaryota - ps6
Amorpheae - ps7
Obazoa - ps8
Opisthokonta - ps9
Holomycota - ps10
Fungi - ps11
Fungi_1 - ps12
Fungi_2 - ps13
Fungi_3 - ps14
Dikarya - ps15
Ascomycota - ps16
Saccharo/Pezizomycotina - ps17
Saccharomycotina - ps18
Saccharomycotina_1 - ps19
Saccharomycotina_2 - ps20
Saccharomycotina_3 - ps21
Phaff./Saccharomycetaceae - ps22
Saccharomyco./mycetaceae - ps23
Saccharomycetaceae - ps24
Saccharomycetaceae_2 - ps25
Saccharomycetaceae_3 - ps26
Kaz./Nau./Saccharomyces - ps27
Saccharomyces - ps28
Arboricola_clade - ps29
Cerevisiae_clade - ps30
Saccharomyces_cerevisiae - ps31
p-value
1e-03
4e-03
1e-02
2e-02
6e-02
Log-odds
0.5
1.0
1.5
2.0
2.5
3.0
3.5
4.0

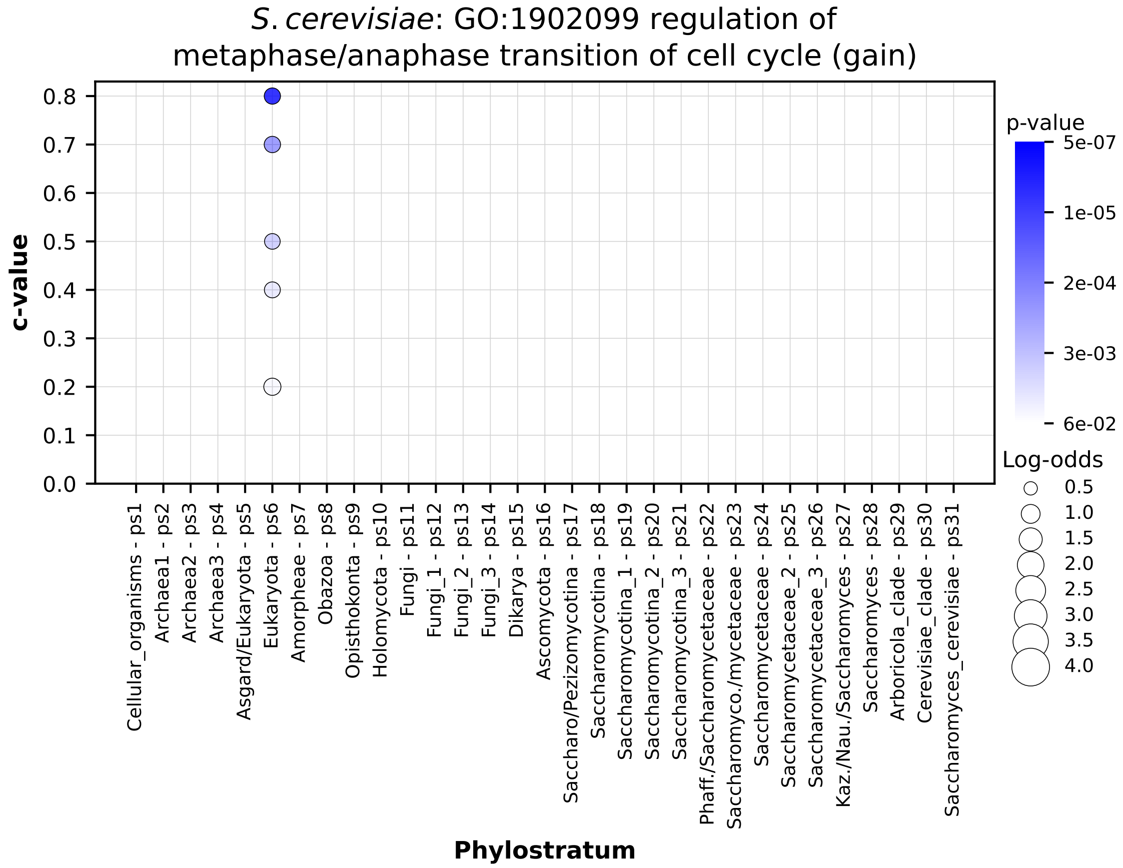

S. cerevisiae: GO:1902099 regulation of metaphase/anaphase transition of cell cycle (gain)
c-value
0.0
0.1
0.2
0.3
0.4
0.5
0.6
0.7
0.8
Phylostratum
Cellular_organisms - ps1
Archaea1 - ps2
Archaea2 - ps3
Archaea3 - ps4
Asgard/Eukaryota - ps5
Eukaryota - ps6
Amorpheae - ps7
Obazoa - ps8
Opisthokonta - ps9
Holomycota - ps10
Fungi - ps11
Fungi_1 - ps12
Fungi_2 - ps13
Fungi_3 - ps14
Dikarya - ps15
Ascomycota - ps16
Saccharo/Pezizomycotina - ps17
Saccharomycotina - ps18
Saccharomycotina_1 - ps19
Saccharomycotina_2 - ps20
Saccharomycotina_3 - ps21
Phaff./Saccharomycetaceae - ps22
Saccharomyco./mycetaceae - ps23
Saccharomycetaceae - ps24
Saccharomycetaceae_2 - ps25
Saccharomycetaceae_3 - ps26
Kaz./Nau./Saccharomyces - ps27
Saccharomyces - ps28
Arboricola_clade - ps29
Cerevisiae_clade - ps30
Saccharomyces_cerevisiae - ps31
p-value
5e-07
1e-05
2e-04
3e-03
6e-02
Log-odds
0.5
1.0
1.5
2.0
2.5
3.0
3.5
4.0

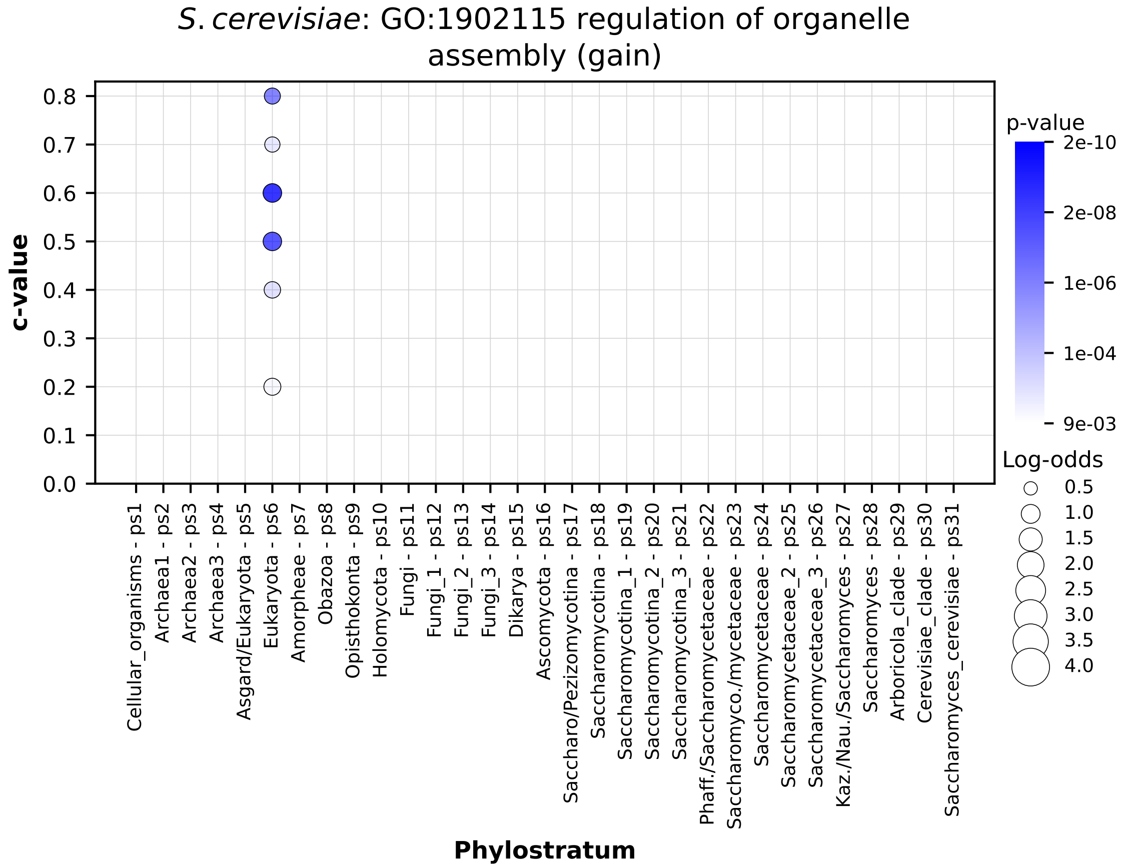

S. cerevisiae: GO:1902115 regulation of organelle assembly (gain)
c-value
0.8
0.7
0.6
0.5
0.4
0.3
0.2
0.1
0.0
Phylostratum
Cellular_organisms - ps1
Archaea1 - ps2
Archaea2 - ps3
Archaea3 - ps4
Asgard/Eukaryota - ps5
Eukaryota - ps6
Amorpheae - ps7
Obazoa - ps8
Opisthokonta - ps9
Holomycota - ps10
Fungi - ps11
Fungi_1 - ps12
Fungi_2 - ps13
Fungi_3 - ps14
Dikarya - ps15
Ascomycota - ps16
Saccharo/Pezizomycotina - ps17
Saccharomycotina - ps18
Saccharomycotina_1 - ps19
Saccharomycotina_2 - ps20
Saccharomycotina_3 - ps21
Phaff./Saccharomycetaceae - ps22
Saccharomyco./mycetaceae - ps23
Saccharomycetaceae - ps24
Saccharomycetaceae_2 - ps25
Saccharomycetaceae_3 - ps26
Kaz./Nau./Saccharomyces - ps27
Saccharomyces - ps28
Arboricola_clade - ps29
Cerevisiae_clade - ps30
Saccharomyces_cerevisiae - ps31
p-value
2e-10
2e-08
1e-06
1e-04
9e-03
Log-odds
0.5
1.0
1.5
2.0
2.5
3.0
3.5
4.0

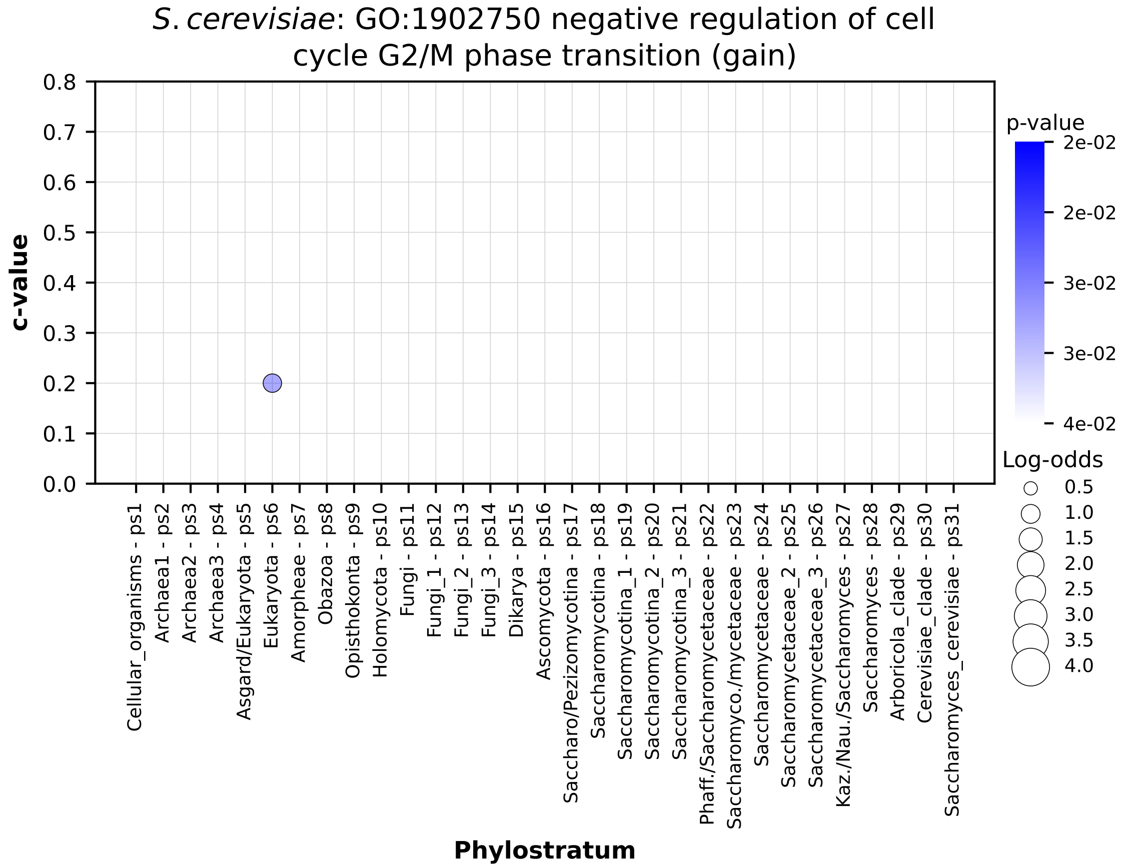
S. cerevisiae: GO:1902750 negative regulation of cell cycle G2/M phase transition (gain)
c-value
0.8
0.7
0.6
0.5
0.4
0.3
0.2
0.1
0.0
p-value
2e-02
2e-02
3e-02
3e-02
4e-02
Log-odds
0.5
1.0
1.5
2.0
2.5
3.0
3.5
4.0
Cellular_organisms - ps1
Archaea1 - ps2
Archaea2 - ps3
Archaea3 - ps4
Asgard/Eukaryota - ps5
Eukaryota - ps6
Amorpheae - ps7
Obazoa - ps8
Opisthokonta - ps9
Holomycota - ps10
Fungi - ps11
Fungi_1 - ps12
Fungi_2 - ps13
Fungi_3 - ps14
Dikarya - ps15
Ascomycota - ps16
Saccharo/Pezizomycotina - ps17
Saccharomycotina - ps18
Saccharomycotina_1 - ps19
Saccharomycotina_2 - ps20
Saccharomycotina_3 - ps21
Phaff./Saccharomycetaceae - ps22
Saccharomyco./mycetaceae - ps23
Saccharomycetaceae - ps24
Saccharomycetaceae_2 - ps25
Saccharomycetaceae_3 - ps26
Kaz./Nau./Saccharomyces - ps27
Saccharomyces - ps28
Arboricola_clade - ps29
Cerevisiae_clade - ps30
Saccharomyces_cerevisiae - ps31
Phylostratum

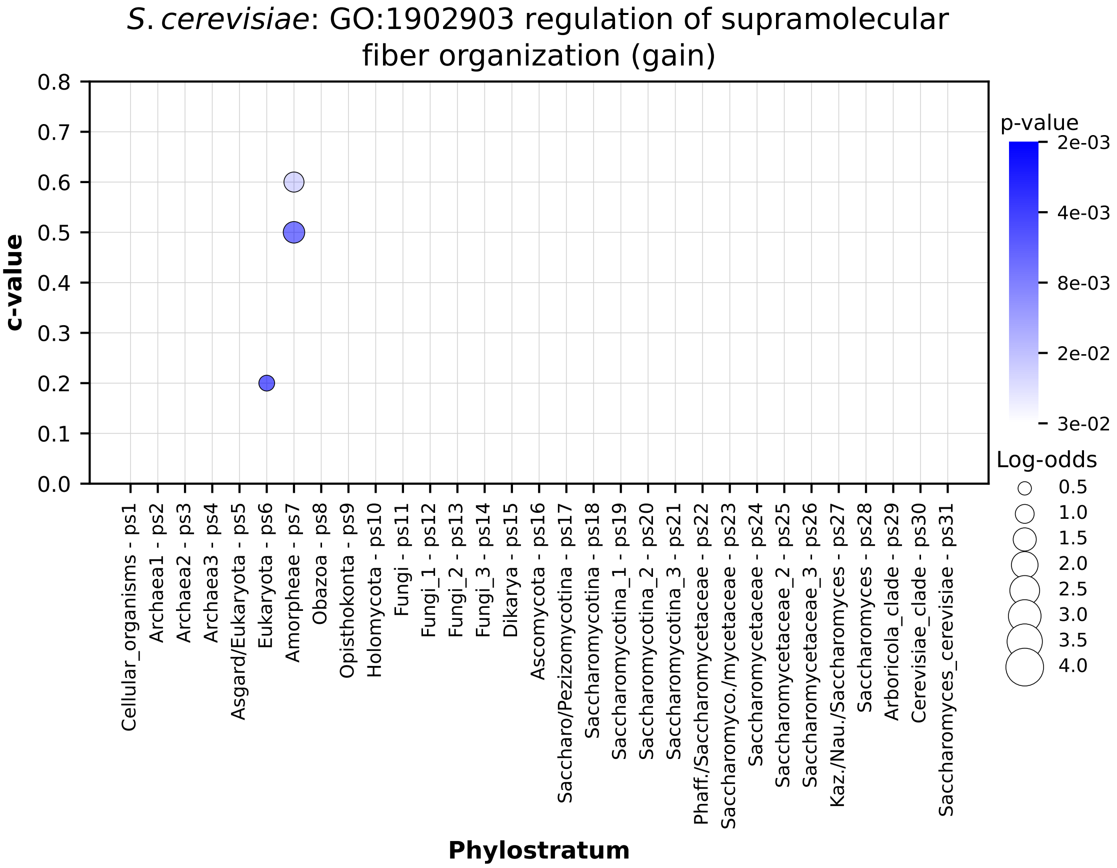

S. cerevisiae: GO:1902903 regulation of supramolecular fiber organization (gain)
c-value
0.8
0.7
0.6
0.5
0.4
0.3
0.2
0.1
0.0
Phylostratum
Cellular_organisms - ps1
Archaea1 - ps2
Archaea2 - ps3
Archaea3 - ps4
Asgard/Eukaryota - ps5
Eukaryota - ps6
Amorpheae - ps7
Obazoa - ps8
Opisthokonta - ps9
Holomycota - ps10
Fungi - ps11
Fungi_1 - ps12
Fungi_2 - ps13
Fungi_3 - ps14
Dikarya - ps15
Ascomycota - ps16
Saccharo/Pezizomycotina - ps17
Saccharomycotina - ps18
Saccharomycotina_1 - ps19
Saccharomycotina_2 - ps20
Saccharomycotina_3 - ps21
Phaff./Saccharomycetaceae - ps22
Saccharomyco./mycetaceae - ps23
Saccharomycetaceae - ps24
Saccharomycetaceae_2 - ps25
Saccharomycetaceae_3 - ps26
Kaz./Nau./Saccharomyces - ps27
Saccharomyces - ps28
Arboricola_clade - ps29
Cerevisiae_clade - ps30
Saccharomyces_cerevisiae - ps31
p-value
2e-03
4e-03
8e-03
2e-02
3e-02
Log-odds
0.5
1.0
1.5
2.0
2.5
3.0
3.5
4.0

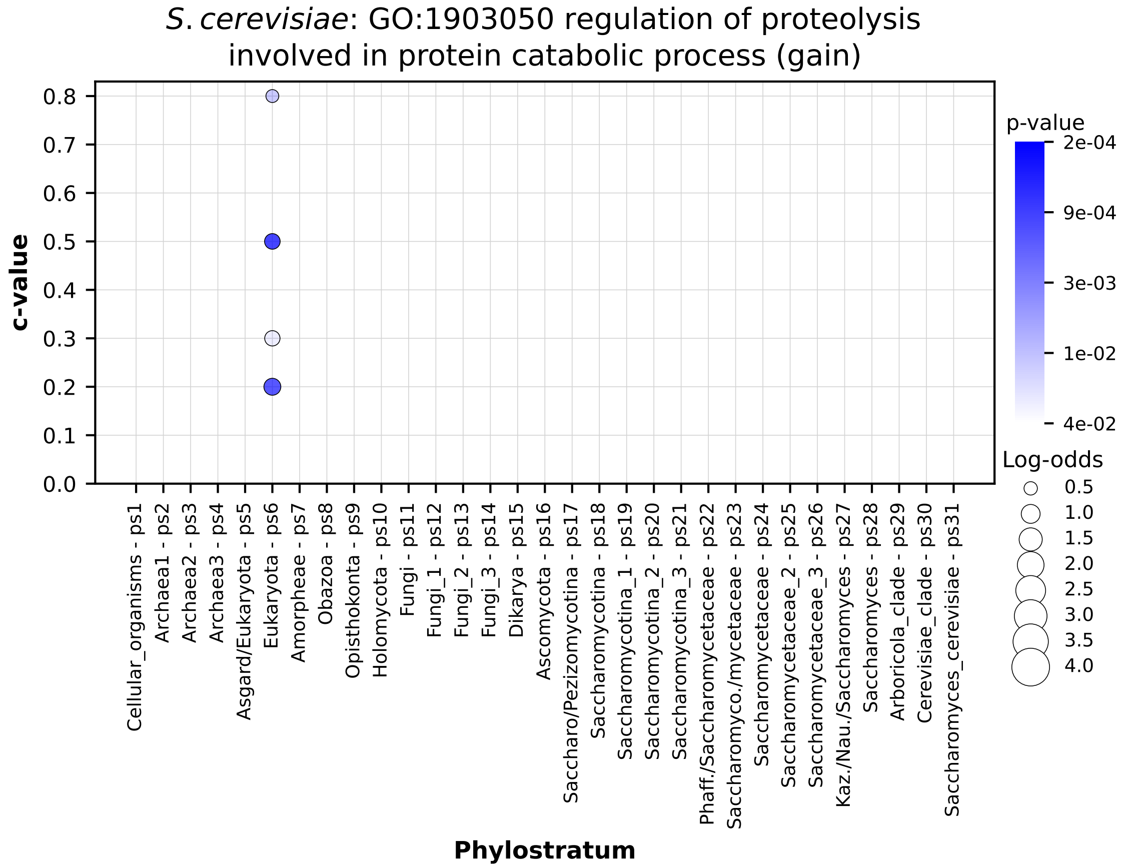

S. cerevisiae: GO:1903050 regulation of proteolysis involved in protein catabolic process (gain)
c-value
0.0
0.1
0.2
0.3
0.4
0.5
0.6
0.7
0.8
Phylostratum
Cellular_organisms - ps1
Archaea1 - ps2
Archaea2 - ps3
Archaea3 - ps4
Asgard/Eukaryota - ps5
Eukaryota - ps6
Amorpheae - ps7
Obazoa - ps8
Opisthokonta - ps9
Holomycota - ps10
Fungi - ps11
Fungi_1 - ps12
Fungi_2 - ps13
Fungi_3 - ps14
Dikarya - ps15
Ascomycota - ps16
Saccharo/Pezizomycotina - ps17
Saccharomycotina - ps18
Saccharomycotina_1 - ps19
Saccharomycotina_2 - ps20
Saccharomycotina_3 - ps21
Phaff./Saccharomycetaceae - ps22
Saccharomyco./mycetaceae - ps23
Saccharomycetaceae - ps24
Saccharomycetaceae_2 - ps25
Saccharomycetaceae_3 - ps26
Kaz./Nau./Saccharomyces - ps27
Saccharomyces - ps28
Arboricola_clade - ps29
Cerevisiae_clade - ps30
Saccharomyces_cerevisiae - ps31
p-value
2e-04
9e-04
3e-03
1e-02
4e-02
Log-odds
0.5
1.0
1.5
2.0
2.5
3.0
3.5
4.0

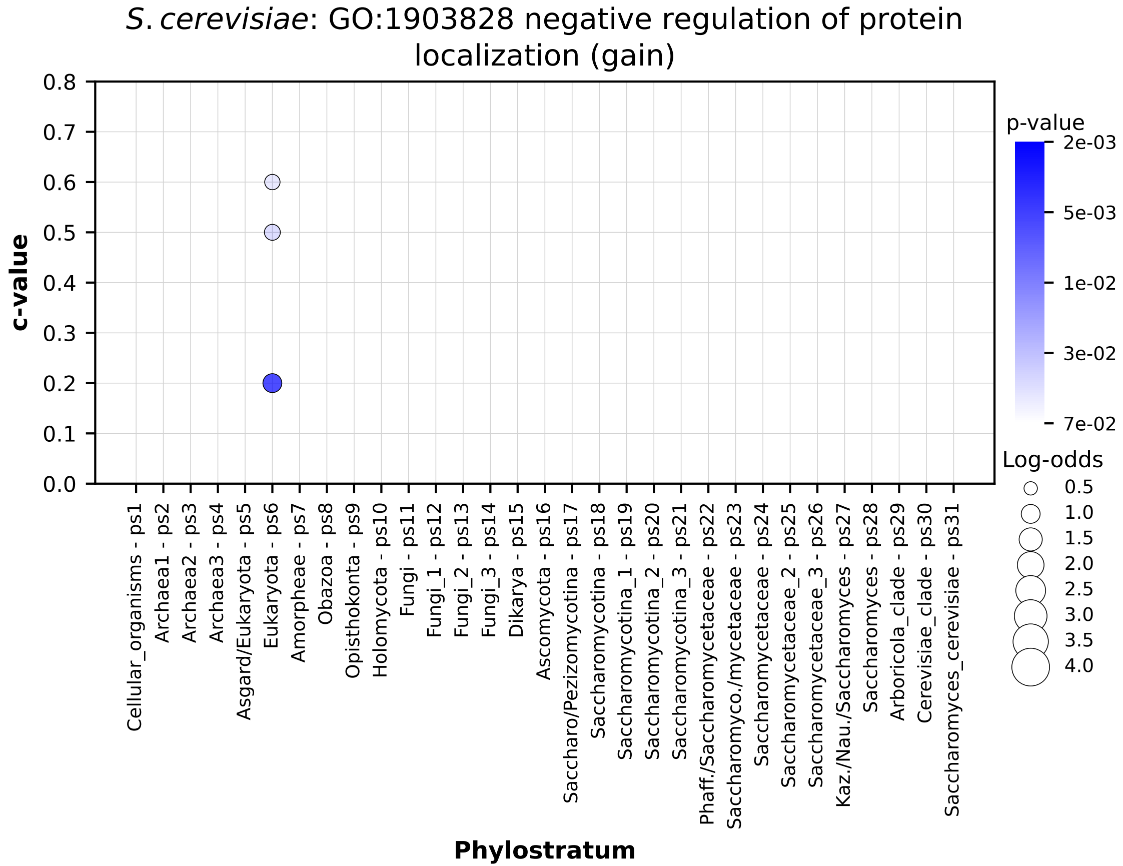
S. cerevisiae: GO:1903828 negative regulation of protein localization (gain)
c-value
0.0
0.1
0.2
0.3
0.4
0.5
0.6
0.7
0.8
Phylostratum
Cellular_organisms - ps1
Archaea1 - ps2
Archaea2 - ps3
Archaea3 - ps4
Asgard/Eukaryota - ps5
Eukaryota - ps6
Amorpheae - ps7
Obazoa - ps8
Opisthokonta - ps9
Holomycota - ps10
Fungi - ps11
Fungi_1 - ps12
Fungi_2 - ps13
Fungi_3 - ps14
Dikarya - ps15
Ascomycota - ps16
Saccharo/Pezizomycotina - ps17
Saccharomycotina - ps18
Saccharomycotina_1 - ps19
Saccharomycotina_2 - ps20
Saccharomycotina_3 - ps21
Phaff./Saccharomycetaceae - ps22
Saccharomyco./mycetaceae - ps23
Saccharomycetaceae - ps24
Saccharomycetaceae_2 - ps25
Saccharomycetaceae_3 - ps26
Kaz./Nau./Saccharomyces - ps27
Saccharomyces - ps28
Arboricola_clade - ps29
Cerevisiae_clade - ps30
Saccharomyces_cerevisiae - ps31
p-value
2e-03
5e-03
1e-02
3e-02
7e-02
Log-odds
0.5
1.0
1.5
2.0
2.5
3.0
3.5
4.0

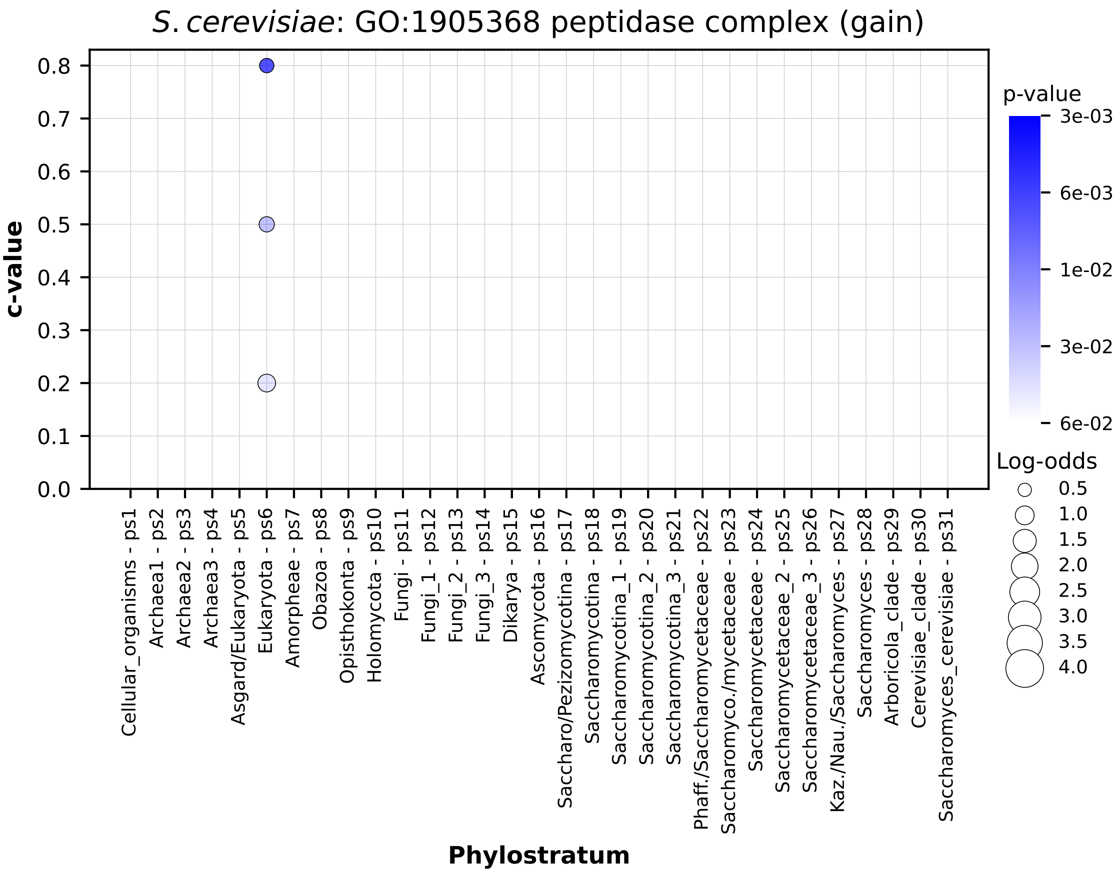

S. cerevisiae: GO:1905368 peptidase complex (gain)
c-value
0.0
0.1
0.2
0.3
0.4
0.5
0.6
0.7
0.8
p-value
3e-03
6e-03
1e-02
3e-02
6e-02
Log-odds
0.5
1.0
1.5
2.0
2.5
3.0
3.5
4.0
Cellular_organisms - ps1
Archaea1 - ps2
Archaea2 - ps3
Archaea3 - ps4
Asgard/Eukaryota - ps5
Eukaryota - ps6
Amorpheae - ps7
Obazoa - ps8
Opisthokonta - ps9
Holomycota - ps10
Fungi - ps11
Fungi_1 - ps12
Fungi_2 - ps13
Fungi_3 - ps14
Dikarya - ps15
Ascomycota - ps16
Saccharo/Pezizomycotina - ps17
Saccharomycotina - ps18
Saccharomycotina_1 - ps19
Saccharomycotina_2 - ps20
Saccharomycotina_3 - ps21
Phaff./Saccharomycetaceae - ps22
Saccharomyco./mycetaceae - ps23
Saccharomycetaceae - ps24
Saccharomycetaceae_2 - ps25
Saccharomycetaceae_3 - ps26
Kaz./Nau./Saccharomyces - ps27
Saccharomyces - ps28
Arboricola_clade - ps29
Cerevisiae_clade - ps30
Saccharomyces_cerevisiae - ps31
Phylostratum

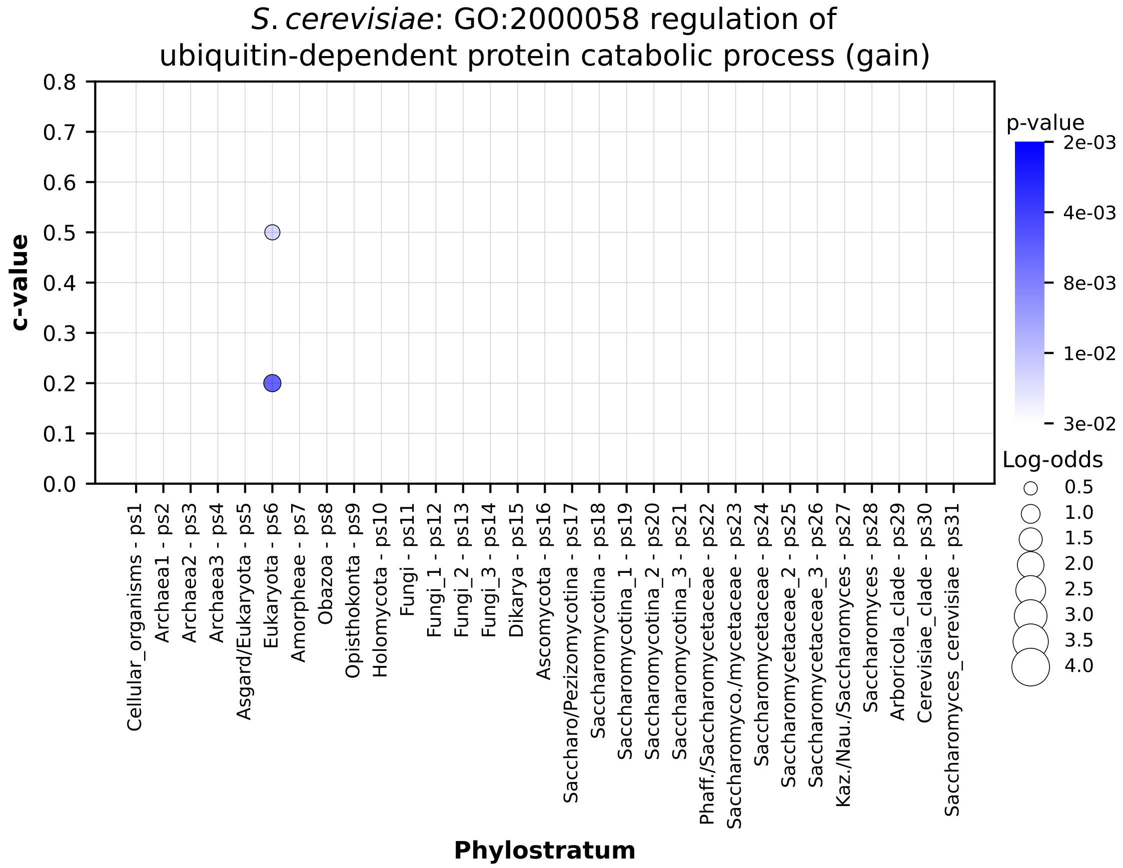
S. cerevisiae: GO:2000058 regulation of
ubiquitin-dependent protein catabolic process (gain)
c-value
0.8
0.7
0.6
0.5
0.4
0.3
0.2
0.1
0.0
p-value
2e-03
4e-03
8e-03
1e-02
3e-02
Log-odds
0.5
1.0
1.5
2.0
2.5
3.0
3.5
4.0
Cellular_organisms - ps1
Archaea1 - ps2
Archaea2 - ps3
Archaea3 - ps4
Asgard/Eukaryota - ps5
Eukaryota - ps6
Amorpheae - ps7
Obazoa - ps8
Opisthokonta - ps9
Holomycota - ps10
Fungi - ps11
Fungi_1 - ps12
Fungi_2 - ps13
Fungi_3 - ps14
Dikarya - ps15
Ascomycota - ps16
Saccharo/Pezizomycotina - ps17
Saccharomycotina - ps18
Saccharomycotina_1 - ps19
Saccharomycotina_2 - ps20
Saccharomycotina_3 - ps21
Phaff./Saccharomycetaceae - ps22
Saccharomyco./mycetaceae - ps23
Saccharomycetaceae - ps24
Saccharomycetaceae_2 - ps25
Saccharomycetaceae_3 - ps26
Kaz./Nau./Saccharomyces - ps27
Saccharomyces - ps28
Arboricola_clade - ps29
Cerevisiae_clade - ps30
Saccharomyces_cerevisiae - ps31
Phylostratum

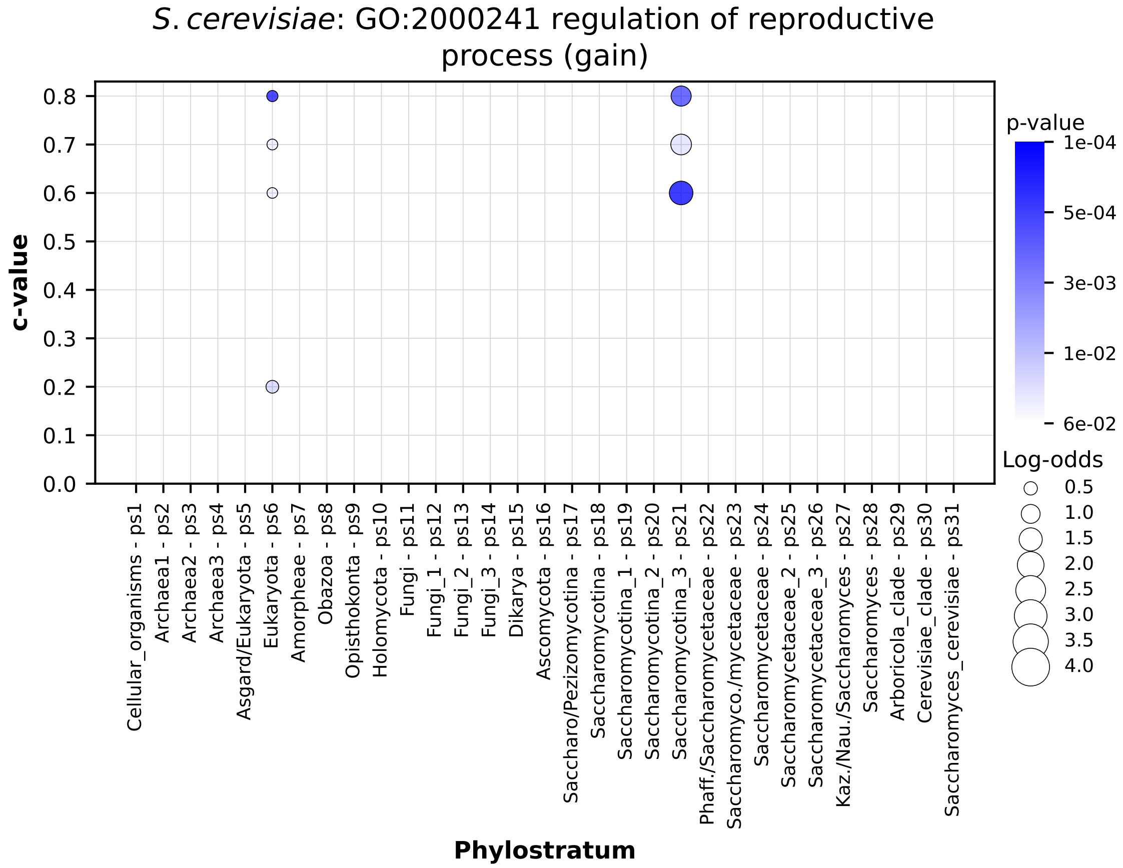

S. cerevisiae: GO:2000241 regulation of reproductive process (gain)
c-value
0.0
0.1
0.2
0.3
0.4
0.5
0.6
0.7
0.8
p-value
1e-04
5e-04
3e-03
1e-02
6e-02
Log-odds
0.5
1.0
1.5
2.0
2.5
3.0
3.5
4.0
Cellular_organisms - ps1
Archaea1 - ps2
Archaea2 - ps3
Archaea3 - ps4
Asgard/Eukaryota - ps5
Eukaryota - ps6
Amorpheae - ps7
Obazoa - ps8
Opisthokonta - ps9
Holomycota - ps10
Fungi - ps11
Fungi_1 - ps12
Fungi_2 - ps13
Fungi_3 - ps14
Dikarya - ps15
Ascomycota - ps16
Saccharo/Pezizomycotina - ps17
Saccharomycotina - ps18
Saccharomycotina_1 - ps19
Saccharomycotina_2 - ps20
Saccharomycotina_3 - ps21
Phaff./Saccharomycetaceae - ps22
Saccharomyco./mycetaceae - ps23
Saccharomycetaceae - ps24
Saccharomycetaceae_2 - ps25
Saccharomycetaceae_3 - ps26
Kaz./Nau./Saccharomyces - ps27
Saccharomyces - ps28
Arboricola_clade - ps29
Cerevisiae_clade - ps30
Saccharomyces_cerevisiae - ps31
Phylostratum

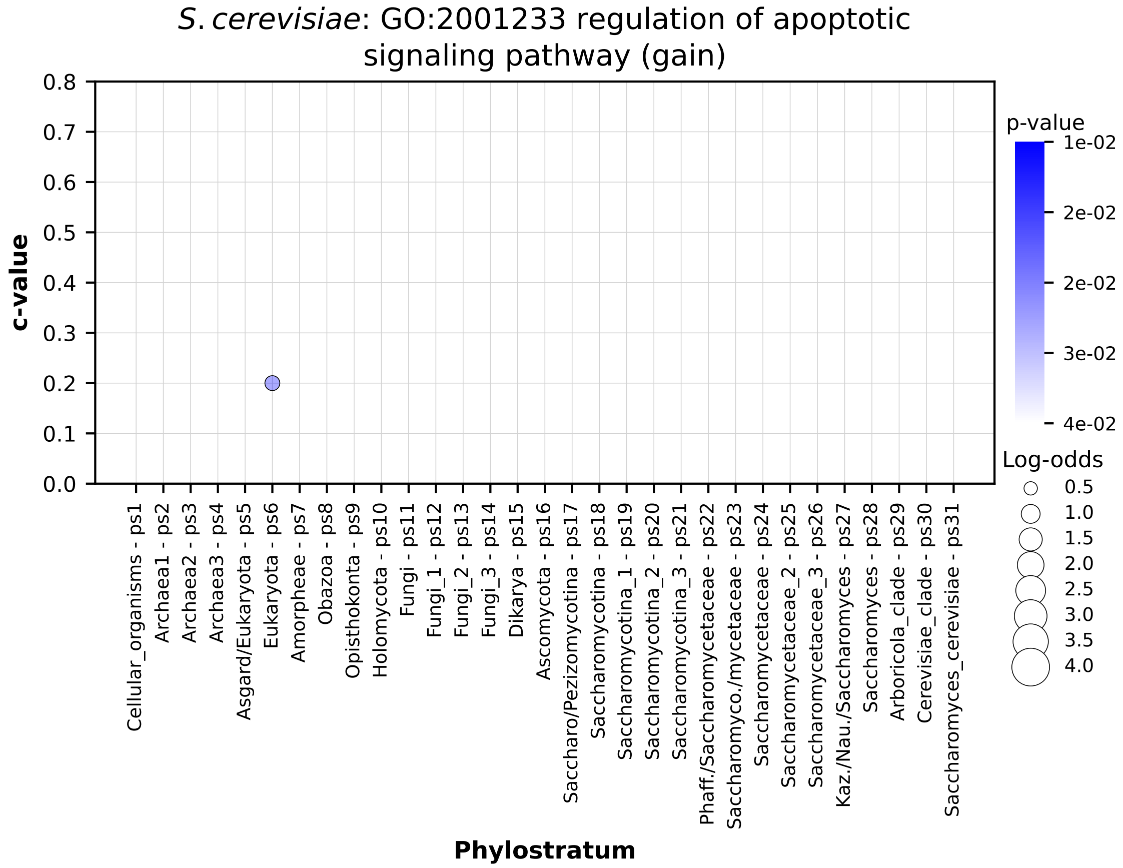

S. cerevisiae: GO:2001233 regulation of apoptotic signaling pathway (gain)
c-value
0.8
0.7
0.6
0.5
0.4
0.3
0.2
0.1
0.0
Phylostratum
Cellular_organisms - ps1
Archaea1 - ps2
Archaea2 - ps3
Archaea3 - ps4
Asgard/Eukaryota - ps5
Eukaryota - ps6
Amorpheae - ps7
Obazoa - ps8
Opisthokonta - ps9
Holomycota - ps10
Fungi - ps11
Fungi_1 - ps12
Fungi_2 - ps13
Fungi_3 - ps14
Dikarya - ps15
Ascomycota - ps16
Saccharo/Pezizomycotina - ps17
Saccharomycotina - ps18
Saccharomycotina_1 - ps19
Saccharomycotina_2 - ps20
Saccharomycotina_3 - ps21
Phaff./Saccharomycetaceae - ps22
Saccharomyco./mycetaceae - ps23
Saccharomycetaceae - ps24
Saccharomycetaceae_2 - ps25
Saccharomycetaceae_3 - ps26
Kaz./Nau./Saccharomyces - ps27
Saccharomyces - ps28
Arboricola_clade - ps29
Cerevisiae_clade - ps30
Saccharomyces_cerevisiae - ps31
p-value
1e-02
2e-02
2e-02
3e-02
4e-02
Log-odds
0.5
1.0
1.5
2.0
2.5
3.0
3.5
4.0

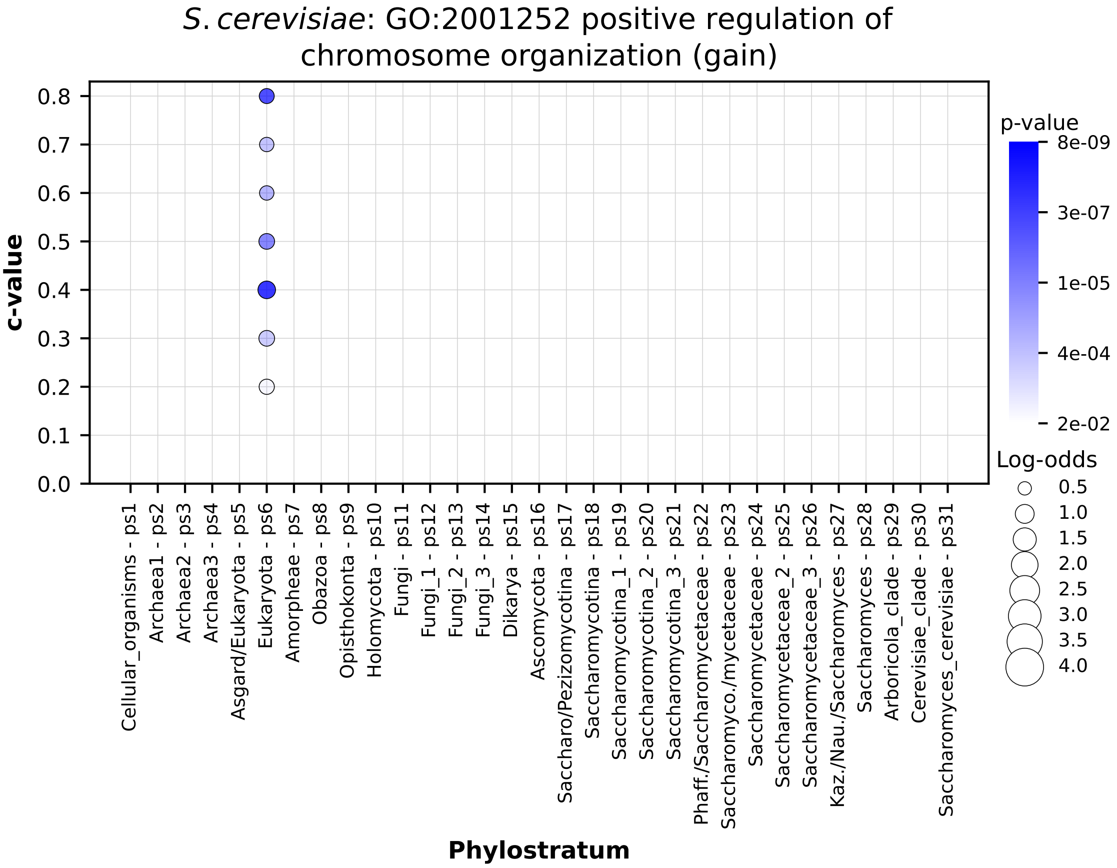
S. cerevisiae: GO:2001252 positive regulation of chromosome organization (gain)
c-value
0.0
0.1
0.2
0.3
0.4
0.5
0.6
0.7
0.8
p-value
8e-09
3e-07
1e-05
4e-04
2e-02
Log-odds
0.5
1.0
1.5
2.0
2.5
3.0
3.5
4.0
Cellular_organisms - ps1
Archaea1 - ps2
Archaea2 - ps3
Archaea3 - ps4
Asgard/Eukaryota - ps5
Eukaryota - ps6
Amorpheae - ps7
Obazoa - ps8
Opisthokonta - ps9
Holomycota - ps10
Fungi - ps11
Fungi_1 - ps12
Fungi_2 - ps13
Fungi_3 - ps14
Dikarya - ps15
Ascomycota - ps16
Saccharo/Pezizomycotina - ps17
Saccharomycotina - ps18
Saccharomycotina_1 - ps19
Saccharomycotina_2 - ps20
Saccharomycotina_3 - ps21
Phaff./Saccharomycetaceae - ps22
Saccharomyco./mycetaceae - ps23
Saccharomycetaceae - ps24
Saccharomycetaceae_2 - ps25
Saccharomycetaceae_3 - ps26
Kaz./Nau./Saccharomyces - ps27
Saccharomyces - ps28
Arboricola_clade - ps29
Cerevisiae_clade - ps30
Saccharomyces_cerevisiae - ps31
Phylostratum

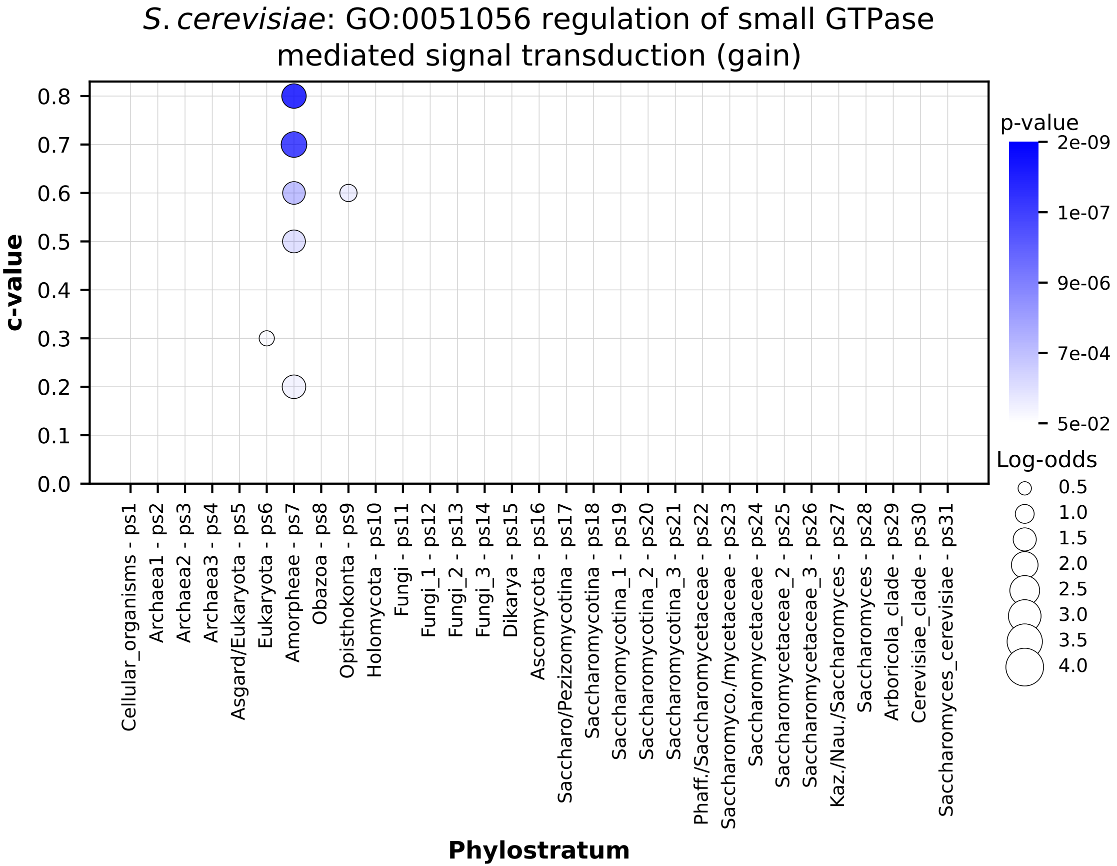

S. cerevisiae: GO:0051056 regulation of small GTPase mediated signal transduction (gain)
c-value
0.0
0.1
0.2
0.3
0.4
0.5
0.6
0.7
0.8
Phylostratum
Cellular_organisms - ps1
Archaea1 - ps2
Archaea2 - ps3
Archaea3 - ps4
Asgard/Eukaryota - ps5
Eukaryota - ps6
Amorpheae - ps7
Obazoa - ps8
Opisthokonta - ps9
Holomycota - ps10
Fungi - ps11
Fungi_1 - ps12
Fungi_2 - ps13
Fungi_3 - ps14
Dikarya - ps15
Ascomycota - ps16
Saccharo/Pezizomycotina - ps17
Saccharomycotina - ps18
Saccharomycotina_1 - ps19
Saccharomycotina_2 - ps20
Saccharomycotina_3 - ps21
Phaff./Saccharomycetaceae - ps22
Saccharomyco./mycetaceae - ps23
Saccharomycetaceae - ps24
Saccharomycetaceae_2 - ps25
Saccharomycetaceae_3 - ps26
Kaz./Nau./Saccharomyces - ps27
Saccharomyces - ps28
Arboricola_clade - ps29
Cerevisiae_clade - ps30
Saccharomyces_cerevisiae - ps31
p-value
2e-09
1e-07
9e-06
7e-04
5e-02
Log-odds
0.5
1.0
1.5
2.0
2.5
3.0
3.5
4.0

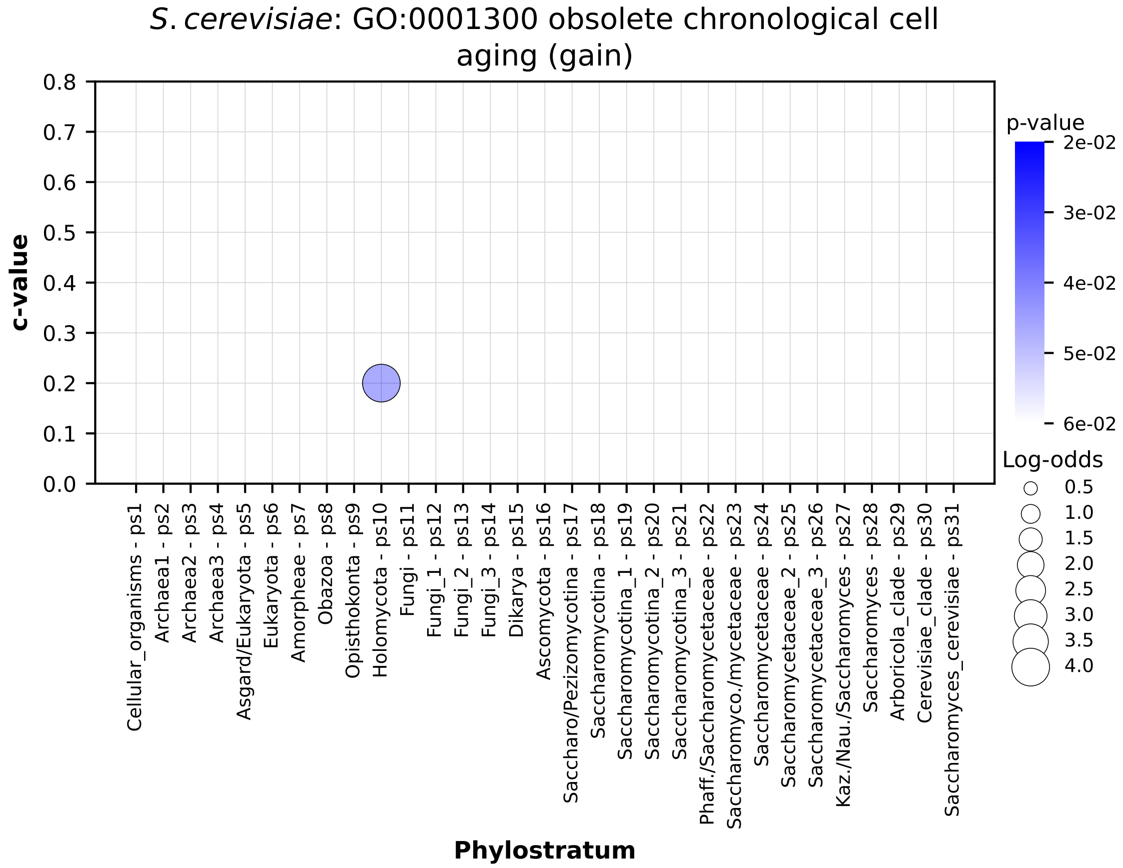

S. cerevisiae: GO:0001300 obsolete chronological cell aging (gain)
c-value
0.8
0.7
0.6
0.5
0.4
0.3
0.2
0.1
0.0
Phylostratum
Cellular_organisms - ps1
Archaea1 - ps2
Archaea2 - ps3
Archaea3 - ps4
Asgard/Eukaryota - ps5
Eukaryota - ps6
Amorpheae - ps7
Obazoa - ps8
Opisthokonta - ps9
Holomycota - ps10
Fungi - ps11
Fungi_1 - ps12
Fungi_2 - ps13
Fungi_3 - ps14
Dikarya - ps15
Ascomycota - ps16
Saccharo/Pezizomycotina - ps17
Saccharomycotina - ps18
Saccharomycotina_1 - ps19
Saccharomycotina_2 - ps20
Saccharomycotina_3 - ps21
Phaff./Saccharomycetaceae - ps22
Saccharomyco./mycetaceae - ps23
Saccharomycetaceae - ps24
Saccharomycetaceae_2 - ps25
Saccharomycetaceae_3 - ps26
Kaz./Nau./Saccharomyces - ps27
Saccharomyces - ps28
Arboricola_clade - ps29
Cerevisiae_clade - ps30
Saccharomyces_cerevisiae - ps31
p-value
2e-02
3e-02
4e-02
5e-02
6e-02
Log-odds
0.5
1.0
1.5
2.0
2.5
3.0
3.5
4.0

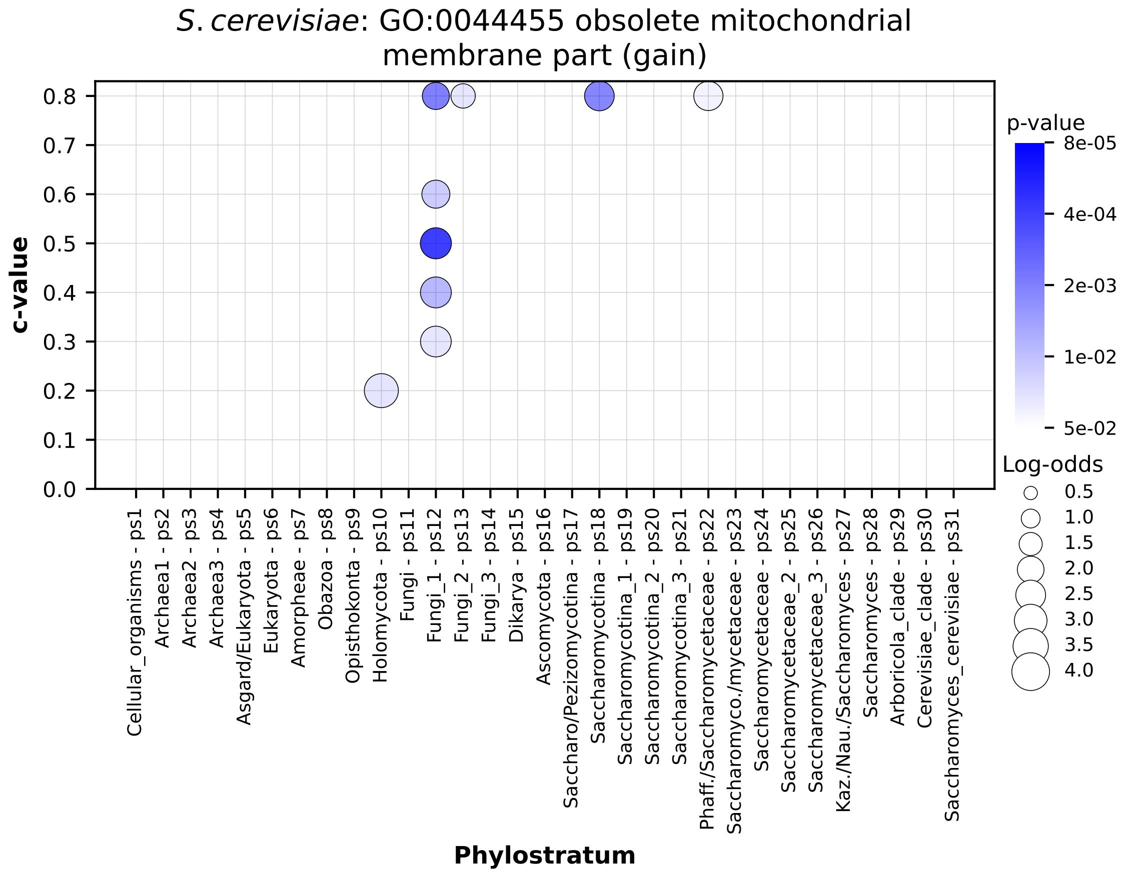

S. cerevisiae: GO:0044455 obsolete mitochondrial membrane part (gain)
c-value
0.0
0.1
0.2
0.3
0.4
0.5
0.6
0.7
0.8
p-value
8e-05
4e-04
2e-03
1e-02
5e-02
Log-odds
0.5
1.0
1.5
2.0
2.5
3.0
3.5
4.0
Cellular_organisms - ps1
Archaea1 - ps2
Archaea2 - ps3
Archaea3 - ps4
Asgard/Eukaryota - ps5
Eukaryota - ps6
Amorpheae - ps7
Obazoa - ps8
Opisthokonta - ps9
Holomycota - ps10
Fungi - ps11
Fungi_1 - ps12
Fungi_2 - ps13
Fungi_3 - ps14
Dikarya - ps15
Ascomycota - ps16
Saccharo/Pezizomycotina - ps17
Saccharomycotina - ps18
Saccharomycotina_1 - ps19
Saccharomycotina_2 - ps20
Saccharomycotina_3 - ps21
Phaff./Saccharomycetaceae - ps22
Saccharomyco./mycetaceae - ps23
Saccharomycetaceae - ps24
Saccharomycetaceae_2 - ps25
Saccharomycetaceae_3 - ps26
Kaz./Nau./Saccharomyces - ps27
Saccharomyces - ps28
Arboricola_clade - ps29
Cerevisiae_clade - ps30
Saccharomyces_cerevisiae - ps31
Phylostratum

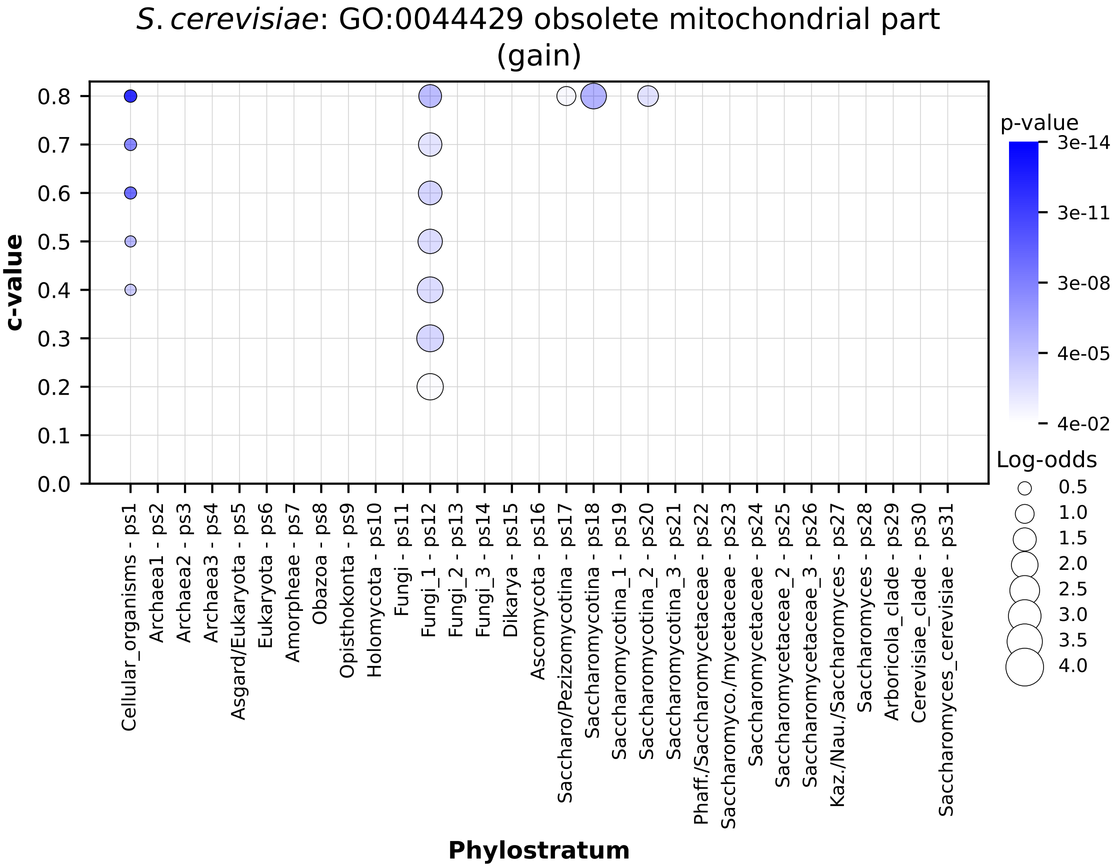

S. cerevisiae: GO:0044429 obsolete mitochondrial part
(gain)
c-value
0.0
0.1
0.2
0.3
0.4
0.5
0.6
0.7
0.8
p-value
3e-14
3e-11
3e-08
4e-05
4e-02
Log-odds
0.5
1.0
1.5
2.0
2.5
3.0
3.5
4.0
Cellular_organisms - ps1
Archaea1 - ps2
Archaea2 - ps3
Archaea3 - ps4
Asgard/Eukaryota - ps5
Eukaryota - ps6
Amorpheae - ps7
Obazoa - ps8
Opisthokonta - ps9
Holomycota - ps10
Fungi - ps11
Fungi_1 - ps12
Fungi_2 - ps13
Fungi_3 - ps14
Dikarya - ps15
Ascomycota - ps16
Saccharo/Pezizomycotina - ps17
Saccharomycotina - ps18
Saccharomycotina_1 - ps19
Saccharomycotina_2 - ps20
Saccharomycotina_3 - ps21
Phaff./Saccharomycetaceae - ps22
Saccharomyco./mycetaceae - ps23
Saccharomycetaceae - ps24
Saccharomycetaceae_2 - ps25
Saccharomycetaceae_3 - ps26
Kaz./Nau./Saccharomyces - ps27
Saccharomyces - ps28
Arboricola_clade - ps29
Cerevisiae_clade - ps30
Saccharomyces_cerevisiae - ps31
Phylostratum

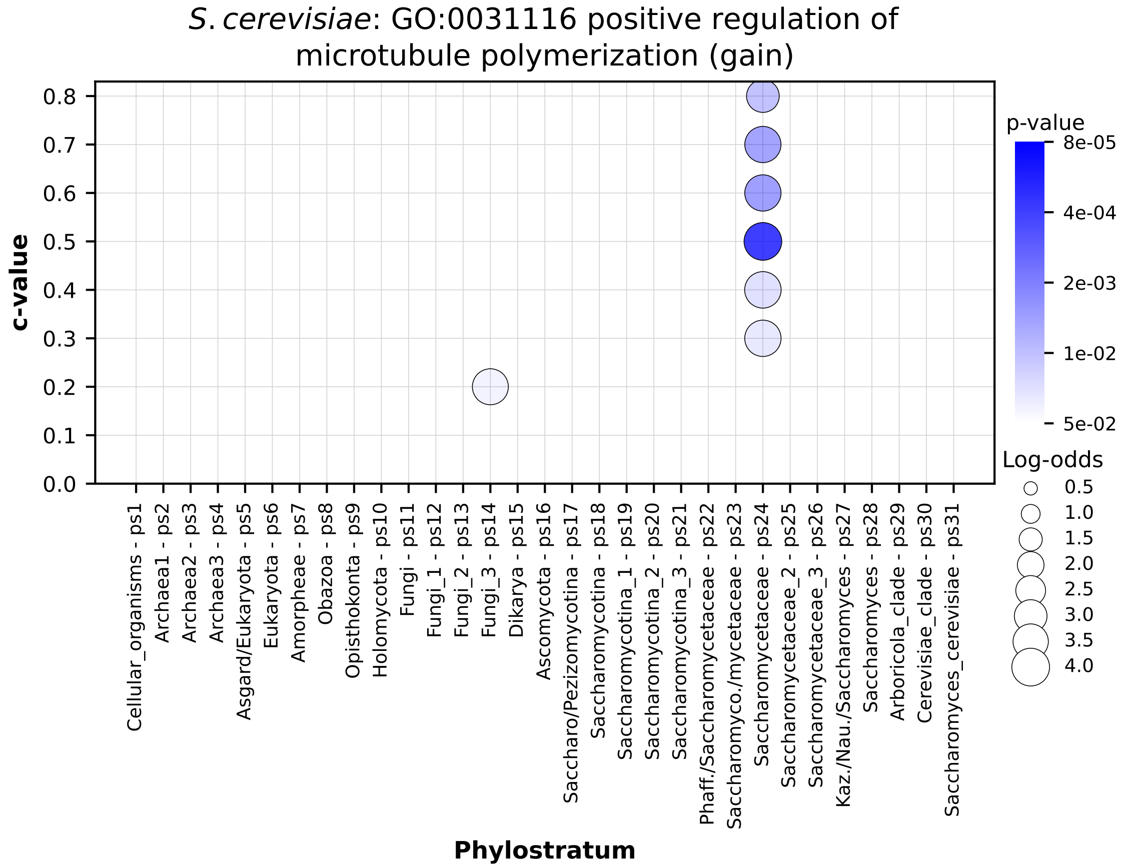

S. cerevisiae: GO:0031116 positive regulation of microtubule polymerization (gain)
c-value
0.8
0.7
0.6
0.5
0.4
0.3
0.2
0.1
0.0
p-value
8e-05
4e-04
2e-03
1e-02
5e-02
Log-odds
0.5
1.0
1.5
2.0
2.5
3.0
3.5
4.0
Cellular_organisms - ps1
Archaea1 - ps2
Archaea2 - ps3
Archaea3 - ps4
Asgard/Eukaryota - ps5
Eukaryota - ps6
Amorpheae - ps7
Obazoa - ps8
Opisthokonta - ps9
Holomycota - ps10
Fungi - ps11
Fungi_1 - ps12
Fungi_2 - ps13
Fungi_3 - ps14
Dikarya - ps15
Ascomycota - ps16
Saccharo/Pezizomycotina - ps17
Saccharomycotina - ps18
Saccharomycotina_1 - ps19
Saccharomycotina_2 - ps20
Saccharomycotina_3 - ps21
Phaff./Saccharomycetaceae - ps22
Saccharomyco./mycetaceae - ps23
Saccharomycetaceae - ps24
Saccharomycetaceae_2 - ps25
Saccharomycetaceae_3 - ps26
Kaz./Nau./Saccharomyces - ps27
Saccharomyces - ps28
Arboricola_clade - ps29
Cerevisiae_clade - ps30
Saccharomyces_cerevisiae - ps31
Phylostratum

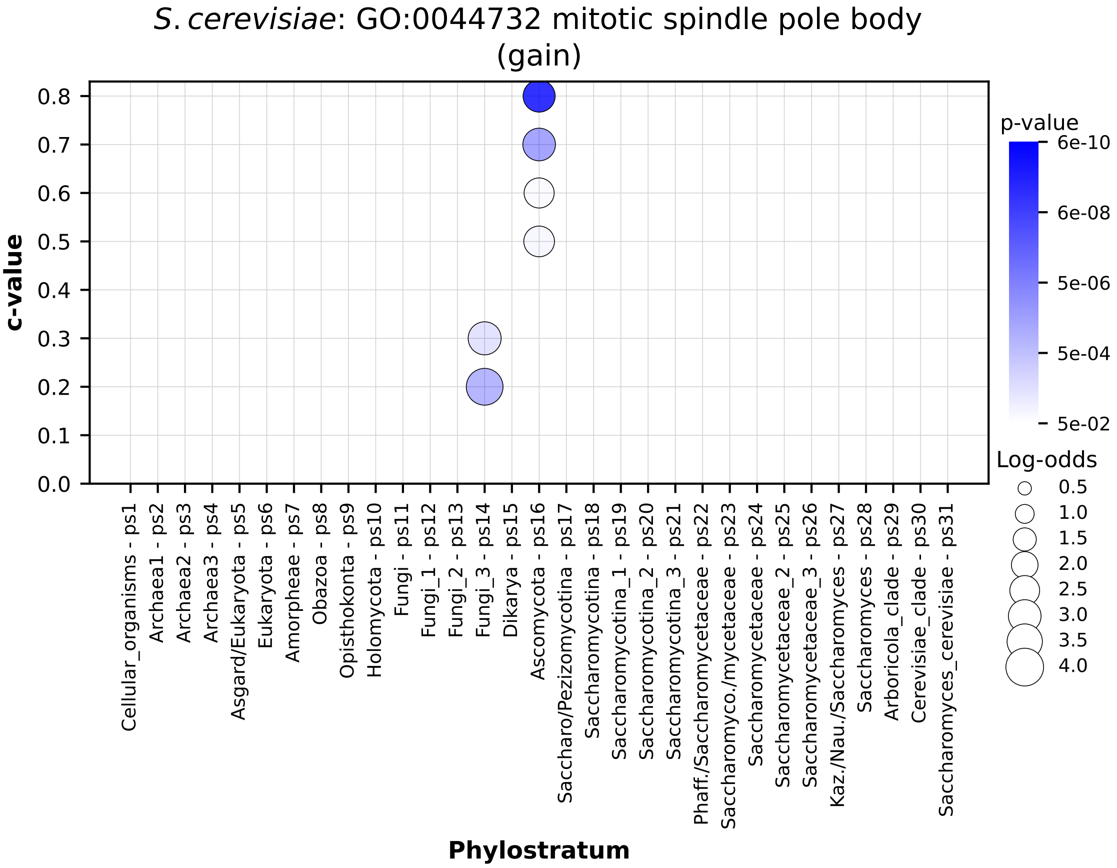

S. cerevisiae: GO:0044732 mitotic spindle pole body
(gain)
c-value
0.0
0.1
0.2
0.3
0.4
0.5
0.6
0.7
0.8
p-value
6e-10
6e-08
5e-06
5e-04
5e-02
Log-odds
0.5
1.0
1.5
2.0
2.5
3.0
3.5
4.0
Cellular_organisms - ps1
Archaea1 - ps2
Archaea2 - ps3
Archaea3 - ps4
Asgard/Eukaryota - ps5
Eukaryota - ps6
Amorpheae - ps7
Obazoa - ps8
Opisthokonta - ps9
Holomycota - ps10
Fungi - ps11
Fungi_1 - ps12
Fungi_2 - ps13
Fungi_3 - ps14
Dikarya - ps15
Ascomycota - ps16
Saccharo/Pezizomycotina - ps17
Saccharomycotina - ps18
Saccharomycotina_1 - ps19
Saccharomycotina_2 - ps20
Saccharomycotina_3 - ps21
Phaff./Saccharomycetaceae - ps22
Saccharomyco./mycetaceae - ps23
Saccharomycetaceae - ps24
Saccharomycetaceae_2 - ps25
Saccharomycetaceae_3 - ps26
Kaz./Nau./Saccharomyces - ps27
Saccharomyces - ps28
Arboricola_clade - ps29
Cerevisiae_clade - ps30
Saccharomyces_cerevisiae - ps31
Phylostratum

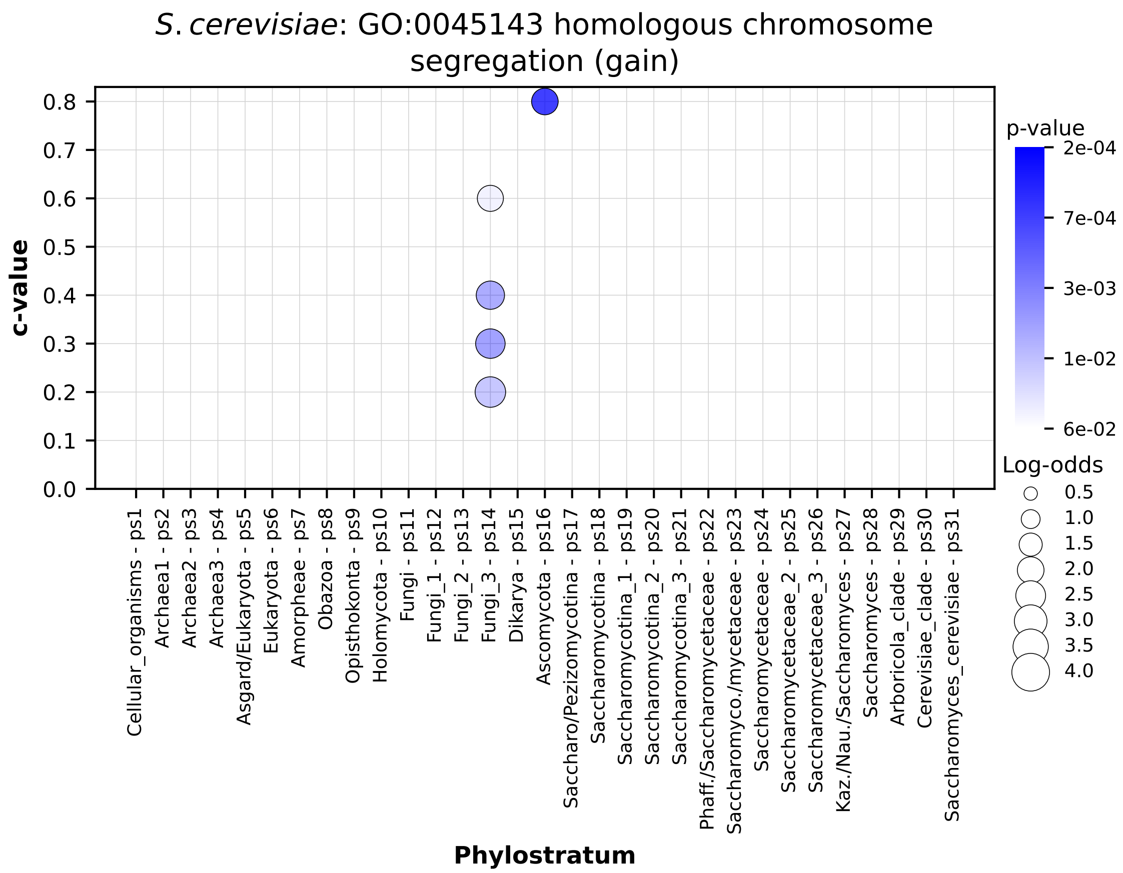

S. cerevisiae: GO:0045143 homologous chromosome segregation (gain)
c-value
0.0
0.1
0.2
0.3
0.4
0.5
0.6
0.7
0.8
Phylostratum
Cellular_organisms - ps1
Archaea1 - ps2
Archaea2 - ps3
Archaea3 - ps4
Asgard/Eukaryota - ps5
Eukaryota - ps6
Amorpheae - ps7
Obazoa - ps8
Opisthokonta - ps9
Holomycota - ps10
Fungi - ps11
Fungi_1 - ps12
Fungi_2 - ps13
Fungi_3 - ps14
Dikarya - ps15
Ascomycota - ps16
Saccharo/Pezizomycotina - ps17
Saccharomycotina - ps18
Saccharomycotina_1 - ps19
Saccharomycotina_2 - ps20
Saccharomycotina_3 - ps21
Phaff./Saccharomycetaceae - ps22
Saccharomyco./mycetaceae - ps23
Saccharomycetaceae - ps24
Saccharomycetaceae_2 - ps25
Saccharomycetaceae_3 - ps26
Kaz./Nau./Saccharomyces - ps27
Saccharomyces - ps28
Arboricola_clade - ps29
Cerevisiae_clade - ps30
Saccharomyces_cerevisiae - ps31
p-value
2e-04
7e-04
3e-03
1e-02
6e-02
Log-odds
0.5
1.0
1.5
2.0
2.5
3.0
3.5
4.0

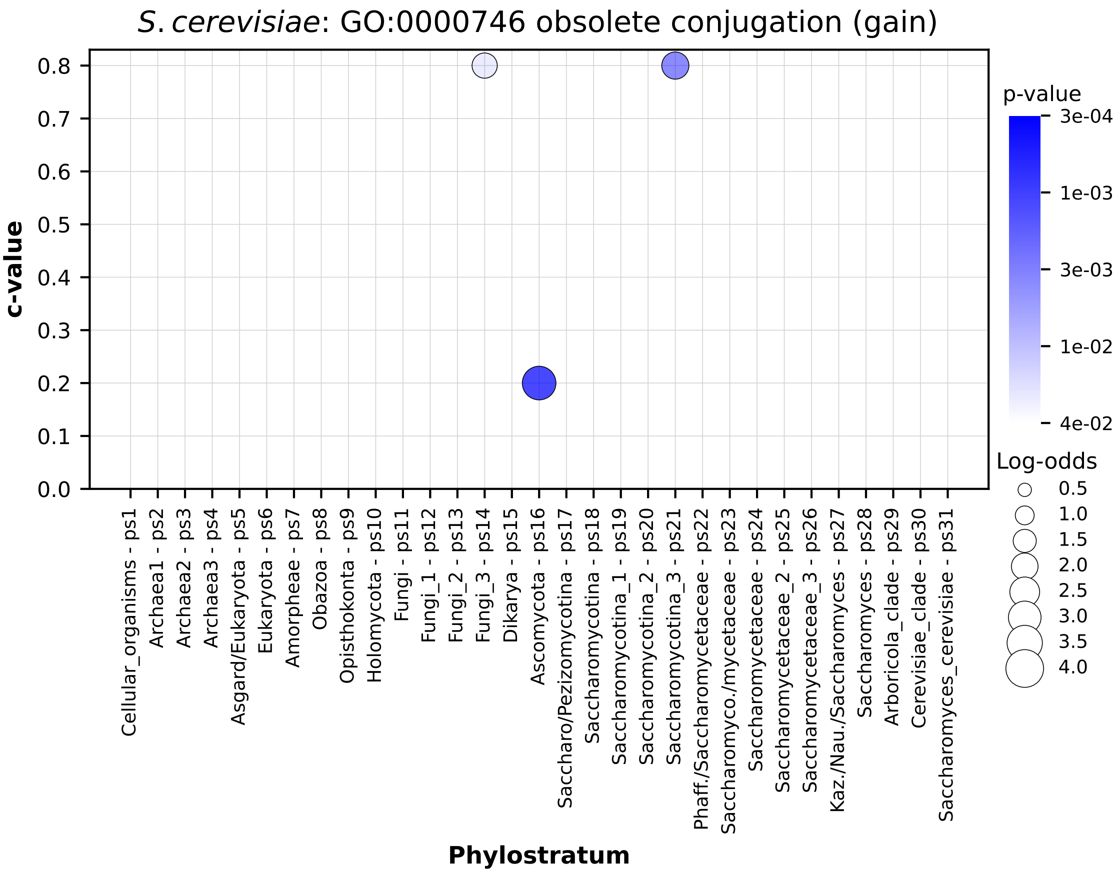

S. cerevisiae: GO:0000746 obsolete conjugation (gain)
c-value
0.0
0.1
0.2
0.3
0.4
0.5
0.6
0.7
0.8
p-value
3e-04
1e-03
3e-03
1e-02
4e-02
Log-odds
0.5
1.0
1.5
2.0
2.5
3.0
3.5
4.0
Cellular_organisms - ps1
Archaea1 - ps2
Archaea2 - ps3
Archaea3 - ps4
Asgard/Eukaryota - ps5
Eukaryota - ps6
Amorpheae - ps7
Obazoa - ps8
Opisthokonta - ps9
Holomycota - ps10
Fungi - ps11
Fungi_1 - ps12
Fungi_2 - ps13
Fungi_3 - ps14
Dikarya - ps15
Ascomycota - ps16
Saccharo/Pezizomycotina - ps17
Saccharomycotina - ps18
Saccharomycotina_1 - ps19
Saccharomycotina_2 - ps20
Saccharomycotina_3 - ps21
Phaff./Saccharomycetaceae - ps22
Saccharomyco./mycetaceae - ps23
Saccharomycetaceae - ps24
Saccharomycetaceae_2 - ps25
Saccharomycetaceae_3 - ps26
Kaz./Nau./Saccharomyces - ps27
Saccharomyces - ps28
Arboricola_clade - ps29
Cerevisiae_clade - ps30
Saccharomyces_cerevisiae - ps31
Phylostratum

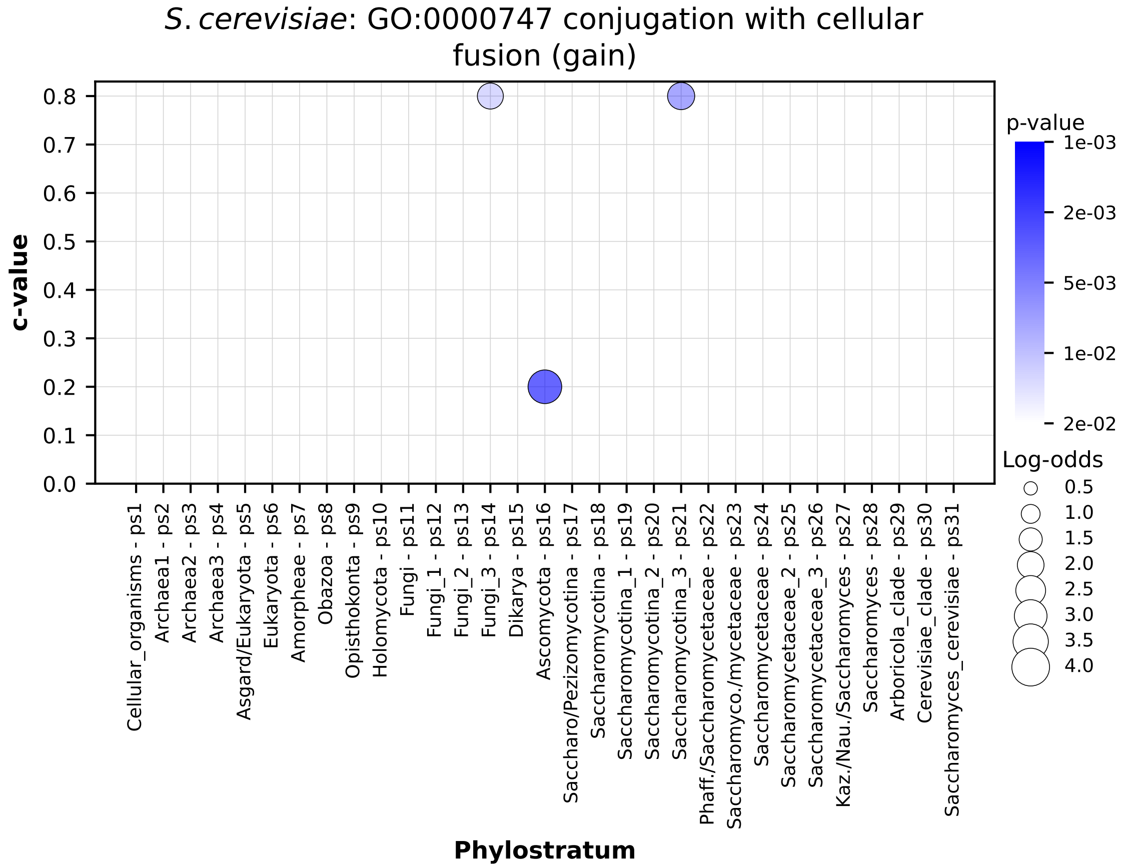

S. cerevisiae: GO:0000747 conjugation with cellular fusion (gain)
c-value
0.0
0.1
0.2
0.3
0.4
0.5
0.6
0.7
0.8
Phylostratum
Cellular_organisms - ps1
Archaea1 - ps2
Archaea2 - ps3
Archaea3 - ps4
Asgard/Eukaryota - ps5
Eukaryota - ps6
Amorpheae - ps7
Obazoa - ps8
Opisthokonta - ps9
Holomycota - ps10
Fungi - ps11
Fungi_1 - ps12
Fungi_2 - ps13
Fungi_3 - ps14
Dikarya - ps15
Ascomycota - ps16
Saccharo/Pezizomycotina - ps17
Saccharomycotina - ps18
Saccharomycotina_1 - ps19
Saccharomycotina_2 - ps20
Saccharomycotina_3 - ps21
Phaff./Saccharomycetaceae - ps22
Saccharomyco./mycetaceae - ps23
Saccharomycetaceae - ps24
Saccharomycetaceae_2 - ps25
Saccharomycetaceae_3 - ps26
Kaz./Nau./Saccharomyces - ps27
Saccharomyces - ps28
Arboricola_clade - ps29
Cerevisiae_clade - ps30
Saccharomyces_cerevisiae - ps31
p-value
1e-03
2e-03
5e-03
1e-02
2e-02
Log-odds
0.5
1.0
1.5
2.0
2.5
3.0
3.5
4.0

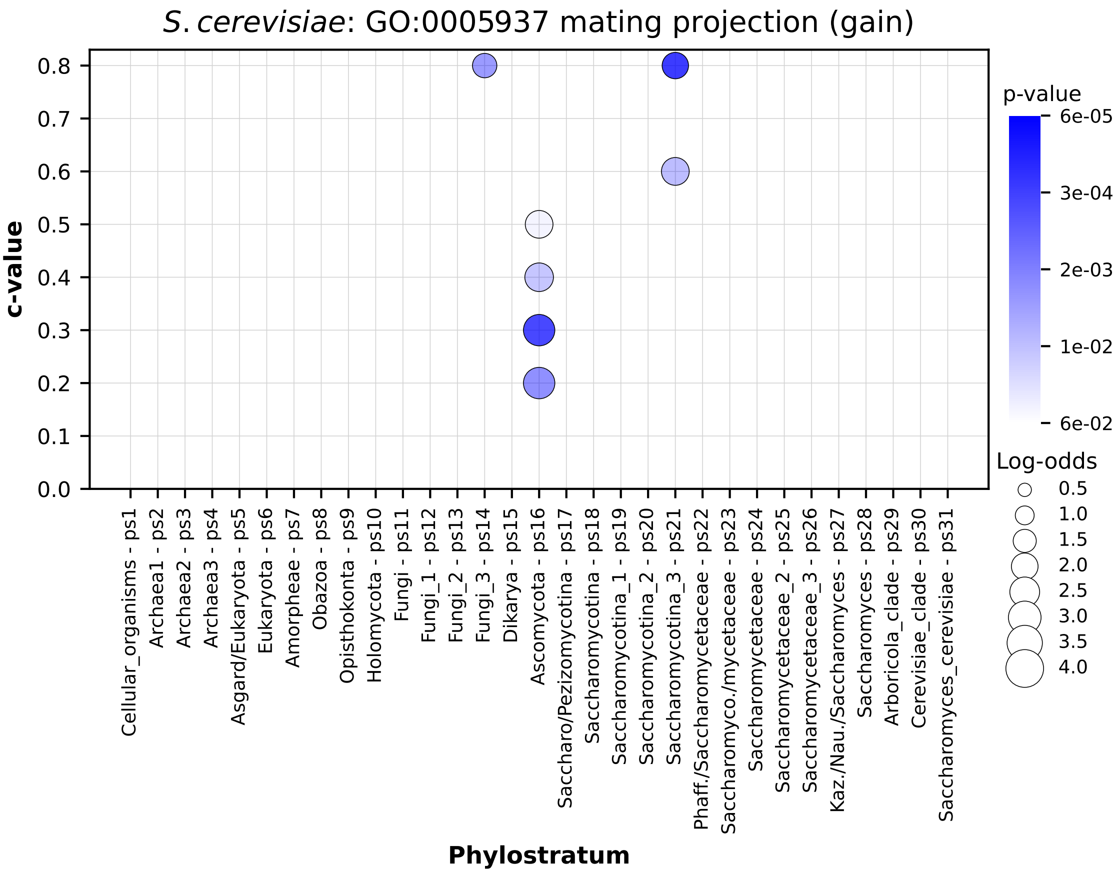

S. cerevisiae: GO:0005937 mating projection (gain)
c-value
0.0
0.1
0.2
0.3
0.4
0.5
0.6
0.7
0.8
p-value
6e-05
3e-04
2e-03
1e-02
6e-02
Log-odds
0.5
1.0
1.5
2.0
2.5
3.0
3.5
4.0
Cellular_organisms - ps1
Archaea1 - ps2
Archaea2 - ps3
Archaea3 - ps4
Asgard/Eukaryota - ps5
Eukaryota - ps6
Amorpheae - ps7
Obazoa - ps8
Opisthokonta - ps9
Holomycota - ps10
Fungi - ps11
Fungi_1 - ps12
Fungi_2 - ps13
Fungi_3 - ps14
Dikarya - ps15
Ascomycota - ps16
Saccharo/Pezizomycotina - ps17
Saccharomycotina - ps18
Saccharomycotina_1 - ps19
Saccharomycotina_2 - ps20
Saccharomycotina_3 - ps21
Phaff./Saccharomycetaceae - ps22
Saccharomyco./mycetaceae - ps23
Saccharomycetaceae - ps24
Saccharomycetaceae_2 - ps25
Saccharomycetaceae_3 - ps26
Kaz./Nau./Saccharomyces - ps27
Saccharomyces - ps28
Arboricola_clade - ps29
Cerevisiae_clade - ps30
Saccharomyces_cerevisiae - ps31
Phylostratum

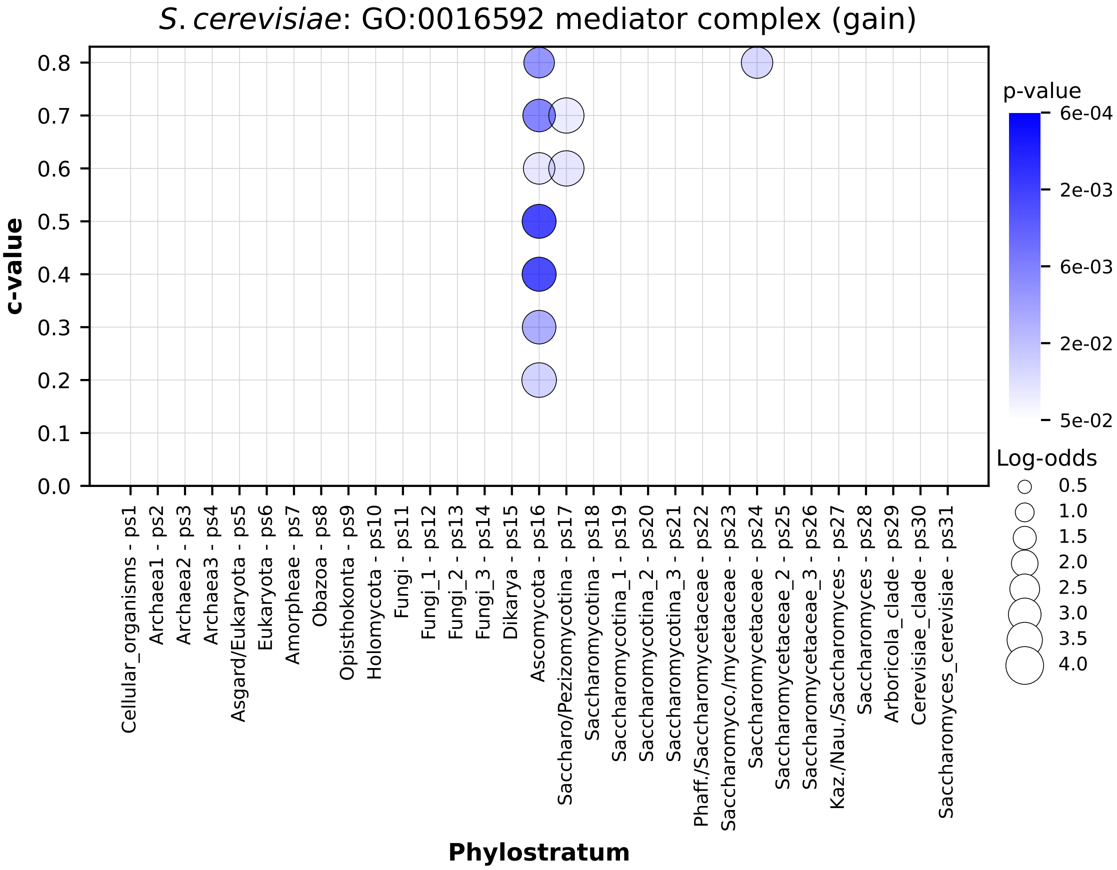

S. cerevisiae: GO:0016592 mediator complex (gain)
c-value
0.0
0.1
0.2
0.3
0.4
0.5
0.6
0.7
0.8
Phylostratum
Cellular_organisms - ps1
Archaea1 - ps2
Archaea2 - ps3
Archaea3 - ps4
Asgard/Eukaryota - ps5
Eukaryota - ps6
Amorpheae - ps7
Obazoa - ps8
Opisthokonta - ps9
Holomycota - ps10
Fungi - ps11
Fungi_1 - ps12
Fungi_2 - ps13
Fungi_3 - ps14
Dikarya - ps15
Ascomycota - ps16
Saccharo/Pezizomycotina - ps17
Saccharomycotina - ps18
Saccharomycotina_1 - ps19
Saccharomycotina_2 - ps20
Saccharomycotina_3 - ps21
Phaff./Saccharomycetaceae - ps22
Saccharomyco./mycetaceae - ps23
Saccharomycetaceae - ps24
Saccharomycetaceae_2 - ps25
Saccharomycetaceae_3 - ps26
Kaz./Nau./Saccharomyces - ps27
Saccharomyces - ps28
Arboricola_clade - ps29
Cerevisiae_clade - ps30
Saccharomyces_cerevisiae - ps31
p-value
6e-04
2e-03
6e-03
2e-02
5e-02
Log-odds
0.5
1.0
1.5
2.0
2.5
3.0
3.5
4.0

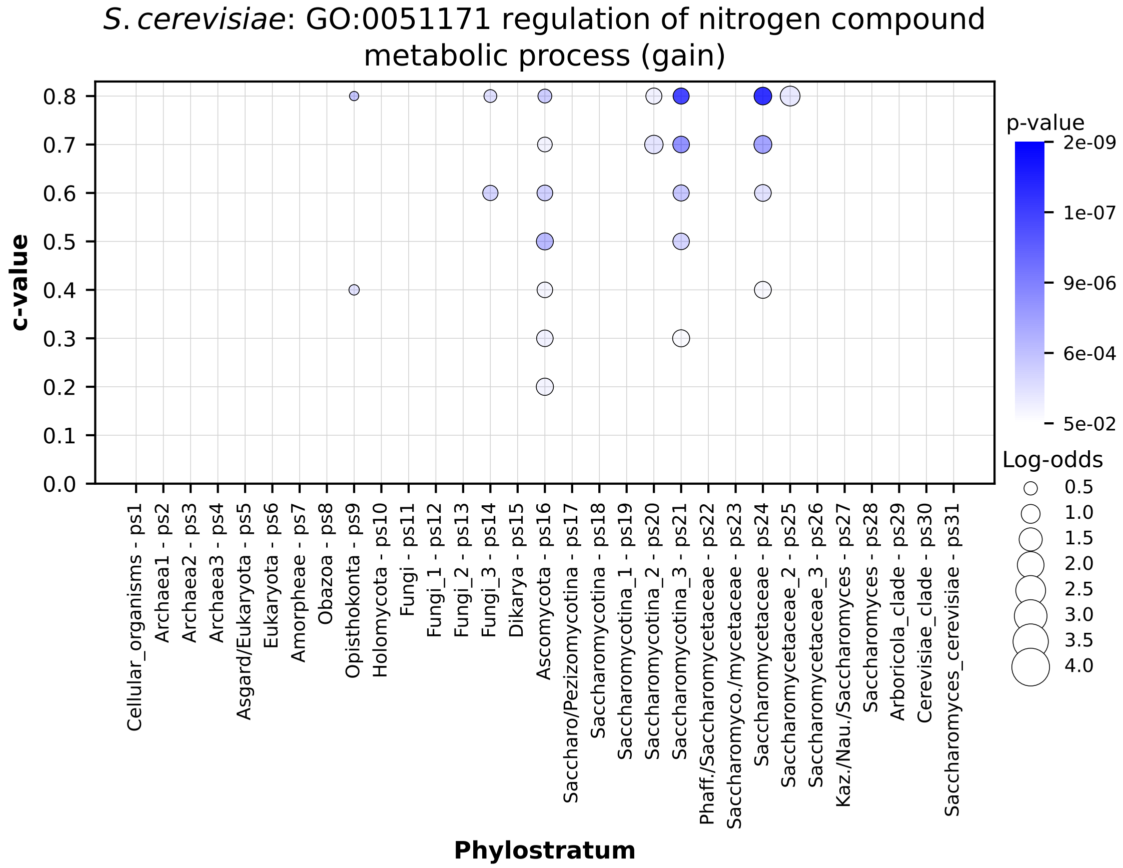

S. cerevisiae: GO:0051171 regulation of nitrogen compound metabolic process (gain)
c-value
0.0
0.1
0.2
0.3
0.4
0.5
0.6
0.7
0.8
p-value
2e-09
1e-07
9e-06
6e-04
5e-02
Log-odds
0.5
1.0
1.5
2.0
2.5
3.0
3.5
4.0
Cellular_organisms - ps1
Archaea1 - ps2
Archaea2 - ps3
Archaea3 - ps4
Asgard/Eukaryota - ps5
Eukaryota - ps6
Amorpheae - ps7
Obazoa - ps8
Opisthokonta - ps9
Holomycota - ps10
Fungi - ps11
Fungi_1 - ps12
Fungi_2 - ps13
Fungi_3 - ps14
Dikarya - ps15
Ascomycota - ps16
Saccharo/Pezizomycotina - ps17
Saccharomycotina - ps18
Saccharomycotina_1 - ps19
Saccharomycotina_2 - ps20
Saccharomycotina_3 - ps21
Phaff./Saccharomycetaceae - ps22
Saccharomyco./mycetaceae - ps23
Saccharomycetaceae - ps24
Saccharomycetaceae_2 - ps25
Saccharomycetaceae_3 - ps26
Kaz./Nau./Saccharomyces - ps27
Saccharomyces - ps28
Arboricola_clade - ps29
Cerevisiae_clade - ps30
Saccharomyces_cerevisiae - ps31
Phylostratum

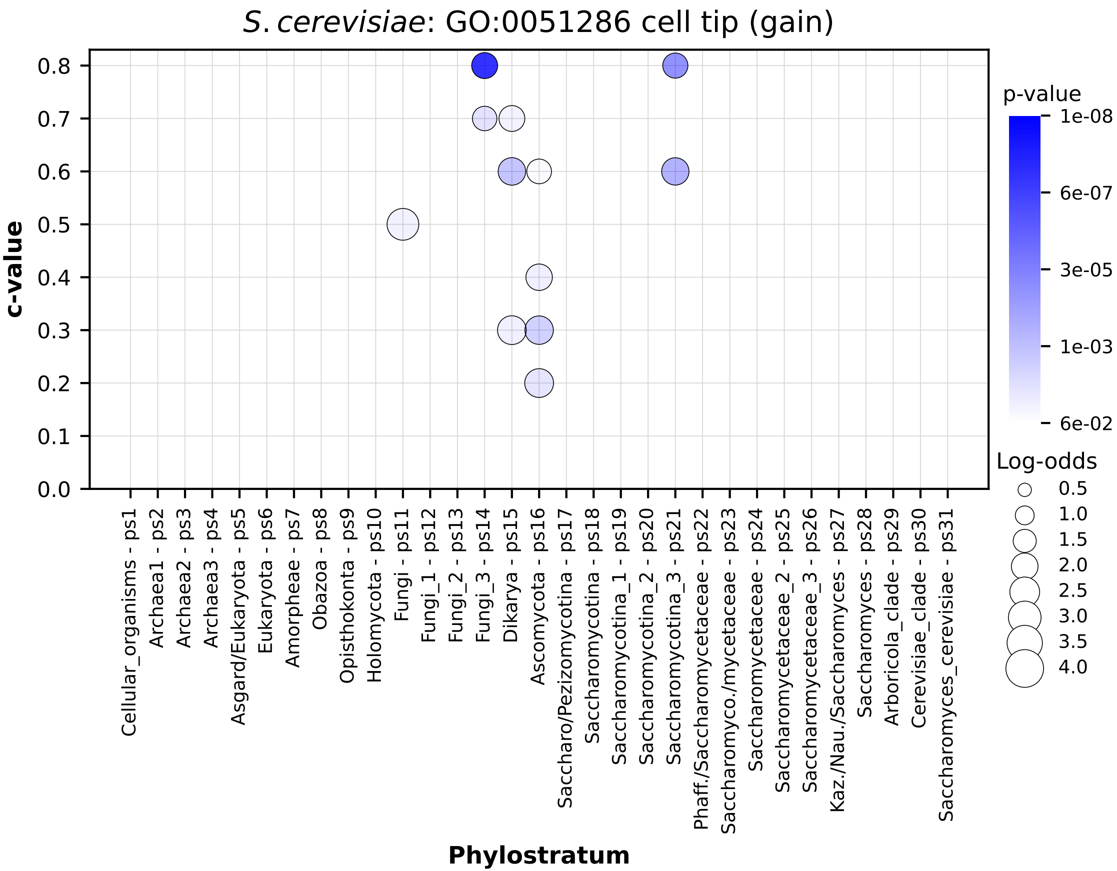

S. cerevisiae: GO:0051286 cell tip (gain)
c-value
0.0
0.1
0.2
0.3
0.4
0.5
0.6
0.7
0.8
Phylostratum
Cellular_organisms - ps1
Archaea1 - ps2
Archaea2 - ps3
Archaea3 - ps4
Asgard/Eukaryota - ps5
Eukaryota - ps6
Amorpheae - ps7
Obazoa - ps8
Opisthokonta - ps9
Holomycota - ps10
Fungi - ps11
Fungi_1 - ps12
Fungi_2 - ps13
Fungi_3 - ps14
Dikarya - ps15
Ascomycota - ps16
Saccharo/Pezizomycotina - ps17
Saccharomycotina - ps18
Saccharomycotina_1 - ps19
Saccharomycotina_2 - ps20
Saccharomycotina_3 - ps21
Phaff./Saccharomycetaceae - ps22
Saccharomyco./mycetaceae - ps23
Saccharomycetaceae - ps24
Saccharomycetaceae_2 - ps25
Saccharomycetaceae_3 - ps26
Kaz./Nau./Saccharomyces - ps27
Saccharomyces - ps28
Arboricola_clade - ps29
Cerevisiae_clade - ps30
Saccharomyces_cerevisiae - ps31
p-value
1e-08
6e-07
3e-05
1e-03
6e-02
Log-odds
0.5
1.0
1.5
2.0
2.5
3.0
3.5
4.0

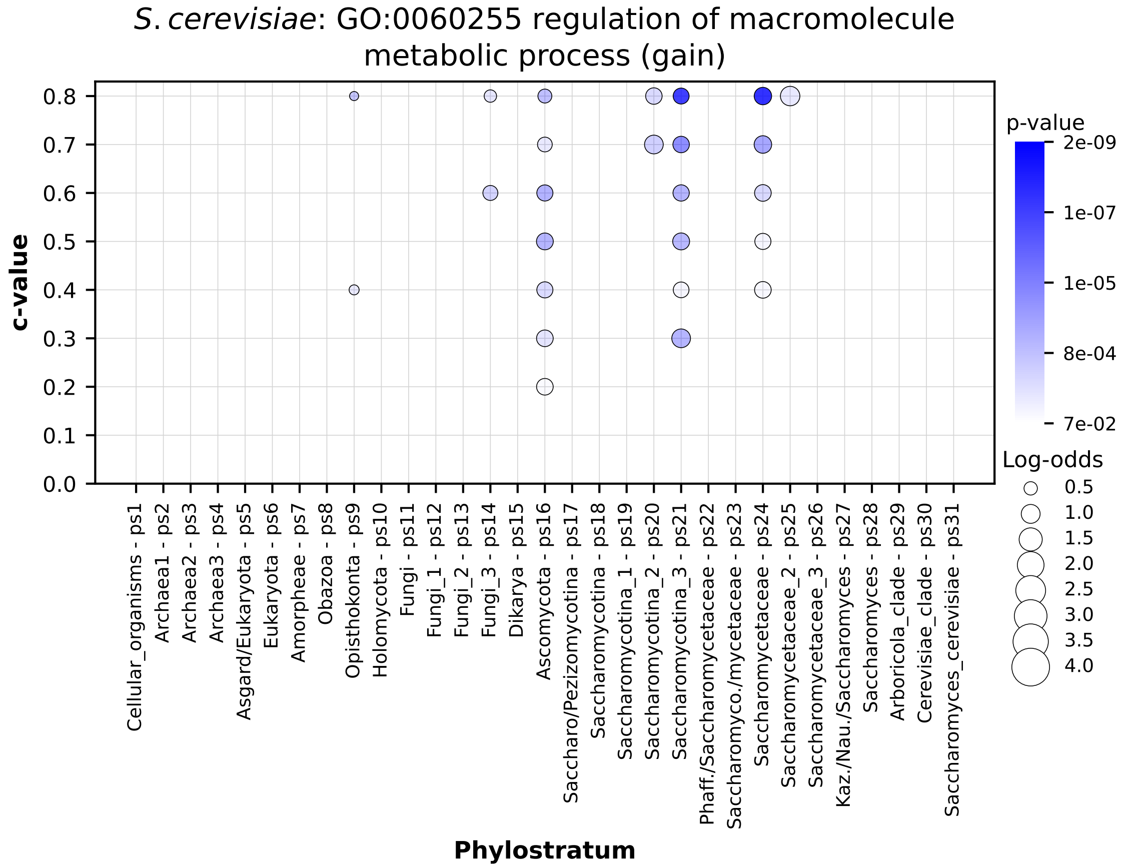
S. cerevisiae: GO:0060255 regulation of macromolecule metabolic process (gain)
c-value
Phylostratum
p-value
2e-09
1e-07
1e-05
8e-04
7e-02
Log-odds
0.5
1.0
1.5
2.0
2.5
3.0
3.5
4.0
Cellular_organisms - ps1
Archaea1 - ps2
Archaea2 - ps3
Archaea3 - ps4
Asgard/Eukaryota - ps5
Eukaryota - ps6
Amorpheae - ps7
Obazoa - ps8
Opisthokonta - ps9
Holomycota - ps10
Fungi - ps11
Fungi_1 - ps12
Fungi_2 - ps13
Fungi_3 - ps14
Dikarya - ps15
Ascomycota - ps16
Saccharo/Pezizomycotina - ps17
Saccharomycotina - ps18
Saccharomycotina_1 - ps19
Saccharomycotina_2 - ps20
Saccharomycotina_3 - ps21
Phaff./Saccharomycetaceae - ps22
Saccharomyco./mycetaceae - ps23
Saccharomycetaceae - ps24
Saccharomycetaceae_2 - ps25
Saccharomycetaceae_3 - ps26
Kaz./Nau./Saccharomyces - ps27
Saccharomyces - ps28
Arboricola_clade - ps29
Cerevisiae_clade - ps30
Saccharomyces_cerevisiae - ps31

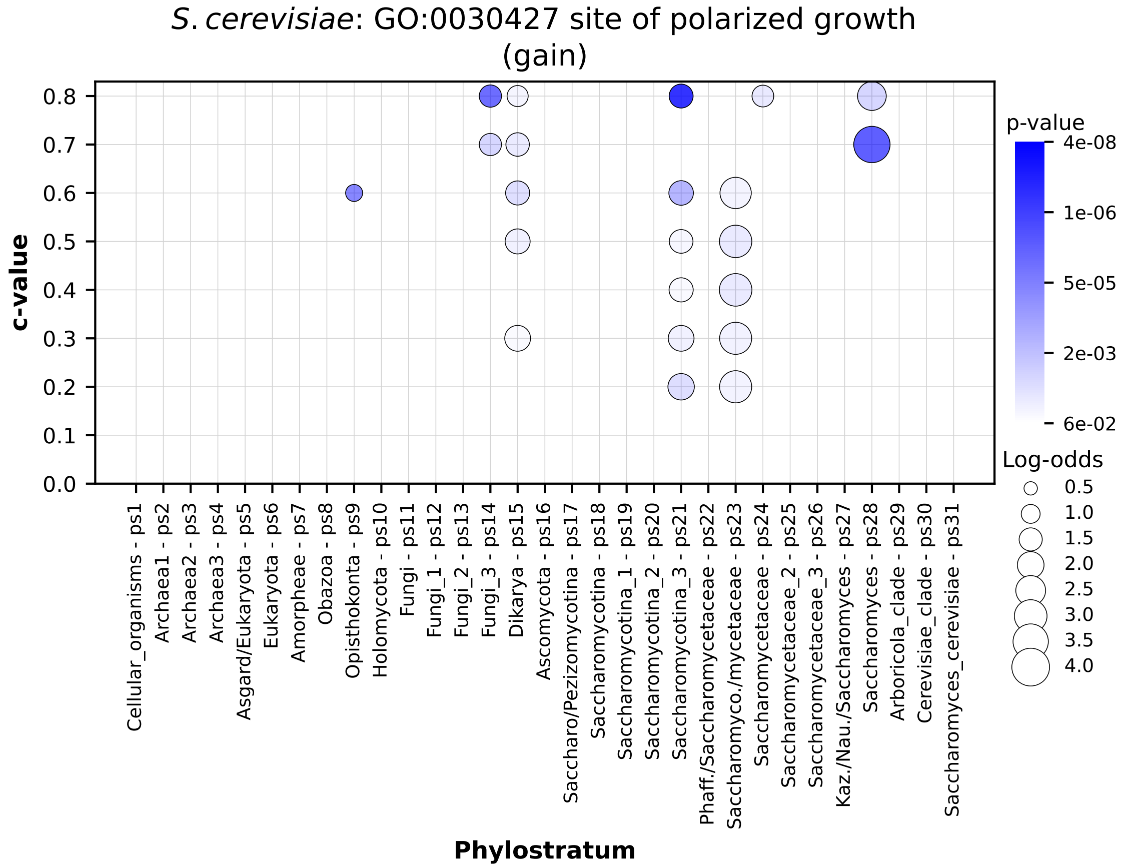

S. cerevisiae: GO:0030427 site of polarized growth
(gain)
c-value
0.0
0.1
0.2
0.3
0.4
0.5
0.6
0.7
0.8
p-value
4e-08
1e-06
5e-05
2e-03
6e-02
Log-odds
0.5
1.0
1.5
2.0
2.5
3.0
3.5
4.0
Cellular_organisms - ps1
Archaea1 - ps2
Archaea2 - ps3
Archaea3 - ps4
Asgard/Eukaryota - ps5
Eukaryota - ps6
Amorpheae - ps7
Obazoa - ps8
Opisthokonta - ps9
Holomycota - ps10
Fungi - ps11
Fungi_1 - ps12
Fungi_2 - ps13
Fungi_3 - ps14
Dikarya - ps15
Ascomycota - ps16
Saccharo/Pezizomycotina - ps17
Saccharomycotina - ps18
Saccharomycotina_1 - ps19
Saccharomycotina_2 - ps20
Saccharomycotina_3 - ps21
Phaff./Saccharomycetaceae - ps22
Saccharomyco./mycetaceae - ps23
Saccharomycetaceae - ps24
Saccharomycetaceae_2 - ps25
Saccharomycetaceae_3 - ps26
Kaz./Nau./Saccharomyces - ps27
Saccharomyces - ps28
Arboricola_clade - ps29
Cerevisiae_clade - ps30
Saccharomyces_cerevisiae - ps31
Phylostratum

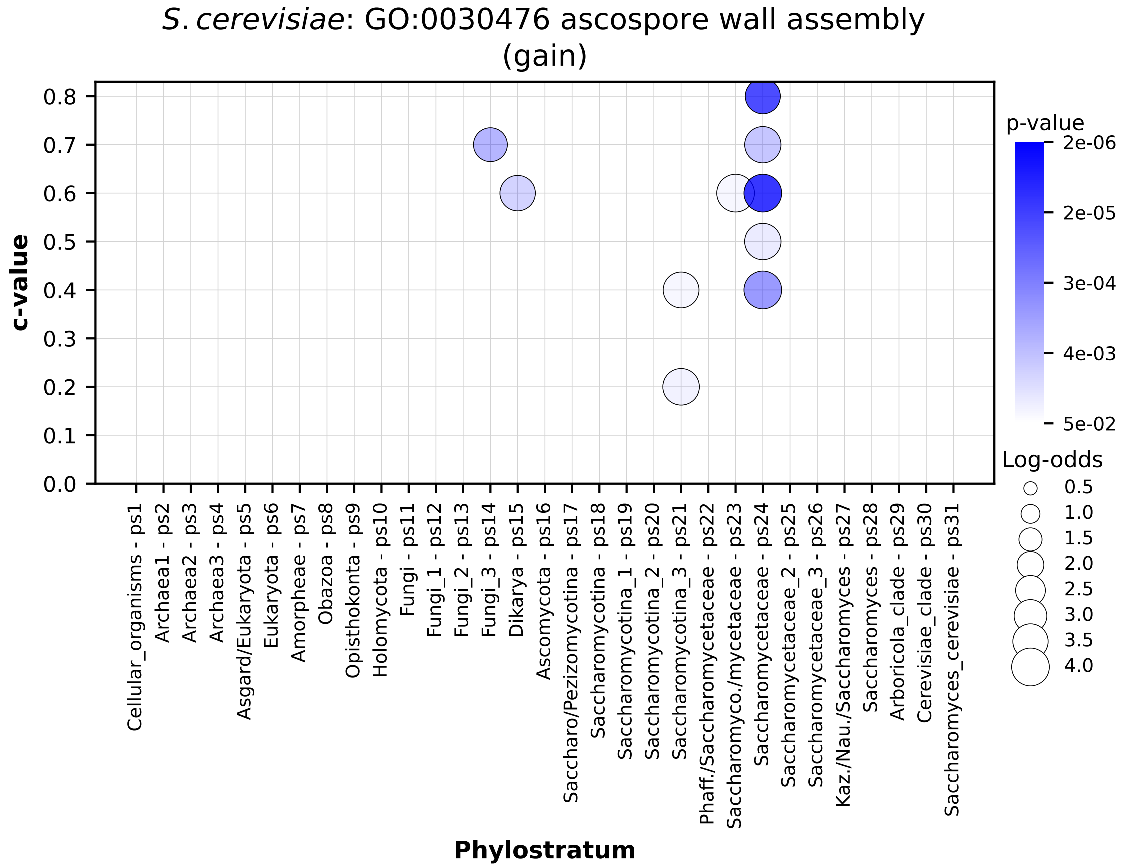

S. cerevisiae: GO:0030476 ascospore wall assembly (gain)
c-value
0.8
0.7
0.6
0.5
0.4
0.3
0.2
0.1
0.0
p-value
2e-06
2e-05
3e-04
4e-03
5e-02
Log-odds
0.5
1.0
1.5
2.0
2.5
3.0
3.5
4.0
Cellular_organisms - ps1
Archaea1 - ps2
Archaea2 - ps3
Archaea3 - ps4
Asgard/Eukaryota - ps5
Eukaryota - ps6
Amorpheae - ps7
Obazoa - ps8
Opisthokonta - ps9
Holomycota - ps10
Fungi - ps11
Fungi_1 - ps12
Fungi_2 - ps13
Fungi_3 - ps14
Dikarya - ps15
Ascomycota - ps16
Saccharo/Pezizomycotina - ps17
Saccharomycotina - ps18
Saccharomycotina_1 - ps19
Saccharomycotina_2 - ps20
Saccharomycotina_3 - ps21
Phaff./Saccharomycetaceae - ps22
Saccharomyco./mycetaceae - ps23
Saccharomycetaceae - ps24
Saccharomycetaceae_2 - ps25
Saccharomycetaceae_3 - ps26
Kaz./Nau./Saccharomyces - ps27
Saccharomyces - ps28
Arboricola_clade - ps29
Cerevisiae_clade - ps30
Saccharomyces_cerevisiae - ps31
Phylostratum

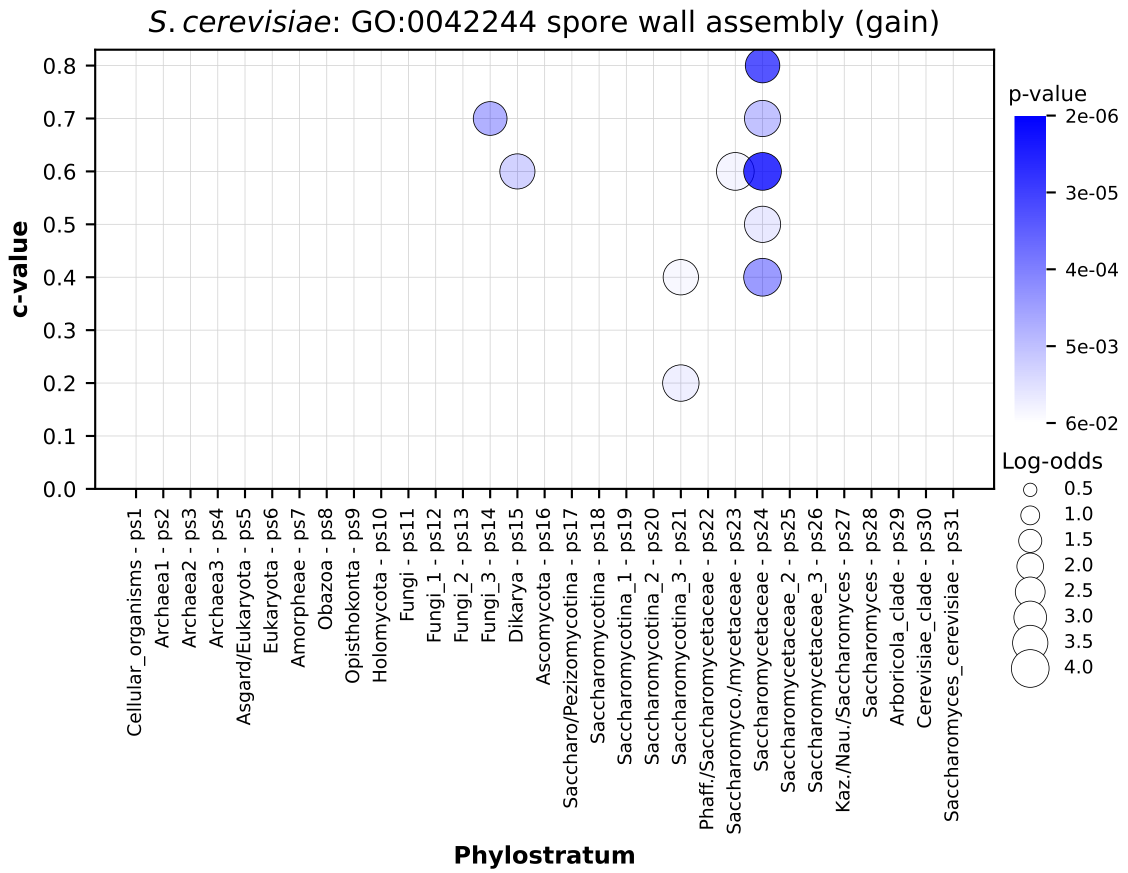

S. cerevisiae: GO:0042244 spore wall assembly (gain)
c-value
0.0
0.1
0.2
0.3
0.4
0.5
0.6
0.7
0.8
p-value
2e-06
3e-05
4e-04
5e-03
6e-02
Log-odds
0.5
1.0
1.5
2.0
2.5
3.0
3.5
4.0
Cellular_organisms - ps1
Archaea1 - ps2
Archaea2 - ps3
Archaea3 - ps4
Asgard/Eukaryota - ps5
Eukaryota - ps6
Amorpheae - ps7
Obazoa - ps8
Opisthokonta - ps9
Holomycota - ps10
Fungi - ps11
Fungi_1 - ps12
Fungi_2 - ps13
Fungi_3 - ps14
Dikarya - ps15
Ascomycota - ps16
Saccharo/Pezizomycotina - ps17
Saccharomycotina - ps18
Saccharomycotina_1 - ps19
Saccharomycotina_2 - ps20
Saccharomycotina_3 - ps21
Phaff./Saccharomycetaceae - ps22
Saccharomyco./mycetaceae - ps23
Saccharomycetaceae - ps24
Saccharomycetaceae_2 - ps25
Saccharomycetaceae_3 - ps26
Kaz./Nau./Saccharomyces - ps27
Saccharomyces - ps28
Arboricola_clade - ps29
Cerevisiae_clade - ps30
Saccharomyces_cerevisiae - ps31
Phylostratum

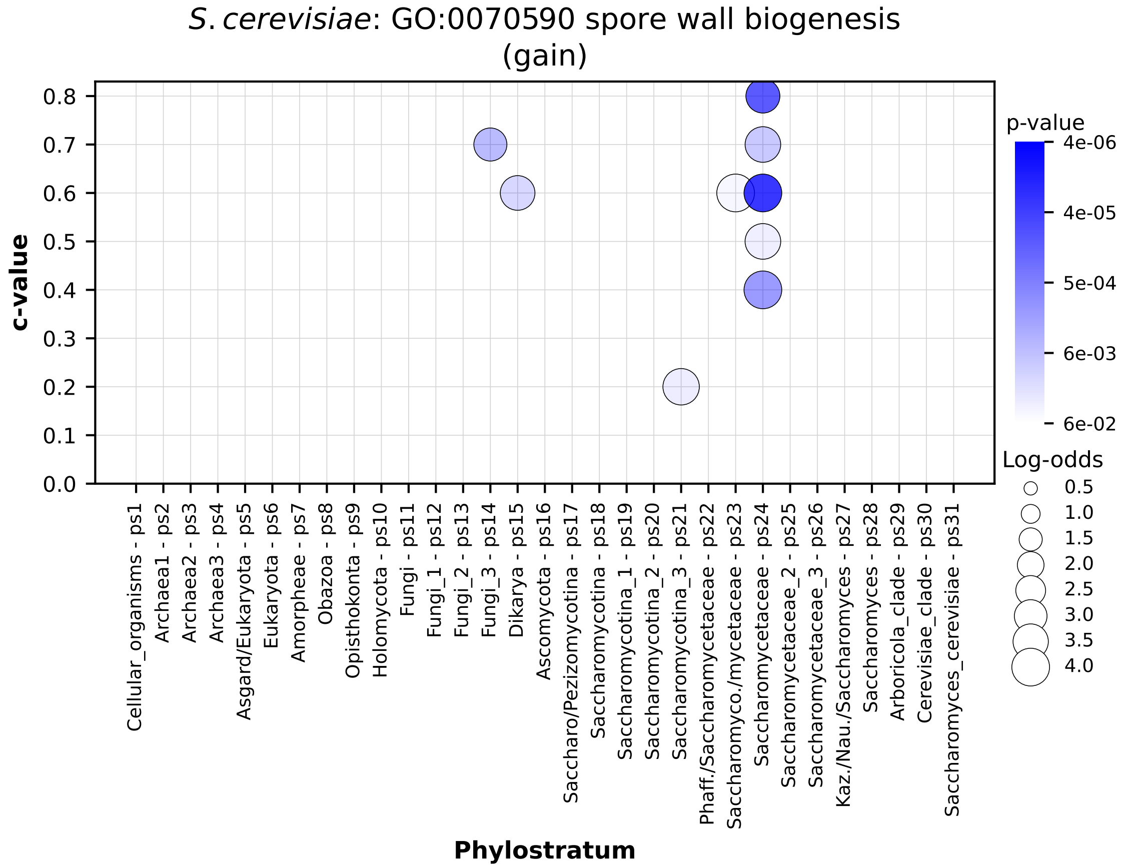

S. cerevisiae: GO:0070590 spore wall biogenesis (gain)
c-value
0.0
0.1
0.2
0.3
0.4
0.5
0.6
0.7
0.8
p-value
4e-06
4e-05
5e-04
6e-03
6e-02
Log-odds
0.5
1.0
1.5
2.0
2.5
3.0
3.5
4.0
Cellular_organisms - ps1
Archaea1 - ps2
Archaea2 - ps3
Archaea3 - ps4
Asgard/Eukaryota - ps5
Eukaryota - ps6
Amorpheae - ps7
Obazoa - ps8
Opisthokonta - ps9
Holomycota - ps10
Fungi - ps11
Fungi_1 - ps12
Fungi_2 - ps13
Fungi_3 - ps14
Dikarya - ps15
Ascomycota - ps16
Saccharo/Pezizomycotina - ps17
Saccharomycotina - ps18
Saccharomycotina_1 - ps19
Saccharomycotina_2 - ps20
Saccharomycotina_3 - ps21
Phaff./Saccharomycetaceae - ps22
Saccharomyco./mycetaceae - ps23
Saccharomycetaceae - ps24
Saccharomycetaceae_2 - ps25
Saccharomycetaceae_3 - ps26
Kaz./Nau./Saccharomyces - ps27
Saccharomyces - ps28
Arboricola_clade - ps29
Cerevisiae_clade - ps30
Saccharomyces_cerevisiae - ps31
Phylostratum

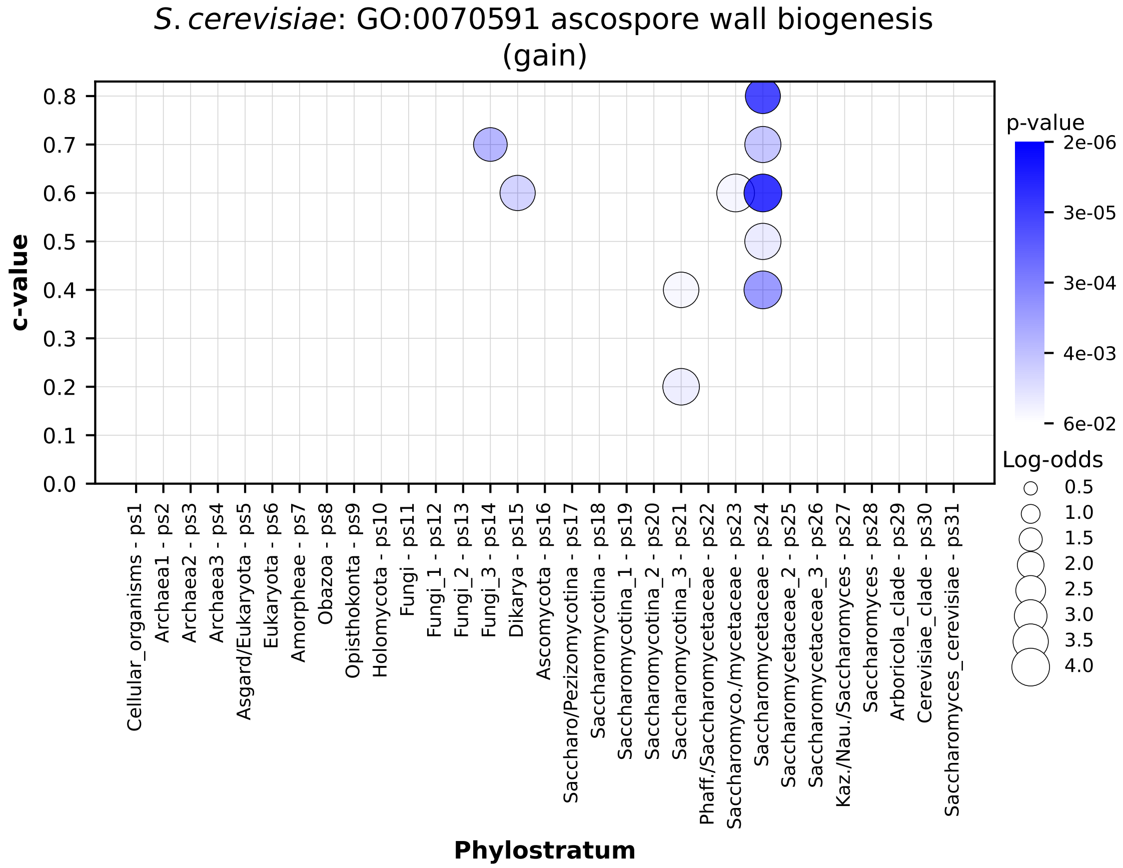

S. cerevisiae: GO:0070591 ascospore wall biogenesis (gain)
c-value
0.0
0.1
0.2
0.3
0.4
0.5
0.6
0.7
0.8
Phylostratum
Cellular_organisms - ps1
Archaea1 - ps2
Archaea2 - ps3
Archaea3 - ps4
Asgard/Eukaryota - ps5
Eukaryota - ps6
Amorpheae - ps7
Obazoa - ps8
Opisthokonta - ps9
Holomycota - ps10
Fungi - ps11
Fungi_1 - ps12
Fungi_2 - ps13
Fungi_3 - ps14
Dikarya - ps15
Ascomycota - ps16
Saccharo/Pezizomycotina - ps17
Saccharomycotina - ps18
Saccharomycotina_1 - ps19
Saccharomycotina_2 - ps20
Saccharomycotina_3 - ps21
Phaff./Saccharomycetaceae - ps22
Saccharomyco./mycetaceae - ps23
Saccharomycetaceae - ps24
Saccharomycetaceae_2 - ps25
Saccharomycetaceae_3 - ps26
Kaz./Nau./Saccharomyces - ps27
Saccharomyces - ps28
Arboricola_clade - ps29
Cerevisiae_clade - ps30
Saccharomyces_cerevisiae - ps31
p-value
2e-06
3e-05
3e-04
4e-03
6e-02
Log-odds
0.5
1.0
1.5
2.0
2.5
3.0
3.5
4.0

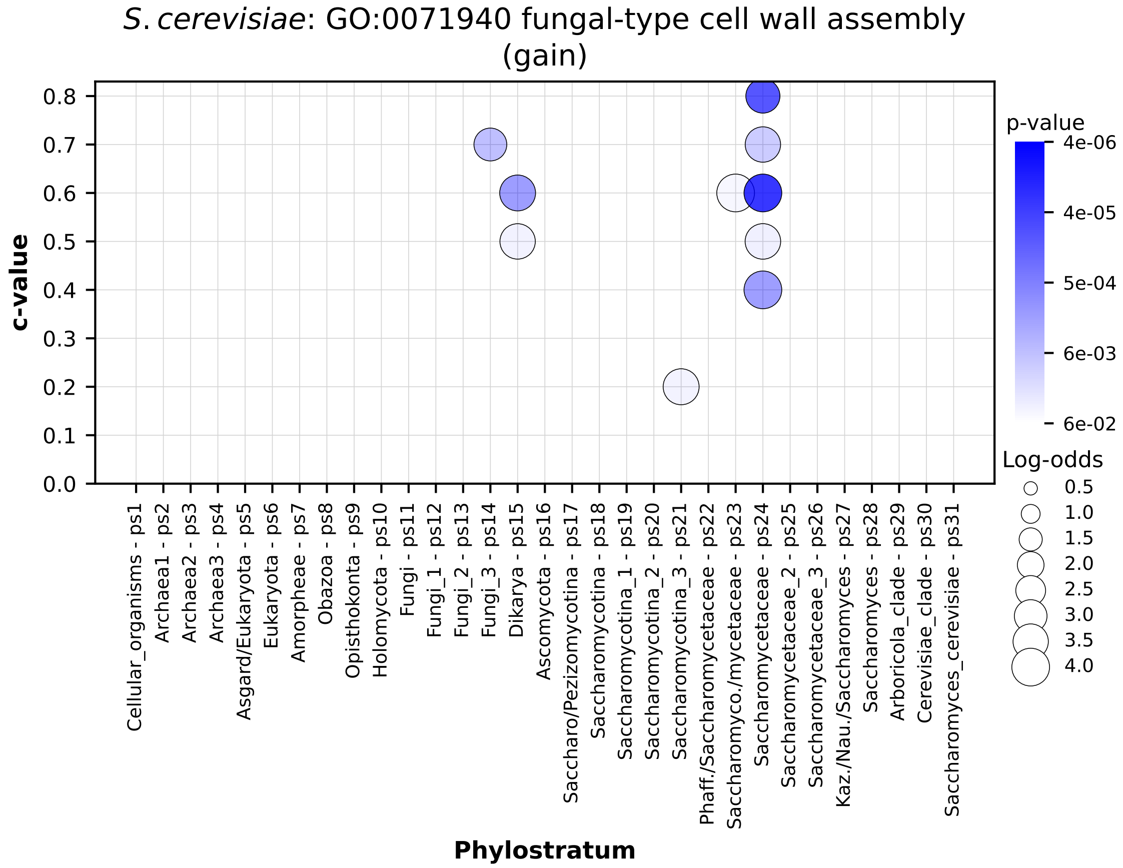

S. cerevisiae: GO:0071940 fungal-type cell wall assembly
(gain)
c-value
0.0
0.1
0.2
0.3
0.4
0.5
0.6
0.7
0.8
Phylostratum
Cellular_organisms - ps1
Archaea1 - ps2
Archaea2 - ps3
Archaea3 - ps4
Asgard/Eukaryota - ps5
Eukaryota - ps6
Amorpheae - ps7
Obazoa - ps8
Opisthokonta - ps9
Holomycota - ps10
Fungi - ps11
Fungi_1 - ps12
Fungi_2 - ps13
Fungi_3 - ps14
Dikarya - ps15
Ascomycota - ps16
Saccharo/Pezizomycotina - ps17
Saccharomycotina - ps18
Saccharomycotina_1 - ps19
Saccharomycotina_2 - ps20
Saccharomycotina_3 - ps21
Phaff./Saccharomycetaceae - ps22
Saccharomyco./mycetaceae - ps23
Saccharomycetaceae - ps24
Saccharomycetaceae_2 - ps25
Saccharomycetaceae_3 - ps26
Kaz./Nau./Saccharomyces - ps27
Saccharomyces - ps28
Arboricola_clade - ps29
Cerevisiae_clade - ps30
Saccharomyces_cerevisiae - ps31
p-value
4e-06
4e-05
5e-04
6e-03
6e-02
Log-odds
0.5
1.0
1.5
2.0
2.5
3.0
3.5
4.0

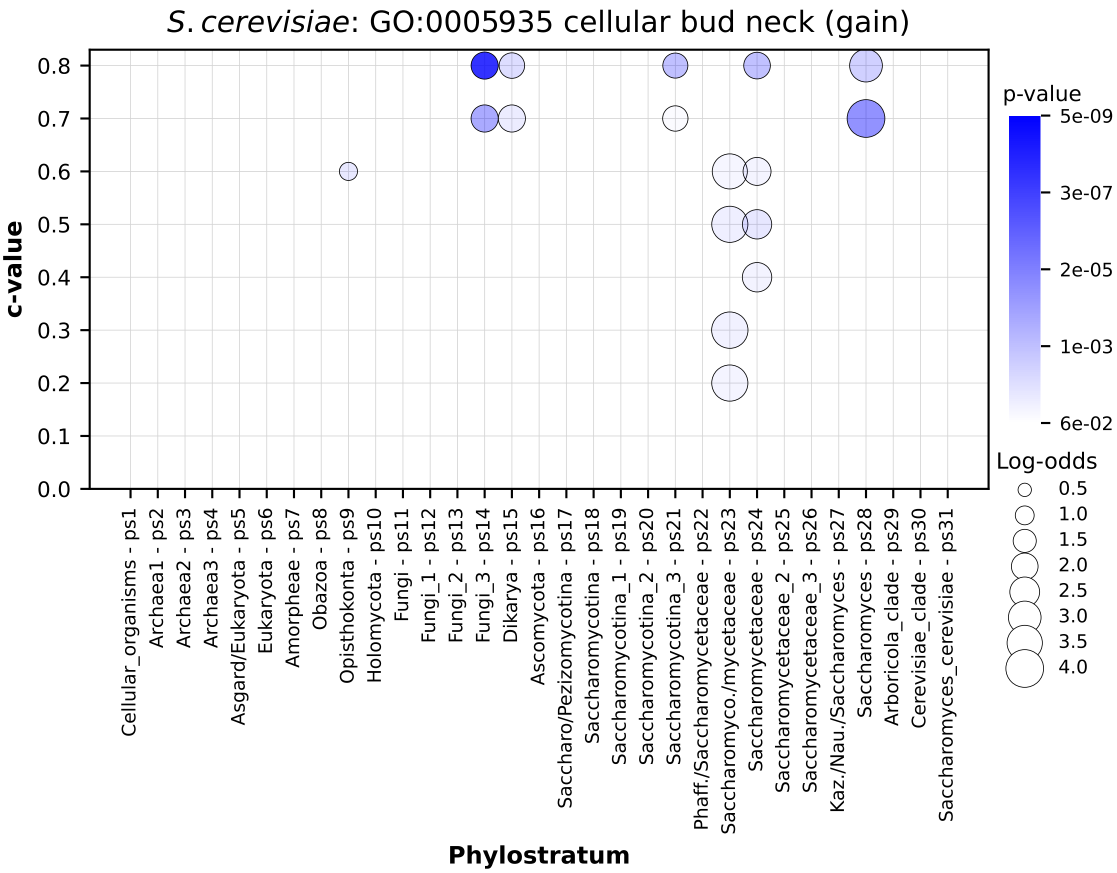

S. cerevisiae: GO:0005935 cellular bud neck (gain)
c-value
0.0
0.1
0.2
0.3
0.4
0.5
0.6
0.7
0.8
p-value
5e-09
3e-07
2e-05
1e-03
6e-02
Log-odds
0.5
1.0
1.5
2.0
2.5
3.0
3.5
4.0
Cellular_organisms - ps1
Archaea1 - ps2
Archaea2 - ps3
Archaea3 - ps4
Asgard/Eukaryota - ps5
Eukaryota - ps6
Amorpheae - ps7
Obazoa - ps8
Opisthokonta - ps9
Holomycota - ps10
Fungi - ps11
Fungi_1 - ps12
Fungi_2 - ps13
Fungi_3 - ps14
Dikarya - ps15
Ascomycota - ps16
Saccharo/Pezizomycotina - ps17
Saccharomycotina - ps18
Saccharomycotina_1 - ps19
Saccharomycotina_2 - ps20
Saccharomycotina_3 - ps21
Phaff./Saccharomycetaceae - ps22
Saccharomyco./mycetaceae - ps23
Saccharomycetaceae - ps24
Saccharomycetaceae_2 - ps25
Saccharomycetaceae_3 - ps26
Kaz./Nau./Saccharomyces - ps27
Saccharomyces - ps28
Arboricola_clade - ps29
Cerevisiae_clade - ps30
Saccharomyces_cerevisiae - ps31
Phylostratum

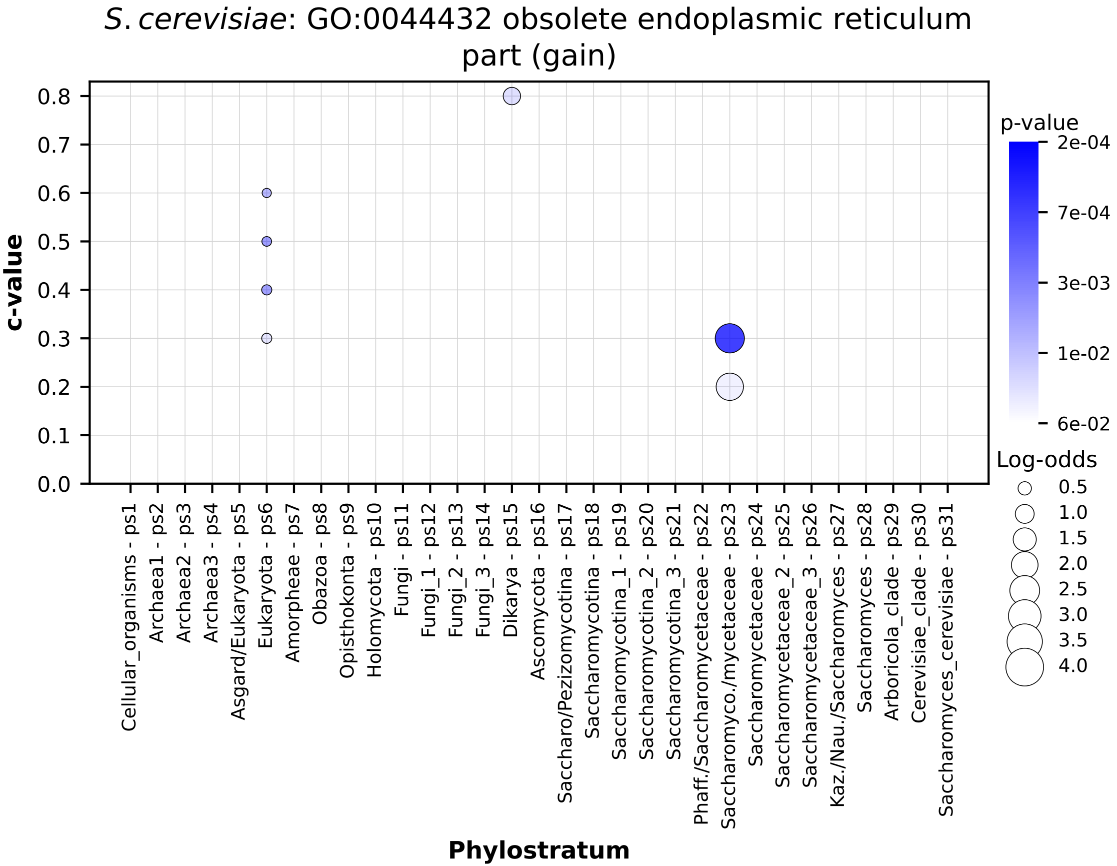

S. cerevisiae: GO:0044432 obsolete endoplasmic reticulum part (gain)
c-value
0.0
0.1
0.2
0.3
0.4
0.5
0.6
0.7
0.8
Phylostratum
Cellular_organisms - ps1
Archaea1 - ps2
Archaea2 - ps3
Archaea3 - ps4
Asgard/Eukaryota - ps5
Eukaryota - ps6
Amorpheae - ps7
Obazoa - ps8
Opisthokonta - ps9
Holomycota - ps10
Fungi - ps11
Fungi_1 - ps12
Fungi_2 - ps13
Fungi_3 - ps14
Dikarya - ps15
Ascomycota - ps16
Saccharo/Pezizomycotina - ps17
Saccharomycotina - ps18
Saccharomycotina_1 - ps19
Saccharomycotina_2 - ps20
Saccharomycotina_3 - ps21
Phaff./Saccharomycetaceae - ps22
Saccharomyco./mycetaceae - ps23
Saccharomycetaceae - ps24
Saccharomycetaceae_2 - ps25
Saccharomycetaceae_3 - ps26
Kaz./Nau./Saccharomyces - ps27
Saccharomyces - ps28
Arboricola_clade - ps29
Cerevisiae_clade - ps30
Saccharomyces_cerevisiae - ps31
p-value
2e-04
7e-04
3e-03
1e-02
6e-02
Log-odds
0.5
1.0
1.5
2.0
2.5
3.0
3.5
4.0

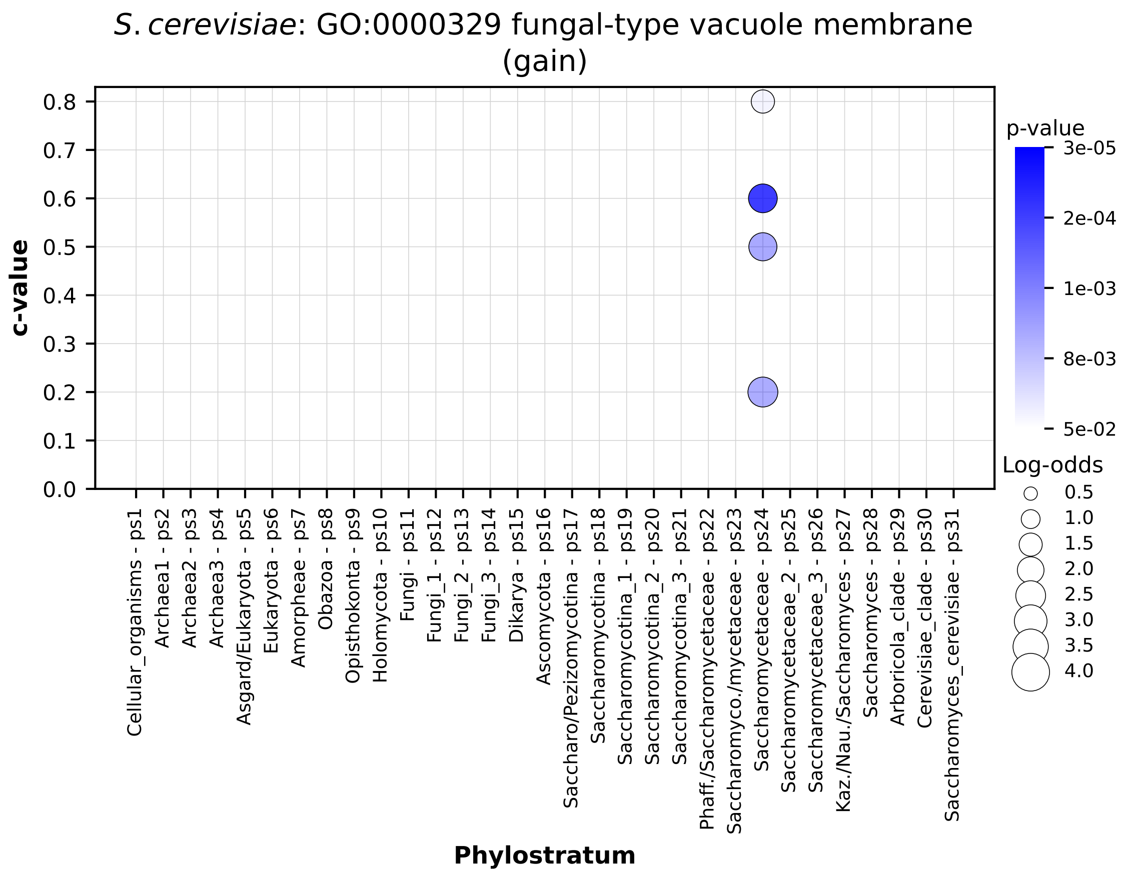
S. cerevisiae: GO:0000329 fungal-type vacuole membrane (gain)
c-value
0.0
0.1
0.2
0.3
0.4
0.5
0.6
0.7
0.8
p-value
3e-05
2e-04
1e-03
8e-03
5e-02
Log-odds
0.5
1.0
1.5
2.0
2.5
3.0
3.5
4.0
Cellular_organisms - ps1
Archaea1 - ps2
Archaea2 - ps3
Archaea3 - ps4
Asgard/Eukaryota - ps5
Eukaryota - ps6
Amorpheae - ps7
Obazoa - ps8
Opisthokonta - ps9
Holomycota - ps10
Fungi - ps11
Fungi_1 - ps12
Fungi_2 - ps13
Fungi_3 - ps14
Dikarya - ps15
Ascomycota - ps16
Saccharo/Pezizomycotina - ps17
Saccharomycotina - ps18
Saccharomycotina_1 - ps19
Saccharomycotina_2 - ps20
Saccharomycotina_3 - ps21
Phaff./Saccharomycetaceae - ps22
Saccharomyco./mycetaceae - ps23
Saccharomycetaceae - ps24
Saccharomycetaceae_2 - ps25
Saccharomycetaceae_3 - ps26
Kaz./Nau./Saccharomyces - ps27
Saccharomyces - ps28
Arboricola_clade - ps29
Cerevisiae_clade - ps30
Saccharomyces_cerevisiae - ps31
Phylostratum

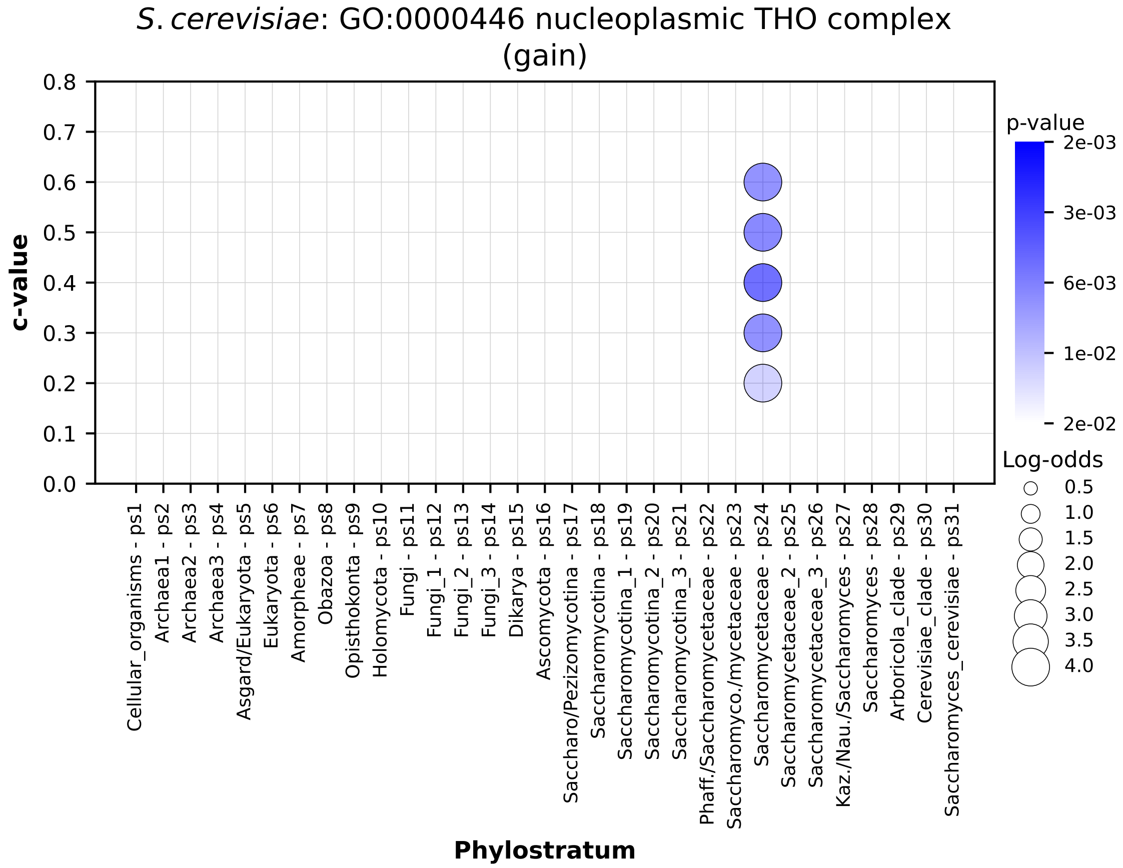
S. cerevisiae: GO:0000446 nucleoplasmic THO complex
(gain)
c-value
0.8
0.7
0.6
0.5
0.4
0.3
0.2
0.1
0.0
p-value
2e-03
3e-03
6e-03
1e-02
2e-02
Log-odds
0.5
1.0
1.5
2.0
2.5
3.0
3.5
4.0
Cellular_organisms - ps1
Archaea1 - ps2
Archaea2 - ps3
Archaea3 - ps4
Asgard/Eukaryota - ps5
Eukaryota - ps6
Amorpheae - ps7
Obazoa - ps8
Opisthokonta - ps9
Holomycota - ps10
Fungi - ps11
Fungi_1 - ps12
Fungi_2 - ps13
Fungi_3 - ps14
Dikarya - ps15
Ascomycota - ps16
Saccharo/Pezizomycotina - ps17
Saccharomycotina - ps18
Saccharomycotina_1 - ps19
Saccharomycotina_2 - ps20
Saccharomycotina_3 - ps21
Phaff./Saccharomycetaceae - ps22
Saccharomyco./mycetaceae - ps23
Saccharomycetaceae - ps24
Saccharomycetaceae_2 - ps25
Saccharomycetaceae_3 - ps26
Kaz./Nau./Saccharomyces - ps27
Saccharomyces - ps28
Arboricola_clade - ps29
Cerevisiae_clade - ps30
Saccharomyces_cerevisiae - ps31
Phylostratum

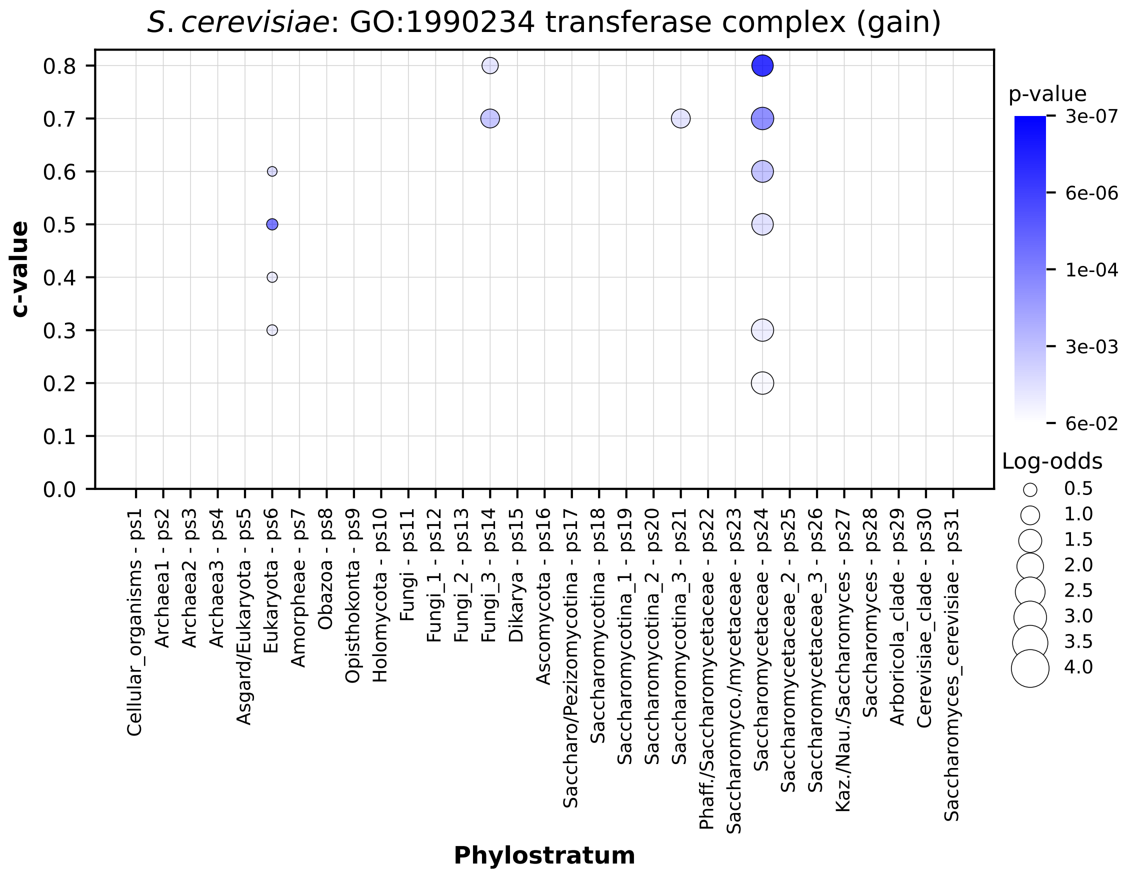

S. cerevisiae: GO:1990234 transferase complex (gain)
c-value
0.0
0.1
0.2
0.3
0.4
0.5
0.6
0.7
0.8
Phylostratum
Cellular_organisms - ps1
Archaea1 - ps2
Archaea2 - ps3
Archaea3 - ps4
Asgard/Eukaryota - ps5
Eukaryota - ps6
Amorpheae - ps7
Obazoa - ps8
Opisthokonta - ps9
Holomycota - ps10
Fungi - ps11
Fungi_1 - ps12
Fungi_2 - ps13
Fungi_3 - ps14
Dikarya - ps15
Ascomycota - ps16
Saccharo/Pezizomycotina - ps17
Saccharomycotina - ps18
[truncated: 513,142 more chars]
