# Supplementary material for: Macroevolutionary dynamics of gene family gain and loss along multicellular eukaryotic lineages
Source: Nat Commun. 2024 Mar 26;15:2663. doi: 10.1038/s41467-024-47017-w (PMC10966110; doi:10.1038/s41467-024-47017-w)
Supplement: Supplementary file 14 — Supplementary Dataset 9 [file 41467_2024_47017_MOESM14_ESM.pdf]

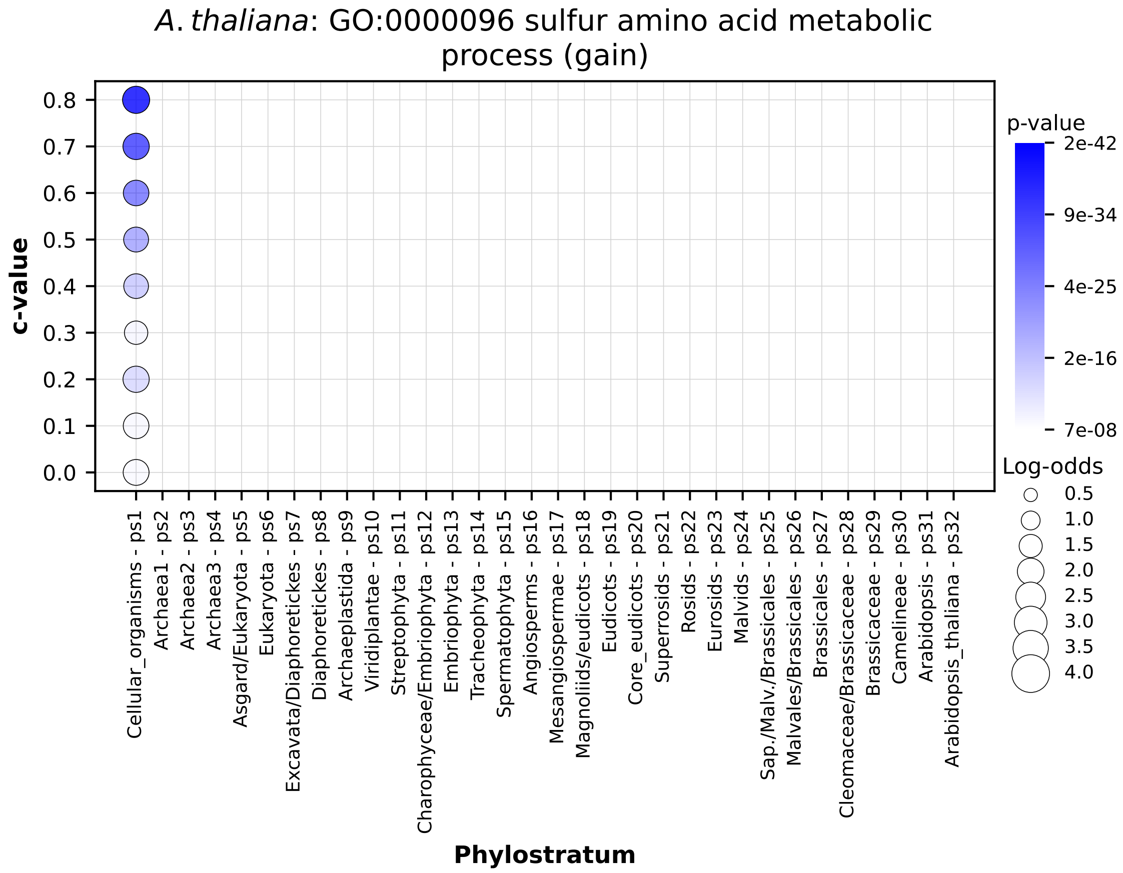

A. thaliana: GO:0000096 sulfur amino acid metabolic process (gain)
c-value
0.8
0.7
0.6
0.5
0.4
0.3
0.2
0.1
0.0
p-value
2e-42
9e-34
4e-25
2e-16
7e-08
Log-odds
0.5
1.0
1.5
2.0
2.5
3.0
3.5
4.0
Cellular_organisms - ps1
Archaea1 - ps2
Archaea2 - ps3
Archaea3 - ps4
Asgard/Eukaryota - ps5
Eukaryota - ps6
Excavata/Diaphoretickes - ps7
Diaphoretickes - ps8
Archaeplastida - ps9
Viridiplantae - ps10
Streptophyta - ps11
Charophyceae/Embriophyta - ps12
Embriophyta - ps13
Tracheophyta - ps14
Spermatophyta - ps15
Angiosperms - ps16
Mesangiospermae - ps17
Magnoliids/eudicots - ps18
Eudicots - ps19
Core_eudicots - ps20
Superrosids - ps21
Rosids - ps22
Eurosids - ps23
Malvids - ps24
Sap./Malv./Brassicales - ps25
Malvales/Brassicales - ps26
Brassicales - ps27
Cleomaceae/Brassicaceae - ps28
Brassicaceae - ps29
Camelineae - ps30
Arabidopsis - ps31
Arabidopsis_thaliana - ps32
Phylostratum

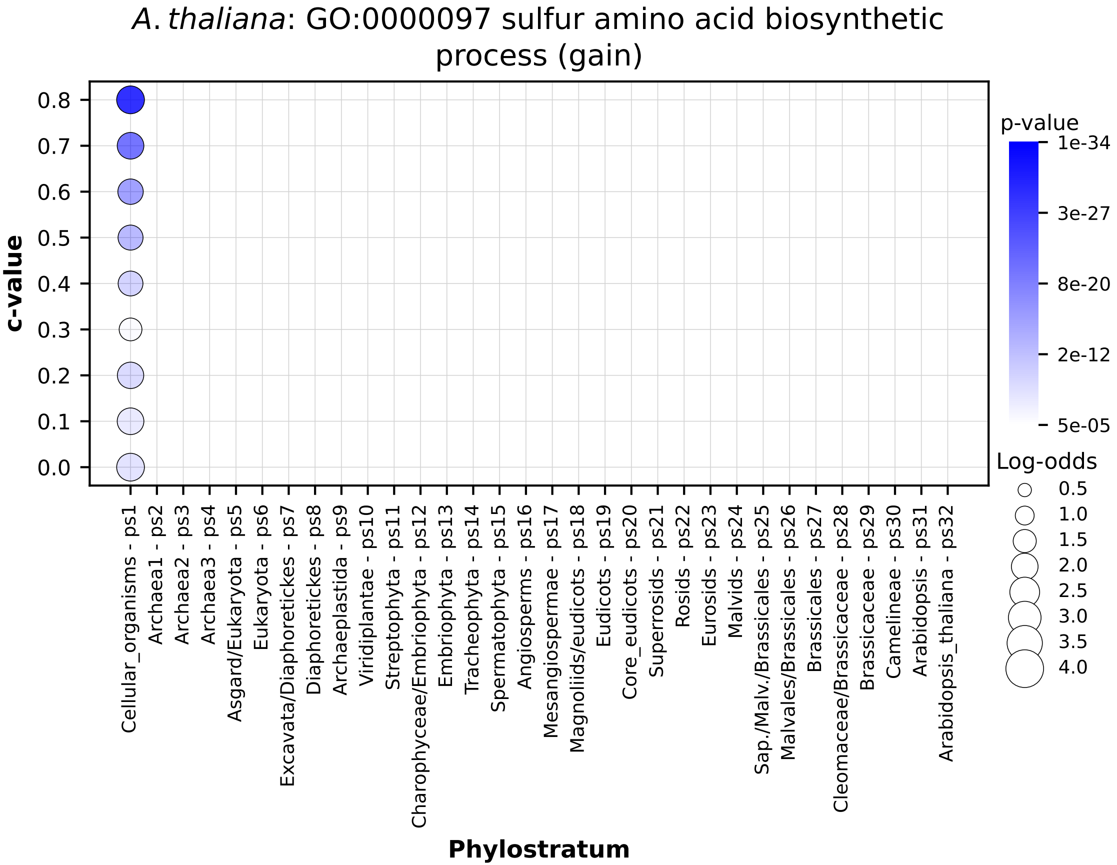
A. thaliana: GO:0000097 sulfur amino acid biosynthetic process (gain)
c-value
0.8
0.7
0.6
0.5
0.4
0.3
0.2
0.1
0.0
Phylostratum
Cellular_organisms - ps1
Archaea1 - ps2
Archaea2 - ps3
Archaea3 - ps4
Asgard/Eukaryota - ps5
Eukaryota - ps6
Excavata/Diaphoretickes - ps7
Diaphoretickes - ps8
Archaeplastida - ps9
Viridiplantae - ps10
Streptophyta - ps11
Charophyceae/Embriophyta - ps12
Embriophyta - ps13
Tracheophyta - ps14
Spermatophyta - ps15
Angiosperms - ps16
Mesangiospermae - ps17
Magnoliids/eudicots - ps18
Eudicots - ps19
Core_eudicots - ps20
Superrosids - ps21
Rosids - ps22
Eurosids - ps23
Malvids - ps24
Sap./Malv./Brassicales - ps25
Malvales/Brassicales - ps26
Brassicales - ps27
Cleomaceae/Brassicaceae - ps28
Brassicaceae - ps29
Camelineae - ps30
Arabidopsis - ps31
Arabidopsis_thaliana - ps32
p-value
1e-34
3e-27
8e-20
2e-12
5e-05
Log-odds
0.5
1.0
1.5
2.0
2.5
3.0
3.5
4.0

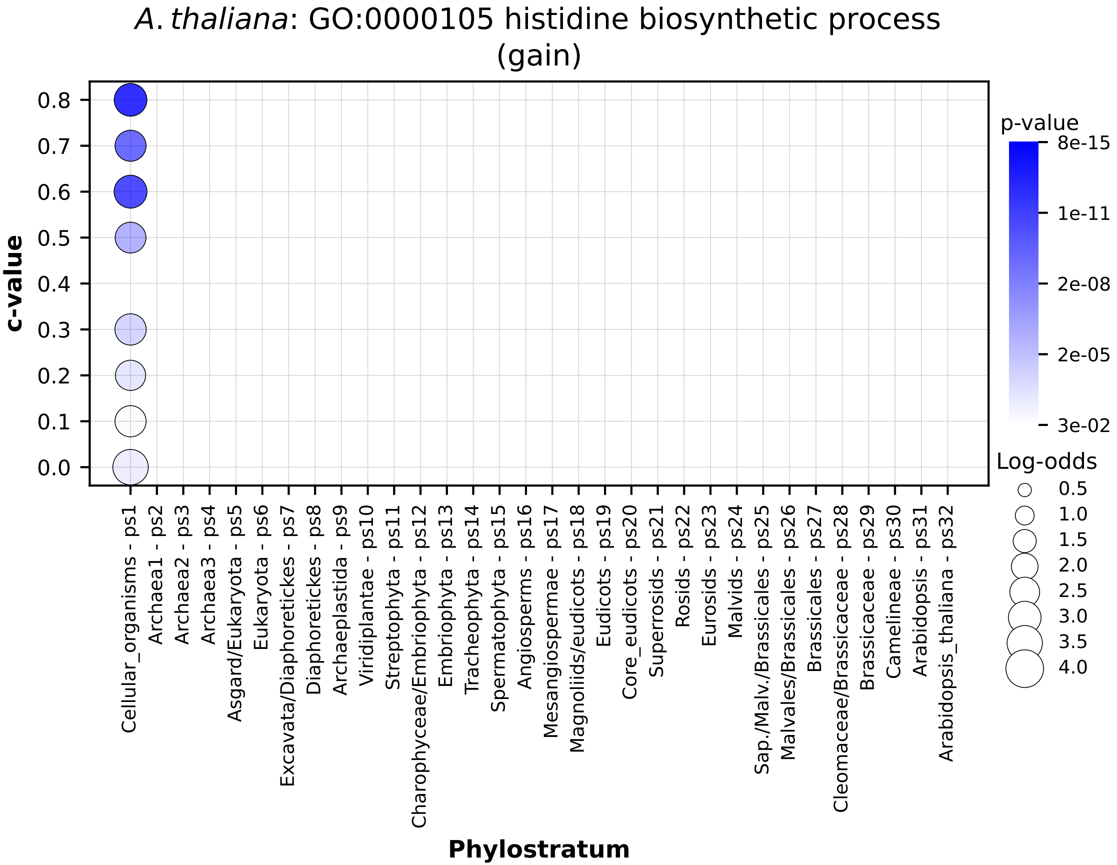

A. thaliana: GO:0000105 histidine biosynthetic process
(gain)
c-value
0.8
0.7
0.6
0.5
0.4
0.3
0.2
0.1
0.0
p-value
8e-15
1e-11
2e-08
2e-05
3e-02
Log-odds
0.5
1.0
1.5
2.0
2.5
3.0
3.5
4.0
Cellular_organisms - ps1
Archaea1 - ps2
Archaea2 - ps3
Archaea3 - ps4
Asgard/Eukaryota - ps5
Eukaryota - ps6
Excavata/Diaphoretickes - ps7
Diaphoretickes - ps8
Archaeplastida - ps9
Viridiplantae - ps10
Streptophyta - ps11
Charophyceae/Embriophyta - ps12
Embriophyta - ps13
Tracheophyta - ps14
Spermatophyta - ps15
Angiosperms - ps16
Mesangiospermae - ps17
Magnoliids/eudicots - ps18
Eudicots - ps19
Core_eudicots - ps20
Superrosids - ps21
Rosids - ps22
Eurosids - ps23
Malvids - ps24
Sap./Malv./Brassicales - ps25
Malvales/Brassicales - ps26
Brassicales - ps27
Cleomaceae/Brassicaceae - ps28
Brassicaceae - ps29
Camelineae - ps30
Arabidopsis - ps31
Arabidopsis_thaliana - ps32
Phylostratum

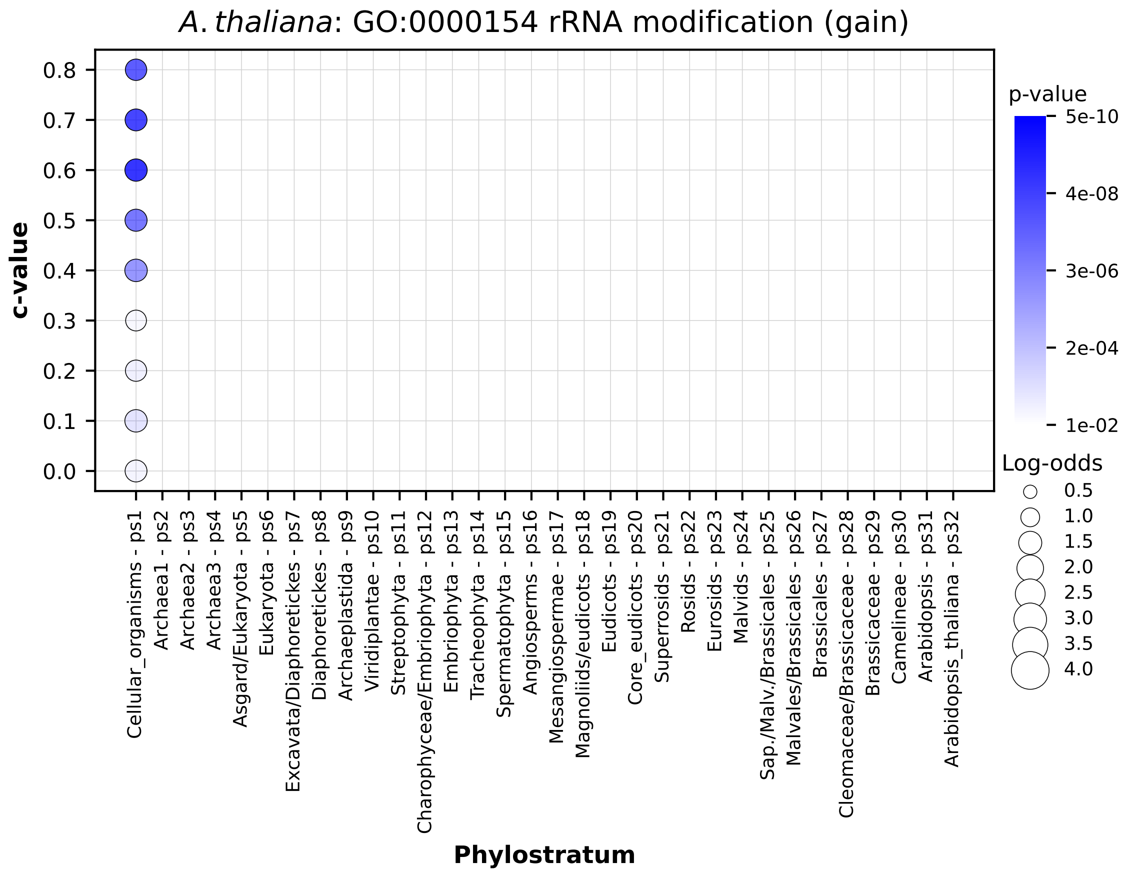

A. thaliana: GO:0000154 rRNA modification (gain)
c-value
0.0
0.1
0.2
0.3
0.4
0.5
0.6
0.7
0.8
p-value
5e-10
4e-08
3e-06
2e-04
1e-02
Log-odds
0.5
1.0
1.5
2.0
2.5
3.0
3.5
4.0
Cellular_organisms - ps1
Archaea1 - ps2
Archaea2 - ps3
Archaea3 - ps4
Asgard/Eukaryota - ps5
Eukaryota - ps6
Excavata/Diaphoretickes - ps7
Diaphoretickes - ps8
Archaeplastida - ps9
Viridiplantae - ps10
Streptophyta - ps11
Charophyceae/Embriophyta - ps12
Embriophyta - ps13
Tracheophyta - ps14
Spermatophyta - ps15
Angiosperms - ps16
Mesangiospermae - ps17
Magnoliids/eudicots - ps18
Eudicots - ps19
Core_eudicots - ps20
Superrosids - ps21
Rosids - ps22
Eurosids - ps23
Malvids - ps24
Sap./Malv./Brassicales - ps25
Malvales/Brassicales - ps26
Brassicales - ps27
Cleomaceae/Brassicaceae - ps28
Brassicaceae - ps29
Camelineae - ps30
Arabidopsis - ps31
Arabidopsis_thaliana - ps32
Phylostratum

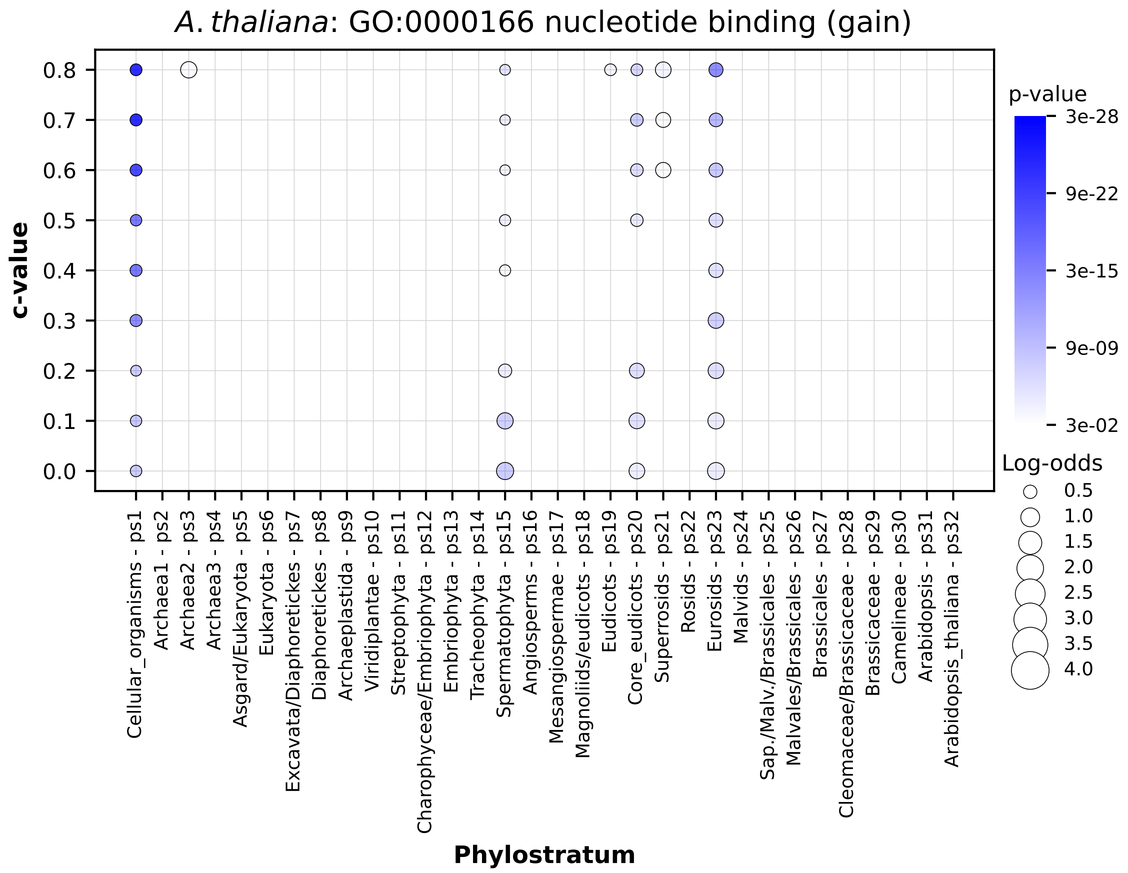
A. thaliana: GO:0000166 nucleotide binding (gain)
c-value
0.8
0.7
0.6
0.5
0.4
0.3
0.2
0.1
0.0
Phylostratum
Cellular_organisms - ps1
Archaea1 - ps2
Archaea2 - ps3
Archaea3 - ps4
Asgard/Eukaryota - ps5
Eukaryota - ps6
Excavata/Diaphoretickes - ps7
Diaphoretickes - ps8
Archaeplastida - ps9
Viridiplantae - ps10
Streptophyta - ps11
Charophyceae/Embriophyta - ps12
Embriophyta - ps13
Tracheophyta - ps14
Spermatophyta - ps15
Angiosperms - ps16
Mesangiospermae - ps17
Magnoliids/eudicots - ps18
Eudicots - ps19
Core_eudicots - ps20
Superrosids - ps21
Rosids - ps22
Eurosids - ps23
Malvids - ps24
Sap./Malv./Brassicales - ps25
Malvales/Brassicales - ps26
Brassicales - ps27
Cleomaceae/Brassicaceae - ps28
Brassicaceae - ps29
Camelineae - ps30
Arabidopsis - ps31
Arabidopsis_thaliana - ps32
p-value
3e-28
9e-22
3e-15
9e-09
3e-02
Log-odds
0.5
1.0
1.5
2.0
2.5
3.0
3.5
4.0

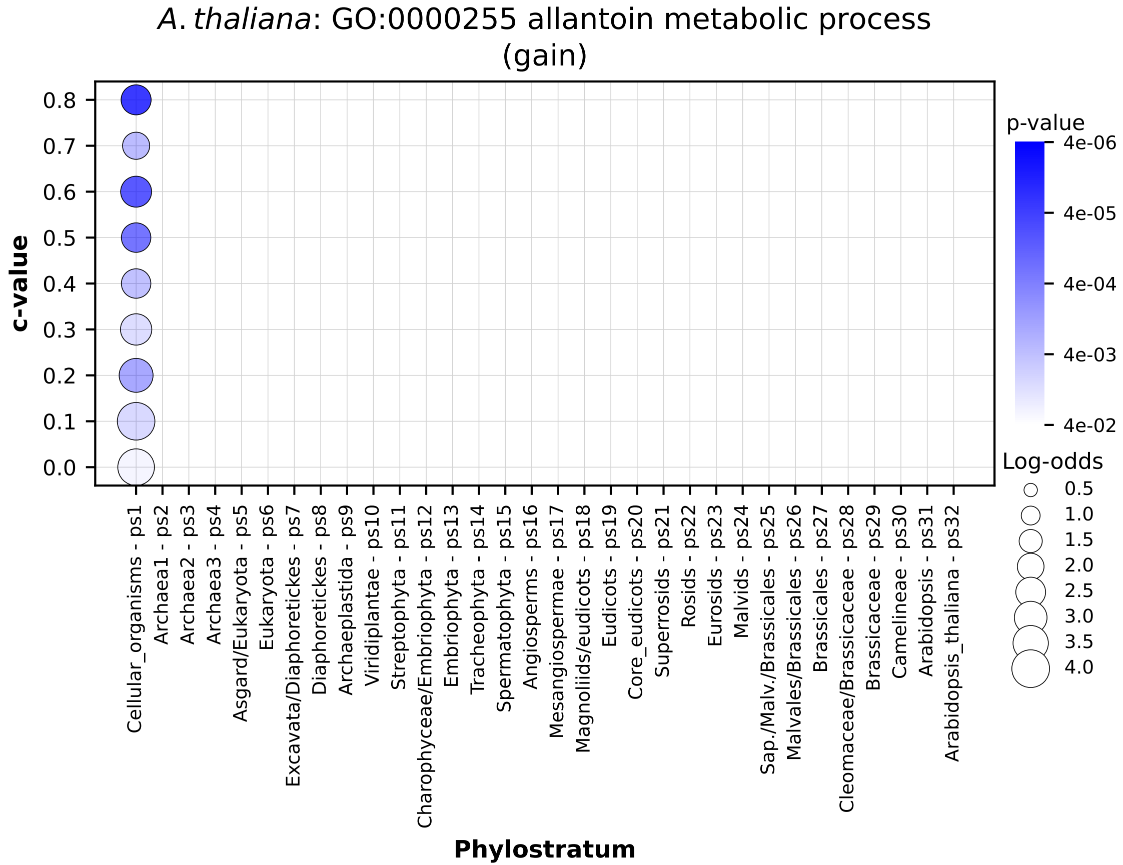

A. thaliana: GO:0000255 allantoin metabolic process
(gain)
c-value
0.8
0.7
0.6
0.5
0.4
0.3
0.2
0.1
0.0
p-value
4e-06
4e-05
4e-04
4e-03
4e-02
Log-odds
0.5
1.0
1.5
2.0
2.5
3.0
3.5
4.0
Cellular_organisms - ps1
Archaea1 - ps2
Archaea2 - ps3
Archaea3 - ps4
Asgard/Eukaryota - ps5
Eukaryota - ps6
Excavata/Diaphoretickes - ps7
Diaphoretickes - ps8
Archaeplastida - ps9
Viridiplantae - ps10
Streptophyta - ps11
Charophyceae/Embriophyta - ps12
Embriophyta - ps13
Tracheophyta - ps14
Spermatophyta - ps15
Angiosperms - ps16
Mesangiospermae - ps17
Magnoliids/eudicots - ps18
Eudicots - ps19
Core_eudicots - ps20
Superrosids - ps21
Rosids - ps22
Eurosids - ps23
Malvids - ps24
Sap./Malv./Brassicales - ps25
Malvales/Brassicales - ps26
Brassicales - ps27
Cleomaceae/Brassicaceae - ps28
Brassicaceae - ps29
Camelineae - ps30
Arabidopsis - ps31
Arabidopsis_thaliana - ps32
Phylostratum

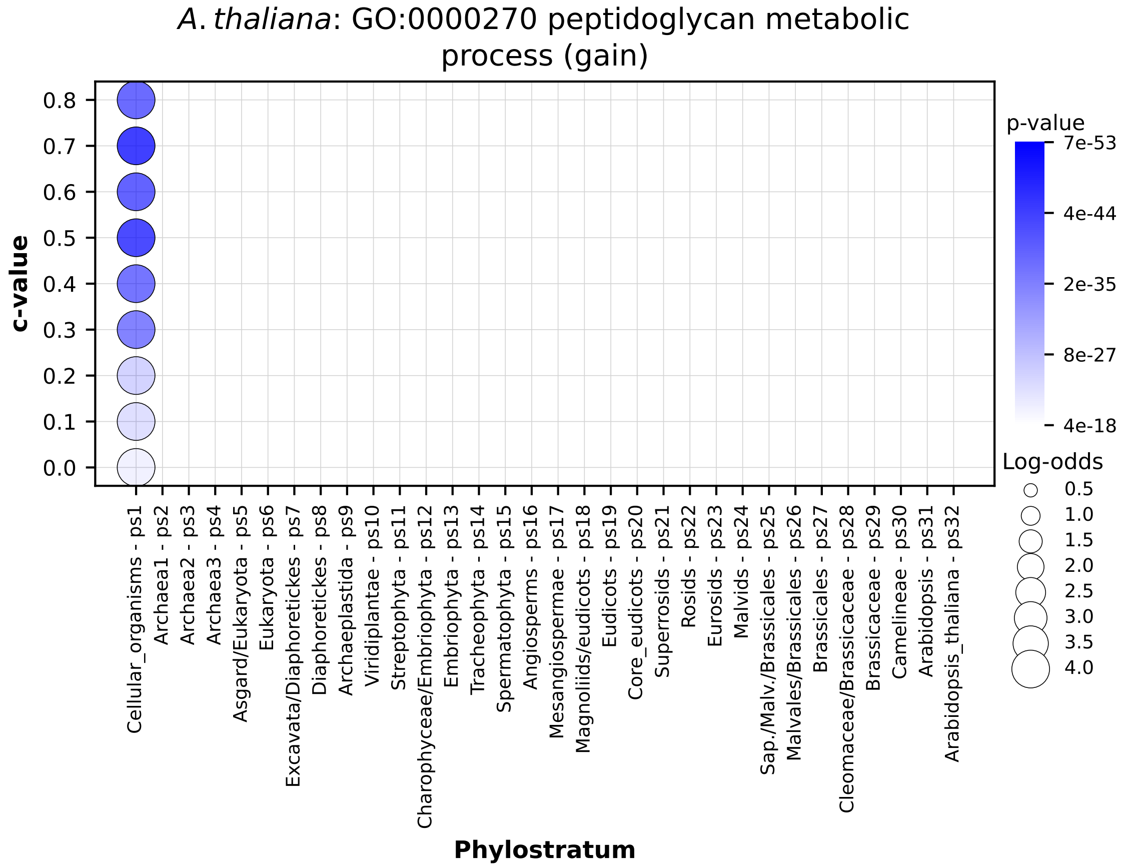

A. thaliana: GO:0000270 peptidoglycan metabolic process (gain)
c-value
0.0
0.1
0.2
0.3
0.4
0.5
0.6
0.7
0.8
p-value
7e-53
4e-44
2e-35
8e-27
4e-18
Log-odds
0.5
1.0
1.5
2.0
2.5
3.0
3.5
4.0
Cellular_organisms - ps1
Archaea1 - ps2
Archaea2 - ps3
Archaea3 - ps4
Asgard/Eukaryota - ps5
Eukaryota - ps6
Excavata/Diaphoretickes - ps7
Diaphoretickes - ps8
Archaeplastida - ps9
Viridiplantae - ps10
Streptophyta - ps11
Charophyceae/Embriophyta - ps12
Embriophyta - ps13
Tracheophyta - ps14
Spermatophyta - ps15
Angiosperms - ps16
Mesangiospermae - ps17
Magnoliids/eudicots - ps18
Eudicots - ps19
Core_eudicots - ps20
Superrosids - ps21
Rosids - ps22
Eurosids - ps23
Malvids - ps24
Sap./Malv./Brassicales - ps25
Malvales/Brassicales - ps26
Brassicales - ps27
Cleomaceae/Brassicaceae - ps28
Brassicaceae - ps29
Camelineae - ps30
Arabidopsis - ps31
Arabidopsis_thaliana - ps32
Phylostratum

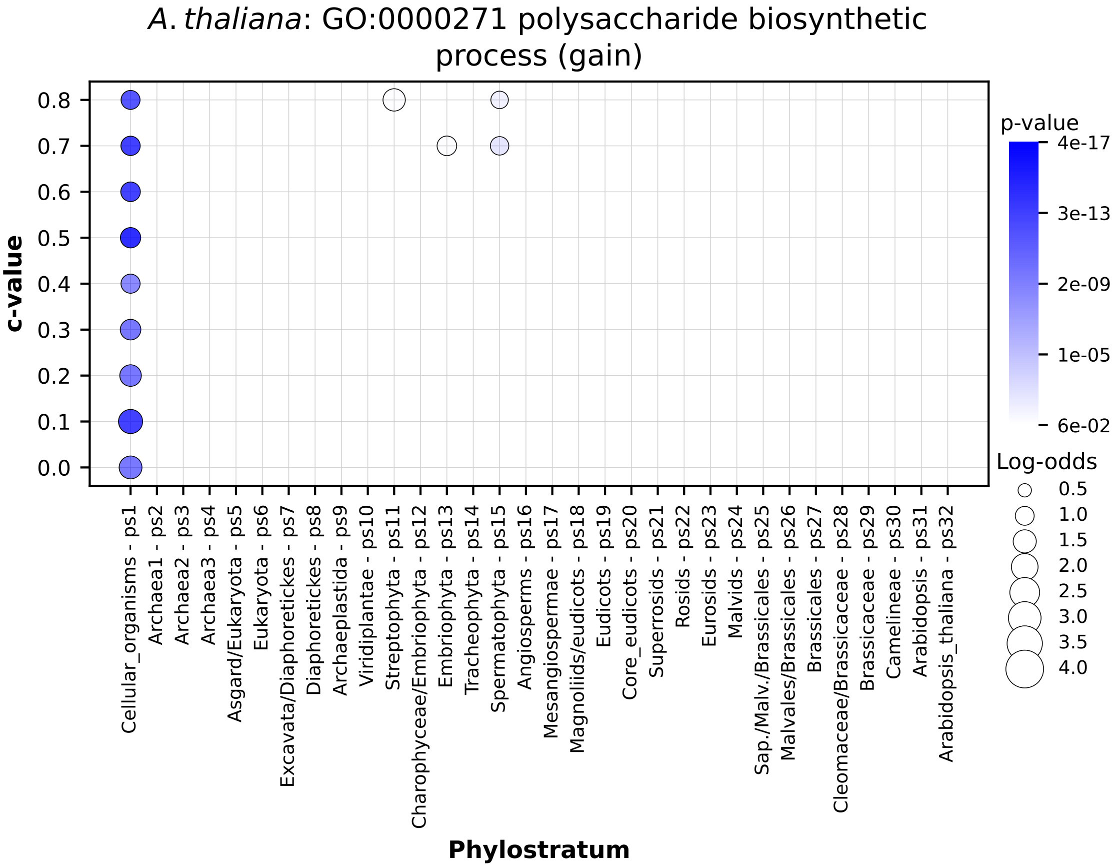

A. thaliana: GO:0000271 polysaccharide biosynthetic process (gain)
c-value
0.8
0.7
0.6
0.5
0.4
0.3
0.2
0.1
0.0
p-value
4e-17
3e-13
2e-09
1e-05
6e-02
Log-odds
0.5
1.0
1.5
2.0
2.5
3.0
3.5
4.0
Cellular_organisms - ps1
Archaea1 - ps2
Archaea2 - ps3
Archaea3 - ps4
Asgard/Eukaryota - ps5
Eukaryota - ps6
Excavata/Diaphoretickes - ps7
Diaphoretickes - ps8
Archaeplastida - ps9
Viridiplantae - ps10
Streptophyta - ps11
Charophyceae/Embriophyta - ps12
Embriophyta - ps13
Tracheophyta - ps14
Spermatophyta - ps15
Angiosperms - ps16
Mesangiospermae - ps17
Magnoliids/eudicots - ps18
Eudicots - ps19
Core_eudicots - ps20
Superrosids - ps21
Rosids - ps22
Eurosids - ps23
Malvids - ps24
Sap./Malv./Brassicales - ps25
Malvales/Brassicales - ps26
Brassicales - ps27
Cleomaceae/Brassicaceae - ps28
Brassicaceae - ps29
Camelineae - ps30
Arabidopsis - ps31
Arabidopsis_thaliana - ps32
Phylostratum

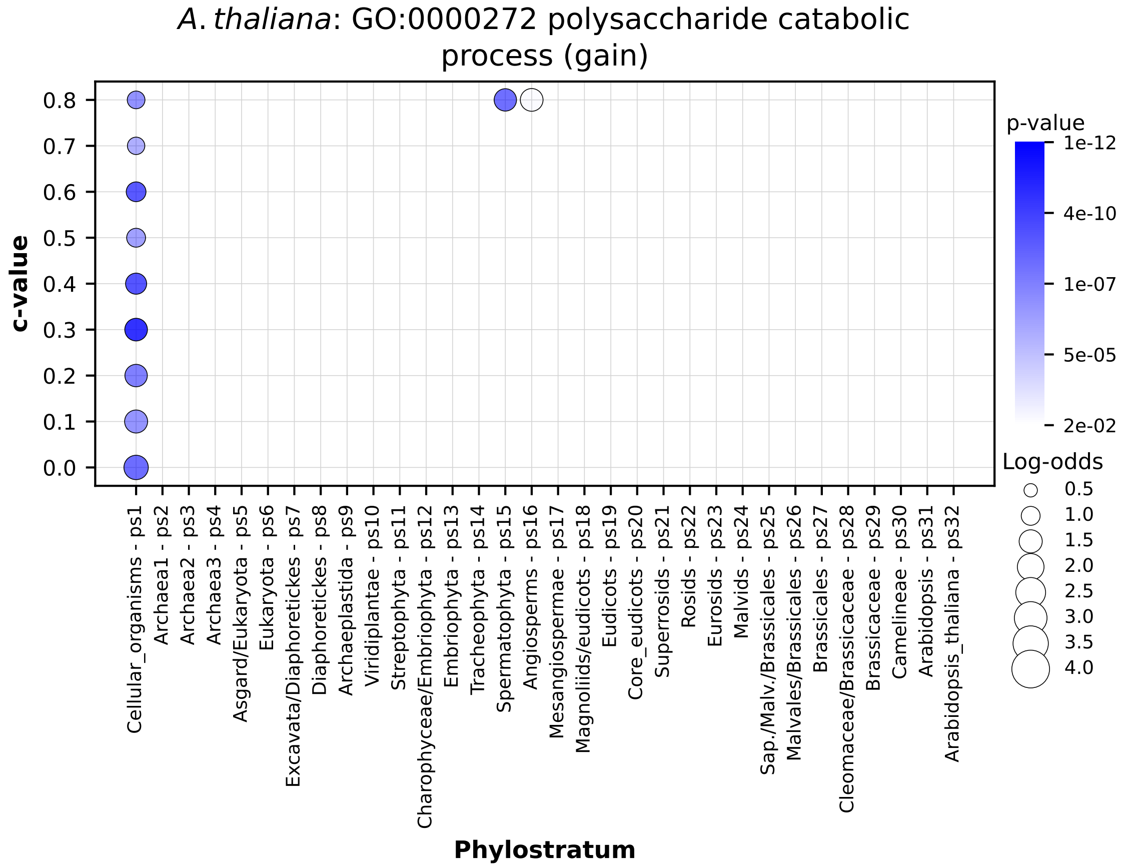

A. thaliana: GO:0000272 polysaccharide catabolic process (gain)
c-value
0.8
0.7
0.6
0.5
0.4
0.3
0.2
0.1
0.0
p-value
1e-12
4e-10
1e-07
5e-05
2e-02
Log-odds
0.5
1.0
1.5
2.0
2.5
3.0
3.5
4.0
Cellular_organisms - ps1
Archaea1 - ps2
Archaea2 - ps3
Archaea3 - ps4
Asgard/Eukaryota - ps5
Eukaryota - ps6
Excavata/Diaphoretickes - ps7
Diaphoretickes - ps8
Archaeplastida - ps9
Viridiplantae - ps10
Streptophyta - ps11
Charophyceae/Embriophyta - ps12
Embriophyta - ps13
Tracheophyta - ps14
Spermatophyta - ps15
Angiosperms - ps16
Mesangiospermae - ps17
Magnoliids/eudicots - ps18
Eudicots - ps19
Core_eudicots - ps20
Superrosids - ps21
Rosids - ps22
Eurosids - ps23
Malvids - ps24
Sap./Malv./Brassicales - ps25
Malvales/Brassicales - ps26
Brassicales - ps27
Cleomaceae/Brassicaceae - ps28
Brassicaceae - ps29
Camelineae - ps30
Arabidopsis - ps31
Arabidopsis_thaliana - ps32
Phylostratum

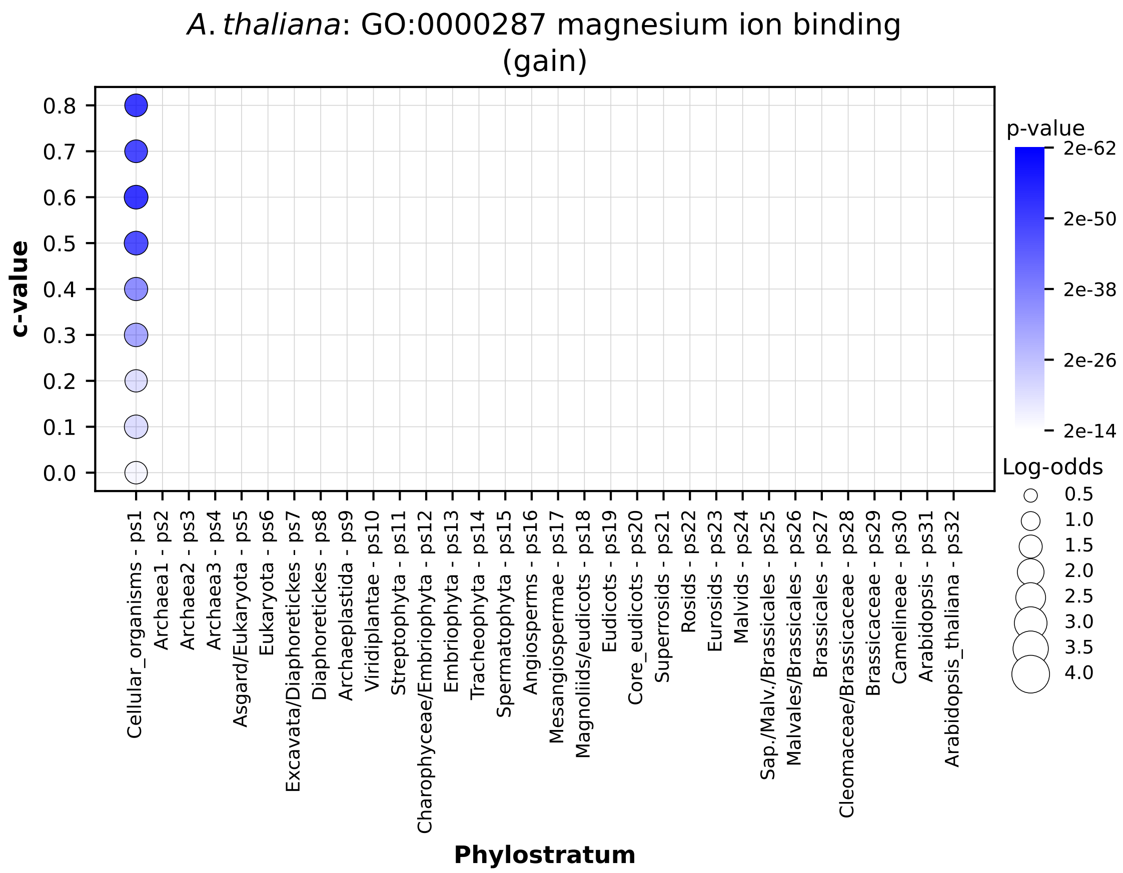

A. thaliana: GO:0000287 magnesium ion binding
(gain)
c-value
0.8
0.7
0.6
0.5
0.4
0.3
0.2
0.1
0.0
p-value
2e-62
2e-50
2e-38
2e-26
2e-14
Log-odds
0.5
1.0
1.5
2.0
2.5
3.0
3.5
4.0
Cellular_organisms - ps1
Archaea1 - ps2
Archaea2 - ps3
Archaea3 - ps4
Asgard/Eukaryota - ps5
Eukaryota - ps6
Excavata/Diaphoretickes - ps7
Diaphoretickes - ps8
Archaeplastida - ps9
Viridiplantae - ps10
Streptophyta - ps11
Charophyceae/Embriophyta - ps12
Embriophyta - ps13
Tracheophyta - ps14
Spermatophyta - ps15
Angiosperms - ps16
Mesangiospermae - ps17
Magnoliids/eudicots - ps18
Eudicots - ps19
Core_eudicots - ps20
Superrosids - ps21
Rosids - ps22
Eurosids - ps23
Malvids - ps24
Sap./Malv./Brassicales - ps25
Malvales/Brassicales - ps26
Brassicales - ps27
Cleomaceae/Brassicaceae - ps28
Brassicaceae - ps29
Camelineae - ps30
Arabidopsis - ps31
Arabidopsis_thaliana - ps32
Phylostratum

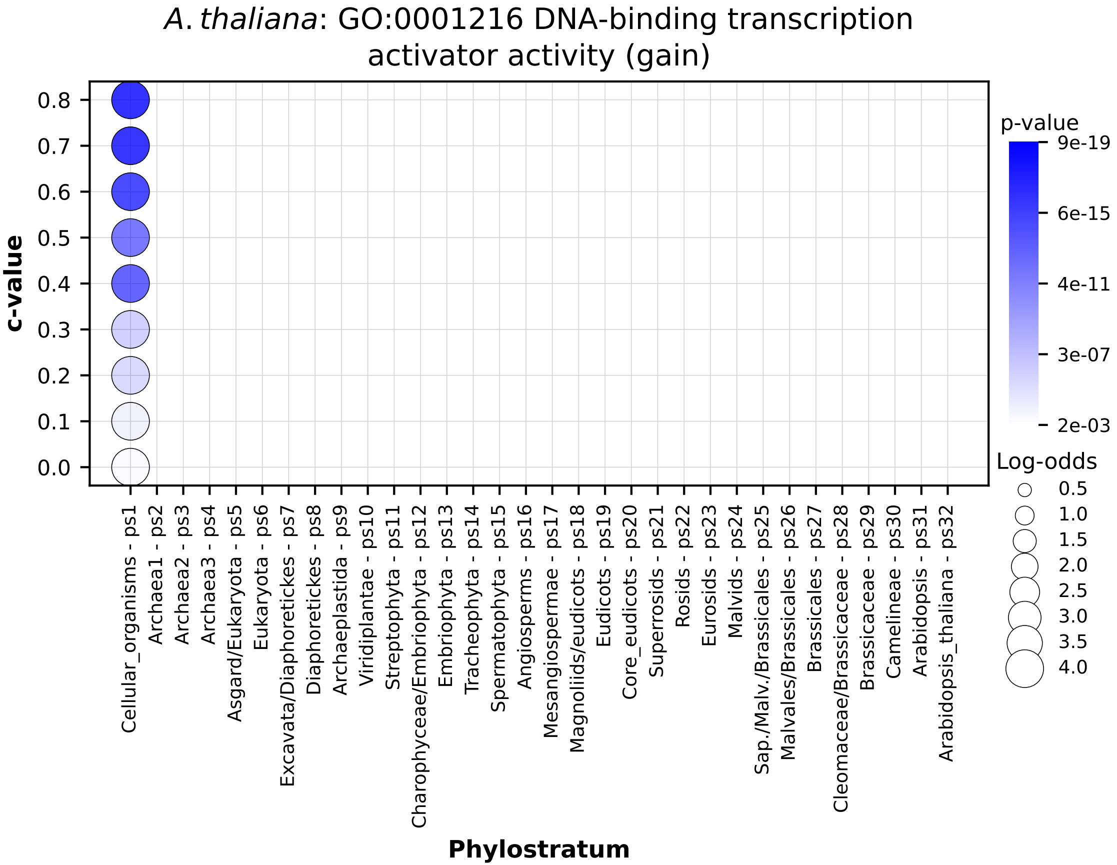

A. thaliana: GO:0001216 DNA-binding transcription activator activity (gain)
c-value
0.0
0.1
0.2
0.3
0.4
0.5
0.6
0.7
0.8
p-value
9e-19
6e-15
4e-11
3e-07
2e-03
Log-odds
0.5
1.0
1.5
2.0
2.5
3.0
3.5
4.0
Cellular_organisms - ps1
Archaea1 - ps2
Archaea2 - ps3
Archaea3 - ps4
Asgard/Eukaryota - ps5
Eukaryota - ps6
Excavata/Diaphoretickes - ps7
Diaphoretickes - ps8
Archaeplastida - ps9
Viridiplantae - ps10
Streptophyta - ps11
Charophyceae/Embriophyta - ps12
Embriophyta - ps13
Tracheophyta - ps14
Spermatophyta - ps15
Angiosperms - ps16
Mesangiospermae - ps17
Magnoliids/eudicots - ps18
Eudicots - ps19
Core_eudicots - ps20
Superrosids - ps21
Rosids - ps22
Eurosids - ps23
Malvids - ps24
Sap./Malv./Brassicales - ps25
Malvales/Brassicales - ps26
Brassicales - ps27
Cleomaceae/Brassicaceae - ps28
Brassicaceae - ps29
Camelineae - ps30
Arabidopsis - ps31
Arabidopsis_thaliana - ps32
Phylostratum

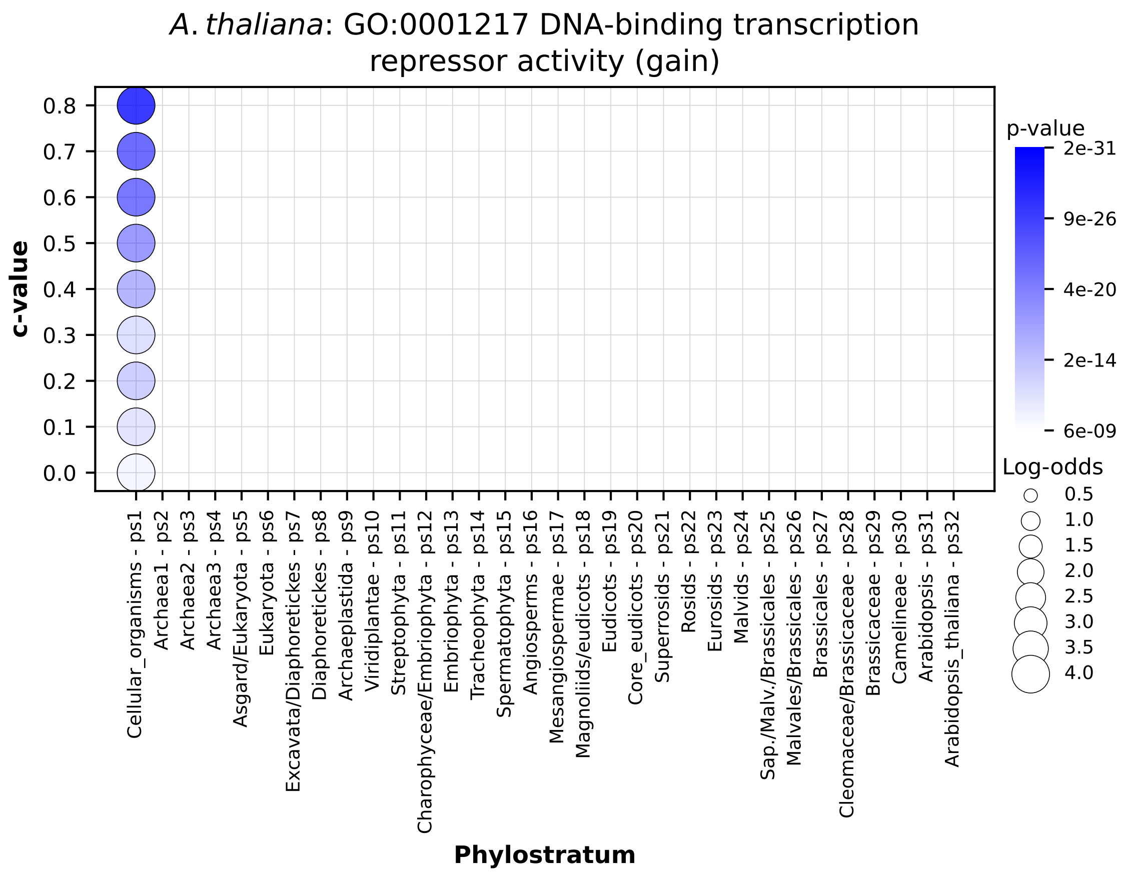

A. thaliana: GO:0001217 DNA-binding transcription repressor activity (gain)
c-value
0.8
0.7
0.6
0.5
0.4
0.3
0.2
0.1
0.0
p-value
2e-31
9e-26
4e-20
2e-14
6e-09
Log-odds
0.5
1.0
1.5
2.0
2.5
3.0
3.5
4.0
Cellular_organisms - ps1
Archaea1 - ps2
Archaea2 - ps3
Archaea3 - ps4
Asgard/Eukaryota - ps5
Eukaryota - ps6
Excavata/Diaphoretickes - ps7
Diaphoretickes - ps8
Archaeplastida - ps9
Viridiplantae - ps10
Streptophyta - ps11
Charophyceae/Embriophyta - ps12
Embriophyta - ps13
Tracheophyta - ps14
Spermatophyta - ps15
Angiosperms - ps16
Mesangiospermae - ps17
Magnoliids/eudicots - ps18
Eudicots - ps19
Core_eudicots - ps20
Superrosids - ps21
Rosids - ps22
Eurosids - ps23
Malvids - ps24
Sap./Malv./Brassicales - ps25
Malvales/Brassicales - ps26
Brassicales - ps27
Cleomaceae/Brassicaceae - ps28
Brassicaceae - ps29
Camelineae - ps30
Arabidopsis - ps31
Arabidopsis_thaliana - ps32
Phylostratum

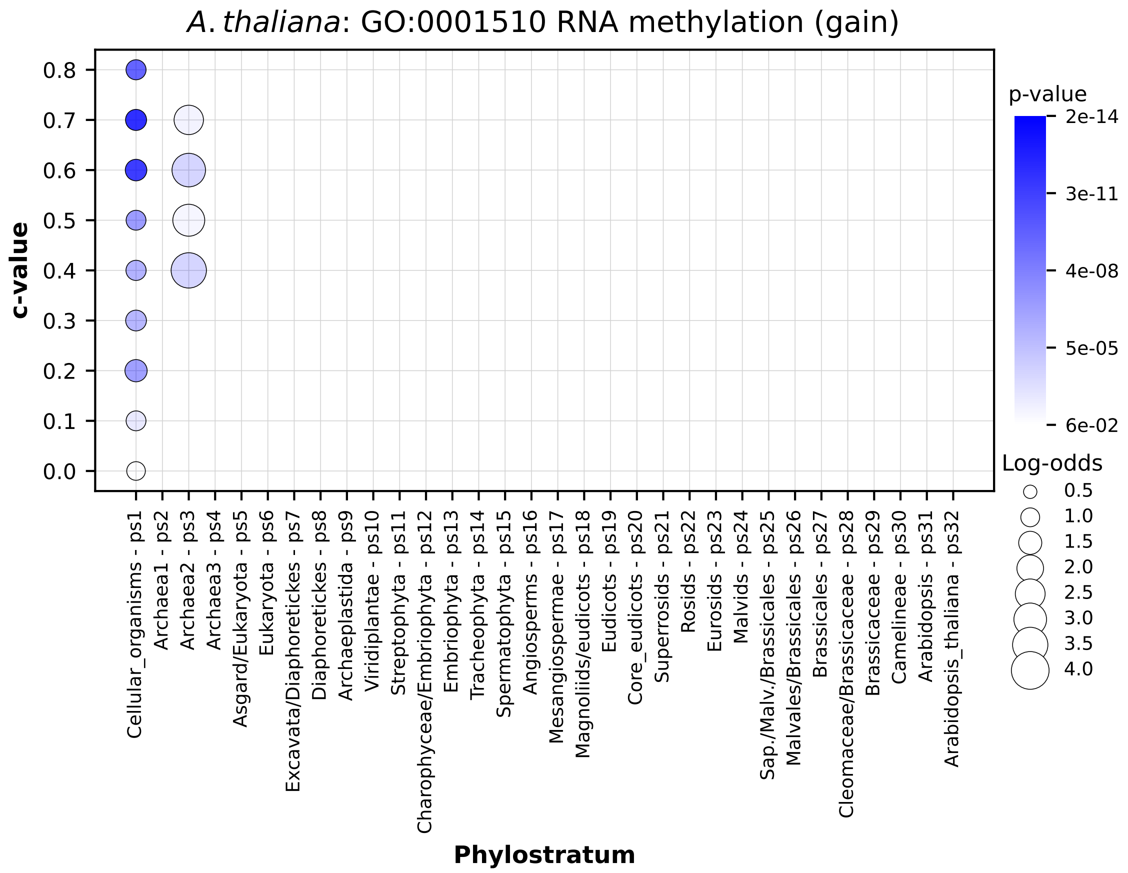
A. thaliana: GO:0001510 RNA methylation (gain)
c-value
0.8
0.7
0.6
0.5
0.4
0.3
0.2
0.1
0.0
p-value
2e-14
3e-11
4e-08
5e-05
6e-02
Log-odds
0.5
1.0
1.5
2.0
2.5
3.0
3.5
4.0
Cellular_organisms - ps1
Archaea1 - ps2
Archaea2 - ps3
Archaea3 - ps4
Asgard/Eukaryota - ps5
Eukaryota - ps6
Excavata/Diaphoretickes - ps7
Diaphoretickes - ps8
Archaeplastida - ps9
Viridiplantae - ps10
Streptophyta - ps11
Charophyceae/Embriophyta - ps12
Embriophyta - ps13
Tracheophyta - ps14
Spermatophyta - ps15
Angiosperms - ps16
Mesangiospermae - ps17
Magnoliids/eudicots - ps18
Eudicots - ps19
Core_eudicots - ps20
Superrosids - ps21
Rosids - ps22
Eurosids - ps23
Malvids - ps24
Sap./Malv./Brassicales - ps25
Malvales/Brassicales - ps26
Brassicales - ps27
Cleomaceae/Brassicaceae - ps28
Brassicaceae - ps29
Camelineae - ps30
Arabidopsis - ps31
Arabidopsis_thaliana - ps32
Phylostratum

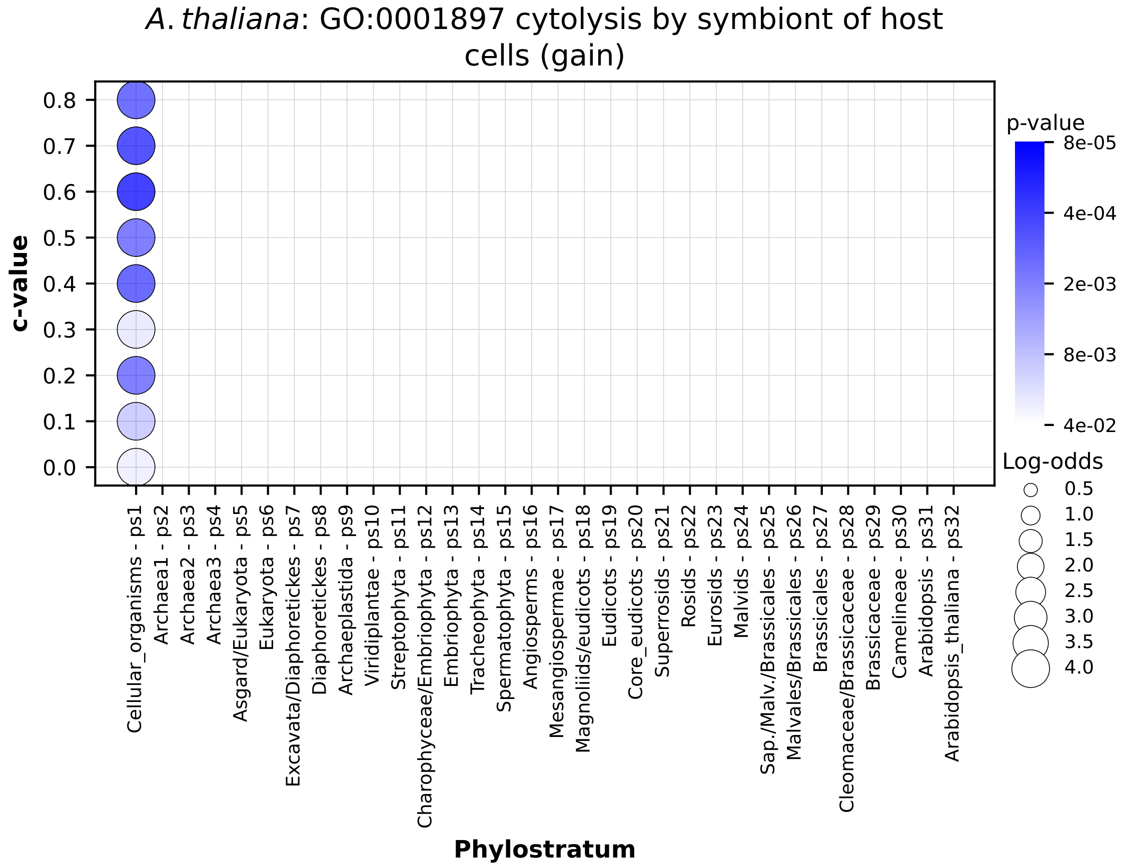
A. thaliana: GO:0001897 cytolysis by symbiont of host cells (gain)
c-value
0.8
0.7
0.6
0.5
0.4
0.3
0.2
0.1
0.0
p-value
8e-05
4e-04
2e-03
8e-03
4e-02
Log-odds
0.5
1.0
1.5
2.0
2.5
3.0
3.5
4.0
Cellular_organisms - ps1
Archaea1 - ps2
Archaea2 - ps3
Archaea3 - ps4
Asgard/Eukaryota - ps5
Eukaryota - ps6
Excavata/Diaphoretickes - ps7
Diaphoretickes - ps8
Archaeplastida - ps9
Viridiplantae - ps10
Streptophyta - ps11
Charophyceae/Embriophyta - ps12
Embriophyta - ps13
Tracheophyta - ps14
Spermatophyta - ps15
Angiosperms - ps16
Mesangiospermae - ps17
Magnoliids/eudicots - ps18
Eudicots - ps19
Core_eudicots - ps20
Superrosids - ps21
Rosids - ps22
Eurosids - ps23
Malvids - ps24
Sap./Malv./Brassicales - ps25
Malvales/Brassicales - ps26
Brassicales - ps27
Cleomaceae/Brassicaceae - ps28
Brassicaceae - ps29
Camelineae - ps30
Arabidopsis - ps31
Arabidopsis_thaliana - ps32
Phylostratum

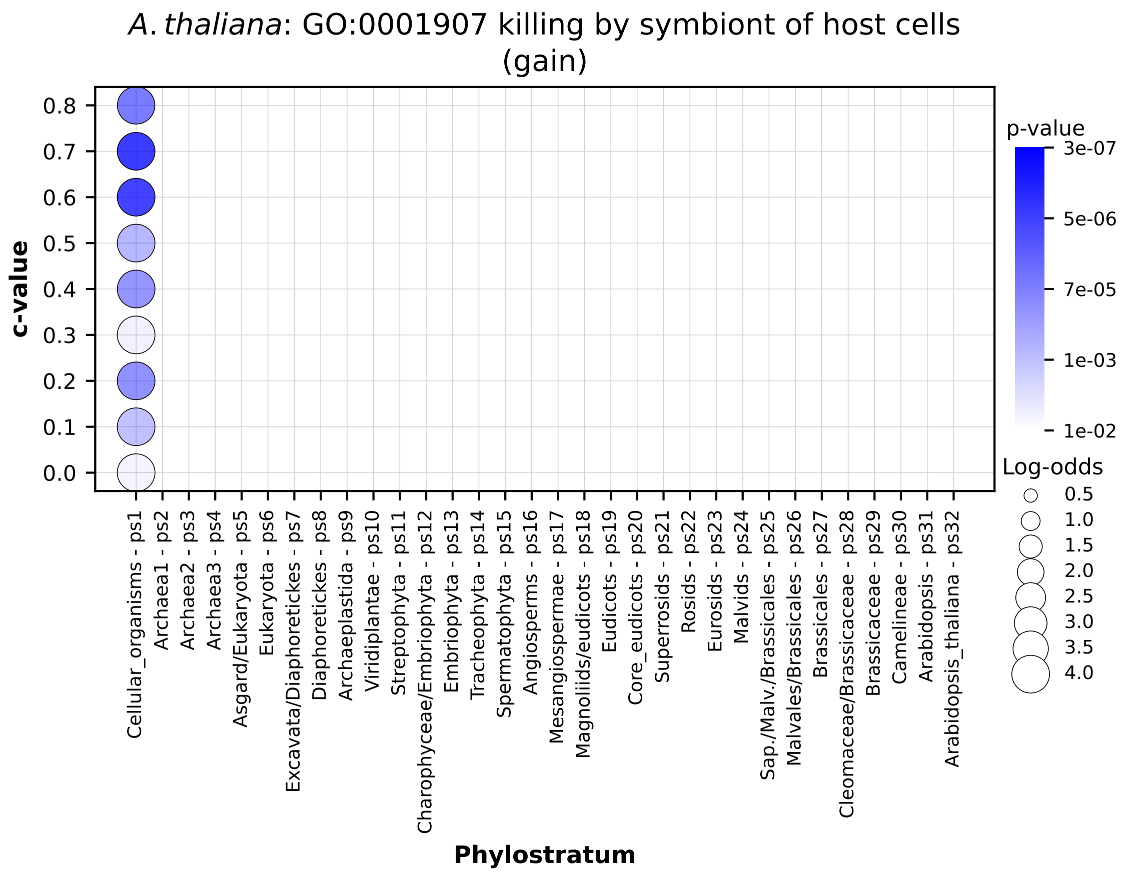
A. thaliana: GO:0001907 killing by symbiont of host cells
(gain)
c-value
0.8
0.7
0.6
0.5
0.4
0.3
0.2
0.1
0.0
p-value
3e-07
5e-06
7e-05
1e-03
1e-02
Log-odds
0.5
1.0
1.5
2.0
2.5
3.0
3.5
4.0
Cellular_organisms - ps1
Archaea1 - ps2
Archaea2 - ps3
Archaea3 - ps4
Asgard/Eukaryota - ps5
Eukaryota - ps6
Excavata/Diaphoretickes - ps7
Diaphoretickes - ps8
Archaeplastida - ps9
Viridiplantae - ps10
Streptophyta - ps11
Charophyceae/Embriophyta - ps12
Embriophyta - ps13
Tracheophyta - ps14
Spermatophyta - ps15
Angiosperms - ps16
Mesangiospermae - ps17
Magnoliids/eudicots - ps18
Eudicots - ps19
Core_eudicots - ps20
Superrosids - ps21
Rosids - ps22
Eurosids - ps23
Malvids - ps24
Sap./Malv./Brassicales - ps25
Malvales/Brassicales - ps26
Brassicales - ps27
Cleomaceae/Brassicaceae - ps28
Brassicaceae - ps29
Camelineae - ps30
Arabidopsis - ps31
Arabidopsis_thaliana - ps32
Phylostratum

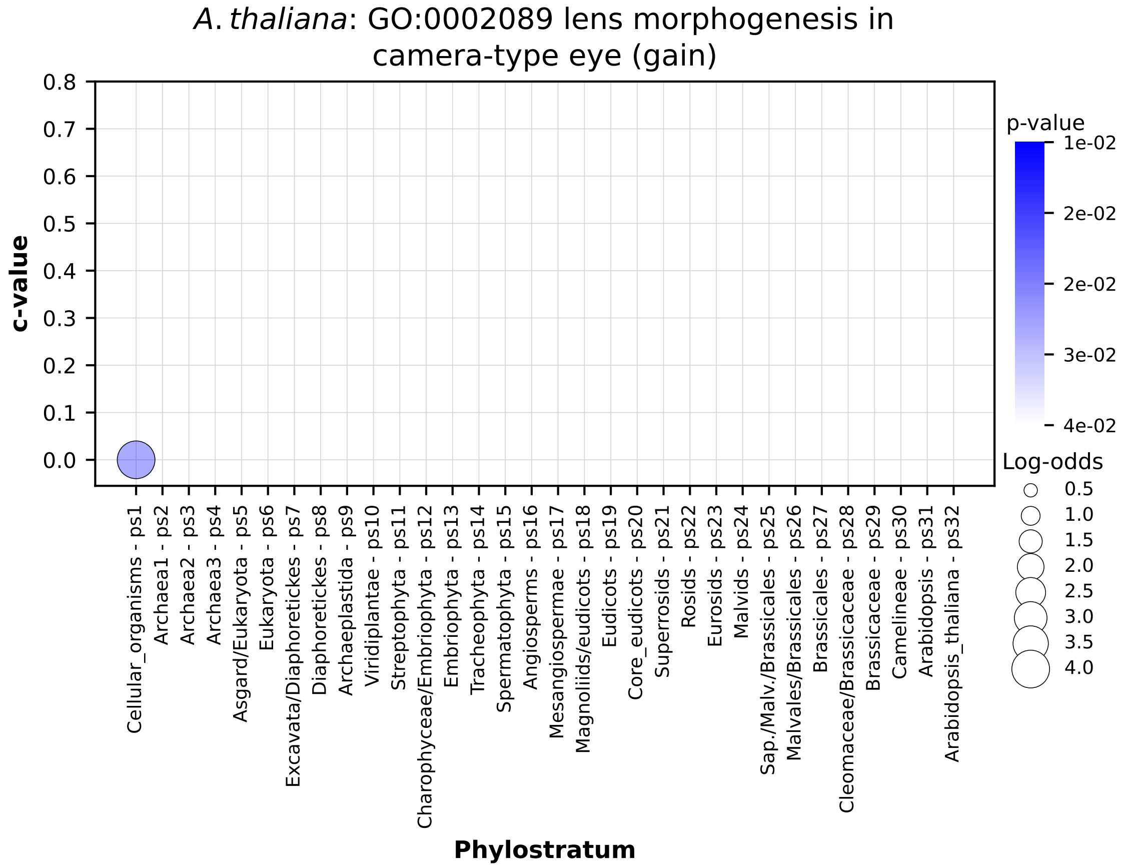
A. thaliana: GO:0002089 lens morphogenesis in camera-type eye (gain)
c-value
0.8
0.7
0.6
0.5
0.4
0.3
0.2
0.1
0.0
Phylostratum
Cellular_organisms - ps1
Archaea1 - ps2
Archaea2 - ps3
Archaea3 - ps4
Asgard/Eukaryota - ps5
Eukaryota - ps6
Excavata/Diaphoretickes - ps7
Diaphoretickes - ps8
Archaeplastida - ps9
Viridiplantae - ps10
Streptophyta - ps11
Charophyceae/Embriophyta - ps12
Embriophyta - ps13
Tracheophyta - ps14
Spermatophyta - ps15
Angiosperms - ps16
Mesangiospermae - ps17
Magnoliids/eudicots - ps18
Eudicots - ps19
Core_eudicots - ps20
Superrosids - ps21
Rosids - ps22
Eurosids - ps23
Malvids - ps24
Sap./Malv./Brassicales - ps25
Malvales/Brassicales - ps26
Brassicales - ps27
Cleomaceae/Brassicaceae - ps28
Brassicaceae - ps29
Camelineae - ps30
Arabidopsis - ps31
Arabidopsis_thaliana - ps32
p-value
1e-02
2e-02
2e-02
3e-02
4e-02
Log-odds
0.5
1.0
1.5
2.0
2.5
3.0
3.5
4.0

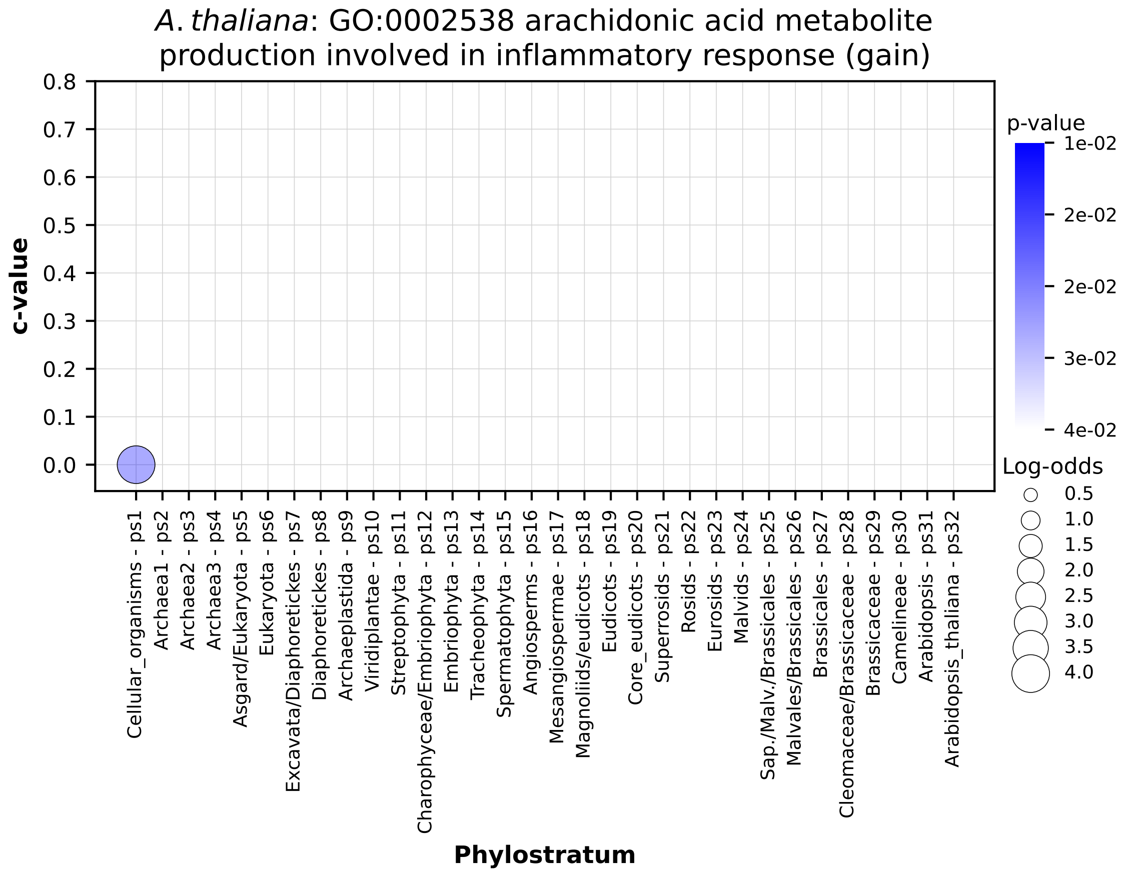

A. thaliana: GO:0002538 arachidonic acid metabolite production involved in inflammatory response (gain)
c-value
0.8
0.7
0.6
0.5
0.4
0.3
0.2
0.1
0.0
p-value
1e-02
2e-02
2e-02
3e-02
4e-02
Log-odds
0.5
1.0
1.5
2.0
2.5
3.0
3.5
4.0
Cellular_organisms - ps1
Archaea1 - ps2
Archaea2 - ps3
Archaea3 - ps4
Asgard/Eukaryota - ps5
Eukaryota - ps6
Excavata/Diaphoretickes - ps7
Diaphoretickes - ps8
Archaeplastida - ps9
Viridiplantae - ps10
Streptophyta - ps11
Charophyceae/Embriophyta - ps12
Embriophyta - ps13
Tracheophyta - ps14
Spermatophyta - ps15
Angiosperms - ps16
Mesangiospermae - ps17
Magnoliids/eudicots - ps18
Eudicots - ps19
Core_eudicots - ps20
Superrosids - ps21
Rosids - ps22
Eurosids - ps23
Malvids - ps24
Sap./Malv./Brassicales - ps25
Malvales/Brassicales - ps26
Brassicales - ps27
Cleomaceae/Brassicaceae - ps28
Brassicaceae - ps29
Camelineae - ps30
Arabidopsis - ps31
Arabidopsis_thaliana - ps32
Phylostratum

A. thaliana: GO:0002540 leukotriene production involved in inflammatory response (gain)

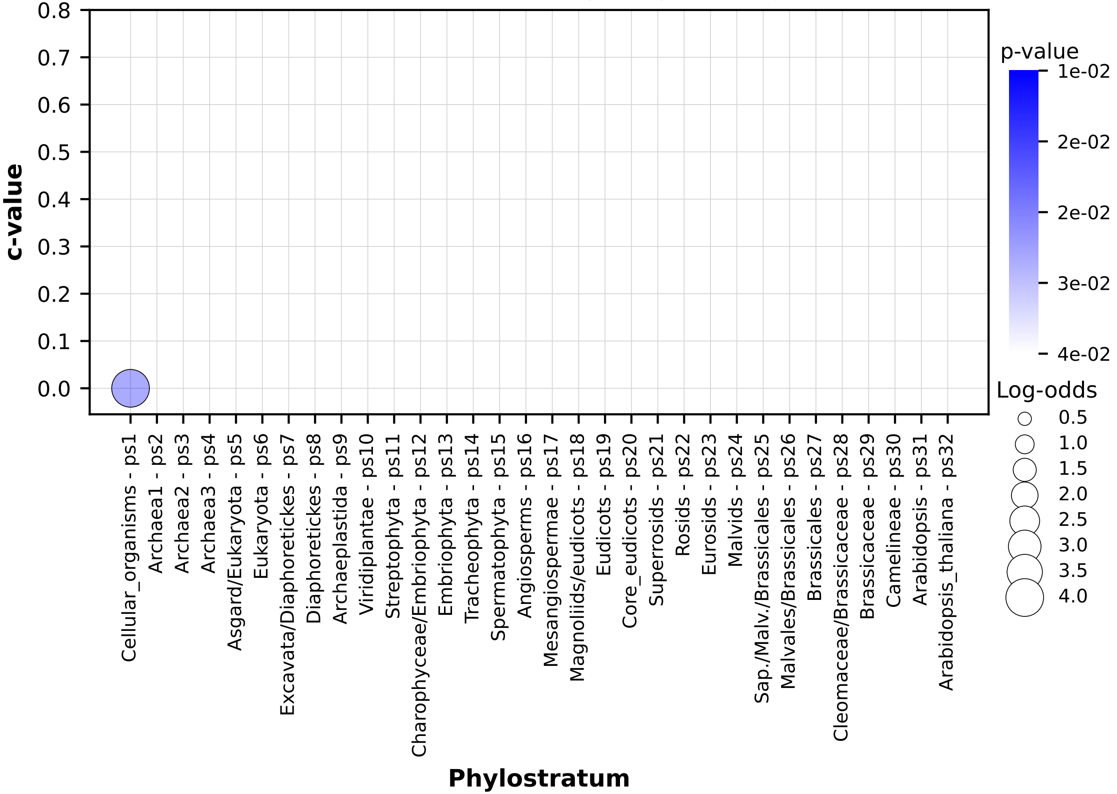

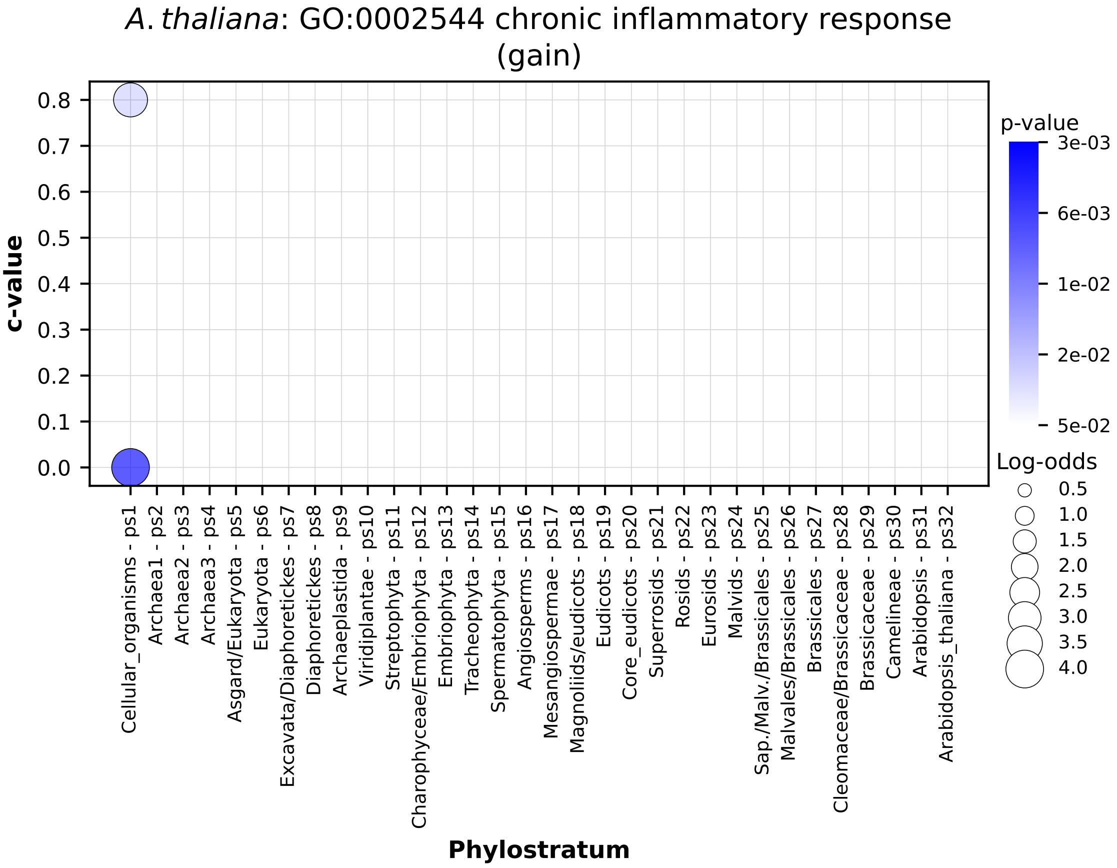

A. thaliana: GO:0002544 chronic inflammatory response (gain)
c-value
0.0
0.1
0.2
0.3
0.4
0.5
0.6
0.7
0.8
Phylostratum
Cellular_organisms - ps1
Archaea1 - ps2
Archaea2 - ps3
Archaea3 - ps4
Asgard/Eukaryota - ps5
Eukaryota - ps6
Excavata/Diaphoretickes - ps7
Diaphoretickes - ps8
Archaeplastida - ps9
Viridiplantae - ps10
Streptophyta - ps11
Charophyceae/Embriophyta - ps12
Embriophyta - ps13
Tracheophyta - ps14
Spermatophyta - ps15
Angiosperms - ps16
Mesangiospermae - ps17
Magnoliids/eudicots - ps18
Eudicots - ps19
Core_eudicots - ps20
Superrosids - ps21
Rosids - ps22
Eurosids - ps23
Malvids - ps24
Sap./Malv./Brassicales - ps25
Malvales/Brassicales - ps26
Brassicales - ps27
Cleomaceae/Brassicaceae - ps28
Brassicaceae - ps29
Camelineae - ps30
Arabidopsis - ps31
Arabidopsis_thaliana - ps32
p-value
3e-03
6e-03
1e-02
2e-02
5e-02
Log-odds
0.5
1.0
1.5
2.0
2.5
3.0
3.5
4.0

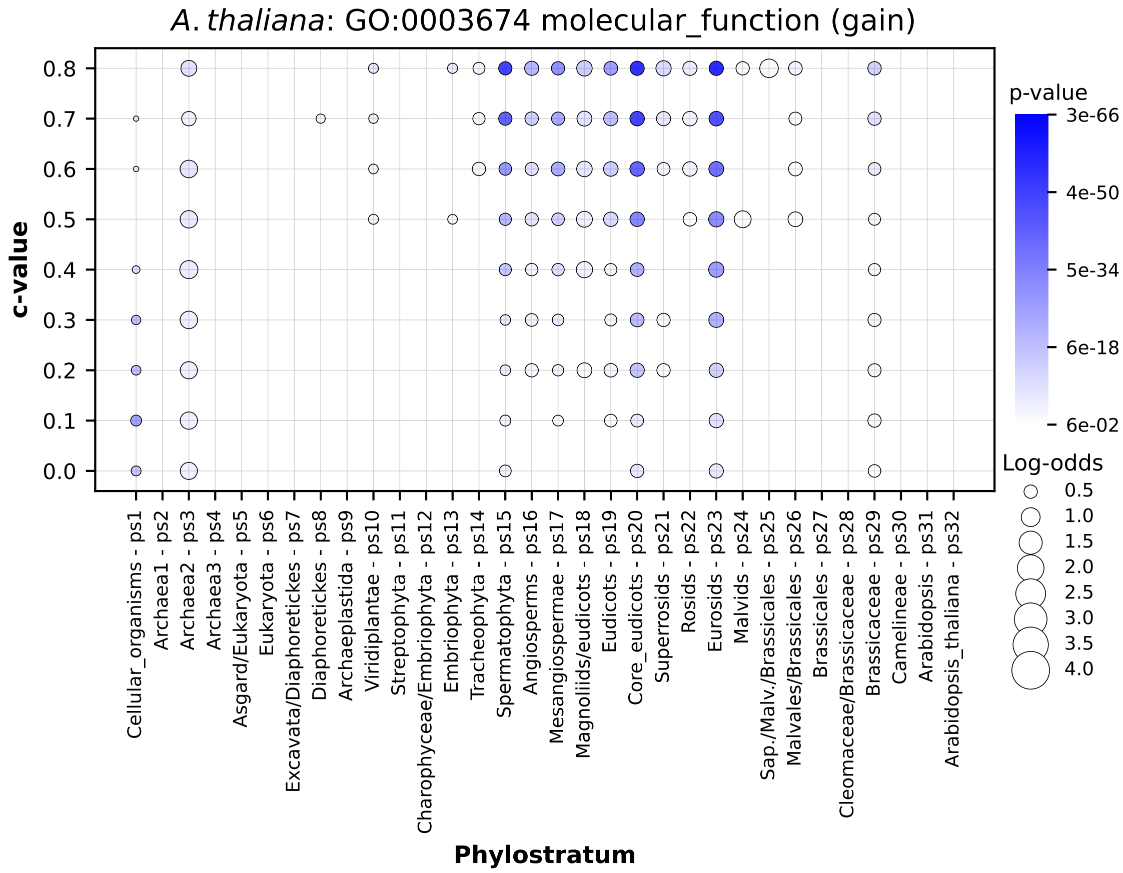

A. thaliana: GO:0003674 molecular_function (gain)
c-value
0.8
0.7
0.6
0.5
0.4
0.3
0.2
0.1
0.0
Phylostratum
Cellular_organisms - ps1
Archaea1 - ps2
Archaea2 - ps3
Archaea3 - ps4
Asgard/Eukaryota - ps5
Eukaryota - ps6
Excavata/Diaphoretickes - ps7
Diaphoretickes - ps8
Archaeplastida - ps9
Viridiplantae - ps10
Streptophyta - ps11
Charophyceae/Embriophyta - ps12
Embriophyta - ps13
Tracheophyta - ps14
Spermatophyta - ps15
Angiosperms - ps16
Mesangiospermae - ps17
Magnoliids/eudicots - ps18
Eudicots - ps19
Core_eudicots - ps20
Superrosids - ps21
Rosids - ps22
Eurosids - ps23
Malvids - ps24
Sap./Malv./Brassicales - ps25
Malvales/Brassicales - ps26
Brassicales - ps27
Cleomaceae/Brassicaceae - ps28
Brassicaceae - ps29
Camelineae - ps30
Arabidopsis - ps31
Arabidopsis_thaliana - ps32
p-value
3e-66
4e-50
5e-34
6e-18
6e-02
Log-odds
0.5
1.0
1.5
2.0
2.5
3.0
3.5
4.0

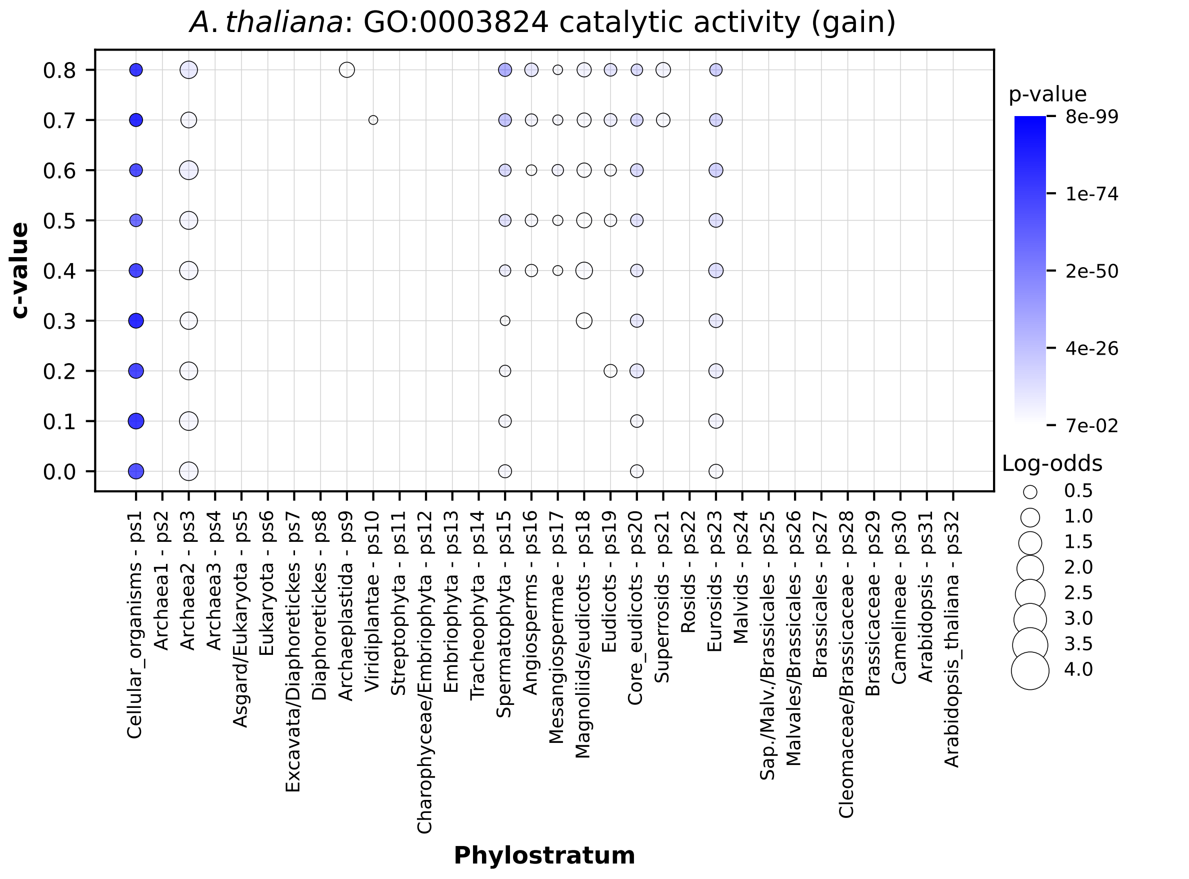

A. thaliana: GO:0003824 catalytic activity (gain)
c-value
0.8
0.7
0.6
0.5
0.4
0.3
0.2
0.1
0.0
Phylostratum
Cellular_organisms - ps1
Archaea1 - ps2
Archaea2 - ps3
Archaea3 - ps4
Asgard/Eukaryota - ps5
Eukaryota - ps6
Excavata/Diaphoretickes - ps7
Diaphoretickes - ps8
Archaeplastida - ps9
Viridiplantae - ps10
Streptophyta - ps11
Charophyceae/Embriophyta - ps12
Embriophyta - ps13
Tracheophyta - ps14
Spermatophyta - ps15
Angiosperms - ps16
Mesangiospermae - ps17
Magnoliids/eudicots - ps18
Eudicots - ps19
Core_eudicots - ps20
Superrosids - ps21
Rosids - ps22
Eurosids - ps23
Malvids - ps24
Sap./Malv./Brassicales - ps25
Malvales/Brassicales - ps26
Brassicales - ps27
Cleomaceae/Brassicaceae - ps28
Brassicaceae - ps29
Camelineae - ps30
Arabidopsis - ps31
Arabidopsis_thaliana - ps32
p-value
8e-99
1e-74
2e-50
4e-26
7e-02
Log-odds
0.5
1.0
1.5
2.0
2.5
3.0
3.5
4.0

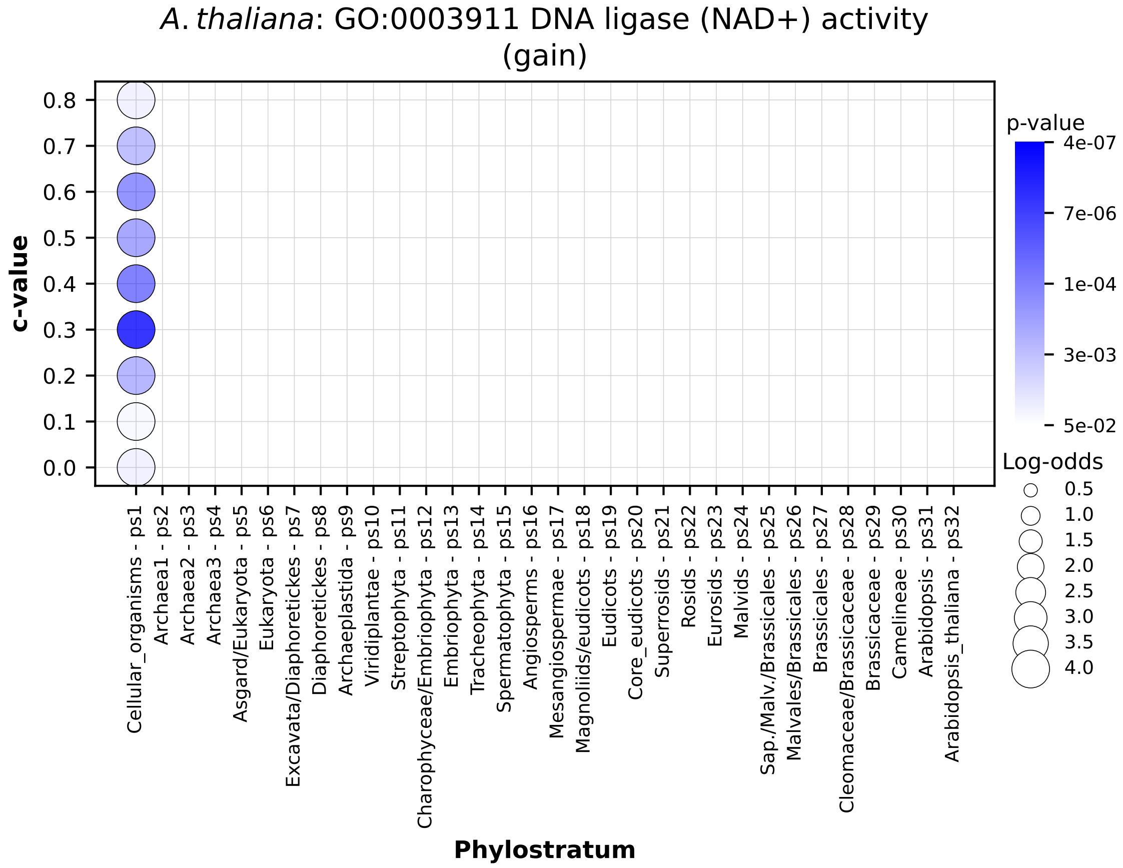

A. thaliana: GO:0003911 DNA ligase (NAD+) activity
(gain)
c-value
0.8
0.7
0.6
0.5
0.4
0.3
0.2
0.1
0.0
p-value
4e-07
7e-06
1e-04
3e-03
5e-02
Log-odds
0.5
1.0
1.5
2.0
2.5
3.0
3.5
4.0
Cellular_organisms - ps1
Archaea1 - ps2
Archaea2 - ps3
Archaea3 - ps4
Asgard/Eukaryota - ps5
Eukaryota - ps6
Excavata/Diaphoretickes - ps7
Diaphoretickes - ps8
Archaeplastida - ps9
Viridiplantae - ps10
Streptophyta - ps11
Charophyceae/Embriophyta - ps12
Embriophyta - ps13
Tracheophyta - ps14
Spermatophyta - ps15
Angiosperms - ps16
Mesangiospermae - ps17
Magnoliids/eudicots - ps18
Eudicots - ps19
Core_eudicots - ps20
Superrosids - ps21
Rosids - ps22
Eurosids - ps23
Malvids - ps24
Sap./Malv./Brassicales - ps25
Malvales/Brassicales - ps26
Brassicales - ps27
Cleomaceae/Brassicaceae - ps28
Brassicaceae - ps29
Camelineae - ps30
Arabidopsis - ps31
Arabidopsis_thaliana - ps32
Phylostratum

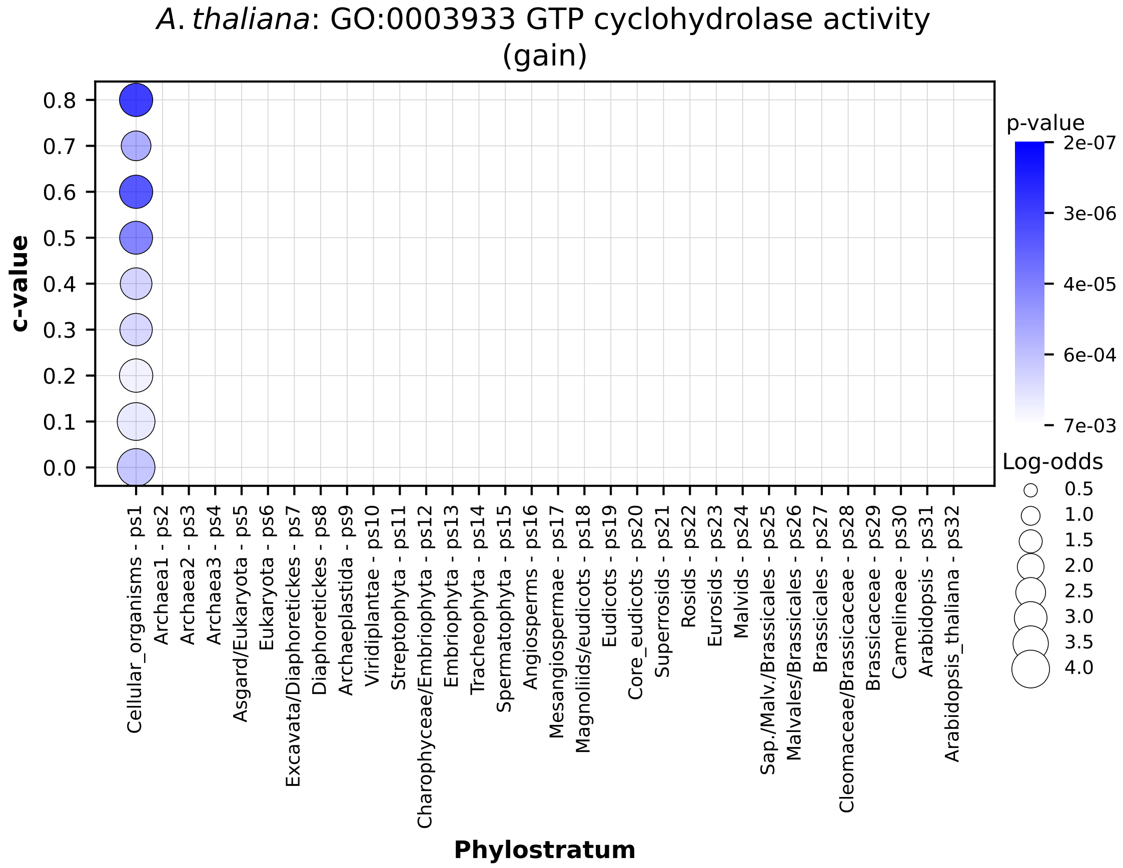

A. thaliana: GO:0003933 GTP cyclohydrolase activity (gain)
c-value
0.8
0.7
0.6
0.5
0.4
0.3
0.2
0.1
0.0
p-value
2e-07
3e-06
4e-05
6e-04
7e-03
Log-odds
0.5
1.0
1.5
2.0
2.5
3.0
3.5
4.0
Cellular_organisms - ps1
Archaea1 - ps2
Archaea2 - ps3
Archaea3 - ps4
Asgard/Eukaryota - ps5
Eukaryota - ps6
Excavata/Diaphoretickes - ps7
Diaphoretickes - ps8
Archaeplastida - ps9
Viridiplantae - ps10
Streptophyta - ps11
Charophyceae/Embriophyta - ps12
Embriophyta - ps13
Tracheophyta - ps14
Spermatophyta - ps15
Angiosperms - ps16
Mesangiospermae - ps17
Magnoliids/eudicots - ps18
Eudicots - ps19
Core_eudicots - ps20
Superrosids - ps21
Rosids - ps22
Eurosids - ps23
Malvids - ps24
Sap./Malv./Brassicales - ps25
Malvales/Brassicales - ps26
Brassicales - ps27
Cleomaceae/Brassicaceae - ps28
Brassicaceae - ps29
Camelineae - ps30
Arabidopsis - ps31
Arabidopsis_thaliana - ps32
Phylostratum

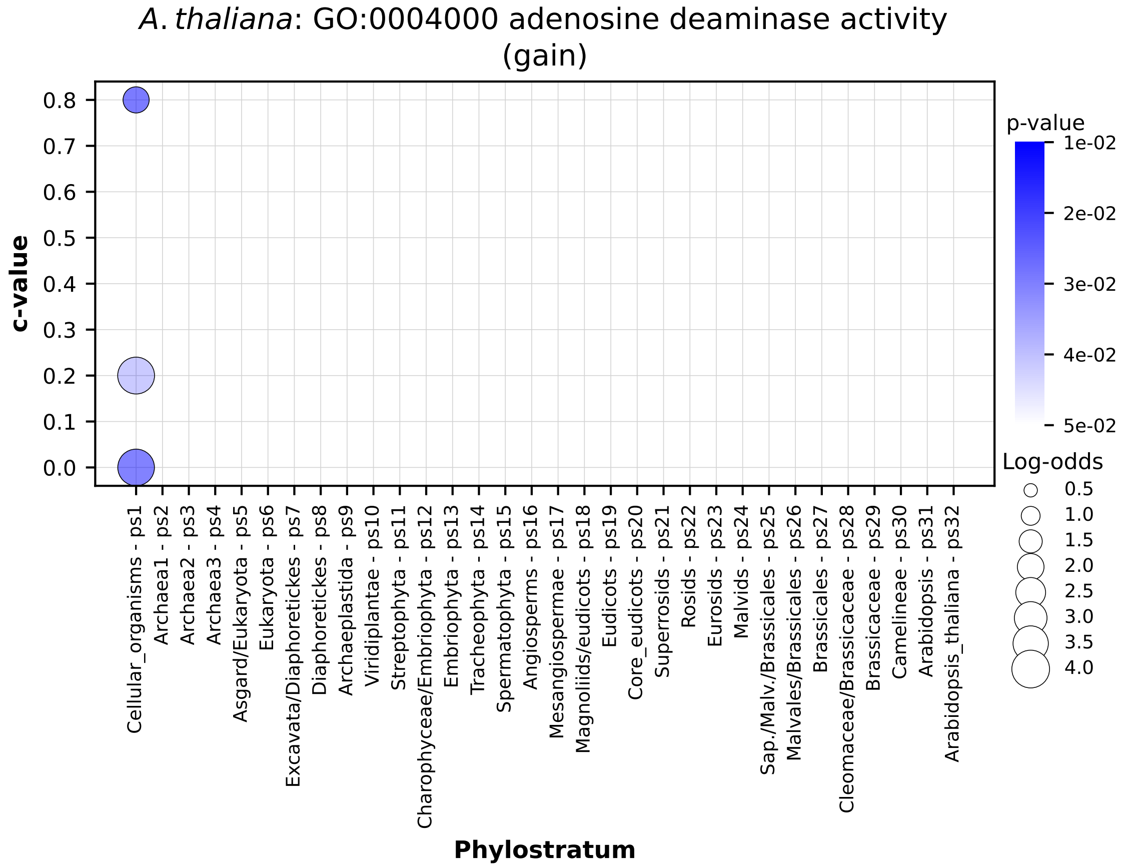
A. thaliana: GO:0004000 adenosine deaminase activity
(gain)
c-value
0.0
0.1
0.2
0.3
0.4
0.5
0.6
0.7
0.8
p-value
1e-02
2e-02
3e-02
4e-02
5e-02
Log-odds
0.5
1.0
1.5
2.0
2.5
3.0
3.5
4.0
Cellular_organisms - ps1
Archaea1 - ps2
Archaea2 - ps3
Archaea3 - ps4
Asgard/Eukaryota - ps5
Eukaryota - ps6
Excavata/Diaphoretickes - ps7
Diaphoretickes - ps8
Archaeplastida - ps9
Viridiplantae - ps10
Streptophyta - ps11
Charophyceae/Embriophyta - ps12
Embriophyta - ps13
Tracheophyta - ps14
Spermatophyta - ps15
Angiosperms - ps16
Mesangiospermae - ps17
Magnoliids/eudicots - ps18
Eudicots - ps19
Core_eudicots - ps20
Superrosids - ps21
Rosids - ps22
Eurosids - ps23
Malvids - ps24
Sap./Malv./Brassicales - ps25
Malvales/Brassicales - ps26
Brassicales - ps27
Cleomaceae/Brassicaceae - ps28
Brassicaceae - ps29
Camelineae - ps30
Arabidopsis - ps31
Arabidopsis_thaliana - ps32
Phylostratum

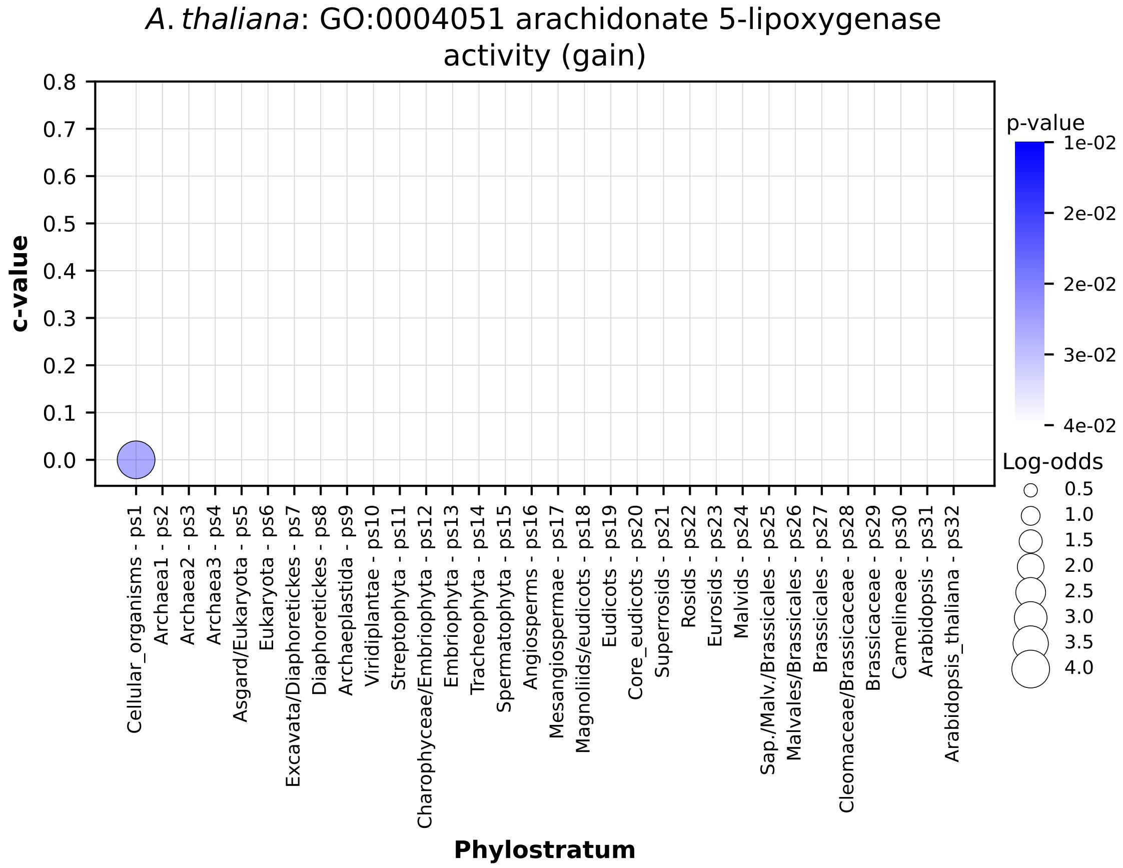

A. thaliana: GO:0004051 arachidonate 5-lipoxygenase activity (gain)
c-value
0.8
0.7
0.6
0.5
0.4
0.3
0.2
0.1
0.0
Phylostratum
Cellular_organisms - ps1
Archaea1 - ps2
Archaea2 - ps3
Archaea3 - ps4
Asgard/Eukaryota - ps5
Eukaryota - ps6
Excavata/Diaphoretickes - ps7
Diaphoretickes - ps8
Archaeplastida - ps9
Viridiplantae - ps10
Streptophyta - ps11
Charophyceae/Embriophyta - ps12
Embriophyta - ps13
Tracheophyta - ps14
Spermatophyta - ps15
Angiosperms - ps16
Mesangiospermae - ps17
Magnoliids/eudicots - ps18
Eudicots - ps19
Core_eudicots - ps20
Superrosids - ps21
Rosids - ps22
Eurosids - ps23
Malvids - ps24
Sap./Malv./Brassicales - ps25
Malvales/Brassicales - ps26
Brassicales - ps27
Cleomaceae/Brassicaceae - ps28
Brassicaceae - ps29
Camelineae - ps30
Arabidopsis - ps31
Arabidopsis_thaliana - ps32
p-value
1e-02
2e-02
2e-02
3e-02
4e-02
Log-odds
0.5
1.0
1.5
2.0
2.5
3.0
3.5
4.0

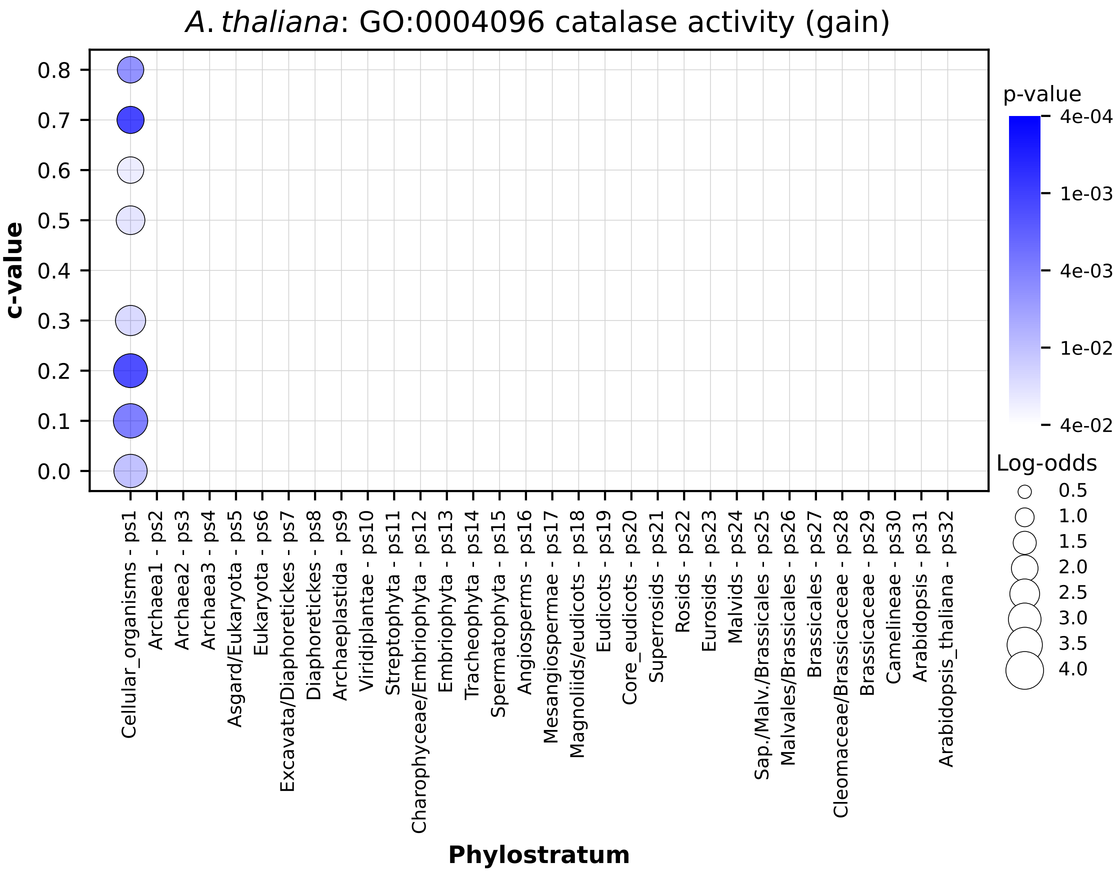

A. thaliana: GO:0004096 catalase activity (gain)
c-value
0.8
0.7
0.6
0.5
0.4
0.3
0.2
0.1
0.0
p-value
4e-04
1e-03
4e-03
1e-02
4e-02
Log-odds
0.5
1.0
1.5
2.0
2.5
3.0
3.5
4.0
Cellular_organisms - ps1
Archaea1 - ps2
Archaea2 - ps3
Archaea3 - ps4
Asgard/Eukaryota - ps5
Eukaryota - ps6
Excavata/Diaphoretickes - ps7
Diaphoretickes - ps8
Archaeplastida - ps9
Viridiplantae - ps10
Streptophyta - ps11
Charophyceae/Embriophyta - ps12
Embriophyta - ps13
Tracheophyta - ps14
Spermatophyta - ps15
Angiosperms - ps16
Mesangiospermae - ps17
Magnoliids/eudicots - ps18
Eudicots - ps19
Core_eudicots - ps20
Superrosids - ps21
Rosids - ps22
Eurosids - ps23
Malvids - ps24
Sap./Malv./Brassicales - ps25
Malvales/Brassicales - ps26
Brassicales - ps27
Cleomaceae/Brassicaceae - ps28
Brassicaceae - ps29
Camelineae - ps30
Arabidopsis - ps31
Arabidopsis_thaliana - ps32
Phylostratum

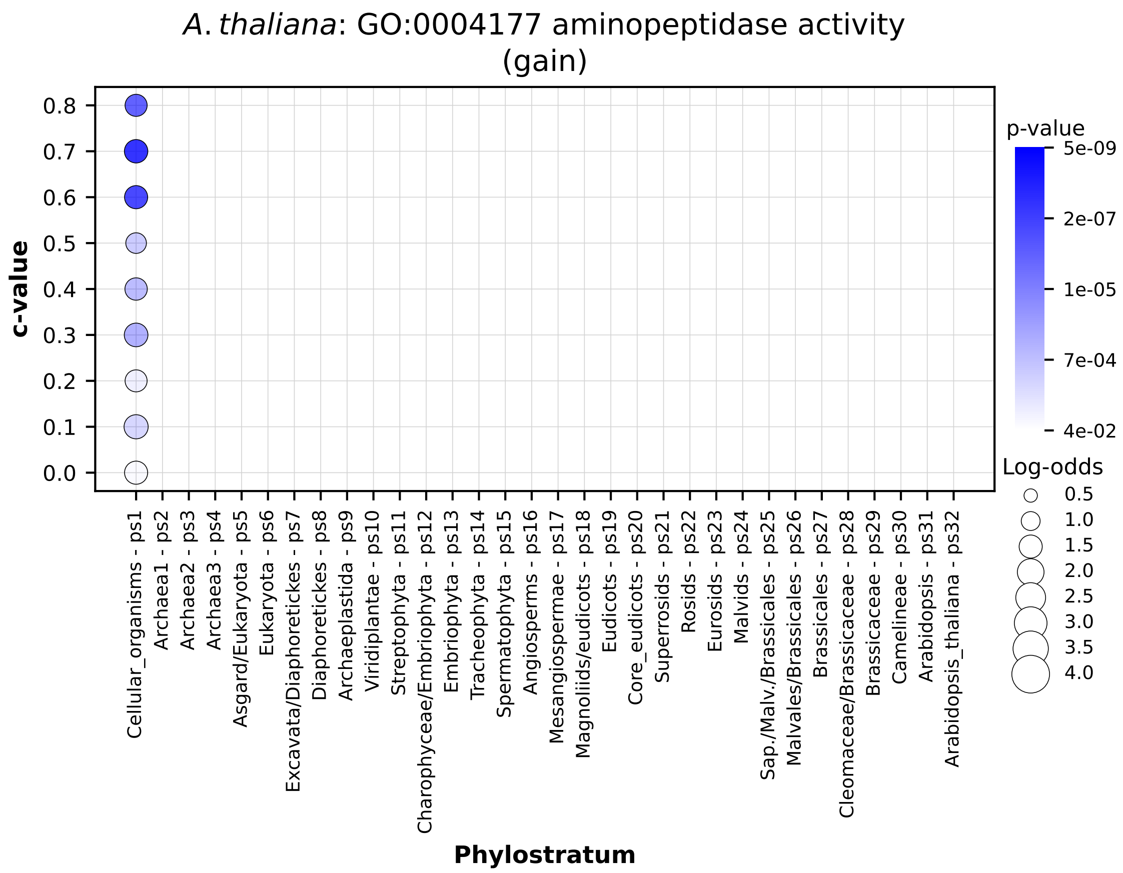

A. thaliana: GO:0004177 aminopeptidase activity (gain)
c-value
0.0
0.1
0.2
0.3
0.4
0.5
0.6
0.7
0.8
p-value
5e-09
2e-07
1e-05
7e-04
4e-02
Log-odds
0.5
1.0
1.5
2.0
2.5
3.0
3.5
4.0
Cellular_organisms - ps1
Archaea1 - ps2
Archaea2 - ps3
Archaea3 - ps4
Asgard/Eukaryota - ps5
Eukaryota - ps6
Excavata/Diaphoretickes - ps7
Diaphoretickes - ps8
Archaeplastida - ps9
Viridiplantae - ps10
Streptophyta - ps11
Charophyceae/Embriophyta - ps12
Embriophyta - ps13
Tracheophyta - ps14
Spermatophyta - ps15
Angiosperms - ps16
Mesangiospermae - ps17
Magnoliids/eudicots - ps18
Eudicots - ps19
Core_eudicots - ps20
Superrosids - ps21
Rosids - ps22
Eurosids - ps23
Malvids - ps24
Sap./Malv./Brassicales - ps25
Malvales/Brassicales - ps26
Brassicales - ps27
Cleomaceae/Brassicaceae - ps28
Brassicaceae - ps29
Camelineae - ps30
Arabidopsis - ps31
Arabidopsis_thaliana - ps32
Phylostratum

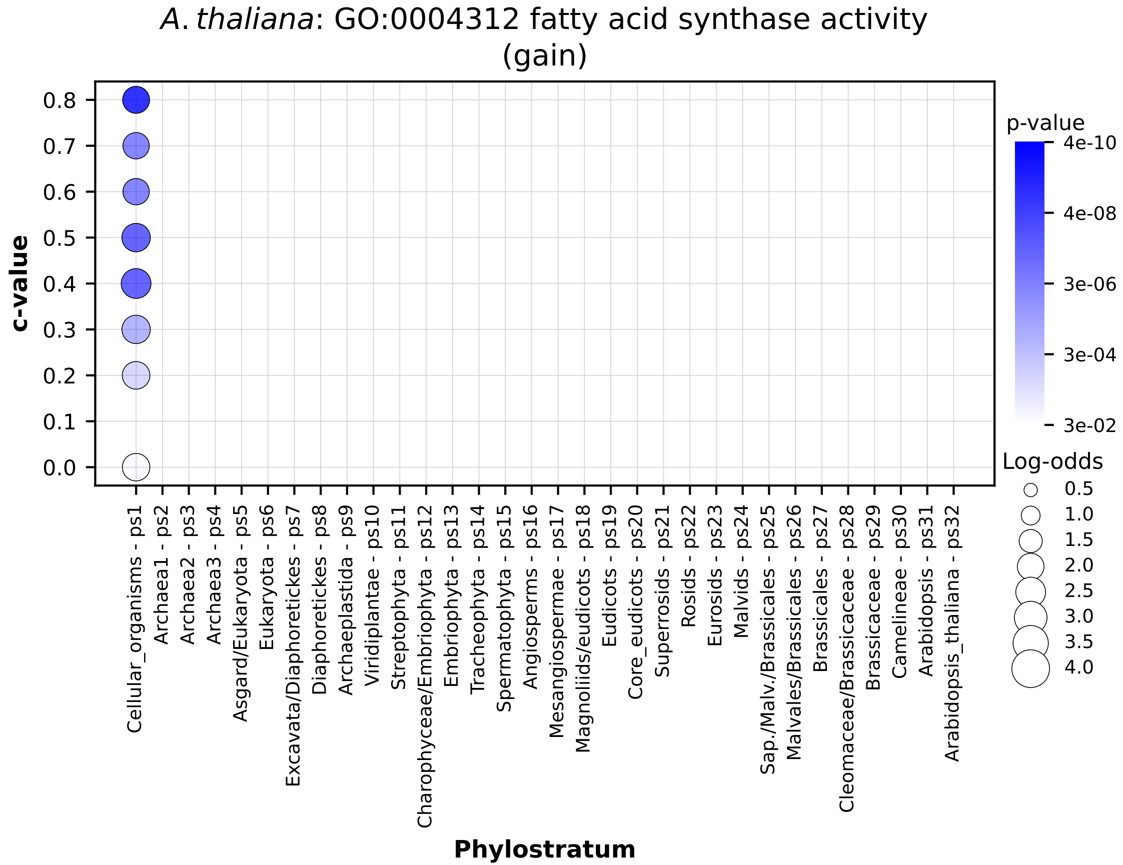

A. thaliana: GO:0004312 fatty acid synthase activity (gain)
c-value
0.0
0.1
0.2
0.3
0.4
0.5
0.6
0.7
0.8
p-value
4e-10
4e-08
3e-06
3e-04
3e-02
Log-odds
0.5
1.0
1.5
2.0
2.5
3.0
3.5
4.0
Cellular_organisms - ps1
Archaea1 - ps2
Archaea2 - ps3
Archaea3 - ps4
Asgard/Eukaryota - ps5
Eukaryota - ps6
Excavata/Diaphoretickes - ps7
Diaphoretickes - ps8
Archaeplastida - ps9
Viridiplantae - ps10
Streptophyta - ps11
Charophyceae/Embriophyta - ps12
Embriophyta - ps13
Tracheophyta - ps14
Spermatophyta - ps15
Angiosperms - ps16
Mesangiospermae - ps17
Magnoliids/eudicots - ps18
Eudicots - ps19
Core_eudicots - ps20
Superrosids - ps21
Rosids - ps22
Eurosids - ps23
Malvids - ps24
Sap./Malv./Brassicales - ps25
Malvales/Brassicales - ps26
Brassicales - ps27
Cleomaceae/Brassicaceae - ps28
Brassicaceae - ps29
Camelineae - ps30
Arabidopsis - ps31
Arabidopsis_thaliana - ps32
Phylostratum

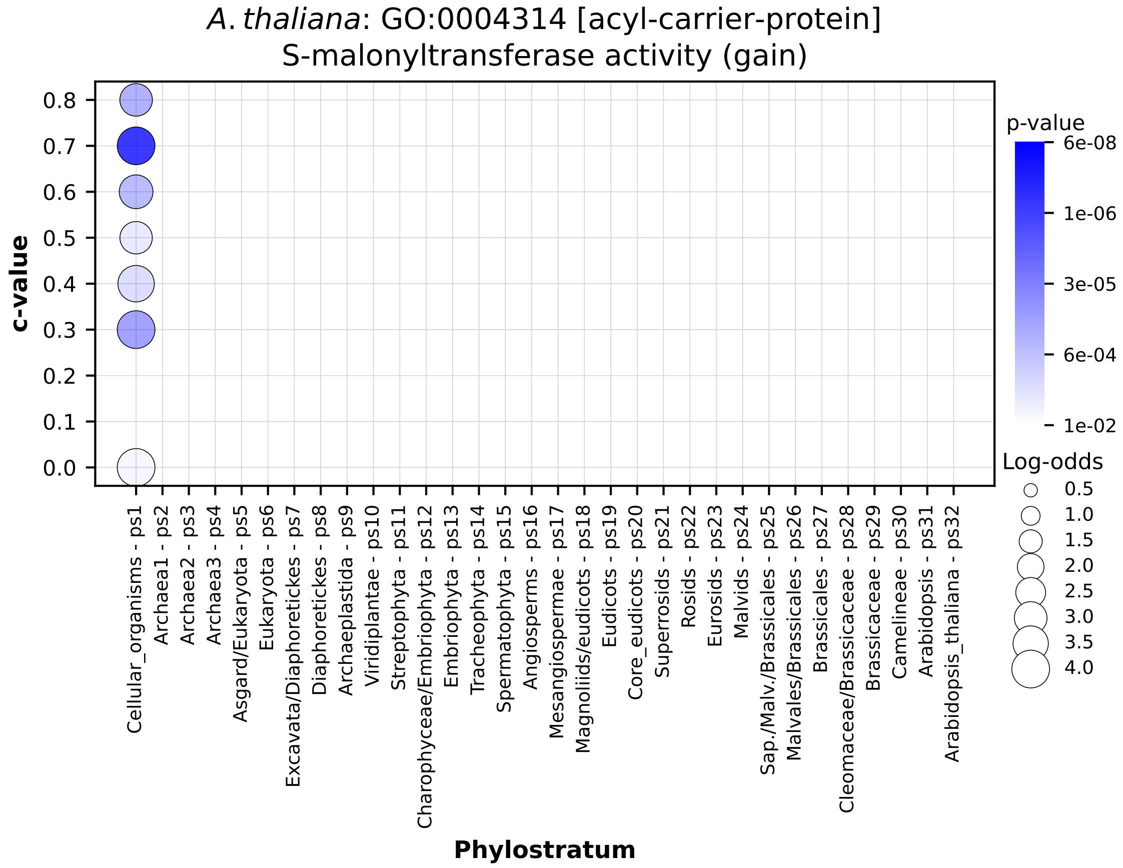

A. thaliana: GO:0004314 [acyl-carrier-protein] S-malonyltransferase activity (gain)
c-value
0.8
0.7
0.6
0.5
0.4
0.3
0.2
0.1
0.0
p-value
6e-08
1e-06
3e-05
6e-04
1e-02
Log-odds
0.5
1.0
1.5
2.0
2.5
3.0
3.5
4.0
Cellular_organisms - ps1
Archaea1 - ps2
Archaea2 - ps3
Archaea3 - ps4
Asgard/Eukaryota - ps5
Eukaryota - ps6
Excavata/Diaphoretickes - ps7
Diaphoretickes - ps8
Archaeplastida - ps9
Viridiplantae - ps10
Streptophyta - ps11
Charophyceae/Embriophyta - ps12
Embriophyta - ps13
Tracheophyta - ps14
Spermatophyta - ps15
Angiosperms - ps16
Mesangiospermae - ps17
Magnoliids/eudicots - ps18
Eudicots - ps19
Core_eudicots - ps20
Superrosids - ps21
Rosids - ps22
Eurosids - ps23
Malvids - ps24
Sap./Malv./Brassicales - ps25
Malvales/Brassicales - ps26
Brassicales - ps27
Cleomaceae/Brassicaceae - ps28
Brassicaceae - ps29
Camelineae - ps30
Arabidopsis - ps31
Arabidopsis_thaliana - ps32
Phylostratum

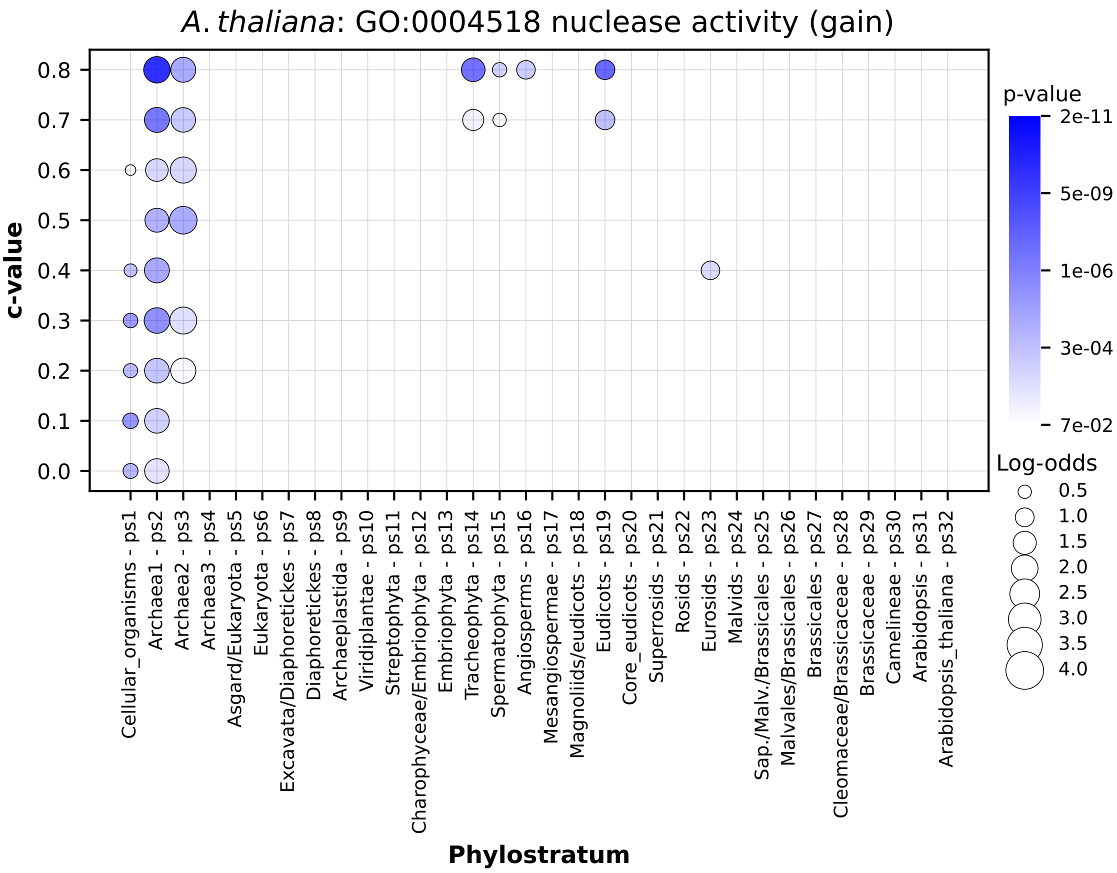

A. thaliana: GO:0004518 nuclease activity (gain)
c-value
0.0
0.1
0.2
0.3
0.4
0.5
0.6
0.7
0.8
Phylostratum
Cellular_organisms - ps1
Archaea1 - ps2
Archaea2 - ps3
Archaea3 - ps4
Asgard/Eukaryota - ps5
Eukaryota - ps6
Excavata/Diaphoretickes - ps7
Diaphoretickes - ps8
Archaeplastida - ps9
Viridiplantae - ps10
Streptophyta - ps11
Charophyceae/Embriophyta - ps12
Embriophyta - ps13
Tracheophyta - ps14
Spermatophyta - ps15
Angiosperms - ps16
Mesangiospermae - ps17
Magnoliids/eudicots - ps18
Eudicots - ps19
Core_eudicots - ps20
Superrosids - ps21
Rosids - ps22
Eurosids - ps23
Malvids - ps24
Sap./Malv./Brassicales - ps25
Malvales/Brassicales - ps26
Brassicales - ps27
Cleomaceae/Brassicaceae - ps28
Brassicaceae - ps29
Camelineae - ps30
Arabidopsis - ps31
Arabidopsis_thaliana - ps32
p-value
2e-11
5e-09
1e-06
3e-04
7e-02
Log-odds
0.5
1.0
1.5
2.0
2.5
3.0
3.5
4.0

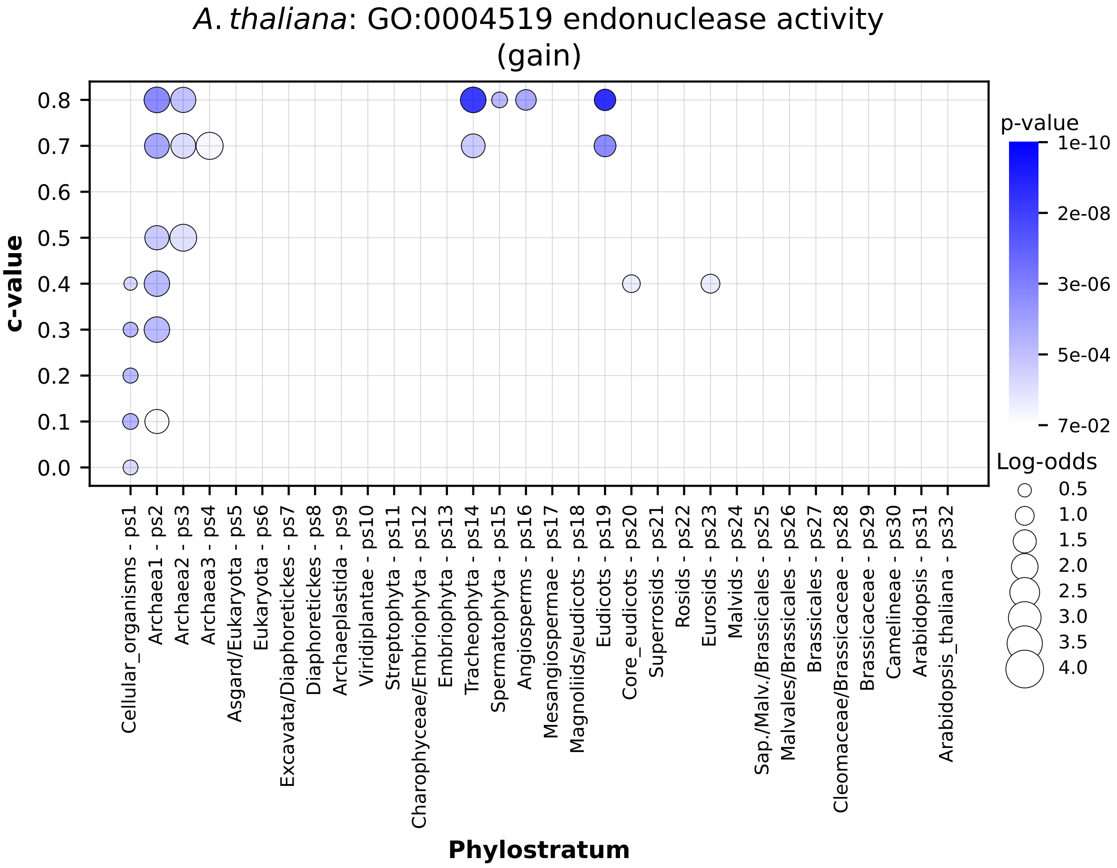
A. thaliana: GO:0004519 endonuclease activity
(gain)
c-value
0.8
0.7
0.6
0.5
0.4
0.3
0.2
0.1
0.0
p-value
1e-10
2e-08
3e-06
5e-04
7e-02
Log-odds
0.5
1.0
1.5
2.0
2.5
3.0
3.5
4.0
Cellular_organisms - ps1
Archaea1 - ps2
Archaea2 - ps3
Archaea3 - ps4
Asgard/Eukaryota - ps5
Eukaryota - ps6
Excavata/Diaphoretickes - ps7
Diaphoretickes - ps8
Archaeplastida - ps9
Viridiplantae - ps10
Streptophyta - ps11
Charophyceae/Embriophyta - ps12
Embriophyta - ps13
Tracheophyta - ps14
Spermatophyta - ps15
Angiosperms - ps16
Mesangiospermae - ps17
Magnoliids/eudicots - ps18
Eudicots - ps19
Core_eudicots - ps20
Superrosids - ps21
Rosids - ps22
Eurosids - ps23
Malvids - ps24
Sap./Malv./Brassicales - ps25
Malvales/Brassicales - ps26
Brassicales - ps27
Cleomaceae/Brassicaceae - ps28
Brassicaceae - ps29
Camelineae - ps30
Arabidopsis - ps31
Arabidopsis_thaliana - ps32
Phylostratum

A. thaliana: GO:0004520 DNA endonuclease activity (gain)

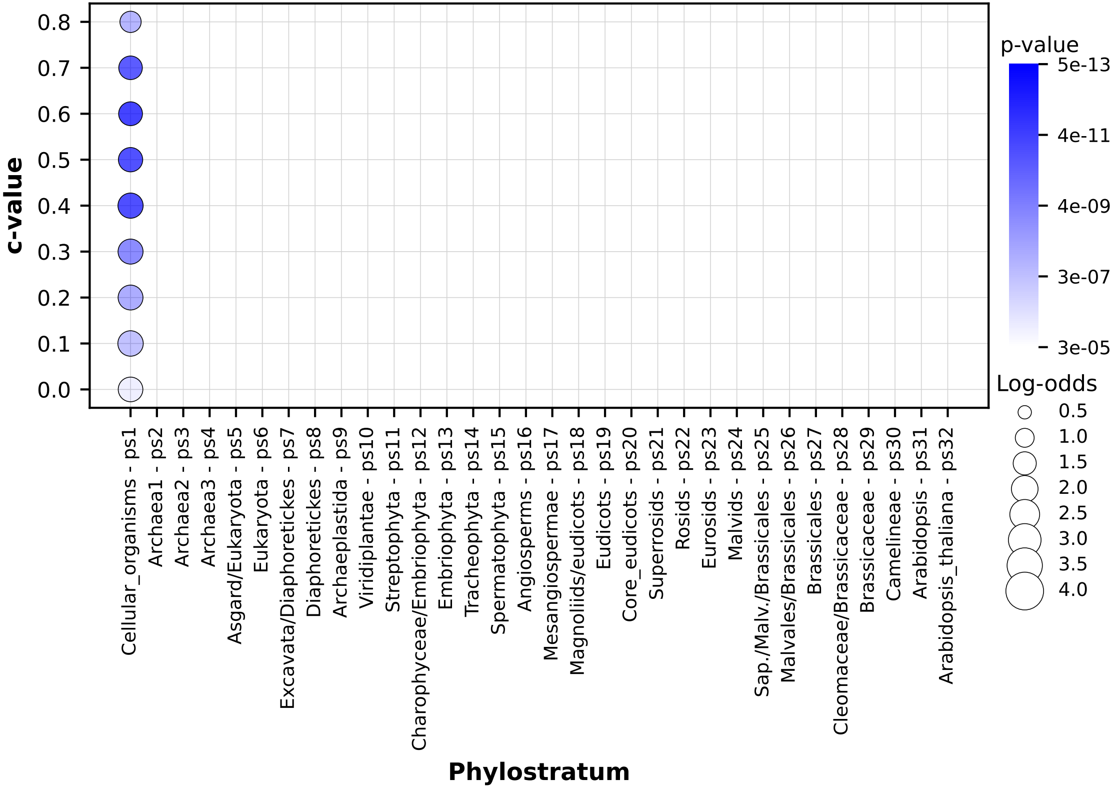

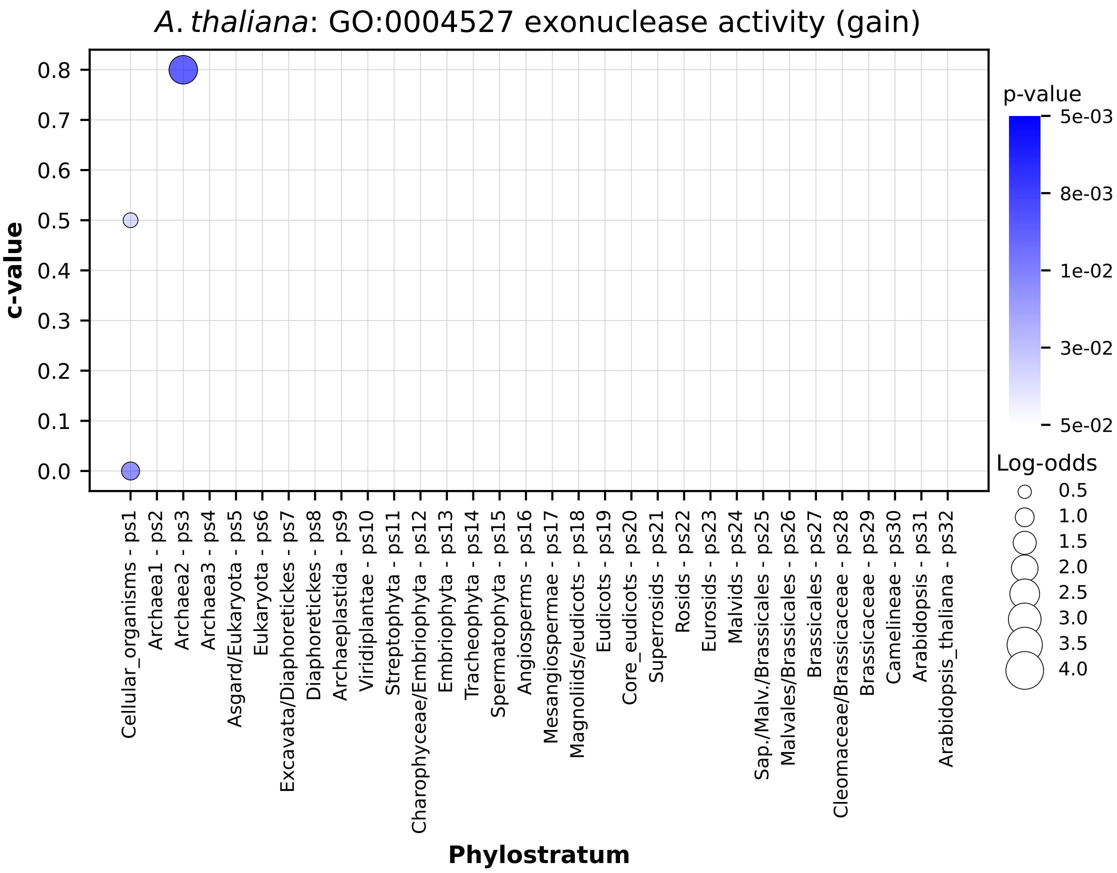

A. thaliana: GO:0004527 exonuclease activity (gain)
c-value
0.8
0.7
0.6
0.5
0.4
0.3
0.2
0.1
0.0
p-value
5e-03
8e-03
1e-02
3e-02
5e-02
Log-odds
0.5
1.0
1.5
2.0
2.5
3.0
3.5
4.0
Cellular_organisms - ps1
Archaea1 - ps2
Archaea2 - ps3
Archaea3 - ps4
Asgard/Eukaryota - ps5
Eukaryota - ps6
Excavata/Diaphoretickes - ps7
Diaphoretickes - ps8
Archaeplastida - ps9
Viridiplantae - ps10
Streptophyta - ps11
Charophyceae/Embriophyta - ps12
Embriophyta - ps13
Tracheophyta - ps14
Spermatophyta - ps15
Angiosperms - ps16
Mesangiospermae - ps17
Magnoliids/eudicots - ps18
Eudicots - ps19
Core_eudicots - ps20
Superrosids - ps21
Rosids - ps22
Eurosids - ps23
Malvids - ps24
Sap./Malv./Brassicales - ps25
Malvales/Brassicales - ps26
Brassicales - ps27
Cleomaceae/Brassicaceae - ps28
Brassicaceae - ps29
Camelineae - ps30
Arabidopsis - ps31
Arabidopsis_thaliana - ps32
Phylostratum

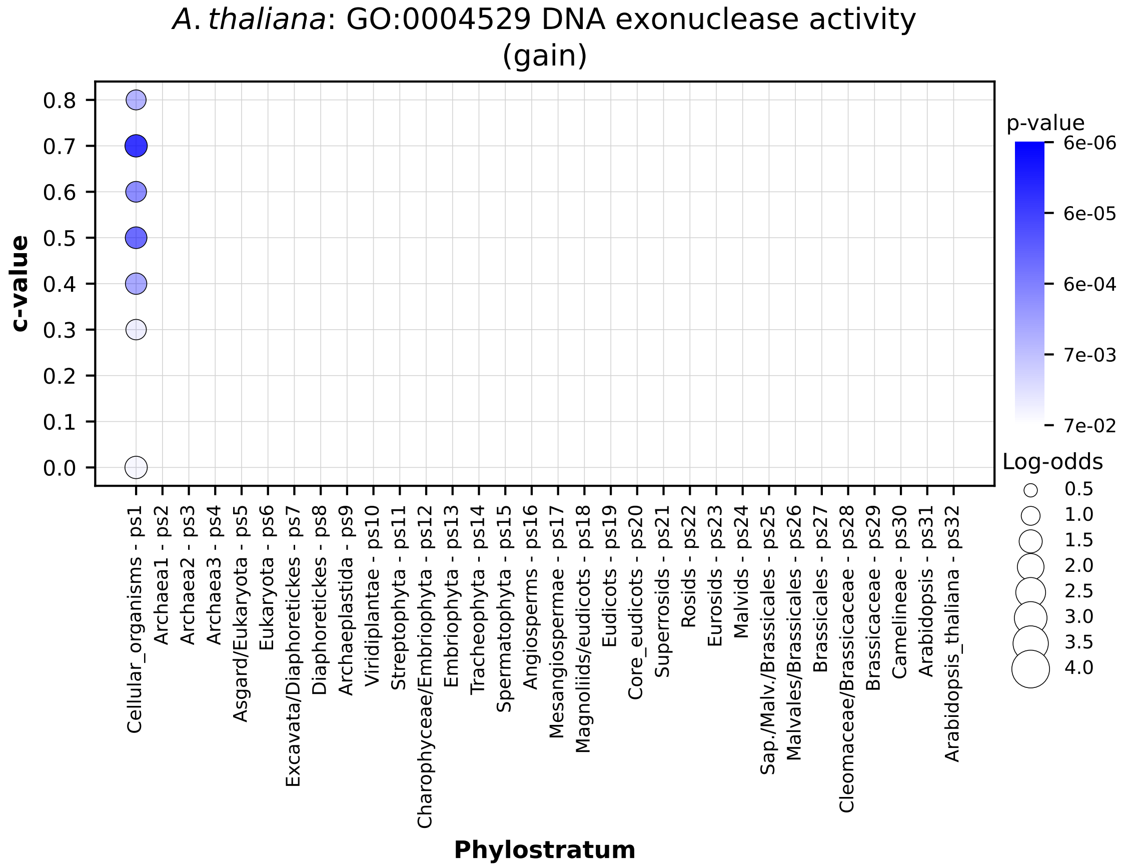

A. thaliana: GO:0004529 DNA exonuclease activity
(gain)
c-value
0.8
0.7
0.6
0.5
0.4
0.3
0.2
0.1
0.0
p-value
6e-06
6e-05
6e-04
7e-03
7e-02
Log-odds
0.5
1.0
1.5
2.0
2.5
3.0
3.5
4.0
Cellular_organisms - ps1
Archaea1 - ps2
Archaea2 - ps3
Archaea3 - ps4
Asgard/Eukaryota - ps5
Eukaryota - ps6
Excavata/Diaphoretickes - ps7
Diaphoretickes - ps8
Archaeplastida - ps9
Viridiplantae - ps10
Streptophyta - ps11
Charophyceae/Embriophyta - ps12
Embriophyta - ps13
Tracheophyta - ps14
Spermatophyta - ps15
Angiosperms - ps16
Mesangiospermae - ps17
Magnoliids/eudicots - ps18
Eudicots - ps19
Core_eudicots - ps20
Superrosids - ps21
Rosids - ps22
Eurosids - ps23
Malvids - ps24
Sap./Malv./Brassicales - ps25
Malvales/Brassicales - ps26
Brassicales - ps27
Cleomaceae/Brassicaceae - ps28
Brassicaceae - ps29
Camelineae - ps30
Arabidopsis - ps31
Arabidopsis_thaliana - ps32
Phylostratum

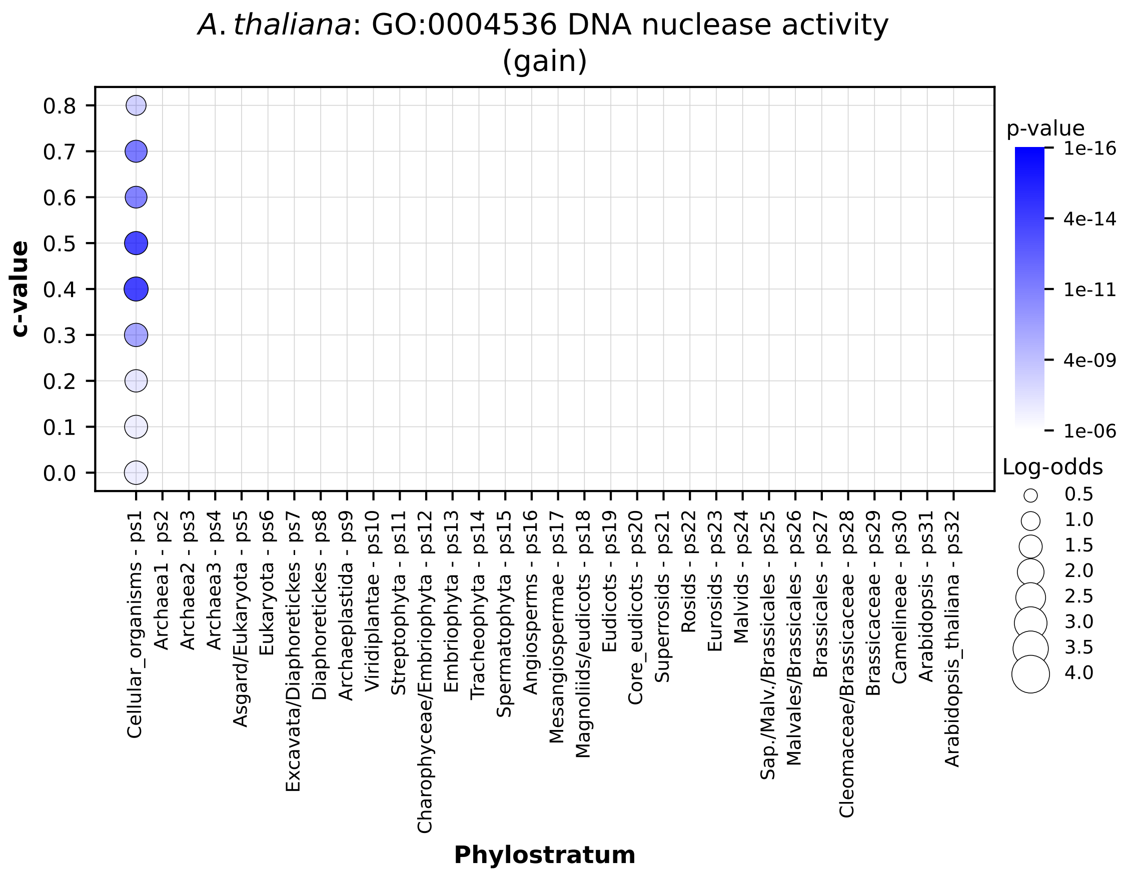

A. thaliana: GO:0004536 DNA nuclease activity
(gain)
c-value
0.0
0.1
0.2
0.3
0.4
0.5
0.6
0.7
0.8
p-value
1e-16
4e-14
1e-11
4e-09
1e-06
Log-odds
0.5
1.0
1.5
2.0
2.5
3.0
3.5
4.0
Cellular_organisms - ps1
Archaea1 - ps2
Archaea2 - ps3
Archaea3 - ps4
Asgard/Eukaryota - ps5
Eukaryota - ps6
Excavata/Diaphoretickes - ps7
Diaphoretickes - ps8
Archaeplastida - ps9
Viridiplantae - ps10
Streptophyta - ps11
Charophyceae/Embriophyta - ps12
Embriophyta - ps13
Tracheophyta - ps14
Spermatophyta - ps15
Angiosperms - ps16
Mesangiospermae - ps17
Magnoliids/eudicots - ps18
Eudicots - ps19
Core_eudicots - ps20
Superrosids - ps21
Rosids - ps22
Eurosids - ps23
Malvids - ps24
Sap./Malv./Brassicales - ps25
Malvales/Brassicales - ps26
Brassicales - ps27
Cleomaceae/Brassicaceae - ps28
Brassicaceae - ps29
Camelineae - ps30
Arabidopsis - ps31
Arabidopsis_thaliana - ps32
Phylostratum

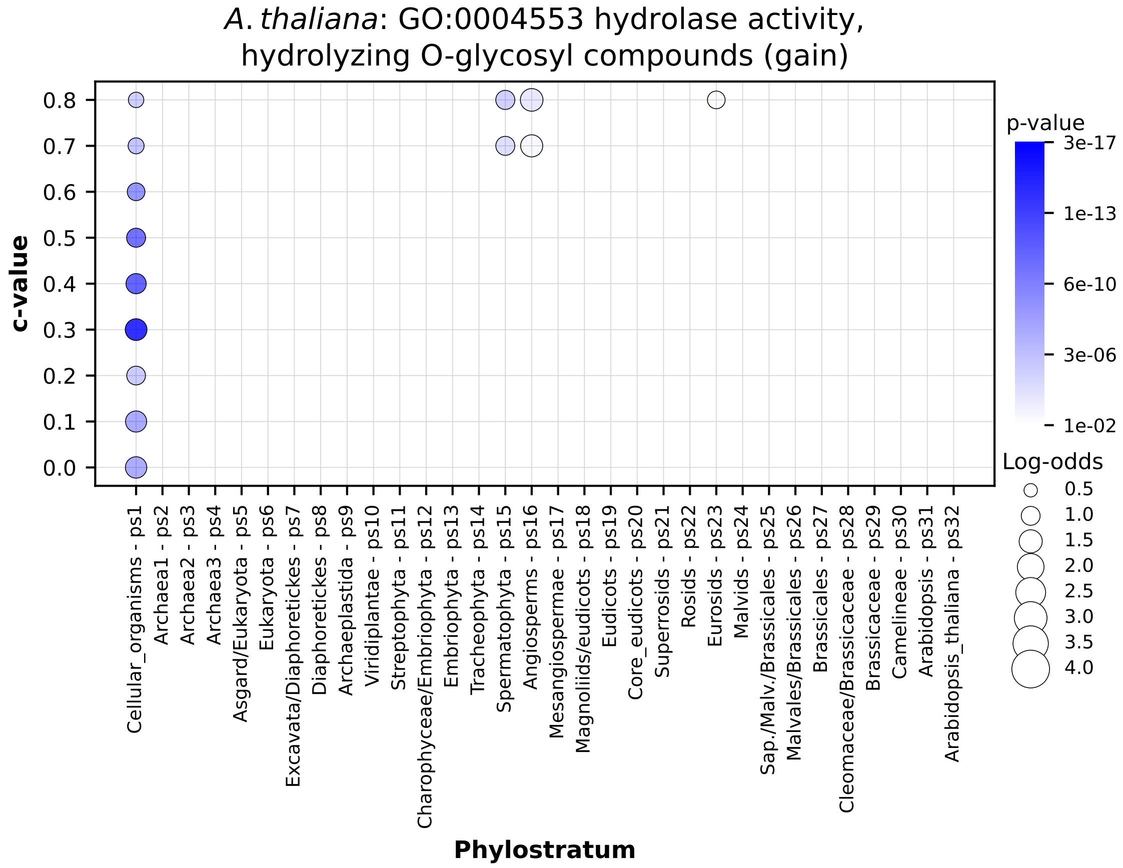

A. thaliana: GO:0004553 hydrolase activity, hydrolyzing O-glycosyl compounds (gain)
c-value
0.8
0.7
0.6
0.5
0.4
0.3
0.2
0.1
0.0
p-value
3e-17
1e-13
6e-10
3e-06
1e-02
Log-odds
0.5
1.0
1.5
2.0
2.5
3.0
3.5
4.0
Cellular_organisms - ps1
Archaea1 - ps2
Archaea2 - ps3
Archaea3 - ps4
Asgard/Eukaryota - ps5
Eukaryota - ps6
Excavata/Diaphoretickes - ps7
Diaphoretickes - ps8
Archaeplastida - ps9
Viridiplantae - ps10
Streptophyta - ps11
Charophyceae/Embriophyta - ps12
Embriophyta - ps13
Tracheophyta - ps14
Spermatophyta - ps15
Angiosperms - ps16
Mesangiospermae - ps17
Magnoliids/eudicots - ps18
Eudicots - ps19
Core_eudicots - ps20
Superrosids - ps21
Rosids - ps22
Eurosids - ps23
Malvids - ps24
Sap./Malv./Brassicales - ps25
Malvales/Brassicales - ps26
Brassicales - ps27
Cleomaceae/Brassicaceae - ps28
Brassicaceae - ps29
Camelineae - ps30
Arabidopsis - ps31
Arabidopsis_thaliana - ps32
Phylostratum

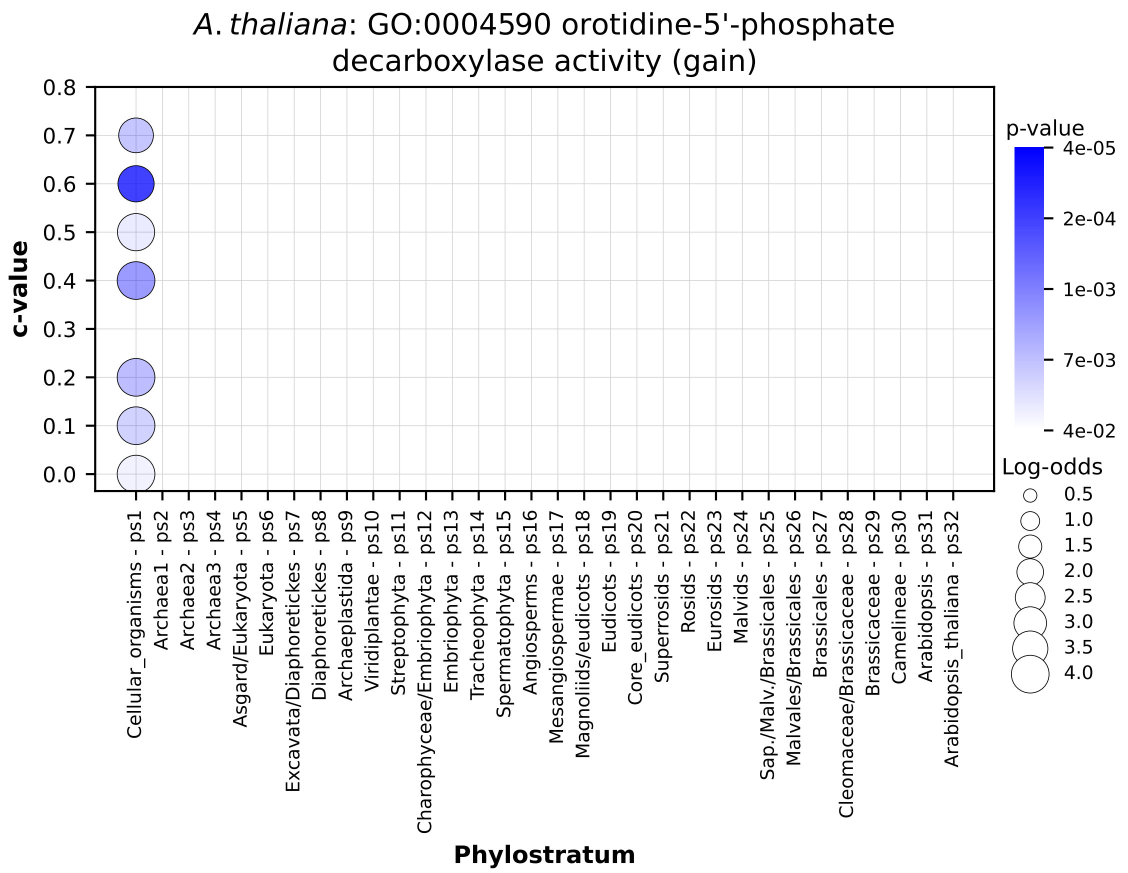

A. thaliana: GO:0004590 orotidine-5'-phosphate decarboxylase activity (gain)
c-value
0.8
0.7
0.6
0.5
0.4
0.3
0.2
0.1
0.0
p-value
4e-05
2e-04
1e-03
7e-03
4e-02
Log-odds
0.5
1.0
1.5
2.0
2.5
3.0
3.5
4.0
Cellular_organisms - ps1
Archaea1 - ps2
Archaea2 - ps3
Archaea3 - ps4
Asgard/Eukaryota - ps5
Eukaryota - ps6
Excavata/Diaphoretickes - ps7
Diaphoretickes - ps8
Archaeplastida - ps9
Viridiplantae - ps10
Streptophyta - ps11
Charophyceae/Embriophyta - ps12
Embriophyta - ps13
Tracheophyta - ps14
Spermatophyta - ps15
Angiosperms - ps16
Mesangiospermae - ps17
Magnoliids/eudicots - ps18
Eudicots - ps19
Core_eudicots - ps20
Superrosids - ps21
Rosids - ps22
Eurosids - ps23
Malvids - ps24
Sap./Malv./Brassicales - ps25
Malvales/Brassicales - ps26
Brassicales - ps27
Cleomaceae/Brassicaceae - ps28
Brassicaceae - ps29
Camelineae - ps30
Arabidopsis - ps31
Arabidopsis_thaliana - ps32
Phylostratum

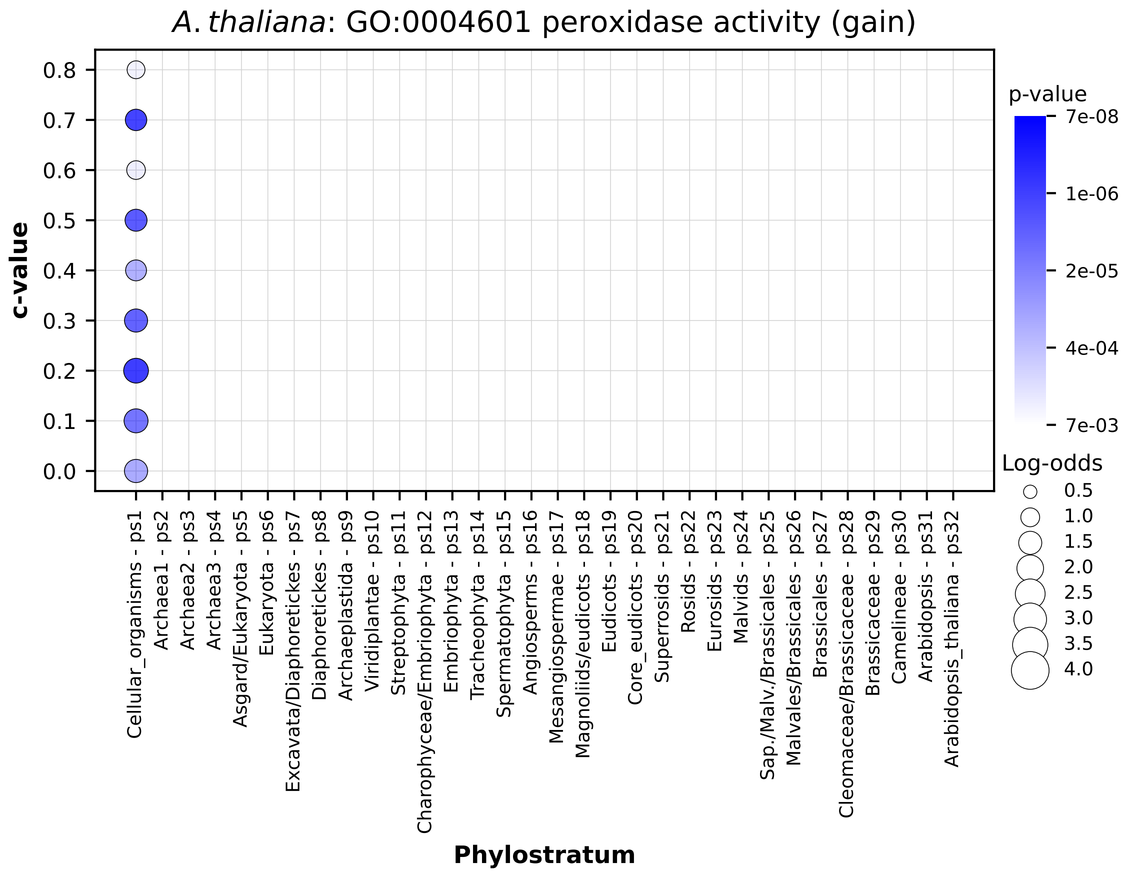
A. thaliana: GO:0004601 peroxidase activity (gain)
c-value
0.0
0.1
0.2
0.3
0.4
0.5
0.6
0.7
0.8
Phylostratum
Cellular_organisms - ps1
Archaea1 - ps2
Archaea2 - ps3
Archaea3 - ps4
Asgard/Eukaryota - ps5
Eukaryota - ps6
Excavata/Diaphoretickes - ps7
Diaphoretickes - ps8
Archaeplastida - ps9
Viridiplantae - ps10
Streptophyta - ps11
Charophyceae/Embriophyta - ps12
Embriophyta - ps13
Tracheophyta - ps14
Spermatophyta - ps15
Angiosperms - ps16
Mesangiospermae - ps17
Magnoliids/eudicots - ps18
Eudicots - ps19
Core_eudicots - ps20
Superrosids - ps21
Rosids - ps22
Eurosids - ps23
Malvids - ps24
Sap./Malv./Brassicales - ps25
Malvales/Brassicales - ps26
Brassicales - ps27
Cleomaceae/Brassicaceae - ps28
Brassicaceae - ps29
Camelineae - ps30
Arabidopsis - ps31
Arabidopsis_thaliana - ps32
p-value
7e-08
1e-06
2e-05
4e-04
7e-03
Log-odds
0.5
1.0
1.5
2.0
2.5
3.0
3.5
4.0

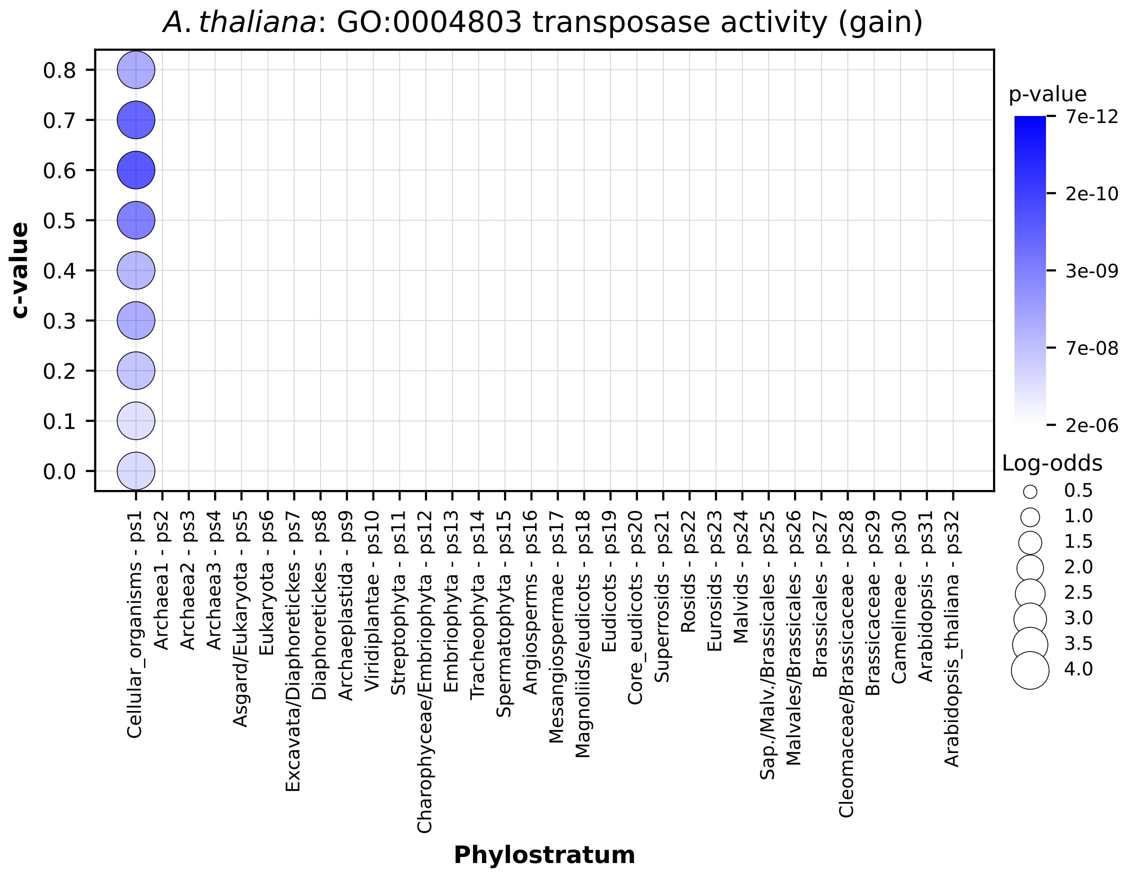

A. thaliana: GO:0004803 transposase activity (gain)
c-value
0.8
0.7
0.6
0.5
0.4
0.3
0.2
0.1
0.0
p-value
7e-12
2e-10
3e-09
7e-08
2e-06
Log-odds
0.5
1.0
1.5
2.0
2.5
3.0
3.5
4.0
Cellular_organisms - ps1
Archaea1 - ps2
Archaea2 - ps3
Archaea3 - ps4
Asgard/Eukaryota - ps5
Eukaryota - ps6
Excavata/Diaphoretickes - ps7
Diaphoretickes - ps8
Archaeplastida - ps9
Viridiplantae - ps10
Streptophyta - ps11
Charophyceae/Embriophyta - ps12
Embriophyta - ps13
Tracheophyta - ps14
Spermatophyta - ps15
Angiosperms - ps16
Mesangiospermae - ps17
Magnoliids/eudicots - ps18
Eudicots - ps19
Core_eudicots - ps20
Superrosids - ps21
Rosids - ps22
Eurosids - ps23
Malvids - ps24
Sap./Malv./Brassicales - ps25
Malvales/Brassicales - ps26
Brassicales - ps27
Cleomaceae/Brassicaceae - ps28
Brassicaceae - ps29
Camelineae - ps30
Arabidopsis - ps31
Arabidopsis_thaliana - ps32
Phylostratum

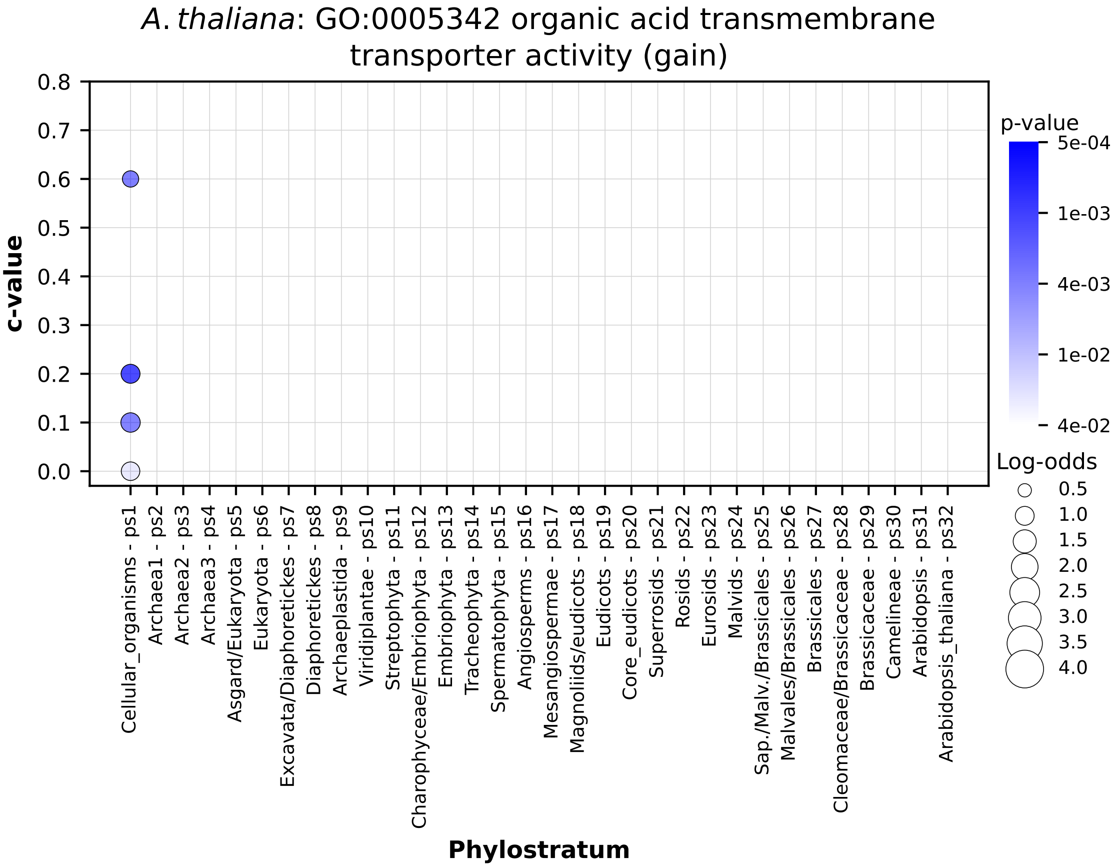

A. thaliana: GO:0005342 organic acid transmembrane transporter activity (gain)
c-value
0.8
0.7
0.6
0.5
0.4
0.3
0.2
0.1
0.0
p-value
5e-04
1e-03
4e-03
1e-02
4e-02
Log-odds
0.5
1.0
1.5
2.0
2.5
3.0
3.5
4.0
Cellular_organisms - ps1
Archaea1 - ps2
Archaea2 - ps3
Archaea3 - ps4
Asgard/Eukaryota - ps5
Eukaryota - ps6
Excavata/Diaphoretickes - ps7
Diaphoretickes - ps8
Archaeplastida - ps9
Viridiplantae - ps10
Streptophyta - ps11
Charophyceae/Embriophyta - ps12
Embriophyta - ps13
Tracheophyta - ps14
Spermatophyta - ps15
Angiosperms - ps16
Mesangiospermae - ps17
Magnoliids/eudicots - ps18
Eudicots - ps19
Core_eudicots - ps20
Superrosids - ps21
Rosids - ps22
Eurosids - ps23
Malvids - ps24
Sap./Malv./Brassicales - ps25
Malvales/Brassicales - ps26
Brassicales - ps27
Cleomaceae/Brassicaceae - ps28
Brassicaceae - ps29
Camelineae - ps30
Arabidopsis - ps31
Arabidopsis_thaliana - ps32
Phylostratum

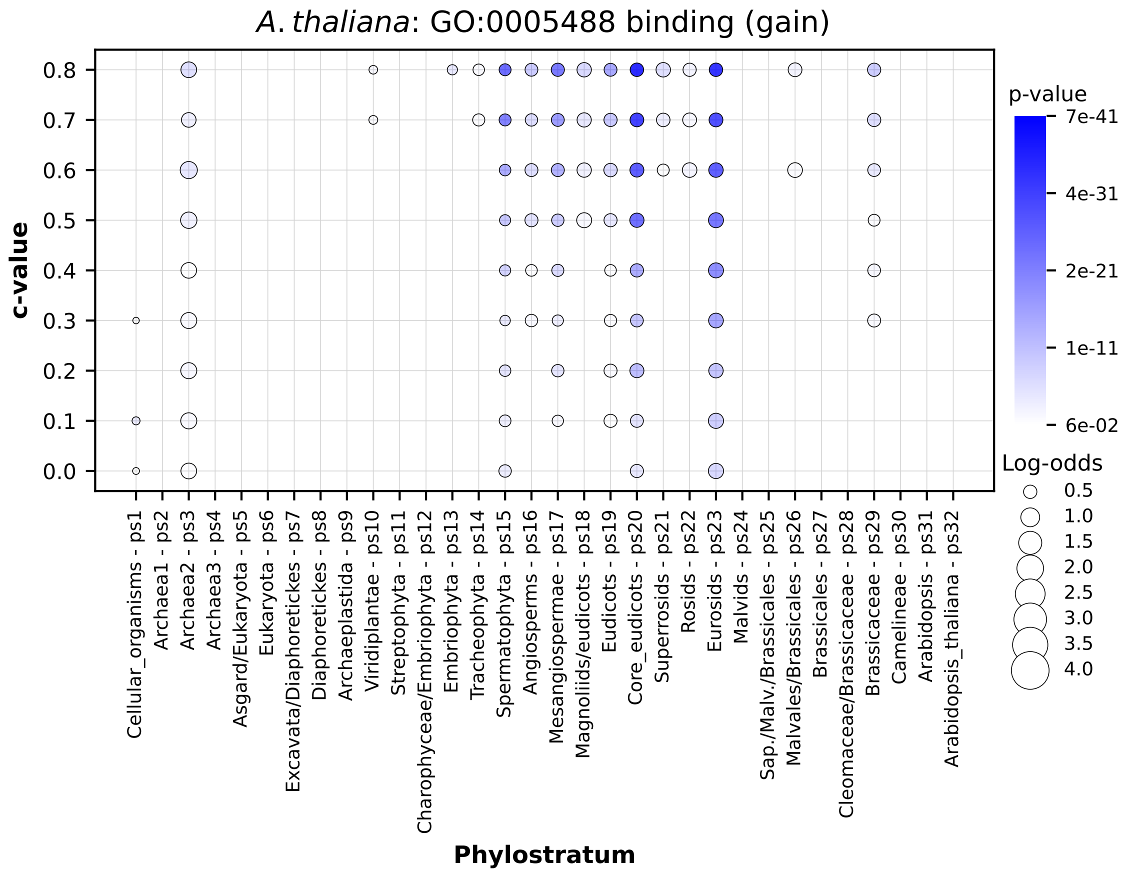
A. thaliana: GO:0005488 binding (gain)
c-value
Phylostratum
p-value
7e-41
4e-31
2e-21
1e-11
6e-02
Log-odds
0.5
1.0
1.5
2.0
2.5
3.0
3.5
4.0
Cellular_organisms - ps1
Archaea1 - ps2
Archaea2 - ps3
Archaea3 - ps4
Asgard/Eukaryota - ps5
Eukaryota - ps6
Excavata/Diaphoretickes - ps7
Diaphoretickes - ps8
Archaeplastida - ps9
Viridiplantae - ps10
Streptophyta - ps11
Charophyceae/Embriophyta - ps12
Embriophyta - ps13
Tracheophyta - ps14
Spermatophyta - ps15
Angiosperms - ps16
Mesangiospermae - ps17
Magnoliids/eudicots - ps18
Eudicots - ps19
Core_eudicots - ps20
Superrosids - ps21
Rosids - ps22
Eurosids - ps23
Malvids - ps24
Sap./Malv./Brassicales - ps25
Malvales/Brassicales - ps26
Brassicales - ps27
Cleomaceae/Brassicaceae - ps28
Brassicaceae - ps29
Camelineae - ps30
Arabidopsis - ps31
Arabidopsis_thaliana - ps32

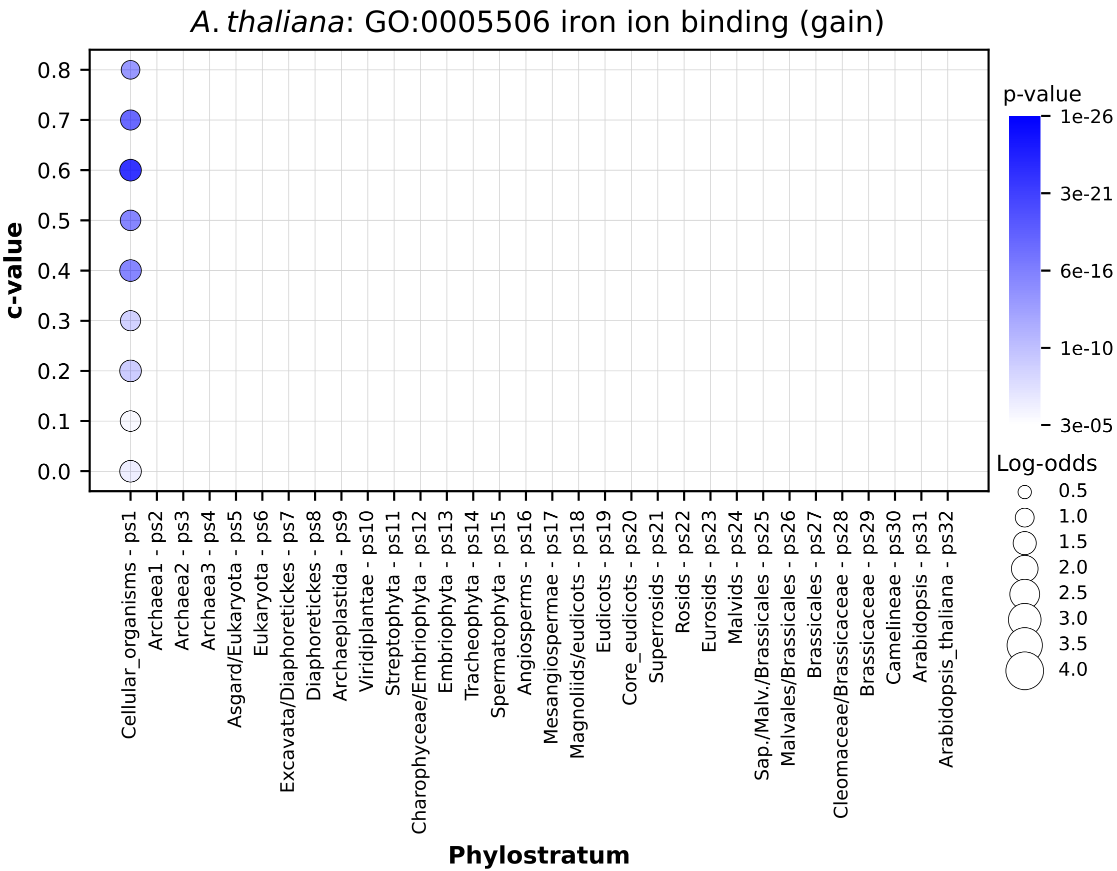

A. thaliana: GO:0005506 iron ion binding (gain)
c-value
0.8
0.7
0.6
0.5
0.4
0.3
0.2
0.1
0.0
Phylostratum
Cellular_organisms - ps1
Archaea1 - ps2
Archaea2 - ps3
Archaea3 - ps4
Asgard/Eukaryota - ps5
Eukaryota - ps6
Excavata/Diaphoretickes - ps7
Diaphoretickes - ps8
Archaeplastida - ps9
Viridiplantae - ps10
Streptophyta - ps11
Charophyceae/Embriophyta - ps12
Embriophyta - ps13
Tracheophyta - ps14
Spermatophyta - ps15
Angiosperms - ps16
Mesangiospermae - ps17
Magnoliids/eudicots - ps18
Eudicots - ps19
Core_eudicots - ps20
Superrosids - ps21
Rosids - ps22
Eurosids - ps23
Malvids - ps24
Sap./Malv./Brassicales - ps25
Malvales/Brassicales - ps26
Brassicales - ps27
Cleomaceae/Brassicaceae - ps28
Brassicaceae - ps29
Camelineae - ps30
Arabidopsis - ps31
Arabidopsis_thaliana - ps32
p-value
1e-26
3e-21
6e-16
1e-10
3e-05
Log-odds
0.5
1.0
1.5
2.0
2.5
3.0
3.5
4.0

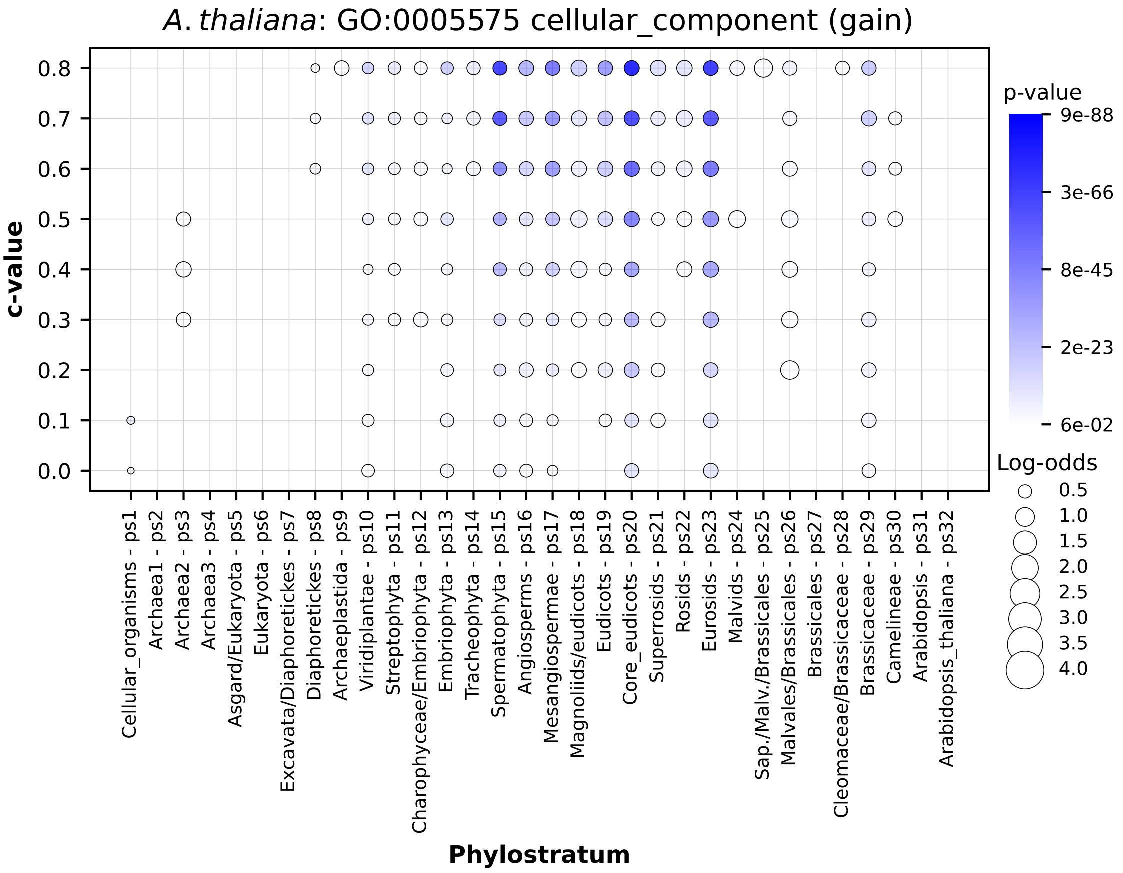
A. thaliana: GO:0005575 cellular_component (gain)
c-value
0.8
0.7
0.6
0.5
0.4
0.3
0.2
0.1
0.0
p-value
9e-88
3e-66
8e-45
2e-23
6e-02
Log-odds
0.5
1.0
1.5
2.0
2.5
3.0
3.5
4.0
Cellular_organisms - ps1
Archaea1 - ps2
Archaea2 - ps3
Archaea3 - ps4
Asgard/Eukaryota - ps5
Eukaryota - ps6
Excavata/Diaphoretickes - ps7
Diaphoretickes - ps8
Archaeplastida - ps9
Viridiplantae - ps10
Streptophyta - ps11
Charophyceae/Embriophyta - ps12
Embriophyta - ps13
Tracheophyta - ps14
Spermatophyta - ps15
Angiosperms - ps16
Mesangiospermae - ps17
Magnoliids/eudicots - ps18
Eudicots - ps19
Core_eudicots - ps20
Superrosids - ps21
Rosids - ps22
Eurosids - ps23
Malvids - ps24
Sap./Malv./Brassicales - ps25
Malvales/Brassicales - ps26
Brassicales - ps27
Cleomaceae/Brassicaceae - ps28
Brassicaceae - ps29
Camelineae - ps30
Arabidopsis - ps31
Arabidopsis_thaliana - ps32
Phylostratum

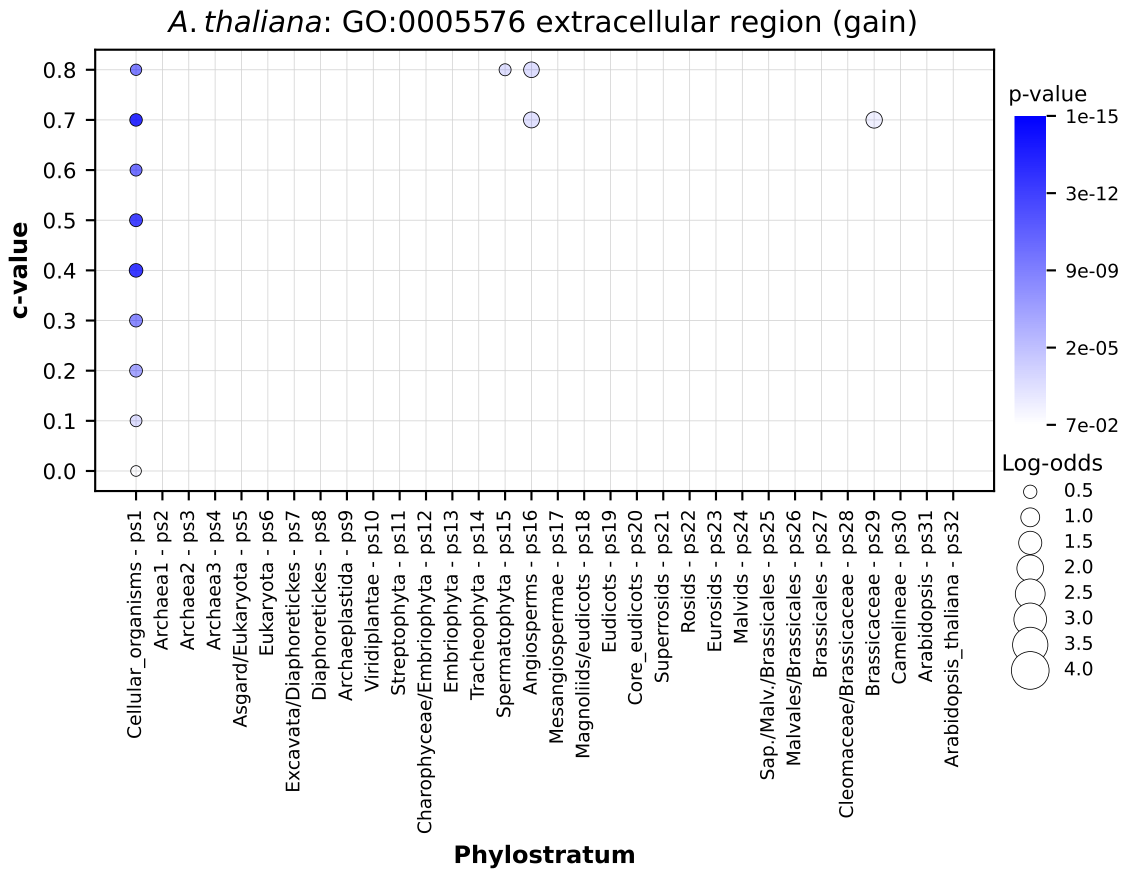

A. thaliana: GO:0005576 extracellular region (gain)
c-value
Phylostratum
p-value
1e-15
3e-12
9e-09
2e-05
7e-02
Log-odds
0.5
1.0
1.5
2.0
2.5
3.0
3.5
4.0
Cellular_organisms - ps1
Archaea1 - ps2
Archaea2 - ps3
Archaea3 - ps4
Asgard/Eukaryota - ps5
Eukaryota - ps6
Excavata/Diaphoretickes - ps7
Diaphoretickes - ps8
Archaeplastida - ps9
Viridiplantae - ps10
Streptophyta - ps11
Charophyceae/Embriophyta - ps12
Embriophyta - ps13
Tracheophyta - ps14
Spermatophyta - ps15
Angiosperms - ps16
Mesangiospermae - ps17
Magnoliids/eudicots - ps18
Eudicots - ps19
Core_eudicots - ps20
Superrosids - ps21
Rosids - ps22
Eurosids - ps23
Malvids - ps24
Sap./Malv./Brassicales - ps25
Malvales/Brassicales - ps26
Brassicales - ps27
Cleomaceae/Brassicaceae - ps28
Brassicaceae - ps29
Camelineae - ps30
Arabidopsis - ps31
Arabidopsis_thaliana - ps32

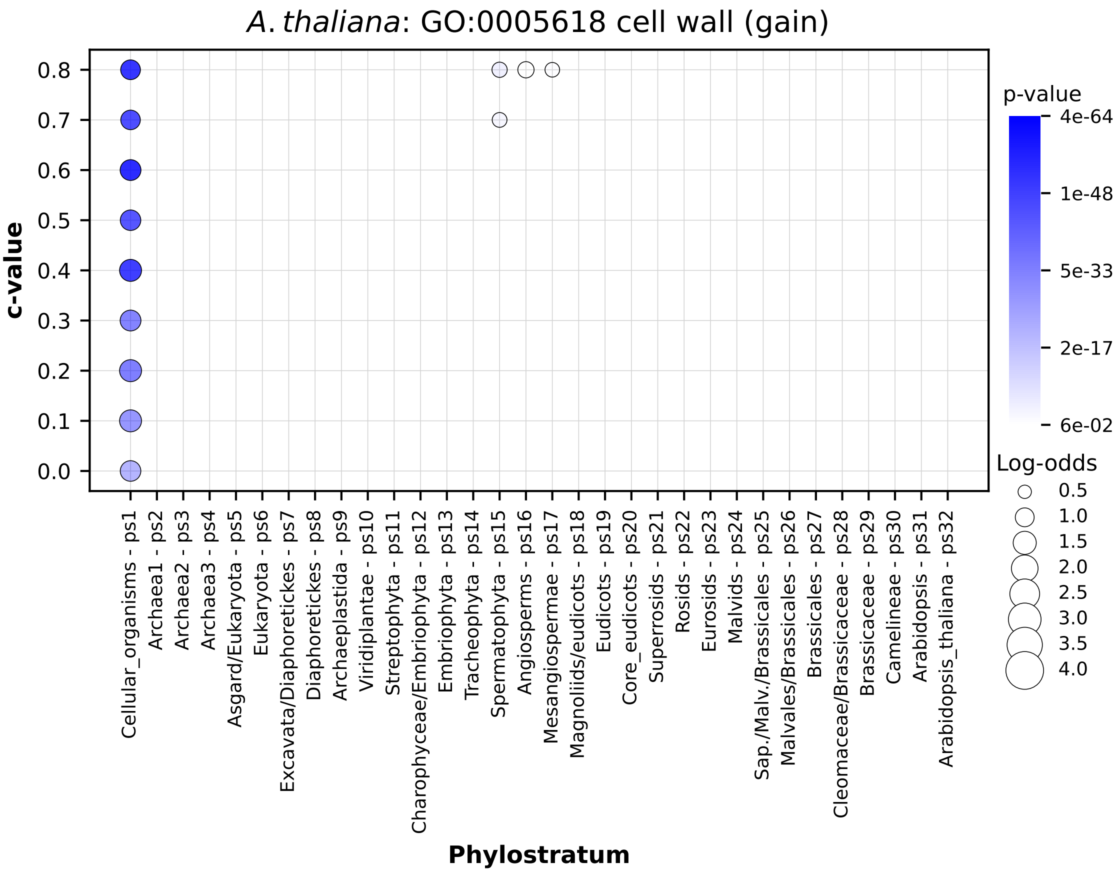

A. thaliana: GO:0005618 cell wall (gain)
c-value
0.0
0.1
0.2
0.3
0.4
0.5
0.6
0.7
0.8
Phylostratum
Cellular_organisms - ps1
Archaea1 - ps2
Archaea2 - ps3
Archaea3 - ps4
Asgard/Eukaryota - ps5
Eukaryota - ps6
Excavata/Diaphoretickes - ps7
Diaphoretickes - ps8
Archaeplastida - ps9
Viridiplantae - ps10
Streptophyta - ps11
Charophyceae/Embriophyta - ps12
Embriophyta - ps13
Tracheophyta - ps14
Spermatophyta - ps15
Angiosperms - ps16
Mesangiospermae - ps17
Magnoliids/eudicots - ps18
Eudicots - ps19
Core_eudicots - ps20
Superrosids - ps21
Rosids - ps22
Eurosids - ps23
Malvids - ps24
Sap./Malv./Brassicales - ps25
Malvales/Brassicales - ps26
Brassicales - ps27
Cleomaceae/Brassicaceae - ps28
Brassicaceae - ps29
Camelineae - ps30
Arabidopsis - ps31
Arabidopsis_thaliana - ps32
p-value
4e-64
1e-48
5e-33
2e-17
6e-02
Log-odds
0.5
1.0
1.5
2.0
2.5
3.0
3.5
4.0

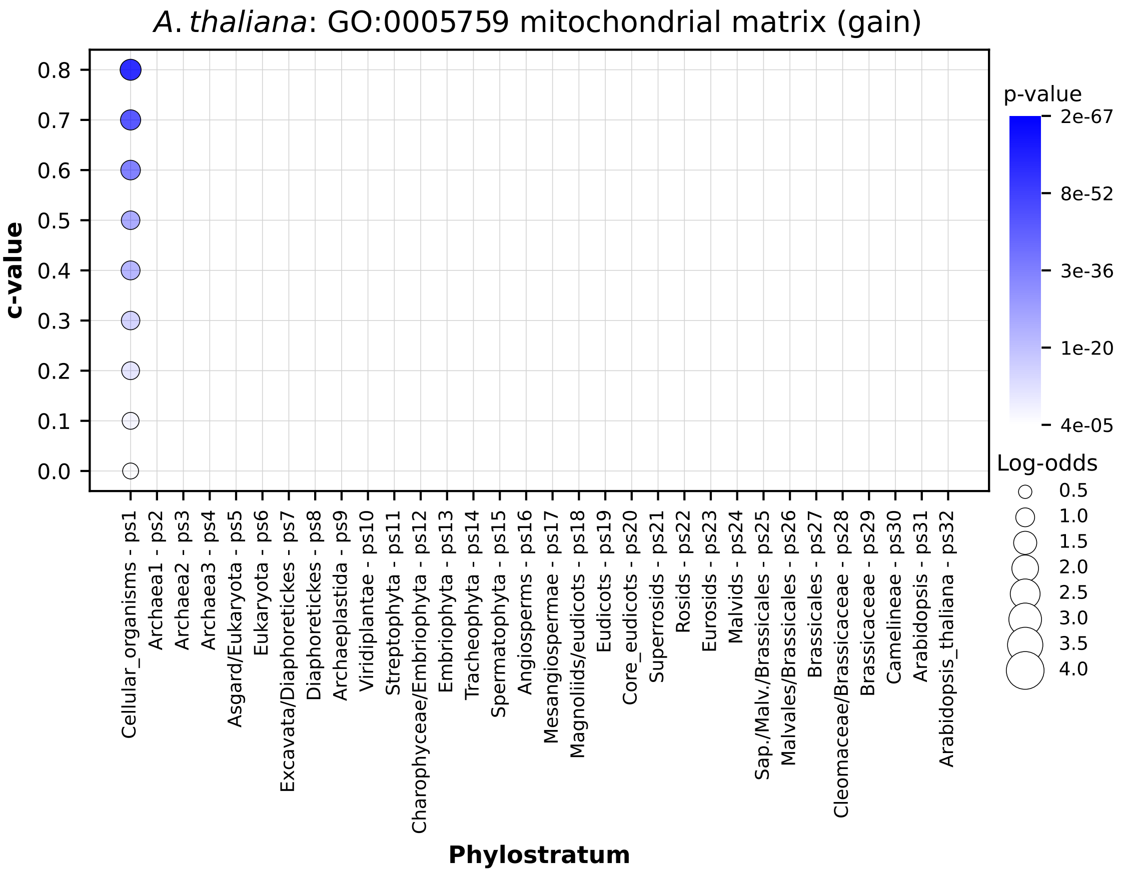

A. thaliana: GO:0005759 mitochondrial matrix (gain)
c-value
0.8
0.7
0.6
0.5
0.4
0.3
0.2
0.1
0.0
p-value
2e-67
8e-52
3e-36
1e-20
4e-05
Log-odds
0.5
1.0
1.5
2.0
2.5
3.0
3.5
4.0
Cellular_organisms - ps1
Archaea1 - ps2
Archaea2 - ps3
Archaea3 - ps4
Asgard/Eukaryota - ps5
Eukaryota - ps6
Excavata/Diaphoretickes - ps7
Diaphoretickes - ps8
Archaeplastida - ps9
Viridiplantae - ps10
Streptophyta - ps11
Charophyceae/Embriophyta - ps12
Embriophyta - ps13
Tracheophyta - ps14
Spermatophyta - ps15
Angiosperms - ps16
Mesangiospermae - ps17
Magnoliids/eudicots - ps18
Eudicots - ps19
Core_eudicots - ps20
Superrosids - ps21
Rosids - ps22
Eurosids - ps23
Malvids - ps24
Sap./Malv./Brassicales - ps25
Malvales/Brassicales - ps26
Brassicales - ps27
Cleomaceae/Brassicaceae - ps28
Brassicaceae - ps29
Camelineae - ps30
Arabidopsis - ps31
Arabidopsis_thaliana - ps32
Phylostratum

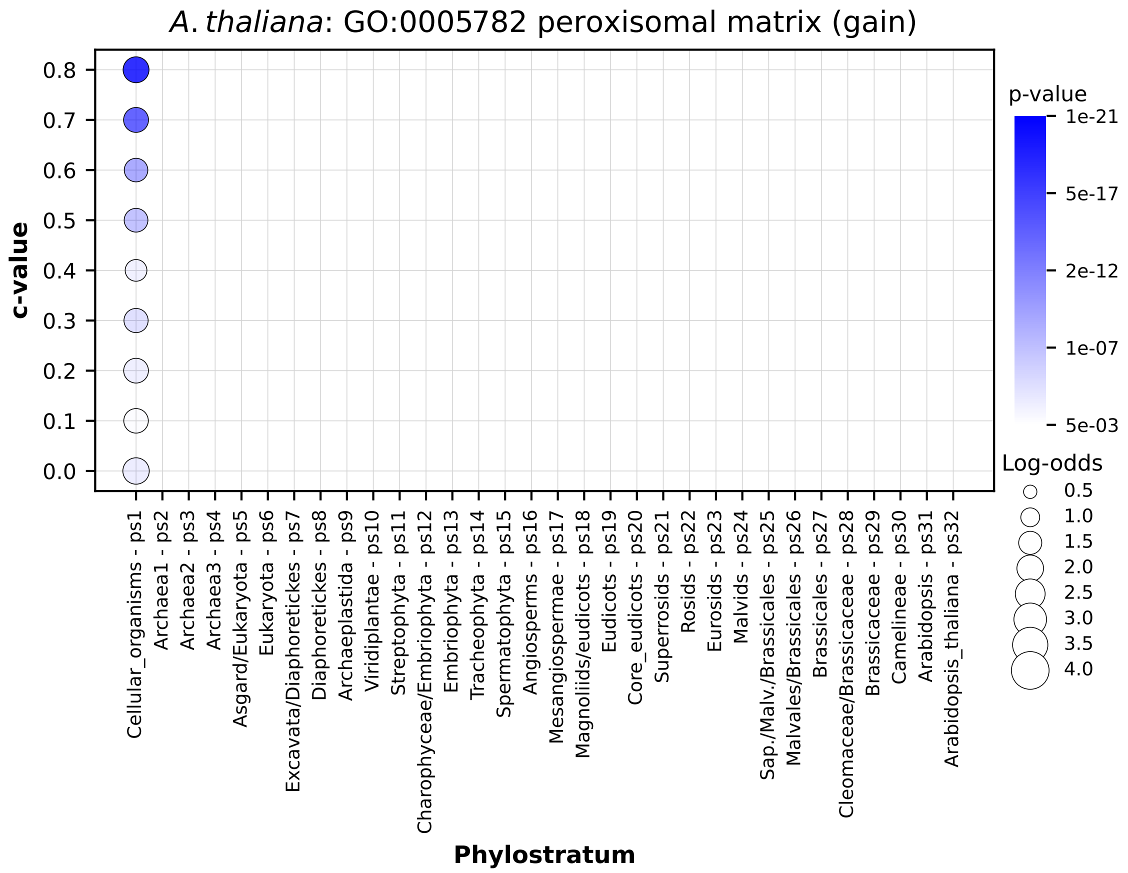

A. thaliana: GO:0005782 peroxisomal matrix (gain)
c-value
0.8
0.7
0.6
0.5
0.4
0.3
0.2
0.1
0.0
p-value
1e-21
5e-17
2e-12
1e-07
5e-03
Log-odds
0.5
1.0
1.5
2.0
2.5
3.0
3.5
4.0
Cellular_organisms - ps1
Archaea1 - ps2
Archaea2 - ps3
Archaea3 - ps4
Asgard/Eukaryota - ps5
Eukaryota - ps6
Excavata/Diaphoretickes - ps7
Diaphoretickes - ps8
Archaeplastida - ps9
Viridiplantae - ps10
Streptophyta - ps11
Charophyceae/Embriophyta - ps12
Embriophyta - ps13
Tracheophyta - ps14
Spermatophyta - ps15
Angiosperms - ps16
Mesangiospermae - ps17
Magnoliids/eudicots - ps18
Eudicots - ps19
Core_eudicots - ps20
Superrosids - ps21
Rosids - ps22
Eurosids - ps23
Malvids - ps24
Sap./Malv./Brassicales - ps25
Malvales/Brassicales - ps26
Brassicales - ps27
Cleomaceae/Brassicaceae - ps28
Brassicaceae - ps29
Camelineae - ps30
Arabidopsis - ps31
Arabidopsis_thaliana - ps32
Phylostratum

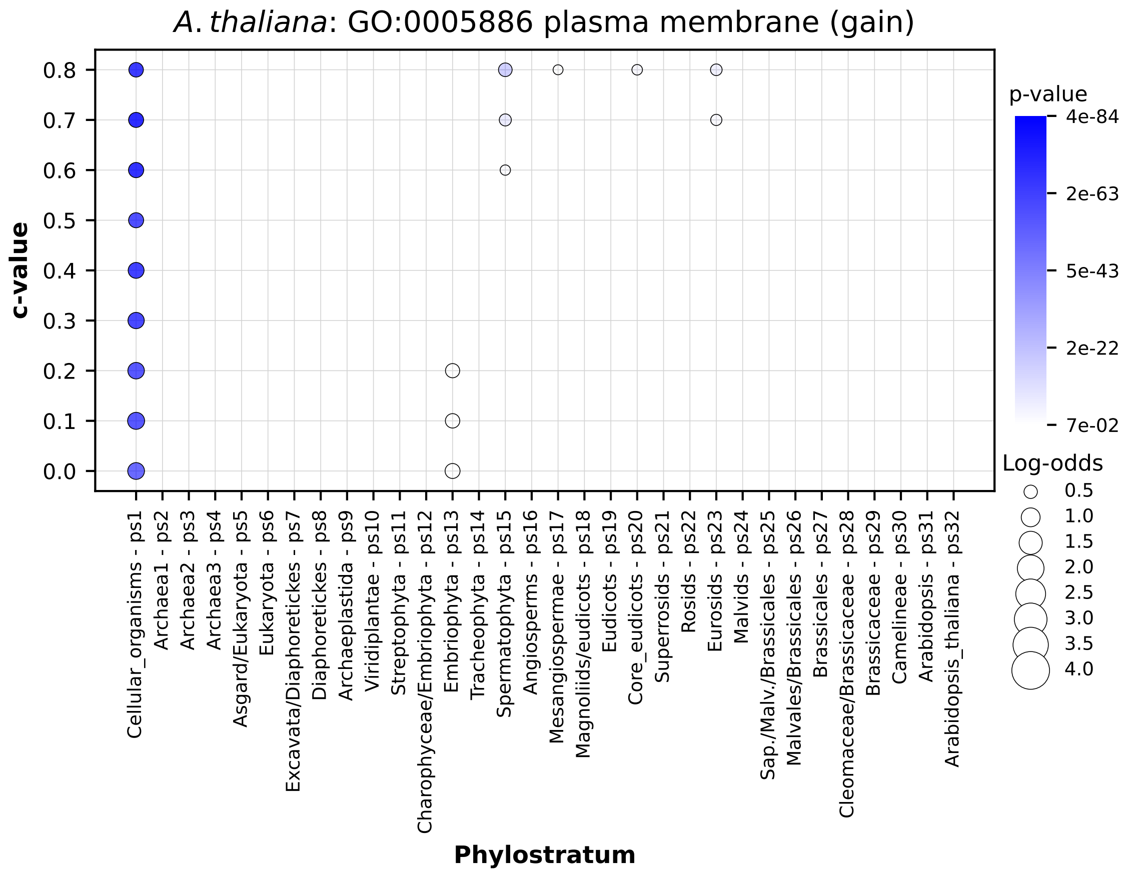
A. thaliana: GO:0005886 plasma membrane (gain)
c-value
0.8
0.7
0.6
0.5
0.4
0.3
0.2
0.1
0.0
p-value
4e-84
2e-63
5e-43
2e-22
7e-02
Log-odds
0.5
1.0
1.5
2.0
2.5
3.0
3.5
4.0
Cellular_organisms - ps1
Archaea1 - ps2
Archaea2 - ps3
Archaea3 - ps4
Asgard/Eukaryota - ps5
Eukaryota - ps6
Excavata/Diaphoretickes - ps7
Diaphoretickes - ps8
Archaeplastida - ps9
Viridiplantae - ps10
Streptophyta - ps11
Charophyceae/Embriophyta - ps12
Embriophyta - ps13
Tracheophyta - ps14
Spermatophyta - ps15
Angiosperms - ps16
Mesangiospermae - ps17
Magnoliids/eudicots - ps18
Eudicots - ps19
Core_eudicots - ps20
Superrosids - ps21
Rosids - ps22
Eurosids - ps23
Malvids - ps24
Sap./Malv./Brassicales - ps25
Malvales/Brassicales - ps26
Brassicales - ps27
Cleomaceae/Brassicaceae - ps28
Brassicaceae - ps29
Camelineae - ps30
Arabidopsis - ps31
Arabidopsis_thaliana - ps32
Phylostratum

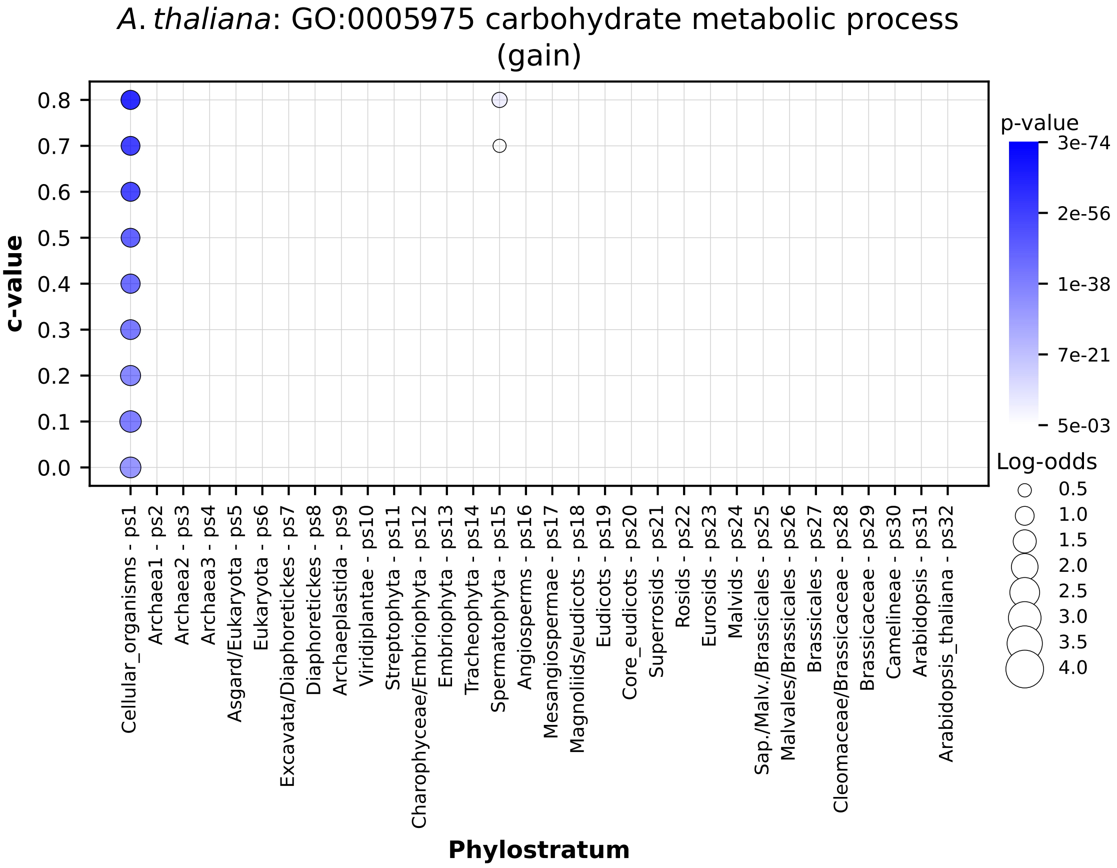

A. thaliana: GO:0005975 carbohydrate metabolic process
(gain)
c-value
0.8
0.7
0.6
0.5
0.4
0.3
0.2
0.1
0.0
p-value
3e-74
2e-56
1e-38
7e-21
5e-03
Log-odds
0.5
1.0
1.5
2.0
2.5
3.0
3.5
4.0
Cellular_organisms - ps1
Archaea1 - ps2
Archaea2 - ps3
Archaea3 - ps4
Asgard/Eukaryota - ps5
Eukaryota - ps6
Excavata/Diaphoretickes - ps7
Diaphoretickes - ps8
Archaeplastida - ps9
Viridiplantae - ps10
Streptophyta - ps11
Charophyceae/Embriophyta - ps12
Embriophyta - ps13
Tracheophyta - ps14
Spermatophyta - ps15
Angiosperms - ps16
Mesangiospermae - ps17
Magnoliids/eudicots - ps18
Eudicots - ps19
Core_eudicots - ps20
Superrosids - ps21
Rosids - ps22
Eurosids - ps23
Malvids - ps24
Sap./Malv./Brassicales - ps25
Malvales/Brassicales - ps26
Brassicales - ps27
Cleomaceae/Brassicaceae - ps28
Brassicaceae - ps29
Camelineae - ps30
Arabidopsis - ps31
Arabidopsis_thaliana - ps32
Phylostratum

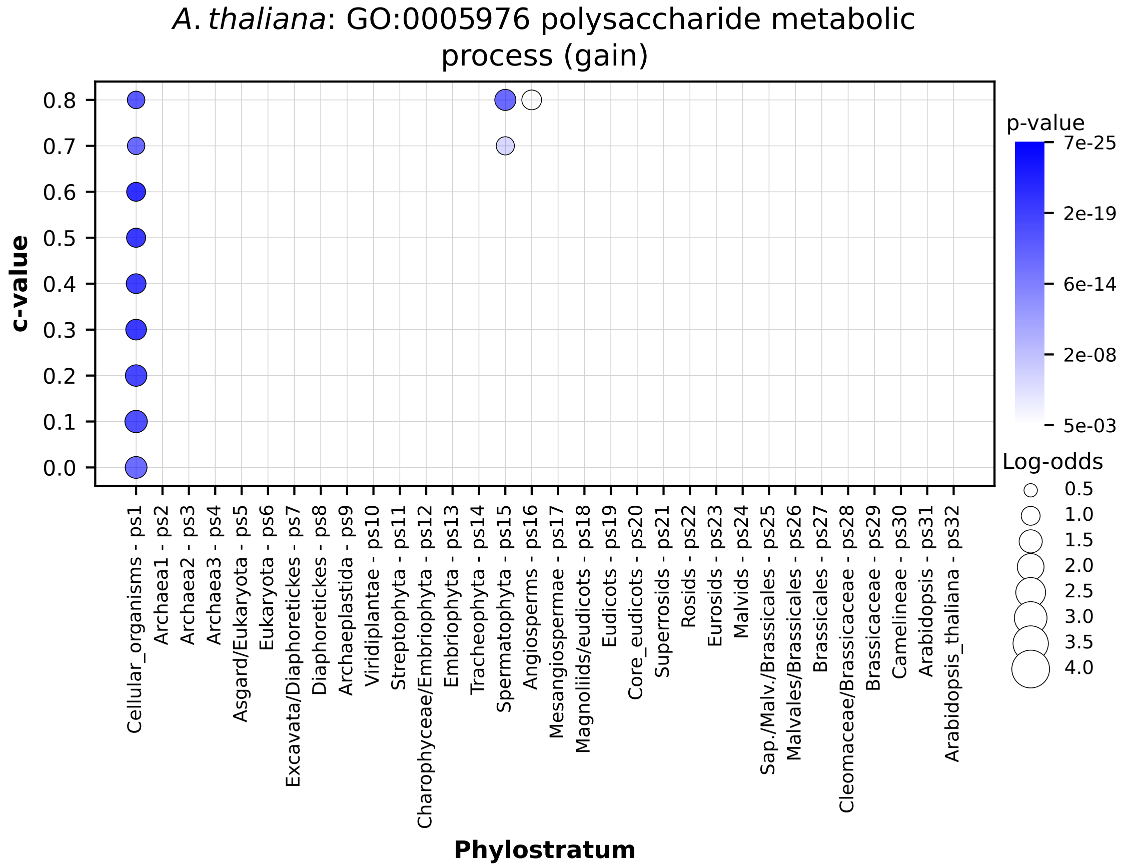

A. thaliana: GO:0005976 polysaccharide metabolic process (gain)
c-value
0.8
0.7
0.6
0.5
0.4
0.3
0.2
0.1
0.0
Phylostratum
Cellular_organisms - ps1
Archaea1 - ps2
Archaea2 - ps3
Archaea3 - ps4
Asgard/Eukaryota - ps5
Eukaryota - ps6
Excavata/Diaphoretickes - ps7
Diaphoretickes - ps8
Archaeplastida - ps9
Viridiplantae - ps10
Streptophyta - ps11
Charophyceae/Embriophyta - ps12
Embriophyta - ps13
Tracheophyta - ps14
Spermatophyta - ps15
Angiosperms - ps16
Mesangiospermae - ps17
Magnoliids/eudicots - ps18
Eudicots - ps19
Core_eudicots - ps20
Superrosids - ps21
Rosids - ps22
Eurosids - ps23
Malvids - ps24
Sap./Malv./Brassicales - ps25
Malvales/Brassicales - ps26
Brassicales - ps27
Cleomaceae/Brassicaceae - ps28
Brassicaceae - ps29
Camelineae - ps30
Arabidopsis - ps31
Arabidopsis_thaliana - ps32
p-value
7e-25
2e-19
6e-14
2e-08
5e-03
Log-odds
0.5
1.0
1.5
2.0
2.5
3.0
3.5
4.0

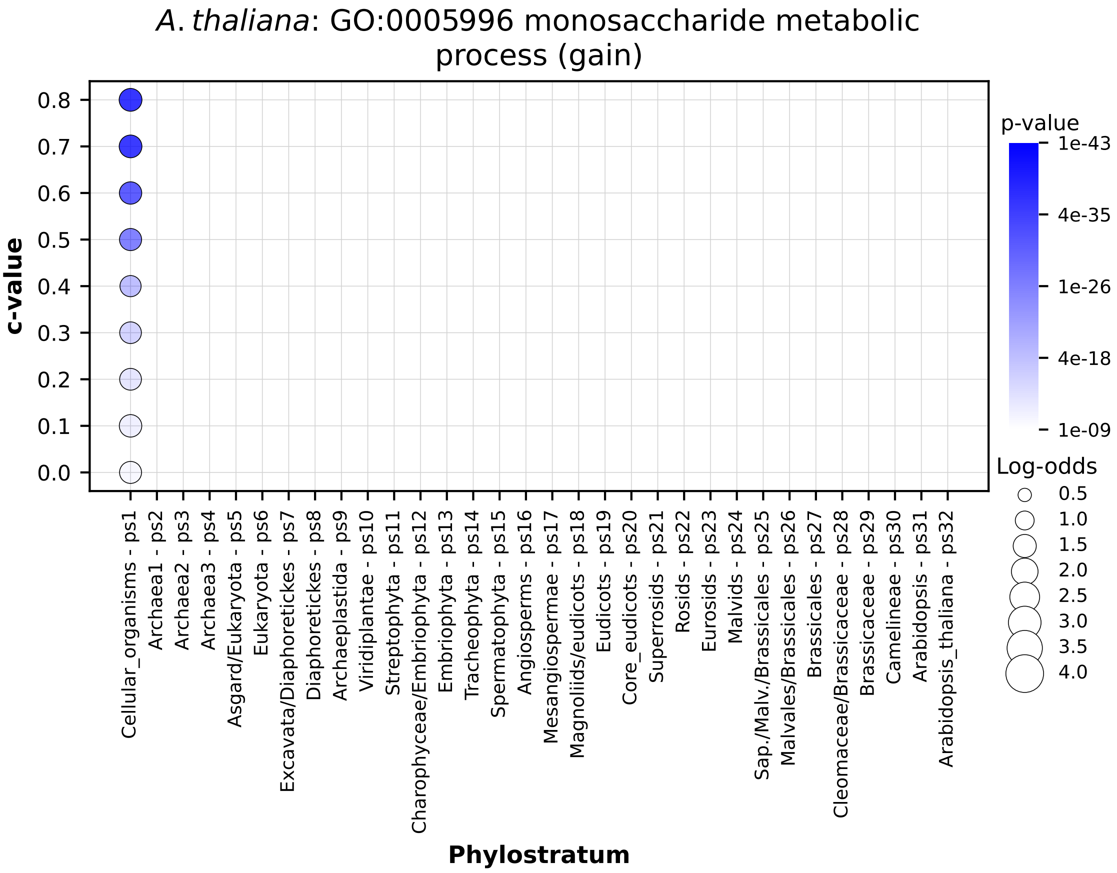
A. thaliana: GO:0005996 monosaccharide metabolic process (gain)
c-value
0.8
0.7
0.6
0.5
0.4
0.3
0.2
0.1
0.0
p-value
1e-43
4e-35
1e-26
4e-18
1e-09
Log-odds
0.5
1.0
1.5
2.0
2.5
3.0
3.5
4.0
Cellular_organisms - ps1
Archaea1 - ps2
Archaea2 - ps3
Archaea3 - ps4
Asgard/Eukaryota - ps5
Eukaryota - ps6
Excavata/Diaphoretickes - ps7
Diaphoretickes - ps8
Archaeplastida - ps9
Viridiplantae - ps10
Streptophyta - ps11
Charophyceae/Embriophyta - ps12
Embriophyta - ps13
Tracheophyta - ps14
Spermatophyta - ps15
Angiosperms - ps16
Mesangiospermae - ps17
Magnoliids/eudicots - ps18
Eudicots - ps19
Core_eudicots - ps20
Superrosids - ps21
Rosids - ps22
Eurosids - ps23
Malvids - ps24
Sap./Malv./Brassicales - ps25
Malvales/Brassicales - ps26
Brassicales - ps27
Cleomaceae/Brassicaceae - ps28
Brassicaceae - ps29
Camelineae - ps30
Arabidopsis - ps31
Arabidopsis_thaliana - ps32
Phylostratum

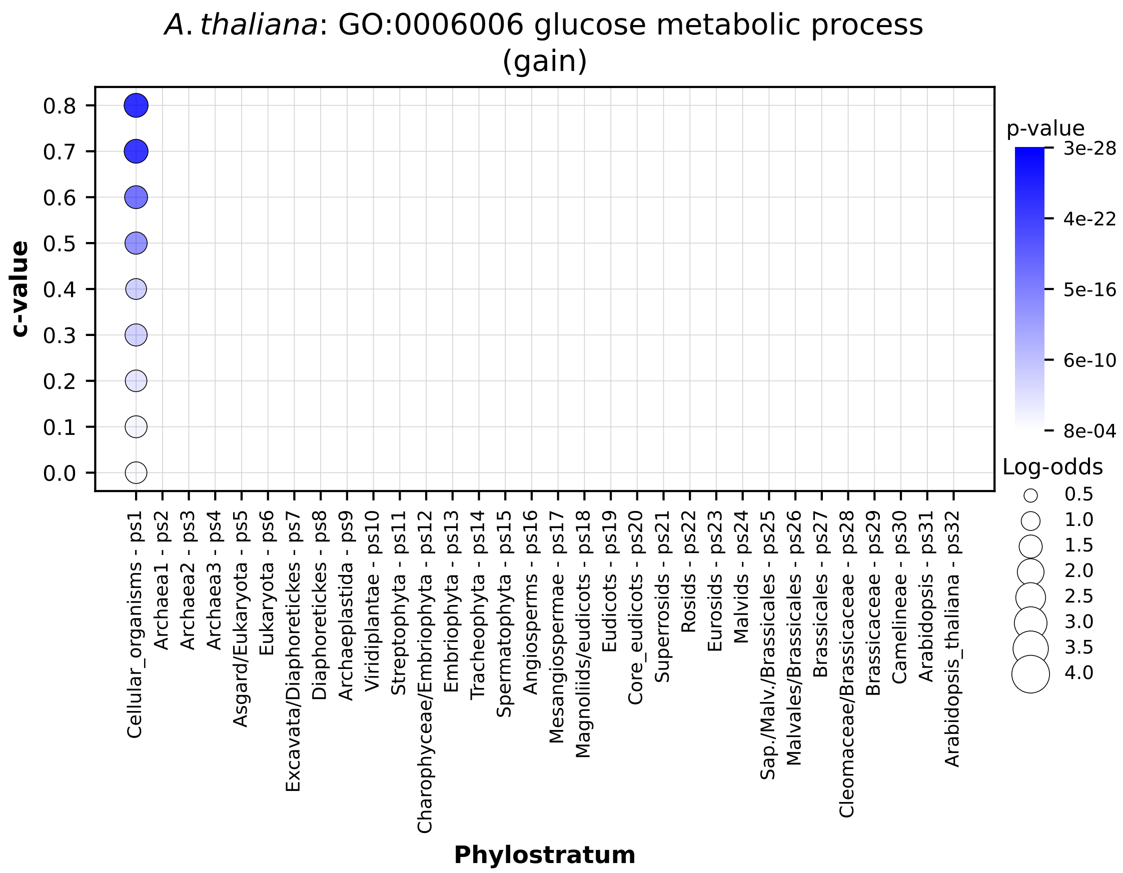
A. thaliana: GO:0006006 glucose metabolic process
(gain)
c-value
0.8
0.7
0.6
0.5
0.4
0.3
0.2
0.1
0.0
p-value
3e-28
4e-22
5e-16
6e-10
8e-04
Log-odds
0.5
1.0
1.5
2.0
2.5
3.0
3.5
4.0
Cellular_organisms - ps1
Archaea1 - ps2
Archaea2 - ps3
Archaea3 - ps4
Asgard/Eukaryota - ps5
Eukaryota - ps6
Excavata/Diaphoretickes - ps7
Diaphoretickes - ps8
Archaeplastida - ps9
Viridiplantae - ps10
Streptophyta - ps11
Charophyceae/Embriophyta - ps12
Embriophyta - ps13
Tracheophyta - ps14
Spermatophyta - ps15
Angiosperms - ps16
Mesangiospermae - ps17
Magnoliids/eudicots - ps18
Eudicots - ps19
Core_eudicots - ps20
Superrosids - ps21
Rosids - ps22
Eurosids - ps23
Malvids - ps24
Sap./Malv./Brassicales - ps25
Malvales/Brassicales - ps26
Brassicales - ps27
Cleomaceae/Brassicaceae - ps28
Brassicaceae - ps29
Camelineae - ps30
Arabidopsis - ps31
Arabidopsis_thaliana - ps32
Phylostratum

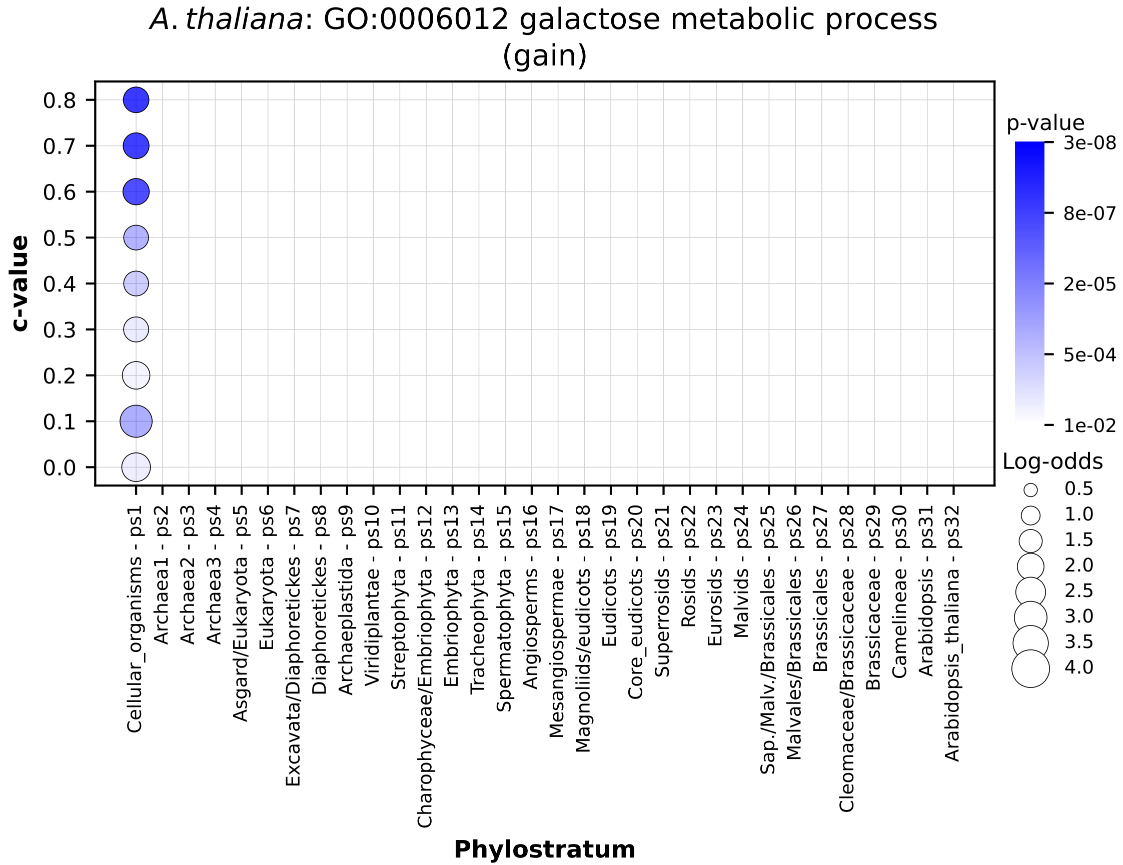
A. thaliana: GO:0006012 galactose metabolic process
(gain)
c-value
0.0
0.1
0.2
0.3
0.4
0.5
0.6
0.7
0.8
Phylostratum
Cellular_organisms - ps1
Archaea1 - ps2
Archaea2 - ps3
Archaea3 - ps4
Asgard/Eukaryota - ps5
Eukaryota - ps6
Excavata/Diaphoretickes - ps7
Diaphoretickes - ps8
Archaeplastida - ps9
Viridiplantae - ps10
Streptophyta - ps11
Charophyceae/Embriophyta - ps12
Embriophyta - ps13
Tracheophyta - ps14
Spermatophyta - ps15
Angiosperms - ps16
Mesangiospermae - ps17
Magnoliids/eudicots - ps18
Eudicots - ps19
Core_eudicots - ps20
Superrosids - ps21
Rosids - ps22
Eurosids - ps23
Malvids - ps24
Sap./Malv./Brassicales - ps25
Malvales/Brassicales - ps26
Brassicales - ps27
Cleomaceae/Brassicaceae - ps28
Brassicaceae - ps29
Camelineae - ps30
Arabidopsis - ps31
Arabidopsis_thaliana - ps32
p-value
3e-08
8e-07
2e-05
5e-04
1e-02
Log-odds
0.5
1.0
1.5
2.0
2.5
3.0
3.5
4.0

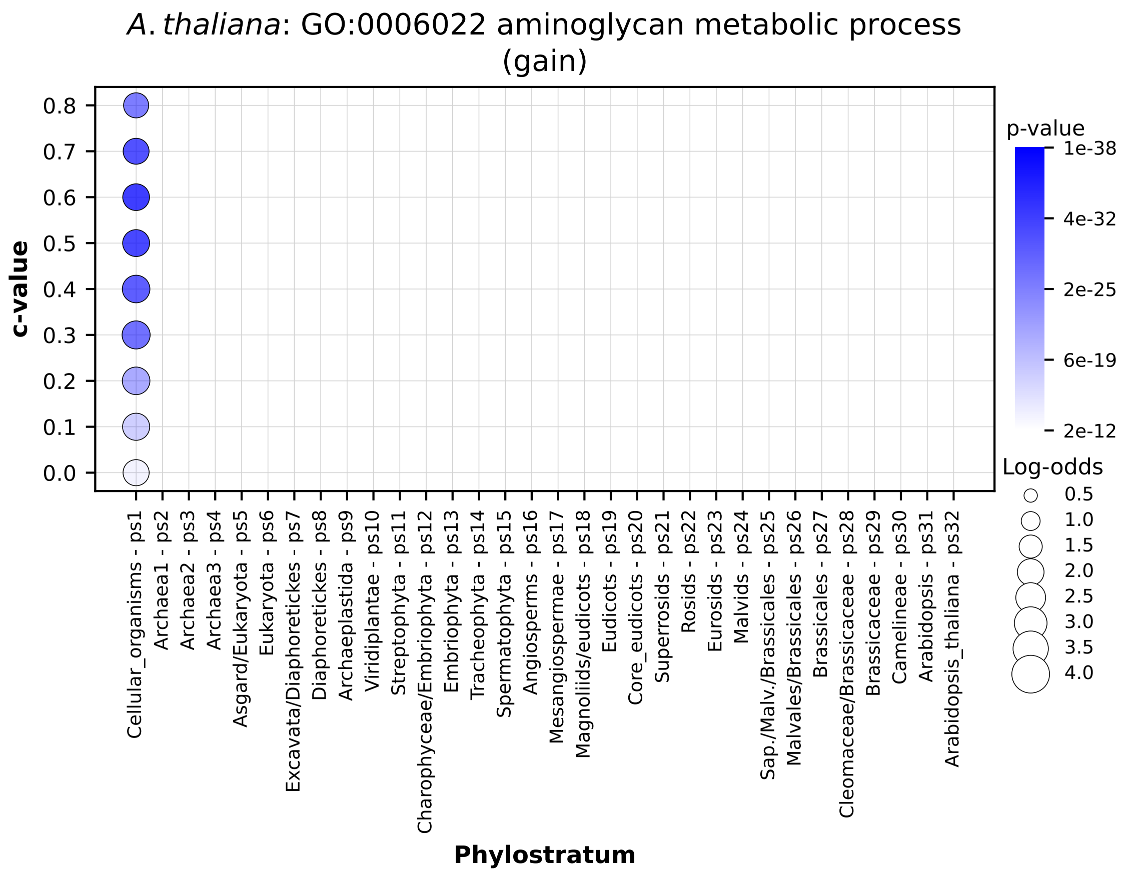
A. thaliana: GO:0006022 aminoglycan metabolic process
(gain)
c-value
0.8
0.7
0.6
0.5
0.4
0.3
0.2
0.1
0.0
p-value
1e-38
4e-32
2e-25
6e-19
2e-12
Log-odds
0.5
1.0
1.5
2.0
2.5
3.0
3.5
4.0
Cellular_organisms - ps1
Archaea1 - ps2
Archaea2 - ps3
Archaea3 - ps4
Asgard/Eukaryota - ps5
Eukaryota - ps6
Excavata/Diaphoretickes - ps7
Diaphoretickes - ps8
Archaeplastida - ps9
Viridiplantae - ps10
Streptophyta - ps11
Charophyceae/Embriophyta - ps12
Embriophyta - ps13
Tracheophyta - ps14
Spermatophyta - ps15
Angiosperms - ps16
Mesangiospermae - ps17
Magnoliids/eudicots - ps18
Eudicots - ps19
Core_eudicots - ps20
Superrosids - ps21
Rosids - ps22
Eurosids - ps23
Malvids - ps24
Sap./Malv./Brassicales - ps25
Malvales/Brassicales - ps26
Brassicales - ps27
Cleomaceae/Brassicaceae - ps28
Brassicaceae - ps29
Camelineae - ps30
Arabidopsis - ps31
Arabidopsis_thaliana - ps32
Phylostratum

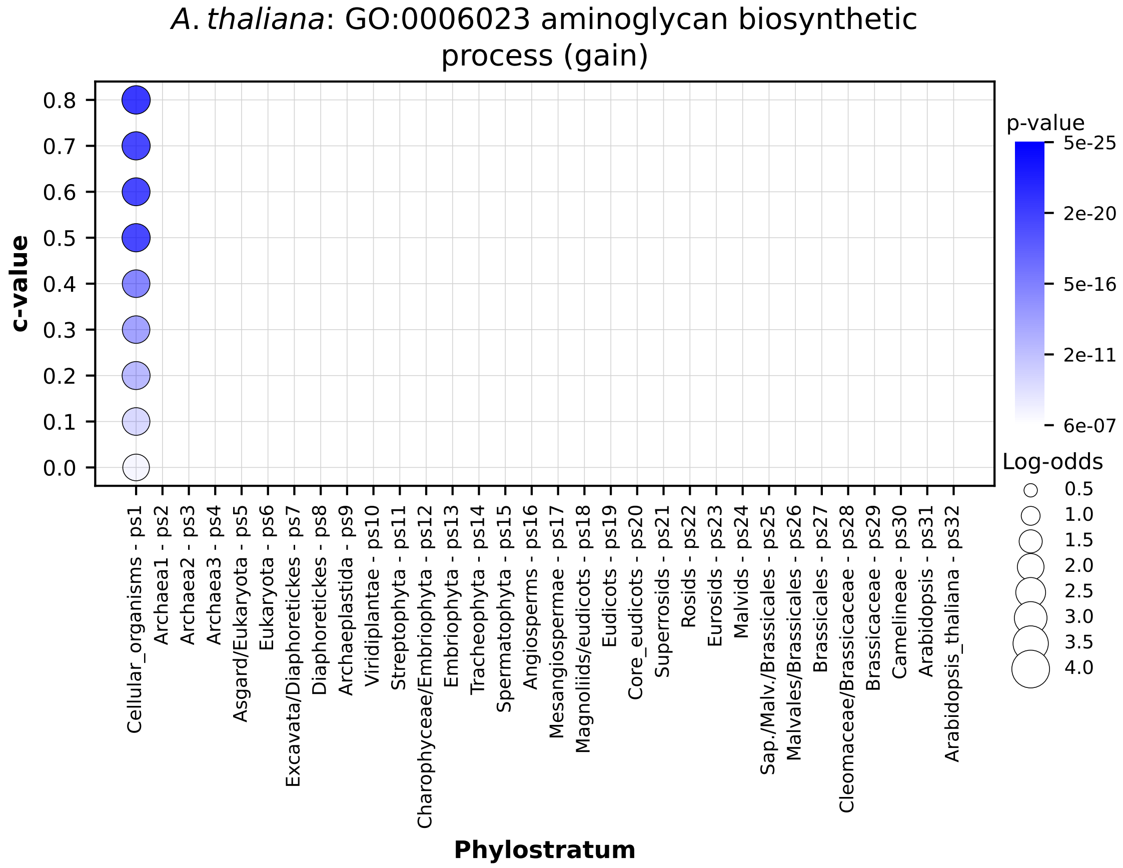
A. thaliana: GO:0006023 aminoglycan biosynthetic process (gain)
c-value
0.0
0.1
0.2
0.3
0.4
0.5
0.6
0.7
0.8
p-value
5e-25
2e-20
5e-16
2e-11
6e-07
Log-odds
0.5
1.0
1.5
2.0
2.5
3.0
3.5
4.0
Cellular_organisms - ps1
Archaea1 - ps2
Archaea2 - ps3
Archaea3 - ps4
Asgard/Eukaryota - ps5
Eukaryota - ps6
Excavata/Diaphoretickes - ps7
Diaphoretickes - ps8
Archaeplastida - ps9
Viridiplantae - ps10
Streptophyta - ps11
Charophyceae/Embriophyta - ps12
Embriophyta - ps13
Tracheophyta - ps14
Spermatophyta - ps15
Angiosperms - ps16
Mesangiospermae - ps17
Magnoliids/eudicots - ps18
Eudicots - ps19
Core_eudicots - ps20
Superrosids - ps21
Rosids - ps22
Eurosids - ps23
Malvids - ps24
Sap./Malv./Brassicales - ps25
Malvales/Brassicales - ps26
Brassicales - ps27
Cleomaceae/Brassicaceae - ps28
Brassicaceae - ps29
Camelineae - ps30
Arabidopsis - ps31
Arabidopsis_thaliana - ps32
Phylostratum

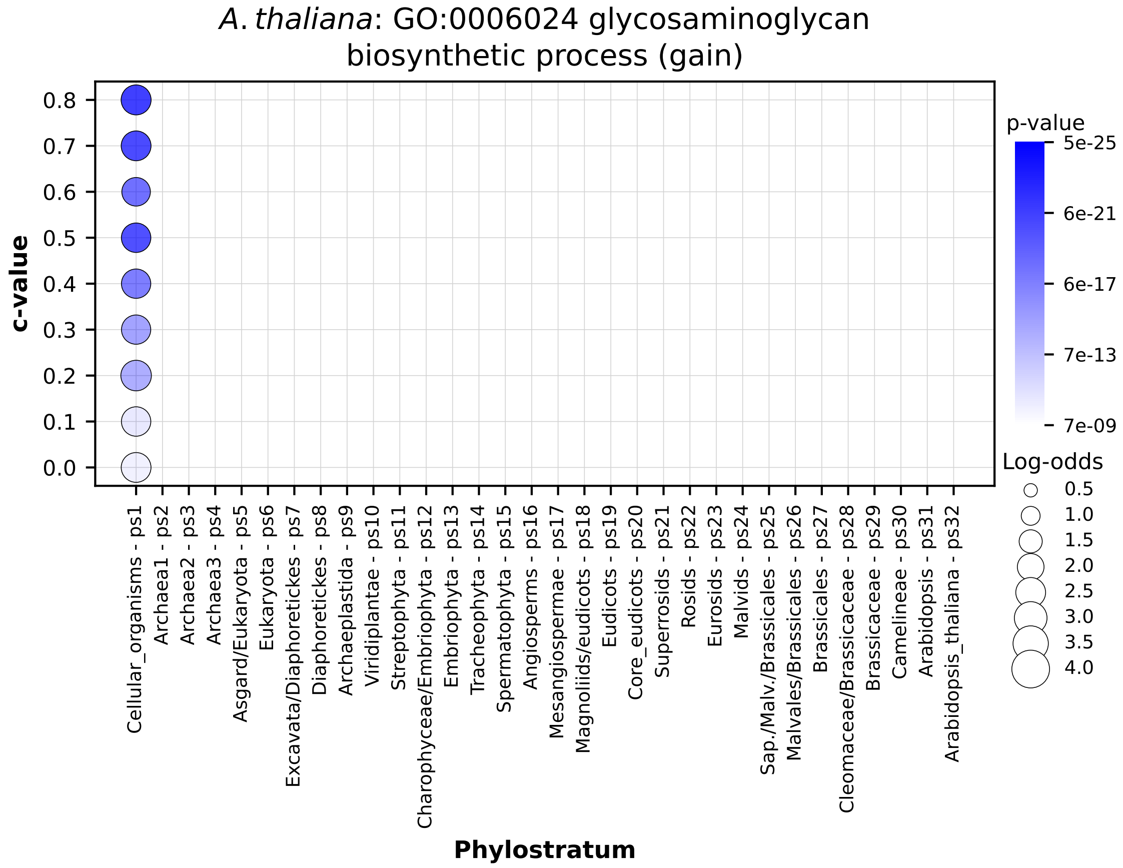

A. thaliana: GO:0006024 glycosaminoglycan biosynthetic process (gain)
c-value
0.0
0.1
0.2
0.3
0.4
0.5
0.6
0.7
0.8
p-value
5e-25
6e-21
6e-17
7e-13
7e-09
Log-odds
0.5
1.0
1.5
2.0
2.5
3.0
3.5
4.0
Cellular_organisms - ps1
Archaea1 - ps2
Archaea2 - ps3
Archaea3 - ps4
Asgard/Eukaryota - ps5
Eukaryota - ps6
Excavata/Diaphoretickes - ps7
Diaphoretickes - ps8
Archaeplastida - ps9
Viridiplantae - ps10
Streptophyta - ps11
Charophyceae/Embriophyta - ps12
Embriophyta - ps13
Tracheophyta - ps14
Spermatophyta - ps15
Angiosperms - ps16
Mesangiospermae - ps17
Magnoliids/eudicots - ps18
Eudicots - ps19
Core_eudicots - ps20
Superrosids - ps21
Rosids - ps22
Eurosids - ps23
Malvids - ps24
Sap./Malv./Brassicales - ps25
Malvales/Brassicales - ps26
Brassicales - ps27
Cleomaceae/Brassicaceae - ps28
Brassicaceae - ps29
Camelineae - ps30
Arabidopsis - ps31
Arabidopsis_thaliana - ps32
Phylostratum

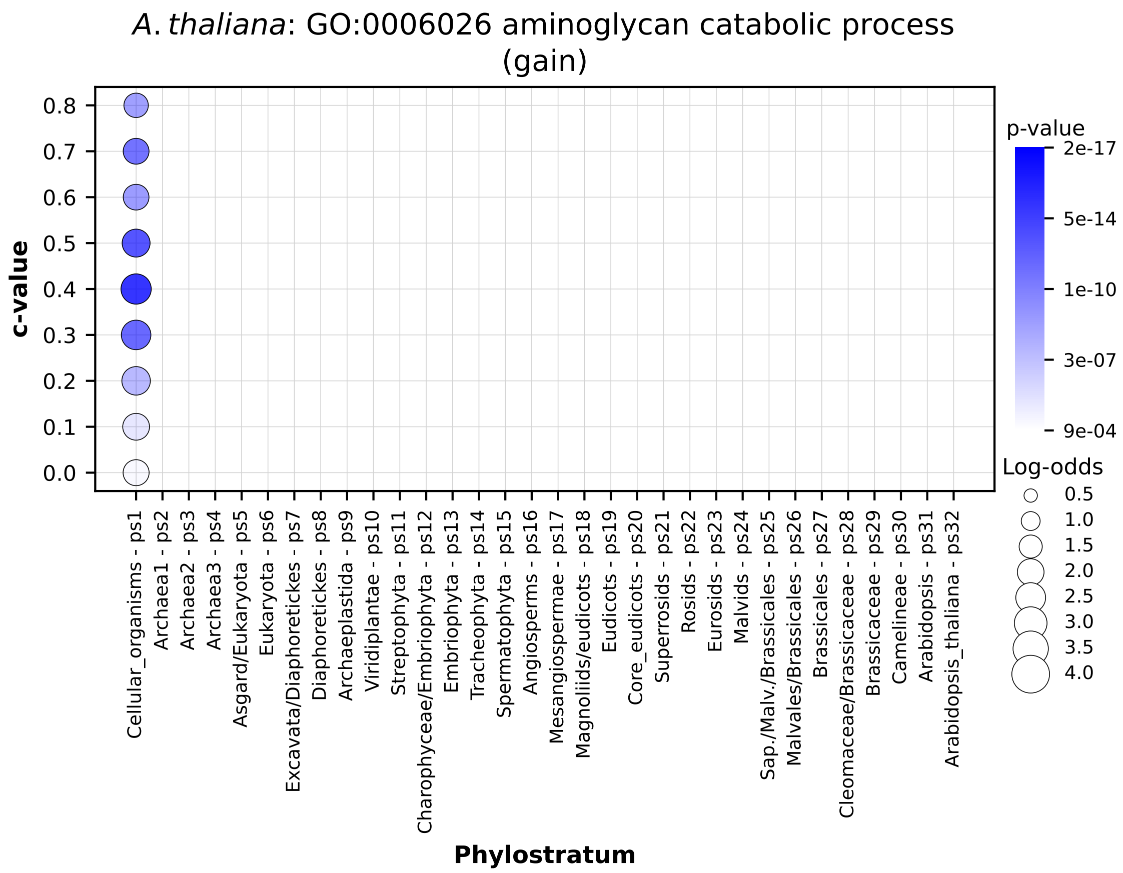

A. thaliana: GO:0006026 aminoglycan catabolic process (gain)
c-value
0.0
0.1
0.2
0.3
0.4
0.5
0.6
0.7
0.8
Phylostratum
Cellular_organisms - ps1
Archaea1 - ps2
Archaea2 - ps3
Archaea3 - ps4
Asgard/Eukaryota - ps5
Eukaryota - ps6
Excavata/Diaphoretickes - ps7
Diaphoretickes - ps8
Archaeplastida - ps9
Viridiplantae - ps10
Streptophyta - ps11
Charophyceae/Embriophyta - ps12
Embriophyta - ps13
Tracheophyta - ps14
Spermatophyta - ps15
Angiosperms - ps16
Mesangiospermae - ps17
Magnoliids/eudicots - ps18
Eudicots - ps19
Core_eudicots - ps20
Superrosids - ps21
Rosids - ps22
Eurosids - ps23
Malvids - ps24
Sap./Malv./Brassicales - ps25
Malvales/Brassicales - ps26
Brassicales - ps27
Cleomaceae/Brassicaceae - ps28
Brassicaceae - ps29
Camelineae - ps30
Arabidopsis - ps31
Arabidopsis_thaliana - ps32
p-value
2e-17
5e-14
1e-10
3e-07
9e-04
Log-odds
0.5
1.0
1.5
2.0
2.5
3.0
3.5
4.0

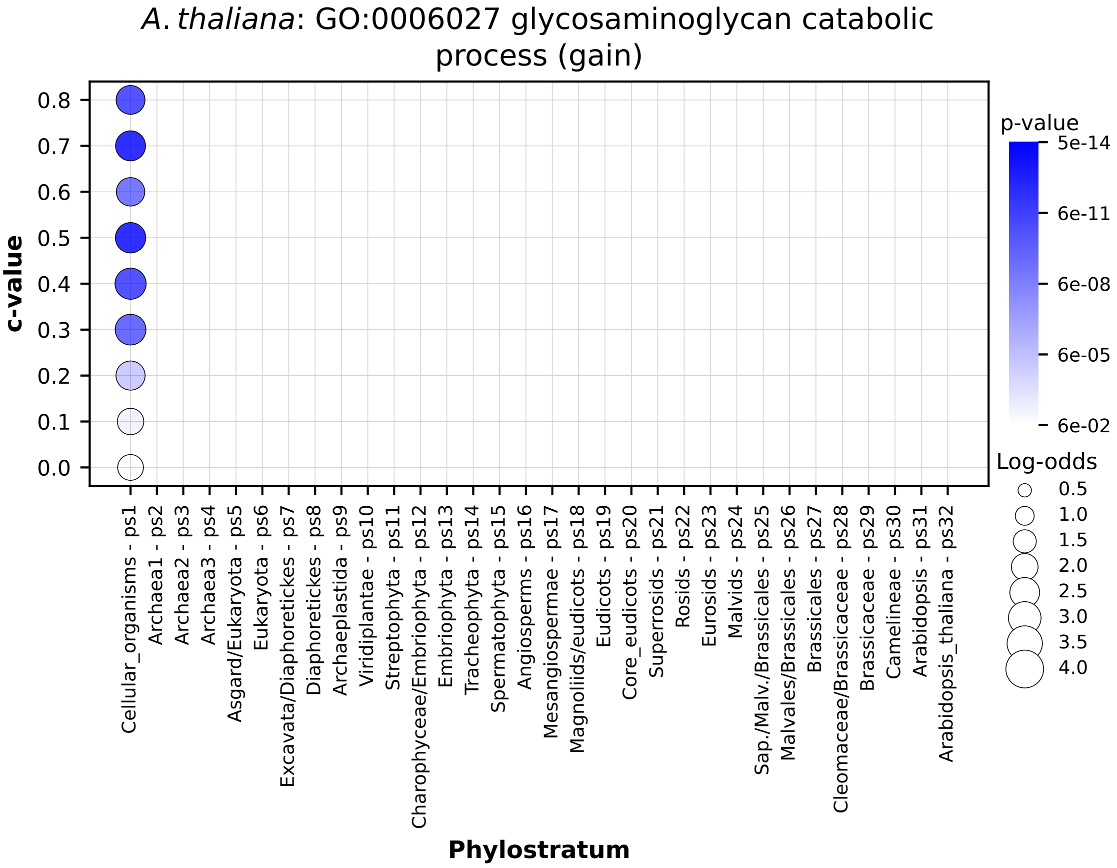
A. thaliana: GO:0006027 glycosaminoglycan catabolic process (gain)
c-value
0.8
0.7
0.6
0.5
0.4
0.3
0.2
0.1
0.0
p-value
5e-14
6e-11
6e-08
6e-05
6e-02
Log-odds
0.5
1.0
1.5
2.0
2.5
3.0
3.5
4.0
Cellular_organisms - ps1
Archaea1 - ps2
Archaea2 - ps3
Archaea3 - ps4
Asgard/Eukaryota - ps5
Eukaryota - ps6
Excavata/Diaphoretickes - ps7
Diaphoretickes - ps8
Archaeplastida - ps9
Viridiplantae - ps10
Streptophyta - ps11
Charophyceae/Embriophyta - ps12
Embriophyta - ps13
Tracheophyta - ps14
Spermatophyta - ps15
Angiosperms - ps16
Mesangiospermae - ps17
Magnoliids/eudicots - ps18
Eudicots - ps19
Core_eudicots - ps20
Superrosids - ps21
Rosids - ps22
Eurosids - ps23
Malvids - ps24
Sap./Malv./Brassicales - ps25
Malvales/Brassicales - ps26
Brassicales - ps27
Cleomaceae/Brassicaceae - ps28
Brassicaceae - ps29
Camelineae - ps30
Arabidopsis - ps31
Arabidopsis_thaliana - ps32
Phylostratum

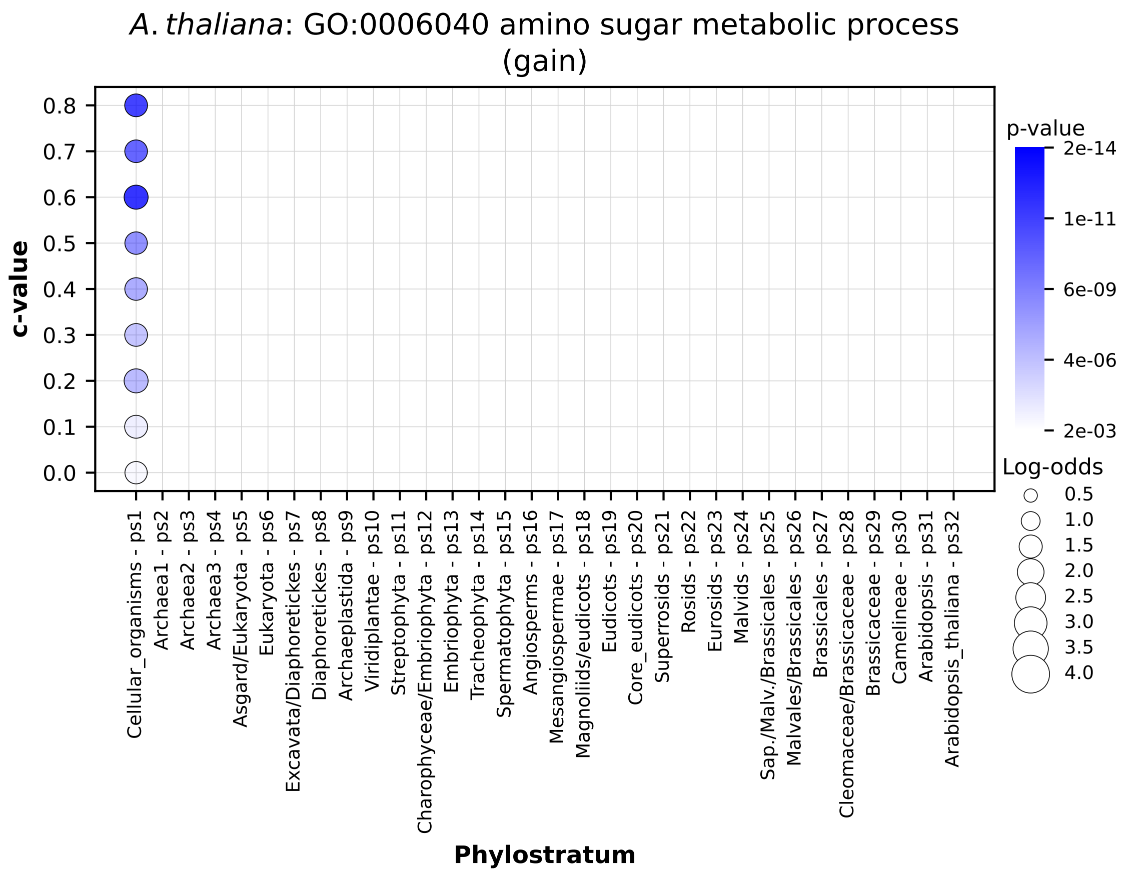

A. thaliana: GO:0006040 amino sugar metabolic process (gain)
c-value
0.0
0.1
0.2
0.3
0.4
0.5
0.6
0.7
0.8
Phylostratum
Cellular_organisms - ps1
Archaea1 - ps2
Archaea2 - ps3
Archaea3 - ps4
Asgard/Eukaryota - ps5
Eukaryota - ps6
Excavata/Diaphoretickes - ps7
Diaphoretickes - ps8
Archaeplastida - ps9
Viridiplantae - ps10
Streptophyta - ps11
Charophyceae/Embriophyta - ps12
Embriophyta - ps13
Tracheophyta - ps14
Spermatophyta - ps15
Angiosperms - ps16
Mesangiospermae - ps17
Magnoliids/eudicots - ps18
Eudicots - ps19
Core_eudicots - ps20
Superrosids - ps21
Rosids - ps22
Eurosids - ps23
Malvids - ps24
Sap./Malv./Brassicales - ps25
Malvales/Brassicales - ps26
Brassicales - ps27
Cleomaceae/Brassicaceae - ps28
Brassicaceae - ps29
Camelineae - ps30
Arabidopsis - ps31
Arabidopsis_thaliana - ps32
p-value
2e-14
1e-11
6e-09
4e-06
2e-03
Log-odds
0.5
1.0
1.5
2.0
2.5
3.0
3.5
4.0

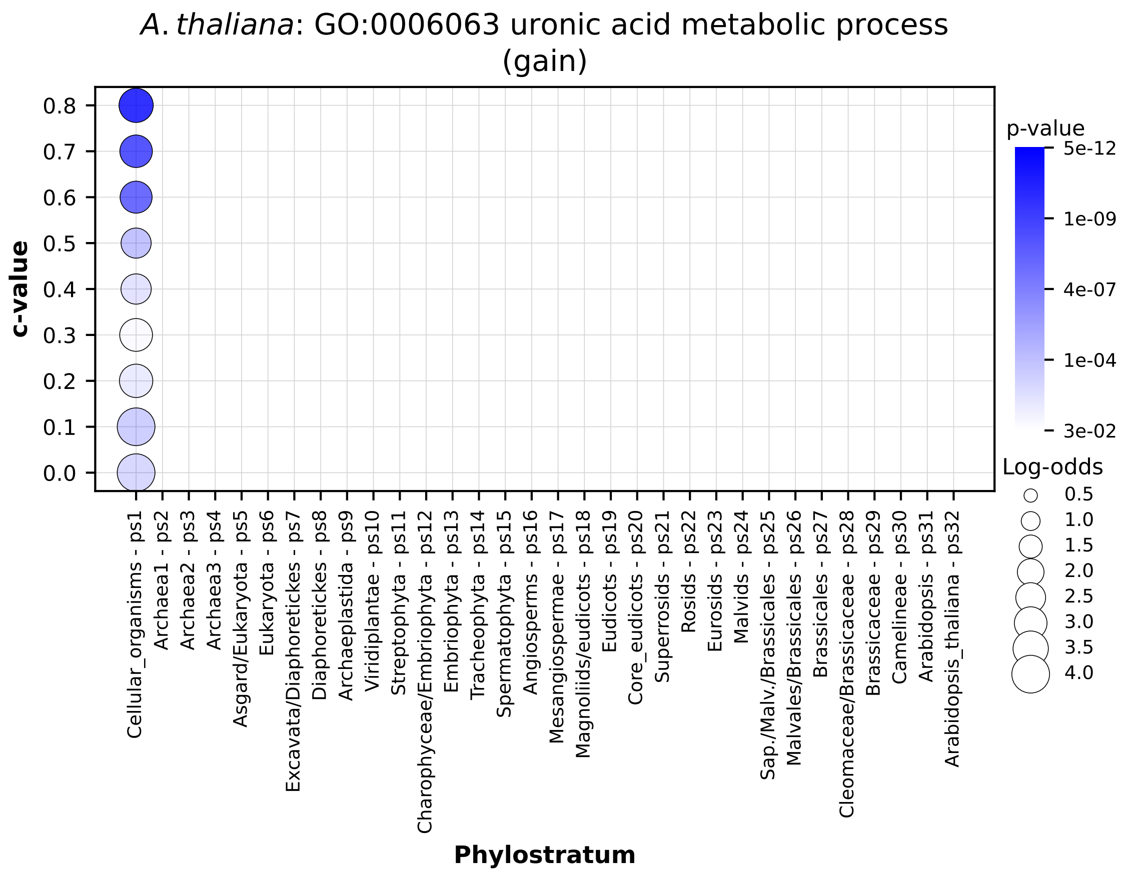

A. thaliana: GO:0006063 uronic acid metabolic process
(gain)
c-value
0.0
0.1
0.2
0.3
0.4
0.5
0.6
0.7
0.8
p-value
5e-12
1e-09
4e-07
1e-04
3e-02
Log-odds
0.5
1.0
1.5
2.0
2.5
3.0
3.5
4.0
Cellular_organisms - ps1
Archaea1 - ps2
Archaea2 - ps3
Archaea3 - ps4
Asgard/Eukaryota - ps5
Eukaryota - ps6
Excavata/Diaphoretickes - ps7
Diaphoretickes - ps8
Archaeplastida - ps9
Viridiplantae - ps10
Streptophyta - ps11
Charophyceae/Embriophyta - ps12
Embriophyta - ps13
Tracheophyta - ps14
Spermatophyta - ps15
Angiosperms - ps16
Mesangiospermae - ps17
Magnoliids/eudicots - ps18
Eudicots - ps19
Core_eudicots - ps20
Superrosids - ps21
Rosids - ps22
Eurosids - ps23
Malvids - ps24
Sap./Malv./Brassicales - ps25
Malvales/Brassicales - ps26
Brassicales - ps27
Cleomaceae/Brassicaceae - ps28
Brassicaceae - ps29
Camelineae - ps30
Arabidopsis - ps31
Arabidopsis_thaliana - ps32
Phylostratum

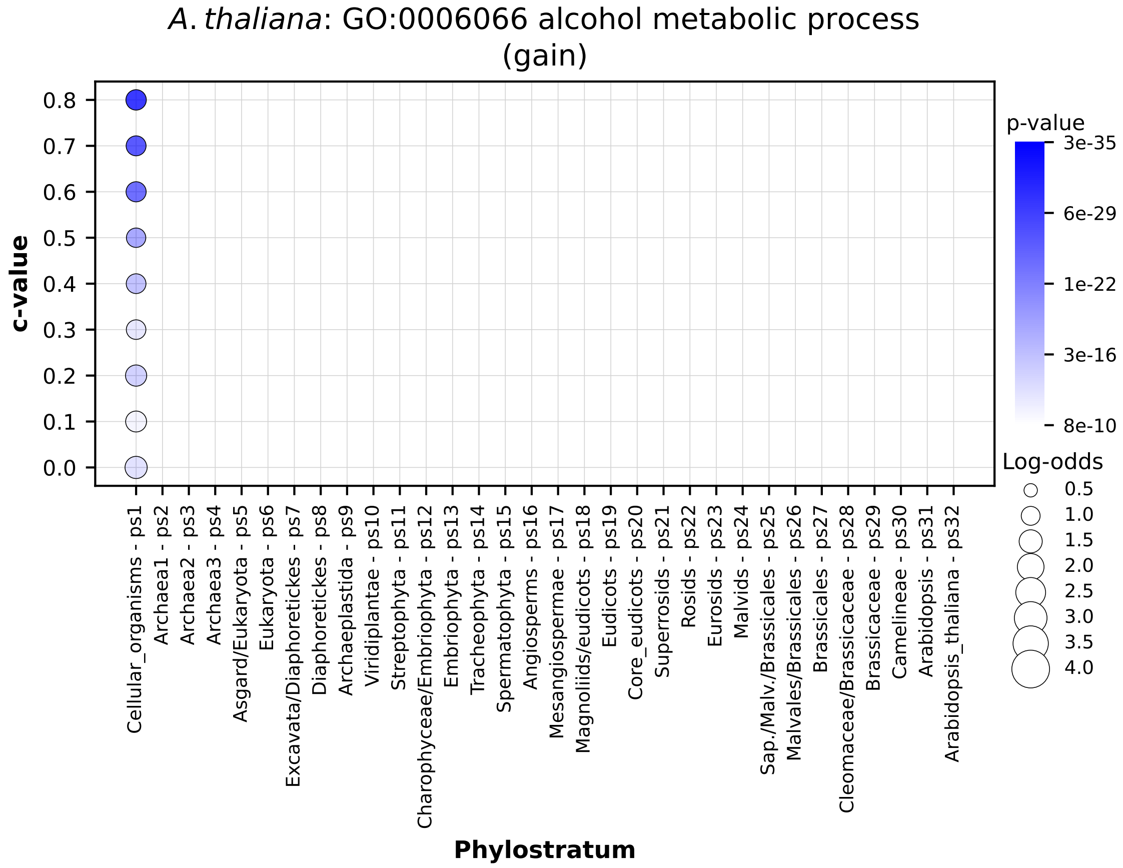

A. thaliana: GO:0006066 alcohol metabolic process
(gain)
c-value
0.8
0.7
0.6
0.5
0.4
0.3
0.2
0.1
0.0
p-value
3e-35
6e-29
1e-22
3e-16
8e-10
Log-odds
0.5
1.0
1.5
2.0
2.5
3.0
3.5
4.0
Cellular_organisms - ps1
Archaea1 - ps2
Archaea2 - ps3
Archaea3 - ps4
Asgard/Eukaryota - ps5
Eukaryota - ps6
Excavata/Diaphoretickes - ps7
Diaphoretickes - ps8
Archaeplastida - ps9
Viridiplantae - ps10
Streptophyta - ps11
Charophyceae/Embriophyta - ps12
Embriophyta - ps13
Tracheophyta - ps14
Spermatophyta - ps15
Angiosperms - ps16
Mesangiospermae - ps17
Magnoliids/eudicots - ps18
Eudicots - ps19
Core_eudicots - ps20
Superrosids - ps21
Rosids - ps22
Eurosids - ps23
Malvids - ps24
Sap./Malv./Brassicales - ps25
Malvales/Brassicales - ps26
Brassicales - ps27
Cleomaceae/Brassicaceae - ps28
Brassicaceae - ps29
Camelineae - ps30
Arabidopsis - ps31
Arabidopsis_thaliana - ps32
Phylostratum

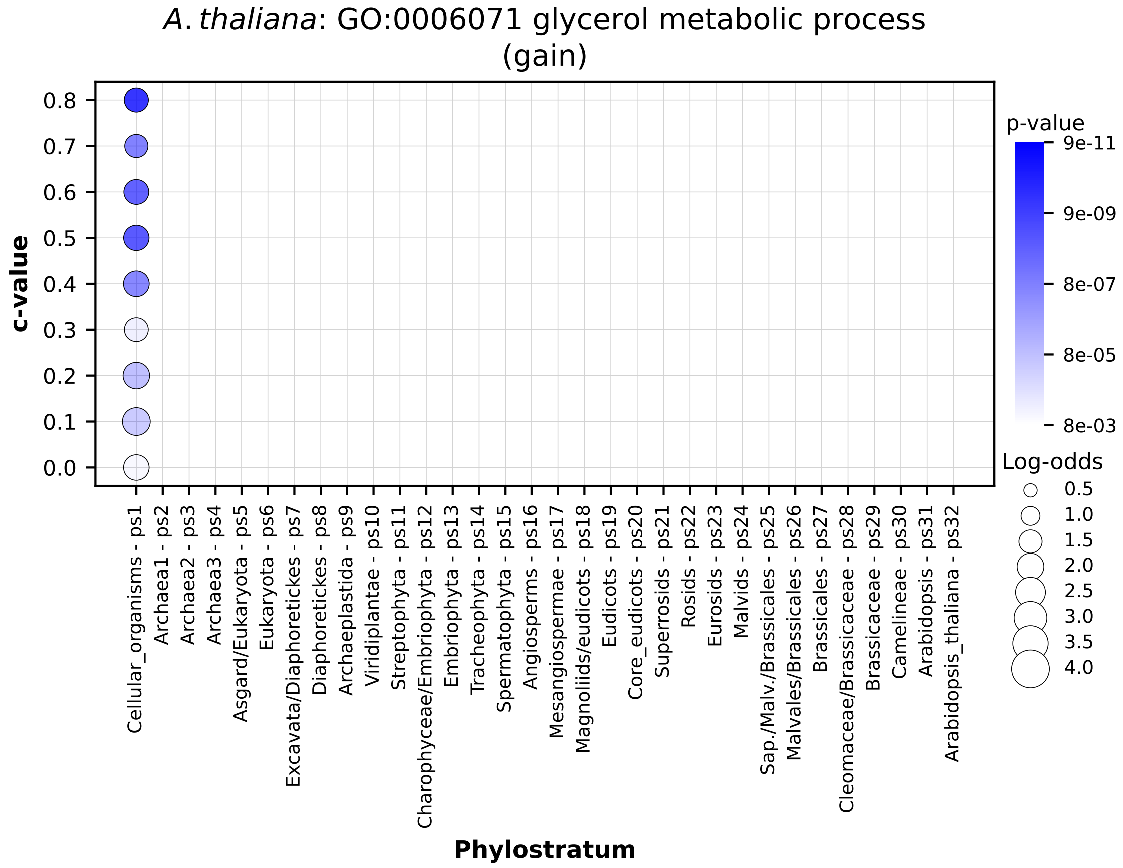
A. thaliana: GO:0006071 glycerol metabolic process
(gain)
c-value
0.8
0.7
0.6
0.5
0.4
0.3
0.2
0.1
0.0
p-value
9e-11
9e-09
8e-07
8e-05
8e-03
Log-odds
0.5
1.0
1.5
2.0
2.5
3.0
3.5
4.0
Cellular_organisms - ps1
Archaea1 - ps2
Archaea2 - ps3
Archaea3 - ps4
Asgard/Eukaryota - ps5
Eukaryota - ps6
Excavata/Diaphoretickes - ps7
Diaphoretickes - ps8
Archaeplastida - ps9
Viridiplantae - ps10
Streptophyta - ps11
Charophyceae/Embriophyta - ps12
Embriophyta - ps13
Tracheophyta - ps14
Spermatophyta - ps15
Angiosperms - ps16
Mesangiospermae - ps17
Magnoliids/eudicots - ps18
Eudicots - ps19
Core_eudicots - ps20
Superrosids - ps21
Rosids - ps22
Eurosids - ps23
Malvids - ps24
Sap./Malv./Brassicales - ps25
Malvales/Brassicales - ps26
Brassicales - ps27
Cleomaceae/Brassicaceae - ps28
Brassicaceae - ps29
Camelineae - ps30
Arabidopsis - ps31
Arabidopsis_thaliana - ps32
Phylostratum

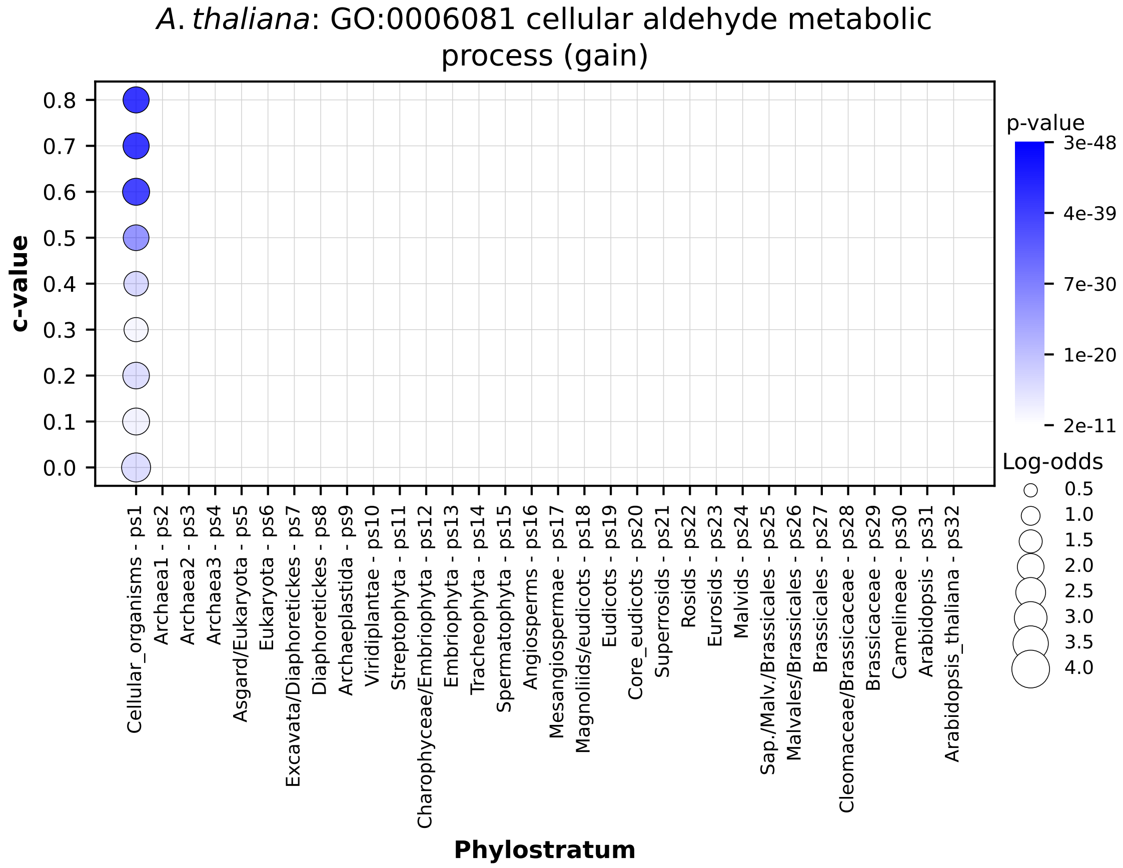
A. thaliana: GO:0006081 cellular aldehyde metabolic process (gain)
c-value
0.8
0.7
0.6
0.5
0.4
0.3
0.2
0.1
0.0
p-value
3e-48
4e-39
7e-30
1e-20
2e-11
Log-odds
0.5
1.0
1.5
2.0
2.5
3.0
3.5
4.0
Cellular_organisms - ps1
Archaea1 - ps2
Archaea2 - ps3
Archaea3 - ps4
Asgard/Eukaryota - ps5
Eukaryota - ps6
Excavata/Diaphoretickes - ps7
Diaphoretickes - ps8
Archaeplastida - ps9
Viridiplantae - ps10
Streptophyta - ps11
Charophyceae/Embriophyta - ps12
Embriophyta - ps13
Tracheophyta - ps14
Spermatophyta - ps15
Angiosperms - ps16
Mesangiospermae - ps17
Magnoliids/eudicots - ps18
Eudicots - ps19
Core_eudicots - ps20
Superrosids - ps21
Rosids - ps22
Eurosids - ps23
Malvids - ps24
Sap./Malv./Brassicales - ps25
Malvales/Brassicales - ps26
Brassicales - ps27
Cleomaceae/Brassicaceae - ps28
Brassicaceae - ps29
Camelineae - ps30
Arabidopsis - ps31
Arabidopsis_thaliana - ps32
Phylostratum

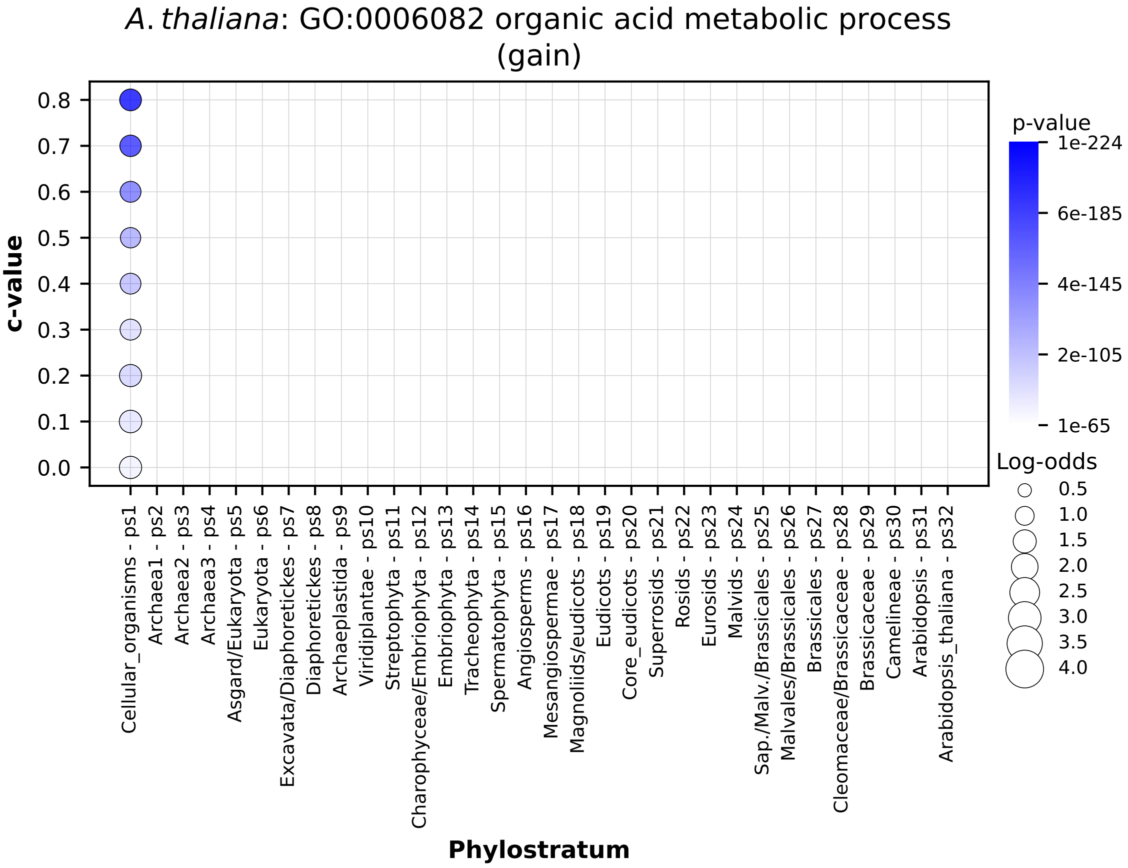

A. thaliana: GO:0006082 organic acid metabolic process
(gain)
c-value
0.8
0.7
0.6
0.5
0.4
0.3
0.2
0.1
0.0
p-value
1e-224
6e-185
4e-145
2e-105
1e-65
Log-odds
0.5
1.0
1.5
2.0
2.5
3.0
3.5
4.0
Cellular_organisms - ps1
Archaea1 - ps2
Archaea2 - ps3
Archaea3 - ps4
Asgard/Eukaryota - ps5
Eukaryota - ps6
Excavata/Diaphoretickes - ps7
Diaphoretickes - ps8
Archaeplastida - ps9
Viridiplantae - ps10
Streptophyta - ps11
Charophyceae/Embriophyta - ps12
Embriophyta - ps13
Tracheophyta - ps14
Spermatophyta - ps15
Angiosperms - ps16
Mesangiospermae - ps17
Magnoliids/eudicots - ps18
Eudicots - ps19
Core_eudicots - ps20
Superrosids - ps21
Rosids - ps22
Eurosids - ps23
Malvids - ps24
Sap./Malv./Brassicales - ps25
Malvales/Brassicales - ps26
Brassicales - ps27
Cleomaceae/Brassicaceae - ps28
Brassicaceae - ps29
Camelineae - ps30
Arabidopsis - ps31
Arabidopsis_thaliana - ps32
Phylostratum

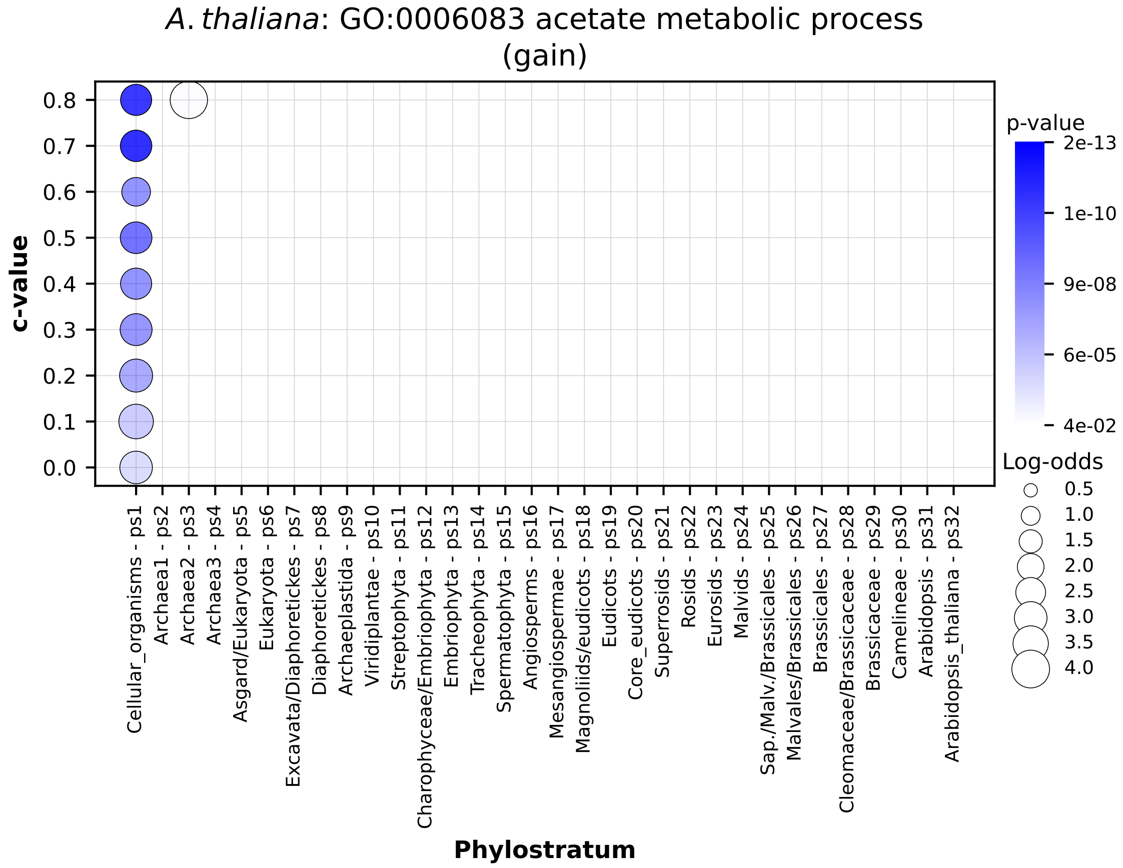

A. thaliana: GO:0006083 acetate metabolic process
(gain)
c-value
0.8
0.7
0.6
0.5
0.4
0.3
0.2
0.1
0.0
Phylostratum
Cellular_organisms - ps1
Archaea1 - ps2
Archaea2 - ps3
Archaea3 - ps4
Asgard/Eukaryota - ps5
Eukaryota - ps6
Excavata/Diaphoretickes - ps7
Diaphoretickes - ps8
Archaeplastida - ps9
Viridiplantae - ps10
Streptophyta - ps11
Charophyceae/Embriophyta - ps12
Embriophyta - ps13
Tracheophyta - ps14
Spermatophyta - ps15
Angiosperms - ps16
Mesangiospermae - ps17
Magnoliids/eudicots - ps18
Eudicots - ps19
Core_eudicots - ps20
Superrosids - ps21
Rosids - ps22
Eurosids - ps23
Malvids - ps24
Sap./Malv./Brassicales - ps25
Malvales/Brassicales - ps26
Brassicales - ps27
Cleomaceae/Brassicaceae - ps28
Brassicaceae - ps29
Camelineae - ps30
Arabidopsis - ps31
Arabidopsis_thaliana - ps32
p-value
2e-13
1e-10
9e-08
6e-05
4e-02
Log-odds
0.5
1.0
1.5
2.0
2.5
3.0
3.5
4.0

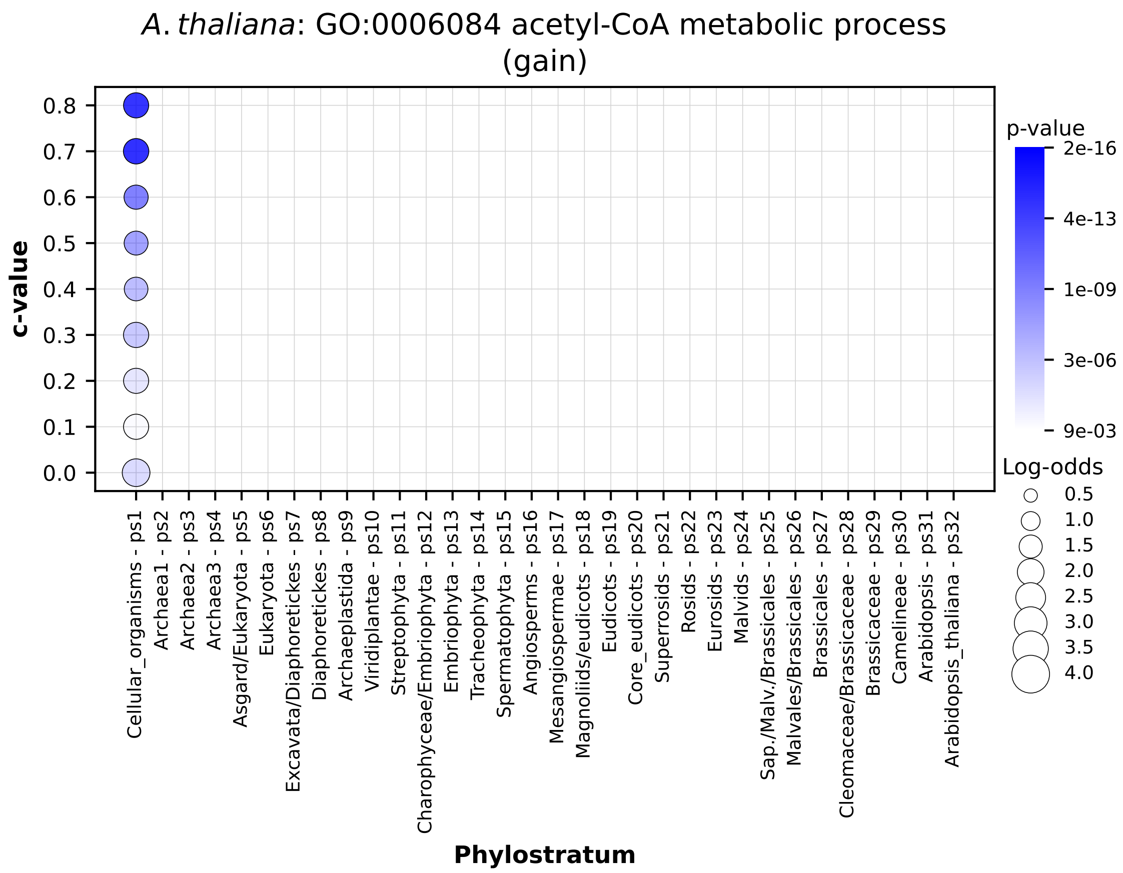

A. thaliana: GO:0006084 acetyl-CoA metabolic process
(gain)
c-value
0.8
0.7
0.6
0.5
0.4
0.3
0.2
0.1
0.0
p-value
2e-16
4e-13
1e-09
3e-06
9e-03
Log-odds
0.5
1.0
1.5
2.0
2.5
3.0
3.5
4.0
Cellular_organisms - ps1
Archaea1 - ps2
Archaea2 - ps3
Archaea3 - ps4
Asgard/Eukaryota - ps5
Eukaryota - ps6
Excavata/Diaphoretickes - ps7
Diaphoretickes - ps8
Archaeplastida - ps9
Viridiplantae - ps10
Streptophyta - ps11
Charophyceae/Embriophyta - ps12
Embriophyta - ps13
Tracheophyta - ps14
Spermatophyta - ps15
Angiosperms - ps16
Mesangiospermae - ps17
Magnoliids/eudicots - ps18
Eudicots - ps19
Core_eudicots - ps20
Superrosids - ps21
Rosids - ps22
Eurosids - ps23
Malvids - ps24
Sap./Malv./Brassicales - ps25
Malvales/Brassicales - ps26
Brassicales - ps27
Cleomaceae/Brassicaceae - ps28
Brassicaceae - ps29
Camelineae - ps30
Arabidopsis - ps31
Arabidopsis_thaliana - ps32
Phylostratum

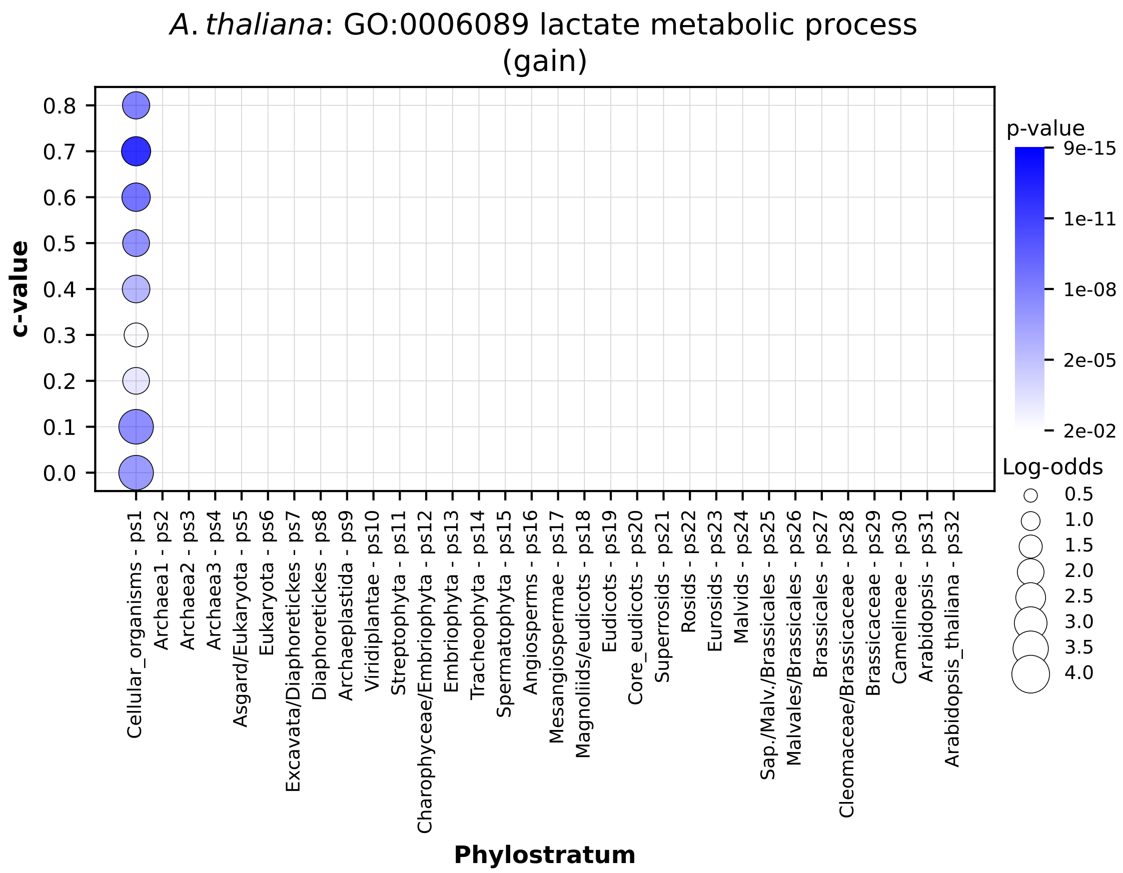

A. thaliana: GO:0006089 lactate metabolic process (gain)
c-value
0.8
0.7
0.6
0.5
0.4
0.3
0.2
0.1
0.0
p-value
9e-15
1e-11
1e-08
2e-05
2e-02
Log-odds
0.5
1.0
1.5
2.0
2.5
3.0
3.5
4.0
Cellular_organisms - ps1
Archaea1 - ps2
Archaea2 - ps3
Archaea3 - ps4
Asgard/Eukaryota - ps5
Eukaryota - ps6
Excavata/Diaphoretickes - ps7
Diaphoretickes - ps8
Archaeplastida - ps9
Viridiplantae - ps10
Streptophyta - ps11
Charophyceae/Embriophyta - ps12
Embriophyta - ps13
Tracheophyta - ps14
Spermatophyta - ps15
Angiosperms - ps16
Mesangiospermae - ps17
Magnoliids/eudicots - ps18
Eudicots - ps19
Core_eudicots - ps20
Superrosids - ps21
Rosids - ps22
Eurosids - ps23
Malvids - ps24
Sap./Malv./Brassicales - ps25
Malvales/Brassicales - ps26
Brassicales - ps27
Cleomaceae/Brassicaceae - ps28
Brassicaceae - ps29
Camelineae - ps30
Arabidopsis - ps31
Arabidopsis_thaliana - ps32
Phylostratum

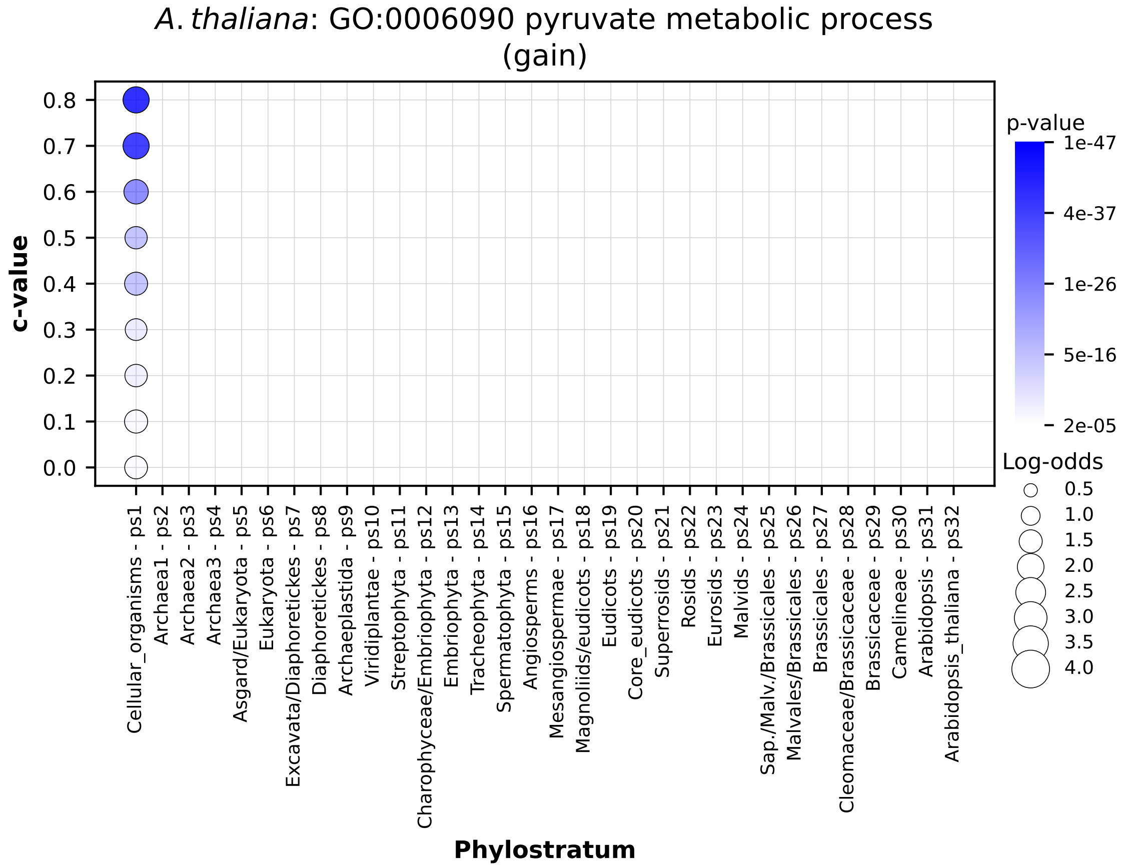

A. thaliana: GO:0006090 pyruvate metabolic process
(gain)
c-value
0.8
0.7
0.6
0.5
0.4
0.3
0.2
0.1
0.0
p-value
1e-47
4e-37
1e-26
5e-16
2e-05
Log-odds
0.5
1.0
1.5
2.0
2.5
3.0
3.5
4.0
Cellular_organisms - ps1
Archaea1 - ps2
Archaea2 - ps3
Archaea3 - ps4
Asgard/Eukaryota - ps5
Eukaryota - ps6
Excavata/Diaphoretickes - ps7
Diaphoretickes - ps8
Archaeplastida - ps9
Viridiplantae - ps10
Streptophyta - ps11
Charophyceae/Embriophyta - ps12
Embriophyta - ps13
Tracheophyta - ps14
Spermatophyta - ps15
Angiosperms - ps16
Mesangiospermae - ps17
Magnoliids/eudicots - ps18
Eudicots - ps19
Core_eudicots - ps20
Superrosids - ps21
Rosids - ps22
Eurosids - ps23
Malvids - ps24
Sap./Malv./Brassicales - ps25
Malvales/Brassicales - ps26
Brassicales - ps27
Cleomaceae/Brassicaceae - ps28
Brassicaceae - ps29
Camelineae - ps30
Arabidopsis - ps31
Arabidopsis_thaliana - ps32
Phylostratum

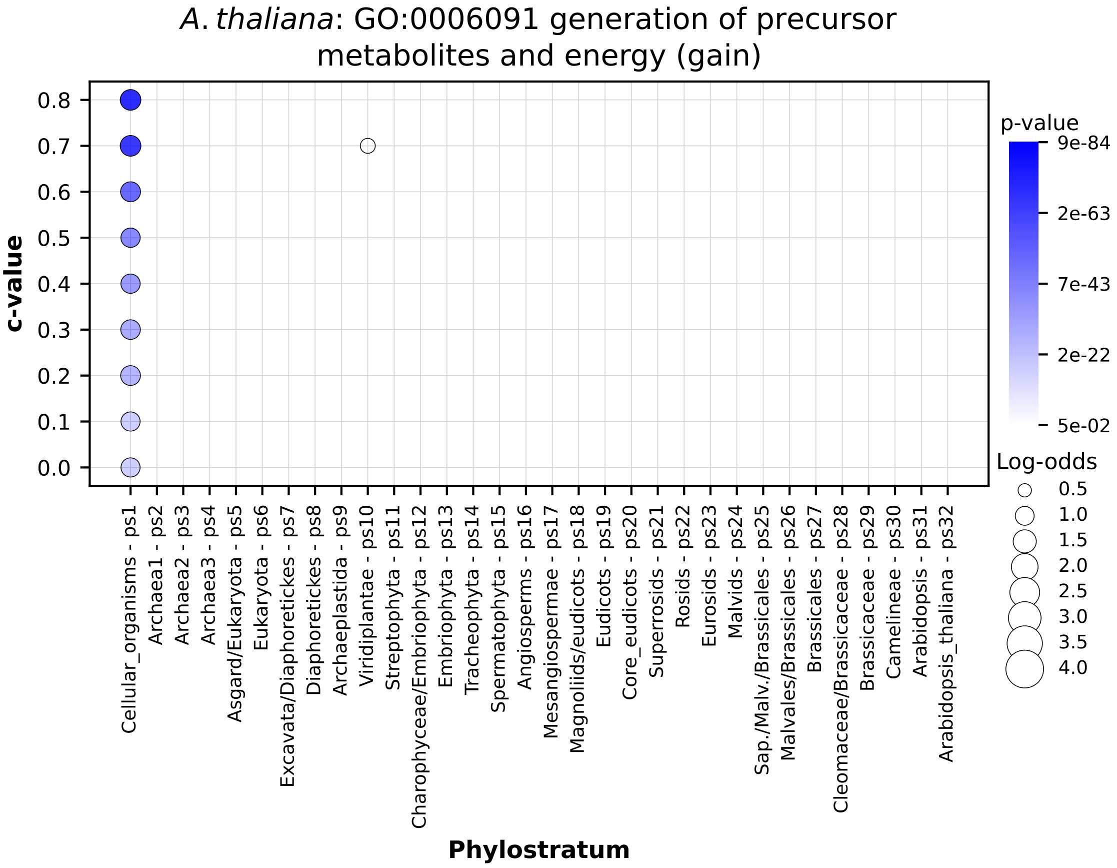
A. thaliana: GO:0006091 generation of precursor metabolites and energy (gain)
c-value
0.8
0.7
0.6
0.5
0.4
0.3
0.2
0.1
0.0
p-value
9e-84
2e-63
7e-43
2e-22
5e-02
Log-odds
0.5
1.0
1.5
2.0
2.5
3.0
3.5
4.0
Cellular_organisms - ps1
Archaea1 - ps2
Archaea2 - ps3
Archaea3 - ps4
Asgard/Eukaryota - ps5
Eukaryota - ps6
Excavata/Diaphoretickes - ps7
Diaphoretickes - ps8
Archaeplastida - ps9
Viridiplantae - ps10
Streptophyta - ps11
Charophyceae/Embriophyta - ps12
Embriophyta - ps13
Tracheophyta - ps14
Spermatophyta - ps15
Angiosperms - ps16
Mesangiospermae - ps17
Magnoliids/eudicots - ps18
Eudicots - ps19
Core_eudicots - ps20
Superrosids - ps21
Rosids - ps22
Eurosids - ps23
Malvids - ps24
Sap./Malv./Brassicales - ps25
Malvales/Brassicales - ps26
Brassicales - ps27
Cleomaceae/Brassicaceae - ps28
Brassicaceae - ps29
Camelineae - ps30
Arabidopsis - ps31
Arabidopsis_thaliana - ps32
Phylostratum

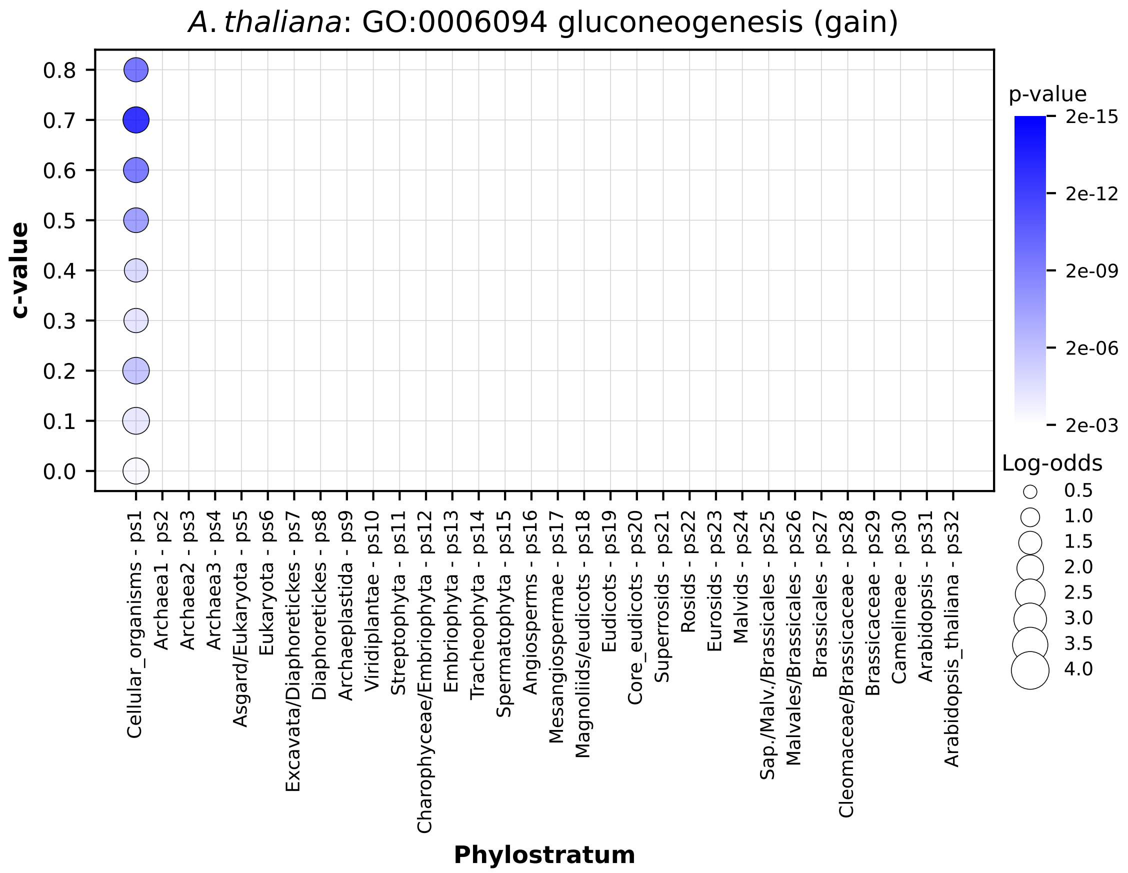

A. thaliana: GO:0006094 gluconeogenesis (gain)
c-value
0.0
0.1
0.2
0.3
0.4
0.5
0.6
0.7
0.8
p-value
2e-15
2e-12
2e-09
2e-06
2e-03
Log-odds
0.5
1.0
1.5
2.0
2.5
3.0
3.5
4.0
Cellular_organisms - ps1
Archaea1 - ps2
Archaea2 - ps3
Archaea3 - ps4
Asgard/Eukaryota - ps5
Eukaryota - ps6
Excavata/Diaphoretickes - ps7
Diaphoretickes - ps8
Archaeplastida - ps9
Viridiplantae - ps10
Streptophyta - ps11
Charophyceae/Embriophyta - ps12
Embriophyta - ps13
Tracheophyta - ps14
Spermatophyta - ps15
Angiosperms - ps16
Mesangiospermae - ps17
Magnoliids/eudicots - ps18
Eudicots - ps19
Core_eudicots - ps20
Superrosids - ps21
Rosids - ps22
Eurosids - ps23
Malvids - ps24
Sap./Malv./Brassicales - ps25
Malvales/Brassicales - ps26
Brassicales - ps27
Cleomaceae/Brassicaceae - ps28
Brassicaceae - ps29
Camelineae - ps30
Arabidopsis - ps31
Arabidopsis_thaliana - ps32
Phylostratum

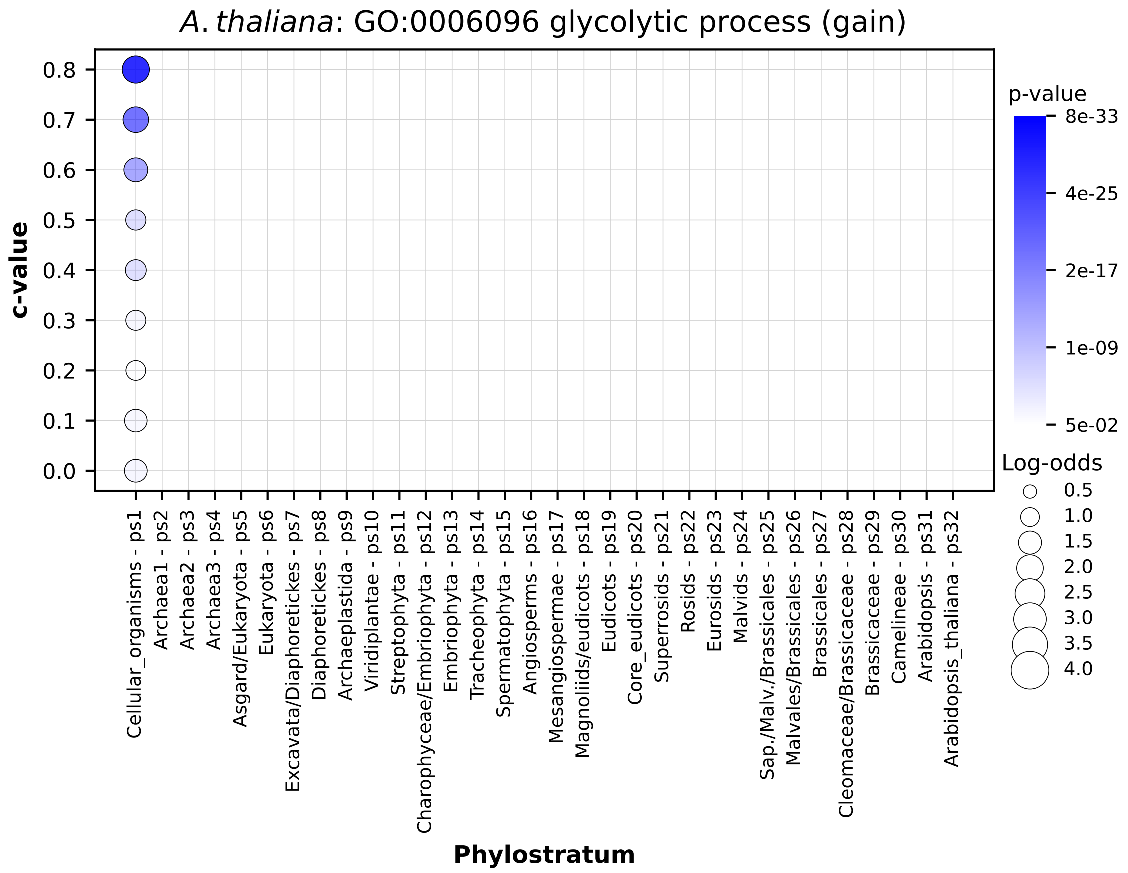

A. thaliana: GO:0006096 glycolytic process (gain)
c-value
0.0
0.1
0.2
0.3
0.4
0.5
0.6
0.7
0.8
p-value
8e-33
4e-25
2e-17
1e-09
5e-02
Log-odds
0.5
1.0
1.5
2.0
2.5
3.0
3.5
4.0
Cellular_organisms - ps1
Archaea1 - ps2
Archaea2 - ps3
Archaea3 - ps4
Asgard/Eukaryota - ps5
Eukaryota - ps6
Excavata/Diaphoretickes - ps7
Diaphoretickes - ps8
Archaeplastida - ps9
Viridiplantae - ps10
Streptophyta - ps11
Charophyceae/Embriophyta - ps12
Embriophyta - ps13
Tracheophyta - ps14
Spermatophyta - ps15
Angiosperms - ps16
Mesangiospermae - ps17
Magnoliids/eudicots - ps18
Eudicots - ps19
Core_eudicots - ps20
Superrosids - ps21
Rosids - ps22
Eurosids - ps23
Malvids - ps24
Sap./Malv./Brassicales - ps25
Malvales/Brassicales - ps26
Brassicales - ps27
Cleomaceae/Brassicaceae - ps28
Brassicaceae - ps29
Camelineae - ps30
Arabidopsis - ps31
Arabidopsis_thaliana - ps32
Phylostratum

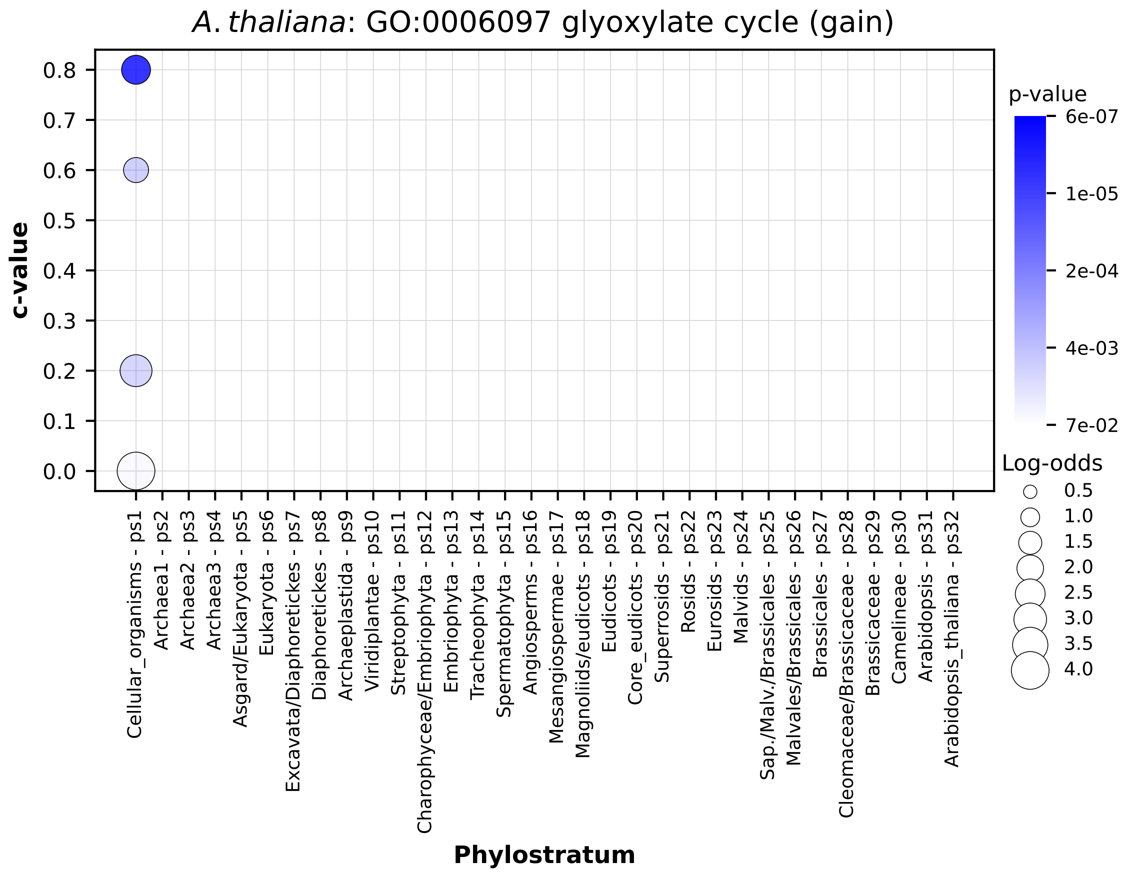
A. thaliana: GO:0006097 glyoxylate cycle (gain)
c-value
0.8
0.7
0.6
0.5
0.4
0.3
0.2
0.1
0.0
p-value
6e-07
1e-05
2e-04
4e-03
7e-02
Log-odds
0.5
1.0
1.5
2.0
2.5
3.0
3.5
4.0
Cellular_organisms - ps1
Archaea1 - ps2
Archaea2 - ps3
Archaea3 - ps4
Asgard/Eukaryota - ps5
Eukaryota - ps6
Excavata/Diaphoretickes - ps7
Diaphoretickes - ps8
Archaeplastida - ps9
Viridiplantae - ps10
Streptophyta - ps11
Charophyceae/Embriophyta - ps12
Embriophyta - ps13
Tracheophyta - ps14
Spermatophyta - ps15
Angiosperms - ps16
Mesangiospermae - ps17
Magnoliids/eudicots - ps18
Eudicots - ps19
Core_eudicots - ps20
Superrosids - ps21
Rosids - ps22
Eurosids - ps23
Malvids - ps24
Sap./Malv./Brassicales - ps25
Malvales/Brassicales - ps26
Brassicales - ps27
Cleomaceae/Brassicaceae - ps28
Brassicaceae - ps29
Camelineae - ps30
Arabidopsis - ps31
Arabidopsis_thaliana - ps32
Phylostratum

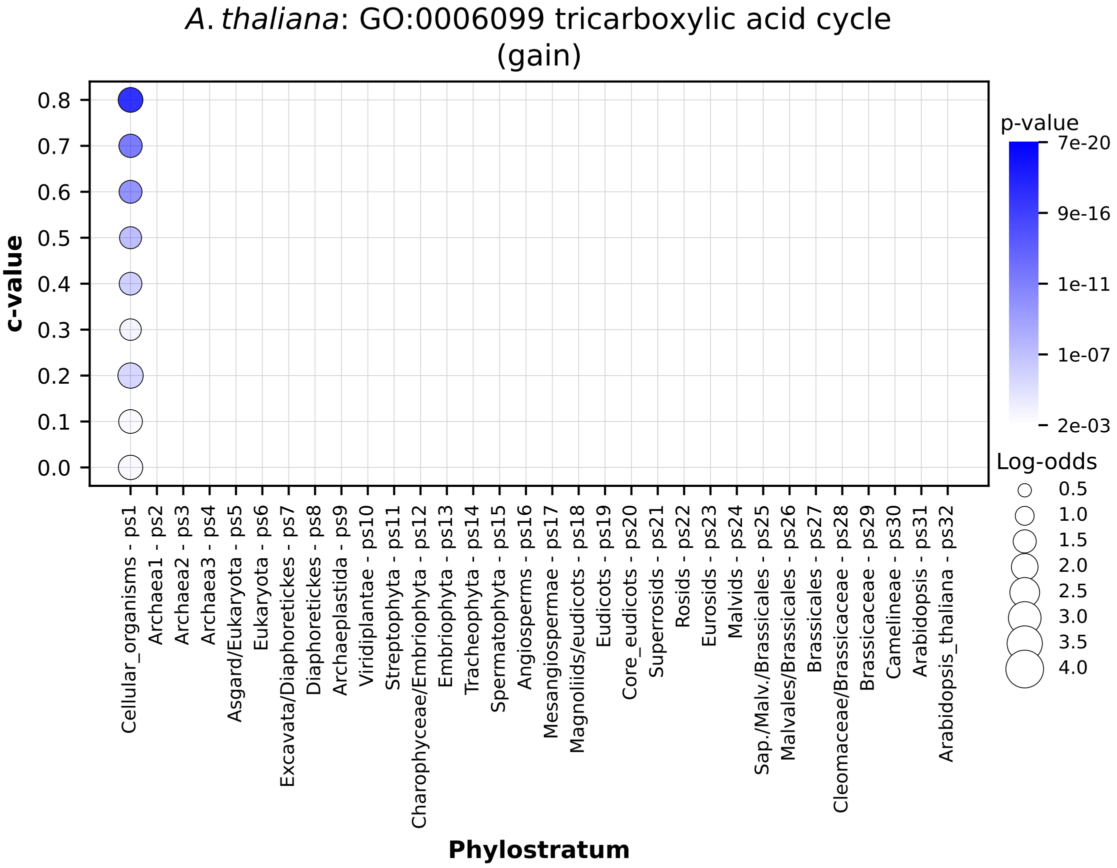

A. thaliana: GO:0006099 tricarboxylic acid cycle (gain)
c-value
0.0
0.1
0.2
0.3
0.4
0.5
0.6
0.7
0.8
p-value
7e-20
9e-16
1e-11
1e-07
2e-03
Log-odds
0.5
1.0
1.5
2.0
2.5
3.0
3.5
4.0
Cellular_organisms - ps1
Archaea1 - ps2
Archaea2 - ps3
Archaea3 - ps4
Asgard/Eukaryota - ps5
Eukaryota - ps6
Excavata/Diaphoretickes - ps7
Diaphoretickes - ps8
Archaeplastida - ps9
Viridiplantae - ps10
Streptophyta - ps11
Charophyceae/Embriophyta - ps12
Embriophyta - ps13
Tracheophyta - ps14
Spermatophyta - ps15
Angiosperms - ps16
Mesangiospermae - ps17
Magnoliids/eudicots - ps18
Eudicots - ps19
Core_eudicots - ps20
Superrosids - ps21
Rosids - ps22
Eurosids - ps23
Malvids - ps24
Sap./Malv./Brassicales - ps25
Malvales/Brassicales - ps26
Brassicales - ps27
Cleomaceae/Brassicaceae - ps28
Brassicaceae - ps29
Camelineae - ps30
Arabidopsis - ps31
Arabidopsis_thaliana - ps32
Phylostratum

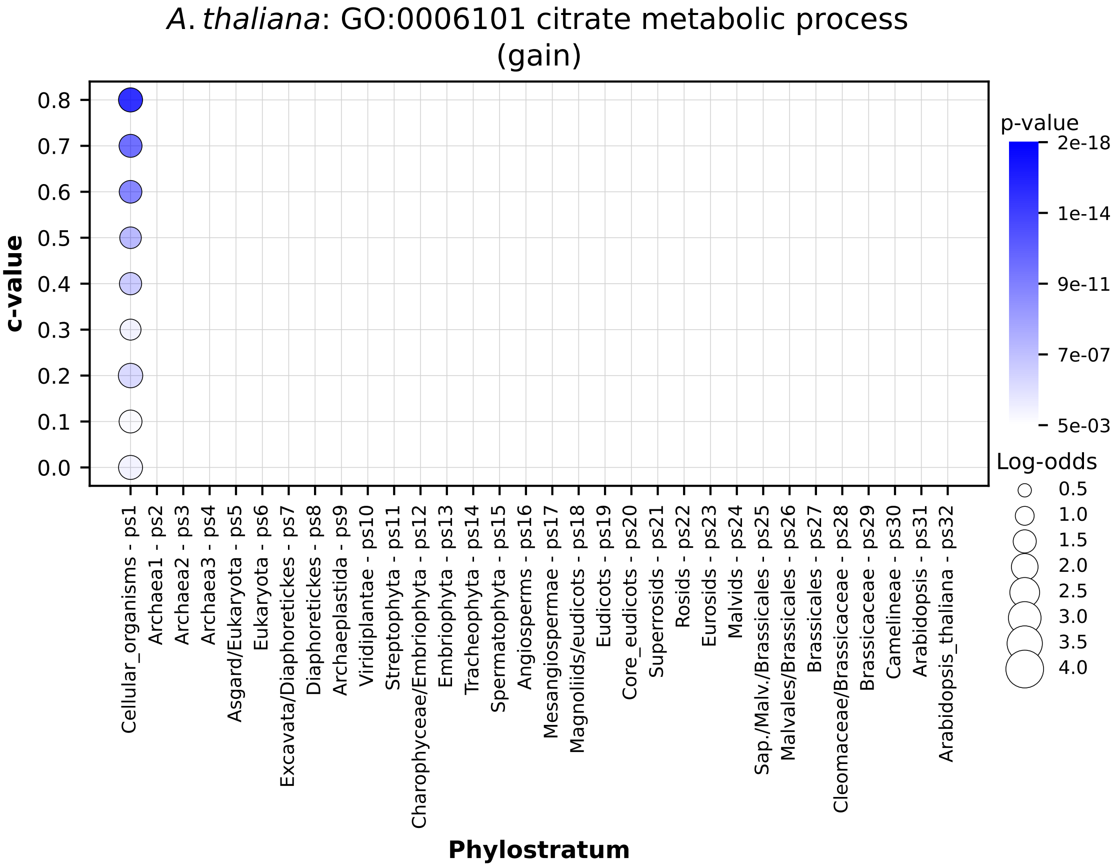
A. thaliana: GO:0006101 citrate metabolic process
(gain)
c-value
0.8
0.7
0.6
0.5
0.4
0.3
0.2
0.1
0.0
p-value
2e-18
1e-14
9e-11
7e-07
5e-03
Log-odds
0.5
1.0
1.5
2.0
2.5
3.0
3.5
4.0
Cellular_organisms - ps1
Archaea1 - ps2
Archaea2 - ps3
Archaea3 - ps4
Asgard/Eukaryota - ps5
Eukaryota - ps6
Excavata/Diaphoretickes - ps7
Diaphoretickes - ps8
Archaeplastida - ps9
Viridiplantae - ps10
Streptophyta - ps11
Charophyceae/Embriophyta - ps12
Embriophyta - ps13
Tracheophyta - ps14
Spermatophyta - ps15
Angiosperms - ps16
Mesangiospermae - ps17
Magnoliids/eudicots - ps18
Eudicots - ps19
Core_eudicots - ps20
Superrosids - ps21
Rosids - ps22
Eurosids - ps23
Malvids - ps24
Sap./Malv./Brassicales - ps25
Malvales/Brassicales - ps26
Brassicales - ps27
Cleomaceae/Brassicaceae - ps28
Brassicaceae - ps29
Camelineae - ps30
Arabidopsis - ps31
Arabidopsis_thaliana - ps32
Phylostratum

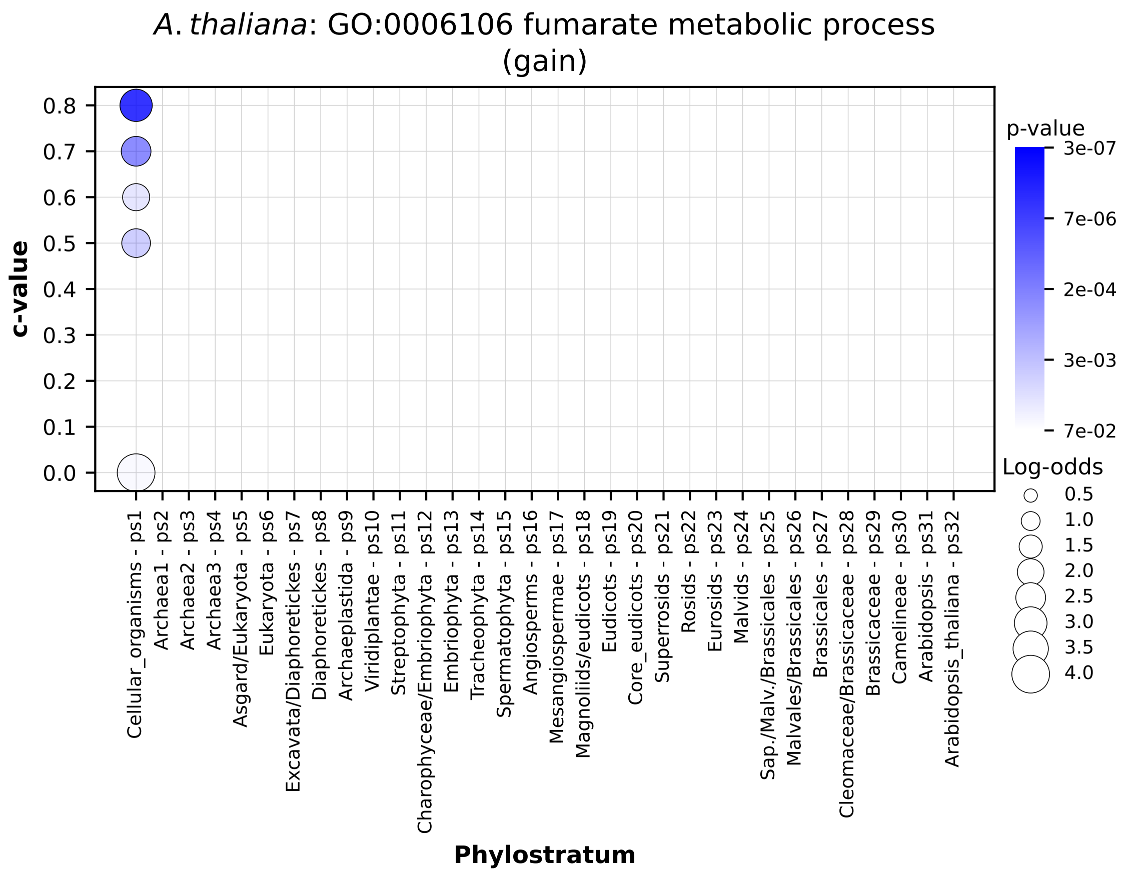
A. thaliana: GO:0006106 fumarate metabolic process
(gain)
c-value
0.8
0.7
0.6
0.5
0.4
0.3
0.2
0.1
0.0
p-value
3e-07
7e-06
2e-04
3e-03
7e-02
Log-odds
0.5
1.0
1.5
2.0
2.5
3.0
3.5
4.0
Cellular_organisms - ps1
Archaea1 - ps2
Archaea2 - ps3
Archaea3 - ps4
Asgard/Eukaryota - ps5
Eukaryota - ps6
Excavata/Diaphoretickes - ps7
Diaphoretickes - ps8
Archaeplastida - ps9
Viridiplantae - ps10
Streptophyta - ps11
Charophyceae/Embriophyta - ps12
Embriophyta - ps13
Tracheophyta - ps14
Spermatophyta - ps15
Angiosperms - ps16
Mesangiospermae - ps17
Magnoliids/eudicots - ps18
Eudicots - ps19
Core_eudicots - ps20
Superrosids - ps21
Rosids - ps22
Eurosids - ps23
Malvids - ps24
Sap./Malv./Brassicales - ps25
Malvales/Brassicales - ps26
Brassicales - ps27
Cleomaceae/Brassicaceae - ps28
Brassicaceae - ps29
Camelineae - ps30
Arabidopsis - ps31
Arabidopsis_thaliana - ps32
Phylostratum

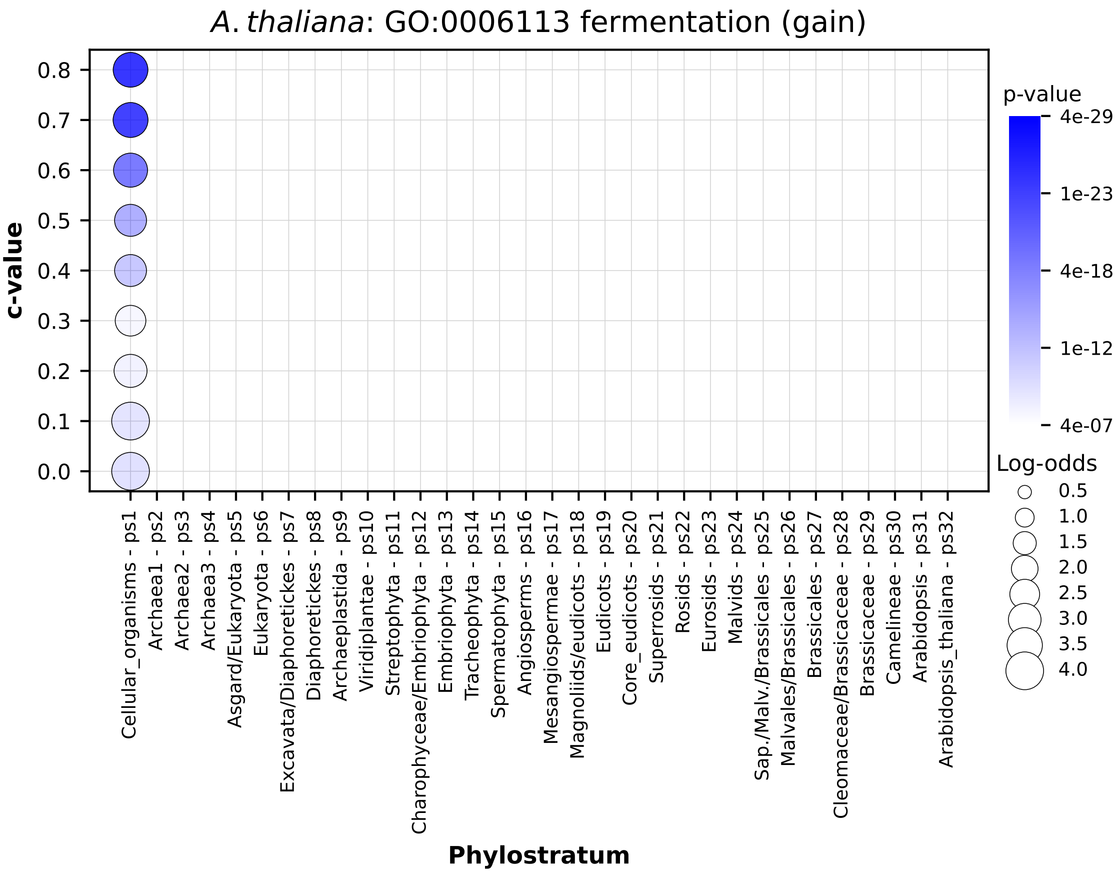

A. thaliana: GO:0006113 fermentation (gain)
c-value
0.0
0.1
0.2
0.3
0.4
0.5
0.6
0.7
0.8
p-value
4e-29
1e-23
4e-18
1e-12
4e-07
Log-odds
0.5
1.0
1.5
2.0
2.5
3.0
3.5
4.0
Cellular_organisms - ps1
Archaea1 - ps2
Archaea2 - ps3
Archaea3 - ps4
Asgard/Eukaryota - ps5
Eukaryota - ps6
Excavata/Diaphoretickes - ps7
Diaphoretickes - ps8
Archaeplastida - ps9
Viridiplantae - ps10
Streptophyta - ps11
Charophyceae/Embriophyta - ps12
Embriophyta - ps13
Tracheophyta - ps14
Spermatophyta - ps15
Angiosperms - ps16
Mesangiospermae - ps17
Magnoliids/eudicots - ps18
Eudicots - ps19
Core_eudicots - ps20
Superrosids - ps21
Rosids - ps22
Eurosids - ps23
Malvids - ps24
Sap./Malv./Brassicales - ps25
Malvales/Brassicales - ps26
Brassicales - ps27
Cleomaceae/Brassicaceae - ps28
Brassicaceae - ps29
Camelineae - ps30
Arabidopsis - ps31
Arabidopsis_thaliana - ps32
Phylostratum

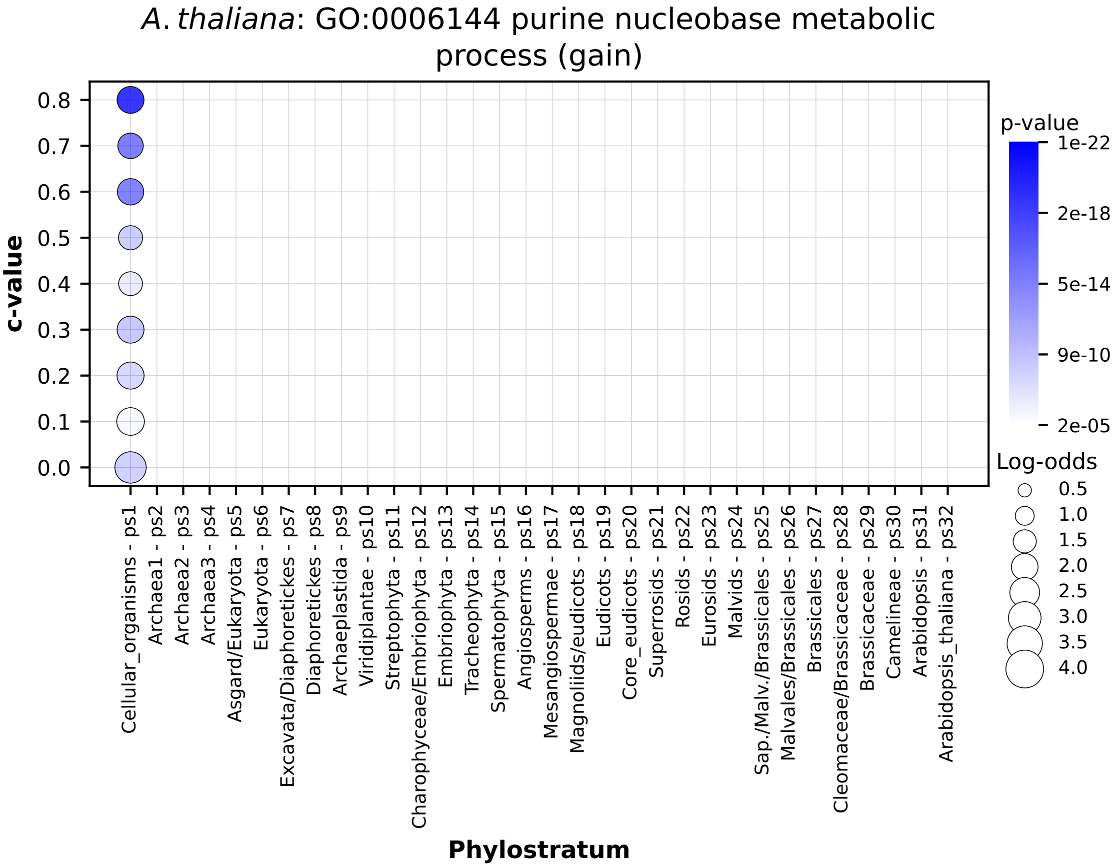

A. thaliana: GO:0006144 purine nucleobase metabolic process (gain)
c-value
0.8
0.7
0.6
0.5
0.4
0.3
0.2
0.1
0.0
Phylostratum
Cellular_organisms - ps1
Archaea1 - ps2
Archaea2 - ps3
Archaea3 - ps4
Asgard/Eukaryota - ps5
Eukaryota - ps6
Excavata/Diaphoretickes - ps7
Diaphoretickes - ps8
Archaeplastida - ps9
Viridiplantae - ps10
Streptophyta - ps11
Charophyceae/Embriophyta - ps12
Embriophyta - ps13
Tracheophyta - ps14
Spermatophyta - ps15
Angiosperms - ps16
Mesangiospermae - ps17
Magnoliids/eudicots - ps18
Eudicots - ps19
Core_eudicots - ps20
Superrosids - ps21
Rosids - ps22
Eurosids - ps23
Malvids - ps24
Sap./Malv./Brassicales - ps25
Malvales/Brassicales - ps26
Brassicales - ps27
Cleomaceae/Brassicaceae - ps28
Brassicaceae - ps29
Camelineae - ps30
Arabidopsis - ps31
Arabidopsis_thaliana - ps32
p-value
1e-22
2e-18
5e-14
9e-10
2e-05
Log-odds
0.5
1.0
1.5
2.0
2.5
3.0
3.5
4.0

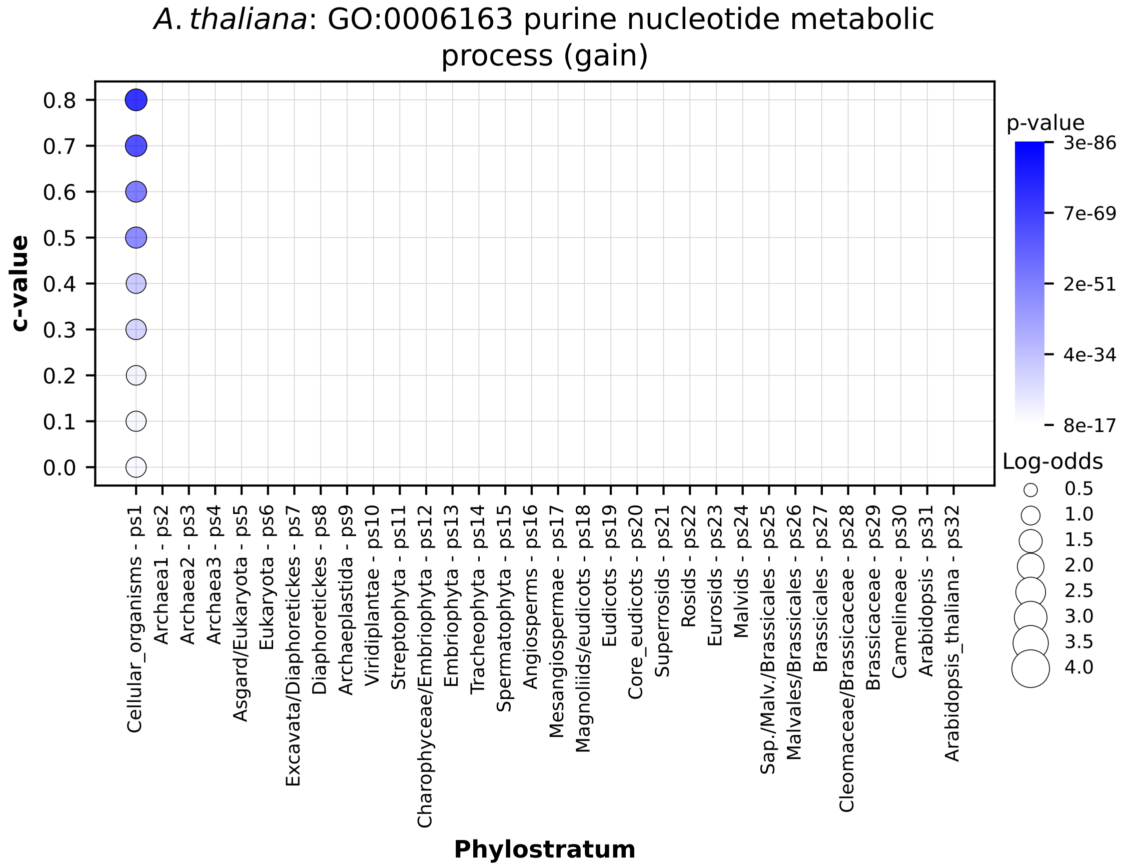

A. thaliana: GO:0006163 purine nucleotide metabolic process (gain)
c-value
0.8
0.7
0.6
0.5
0.4
0.3
0.2
0.1
0.0
p-value
3e-86
7e-69
2e-51
4e-34
8e-17
Log-odds
0.5
1.0
1.5
2.0
2.5
3.0
3.5
4.0
Cellular_organisms - ps1
Archaea1 - ps2
Archaea2 - ps3
Archaea3 - ps4
Asgard/Eukaryota - ps5
Eukaryota - ps6
Excavata/Diaphoretickes - ps7
Diaphoretickes - ps8
Archaeplastida - ps9
Viridiplantae - ps10
Streptophyta - ps11
Charophyceae/Embriophyta - ps12
Embriophyta - ps13
Tracheophyta - ps14
Spermatophyta - ps15
Angiosperms - ps16
Mesangiospermae - ps17
Magnoliids/eudicots - ps18
Eudicots - ps19
Core_eudicots - ps20
Superrosids - ps21
Rosids - ps22
Eurosids - ps23
Malvids - ps24
Sap./Malv./Brassicales - ps25
Malvales/Brassicales - ps26
Brassicales - ps27
Cleomaceae/Brassicaceae - ps28
Brassicaceae - ps29
Camelineae - ps30
Arabidopsis - ps31
Arabidopsis_thaliana - ps32
Phylostratum

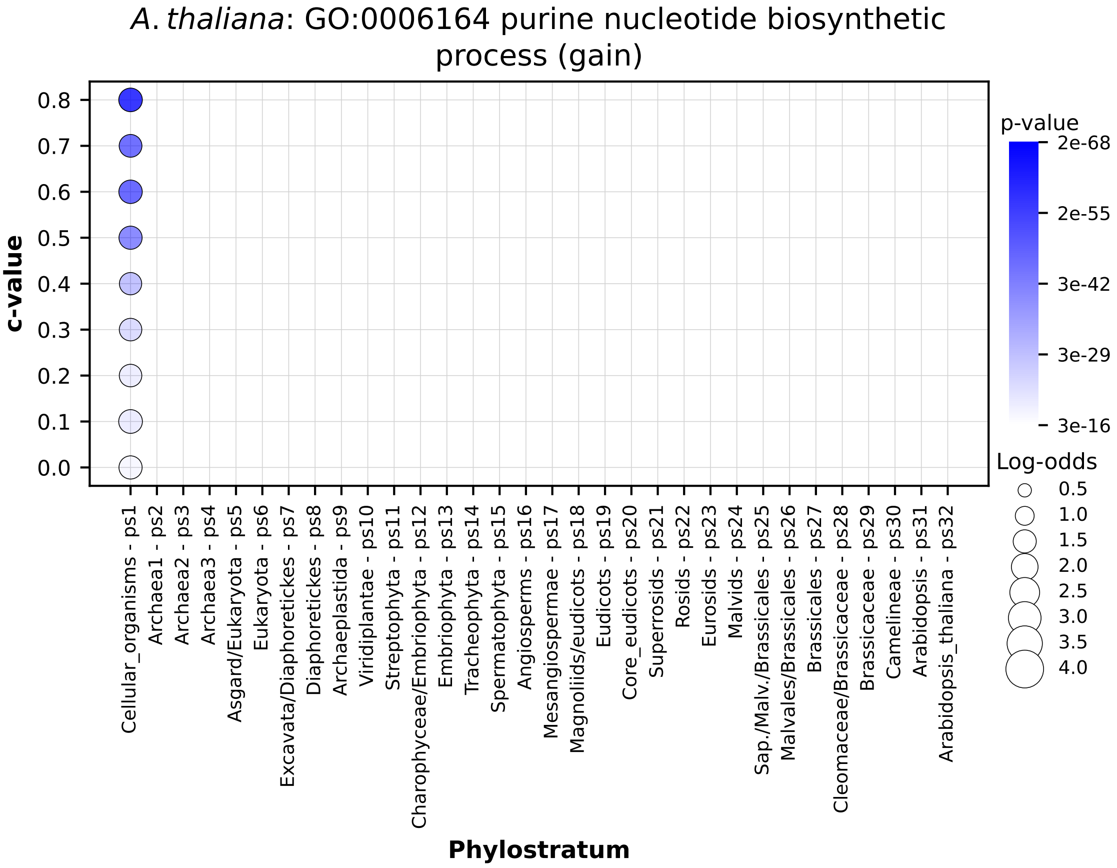

A. thaliana: GO:0006164 purine nucleotide biosynthetic process (gain)
c-value
0.8
0.7
0.6
0.5
0.4
0.3
0.2
0.1
0.0
p-value
2e-68
2e-55
3e-42
3e-29
3e-16
Log-odds
0.5
1.0
1.5
2.0
2.5
3.0
3.5
4.0
Cellular_organisms - ps1
Archaea1 - ps2
Archaea2 - ps3
Archaea3 - ps4
Asgard/Eukaryota - ps5
Eukaryota - ps6
Excavata/Diaphoretickes - ps7
Diaphoretickes - ps8
Archaeplastida - ps9
Viridiplantae - ps10
Streptophyta - ps11
Charophyceae/Embriophyta - ps12
Embriophyta - ps13
Tracheophyta - ps14
Spermatophyta - ps15
Angiosperms - ps16
Mesangiospermae - ps17
Magnoliids/eudicots - ps18
Eudicots - ps19
Core_eudicots - ps20
Superrosids - ps21
Rosids - ps22
Eurosids - ps23
Malvids - ps24
Sap./Malv./Brassicales - ps25
Malvales/Brassicales - ps26
Brassicales - ps27
Cleomaceae/Brassicaceae - ps28
Brassicaceae - ps29
Camelineae - ps30
Arabidopsis - ps31
Arabidopsis_thaliana - ps32
Phylostratum

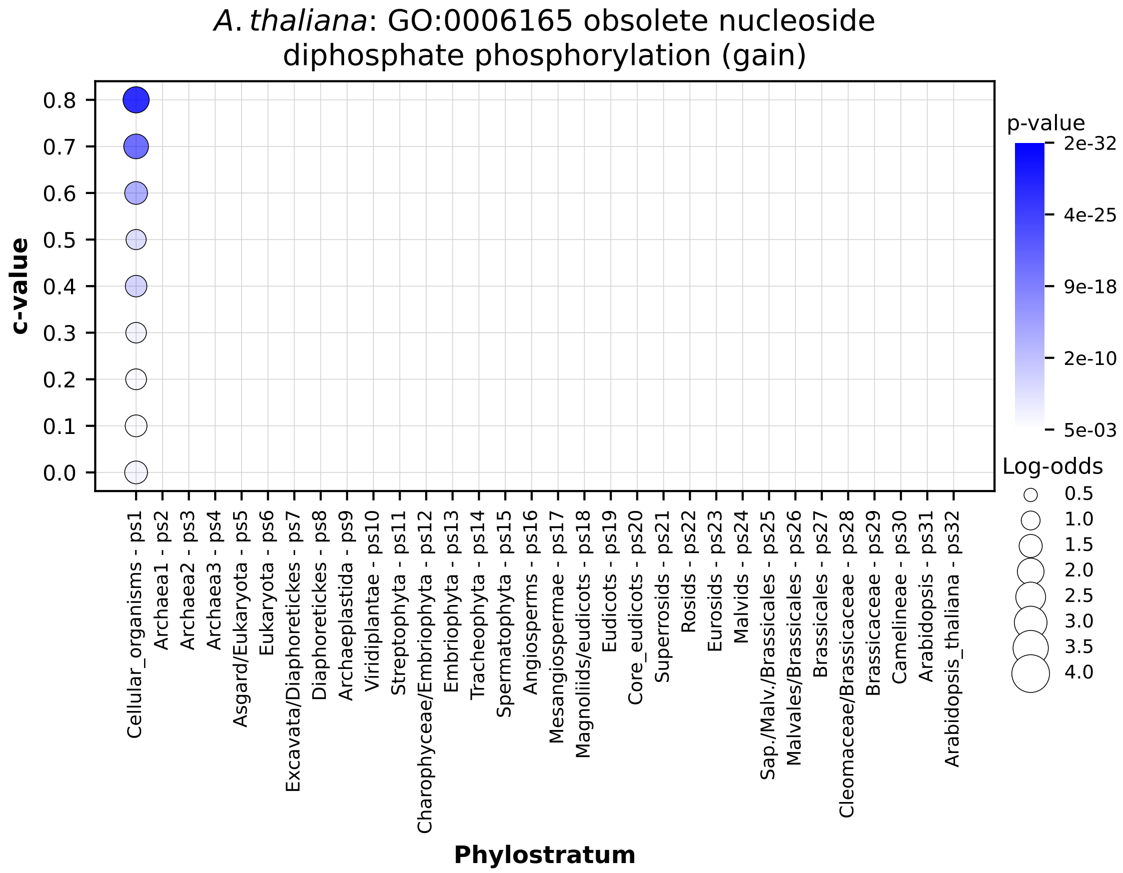
A. thaliana: GO:0006165 obsolete nucleoside diphosphate phosphorylation (gain)
c-value
Phylostratum
p-value
2e-32
4e-25
9e-18
2e-10
5e-03
Log-odds
0.5
1.0
1.5
2.0
2.5
3.0
3.5
4.0
Cellular_organisms - ps1
Archaea1 - ps2
Archaea2 - ps3
Archaea3 - ps4
Asgard/Eukaryota - ps5
Eukaryota - ps6
Excavata/Diaphoretickes - ps7
Diaphoretickes - ps8
Archaeplastida - ps9
Viridiplantae - ps10
Streptophyta - ps11
Charophyceae/Embriophyta - ps12
Embriophyta - ps13
Tracheophyta - ps14
Spermatophyta - ps15
Angiosperms - ps16
Mesangiospermae - ps17
Magnoliids/eudicots - ps18
Eudicots - ps19
Core_eudicots - ps20
Superrosids - ps21
Rosids - ps22
Eurosids - ps23
Malvids - ps24
Sap./Malv./Brassicales - ps25
Malvales/Brassicales - ps26
Brassicales - ps27
Cleomaceae/Brassicaceae - ps28
Brassicaceae - ps29
Camelineae - ps30
Arabidopsis - ps31
Arabidopsis_thaliana - ps32

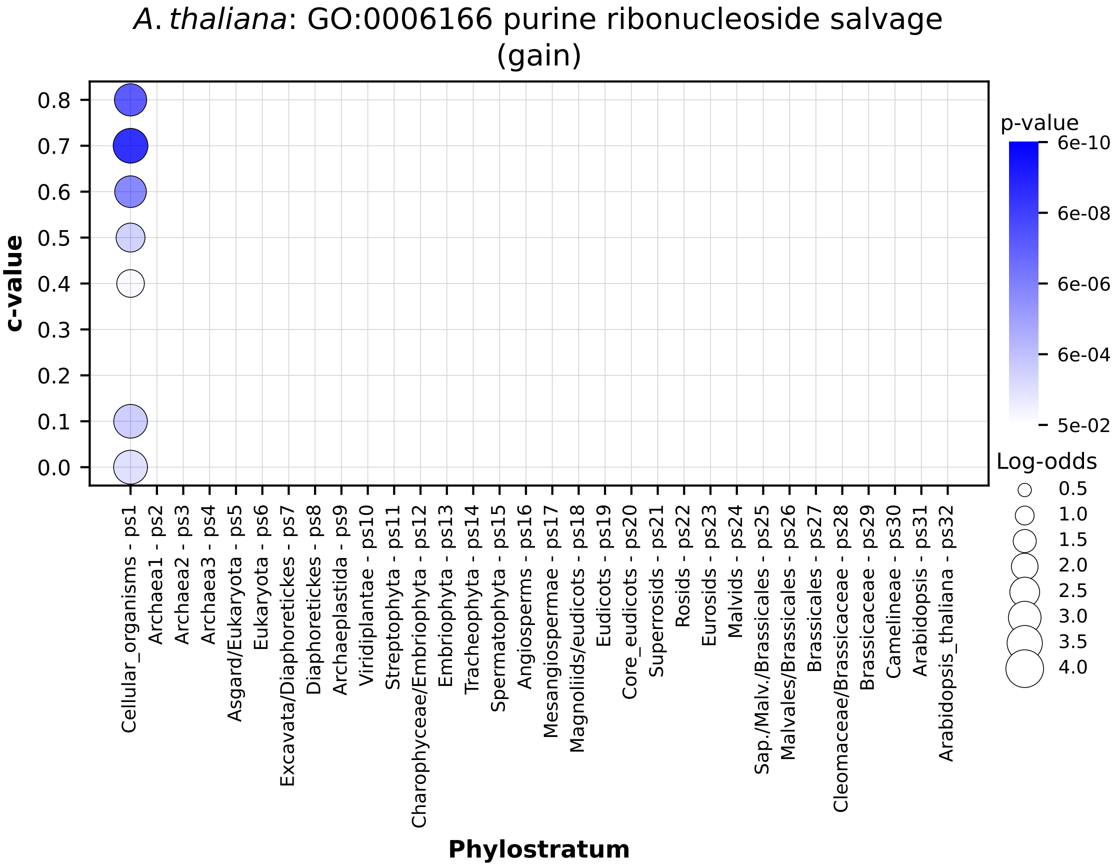

A. thaliana: GO:0006166 purine ribonucleoside salvage
(gain)
c-value
0.8
0.7
0.6
0.5
0.4
0.3
0.2
0.1
0.0
p-value
6e-10
6e-08
6e-06
6e-04
5e-02
Log-odds
0.5
1.0
1.5
2.0
2.5
3.0
3.5
4.0
Cellular_organisms - ps1
Archaea1 - ps2
Archaea2 - ps3
Archaea3 - ps4
Asgard/Eukaryota - ps5
Eukaryota - ps6
Excavata/Diaphoretickes - ps7
Diaphoretickes - ps8
Archaeplastida - ps9
Viridiplantae - ps10
Streptophyta - ps11
Charophyceae/Embriophyta - ps12
Embriophyta - ps13
Tracheophyta - ps14
Spermatophyta - ps15
Angiosperms - ps16
Mesangiospermae - ps17
Magnoliids/eudicots - ps18
Eudicots - ps19
Core_eudicots - ps20
Superrosids - ps21
Rosids - ps22
Eurosids - ps23
Malvids - ps24
Sap./Malv./Brassicales - ps25
Malvales/Brassicales - ps26
Brassicales - ps27
Cleomaceae/Brassicaceae - ps28
Brassicaceae - ps29
Camelineae - ps30
Arabidopsis - ps31
Arabidopsis_thaliana - ps32
Phylostratum

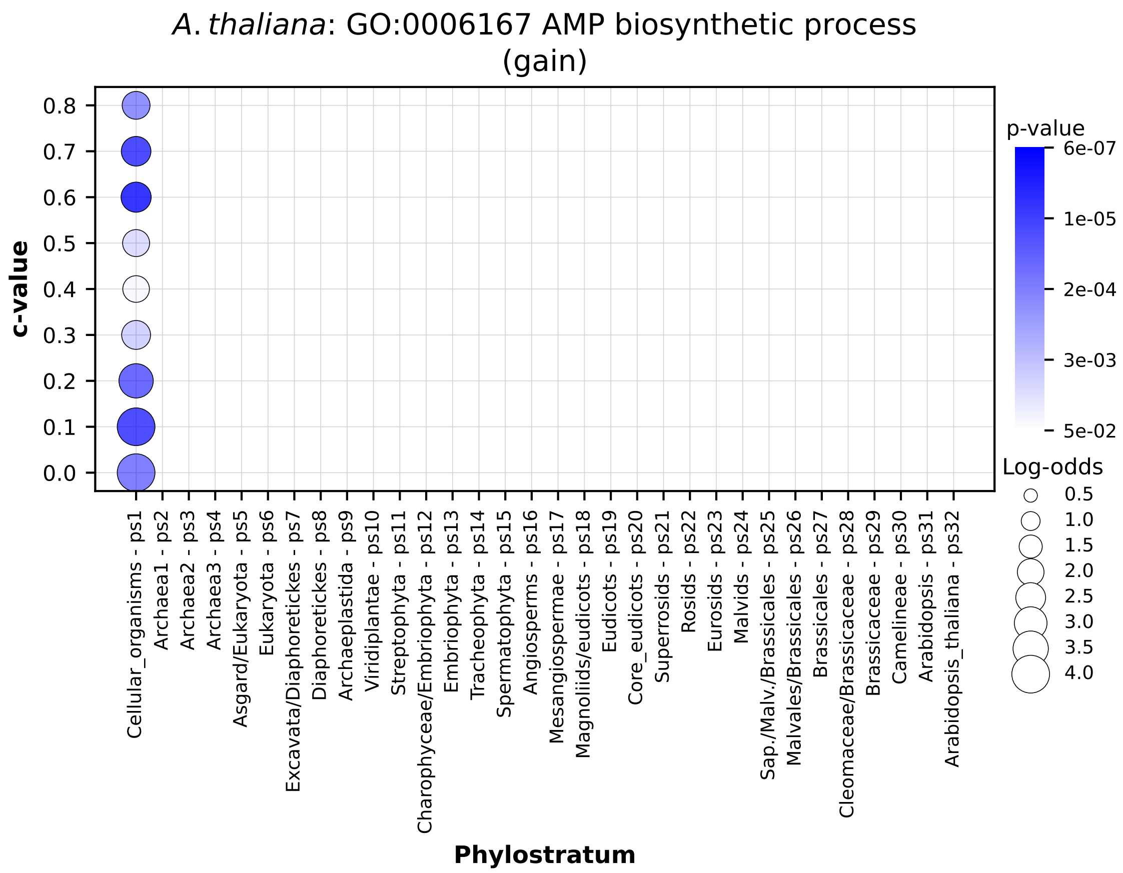

A. thaliana: GO:0006167 AMP biosynthetic process (gain)
c-value
0.0
0.1
0.2
0.3
0.4
0.5
0.6
0.7
0.8
Phylostratum
Cellular_organisms - ps1
Archaea1 - ps2
Archaea2 - ps3
Archaea3 - ps4
Asgard/Eukaryota - ps5
Eukaryota - ps6
Excavata/Diaphoretickes - ps7
Diaphoretickes - ps8
Archaeplastida - ps9
Viridiplantae - ps10
Streptophyta - ps11
Charophyceae/Embriophyta - ps12
Embriophyta - ps13
Tracheophyta - ps14
Spermatophyta - ps15
Angiosperms - ps16
Mesangiospermae - ps17
Magnoliids/eudicots - ps18
Eudicots - ps19
Core_eudicots - ps20
Superrosids - ps21
Rosids - ps22
Eurosids - ps23
Malvids - ps24
Sap./Malv./Brassicales - ps25
Malvales/Brassicales - ps26
Brassicales - ps27
Cleomaceae/Brassicaceae - ps28
Brassicaceae - ps29
Camelineae - ps30
Arabidopsis - ps31
Arabidopsis_thaliana - ps32
p-value
6e-07
1e-05
2e-04
3e-03
5e-02
Log-odds
0.5
1.0
1.5
2.0
2.5
3.0
3.5
4.0

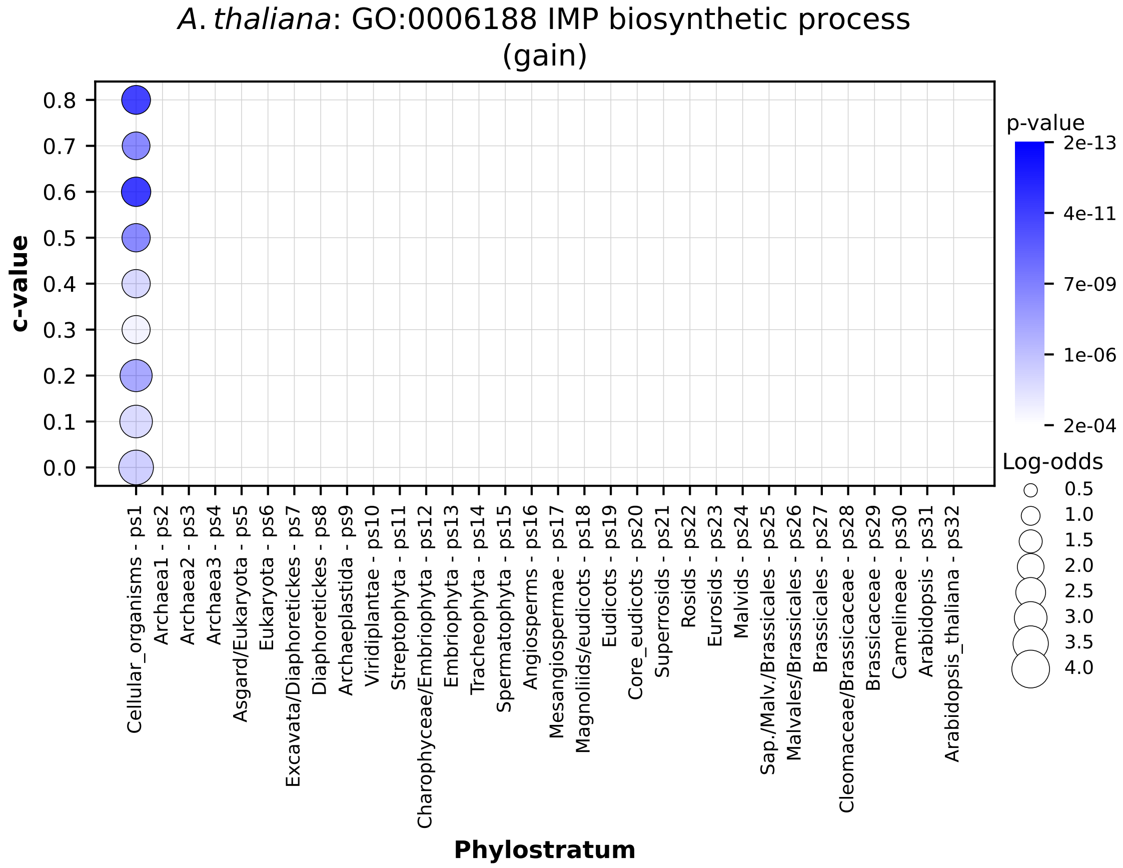

A. thaliana: GO:0006188 IMP biosynthetic process
(gain)
c-value
0.8
0.7
0.6
0.5
0.4
0.3
0.2
0.1
0.0
p-value
2e-13
4e-11
7e-09
1e-06
2e-04
Log-odds
0.5
1.0
1.5
2.0
2.5
3.0
3.5
4.0
Cellular_organisms - ps1
Archaea1 - ps2
Archaea2 - ps3
Archaea3 - ps4
Asgard/Eukaryota - ps5
Eukaryota - ps6
Excavata/Diaphoretickes - ps7
Diaphoretickes - ps8
Archaeplastida - ps9
Viridiplantae - ps10
Streptophyta - ps11
Charophyceae/Embriophyta - ps12
Embriophyta - ps13
Tracheophyta - ps14
Spermatophyta - ps15
Angiosperms - ps16
Mesangiospermae - ps17
Magnoliids/eudicots - ps18
Eudicots - ps19
Core_eudicots - ps20
Superrosids - ps21
Rosids - ps22
Eurosids - ps23
Malvids - ps24
Sap./Malv./Brassicales - ps25
Malvales/Brassicales - ps26
Brassicales - ps27
Cleomaceae/Brassicaceae - ps28
Brassicaceae - ps29
Camelineae - ps30
Arabidopsis - ps31
Arabidopsis_thaliana - ps32
Phylostratum

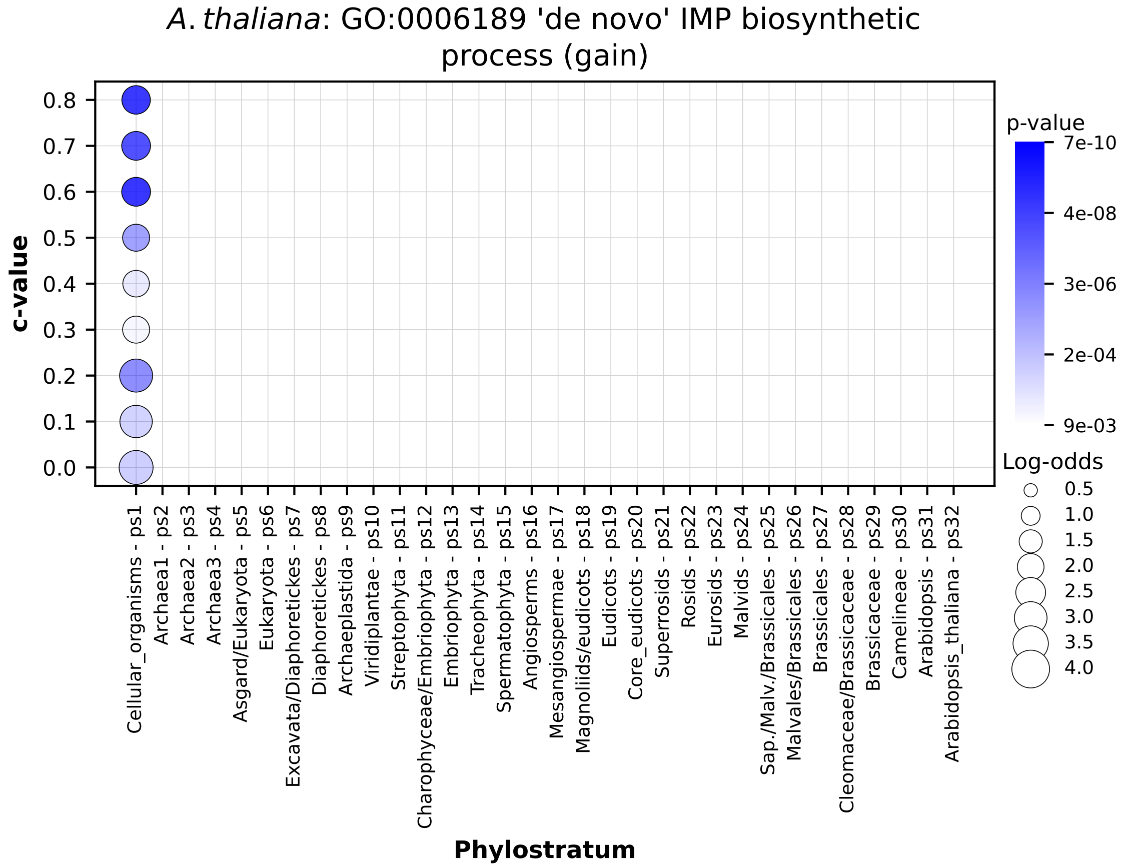
A. thaliana: GO:0006189 'de novo' IMP biosynthetic process (gain)
c-value
0.8
0.7
0.6
0.5
0.4
0.3
0.2
0.1
0.0
p-value
7e-10
4e-08
3e-06
2e-04
9e-03
Log-odds
0.5
1.0
1.5
2.0
2.5
3.0
3.5
4.0
Cellular_organisms - ps1
Archaea1 - ps2
Archaea2 - ps3
Archaea3 - ps4
Asgard/Eukaryota - ps5
Eukaryota - ps6
Excavata/Diaphoretickes - ps7
Diaphoretickes - ps8
Archaeplastida - ps9
Viridiplantae - ps10
Streptophyta - ps11
Charophyceae/Embriophyta - ps12
Embriophyta - ps13
Tracheophyta - ps14
Spermatophyta - ps15
Angiosperms - ps16
Mesangiospermae - ps17
Magnoliids/eudicots - ps18
Eudicots - ps19
Core_eudicots - ps20
Superrosids - ps21
Rosids - ps22
Eurosids - ps23
Malvids - ps24
Sap./Malv./Brassicales - ps25
Malvales/Brassicales - ps26
Brassicales - ps27
Cleomaceae/Brassicaceae - ps28
Brassicaceae - ps29
Camelineae - ps30
Arabidopsis - ps31
Arabidopsis_thaliana - ps32
Phylostratum

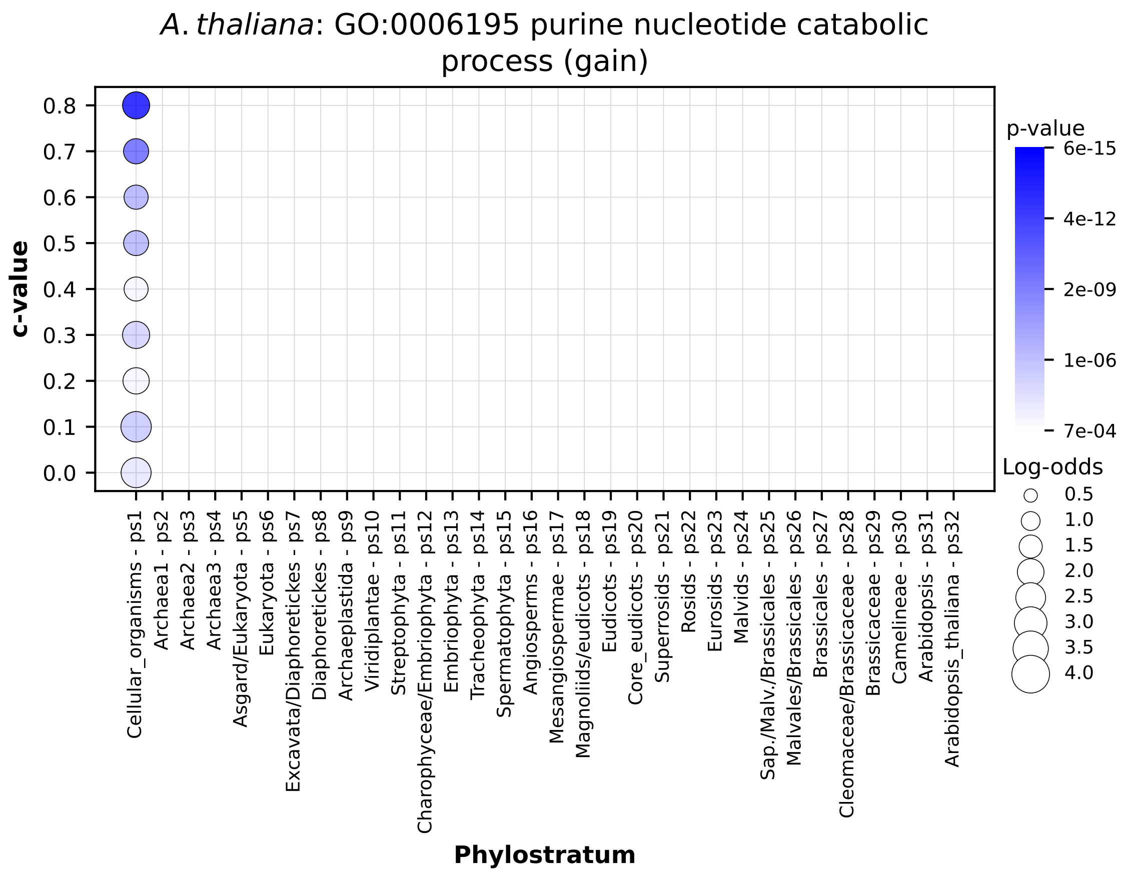

A. thaliana: GO:0006195 purine nucleotide catabolic process (gain)
c-value
0.8
0.7
0.6
0.5
0.4
0.3
0.2
0.1
0.0
p-value
6e-15
4e-12
2e-09
1e-06
7e-04
Log-odds
0.5
1.0
1.5
2.0
2.5
3.0
3.5
4.0
Cellular_organisms - ps1
Archaea1 - ps2
Archaea2 - ps3
Archaea3 - ps4
Asgard/Eukaryota - ps5
Eukaryota - ps6
Excavata/Diaphoretickes - ps7
Diaphoretickes - ps8
Archaeplastida - ps9
Viridiplantae - ps10
Streptophyta - ps11
Charophyceae/Embriophyta - ps12
Embriophyta - ps13
Tracheophyta - ps14
Spermatophyta - ps15
Angiosperms - ps16
Mesangiospermae - ps17
Magnoliids/eudicots - ps18
Eudicots - ps19
Core_eudicots - ps20
Superrosids - ps21
Rosids - ps22
Eurosids - ps23
Malvids - ps24
Sap./Malv./Brassicales - ps25
Malvales/Brassicales - ps26
Brassicales - ps27
Cleomaceae/Brassicaceae - ps28
Brassicaceae - ps29
Camelineae - ps30
Arabidopsis - ps31
Arabidopsis_thaliana - ps32
Phylostratum

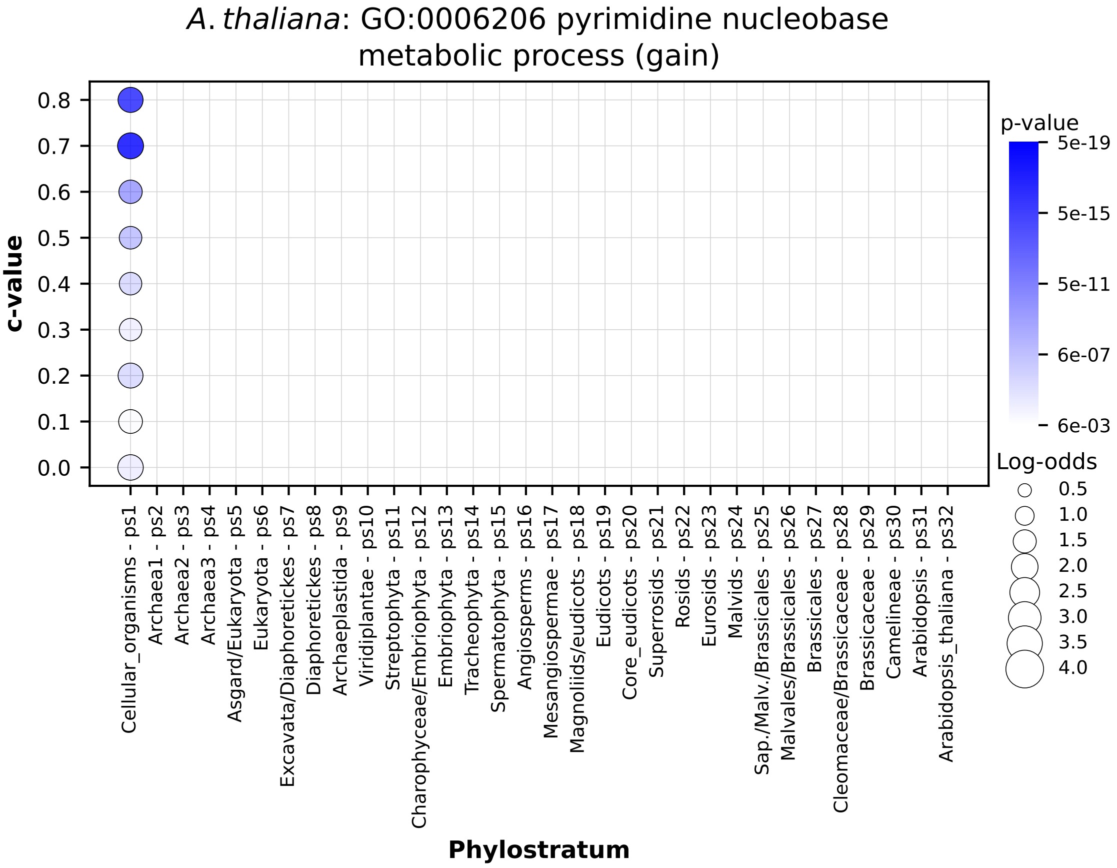

A. thaliana: GO:0006206 pyrimidine nucleobase metabolic process (gain)
c-value
0.8
0.7
0.6
0.5
0.4
0.3
0.2
0.1
0.0
p-value
5e-19
5e-15
5e-11
6e-07
6e-03
Log-odds
0.5
1.0
1.5
2.0
2.5
3.0
3.5
4.0
Cellular_organisms - ps1
Archaea1 - ps2
Archaea2 - ps3
Archaea3 - ps4
Asgard/Eukaryota - ps5
Eukaryota - ps6
Excavata/Diaphoretickes - ps7
Diaphoretickes - ps8
Archaeplastida - ps9
Viridiplantae - ps10
Streptophyta - ps11
Charophyceae/Embriophyta - ps12
Embriophyta - ps13
Tracheophyta - ps14
Spermatophyta - ps15
Angiosperms - ps16
Mesangiospermae - ps17
Magnoliids/eudicots - ps18
Eudicots - ps19
Core_eudicots - ps20
Superrosids - ps21
Rosids - ps22
Eurosids - ps23
Malvids - ps24
Sap./Malv./Brassicales - ps25
Malvales/Brassicales - ps26
Brassicales - ps27
Cleomaceae/Brassicaceae - ps28
Brassicaceae - ps29
Camelineae - ps30
Arabidopsis - ps31
Arabidopsis_thaliana - ps32
Phylostratum

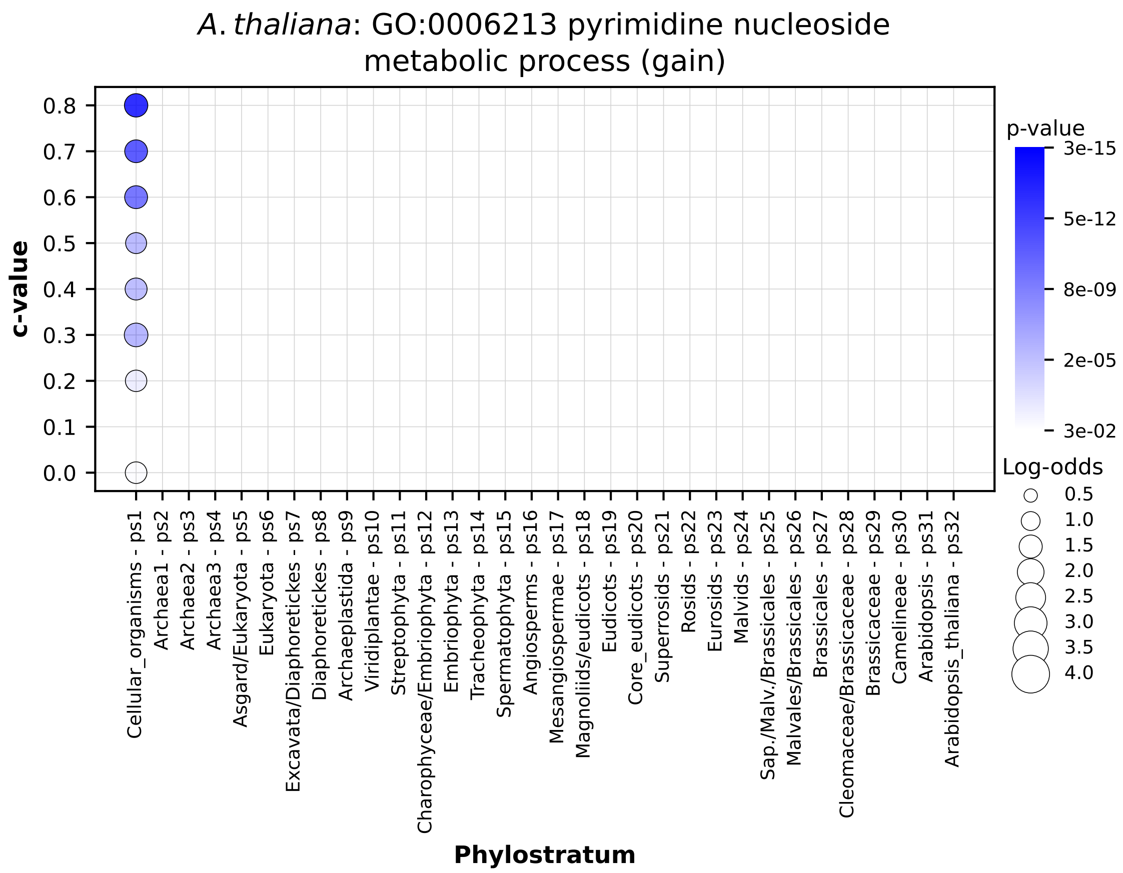

A. thaliana: GO:0006213 pyrimidine nucleoside metabolic process (gain)
c-value
0.8
0.7
0.6
0.5
0.4
0.3
0.2
0.1
0.0
p-value
3e-15
5e-12
8e-09
2e-05
3e-02
Log-odds
0.5
1.0
1.5
2.0
2.5
3.0
3.5
4.0
Cellular_organisms - ps1
Archaea1 - ps2
Archaea2 - ps3
Archaea3 - ps4
Asgard/Eukaryota - ps5
Eukaryota - ps6
Excavata/Diaphoretickes - ps7
Diaphoretickes - ps8
Archaeplastida - ps9
Viridiplantae - ps10
Streptophyta - ps11
Charophyceae/Embriophyta - ps12
Embriophyta - ps13
Tracheophyta - ps14
Spermatophyta - ps15
Angiosperms - ps16
Mesangiospermae - ps17
Magnoliids/eudicots - ps18
Eudicots - ps19
Core_eudicots - ps20
Superrosids - ps21
Rosids - ps22
Eurosids - ps23
Malvids - ps24
Sap./Malv./Brassicales - ps25
Malvales/Brassicales - ps26
Brassicales - ps27
Cleomaceae/Brassicaceae - ps28
Brassicaceae - ps29
Camelineae - ps30
Arabidopsis - ps31
Arabidopsis_thaliana - ps32
Phylostratum

A. thaliana: GO:0006220 pyrimidine nucleotide metabolic process (gain)

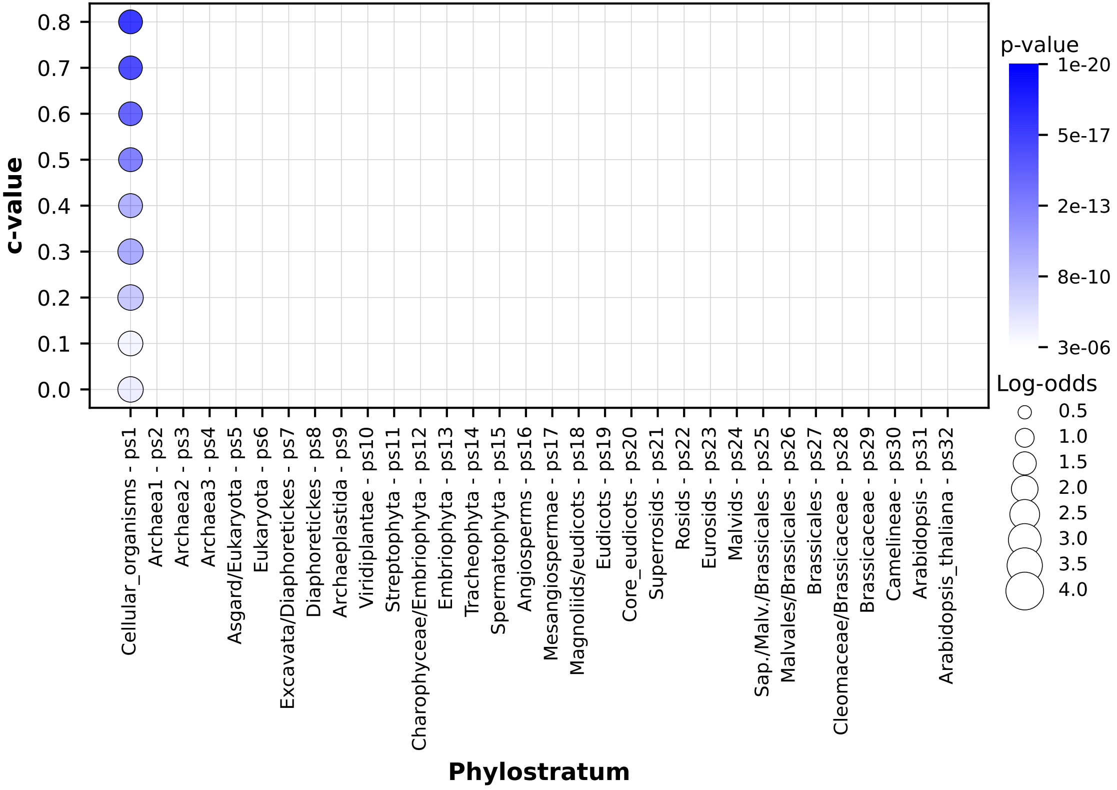

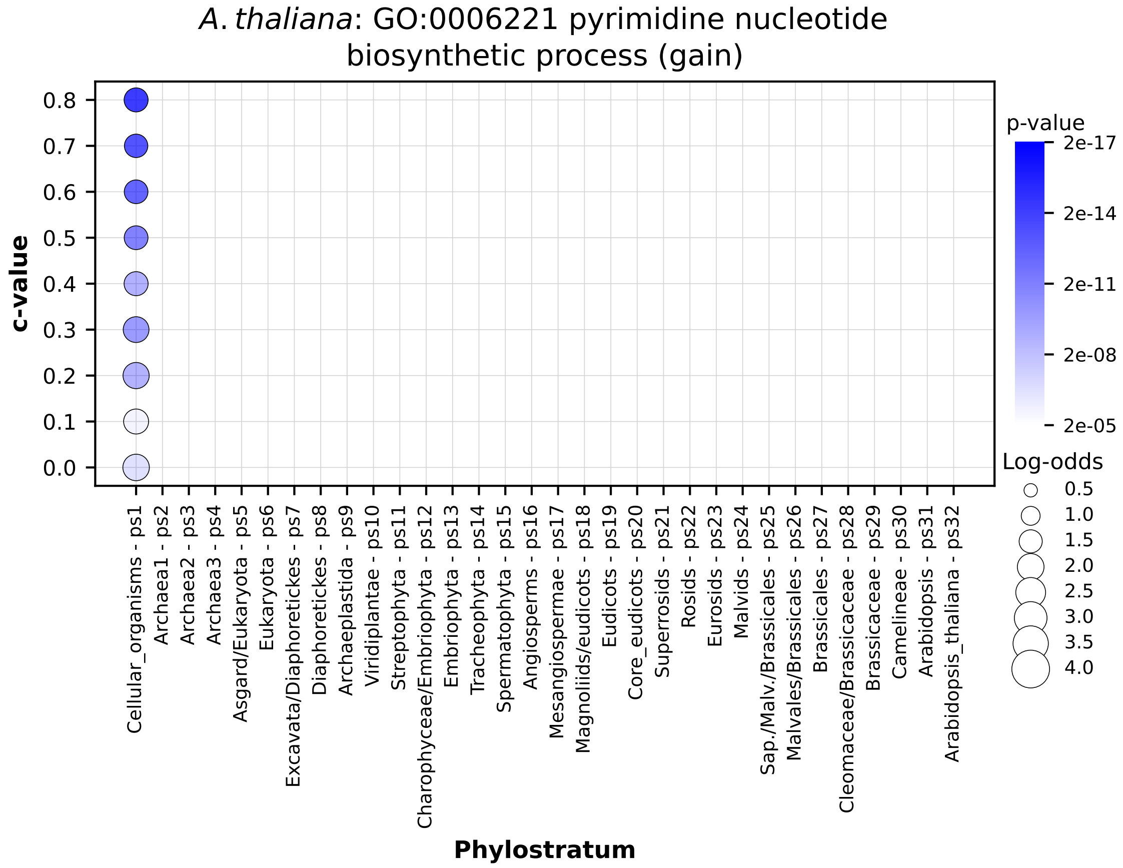

A. thaliana: GO:0006221 pyrimidine nucleotide biosynthetic process (gain)
c-value
0.8
0.7
0.6
0.5
0.4
0.3
0.2
0.1
0.0
p-value
2e-17
2e-14
2e-11
2e-08
2e-05
Log-odds
0.5
1.0
1.5
2.0
2.5
3.0
3.5
4.0
Cellular_organisms - ps1
Archaea1 - ps2
Archaea2 - ps3
Archaea3 - ps4
Asgard/Eukaryota - ps5
Eukaryota - ps6
Excavata/Diaphoretickes - ps7
Diaphoretickes - ps8
Archaeplastida - ps9
Viridiplantae - ps10
Streptophyta - ps11
Charophyceae/Embriophyta - ps12
Embriophyta - ps13
Tracheophyta - ps14
Spermatophyta - ps15
Angiosperms - ps16
Mesangiospermae - ps17
Magnoliids/eudicots - ps18
Eudicots - ps19
Core_eudicots - ps20
Superrosids - ps21
Rosids - ps22
Eurosids - ps23
Malvids - ps24
Sap./Malv./Brassicales - ps25
Malvales/Brassicales - ps26
Brassicales - ps27
Cleomaceae/Brassicaceae - ps28
Brassicaceae - ps29
Camelineae - ps30
Arabidopsis - ps31
Arabidopsis_thaliana - ps32
Phylostratum

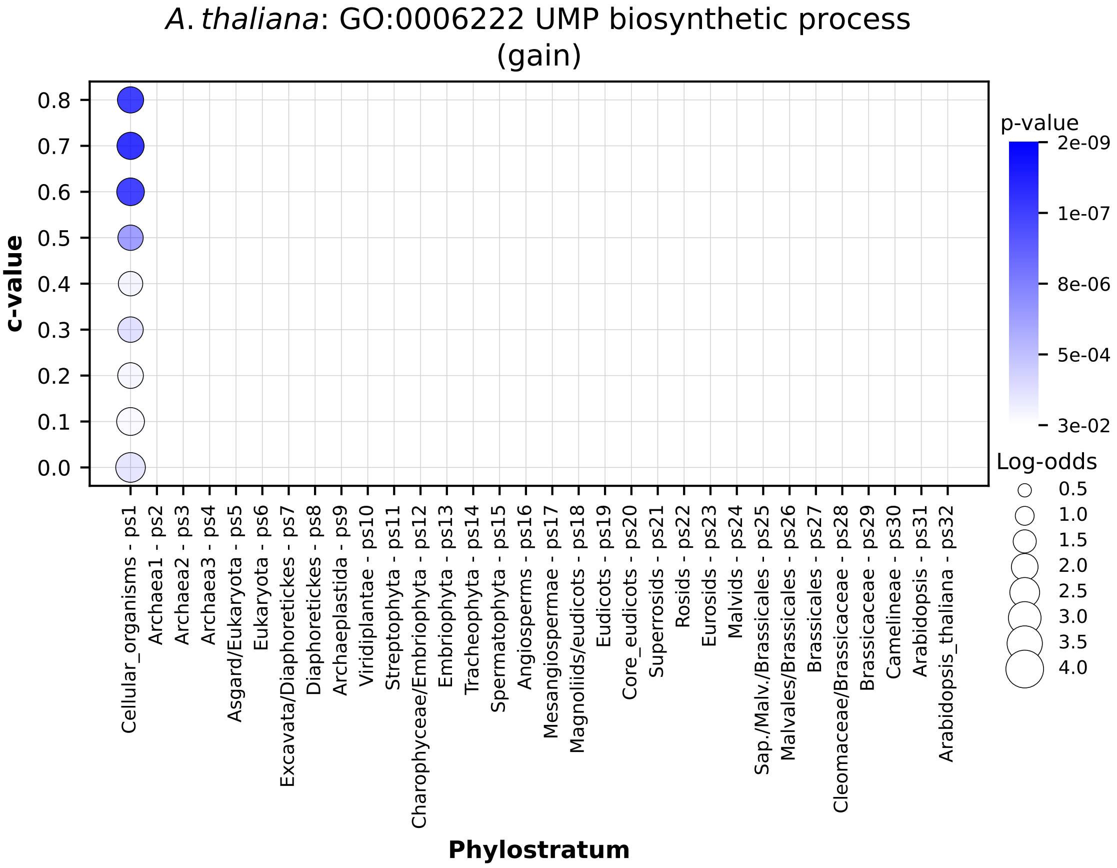
A. thaliana: GO:0006222 UMP biosynthetic process
(gain)
c-value
0.8
0.7
0.6
0.5
0.4
0.3
0.2
0.1
0.0
p-value
2e-09
1e-07
8e-06
5e-04
3e-02
Log-odds
0.5
1.0
1.5
2.0
2.5
3.0
3.5
4.0
Cellular_organisms - ps1
Archaea1 - ps2
Archaea2 - ps3
Archaea3 - ps4
Asgard/Eukaryota - ps5
Eukaryota - ps6
Excavata/Diaphoretickes - ps7
Diaphoretickes - ps8
Archaeplastida - ps9
Viridiplantae - ps10
Streptophyta - ps11
Charophyceae/Embriophyta - ps12
Embriophyta - ps13
Tracheophyta - ps14
Spermatophyta - ps15
Angiosperms - ps16
Mesangiospermae - ps17
Magnoliids/eudicots - ps18
Eudicots - ps19
Core_eudicots - ps20
Superrosids - ps21
Rosids - ps22
Eurosids - ps23
Malvids - ps24
Sap./Malv./Brassicales - ps25
Malvales/Brassicales - ps26
Brassicales - ps27
Cleomaceae/Brassicaceae - ps28
Brassicaceae - ps29
Camelineae - ps30
Arabidopsis - ps31
Arabidopsis_thaliana - ps32
Phylostratum

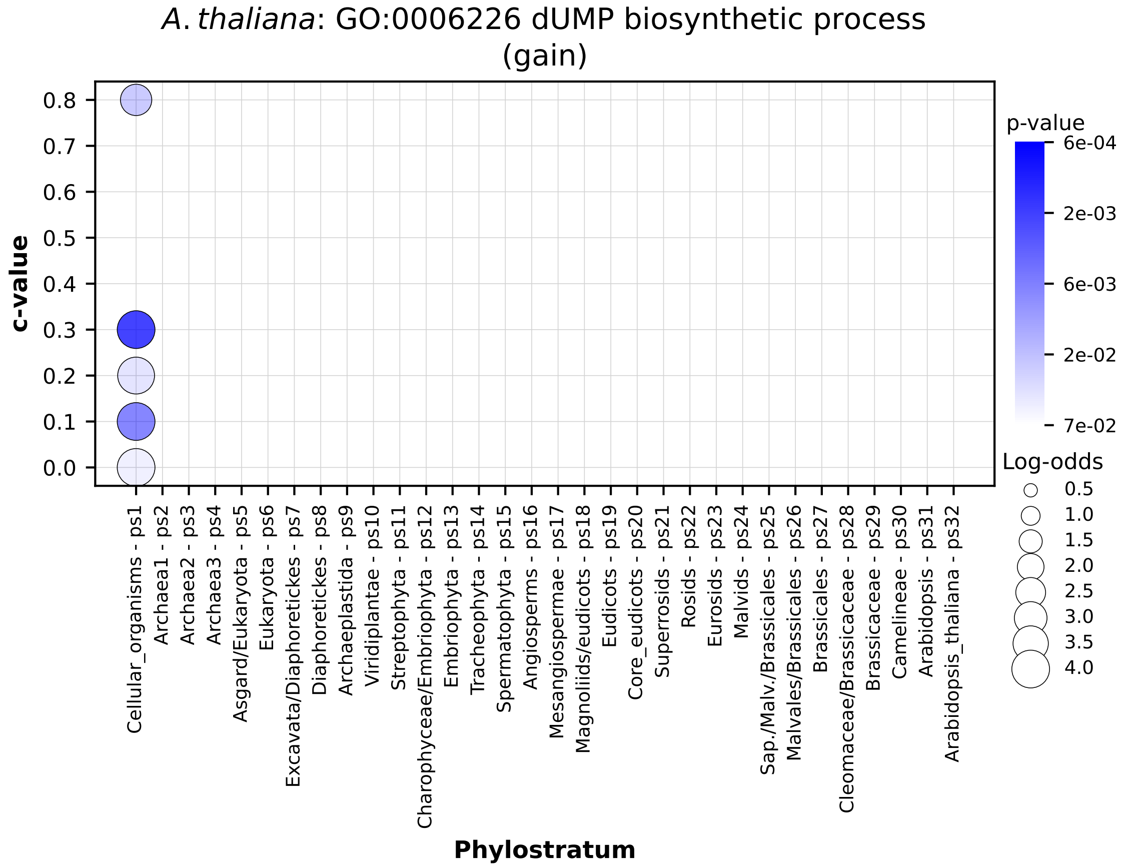

A. thaliana: GO:0006226 dUMP biosynthetic process
(gain)
c-value
0.0
0.1
0.2
0.3
0.4
0.5
0.6
0.7
0.8
Phylostratum
Cellular_organisms - ps1
Archaea1 - ps2
Archaea2 - ps3
Archaea3 - ps4
Asgard/Eukaryota - ps5
Eukaryota - ps6
Excavata/Diaphoretickes - ps7
Diaphoretickes - ps8
Archaeplastida - ps9
Viridiplantae - ps10
Streptophyta - ps11
Charophyceae/Embriophyta - ps12
Embriophyta - ps13
Tracheophyta - ps14
Spermatophyta - ps15
Angiosperms - ps16
Mesangiospermae - ps17
Magnoliids/eudicots - ps18
Eudicots - ps19
Core_eudicots - ps20
Superrosids - ps21
Rosids - ps22
Eurosids - ps23
Malvids - ps24
Sap./Malv./Brassicales - ps25
Malvales/Brassicales - ps26
Brassicales - ps27
Cleomaceae/Brassicaceae - ps28
Brassicaceae - ps29
Camelineae - ps30
Arabidopsis - ps31
Arabidopsis_thaliana - ps32
p-value
6e-04
2e-03
6e-03
2e-02
7e-02
Log-odds
0.5
1.0
1.5
2.0
2.5
3.0
3.5
4.0

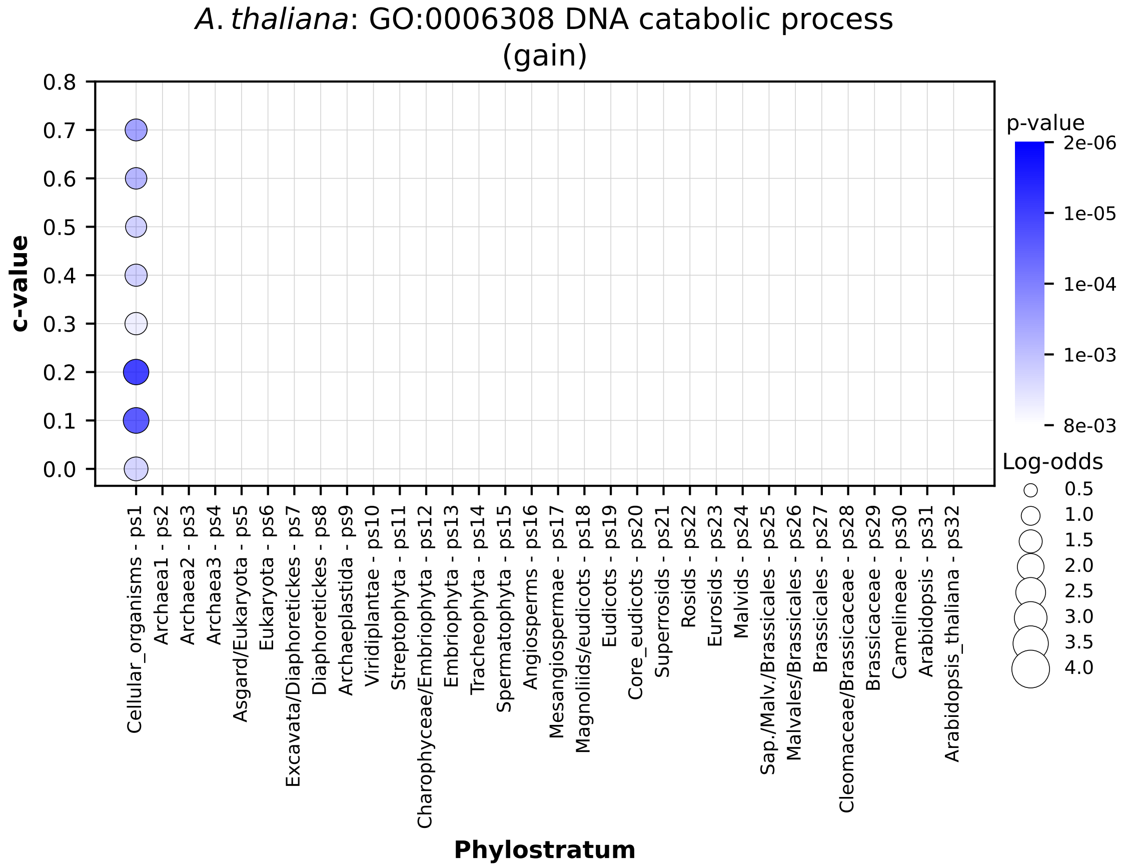

A. thaliana: GO:0006308 DNA catabolic process (gain)
c-value
0.0
0.1
0.2
0.3
0.4
0.5
0.6
0.7
0.8
p-value
2e-06
1e-05
1e-04
1e-03
8e-03
Log-odds
0.5
1.0
1.5
2.0
2.5
3.0
3.5
4.0
Cellular_organisms - ps1
Archaea1 - ps2
Archaea2 - ps3
Archaea3 - ps4
Asgard/Eukaryota - ps5
Eukaryota - ps6
Excavata/Diaphoretickes - ps7
Diaphoretickes - ps8
Archaeplastida - ps9
Viridiplantae - ps10
Streptophyta - ps11
Charophyceae/Embriophyta - ps12
Embriophyta - ps13
Tracheophyta - ps14
Spermatophyta - ps15
Angiosperms - ps16
Mesangiospermae - ps17
Magnoliids/eudicots - ps18
Eudicots - ps19
Core_eudicots - ps20
Superrosids - ps21
Rosids - ps22
Eurosids - ps23
Malvids - ps24
Sap./Malv./Brassicales - ps25
Malvales/Brassicales - ps26
Brassicales - ps27
Cleomaceae/Brassicaceae - ps28
Brassicaceae - ps29
Camelineae - ps30
Arabidopsis - ps31
Arabidopsis_thaliana - ps32
Phylostratum

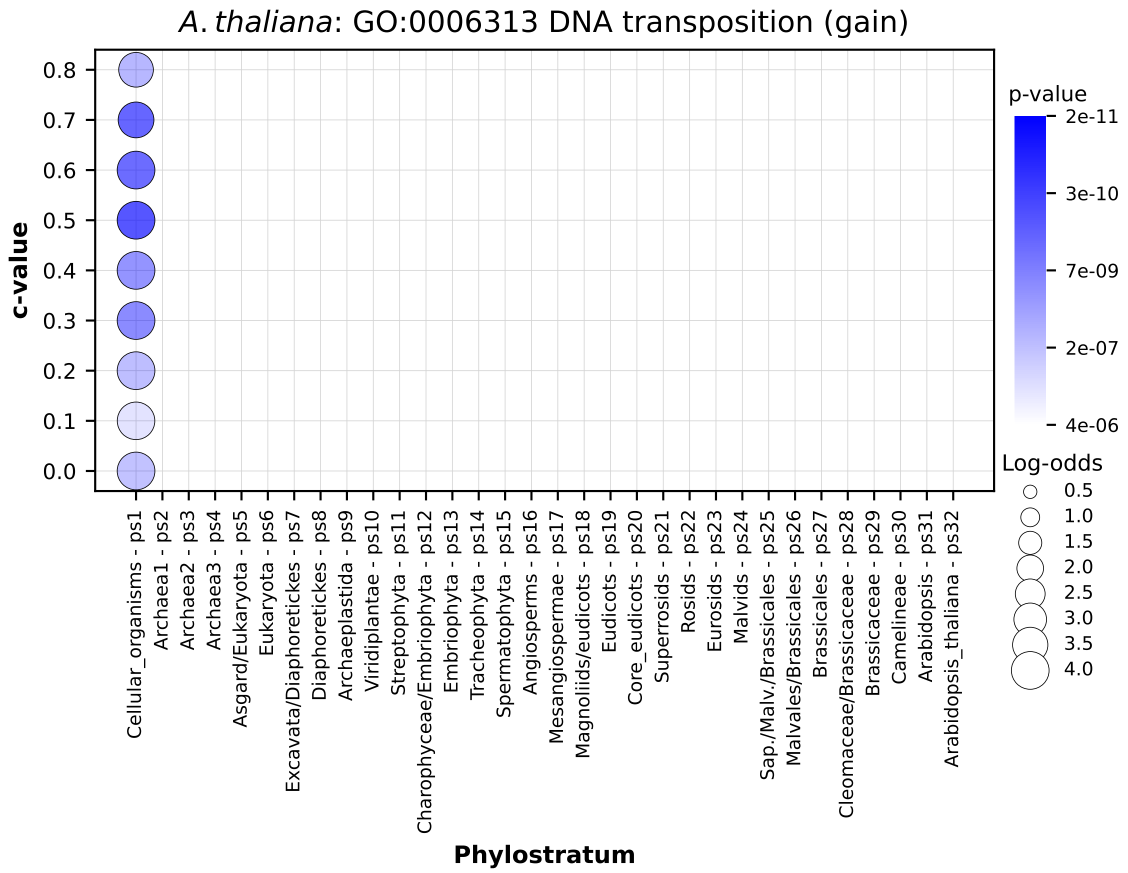

A. thaliana: GO:0006313 DNA transposition (gain)
c-value
0.8
0.7
0.6
0.5
0.4
0.3
0.2
0.1
0.0
p-value
2e-11
3e-10
7e-09
2e-07
4e-06
Log-odds
0.5
1.0
1.5
2.0
2.5
3.0
3.5
4.0
Cellular_organisms - ps1
Archaea1 - ps2
Archaea2 - ps3
Archaea3 - ps4
Asgard/Eukaryota - ps5
Eukaryota - ps6
Excavata/Diaphoretickes - ps7
Diaphoretickes - ps8
Archaeplastida - ps9
Viridiplantae - ps10
Streptophyta - ps11
Charophyceae/Embriophyta - ps12
Embriophyta - ps13
Tracheophyta - ps14
Spermatophyta - ps15
Angiosperms - ps16
Mesangiospermae - ps17
Magnoliids/eudicots - ps18
Eudicots - ps19
Core_eudicots - ps20
Superrosids - ps21
Rosids - ps22
Eurosids - ps23
Malvids - ps24
Sap./Malv./Brassicales - ps25
Malvales/Brassicales - ps26
Brassicales - ps27
Cleomaceae/Brassicaceae - ps28
Brassicaceae - ps29
Camelineae - ps30
Arabidopsis - ps31
Arabidopsis_thaliana - ps32
Phylostratum

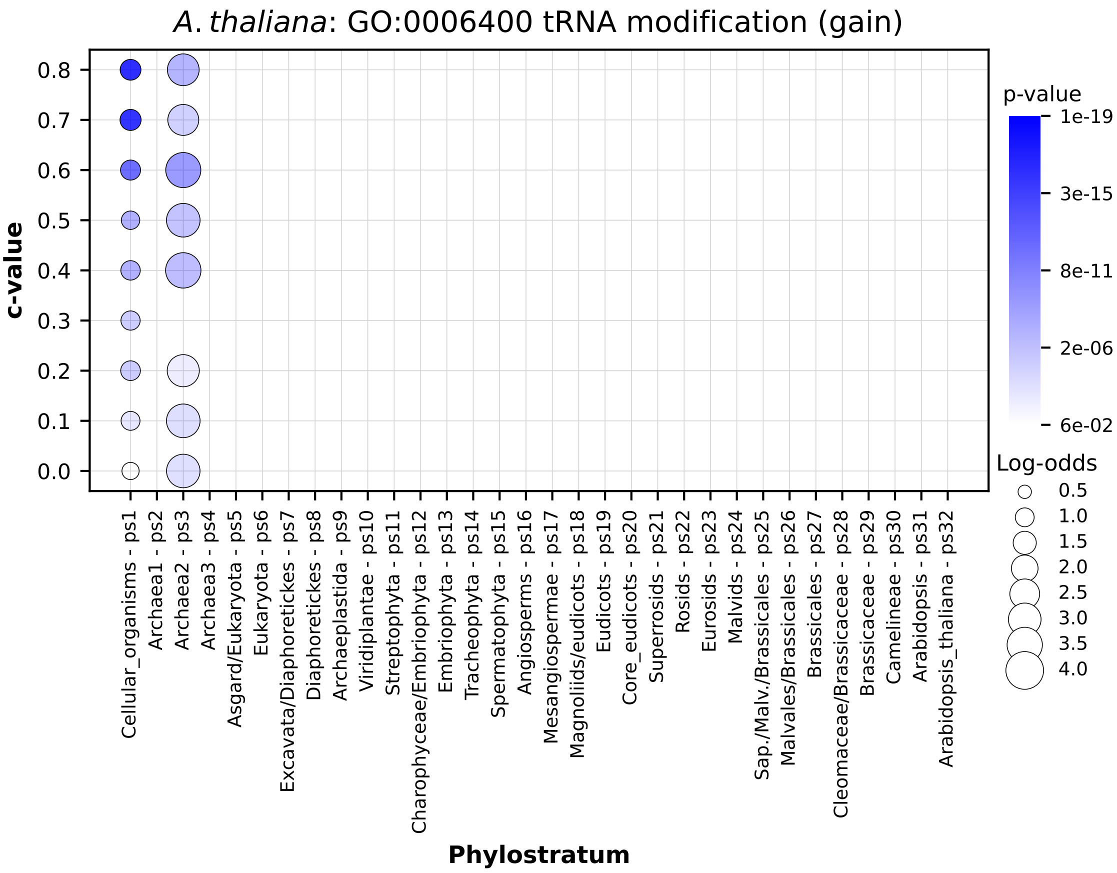
A. thaliana: GO:0006400 tRNA modification (gain)
c-value
0.8
0.7
0.6
0.5
0.4
0.3
0.2
0.1
0.0
Phylostratum
Cellular_organisms - ps1
Archaea1 - ps2
Archaea2 - ps3
Archaea3 - ps4
Asgard/Eukaryota - ps5
Eukaryota - ps6
Excavata/Diaphoretickes - ps7
Diaphoretickes - ps8
Archaeplastida - ps9
Viridiplantae - ps10
Streptophyta - ps11
Charophyceae/Embriophyta - ps12
Embriophyta - ps13
Tracheophyta - ps14
Spermatophyta - ps15
Angiosperms - ps16
Mesangiospermae - ps17
Magnoliids/eudicots - ps18
Eudicots - ps19
Core_eudicots - ps20
Superrosids - ps21
Rosids - ps22
Eurosids - ps23
Malvids - ps24
Sap./Malv./Brassicales - ps25
Malvales/Brassicales - ps26
Brassicales - ps27
Cleomaceae/Brassicaceae - ps28
Brassicaceae - ps29
Camelineae - ps30
Arabidopsis - ps31
Arabidopsis_thaliana - ps32
p-value
1e-19
3e-15
8e-11
2e-06
6e-02
Log-odds
0.5
1.0
1.5
2.0
2.5
3.0
3.5
4.0

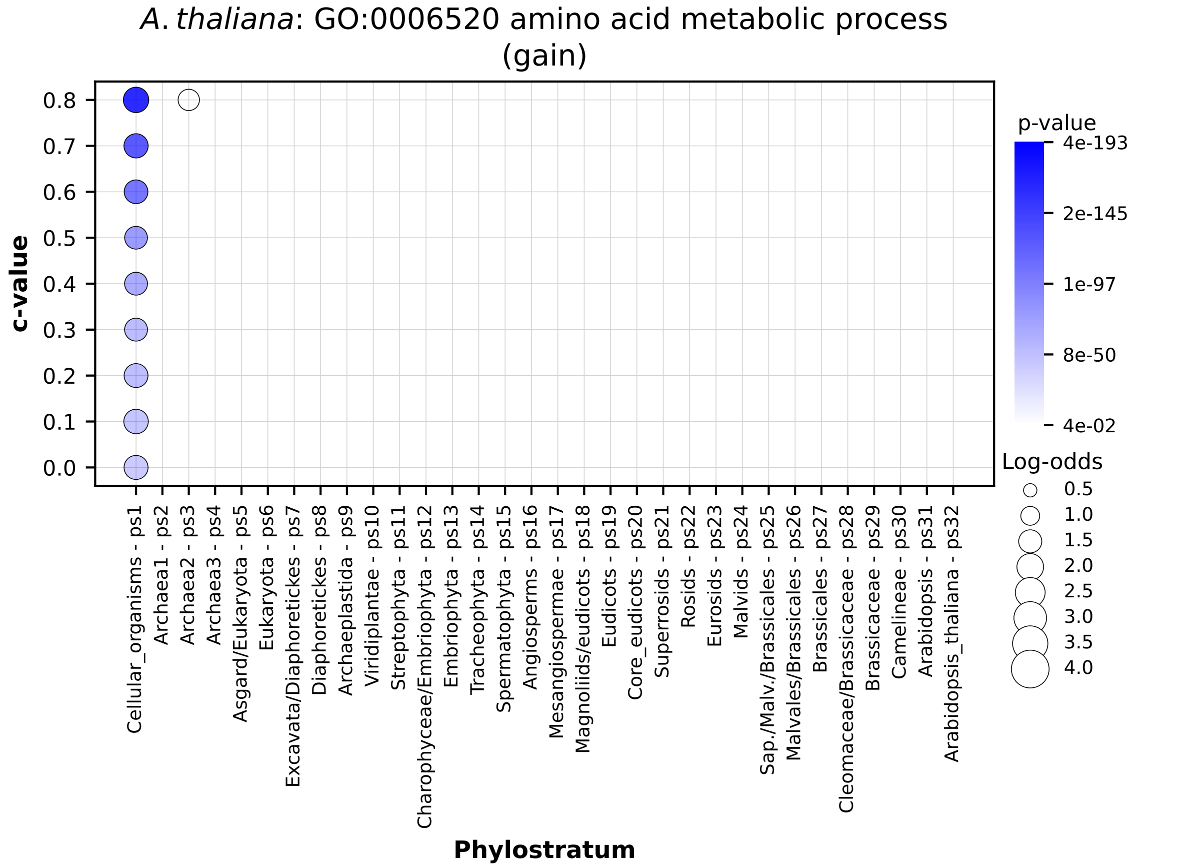

A. thaliana: GO:0006520 amino acid metabolic process
(gain)
c-value
0.8
0.7
0.6
0.5
0.4
0.3
0.2
0.1
0.0
p-value
4e-193
2e-145
1e-97
8e-50
4e-02
Log-odds
0.5
1.0
1.5
2.0
2.5
3.0
3.5
4.0
Cellular_organisms - ps1
Archaea1 - ps2
Archaea2 - ps3
Archaea3 - ps4
Asgard/Eukaryota - ps5
Eukaryota - ps6
Excavata/Diaphoretickes - ps7
Diaphoretickes - ps8
Archaeplastida - ps9
Viridiplantae - ps10
Streptophyta - ps11
Charophyceae/Embriophyta - ps12
Embriophyta - ps13
Tracheophyta - ps14
Spermatophyta - ps15
Angiosperms - ps16
Mesangiospermae - ps17
Magnoliids/eudicots - ps18
Eudicots - ps19
Core_eudicots - ps20
Superrosids - ps21
Rosids - ps22
Eurosids - ps23
Malvids - ps24
Sap./Malv./Brassicales - ps25
Malvales/Brassicales - ps26
Brassicales - ps27
Cleomaceae/Brassicaceae - ps28
Brassicaceae - ps29
Camelineae - ps30
Arabidopsis - ps31
Arabidopsis_thaliana - ps32
Phylostratum

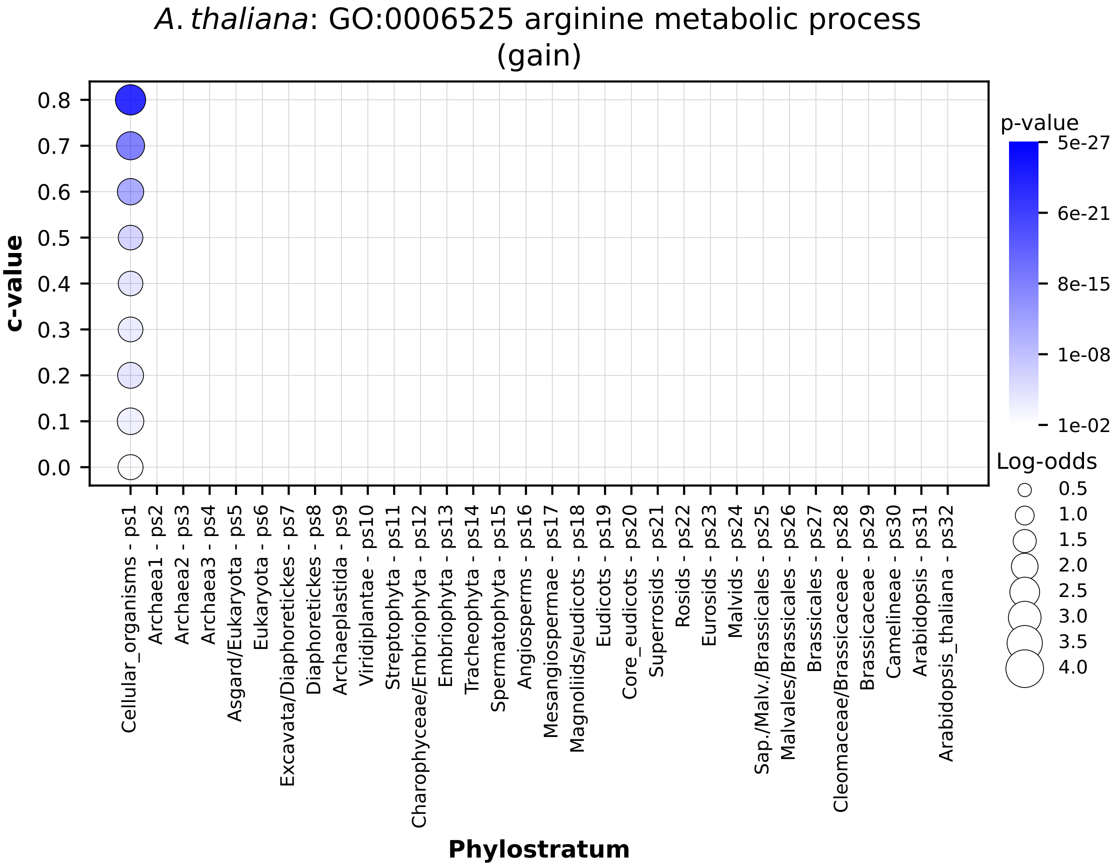

A. thaliana: GO:0006525 arginine metabolic process (gain)
c-value
0.0
0.1
0.2
0.3
0.4
0.5
0.6
0.7
0.8
p-value
5e-27
6e-21
8e-15
1e-08
1e-02
Log-odds
0.5
1.0
1.5
2.0
2.5
3.0
3.5
4.0
Cellular_organisms - ps1
Archaea1 - ps2
Archaea2 - ps3
Archaea3 - ps4
Asgard/Eukaryota - ps5
Eukaryota - ps6
Excavata/Diaphoretickes - ps7
Diaphoretickes - ps8
Archaeplastida - ps9
Viridiplantae - ps10
Streptophyta - ps11
Charophyceae/Embriophyta - ps12
Embriophyta - ps13
Tracheophyta - ps14
Spermatophyta - ps15
Angiosperms - ps16
Mesangiospermae - ps17
Magnoliids/eudicots - ps18
Eudicots - ps19
Core_eudicots - ps20
Superrosids - ps21
Rosids - ps22
Eurosids - ps23
Malvids - ps24
Sap./Malv./Brassicales - ps25
Malvales/Brassicales - ps26
Brassicales - ps27
Cleomaceae/Brassicaceae - ps28
Brassicaceae - ps29
Camelineae - ps30
Arabidopsis - ps31
Arabidopsis_thaliana - ps32
Phylostratum

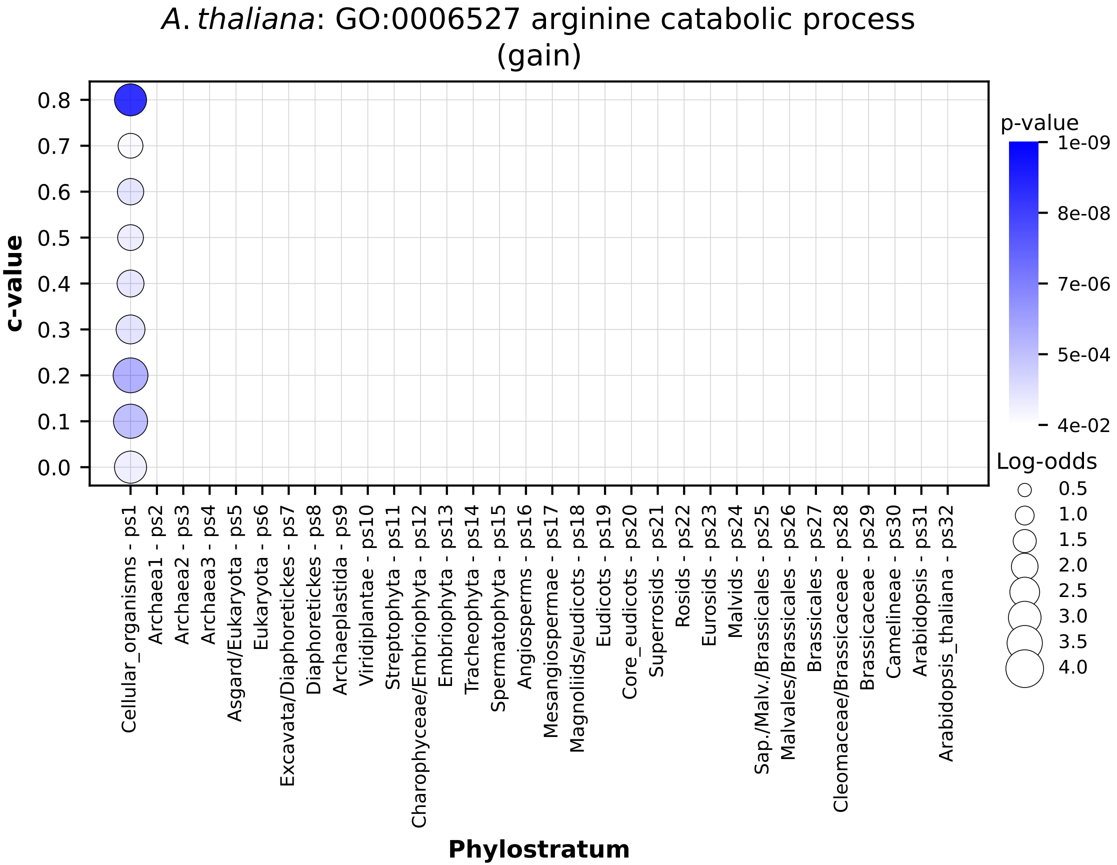

A. thaliana: GO:0006527 arginine catabolic process
(gain)
c-value
0.0
0.1
0.2
0.3
0.4
0.5
0.6
0.7
0.8
p-value
1e-09
8e-08
7e-06
5e-04
4e-02
Log-odds
0.5
1.0
1.5
2.0
2.5
3.0
3.5
4.0
Cellular_organisms - ps1
Archaea1 - ps2
Archaea2 - ps3
Archaea3 - ps4
Asgard/Eukaryota - ps5
Eukaryota - ps6
Excavata/Diaphoretickes - ps7
Diaphoretickes - ps8
Archaeplastida - ps9
Viridiplantae - ps10
Streptophyta - ps11
Charophyceae/Embriophyta - ps12
Embriophyta - ps13
Tracheophyta - ps14
Spermatophyta - ps15
Angiosperms - ps16
Mesangiospermae - ps17
Magnoliids/eudicots - ps18
Eudicots - ps19
Core_eudicots - ps20
Superrosids - ps21
Rosids - ps22
Eurosids - ps23
Malvids - ps24
Sap./Malv./Brassicales - ps25
Malvales/Brassicales - ps26
Brassicales - ps27
Cleomaceae/Brassicaceae - ps28
Brassicaceae - ps29
Camelineae - ps30
Arabidopsis - ps31
Arabidopsis_thaliana - ps32
Phylostratum

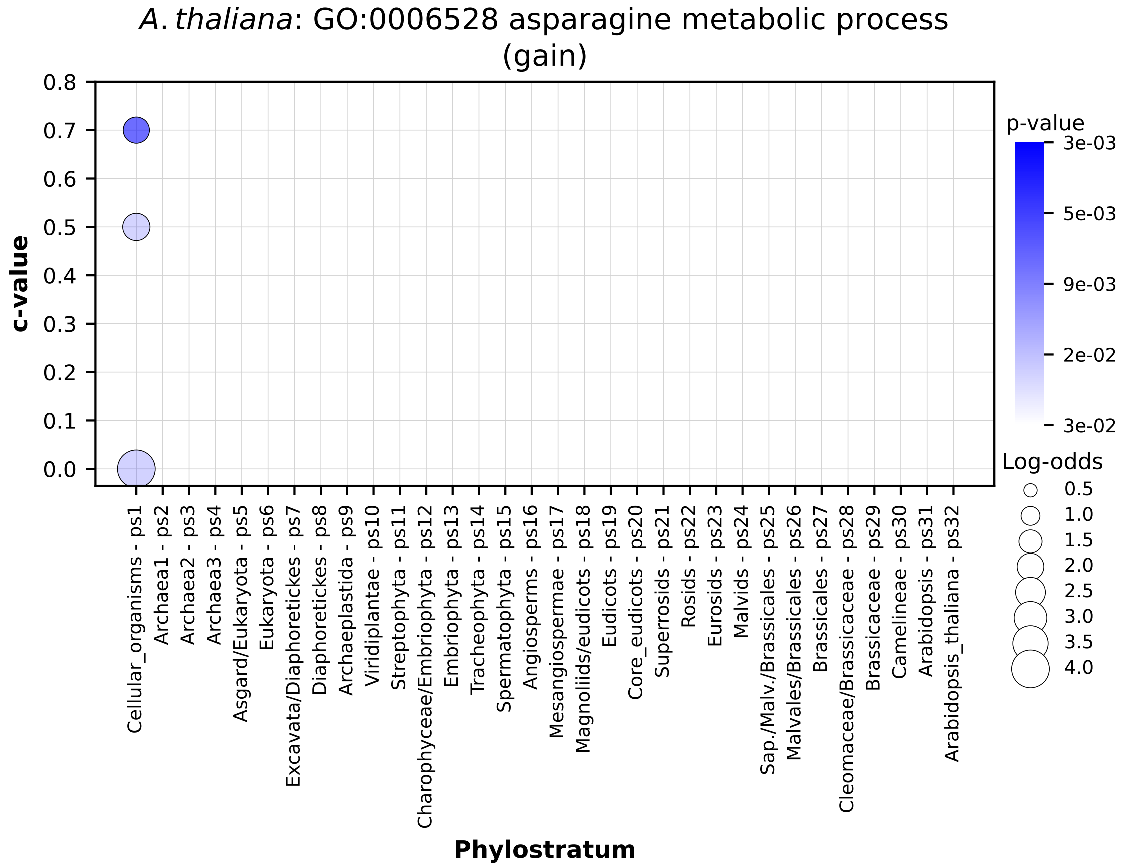

A. thaliana: GO:0006528 asparagine metabolic process
(gain)
c-value
0.8
0.7
0.6
0.5
0.4
0.3
0.2
0.1
0.0
Phylostratum
Cellular_organisms - ps1
Archaea1 - ps2
Archaea2 - ps3
Archaea3 - ps4
Asgard/Eukaryota - ps5
Eukaryota - ps6
Excavata/Diaphoretickes - ps7
Diaphoretickes - ps8
Archaeplastida - ps9
Viridiplantae - ps10
Streptophyta - ps11
Charophyceae/Embriophyta - ps12
Embriophyta - ps13
Tracheophyta - ps14
Spermatophyta - ps15
Angiosperms - ps16
Mesangiospermae - ps17
Magnoliids/eudicots - ps18
Eudicots - ps19
Core_eudicots - ps20
Superrosids - ps21
Rosids - ps22
Eurosids - ps23
Malvids - ps24
Sap./Malv./Brassicales - ps25
Malvales/Brassicales - ps26
Brassicales - ps27
Cleomaceae/Brassicaceae - ps28
Brassicaceae - ps29
Camelineae - ps30
Arabidopsis - ps31
Arabidopsis_thaliana - ps32
p-value
3e-03
5e-03
9e-03
2e-02
3e-02
Log-odds
0.5
1.0
1.5
2.0
2.5
3.0
3.5
4.0

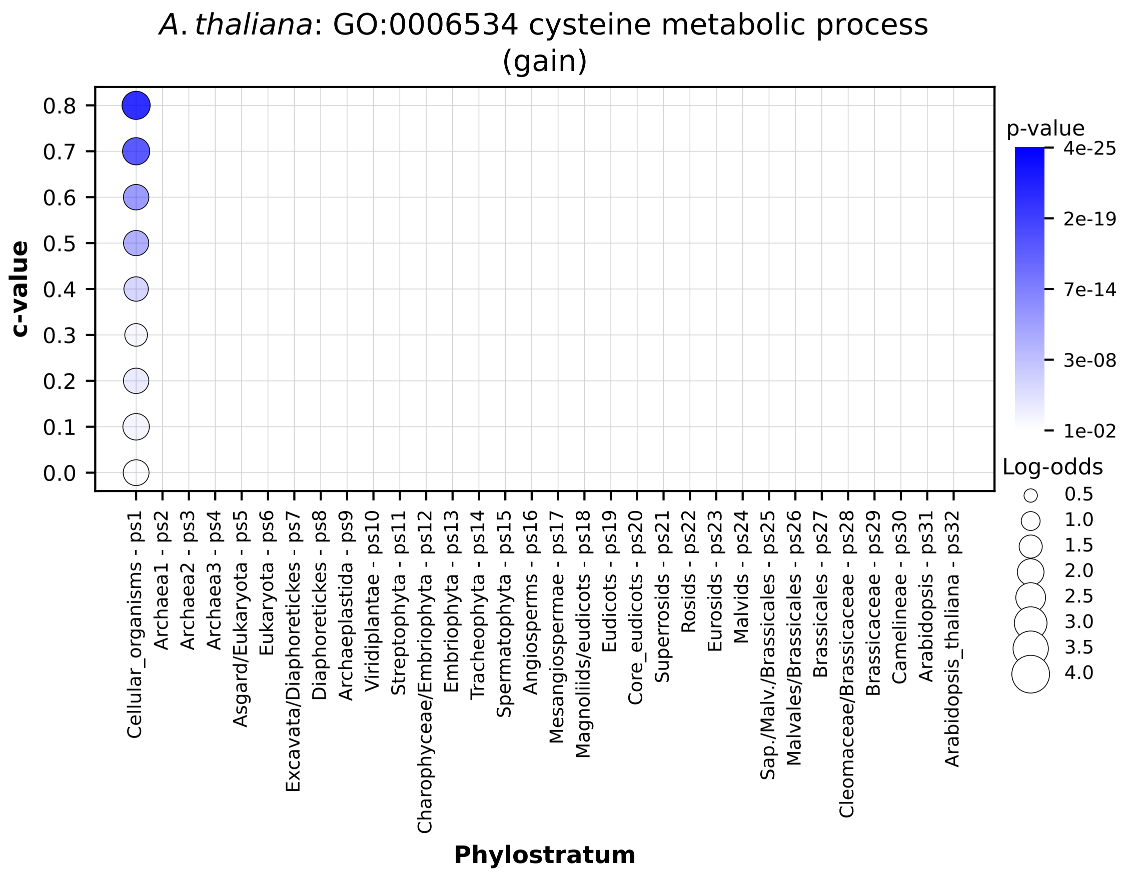

A. thaliana: GO:0006534 cysteine metabolic process
(gain)
c-value
0.8
0.7
0.6
0.5
0.4
0.3
0.2
0.1
0.0
p-value
4e-25
2e-19
7e-14
3e-08
1e-02
Log-odds
0.5
1.0
1.5
2.0
2.5
3.0
3.5
4.0
Cellular_organisms - ps1
Archaea1 - ps2
Archaea2 - ps3
Archaea3 - ps4
Asgard/Eukaryota - ps5
Eukaryota - ps6
Excavata/Diaphoretickes - ps7
Diaphoretickes - ps8
Archaeplastida - ps9
Viridiplantae - ps10
Streptophyta - ps11
Charophyceae/Embriophyta - ps12
Embriophyta - ps13
Tracheophyta - ps14
Spermatophyta - ps15
Angiosperms - ps16
Mesangiospermae - ps17
Magnoliids/eudicots - ps18
Eudicots - ps19
Core_eudicots - ps20
Superrosids - ps21
Rosids - ps22
Eurosids - ps23
Malvids - ps24
Sap./Malv./Brassicales - ps25
Malvales/Brassicales - ps26
Brassicales - ps27
Cleomaceae/Brassicaceae - ps28
Brassicaceae - ps29
Camelineae - ps30
Arabidopsis - ps31
Arabidopsis_thaliana - ps32
Phylostratum

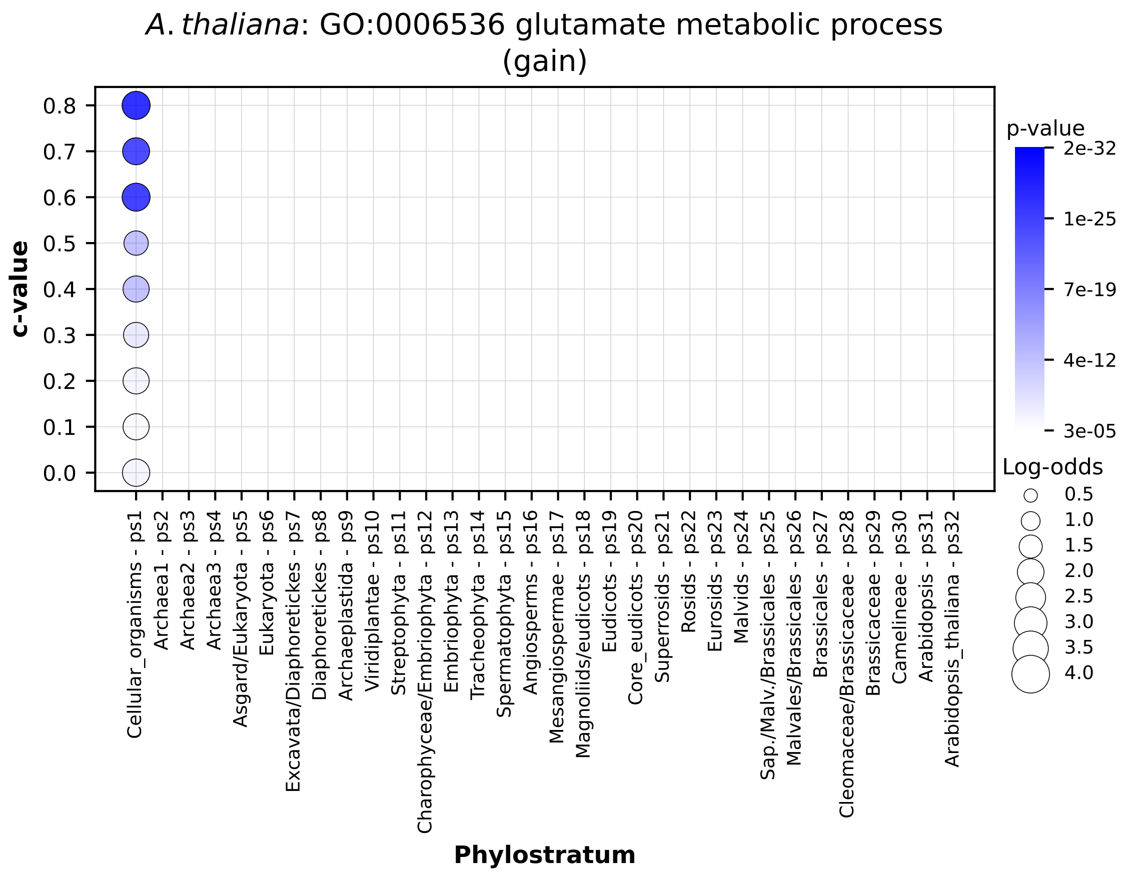

A. thaliana: GO:0006536 glutamate metabolic process (gain)
c-value
0.8
0.7
0.6
0.5
0.4
0.3
0.2
0.1
0.0
p-value
2e-32
1e-25
7e-19
4e-12
3e-05
Log-odds
0.5
1.0
1.5
2.0
2.5
3.0
3.5
4.0
Cellular_organisms - ps1
Archaea1 - ps2
Archaea2 - ps3
Archaea3 - ps4
Asgard/Eukaryota - ps5
Eukaryota - ps6
Excavata/Diaphoretickes - ps7
Diaphoretickes - ps8
Archaeplastida - ps9
Viridiplantae - ps10
Streptophyta - ps11
Charophyceae/Embriophyta - ps12
Embriophyta - ps13
Tracheophyta - ps14
Spermatophyta - ps15
Angiosperms - ps16
Mesangiospermae - ps17
Magnoliids/eudicots - ps18
Eudicots - ps19
Core_eudicots - ps20
Superrosids - ps21
Rosids - ps22
Eurosids - ps23
Malvids - ps24
Sap./Malv./Brassicales - ps25
Malvales/Brassicales - ps26
Brassicales - ps27
Cleomaceae/Brassicaceae - ps28
Brassicaceae - ps29
Camelineae - ps30
Arabidopsis - ps31
Arabidopsis_thaliana - ps32
Phylostratum

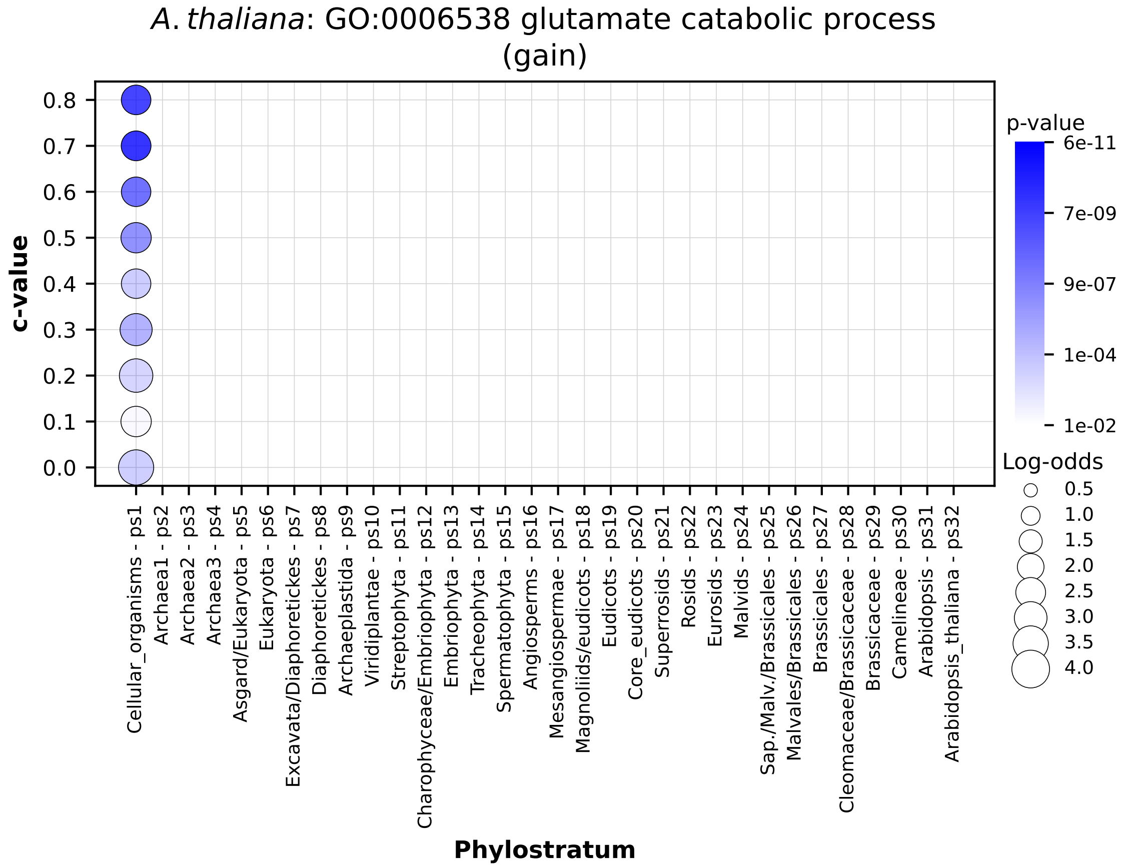
A. thaliana: GO:0006538 glutamate catabolic process
(gain)
c-value
0.8
0.7
0.6
0.5
0.4
0.3
0.2
0.1
0.0
Phylostratum
Cellular_organisms - ps1
Archaea1 - ps2
Archaea2 - ps3
Archaea3 - ps4
Asgard/Eukaryota - ps5
Eukaryota - ps6
Excavata/Diaphoretickes - ps7
Diaphoretickes - ps8
Archaeplastida - ps9
Viridiplantae - ps10
Streptophyta - ps11
Charophyceae/Embriophyta - ps12
Embriophyta - ps13
Tracheophyta - ps14
Spermatophyta - ps15
Angiosperms - ps16
Mesangiospermae - ps17
Magnoliids/eudicots - ps18
Eudicots - ps19
Core_eudicots - ps20
Superrosids - ps21
Rosids - ps22
Eurosids - ps23
Malvids - ps24
Sap./Malv./Brassicales - ps25
Malvales/Brassicales - ps26
Brassicales - ps27
Cleomaceae/Brassicaceae - ps28
Brassicaceae - ps29
Camelineae - ps30
Arabidopsis - ps31
Arabidopsis_thaliana - ps32
p-value
6e-11
7e-09
9e-07
1e-04
1e-02
Log-odds
0.5
1.0
1.5
2.0
2.5
3.0
3.5
4.0

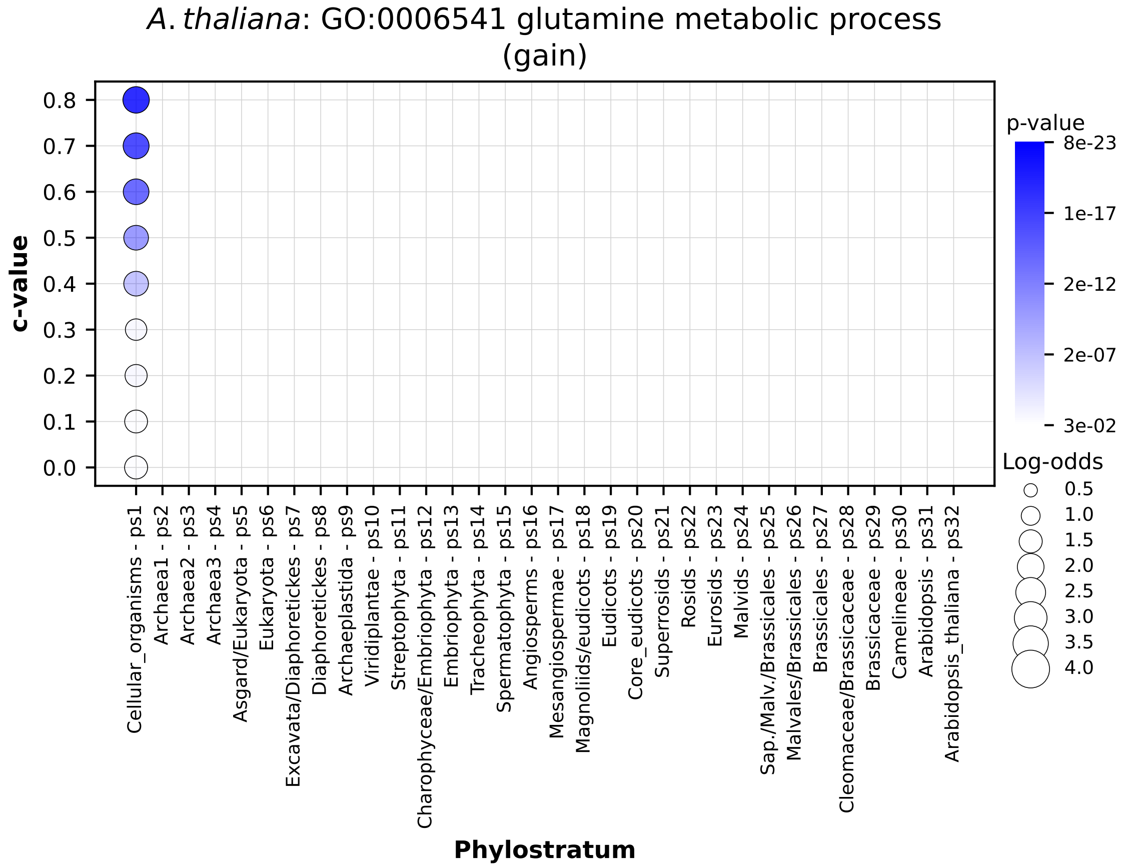

A. thaliana: GO:0006541 glutamine metabolic process
(gain)
c-value
0.0
0.1
0.2
0.3
0.4
0.5
0.6
0.7
0.8
Phylostratum
Cellular_organisms - ps1
Archaea1 - ps2
Archaea2 - ps3
Archaea3 - ps4
Asgard/Eukaryota - ps5
Eukaryota - ps6
Excavata/Diaphoretickes - ps7
Diaphoretickes - ps8
Archaeplastida - ps9
Viridiplantae - ps10
Streptophyta - ps11
Charophyceae/Embriophyta - ps12
Embriophyta - ps13
Tracheophyta - ps14
Spermatophyta - ps15
Angiosperms - ps16
Mesangiospermae - ps17
Magnoliids/eudicots - ps18
Eudicots - ps19
Core_eudicots - ps20
Superrosids - ps21
Rosids - ps22
Eurosids - ps23
Malvids - ps24
Sap./Malv./Brassicales - ps25
Malvales/Brassicales - ps26
Brassicales - ps27
Cleomaceae/Brassicaceae - ps28
Brassicaceae - ps29
Camelineae - ps30
Arabidopsis - ps31
Arabidopsis_thaliana - ps32
p-value
8e-23
1e-17
2e-12
2e-07
3e-02
Log-odds
0.5
1.0
1.5
2.0
2.5
3.0
3.5
4.0

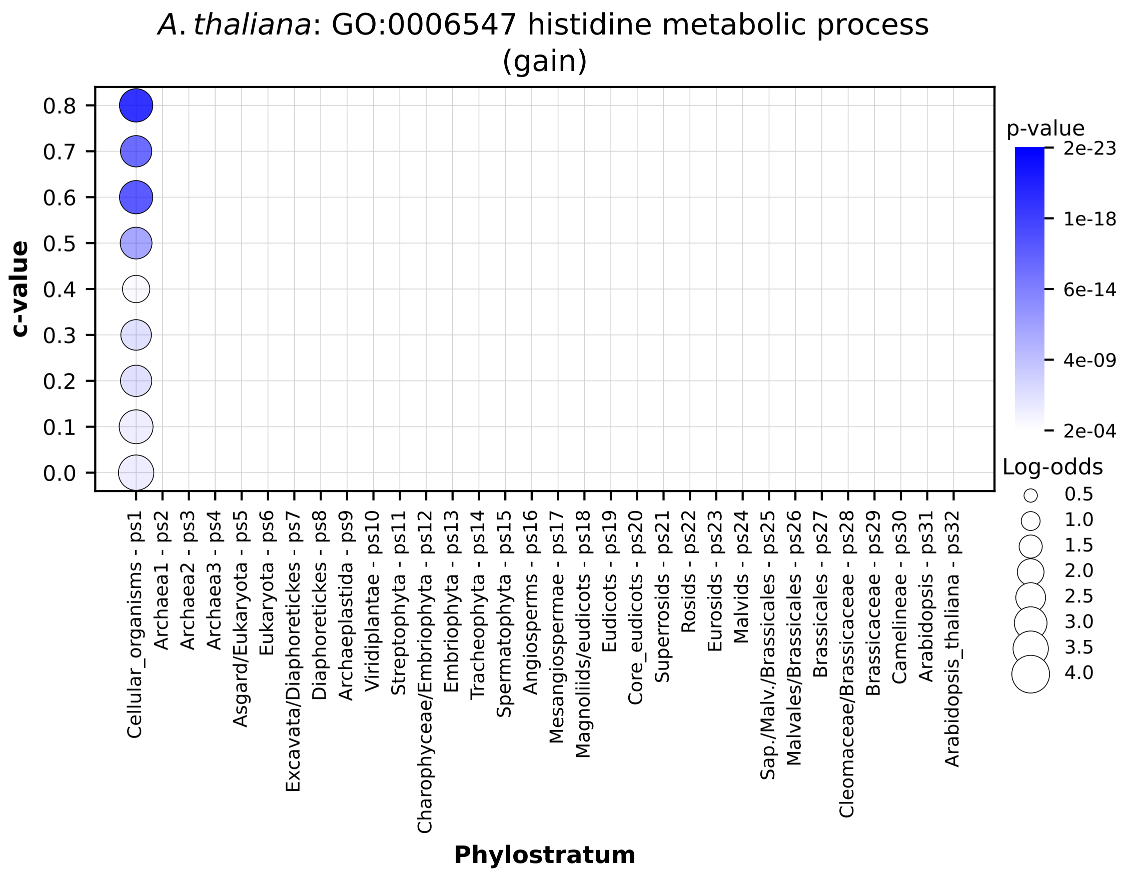

A. thaliana: GO:0006547 histidine metabolic process
(gain)
c-value
0.8
0.7
0.6
0.5
0.4
0.3
0.2
0.1
0.0
p-value
2e-23
1e-18
6e-14
4e-09
2e-04
Log-odds
0.5
1.0
1.5
2.0
2.5
3.0
3.5
4.0
Cellular_organisms - ps1
Archaea1 - ps2
Archaea2 - ps3
Archaea3 - ps4
Asgard/Eukaryota - ps5
Eukaryota - ps6
Excavata/Diaphoretickes - ps7
Diaphoretickes - ps8
Archaeplastida - ps9
Viridiplantae - ps10
Streptophyta - ps11
Charophyceae/Embriophyta - ps12
Embriophyta - ps13
Tracheophyta - ps14
Spermatophyta - ps15
Angiosperms - ps16
Mesangiospermae - ps17
Magnoliids/eudicots - ps18
Eudicots - ps19
Core_eudicots - ps20
Superrosids - ps21
Rosids - ps22
Eurosids - ps23
Malvids - ps24
Sap./Malv./Brassicales - ps25
Malvales/Brassicales - ps26
Brassicales - ps27
Cleomaceae/Brassicaceae - ps28
Brassicaceae - ps29
Camelineae - ps30
Arabidopsis - ps31
Arabidopsis_thaliana - ps32
Phylostratum

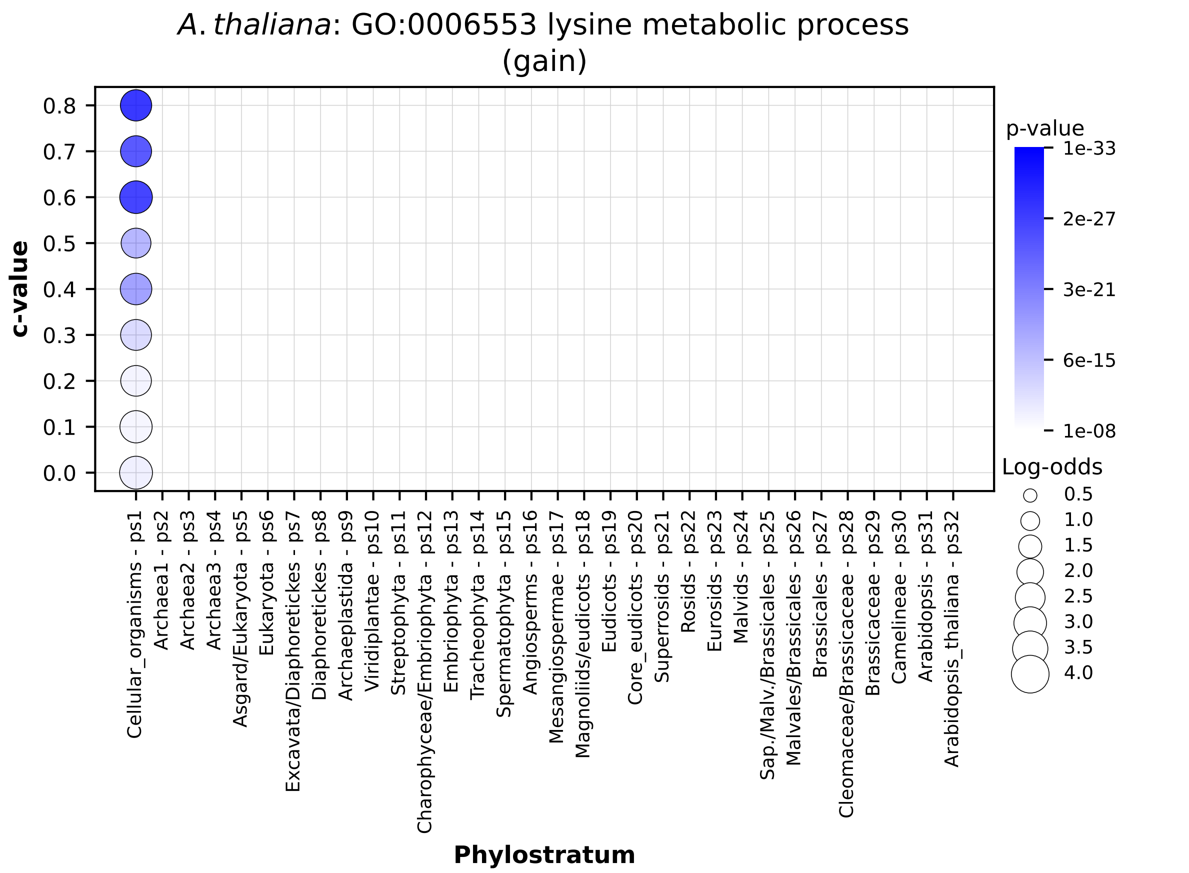

A. thaliana: GO:0006553 lysine metabolic process
(gain)
c-value
0.8
0.7
0.6
0.5
0.4
0.3
0.2
0.1
0.0
p-value
1e-33
2e-27
3e-21
6e-15
1e-08
Log-odds
0.5
1.0
1.5
2.0
2.5
3.0
3.5
4.0
Cellular_organisms - ps1
Archaea1 - ps2
Archaea2 - ps3
Archaea3 - ps4
Asgard/Eukaryota - ps5
Eukaryota - ps6
Excavata/Diaphoretickes - ps7
Diaphoretickes - ps8
Archaeplastida - ps9
Viridiplantae - ps10
Streptophyta - ps11
Charophyceae/Embriophyta - ps12
Embriophyta - ps13
Tracheophyta - ps14
Spermatophyta - ps15
Angiosperms - ps16
Mesangiospermae - ps17
Magnoliids/eudicots - ps18
Eudicots - ps19
Core_eudicots - ps20
Superrosids - ps21
Rosids - ps22
Eurosids - ps23
Malvids - ps24
Sap./Malv./Brassicales - ps25
Malvales/Brassicales - ps26
Brassicales - ps27
Cleomaceae/Brassicaceae - ps28
Brassicaceae - ps29
Camelineae - ps30
Arabidopsis - ps31
Arabidopsis_thaliana - ps32
Phylostratum

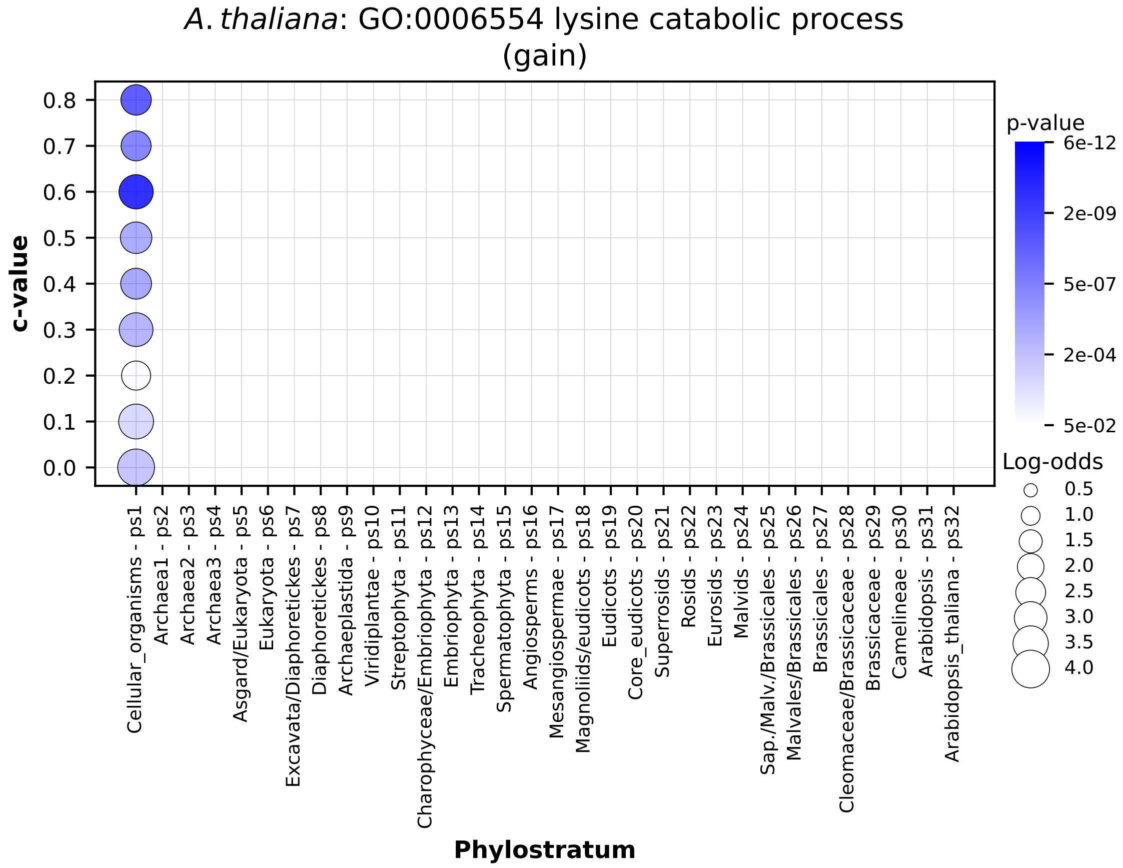

A. thaliana: GO:0006554 lysine catabolic process
(gain)
c-value
0.8
0.7
0.6
0.5
0.4
0.3
0.2
0.1
0.0
p-value
6e-12
2e-09
5e-07
2e-04
5e-02
Log-odds
0.5
1.0
1.5
2.0
2.5
3.0
3.5
4.0
Cellular_organisms - ps1
Archaea1 - ps2
Archaea2 - ps3
Archaea3 - ps4
Asgard/Eukaryota - ps5
Eukaryota - ps6
Excavata/Diaphoretickes - ps7
Diaphoretickes - ps8
Archaeplastida - ps9
Viridiplantae - ps10
Streptophyta - ps11
Charophyceae/Embriophyta - ps12
Embriophyta - ps13
Tracheophyta - ps14
Spermatophyta - ps15
Angiosperms - ps16
Mesangiospermae - ps17
Magnoliids/eudicots - ps18
Eudicots - ps19
Core_eudicots - ps20
Superrosids - ps21
Rosids - ps22
Eurosids - ps23
Malvids - ps24
Sap./Malv./Brassicales - ps25
Malvales/Brassicales - ps26
Brassicales - ps27
Cleomaceae/Brassicaceae - ps28
Brassicaceae - ps29
Camelineae - ps30
Arabidopsis - ps31
Arabidopsis_thaliana - ps32
Phylostratum

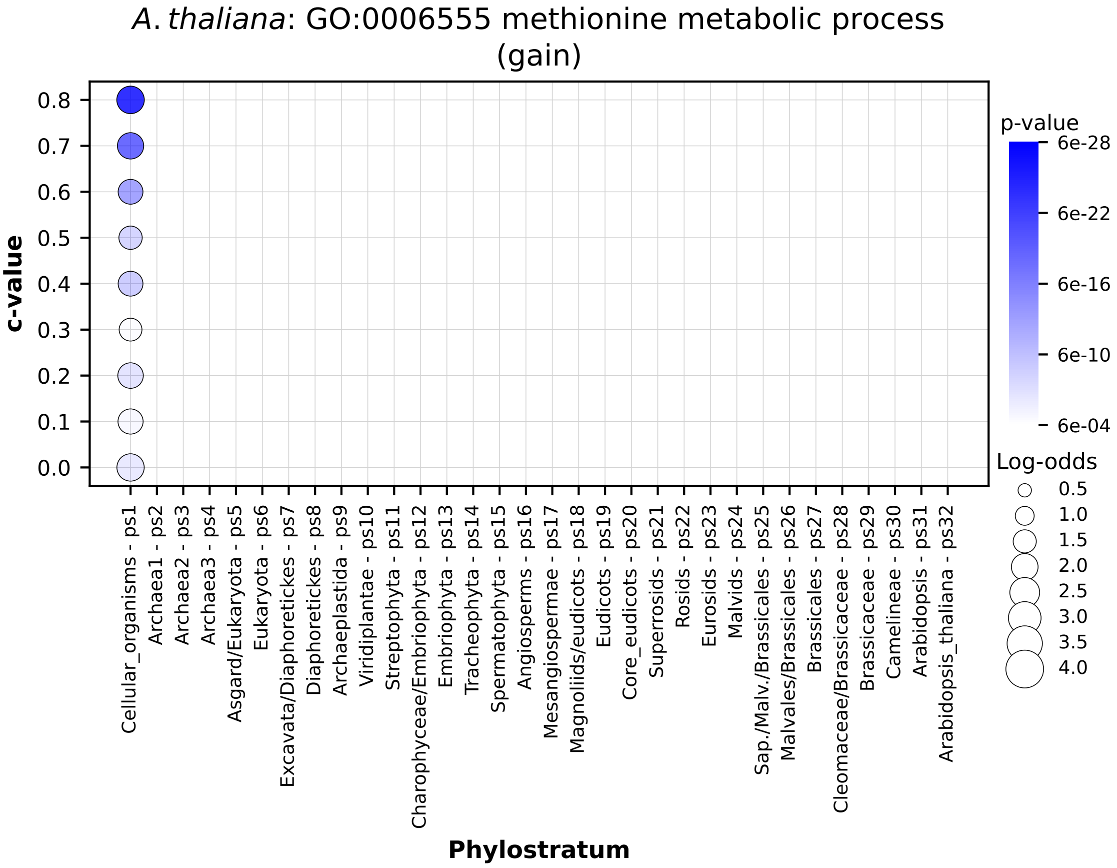

A. thaliana: GO:0006555 methionine metabolic process (gain)
c-value
0.8
0.7
0.6
0.5
0.4
0.3
0.2
0.1
0.0
p-value
6e-28
6e-22
6e-16
6e-10
6e-04
Log-odds
0.5
1.0
1.5
2.0
2.5
3.0
3.5
4.0
Cellular_organisms - ps1
Archaea1 - ps2
Archaea2 - ps3
Archaea3 - ps4
Asgard/Eukaryota - ps5
Eukaryota - ps6
Excavata/Diaphoretickes - ps7
Diaphoretickes - ps8
Archaeplastida - ps9
Viridiplantae - ps10
Streptophyta - ps11
Charophyceae/Embriophyta - ps12
Embriophyta - ps13
Tracheophyta - ps14
Spermatophyta - ps15
Angiosperms - ps16
Mesangiospermae - ps17
Magnoliids/eudicots - ps18
Eudicots - ps19
Core_eudicots - ps20
Superrosids - ps21
Rosids - ps22
Eurosids - ps23
Malvids - ps24
Sap./Malv./Brassicales - ps25
Malvales/Brassicales - ps26
Brassicales - ps27
Cleomaceae/Brassicaceae - ps28
Brassicaceae - ps29
Camelineae - ps30
Arabidopsis - ps31
Arabidopsis_thaliana - ps32
Phylostratum

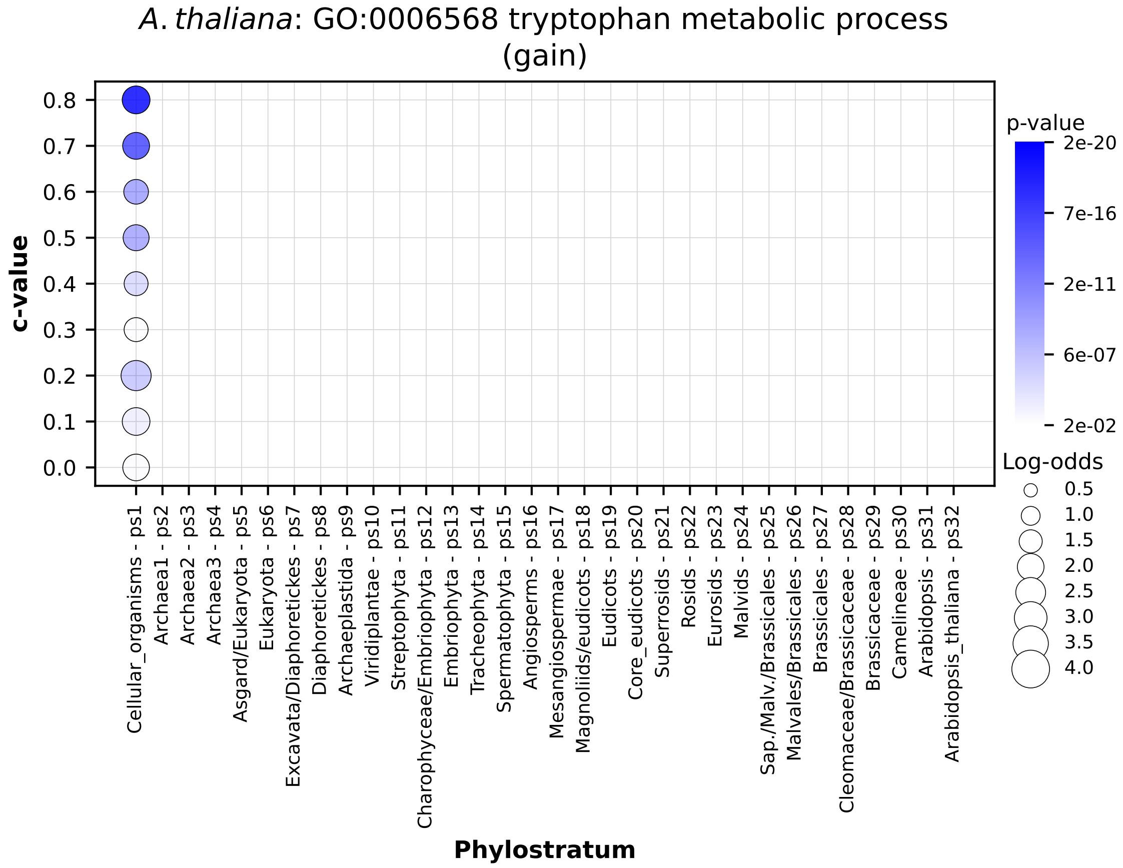

A. thaliana: GO:0006568 tryptophan metabolic process (gain)
c-value
0.0
0.1
0.2
0.3
0.4
0.5
0.6
0.7
0.8
Phylostratum
Cellular_organisms - ps1
Archaea1 - ps2
Archaea2 - ps3
Archaea3 - ps4
Asgard/Eukaryota - ps5
Eukaryota - ps6
Excavata/Diaphoretickes - ps7
Diaphoretickes - ps8
Archaeplastida - ps9
Viridiplantae - ps10
Streptophyta - ps11
Charophyceae/Embriophyta - ps12
Embriophyta - ps13
Tracheophyta - ps14
Spermatophyta - ps15
Angiosperms - ps16
Mesangiospermae - ps17
Magnoliids/eudicots - ps18
Eudicots - ps19
Core_eudicots - ps20
Superrosids - ps21
Rosids - ps22
Eurosids - ps23
Malvids - ps24
Sap./Malv./Brassicales - ps25
Malvales/Brassicales - ps26
Brassicales - ps27
Cleomaceae/Brassicaceae - ps28
Brassicaceae - ps29
Camelineae - ps30
Arabidopsis - ps31
Arabidopsis_thaliana - ps32
p-value
2e-20
7e-16
2e-11
6e-07
2e-02
Log-odds
0.5
1.0
1.5
2.0
2.5
3.0
3.5
4.0

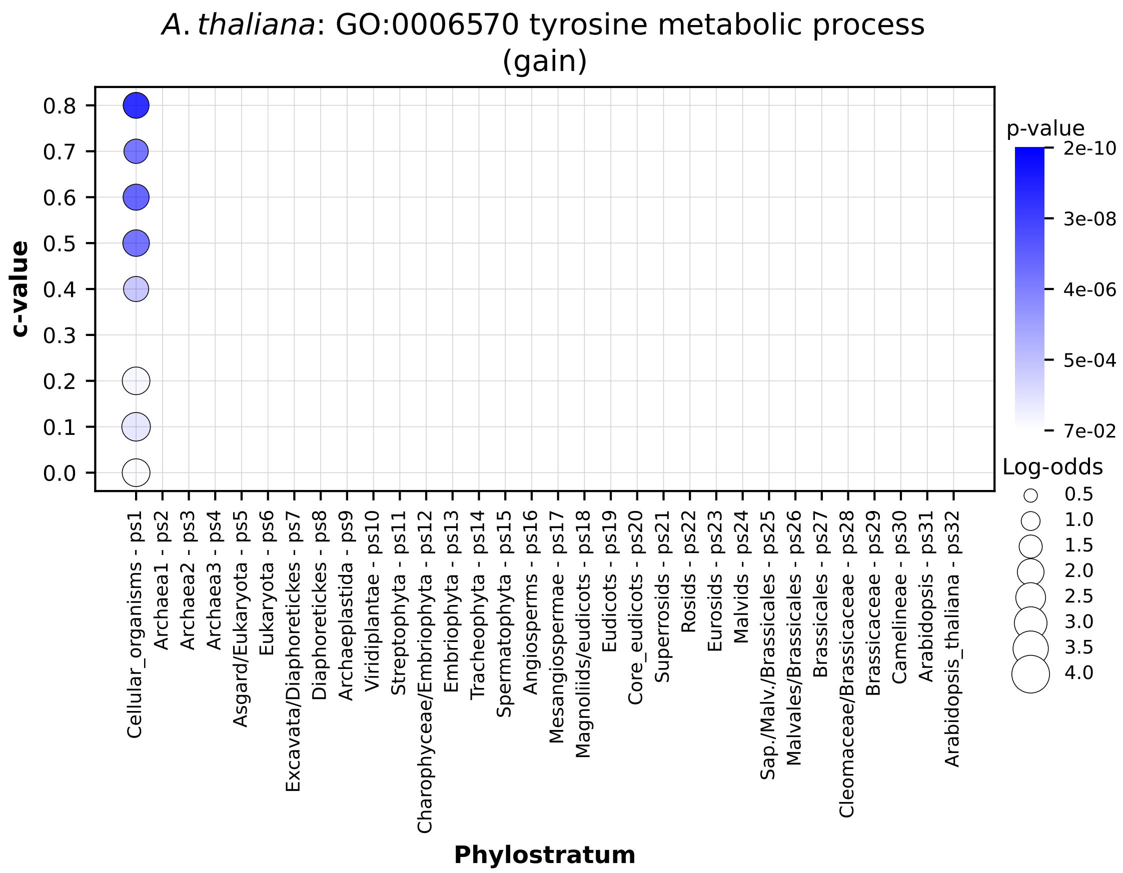

A. thaliana: GO:0006570 tyrosine metabolic process
(gain)
c-value
0.8
0.7
0.6
0.5
0.4
0.3
0.2
0.1
0.0
p-value
2e-10
3e-08
4e-06
5e-04
7e-02
Log-odds
0.5
1.0
1.5
2.0
2.5
3.0
3.5
4.0
Cellular_organisms - ps1
Archaea1 - ps2
Archaea2 - ps3
Archaea3 - ps4
Asgard/Eukaryota - ps5
Eukaryota - ps6
Excavata/Diaphoretickes - ps7
Diaphoretickes - ps8
Archaeplastida - ps9
Viridiplantae - ps10
Streptophyta - ps11
Charophyceae/Embriophyta - ps12
Embriophyta - ps13
Tracheophyta - ps14
Spermatophyta - ps15
Angiosperms - ps16
Mesangiospermae - ps17
Magnoliids/eudicots - ps18
Eudicots - ps19
Core_eudicots - ps20
Superrosids - ps21
Rosids - ps22
Eurosids - ps23
Malvids - ps24
Sap./Malv./Brassicales - ps25
Malvales/Brassicales - ps26
Brassicales - ps27
Cleomaceae/Brassicaceae - ps28
Brassicaceae - ps29
Camelineae - ps30
Arabidopsis - ps31
Arabidopsis_thaliana - ps32
Phylostratum

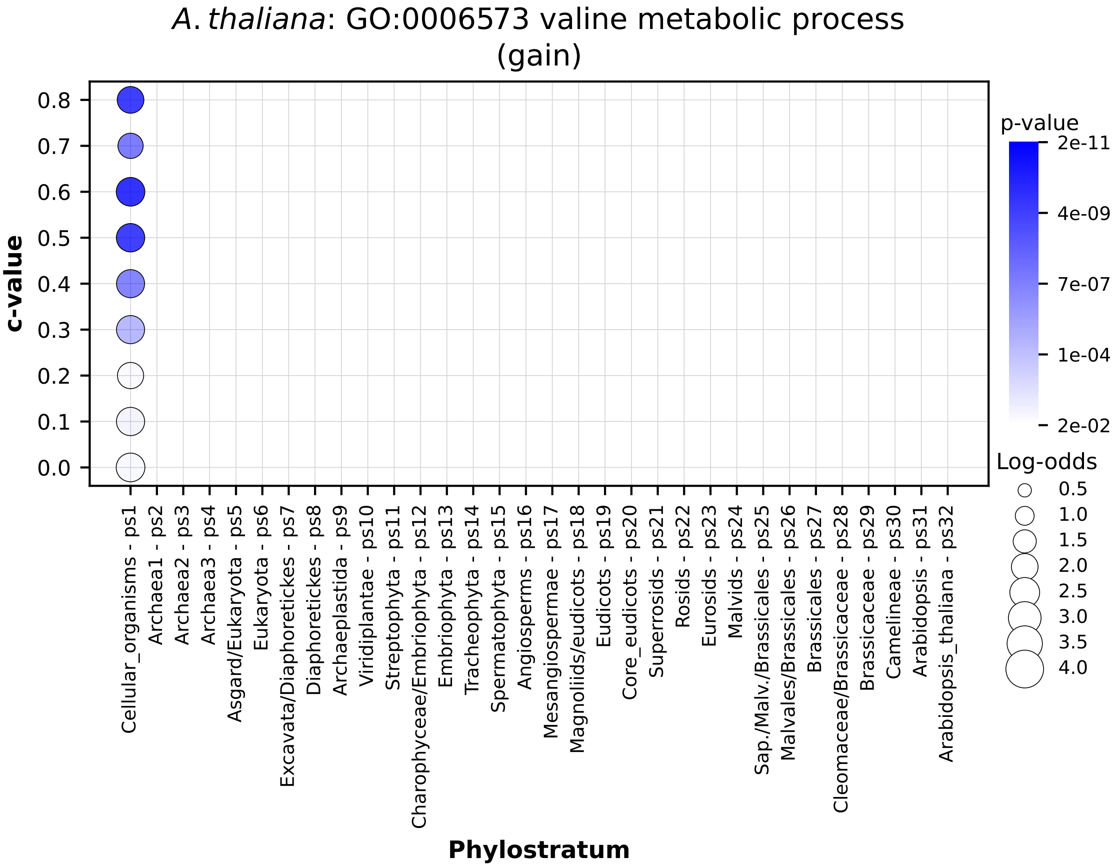

A. thaliana: GO:0006573 valine metabolic process
(gain)
c-value
0.8
0.7
0.6
0.5
0.4
0.3
0.2
0.1
0.0
p-value
2e-11
4e-09
7e-07
1e-04
2e-02
Log-odds
0.5
1.0
1.5
2.0
2.5
3.0
3.5
4.0
Cellular_organisms - ps1
Archaea1 - ps2
Archaea2 - ps3
Archaea3 - ps4
Asgard/Eukaryota - ps5
Eukaryota - ps6
Excavata/Diaphoretickes - ps7
Diaphoretickes - ps8
Archaeplastida - ps9
Viridiplantae - ps10
Streptophyta - ps11
Charophyceae/Embriophyta - ps12
Embriophyta - ps13
Tracheophyta - ps14
Spermatophyta - ps15
Angiosperms - ps16
Mesangiospermae - ps17
Magnoliids/eudicots - ps18
Eudicots - ps19
Core_eudicots - ps20
Superrosids - ps21
Rosids - ps22
Eurosids - ps23
Malvids - ps24
Sap./Malv./Brassicales - ps25
Malvales/Brassicales - ps26
Brassicales - ps27
Cleomaceae/Brassicaceae - ps28
Brassicaceae - ps29
Camelineae - ps30
Arabidopsis - ps31
Arabidopsis_thaliana - ps32
Phylostratum

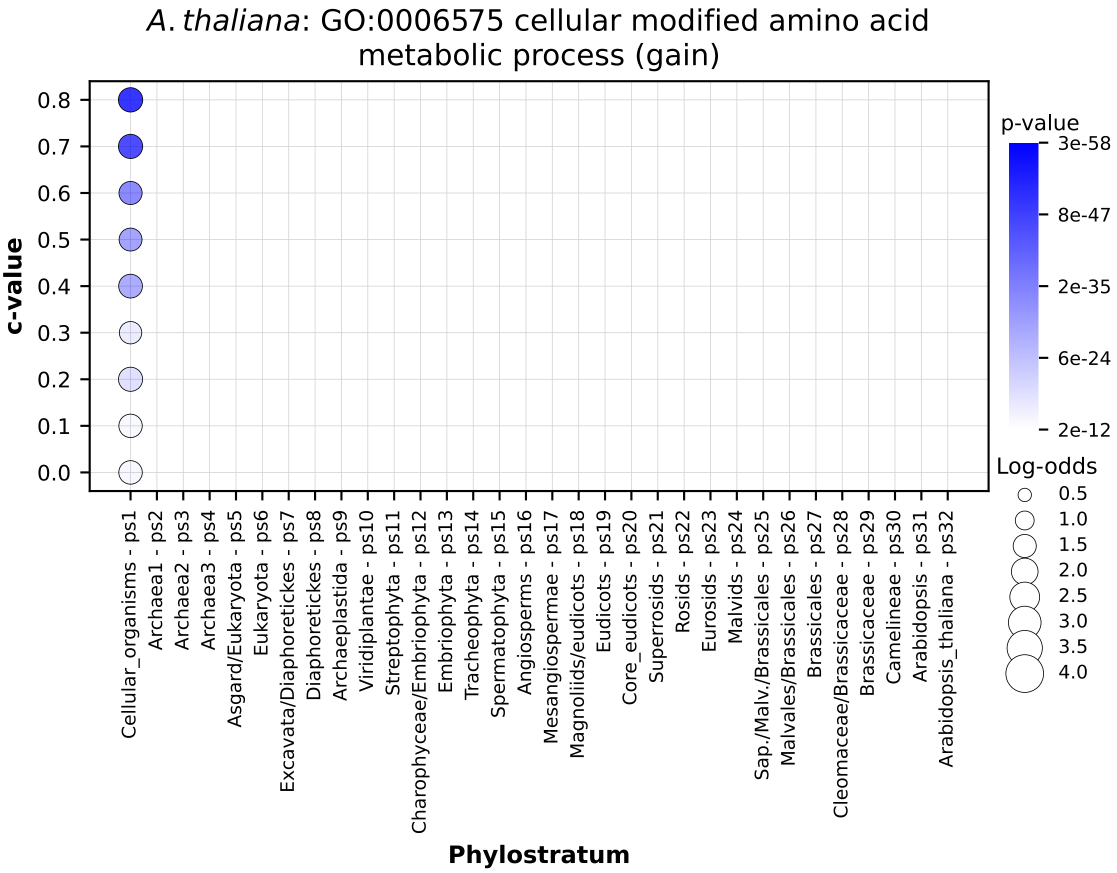

A. thaliana: GO:0006575 cellular modified amino acid metabolic process (gain)
c-value
0.8
0.7
0.6
0.5
0.4
0.3
0.2
0.1
0.0
p-value
3e-58
8e-47
2e-35
6e-24
2e-12
Log-odds
0.5
1.0
1.5
2.0
2.5
3.0
3.5
4.0
Cellular_organisms - ps1
Archaea1 - ps2
Archaea2 - ps3
Archaea3 - ps4
Asgard/Eukaryota - ps5
Eukaryota - ps6
Excavata/Diaphoretickes - ps7
Diaphoretickes - ps8
Archaeplastida - ps9
Viridiplantae - ps10
Streptophyta - ps11
Charophyceae/Embriophyta - ps12
Embriophyta - ps13
Tracheophyta - ps14
Spermatophyta - ps15
Angiosperms - ps16
Mesangiospermae - ps17
Magnoliids/eudicots - ps18
Eudicots - ps19
Core_eudicots - ps20
Superrosids - ps21
Rosids - ps22
Eurosids - ps23
Malvids - ps24
Sap./Malv./Brassicales - ps25
Malvales/Brassicales - ps26
Brassicales - ps27
Cleomaceae/Brassicaceae - ps28
Brassicaceae - ps29
Camelineae - ps30
Arabidopsis - ps31
Arabidopsis_thaliana - ps32
Phylostratum

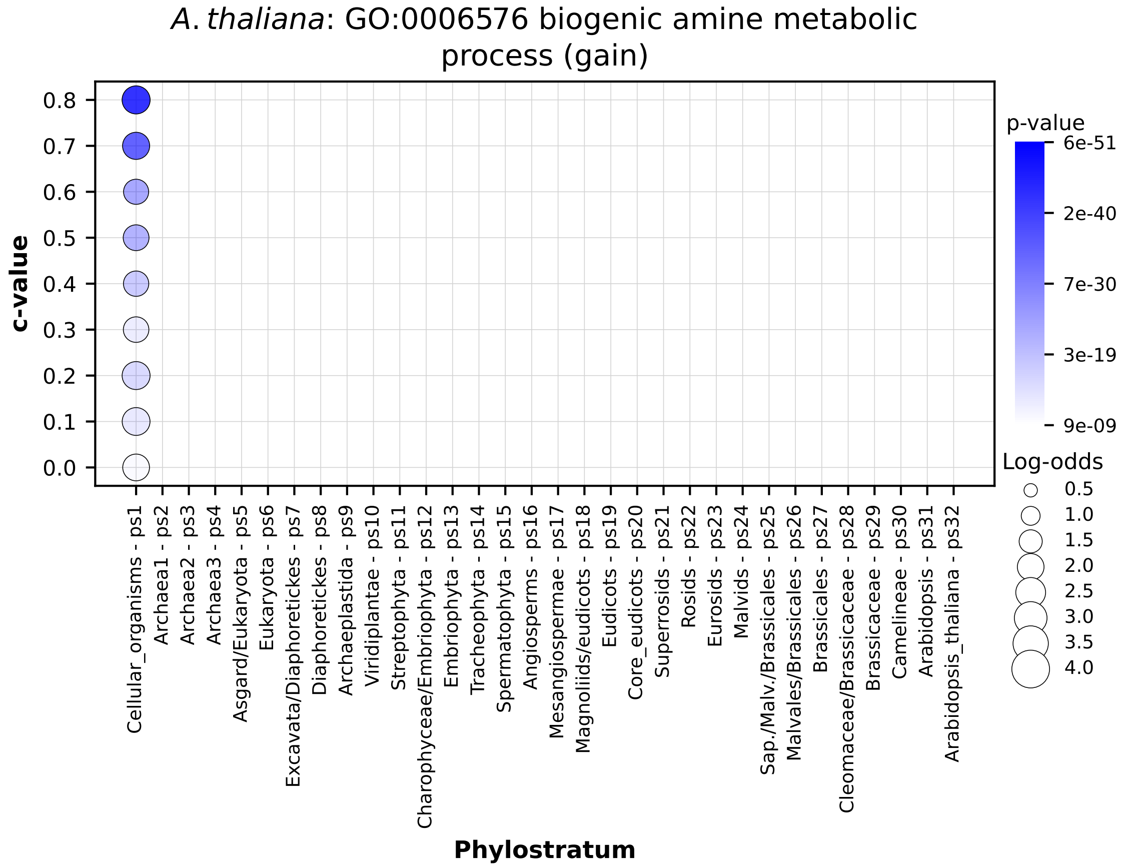

A. thaliana: GO:0006576 biogenic amine metabolic process (gain)
c-value
0.8
0.7
0.6
0.5
0.4
0.3
0.2
0.1
0.0
p-value
6e-51
2e-40
7e-30
3e-19
9e-09
Log-odds
0.5
1.0
1.5
2.0
2.5
3.0
3.5
4.0
Cellular_organisms - ps1
Archaea1 - ps2
Archaea2 - ps3
Archaea3 - ps4
Asgard/Eukaryota - ps5
Eukaryota - ps6
Excavata/Diaphoretickes - ps7
Diaphoretickes - ps8
Archaeplastida - ps9
Viridiplantae - ps10
Streptophyta - ps11
Charophyceae/Embriophyta - ps12
Embriophyta - ps13
Tracheophyta - ps14
Spermatophyta - ps15
Angiosperms - ps16
Mesangiospermae - ps17
Magnoliids/eudicots - ps18
Eudicots - ps19
Core_eudicots - ps20
Superrosids - ps21
Rosids - ps22
Eurosids - ps23
Malvids - ps24
Sap./Malv./Brassicales - ps25
Malvales/Brassicales - ps26
Brassicales - ps27
Cleomaceae/Brassicaceae - ps28
Brassicaceae - ps29
Camelineae - ps30
Arabidopsis - ps31
Arabidopsis_thaliana - ps32
Phylostratum

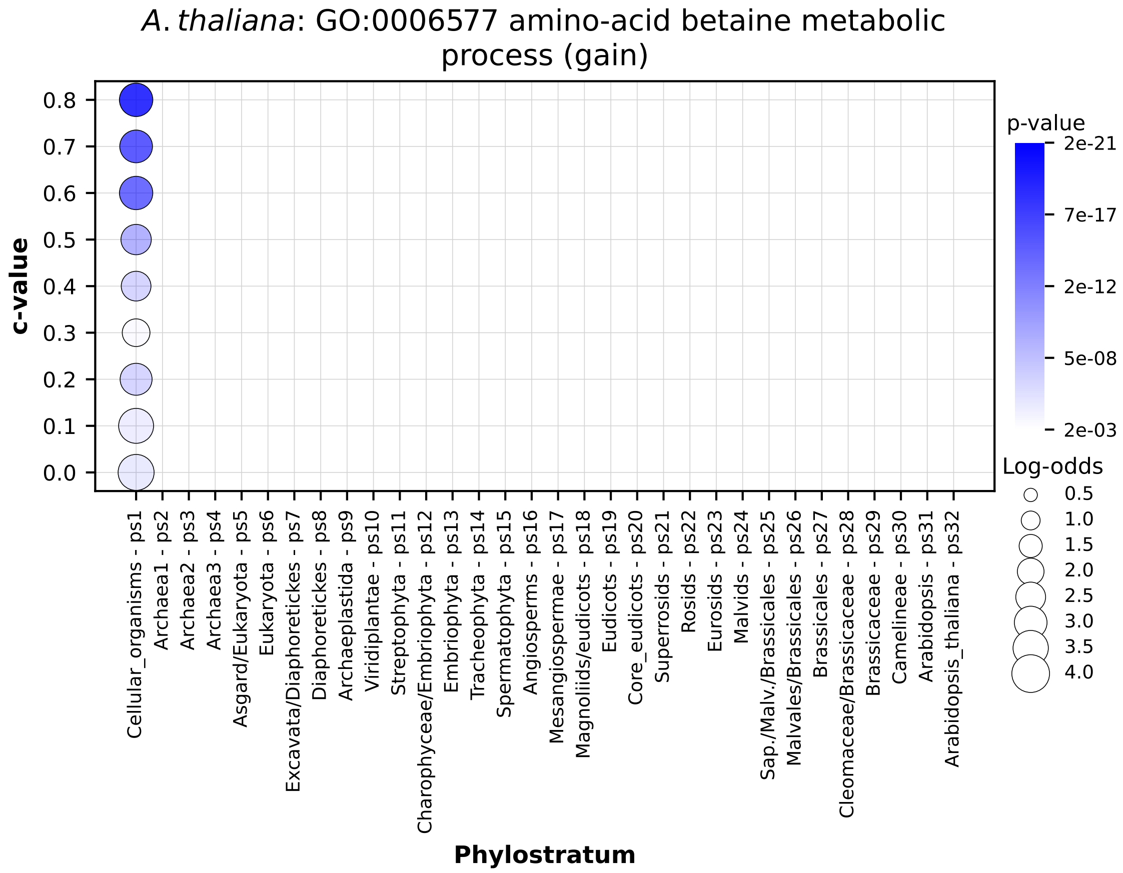

A. thaliana: GO:0006577 amino-acid betaine metabolic process (gain)
c-value
0.8
0.7
0.6
0.5
0.4
0.3
0.2
0.1
0.0
p-value
2e-21
7e-17
2e-12
5e-08
2e-03
Log-odds
0.5
1.0
1.5
2.0
2.5
3.0
3.5
4.0
Cellular_organisms - ps1
Archaea1 - ps2
Archaea2 - ps3
Archaea3 - ps4
Asgard/Eukaryota - ps5
Eukaryota - ps6
Excavata/Diaphoretickes - ps7
Diaphoretickes - ps8
Archaeplastida - ps9
Viridiplantae - ps10
Streptophyta - ps11
Charophyceae/Embriophyta - ps12
Embriophyta - ps13
Tracheophyta - ps14
Spermatophyta - ps15
Angiosperms - ps16
Mesangiospermae - ps17
Magnoliids/eudicots - ps18
Eudicots - ps19
Core_eudicots - ps20
Superrosids - ps21
Rosids - ps22
Eurosids - ps23
Malvids - ps24
Sap./Malv./Brassicales - ps25
Malvales/Brassicales - ps26
Brassicales - ps27
Cleomaceae/Brassicaceae - ps28
Brassicaceae - ps29
Camelineae - ps30
Arabidopsis - ps31
Arabidopsis_thaliana - ps32
Phylostratum

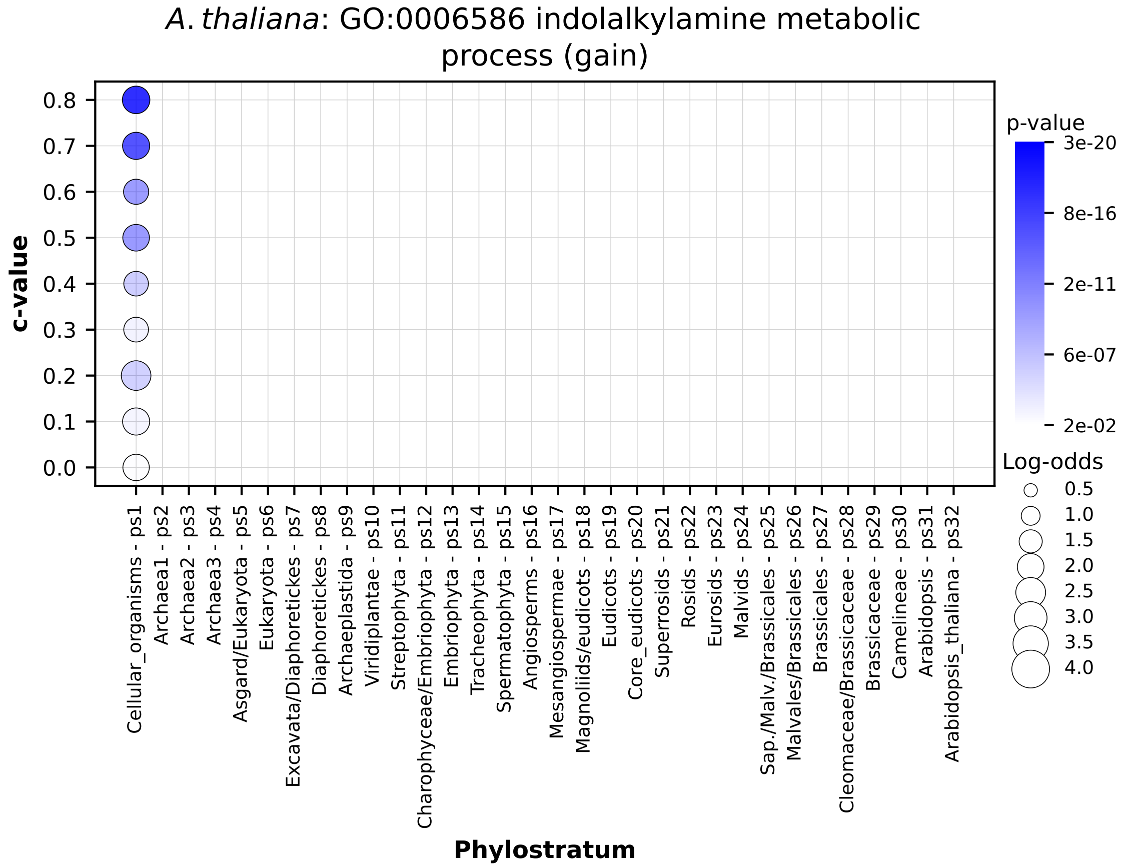

A. thaliana: GO:0006586 indolalkylamine metabolic process (gain)
c-value
0.8
0.7
0.6
0.5
0.4
0.3
0.2
0.1
0.0
p-value
3e-20
8e-16
2e-11
6e-07
2e-02
Log-odds
0.5
1.0
1.5
2.0
2.5
3.0
3.5
4.0
Cellular_organisms - ps1
Archaea1 - ps2
Archaea2 - ps3
Archaea3 - ps4
Asgard/Eukaryota - ps5
Eukaryota - ps6
Excavata/Diaphoretickes - ps7
Diaphoretickes - ps8
Archaeplastida - ps9
Viridiplantae - ps10
Streptophyta - ps11
Charophyceae/Embriophyta - ps12
Embriophyta - ps13
Tracheophyta - ps14
Spermatophyta - ps15
Angiosperms - ps16
Mesangiospermae - ps17
Magnoliids/eudicots - ps18
Eudicots - ps19
Core_eudicots - ps20
Superrosids - ps21
Rosids - ps22
Eurosids - ps23
Malvids - ps24
Sap./Malv./Brassicales - ps25
Malvales/Brassicales - ps26
Brassicales - ps27
Cleomaceae/Brassicaceae - ps28
Brassicaceae - ps29
Camelineae - ps30
Arabidopsis - ps31
Arabidopsis_thaliana - ps32
Phylostratum

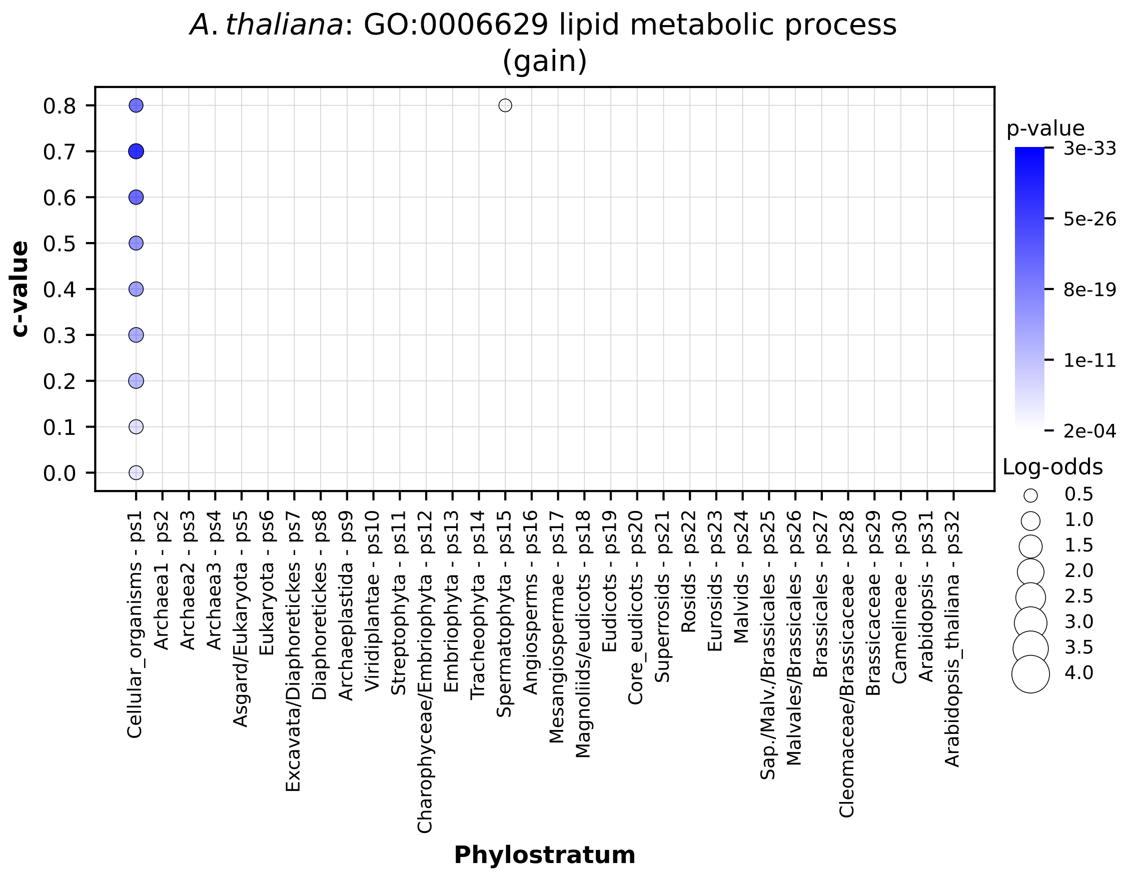

A. thaliana: GO:0006629 lipid metabolic process
(gain)
c-value
0.8
0.7
0.6
0.5
0.4
0.3
0.2
0.1
0.0
p-value
3e-33
5e-26
8e-19
1e-11
2e-04
Log-odds
0.5
1.0
1.5
2.0
2.5
3.0
3.5
4.0
Cellular_organisms - ps1
Archaea1 - ps2
Archaea2 - ps3
Archaea3 - ps4
Asgard/Eukaryota - ps5
Eukaryota - ps6
Excavata/Diaphoretickes - ps7
Diaphoretickes - ps8
Archaeplastida - ps9
Viridiplantae - ps10
Streptophyta - ps11
Charophyceae/Embriophyta - ps12
Embriophyta - ps13
Tracheophyta - ps14
Spermatophyta - ps15
Angiosperms - ps16
Mesangiospermae - ps17
Magnoliids/eudicots - ps18
Eudicots - ps19
Core_eudicots - ps20
Superrosids - ps21
Rosids - ps22
Eurosids - ps23
Malvids - ps24
Sap./Malv./Brassicales - ps25
Malvales/Brassicales - ps26
Brassicales - ps27
Cleomaceae/Brassicaceae - ps28
Brassicaceae - ps29
Camelineae - ps30
Arabidopsis - ps31
Arabidopsis_thaliana - ps32
Phylostratum

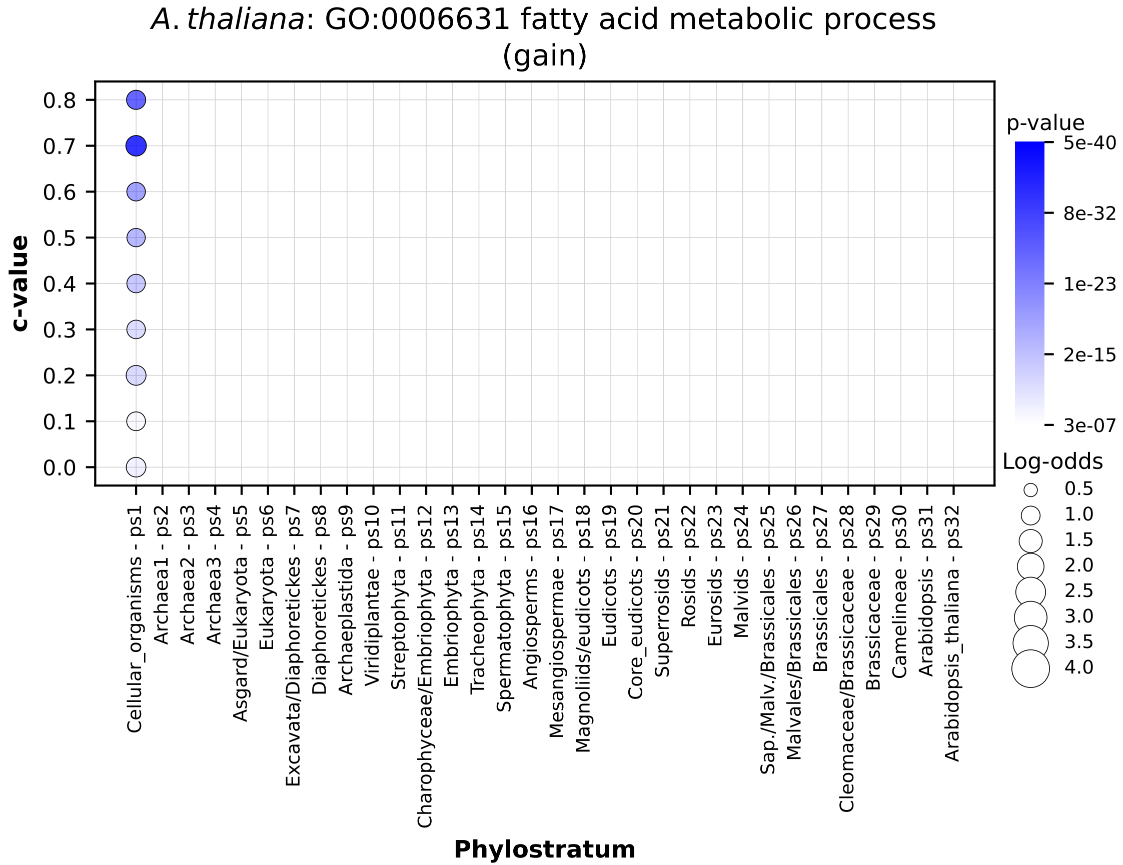

A. thaliana: GO:0006631 fatty acid metabolic process
(gain)
c-value
0.8
0.7
0.6
0.5
0.4
0.3
0.2
0.1
0.0
Phylostratum
Cellular_organisms - ps1
Archaea1 - ps2
Archaea2 - ps3
Archaea3 - ps4
Asgard/Eukaryota - ps5
Eukaryota - ps6
Excavata/Diaphoretickes - ps7
Diaphoretickes - ps8
Archaeplastida - ps9
Viridiplantae - ps10
Streptophyta - ps11
Charophyceae/Embriophyta - ps12
Embriophyta - ps13
Tracheophyta - ps14
Spermatophyta - ps15
Angiosperms - ps16
Mesangiospermae - ps17
Magnoliids/eudicots - ps18
Eudicots - ps19
Core_eudicots - ps20
Superrosids - ps21
Rosids - ps22
Eurosids - ps23
Malvids - ps24
Sap./Malv./Brassicales - ps25
Malvales/Brassicales - ps26
Brassicales - ps27
Cleomaceae/Brassicaceae - ps28
Brassicaceae - ps29
Camelineae - ps30
Arabidopsis - ps31
Arabidopsis_thaliana - ps32
p-value
5e-40
8e-32
1e-23
2e-15
3e-07
Log-odds
0.5
1.0
1.5
2.0
2.5
3.0
3.5
4.0

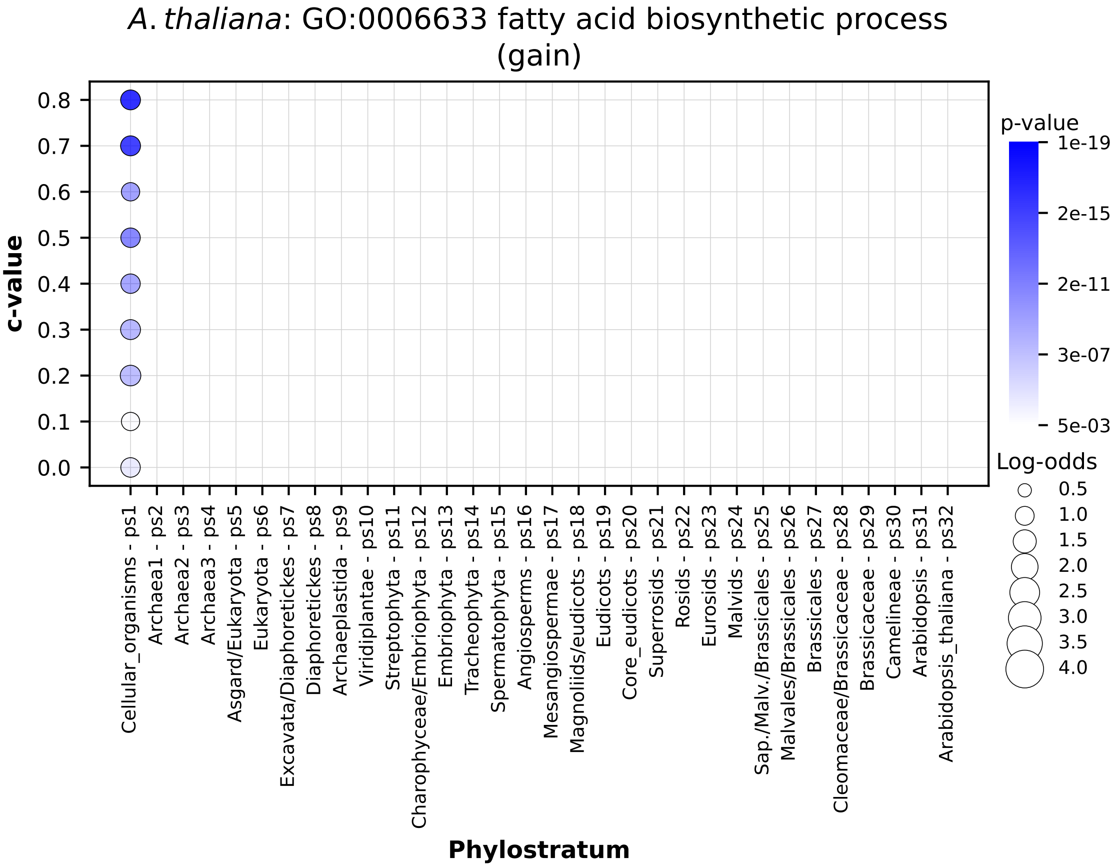

A. thaliana: GO:0006633 fatty acid biosynthetic process
(gain)
c-value
0.8
0.7
0.6
0.5
0.4
0.3
0.2
0.1
0.0
Phylostratum
Cellular_organisms - ps1
Archaea1 - ps2
Archaea2 - ps3
Archaea3 - ps4
Asgard/Eukaryota - ps5
Eukaryota - ps6
Excavata/Diaphoretickes - ps7
Diaphoretickes - ps8
Archaeplastida - ps9
Viridiplantae - ps10
Streptophyta - ps11
Charophyceae/Embriophyta - ps12
Embriophyta - ps13
Tracheophyta - ps14
Spermatophyta - ps15
Angiosperms - ps16
Mesangiospermae - ps17
Magnoliids/eudicots - ps18
Eudicots - ps19
Core_eudicots - ps20
Superrosids - ps21
Rosids - ps22
Eurosids - ps23
Malvids - ps24
Sap./Malv./Brassicales - ps25
Malvales/Brassicales - ps26
Brassicales - ps27
Cleomaceae/Brassicaceae - ps28
Brassicaceae - ps29
Camelineae - ps30
Arabidopsis - ps31
Arabidopsis_thaliana - ps32
p-value
1e-19
2e-15
2e-11
3e-07
5e-03
Log-odds
0.5
1.0
1.5
2.0
2.5
3.0
3.5
4.0

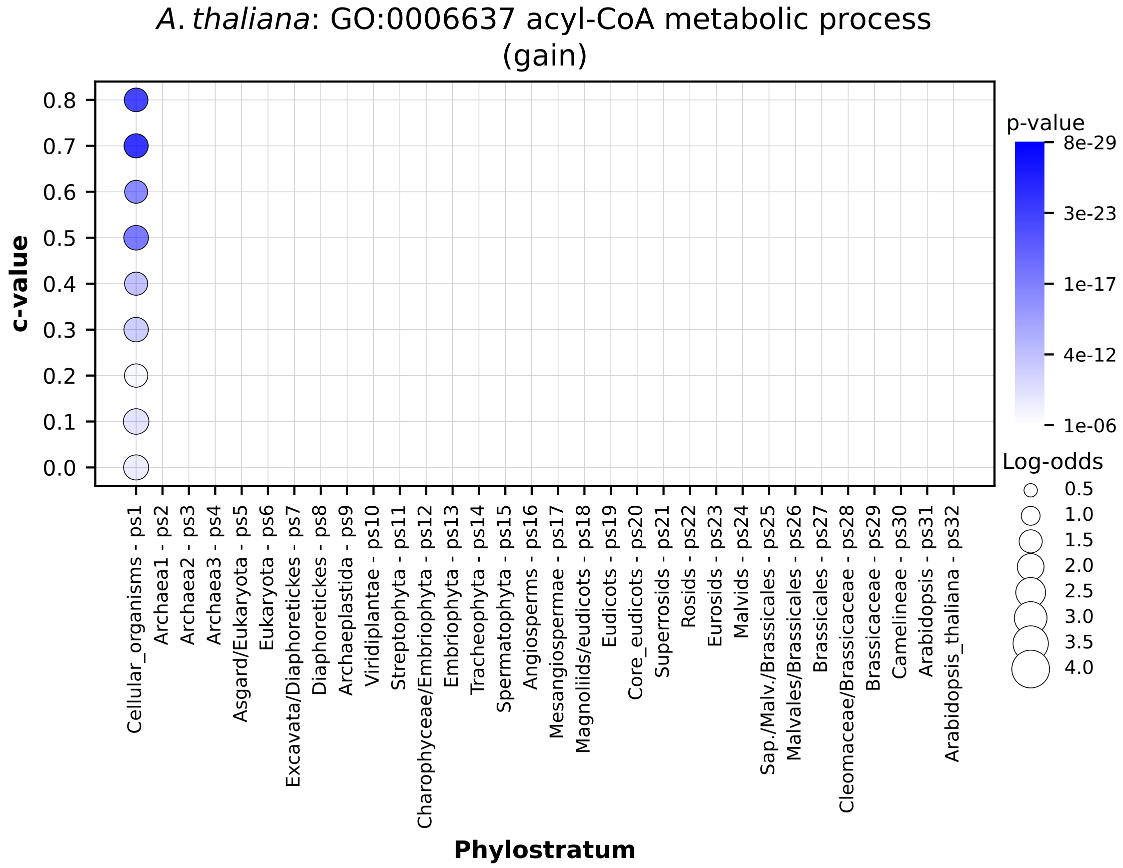
A. thaliana: GO:0006637 acyl-CoA metabolic process
(gain)
c-value
0.0
0.1
0.2
0.3
0.4
0.5
0.6
0.7
0.8
p-value
8e-29
3e-23
1e-17
4e-12
1e-06
Log-odds
0.5
1.0
1.5
2.0
2.5
3.0
3.5
4.0
Cellular_organisms - ps1
Archaea1 - ps2
Archaea2 - ps3
Archaea3 - ps4
Asgard/Eukaryota - ps5
Eukaryota - ps6
Excavata/Diaphoretickes - ps7
Diaphoretickes - ps8
Archaeplastida - ps9
Viridiplantae - ps10
Streptophyta - ps11
Charophyceae/Embriophyta - ps12
Embriophyta - ps13
Tracheophyta - ps14
Spermatophyta - ps15
Angiosperms - ps16
Mesangiospermae - ps17
Magnoliids/eudicots - ps18
Eudicots - ps19
Core_eudicots - ps20
Superrosids - ps21
Rosids - ps22
Eurosids - ps23
Malvids - ps24
Sap./Malv./Brassicales - ps25
Malvales/Brassicales - ps26
Brassicales - ps27
Cleomaceae/Brassicaceae - ps28
Brassicaceae - ps29
Camelineae - ps30
Arabidopsis - ps31
Arabidopsis_thaliana - ps32
Phylostratum

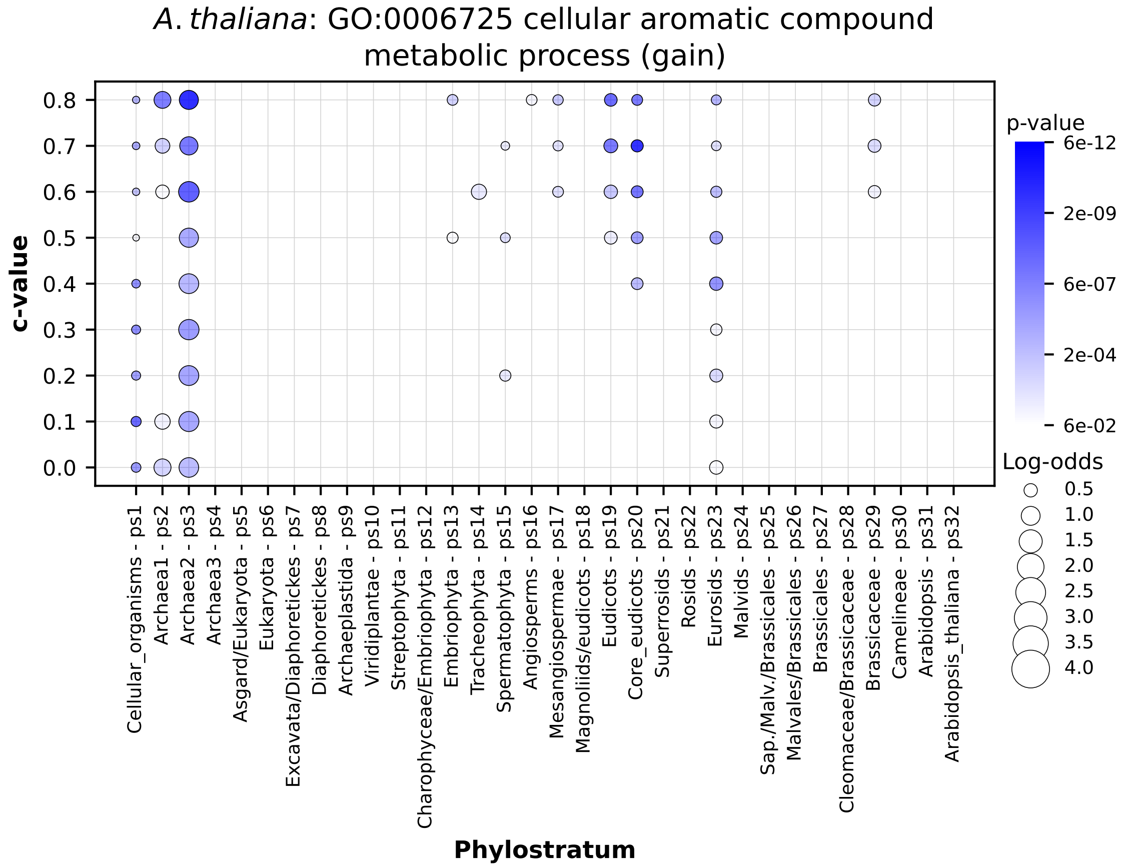

A. thaliana: GO:0006725 cellular aromatic compound metabolic process (gain)
c-value
0.8
0.7
0.6
0.5
0.4
0.3
0.2
0.1
0.0
p-value
6e-12
2e-09
6e-07
2e-04
6e-02
Log-odds
0.5
1.0
1.5
2.0
2.5
3.0
3.5
4.0
Cellular_organisms - ps1
Archaea1 - ps2
Archaea2 - ps3
Archaea3 - ps4
Asgard/Eukaryota - ps5
Eukaryota - ps6
Excavata/Diaphoretickes - ps7
Diaphoretickes - ps8
Archaeplastida - ps9
Viridiplantae - ps10
Streptophyta - ps11
Charophyceae/Embriophyta - ps12
Embriophyta - ps13
Tracheophyta - ps14
Spermatophyta - ps15
Angiosperms - ps16
Mesangiospermae - ps17
Magnoliids/eudicots - ps18
Eudicots - ps19
Core_eudicots - ps20
Superrosids - ps21
Rosids - ps22
Eurosids - ps23
Malvids - ps24
Sap./Malv./Brassicales - ps25
Malvales/Brassicales - ps26
Brassicales - ps27
Cleomaceae/Brassicaceae - ps28
Brassicaceae - ps29
Camelineae - ps30
Arabidopsis - ps31
Arabidopsis_thaliana - ps32
Phylostratum

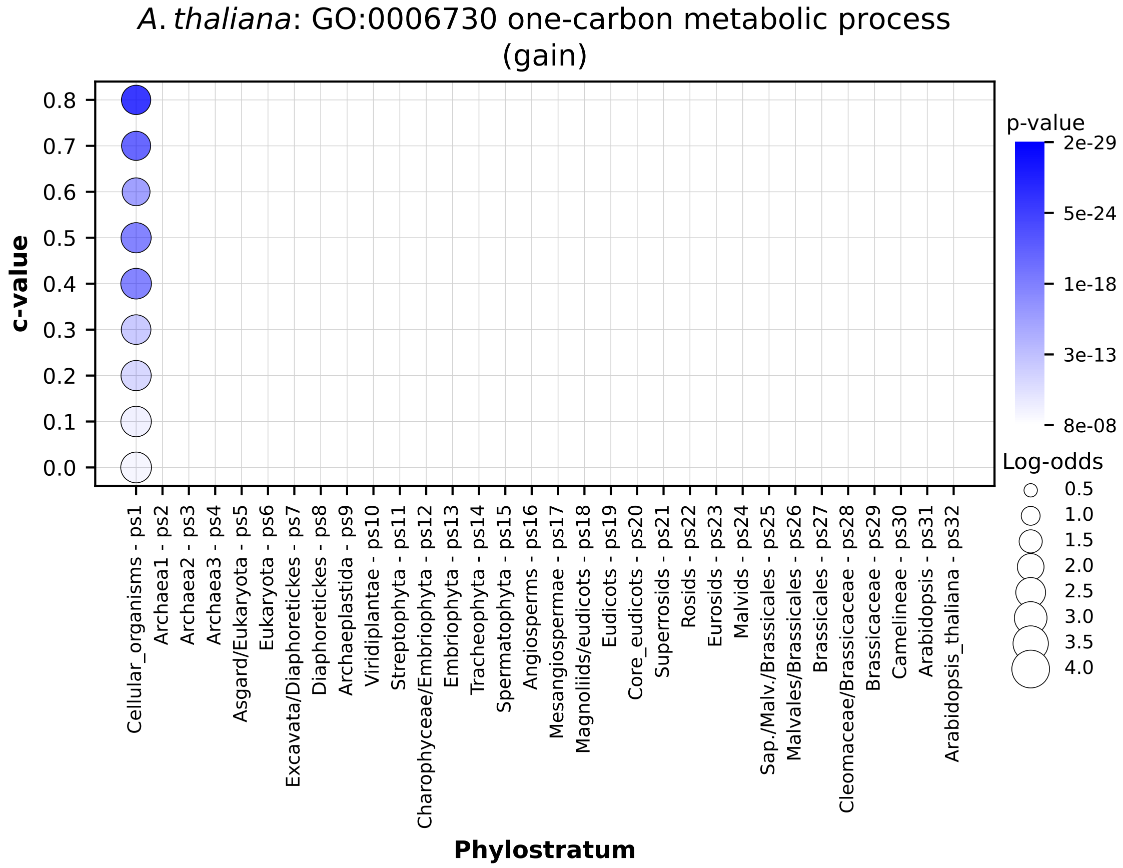

A. thaliana: GO:0006730 one-carbon metabolic process
(gain)
c-value
0.8
0.7
0.6
0.5
0.4
0.3
0.2
0.1
0.0
p-value
2e-29
5e-24
1e-18
3e-13
8e-08
Log-odds
0.5
1.0
1.5
2.0
2.5
3.0
3.5
4.0
Cellular_organisms - ps1
Archaea1 - ps2
Archaea2 - ps3
Archaea3 - ps4
Asgard/Eukaryota - ps5
Eukaryota - ps6
Excavata/Diaphoretickes - ps7
Diaphoretickes - ps8
Archaeplastida - ps9
Viridiplantae - ps10
Streptophyta - ps11
Charophyceae/Embriophyta - ps12
Embriophyta - ps13
Tracheophyta - ps14
Spermatophyta - ps15
Angiosperms - ps16
Mesangiospermae - ps17
Magnoliids/eudicots - ps18
Eudicots - ps19
Core_eudicots - ps20
Superrosids - ps21
Rosids - ps22
Eurosids - ps23
Malvids - ps24
Sap./Malv./Brassicales - ps25
Malvales/Brassicales - ps26
Brassicales - ps27
Cleomaceae/Brassicaceae - ps28
Brassicaceae - ps29
Camelineae - ps30
Arabidopsis - ps31
Arabidopsis_thaliana - ps32
Phylostratum

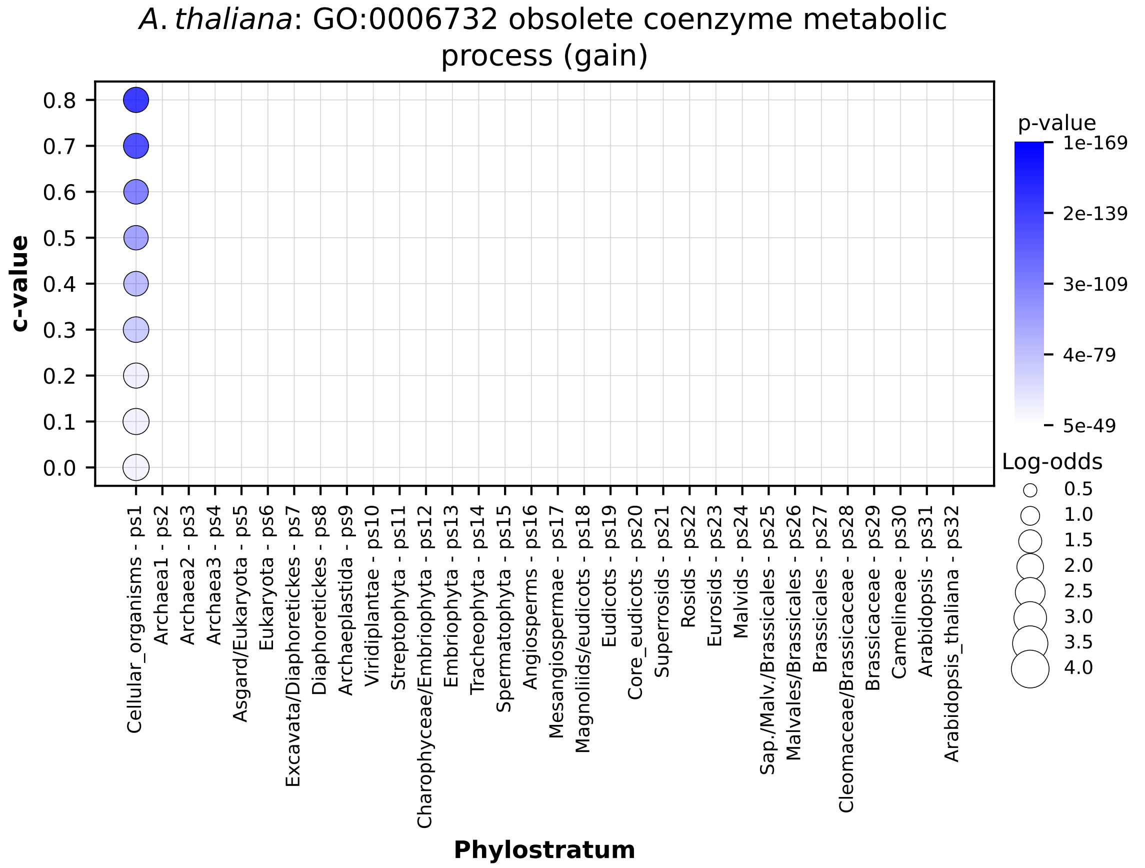
A. thaliana: GO:0006732 obsolete coenzyme metabolic process (gain)
c-value
0.0
0.1
0.2
0.3
0.4
0.5
0.6
0.7
0.8
Phylostratum
Cellular_organisms - ps1
Archaea1 - ps2
Archaea2 - ps3
Archaea3 - ps4
Asgard/Eukaryota - ps5
Eukaryota - ps6
Excavata/Diaphoretickes - ps7
Diaphoretickes - ps8
Archaeplastida - ps9
Viridiplantae - ps10
Streptophyta - ps11
Charophyceae/Embriophyta - ps12
Embriophyta - ps13
Tracheophyta - ps14
Spermatophyta - ps15
Angiosperms - ps16
Mesangiospermae - ps17
Magnoliids/eudicots - ps18
Eudicots - ps19
Core_eudicots - ps20
Superrosids - ps21
Rosids - ps22
Eurosids - ps23
Malvids - ps24
Sap./Malv./Brassicales - ps25
Malvales/Brassicales - ps26
Brassicales - ps27
Cleomaceae/Brassicaceae - ps28
Brassicaceae - ps29
Camelineae - ps30
Arabidopsis - ps31
Arabidopsis_thaliana - ps32
p-value
1e-169
2e-139
3e-109
4e-79
5e-49
Log-odds
0.5
1.0
1.5
2.0
2.5
3.0
3.5
4.0

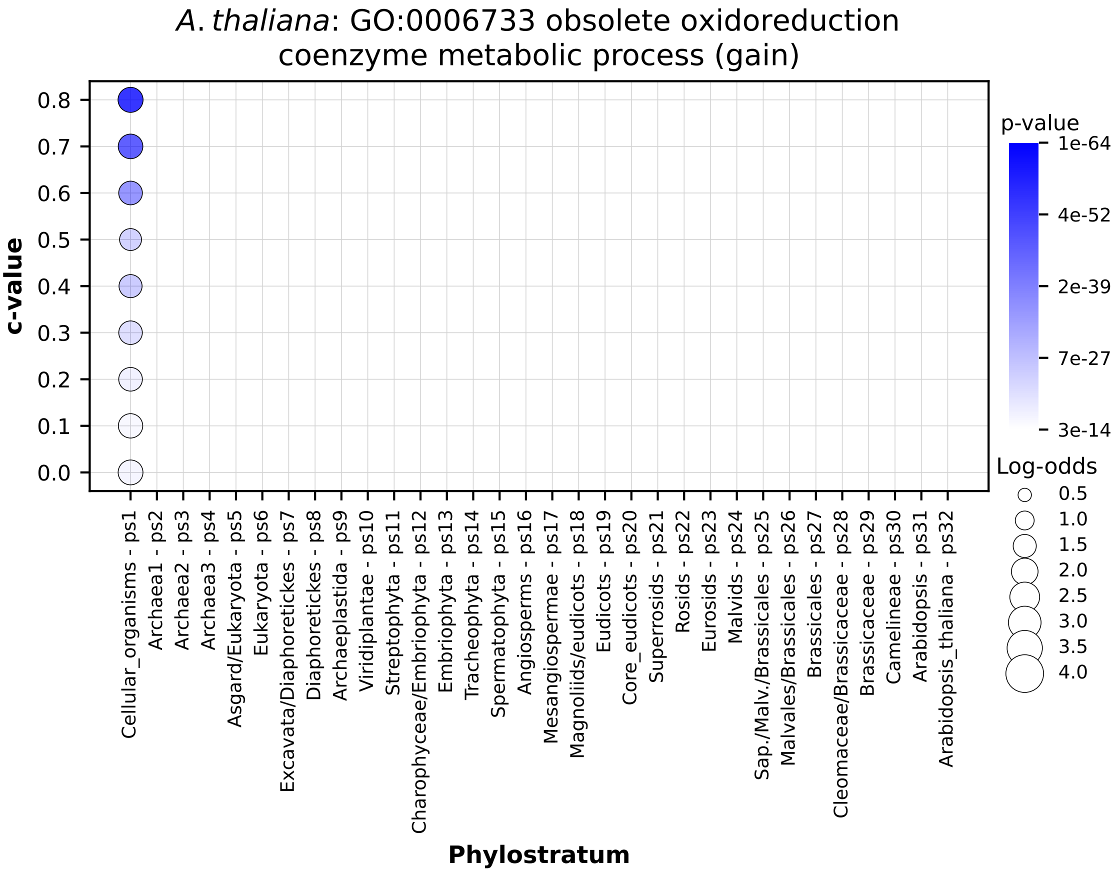

A. thaliana: GO:0006733 obsolete oxidoreduction coenzyme metabolic process (gain)
c-value
0.8
0.7
0.6
0.5
0.4
0.3
0.2
0.1
0.0
p-value
1e-64
4e-52
2e-39
7e-27
3e-14
Log-odds
0.5
1.0
1.5
2.0
2.5
3.0
3.5
4.0
Cellular_organisms - ps1
Archaea1 - ps2
Archaea2 - ps3
Archaea3 - ps4
Asgard/Eukaryota - ps5
Eukaryota - ps6
Excavata/Diaphoretickes - ps7
Diaphoretickes - ps8
Archaeplastida - ps9
Viridiplantae - ps10
Streptophyta - ps11
Charophyceae/Embriophyta - ps12
Embriophyta - ps13
Tracheophyta - ps14
Spermatophyta - ps15
Angiosperms - ps16
Mesangiospermae - ps17
Magnoliids/eudicots - ps18
Eudicots - ps19
Core_eudicots - ps20
Superrosids - ps21
Rosids - ps22
Eurosids - ps23
Malvids - ps24
Sap./Malv./Brassicales - ps25
Malvales/Brassicales - ps26
Brassicales - ps27
Cleomaceae/Brassicaceae - ps28
Brassicaceae - ps29
Camelineae - ps30
Arabidopsis - ps31
Arabidopsis_thaliana - ps32
Phylostratum

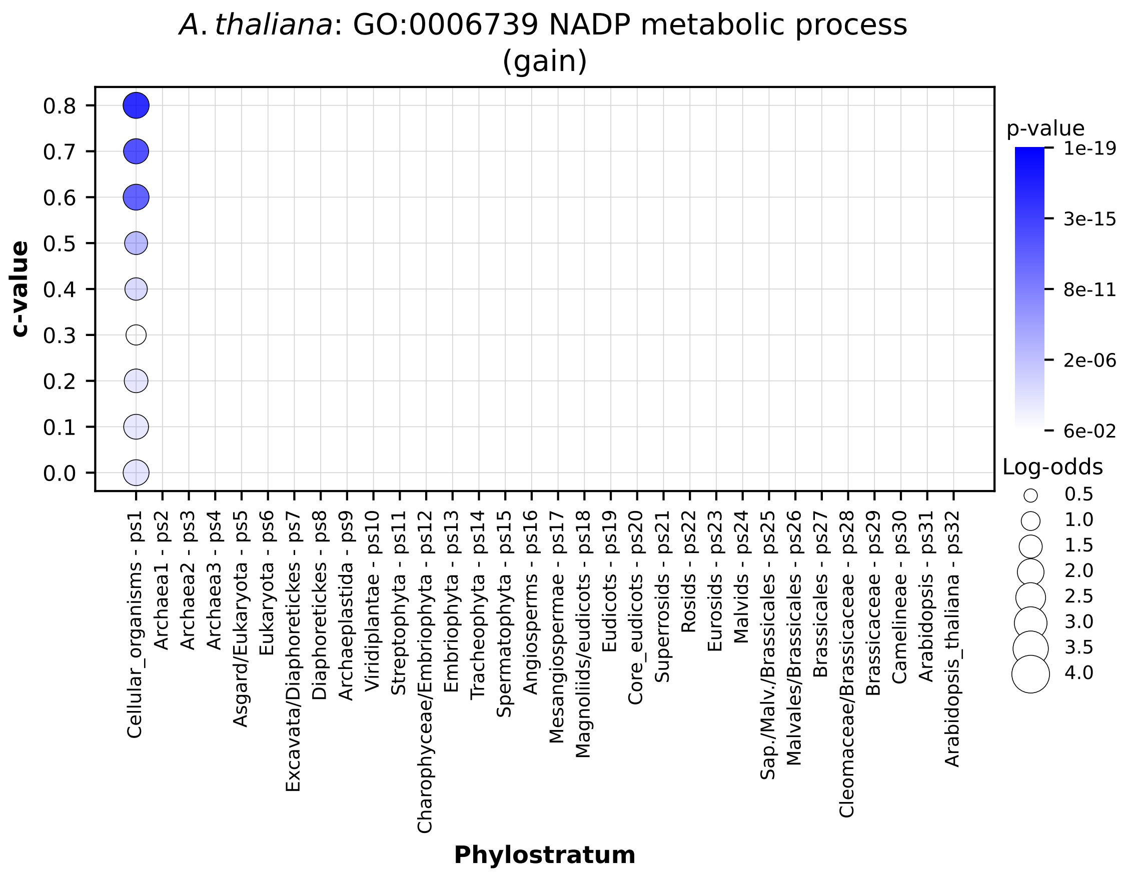

A. thaliana: GO:0006739 NADP metabolic process (gain)
c-value
0.8
0.7
0.6
0.5
0.4
0.3
0.2
0.1
0.0
p-value
1e-19
3e-15
8e-11
2e-06
6e-02
Log-odds
0.5
1.0
1.5
2.0
2.5
3.0
3.5
4.0
Cellular_organisms - ps1
Archaea1 - ps2
Archaea2 - ps3
Archaea3 - ps4
Asgard/Eukaryota - ps5
Eukaryota - ps6
Excavata/Diaphoretickes - ps7
Diaphoretickes - ps8
Archaeplastida - ps9
Viridiplantae - ps10
Streptophyta - ps11
Charophyceae/Embriophyta - ps12
Embriophyta - ps13
Tracheophyta - ps14
Spermatophyta - ps15
Angiosperms - ps16
Mesangiospermae - ps17
Magnoliids/eudicots - ps18
Eudicots - ps19
Core_eudicots - ps20
Superrosids - ps21
Rosids - ps22
Eurosids - ps23
Malvids - ps24
Sap./Malv./Brassicales - ps25
Malvales/Brassicales - ps26
Brassicales - ps27
Cleomaceae/Brassicaceae - ps28
Brassicaceae - ps29
Camelineae - ps30
Arabidopsis - ps31
Arabidopsis_thaliana - ps32
Phylostratum

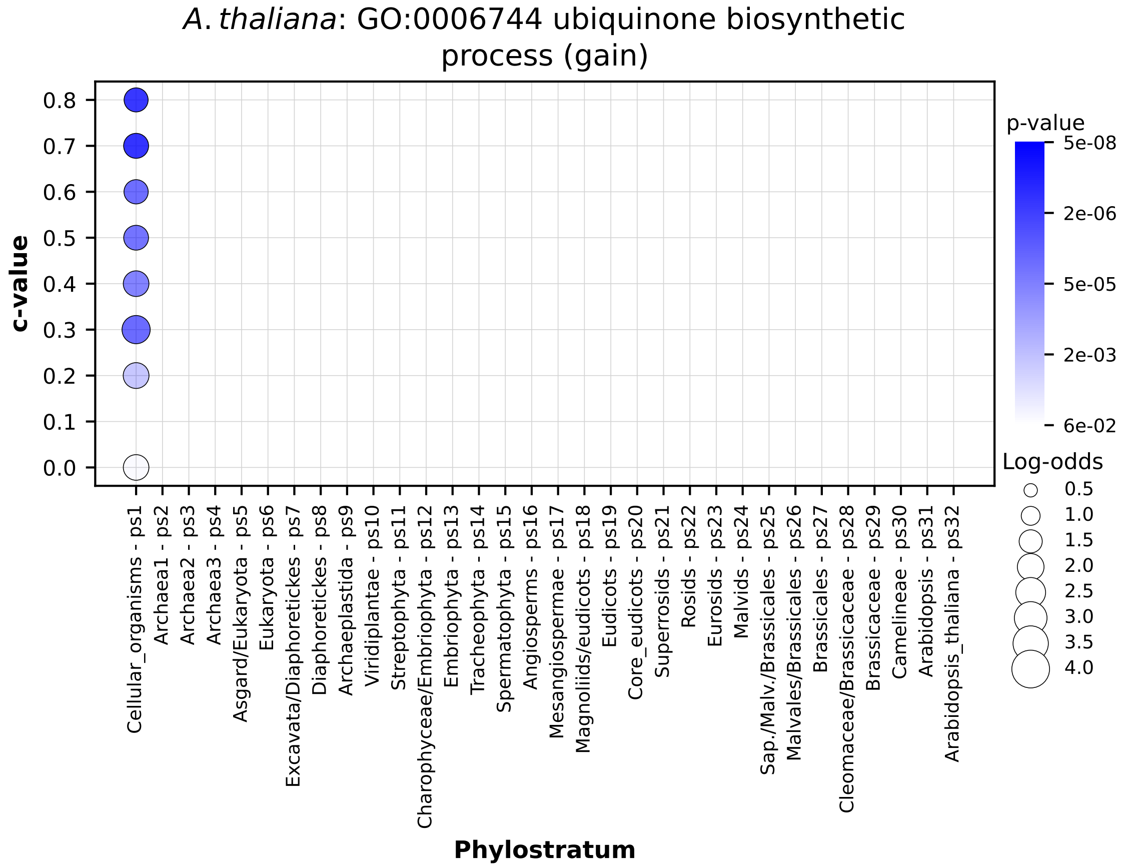

A. thaliana: GO:0006744 ubiquinone biosynthetic process (gain)
c-value
0.8
0.7
0.6
0.5
0.4
0.3
0.2
0.1
0.0
p-value
5e-08
2e-06
5e-05
2e-03
6e-02
Log-odds
0.5
1.0
1.5
2.0
2.5
3.0
3.5
4.0
Cellular_organisms - ps1
Archaea1 - ps2
Archaea2 - ps3
Archaea3 - ps4
Asgard/Eukaryota - ps5
Eukaryota - ps6
Excavata/Diaphoretickes - ps7
Diaphoretickes - ps8
Archaeplastida - ps9
Viridiplantae - ps10
Streptophyta - ps11
Charophyceae/Embriophyta - ps12
Embriophyta - ps13
Tracheophyta - ps14
Spermatophyta - ps15
Angiosperms - ps16
Mesangiospermae - ps17
Magnoliids/eudicots - ps18
Eudicots - ps19
Core_eudicots - ps20
Superrosids - ps21
Rosids - ps22
Eurosids - ps23
Malvids - ps24
Sap./Malv./Brassicales - ps25
Malvales/Brassicales - ps26
Brassicales - ps27
Cleomaceae/Brassicaceae - ps28
Brassicaceae - ps29
Camelineae - ps30
Arabidopsis - ps31
Arabidopsis_thaliana - ps32
Phylostratum

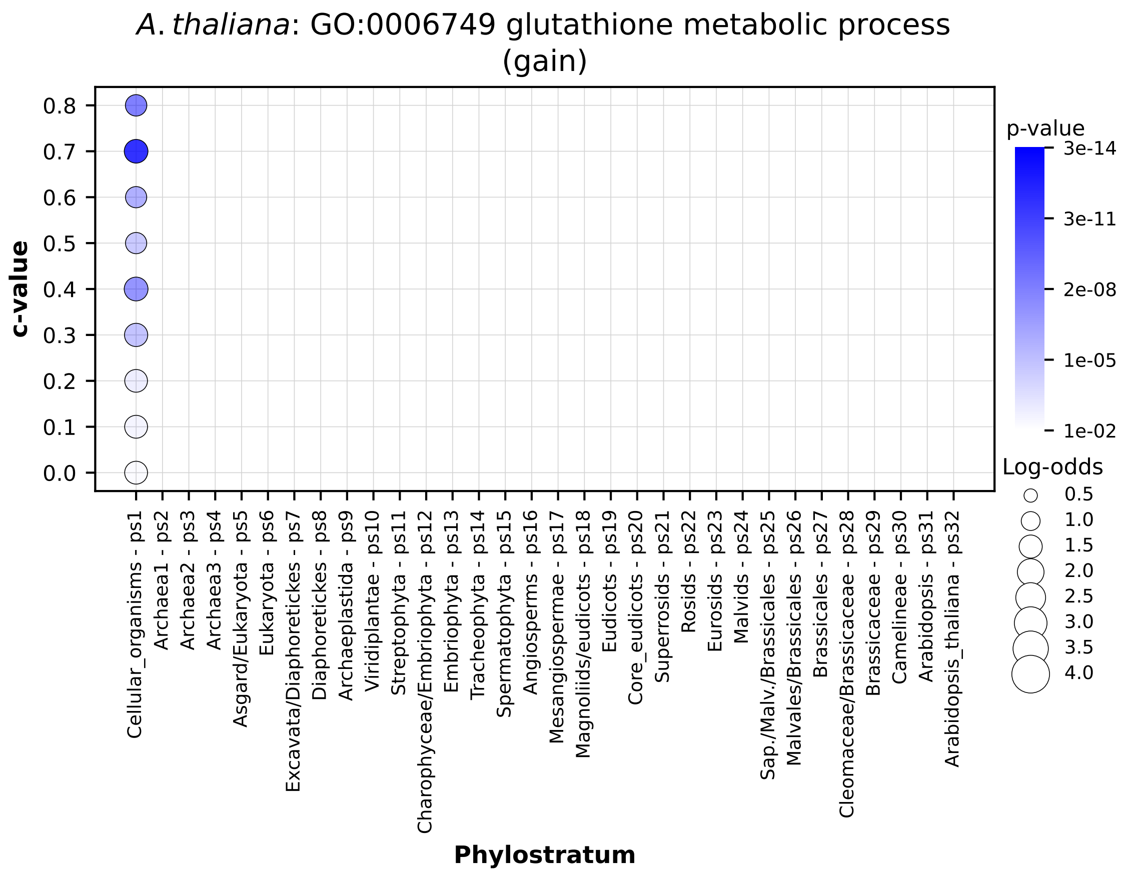

A. thaliana: GO:0006749 glutathione metabolic process
(gain)
c-value
0.0
0.1
0.2
0.3
0.4
0.5
0.6
0.7
0.8
Phylostratum
Cellular_organisms - ps1
Archaea1 - ps2
Archaea2 - ps3
Archaea3 - ps4
Asgard/Eukaryota - ps5
Eukaryota - ps6
Excavata/Diaphoretickes - ps7
Diaphoretickes - ps8
Archaeplastida - ps9
Viridiplantae - ps10
Streptophyta - ps11
Charophyceae/Embriophyta - ps12
Embriophyta - ps13
Tracheophyta - ps14
Spermatophyta - ps15
Angiosperms - ps16
Mesangiospermae - ps17
Magnoliids/eudicots - ps18
Eudicots - ps19
Core_eudicots - ps20
Superrosids - ps21
Rosids - ps22
Eurosids - ps23
Malvids - ps24
Sap./Malv./Brassicales - ps25
Malvales/Brassicales - ps26
Brassicales - ps27
Cleomaceae/Brassicaceae - ps28
Brassicaceae - ps29
Camelineae - ps30
Arabidopsis - ps31
Arabidopsis_thaliana - ps32
p-value
3e-14
3e-11
2e-08
1e-05
1e-02
Log-odds
0.5
1.0
1.5
2.0
2.5
3.0
3.5
4.0

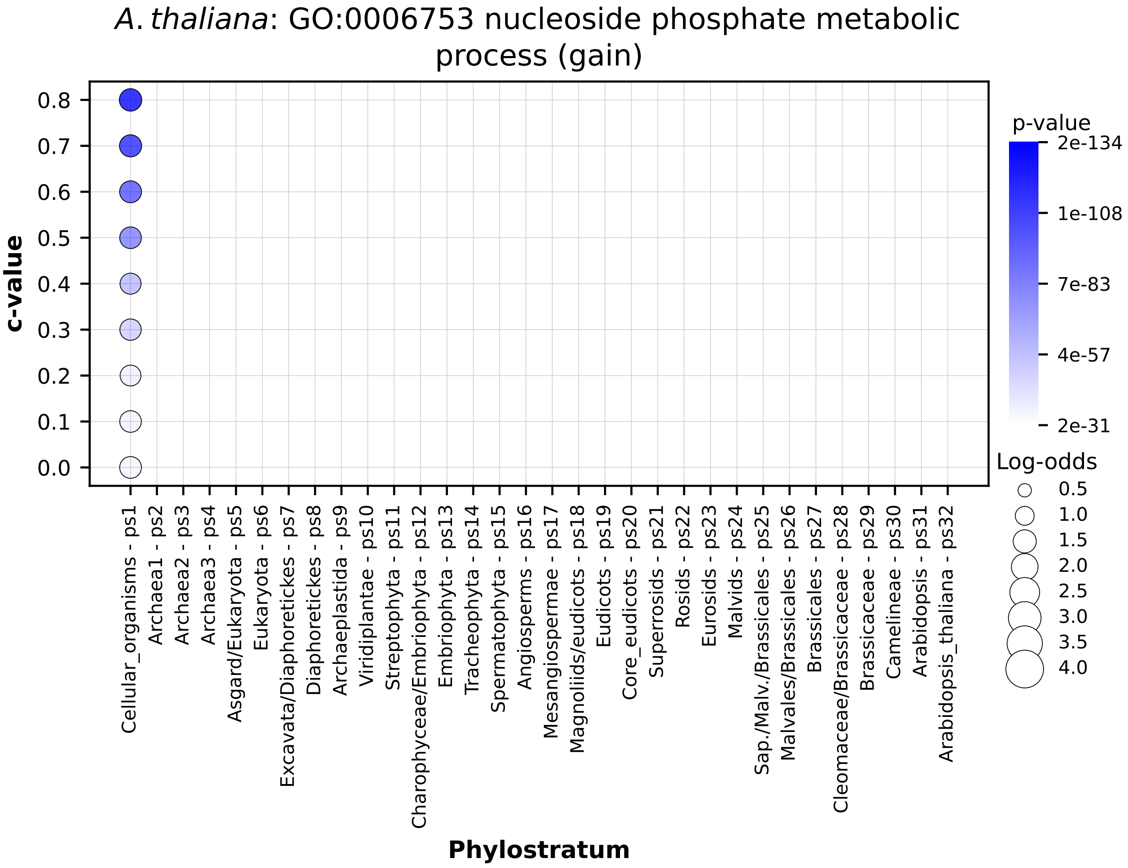
A. thaliana: GO:0006753 nucleoside phosphate metabolic process (gain)
c-value
0.0
0.1
0.2
0.3
0.4
0.5
0.6
0.7
0.8
Phylostratum
Cellular_organisms - ps1
Archaea1 - ps2
Archaea2 - ps3
Archaea3 - ps4
Asgard/Eukaryota - ps5
Eukaryota - ps6
Excavata/Diaphoretickes - ps7
Diaphoretickes - ps8
Archaeplastida - ps9
Viridiplantae - ps10
Streptophyta - ps11
Charophyceae/Embriophyta - ps12
Embriophyta - ps13
Tracheophyta - ps14
Spermatophyta - ps15
Angiosperms - ps16
Mesangiospermae - ps17
Magnoliids/eudicots - ps18
Eudicots - ps19
Core_eudicots - ps20
Superrosids - ps21
Rosids - ps22
Eurosids - ps23
Malvids - ps24
Sap./Malv./Brassicales - ps25
Malvales/Brassicales - ps26
Brassicales - ps27
Cleomaceae/Brassicaceae - ps28
Brassicaceae - ps29
Camelineae - ps30
Arabidopsis - ps31
Arabidopsis_thaliana - ps32
p-value
2e-134
1e-108
7e-83
4e-57
2e-31
Log-odds
0.5
1.0
1.5
2.0
2.5
3.0
3.5
4.0

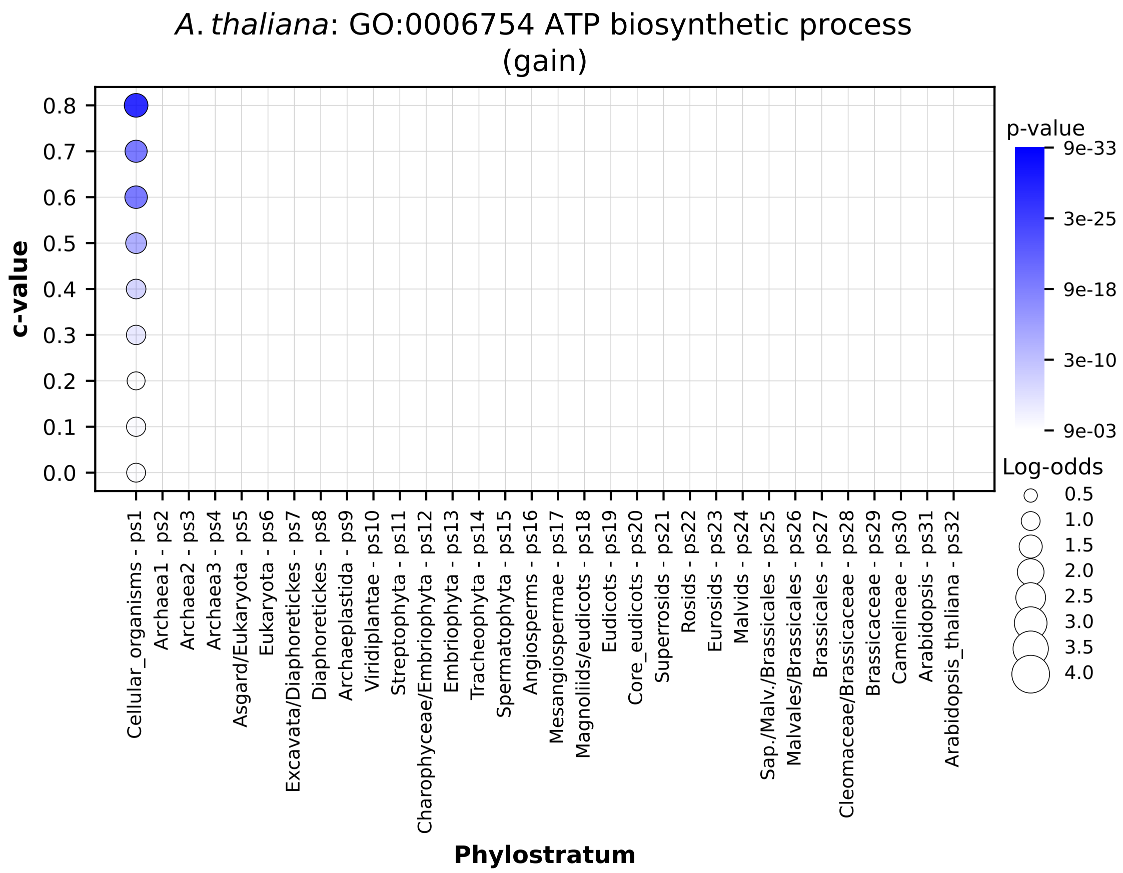

A. thaliana: GO:0006754 ATP biosynthetic process
(gain)
c-value
0.8
0.7
0.6
0.5
0.4
0.3
0.2
0.1
0.0
p-value
9e-33
3e-25
9e-18
3e-10
9e-03
Log-odds
0.5
1.0
1.5
2.0
2.5
3.0
3.5
4.0
Cellular_organisms - ps1
Archaea1 - ps2
Archaea2 - ps3
Archaea3 - ps4
Asgard/Eukaryota - ps5
Eukaryota - ps6
Excavata/Diaphoretickes - ps7
Diaphoretickes - ps8
Archaeplastida - ps9
Viridiplantae - ps10
Streptophyta - ps11
Charophyceae/Embriophyta - ps12
Embriophyta - ps13
Tracheophyta - ps14
Spermatophyta - ps15
Angiosperms - ps16
Mesangiospermae - ps17
Magnoliids/eudicots - ps18
Eudicots - ps19
Core_eudicots - ps20
Superrosids - ps21
Rosids - ps22
Eurosids - ps23
Malvids - ps24
Sap./Malv./Brassicales - ps25
Malvales/Brassicales - ps26
Brassicales - ps27
Cleomaceae/Brassicaceae - ps28
Brassicaceae - ps29
Camelineae - ps30
Arabidopsis - ps31
Arabidopsis_thaliana - ps32
Phylostratum

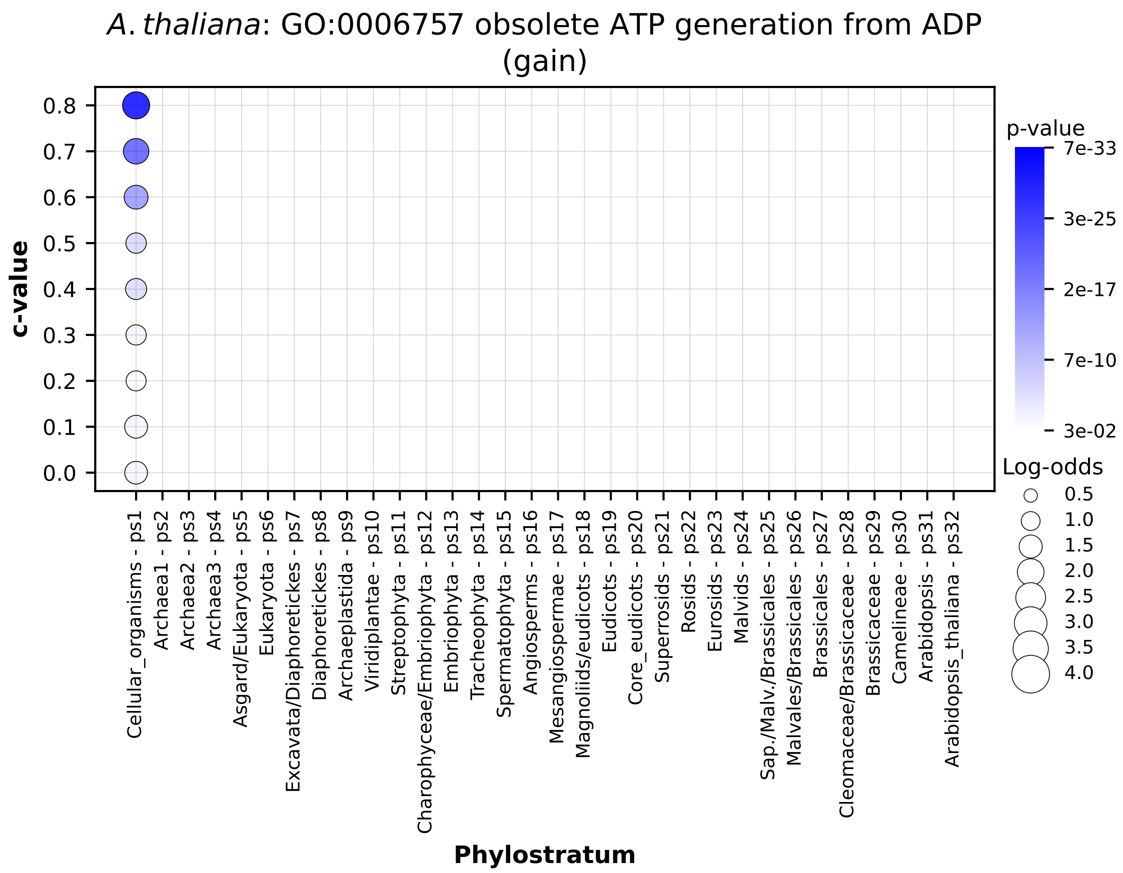

A. thaliana: GO:0006757 obsolete ATP generation from ADP (gain)
c-value
0.8
0.7
0.6
0.5
0.4
0.3
0.2
0.1
0.0
p-value
7e-33
3e-25
2e-17
7e-10
3e-02
Log-odds
0.5
1.0
1.5
2.0
2.5
3.0
3.5
4.0
Cellular_organisms - ps1
Archaea1 - ps2
Archaea2 - ps3
Archaea3 - ps4
Asgard/Eukaryota - ps5
Eukaryota - ps6
Excavata/Diaphoretickes - ps7
Diaphoretickes - ps8
Archaeplastida - ps9
Viridiplantae - ps10
Streptophyta - ps11
Charophyceae/Embriophyta - ps12
Embriophyta - ps13
Tracheophyta - ps14
Spermatophyta - ps15
Angiosperms - ps16
Mesangiospermae - ps17
Magnoliids/eudicots - ps18
Eudicots - ps19
Core_eudicots - ps20
Superrosids - ps21
Rosids - ps22
Eurosids - ps23
Malvids - ps24
Sap./Malv./Brassicales - ps25
Malvales/Brassicales - ps26
Brassicales - ps27
Cleomaceae/Brassicaceae - ps28
Brassicaceae - ps29
Camelineae - ps30
Arabidopsis - ps31
Arabidopsis_thaliana - ps32
Phylostratum

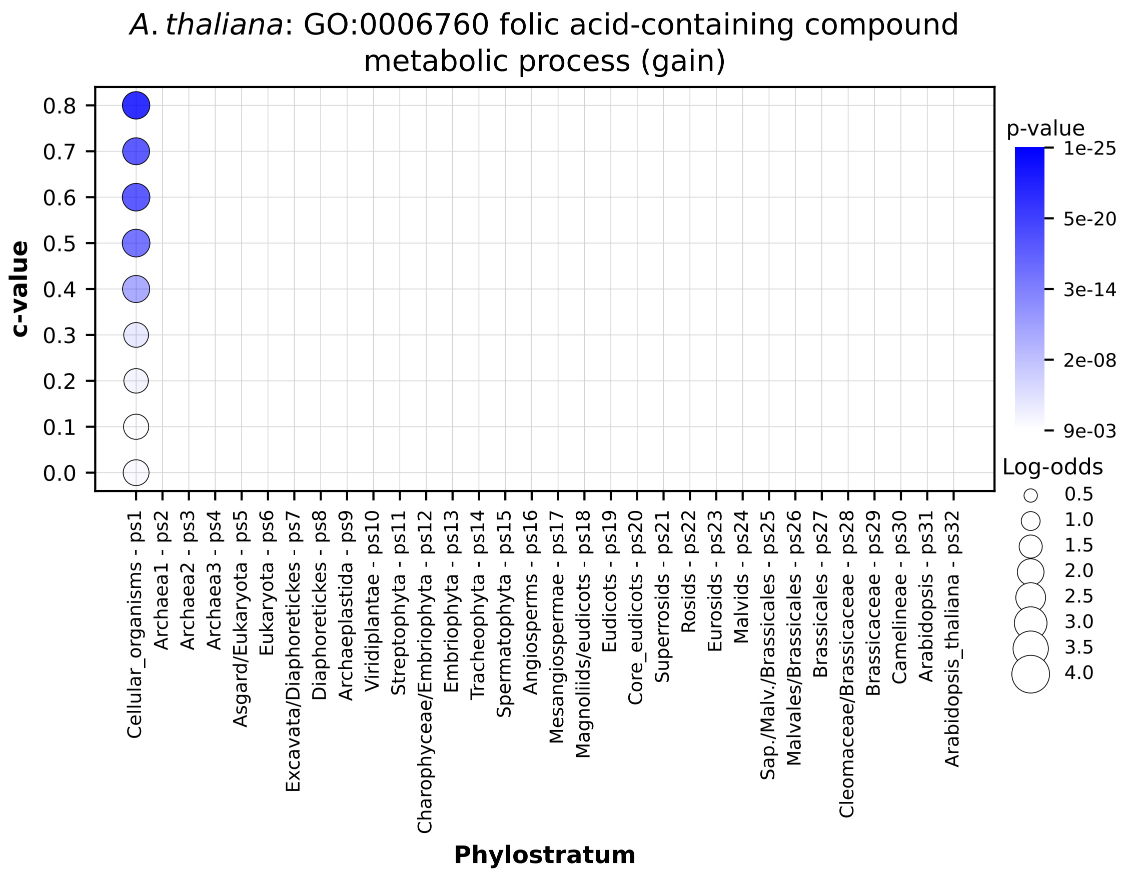

A. thaliana: GO:0006760 folic acid-containing compound metabolic process (gain)
c-value
0.8
0.7
0.6
0.5
0.4
0.3
0.2
0.1
0.0
p-value
1e-25
5e-20
3e-14
2e-08
9e-03
Log-odds
0.5
1.0
1.5
2.0
2.5
3.0
3.5
4.0
Cellular_organisms - ps1
Archaea1 - ps2
Archaea2 - ps3
Archaea3 - ps4
Asgard/Eukaryota - ps5
Eukaryota - ps6
Excavata/Diaphoretickes - ps7
Diaphoretickes - ps8
Archaeplastida - ps9
Viridiplantae - ps10
Streptophyta - ps11
Charophyceae/Embriophyta - ps12
Embriophyta - ps13
Tracheophyta - ps14
Spermatophyta - ps15
Angiosperms - ps16
Mesangiospermae - ps17
Magnoliids/eudicots - ps18
Eudicots - ps19
Core_eudicots - ps20
Superrosids - ps21
Rosids - ps22
Eurosids - ps23
Malvids - ps24
Sap./Malv./Brassicales - ps25
Malvales/Brassicales - ps26
Brassicales - ps27
Cleomaceae/Brassicaceae - ps28
Brassicaceae - ps29
Camelineae - ps30
Arabidopsis - ps31
Arabidopsis_thaliana - ps32
Phylostratum

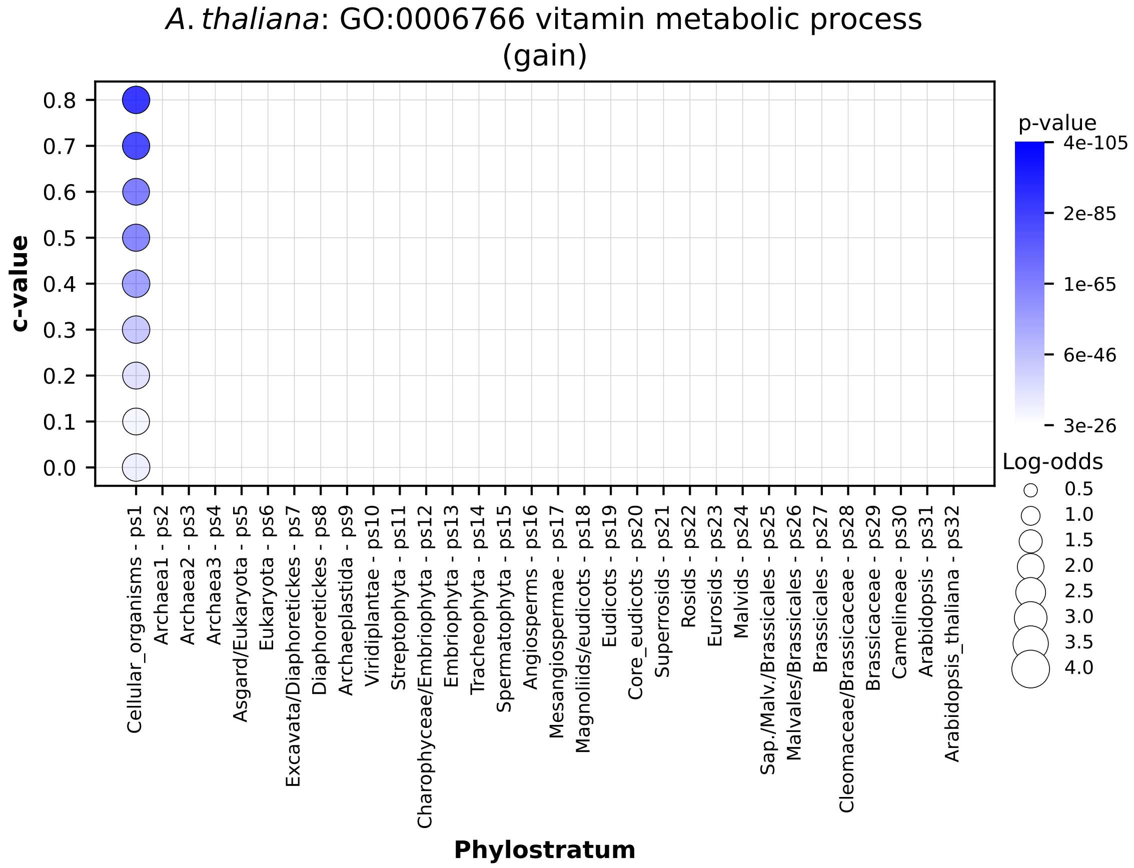
A. thaliana: GO:0006766 vitamin metabolic process
(gain)
c-value
0.8
0.7
0.6
0.5
0.4
0.3
0.2
0.1
0.0
p-value
4e-105
2e-85
1e-65
6e-46
3e-26
Log-odds
0.5
1.0
1.5
2.0
2.5
3.0
3.5
4.0
Cellular_organisms - ps1
Archaea1 - ps2
Archaea2 - ps3
Archaea3 - ps4
Asgard/Eukaryota - ps5
Eukaryota - ps6
Excavata/Diaphoretickes - ps7
Diaphoretickes - ps8
Archaeplastida - ps9
Viridiplantae - ps10
Streptophyta - ps11
Charophyceae/Embriophyta - ps12
Embriophyta - ps13
Tracheophyta - ps14
Spermatophyta - ps15
Angiosperms - ps16
Mesangiospermae - ps17
Magnoliids/eudicots - ps18
Eudicots - ps19
Core_eudicots - ps20
Superrosids - ps21
Rosids - ps22
Eurosids - ps23
Malvids - ps24
Sap./Malv./Brassicales - ps25
Malvales/Brassicales - ps26
Brassicales - ps27
Cleomaceae/Brassicaceae - ps28
Brassicaceae - ps29
Camelineae - ps30
Arabidopsis - ps31
Arabidopsis_thaliana - ps32
Phylostratum

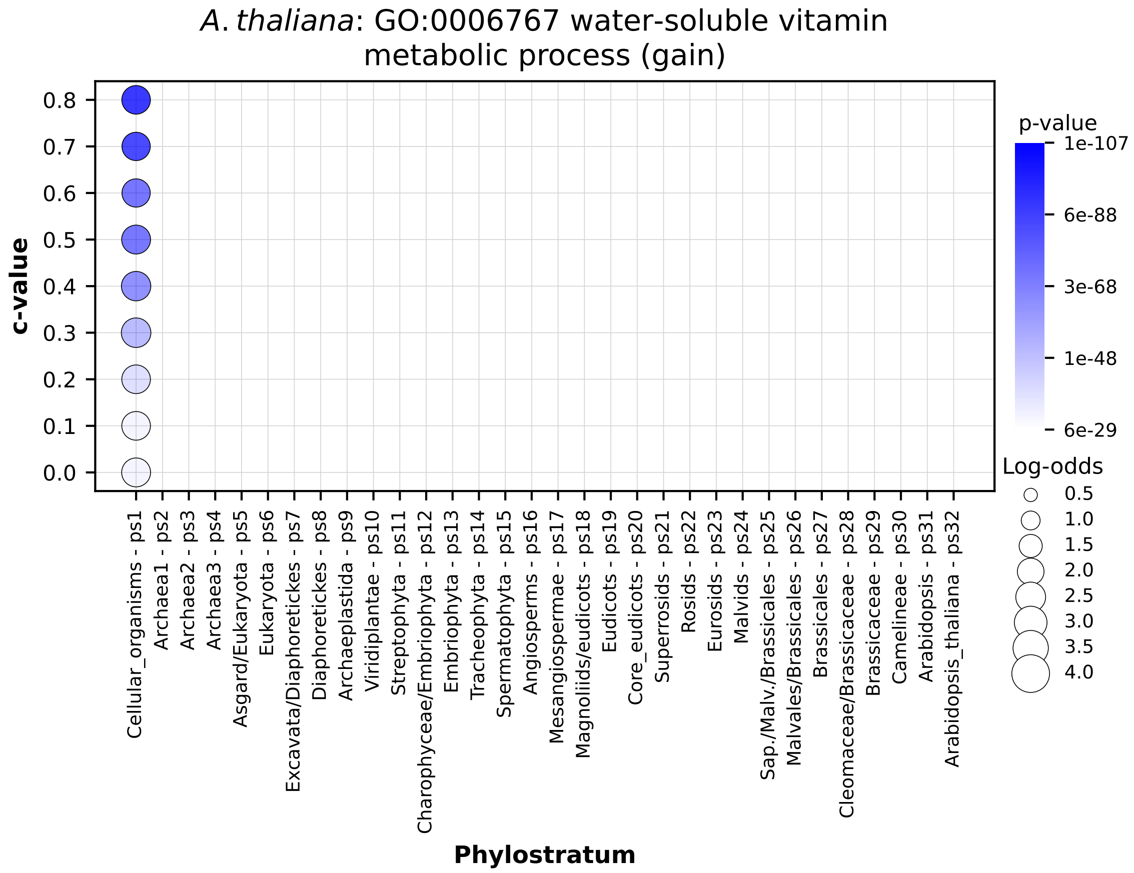

A. thaliana: GO:0006767 water-soluble vitamin metabolic process (gain)
c-value
0.0
0.1
0.2
0.3
0.4
0.5
0.6
0.7
0.8
p-value
1e-107
6e-88
3e-68
1e-48
6e-29
Log-odds
0.5
1.0
1.5
2.0
2.5
3.0
3.5
4.0
Cellular_organisms - ps1
Archaea1 - ps2
Archaea2 - ps3
Archaea3 - ps4
Asgard/Eukaryota - ps5
Eukaryota - ps6
Excavata/Diaphoretickes - ps7
Diaphoretickes - ps8
Archaeplastida - ps9
Viridiplantae - ps10
Streptophyta - ps11
Charophyceae/Embriophyta - ps12
Embriophyta - ps13
Tracheophyta - ps14
Spermatophyta - ps15
Angiosperms - ps16
Mesangiospermae - ps17
Magnoliids/eudicots - ps18
Eudicots - ps19
Core_eudicots - ps20
Superrosids - ps21
Rosids - ps22
Eurosids - ps23
Malvids - ps24
Sap./Malv./Brassicales - ps25
Malvales/Brassicales - ps26
Brassicales - ps27
Cleomaceae/Brassicaceae - ps28
Brassicaceae - ps29
Camelineae - ps30
Arabidopsis - ps31
Arabidopsis_thaliana - ps32
Phylostratum

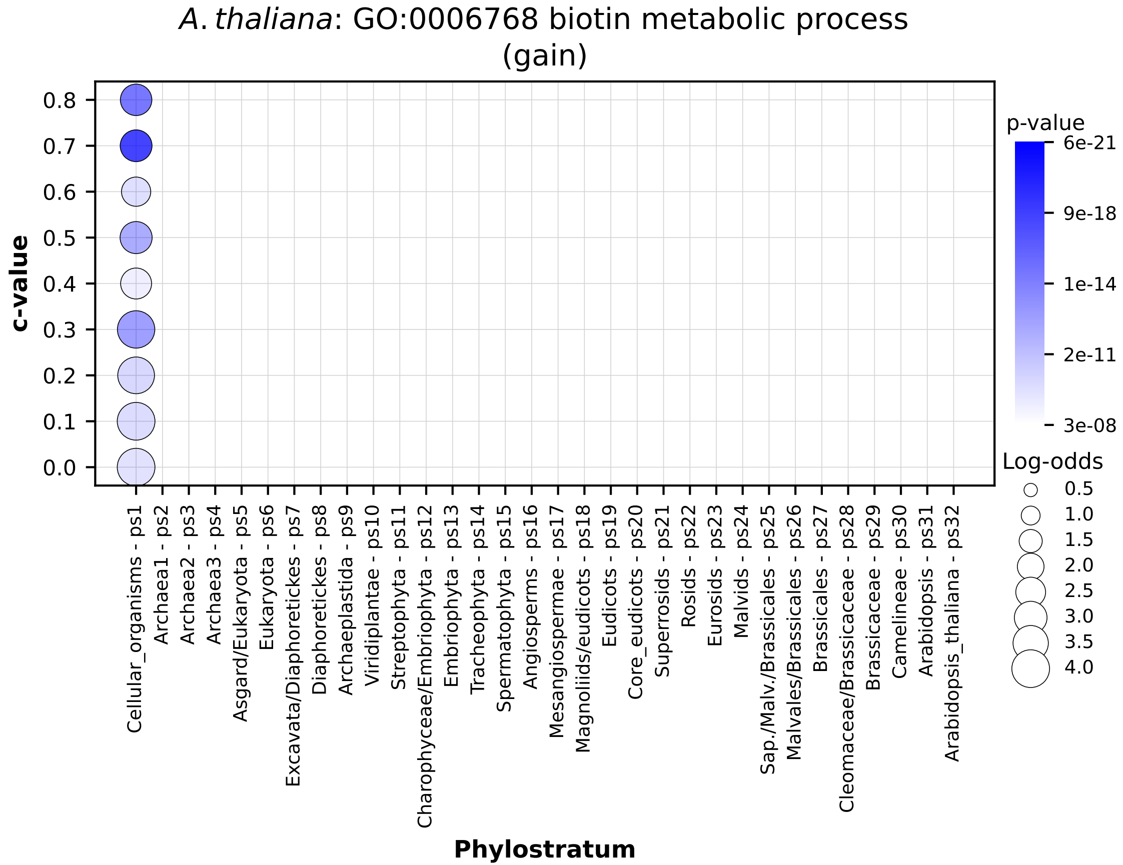

A. thaliana: GO:0006768 biotin metabolic process (gain)
c-value
0.8
0.7
0.6
0.5
0.4
0.3
0.2
0.1
0.0
p-value
6e-21
9e-18
1e-14
2e-11
3e-08
Log-odds
0.5
1.0
1.5
2.0
2.5
3.0
3.5
4.0
Cellular_organisms - ps1
Archaea1 - ps2
Archaea2 - ps3
Archaea3 - ps4
Asgard/Eukaryota - ps5
Eukaryota - ps6
Excavata/Diaphoretickes - ps7
Diaphoretickes - ps8
Archaeplastida - ps9
Viridiplantae - ps10
Streptophyta - ps11
Charophyceae/Embriophyta - ps12
Embriophyta - ps13
Tracheophyta - ps14
Spermatophyta - ps15
Angiosperms - ps16
Mesangiospermae - ps17
Magnoliids/eudicots - ps18
Eudicots - ps19
Core_eudicots - ps20
Superrosids - ps21
Rosids - ps22
Eurosids - ps23
Malvids - ps24
Sap./Malv./Brassicales - ps25
Malvales/Brassicales - ps26
Brassicales - ps27
Cleomaceae/Brassicaceae - ps28
Brassicaceae - ps29
Camelineae - ps30
Arabidopsis - ps31
Arabidopsis_thaliana - ps32
Phylostratum

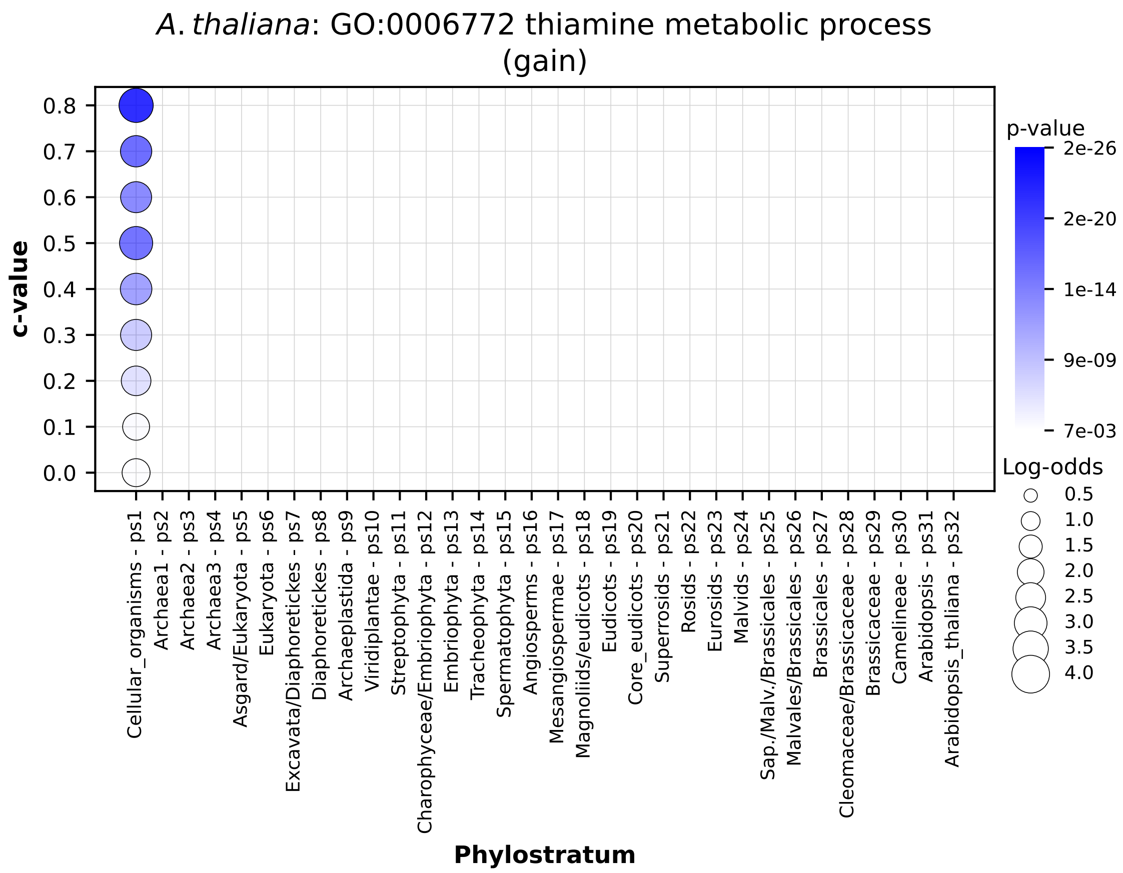

A. thaliana: GO:0006772 thiamine metabolic process
(gain)
c-value
0.8
0.7
0.6
0.5
0.4
0.3
0.2
0.1
0.0
p-value
2e-26
2e-20
1e-14
9e-09
7e-03
Log-odds
0.5
1.0
1.5
2.0
2.5
3.0
3.5
4.0
Cellular_organisms - ps1
Archaea1 - ps2
Archaea2 - ps3
Archaea3 - ps4
Asgard/Eukaryota - ps5
Eukaryota - ps6
Excavata/Diaphoretickes - ps7
Diaphoretickes - ps8
Archaeplastida - ps9
Viridiplantae - ps10
Streptophyta - ps11
Charophyceae/Embriophyta - ps12
Embriophyta - ps13
Tracheophyta - ps14
Spermatophyta - ps15
Angiosperms - ps16
Mesangiospermae - ps17
Magnoliids/eudicots - ps18
Eudicots - ps19
Core_eudicots - ps20
Superrosids - ps21
Rosids - ps22
Eurosids - ps23
Malvids - ps24
Sap./Malv./Brassicales - ps25
Malvales/Brassicales - ps26
Brassicales - ps27
Cleomaceae/Brassicaceae - ps28
Brassicaceae - ps29
Camelineae - ps30
Arabidopsis - ps31
Arabidopsis_thaliana - ps32
Phylostratum

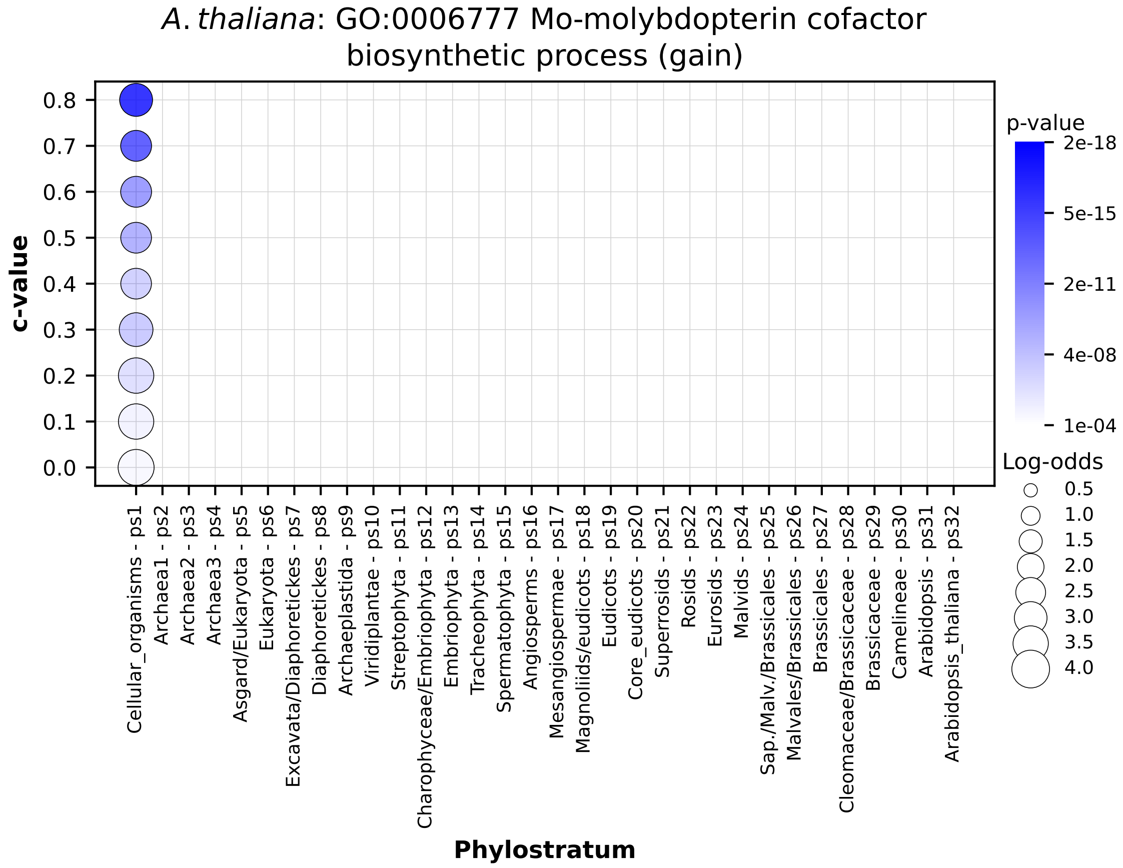

A. thaliana: GO:0006777 Mo-molybdopterin cofactor biosynthetic process (gain)
c-value
0.8
0.7
0.6
0.5
0.4
0.3
0.2
0.1
0.0
p-value
2e-18
5e-15
2e-11
4e-08
1e-04
Log-odds
0.5
1.0
1.5
2.0
2.5
3.0
3.5
4.0
Cellular_organisms - ps1
Archaea1 - ps2
Archaea2 - ps3
Archaea3 - ps4
Asgard/Eukaryota - ps5
Eukaryota - ps6
Excavata/Diaphoretickes - ps7
Diaphoretickes - ps8
Archaeplastida - ps9
Viridiplantae - ps10
Streptophyta - ps11
Charophyceae/Embriophyta - ps12
Embriophyta - ps13
Tracheophyta - ps14
Spermatophyta - ps15
Angiosperms - ps16
Mesangiospermae - ps17
Magnoliids/eudicots - ps18
Eudicots - ps19
Core_eudicots - ps20
Superrosids - ps21
Rosids - ps22
Eurosids - ps23
Malvids - ps24
Sap./Malv./Brassicales - ps25
Malvales/Brassicales - ps26
Brassicales - ps27
Cleomaceae/Brassicaceae - ps28
Brassicaceae - ps29
Camelineae - ps30
Arabidopsis - ps31
Arabidopsis_thaliana - ps32
Phylostratum

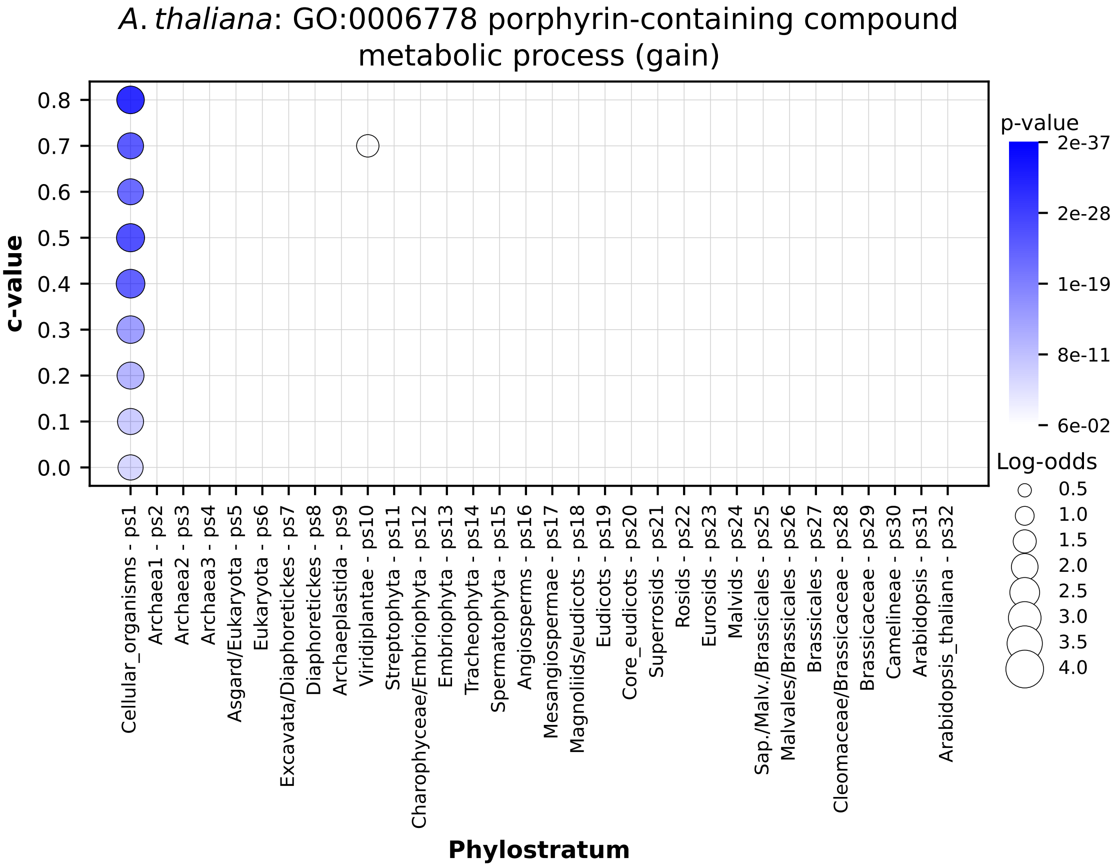

A. thaliana: GO:0006778 porphyrin-containing compound metabolic process (gain)
c-value
0.8
0.7
0.6
0.5
0.4
0.3
0.2
0.1
0.0
p-value
2e-37
2e-28
1e-19
8e-11
6e-02
Log-odds
0.5
1.0
1.5
2.0
2.5
3.0
3.5
4.0
Cellular_organisms - ps1
Archaea1 - ps2
Archaea2 - ps3
Archaea3 - ps4
Asgard/Eukaryota - ps5
Eukaryota - ps6
Excavata/Diaphoretickes - ps7
Diaphoretickes - ps8
Archaeplastida - ps9
Viridiplantae - ps10
Streptophyta - ps11
Charophyceae/Embriophyta - ps12
Embriophyta - ps13
Tracheophyta - ps14
Spermatophyta - ps15
Angiosperms - ps16
Mesangiospermae - ps17
Magnoliids/eudicots - ps18
Eudicots - ps19
Core_eudicots - ps20
Superrosids - ps21
Rosids - ps22
Eurosids - ps23
Malvids - ps24
Sap./Malv./Brassicales - ps25
Malvales/Brassicales - ps26
Brassicales - ps27
Cleomaceae/Brassicaceae - ps28
Brassicaceae - ps29
Camelineae - ps30
Arabidopsis - ps31
Arabidopsis_thaliana - ps32
Phylostratum

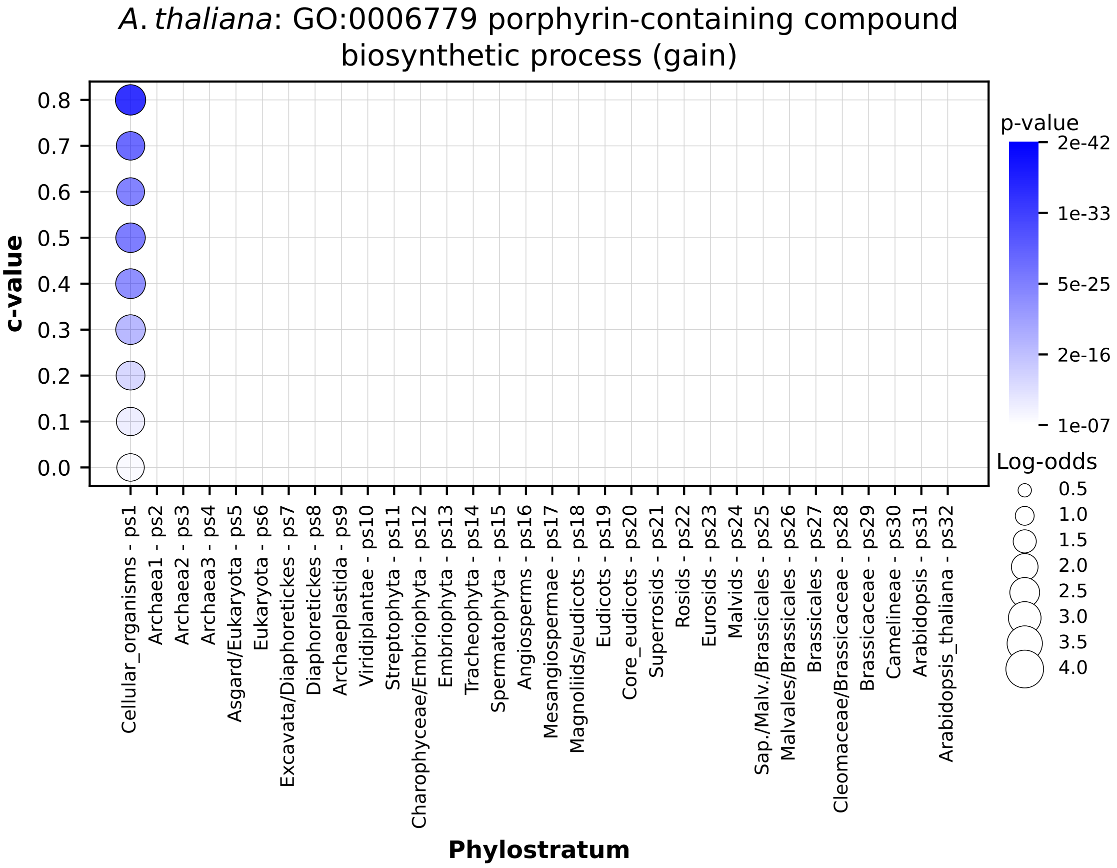
A. thaliana: GO:0006779 porphyrin-containing compound biosynthetic process (gain)
c-value
0.8
0.7
0.6
0.5
0.4
0.3
0.2
0.1
0.0
p-value
2e-42
1e-33
5e-25
2e-16
1e-07
Log-odds
0.5
1.0
1.5
2.0
2.5
3.0
3.5
4.0
Cellular_organisms - ps1
Archaea1 - ps2
Archaea2 - ps3
Archaea3 - ps4
Asgard/Eukaryota - ps5
Eukaryota - ps6
Excavata/Diaphoretickes - ps7
Diaphoretickes - ps8
Archaeplastida - ps9
Viridiplantae - ps10
Streptophyta - ps11
Charophyceae/Embriophyta - ps12
Embriophyta - ps13
Tracheophyta - ps14
Spermatophyta - ps15
Angiosperms - ps16
Mesangiospermae - ps17
Magnoliids/eudicots - ps18
Eudicots - ps19
Core_eudicots - ps20
Superrosids - ps21
Rosids - ps22
Eurosids - ps23
Malvids - ps24
Sap./Malv./Brassicales - ps25
Malvales/Brassicales - ps26
Brassicales - ps27
Cleomaceae/Brassicaceae - ps28
Brassicaceae - ps29
Camelineae - ps30
Arabidopsis - ps31
Arabidopsis_thaliana - ps32
Phylostratum

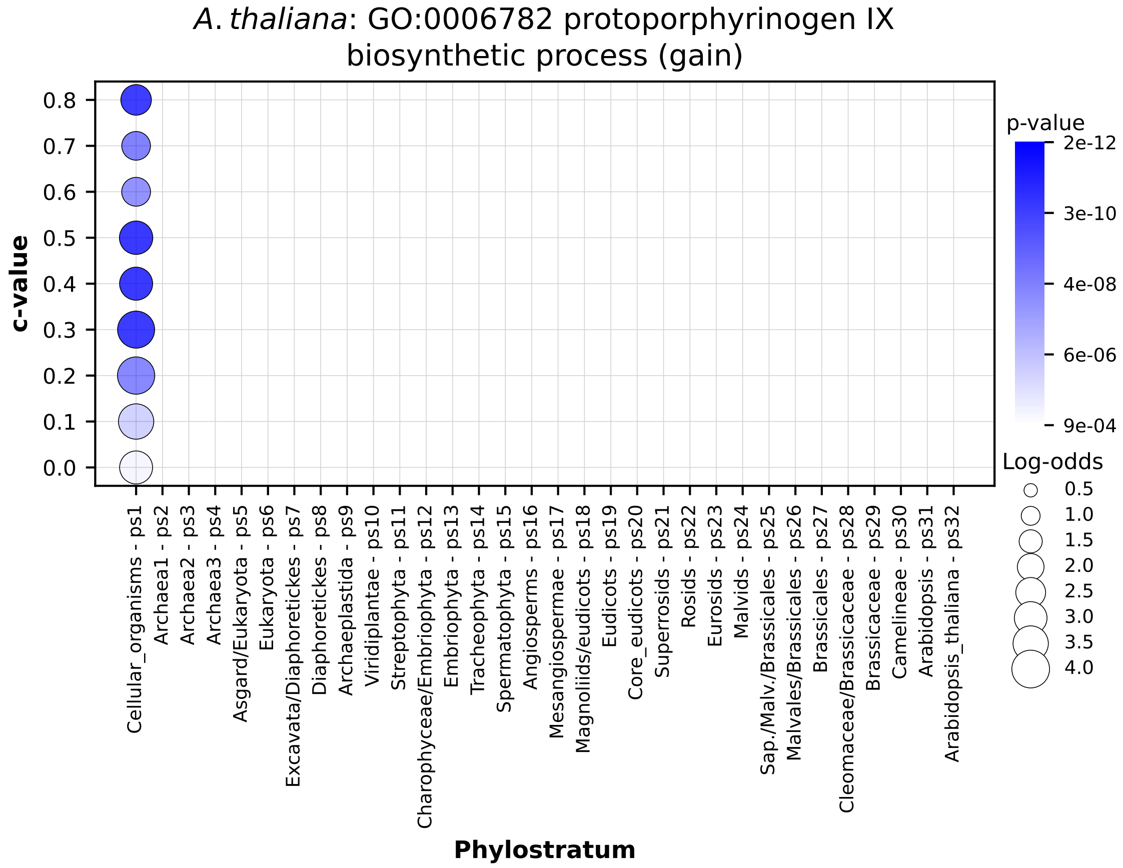
A. thaliana: GO:0006782 protoporphyrinogen IX biosynthetic process (gain)
c-value
0.8
0.7
0.6
0.5
0.4
0.3
0.2
0.1
0.0
p-value
2e-12
3e-10
4e-08
6e-06
9e-04
Log-odds
0.5
1.0
1.5
2.0
2.5
3.0
3.5
4.0
Cellular_organisms - ps1
Archaea1 - ps2
Archaea2 - ps3
Archaea3 - ps4
Asgard/Eukaryota - ps5
Eukaryota - ps6
Excavata/Diaphoretickes - ps7
Diaphoretickes - ps8
Archaeplastida - ps9
Viridiplantae - ps10
Streptophyta - ps11
Charophyceae/Embriophyta - ps12
Embriophyta - ps13
Tracheophyta - ps14
Spermatophyta - ps15
Angiosperms - ps16
Mesangiospermae - ps17
Magnoliids/eudicots - ps18
Eudicots - ps19
Core_eudicots - ps20
Superrosids - ps21
Rosids - ps22
Eurosids - ps23
Malvids - ps24
Sap./Malv./Brassicales - ps25
Malvales/Brassicales - ps26
Brassicales - ps27
Cleomaceae/Brassicaceae - ps28
Brassicaceae - ps29
Camelineae - ps30
Arabidopsis - ps31
Arabidopsis_thaliana - ps32
Phylostratum

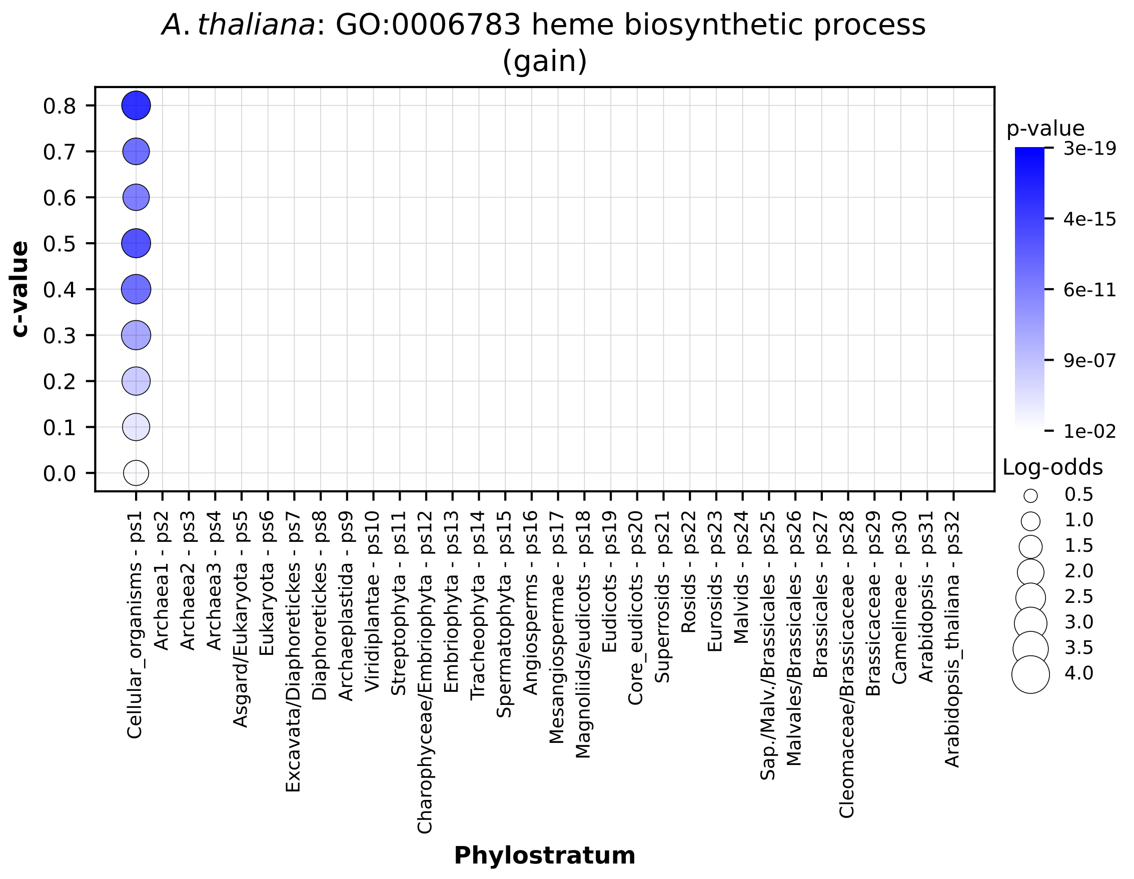

A. thaliana: GO:0006783 heme biosynthetic process (gain)
c-value
0.8
0.7
0.6
0.5
0.4
0.3
0.2
0.1
0.0
p-value
3e-19
4e-15
6e-11
9e-07
1e-02
Log-odds
0.5
1.0
1.5
2.0
2.5
3.0
3.5
4.0
Cellular_organisms - ps1
Archaea1 - ps2
Archaea2 - ps3
Archaea3 - ps4
Asgard/Eukaryota - ps5
Eukaryota - ps6
Excavata/Diaphoretickes - ps7
Diaphoretickes - ps8
Archaeplastida - ps9
Viridiplantae - ps10
Streptophyta - ps11
Charophyceae/Embriophyta - ps12
Embriophyta - ps13
Tracheophyta - ps14
Spermatophyta - ps15
Angiosperms - ps16
Mesangiospermae - ps17
Magnoliids/eudicots - ps18
Eudicots - ps19
Core_eudicots - ps20
Superrosids - ps21
Rosids - ps22
Eurosids - ps23
Malvids - ps24
Sap./Malv./Brassicales - ps25
Malvales/Brassicales - ps26
Brassicales - ps27
Cleomaceae/Brassicaceae - ps28
Brassicaceae - ps29
Camelineae - ps30
Arabidopsis - ps31
Arabidopsis_thaliana - ps32
Phylostratum

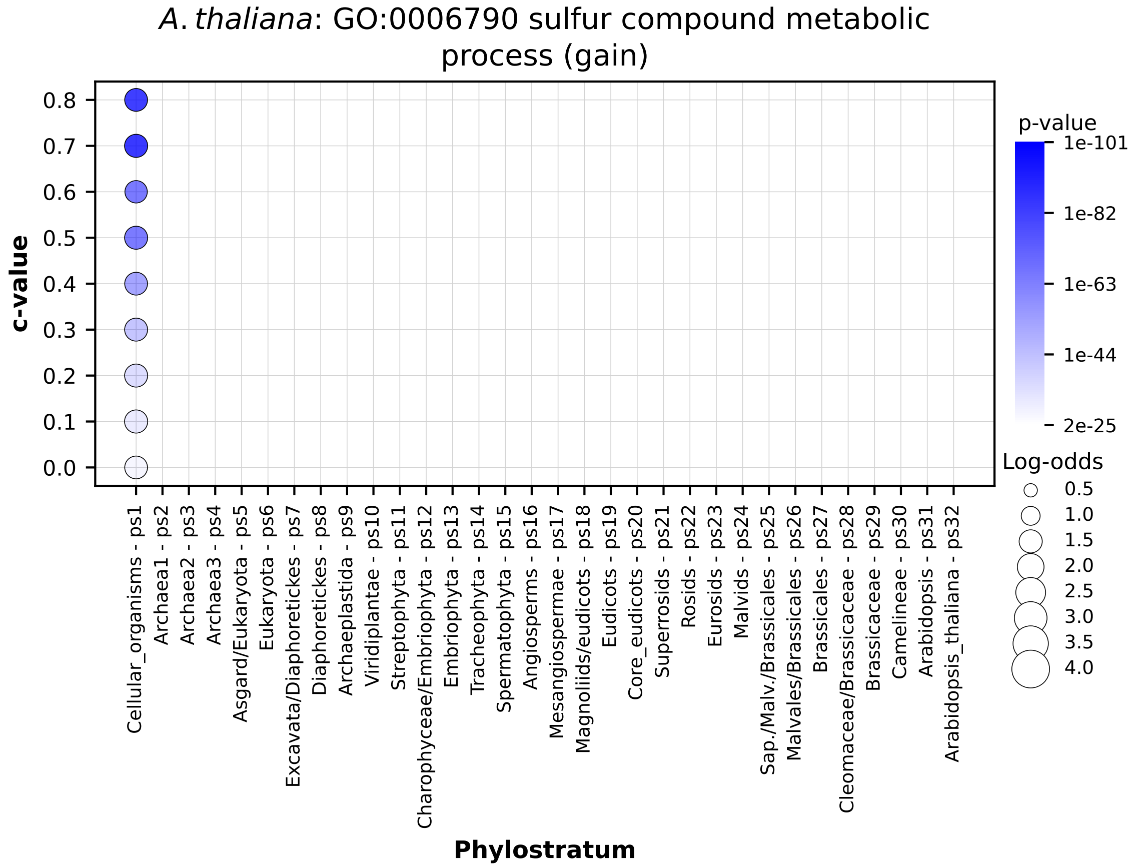

A. thaliana: GO:0006790 sulfur compound metabolic process (gain)
c-value
0.0
0.1
0.2
0.3
0.4
0.5
0.6
0.7
0.8
p-value
1e-101
1e-82
1e-63
1e-44
2e-25
Log-odds
0.5
1.0
1.5
2.0
2.5
3.0
3.5
4.0
Cellular_organisms - ps1
Archaea1 - ps2
Archaea2 - ps3
Archaea3 - ps4
Asgard/Eukaryota - ps5
Eukaryota - ps6
Excavata/Diaphoretickes - ps7
Diaphoretickes - ps8
Archaeplastida - ps9
Viridiplantae - ps10
Streptophyta - ps11
Charophyceae/Embriophyta - ps12
Embriophyta - ps13
Tracheophyta - ps14
Spermatophyta - ps15
Angiosperms - ps16
Mesangiospermae - ps17
Magnoliids/eudicots - ps18
Eudicots - ps19
Core_eudicots - ps20
Superrosids - ps21
Rosids - ps22
Eurosids - ps23
Malvids - ps24
Sap./Malv./Brassicales - ps25
Malvales/Brassicales - ps26
Brassicales - ps27
Cleomaceae/Brassicaceae - ps28
Brassicaceae - ps29
Camelineae - ps30
Arabidopsis - ps31
Arabidopsis_thaliana - ps32
Phylostratum

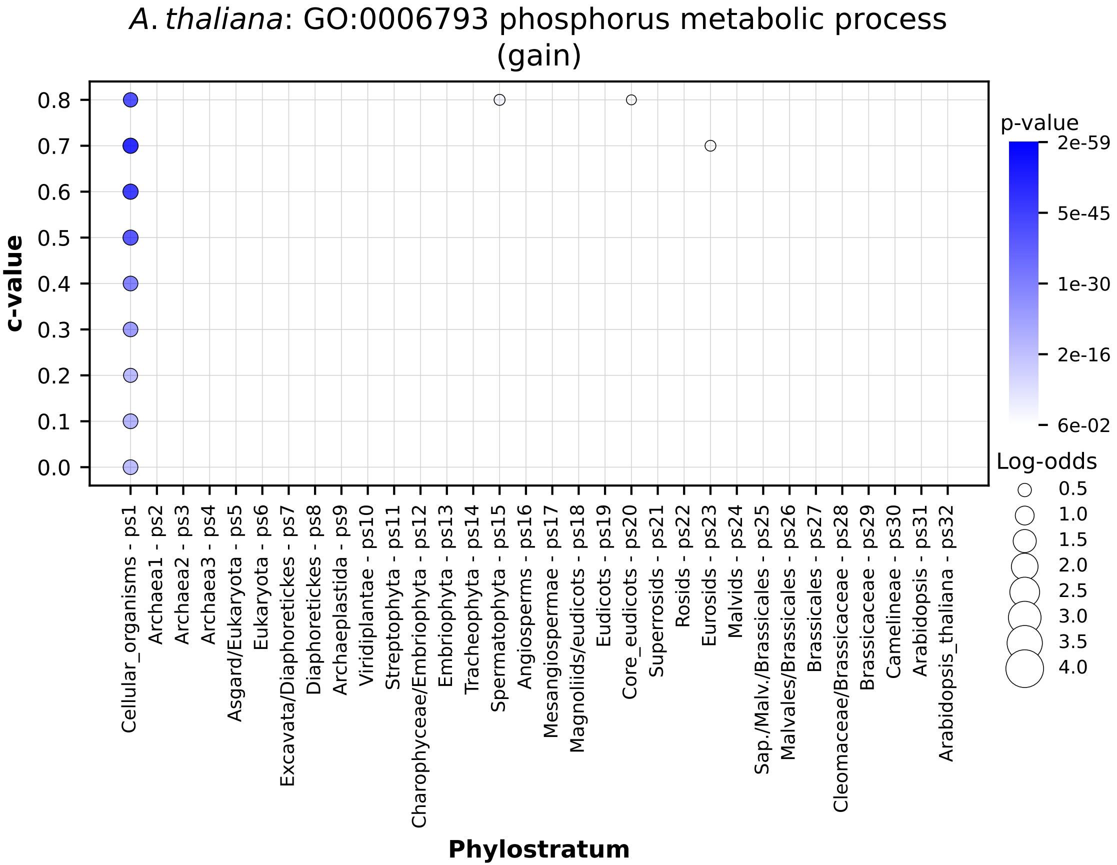

A. thaliana: GO:0006793 phosphorus metabolic process (gain)
c-value
0.8
0.7
0.6
0.5
0.4
0.3
0.2
0.1
0.0
p-value
2e-59
5e-45
1e-30
2e-16
6e-02
Log-odds
0.5
1.0
1.5
2.0
2.5
3.0
3.5
4.0
Cellular_organisms - ps1
Archaea1 - ps2
Archaea2 - ps3
Archaea3 - ps4
Asgard/Eukaryota - ps5
Eukaryota - ps6
Excavata/Diaphoretickes - ps7
Diaphoretickes - ps8
Archaeplastida - ps9
Viridiplantae - ps10
Streptophyta - ps11
Charophyceae/Embriophyta - ps12
Embriophyta - ps13
Tracheophyta - ps14
Spermatophyta - ps15
Angiosperms - ps16
Mesangiospermae - ps17
Magnoliids/eudicots - ps18
Eudicots - ps19
Core_eudicots - ps20
Superrosids - ps21
Rosids - ps22
Eurosids - ps23
Malvids - ps24
Sap./Malv./Brassicales - ps25
Malvales/Brassicales - ps26
Brassicales - ps27
Cleomaceae/Brassicaceae - ps28
Brassicaceae - ps29
Camelineae - ps30
Arabidopsis - ps31
Arabidopsis_thaliana - ps32
Phylostratum

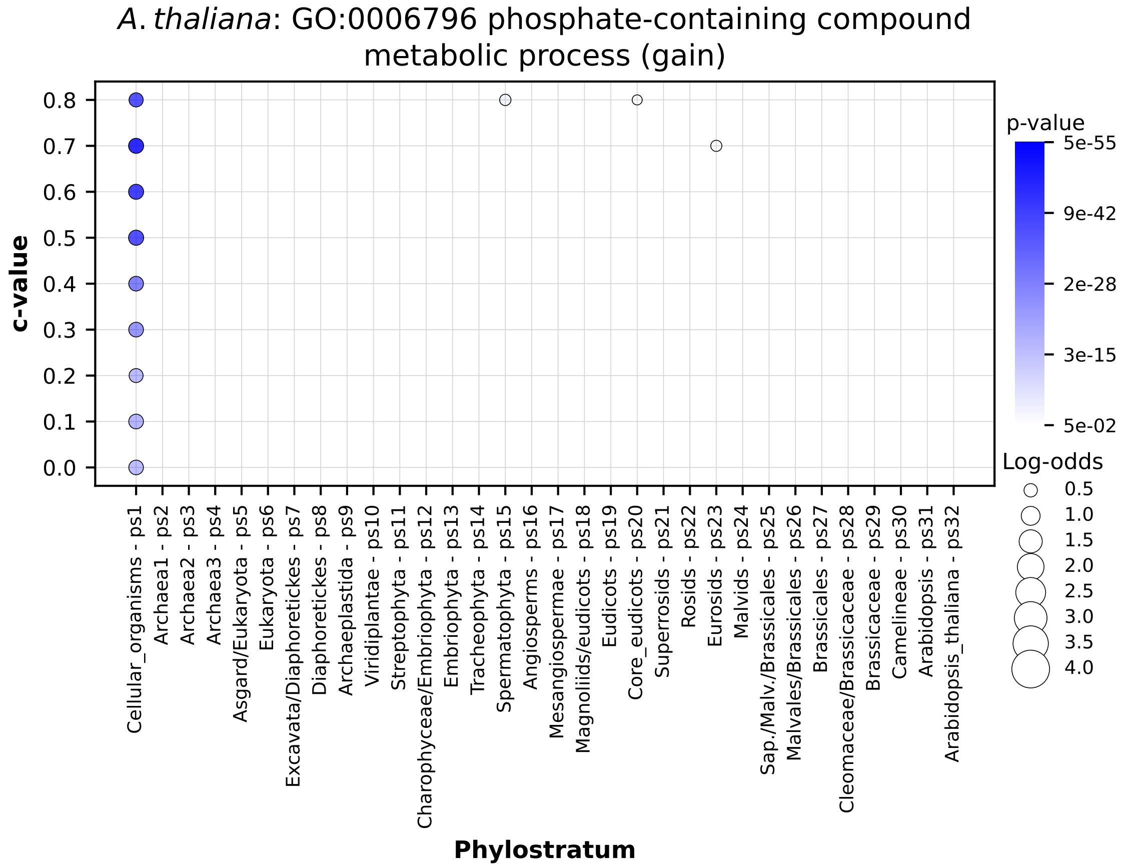

A. thaliana: GO:0006796 phosphate-containing compound metabolic process (gain)
c-value
0.0
0.1
0.2
0.3
0.4
0.5
0.6
0.7
0.8
Phylostratum
Cellular_organisms - ps1
Archaea1 - ps2
Archaea2 - ps3
Archaea3 - ps4
Asgard/Eukaryota - ps5
Eukaryota - ps6
Excavata/Diaphoretickes - ps7
Diaphoretickes - ps8
Archaeplastida - ps9
Viridiplantae - ps10
Streptophyta - ps11
Charophyceae/Embriophyta - ps12
Embriophyta - ps13
Tracheophyta - ps14
Spermatophyta - ps15
Angiosperms - ps16
Mesangiospermae - ps17
Magnoliids/eudicots - ps18
Eudicots - ps19
Core_eudicots - ps20
Superrosids - ps21
Rosids - ps22
Eurosids - ps23
Malvids - ps24
Sap./Malv./Brassicales - ps25
Malvales/Brassicales - ps26
Brassicales - ps27
Cleomaceae/Brassicaceae - ps28
Brassicaceae - ps29
Camelineae - ps30
Arabidopsis - ps31
Arabidopsis_thaliana - ps32
p-value
5e-55
9e-42
2e-28
3e-15
5e-02
Log-odds
0.5
1.0
1.5
2.0
2.5
3.0
3.5
4.0

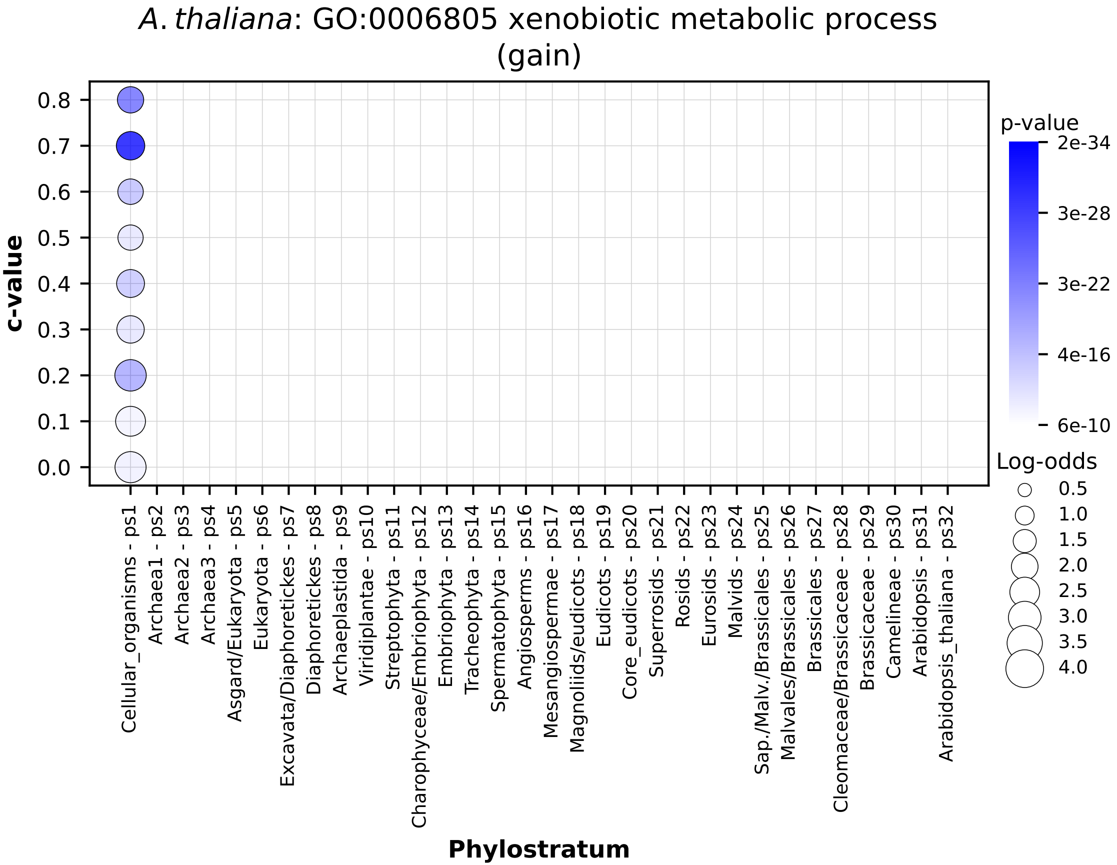
A. thaliana: GO:0006805 xenobiotic metabolic process
(gain)
c-value
0.8
0.7
0.6
0.5
0.4
0.3
0.2
0.1
0.0
p-value
2e-34
3e-28
3e-22
4e-16
6e-10
Log-odds
0.5
1.0
1.5
2.0
2.5
3.0
3.5
4.0
Cellular_organisms - ps1
Archaea1 - ps2
Archaea2 - ps3
Archaea3 - ps4
Asgard/Eukaryota - ps5
Eukaryota - ps6
Excavata/Diaphoretickes - ps7
Diaphoretickes - ps8
Archaeplastida - ps9
Viridiplantae - ps10
Streptophyta - ps11
Charophyceae/Embriophyta - ps12
Embriophyta - ps13
Tracheophyta - ps14
Spermatophyta - ps15
Angiosperms - ps16
Mesangiospermae - ps17
Magnoliids/eudicots - ps18
Eudicots - ps19
Core_eudicots - ps20
Superrosids - ps21
Rosids - ps22
Eurosids - ps23
Malvids - ps24
Sap./Malv./Brassicales - ps25
Malvales/Brassicales - ps26
Brassicales - ps27
Cleomaceae/Brassicaceae - ps28
Brassicaceae - ps29
Camelineae - ps30
Arabidopsis - ps31
Arabidopsis_thaliana - ps32
Phylostratum

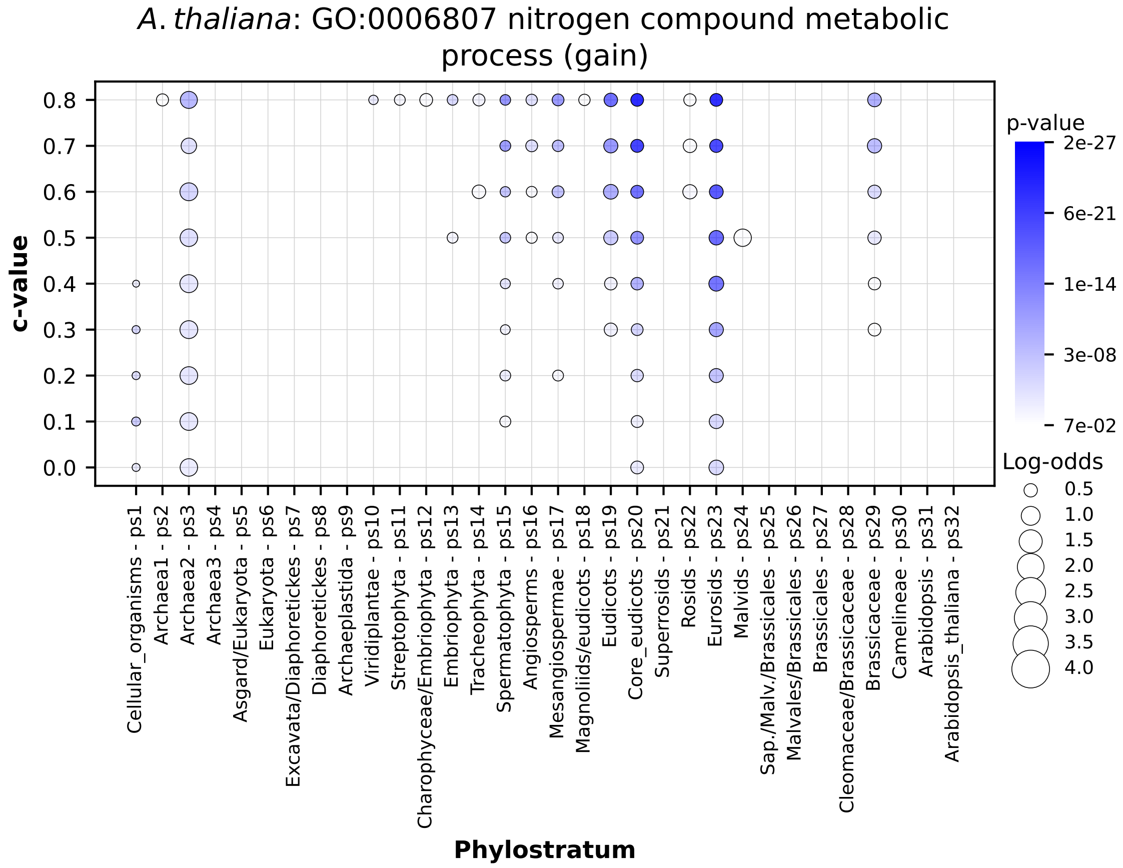

A. thaliana: GO:0006807 nitrogen compound metabolic process (gain)
c-value
0.8
0.7
0.6
0.5
0.4
0.3
0.2
0.1
0.0
p-value
2e-27
6e-21
1e-14
3e-08
7e-02
Log-odds
0.5
1.0
1.5
2.0
2.5
3.0
3.5
4.0
Cellular_organisms - ps1
Archaea1 - ps2
Archaea2 - ps3
Archaea3 - ps4
Asgard/Eukaryota - ps5
Eukaryota - ps6
Excavata/Diaphoretickes - ps7
Diaphoretickes - ps8
Archaeplastida - ps9
Viridiplantae - ps10
Streptophyta - ps11
Charophyceae/Embriophyta - ps12
Embriophyta - ps13
Tracheophyta - ps14
Spermatophyta - ps15
Angiosperms - ps16
Mesangiospermae - ps17
Magnoliids/eudicots - ps18
Eudicots - ps19
Core_eudicots - ps20
Superrosids - ps21
Rosids - ps22
Eurosids - ps23
Malvids - ps24
Sap./Malv./Brassicales - ps25
Malvales/Brassicales - ps26
Brassicales - ps27
Cleomaceae/Brassicaceae - ps28
Brassicaceae - ps29
Camelineae - ps30
Arabidopsis - ps31
Arabidopsis_thaliana - ps32
Phylostratum

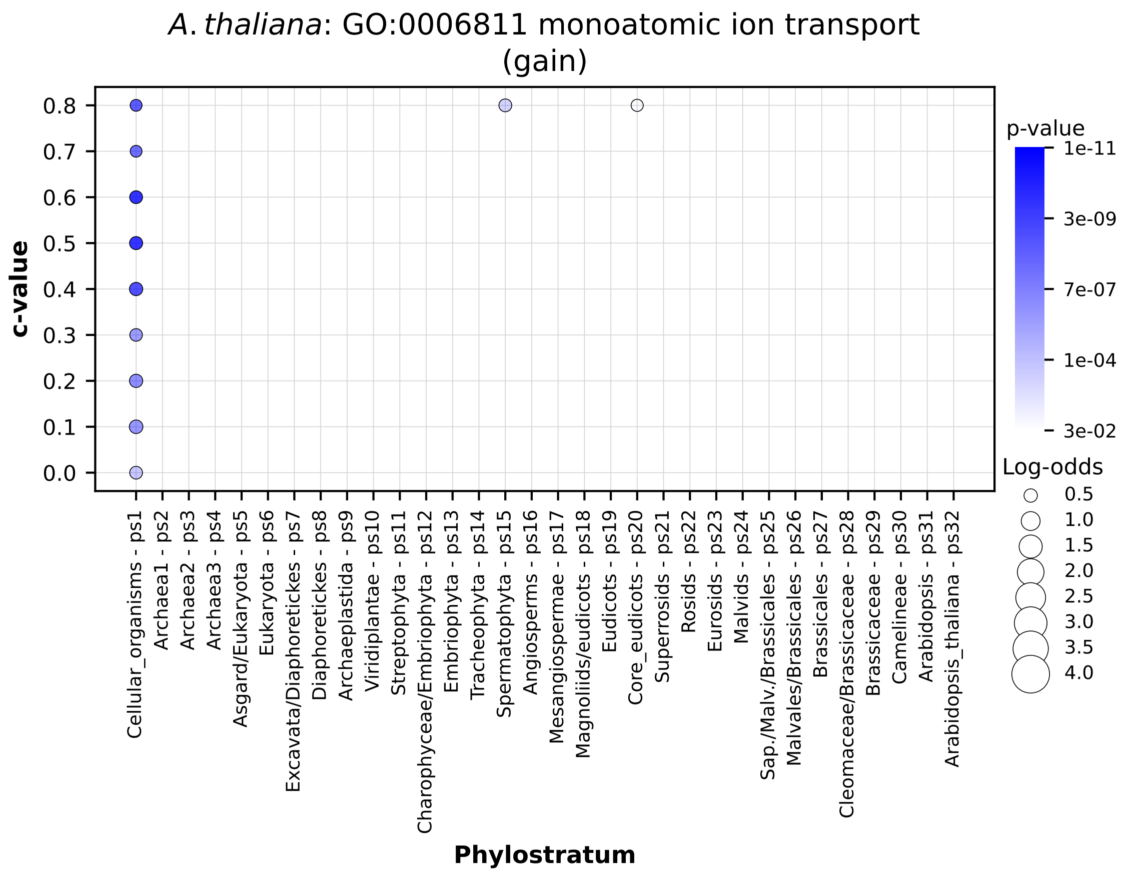

A. thaliana: GO:0006811 monoatomic ion transport
(gain)
c-value
0.8
0.7
0.6
0.5
0.4
0.3
0.2
0.1
0.0
p-value
1e-11
3e-09
7e-07
1e-04
3e-02
Log-odds
0.5
1.0
1.5
2.0
2.5
3.0
3.5
4.0
Cellular_organisms - ps1
Archaea1 - ps2
Archaea2 - ps3
Archaea3 - ps4
Asgard/Eukaryota - ps5
Eukaryota - ps6
Excavata/Diaphoretickes - ps7
Diaphoretickes - ps8
Archaeplastida - ps9
Viridiplantae - ps10
Streptophyta - ps11
Charophyceae/Embriophyta - ps12
Embriophyta - ps13
Tracheophyta - ps14
Spermatophyta - ps15
Angiosperms - ps16
Mesangiospermae - ps17
Magnoliids/eudicots - ps18
Eudicots - ps19
Core_eudicots - ps20
Superrosids - ps21
Rosids - ps22
Eurosids - ps23
Malvids - ps24
Sap./Malv./Brassicales - ps25
Malvales/Brassicales - ps26
Brassicales - ps27
Cleomaceae/Brassicaceae - ps28
Brassicaceae - ps29
Camelineae - ps30
Arabidopsis - ps31
Arabidopsis_thaliana - ps32
Phylostratum

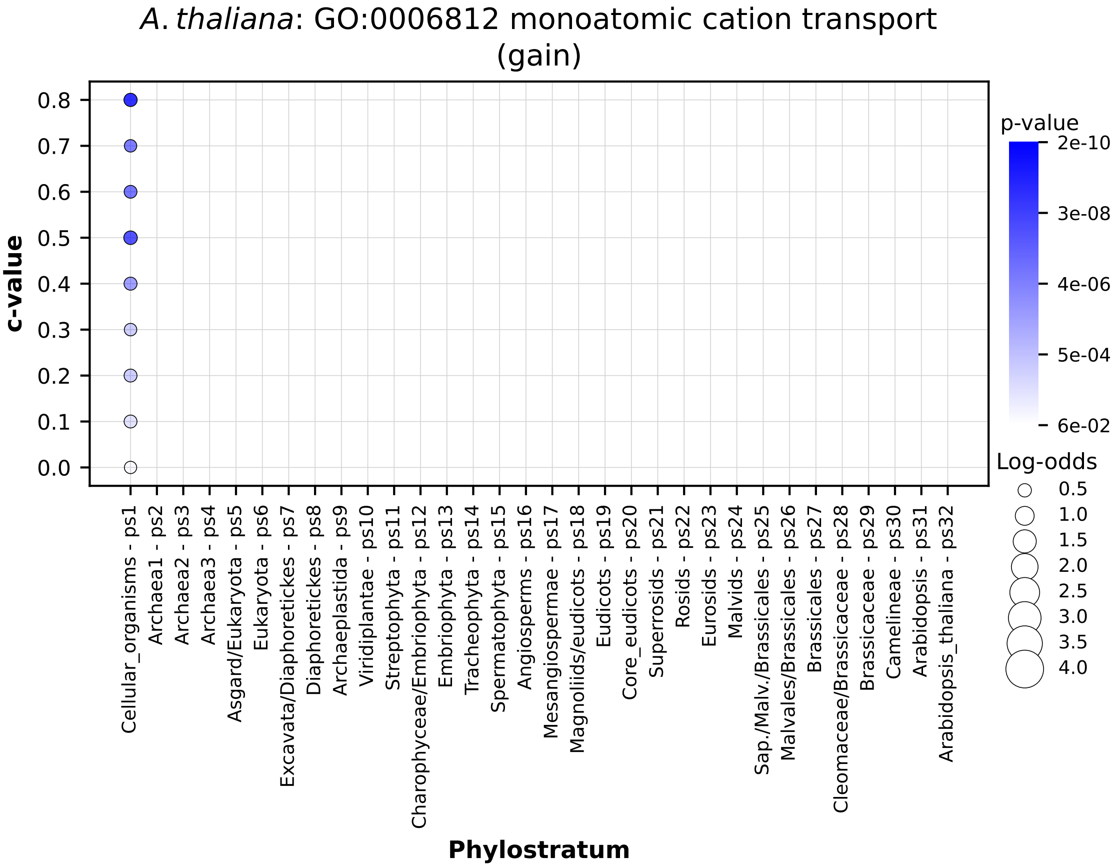

A. thaliana: GO:0006812 monoatomic cation transport
(gain)
c-value
0.8
0.7
0.6
0.5
0.4
0.3
0.2
0.1
0.0
p-value
2e-10
3e-08
4e-06
5e-04
6e-02
Log-odds
0.5
1.0
1.5
2.0
2.5
3.0
3.5
4.0
Cellular_organisms - ps1
Archaea1 - ps2
Archaea2 - ps3
Archaea3 - ps4
Asgard/Eukaryota - ps5
Eukaryota - ps6
Excavata/Diaphoretickes - ps7
Diaphoretickes - ps8
Archaeplastida - ps9
Viridiplantae - ps10
Streptophyta - ps11
Charophyceae/Embriophyta - ps12
Embriophyta - ps13
Tracheophyta - ps14
Spermatophyta - ps15
Angiosperms - ps16
Mesangiospermae - ps17
Magnoliids/eudicots - ps18
Eudicots - ps19
Core_eudicots - ps20
Superrosids - ps21
Rosids - ps22
Eurosids - ps23
Malvids - ps24
Sap./Malv./Brassicales - ps25
Malvales/Brassicales - ps26
Brassicales - ps27
Cleomaceae/Brassicaceae - ps28
Brassicaceae - ps29
Camelineae - ps30
Arabidopsis - ps31
Arabidopsis_thaliana - ps32
Phylostratum

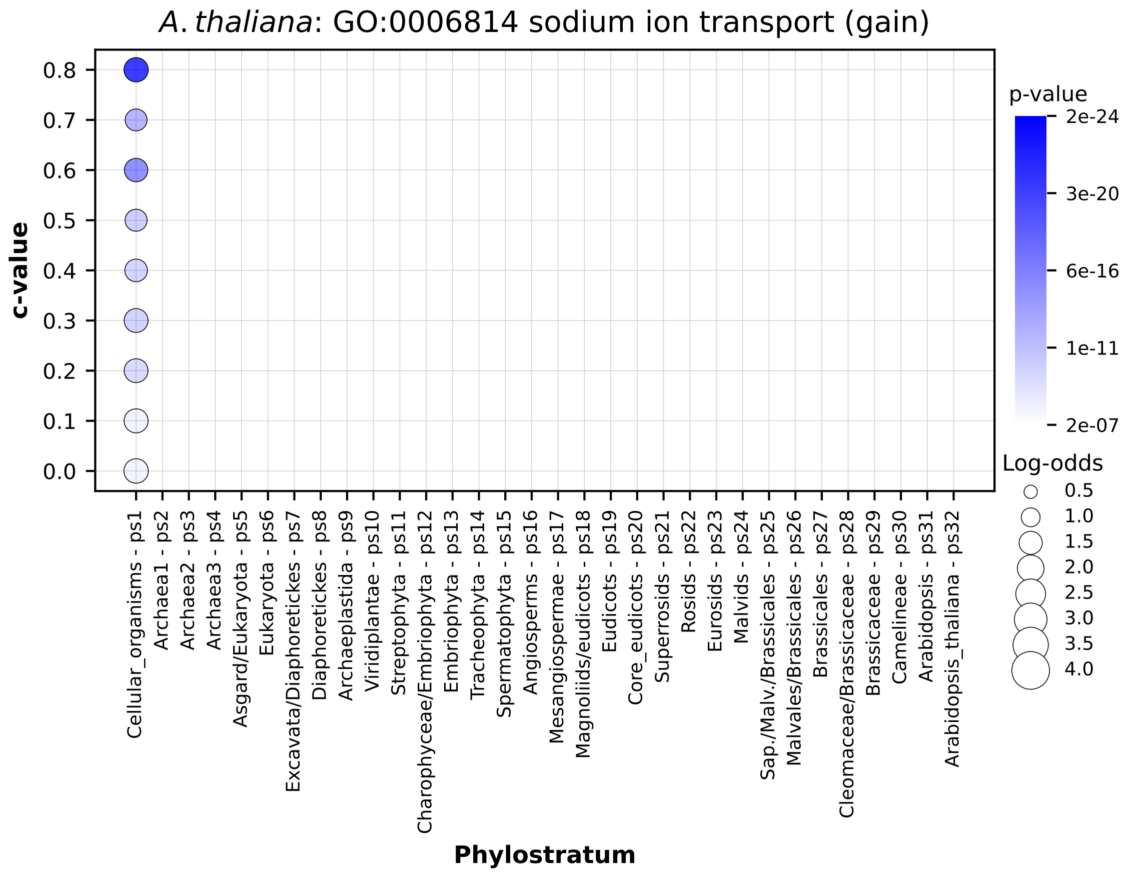

A. thaliana: GO:0006814 sodium ion transport (gain)
c-value
0.0
0.1
0.2
0.3
0.4
0.5
0.6
0.7
0.8
Phylostratum
Cellular_organisms - ps1
Archaea1 - ps2
Archaea2 - ps3
Archaea3 - ps4
Asgard/Eukaryota - ps5
Eukaryota - ps6
Excavata/Diaphoretickes - ps7
Diaphoretickes - ps8
Archaeplastida - ps9
Viridiplantae - ps10
Streptophyta - ps11
Charophyceae/Embriophyta - ps12
Embriophyta - ps13
Tracheophyta - ps14
Spermatophyta - ps15
Angiosperms - ps16
Mesangiospermae - ps17
Magnoliids/eudicots - ps18
Eudicots - ps19
Core_eudicots - ps20
Superrosids - ps21
Rosids - ps22
Eurosids - ps23
Malvids - ps24
Sap./Malv./Brassicales - ps25
Malvales/Brassicales - ps26
Brassicales - ps27
Cleomaceae/Brassicaceae - ps28
Brassicaceae - ps29
Camelineae - ps30
Arabidopsis - ps31
Arabidopsis_thaliana - ps32
p-value
2e-24
3e-20
6e-16
1e-11
2e-07
Log-odds
0.5
1.0
1.5
2.0
2.5
3.0
3.5
4.0

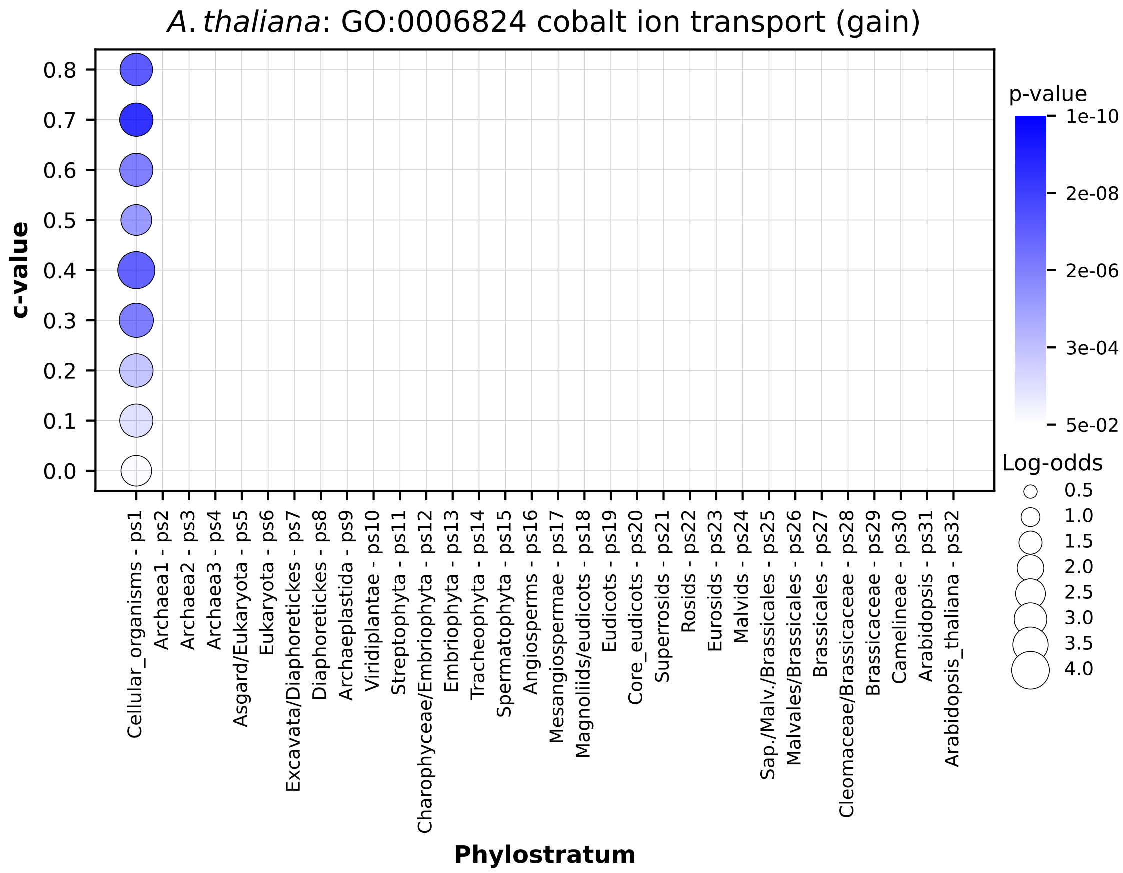

A. thaliana: GO:0006824 cobalt ion transport (gain)
c-value
0.8
0.7
0.6
0.5
0.4
0.3
0.2
0.1
0.0
p-value
1e-10
2e-08
2e-06
3e-04
5e-02
Log-odds
0.5
1.0
1.5
2.0
2.5
3.0
3.5
4.0
Cellular_organisms - ps1
Archaea1 - ps2
Archaea2 - ps3
Archaea3 - ps4
Asgard/Eukaryota - ps5
Eukaryota - ps6
Excavata/Diaphoretickes - ps7
Diaphoretickes - ps8
Archaeplastida - ps9
Viridiplantae - ps10
Streptophyta - ps11
Charophyceae/Embriophyta - ps12
Embriophyta - ps13
Tracheophyta - ps14
Spermatophyta - ps15
Angiosperms - ps16
Mesangiospermae - ps17
Magnoliids/eudicots - ps18
Eudicots - ps19
Core_eudicots - ps20
Superrosids - ps21
Rosids - ps22
Eurosids - ps23
Malvids - ps24
Sap./Malv./Brassicales - ps25
Malvales/Brassicales - ps26
Brassicales - ps27
Cleomaceae/Brassicaceae - ps28
Brassicaceae - ps29
Camelineae - ps30
Arabidopsis - ps31
Arabidopsis_thaliana - ps32
Phylostratum

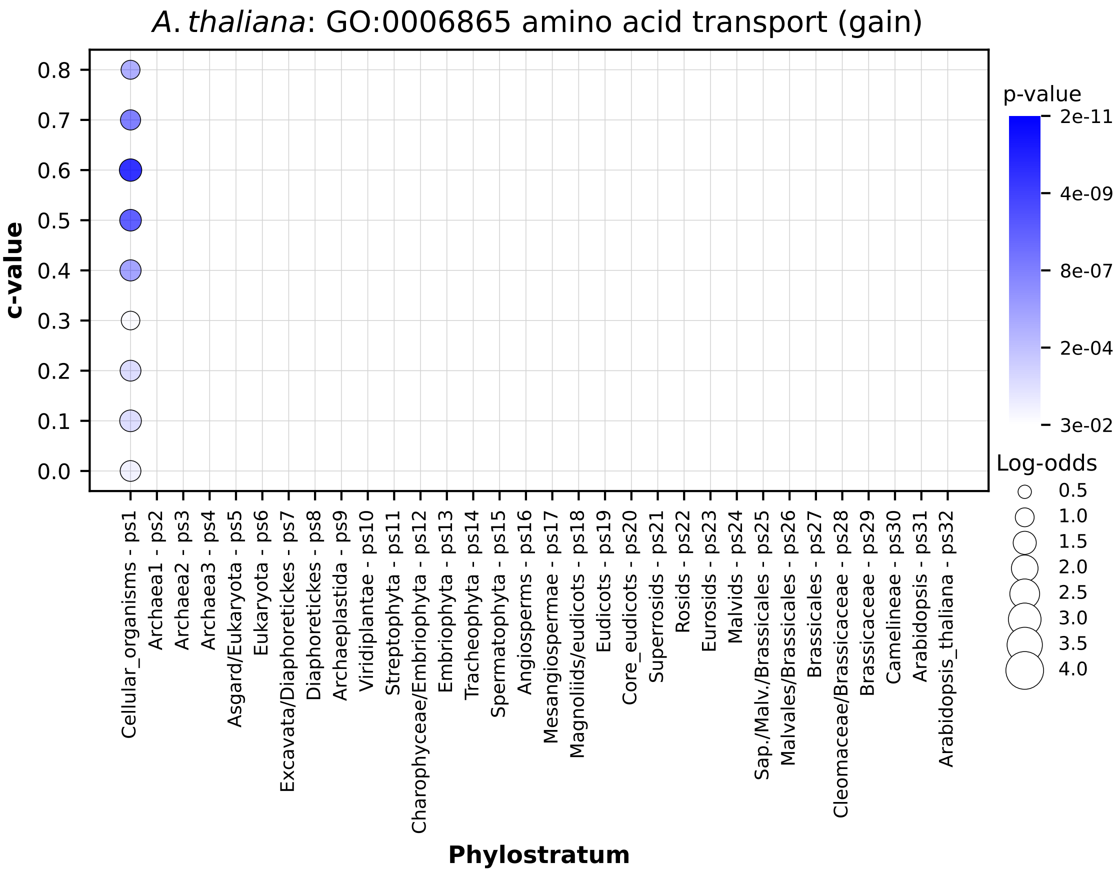
A. thaliana: GO:0006865 amino acid transport (gain)
c-value
0.8
0.7
0.6
0.5
0.4
0.3
0.2
0.1
0.0
Phylostratum
Cellular_organisms - ps1
Archaea1 - ps2
Archaea2 - ps3
Archaea3 - ps4
Asgard/Eukaryota - ps5
Eukaryota - ps6
Excavata/Diaphoretickes - ps7
Diaphoretickes - ps8
Archaeplastida - ps9
Viridiplantae - ps10
Streptophyta - ps11
Charophyceae/Embriophyta - ps12
Embriophyta - ps13
Tracheophyta - ps14
Spermatophyta - ps15
Angiosperms - ps16
Mesangiospermae - ps17
Magnoliids/eudicots - ps18
Eudicots - ps19
Core_eudicots - ps20
Superrosids - ps21
Rosids - ps22
Eurosids - ps23
Malvids - ps24
Sap./Malv./Brassicales - ps25
Malvales/Brassicales - ps26
Brassicales - ps27
Cleomaceae/Brassicaceae - ps28
Brassicaceae - ps29
Camelineae - ps30
Arabidopsis - ps31
Arabidopsis_thaliana - ps32
p-value
2e-11
4e-09
8e-07
2e-04
3e-02
Log-odds
0.5
1.0
1.5
2.0
2.5
3.0
3.5
4.0

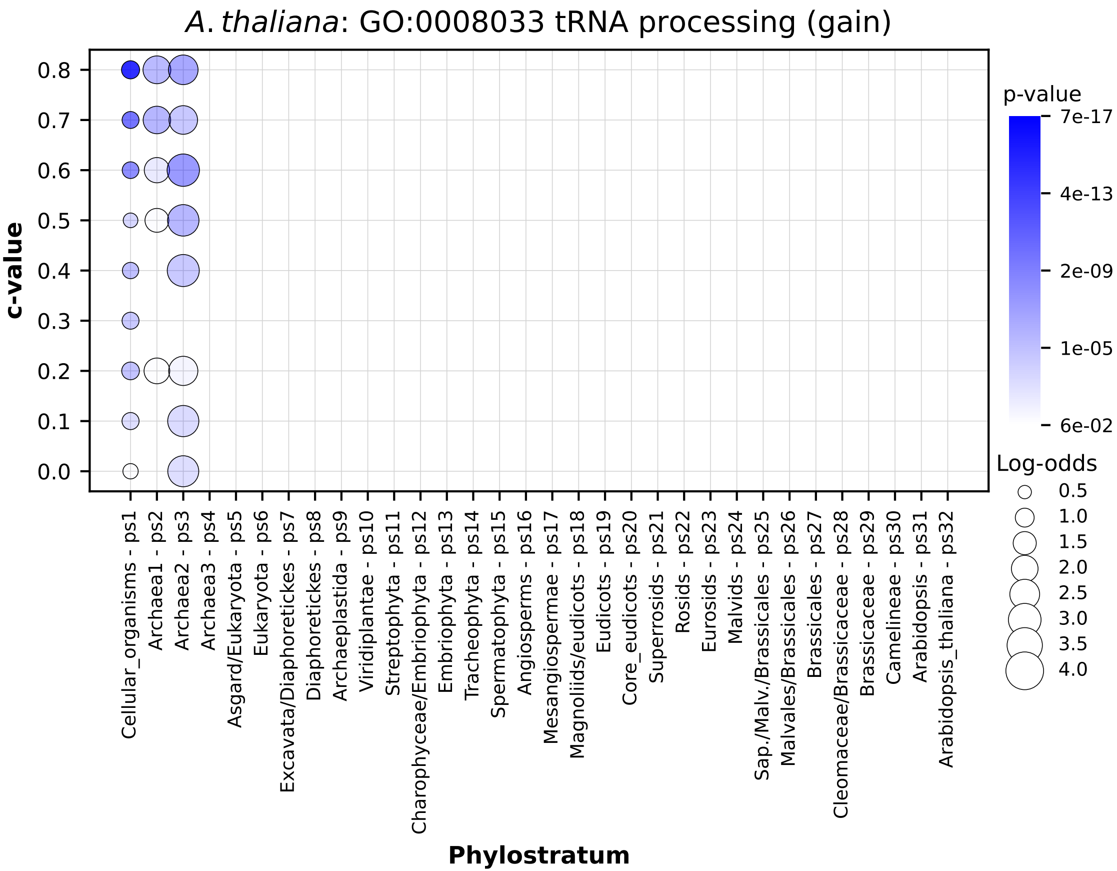

A. thaliana: GO:0008033 tRNA processing (gain)
c-value
0.8
0.7
0.6
0.5
0.4
0.3
0.2
0.1
0.0
p-value
7e-17
4e-13
2e-09
1e-05
6e-02
Log-odds
0.5
1.0
1.5
2.0
2.5
3.0
3.5
4.0
Cellular_organisms - ps1
Archaea1 - ps2
Archaea2 - ps3
Archaea3 - ps4
Asgard/Eukaryota - ps5
Eukaryota - ps6
Excavata/Diaphoretickes - ps7
Diaphoretickes - ps8
Archaeplastida - ps9
Viridiplantae - ps10
Streptophyta - ps11
Charophyceae/Embriophyta - ps12
Embriophyta - ps13
Tracheophyta - ps14
Spermatophyta - ps15
Angiosperms - ps16
Mesangiospermae - ps17
Magnoliids/eudicots - ps18
Eudicots - ps19
Core_eudicots - ps20
Superrosids - ps21
Rosids - ps22
Eurosids - ps23
Malvids - ps24
Sap./Malv./Brassicales - ps25
Malvales/Brassicales - ps26
Brassicales - ps27
Cleomaceae/Brassicaceae - ps28
Brassicaceae - ps29
Camelineae - ps30
Arabidopsis - ps31
Arabidopsis_thaliana - ps32
Phylostratum

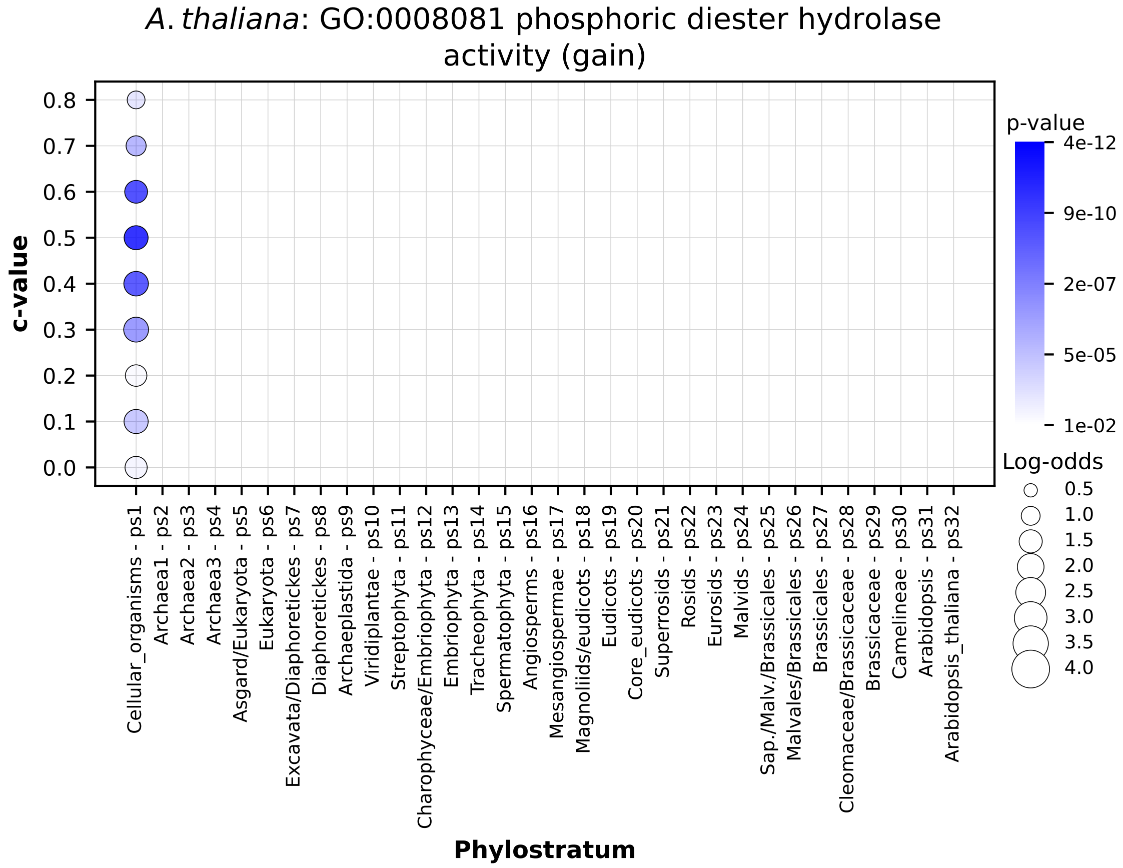

A. thaliana: GO:0008081 phosphoric diester hydrolase activity (gain)
c-value
0.8
0.7
0.6
0.5
0.4
0.3
0.2
0.1
0.0
p-value
4e-12
9e-10
2e-07
5e-05
1e-02
Log-odds
0.5
1.0
1.5
2.0
2.5
3.0
3.5
4.0
Cellular_organisms - ps1
Archaea1 - ps2
Archaea2 - ps3
Archaea3 - ps4
Asgard/Eukaryota - ps5
Eukaryota - ps6
Excavata/Diaphoretickes - ps7
Diaphoretickes - ps8
Archaeplastida - ps9
Viridiplantae - ps10
Streptophyta - ps11
Charophyceae/Embriophyta - ps12
Embriophyta - ps13
Tracheophyta - ps14
Spermatophyta - ps15
Angiosperms - ps16
Mesangiospermae - ps17
Magnoliids/eudicots - ps18
Eudicots - ps19
Core_eudicots - ps20
Superrosids - ps21
Rosids - ps22
Eurosids - ps23
Malvids - ps24
Sap./Malv./Brassicales - ps25
Malvales/Brassicales - ps26
Brassicales - ps27
Cleomaceae/Brassicaceae - ps28
Brassicaceae - ps29
Camelineae - ps30
Arabidopsis - ps31
Arabidopsis_thaliana - ps32
Phylostratum

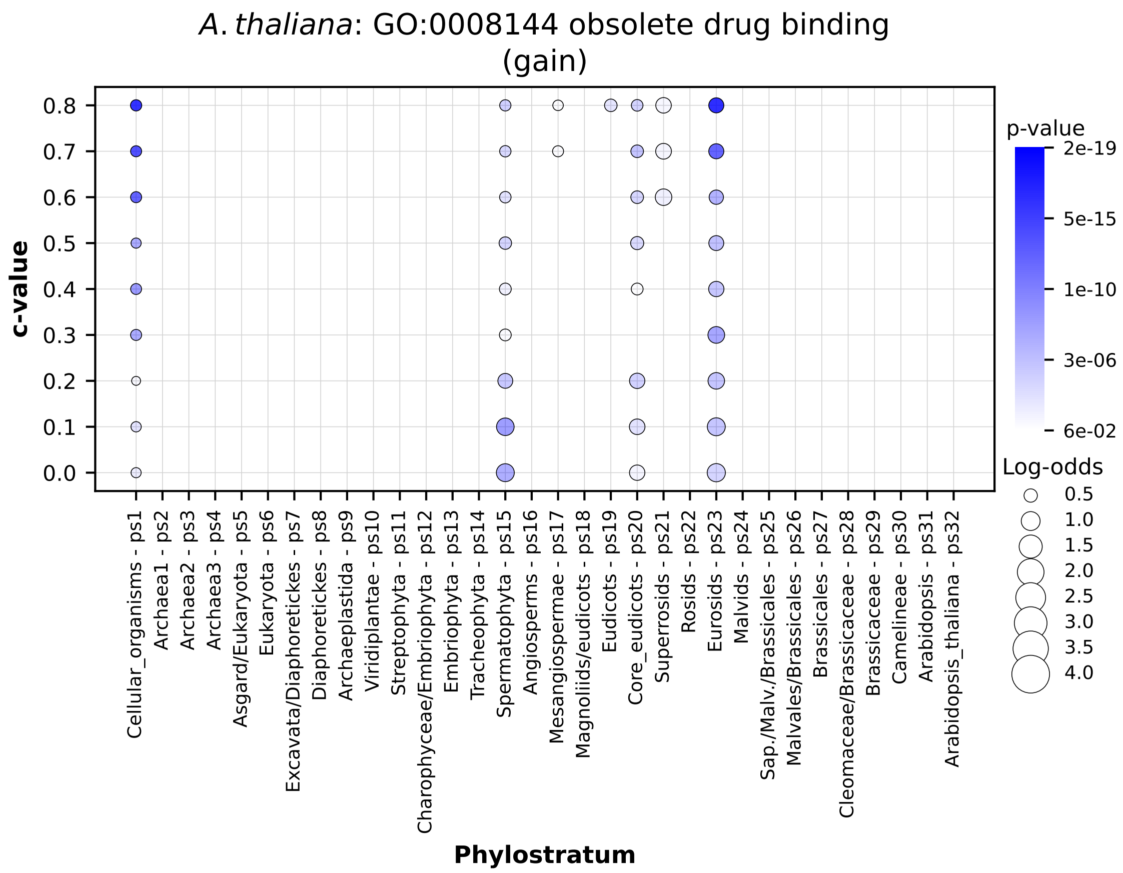
A. thaliana: GO:0008144 obsolete drug binding
(gain)
c-value
0.8
0.7
0.6
0.5
0.4
0.3
0.2
0.1
0.0
Phylostratum
Cellular_organisms - ps1
Archaea1 - ps2
Archaea2 - ps3
Archaea3 - ps4
Asgard/Eukaryota - ps5
Eukaryota - ps6
Excavata/Diaphoretickes - ps7
Diaphoretickes - ps8
Archaeplastida - ps9
Viridiplantae - ps10
Streptophyta - ps11
Charophyceae/Embriophyta - ps12
Embriophyta - ps13
Tracheophyta - ps14
Spermatophyta - ps15
Angiosperms - ps16
Mesangiospermae - ps17
Magnoliids/eudicots - ps18
Eudicots - ps19
Core_eudicots - ps20
Superrosids - ps21
Rosids - ps22
Eurosids - ps23
Malvids - ps24
Sap./Malv./Brassicales - ps25
Malvales/Brassicales - ps26
Brassicales - ps27
Cleomaceae/Brassicaceae - ps28
Brassicaceae - ps29
Camelineae - ps30
Arabidopsis - ps31
Arabidopsis_thaliana - ps32
p-value
2e-19
5e-15
1e-10
3e-06
6e-02
Log-odds
0.5
1.0
1.5
2.0
2.5
3.0
3.5
4.0

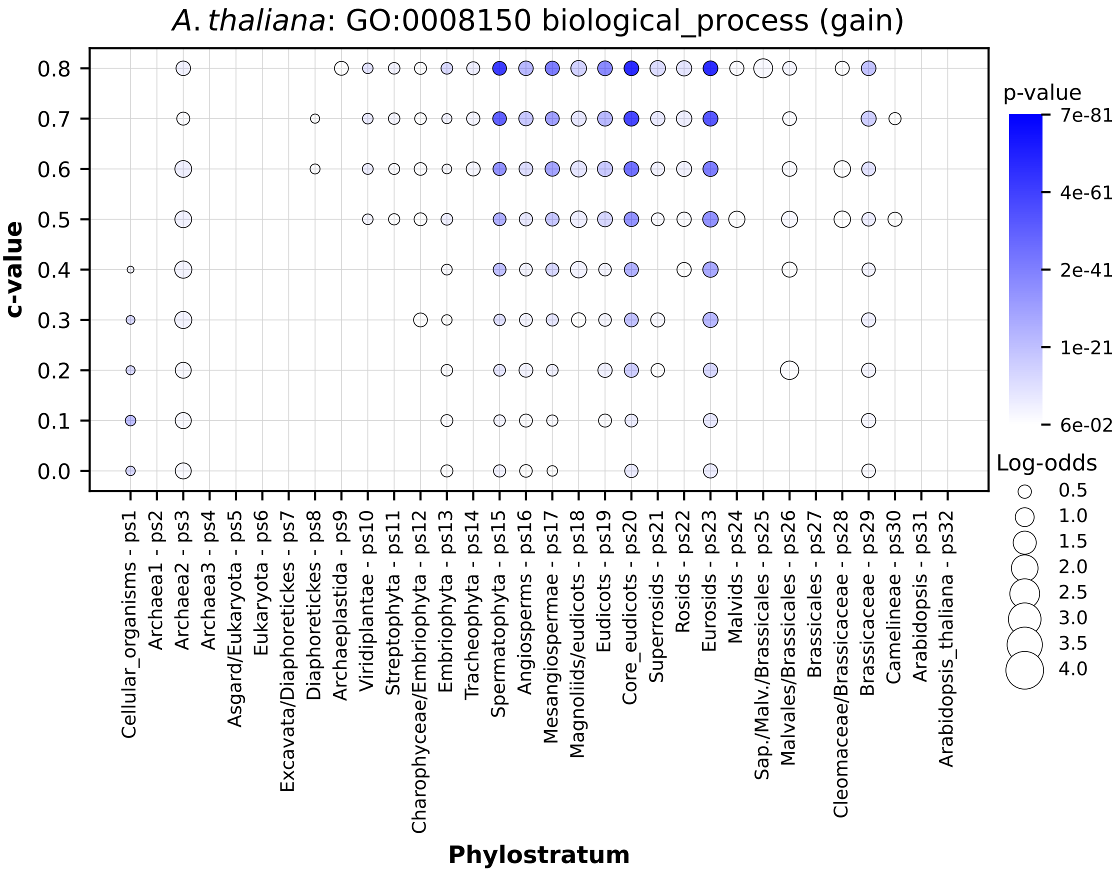
A. thaliana: GO:0008150 biological_process (gain)
c-value
0.8
0.7
0.6
0.5
0.4
0.3
0.2
0.1
0.0
Phylostratum
Cellular_organisms - ps1
Archaea1 - ps2
Archaea2 - ps3
Archaea3 - ps4
Asgard/Eukaryota - ps5
Eukaryota - ps6
Excavata/Diaphoretickes - ps7
Diaphoretickes - ps8
Archaeplastida - ps9
Viridiplantae - ps10
Streptophyta - ps11
Charophyceae/Embriophyta - ps12
Embriophyta - ps13
Tracheophyta - ps14
Spermatophyta - ps15
Angiosperms - ps16
Mesangiospermae - ps17
Magnoliids/eudicots - ps18
Eudicots - ps19
Core_eudicots - ps20
Superrosids - ps21
Rosids - ps22
Eurosids - ps23
Malvids - ps24
Sap./Malv./Brassicales - ps25
Malvales/Brassicales - ps26
Brassicales - ps27
Cleomaceae/Brassicaceae - ps28
Brassicaceae - ps29
Camelineae - ps30
Arabidopsis - ps31
Arabidopsis_thaliana - ps32
p-value
7e-81
4e-61
2e-41
1e-21
6e-02
Log-odds
0.5
1.0
1.5
2.0
2.5
3.0
3.5
4.0

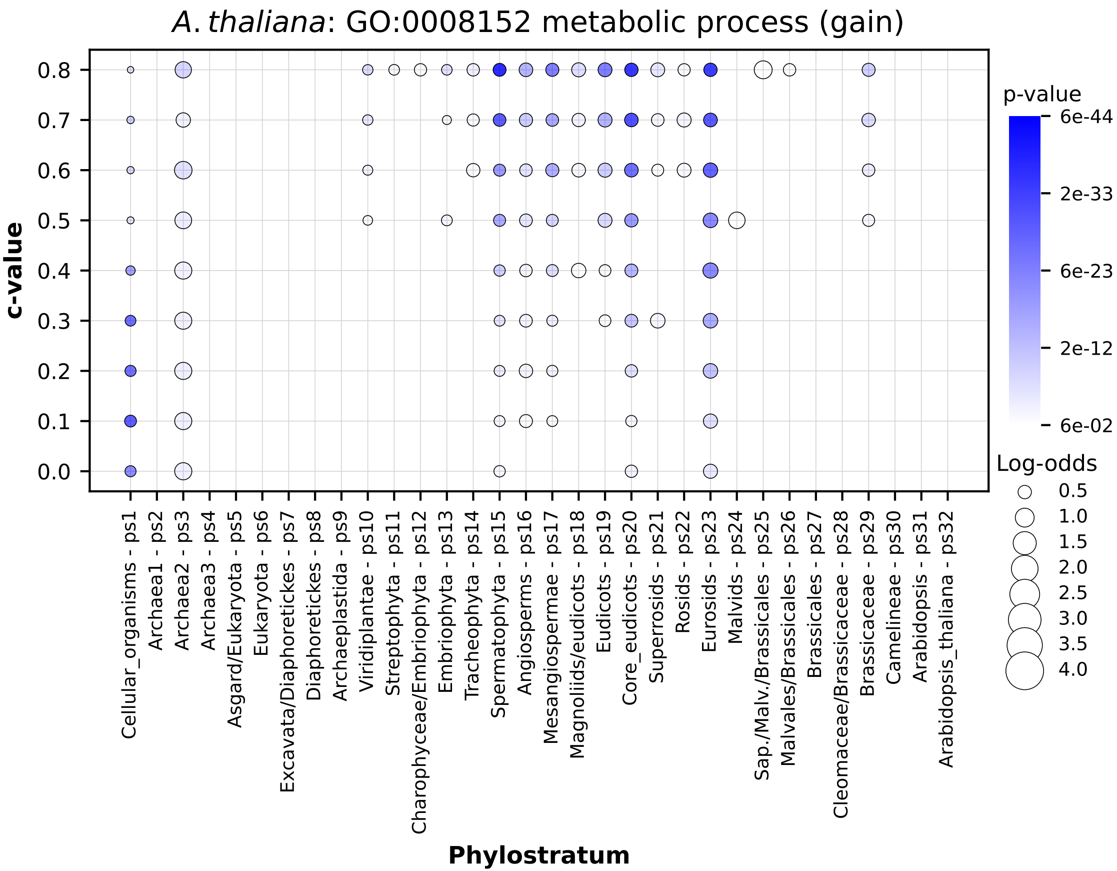

A. thaliana: GO:0008152 metabolic process (gain)
c-value
0.8
0.7
0.6
0.5
0.4
0.3
0.2
0.1
0.0
Phylostratum
Cellular_organisms - ps1
Archaea1 - ps2
Archaea2 - ps3
Archaea3 - ps4
Asgard/Eukaryota - ps5
Eukaryota - ps6
Excavata/Diaphoretickes - ps7
Diaphoretickes - ps8
Archaeplastida - ps9
Viridiplantae - ps10
Streptophyta - ps11
Charophyceae/Embriophyta - ps12
Embriophyta - ps13
Tracheophyta - ps14
Spermatophyta - ps15
Angiosperms - ps16
Mesangiospermae - ps17
Magnoliids/eudicots - ps18
Eudicots - ps19
Core_eudicots - ps20
Superrosids - ps21
Rosids - ps22
Eurosids - ps23
Malvids - ps24
Sap./Malv./Brassicales - ps25
Malvales/Brassicales - ps26
Brassicales - ps27
Cleomaceae/Brassicaceae - ps28
Brassicaceae - ps29
Camelineae - ps30
Arabidopsis - ps31
Arabidopsis_thaliana - ps32
p-value
6e-44
2e-33
6e-23
2e-12
6e-02
Log-odds
0.5
1.0
1.5
2.0
2.5
3.0
3.5
4.0

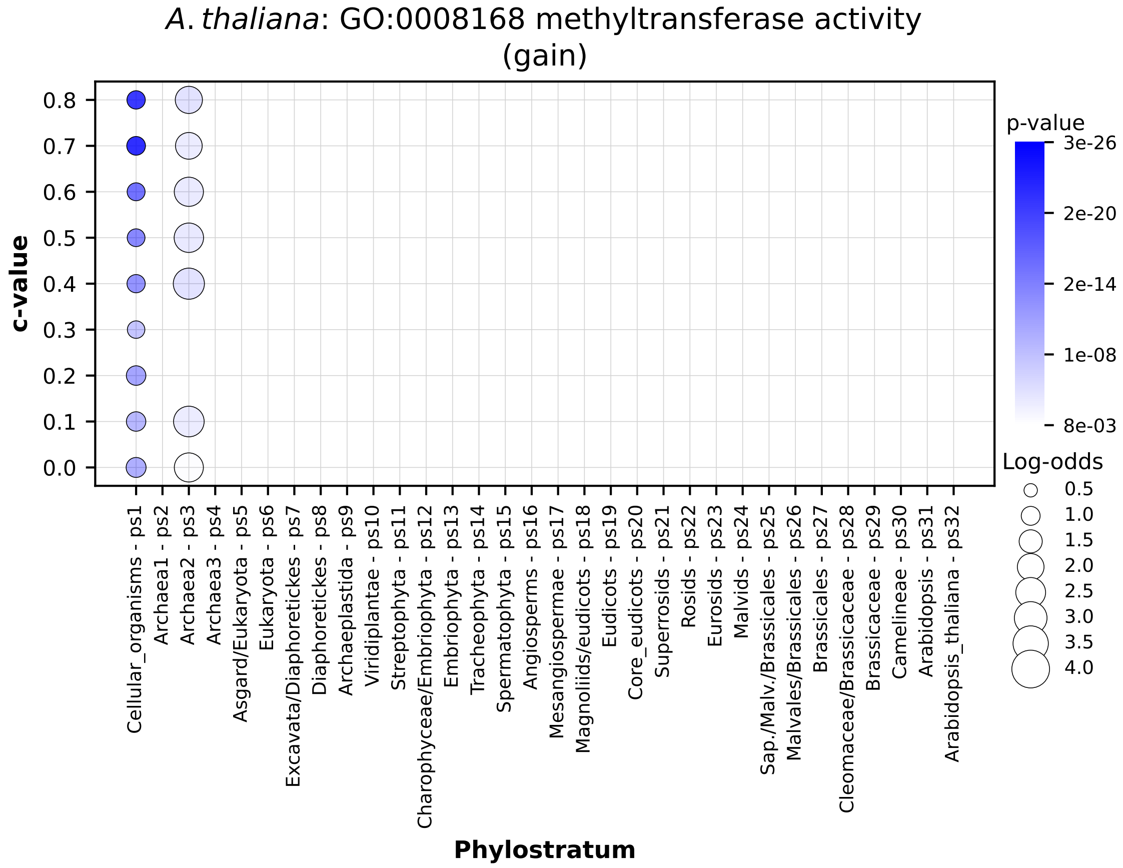

A. thaliana: GO:0008168 methyltransferase activity (gain)
c-value
0.8
0.7
0.6
0.5
0.4
0.3
0.2
0.1
0.0
p-value
3e-26
2e-20
2e-14
1e-08
8e-03
Log-odds
0.5
1.0
1.5
2.0
2.5
3.0
3.5
4.0
Cellular_organisms - ps1
Archaea1 - ps2
Archaea2 - ps3
Archaea3 - ps4
Asgard/Eukaryota - ps5
Eukaryota - ps6
Excavata/Diaphoretickes - ps7
Diaphoretickes - ps8
Archaeplastida - ps9
Viridiplantae - ps10
Streptophyta - ps11
Charophyceae/Embriophyta - ps12
Embriophyta - ps13
Tracheophyta - ps14
Spermatophyta - ps15
Angiosperms - ps16
Mesangiospermae - ps17
Magnoliids/eudicots - ps18
Eudicots - ps19
Core_eudicots - ps20
Superrosids - ps21
Rosids - ps22
Eurosids - ps23
Malvids - ps24
Sap./Malv./Brassicales - ps25
Malvales/Brassicales - ps26
Brassicales - ps27
Cleomaceae/Brassicaceae - ps28
Brassicaceae - ps29
Camelineae - ps30
Arabidopsis - ps31
Arabidopsis_thaliana - ps32
Phylostratum

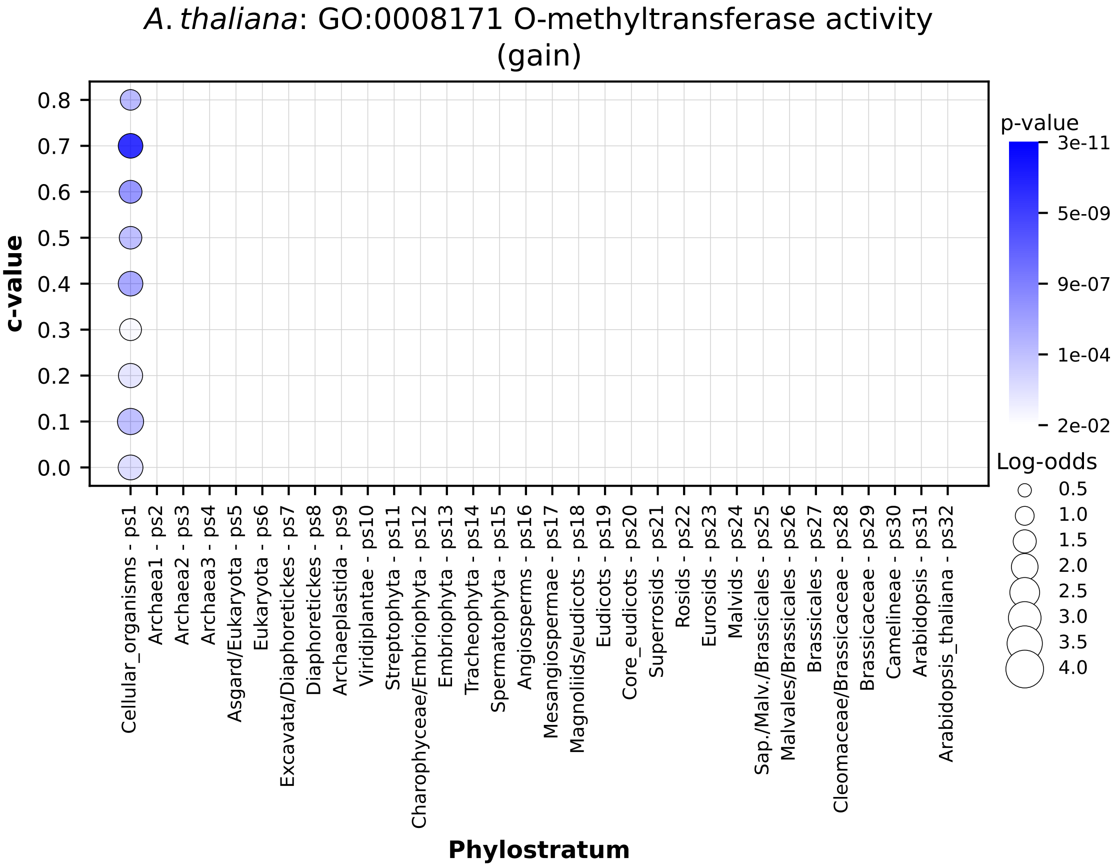

A. thaliana: GO:0008171 O-methyltransferase activity (gain)
c-value
0.0
0.1
0.2
0.3
0.4
0.5
0.6
0.7
0.8
p-value
3e-11
5e-09
9e-07
1e-04
2e-02
Log-odds
0.5
1.0
1.5
2.0
2.5
3.0
3.5
4.0
Cellular_organisms - ps1
Archaea1 - ps2
Archaea2 - ps3
Archaea3 - ps4
Asgard/Eukaryota - ps5
Eukaryota - ps6
Excavata/Diaphoretickes - ps7
Diaphoretickes - ps8
Archaeplastida - ps9
Viridiplantae - ps10
Streptophyta - ps11
Charophyceae/Embriophyta - ps12
Embriophyta - ps13
Tracheophyta - ps14
Spermatophyta - ps15
Angiosperms - ps16
Mesangiospermae - ps17
Magnoliids/eudicots - ps18
Eudicots - ps19
Core_eudicots - ps20
Superrosids - ps21
Rosids - ps22
Eurosids - ps23
Malvids - ps24
Sap./Malv./Brassicales - ps25
Malvales/Brassicales - ps26
Brassicales - ps27
Cleomaceae/Brassicaceae - ps28
Brassicaceae - ps29
Camelineae - ps30
Arabidopsis - ps31
Arabidopsis_thaliana - ps32
Phylostratum

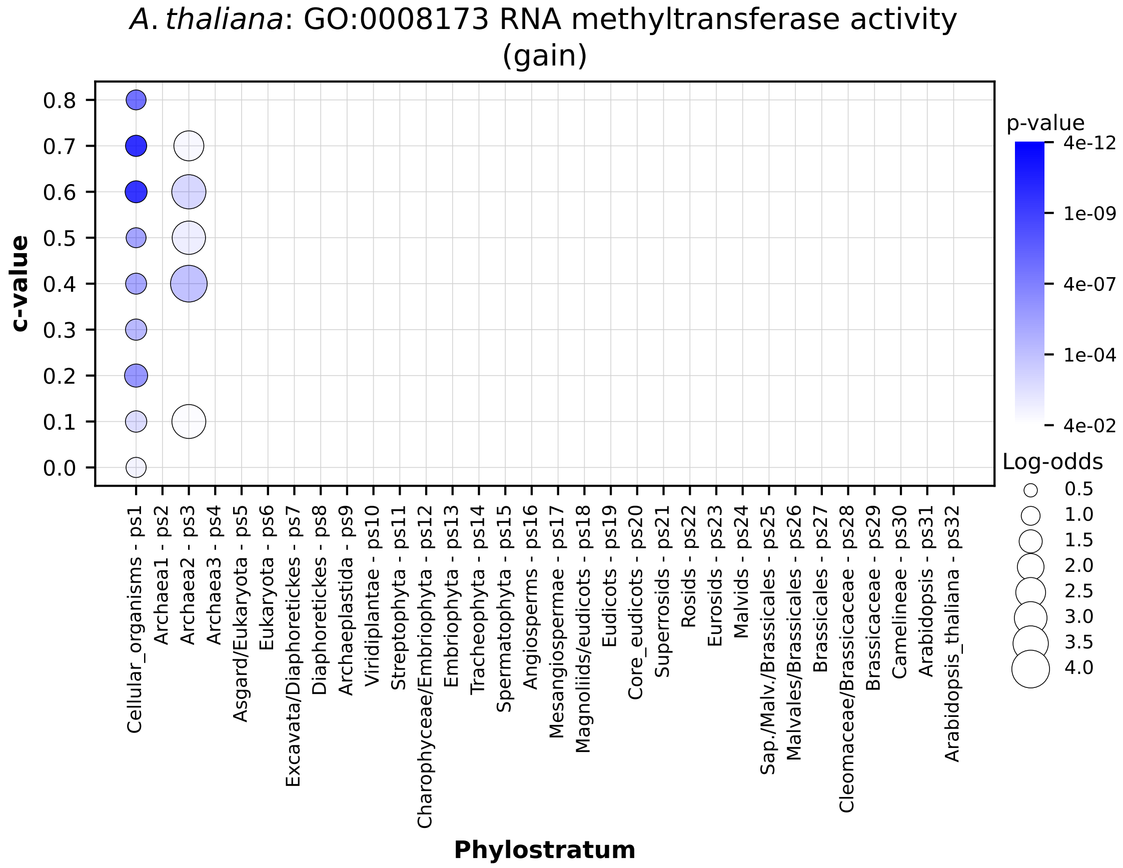

A. thaliana: GO:0008173 RNA methyltransferase activity (gain)
c-value
0.8
0.7
0.6
0.5
0.4
0.3
0.2
0.1
0.0
p-value
4e-12
1e-09
4e-07
1e-04
4e-02
Log-odds
0.5
1.0
1.5
2.0
2.5
3.0
3.5
4.0
Cellular_organisms - ps1
Archaea1 - ps2
Archaea2 - ps3
Archaea3 - ps4
Asgard/Eukaryota - ps5
Eukaryota - ps6
Excavata/Diaphoretickes - ps7
Diaphoretickes - ps8
Archaeplastida - ps9
Viridiplantae - ps10
Streptophyta - ps11
Charophyceae/Embriophyta - ps12
Embriophyta - ps13
Tracheophyta - ps14
Spermatophyta - ps15
Angiosperms - ps16
Mesangiospermae - ps17
Magnoliids/eudicots - ps18
Eudicots - ps19
Core_eudicots - ps20
Superrosids - ps21
Rosids - ps22
Eurosids - ps23
Malvids - ps24
Sap./Malv./Brassicales - ps25
Malvales/Brassicales - ps26
Brassicales - ps27
Cleomaceae/Brassicaceae - ps28
Brassicaceae - ps29
Camelineae - ps30
Arabidopsis - ps31
Arabidopsis_thaliana - ps32
Phylostratum

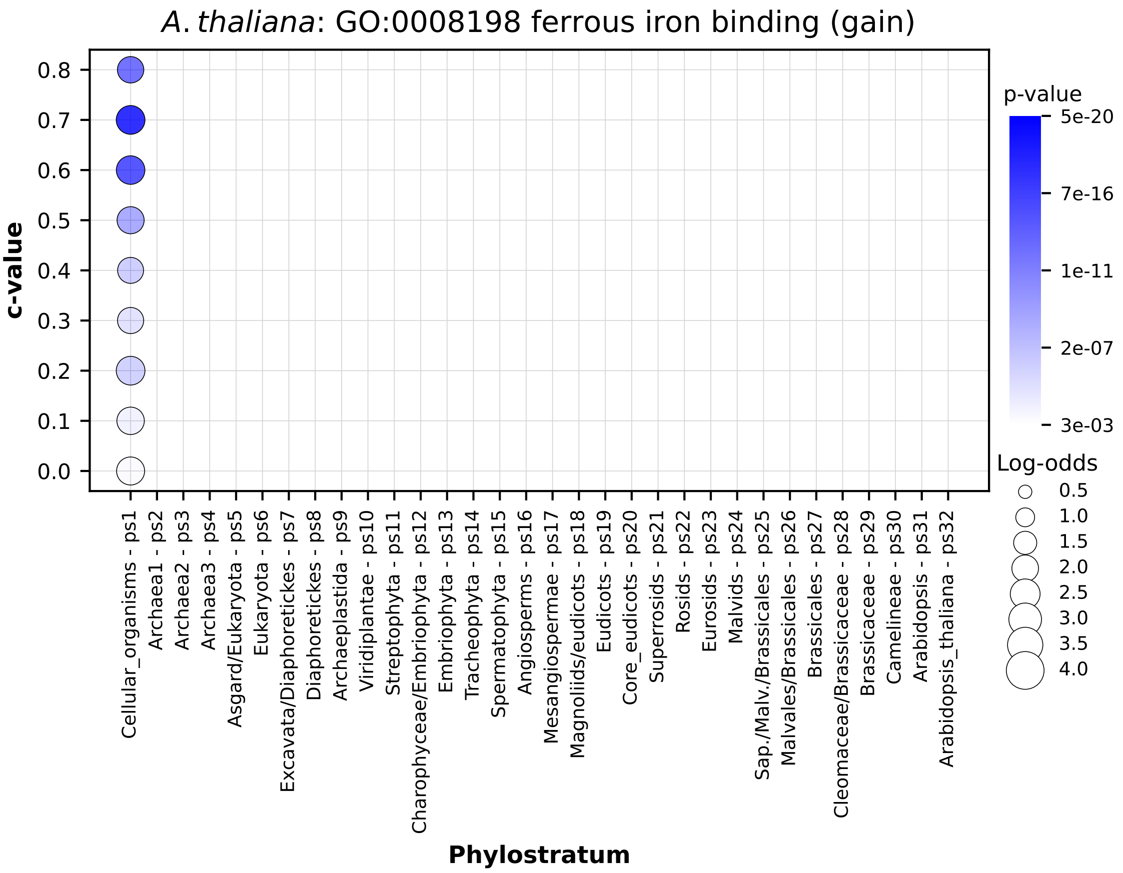
A. thaliana: GO:0008198 ferrous iron binding (gain)
c-value
0.8
0.7
0.6
0.5
0.4
0.3
0.2
0.1
0.0
p-value
5e-20
7e-16
1e-11
2e-07
3e-03
Log-odds
0.5
1.0
1.5
2.0
2.5
3.0
3.5
4.0
Cellular_organisms - ps1
Archaea1 - ps2
Archaea2 - ps3
Archaea3 - ps4
Asgard/Eukaryota - ps5
Eukaryota - ps6
Excavata/Diaphoretickes - ps7
Diaphoretickes - ps8
Archaeplastida - ps9
Viridiplantae - ps10
Streptophyta - ps11
Charophyceae/Embriophyta - ps12
Embriophyta - ps13
Tracheophyta - ps14
Spermatophyta - ps15
Angiosperms - ps16
Mesangiospermae - ps17
Magnoliids/eudicots - ps18
Eudicots - ps19
Core_eudicots - ps20
Superrosids - ps21
Rosids - ps22
Eurosids - ps23
Malvids - ps24
Sap./Malv./Brassicales - ps25
Malvales/Brassicales - ps26
Brassicales - ps27
Cleomaceae/Brassicaceae - ps28
Brassicaceae - ps29
Camelineae - ps30
Arabidopsis - ps31
Arabidopsis_thaliana - ps32
Phylostratum

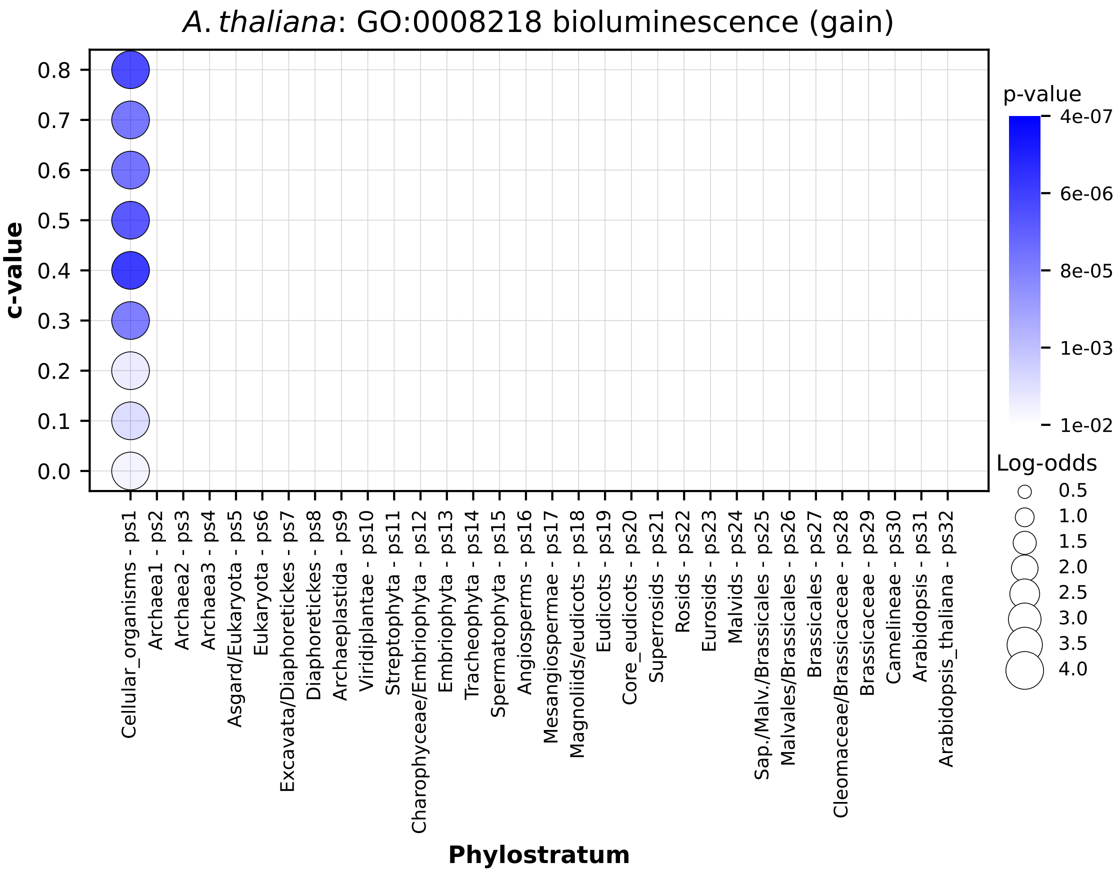

A. thaliana: GO:0008218 bioluminescence (gain)
c-value
0.8
0.7
0.6
0.5
0.4
0.3
0.2
0.1
0.0
p-value
4e-07
6e-06
8e-05
1e-03
1e-02
Log-odds
0.5
1.0
1.5
2.0
2.5
3.0
3.5
4.0
Cellular_organisms - ps1
Archaea1 - ps2
Archaea2 - ps3
Archaea3 - ps4
Asgard/Eukaryota - ps5
Eukaryota - ps6
Excavata/Diaphoretickes - ps7
Diaphoretickes - ps8
Archaeplastida - ps9
Viridiplantae - ps10
Streptophyta - ps11
Charophyceae/Embriophyta - ps12
Embriophyta - ps13
Tracheophyta - ps14
Spermatophyta - ps15
Angiosperms - ps16
Mesangiospermae - ps17
Magnoliids/eudicots - ps18
Eudicots - ps19
Core_eudicots - ps20
Superrosids - ps21
Rosids - ps22
Eurosids - ps23
Malvids - ps24
Sap./Malv./Brassicales - ps25
Malvales/Brassicales - ps26
Brassicales - ps27
Cleomaceae/Brassicaceae - ps28
Brassicaceae - ps29
Camelineae - ps30
Arabidopsis - ps31
Arabidopsis_thaliana - ps32
Phylostratum

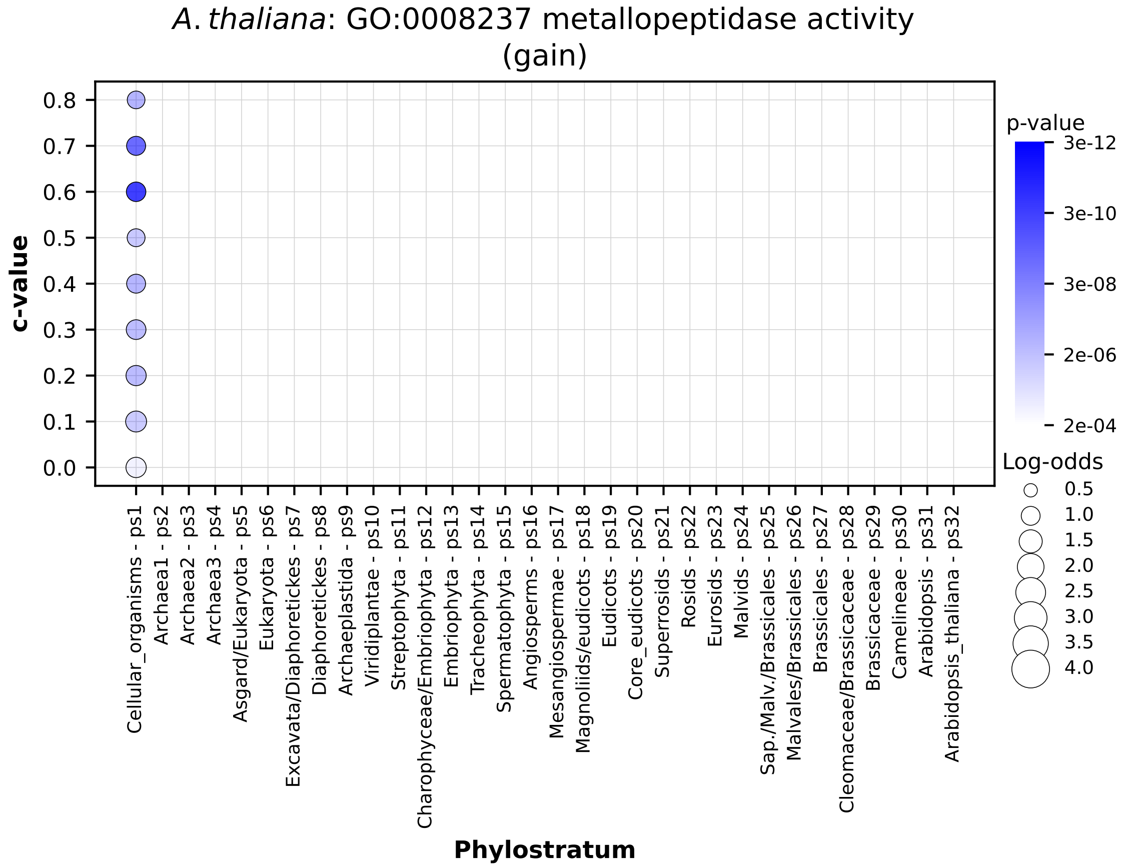
A. thaliana: GO:0008237 metallopeptidase activity
(gain)
c-value
0.8
0.7
0.6
0.5
0.4
0.3
0.2
0.1
0.0
p-value
3e-12
3e-10
3e-08
2e-06
2e-04
Log-odds
0.5
1.0
1.5
2.0
2.5
3.0
3.5
4.0
Cellular_organisms - ps1
Archaea1 - ps2
Archaea2 - ps3
Archaea3 - ps4
Asgard/Eukaryota - ps5
Eukaryota - ps6
Excavata/Diaphoretickes - ps7
Diaphoretickes - ps8
Archaeplastida - ps9
Viridiplantae - ps10
Streptophyta - ps11
Charophyceae/Embriophyta - ps12
Embriophyta - ps13
Tracheophyta - ps14
Spermatophyta - ps15
Angiosperms - ps16
Mesangiospermae - ps17
Magnoliids/eudicots - ps18
Eudicots - ps19
Core_eudicots - ps20
Superrosids - ps21
Rosids - ps22
Eurosids - ps23
Malvids - ps24
Sap./Malv./Brassicales - ps25
Malvales/Brassicales - ps26
Brassicales - ps27
Cleomaceae/Brassicaceae - ps28
Brassicaceae - ps29
Camelineae - ps30
Arabidopsis - ps31
Arabidopsis_thaliana - ps32
Phylostratum

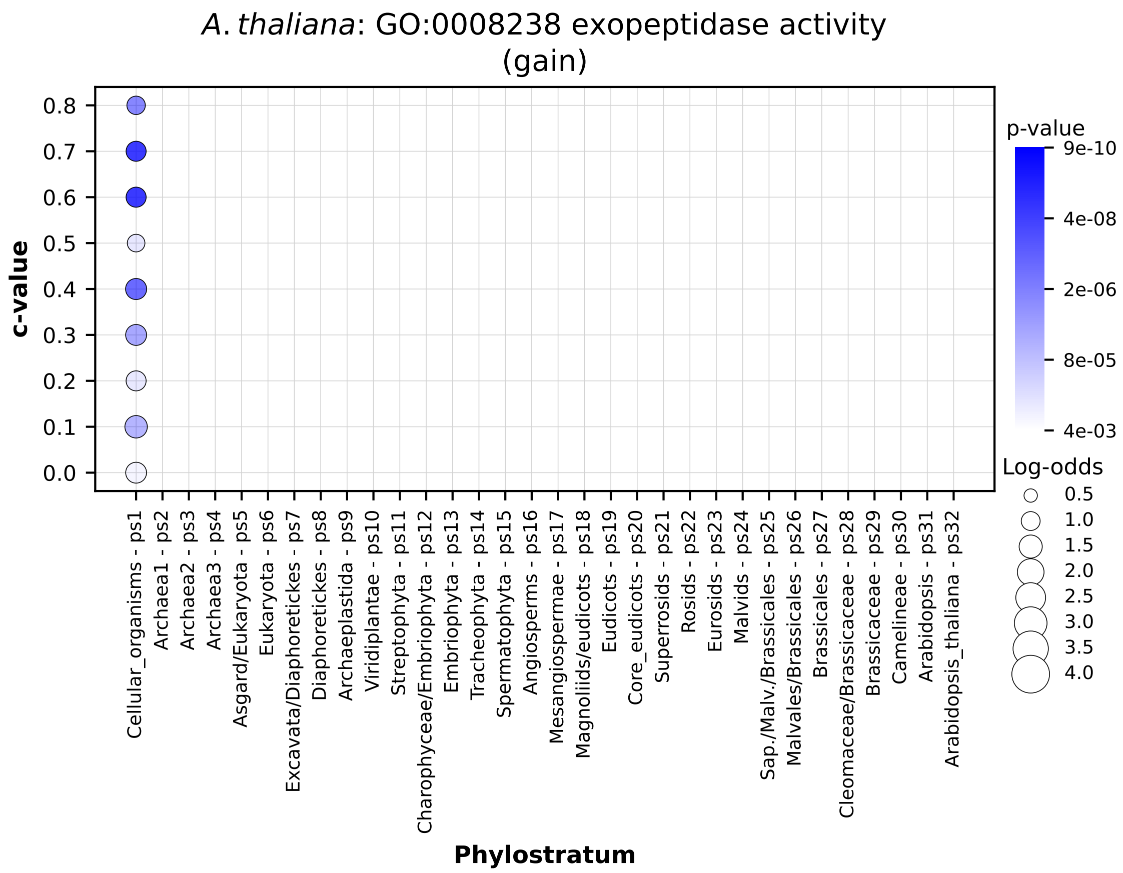

A. thaliana: GO:0008238 exopeptidase activity
(gain)
c-value
0.8
0.7
0.6
0.5
0.4
0.3
0.2
0.1
0.0
p-value
9e-10
4e-08
2e-06
8e-05
4e-03
Log-odds
0.5
1.0
1.5
2.0
2.5
3.0
3.5
4.0
Cellular_organisms - ps1
Archaea1 - ps2
Archaea2 - ps3
Archaea3 - ps4
Asgard/Eukaryota - ps5
Eukaryota - ps6
Excavata/Diaphoretickes - ps7
Diaphoretickes - ps8
Archaeplastida - ps9
Viridiplantae - ps10
Streptophyta - ps11
Charophyceae/Embriophyta - ps12
Embriophyta - ps13
Tracheophyta - ps14
Spermatophyta - ps15
Angiosperms - ps16
Mesangiospermae - ps17
Magnoliids/eudicots - ps18
Eudicots - ps19
Core_eudicots - ps20
Superrosids - ps21
Rosids - ps22
Eurosids - ps23
Malvids - ps24
Sap./Malv./Brassicales - ps25
Malvales/Brassicales - ps26
Brassicales - ps27
Cleomaceae/Brassicaceae - ps28
Brassicaceae - ps29
Camelineae - ps30
Arabidopsis - ps31
Arabidopsis_thaliana - ps32
Phylostratum

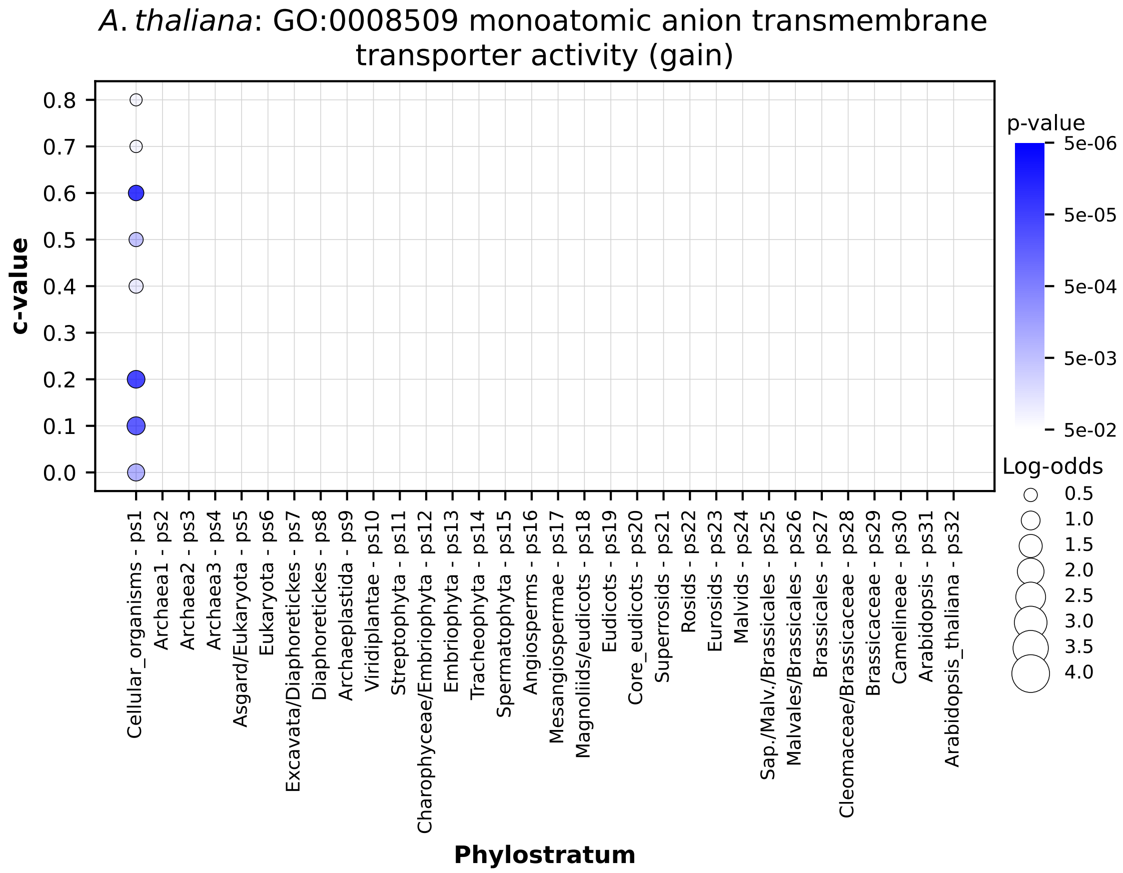
A. thaliana: GO:0008509 monoatomic anion transmembrane transporter activity (gain)
c-value
0.8
0.7
0.6
0.5
0.4
0.3
0.2
0.1
0.0
p-value
5e-06
5e-05
5e-04
5e-03
5e-02
Log-odds
0.5
1.0
1.5
2.0
2.5
3.0
3.5
4.0
Cellular_organisms - ps1
Archaea1 - ps2
Archaea2 - ps3
Archaea3 - ps4
Asgard/Eukaryota - ps5
Eukaryota - ps6
Excavata/Diaphoretickes - ps7
Diaphoretickes - ps8
Archaeplastida - ps9
Viridiplantae - ps10
Streptophyta - ps11
Charophyceae/Embriophyta - ps12
Embriophyta - ps13
Tracheophyta - ps14
Spermatophyta - ps15
Angiosperms - ps16
Mesangiospermae - ps17
Magnoliids/eudicots - ps18
Eudicots - ps19
Core_eudicots - ps20
Superrosids - ps21
Rosids - ps22
Eurosids - ps23
Malvids - ps24
Sap./Malv./Brassicales - ps25
Malvales/Brassicales - ps26
Brassicales - ps27
Cleomaceae/Brassicaceae - ps28
Brassicaceae - ps29
Camelineae - ps30
Arabidopsis - ps31
Arabidopsis_thaliana - ps32
Phylostratum

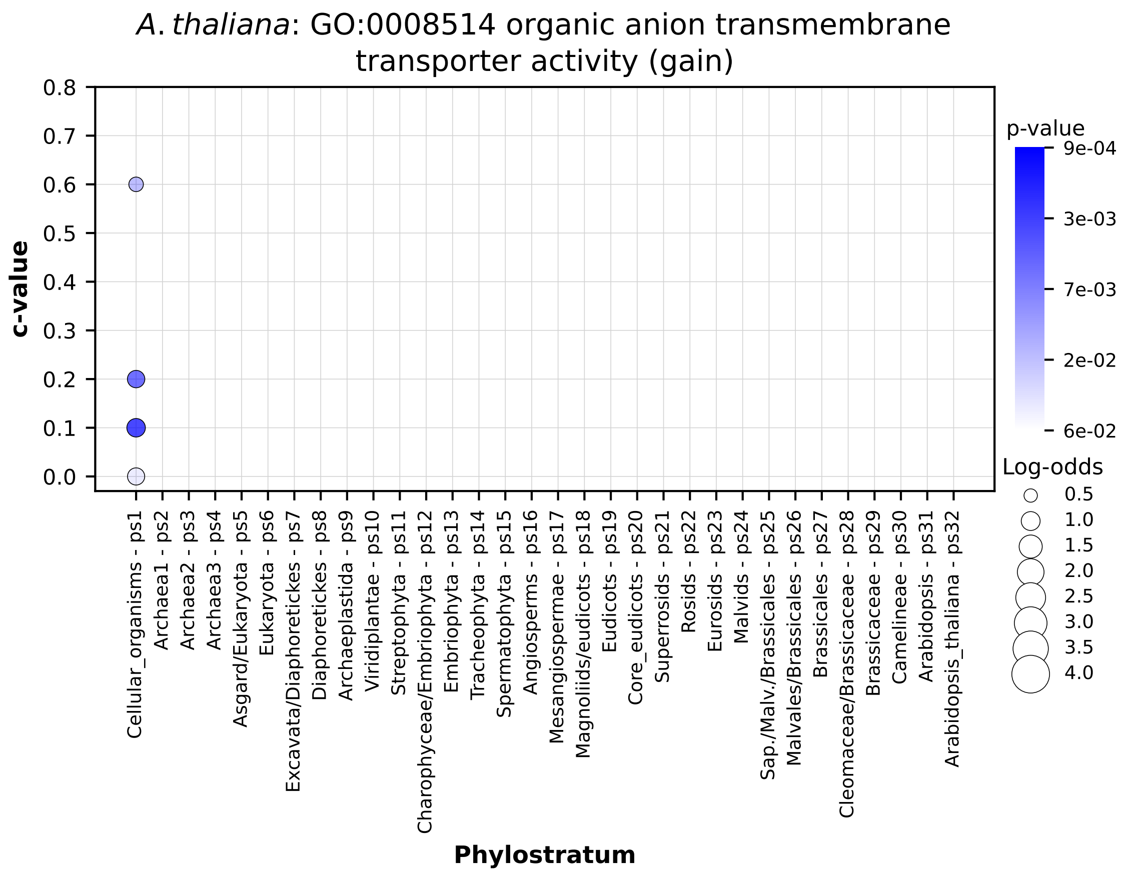
A. thaliana: GO:0008514 organic anion transmembrane transporter activity (gain)
c-value
0.0
0.1
0.2
0.3
0.4
0.5
0.6
0.7
0.8
Phylostratum
Cellular_organisms - ps1
Archaea1 - ps2
Archaea2 - ps3
Archaea3 - ps4
Asgard/Eukaryota - ps5
Eukaryota - ps6
Excavata/Diaphoretickes - ps7
Diaphoretickes - ps8
Archaeplastida - ps9
Viridiplantae - ps10
Streptophyta - ps11
Charophyceae/Embriophyta - ps12
Embriophyta - ps13
Tracheophyta - ps14
Spermatophyta - ps15
Angiosperms - ps16
Mesangiospermae - ps17
Magnoliids/eudicots - ps18
Eudicots - ps19
Core_eudicots - ps20
Superrosids - ps21
Rosids - ps22
Eurosids - ps23
Malvids - ps24
Sap./Malv./Brassicales - ps25
Malvales/Brassicales - ps26
Brassicales - ps27
Cleomaceae/Brassicaceae - ps28
Brassicaceae - ps29
Camelineae - ps30
Arabidopsis - ps31
Arabidopsis_thaliana - ps32
p-value
9e-04
3e-03
7e-03
2e-02
6e-02
Log-odds
0.5
1.0
1.5
2.0
2.5
3.0
3.5
4.0

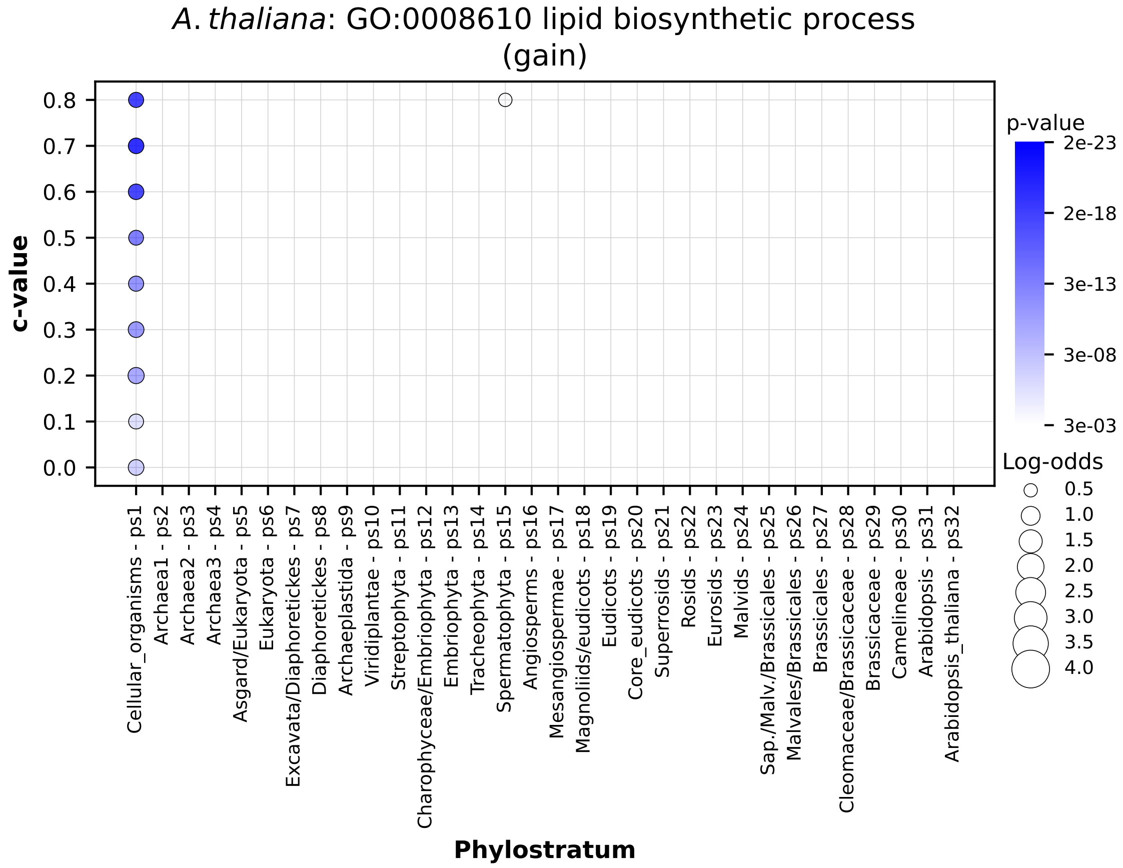

A. thaliana: GO:0008610 lipid biosynthetic process
(gain)
c-value
0.8
0.7
0.6
0.5
0.4
0.3
0.2
0.1
0.0
p-value
2e-23
2e-18
3e-13
3e-08
3e-03
Log-odds
0.5
1.0
1.5
2.0
2.5
3.0
3.5
4.0
Cellular_organisms - ps1
Archaea1 - ps2
Archaea2 - ps3
Archaea3 - ps4
Asgard/Eukaryota - ps5
Eukaryota - ps6
Excavata/Diaphoretickes - ps7
Diaphoretickes - ps8
Archaeplastida - ps9
Viridiplantae - ps10
Streptophyta - ps11
Charophyceae/Embriophyta - ps12
Embriophyta - ps13
Tracheophyta - ps14
Spermatophyta - ps15
Angiosperms - ps16
Mesangiospermae - ps17
Magnoliids/eudicots - ps18
Eudicots - ps19
Core_eudicots - ps20
Superrosids - ps21
Rosids - ps22
Eurosids - ps23
Malvids - ps24
Sap./Malv./Brassicales - ps25
Malvales/Brassicales - ps26
Brassicales - ps27
Cleomaceae/Brassicaceae - ps28
Brassicaceae - ps29
Camelineae - ps30
Arabidopsis - ps31
Arabidopsis_thaliana - ps32
Phylostratum

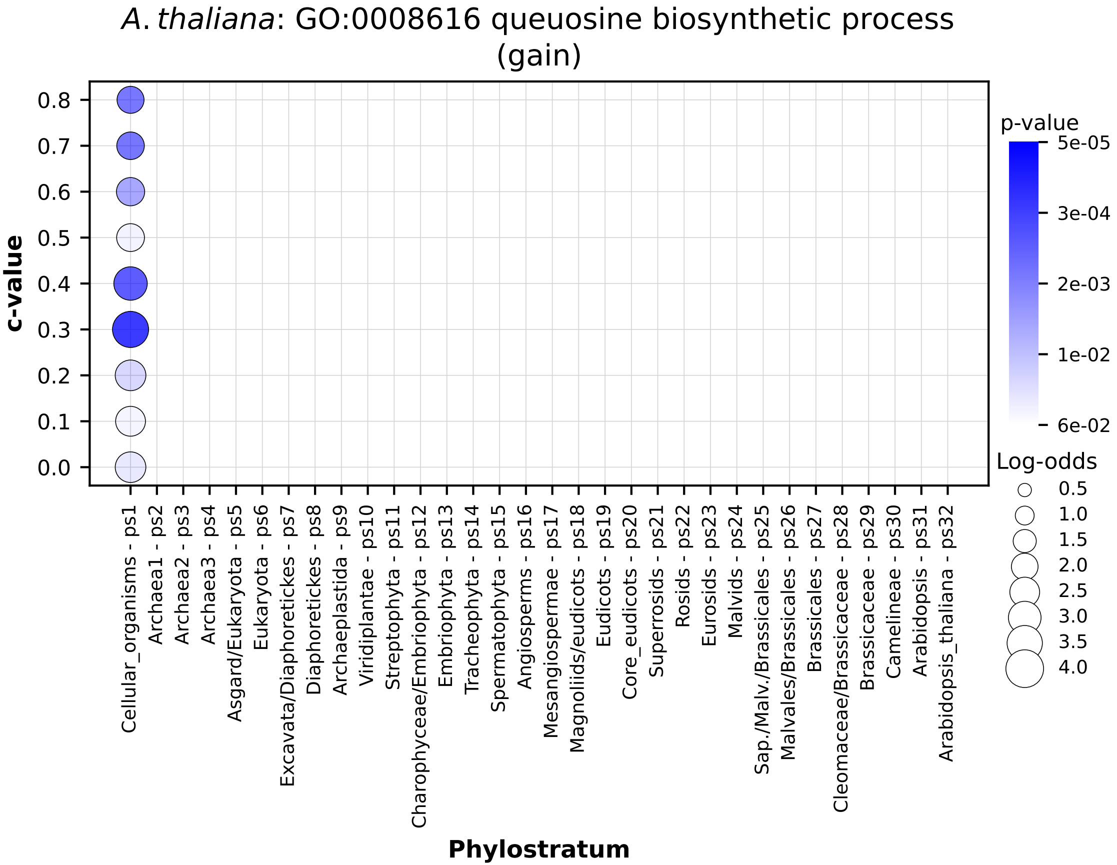

A. thaliana: GO:0008616 queuosine biosynthetic process (gain)
c-value
0.8
0.7
0.6
0.5
0.4
0.3
0.2
0.1
0.0
Phylostratum
Cellular_organisms - ps1
Archaea1 - ps2
Archaea2 - ps3
Archaea3 - ps4
Asgard/Eukaryota - ps5
Eukaryota - ps6
Excavata/Diaphoretickes - ps7
Diaphoretickes - ps8
Archaeplastida - ps9
Viridiplantae - ps10
Streptophyta - ps11
Charophyceae/Embriophyta - ps12
Embriophyta - ps13
Tracheophyta - ps14
Spermatophyta - ps15
Angiosperms - ps16
Mesangiospermae - ps17
Magnoliids/eudicots - ps18
Eudicots - ps19
Core_eudicots - ps20
Superrosids - ps21
Rosids - ps22
Eurosids - ps23
Malvids - ps24
Sap./Malv./Brassicales - ps25
Malvales/Brassicales - ps26
Brassicales - ps27
Cleomaceae/Brassicaceae - ps28
Brassicaceae - ps29
Camelineae - ps30
Arabidopsis - ps31
Arabidopsis_thaliana - ps32
p-value
5e-05
3e-04
2e-03
1e-02
6e-02
Log-odds
0.5
1.0
1.5
2.0
2.5
3.0
3.5
4.0

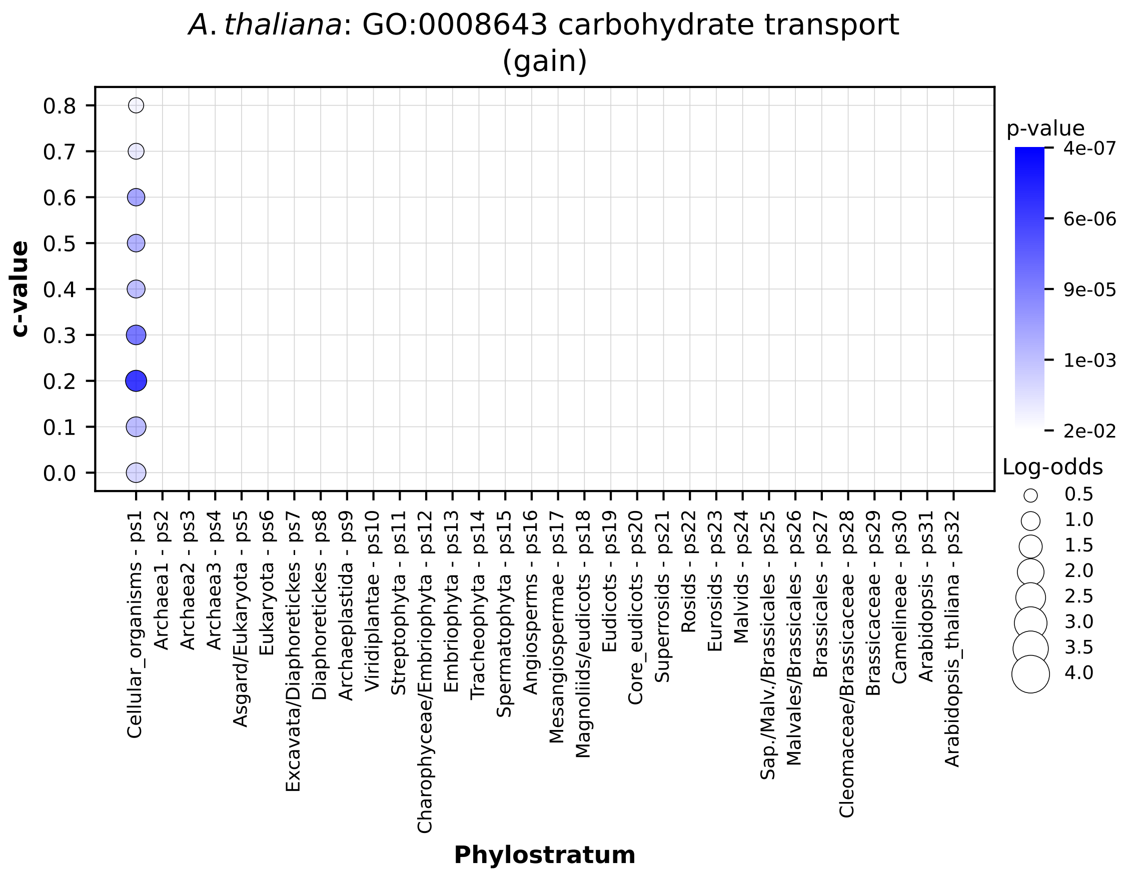

A. thaliana: GO:0008643 carbohydrate transport (gain)
c-value
0.8
0.7
0.6
0.5
0.4
0.3
0.2
0.1
0.0
p-value
4e-07
6e-06
9e-05
1e-03
2e-02
Log-odds
0.5
1.0
1.5
2.0
2.5
3.0
3.5
4.0
Cellular_organisms - ps1
Archaea1 - ps2
Archaea2 - ps3
Archaea3 - ps4
Asgard/Eukaryota - ps5
Eukaryota - ps6
Excavata/Diaphoretickes - ps7
Diaphoretickes - ps8
Archaeplastida - ps9
Viridiplantae - ps10
Streptophyta - ps11
Charophyceae/Embriophyta - ps12
Embriophyta - ps13
Tracheophyta - ps14
Spermatophyta - ps15
Angiosperms - ps16
Mesangiospermae - ps17
Magnoliids/eudicots - ps18
Eudicots - ps19
Core_eudicots - ps20
Superrosids - ps21
Rosids - ps22
Eurosids - ps23
Malvids - ps24
Sap./Malv./Brassicales - ps25
Malvales/Brassicales - ps26
Brassicales - ps27
Cleomaceae/Brassicaceae - ps28
Brassicaceae - ps29
Camelineae - ps30
Arabidopsis - ps31
Arabidopsis_thaliana - ps32
Phylostratum

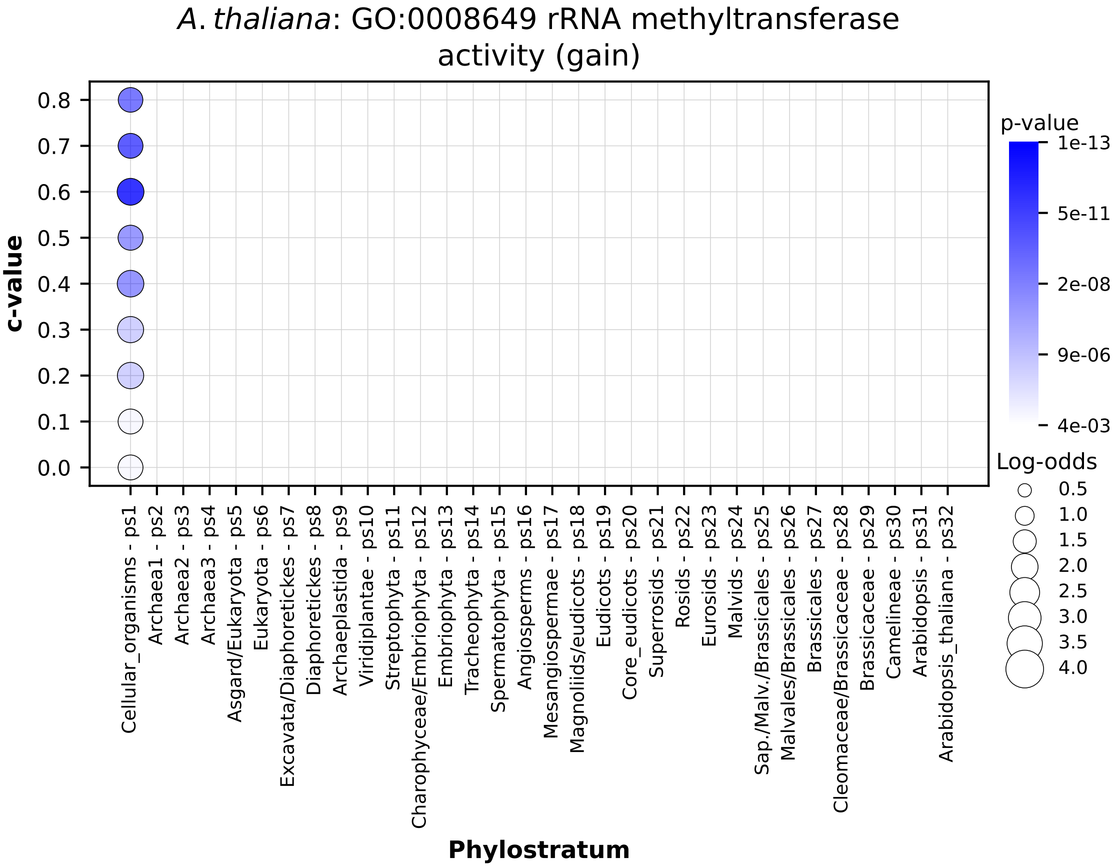

A. thaliana: GO:0008649 rRNA methyltransferase activity (gain)
c-value
0.8
0.7
0.6
0.5
0.4
0.3
0.2
0.1
0.0
p-value
1e-13
5e-11
2e-08
9e-06
4e-03
Log-odds
0.5
1.0
1.5
2.0
2.5
3.0
3.5
4.0
Cellular_organisms - ps1
Archaea1 - ps2
Archaea2 - ps3
Archaea3 - ps4
Asgard/Eukaryota - ps5
Eukaryota - ps6
Excavata/Diaphoretickes - ps7
Diaphoretickes - ps8
Archaeplastida - ps9
Viridiplantae - ps10
Streptophyta - ps11
Charophyceae/Embriophyta - ps12
Embriophyta - ps13
Tracheophyta - ps14
Spermatophyta - ps15
Angiosperms - ps16
Mesangiospermae - ps17
Magnoliids/eudicots - ps18
Eudicots - ps19
Core_eudicots - ps20
Superrosids - ps21
Rosids - ps22
Eurosids - ps23
Malvids - ps24
Sap./Malv./Brassicales - ps25
Malvales/Brassicales - ps26
Brassicales - ps27
Cleomaceae/Brassicaceae - ps28
Brassicaceae - ps29
Camelineae - ps30
Arabidopsis - ps31
Arabidopsis_thaliana - ps32
Phylostratum

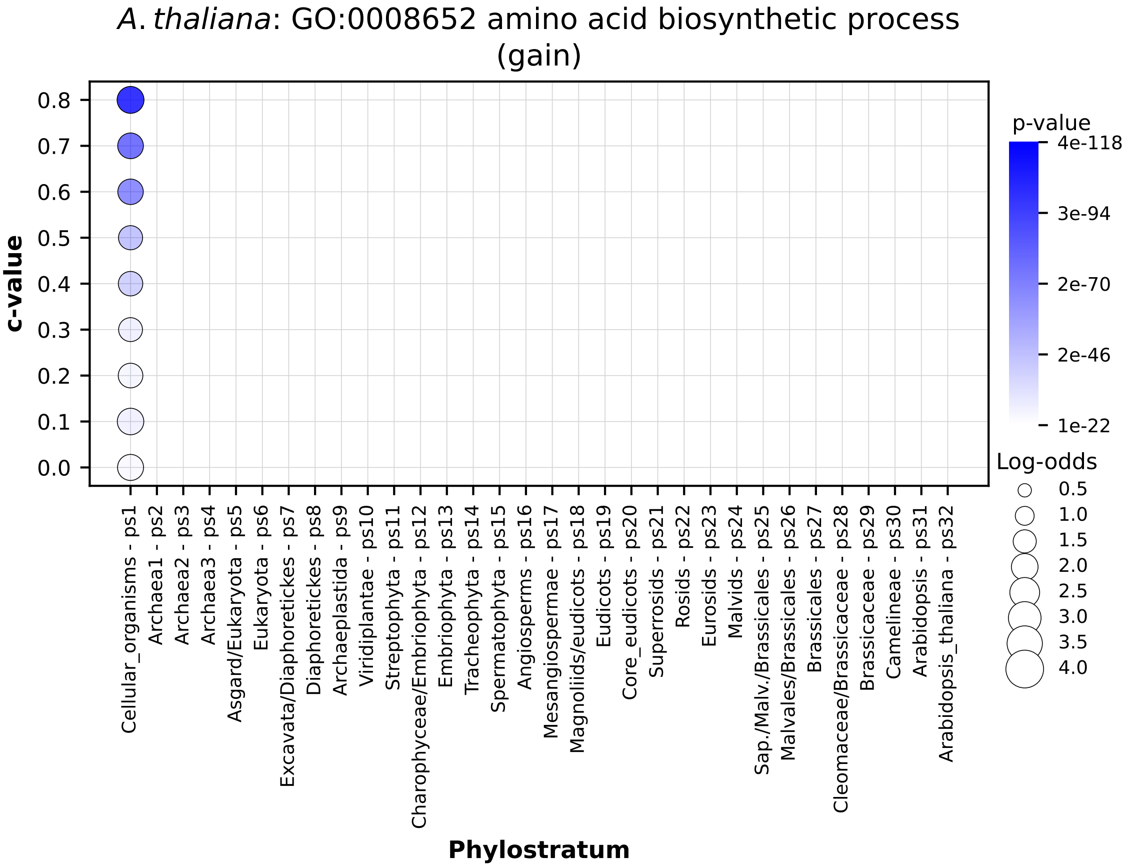

A. thaliana: GO:0008652 amino acid biosynthetic process
(gain)
c-value
0.8
0.7
0.6
0.5
0.4
0.3
0.2
0.1
0.0
p-value
4e-118
3e-94
2e-70
2e-46
1e-22
Log-odds
0.5
1.0
1.5
2.0
2.5
3.0
3.5
4.0
Cellular_organisms - ps1
Archaea1 - ps2
Archaea2 - ps3
Archaea3 - ps4
Asgard/Eukaryota - ps5
Eukaryota - ps6
Excavata/Diaphoretickes - ps7
Diaphoretickes - ps8
Archaeplastida - ps9
Viridiplantae - ps10
Streptophyta - ps11
Charophyceae/Embriophyta - ps12
Embriophyta - ps13
Tracheophyta - ps14
Spermatophyta - ps15
Angiosperms - ps16
Mesangiospermae - ps17
Magnoliids/eudicots - ps18
Eudicots - ps19
Core_eudicots - ps20
Superrosids - ps21
Rosids - ps22
Eurosids - ps23
Malvids - ps24
Sap./Malv./Brassicales - ps25
Malvales/Brassicales - ps26
Brassicales - ps27
Cleomaceae/Brassicaceae - ps28
Brassicaceae - ps29
Camelineae - ps30
Arabidopsis - ps31
Arabidopsis_thaliana - ps32
Phylostratum

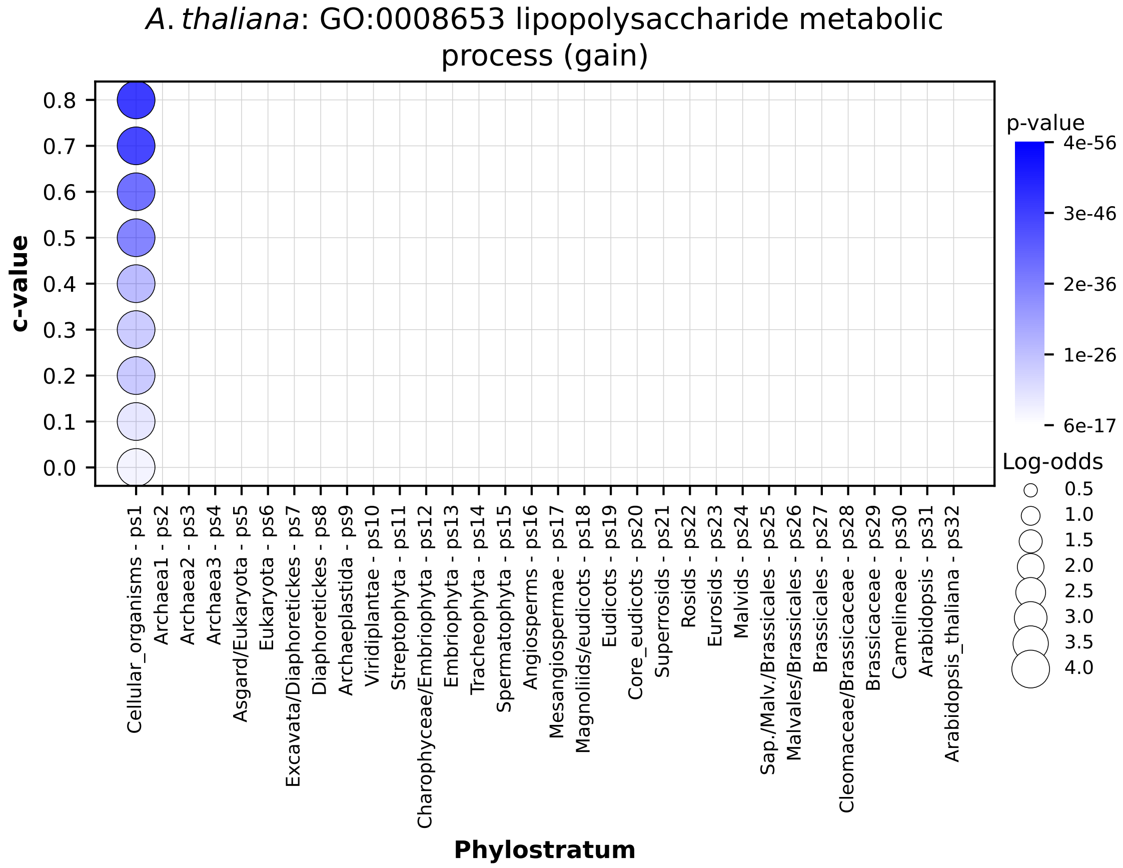
A. thaliana: GO:0008653 lipopolysaccharide metabolic process (gain)
c-value
0.8
0.7
0.6
0.5
0.4
0.3
0.2
0.1
0.0
p-value
4e-56
3e-46
2e-36
1e-26
6e-17
Log-odds
0.5
1.0
1.5
2.0
2.5
3.0
3.5
4.0
Cellular_organisms - ps1
Archaea1 - ps2
Archaea2 - ps3
Archaea3 - ps4
Asgard/Eukaryota - ps5
Eukaryota - ps6
Excavata/Diaphoretickes - ps7
Diaphoretickes - ps8
Archaeplastida - ps9
Viridiplantae - ps10
Streptophyta - ps11
Charophyceae/Embriophyta - ps12
Embriophyta - ps13
Tracheophyta - ps14
Spermatophyta - ps15
Angiosperms - ps16
Mesangiospermae - ps17
Magnoliids/eudicots - ps18
Eudicots - ps19
Core_eudicots - ps20
Superrosids - ps21
Rosids - ps22
Eurosids - ps23
Malvids - ps24
Sap./Malv./Brassicales - ps25
Malvales/Brassicales - ps26
Brassicales - ps27
Cleomaceae/Brassicaceae - ps28
Brassicaceae - ps29
Camelineae - ps30
Arabidopsis - ps31
Arabidopsis_thaliana - ps32
Phylostratum

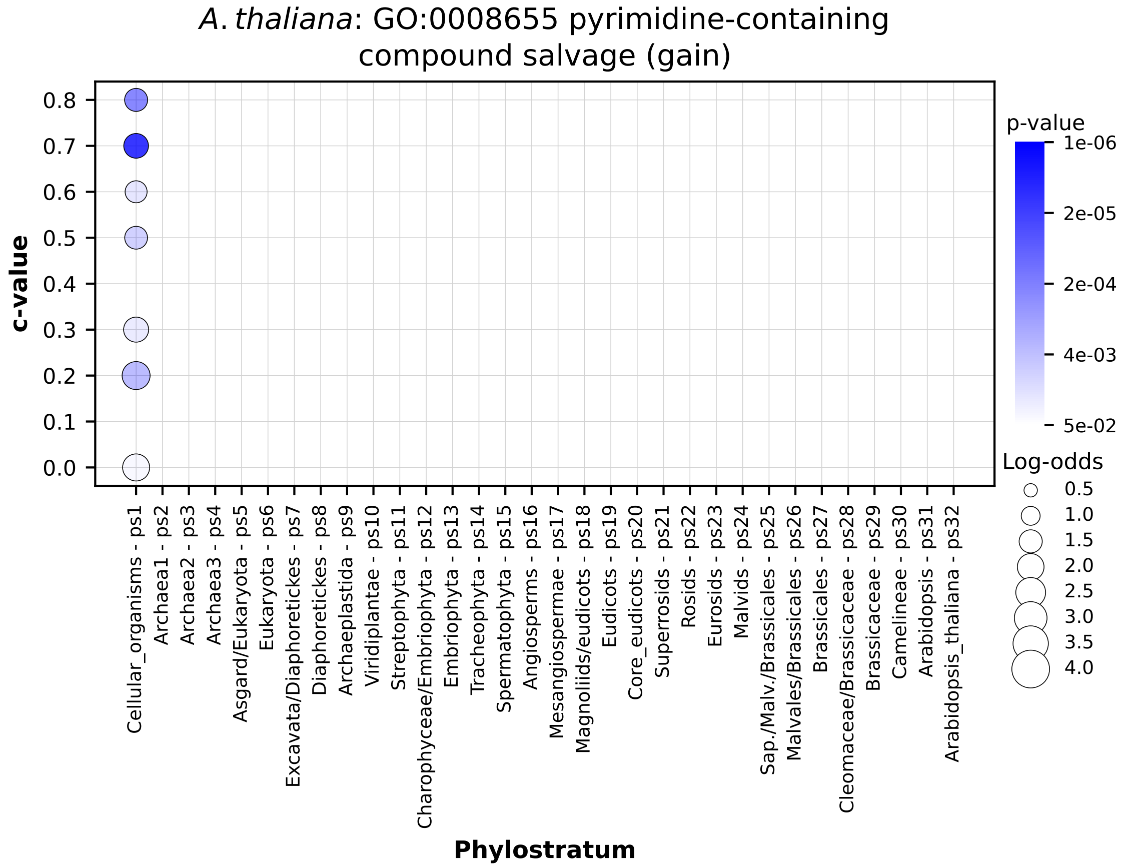
A. thaliana: GO:0008655 pyrimidine-containing compound salvage (gain)
c-value
0.8
0.7
0.6
0.5
0.4
0.3
0.2
0.1
0.0
Phylostratum
Cellular_organisms - ps1
Archaea1 - ps2
Archaea2 - ps3
Archaea3 - ps4
Asgard/Eukaryota - ps5
Eukaryota - ps6
Excavata/Diaphoretickes - ps7
Diaphoretickes - ps8
Archaeplastida - ps9
Viridiplantae - ps10
Streptophyta - ps11
Charophyceae/Embriophyta - ps12
Embriophyta - ps13
Tracheophyta - ps14
Spermatophyta - ps15
Angiosperms - ps16
Mesangiospermae - ps17
Magnoliids/eudicots - ps18
Eudicots - ps19
Core_eudicots - ps20
Superrosids - ps21
Rosids - ps22
Eurosids - ps23
Malvids - ps24
Sap./Malv./Brassicales - ps25
Malvales/Brassicales - ps26
Brassicales - ps27
Cleomaceae/Brassicaceae - ps28
Brassicaceae - ps29
Camelineae - ps30
Arabidopsis - ps31
Arabidopsis_thaliana - ps32
p-value
1e-06
2e-05
2e-04
4e-03
5e-02
Log-odds
0.5
1.0
1.5
2.0
2.5
3.0
3.5
4.0

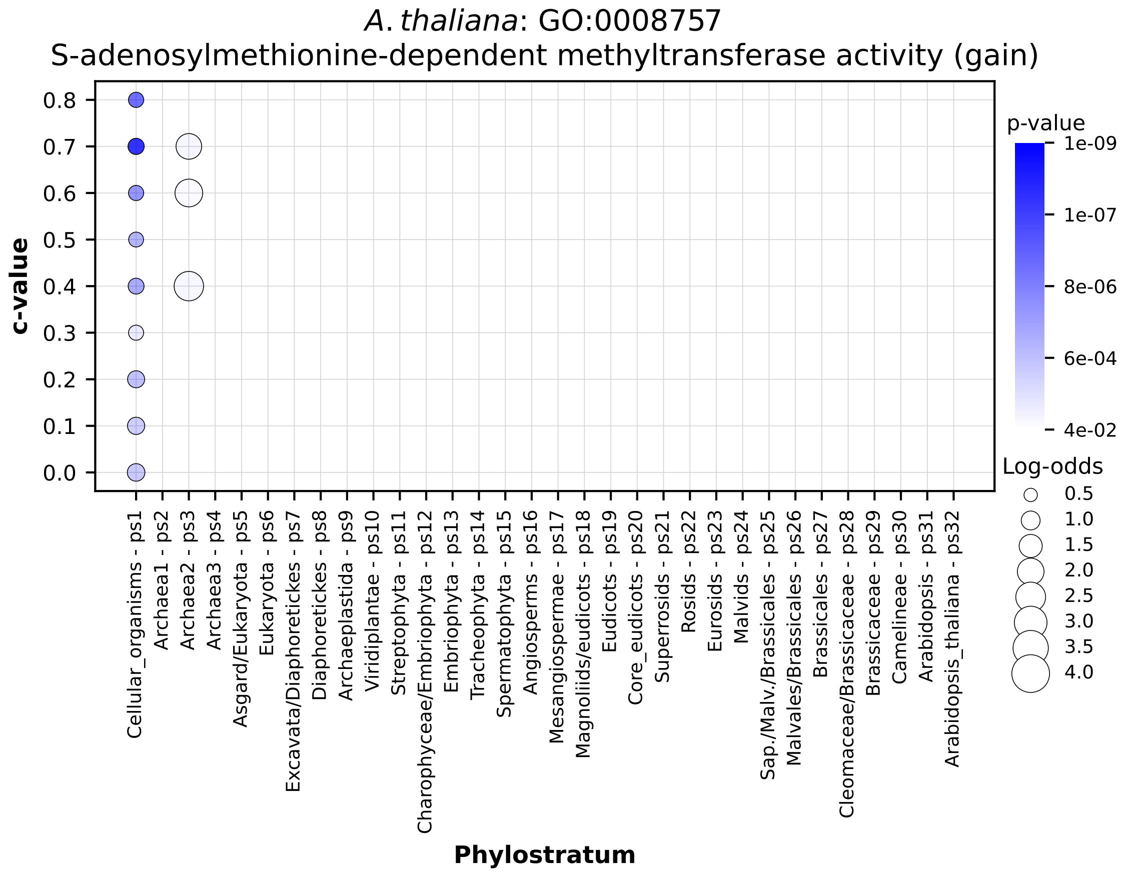
A. thaliana: GO:0008757
S-adenosylmethionine-dependent methyltransferase activity (gain)
c-value
0.8
0.7
0.6
0.5
0.4
0.3
0.2
0.1
0.0
p-value
1e-09
1e-07
8e-06
6e-04
4e-02
Log-odds
0.5
1.0
1.5
2.0
2.5
3.0
3.5
4.0
Cellular_organisms - ps1
Archaea1 - ps2
Archaea2 - ps3
Archaea3 - ps4
Asgard/Eukaryota - ps5
Eukaryota - ps6
Excavata/Diaphoretickes - ps7
Diaphoretickes - ps8
Archaeplastida - ps9
Viridiplantae - ps10
Streptophyta - ps11
Charophyceae/Embriophyta - ps12
Embriophyta - ps13
Tracheophyta - ps14
Spermatophyta - ps15
Angiosperms - ps16
Mesangiospermae - ps17
Magnoliids/eudicots - ps18
Eudicots - ps19
Core_eudicots - ps20
Superrosids - ps21
Rosids - ps22
Eurosids - ps23
Malvids - ps24
Sap./Malv./Brassicales - ps25
Malvales/Brassicales - ps26
Brassicales - ps27
Cleomaceae/Brassicaceae - ps28
Brassicaceae - ps29
Camelineae - ps30
Arabidopsis - ps31
Arabidopsis_thaliana - ps32
Phylostratum

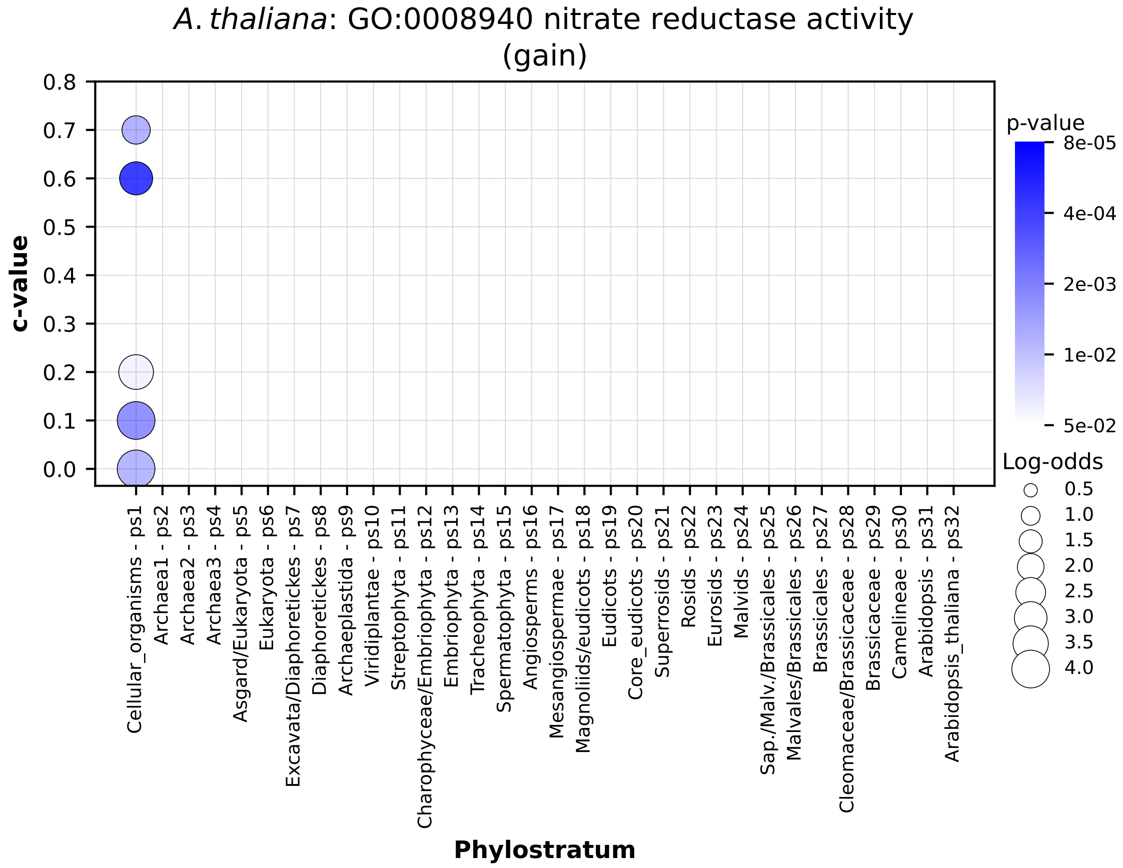
A. thaliana: GO:0008940 nitrate reductase activity (gain)
c-value
0.8
0.7
0.6
0.5
0.4
0.3
0.2
0.1
0.0
p-value
8e-05
4e-04
2e-03
1e-02
5e-02
Log-odds
0.5
1.0
1.5
2.0
2.5
3.0
3.5
4.0
Cellular_organisms - ps1
Archaea1 - ps2
Archaea2 - ps3
Archaea3 - ps4
Asgard/Eukaryota - ps5
Eukaryota - ps6
Excavata/Diaphoretickes - ps7
Diaphoretickes - ps8
Archaeplastida - ps9
Viridiplantae - ps10
Streptophyta - ps11
Charophyceae/Embriophyta - ps12
Embriophyta - ps13
Tracheophyta - ps14
Spermatophyta - ps15
Angiosperms - ps16
Mesangiospermae - ps17
Magnoliids/eudicots - ps18
Eudicots - ps19
Core_eudicots - ps20
Superrosids - ps21
Rosids - ps22
Eurosids - ps23
Malvids - ps24
Sap./Malv./Brassicales - ps25
Malvales/Brassicales - ps26
Brassicales - ps27
Cleomaceae/Brassicaceae - ps28
Brassicaceae - ps29
Camelineae - ps30
Arabidopsis - ps31
Arabidopsis_thaliana - ps32
Phylostratum

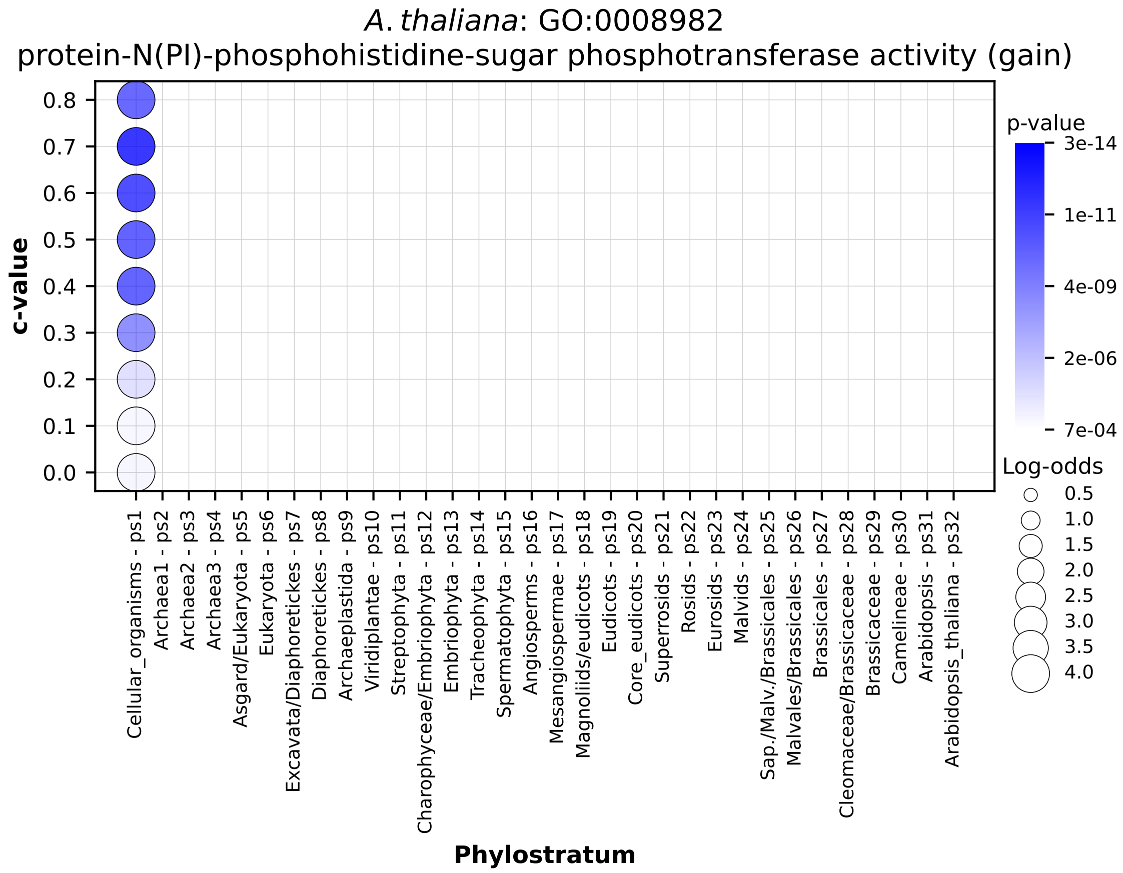

A. thaliana: GO:0008982
protein-N(PI)-phosphohistidine-sugar phosphotransferase activity (gain)
c-value
0.8
0.7
0.6
0.5
0.4
0.3
0.2
0.1
0.0
p-value
3e-14
1e-11
4e-09
2e-06
7e-04
Log-odds
0.5
1.0
1.5
2.0
2.5
3.0
3.5
4.0
Cellular_organisms - ps1
Archaea1 - ps2
Archaea2 - ps3
Archaea3 - ps4
Asgard/Eukaryota - ps5
Eukaryota - ps6
Excavata/Diaphoretickes - ps7
Diaphoretickes - ps8
Archaeplastida - ps9
Viridiplantae - ps10
Streptophyta - ps11
Charophyceae/Embriophyta - ps12
Embriophyta - ps13
Tracheophyta - ps14
Spermatophyta - ps15
Angiosperms - ps16
Mesangiospermae - ps17
Magnoliids/eudicots - ps18
Eudicots - ps19
Core_eudicots - ps20
Superrosids - ps21
Rosids - ps22
Eurosids - ps23
Malvids - ps24
Sap./Malv./Brassicales - ps25
Malvales/Brassicales - ps26
Brassicales - ps27
Cleomaceae/Brassicaceae - ps28
Brassicaceae - ps29
Camelineae - ps30
Arabidopsis - ps31
Arabidopsis_thaliana - ps32
Phylostratum

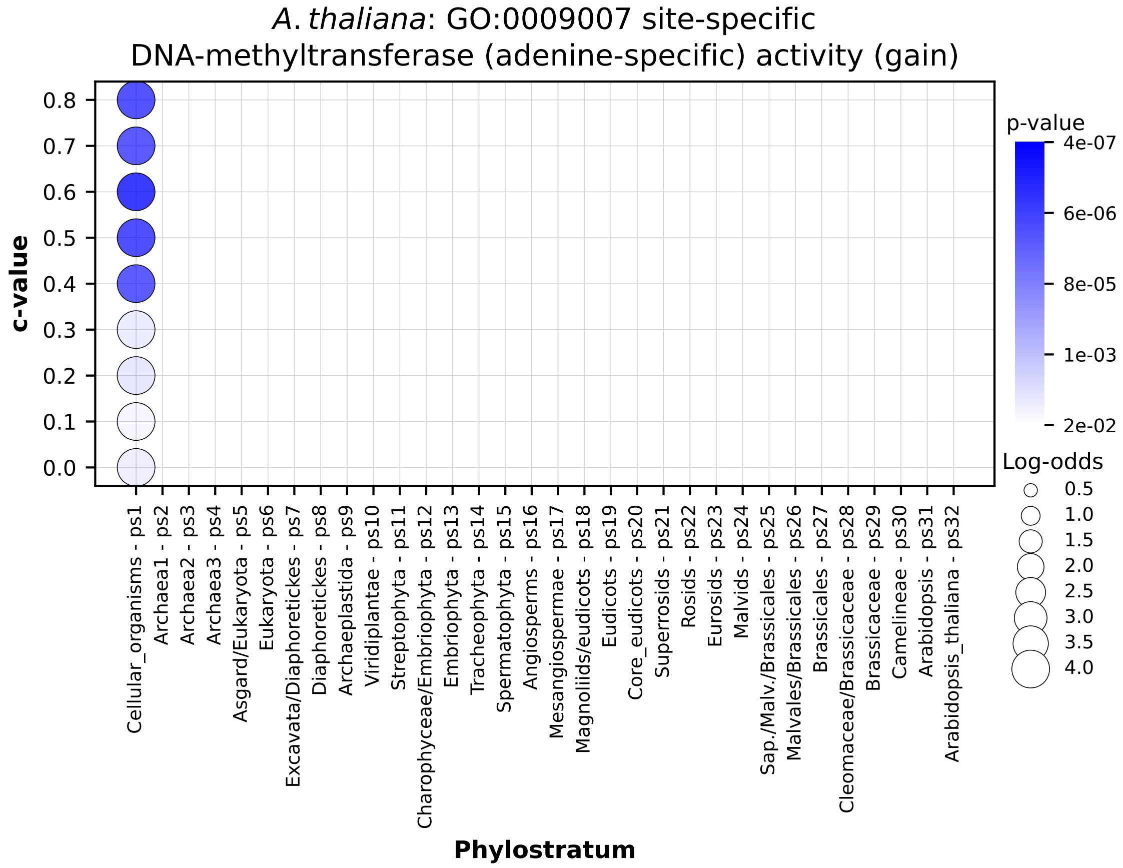

A. thaliana: GO:0009007 site-specific DNA-methyltransferase (adenine-specific) activity (gain)
c-value
0.0
0.1
0.2
0.3
0.4
0.5
0.6
0.7
0.8
p-value
4e-07
6e-06
8e-05
1e-03
2e-02
Log-odds
0.5
1.0
1.5
2.0
2.5
3.0
3.5
4.0
Cellular_organisms - ps1
Archaea1 - ps2
Archaea2 - ps3
Archaea3 - ps4
Asgard/Eukaryota - ps5
Eukaryota - ps6
Excavata/Diaphoretickes - ps7
Diaphoretickes - ps8
Archaeplastida - ps9
Viridiplantae - ps10
Streptophyta - ps11
Charophyceae/Embriophyta - ps12
Embriophyta - ps13
Tracheophyta - ps14
Spermatophyta - ps15
Angiosperms - ps16
Mesangiospermae - ps17
Magnoliids/eudicots - ps18
Eudicots - ps19
Core_eudicots - ps20
Superrosids - ps21
Rosids - ps22
Eurosids - ps23
Malvids - ps24
Sap./Malv./Brassicales - ps25
Malvales/Brassicales - ps26
Brassicales - ps27
Cleomaceae/Brassicaceae - ps28
Brassicaceae - ps29
Camelineae - ps30
Arabidopsis - ps31
Arabidopsis_thaliana - ps32
Phylostratum

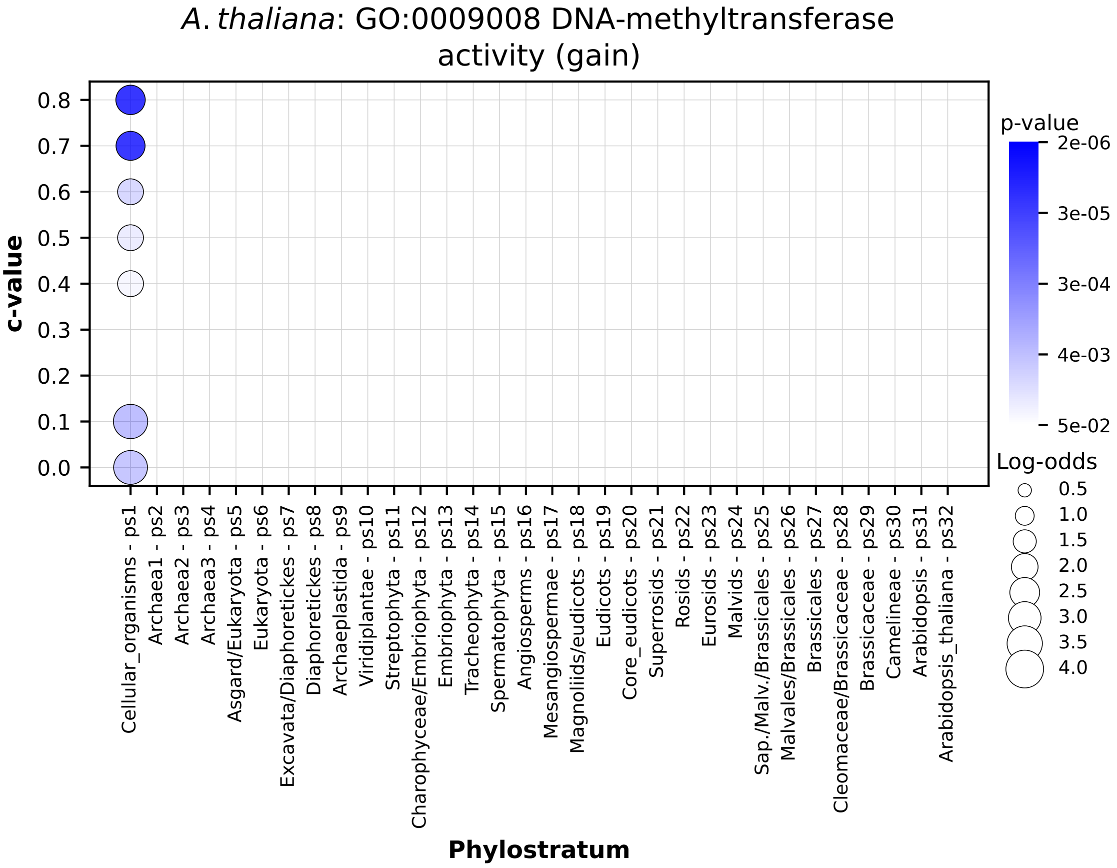

A. thaliana: GO:0009008 DNA-methyltransferase activity (gain)
c-value
0.8
0.7
0.6
0.5
0.4
0.3
0.2
0.1
0.0
p-value
2e-06
3e-05
3e-04
4e-03
5e-02
Log-odds
0.5
1.0
1.5
2.0
2.5
3.0
3.5
4.0
Cellular_organisms - ps1
Archaea1 - ps2
Archaea2 - ps3
Archaea3 - ps4
Asgard/Eukaryota - ps5
Eukaryota - ps6
Excavata/Diaphoretickes - ps7
Diaphoretickes - ps8
Archaeplastida - ps9
Viridiplantae - ps10
Streptophyta - ps11
Charophyceae/Embriophyta - ps12
Embriophyta - ps13
Tracheophyta - ps14
Spermatophyta - ps15
Angiosperms - ps16
Mesangiospermae - ps17
Magnoliids/eudicots - ps18
Eudicots - ps19
Core_eudicots - ps20
Superrosids - ps21
Rosids - ps22
Eurosids - ps23
Malvids - ps24
Sap./Malv./Brassicales - ps25
Malvales/Brassicales - ps26
Brassicales - ps27
Cleomaceae/Brassicaceae - ps28
Brassicaceae - ps29
Camelineae - ps30
Arabidopsis - ps31
Arabidopsis_thaliana - ps32
Phylostratum

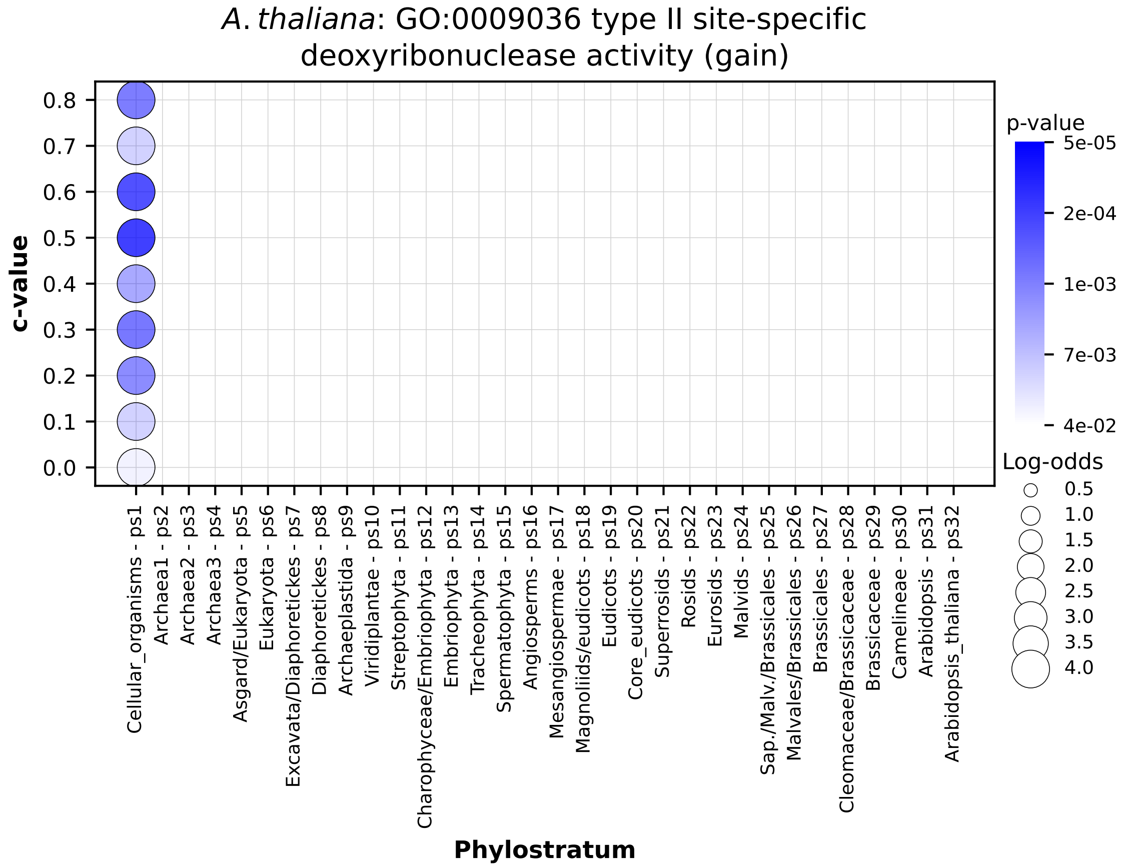

A. thaliana: GO:0009036 type II site-specific deoxyribonuclease activity (gain)
c-value
0.8
0.7
0.6
0.5
0.4
0.3
0.2
0.1
0.0
p-value
5e-05
2e-04
1e-03
7e-03
4e-02
Log-odds
0.5
1.0
1.5
2.0
2.5
3.0
3.5
4.0
Cellular_organisms - ps1
Archaea1 - ps2
Archaea2 - ps3
Archaea3 - ps4
Asgard/Eukaryota - ps5
Eukaryota - ps6
Excavata/Diaphoretickes - ps7
Diaphoretickes - ps8
Archaeplastida - ps9
Viridiplantae - ps10
Streptophyta - ps11
Charophyceae/Embriophyta - ps12
Embriophyta - ps13
Tracheophyta - ps14
Spermatophyta - ps15
Angiosperms - ps16
Mesangiospermae - ps17
Magnoliids/eudicots - ps18
Eudicots - ps19
Core_eudicots - ps20
Superrosids - ps21
Rosids - ps22
Eurosids - ps23
Malvids - ps24
Sap./Malv./Brassicales - ps25
Malvales/Brassicales - ps26
Brassicales - ps27
Cleomaceae/Brassicaceae - ps28
Brassicaceae - ps29
Camelineae - ps30
Arabidopsis - ps31
Arabidopsis_thaliana - ps32
Phylostratum

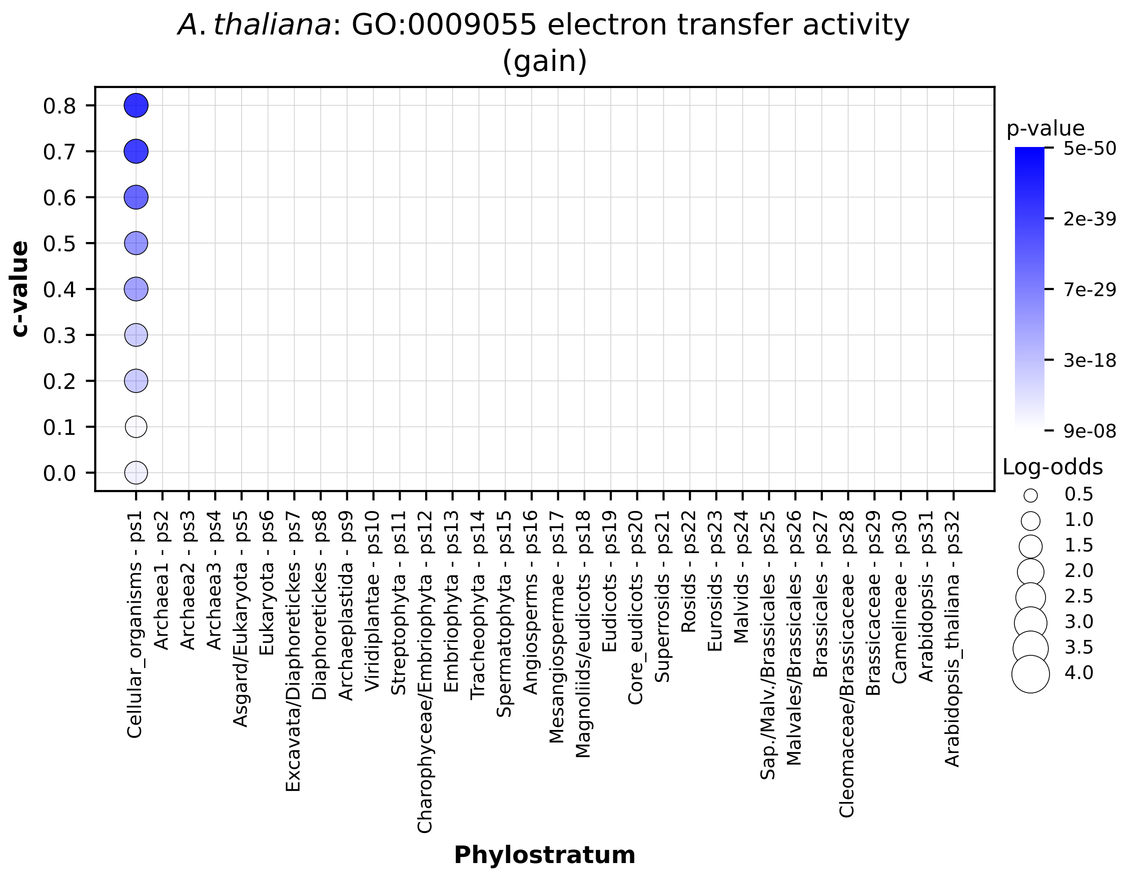

A. thaliana: GO:0009055 electron transfer activity (gain)
c-value
0.0
0.1
0.2
0.3
0.4
0.5
0.6
0.7
0.8
p-value
5e-50
2e-39
7e-29
3e-18
9e-08
Log-odds
0.5
1.0
1.5
2.0
2.5
3.0
3.5
4.0
Cellular_organisms - ps1
Archaea1 - ps2
Archaea2 - ps3
Archaea3 - ps4
Asgard/Eukaryota - ps5
Eukaryota - ps6
Excavata/Diaphoretickes - ps7
Diaphoretickes - ps8
Archaeplastida - ps9
Viridiplantae - ps10
Streptophyta - ps11
Charophyceae/Embriophyta - ps12
Embriophyta - ps13
Tracheophyta - ps14
Spermatophyta - ps15
Angiosperms - ps16
Mesangiospermae - ps17
Magnoliids/eudicots - ps18
Eudicots - ps19
Core_eudicots - ps20
Superrosids - ps21
Rosids - ps22
Eurosids - ps23
Malvids - ps24
Sap./Malv./Brassicales - ps25
Malvales/Brassicales - ps26
Brassicales - ps27
Cleomaceae/Brassicaceae - ps28
Brassicaceae - ps29
Camelineae - ps30
Arabidopsis - ps31
Arabidopsis_thaliana - ps32
Phylostratum

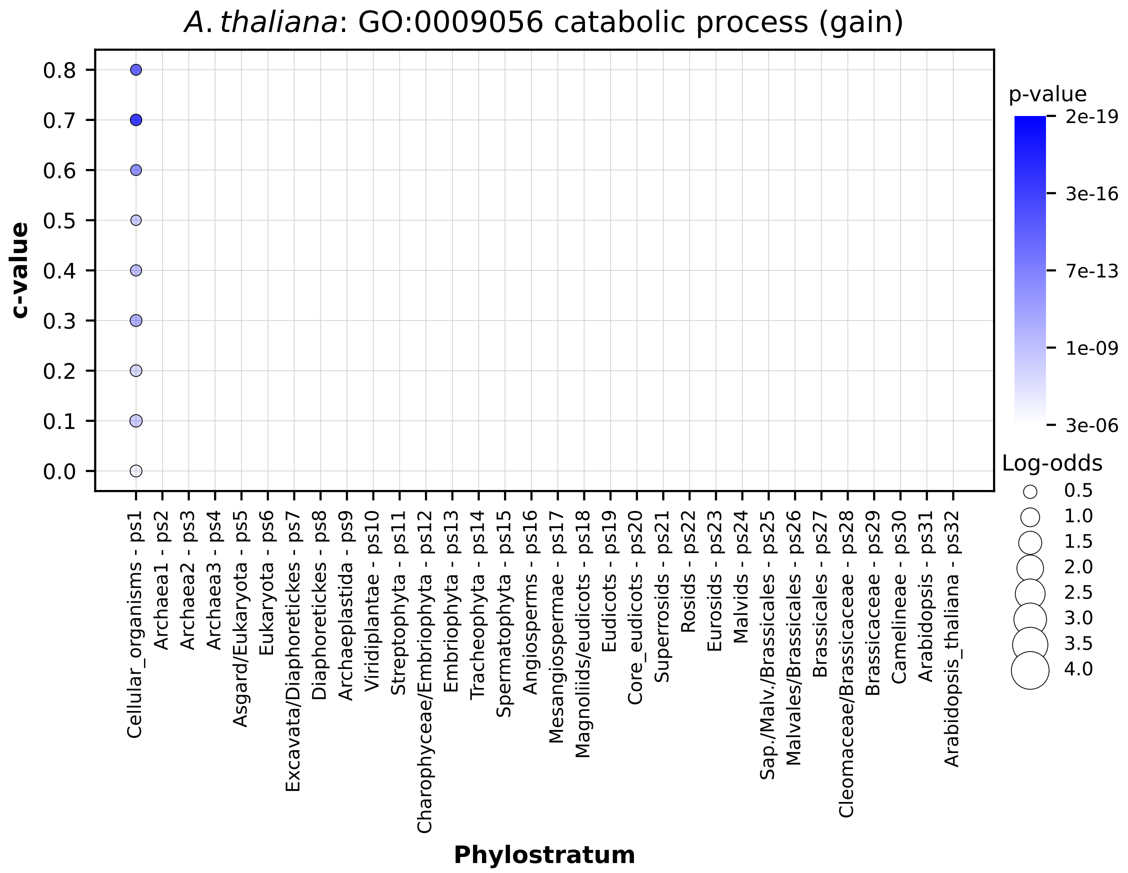
A. thaliana: GO:0009056 catabolic process (gain)
c-value
0.8
0.7
0.6
0.5
0.4
0.3
0.2
0.1
0.0
Phylostratum
Cellular_organisms - ps1
Archaea1 - ps2
Archaea2 - ps3
Archaea3 - ps4
Asgard/Eukaryota - ps5
Eukaryota - ps6
Excavata/Diaphoretickes - ps7
Diaphoretickes - ps8
Archaeplastida - ps9
Viridiplantae - ps10
Streptophyta - ps11
Charophyceae/Embriophyta - ps12
Embriophyta - ps13
Tracheophyta - ps14
Spermatophyta - ps15
Angiosperms - ps16
Mesangiospermae - ps17
Magnoliids/eudicots - ps18
Eudicots - ps19
Core_eudicots - ps20
Superrosids - ps21
Rosids - ps22
Eurosids - ps23
Malvids - ps24
Sap./Malv./Brassicales - ps25
Malvales/Brassicales - ps26
Brassicales - ps27
Cleomaceae/Brassicaceae - ps28
Brassicaceae - ps29
Camelineae - ps30
Arabidopsis - ps31
Arabidopsis_thaliana - ps32
p-value
2e-19
3e-16
7e-13
1e-09
3e-06
Log-odds
0.5
1.0
1.5
2.0
2.5
3.0
3.5
4.0

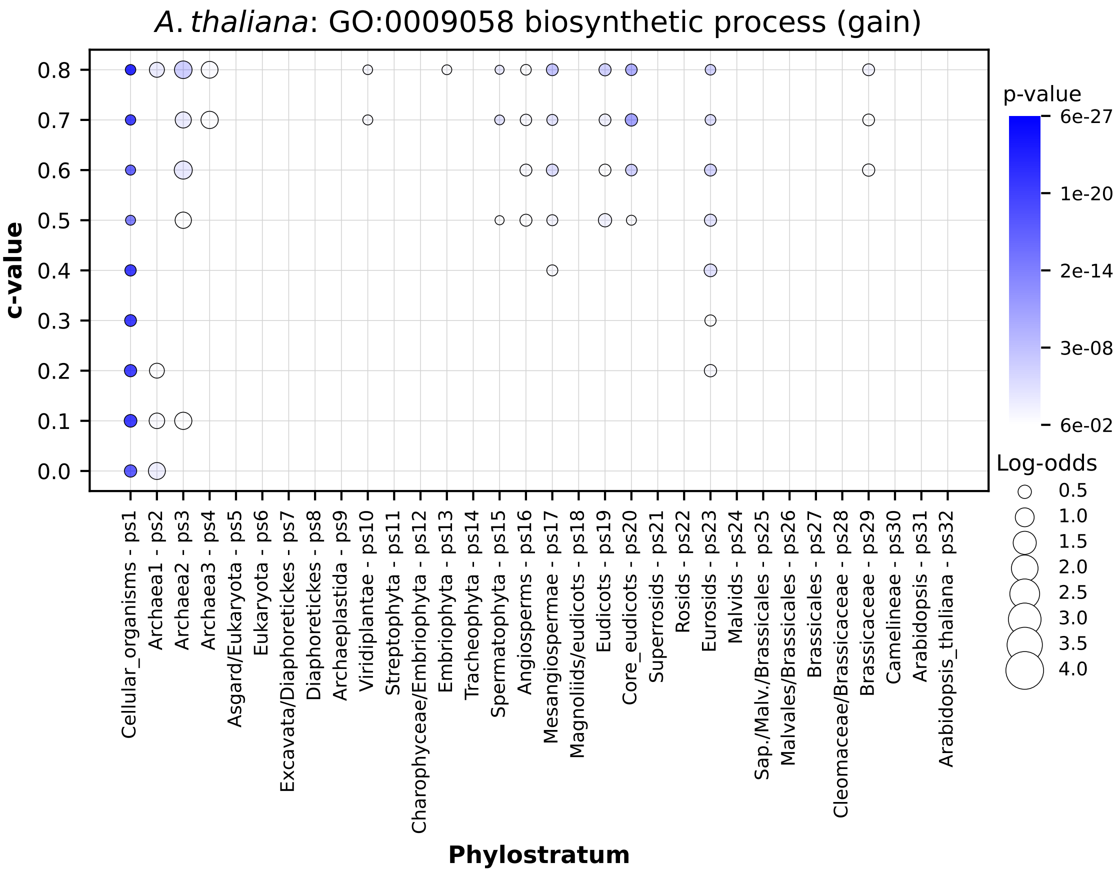

A. thaliana: GO:0009058 biosynthetic process (gain)
c-value
0.8
0.7
0.6
0.5
0.4
0.3
0.2
0.1
0.0
Phylostratum
Cellular_organisms - ps1
Archaea1 - ps2
Archaea2 - ps3
Archaea3 - ps4
Asgard/Eukaryota - ps5
Eukaryota - ps6
Excavata/Diaphoretickes - ps7
Diaphoretickes - ps8
Archaeplastida - ps9
Viridiplantae - ps10
Streptophyta - ps11
Charophyceae/Embriophyta - ps12
Embriophyta - ps13
Tracheophyta - ps14
Spermatophyta - ps15
Angiosperms - ps16
Mesangiospermae - ps17
Magnoliids/eudicots - ps18
Eudicots - ps19
Core_eudicots - ps20
Superrosids - ps21
Rosids - ps22
Eurosids - ps23
Malvids - ps24
Sap./Malv./Brassicales - ps25
Malvales/Brassicales - ps26
Brassicales - ps27
Cleomaceae/Brassicaceae - ps28
Brassicaceae - ps29
Camelineae - ps30
Arabidopsis - ps31
Arabidopsis_thaliana - ps32
p-value
6e-27
1e-20
2e-14
3e-08
6e-02
Log-odds
0.5
1.0
1.5
2.0
2.5
3.0
3.5
4.0

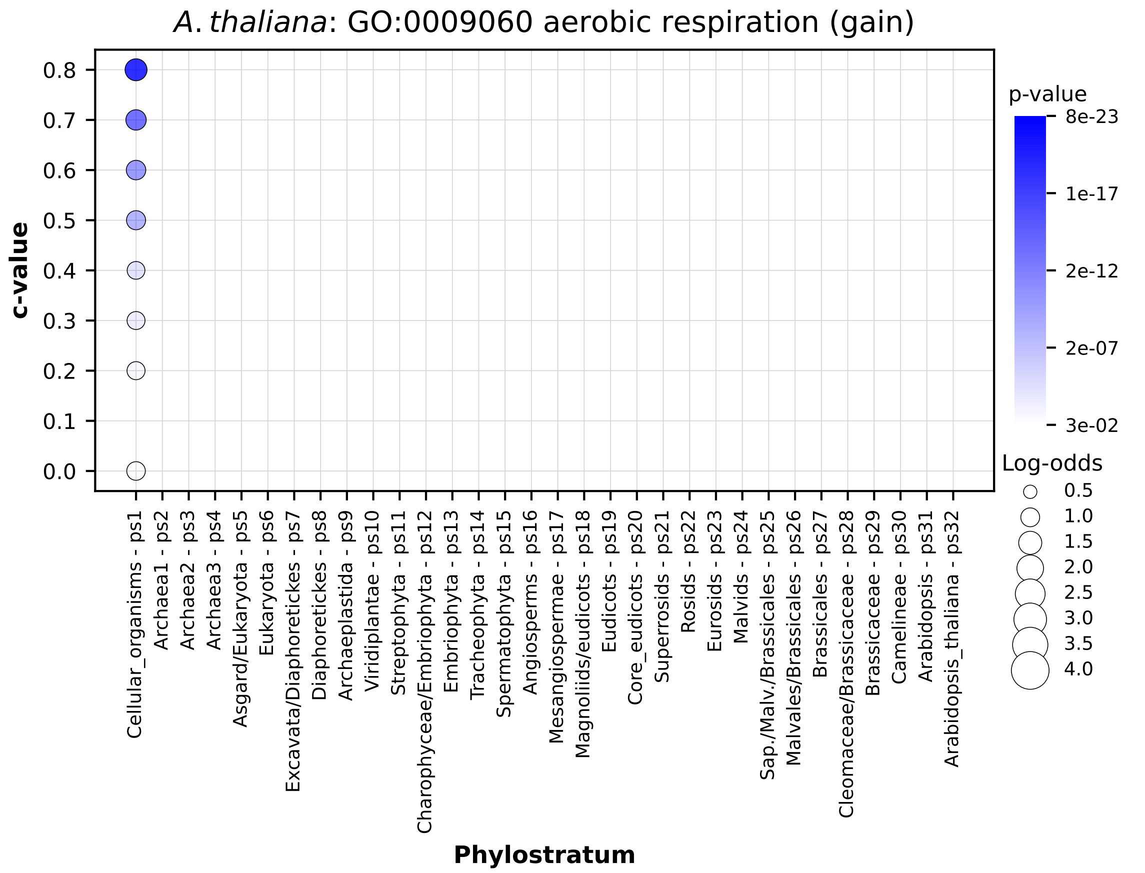
A. thaliana: GO:0009060 aerobic respiration (gain)
c-value
0.0
0.1
0.2
0.3
0.4
0.5
0.6
0.7
0.8
p-value
8e-23
1e-17
2e-12
2e-07
3e-02
Log-odds
0.5
1.0
1.5
2.0
2.5
3.0
3.5
4.0
Cellular_organisms - ps1
Archaea1 - ps2
Archaea2 - ps3
Archaea3 - ps4
Asgard/Eukaryota - ps5
Eukaryota - ps6
Excavata/Diaphoretickes - ps7
Diaphoretickes - ps8
Archaeplastida - ps9
Viridiplantae - ps10
Streptophyta - ps11
Charophyceae/Embriophyta - ps12
Embriophyta - ps13
Tracheophyta - ps14
Spermatophyta - ps15
Angiosperms - ps16
Mesangiospermae - ps17
Magnoliids/eudicots - ps18
Eudicots - ps19
Core_eudicots - ps20
Superrosids - ps21
Rosids - ps22
Eurosids - ps23
Malvids - ps24
Sap./Malv./Brassicales - ps25
Malvales/Brassicales - ps26
Brassicales - ps27
Cleomaceae/Brassicaceae - ps28
Brassicaceae - ps29
Camelineae - ps30
Arabidopsis - ps31
Arabidopsis_thaliana - ps32
Phylostratum

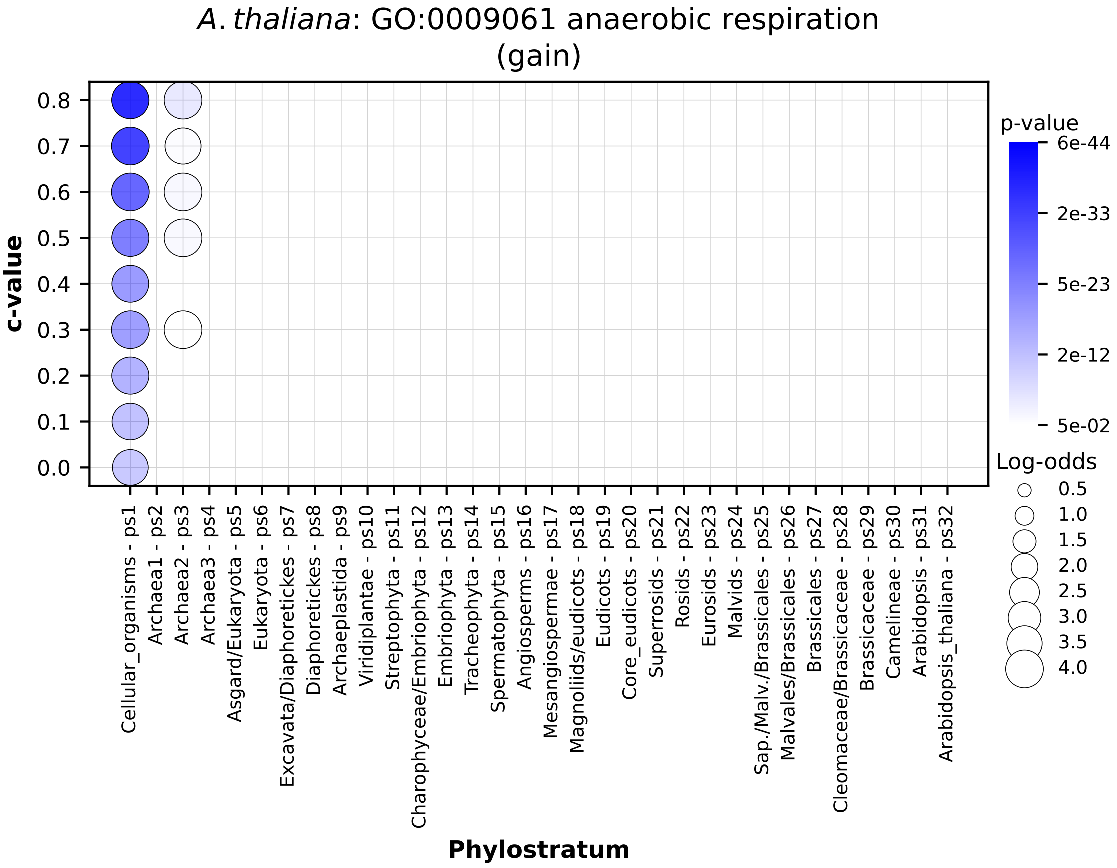

A. thaliana: GO:0009061 anaerobic respiration
(gain)
c-value
0.8
0.7
0.6
0.5
0.4
0.3
0.2
0.1
0.0
Phylostratum
Cellular_organisms - ps1
Archaea1 - ps2
Archaea2 - ps3
Archaea3 - ps4
Asgard/Eukaryota - ps5
Eukaryota - ps6
Excavata/Diaphoretickes - ps7
Diaphoretickes - ps8
Archaeplastida - ps9
Viridiplantae - ps10
Streptophyta - ps11
Charophyceae/Embriophyta - ps12
Embriophyta - ps13
Tracheophyta - ps14
Spermatophyta - ps15
Angiosperms - ps16
Mesangiospermae - ps17
Magnoliids/eudicots - ps18
Eudicots - ps19
Core_eudicots - ps20
Superrosids - ps21
Rosids - ps22
Eurosids - ps23
Malvids - ps24
Sap./Malv./Brassicales - ps25
Malvales/Brassicales - ps26
Brassicales - ps27
Cleomaceae/Brassicaceae - ps28
Brassicaceae - ps29
Camelineae - ps30
Arabidopsis - ps31
Arabidopsis_thaliana - ps32
p-value
6e-44
2e-33
5e-23
2e-12
5e-02
Log-odds
0.5
1.0
1.5
2.0
2.5
3.0
3.5
4.0

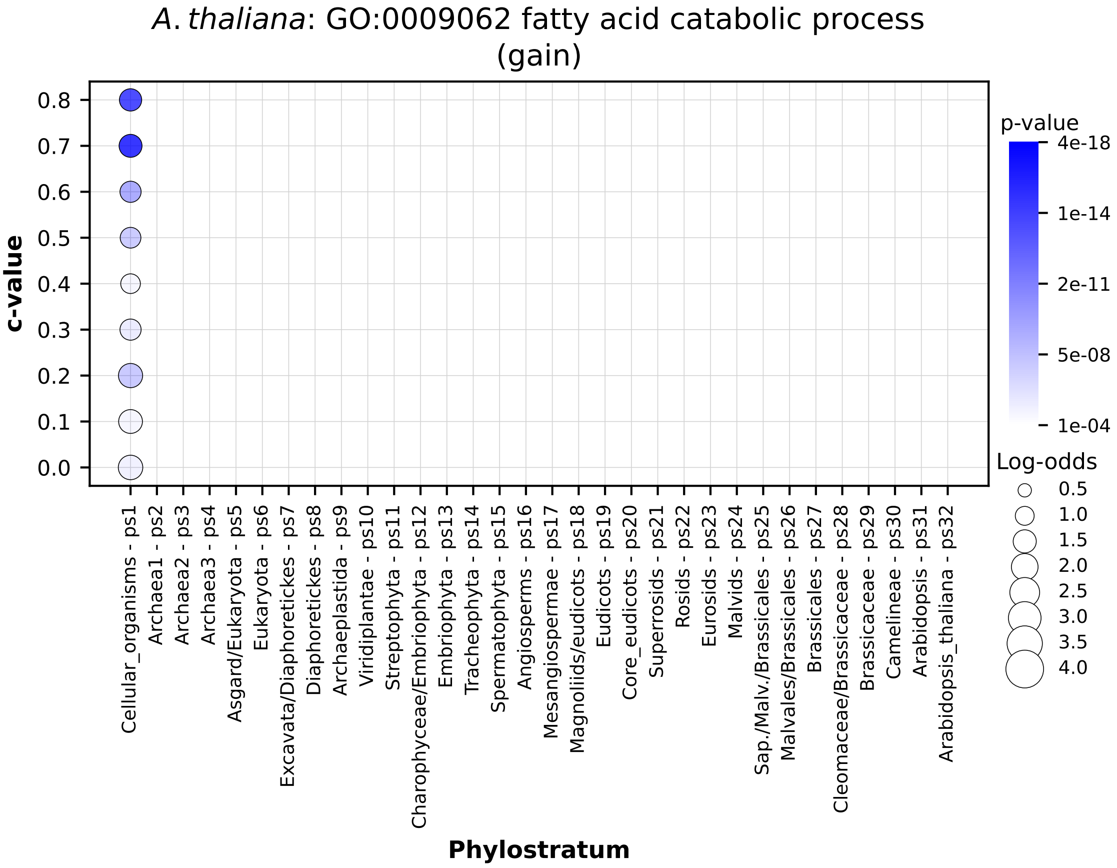

A. thaliana: GO:0009062 fatty acid catabolic process
(gain)
c-value
0.0
0.1
0.2
0.3
0.4
0.5
0.6
0.7
0.8
p-value
4e-18
1e-14
2e-11
5e-08
1e-04
Log-odds
0.5
1.0
1.5
2.0
2.5
3.0
3.5
4.0
Cellular_organisms - ps1
Archaea1 - ps2
Archaea2 - ps3
Archaea3 - ps4
Asgard/Eukaryota - ps5
Eukaryota - ps6
Excavata/Diaphoretickes - ps7
Diaphoretickes - ps8
Archaeplastida - ps9
Viridiplantae - ps10
Streptophyta - ps11
Charophyceae/Embriophyta - ps12
Embriophyta - ps13
Tracheophyta - ps14
Spermatophyta - ps15
Angiosperms - ps16
Mesangiospermae - ps17
Magnoliids/eudicots - ps18
Eudicots - ps19
Core_eudicots - ps20
Superrosids - ps21
Rosids - ps22
Eurosids - ps23
Malvids - ps24
Sap./Malv./Brassicales - ps25
Malvales/Brassicales - ps26
Brassicales - ps27
Cleomaceae/Brassicaceae - ps28
Brassicaceae - ps29
Camelineae - ps30
Arabidopsis - ps31
Arabidopsis_thaliana - ps32
Phylostratum

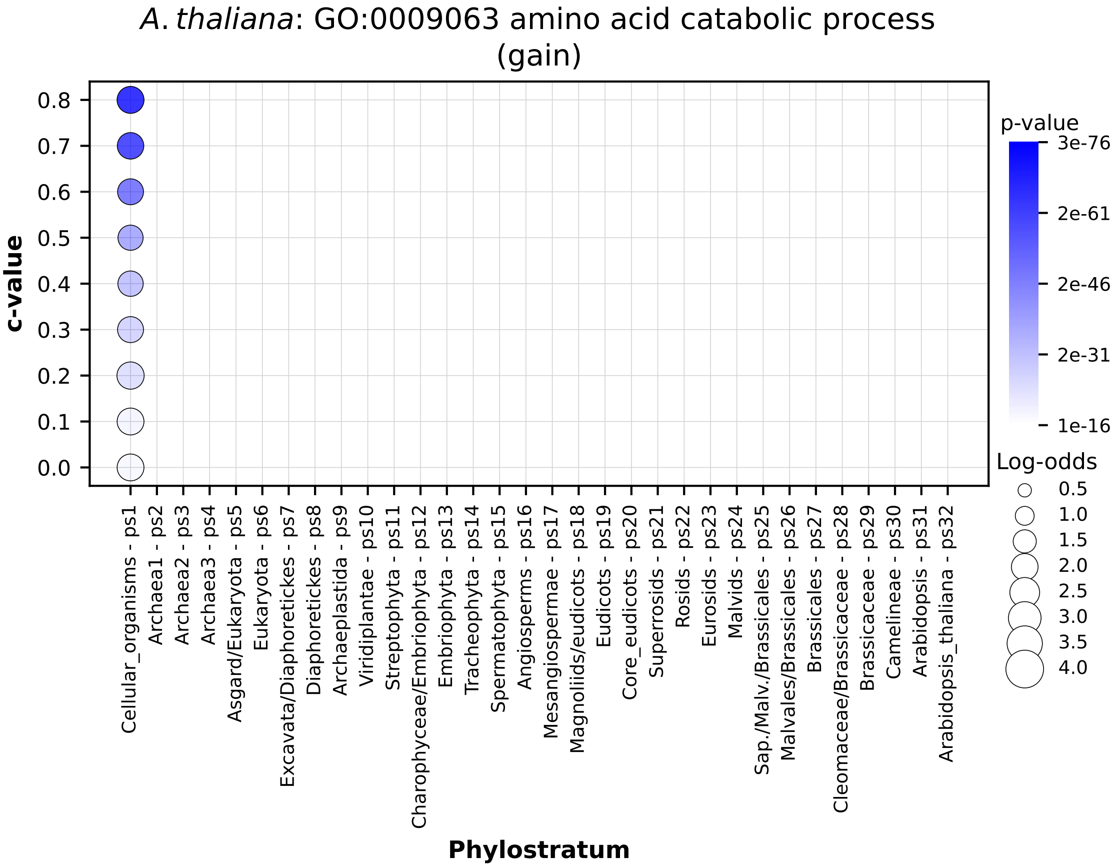

A. thaliana: GO:0009063 amino acid catabolic process (gain)
c-value
0.8
0.7
0.6
0.5
0.4
0.3
0.2
0.1
0.0
p-value
3e-76
2e-61
2e-46
2e-31
1e-16
Log-odds
0.5
1.0
1.5
2.0
2.5
3.0
3.5
4.0
Cellular_organisms - ps1
Archaea1 - ps2
Archaea2 - ps3
Archaea3 - ps4
Asgard/Eukaryota - ps5
Eukaryota - ps6
Excavata/Diaphoretickes - ps7
Diaphoretickes - ps8
Archaeplastida - ps9
Viridiplantae - ps10
Streptophyta - ps11
Charophyceae/Embriophyta - ps12
Embriophyta - ps13
Tracheophyta - ps14
Spermatophyta - ps15
Angiosperms - ps16
Mesangiospermae - ps17
Magnoliids/eudicots - ps18
Eudicots - ps19
Core_eudicots - ps20
Superrosids - ps21
Rosids - ps22
Eurosids - ps23
Malvids - ps24
Sap./Malv./Brassicales - ps25
Malvales/Brassicales - ps26
Brassicales - ps27
Cleomaceae/Brassicaceae - ps28
Brassicaceae - ps29
Camelineae - ps30
Arabidopsis - ps31
Arabidopsis_thaliana - ps32
Phylostratum

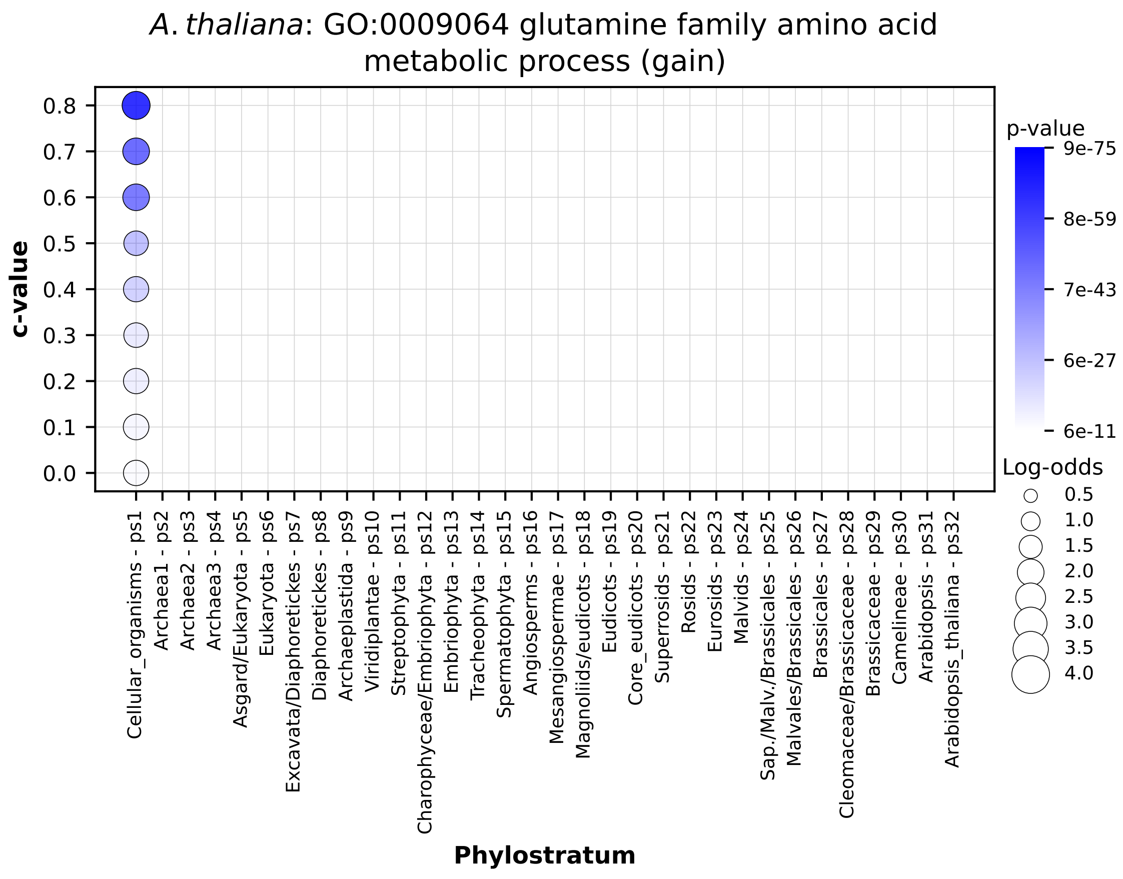

A. thaliana: GO:0009064 glutamine family amino acid metabolic process (gain)
c-value
0.0
0.1
0.2
0.3
0.4
0.5
0.6
0.7
0.8
Phylostratum
Cellular_organisms - ps1
Archaea1 - ps2
Archaea2 - ps3
Archaea3 - ps4
Asgard/Eukaryota - ps5
Eukaryota - ps6
Excavata/Diaphoretickes - ps7
Diaphoretickes - ps8
Archaeplastida - ps9
Viridiplantae - ps10
Streptophyta - ps11
Charophyceae/Embriophyta - ps12
Embriophyta - ps13
Tracheophyta - ps14
Spermatophyta - ps15
Angiosperms - ps16
Mesangiospermae - ps17
Magnoliids/eudicots - ps18
Eudicots - ps19
Core_eudicots - ps20
Superrosids - ps21
Rosids - ps22
Eurosids - ps23
Malvids - ps24
Sap./Malv./Brassicales - ps25
Malvales/Brassicales - ps26
Brassicales - ps27
Cleomaceae/Brassicaceae - ps28
Brassicaceae - ps29
Camelineae - ps30
Arabidopsis - ps31
Arabidopsis_thaliana - ps32
p-value
9e-75
8e-59
7e-43
6e-27
6e-11
Log-odds
0.5
1.0
1.5
2.0
2.5
3.0
3.5
4.0

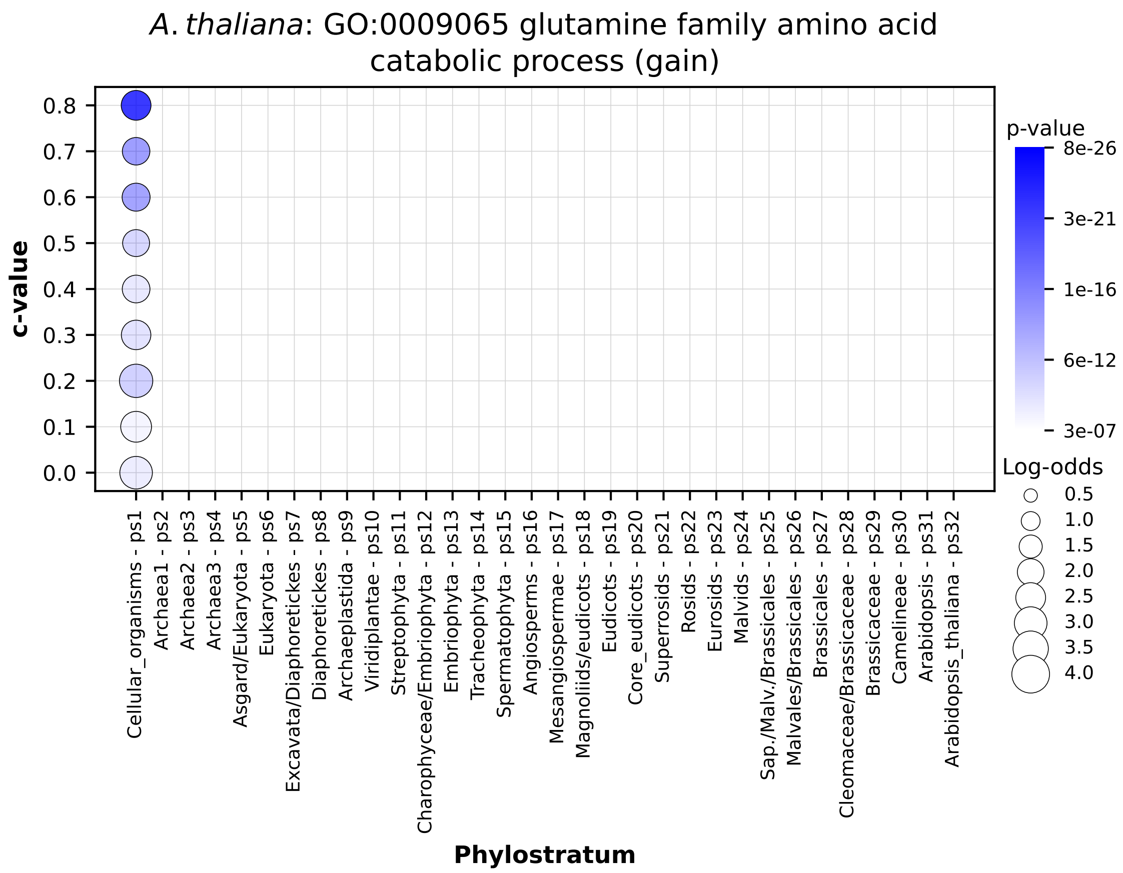
A. thaliana: GO:0009065 glutamine family amino acid catabolic process (gain)
c-value
0.0
0.1
0.2
0.3
0.4
0.5
0.6
0.7
0.8
p-value
8e-26
3e-21
1e-16
6e-12
3e-07
Log-odds
0.5
1.0
1.5
2.0
2.5
3.0
3.5
4.0
Cellular_organisms - ps1
Archaea1 - ps2
Archaea2 - ps3
Archaea3 - ps4
Asgard/Eukaryota - ps5
Eukaryota - ps6
Excavata/Diaphoretickes - ps7
Diaphoretickes - ps8
Archaeplastida - ps9
Viridiplantae - ps10
Streptophyta - ps11
Charophyceae/Embriophyta - ps12
Embriophyta - ps13
Tracheophyta - ps14
Spermatophyta - ps15
Angiosperms - ps16
Mesangiospermae - ps17
Magnoliids/eudicots - ps18
Eudicots - ps19
Core_eudicots - ps20
Superrosids - ps21
Rosids - ps22
Eurosids - ps23
Malvids - ps24
Sap./Malv./Brassicales - ps25
Malvales/Brassicales - ps26
Brassicales - ps27
Cleomaceae/Brassicaceae - ps28
Brassicaceae - ps29
Camelineae - ps30
Arabidopsis - ps31
Arabidopsis_thaliana - ps32
Phylostratum

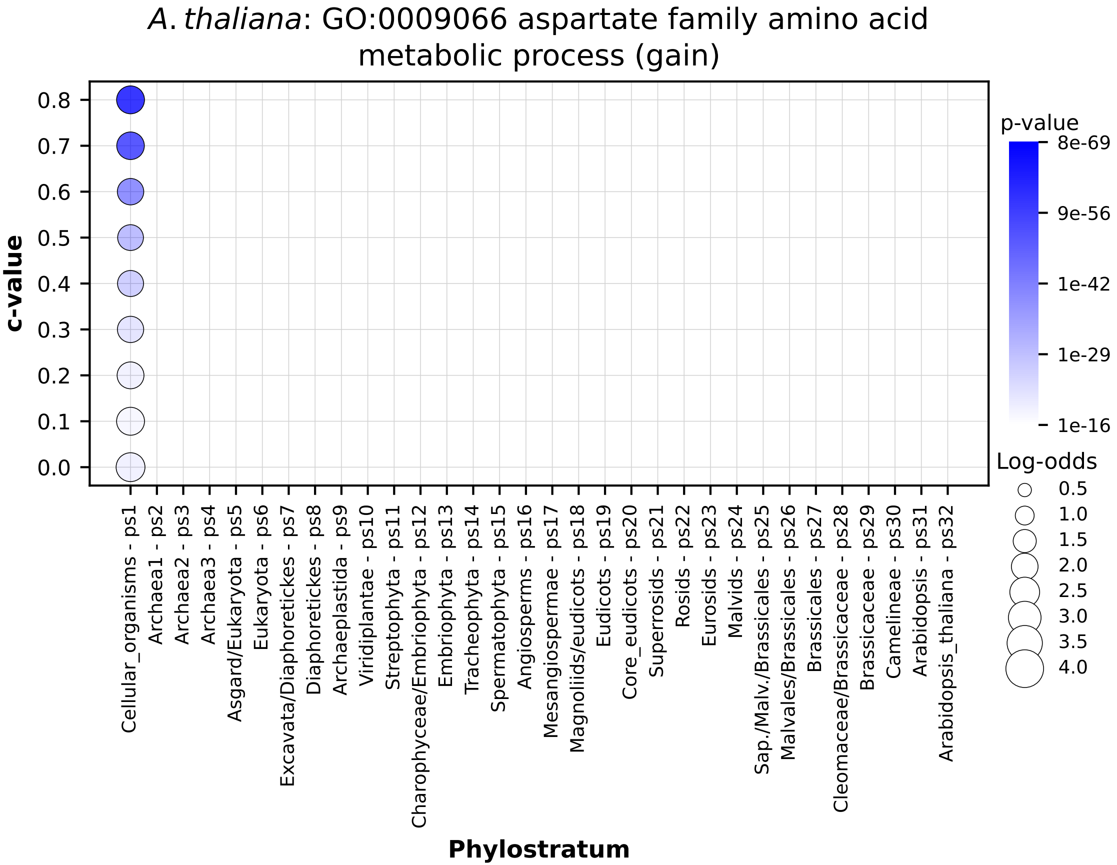
A. thaliana: GO:0009066 aspartate family amino acid metabolic process (gain)
c-value
0.0
0.1
0.2
0.3
0.4
0.5
0.6
0.7
0.8
p-value
8e-69
9e-56
1e-42
1e-29
1e-16
Log-odds
0.5
1.0
1.5
2.0
2.5
3.0
3.5
4.0
Cellular_organisms - ps1
Archaea1 - ps2
Archaea2 - ps3
Archaea3 - ps4
Asgard/Eukaryota - ps5
Eukaryota - ps6
Excavata/Diaphoretickes - ps7
Diaphoretickes - ps8
Archaeplastida - ps9
Viridiplantae - ps10
Streptophyta - ps11
Charophyceae/Embriophyta - ps12
Embriophyta - ps13
Tracheophyta - ps14
Spermatophyta - ps15
Angiosperms - ps16
Mesangiospermae - ps17
Magnoliids/eudicots - ps18
Eudicots - ps19
Core_eudicots - ps20
Superrosids - ps21
Rosids - ps22
Eurosids - ps23
Malvids - ps24
Sap./Malv./Brassicales - ps25
Malvales/Brassicales - ps26
Brassicales - ps27
Cleomaceae/Brassicaceae - ps28
Brassicaceae - ps29
Camelineae - ps30
Arabidopsis - ps31
Arabidopsis_thaliana - ps32
Phylostratum

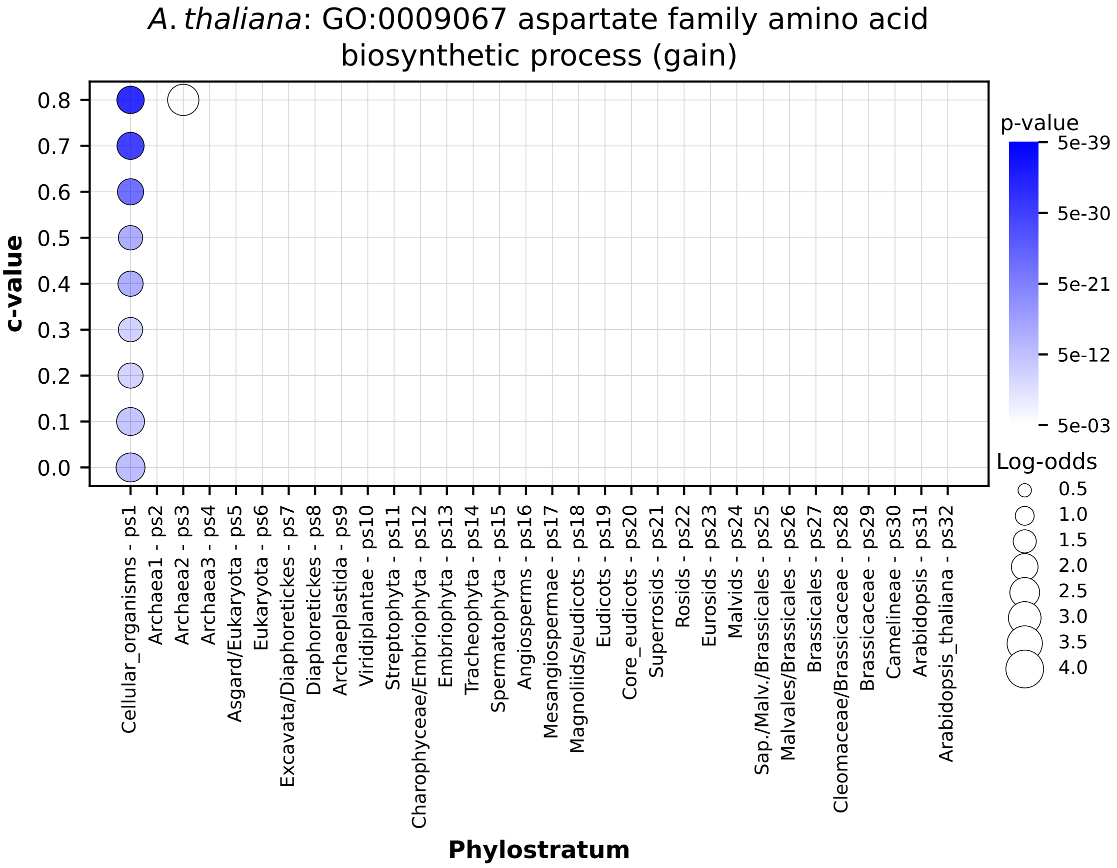

A. thaliana: GO:0009067 aspartate family amino acid biosynthetic process (gain)
c-value
0.8
0.7
0.6
0.5
0.4
0.3
0.2
0.1
0.0
p-value
5e-39
5e-30
5e-21
5e-12
5e-03
Log-odds
0.5
1.0
1.5
2.0
2.5
3.0
3.5
4.0
Cellular_organisms - ps1
Archaea1 - ps2
Archaea2 - ps3
Archaea3 - ps4
Asgard/Eukaryota - ps5
Eukaryota - ps6
Excavata/Diaphoretickes - ps7
Diaphoretickes - ps8
Archaeplastida - ps9
Viridiplantae - ps10
Streptophyta - ps11
Charophyceae/Embriophyta - ps12
Embriophyta - ps13
Tracheophyta - ps14
Spermatophyta - ps15
Angiosperms - ps16
Mesangiospermae - ps17
Magnoliids/eudicots - ps18
Eudicots - ps19
Core_eudicots - ps20
Superrosids - ps21
Rosids - ps22
Eurosids - ps23
Malvids - ps24
Sap./Malv./Brassicales - ps25
Malvales/Brassicales - ps26
Brassicales - ps27
Cleomaceae/Brassicaceae - ps28
Brassicaceae - ps29
Camelineae - ps30
Arabidopsis - ps31
Arabidopsis_thaliana - ps32
Phylostratum

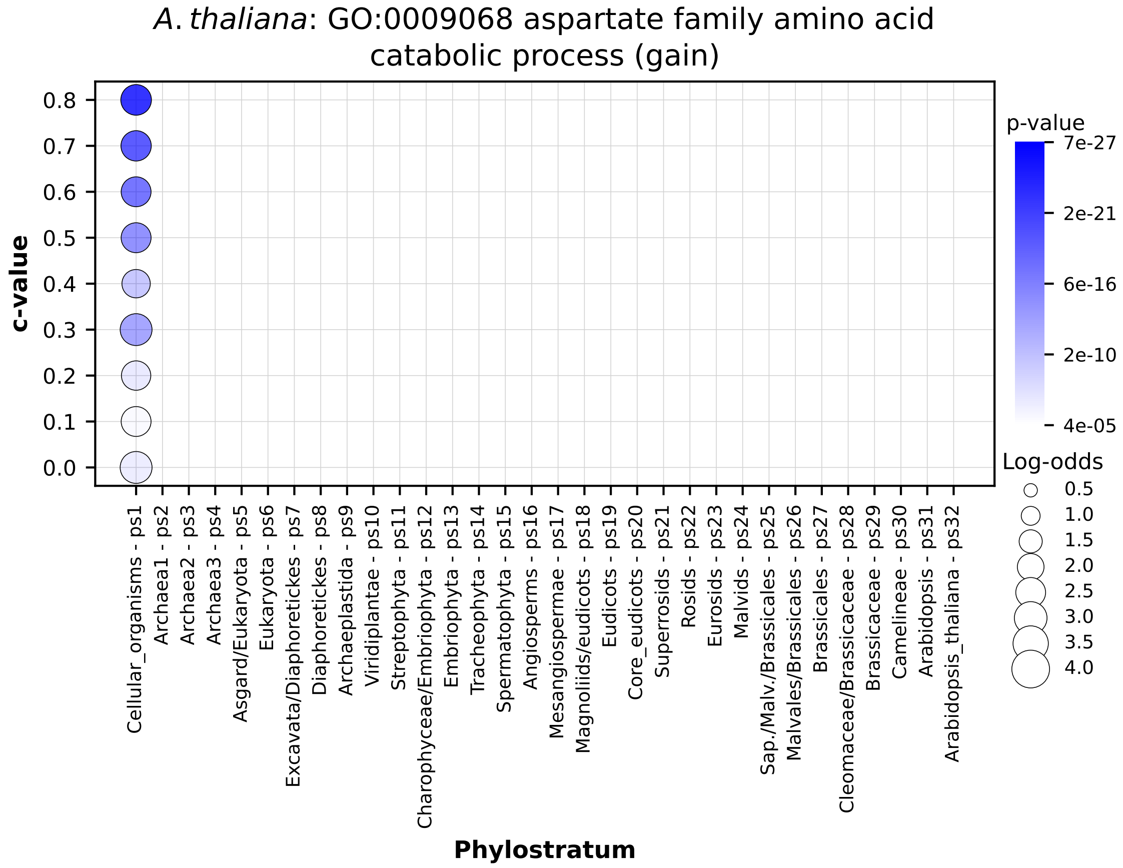

A. thaliana: GO:0009068 aspartate family amino acid catabolic process (gain)
c-value
0.8
0.7
0.6
0.5
0.4
0.3
0.2
0.1
0.0
Phylostratum
Cellular_organisms - ps1
Archaea1 - ps2
Archaea2 - ps3
Archaea3 - ps4
Asgard/Eukaryota - ps5
Eukaryota - ps6
Excavata/Diaphoretickes - ps7
Diaphoretickes - ps8
Archaeplastida - ps9
Viridiplantae - ps10
Streptophyta - ps11
Charophyceae/Embriophyta - ps12
Embriophyta - ps13
Tracheophyta - ps14
Spermatophyta - ps15
Angiosperms - ps16
Mesangiospermae - ps17
Magnoliids/eudicots - ps18
Eudicots - ps19
Core_eudicots - ps20
Superrosids - ps21
Rosids - ps22
Eurosids - ps23
Malvids - ps24
Sap./Malv./Brassicales - ps25
Malvales/Brassicales - ps26
Brassicales - ps27
Cleomaceae/Brassicaceae - ps28
Brassicaceae - ps29
Camelineae - ps30
Arabidopsis - ps31
Arabidopsis_thaliana - ps32
p-value
7e-27
2e-21
6e-16
2e-10
4e-05
Log-odds
0.5
1.0
1.5
2.0
2.5
3.0
3.5
4.0

A. thaliana: GO:0009069 serine family amino acid metabolic process (gain)

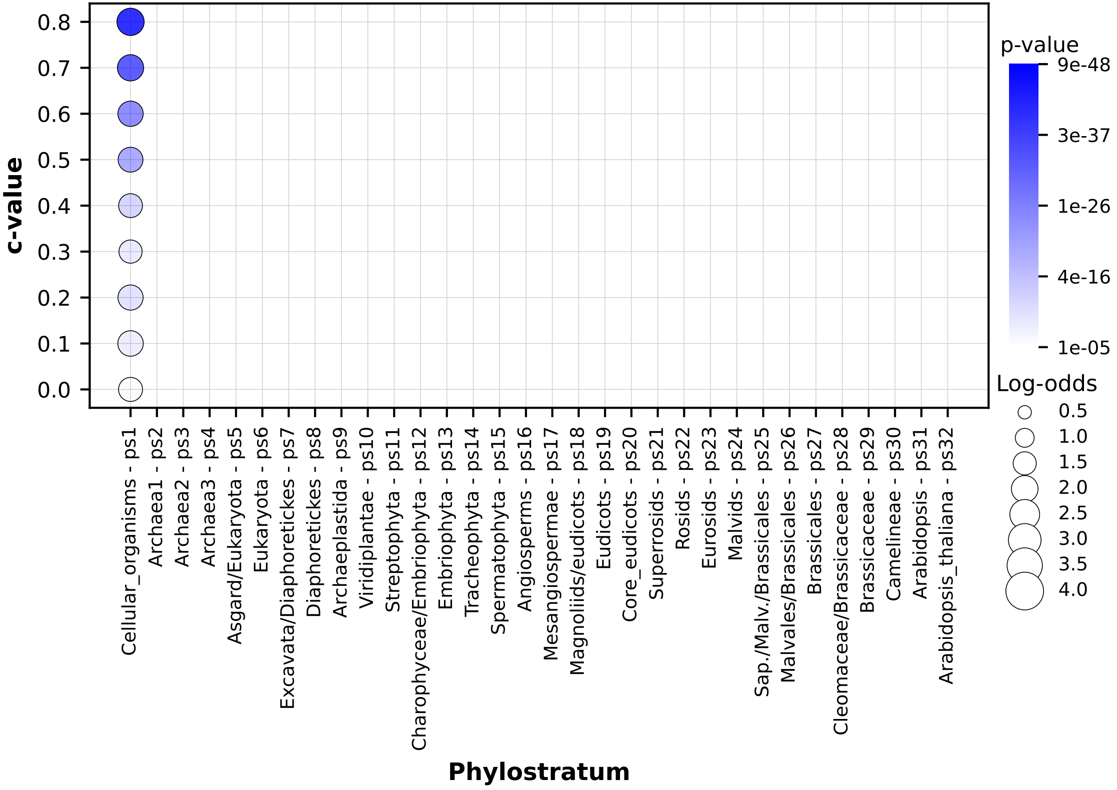

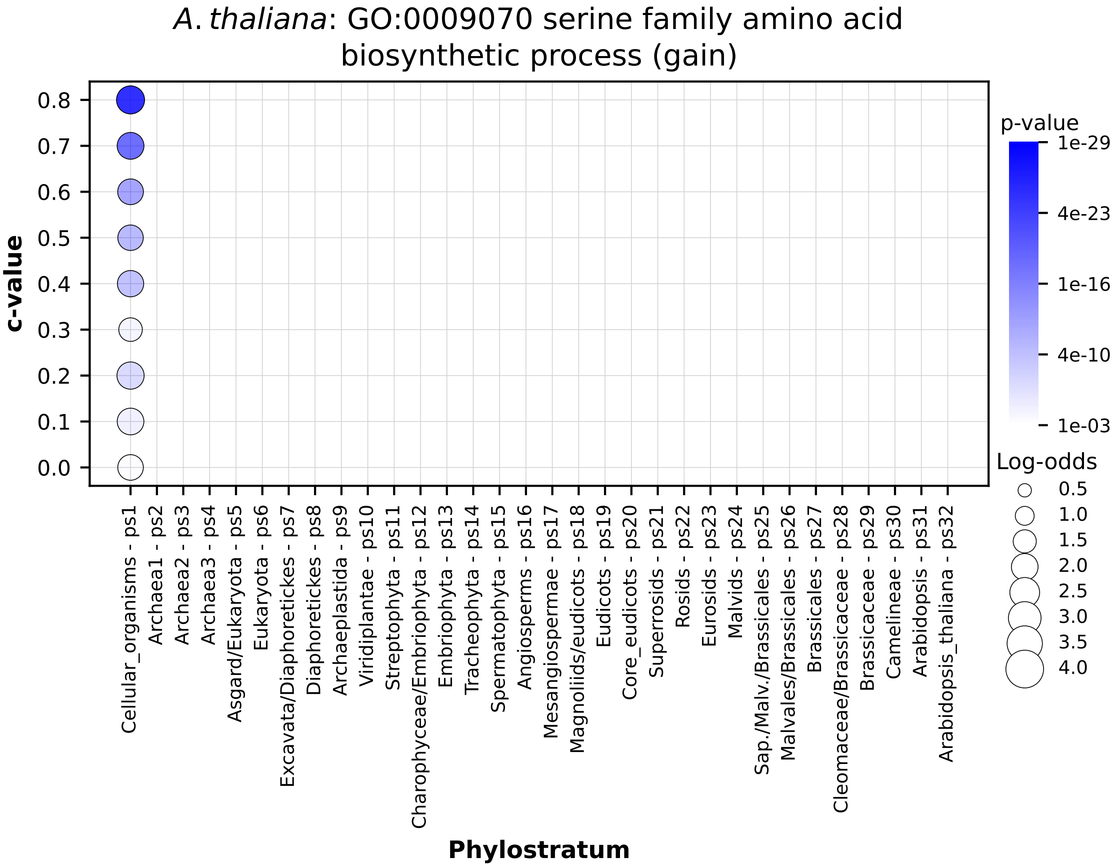
A. thaliana: GO:0009070 serine family amino acid biosynthetic process (gain)
c-value
0.8
0.7
0.6
0.5
0.4
0.3
0.2
0.1
0.0
p-value
1e-29
4e-23
1e-16
4e-10
1e-03
Log-odds
0.5
1.0
1.5
2.0
2.5
3.0
3.5
4.0
Cellular_organisms - ps1
Archaea1 - ps2
Archaea2 - ps3
Archaea3 - ps4
Asgard/Eukaryota - ps5
Eukaryota - ps6
Excavata/Diaphoretickes - ps7
Diaphoretickes - ps8
Archaeplastida - ps9
Viridiplantae - ps10
Streptophyta - ps11
Charophyceae/Embriophyta - ps12
Embriophyta - ps13
Tracheophyta - ps14
Spermatophyta - ps15
Angiosperms - ps16
Mesangiospermae - ps17
Magnoliids/eudicots - ps18
Eudicots - ps19
Core_eudicots - ps20
Superrosids - ps21
Rosids - ps22
Eurosids - ps23
Malvids - ps24
Sap./Malv./Brassicales - ps25
Malvales/Brassicales - ps26
Brassicales - ps27
Cleomaceae/Brassicaceae - ps28
Brassicaceae - ps29
Camelineae - ps30
Arabidopsis - ps31
Arabidopsis_thaliana - ps32
Phylostratum

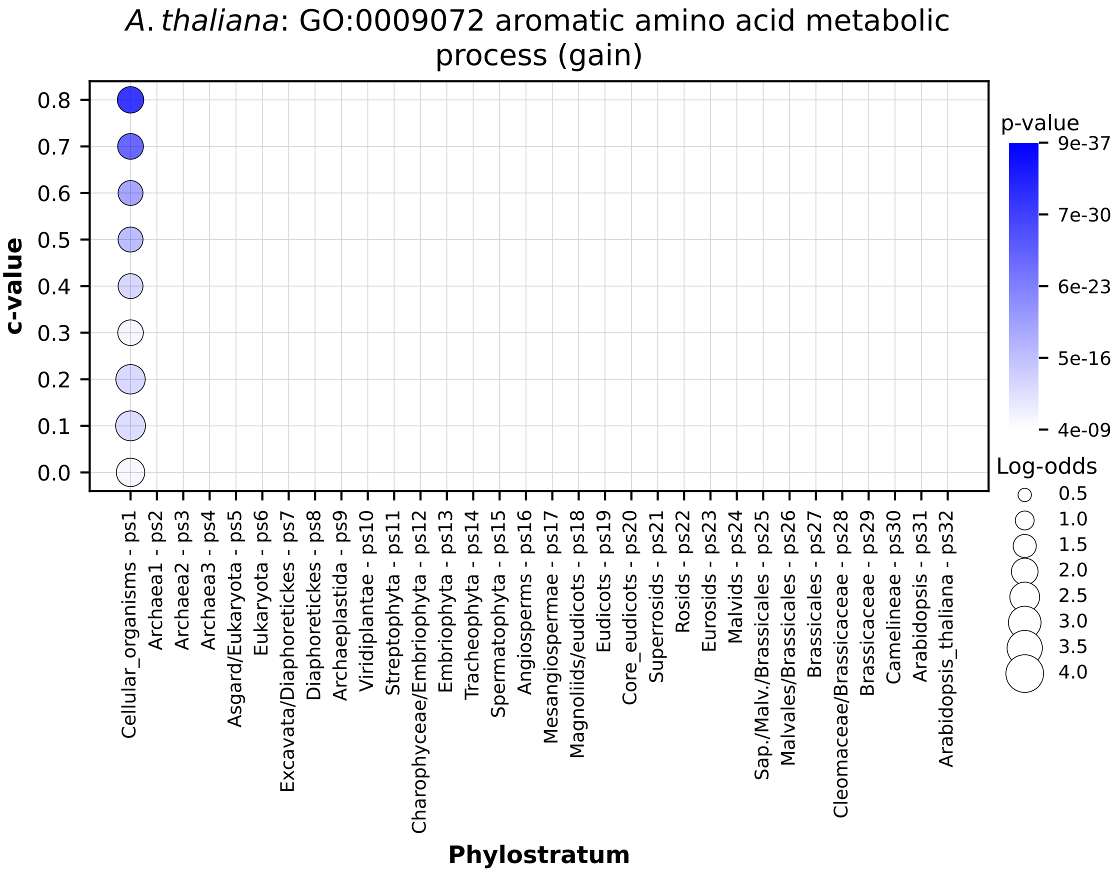

A. thaliana: GO:0009072 aromatic amino acid metabolic process (gain)
c-value
0.8
0.7
0.6
0.5
0.4
0.3
0.2
0.1
0.0
p-value
9e-37
7e-30
6e-23
5e-16
4e-09
Log-odds
0.5
1.0
1.5
2.0
2.5
3.0
3.5
4.0
Cellular_organisms - ps1
Archaea1 - ps2
Archaea2 - ps3
Archaea3 - ps4
Asgard/Eukaryota - ps5
Eukaryota - ps6
Excavata/Diaphoretickes - ps7
Diaphoretickes - ps8
Archaeplastida - ps9
Viridiplantae - ps10
Streptophyta - ps11
Charophyceae/Embriophyta - ps12
Embriophyta - ps13
Tracheophyta - ps14
Spermatophyta - ps15
Angiosperms - ps16
Mesangiospermae - ps17
Magnoliids/eudicots - ps18
Eudicots - ps19
Core_eudicots - ps20
Superrosids - ps21
Rosids - ps22
Eurosids - ps23
Malvids - ps24
Sap./Malv./Brassicales - ps25
Malvales/Brassicales - ps26
Brassicales - ps27
Cleomaceae/Brassicaceae - ps28
Brassicaceae - ps29
Camelineae - ps30
Arabidopsis - ps31
Arabidopsis_thaliana - ps32
Phylostratum

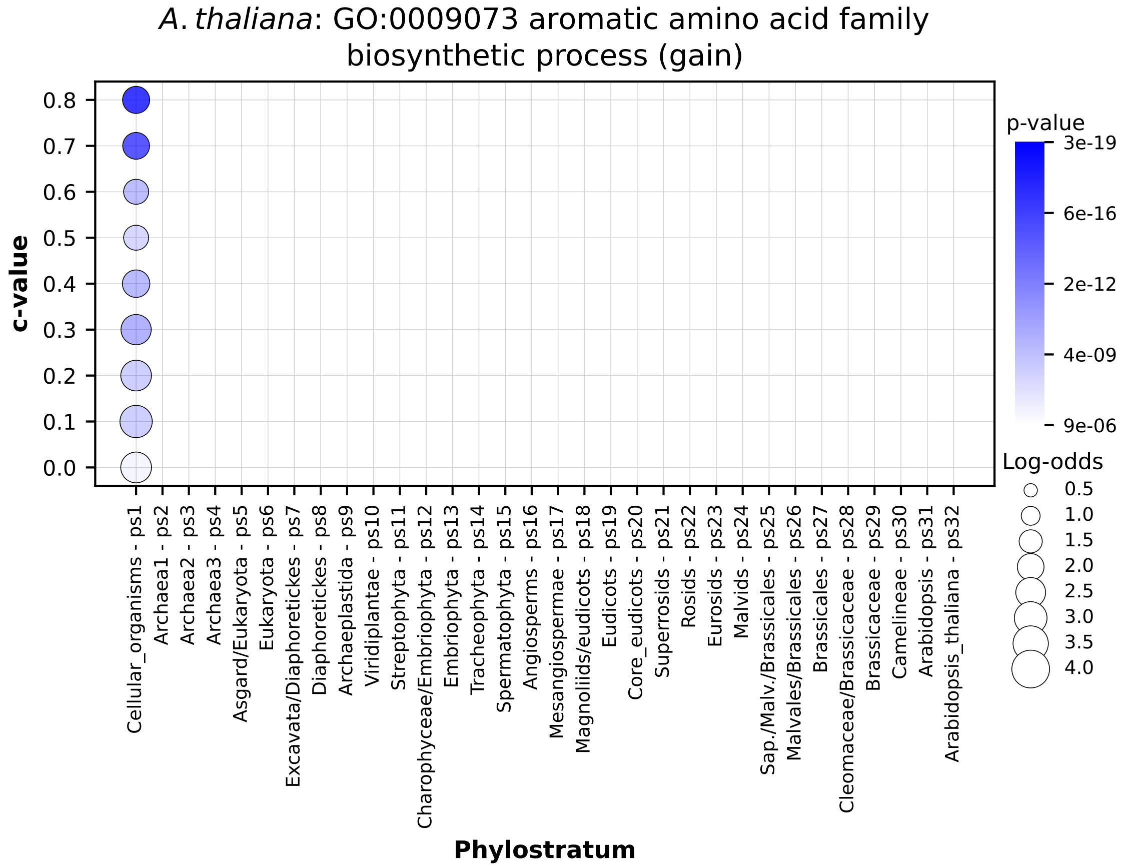
A. thaliana: GO:0009073 aromatic amino acid family biosynthetic process (gain)
c-value
0.8
0.7
0.6
0.5
0.4
0.3
0.2
0.1
0.0
Phylostratum
Cellular_organisms - ps1
Archaea1 - ps2
Archaea2 - ps3
Archaea3 - ps4
Asgard/Eukaryota - ps5
Eukaryota - ps6
Excavata/Diaphoretickes - ps7
Diaphoretickes - ps8
Archaeplastida - ps9
Viridiplantae - ps10
Streptophyta - ps11
Charophyceae/Embriophyta - ps12
Embriophyta - ps13
Tracheophyta - ps14
Spermatophyta - ps15
Angiosperms - ps16
Mesangiospermae - ps17
Magnoliids/eudicots - ps18
Eudicots - ps19
Core_eudicots - ps20
Superrosids - ps21
Rosids - ps22
Eurosids - ps23
Malvids - ps24
Sap./Malv./Brassicales - ps25
Malvales/Brassicales - ps26
Brassicales - ps27
Cleomaceae/Brassicaceae - ps28
Brassicaceae - ps29
Camelineae - ps30
Arabidopsis - ps31
Arabidopsis_thaliana - ps32
p-value
3e-19
6e-16
2e-12
4e-09
9e-06
Log-odds
0.5
1.0
1.5
2.0
2.5
3.0
3.5
4.0

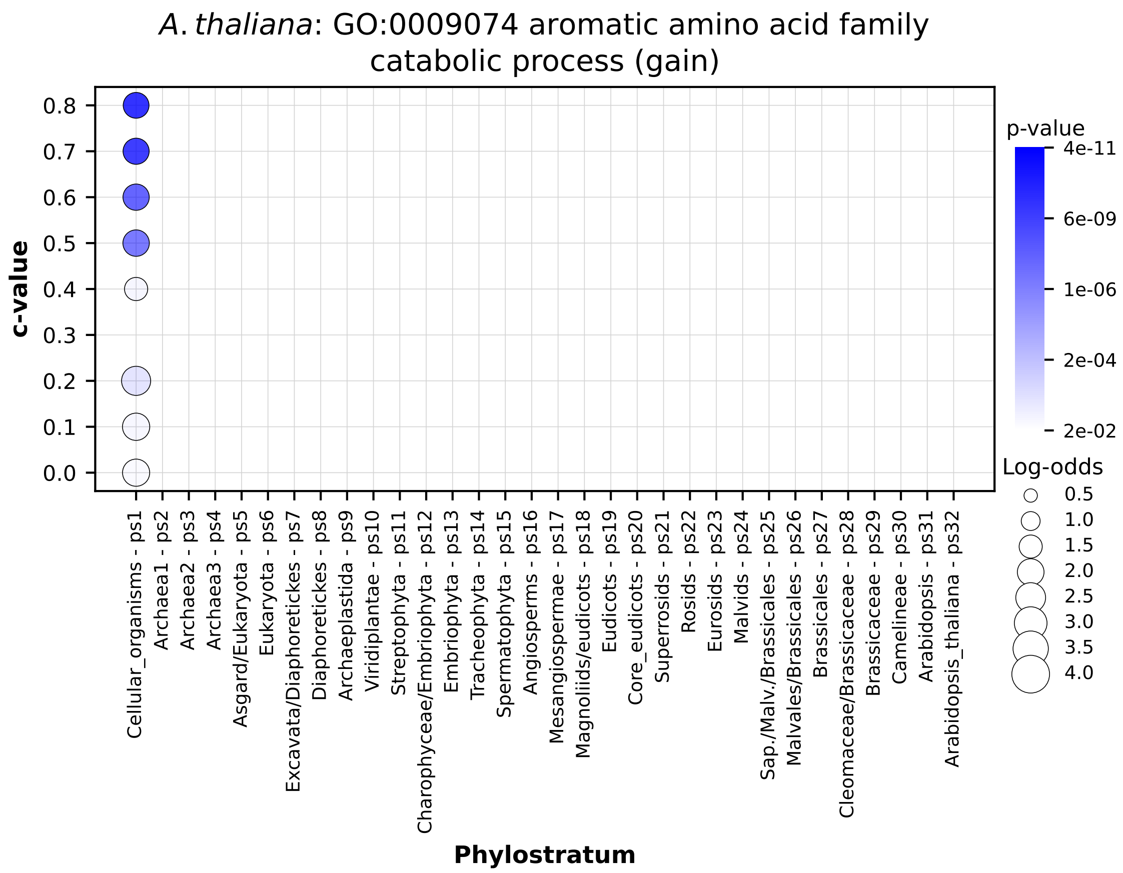

A. thaliana: GO:0009074 aromatic amino acid family catabolic process (gain)
c-value
0.8
0.7
0.6
0.5
0.4
0.3
0.2
0.1
0.0
p-value
4e-11
6e-09
1e-06
2e-04
2e-02
Log-odds
0.5
1.0
1.5
2.0
2.5
3.0
3.5
4.0
Cellular_organisms - ps1
Archaea1 - ps2
Archaea2 - ps3
Archaea3 - ps4
Asgard/Eukaryota - ps5
Eukaryota - ps6
Excavata/Diaphoretickes - ps7
Diaphoretickes - ps8
Archaeplastida - ps9
Viridiplantae - ps10
Streptophyta - ps11
Charophyceae/Embriophyta - ps12
Embriophyta - ps13
Tracheophyta - ps14
Spermatophyta - ps15
Angiosperms - ps16
Mesangiospermae - ps17
Magnoliids/eudicots - ps18
Eudicots - ps19
Core_eudicots - ps20
Superrosids - ps21
Rosids - ps22
Eurosids - ps23
Malvids - ps24
Sap./Malv./Brassicales - ps25
Malvales/Brassicales - ps26
Brassicales - ps27
Cleomaceae/Brassicaceae - ps28
Brassicaceae - ps29
Camelineae - ps30
Arabidopsis - ps31
Arabidopsis_thaliana - ps32
Phylostratum

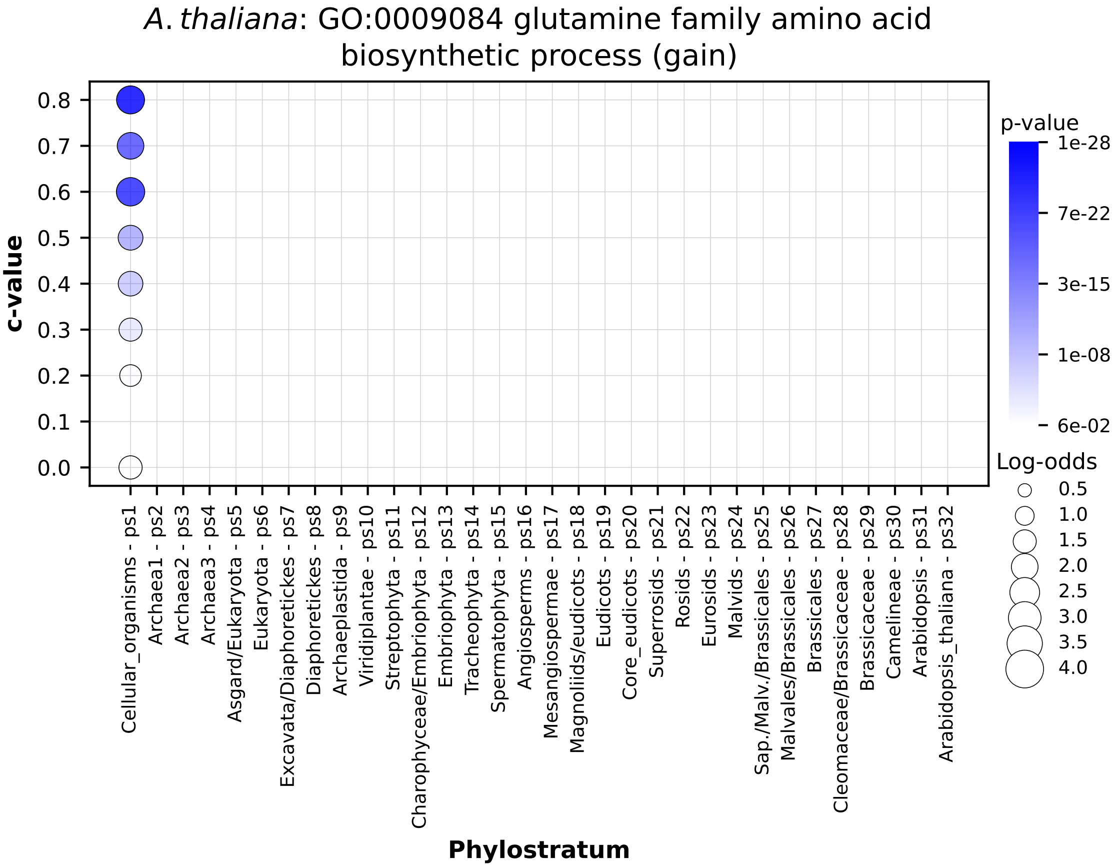

A. thaliana: GO:0009084 glutamine family amino acid biosynthetic process (gain)
c-value
0.0
0.1
0.2
0.3
0.4
0.5
0.6
0.7
0.8
p-value
1e-28
7e-22
3e-15
1e-08
6e-02
Log-odds
0.5
1.0
1.5
2.0
2.5
3.0
3.5
4.0
Cellular_organisms - ps1
Archaea1 - ps2
Archaea2 - ps3
Archaea3 - ps4
Asgard/Eukaryota - ps5
Eukaryota - ps6
Excavata/Diaphoretickes - ps7
Diaphoretickes - ps8
Archaeplastida - ps9
Viridiplantae - ps10
Streptophyta - ps11
Charophyceae/Embriophyta - ps12
Embriophyta - ps13
Tracheophyta - ps14
Spermatophyta - ps15
Angiosperms - ps16
Mesangiospermae - ps17
Magnoliids/eudicots - ps18
Eudicots - ps19
Core_eudicots - ps20
Superrosids - ps21
Rosids - ps22
Eurosids - ps23
Malvids - ps24
Sap./Malv./Brassicales - ps25
Malvales/Brassicales - ps26
Brassicales - ps27
Cleomaceae/Brassicaceae - ps28
Brassicaceae - ps29
Camelineae - ps30
Arabidopsis - ps31
Arabidopsis_thaliana - ps32
Phylostratum

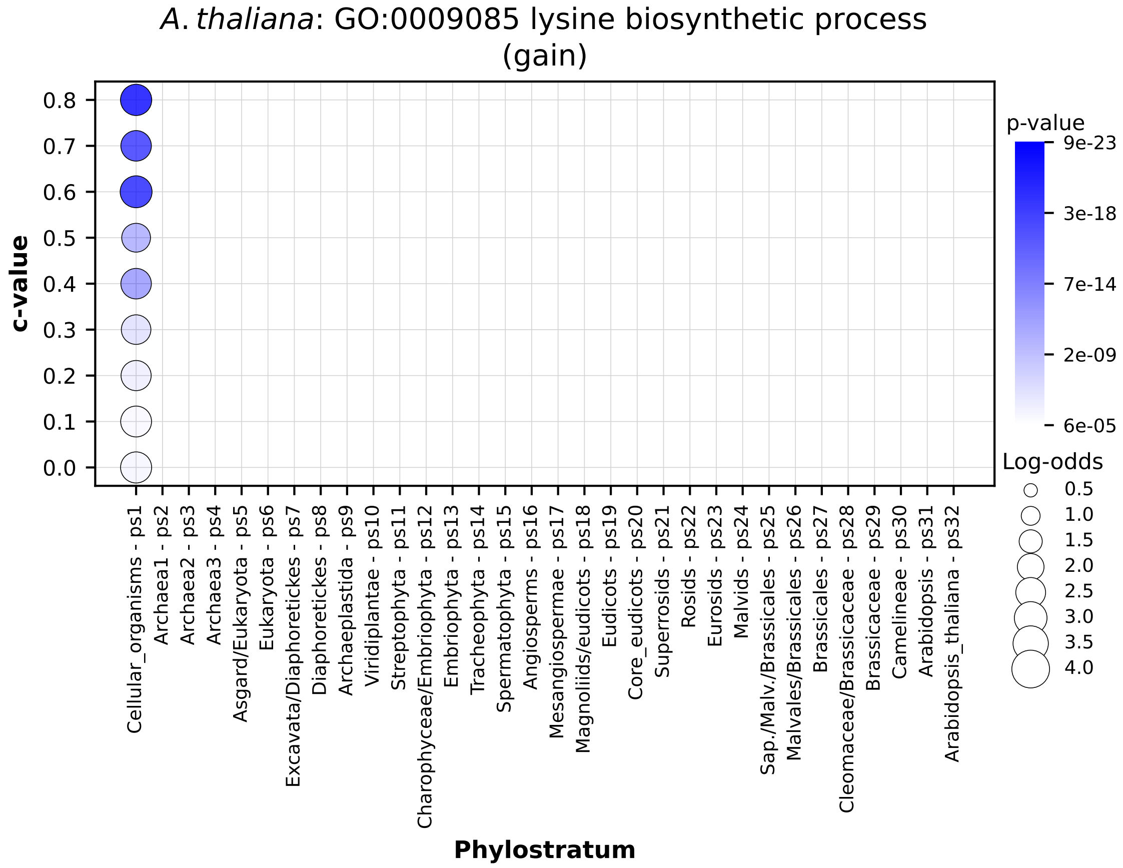

A. thaliana: GO:0009085 lysine biosynthetic process
(gain)
c-value
0.8
0.7
0.6
0.5
0.4
0.3
0.2
0.1
0.0
p-value
9e-23
3e-18
7e-14
2e-09
6e-05
Log-odds
0.5
1.0
1.5
2.0
2.5
3.0
3.5
4.0
Cellular_organisms - ps1
Archaea1 - ps2
Archaea2 - ps3
Archaea3 - ps4
Asgard/Eukaryota - ps5
Eukaryota - ps6
Excavata/Diaphoretickes - ps7
Diaphoretickes - ps8
Archaeplastida - ps9
Viridiplantae - ps10
Streptophyta - ps11
Charophyceae/Embriophyta - ps12
Embriophyta - ps13
Tracheophyta - ps14
Spermatophyta - ps15
Angiosperms - ps16
Mesangiospermae - ps17
Magnoliids/eudicots - ps18
Eudicots - ps19
Core_eudicots - ps20
Superrosids - ps21
Rosids - ps22
Eurosids - ps23
Malvids - ps24
Sap./Malv./Brassicales - ps25
Malvales/Brassicales - ps26
Brassicales - ps27
Cleomaceae/Brassicaceae - ps28
Brassicaceae - ps29
Camelineae - ps30
Arabidopsis - ps31
Arabidopsis_thaliana - ps32
Phylostratum

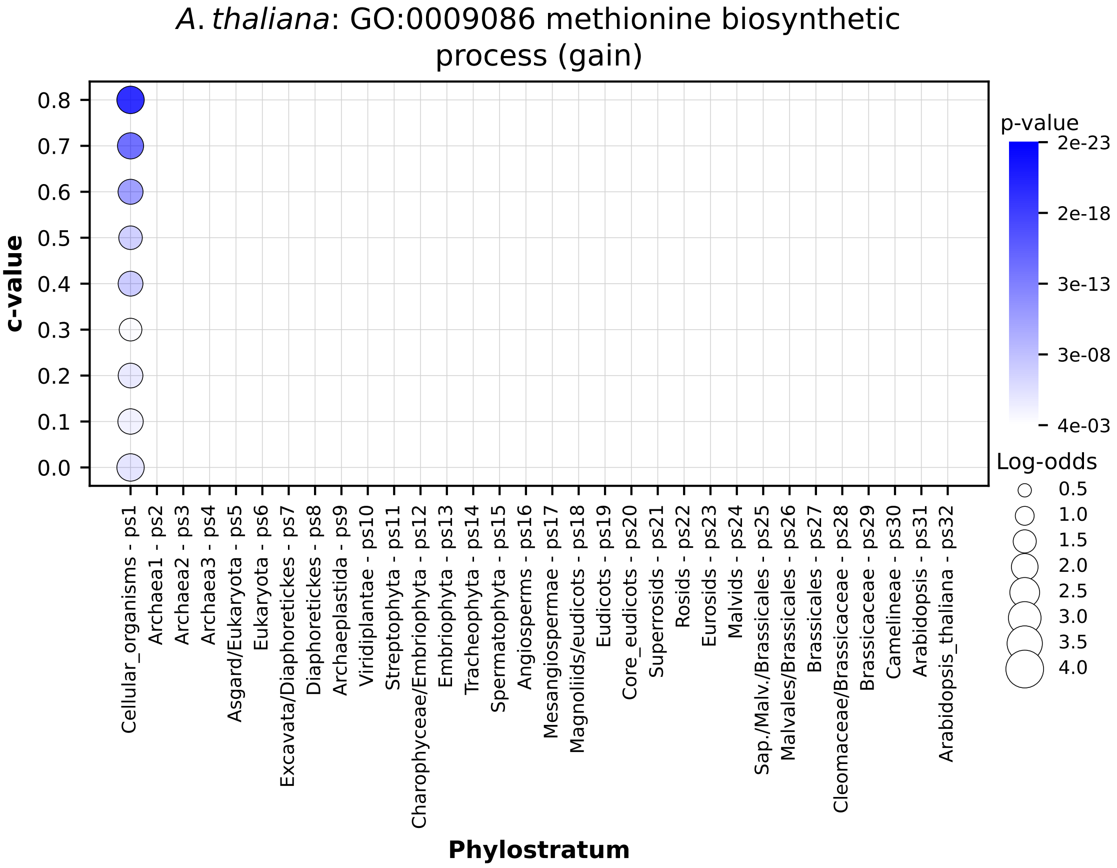

A. thaliana: GO:0009086 methionine biosynthetic process (gain)
c-value
0.8
0.7
0.6
0.5
0.4
0.3
0.2
0.1
0.0
p-value
2e-23
2e-18
3e-13
3e-08
4e-03
Log-odds
0.5
1.0
1.5
2.0
2.5
3.0
3.5
4.0
Cellular_organisms - ps1
Archaea1 - ps2
Archaea2 - ps3
Archaea3 - ps4
Asgard/Eukaryota - ps5
Eukaryota - ps6
Excavata/Diaphoretickes - ps7
Diaphoretickes - ps8
Archaeplastida - ps9
Viridiplantae - ps10
Streptophyta - ps11
Charophyceae/Embriophyta - ps12
Embriophyta - ps13
Tracheophyta - ps14
Spermatophyta - ps15
Angiosperms - ps16
Mesangiospermae - ps17
Magnoliids/eudicots - ps18
Eudicots - ps19
Core_eudicots - ps20
Superrosids - ps21
Rosids - ps22
Eurosids - ps23
Malvids - ps24
Sap./Malv./Brassicales - ps25
Malvales/Brassicales - ps26
Brassicales - ps27
Cleomaceae/Brassicaceae - ps28
Brassicaceae - ps29
Camelineae - ps30
Arabidopsis - ps31
Arabidopsis_thaliana - ps32
Phylostratum

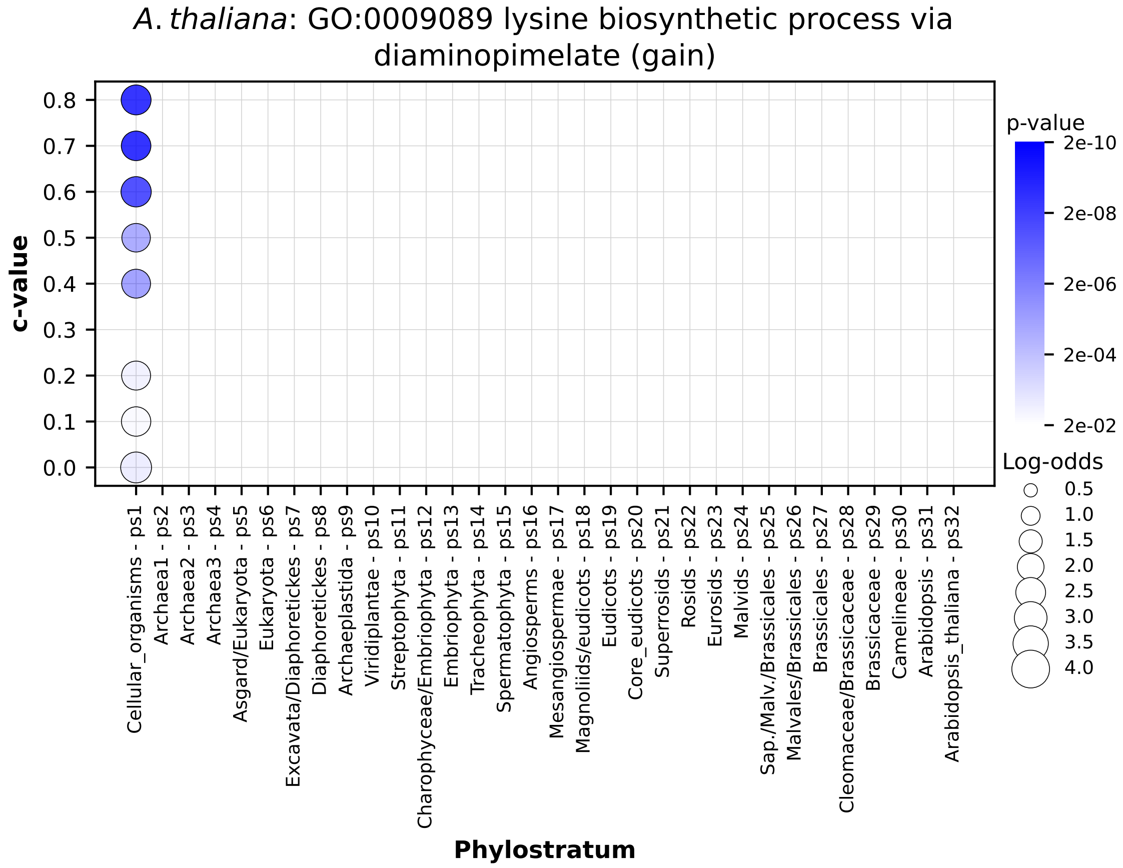

A. thaliana: GO:0009089 lysine biosynthetic process via diaminopimelate (gain)
c-value
0.8
0.7
0.6
0.5
0.4
0.3
0.2
0.1
0.0
p-value
2e-10
2e-08
2e-06
2e-04
2e-02
Log-odds
0.5
1.0
1.5
2.0
2.5
3.0
3.5
4.0
Cellular_organisms - ps1
Archaea1 - ps2
Archaea2 - ps3
Archaea3 - ps4
Asgard/Eukaryota - ps5
Eukaryota - ps6
Excavata/Diaphoretickes - ps7
Diaphoretickes - ps8
Archaeplastida - ps9
Viridiplantae - ps10
Streptophyta - ps11
Charophyceae/Embriophyta - ps12
Embriophyta - ps13
Tracheophyta - ps14
Spermatophyta - ps15
Angiosperms - ps16
Mesangiospermae - ps17
Magnoliids/eudicots - ps18
Eudicots - ps19
Core_eudicots - ps20
Superrosids - ps21
Rosids - ps22
Eurosids - ps23
Malvids - ps24
Sap./Malv./Brassicales - ps25
Malvales/Brassicales - ps26
Brassicales - ps27
Cleomaceae/Brassicaceae - ps28
Brassicaceae - ps29
Camelineae - ps30
Arabidopsis - ps31
Arabidopsis_thaliana - ps32
Phylostratum

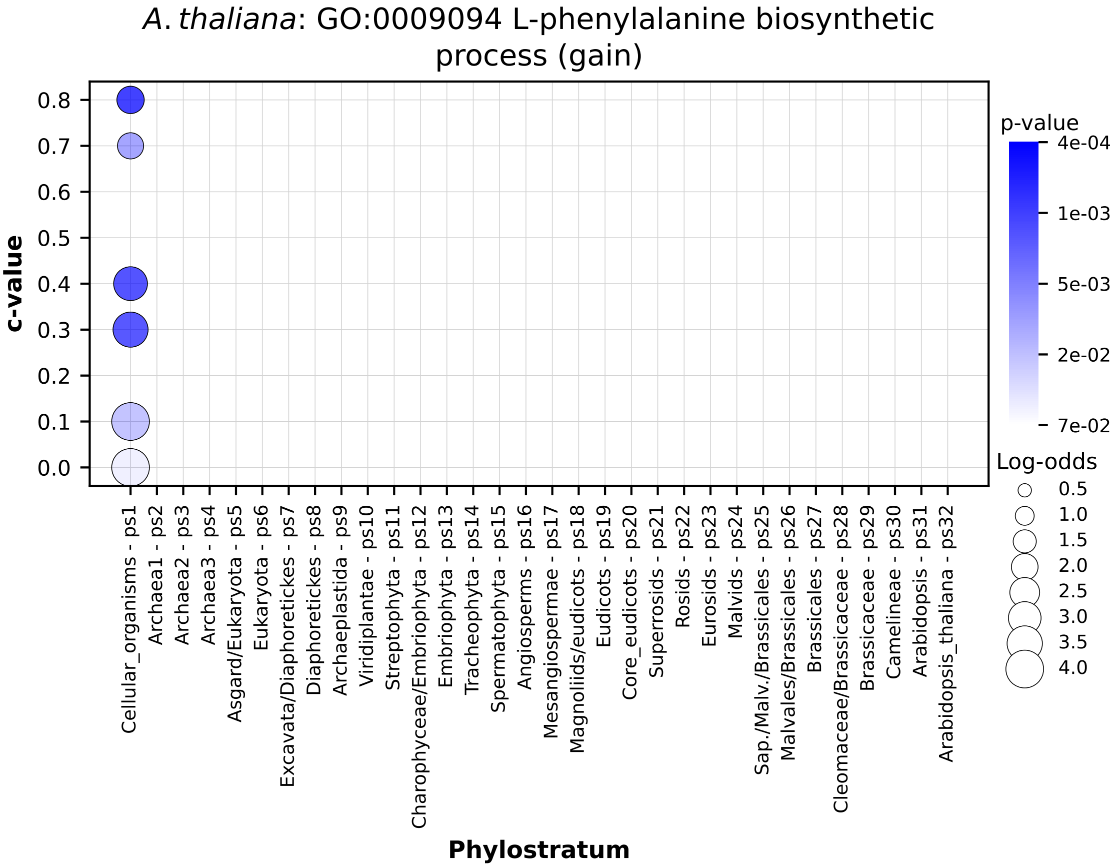

A. thaliana: GO:0009094 L-phenylalanine biosynthetic process (gain)
c-value
0.8
0.7
0.6
0.5
0.4
0.3
0.2
0.1
0.0
p-value
4e-04
1e-03
5e-03
2e-02
7e-02
Log-odds
0.5
1.0
1.5
2.0
2.5
3.0
3.5
4.0
Cellular_organisms - ps1
Archaea1 - ps2
Archaea2 - ps3
Archaea3 - ps4
Asgard/Eukaryota - ps5
Eukaryota - ps6
Excavata/Diaphoretickes - ps7
Diaphoretickes - ps8
Archaeplastida - ps9
Viridiplantae - ps10
Streptophyta - ps11
Charophyceae/Embriophyta - ps12
Embriophyta - ps13
Tracheophyta - ps14
Spermatophyta - ps15
Angiosperms - ps16
Mesangiospermae - ps17
Magnoliids/eudicots - ps18
Eudicots - ps19
Core_eudicots - ps20
Superrosids - ps21
Rosids - ps22
Eurosids - ps23
Malvids - ps24
Sap./Malv./Brassicales - ps25
Malvales/Brassicales - ps26
Brassicales - ps27
Cleomaceae/Brassicaceae - ps28
Brassicaceae - ps29
Camelineae - ps30
Arabidopsis - ps31
Arabidopsis_thaliana - ps32
Phylostratum

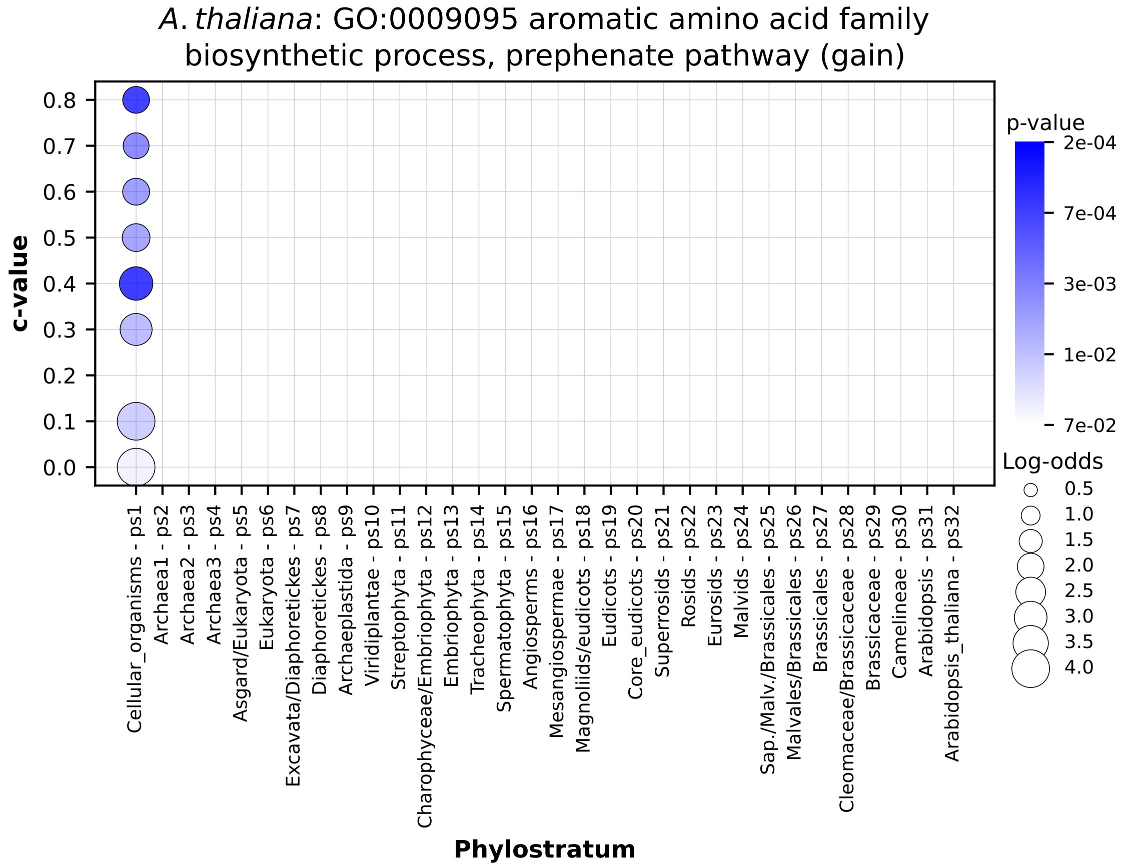

A. thaliana: GO:0009095 aromatic amino acid family biosynthetic process, prephenate pathway (gain)
c-value
0.8
0.7
0.6
0.5
0.4
0.3
0.2
0.1
0.0
p-value
2e-04
7e-04
3e-03
1e-02
7e-02
Log-odds
0.5
1.0
1.5
2.0
2.5
3.0
3.5
4.0
Cellular_organisms - ps1
Archaea1 - ps2
Archaea2 - ps3
Archaea3 - ps4
Asgard/Eukaryota - ps5
Eukaryota - ps6
Excavata/Diaphoretickes - ps7
Diaphoretickes - ps8
Archaeplastida - ps9
Viridiplantae - ps10
Streptophyta - ps11
Charophyceae/Embriophyta - ps12
Embriophyta - ps13
Tracheophyta - ps14
Spermatophyta - ps15
Angiosperms - ps16
Mesangiospermae - ps17
Magnoliids/eudicots - ps18
Eudicots - ps19
Core_eudicots - ps20
Superrosids - ps21
Rosids - ps22
Eurosids - ps23
Malvids - ps24
Sap./Malv./Brassicales - ps25
Malvales/Brassicales - ps26
Brassicales - ps27
Cleomaceae/Brassicaceae - ps28
Brassicaceae - ps29
Camelineae - ps30
Arabidopsis - ps31
Arabidopsis_thaliana - ps32
Phylostratum

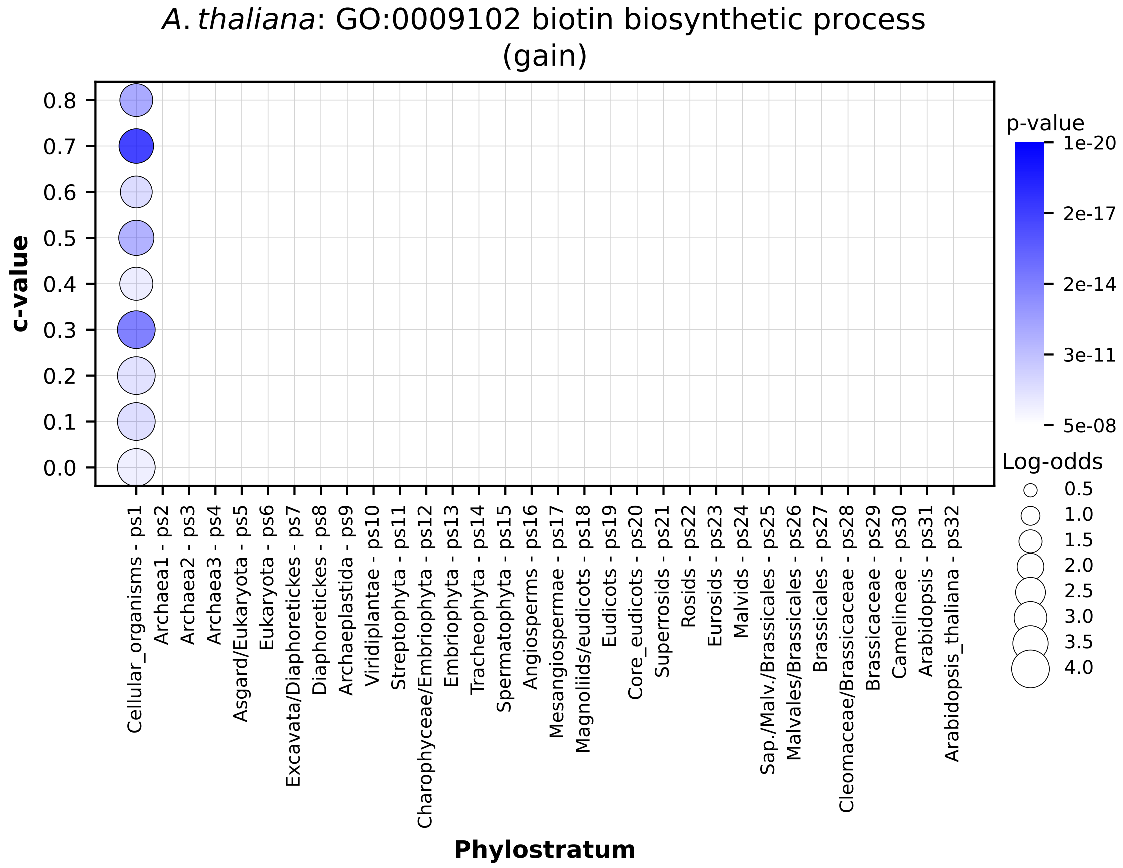

A. thaliana: GO:0009102 biotin biosynthetic process
(gain)
c-value
0.8
0.7
0.6
0.5
0.4
0.3
0.2
0.1
0.0
p-value
1e-20
2e-17
2e-14
3e-11
5e-08
Log-odds
0.5
1.0
1.5
2.0
2.5
3.0
3.5
4.0
Cellular_organisms - ps1
Archaea1 - ps2
Archaea2 - ps3
Archaea3 - ps4
Asgard/Eukaryota - ps5
Eukaryota - ps6
Excavata/Diaphoretickes - ps7
Diaphoretickes - ps8
Archaeplastida - ps9
Viridiplantae - ps10
Streptophyta - ps11
Charophyceae/Embriophyta - ps12
Embriophyta - ps13
Tracheophyta - ps14
Spermatophyta - ps15
Angiosperms - ps16
Mesangiospermae - ps17
Magnoliids/eudicots - ps18
Eudicots - ps19
Core_eudicots - ps20
Superrosids - ps21
Rosids - ps22
Eurosids - ps23
Malvids - ps24
Sap./Malv./Brassicales - ps25
Malvales/Brassicales - ps26
Brassicales - ps27
Cleomaceae/Brassicaceae - ps28
Brassicaceae - ps29
Camelineae - ps30
Arabidopsis - ps31
Arabidopsis_thaliana - ps32
Phylostratum

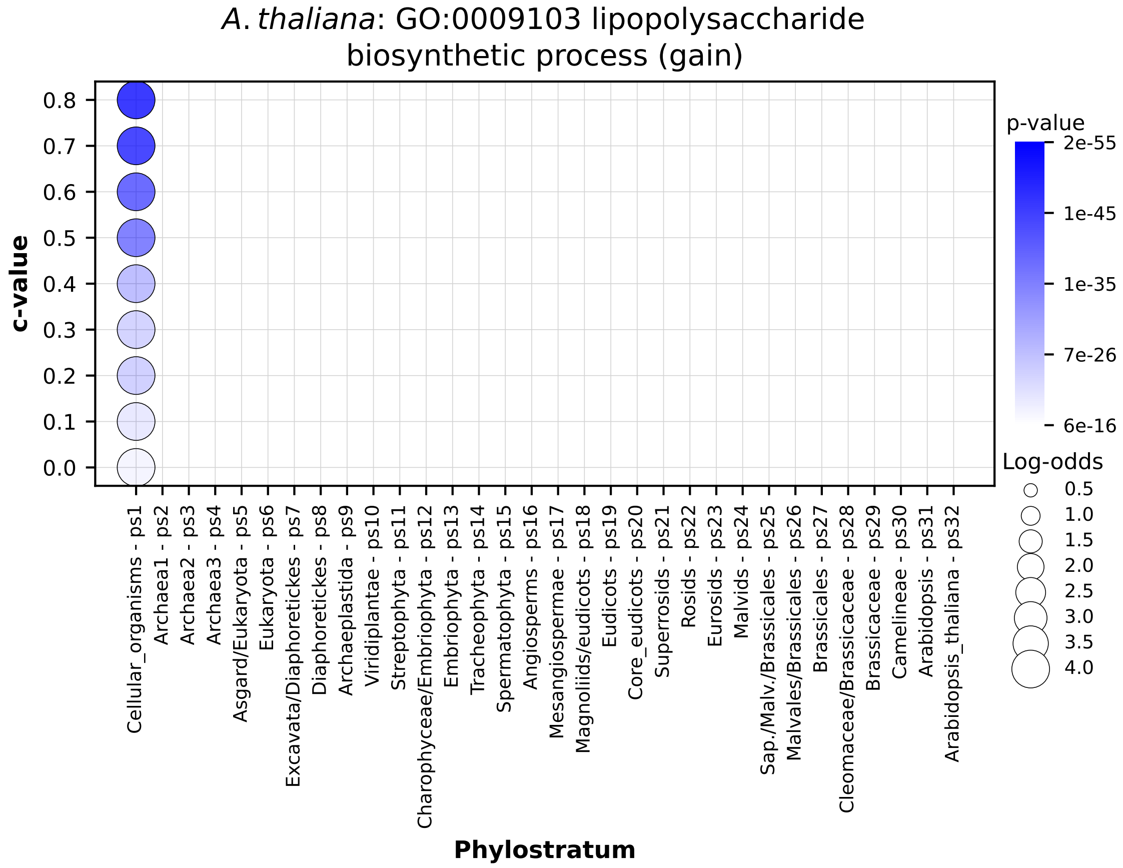

A. thaliana: GO:0009103 lipopolysaccharide biosynthetic process (gain)
c-value
0.8
0.7
0.6
0.5
0.4
0.3
0.2
0.1
0.0
p-value
2e-55
1e-45
1e-35
7e-26
6e-16
Log-odds
0.5
1.0
1.5
2.0
2.5
3.0
3.5
4.0
Cellular_organisms - ps1
Archaea1 - ps2
Archaea2 - ps3
Archaea3 - ps4
Asgard/Eukaryota - ps5
Eukaryota - ps6
Excavata/Diaphoretickes - ps7
Diaphoretickes - ps8
Archaeplastida - ps9
Viridiplantae - ps10
Streptophyta - ps11
Charophyceae/Embriophyta - ps12
Embriophyta - ps13
Tracheophyta - ps14
Spermatophyta - ps15
Angiosperms - ps16
Mesangiospermae - ps17
Magnoliids/eudicots - ps18
Eudicots - ps19
Core_eudicots - ps20
Superrosids - ps21
Rosids - ps22
Eurosids - ps23
Malvids - ps24
Sap./Malv./Brassicales - ps25
Malvales/Brassicales - ps26
Brassicales - ps27
Cleomaceae/Brassicaceae - ps28
Brassicaceae - ps29
Camelineae - ps30
Arabidopsis - ps31
Arabidopsis_thaliana - ps32
Phylostratum

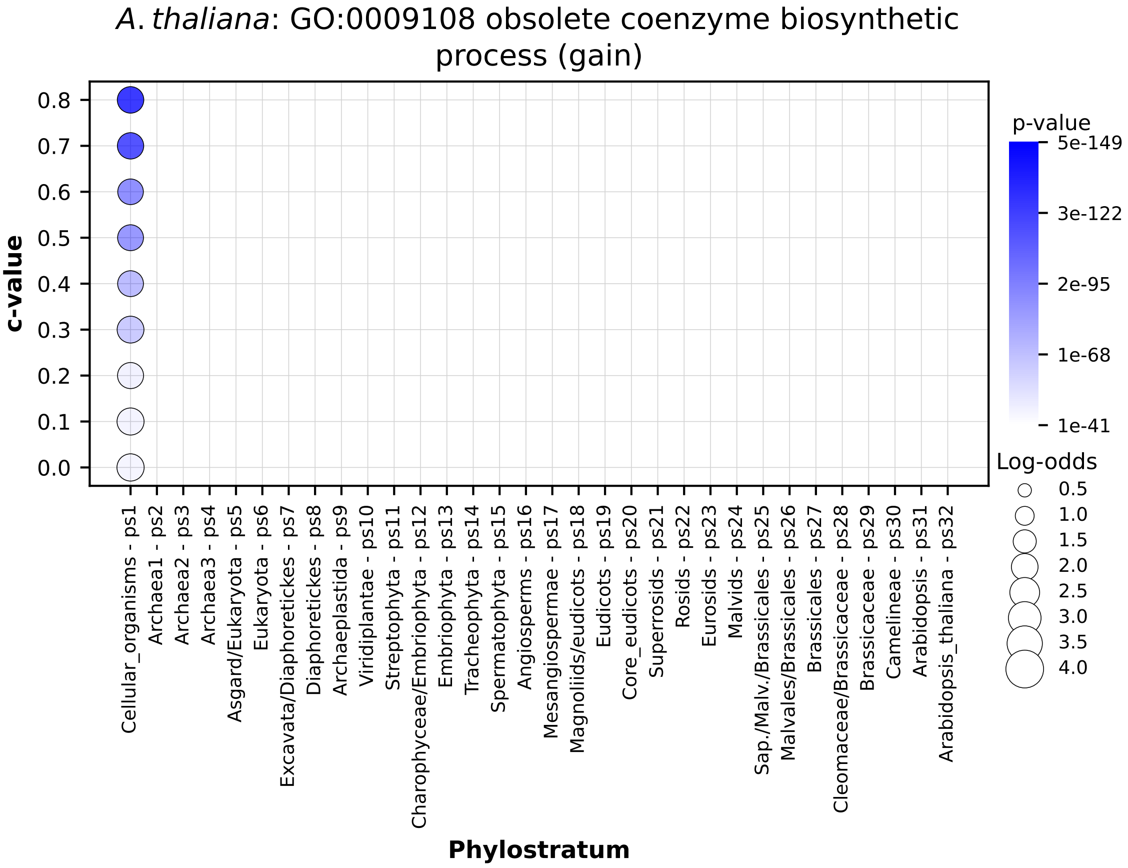

A. thaliana: GO:0009108 obsolete coenzyme biosynthetic process (gain)
c-value
0.8
0.7
0.6
0.5
0.4
0.3
0.2
0.1
0.0
p-value
5e-149
3e-122
2e-95
1e-68
1e-41
Log-odds
0.5
1.0
1.5
2.0
2.5
3.0
3.5
4.0
Cellular_organisms - ps1
Archaea1 - ps2
Archaea2 - ps3
Archaea3 - ps4
Asgard/Eukaryota - ps5
Eukaryota - ps6
Excavata/Diaphoretickes - ps7
Diaphoretickes - ps8
Archaeplastida - ps9
Viridiplantae - ps10
Streptophyta - ps11
Charophyceae/Embriophyta - ps12
Embriophyta - ps13
Tracheophyta - ps14
Spermatophyta - ps15
Angiosperms - ps16
Mesangiospermae - ps17
Magnoliids/eudicots - ps18
Eudicots - ps19
Core_eudicots - ps20
Superrosids - ps21
Rosids - ps22
Eurosids - ps23
Malvids - ps24
Sap./Malv./Brassicales - ps25
Malvales/Brassicales - ps26
Brassicales - ps27
Cleomaceae/Brassicaceae - ps28
Brassicaceae - ps29
Camelineae - ps30
Arabidopsis - ps31
Arabidopsis_thaliana - ps32
Phylostratum

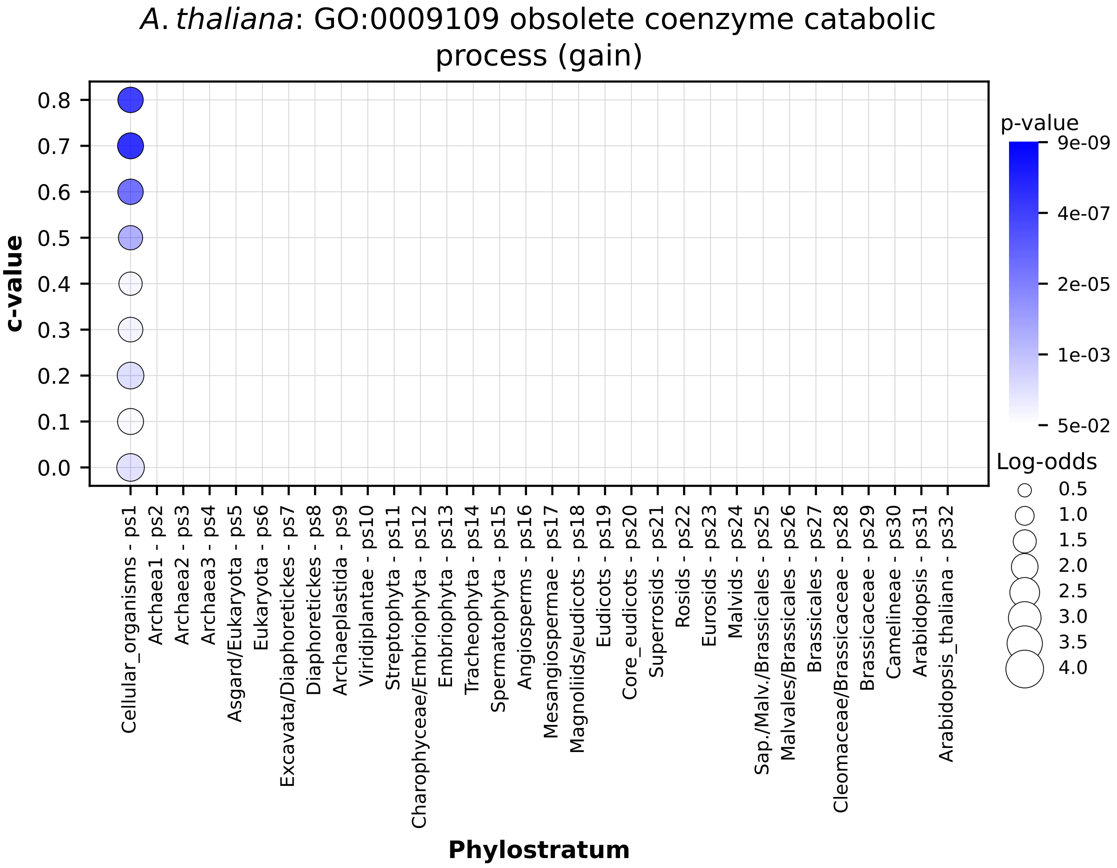

A. thaliana: GO:0009109 obsolete coenzyme catabolic process (gain)
c-value
0.8
0.7
0.6
0.5
0.4
0.3
0.2
0.1
0.0
p-value
9e-09
4e-07
2e-05
1e-03
5e-02
Log-odds
0.5
1.0
1.5
2.0
2.5
3.0
3.5
4.0
Cellular_organisms - ps1
Archaea1 - ps2
Archaea2 - ps3
Archaea3 - ps4
Asgard/Eukaryota - ps5
Eukaryota - ps6
Excavata/Diaphoretickes - ps7
Diaphoretickes - ps8
Archaeplastida - ps9
Viridiplantae - ps10
Streptophyta - ps11
Charophyceae/Embriophyta - ps12
Embriophyta - ps13
Tracheophyta - ps14
Spermatophyta - ps15
Angiosperms - ps16
Mesangiospermae - ps17
Magnoliids/eudicots - ps18
Eudicots - ps19
Core_eudicots - ps20
Superrosids - ps21
Rosids - ps22
Eurosids - ps23
Malvids - ps24
Sap./Malv./Brassicales - ps25
Malvales/Brassicales - ps26
Brassicales - ps27
Cleomaceae/Brassicaceae - ps28
Brassicaceae - ps29
Camelineae - ps30
Arabidopsis - ps31
Arabidopsis_thaliana - ps32
Phylostratum

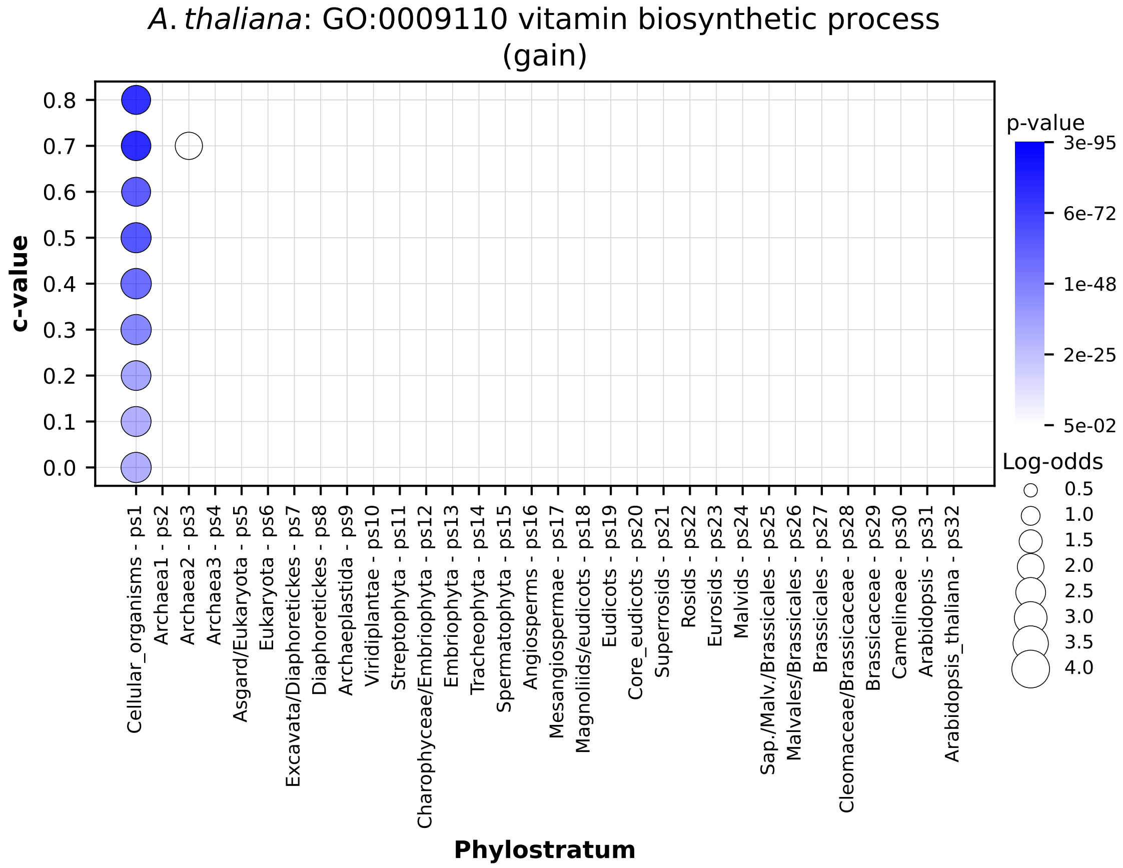

A. thaliana: GO:0009110 vitamin biosynthetic process
(gain)
c-value
0.8
0.7
0.6
0.5
0.4
0.3
0.2
0.1
0.0
p-value
3e-95
6e-72
1e-48
2e-25
5e-02
Log-odds
0.5
1.0
1.5
2.0
2.5
3.0
3.5
4.0
Cellular_organisms - ps1
Archaea1 - ps2
Archaea2 - ps3
Archaea3 - ps4
Asgard/Eukaryota - ps5
Eukaryota - ps6
Excavata/Diaphoretickes - ps7
Diaphoretickes - ps8
Archaeplastida - ps9
Viridiplantae - ps10
Streptophyta - ps11
Charophyceae/Embriophyta - ps12
Embriophyta - ps13
Tracheophyta - ps14
Spermatophyta - ps15
Angiosperms - ps16
Mesangiospermae - ps17
Magnoliids/eudicots - ps18
Eudicots - ps19
Core_eudicots - ps20
Superrosids - ps21
Rosids - ps22
Eurosids - ps23
Malvids - ps24
Sap./Malv./Brassicales - ps25
Malvales/Brassicales - ps26
Brassicales - ps27
Cleomaceae/Brassicaceae - ps28
Brassicaceae - ps29
Camelineae - ps30
Arabidopsis - ps31
Arabidopsis_thaliana - ps32
Phylostratum

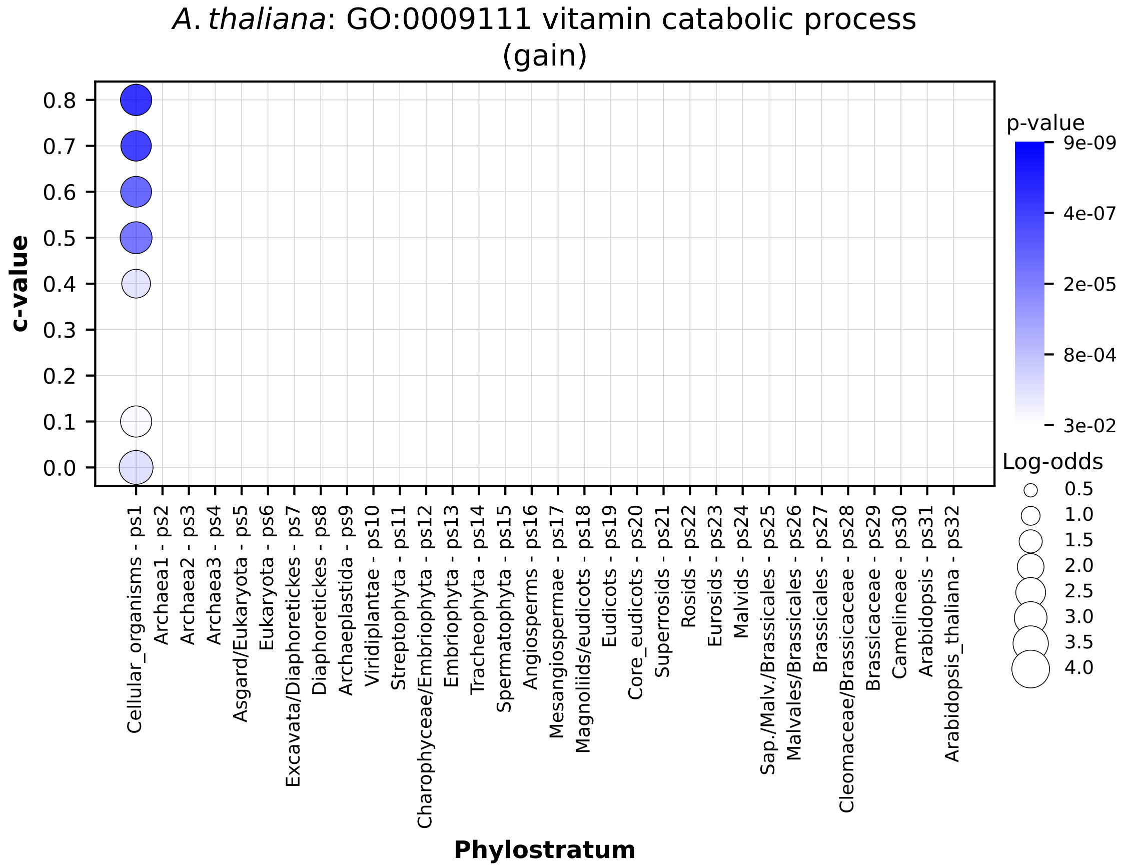

A. thaliana: GO:0009111 vitamin catabolic process
(gain)
c-value
0.8
0.7
0.6
0.5
0.4
0.3
0.2
0.1
0.0
p-value
9e-09
4e-07
2e-05
8e-04
3e-02
Log-odds
0.5
1.0
1.5
2.0
2.5
3.0
3.5
4.0
Cellular_organisms - ps1
Archaea1 - ps2
Archaea2 - ps3
Archaea3 - ps4
Asgard/Eukaryota - ps5
Eukaryota - ps6
Excavata/Diaphoretickes - ps7
Diaphoretickes - ps8
Archaeplastida - ps9
Viridiplantae - ps10
Streptophyta - ps11
Charophyceae/Embriophyta - ps12
Embriophyta - ps13
Tracheophyta - ps14
Spermatophyta - ps15
Angiosperms - ps16
Mesangiospermae - ps17
Magnoliids/eudicots - ps18
Eudicots - ps19
Core_eudicots - ps20
Superrosids - ps21
Rosids - ps22
Eurosids - ps23
Malvids - ps24
Sap./Malv./Brassicales - ps25
Malvales/Brassicales - ps26
Brassicales - ps27
Cleomaceae/Brassicaceae - ps28
Brassicaceae - ps29
Camelineae - ps30
Arabidopsis - ps31
Arabidopsis_thaliana - ps32
Phylostratum

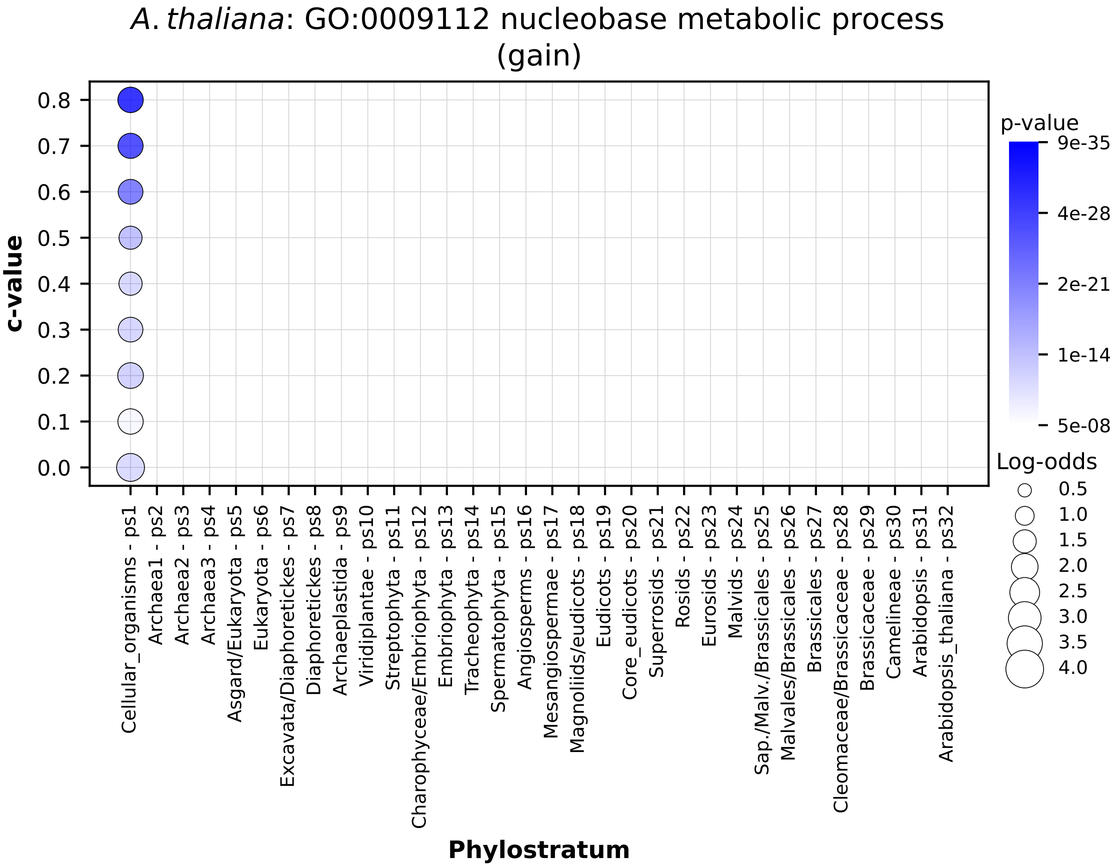
A. thaliana: GO:0009112 nucleobase metabolic process
(gain)
c-value
0.8
0.7
0.6
0.5
0.4
0.3
0.2
0.1
0.0
p-value
9e-35
4e-28
2e-21
1e-14
5e-08
Log-odds
0.5
1.0
1.5
2.0
2.5
3.0
3.5
4.0
Cellular_organisms - ps1
Archaea1 - ps2
Archaea2 - ps3
Archaea3 - ps4
Asgard/Eukaryota - ps5
Eukaryota - ps6
Excavata/Diaphoretickes - ps7
Diaphoretickes - ps8
Archaeplastida - ps9
Viridiplantae - ps10
Streptophyta - ps11
Charophyceae/Embriophyta - ps12
Embriophyta - ps13
Tracheophyta - ps14
Spermatophyta - ps15
Angiosperms - ps16
Mesangiospermae - ps17
Magnoliids/eudicots - ps18
Eudicots - ps19
Core_eudicots - ps20
Superrosids - ps21
Rosids - ps22
Eurosids - ps23
Malvids - ps24
Sap./Malv./Brassicales - ps25
Malvales/Brassicales - ps26
Brassicales - ps27
Cleomaceae/Brassicaceae - ps28
Brassicaceae - ps29
Camelineae - ps30
Arabidopsis - ps31
Arabidopsis_thaliana - ps32
Phylostratum

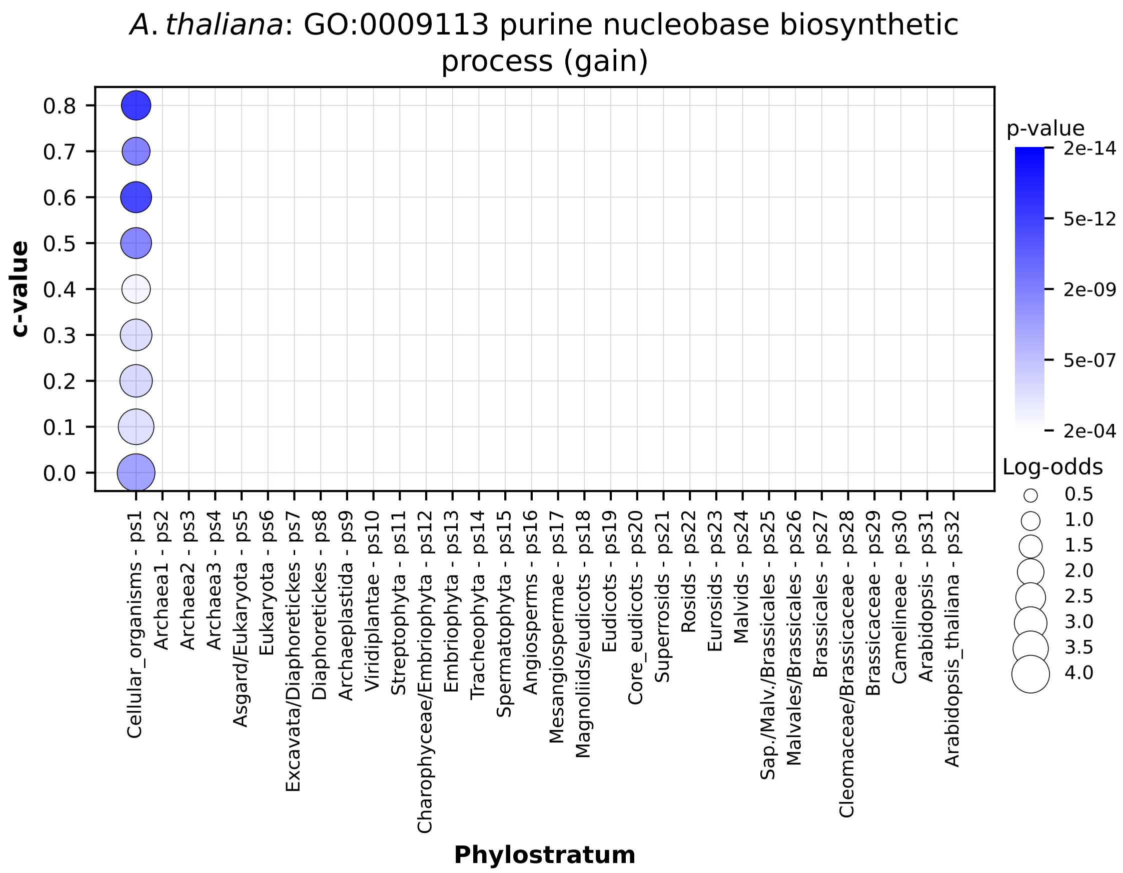

A. thaliana: GO:0009113 purine nucleobase biosynthetic process (gain)
c-value
0.0
0.1
0.2
0.3
0.4
0.5
0.6
0.7
0.8
p-value
2e-14
5e-12
2e-09
5e-07
2e-04
Log-odds
0.5
1.0
1.5
2.0
2.5
3.0
3.5
4.0
Cellular_organisms - ps1
Archaea1 - ps2
Archaea2 - ps3
Archaea3 - ps4
Asgard/Eukaryota - ps5
Eukaryota - ps6
Excavata/Diaphoretickes - ps7
Diaphoretickes - ps8
Archaeplastida - ps9
Viridiplantae - ps10
Streptophyta - ps11
Charophyceae/Embriophyta - ps12
Embriophyta - ps13
Tracheophyta - ps14
Spermatophyta - ps15
Angiosperms - ps16
Mesangiospermae - ps17
Magnoliids/eudicots - ps18
Eudicots - ps19
Core_eudicots - ps20
Superrosids - ps21
Rosids - ps22
Eurosids - ps23
Malvids - ps24
Sap./Malv./Brassicales - ps25
Malvales/Brassicales - ps26
Brassicales - ps27
Cleomaceae/Brassicaceae - ps28
Brassicaceae - ps29
Camelineae - ps30
Arabidopsis - ps31
Arabidopsis_thaliana - ps32
Phylostratum

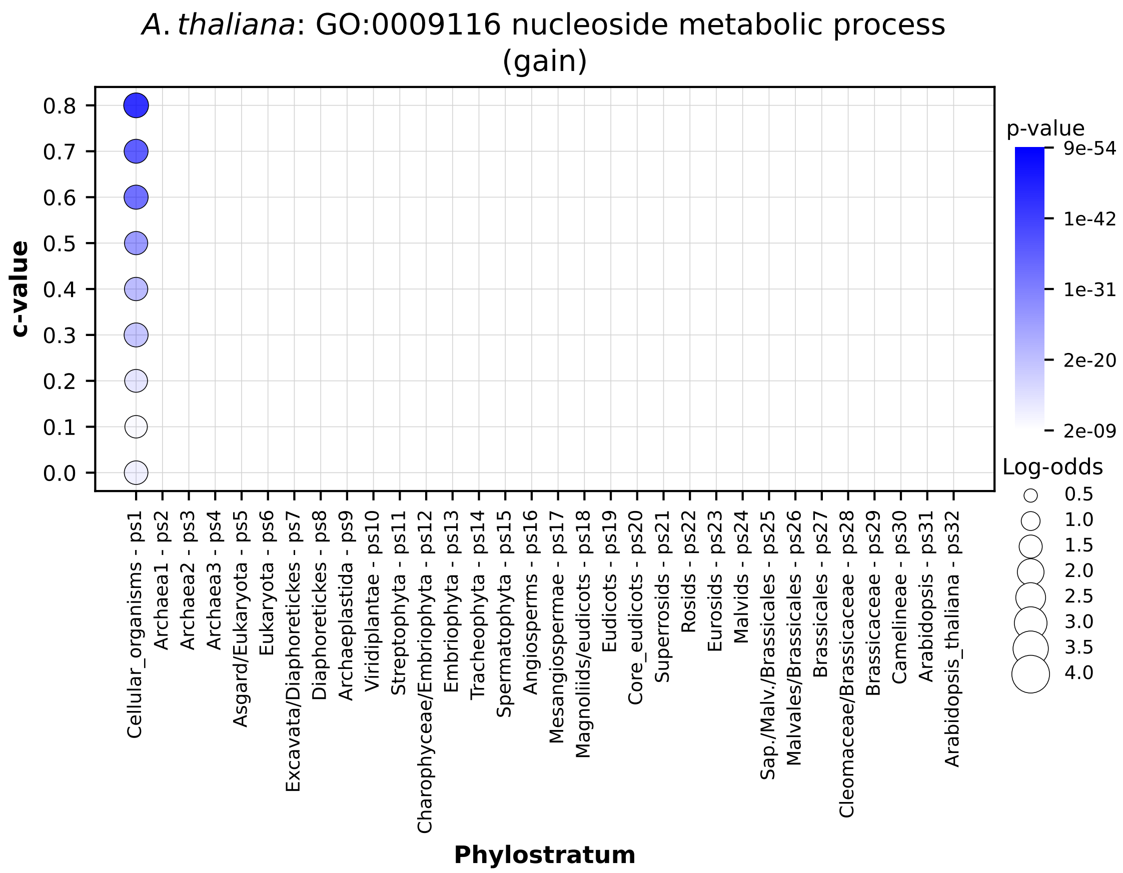

A. thaliana: GO:0009116 nucleoside metabolic process
(gain)
c-value
0.8
0.7
0.6
0.5
0.4
0.3
0.2
0.1
0.0
p-value
9e-54
1e-42
1e-31
2e-20
2e-09
Log-odds
0.5
1.0
1.5
2.0
2.5
3.0
3.5
4.0
Cellular_organisms - ps1
Archaea1 - ps2
Archaea2 - ps3
Archaea3 - ps4
Asgard/Eukaryota - ps5
Eukaryota - ps6
Excavata/Diaphoretickes - ps7
Diaphoretickes - ps8
Archaeplastida - ps9
Viridiplantae - ps10
Streptophyta - ps11
Charophyceae/Embriophyta - ps12
Embriophyta - ps13
Tracheophyta - ps14
Spermatophyta - ps15
Angiosperms - ps16
Mesangiospermae - ps17
Magnoliids/eudicots - ps18
Eudicots - ps19
Core_eudicots - ps20
Superrosids - ps21
Rosids - ps22
Eurosids - ps23
Malvids - ps24
Sap./Malv./Brassicales - ps25
Malvales/Brassicales - ps26
Brassicales - ps27
Cleomaceae/Brassicaceae - ps28
Brassicaceae - ps29
Camelineae - ps30
Arabidopsis - ps31
Arabidopsis_thaliana - ps32
Phylostratum

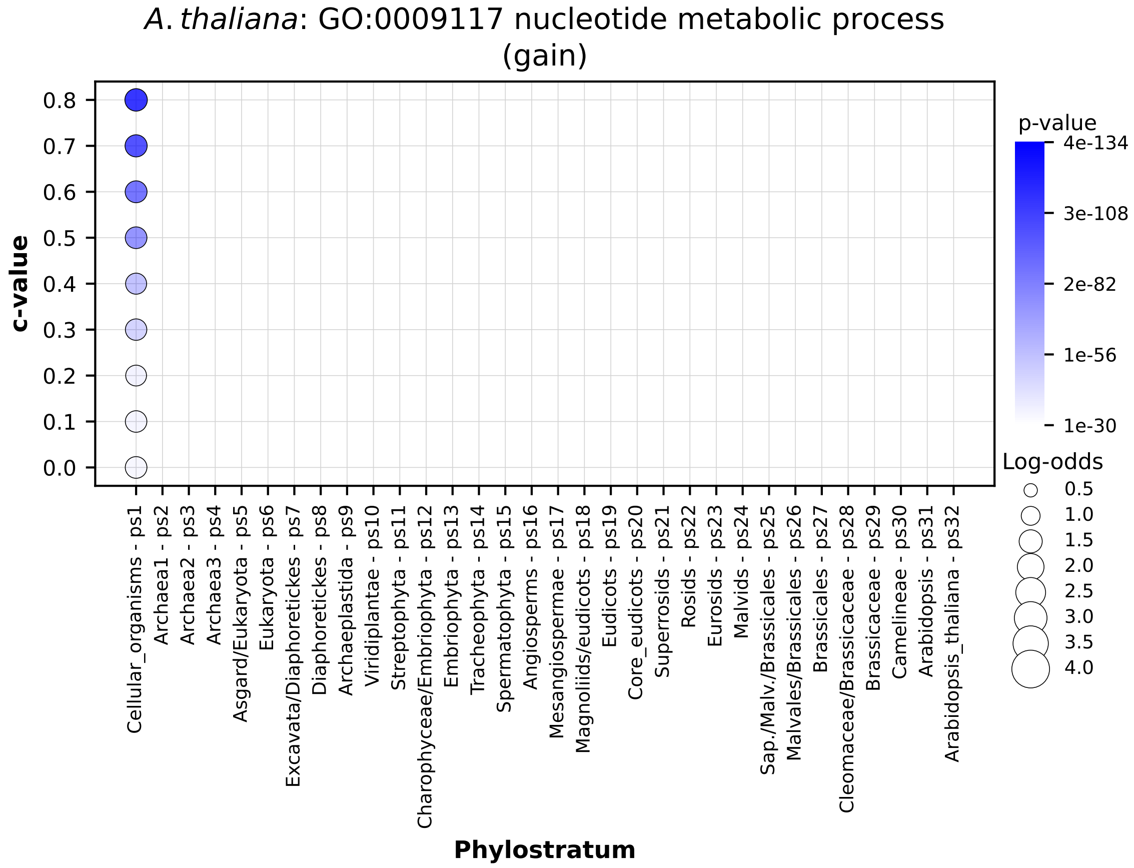

A. thaliana: GO:0009117 nucleotide metabolic process
(gain)
c-value
0.8
0.7
0.6
0.5
0.4
0.3
0.2
0.1
0.0
p-value
4e-134
3e-108
2e-82
1e-56
1e-30
Log-odds
0.5
1.0
1.5
2.0
2.5
3.0
3.5
4.0
Cellular_organisms - ps1
Archaea1 - ps2
Archaea2 - ps3
Archaea3 - ps4
Asgard/Eukaryota - ps5
Eukaryota - ps6
Excavata/Diaphoretickes - ps7
Diaphoretickes - ps8
Archaeplastida - ps9
Viridiplantae - ps10
Streptophyta - ps11
Charophyceae/Embriophyta - ps12
Embriophyta - ps13
Tracheophyta - ps14
Spermatophyta - ps15
Angiosperms - ps16
Mesangiospermae - ps17
Magnoliids/eudicots - ps18
Eudicots - ps19
Core_eudicots - ps20
Superrosids - ps21
Rosids - ps22
Eurosids - ps23
Malvids - ps24
Sap./Malv./Brassicales - ps25
Malvales/Brassicales - ps26
Brassicales - ps27
Cleomaceae/Brassicaceae - ps28
Brassicaceae - ps29
Camelineae - ps30
Arabidopsis - ps31
Arabidopsis_thaliana - ps32
Phylostratum

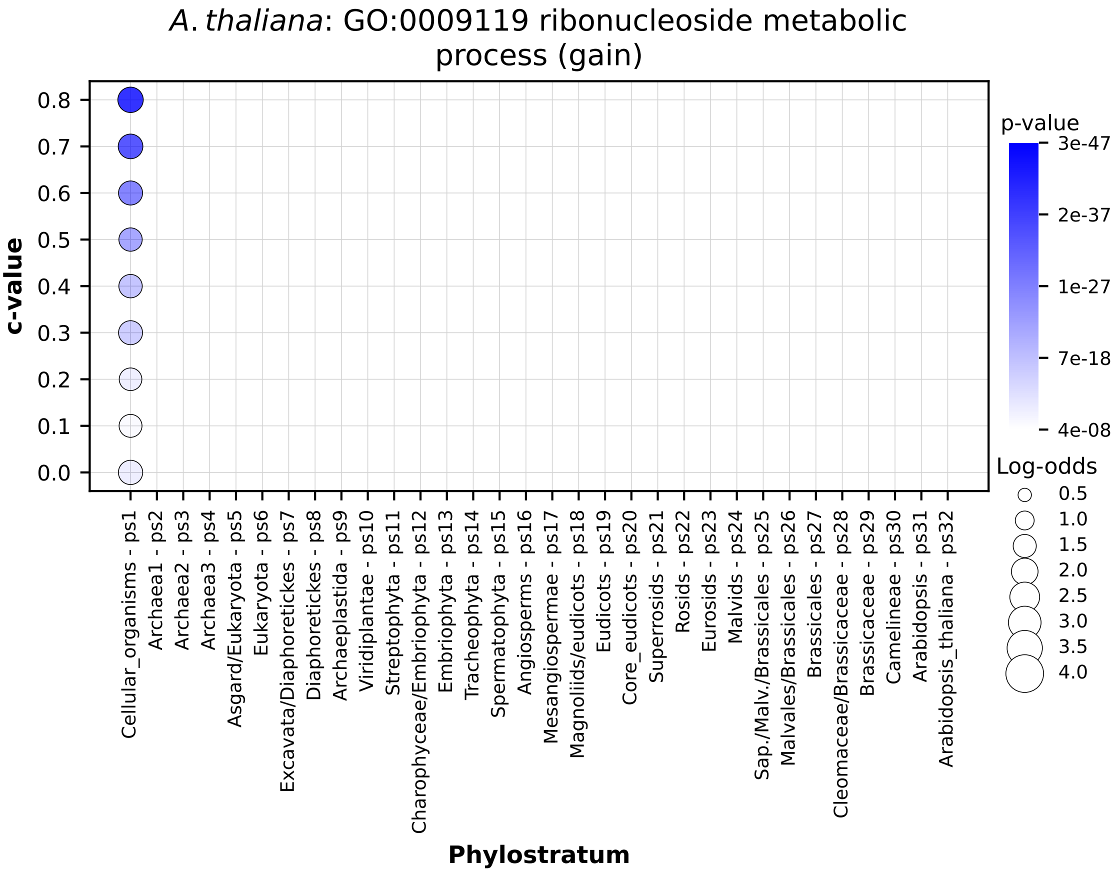
A. thaliana: GO:0009119 ribonucleoside metabolic process (gain)
c-value
0.8
0.7
0.6
0.5
0.4
0.3
0.2
0.1
0.0
p-value
3e-47
2e-37
1e-27
7e-18
4e-08
Log-odds
0.5
1.0
1.5
2.0
2.5
3.0
3.5
4.0
Cellular_organisms - ps1
Archaea1 - ps2
Archaea2 - ps3
Archaea3 - ps4
Asgard/Eukaryota - ps5
Eukaryota - ps6
Excavata/Diaphoretickes - ps7
Diaphoretickes - ps8
Archaeplastida - ps9
Viridiplantae - ps10
Streptophyta - ps11
Charophyceae/Embriophyta - ps12
Embriophyta - ps13
Tracheophyta - ps14
Spermatophyta - ps15
Angiosperms - ps16
Mesangiospermae - ps17
Magnoliids/eudicots - ps18
Eudicots - ps19
Core_eudicots - ps20
Superrosids - ps21
Rosids - ps22
Eurosids - ps23
Malvids - ps24
Sap./Malv./Brassicales - ps25
Malvales/Brassicales - ps26
Brassicales - ps27
Cleomaceae/Brassicaceae - ps28
Brassicaceae - ps29
Camelineae - ps30
Arabidopsis - ps31
Arabidopsis_thaliana - ps32
Phylostratum

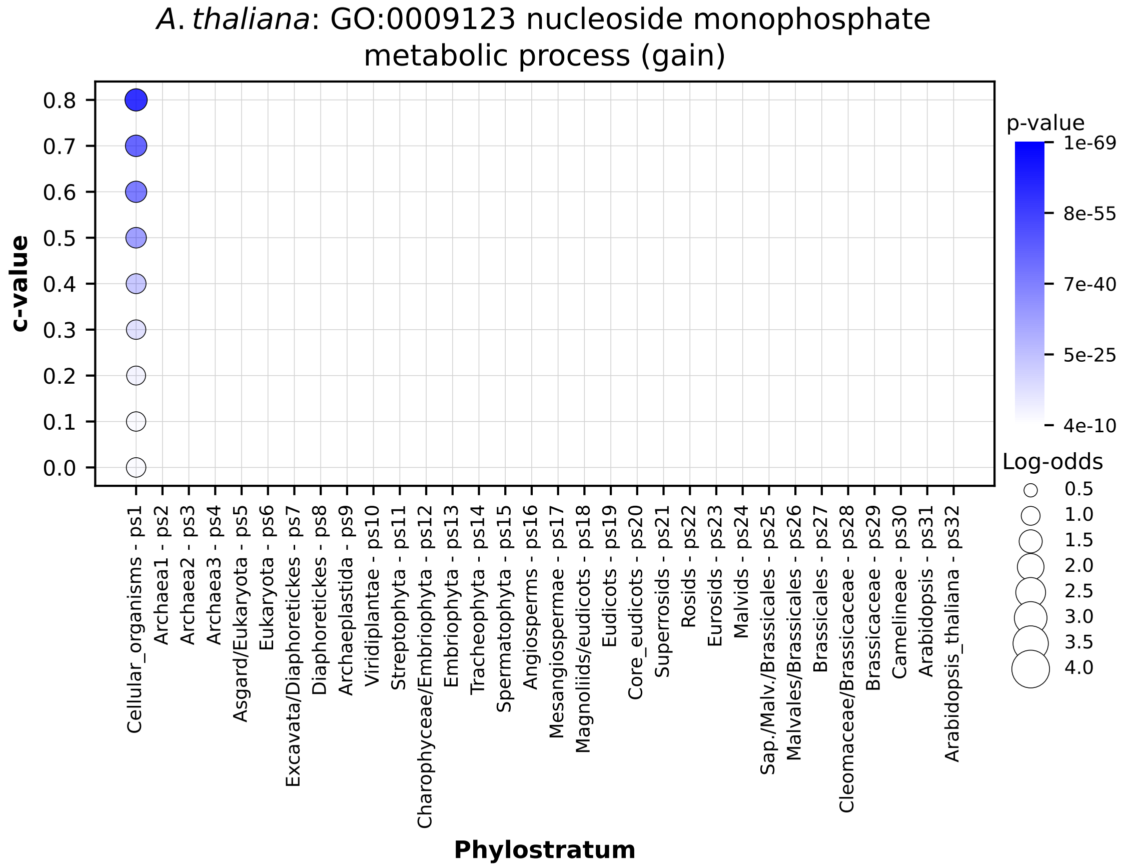
A. thaliana: GO:0009123 nucleoside monophosphate metabolic process (gain)
c-value
0.8
0.7
0.6
0.5
0.4
0.3
0.2
0.1
0.0
p-value
1e-69
8e-55
7e-40
5e-25
4e-10
Log-odds
0.5
1.0
1.5
2.0
2.5
3.0
3.5
4.0
Cellular_organisms - ps1
Archaea1 - ps2
Archaea2 - ps3
Archaea3 - ps4
Asgard/Eukaryota - ps5
Eukaryota - ps6
Excavata/Diaphoretickes - ps7
Diaphoretickes - ps8
Archaeplastida - ps9
Viridiplantae - ps10
Streptophyta - ps11
Charophyceae/Embriophyta - ps12
Embriophyta - ps13
Tracheophyta - ps14
Spermatophyta - ps15
Angiosperms - ps16
Mesangiospermae - ps17
Magnoliids/eudicots - ps18
Eudicots - ps19
Core_eudicots - ps20
Superrosids - ps21
Rosids - ps22
Eurosids - ps23
Malvids - ps24
Sap./Malv./Brassicales - ps25
Malvales/Brassicales - ps26
Brassicales - ps27
Cleomaceae/Brassicaceae - ps28
Brassicaceae - ps29
Camelineae - ps30
Arabidopsis - ps31
Arabidopsis_thaliana - ps32
Phylostratum

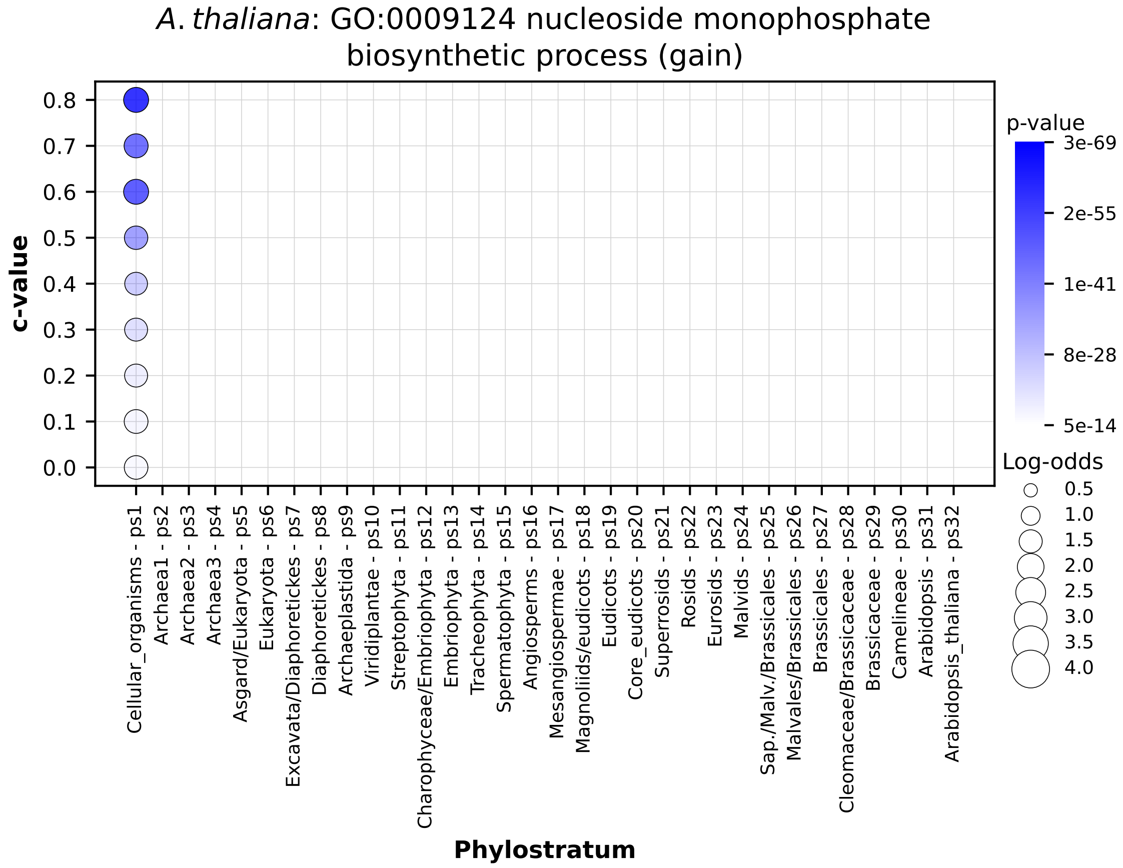
A. thaliana: GO:0009124 nucleoside monophosphate biosynthetic process (gain)
c-value
0.8
0.7
0.6
0.5
0.4
0.3
0.2
0.1
0.0
p-value
3e-69
2e-55
1e-41
8e-28
5e-14
Log-odds
0.5
1.0
1.5
2.0
2.5
3.0
3.5
4.0
Cellular_organisms - ps1
Archaea1 - ps2
Archaea2 - ps3
Archaea3 - ps4
Asgard/Eukaryota - ps5
Eukaryota - ps6
Excavata/Diaphoretickes - ps7
Diaphoretickes - ps8
Archaeplastida - ps9
Viridiplantae - ps10
Streptophyta - ps11
Charophyceae/Embriophyta - ps12
Embriophyta - ps13
Tracheophyta - ps14
Spermatophyta - ps15
Angiosperms - ps16
Mesangiospermae - ps17
Magnoliids/eudicots - ps18
Eudicots - ps19
Core_eudicots - ps20
Superrosids - ps21
Rosids - ps22
Eurosids - ps23
Malvids - ps24
Sap./Malv./Brassicales - ps25
Malvales/Brassicales - ps26
Brassicales - ps27
Cleomaceae/Brassicaceae - ps28
Brassicaceae - ps29
Camelineae - ps30
Arabidopsis - ps31
Arabidopsis_thaliana - ps32
Phylostratum

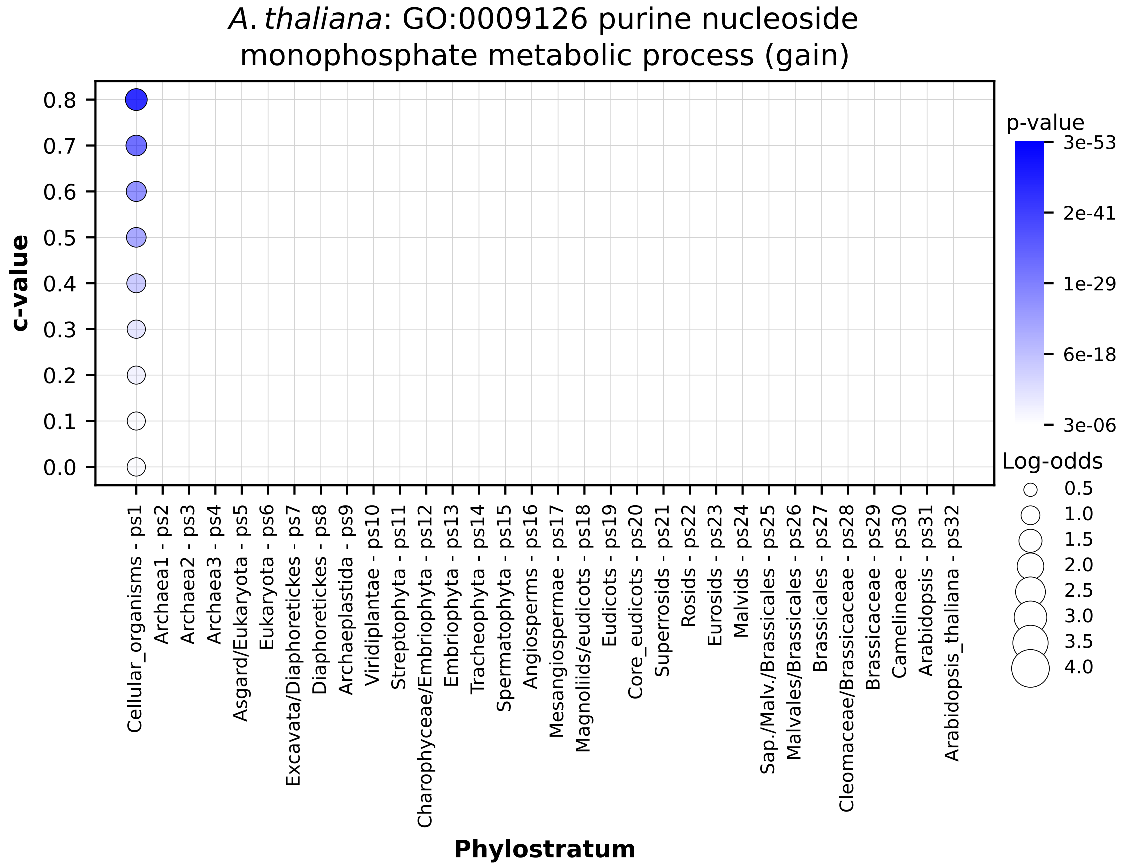

A. thaliana: GO:0009126 purine nucleoside monophosphate metabolic process (gain)
c-value
0.8
0.7
0.6
0.5
0.4
0.3
0.2
0.1
0.0
p-value
3e-53
2e-41
1e-29
6e-18
3e-06
Log-odds
0.5
1.0
1.5
2.0
2.5
3.0
3.5
4.0
Cellular_organisms - ps1
Archaea1 - ps2
Archaea2 - ps3
Archaea3 - ps4
Asgard/Eukaryota - ps5
Eukaryota - ps6
Excavata/Diaphoretickes - ps7
Diaphoretickes - ps8
Archaeplastida - ps9
Viridiplantae - ps10
Streptophyta - ps11
Charophyceae/Embriophyta - ps12
Embriophyta - ps13
Tracheophyta - ps14
Spermatophyta - ps15
Angiosperms - ps16
Mesangiospermae - ps17
Magnoliids/eudicots - ps18
Eudicots - ps19
Core_eudicots - ps20
Superrosids - ps21
Rosids - ps22
Eurosids - ps23
Malvids - ps24
Sap./Malv./Brassicales - ps25
Malvales/Brassicales - ps26
Brassicales - ps27
Cleomaceae/Brassicaceae - ps28
Brassicaceae - ps29
Camelineae - ps30
Arabidopsis - ps31
Arabidopsis_thaliana - ps32
Phylostratum

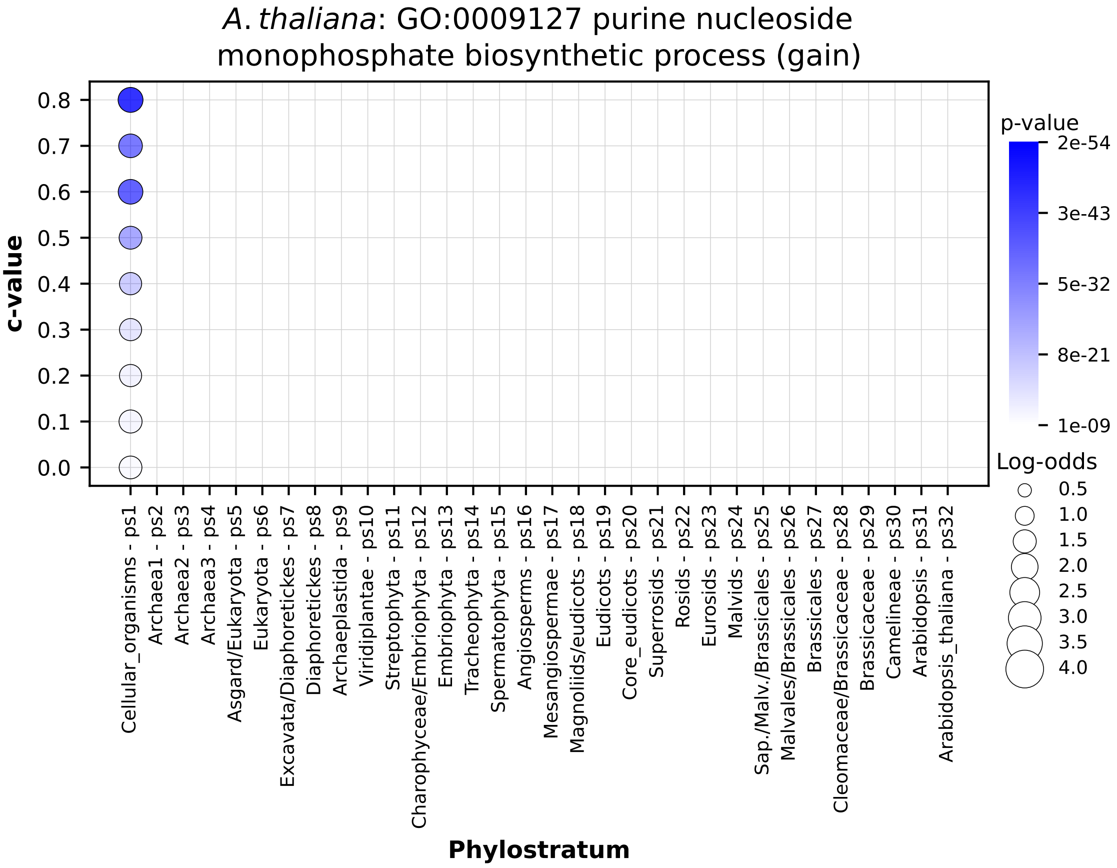

A. thaliana: GO:0009127 purine nucleoside monophosphate biosynthetic process (gain)
c-value
0.8
0.7
0.6
0.5
0.4
0.3
0.2
0.1
0.0
p-value
2e-54
3e-43
5e-32
8e-21
1e-09
Log-odds
0.5
1.0
1.5
2.0
2.5
3.0
3.5
4.0
Cellular_organisms - ps1
Archaea1 - ps2
Archaea2 - ps3
Archaea3 - ps4
Asgard/Eukaryota - ps5
Eukaryota - ps6
Excavata/Diaphoretickes - ps7
Diaphoretickes - ps8
Archaeplastida - ps9
Viridiplantae - ps10
Streptophyta - ps11
Charophyceae/Embriophyta - ps12
Embriophyta - ps13
Tracheophyta - ps14
Spermatophyta - ps15
Angiosperms - ps16
Mesangiospermae - ps17
Magnoliids/eudicots - ps18
Eudicots - ps19
Core_eudicots - ps20
Superrosids - ps21
Rosids - ps22
Eurosids - ps23
Malvids - ps24
Sap./Malv./Brassicales - ps25
Malvales/Brassicales - ps26
Brassicales - ps27
Cleomaceae/Brassicaceae - ps28
Brassicaceae - ps29
Camelineae - ps30
Arabidopsis - ps31
Arabidopsis_thaliana - ps32
Phylostratum

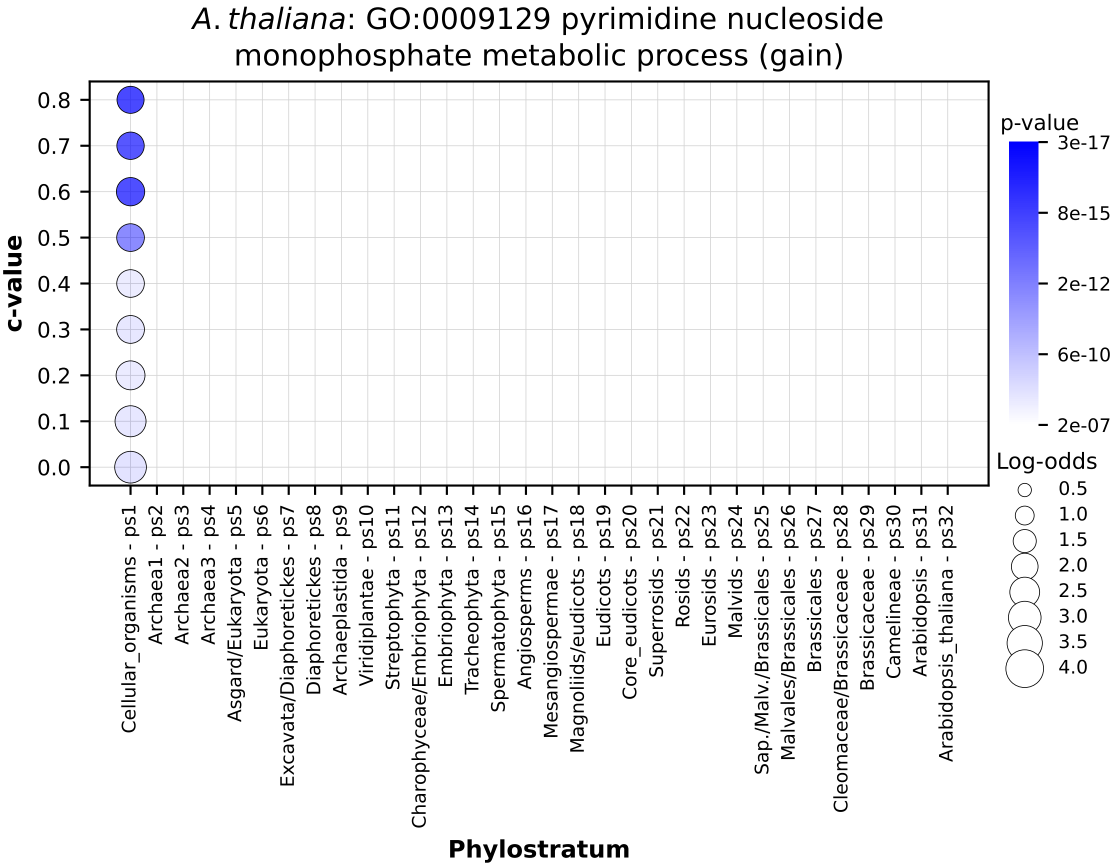

A. thaliana: GO:0009129 pyrimidine nucleoside monophosphate metabolic process (gain)
c-value
0.8
0.7
0.6
0.5
0.4
0.3
0.2
0.1
0.0
p-value
3e-17
8e-15
2e-12
6e-10
2e-07
Log-odds
0.5
1.0
1.5
2.0
2.5
3.0
3.5
4.0
Cellular_organisms - ps1
Archaea1 - ps2
Archaea2 - ps3
Archaea3 - ps4
Asgard/Eukaryota - ps5
Eukaryota - ps6
Excavata/Diaphoretickes - ps7
Diaphoretickes - ps8
Archaeplastida - ps9
Viridiplantae - ps10
Streptophyta - ps11
Charophyceae/Embriophyta - ps12
Embriophyta - ps13
Tracheophyta - ps14
Spermatophyta - ps15
Angiosperms - ps16
Mesangiospermae - ps17
Magnoliids/eudicots - ps18
Eudicots - ps19
Core_eudicots - ps20
Superrosids - ps21
Rosids - ps22
Eurosids - ps23
Malvids - ps24
Sap./Malv./Brassicales - ps25
Malvales/Brassicales - ps26
Brassicales - ps27
Cleomaceae/Brassicaceae - ps28
Brassicaceae - ps29
Camelineae - ps30
Arabidopsis - ps31
Arabidopsis_thaliana - ps32
Phylostratum

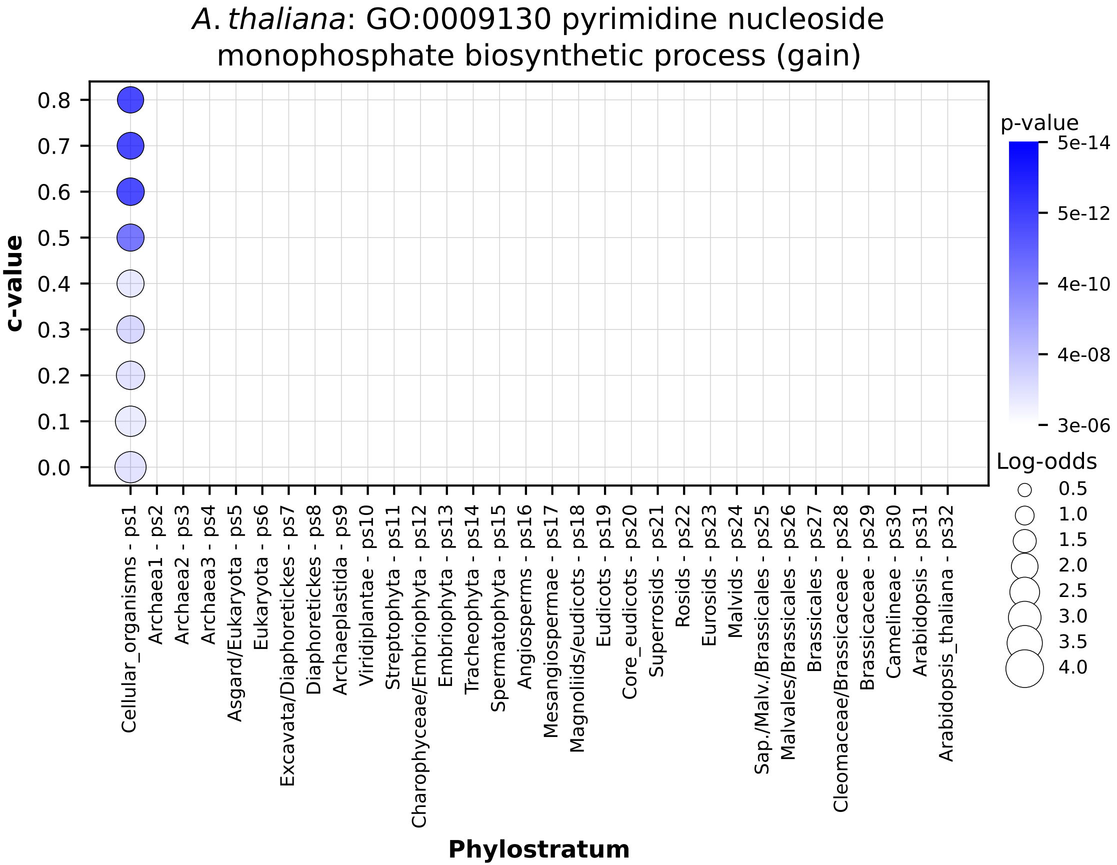

A. thaliana: GO:0009130 pyrimidine nucleoside monophosphate biosynthetic process (gain)
c-value
0.8
0.7
0.6
0.5
0.4
0.3
0.2
0.1
0.0
Phylostratum
Cellular_organisms - ps1
Archaea1 - ps2
Archaea2 - ps3
Archaea3 - ps4
Asgard/Eukaryota - ps5
Eukaryota - ps6
Excavata/Diaphoretickes - ps7
Diaphoretickes - ps8
Archaeplastida - ps9
Viridiplantae - ps10
Streptophyta - ps11
Charophyceae/Embriophyta - ps12
Embriophyta - ps13
Tracheophyta - ps14
Spermatophyta - ps15
Angiosperms - ps16
Mesangiospermae - ps17
Magnoliids/eudicots - ps18
Eudicots - ps19
Core_eudicots - ps20
Superrosids - ps21
Rosids - ps22
Eurosids - ps23
Malvids - ps24
Sap./Malv./Brassicales - ps25
Malvales/Brassicales - ps26
Brassicales - ps27
Cleomaceae/Brassicaceae - ps28
Brassicaceae - ps29
Camelineae - ps30
Arabidopsis - ps31
Arabidopsis_thaliana - ps32
p-value
5e-14
5e-12
4e-10
4e-08
3e-06
Log-odds
0.5
1.0
1.5
2.0
2.5
3.0
3.5
4.0

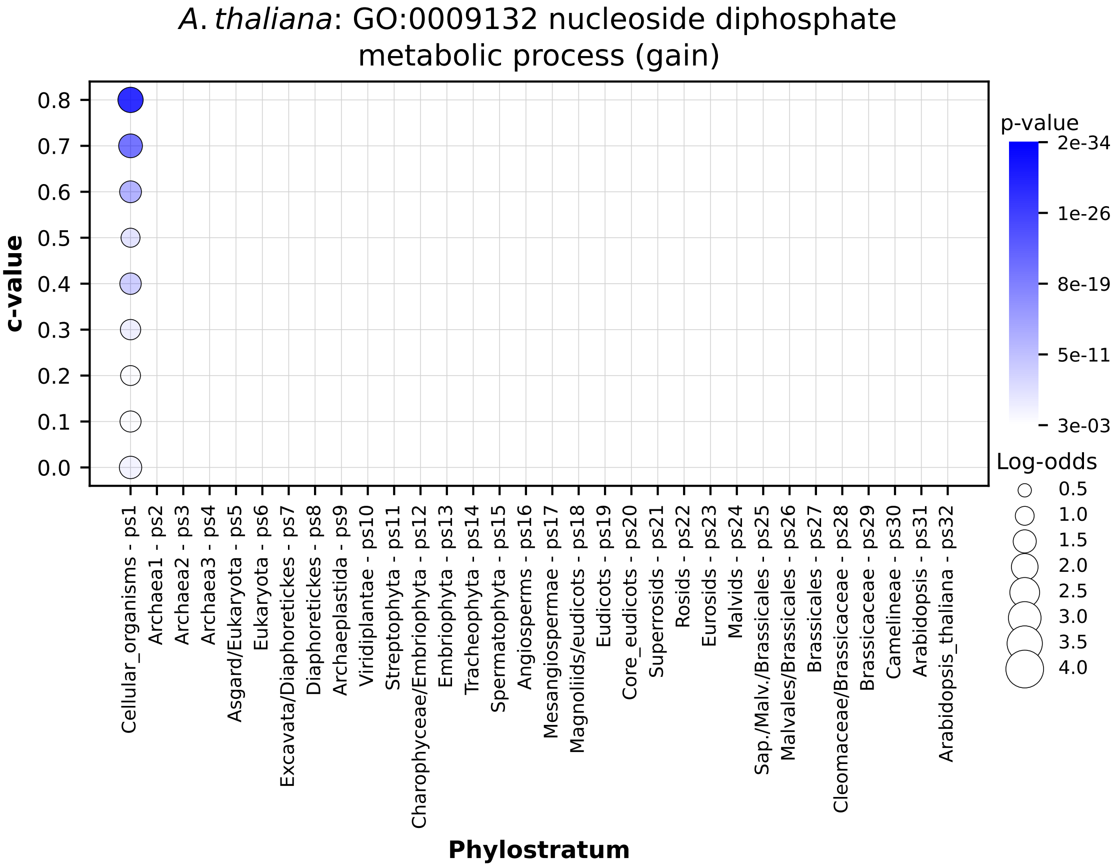

A. thaliana: GO:0009132 nucleoside diphosphate metabolic process (gain)
c-value
0.8
0.7
0.6
0.5
0.4
0.3
0.2
0.1
0.0
p-value
2e-34
1e-26
8e-19
5e-11
3e-03
Log-odds
0.5
1.0
1.5
2.0
2.5
3.0
3.5
4.0
Cellular_organisms - ps1
Archaea1 - ps2
Archaea2 - ps3
Archaea3 - ps4
Asgard/Eukaryota - ps5
Eukaryota - ps6
Excavata/Diaphoretickes - ps7
Diaphoretickes - ps8
Archaeplastida - ps9
Viridiplantae - ps10
Streptophyta - ps11
Charophyceae/Embriophyta - ps12
Embriophyta - ps13
Tracheophyta - ps14
Spermatophyta - ps15
Angiosperms - ps16
Mesangiospermae - ps17
Magnoliids/eudicots - ps18
Eudicots - ps19
Core_eudicots - ps20
Superrosids - ps21
Rosids - ps22
Eurosids - ps23
Malvids - ps24
Sap./Malv./Brassicales - ps25
Malvales/Brassicales - ps26
Brassicales - ps27
Cleomaceae/Brassicaceae - ps28
Brassicaceae - ps29
Camelineae - ps30
Arabidopsis - ps31
Arabidopsis_thaliana - ps32
Phylostratum

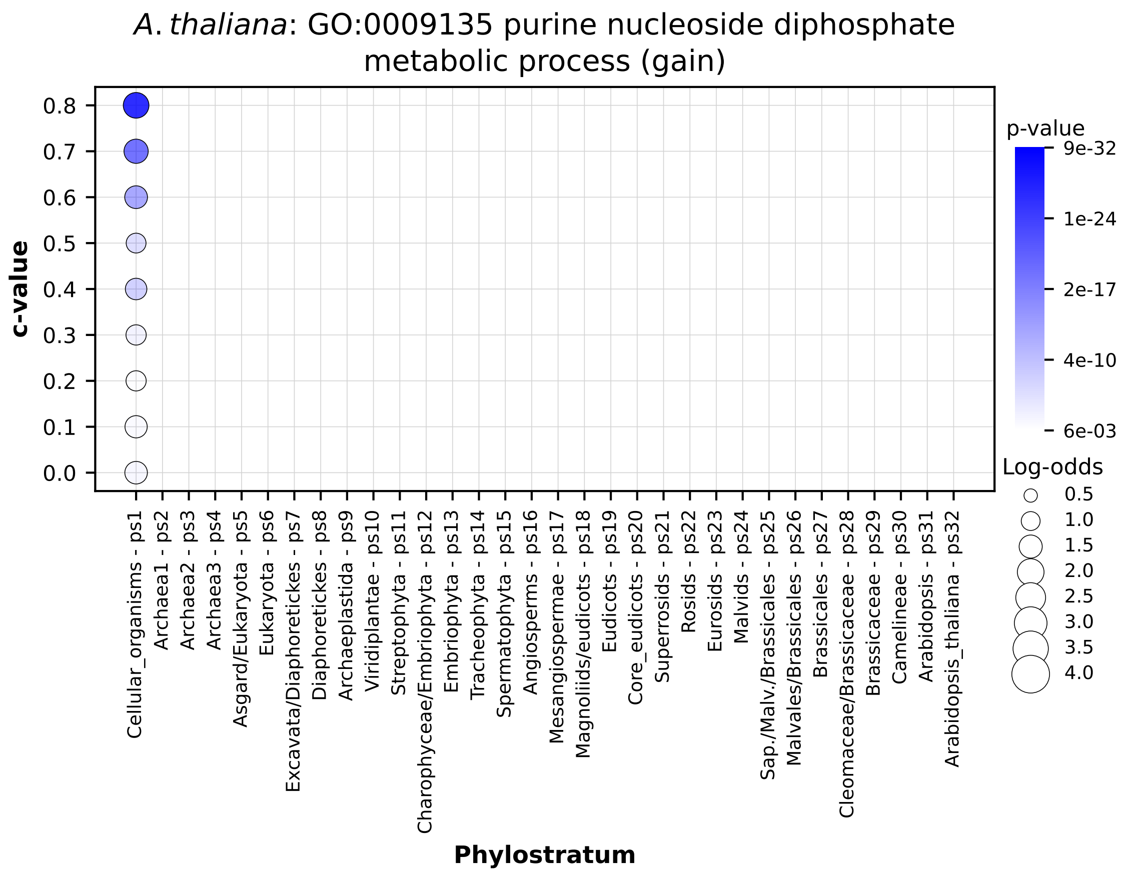
A. thaliana: GO:0009135 purine nucleoside diphosphate metabolic process (gain)
c-value
0.8
0.7
0.6
0.5
0.4
0.3
0.2
0.1
0.0
p-value
9e-32
1e-24
2e-17
4e-10
6e-03
Log-odds
0.5
1.0
1.5
2.0
2.5
3.0
3.5
4.0
Cellular_organisms - ps1
Archaea1 - ps2
Archaea2 - ps3
Archaea3 - ps4
Asgard/Eukaryota - ps5
Eukaryota - ps6
Excavata/Diaphoretickes - ps7
Diaphoretickes - ps8
Archaeplastida - ps9
Viridiplantae - ps10
Streptophyta - ps11
Charophyceae/Embriophyta - ps12
Embriophyta - ps13
Tracheophyta - ps14
Spermatophyta - ps15
Angiosperms - ps16
Mesangiospermae - ps17
Magnoliids/eudicots - ps18
Eudicots - ps19
Core_eudicots - ps20
Superrosids - ps21
Rosids - ps22
Eurosids - ps23
Malvids - ps24
Sap./Malv./Brassicales - ps25
Malvales/Brassicales - ps26
Brassicales - ps27
Cleomaceae/Brassicaceae - ps28
Brassicaceae - ps29
Camelineae - ps30
Arabidopsis - ps31
Arabidopsis_thaliana - ps32
Phylostratum

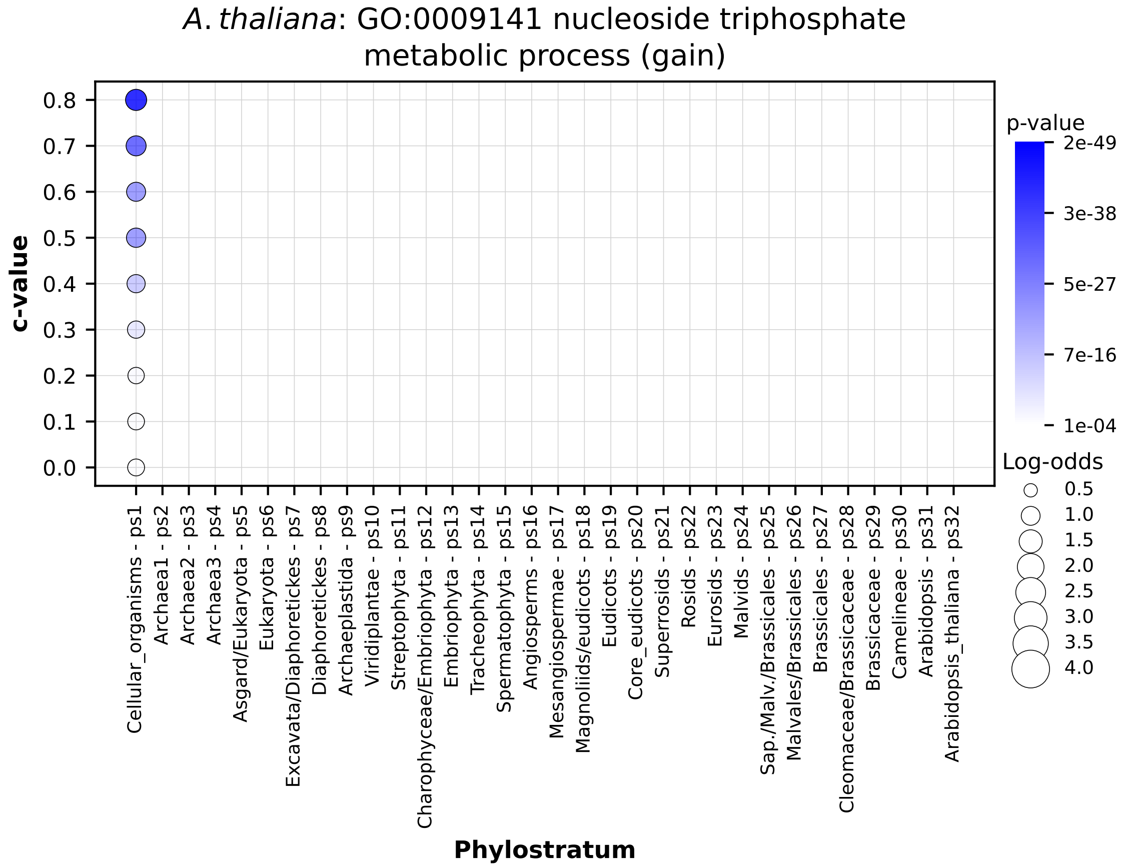

A. thaliana: GO:0009141 nucleoside triphosphate metabolic process (gain)
c-value
0.8
0.7
0.6
0.5
0.4
0.3
0.2
0.1
0.0
p-value
2e-49
3e-38
5e-27
7e-16
1e-04
Log-odds
0.5
1.0
1.5
2.0
2.5
3.0
3.5
4.0
Cellular_organisms - ps1
Archaea1 - ps2
Archaea2 - ps3
Archaea3 - ps4
Asgard/Eukaryota - ps5
Eukaryota - ps6
Excavata/Diaphoretickes - ps7
Diaphoretickes - ps8
Archaeplastida - ps9
Viridiplantae - ps10
Streptophyta - ps11
Charophyceae/Embriophyta - ps12
Embriophyta - ps13
Tracheophyta - ps14
Spermatophyta - ps15
Angiosperms - ps16
Mesangiospermae - ps17
Magnoliids/eudicots - ps18
Eudicots - ps19
Core_eudicots - ps20
Superrosids - ps21
Rosids - ps22
Eurosids - ps23
Malvids - ps24
Sap./Malv./Brassicales - ps25
Malvales/Brassicales - ps26
Brassicales - ps27
Cleomaceae/Brassicaceae - ps28
Brassicaceae - ps29
Camelineae - ps30
Arabidopsis - ps31
Arabidopsis_thaliana - ps32
Phylostratum

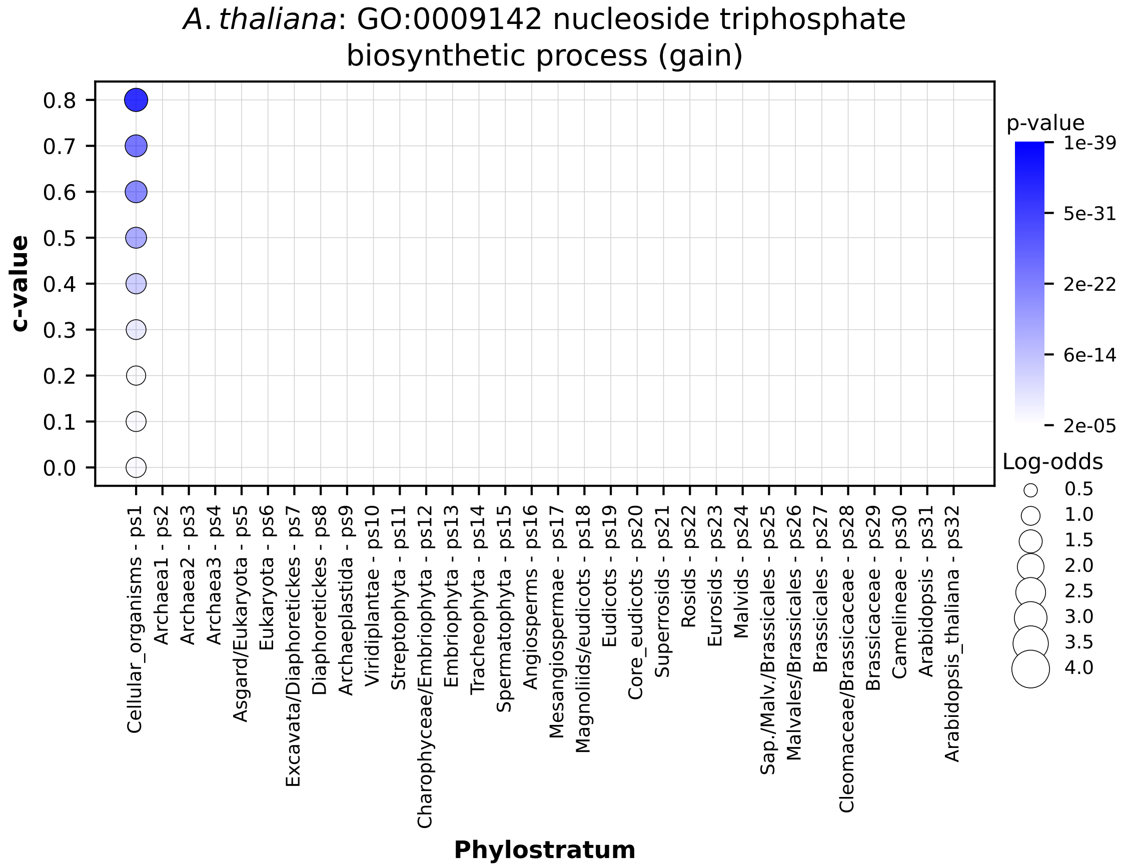

A. thaliana: GO:0009142 nucleoside triphosphate biosynthetic process (gain)
c-value
0.8
0.7
0.6
0.5
0.4
0.3
0.2
0.1
0.0
p-value
1e-39
5e-31
2e-22
6e-14
2e-05
Log-odds
0.5
1.0
1.5
2.0
2.5
3.0
3.5
4.0
Cellular_organisms - ps1
Archaea1 - ps2
Archaea2 - ps3
Archaea3 - ps4
Asgard/Eukaryota - ps5
Eukaryota - ps6
Excavata/Diaphoretickes - ps7
Diaphoretickes - ps8
Archaeplastida - ps9
Viridiplantae - ps10
Streptophyta - ps11
Charophyceae/Embriophyta - ps12
Embriophyta - ps13
Tracheophyta - ps14
Spermatophyta - ps15
Angiosperms - ps16
Mesangiospermae - ps17
Magnoliids/eudicots - ps18
Eudicots - ps19
Core_eudicots - ps20
Superrosids - ps21
Rosids - ps22
Eurosids - ps23
Malvids - ps24
Sap./Malv./Brassicales - ps25
Malvales/Brassicales - ps26
Brassicales - ps27
Cleomaceae/Brassicaceae - ps28
Brassicaceae - ps29
Camelineae - ps30
Arabidopsis - ps31
Arabidopsis_thaliana - ps32
Phylostratum

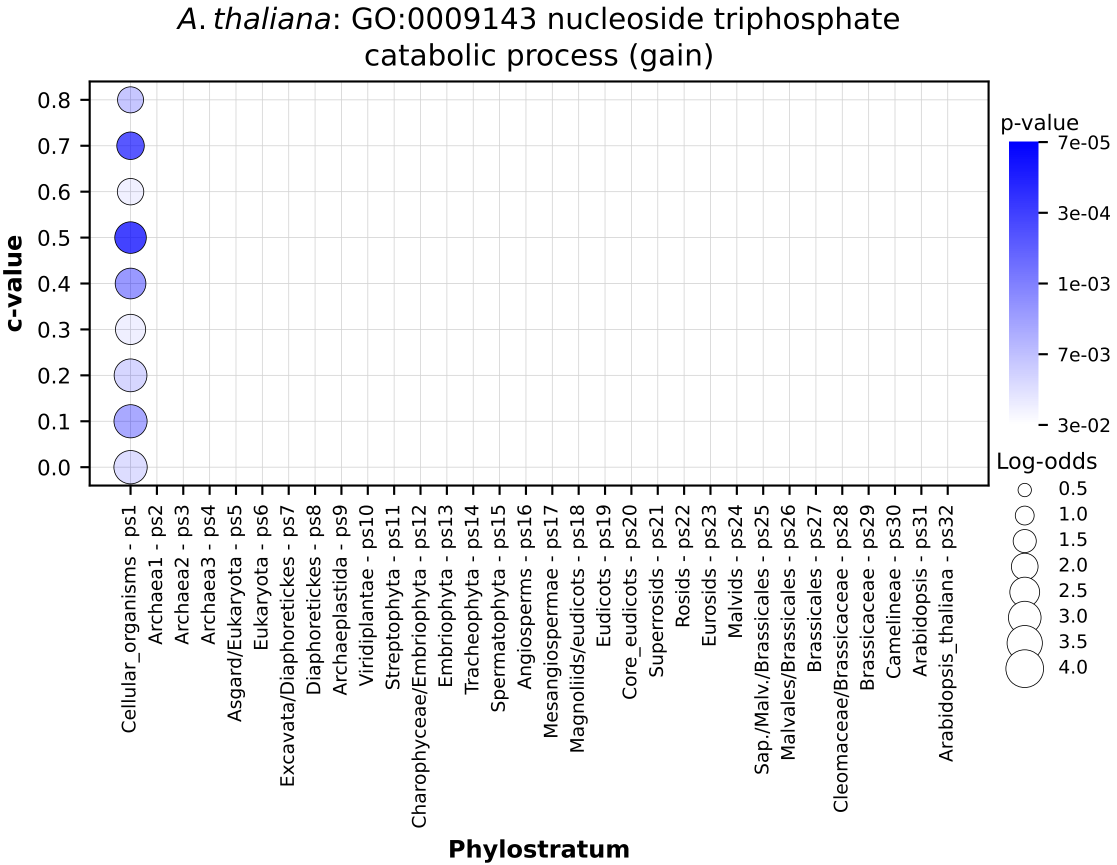

A. thaliana: GO:0009143 nucleoside triphosphate catabolic process (gain)
c-value
0.8
0.7
0.6
0.5
0.4
0.3
0.2
0.1
0.0
p-value
7e-05
3e-04
1e-03
7e-03
3e-02
Log-odds
0.5
1.0
1.5
2.0
2.5
3.0
3.5
4.0
Cellular_organisms - ps1
Archaea1 - ps2
Archaea2 - ps3
Archaea3 - ps4
Asgard/Eukaryota - ps5
Eukaryota - ps6
Excavata/Diaphoretickes - ps7
Diaphoretickes - ps8
Archaeplastida - ps9
Viridiplantae - ps10
Streptophyta - ps11
Charophyceae/Embriophyta - ps12
Embriophyta - ps13
Tracheophyta - ps14
Spermatophyta - ps15
Angiosperms - ps16
Mesangiospermae - ps17
Magnoliids/eudicots - ps18
Eudicots - ps19
Core_eudicots - ps20
Superrosids - ps21
Rosids - ps22
Eurosids - ps23
Malvids - ps24
Sap./Malv./Brassicales - ps25
Malvales/Brassicales - ps26
Brassicales - ps27
Cleomaceae/Brassicaceae - ps28
Brassicaceae - ps29
Camelineae - ps30
Arabidopsis - ps31
Arabidopsis_thaliana - ps32
Phylostratum

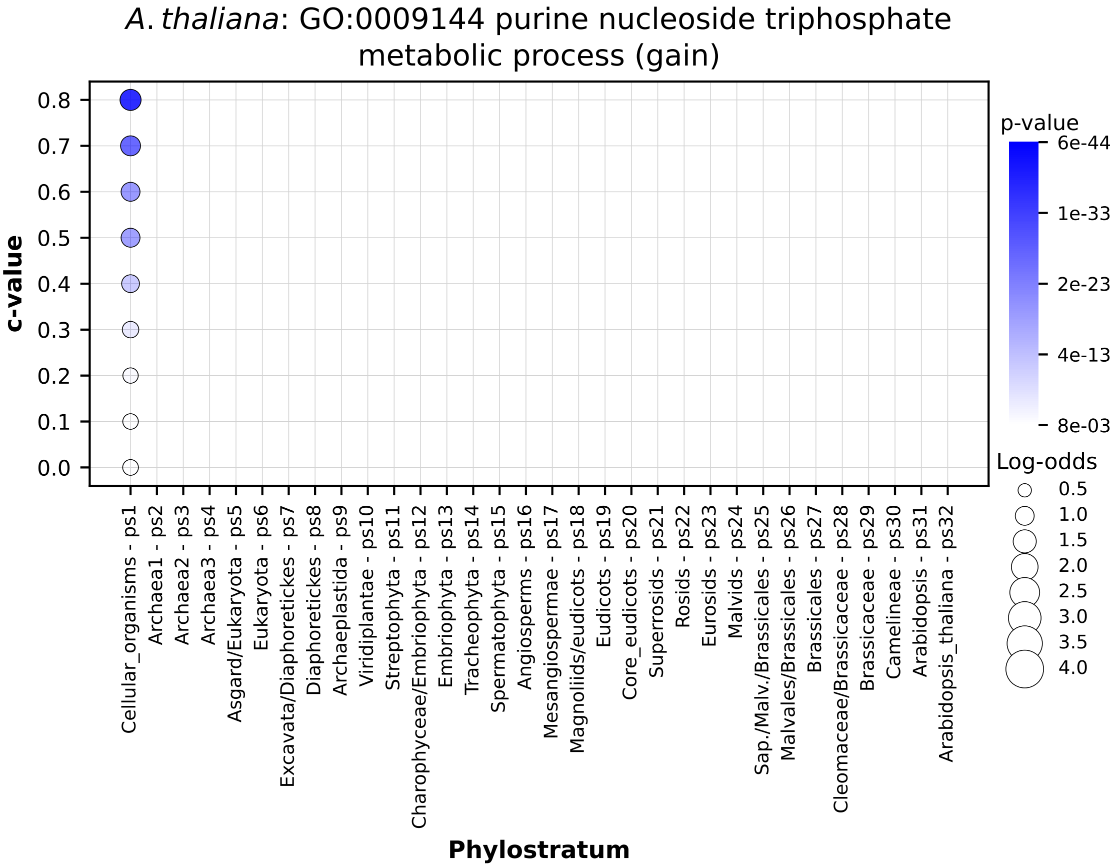

A. thaliana: GO:0009144 purine nucleoside triphosphate metabolic process (gain)
c-value
0.8
0.7
0.6
0.5
0.4
0.3
0.2
0.1
0.0
p-value
6e-44
1e-33
2e-23
4e-13
8e-03
Log-odds
0.5
1.0
1.5
2.0
2.5
3.0
3.5
4.0
Cellular_organisms - ps1
Archaea1 - ps2
Archaea2 - ps3
Archaea3 - ps4
Asgard/Eukaryota - ps5
Eukaryota - ps6
Excavata/Diaphoretickes - ps7
Diaphoretickes - ps8
Archaeplastida - ps9
Viridiplantae - ps10
Streptophyta - ps11
Charophyceae/Embriophyta - ps12
Embriophyta - ps13
Tracheophyta - ps14
Spermatophyta - ps15
Angiosperms - ps16
Mesangiospermae - ps17
Magnoliids/eudicots - ps18
Eudicots - ps19
Core_eudicots - ps20
Superrosids - ps21
Rosids - ps22
Eurosids - ps23
Malvids - ps24
Sap./Malv./Brassicales - ps25
Malvales/Brassicales - ps26
Brassicales - ps27
Cleomaceae/Brassicaceae - ps28
Brassicaceae - ps29
Camelineae - ps30
Arabidopsis - ps31
Arabidopsis_thaliana - ps32
Phylostratum

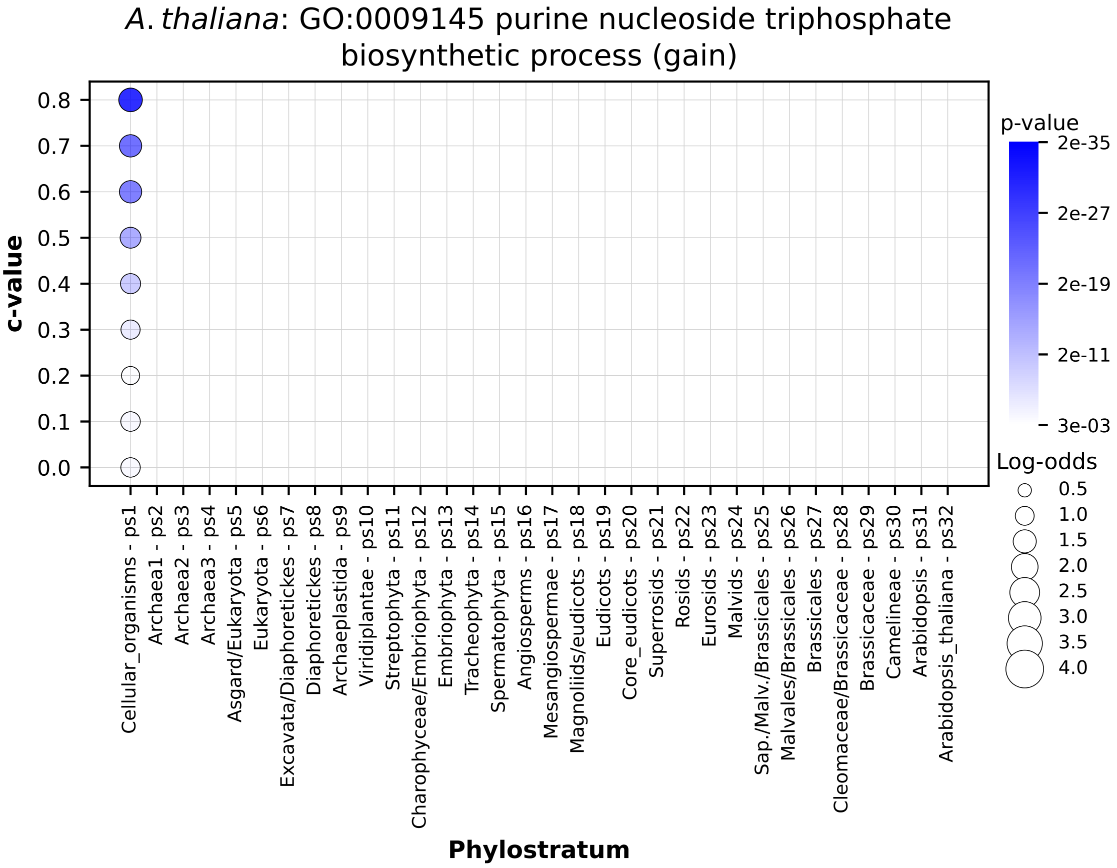

A. thaliana: GO:0009145 purine nucleoside triphosphate biosynthetic process (gain)
c-value
0.8
0.7
0.6
0.5
0.4
0.3
0.2
0.1
0.0
p-value
2e-35
2e-27
2e-19
2e-11
3e-03
Log-odds
0.5
1.0
1.5
2.0
2.5
3.0
3.5
4.0
Cellular_organisms - ps1
Archaea1 - ps2
Archaea2 - ps3
Archaea3 - ps4
Asgard/Eukaryota - ps5
Eukaryota - ps6
Excavata/Diaphoretickes - ps7
Diaphoretickes - ps8
Archaeplastida - ps9
Viridiplantae - ps10
Streptophyta - ps11
Charophyceae/Embriophyta - ps12
Embriophyta - ps13
Tracheophyta - ps14
Spermatophyta - ps15
Angiosperms - ps16
Mesangiospermae - ps17
Magnoliids/eudicots - ps18
Eudicots - ps19
Core_eudicots - ps20
Superrosids - ps21
Rosids - ps22
Eurosids - ps23
Malvids - ps24
Sap./Malv./Brassicales - ps25
Malvales/Brassicales - ps26
Brassicales - ps27
Cleomaceae/Brassicaceae - ps28
Brassicaceae - ps29
Camelineae - ps30
Arabidopsis - ps31
Arabidopsis_thaliana - ps32
Phylostratum

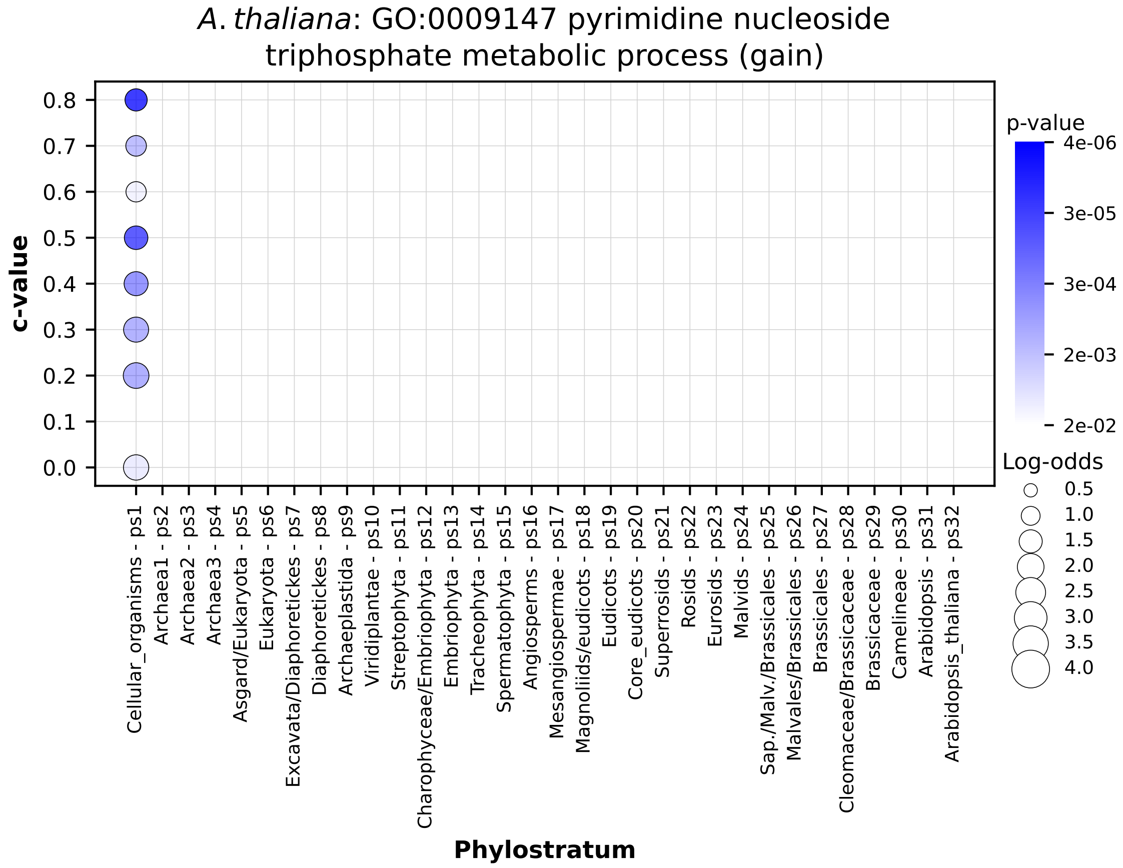

A. thaliana: GO:0009147 pyrimidine nucleoside triphosphate metabolic process (gain)
c-value
0.0
0.1
0.2
0.3
0.4
0.5
0.6
0.7
0.8
p-value
4e-06
3e-05
3e-04
2e-03
2e-02
Log-odds
0.5
1.0
1.5
2.0
2.5
3.0
3.5
4.0
Cellular_organisms - ps1
Archaea1 - ps2
Archaea2 - ps3
Archaea3 - ps4
Asgard/Eukaryota - ps5
Eukaryota - ps6
Excavata/Diaphoretickes - ps7
Diaphoretickes - ps8
Archaeplastida - ps9
Viridiplantae - ps10
Streptophyta - ps11
Charophyceae/Embriophyta - ps12
Embriophyta - ps13
Tracheophyta - ps14
Spermatophyta - ps15
Angiosperms - ps16
Mesangiospermae - ps17
Magnoliids/eudicots - ps18
Eudicots - ps19
Core_eudicots - ps20
Superrosids - ps21
Rosids - ps22
Eurosids - ps23
Malvids - ps24
Sap./Malv./Brassicales - ps25
Malvales/Brassicales - ps26
Brassicales - ps27
Cleomaceae/Brassicaceae - ps28
Brassicaceae - ps29
Camelineae - ps30
Arabidopsis - ps31
Arabidopsis_thaliana - ps32
Phylostratum

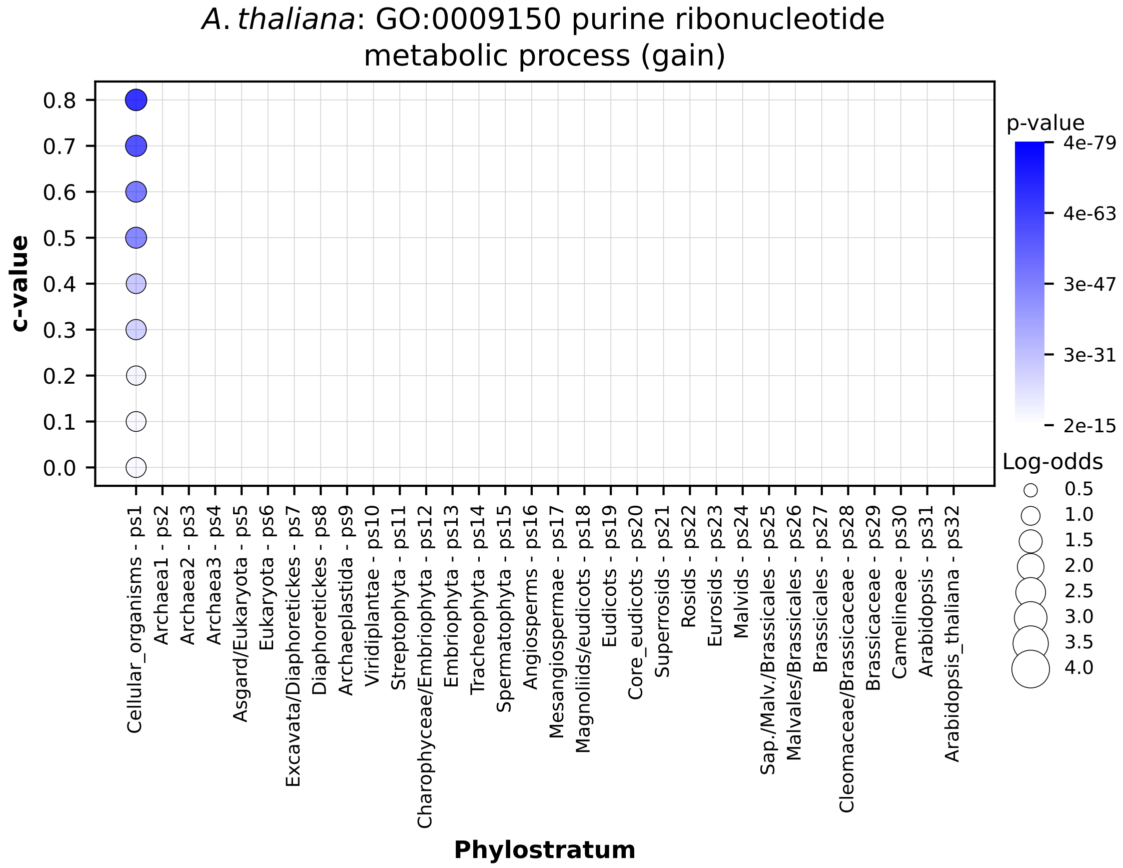
A. thaliana: GO:0009150 purine ribonucleotide metabolic process (gain)
c-value
0.8
0.7
0.6
0.5
0.4
0.3
0.2
0.1
0.0
p-value
4e-79
4e-63
3e-47
3e-31
2e-15
Log-odds
0.5
1.0
1.5
2.0
2.5
3.0
3.5
4.0
Cellular_organisms - ps1
Archaea1 - ps2
Archaea2 - ps3
Archaea3 - ps4
Asgard/Eukaryota - ps5
Eukaryota - ps6
Excavata/Diaphoretickes - ps7
Diaphoretickes - ps8
Archaeplastida - ps9
Viridiplantae - ps10
Streptophyta - ps11
Charophyceae/Embriophyta - ps12
Embriophyta - ps13
Tracheophyta - ps14
Spermatophyta - ps15
Angiosperms - ps16
Mesangiospermae - ps17
Magnoliids/eudicots - ps18
Eudicots - ps19
Core_eudicots - ps20
Superrosids - ps21
Rosids - ps22
Eurosids - ps23
Malvids - ps24
Sap./Malv./Brassicales - ps25
Malvales/Brassicales - ps26
Brassicales - ps27
Cleomaceae/Brassicaceae - ps28
Brassicaceae - ps29
Camelineae - ps30
Arabidopsis - ps31
Arabidopsis_thaliana - ps32
Phylostratum

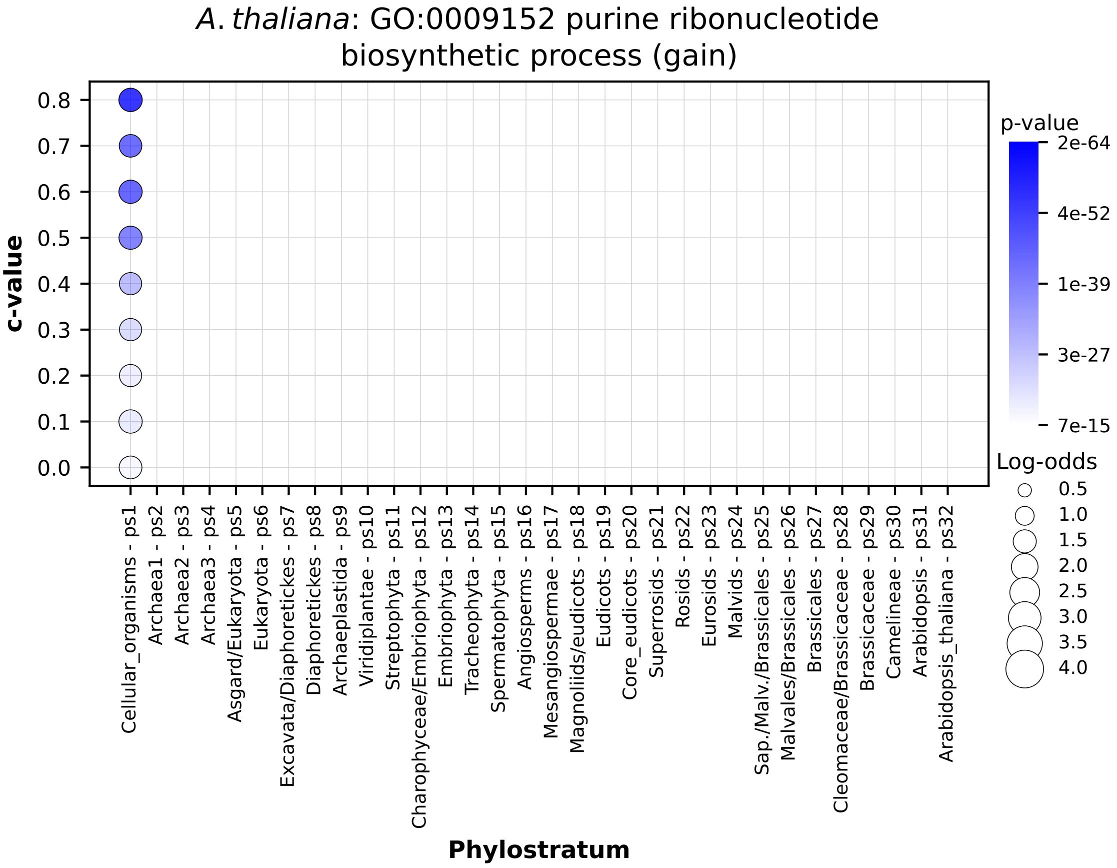

A. thaliana: GO:0009152 purine ribonucleotide biosynthetic process (gain)
c-value
0.8
0.7
0.6
0.5
0.4
0.3
0.2
0.1
0.0
p-value
2e-64
4e-52
1e-39
3e-27
7e-15
Log-odds
0.5
1.0
1.5
2.0
2.5
3.0
3.5
4.0
Cellular_organisms - ps1
Archaea1 - ps2
Archaea2 - ps3
Archaea3 - ps4
Asgard/Eukaryota - ps5
Eukaryota - ps6
Excavata/Diaphoretickes - ps7
Diaphoretickes - ps8
Archaeplastida - ps9
Viridiplantae - ps10
Streptophyta - ps11
Charophyceae/Embriophyta - ps12
Embriophyta - ps13
Tracheophyta - ps14
Spermatophyta - ps15
Angiosperms - ps16
Mesangiospermae - ps17
Magnoliids/eudicots - ps18
Eudicots - ps19
Core_eudicots - ps20
Superrosids - ps21
Rosids - ps22
Eurosids - ps23
Malvids - ps24
Sap./Malv./Brassicales - ps25
Malvales/Brassicales - ps26
Brassicales - ps27
Cleomaceae/Brassicaceae - ps28
Brassicaceae - ps29
Camelineae - ps30
Arabidopsis - ps31
Arabidopsis_thaliana - ps32
Phylostratum

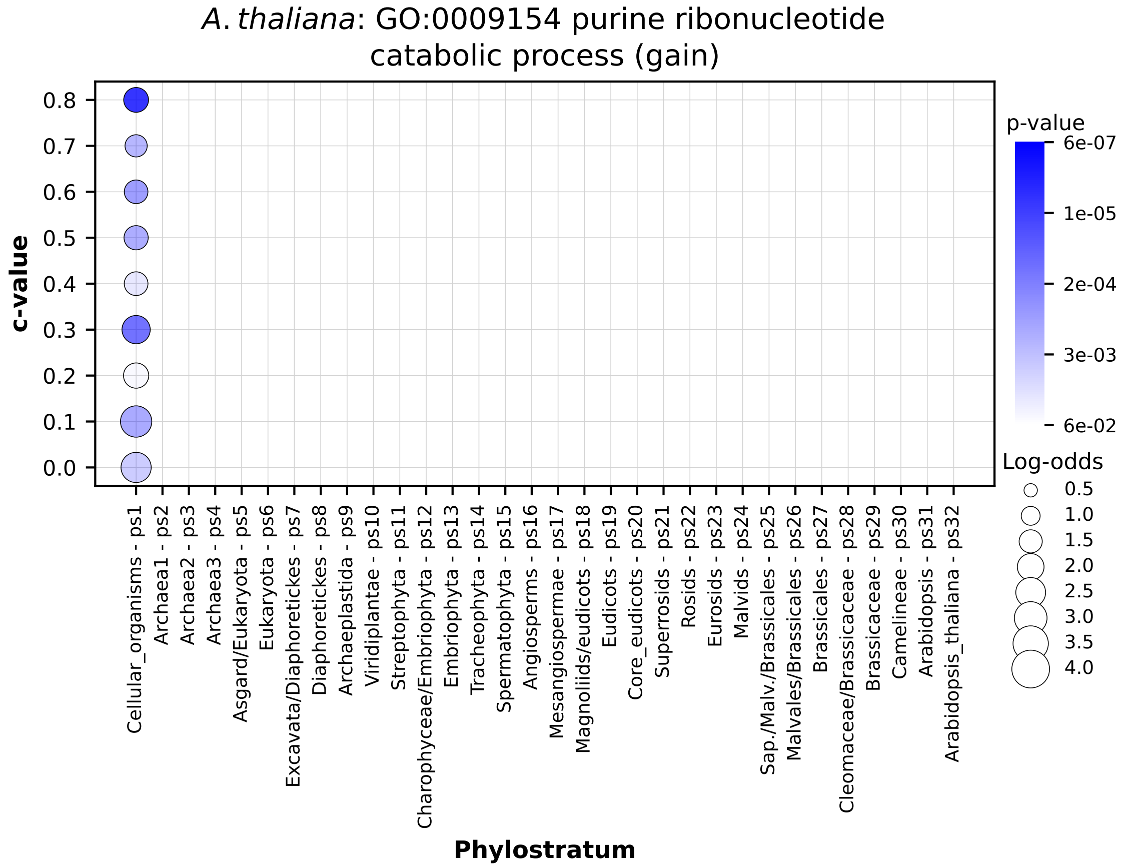

A. thaliana: GO:0009154 purine ribonucleotide catabolic process (gain)
c-value
0.8
0.7
0.6
0.5
0.4
0.3
0.2
0.1
0.0
p-value
6e-07
1e-05
2e-04
3e-03
6e-02
Log-odds
0.5
1.0
1.5
2.0
2.5
3.0
3.5
4.0
Cellular_organisms - ps1
Archaea1 - ps2
Archaea2 - ps3
Archaea3 - ps4
Asgard/Eukaryota - ps5
Eukaryota - ps6
Excavata/Diaphoretickes - ps7
Diaphoretickes - ps8
Archaeplastida - ps9
Viridiplantae - ps10
Streptophyta - ps11
Charophyceae/Embriophyta - ps12
Embriophyta - ps13
Tracheophyta - ps14
Spermatophyta - ps15
Angiosperms - ps16
Mesangiospermae - ps17
Magnoliids/eudicots - ps18
Eudicots - ps19
Core_eudicots - ps20
Superrosids - ps21
Rosids - ps22
Eurosids - ps23
Malvids - ps24
Sap./Malv./Brassicales - ps25
Malvales/Brassicales - ps26
Brassicales - ps27
Cleomaceae/Brassicaceae - ps28
Brassicaceae - ps29
Camelineae - ps30
Arabidopsis - ps31
Arabidopsis_thaliana - ps32
Phylostratum

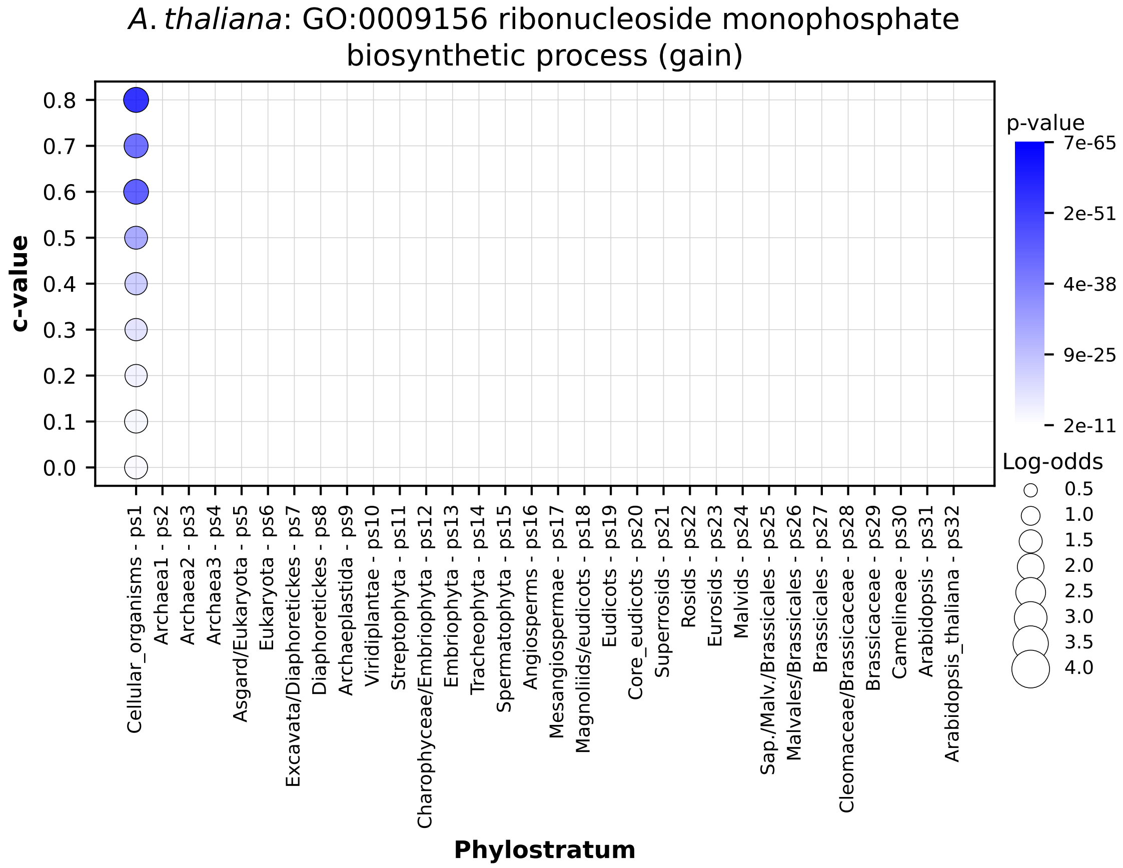
A. thaliana: GO:0009156 ribonucleoside monophosphate biosynthetic process (gain)
c-value
0.8
0.7
0.6
0.5
0.4
0.3
0.2
0.1
0.0
p-value
7e-65
2e-51
4e-38
9e-25
2e-11
Log-odds
0.5
1.0
1.5
2.0
2.5
3.0
3.5
4.0
Cellular_organisms - ps1
Archaea1 - ps2
Archaea2 - ps3
Archaea3 - ps4
Asgard/Eukaryota - ps5
Eukaryota - ps6
Excavata/Diaphoretickes - ps7
Diaphoretickes - ps8
Archaeplastida - ps9
Viridiplantae - ps10
Streptophyta - ps11
Charophyceae/Embriophyta - ps12
Embriophyta - ps13
Tracheophyta - ps14
Spermatophyta - ps15
Angiosperms - ps16
Mesangiospermae - ps17
Magnoliids/eudicots - ps18
Eudicots - ps19
Core_eudicots - ps20
Superrosids - ps21
Rosids - ps22
Eurosids - ps23
Malvids - ps24
Sap./Malv./Brassicales - ps25
Malvales/Brassicales - ps26
Brassicales - ps27
Cleomaceae/Brassicaceae - ps28
Brassicaceae - ps29
Camelineae - ps30
Arabidopsis - ps31
Arabidopsis_thaliana - ps32
Phylostratum

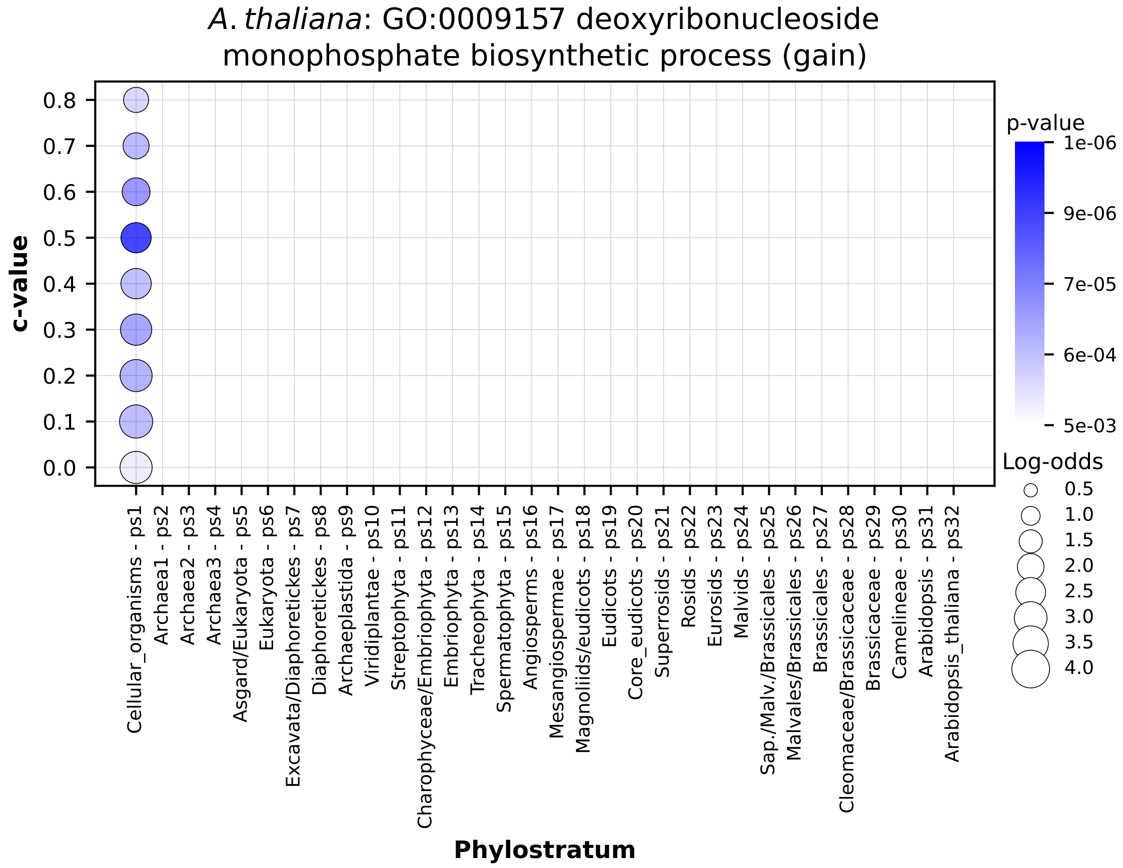

$A. thaliana$: GO:0009157 deoxyribonucleoside monophosphate biosynthetic process (gain)
c-value
0.8
0.7
0.6
0.5
0.4
0.3
0.2
0.1
0.0
Phylostratum
Cellular_organisms - ps1
Archaea1 - ps2
Archaea2 - ps3
Archaea3 - ps4
Asgard/Eukaryota - ps5
Eukaryota - ps6
Excavata/Diaphoretickes - ps7
Diaphoretickes - ps8
Archaeplastida - ps9
Viridiplantae - ps10
Streptophyta - ps11
Charophyceae/Embriophyta - ps12
Embriophyta - ps13
Tracheophyta - ps14
Spermatophyta - ps15
Angiosperms - ps16
Mesangiospermae - ps17
Magnoliids/eudicots - ps18
Eudicots - ps19
Core_eudicots - ps20
Superrosids - ps21
Rosids - ps22
Eurosids - ps23
Malvids - ps24
Sap./Malv./Brassicales - ps25
Malvales/Brassicales - ps26
Brassicales - ps27
Cleomaceae/Brassicaceae - ps28
Brassicaceae - ps29
Camelineae - ps30
Arabidopsis - ps31
Arabidopsis_thaliana - ps32
p-value
1e-06
9e-06
7e-05
6e-04
5e-03
Log-odds
0.5
1.0
1.5
2.0
2.5
3.0
3.5
4.0

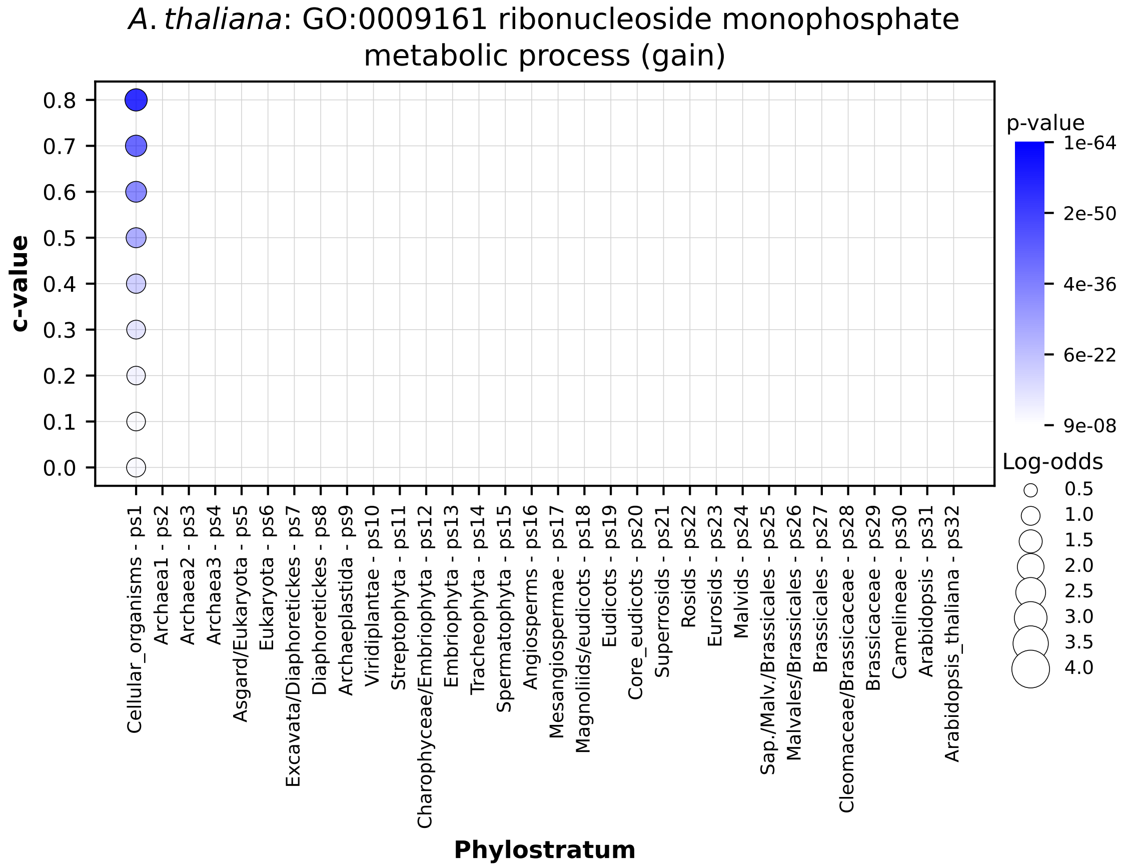

A. thaliana: GO:0009161 ribonucleoside monophosphate metabolic process (gain)
c-value
0.8
0.7
0.6
0.5
0.4
0.3
0.2
0.1
0.0
p-value
1e-64
2e-50
4e-36
6e-22
9e-08
Log-odds
0.5
1.0
1.5
2.0
2.5
3.0
3.5
4.0
Cellular_organisms - ps1
Archaea1 - ps2
Archaea2 - ps3
Archaea3 - ps4
Asgard/Eukaryota - ps5
Eukaryota - ps6
Excavata/Diaphoretickes - ps7
Diaphoretickes - ps8
Archaeplastida - ps9
Viridiplantae - ps10
Streptophyta - ps11
Charophyceae/Embriophyta - ps12
Embriophyta - ps13
Tracheophyta - ps14
Spermatophyta - ps15
Angiosperms - ps16
Mesangiospermae - ps17
Magnoliids/eudicots - ps18
Eudicots - ps19
Core_eudicots - ps20
Superrosids - ps21
Rosids - ps22
Eurosids - ps23
Malvids - ps24
Sap./Malv./Brassicales - ps25
Malvales/Brassicales - ps26
Brassicales - ps27
Cleomaceae/Brassicaceae - ps28
Brassicaceae - ps29
Camelineae - ps30
Arabidopsis - ps31
Arabidopsis_thaliana - ps32
Phylostratum

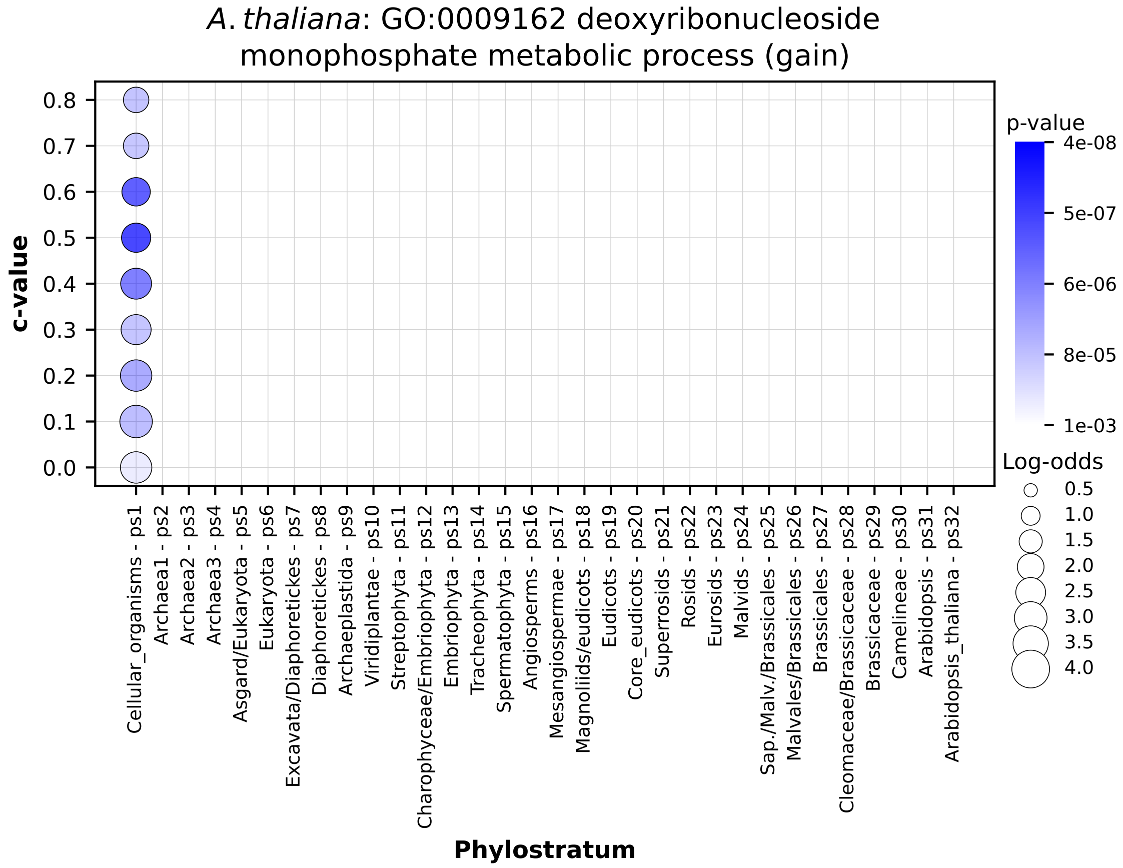

A. thaliana: GO:0009162 deoxyribonucleoside monophosphate metabolic process (gain)
c-value
0.8
0.7
0.6
0.5
0.4
0.3
0.2
0.1
0.0
p-value
4e-08
5e-07
6e-06
8e-05
1e-03
Log-odds
0.5
1.0
1.5
2.0
2.5
3.0
3.5
4.0
Cellular_organisms - ps1
Archaea1 - ps2
Archaea2 - ps3
Archaea3 - ps4
Asgard/Eukaryota - ps5
Eukaryota - ps6
Excavata/Diaphoretickes - ps7
Diaphoretickes - ps8
Archaeplastida - ps9
Viridiplantae - ps10
Streptophyta - ps11
Charophyceae/Embriophyta - ps12
Embriophyta - ps13
Tracheophyta - ps14
Spermatophyta - ps15
Angiosperms - ps16
Mesangiospermae - ps17
Magnoliids/eudicots - ps18
Eudicots - ps19
Core_eudicots - ps20
Superrosids - ps21
Rosids - ps22
Eurosids - ps23
Malvids - ps24
Sap./Malv./Brassicales - ps25
Malvales/Brassicales - ps26
Brassicales - ps27
Cleomaceae/Brassicaceae - ps28
Brassicaceae - ps29
Camelineae - ps30
Arabidopsis - ps31
Arabidopsis_thaliana - ps32
Phylostratum

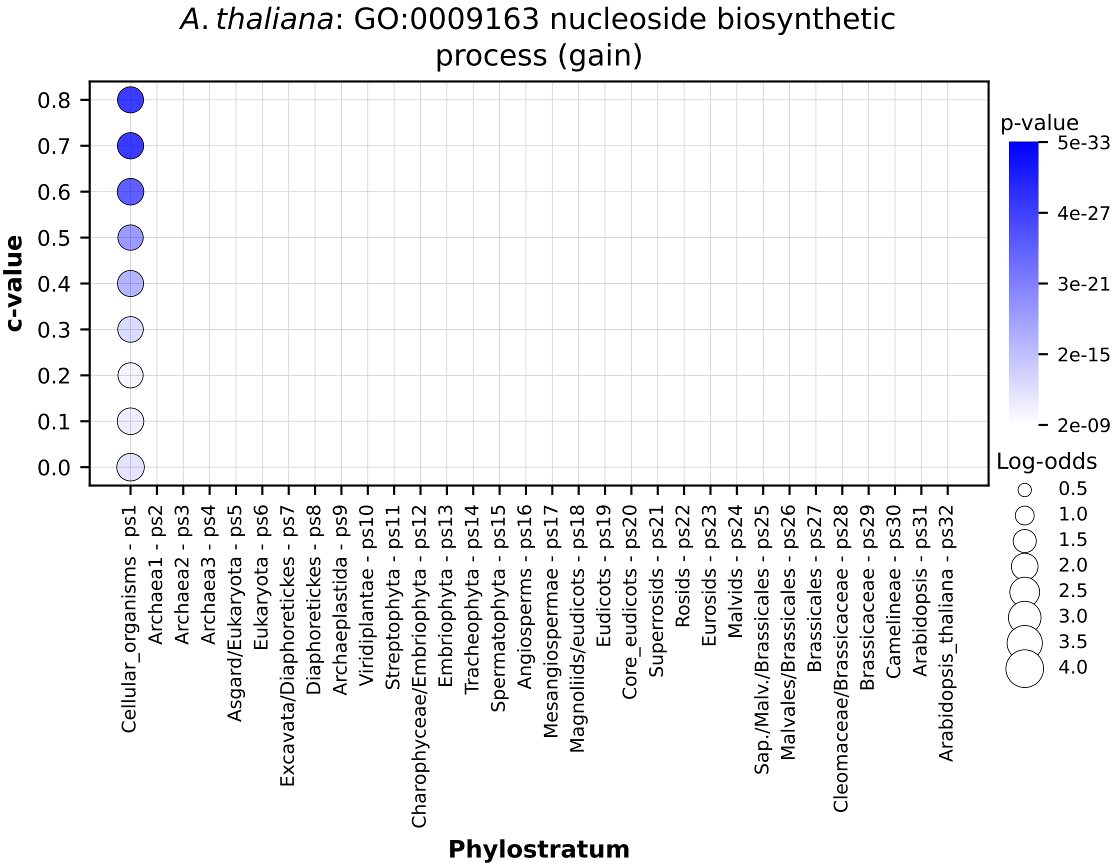

A. thaliana: GO:0009163 nucleoside biosynthetic process (gain)
c-value
0.8
0.7
0.6
0.5
0.4
0.3
0.2
0.1
0.0
p-value
5e-33
4e-27
3e-21
2e-15
2e-09
Log-odds
0.5
1.0
1.5
2.0
2.5
3.0
3.5
4.0
Cellular_organisms - ps1
Archaea1 - ps2
Archaea2 - ps3
Archaea3 - ps4
Asgard/Eukaryota - ps5
Eukaryota - ps6
Excavata/Diaphoretickes - ps7
Diaphoretickes - ps8
Archaeplastida - ps9
Viridiplantae - ps10
Streptophyta - ps11
Charophyceae/Embriophyta - ps12
Embriophyta - ps13
Tracheophyta - ps14
Spermatophyta - ps15
Angiosperms - ps16
Mesangiospermae - ps17
Magnoliids/eudicots - ps18
Eudicots - ps19
Core_eudicots - ps20
Superrosids - ps21
Rosids - ps22
Eurosids - ps23
Malvids - ps24
Sap./Malv./Brassicales - ps25
Malvales/Brassicales - ps26
Brassicales - ps27
Cleomaceae/Brassicaceae - ps28
Brassicaceae - ps29
Camelineae - ps30
Arabidopsis - ps31
Arabidopsis_thaliana - ps32
Phylostratum

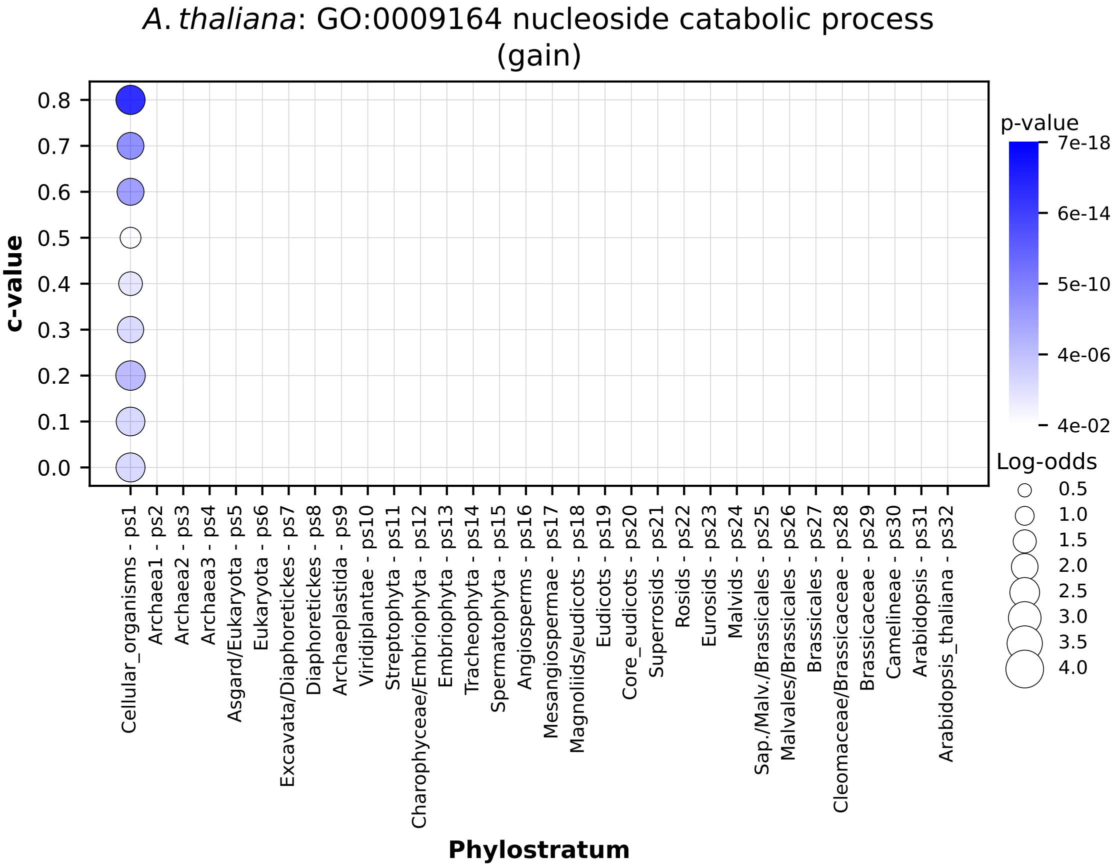

A. thaliana: GO:0009164 nucleoside catabolic process
(gain)
c-value
0.8
0.7
0.6
0.5
0.4
0.3
0.2
0.1
0.0
p-value
7e-18
6e-14
5e-10
4e-06
4e-02
Log-odds
0.5
1.0
1.5
2.0
2.5
3.0
3.5
4.0
Cellular_organisms - ps1
Archaea1 - ps2
Archaea2 - ps3
Archaea3 - ps4
Asgard/Eukaryota - ps5
Eukaryota - ps6
Excavata/Diaphoretickes - ps7
Diaphoretickes - ps8
Archaeplastida - ps9
Viridiplantae - ps10
Streptophyta - ps11
Charophyceae/Embriophyta - ps12
Embriophyta - ps13
Tracheophyta - ps14
Spermatophyta - ps15
Angiosperms - ps16
Mesangiospermae - ps17
Magnoliids/eudicots - ps18
Eudicots - ps19
Core_eudicots - ps20
Superrosids - ps21
Rosids - ps22
Eurosids - ps23
Malvids - ps24
Sap./Malv./Brassicales - ps25
Malvales/Brassicales - ps26
Brassicales - ps27
Cleomaceae/Brassicaceae - ps28
Brassicaceae - ps29
Camelineae - ps30
Arabidopsis - ps31
Arabidopsis_thaliana - ps32
Phylostratum

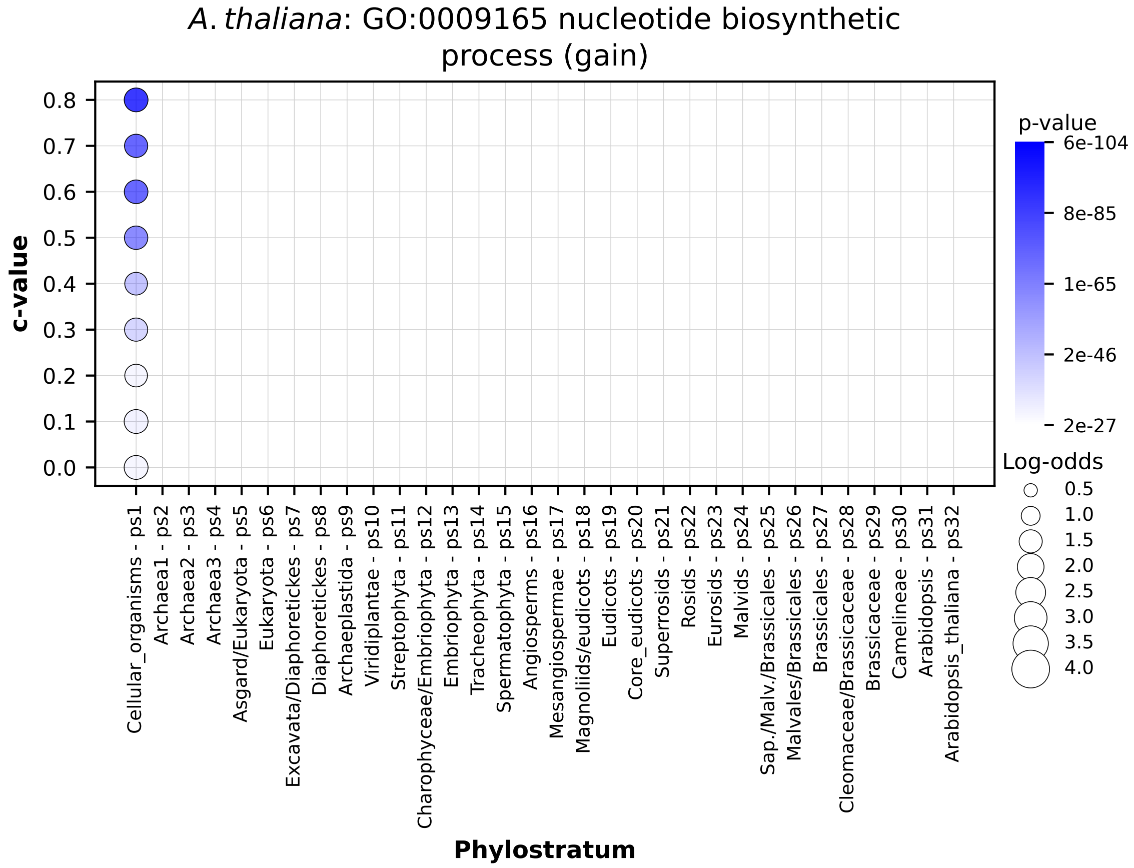

A. thaliana: GO:0009165 nucleotide biosynthetic process (gain)
c-value
0.8
0.7
0.6
0.5
0.4
0.3
0.2
0.1
0.0
p-value
6e-104
8e-85
1e-65
2e-46
2e-27
Log-odds
0.5
1.0
1.5
2.0
2.5
3.0
3.5
4.0
Cellular_organisms - ps1
Archaea1 - ps2
Archaea2 - ps3
Archaea3 - ps4
Asgard/Eukaryota - ps5
Eukaryota - ps6
Excavata/Diaphoretickes - ps7
Diaphoretickes - ps8
Archaeplastida - ps9
Viridiplantae - ps10
Streptophyta - ps11
Charophyceae/Embriophyta - ps12
Embriophyta - ps13
Tracheophyta - ps14
Spermatophyta - ps15
Angiosperms - ps16
Mesangiospermae - ps17
Magnoliids/eudicots - ps18
Eudicots - ps19
Core_eudicots - ps20
Superrosids - ps21
Rosids - ps22
Eurosids - ps23
Malvids - ps24
Sap./Malv./Brassicales - ps25
Malvales/Brassicales - ps26
Brassicales - ps27
Cleomaceae/Brassicaceae - ps28
Brassicaceae - ps29
Camelineae - ps30
Arabidopsis - ps31
Arabidopsis_thaliana - ps32
Phylostratum

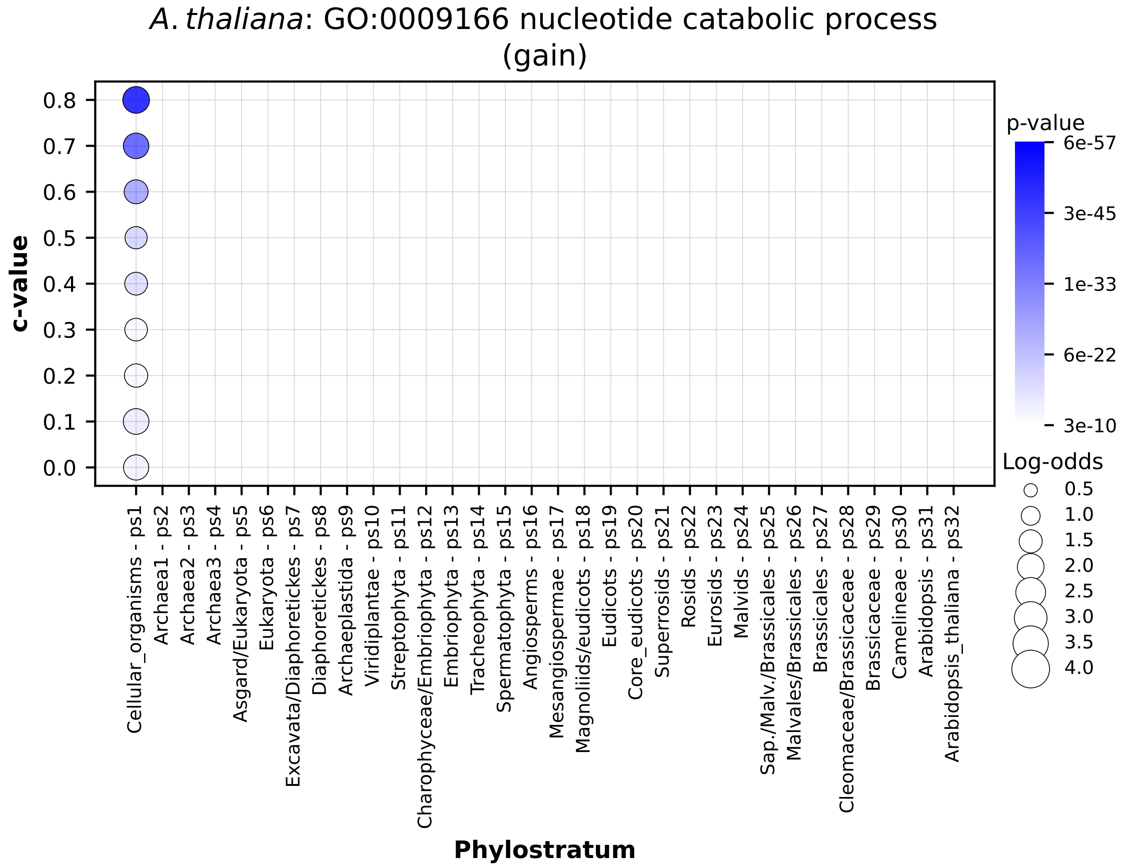

A. thaliana: GO:0009166 nucleotide catabolic process (gain)
c-value
0.8
0.7
0.6
0.5
0.4
0.3
0.2
0.1
0.0
p-value
6e-57
3e-45
1e-33
6e-22
3e-10
Log-odds
0.5
1.0
1.5
2.0
2.5
3.0
3.5
4.0
Cellular_organisms - ps1
Archaea1 - ps2
Archaea2 - ps3
Archaea3 - ps4
Asgard/Eukaryota - ps5
Eukaryota - ps6
Excavata/Diaphoretickes - ps7
Diaphoretickes - ps8
Archaeplastida - ps9
Viridiplantae - ps10
Streptophyta - ps11
Charophyceae/Embriophyta - ps12
Embriophyta - ps13
Tracheophyta - ps14
Spermatophyta - ps15
Angiosperms - ps16
Mesangiospermae - ps17
Magnoliids/eudicots - ps18
Eudicots - ps19
Core_eudicots - ps20
Superrosids - ps21
Rosids - ps22
Eurosids - ps23
Malvids - ps24
Sap./Malv./Brassicales - ps25
Malvales/Brassicales - ps26
Brassicales - ps27
Cleomaceae/Brassicaceae - ps28
Brassicaceae - ps29
Camelineae - ps30
Arabidopsis - ps31
Arabidopsis_thaliana - ps32
Phylostratum

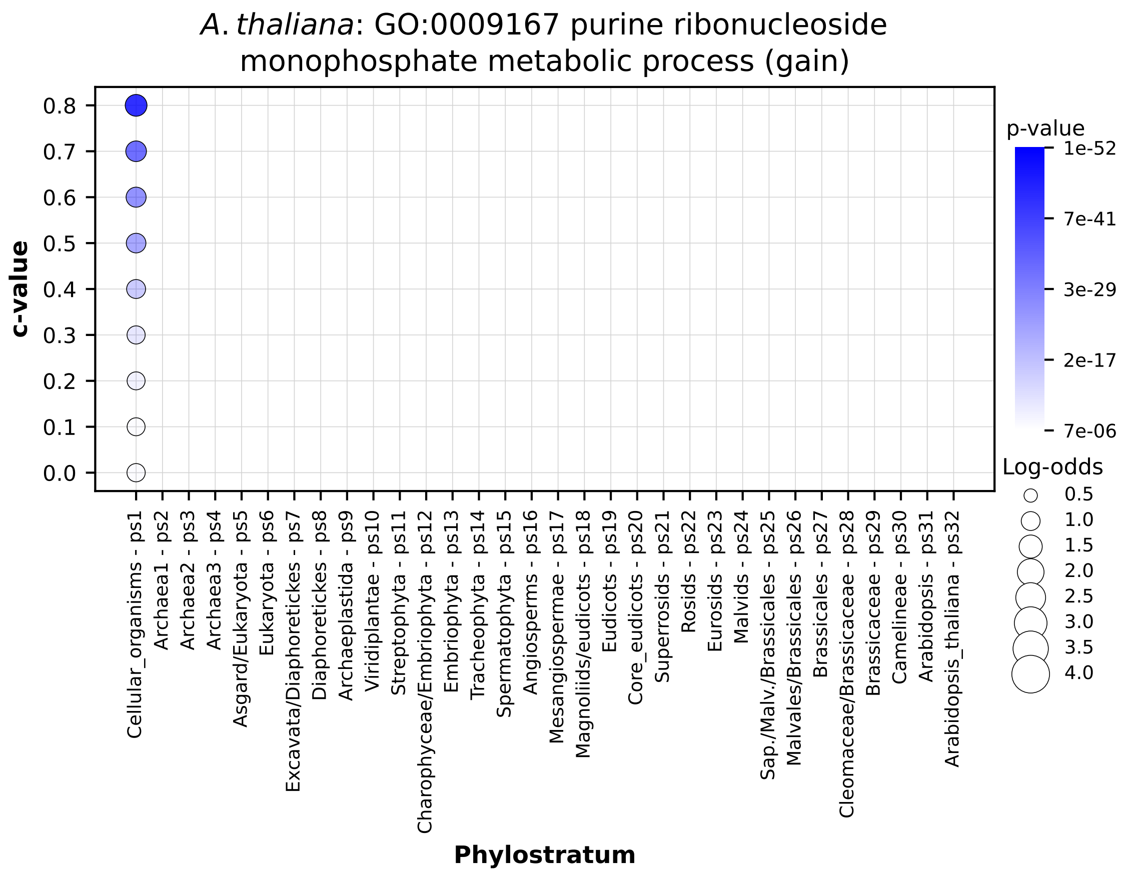

A. thaliana: GO:0009167 purine ribonucleoside monophosphate metabolic process (gain)
c-value
0.8
0.7
0.6
0.5
0.4
0.3
0.2
0.1
0.0
p-value
1e-52
7e-41
3e-29
2e-17
7e-06
Log-odds
0.5
1.0
1.5
2.0
2.5
3.0
3.5
4.0
Cellular_organisms - ps1
Archaea1 - ps2
Archaea2 - ps3
Archaea3 - ps4
Asgard/Eukaryota - ps5
Eukaryota - ps6
Excavata/Diaphoretickes - ps7
Diaphoretickes - ps8
Archaeplastida - ps9
Viridiplantae - ps10
Streptophyta - ps11
Charophyceae/Embriophyta - ps12
Embriophyta - ps13
Tracheophyta - ps14
Spermatophyta - ps15
Angiosperms - ps16
Mesangiospermae - ps17
Magnoliids/eudicots - ps18
Eudicots - ps19
Core_eudicots - ps20
Superrosids - ps21
Rosids - ps22
Eurosids - ps23
Malvids - ps24
Sap./Malv./Brassicales - ps25
Malvales/Brassicales - ps26
Brassicales - ps27
Cleomaceae/Brassicaceae - ps28
Brassicaceae - ps29
Camelineae - ps30
Arabidopsis - ps31
Arabidopsis_thaliana - ps32
Phylostratum

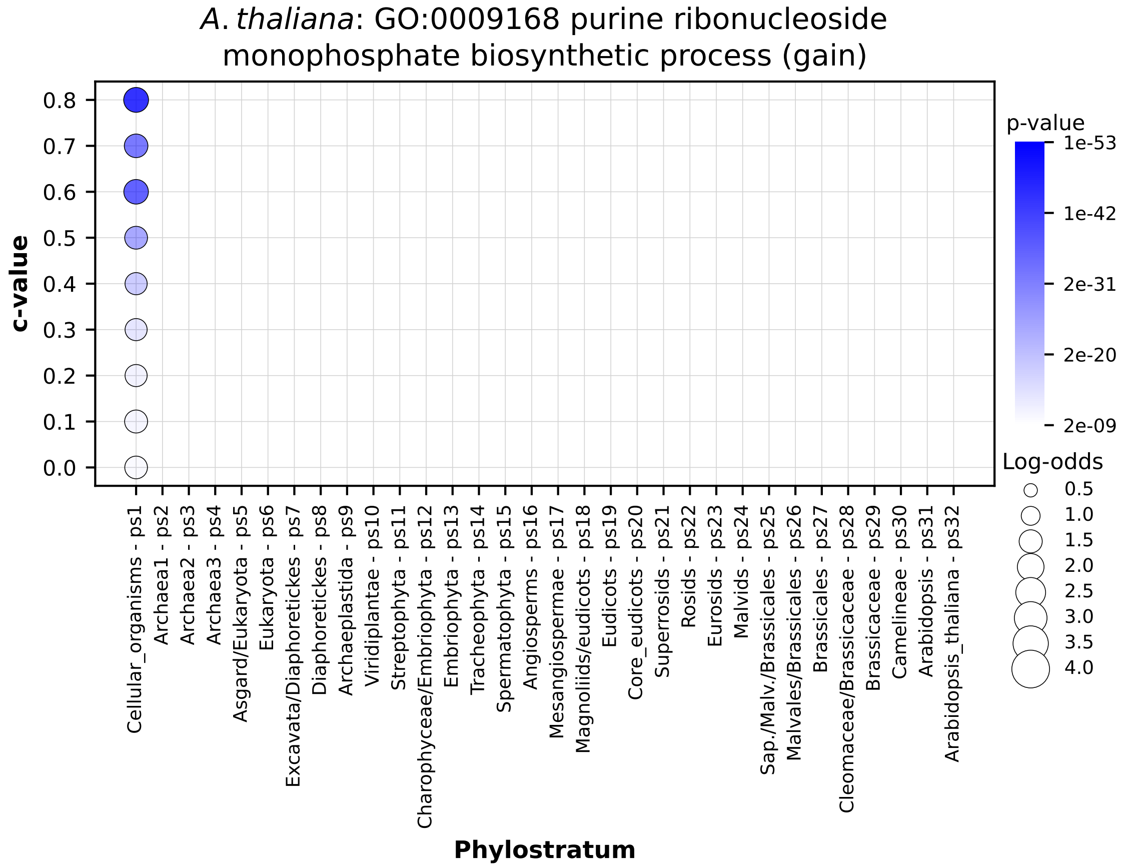

A. thaliana: GO:0009168 purine ribonucleoside monophosphate biosynthetic process (gain)
c-value
0.8
0.7
0.6
0.5
0.4
0.3
0.2
0.1
0.0
p-value
1e-53
1e-42
2e-31
2e-20
2e-09
Log-odds
0.5
1.0
1.5
2.0
2.5
3.0
3.5
4.0
Cellular_organisms - ps1
Archaea1 - ps2
Archaea2 - ps3
Archaea3 - ps4
Asgard/Eukaryota - ps5
Eukaryota - ps6
Excavata/Diaphoretickes - ps7
Diaphoretickes - ps8
Archaeplastida - ps9
Viridiplantae - ps10
Streptophyta - ps11
Charophyceae/Embriophyta - ps12
Embriophyta - ps13
Tracheophyta - ps14
Spermatophyta - ps15
Angiosperms - ps16
Mesangiospermae - ps17
Magnoliids/eudicots - ps18
Eudicots - ps19
Core_eudicots - ps20
Superrosids - ps21
Rosids - ps22
Eurosids - ps23
Malvids - ps24
Sap./Malv./Brassicales - ps25
Malvales/Brassicales - ps26
Brassicales - ps27
Cleomaceae/Brassicaceae - ps28
Brassicaceae - ps29
Camelineae - ps30
Arabidopsis - ps31
Arabidopsis_thaliana - ps32
Phylostratum

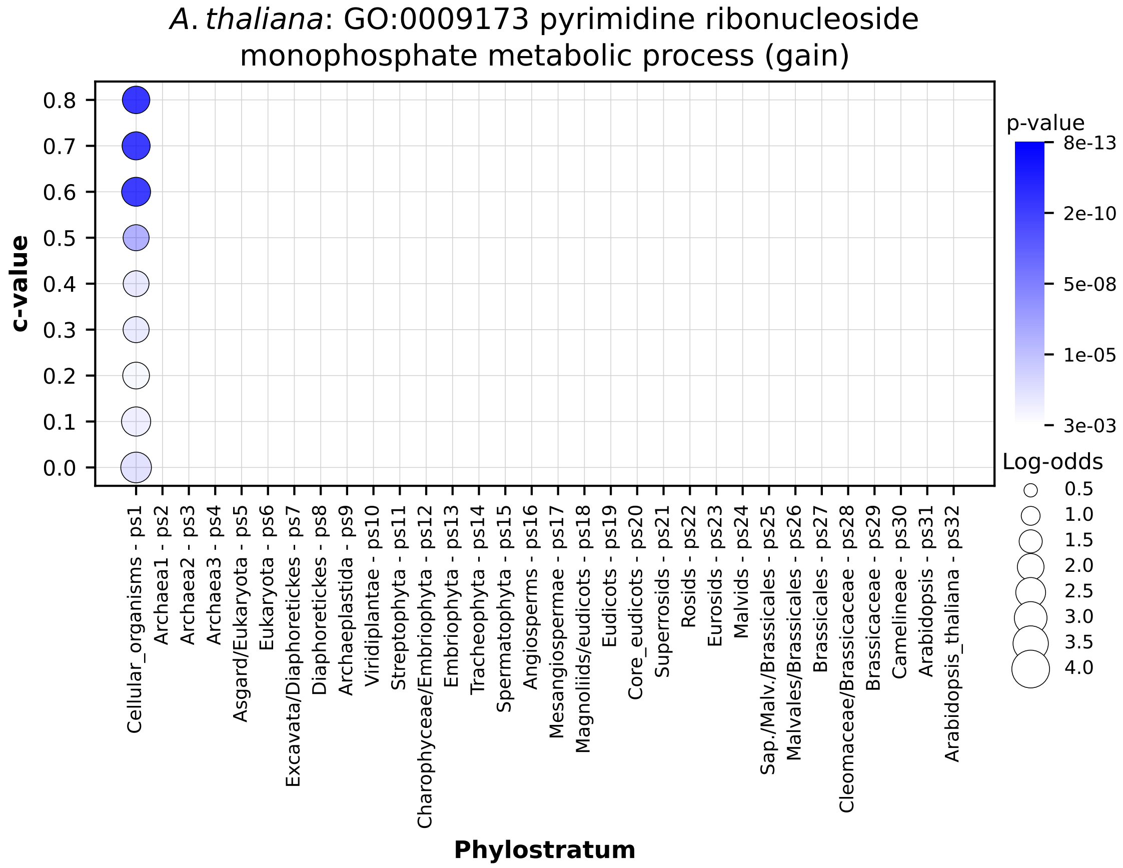
A. thaliana: GO:0009173 pyrimidine ribonucleoside monophosphate metabolic process (gain)
c-value
0.8
0.7
0.6
0.5
0.4
0.3
0.2
0.1
0.0
p-value
8e-13
2e-10
5e-08
1e-05
3e-03
Log-odds
0.5
1.0
1.5
2.0
2.5
3.0
3.5
4.0
Cellular_organisms - ps1
Archaea1 - ps2
Archaea2 - ps3
Archaea3 - ps4
Asgard/Eukaryota - ps5
Eukaryota - ps6
Excavata/Diaphoretickes - ps7
Diaphoretickes - ps8
Archaeplastida - ps9
Viridiplantae - ps10
Streptophyta - ps11
Charophyceae/Embriophyta - ps12
Embriophyta - ps13
Tracheophyta - ps14
Spermatophyta - ps15
Angiosperms - ps16
Mesangiospermae - ps17
Magnoliids/eudicots - ps18
Eudicots - ps19
Core_eudicots - ps20
Superrosids - ps21
Rosids - ps22
Eurosids - ps23
Malvids - ps24
Sap./Malv./Brassicales - ps25
Malvales/Brassicales - ps26
Brassicales - ps27
Cleomaceae/Brassicaceae - ps28
Brassicaceae - ps29
Camelineae - ps30
Arabidopsis - ps31
Arabidopsis_thaliana - ps32
Phylostratum

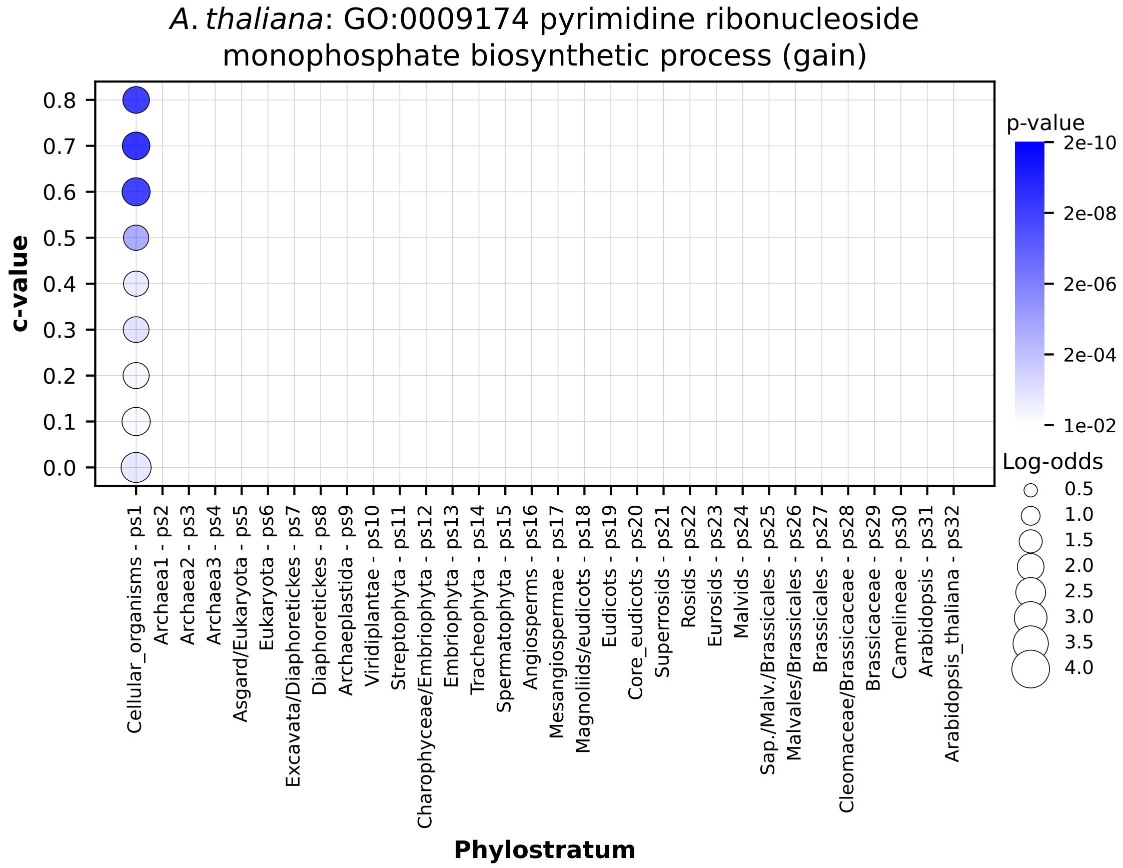

A. thaliana: GO:0009174 pyrimidine ribonucleoside monophosphate biosynthetic process (gain)
c-value
0.0
0.1
0.2
0.3
0.4
0.5
0.6
0.7
0.8
Phylostratum
Cellular_organisms - ps1
Archaea1 - ps2
Archaea2 - ps3
Archaea3 - ps4
Asgard/Eukaryota - ps5
Eukaryota - ps6
Excavata/Diaphoretickes - ps7
Diaphoretickes - ps8
Archaeplastida - ps9
Viridiplantae - ps10
Streptophyta - ps11
Charophyceae/Embriophyta - ps12
Embriophyta - ps13
Tracheophyta - ps14
Spermatophyta - ps15
Angiosperms - ps16
Mesangiospermae - ps17
Magnoliids/eudicots - ps18
Eudicots - ps19
Core_eudicots - ps20
Superrosids - ps21
Rosids - ps22
Eurosids - ps23
Malvids - ps24
Sap./Malv./Brassicales - ps25
Malvales/Brassicales - ps26
Brassicales - ps27
Cleomaceae/Brassicaceae - ps28
Brassicaceae - ps29
Camelineae - ps30
Arabidopsis - ps31
Arabidopsis_thaliana - ps32
p-value
2e-10
2e-08
2e-06
2e-04
1e-02
Log-odds
0.5
1.0
1.5
2.0
2.5
3.0
3.5
4.0

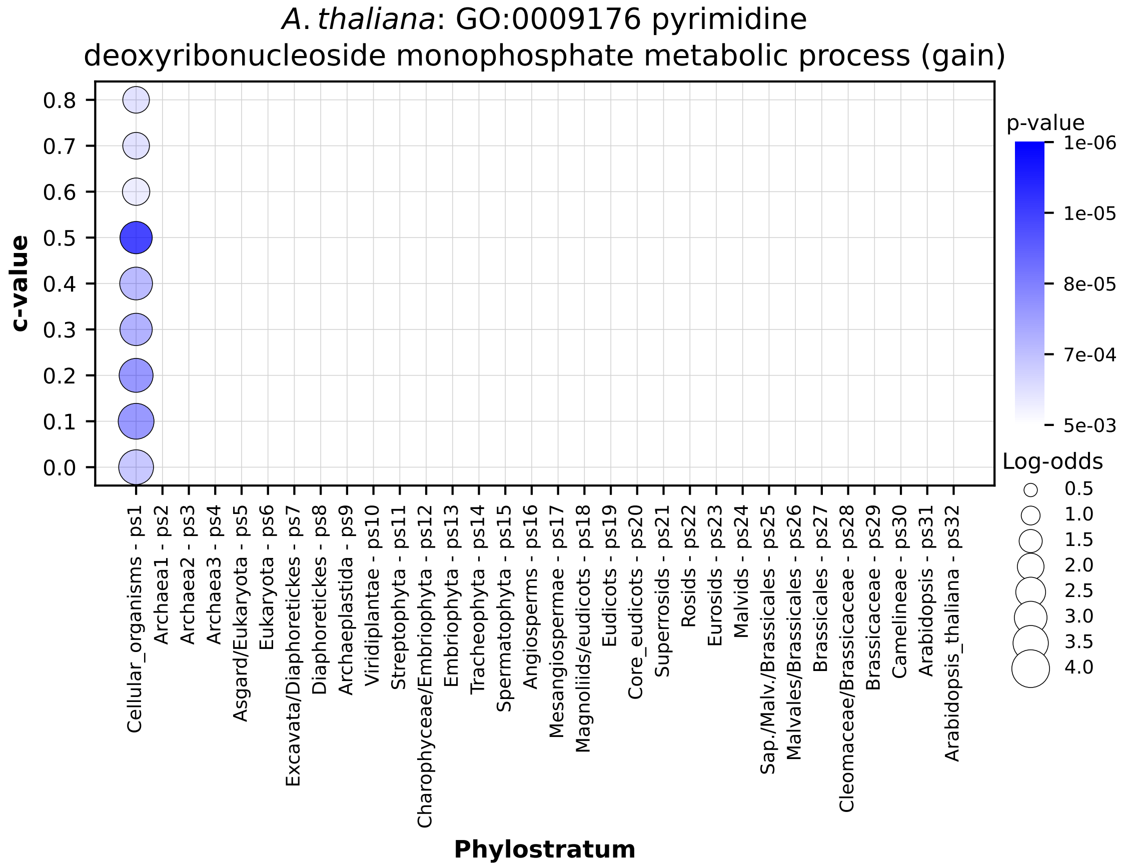

A. thaliana: GO:0009176 pyrimidine deoxyribonucleoside monophosphate metabolic process (gain)
c-value
0.8
0.7
0.6
0.5
0.4
0.3
0.2
0.1
0.0
Phylostratum
Cellular_organisms - ps1
Archaea1 - ps2
Archaea2 - ps3
Archaea3 - ps4
Asgard/Eukaryota - ps5
Eukaryota - ps6
Excavata/Diaphoretickes - ps7
Diaphoretickes - ps8
Archaeplastida - ps9
Viridiplantae - ps10
Streptophyta - ps11
Charophyceae/Embriophyta - ps12
Embriophyta - ps13
Tracheophyta - ps14
Spermatophyta - ps15
Angiosperms - ps16
Mesangiospermae - ps17
Magnoliids/eudicots - ps18
Eudicots - ps19
Core_eudicots - ps20
Superrosids - ps21
Rosids - ps22
Eurosids - ps23
Malvids - ps24
Sap./Malv./Brassicales - ps25
Malvales/Brassicales - ps26
Brassicales - ps27
Cleomaceae/Brassicaceae - ps28
Brassicaceae - ps29
Camelineae - ps30
Arabidopsis - ps31
Arabidopsis_thaliana - ps32
p-value
1e-06
1e-05
8e-05
7e-04
5e-03
Log-odds
0.5
1.0
1.5
2.0
2.5
3.0
3.5
4.0

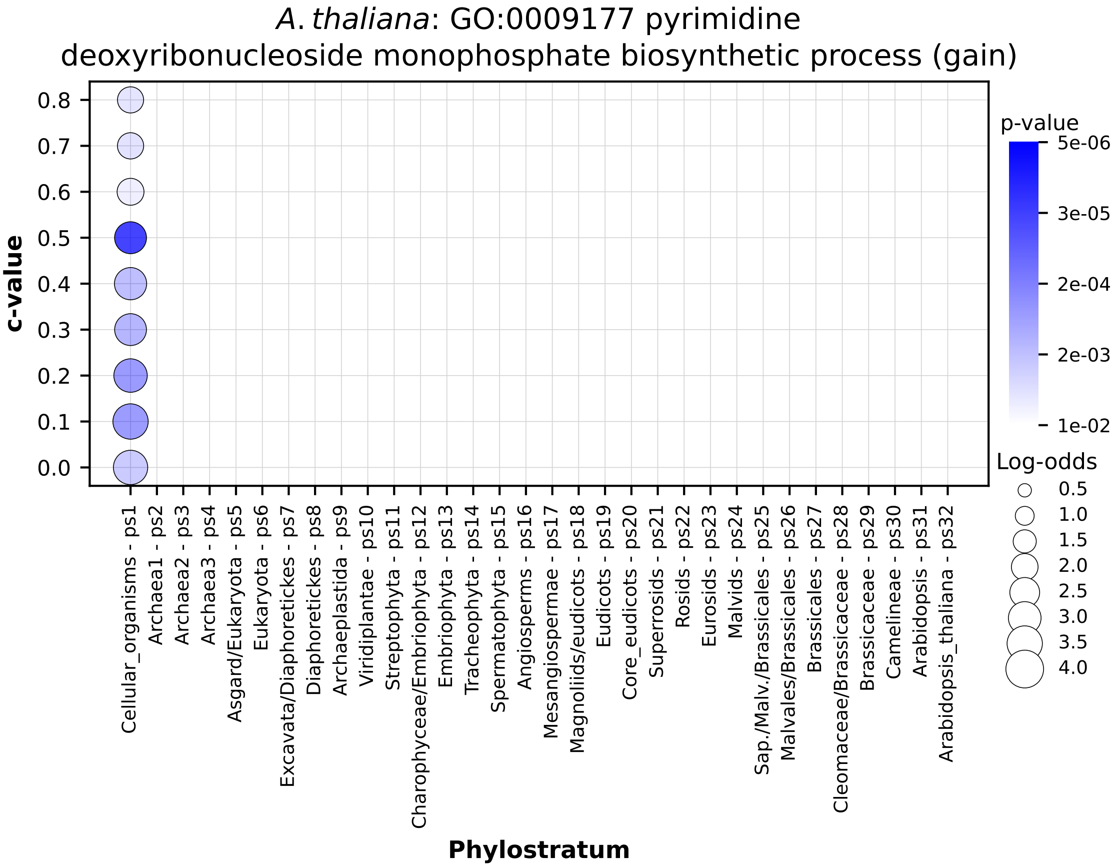

A. thaliana: GO:0009177 pyrimidine deoxyribonucleoside monophosphate biosynthetic process (gain)
c-value
0.0
0.1
0.2
0.3
0.4
0.5
0.6
0.7
0.8
p-value
5e-06
3e-05
2e-04
2e-03
1e-02
Log-odds
0.5
1.0
1.5
2.0
2.5
3.0
3.5
4.0
Cellular_organisms - ps1
Archaea1 - ps2
Archaea2 - ps3
Archaea3 - ps4
Asgard/Eukaryota - ps5
Eukaryota - ps6
Excavata/Diaphoretickes - ps7
Diaphoretickes - ps8
Archaeplastida - ps9
Viridiplantae - ps10
Streptophyta - ps11
Charophyceae/Embriophyta - ps12
Embriophyta - ps13
Tracheophyta - ps14
Spermatophyta - ps15
Angiosperms - ps16
Mesangiospermae - ps17
Magnoliids/eudicots - ps18
Eudicots - ps19
Core_eudicots - ps20
Superrosids - ps21
Rosids - ps22
Eurosids - ps23
Malvids - ps24
Sap./Malv./Brassicales - ps25
Malvales/Brassicales - ps26
Brassicales - ps27
Cleomaceae/Brassicaceae - ps28
Brassicaceae - ps29
Camelineae - ps30
Arabidopsis - ps31
Arabidopsis_thaliana - ps32
Phylostratum

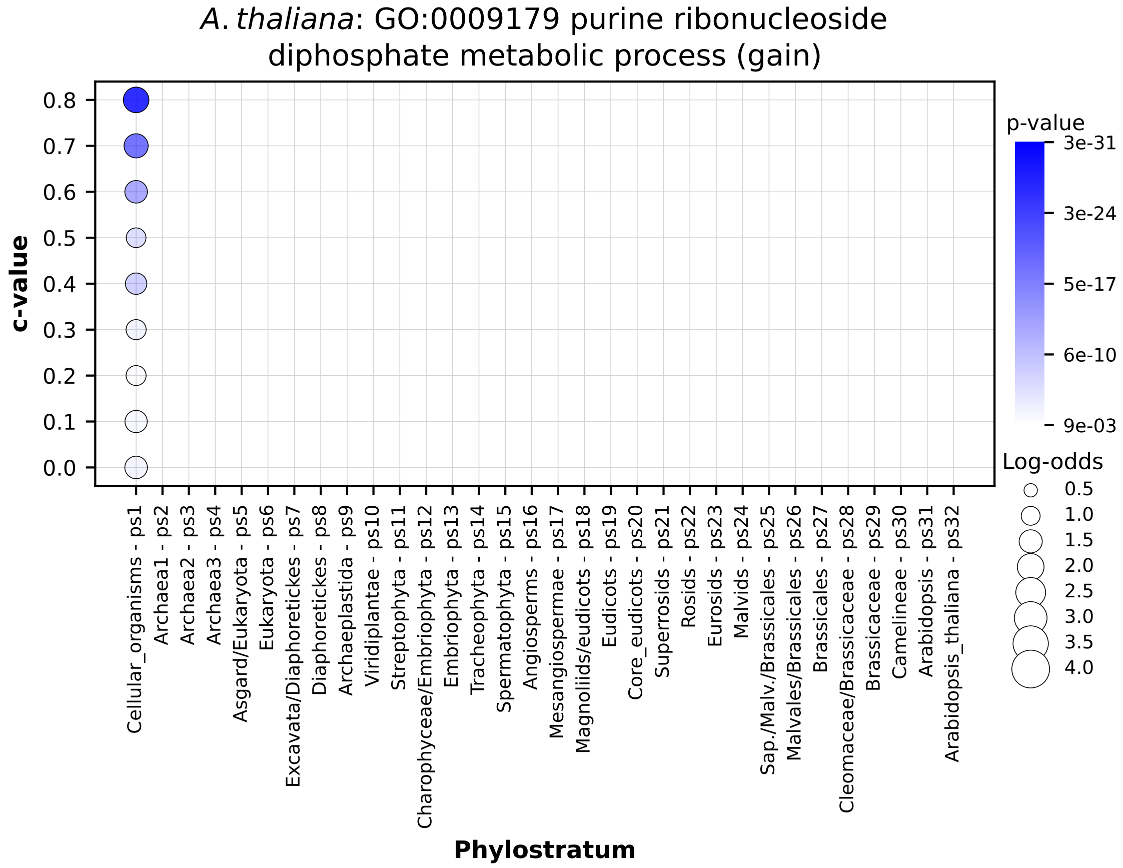

A. thaliana: GO:0009179 purine ribonucleoside diphosphate metabolic process (gain)
c-value
0.8
0.7
0.6
0.5
0.4
0.3
0.2
0.1
0.0
p-value
3e-31
3e-24
5e-17
6e-10
9e-03
Log-odds
0.5
1.0
1.5
2.0
2.5
3.0
3.5
4.0
Cellular_organisms - ps1
Archaea1 - ps2
Archaea2 - ps3
Archaea3 - ps4
Asgard/Eukaryota - ps5
Eukaryota - ps6
Excavata/Diaphoretickes - ps7
Diaphoretickes - ps8
Archaeplastida - ps9
Viridiplantae - ps10
Streptophyta - ps11
Charophyceae/Embriophyta - ps12
Embriophyta - ps13
Tracheophyta - ps14
Spermatophyta - ps15
Angiosperms - ps16
Mesangiospermae - ps17
Magnoliids/eudicots - ps18
Eudicots - ps19
Core_eudicots - ps20
Superrosids - ps21
Rosids - ps22
Eurosids - ps23
Malvids - ps24
Sap./Malv./Brassicales - ps25
Malvales/Brassicales - ps26
Brassicales - ps27
Cleomaceae/Brassicaceae - ps28
Brassicaceae - ps29
Camelineae - ps30
Arabidopsis - ps31
Arabidopsis_thaliana - ps32
Phylostratum

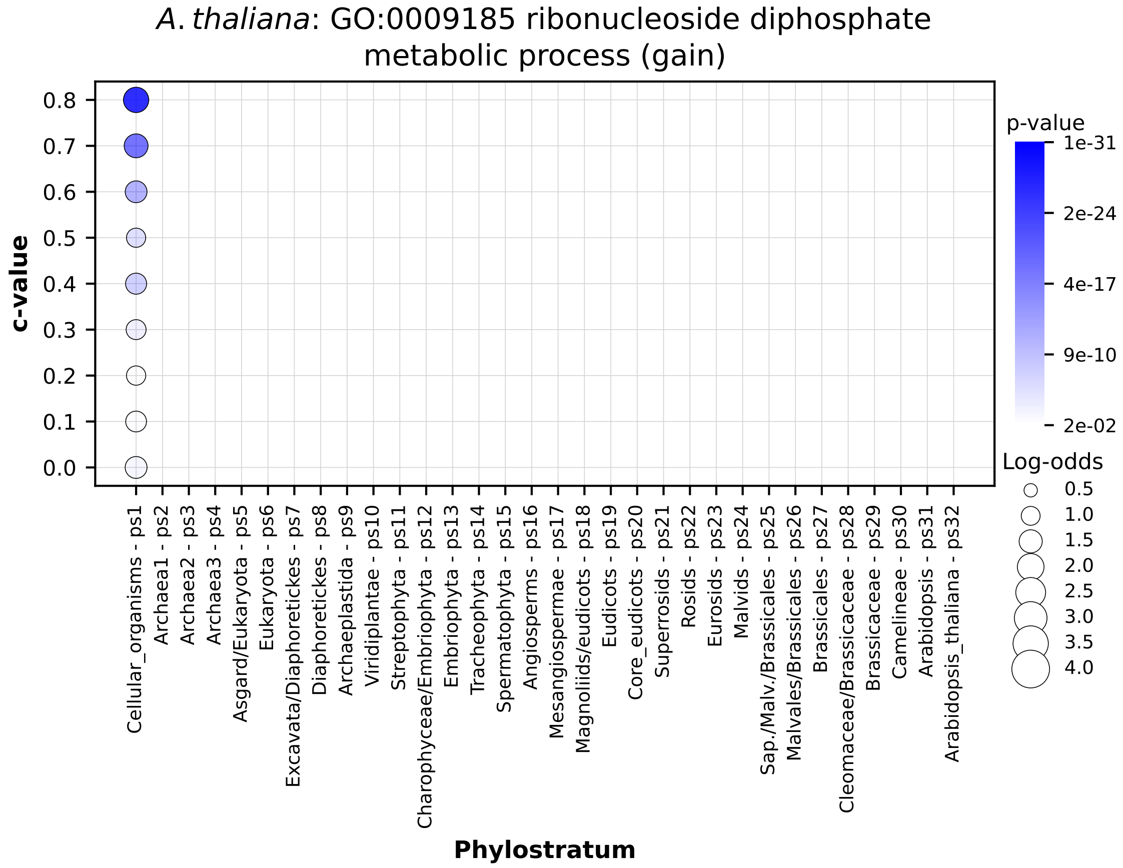

A. thaliana: GO:0009185 ribonucleoside diphosphate metabolic process (gain)
c-value
0.0
0.1
0.2
0.3
0.4
0.5
0.6
0.7
0.8
Phylostratum
Cellular_organisms - ps1
Archaea1 - ps2
Archaea2 - ps3
Archaea3 - ps4
Asgard/Eukaryota - ps5
Eukaryota - ps6
Excavata/Diaphoretickes - ps7
Diaphoretickes - ps8
Archaeplastida - ps9
Viridiplantae - ps10
Streptophyta - ps11
Charophyceae/Embriophyta - ps12
Embriophyta - ps13
Tracheophyta - ps14
Spermatophyta - ps15
Angiosperms - ps16
Mesangiospermae - ps17
Magnoliids/eudicots - ps18
Eudicots - ps19
Core_eudicots - ps20
Superrosids - ps21
Rosids - ps22
Eurosids - ps23
Malvids - ps24
Sap./Malv./Brassicales - ps25
Malvales/Brassicales - ps26
Brassicales - ps27
Cleomaceae/Brassicaceae - ps28
Brassicaceae - ps29
Camelineae - ps30
Arabidopsis - ps31
Arabidopsis_thaliana - ps32
p-value
1e-31
2e-24
4e-17
9e-10
2e-02
Log-odds
0.5
1.0
1.5
2.0
2.5
3.0
3.5
4.0

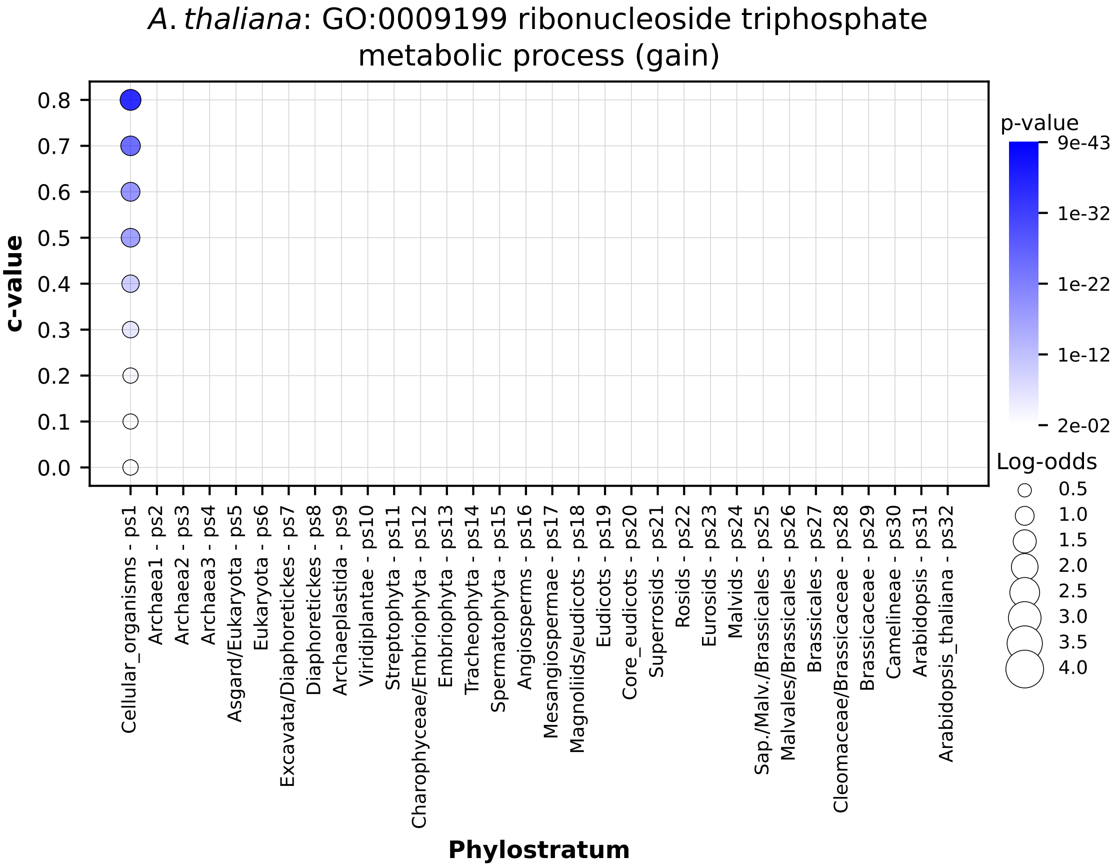
A. thaliana: GO:0009199 ribonucleoside triphosphate metabolic process (gain)
c-value
0.8
0.7
0.6
0.5
0.4
0.3
0.2
0.1
0.0
p-value
9e-43
1e-32
1e-22
1e-12
2e-02
Log-odds
0.5
1.0
1.5
2.0
2.5
3.0
3.5
4.0
Cellular_organisms - ps1
Archaea1 - ps2
Archaea2 - ps3
Archaea3 - ps4
Asgard/Eukaryota - ps5
Eukaryota - ps6
Excavata/Diaphoretickes - ps7
Diaphoretickes - ps8
Archaeplastida - ps9
Viridiplantae - ps10
Streptophyta - ps11
Charophyceae/Embriophyta - ps12
Embriophyta - ps13
Tracheophyta - ps14
Spermatophyta - ps15
Angiosperms - ps16
Mesangiospermae - ps17
Magnoliids/eudicots - ps18
Eudicots - ps19
Core_eudicots - ps20
Superrosids - ps21
Rosids - ps22
Eurosids - ps23
Malvids - ps24
Sap./Malv./Brassicales - ps25
Malvales/Brassicales - ps26
Brassicales - ps27
Cleomaceae/Brassicaceae - ps28
Brassicaceae - ps29
Camelineae - ps30
Arabidopsis - ps31
Arabidopsis_thaliana - ps32
Phylostratum

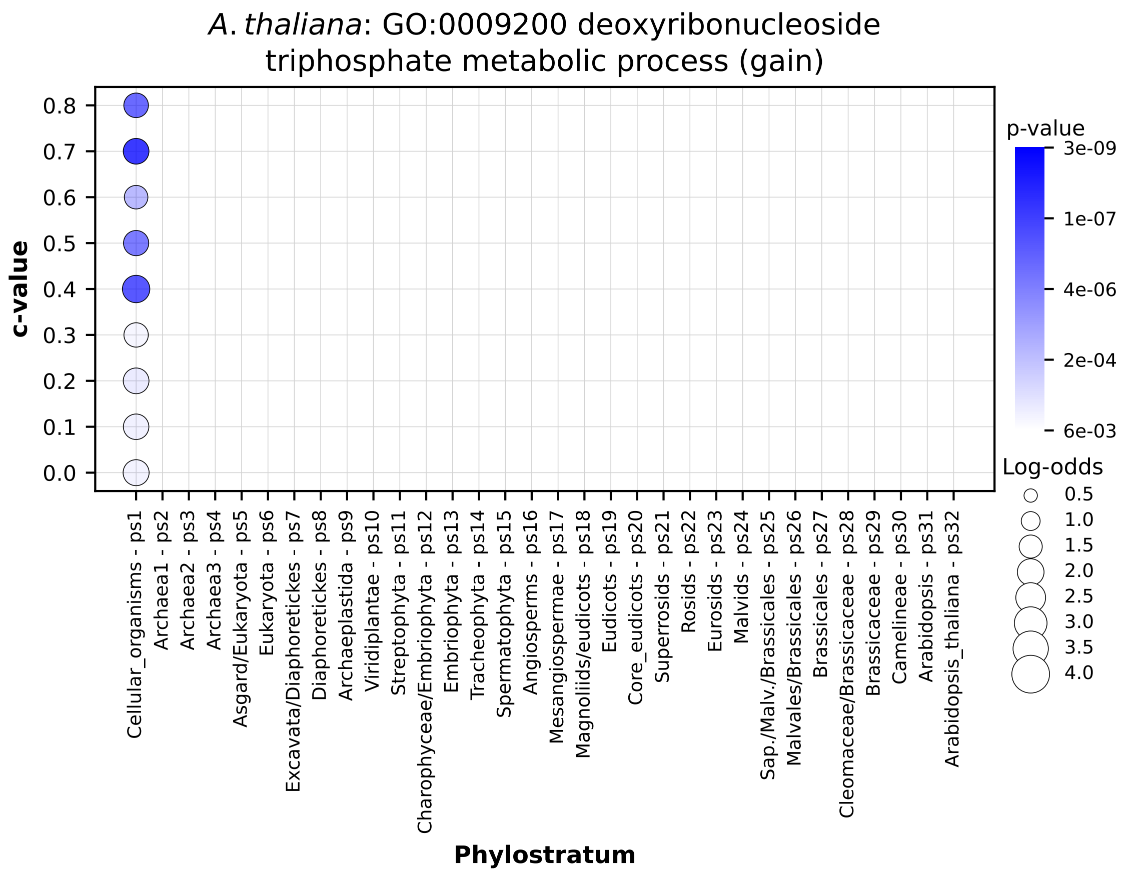

A. thaliana: GO:0009200 deoxyribonucleoside triphosphate metabolic process (gain)
c-value
0.8
0.7
0.6
0.5
0.4
0.3
0.2
0.1
0.0
p-value
3e-09
1e-07
4e-06
2e-04
6e-03
Log-odds
0.5
1.0
1.5
2.0
2.5
3.0
3.5
4.0
Cellular_organisms - ps1
Archaea1 - ps2
Archaea2 - ps3
Archaea3 - ps4
Asgard/Eukaryota - ps5
Eukaryota - ps6
Excavata/Diaphoretickes - ps7
Diaphoretickes - ps8
Archaeplastida - ps9
Viridiplantae - ps10
Streptophyta - ps11
Charophyceae/Embriophyta - ps12
Embriophyta - ps13
Tracheophyta - ps14
Spermatophyta - ps15
Angiosperms - ps16
Mesangiospermae - ps17
Magnoliids/eudicots - ps18
Eudicots - ps19
Core_eudicots - ps20
Superrosids - ps21
Rosids - ps22
Eurosids - ps23
Malvids - ps24
Sap./Malv./Brassicales - ps25
Malvales/Brassicales - ps26
Brassicales - ps27
Cleomaceae/Brassicaceae - ps28
Brassicaceae - ps29
Camelineae - ps30
Arabidopsis - ps31
Arabidopsis_thaliana - ps32
Phylostratum

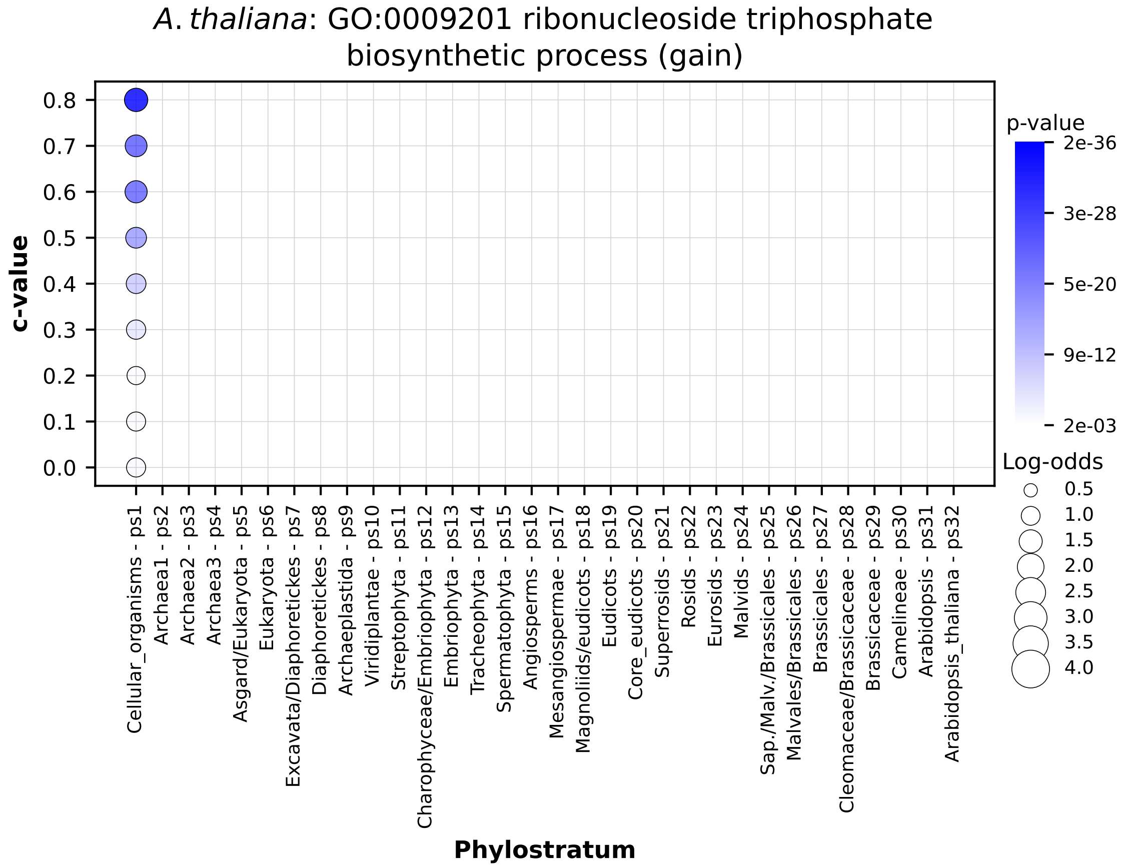

A. thaliana: GO:0009201 ribonucleoside triphosphate biosynthetic process (gain)
c-value
0.0
0.1
0.2
0.3
0.4
0.5
0.6
0.7
0.8
p-value
2e-36
3e-28
5e-20
9e-12
2e-03
Log-odds
0.5
1.0
1.5
2.0
2.5
3.0
3.5
4.0
Cellular_organisms - ps1
Archaea1 - ps2
Archaea2 - ps3
Archaea3 - ps4
Asgard/Eukaryota - ps5
Eukaryota - ps6
Excavata/Diaphoretickes - ps7
Diaphoretickes - ps8
Archaeplastida - ps9
Viridiplantae - ps10
Streptophyta - ps11
Charophyceae/Embriophyta - ps12
Embriophyta - ps13
Tracheophyta - ps14
Spermatophyta - ps15
Angiosperms - ps16
Mesangiospermae - ps17
Magnoliids/eudicots - ps18
Eudicots - ps19
Core_eudicots - ps20
Superrosids - ps21
Rosids - ps22
Eurosids - ps23
Malvids - ps24
Sap./Malv./Brassicales - ps25
Malvales/Brassicales - ps26
Brassicales - ps27
Cleomaceae/Brassicaceae - ps28
Brassicaceae - ps29
Camelineae - ps30
Arabidopsis - ps31
Arabidopsis_thaliana - ps32
Phylostratum

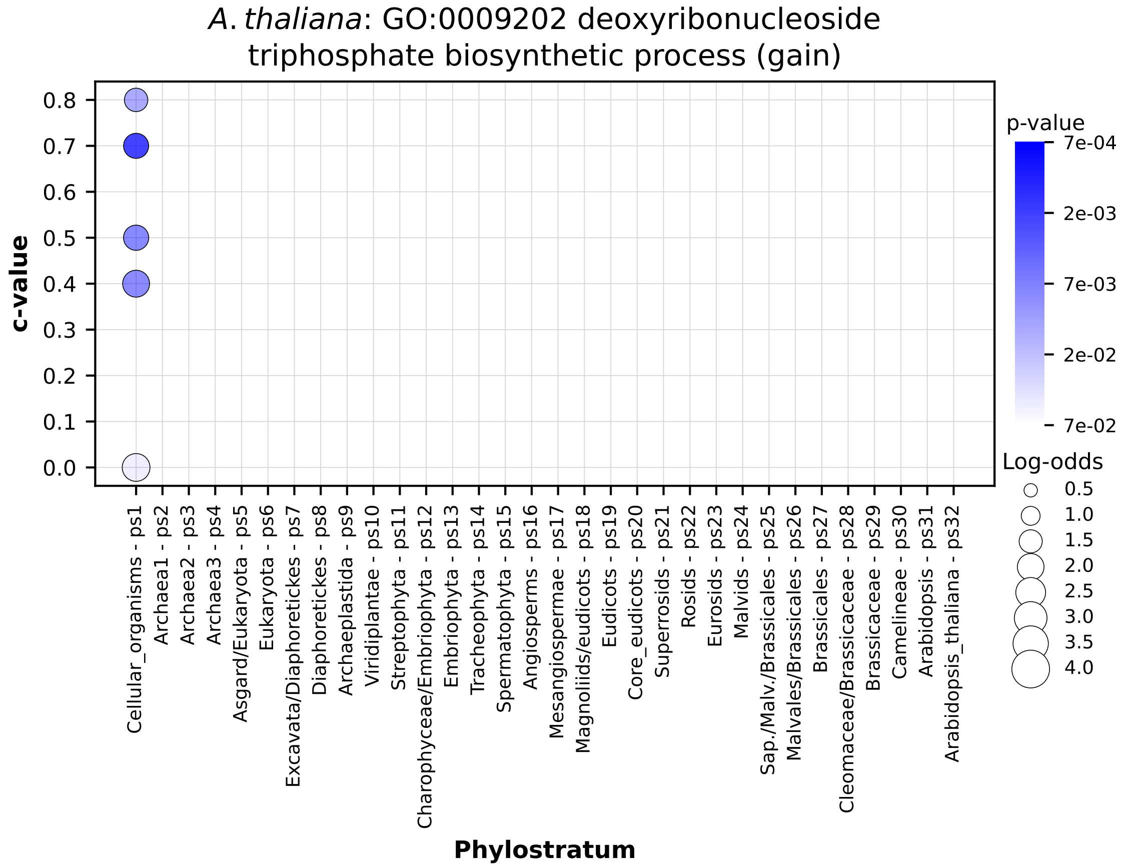

A. thaliana: GO:0009202 deoxyribonucleoside triphosphate biosynthetic process (gain)
c-value
0.8
0.7
0.6
0.5
0.4
0.3
0.2
0.1
0.0
p-value
7e-04
2e-03
7e-03
2e-02
7e-02
Log-odds
0.5
1.0
1.5
2.0
2.5
3.0
3.5
4.0
Cellular_organisms - ps1
Archaea1 - ps2
Archaea2 - ps3
Archaea3 - ps4
Asgard/Eukaryota - ps5
Eukaryota - ps6
Excavata/Diaphoretickes - ps7
Diaphoretickes - ps8
Archaeplastida - ps9
Viridiplantae - ps10
Streptophyta - ps11
Charophyceae/Embriophyta - ps12
Embriophyta - ps13
Tracheophyta - ps14
Spermatophyta - ps15
Angiosperms - ps16
Mesangiospermae - ps17
Magnoliids/eudicots - ps18
Eudicots - ps19
Core_eudicots - ps20
Superrosids - ps21
Rosids - ps22
Eurosids - ps23
Malvids - ps24
Sap./Malv./Brassicales - ps25
Malvales/Brassicales - ps26
Brassicales - ps27
Cleomaceae/Brassicaceae - ps28
Brassicaceae - ps29
Camelineae - ps30
Arabidopsis - ps31
Arabidopsis_thaliana - ps32
Phylostratum

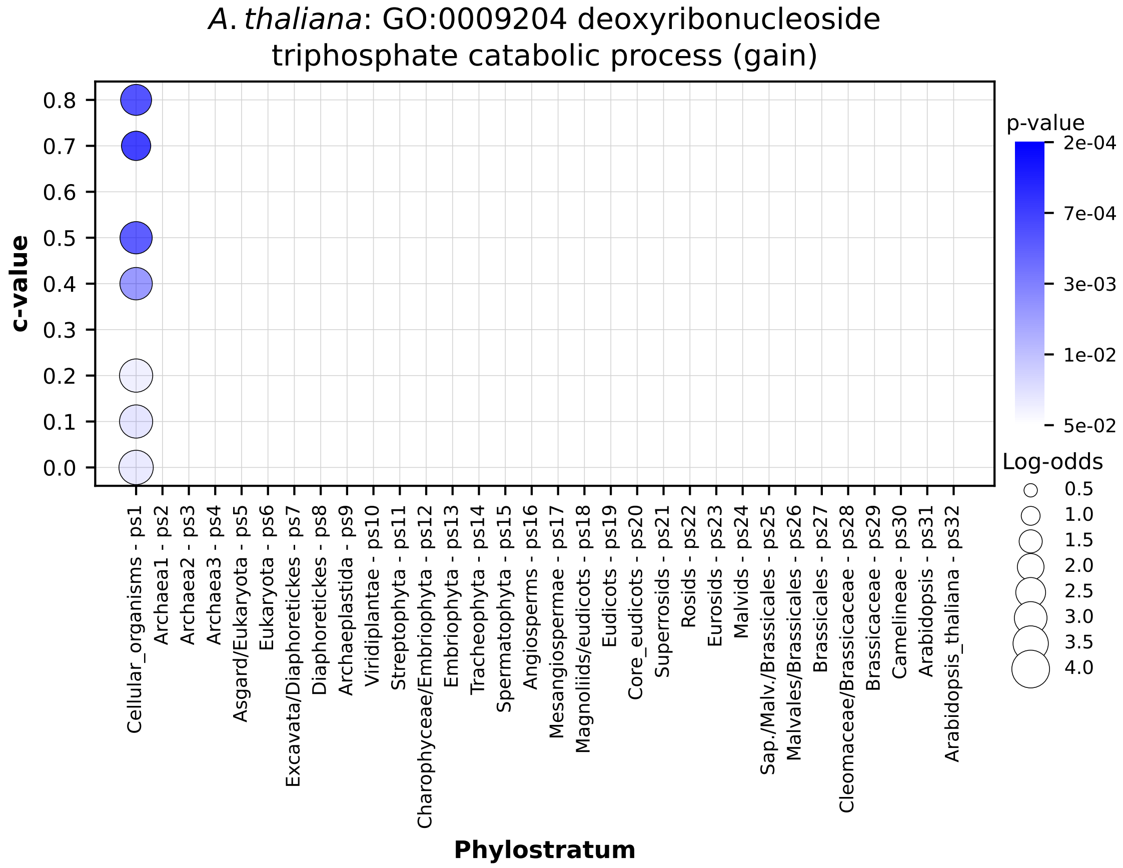

A. thaliana: GO:0009204 deoxyribonucleoside triphosphate catabolic process (gain)
c-value
0.0
0.1
0.2
0.3
0.4
0.5
0.6
0.7
0.8
Phylostratum
Cellular_organisms - ps1
Archaea1 - ps2
Archaea2 - ps3
Archaea3 - ps4
Asgard/Eukaryota - ps5
Eukaryota - ps6
Excavata/Diaphoretickes - ps7
Diaphoretickes - ps8
Archaeplastida - ps9
Viridiplantae - ps10
Streptophyta - ps11
Charophyceae/Embriophyta - ps12
Embriophyta - ps13
Tracheophyta - ps14
Spermatophyta - ps15
Angiosperms - ps16
Mesangiospermae - ps17
Magnoliids/eudicots - ps18
Eudicots - ps19
Core_eudicots - ps20
Superrosids - ps21
Rosids - ps22
Eurosids - ps23
Malvids - ps24
Sap./Malv./Brassicales - ps25
Malvales/Brassicales - ps26
Brassicales - ps27
Cleomaceae/Brassicaceae - ps28
Brassicaceae - ps29
Camelineae - ps30
Arabidopsis - ps31
Arabidopsis_thaliana - ps32
p-value
2e-04
7e-04
3e-03
1e-02
5e-02
Log-odds
0.5
1.0
1.5
2.0
2.5
3.0
3.5
4.0

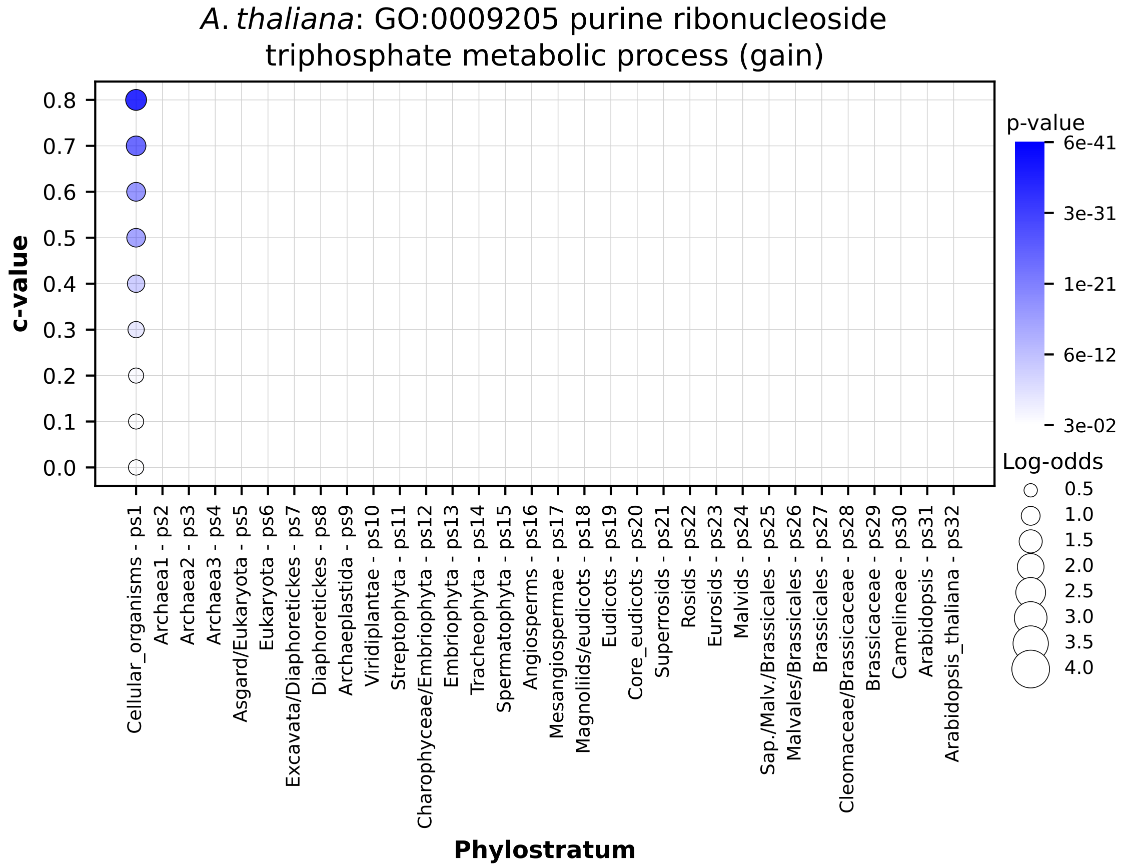

A. thaliana: GO:0009205 purine ribonucleoside triphosphate metabolic process (gain)
c-value
0.8
0.7
0.6
0.5
0.4
0.3
0.2
0.1
0.0
p-value
6e-41
3e-31
1e-21
6e-12
3e-02
Log-odds
0.5
1.0
1.5
2.0
2.5
3.0
3.5
4.0
Cellular_organisms - ps1
Archaea1 - ps2
Archaea2 - ps3
Archaea3 - ps4
Asgard/Eukaryota - ps5
Eukaryota - ps6
Excavata/Diaphoretickes - ps7
Diaphoretickes - ps8
Archaeplastida - ps9
Viridiplantae - ps10
Streptophyta - ps11
Charophyceae/Embriophyta - ps12
Embriophyta - ps13
Tracheophyta - ps14
Spermatophyta - ps15
Angiosperms - ps16
Mesangiospermae - ps17
Magnoliids/eudicots - ps18
Eudicots - ps19
Core_eudicots - ps20
Superrosids - ps21
Rosids - ps22
Eurosids - ps23
Malvids - ps24
Sap./Malv./Brassicales - ps25
Malvales/Brassicales - ps26
Brassicales - ps27
Cleomaceae/Brassicaceae - ps28
Brassicaceae - ps29
Camelineae - ps30
Arabidopsis - ps31
Arabidopsis_thaliana - ps32
Phylostratum

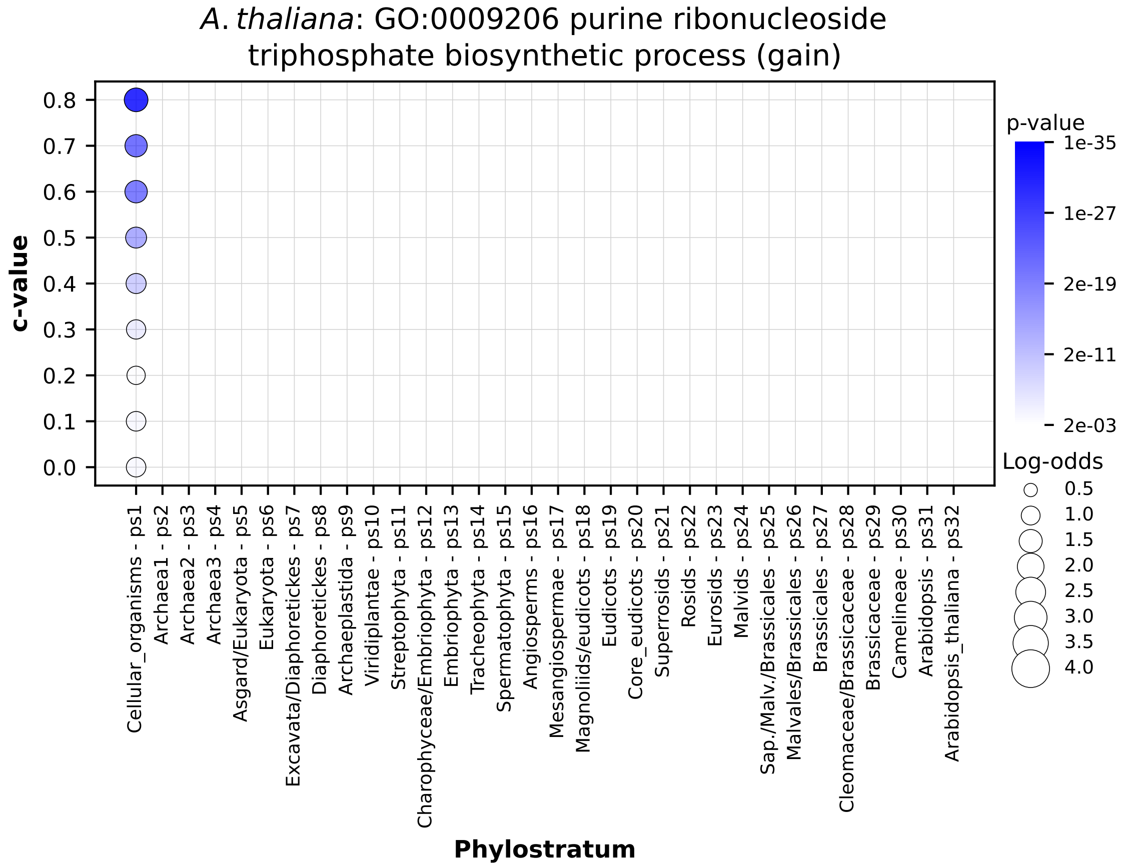

A. thaliana: GO:0009206 purine ribonucleoside triphosphate biosynthetic process (gain)
c-value
0.8
0.7
0.6
0.5
0.4
0.3
0.2
0.1
0.0
p-value
1e-35
1e-27
2e-19
2e-11
2e-03
Log-odds
0.5
1.0
1.5
2.0
2.5
3.0
3.5
4.0
Cellular_organisms - ps1
Archaea1 - ps2
Archaea2 - ps3
Archaea3 - ps4
Asgard/Eukaryota - ps5
Eukaryota - ps6
Excavata/Diaphoretickes - ps7
Diaphoretickes - ps8
Archaeplastida - ps9
Viridiplantae - ps10
Streptophyta - ps11
Charophyceae/Embriophyta - ps12
Embriophyta - ps13
Tracheophyta - ps14
Spermatophyta - ps15
Angiosperms - ps16
Mesangiospermae - ps17
Magnoliids/eudicots - ps18
Eudicots - ps19
Core_eudicots - ps20
Superrosids - ps21
Rosids - ps22
Eurosids - ps23
Malvids - ps24
Sap./Malv./Brassicales - ps25
Malvales/Brassicales - ps26
Brassicales - ps27
Cleomaceae/Brassicaceae - ps28
Brassicaceae - ps29
Camelineae - ps30
Arabidopsis - ps31
Arabidopsis_thaliana - ps32
Phylostratum

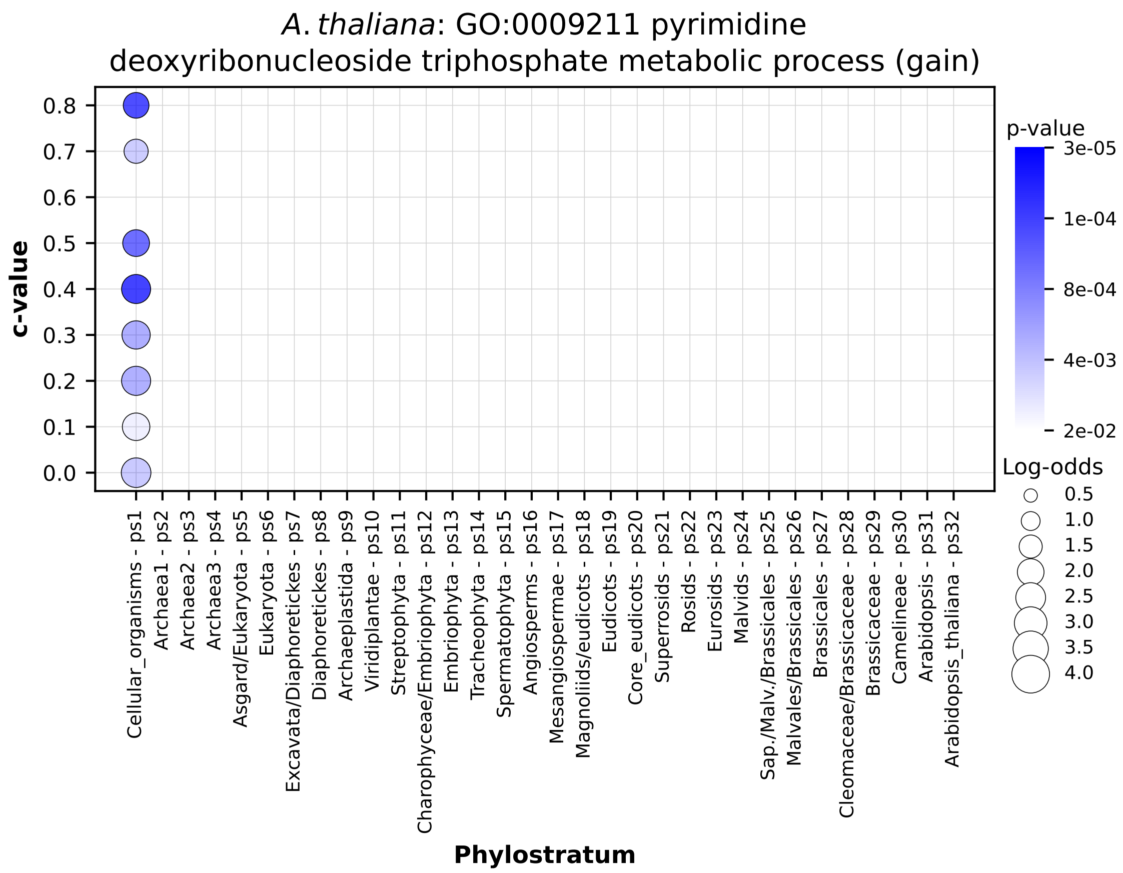
A. thaliana: GO:0009211 pyrimidine deoxyribonucleoside triphosphate metabolic process (gain)
c-value
0.8
0.7
0.6
0.5
0.4
0.3
0.2
0.1
0.0
p-value
3e-05
1e-04
8e-04
4e-03
2e-02
Log-odds
0.5
1.0
1.5
2.0
2.5
3.0
3.5
4.0
Cellular_organisms - ps1
Archaea1 - ps2
Archaea2 - ps3
Archaea3 - ps4
Asgard/Eukaryota - ps5
Eukaryota - ps6
Excavata/Diaphoretickes - ps7
Diaphoretickes - ps8
Archaeplastida - ps9
Viridiplantae - ps10
Streptophyta - ps11
Charophyceae/Embriophyta - ps12
Embriophyta - ps13
Tracheophyta - ps14
Spermatophyta - ps15
Angiosperms - ps16
Mesangiospermae - ps17
Magnoliids/eudicots - ps18
Eudicots - ps19
Core_eudicots - ps20
Superrosids - ps21
Rosids - ps22
Eurosids - ps23
Malvids - ps24
Sap./Malv./Brassicales - ps25
Malvales/Brassicales - ps26
Brassicales - ps27
Cleomaceae/Brassicaceae - ps28
Brassicaceae - ps29
Camelineae - ps30
Arabidopsis - ps31
Arabidopsis_thaliana - ps32
Phylostratum

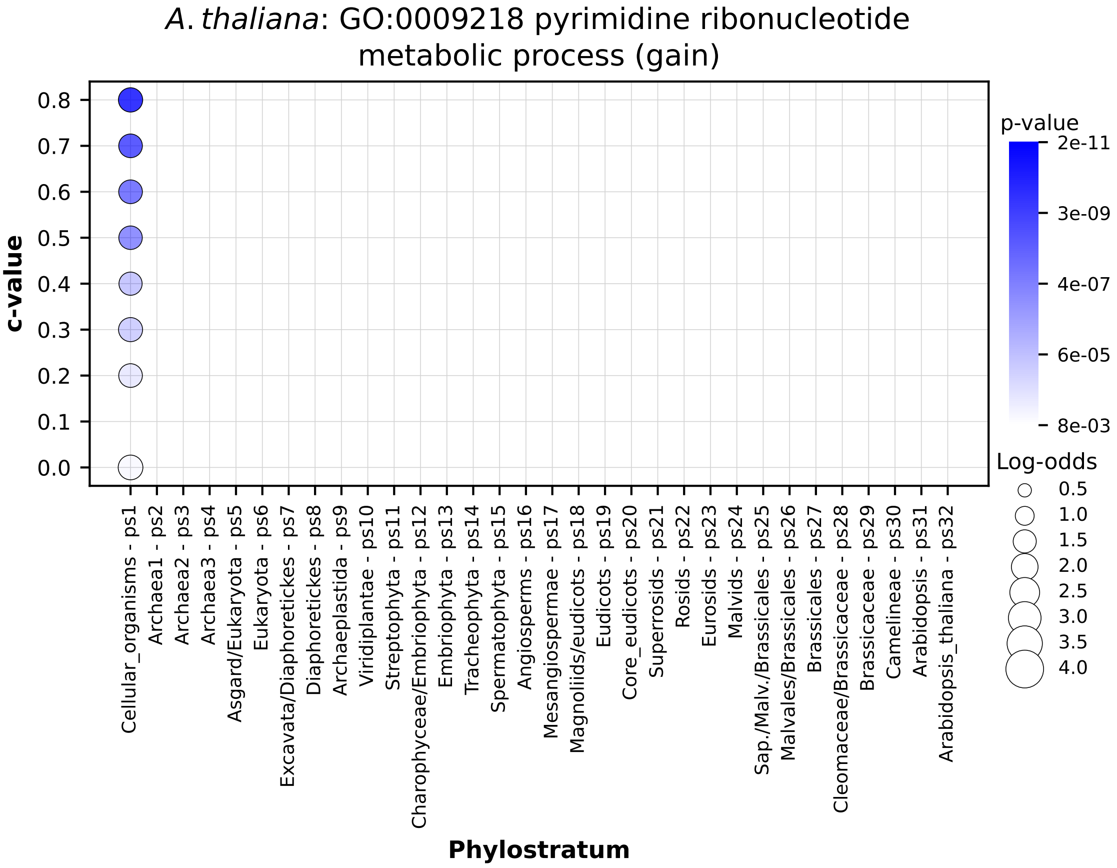

A. thaliana: GO:0009218 pyrimidine ribonucleotide metabolic process (gain)
c-value
0.8
0.7
0.6
0.5
0.4
0.3
0.2
0.1
0.0
p-value
2e-11
3e-09
4e-07
6e-05
8e-03
Log-odds
0.5
1.0
1.5
2.0
2.5
3.0
3.5
4.0
Cellular_organisms - ps1
Archaea1 - ps2
Archaea2 - ps3
Archaea3 - ps4
Asgard/Eukaryota - ps5
Eukaryota - ps6
Excavata/Diaphoretickes - ps7
Diaphoretickes - ps8
Archaeplastida - ps9
Viridiplantae - ps10
Streptophyta - ps11
Charophyceae/Embriophyta - ps12
Embriophyta - ps13
Tracheophyta - ps14
Spermatophyta - ps15
Angiosperms - ps16
Mesangiospermae - ps17
Magnoliids/eudicots - ps18
Eudicots - ps19
Core_eudicots - ps20
Superrosids - ps21
Rosids - ps22
Eurosids - ps23
Malvids - ps24
Sap./Malv./Brassicales - ps25
Malvales/Brassicales - ps26
Brassicales - ps27
Cleomaceae/Brassicaceae - ps28
Brassicaceae - ps29
Camelineae - ps30
Arabidopsis - ps31
Arabidopsis_thaliana - ps32
Phylostratum

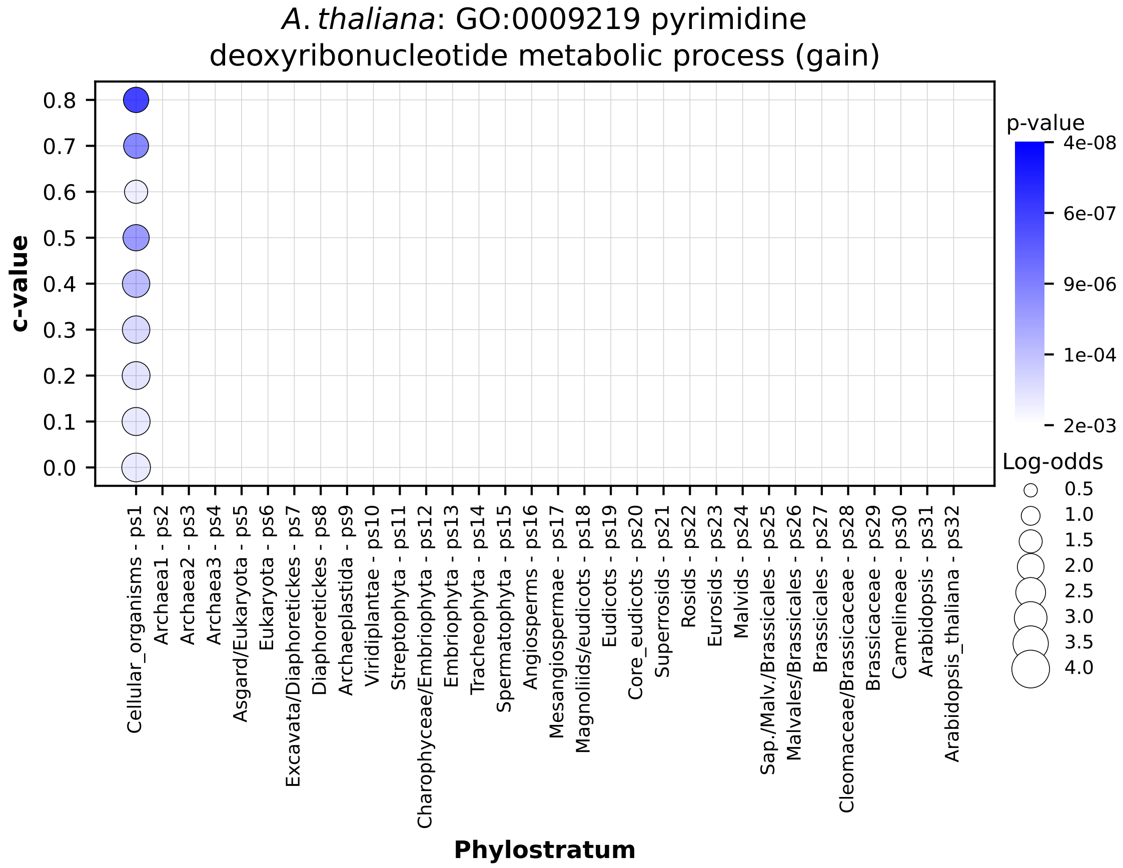
A. thaliana: GO:0009219 pyrimidine deoxyribonucleotide metabolic process (gain)
c-value
0.8
0.7
0.6
0.5
0.4
0.3
0.2
0.1
0.0
p-value
4e-08
6e-07
9e-06
1e-04
2e-03
Log-odds
0.5
1.0
1.5
2.0
2.5
3.0
3.5
4.0
Cellular_organisms - ps1
Archaea1 - ps2
Archaea2 - ps3
Archaea3 - ps4
Asgard/Eukaryota - ps5
Eukaryota - ps6
Excavata/Diaphoretickes - ps7
Diaphoretickes - ps8
Archaeplastida - ps9
Viridiplantae - ps10
Streptophyta - ps11
Charophyceae/Embriophyta - ps12
Embriophyta - ps13
Tracheophyta - ps14
Spermatophyta - ps15
Angiosperms - ps16
Mesangiospermae - ps17
Magnoliids/eudicots - ps18
Eudicots - ps19
Core_eudicots - ps20
Superrosids - ps21
Rosids - ps22
Eurosids - ps23
Malvids - ps24
Sap./Malv./Brassicales - ps25
Malvales/Brassicales - ps26
Brassicales - ps27
Cleomaceae/Brassicaceae - ps28
Brassicaceae - ps29
Camelineae - ps30
Arabidopsis - ps31
Arabidopsis_thaliana - ps32
Phylostratum

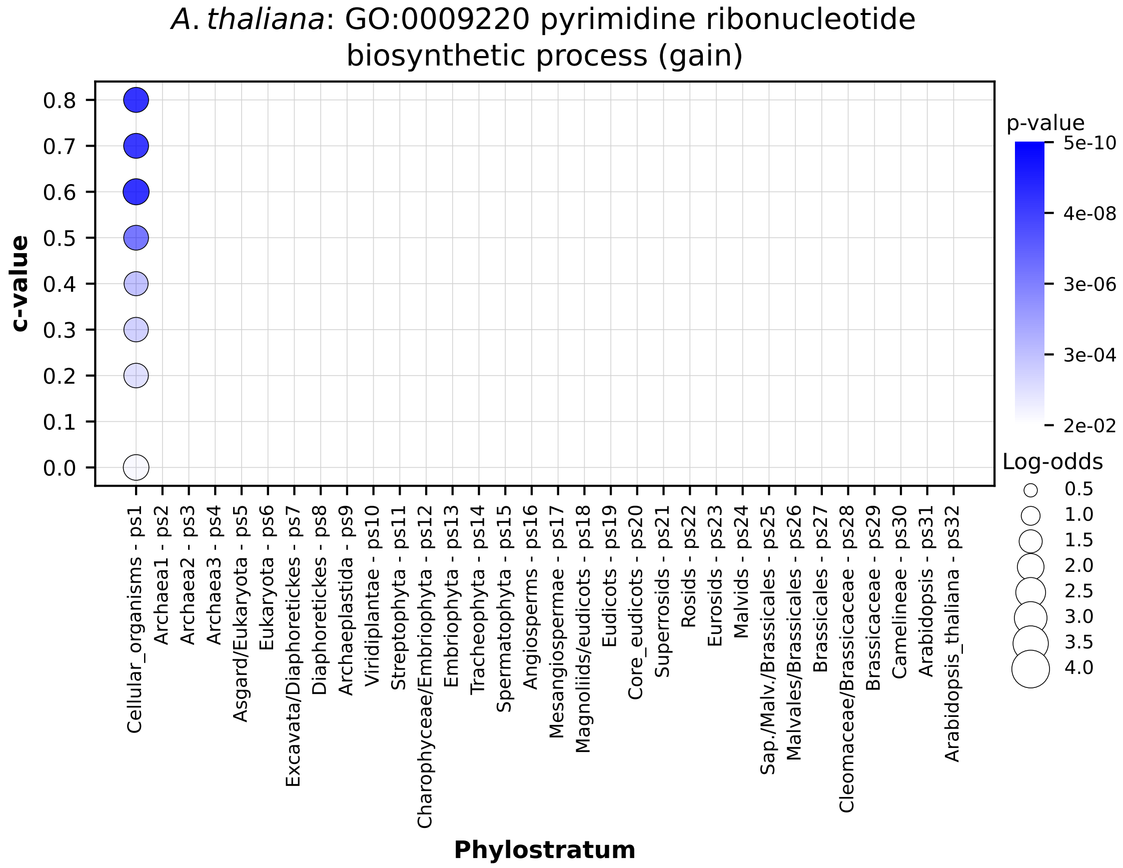

A. thaliana: GO:0009220 pyrimidine ribonucleotide biosynthetic process (gain)
c-value
0.8
0.7
0.6
0.5
0.4
0.3
0.2
0.1
0.0
p-value
5e-10
4e-08
3e-06
3e-04
2e-02
Log-odds
0.5
1.0
1.5
2.0
2.5
3.0
3.5
4.0
Cellular_organisms - ps1
Archaea1 - ps2
Archaea2 - ps3
Archaea3 - ps4
Asgard/Eukaryota - ps5
Eukaryota - ps6
Excavata/Diaphoretickes - ps7
Diaphoretickes - ps8
Archaeplastida - ps9
Viridiplantae - ps10
Streptophyta - ps11
Charophyceae/Embriophyta - ps12
Embriophyta - ps13
Tracheophyta - ps14
Spermatophyta - ps15
Angiosperms - ps16
Mesangiospermae - ps17
Magnoliids/eudicots - ps18
Eudicots - ps19
Core_eudicots - ps20
Superrosids - ps21
Rosids - ps22
Eurosids - ps23
Malvids - ps24
Sap./Malv./Brassicales - ps25
Malvales/Brassicales - ps26
Brassicales - ps27
Cleomaceae/Brassicaceae - ps28
Brassicaceae - ps29
Camelineae - ps30
Arabidopsis - ps31
Arabidopsis_thaliana - ps32
Phylostratum

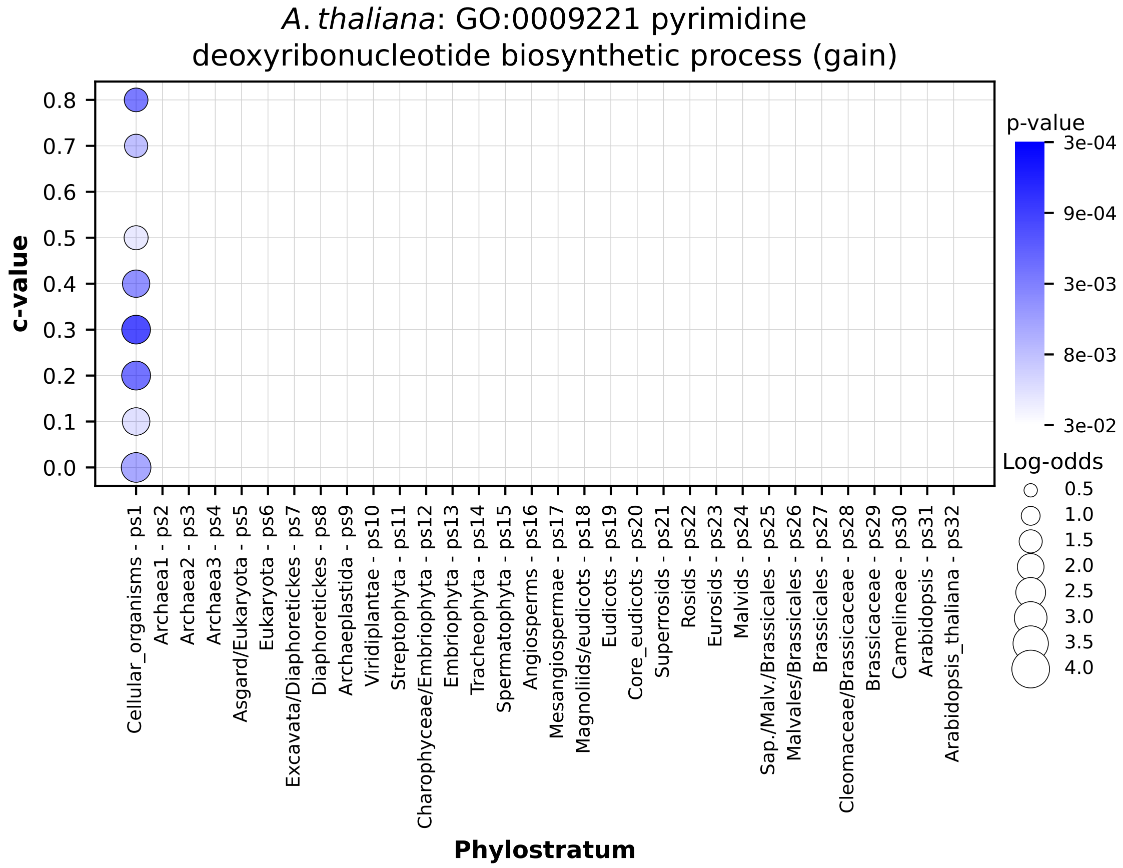

A. thaliana: GO:0009221 pyrimidine deoxyribonucleotide biosynthetic process (gain)
c-value
0.8
0.7
0.6
0.5
0.4
0.3
0.2
0.1
0.0
p-value
3e-04
9e-04
3e-03
8e-03
3e-02
Log-odds
0.5
1.0
1.5
2.0
2.5
3.0
3.5
4.0
Cellular_organisms - ps1
Archaea1 - ps2
Archaea2 - ps3
Archaea3 - ps4
Asgard/Eukaryota - ps5
Eukaryota - ps6
Excavata/Diaphoretickes - ps7
Diaphoretickes - ps8
Archaeplastida - ps9
Viridiplantae - ps10
Streptophyta - ps11
Charophyceae/Embriophyta - ps12
Embriophyta - ps13
Tracheophyta - ps14
Spermatophyta - ps15
Angiosperms - ps16
Mesangiospermae - ps17
Magnoliids/eudicots - ps18
Eudicots - ps19
Core_eudicots - ps20
Superrosids - ps21
Rosids - ps22
Eurosids - ps23
Malvids - ps24
Sap./Malv./Brassicales - ps25
Malvales/Brassicales - ps26
Brassicales - ps27
Cleomaceae/Brassicaceae - ps28
Brassicaceae - ps29
Camelineae - ps30
Arabidopsis - ps31
Arabidopsis_thaliana - ps32
Phylostratum

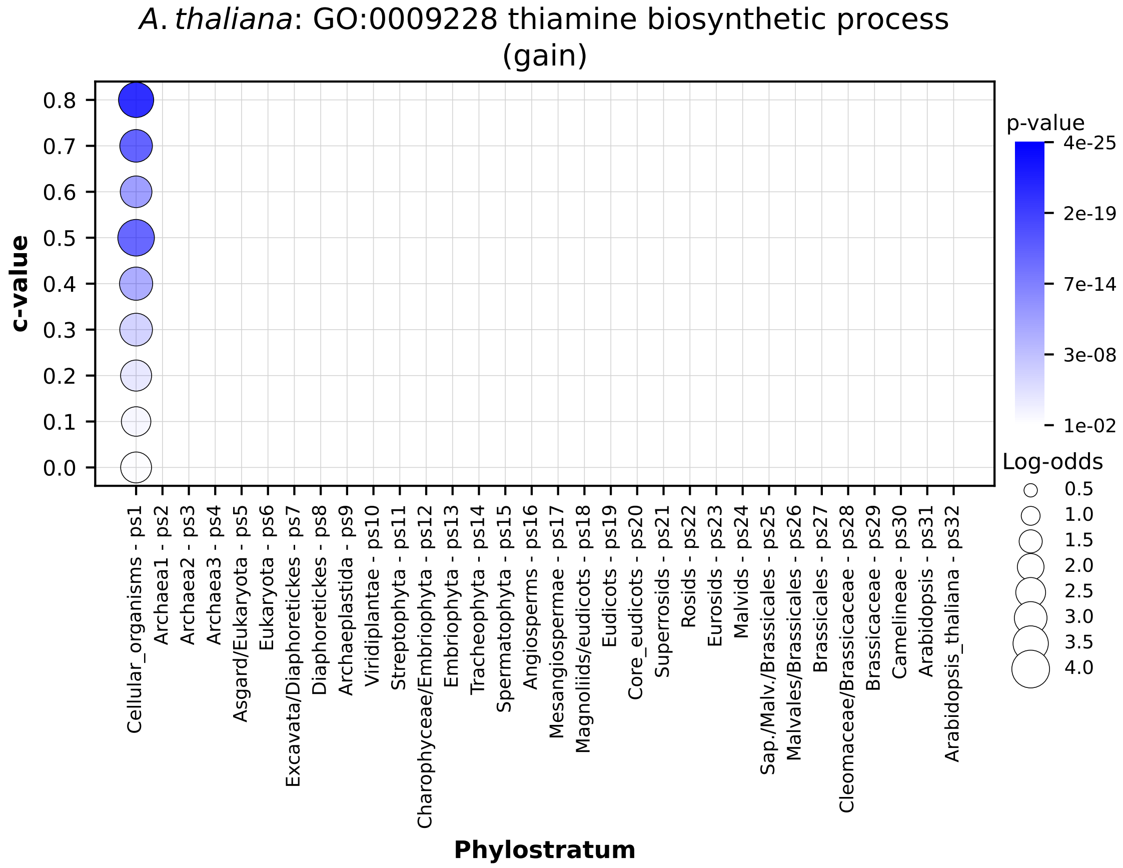

A. thaliana: GO:0009228 thiamine biosynthetic process
(gain)
c-value
0.8
0.7
0.6
0.5
0.4
0.3
0.2
0.1
0.0
p-value
4e-25
2e-19
7e-14
3e-08
1e-02
Log-odds
0.5
1.0
1.5
2.0
2.5
3.0
3.5
4.0
Cellular_organisms - ps1
Archaea1 - ps2
Archaea2 - ps3
Archaea3 - ps4
Asgard/Eukaryota - ps5
Eukaryota - ps6
Excavata/Diaphoretickes - ps7
Diaphoretickes - ps8
Archaeplastida - ps9
Viridiplantae - ps10
Streptophyta - ps11
Charophyceae/Embriophyta - ps12
Embriophyta - ps13
Tracheophyta - ps14
Spermatophyta - ps15
Angiosperms - ps16
Mesangiospermae - ps17
Magnoliids/eudicots - ps18
Eudicots - ps19
Core_eudicots - ps20
Superrosids - ps21
Rosids - ps22
Eurosids - ps23
Malvids - ps24
Sap./Malv./Brassicales - ps25
Malvales/Brassicales - ps26
Brassicales - ps27
Cleomaceae/Brassicaceae - ps28
Brassicaceae - ps29
Camelineae - ps30
Arabidopsis - ps31
Arabidopsis_thaliana - ps32
Phylostratum

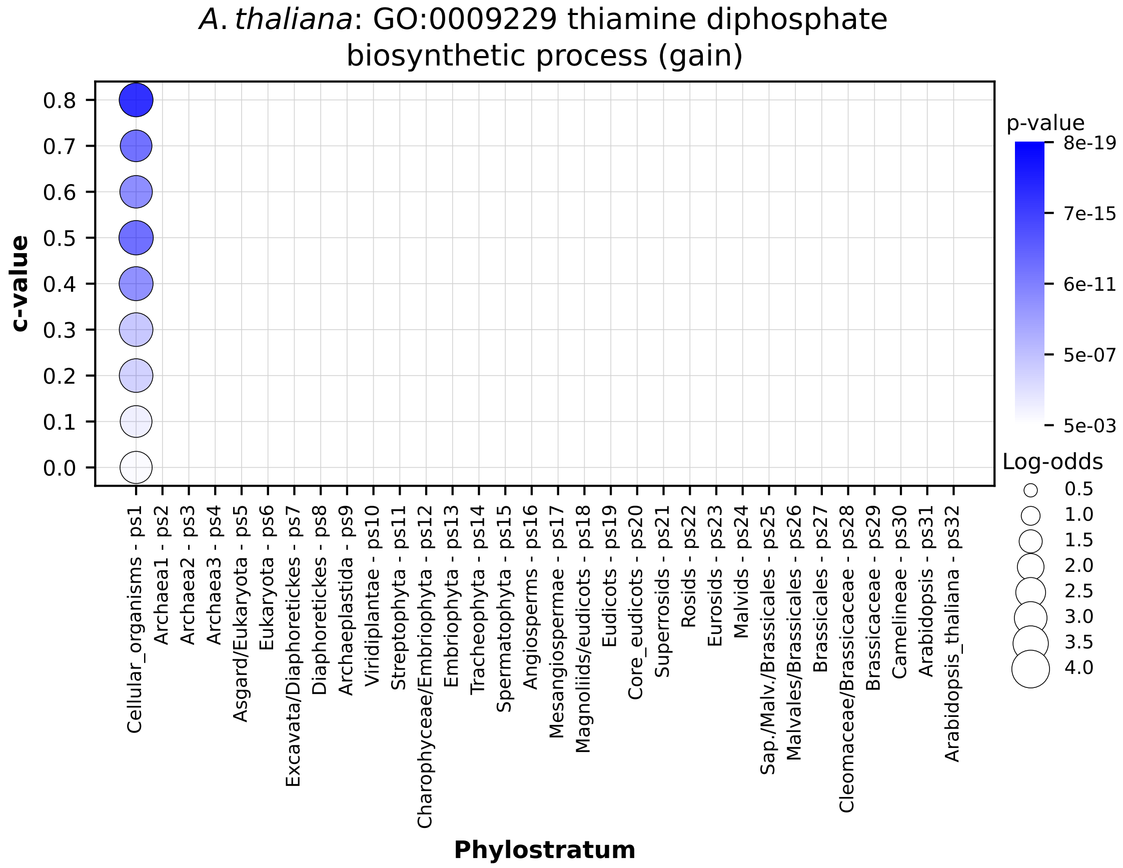

A. thaliana: GO:0009229 thiamine diphosphate biosynthetic process (gain)
c-value
0.8
0.7
0.6
0.5
0.4
0.3
0.2
0.1
0.0
p-value
8e-19
7e-15
6e-11
5e-07
5e-03
Log-odds
0.5
1.0
1.5
2.0
2.5
3.0
3.5
4.0
Cellular_organisms - ps1
Archaea1 - ps2
Archaea2 - ps3
Archaea3 - ps4
Asgard/Eukaryota - ps5
Eukaryota - ps6
Excavata/Diaphoretickes - ps7
Diaphoretickes - ps8
Archaeplastida - ps9
Viridiplantae - ps10
Streptophyta - ps11
Charophyceae/Embriophyta - ps12
Embriophyta - ps13
Tracheophyta - ps14
Spermatophyta - ps15
Angiosperms - ps16
Mesangiospermae - ps17
Magnoliids/eudicots - ps18
Eudicots - ps19
Core_eudicots - ps20
Superrosids - ps21
Rosids - ps22
Eurosids - ps23
Malvids - ps24
Sap./Malv./Brassicales - ps25
Malvales/Brassicales - ps26
Brassicales - ps27
Cleomaceae/Brassicaceae - ps28
Brassicaceae - ps29
Camelineae - ps30
Arabidopsis - ps31
Arabidopsis_thaliana - ps32
Phylostratum

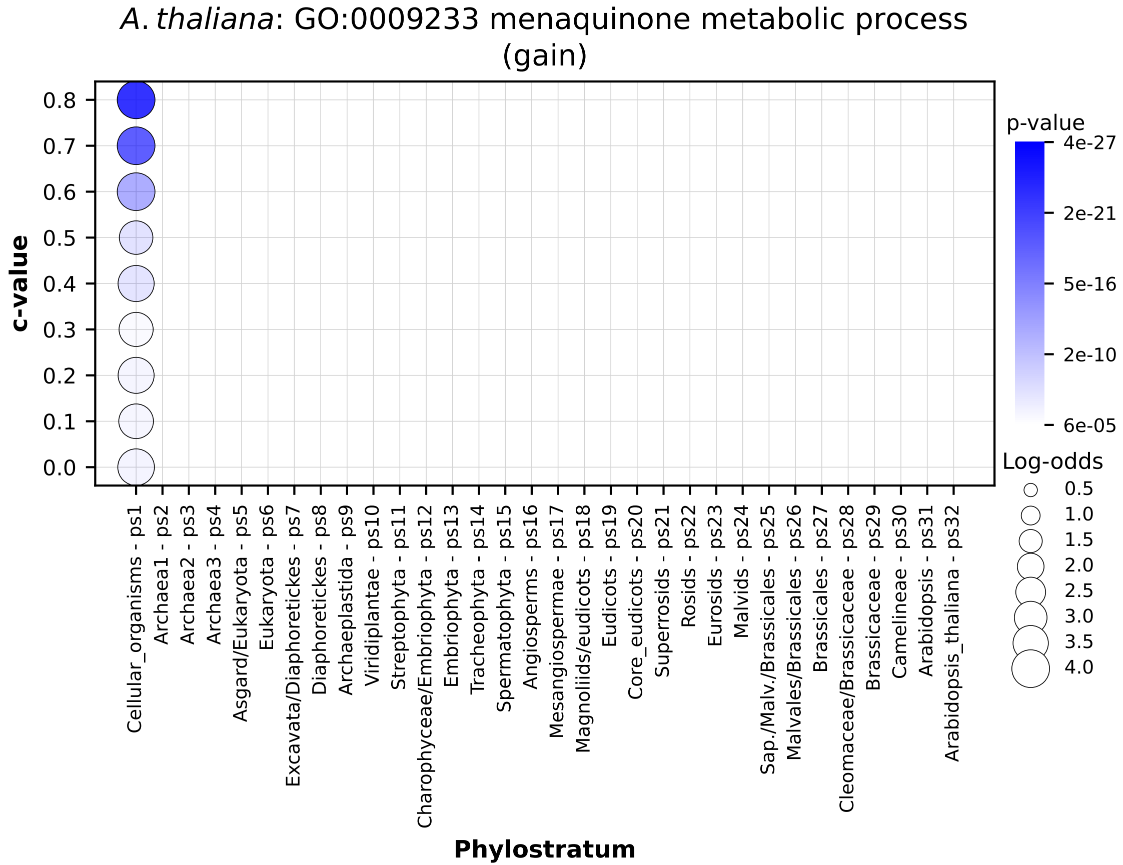

A. thaliana: GO:0009233 menaquinone metabolic process (gain)
c-value
0.8
0.7
0.6
0.5
0.4
0.3
0.2
0.1
0.0
Phylostratum
Cellular_organisms - ps1
Archaea1 - ps2
Archaea2 - ps3
Archaea3 - ps4
Asgard/Eukaryota - ps5
Eukaryota - ps6
Excavata/Diaphoretickes - ps7
Diaphoretickes - ps8
Archaeplastida - ps9
Viridiplantae - ps10
Streptophyta - ps11
Charophyceae/Embriophyta - ps12
Embriophyta - ps13
Tracheophyta - ps14
Spermatophyta - ps15
Angiosperms - ps16
Mesangiospermae - ps17
Magnoliids/eudicots - ps18
Eudicots - ps19
Core_eudicots - ps20
Superrosids - ps21
Rosids - ps22
Eurosids - ps23
Malvids - ps24
Sap./Malv./Brassicales - ps25
Malvales/Brassicales - ps26
Brassicales - ps27
Cleomaceae/Brassicaceae - ps28
Brassicaceae - ps29
Camelineae - ps30
Arabidopsis - ps31
Arabidopsis_thaliana - ps32
p-value
4e-27
2e-21
5e-16
2e-10
6e-05
Log-odds
0.5
1.0
1.5
2.0
2.5
3.0
3.5
4.0

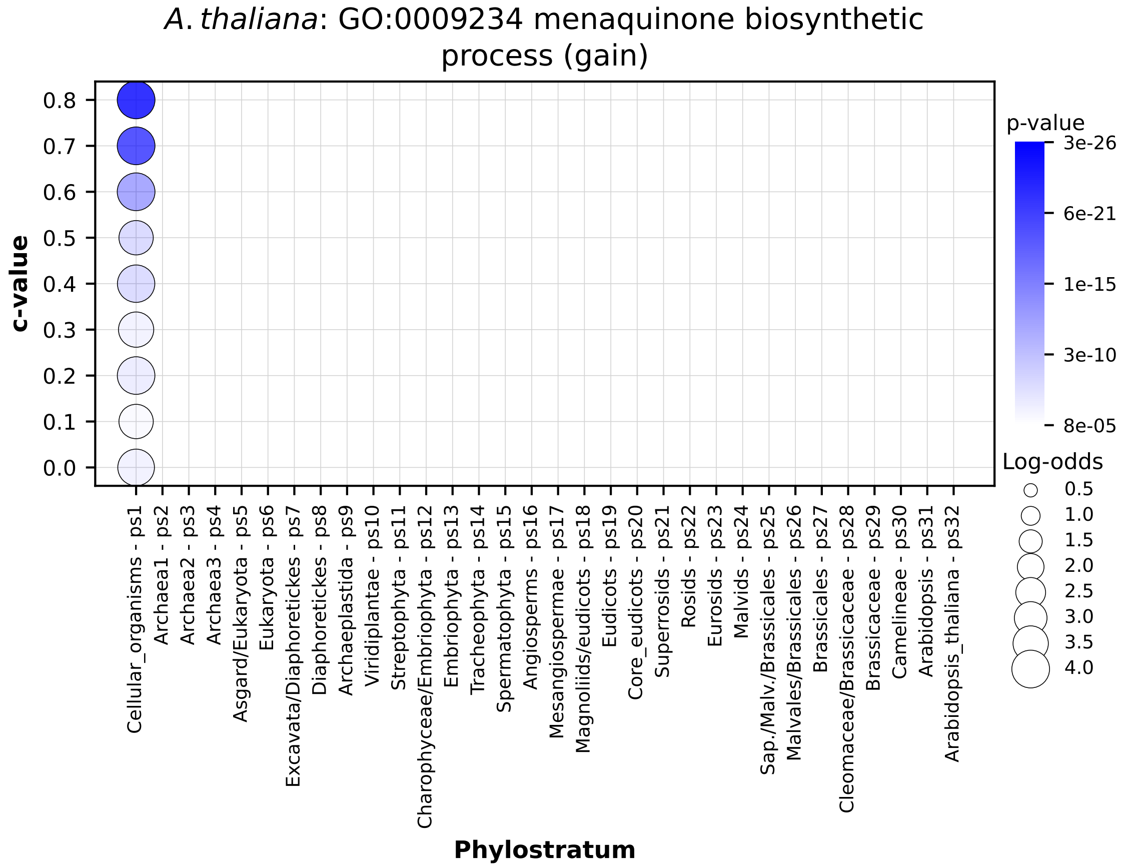
A. thaliana: GO:0009234 menaquinone biosynthetic process (gain)
c-value
0.0
0.1
0.2
0.3
0.4
0.5
0.6
0.7
0.8
Phylostratum
Cellular_organisms - ps1
Archaea1 - ps2
Archaea2 - ps3
Archaea3 - ps4
Asgard/Eukaryota - ps5
Eukaryota - ps6
Excavata/Diaphoretickes - ps7
Diaphoretickes - ps8
Archaeplastida - ps9
Viridiplantae - ps10
Streptophyta - ps11
Charophyceae/Embriophyta - ps12
Embriophyta - ps13
Tracheophyta - ps14
Spermatophyta - ps15
Angiosperms - ps16
Mesangiospermae - ps17
Magnoliids/eudicots - ps18
Eudicots - ps19
Core_eudicots - ps20
Superrosids - ps21
Rosids - ps22
Eurosids - ps23
Malvids - ps24
Sap./Malv./Brassicales - ps25
Malvales/Brassicales - ps26
Brassicales - ps27
Cleomaceae/Brassicaceae - ps28
Brassicaceae - ps29
Camelineae - ps30
Arabidopsis - ps31
Arabidopsis_thaliana - ps32
p-value
3e-26
6e-21
1e-15
3e-10
8e-05
Log-odds
0.5
1.0
1.5
2.0
2.5
3.0
3.5
4.0

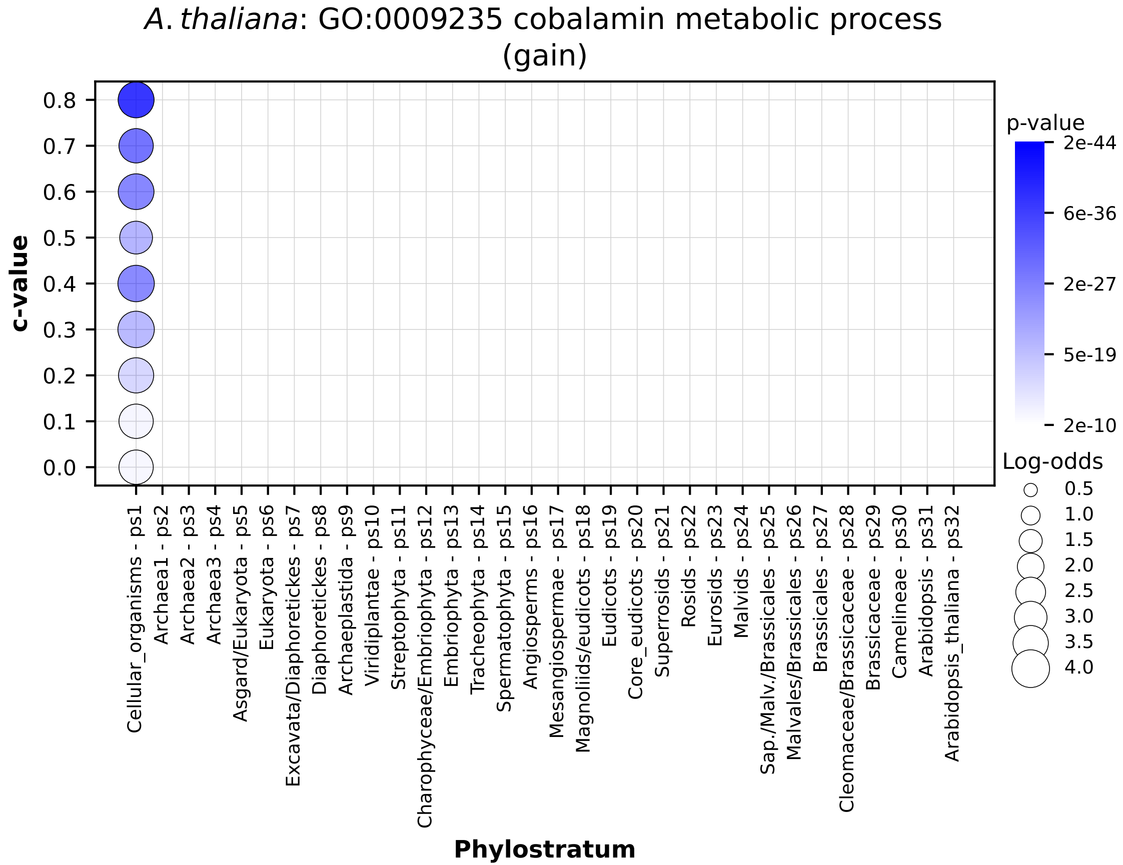

A. thaliana: GO:0009235 cobalamin metabolic process
(gain)
c-value
0.8
0.7
0.6
0.5
0.4
0.3
0.2
0.1
0.0
p-value
2e-44
6e-36
2e-27
5e-19
2e-10
Log-odds
0.5
1.0
1.5
2.0
2.5
3.0
3.5
4.0
Cellular_organisms - ps1
Archaea1 - ps2
Archaea2 - ps3
Archaea3 - ps4
Asgard/Eukaryota - ps5
Eukaryota - ps6
Excavata/Diaphoretickes - ps7
Diaphoretickes - ps8
Archaeplastida - ps9
Viridiplantae - ps10
Streptophyta - ps11
Charophyceae/Embriophyta - ps12
Embriophyta - ps13
Tracheophyta - ps14
Spermatophyta - ps15
Angiosperms - ps16
Mesangiospermae - ps17
Magnoliids/eudicots - ps18
Eudicots - ps19
Core_eudicots - ps20
Superrosids - ps21
Rosids - ps22
Eurosids - ps23
Malvids - ps24
Sap./Malv./Brassicales - ps25
Malvales/Brassicales - ps26
Brassicales - ps27
Cleomaceae/Brassicaceae - ps28
Brassicaceae - ps29
Camelineae - ps30
Arabidopsis - ps31
Arabidopsis_thaliana - ps32
Phylostratum

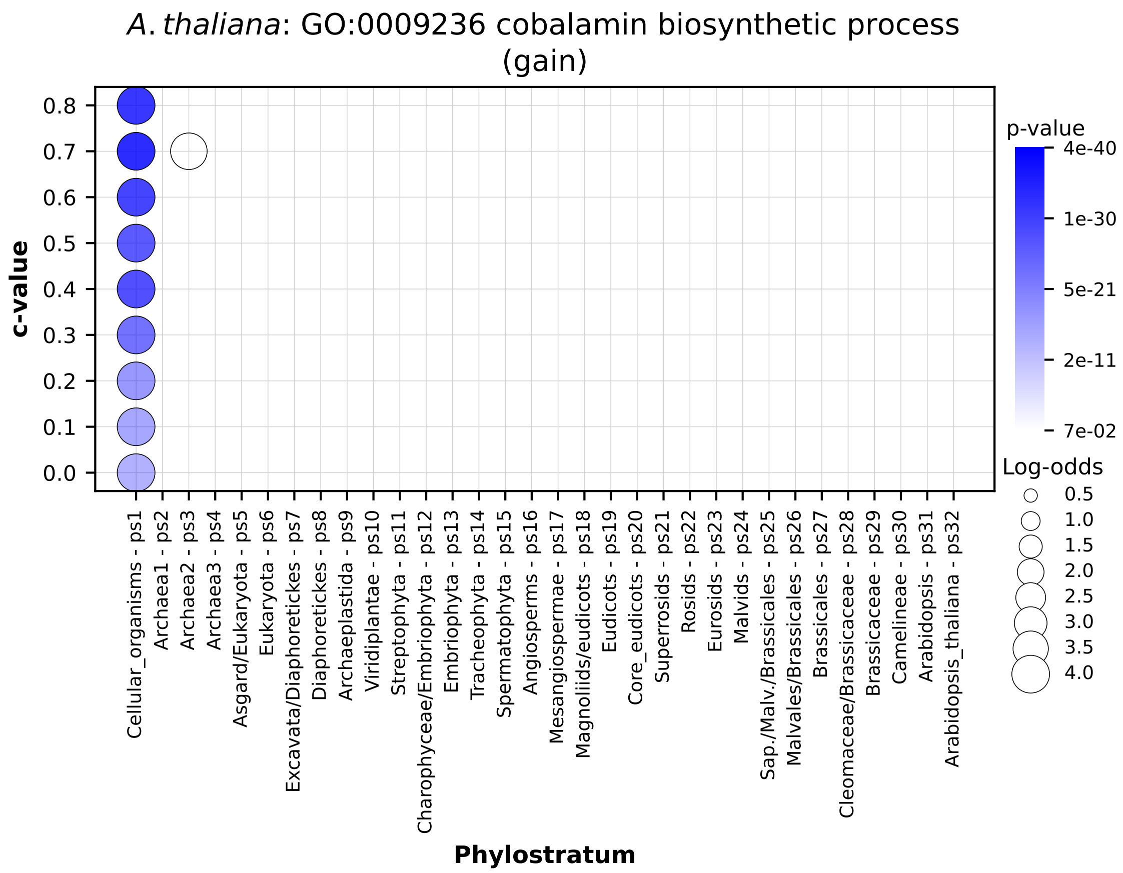

A. thaliana: GO:0009236 cobalamin biosynthetic process (gain)
c-value
0.8
0.7
0.6
0.5
0.4
0.3
0.2
0.1
0.0
p-value
4e-40
1e-30
5e-21
2e-11
7e-02
Log-odds
0.5
1.0
1.5
2.0
2.5
3.0
3.5
4.0
Cellular_organisms - ps1
Archaea1 - ps2
Archaea2 - ps3
Archaea3 - ps4
Asgard/Eukaryota - ps5
Eukaryota - ps6
Excavata/Diaphoretickes - ps7
Diaphoretickes - ps8
Archaeplastida - ps9
Viridiplantae - ps10
Streptophyta - ps11
Charophyceae/Embriophyta - ps12
Embriophyta - ps13
Tracheophyta - ps14
Spermatophyta - ps15
Angiosperms - ps16
Mesangiospermae - ps17
Magnoliids/eudicots - ps18
Eudicots - ps19
Core_eudicots - ps20
Superrosids - ps21
Rosids - ps22
Eurosids - ps23
Malvids - ps24
Sap./Malv./Brassicales - ps25
Malvales/Brassicales - ps26
Brassicales - ps27
Cleomaceae/Brassicaceae - ps28
Brassicaceae - ps29
Camelineae - ps30
Arabidopsis - ps31
Arabidopsis_thaliana - ps32
Phylostratum

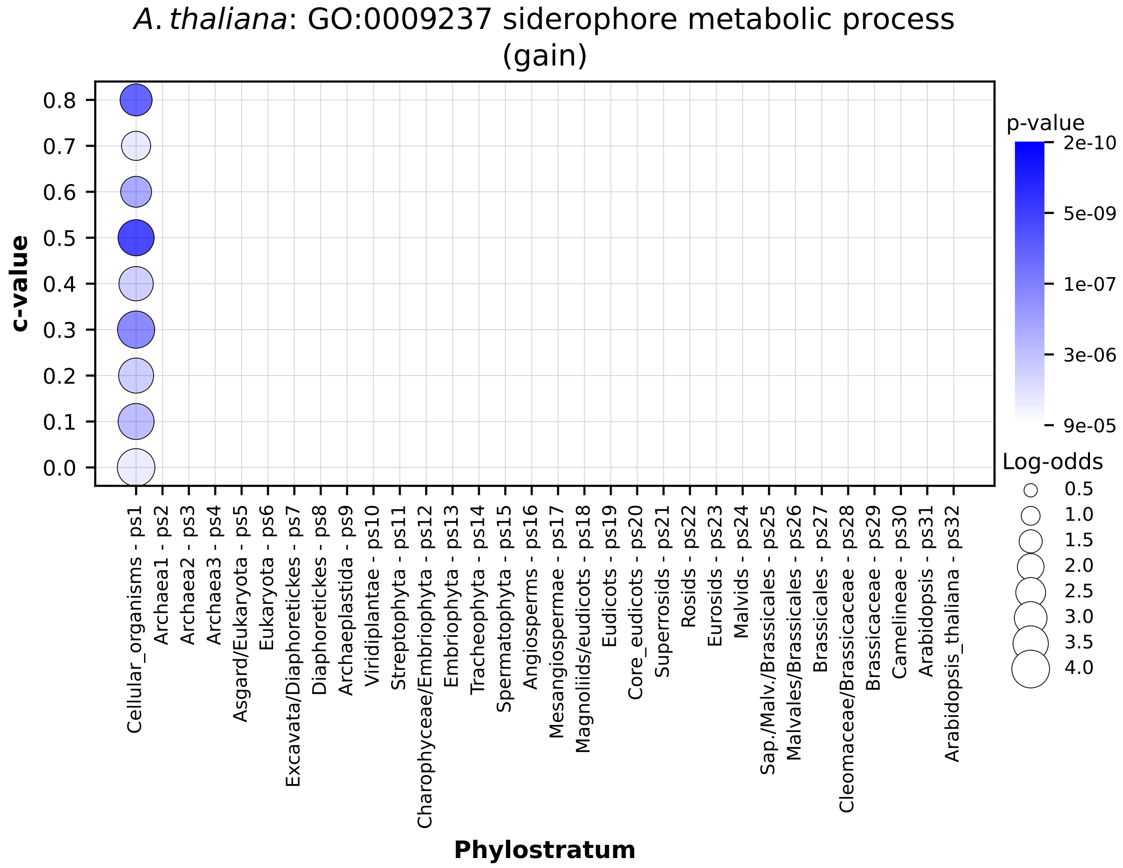

A. thaliana: GO:0009237 siderophore metabolic process (gain)
c-value
0.8
0.7
0.6
0.5
0.4
0.3
0.2
0.1
0.0
p-value
2e-10
5e-09
1e-07
3e-06
9e-05
Log-odds
0.5
1.0
1.5
2.0
2.5
3.0
3.5
4.0
Cellular_organisms - ps1
Archaea1 - ps2
Archaea2 - ps3
Archaea3 - ps4
Asgard/Eukaryota - ps5
Eukaryota - ps6
Excavata/Diaphoretickes - ps7
Diaphoretickes - ps8
Archaeplastida - ps9
Viridiplantae - ps10
Streptophyta - ps11
Charophyceae/Embriophyta - ps12
Embriophyta - ps13
Tracheophyta - ps14
Spermatophyta - ps15
Angiosperms - ps16
Mesangiospermae - ps17
Magnoliids/eudicots - ps18
Eudicots - ps19
Core_eudicots - ps20
Superrosids - ps21
Rosids - ps22
Eurosids - ps23
Malvids - ps24
Sap./Malv./Brassicales - ps25
Malvales/Brassicales - ps26
Brassicales - ps27
Cleomaceae/Brassicaceae - ps28
Brassicaceae - ps29
Camelineae - ps30
Arabidopsis - ps31
Arabidopsis_thaliana - ps32
Phylostratum

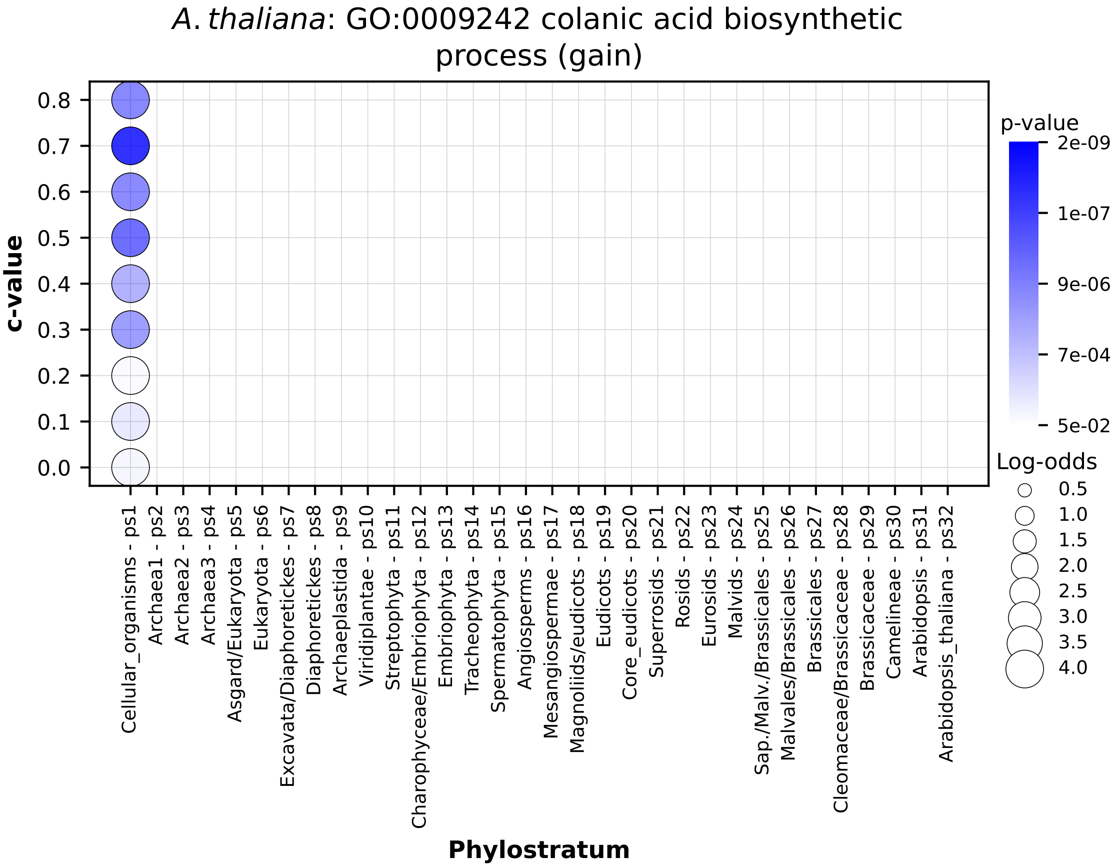

A. thaliana: GO:0009242 colanic acid biosynthetic process (gain)
c-value
0.0
0.1
0.2
0.3
0.4
0.5
0.6
0.7
0.8
p-value
2e-09
1e-07
9e-06
7e-04
5e-02
Log-odds
0.5
1.0
1.5
2.0
2.5
3.0
3.5
4.0
Cellular_organisms - ps1
Archaea1 - ps2
Archaea2 - ps3
Archaea3 - ps4
Asgard/Eukaryota - ps5
Eukaryota - ps6
Excavata/Diaphoretickes - ps7
Diaphoretickes - ps8
Archaeplastida - ps9
Viridiplantae - ps10
Streptophyta - ps11
Charophyceae/Embriophyta - ps12
Embriophyta - ps13
Tracheophyta - ps14
Spermatophyta - ps15
Angiosperms - ps16
Mesangiospermae - ps17
Magnoliids/eudicots - ps18
Eudicots - ps19
Core_eudicots - ps20
Superrosids - ps21
Rosids - ps22
Eurosids - ps23
Malvids - ps24
Sap./Malv./Brassicales - ps25
Malvales/Brassicales - ps26
Brassicales - ps27
Cleomaceae/Brassicaceae - ps28
Brassicaceae - ps29
Camelineae - ps30
Arabidopsis - ps31
Arabidopsis_thaliana - ps32
Phylostratum

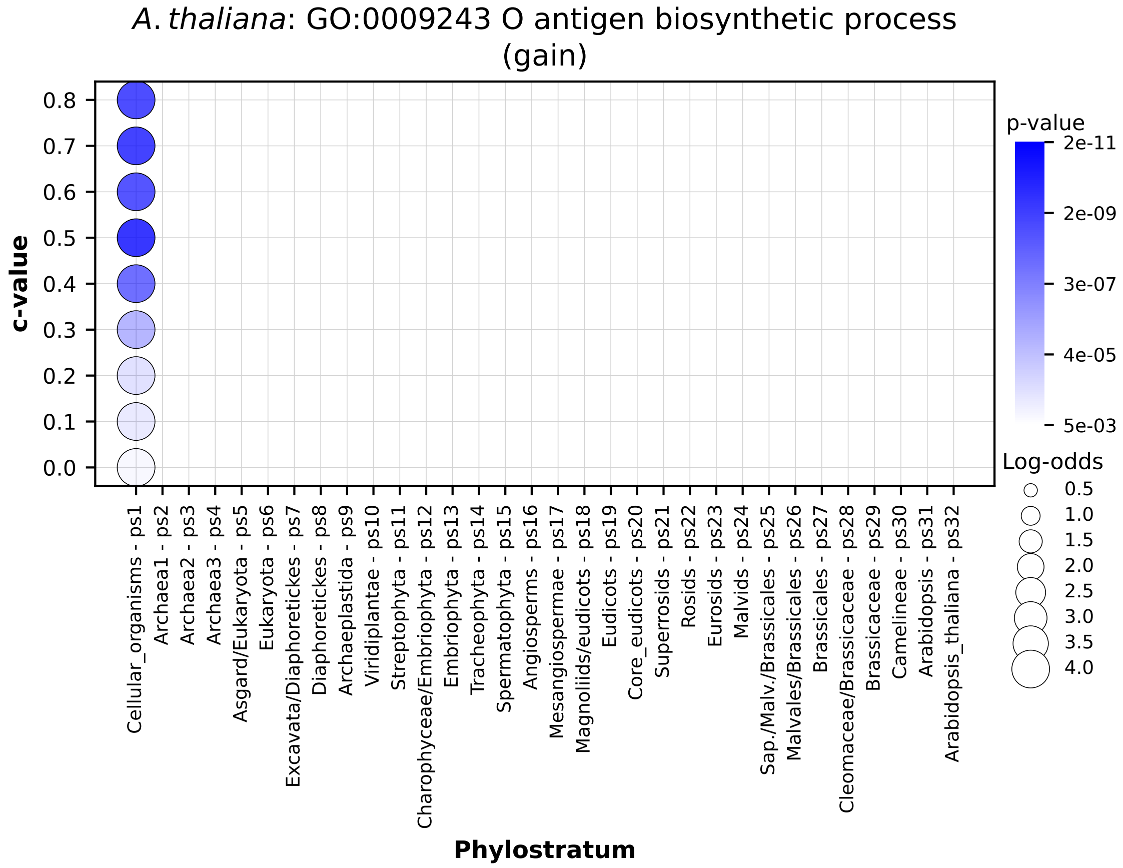
A. thaliana: GO:0009243 O antigen biosynthetic process (gain)
c-value
0.8
0.7
0.6
0.5
0.4
0.3
0.2
0.1
0.0
p-value
2e-11
2e-09
3e-07
4e-05
5e-03
Log-odds
0.5
1.0
1.5
2.0
2.5
3.0
3.5
4.0
Cellular_organisms - ps1
Archaea1 - ps2
Archaea2 - ps3
Archaea3 - ps4
Asgard/Eukaryota - ps5
Eukaryota - ps6
Excavata/Diaphoretickes - ps7
Diaphoretickes - ps8
Archaeplastida - ps9
Viridiplantae - ps10
Streptophyta - ps11
Charophyceae/Embriophyta - ps12
Embriophyta - ps13
Tracheophyta - ps14
Spermatophyta - ps15
Angiosperms - ps16
Mesangiospermae - ps17
Magnoliids/eudicots - ps18
Eudicots - ps19
Core_eudicots - ps20
Superrosids - ps21
Rosids - ps22
Eurosids - ps23
Malvids - ps24
Sap./Malv./Brassicales - ps25
Malvales/Brassicales - ps26
Brassicales - ps27
Cleomaceae/Brassicaceae - ps28
Brassicaceae - ps29
Camelineae - ps30
Arabidopsis - ps31
Arabidopsis_thaliana - ps32
Phylostratum

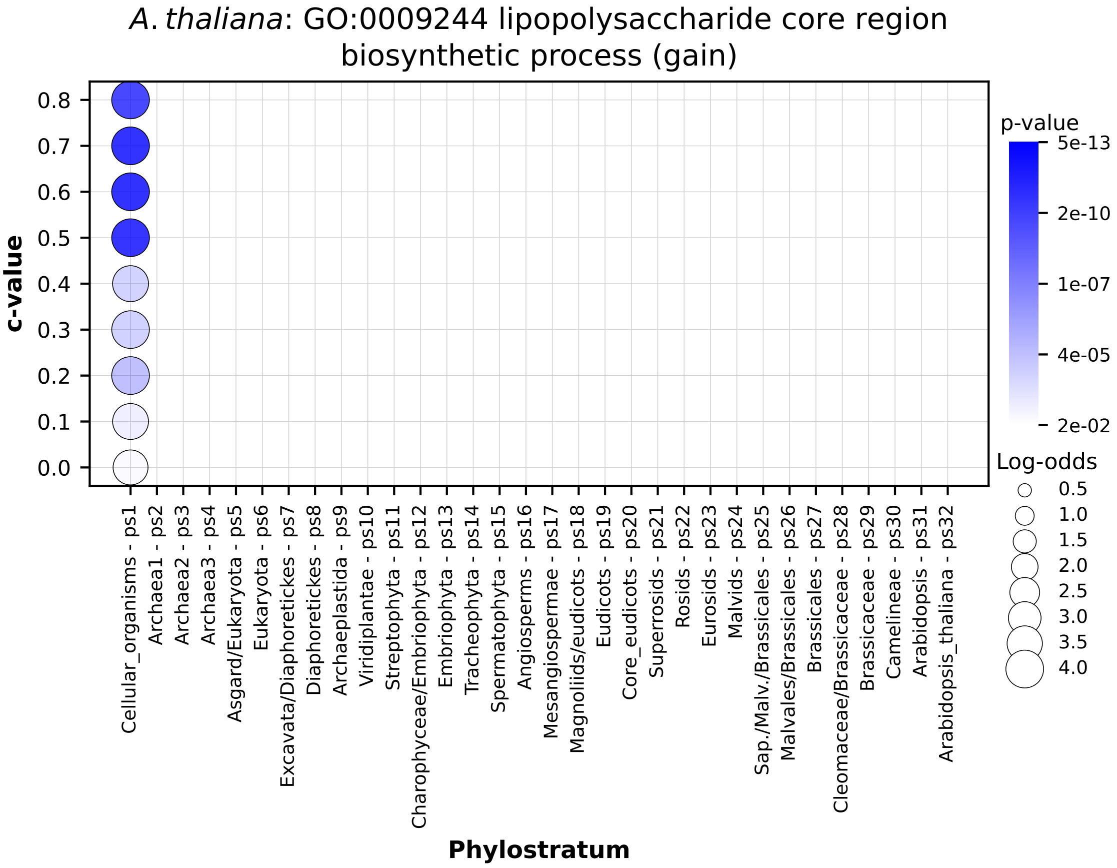
A. thaliana: GO:0009244 lipopolysaccharide core region biosynthetic process (gain)
c-value
0.8
0.7
0.6
0.5
0.4
0.3
0.2
0.1
0.0
p-value
5e-13
2e-10
1e-07
4e-05
2e-02
Log-odds
0.5
1.0
1.5
2.0
2.5
3.0
3.5
4.0
Cellular_organisms - ps1
Archaea1 - ps2
Archaea2 - ps3
Archaea3 - ps4
Asgard/Eukaryota - ps5
Eukaryota - ps6
Excavata/Diaphoretickes - ps7
Diaphoretickes - ps8
Archaeplastida - ps9
Viridiplantae - ps10
Streptophyta - ps11
Charophyceae/Embriophyta - ps12
Embriophyta - ps13
Tracheophyta - ps14
Spermatophyta - ps15
Angiosperms - ps16
Mesangiospermae - ps17
Magnoliids/eudicots - ps18
Eudicots - ps19
Core_eudicots - ps20
Superrosids - ps21
Rosids - ps22
Eurosids - ps23
Malvids - ps24
Sap./Malv./Brassicales - ps25
Malvales/Brassicales - ps26
Brassicales - ps27
Cleomaceae/Brassicaceae - ps28
Brassicaceae - ps29
Camelineae - ps30
Arabidopsis - ps31
Arabidopsis_thaliana - ps32
Phylostratum

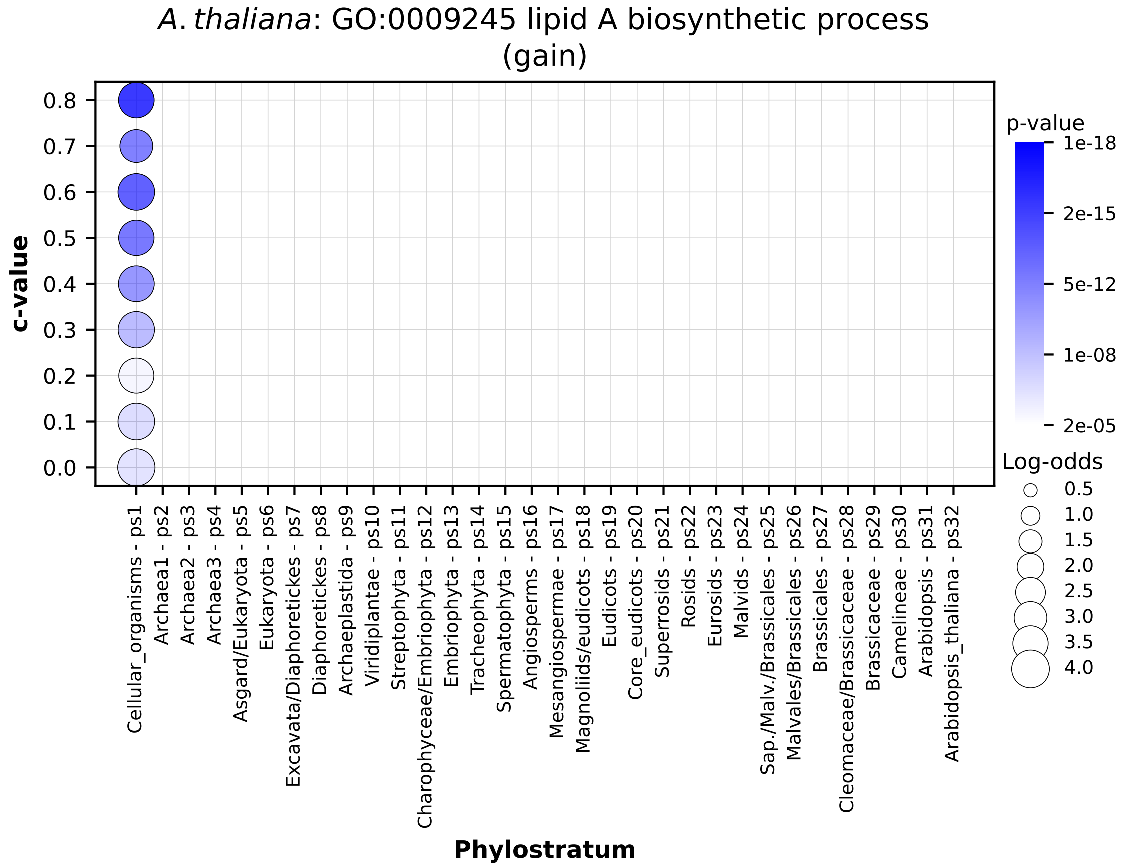

A. thaliana: GO:0009245 lipid A biosynthetic process (gain)
c-value
0.8
0.7
0.6
0.5
0.4
0.3
0.2
0.1
0.0
p-value
1e-18
2e-15
5e-12
1e-08
2e-05
Log-odds
0.5
1.0
1.5
2.0
2.5
3.0
3.5
4.0
Cellular_organisms - ps1
Archaea1 - ps2
Archaea2 - ps3
Archaea3 - ps4
Asgard/Eukaryota - ps5
Eukaryota - ps6
Excavata/Diaphoretickes - ps7
Diaphoretickes - ps8
Archaeplastida - ps9
Viridiplantae - ps10
Streptophyta - ps11
Charophyceae/Embriophyta - ps12
Embriophyta - ps13
Tracheophyta - ps14
Spermatophyta - ps15
Angiosperms - ps16
Mesangiospermae - ps17
Magnoliids/eudicots - ps18
Eudicots - ps19
Core_eudicots - ps20
Superrosids - ps21
Rosids - ps22
Eurosids - ps23
Malvids - ps24
Sap./Malv./Brassicales - ps25
Malvales/Brassicales - ps26
Brassicales - ps27
Cleomaceae/Brassicaceae - ps28
Brassicaceae - ps29
Camelineae - ps30
Arabidopsis - ps31
Arabidopsis_thaliana - ps32
Phylostratum

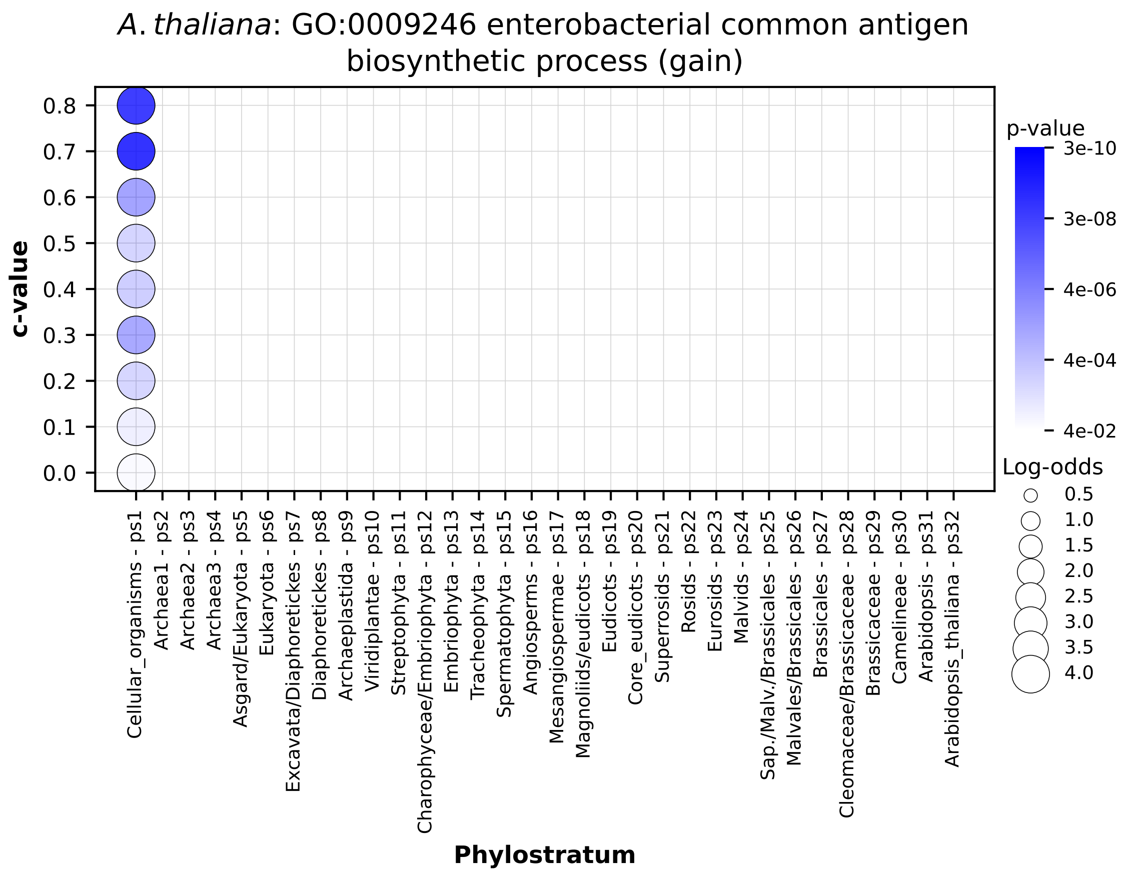

A. thaliana: GO:0009246 enterobacterial common antigen biosynthetic process (gain)
c-value
0.0
0.1
0.2
0.3
0.4
0.5
0.6
0.7
0.8
p-value
3e-10
3e-08
4e-06
4e-04
4e-02
Log-odds
0.5
1.0
1.5
2.0
2.5
3.0
3.5
4.0
Cellular_organisms - ps1
Archaea1 - ps2
Archaea2 - ps3
Archaea3 - ps4
Asgard/Eukaryota - ps5
Eukaryota - ps6
Excavata/Diaphoretickes - ps7
Diaphoretickes - ps8
Archaeplastida - ps9
Viridiplantae - ps10
Streptophyta - ps11
Charophyceae/Embriophyta - ps12
Embriophyta - ps13
Tracheophyta - ps14
Spermatophyta - ps15
Angiosperms - ps16
Mesangiospermae - ps17
Magnoliids/eudicots - ps18
Eudicots - ps19
Core_eudicots - ps20
Superrosids - ps21
Rosids - ps22
Eurosids - ps23
Malvids - ps24
Sap./Malv./Brassicales - ps25
Malvales/Brassicales - ps26
Brassicales - ps27
Cleomaceae/Brassicaceae - ps28
Brassicaceae - ps29
Camelineae - ps30
Arabidopsis - ps31
Arabidopsis_thaliana - ps32
Phylostratum

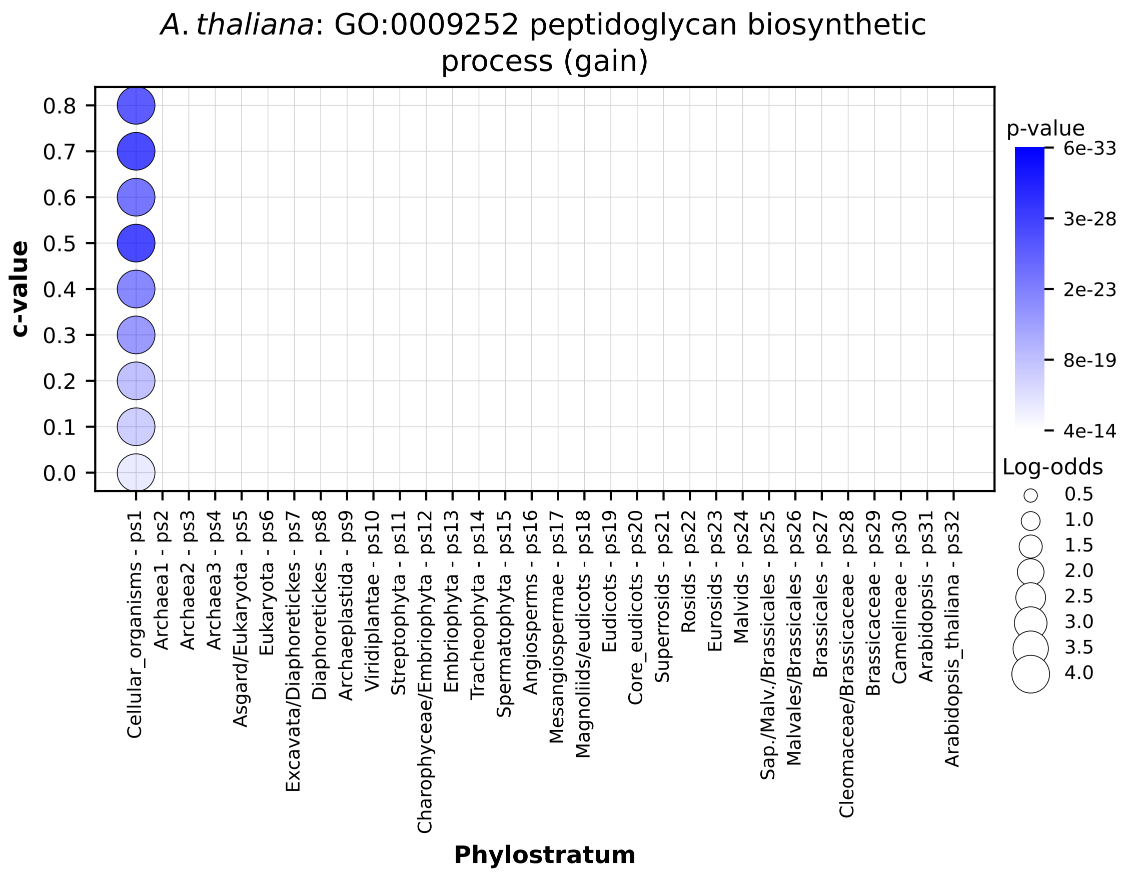

A. thaliana: GO:0009252 peptidoglycan biosynthetic process (gain)
c-value
0.8
0.7
0.6
0.5
0.4
0.3
0.2
0.1
0.0
p-value
6e-33
3e-28
2e-23
8e-19
4e-14
Log-odds
0.5
1.0
1.5
2.0
2.5
3.0
3.5
4.0
Cellular_organisms - ps1
Archaea1 - ps2
Archaea2 - ps3
Archaea3 - ps4
Asgard/Eukaryota - ps5
Eukaryota - ps6
Excavata/Diaphoretickes - ps7
Diaphoretickes - ps8
Archaeplastida - ps9
Viridiplantae - ps10
Streptophyta - ps11
Charophyceae/Embriophyta - ps12
Embriophyta - ps13
Tracheophyta - ps14
Spermatophyta - ps15
Angiosperms - ps16
Mesangiospermae - ps17
Magnoliids/eudicots - ps18
Eudicots - ps19
Core_eudicots - ps20
Superrosids - ps21
Rosids - ps22
Eurosids - ps23
Malvids - ps24
Sap./Malv./Brassicales - ps25
Malvales/Brassicales - ps26
Brassicales - ps27
Cleomaceae/Brassicaceae - ps28
Brassicaceae - ps29
Camelineae - ps30
Arabidopsis - ps31
Arabidopsis_thaliana - ps32
Phylostratum

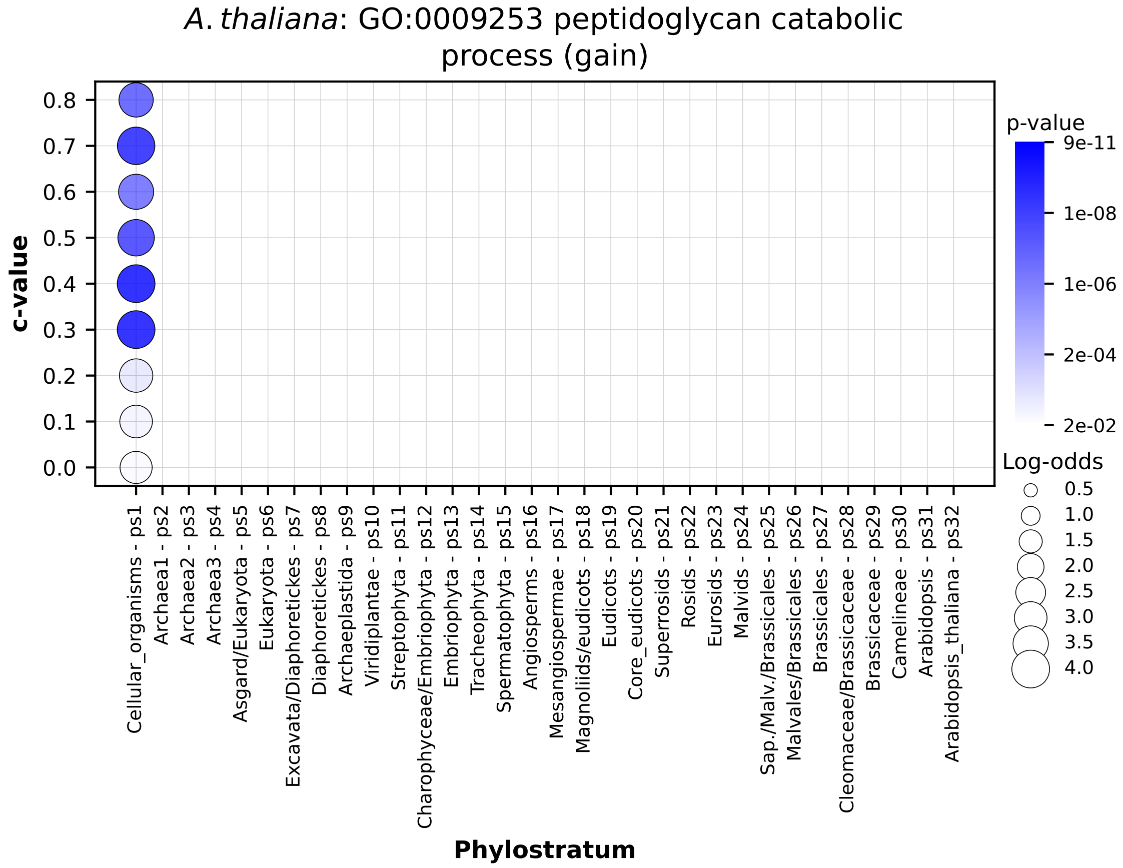

A. thaliana: GO:0009253 peptidoglycan catabolic process (gain)
c-value
0.8
0.7
0.6
0.5
0.4
0.3
0.2
0.1
0.0
p-value
9e-11
1e-08
1e-06
2e-04
2e-02
Log-odds
0.5
1.0
1.5
2.0
2.5
3.0
3.5
4.0
Cellular_organisms - ps1
Archaea1 - ps2
Archaea2 - ps3
Archaea3 - ps4
Asgard/Eukaryota - ps5
Eukaryota - ps6
Excavata/Diaphoretickes - ps7
Diaphoretickes - ps8
Archaeplastida - ps9
Viridiplantae - ps10
Streptophyta - ps11
Charophyceae/Embriophyta - ps12
Embriophyta - ps13
Tracheophyta - ps14
Spermatophyta - ps15
Angiosperms - ps16
Mesangiospermae - ps17
Magnoliids/eudicots - ps18
Eudicots - ps19
Core_eudicots - ps20
Superrosids - ps21
Rosids - ps22
Eurosids - ps23
Malvids - ps24
Sap./Malv./Brassicales - ps25
Malvales/Brassicales - ps26
Brassicales - ps27
Cleomaceae/Brassicaceae - ps28
Brassicaceae - ps29
Camelineae - ps30
Arabidopsis - ps31
Arabidopsis_thaliana - ps32
Phylostratum

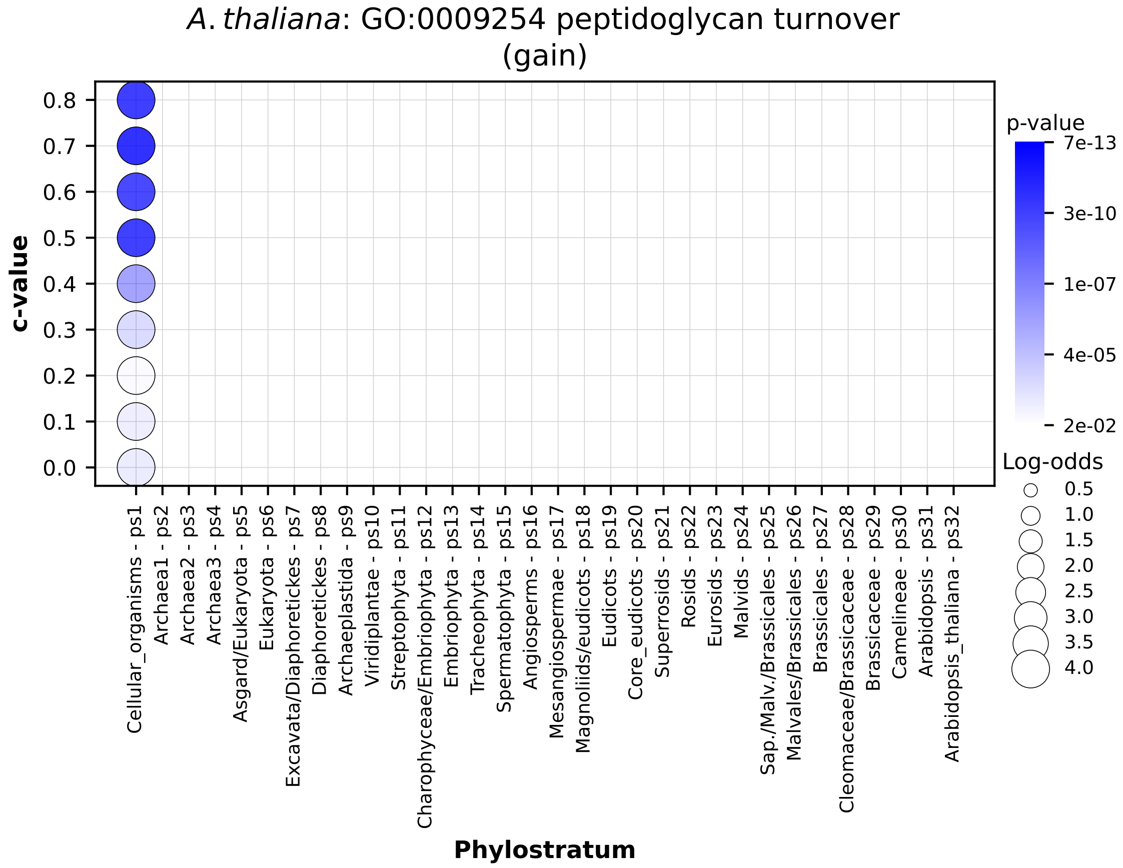

A. thaliana: GO:0009254 peptidoglycan turnover
(gain)
c-value
0.8
0.7
0.6
0.5
0.4
0.3
0.2
0.1
0.0
p-value
7e-13
3e-10
1e-07
4e-05
2e-02
Log-odds
0.5
1.0
1.5
2.0
2.5
3.0
3.5
4.0
Cellular_organisms - ps1
Archaea1 - ps2
Archaea2 - ps3
Archaea3 - ps4
Asgard/Eukaryota - ps5
Eukaryota - ps6
Excavata/Diaphoretickes - ps7
Diaphoretickes - ps8
Archaeplastida - ps9
Viridiplantae - ps10
Streptophyta - ps11
Charophyceae/Embriophyta - ps12
Embriophyta - ps13
Tracheophyta - ps14
Spermatophyta - ps15
Angiosperms - ps16
Mesangiospermae - ps17
Magnoliids/eudicots - ps18
Eudicots - ps19
Core_eudicots - ps20
Superrosids - ps21
Rosids - ps22
Eurosids - ps23
Malvids - ps24
Sap./Malv./Brassicales - ps25
Malvales/Brassicales - ps26
Brassicales - ps27
Cleomaceae/Brassicaceae - ps28
Brassicaceae - ps29
Camelineae - ps30
Arabidopsis - ps31
Arabidopsis_thaliana - ps32
Phylostratum

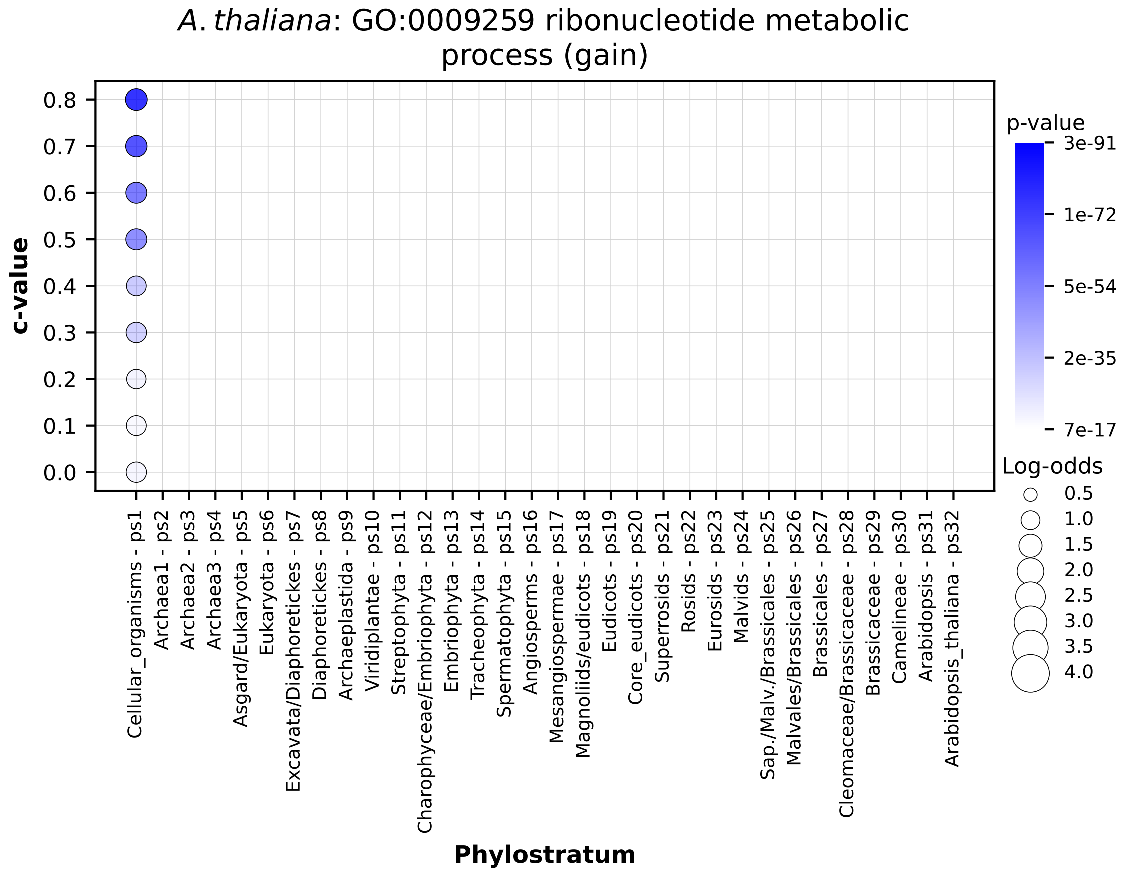

A. thaliana: GO:0009259 ribonucleotide metabolic process (gain)
c-value
0.0
0.1
0.2
0.3
0.4
0.5
0.6
0.7
0.8
p-value
3e-91
1e-72
5e-54
2e-35
7e-17
Log-odds
0.5
1.0
1.5
2.0
2.5
3.0
3.5
4.0
Cellular_organisms - ps1
Archaea1 - ps2
Archaea2 - ps3
Archaea3 - ps4
Asgard/Eukaryota - ps5
Eukaryota - ps6
Excavata/Diaphoretickes - ps7
Diaphoretickes - ps8
Archaeplastida - ps9
Viridiplantae - ps10
Streptophyta - ps11
Charophyceae/Embriophyta - ps12
Embriophyta - ps13
Tracheophyta - ps14
Spermatophyta - ps15
Angiosperms - ps16
Mesangiospermae - ps17
Magnoliids/eudicots - ps18
Eudicots - ps19
Core_eudicots - ps20
Superrosids - ps21
Rosids - ps22
Eurosids - ps23
Malvids - ps24
Sap./Malv./Brassicales - ps25
Malvales/Brassicales - ps26
Brassicales - ps27
Cleomaceae/Brassicaceae - ps28
Brassicaceae - ps29
Camelineae - ps30
Arabidopsis - ps31
Arabidopsis_thaliana - ps32
Phylostratum

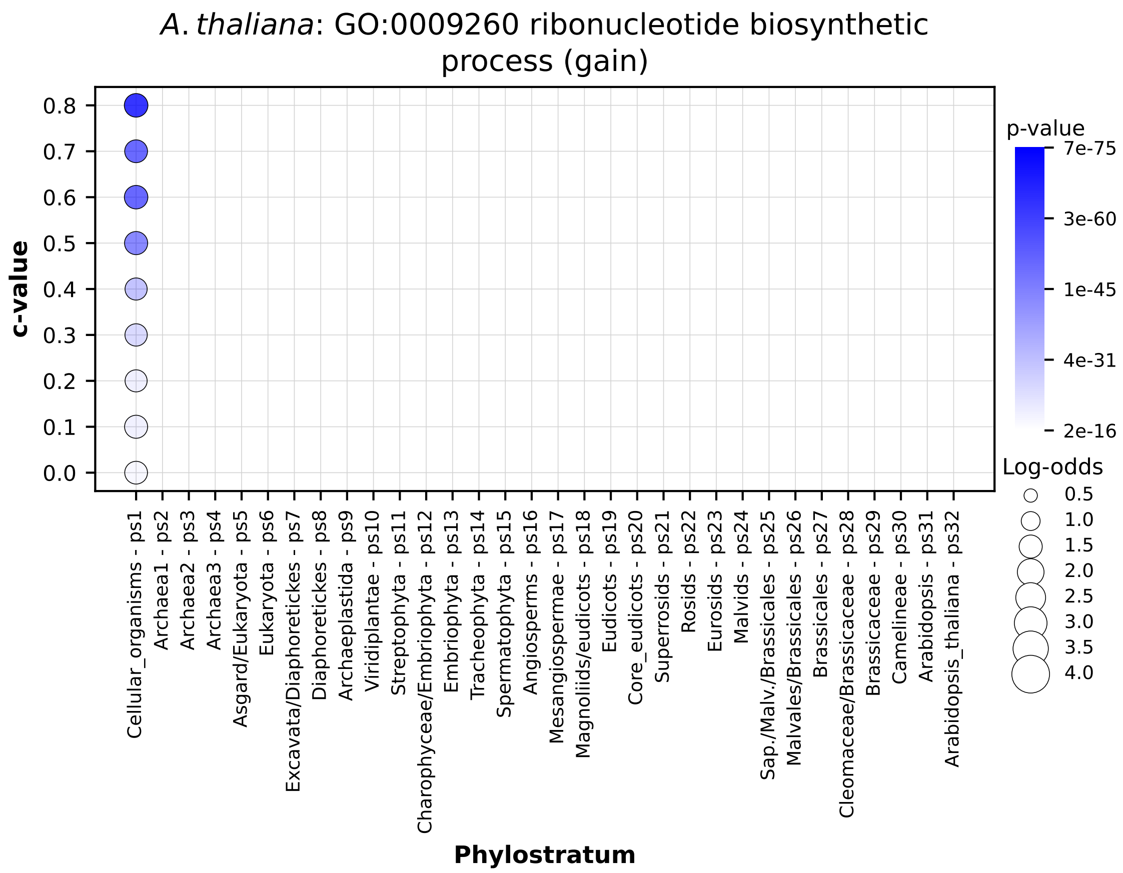
A. thaliana: GO:0009260 ribonucleotide biosynthetic process (gain)
c-value
0.0
0.1
0.2
0.3
0.4
0.5
0.6
0.7
0.8
p-value
7e-75
3e-60
1e-45
4e-31
2e-16
Log-odds
0.5
1.0
1.5
2.0
2.5
3.0
3.5
4.0
Cellular_organisms - ps1
Archaea1 - ps2
Archaea2 - ps3
Archaea3 - ps4
Asgard/Eukaryota - ps5
Eukaryota - ps6
Excavata/Diaphoretickes - ps7
Diaphoretickes - ps8
Archaeplastida - ps9
Viridiplantae - ps10
Streptophyta - ps11
Charophyceae/Embriophyta - ps12
Embriophyta - ps13
Tracheophyta - ps14
Spermatophyta - ps15
Angiosperms - ps16
Mesangiospermae - ps17
Magnoliids/eudicots - ps18
Eudicots - ps19
Core_eudicots - ps20
Superrosids - ps21
Rosids - ps22
Eurosids - ps23
Malvids - ps24
Sap./Malv./Brassicales - ps25
Malvales/Brassicales - ps26
Brassicales - ps27
Cleomaceae/Brassicaceae - ps28
Brassicaceae - ps29
Camelineae - ps30
Arabidopsis - ps31
Arabidopsis_thaliana - ps32
Phylostratum

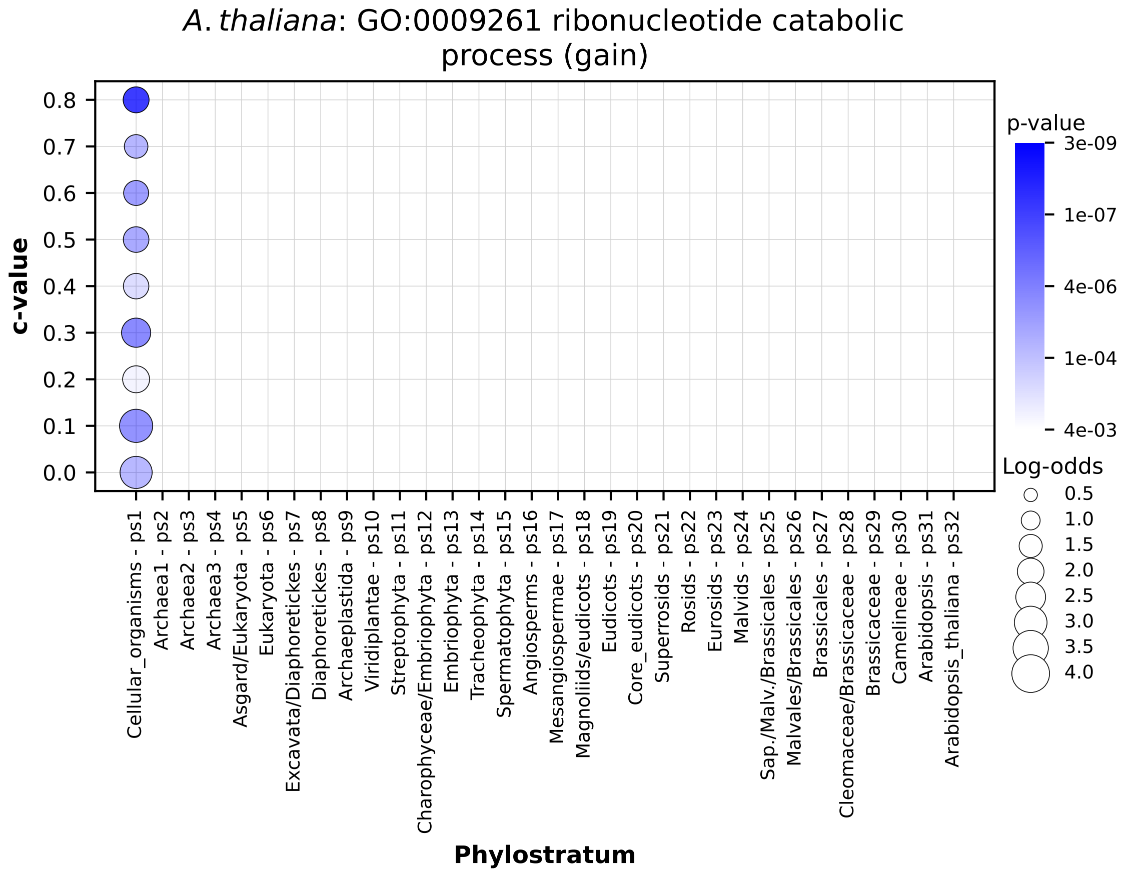

A. thaliana: GO:0009261 ribonucleotide catabolic process (gain)
c-value
0.8
0.7
0.6
0.5
0.4
0.3
0.2
0.1
0.0
p-value
3e-09
1e-07
4e-06
1e-04
4e-03
Log-odds
0.5
1.0
1.5
2.0
2.5
3.0
3.5
4.0
Cellular_organisms - ps1
Archaea1 - ps2
Archaea2 - ps3
Archaea3 - ps4
Asgard/Eukaryota - ps5
Eukaryota - ps6
Excavata/Diaphoretickes - ps7
Diaphoretickes - ps8
Archaeplastida - ps9
Viridiplantae - ps10
Streptophyta - ps11
Charophyceae/Embriophyta - ps12
Embriophyta - ps13
Tracheophyta - ps14
Spermatophyta - ps15
Angiosperms - ps16
Mesangiospermae - ps17
Magnoliids/eudicots - ps18
Eudicots - ps19
Core_eudicots - ps20
Superrosids - ps21
Rosids - ps22
Eurosids - ps23
Malvids - ps24
Sap./Malv./Brassicales - ps25
Malvales/Brassicales - ps26
Brassicales - ps27
Cleomaceae/Brassicaceae - ps28
Brassicaceae - ps29
Camelineae - ps30
Arabidopsis - ps31
Arabidopsis_thaliana - ps32
Phylostratum

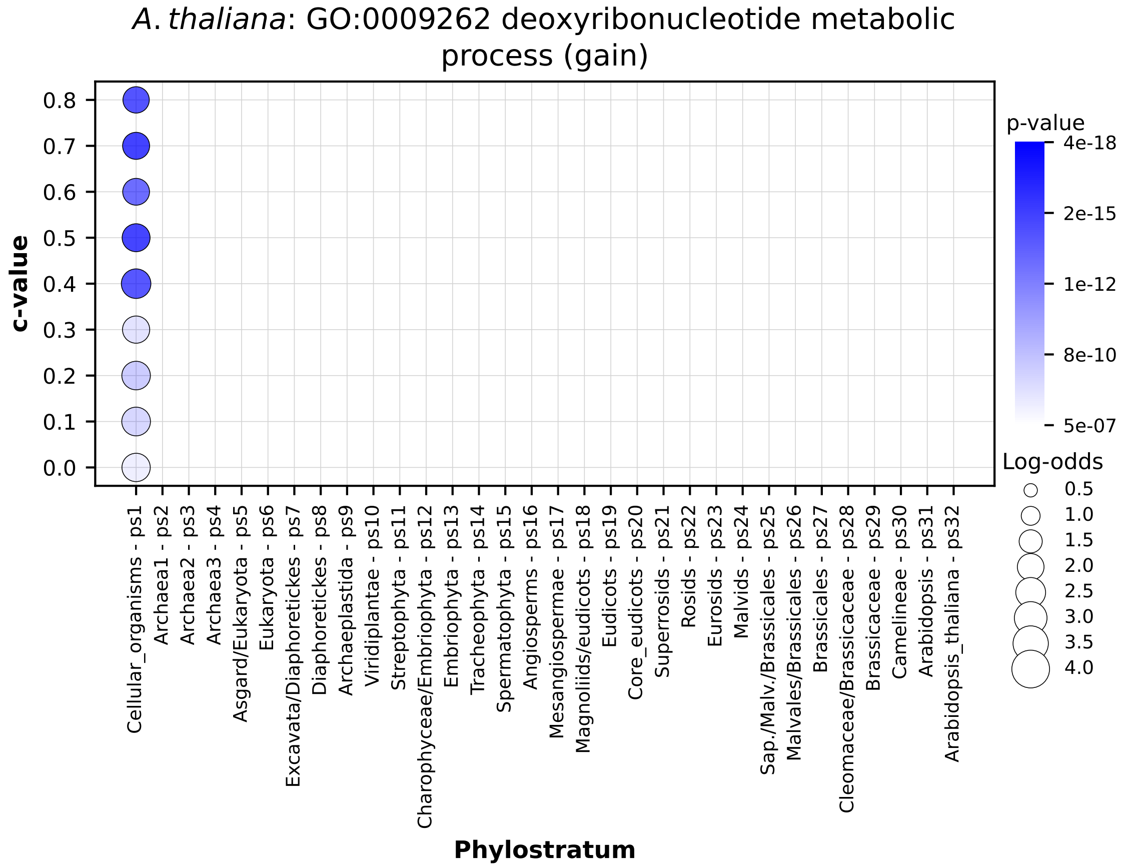

A. thaliana: GO:0009262 deoxyribonucleotide metabolic process (gain)
c-value
0.0
0.1
0.2
0.3
0.4
0.5
0.6
0.7
0.8
Phylostratum
Cellular_organisms - ps1
Archaea1 - ps2
Archaea2 - ps3
Archaea3 - ps4
Asgard/Eukaryota - ps5
Eukaryota - ps6
Excavata/Diaphoretickes - ps7
Diaphoretickes - ps8
Archaeplastida - ps9
Viridiplantae - ps10
Streptophyta - ps11
Charophyceae/Embriophyta - ps12
Embriophyta - ps13
Tracheophyta - ps14
Spermatophyta - ps15
Angiosperms - ps16
Mesangiospermae - ps17
Magnoliids/eudicots - ps18
Eudicots - ps19
Core_eudicots - ps20
Superrosids - ps21
Rosids - ps22
Eurosids - ps23
Malvids - ps24
Sap./Malv./Brassicales - ps25
Malvales/Brassicales - ps26
Brassicales - ps27
Cleomaceae/Brassicaceae - ps28
Brassicaceae - ps29
Camelineae - ps30
Arabidopsis - ps31
Arabidopsis_thaliana - ps32
p-value
4e-18
2e-15
1e-12
8e-10
5e-07
Log-odds
0.5
1.0
1.5
2.0
2.5
3.0
3.5
4.0

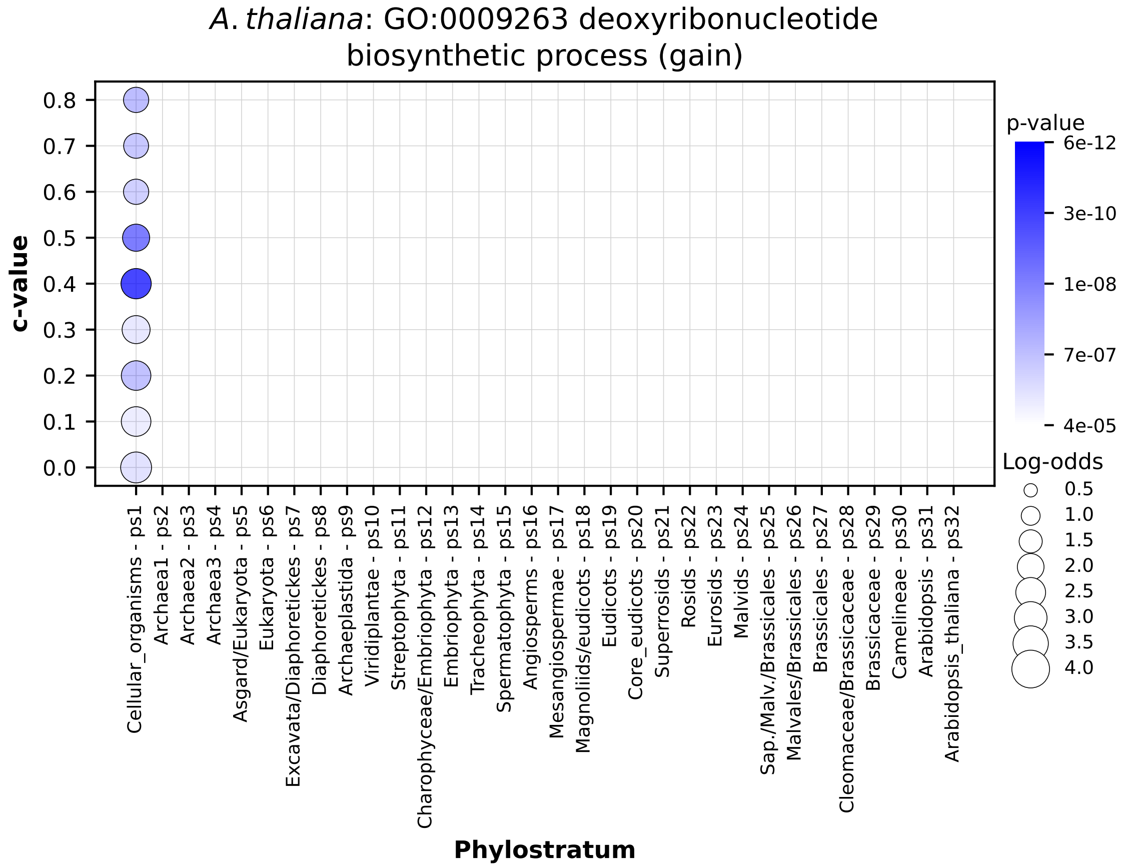

A. thaliana: GO:0009263 deoxyribonucleotide biosynthetic process (gain)
c-value
0.8
0.7
0.6
0.5
0.4
0.3
0.2
0.1
0.0
p-value
6e-12
3e-10
1e-08
7e-07
4e-05
Log-odds
0.5
1.0
1.5
2.0
2.5
3.0
3.5
4.0
Cellular_organisms - ps1
Archaea1 - ps2
Archaea2 - ps3
Archaea3 - ps4
Asgard/Eukaryota - ps5
Eukaryota - ps6
Excavata/Diaphoretickes - ps7
Diaphoretickes - ps8
Archaeplastida - ps9
Viridiplantae - ps10
Streptophyta - ps11
Charophyceae/Embriophyta - ps12
Embriophyta - ps13
Tracheophyta - ps14
Spermatophyta - ps15
Angiosperms - ps16
Mesangiospermae - ps17
Magnoliids/eudicots - ps18
Eudicots - ps19
Core_eudicots - ps20
Superrosids - ps21
Rosids - ps22
Eurosids - ps23
Malvids - ps24
Sap./Malv./Brassicales - ps25
Malvales/Brassicales - ps26
Brassicales - ps27
Cleomaceae/Brassicaceae - ps28
Brassicaceae - ps29
Camelineae - ps30
Arabidopsis - ps31
Arabidopsis_thaliana - ps32
Phylostratum

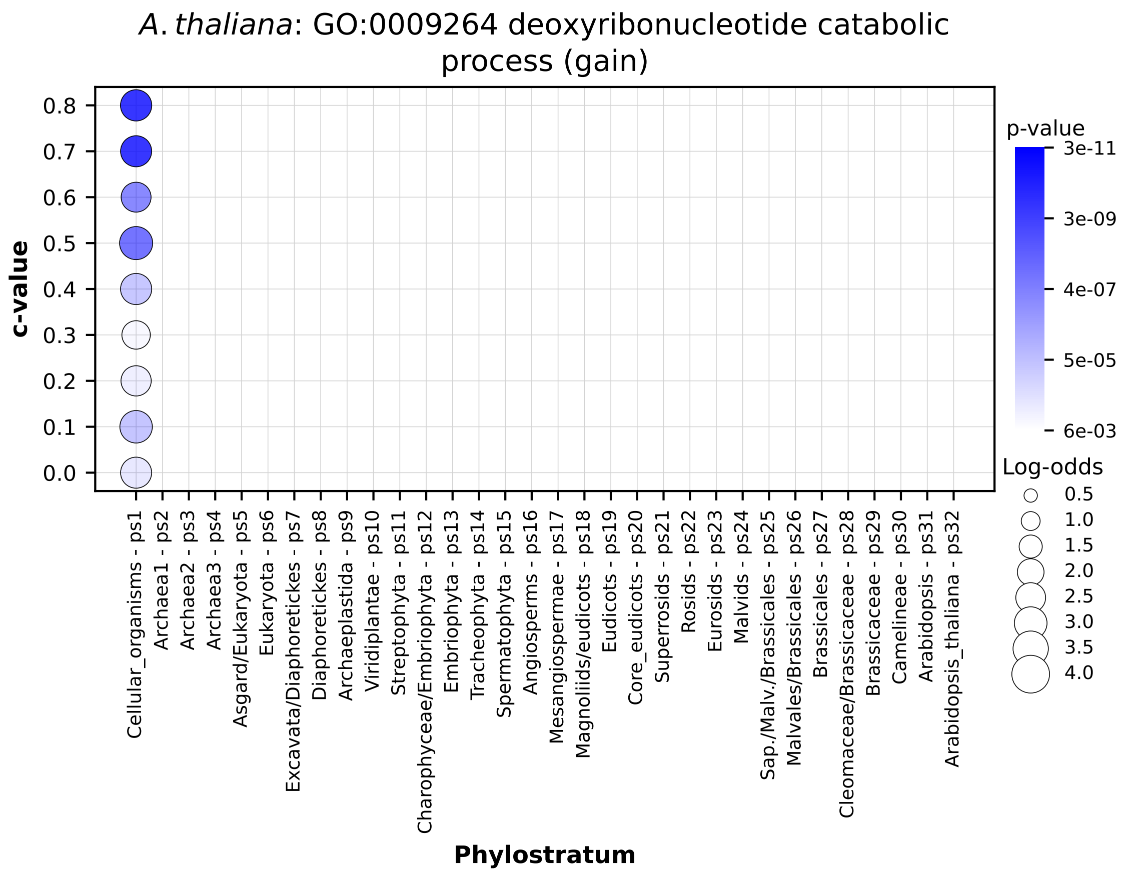

A. thaliana: GO:0009264 deoxyribonucleotide catabolic process (gain)
c-value
0.8
0.7
0.6
0.5
0.4
0.3
0.2
0.1
0.0
Phylostratum
Cellular_organisms - ps1
Archaea1 - ps2
Archaea2 - ps3
Archaea3 - ps4
Asgard/Eukaryota - ps5
Eukaryota - ps6
Excavata/Diaphoretickes - ps7
Diaphoretickes - ps8
Archaeplastida - ps9
Viridiplantae - ps10
Streptophyta - ps11
Charophyceae/Embriophyta - ps12
Embriophyta - ps13
Tracheophyta - ps14
Spermatophyta - ps15
Angiosperms - ps16
Mesangiospermae - ps17
Magnoliids/eudicots - ps18
Eudicots - ps19
Core_eudicots - ps20
Superrosids - ps21
Rosids - ps22
Eurosids - ps23
Malvids - ps24
Sap./Malv./Brassicales - ps25
Malvales/Brassicales - ps26
Brassicales - ps27
Cleomaceae/Brassicaceae - ps28
Brassicaceae - ps29
Camelineae - ps30
Arabidopsis - ps31
Arabidopsis_thaliana - ps32
p-value
3e-11
3e-09
4e-07
5e-05
6e-03
Log-odds
0.5
1.0
1.5
2.0
2.5
3.0
3.5
4.0

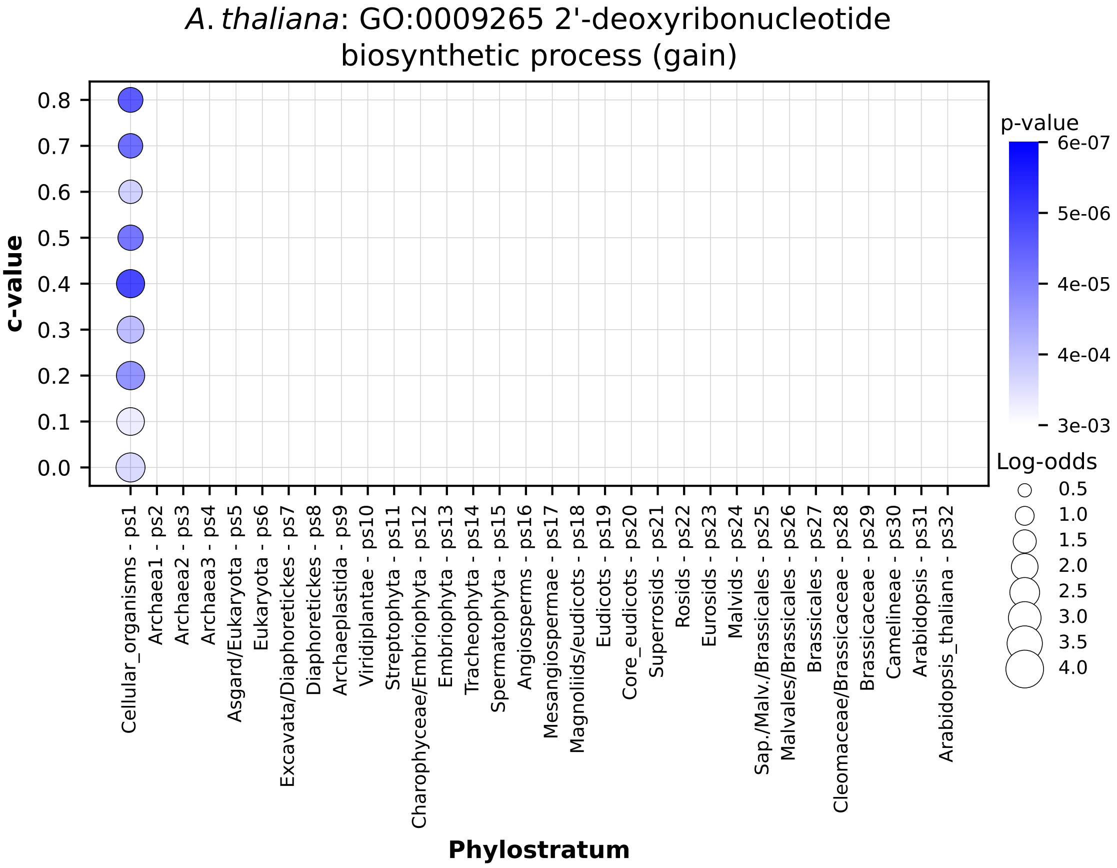

A. thaliana: GO:0009265 2'-deoxyribonucleotide biosynthetic process (gain)
c-value
0.0
0.1
0.2
0.3
0.4
0.5
0.6
0.7
0.8
Phylostratum
Cellular_organisms - ps1
Archaea1 - ps2
Archaea2 - ps3
Archaea3 - ps4
Asgard/Eukaryota - ps5
Eukaryota - ps6
Excavata/Diaphoretickes - ps7
Diaphoretickes - ps8
Archaeplastida - ps9
Viridiplantae - ps10
Streptophyta - ps11
Charophyceae/Embriophyta - ps12
Embriophyta - ps13
Tracheophyta - ps14
Spermatophyta - ps15
Angiosperms - ps16
Mesangiospermae - ps17
Magnoliids/eudicots - ps18
Eudicots - ps19
Core_eudicots - ps20
Superrosids - ps21
Rosids - ps22
Eurosids - ps23
Malvids - ps24
Sap./Malv./Brassicales - ps25
Malvales/Brassicales - ps26
Brassicales - ps27
Cleomaceae/Brassicaceae - ps28
Brassicaceae - ps29
Camelineae - ps30
Arabidopsis - ps31
Arabidopsis_thaliana - ps32
p-value
6e-07
5e-06
4e-05
4e-04
3e-03
Log-odds
0.5
1.0
1.5
2.0
2.5
3.0
3.5
4.0

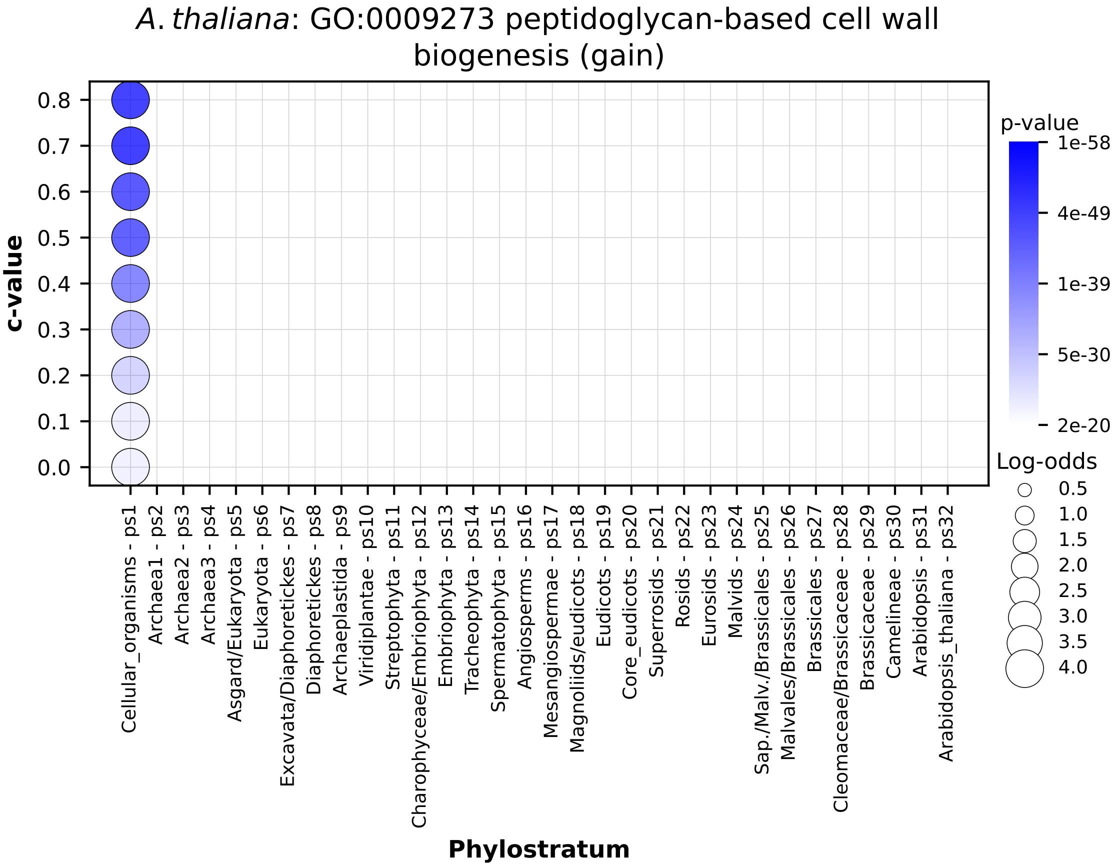

A. thaliana: GO:0009273 peptidoglycan-based cell wall biogenesis (gain)
c-value
0.8
0.7
0.6
0.5
0.4
0.3
0.2
0.1
0.0
p-value
1e-58
4e-49
1e-39
5e-30
2e-20
Log-odds
0.5
1.0
1.5
2.0
2.5
3.0
3.5
4.0
Cellular_organisms - ps1
Archaea1 - ps2
Archaea2 - ps3
Archaea3 - ps4
Asgard/Eukaryota - ps5
Eukaryota - ps6
Excavata/Diaphoretickes - ps7
Diaphoretickes - ps8
Archaeplastida - ps9
Viridiplantae - ps10
Streptophyta - ps11
Charophyceae/Embriophyta - ps12
Embriophyta - ps13
Tracheophyta - ps14
Spermatophyta - ps15
Angiosperms - ps16
Mesangiospermae - ps17
Magnoliids/eudicots - ps18
Eudicots - ps19
Core_eudicots - ps20
Superrosids - ps21
Rosids - ps22
Eurosids - ps23
Malvids - ps24
Sap./Malv./Brassicales - ps25
Malvales/Brassicales - ps26
Brassicales - ps27
Cleomaceae/Brassicaceae - ps28
Brassicaceae - ps29
Camelineae - ps30
Arabidopsis - ps31
Arabidopsis_thaliana - ps32
Phylostratum

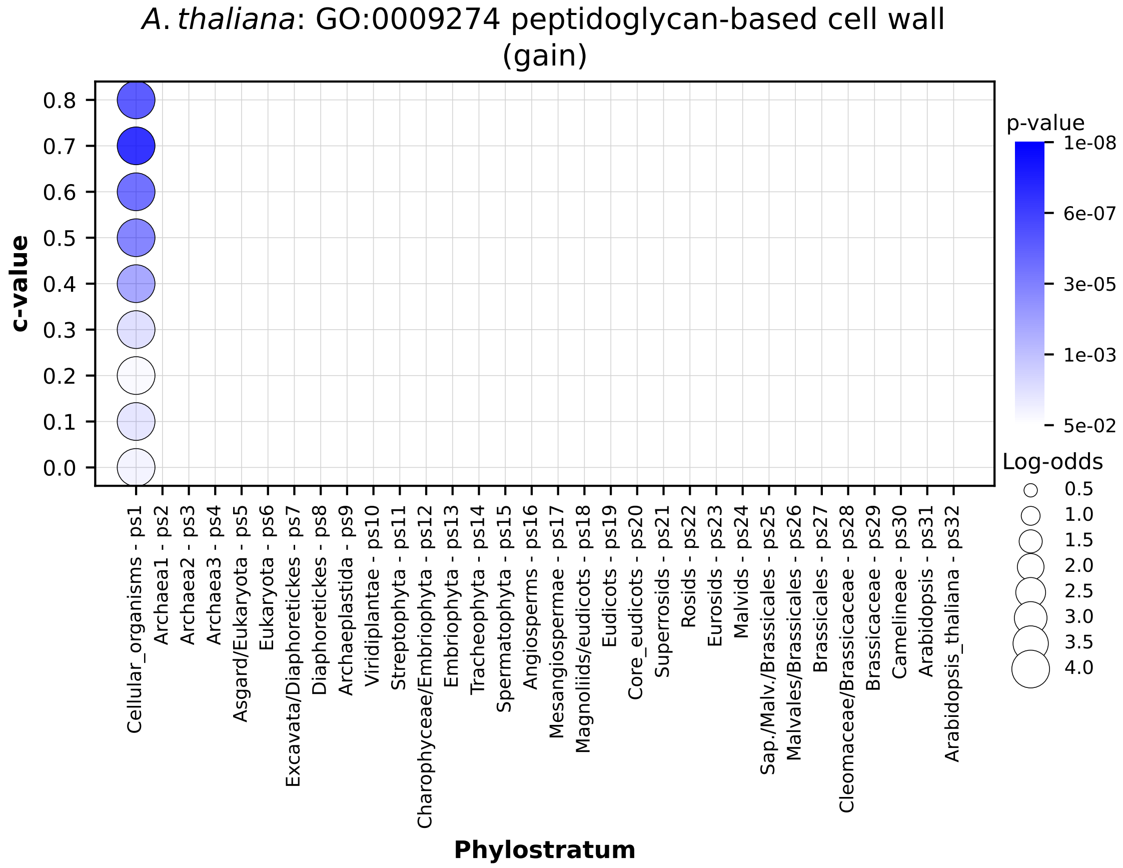

A. thaliana: GO:0009274 peptidoglycan-based cell wall (gain)
c-value
0.8
0.7
0.6
0.5
0.4
0.3
0.2
0.1
0.0
p-value
1e-08
6e-07
3e-05
1e-03
5e-02
Log-odds
0.5
1.0
1.5
2.0
2.5
3.0
3.5
4.0
Cellular_organisms - ps1
Archaea1 - ps2
Archaea2 - ps3
Archaea3 - ps4
Asgard/Eukaryota - ps5
Eukaryota - ps6
Excavata/Diaphoretickes - ps7
Diaphoretickes - ps8
Archaeplastida - ps9
Viridiplantae - ps10
Streptophyta - ps11
Charophyceae/Embriophyta - ps12
Embriophyta - ps13
Tracheophyta - ps14
Spermatophyta - ps15
Angiosperms - ps16
Mesangiospermae - ps17
Magnoliids/eudicots - ps18
Eudicots - ps19
Core_eudicots - ps20
Superrosids - ps21
Rosids - ps22
Eurosids - ps23
Malvids - ps24
Sap./Malv./Brassicales - ps25
Malvales/Brassicales - ps26
Brassicales - ps27
Cleomaceae/Brassicaceae - ps28
Brassicaceae - ps29
Camelineae - ps30
Arabidopsis - ps31
Arabidopsis_thaliana - ps32
Phylostratum

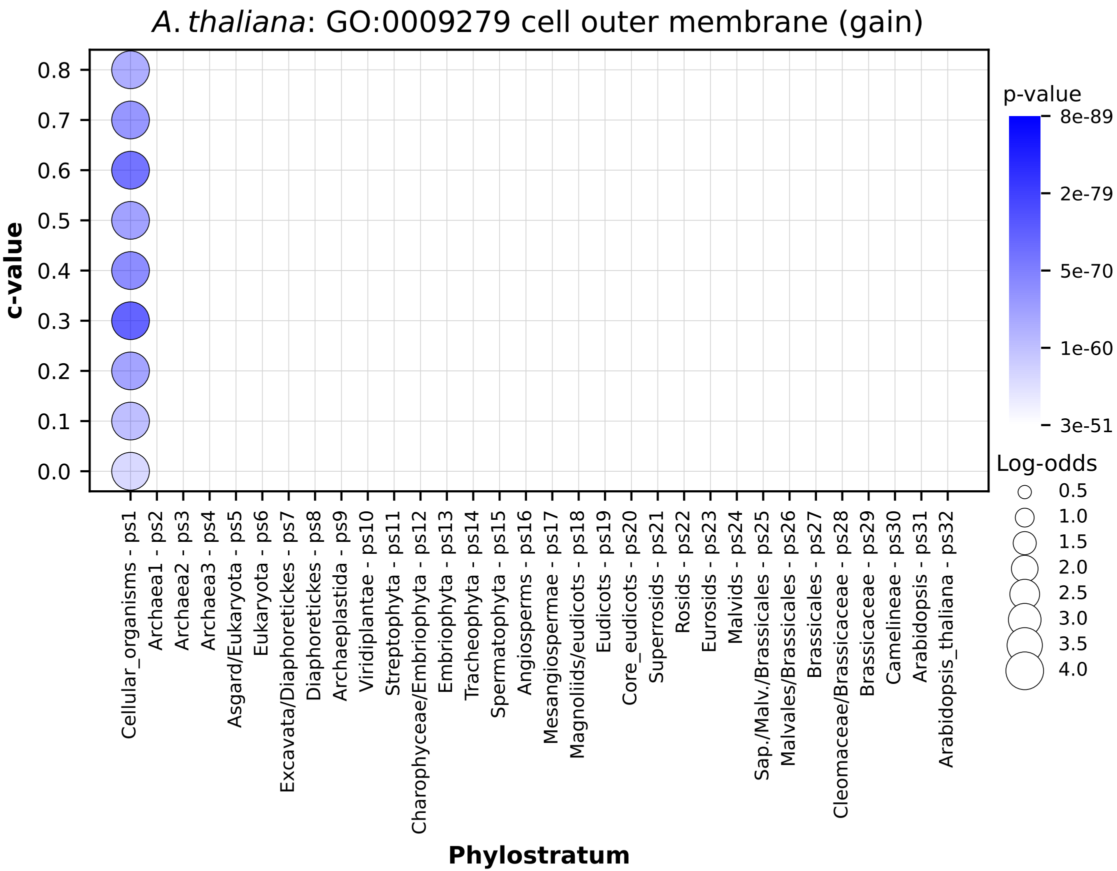

A. thaliana: GO:0009279 cell outer membrane (gain)
c-value
0.8
0.7
0.6
0.5
0.4
0.3
0.2
0.1
0.0
p-value
8e-89
2e-79
5e-70
1e-60
3e-51
Log-odds
0.5
1.0
1.5
2.0
2.5
3.0
3.5
4.0
Cellular_organisms - ps1
Archaea1 - ps2
Archaea2 - ps3
Archaea3 - ps4
Asgard/Eukaryota - ps5
Eukaryota - ps6
Excavata/Diaphoretickes - ps7
Diaphoretickes - ps8
Archaeplastida - ps9
Viridiplantae - ps10
Streptophyta - ps11
Charophyceae/Embriophyta - ps12
Embriophyta - ps13
Tracheophyta - ps14
Spermatophyta - ps15
Angiosperms - ps16
Mesangiospermae - ps17
Magnoliids/eudicots - ps18
Eudicots - ps19
Core_eudicots - ps20
Superrosids - ps21
Rosids - ps22
Eurosids - ps23
Malvids - ps24
Sap./Malv./Brassicales - ps25
Malvales/Brassicales - ps26
Brassicales - ps27
Cleomaceae/Brassicaceae - ps28
Brassicaceae - ps29
Camelineae - ps30
Arabidopsis - ps31
Arabidopsis_thaliana - ps32
Phylostratum

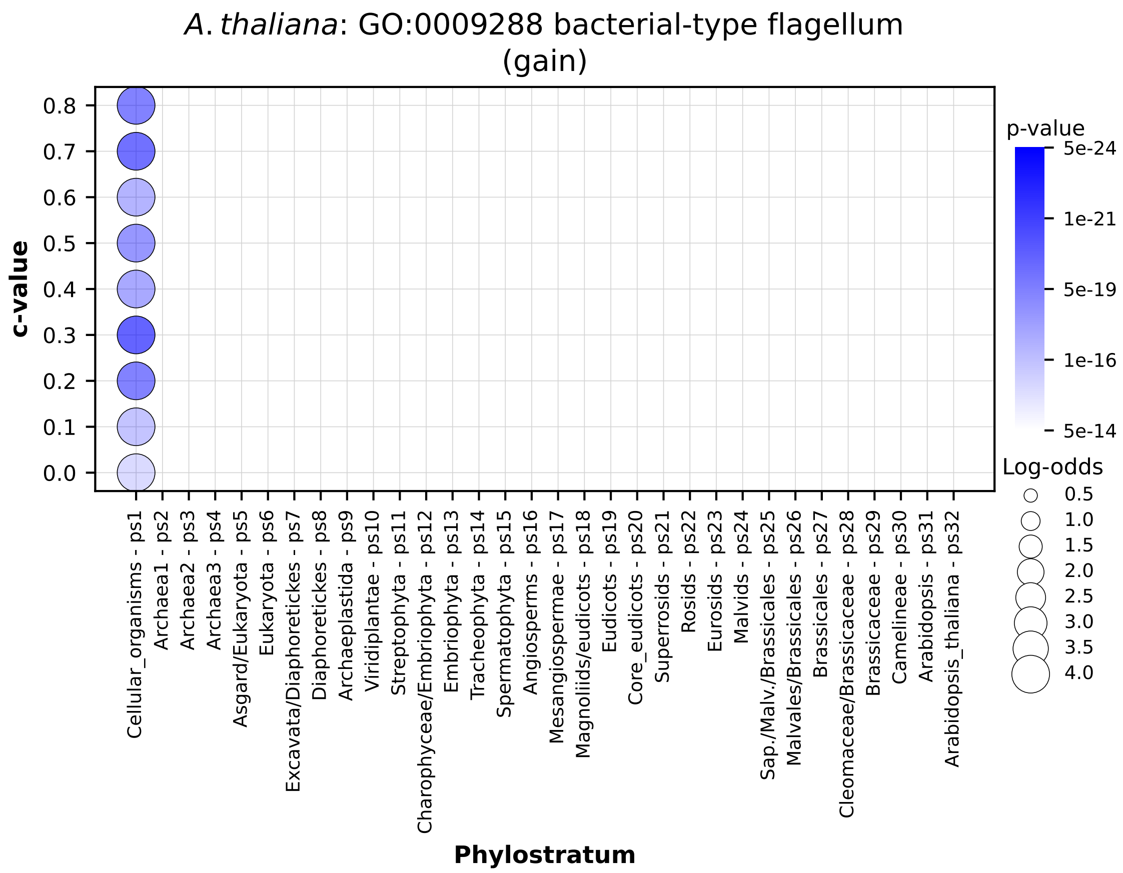

A. thaliana: GO:0009288 bacterial-type flagellum
(gain)
c-value
0.8
0.7
0.6
0.5
0.4
0.3
0.2
0.1
0.0
p-value
5e-24
1e-21
5e-19
1e-16
5e-14
Log-odds
0.5
1.0
1.5
2.0
2.5
3.0
3.5
4.0
Cellular_organisms - ps1
Archaea1 - ps2
Archaea2 - ps3
Archaea3 - ps4
Asgard/Eukaryota - ps5
Eukaryota - ps6
Excavata/Diaphoretickes - ps7
Diaphoretickes - ps8
Archaeplastida - ps9
Viridiplantae - ps10
Streptophyta - ps11
Charophyceae/Embriophyta - ps12
Embriophyta - ps13
Tracheophyta - ps14
Spermatophyta - ps15
Angiosperms - ps16
Mesangiospermae - ps17
Magnoliids/eudicots - ps18
Eudicots - ps19
Core_eudicots - ps20
Superrosids - ps21
Rosids - ps22
Eurosids - ps23
Malvids - ps24
Sap./Malv./Brassicales - ps25
Malvales/Brassicales - ps26
Brassicales - ps27
Cleomaceae/Brassicaceae - ps28
Brassicaceae - ps29
Camelineae - ps30
Arabidopsis - ps31
Arabidopsis_thaliana - ps32
Phylostratum

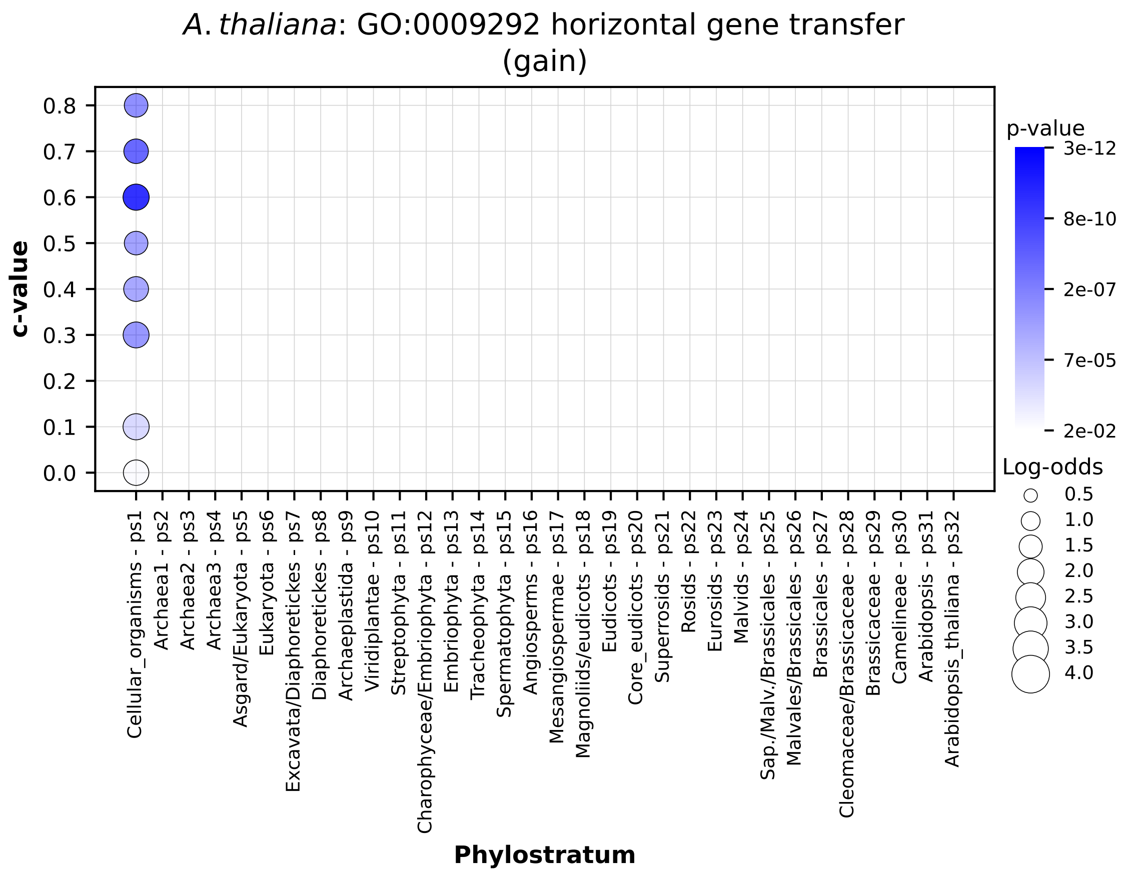
A. thaliana: GO:0009292 horizontal gene transfer
(gain)
c-value
0.8
0.7
0.6
0.5
0.4
0.3
0.2
0.1
0.0
p-value
3e-12
8e-10
2e-07
7e-05
2e-02
Log-odds
0.5
1.0
1.5
2.0
2.5
3.0
3.5
4.0
Cellular_organisms - ps1
Archaea1 - ps2
Archaea2 - ps3
Archaea3 - ps4
Asgard/Eukaryota - ps5
Eukaryota - ps6
Excavata/Diaphoretickes - ps7
Diaphoretickes - ps8
Archaeplastida - ps9
Viridiplantae - ps10
Streptophyta - ps11
Charophyceae/Embriophyta - ps12
Embriophyta - ps13
Tracheophyta - ps14
Spermatophyta - ps15
Angiosperms - ps16
Mesangiospermae - ps17
Magnoliids/eudicots - ps18
Eudicots - ps19
Core_eudicots - ps20
Superrosids - ps21
Rosids - ps22
Eurosids - ps23
Malvids - ps24
Sap./Malv./Brassicales - ps25
Malvales/Brassicales - ps26
Brassicales - ps27
Cleomaceae/Brassicaceae - ps28
Brassicaceae - ps29
Camelineae - ps30
Arabidopsis - ps31
Arabidopsis_thaliana - ps32
Phylostratum

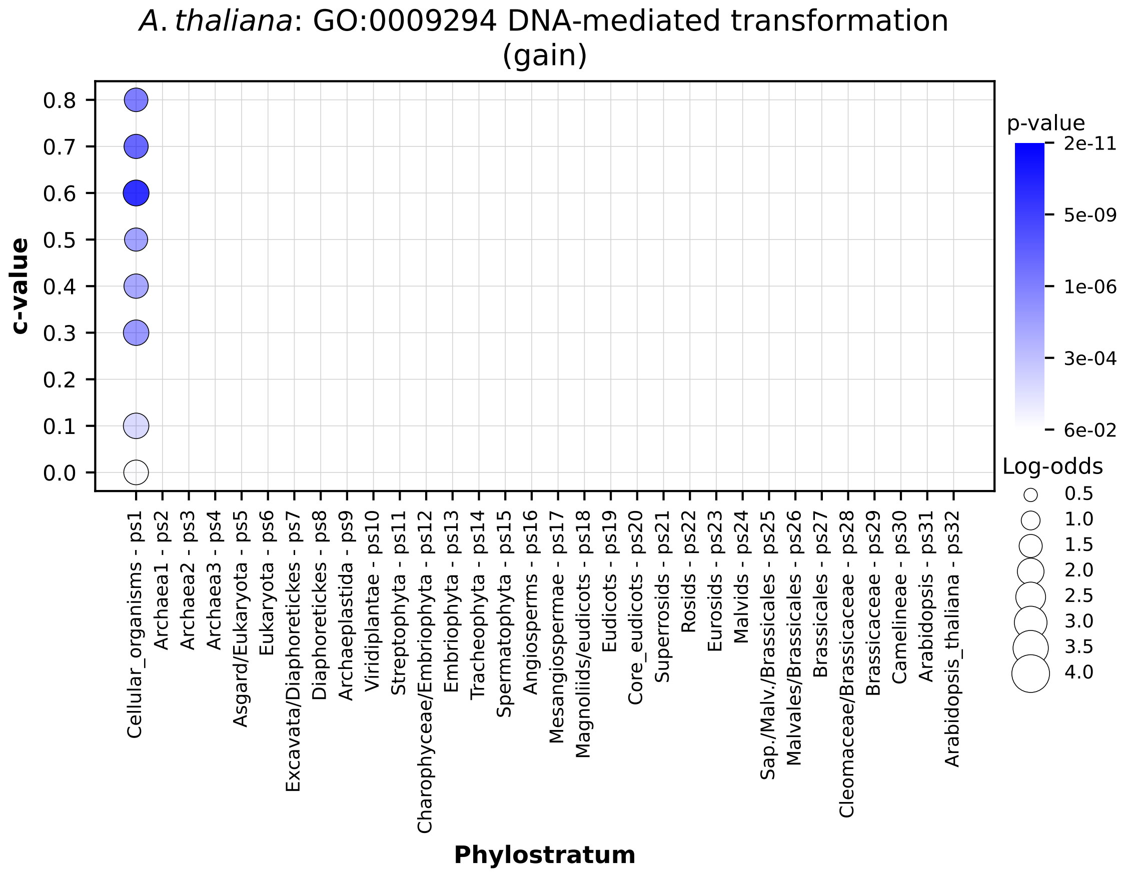

A. thaliana: GO:0009294 DNA-mediated transformation (gain)
c-value
0.0
0.1
0.2
0.3
0.4
0.5
0.6
0.7
0.8
Phylostratum
Cellular_organisms - ps1
Archaea1 - ps2
Archaea2 - ps3
Archaea3 - ps4
Asgard/Eukaryota - ps5
Eukaryota - ps6
Excavata/Diaphoretickes - ps7
Diaphoretickes - ps8
Archaeplastida - ps9
Viridiplantae - ps10
Streptophyta - ps11
Charophyceae/Embriophyta - ps12
Embriophyta - ps13
Tracheophyta - ps14
Spermatophyta - ps15
Angiosperms - ps16
Mesangiospermae - ps17
Magnoliids/eudicots - ps18
Eudicots - ps19
Core_eudicots - ps20
Superrosids - ps21
Rosids - ps22
Eurosids - ps23
Malvids - ps24
Sap./Malv./Brassicales - ps25
Malvales/Brassicales - ps26
Brassicales - ps27
Cleomaceae/Brassicaceae - ps28
Brassicaceae - ps29
Camelineae - ps30
Arabidopsis - ps31
Arabidopsis_thaliana - ps32
p-value
2e-11
5e-09
1e-06
3e-04
6e-02
Log-odds
0.5
1.0
1.5
2.0
2.5
3.0
3.5
4.0

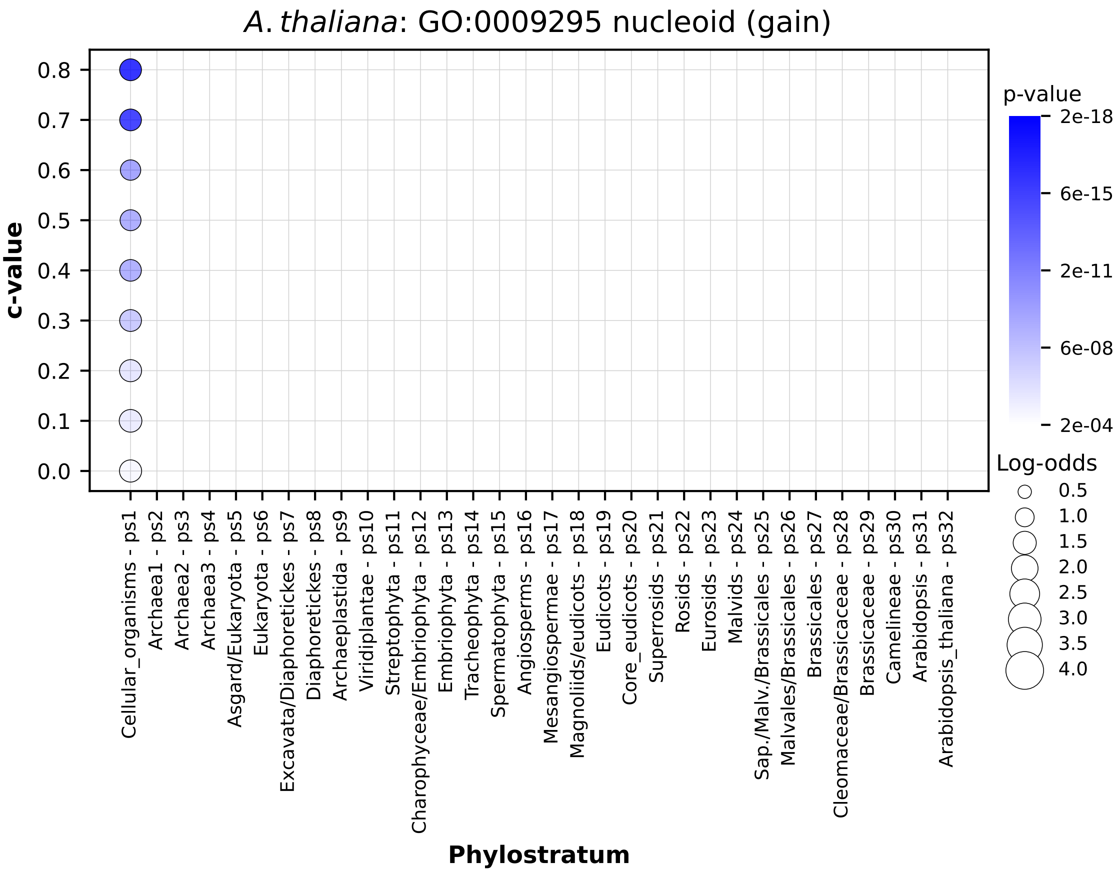

A. thaliana: GO:0009295 nucleoid (gain)
c-value
0.8
0.7
0.6
0.5
0.4
0.3
0.2
0.1
0.0
p-value
2e-18
6e-15
2e-11
6e-08
2e-04
Log-odds
0.5
1.0
1.5
2.0
2.5
3.0
3.5
4.0
Cellular_organisms - ps1
Archaea1 - ps2
Archaea2 - ps3
Archaea3 - ps4
Asgard/Eukaryota - ps5
Eukaryota - ps6
Excavata/Diaphoretickes - ps7
Diaphoretickes - ps8
Archaeplastida - ps9
Viridiplantae - ps10
Streptophyta - ps11
Charophyceae/Embriophyta - ps12
Embriophyta - ps13
Tracheophyta - ps14
Spermatophyta - ps15
Angiosperms - ps16
Mesangiospermae - ps17
Magnoliids/eudicots - ps18
Eudicots - ps19
Core_eudicots - ps20
Superrosids - ps21
Rosids - ps22
Eurosids - ps23
Malvids - ps24
Sap./Malv./Brassicales - ps25
Malvales/Brassicales - ps26
Brassicales - ps27
Cleomaceae/Brassicaceae - ps28
Brassicaceae - ps29
Camelineae - ps30
Arabidopsis - ps31
Arabidopsis_thaliana - ps32
Phylostratum

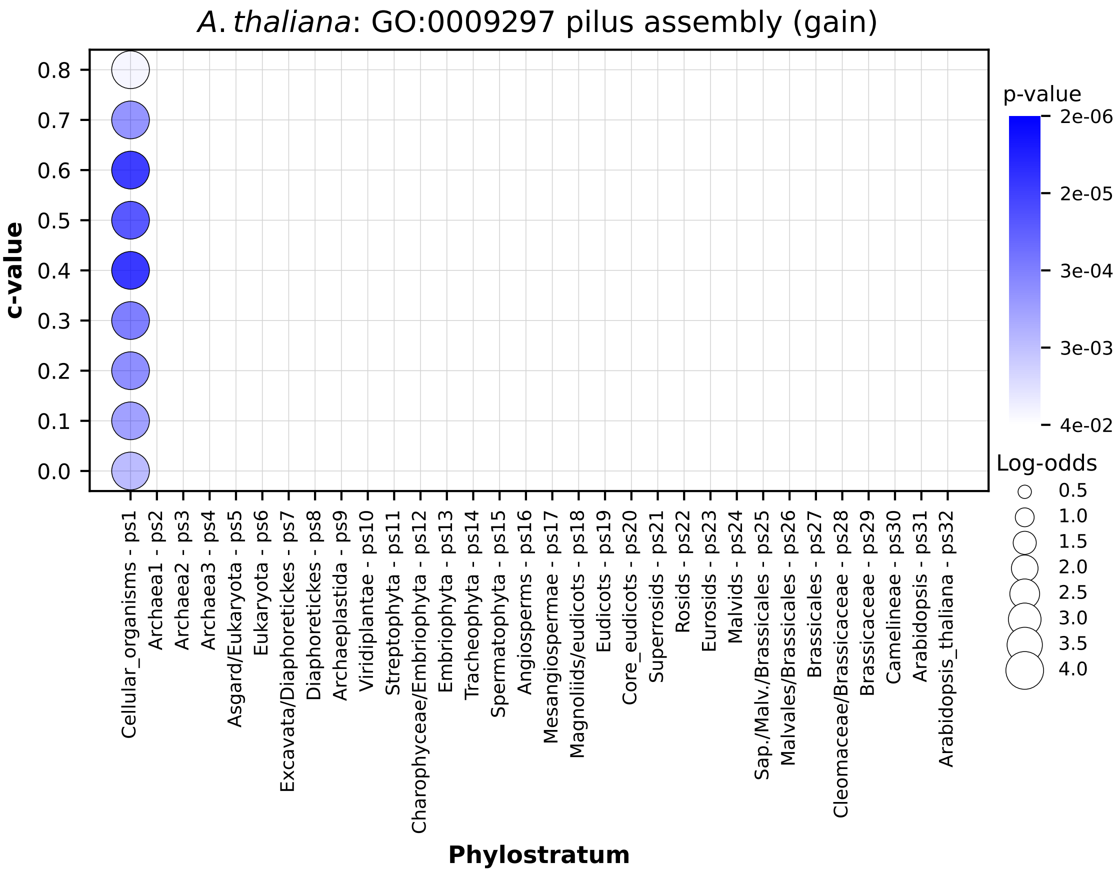
A. thaliana: GO:0009297 pilus assembly (gain)
c-value
0.8
0.7
0.6
0.5
0.4
0.3
0.2
0.1
0.0
Phylostratum
Cellular_organisms - ps1
Archaea1 - ps2
Archaea2 - ps3
Archaea3 - ps4
Asgard/Eukaryota - ps5
Eukaryota - ps6
Excavata/Diaphoretickes - ps7
Diaphoretickes - ps8
Archaeplastida - ps9
Viridiplantae - ps10
Streptophyta - ps11
Charophyceae/Embriophyta - ps12
Embriophyta - ps13
Tracheophyta - ps14
Spermatophyta - ps15
Angiosperms - ps16
Mesangiospermae - ps17
Magnoliids/eudicots - ps18
Eudicots - ps19
Core_eudicots - ps20
Superrosids - ps21
Rosids - ps22
Eurosids - ps23
Malvids - ps24
Sap./Malv./Brassicales - ps25
Malvales/Brassicales - ps26
Brassicales - ps27
Cleomaceae/Brassicaceae - ps28
Brassicaceae - ps29
Camelineae - ps30
Arabidopsis - ps31
Arabidopsis_thaliana - ps32
p-value
2e-06
2e-05
3e-04
3e-03
4e-02
Log-odds
0.5
1.0
1.5
2.0
2.5
3.0
3.5
4.0

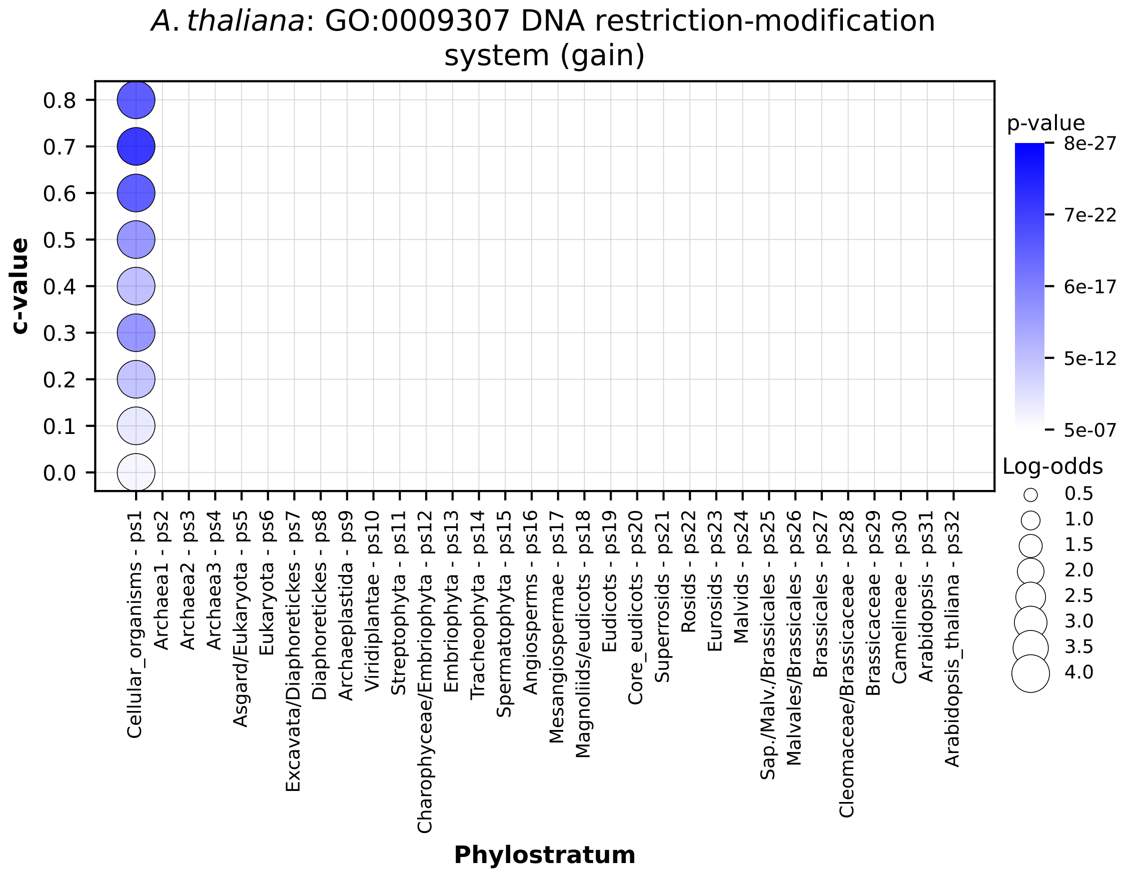
A. thaliana: GO:0009307 DNA restriction-modification system (gain)
c-value
0.8
0.7
0.6
0.5
0.4
0.3
0.2
0.1
0.0
p-value
8e-27
7e-22
6e-17
5e-12
5e-07
Log-odds
0.5
1.0
1.5
2.0
2.5
3.0
3.5
4.0
Cellular_organisms - ps1
Archaea1 - ps2
Archaea2 - ps3
Archaea3 - ps4
Asgard/Eukaryota - ps5
Eukaryota - ps6
Excavata/Diaphoretickes - ps7
Diaphoretickes - ps8
Archaeplastida - ps9
Viridiplantae - ps10
Streptophyta - ps11
Charophyceae/Embriophyta - ps12
Embriophyta - ps13
Tracheophyta - ps14
Spermatophyta - ps15
Angiosperms - ps16
Mesangiospermae - ps17
Magnoliids/eudicots - ps18
Eudicots - ps19
Core_eudicots - ps20
Superrosids - ps21
Rosids - ps22
Eurosids - ps23
Malvids - ps24
Sap./Malv./Brassicales - ps25
Malvales/Brassicales - ps26
Brassicales - ps27
Cleomaceae/Brassicaceae - ps28
Brassicaceae - ps29
Camelineae - ps30
Arabidopsis - ps31
Arabidopsis_thaliana - ps32
Phylostratum

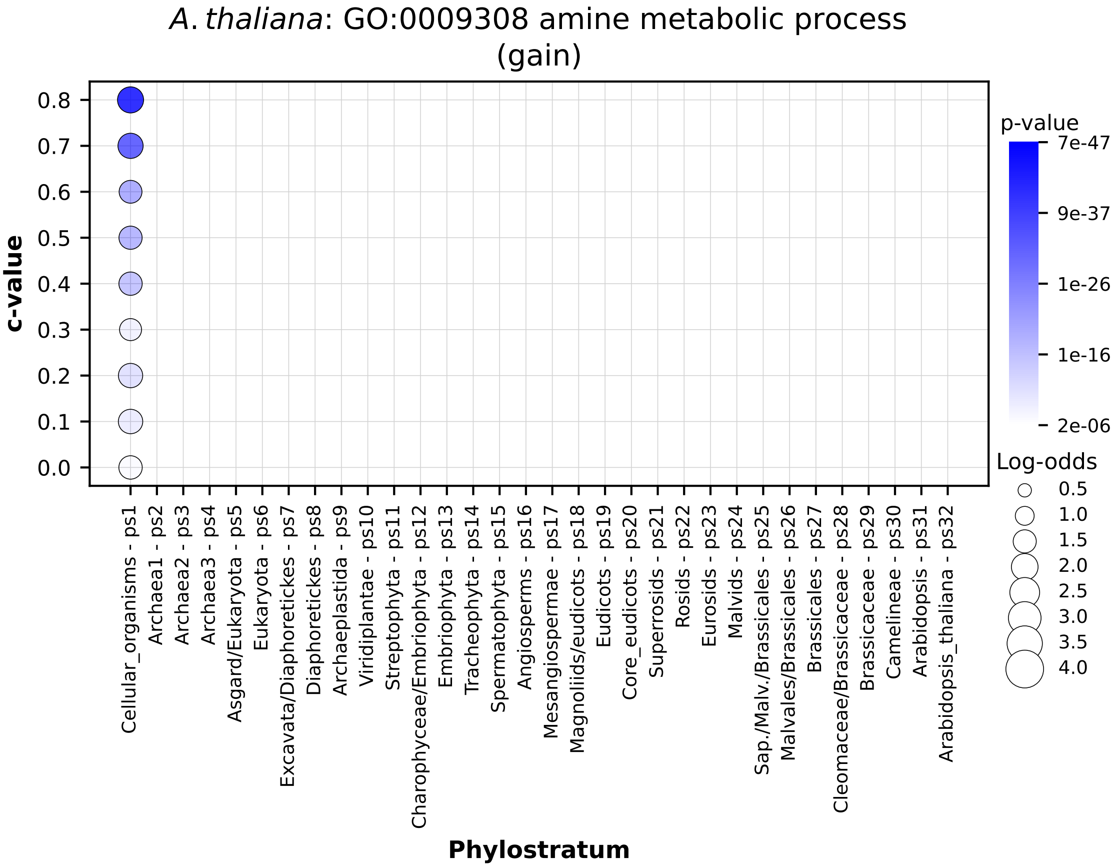

A. thaliana: GO:0009308 amine metabolic process
(gain)
c-value
0.8
0.7
0.6
0.5
0.4
0.3
0.2
0.1
0.0
p-value
7e-47
9e-37
1e-26
1e-16
2e-06
Log-odds
0.5
1.0
1.5
2.0
2.5
3.0
3.5
4.0
Cellular_organisms - ps1
Archaea1 - ps2
Archaea2 - ps3
Archaea3 - ps4
Asgard/Eukaryota - ps5
Eukaryota - ps6
Excavata/Diaphoretickes - ps7
Diaphoretickes - ps8
Archaeplastida - ps9
Viridiplantae - ps10
Streptophyta - ps11
Charophyceae/Embriophyta - ps12
Embriophyta - ps13
Tracheophyta - ps14
Spermatophyta - ps15
Angiosperms - ps16
Mesangiospermae - ps17
Magnoliids/eudicots - ps18
Eudicots - ps19
Core_eudicots - ps20
Superrosids - ps21
Rosids - ps22
Eurosids - ps23
Malvids - ps24
Sap./Malv./Brassicales - ps25
Malvales/Brassicales - ps26
Brassicales - ps27
Cleomaceae/Brassicaceae - ps28
Brassicaceae - ps29
Camelineae - ps30
Arabidopsis - ps31
Arabidopsis_thaliana - ps32
Phylostratum

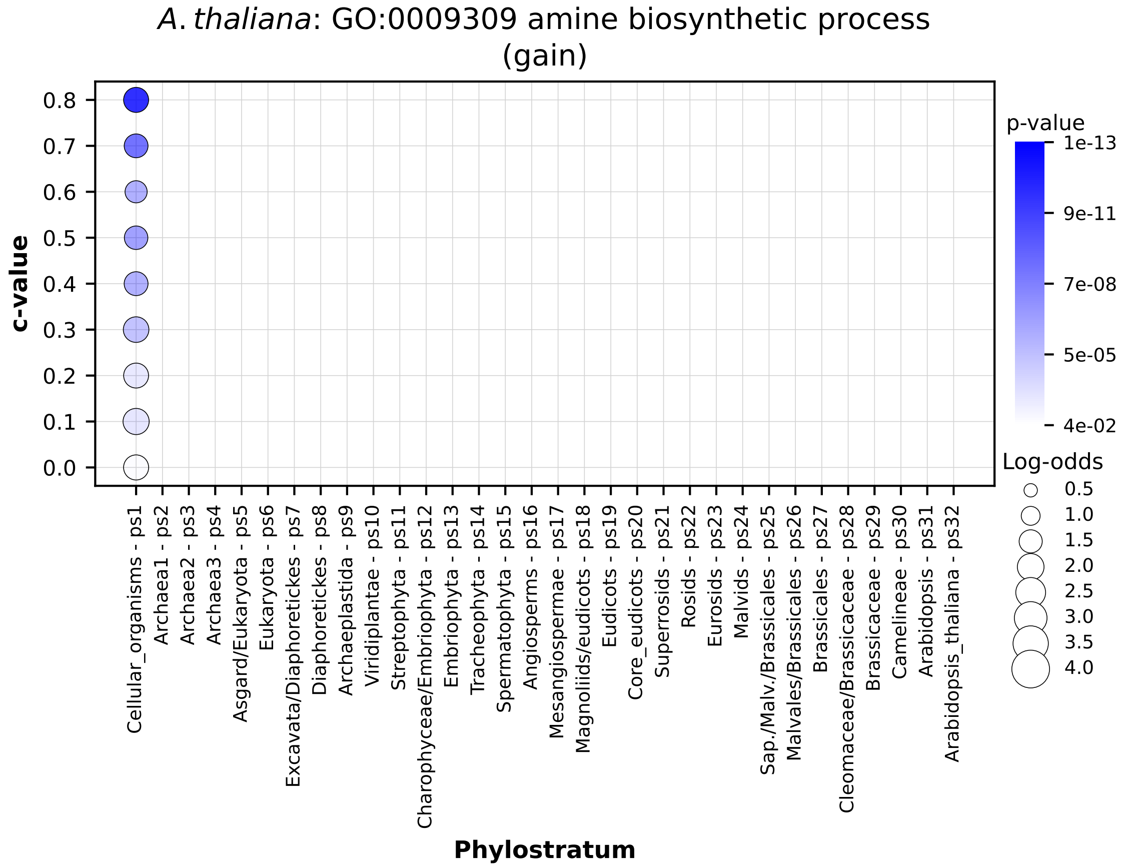

A. thaliana: GO:0009309 amine biosynthetic process (gain)
c-value
0.8
0.7
0.6
0.5
0.4
0.3
0.2
0.1
0.0
p-value
1e-13
9e-11
7e-08
5e-05
4e-02
Log-odds
0.5
1.0
1.5
2.0
2.5
3.0
3.5
4.0
Cellular_organisms - ps1
Archaea1 - ps2
Archaea2 - ps3
Archaea3 - ps4
Asgard/Eukaryota - ps5
Eukaryota - ps6
Excavata/Diaphoretickes - ps7
Diaphoretickes - ps8
Archaeplastida - ps9
Viridiplantae - ps10
Streptophyta - ps11
Charophyceae/Embriophyta - ps12
Embriophyta - ps13
Tracheophyta - ps14
Spermatophyta - ps15
Angiosperms - ps16
Mesangiospermae - ps17
Magnoliids/eudicots - ps18
Eudicots - ps19
Core_eudicots - ps20
Superrosids - ps21
Rosids - ps22
Eurosids - ps23
Malvids - ps24
Sap./Malv./Brassicales - ps25
Malvales/Brassicales - ps26
Brassicales - ps27
Cleomaceae/Brassicaceae - ps28
Brassicaceae - ps29
Camelineae - ps30
Arabidopsis - ps31
Arabidopsis_thaliana - ps32
Phylostratum

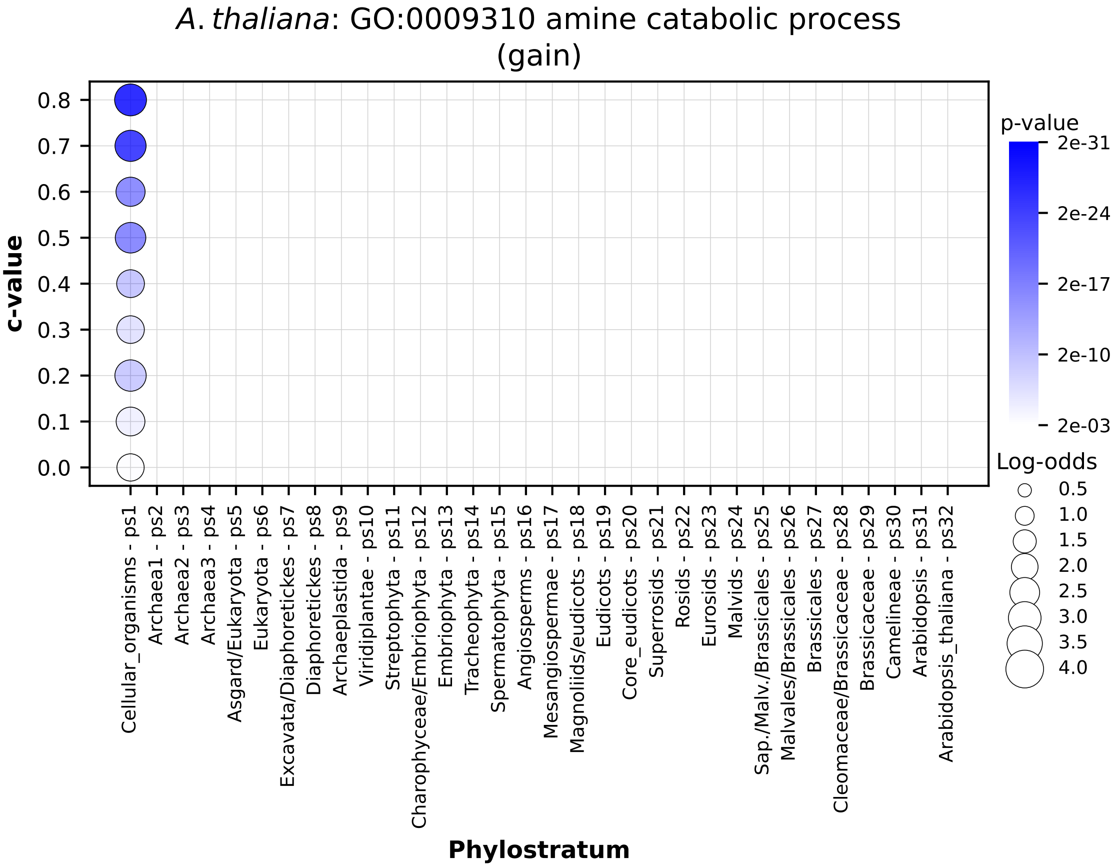

A. thaliana: GO:0009310 amine catabolic process
(gain)
c-value
0.0
0.1
0.2
0.3
0.4
0.5
0.6
0.7
0.8
Phylostratum
Cellular_organisms - ps1
Archaea1 - ps2
Archaea2 - ps3
Archaea3 - ps4
Asgard/Eukaryota - ps5
Eukaryota - ps6
Excavata/Diaphoretickes - ps7
Diaphoretickes - ps8
Archaeplastida - ps9
Viridiplantae - ps10
Streptophyta - ps11
Charophyceae/Embriophyta - ps12
Embriophyta - ps13
Tracheophyta - ps14
Spermatophyta - ps15
Angiosperms - ps16
Mesangiospermae - ps17
Magnoliids/eudicots - ps18
Eudicots - ps19
Core_eudicots - ps20
Superrosids - ps21
Rosids - ps22
Eurosids - ps23
Malvids - ps24
Sap./Malv./Brassicales - ps25
Malvales/Brassicales - ps26
Brassicales - ps27
Cleomaceae/Brassicaceae - ps28
Brassicaceae - ps29
Camelineae - ps30
Arabidopsis - ps31
Arabidopsis_thaliana - ps32
p-value
2e-31
2e-24
2e-17
2e-10
2e-03
Log-odds
0.5
1.0
1.5
2.0
2.5
3.0
3.5
4.0

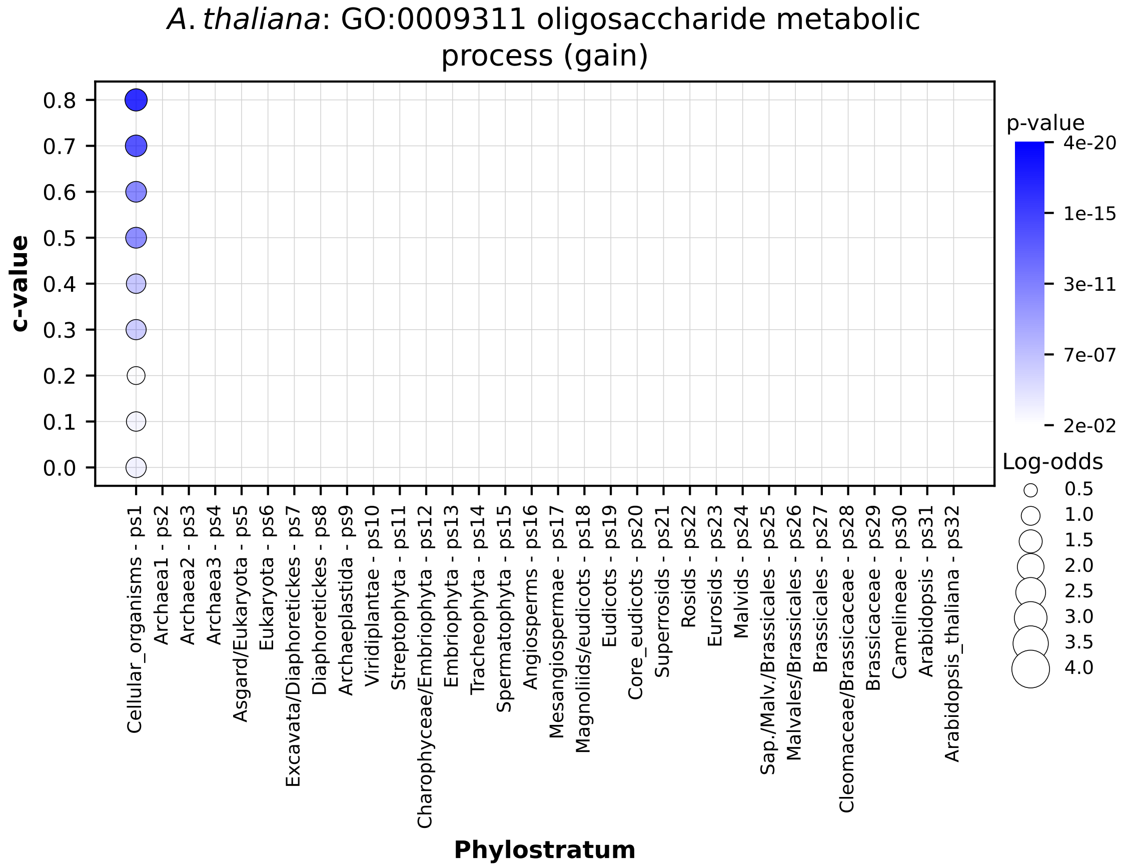
A. thaliana: GO:0009311 oligosaccharide metabolic process (gain)
c-value
0.8
0.7
0.6
0.5
0.4
0.3
0.2
0.1
0.0
p-value
4e-20
1e-15
3e-11
7e-07
2e-02
Log-odds
0.5
1.0
1.5
2.0
2.5
3.0
3.5
4.0
Cellular_organisms - ps1
Archaea1 - ps2
Archaea2 - ps3
Archaea3 - ps4
Asgard/Eukaryota - ps5
Eukaryota - ps6
Excavata/Diaphoretickes - ps7
Diaphoretickes - ps8
Archaeplastida - ps9
Viridiplantae - ps10
Streptophyta - ps11
Charophyceae/Embriophyta - ps12
Embriophyta - ps13
Tracheophyta - ps14
Spermatophyta - ps15
Angiosperms - ps16
Mesangiospermae - ps17
Magnoliids/eudicots - ps18
Eudicots - ps19
Core_eudicots - ps20
Superrosids - ps21
Rosids - ps22
Eurosids - ps23
Malvids - ps24
Sap./Malv./Brassicales - ps25
Malvales/Brassicales - ps26
Brassicales - ps27
Cleomaceae/Brassicaceae - ps28
Brassicaceae - ps29
Camelineae - ps30
Arabidopsis - ps31
Arabidopsis_thaliana - ps32
Phylostratum

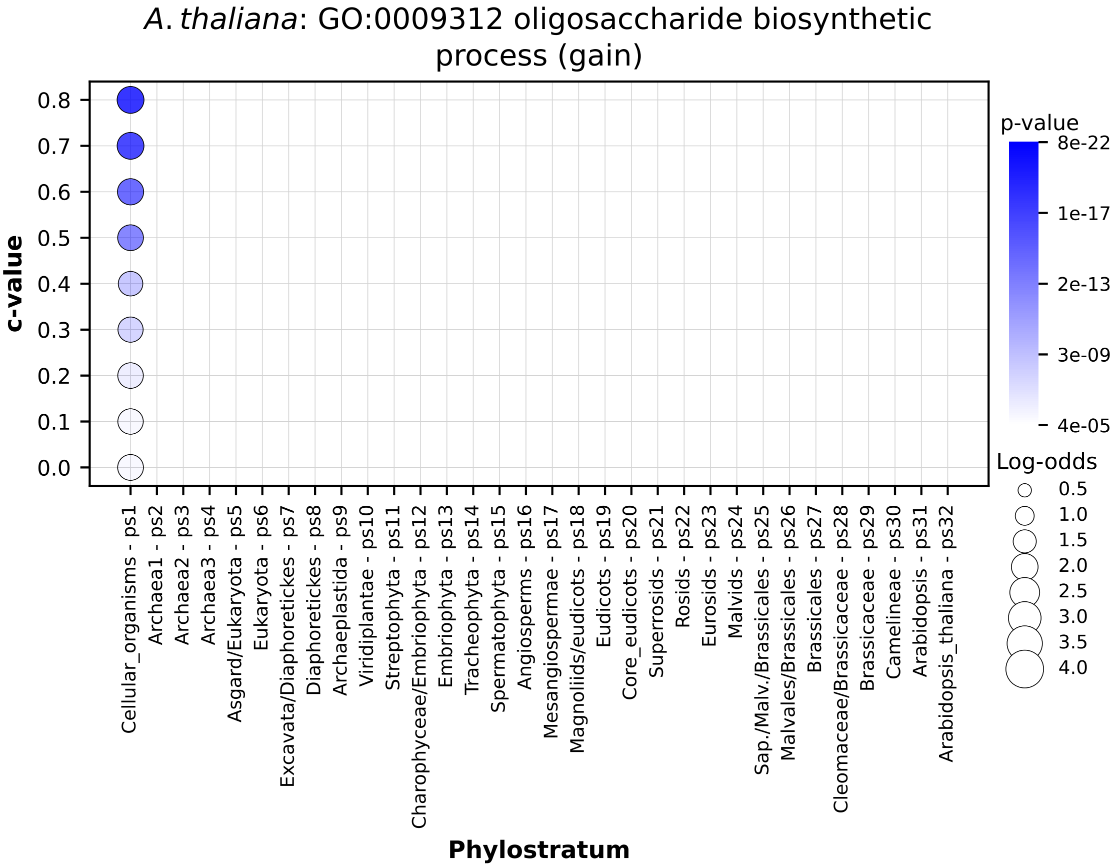

A. thaliana: GO:0009312 oligosaccharide biosynthetic process (gain)
c-value
0.8
0.7
0.6
0.5
0.4
0.3
0.2
0.1
0.0
p-value
8e-22
1e-17
2e-13
3e-09
4e-05
Log-odds
0.5
1.0
1.5
2.0
2.5
3.0
3.5
4.0
Cellular_organisms - ps1
Archaea1 - ps2
Archaea2 - ps3
Archaea3 - ps4
Asgard/Eukaryota - ps5
Eukaryota - ps6
Excavata/Diaphoretickes - ps7
Diaphoretickes - ps8
Archaeplastida - ps9
Viridiplantae - ps10
Streptophyta - ps11
Charophyceae/Embriophyta - ps12
Embriophyta - ps13
Tracheophyta - ps14
Spermatophyta - ps15
Angiosperms - ps16
Mesangiospermae - ps17
Magnoliids/eudicots - ps18
Eudicots - ps19
Core_eudicots - ps20
Superrosids - ps21
Rosids - ps22
Eurosids - ps23
Malvids - ps24
Sap./Malv./Brassicales - ps25
Malvales/Brassicales - ps26
Brassicales - ps27
Cleomaceae/Brassicaceae - ps28
Brassicaceae - ps29
Camelineae - ps30
Arabidopsis - ps31
Arabidopsis_thaliana - ps32
Phylostratum

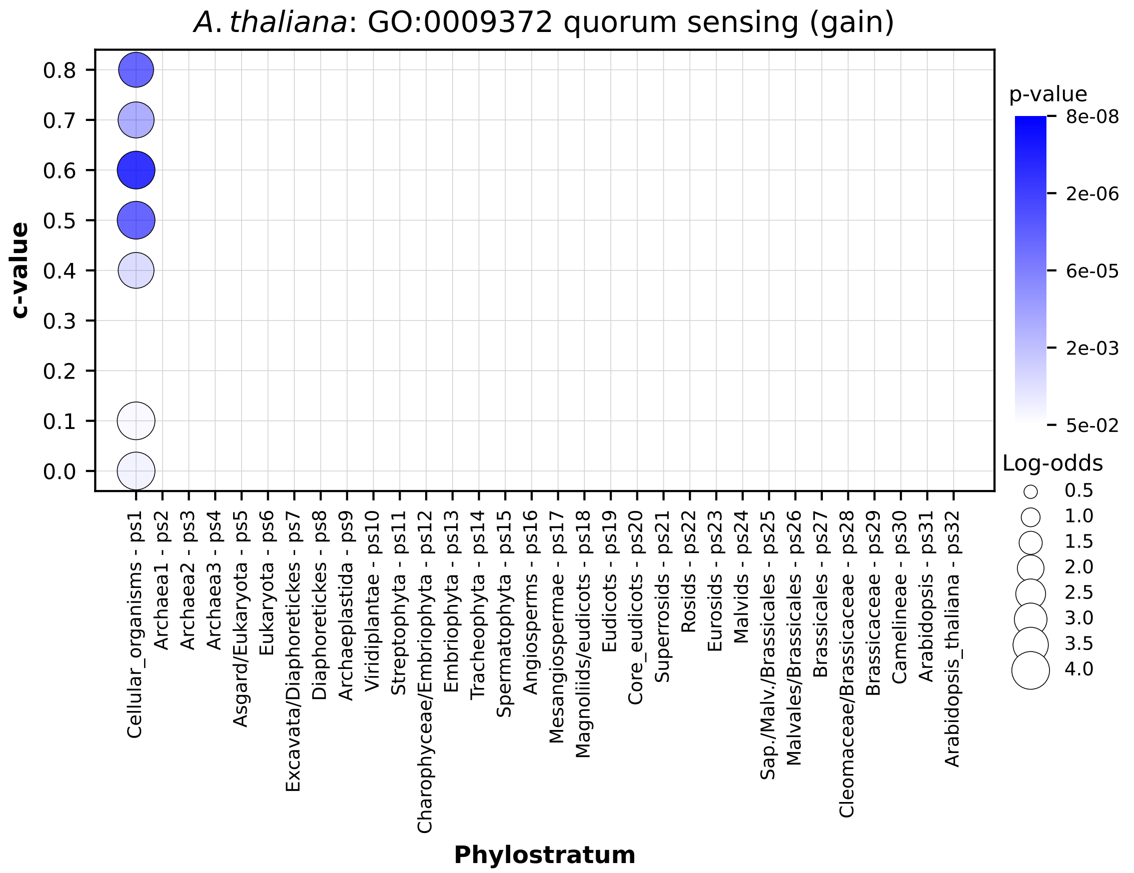

A. thaliana: GO:0009372 quorum sensing (gain)
c-value
0.8
0.7
0.6
0.5
0.4
0.3
0.2
0.1
0.0
p-value
8e-08
2e-06
6e-05
2e-03
5e-02
Log-odds
0.5
1.0
1.5
2.0
2.5
3.0
3.5
4.0
Cellular_organisms - ps1
Archaea1 - ps2
Archaea2 - ps3
Archaea3 - ps4
Asgard/Eukaryota - ps5
Eukaryota - ps6
Excavata/Diaphoretickes - ps7
Diaphoretickes - ps8
Archaeplastida - ps9
Viridiplantae - ps10
Streptophyta - ps11
Charophyceae/Embriophyta - ps12
Embriophyta - ps13
Tracheophyta - ps14
Spermatophyta - ps15
Angiosperms - ps16
Mesangiospermae - ps17
Magnoliids/eudicots - ps18
Eudicots - ps19
Core_eudicots - ps20
Superrosids - ps21
Rosids - ps22
Eurosids - ps23
Malvids - ps24
Sap./Malv./Brassicales - ps25
Malvales/Brassicales - ps26
Brassicales - ps27
Cleomaceae/Brassicaceae - ps28
Brassicaceae - ps29
Camelineae - ps30
Arabidopsis - ps31
Arabidopsis_thaliana - ps32
Phylostratum

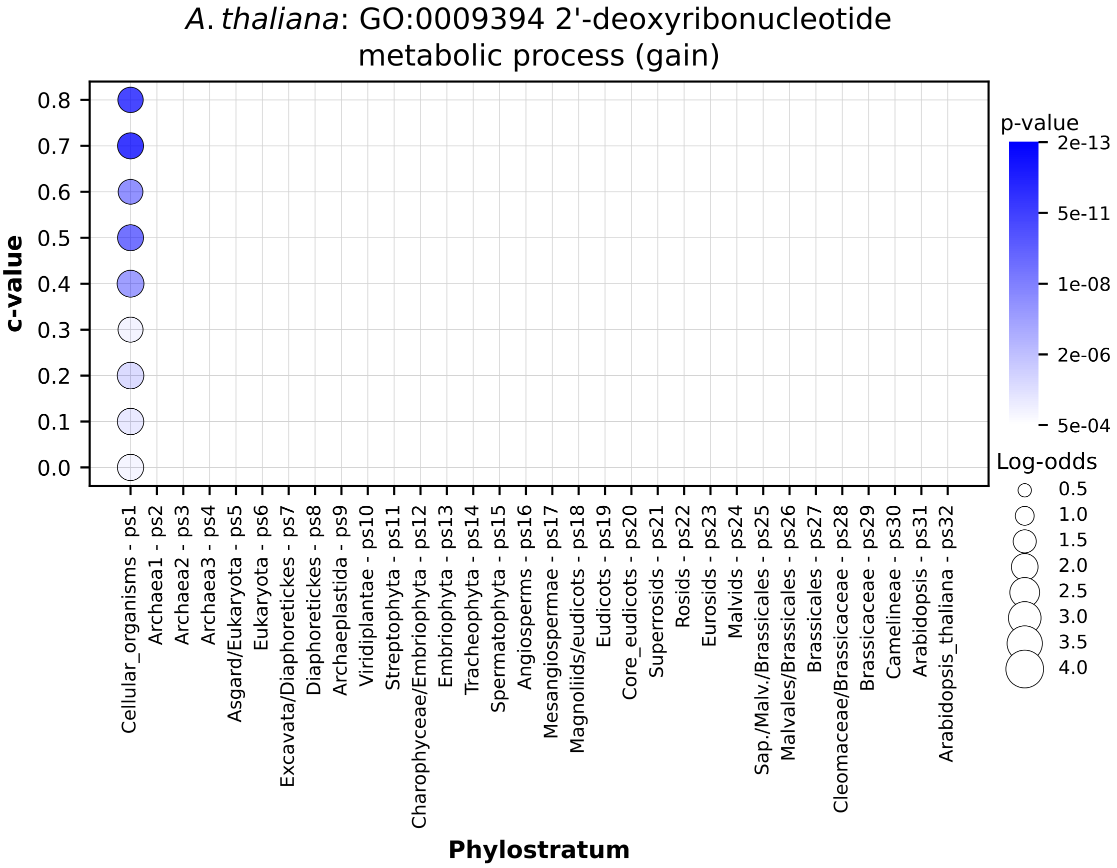

A. thaliana: GO:0009394 2'-deoxyribonucleotide metabolic process (gain)
c-value
0.0
0.1
0.2
0.3
0.4
0.5
0.6
0.7
0.8
Phylostratum
Cellular_organisms - ps1
Archaea1 - ps2
Archaea2 - ps3
Archaea3 - ps4
Asgard/Eukaryota - ps5
Eukaryota - ps6
Excavata/Diaphoretickes - ps7
Diaphoretickes - ps8
Archaeplastida - ps9
Viridiplantae - ps10
Streptophyta - ps11
Charophyceae/Embriophyta - ps12
Embriophyta - ps13
Tracheophyta - ps14
Spermatophyta - ps15
Angiosperms - ps16
Mesangiospermae - ps17
Magnoliids/eudicots - ps18
Eudicots - ps19
Core_eudicots - ps20
Superrosids - ps21
Rosids - ps22
Eurosids - ps23
Malvids - ps24
Sap./Malv./Brassicales - ps25
Malvales/Brassicales - ps26
Brassicales - ps27
Cleomaceae/Brassicaceae - ps28
Brassicaceae - ps29
Camelineae - ps30
Arabidopsis - ps31
Arabidopsis_thaliana - ps32
p-value
2e-13
5e-11
1e-08
2e-06
5e-04
Log-odds
0.5
1.0
1.5
2.0
2.5
3.0
3.5
4.0

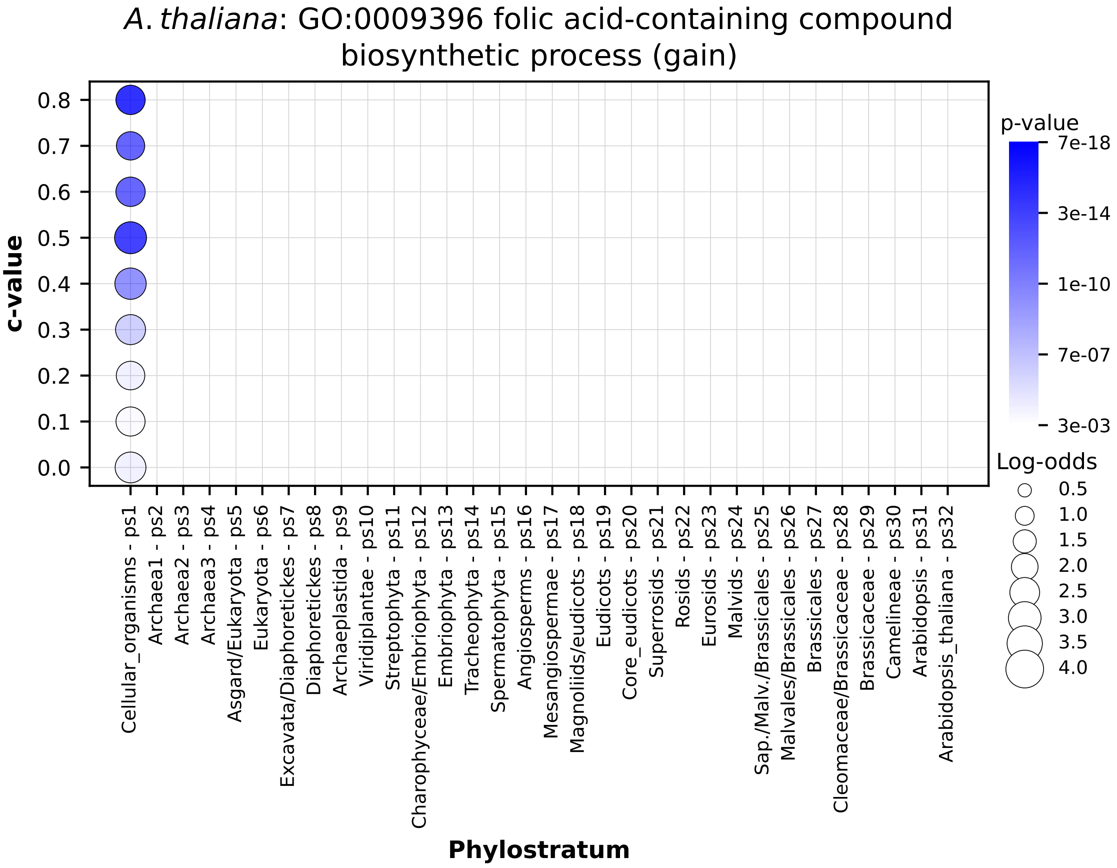

A. thaliana: GO:0009396 folic acid-containing compound biosynthetic process (gain)
c-value
0.8
0.7
0.6
0.5
0.4
0.3
0.2
0.1
0.0
p-value
7e-18
3e-14
1e-10
7e-07
3e-03
Log-odds
0.5
1.0
1.5
2.0
2.5
3.0
3.5
4.0
Cellular_organisms - ps1
Archaea1 - ps2
Archaea2 - ps3
Archaea3 - ps4
Asgard/Eukaryota - ps5
Eukaryota - ps6
Excavata/Diaphoretickes - ps7
Diaphoretickes - ps8
Archaeplastida - ps9
Viridiplantae - ps10
Streptophyta - ps11
Charophyceae/Embriophyta - ps12
Embriophyta - ps13
Tracheophyta - ps14
Spermatophyta - ps15
Angiosperms - ps16
Mesangiospermae - ps17
Magnoliids/eudicots - ps18
Eudicots - ps19
Core_eudicots - ps20
Superrosids - ps21
Rosids - ps22
Eurosids - ps23
Malvids - ps24
Sap./Malv./Brassicales - ps25
Malvales/Brassicales - ps26
Brassicales - ps27
Cleomaceae/Brassicaceae - ps28
Brassicaceae - ps29
Camelineae - ps30
Arabidopsis - ps31
Arabidopsis_thaliana - ps32
Phylostratum

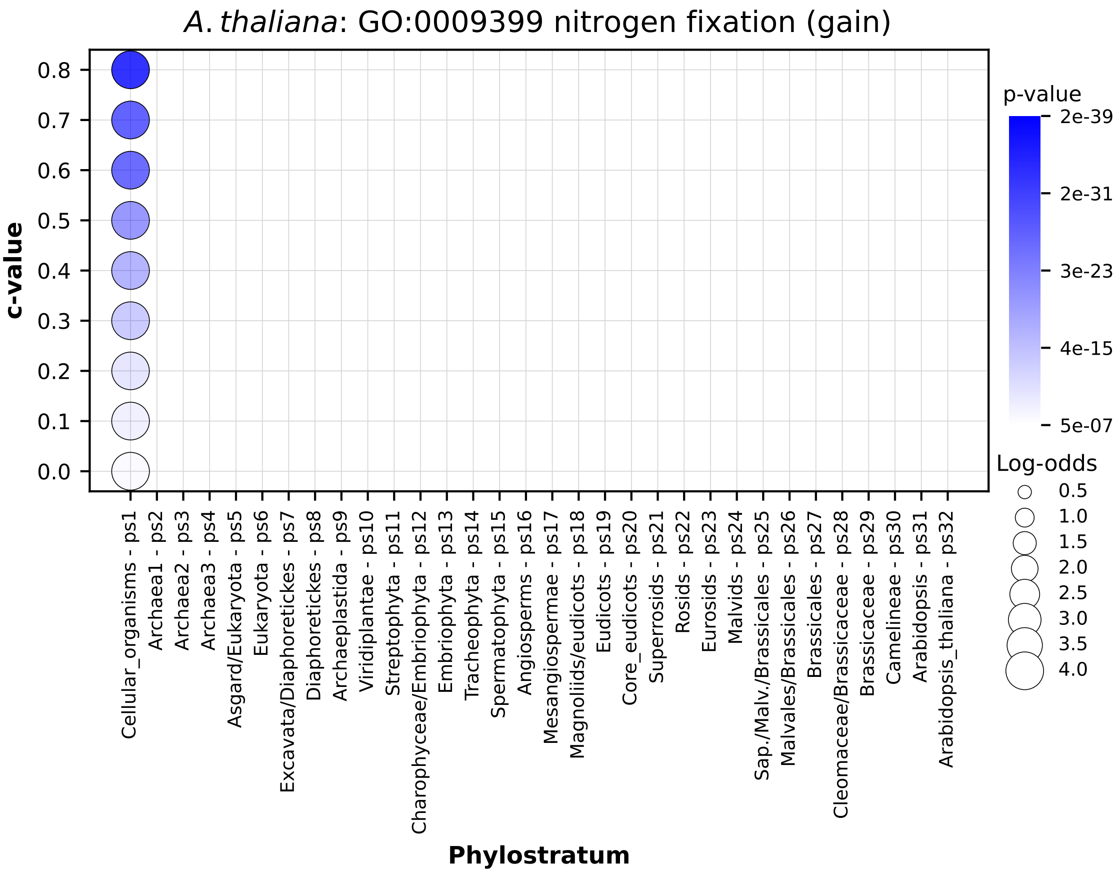

A. thaliana: GO:0009399 nitrogen fixation (gain)
c-value
0.0
0.1
0.2
0.3
0.4
0.5
0.6
0.7
0.8
p-value
2e-39
2e-31
3e-23
4e-15
5e-07
Log-odds
0.5
1.0
1.5
2.0
2.5
3.0
3.5
4.0
Cellular_organisms - ps1
Archaea1 - ps2
Archaea2 - ps3
Archaea3 - ps4
Asgard/Eukaryota - ps5
Eukaryota - ps6
Excavata/Diaphoretickes - ps7
Diaphoretickes - ps8
Archaeplastida - ps9
Viridiplantae - ps10
Streptophyta - ps11
Charophyceae/Embriophyta - ps12
Embriophyta - ps13
Tracheophyta - ps14
Spermatophyta - ps15
Angiosperms - ps16
Mesangiospermae - ps17
Magnoliids/eudicots - ps18
Eudicots - ps19
Core_eudicots - ps20
Superrosids - ps21
Rosids - ps22
Eurosids - ps23
Malvids - ps24
Sap./Malv./Brassicales - ps25
Malvales/Brassicales - ps26
Brassicales - ps27
Cleomaceae/Brassicaceae - ps28
Brassicaceae - ps29
Camelineae - ps30
Arabidopsis - ps31
Arabidopsis_thaliana - ps32
Phylostratum

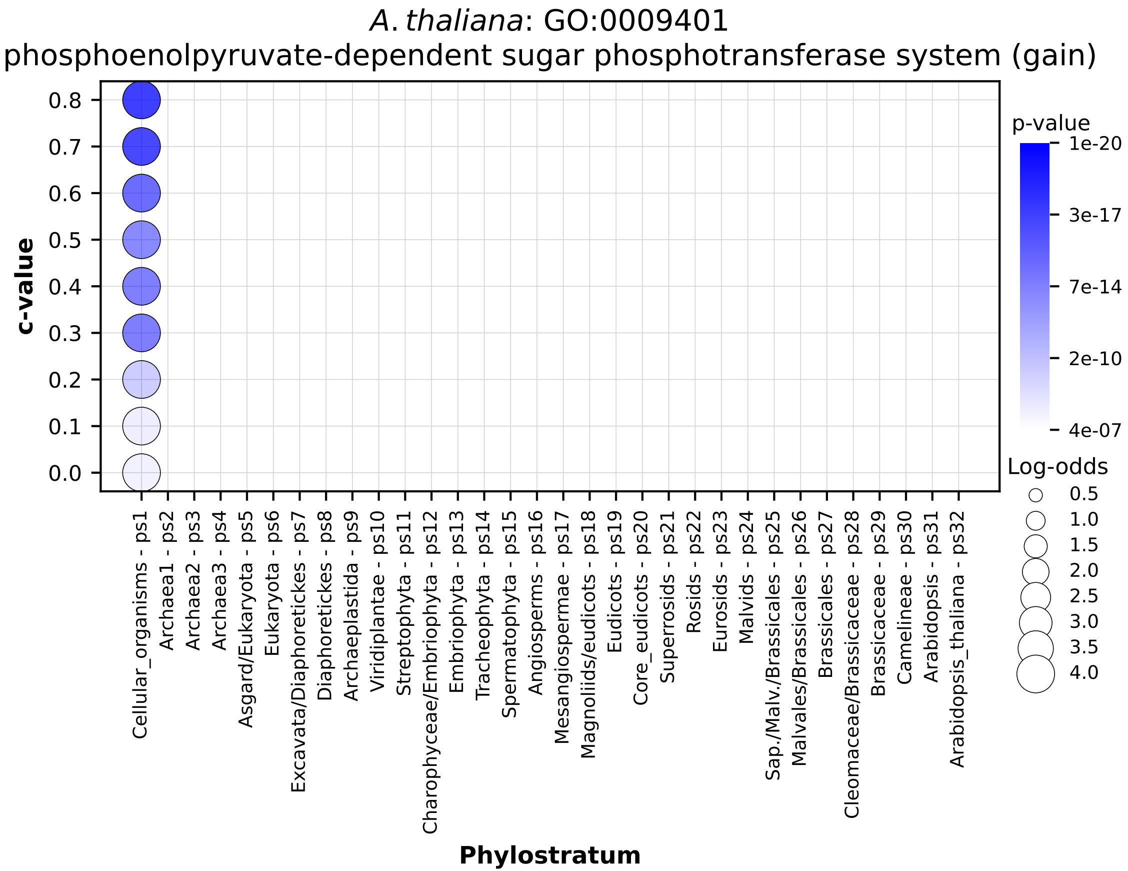
A. thaliana: GO:0009401
phosphoenolpyruvate-dependent sugar phosphotransferase system (gain)
c-value
0.8
0.7
0.6
0.5
0.4
0.3
0.2
0.1
0.0
p-value
1e-20
3e-17
7e-14
2e-10
4e-07
Log-odds
0.5
1.0
1.5
2.0
2.5
3.0
3.5
4.0
Cellular_organisms - ps1
Archaea1 - ps2
Archaea2 - ps3
Archaea3 - ps4
Asgard/Eukaryota - ps5
Eukaryota - ps6
Excavata/Diaphoretickes - ps7
Diaphoretickes - ps8
Archaeplastida - ps9
Viridiplantae - ps10
Streptophyta - ps11
Charophyceae/Embriophyta - ps12
Embriophyta - ps13
Tracheophyta - ps14
Spermatophyta - ps15
Angiosperms - ps16
Mesangiospermae - ps17
Magnoliids/eudicots - ps18
Eudicots - ps19
Core_eudicots - ps20
Superrosids - ps21
Rosids - ps22
Eurosids - ps23
Malvids - ps24
Sap./Malv./Brassicales - ps25
Malvales/Brassicales - ps26
Brassicales - ps27
Cleomaceae/Brassicaceae - ps28
Brassicaceae - ps29
Camelineae - ps30
Arabidopsis - ps31
Arabidopsis_thaliana - ps32
Phylostratum

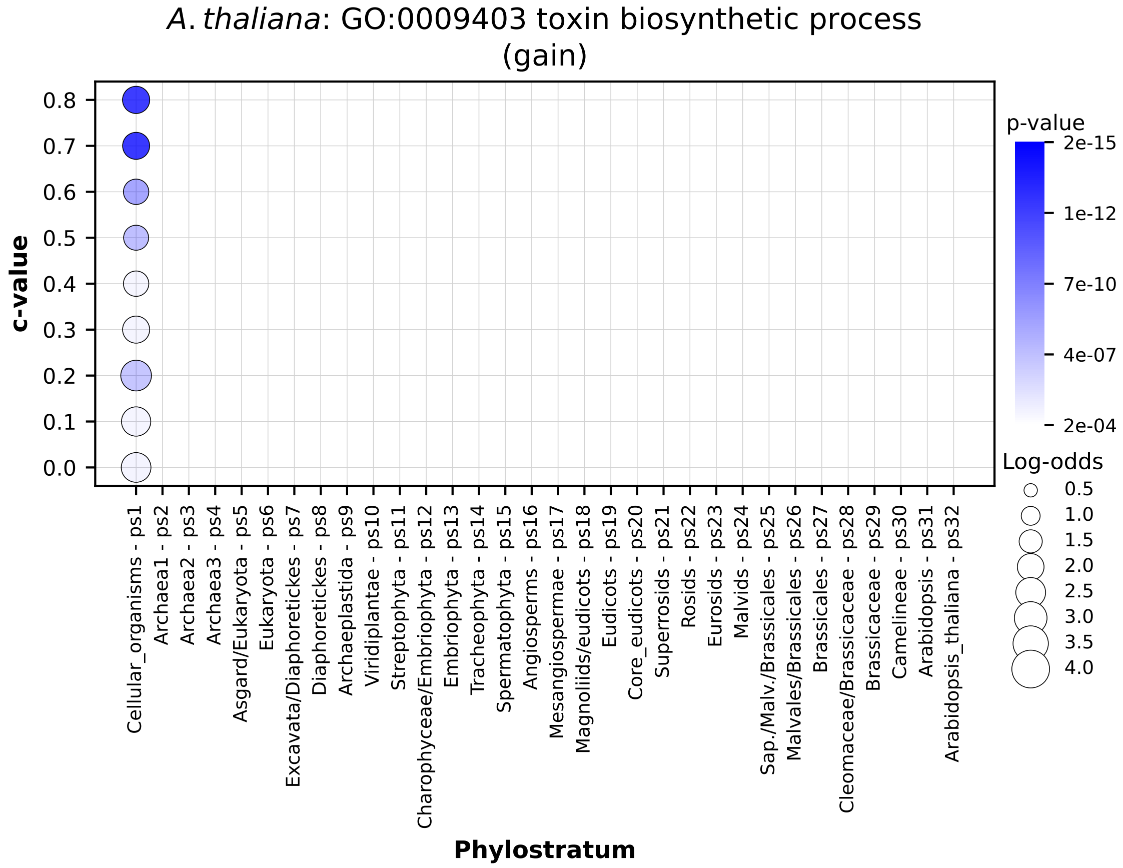

A. thaliana: GO:0009403 toxin biosynthetic process (gain)
c-value
0.0
0.1
0.2
0.3
0.4
0.5
0.6
0.7
0.8
p-value
2e-15
1e-12
7e-10
4e-07
2e-04
Log-odds
0.5
1.0
1.5
2.0
2.5
3.0
3.5
4.0
Cellular_organisms - ps1
Archaea1 - ps2
Archaea2 - ps3
Archaea3 - ps4
Asgard/Eukaryota - ps5
Eukaryota - ps6
Excavata/Diaphoretickes - ps7
Diaphoretickes - ps8
Archaeplastida - ps9
Viridiplantae - ps10
Streptophyta - ps11
Charophyceae/Embriophyta - ps12
Embriophyta - ps13
Tracheophyta - ps14
Spermatophyta - ps15
Angiosperms - ps16
Mesangiospermae - ps17
Magnoliids/eudicots - ps18
Eudicots - ps19
Core_eudicots - ps20
Superrosids - ps21
Rosids - ps22
Eurosids - ps23
Malvids - ps24
Sap./Malv./Brassicales - ps25
Malvales/Brassicales - ps26
Brassicales - ps27
Cleomaceae/Brassicaceae - ps28
Brassicaceae - ps29
Camelineae - ps30
Arabidopsis - ps31
Arabidopsis_thaliana - ps32
Phylostratum

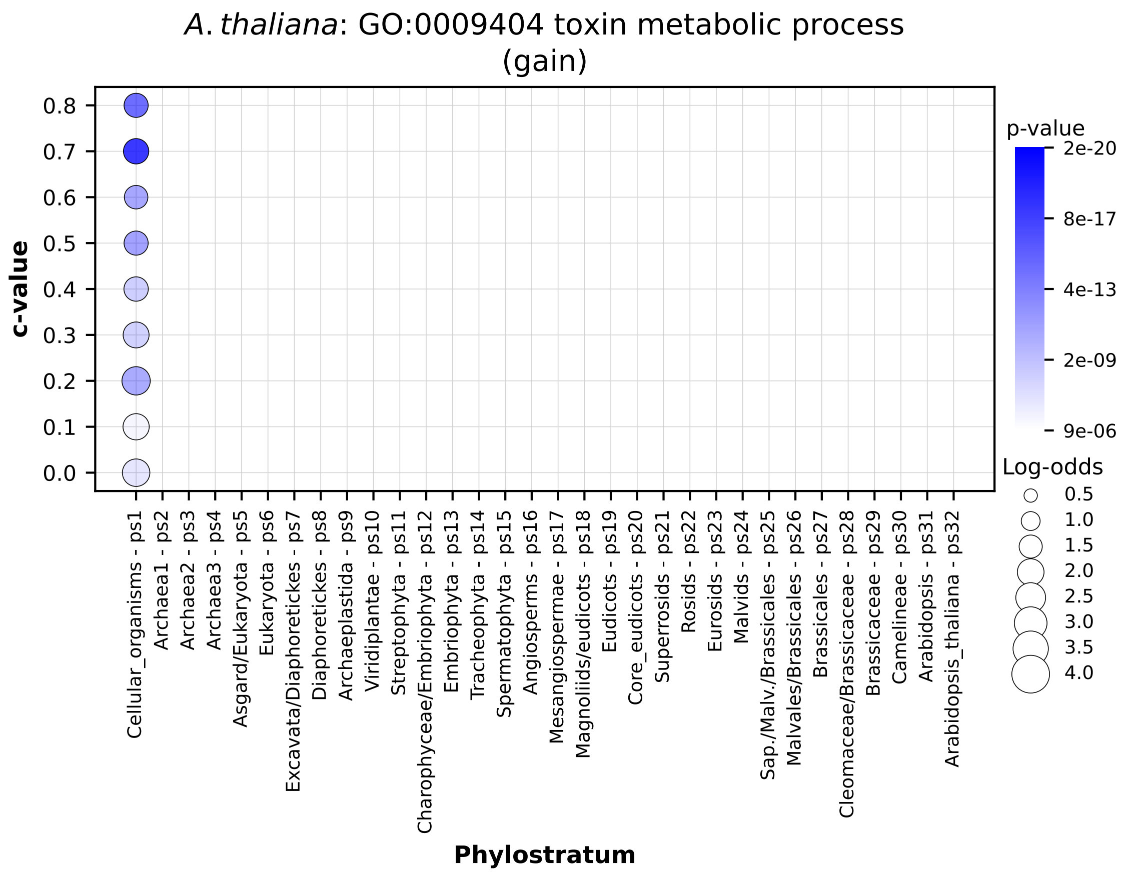

A. thaliana: GO:0009404 toxin metabolic process
(gain)
c-value
0.0
0.1
0.2
0.3
0.4
0.5
0.6
0.7
0.8
p-value
2e-20
8e-17
4e-13
2e-09
9e-06
Log-odds
0.5
1.0
1.5
2.0
2.5
3.0
3.5
4.0
Cellular_organisms - ps1
Archaea1 - ps2
Archaea2 - ps3
Archaea3 - ps4
Asgard/Eukaryota - ps5
Eukaryota - ps6
Excavata/Diaphoretickes - ps7
Diaphoretickes - ps8
Archaeplastida - ps9
Viridiplantae - ps10
Streptophyta - ps11
Charophyceae/Embriophyta - ps12
Embriophyta - ps13
Tracheophyta - ps14
Spermatophyta - ps15
Angiosperms - ps16
Mesangiospermae - ps17
Magnoliids/eudicots - ps18
Eudicots - ps19
Core_eudicots - ps20
Superrosids - ps21
Rosids - ps22
Eurosids - ps23
Malvids - ps24
Sap./Malv./Brassicales - ps25
Malvales/Brassicales - ps26
Brassicales - ps27
Cleomaceae/Brassicaceae - ps28
Brassicaceae - ps29
Camelineae - ps30
Arabidopsis - ps31
Arabidopsis_thaliana - ps32
Phylostratum

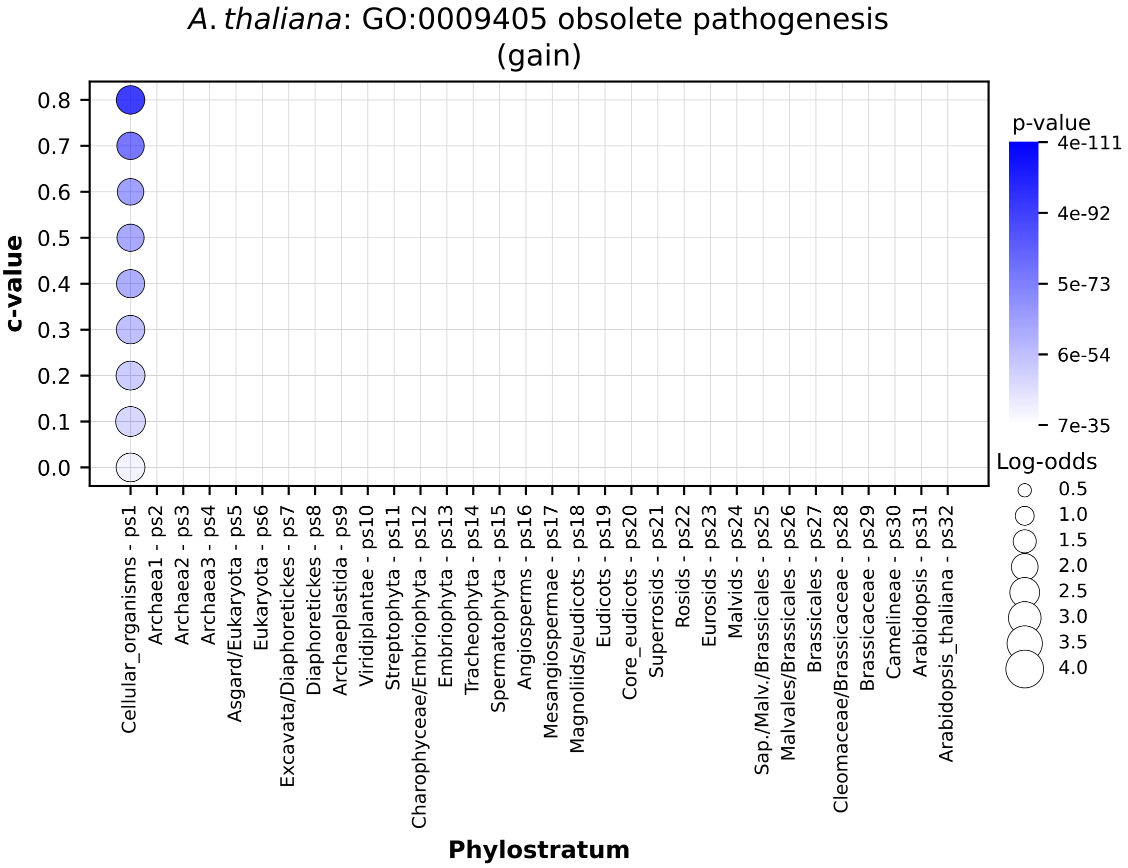
A. thaliana: GO:0009405 obsolete pathogenesis
(gain)
c-value
0.8
0.7
0.6
0.5
0.4
0.3
0.2
0.1
0.0
p-value
4e-111
4e-92
5e-73
6e-54
7e-35
Log-odds
0.5
1.0
1.5
2.0
2.5
3.0
3.5
4.0
Cellular_organisms - ps1
Archaea1 - ps2
Archaea2 - ps3
Archaea3 - ps4
Asgard/Eukaryota - ps5
Eukaryota - ps6
Excavata/Diaphoretickes - ps7
Diaphoretickes - ps8
Archaeplastida - ps9
Viridiplantae - ps10
Streptophyta - ps11
Charophyceae/Embriophyta - ps12
Embriophyta - ps13
Tracheophyta - ps14
Spermatophyta - ps15
Angiosperms - ps16
Mesangiospermae - ps17
Magnoliids/eudicots - ps18
Eudicots - ps19
Core_eudicots - ps20
Superrosids - ps21
Rosids - ps22
Eurosids - ps23
Malvids - ps24
Sap./Malv./Brassicales - ps25
Malvales/Brassicales - ps26
Brassicales - ps27
Cleomaceae/Brassicaceae - ps28
Brassicaceae - ps29
Camelineae - ps30
Arabidopsis - ps31
Arabidopsis_thaliana - ps32
Phylostratum

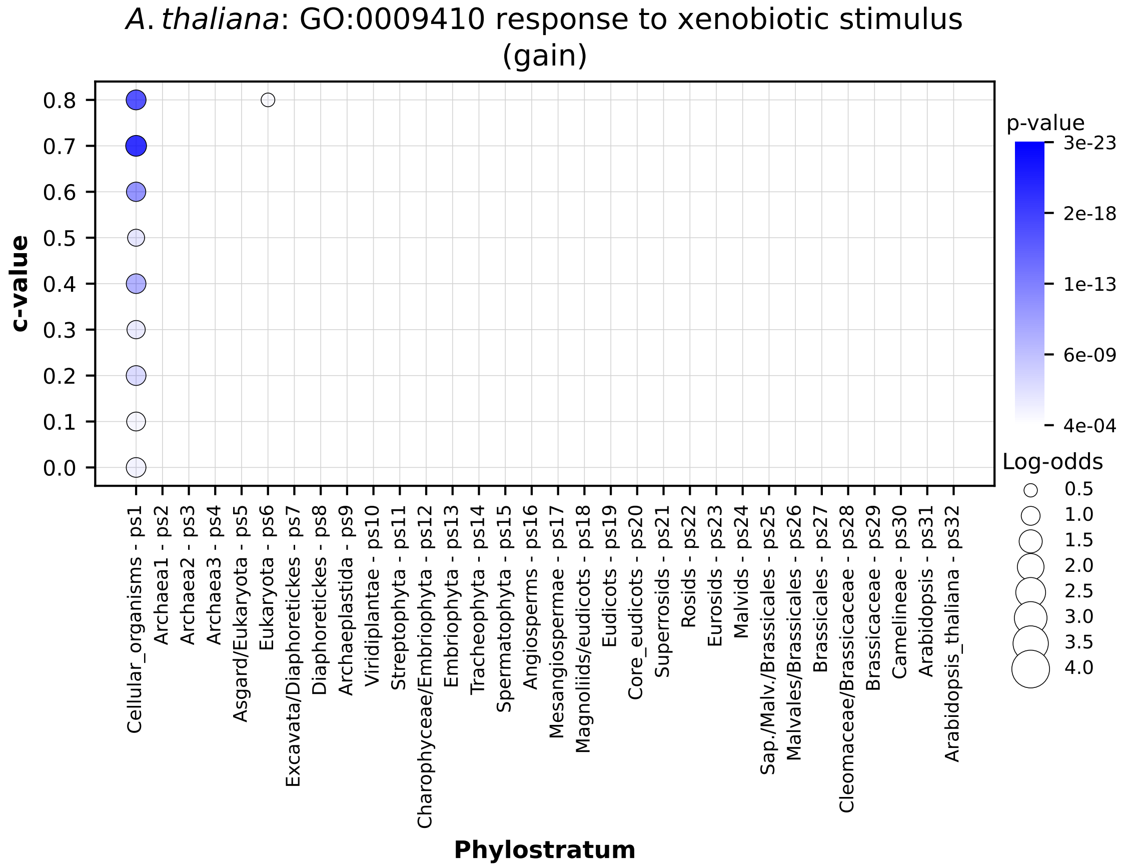

A. thaliana: GO:0009410 response to xenobiotic stimulus
(gain)
c-value
0.8
0.7
0.6
0.5
0.4
0.3
0.2
0.1
0.0
p-value
3e-23
2e-18
1e-13
6e-09
4e-04
Log-odds
0.5
1.0
1.5
2.0
2.5
3.0
3.5
4.0
Cellular_organisms - ps1
Archaea1 - ps2
Archaea2 - ps3
Archaea3 - ps4
Asgard/Eukaryota - ps5
Eukaryota - ps6
Excavata/Diaphoretickes - ps7
Diaphoretickes - ps8
Archaeplastida - ps9
Viridiplantae - ps10
Streptophyta - ps11
Charophyceae/Embriophyta - ps12
Embriophyta - ps13
Tracheophyta - ps14
Spermatophyta - ps15
Angiosperms - ps16
Mesangiospermae - ps17
Magnoliids/eudicots - ps18
Eudicots - ps19
Core_eudicots - ps20
Superrosids - ps21
Rosids - ps22
Eurosids - ps23
Malvids - ps24
Sap./Malv./Brassicales - ps25
Malvales/Brassicales - ps26
Brassicales - ps27
Cleomaceae/Brassicaceae - ps28
Brassicaceae - ps29
Camelineae - ps30
Arabidopsis - ps31
Arabidopsis_thaliana - ps32
Phylostratum

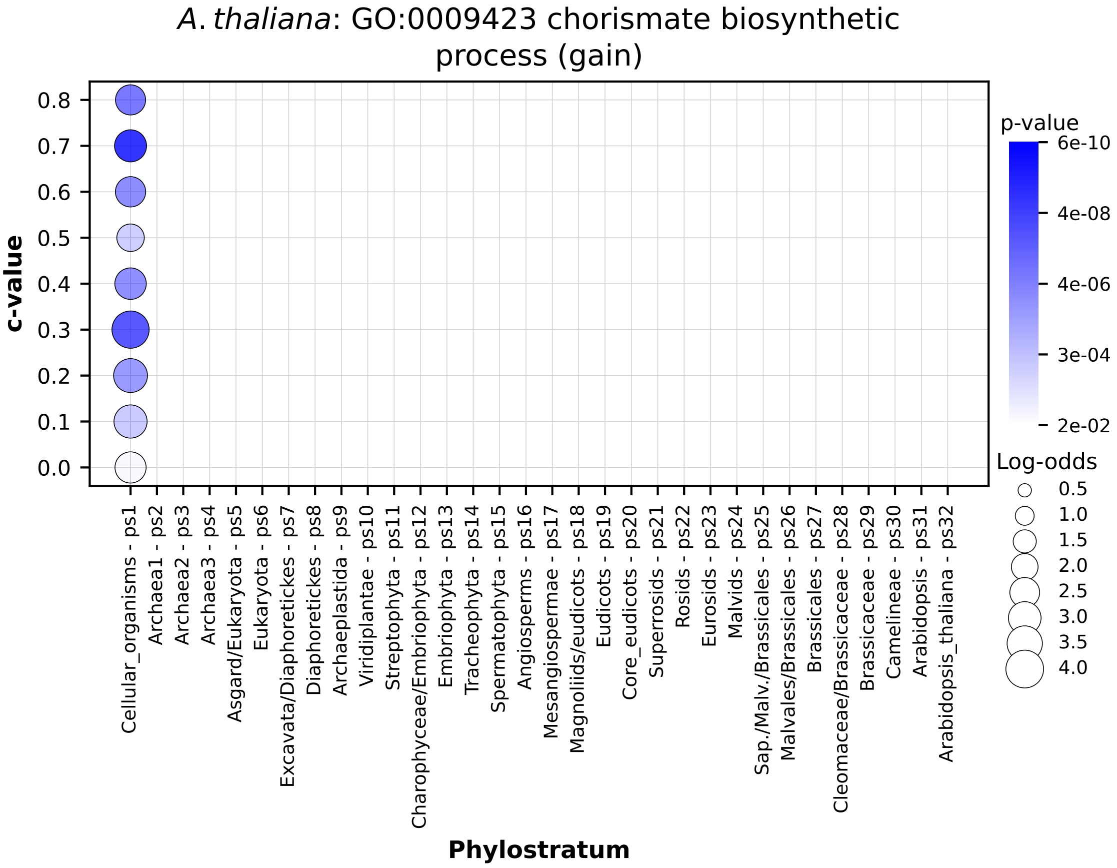

A. thaliana: GO:0009423 chorismate biosynthetic process (gain)
c-value
0.8
0.7
0.6
0.5
0.4
0.3
0.2
0.1
0.0
p-value
6e-10
4e-08
4e-06
3e-04
2e-02
Log-odds
0.5
1.0
1.5
2.0
2.5
3.0
3.5
4.0
Cellular_organisms - ps1
Archaea1 - ps2
Archaea2 - ps3
Archaea3 - ps4
Asgard/Eukaryota - ps5
Eukaryota - ps6
Excavata/Diaphoretickes - ps7
Diaphoretickes - ps8
Archaeplastida - ps9
Viridiplantae - ps10
Streptophyta - ps11
Charophyceae/Embriophyta - ps12
Embriophyta - ps13
Tracheophyta - ps14
Spermatophyta - ps15
Angiosperms - ps16
Mesangiospermae - ps17
Magnoliids/eudicots - ps18
Eudicots - ps19
Core_eudicots - ps20
Superrosids - ps21
Rosids - ps22
Eurosids - ps23
Malvids - ps24
Sap./Malv./Brassicales - ps25
Malvales/Brassicales - ps26
Brassicales - ps27
Cleomaceae/Brassicaceae - ps28
Brassicaceae - ps29
Camelineae - ps30
Arabidopsis - ps31
Arabidopsis_thaliana - ps32
Phylostratum

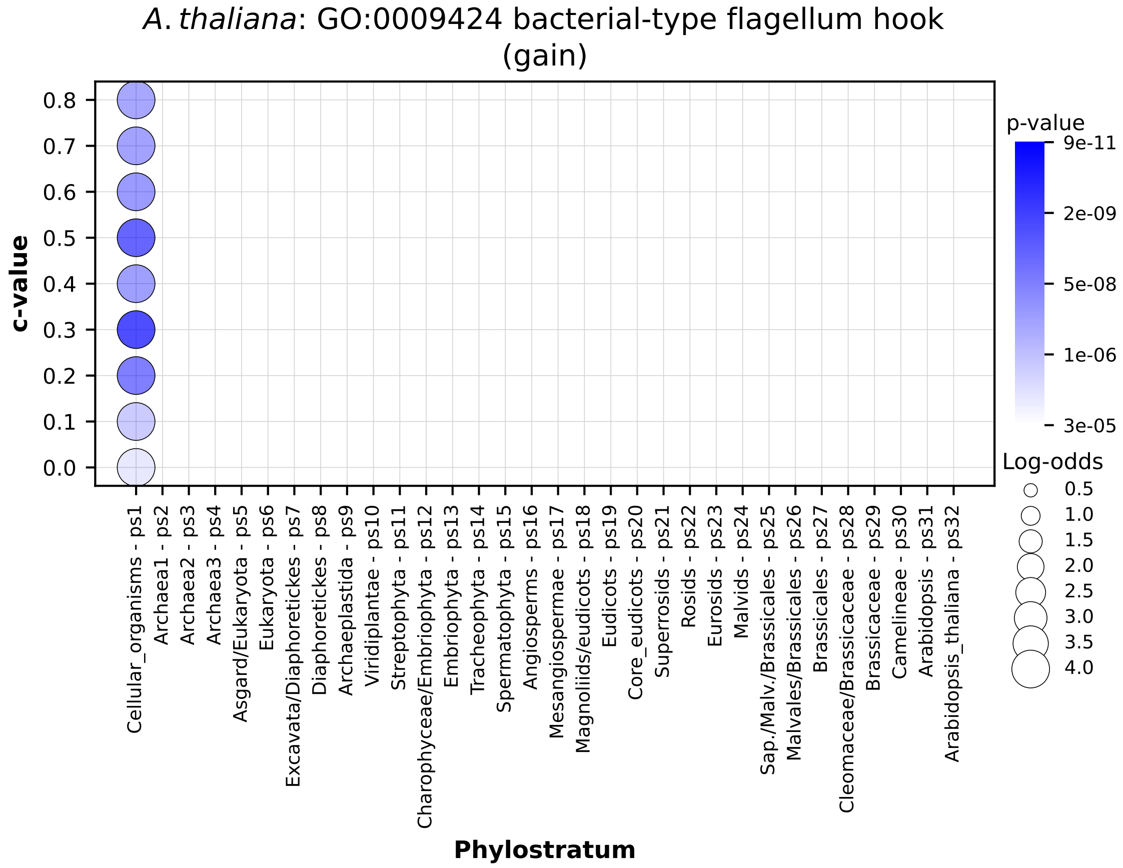

A. thaliana: GO:0009424 bacterial-type flagellum hook (gain)
c-value
0.8
0.7
0.6
0.5
0.4
0.3
0.2
0.1
0.0
p-value
9e-11
2e-09
5e-08
1e-06
3e-05
Log-odds
0.5
1.0
1.5
2.0
2.5
3.0
3.5
4.0
Cellular_organisms - ps1
Archaea1 - ps2
Archaea2 - ps3
Archaea3 - ps4
Asgard/Eukaryota - ps5
Eukaryota - ps6
Excavata/Diaphoretickes - ps7
Diaphoretickes - ps8
Archaeplastida - ps9
Viridiplantae - ps10
Streptophyta - ps11
Charophyceae/Embriophyta - ps12
Embriophyta - ps13
Tracheophyta - ps14
Spermatophyta - ps15
Angiosperms - ps16
Mesangiospermae - ps17
Magnoliids/eudicots - ps18
Eudicots - ps19
Core_eudicots - ps20
Superrosids - ps21
Rosids - ps22
Eurosids - ps23
Malvids - ps24
Sap./Malv./Brassicales - ps25
Malvales/Brassicales - ps26
Brassicales - ps27
Cleomaceae/Brassicaceae - ps28
Brassicaceae - ps29
Camelineae - ps30
Arabidopsis - ps31
Arabidopsis_thaliana - ps32
Phylostratum

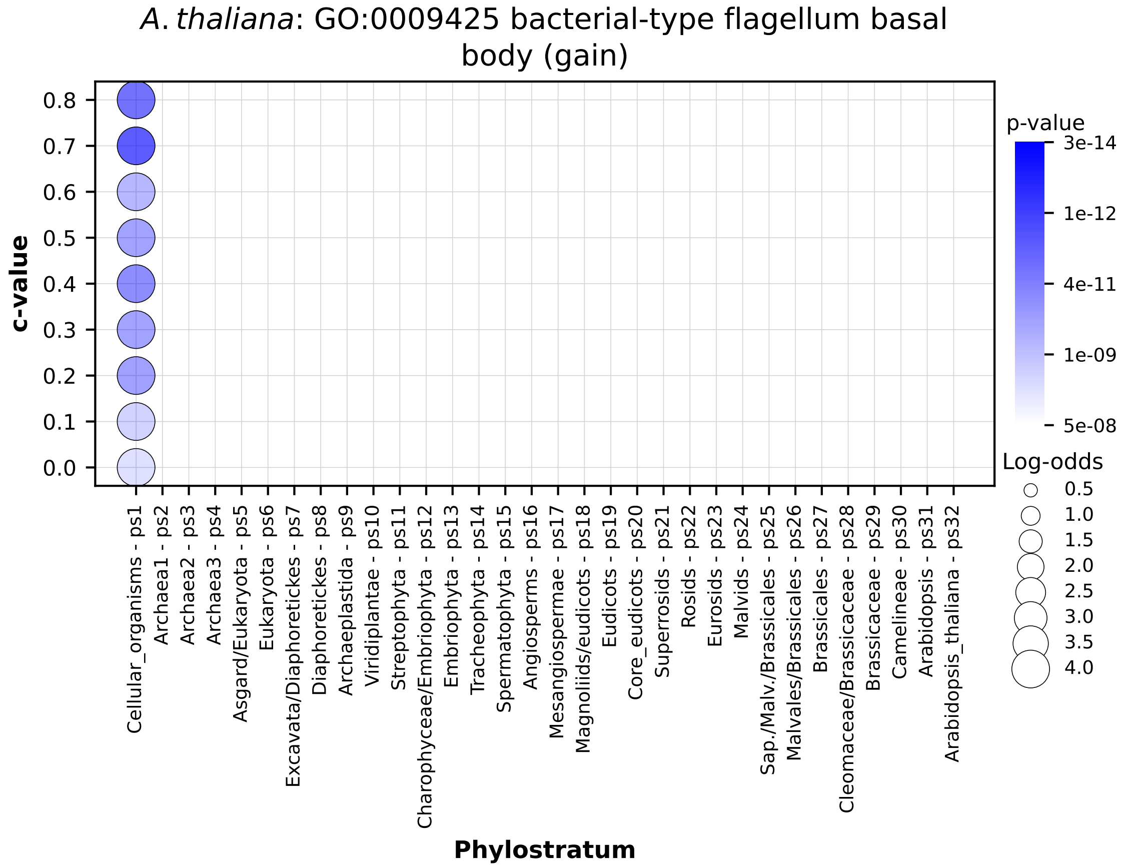
A. thaliana: GO:0009425 bacterial-type flagellum basal body (gain)
c-value
0.8
0.7
0.6
0.5
0.4
0.3
0.2
0.1
0.0
p-value
3e-14
1e-12
4e-11
1e-09
5e-08
Log-odds
0.5
1.0
1.5
2.0
2.5
3.0
3.5
4.0
Cellular_organisms - ps1
Archaea1 - ps2
Archaea2 - ps3
Archaea3 - ps4
Asgard/Eukaryota - ps5
Eukaryota - ps6
Excavata/Diaphoretickes - ps7
Diaphoretickes - ps8
Archaeplastida - ps9
Viridiplantae - ps10
Streptophyta - ps11
Charophyceae/Embriophyta - ps12
Embriophyta - ps13
Tracheophyta - ps14
Spermatophyta - ps15
Angiosperms - ps16
Mesangiospermae - ps17
Magnoliids/eudicots - ps18
Eudicots - ps19
Core_eudicots - ps20
Superrosids - ps21
Rosids - ps22
Eurosids - ps23
Malvids - ps24
Sap./Malv./Brassicales - ps25
Malvales/Brassicales - ps26
Brassicales - ps27
Cleomaceae/Brassicaceae - ps28
Brassicaceae - ps29
Camelineae - ps30
Arabidopsis - ps31
Arabidopsis_thaliana - ps32
Phylostratum

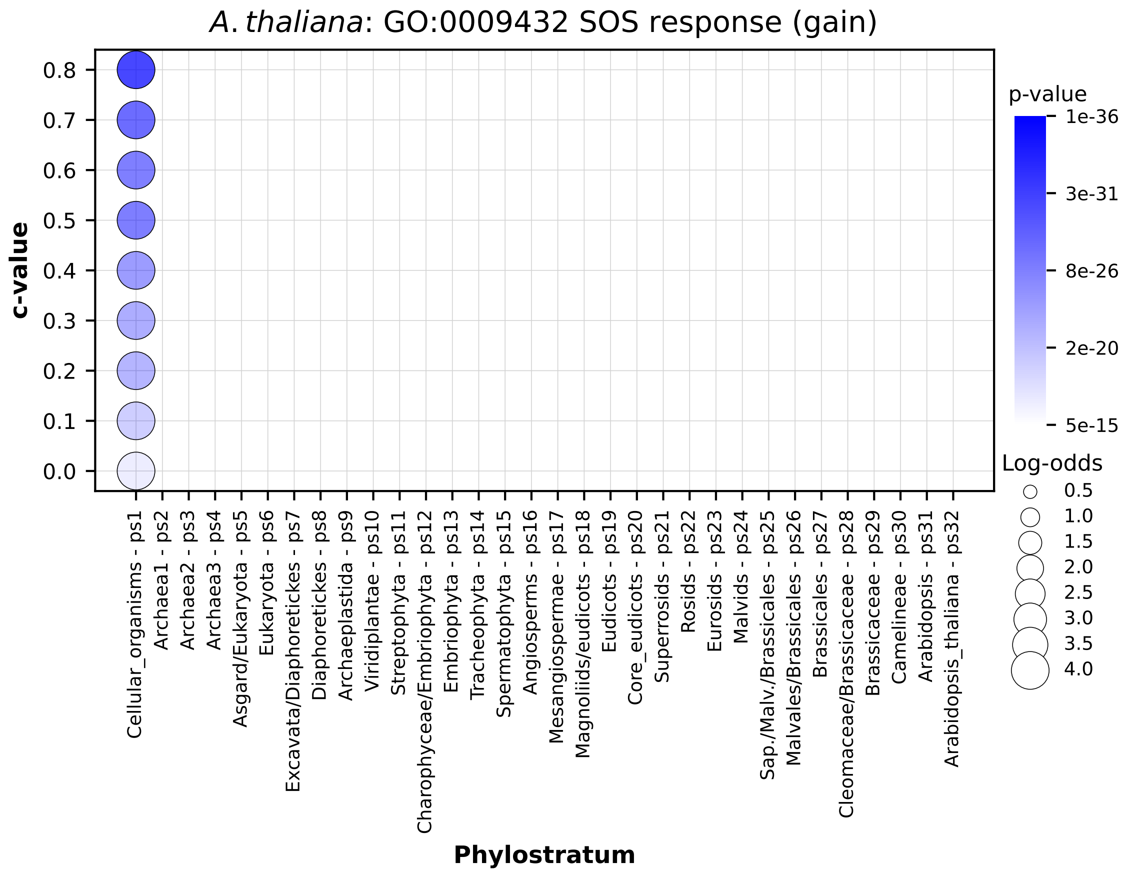

A. thaliana: GO:0009432 SOS response (gain)
c-value
0.8
0.7
0.6
0.5
0.4
0.3
0.2
0.1
0.0
Phylostratum
Cellular_organisms - ps1
Archaea1 - ps2
Archaea2 - ps3
Archaea3 - ps4
Asgard/Eukaryota - ps5
Eukaryota - ps6
Excavata/Diaphoretickes - ps7
Diaphoretickes - ps8
Archaeplastida - ps9
Viridiplantae - ps10
Streptophyta - ps11
Charophyceae/Embriophyta - ps12
Embriophyta - ps13
Tracheophyta - ps14
Spermatophyta - ps15
Angiosperms - ps16
Mesangiospermae - ps17
Magnoliids/eudicots - ps18
Eudicots - ps19
Core_eudicots - ps20
Superrosids - ps21
Rosids - ps22
Eurosids - ps23
Malvids - ps24
Sap./Malv./Brassicales - ps25
Malvales/Brassicales - ps26
Brassicales - ps27
Cleomaceae/Brassicaceae - ps28
Brassicaceae - ps29
Camelineae - ps30
Arabidopsis - ps31
Arabidopsis_thaliana - ps32
p-value
1e-36
3e-31
8e-26
2e-20
5e-15
Log-odds
0.5
1.0
1.5
2.0
2.5
3.0
3.5
4.0

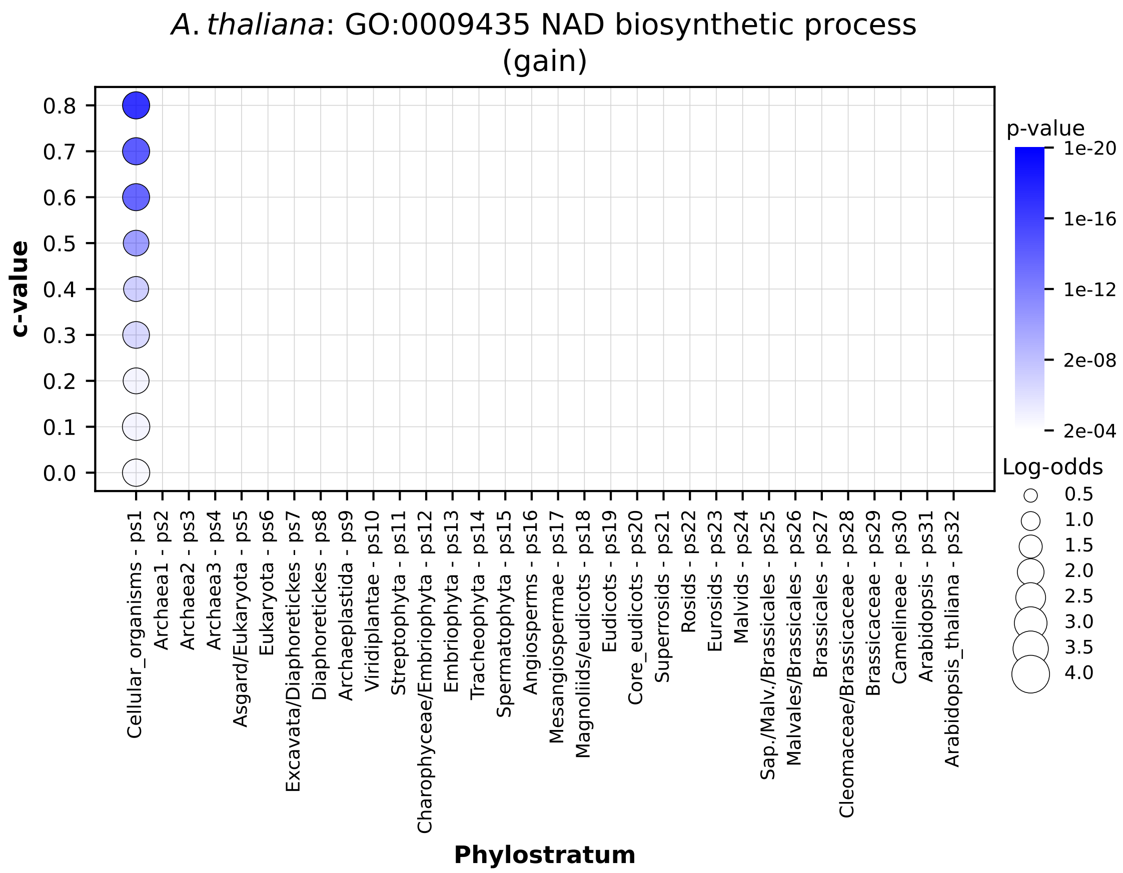

$A. thaliana$: GO:0009435 NAD biosynthetic process
(gain)
c-value
0.0
0.1
0.2
0.3
0.4
0.5
0.6
0.7
0.8
p-value
1e-20
1e-16
1e-12
2e-08
2e-04
Log-odds
0.5
1.0
1.5
2.0
2.5
3.0
3.5
4.0
Cellular_organisms - ps1
Archaea1 - ps2
Archaea2 - ps3
Archaea3 - ps4
Asgard/Eukaryota - ps5
Eukaryota - ps6
Excavata/Diaphoretickes - ps7
Diaphoretickes - ps8
Archaeplastida - ps9
Viridiplantae - ps10
Streptophyta - ps11
Charophyceae/Embriophyta - ps12
Embriophyta - ps13
Tracheophyta - ps14
Spermatophyta - ps15
Angiosperms - ps16
Mesangiospermae - ps17
Magnoliids/eudicots - ps18
Eudicots - ps19
Core_eudicots - ps20
Superrosids - ps21
Rosids - ps22
Eurosids - ps23
Malvids - ps24
Sap./Malv./Brassicales - ps25
Malvales/Brassicales - ps26
Brassicales - ps27
Cleomaceae/Brassicaceae - ps28
Brassicaceae - ps29
Camelineae - ps30
Arabidopsis - ps31
Arabidopsis_thaliana - ps32
Phylostratum

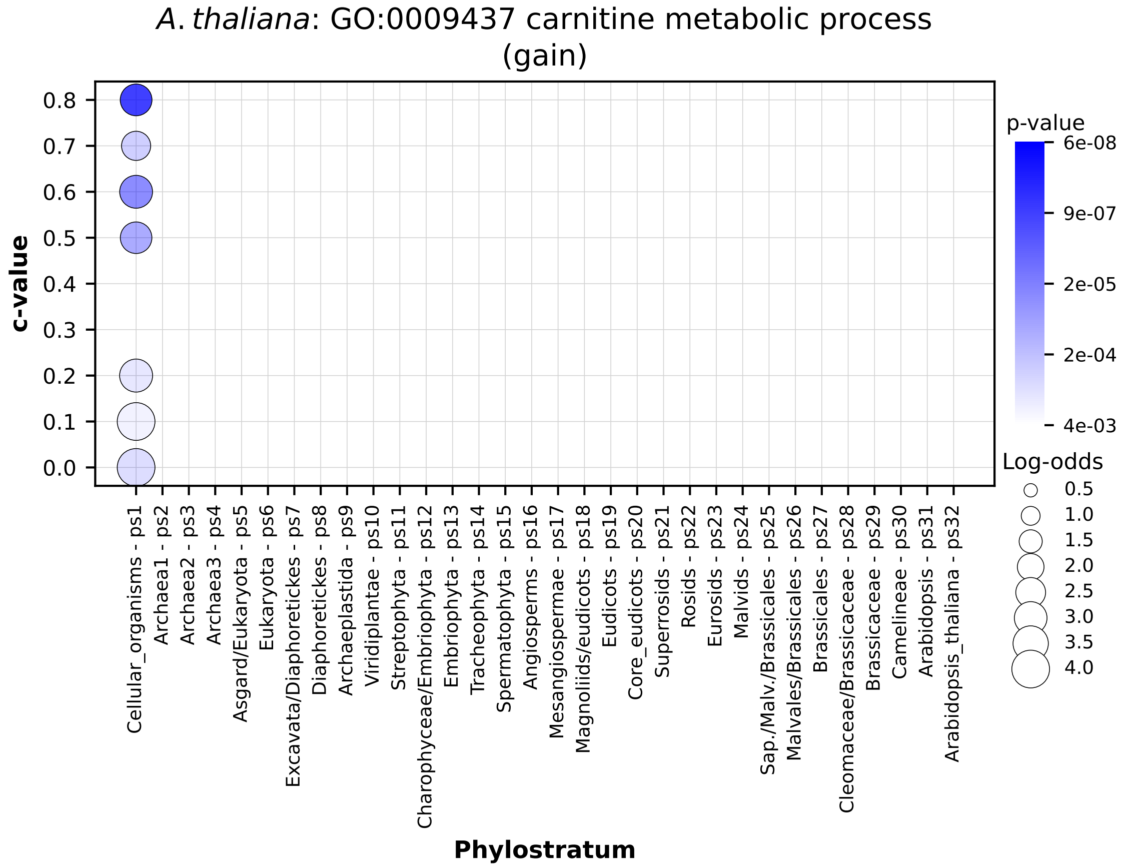

A. thaliana: GO:0009437 carnitine metabolic process (gain)
c-value
0.8
0.7
0.6
0.5
0.4
0.3
0.2
0.1
0.0
p-value
6e-08
9e-07
2e-05
2e-04
4e-03
Log-odds
0.5
1.0
1.5
2.0
2.5
3.0
3.5
4.0
Cellular_organisms - ps1
Archaea1 - ps2
Archaea2 - ps3
Archaea3 - ps4
Asgard/Eukaryota - ps5
Eukaryota - ps6
Excavata/Diaphoretickes - ps7
Diaphoretickes - ps8
Archaeplastida - ps9
Viridiplantae - ps10
Streptophyta - ps11
Charophyceae/Embriophyta - ps12
Embriophyta - ps13
Tracheophyta - ps14
Spermatophyta - ps15
Angiosperms - ps16
Mesangiospermae - ps17
Magnoliids/eudicots - ps18
Eudicots - ps19
Core_eudicots - ps20
Superrosids - ps21
Rosids - ps22
Eurosids - ps23
Malvids - ps24
Sap./Malv./Brassicales - ps25
Malvales/Brassicales - ps26
Brassicales - ps27
Cleomaceae/Brassicaceae - ps28
Brassicaceae - ps29
Camelineae - ps30
Arabidopsis - ps31
Arabidopsis_thaliana - ps32
Phylostratum

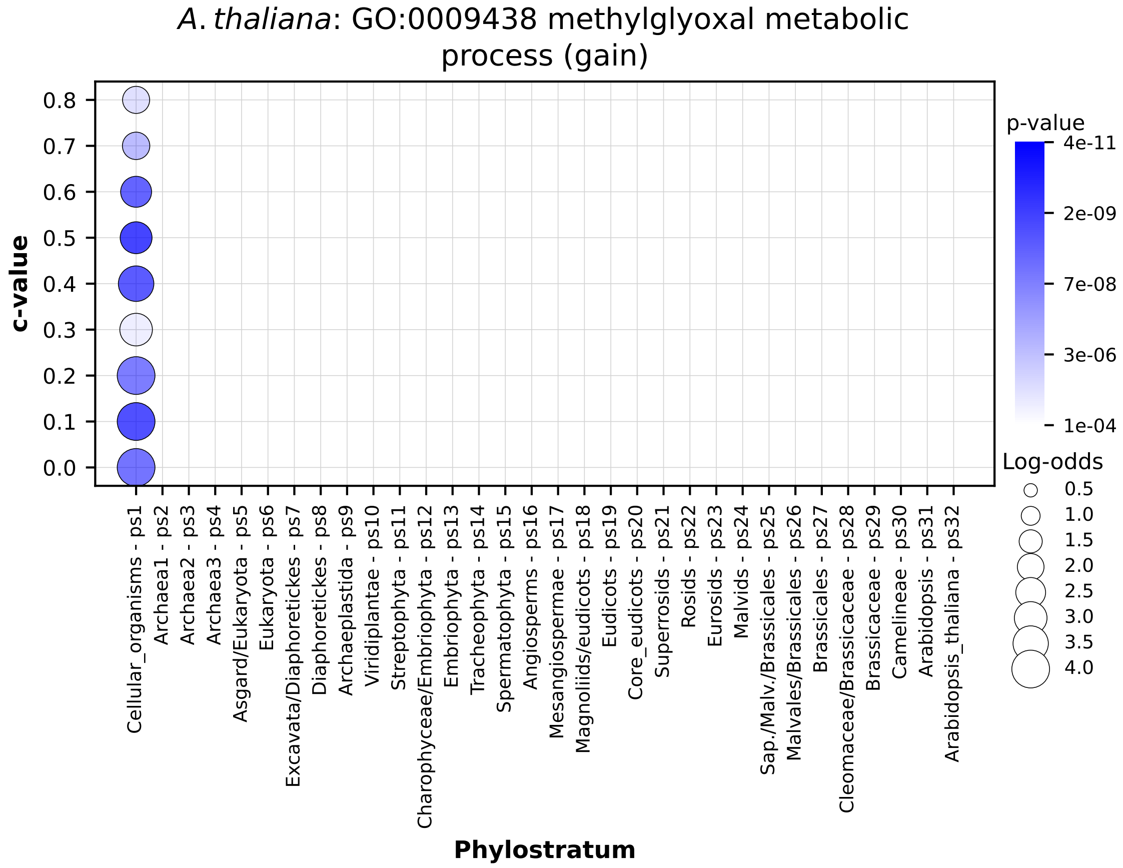
A. thaliana: GO:0009438 methylglyoxal metabolic process (gain)
c-value
0.8
0.7
0.6
0.5
0.4
0.3
0.2
0.1
0.0
p-value
4e-11
2e-09
7e-08
3e-06
1e-04
Log-odds
0.5
1.0
1.5
2.0
2.5
3.0
3.5
4.0
Cellular_organisms - ps1
Archaea1 - ps2
Archaea2 - ps3
Archaea3 - ps4
Asgard/Eukaryota - ps5
Eukaryota - ps6
Excavata/Diaphoretickes - ps7
Diaphoretickes - ps8
Archaeplastida - ps9
Viridiplantae - ps10
Streptophyta - ps11
Charophyceae/Embriophyta - ps12
Embriophyta - ps13
Tracheophyta - ps14
Spermatophyta - ps15
Angiosperms - ps16
Mesangiospermae - ps17
Magnoliids/eudicots - ps18
Eudicots - ps19
Core_eudicots - ps20
Superrosids - ps21
Rosids - ps22
Eurosids - ps23
Malvids - ps24
Sap./Malv./Brassicales - ps25
Malvales/Brassicales - ps26
Brassicales - ps27
Cleomaceae/Brassicaceae - ps28
Brassicaceae - ps29
Camelineae - ps30
Arabidopsis - ps31
Arabidopsis_thaliana - ps32
Phylostratum

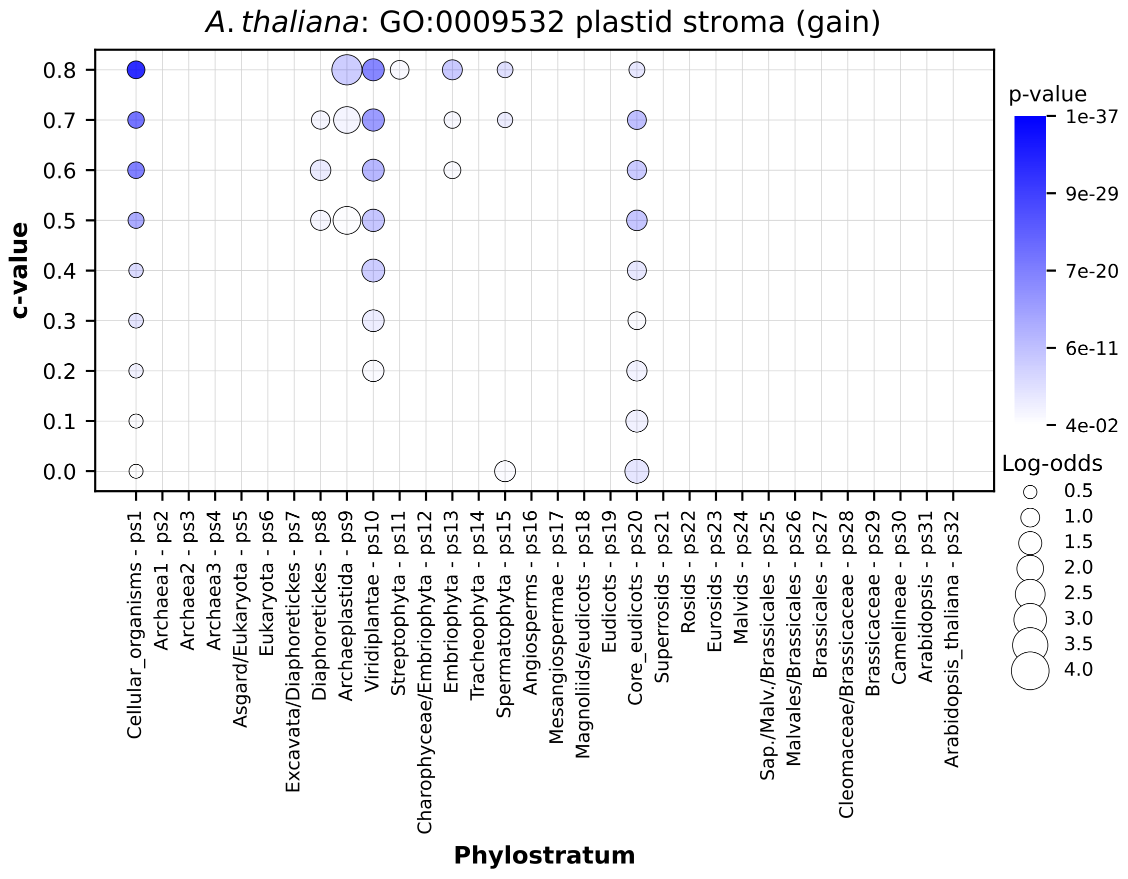

A. thaliana: GO:0009532 plastid stroma (gain)
c-value
0.8
0.7
0.6
0.5
0.4
0.3
0.2
0.1
0.0
p-value
1e-37
9e-29
7e-20
6e-11
4e-02
Log-odds
0.5
1.0
1.5
2.0
2.5
3.0
3.5
4.0
Cellular_organisms - ps1
Archaea1 - ps2
Archaea2 - ps3
Archaea3 - ps4
Asgard/Eukaryota - ps5
Eukaryota - ps6
Excavata/Diaphoretickes - ps7
Diaphoretickes - ps8
Archaeplastida - ps9
Viridiplantae - ps10
Streptophyta - ps11
Charophyceae/Embriophyta - ps12
Embriophyta - ps13
Tracheophyta - ps14
Spermatophyta - ps15
Angiosperms - ps16
Mesangiospermae - ps17
Magnoliids/eudicots - ps18
Eudicots - ps19
Core_eudicots - ps20
Superrosids - ps21
Rosids - ps22
Eurosids - ps23
Malvids - ps24
Sap./Malv./Brassicales - ps25
Malvales/Brassicales - ps26
Brassicales - ps27
Cleomaceae/Brassicaceae - ps28
Brassicaceae - ps29
Camelineae - ps30
Arabidopsis - ps31
Arabidopsis_thaliana - ps32
Phylostratum

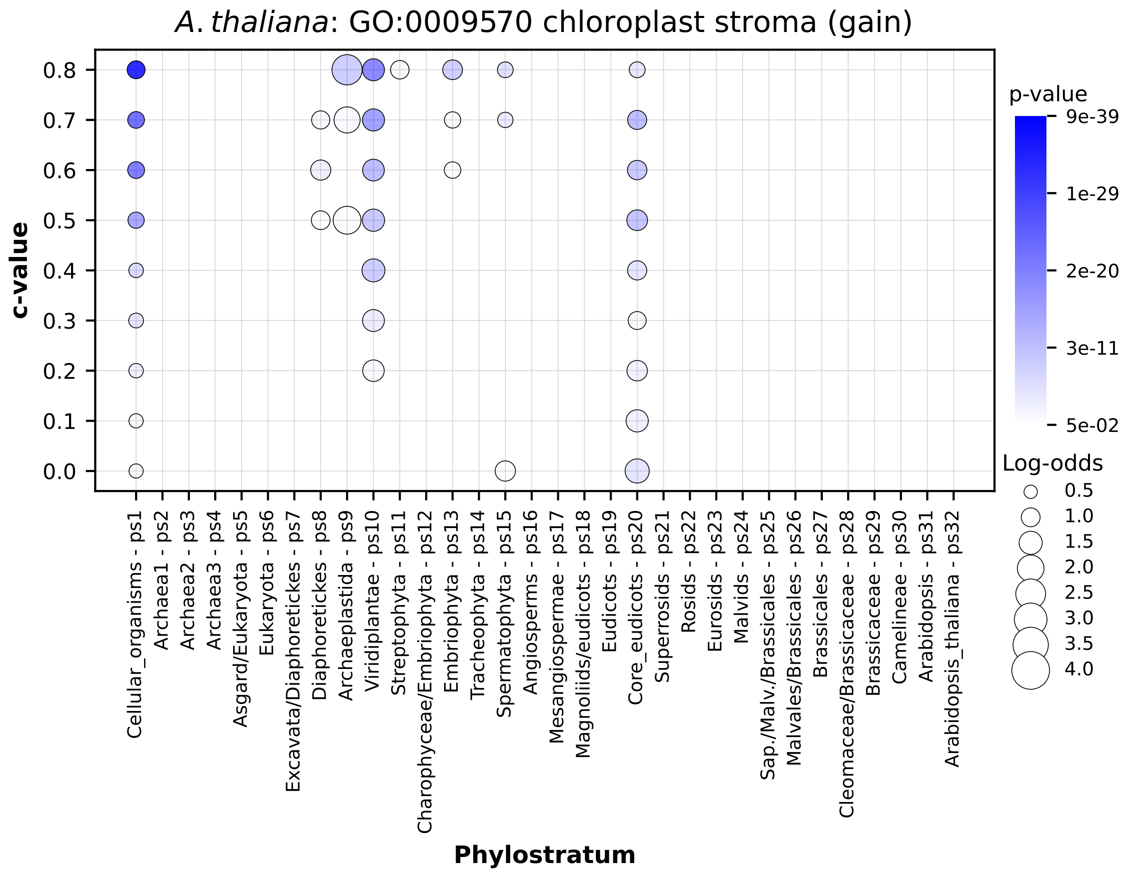

A. thaliana: GO:0009570 chloroplast stroma (gain)
c-value
0.8
0.7
0.6
0.5
0.4
0.3
0.2
0.1
0.0
p-value
9e-39
1e-29
2e-20
3e-11
5e-02
Log-odds
0.5
1.0
1.5
2.0
2.5
3.0
3.5
4.0
Cellular_organisms - ps1
Archaea1 - ps2
Archaea2 - ps3
Archaea3 - ps4
Asgard/Eukaryota - ps5
Eukaryota - ps6
Excavata/Diaphoretickes - ps7
Diaphoretickes - ps8
Archaeplastida - ps9
Viridiplantae - ps10
Streptophyta - ps11
Charophyceae/Embriophyta - ps12
Embriophyta - ps13
Tracheophyta - ps14
Spermatophyta - ps15
Angiosperms - ps16
Mesangiospermae - ps17
Magnoliids/eudicots - ps18
Eudicots - ps19
Core_eudicots - ps20
Superrosids - ps21
Rosids - ps22
Eurosids - ps23
Malvids - ps24
Sap./Malv./Brassicales - ps25
Malvales/Brassicales - ps26
Brassicales - ps27
Cleomaceae/Brassicaceae - ps28
Brassicaceae - ps29
Camelineae - ps30
Arabidopsis - ps31
Arabidopsis_thaliana - ps32
Phylostratum

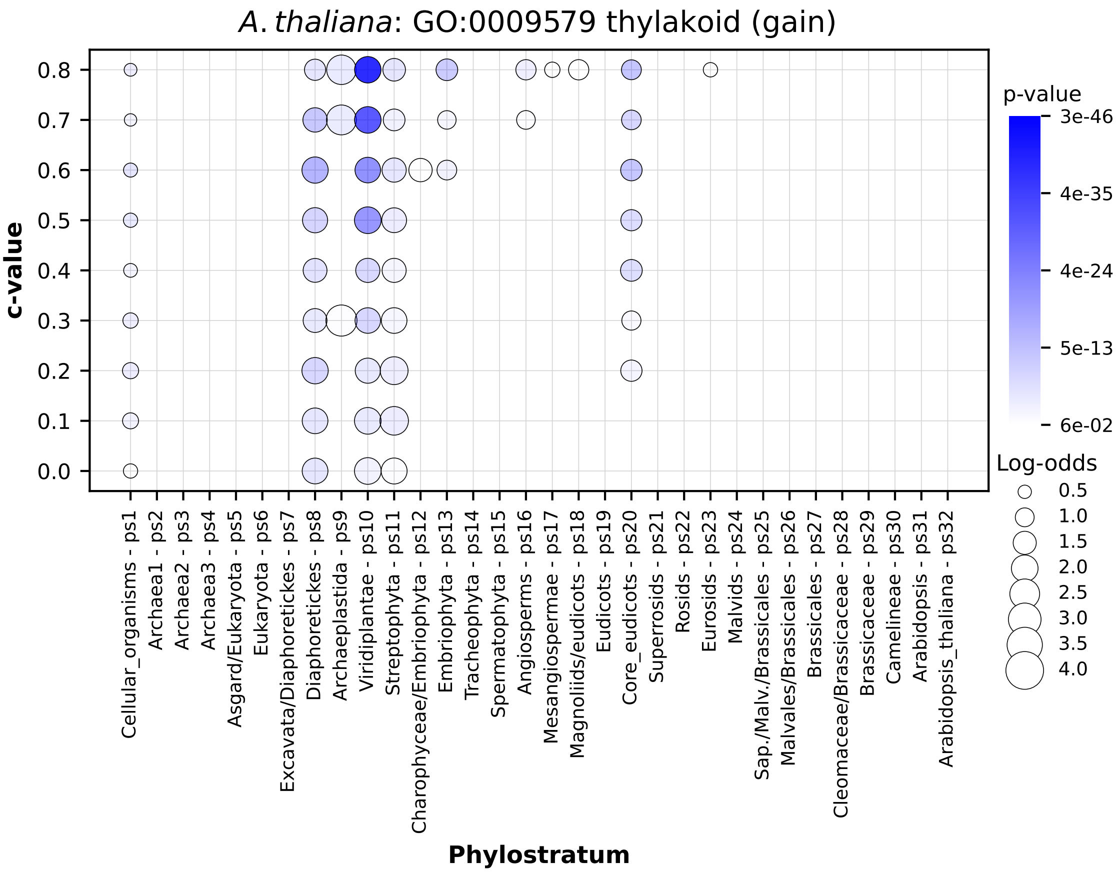

A. thaliana: GO:0009579 thylakoid (gain)
c-value
0.8
0.7
0.6
0.5
0.4
0.3
0.2
0.1
0.0
Phylostratum
Cellular_organisms - ps1
Archaea1 - ps2
Archaea2 - ps3
Archaea3 - ps4
Asgard/Eukaryota - ps5
Eukaryota - ps6
Excavata/Diaphoretickes - ps7
Diaphoretickes - ps8
Archaeplastida - ps9
Viridiplantae - ps10
Streptophyta - ps11
Charophyceae/Embriophyta - ps12
Embriophyta - ps13
Tracheophyta - ps14
Spermatophyta - ps15
Angiosperms - ps16
Mesangiospermae - ps17
Magnoliids/eudicots - ps18
Eudicots - ps19
Core_eudicots - ps20
Superrosids - ps21
Rosids - ps22
Eurosids - ps23
Malvids - ps24
Sap./Malv./Brassicales - ps25
Malvales/Brassicales - ps26
Brassicales - ps27
Cleomaceae/Brassicaceae - ps28
Brassicaceae - ps29
Camelineae - ps30
Arabidopsis - ps31
Arabidopsis_thaliana - ps32
p-value
3e-46
4e-35
4e-24
5e-13
6e-02
Log-odds
0.5
1.0
1.5
2.0
2.5
3.0
3.5
4.0

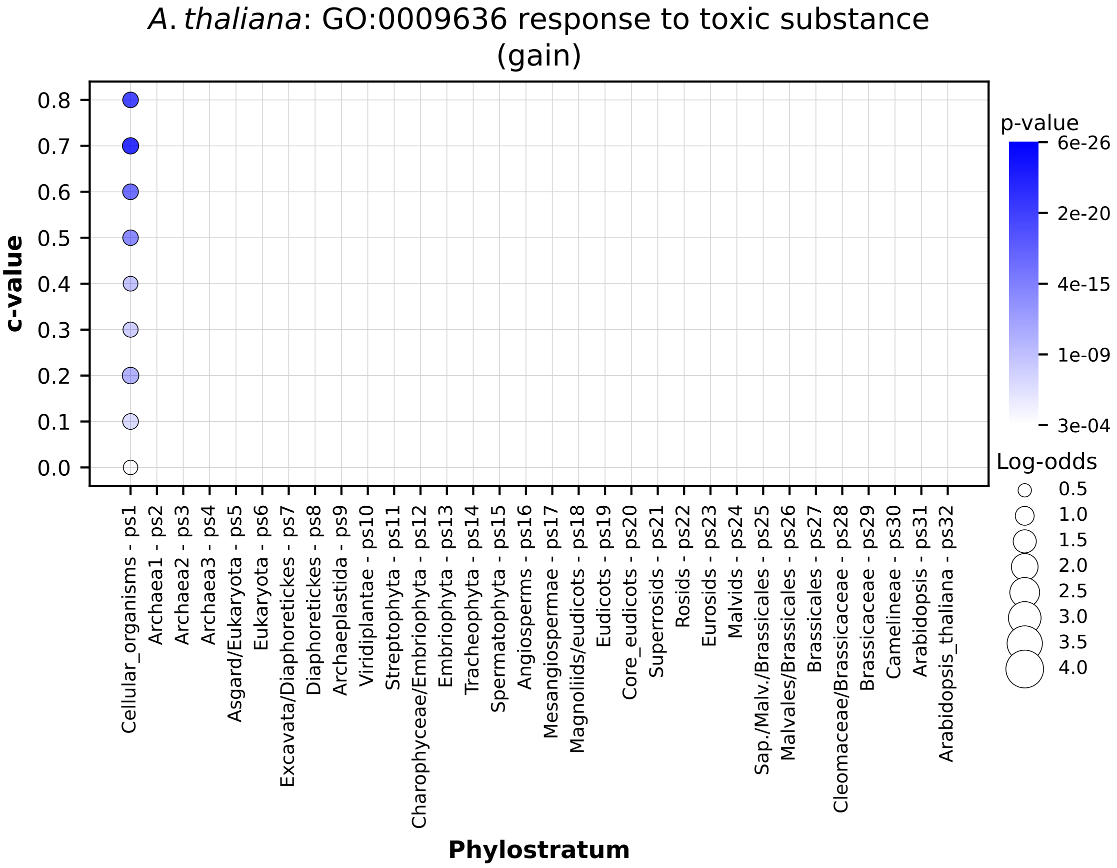

A. thaliana: GO:0009636 response to toxic substance (gain)
c-value
0.8
0.7
0.6
0.5
0.4
0.3
0.2
0.1
0.0
p-value
6e-26
2e-20
4e-15
1e-09
3e-04
Log-odds
0.5
1.0
1.5
2.0
2.5
3.0
3.5
4.0
Cellular_organisms - ps1
Archaea1 - ps2
Archaea2 - ps3
Archaea3 - ps4
Asgard/Eukaryota - ps5
Eukaryota - ps6
Excavata/Diaphoretickes - ps7
Diaphoretickes - ps8
Archaeplastida - ps9
Viridiplantae - ps10
Streptophyta - ps11
Charophyceae/Embriophyta - ps12
Embriophyta - ps13
Tracheophyta - ps14
Spermatophyta - ps15
Angiosperms - ps16
Mesangiospermae - ps17
Magnoliids/eudicots - ps18
Eudicots - ps19
Core_eudicots - ps20
Superrosids - ps21
Rosids - ps22
Eurosids - ps23
Malvids - ps24
Sap./Malv./Brassicales - ps25
Malvales/Brassicales - ps26
Brassicales - ps27
Cleomaceae/Brassicaceae - ps28
Brassicaceae - ps29
Camelineae - ps30
Arabidopsis - ps31
Arabidopsis_thaliana - ps32
Phylostratum

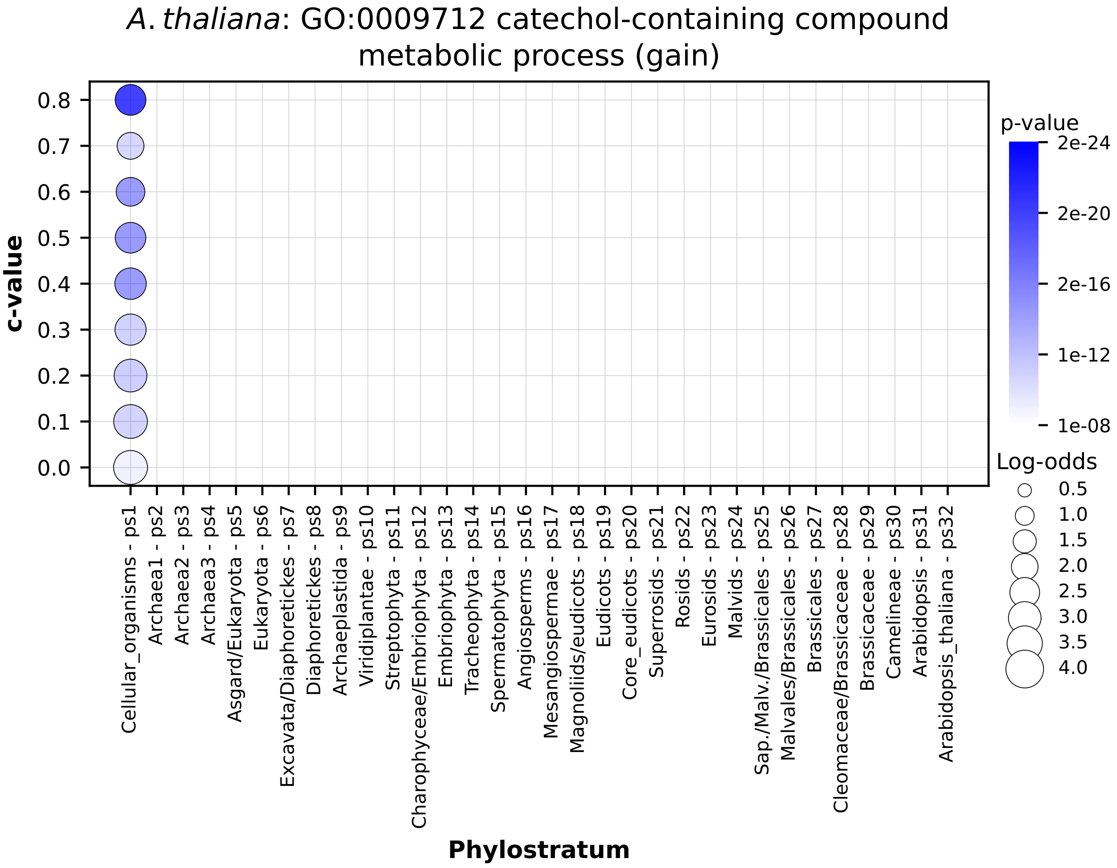

A. thaliana: GO:0009712 catechol-containing compound metabolic process (gain)
c-value
0.8
0.7
0.6
0.5
0.4
0.3
0.2
0.1
0.0
p-value
2e-24
2e-20
2e-16
1e-12
1e-08
Log-odds
0.5
1.0
1.5
2.0
2.5
3.0
3.5
4.0
Cellular_organisms - ps1
Archaea1 - ps2
Archaea2 - ps3
Archaea3 - ps4
Asgard/Eukaryota - ps5
Eukaryota - ps6
Excavata/Diaphoretickes - ps7
Diaphoretickes - ps8
Archaeplastida - ps9
Viridiplantae - ps10
Streptophyta - ps11
Charophyceae/Embriophyta - ps12
Embriophyta - ps13
Tracheophyta - ps14
Spermatophyta - ps15
Angiosperms - ps16
Mesangiospermae - ps17
Magnoliids/eudicots - ps18
Eudicots - ps19
Core_eudicots - ps20
Superrosids - ps21
Rosids - ps22
Eurosids - ps23
Malvids - ps24
Sap./Malv./Brassicales - ps25
Malvales/Brassicales - ps26
Brassicales - ps27
Cleomaceae/Brassicaceae - ps28
Brassicaceae - ps29
Camelineae - ps30
Arabidopsis - ps31
Arabidopsis_thaliana - ps32
Phylostratum

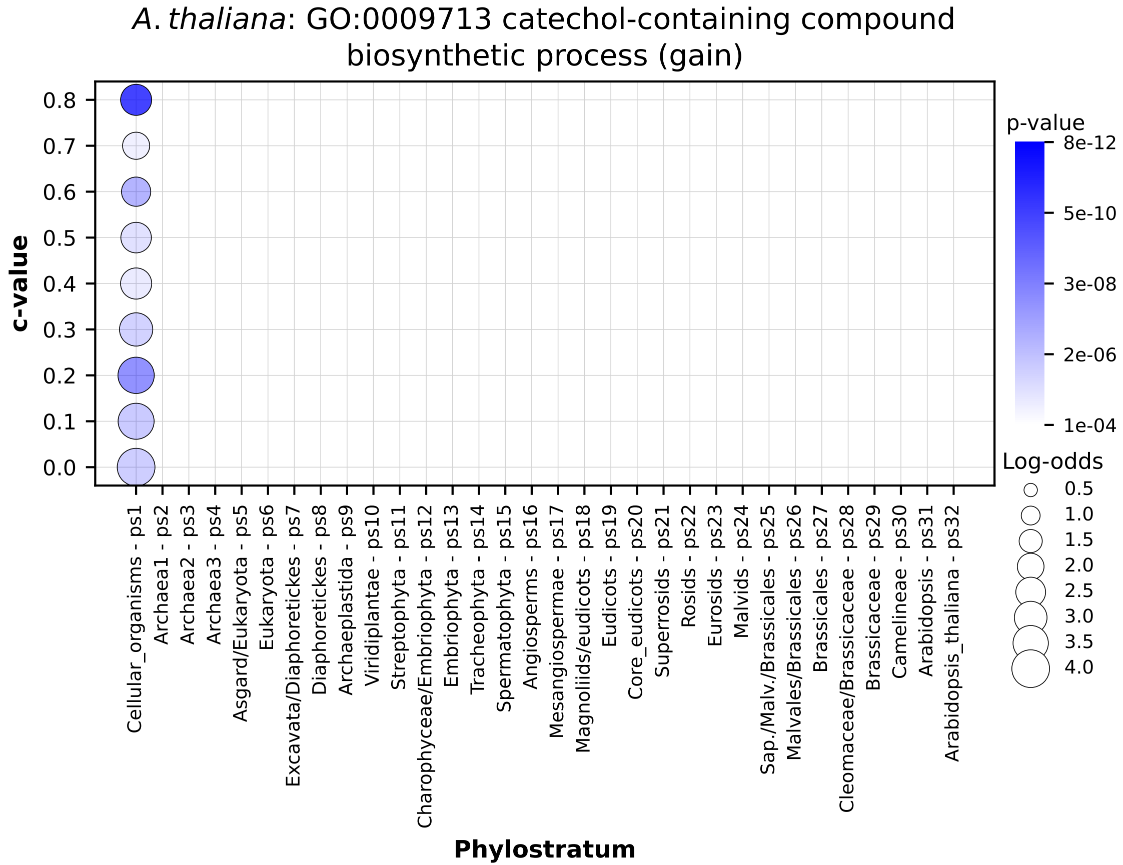

A. thaliana: GO:0009713 catechol-containing compound biosynthetic process (gain)
c-value
0.8
0.7
0.6
0.5
0.4
0.3
0.2
0.1
0.0
Phylostratum
Cellular_organisms - ps1
Archaea1 - ps2
Archaea2 - ps3
Archaea3 - ps4
Asgard/Eukaryota - ps5
Eukaryota - ps6
Excavata/Diaphoretickes - ps7
Diaphoretickes - ps8
Archaeplastida - ps9
Viridiplantae - ps10
Streptophyta - ps11
Charophyceae/Embriophyta - ps12
Embriophyta - ps13
Tracheophyta - ps14
Spermatophyta - ps15
Angiosperms - ps16
Mesangiospermae - ps17
Magnoliids/eudicots - ps18
Eudicots - ps19
Core_eudicots - ps20
Superrosids - ps21
Rosids - ps22
Eurosids - ps23
Malvids - ps24
Sap./Malv./Brassicales - ps25
Malvales/Brassicales - ps26
Brassicales - ps27
Cleomaceae/Brassicaceae - ps28
Brassicaceae - ps29
Camelineae - ps30
Arabidopsis - ps31
Arabidopsis_thaliana - ps32
p-value
8e-12
5e-10
3e-08
2e-06
1e-04
Log-odds
0.5
1.0
1.5
2.0
2.5
3.0
3.5
4.0

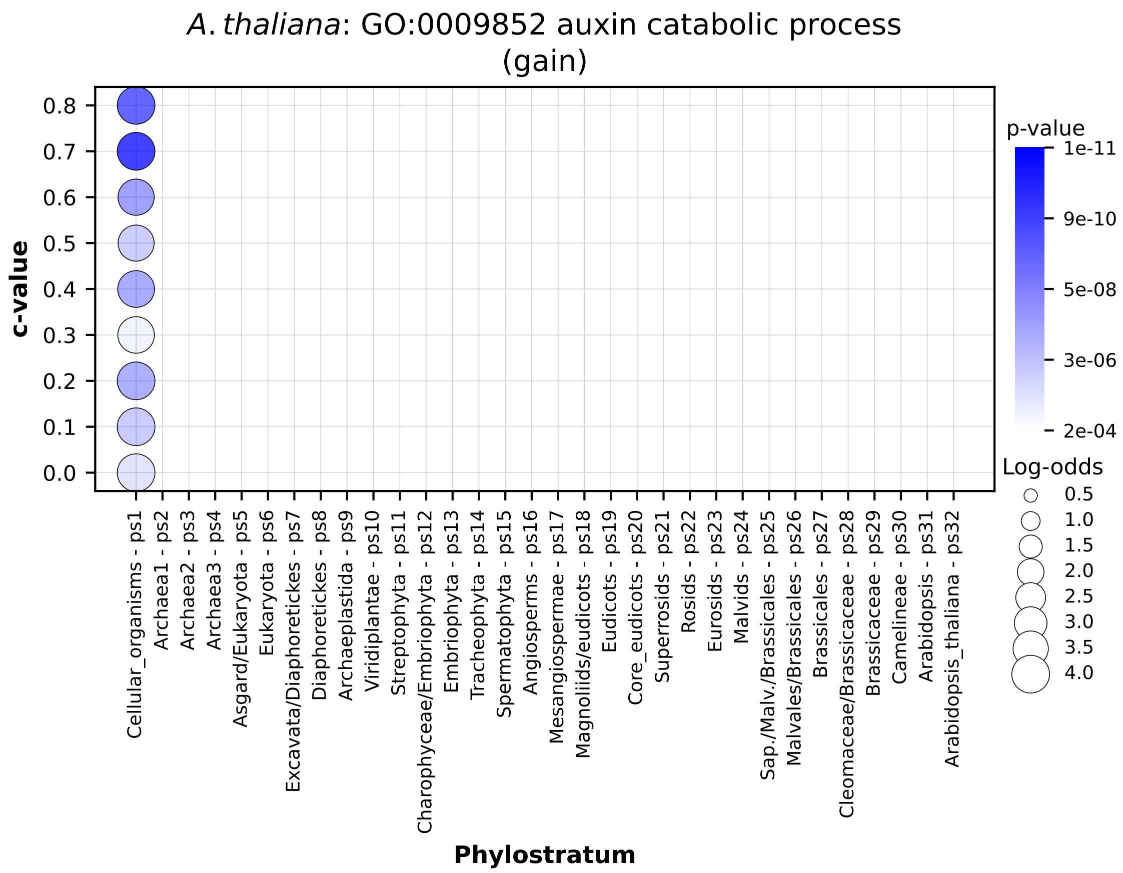

A. thaliana: GO:0009852 auxin catabolic process
(gain)
c-value
0.8
0.7
0.6
0.5
0.4
0.3
0.2
0.1
0.0
p-value
1e-11
9e-10
5e-08
3e-06
2e-04
Log-odds
0.5
1.0
1.5
2.0
2.5
3.0
3.5
4.0
Cellular_organisms - ps1
Archaea1 - ps2
Archaea2 - ps3
Archaea3 - ps4
Asgard/Eukaryota - ps5
Eukaryota - ps6
Excavata/Diaphoretickes - ps7
Diaphoretickes - ps8
Archaeplastida - ps9
Viridiplantae - ps10
Streptophyta - ps11
Charophyceae/Embriophyta - ps12
Embriophyta - ps13
Tracheophyta - ps14
Spermatophyta - ps15
Angiosperms - ps16
Mesangiospermae - ps17
Magnoliids/eudicots - ps18
Eudicots - ps19
Core_eudicots - ps20
Superrosids - ps21
Rosids - ps22
Eurosids - ps23
Malvids - ps24
Sap./Malv./Brassicales - ps25
Malvales/Brassicales - ps26
Brassicales - ps27
Cleomaceae/Brassicaceae - ps28
Brassicaceae - ps29
Camelineae - ps30
Arabidopsis - ps31
Arabidopsis_thaliana - ps32
Phylostratum

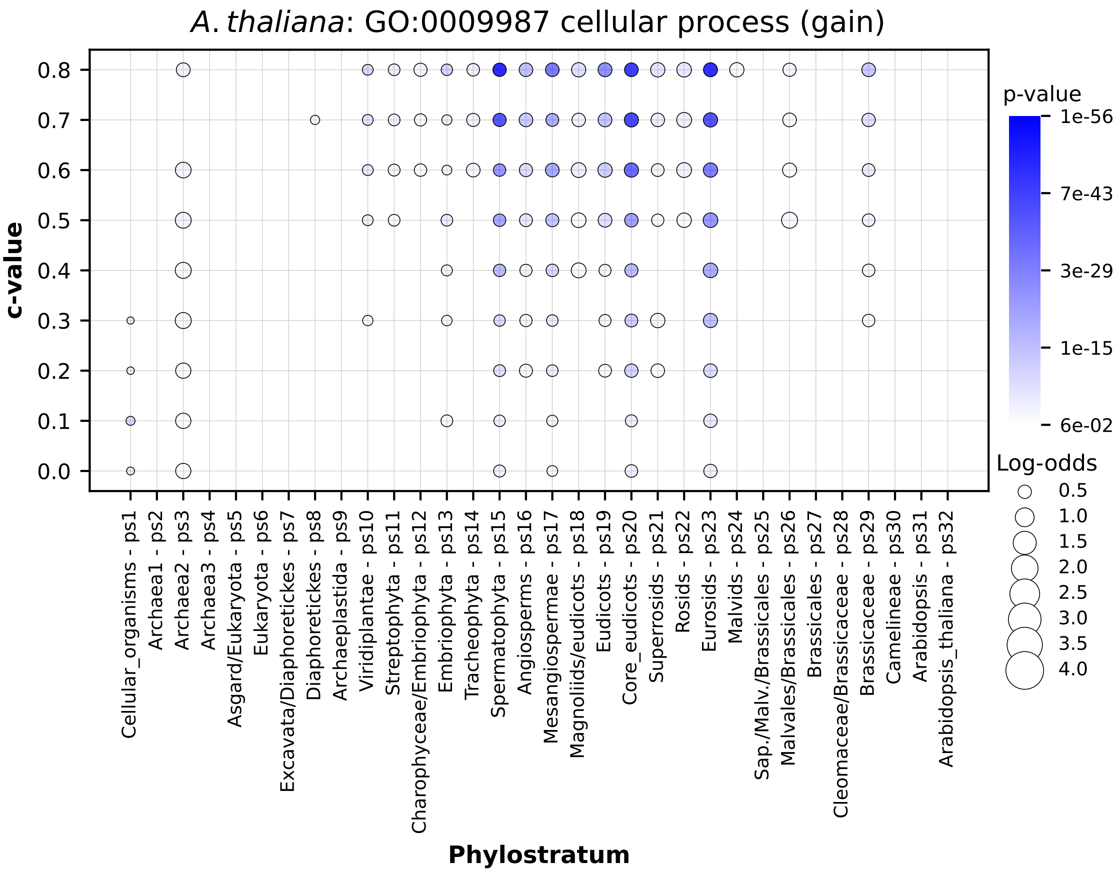

A. thaliana: GO:0009987 cellular process (gain)
c-value
0.8
0.7
0.6
0.5
0.4
0.3
0.2
0.1
0.0
Phylostratum
Cellular_organisms - ps1
Archaea1 - ps2
Archaea2 - ps3
Archaea3 - ps4
Asgard/Eukaryota - ps5
Eukaryota - ps6
Excavata/Diaphoretickes - ps7
Diaphoretickes - ps8
Archaeplastida - ps9
Viridiplantae - ps10
Streptophyta - ps11
Charophyceae/Embriophyta - ps12
Embriophyta - ps13
Tracheophyta - ps14
Spermatophyta - ps15
Angiosperms - ps16
Mesangiospermae - ps17
Magnoliids/eudicots - ps18
Eudicots - ps19
Core_eudicots - ps20
Superrosids - ps21
Rosids - ps22
Eurosids - ps23
Malvids - ps24
Sap./Malv./Brassicales - ps25
Malvales/Brassicales - ps26
Brassicales - ps27
Cleomaceae/Brassicaceae - ps28
Brassicaceae - ps29
Camelineae - ps30
Arabidopsis - ps31
Arabidopsis_thaliana - ps32
p-value
1e-56
7e-43
3e-29
1e-15
6e-02
Log-odds
0.5
1.0
1.5
2.0
2.5
3.0
3.5
4.0

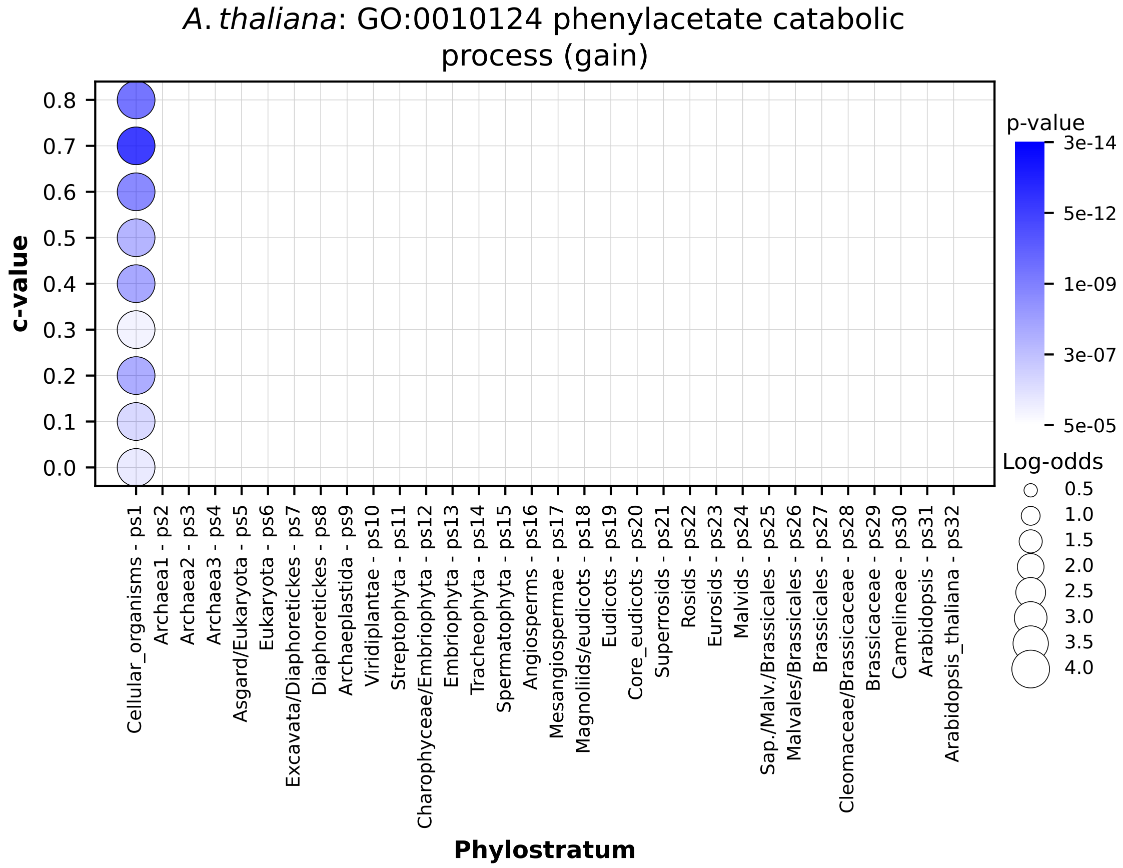
A. thaliana: GO:0010124 phenylacetate catabolic process (gain)
c-value
0.0
0.1
0.2
0.3
0.4
0.5
0.6
0.7
0.8
p-value
3e-14
5e-12
1e-09
3e-07
5e-05
Log-odds
0.5
1.0
1.5
2.0
2.5
3.0
3.5
4.0
Cellular_organisms - ps1
Archaea1 - ps2
Archaea2 - ps3
Archaea3 - ps4
Asgard/Eukaryota - ps5
Eukaryota - ps6
Excavata/Diaphoretickes - ps7
Diaphoretickes - ps8
Archaeplastida - ps9
Viridiplantae - ps10
Streptophyta - ps11
Charophyceae/Embriophyta - ps12
Embriophyta - ps13
Tracheophyta - ps14
Spermatophyta - ps15
Angiosperms - ps16
Mesangiospermae - ps17
Magnoliids/eudicots - ps18
Eudicots - ps19
Core_eudicots - ps20
Superrosids - ps21
Rosids - ps22
Eurosids - ps23
Malvids - ps24
Sap./Malv./Brassicales - ps25
Malvales/Brassicales - ps26
Brassicales - ps27
Cleomaceae/Brassicaceae - ps28
Brassicaceae - ps29
Camelineae - ps30
Arabidopsis - ps31
Arabidopsis_thaliana - ps32
Phylostratum

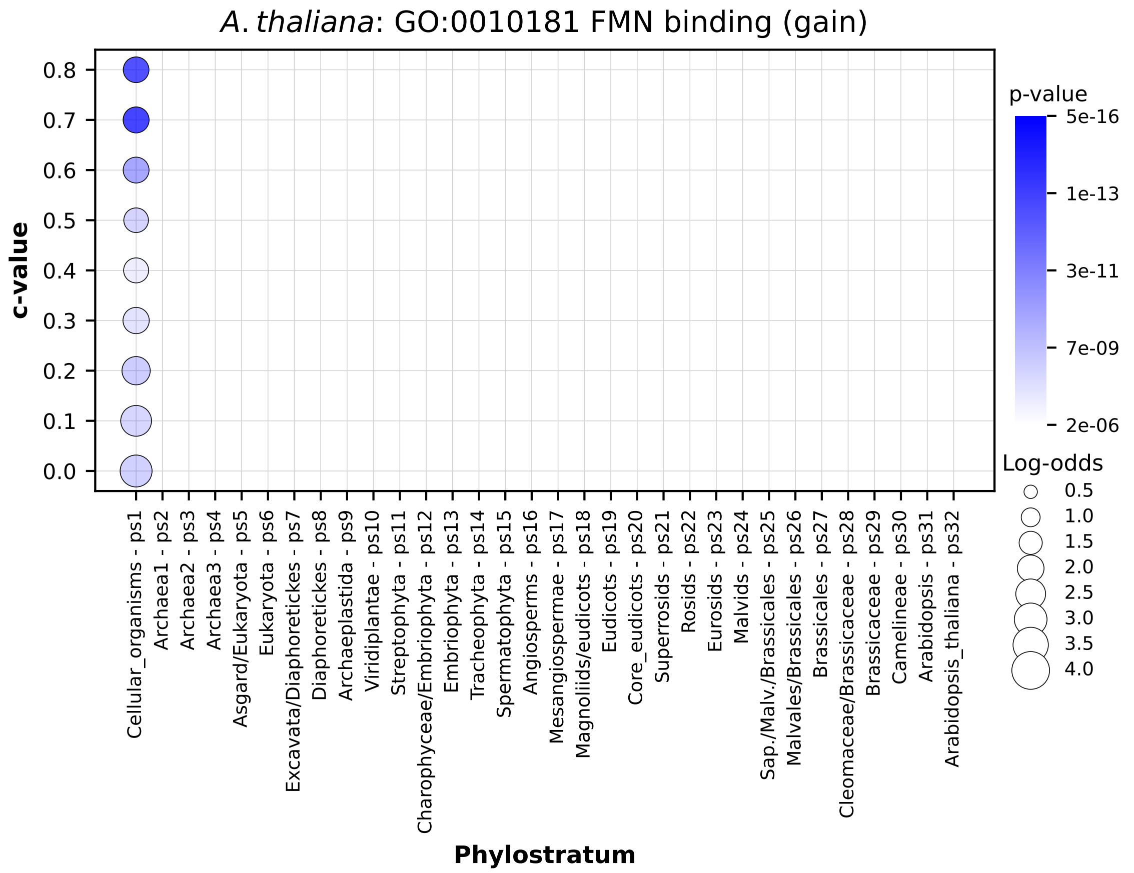

A. thaliana: GO:0010181 FMN binding (gain)
c-value
0.0
0.1
0.2
0.3
0.4
0.5
0.6
0.7
0.8
Phylostratum
Cellular_organisms - ps1
Archaea1 - ps2
Archaea2 - ps3
Archaea3 - ps4
Asgard/Eukaryota - ps5
Eukaryota - ps6
Excavata/Diaphoretickes - ps7
Diaphoretickes - ps8
Archaeplastida - ps9
Viridiplantae - ps10
Streptophyta - ps11
Charophyceae/Embriophyta - ps12
Embriophyta - ps13
Tracheophyta - ps14
Spermatophyta - ps15
Angiosperms - ps16
Mesangiospermae - ps17
Magnoliids/eudicots - ps18
Eudicots - ps19
Core_eudicots - ps20
Superrosids - ps21
Rosids - ps22
Eurosids - ps23
Malvids - ps24
Sap./Malv./Brassicales - ps25
Malvales/Brassicales - ps26
Brassicales - ps27
Cleomaceae/Brassicaceae - ps28
Brassicaceae - ps29
Camelineae - ps30
Arabidopsis - ps31
Arabidopsis_thaliana - ps32
p-value
5e-16
1e-13
3e-11
7e-09
2e-06
Log-odds
0.5
1.0
1.5
2.0
2.5
3.0
3.5
4.0

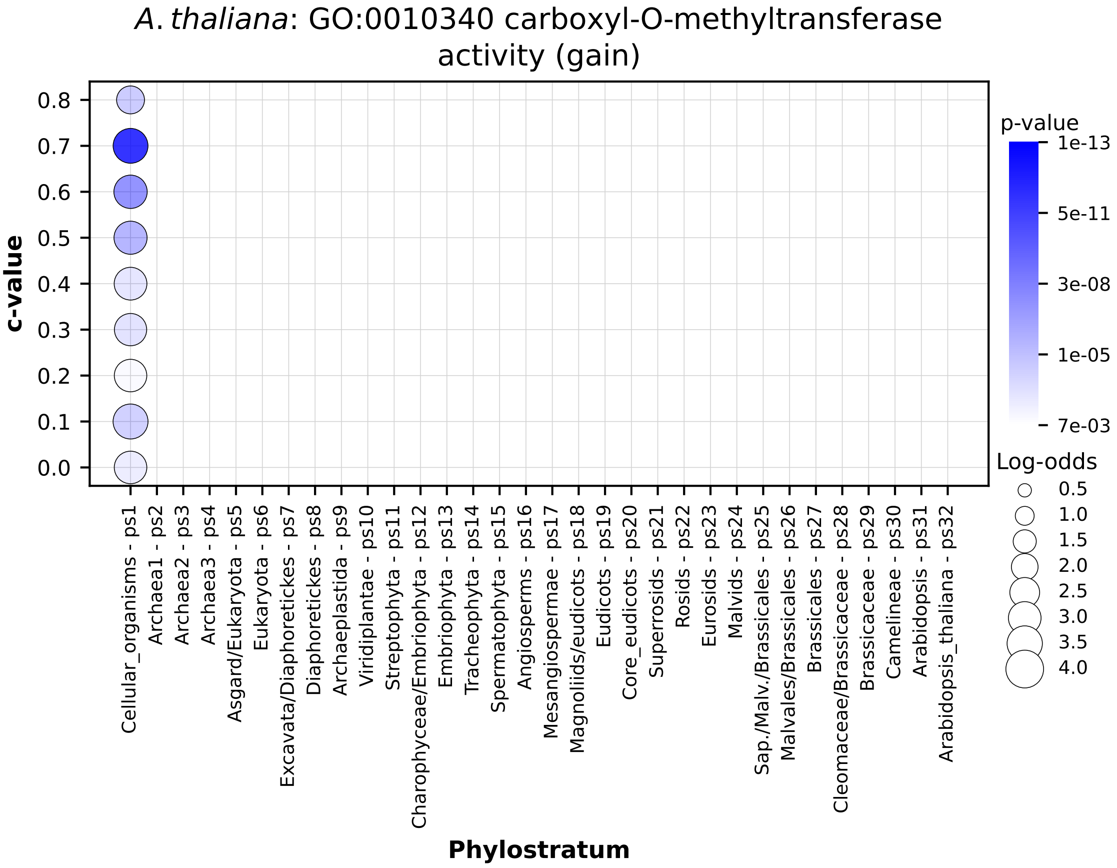
A. thaliana: GO:0010340 carboxyl-O-methyltransferase activity (gain)
c-value
0.0
0.1
0.2
0.3
0.4
0.5
0.6
0.7
0.8
Phylostratum
Cellular_organisms - ps1
Archaea1 - ps2
Archaea2 - ps3
Archaea3 - ps4
Asgard/Eukaryota - ps5
Eukaryota - ps6
Excavata/Diaphoretickes - ps7
Diaphoretickes - ps8
Archaeplastida - ps9
Viridiplantae - ps10
Streptophyta - ps11
Charophyceae/Embriophyta - ps12
Embriophyta - ps13
Tracheophyta - ps14
Spermatophyta - ps15
Angiosperms - ps16
Mesangiospermae - ps17
Magnoliids/eudicots - ps18
Eudicots - ps19
Core_eudicots - ps20
Superrosids - ps21
Rosids - ps22
Eurosids - ps23
Malvids - ps24
Sap./Malv./Brassicales - ps25
Malvales/Brassicales - ps26
Brassicales - ps27
Cleomaceae/Brassicaceae - ps28
Brassicaceae - ps29
Camelineae - ps30
Arabidopsis - ps31
Arabidopsis_thaliana - ps32
p-value
1e-13
5e-11
3e-08
1e-05
7e-03
Log-odds
0.5
1.0
1.5
2.0
2.5
3.0
3.5
4.0

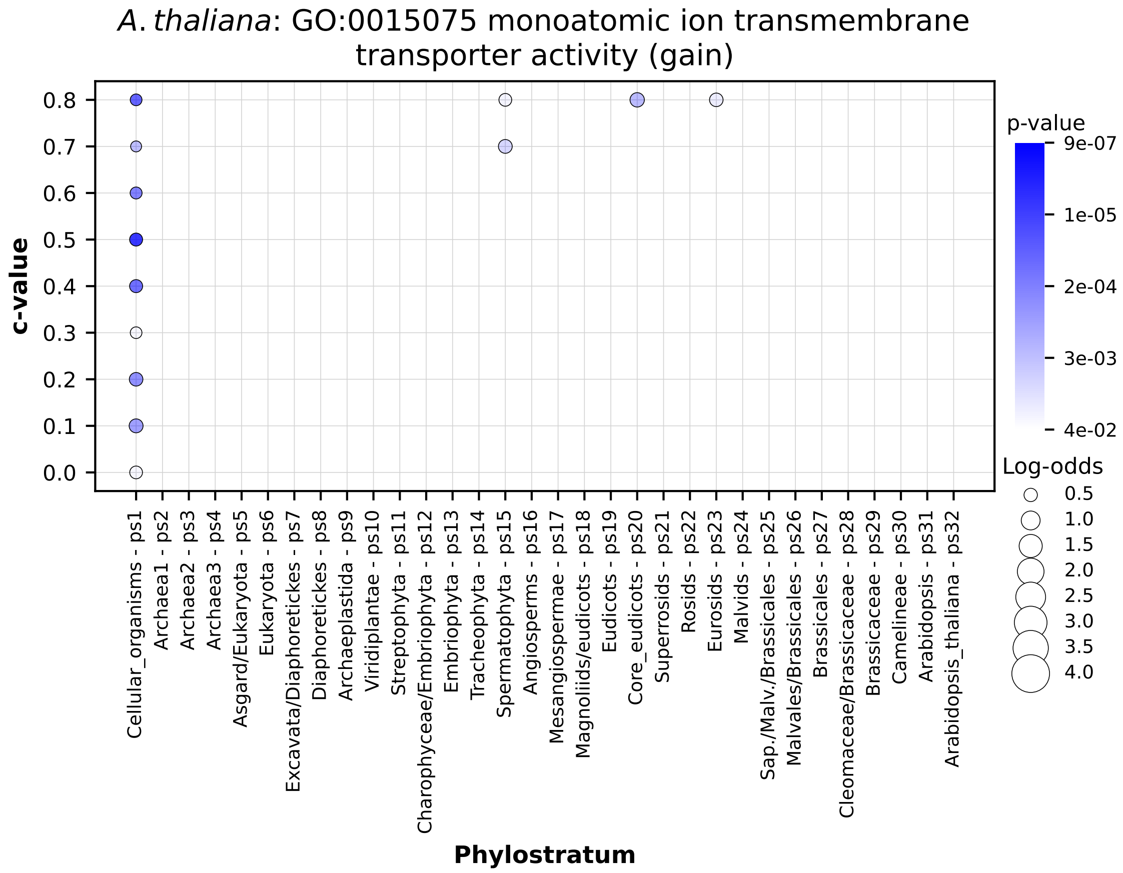
A. thaliana: GO:0015075 monoatomic ion transmembrane transporter activity (gain)
c-value
0.0
0.1
0.2
0.3
0.4
0.5
0.6
0.7
0.8
Phylostratum
Cellular_organisms - ps1
Archaea1 - ps2
Archaea2 - ps3
Archaea3 - ps4
Asgard/Eukaryota - ps5
Eukaryota - ps6
Excavata/Diaphoretickes - ps7
Diaphoretickes - ps8
Archaeplastida - ps9
Viridiplantae - ps10
Streptophyta - ps11
Charophyceae/Embriophyta - ps12
Embriophyta - ps13
Tracheophyta - ps14
Spermatophyta - ps15
Angiosperms - ps16
Mesangiospermae - ps17
Magnoliids/eudicots - ps18
Eudicots - ps19
Core_eudicots - ps20
Superrosids - ps21
Rosids - ps22
Eurosids - ps23
Malvids - ps24
Sap./Malv./Brassicales - ps25
Malvales/Brassicales - ps26
Brassicales - ps27
Cleomaceae/Brassicaceae - ps28
Brassicaceae - ps29
Camelineae - ps30
Arabidopsis - ps31
Arabidopsis_thaliana - ps32
p-value
9e-07
1e-05
2e-04
3e-03
4e-02
Log-odds
0.5
1.0
1.5
2.0
2.5
3.0
3.5
4.0

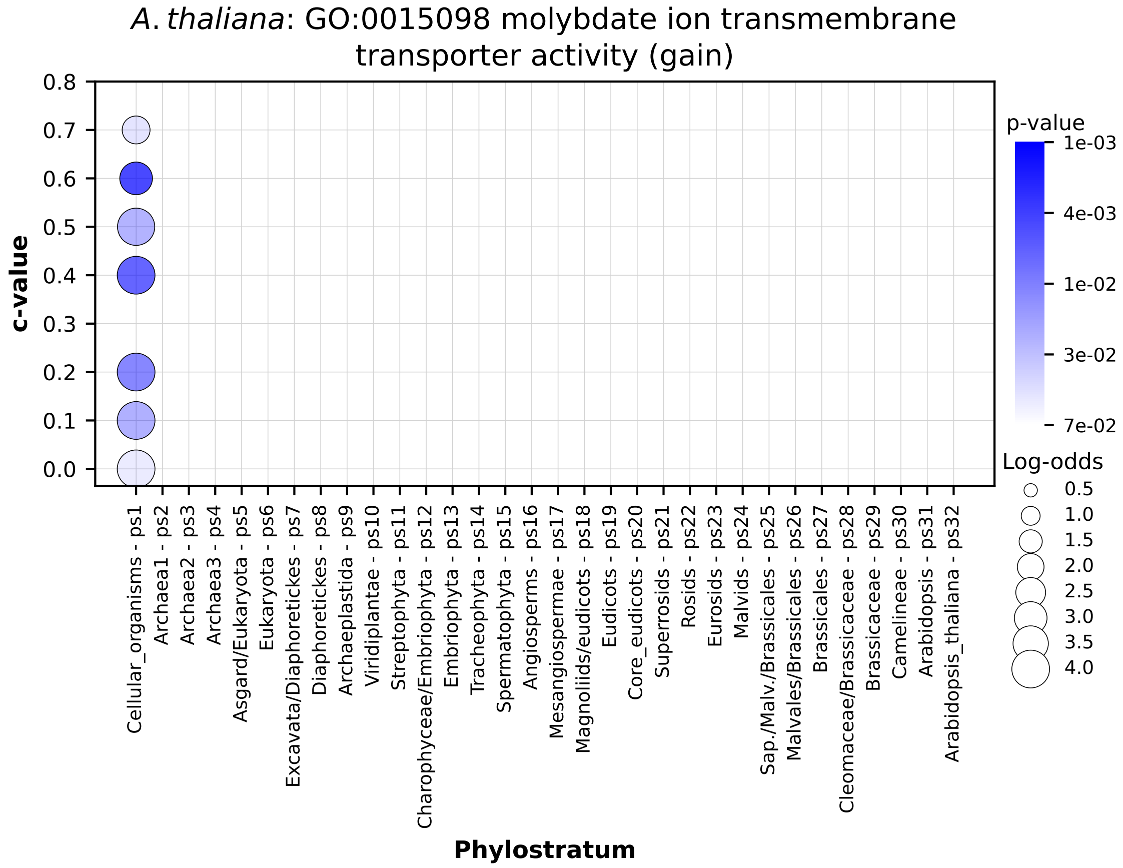

A. thaliana: GO:0015098 molybdate ion transmembrane transporter activity (gain)
c-value
0.8
0.7
0.6
0.5
0.4
0.3
0.2
0.1
0.0
p-value
1e-03
4e-03
1e-02
3e-02
7e-02
Log-odds
0.5
1.0
1.5
2.0
2.5
3.0
3.5
4.0
Cellular_organisms - ps1
Archaea1 - ps2
Archaea2 - ps3
Archaea3 - ps4
Asgard/Eukaryota - ps5
Eukaryota - ps6
Excavata/Diaphoretickes - ps7
Diaphoretickes - ps8
Archaeplastida - ps9
Viridiplantae - ps10
Streptophyta - ps11
Charophyceae/Embriophyta - ps12
Embriophyta - ps13
Tracheophyta - ps14
Spermatophyta - ps15
Angiosperms - ps16
Mesangiospermae - ps17
Magnoliids/eudicots - ps18
Eudicots - ps19
Core_eudicots - ps20
Superrosids - ps21
Rosids - ps22
Eurosids - ps23
Malvids - ps24
Sap./Malv./Brassicales - ps25
Malvales/Brassicales - ps26
Brassicales - ps27
Cleomaceae/Brassicaceae - ps28
Brassicaceae - ps29
Camelineae - ps30
Arabidopsis - ps31
Arabidopsis_thaliana - ps32
Phylostratum

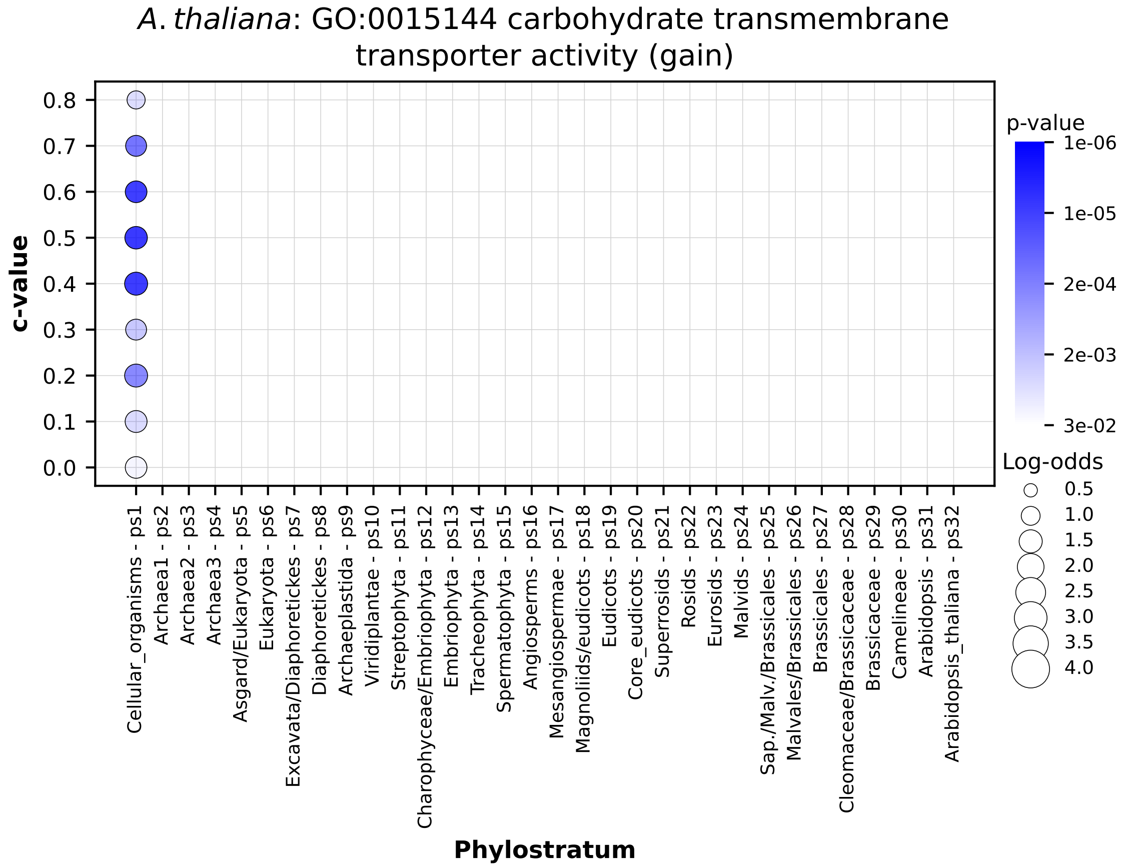

A. thaliana: GO:0015144 carbohydrate transmembrane transporter activity (gain)
c-value
0.8
0.7
0.6
0.5
0.4
0.3
0.2
0.1
0.0
p-value
1e-06
1e-05
2e-04
2e-03
3e-02
Log-odds
0.5
1.0
1.5
2.0
2.5
3.0
3.5
4.0
Cellular_organisms - ps1
Archaea1 - ps2
Archaea2 - ps3
Archaea3 - ps4
Asgard/Eukaryota - ps5
Eukaryota - ps6
Excavata/Diaphoretickes - ps7
Diaphoretickes - ps8
Archaeplastida - ps9
Viridiplantae - ps10
Streptophyta - ps11
Charophyceae/Embriophyta - ps12
Embriophyta - ps13
Tracheophyta - ps14
Spermatophyta - ps15
Angiosperms - ps16
Mesangiospermae - ps17
Magnoliids/eudicots - ps18
Eudicots - ps19
Core_eudicots - ps20
Superrosids - ps21
Rosids - ps22
Eurosids - ps23
Malvids - ps24
Sap./Malv./Brassicales - ps25
Malvales/Brassicales - ps26
Brassicales - ps27
Cleomaceae/Brassicaceae - ps28
Brassicaceae - ps29
Camelineae - ps30
Arabidopsis - ps31
Arabidopsis_thaliana - ps32
Phylostratum

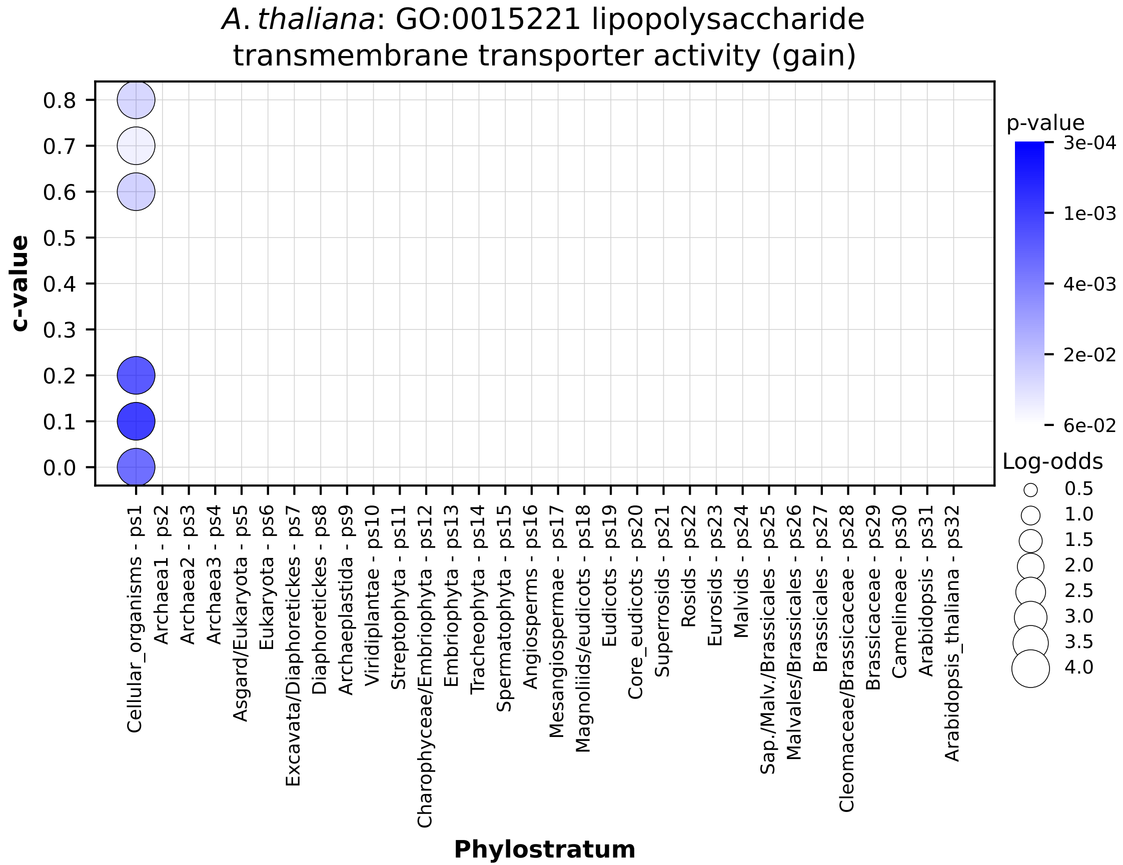

A. thaliana: GO:0015221 lipopolysaccharide transmembrane transporter activity (gain)
c-value
0.8
0.7
0.6
0.5
0.4
0.3
0.2
0.1
0.0
p-value
3e-04
1e-03
4e-03
2e-02
6e-02
Log-odds
0.5
1.0
1.5
2.0
2.5
3.0
3.5
4.0
Cellular_organisms - ps1
Archaea1 - ps2
Archaea2 - ps3
Archaea3 - ps4
Asgard/Eukaryota - ps5
Eukaryota - ps6
Excavata/Diaphoretickes - ps7
Diaphoretickes - ps8
Archaeplastida - ps9
Viridiplantae - ps10
Streptophyta - ps11
Charophyceae/Embriophyta - ps12
Embriophyta - ps13
Tracheophyta - ps14
Spermatophyta - ps15
Angiosperms - ps16
Mesangiospermae - ps17
Magnoliids/eudicots - ps18
Eudicots - ps19
Core_eudicots - ps20
Superrosids - ps21
Rosids - ps22
Eurosids - ps23
Malvids - ps24
Sap./Malv./Brassicales - ps25
Malvales/Brassicales - ps26
Brassicales - ps27
Cleomaceae/Brassicaceae - ps28
Brassicaceae - ps29
Camelineae - ps30
Arabidopsis - ps31
Arabidopsis_thaliana - ps32
Phylostratum

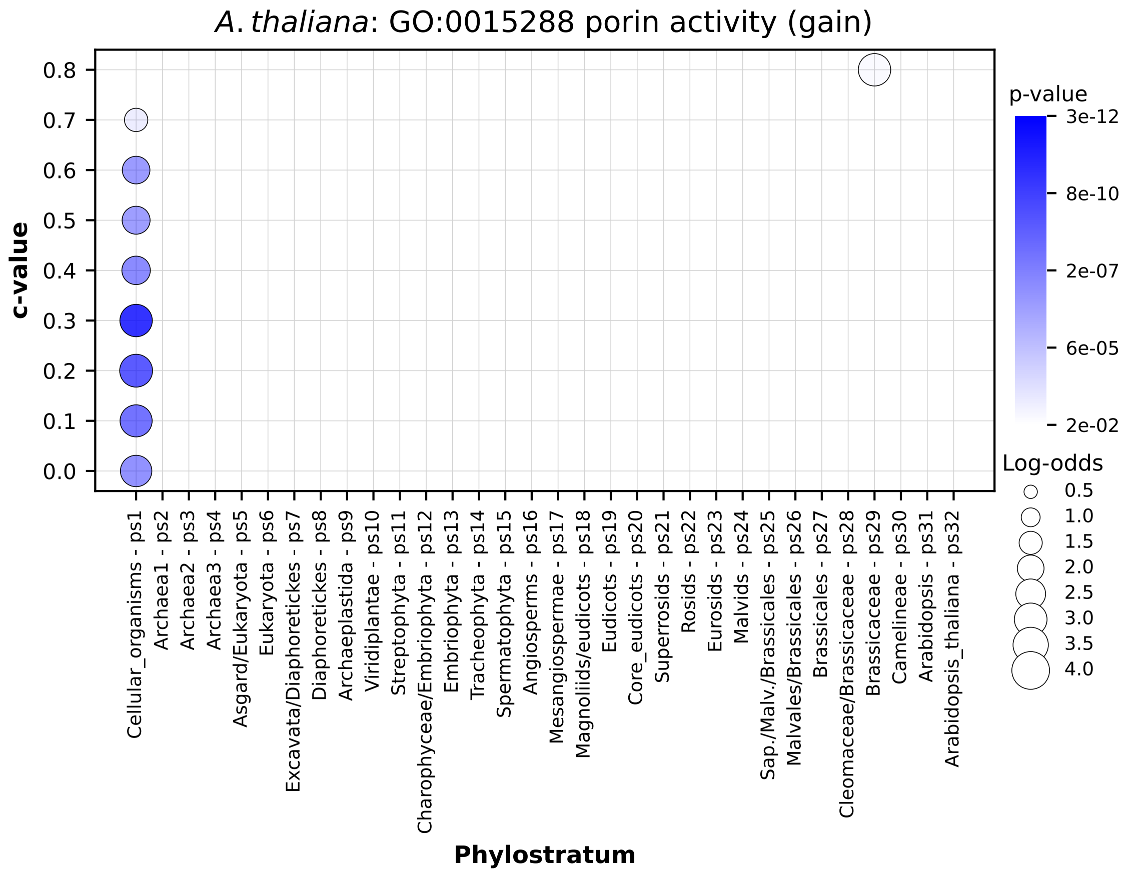

A. thaliana: GO:0015288 porin activity (gain)
c-value
0.0
0.1
0.2
0.3
0.4
0.5
0.6
0.7
0.8
Phylostratum
Cellular_organisms - ps1
Archaea1 - ps2
Archaea2 - ps3
Archaea3 - ps4
Asgard/Eukaryota - ps5
Eukaryota - ps6
Excavata/Diaphoretickes - ps7
Diaphoretickes - ps8
Archaeplastida - ps9
Viridiplantae - ps10
Streptophyta - ps11
Charophyceae/Embriophyta - ps12
Embriophyta - ps13
Tracheophyta - ps14
Spermatophyta - ps15
Angiosperms - ps16
Mesangiospermae - ps17
Magnoliids/eudicots - ps18
Eudicots - ps19
Core_eudicots - ps20
Superrosids - ps21
Rosids - ps22
Eurosids - ps23
Malvids - ps24
Sap./Malv./Brassicales - ps25
Malvales/Brassicales - ps26
Brassicales - ps27
Cleomaceae/Brassicaceae - ps28
Brassicaceae - ps29
Camelineae - ps30
Arabidopsis - ps31
Arabidopsis_thaliana - ps32
p-value
3e-12
8e-10
2e-07
6e-05
2e-02
Log-odds
0.5
1.0
1.5
2.0
2.5
3.0
3.5
4.0

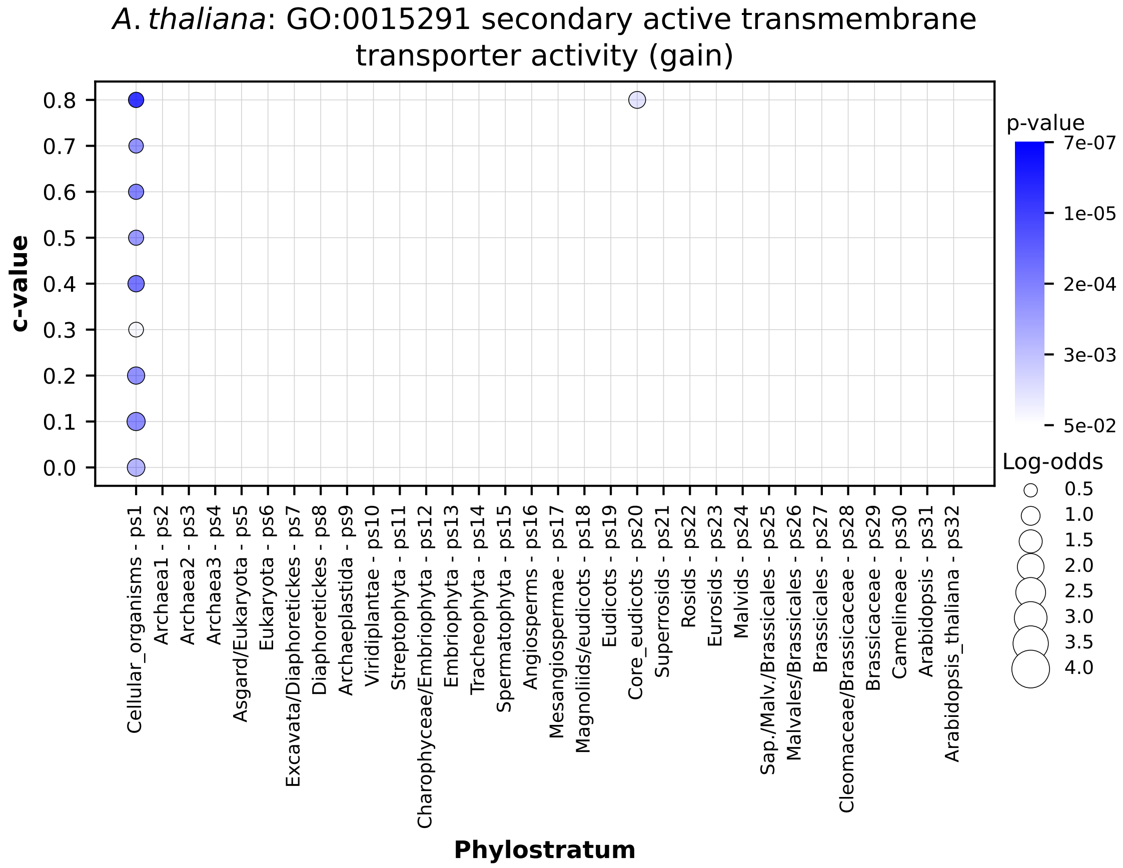

A. thaliana: GO:0015291 secondary active transmembrane transporter activity (gain)
c-value
0.0
0.1
0.2
0.3
0.4
0.5
0.6
0.7
0.8
Phylostratum
Cellular_organisms - ps1
Archaea1 - ps2
Archaea2 - ps3
Archaea3 - ps4
Asgard/Eukaryota - ps5
Eukaryota - ps6
Excavata/Diaphoretickes - ps7
Diaphoretickes - ps8
Archaeplastida - ps9
Viridiplantae - ps10
Streptophyta - ps11
Charophyceae/Embriophyta - ps12
Embriophyta - ps13
Tracheophyta - ps14
Spermatophyta - ps15
Angiosperms - ps16
Mesangiospermae - ps17
Magnoliids/eudicots - ps18
Eudicots - ps19
Core_eudicots - ps20
Superrosids - ps21
Rosids - ps22
Eurosids - ps23
Malvids - ps24
Sap./Malv./Brassicales - ps25
Malvales/Brassicales - ps26
Brassicales - ps27
Cleomaceae/Brassicaceae - ps28
Brassicaceae - ps29
Camelineae - ps30
Arabidopsis - ps31
Arabidopsis_thaliana - ps32
p-value
7e-07
1e-05
2e-04
3e-03
5e-02
Log-odds
0.5
1.0
1.5
2.0
2.5
3.0
3.5
4.0

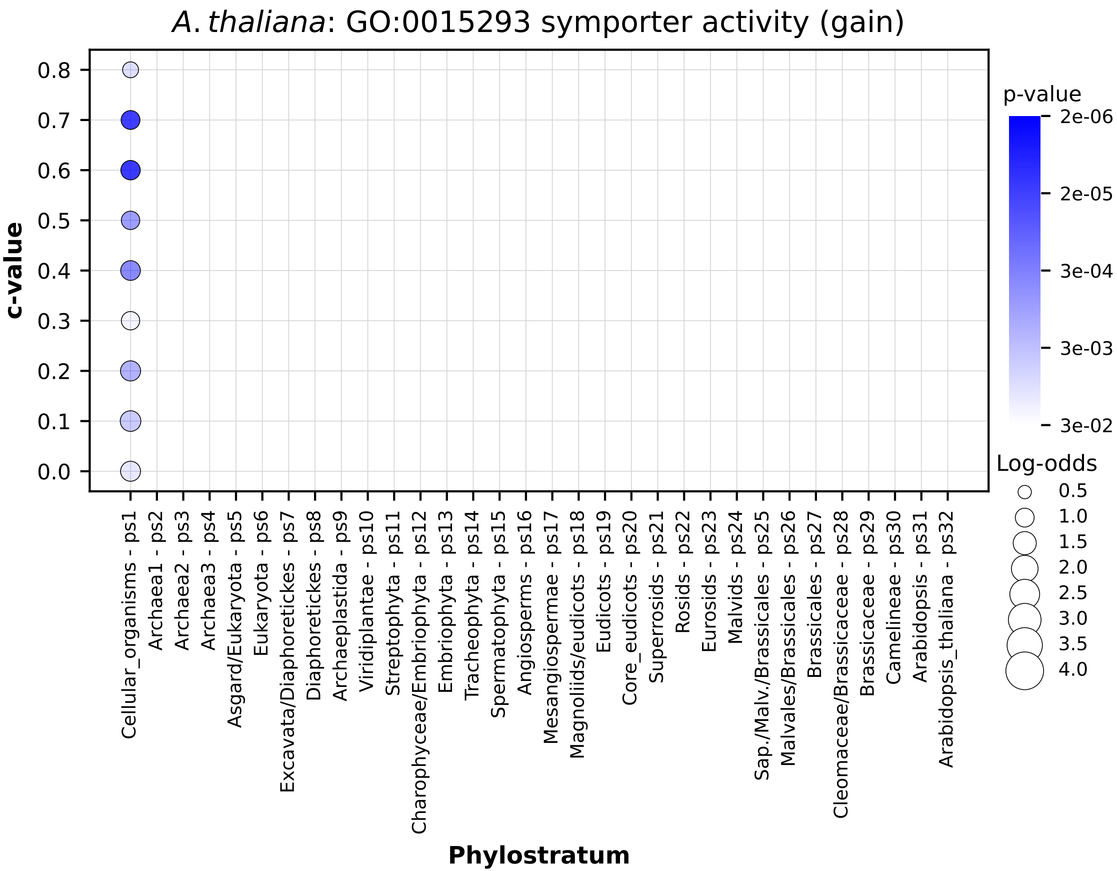

A. thaliana: GO:0015293 symporter activity (gain)
c-value
0.8
0.7
0.6
0.5
0.4
0.3
0.2
0.1
0.0
p-value
2e-06
2e-05
3e-04
3e-03
3e-02
Log-odds
0.5
1.0
1.5
2.0
2.5
3.0
3.5
4.0
Cellular_organisms - ps1
Archaea1 - ps2
Archaea2 - ps3
Archaea3 - ps4
Asgard/Eukaryota - ps5
Eukaryota - ps6
Excavata/Diaphoretickes - ps7
Diaphoretickes - ps8
Archaeplastida - ps9
Viridiplantae - ps10
Streptophyta - ps11
Charophyceae/Embriophyta - ps12
Embriophyta - ps13
Tracheophyta - ps14
Spermatophyta - ps15
Angiosperms - ps16
Mesangiospermae - ps17
Magnoliids/eudicots - ps18
Eudicots - ps19
Core_eudicots - ps20
Superrosids - ps21
Rosids - ps22
Eurosids - ps23
Malvids - ps24
Sap./Malv./Brassicales - ps25
Malvales/Brassicales - ps26
Brassicales - ps27
Cleomaceae/Brassicaceae - ps28
Brassicaceae - ps29
Camelineae - ps30
Arabidopsis - ps31
Arabidopsis_thaliana - ps32
Phylostratum

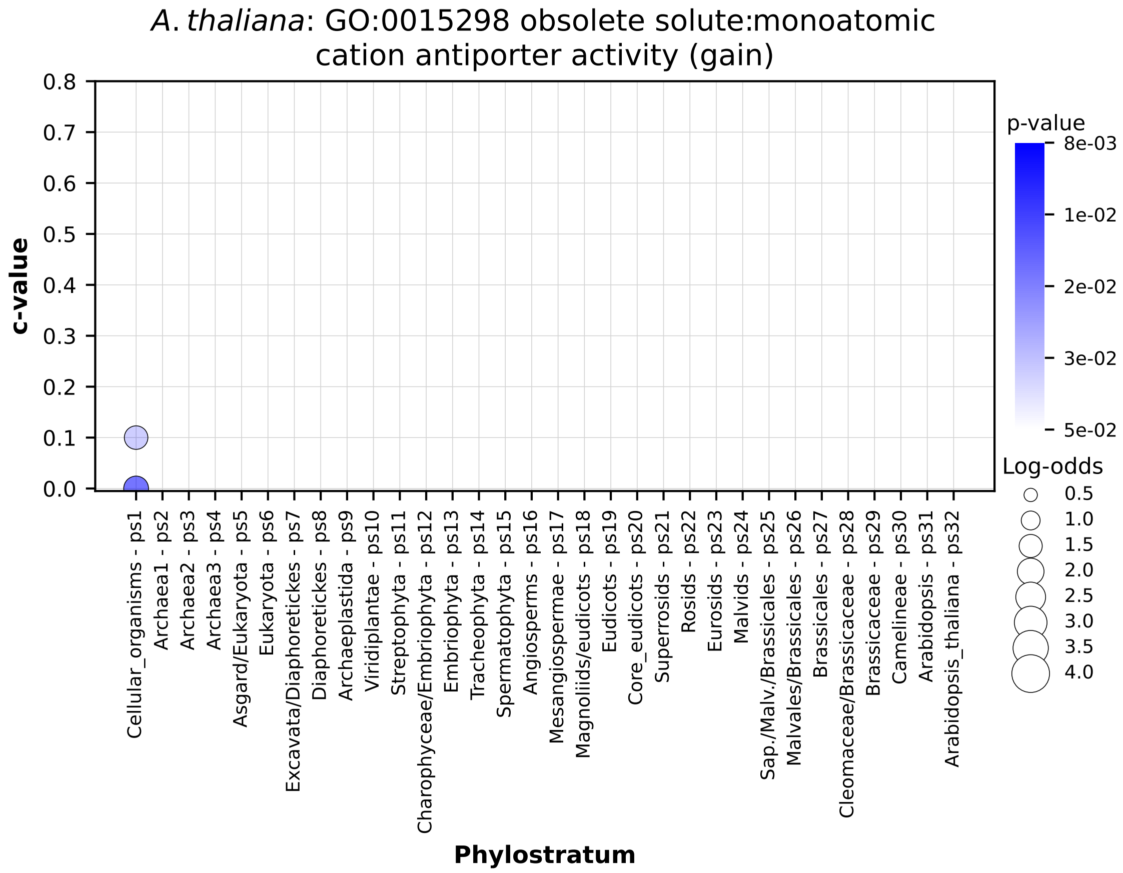

A. thaliana: GO:0015298 obsolete solute:monoatomic cation antiporter activity (gain)
c-value
0.8
0.7
0.6
0.5
0.4
0.3
0.2
0.1
0.0
p-value
8e-03
1e-02
2e-02
3e-02
5e-02
Log-odds
0.5
1.0
1.5
2.0
2.5
3.0
3.5
4.0
Cellular_organisms - ps1
Archaea1 - ps2
Archaea2 - ps3
Archaea3 - ps4
Asgard/Eukaryota - ps5
Eukaryota - ps6
Excavata/Diaphoretickes - ps7
Diaphoretickes - ps8
Archaeplastida - ps9
Viridiplantae - ps10
Streptophyta - ps11
Charophyceae/Embriophyta - ps12
Embriophyta - ps13
Tracheophyta - ps14
Spermatophyta - ps15
Angiosperms - ps16
Mesangiospermae - ps17
Magnoliids/eudicots - ps18
Eudicots - ps19
Core_eudicots - ps20
Superrosids - ps21
Rosids - ps22
Eurosids - ps23
Malvids - ps24
Sap./Malv./Brassicales - ps25
Malvales/Brassicales - ps26
Brassicales - ps27
Cleomaceae/Brassicaceae - ps28
Brassicaceae - ps29
Camelineae - ps30
Arabidopsis - ps31
Arabidopsis_thaliana - ps32
Phylostratum

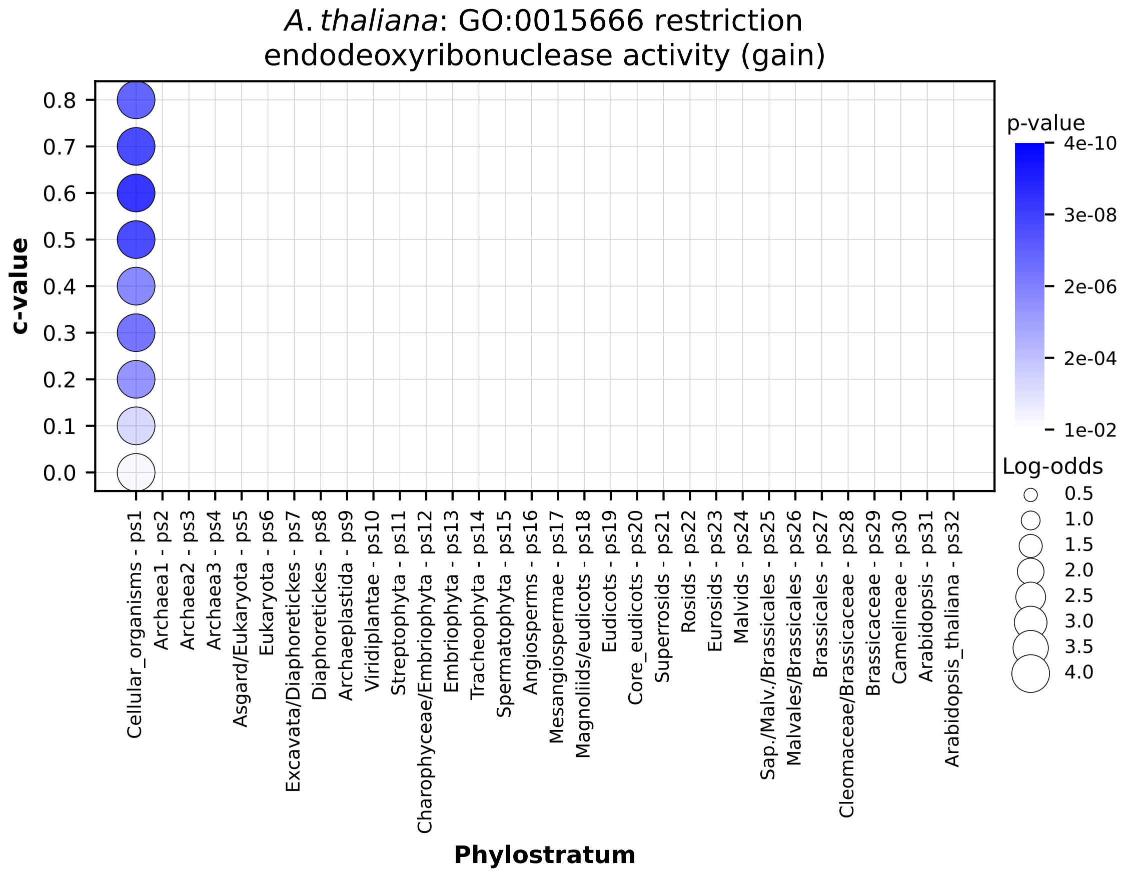

A. thaliana: GO:0015666 restriction endodeoxyribonuclease activity (gain)
c-value
0.0
0.1
0.2
0.3
0.4
0.5
0.6
0.7
0.8
p-value
4e-10
3e-08
2e-06
2e-04
1e-02
Log-odds
0.5
1.0
1.5
2.0
2.5
3.0
3.5
4.0
Cellular_organisms - ps1
Archaea1 - ps2
Archaea2 - ps3
Archaea3 - ps4
Asgard/Eukaryota - ps5
Eukaryota - ps6
Excavata/Diaphoretickes - ps7
Diaphoretickes - ps8
Archaeplastida - ps9
Viridiplantae - ps10
Streptophyta - ps11
Charophyceae/Embriophyta - ps12
Embriophyta - ps13
Tracheophyta - ps14
Spermatophyta - ps15
Angiosperms - ps16
Mesangiospermae - ps17
Magnoliids/eudicots - ps18
Eudicots - ps19
Core_eudicots - ps20
Superrosids - ps21
Rosids - ps22
Eurosids - ps23
Malvids - ps24
Sap./Malv./Brassicales - ps25
Malvales/Brassicales - ps26
Brassicales - ps27
Cleomaceae/Brassicaceae - ps28
Brassicaceae - ps29
Camelineae - ps30
Arabidopsis - ps31
Arabidopsis_thaliana - ps32
Phylostratum

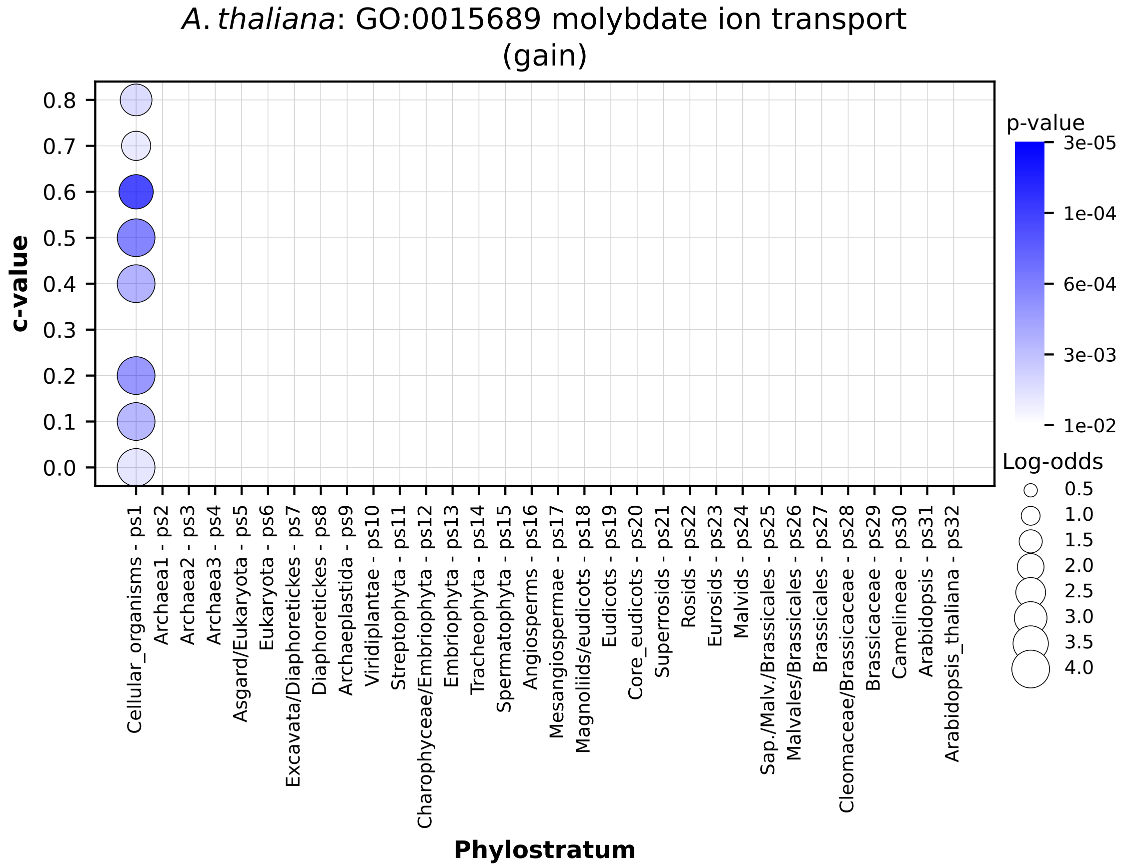

A. thaliana: GO:0015689 molybdate ion transport
(gain)
c-value
0.8
0.7
0.6
0.5
0.4
0.3
0.2
0.1
0.0
p-value
3e-05
1e-04
6e-04
3e-03
1e-02
Log-odds
0.5
1.0
1.5
2.0
2.5
3.0
3.5
4.0
Cellular_organisms - ps1
Archaea1 - ps2
Archaea2 - ps3
Archaea3 - ps4
Asgard/Eukaryota - ps5
Eukaryota - ps6
Excavata/Diaphoretickes - ps7
Diaphoretickes - ps8
Archaeplastida - ps9
Viridiplantae - ps10
Streptophyta - ps11
Charophyceae/Embriophyta - ps12
Embriophyta - ps13
Tracheophyta - ps14
Spermatophyta - ps15
Angiosperms - ps16
Mesangiospermae - ps17
Magnoliids/eudicots - ps18
Eudicots - ps19
Core_eudicots - ps20
Superrosids - ps21
Rosids - ps22
Eurosids - ps23
Malvids - ps24
Sap./Malv./Brassicales - ps25
Malvales/Brassicales - ps26
Brassicales - ps27
Cleomaceae/Brassicaceae - ps28
Brassicaceae - ps29
Camelineae - ps30
Arabidopsis - ps31
Arabidopsis_thaliana - ps32
Phylostratum

A. thaliana: GO:0015774 polysaccharide transport (gain)

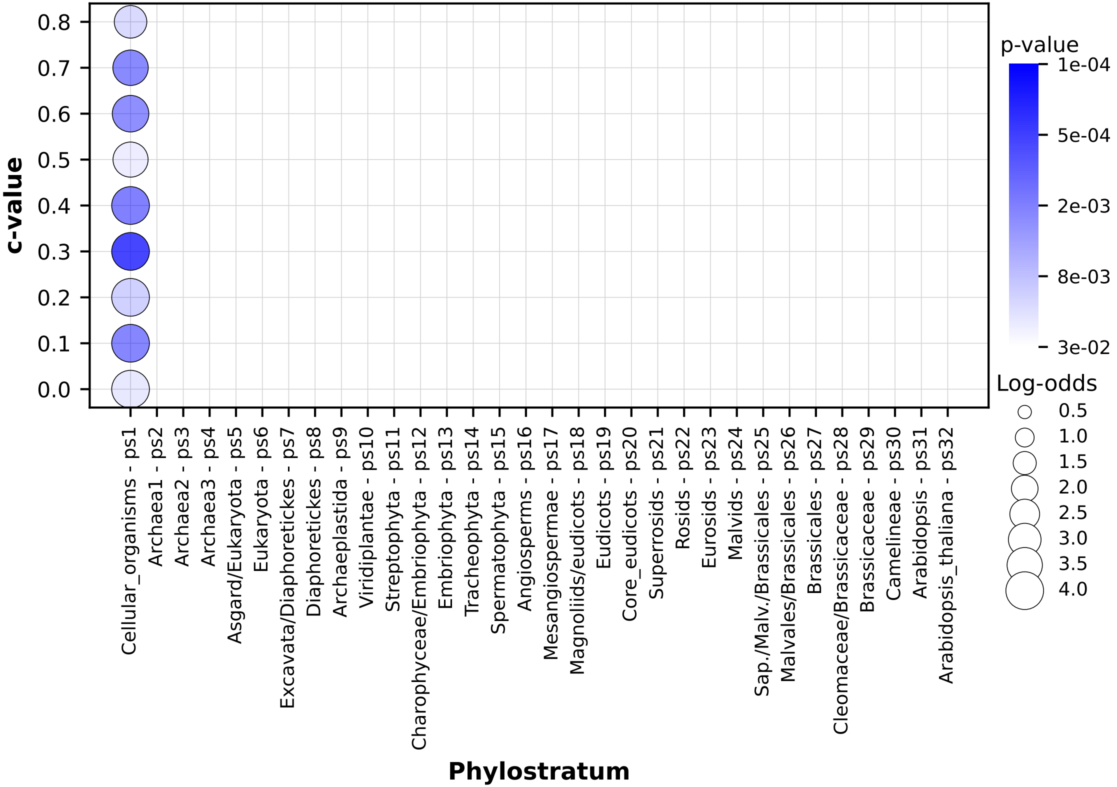

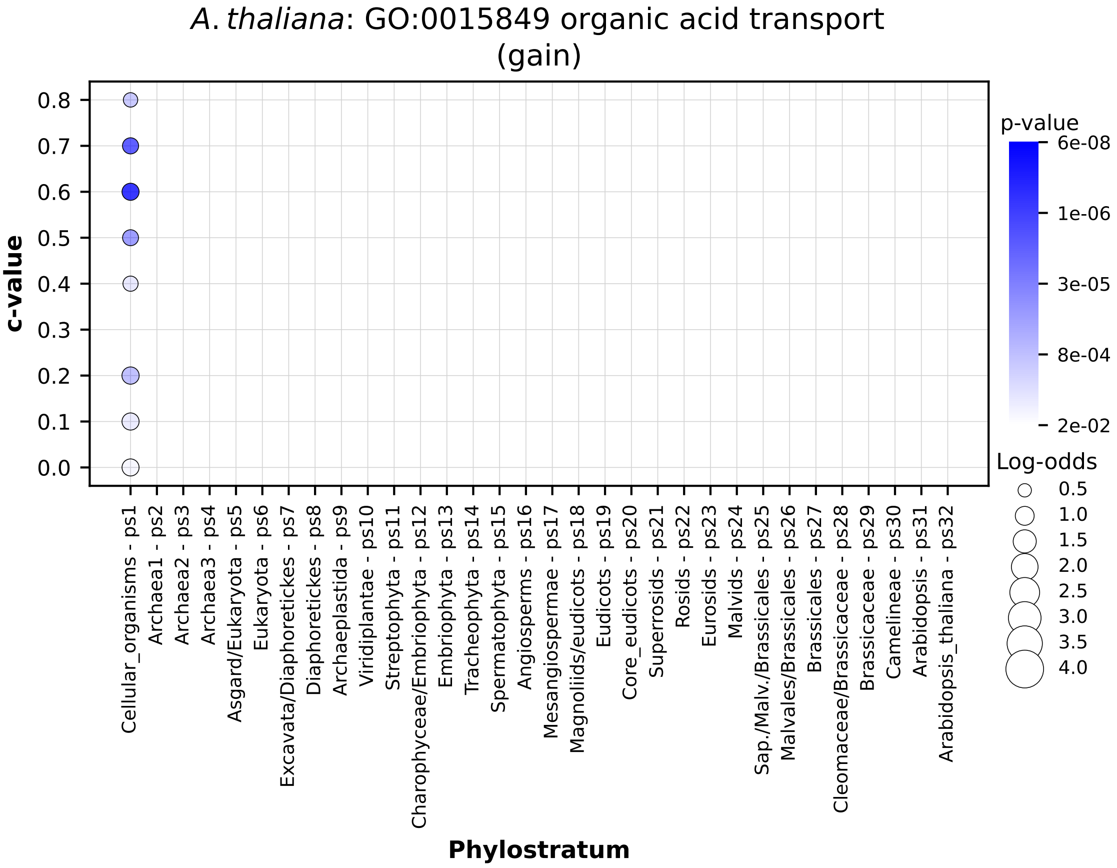

A. thaliana: GO:0015849 organic acid transport
(gain)
c-value
0.8
0.7
0.6
0.5
0.4
0.3
0.2
0.1
0.0
Phylostratum
Cellular_organisms - ps1
Archaea1 - ps2
Archaea2 - ps3
Archaea3 - ps4
Asgard/Eukaryota - ps5
Eukaryota - ps6
Excavata/Diaphoretickes - ps7
Diaphoretickes - ps8
Archaeplastida - ps9
Viridiplantae - ps10
Streptophyta - ps11
Charophyceae/Embriophyta - ps12
Embriophyta - ps13
Tracheophyta - ps14
Spermatophyta - ps15
Angiosperms - ps16
Mesangiospermae - ps17
Magnoliids/eudicots - ps18
Eudicots - ps19
Core_eudicots - ps20
Superrosids - ps21
Rosids - ps22
Eurosids - ps23
Malvids - ps24
Sap./Malv./Brassicales - ps25
Malvales/Brassicales - ps26
Brassicales - ps27
Cleomaceae/Brassicaceae - ps28
Brassicaceae - ps29
Camelineae - ps30
Arabidopsis - ps31
Arabidopsis_thaliana - ps32
p-value
6e-08
1e-06
3e-05
8e-04
2e-02
Log-odds
0.5
1.0
1.5
2.0
2.5
3.0
3.5
4.0

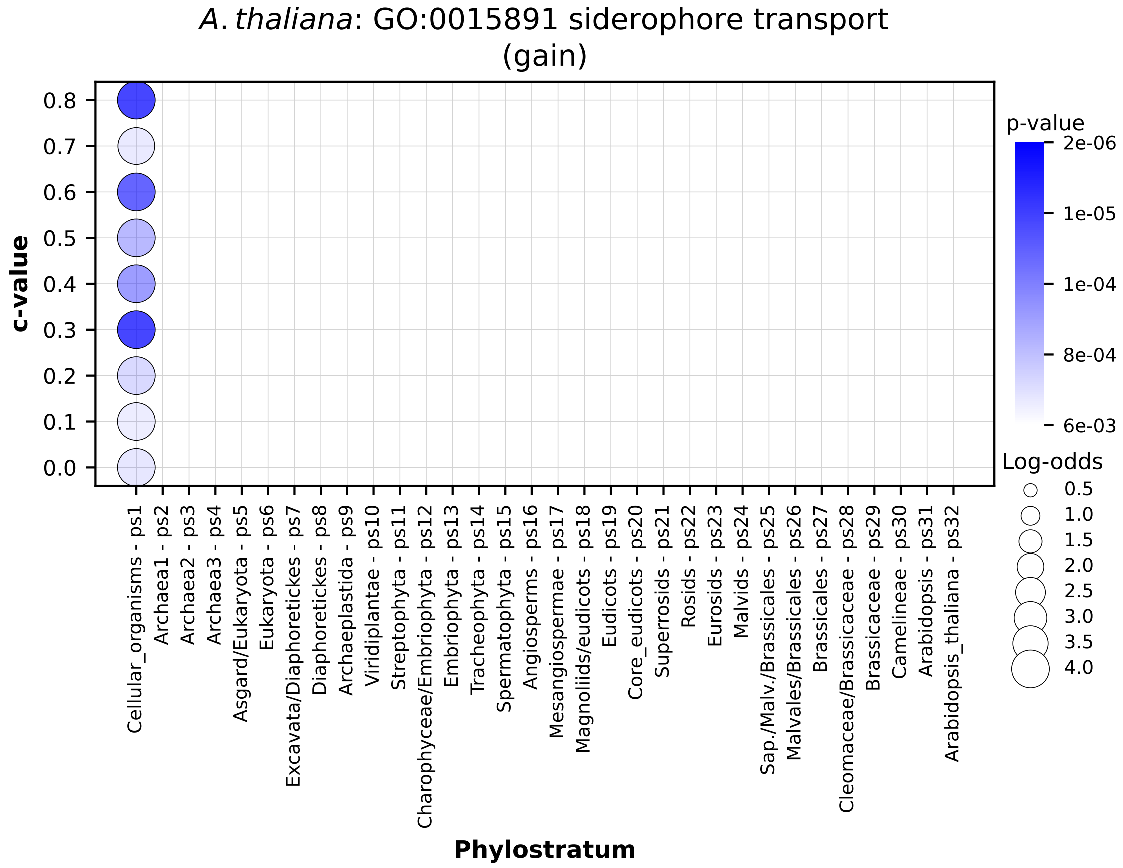

A. thaliana: GO:0015891 siderophore transport
(gain)
c-value
0.8
0.7
0.6
0.5
0.4
0.3
0.2
0.1
0.0
p-value
2e-06
1e-05
1e-04
8e-04
6e-03
Log-odds
0.5
1.0
1.5
2.0
2.5
3.0
3.5
4.0
Cellular_organisms - ps1
Archaea1 - ps2
Archaea2 - ps3
Archaea3 - ps4
Asgard/Eukaryota - ps5
Eukaryota - ps6
Excavata/Diaphoretickes - ps7
Diaphoretickes - ps8
Archaeplastida - ps9
Viridiplantae - ps10
Streptophyta - ps11
Charophyceae/Embriophyta - ps12
Embriophyta - ps13
Tracheophyta - ps14
Spermatophyta - ps15
Angiosperms - ps16
Mesangiospermae - ps17
Magnoliids/eudicots - ps18
Eudicots - ps19
Core_eudicots - ps20
Superrosids - ps21
Rosids - ps22
Eurosids - ps23
Malvids - ps24
Sap./Malv./Brassicales - ps25
Malvales/Brassicales - ps26
Brassicales - ps27
Cleomaceae/Brassicaceae - ps28
Brassicaceae - ps29
Camelineae - ps30
Arabidopsis - ps31
Arabidopsis_thaliana - ps32
Phylostratum

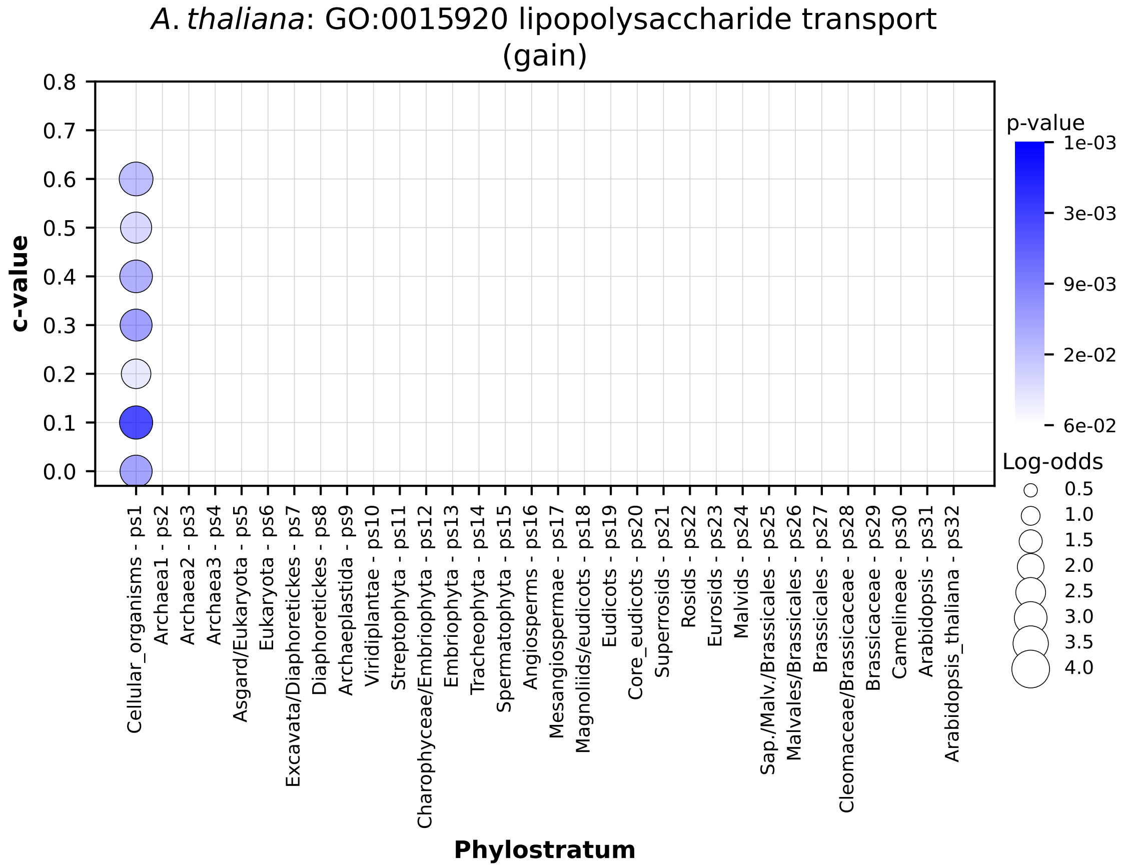
A. thaliana: GO:0015920 lipopolysaccharide transport (gain)
c-value
0.8
0.7
0.6
0.5
0.4
0.3
0.2
0.1
0.0
Phylostratum
Cellular_organisms - ps1
Archaea1 - ps2
Archaea2 - ps3
Archaea3 - ps4
Asgard/Eukaryota - ps5
Eukaryota - ps6
Excavata/Diaphoretickes - ps7
Diaphoretickes - ps8
Archaeplastida - ps9
Viridiplantae - ps10
Streptophyta - ps11
Charophyceae/Embriophyta - ps12
Embriophyta - ps13
Tracheophyta - ps14
Spermatophyta - ps15
Angiosperms - ps16
Mesangiospermae - ps17
Magnoliids/eudicots - ps18
Eudicots - ps19
Core_eudicots - ps20
Superrosids - ps21
Rosids - ps22
Eurosids - ps23
Malvids - ps24
Sap./Malv./Brassicales - ps25
Malvales/Brassicales - ps26
Brassicales - ps27
Cleomaceae/Brassicaceae - ps28
Brassicaceae - ps29
Camelineae - ps30
Arabidopsis - ps31
Arabidopsis_thaliana - ps32
p-value
1e-03
3e-03
9e-03
2e-02
6e-02
Log-odds
0.5
1.0
1.5
2.0
2.5
3.0
3.5
4.0

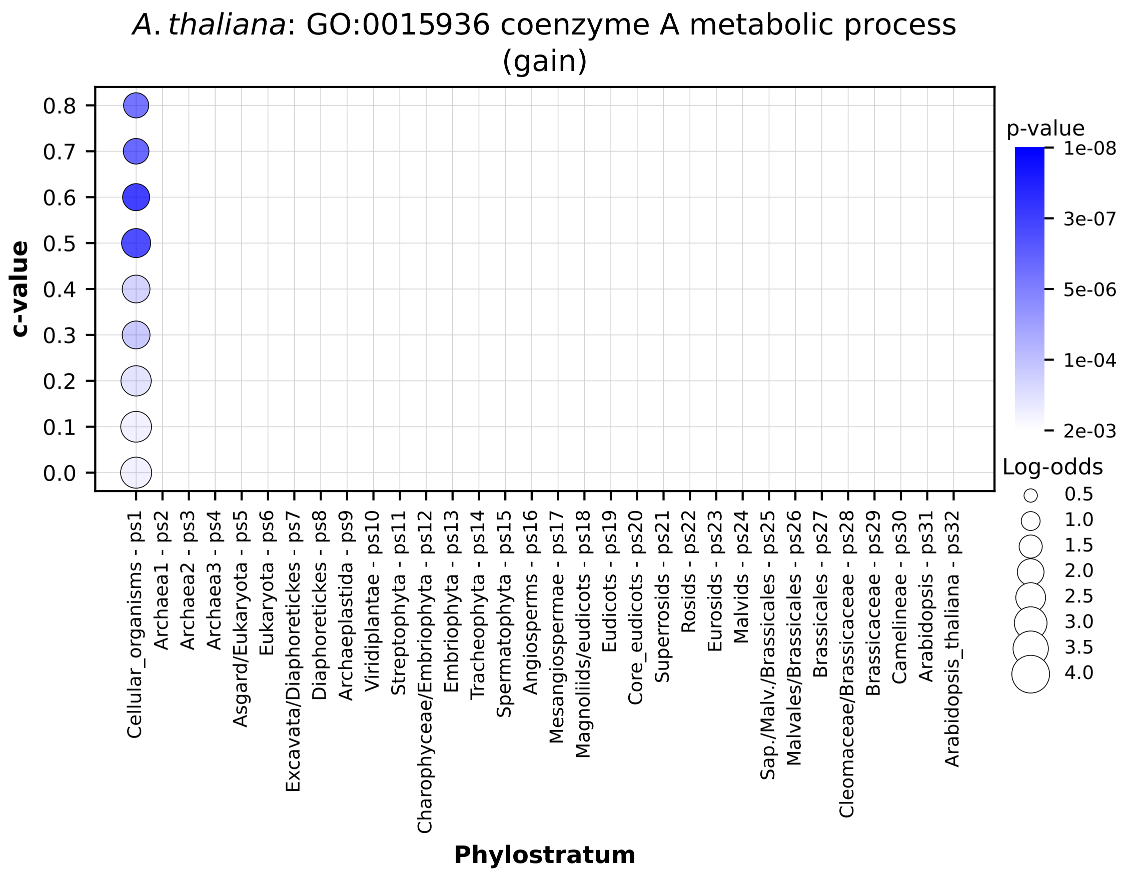

A. thaliana: GO:0015936 coenzyme A metabolic process (gain)
c-value
0.0
0.1
0.2
0.3
0.4
0.5
0.6
0.7
0.8
Phylostratum
Cellular_organisms - ps1
Archaea1 - ps2
Archaea2 - ps3
Archaea3 - ps4
Asgard/Eukaryota - ps5
Eukaryota - ps6
Excavata/Diaphoretickes - ps7
Diaphoretickes - ps8
Archaeplastida - ps9
Viridiplantae - ps10
Streptophyta - ps11
Charophyceae/Embriophyta - ps12
Embriophyta - ps13
Tracheophyta - ps14
Spermatophyta - ps15
Angiosperms - ps16
Mesangiospermae - ps17
Magnoliids/eudicots - ps18
Eudicots - ps19
Core_eudicots - ps20
Superrosids - ps21
Rosids - ps22
Eurosids - ps23
Malvids - ps24
Sap./Malv./Brassicales - ps25
Malvales/Brassicales - ps26
Brassicales - ps27
Cleomaceae/Brassicaceae - ps28
Brassicaceae - ps29
Camelineae - ps30
Arabidopsis - ps31
Arabidopsis_thaliana - ps32
p-value
1e-08
3e-07
5e-06
1e-04
2e-03
Log-odds
0.5
1.0
1.5
2.0
2.5
3.0
3.5
4.0

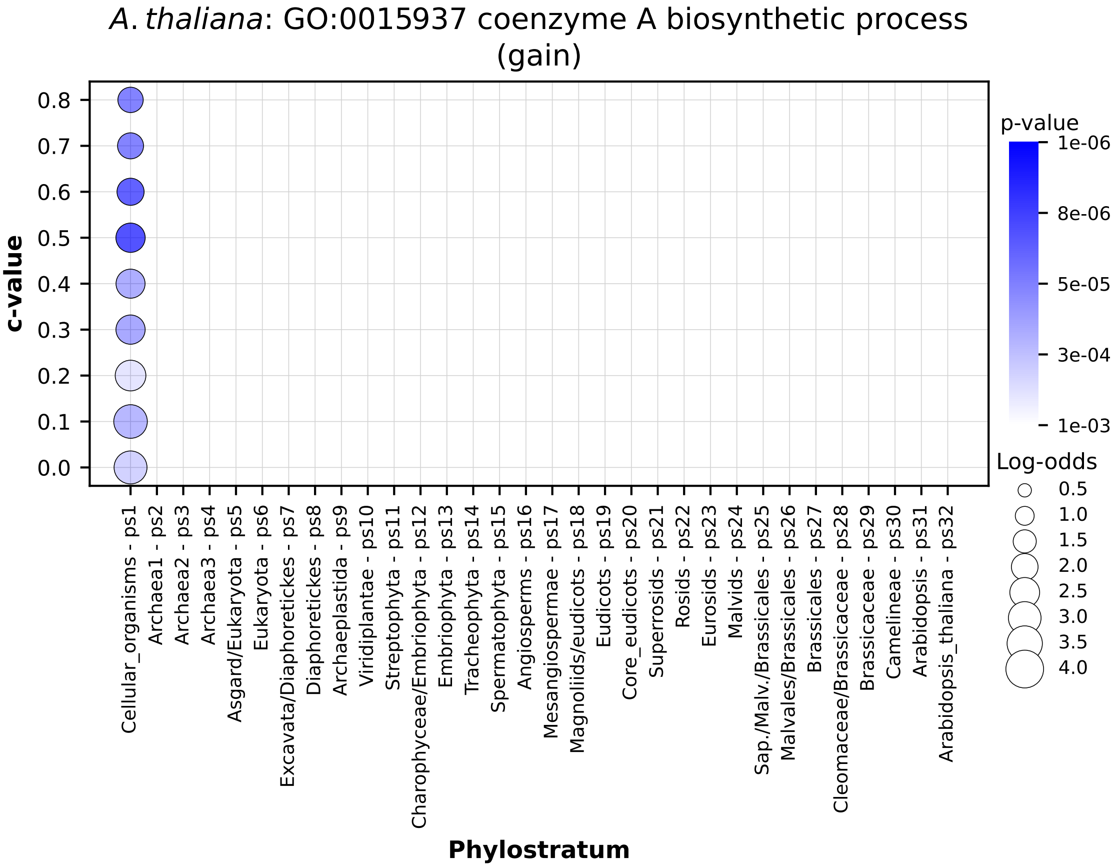

A. thaliana: GO:0015937 coenzyme A biosynthetic process (gain)
c-value
0.8
0.7
0.6
0.5
0.4
0.3
0.2
0.1
0.0
Phylostratum
Cellular_organisms - ps1
Archaea1 - ps2
Archaea2 - ps3
Archaea3 - ps4
Asgard/Eukaryota - ps5
Eukaryota - ps6
Excavata/Diaphoretickes - ps7
Diaphoretickes - ps8
Archaeplastida - ps9
Viridiplantae - ps10
Streptophyta - ps11
Charophyceae/Embriophyta - ps12
Embriophyta - ps13
Tracheophyta - ps14
Spermatophyta - ps15
Angiosperms - ps16
Mesangiospermae - ps17
Magnoliids/eudicots - ps18
Eudicots - ps19
Core_eudicots - ps20
Superrosids - ps21
Rosids - ps22
Eurosids - ps23
Malvids - ps24
Sap./Malv./Brassicales - ps25
Malvales/Brassicales - ps26
Brassicales - ps27
Cleomaceae/Brassicaceae - ps28
Brassicaceae - ps29
Camelineae - ps30
Arabidopsis - ps31
Arabidopsis_thaliana - ps32
p-value
1e-06
8e-06
5e-05
3e-04
1e-03
Log-odds
0.5
1.0
1.5
2.0
2.5
3.0
3.5
4.0

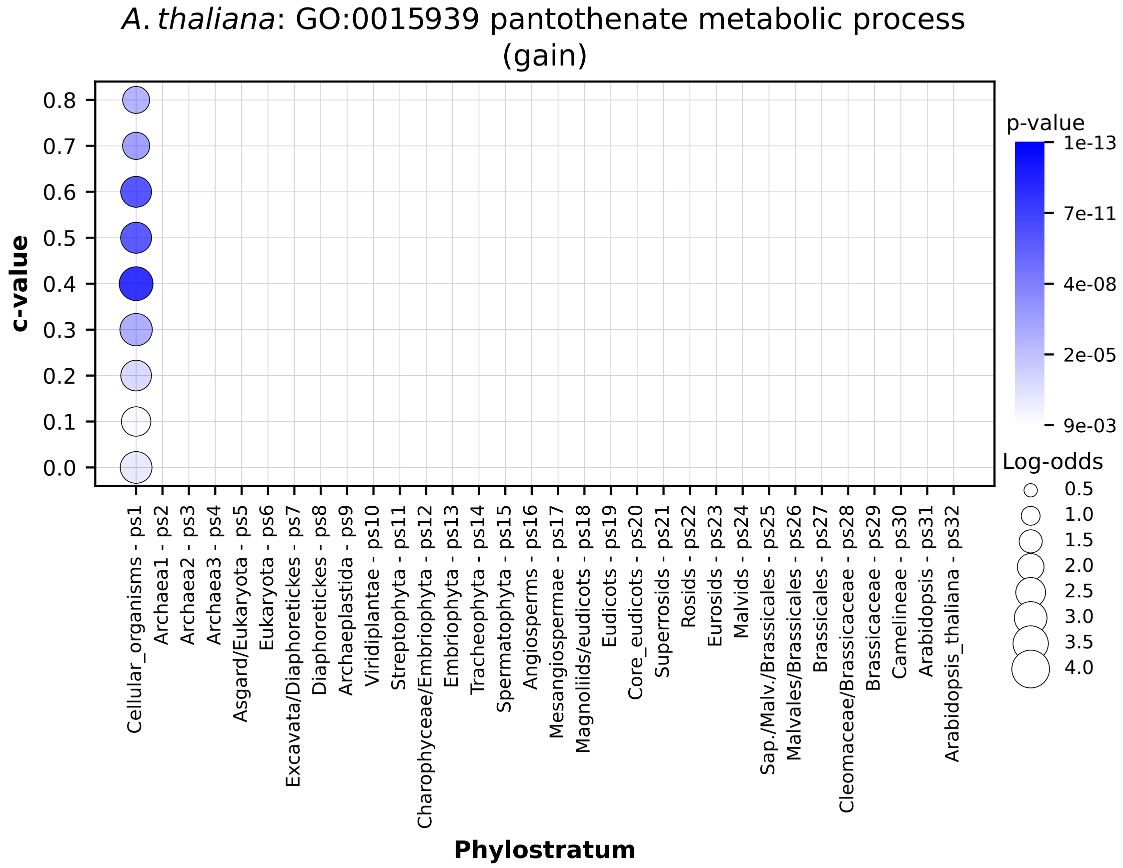

A. thaliana: GO:0015939 pantothenate metabolic process (gain)
c-value
0.8
0.7
0.6
0.5
0.4
0.3
0.2
0.1
0.0
p-value
1e-13
7e-11
4e-08
2e-05
9e-03
Log-odds
0.5
1.0
1.5
2.0
2.5
3.0
3.5
4.0
Cellular_organisms - ps1
Archaea1 - ps2
Archaea2 - ps3
Archaea3 - ps4
Asgard/Eukaryota - ps5
Eukaryota - ps6
Excavata/Diaphoretickes - ps7
Diaphoretickes - ps8
Archaeplastida - ps9
Viridiplantae - ps10
Streptophyta - ps11
Charophyceae/Embriophyta - ps12
Embriophyta - ps13
Tracheophyta - ps14
Spermatophyta - ps15
Angiosperms - ps16
Mesangiospermae - ps17
Magnoliids/eudicots - ps18
Eudicots - ps19
Core_eudicots - ps20
Superrosids - ps21
Rosids - ps22
Eurosids - ps23
Malvids - ps24
Sap./Malv./Brassicales - ps25
Malvales/Brassicales - ps26
Brassicales - ps27
Cleomaceae/Brassicaceae - ps28
Brassicaceae - ps29
Camelineae - ps30
Arabidopsis - ps31
Arabidopsis_thaliana - ps32
Phylostratum

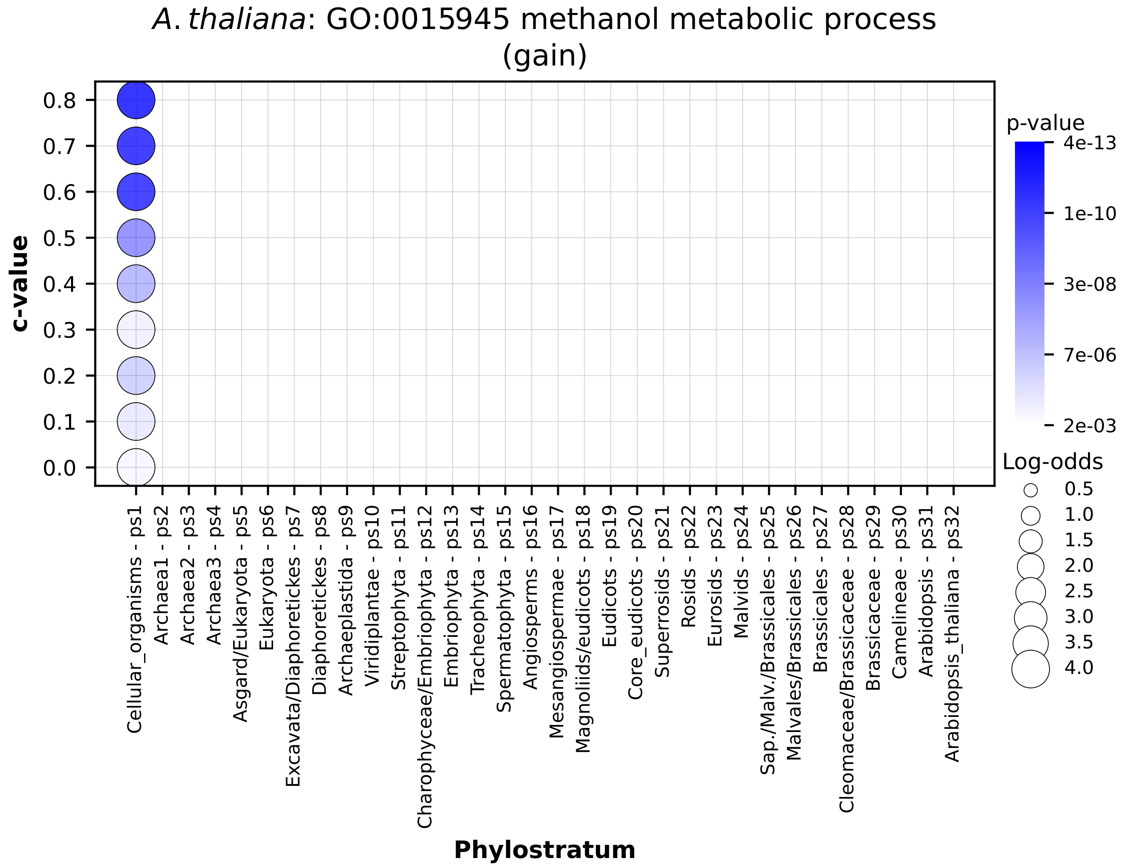
A. thaliana: GO:0015945 methanol metabolic process
(gain)
c-value
0.8
0.7
0.6
0.5
0.4
0.3
0.2
0.1
0.0
p-value
4e-13
1e-10
3e-08
7e-06
2e-03
Log-odds
0.5
1.0
1.5
2.0
2.5
3.0
3.5
4.0
Cellular_organisms - ps1
Archaea1 - ps2
Archaea2 - ps3
Archaea3 - ps4
Asgard/Eukaryota - ps5
Eukaryota - ps6
Excavata/Diaphoretickes - ps7
Diaphoretickes - ps8
Archaeplastida - ps9
Viridiplantae - ps10
Streptophyta - ps11
Charophyceae/Embriophyta - ps12
Embriophyta - ps13
Tracheophyta - ps14
Spermatophyta - ps15
Angiosperms - ps16
Mesangiospermae - ps17
Magnoliids/eudicots - ps18
Eudicots - ps19
Core_eudicots - ps20
Superrosids - ps21
Rosids - ps22
Eurosids - ps23
Malvids - ps24
Sap./Malv./Brassicales - ps25
Malvales/Brassicales - ps26
Brassicales - ps27
Cleomaceae/Brassicaceae - ps28
Brassicaceae - ps29
Camelineae - ps30
Arabidopsis - ps31
Arabidopsis_thaliana - ps32
Phylostratum

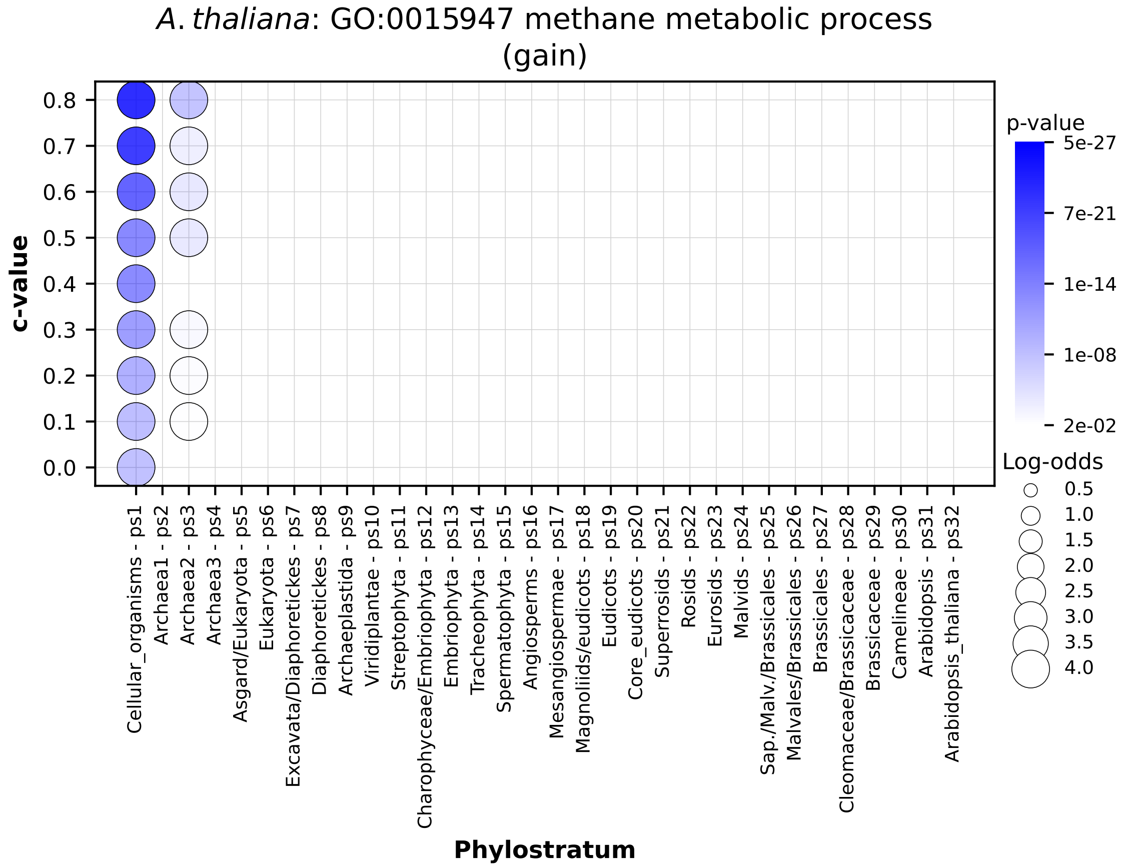

A. thaliana: GO:0015947 methane metabolic process (gain)
c-value
0.8
0.7
0.6
0.5
0.4
0.3
0.2
0.1
0.0
Phylostratum
Cellular_organisms - ps1
Archaea1 - ps2
Archaea2 - ps3
Archaea3 - ps4
Asgard/Eukaryota - ps5
Eukaryota - ps6
Excavata/Diaphoretickes - ps7
Diaphoretickes - ps8
Archaeplastida - ps9
Viridiplantae - ps10
Streptophyta - ps11
Charophyceae/Embriophyta - ps12
Embriophyta - ps13
Tracheophyta - ps14
Spermatophyta - ps15
Angiosperms - ps16
Mesangiospermae - ps17
Magnoliids/eudicots - ps18
Eudicots - ps19
Core_eudicots - ps20
Superrosids - ps21
Rosids - ps22
Eurosids - ps23
Malvids - ps24
Sap./Malv./Brassicales - ps25
Malvales/Brassicales - ps26
Brassicales - ps27
Cleomaceae/Brassicaceae - ps28
Brassicaceae - ps29
Camelineae - ps30
Arabidopsis - ps31
Arabidopsis_thaliana - ps32
p-value
5e-27
7e-21
1e-14
1e-08
2e-02
Log-odds
0.5
1.0
1.5
2.0
2.5
3.0
3.5
4.0

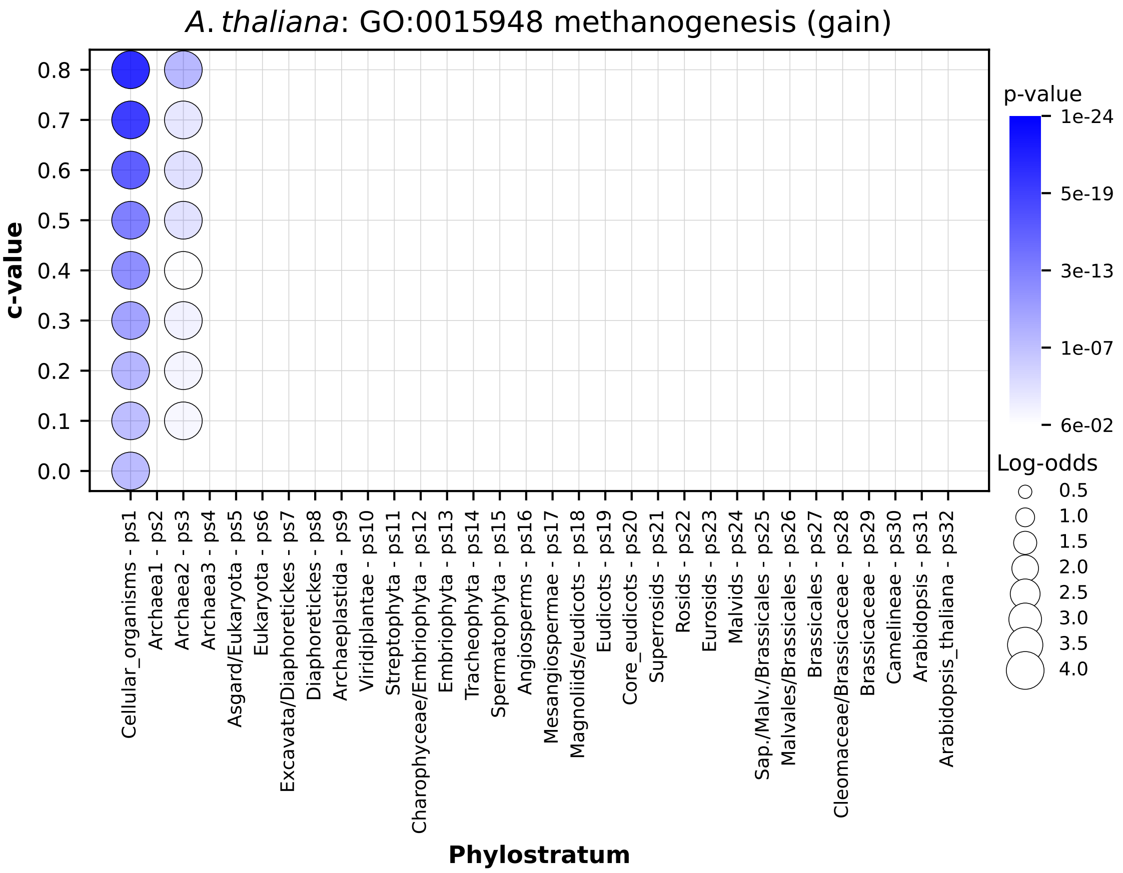

A. thaliana: GO:0015948 methanogenesis (gain)
c-value
0.8
0.7
0.6
0.5
0.4
0.3
0.2
0.1
0.0
p-value
1e-24
5e-19
3e-13
1e-07
6e-02
Log-odds
0.5
1.0
1.5
2.0
2.5
3.0
3.5
4.0
Cellular_organisms - ps1
Archaea1 - ps2
Archaea2 - ps3
Archaea3 - ps4
Asgard/Eukaryota - ps5
Eukaryota - ps6
Excavata/Diaphoretickes - ps7
Diaphoretickes - ps8
Archaeplastida - ps9
Viridiplantae - ps10
Streptophyta - ps11
Charophyceae/Embriophyta - ps12
Embriophyta - ps13
Tracheophyta - ps14
Spermatophyta - ps15
Angiosperms - ps16
Mesangiospermae - ps17
Magnoliids/eudicots - ps18
Eudicots - ps19
Core_eudicots - ps20
Superrosids - ps21
Rosids - ps22
Eurosids - ps23
Malvids - ps24
Sap./Malv./Brassicales - ps25
Malvales/Brassicales - ps26
Brassicales - ps27
Cleomaceae/Brassicaceae - ps28
Brassicaceae - ps29
Camelineae - ps30
Arabidopsis - ps31
Arabidopsis_thaliana - ps32
Phylostratum

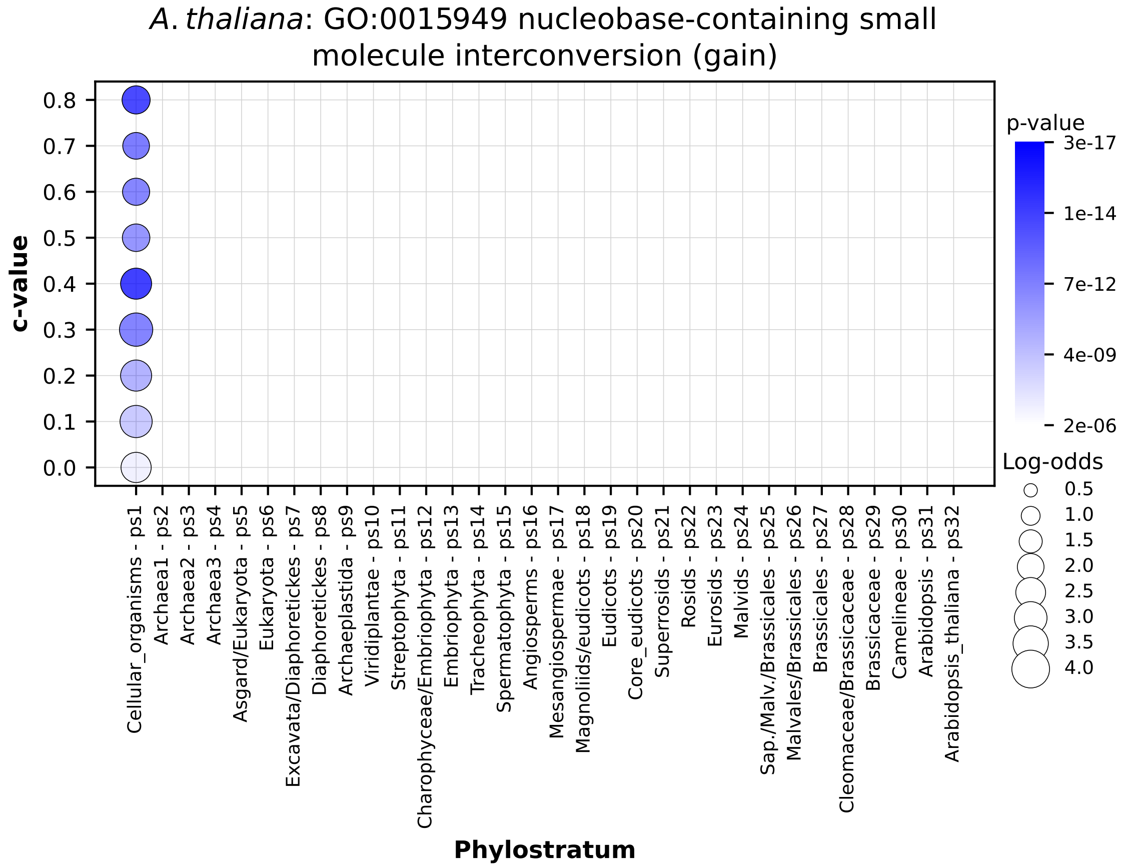
A. thaliana: GO:0015949 nucleobase-containing small molecule interconversion (gain)
c-value
0.8
0.7
0.6
0.5
0.4
0.3
0.2
0.1
0.0
p-value
3e-17
1e-14
7e-12
4e-09
2e-06
Log-odds
0.5
1.0
1.5
2.0
2.5
3.0
3.5
4.0
Cellular_organisms - ps1
Archaea1 - ps2
Archaea2 - ps3
Archaea3 - ps4
Asgard/Eukaryota - ps5
Eukaryota - ps6
Excavata/Diaphoretickes - ps7
Diaphoretickes - ps8
Archaeplastida - ps9
Viridiplantae - ps10
Streptophyta - ps11
Charophyceae/Embriophyta - ps12
Embriophyta - ps13
Tracheophyta - ps14
Spermatophyta - ps15
Angiosperms - ps16
Mesangiospermae - ps17
Magnoliids/eudicots - ps18
Eudicots - ps19
Core_eudicots - ps20
Superrosids - ps21
Rosids - ps22
Eurosids - ps23
Malvids - ps24
Sap./Malv./Brassicales - ps25
Malvales/Brassicales - ps26
Brassicales - ps27
Cleomaceae/Brassicaceae - ps28
Brassicaceae - ps29
Camelineae - ps30
Arabidopsis - ps31
Arabidopsis_thaliana - ps32
Phylostratum

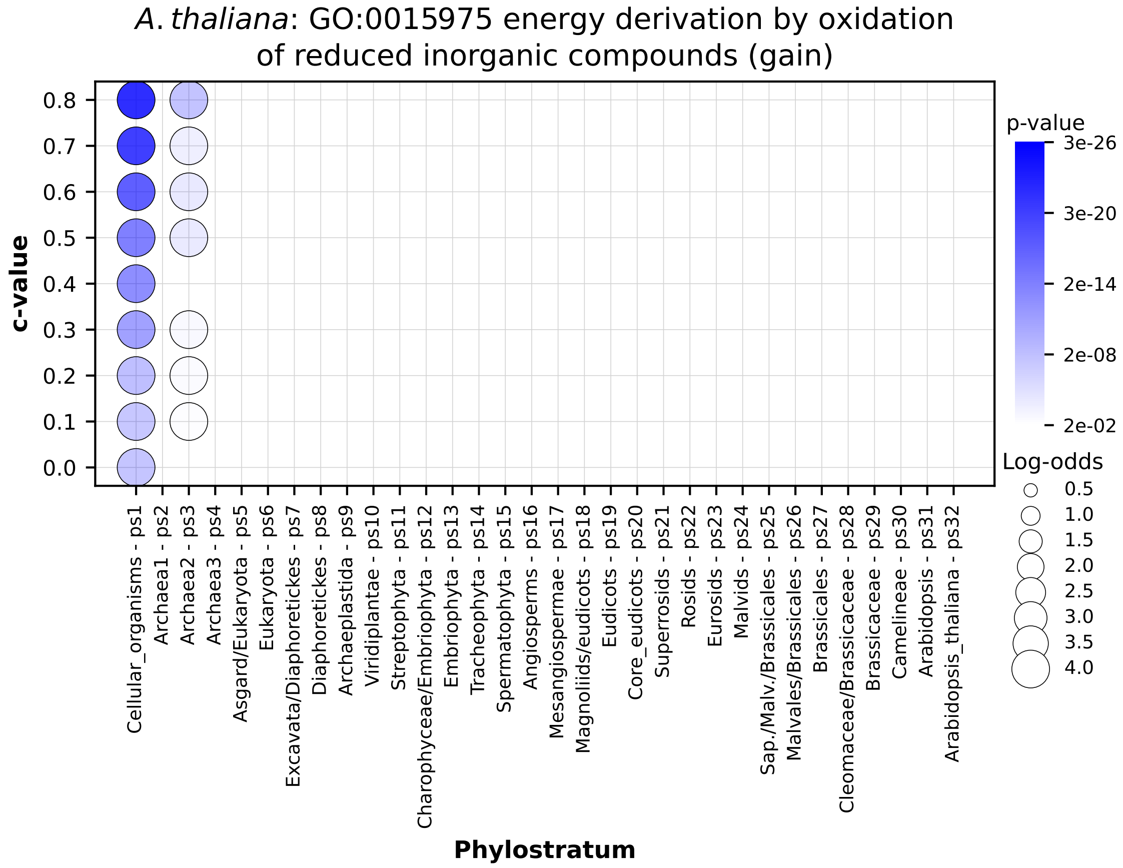

A. thaliana: GO:0015975 energy derivation by oxidation of reduced inorganic compounds (gain)
c-value
0.8
0.7
0.6
0.5
0.4
0.3
0.2
0.1
0.0
Phylostratum
Cellular_organisms - ps1
Archaea1 - ps2
Archaea2 - ps3
Archaea3 - ps4
Asgard/Eukaryota - ps5
Eukaryota - ps6
Excavata/Diaphoretickes - ps7
Diaphoretickes - ps8
Archaeplastida - ps9
Viridiplantae - ps10
Streptophyta - ps11
Charophyceae/Embriophyta - ps12
Embriophyta - ps13
Tracheophyta - ps14
Spermatophyta - ps15
Angiosperms - ps16
Mesangiospermae - ps17
Magnoliids/eudicots - ps18
Eudicots - ps19
Core_eudicots - ps20
Superrosids - ps21
Rosids - ps22
Eurosids - ps23
Malvids - ps24
Sap./Malv./Brassicales - ps25
Malvales/Brassicales - ps26
Brassicales - ps27
Cleomaceae/Brassicaceae - ps28
Brassicaceae - ps29
Camelineae - ps30
Arabidopsis - ps31
Arabidopsis_thaliana - ps32
p-value
3e-26
3e-20
2e-14
2e-08
2e-02
Log-odds
0.5
1.0
1.5
2.0
2.5
3.0
3.5
4.0

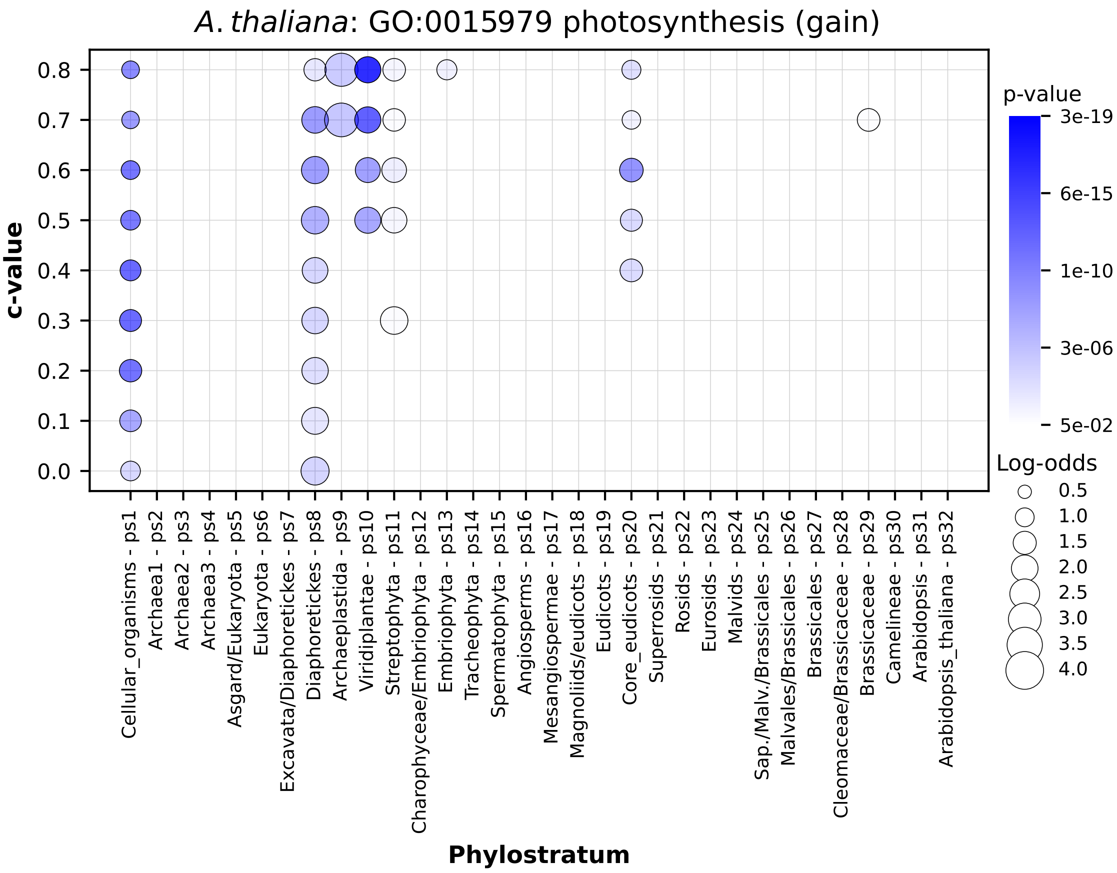

A. thaliana: GO:0015979 photosynthesis (gain)
c-value
0.8
0.7
0.6
0.5
0.4
0.3
0.2
0.1
0.0
Phylostratum
Cellular_organisms - ps1
Archaea1 - ps2
Archaea2 - ps3
Archaea3 - ps4
Asgard/Eukaryota - ps5
Eukaryota - ps6
Excavata/Diaphoretickes - ps7
Diaphoretickes - ps8
Archaeplastida - ps9
Viridiplantae - ps10
Streptophyta - ps11
Charophyceae/Embriophyta - ps12
Embriophyta - ps13
Tracheophyta - ps14
Spermatophyta - ps15
Angiosperms - ps16
Mesangiospermae - ps17
Magnoliids/eudicots - ps18
Eudicots - ps19
Core_eudicots - ps20
Superrosids - ps21
Rosids - ps22
Eurosids - ps23
Malvids - ps24
Sap./Malv./Brassicales - ps25
Malvales/Brassicales - ps26
Brassicales - ps27
Cleomaceae/Brassicaceae - ps28
Brassicaceae - ps29
Camelineae - ps30
Arabidopsis - ps31
Arabidopsis_thaliana - ps32
p-value
3e-19
6e-15
1e-10
3e-06
5e-02
Log-odds
0.5
1.0
1.5
2.0
2.5
3.0
3.5
4.0

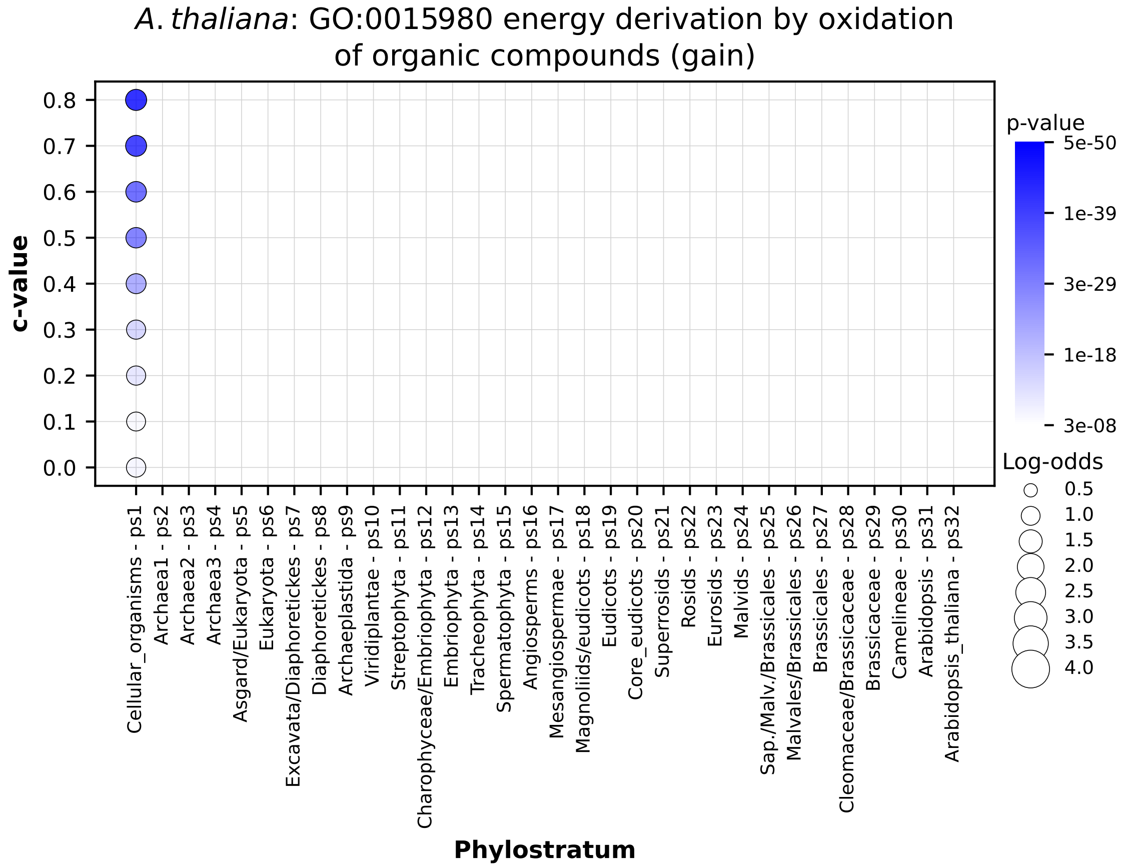

A. thaliana: GO:0015980 energy derivation by oxidation of organic compounds (gain)
c-value
0.8
0.7
0.6
0.5
0.4
0.3
0.2
0.1
0.0
p-value
5e-50
1e-39
3e-29
1e-18
3e-08
Log-odds
0.5
1.0
1.5
2.0
2.5
3.0
3.5
4.0
Cellular_organisms - ps1
Archaea1 - ps2
Archaea2 - ps3
Archaea3 - ps4
Asgard/Eukaryota - ps5
Eukaryota - ps6
Excavata/Diaphoretickes - ps7
Diaphoretickes - ps8
Archaeplastida - ps9
Viridiplantae - ps10
Streptophyta - ps11
Charophyceae/Embriophyta - ps12
Embriophyta - ps13
Tracheophyta - ps14
Spermatophyta - ps15
Angiosperms - ps16
Mesangiospermae - ps17
Magnoliids/eudicots - ps18
Eudicots - ps19
Core_eudicots - ps20
Superrosids - ps21
Rosids - ps22
Eurosids - ps23
Malvids - ps24
Sap./Malv./Brassicales - ps25
Malvales/Brassicales - ps26
Brassicales - ps27
Cleomaceae/Brassicaceae - ps28
Brassicaceae - ps29
Camelineae - ps30
Arabidopsis - ps31
Arabidopsis_thaliana - ps32
Phylostratum

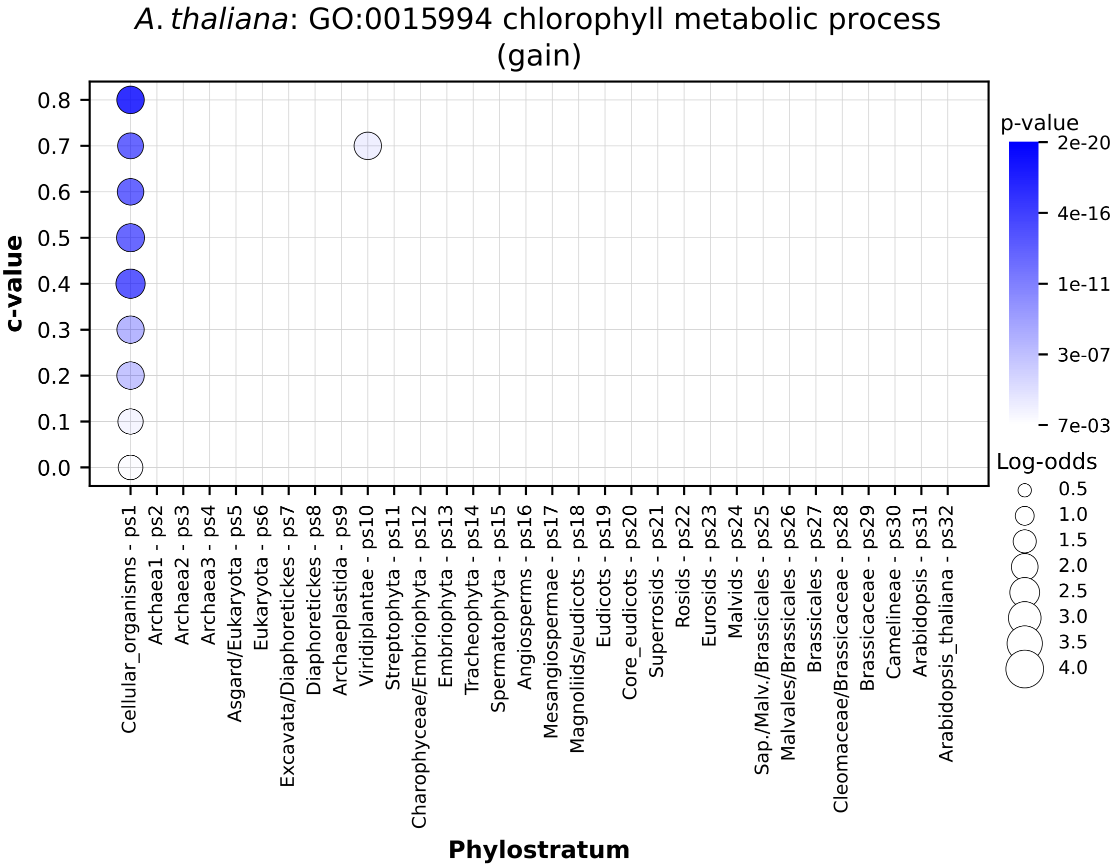

A. thaliana: GO:0015994 chlorophyll metabolic process (gain)
c-value
0.8
0.7
0.6
0.5
0.4
0.3
0.2
0.1
0.0
p-value
2e-20
4e-16
1e-11
3e-07
7e-03
Log-odds
0.5
1.0
1.5
2.0
2.5
3.0
3.5
4.0
Cellular_organisms - ps1
Archaea1 - ps2
Archaea2 - ps3
Archaea3 - ps4
Asgard/Eukaryota - ps5
Eukaryota - ps6
Excavata/Diaphoretickes - ps7
Diaphoretickes - ps8
Archaeplastida - ps9
Viridiplantae - ps10
Streptophyta - ps11
Charophyceae/Embriophyta - ps12
Embriophyta - ps13
Tracheophyta - ps14
Spermatophyta - ps15
Angiosperms - ps16
Mesangiospermae - ps17
Magnoliids/eudicots - ps18
Eudicots - ps19
Core_eudicots - ps20
Superrosids - ps21
Rosids - ps22
Eurosids - ps23
Malvids - ps24
Sap./Malv./Brassicales - ps25
Malvales/Brassicales - ps26
Brassicales - ps27
Cleomaceae/Brassicaceae - ps28
Brassicaceae - ps29
Camelineae - ps30
Arabidopsis - ps31
Arabidopsis_thaliana - ps32
Phylostratum

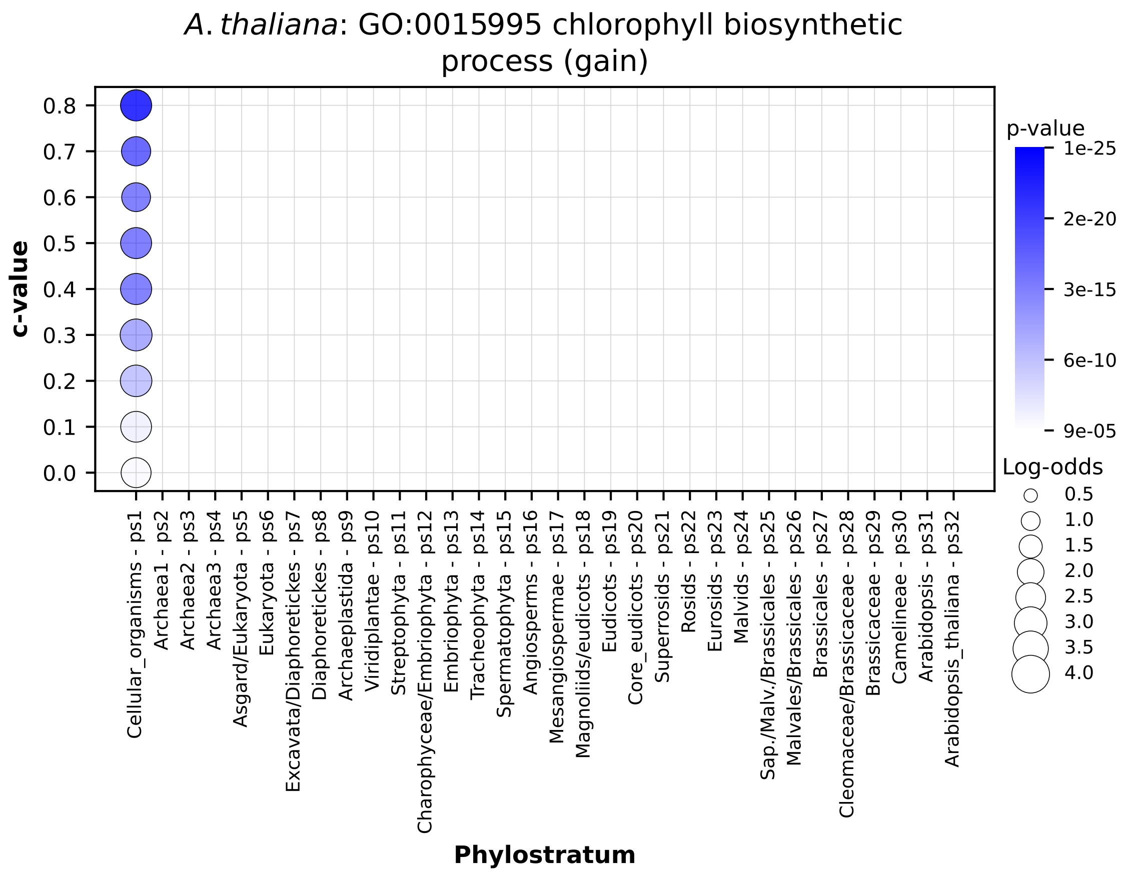

A. thaliana: GO:0015995 chlorophyll biosynthetic process (gain)
c-value
0.0
0.1
0.2
0.3
0.4
0.5
0.6
0.7
0.8
p-value
1e-25
2e-20
3e-15
6e-10
9e-05
Log-odds
0.5
1.0
1.5
2.0
2.5
3.0
3.5
4.0
Cellular_organisms - ps1
Archaea1 - ps2
Archaea2 - ps3
Archaea3 - ps4
Asgard/Eukaryota - ps5
Eukaryota - ps6
Excavata/Diaphoretickes - ps7
Diaphoretickes - ps8
Archaeplastida - ps9
Viridiplantae - ps10
Streptophyta - ps11
Charophyceae/Embriophyta - ps12
Embriophyta - ps13
Tracheophyta - ps14
Spermatophyta - ps15
Angiosperms - ps16
Mesangiospermae - ps17
Magnoliids/eudicots - ps18
Eudicots - ps19
Core_eudicots - ps20
Superrosids - ps21
Rosids - ps22
Eurosids - ps23
Malvids - ps24
Sap./Malv./Brassicales - ps25
Malvales/Brassicales - ps26
Brassicales - ps27
Cleomaceae/Brassicaceae - ps28
Brassicaceae - ps29
Camelineae - ps30
Arabidopsis - ps31
Arabidopsis_thaliana - ps32
Phylostratum

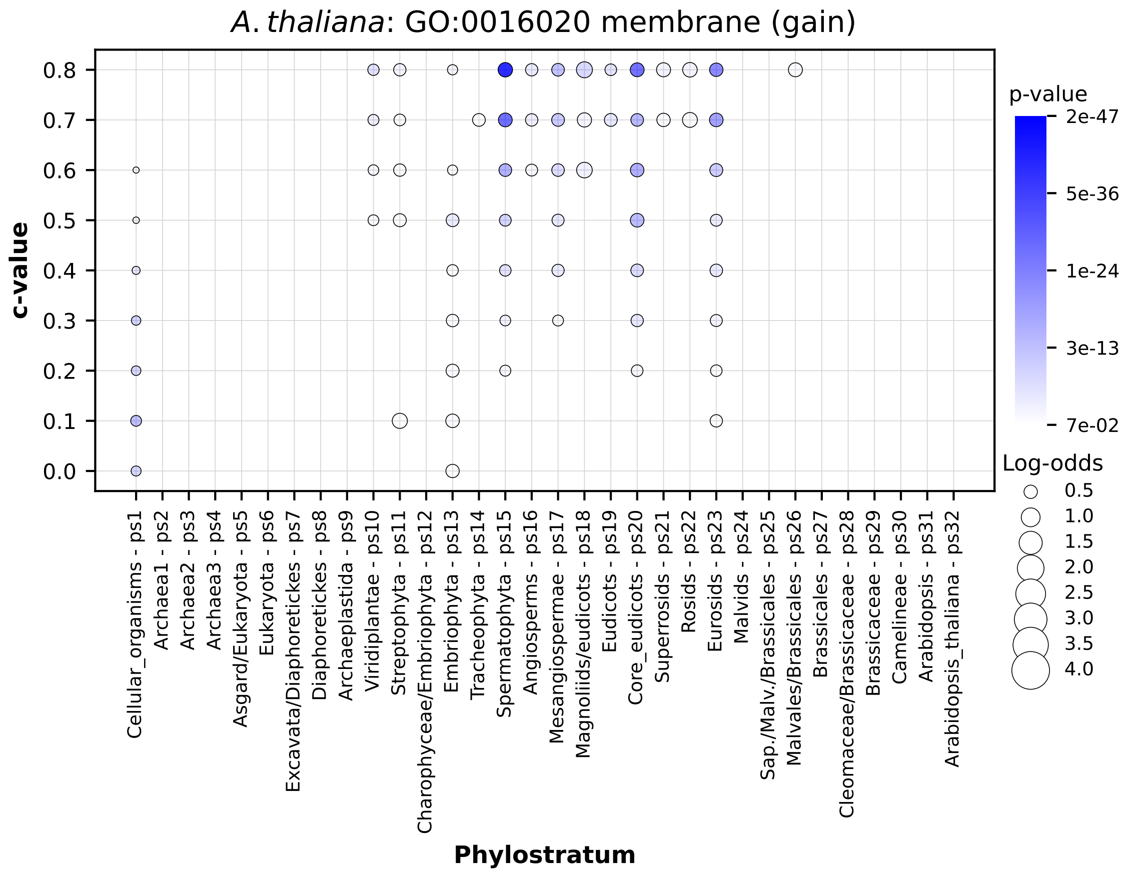
A. thaliana: GO:0016020 membrane (gain)
c-value
0.8
0.7
0.6
0.5
0.4
0.3
0.2
0.1
0.0
p-value
2e-47
5e-36
1e-24
3e-13
7e-02
Log-odds
0.5
1.0
1.5
2.0
2.5
3.0
3.5
4.0
Cellular_organisms - ps1
Archaea1 - ps2
Archaea2 - ps3
Archaea3 - ps4
Asgard/Eukaryota - ps5
Eukaryota - ps6
Excavata/Diaphoretickes - ps7
Diaphoretickes - ps8
Archaeplastida - ps9
Viridiplantae - ps10
Streptophyta - ps11
Charophyceae/Embriophyta - ps12
Embriophyta - ps13
Tracheophyta - ps14
Spermatophyta - ps15
Angiosperms - ps16
Mesangiospermae - ps17
Magnoliids/eudicots - ps18
Eudicots - ps19
Core_eudicots - ps20
Superrosids - ps21
Rosids - ps22
Eurosids - ps23
Malvids - ps24
Sap./Malv./Brassicales - ps25
Malvales/Brassicales - ps26
Brassicales - ps27
Cleomaceae/Brassicaceae - ps28
Brassicaceae - ps29
Camelineae - ps30
Arabidopsis - ps31
Arabidopsis_thaliana - ps32
Phylostratum

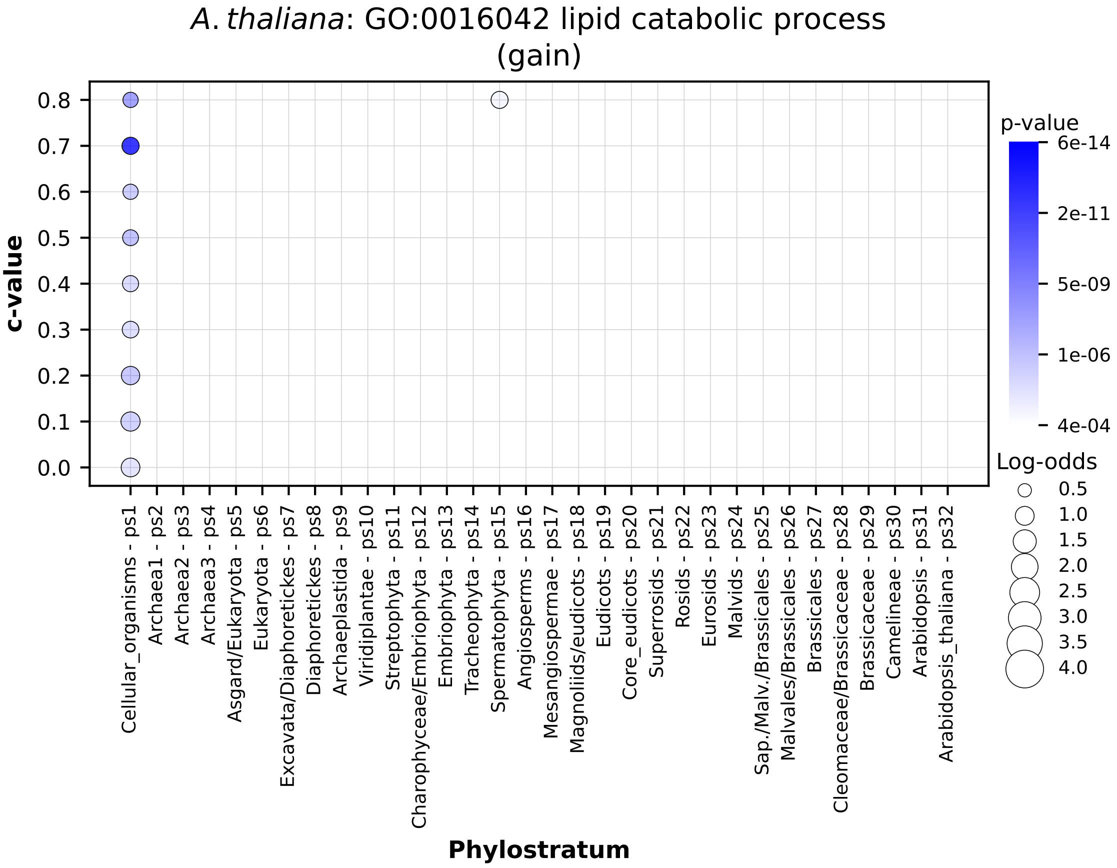

A. thaliana: GO:0016042 lipid catabolic process
(gain)
c-value
0.0
0.1
0.2
0.3
0.4
0.5
0.6
0.7
0.8
p-value
6e-14
2e-11
5e-09
1e-06
4e-04
Log-odds
0.5
1.0
1.5
2.0
2.5
3.0
3.5
4.0
Cellular_organisms - ps1
Archaea1 - ps2
Archaea2 - ps3
Archaea3 - ps4
Asgard/Eukaryota - ps5
Eukaryota - ps6
Excavata/Diaphoretickes - ps7
Diaphoretickes - ps8
Archaeplastida - ps9
Viridiplantae - ps10
Streptophyta - ps11
Charophyceae/Embriophyta - ps12
Embriophyta - ps13
Tracheophyta - ps14
Spermatophyta - ps15
Angiosperms - ps16
Mesangiospermae - ps17
Magnoliids/eudicots - ps18
Eudicots - ps19
Core_eudicots - ps20
Superrosids - ps21
Rosids - ps22
Eurosids - ps23
Malvids - ps24
Sap./Malv./Brassicales - ps25
Malvales/Brassicales - ps26
Brassicales - ps27
Cleomaceae/Brassicaceae - ps28
Brassicaceae - ps29
Camelineae - ps30
Arabidopsis - ps31
Arabidopsis_thaliana - ps32
Phylostratum

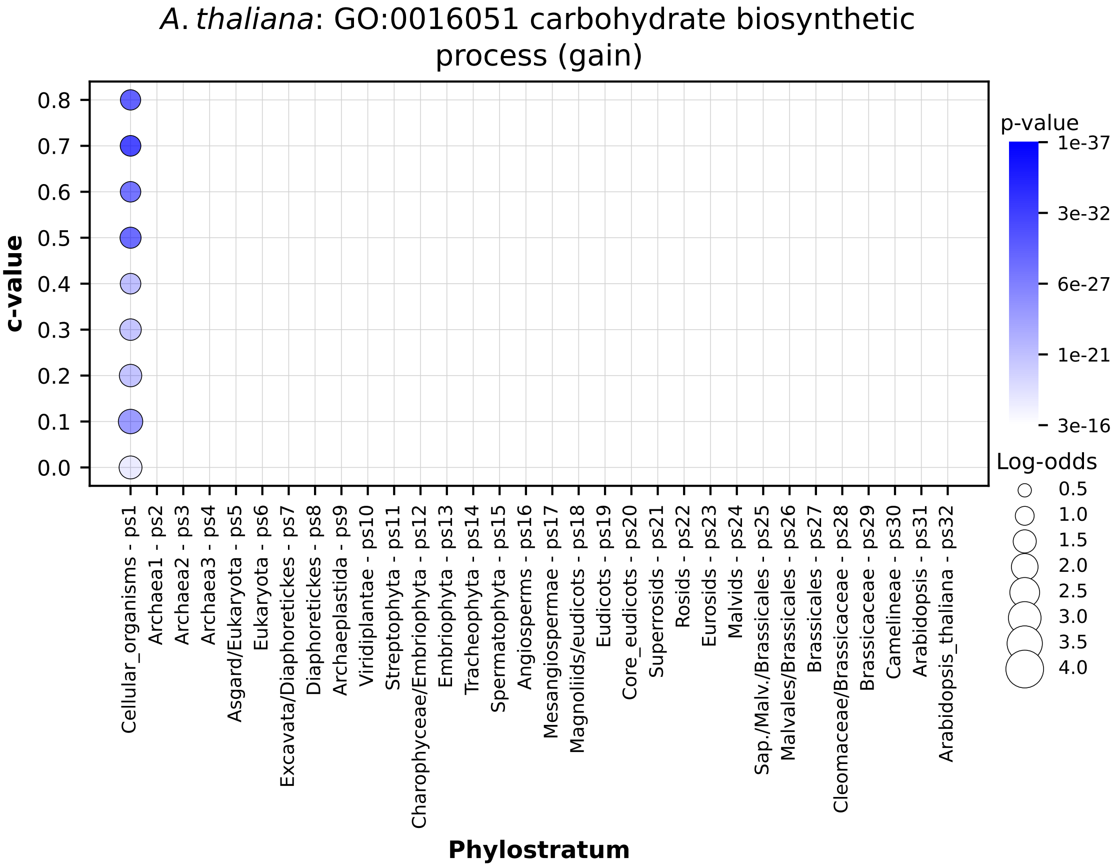

A. thaliana: GO:0016051 carbohydrate biosynthetic process (gain)
c-value
0.8
0.7
0.6
0.5
0.4
0.3
0.2
0.1
0.0
Phylostratum
Cellular_organisms - ps1
Archaea1 - ps2
Archaea2 - ps3
Archaea3 - ps4
Asgard/Eukaryota - ps5
Eukaryota - ps6
Excavata/Diaphoretickes - ps7
Diaphoretickes - ps8
Archaeplastida - ps9
Viridiplantae - ps10
Streptophyta - ps11
Charophyceae/Embriophyta - ps12
Embriophyta - ps13
Tracheophyta - ps14
Spermatophyta - ps15
Angiosperms - ps16
Mesangiospermae - ps17
Magnoliids/eudicots - ps18
Eudicots - ps19
Core_eudicots - ps20
Superrosids - ps21
Rosids - ps22
Eurosids - ps23
Malvids - ps24
Sap./Malv./Brassicales - ps25
Malvales/Brassicales - ps26
Brassicales - ps27
Cleomaceae/Brassicaceae - ps28
Brassicaceae - ps29
Camelineae - ps30
Arabidopsis - ps31
Arabidopsis_thaliana - ps32
p-value
1e-37
3e-32
6e-27
1e-21
3e-16
Log-odds
0.5
1.0
1.5
2.0
2.5
3.0
3.5
4.0

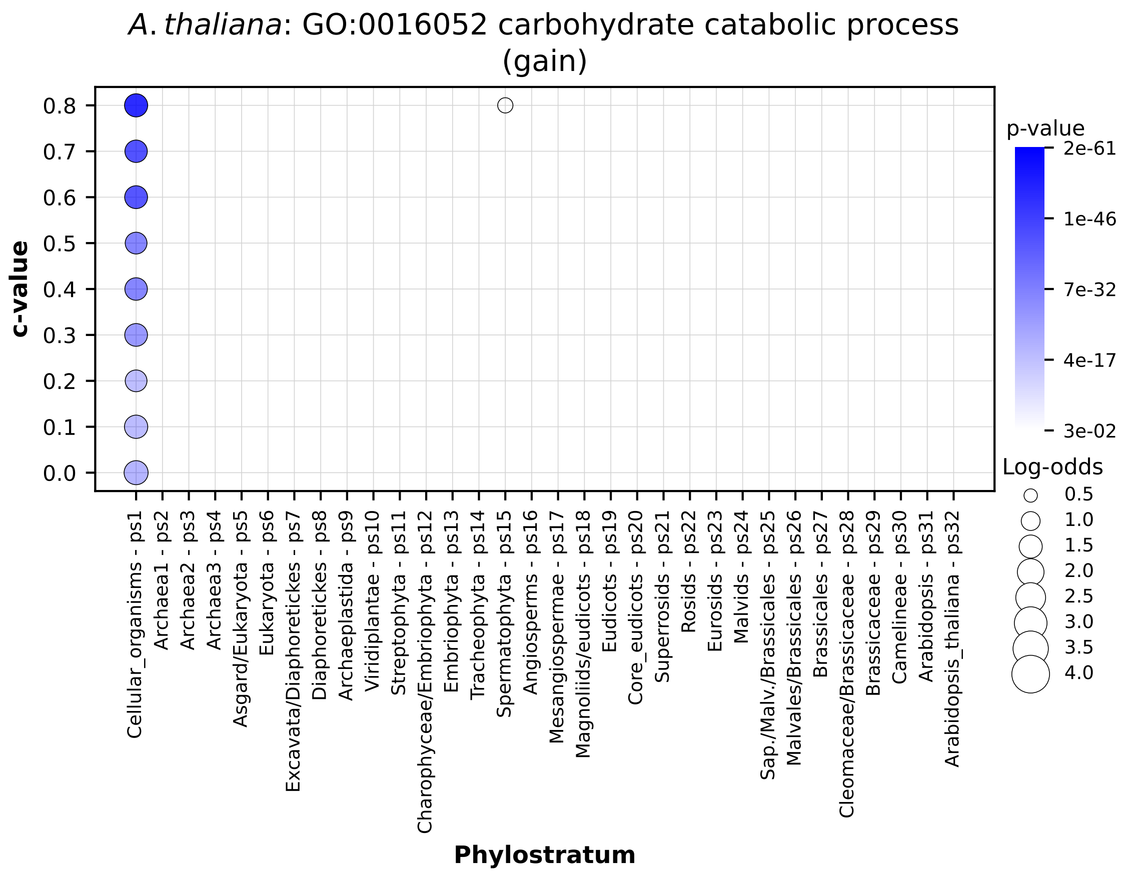

A. thaliana: GO:0016052 carbohydrate catabolic process (gain)
c-value
0.0
0.1
0.2
0.3
0.4
0.5
0.6
0.7
0.8
p-value
2e-61
1e-46
7e-32
4e-17
3e-02
Log-odds
0.5
1.0
1.5
2.0
2.5
3.0
3.5
4.0
Cellular_organisms - ps1
Archaea1 - ps2
Archaea2 - ps3
Archaea3 - ps4
Asgard/Eukaryota - ps5
Eukaryota - ps6
Excavata/Diaphoretickes - ps7
Diaphoretickes - ps8
Archaeplastida - ps9
Viridiplantae - ps10
Streptophyta - ps11
Charophyceae/Embriophyta - ps12
Embriophyta - ps13
Tracheophyta - ps14
Spermatophyta - ps15
Angiosperms - ps16
Mesangiospermae - ps17
Magnoliids/eudicots - ps18
Eudicots - ps19
Core_eudicots - ps20
Superrosids - ps21
Rosids - ps22
Eurosids - ps23
Malvids - ps24
Sap./Malv./Brassicales - ps25
Malvales/Brassicales - ps26
Brassicales - ps27
Cleomaceae/Brassicaceae - ps28
Brassicaceae - ps29
Camelineae - ps30
Arabidopsis - ps31
Arabidopsis_thaliana - ps32
Phylostratum

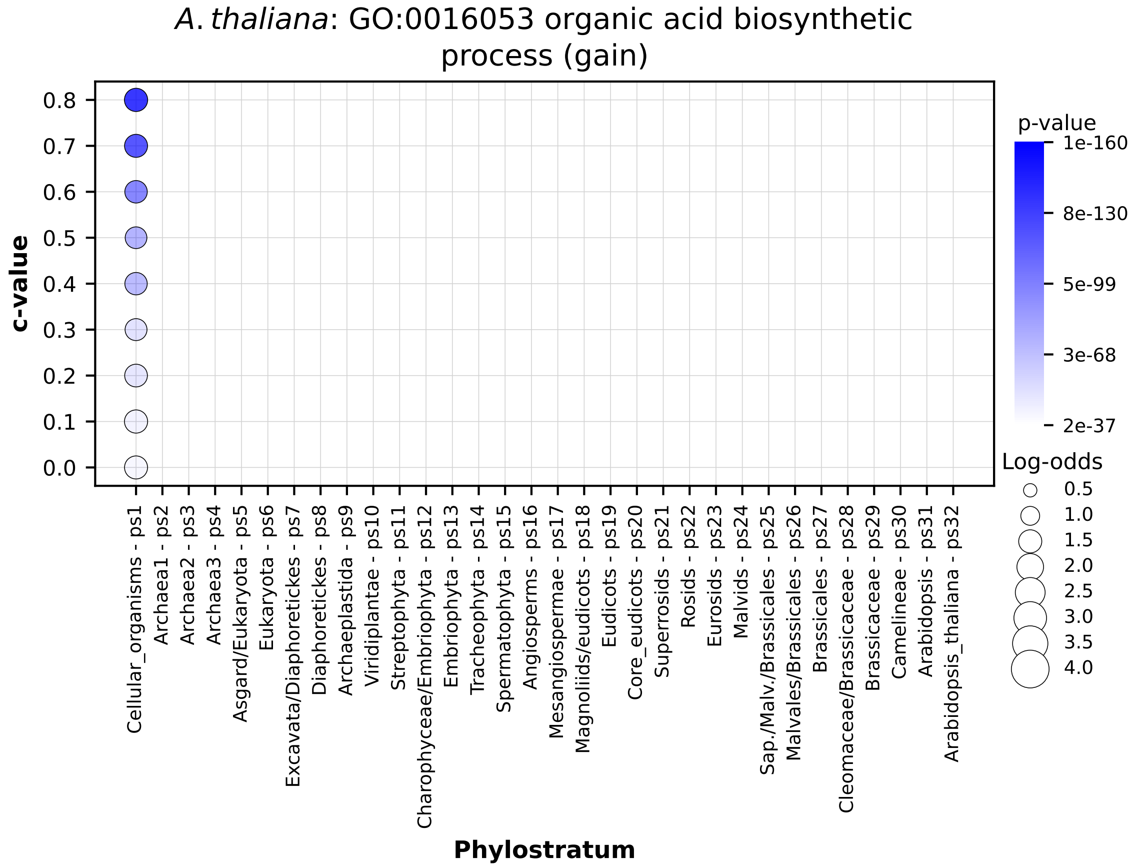

A. thaliana: GO:0016053 organic acid biosynthetic process (gain)
c-value
0.8
0.7
0.6
0.5
0.4
0.3
0.2
0.1
0.0
p-value
1e-160
8e-130
5e-99
3e-68
2e-37
Log-odds
0.5
1.0
1.5
2.0
2.5
3.0
3.5
4.0
Cellular_organisms - ps1
Archaea1 - ps2
Archaea2 - ps3
Archaea3 - ps4
Asgard/Eukaryota - ps5
Eukaryota - ps6
Excavata/Diaphoretickes - ps7
Diaphoretickes - ps8
Archaeplastida - ps9
Viridiplantae - ps10
Streptophyta - ps11
Charophyceae/Embriophyta - ps12
Embriophyta - ps13
Tracheophyta - ps14
Spermatophyta - ps15
Angiosperms - ps16
Mesangiospermae - ps17
Magnoliids/eudicots - ps18
Eudicots - ps19
Core_eudicots - ps20
Superrosids - ps21
Rosids - ps22
Eurosids - ps23
Malvids - ps24
Sap./Malv./Brassicales - ps25
Malvales/Brassicales - ps26
Brassicales - ps27
Cleomaceae/Brassicaceae - ps28
Brassicaceae - ps29
Camelineae - ps30
Arabidopsis - ps31
Arabidopsis_thaliana - ps32
Phylostratum

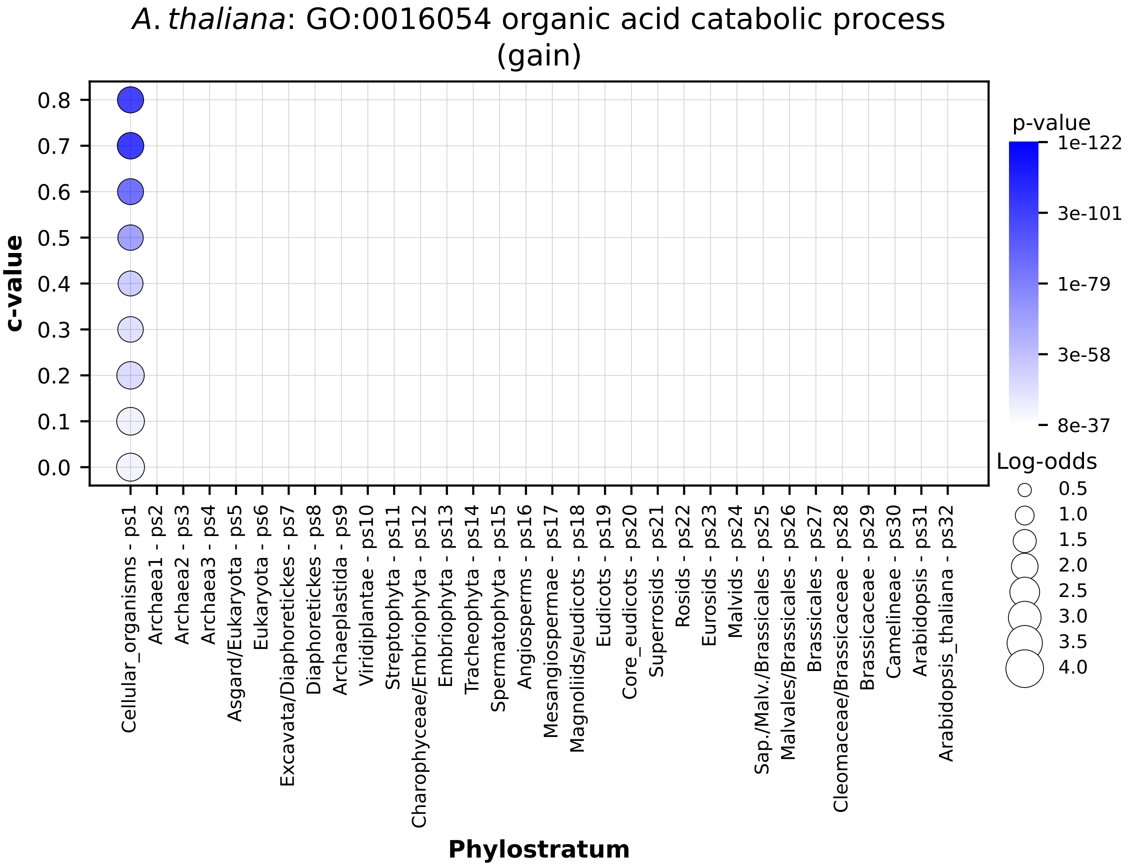

A. thaliana: GO:0016054 organic acid catabolic process
(gain)
c-value
0.0
0.1
0.2
0.3
0.4
0.5
0.6
0.7
0.8
p-value
1e-122
3e-101
1e-79
3e-58
8e-37
Log-odds
0.5
1.0
1.5
2.0
2.5
3.0
3.5
4.0
Cellular_organisms - ps1
Archaea1 - ps2
Archaea2 - ps3
Archaea3 - ps4
Asgard/Eukaryota - ps5
Eukaryota - ps6
Excavata/Diaphoretickes - ps7
Diaphoretickes - ps8
Archaeplastida - ps9
Viridiplantae - ps10
Streptophyta - ps11
Charophyceae/Embriophyta - ps12
Embriophyta - ps13
Tracheophyta - ps14
Spermatophyta - ps15
Angiosperms - ps16
Mesangiospermae - ps17
Magnoliids/eudicots - ps18
Eudicots - ps19
Core_eudicots - ps20
Superrosids - ps21
Rosids - ps22
Eurosids - ps23
Malvids - ps24
Sap./Malv./Brassicales - ps25
Malvales/Brassicales - ps26
Brassicales - ps27
Cleomaceae/Brassicaceae - ps28
Brassicaceae - ps29
Camelineae - ps30
Arabidopsis - ps31
Arabidopsis_thaliana - ps32
Phylostratum

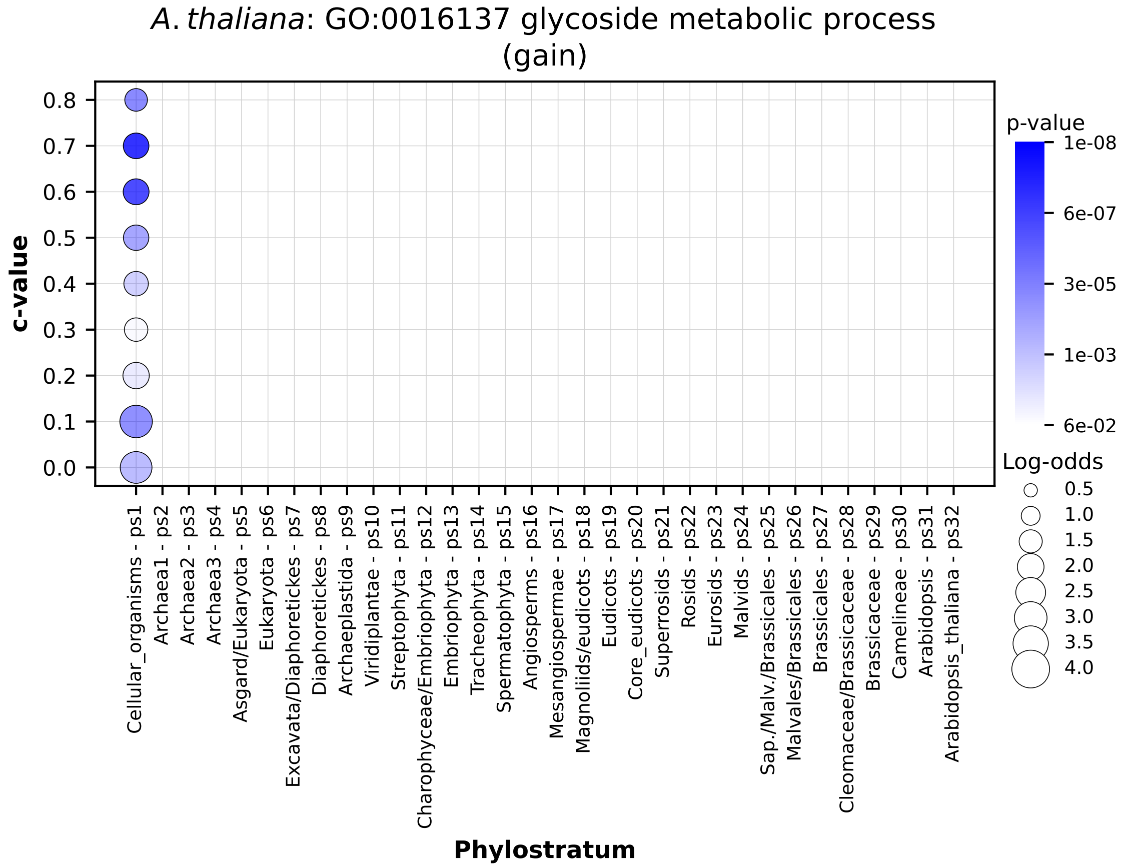
A. thaliana: GO:0016137 glycoside metabolic process (gain)
c-value
0.0
0.1
0.2
0.3
0.4
0.5
0.6
0.7
0.8
Phylostratum
Cellular_organisms - ps1
Archaea1 - ps2
Archaea2 - ps3
Archaea3 - ps4
Asgard/Eukaryota - ps5
Eukaryota - ps6
Excavata/Diaphoretickes - ps7
Diaphoretickes - ps8
Archaeplastida - ps9
Viridiplantae - ps10
Streptophyta - ps11
Charophyceae/Embriophyta - ps12
Embriophyta - ps13
Tracheophyta - ps14
Spermatophyta - ps15
Angiosperms - ps16
Mesangiospermae - ps17
Magnoliids/eudicots - ps18
Eudicots - ps19
Core_eudicots - ps20
Superrosids - ps21
Rosids - ps22
Eurosids - ps23
Malvids - ps24
Sap./Malv./Brassicales - ps25
Malvales/Brassicales - ps26
Brassicales - ps27
Cleomaceae/Brassicaceae - ps28
Brassicaceae - ps29
Camelineae - ps30
Arabidopsis - ps31
Arabidopsis_thaliana - ps32
p-value
1e-08
6e-07
3e-05
1e-03
6e-02
Log-odds
0.5
1.0
1.5
2.0
2.5
3.0
3.5
4.0

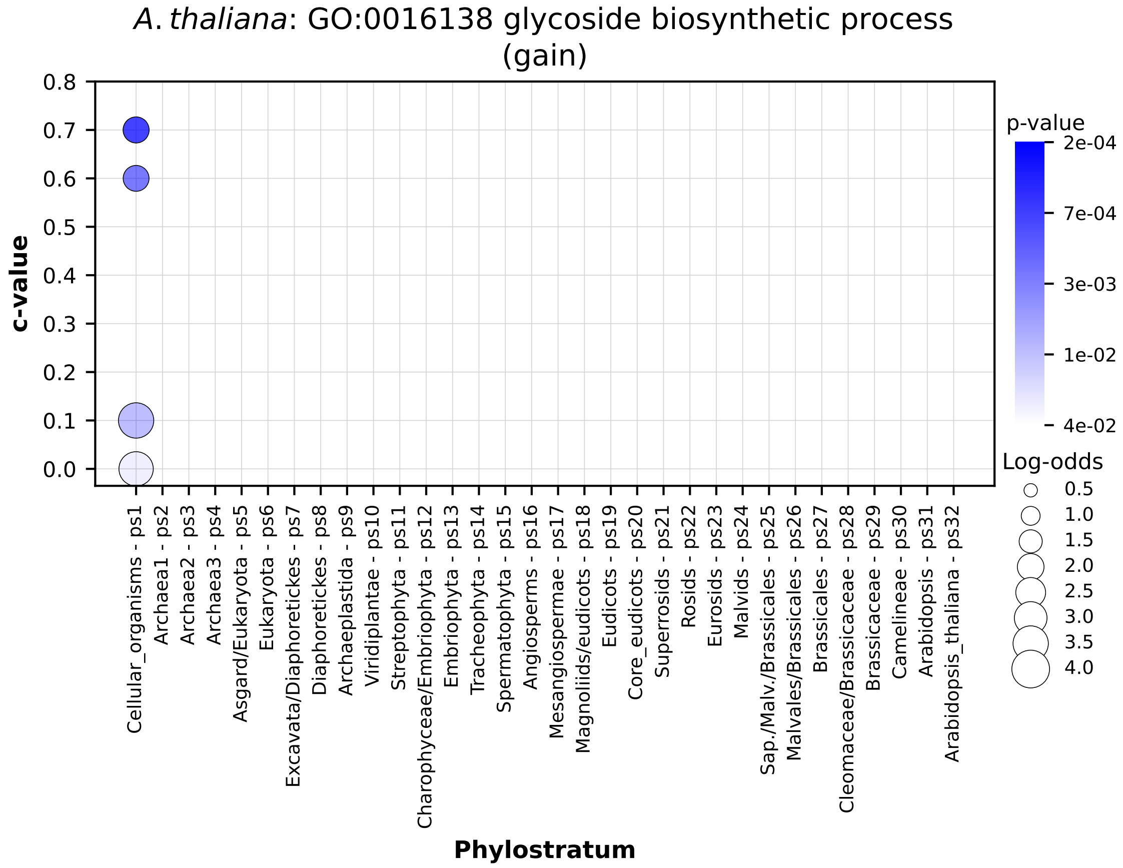

A. thaliana: GO:0016138 glycoside biosynthetic process (gain)
c-value
0.8
0.7
0.6
0.5
0.4
0.3
0.2
0.1
0.0
p-value
2e-04
7e-04
3e-03
1e-02
4e-02
Log-odds
0.5
1.0
1.5
2.0
2.5
3.0
3.5
4.0
Cellular_organisms - ps1
Archaea1 - ps2
Archaea2 - ps3
Archaea3 - ps4
Asgard/Eukaryota - ps5
Eukaryota - ps6
Excavata/Diaphoretickes - ps7
Diaphoretickes - ps8
Archaeplastida - ps9
Viridiplantae - ps10
Streptophyta - ps11
Charophyceae/Embriophyta - ps12
Embriophyta - ps13
Tracheophyta - ps14
Spermatophyta - ps15
Angiosperms - ps16
Mesangiospermae - ps17
Magnoliids/eudicots - ps18
Eudicots - ps19
Core_eudicots - ps20
Superrosids - ps21
Rosids - ps22
Eurosids - ps23
Malvids - ps24
Sap./Malv./Brassicales - ps25
Malvales/Brassicales - ps26
Brassicales - ps27
Cleomaceae/Brassicaceae - ps28
Brassicaceae - ps29
Camelineae - ps30
Arabidopsis - ps31
Arabidopsis_thaliana - ps32
Phylostratum

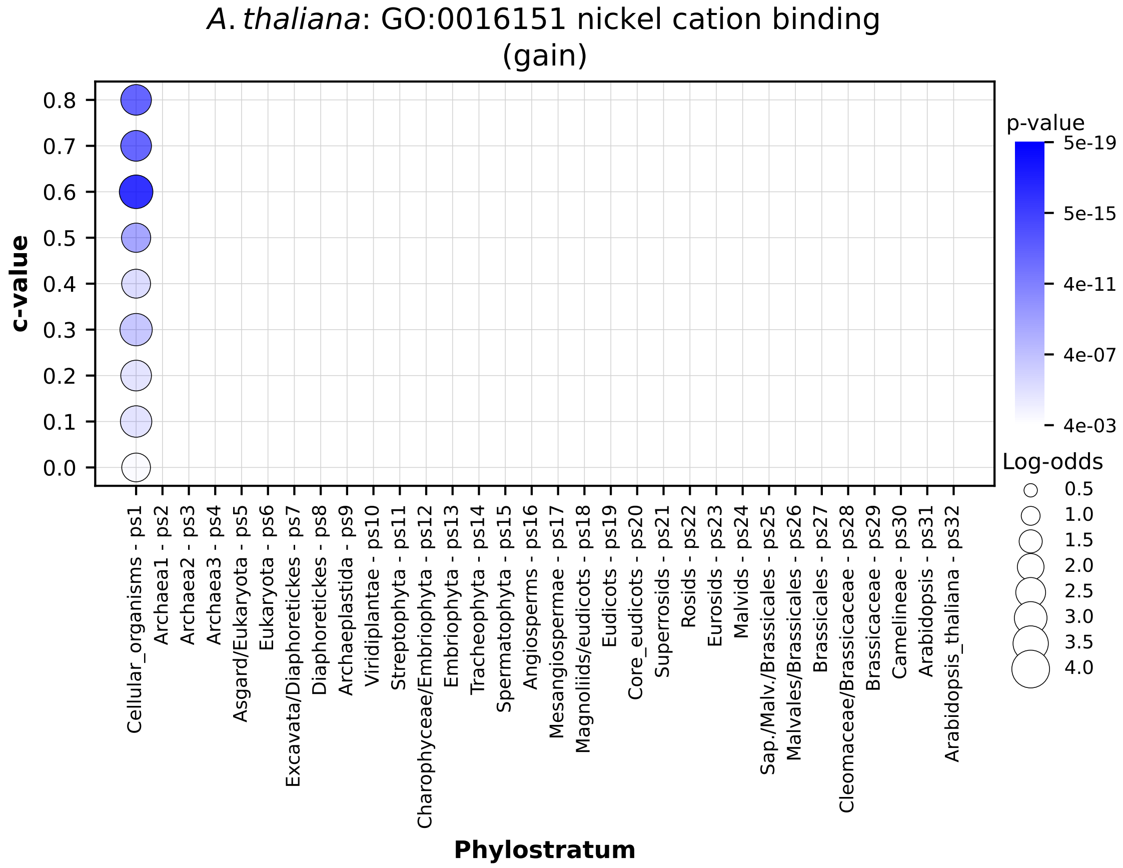

A. thaliana: GO:0016151 nickel cation binding
(gain)
c-value
0.8
0.7
0.6
0.5
0.4
0.3
0.2
0.1
0.0
Phylostratum
Cellular_organisms - ps1
Archaea1 - ps2
Archaea2 - ps3
Archaea3 - ps4
Asgard/Eukaryota - ps5
Eukaryota - ps6
Excavata/Diaphoretickes - ps7
Diaphoretickes - ps8
Archaeplastida - ps9
Viridiplantae - ps10
Streptophyta - ps11
Charophyceae/Embriophyta - ps12
Embriophyta - ps13
Tracheophyta - ps14
Spermatophyta - ps15
Angiosperms - ps16
Mesangiospermae - ps17
Magnoliids/eudicots - ps18
Eudicots - ps19
Core_eudicots - ps20
Superrosids - ps21
Rosids - ps22
Eurosids - ps23
Malvids - ps24
Sap./Malv./Brassicales - ps25
Malvales/Brassicales - ps26
Brassicales - ps27
Cleomaceae/Brassicaceae - ps28
Brassicaceae - ps29
Camelineae - ps30
Arabidopsis - ps31
Arabidopsis_thaliana - ps32
p-value
5e-19
5e-15
4e-11
4e-07
4e-03
Log-odds
0.5
1.0
1.5
2.0
2.5
3.0
3.5
4.0

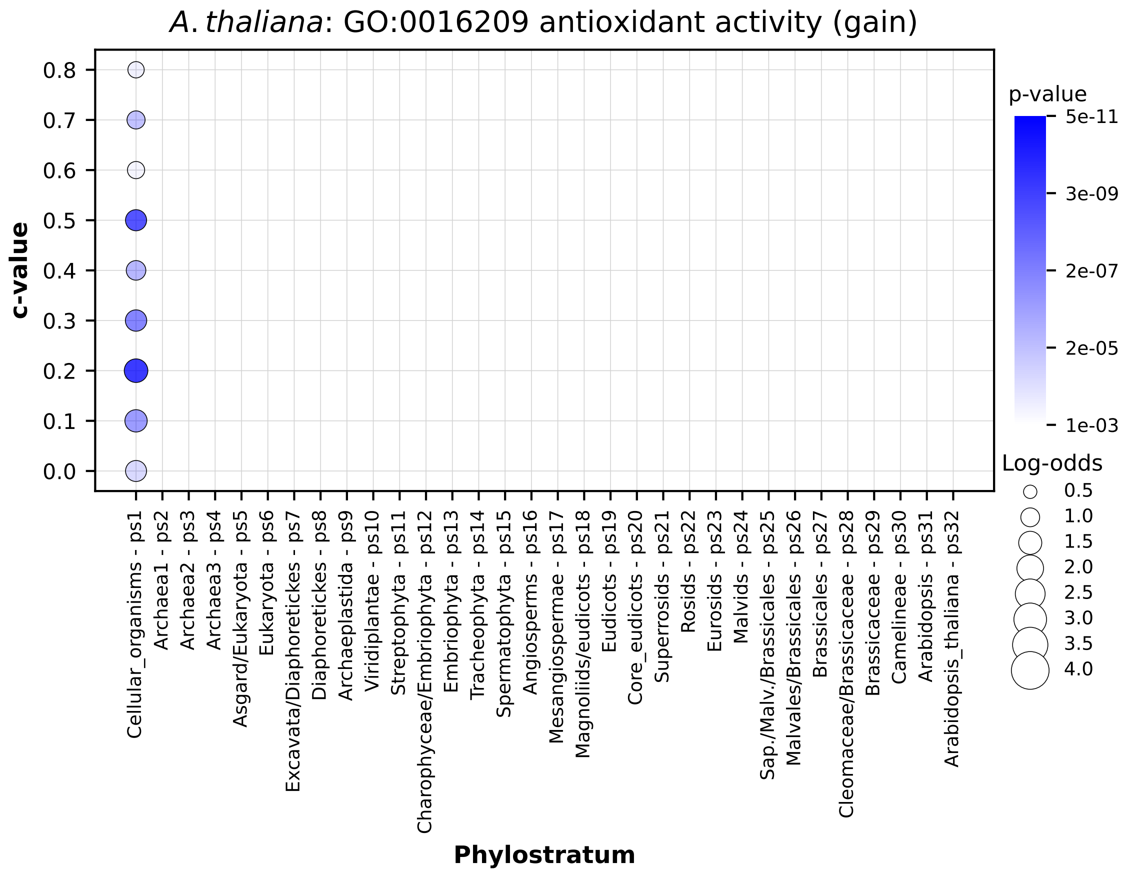

A. thaliana: GO:0016209 antioxidant activity (gain)
c-value
0.0
0.1
0.2
0.3
0.4
0.5
0.6
0.7
0.8
Phylostratum
Cellular_organisms - ps1
Archaea1 - ps2
Archaea2 - ps3
Archaea3 - ps4
Asgard/Eukaryota - ps5
Eukaryota - ps6
Excavata/Diaphoretickes - ps7
Diaphoretickes - ps8
Archaeplastida - ps9
Viridiplantae - ps10
Streptophyta - ps11
Charophyceae/Embriophyta - ps12
Embriophyta - ps13
Tracheophyta - ps14
Spermatophyta - ps15
Angiosperms - ps16
Mesangiospermae - ps17
Magnoliids/eudicots - ps18
Eudicots - ps19
Core_eudicots - ps20
Superrosids - ps21
Rosids - ps22
Eurosids - ps23
Malvids - ps24
Sap./Malv./Brassicales - ps25
Malvales/Brassicales - ps26
Brassicales - ps27
Cleomaceae/Brassicaceae - ps28
Brassicaceae - ps29
Camelineae - ps30
Arabidopsis - ps31
Arabidopsis_thaliana - ps32
p-value
5e-11
3e-09
2e-07
2e-05
1e-03
Log-odds
0.5
1.0
1.5
2.0
2.5
3.0
3.5
4.0

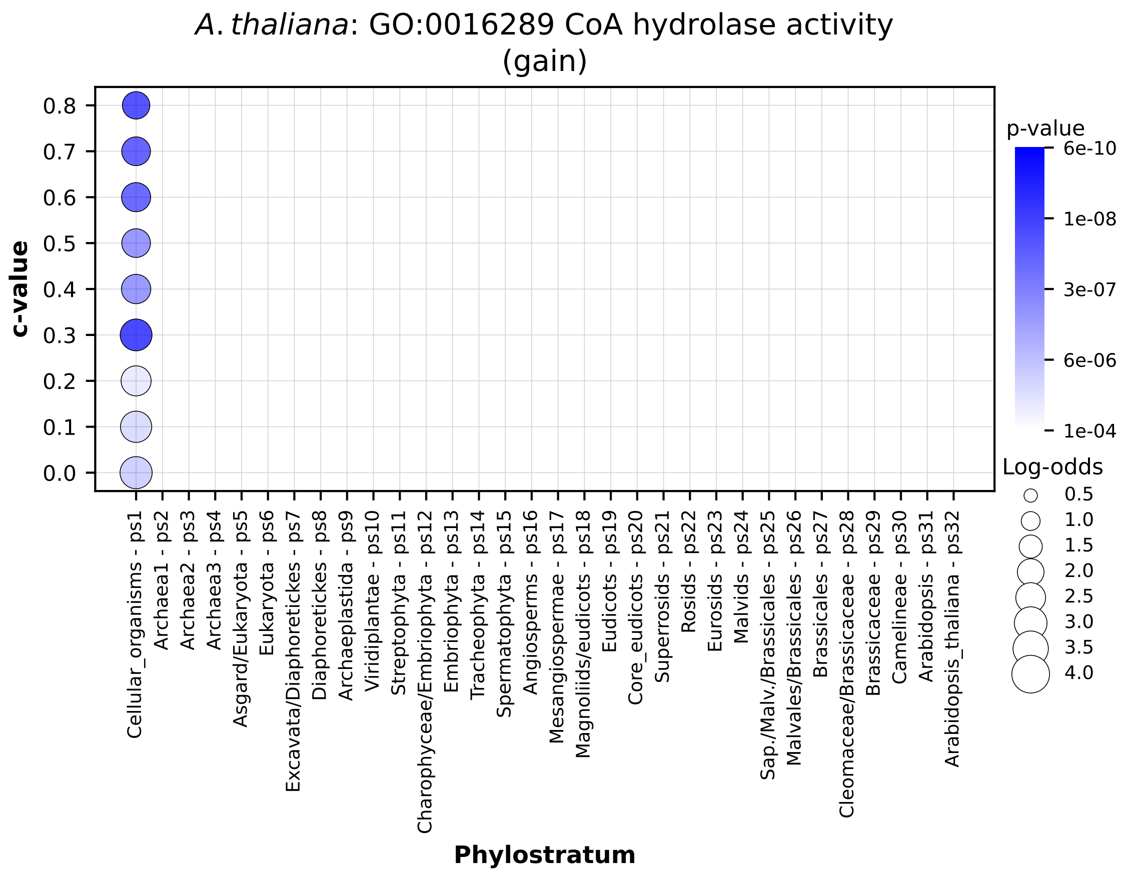
A. thaliana: GO:0016289 CoA hydrolase activity
(gain)
c-value
0.0
0.1
0.2
0.3
0.4
0.5
0.6
0.7
0.8
p-value
6e-10
1e-08
3e-07
6e-06
1e-04
Log-odds
0.5
1.0
1.5
2.0
2.5
3.0
3.5
4.0
Cellular_organisms - ps1
Archaea1 - ps2
Archaea2 - ps3
Archaea3 - ps4
Asgard/Eukaryota - ps5
Eukaryota - ps6
Excavata/Diaphoretickes - ps7
Diaphoretickes - ps8
Archaeplastida - ps9
Viridiplantae - ps10
Streptophyta - ps11
Charophyceae/Embriophyta - ps12
Embriophyta - ps13
Tracheophyta - ps14
Spermatophyta - ps15
Angiosperms - ps16
Mesangiospermae - ps17
Magnoliids/eudicots - ps18
Eudicots - ps19
Core_eudicots - ps20
Superrosids - ps21
Rosids - ps22
Eurosids - ps23
Malvids - ps24
Sap./Malv./Brassicales - ps25
Malvales/Brassicales - ps26
Brassicales - ps27
Cleomaceae/Brassicaceae - ps28
Brassicaceae - ps29
Camelineae - ps30
Arabidopsis - ps31
Arabidopsis_thaliana - ps32
Phylostratum

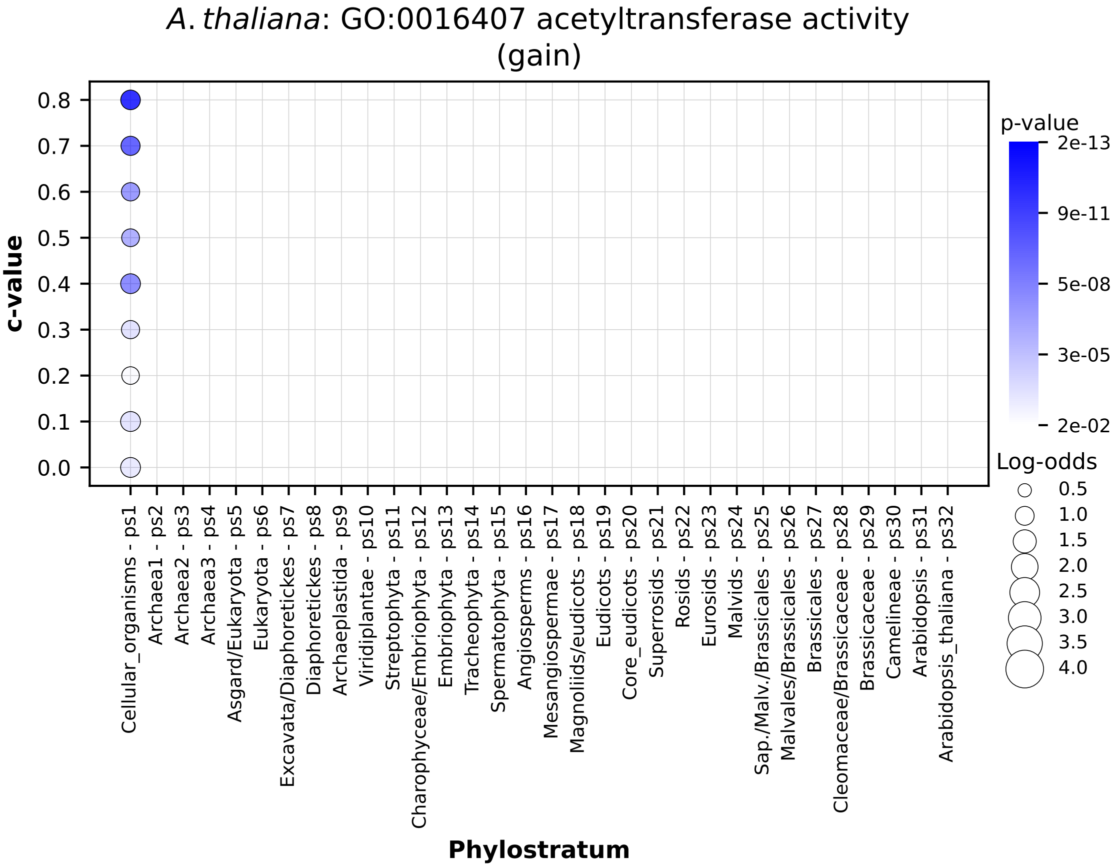
A. thaliana: GO:0016407 acetyltransferase activity
(gain)
c-value
0.0
0.1
0.2
0.3
0.4
0.5
0.6
0.7
0.8
p-value
2e-13
9e-11
5e-08
3e-05
2e-02
Log-odds
0.5
1.0
1.5
2.0
2.5
3.0
3.5
4.0
Cellular_organisms - ps1
Archaea1 - ps2
Archaea2 - ps3
Archaea3 - ps4
Asgard/Eukaryota - ps5
Eukaryota - ps6
Excavata/Diaphoretickes - ps7
Diaphoretickes - ps8
Archaeplastida - ps9
Viridiplantae - ps10
Streptophyta - ps11
Charophyceae/Embriophyta - ps12
Embriophyta - ps13
Tracheophyta - ps14
Spermatophyta - ps15
Angiosperms - ps16
Mesangiospermae - ps17
Magnoliids/eudicots - ps18
Eudicots - ps19
Core_eudicots - ps20
Superrosids - ps21
Rosids - ps22
Eurosids - ps23
Malvids - ps24
Sap./Malv./Brassicales - ps25
Malvales/Brassicales - ps26
Brassicales - ps27
Cleomaceae/Brassicaceae - ps28
Brassicaceae - ps29
Camelineae - ps30
Arabidopsis - ps31
Arabidopsis_thaliana - ps32
Phylostratum

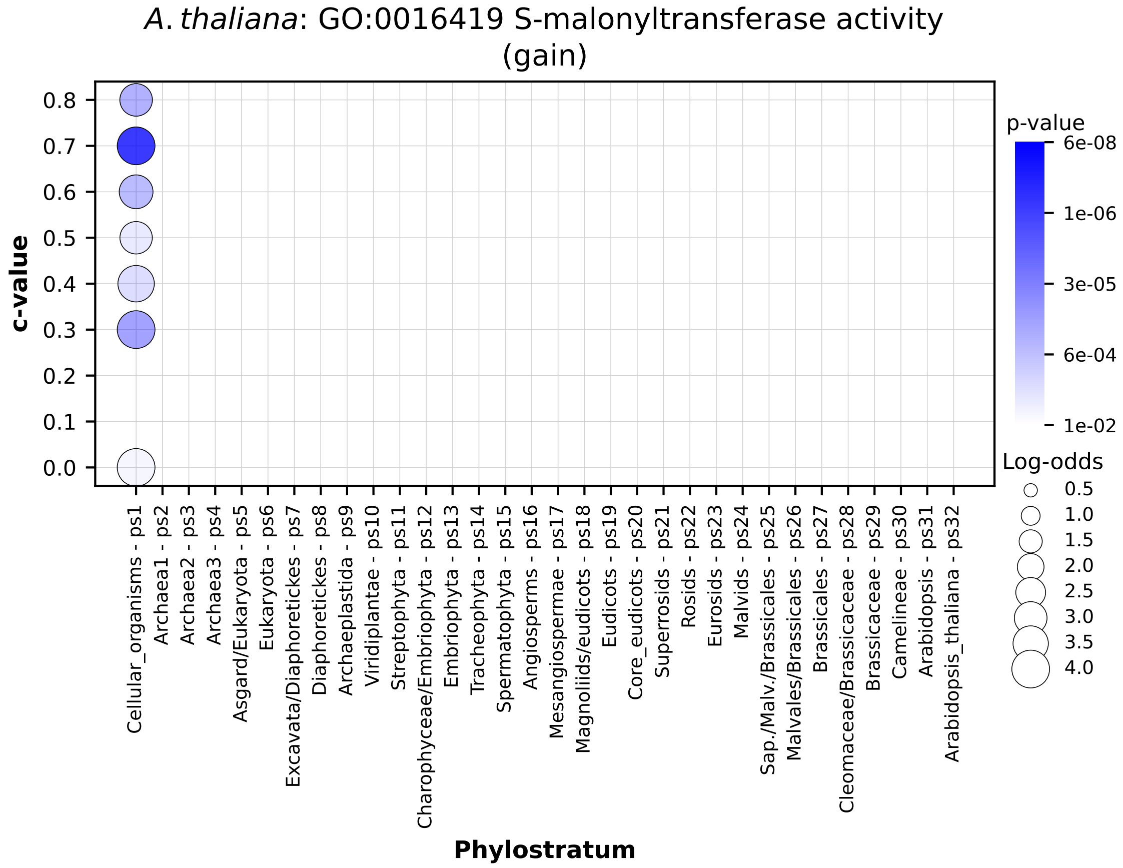
A. thaliana: GO:0016419 S-malonyltransferase activity (gain)
c-value
0.8
0.7
0.6
0.5
0.4
0.3
0.2
0.1
0.0
p-value
6e-08
1e-06
3e-05
6e-04
1e-02
Log-odds
0.5
1.0
1.5
2.0
2.5
3.0
3.5
4.0
Cellular_organisms - ps1
Archaea1 - ps2
Archaea2 - ps3
Archaea3 - ps4
Asgard/Eukaryota - ps5
Eukaryota - ps6
Excavata/Diaphoretickes - ps7
Diaphoretickes - ps8
Archaeplastida - ps9
Viridiplantae - ps10
Streptophyta - ps11
Charophyceae/Embriophyta - ps12
Embriophyta - ps13
Tracheophyta - ps14
Spermatophyta - ps15
Angiosperms - ps16
Mesangiospermae - ps17
Magnoliids/eudicots - ps18
Eudicots - ps19
Core_eudicots - ps20
Superrosids - ps21
Rosids - ps22
Eurosids - ps23
Malvids - ps24
Sap./Malv./Brassicales - ps25
Malvales/Brassicales - ps26
Brassicales - ps27
Cleomaceae/Brassicaceae - ps28
Brassicaceae - ps29
Camelineae - ps30
Arabidopsis - ps31
Arabidopsis_thaliana - ps32
Phylostratum

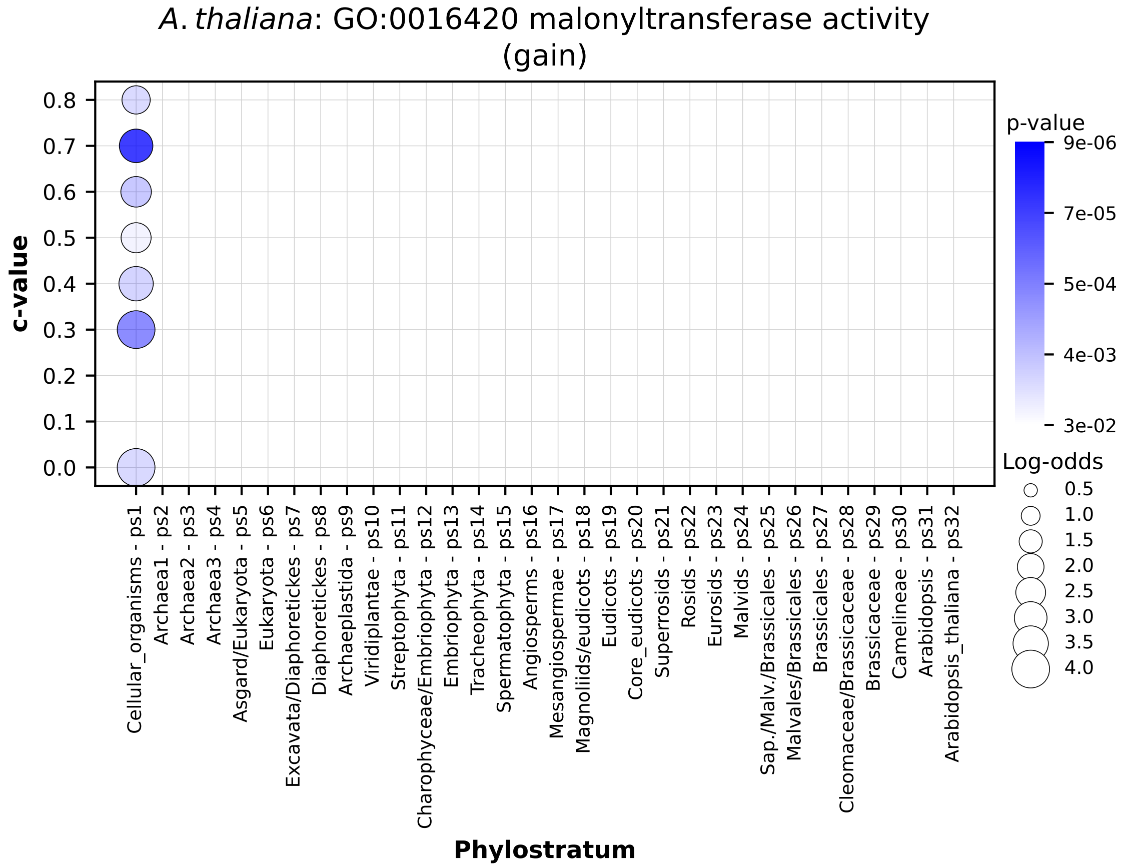

A. thaliana: GO:0016420 malonyltransferase activity (gain)
c-value
0.8
0.7
0.6
0.5
0.4
0.3
0.2
0.1
0.0
Phylostratum
Cellular_organisms - ps1
Archaea1 - ps2
Archaea2 - ps3
Archaea3 - ps4
Asgard/Eukaryota - ps5
Eukaryota - ps6
Excavata/Diaphoretickes - ps7
Diaphoretickes - ps8
Archaeplastida - ps9
Viridiplantae - ps10
Streptophyta - ps11
Charophyceae/Embriophyta - ps12
Embriophyta - ps13
Tracheophyta - ps14
Spermatophyta - ps15
Angiosperms - ps16
Mesangiospermae - ps17
Magnoliids/eudicots - ps18
Eudicots - ps19
Core_eudicots - ps20
Superrosids - ps21
Rosids - ps22
Eurosids - ps23
Malvids - ps24
Sap./Malv./Brassicales - ps25
Malvales/Brassicales - ps26
Brassicales - ps27
Cleomaceae/Brassicaceae - ps28
Brassicaceae - ps29
Camelineae - ps30
Arabidopsis - ps31
Arabidopsis_thaliana - ps32
p-value
9e-06
7e-05
5e-04
4e-03
3e-02
Log-odds
0.5
1.0
1.5
2.0
2.5
3.0
3.5
4.0

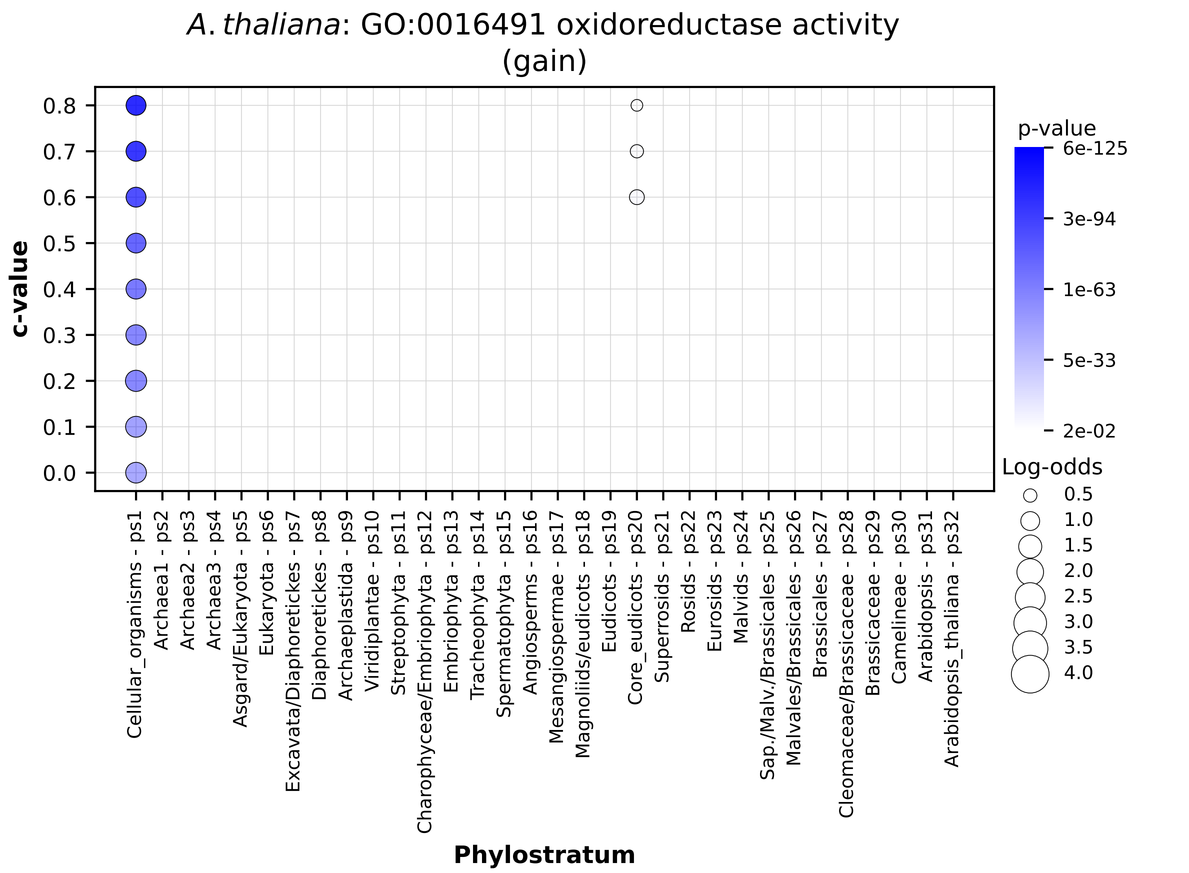

A. thaliana: GO:0016491 oxidoreductase activity
(gain)
c-value
0.8
0.7
0.6
0.5
0.4
0.3
0.2
0.1
0.0
p-value
6e-125
3e-94
1e-63
5e-33
2e-02
Log-odds
0.5
1.0
1.5
2.0
2.5
3.0
3.5
4.0
Cellular_organisms - ps1
Archaea1 - ps2
Archaea2 - ps3
Archaea3 - ps4
Asgard/Eukaryota - ps5
Eukaryota - ps6
Excavata/Diaphoretickes - ps7
Diaphoretickes - ps8
Archaeplastida - ps9
Viridiplantae - ps10
Streptophyta - ps11
Charophyceae/Embriophyta - ps12
Embriophyta - ps13
Tracheophyta - ps14
Spermatophyta - ps15
Angiosperms - ps16
Mesangiospermae - ps17
Magnoliids/eudicots - ps18
Eudicots - ps19
Core_eudicots - ps20
Superrosids - ps21
Rosids - ps22
Eurosids - ps23
Malvids - ps24
Sap./Malv./Brassicales - ps25
Malvales/Brassicales - ps26
Brassicales - ps27
Cleomaceae/Brassicaceae - ps28
Brassicaceae - ps29
Camelineae - ps30
Arabidopsis - ps31
Arabidopsis_thaliana - ps32
Phylostratum

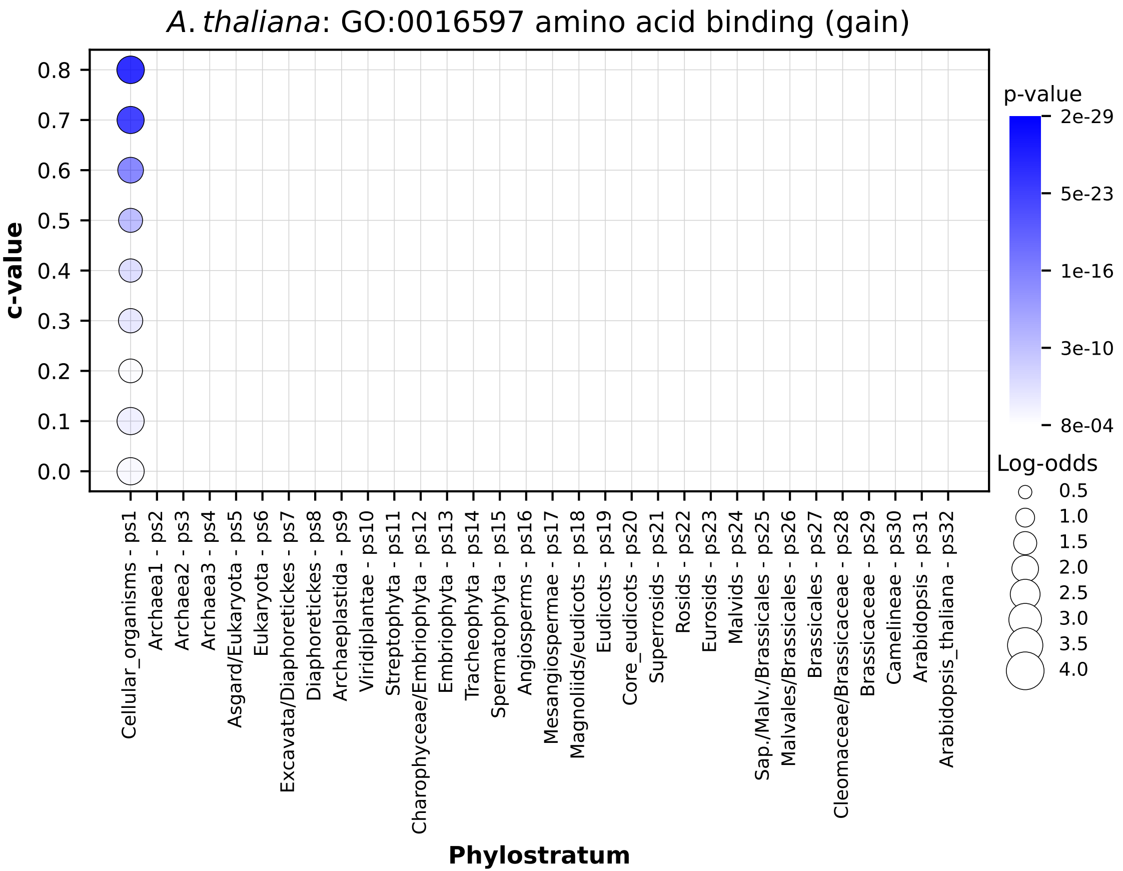

A. thaliana: GO:0016597 amino acid binding (gain)
c-value
0.8
0.7
0.6
0.5
0.4
0.3
0.2
0.1
0.0
p-value
2e-29
5e-23
1e-16
3e-10
8e-04
Log-odds
0.5
1.0
1.5
2.0
2.5
3.0
3.5
4.0
Cellular_organisms - ps1
Archaea1 - ps2
Archaea2 - ps3
Archaea3 - ps4
Asgard/Eukaryota - ps5
Eukaryota - ps6
Excavata/Diaphoretickes - ps7
Diaphoretickes - ps8
Archaeplastida - ps9
Viridiplantae - ps10
Streptophyta - ps11
Charophyceae/Embriophyta - ps12
Embriophyta - ps13
Tracheophyta - ps14
Spermatophyta - ps15
Angiosperms - ps16
Mesangiospermae - ps17
Magnoliids/eudicots - ps18
Eudicots - ps19
Core_eudicots - ps20
Superrosids - ps21
Rosids - ps22
Eurosids - ps23
Malvids - ps24
Sap./Malv./Brassicales - ps25
Malvales/Brassicales - ps26
Brassicales - ps27
Cleomaceae/Brassicaceae - ps28
Brassicaceae - ps29
Camelineae - ps30
Arabidopsis - ps31
Arabidopsis_thaliana - ps32
Phylostratum

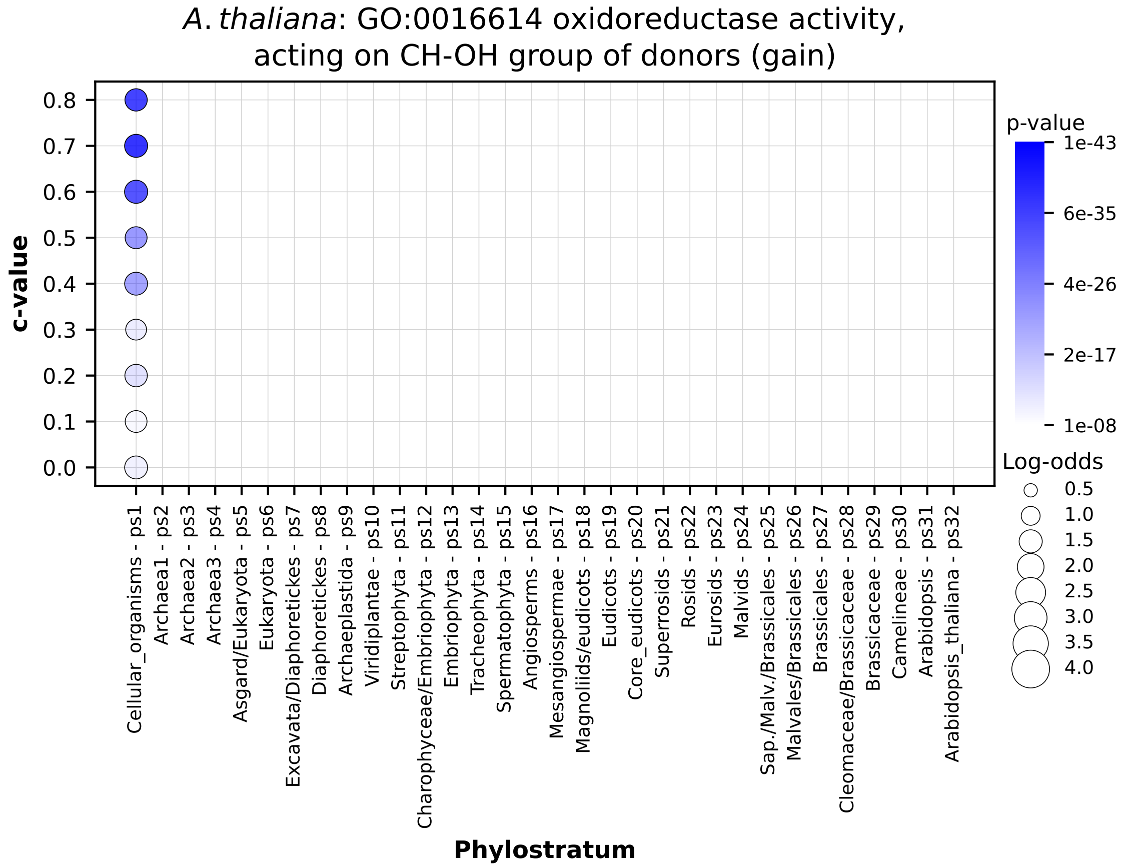
A. thaliana: GO:0016614 oxidoreductase activity, acting on CH-OH group of donors (gain)
c-value
0.0
0.1
0.2
0.3
0.4
0.5
0.6
0.7
0.8
p-value
1e-43
6e-35
4e-26
2e-17
1e-08
Log-odds
0.5
1.0
1.5
2.0
2.5
3.0
3.5
4.0
Cellular_organisms - ps1
Archaea1 - ps2
Archaea2 - ps3
Archaea3 - ps4
Asgard/Eukaryota - ps5
Eukaryota - ps6
Excavata/Diaphoretickes - ps7
Diaphoretickes - ps8
Archaeplastida - ps9
Viridiplantae - ps10
Streptophyta - ps11
Charophyceae/Embriophyta - ps12
Embriophyta - ps13
Tracheophyta - ps14
Spermatophyta - ps15
Angiosperms - ps16
Mesangiospermae - ps17
Magnoliids/eudicots - ps18
Eudicots - ps19
Core_eudicots - ps20
Superrosids - ps21
Rosids - ps22
Eurosids - ps23
Malvids - ps24
Sap./Malv./Brassicales - ps25
Malvales/Brassicales - ps26
Brassicales - ps27
Cleomaceae/Brassicaceae - ps28
Brassicaceae - ps29
Camelineae - ps30
Arabidopsis - ps31
Arabidopsis_thaliana - ps32
Phylostratum

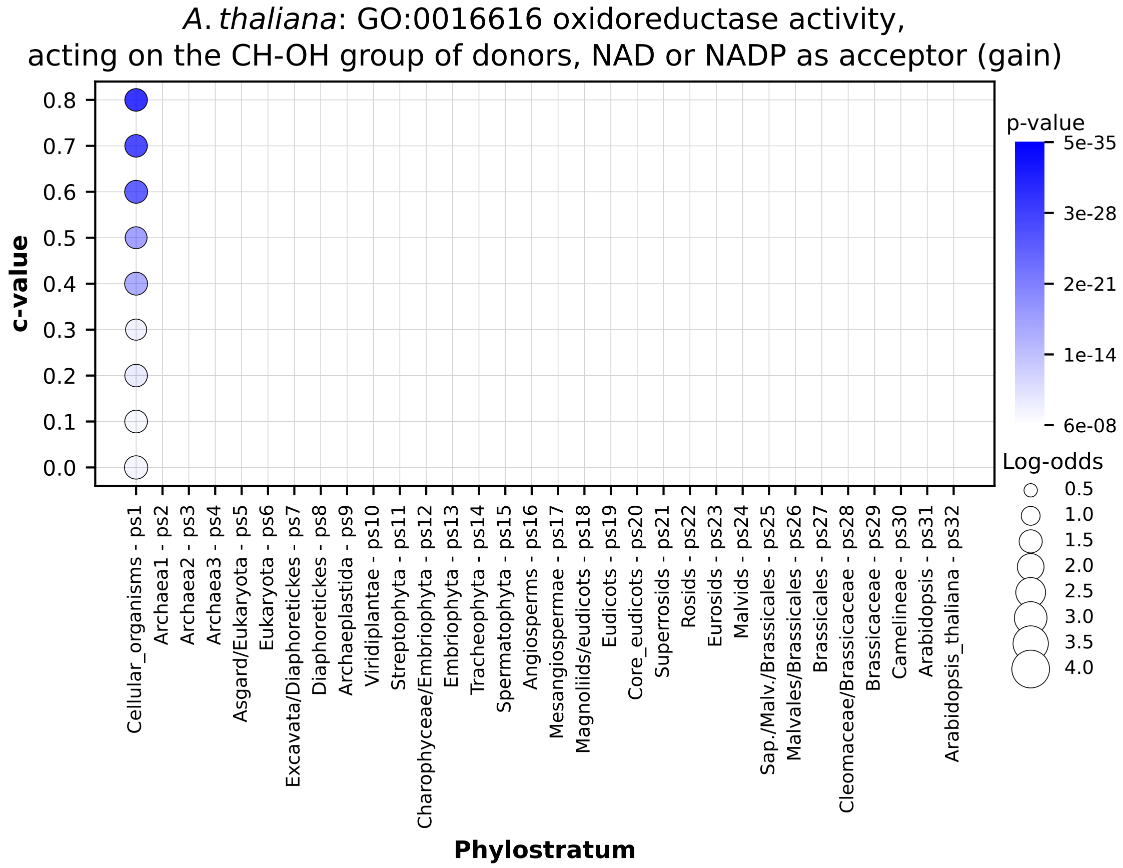

A. thaliana: GO:0016616 oxidoreductase activity,
acting on the CH-OH group of donors, NAD or NADP as acceptor (gain)
c-value
0.8
0.7
0.6
0.5
0.4
0.3
0.2
0.1
0.0
p-value
5e-35
3e-28
2e-21
1e-14
6e-08
Log-odds
0.5
1.0
1.5
2.0
2.5
3.0
3.5
4.0
Cellular_organisms - ps1
Archaea1 - ps2
Archaea2 - ps3
Archaea3 - ps4
Asgard/Eukaryota - ps5
Eukaryota - ps6
Excavata/Diaphoretickes - ps7
Diaphoretickes - ps8
Archaeplastida - ps9
Viridiplantae - ps10
Streptophyta - ps11
Charophyceae/Embriophyta - ps12
Embriophyta - ps13
Tracheophyta - ps14
Spermatophyta - ps15
Angiosperms - ps16
Mesangiospermae - ps17
Magnoliids/eudicots - ps18
Eudicots - ps19
Core_eudicots - ps20
Superrosids - ps21
Rosids - ps22
Eurosids - ps23
Malvids - ps24
Sap./Malv./Brassicales - ps25
Malvales/Brassicales - ps26
Brassicales - ps27
Cleomaceae/Brassicaceae - ps28
Brassicaceae - ps29
Camelineae - ps30
Arabidopsis - ps31
Arabidopsis_thaliana - ps32
Phylostratum

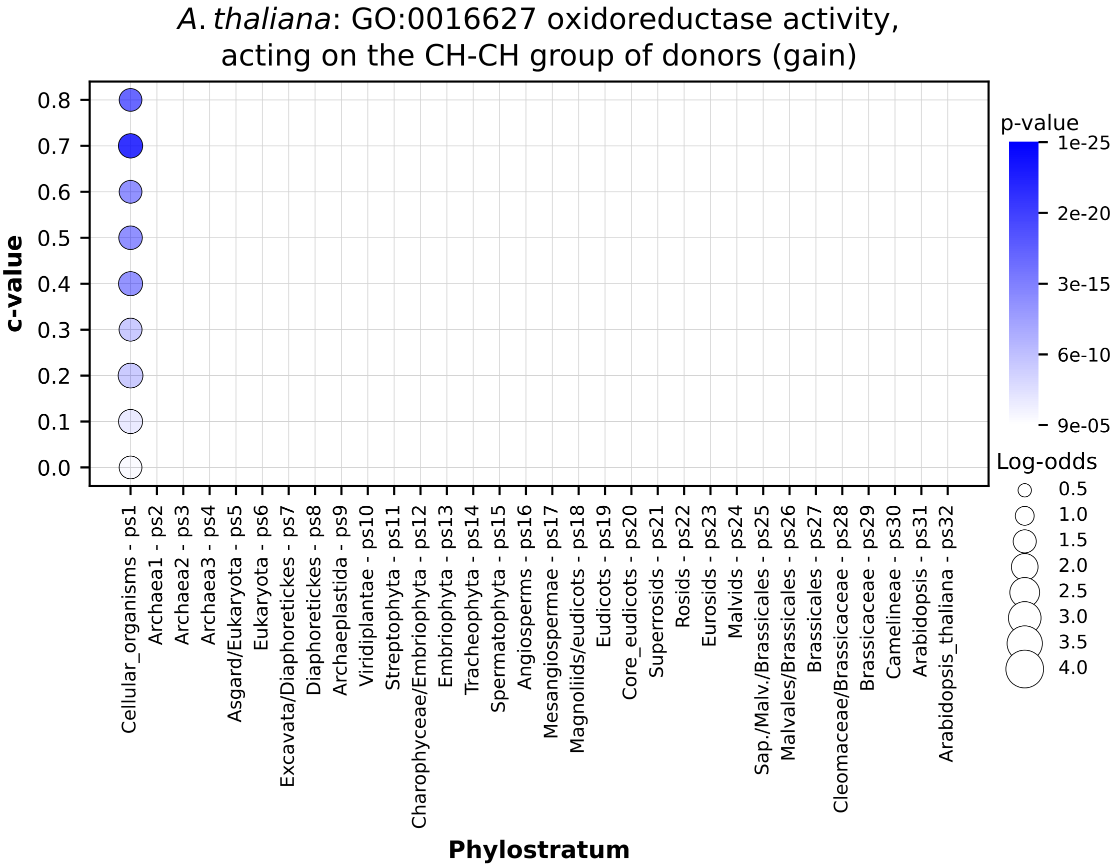
A. thaliana: GO:0016627 oxidoreductase activity, acting on the CH-CH group of donors (gain)
c-value
0.8
0.7
0.6
0.5
0.4
0.3
0.2
0.1
0.0
p-value
1e-25
2e-20
3e-15
6e-10
9e-05
Log-odds
0.5
1.0
1.5
2.0
2.5
3.0
3.5
4.0
Cellular_organisms - ps1
Archaea1 - ps2
Archaea2 - ps3
Archaea3 - ps4
Asgard/Eukaryota - ps5
Eukaryota - ps6
Excavata/Diaphoretickes - ps7
Diaphoretickes - ps8
Archaeplastida - ps9
Viridiplantae - ps10
Streptophyta - ps11
Charophyceae/Embriophyta - ps12
Embriophyta - ps13
Tracheophyta - ps14
Spermatophyta - ps15
Angiosperms - ps16
Mesangiospermae - ps17
Magnoliids/eudicots - ps18
Eudicots - ps19
Core_eudicots - ps20
Superrosids - ps21
Rosids - ps22
Eurosids - ps23
Malvids - ps24
Sap./Malv./Brassicales - ps25
Malvales/Brassicales - ps26
Brassicales - ps27
Cleomaceae/Brassicaceae - ps28
Brassicaceae - ps29
Camelineae - ps30
Arabidopsis - ps31
Arabidopsis_thaliana - ps32
Phylostratum

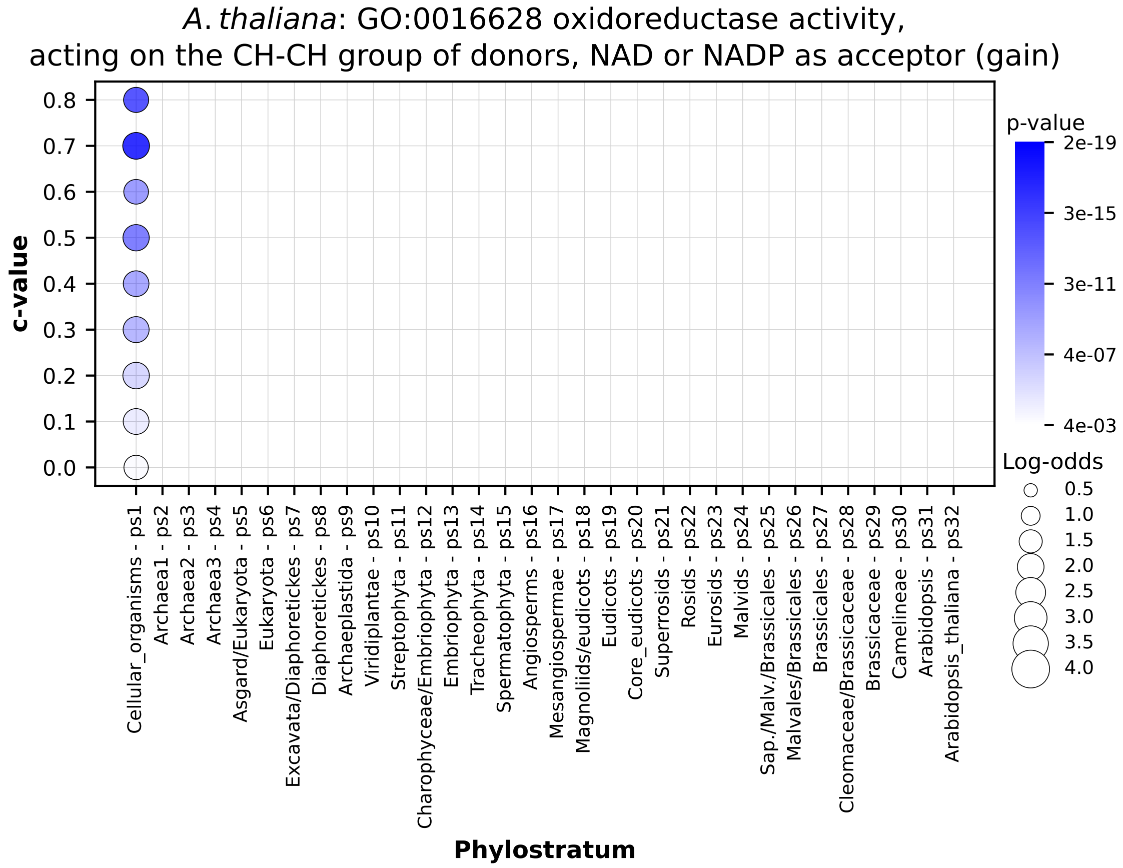
A. thaliana: GO:0016628 oxidoreductase activity,
acting on the CH-CH group of donors, NAD or NADP as acceptor (gain)
c-value
0.8
0.7
0.6
0.5
0.4
0.3
0.2
0.1
0.0
p-value
2e-19
3e-15
3e-11
4e-07
4e-03
Log-odds
0.5
1.0
1.5
2.0
2.5
3.0
3.5
4.0
Cellular_organisms - ps1
Archaea1 - ps2
Archaea2 - ps3
Archaea3 - ps4
Asgard/Eukaryota - ps5
Eukaryota - ps6
Excavata/Diaphoretickes - ps7
Diaphoretickes - ps8
Archaeplastida - ps9
Viridiplantae - ps10
Streptophyta - ps11
Charophyceae/Embriophyta - ps12
Embriophyta - ps13
Tracheophyta - ps14
Spermatophyta - ps15
Angiosperms - ps16
Mesangiospermae - ps17
Magnoliids/eudicots - ps18
Eudicots - ps19
Core_eudicots - ps20
Superrosids - ps21
Rosids - ps22
Eurosids - ps23
Malvids - ps24
Sap./Malv./Brassicales - ps25
Malvales/Brassicales - ps26
Brassicales - ps27
Cleomaceae/Brassicaceae - ps28
Brassicaceae - ps29
Camelineae - ps30
Arabidopsis - ps31
Arabidopsis_thaliana - ps32
Phylostratum

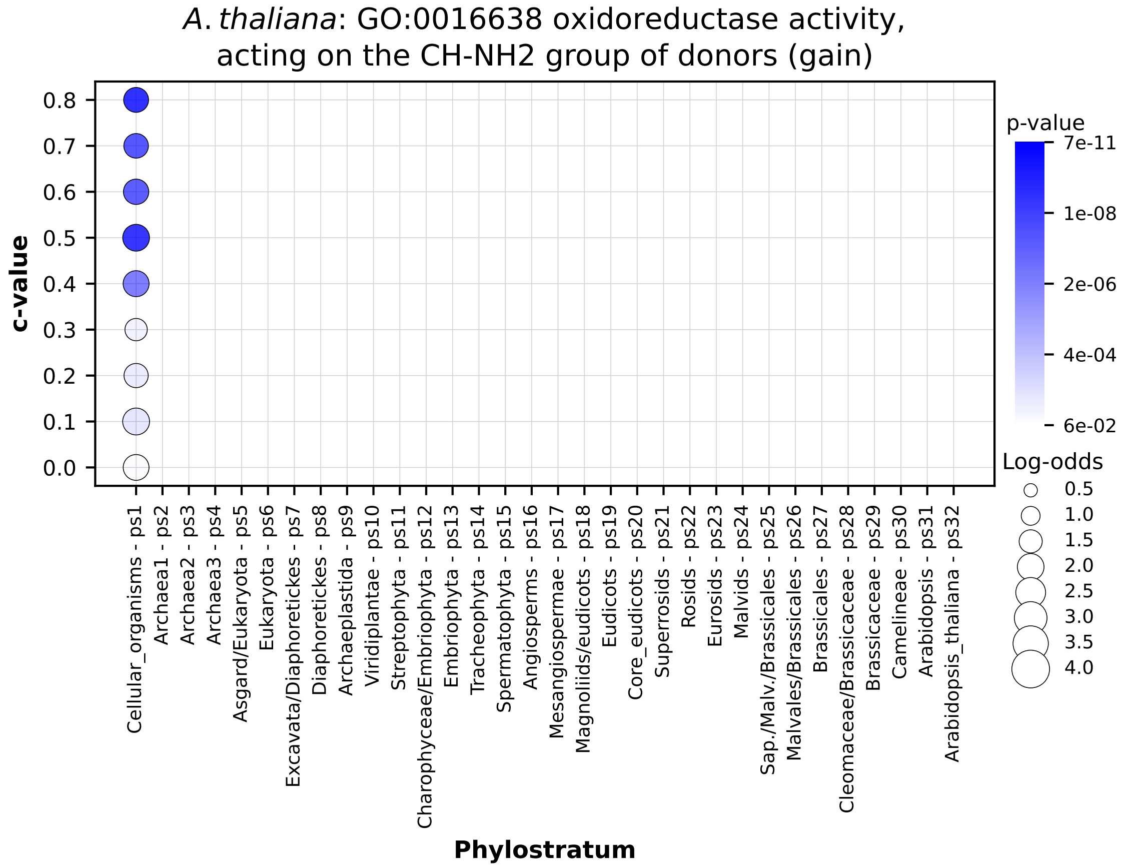
A. thaliana: GO:0016638 oxidoreductase activity, acting on the CH-NH2 group of donors (gain)
c-value
0.0
0.1
0.2
0.3
0.4
0.5
0.6
0.7
0.8
Phylostratum
Cellular_organisms - ps1
Archaea1 - ps2
Archaea2 - ps3
Archaea3 - ps4
Asgard/Eukaryota - ps5
Eukaryota - ps6
Excavata/Diaphoretickes - ps7
Diaphoretickes - ps8
Archaeplastida - ps9
Viridiplantae - ps10
Streptophyta - ps11
Charophyceae/Embriophyta - ps12
Embriophyta - ps13
Tracheophyta - ps14
Spermatophyta - ps15
Angiosperms - ps16
Mesangiospermae - ps17
Magnoliids/eudicots - ps18
Eudicots - ps19
Core_eudicots - ps20
Superrosids - ps21
Rosids - ps22
Eurosids - ps23
Malvids - ps24
Sap./Malv./Brassicales - ps25
Malvales/Brassicales - ps26
Brassicales - ps27
Cleomaceae/Brassicaceae - ps28
Brassicaceae - ps29
Camelineae - ps30
Arabidopsis - ps31
Arabidopsis_thaliana - ps32
p-value
7e-11
1e-08
2e-06
4e-04
6e-02
Log-odds
0.5
1.0
1.5
2.0
2.5
3.0
3.5
4.0

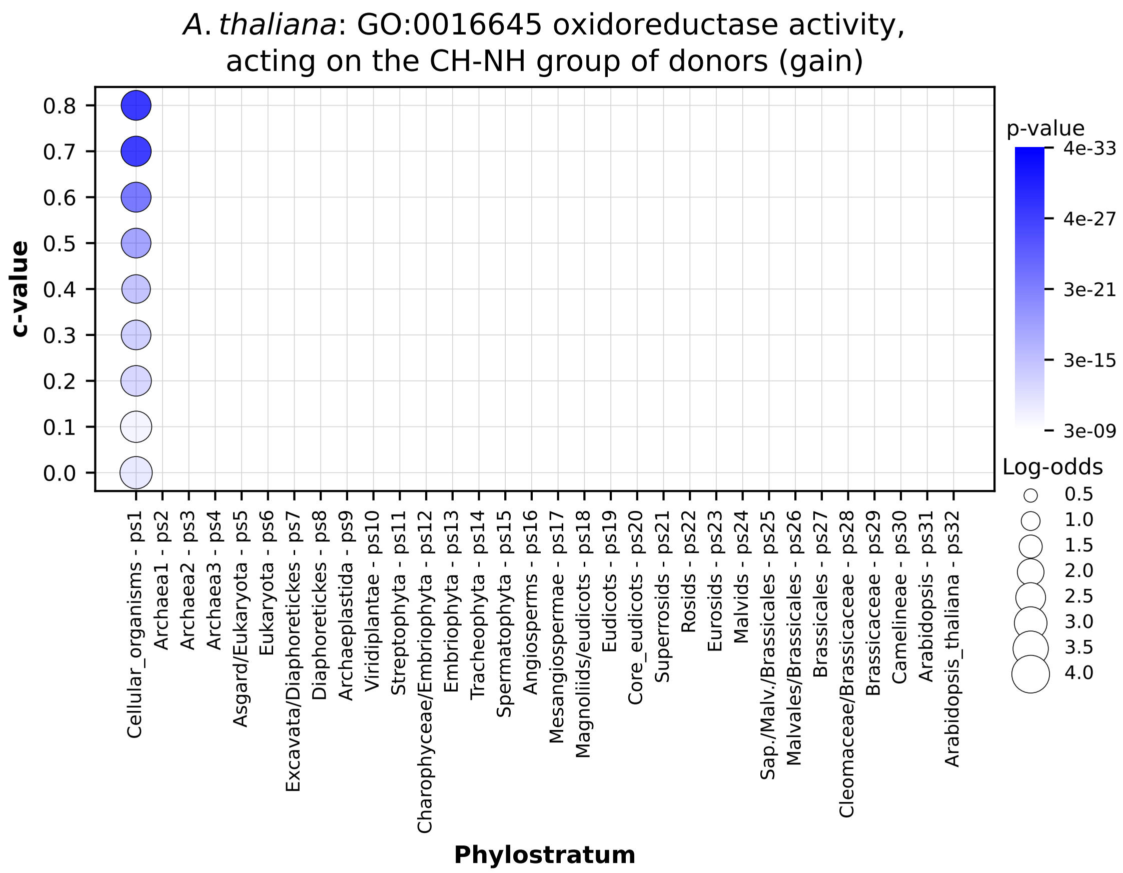

A. thaliana: GO:0016645 oxidoreductase activity, acting on the CH-NH group of donors (gain)
c-value
0.8
0.7
0.6
0.5
0.4
0.3
0.2
0.1
0.0
Phylostratum
Cellular_organisms - ps1
Archaea1 - ps2
Archaea2 - ps3
Archaea3 - ps4
Asgard/Eukaryota - ps5
Eukaryota - ps6
Excavata/Diaphoretickes - ps7
Diaphoretickes - ps8
Archaeplastida - ps9
Viridiplantae - ps10
Streptophyta - ps11
Charophyceae/Embriophyta - ps12
Embriophyta - ps13
Tracheophyta - ps14
Spermatophyta - ps15
Angiosperms - ps16
Mesangiospermae - ps17
Magnoliids/eudicots - ps18
Eudicots - ps19
Core_eudicots - ps20
Superrosids - ps21
Rosids - ps22
Eurosids - ps23
Malvids - ps24
Sap./Malv./Brassicales - ps25
Malvales/Brassicales - ps26
Brassicales - ps27
Cleomaceae/Brassicaceae - ps28
Brassicaceae - ps29
Camelineae - ps30
Arabidopsis - ps31
Arabidopsis_thaliana - ps32
p-value
4e-33
4e-27
3e-21
3e-15
3e-09
Log-odds
0.5
1.0
1.5
2.0
2.5
3.0
3.5
4.0

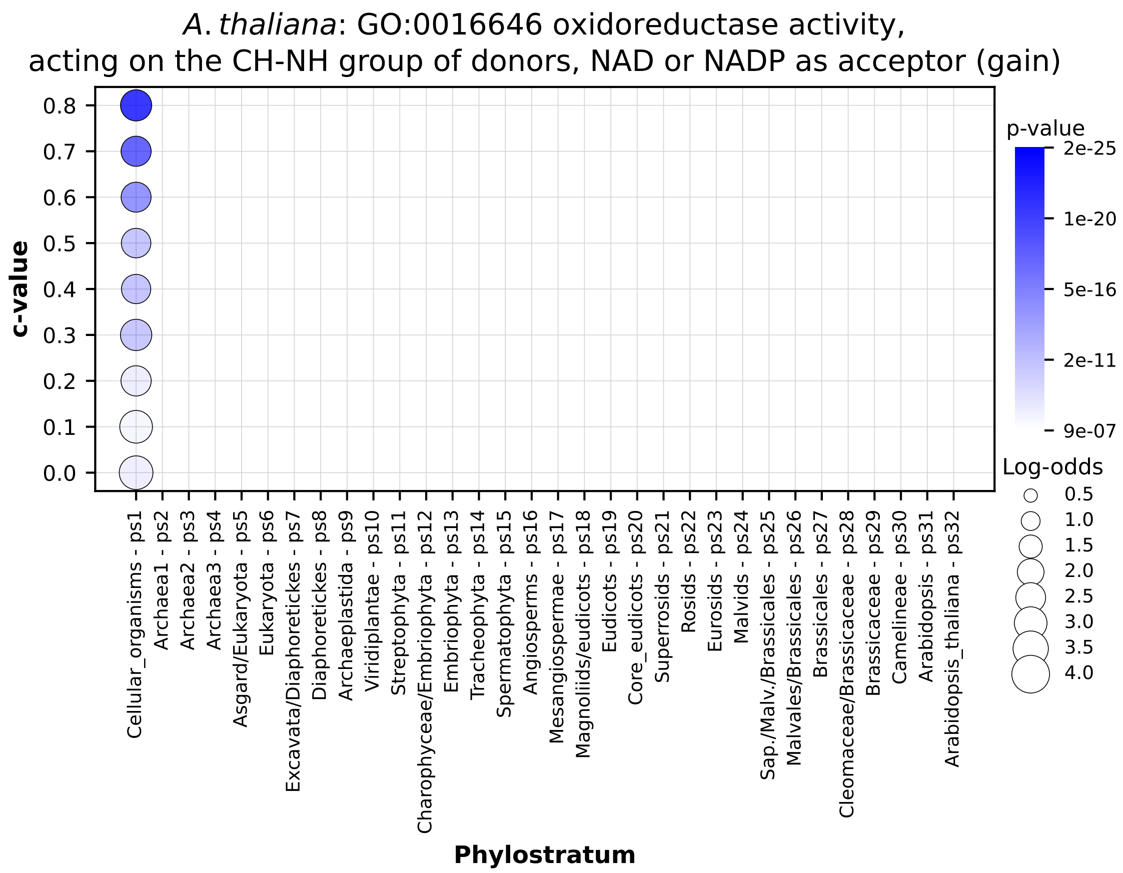
A. thaliana: GO:0016646 oxidoreductase activity,
acting on the CH-NH group of donors, NAD or NADP as acceptor (gain)
c-value
0.8
0.7
0.6
0.5
0.4
0.3
0.2
0.1
0.0
p-value
2e-25
1e-20
5e-16
2e-11
9e-07
Log-odds
0.5
1.0
1.5
2.0
2.5
3.0
3.5
4.0
Cellular_organisms - ps1
Archaea1 - ps2
Archaea2 - ps3
Archaea3 - ps4
Asgard/Eukaryota - ps5
Eukaryota - ps6
Excavata/Diaphoretickes - ps7
Diaphoretickes - ps8
Archaeplastida - ps9
Viridiplantae - ps10
Streptophyta - ps11
Charophyceae/Embriophyta - ps12
Embriophyta - ps13
Tracheophyta - ps14
Spermatophyta - ps15
Angiosperms - ps16
Mesangiospermae - ps17
Magnoliids/eudicots - ps18
Eudicots - ps19
Core_eudicots - ps20
Superrosids - ps21
Rosids - ps22
Eurosids - ps23
Malvids - ps24
Sap./Malv./Brassicales - ps25
Malvales/Brassicales - ps26
Brassicales - ps27
Cleomaceae/Brassicaceae - ps28
Brassicaceae - ps29
Camelineae - ps30
Arabidopsis - ps31
Arabidopsis_thaliana - ps32
Phylostratum

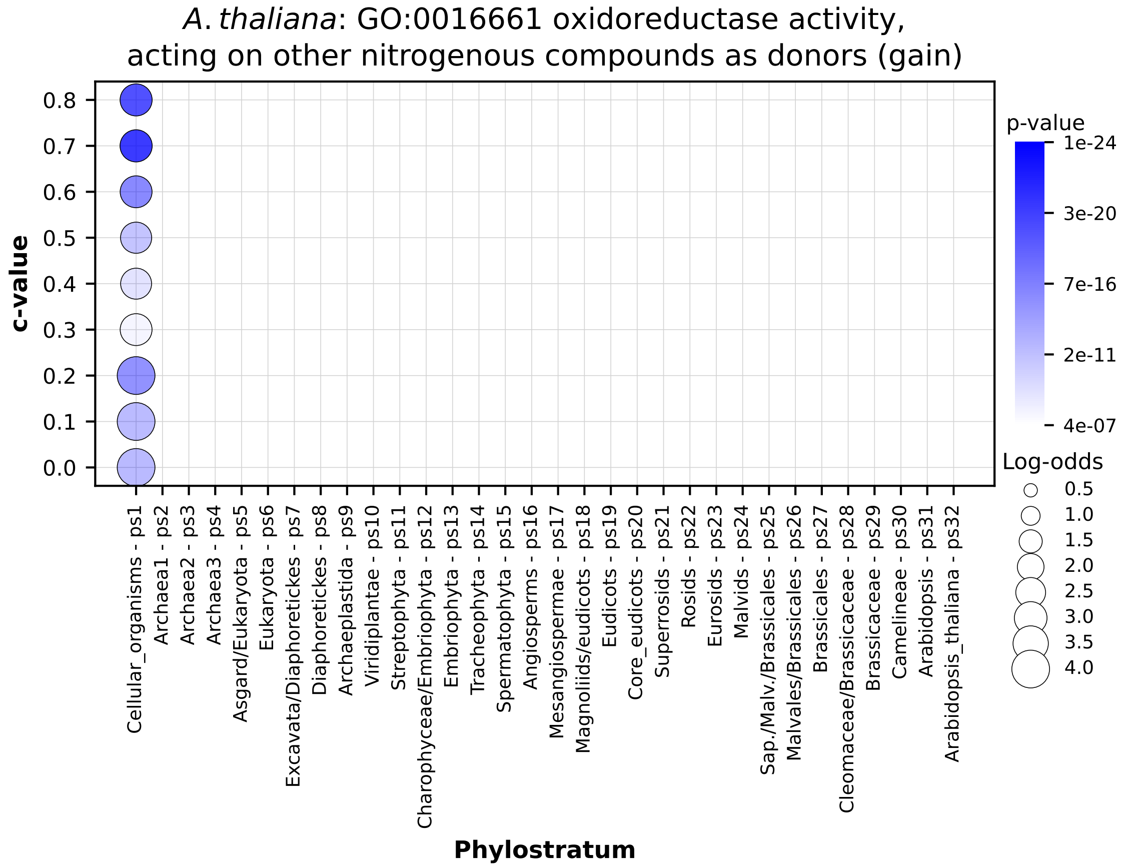
A. thaliana: GO:0016661 oxidoreductase activity, acting on other nitrogenous compounds as donors (gain)
c-value
0.0
0.1
0.2
0.3
0.4
0.5
0.6
0.7
0.8
p-value
1e-24
3e-20
7e-16
2e-11
4e-07
Log-odds
0.5
1.0
1.5
2.0
2.5
3.0
3.5
4.0
Cellular_organisms - ps1
Archaea1 - ps2
Archaea2 - ps3
Archaea3 - ps4
Asgard/Eukaryota - ps5
Eukaryota - ps6
Excavata/Diaphoretickes - ps7
Diaphoretickes - ps8
Archaeplastida - ps9
Viridiplantae - ps10
Streptophyta - ps11
Charophyceae/Embriophyta - ps12
Embriophyta - ps13
Tracheophyta - ps14
Spermatophyta - ps15
Angiosperms - ps16
Mesangiospermae - ps17
Magnoliids/eudicots - ps18
Eudicots - ps19
Core_eudicots - ps20
Superrosids - ps21
Rosids - ps22
Eurosids - ps23
Malvids - ps24
Sap./Malv./Brassicales - ps25
Malvales/Brassicales - ps26
Brassicales - ps27
Cleomaceae/Brassicaceae - ps28
Brassicaceae - ps29
Camelineae - ps30
Arabidopsis - ps31
Arabidopsis_thaliana - ps32
Phylostratum

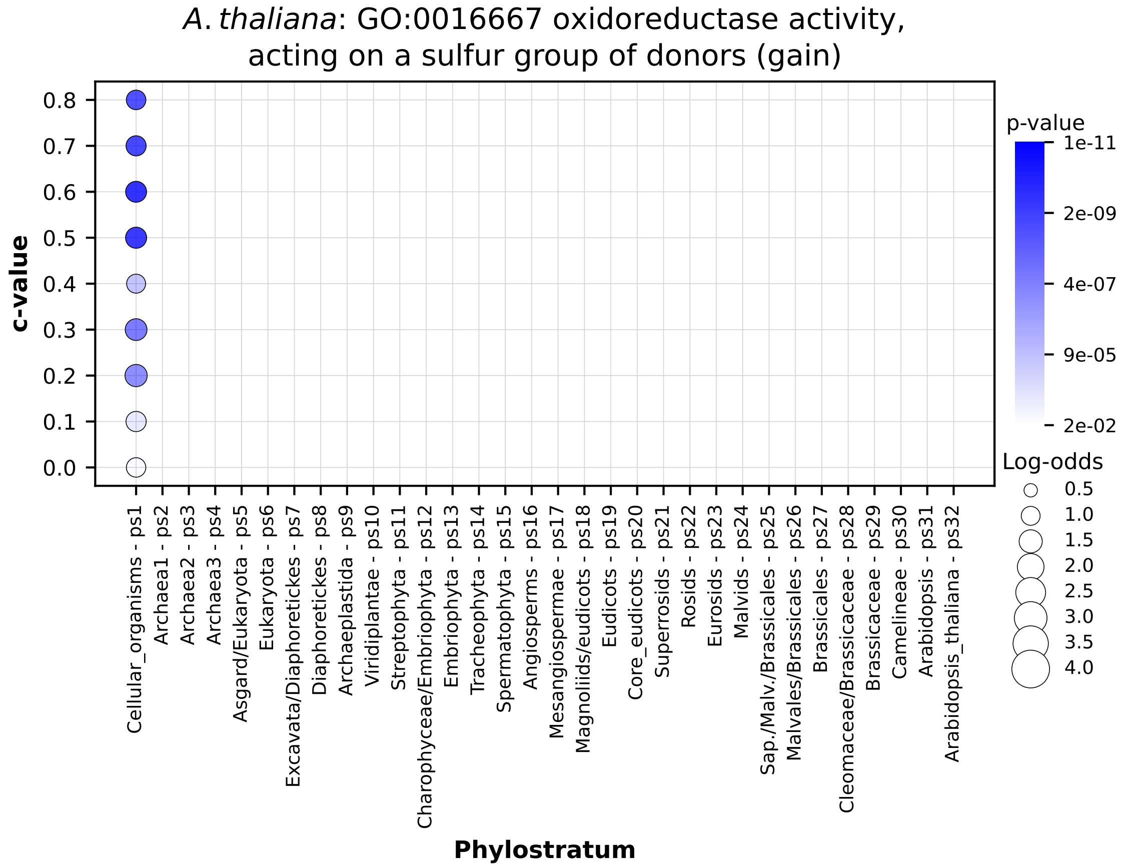

A. thaliana: GO:0016667 oxidoreductase activity, acting on a sulfur group of donors (gain)
c-value
0.0
0.1
0.2
0.3
0.4
0.5
0.6
0.7
0.8
p-value
1e-11
2e-09
4e-07
9e-05
2e-02
Log-odds
0.5
1.0
1.5
2.0
2.5
3.0
3.5
4.0
Cellular_organisms - ps1
Archaea1 - ps2
Archaea2 - ps3
Archaea3 - ps4
Asgard/Eukaryota - ps5
Eukaryota - ps6
Excavata/Diaphoretickes - ps7
Diaphoretickes - ps8
Archaeplastida - ps9
Viridiplantae - ps10
Streptophyta - ps11
Charophyceae/Embriophyta - ps12
Embriophyta - ps13
Tracheophyta - ps14
Spermatophyta - ps15
Angiosperms - ps16
Mesangiospermae - ps17
Magnoliids/eudicots - ps18
Eudicots - ps19
Core_eudicots - ps20
Superrosids - ps21
Rosids - ps22
Eurosids - ps23
Malvids - ps24
Sap./Malv./Brassicales - ps25
Malvales/Brassicales - ps26
Brassicales - ps27
Cleomaceae/Brassicaceae - ps28
Brassicaceae - ps29
Camelineae - ps30
Arabidopsis - ps31
Arabidopsis_thaliana - ps32
Phylostratum

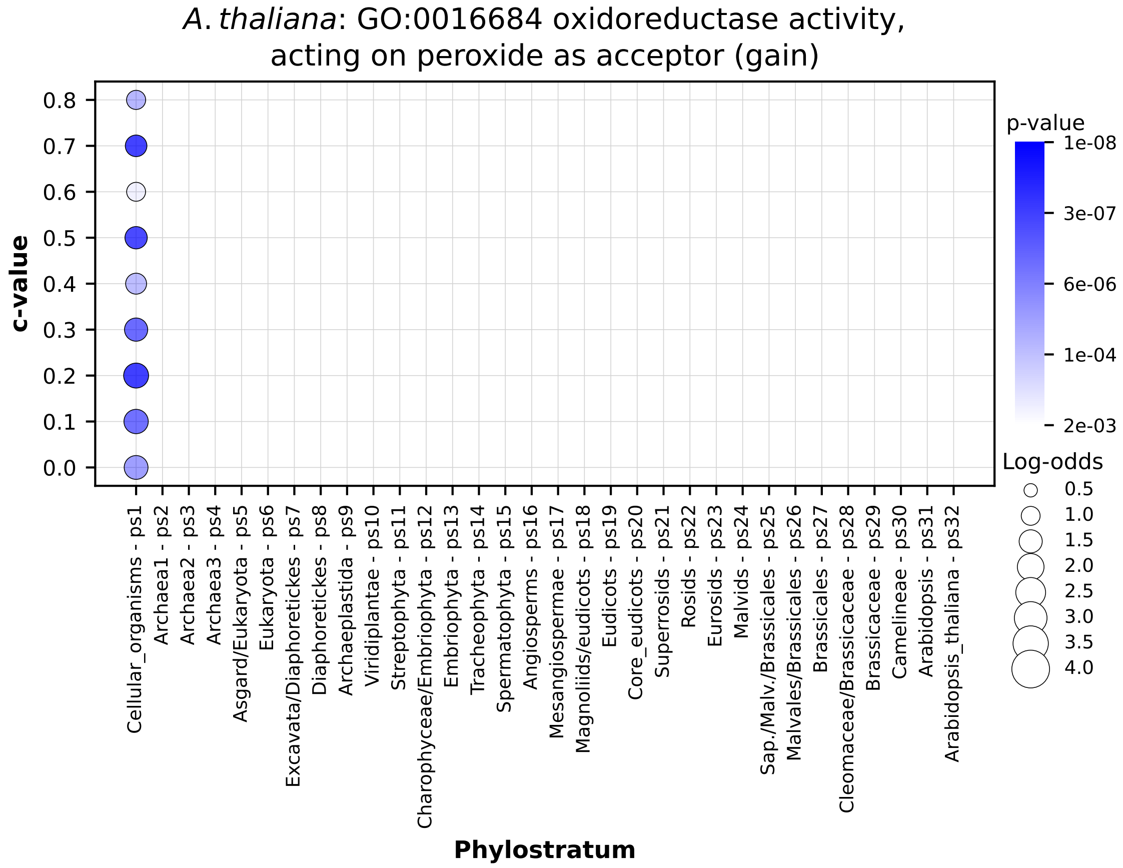
A. thaliana: GO:0016684 oxidoreductase activity, acting on peroxide as acceptor (gain)
c-value
0.8
0.7
0.6
0.5
0.4
0.3
0.2
0.1
0.0
p-value
1e-08
3e-07
6e-06
1e-04
2e-03
Log-odds
0.5
1.0
1.5
2.0
2.5
3.0
3.5
4.0
Cellular_organisms - ps1
Archaea1 - ps2
Archaea2 - ps3
Archaea3 - ps4
Asgard/Eukaryota - ps5
Eukaryota - ps6
Excavata/Diaphoretickes - ps7
Diaphoretickes - ps8
Archaeplastida - ps9
Viridiplantae - ps10
Streptophyta - ps11
Charophyceae/Embriophyta - ps12
Embriophyta - ps13
Tracheophyta - ps14
Spermatophyta - ps15
Angiosperms - ps16
Mesangiospermae - ps17
Magnoliids/eudicots - ps18
Eudicots - ps19
Core_eudicots - ps20
Superrosids - ps21
Rosids - ps22
Eurosids - ps23
Malvids - ps24
Sap./Malv./Brassicales - ps25
Malvales/Brassicales - ps26
Brassicales - ps27
Cleomaceae/Brassicaceae - ps28
Brassicaceae - ps29
Camelineae - ps30
Arabidopsis - ps31
Arabidopsis_thaliana - ps32
Phylostratum

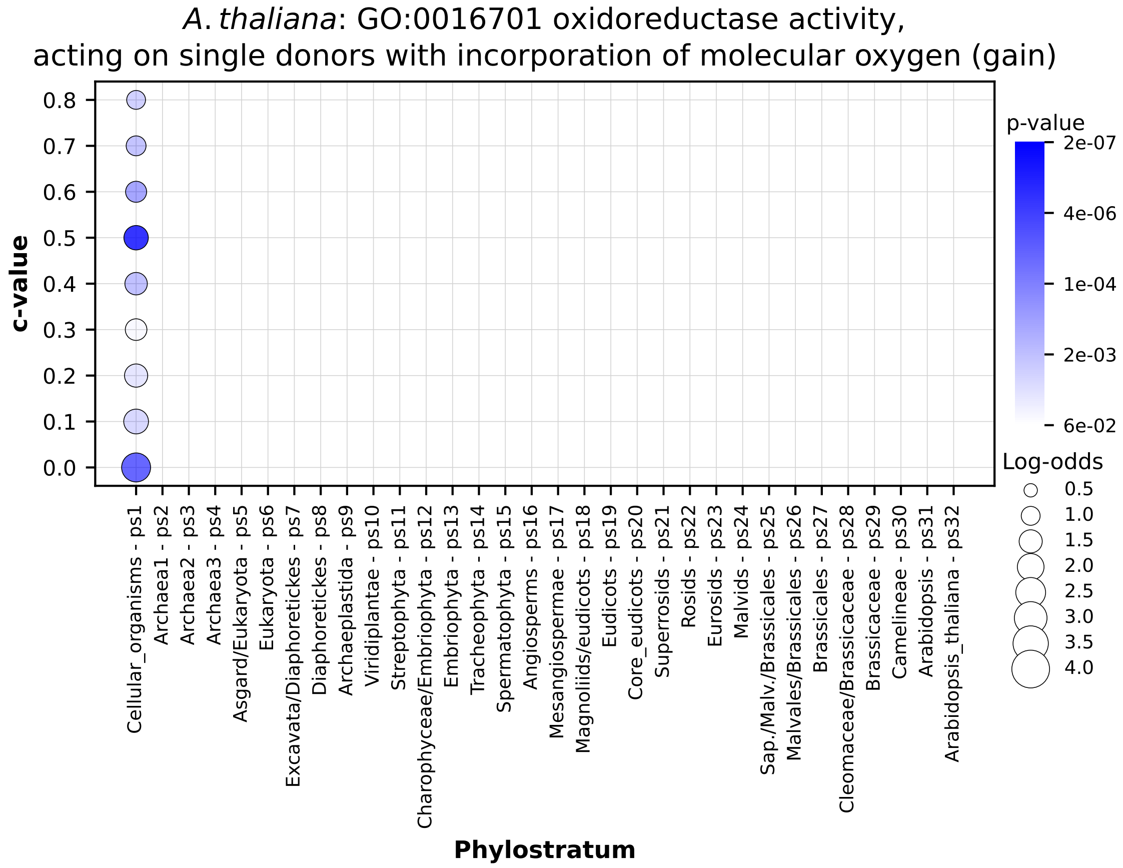

A. thaliana: GO:0016701 oxidoreductase activity,
acting on single donors with incorporation of molecular oxygen (gain)
c-value
0.8
0.7
0.6
0.5
0.4
0.3
0.2
0.1
0.0
p-value
2e-07
4e-06
1e-04
2e-03
6e-02
Log-odds
0.5
1.0
1.5
2.0
2.5
3.0
3.5
4.0
Cellular_organisms - ps1
Archaea1 - ps2
Archaea2 - ps3
Archaea3 - ps4
Asgard/Eukaryota - ps5
Eukaryota - ps6
Excavata/Diaphoretickes - ps7
Diaphoretickes - ps8
Archaeplastida - ps9
Viridiplantae - ps10
Streptophyta - ps11
Charophyceae/Embriophyta - ps12
Embriophyta - ps13
Tracheophyta - ps14
Spermatophyta - ps15
Angiosperms - ps16
Mesangiospermae - ps17
Magnoliids/eudicots - ps18
Eudicots - ps19
Core_eudicots - ps20
Superrosids - ps21
Rosids - ps22
Eurosids - ps23
Malvids - ps24
Sap./Malv./Brassicales - ps25
Malvales/Brassicales - ps26
Brassicales - ps27
Cleomaceae/Brassicaceae - ps28
Brassicaceae - ps29
Camelineae - ps30
Arabidopsis - ps31
Arabidopsis_thaliana - ps32
Phylostratum

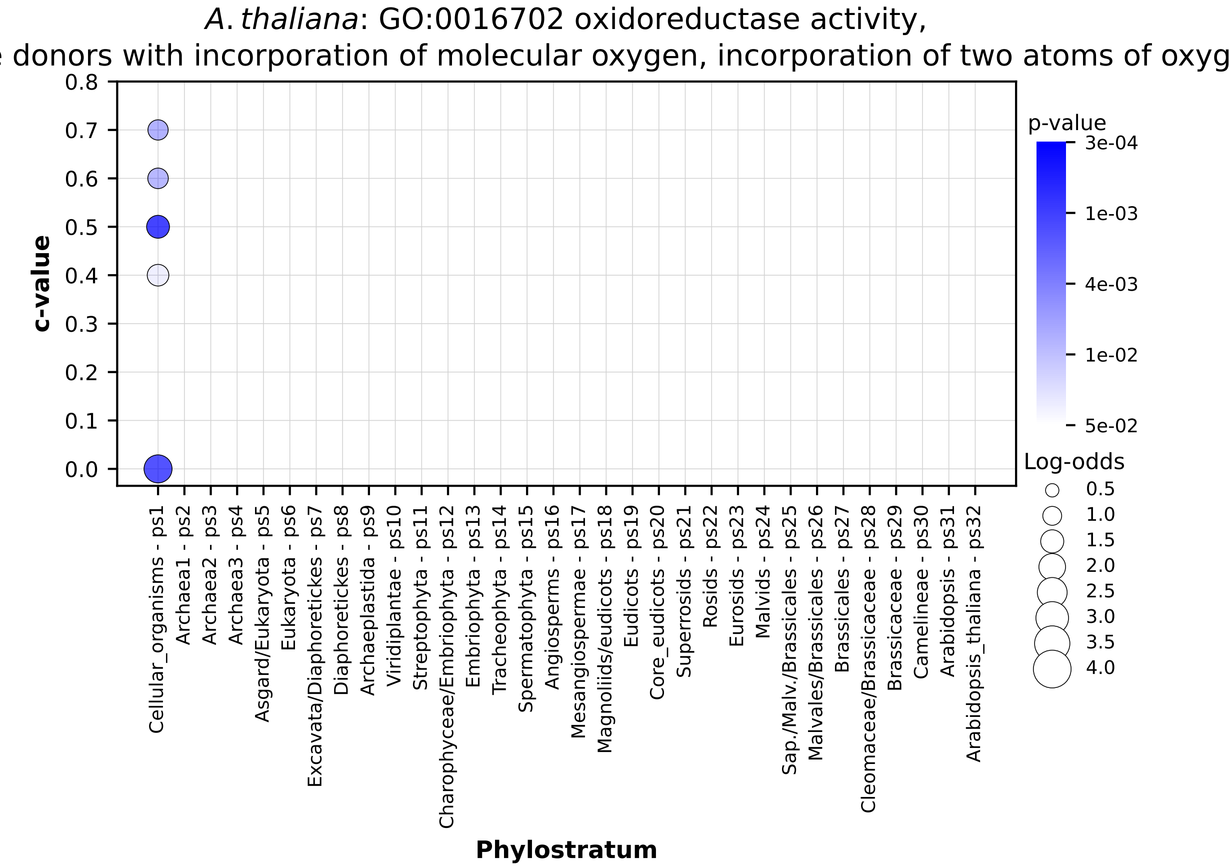

A. thaliana: GO:0016702 oxidoreductase activity,
e donors with incorporation of molecular oxygen, incorporation of two atoms of oxyg
c-value
Phylostratum
p-value
3e-04
1e-03
4e-03
1e-02
5e-02
Log-odds
0.5
1.0
1.5
2.0
2.5
3.0
3.5
4.0
Cellular_organisms - ps1
Archaea1 - ps2
Archaea2 - ps3
Archaea3 - ps4
Asgard/Eukaryota - ps5
Eukaryota - ps6
Excavata/Diaphoretickes - ps7
Diaphoretickes - ps8
Archaeplastida - ps9
Viridiplantae - ps10
Streptophyta - ps11
Charophyceae/Embriophyta - ps12
Embriophyta - ps13
Tracheophyta - ps14
Spermatophyta - ps15
Angiosperms - ps16
Mesangiospermae - ps17
Magnoliids/eudicots - ps18
Eudicots - ps19
Core_eudicots - ps20
Superrosids - ps21
Rosids - ps22
Eurosids - ps23
Malvids - ps24
Sap./Malv./Brassicales - ps25
Malvales/Brassicales - ps26
Brassicales - ps27
Cleomaceae/Brassicaceae - ps28
Brassicaceae - ps29
Camelineae - ps30
Arabidopsis - ps31
Arabidopsis_thaliana - ps32

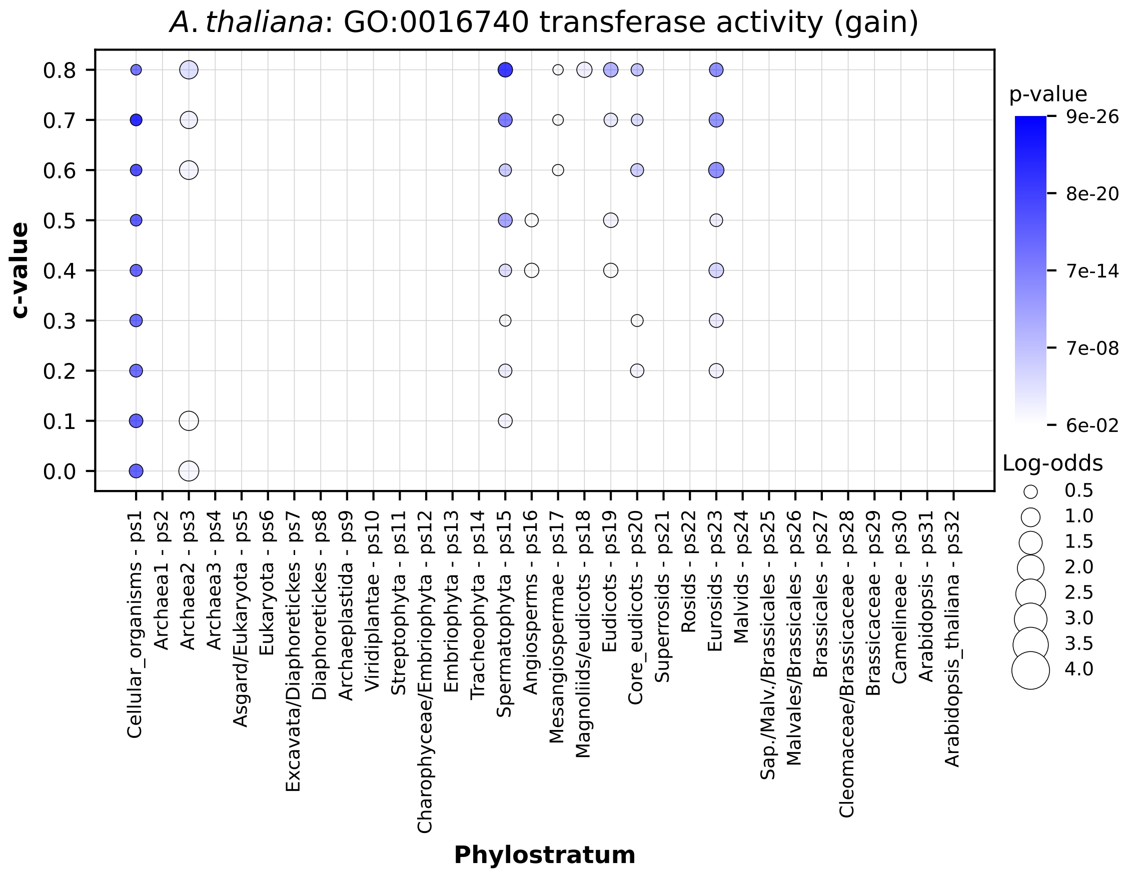
A. thaliana: GO:0016740 transferase activity (gain)
c-value
0.8
0.7
0.6
0.5
0.4
0.3
0.2
0.1
0.0
Cellular_organisms - ps1
Archaea1 - ps2
Archaea2 - ps3
Archaea3 - ps4
Asgard/Eukaryota - ps5
Eukaryota - ps6
Excavata/Diaphoretickes - ps7
Diaphoretickes - ps8
Archaeplastida - ps9
Viridiplantae - ps10
Streptophyta - ps11
Charophyceae/Embriophyta - ps12
Embriophyta - ps13
Tracheophyta - ps14
Spermatophyta - ps15
Angiosperms - ps16
Mesangiospermae - ps17
Magnoliids/eudicots - ps18
Eudicots - ps19
Core_eudicots - ps20
Superrosids - ps21
Rosids - ps22
Eurosids - ps23
Malvids - ps24
Sap./Malv./Brassicales - ps25
Malvales/Brassicales - ps26
Brassicales - ps27
Cleomaceae/Brassicaceae - ps28
Brassicaceae - ps29
Camelineae - ps30
Arabidopsis - ps31
Arabidopsis_thaliana - ps32
Phylostratum
p-value
9e-26
8e-20
7e-14
7e-08
6e-02
Log-odds
0.5
1.0
1.5
2.0
2.5
3.0
3.5
4.0

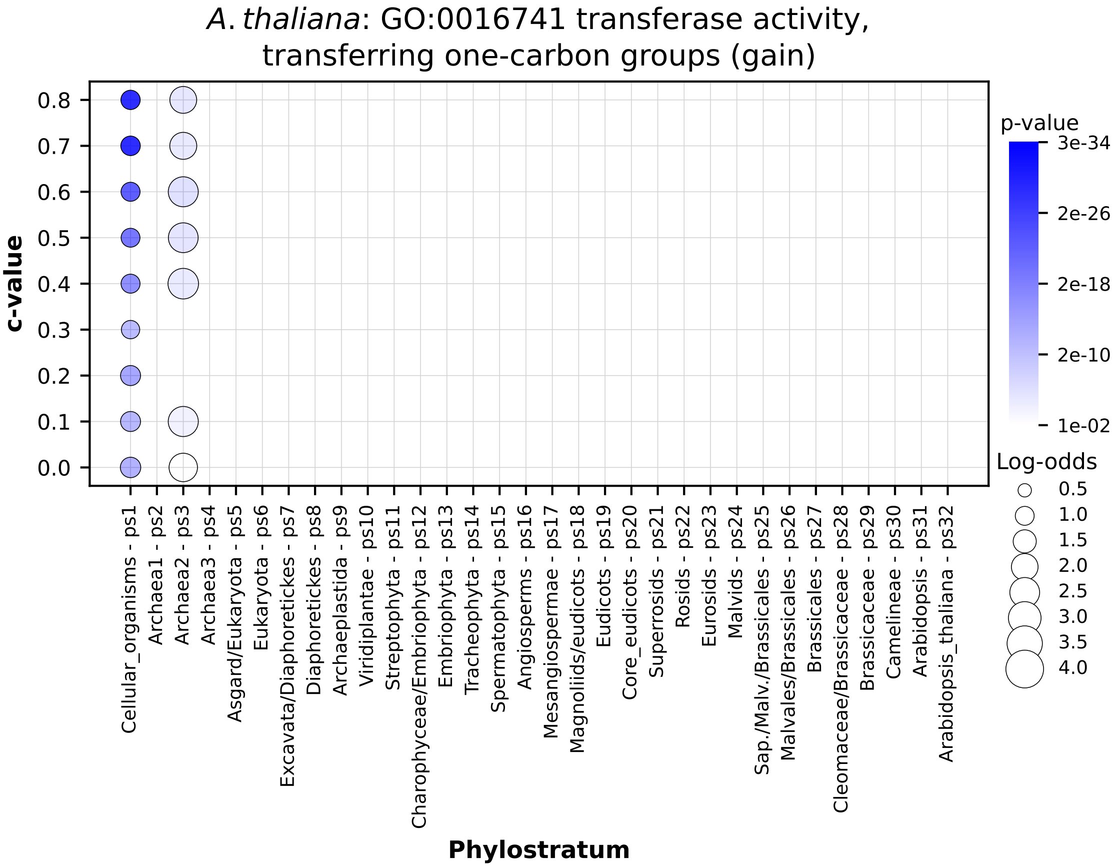

A. thaliana: GO:0016741 transferase activity, transferring one-carbon groups (gain)
c-value
0.8
0.7
0.6
0.5
0.4
0.3
0.2
0.1
0.0
p-value
3e-34
2e-26
2e-18
2e-10
1e-02
Log-odds
0.5
1.0
1.5
2.0
2.5
3.0
3.5
4.0
Cellular_organisms - ps1
Archaea1 - ps2
Archaea2 - ps3
Archaea3 - ps4
Asgard/Eukaryota - ps5
Eukaryota - ps6
Excavata/Diaphoretickes - ps7
Diaphoretickes - ps8
Archaeplastida - ps9
Viridiplantae - ps10
Streptophyta - ps11
Charophyceae/Embriophyta - ps12
Embriophyta - ps13
Tracheophyta - ps14
Spermatophyta - ps15
Angiosperms - ps16
Mesangiospermae - ps17
Magnoliids/eudicots - ps18
Eudicots - ps19
Core_eudicots - ps20
Superrosids - ps21
Rosids - ps22
Eurosids - ps23
Malvids - ps24
Sap./Malv./Brassicales - ps25
Malvales/Brassicales - ps26
Brassicales - ps27
Cleomaceae/Brassicaceae - ps28
Brassicaceae - ps29
Camelineae - ps30
Arabidopsis - ps31
Arabidopsis_thaliana - ps32
Phylostratum

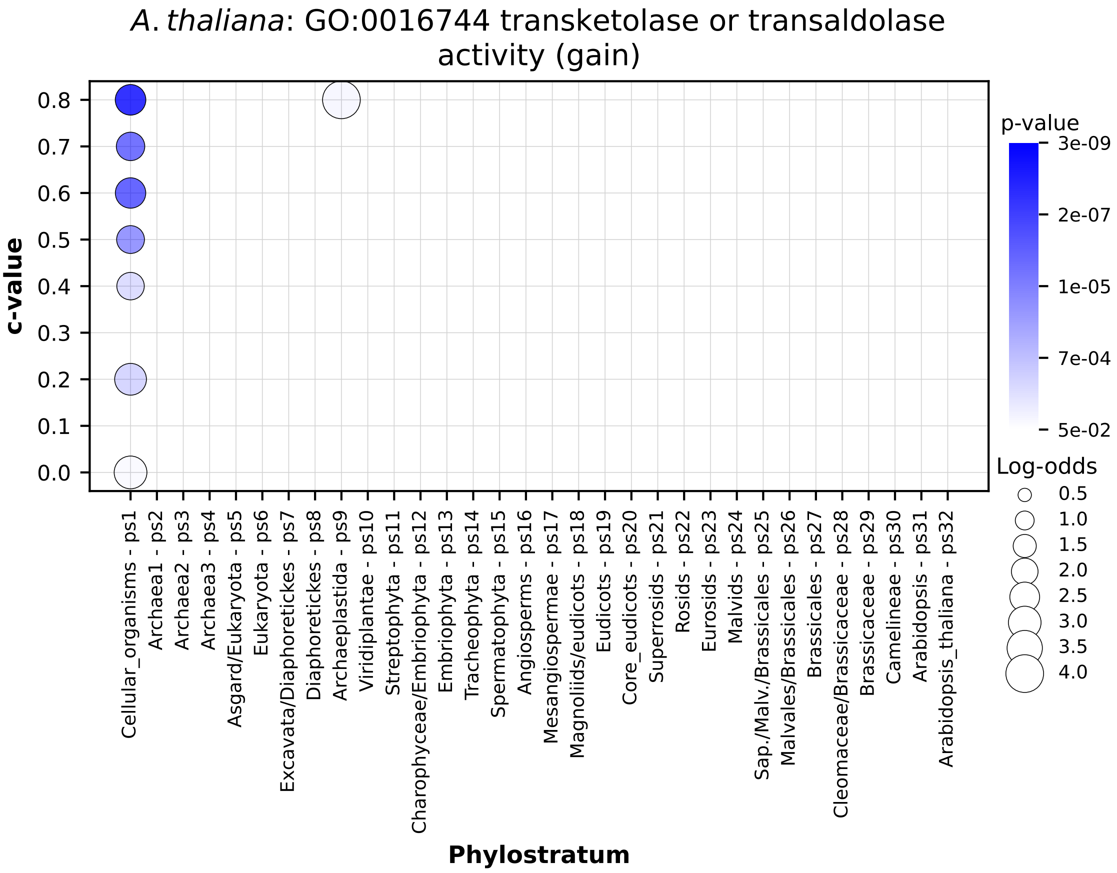

A. thaliana: GO:0016744 transketolase or transaldolase activity (gain)
c-value
0.0
0.1
0.2
0.3
0.4
0.5
0.6
0.7
0.8
Phylostratum
Cellular_organisms - ps1
Archaea1 - ps2
Archaea2 - ps3
Archaea3 - ps4
Asgard/Eukaryota - ps5
Eukaryota - ps6
Excavata/Diaphoretickes - ps7
Diaphoretickes - ps8
Archaeplastida - ps9
Viridiplantae - ps10
Streptophyta - ps11
Charophyceae/Embriophyta - ps12
Embriophyta - ps13
Tracheophyta - ps14
Spermatophyta - ps15
Angiosperms - ps16
Mesangiospermae - ps17
Magnoliids/eudicots - ps18
Eudicots - ps19
Core_eudicots - ps20
Superrosids - ps21
Rosids - ps22
Eurosids - ps23
Malvids - ps24
Sap./Malv./Brassicales - ps25
Malvales/Brassicales - ps26
Brassicales - ps27
Cleomaceae/Brassicaceae - ps28
Brassicaceae - ps29
Camelineae - ps30
Arabidopsis - ps31
Arabidopsis_thaliana - ps32
p-value
3e-09
2e-07
1e-05
7e-04
5e-02
Log-odds
0.5
1.0
1.5
2.0
2.5
3.0
3.5
4.0

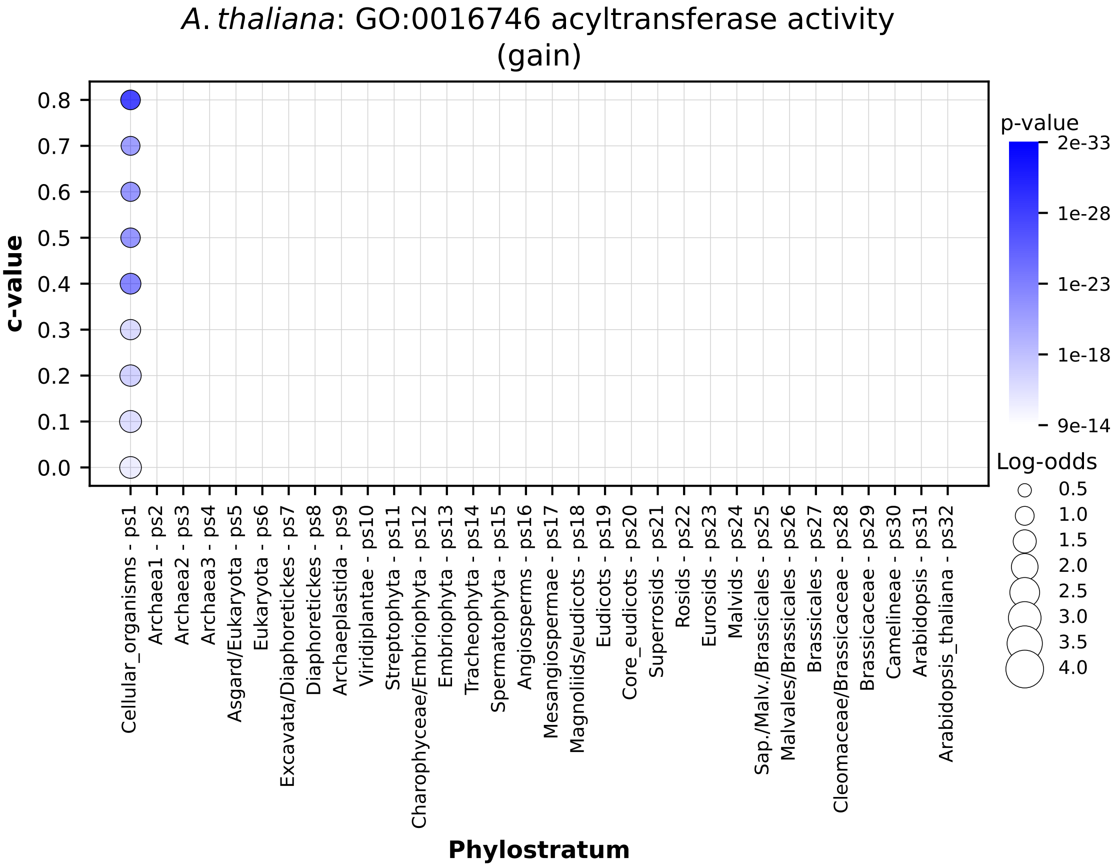

A. thaliana: GO:0016746 acyltransferase activity (gain)
c-value
0.8
0.7
0.6
0.5
0.4
0.3
0.2
0.1
0.0
p-value
2e-33
1e-28
1e-23
1e-18
9e-14
Log-odds
0.5
1.0
1.5
2.0
2.5
3.0
3.5
4.0
Cellular_organisms - ps1
Archaea1 - ps2
Archaea2 - ps3
Archaea3 - ps4
Asgard/Eukaryota - ps5
Eukaryota - ps6
Excavata/Diaphoretickes - ps7
Diaphoretickes - ps8
Archaeplastida - ps9
Viridiplantae - ps10
Streptophyta - ps11
Charophyceae/Embriophyta - ps12
Embriophyta - ps13
Tracheophyta - ps14
Spermatophyta - ps15
Angiosperms - ps16
Mesangiospermae - ps17
Magnoliids/eudicots - ps18
Eudicots - ps19
Core_eudicots - ps20
Superrosids - ps21
Rosids - ps22
Eurosids - ps23
Malvids - ps24
Sap./Malv./Brassicales - ps25
Malvales/Brassicales - ps26
Brassicales - ps27
Cleomaceae/Brassicaceae - ps28
Brassicaceae - ps29
Camelineae - ps30
Arabidopsis - ps31
Arabidopsis_thaliana - ps32
Phylostratum

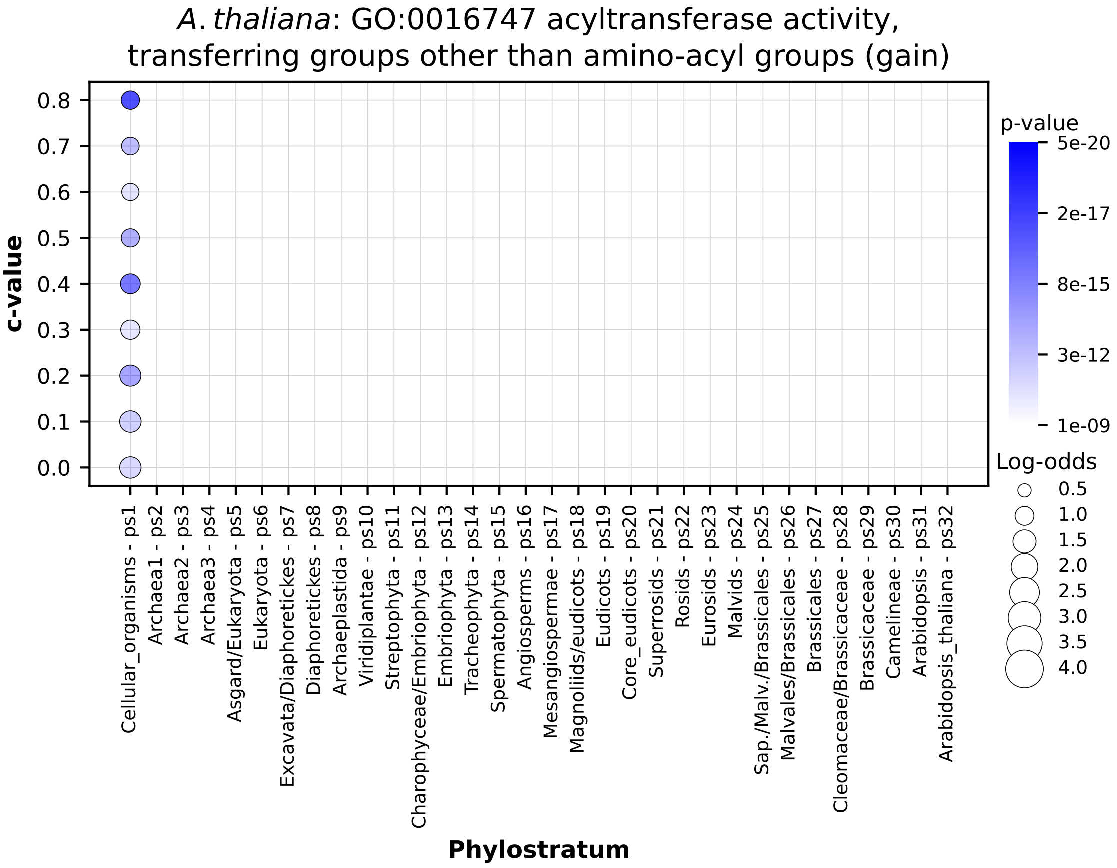

A. thaliana: GO:0016747 acyltransferase activity, transferring groups other than amino-acyl groups (gain)
c-value
0.8
0.7
0.6
0.5
0.4
0.3
0.2
0.1
0.0
p-value
5e-20
2e-17
8e-15
3e-12
1e-09
Log-odds
0.5
1.0
1.5
2.0
2.5
3.0
3.5
4.0
Cellular_organisms - ps1
Archaea1 - ps2
Archaea2 - ps3
Archaea3 - ps4
Asgard/Eukaryota - ps5
Eukaryota - ps6
Excavata/Diaphoretickes - ps7
Diaphoretickes - ps8
Archaeplastida - ps9
Viridiplantae - ps10
Streptophyta - ps11
Charophyceae/Embriophyta - ps12
Embriophyta - ps13
Tracheophyta - ps14
Spermatophyta - ps15
Angiosperms - ps16
Mesangiospermae - ps17
Magnoliids/eudicots - ps18
Eudicots - ps19
Core_eudicots - ps20
Superrosids - ps21
Rosids - ps22
Eurosids - ps23
Malvids - ps24
Sap./Malv./Brassicales - ps25
Malvales/Brassicales - ps26
Brassicales - ps27
Cleomaceae/Brassicaceae - ps28
Brassicaceae - ps29
Camelineae - ps30
Arabidopsis - ps31
Arabidopsis_thaliana - ps32
Phylostratum

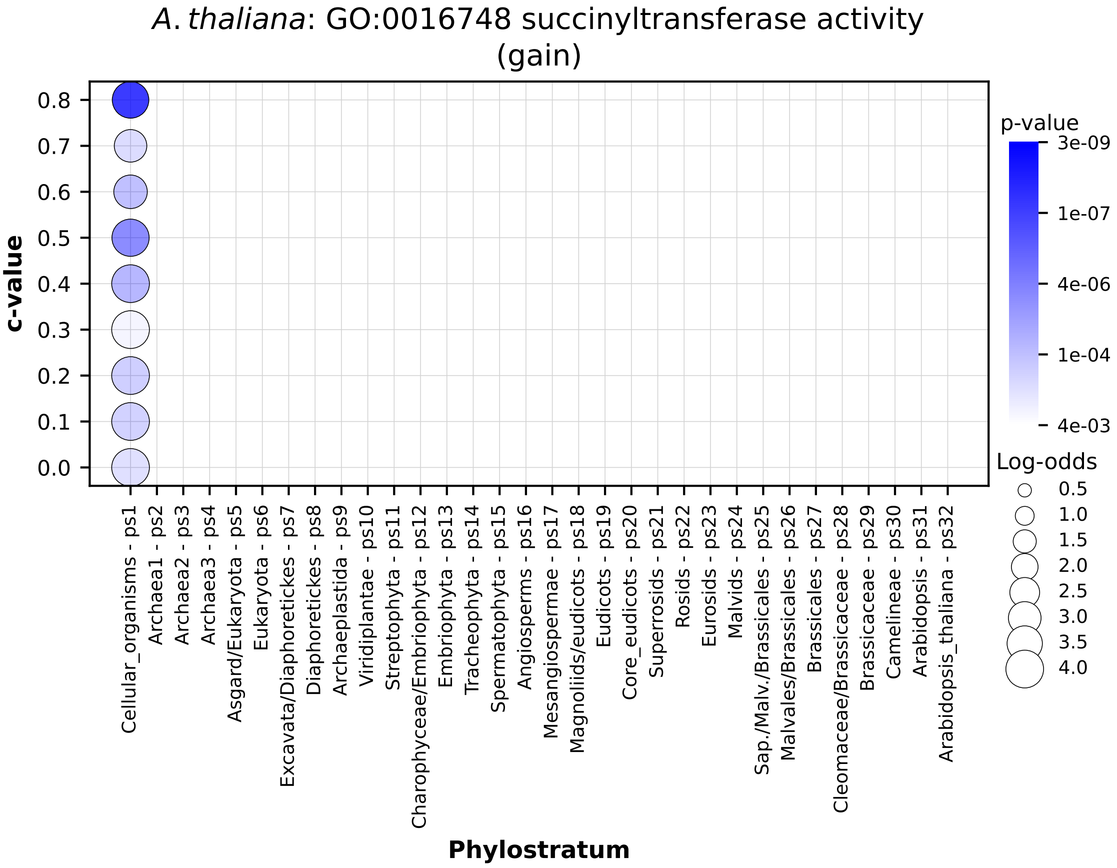
A. thaliana: GO:0016748 succinyltransferase activity
(gain)
c-value
0.0
0.1
0.2
0.3
0.4
0.5
0.6
0.7
0.8
p-value
3e-09
1e-07
4e-06
1e-04
4e-03
Log-odds
0.5
1.0
1.5
2.0
2.5
3.0
3.5
4.0
Cellular_organisms - ps1
Archaea1 - ps2
Archaea2 - ps3
Archaea3 - ps4
Asgard/Eukaryota - ps5
Eukaryota - ps6
Excavata/Diaphoretickes - ps7
Diaphoretickes - ps8
Archaeplastida - ps9
Viridiplantae - ps10
Streptophyta - ps11
Charophyceae/Embriophyta - ps12
Embriophyta - ps13
Tracheophyta - ps14
Spermatophyta - ps15
Angiosperms - ps16
Mesangiospermae - ps17
Magnoliids/eudicots - ps18
Eudicots - ps19
Core_eudicots - ps20
Superrosids - ps21
Rosids - ps22
Eurosids - ps23
Malvids - ps24
Sap./Malv./Brassicales - ps25
Malvales/Brassicales - ps26
Brassicales - ps27
Cleomaceae/Brassicaceae - ps28
Brassicaceae - ps29
Camelineae - ps30
Arabidopsis - ps31
Arabidopsis_thaliana - ps32
Phylostratum

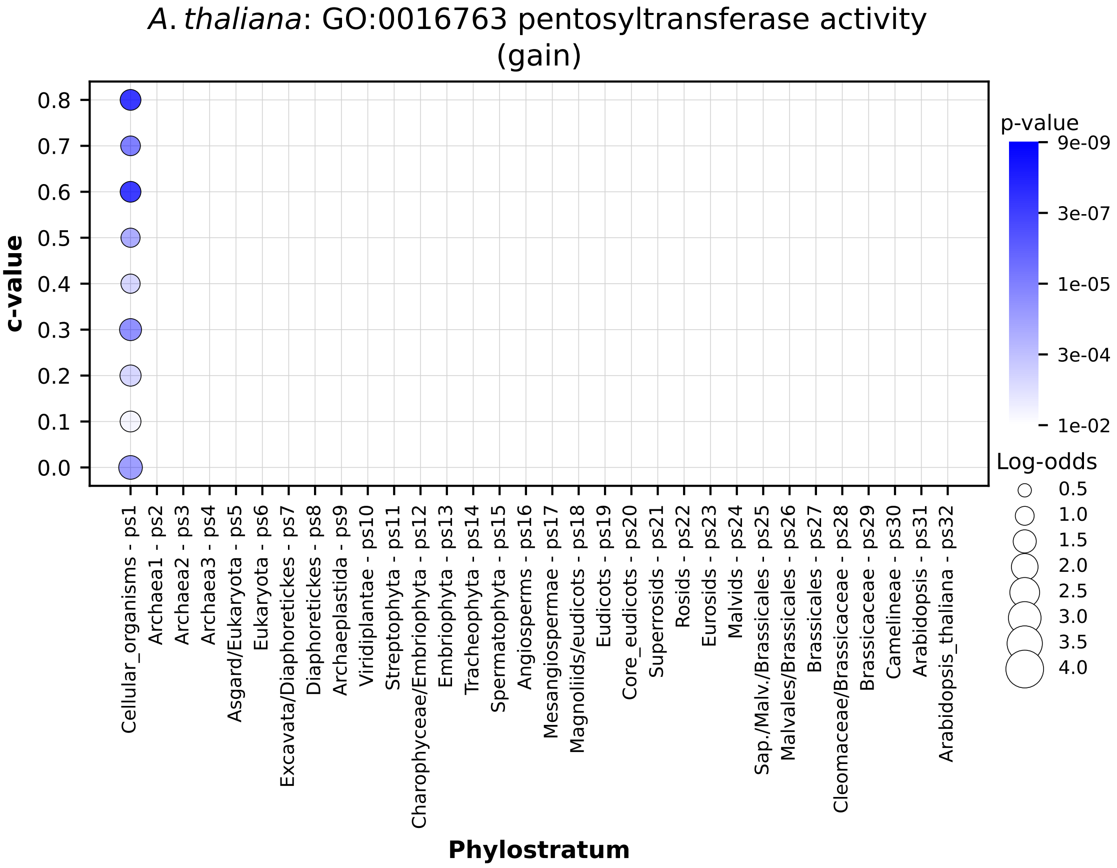
A. thaliana: GO:0016763 pentosyltransferase activity
(gain)
c-value
0.8
0.7
0.6
0.5
0.4
0.3
0.2
0.1
0.0
p-value
9e-09
3e-07
1e-05
3e-04
1e-02
Log-odds
0.5
1.0
1.5
2.0
2.5
3.0
3.5
4.0
Cellular_organisms - ps1
Archaea1 - ps2
Archaea2 - ps3
Archaea3 - ps4
Asgard/Eukaryota - ps5
Eukaryota - ps6
Excavata/Diaphoretickes - ps7
Diaphoretickes - ps8
Archaeplastida - ps9
Viridiplantae - ps10
Streptophyta - ps11
Charophyceae/Embriophyta - ps12
Embriophyta - ps13
Tracheophyta - ps14
Spermatophyta - ps15
Angiosperms - ps16
Mesangiospermae - ps17
Magnoliids/eudicots - ps18
Eudicots - ps19
Core_eudicots - ps20
Superrosids - ps21
Rosids - ps22
Eurosids - ps23
Malvids - ps24
Sap./Malv./Brassicales - ps25
Malvales/Brassicales - ps26
Brassicales - ps27
Cleomaceae/Brassicaceae - ps28
Brassicaceae - ps29
Camelineae - ps30
Arabidopsis - ps31
Arabidopsis_thaliana - ps32
Phylostratum

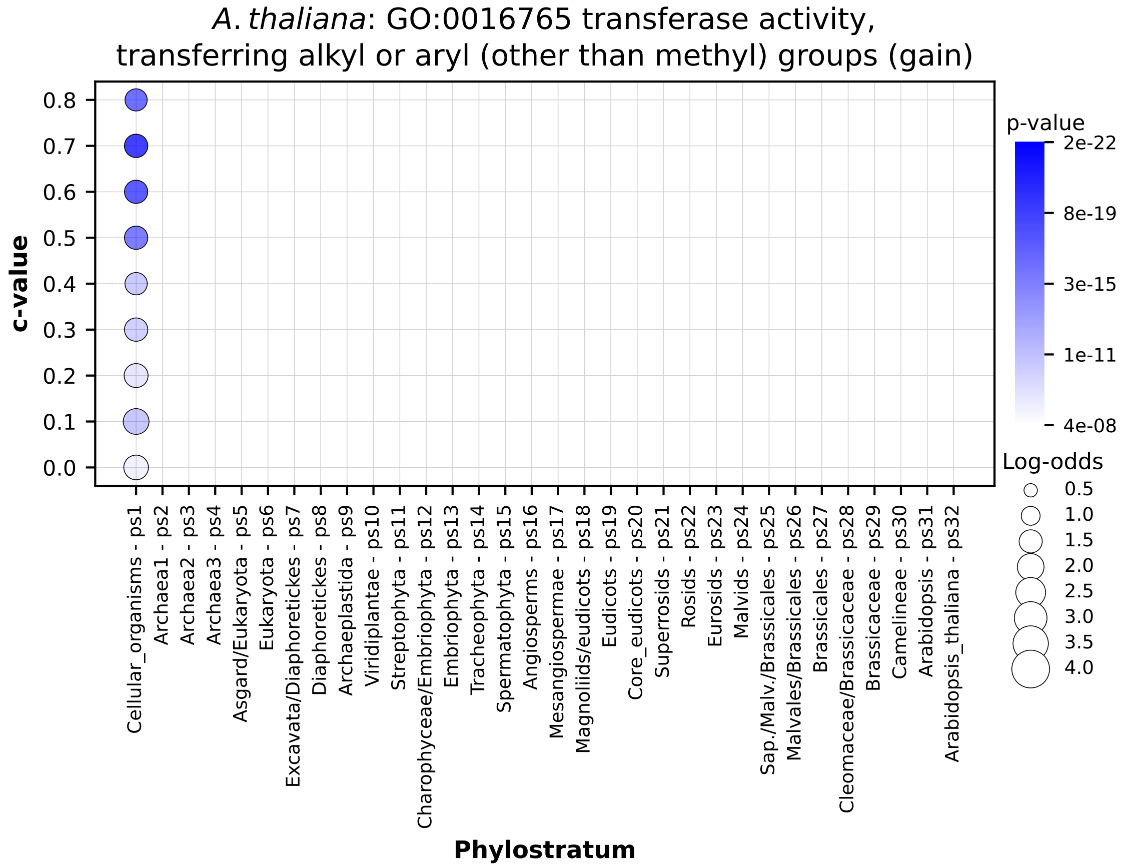

A. thaliana: GO:0016765 transferase activity,
transferring alkyl or aryl (other than methyl) groups (gain)
c-value
0.8
0.7
0.6
0.5
0.4
0.3
0.2
0.1
0.0
p-value
2e-22
8e-19
3e-15
1e-11
4e-08
Log-odds
0.5
1.0
1.5
2.0
2.5
3.0
3.5
4.0
Cellular_organisms - ps1
Archaea1 - ps2
Archaea2 - ps3
Archaea3 - ps4
Asgard/Eukaryota - ps5
Eukaryota - ps6
Excavata/Diaphoretickes - ps7
Diaphoretickes - ps8
Archaeplastida - ps9
Viridiplantae - ps10
Streptophyta - ps11
Charophyceae/Embriophyta - ps12
Embriophyta - ps13
Tracheophyta - ps14
Spermatophyta - ps15
Angiosperms - ps16
Mesangiospermae - ps17
Magnoliids/eudicots - ps18
Eudicots - ps19
Core_eudicots - ps20
Superrosids - ps21
Rosids - ps22
Eurosids - ps23
Malvids - ps24
Sap./Malv./Brassicales - ps25
Malvales/Brassicales - ps26
Brassicales - ps27
Cleomaceae/Brassicaceae - ps28
Brassicaceae - ps29
Camelineae - ps30
Arabidopsis - ps31
Arabidopsis_thaliana - ps32
Phylostratum

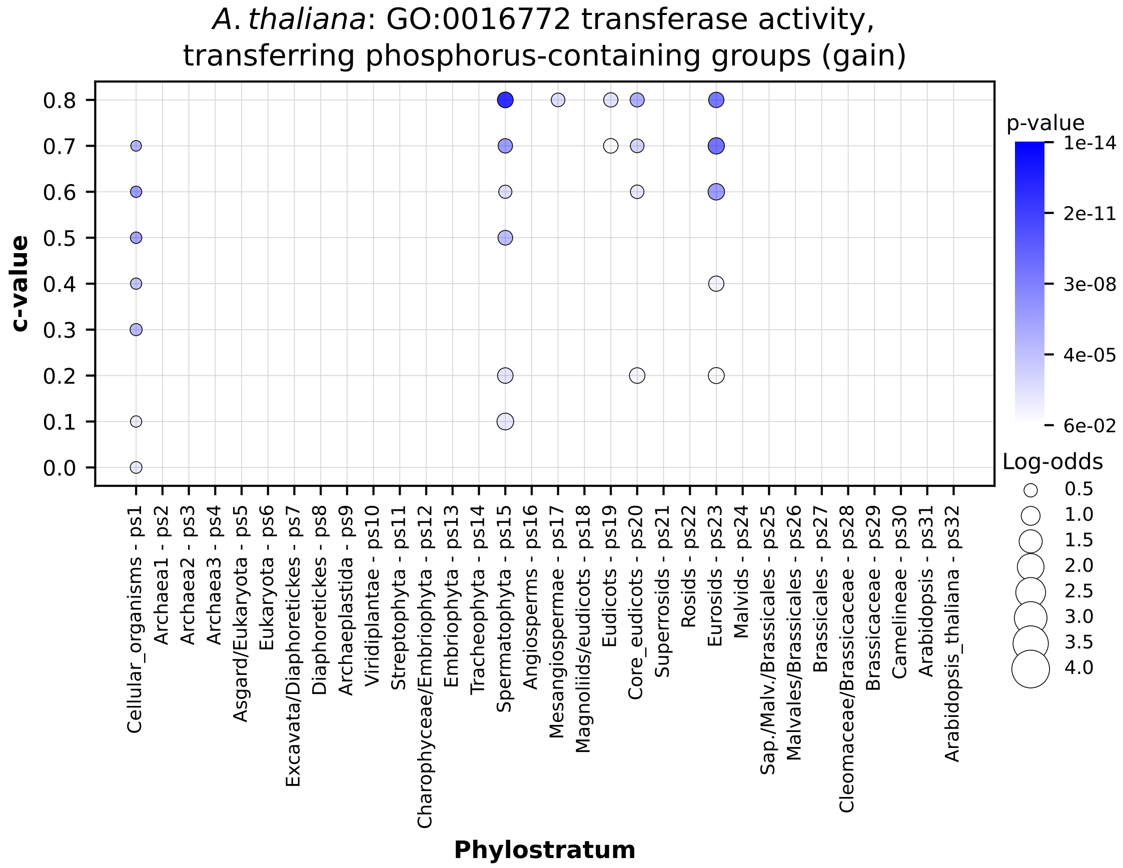

A. thaliana: GO:0016772 transferase activity, transferring phosphorus-containing groups (gain)
c-value
0.0
0.1
0.2
0.3
0.4
0.5
0.6
0.7
0.8
Phylostratum
Cellular_organisms - ps1
Archaea1 - ps2
Archaea2 - ps3
Archaea3 - ps4
Asgard/Eukaryota - ps5
Eukaryota - ps6
Excavata/Diaphoretickes - ps7
Diaphoretickes - ps8
Archaeplastida - ps9
Viridiplantae - ps10
Streptophyta - ps11
Charophyceae/Embriophyta - ps12
Embriophyta - ps13
Tracheophyta - ps14
Spermatophyta - ps15
Angiosperms - ps16
Mesangiospermae - ps17
Magnoliids/eudicots - ps18
Eudicots - ps19
Core_eudicots - ps20
Superrosids - ps21
Rosids - ps22
Eurosids - ps23
Malvids - ps24
Sap./Malv./Brassicales - ps25
Malvales/Brassicales - ps26
Brassicales - ps27
Cleomaceae/Brassicaceae - ps28
Brassicaceae - ps29
Camelineae - ps30
Arabidopsis - ps31
Arabidopsis_thaliana - ps32
p-value
1e-14
2e-11
3e-08
4e-05
6e-02
Log-odds
0.5
1.0
1.5
2.0
2.5
3.0
3.5
4.0

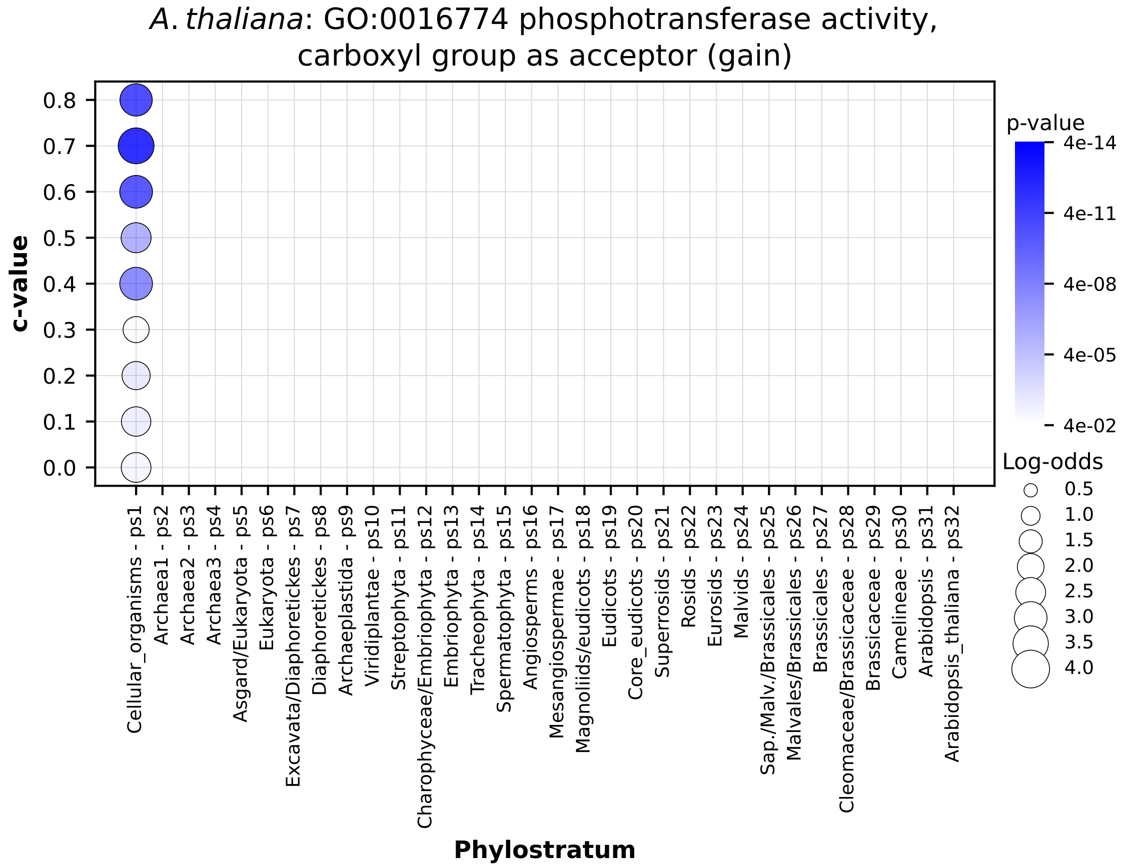

A. thaliana: GO:0016774 phosphotransferase activity, carboxyl group as acceptor (gain)
c-value
0.8
0.7
0.6
0.5
0.4
0.3
0.2
0.1
0.0
p-value
4e-14
4e-11
4e-08
4e-05
4e-02
Log-odds
0.5
1.0
1.5
2.0
2.5
3.0
3.5
4.0
Cellular_organisms - ps1
Archaea1 - ps2
Archaea2 - ps3
Archaea3 - ps4
Asgard/Eukaryota - ps5
Eukaryota - ps6
Excavata/Diaphoretickes - ps7
Diaphoretickes - ps8
Archaeplastida - ps9
Viridiplantae - ps10
Streptophyta - ps11
Charophyceae/Embriophyta - ps12
Embriophyta - ps13
Tracheophyta - ps14
Spermatophyta - ps15
Angiosperms - ps16
Mesangiospermae - ps17
Magnoliids/eudicots - ps18
Eudicots - ps19
Core_eudicots - ps20
Superrosids - ps21
Rosids - ps22
Eurosids - ps23
Malvids - ps24
Sap./Malv./Brassicales - ps25
Malvales/Brassicales - ps26
Brassicales - ps27
Cleomaceae/Brassicaceae - ps28
Brassicaceae - ps29
Camelineae - ps30
Arabidopsis - ps31
Arabidopsis_thaliana - ps32
Phylostratum

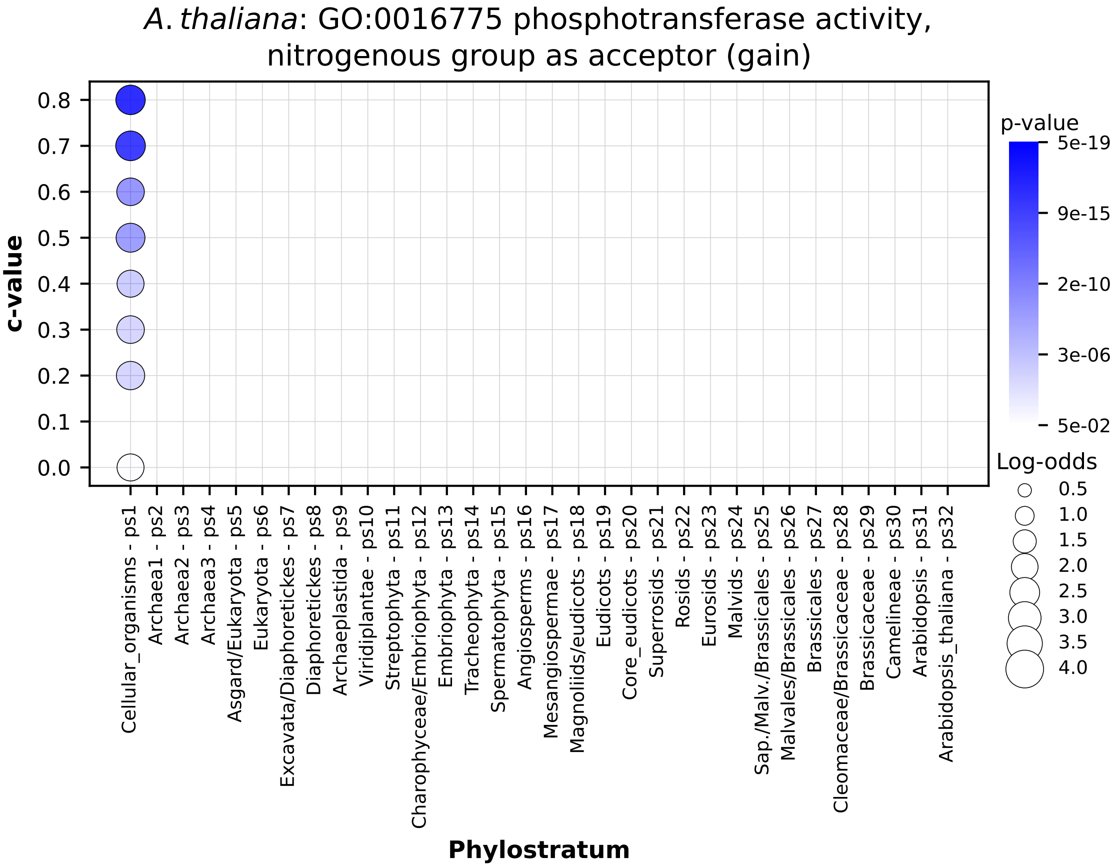

A. thaliana: GO:0016775 phosphotransferase activity, nitrogenous group as acceptor (gain)
c-value
0.8
0.7
0.6
0.5
0.4
0.3
0.2
0.1
0.0
Phylostratum
Cellular_organisms - ps1
Archaea1 - ps2
Archaea2 - ps3
Archaea3 - ps4
Asgard/Eukaryota - ps5
Eukaryota - ps6
Excavata/Diaphoretickes - ps7
Diaphoretickes - ps8
Archaeplastida - ps9
Viridiplantae - ps10
Streptophyta - ps11
Charophyceae/Embriophyta - ps12
Embriophyta - ps13
Tracheophyta - ps14
Spermatophyta - ps15
Angiosperms - ps16
Mesangiospermae - ps17
Magnoliids/eudicots - ps18
Eudicots - ps19
Core_eudicots - ps20
Superrosids - ps21
Rosids - ps22
Eurosids - ps23
Malvids - ps24
Sap./Malv./Brassicales - ps25
Malvales/Brassicales - ps26
Brassicales - ps27
Cleomaceae/Brassicaceae - ps28
Brassicaceae - ps29
Camelineae - ps30
Arabidopsis - ps31
Arabidopsis_thaliana - ps32
p-value
5e-19
9e-15
2e-10
3e-06
5e-02
Log-odds
0.5
1.0
1.5
2.0
2.5
3.0
3.5
4.0

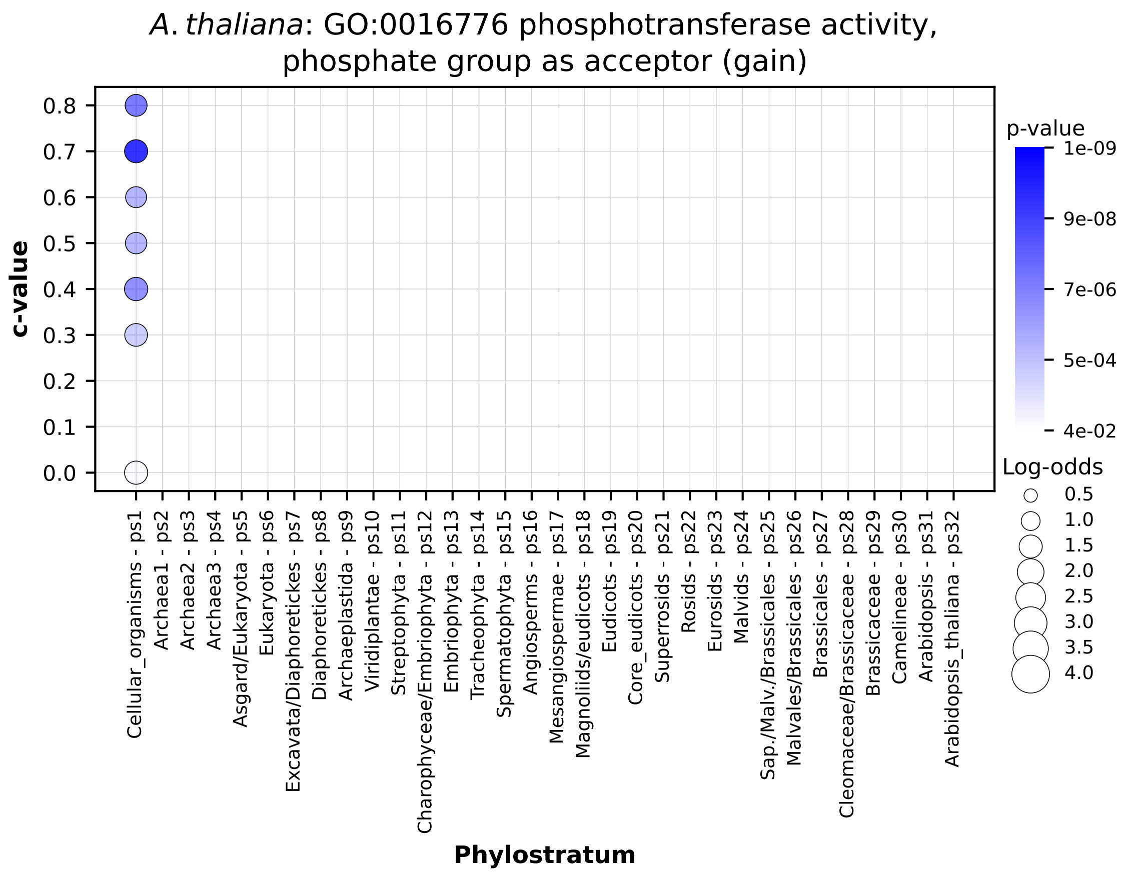

A. thaliana: GO:0016776 phosphotransferase activity, phosphate group as acceptor (gain)
c-value
0.8
0.7
0.6
0.5
0.4
0.3
0.2
0.1
0.0
Phylostratum
Cellular_organisms - ps1
Archaea1 - ps2
Archaea2 - ps3
Archaea3 - ps4
Asgard/Eukaryota - ps5
Eukaryota - ps6
Excavata/Diaphoretickes - ps7
Diaphoretickes - ps8
Archaeplastida - ps9
Viridiplantae - ps10
Streptophyta - ps11
Charophyceae/Embriophyta - ps12
Embriophyta - ps13
Tracheophyta - ps14
Spermatophyta - ps15
Angiosperms - ps16
Mesangiospermae - ps17
Magnoliids/eudicots - ps18
Eudicots - ps19
Core_eudicots - ps20
Superrosids - ps21
Rosids - ps22
Eurosids - ps23
Malvids - ps24
Sap./Malv./Brassicales - ps25
Malvales/Brassicales - ps26
Brassicales - ps27
Cleomaceae/Brassicaceae - ps28
Brassicaceae - ps29
Camelineae - ps30
Arabidopsis - ps31
Arabidopsis_thaliana - ps32
p-value
1e-09
9e-08
7e-06
5e-04
4e-02
Log-odds
0.5
1.0
1.5
2.0
2.5
3.0
3.5
4.0

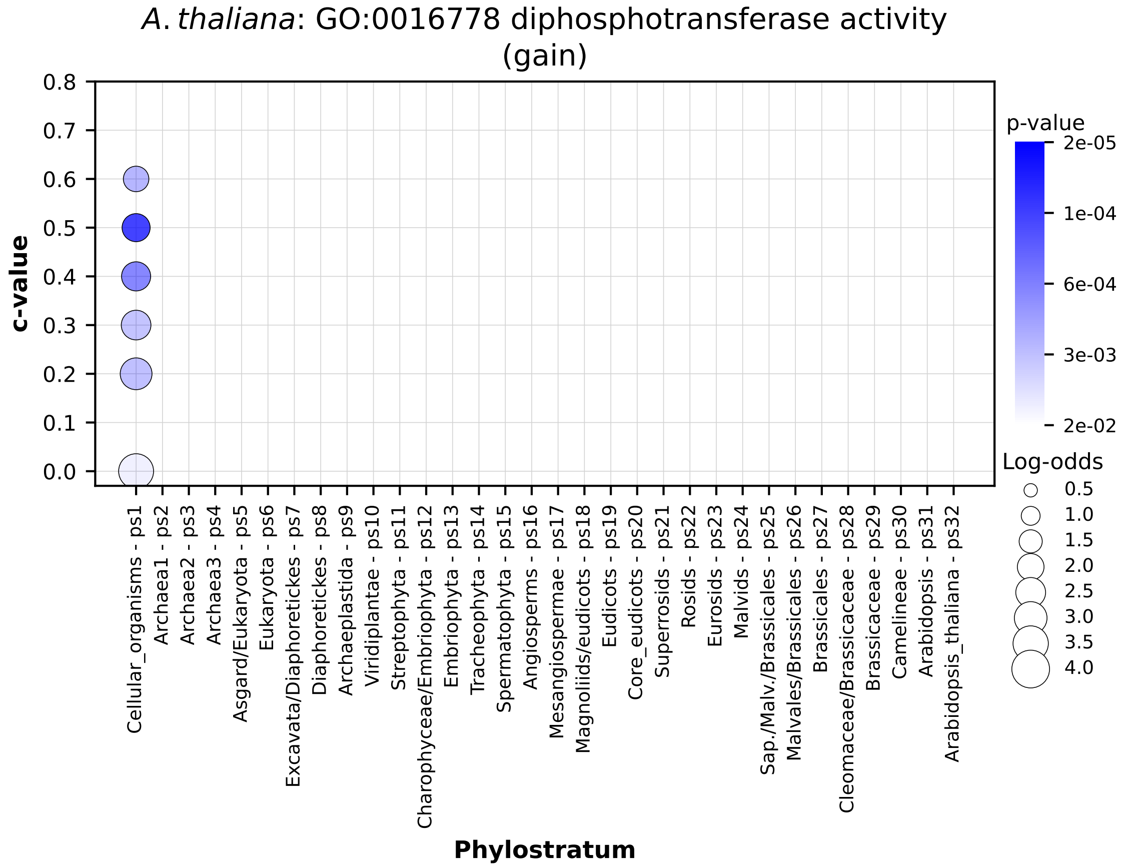

A. thaliana: GO:0016778 diphosphotransferase activity
(gain)
c-value
0.8
0.7
0.6
0.5
0.4
0.3
0.2
0.1
0.0
Phylostratum
Cellular_organisms - ps1
Archaea1 - ps2
Archaea2 - ps3
Archaea3 - ps4
Asgard/Eukaryota - ps5
Eukaryota - ps6
Excavata/Diaphoretickes - ps7
Diaphoretickes - ps8
Archaeplastida - ps9
Viridiplantae - ps10
Streptophyta - ps11
Charophyceae/Embriophyta - ps12
Embriophyta - ps13
Tracheophyta - ps14
Spermatophyta - ps15
Angiosperms - ps16
Mesangiospermae - ps17
Magnoliids/eudicots - ps18
Eudicots - ps19
Core_eudicots - ps20
Superrosids - ps21
Rosids - ps22
Eurosids - ps23
Malvids - ps24
Sap./Malv./Brassicales - ps25
Malvales/Brassicales - ps26
Brassicales - ps27
Cleomaceae/Brassicaceae - ps28
Brassicaceae - ps29
Camelineae - ps30
Arabidopsis - ps31
Arabidopsis_thaliana - ps32
p-value
2e-05
1e-04
6e-04
3e-03
2e-02
Log-odds
0.5
1.0
1.5
2.0
2.5
3.0
3.5
4.0

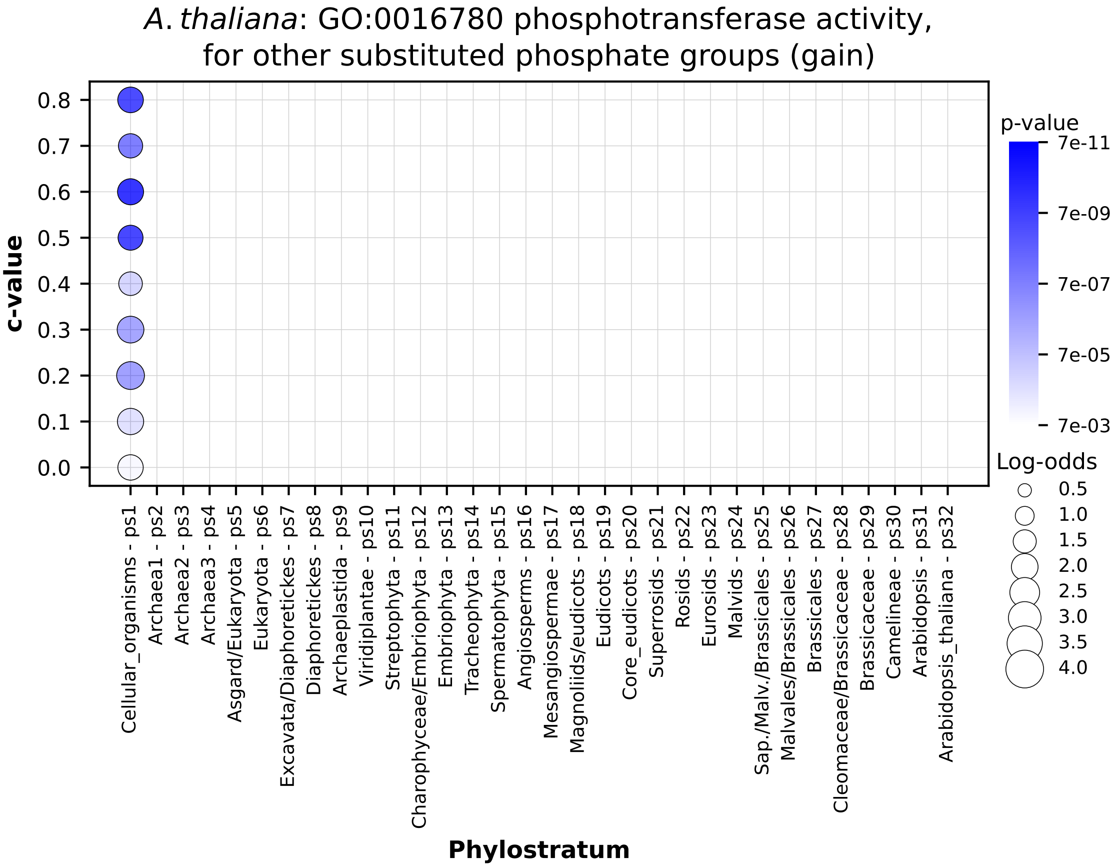

A. thaliana: GO:0016780 phosphotransferase activity, for other substituted phosphate groups (gain)
c-value
0.8
0.7
0.6
0.5
0.4
0.3
0.2
0.1
0.0
p-value
7e-11
7e-09
7e-07
7e-05
7e-03
Log-odds
0.5
1.0
1.5
2.0
2.5
3.0
3.5
4.0
Cellular_organisms - ps1
Archaea1 - ps2
Archaea2 - ps3
Archaea3 - ps4
Asgard/Eukaryota - ps5
Eukaryota - ps6
Excavata/Diaphoretickes - ps7
Diaphoretickes - ps8
Archaeplastida - ps9
Viridiplantae - ps10
Streptophyta - ps11
Charophyceae/Embriophyta - ps12
Embriophyta - ps13
Tracheophyta - ps14
Spermatophyta - ps15
Angiosperms - ps16
Mesangiospermae - ps17
Magnoliids/eudicots - ps18
Eudicots - ps19
Core_eudicots - ps20
Superrosids - ps21
Rosids - ps22
Eurosids - ps23
Malvids - ps24
Sap./Malv./Brassicales - ps25
Malvales/Brassicales - ps26
Brassicales - ps27
Cleomaceae/Brassicaceae - ps28
Brassicaceae - ps29
Camelineae - ps30
Arabidopsis - ps31
Arabidopsis_thaliana - ps32
Phylostratum

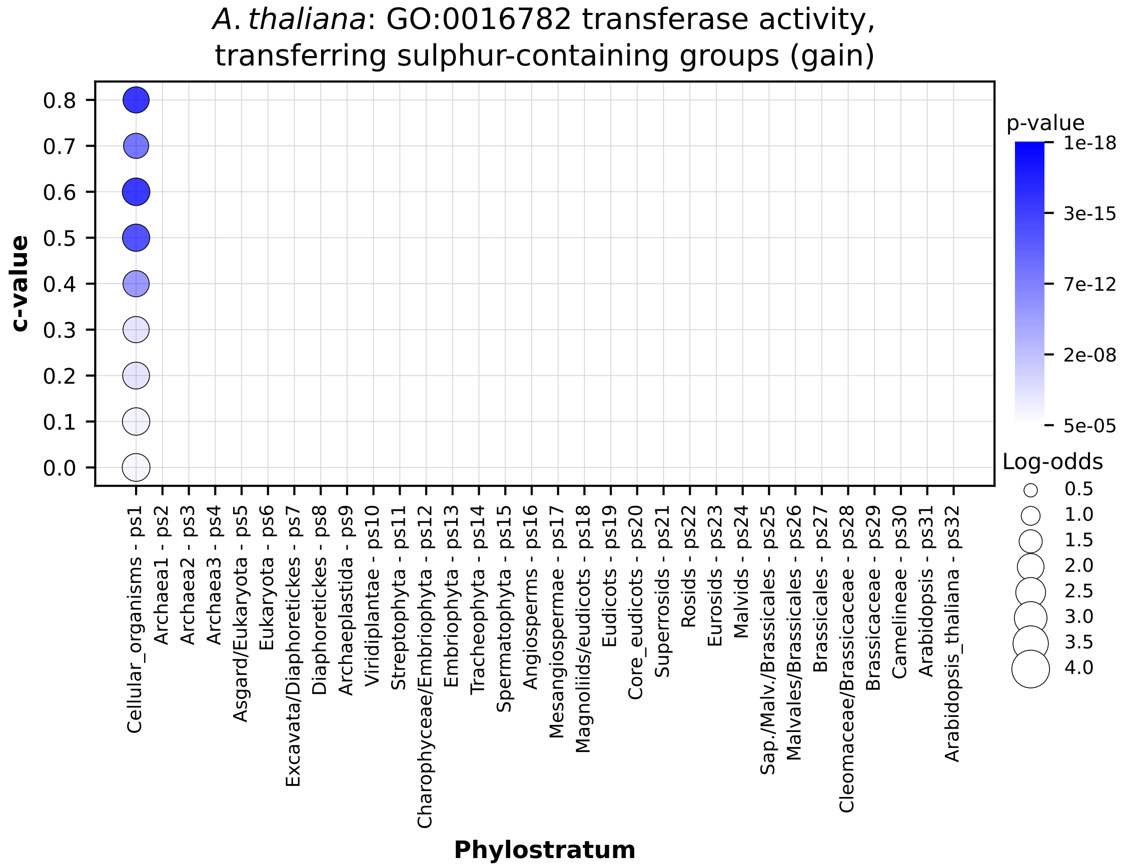

A. thaliana: GO:0016782 transferase activity,
transferring sulphur-containing groups (gain)
c-value
0.8
0.7
0.6
0.5
0.4
0.3
0.2
0.1
0.0
p-value
1e-18
3e-15
7e-12
2e-08
5e-05
Log-odds
0.5
1.0
1.5
2.0
2.5
3.0
3.5
4.0
Cellular_organisms - ps1
Archaea1 - ps2
Archaea2 - ps3
Archaea3 - ps4
Asgard/Eukaryota - ps5
Eukaryota - ps6
Excavata/Diaphoretickes - ps7
Diaphoretickes - ps8
Archaeplastida - ps9
Viridiplantae - ps10
Streptophyta - ps11
Charophyceae/Embriophyta - ps12
Embriophyta - ps13
Tracheophyta - ps14
Spermatophyta - ps15
Angiosperms - ps16
Mesangiospermae - ps17
Magnoliids/eudicots - ps18
Eudicots - ps19
Core_eudicots - ps20
Superrosids - ps21
Rosids - ps22
Eurosids - ps23
Malvids - ps24
Sap./Malv./Brassicales - ps25
Malvales/Brassicales - ps26
Brassicales - ps27
Cleomaceae/Brassicaceae - ps28
Brassicaceae - ps29
Camelineae - ps30
Arabidopsis - ps31
Arabidopsis_thaliana - ps32
Phylostratum

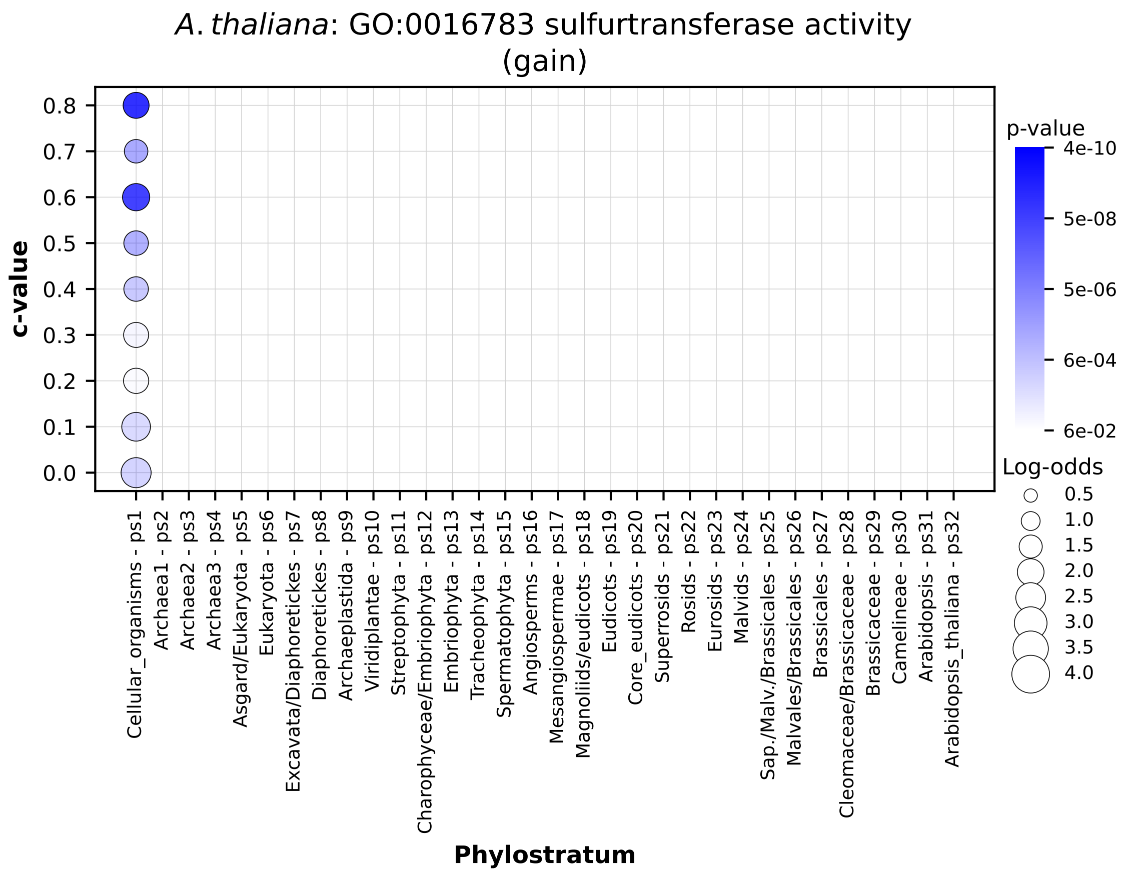

A. thaliana: GO:0016783 sulfurtransferase activity
(gain)
c-value
0.8
0.7
0.6
0.5
0.4
0.3
0.2
0.1
0.0
p-value
4e-10
5e-08
5e-06
6e-04
6e-02
Log-odds
0.5
1.0
1.5
2.0
2.5
3.0
3.5
4.0
Cellular_organisms - ps1
Archaea1 - ps2
Archaea2 - ps3
Archaea3 - ps4
Asgard/Eukaryota - ps5
Eukaryota - ps6
Excavata/Diaphoretickes - ps7
Diaphoretickes - ps8
Archaeplastida - ps9
Viridiplantae - ps10
Streptophyta - ps11
Charophyceae/Embriophyta - ps12
Embriophyta - ps13
Tracheophyta - ps14
Spermatophyta - ps15
Angiosperms - ps16
Mesangiospermae - ps17
Magnoliids/eudicots - ps18
Eudicots - ps19
Core_eudicots - ps20
Superrosids - ps21
Rosids - ps22
Eurosids - ps23
Malvids - ps24
Sap./Malv./Brassicales - ps25
Malvales/Brassicales - ps26
Brassicales - ps27
Cleomaceae/Brassicaceae - ps28
Brassicaceae - ps29
Camelineae - ps30
Arabidopsis - ps31
Arabidopsis_thaliana - ps32
Phylostratum

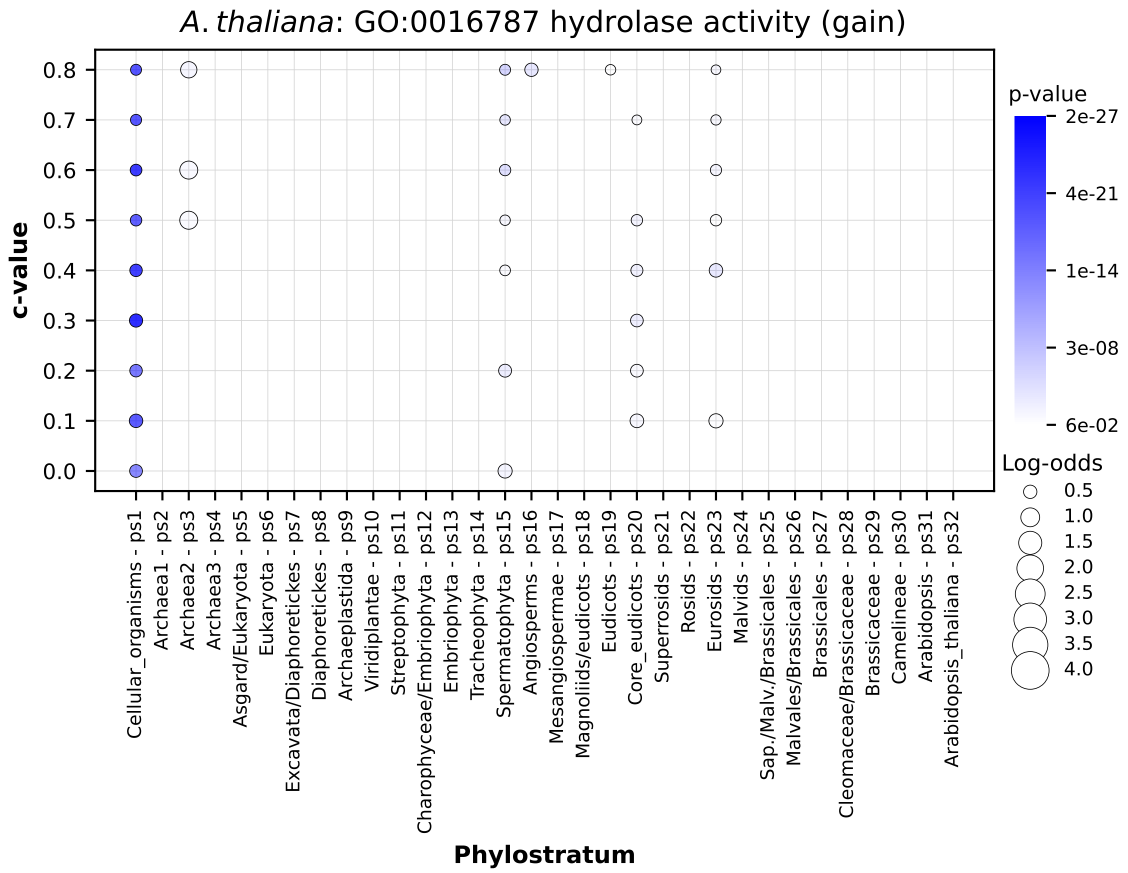

A. thaliana: GO:0016787 hydrolase activity (gain)
c-value
0.8
0.7
0.6
0.5
0.4
0.3
0.2
0.1
0.0
p-value
2e-27
4e-21
1e-14
3e-08
6e-02
Log-odds
0.5
1.0
1.5
2.0
2.5
3.0
3.5
4.0
Cellular_organisms - ps1
Archaea1 - ps2
Archaea2 - ps3
Archaea3 - ps4
Asgard/Eukaryota - ps5
Eukaryota - ps6
Excavata/Diaphoretickes - ps7
Diaphoretickes - ps8
Archaeplastida - ps9
Viridiplantae - ps10
Streptophyta - ps11
Charophyceae/Embriophyta - ps12
Embriophyta - ps13
Tracheophyta - ps14
Spermatophyta - ps15
Angiosperms - ps16
Mesangiospermae - ps17
Magnoliids/eudicots - ps18
Eudicots - ps19
Core_eudicots - ps20
Superrosids - ps21
Rosids - ps22
Eurosids - ps23
Malvids - ps24
Sap./Malv./Brassicales - ps25
Malvales/Brassicales - ps26
Brassicales - ps27
Cleomaceae/Brassicaceae - ps28
Brassicaceae - ps29
Camelineae - ps30
Arabidopsis - ps31
Arabidopsis_thaliana - ps32
Phylostratum

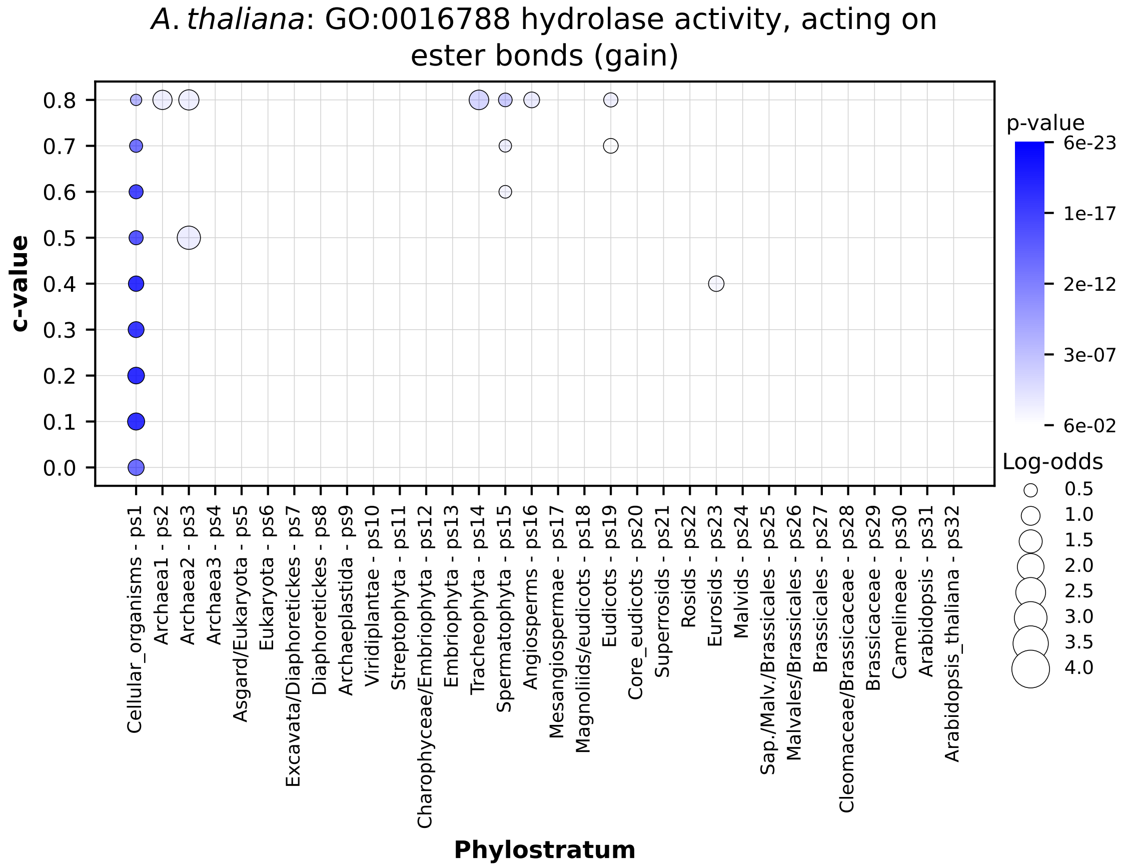
A. thaliana: GO:0016788 hydrolase activity, acting on ester bonds (gain)
c-value
Phylostratum
p-value
6e-23
1e-17
2e-12
3e-07
6e-02
Log-odds
0.5
1.0
1.5
2.0
2.5
3.0
3.5
4.0
0.0
0.1
0.2
0.3
0.4
0.5
0.6
0.7
0.8
Cellular_organisms - ps1
Archaea1 - ps2
Archaea2 - ps3
Archaea3 - ps4
Asgard/Eukaryota - ps5
Eukaryota - ps6
Excavata/Diaphoretickes - ps7
Diaphoretickes - ps8
Archaeplastida - ps9
Viridiplantae - ps10
Streptophyta - ps11
Charophyceae/Embriophyta - ps12
Embriophyta - ps13
Tracheophyta - ps14
Spermatophyta - ps15
Angiosperms - ps16
Mesangiospermae - ps17
Magnoliids/eudicots - ps18
Eudicots - ps19
Core_eudicots - ps20
Superrosids - ps21
Rosids - ps22
Eurosids - ps23
Malvids - ps24
Sap./Malv./Brassicales - ps25
Malvales/Brassicales - ps26
Brassicales - ps27
Cleomaceae/Brassicaceae - ps28
Brassicaceae - ps29
Camelineae - ps30
Arabidopsis - ps31
Arabidopsis_thaliana - ps32

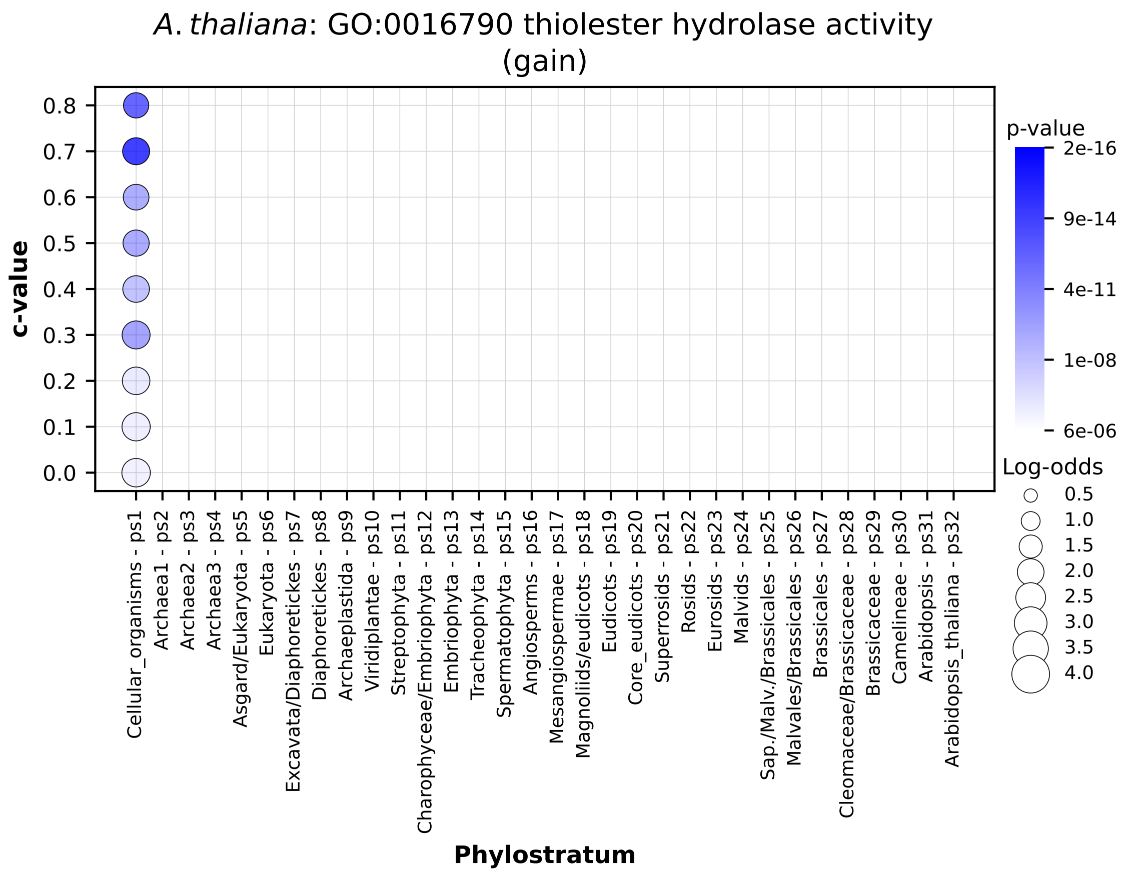

A. thaliana: GO:0016790 thiolester hydrolase activity (gain)
c-value
0.0
0.1
0.2
0.3
0.4
0.5
0.6
0.7
0.8
p-value
2e-16
9e-14
4e-11
1e-08
6e-06
Log-odds
0.5
1.0
1.5
2.0
2.5
3.0
3.5
4.0
Cellular_organisms - ps1
Archaea1 - ps2
Archaea2 - ps3
Archaea3 - ps4
Asgard/Eukaryota - ps5
Eukaryota - ps6
Excavata/Diaphoretickes - ps7
Diaphoretickes - ps8
Archaeplastida - ps9
Viridiplantae - ps10
Streptophyta - ps11
Charophyceae/Embriophyta - ps12
Embriophyta - ps13
Tracheophyta - ps14
Spermatophyta - ps15
Angiosperms - ps16
Mesangiospermae - ps17
Magnoliids/eudicots - ps18
Eudicots - ps19
Core_eudicots - ps20
Superrosids - ps21
Rosids - ps22
Eurosids - ps23
Malvids - ps24
Sap./Malv./Brassicales - ps25
Malvales/Brassicales - ps26
Brassicales - ps27
Cleomaceae/Brassicaceae - ps28
Brassicaceae - ps29
Camelineae - ps30
Arabidopsis - ps31
Arabidopsis_thaliana - ps32
Phylostratum

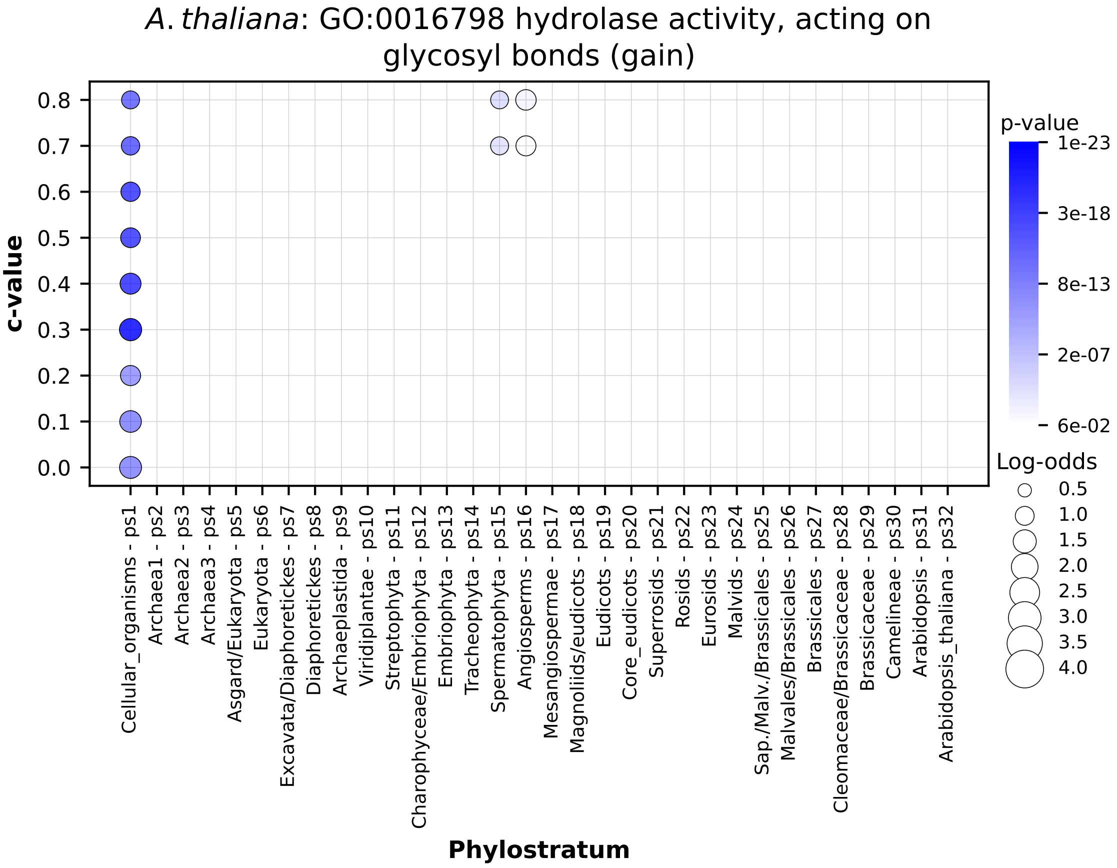
A. thaliana: GO:0016798 hydrolase activity, acting on glycosyl bonds (gain)
c-value
0.8
0.7
0.6
0.5
0.4
0.3
0.2
0.1
0.0
p-value
1e-23
3e-18
8e-13
2e-07
6e-02
Log-odds
0.5
1.0
1.5
2.0
2.5
3.0
3.5
4.0
Cellular_organisms - ps1
Archaea1 - ps2
Archaea2 - ps3
Archaea3 - ps4
Asgard/Eukaryota - ps5
Eukaryota - ps6
Excavata/Diaphoretickes - ps7
Diaphoretickes - ps8
Archaeplastida - ps9
Viridiplantae - ps10
Streptophyta - ps11
Charophyceae/Embriophyta - ps12
Embriophyta - ps13
Tracheophyta - ps14
Spermatophyta - ps15
Angiosperms - ps16
Mesangiospermae - ps17
Magnoliids/eudicots - ps18
Eudicots - ps19
Core_eudicots - ps20
Superrosids - ps21
Rosids - ps22
Eurosids - ps23
Malvids - ps24
Sap./Malv./Brassicales - ps25
Malvales/Brassicales - ps26
Brassicales - ps27
Cleomaceae/Brassicaceae - ps28
Brassicaceae - ps29
Camelineae - ps30
Arabidopsis - ps31
Arabidopsis_thaliana - ps32
Phylostratum

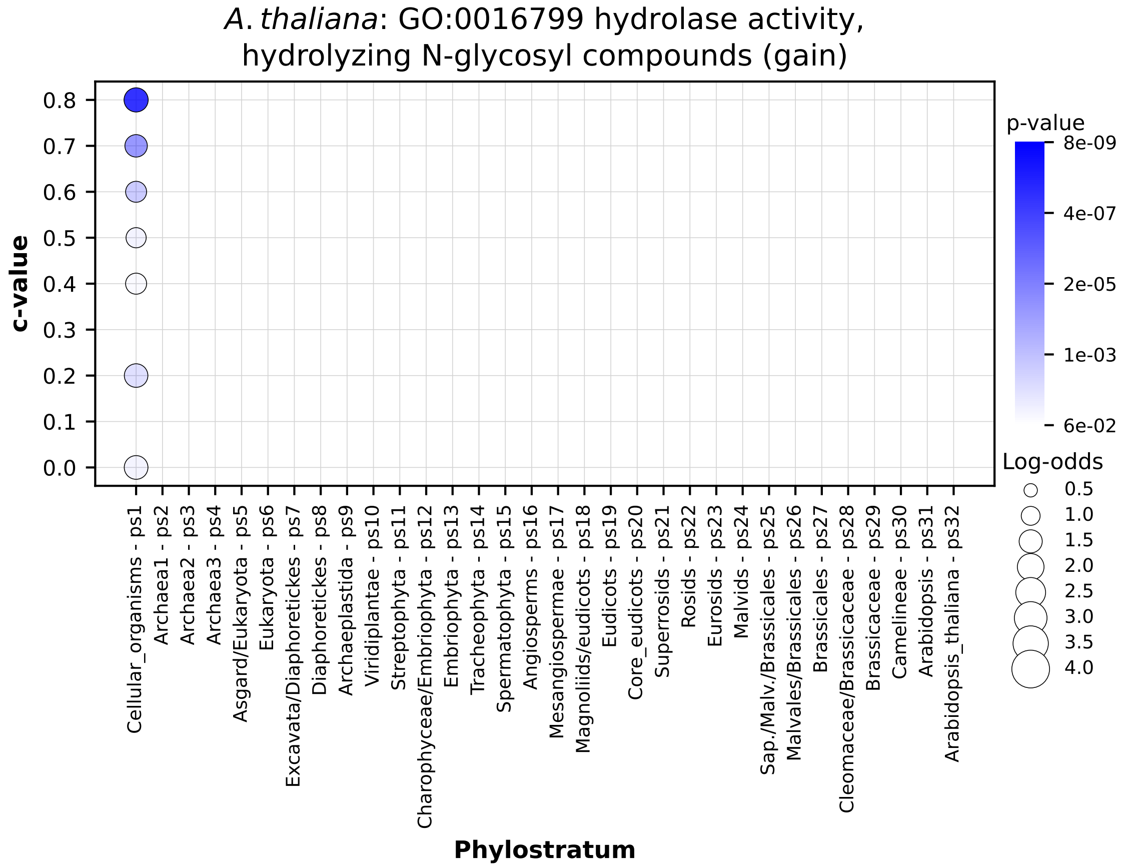

$A. thaliana$: GO:0016799 hydrolase activity, hydrolyzing N-glycosyl compounds (gain)
c-value
0.0
0.1
0.2
0.3
0.4
0.5
0.6
0.7
0.8
p-value
8e-09
4e-07
2e-05
1e-03
6e-02
Log-odds
0.5
1.0
1.5
2.0
2.5
3.0
3.5
4.0
Cellular_organisms - ps1
Archaea1 - ps2
Archaea2 - ps3
Archaea3 - ps4
Asgard/Eukaryota - ps5
Eukaryota - ps6
Excavata/Diaphoretickes - ps7
Diaphoretickes - ps8
Archaeplastida - ps9
Viridiplantae - ps10
Streptophyta - ps11
Charophyceae/Embriophyta - ps12
Embriophyta - ps13
Tracheophyta - ps14
Spermatophyta - ps15
Angiosperms - ps16
Mesangiospermae - ps17
Magnoliids/eudicots - ps18
Eudicots - ps19
Core_eudicots - ps20
Superrosids - ps21
Rosids - ps22
Eurosids - ps23
Malvids - ps24
Sap./Malv./Brassicales - ps25
Malvales/Brassicales - ps26
Brassicales - ps27
Cleomaceae/Brassicaceae - ps28
Brassicaceae - ps29
Camelineae - ps30
Arabidopsis - ps31
Arabidopsis_thaliana - ps32
Phylostratum

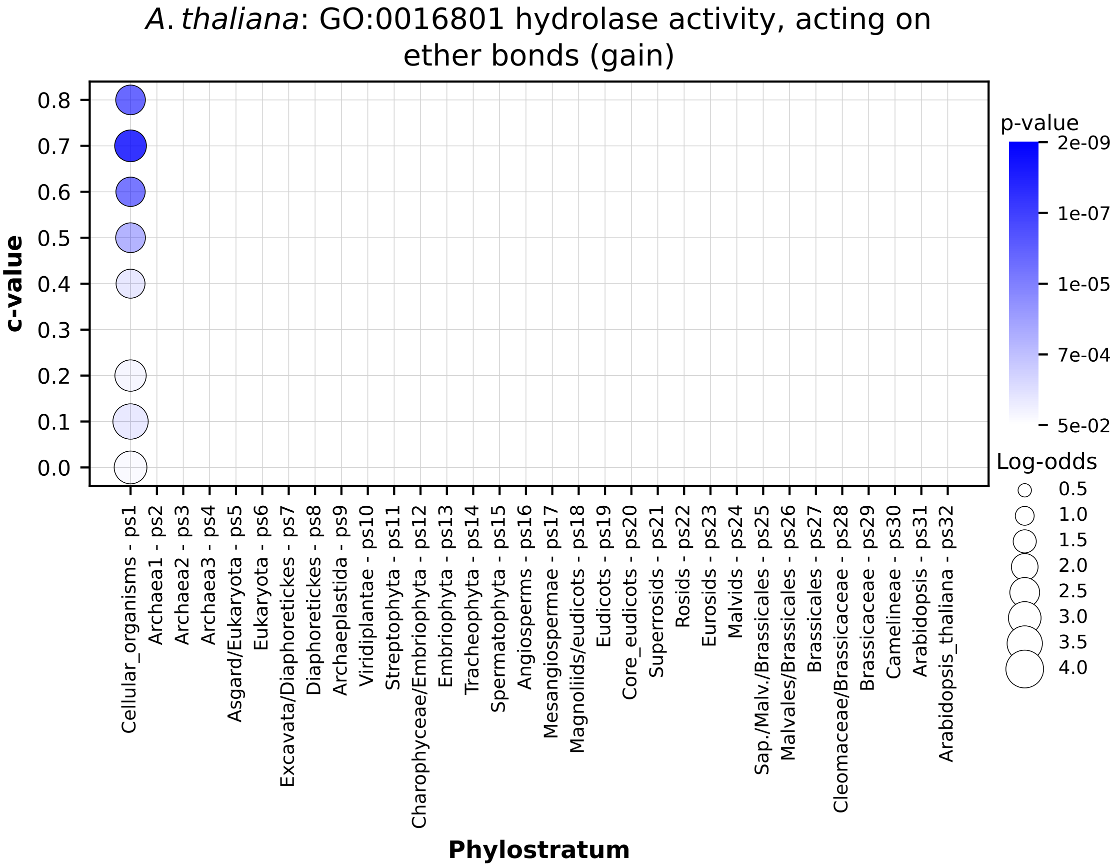

A. thaliana: GO:0016801 hydrolase activity, acting on ether bonds (gain)
c-value
0.8
0.7
0.6
0.5
0.4
0.3
0.2
0.1
0.0
p-value
2e-09
1e-07
1e-05
7e-04
5e-02
Log-odds
0.5
1.0
1.5
2.0
2.5
3.0
3.5
4.0
Cellular_organisms - ps1
Archaea1 - ps2
Archaea2 - ps3
Archaea3 - ps4
Asgard/Eukaryota - ps5
Eukaryota - ps6
Excavata/Diaphoretickes - ps7
Diaphoretickes - ps8
Archaeplastida - ps9
Viridiplantae - ps10
Streptophyta - ps11
Charophyceae/Embriophyta - ps12
Embriophyta - ps13
Tracheophyta - ps14
Spermatophyta - ps15
Angiosperms - ps16
Mesangiospermae - ps17
Magnoliids/eudicots - ps18
Eudicots - ps19
Core_eudicots - ps20
Superrosids - ps21
Rosids - ps22
Eurosids - ps23
Malvids - ps24
Sap./Malv./Brassicales - ps25
Malvales/Brassicales - ps26
Brassicales - ps27
Cleomaceae/Brassicaceae - ps28
Brassicaceae - ps29
Camelineae - ps30
Arabidopsis - ps31
Arabidopsis_thaliana - ps32
Phylostratum

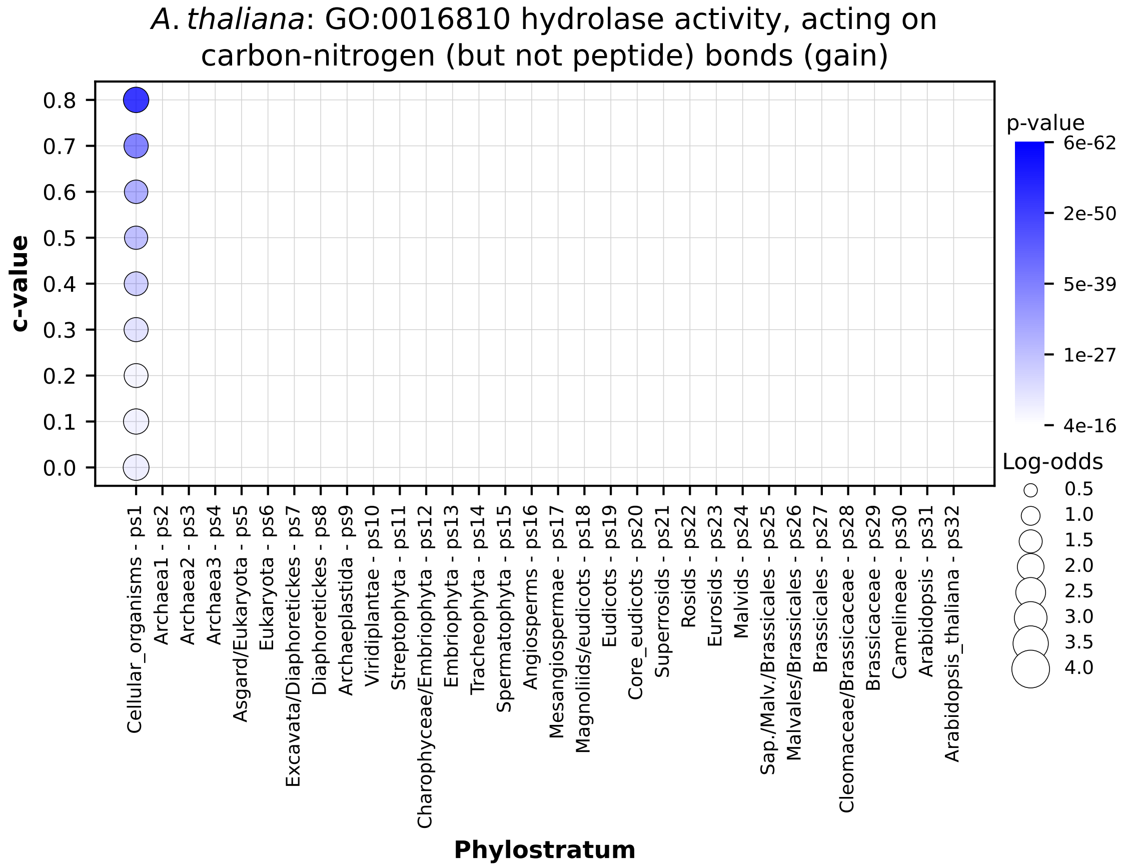

A. thaliana: GO:0016810 hydrolase activity, acting on carbon-nitrogen (but not peptide) bonds (gain)
c-value
0.8
0.7
0.6
0.5
0.4
0.3
0.2
0.1
0.0
p-value
6e-62
2e-50
5e-39
1e-27
4e-16
Log-odds
0.5
1.0
1.5
2.0
2.5
3.0
3.5
4.0
Cellular_organisms - ps1
Archaea1 - ps2
Archaea2 - ps3
Archaea3 - ps4
Asgard/Eukaryota - ps5
Eukaryota - ps6
Excavata/Diaphoretickes - ps7
Diaphoretickes - ps8
Archaeplastida - ps9
Viridiplantae - ps10
Streptophyta - ps11
Charophyceae/Embriophyta - ps12
Embriophyta - ps13
Tracheophyta - ps14
Spermatophyta - ps15
Angiosperms - ps16
Mesangiospermae - ps17
Magnoliids/eudicots - ps18
Eudicots - ps19
Core_eudicots - ps20
Superrosids - ps21
Rosids - ps22
Eurosids - ps23
Malvids - ps24
Sap./Malv./Brassicales - ps25
Malvales/Brassicales - ps26
Brassicales - ps27
Cleomaceae/Brassicaceae - ps28
Brassicaceae - ps29
Camelineae - ps30
Arabidopsis - ps31
Arabidopsis_thaliana - ps32
Phylostratum

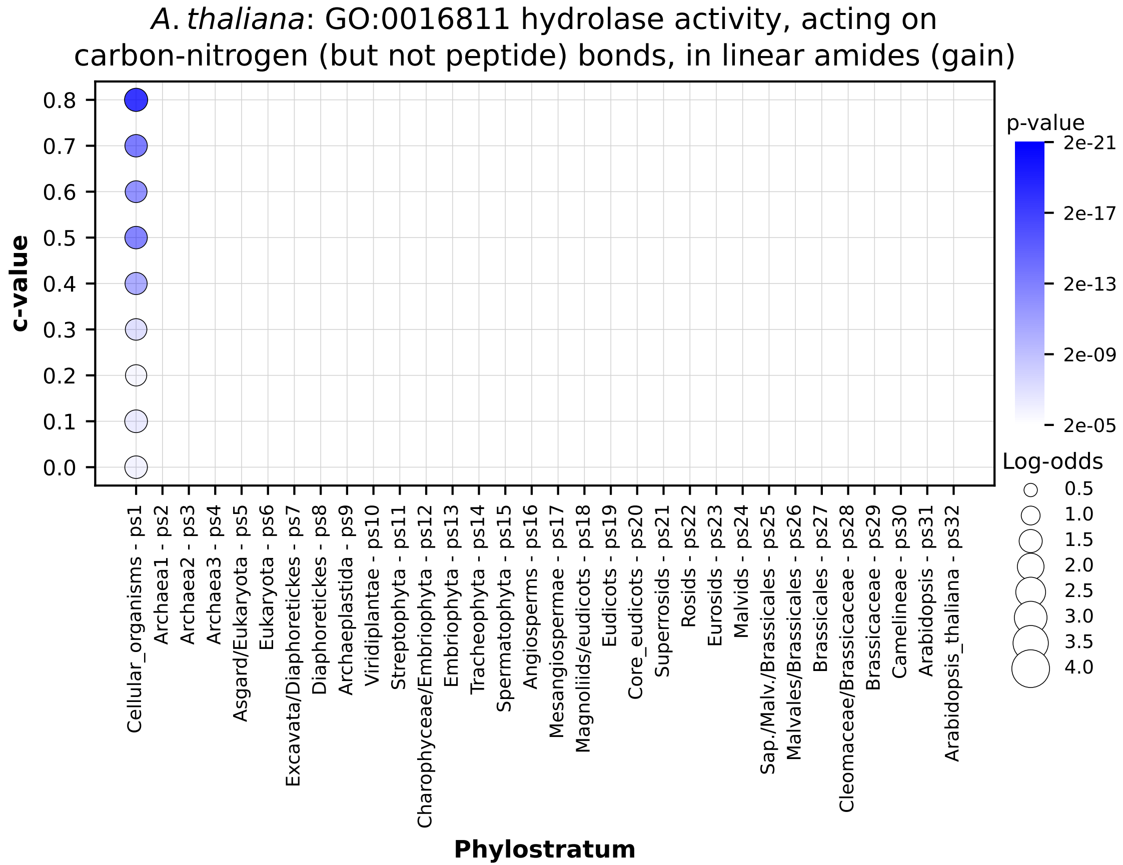

A. thaliana: GO:0016811 hydrolase activity, acting on carbon-nitrogen (but not peptide) bonds, in linear amides (gain)
c-value
0.8
0.7
0.6
0.5
0.4
0.3
0.2
0.1
0.0
p-value
2e-21
2e-17
2e-13
2e-09
2e-05
Log-odds
0.5
1.0
1.5
2.0
2.5
3.0
3.5
4.0
Cellular_organisms - ps1
Archaea1 - ps2
Archaea2 - ps3
Archaea3 - ps4
Asgard/Eukaryota - ps5
Eukaryota - ps6
Excavata/Diaphoretickes - ps7
Diaphoretickes - ps8
Archaeplastida - ps9
Viridiplantae - ps10
Streptophyta - ps11
Charophyceae/Embriophyta - ps12
Embriophyta - ps13
Tracheophyta - ps14
Spermatophyta - ps15
Angiosperms - ps16
Mesangiospermae - ps17
Magnoliids/eudicots - ps18
Eudicots - ps19
Core_eudicots - ps20
Superrosids - ps21
Rosids - ps22
Eurosids - ps23
Malvids - ps24
Sap./Malv./Brassicales - ps25
Malvales/Brassicales - ps26
Brassicales - ps27
Cleomaceae/Brassicaceae - ps28
Brassicaceae - ps29
Camelineae - ps30
Arabidopsis - ps31
Arabidopsis_thaliana - ps32
Phylostratum

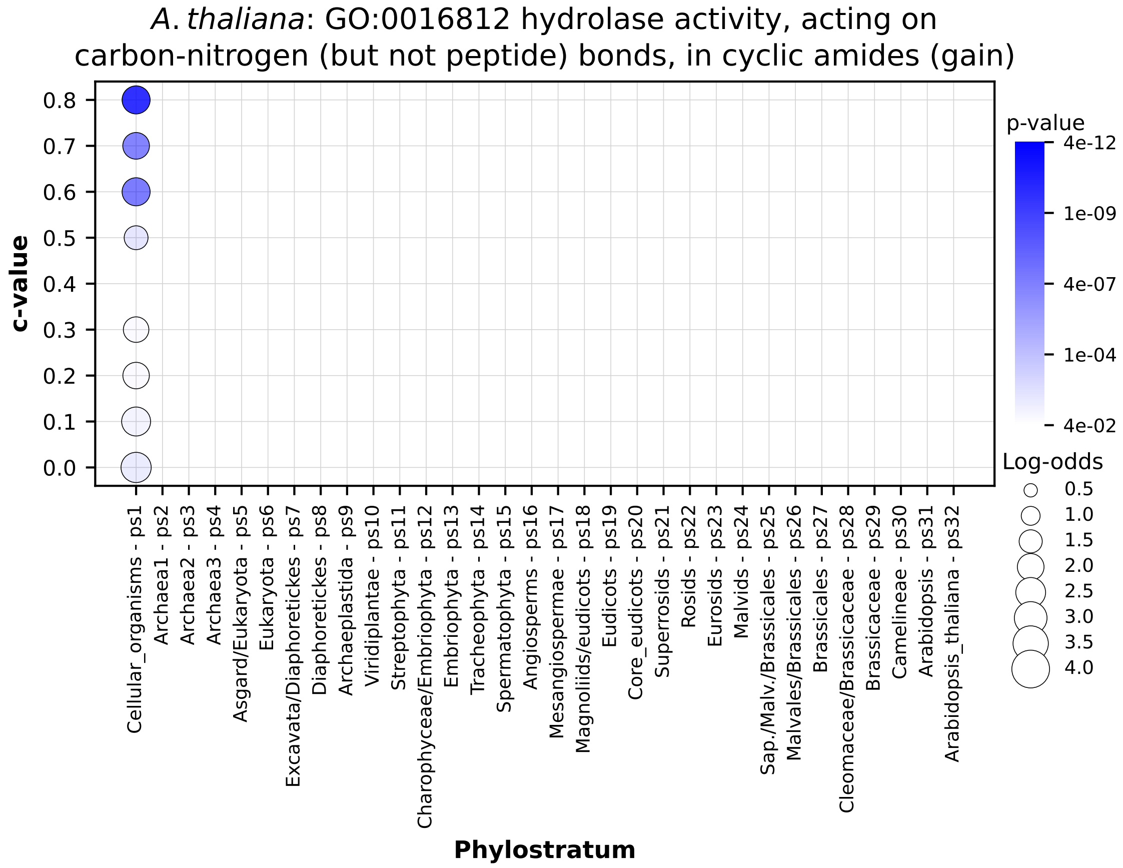

A. thaliana: GO:0016812 hydrolase activity, acting on carbon-nitrogen (but not peptide) bonds, in cyclic amides (gain)
c-value
0.8
0.7
0.6
0.5
0.4
0.3
0.2
0.1
0.0
p-value
4e-12
1e-09
4e-07
1e-04
4e-02
Log-odds
0.5
1.0
1.5
2.0
2.5
3.0
3.5
4.0
Cellular_organisms - ps1
Archaea1 - ps2
Archaea2 - ps3
Archaea3 - ps4
Asgard/Eukaryota - ps5
Eukaryota - ps6
Excavata/Diaphoretickes - ps7
Diaphoretickes - ps8
Archaeplastida - ps9
Viridiplantae - ps10
Streptophyta - ps11
Charophyceae/Embriophyta - ps12
Embriophyta - ps13
Tracheophyta - ps14
Spermatophyta - ps15
Angiosperms - ps16
Mesangiospermae - ps17
Magnoliids/eudicots - ps18
Eudicots - ps19
Core_eudicots - ps20
Superrosids - ps21
Rosids - ps22
Eurosids - ps23
Malvids - ps24
Sap./Malv./Brassicales - ps25
Malvales/Brassicales - ps26
Brassicales - ps27
Cleomaceae/Brassicaceae - ps28
Brassicaceae - ps29
Camelineae - ps30
Arabidopsis - ps31
Arabidopsis_thaliana - ps32
Phylostratum

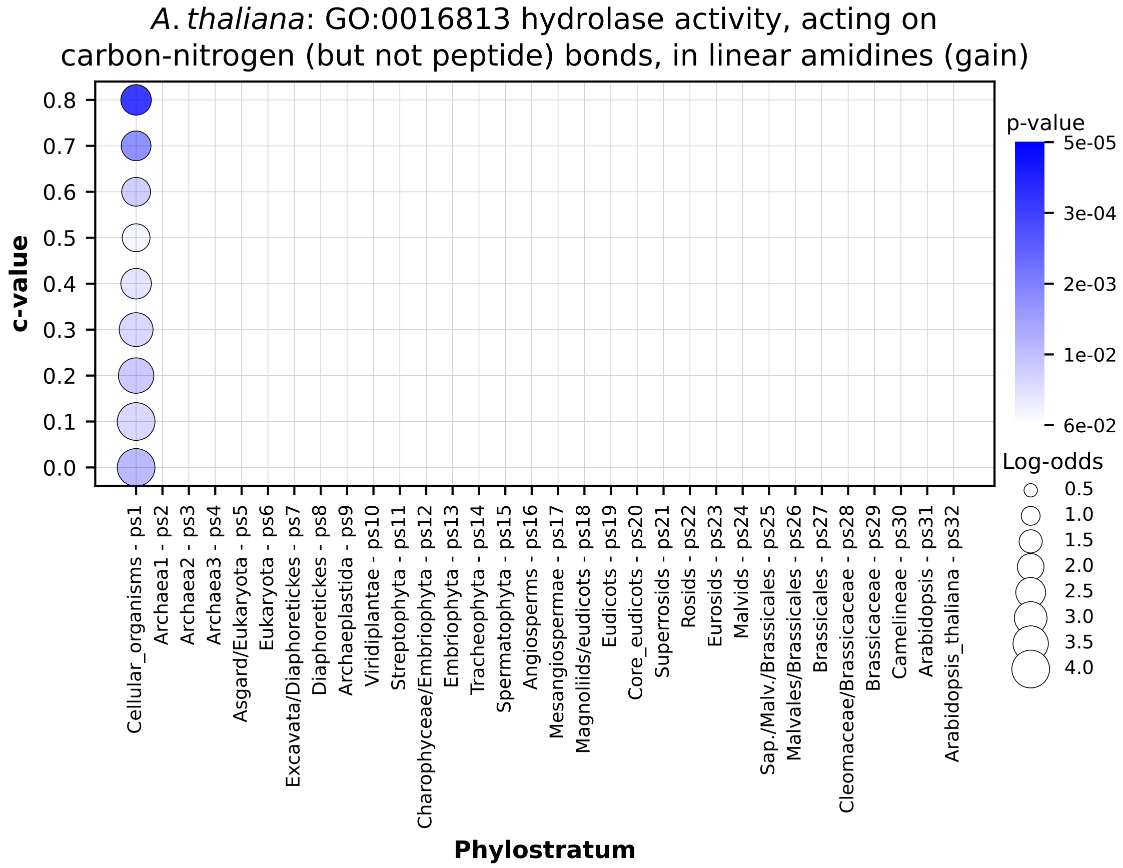
A. thaliana: GO:0016813 hydrolase activity, acting on carbon-nitrogen (but not peptide) bonds, in linear amidines (gain)
c-value
0.0
0.1
0.2
0.3
0.4
0.5
0.6
0.7
0.8
p-value
5e-05
3e-04
2e-03
1e-02
6e-02
Log-odds
0.5
1.0
1.5
2.0
2.5
3.0
3.5
4.0
Cellular_organisms - ps1
Archaea1 - ps2
Archaea2 - ps3
Archaea3 - ps4
Asgard/Eukaryota - ps5
Eukaryota - ps6
Excavata/Diaphoretickes - ps7
Diaphoretickes - ps8
Archaeplastida - ps9
Viridiplantae - ps10
Streptophyta - ps11
Charophyceae/Embriophyta - ps12
Embriophyta - ps13
Tracheophyta - ps14
Spermatophyta - ps15
Angiosperms - ps16
Mesangiospermae - ps17
Magnoliids/eudicots - ps18
Eudicots - ps19
Core_eudicots - ps20
Superrosids - ps21
Rosids - ps22
Eurosids - ps23
Malvids - ps24
Sap./Malv./Brassicales - ps25
Malvales/Brassicales - ps26
Brassicales - ps27
Cleomaceae/Brassicaceae - ps28
Brassicaceae - ps29
Camelineae - ps30
Arabidopsis - ps31
Arabidopsis_thaliana - ps32
Phylostratum

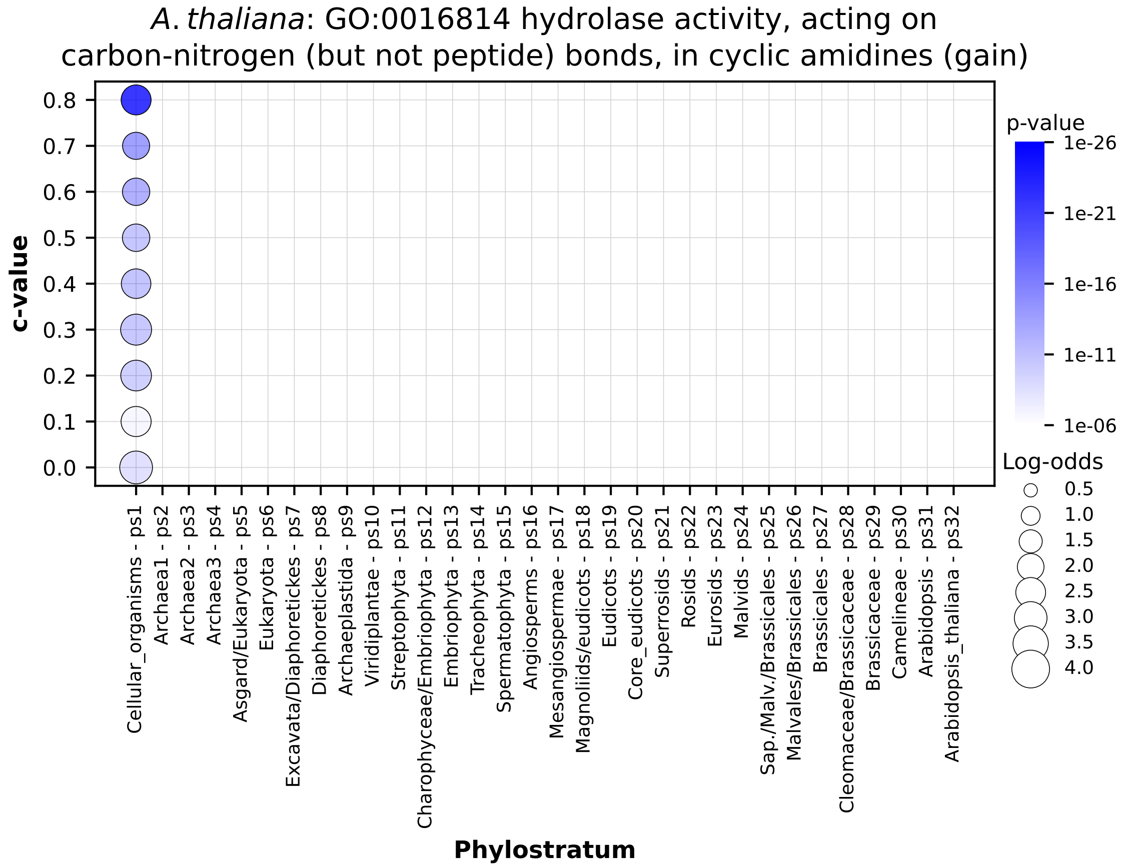

A. thaliana: GO:0016814 hydrolase activity, acting on carbon-nitrogen (but not peptide) bonds, in cyclic amidines (gain)
c-value
0.0
0.1
0.2
0.3
0.4
0.5
0.6
0.7
0.8
Phylostratum
Cellular_organisms - ps1
Archaea1 - ps2
Archaea2 - ps3
Archaea3 - ps4
Asgard/Eukaryota - ps5
Eukaryota - ps6
Excavata/Diaphoretickes - ps7
Diaphoretickes - ps8
Archaeplastida - ps9
Viridiplantae - ps10
Streptophyta - ps11
Charophyceae/Embriophyta - ps12
Embriophyta - ps13
Tracheophyta - ps14
Spermatophyta - ps15
Angiosperms - ps16
Mesangiospermae - ps17
Magnoliids/eudicots - ps18
Eudicots - ps19
Core_eudicots - ps20
Superrosids - ps21
Rosids - ps22
Eurosids - ps23
Malvids - ps24
Sap./Malv./Brassicales - ps25
Malvales/Brassicales - ps26
Brassicales - ps27
Cleomaceae/Brassicaceae - ps28
Brassicaceae - ps29
Camelineae - ps30
Arabidopsis - ps31
Arabidopsis_thaliana - ps32
p-value
1e-26
1e-21
1e-16
1e-11
1e-06
Log-odds
0.5
1.0
1.5
2.0
2.5
3.0
3.5
4.0

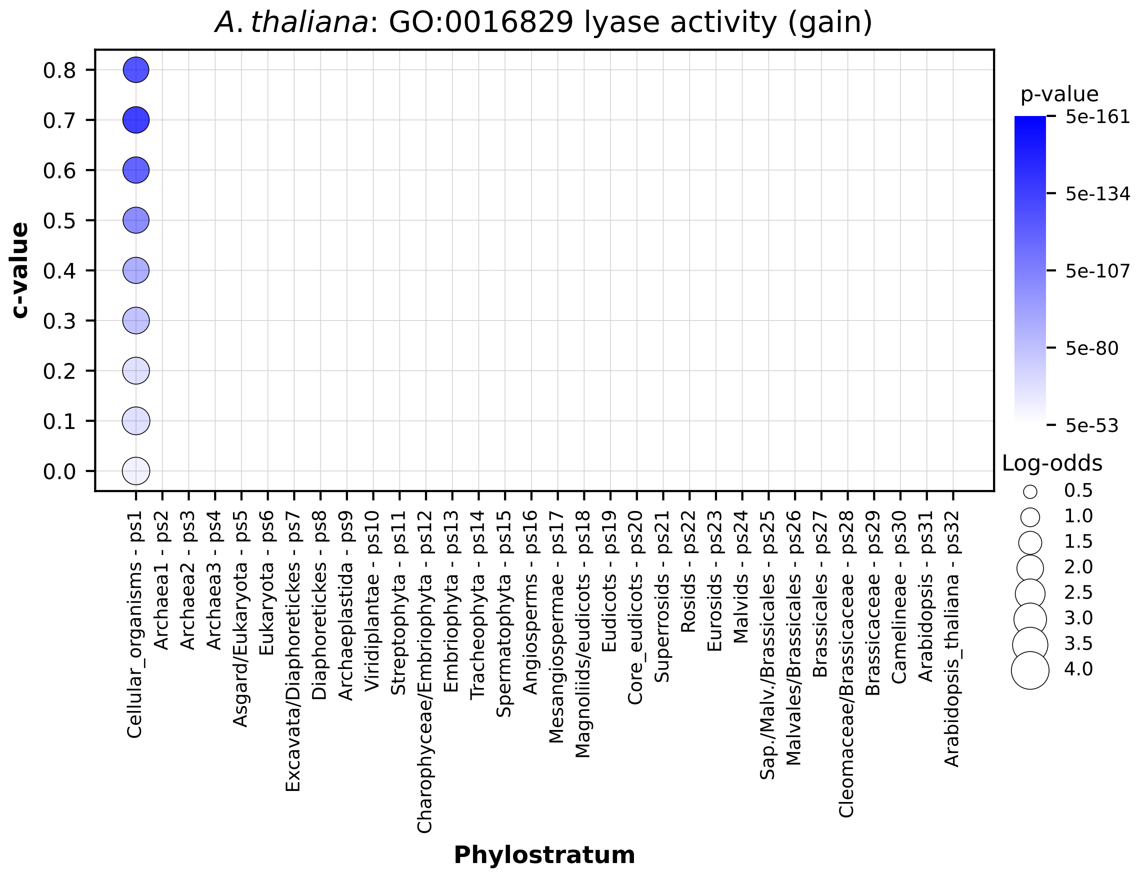

A. thaliana: GO:0016829 lyase activity (gain)
c-value
0.0
0.1
0.2
0.3
0.4
0.5
0.6
0.7
0.8
p-value
5e-161
5e-134
5e-107
5e-80
5e-53
Log-odds
0.5
1.0
1.5
2.0
2.5
3.0
3.5
4.0
Cellular_organisms - ps1
Archaea1 - ps2
Archaea2 - ps3
Archaea3 - ps4
Asgard/Eukaryota - ps5
Eukaryota - ps6
Excavata/Diaphoretickes - ps7
Diaphoretickes - ps8
Archaeplastida - ps9
Viridiplantae - ps10
Streptophyta - ps11
Charophyceae/Embriophyta - ps12
Embriophyta - ps13
Tracheophyta - ps14
Spermatophyta - ps15
Angiosperms - ps16
Mesangiospermae - ps17
Magnoliids/eudicots - ps18
Eudicots - ps19
Core_eudicots - ps20
Superrosids - ps21
Rosids - ps22
Eurosids - ps23
Malvids - ps24
Sap./Malv./Brassicales - ps25
Malvales/Brassicales - ps26
Brassicales - ps27
Cleomaceae/Brassicaceae - ps28
Brassicaceae - ps29
Camelineae - ps30
Arabidopsis - ps31
Arabidopsis_thaliana - ps32
Phylostratum

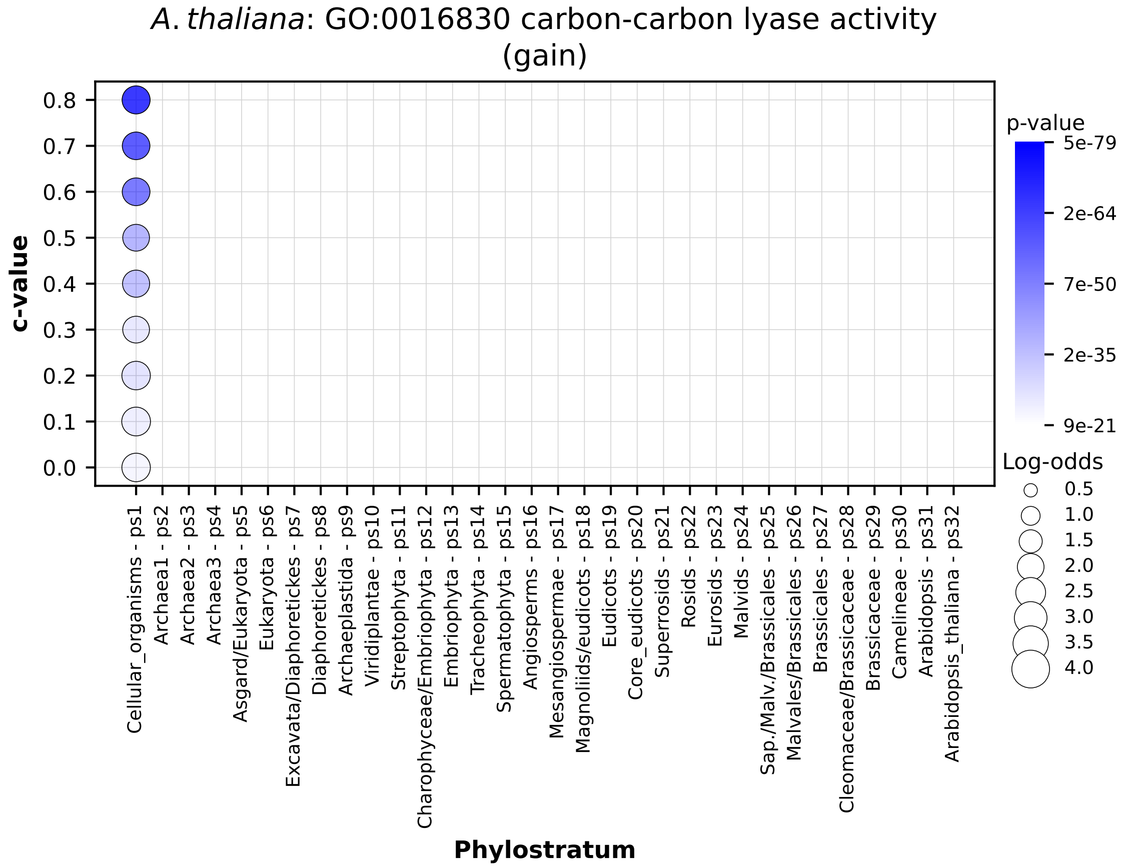

A. thaliana: GO:0016830 carbon-carbon lyase activity (gain)
c-value
0.8
0.7
0.6
0.5
0.4
0.3
0.2
0.1
0.0
p-value
5e-79
2e-64
7e-50
2e-35
9e-21
Log-odds
0.5
1.0
1.5
2.0
2.5
3.0
3.5
4.0
Cellular_organisms - ps1
Archaea1 - ps2
Archaea2 - ps3
Archaea3 - ps4
Asgard/Eukaryota - ps5
Eukaryota - ps6
Excavata/Diaphoretickes - ps7
Diaphoretickes - ps8
Archaeplastida - ps9
Viridiplantae - ps10
Streptophyta - ps11
Charophyceae/Embriophyta - ps12
Embriophyta - ps13
Tracheophyta - ps14
Spermatophyta - ps15
Angiosperms - ps16
Mesangiospermae - ps17
Magnoliids/eudicots - ps18
Eudicots - ps19
Core_eudicots - ps20
Superrosids - ps21
Rosids - ps22
Eurosids - ps23
Malvids - ps24
Sap./Malv./Brassicales - ps25
Malvales/Brassicales - ps26
Brassicales - ps27
Cleomaceae/Brassicaceae - ps28
Brassicaceae - ps29
Camelineae - ps30
Arabidopsis - ps31
Arabidopsis_thaliana - ps32
Phylostratum

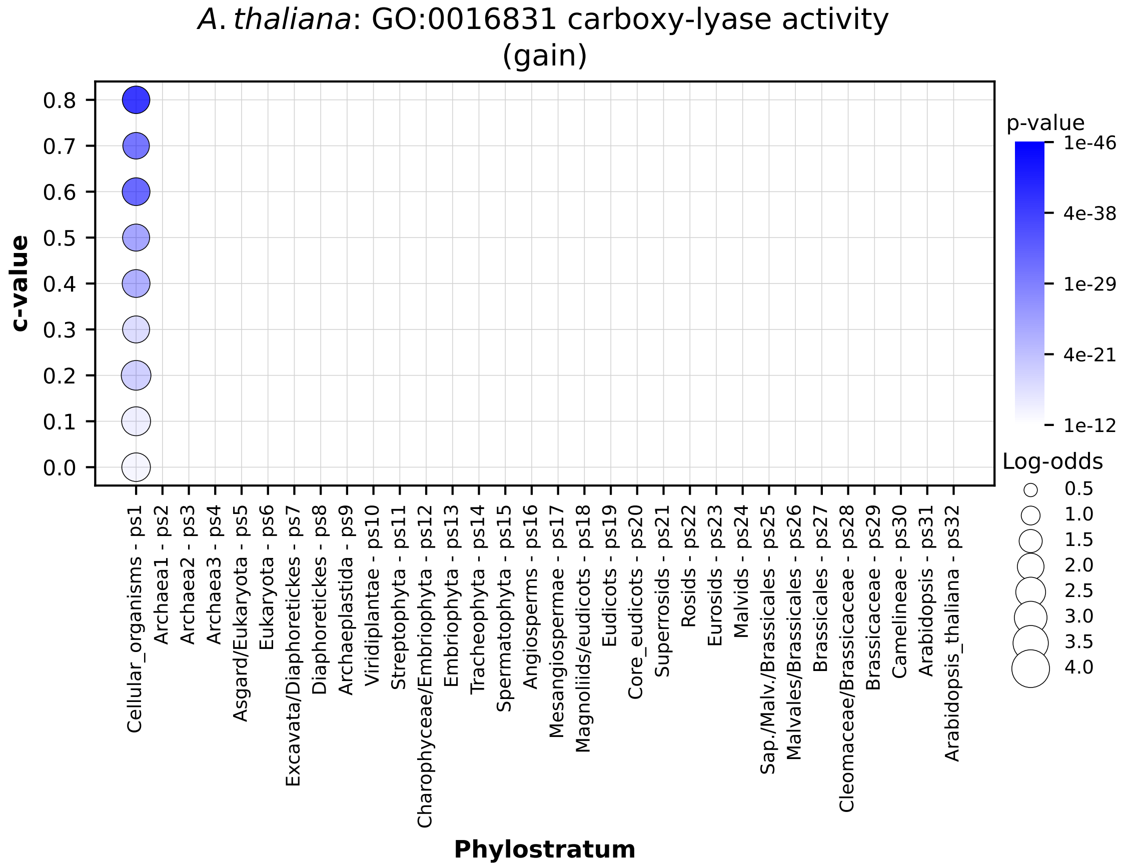
A. thaliana: GO:0016831 carboxy-lyase activity
(gain)
c-value
0.8
0.7
0.6
0.5
0.4
0.3
0.2
0.1
0.0
p-value
1e-46
4e-38
1e-29
4e-21
1e-12
Log-odds
0.5
1.0
1.5
2.0
2.5
3.0
3.5
4.0
Cellular_organisms - ps1
Archaea1 - ps2
Archaea2 - ps3
Archaea3 - ps4
Asgard/Eukaryota - ps5
Eukaryota - ps6
Excavata/Diaphoretickes - ps7
Diaphoretickes - ps8
Archaeplastida - ps9
Viridiplantae - ps10
Streptophyta - ps11
Charophyceae/Embriophyta - ps12
Embriophyta - ps13
Tracheophyta - ps14
Spermatophyta - ps15
Angiosperms - ps16
Mesangiospermae - ps17
Magnoliids/eudicots - ps18
Eudicots - ps19
Core_eudicots - ps20
Superrosids - ps21
Rosids - ps22
Eurosids - ps23
Malvids - ps24
Sap./Malv./Brassicales - ps25
Malvales/Brassicales - ps26
Brassicales - ps27
Cleomaceae/Brassicaceae - ps28
Brassicaceae - ps29
Camelineae - ps30
Arabidopsis - ps31
Arabidopsis_thaliana - ps32
Phylostratum

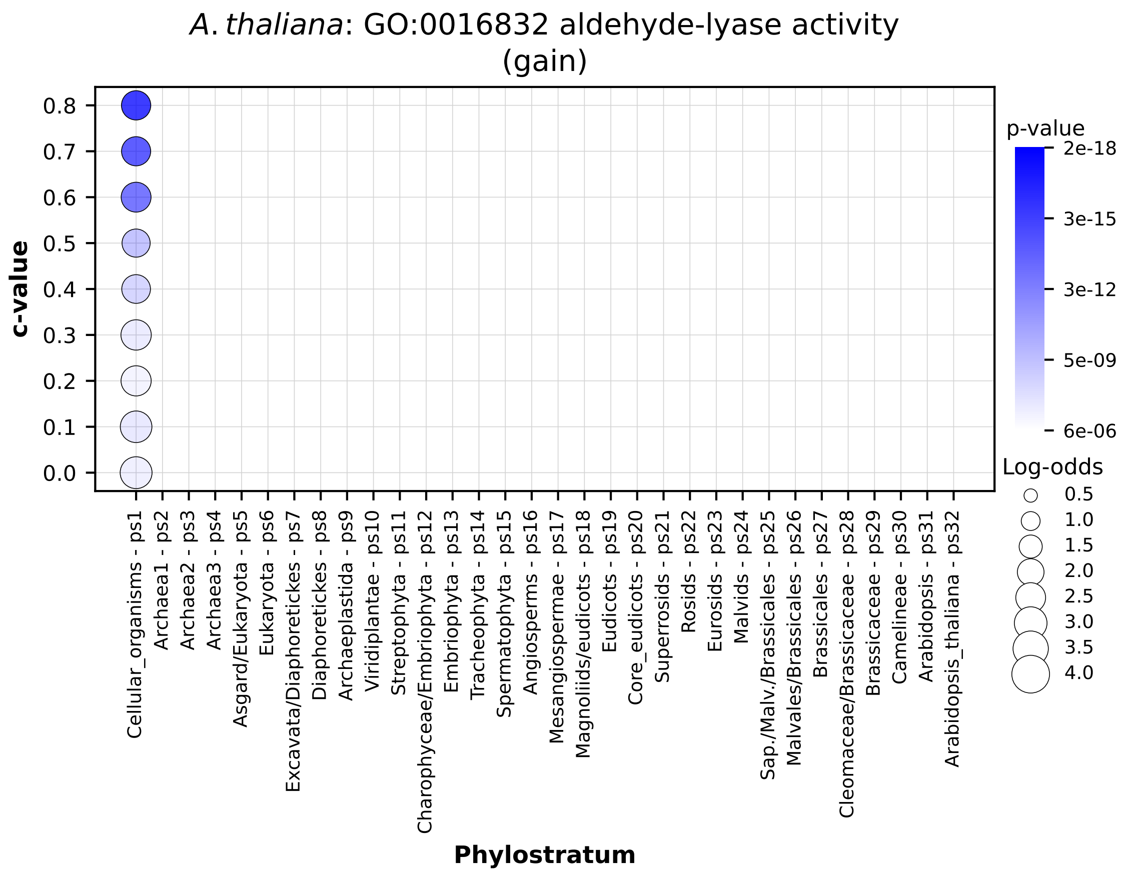

A. thaliana: GO:0016832 aldehyde-lyase activity
(gain)
c-value
0.8
0.7
0.6
0.5
0.4
0.3
0.2
0.1
0.0
p-value
2e-18
3e-15
3e-12
5e-09
6e-06
Log-odds
0.5
1.0
1.5
2.0
2.5
3.0
3.5
4.0
Cellular_organisms - ps1
Archaea1 - ps2
Archaea2 - ps3
Archaea3 - ps4
Asgard/Eukaryota - ps5
Eukaryota - ps6
Excavata/Diaphoretickes - ps7
Diaphoretickes - ps8
Archaeplastida - ps9
Viridiplantae - ps10
Streptophyta - ps11
Charophyceae/Embriophyta - ps12
Embriophyta - ps13
Tracheophyta - ps14
Spermatophyta - ps15
Angiosperms - ps16
Mesangiospermae - ps17
Magnoliids/eudicots - ps18
Eudicots - ps19
Core_eudicots - ps20
Superrosids - ps21
Rosids - ps22
Eurosids - ps23
Malvids - ps24
Sap./Malv./Brassicales - ps25
Malvales/Brassicales - ps26
Brassicales - ps27
Cleomaceae/Brassicaceae - ps28
Brassicaceae - ps29
Camelineae - ps30
Arabidopsis - ps31
Arabidopsis_thaliana - ps32
Phylostratum

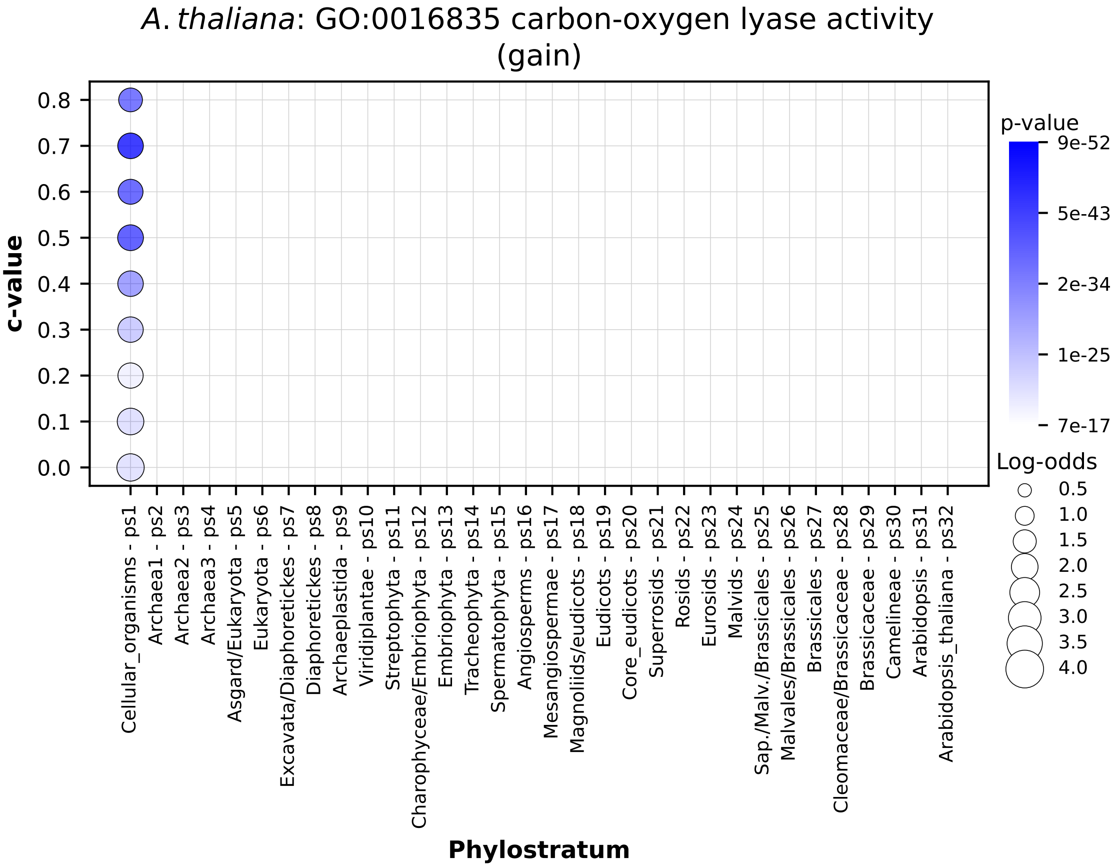

A. thaliana: GO:0016835 carbon-oxygen lyase activity (gain)
c-value
0.8
0.7
0.6
0.5
0.4
0.3
0.2
0.1
0.0
p-value
9e-52
5e-43
2e-34
1e-25
7e-17
Log-odds
0.5
1.0
1.5
2.0
2.5
3.0
3.5
4.0
Cellular_organisms - ps1
Archaea1 - ps2
Archaea2 - ps3
Archaea3 - ps4
Asgard/Eukaryota - ps5
Eukaryota - ps6
Excavata/Diaphoretickes - ps7
Diaphoretickes - ps8
Archaeplastida - ps9
Viridiplantae - ps10
Streptophyta - ps11
Charophyceae/Embriophyta - ps12
Embriophyta - ps13
Tracheophyta - ps14
Spermatophyta - ps15
Angiosperms - ps16
Mesangiospermae - ps17
Magnoliids/eudicots - ps18
Eudicots - ps19
Core_eudicots - ps20
Superrosids - ps21
Rosids - ps22
Eurosids - ps23
Malvids - ps24
Sap./Malv./Brassicales - ps25
Malvales/Brassicales - ps26
Brassicales - ps27
Cleomaceae/Brassicaceae - ps28
Brassicaceae - ps29
Camelineae - ps30
Arabidopsis - ps31
Arabidopsis_thaliana - ps32
Phylostratum

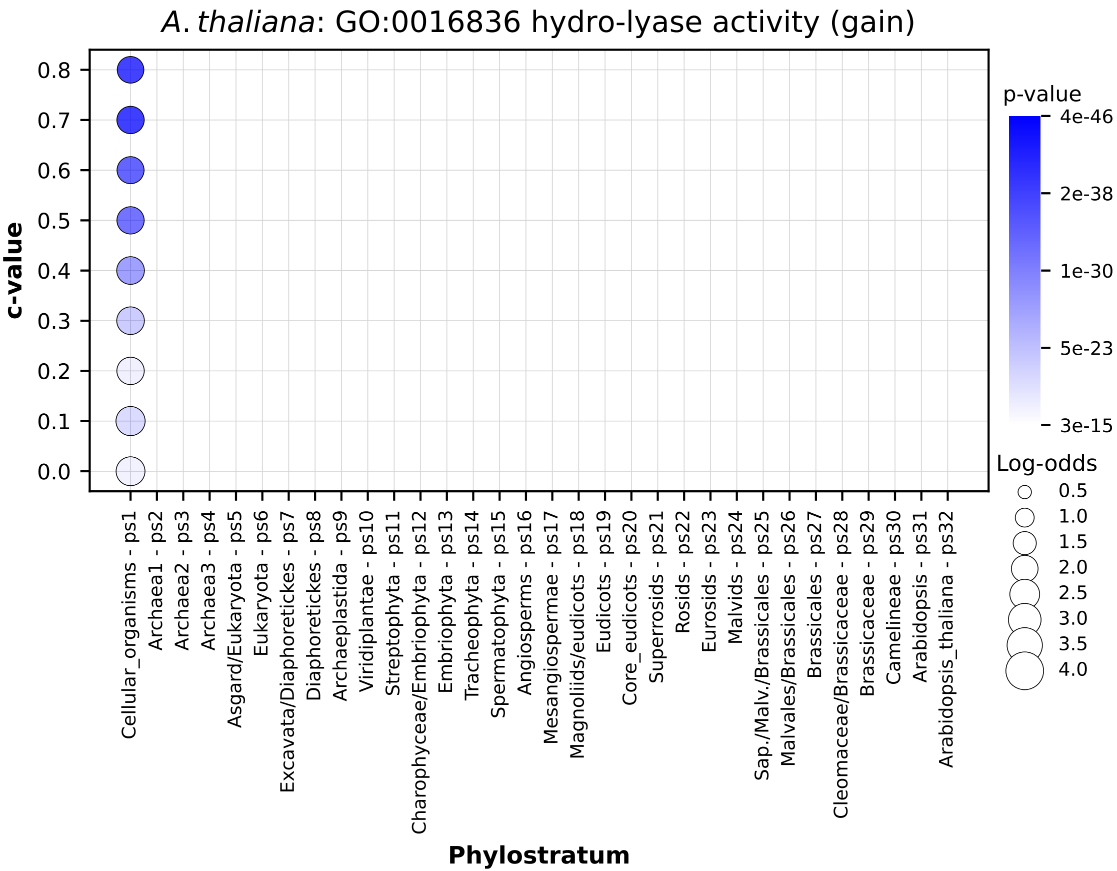
A. thaliana: GO:0016836 hydro-lyase activity (gain)
c-value
0.8
0.7
0.6
0.5
0.4
0.3
0.2
0.1
0.0
p-value
4e-46
2e-38
1e-30
5e-23
3e-15
Log-odds
0.5
1.0
1.5
2.0
2.5
3.0
3.5
4.0
Cellular_organisms - ps1
Archaea1 - ps2
Archaea2 - ps3
Archaea3 - ps4
Asgard/Eukaryota - ps5
Eukaryota - ps6
Excavata/Diaphoretickes - ps7
Diaphoretickes - ps8
Archaeplastida - ps9
Viridiplantae - ps10
Streptophyta - ps11
Charophyceae/Embriophyta - ps12
Embriophyta - ps13
Tracheophyta - ps14
Spermatophyta - ps15
Angiosperms - ps16
Mesangiospermae - ps17
Magnoliids/eudicots - ps18
Eudicots - ps19
Core_eudicots - ps20
Superrosids - ps21
Rosids - ps22
Eurosids - ps23
Malvids - ps24
Sap./Malv./Brassicales - ps25
Malvales/Brassicales - ps26
Brassicales - ps27
Cleomaceae/Brassicaceae - ps28
Brassicaceae - ps29
Camelineae - ps30
Arabidopsis - ps31
Arabidopsis_thaliana - ps32
Phylostratum

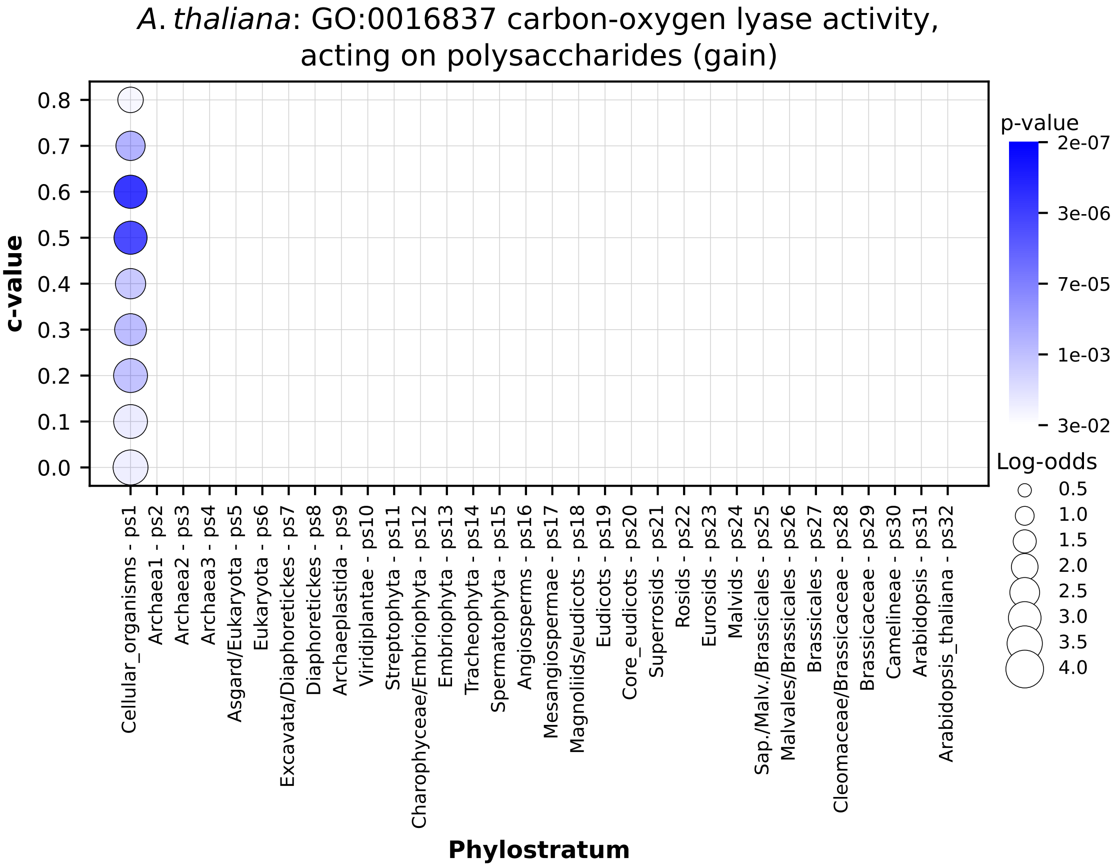

A. thaliana: GO:0016837 carbon-oxygen lyase activity, acting on polysaccharides (gain)
c-value
0.8
0.7
0.6
0.5
0.4
0.3
0.2
0.1
0.0
p-value
2e-07
3e-06
7e-05
1e-03
3e-02
Log-odds
0.5
1.0
1.5
2.0
2.5
3.0
3.5
4.0
Cellular_organisms - ps1
Archaea1 - ps2
Archaea2 - ps3
Archaea3 - ps4
Asgard/Eukaryota - ps5
Eukaryota - ps6
Excavata/Diaphoretickes - ps7
Diaphoretickes - ps8
Archaeplastida - ps9
Viridiplantae - ps10
Streptophyta - ps11
Charophyceae/Embriophyta - ps12
Embriophyta - ps13
Tracheophyta - ps14
Spermatophyta - ps15
Angiosperms - ps16
Mesangiospermae - ps17
Magnoliids/eudicots - ps18
Eudicots - ps19
Core_eudicots - ps20
Superrosids - ps21
Rosids - ps22
Eurosids - ps23
Malvids - ps24
Sap./Malv./Brassicales - ps25
Malvales/Brassicales - ps26
Brassicales - ps27
Cleomaceae/Brassicaceae - ps28
Brassicaceae - ps29
Camelineae - ps30
Arabidopsis - ps31
Arabidopsis_thaliana - ps32
Phylostratum

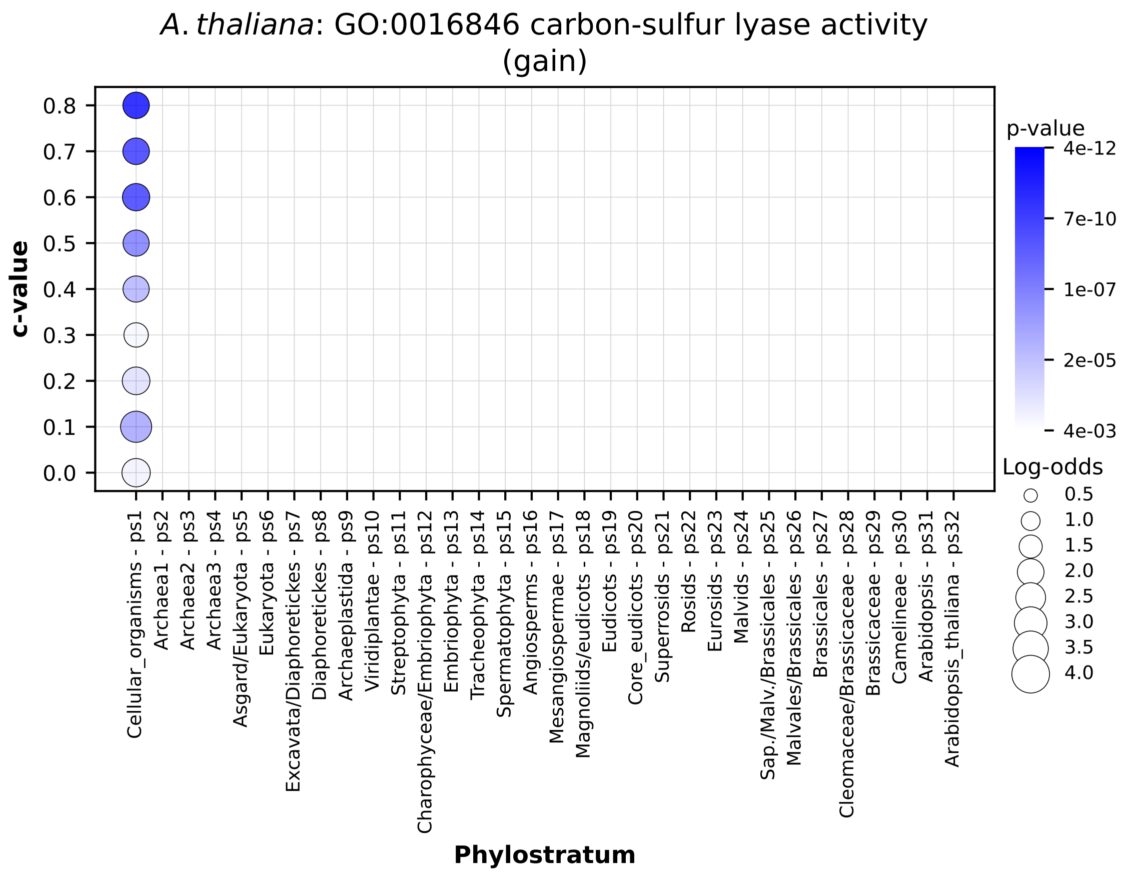
A. thaliana: GO:0016846 carbon-sulfur lyase activity
(gain)
c-value
0.8
0.7
0.6
0.5
0.4
0.3
0.2
0.1
0.0
p-value
4e-12
7e-10
1e-07
2e-05
4e-03
Log-odds
0.5
1.0
1.5
2.0
2.5
3.0
3.5
4.0
Cellular_organisms - ps1
Archaea1 - ps2
Archaea2 - ps3
Archaea3 - ps4
Asgard/Eukaryota - ps5
Eukaryota - ps6
Excavata/Diaphoretickes - ps7
Diaphoretickes - ps8
Archaeplastida - ps9
Viridiplantae - ps10
Streptophyta - ps11
Charophyceae/Embriophyta - ps12
Embriophyta - ps13
Tracheophyta - ps14
Spermatophyta - ps15
Angiosperms - ps16
Mesangiospermae - ps17
Magnoliids/eudicots - ps18
Eudicots - ps19
Core_eudicots - ps20
Superrosids - ps21
Rosids - ps22
Eurosids - ps23
Malvids - ps24
Sap./Malv./Brassicales - ps25
Malvales/Brassicales - ps26
Brassicales - ps27
Cleomaceae/Brassicaceae - ps28
Brassicaceae - ps29
Camelineae - ps30
Arabidopsis - ps31
Arabidopsis_thaliana - ps32
Phylostratum

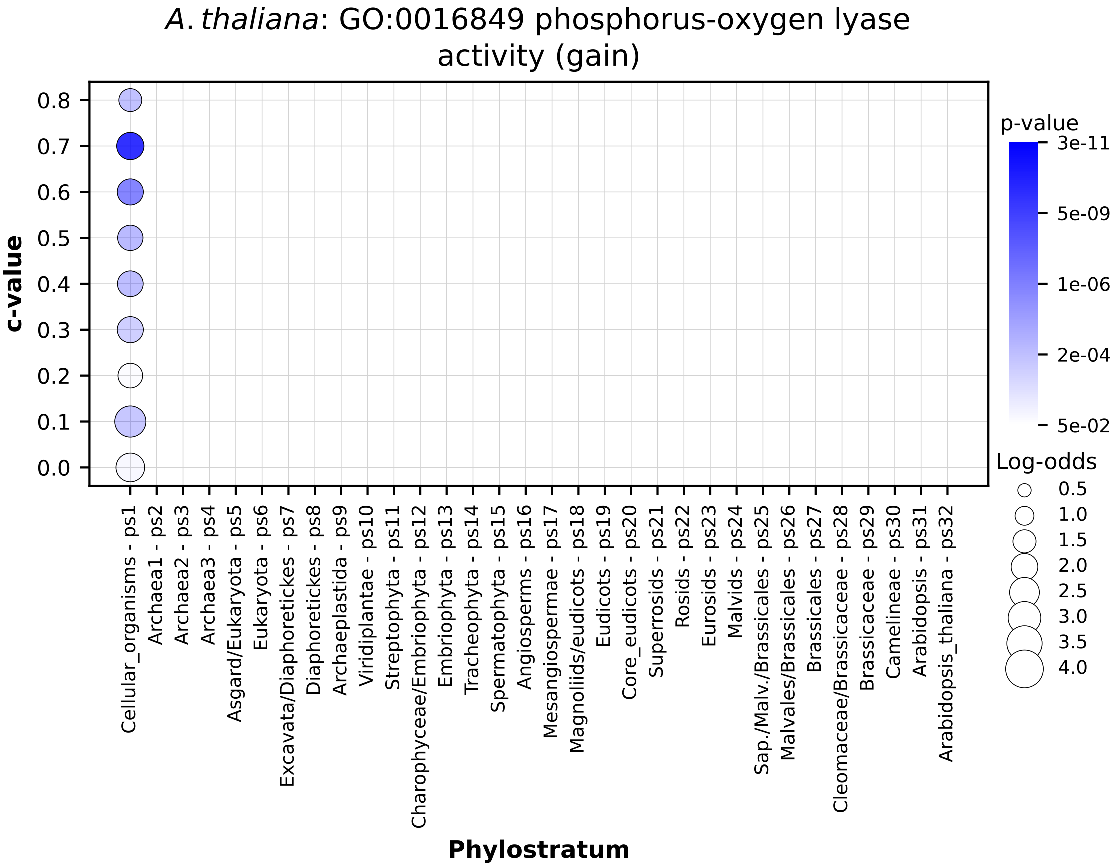
A. thaliana: GO:0016849 phosphorus-oxygen lyase activity (gain)
c-value
0.8
0.7
0.6
0.5
0.4
0.3
0.2
0.1
0.0
p-value
3e-11
5e-09
1e-06
2e-04
5e-02
Log-odds
0.5
1.0
1.5
2.0
2.5
3.0
3.5
4.0
Cellular_organisms - ps1
Archaea1 - ps2
Archaea2 - ps3
Archaea3 - ps4
Asgard/Eukaryota - ps5
Eukaryota - ps6
Excavata/Diaphoretickes - ps7
Diaphoretickes - ps8
Archaeplastida - ps9
Viridiplantae - ps10
Streptophyta - ps11
Charophyceae/Embriophyta - ps12
Embriophyta - ps13
Tracheophyta - ps14
Spermatophyta - ps15
Angiosperms - ps16
Mesangiospermae - ps17
Magnoliids/eudicots - ps18
Eudicots - ps19
Core_eudicots - ps20
Superrosids - ps21
Rosids - ps22
Eurosids - ps23
Malvids - ps24
Sap./Malv./Brassicales - ps25
Malvales/Brassicales - ps26
Brassicales - ps27
Cleomaceae/Brassicaceae - ps28
Brassicaceae - ps29
Camelineae - ps30
Arabidopsis - ps31
Arabidopsis_thaliana - ps32
Phylostratum

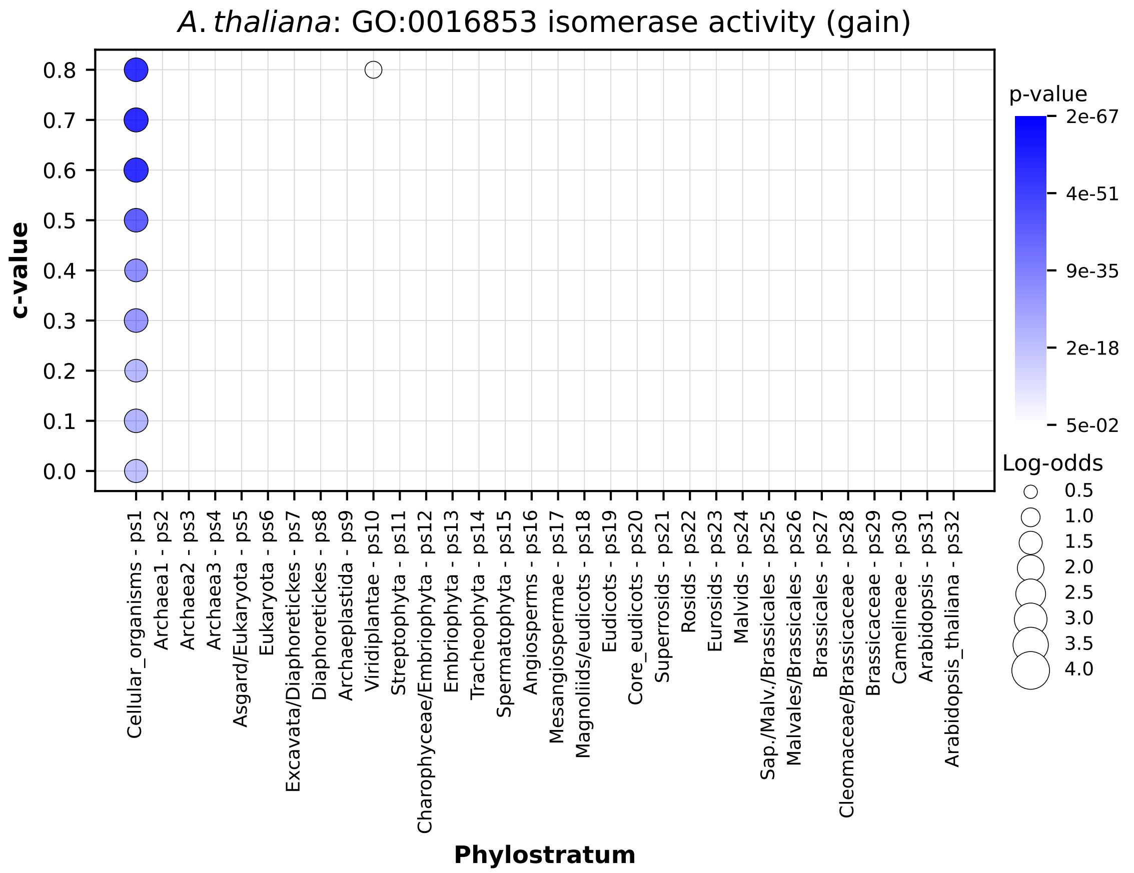

A. thaliana: GO:0016853 isomerase activity (gain)
c-value
0.8
0.7
0.6
0.5
0.4
0.3
0.2
0.1
0.0
p-value
2e-67
4e-51
9e-35
2e-18
5e-02
Log-odds
0.5
1.0
1.5
2.0
2.5
3.0
3.5
4.0
Cellular_organisms - ps1
Archaea1 - ps2
Archaea2 - ps3
Archaea3 - ps4
Asgard/Eukaryota - ps5
Eukaryota - ps6
Excavata/Diaphoretickes - ps7
Diaphoretickes - ps8
Archaeplastida - ps9
Viridiplantae - ps10
Streptophyta - ps11
Charophyceae/Embriophyta - ps12
Embriophyta - ps13
Tracheophyta - ps14
Spermatophyta - ps15
Angiosperms - ps16
Mesangiospermae - ps17
Magnoliids/eudicots - ps18
Eudicots - ps19
Core_eudicots - ps20
Superrosids - ps21
Rosids - ps22
Eurosids - ps23
Malvids - ps24
Sap./Malv./Brassicales - ps25
Malvales/Brassicales - ps26
Brassicales - ps27
Cleomaceae/Brassicaceae - ps28
Brassicaceae - ps29
Camelineae - ps30
Arabidopsis - ps31
Arabidopsis_thaliana - ps32
Phylostratum

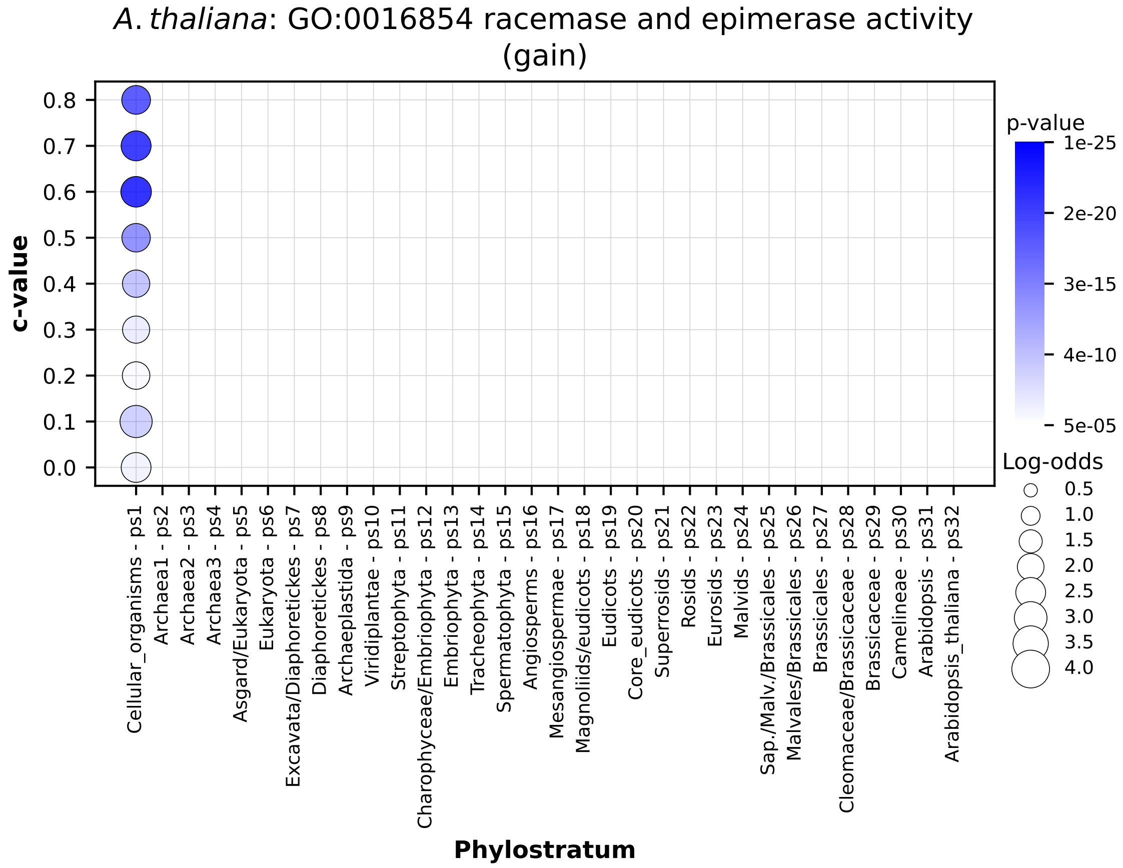

A. thaliana: GO:0016854 racemase and epimerase activity
(gain)
c-value
0.8
0.7
0.6
0.5
0.4
0.3
0.2
0.1
0.0
p-value
1e-25
2e-20
3e-15
4e-10
5e-05
Log-odds
0.5
1.0
1.5
2.0
2.5
3.0
3.5
4.0
Cellular_organisms - ps1
Archaea1 - ps2
Archaea2 - ps3
Archaea3 - ps4
Asgard/Eukaryota - ps5
Eukaryota - ps6
Excavata/Diaphoretickes - ps7
Diaphoretickes - ps8
Archaeplastida - ps9
Viridiplantae - ps10
Streptophyta - ps11
Charophyceae/Embriophyta - ps12
Embriophyta - ps13
Tracheophyta - ps14
Spermatophyta - ps15
Angiosperms - ps16
Mesangiospermae - ps17
Magnoliids/eudicots - ps18
Eudicots - ps19
Core_eudicots - ps20
Superrosids - ps21
Rosids - ps22
Eurosids - ps23
Malvids - ps24
Sap./Malv./Brassicales - ps25
Malvales/Brassicales - ps26
Brassicales - ps27
Cleomaceae/Brassicaceae - ps28
Brassicaceae - ps29
Camelineae - ps30
Arabidopsis - ps31
Arabidopsis_thaliana - ps32
Phylostratum

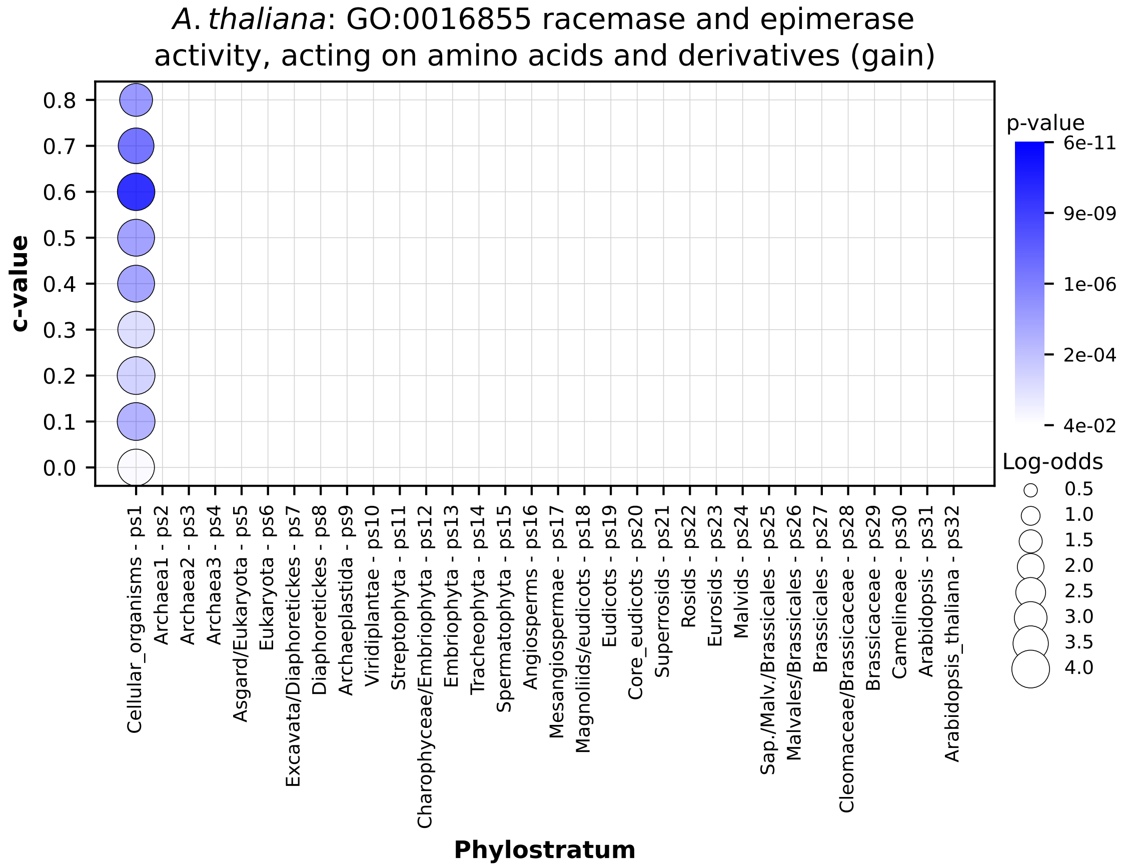

A. thaliana: GO:0016855 racemase and epimerase activity, acting on amino acids and derivatives (gain)
c-value
0.0
0.1
0.2
0.3
0.4
0.5
0.6
0.7
0.8
p-value
6e-11
9e-09
1e-06
2e-04
4e-02
Log-odds
0.5
1.0
1.5
2.0
2.5
3.0
3.5
4.0
Cellular_organisms - ps1
Archaea1 - ps2
Archaea2 - ps3
Archaea3 - ps4
Asgard/Eukaryota - ps5
Eukaryota - ps6
Excavata/Diaphoretickes - ps7
Diaphoretickes - ps8
Archaeplastida - ps9
Viridiplantae - ps10
Streptophyta - ps11
Charophyceae/Embriophyta - ps12
Embriophyta - ps13
Tracheophyta - ps14
Spermatophyta - ps15
Angiosperms - ps16
Mesangiospermae - ps17
Magnoliids/eudicots - ps18
Eudicots - ps19
Core_eudicots - ps20
Superrosids - ps21
Rosids - ps22
Eurosids - ps23
Malvids - ps24
Sap./Malv./Brassicales - ps25
Malvales/Brassicales - ps26
Brassicales - ps27
Cleomaceae/Brassicaceae - ps28
Brassicaceae - ps29
Camelineae - ps30
Arabidopsis - ps31
Arabidopsis_thaliana - ps32
Phylostratum

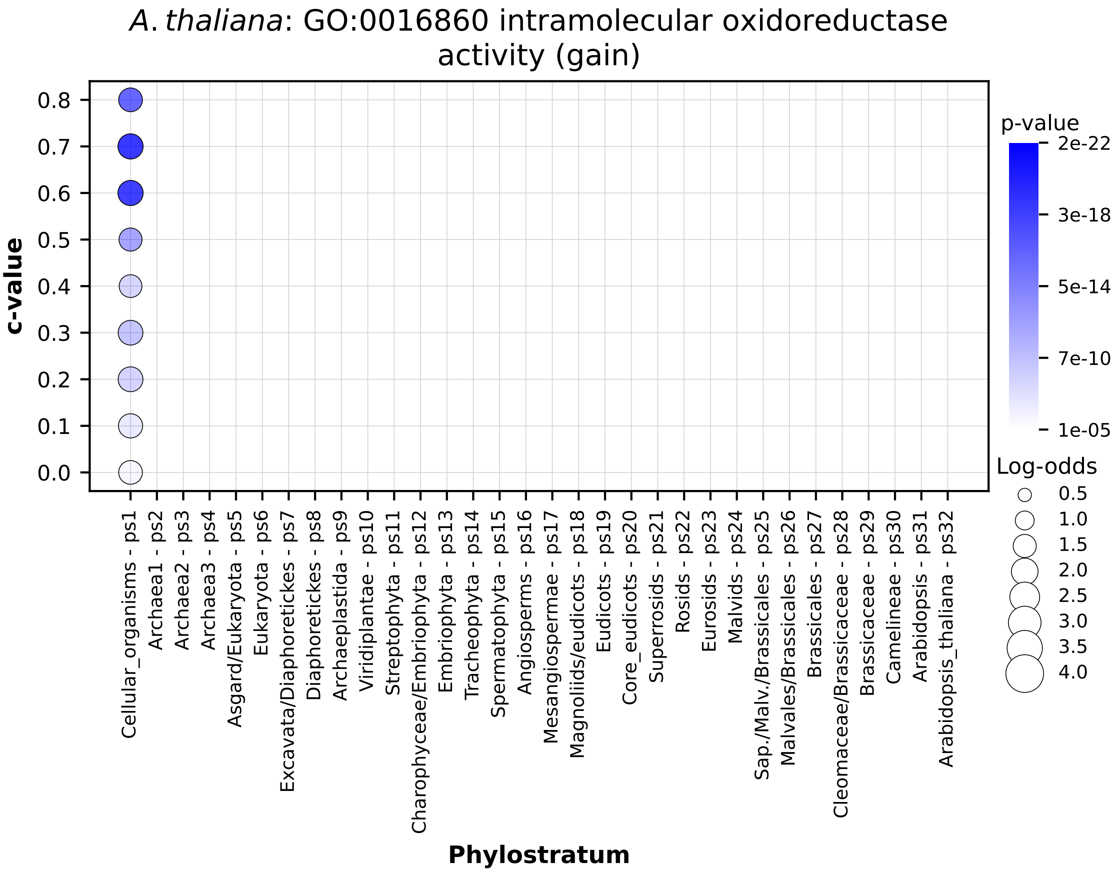

A. thaliana: GO:0016860 intramolecular oxidoreductase activity (gain)
c-value
0.8
0.7
0.6
0.5
0.4
0.3
0.2
0.1
0.0
p-value
2e-22
3e-18
5e-14
7e-10
1e-05
Log-odds
0.5
1.0
1.5
2.0
2.5
3.0
3.5
4.0
Cellular_organisms - ps1
Archaea1 - ps2
Archaea2 - ps3
Archaea3 - ps4
Asgard/Eukaryota - ps5
Eukaryota - ps6
Excavata/Diaphoretickes - ps7
Diaphoretickes - ps8
Archaeplastida - ps9
Viridiplantae - ps10
Streptophyta - ps11
Charophyceae/Embriophyta - ps12
Embriophyta - ps13
Tracheophyta - ps14
Spermatophyta - ps15
Angiosperms - ps16
Mesangiospermae - ps17
Magnoliids/eudicots - ps18
Eudicots - ps19
Core_eudicots - ps20
Superrosids - ps21
Rosids - ps22
Eurosids - ps23
Malvids - ps24
Sap./Malv./Brassicales - ps25
Malvales/Brassicales - ps26
Brassicales - ps27
Cleomaceae/Brassicaceae - ps28
Brassicaceae - ps29
Camelineae - ps30
Arabidopsis - ps31
Arabidopsis_thaliana - ps32
Phylostratum

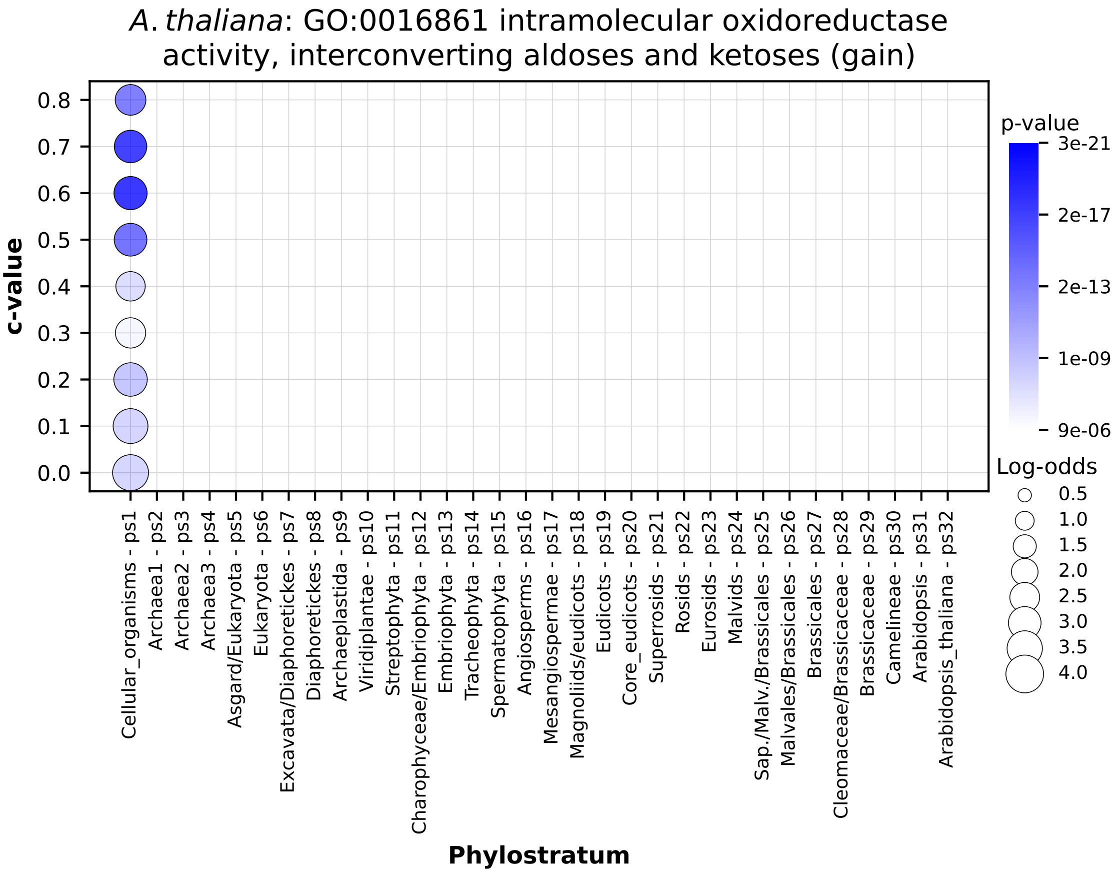

A. thaliana: GO:0016861 intramolecular oxidoreductase activity, interconverting aldoses and ketoses (gain)
c-value
0.8
0.7
0.6
0.5
0.4
0.3
0.2
0.1
0.0
Phylostratum
Cellular_organisms - ps1
Archaea1 - ps2
Archaea2 - ps3
Archaea3 - ps4
Asgard/Eukaryota - ps5
Eukaryota - ps6
Excavata/Diaphoretickes - ps7
Diaphoretickes - ps8
Archaeplastida - ps9
Viridiplantae - ps10
Streptophyta - ps11
Charophyceae/Embriophyta - ps12
Embriophyta - ps13
Tracheophyta - ps14
Spermatophyta - ps15
Angiosperms - ps16
Mesangiospermae - ps17
Magnoliids/eudicots - ps18
Eudicots - ps19
Core_eudicots - ps20
Superrosids - ps21
Rosids - ps22
Eurosids - ps23
Malvids - ps24
Sap./Malv./Brassicales - ps25
Malvales/Brassicales - ps26
Brassicales - ps27
Cleomaceae/Brassicaceae - ps28
Brassicaceae - ps29
Camelineae - ps30
Arabidopsis - ps31
Arabidopsis_thaliana - ps32
p-value
3e-21
2e-17
2e-13
1e-09
9e-06
Log-odds
0.5
1.0
1.5
2.0
2.5
3.0
3.5
4.0

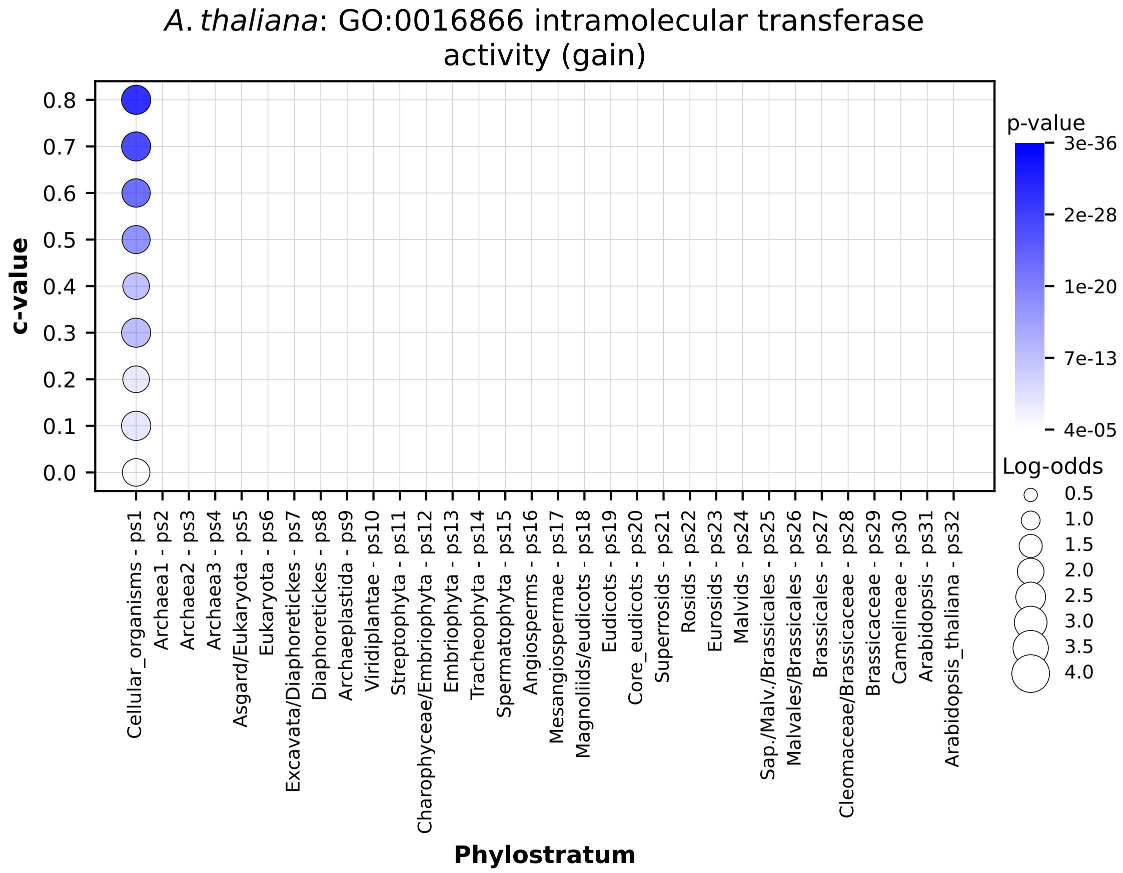

A. thaliana: GO:0016866 intramolecular transferase activity (gain)
c-value
0.8
0.7
0.6
0.5
0.4
0.3
0.2
0.1
0.0
p-value
3e-36
2e-28
1e-20
7e-13
4e-05
Log-odds
0.5
1.0
1.5
2.0
2.5
3.0
3.5
4.0
Cellular_organisms - ps1
Archaea1 - ps2
Archaea2 - ps3
Archaea3 - ps4
Asgard/Eukaryota - ps5
Eukaryota - ps6
Excavata/Diaphoretickes - ps7
Diaphoretickes - ps8
Archaeplastida - ps9
Viridiplantae - ps10
Streptophyta - ps11
Charophyceae/Embriophyta - ps12
Embriophyta - ps13
Tracheophyta - ps14
Spermatophyta - ps15
Angiosperms - ps16
Mesangiospermae - ps17
Magnoliids/eudicots - ps18
Eudicots - ps19
Core_eudicots - ps20
Superrosids - ps21
Rosids - ps22
Eurosids - ps23
Malvids - ps24
Sap./Malv./Brassicales - ps25
Malvales/Brassicales - ps26
Brassicales - ps27
Cleomaceae/Brassicaceae - ps28
Brassicaceae - ps29
Camelineae - ps30
Arabidopsis - ps31
Arabidopsis_thaliana - ps32
Phylostratum

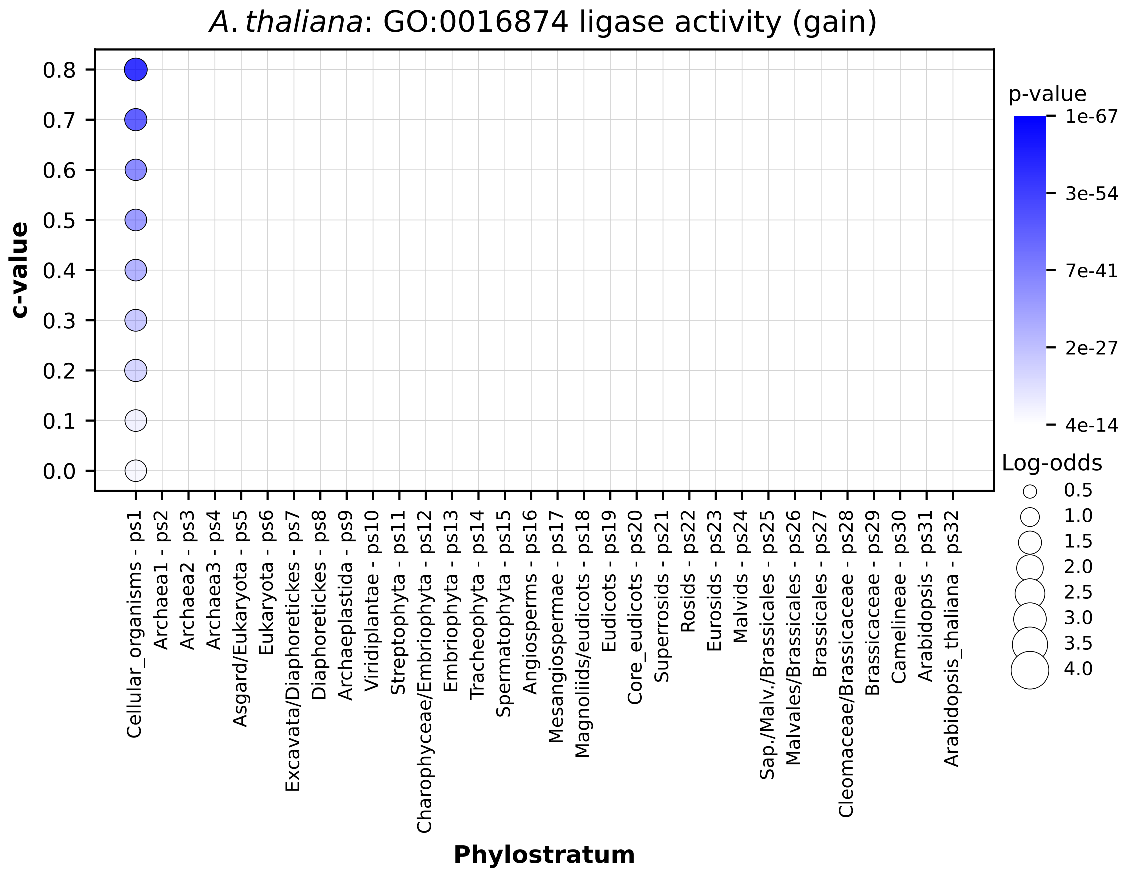

A. thaliana: GO:0016874 ligase activity (gain)
c-value
0.0
0.1
0.2
0.3
0.4
0.5
0.6
0.7
0.8
p-value
1e-67
3e-54
7e-41
2e-27
4e-14
Log-odds
0.5
1.0
1.5
2.0
2.5
3.0
3.5
4.0
Cellular_organisms - ps1
Archaea1 - ps2
Archaea2 - ps3
Archaea3 - ps4
Asgard/Eukaryota - ps5
Eukaryota - ps6
Excavata/Diaphoretickes - ps7
Diaphoretickes - ps8
Archaeplastida - ps9
Viridiplantae - ps10
Streptophyta - ps11
Charophyceae/Embriophyta - ps12
Embriophyta - ps13
Tracheophyta - ps14
Spermatophyta - ps15
Angiosperms - ps16
Mesangiospermae - ps17
Magnoliids/eudicots - ps18
Eudicots - ps19
Core_eudicots - ps20
Superrosids - ps21
Rosids - ps22
Eurosids - ps23
Malvids - ps24
Sap./Malv./Brassicales - ps25
Malvales/Brassicales - ps26
Brassicales - ps27
Cleomaceae/Brassicaceae - ps28
Brassicaceae - ps29
Camelineae - ps30
Arabidopsis - ps31
Arabidopsis_thaliana - ps32
Phylostratum

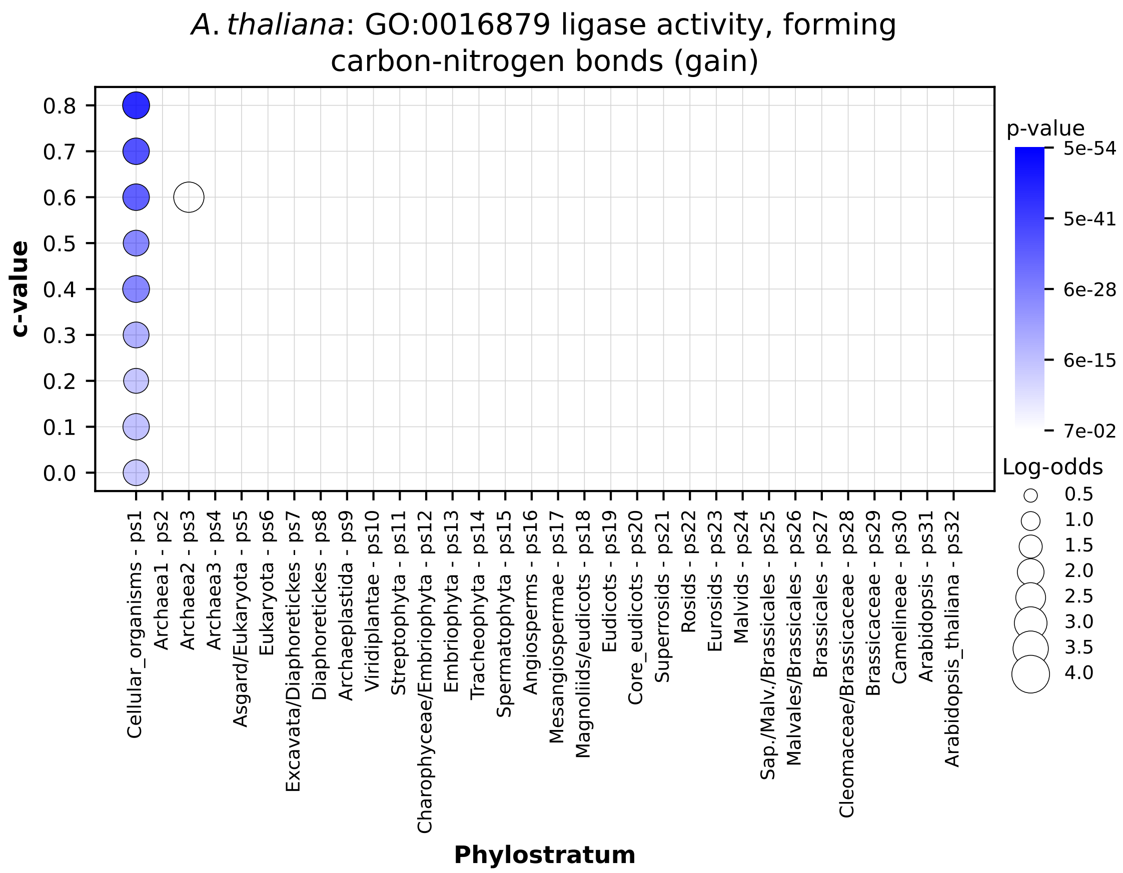
A. thaliana: GO:0016879 ligase activity, forming carbon-nitrogen bonds (gain)
c-value
0.8
0.7
0.6
0.5
0.4
0.3
0.2
0.1
0.0
p-value
5e-54
5e-41
6e-28
6e-15
7e-02
Log-odds
0.5
1.0
1.5
2.0
2.5
3.0
3.5
4.0
Cellular_organisms - ps1
Archaea1 - ps2
Archaea2 - ps3
Archaea3 - ps4
Asgard/Eukaryota - ps5
Eukaryota - ps6
Excavata/Diaphoretickes - ps7
Diaphoretickes - ps8
Archaeplastida - ps9
Viridiplantae - ps10
Streptophyta - ps11
Charophyceae/Embriophyta - ps12
Embriophyta - ps13
Tracheophyta - ps14
Spermatophyta - ps15
Angiosperms - ps16
Mesangiospermae - ps17
Magnoliids/eudicots - ps18
Eudicots - ps19
Core_eudicots - ps20
Superrosids - ps21
Rosids - ps22
Eurosids - ps23
Malvids - ps24
Sap./Malv./Brassicales - ps25
Malvales/Brassicales - ps26
Brassicales - ps27
Cleomaceae/Brassicaceae - ps28
Brassicaceae - ps29
Camelineae - ps30
Arabidopsis - ps31
Arabidopsis_thaliana - ps32
Phylostratum

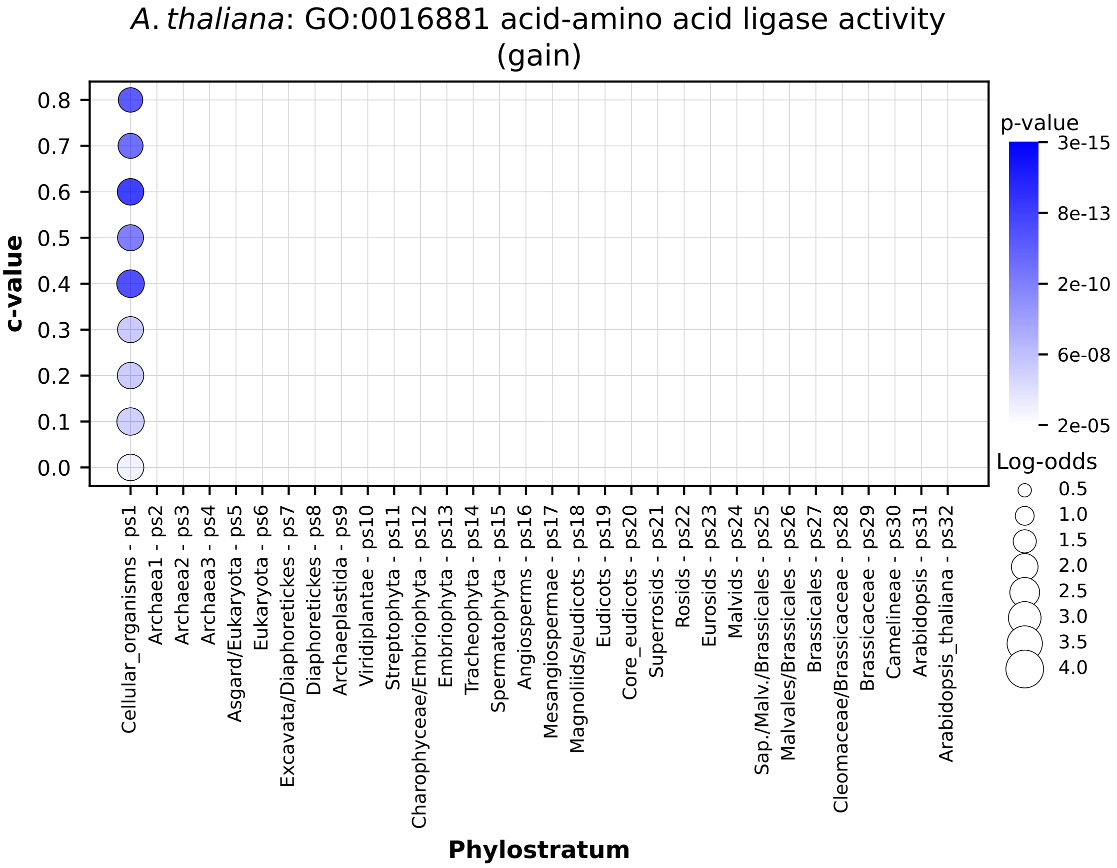

A. thaliana: GO:0016881 acid-amino acid ligase activity
(gain)
c-value
0.8
0.7
0.6
0.5
0.4
0.3
0.2
0.1
0.0
p-value
3e-15
8e-13
2e-10
6e-08
2e-05
Log-odds
0.5
1.0
1.5
2.0
2.5
3.0
3.5
4.0
Cellular_organisms - ps1
Archaea1 - ps2
Archaea2 - ps3
Archaea3 - ps4
Asgard/Eukaryota - ps5
Eukaryota - ps6
Excavata/Diaphoretickes - ps7
Diaphoretickes - ps8
Archaeplastida - ps9
Viridiplantae - ps10
Streptophyta - ps11
Charophyceae/Embriophyta - ps12
Embriophyta - ps13
Tracheophyta - ps14
Spermatophyta - ps15
Angiosperms - ps16
Mesangiospermae - ps17
Magnoliids/eudicots - ps18
Eudicots - ps19
Core_eudicots - ps20
Superrosids - ps21
Rosids - ps22
Eurosids - ps23
Malvids - ps24
Sap./Malv./Brassicales - ps25
Malvales/Brassicales - ps26
Brassicales - ps27
Cleomaceae/Brassicaceae - ps28
Brassicaceae - ps29
Camelineae - ps30
Arabidopsis - ps31
Arabidopsis_thaliana - ps32
Phylostratum

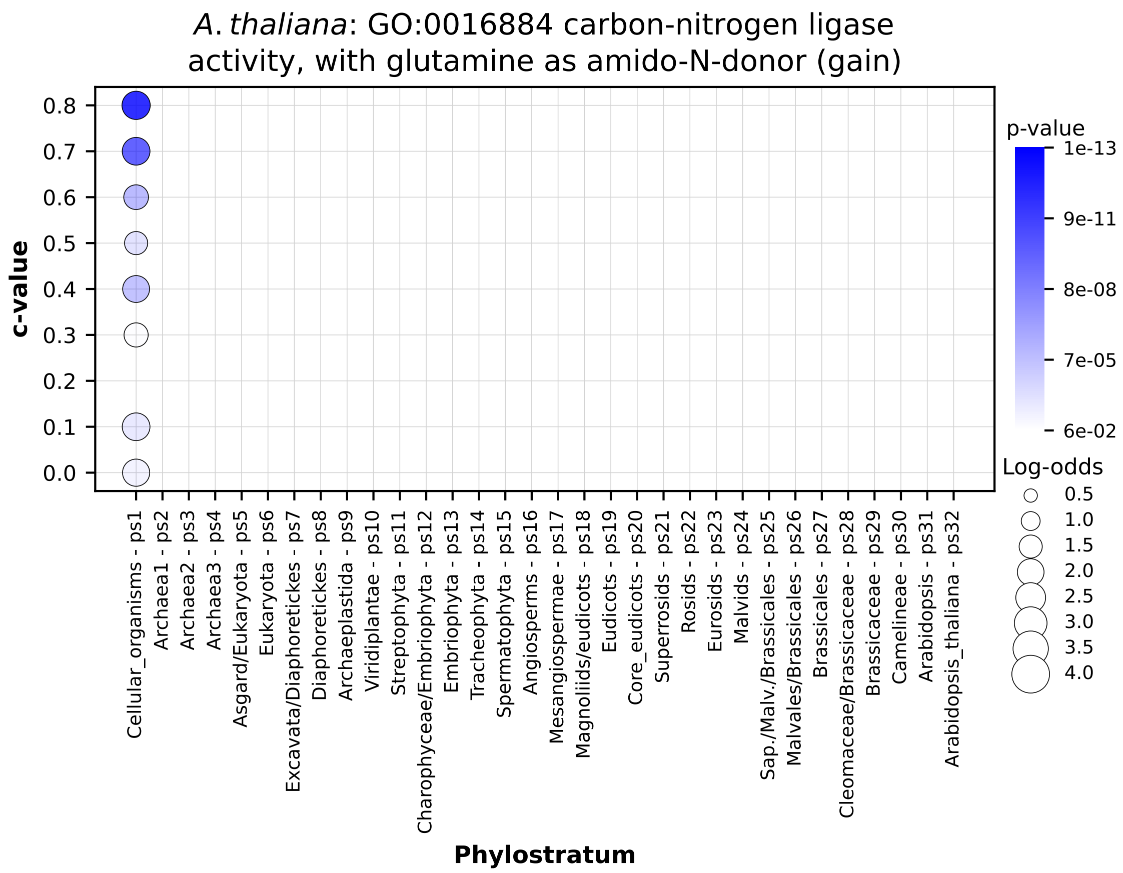

A. thaliana: GO:0016884 carbon-nitrogen ligase activity, with glutamine as amido-N-donor (gain)
c-value
0.8
0.7
0.6
0.5
0.4
0.3
0.2
0.1
0.0
p-value
1e-13
9e-11
8e-08
7e-05
6e-02
Log-odds
0.5
1.0
1.5
2.0
2.5
3.0
3.5
4.0
Cellular_organisms - ps1
Archaea1 - ps2
Archaea2 - ps3
Archaea3 - ps4
Asgard/Eukaryota - ps5
Eukaryota - ps6
Excavata/Diaphoretickes - ps7
Diaphoretickes - ps8
Archaeplastida - ps9
Viridiplantae - ps10
Streptophyta - ps11
Charophyceae/Embriophyta - ps12
Embriophyta - ps13
Tracheophyta - ps14
Spermatophyta - ps15
Angiosperms - ps16
Mesangiospermae - ps17
Magnoliids/eudicots - ps18
Eudicots - ps19
Core_eudicots - ps20
Superrosids - ps21
Rosids - ps22
Eurosids - ps23
Malvids - ps24
Sap./Malv./Brassicales - ps25
Malvales/Brassicales - ps26
Brassicales - ps27
Cleomaceae/Brassicaceae - ps28
Brassicaceae - ps29
Camelineae - ps30
Arabidopsis - ps31
Arabidopsis_thaliana - ps32
Phylostratum

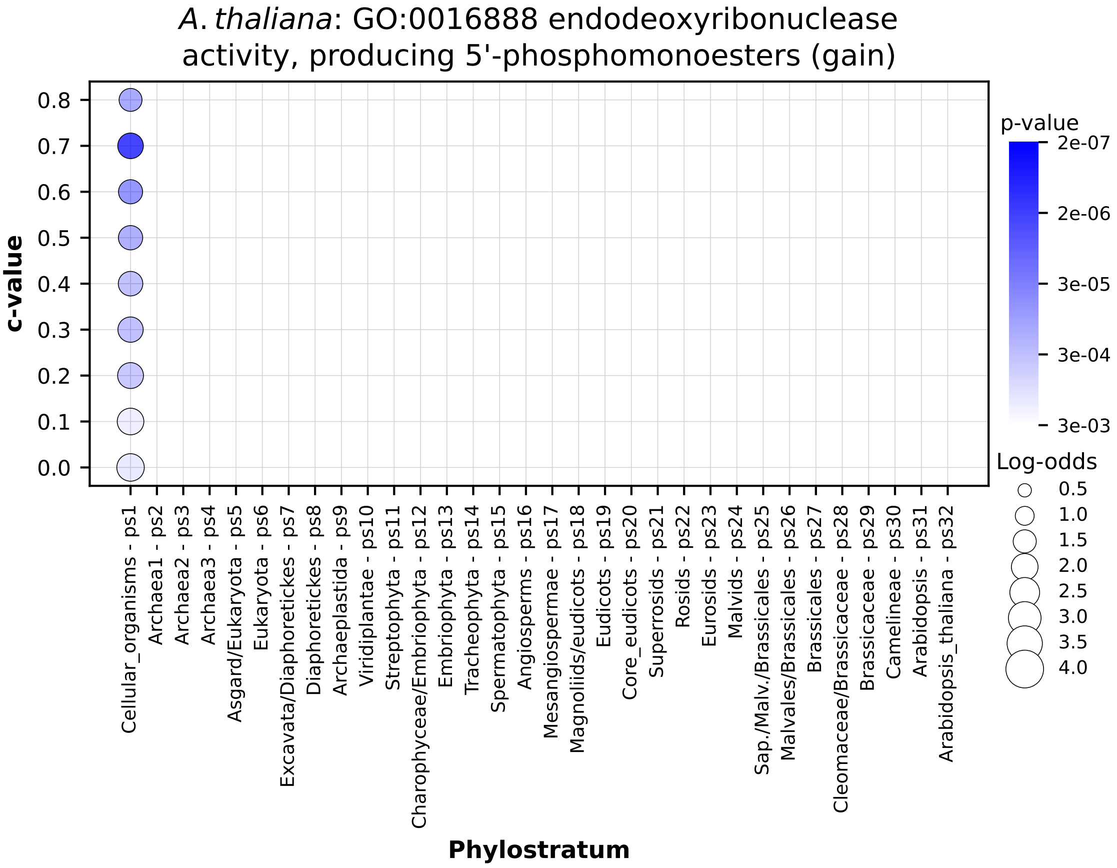

A. thaliana: GO:0016888 endodeoxyribonuclease activity, producing 5'-phosphomonoesters (gain)
c-value
0.8
0.7
0.6
0.5
0.4
0.3
0.2
0.1
0.0
p-value
2e-07
2e-06
3e-05
3e-04
3e-03
Log-odds
0.5
1.0
1.5
2.0
2.5
3.0
3.5
4.0
Cellular_organisms - ps1
Archaea1 - ps2
Archaea2 - ps3
Archaea3 - ps4
Asgard/Eukaryota - ps5
Eukaryota - ps6
Excavata/Diaphoretickes - ps7
Diaphoretickes - ps8
Archaeplastida - ps9
Viridiplantae - ps10
Streptophyta - ps11
Charophyceae/Embriophyta - ps12
Embriophyta - ps13
Tracheophyta - ps14
Spermatophyta - ps15
Angiosperms - ps16
Mesangiospermae - ps17
Magnoliids/eudicots - ps18
Eudicots - ps19
Core_eudicots - ps20
Superrosids - ps21
Rosids - ps22
Eurosids - ps23
Malvids - ps24
Sap./Malv./Brassicales - ps25
Malvales/Brassicales - ps26
Brassicales - ps27
Cleomaceae/Brassicaceae - ps28
Brassicaceae - ps29
Camelineae - ps30
Arabidopsis - ps31
Arabidopsis_thaliana - ps32
Phylostratum

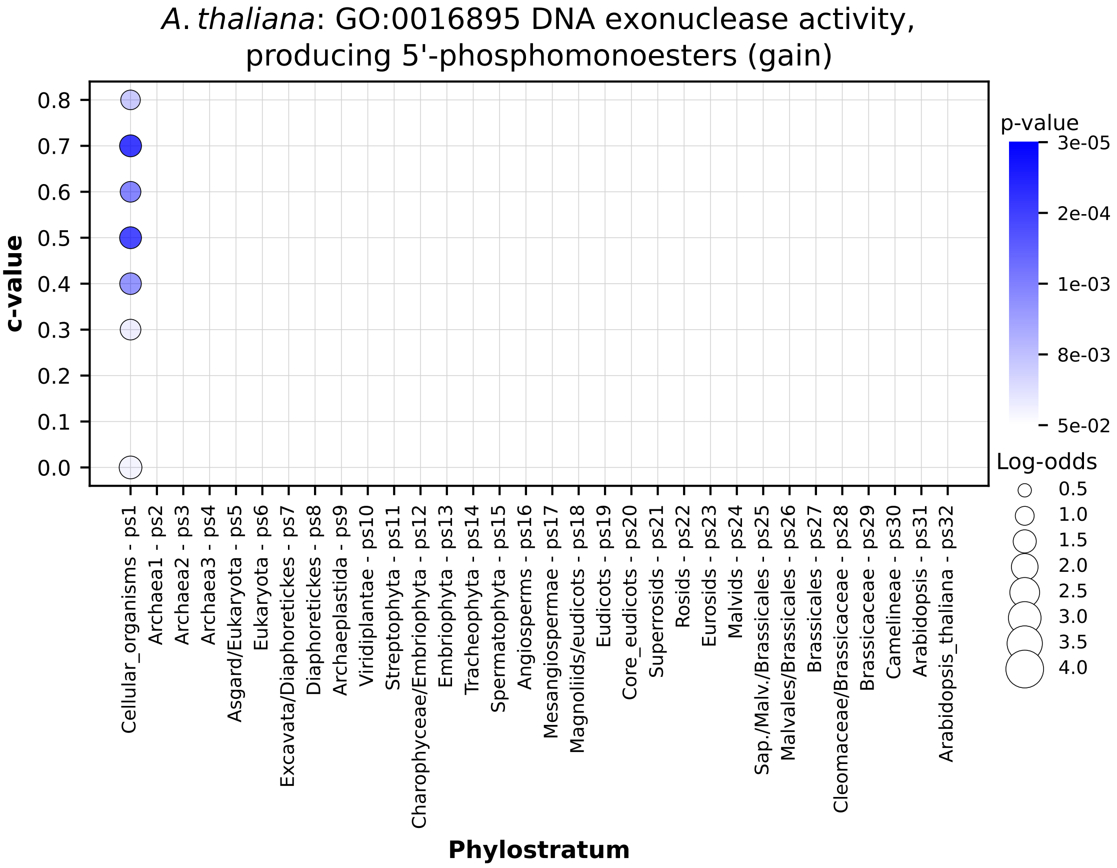

A. thaliana: GO:0016895 DNA exonuclease activity, producing 5'-phosphomonoesters (gain)
c-value
0.8
0.7
0.6
0.5
0.4
0.3
0.2
0.1
0.0
p-value
3e-05
2e-04
1e-03
8e-03
5e-02
Log-odds
0.5
1.0
1.5
2.0
2.5
3.0
3.5
4.0
Cellular_organisms - ps1
Archaea1 - ps2
Archaea2 - ps3
Archaea3 - ps4
Asgard/Eukaryota - ps5
Eukaryota - ps6
Excavata/Diaphoretickes - ps7
Diaphoretickes - ps8
Archaeplastida - ps9
Viridiplantae - ps10
Streptophyta - ps11
Charophyceae/Embriophyta - ps12
Embriophyta - ps13
Tracheophyta - ps14
Spermatophyta - ps15
Angiosperms - ps16
Mesangiospermae - ps17
Magnoliids/eudicots - ps18
Eudicots - ps19
Core_eudicots - ps20
Superrosids - ps21
Rosids - ps22
Eurosids - ps23
Malvids - ps24
Sap./Malv./Brassicales - ps25
Malvales/Brassicales - ps26
Brassicales - ps27
Cleomaceae/Brassicaceae - ps28
Brassicaceae - ps29
Camelineae - ps30
Arabidopsis - ps31
Arabidopsis_thaliana - ps32
Phylostratum

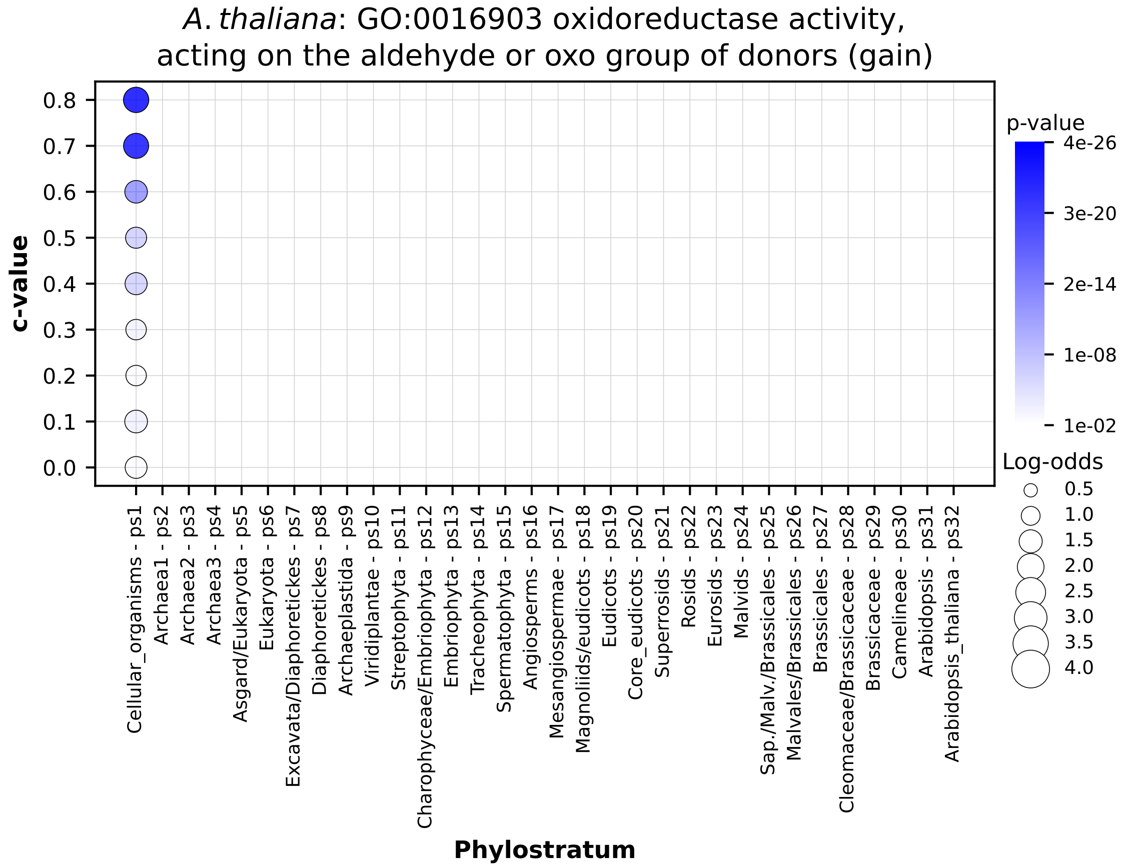

A. thaliana: GO:0016903 oxidoreductase activity, acting on the aldehyde or oxo group of donors (gain)
c-value
0.8
0.7
0.6
0.5
0.4
0.3
0.2
0.1
0.0
p-value
4e-26
3e-20
2e-14
1e-08
1e-02
Log-odds
0.5
1.0
1.5
2.0
2.5
3.0
3.5
4.0
Cellular_organisms - ps1
Archaea1 - ps2
Archaea2 - ps3
Archaea3 - ps4
Asgard/Eukaryota - ps5
Eukaryota - ps6
Excavata/Diaphoretickes - ps7
Diaphoretickes - ps8
Archaeplastida - ps9
Viridiplantae - ps10
Streptophyta - ps11
Charophyceae/Embriophyta - ps12
Embriophyta - ps13
Tracheophyta - ps14
Spermatophyta - ps15
Angiosperms - ps16
Mesangiospermae - ps17
Magnoliids/eudicots - ps18
Eudicots - ps19
Core_eudicots - ps20
Superrosids - ps21
Rosids - ps22
Eurosids - ps23
Malvids - ps24
Sap./Malv./Brassicales - ps25
Malvales/Brassicales - ps26
Brassicales - ps27
Cleomaceae/Brassicaceae - ps28
Brassicaceae - ps29
Camelineae - ps30
Arabidopsis - ps31
Arabidopsis_thaliana - ps32
Phylostratum

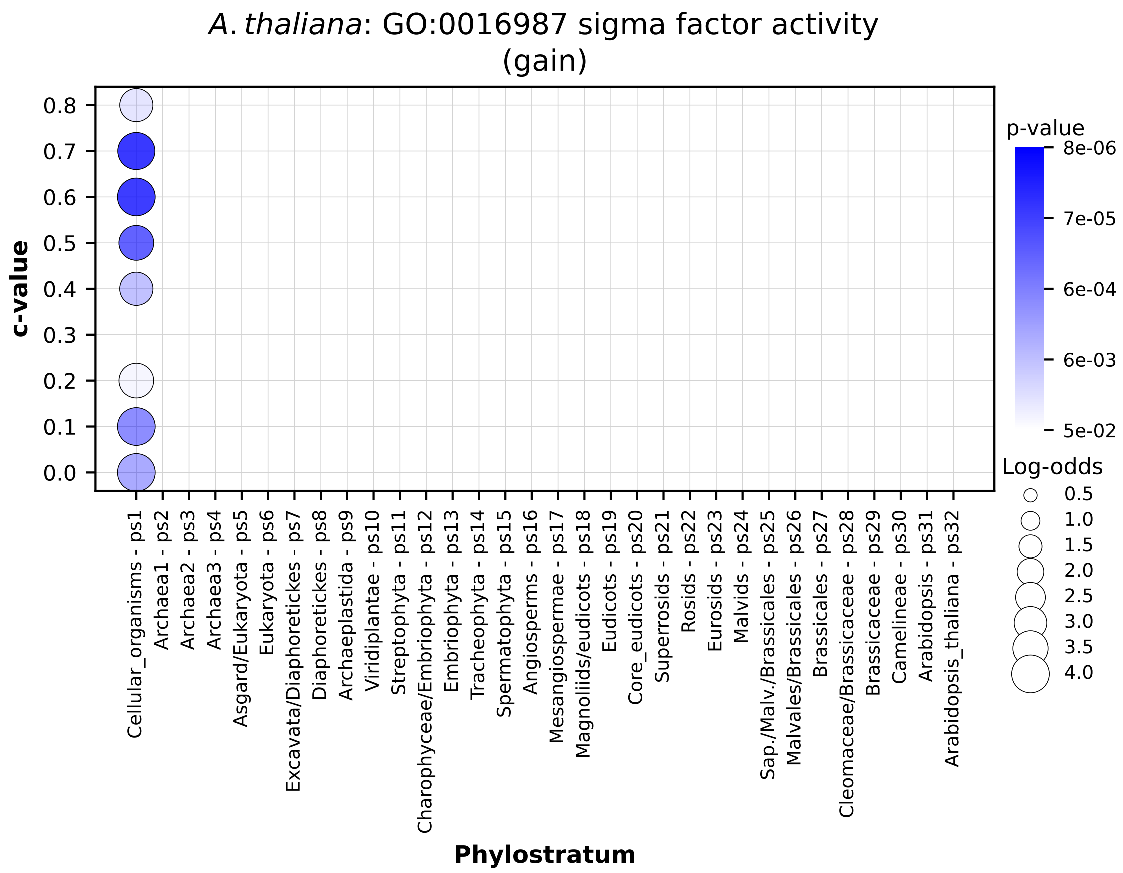
A. thaliana: GO:0016987 sigma factor activity
(gain)
c-value
0.8
0.7
0.6
0.5
0.4
0.3
0.2
0.1
0.0
p-value
8e-06
7e-05
6e-04
6e-03
5e-02
Log-odds
0.5
1.0
1.5
2.0
2.5
3.0
3.5
4.0
Cellular_organisms - ps1
Archaea1 - ps2
Archaea2 - ps3
Archaea3 - ps4
Asgard/Eukaryota - ps5
Eukaryota - ps6
Excavata/Diaphoretickes - ps7
Diaphoretickes - ps8
Archaeplastida - ps9
Viridiplantae - ps10
Streptophyta - ps11
Charophyceae/Embriophyta - ps12
Embriophyta - ps13
Tracheophyta - ps14
Spermatophyta - ps15
Angiosperms - ps16
Mesangiospermae - ps17
Magnoliids/eudicots - ps18
Eudicots - ps19
Core_eudicots - ps20
Superrosids - ps21
Rosids - ps22
Eurosids - ps23
Malvids - ps24
Sap./Malv./Brassicales - ps25
Malvales/Brassicales - ps26
Brassicales - ps27
Cleomaceae/Brassicaceae - ps28
Brassicaceae - ps29
Camelineae - ps30
Arabidopsis - ps31
Arabidopsis_thaliana - ps32
Phylostratum

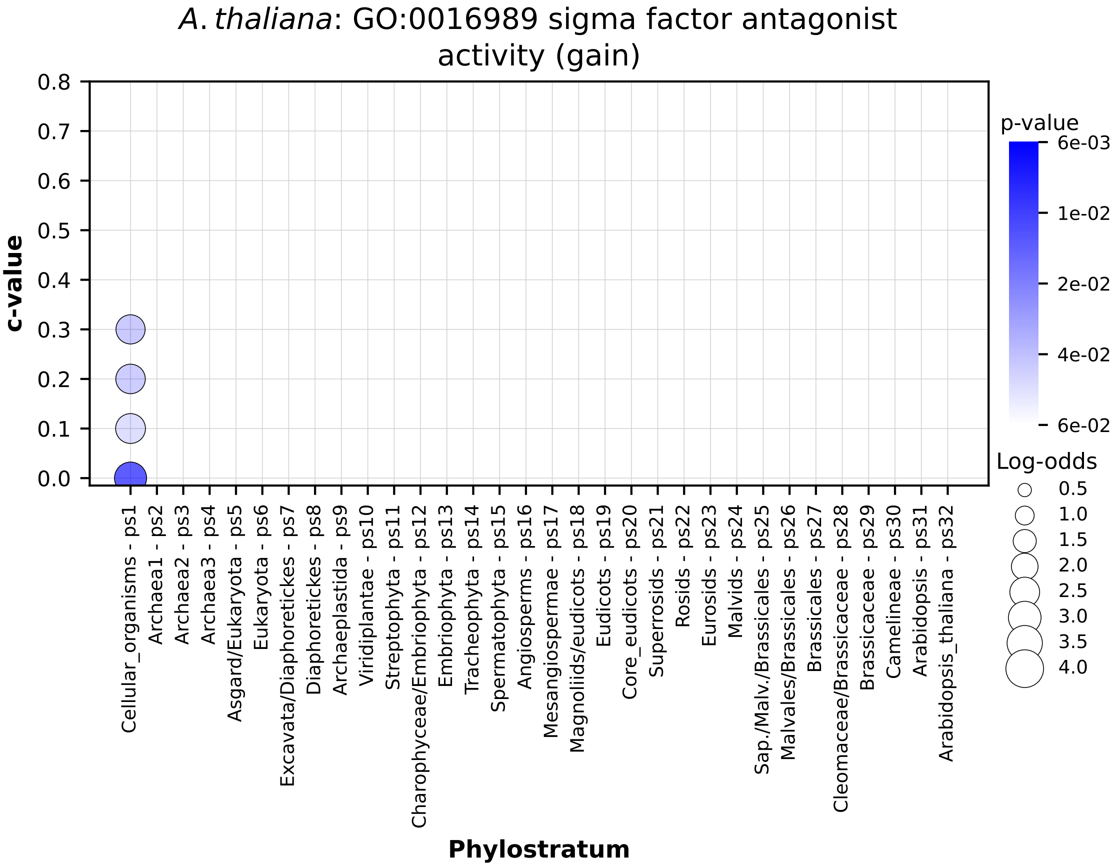

A. thaliana: GO:0016989 sigma factor antagonist activity (gain)
c-value
0.8
0.7
0.6
0.5
0.4
0.3
0.2
0.1
0.0
p-value
6e-03
1e-02
2e-02
4e-02
6e-02
Log-odds
0.5
1.0
1.5
2.0
2.5
3.0
3.5
4.0
Cellular_organisms - ps1
Archaea1 - ps2
Archaea2 - ps3
Archaea3 - ps4
Asgard/Eukaryota - ps5
Eukaryota - ps6
Excavata/Diaphoretickes - ps7
Diaphoretickes - ps8
Archaeplastida - ps9
Viridiplantae - ps10
Streptophyta - ps11
Charophyceae/Embriophyta - ps12
Embriophyta - ps13
Tracheophyta - ps14
Spermatophyta - ps15
Angiosperms - ps16
Mesangiospermae - ps17
Magnoliids/eudicots - ps18
Eudicots - ps19
Core_eudicots - ps20
Superrosids - ps21
Rosids - ps22
Eurosids - ps23
Malvids - ps24
Sap./Malv./Brassicales - ps25
Malvales/Brassicales - ps26
Brassicales - ps27
Cleomaceae/Brassicaceae - ps28
Brassicaceae - ps29
Camelineae - ps30
Arabidopsis - ps31
Arabidopsis_thaliana - ps32
Phylostratum

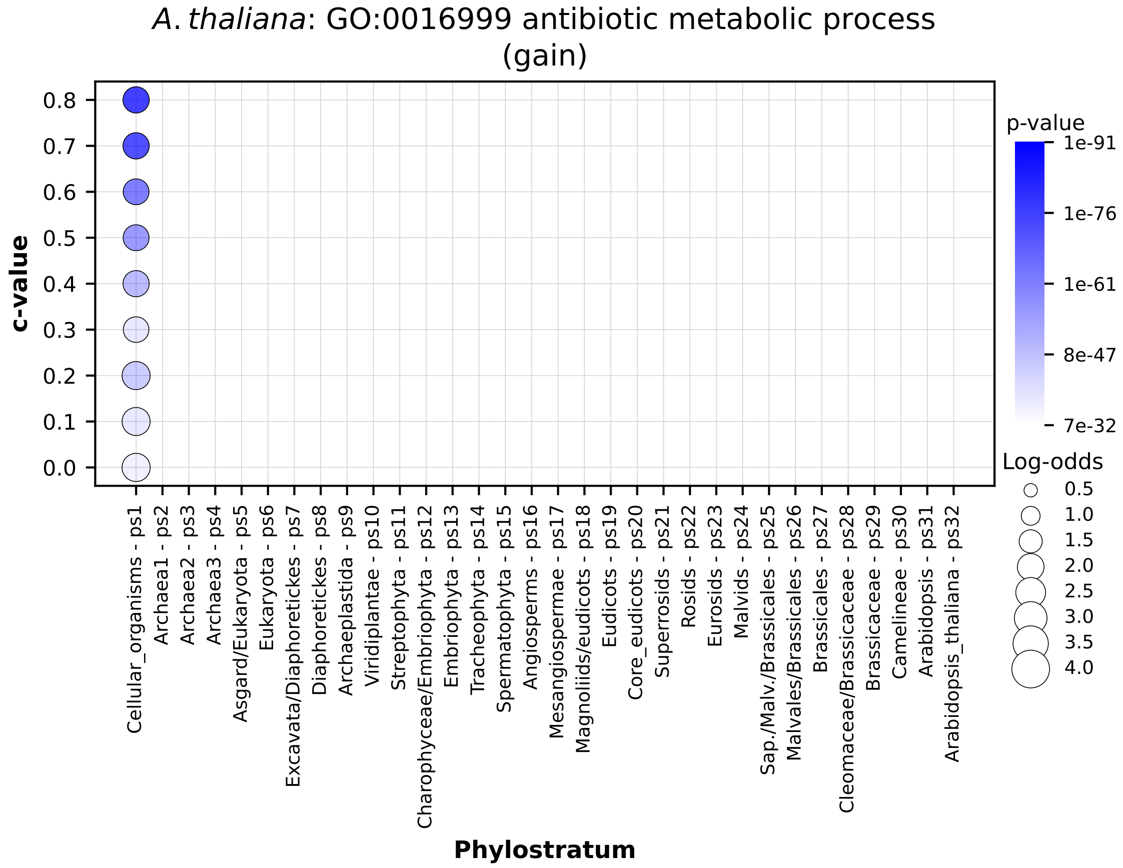
A. thaliana: GO:0016999 antibiotic metabolic process
(gain)
c-value
0.8
0.7
0.6
0.5
0.4
0.3
0.2
0.1
0.0
p-value
1e-91
1e-76
1e-61
8e-47
7e-32
Log-odds
0.5
1.0
1.5
2.0
2.5
3.0
3.5
4.0
Cellular_organisms - ps1
Archaea1 - ps2
Archaea2 - ps3
Archaea3 - ps4
Asgard/Eukaryota - ps5
Eukaryota - ps6
Excavata/Diaphoretickes - ps7
Diaphoretickes - ps8
Archaeplastida - ps9
Viridiplantae - ps10
Streptophyta - ps11
Charophyceae/Embriophyta - ps12
Embriophyta - ps13
Tracheophyta - ps14
Spermatophyta - ps15
Angiosperms - ps16
Mesangiospermae - ps17
Magnoliids/eudicots - ps18
Eudicots - ps19
Core_eudicots - ps20
Superrosids - ps21
Rosids - ps22
Eurosids - ps23
Malvids - ps24
Sap./Malv./Brassicales - ps25
Malvales/Brassicales - ps26
Brassicales - ps27
Cleomaceae/Brassicaceae - ps28
Brassicaceae - ps29
Camelineae - ps30
Arabidopsis - ps31
Arabidopsis_thaliana - ps32
Phylostratum

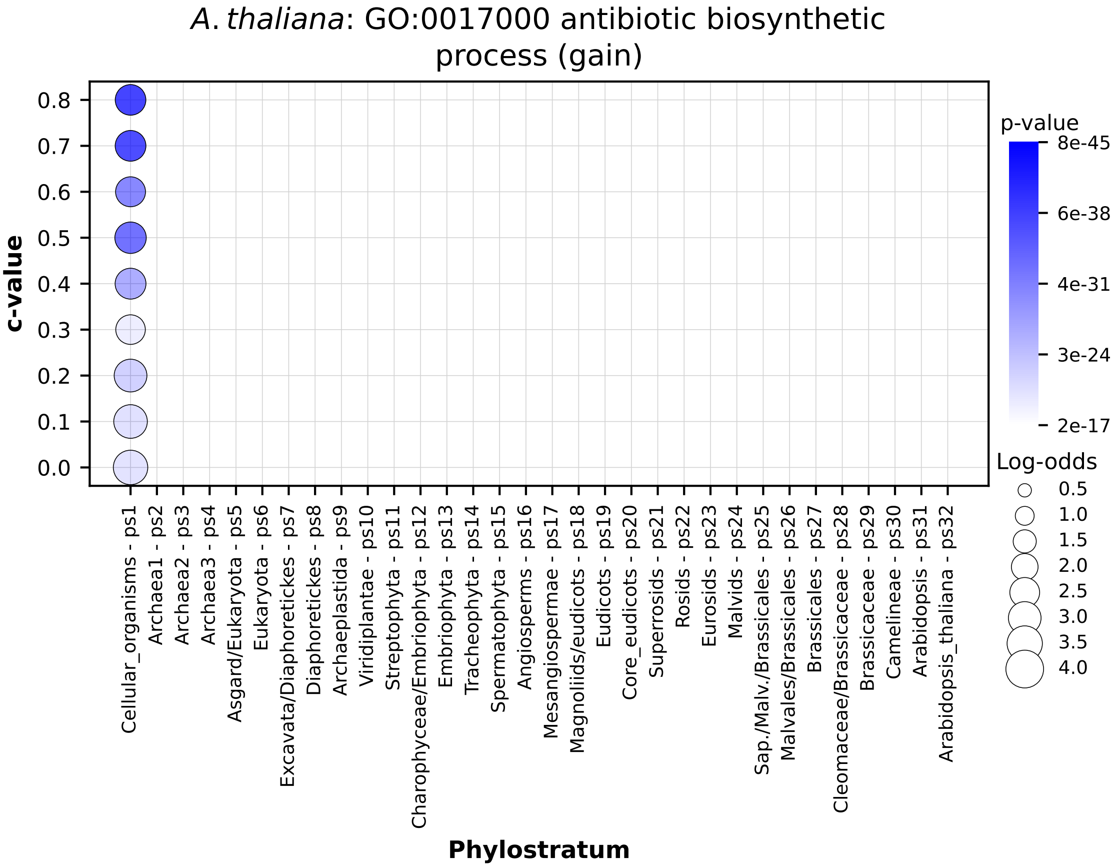

A. thaliana: GO:0017000 antibiotic biosynthetic process (gain)
c-value
0.8
0.7
0.6
0.5
0.4
0.3
0.2
0.1
0.0
p-value
8e-45
6e-38
4e-31
3e-24
2e-17
Log-odds
0.5
1.0
1.5
2.0
2.5
3.0
3.5
4.0
Cellular_organisms - ps1
Archaea1 - ps2
Archaea2 - ps3
Archaea3 - ps4
Asgard/Eukaryota - ps5
Eukaryota - ps6
Excavata/Diaphoretickes - ps7
Diaphoretickes - ps8
Archaeplastida - ps9
Viridiplantae - ps10
Streptophyta - ps11
Charophyceae/Embriophyta - ps12
Embriophyta - ps13
Tracheophyta - ps14
Spermatophyta - ps15
Angiosperms - ps16
Mesangiospermae - ps17
Magnoliids/eudicots - ps18
Eudicots - ps19
Core_eudicots - ps20
Superrosids - ps21
Rosids - ps22
Eurosids - ps23
Malvids - ps24
Sap./Malv./Brassicales - ps25
Malvales/Brassicales - ps26
Brassicales - ps27
Cleomaceae/Brassicaceae - ps28
Brassicaceae - ps29
Camelineae - ps30
Arabidopsis - ps31
Arabidopsis_thaliana - ps32
Phylostratum

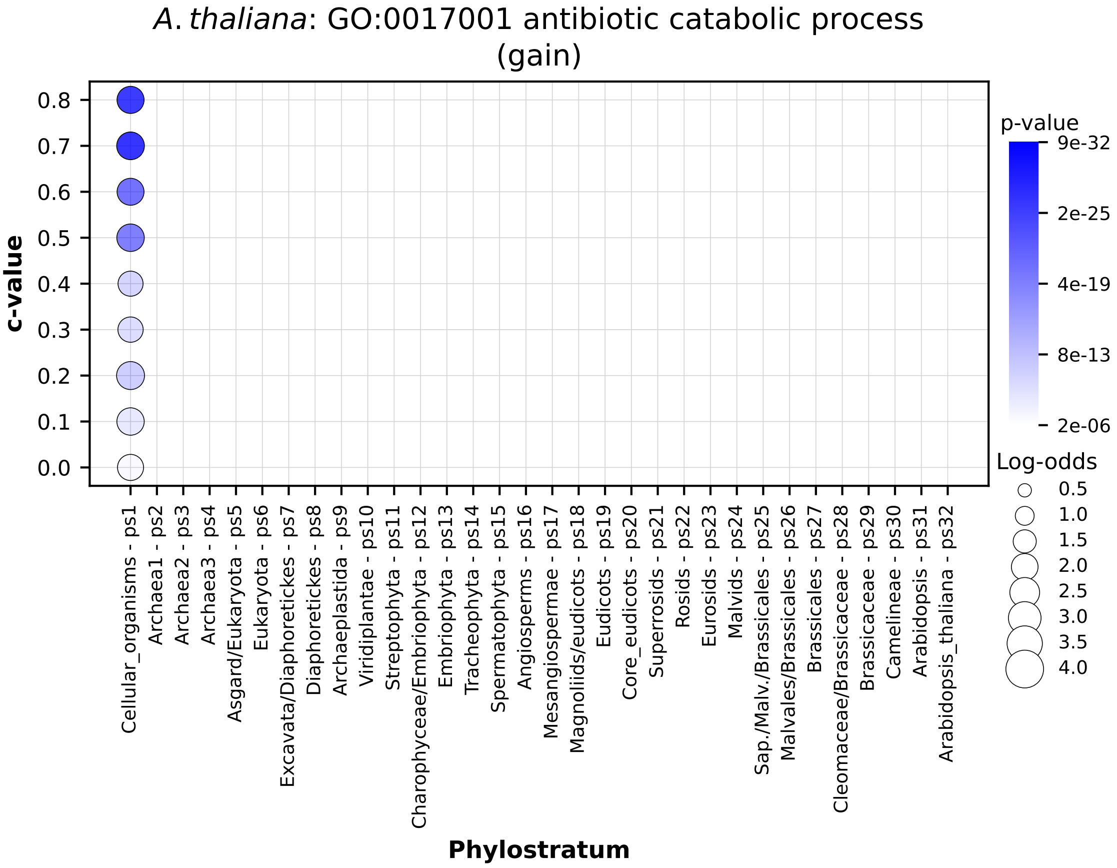
A. thaliana: GO:0017001 antibiotic catabolic process
(gain)
c-value
0.0
0.1
0.2
0.3
0.4
0.5
0.6
0.7
0.8
p-value
9e-32
2e-25
4e-19
8e-13
2e-06
Log-odds
0.5
1.0
1.5
2.0
2.5
3.0
3.5
4.0
Cellular_organisms - ps1
Archaea1 - ps2
Archaea2 - ps3
Archaea3 - ps4
Asgard/Eukaryota - ps5
Eukaryota - ps6
Excavata/Diaphoretickes - ps7
Diaphoretickes - ps8
Archaeplastida - ps9
Viridiplantae - ps10
Streptophyta - ps11
Charophyceae/Embriophyta - ps12
Embriophyta - ps13
Tracheophyta - ps14
Spermatophyta - ps15
Angiosperms - ps16
Mesangiospermae - ps17
Magnoliids/eudicots - ps18
Eudicots - ps19
Core_eudicots - ps20
Superrosids - ps21
Rosids - ps22
Eurosids - ps23
Malvids - ps24
Sap./Malv./Brassicales - ps25
Malvales/Brassicales - ps26
Brassicales - ps27
Cleomaceae/Brassicaceae - ps28
Brassicaceae - ps29
Camelineae - ps30
Arabidopsis - ps31
Arabidopsis_thaliana - ps32
Phylostratum

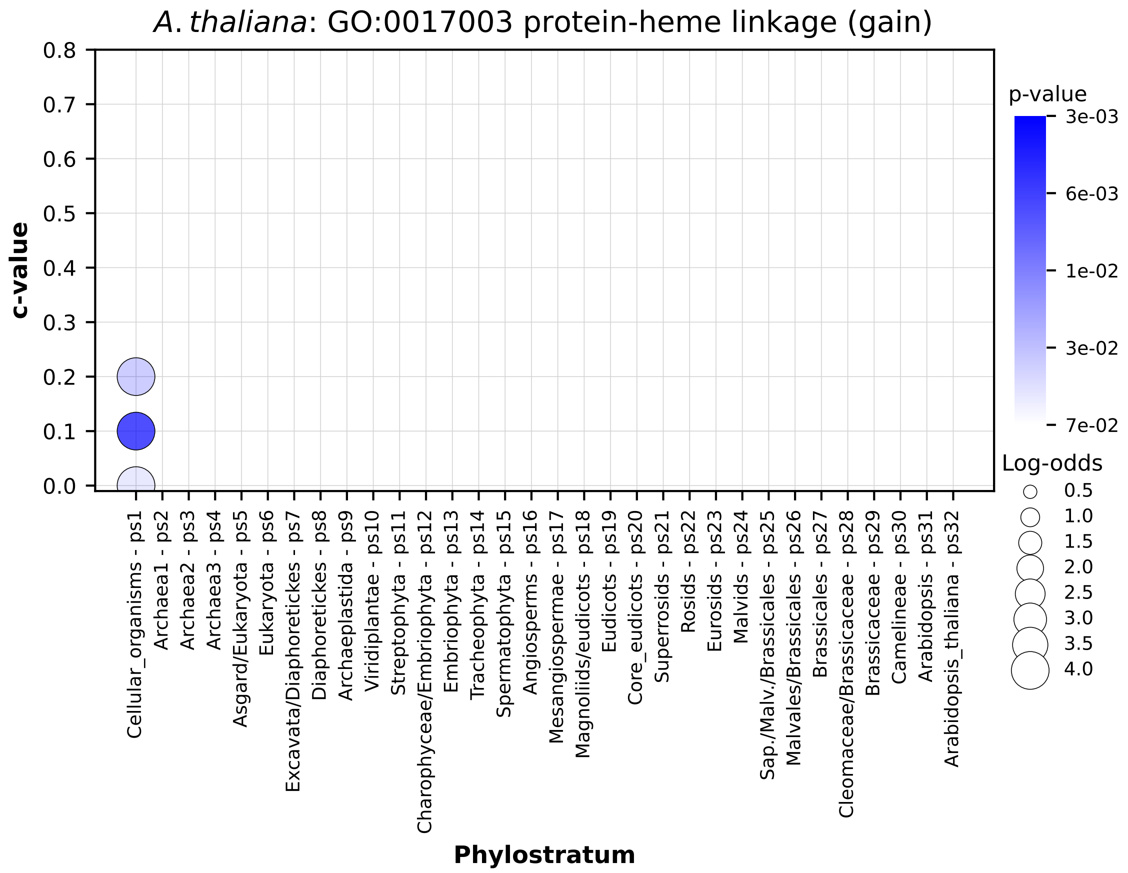
A. thaliana: GO:0017003 protein-heme linkage (gain)
c-value
0.8
0.7
0.6
0.5
0.4
0.3
0.2
0.1
0.0
Phylostratum
Cellular_organisms - ps1
Archaea1 - ps2
Archaea2 - ps3
Archaea3 - ps4
Asgard/Eukaryota - ps5
Eukaryota - ps6
Excavata/Diaphoretickes - ps7
Diaphoretickes - ps8
Archaeplastida - ps9
Viridiplantae - ps10
Streptophyta - ps11
Charophyceae/Embriophyta - ps12
Embriophyta - ps13
Tracheophyta - ps14
Spermatophyta - ps15
Angiosperms - ps16
Mesangiospermae - ps17
Magnoliids/eudicots - ps18
Eudicots - ps19
Core_eudicots - ps20
Superrosids - ps21
Rosids - ps22
Eurosids - ps23
Malvids - ps24
Sap./Malv./Brassicales - ps25
Malvales/Brassicales - ps26
Brassicales - ps27
Cleomaceae/Brassicaceae - ps28
Brassicaceae - ps29
Camelineae - ps30
Arabidopsis - ps31
Arabidopsis_thaliana - ps32
p-value
3e-03
6e-03
1e-02
3e-02
7e-02
Log-odds
0.5
1.0
1.5
2.0
2.5
3.0
3.5
4.0

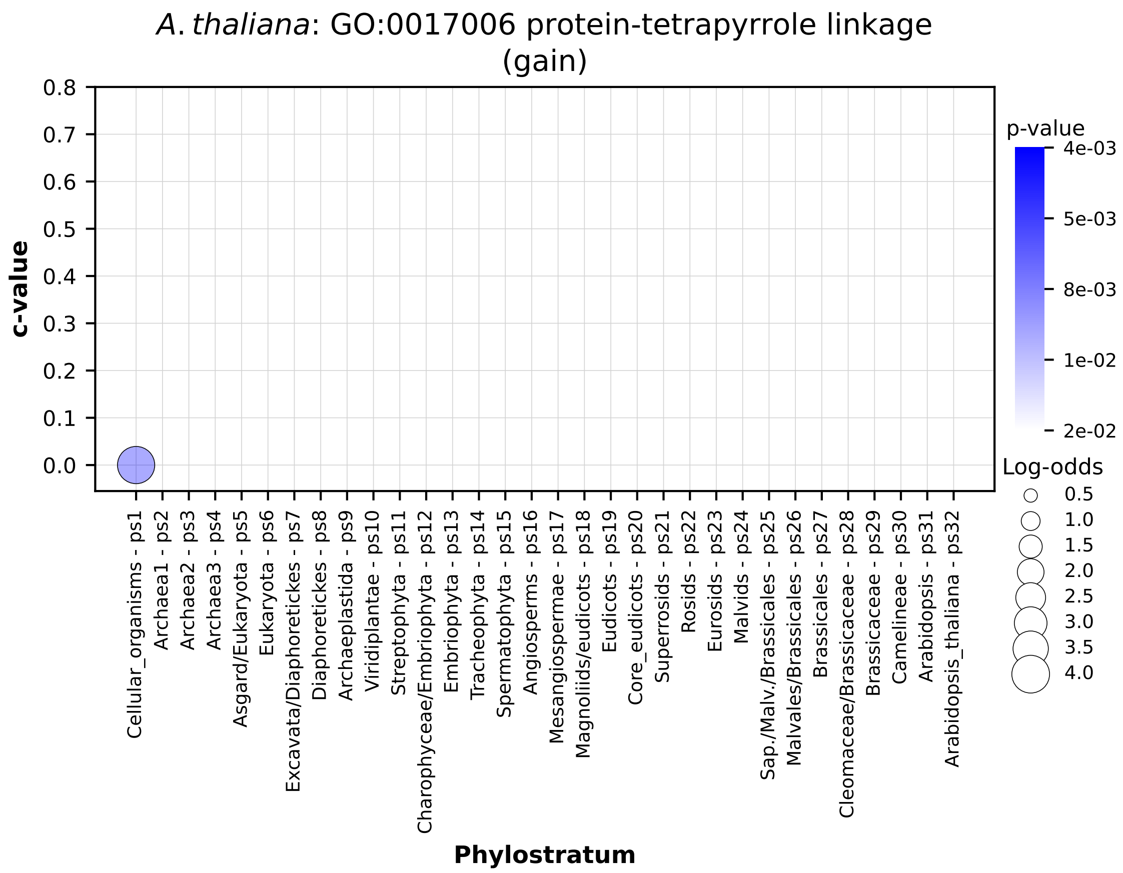
A. thaliana: GO:0017006 protein-tetrapyrrole linkage
(gain)
c-value
0.8
0.7
0.6
0.5
0.4
0.3
0.2
0.1
0.0
Phylostratum
Cellular_organisms - ps1
Archaea1 - ps2
Archaea2 - ps3
Archaea3 - ps4
Asgard/Eukaryota - ps5
Eukaryota - ps6
Excavata/Diaphoretickes - ps7
Diaphoretickes - ps8
Archaeplastida - ps9
Viridiplantae - ps10
Streptophyta - ps11
Charophyceae/Embriophyta - ps12
Embriophyta - ps13
Tracheophyta - ps14
Spermatophyta - ps15
Angiosperms - ps16
Mesangiospermae - ps17
Magnoliids/eudicots - ps18
Eudicots - ps19
Core_eudicots - ps20
Superrosids - ps21
Rosids - ps22
Eurosids - ps23
Malvids - ps24
Sap./Malv./Brassicales - ps25
Malvales/Brassicales - ps26
Brassicales - ps27
Cleomaceae/Brassicaceae - ps28
Brassicaceae - ps29
Camelineae - ps30
Arabidopsis - ps31
Arabidopsis_thaliana - ps32
p-value
4e-03
5e-03
8e-03
1e-02
2e-02
Log-odds
0.5
1.0
1.5
2.0
2.5
3.0
3.5
4.0

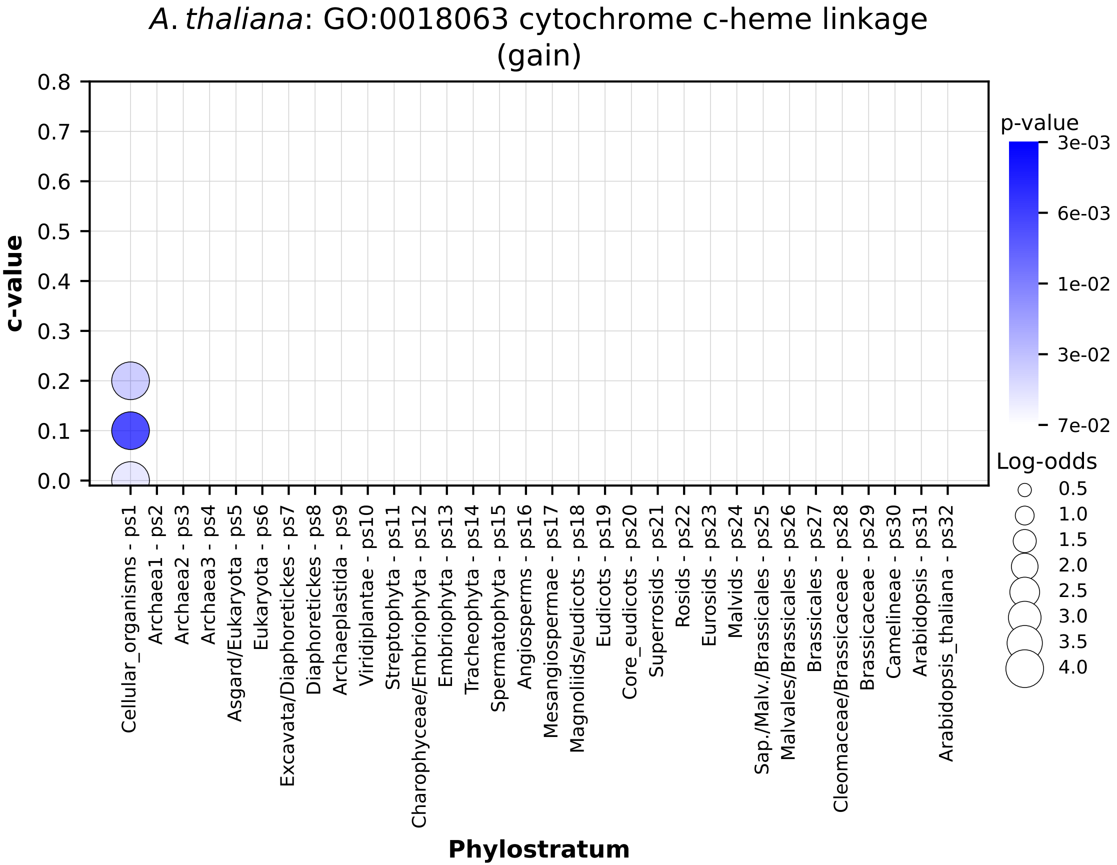

A. thaliana: GO:0018063 cytochrome c-heme linkage (gain)
c-value
0.8
0.7
0.6
0.5
0.4
0.3
0.2
0.1
0.0
p-value
3e-03
6e-03
1e-02
3e-02
7e-02
Log-odds
0.5
1.0
1.5
2.0
2.5
3.0
3.5
4.0
Cellular_organisms - ps1
Archaea1 - ps2
Archaea2 - ps3
Archaea3 - ps4
Asgard/Eukaryota - ps5
Eukaryota - ps6
Excavata/Diaphoretickes - ps7
Diaphoretickes - ps8
Archaeplastida - ps9
Viridiplantae - ps10
Streptophyta - ps11
Charophyceae/Embriophyta - ps12
Embriophyta - ps13
Tracheophyta - ps14
Spermatophyta - ps15
Angiosperms - ps16
Mesangiospermae - ps17
Magnoliids/eudicots - ps18
Eudicots - ps19
Core_eudicots - ps20
Superrosids - ps21
Rosids - ps22
Eurosids - ps23
Malvids - ps24
Sap./Malv./Brassicales - ps25
Malvales/Brassicales - ps26
Brassicales - ps27
Cleomaceae/Brassicaceae - ps28
Brassicaceae - ps29
Camelineae - ps30
Arabidopsis - ps31
Arabidopsis_thaliana - ps32
Phylostratum

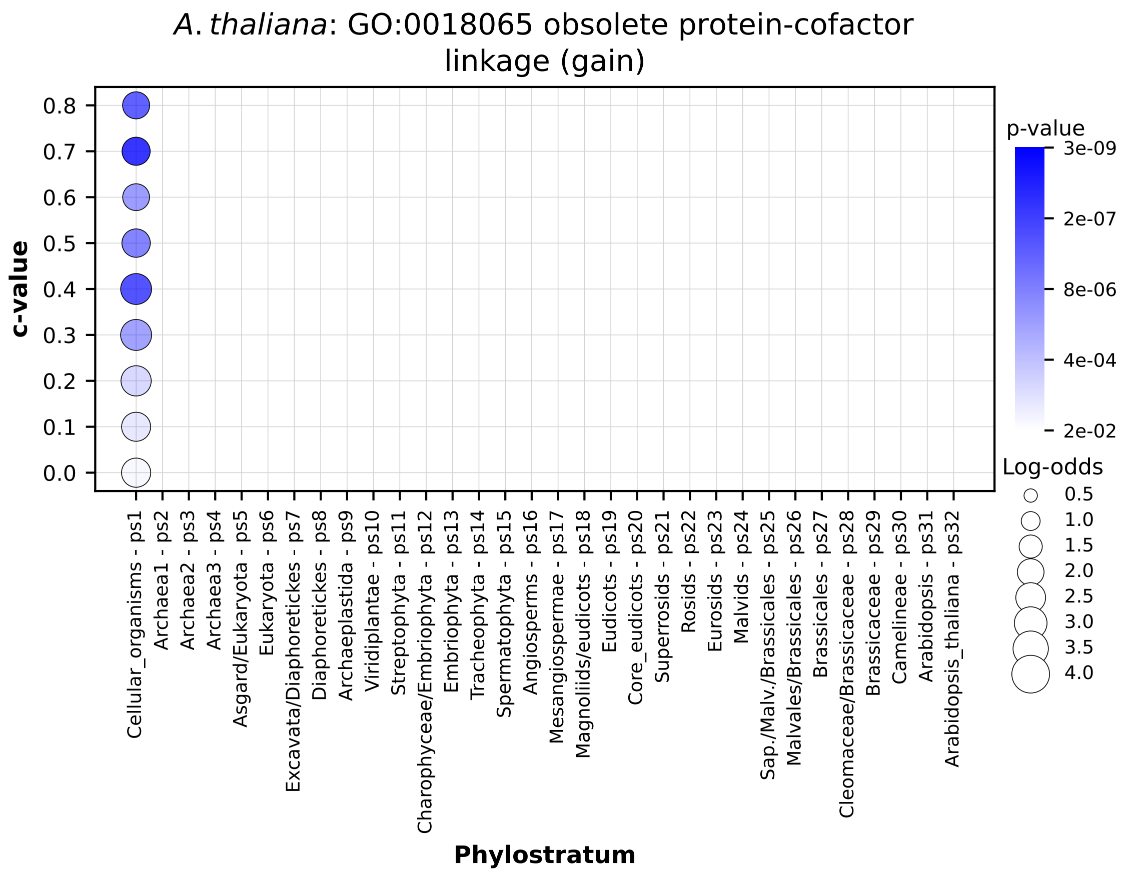

A. thaliana: GO:0018065 obsolete protein-cofactor linkage (gain)
c-value
0.8
0.7
0.6
0.5
0.4
0.3
0.2
0.1
0.0
p-value
3e-09
2e-07
8e-06
4e-04
2e-02
Log-odds
0.5
1.0
1.5
2.0
2.5
3.0
3.5
4.0
Cellular_organisms - ps1
Archaea1 - ps2
Archaea2 - ps3
Archaea3 - ps4
Asgard/Eukaryota - ps5
Eukaryota - ps6
Excavata/Diaphoretickes - ps7
Diaphoretickes - ps8
Archaeplastida - ps9
Viridiplantae - ps10
Streptophyta - ps11
Charophyceae/Embriophyta - ps12
Embriophyta - ps13
Tracheophyta - ps14
Spermatophyta - ps15
Angiosperms - ps16
Mesangiospermae - ps17
Magnoliids/eudicots - ps18
Eudicots - ps19
Core_eudicots - ps20
Superrosids - ps21
Rosids - ps22
Eurosids - ps23
Malvids - ps24
Sap./Malv./Brassicales - ps25
Malvales/Brassicales - ps26
Brassicales - ps27
Cleomaceae/Brassicaceae - ps28
Brassicaceae - ps29
Camelineae - ps30
Arabidopsis - ps31
Arabidopsis_thaliana - ps32
Phylostratum

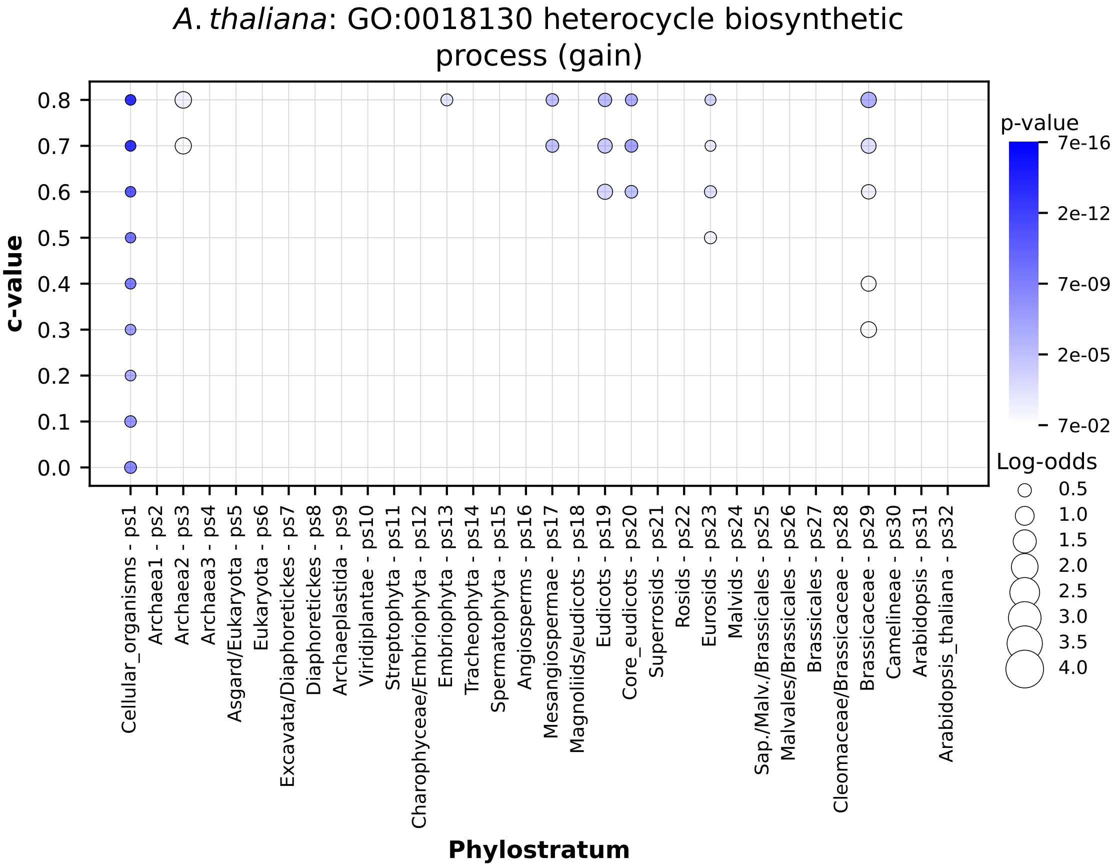
A. thaliana: GO:0018130 heterocycle biosynthetic process (gain)
c-value
0.8
0.7
0.6
0.5
0.4
0.3
0.2
0.1
0.0
p-value
7e-16
2e-12
7e-09
2e-05
7e-02
Log-odds
0.5
1.0
1.5
2.0
2.5
3.0
3.5
4.0
Cellular_organisms - ps1
Archaea1 - ps2
Archaea2 - ps3
Archaea3 - ps4
Asgard/Eukaryota - ps5
Eukaryota - ps6
Excavata/Diaphoretickes - ps7
Diaphoretickes - ps8
Archaeplastida - ps9
Viridiplantae - ps10
Streptophyta - ps11
Charophyceae/Embriophyta - ps12
Embriophyta - ps13
Tracheophyta - ps14
Spermatophyta - ps15
Angiosperms - ps16
Mesangiospermae - ps17
Magnoliids/eudicots - ps18
Eudicots - ps19
Core_eudicots - ps20
Superrosids - ps21
Rosids - ps22
Eurosids - ps23
Malvids - ps24
Sap./Malv./Brassicales - ps25
Malvales/Brassicales - ps26
Brassicales - ps27
Cleomaceae/Brassicaceae - ps28
Brassicaceae - ps29
Camelineae - ps30
Arabidopsis - ps31
Arabidopsis_thaliana - ps32
Phylostratum

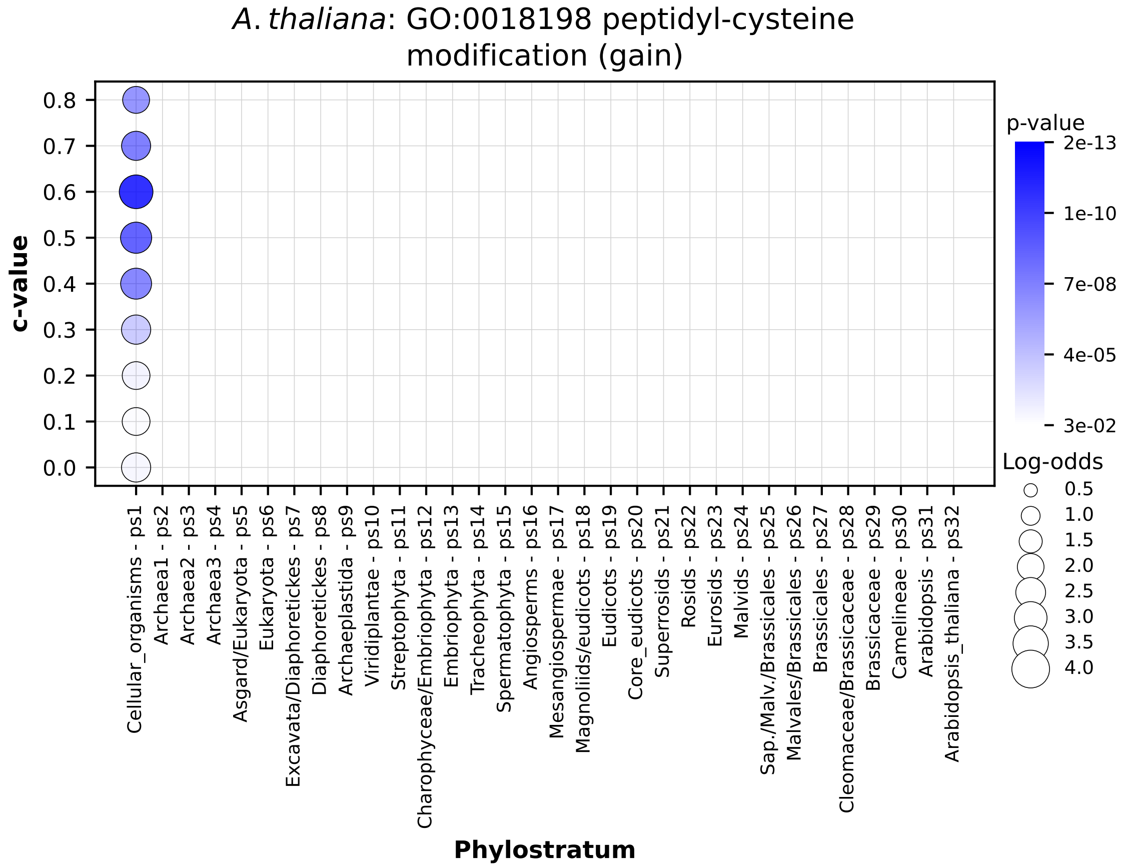

A. thaliana: GO:0018198 peptidyl-cysteine modification (gain)
c-value
0.8
0.7
0.6
0.5
0.4
0.3
0.2
0.1
0.0
Phylostratum
Cellular_organisms - ps1
Archaea1 - ps2
Archaea2 - ps3
Archaea3 - ps4
Asgard/Eukaryota - ps5
Eukaryota - ps6
Excavata/Diaphoretickes - ps7
Diaphoretickes - ps8
Archaeplastida - ps9
Viridiplantae - ps10
Streptophyta - ps11
Charophyceae/Embriophyta - ps12
Embriophyta - ps13
Tracheophyta - ps14
Spermatophyta - ps15
Angiosperms - ps16
Mesangiospermae - ps17
Magnoliids/eudicots - ps18
Eudicots - ps19
Core_eudicots - ps20
Superrosids - ps21
Rosids - ps22
Eurosids - ps23
Malvids - ps24
Sap./Malv./Brassicales - ps25
Malvales/Brassicales - ps26
Brassicales - ps27
Cleomaceae/Brassicaceae - ps28
Brassicaceae - ps29
Camelineae - ps30
Arabidopsis - ps31
Arabidopsis_thaliana - ps32
p-value
2e-13
1e-10
7e-08
4e-05
3e-02
Log-odds
0.5
1.0
1.5
2.0
2.5
3.0
3.5
4.0

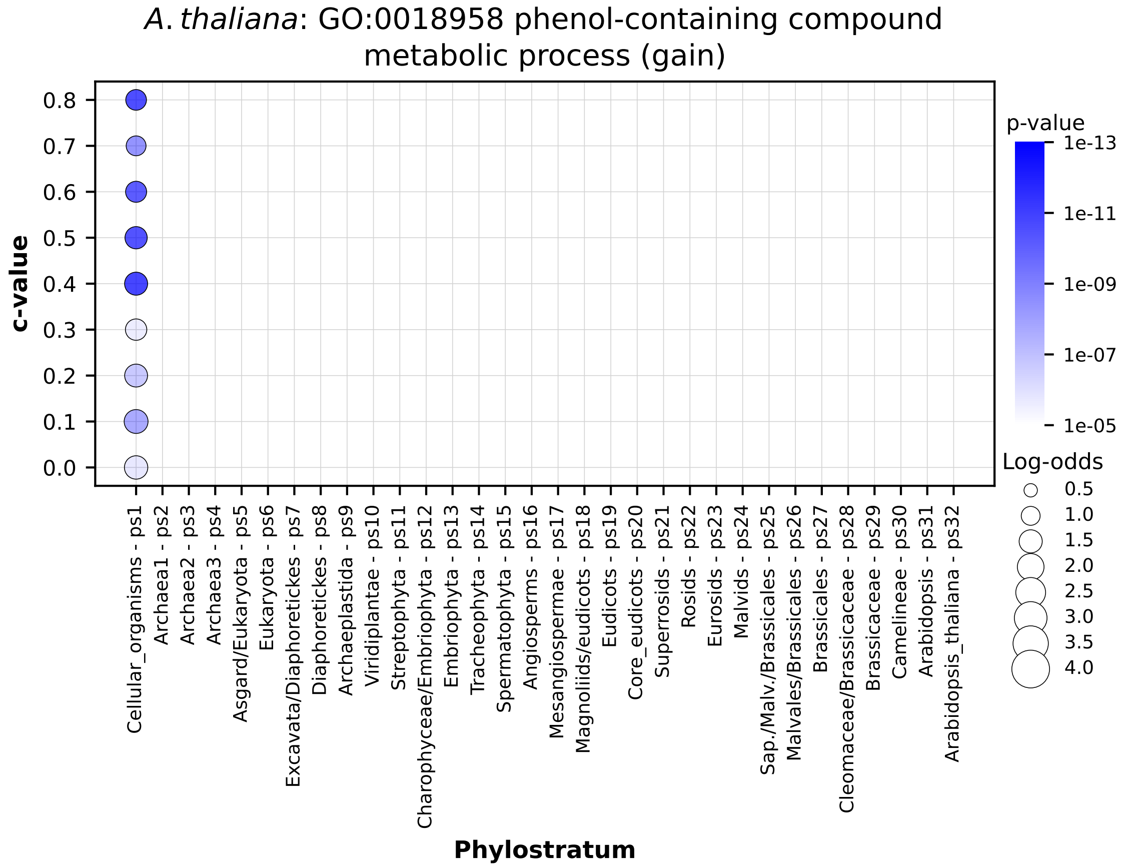
A. thaliana: GO:0018958 phenol-containing compound metabolic process (gain)
c-value
0.8
0.7
0.6
0.5
0.4
0.3
0.2
0.1
0.0
p-value
1e-13
1e-11
1e-09
1e-07
1e-05
Log-odds
0.5
1.0
1.5
2.0
2.5
3.0
3.5
4.0
Cellular_organisms - ps1
Archaea1 - ps2
Archaea2 - ps3
Archaea3 - ps4
Asgard/Eukaryota - ps5
Eukaryota - ps6
Excavata/Diaphoretickes - ps7
Diaphoretickes - ps8
Archaeplastida - ps9
Viridiplantae - ps10
Streptophyta - ps11
Charophyceae/Embriophyta - ps12
Embriophyta - ps13
Tracheophyta - ps14
Spermatophyta - ps15
Angiosperms - ps16
Mesangiospermae - ps17
Magnoliids/eudicots - ps18
Eudicots - ps19
Core_eudicots - ps20
Superrosids - ps21
Rosids - ps22
Eurosids - ps23
Malvids - ps24
Sap./Malv./Brassicales - ps25
Malvales/Brassicales - ps26
Brassicales - ps27
Cleomaceae/Brassicaceae - ps28
Brassicaceae - ps29
Camelineae - ps30
Arabidopsis - ps31
Arabidopsis_thaliana - ps32
Phylostratum

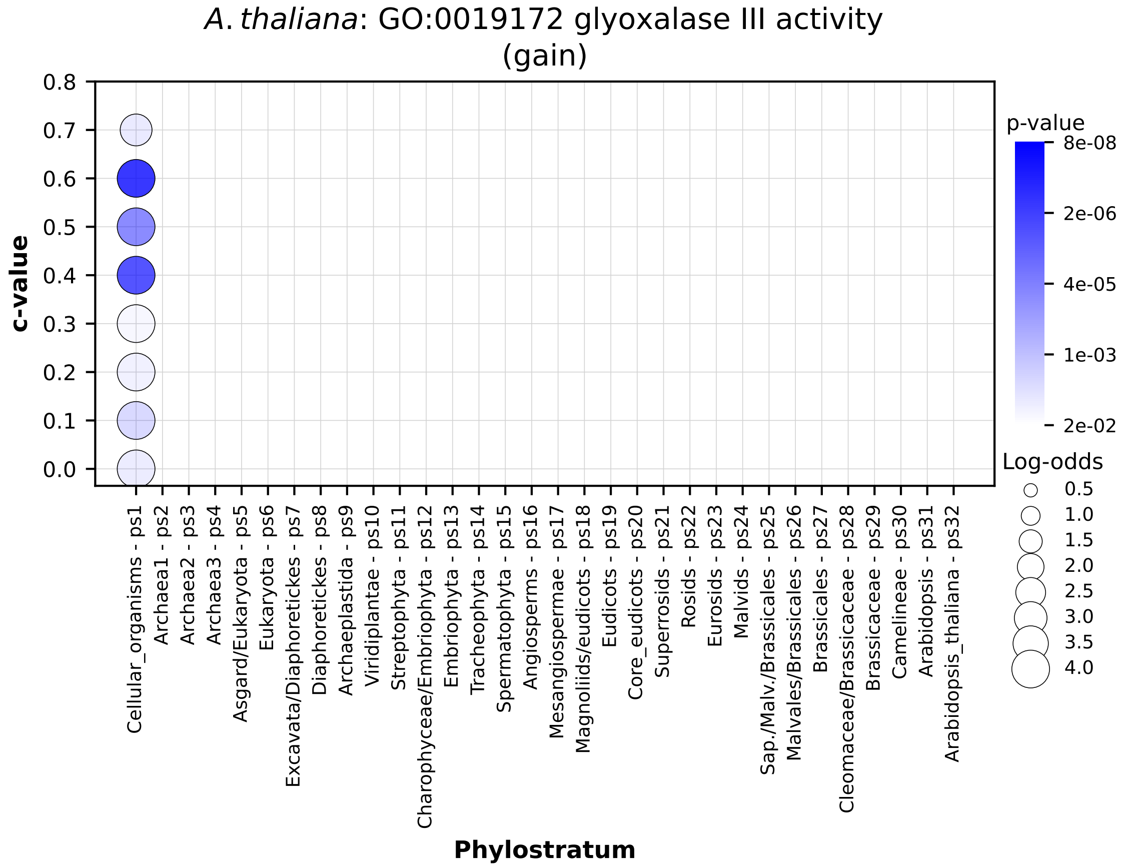

A. thaliana: GO:0019172 glyoxalase III activity
(gain)
c-value
0.8
0.7
0.6
0.5
0.4
0.3
0.2
0.1
0.0
p-value
8e-08
2e-06
4e-05
1e-03
2e-02
Log-odds
0.5
1.0
1.5
2.0
2.5
3.0
3.5
4.0
Cellular_organisms - ps1
Archaea1 - ps2
Archaea2 - ps3
Archaea3 - ps4
Asgard/Eukaryota - ps5
Eukaryota - ps6
Excavata/Diaphoretickes - ps7
Diaphoretickes - ps8
Archaeplastida - ps9
Viridiplantae - ps10
Streptophyta - ps11
Charophyceae/Embriophyta - ps12
Embriophyta - ps13
Tracheophyta - ps14
Spermatophyta - ps15
Angiosperms - ps16
Mesangiospermae - ps17
Magnoliids/eudicots - ps18
Eudicots - ps19
Core_eudicots - ps20
Superrosids - ps21
Rosids - ps22
Eurosids - ps23
Malvids - ps24
Sap./Malv./Brassicales - ps25
Malvales/Brassicales - ps26
Brassicales - ps27
Cleomaceae/Brassicaceae - ps28
Brassicaceae - ps29
Camelineae - ps30
Arabidopsis - ps31
Arabidopsis_thaliana - ps32
Phylostratum

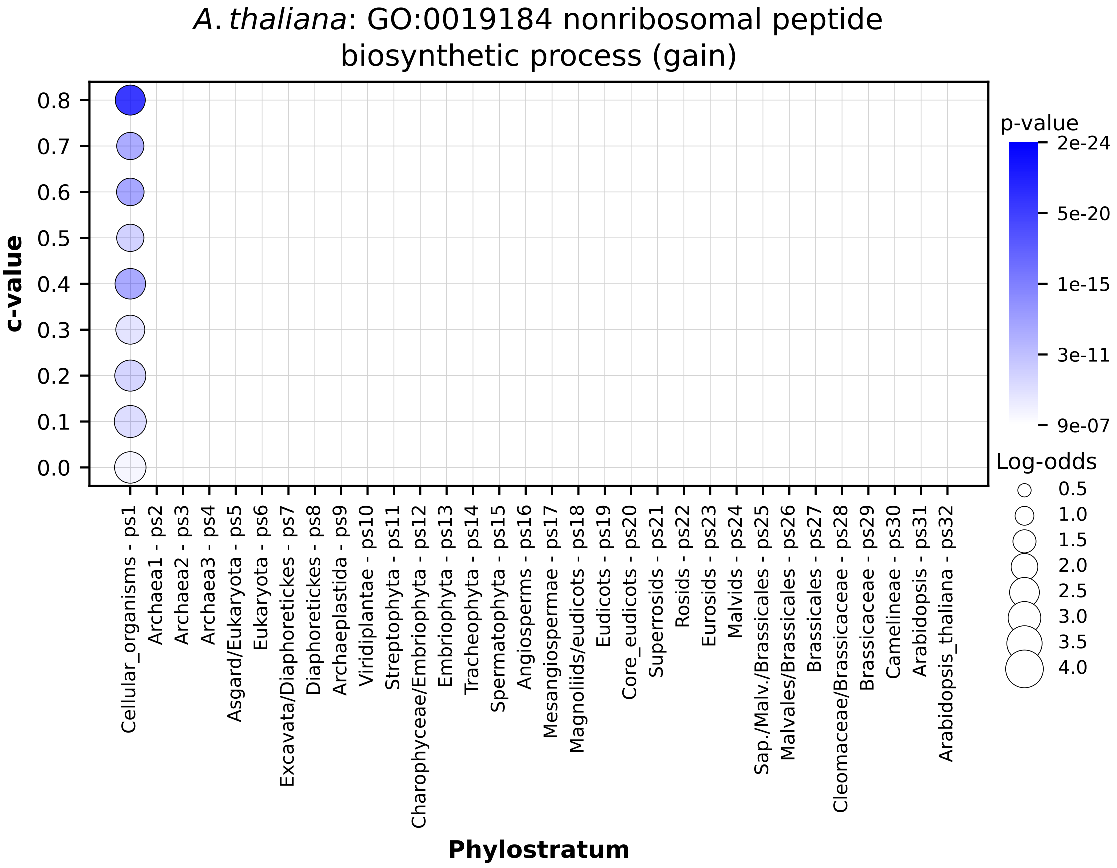
A. thaliana: GO:0019184 nonribosomal peptide biosynthetic process (gain)
c-value
0.8
0.7
0.6
0.5
0.4
0.3
0.2
0.1
0.0
p-value
2e-24
5e-20
1e-15
3e-11
9e-07
Log-odds
0.5
1.0
1.5
2.0
2.5
3.0
3.5
4.0
Cellular_organisms - ps1
Archaea1 - ps2
Archaea2 - ps3
Archaea3 - ps4
Asgard/Eukaryota - ps5
Eukaryota - ps6
Excavata/Diaphoretickes - ps7
Diaphoretickes - ps8
Archaeplastida - ps9
Viridiplantae - ps10
Streptophyta - ps11
Charophyceae/Embriophyta - ps12
Embriophyta - ps13
Tracheophyta - ps14
Spermatophyta - ps15
Angiosperms - ps16
Mesangiospermae - ps17
Magnoliids/eudicots - ps18
Eudicots - ps19
Core_eudicots - ps20
Superrosids - ps21
Rosids - ps22
Eurosids - ps23
Malvids - ps24
Sap./Malv./Brassicales - ps25
Malvales/Brassicales - ps26
Brassicales - ps27
Cleomaceae/Brassicaceae - ps28
Brassicaceae - ps29
Camelineae - ps30
Arabidopsis - ps31
Arabidopsis_thaliana - ps32
Phylostratum

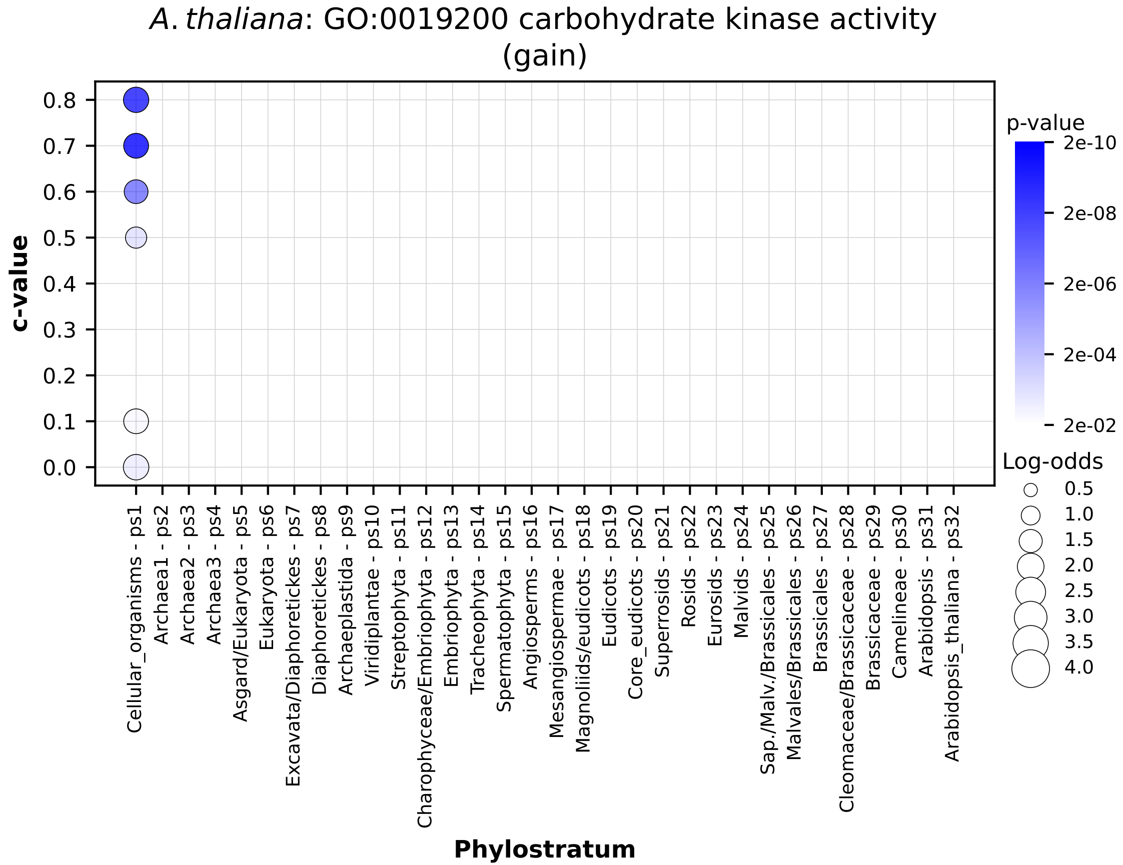

A. thaliana: GO:0019200 carbohydrate kinase activity (gain)
c-value
0.8
0.7
0.6
0.5
0.4
0.3
0.2
0.1
0.0
p-value
2e-10
2e-08
2e-06
2e-04
2e-02
Log-odds
0.5
1.0
1.5
2.0
2.5
3.0
3.5
4.0
Cellular_organisms - ps1
Archaea1 - ps2
Archaea2 - ps3
Archaea3 - ps4
Asgard/Eukaryota - ps5
Eukaryota - ps6
Excavata/Diaphoretickes - ps7
Diaphoretickes - ps8
Archaeplastida - ps9
Viridiplantae - ps10
Streptophyta - ps11
Charophyceae/Embriophyta - ps12
Embriophyta - ps13
Tracheophyta - ps14
Spermatophyta - ps15
Angiosperms - ps16
Mesangiospermae - ps17
Magnoliids/eudicots - ps18
Eudicots - ps19
Core_eudicots - ps20
Superrosids - ps21
Rosids - ps22
Eurosids - ps23
Malvids - ps24
Sap./Malv./Brassicales - ps25
Malvales/Brassicales - ps26
Brassicales - ps27
Cleomaceae/Brassicaceae - ps28
Brassicaceae - ps29
Camelineae - ps30
Arabidopsis - ps31
Arabidopsis_thaliana - ps32
Phylostratum

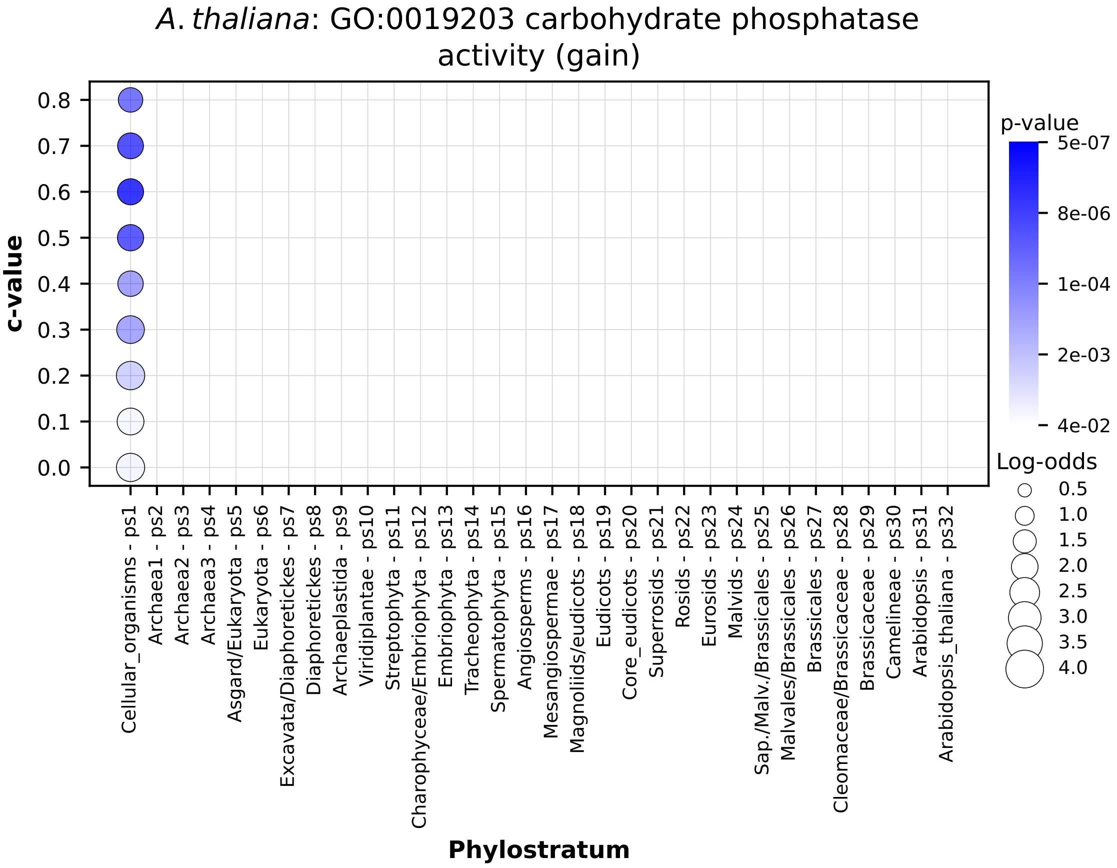

A. thaliana: GO:0019203 carbohydrate phosphatase activity (gain)
c-value
0.8
0.7
0.6
0.5
0.4
0.3
0.2
0.1
0.0
Phylostratum
Cellular_organisms - ps1
Archaea1 - ps2
Archaea2 - ps3
Archaea3 - ps4
Asgard/Eukaryota - ps5
Eukaryota - ps6
Excavata/Diaphoretickes - ps7
Diaphoretickes - ps8
Archaeplastida - ps9
Viridiplantae - ps10
Streptophyta - ps11
Charophyceae/Embriophyta - ps12
Embriophyta - ps13
Tracheophyta - ps14
Spermatophyta - ps15
Angiosperms - ps16
Mesangiospermae - ps17
Magnoliids/eudicots - ps18
Eudicots - ps19
Core_eudicots - ps20
Superrosids - ps21
Rosids - ps22
Eurosids - ps23
Malvids - ps24
Sap./Malv./Brassicales - ps25
Malvales/Brassicales - ps26
Brassicales - ps27
Cleomaceae/Brassicaceae - ps28
Brassicaceae - ps29
Camelineae - ps30
Arabidopsis - ps31
Arabidopsis_thaliana - ps32
p-value
5e-07
8e-06
1e-04
2e-03
4e-02
Log-odds
0.5
1.0
1.5
2.0
2.5
3.0
3.5
4.0

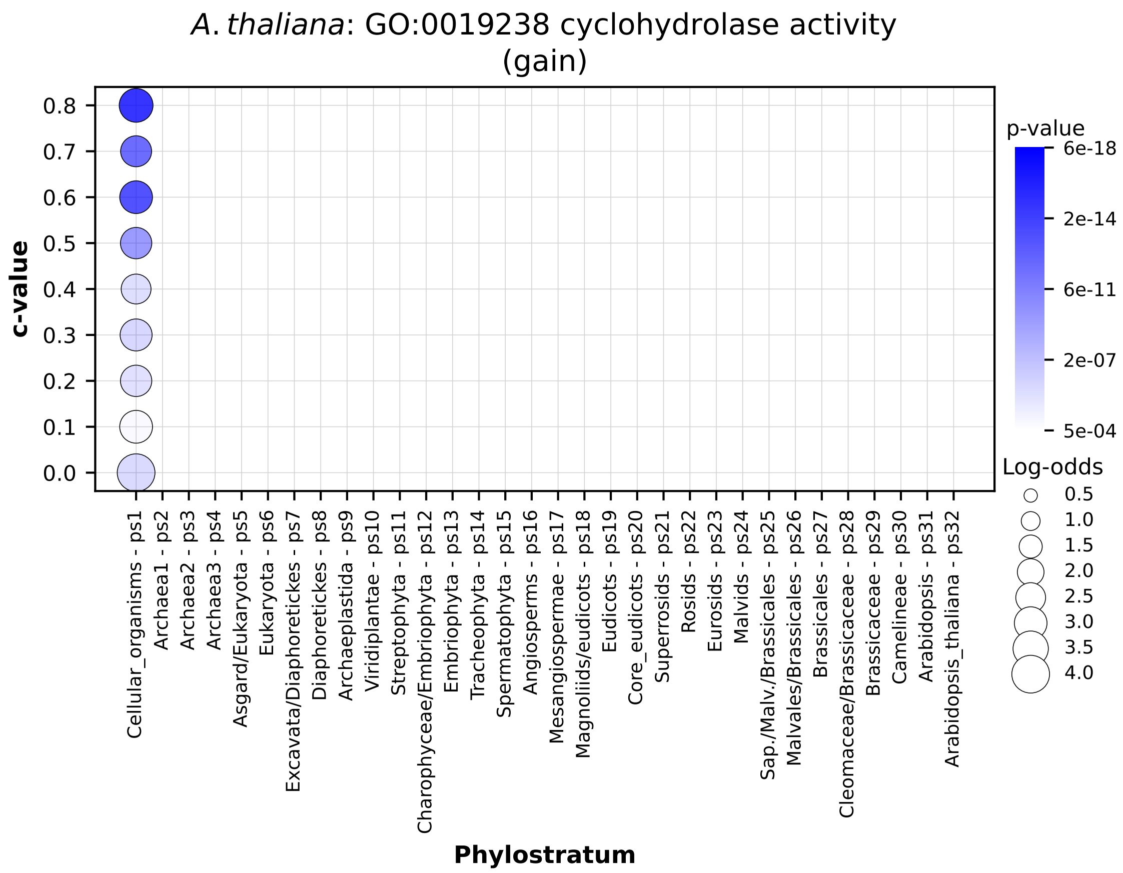

A. thaliana: GO:0019238 cyclohydrolase activity (gain)
c-value
0.8
0.7
0.6
0.5
0.4
0.3
0.2
0.1
0.0
p-value
6e-18
2e-14
6e-11
2e-07
5e-04
Log-odds
0.5
1.0
1.5
2.0
2.5
3.0
3.5
4.0
Cellular_organisms - ps1
Archaea1 - ps2
Archaea2 - ps3
Archaea3 - ps4
Asgard/Eukaryota - ps5
Eukaryota - ps6
Excavata/Diaphoretickes - ps7
Diaphoretickes - ps8
Archaeplastida - ps9
Viridiplantae - ps10
Streptophyta - ps11
Charophyceae/Embriophyta - ps12
Embriophyta - ps13
Tracheophyta - ps14
Spermatophyta - ps15
Angiosperms - ps16
Mesangiospermae - ps17
Magnoliids/eudicots - ps18
Eudicots - ps19
Core_eudicots - ps20
Superrosids - ps21
Rosids - ps22
Eurosids - ps23
Malvids - ps24
Sap./Malv./Brassicales - ps25
Malvales/Brassicales - ps26
Brassicales - ps27
Cleomaceae/Brassicaceae - ps28
Brassicaceae - ps29
Camelineae - ps30
Arabidopsis - ps31
Arabidopsis_thaliana - ps32
Phylostratum

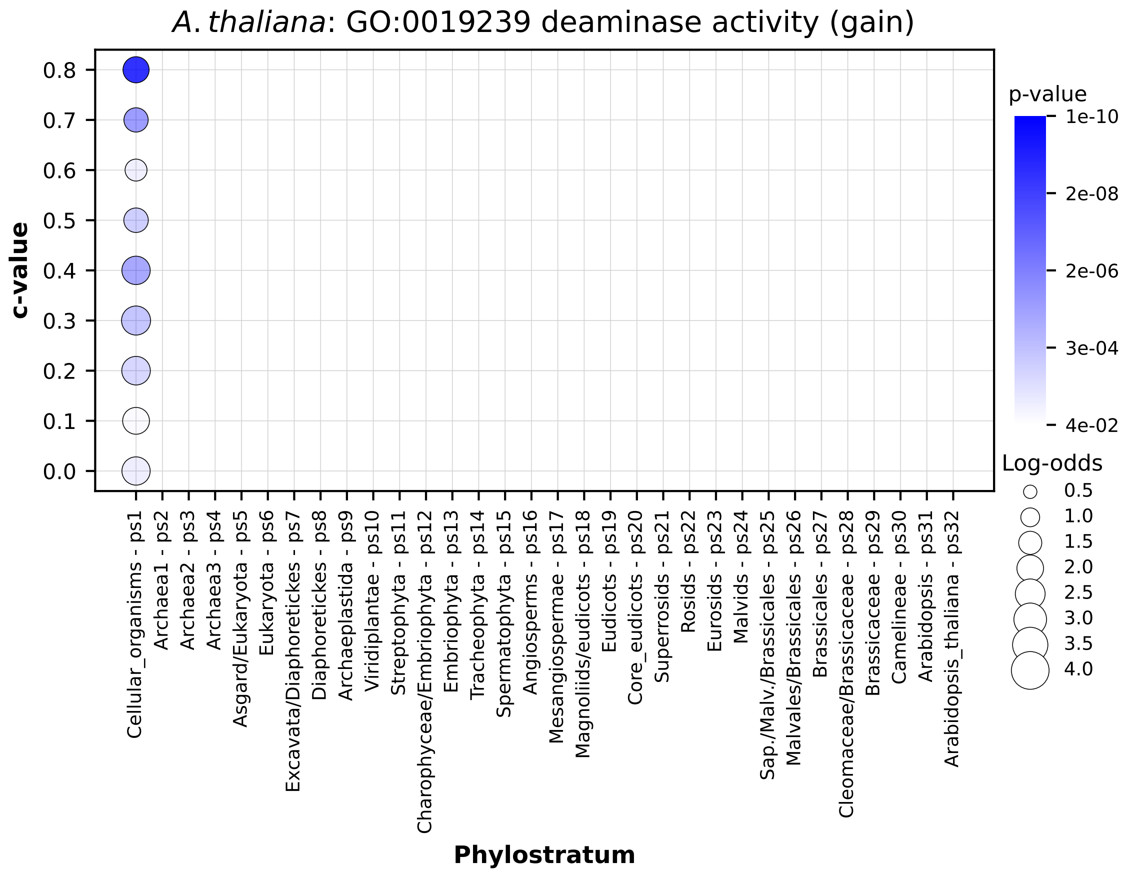

A. thaliana: GO:0019239 deaminase activity (gain)
c-value
0.8
0.7
0.6
0.5
0.4
0.3
0.2
0.1
0.0
p-value
1e-10
2e-08
2e-06
3e-04
4e-02
Log-odds
0.5
1.0
1.5
2.0
2.5
3.0
3.5
4.0
Cellular_organisms - ps1
Archaea1 - ps2
Archaea2 - ps3
Archaea3 - ps4
Asgard/Eukaryota - ps5
Eukaryota - ps6
Excavata/Diaphoretickes - ps7
Diaphoretickes - ps8
Archaeplastida - ps9
Viridiplantae - ps10
Streptophyta - ps11
Charophyceae/Embriophyta - ps12
Embriophyta - ps13
Tracheophyta - ps14
Spermatophyta - ps15
Angiosperms - ps16
Mesangiospermae - ps17
Magnoliids/eudicots - ps18
Eudicots - ps19
Core_eudicots - ps20
Superrosids - ps21
Rosids - ps22
Eurosids - ps23
Malvids - ps24
Sap./Malv./Brassicales - ps25
Malvales/Brassicales - ps26
Brassicales - ps27
Cleomaceae/Brassicaceae - ps28
Brassicaceae - ps29
Camelineae - ps30
Arabidopsis - ps31
Arabidopsis_thaliana - ps32
Phylostratum

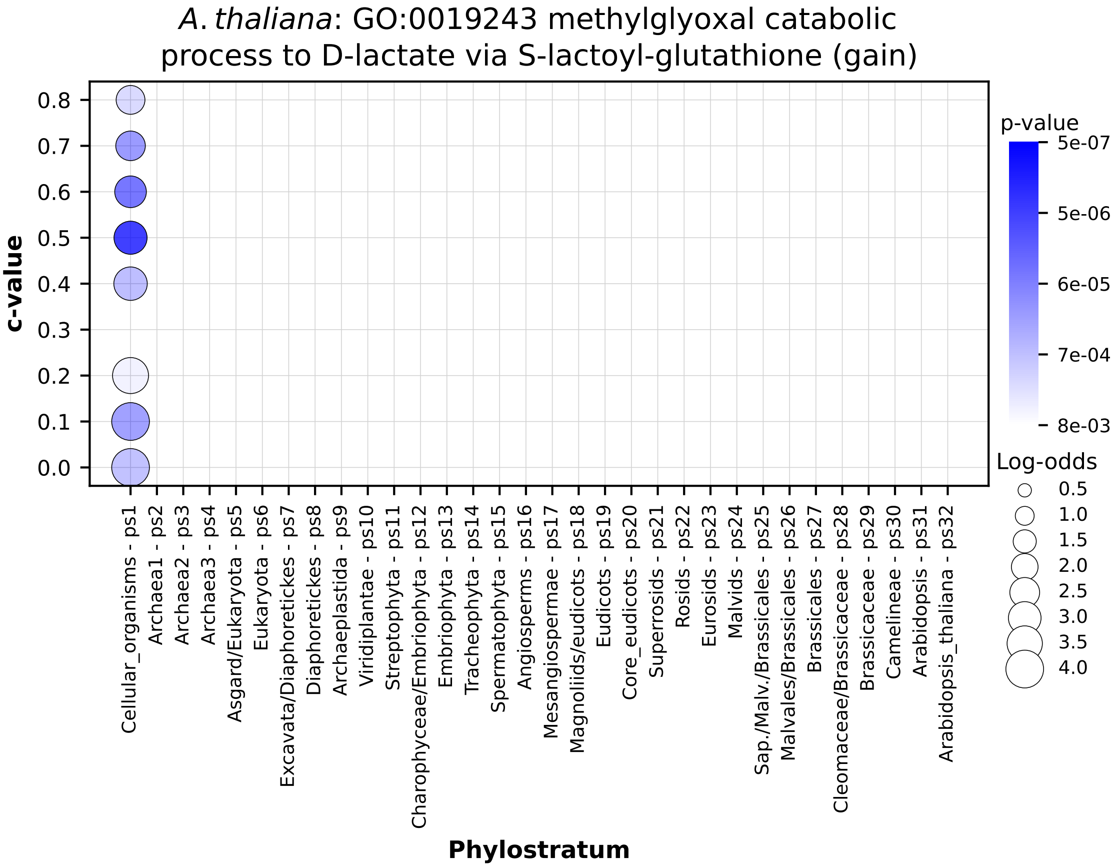

A. thaliana: GO:0019243 methylglyoxal catabolic process to D-lactate via S-lactoyl-glutathione (gain)
c-value
0.0
0.1
0.2
0.3
0.4
0.5
0.6
0.7
0.8
p-value
5e-07
5e-06
6e-05
7e-04
8e-03
Log-odds
0.5
1.0
1.5
2.0
2.5
3.0
3.5
4.0
Cellular_organisms - ps1
Archaea1 - ps2
Archaea2 - ps3
Archaea3 - ps4
Asgard/Eukaryota - ps5
Eukaryota - ps6
Excavata/Diaphoretickes - ps7
Diaphoretickes - ps8
Archaeplastida - ps9
Viridiplantae - ps10
Streptophyta - ps11
Charophyceae/Embriophyta - ps12
Embriophyta - ps13
Tracheophyta - ps14
Spermatophyta - ps15
Angiosperms - ps16
Mesangiospermae - ps17
Magnoliids/eudicots - ps18
Eudicots - ps19
Core_eudicots - ps20
Superrosids - ps21
Rosids - ps22
Eurosids - ps23
Malvids - ps24
Sap./Malv./Brassicales - ps25
Malvales/Brassicales - ps26
Brassicales - ps27
Cleomaceae/Brassicaceae - ps28
Brassicaceae - ps29
Camelineae - ps30
Arabidopsis - ps31
Arabidopsis_thaliana - ps32
Phylostratum

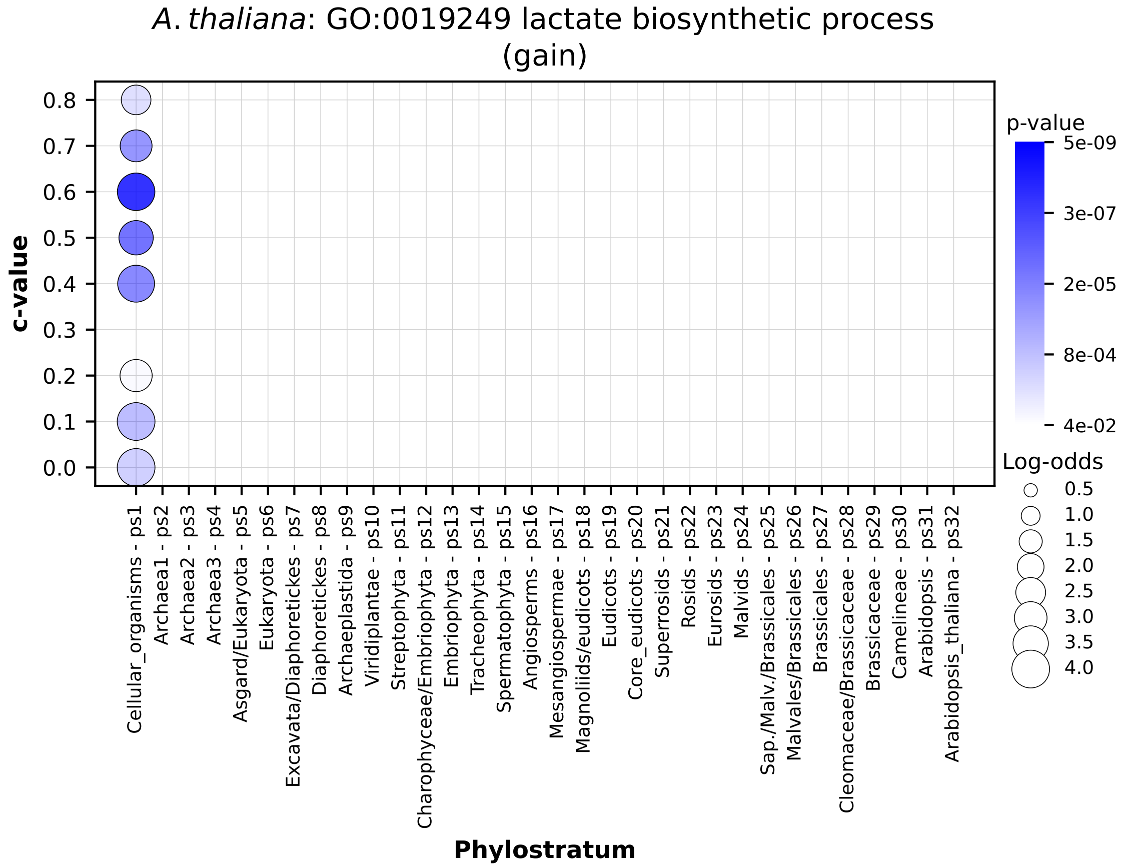

A. thaliana: GO:0019249 lactate biosynthetic process
(gain)
c-value
0.8
0.7
0.6
0.5
0.4
0.3
0.2
0.1
0.0
p-value
5e-09
3e-07
2e-05
8e-04
4e-02
Log-odds
0.5
1.0
1.5
2.0
2.5
3.0
3.5
4.0
Cellular_organisms - ps1
Archaea1 - ps2
Archaea2 - ps3
Archaea3 - ps4
Asgard/Eukaryota - ps5
Eukaryota - ps6
Excavata/Diaphoretickes - ps7
Diaphoretickes - ps8
Archaeplastida - ps9
Viridiplantae - ps10
Streptophyta - ps11
Charophyceae/Embriophyta - ps12
Embriophyta - ps13
Tracheophyta - ps14
Spermatophyta - ps15
Angiosperms - ps16
Mesangiospermae - ps17
Magnoliids/eudicots - ps18
Eudicots - ps19
Core_eudicots - ps20
Superrosids - ps21
Rosids - ps22
Eurosids - ps23
Malvids - ps24
Sap./Malv./Brassicales - ps25
Malvales/Brassicales - ps26
Brassicales - ps27
Cleomaceae/Brassicaceae - ps28
Brassicaceae - ps29
Camelineae - ps30
Arabidopsis - ps31
Arabidopsis_thaliana - ps32
Phylostratum

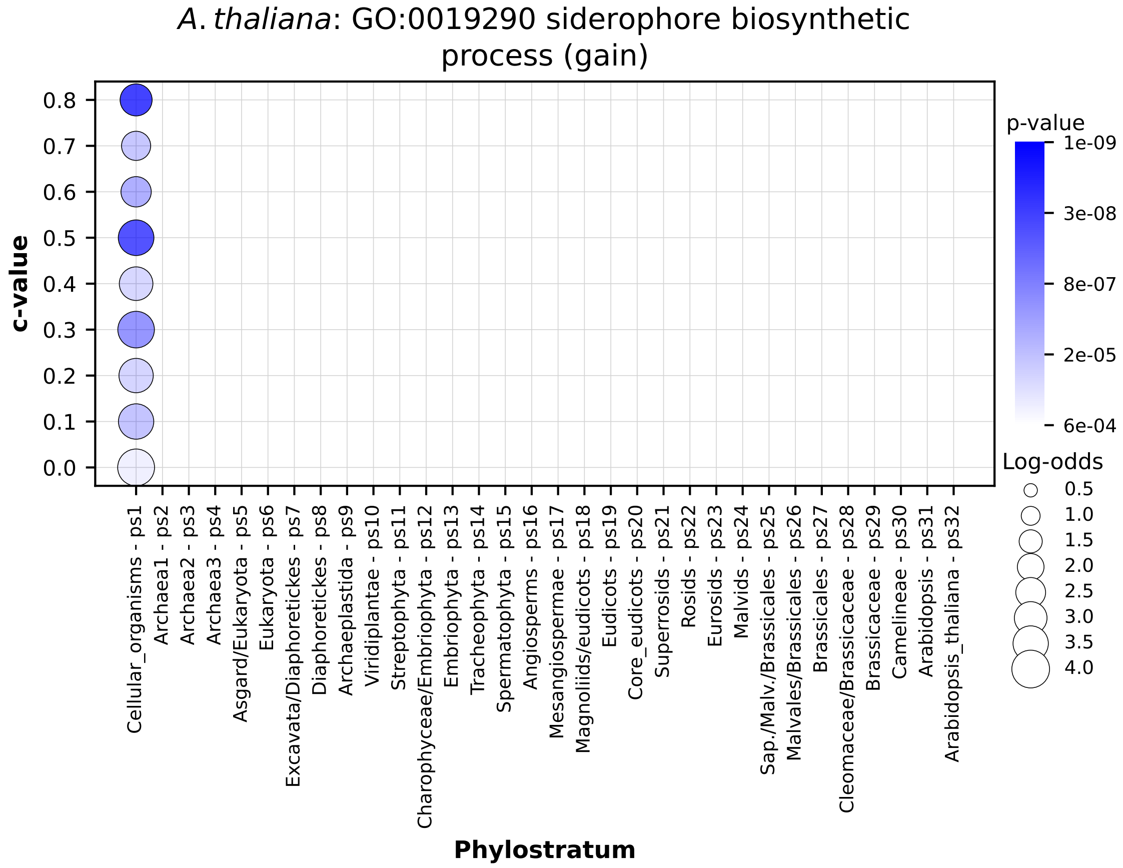

A. thaliana: GO:0019290 siderophore biosynthetic process (gain)
c-value
0.8
0.7
0.6
0.5
0.4
0.3
0.2
0.1
0.0
p-value
1e-09
3e-08
8e-07
2e-05
6e-04
Log-odds
0.5
1.0
1.5
2.0
2.5
3.0
3.5
4.0
Cellular_organisms - ps1
Archaea1 - ps2
Archaea2 - ps3
Archaea3 - ps4
Asgard/Eukaryota - ps5
Eukaryota - ps6
Excavata/Diaphoretickes - ps7
Diaphoretickes - ps8
Archaeplastida - ps9
Viridiplantae - ps10
Streptophyta - ps11
Charophyceae/Embriophyta - ps12
Embriophyta - ps13
Tracheophyta - ps14
Spermatophyta - ps15
Angiosperms - ps16
Mesangiospermae - ps17
Magnoliids/eudicots - ps18
Eudicots - ps19
Core_eudicots - ps20
Superrosids - ps21
Rosids - ps22
Eurosids - ps23
Malvids - ps24
Sap./Malv./Brassicales - ps25
Malvales/Brassicales - ps26
Brassicales - ps27
Cleomaceae/Brassicaceae - ps28
Brassicaceae - ps29
Camelineae - ps30
Arabidopsis - ps31
Arabidopsis_thaliana - ps32
Phylostratum

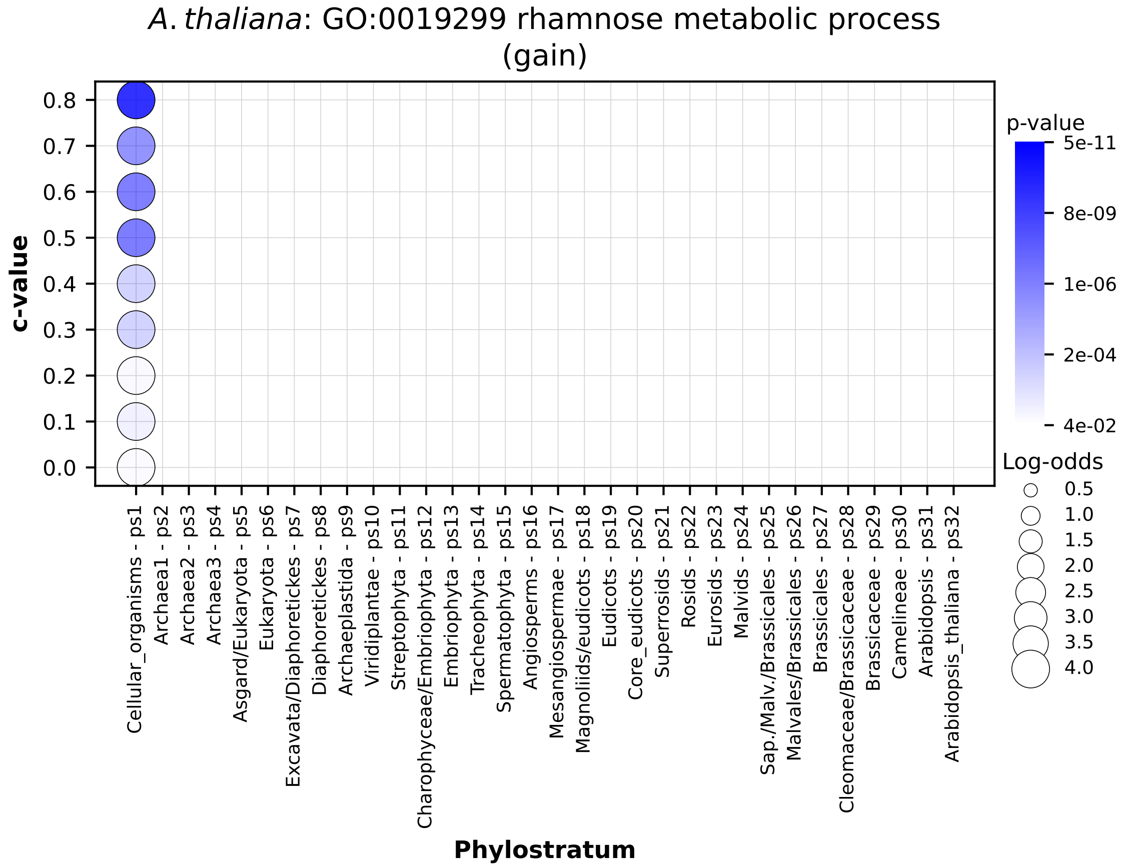

A. thaliana: GO:0019299 rhamnose metabolic process
(gain)
c-value
0.8
0.7
0.6
0.5
0.4
0.3
0.2
0.1
0.0
Phylostratum
Cellular_organisms - ps1
Archaea1 - ps2
Archaea2 - ps3
Archaea3 - ps4
Asgard/Eukaryota - ps5
Eukaryota - ps6
Excavata/Diaphoretickes - ps7
Diaphoretickes - ps8
Archaeplastida - ps9
Viridiplantae - ps10
Streptophyta - ps11
Charophyceae/Embriophyta - ps12
Embriophyta - ps13
Tracheophyta - ps14
Spermatophyta - ps15
Angiosperms - ps16
Mesangiospermae - ps17
Magnoliids/eudicots - ps18
Eudicots - ps19
Core_eudicots - ps20
Superrosids - ps21
Rosids - ps22
Eurosids - ps23
Malvids - ps24
Sap./Malv./Brassicales - ps25
Malvales/Brassicales - ps26
Brassicales - ps27
Cleomaceae/Brassicaceae - ps28
Brassicaceae - ps29
Camelineae - ps30
Arabidopsis - ps31
Arabidopsis_thaliana - ps32
p-value
5e-11
8e-09
1e-06
2e-04
4e-02
Log-odds
0.5
1.0
1.5
2.0
2.5
3.0
3.5
4.0

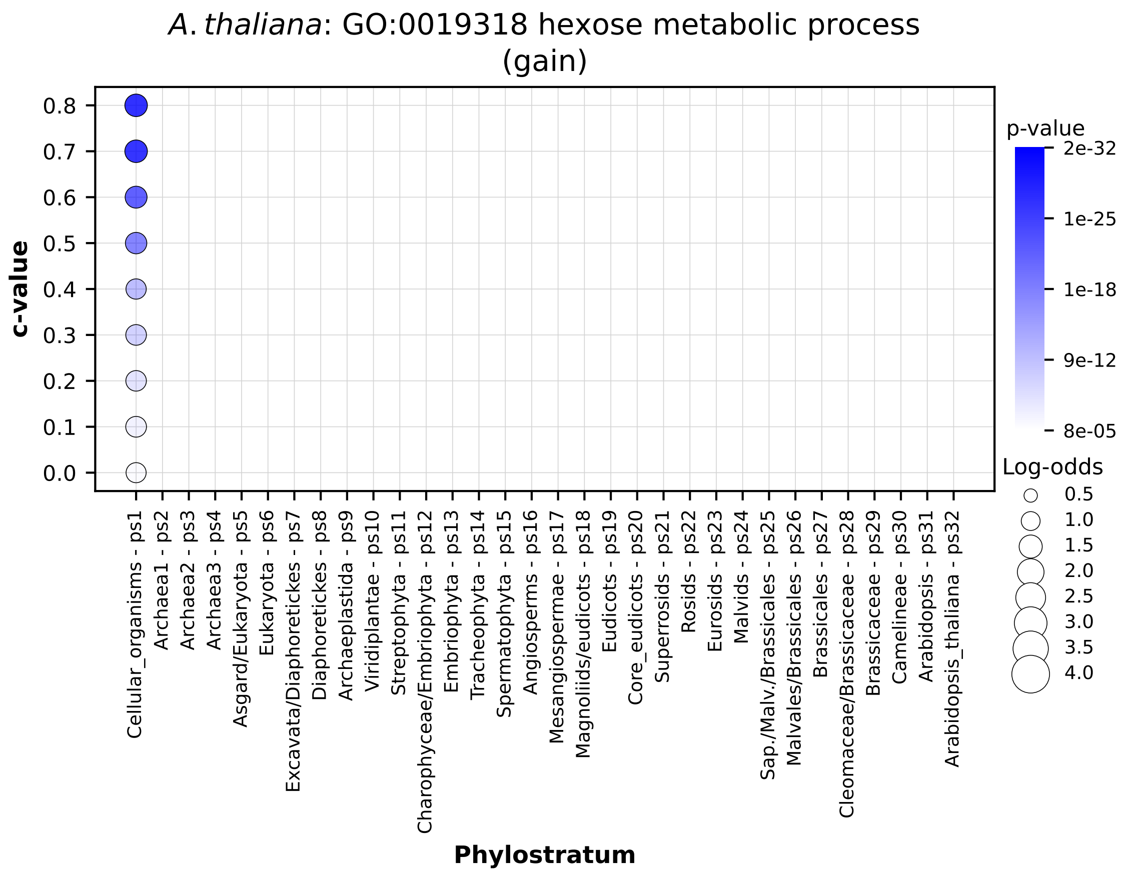
A. thaliana: GO:0019318 hexose metabolic process
(gain)
c-value
0.8
0.7
0.6
0.5
0.4
0.3
0.2
0.1
0.0
p-value
2e-32
1e-25
1e-18
9e-12
8e-05
Log-odds
0.5
1.0
1.5
2.0
2.5
3.0
3.5
4.0
Cellular_organisms - ps1
Archaea1 - ps2
Archaea2 - ps3
Archaea3 - ps4
Asgard/Eukaryota - ps5
Eukaryota - ps6
Excavata/Diaphoretickes - ps7
Diaphoretickes - ps8
Archaeplastida - ps9
Viridiplantae - ps10
Streptophyta - ps11
Charophyceae/Embriophyta - ps12
Embriophyta - ps13
Tracheophyta - ps14
Spermatophyta - ps15
Angiosperms - ps16
Mesangiospermae - ps17
Magnoliids/eudicots - ps18
Eudicots - ps19
Core_eudicots - ps20
Superrosids - ps21
Rosids - ps22
Eurosids - ps23
Malvids - ps24
Sap./Malv./Brassicales - ps25
Malvales/Brassicales - ps26
Brassicales - ps27
Cleomaceae/Brassicaceae - ps28
Brassicaceae - ps29
Camelineae - ps30
Arabidopsis - ps31
Arabidopsis_thaliana - ps32
Phylostratum

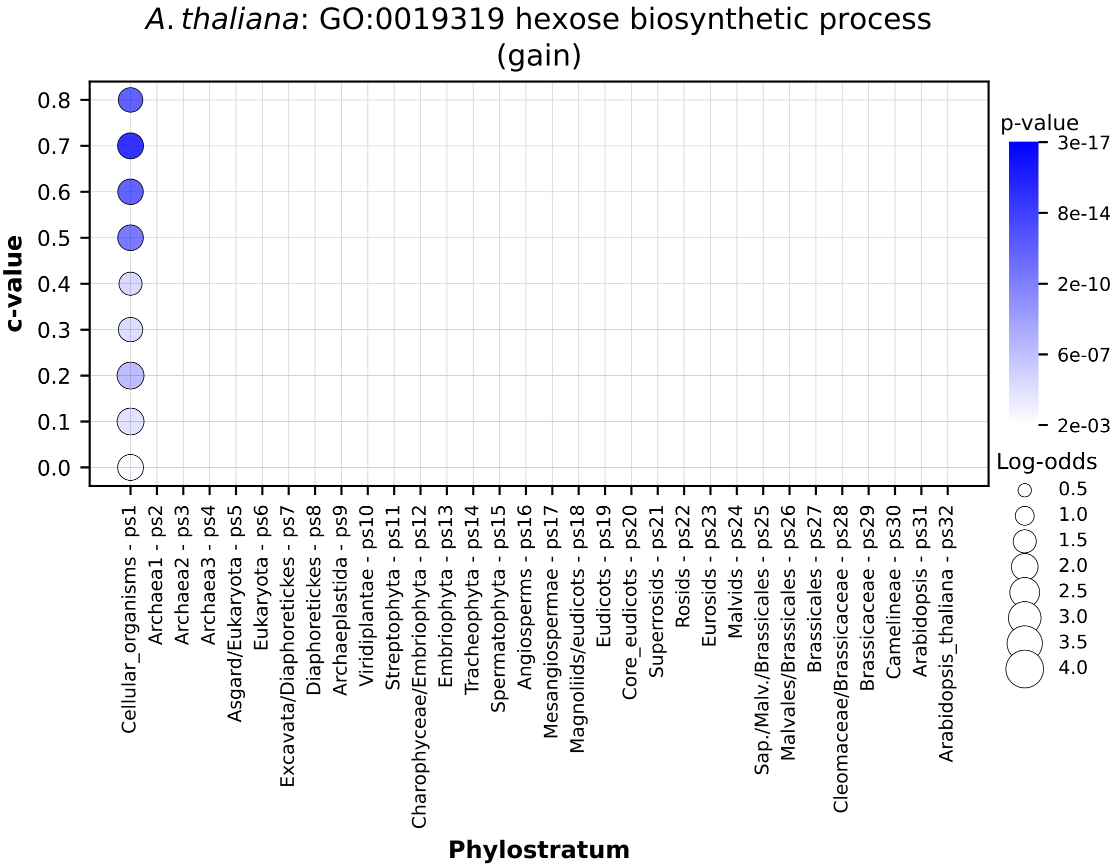

A. thaliana: GO:0019319 hexose biosynthetic process
(gain)
c-value
0.8
0.7
0.6
0.5
0.4
0.3
0.2
0.1
0.0
p-value
3e-17
8e-14
2e-10
6e-07
2e-03
Log-odds
0.5
1.0
1.5
2.0
2.5
3.0
3.5
4.0
Cellular_organisms - ps1
Archaea1 - ps2
Archaea2 - ps3
Archaea3 - ps4
Asgard/Eukaryota - ps5
Eukaryota - ps6
Excavata/Diaphoretickes - ps7
Diaphoretickes - ps8
Archaeplastida - ps9
Viridiplantae - ps10
Streptophyta - ps11
Charophyceae/Embriophyta - ps12
Embriophyta - ps13
Tracheophyta - ps14
Spermatophyta - ps15
Angiosperms - ps16
Mesangiospermae - ps17
Magnoliids/eudicots - ps18
Eudicots - ps19
Core_eudicots - ps20
Superrosids - ps21
Rosids - ps22
Eurosids - ps23
Malvids - ps24
Sap./Malv./Brassicales - ps25
Malvales/Brassicales - ps26
Brassicales - ps27
Cleomaceae/Brassicaceae - ps28
Brassicaceae - ps29
Camelineae - ps30
Arabidopsis - ps31
Arabidopsis_thaliana - ps32
Phylostratum

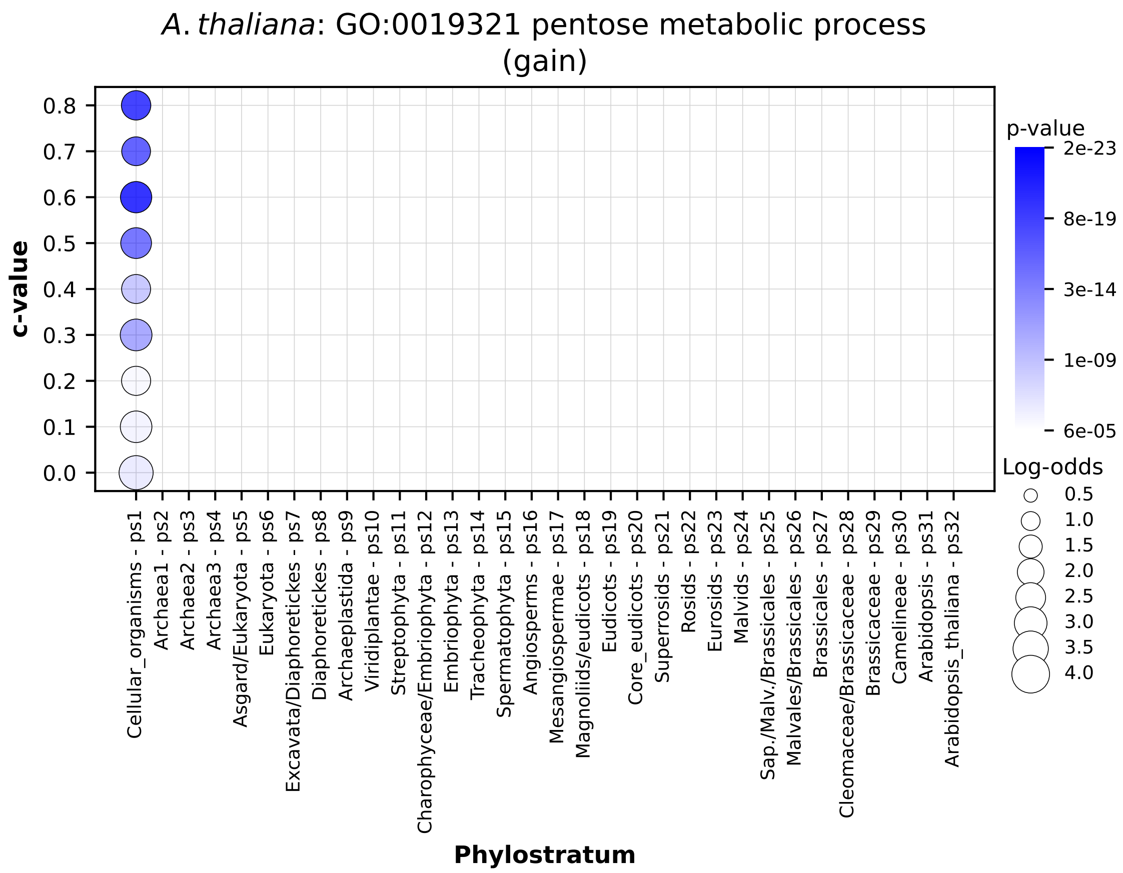

A. thaliana: GO:0019321 pentose metabolic process (gain)
c-value
0.8
0.7
0.6
0.5
0.4
0.3
0.2
0.1
0.0
p-value
2e-23
8e-19
3e-14
1e-09
6e-05
Log-odds
0.5
1.0
1.5
2.0
2.5
3.0
3.5
4.0
Cellular_organisms - ps1
Archaea1 - ps2
Archaea2 - ps3
Archaea3 - ps4
Asgard/Eukaryota - ps5
Eukaryota - ps6
Excavata/Diaphoretickes - ps7
Diaphoretickes - ps8
Archaeplastida - ps9
Viridiplantae - ps10
Streptophyta - ps11
Charophyceae/Embriophyta - ps12
Embriophyta - ps13
Tracheophyta - ps14
Spermatophyta - ps15
Angiosperms - ps16
Mesangiospermae - ps17
Magnoliids/eudicots - ps18
Eudicots - ps19
Core_eudicots - ps20
Superrosids - ps21
Rosids - ps22
Eurosids - ps23
Malvids - ps24
Sap./Malv./Brassicales - ps25
Malvales/Brassicales - ps26
Brassicales - ps27
Cleomaceae/Brassicaceae - ps28
Brassicaceae - ps29
Camelineae - ps30
Arabidopsis - ps31
Arabidopsis_thaliana - ps32
Phylostratum

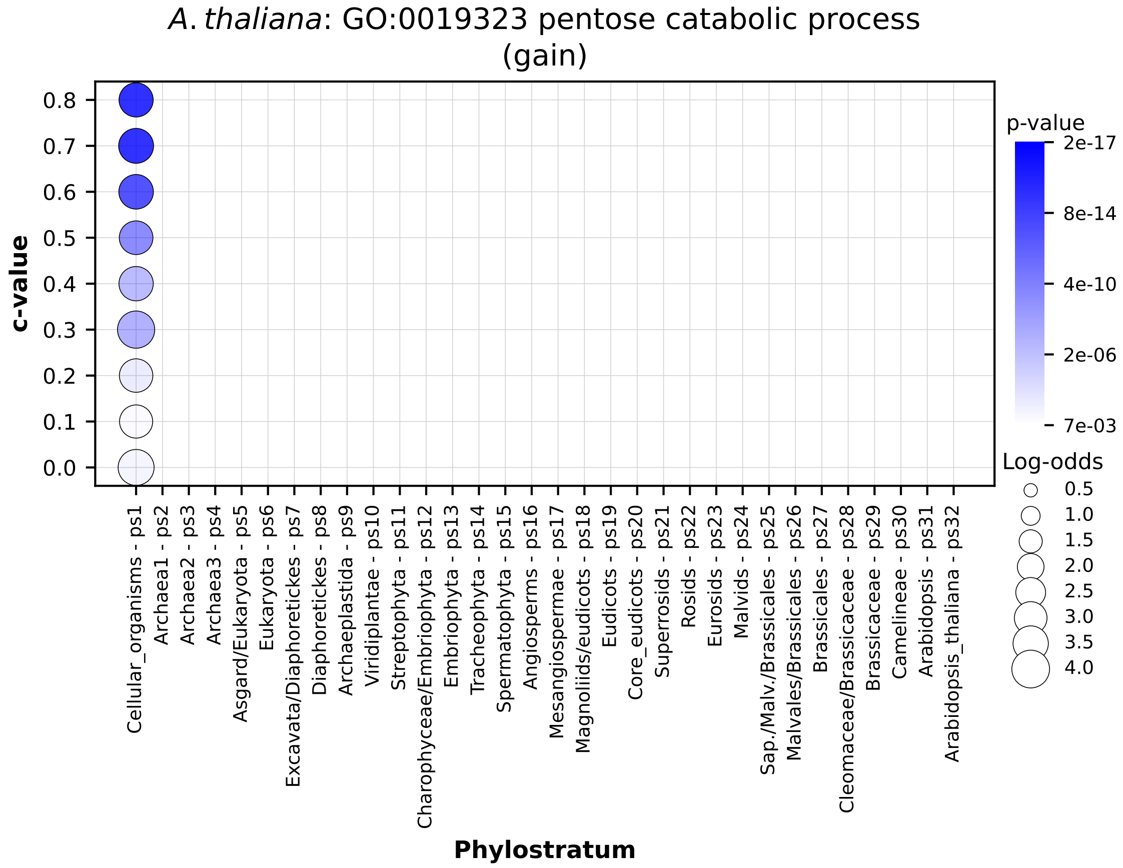

A. thaliana: GO:0019323 pentose catabolic process (gain)
c-value
0.8
0.7
0.6
0.5
0.4
0.3
0.2
0.1
0.0
p-value
2e-17
8e-14
4e-10
2e-06
7e-03
Log-odds
0.5
1.0
1.5
2.0
2.5
3.0
3.5
4.0
Cellular_organisms - ps1
Archaea1 - ps2
Archaea2 - ps3
Archaea3 - ps4
Asgard/Eukaryota - ps5
Eukaryota - ps6
Excavata/Diaphoretickes - ps7
Diaphoretickes - ps8
Archaeplastida - ps9
Viridiplantae - ps10
Streptophyta - ps11
Charophyceae/Embriophyta - ps12
Embriophyta - ps13
Tracheophyta - ps14
Spermatophyta - ps15
Angiosperms - ps16
Mesangiospermae - ps17
Magnoliids/eudicots - ps18
Eudicots - ps19
Core_eudicots - ps20
Superrosids - ps21
Rosids - ps22
Eurosids - ps23
Malvids - ps24
Sap./Malv./Brassicales - ps25
Malvales/Brassicales - ps26
Brassicales - ps27
Cleomaceae/Brassicaceae - ps28
Brassicaceae - ps29
Camelineae - ps30
Arabidopsis - ps31
Arabidopsis_thaliana - ps32
Phylostratum

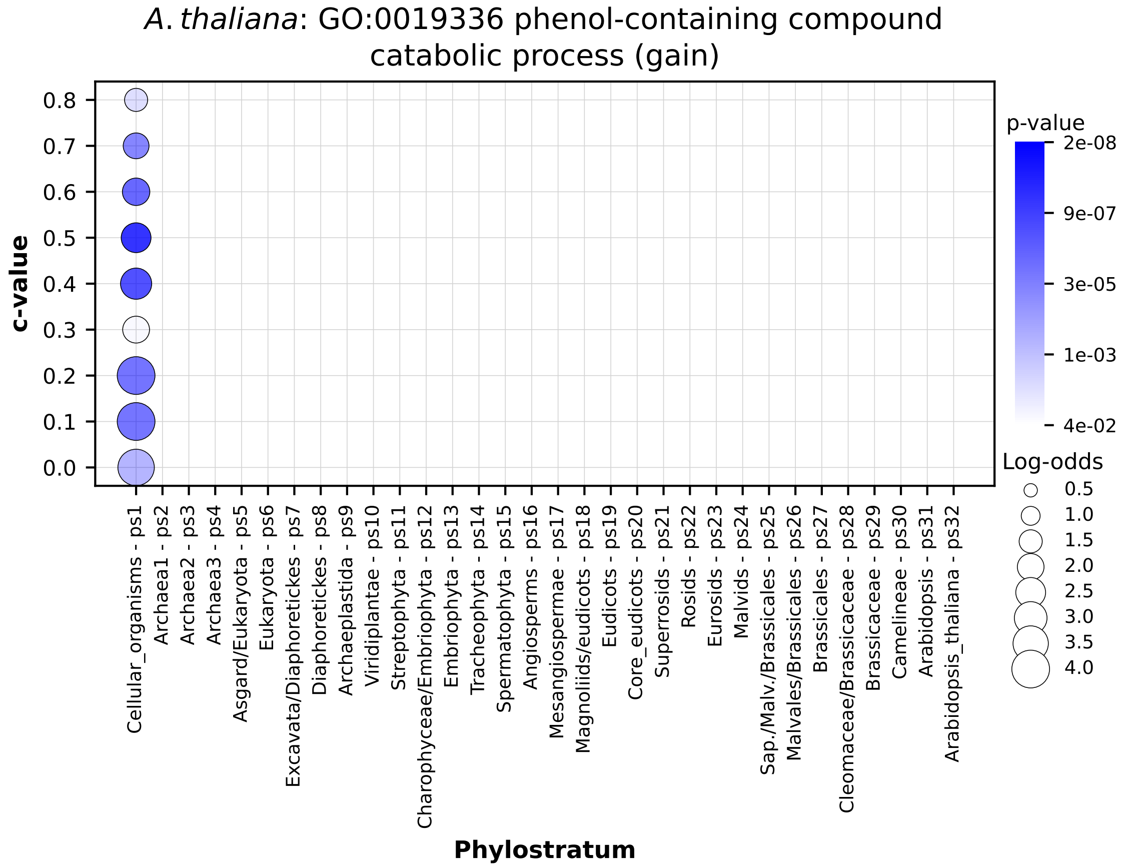
A. thaliana: GO:0019336 phenol-containing compound catabolic process (gain)
c-value
0.8
0.7
0.6
0.5
0.4
0.3
0.2
0.1
0.0
Phylostratum
Cellular_organisms - ps1
Archaea1 - ps2
Archaea2 - ps3
Archaea3 - ps4
Asgard/Eukaryota - ps5
Eukaryota - ps6
Excavata/Diaphoretickes - ps7
Diaphoretickes - ps8
Archaeplastida - ps9
Viridiplantae - ps10
Streptophyta - ps11
Charophyceae/Embriophyta - ps12
Embriophyta - ps13
Tracheophyta - ps14
Spermatophyta - ps15
Angiosperms - ps16
Mesangiospermae - ps17
Magnoliids/eudicots - ps18
Eudicots - ps19
Core_eudicots - ps20
Superrosids - ps21
Rosids - ps22
Eurosids - ps23
Malvids - ps24
Sap./Malv./Brassicales - ps25
Malvales/Brassicales - ps26
Brassicales - ps27
Cleomaceae/Brassicaceae - ps28
Brassicaceae - ps29
Camelineae - ps30
Arabidopsis - ps31
Arabidopsis_thaliana - ps32
p-value
2e-08
9e-07
3e-05
1e-03
4e-02
Log-odds
0.5
1.0
1.5
2.0
2.5
3.0
3.5
4.0

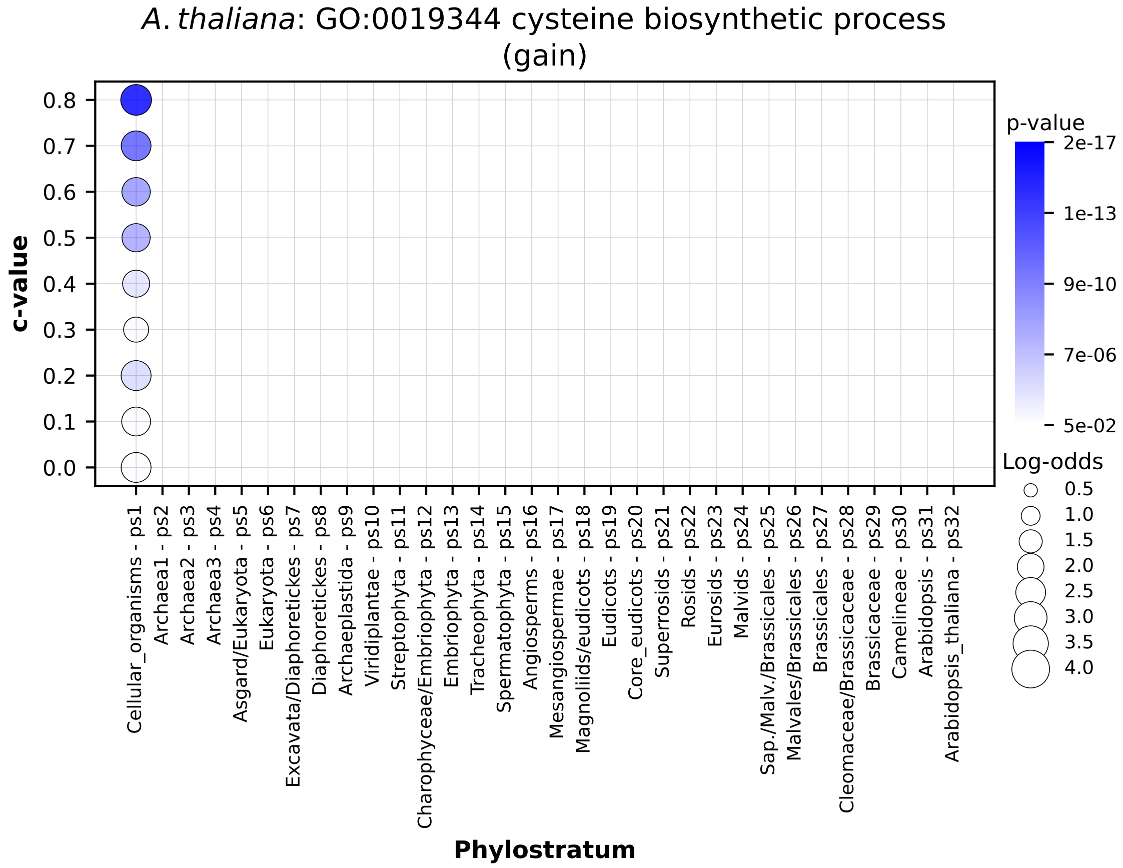
A. thaliana: GO:0019344 cysteine biosynthetic process
(gain)
c-value
0.8
0.7
0.6
0.5
0.4
0.3
0.2
0.1
0.0
p-value
2e-17
1e-13
9e-10
7e-06
5e-02
Log-odds
0.5
1.0
1.5
2.0
2.5
3.0
3.5
4.0
Cellular_organisms - ps1
Archaea1 - ps2
Archaea2 - ps3
Archaea3 - ps4
Asgard/Eukaryota - ps5
Eukaryota - ps6
Excavata/Diaphoretickes - ps7
Diaphoretickes - ps8
Archaeplastida - ps9
Viridiplantae - ps10
Streptophyta - ps11
Charophyceae/Embriophyta - ps12
Embriophyta - ps13
Tracheophyta - ps14
Spermatophyta - ps15
Angiosperms - ps16
Mesangiospermae - ps17
Magnoliids/eudicots - ps18
Eudicots - ps19
Core_eudicots - ps20
Superrosids - ps21
Rosids - ps22
Eurosids - ps23
Malvids - ps24
Sap./Malv./Brassicales - ps25
Malvales/Brassicales - ps26
Brassicales - ps27
Cleomaceae/Brassicaceae - ps28
Brassicaceae - ps29
Camelineae - ps30
Arabidopsis - ps31
Arabidopsis_thaliana - ps32
Phylostratum

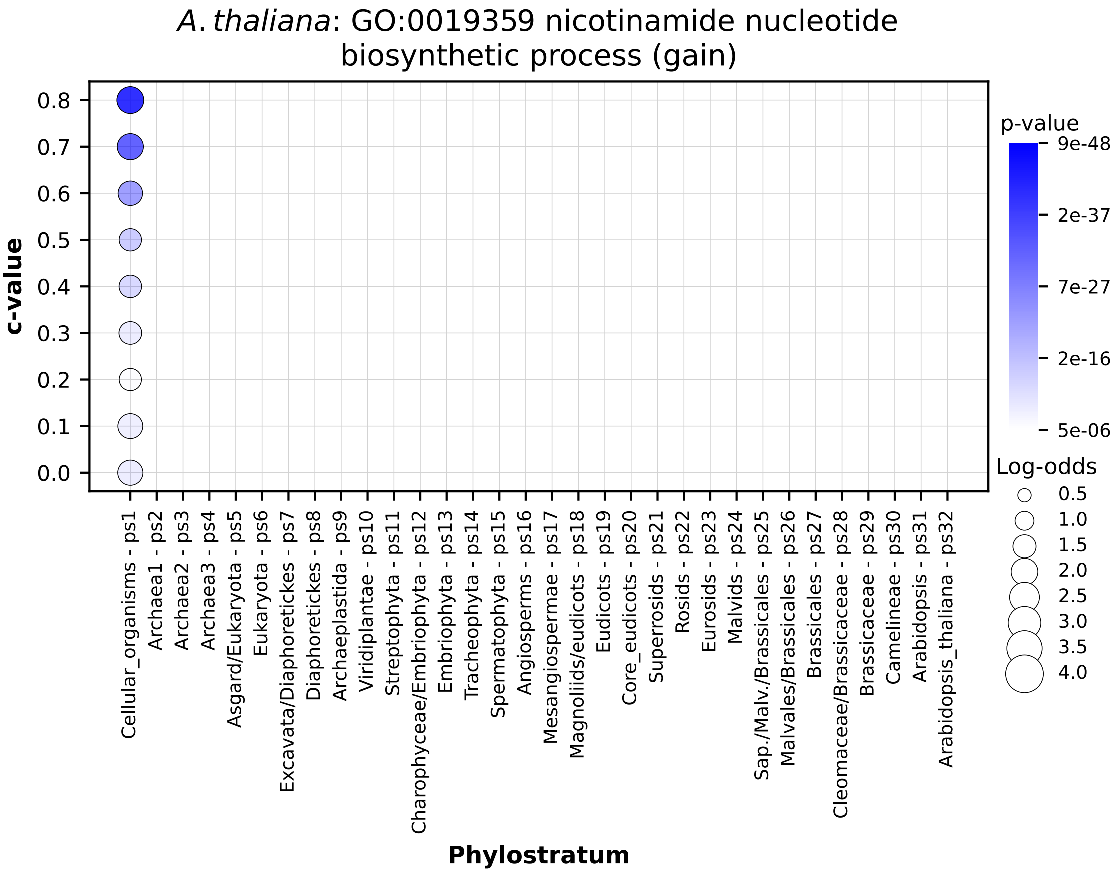

A. thaliana: GO:0019359 nicotinamide nucleotide biosynthetic process (gain)
c-value
0.0
0.1
0.2
0.3
0.4
0.5
0.6
0.7
0.8
Phylostratum
Cellular_organisms - ps1
Archaea1 - ps2
Archaea2 - ps3
Archaea3 - ps4
Asgard/Eukaryota - ps5
Eukaryota - ps6
Excavata/Diaphoretickes - ps7
Diaphoretickes - ps8
Archaeplastida - ps9
Viridiplantae - ps10
Streptophyta - ps11
Charophyceae/Embriophyta - ps12
Embriophyta - ps13
Tracheophyta - ps14
Spermatophyta - ps15
Angiosperms - ps16
Mesangiospermae - ps17
Magnoliids/eudicots - ps18
Eudicots - ps19
Core_eudicots - ps20
Superrosids - ps21
Rosids - ps22
Eurosids - ps23
Malvids - ps24
Sap./Malv./Brassicales - ps25
Malvales/Brassicales - ps26
Brassicales - ps27
Cleomaceae/Brassicaceae - ps28
Brassicaceae - ps29
Camelineae - ps30
Arabidopsis - ps31
Arabidopsis_thaliana - ps32
p-value
9e-48
2e-37
7e-27
2e-16
5e-06
Log-odds
0.5
1.0
1.5
2.0
2.5
3.0
3.5
4.0

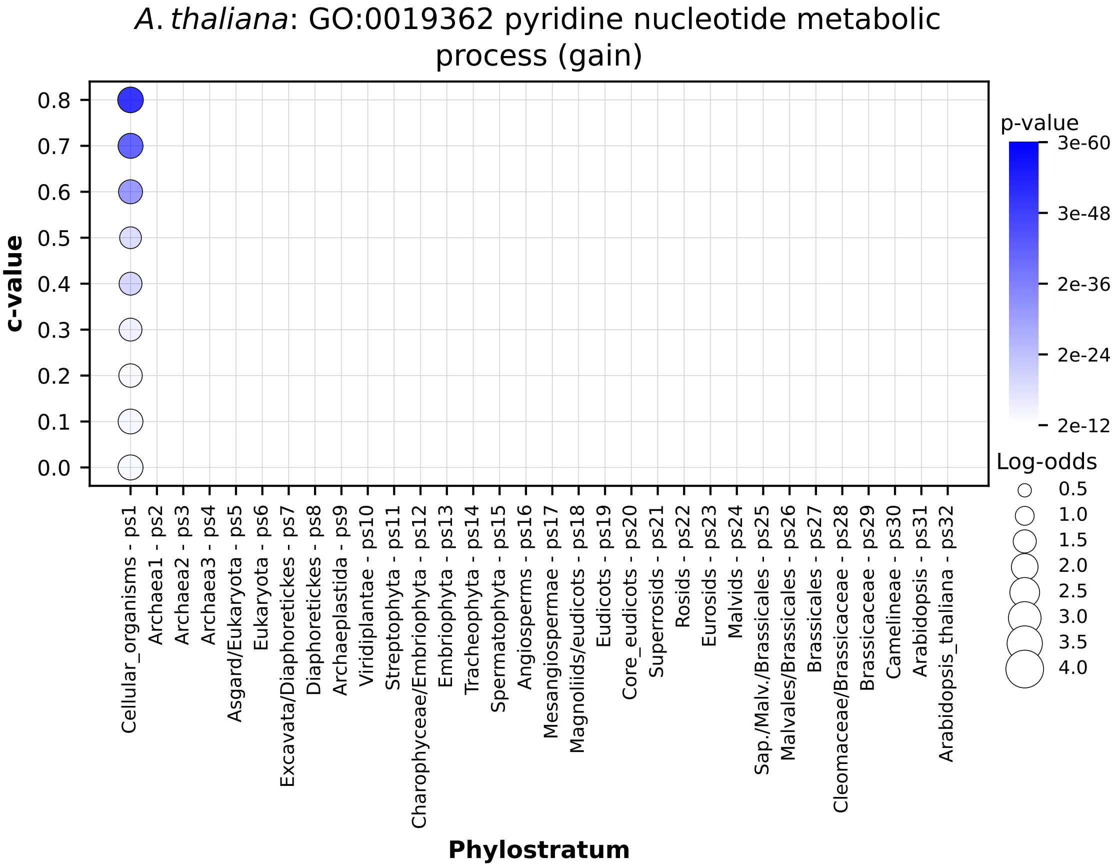

A. thaliana: GO:0019362 pyridine nucleotide metabolic process (gain)
c-value
0.8
0.7
0.6
0.5
0.4
0.3
0.2
0.1
0.0
p-value
3e-60
3e-48
2e-36
2e-24
2e-12
Log-odds
0.5
1.0
1.5
2.0
2.5
3.0
3.5
4.0
Cellular_organisms - ps1
Archaea1 - ps2
Archaea2 - ps3
Archaea3 - ps4
Asgard/Eukaryota - ps5
Eukaryota - ps6
Excavata/Diaphoretickes - ps7
Diaphoretickes - ps8
Archaeplastida - ps9
Viridiplantae - ps10
Streptophyta - ps11
Charophyceae/Embriophyta - ps12
Embriophyta - ps13
Tracheophyta - ps14
Spermatophyta - ps15
Angiosperms - ps16
Mesangiospermae - ps17
Magnoliids/eudicots - ps18
Eudicots - ps19
Core_eudicots - ps20
Superrosids - ps21
Rosids - ps22
Eurosids - ps23
Malvids - ps24
Sap./Malv./Brassicales - ps25
Malvales/Brassicales - ps26
Brassicales - ps27
Cleomaceae/Brassicaceae - ps28
Brassicaceae - ps29
Camelineae - ps30
Arabidopsis - ps31
Arabidopsis_thaliana - ps32
Phylostratum

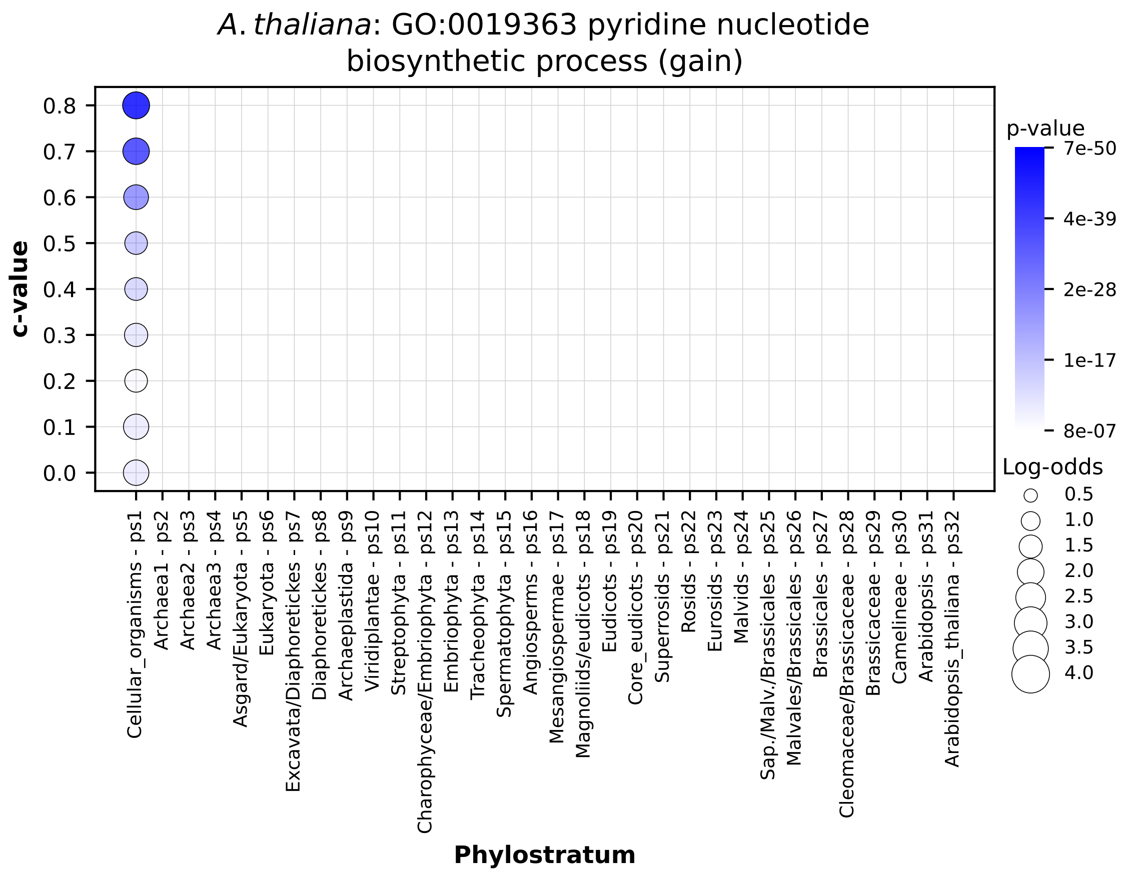
A. thaliana: GO:0019363 pyridine nucleotide biosynthetic process (gain)
c-value
Phylostratum
p-value
7e-50
4e-39
2e-28
1e-17
8e-07
Log-odds
0.5
1.0
1.5
2.0
2.5
3.0
3.5
4.0
Cellular_organisms - ps1
Archaea1 - ps2
Archaea2 - ps3
Archaea3 - ps4
Asgard/Eukaryota - ps5
Eukaryota - ps6
Excavata/Diaphoretickes - ps7
Diaphoretickes - ps8
Archaeplastida - ps9
Viridiplantae - ps10
Streptophyta - ps11
Charophyceae/Embriophyta - ps12
Embriophyta - ps13
Tracheophyta - ps14
Spermatophyta - ps15
Angiosperms - ps16
Mesangiospermae - ps17
Magnoliids/eudicots - ps18
Eudicots - ps19
Core_eudicots - ps20
Superrosids - ps21
Rosids - ps22
Eurosids - ps23
Malvids - ps24
Sap./Malv./Brassicales - ps25
Malvales/Brassicales - ps26
Brassicales - ps27
Cleomaceae/Brassicaceae - ps28
Brassicaceae - ps29
Camelineae - ps30
Arabidopsis - ps31
Arabidopsis_thaliana - ps32

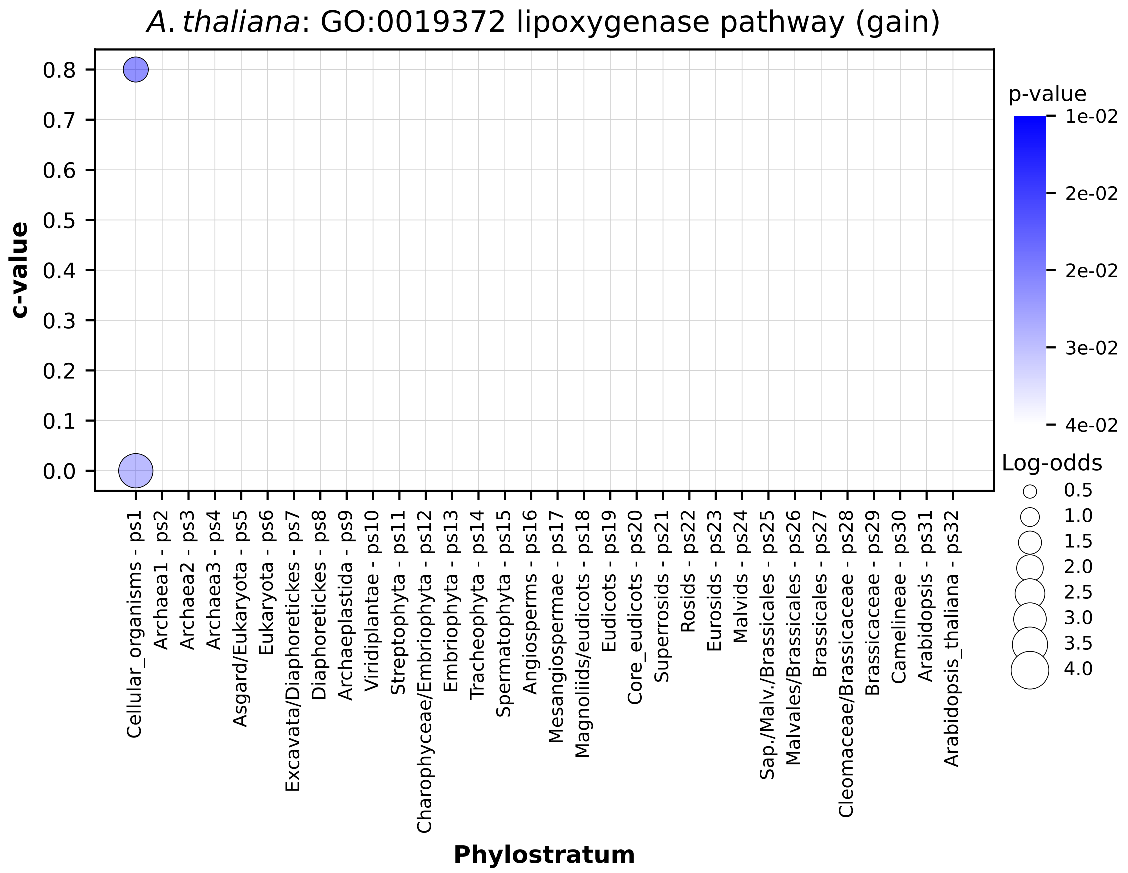

A. thaliana: GO:0019372 lipoxygenase pathway (gain)
c-value
0.0
0.1
0.2
0.3
0.4
0.5
0.6
0.7
0.8
p-value
1e-02
2e-02
2e-02
3e-02
4e-02
Log-odds
0.5
1.0
1.5
2.0
2.5
3.0
3.5
4.0
Cellular_organisms - ps1
Archaea1 - ps2
Archaea2 - ps3
Archaea3 - ps4
Asgard/Eukaryota - ps5
Eukaryota - ps6
Excavata/Diaphoretickes - ps7
Diaphoretickes - ps8
Archaeplastida - ps9
Viridiplantae - ps10
Streptophyta - ps11
Charophyceae/Embriophyta - ps12
Embriophyta - ps13
Tracheophyta - ps14
Spermatophyta - ps15
Angiosperms - ps16
Mesangiospermae - ps17
Magnoliids/eudicots - ps18
Eudicots - ps19
Core_eudicots - ps20
Superrosids - ps21
Rosids - ps22
Eurosids - ps23
Malvids - ps24
Sap./Malv./Brassicales - ps25
Malvales/Brassicales - ps26
Brassicales - ps27
Cleomaceae/Brassicaceae - ps28
Brassicaceae - ps29
Camelineae - ps30
Arabidopsis - ps31
Arabidopsis_thaliana - ps32
Phylostratum

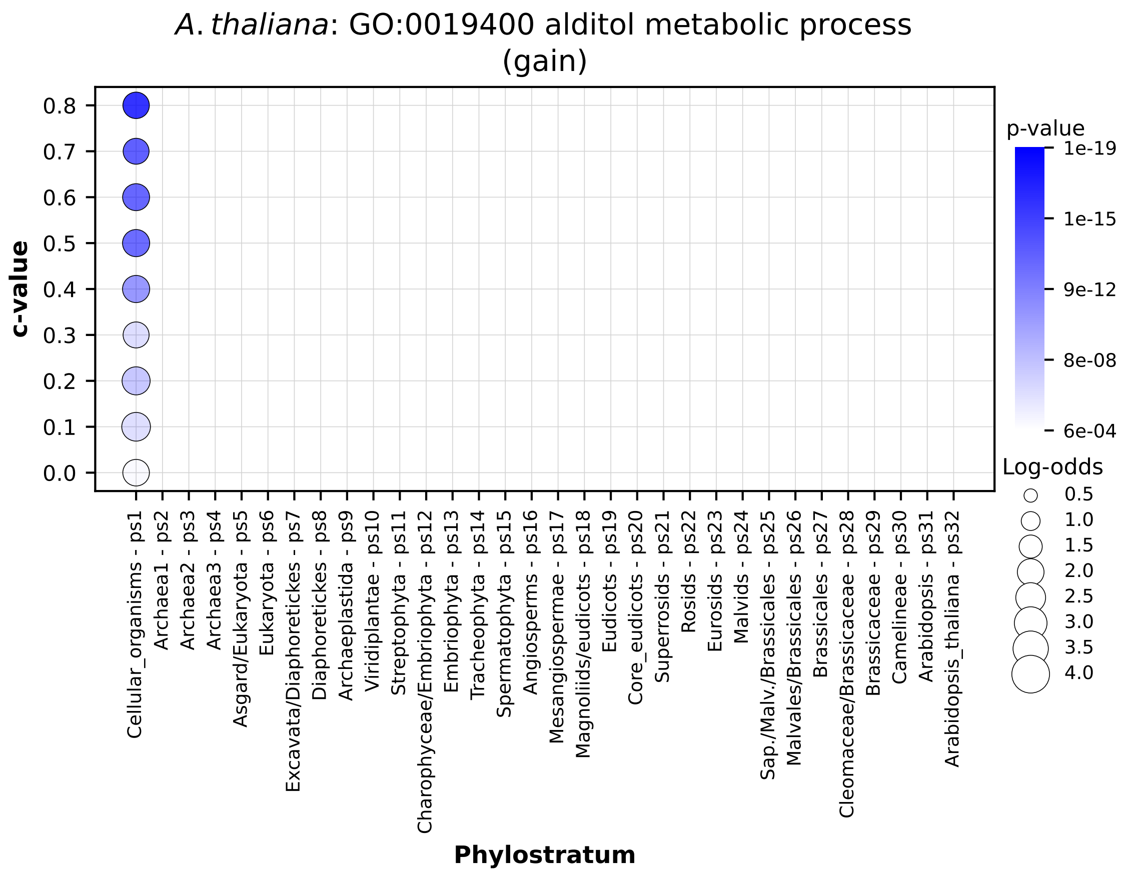

A. thaliana: GO:0019400 alditol metabolic process
(gain)
c-value
0.8
0.7
0.6
0.5
0.4
0.3
0.2
0.1
0.0
p-value
1e-19
1e-15
9e-12
8e-08
6e-04
Log-odds
0.5
1.0
1.5
2.0
2.5
3.0
3.5
4.0
Cellular_organisms - ps1
Archaea1 - ps2
Archaea2 - ps3
Archaea3 - ps4
Asgard/Eukaryota - ps5
Eukaryota - ps6
Excavata/Diaphoretickes - ps7
Diaphoretickes - ps8
Archaeplastida - ps9
Viridiplantae - ps10
Streptophyta - ps11
Charophyceae/Embriophyta - ps12
Embriophyta - ps13
Tracheophyta - ps14
Spermatophyta - ps15
Angiosperms - ps16
Mesangiospermae - ps17
Magnoliids/eudicots - ps18
Eudicots - ps19
Core_eudicots - ps20
Superrosids - ps21
Rosids - ps22
Eurosids - ps23
Malvids - ps24
Sap./Malv./Brassicales - ps25
Malvales/Brassicales - ps26
Brassicales - ps27
Cleomaceae/Brassicaceae - ps28
Brassicaceae - ps29
Camelineae - ps30
Arabidopsis - ps31
Arabidopsis_thaliana - ps32
Phylostratum

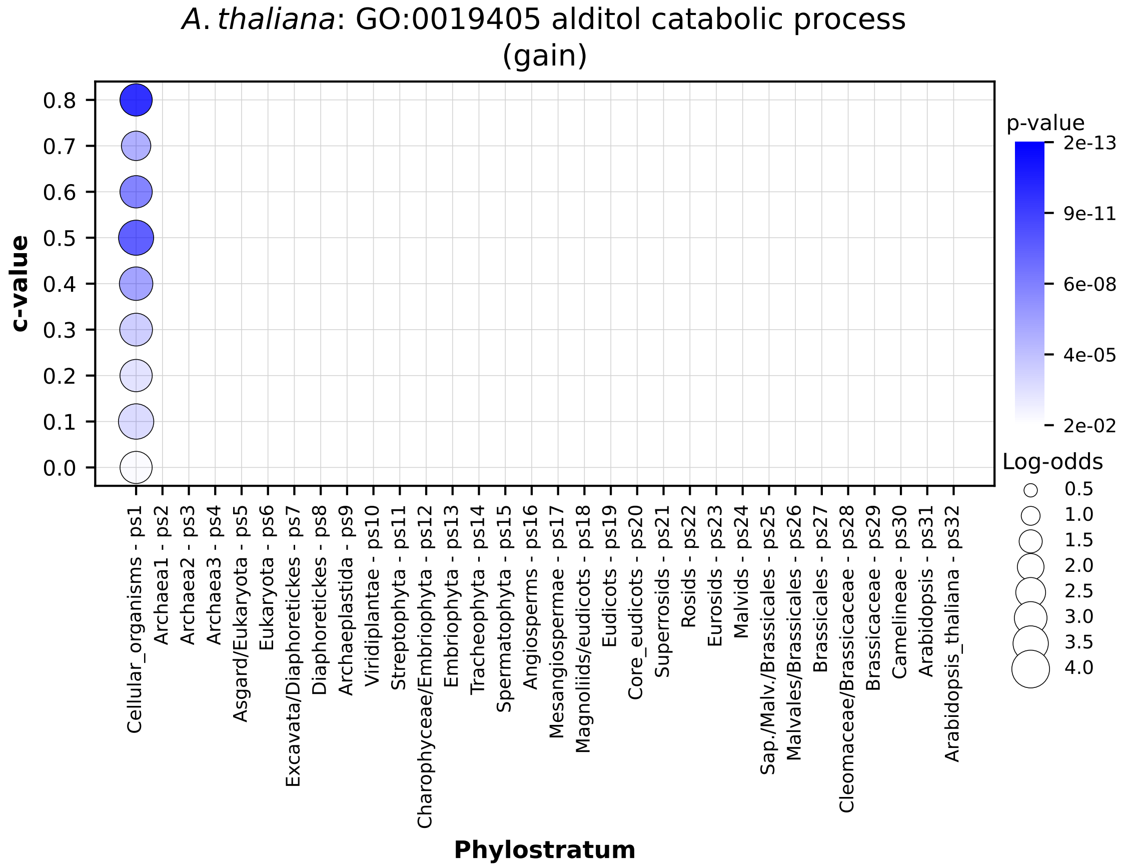
A. thaliana: GO:0019405 alditol catabolic process
(gain)
c-value
0.0
0.1
0.2
0.3
0.4
0.5
0.6
0.7
0.8
p-value
2e-13
9e-11
6e-08
4e-05
2e-02
Log-odds
0.5
1.0
1.5
2.0
2.5
3.0
3.5
4.0
Cellular_organisms - ps1
Archaea1 - ps2
Archaea2 - ps3
Archaea3 - ps4
Asgard/Eukaryota - ps5
Eukaryota - ps6
Excavata/Diaphoretickes - ps7
Diaphoretickes - ps8
Archaeplastida - ps9
Viridiplantae - ps10
Streptophyta - ps11
Charophyceae/Embriophyta - ps12
Embriophyta - ps13
Tracheophyta - ps14
Spermatophyta - ps15
Angiosperms - ps16
Mesangiospermae - ps17
Magnoliids/eudicots - ps18
Eudicots - ps19
Core_eudicots - ps20
Superrosids - ps21
Rosids - ps22
Eurosids - ps23
Malvids - ps24
Sap./Malv./Brassicales - ps25
Malvales/Brassicales - ps26
Brassicales - ps27
Cleomaceae/Brassicaceae - ps28
Brassicaceae - ps29
Camelineae - ps30
Arabidopsis - ps31
Arabidopsis_thaliana - ps32
Phylostratum

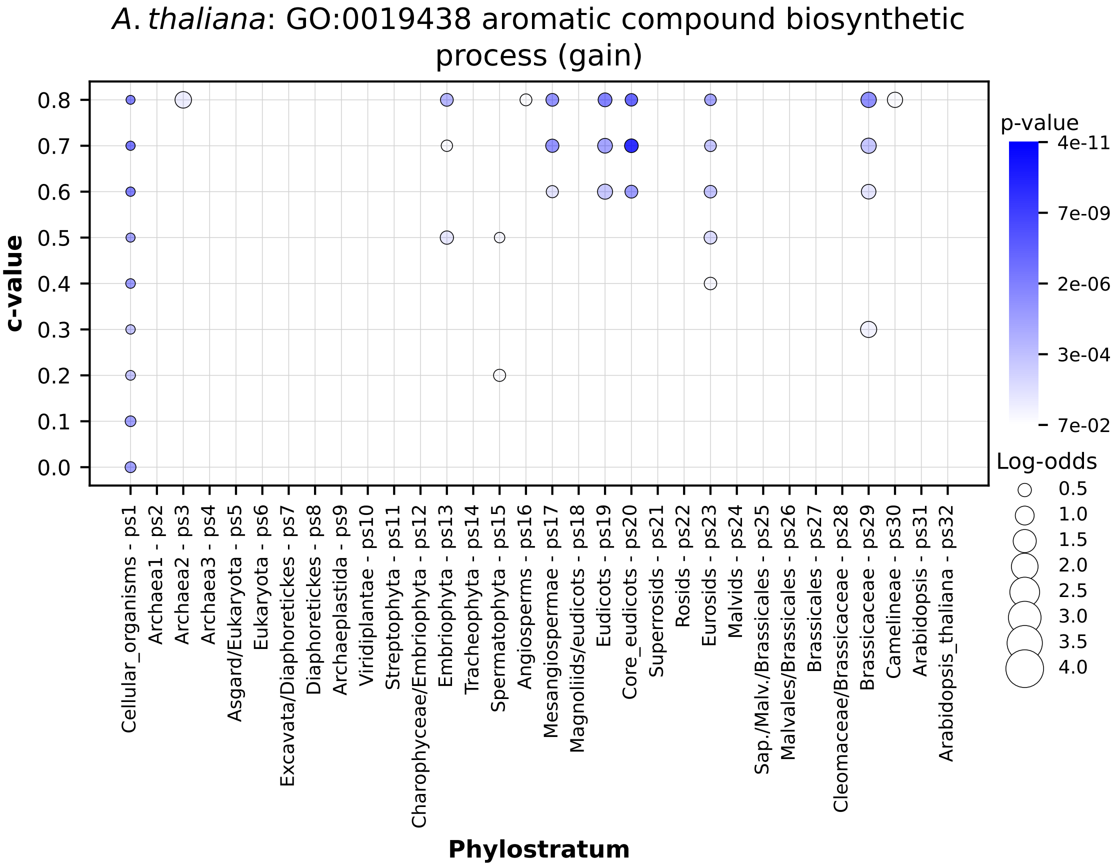

A. thaliana: GO:0019438 aromatic compound biosynthetic process (gain)
c-value
0.8
0.7
0.6
0.5
0.4
0.3
0.2
0.1
0.0
Phylostratum
Cellular_organisms - ps1
Archaea1 - ps2
Archaea2 - ps3
Archaea3 - ps4
Asgard/Eukaryota - ps5
Eukaryota - ps6
Excavata/Diaphoretickes - ps7
Diaphoretickes - ps8
Archaeplastida - ps9
Viridiplantae - ps10
Streptophyta - ps11
Charophyceae/Embriophyta - ps12
Embriophyta - ps13
Tracheophyta - ps14
Spermatophyta - ps15
Angiosperms - ps16
Mesangiospermae - ps17
Magnoliids/eudicots - ps18
Eudicots - ps19
Core_eudicots - ps20
Superrosids - ps21
Rosids - ps22
Eurosids - ps23
Malvids - ps24
Sap./Malv./Brassicales - ps25
Malvales/Brassicales - ps26
Brassicales - ps27
Cleomaceae/Brassicaceae - ps28
Brassicaceae - ps29
Camelineae - ps30
Arabidopsis - ps31
Arabidopsis_thaliana - ps32
p-value
4e-11
7e-09
2e-06
3e-04
7e-02
Log-odds
0.5
1.0
1.5
2.0
2.5
3.0
3.5
4.0

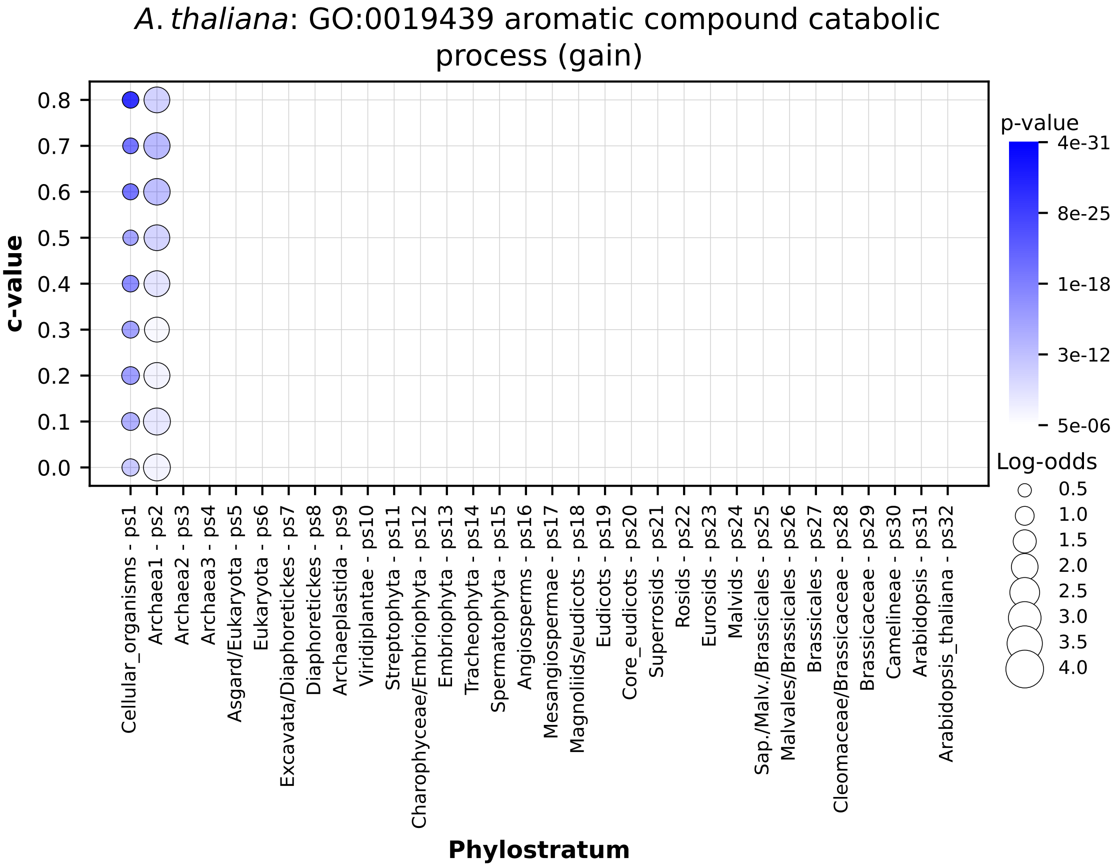

A. thaliana: GO:0019439 aromatic compound catabolic process (gain)
c-value
0.8
0.7
0.6
0.5
0.4
0.3
0.2
0.1
0.0
p-value
4e-31
8e-25
1e-18
3e-12
5e-06
Log-odds
0.5
1.0
1.5
2.0
2.5
3.0
3.5
4.0
Cellular_organisms - ps1
Archaea1 - ps2
Archaea2 - ps3
Archaea3 - ps4
Asgard/Eukaryota - ps5
Eukaryota - ps6
Excavata/Diaphoretickes - ps7
Diaphoretickes - ps8
Archaeplastida - ps9
Viridiplantae - ps10
Streptophyta - ps11
Charophyceae/Embriophyta - ps12
Embriophyta - ps13
Tracheophyta - ps14
Spermatophyta - ps15
Angiosperms - ps16
Mesangiospermae - ps17
Magnoliids/eudicots - ps18
Eudicots - ps19
Core_eudicots - ps20
Superrosids - ps21
Rosids - ps22
Eurosids - ps23
Malvids - ps24
Sap./Malv./Brassicales - ps25
Malvales/Brassicales - ps26
Brassicales - ps27
Cleomaceae/Brassicaceae - ps28
Brassicaceae - ps29
Camelineae - ps30
Arabidopsis - ps31
Arabidopsis_thaliana - ps32
Phylostratum

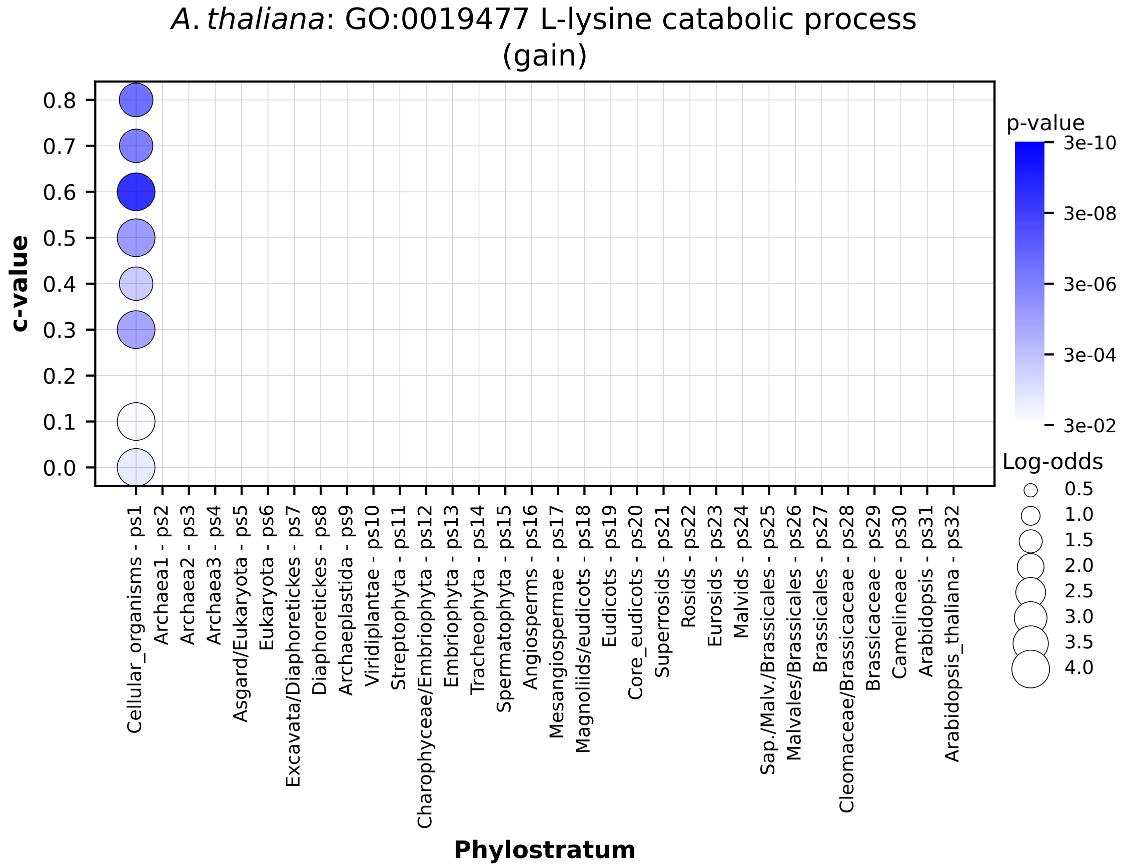
A. thaliana: GO:0019477 L-lysine catabolic process
(gain)
c-value
0.8
0.7
0.6
0.5
0.4
0.3
0.2
0.1
0.0
p-value
3e-10
3e-08
3e-06
3e-04
3e-02
Log-odds
0.5
1.0
1.5
2.0
2.5
3.0
3.5
4.0
Cellular_organisms - ps1
Archaea1 - ps2
Archaea2 - ps3
Archaea3 - ps4
Asgard/Eukaryota - ps5
Eukaryota - ps6
Excavata/Diaphoretickes - ps7
Diaphoretickes - ps8
Archaeplastida - ps9
Viridiplantae - ps10
Streptophyta - ps11
Charophyceae/Embriophyta - ps12
Embriophyta - ps13
Tracheophyta - ps14
Spermatophyta - ps15
Angiosperms - ps16
Mesangiospermae - ps17
Magnoliids/eudicots - ps18
Eudicots - ps19
Core_eudicots - ps20
Superrosids - ps21
Rosids - ps22
Eurosids - ps23
Malvids - ps24
Sap./Malv./Brassicales - ps25
Malvales/Brassicales - ps26
Brassicales - ps27
Cleomaceae/Brassicaceae - ps28
Brassicaceae - ps29
Camelineae - ps30
Arabidopsis - ps31
Arabidopsis_thaliana - ps32
Phylostratum

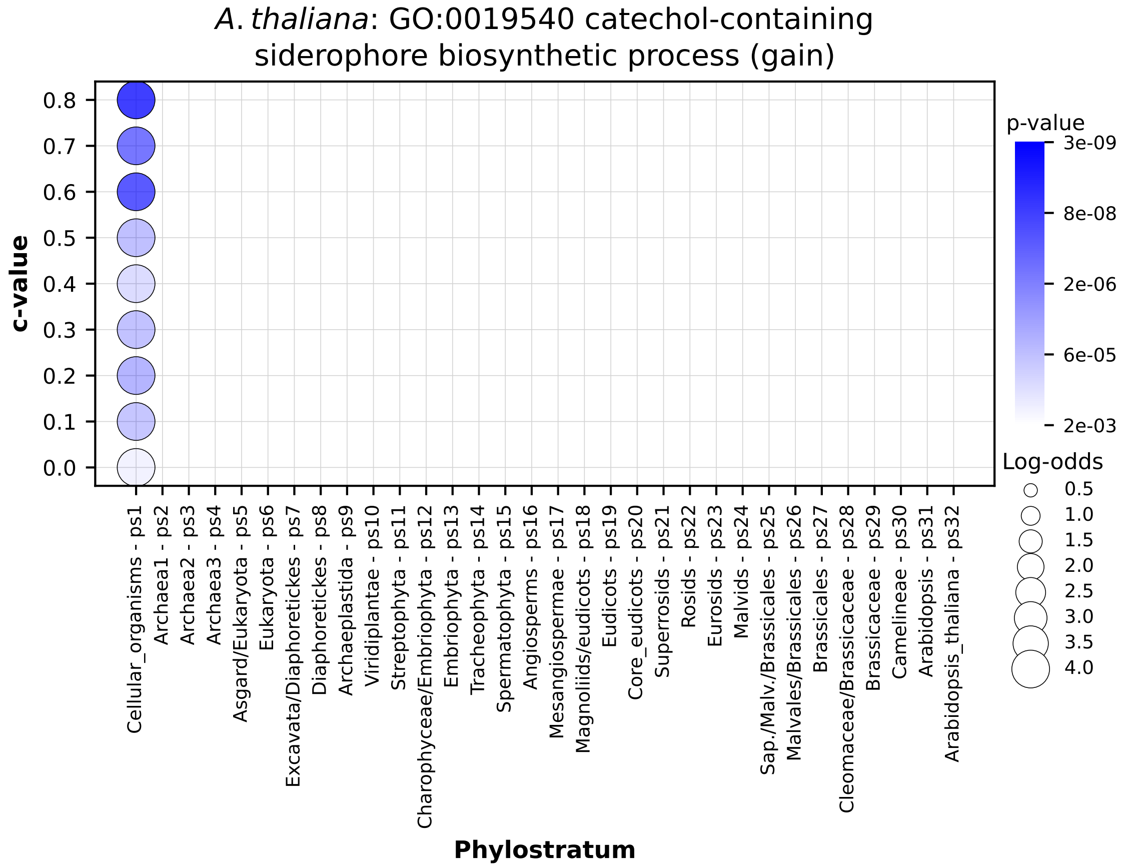

A. thaliana: GO:0019540 catechol-containing siderophore biosynthetic process (gain)
c-value
0.0
0.1
0.2
0.3
0.4
0.5
0.6
0.7
0.8
p-value
3e-09
8e-08
2e-06
6e-05
2e-03
Log-odds
0.5
1.0
1.5
2.0
2.5
3.0
3.5
4.0
Cellular_organisms - ps1
Archaea1 - ps2
Archaea2 - ps3
Archaea3 - ps4
Asgard/Eukaryota - ps5
Eukaryota - ps6
Excavata/Diaphoretickes - ps7
Diaphoretickes - ps8
Archaeplastida - ps9
Viridiplantae - ps10
Streptophyta - ps11
Charophyceae/Embriophyta - ps12
Embriophyta - ps13
Tracheophyta - ps14
Spermatophyta - ps15
Angiosperms - ps16
Mesangiospermae - ps17
Magnoliids/eudicots - ps18
Eudicots - ps19
Core_eudicots - ps20
Superrosids - ps21
Rosids - ps22
Eurosids - ps23
Malvids - ps24
Sap./Malv./Brassicales - ps25
Malvales/Brassicales - ps26
Brassicales - ps27
Cleomaceae/Brassicaceae - ps28
Brassicaceae - ps29
Camelineae - ps30
Arabidopsis - ps31
Arabidopsis_thaliana - ps32
Phylostratum

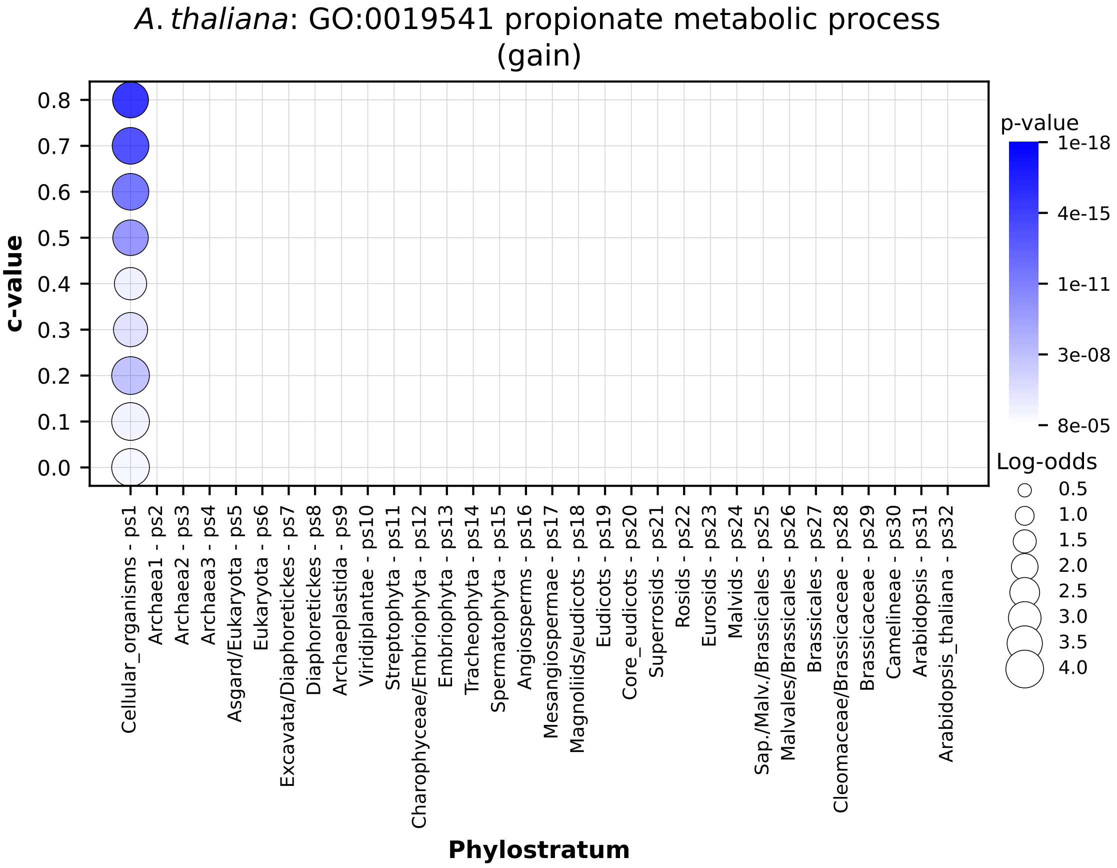

A. thaliana: GO:0019541 propionate metabolic process (gain)
c-value
0.8
0.7
0.6
0.5
0.4
0.3
0.2
0.1
0.0
p-value
1e-18
4e-15
1e-11
3e-08
8e-05
Log-odds
0.5
1.0
1.5
2.0
2.5
3.0
3.5
4.0
Cellular_organisms - ps1
Archaea1 - ps2
Archaea2 - ps3
Archaea3 - ps4
Asgard/Eukaryota - ps5
Eukaryota - ps6
Excavata/Diaphoretickes - ps7
Diaphoretickes - ps8
Archaeplastida - ps9
Viridiplantae - ps10
Streptophyta - ps11
Charophyceae/Embriophyta - ps12
Embriophyta - ps13
Tracheophyta - ps14
Spermatophyta - ps15
Angiosperms - ps16
Mesangiospermae - ps17
Magnoliids/eudicots - ps18
Eudicots - ps19
Core_eudicots - ps20
Superrosids - ps21
Rosids - ps22
Eurosids - ps23
Malvids - ps24
Sap./Malv./Brassicales - ps25
Malvales/Brassicales - ps26
Brassicales - ps27
Cleomaceae/Brassicaceae - ps28
Brassicaceae - ps29
Camelineae - ps30
Arabidopsis - ps31
Arabidopsis_thaliana - ps32
Phylostratum

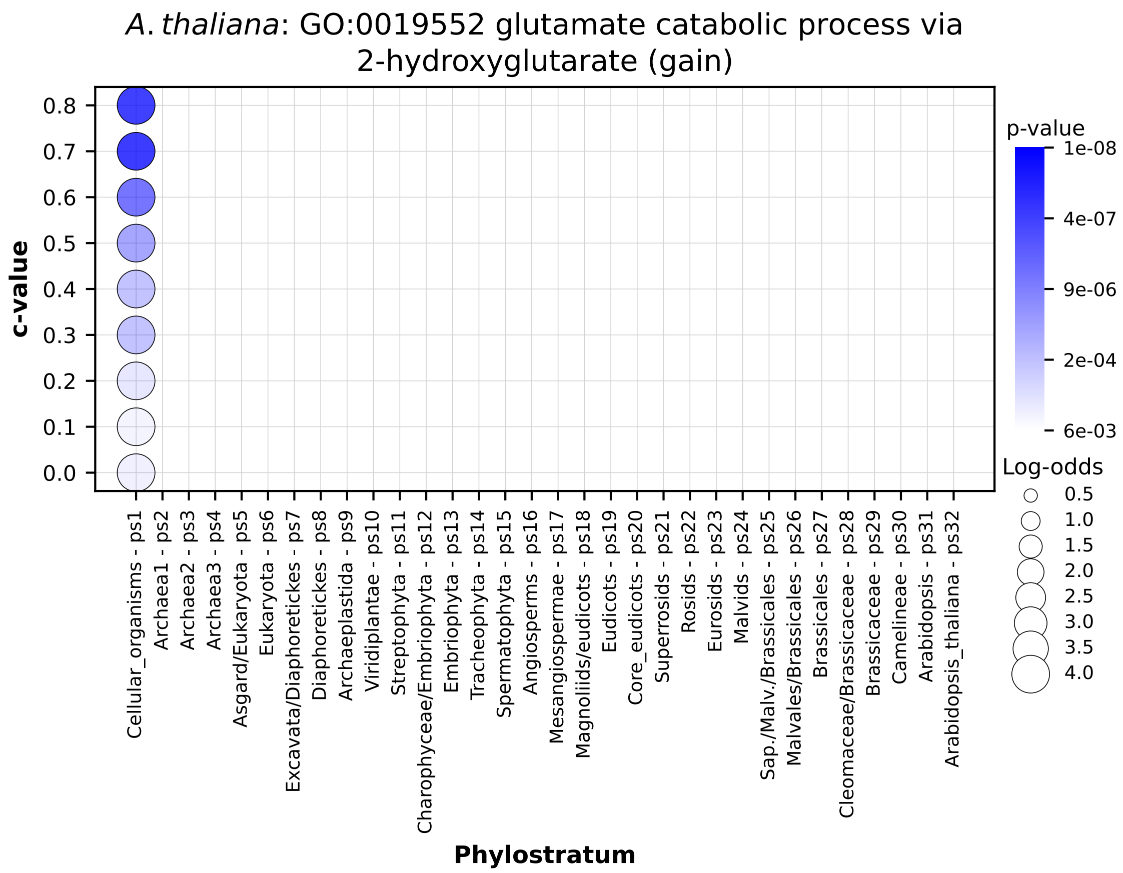

A. thaliana: GO:0019552 glutamate catabolic process via 2-hydroxyglutarate (gain)
c-value
0.8
0.7
0.6
0.5
0.4
0.3
0.2
0.1
0.0
p-value
1e-08
4e-07
9e-06
2e-04
6e-03
Log-odds
0.5
1.0
1.5
2.0
2.5
3.0
3.5
4.0
Cellular_organisms - ps1
Archaea1 - ps2
Archaea2 - ps3
Archaea3 - ps4
Asgard/Eukaryota - ps5
Eukaryota - ps6
Excavata/Diaphoretickes - ps7
Diaphoretickes - ps8
Archaeplastida - ps9
Viridiplantae - ps10
Streptophyta - ps11
Charophyceae/Embriophyta - ps12
Embriophyta - ps13
Tracheophyta - ps14
Spermatophyta - ps15
Angiosperms - ps16
Mesangiospermae - ps17
Magnoliids/eudicots - ps18
Eudicots - ps19
Core_eudicots - ps20
Superrosids - ps21
Rosids - ps22
Eurosids - ps23
Malvids - ps24
Sap./Malv./Brassicales - ps25
Malvales/Brassicales - ps26
Brassicales - ps27
Cleomaceae/Brassicaceae - ps28
Brassicaceae - ps29
Camelineae - ps30
Arabidopsis - ps31
Arabidopsis_thaliana - ps32
Phylostratum

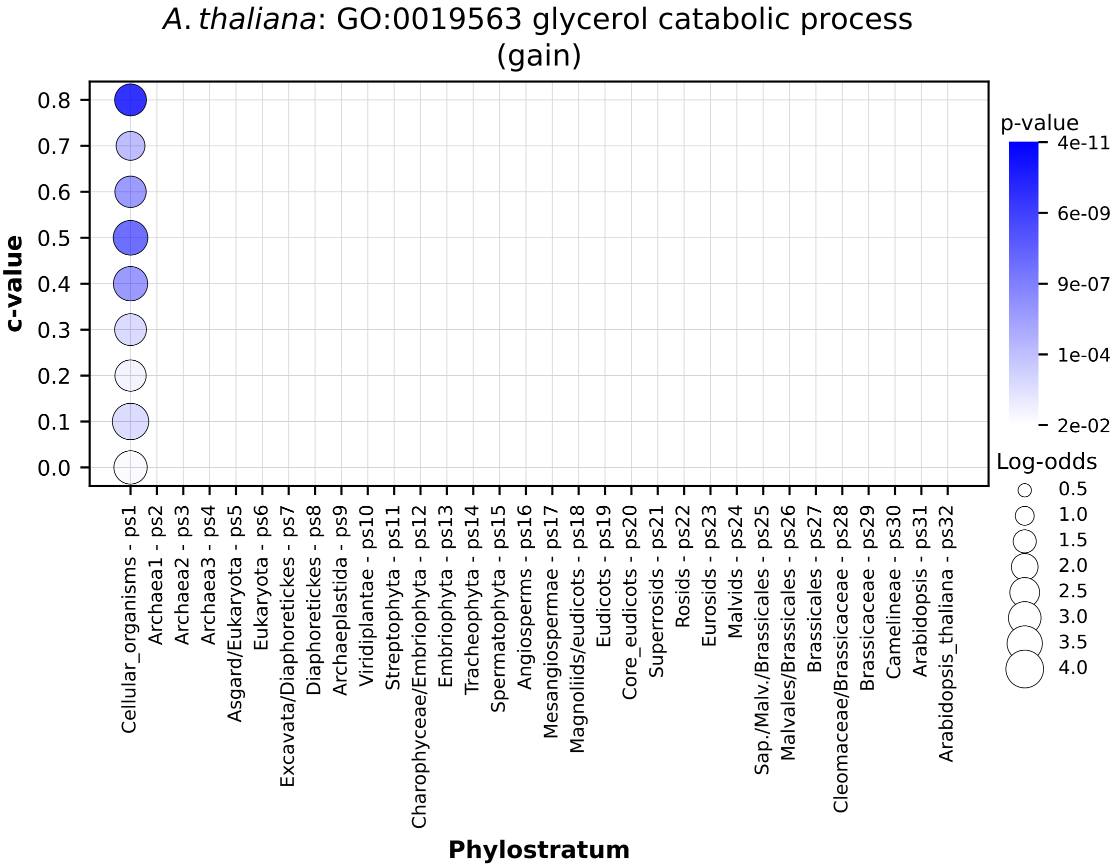

A. thaliana: GO:0019563 glycerol catabolic process
(gain)
c-value
0.8
0.7
0.6
0.5
0.4
0.3
0.2
0.1
0.0
p-value
4e-11
6e-09
9e-07
1e-04
2e-02
Log-odds
0.5
1.0
1.5
2.0
2.5
3.0
3.5
4.0
Cellular_organisms - ps1
Archaea1 - ps2
Archaea2 - ps3
Archaea3 - ps4
Asgard/Eukaryota - ps5
Eukaryota - ps6
Excavata/Diaphoretickes - ps7
Diaphoretickes - ps8
Archaeplastida - ps9
Viridiplantae - ps10
Streptophyta - ps11
Charophyceae/Embriophyta - ps12
Embriophyta - ps13
Tracheophyta - ps14
Spermatophyta - ps15
Angiosperms - ps16
Mesangiospermae - ps17
Magnoliids/eudicots - ps18
Eudicots - ps19
Core_eudicots - ps20
Superrosids - ps21
Rosids - ps22
Eurosids - ps23
Malvids - ps24
Sap./Malv./Brassicales - ps25
Malvales/Brassicales - ps26
Brassicales - ps27
Cleomaceae/Brassicaceae - ps28
Brassicaceae - ps29
Camelineae - ps30
Arabidopsis - ps31
Arabidopsis_thaliana - ps32
Phylostratum

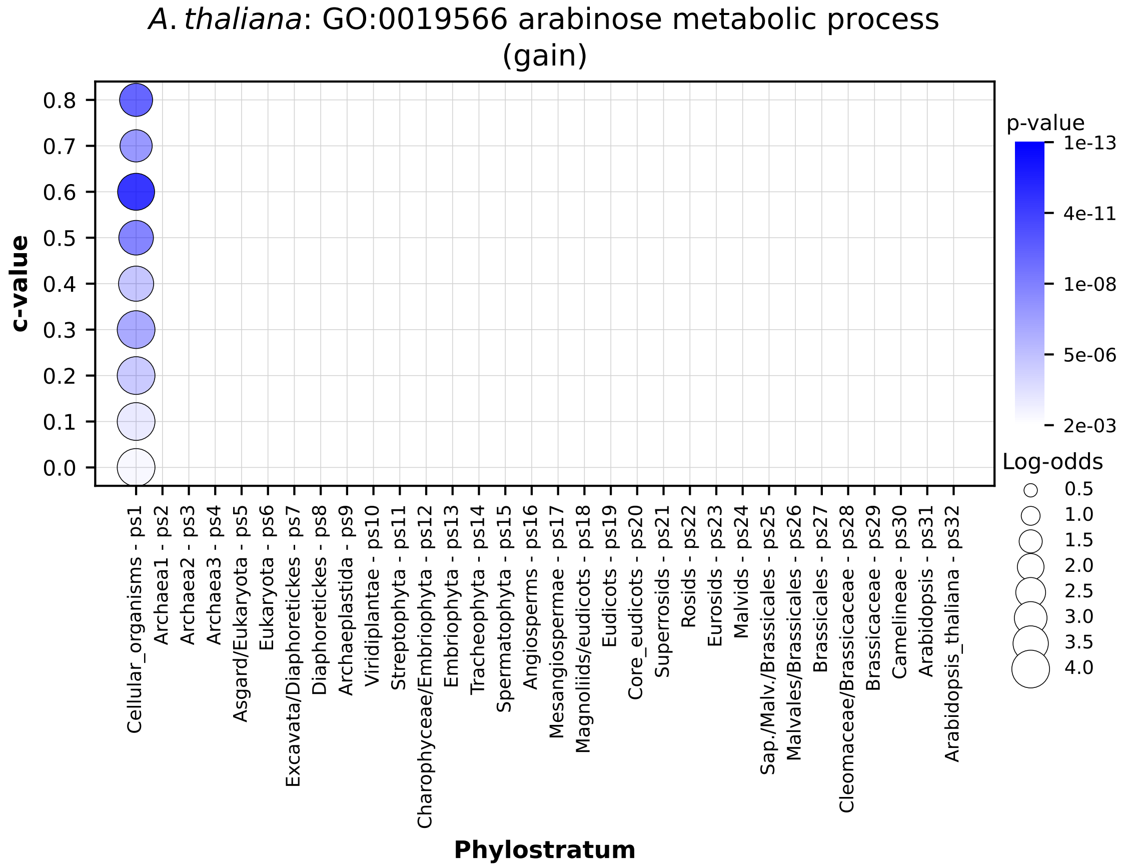
A. thaliana: GO:0019566 arabinose metabolic process
(gain)
c-value
0.8
0.7
0.6
0.5
0.4
0.3
0.2
0.1
0.0
p-value
1e-13
4e-11
1e-08
5e-06
2e-03
Log-odds
0.5
1.0
1.5
2.0
2.5
3.0
3.5
4.0
Cellular_organisms - ps1
Archaea1 - ps2
Archaea2 - ps3
Archaea3 - ps4
Asgard/Eukaryota - ps5
Eukaryota - ps6
Excavata/Diaphoretickes - ps7
Diaphoretickes - ps8
Archaeplastida - ps9
Viridiplantae - ps10
Streptophyta - ps11
Charophyceae/Embriophyta - ps12
Embriophyta - ps13
Tracheophyta - ps14
Spermatophyta - ps15
Angiosperms - ps16
Mesangiospermae - ps17
Magnoliids/eudicots - ps18
Eudicots - ps19
Core_eudicots - ps20
Superrosids - ps21
Rosids - ps22
Eurosids - ps23
Malvids - ps24
Sap./Malv./Brassicales - ps25
Malvales/Brassicales - ps26
Brassicales - ps27
Cleomaceae/Brassicaceae - ps28
Brassicaceae - ps29
Camelineae - ps30
Arabidopsis - ps31
Arabidopsis_thaliana - ps32
Phylostratum

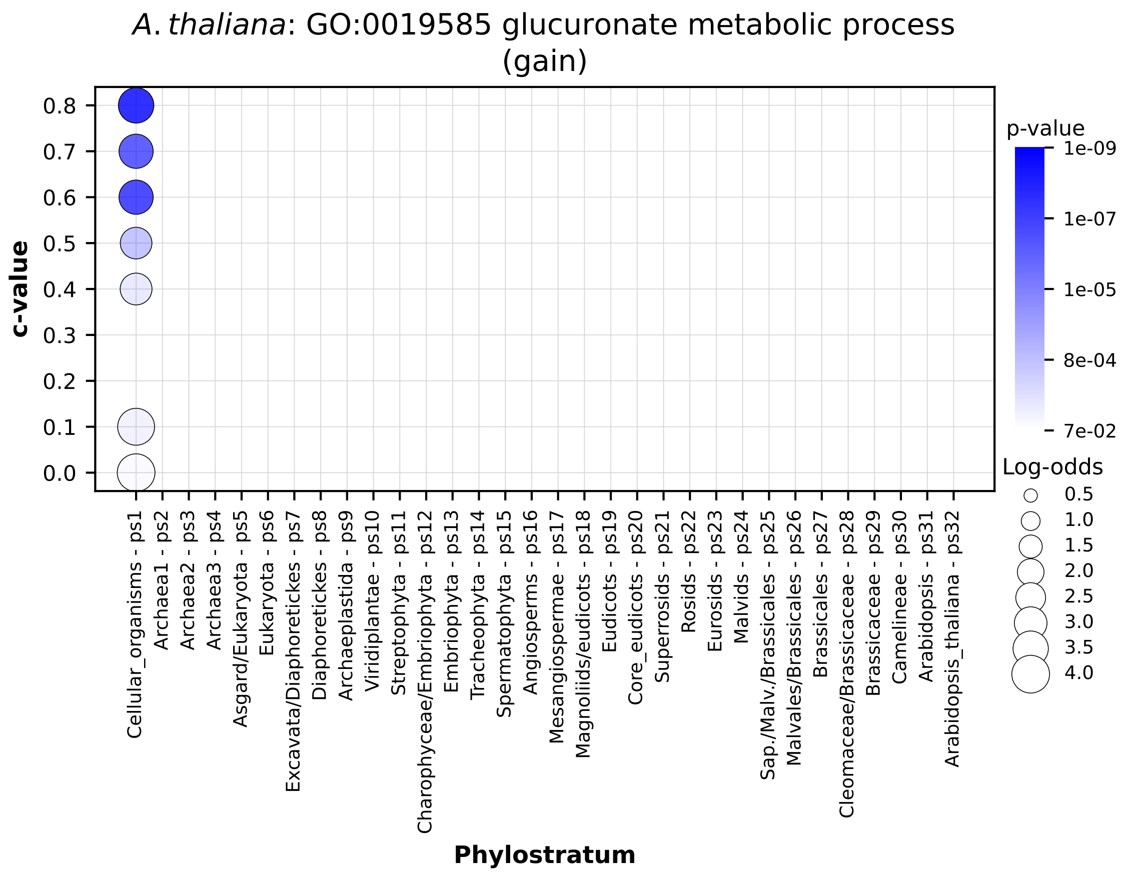
A. thaliana: GO:0019585 glucuronate metabolic process
(gain)
c-value
0.8
0.7
0.6
0.5
0.4
0.3
0.2
0.1
0.0
p-value
1e-09
1e-07
1e-05
8e-04
7e-02
Log-odds
0.5
1.0
1.5
2.0
2.5
3.0
3.5
4.0
Cellular_organisms - ps1
Archaea1 - ps2
Archaea2 - ps3
Archaea3 - ps4
Asgard/Eukaryota - ps5
Eukaryota - ps6
Excavata/Diaphoretickes - ps7
Diaphoretickes - ps8
Archaeplastida - ps9
Viridiplantae - ps10
Streptophyta - ps11
Charophyceae/Embriophyta - ps12
Embriophyta - ps13
Tracheophyta - ps14
Spermatophyta - ps15
Angiosperms - ps16
Mesangiospermae - ps17
Magnoliids/eudicots - ps18
Eudicots - ps19
Core_eudicots - ps20
Superrosids - ps21
Rosids - ps22
Eurosids - ps23
Malvids - ps24
Sap./Malv./Brassicales - ps25
Malvales/Brassicales - ps26
Brassicales - ps27
Cleomaceae/Brassicaceae - ps28
Brassicaceae - ps29
Camelineae - ps30
Arabidopsis - ps31
Arabidopsis_thaliana - ps32
Phylostratum

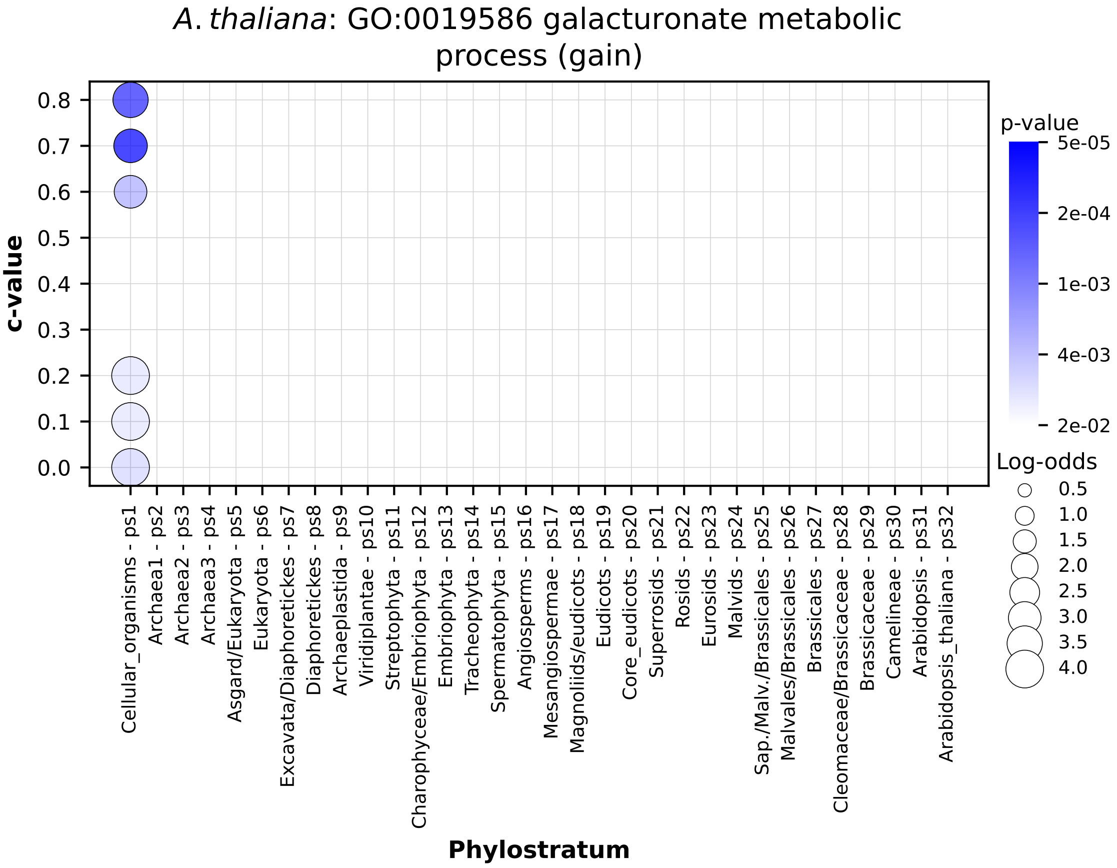

A. thaliana: GO:0019586 galacturonate metabolic process (gain)
c-value
0.8
0.7
0.6
0.5
0.4
0.3
0.2
0.1
0.0
p-value
5e-05
2e-04
1e-03
4e-03
2e-02
Log-odds
0.5
1.0
1.5
2.0
2.5
3.0
3.5
4.0
Cellular_organisms - ps1
Archaea1 - ps2
Archaea2 - ps3
Archaea3 - ps4
Asgard/Eukaryota - ps5
Eukaryota - ps6
Excavata/Diaphoretickes - ps7
Diaphoretickes - ps8
Archaeplastida - ps9
Viridiplantae - ps10
Streptophyta - ps11
Charophyceae/Embriophyta - ps12
Embriophyta - ps13
Tracheophyta - ps14
Spermatophyta - ps15
Angiosperms - ps16
Mesangiospermae - ps17
Magnoliids/eudicots - ps18
Eudicots - ps19
Core_eudicots - ps20
Superrosids - ps21
Rosids - ps22
Eurosids - ps23
Malvids - ps24
Sap./Malv./Brassicales - ps25
Malvales/Brassicales - ps26
Brassicales - ps27
Cleomaceae/Brassicaceae - ps28
Brassicaceae - ps29
Camelineae - ps30
Arabidopsis - ps31
Arabidopsis_thaliana - ps32
Phylostratum

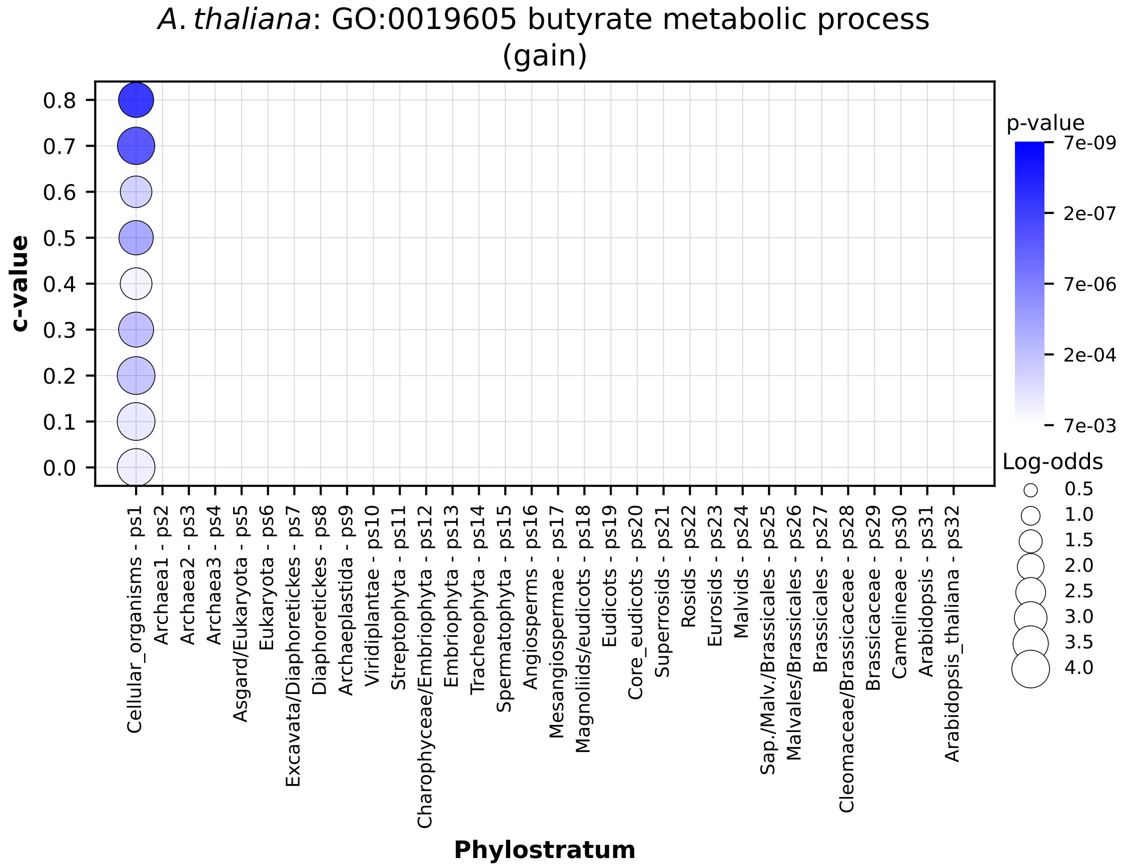

A. thaliana: GO:0019605 butyrate metabolic process
(gain)
c-value
0.8
0.7
0.6
0.5
0.4
0.3
0.2
0.1
0.0
p-value
7e-09
2e-07
7e-06
2e-04
7e-03
Log-odds
0.5
1.0
1.5
2.0
2.5
3.0
3.5
4.0
Cellular_organisms - ps1
Archaea1 - ps2
Archaea2 - ps3
Archaea3 - ps4
Asgard/Eukaryota - ps5
Eukaryota - ps6
Excavata/Diaphoretickes - ps7
Diaphoretickes - ps8
Archaeplastida - ps9
Viridiplantae - ps10
Streptophyta - ps11
Charophyceae/Embriophyta - ps12
Embriophyta - ps13
Tracheophyta - ps14
Spermatophyta - ps15
Angiosperms - ps16
Mesangiospermae - ps17
Magnoliids/eudicots - ps18
Eudicots - ps19
Core_eudicots - ps20
Superrosids - ps21
Rosids - ps22
Eurosids - ps23
Malvids - ps24
Sap./Malv./Brassicales - ps25
Malvales/Brassicales - ps26
Brassicales - ps27
Cleomaceae/Brassicaceae - ps28
Brassicaceae - ps29
Camelineae - ps30
Arabidopsis - ps31
Arabidopsis_thaliana - ps32
Phylostratum

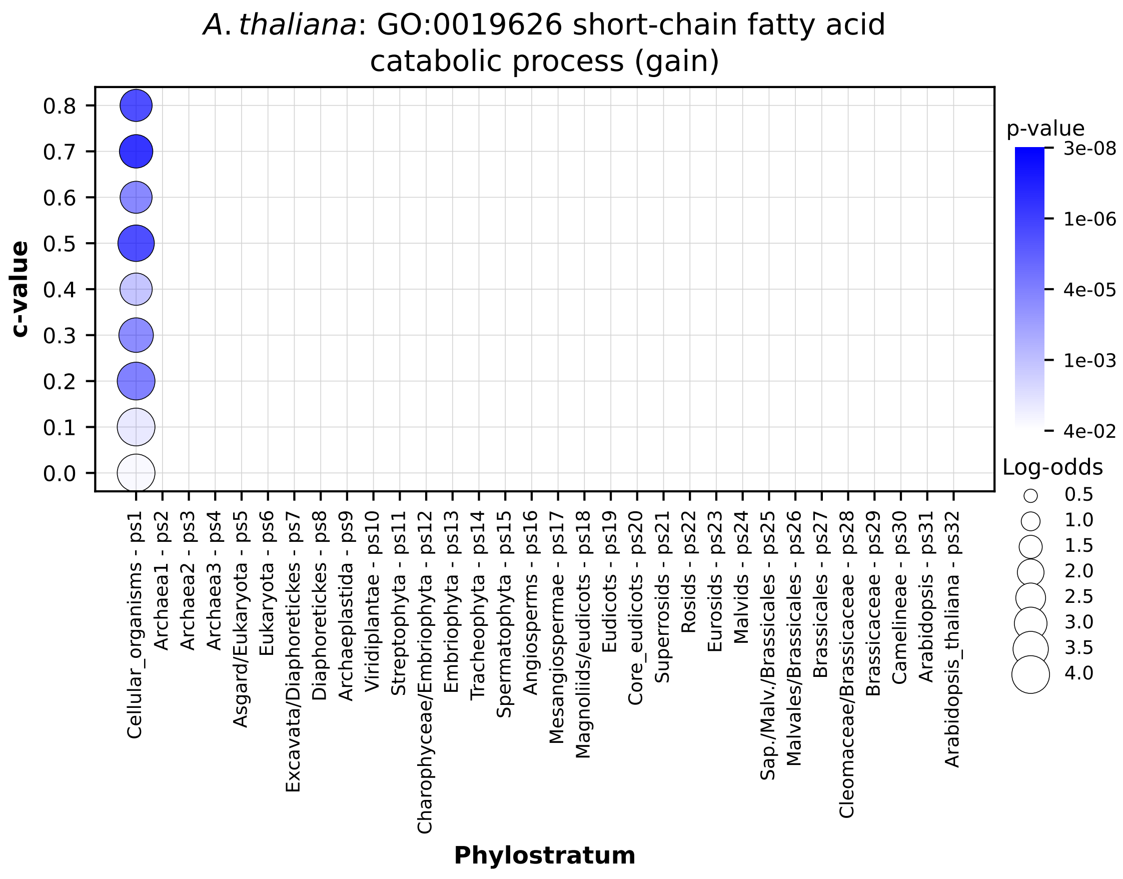

A. thaliana: GO:0019626 short-chain fatty acid catabolic process (gain)
c-value
0.0
0.1
0.2
0.3
0.4
0.5
0.6
0.7
0.8
p-value
3e-08
1e-06
4e-05
1e-03
4e-02
Log-odds
0.5
1.0
1.5
2.0
2.5
3.0
3.5
4.0
Cellular_organisms - ps1
Archaea1 - ps2
Archaea2 - ps3
Archaea3 - ps4
Asgard/Eukaryota - ps5
Eukaryota - ps6
Excavata/Diaphoretickes - ps7
Diaphoretickes - ps8
Archaeplastida - ps9
Viridiplantae - ps10
Streptophyta - ps11
Charophyceae/Embriophyta - ps12
Embriophyta - ps13
Tracheophyta - ps14
Spermatophyta - ps15
Angiosperms - ps16
Mesangiospermae - ps17
Magnoliids/eudicots - ps18
Eudicots - ps19
Core_eudicots - ps20
Superrosids - ps21
Rosids - ps22
Eurosids - ps23
Malvids - ps24
Sap./Malv./Brassicales - ps25
Malvales/Brassicales - ps26
Brassicales - ps27
Cleomaceae/Brassicaceae - ps28
Brassicaceae - ps29
Camelineae - ps30
Arabidopsis - ps31
Arabidopsis_thaliana - ps32
Phylostratum

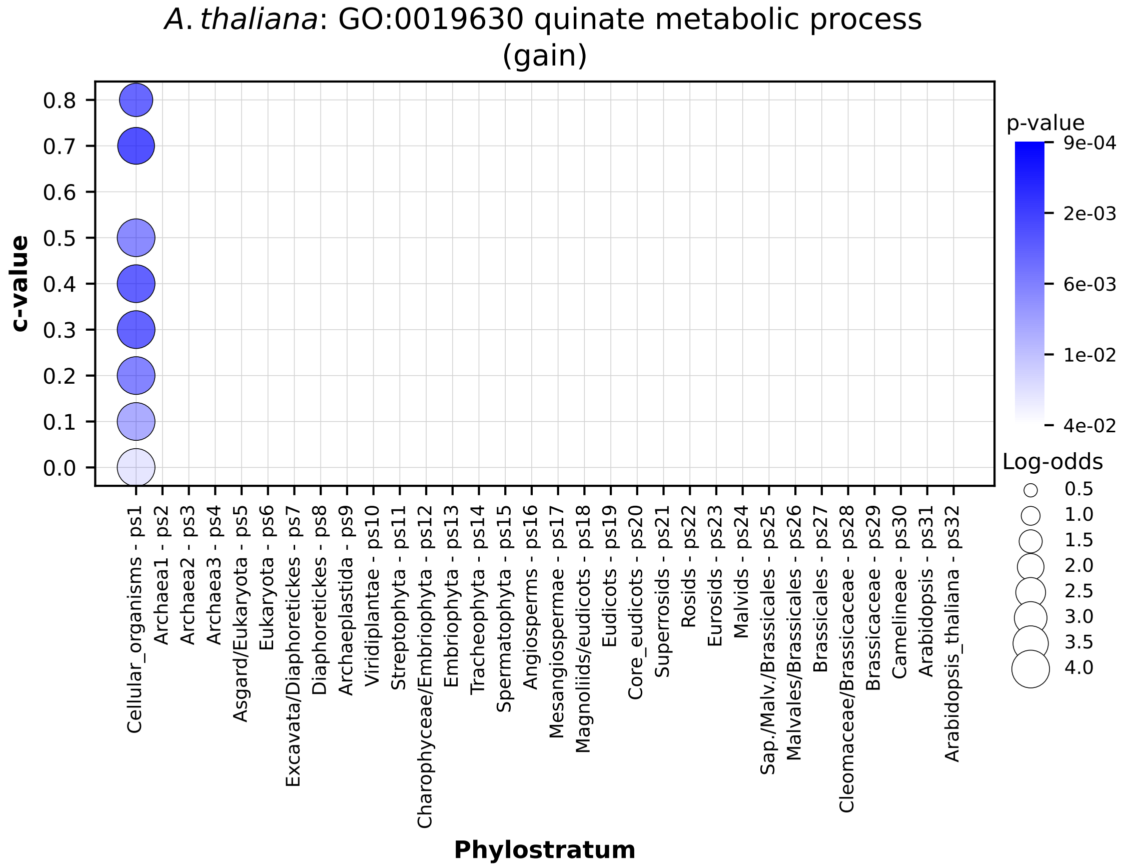

A. thaliana: GO:0019630 quinate metabolic process
(gain)
c-value
0.8
0.7
0.6
0.5
0.4
0.3
0.2
0.1
0.0
p-value
9e-04
2e-03
6e-03
1e-02
4e-02
Log-odds
0.5
1.0
1.5
2.0
2.5
3.0
3.5
4.0
Cellular_organisms - ps1
Archaea1 - ps2
Archaea2 - ps3
Archaea3 - ps4
Asgard/Eukaryota - ps5
Eukaryota - ps6
Excavata/Diaphoretickes - ps7
Diaphoretickes - ps8
Archaeplastida - ps9
Viridiplantae - ps10
Streptophyta - ps11
Charophyceae/Embriophyta - ps12
Embriophyta - ps13
Tracheophyta - ps14
Spermatophyta - ps15
Angiosperms - ps16
Mesangiospermae - ps17
Magnoliids/eudicots - ps18
Eudicots - ps19
Core_eudicots - ps20
Superrosids - ps21
Rosids - ps22
Eurosids - ps23
Malvids - ps24
Sap./Malv./Brassicales - ps25
Malvales/Brassicales - ps26
Brassicales - ps27
Cleomaceae/Brassicaceae - ps28
Brassicaceae - ps29
Camelineae - ps30
Arabidopsis - ps31
Arabidopsis_thaliana - ps32
Phylostratum

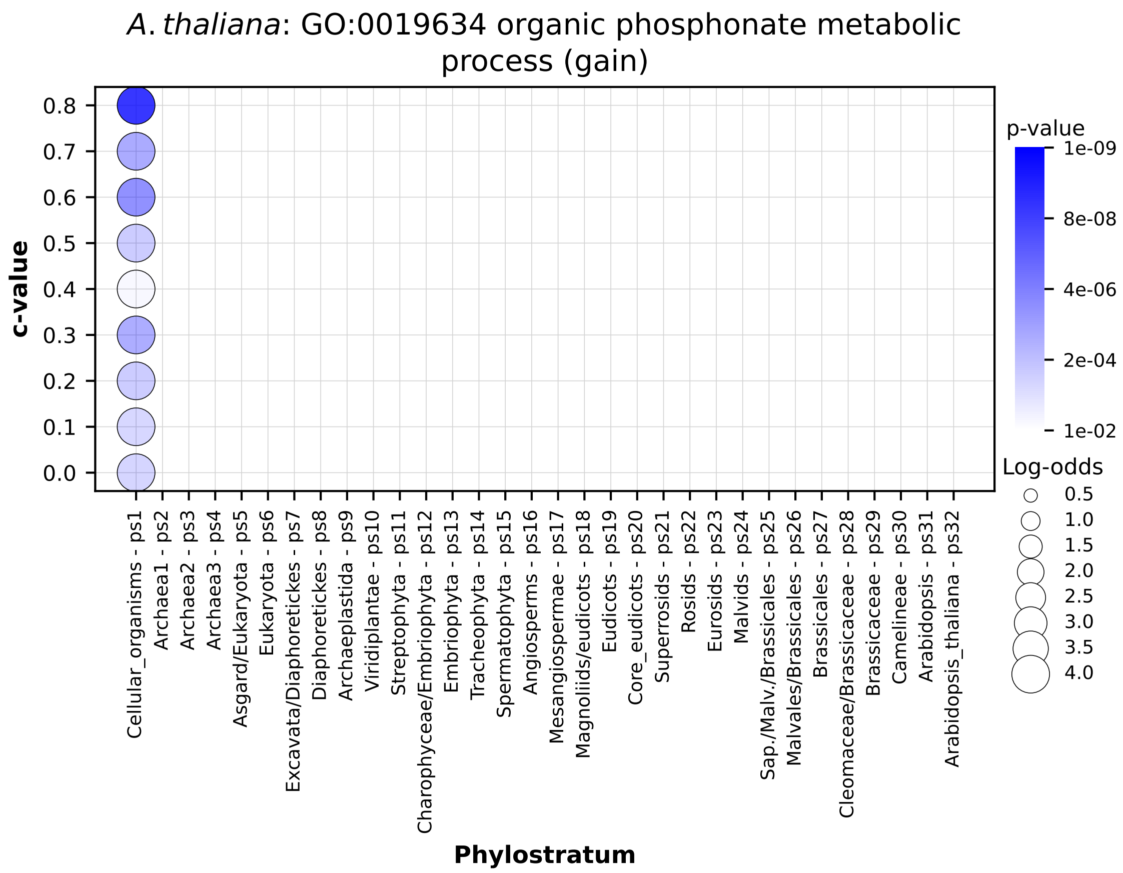

A. thaliana: GO:0019634 organic phosphonate metabolic process (gain)
c-value
0.8
0.7
0.6
0.5
0.4
0.3
0.2
0.1
0.0
Phylostratum
Cellular_organisms - ps1
Archaea1 - ps2
Archaea2 - ps3
Archaea3 - ps4
Asgard/Eukaryota - ps5
Eukaryota - ps6
Excavata/Diaphoretickes - ps7
Diaphoretickes - ps8
Archaeplastida - ps9
Viridiplantae - ps10
Streptophyta - ps11
Charophyceae/Embriophyta - ps12
Embriophyta - ps13
Tracheophyta - ps14
Spermatophyta - ps15
Angiosperms - ps16
Mesangiospermae - ps17
Magnoliids/eudicots - ps18
Eudicots - ps19
Core_eudicots - ps20
Superrosids - ps21
Rosids - ps22
Eurosids - ps23
Malvids - ps24
Sap./Malv./Brassicales - ps25
Malvales/Brassicales - ps26
Brassicales - ps27
Cleomaceae/Brassicaceae - ps28
Brassicaceae - ps29
Camelineae - ps30
Arabidopsis - ps31
Arabidopsis_thaliana - ps32
p-value
1e-09
8e-08
4e-06
2e-04
1e-02
Log-odds
0.5
1.0
1.5
2.0
2.5
3.0
3.5
4.0

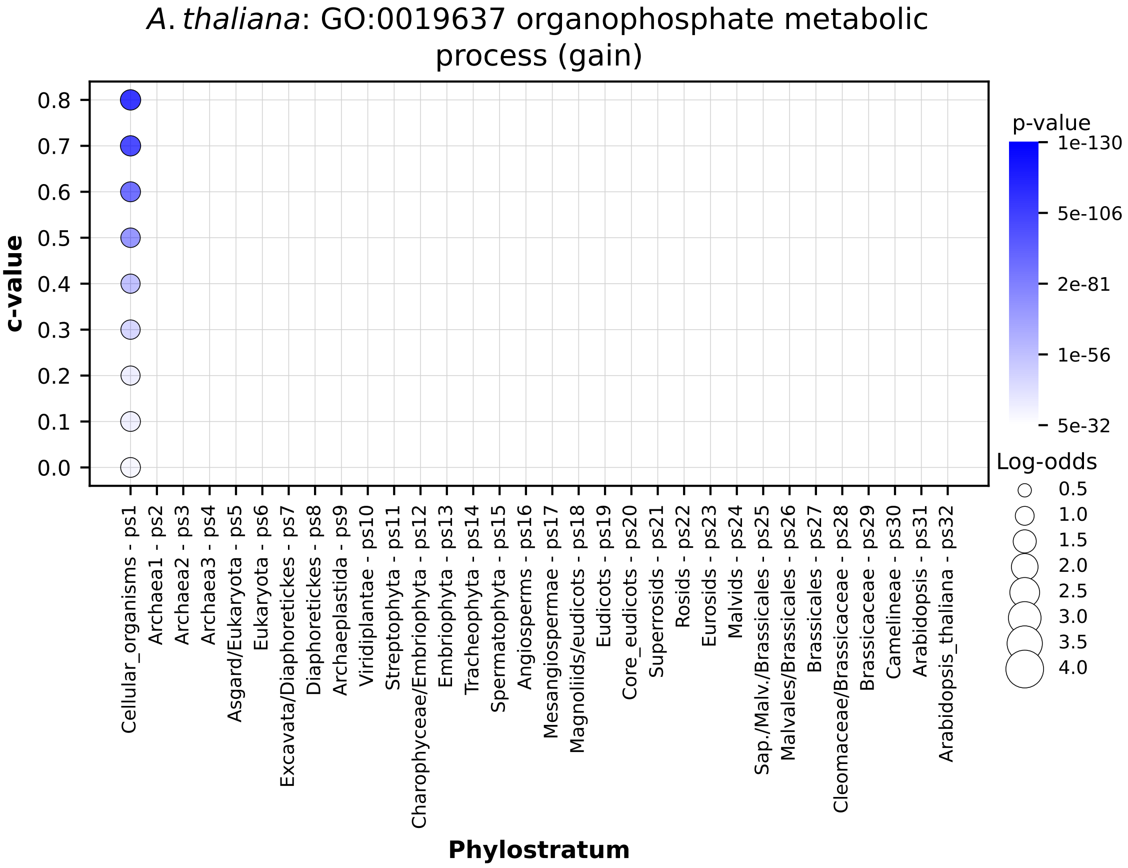
A. thaliana: GO:0019637 organophosphate metabolic process (gain)
c-value
0.8
0.7
0.6
0.5
0.4
0.3
0.2
0.1
0.0
Phylostratum
Cellular_organisms - ps1
Archaea1 - ps2
Archaea2 - ps3
Archaea3 - ps4
Asgard/Eukaryota - ps5
Eukaryota - ps6
Excavata/Diaphoretickes - ps7
Diaphoretickes - ps8
Archaeplastida - ps9
Viridiplantae - ps10
Streptophyta - ps11
Charophyceae/Embriophyta - ps12
Embriophyta - ps13
Tracheophyta - ps14
Spermatophyta - ps15
Angiosperms - ps16
Mesangiospermae - ps17
Magnoliids/eudicots - ps18
Eudicots - ps19
Core_eudicots - ps20
Superrosids - ps21
Rosids - ps22
Eurosids - ps23
Malvids - ps24
Sap./Malv./Brassicales - ps25
Malvales/Brassicales - ps26
Brassicales - ps27
Cleomaceae/Brassicaceae - ps28
Brassicaceae - ps29
Camelineae - ps30
Arabidopsis - ps31
Arabidopsis_thaliana - ps32
p-value
1e-130
5e-106
2e-81
1e-56
5e-32
Log-odds
0.5
1.0
1.5
2.0
2.5
3.0
3.5
4.0

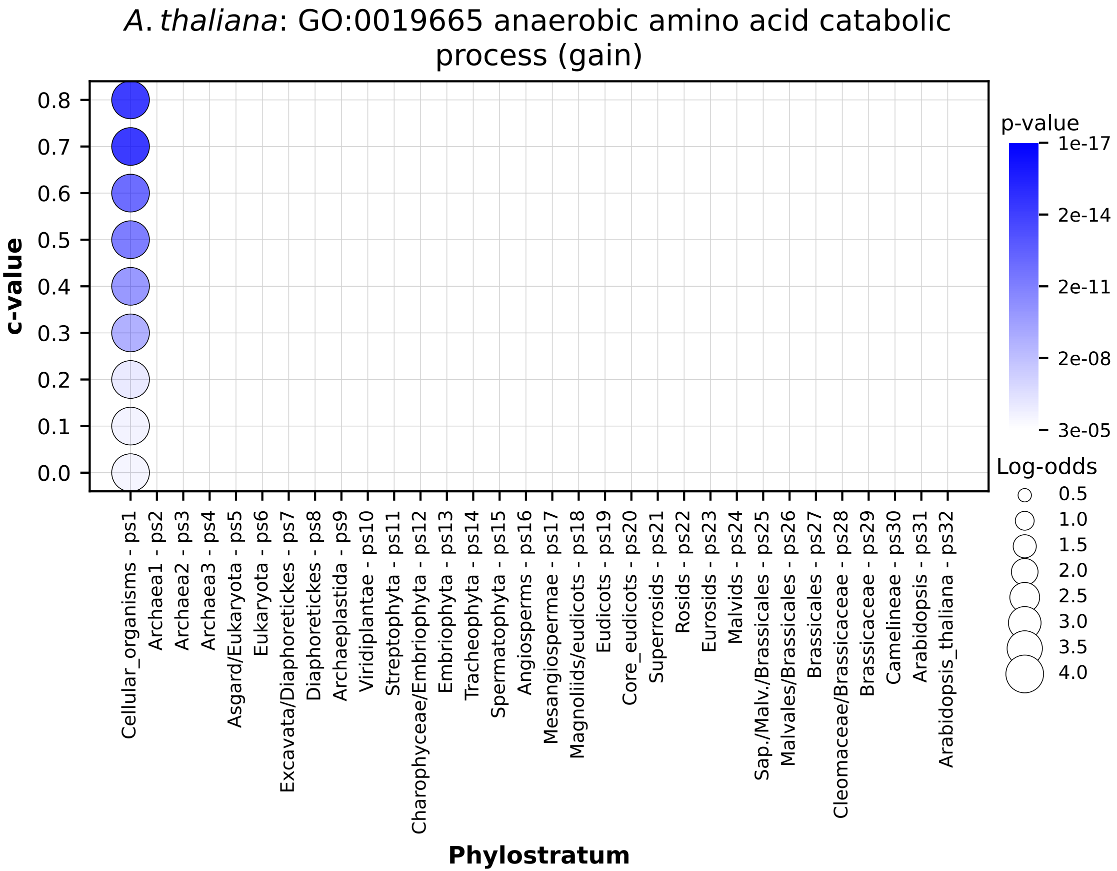
A. thaliana: GO:0019665 anaerobic amino acid catabolic process (gain)
c-value
0.8
0.7
0.6
0.5
0.4
0.3
0.2
0.1
0.0
Phylostratum
Cellular_organisms - ps1
Archaea1 - ps2
Archaea2 - ps3
Archaea3 - ps4
Asgard/Eukaryota - ps5
Eukaryota - ps6
Excavata/Diaphoretickes - ps7
Diaphoretickes - ps8
Archaeplastida - ps9
Viridiplantae - ps10
Streptophyta - ps11
Charophyceae/Embriophyta - ps12
Embriophyta - ps13
Tracheophyta - ps14
Spermatophyta - ps15
Angiosperms - ps16
Mesangiospermae - ps17
Magnoliids/eudicots - ps18
Eudicots - ps19
Core_eudicots - ps20
Superrosids - ps21
Rosids - ps22
Eurosids - ps23
Malvids - ps24
Sap./Malv./Brassicales - ps25
Malvales/Brassicales - ps26
Brassicales - ps27
Cleomaceae/Brassicaceae - ps28
Brassicaceae - ps29
Camelineae - ps30
Arabidopsis - ps31
Arabidopsis_thaliana - ps32
p-value
1e-17
2e-14
2e-11
2e-08
3e-05
Log-odds
0.5
1.0
1.5
2.0
2.5
3.0
3.5
4.0

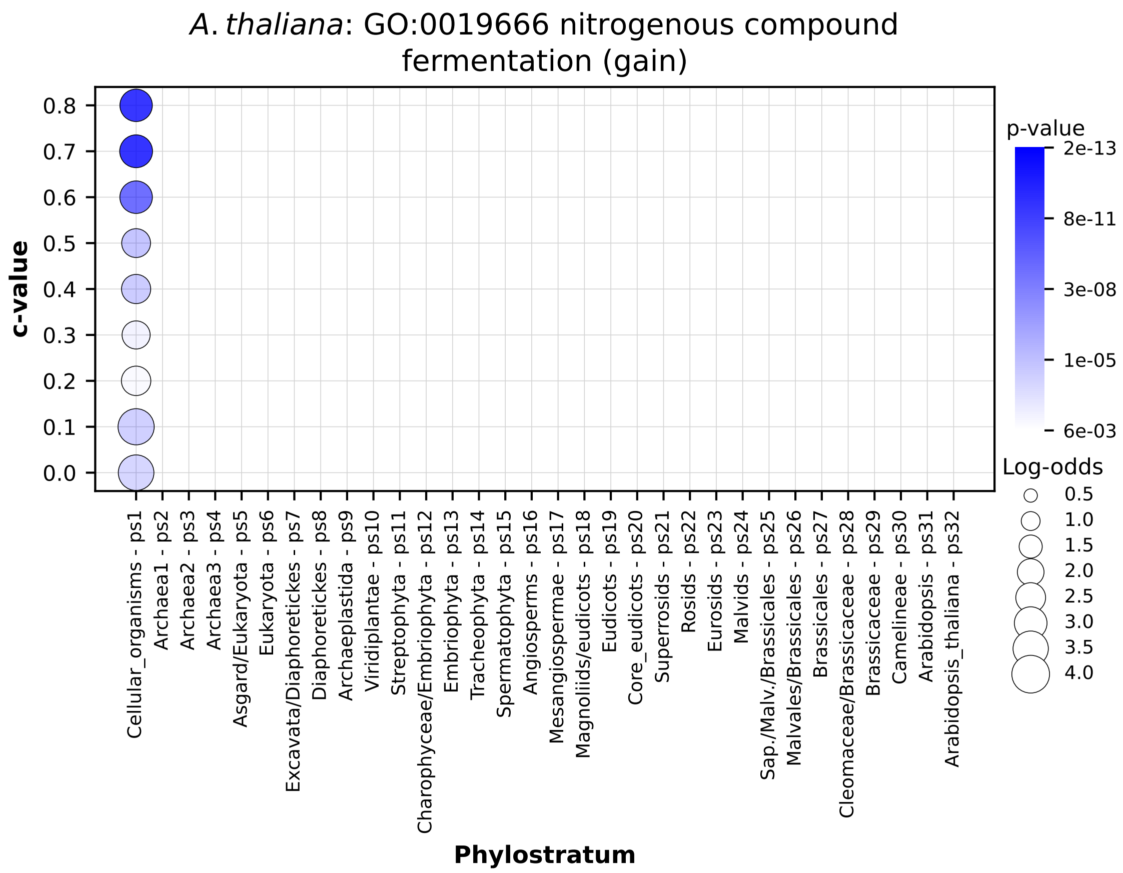

A. thaliana: GO:0019666 nitrogenous compound fermentation (gain)
c-value
0.8
0.7
0.6
0.5
0.4
0.3
0.2
0.1
0.0
p-value
2e-13
8e-11
3e-08
1e-05
6e-03
Log-odds
0.5
1.0
1.5
2.0
2.5
3.0
3.5
4.0
Cellular_organisms - ps1
Archaea1 - ps2
Archaea2 - ps3
Archaea3 - ps4
Asgard/Eukaryota - ps5
Eukaryota - ps6
Excavata/Diaphoretickes - ps7
Diaphoretickes - ps8
Archaeplastida - ps9
Viridiplantae - ps10
Streptophyta - ps11
Charophyceae/Embriophyta - ps12
Embriophyta - ps13
Tracheophyta - ps14
Spermatophyta - ps15
Angiosperms - ps16
Mesangiospermae - ps17
Magnoliids/eudicots - ps18
Eudicots - ps19
Core_eudicots - ps20
Superrosids - ps21
Rosids - ps22
Eurosids - ps23
Malvids - ps24
Sap./Malv./Brassicales - ps25
Malvales/Brassicales - ps26
Brassicales - ps27
Cleomaceae/Brassicaceae - ps28
Brassicaceae - ps29
Camelineae - ps30
Arabidopsis - ps31
Arabidopsis_thaliana - ps32
Phylostratum

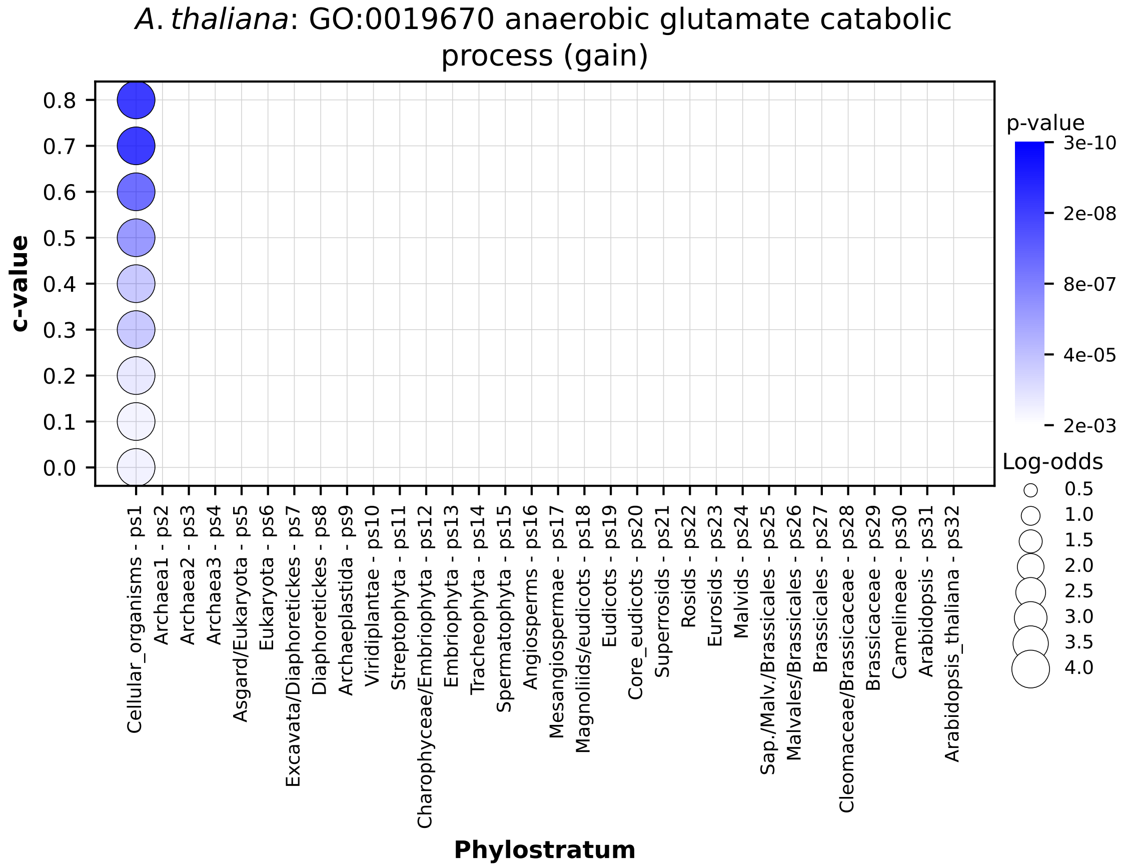

A. thaliana: GO:0019670 anaerobic glutamate catabolic process (gain)
c-value
0.8
0.7
0.6
0.5
0.4
0.3
0.2
0.1
0.0
p-value
3e-10
2e-08
8e-07
4e-05
2e-03
Log-odds
0.5
1.0
1.5
2.0
2.5
3.0
3.5
4.0
Cellular_organisms - ps1
Archaea1 - ps2
Archaea2 - ps3
Archaea3 - ps4
Asgard/Eukaryota - ps5
Eukaryota - ps6
Excavata/Diaphoretickes - ps7
Diaphoretickes - ps8
Archaeplastida - ps9
Viridiplantae - ps10
Streptophyta - ps11
Charophyceae/Embriophyta - ps12
Embriophyta - ps13
Tracheophyta - ps14
Spermatophyta - ps15
Angiosperms - ps16
Mesangiospermae - ps17
Magnoliids/eudicots - ps18
Eudicots - ps19
Core_eudicots - ps20
Superrosids - ps21
Rosids - ps22
Eurosids - ps23
Malvids - ps24
Sap./Malv./Brassicales - ps25
Malvales/Brassicales - ps26
Brassicales - ps27
Cleomaceae/Brassicaceae - ps28
Brassicaceae - ps29
Camelineae - ps30
Arabidopsis - ps31
Arabidopsis_thaliana - ps32
Phylostratum

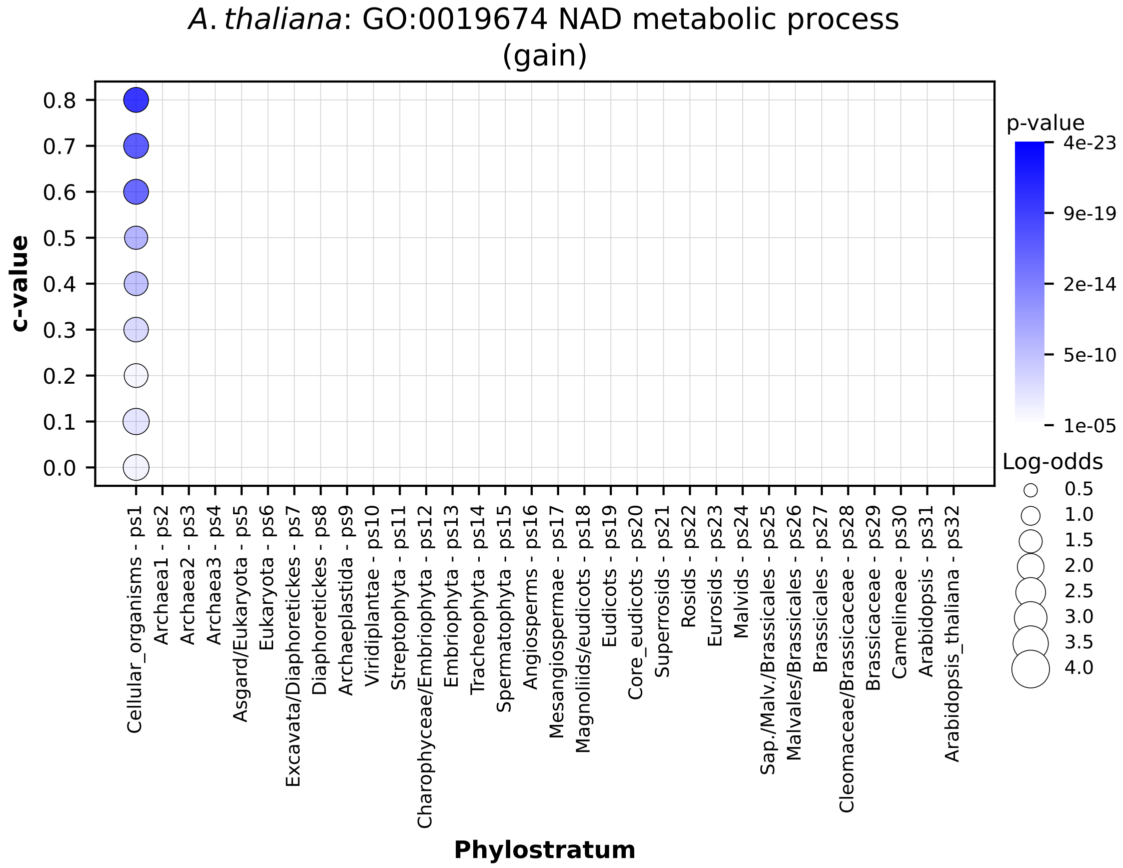

A. thaliana: GO:0019674 NAD metabolic process
(gain)
c-value
0.8
0.7
0.6
0.5
0.4
0.3
0.2
0.1
0.0
p-value
4e-23
9e-19
2e-14
5e-10
1e-05
Log-odds
0.5
1.0
1.5
2.0
2.5
3.0
3.5
4.0
Cellular_organisms - ps1
Archaea1 - ps2
Archaea2 - ps3
Archaea3 - ps4
Asgard/Eukaryota - ps5
Eukaryota - ps6
Excavata/Diaphoretickes - ps7
Diaphoretickes - ps8
Archaeplastida - ps9
Viridiplantae - ps10
Streptophyta - ps11
Charophyceae/Embriophyta - ps12
Embriophyta - ps13
Tracheophyta - ps14
Spermatophyta - ps15
Angiosperms - ps16
Mesangiospermae - ps17
Magnoliids/eudicots - ps18
Eudicots - ps19
Core_eudicots - ps20
Superrosids - ps21
Rosids - ps22
Eurosids - ps23
Malvids - ps24
Sap./Malv./Brassicales - ps25
Malvales/Brassicales - ps26
Brassicales - ps27
Cleomaceae/Brassicaceae - ps28
Brassicaceae - ps29
Camelineae - ps30
Arabidopsis - ps31
Arabidopsis_thaliana - ps32
Phylostratum

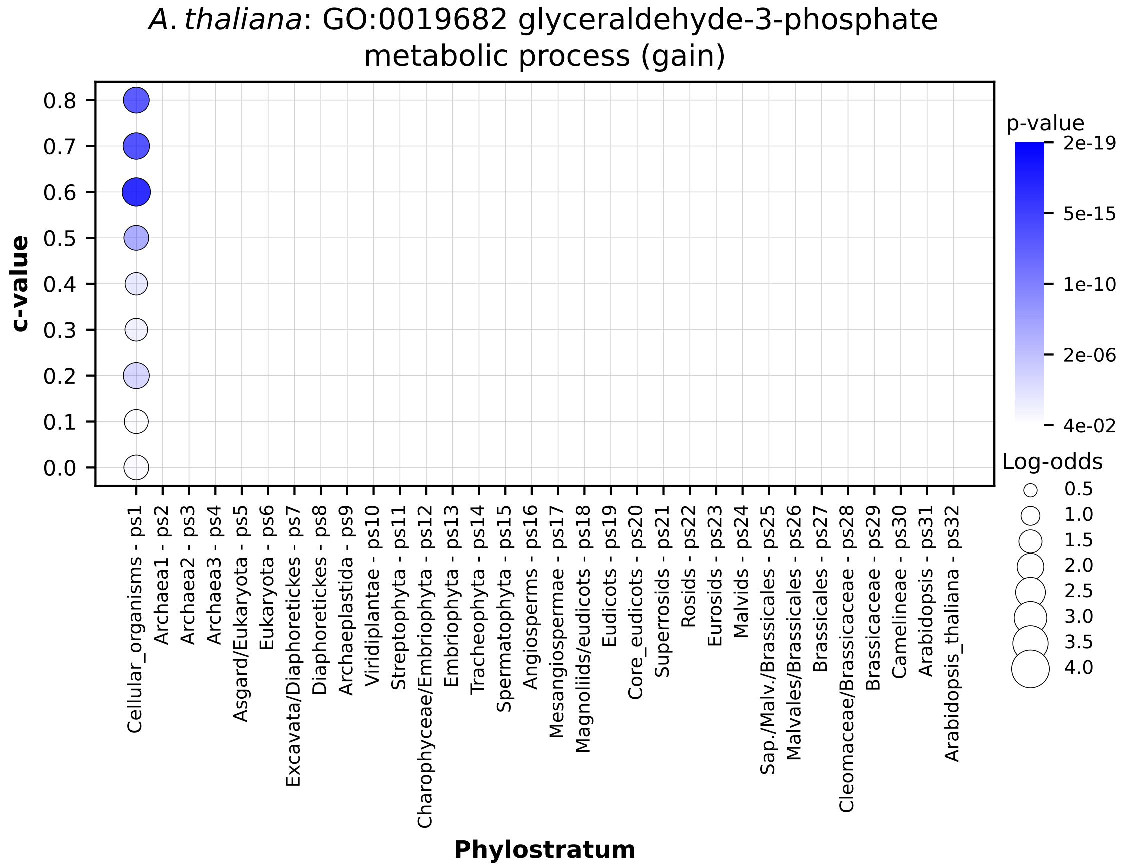

A. thaliana: GO:0019682 glyceraldehyde-3-phosphate metabolic process (gain)
c-value
0.0
0.1
0.2
0.3
0.4
0.5
0.6
0.7
0.8
p-value
2e-19
5e-15
1e-10
2e-06
4e-02
Log-odds
0.5
1.0
1.5
2.0
2.5
3.0
3.5
4.0
Cellular_organisms - ps1
Archaea1 - ps2
Archaea2 - ps3
Archaea3 - ps4
Asgard/Eukaryota - ps5
Eukaryota - ps6
Excavata/Diaphoretickes - ps7
Diaphoretickes - ps8
Archaeplastida - ps9
Viridiplantae - ps10
Streptophyta - ps11
Charophyceae/Embriophyta - ps12
Embriophyta - ps13
Tracheophyta - ps14
Spermatophyta - ps15
Angiosperms - ps16
Mesangiospermae - ps17
Magnoliids/eudicots - ps18
Eudicots - ps19
Core_eudicots - ps20
Superrosids - ps21
Rosids - ps22
Eurosids - ps23
Malvids - ps24
Sap./Malv./Brassicales - ps25
Malvales/Brassicales - ps26
Brassicales - ps27
Cleomaceae/Brassicaceae - ps28
Brassicaceae - ps29
Camelineae - ps30
Arabidopsis - ps31
Arabidopsis_thaliana - ps32
Phylostratum

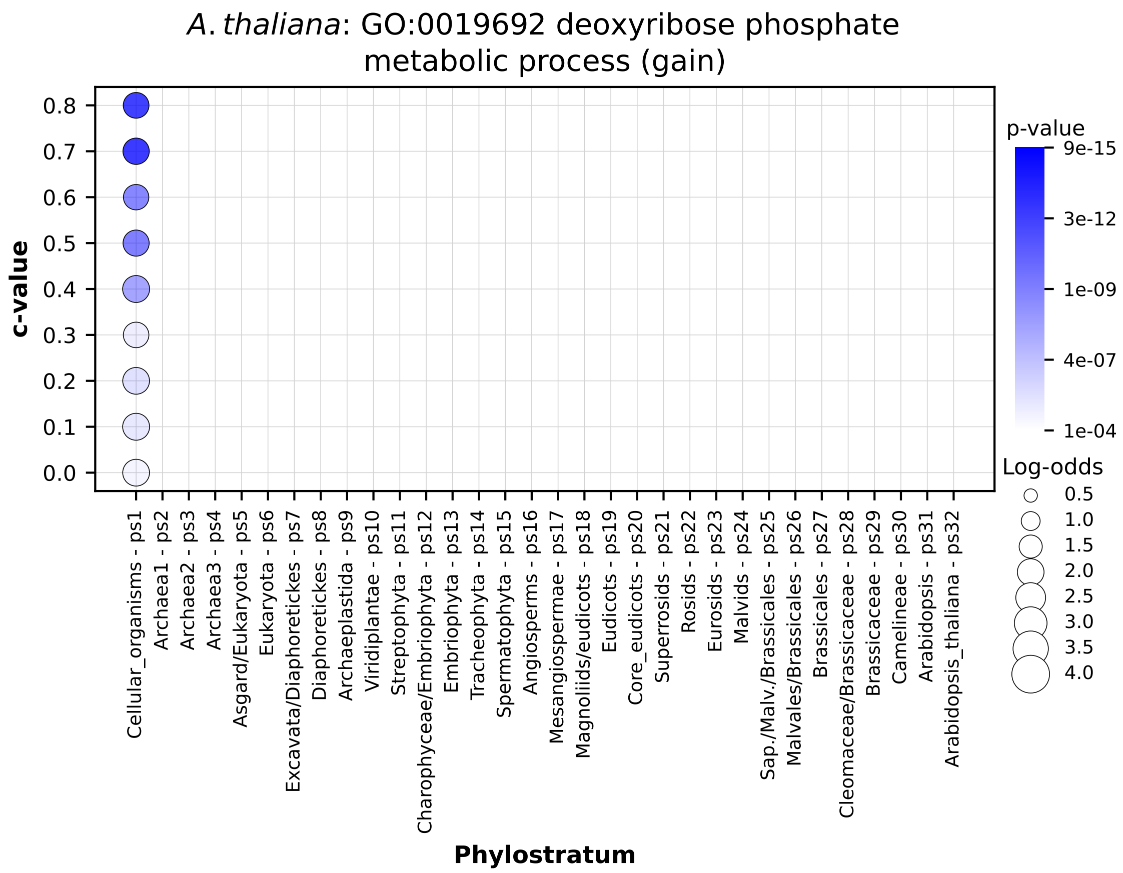

A. thaliana: GO:0019692 deoxyribose phosphate metabolic process (gain)
c-value
0.8
0.7
0.6
0.5
0.4
0.3
0.2
0.1
0.0
p-value
9e-15
3e-12
1e-09
4e-07
1e-04
Log-odds
0.5
1.0
1.5
2.0
2.5
3.0
3.5
4.0
Cellular_organisms - ps1
Archaea1 - ps2
Archaea2 - ps3
Archaea3 - ps4
Asgard/Eukaryota - ps5
Eukaryota - ps6
Excavata/Diaphoretickes - ps7
Diaphoretickes - ps8
Archaeplastida - ps9
Viridiplantae - ps10
Streptophyta - ps11
Charophyceae/Embriophyta - ps12
Embriophyta - ps13
Tracheophyta - ps14
Spermatophyta - ps15
Angiosperms - ps16
Mesangiospermae - ps17
Magnoliids/eudicots - ps18
Eudicots - ps19
Core_eudicots - ps20
Superrosids - ps21
Rosids - ps22
Eurosids - ps23
Malvids - ps24
Sap./Malv./Brassicales - ps25
Malvales/Brassicales - ps26
Brassicales - ps27
Cleomaceae/Brassicaceae - ps28
Brassicaceae - ps29
Camelineae - ps30
Arabidopsis - ps31
Arabidopsis_thaliana - ps32
Phylostratum

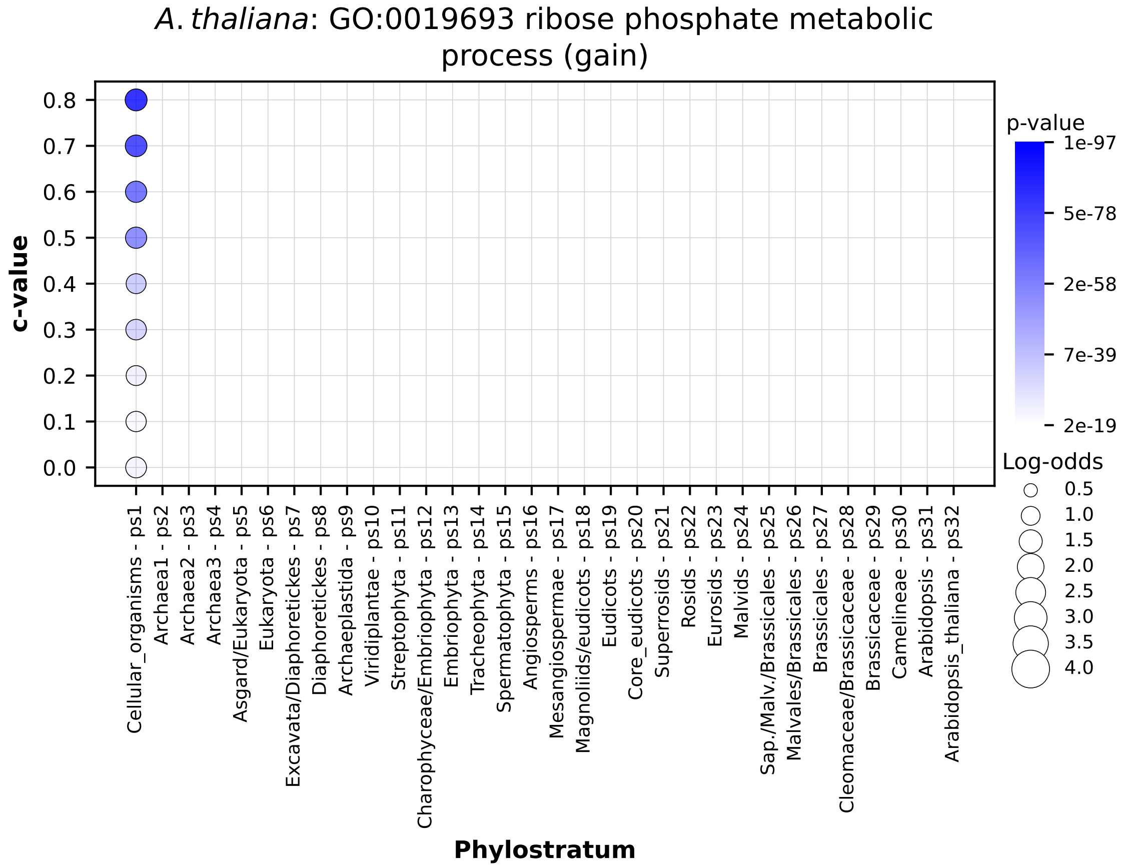
A. thaliana: GO:0019693 ribose phosphate metabolic process (gain)
c-value
0.8
0.7
0.6
0.5
0.4
0.3
0.2
0.1
0.0
p-value
1e-97
5e-78
2e-58
7e-39
2e-19
Log-odds
0.5
1.0
1.5
2.0
2.5
3.0
3.5
4.0
Cellular_organisms - ps1
Archaea1 - ps2
Archaea2 - ps3
Archaea3 - ps4
Asgard/Eukaryota - ps5
Eukaryota - ps6
Excavata/Diaphoretickes - ps7
Diaphoretickes - ps8
Archaeplastida - ps9
Viridiplantae - ps10
Streptophyta - ps11
Charophyceae/Embriophyta - ps12
Embriophyta - ps13
Tracheophyta - ps14
Spermatophyta - ps15
Angiosperms - ps16
Mesangiospermae - ps17
Magnoliids/eudicots - ps18
Eudicots - ps19
Core_eudicots - ps20
Superrosids - ps21
Rosids - ps22
Eurosids - ps23
Malvids - ps24
Sap./Malv./Brassicales - ps25
Malvales/Brassicales - ps26
Brassicales - ps27
Cleomaceae/Brassicaceae - ps28
Brassicaceae - ps29
Camelineae - ps30
Arabidopsis - ps31
Arabidopsis_thaliana - ps32
Phylostratum

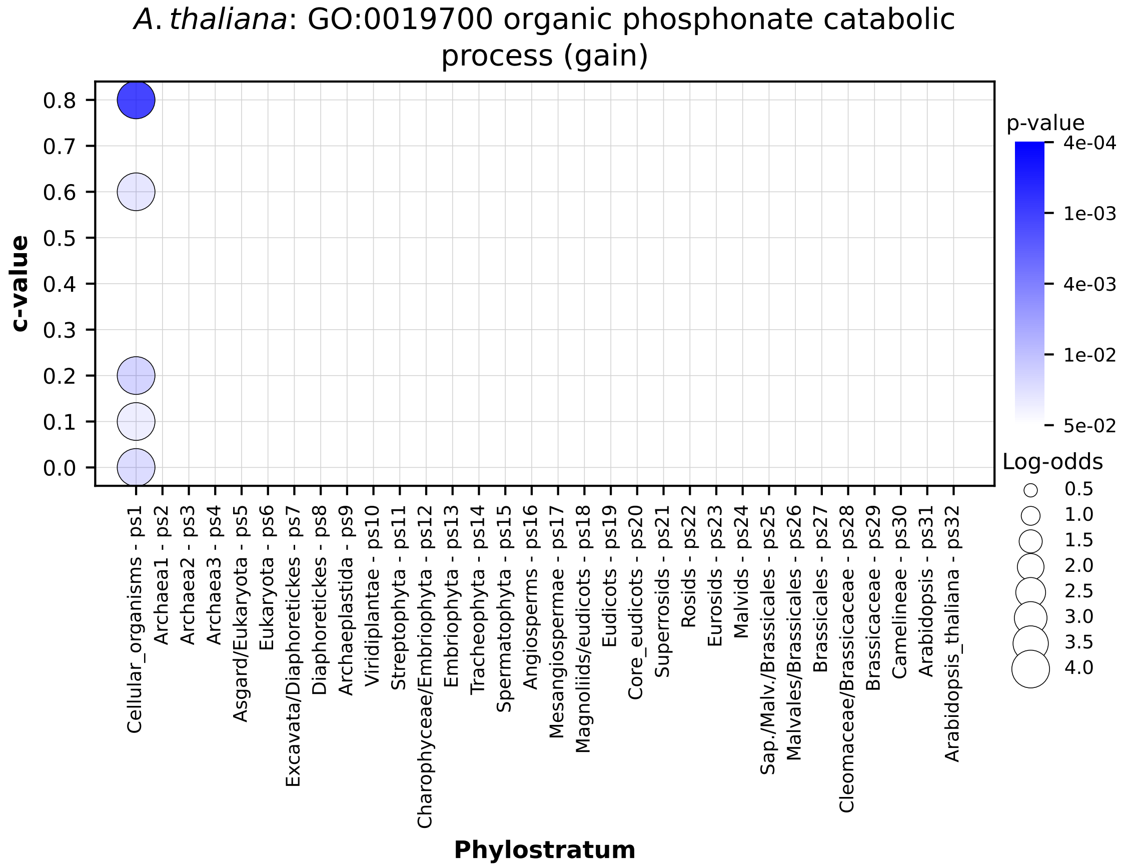

A. thaliana: GO:0019700 organic phosphonate catabolic process (gain)
c-value
0.8
0.7
0.6
0.5
0.4
0.3
0.2
0.1
0.0
p-value
4e-04
1e-03
4e-03
1e-02
5e-02
Log-odds
0.5
1.0
1.5
2.0
2.5
3.0
3.5
4.0
Cellular_organisms - ps1
Archaea1 - ps2
Archaea2 - ps3
Archaea3 - ps4
Asgard/Eukaryota - ps5
Eukaryota - ps6
Excavata/Diaphoretickes - ps7
Diaphoretickes - ps8
Archaeplastida - ps9
Viridiplantae - ps10
Streptophyta - ps11
Charophyceae/Embriophyta - ps12
Embriophyta - ps13
Tracheophyta - ps14
Spermatophyta - ps15
Angiosperms - ps16
Mesangiospermae - ps17
Magnoliids/eudicots - ps18
Eudicots - ps19
Core_eudicots - ps20
Superrosids - ps21
Rosids - ps22
Eurosids - ps23
Malvids - ps24
Sap./Malv./Brassicales - ps25
Malvales/Brassicales - ps26
Brassicales - ps27
Cleomaceae/Brassicaceae - ps28
Brassicaceae - ps29
Camelineae - ps30
Arabidopsis - ps31
Arabidopsis_thaliana - ps32
Phylostratum

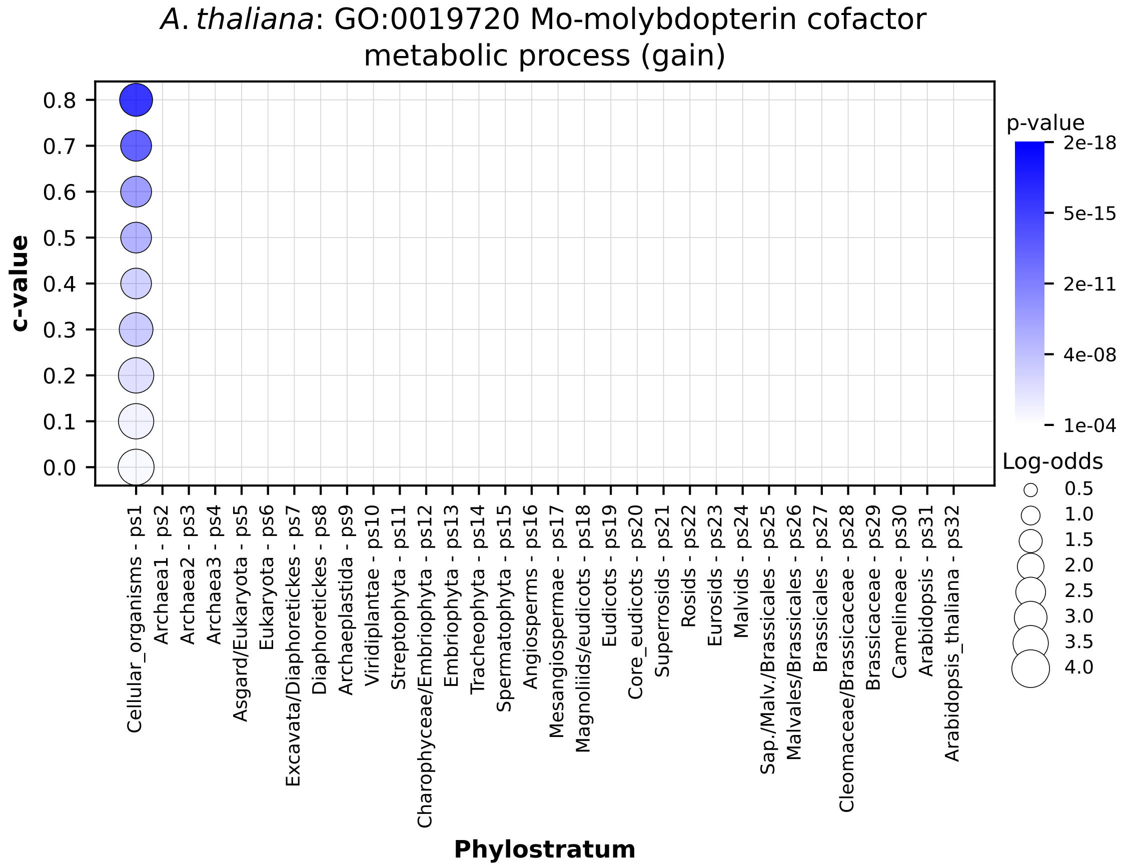
A. thaliana: GO:0019720 Mo-molybdopterin cofactor metabolic process (gain)
c-value
0.8
0.7
0.6
0.5
0.4
0.3
0.2
0.1
0.0
p-value
2e-18
5e-15
2e-11
4e-08
1e-04
Log-odds
0.5
1.0
1.5
2.0
2.5
3.0
3.5
4.0
Cellular_organisms - ps1
Archaea1 - ps2
Archaea2 - ps3
Archaea3 - ps4
Asgard/Eukaryota - ps5
Eukaryota - ps6
Excavata/Diaphoretickes - ps7
Diaphoretickes - ps8
Archaeplastida - ps9
Viridiplantae - ps10
Streptophyta - ps11
Charophyceae/Embriophyta - ps12
Embriophyta - ps13
Tracheophyta - ps14
Spermatophyta - ps15
Angiosperms - ps16
Mesangiospermae - ps17
Magnoliids/eudicots - ps18
Eudicots - ps19
Core_eudicots - ps20
Superrosids - ps21
Rosids - ps22
Eurosids - ps23
Malvids - ps24
Sap./Malv./Brassicales - ps25
Malvales/Brassicales - ps26
Brassicales - ps27
Cleomaceae/Brassicaceae - ps28
Brassicaceae - ps29
Camelineae - ps30
Arabidopsis - ps31
Arabidopsis_thaliana - ps32
Phylostratum

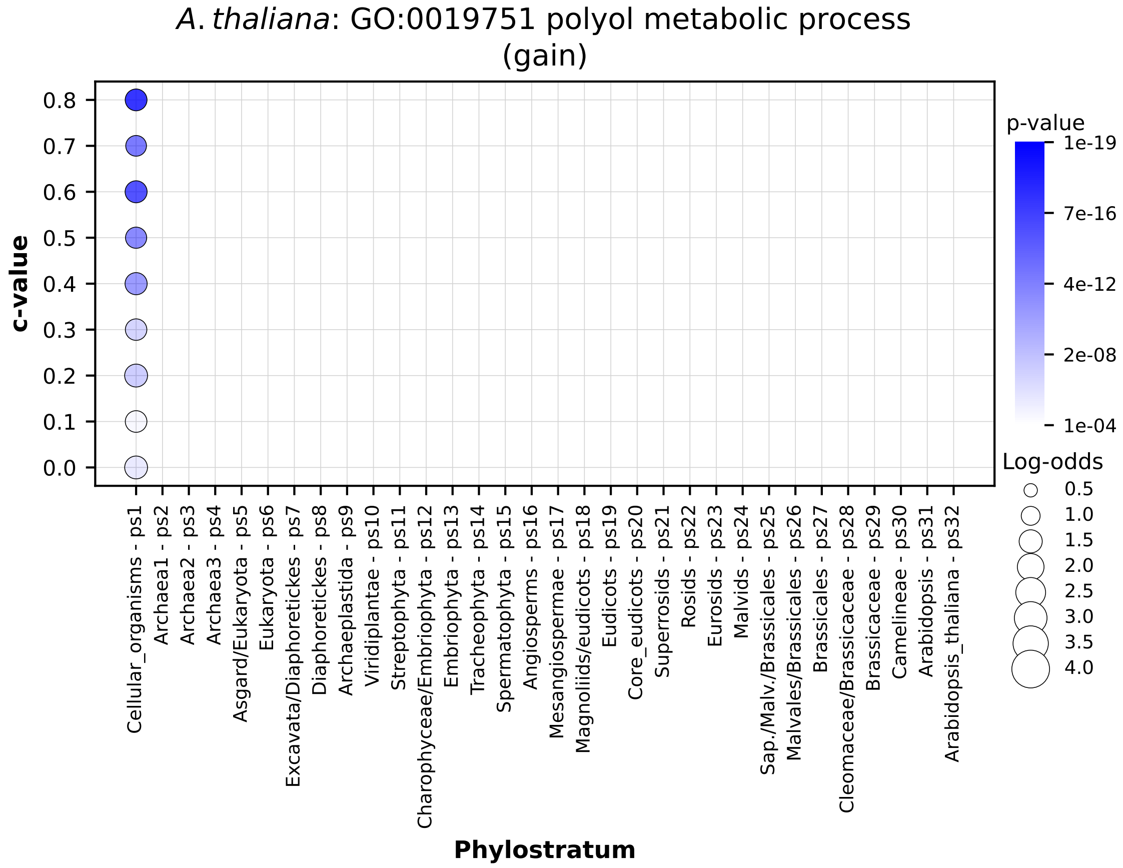

A. thaliana: GO:0019751 polyol metabolic process (gain)
c-value
0.8
0.7
0.6
0.5
0.4
0.3
0.2
0.1
0.0
p-value
1e-19
7e-16
4e-12
2e-08
1e-04
Log-odds
0.5
1.0
1.5
2.0
2.5
3.0
3.5
4.0
Cellular_organisms - ps1
Archaea1 - ps2
Archaea2 - ps3
Archaea3 - ps4
Asgard/Eukaryota - ps5
Eukaryota - ps6
Excavata/Diaphoretickes - ps7
Diaphoretickes - ps8
Archaeplastida - ps9
Viridiplantae - ps10
Streptophyta - ps11
Charophyceae/Embriophyta - ps12
Embriophyta - ps13
Tracheophyta - ps14
Spermatophyta - ps15
Angiosperms - ps16
Mesangiospermae - ps17
Magnoliids/eudicots - ps18
Eudicots - ps19
Core_eudicots - ps20
Superrosids - ps21
Rosids - ps22
Eurosids - ps23
Malvids - ps24
Sap./Malv./Brassicales - ps25
Malvales/Brassicales - ps26
Brassicales - ps27
Cleomaceae/Brassicaceae - ps28
Brassicaceae - ps29
Camelineae - ps30
Arabidopsis - ps31
Arabidopsis_thaliana - ps32
Phylostratum

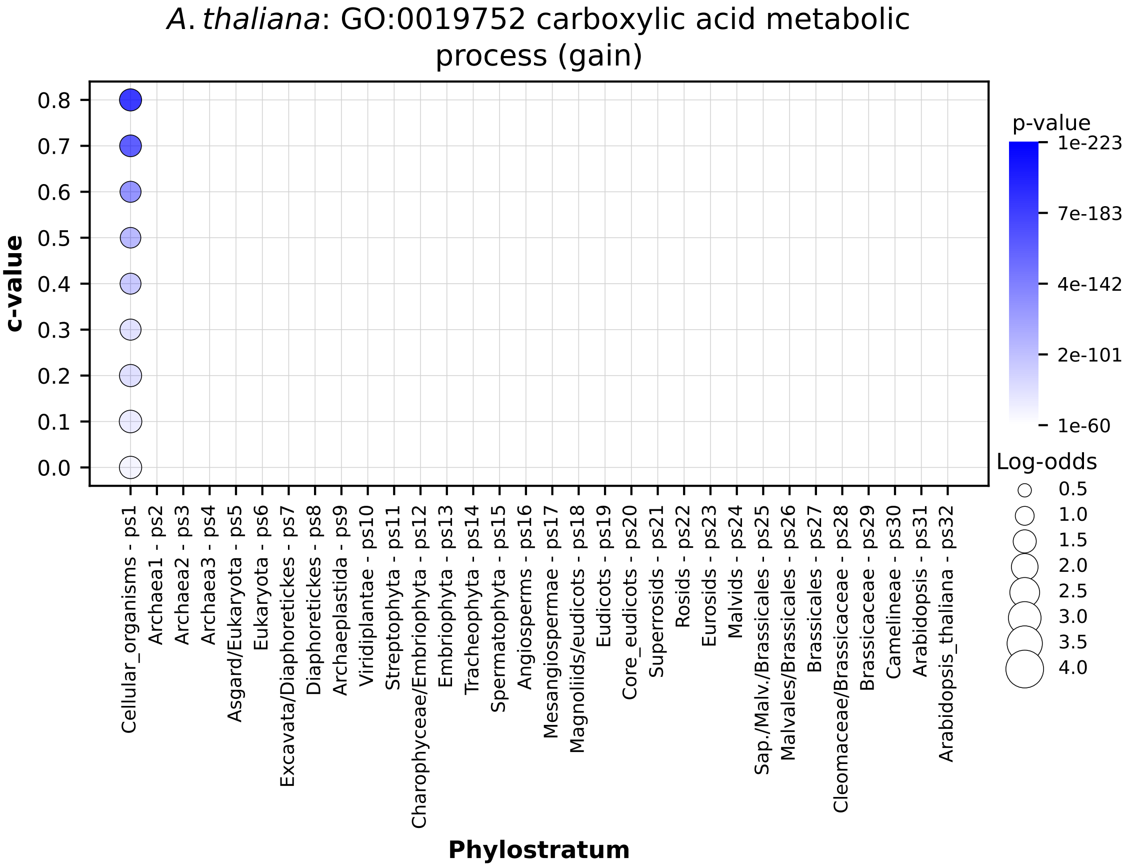

A. thaliana: GO:0019752 carboxylic acid metabolic process (gain)
c-value
0.8
0.7
0.6
0.5
0.4
0.3
0.2
0.1
0.0
p-value
1e-223
7e-183
4e-142
2e-101
1e-60
Log-odds
0.5
1.0
1.5
2.0
2.5
3.0
3.5
4.0
Cellular_organisms - ps1
Archaea1 - ps2
Archaea2 - ps3
Archaea3 - ps4
Asgard/Eukaryota - ps5
Eukaryota - ps6
Excavata/Diaphoretickes - ps7
Diaphoretickes - ps8
Archaeplastida - ps9
Viridiplantae - ps10
Streptophyta - ps11
Charophyceae/Embriophyta - ps12
Embriophyta - ps13
Tracheophyta - ps14
Spermatophyta - ps15
Angiosperms - ps16
Mesangiospermae - ps17
Magnoliids/eudicots - ps18
Eudicots - ps19
Core_eudicots - ps20
Superrosids - ps21
Rosids - ps22
Eurosids - ps23
Malvids - ps24
Sap./Malv./Brassicales - ps25
Malvales/Brassicales - ps26
Brassicales - ps27
Cleomaceae/Brassicaceae - ps28
Brassicaceae - ps29
Camelineae - ps30
Arabidopsis - ps31
Arabidopsis_thaliana - ps32
Phylostratum

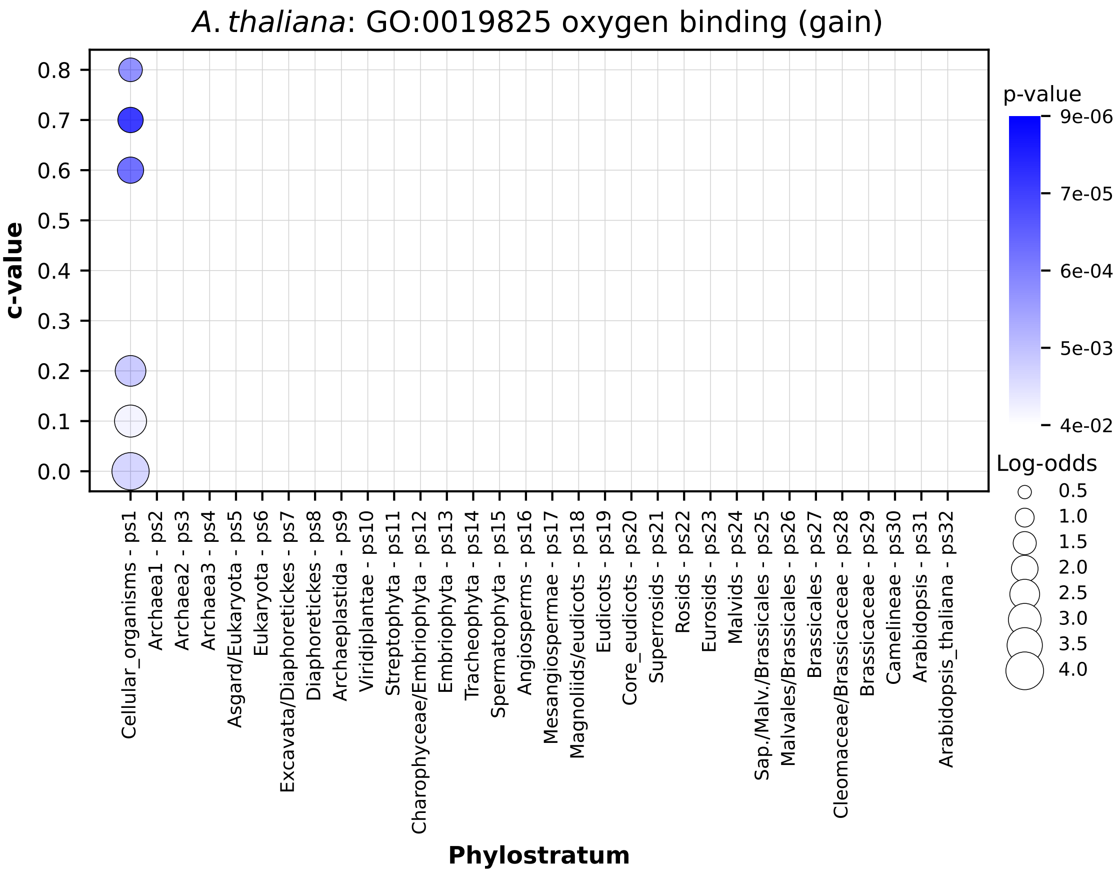

A. thaliana: GO:0019825 oxygen binding (gain)
c-value
0.0
0.1
0.2
0.3
0.4
0.5
0.6
0.7
0.8
p-value
9e-06
7e-05
6e-04
5e-03
4e-02
Log-odds
0.5
1.0
1.5
2.0
2.5
3.0
3.5
4.0
Cellular_organisms - ps1
Archaea1 - ps2
Archaea2 - ps3
Archaea3 - ps4
Asgard/Eukaryota - ps5
Eukaryota - ps6
Excavata/Diaphoretickes - ps7
Diaphoretickes - ps8
Archaeplastida - ps9
Viridiplantae - ps10
Streptophyta - ps11
Charophyceae/Embriophyta - ps12
Embriophyta - ps13
Tracheophyta - ps14
Spermatophyta - ps15
Angiosperms - ps16
Mesangiospermae - ps17
Magnoliids/eudicots - ps18
Eudicots - ps19
Core_eudicots - ps20
Superrosids - ps21
Rosids - ps22
Eurosids - ps23
Malvids - ps24
Sap./Malv./Brassicales - ps25
Malvales/Brassicales - ps26
Brassicales - ps27
Cleomaceae/Brassicaceae - ps28
Brassicaceae - ps29
Camelineae - ps30
Arabidopsis - ps31
Arabidopsis_thaliana - ps32
Phylostratum

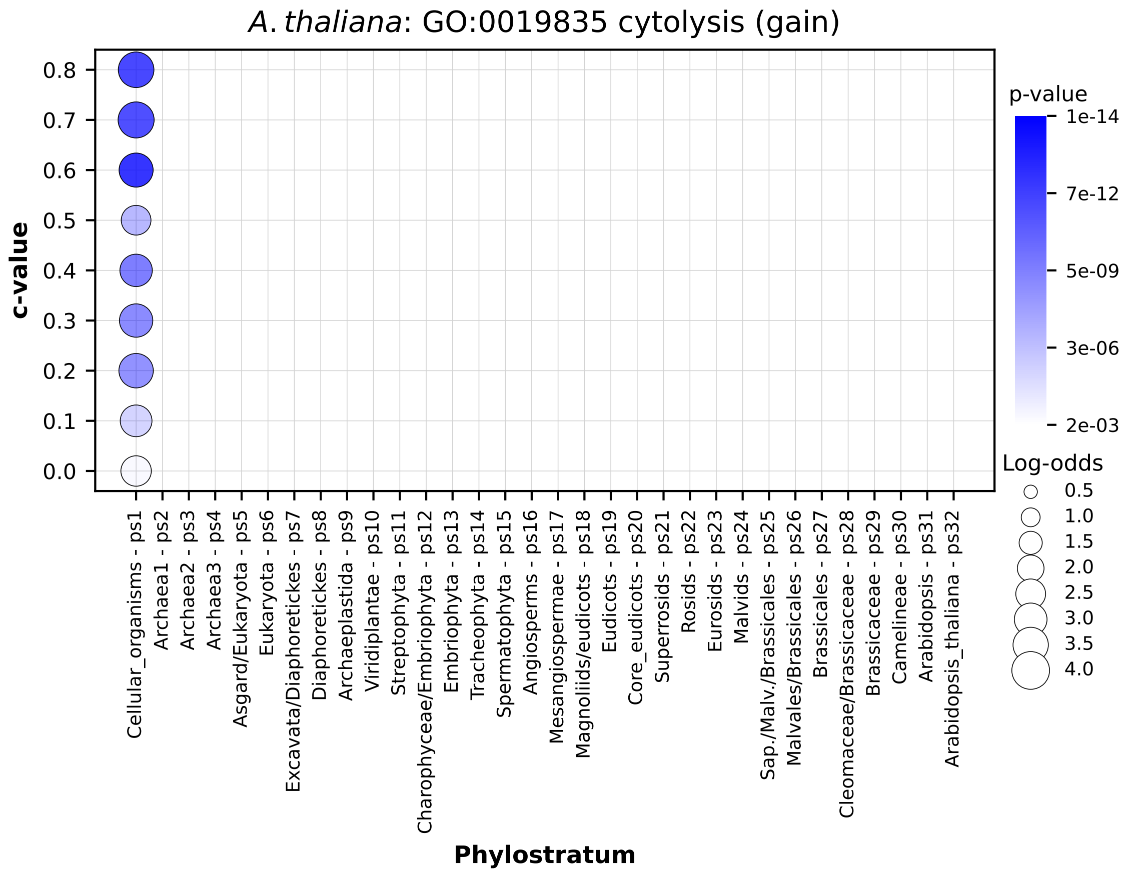

A. thaliana: GO:0019835 cytolysis (gain)
c-value
0.8
0.7
0.6
0.5
0.4
0.3
0.2
0.1
0.0
p-value
1e-14
7e-12
5e-09
3e-06
2e-03
Log-odds
0.5
1.0
1.5
2.0
2.5
3.0
3.5
4.0
Cellular_organisms - ps1
Archaea1 - ps2
Archaea2 - ps3
Archaea3 - ps4
Asgard/Eukaryota - ps5
Eukaryota - ps6
Excavata/Diaphoretickes - ps7
Diaphoretickes - ps8
Archaeplastida - ps9
Viridiplantae - ps10
Streptophyta - ps11
Charophyceae/Embriophyta - ps12
Embriophyta - ps13
Tracheophyta - ps14
Spermatophyta - ps15
Angiosperms - ps16
Mesangiospermae - ps17
Magnoliids/eudicots - ps18
Eudicots - ps19
Core_eudicots - ps20
Superrosids - ps21
Rosids - ps22
Eurosids - ps23
Malvids - ps24
Sap./Malv./Brassicales - ps25
Malvales/Brassicales - ps26
Brassicales - ps27
Cleomaceae/Brassicaceae - ps28
Brassicaceae - ps29
Camelineae - ps30
Arabidopsis - ps31
Arabidopsis_thaliana - ps32
Phylostratum

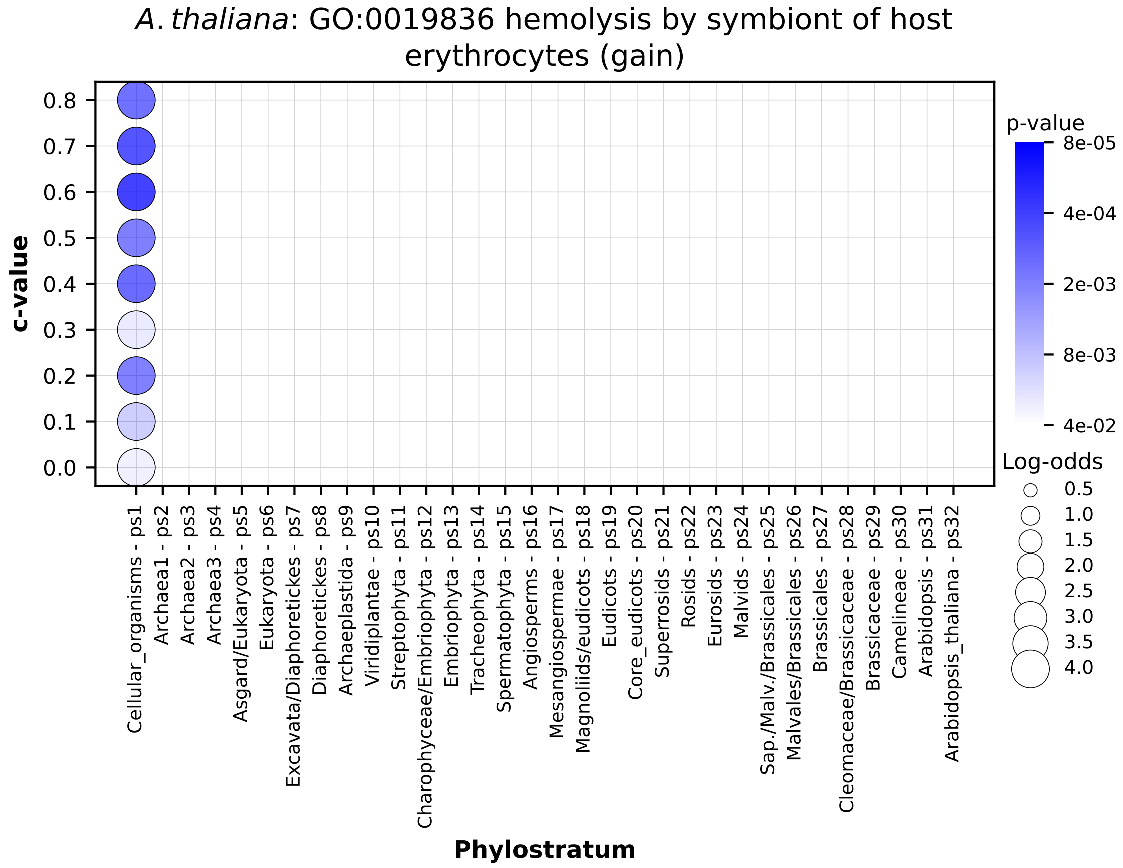
A. thaliana: GO:0019836 hemolysis by symbiont of host erythrocytes (gain)
c-value
0.8
0.7
0.6
0.5
0.4
0.3
0.2
0.1
0.0
Phylostratum
Cellular_organisms - ps1
Archaea1 - ps2
Archaea2 - ps3
Archaea3 - ps4
Asgard/Eukaryota - ps5
Eukaryota - ps6
Excavata/Diaphoretickes - ps7
Diaphoretickes - ps8
Archaeplastida - ps9
Viridiplantae - ps10
Streptophyta - ps11
Charophyceae/Embriophyta - ps12
Embriophyta - ps13
Tracheophyta - ps14
Spermatophyta - ps15
Angiosperms - ps16
Mesangiospermae - ps17
Magnoliids/eudicots - ps18
Eudicots - ps19
Core_eudicots - ps20
Superrosids - ps21
Rosids - ps22
Eurosids - ps23
Malvids - ps24
Sap./Malv./Brassicales - ps25
Malvales/Brassicales - ps26
Brassicales - ps27
Cleomaceae/Brassicaceae - ps28
Brassicaceae - ps29
Camelineae - ps30
Arabidopsis - ps31
Arabidopsis_thaliana - ps32
p-value
8e-05
4e-04
2e-03
8e-03
4e-02
Log-odds
0.5
1.0
1.5
2.0
2.5
3.0
3.5
4.0

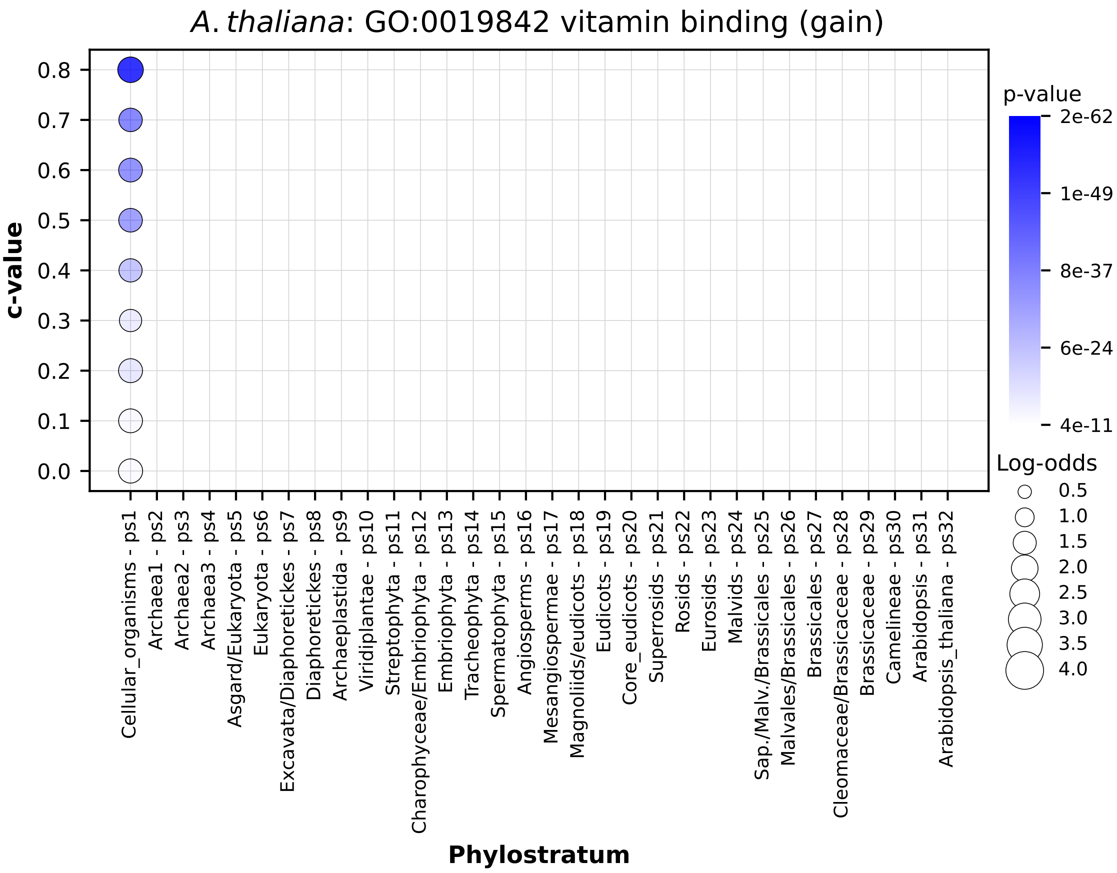

A. thaliana: GO:0019842 vitamin binding (gain)
c-value
0.8
0.7
0.6
0.5
0.4
0.3
0.2
0.1
0.0
p-value
2e-62
1e-49
8e-37
6e-24
4e-11
Log-odds
0.5
1.0
1.5
2.0
2.5
3.0
3.5
4.0
Cellular_organisms - ps1
Archaea1 - ps2
Archaea2 - ps3
Archaea3 - ps4
Asgard/Eukaryota - ps5
Eukaryota - ps6
Excavata/Diaphoretickes - ps7
Diaphoretickes - ps8
Archaeplastida - ps9
Viridiplantae - ps10
Streptophyta - ps11
Charophyceae/Embriophyta - ps12
Embriophyta - ps13
Tracheophyta - ps14
Spermatophyta - ps15
Angiosperms - ps16
Mesangiospermae - ps17
Magnoliids/eudicots - ps18
Eudicots - ps19
Core_eudicots - ps20
Superrosids - ps21
Rosids - ps22
Eurosids - ps23
Malvids - ps24
Sap./Malv./Brassicales - ps25
Malvales/Brassicales - ps26
Brassicales - ps27
Cleomaceae/Brassicaceae - ps28
Brassicaceae - ps29
Camelineae - ps30
Arabidopsis - ps31
Arabidopsis_thaliana - ps32
Phylostratum

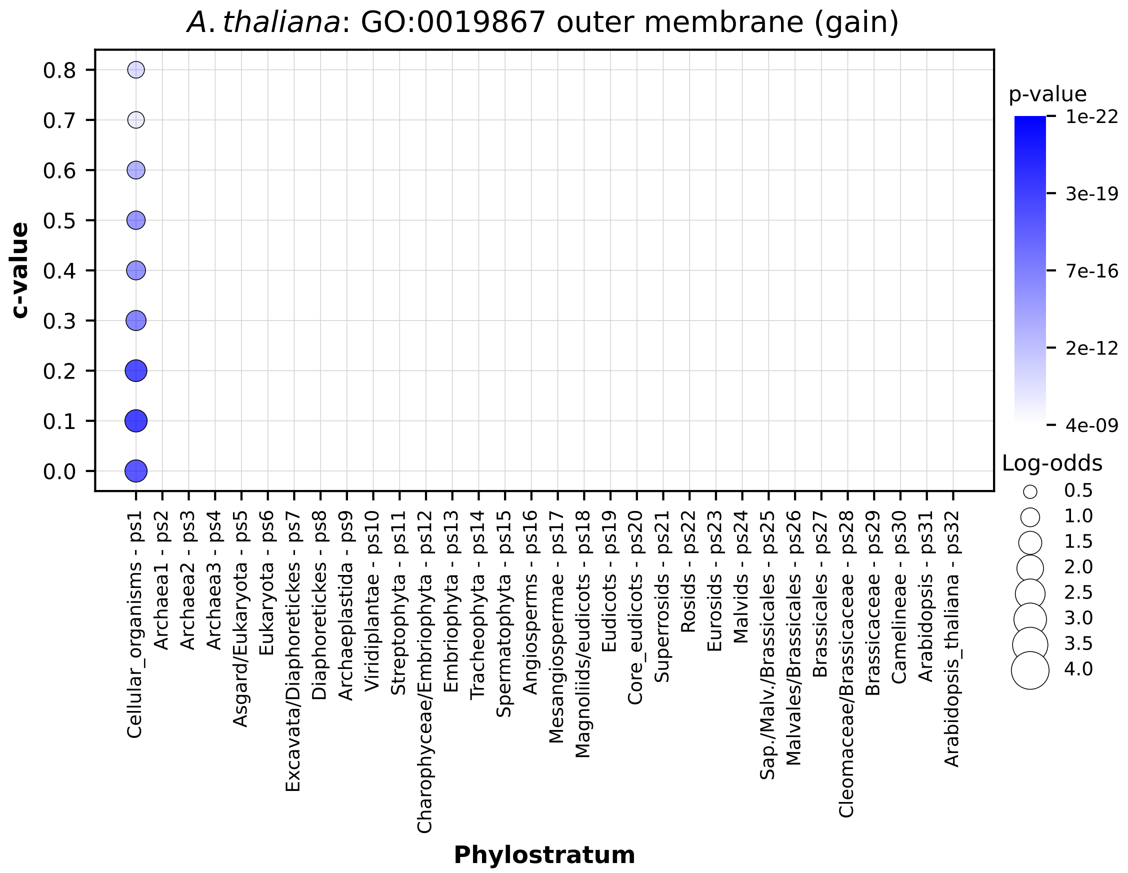
A. thaliana: GO:0019867 outer membrane (gain)
c-value
0.8
0.7
0.6
0.5
0.4
0.3
0.2
0.1
0.0
p-value
1e-22
3e-19
7e-16
2e-12
4e-09
Log-odds
0.5
1.0
1.5
2.0
2.5
3.0
3.5
4.0
Cellular_organisms - ps1
Archaea1 - ps2
Archaea2 - ps3
Archaea3 - ps4
Asgard/Eukaryota - ps5
Eukaryota - ps6
Excavata/Diaphoretickes - ps7
Diaphoretickes - ps8
Archaeplastida - ps9
Viridiplantae - ps10
Streptophyta - ps11
Charophyceae/Embriophyta - ps12
Embriophyta - ps13
Tracheophyta - ps14
Spermatophyta - ps15
Angiosperms - ps16
Mesangiospermae - ps17
Magnoliids/eudicots - ps18
Eudicots - ps19
Core_eudicots - ps20
Superrosids - ps21
Rosids - ps22
Eurosids - ps23
Malvids - ps24
Sap./Malv./Brassicales - ps25
Malvales/Brassicales - ps26
Brassicales - ps27
Cleomaceae/Brassicaceae - ps28
Brassicaceae - ps29
Camelineae - ps30
Arabidopsis - ps31
Arabidopsis_thaliana - ps32
Phylostratum

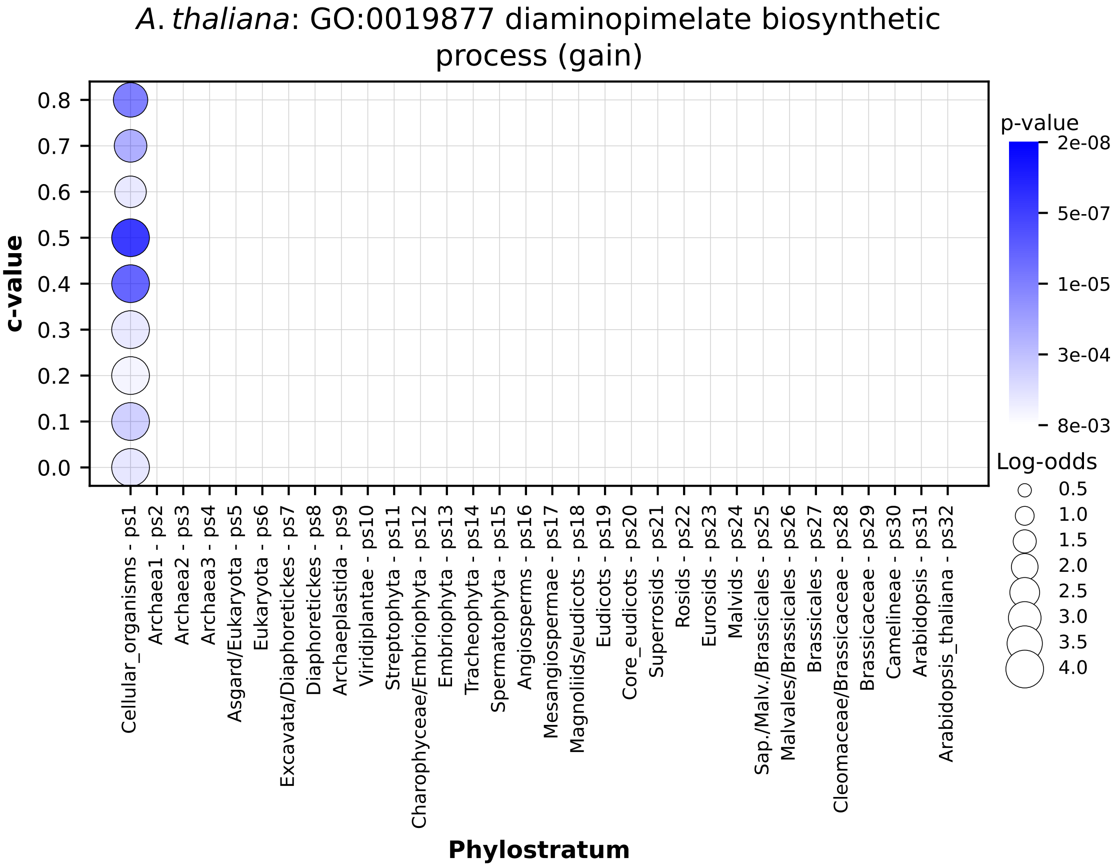
A. thaliana: GO:0019877 diaminopimelate biosynthetic process (gain)
c-value
0.8
0.7
0.6
0.5
0.4
0.3
0.2
0.1
0.0
p-value
2e-08
5e-07
1e-05
3e-04
8e-03
Log-odds
0.5
1.0
1.5
2.0
2.5
3.0
3.5
4.0
Cellular_organisms - ps1
Archaea1 - ps2
Archaea2 - ps3
Archaea3 - ps4
Asgard/Eukaryota - ps5
Eukaryota - ps6
Excavata/Diaphoretickes - ps7
Diaphoretickes - ps8
Archaeplastida - ps9
Viridiplantae - ps10
Streptophyta - ps11
Charophyceae/Embriophyta - ps12
Embriophyta - ps13
Tracheophyta - ps14
Spermatophyta - ps15
Angiosperms - ps16
Mesangiospermae - ps17
Magnoliids/eudicots - ps18
Eudicots - ps19
Core_eudicots - ps20
Superrosids - ps21
Rosids - ps22
Eurosids - ps23
Malvids - ps24
Sap./Malv./Brassicales - ps25
Malvales/Brassicales - ps26
Brassicales - ps27
Cleomaceae/Brassicaceae - ps28
Brassicaceae - ps29
Camelineae - ps30
Arabidopsis - ps31
Arabidopsis_thaliana - ps32
Phylostratum

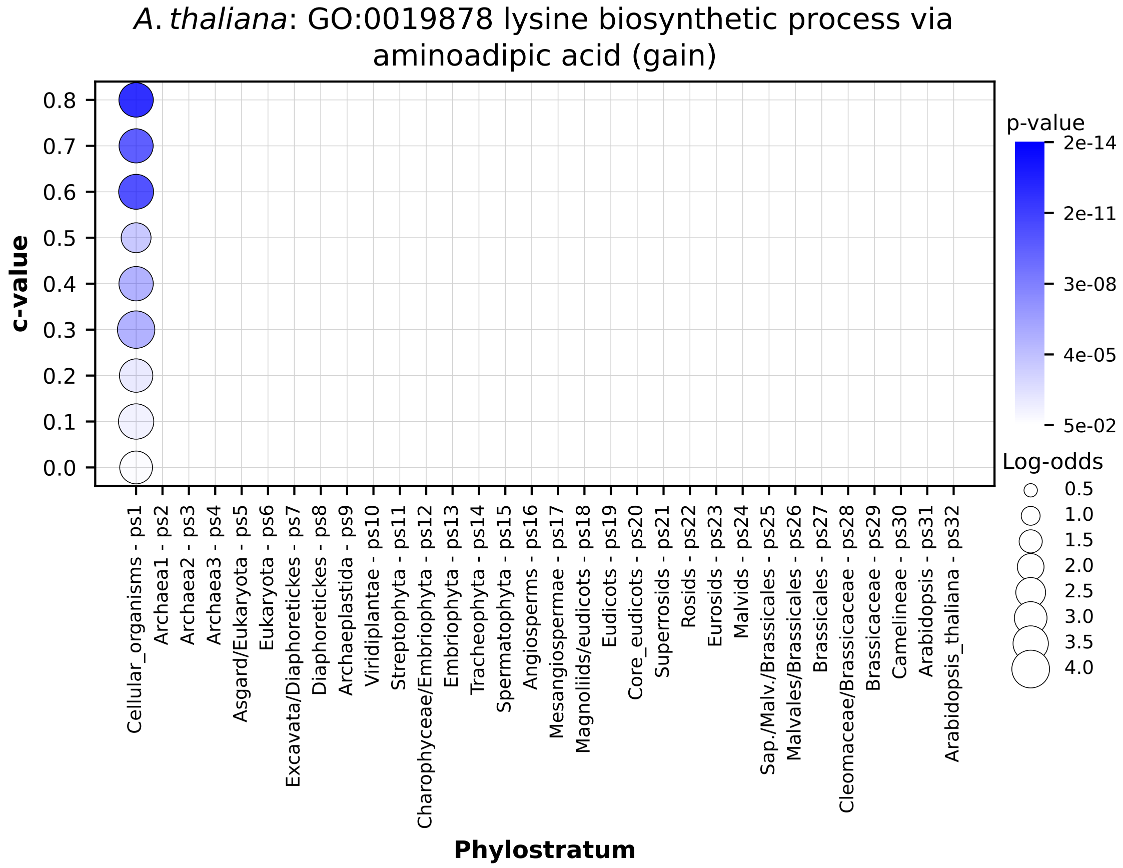
A. thaliana: GO:0019878 lysine biosynthetic process via aminoadipic acid (gain)
c-value
0.8
0.7
0.6
0.5
0.4
0.3
0.2
0.1
0.0
p-value
2e-14
2e-11
3e-08
4e-05
5e-02
Log-odds
0.5
1.0
1.5
2.0
2.5
3.0
3.5
4.0
Cellular_organisms - ps1
Archaea1 - ps2
Archaea2 - ps3
Archaea3 - ps4
Asgard/Eukaryota - ps5
Eukaryota - ps6
Excavata/Diaphoretickes - ps7
Diaphoretickes - ps8
Archaeplastida - ps9
Viridiplantae - ps10
Streptophyta - ps11
Charophyceae/Embriophyta - ps12
Embriophyta - ps13
Tracheophyta - ps14
Spermatophyta - ps15
Angiosperms - ps16
Mesangiospermae - ps17
Magnoliids/eudicots - ps18
Eudicots - ps19
Core_eudicots - ps20
Superrosids - ps21
Rosids - ps22
Eurosids - ps23
Malvids - ps24
Sap./Malv./Brassicales - ps25
Malvales/Brassicales - ps26
Brassicales - ps27
Cleomaceae/Brassicaceae - ps28
Brassicaceae - ps29
Camelineae - ps30
Arabidopsis - ps31
Arabidopsis_thaliana - ps32
Phylostratum

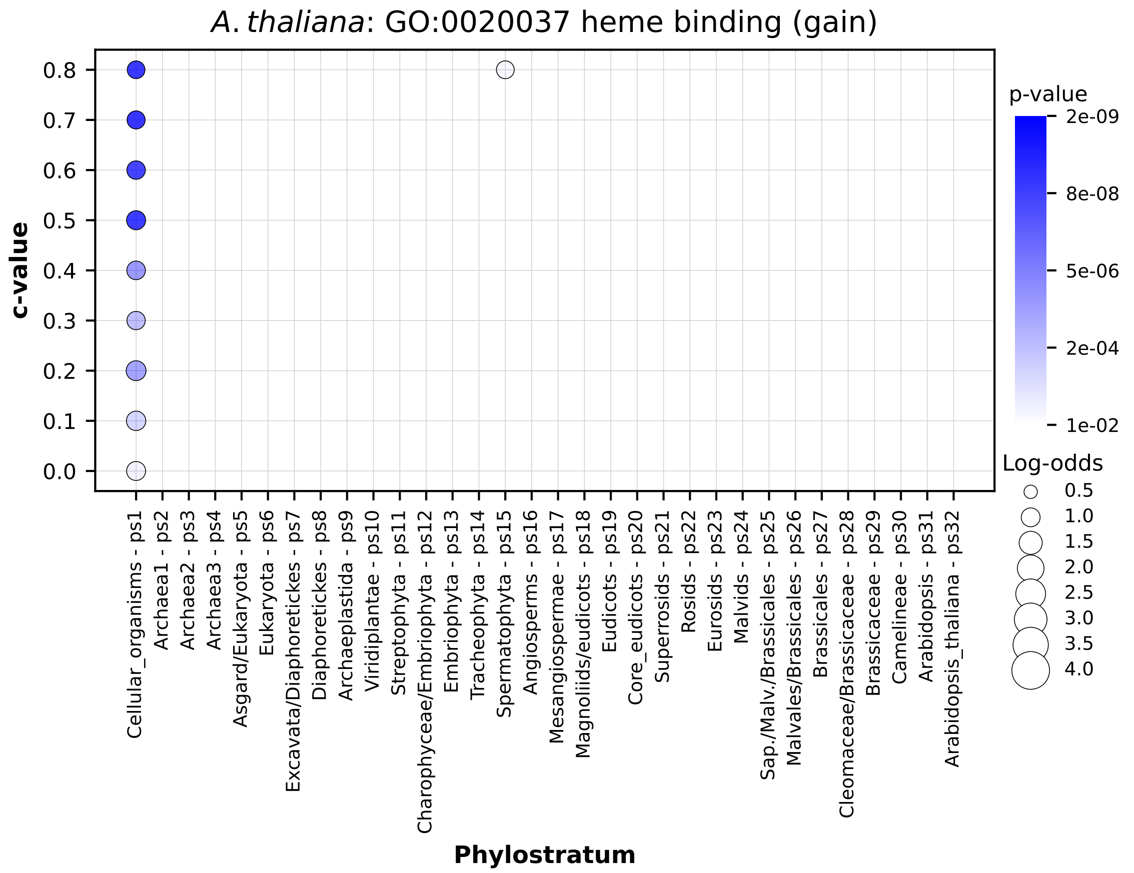

A. thaliana: GO:0020037 heme binding (gain)
c-value
0.0
0.1
0.2
0.3
0.4
0.5
0.6
0.7
0.8
Phylostratum
Cellular_organisms - ps1
Archaea1 - ps2
Archaea2 - ps3
Archaea3 - ps4
Asgard/Eukaryota - ps5
Eukaryota - ps6
Excavata/Diaphoretickes - ps7
Diaphoretickes - ps8
Archaeplastida - ps9
Viridiplantae - ps10
Streptophyta - ps11
Charophyceae/Embriophyta - ps12
Embriophyta - ps13
Tracheophyta - ps14
Spermatophyta - ps15
Angiosperms - ps16
Mesangiospermae - ps17
Magnoliids/eudicots - ps18
Eudicots - ps19
Core_eudicots - ps20
Superrosids - ps21
Rosids - ps22
Eurosids - ps23
Malvids - ps24
Sap./Malv./Brassicales - ps25
Malvales/Brassicales - ps26
Brassicales - ps27
Cleomaceae/Brassicaceae - ps28
Brassicaceae - ps29
Camelineae - ps30
Arabidopsis - ps31
Arabidopsis_thaliana - ps32
p-value
2e-09
8e-08
5e-06
2e-04
1e-02
Log-odds
0.5
1.0
1.5
2.0
2.5
3.0
3.5
4.0

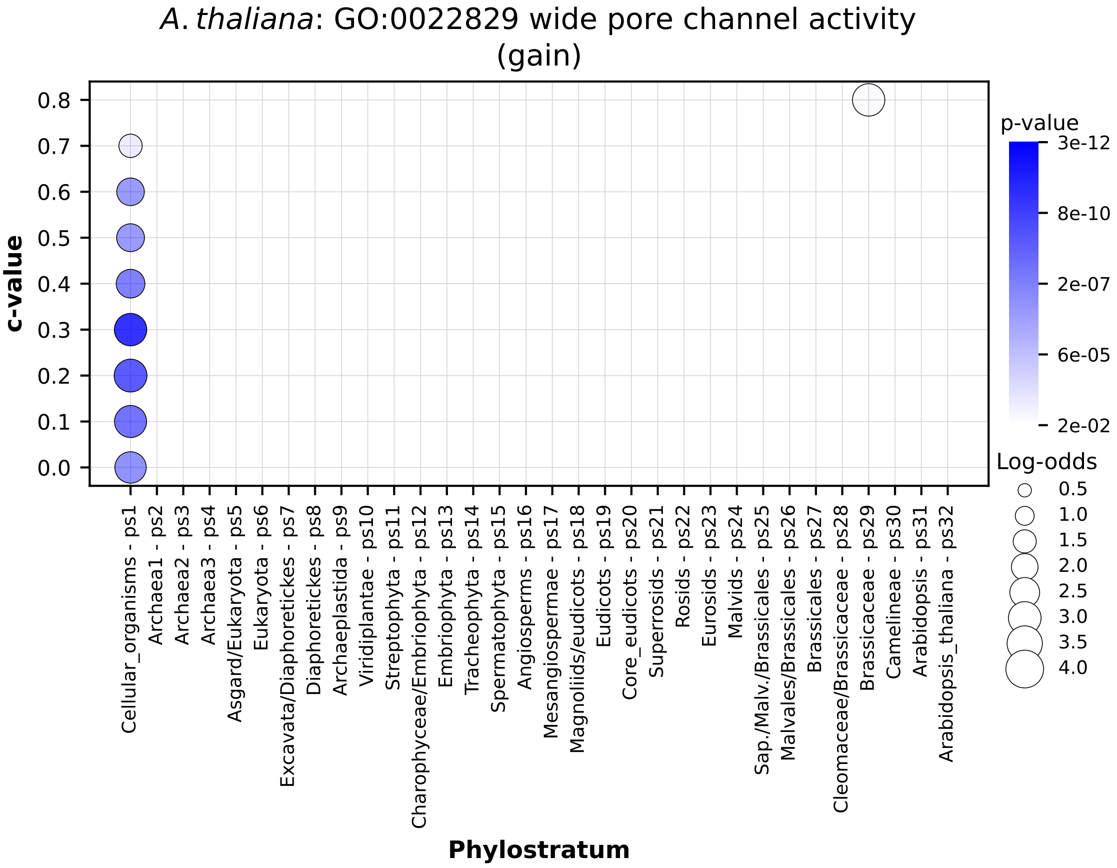

A. thaliana: GO:0022829 wide pore channel activity
(gain)
c-value
0.8
0.7
0.6
0.5
0.4
0.3
0.2
0.1
0.0
p-value
3e-12
8e-10
2e-07
6e-05
2e-02
Log-odds
0.5
1.0
1.5
2.0
2.5
3.0
3.5
4.0
Cellular_organisms - ps1
Archaea1 - ps2
Archaea2 - ps3
Archaea3 - ps4
Asgard/Eukaryota - ps5
Eukaryota - ps6
Excavata/Diaphoretickes - ps7
Diaphoretickes - ps8
Archaeplastida - ps9
Viridiplantae - ps10
Streptophyta - ps11
Charophyceae/Embriophyta - ps12
Embriophyta - ps13
Tracheophyta - ps14
Spermatophyta - ps15
Angiosperms - ps16
Mesangiospermae - ps17
Magnoliids/eudicots - ps18
Eudicots - ps19
Core_eudicots - ps20
Superrosids - ps21
Rosids - ps22
Eurosids - ps23
Malvids - ps24
Sap./Malv./Brassicales - ps25
Malvales/Brassicales - ps26
Brassicales - ps27
Cleomaceae/Brassicaceae - ps28
Brassicaceae - ps29
Camelineae - ps30
Arabidopsis - ps31
Arabidopsis_thaliana - ps32
Phylostratum

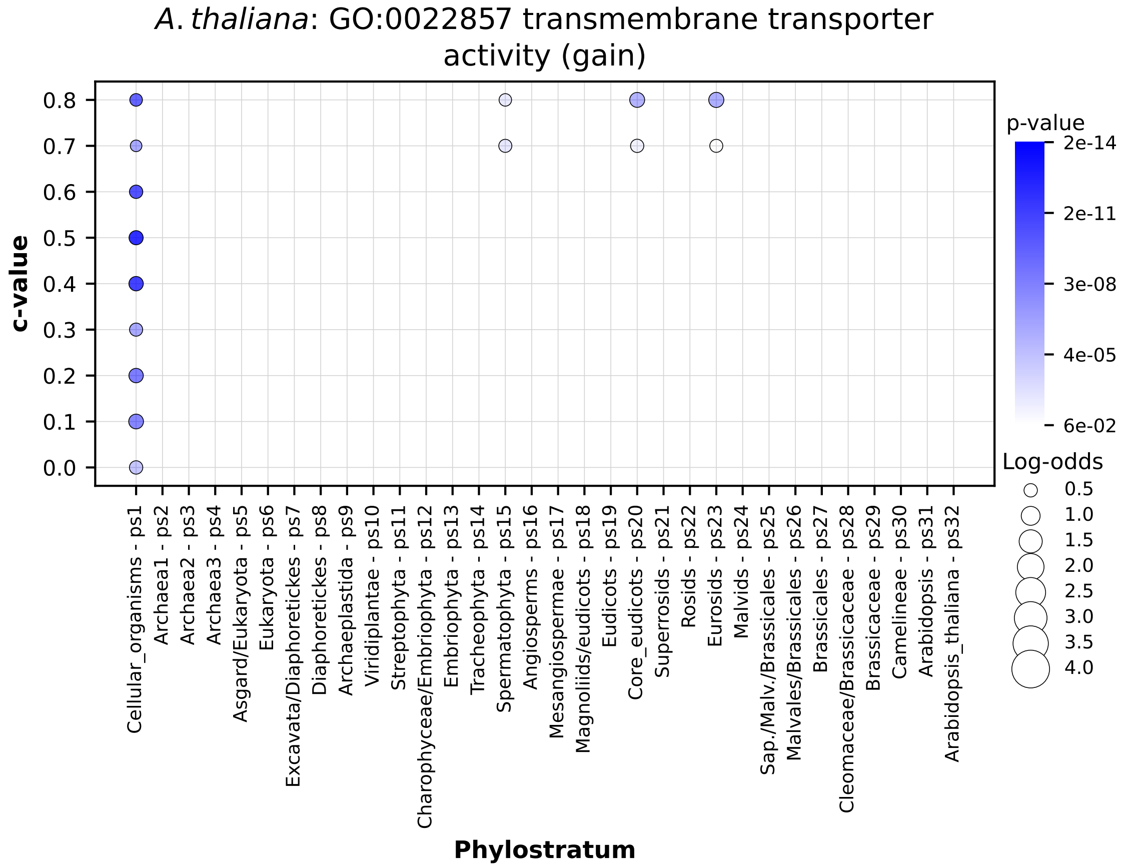

A. thaliana: GO:0022857 transmembrane transporter activity (gain)
c-value
0.8
0.7
0.6
0.5
0.4
0.3
0.2
0.1
0.0
p-value
2e-14
2e-11
3e-08
4e-05
6e-02
Log-odds
0.5
1.0
1.5
2.0
2.5
3.0
3.5
4.0
Cellular_organisms - ps1
Archaea1 - ps2
Archaea2 - ps3
Archaea3 - ps4
Asgard/Eukaryota - ps5
Eukaryota - ps6
Excavata/Diaphoretickes - ps7
Diaphoretickes - ps8
Archaeplastida - ps9
Viridiplantae - ps10
Streptophyta - ps11
Charophyceae/Embriophyta - ps12
Embriophyta - ps13
Tracheophyta - ps14
Spermatophyta - ps15
Angiosperms - ps16
Mesangiospermae - ps17
Magnoliids/eudicots - ps18
Eudicots - ps19
Core_eudicots - ps20
Superrosids - ps21
Rosids - ps22
Eurosids - ps23
Malvids - ps24
Sap./Malv./Brassicales - ps25
Malvales/Brassicales - ps26
Brassicales - ps27
Cleomaceae/Brassicaceae - ps28
Brassicaceae - ps29
Camelineae - ps30
Arabidopsis - ps31
Arabidopsis_thaliana - ps32
Phylostratum

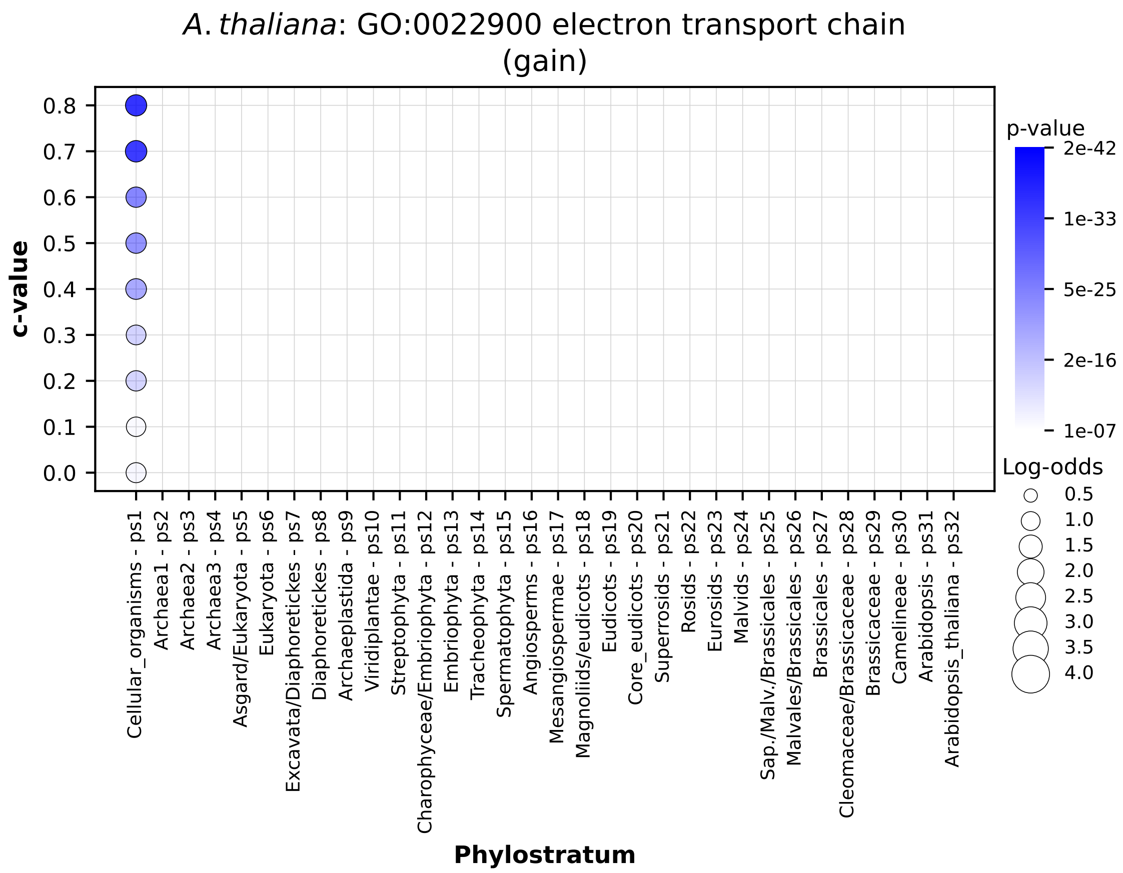
A. thaliana: GO:0022900 electron transport chain
(gain)
c-value
0.8
0.7
0.6
0.5
0.4
0.3
0.2
0.1
0.0
p-value
2e-42
1e-33
5e-25
2e-16
1e-07
Log-odds
0.5
1.0
1.5
2.0
2.5
3.0
3.5
4.0
Cellular_organisms - ps1
Archaea1 - ps2
Archaea2 - ps3
Archaea3 - ps4
Asgard/Eukaryota - ps5
Eukaryota - ps6
Excavata/Diaphoretickes - ps7
Diaphoretickes - ps8
Archaeplastida - ps9
Viridiplantae - ps10
Streptophyta - ps11
Charophyceae/Embriophyta - ps12
Embriophyta - ps13
Tracheophyta - ps14
Spermatophyta - ps15
Angiosperms - ps16
Mesangiospermae - ps17
Magnoliids/eudicots - ps18
Eudicots - ps19
Core_eudicots - ps20
Superrosids - ps21
Rosids - ps22
Eurosids - ps23
Malvids - ps24
Sap./Malv./Brassicales - ps25
Malvales/Brassicales - ps26
Brassicales - ps27
Cleomaceae/Brassicaceae - ps28
Brassicaceae - ps29
Camelineae - ps30
Arabidopsis - ps31
Arabidopsis_thaliana - ps32
Phylostratum

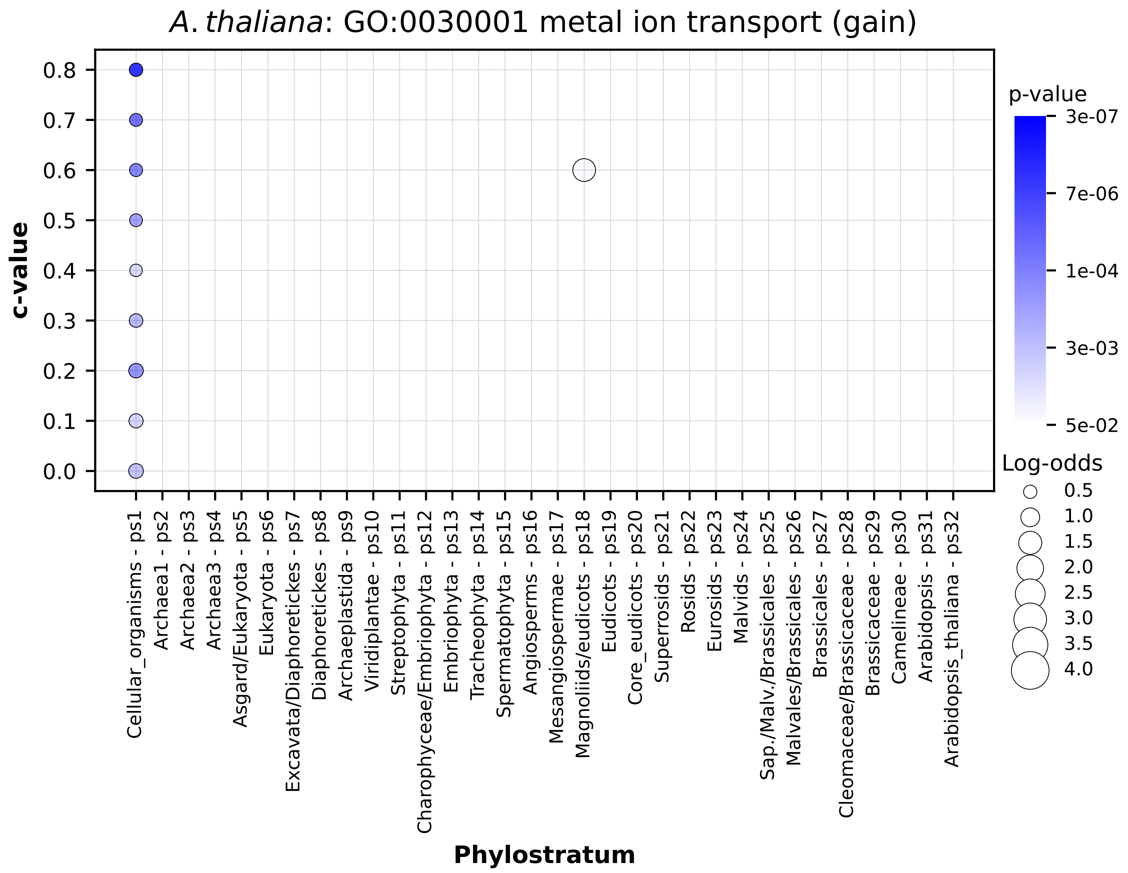

A. thaliana: GO:0030001 metal ion transport (gain)
c-value
0.0
0.1
0.2
0.3
0.4
0.5
0.6
0.7
0.8
p-value
3e-07
7e-06
1e-04
3e-03
5e-02
Log-odds
0.5
1.0
1.5
2.0
2.5
3.0
3.5
4.0
Cellular_organisms - ps1
Archaea1 - ps2
Archaea2 - ps3
Archaea3 - ps4
Asgard/Eukaryota - ps5
Eukaryota - ps6
Excavata/Diaphoretickes - ps7
Diaphoretickes - ps8
Archaeplastida - ps9
Viridiplantae - ps10
Streptophyta - ps11
Charophyceae/Embriophyta - ps12
Embriophyta - ps13
Tracheophyta - ps14
Spermatophyta - ps15
Angiosperms - ps16
Mesangiospermae - ps17
Magnoliids/eudicots - ps18
Eudicots - ps19
Core_eudicots - ps20
Superrosids - ps21
Rosids - ps22
Eurosids - ps23
Malvids - ps24
Sap./Malv./Brassicales - ps25
Malvales/Brassicales - ps26
Brassicales - ps27
Cleomaceae/Brassicaceae - ps28
Brassicaceae - ps29
Camelineae - ps30
Arabidopsis - ps31
Arabidopsis_thaliana - ps32
Phylostratum

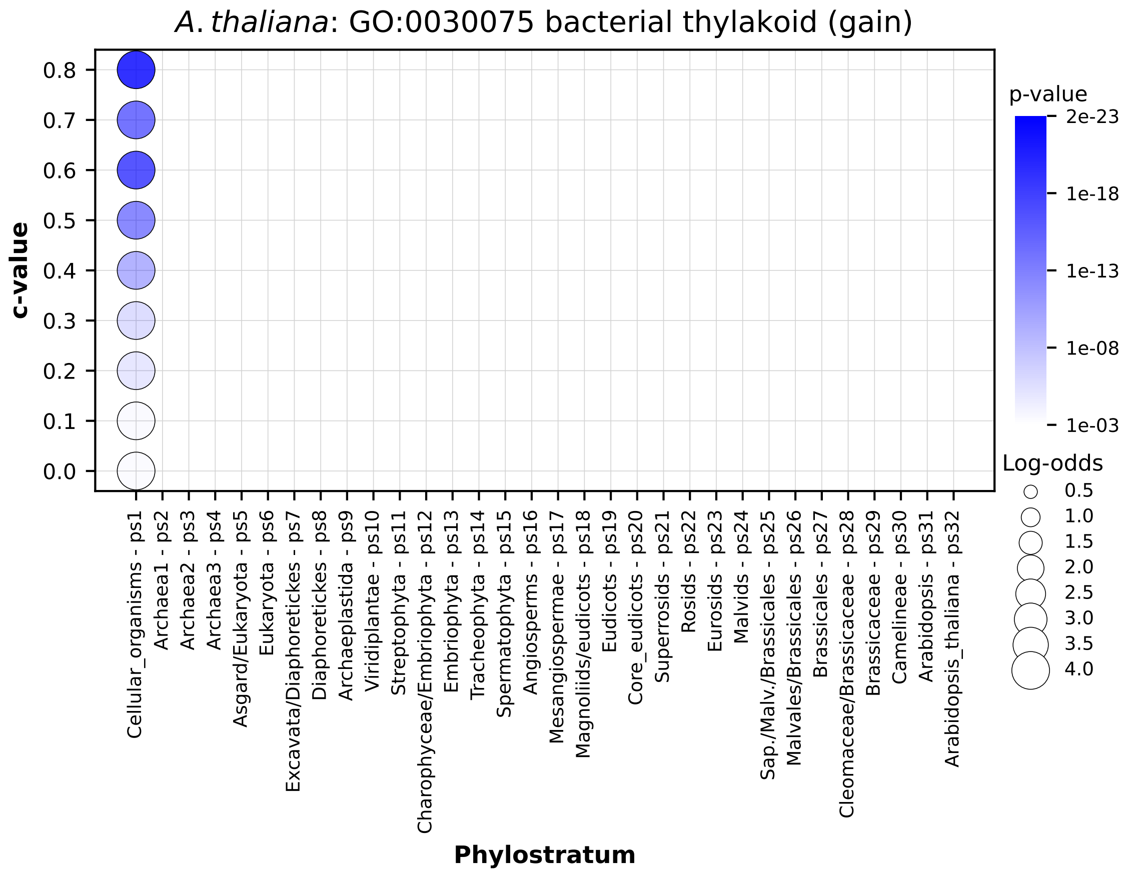

A. thaliana: GO:0030075 bacterial thylakoid (gain)
c-value
0.8
0.7
0.6
0.5
0.4
0.3
0.2
0.1
0.0
Phylostratum
Cellular_organisms - ps1
Archaea1 - ps2
Archaea2 - ps3
Archaea3 - ps4
Asgard/Eukaryota - ps5
Eukaryota - ps6
Excavata/Diaphoretickes - ps7
Diaphoretickes - ps8
Archaeplastida - ps9
Viridiplantae - ps10
Streptophyta - ps11
Charophyceae/Embriophyta - ps12
Embriophyta - ps13
Tracheophyta - ps14
Spermatophyta - ps15
Angiosperms - ps16
Mesangiospermae - ps17
Magnoliids/eudicots - ps18
Eudicots - ps19
Core_eudicots - ps20
Superrosids - ps21
Rosids - ps22
Eurosids - ps23
Malvids - ps24
Sap./Malv./Brassicales - ps25
Malvales/Brassicales - ps26
Brassicales - ps27
Cleomaceae/Brassicaceae - ps28
Brassicaceae - ps29
Camelineae - ps30
Arabidopsis - ps31
Arabidopsis_thaliana - ps32
p-value
2e-23
1e-18
1e-13
1e-08
1e-03
Log-odds
0.5
1.0
1.5
2.0
2.5
3.0
3.5
4.0

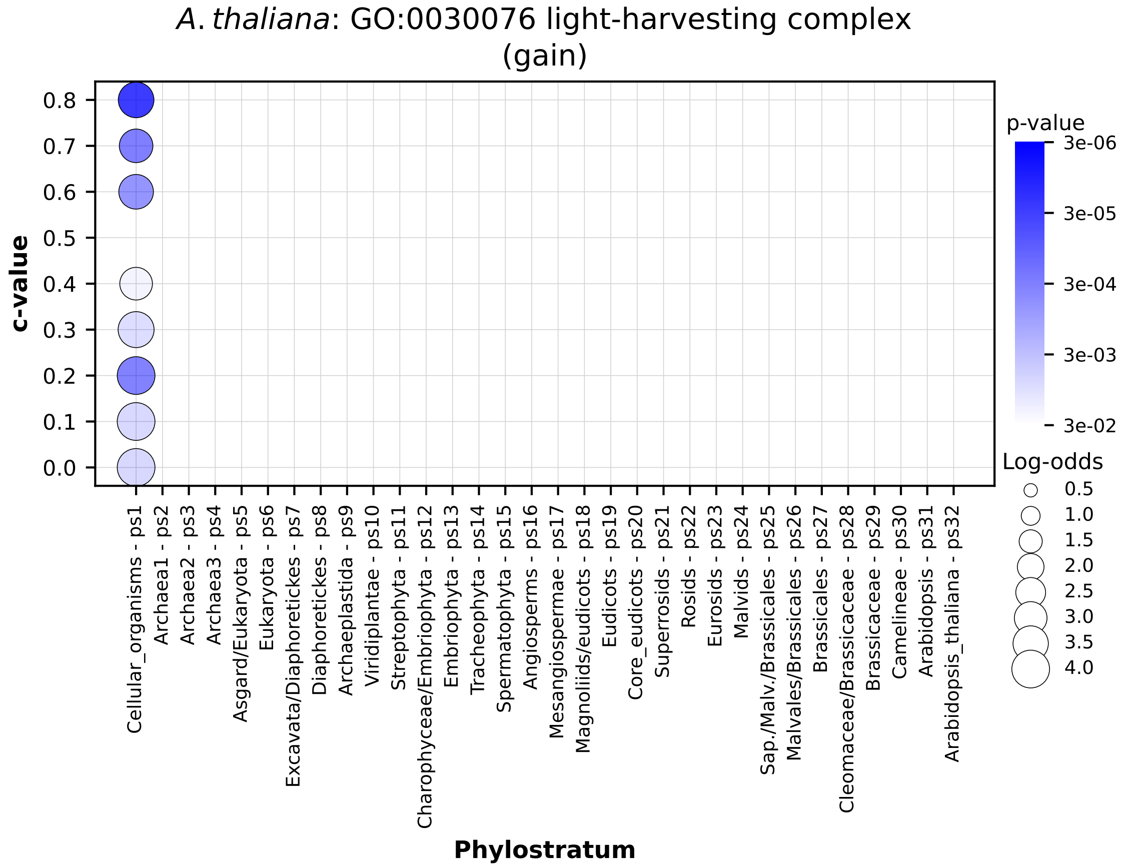

A. thaliana: GO:0030076 light-harvesting complex (gain)
c-value
0.0
0.1
0.2
0.3
0.4
0.5
0.6
0.7
0.8
Phylostratum
Cellular_organisms - ps1
Archaea1 - ps2
Archaea2 - ps3
Archaea3 - ps4
Asgard/Eukaryota - ps5
Eukaryota - ps6
Excavata/Diaphoretickes - ps7
Diaphoretickes - ps8
Archaeplastida - ps9
Viridiplantae - ps10
Streptophyta - ps11
Charophyceae/Embriophyta - ps12
Embriophyta - ps13
Tracheophyta - ps14
Spermatophyta - ps15
Angiosperms - ps16
Mesangiospermae - ps17
Magnoliids/eudicots - ps18
Eudicots - ps19
Core_eudicots - ps20
Superrosids - ps21
Rosids - ps22
Eurosids - ps23
Malvids - ps24
Sap./Malv./Brassicales - ps25
Malvales/Brassicales - ps26
Brassicales - ps27
Cleomaceae/Brassicaceae - ps28
Brassicaceae - ps29
Camelineae - ps30
Arabidopsis - ps31
Arabidopsis_thaliana - ps32
p-value
3e-06
3e-05
3e-04
3e-03
3e-02
Log-odds
0.5
1.0
1.5
2.0
2.5
3.0
3.5
4.0

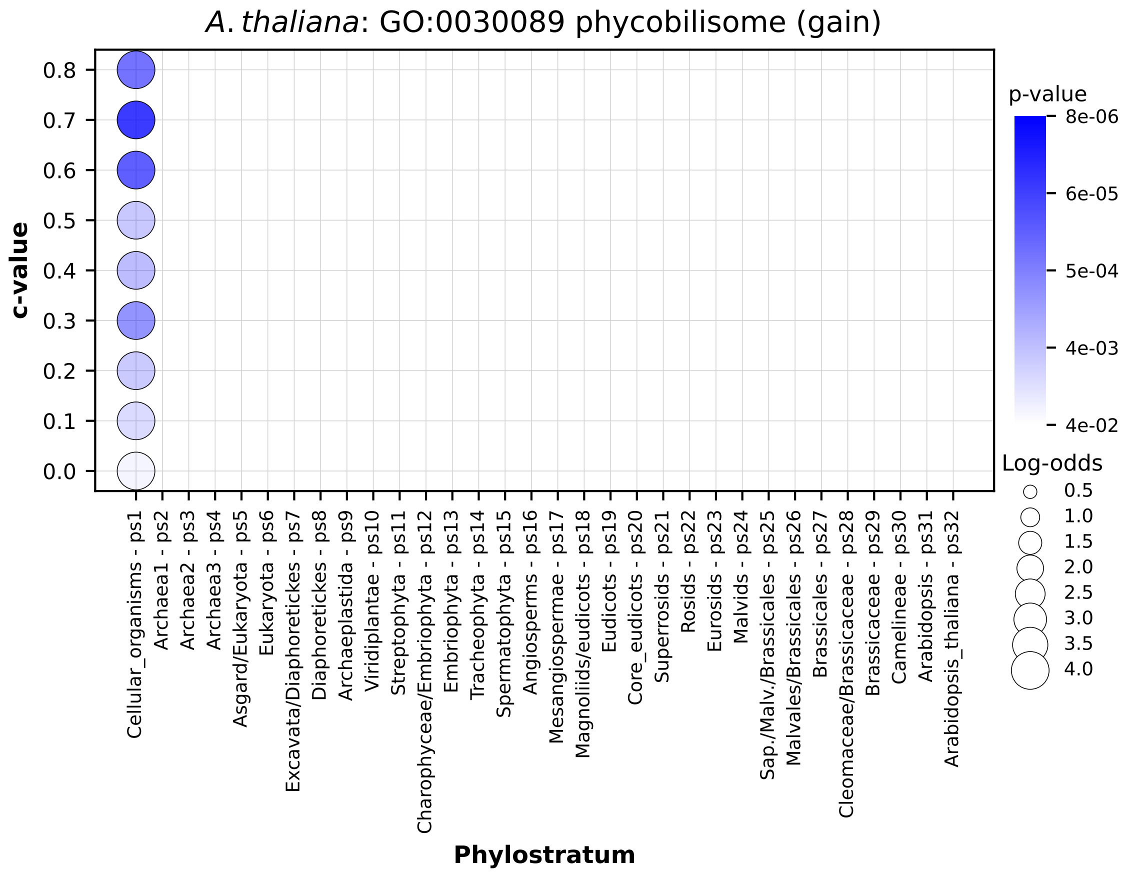
A. thaliana: GO:0030089 phycobilisome (gain)
c-value
0.8
0.7
0.6
0.5
0.4
0.3
0.2
0.1
0.0
Phylostratum
Cellular_organisms - ps1
Archaea1 - ps2
Archaea2 - ps3
Archaea3 - ps4
Asgard/Eukaryota - ps5
Eukaryota - ps6
Excavata/Diaphoretickes - ps7
Diaphoretickes - ps8
Archaeplastida - ps9
Viridiplantae - ps10
Streptophyta - ps11
Charophyceae/Embriophyta - ps12
Embriophyta - ps13
Tracheophyta - ps14
Spermatophyta - ps15
Angiosperms - ps16
Mesangiospermae - ps17
Magnoliids/eudicots - ps18
Eudicots - ps19
Core_eudicots - ps20
Superrosids - ps21
Rosids - ps22
Eurosids - ps23
Malvids - ps24
Sap./Malv./Brassicales - ps25
Malvales/Brassicales - ps26
Brassicales - ps27
Cleomaceae/Brassicaceae - ps28
Brassicaceae - ps29
Camelineae - ps30
Arabidopsis - ps31
Arabidopsis_thaliana - ps32
p-value
8e-06
6e-05
5e-04
4e-03
4e-02
Log-odds
0.5
1.0
1.5
2.0
2.5
3.0
3.5
4.0

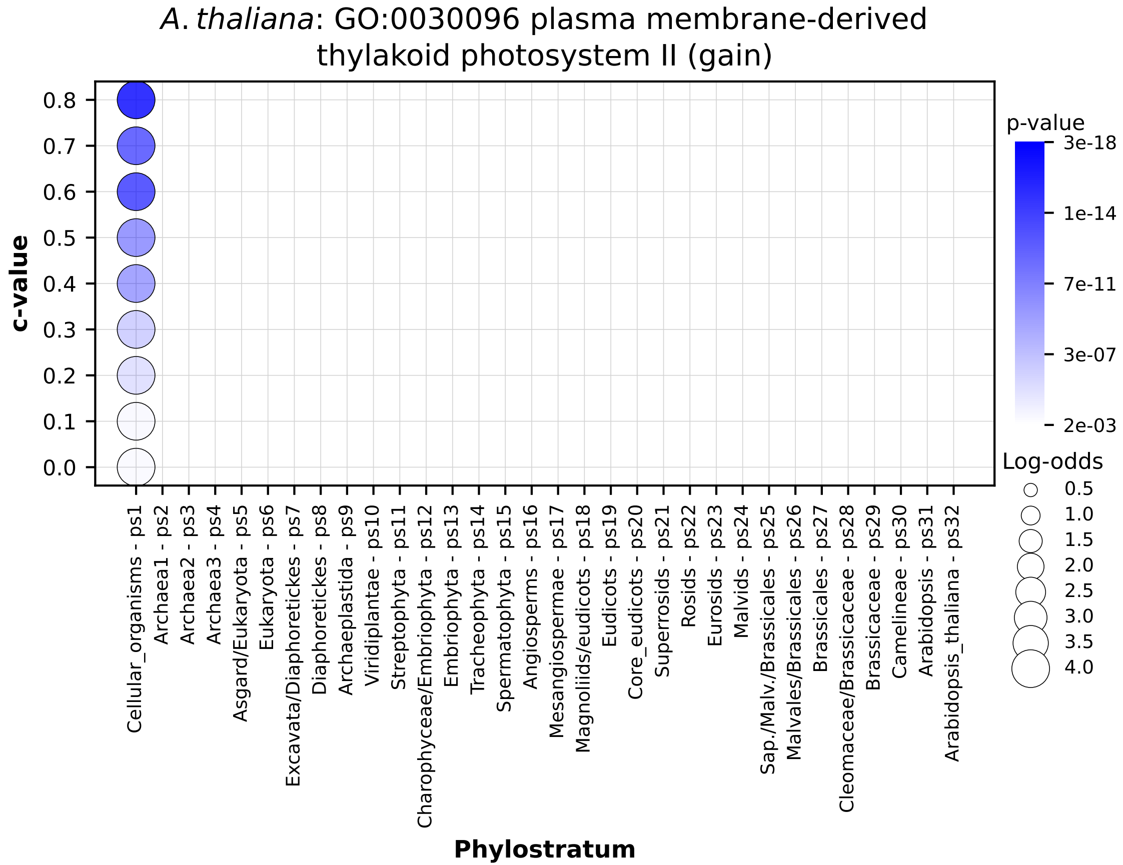

A. thaliana: GO:0030096 plasma membrane-derived thylakoid photosystem II (gain)
c-value
0.8
0.7
0.6
0.5
0.4
0.3
0.2
0.1
0.0
p-value
3e-18
1e-14
7e-11
3e-07
2e-03
Log-odds
0.5
1.0
1.5
2.0
2.5
3.0
3.5
4.0
Cellular_organisms - ps1
Archaea1 - ps2
Archaea2 - ps3
Archaea3 - ps4
Asgard/Eukaryota - ps5
Eukaryota - ps6
Excavata/Diaphoretickes - ps7
Diaphoretickes - ps8
Archaeplastida - ps9
Viridiplantae - ps10
Streptophyta - ps11
Charophyceae/Embriophyta - ps12
Embriophyta - ps13
Tracheophyta - ps14
Spermatophyta - ps15
Angiosperms - ps16
Mesangiospermae - ps17
Magnoliids/eudicots - ps18
Eudicots - ps19
Core_eudicots - ps20
Superrosids - ps21
Rosids - ps22
Eurosids - ps23
Malvids - ps24
Sap./Malv./Brassicales - ps25
Malvales/Brassicales - ps26
Brassicales - ps27
Cleomaceae/Brassicaceae - ps28
Brassicaceae - ps29
Camelineae - ps30
Arabidopsis - ps31
Arabidopsis_thaliana - ps32
Phylostratum

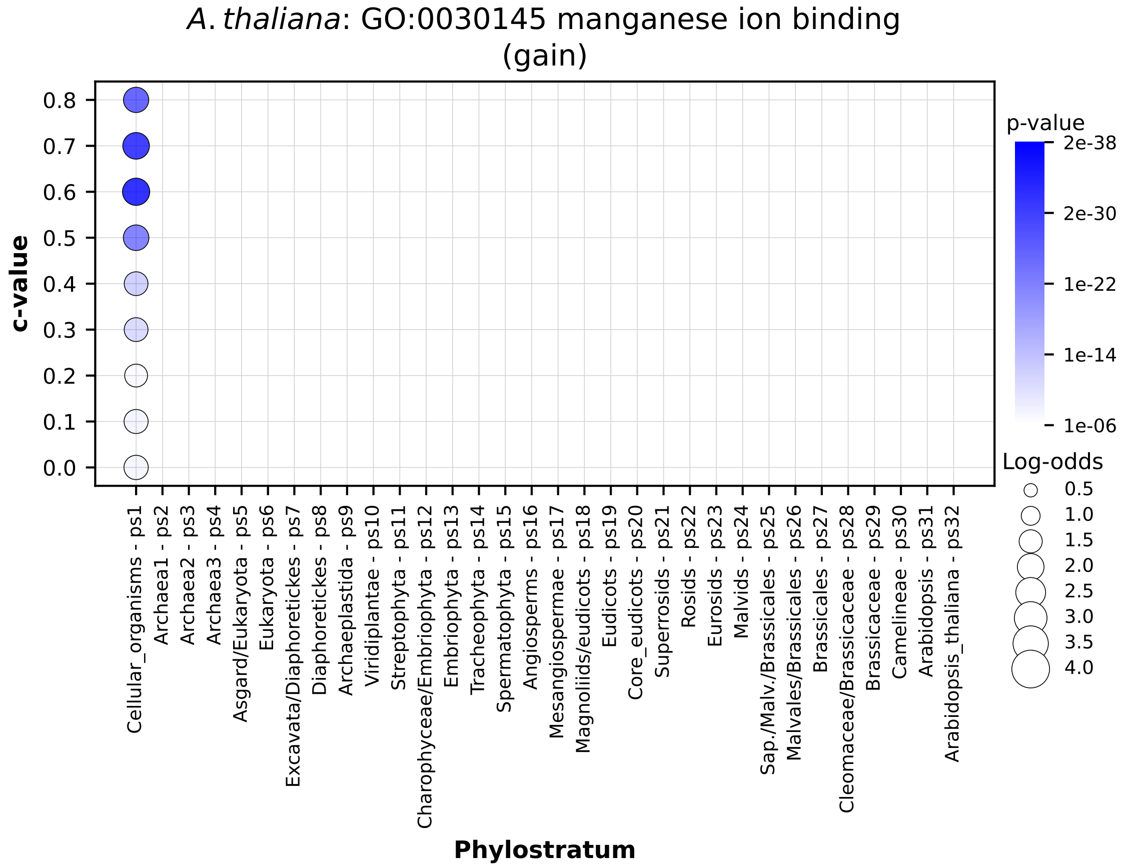

A. thaliana: GO:0030145 manganese ion binding (gain)
c-value
0.8
0.7
0.6
0.5
0.4
0.3
0.2
0.1
0.0
p-value
2e-38
2e-30
1e-22
1e-14
1e-06
Log-odds
0.5
1.0
1.5
2.0
2.5
3.0
3.5
4.0
Cellular_organisms - ps1
Archaea1 - ps2
Archaea2 - ps3
Archaea3 - ps4
Asgard/Eukaryota - ps5
Eukaryota - ps6
Excavata/Diaphoretickes - ps7
Diaphoretickes - ps8
Archaeplastida - ps9
Viridiplantae - ps10
Streptophyta - ps11
Charophyceae/Embriophyta - ps12
Embriophyta - ps13
Tracheophyta - ps14
Spermatophyta - ps15
Angiosperms - ps16
Mesangiospermae - ps17
Magnoliids/eudicots - ps18
Eudicots - ps19
Core_eudicots - ps20
Superrosids - ps21
Rosids - ps22
Eurosids - ps23
Malvids - ps24
Sap./Malv./Brassicales - ps25
Malvales/Brassicales - ps26
Brassicales - ps27
Cleomaceae/Brassicaceae - ps28
Brassicaceae - ps29
Camelineae - ps30
Arabidopsis - ps31
Arabidopsis_thaliana - ps32
Phylostratum

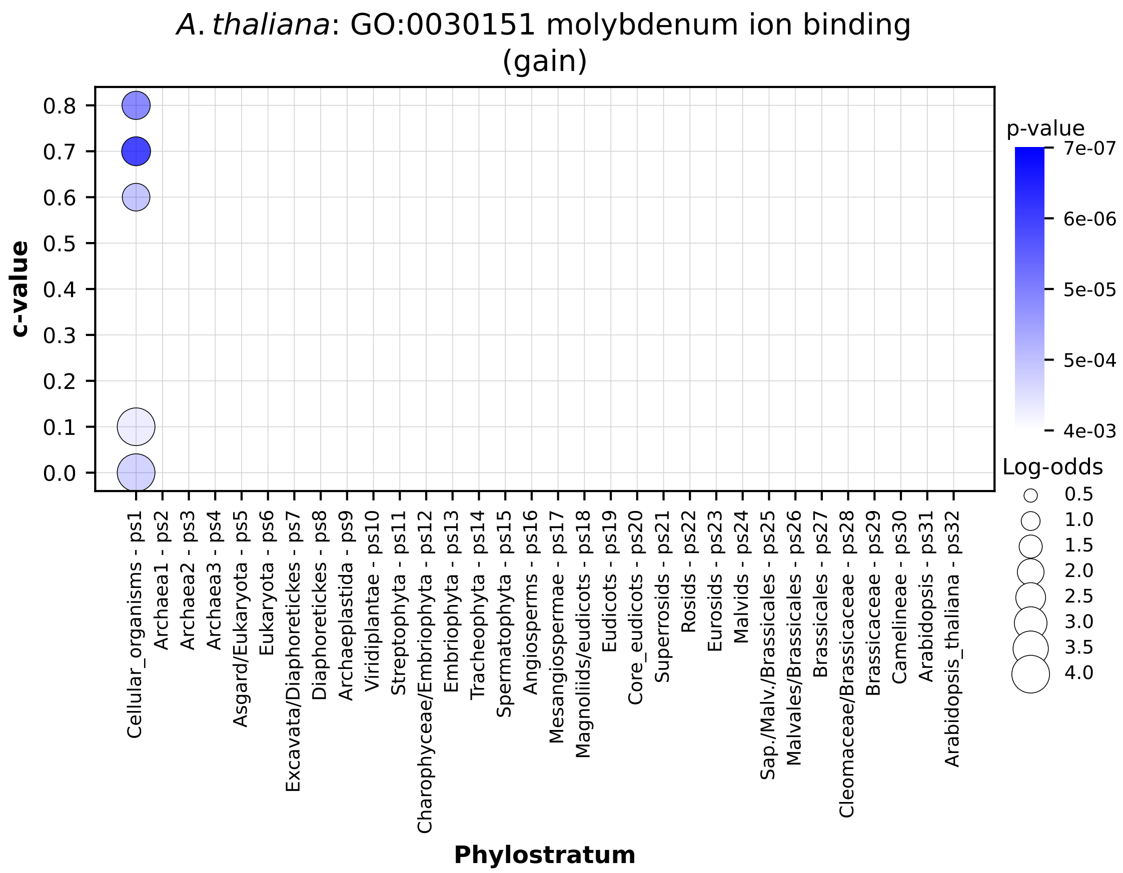

A. thaliana: GO:0030151 molybdenum ion binding (gain)
c-value
0.0
0.1
0.2
0.3
0.4
0.5
0.6
0.7
0.8
p-value
7e-07
6e-06
5e-05
5e-04
4e-03
Log-odds
0.5
1.0
1.5
2.0
2.5
3.0
3.5
4.0
Cellular_organisms - ps1
Archaea1 - ps2
Archaea2 - ps3
Archaea3 - ps4
Asgard/Eukaryota - ps5
Eukaryota - ps6
Excavata/Diaphoretickes - ps7
Diaphoretickes - ps8
Archaeplastida - ps9
Viridiplantae - ps10
Streptophyta - ps11
Charophyceae/Embriophyta - ps12
Embriophyta - ps13
Tracheophyta - ps14
Spermatophyta - ps15
Angiosperms - ps16
Mesangiospermae - ps17
Magnoliids/eudicots - ps18
Eudicots - ps19
Core_eudicots - ps20
Superrosids - ps21
Rosids - ps22
Eurosids - ps23
Malvids - ps24
Sap./Malv./Brassicales - ps25
Malvales/Brassicales - ps26
Brassicales - ps27
Cleomaceae/Brassicaceae - ps28
Brassicaceae - ps29
Camelineae - ps30
Arabidopsis - ps31
Arabidopsis_thaliana - ps32
Phylostratum

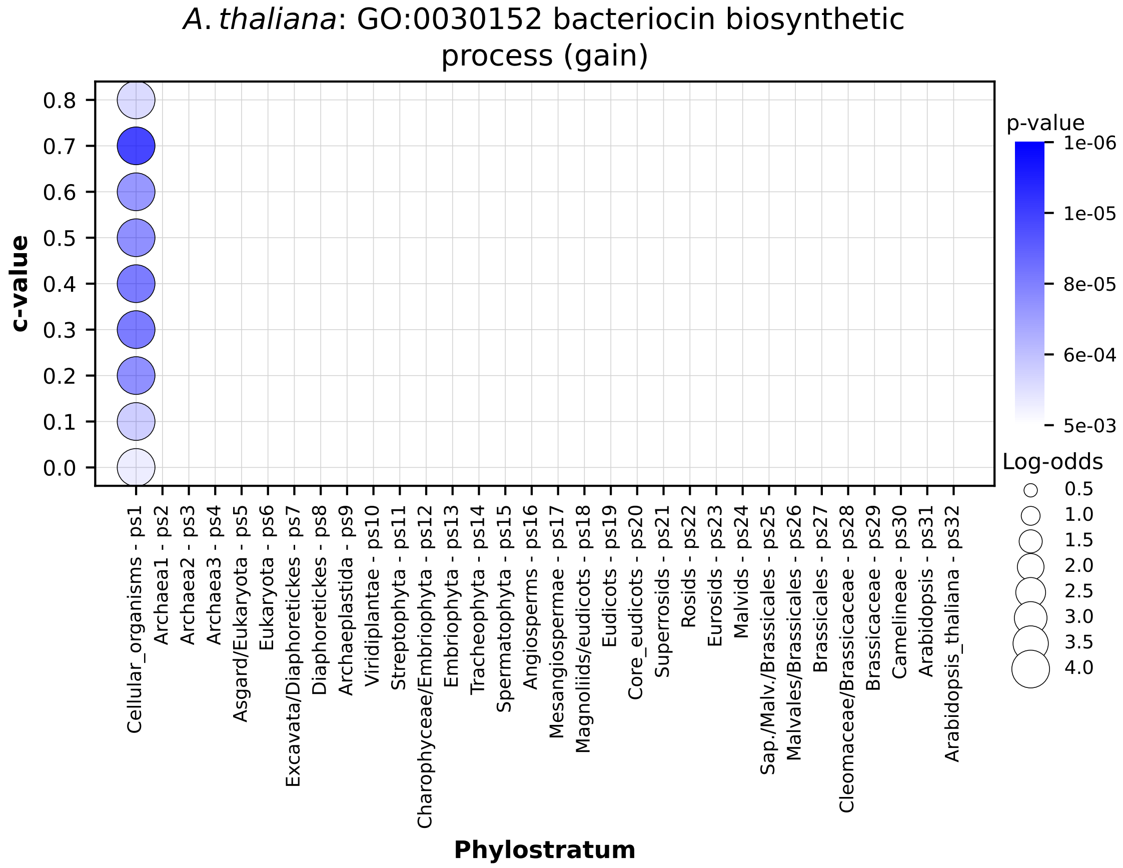
A. thaliana: GO:0030152 bacteriocin biosynthetic process (gain)
c-value
0.8
0.7
0.6
0.5
0.4
0.3
0.2
0.1
0.0
p-value
1e-06
1e-05
8e-05
6e-04
5e-03
Log-odds
0.5
1.0
1.5
2.0
2.5
3.0
3.5
4.0
Cellular_organisms - ps1
Archaea1 - ps2
Archaea2 - ps3
Archaea3 - ps4
Asgard/Eukaryota - ps5
Eukaryota - ps6
Excavata/Diaphoretickes - ps7
Diaphoretickes - ps8
Archaeplastida - ps9
Viridiplantae - ps10
Streptophyta - ps11
Charophyceae/Embriophyta - ps12
Embriophyta - ps13
Tracheophyta - ps14
Spermatophyta - ps15
Angiosperms - ps16
Mesangiospermae - ps17
Magnoliids/eudicots - ps18
Eudicots - ps19
Core_eudicots - ps20
Superrosids - ps21
Rosids - ps22
Eurosids - ps23
Malvids - ps24
Sap./Malv./Brassicales - ps25
Malvales/Brassicales - ps26
Brassicales - ps27
Cleomaceae/Brassicaceae - ps28
Brassicaceae - ps29
Camelineae - ps30
Arabidopsis - ps31
Arabidopsis_thaliana - ps32
Phylostratum

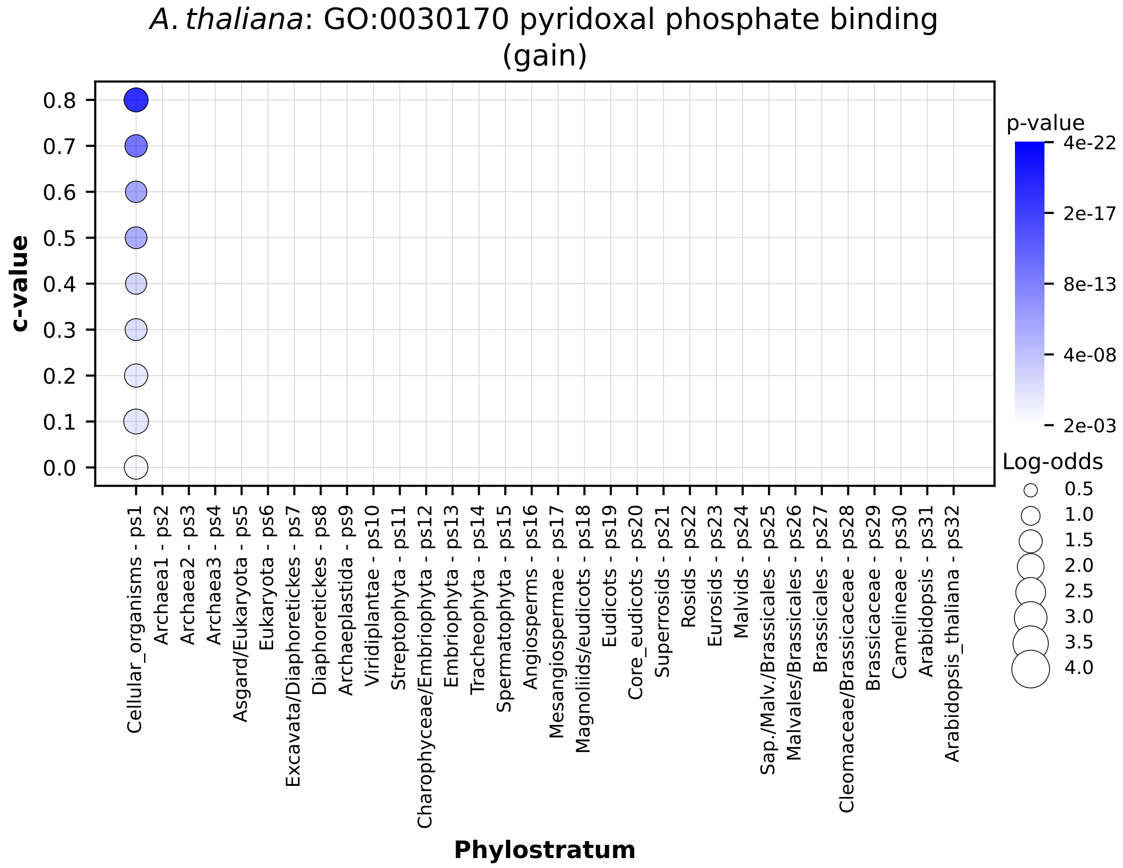

A. thaliana: GO:0030170 pyridoxal phosphate binding (gain)
c-value
0.8
0.7
0.6
0.5
0.4
0.3
0.2
0.1
0.0
p-value
4e-22
2e-17
8e-13
4e-08
2e-03
Log-odds
0.5
1.0
1.5
2.0
2.5
3.0
3.5
4.0
Cellular_organisms - ps1
Archaea1 - ps2
Archaea2 - ps3
Archaea3 - ps4
Asgard/Eukaryota - ps5
Eukaryota - ps6
Excavata/Diaphoretickes - ps7
Diaphoretickes - ps8
Archaeplastida - ps9
Viridiplantae - ps10
Streptophyta - ps11
Charophyceae/Embriophyta - ps12
Embriophyta - ps13
Tracheophyta - ps14
Spermatophyta - ps15
Angiosperms - ps16
Mesangiospermae - ps17
Magnoliids/eudicots - ps18
Eudicots - ps19
Core_eudicots - ps20
Superrosids - ps21
Rosids - ps22
Eurosids - ps23
Malvids - ps24
Sap./Malv./Brassicales - ps25
Malvales/Brassicales - ps26
Brassicales - ps27
Cleomaceae/Brassicaceae - ps28
Brassicaceae - ps29
Camelineae - ps30
Arabidopsis - ps31
Arabidopsis_thaliana - ps32
Phylostratum

A. thaliana: GO:0030203 glycosaminoglycan metabolic process (gain)

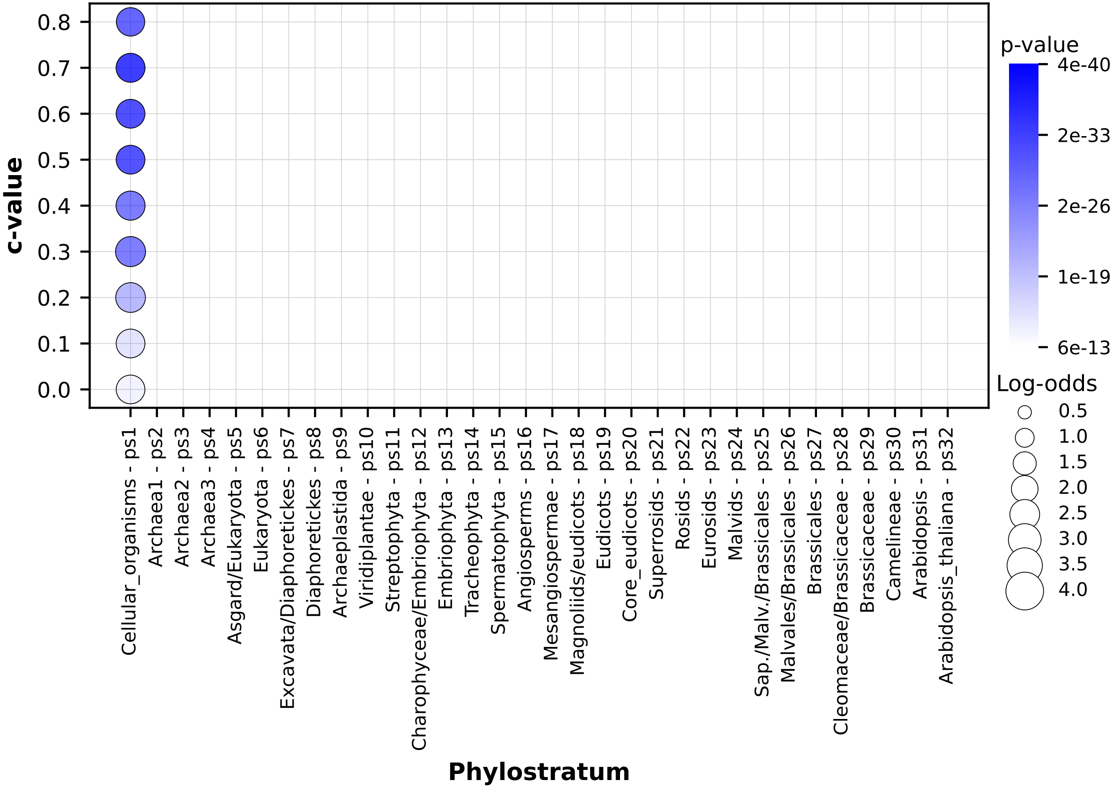

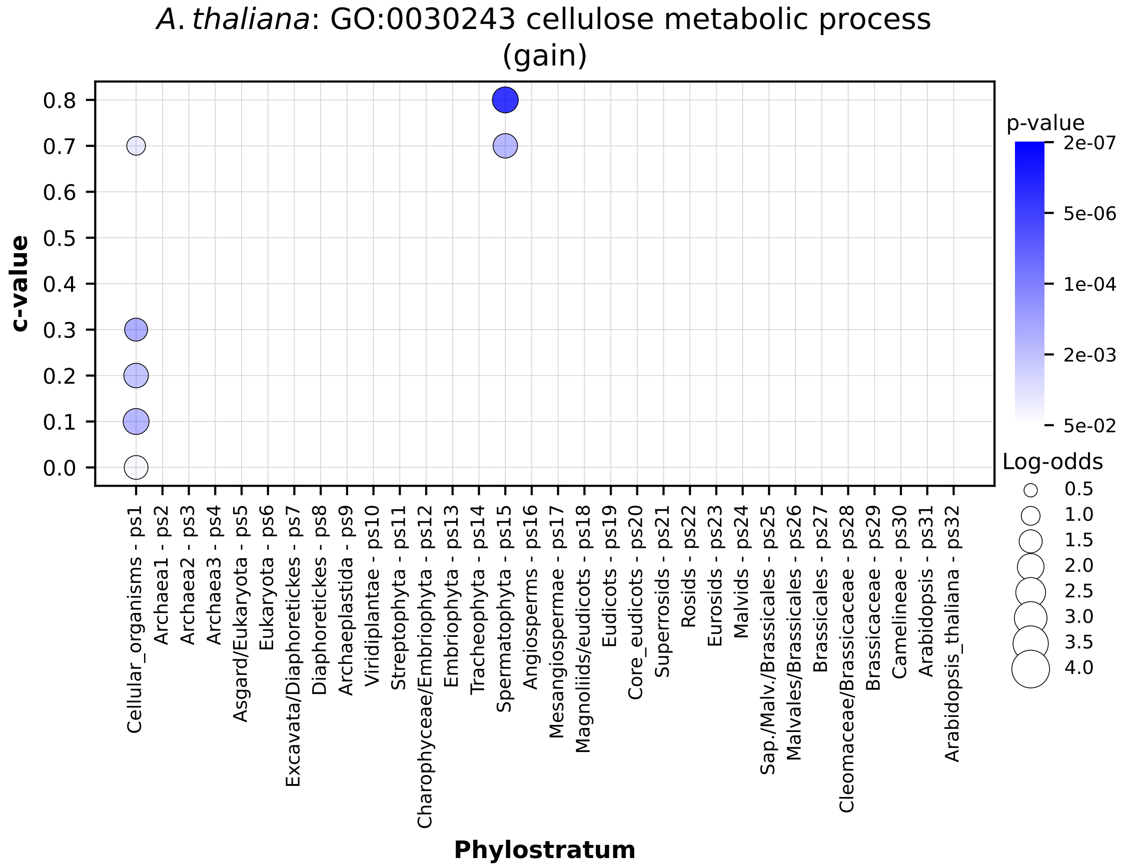
A. thaliana: GO:0030243 cellulose metabolic process
(gain)
c-value
Phylostratum
p-value
2e-07
5e-06
1e-04
2e-03
5e-02
Log-odds
0.5
1.0
1.5
2.0
2.5
3.0
3.5
4.0
Cellular_organisms - ps1
Archaea1 - ps2
Archaea2 - ps3
Archaea3 - ps4
Asgard/Eukaryota - ps5
Eukaryota - ps6
Excavata/Diaphoretickes - ps7
Diaphoretickes - ps8
Archaeplastida - ps9
Viridiplantae - ps10
Streptophyta - ps11
Charophyceae/Embriophyta - ps12
Embriophyta - ps13
Tracheophyta - ps14
Spermatophyta - ps15
Angiosperms - ps16
Mesangiospermae - ps17
Magnoliids/eudicots - ps18
Eudicots - ps19
Core_eudicots - ps20
Superrosids - ps21
Rosids - ps22
Eurosids - ps23
Malvids - ps24
Sap./Malv./Brassicales - ps25
Malvales/Brassicales - ps26
Brassicales - ps27
Cleomaceae/Brassicaceae - ps28
Brassicaceae - ps29
Camelineae - ps30
Arabidopsis - ps31
Arabidopsis_thaliana - ps32

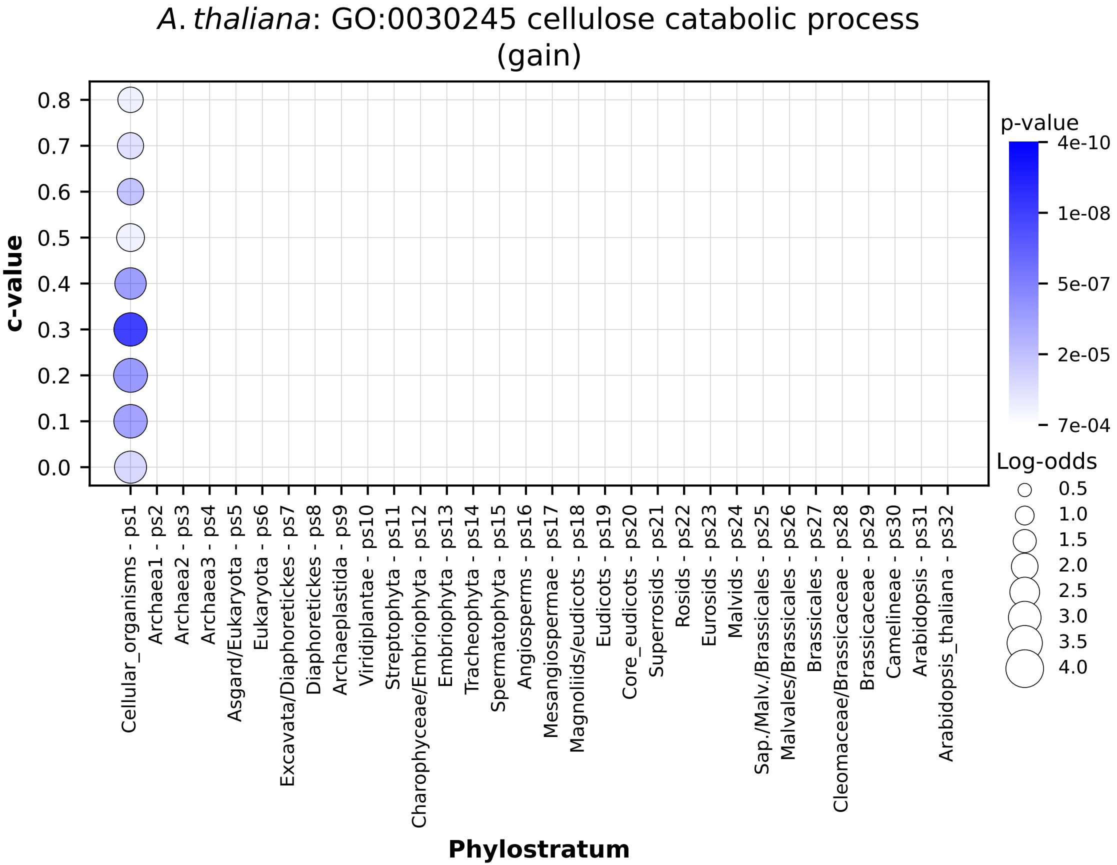

A. thaliana: GO:0030245 cellulose catabolic process
(gain)
c-value
0.8
0.7
0.6
0.5
0.4
0.3
0.2
0.1
0.0
p-value
4e-10
1e-08
5e-07
2e-05
7e-04
Log-odds
0.5
1.0
1.5
2.0
2.5
3.0
3.5
4.0
Cellular_organisms - ps1
Archaea1 - ps2
Archaea2 - ps3
Archaea3 - ps4
Asgard/Eukaryota - ps5
Eukaryota - ps6
Excavata/Diaphoretickes - ps7
Diaphoretickes - ps8
Archaeplastida - ps9
Viridiplantae - ps10
Streptophyta - ps11
Charophyceae/Embriophyta - ps12
Embriophyta - ps13
Tracheophyta - ps14
Spermatophyta - ps15
Angiosperms - ps16
Mesangiospermae - ps17
Magnoliids/eudicots - ps18
Eudicots - ps19
Core_eudicots - ps20
Superrosids - ps21
Rosids - ps22
Eurosids - ps23
Malvids - ps24
Sap./Malv./Brassicales - ps25
Malvales/Brassicales - ps26
Brassicales - ps27
Cleomaceae/Brassicaceae - ps28
Brassicaceae - ps29
Camelineae - ps30
Arabidopsis - ps31
Arabidopsis_thaliana - ps32
Phylostratum

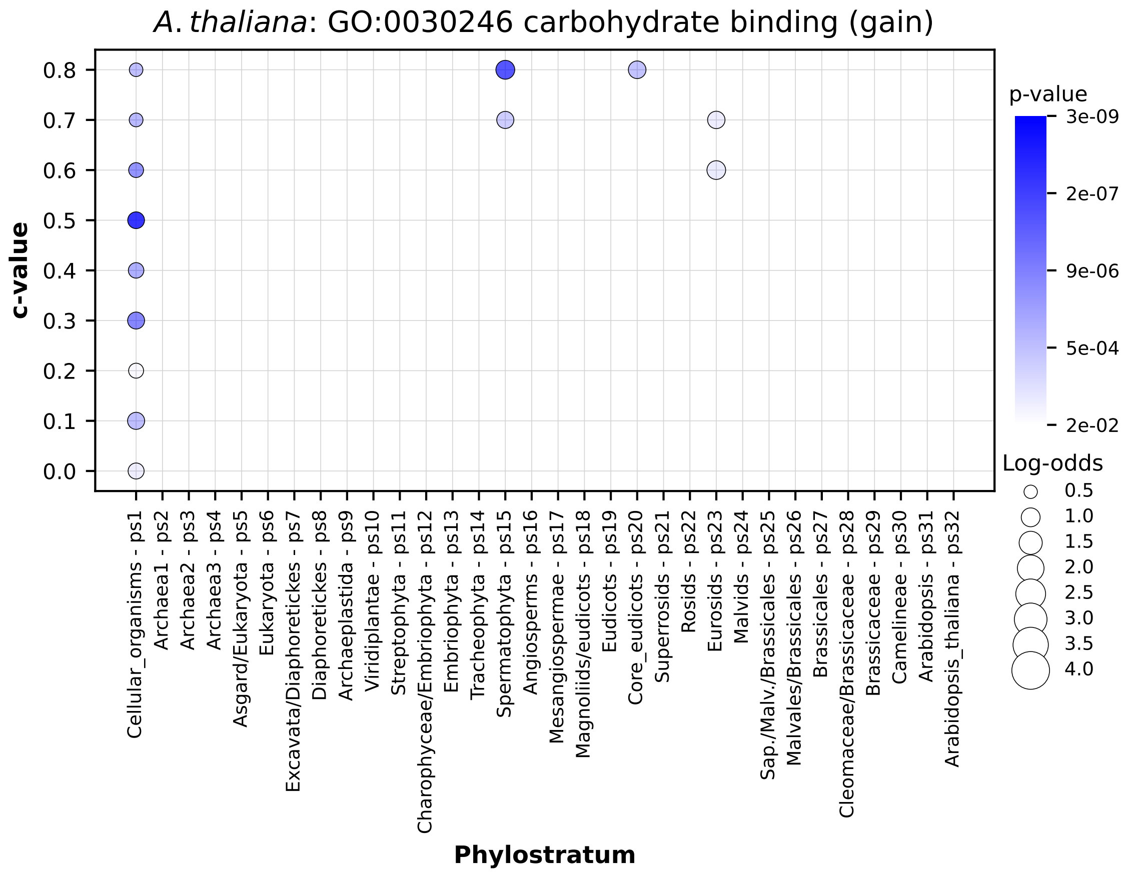

A. thaliana: GO:0030246 carbohydrate binding (gain)
c-value
0.0
0.1
0.2
0.3
0.4
0.5
0.6
0.7
0.8
p-value
3e-09
2e-07
9e-06
5e-04
2e-02
Log-odds
0.5
1.0
1.5
2.0
2.5
3.0
3.5
4.0
Cellular_organisms - ps1
Archaea1 - ps2
Archaea2 - ps3
Archaea3 - ps4
Asgard/Eukaryota - ps5
Eukaryota - ps6
Excavata/Diaphoretickes - ps7
Diaphoretickes - ps8
Archaeplastida - ps9
Viridiplantae - ps10
Streptophyta - ps11
Charophyceae/Embriophyta - ps12
Embriophyta - ps13
Tracheophyta - ps14
Spermatophyta - ps15
Angiosperms - ps16
Mesangiospermae - ps17
Magnoliids/eudicots - ps18
Eudicots - ps19
Core_eudicots - ps20
Superrosids - ps21
Rosids - ps22
Eurosids - ps23
Malvids - ps24
Sap./Malv./Brassicales - ps25
Malvales/Brassicales - ps26
Brassicales - ps27
Cleomaceae/Brassicaceae - ps28
Brassicaceae - ps29
Camelineae - ps30
Arabidopsis - ps31
Arabidopsis_thaliana - ps32
Phylostratum

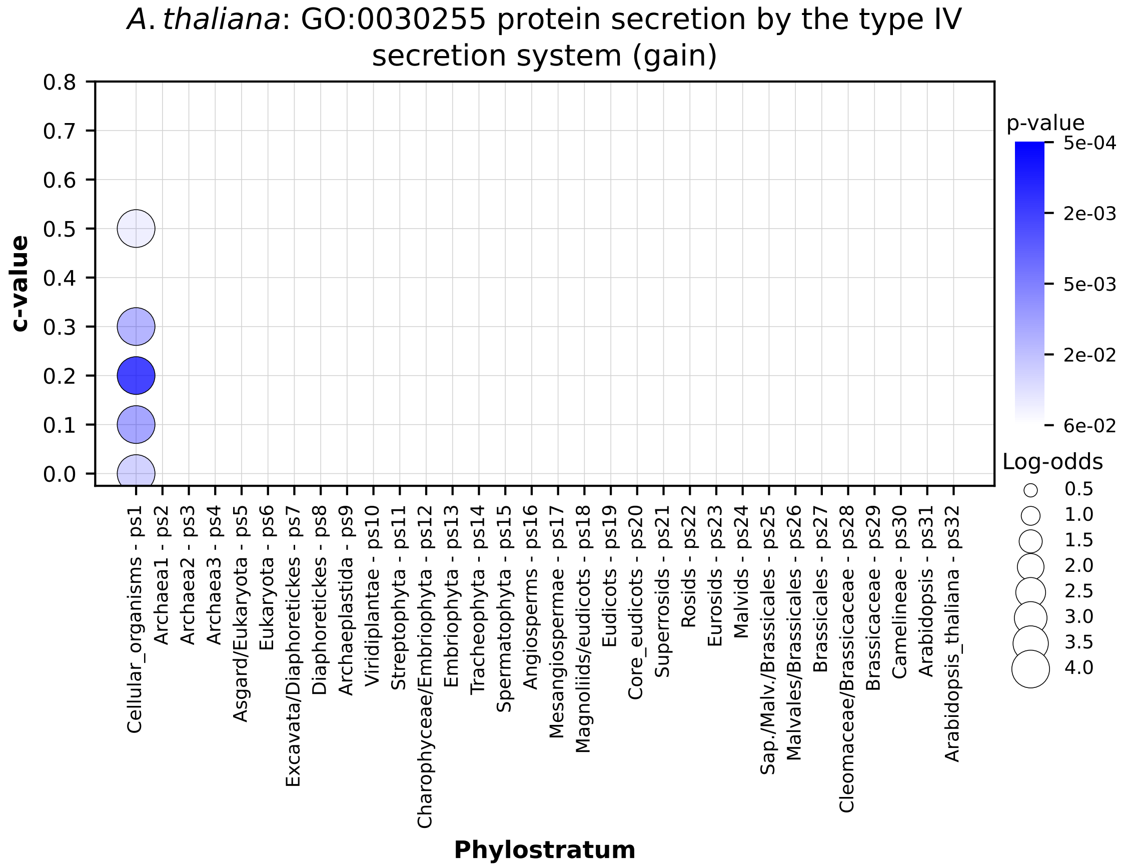
A. thaliana: GO:0030255 protein secretion by the type IV secretion system (gain)
c-value
0.8
0.7
0.6
0.5
0.4
0.3
0.2
0.1
0.0
Phylostratum
Cellular_organisms - ps1
Archaea1 - ps2
Archaea2 - ps3
Archaea3 - ps4
Asgard/Eukaryota - ps5
Eukaryota - ps6
Excavata/Diaphoretickes - ps7
Diaphoretickes - ps8
Archaeplastida - ps9
Viridiplantae - ps10
Streptophyta - ps11
Charophyceae/Embriophyta - ps12
Embriophyta - ps13
Tracheophyta - ps14
Spermatophyta - ps15
Angiosperms - ps16
Mesangiospermae - ps17
Magnoliids/eudicots - ps18
Eudicots - ps19
Core_eudicots - ps20
Superrosids - ps21
Rosids - ps22
Eurosids - ps23
Malvids - ps24
Sap./Malv./Brassicales - ps25
Malvales/Brassicales - ps26
Brassicales - ps27
Cleomaceae/Brassicaceae - ps28
Brassicaceae - ps29
Camelineae - ps30
Arabidopsis - ps31
Arabidopsis_thaliana - ps32
p-value
5e-04
2e-03
5e-03
2e-02
6e-02
Log-odds
0.5
1.0
1.5
2.0
2.5
3.0
3.5
4.0

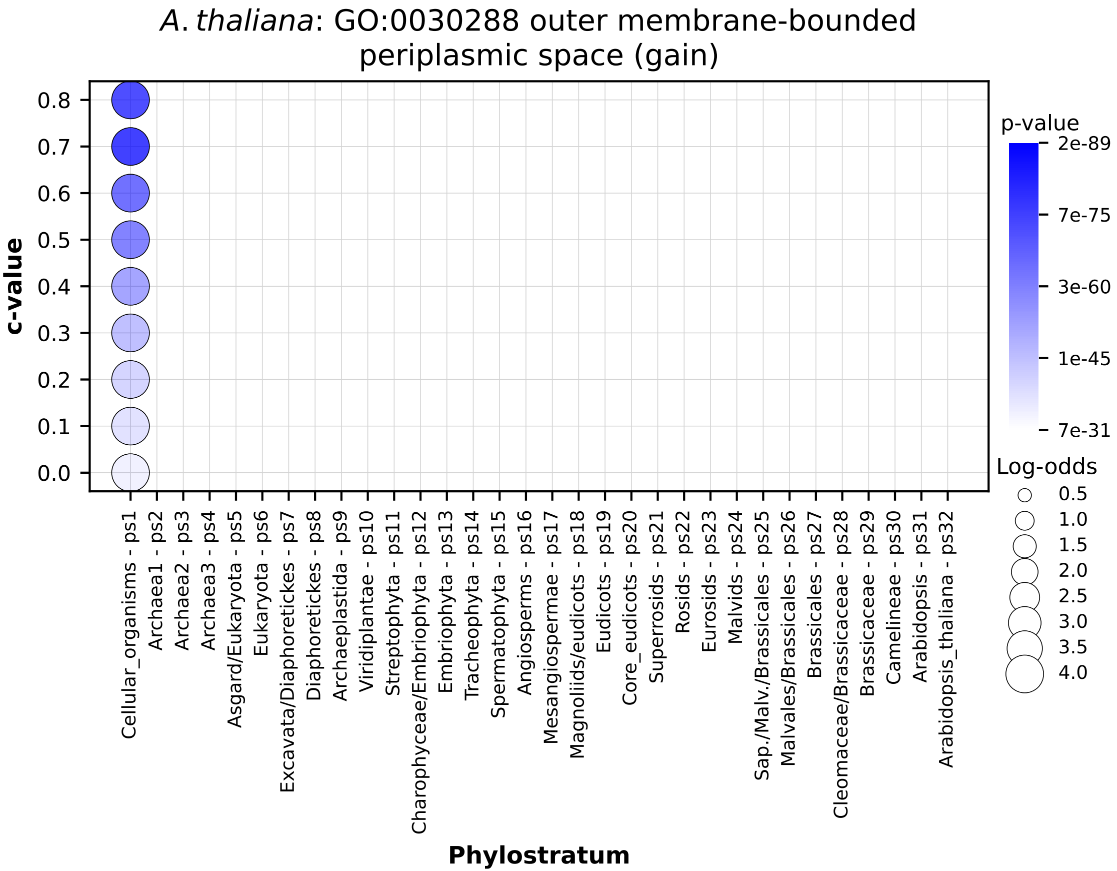
A. thaliana: GO:0030288 outer membrane-bounded periplasmic space (gain)
c-value
0.8
0.7
0.6
0.5
0.4
0.3
0.2
0.1
0.0
p-value
2e-89
7e-75
3e-60
1e-45
7e-31
Log-odds
0.5
1.0
1.5
2.0
2.5
3.0
3.5
4.0
Cellular_organisms - ps1
Archaea1 - ps2
Archaea2 - ps3
Archaea3 - ps4
Asgard/Eukaryota - ps5
Eukaryota - ps6
Excavata/Diaphoretickes - ps7
Diaphoretickes - ps8
Archaeplastida - ps9
Viridiplantae - ps10
Streptophyta - ps11
Charophyceae/Embriophyta - ps12
Embriophyta - ps13
Tracheophyta - ps14
Spermatophyta - ps15
Angiosperms - ps16
Mesangiospermae - ps17
Magnoliids/eudicots - ps18
Eudicots - ps19
Core_eudicots - ps20
Superrosids - ps21
Rosids - ps22
Eurosids - ps23
Malvids - ps24
Sap./Malv./Brassicales - ps25
Malvales/Brassicales - ps26
Brassicales - ps27
Cleomaceae/Brassicaceae - ps28
Brassicaceae - ps29
Camelineae - ps30
Arabidopsis - ps31
Arabidopsis_thaliana - ps32
Phylostratum

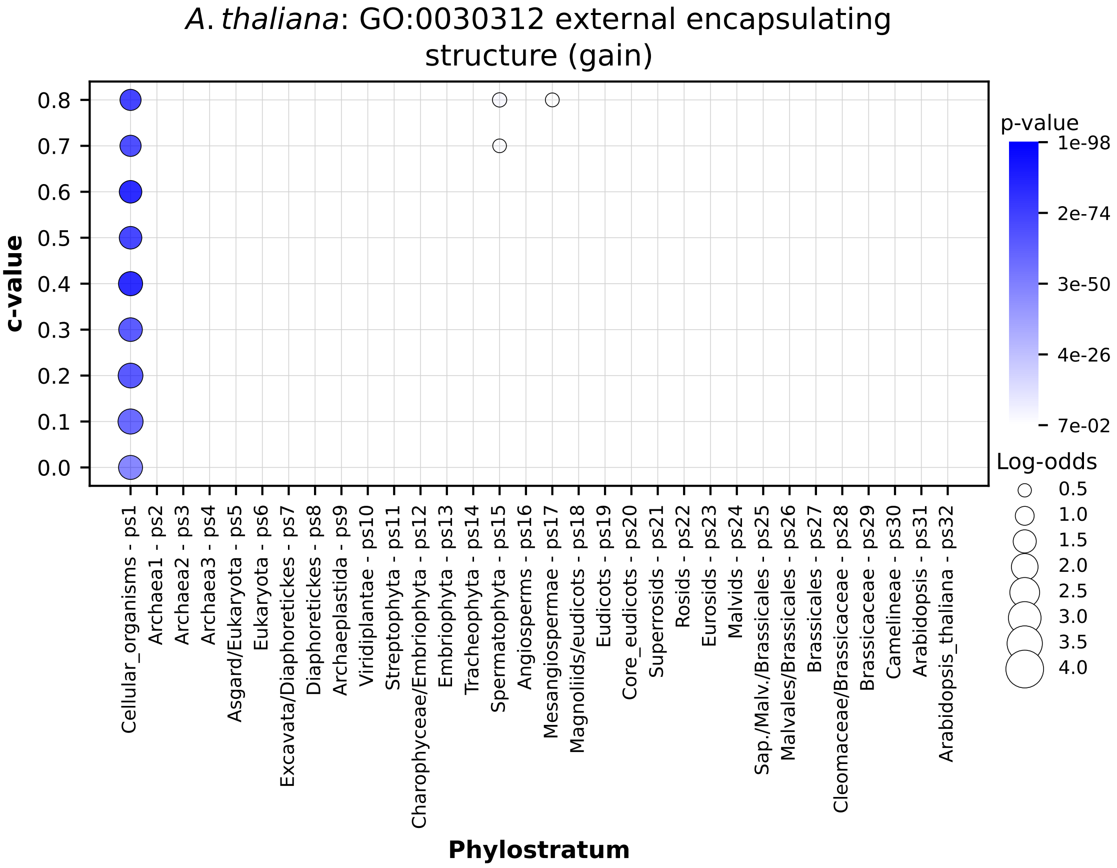

A. thaliana: GO:0030312 external encapsulating structure (gain)
c-value
0.0
0.1
0.2
0.3
0.4
0.5
0.6
0.7
0.8
p-value
1e-98
2e-74
3e-50
4e-26
7e-02
Log-odds
0.5
1.0
1.5
2.0
2.5
3.0
3.5
4.0
Cellular_organisms - ps1
Archaea1 - ps2
Archaea2 - ps3
Archaea3 - ps4
Asgard/Eukaryota - ps5
Eukaryota - ps6
Excavata/Diaphoretickes - ps7
Diaphoretickes - ps8
Archaeplastida - ps9
Viridiplantae - ps10
Streptophyta - ps11
Charophyceae/Embriophyta - ps12
Embriophyta - ps13
Tracheophyta - ps14
Spermatophyta - ps15
Angiosperms - ps16
Mesangiospermae - ps17
Magnoliids/eudicots - ps18
Eudicots - ps19
Core_eudicots - ps20
Superrosids - ps21
Rosids - ps22
Eurosids - ps23
Malvids - ps24
Sap./Malv./Brassicales - ps25
Malvales/Brassicales - ps26
Brassicales - ps27
Cleomaceae/Brassicaceae - ps28
Brassicaceae - ps29
Camelineae - ps30
Arabidopsis - ps31
Arabidopsis_thaliana - ps32
Phylostratum

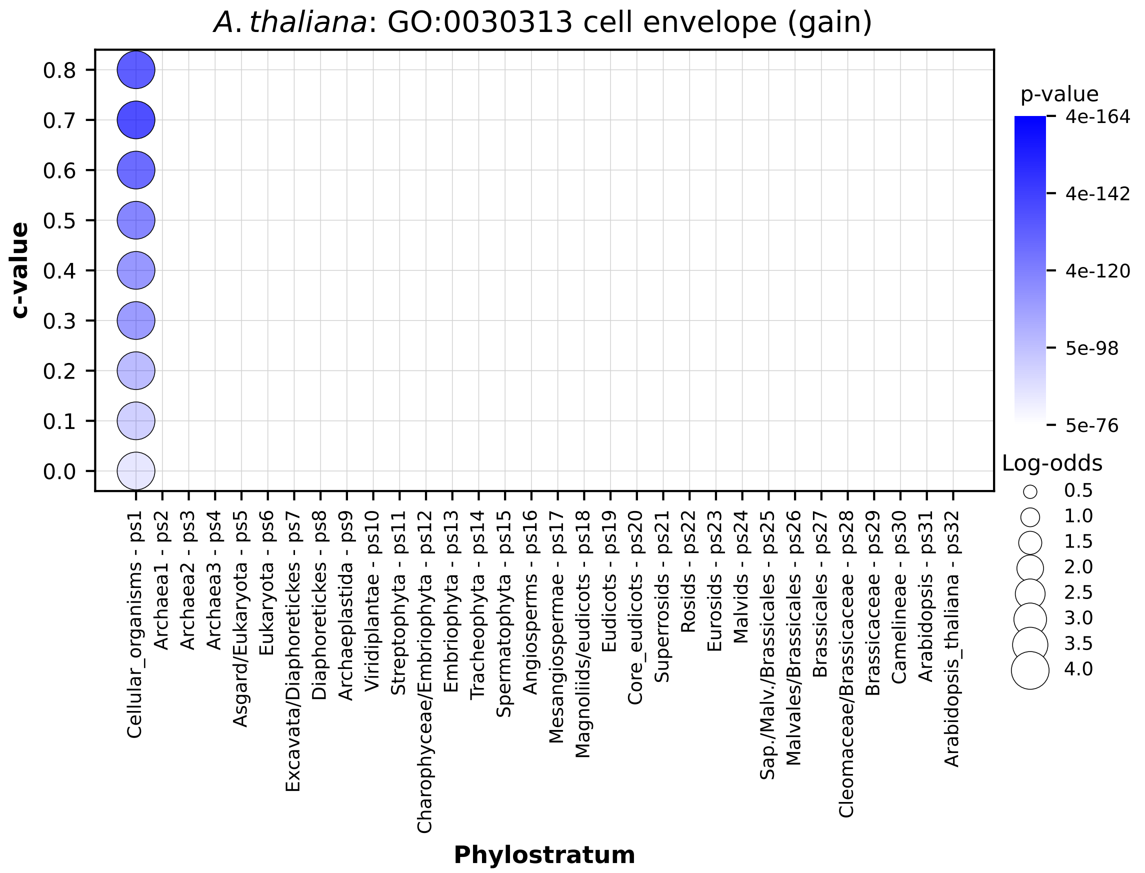
A. thaliana: GO:0030313 cell envelope (gain)
c-value
0.8
0.7
0.6
0.5
0.4
0.3
0.2
0.1
0.0
p-value
4e-164
4e-142
4e-120
5e-98
5e-76
Log-odds
0.5
1.0
1.5
2.0
2.5
3.0
3.5
4.0
Cellular_organisms - ps1
Archaea1 - ps2
Archaea2 - ps3
Archaea3 - ps4
Asgard/Eukaryota - ps5
Eukaryota - ps6
Excavata/Diaphoretickes - ps7
Diaphoretickes - ps8
Archaeplastida - ps9
Viridiplantae - ps10
Streptophyta - ps11
Charophyceae/Embriophyta - ps12
Embriophyta - ps13
Tracheophyta - ps14
Spermatophyta - ps15
Angiosperms - ps16
Mesangiospermae - ps17
Magnoliids/eudicots - ps18
Eudicots - ps19
Core_eudicots - ps20
Superrosids - ps21
Rosids - ps22
Eurosids - ps23
Malvids - ps24
Sap./Malv./Brassicales - ps25
Malvales/Brassicales - ps26
Brassicales - ps27
Cleomaceae/Brassicaceae - ps28
Brassicaceae - ps29
Camelineae - ps30
Arabidopsis - ps31
Arabidopsis_thaliana - ps32
Phylostratum

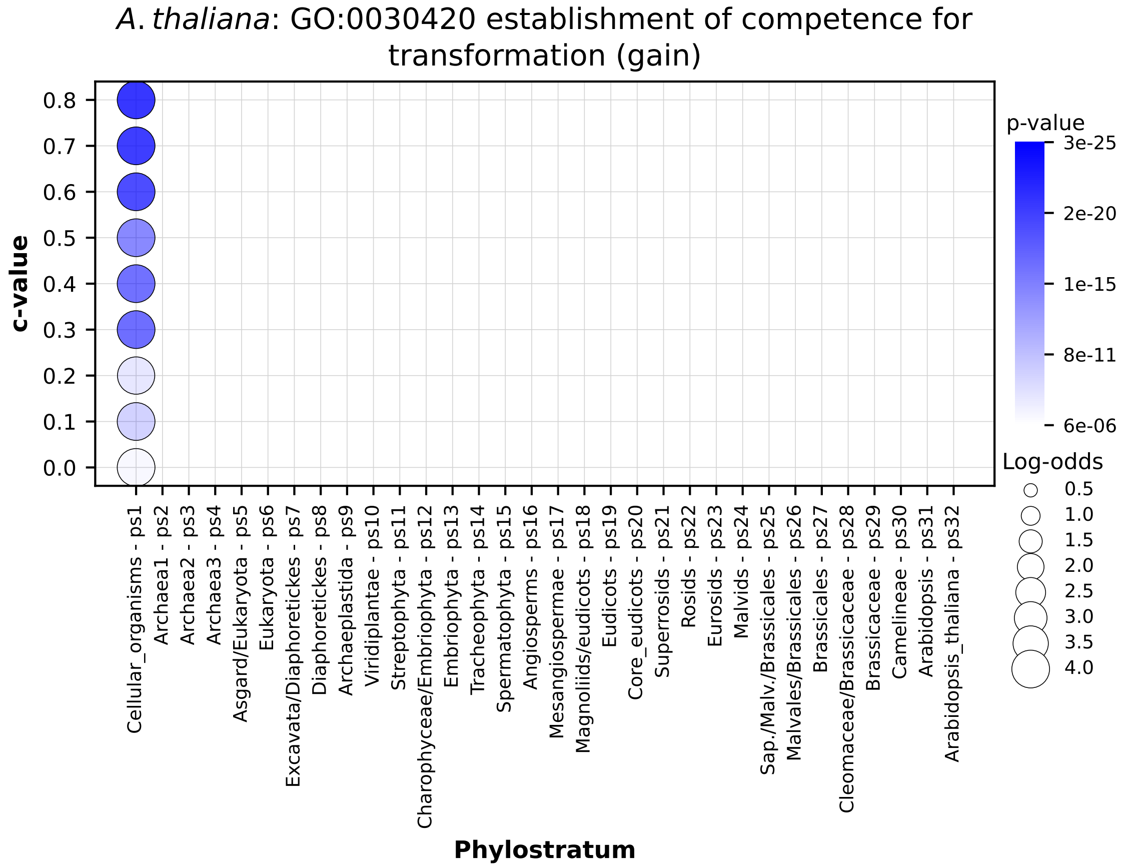

A. thaliana: GO:0030420 establishment of competence for transformation (gain)
c-value
0.0
0.1
0.2
0.3
0.4
0.5
0.6
0.7
0.8
p-value
3e-25
2e-20
1e-15
8e-11
6e-06
Log-odds
0.5
1.0
1.5
2.0
2.5
3.0
3.5
4.0
Cellular_organisms - ps1
Archaea1 - ps2
Archaea2 - ps3
Archaea3 - ps4
Asgard/Eukaryota - ps5
Eukaryota - ps6
Excavata/Diaphoretickes - ps7
Diaphoretickes - ps8
Archaeplastida - ps9
Viridiplantae - ps10
Streptophyta - ps11
Charophyceae/Embriophyta - ps12
Embriophyta - ps13
Tracheophyta - ps14
Spermatophyta - ps15
Angiosperms - ps16
Mesangiospermae - ps17
Magnoliids/eudicots - ps18
Eudicots - ps19
Core_eudicots - ps20
Superrosids - ps21
Rosids - ps22
Eurosids - ps23
Malvids - ps24
Sap./Malv./Brassicales - ps25
Malvales/Brassicales - ps26
Brassicales - ps27
Cleomaceae/Brassicaceae - ps28
Brassicaceae - ps29
Camelineae - ps30
Arabidopsis - ps31
Arabidopsis_thaliana - ps32
Phylostratum

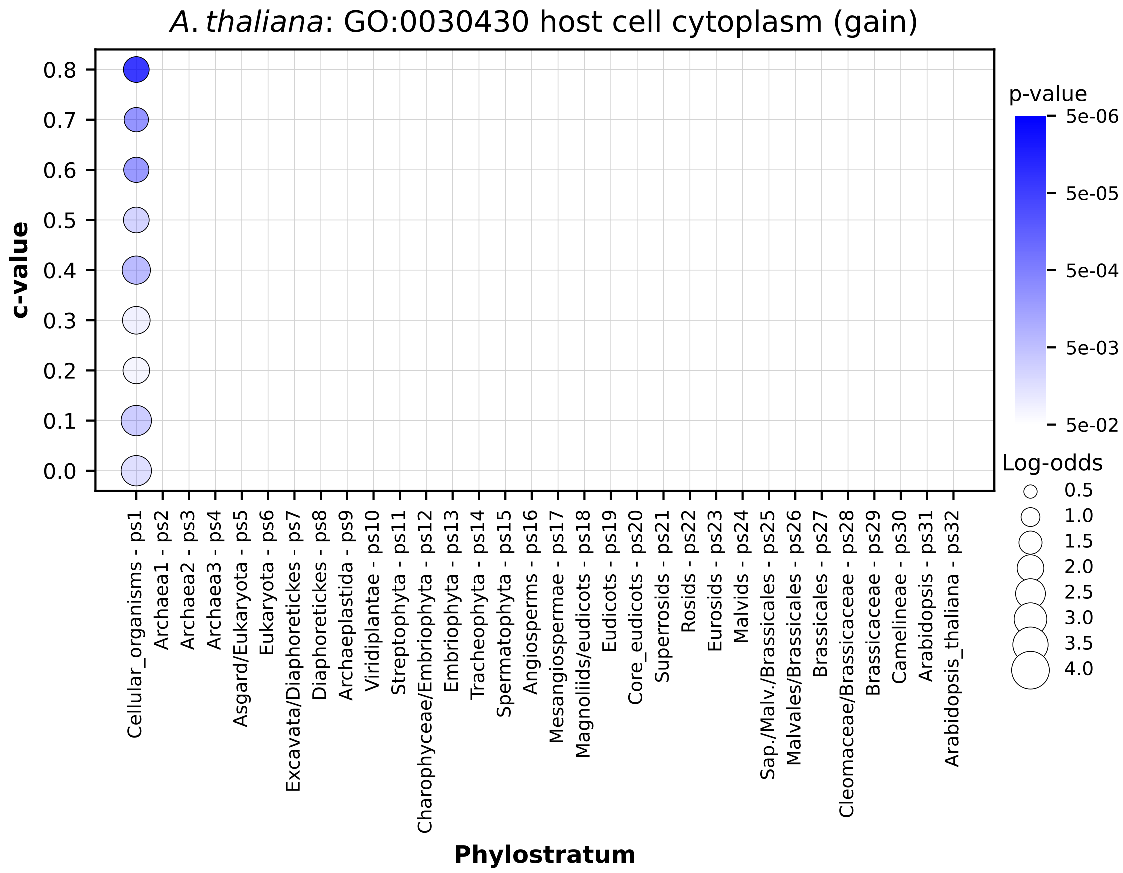

A. thaliana: GO:0030430 host cell cytoplasm (gain)
c-value
0.0
0.1
0.2
0.3
0.4
0.5
0.6
0.7
0.8
Phylostratum
Cellular_organisms - ps1
Archaea1 - ps2
Archaea2 - ps3
Archaea3 - ps4
Asgard/Eukaryota - ps5
Eukaryota - ps6
Excavata/Diaphoretickes - ps7
Diaphoretickes - ps8
Archaeplastida - ps9
Viridiplantae - ps10
Streptophyta - ps11
Charophyceae/Embriophyta - ps12
Embriophyta - ps13
Tracheophyta - ps14
Spermatophyta - ps15
Angiosperms - ps16
Mesangiospermae - ps17
Magnoliids/eudicots - ps18
Eudicots - ps19
Core_eudicots - ps20
Superrosids - ps21
Rosids - ps22
Eurosids - ps23
Malvids - ps24
Sap./Malv./Brassicales - ps25
Malvales/Brassicales - ps26
Brassicales - ps27
Cleomaceae/Brassicaceae - ps28
Brassicaceae - ps29
Camelineae - ps30
Arabidopsis - ps31
Arabidopsis_thaliana - ps32
p-value
5e-06
5e-05
5e-04
5e-03
5e-02
Log-odds
0.5
1.0
1.5
2.0
2.5
3.0
3.5
4.0

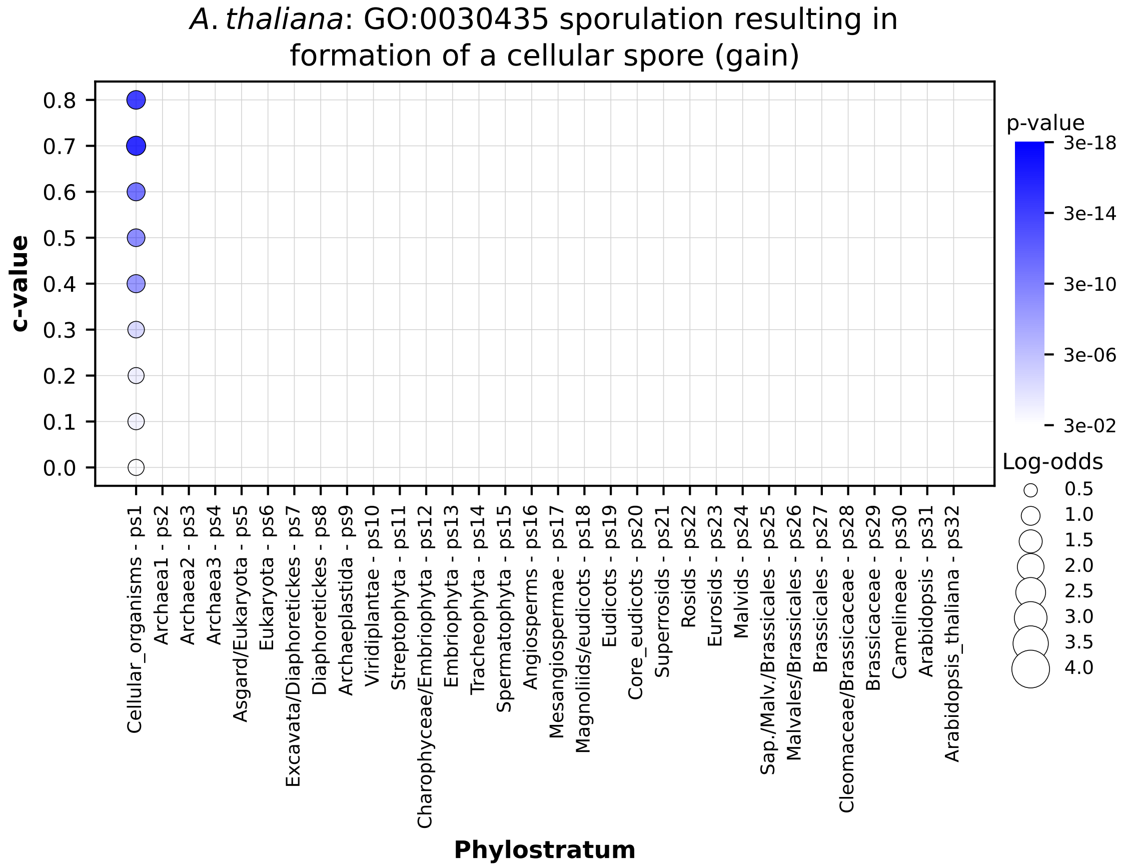

A. thaliana: GO:0030435 sporulation resulting in formation of a cellular spore (gain)
c-value
0.8
0.7
0.6
0.5
0.4
0.3
0.2
0.1
0.0
p-value
3e-18
3e-14
3e-10
3e-06
3e-02
Log-odds
0.5
1.0
1.5
2.0
2.5
3.0
3.5
4.0
Cellular_organisms - ps1
Archaea1 - ps2
Archaea2 - ps3
Archaea3 - ps4
Asgard/Eukaryota - ps5
Eukaryota - ps6
Excavata/Diaphoretickes - ps7
Diaphoretickes - ps8
Archaeplastida - ps9
Viridiplantae - ps10
Streptophyta - ps11
Charophyceae/Embriophyta - ps12
Embriophyta - ps13
Tracheophyta - ps14
Spermatophyta - ps15
Angiosperms - ps16
Mesangiospermae - ps17
Magnoliids/eudicots - ps18
Eudicots - ps19
Core_eudicots - ps20
Superrosids - ps21
Rosids - ps22
Eurosids - ps23
Malvids - ps24
Sap./Malv./Brassicales - ps25
Malvales/Brassicales - ps26
Brassicales - ps27
Cleomaceae/Brassicaceae - ps28
Brassicaceae - ps29
Camelineae - ps30
Arabidopsis - ps31
Arabidopsis_thaliana - ps32
Phylostratum

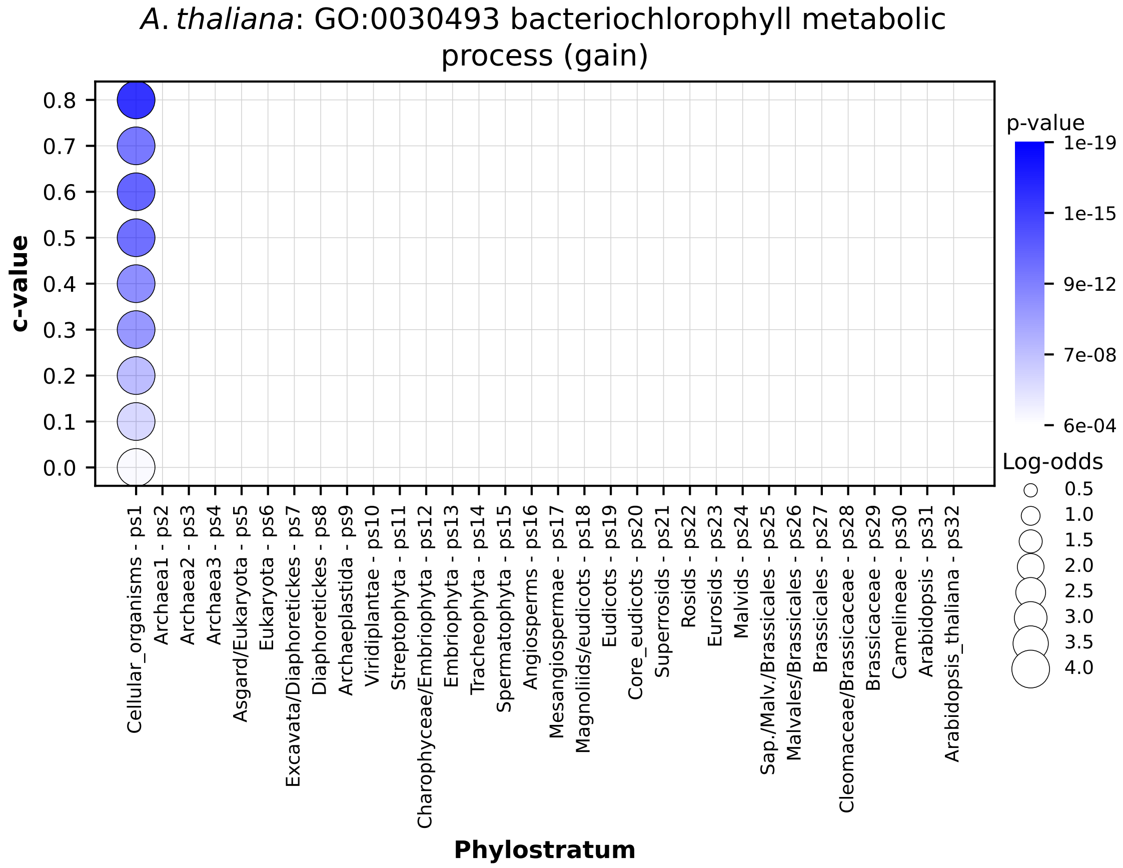

A. thaliana: GO:0030493 bacteriochlorophyll metabolic process (gain)
c-value
0.0
0.1
0.2
0.3
0.4
0.5
0.6
0.7
0.8
p-value
1e-19
1e-15
9e-12
7e-08
6e-04
Log-odds
0.5
1.0
1.5
2.0
2.5
3.0
3.5
4.0
Cellular_organisms - ps1
Archaea1 - ps2
Archaea2 - ps3
Archaea3 - ps4
Asgard/Eukaryota - ps5
Eukaryota - ps6
Excavata/Diaphoretickes - ps7
Diaphoretickes - ps8
Archaeplastida - ps9
Viridiplantae - ps10
Streptophyta - ps11
Charophyceae/Embriophyta - ps12
Embriophyta - ps13
Tracheophyta - ps14
Spermatophyta - ps15
Angiosperms - ps16
Mesangiospermae - ps17
Magnoliids/eudicots - ps18
Eudicots - ps19
Core_eudicots - ps20
Superrosids - ps21
Rosids - ps22
Eurosids - ps23
Malvids - ps24
Sap./Malv./Brassicales - ps25
Malvales/Brassicales - ps26
Brassicales - ps27
Cleomaceae/Brassicaceae - ps28
Brassicaceae - ps29
Camelineae - ps30
Arabidopsis - ps31
Arabidopsis_thaliana - ps32
Phylostratum

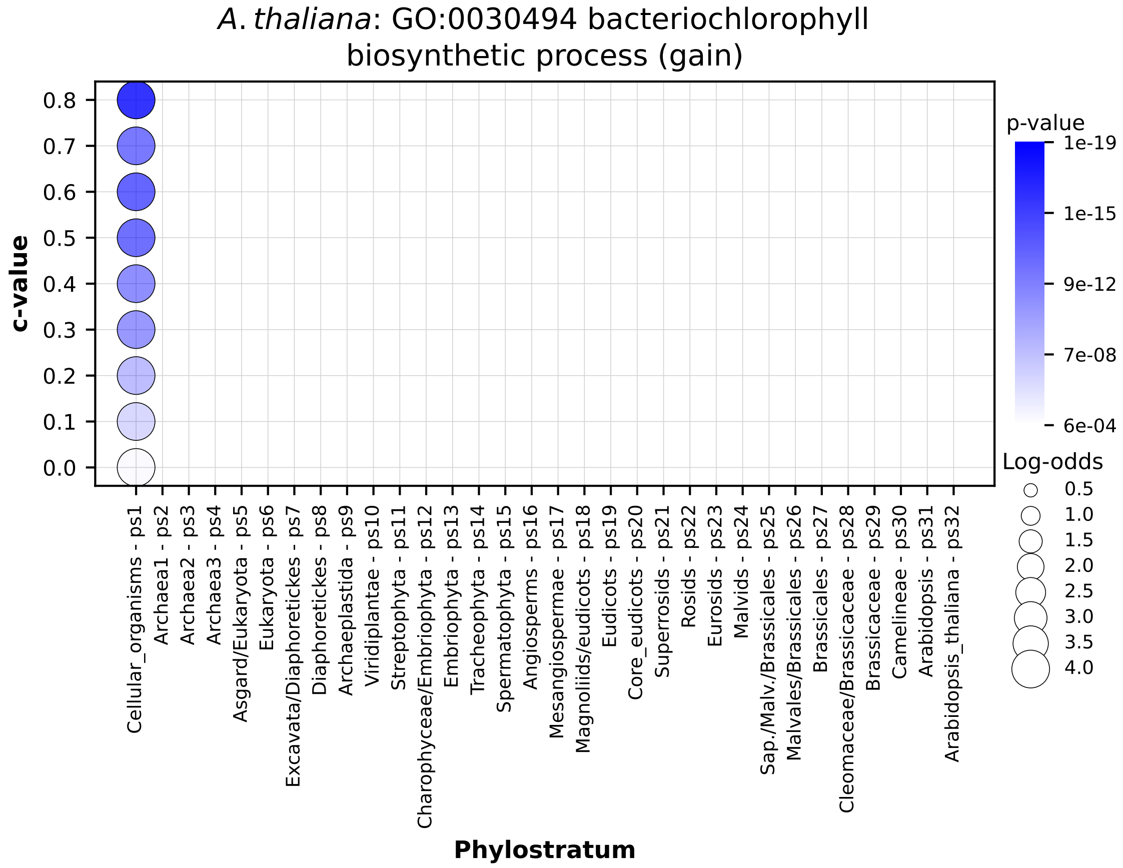

A. thaliana: GO:0030494 bacteriochlorophyll biosynthetic process (gain)
c-value
0.8
0.7
0.6
0.5
0.4
0.3
0.2
0.1
0.0
p-value
1e-19
1e-15
9e-12
7e-08
6e-04
Log-odds
0.5
1.0
1.5
2.0
2.5
3.0
3.5
4.0
Cellular_organisms - ps1
Archaea1 - ps2
Archaea2 - ps3
Archaea3 - ps4
Asgard/Eukaryota - ps5
Eukaryota - ps6
Excavata/Diaphoretickes - ps7
Diaphoretickes - ps8
Archaeplastida - ps9
Viridiplantae - ps10
Streptophyta - ps11
Charophyceae/Embriophyta - ps12
Embriophyta - ps13
Tracheophyta - ps14
Spermatophyta - ps15
Angiosperms - ps16
Mesangiospermae - ps17
Magnoliids/eudicots - ps18
Eudicots - ps19
Core_eudicots - ps20
Superrosids - ps21
Rosids - ps22
Eurosids - ps23
Malvids - ps24
Sap./Malv./Brassicales - ps25
Malvales/Brassicales - ps26
Brassicales - ps27
Cleomaceae/Brassicaceae - ps28
Brassicaceae - ps29
Camelineae - ps30
Arabidopsis - ps31
Arabidopsis_thaliana - ps32
Phylostratum

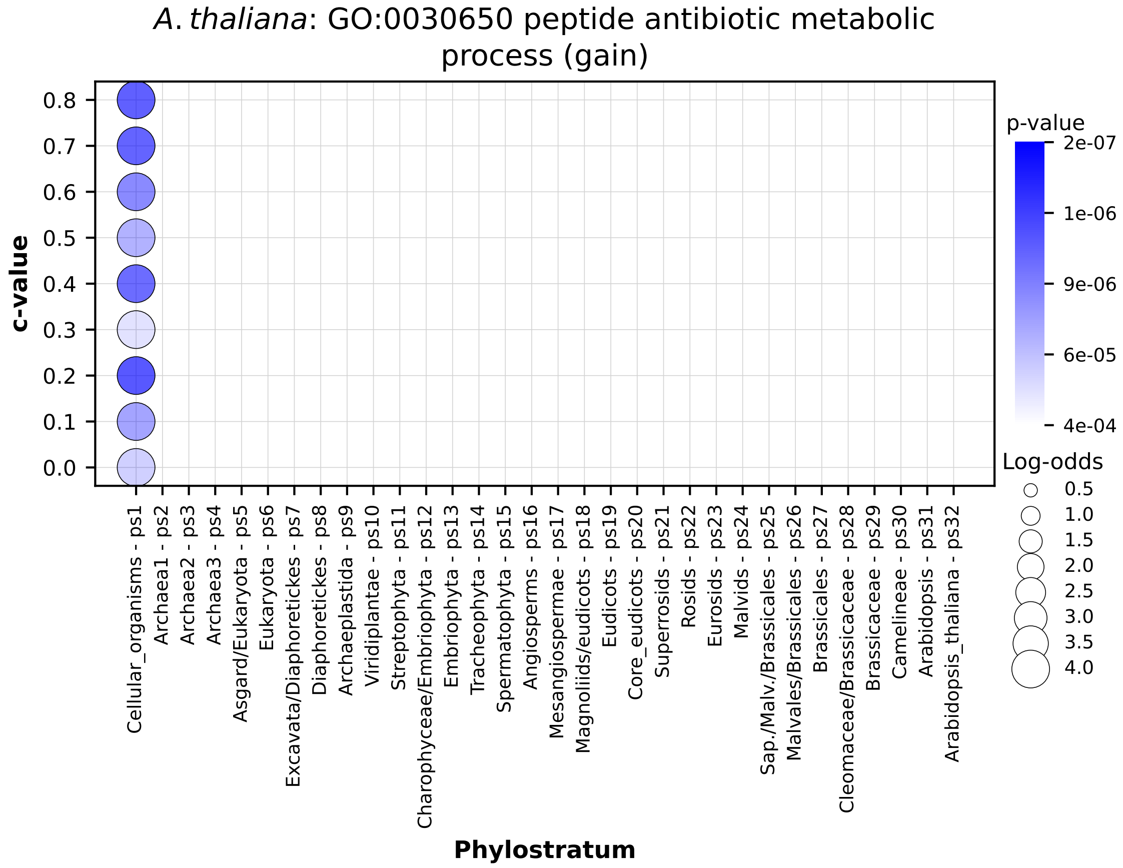
A. thaliana: GO:0030650 peptide antibiotic metabolic process (gain)
c-value
0.8
0.7
0.6
0.5
0.4
0.3
0.2
0.1
0.0
p-value
2e-07
1e-06
9e-06
6e-05
4e-04
Log-odds
0.5
1.0
1.5
2.0
2.5
3.0
3.5
4.0
Cellular_organisms - ps1
Archaea1 - ps2
Archaea2 - ps3
Archaea3 - ps4
Asgard/Eukaryota - ps5
Eukaryota - ps6
Excavata/Diaphoretickes - ps7
Diaphoretickes - ps8
Archaeplastida - ps9
Viridiplantae - ps10
Streptophyta - ps11
Charophyceae/Embriophyta - ps12
Embriophyta - ps13
Tracheophyta - ps14
Spermatophyta - ps15
Angiosperms - ps16
Mesangiospermae - ps17
Magnoliids/eudicots - ps18
Eudicots - ps19
Core_eudicots - ps20
Superrosids - ps21
Rosids - ps22
Eurosids - ps23
Malvids - ps24
Sap./Malv./Brassicales - ps25
Malvales/Brassicales - ps26
Brassicales - ps27
Cleomaceae/Brassicaceae - ps28
Brassicaceae - ps29
Camelineae - ps30
Arabidopsis - ps31
Arabidopsis_thaliana - ps32
Phylostratum

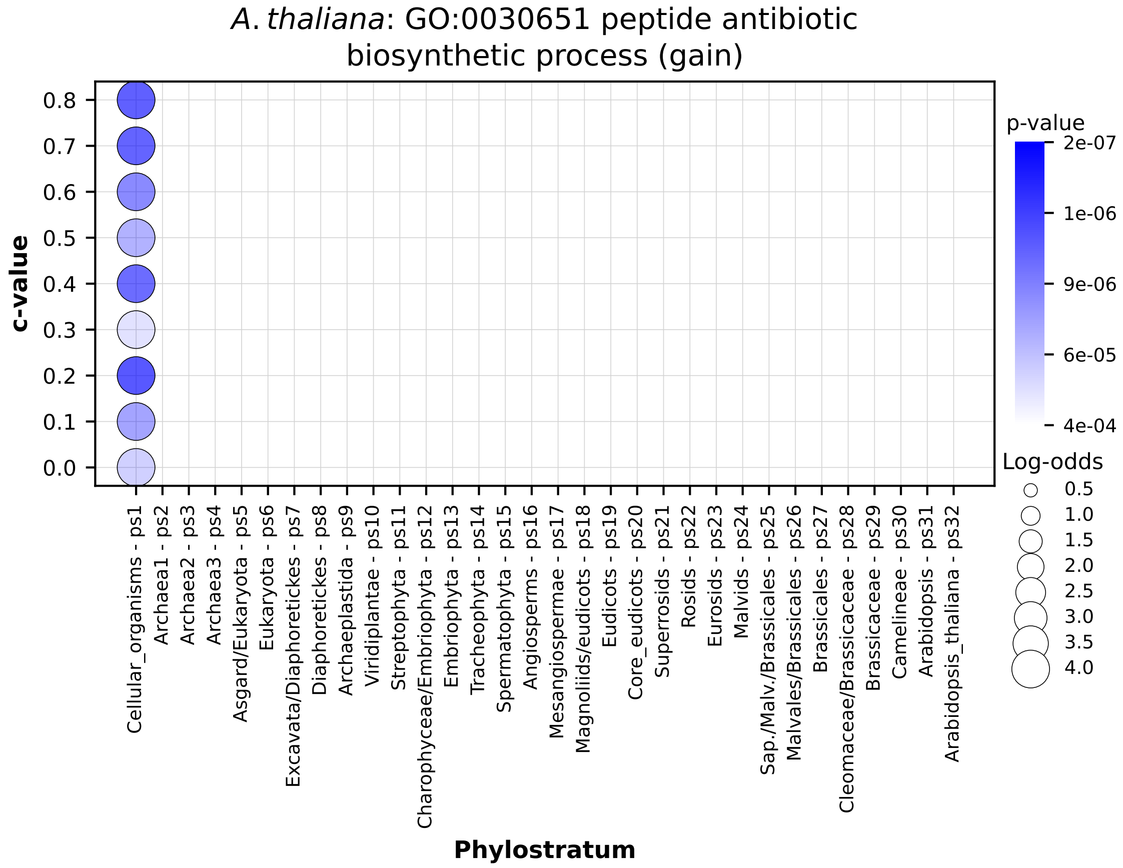
A. thaliana: GO:0030651 peptide antibiotic biosynthetic process (gain)
c-value
0.8
0.7
0.6
0.5
0.4
0.3
0.2
0.1
0.0
p-value
2e-07
1e-06
9e-06
6e-05
4e-04
Log-odds
0.5
1.0
1.5
2.0
2.5
3.0
3.5
4.0
Cellular_organisms - ps1
Archaea1 - ps2
Archaea2 - ps3
Archaea3 - ps4
Asgard/Eukaryota - ps5
Eukaryota - ps6
Excavata/Diaphoretickes - ps7
Diaphoretickes - ps8
Archaeplastida - ps9
Viridiplantae - ps10
Streptophyta - ps11
Charophyceae/Embriophyta - ps12
Embriophyta - ps13
Tracheophyta - ps14
Spermatophyta - ps15
Angiosperms - ps16
Mesangiospermae - ps17
Magnoliids/eudicots - ps18
Eudicots - ps19
Core_eudicots - ps20
Superrosids - ps21
Rosids - ps22
Eurosids - ps23
Malvids - ps24
Sap./Malv./Brassicales - ps25
Malvales/Brassicales - ps26
Brassicales - ps27
Cleomaceae/Brassicaceae - ps28
Brassicaceae - ps29
Camelineae - ps30
Arabidopsis - ps31
Arabidopsis_thaliana - ps32
Phylostratum

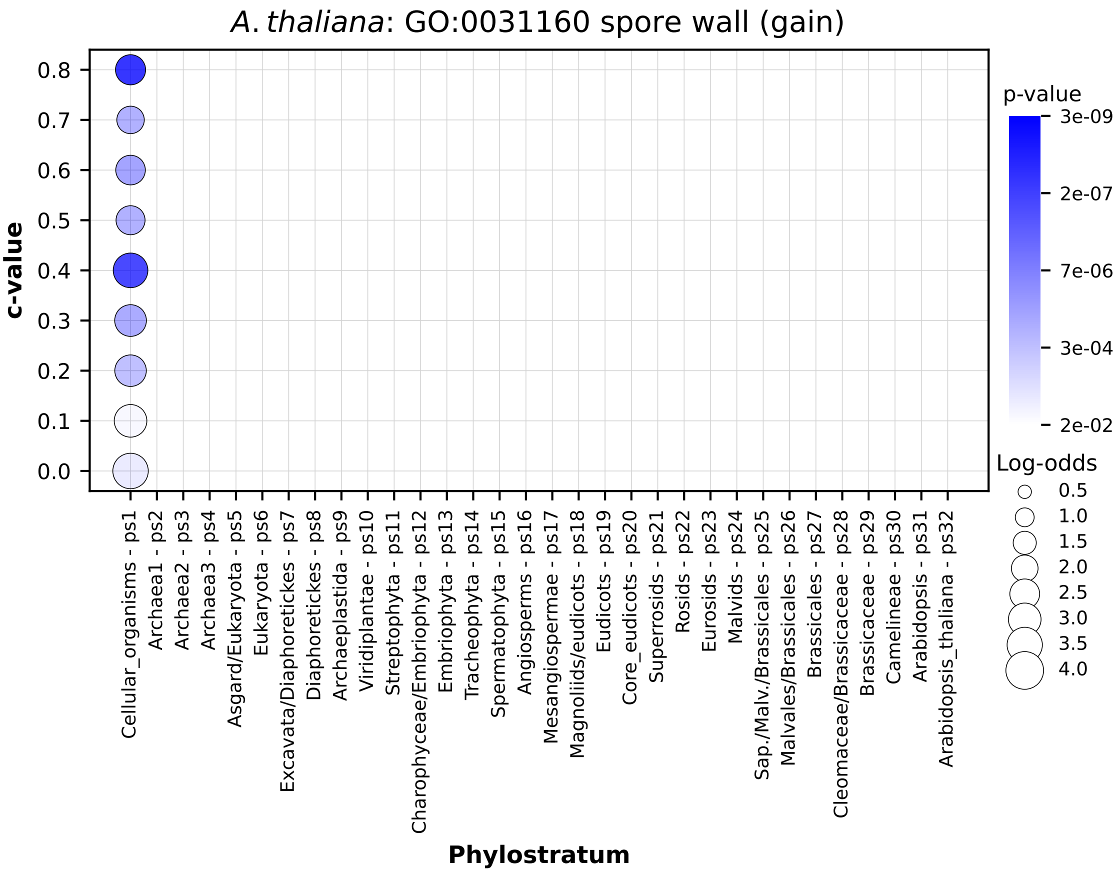

A. thaliana: GO:0031160 spore wall (gain)
c-value
0.8
0.7
0.6
0.5
0.4
0.3
0.2
0.1
0.0
p-value
3e-09
2e-07
7e-06
3e-04
2e-02
Log-odds
0.5
1.0
1.5
2.0
2.5
3.0
3.5
4.0
Cellular_organisms - ps1
Archaea1 - ps2
Archaea2 - ps3
Archaea3 - ps4
Asgard/Eukaryota - ps5
Eukaryota - ps6
Excavata/Diaphoretickes - ps7
Diaphoretickes - ps8
Archaeplastida - ps9
Viridiplantae - ps10
Streptophyta - ps11
Charophyceae/Embriophyta - ps12
Embriophyta - ps13
Tracheophyta - ps14
Spermatophyta - ps15
Angiosperms - ps16
Mesangiospermae - ps17
Magnoliids/eudicots - ps18
Eudicots - ps19
Core_eudicots - ps20
Superrosids - ps21
Rosids - ps22
Eurosids - ps23
Malvids - ps24
Sap./Malv./Brassicales - ps25
Malvales/Brassicales - ps26
Brassicales - ps27
Cleomaceae/Brassicaceae - ps28
Brassicaceae - ps29
Camelineae - ps30
Arabidopsis - ps31
Arabidopsis_thaliana - ps32
Phylostratum

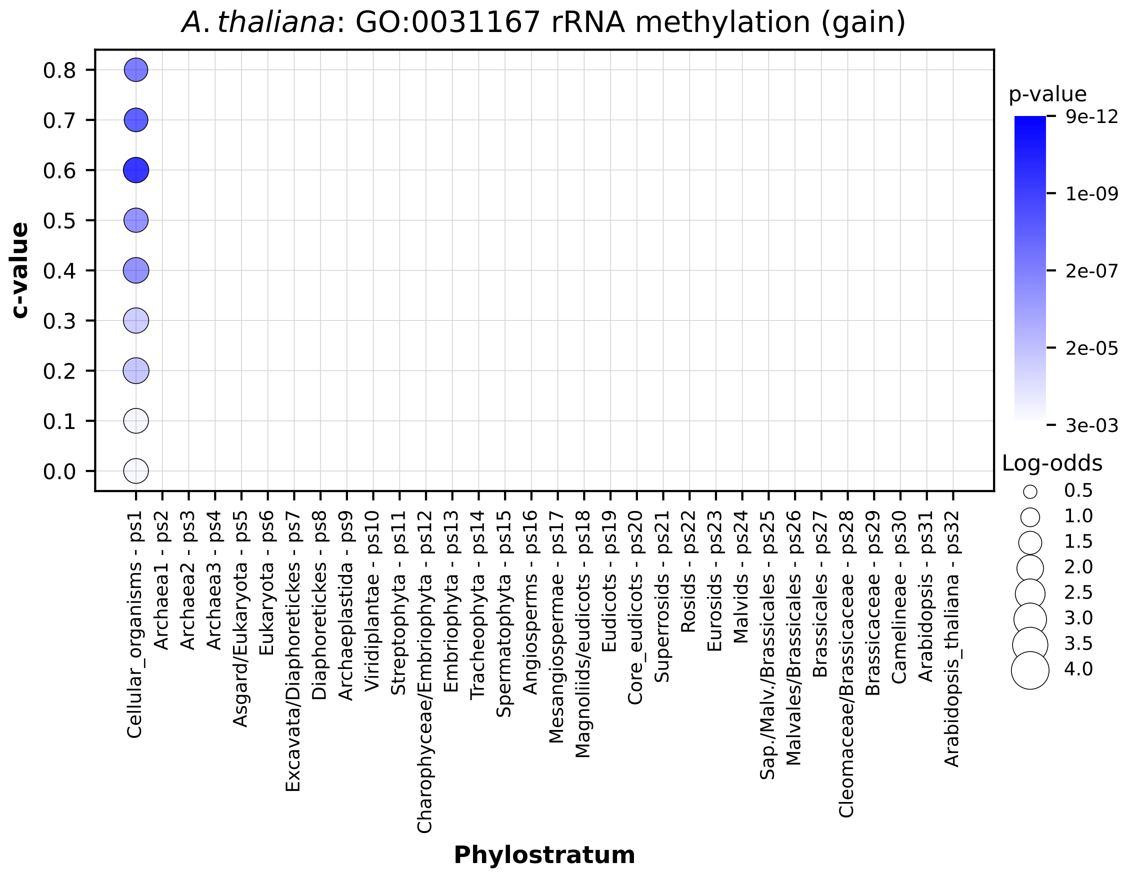

A. thaliana: GO:0031167 rRNA methylation (gain)
c-value
0.8
0.7
0.6
0.5
0.4
0.3
0.2
0.1
0.0
p-value
9e-12
1e-09
2e-07
2e-05
3e-03
Log-odds
0.5
1.0
1.5
2.0
2.5
3.0
3.5
4.0
Cellular_organisms - ps1
Archaea1 - ps2
Archaea2 - ps3
Archaea3 - ps4
Asgard/Eukaryota - ps5
Eukaryota - ps6
Excavata/Diaphoretickes - ps7
Diaphoretickes - ps8
Archaeplastida - ps9
Viridiplantae - ps10
Streptophyta - ps11
Charophyceae/Embriophyta - ps12
Embriophyta - ps13
Tracheophyta - ps14
Spermatophyta - ps15
Angiosperms - ps16
Mesangiospermae - ps17
Magnoliids/eudicots - ps18
Eudicots - ps19
Core_eudicots - ps20
Superrosids - ps21
Rosids - ps22
Eurosids - ps23
Malvids - ps24
Sap./Malv./Brassicales - ps25
Malvales/Brassicales - ps26
Brassicales - ps27
Cleomaceae/Brassicaceae - ps28
Brassicaceae - ps29
Camelineae - ps30
Arabidopsis - ps31
Arabidopsis_thaliana - ps32
Phylostratum

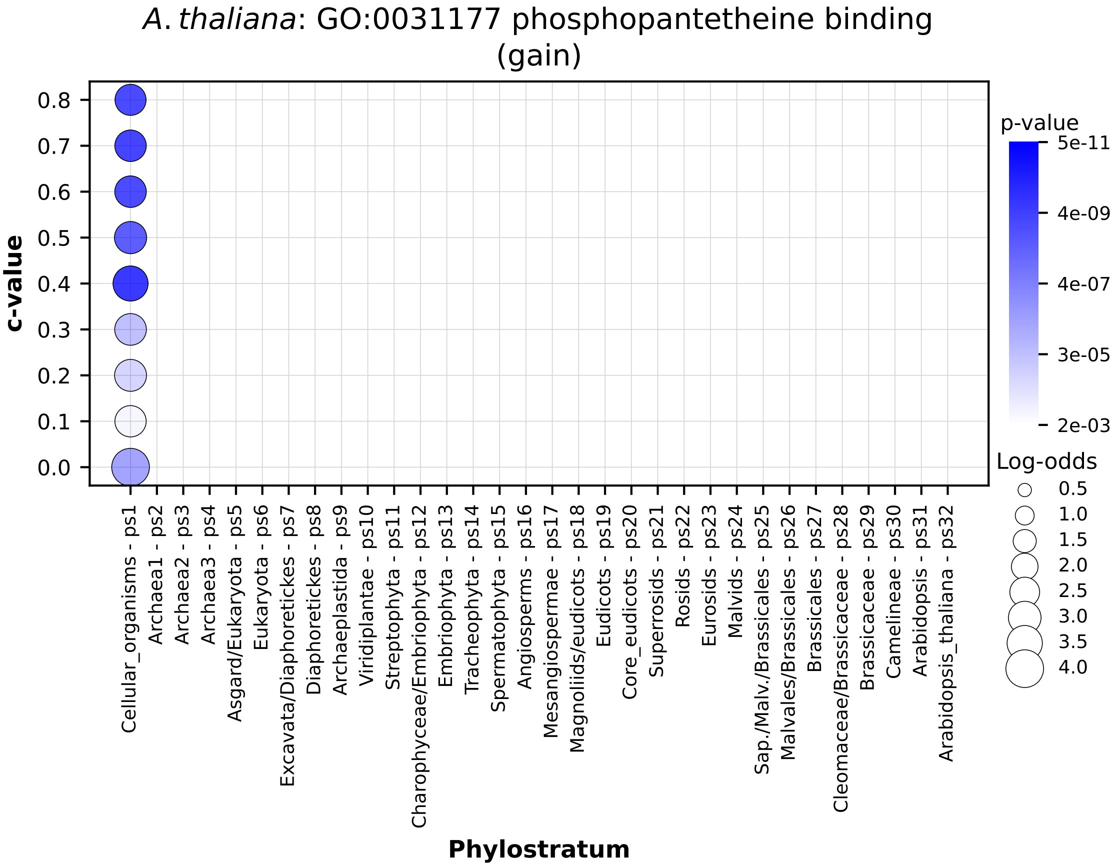
A. thaliana: GO:0031177 phosphopantetheine binding (gain)
c-value
0.8
0.7
0.6
0.5
0.4
0.3
0.2
0.1
0.0
p-value
5e-11
4e-09
4e-07
3e-05
2e-03
Log-odds
0.5
1.0
1.5
2.0
2.5
3.0
3.5
4.0
Cellular_organisms - ps1
Archaea1 - ps2
Archaea2 - ps3
Archaea3 - ps4
Asgard/Eukaryota - ps5
Eukaryota - ps6
Excavata/Diaphoretickes - ps7
Diaphoretickes - ps8
Archaeplastida - ps9
Viridiplantae - ps10
Streptophyta - ps11
Charophyceae/Embriophyta - ps12
Embriophyta - ps13
Tracheophyta - ps14
Spermatophyta - ps15
Angiosperms - ps16
Mesangiospermae - ps17
Magnoliids/eudicots - ps18
Eudicots - ps19
Core_eudicots - ps20
Superrosids - ps21
Rosids - ps22
Eurosids - ps23
Malvids - ps24
Sap./Malv./Brassicales - ps25
Malvales/Brassicales - ps26
Brassicales - ps27
Cleomaceae/Brassicaceae - ps28
Brassicaceae - ps29
Camelineae - ps30
Arabidopsis - ps31
Arabidopsis_thaliana - ps32
Phylostratum

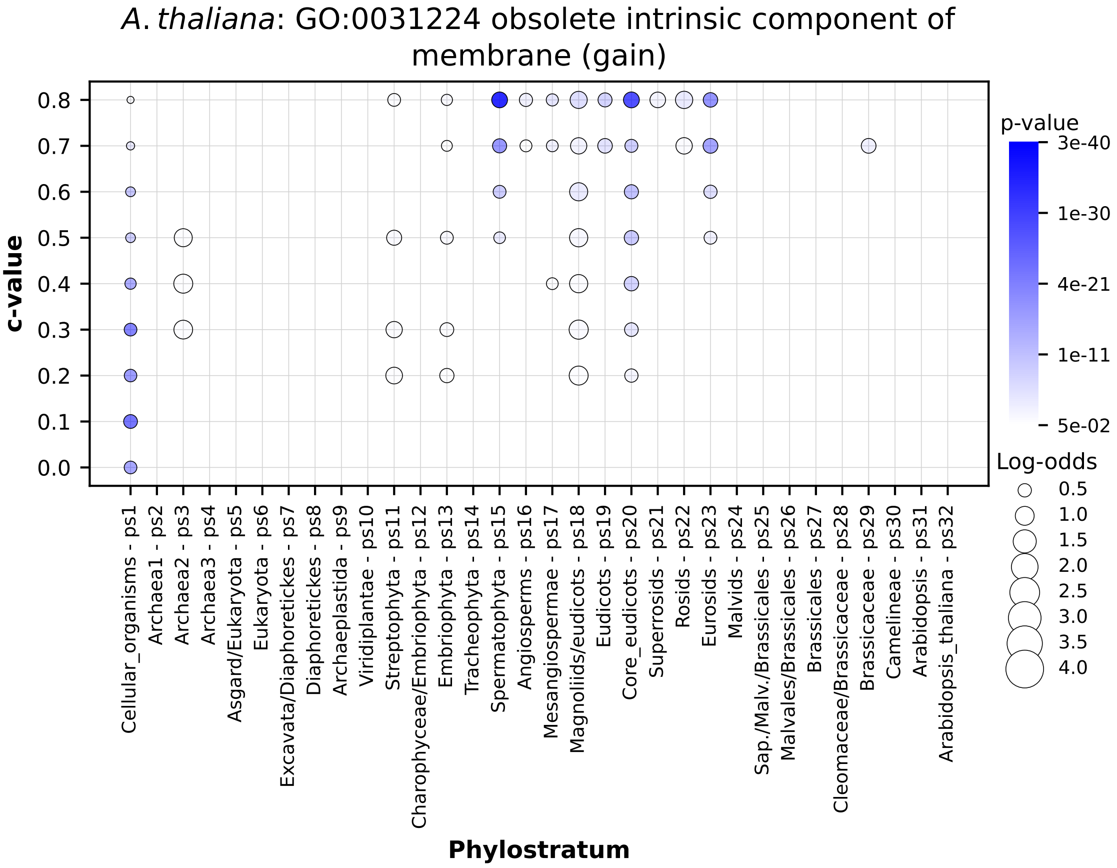

A. thaliana: GO:0031224 obsolete intrinsic component of membrane (gain)
c-value
0.8
0.7
0.6
0.5
0.4
0.3
0.2
0.1
0.0
p-value
3e-40
1e-30
4e-21
1e-11
5e-02
Log-odds
0.5
1.0
1.5
2.0
2.5
3.0
3.5
4.0
Cellular_organisms - ps1
Archaea1 - ps2
Archaea2 - ps3
Archaea3 - ps4
Asgard/Eukaryota - ps5
Eukaryota - ps6
Excavata/Diaphoretickes - ps7
Diaphoretickes - ps8
Archaeplastida - ps9
Viridiplantae - ps10
Streptophyta - ps11
Charophyceae/Embriophyta - ps12
Embriophyta - ps13
Tracheophyta - ps14
Spermatophyta - ps15
Angiosperms - ps16
Mesangiospermae - ps17
Magnoliids/eudicots - ps18
Eudicots - ps19
Core_eudicots - ps20
Superrosids - ps21
Rosids - ps22
Eurosids - ps23
Malvids - ps24
Sap./Malv./Brassicales - ps25
Malvales/Brassicales - ps26
Brassicales - ps27
Cleomaceae/Brassicaceae - ps28
Brassicaceae - ps29
Camelineae - ps30
Arabidopsis - ps31
Arabidopsis_thaliana - ps32
Phylostratum

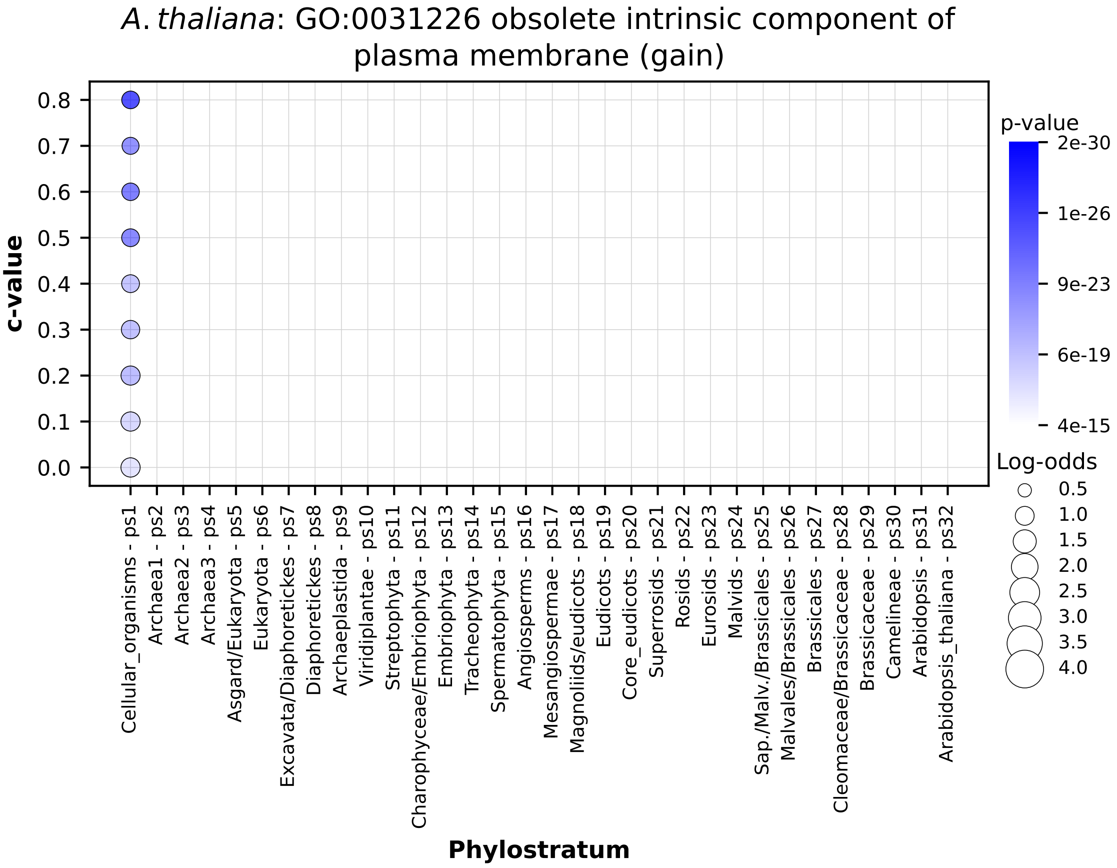

A. thaliana: GO:0031226 obsolete intrinsic component of plasma membrane (gain)
c-value
0.8
0.7
0.6
0.5
0.4
0.3
0.2
0.1
0.0
Phylostratum
Cellular_organisms - ps1
Archaea1 - ps2
Archaea2 - ps3
Archaea3 - ps4
Asgard/Eukaryota - ps5
Eukaryota - ps6
Excavata/Diaphoretickes - ps7
Diaphoretickes - ps8
Archaeplastida - ps9
Viridiplantae - ps10
Streptophyta - ps11
Charophyceae/Embriophyta - ps12
Embriophyta - ps13
Tracheophyta - ps14
Spermatophyta - ps15
Angiosperms - ps16
Mesangiospermae - ps17
Magnoliids/eudicots - ps18
Eudicots - ps19
Core_eudicots - ps20
Superrosids - ps21
Rosids - ps22
Eurosids - ps23
Malvids - ps24
Sap./Malv./Brassicales - ps25
Malvales/Brassicales - ps26
Brassicales - ps27
Cleomaceae/Brassicaceae - ps28
Brassicaceae - ps29
Camelineae - ps30
Arabidopsis - ps31
Arabidopsis_thaliana - ps32
p-value
2e-30
1e-26
9e-23
6e-19
4e-15
Log-odds
0.5
1.0
1.5
2.0
2.5
3.0
3.5
4.0

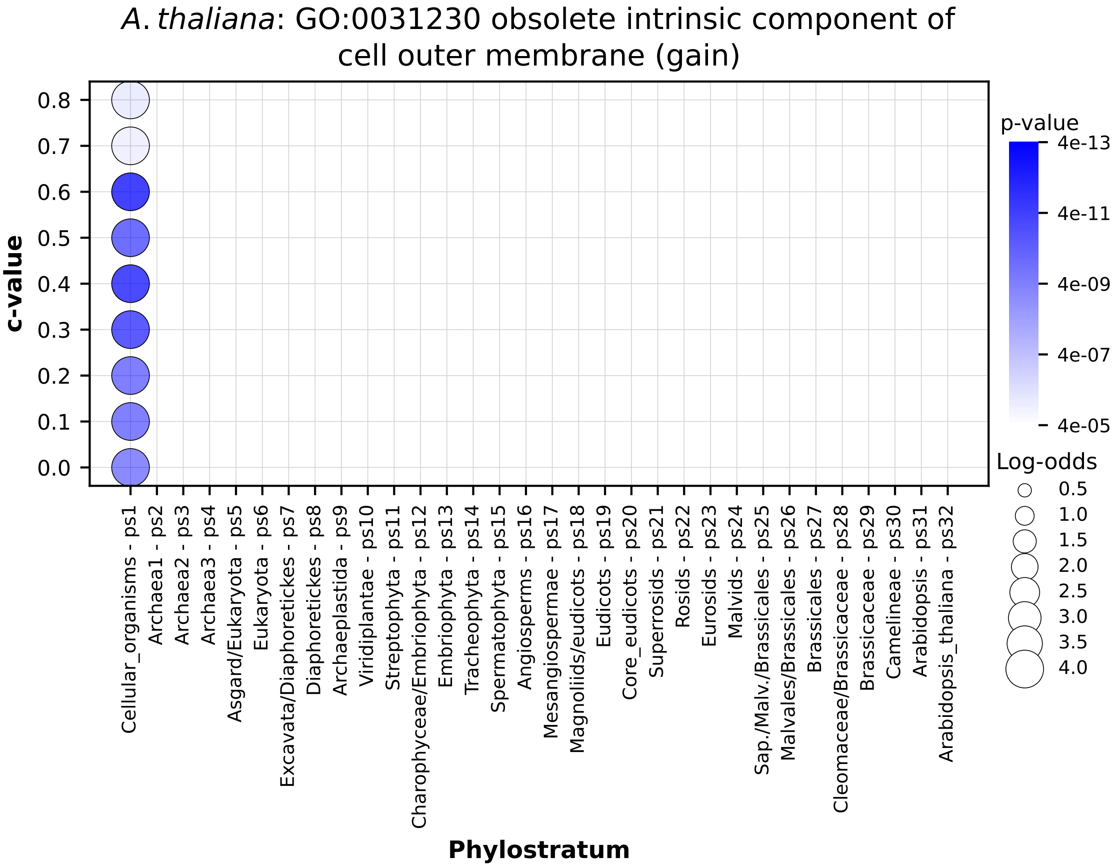
A. thaliana: GO:0031230 obsolete intrinsic component of cell outer membrane (gain)
c-value
0.8
0.7
0.6
0.5
0.4
0.3
0.2
0.1
0.0
p-value
4e-13
4e-11
4e-09
4e-07
4e-05
Log-odds
0.5
1.0
1.5
2.0
2.5
3.0
3.5
4.0
Cellular_organisms - ps1
Archaea1 - ps2
Archaea2 - ps3
Archaea3 - ps4
Asgard/Eukaryota - ps5
Eukaryota - ps6
Excavata/Diaphoretickes - ps7
Diaphoretickes - ps8
Archaeplastida - ps9
Viridiplantae - ps10
Streptophyta - ps11
Charophyceae/Embriophyta - ps12
Embriophyta - ps13
Tracheophyta - ps14
Spermatophyta - ps15
Angiosperms - ps16
Mesangiospermae - ps17
Magnoliids/eudicots - ps18
Eudicots - ps19
Core_eudicots - ps20
Superrosids - ps21
Rosids - ps22
Eurosids - ps23
Malvids - ps24
Sap./Malv./Brassicales - ps25
Malvales/Brassicales - ps26
Brassicales - ps27
Cleomaceae/Brassicaceae - ps28
Brassicaceae - ps29
Camelineae - ps30
Arabidopsis - ps31
Arabidopsis_thaliana - ps32
Phylostratum

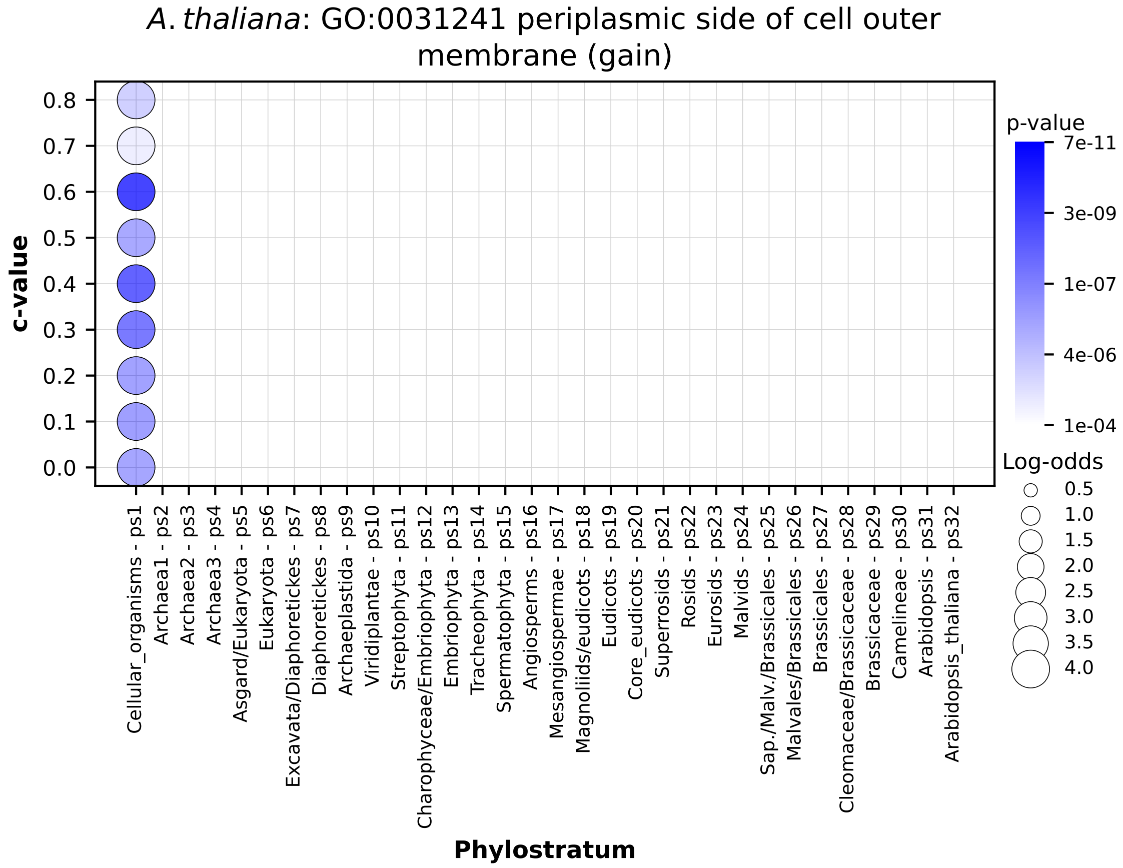

A. thaliana: GO:0031241 periplasmic side of cell outer membrane (gain)
c-value
0.8
0.7
0.6
0.5
0.4
0.3
0.2
0.1
0.0
p-value
7e-11
3e-09
1e-07
4e-06
1e-04
Log-odds
0.5
1.0
1.5
2.0
2.5
3.0
3.5
4.0
Cellular_organisms - ps1
Archaea1 - ps2
Archaea2 - ps3
Archaea3 - ps4
Asgard/Eukaryota - ps5
Eukaryota - ps6
Excavata/Diaphoretickes - ps7
Diaphoretickes - ps8
Archaeplastida - ps9
Viridiplantae - ps10
Streptophyta - ps11
Charophyceae/Embriophyta - ps12
Embriophyta - ps13
Tracheophyta - ps14
Spermatophyta - ps15
Angiosperms - ps16
Mesangiospermae - ps17
Magnoliids/eudicots - ps18
Eudicots - ps19
Core_eudicots - ps20
Superrosids - ps21
Rosids - ps22
Eurosids - ps23
Malvids - ps24
Sap./Malv./Brassicales - ps25
Malvales/Brassicales - ps26
Brassicales - ps27
Cleomaceae/Brassicaceae - ps28
Brassicaceae - ps29
Camelineae - ps30
Arabidopsis - ps31
Arabidopsis_thaliana - ps32
Phylostratum

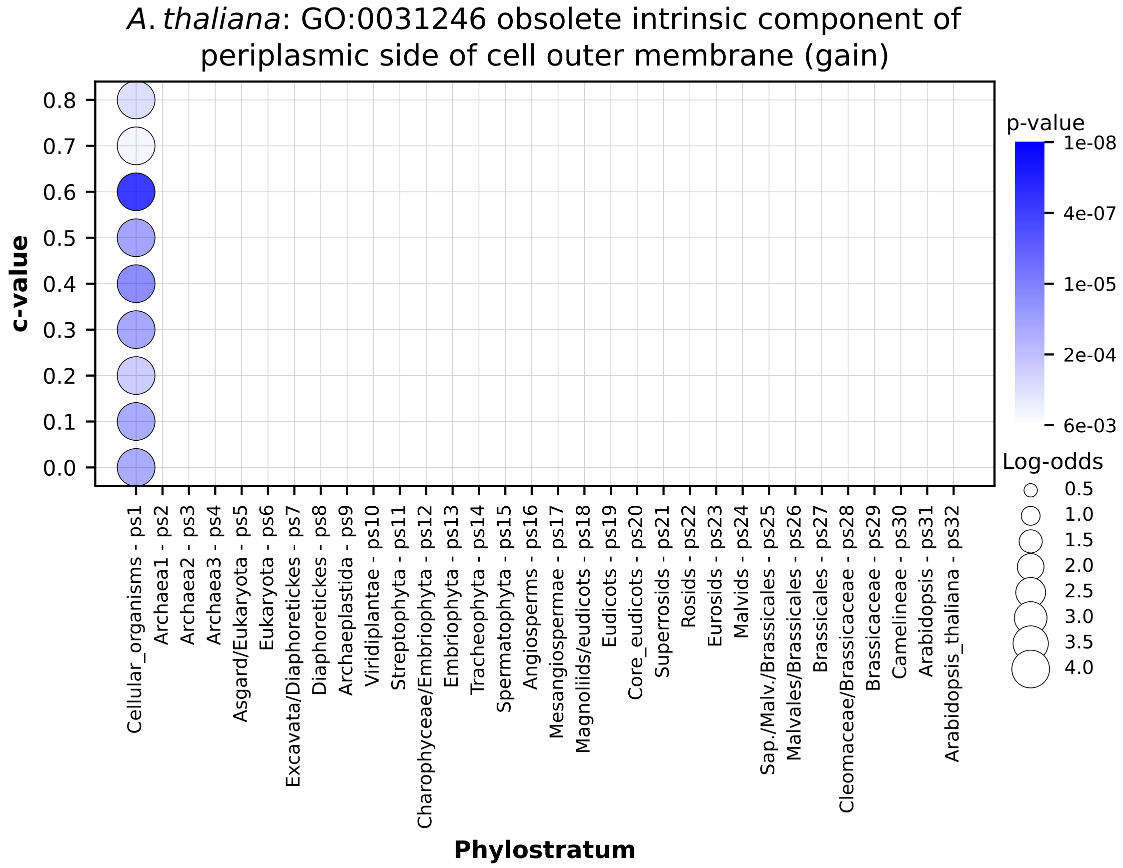

A. thaliana: GO:0031246 obsolete intrinsic component of periplasmic side of cell outer membrane (gain)
c-value
0.8
0.7
0.6
0.5
0.4
0.3
0.2
0.1
0.0
p-value
1e-08
4e-07
1e-05
2e-04
6e-03
Log-odds
0.5
1.0
1.5
2.0
2.5
3.0
3.5
4.0
Cellular_organisms - ps1
Archaea1 - ps2
Archaea2 - ps3
Archaea3 - ps4
Asgard/Eukaryota - ps5
Eukaryota - ps6
Excavata/Diaphoretickes - ps7
Diaphoretickes - ps8
Archaeplastida - ps9
Viridiplantae - ps10
Streptophyta - ps11
Charophyceae/Embriophyta - ps12
Embriophyta - ps13
Tracheophyta - ps14
Spermatophyta - ps15
Angiosperms - ps16
Mesangiospermae - ps17
Magnoliids/eudicots - ps18
Eudicots - ps19
Core_eudicots - ps20
Superrosids - ps21
Rosids - ps22
Eurosids - ps23
Malvids - ps24
Sap./Malv./Brassicales - ps25
Malvales/Brassicales - ps26
Brassicales - ps27
Cleomaceae/Brassicaceae - ps28
Brassicaceae - ps29
Camelineae - ps30
Arabidopsis - ps31
Arabidopsis_thaliana - ps32
Phylostratum

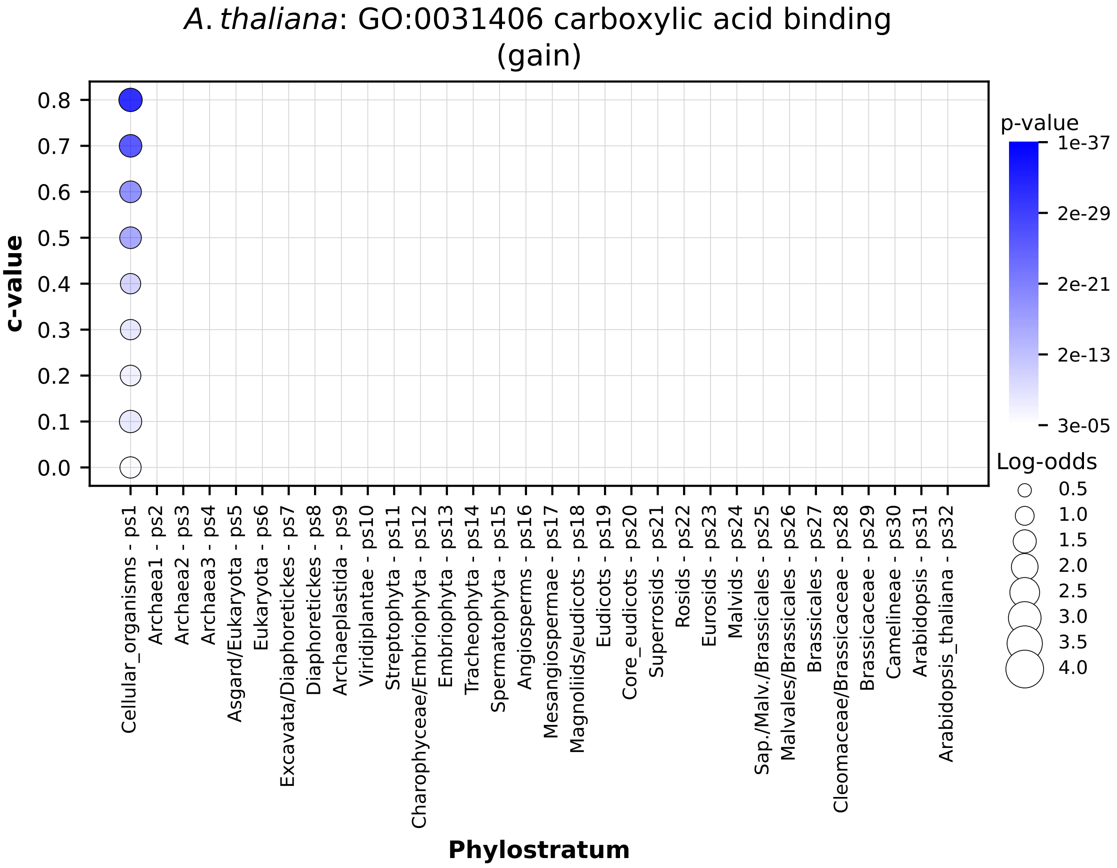

A. thaliana: GO:0031406 carboxylic acid binding (gain)
c-value
0.8
0.7
0.6
0.5
0.4
0.3
0.2
0.1
0.0
p-value
1e-37
2e-29
2e-21
2e-13
3e-05
Log-odds
0.5
1.0
1.5
2.0
2.5
3.0
3.5
4.0
Cellular_organisms - ps1
Archaea1 - ps2
Archaea2 - ps3
Archaea3 - ps4
Asgard/Eukaryota - ps5
Eukaryota - ps6
Excavata/Diaphoretickes - ps7
Diaphoretickes - ps8
Archaeplastida - ps9
Viridiplantae - ps10
Streptophyta - ps11
Charophyceae/Embriophyta - ps12
Embriophyta - ps13
Tracheophyta - ps14
Spermatophyta - ps15
Angiosperms - ps16
Mesangiospermae - ps17
Magnoliids/eudicots - ps18
Eudicots - ps19
Core_eudicots - ps20
Superrosids - ps21
Rosids - ps22
Eurosids - ps23
Malvids - ps24
Sap./Malv./Brassicales - ps25
Malvales/Brassicales - ps26
Brassicales - ps27
Cleomaceae/Brassicaceae - ps28
Brassicaceae - ps29
Camelineae - ps30
Arabidopsis - ps31
Arabidopsis_thaliana - ps32
Phylostratum

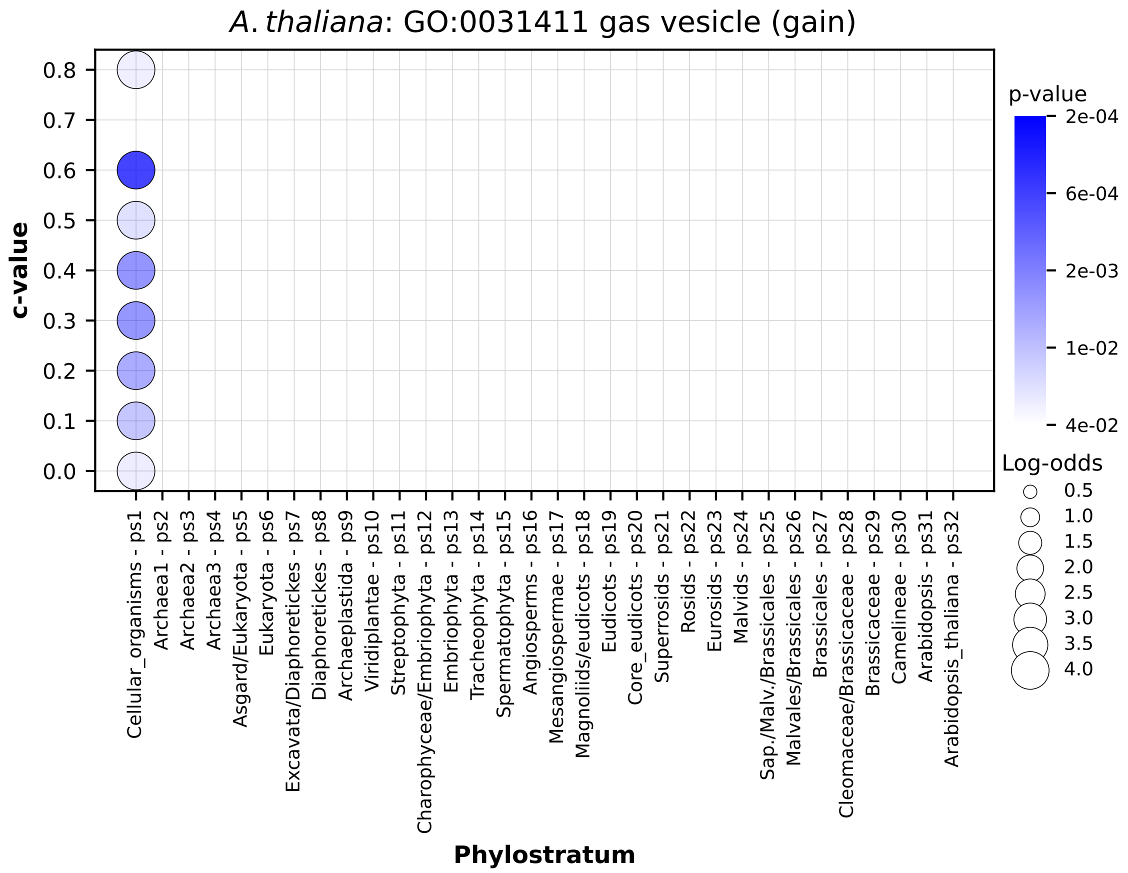

A. thaliana: GO:0031411 gas vesicle (gain)
c-value
0.0
0.1
0.2
0.3
0.4
0.5
0.6
0.7
0.8
p-value
2e-04
6e-04
2e-03
1e-02
4e-02
Log-odds
0.5
1.0
1.5
2.0
2.5
3.0
3.5
4.0
Cellular_organisms - ps1
Archaea1 - ps2
Archaea2 - ps3
Archaea3 - ps4
Asgard/Eukaryota - ps5
Eukaryota - ps6
Excavata/Diaphoretickes - ps7
Diaphoretickes - ps8
Archaeplastida - ps9
Viridiplantae - ps10
Streptophyta - ps11
Charophyceae/Embriophyta - ps12
Embriophyta - ps13
Tracheophyta - ps14
Spermatophyta - ps15
Angiosperms - ps16
Mesangiospermae - ps17
Magnoliids/eudicots - ps18
Eudicots - ps19
Core_eudicots - ps20
Superrosids - ps21
Rosids - ps22
Eurosids - ps23
Malvids - ps24
Sap./Malv./Brassicales - ps25
Malvales/Brassicales - ps26
Brassicales - ps27
Cleomaceae/Brassicaceae - ps28
Brassicaceae - ps29
Camelineae - ps30
Arabidopsis - ps31
Arabidopsis_thaliana - ps32
Phylostratum

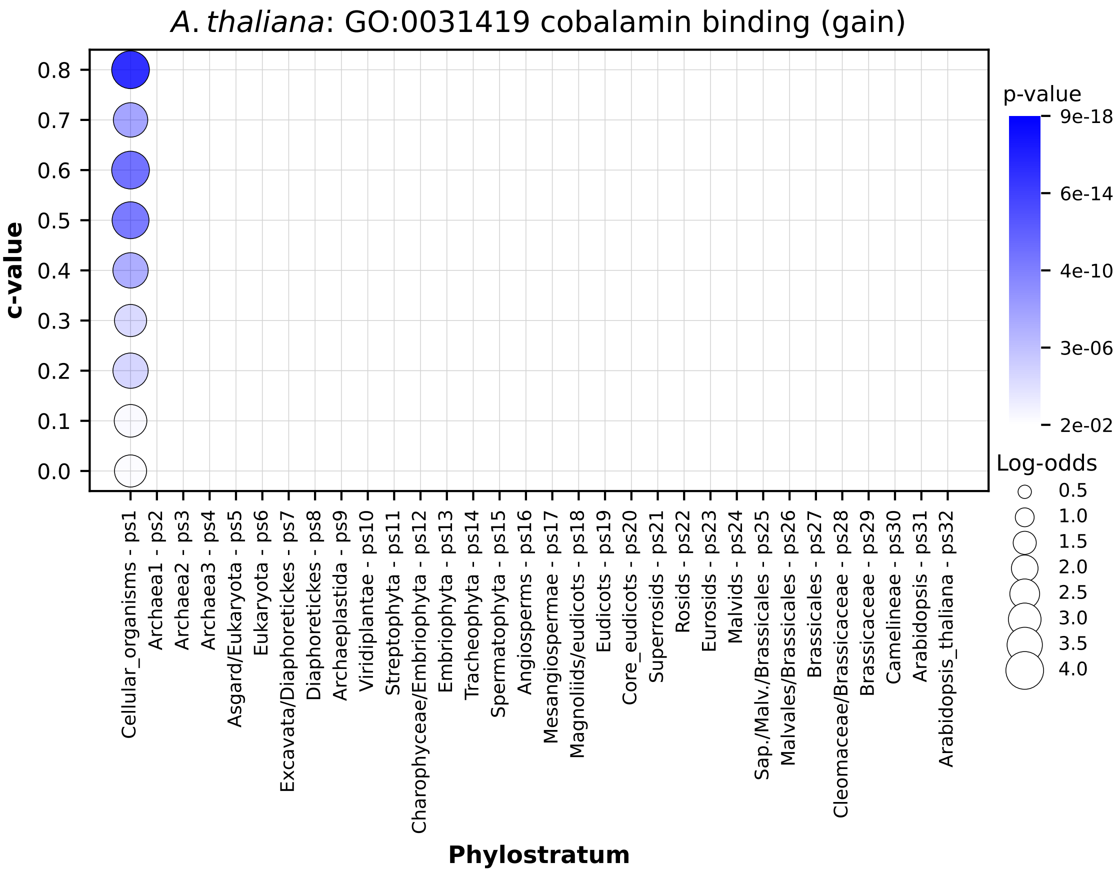
A. thaliana: GO:0031419 cobalamin binding (gain)
c-value
0.0
0.1
0.2
0.3
0.4
0.5
0.6
0.7
0.8
Phylostratum
Cellular_organisms - ps1
Archaea1 - ps2
Archaea2 - ps3
Archaea3 - ps4
Asgard/Eukaryota - ps5
Eukaryota - ps6
Excavata/Diaphoretickes - ps7
Diaphoretickes - ps8
Archaeplastida - ps9
Viridiplantae - ps10
Streptophyta - ps11
Charophyceae/Embriophyta - ps12
Embriophyta - ps13
Tracheophyta - ps14
Spermatophyta - ps15
Angiosperms - ps16
Mesangiospermae - ps17
Magnoliids/eudicots - ps18
Eudicots - ps19
Core_eudicots - ps20
Superrosids - ps21
Rosids - ps22
Eurosids - ps23
Malvids - ps24
Sap./Malv./Brassicales - ps25
Malvales/Brassicales - ps26
Brassicales - ps27
Cleomaceae/Brassicaceae - ps28
Brassicaceae - ps29
Camelineae - ps30
Arabidopsis - ps31
Arabidopsis_thaliana - ps32
p-value
9e-18
6e-14
4e-10
3e-06
2e-02
Log-odds
0.5
1.0
1.5
2.0
2.5
3.0
3.5
4.0

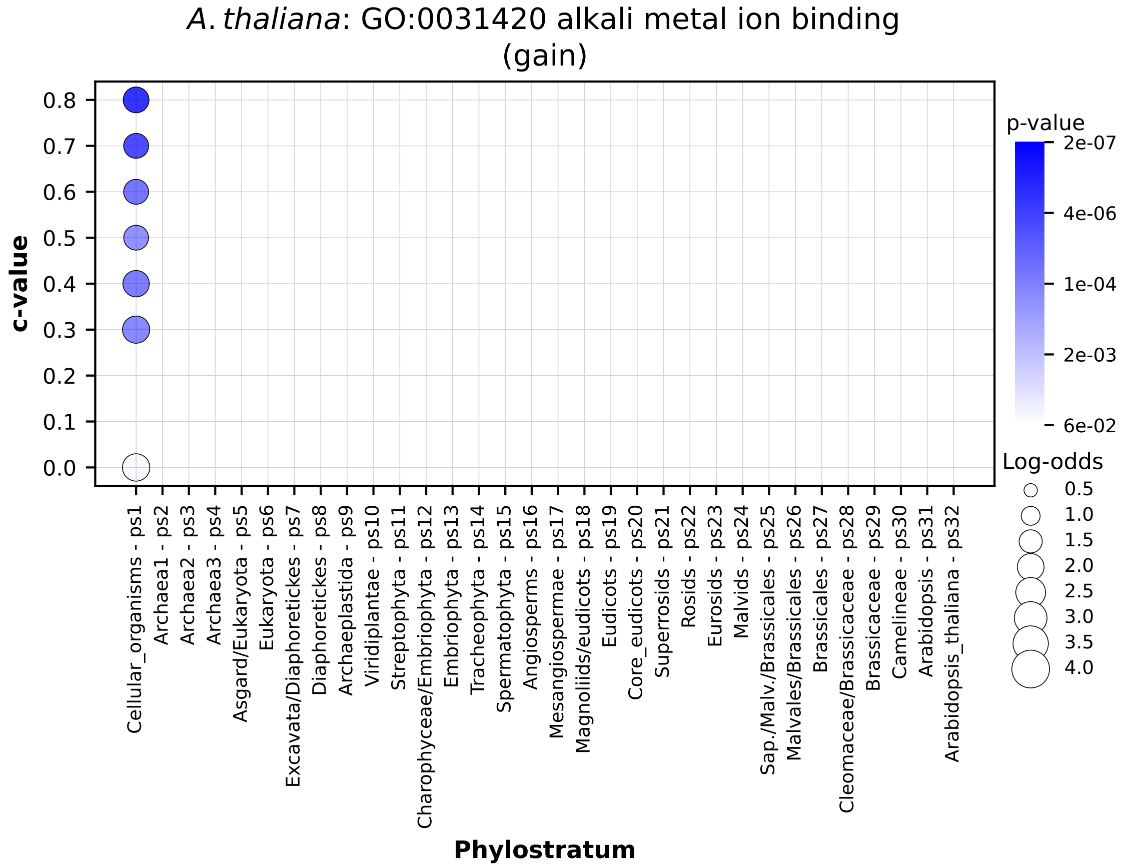

A. thaliana: GO:0031420 alkali metal ion binding (gain)
c-value
0.8
0.7
0.6
0.5
0.4
0.3
0.2
0.1
0.0
p-value
2e-07
4e-06
1e-04
2e-03
6e-02
Log-odds
0.5
1.0
1.5
2.0
2.5
3.0
3.5
4.0
Cellular_organisms - ps1
Archaea1 - ps2
Archaea2 - ps3
Archaea3 - ps4
Asgard/Eukaryota - ps5
Eukaryota - ps6
Excavata/Diaphoretickes - ps7
Diaphoretickes - ps8
Archaeplastida - ps9
Viridiplantae - ps10
Streptophyta - ps11
Charophyceae/Embriophyta - ps12
Embriophyta - ps13
Tracheophyta - ps14
Spermatophyta - ps15
Angiosperms - ps16
Mesangiospermae - ps17
Magnoliids/eudicots - ps18
Eudicots - ps19
Core_eudicots - ps20
Superrosids - ps21
Rosids - ps22
Eurosids - ps23
Malvids - ps24
Sap./Malv./Brassicales - ps25
Malvales/Brassicales - ps26
Brassicales - ps27
Cleomaceae/Brassicaceae - ps28
Brassicaceae - ps29
Camelineae - ps30
Arabidopsis - ps31
Arabidopsis_thaliana - ps32
Phylostratum

A. thaliana: GO:0031554 regulation of termination of DNA-templated transcription (gain)

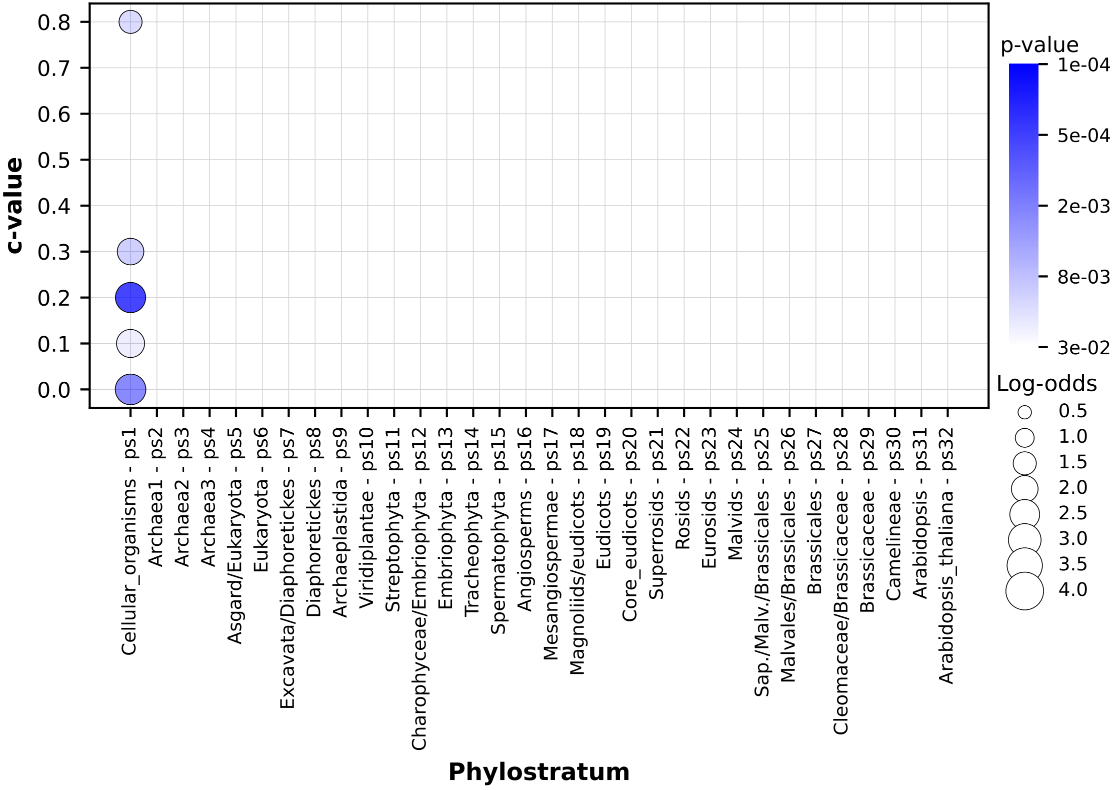

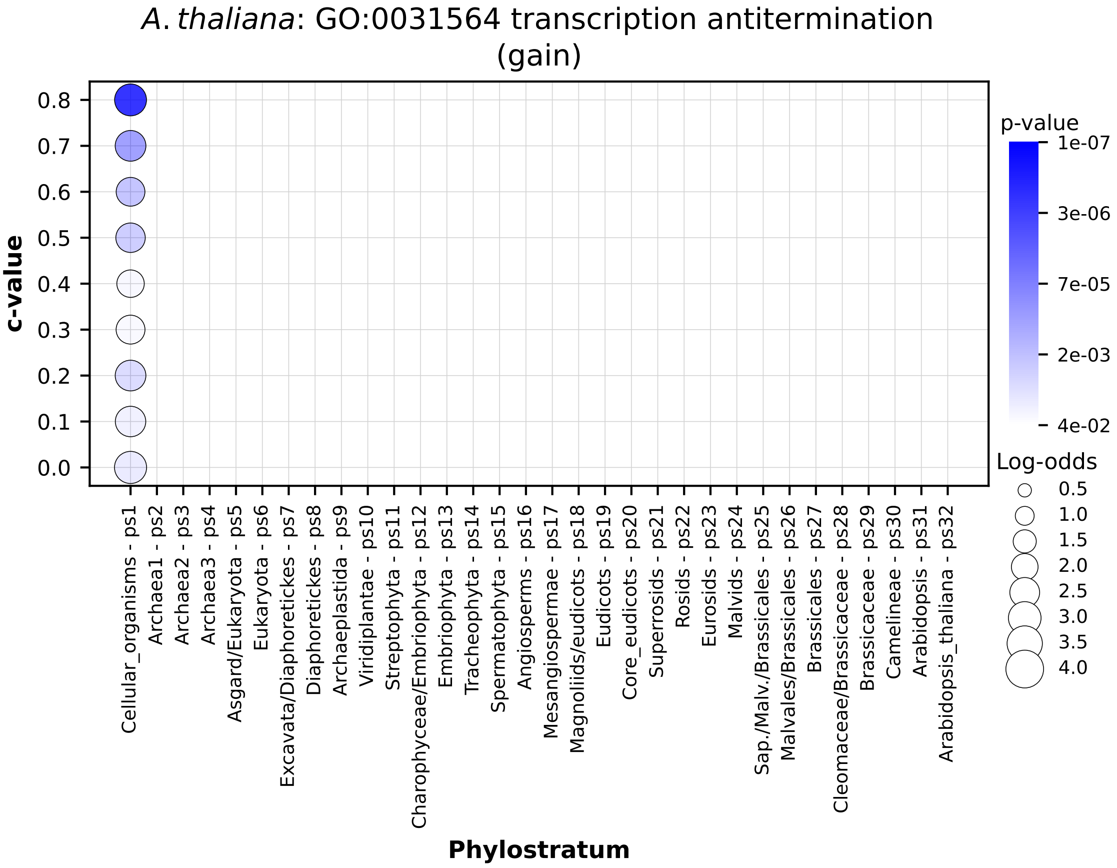

A. thaliana: GO:0031564 transcription antitermination (gain)
c-value
0.0
0.1
0.2
0.3
0.4
0.5
0.6
0.7
0.8
p-value
1e-07
3e-06
7e-05
2e-03
4e-02
Log-odds
0.5
1.0
1.5
2.0
2.5
3.0
3.5
4.0
Cellular_organisms - ps1
Archaea1 - ps2
Archaea2 - ps3
Archaea3 - ps4
Asgard/Eukaryota - ps5
Eukaryota - ps6
Excavata/Diaphoretickes - ps7
Diaphoretickes - ps8
Archaeplastida - ps9
Viridiplantae - ps10
Streptophyta - ps11
Charophyceae/Embriophyta - ps12
Embriophyta - ps13
Tracheophyta - ps14
Spermatophyta - ps15
Angiosperms - ps16
Mesangiospermae - ps17
Magnoliids/eudicots - ps18
Eudicots - ps19
Core_eudicots - ps20
Superrosids - ps21
Rosids - ps22
Eurosids - ps23
Malvids - ps24
Sap./Malv./Brassicales - ps25
Malvales/Brassicales - ps26
Brassicales - ps27
Cleomaceae/Brassicaceae - ps28
Brassicaceae - ps29
Camelineae - ps30
Arabidopsis - ps31
Arabidopsis_thaliana - ps32
Phylostratum

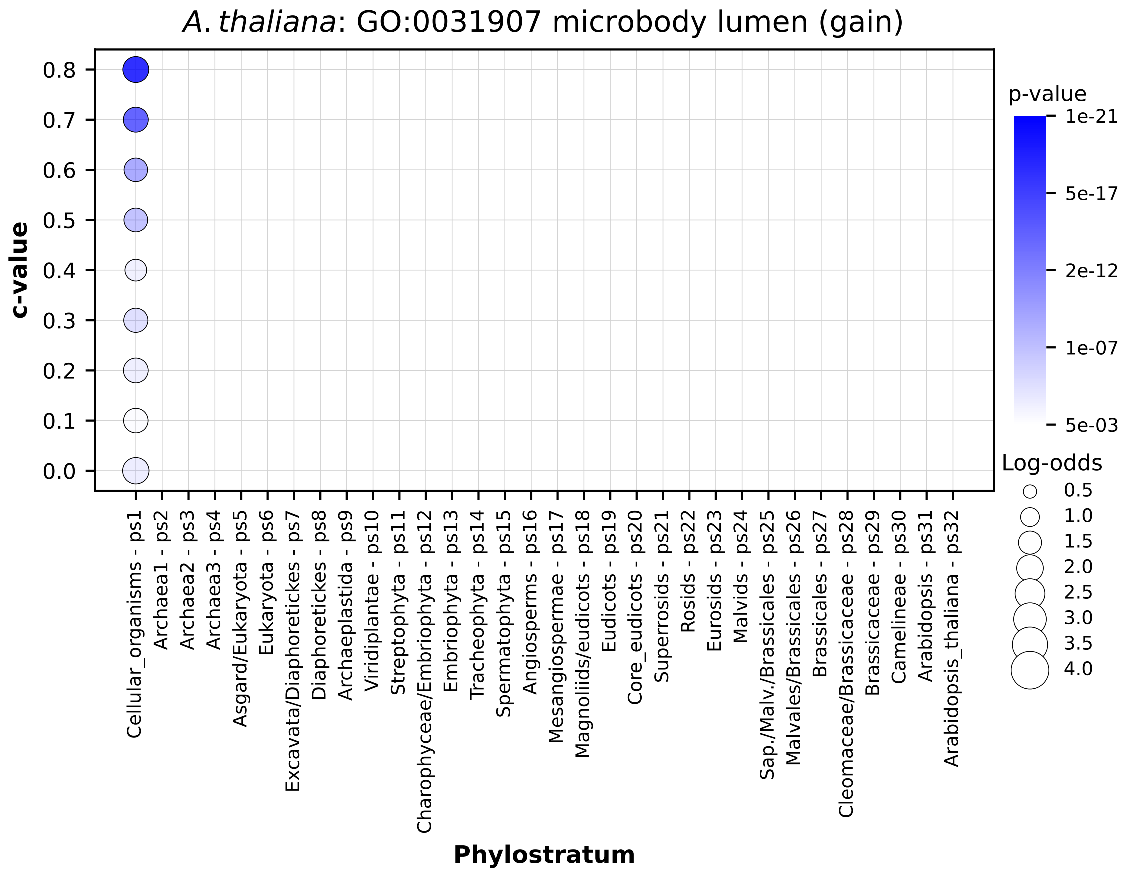
A. thaliana: GO:0031907 microbody lumen (gain)
c-value
0.8
0.7
0.6
0.5
0.4
0.3
0.2
0.1
0.0
p-value
1e-21
5e-17
2e-12
1e-07
5e-03
Log-odds
0.5
1.0
1.5
2.0
2.5
3.0
3.5
4.0
Cellular_organisms - ps1
Archaea1 - ps2
Archaea2 - ps3
Archaea3 - ps4
Asgard/Eukaryota - ps5
Eukaryota - ps6
Excavata/Diaphoretickes - ps7
Diaphoretickes - ps8
Archaeplastida - ps9
Viridiplantae - ps10
Streptophyta - ps11
Charophyceae/Embriophyta - ps12
Embriophyta - ps13
Tracheophyta - ps14
Spermatophyta - ps15
Angiosperms - ps16
Mesangiospermae - ps17
Magnoliids/eudicots - ps18
Eudicots - ps19
Core_eudicots - ps20
Superrosids - ps21
Rosids - ps22
Eurosids - ps23
Malvids - ps24
Sap./Malv./Brassicales - ps25
Malvales/Brassicales - ps26
Brassicales - ps27
Cleomaceae/Brassicaceae - ps28
Brassicaceae - ps29
Camelineae - ps30
Arabidopsis - ps31
Arabidopsis_thaliana - ps32
Phylostratum

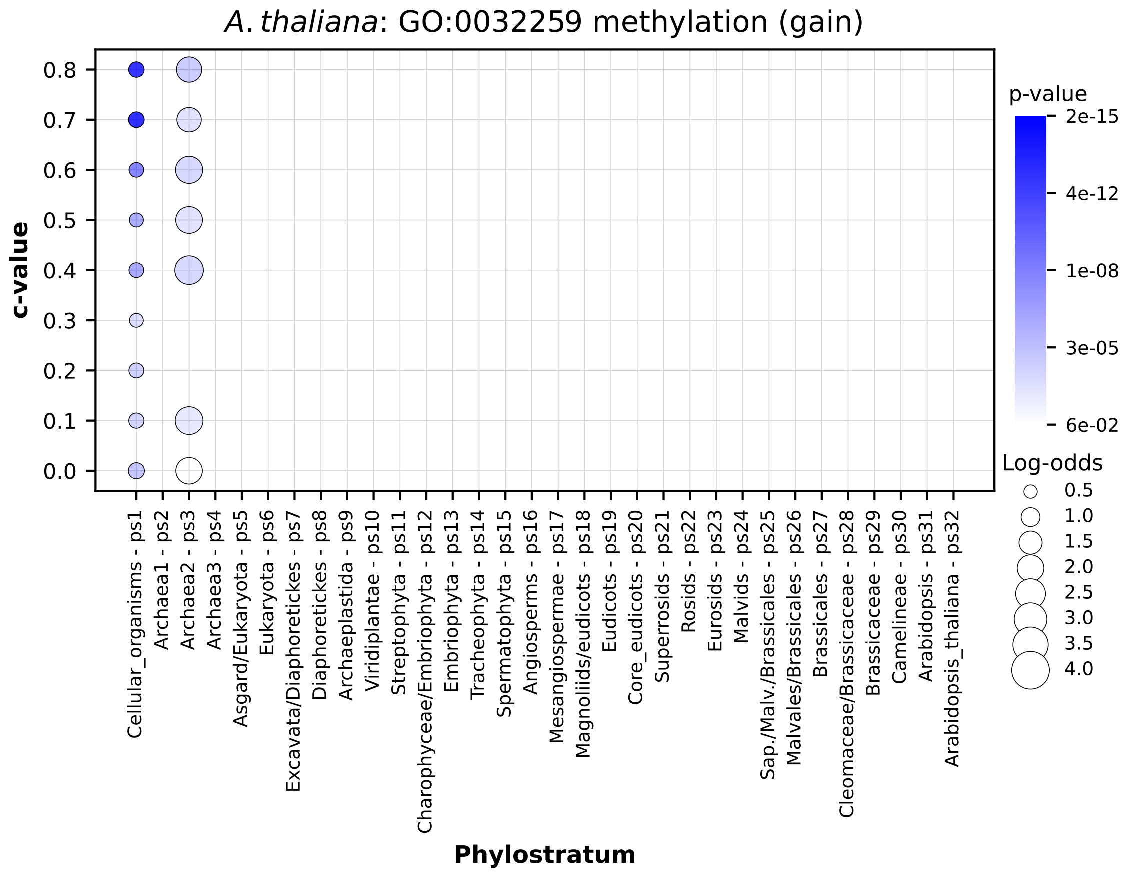

A. thaliana: GO:0032259 methylation (gain)
c-value
0.8
0.7
0.6
0.5
0.4
0.3
0.2
0.1
0.0
Phylostratum
Cellular_organisms - ps1
Archaea1 - ps2
Archaea2 - ps3
Archaea3 - ps4
Asgard/Eukaryota - ps5
Eukaryota - ps6
Excavata/Diaphoretickes - ps7
Diaphoretickes - ps8
Archaeplastida - ps9
Viridiplantae - ps10
Streptophyta - ps11
Charophyceae/Embriophyta - ps12
Embriophyta - ps13
Tracheophyta - ps14
Spermatophyta - ps15
Angiosperms - ps16
Mesangiospermae - ps17
Magnoliids/eudicots - ps18
Eudicots - ps19
Core_eudicots - ps20
Superrosids - ps21
Rosids - ps22
Eurosids - ps23
Malvids - ps24
Sap./Malv./Brassicales - ps25
Malvales/Brassicales - ps26
Brassicales - ps27
Cleomaceae/Brassicaceae - ps28
Brassicaceae - ps29
Camelineae - ps30
Arabidopsis - ps31
Arabidopsis_thaliana - ps32
p-value
2e-15
4e-12
1e-08
3e-05
6e-02
Log-odds
0.5
1.0
1.5
2.0
2.5
3.0
3.5
4.0

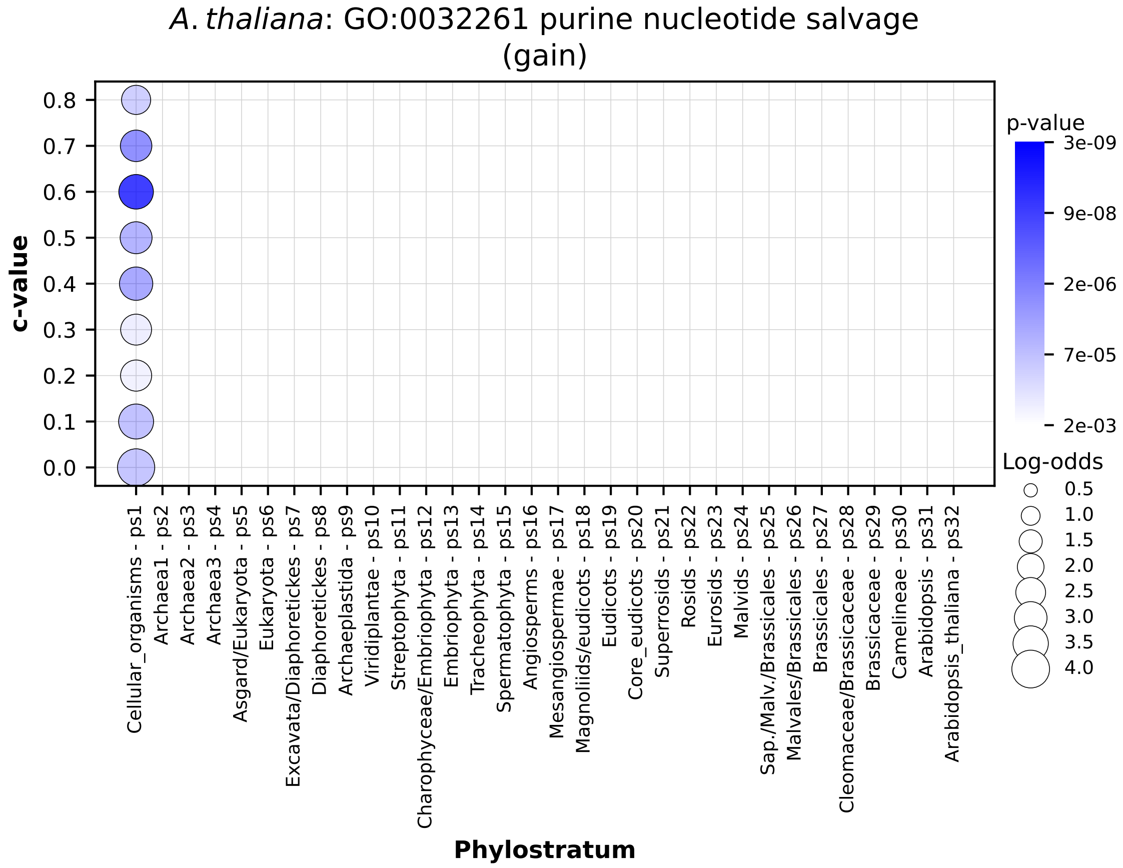

A. thaliana: GO:0032261 purine nucleotide salvage
(gain)
c-value
0.8
0.7
0.6
0.5
0.4
0.3
0.2
0.1
0.0
p-value
3e-09
9e-08
2e-06
7e-05
2e-03
Log-odds
0.5
1.0
1.5
2.0
2.5
3.0
3.5
4.0
Cellular_organisms - ps1
Archaea1 - ps2
Archaea2 - ps3
Archaea3 - ps4
Asgard/Eukaryota - ps5
Eukaryota - ps6
Excavata/Diaphoretickes - ps7
Diaphoretickes - ps8
Archaeplastida - ps9
Viridiplantae - ps10
Streptophyta - ps11
Charophyceae/Embriophyta - ps12
Embriophyta - ps13
Tracheophyta - ps14
Spermatophyta - ps15
Angiosperms - ps16
Mesangiospermae - ps17
Magnoliids/eudicots - ps18
Eudicots - ps19
Core_eudicots - ps20
Superrosids - ps21
Rosids - ps22
Eurosids - ps23
Malvids - ps24
Sap./Malv./Brassicales - ps25
Malvales/Brassicales - ps26
Brassicales - ps27
Cleomaceae/Brassicaceae - ps28
Brassicaceae - ps29
Camelineae - ps30
Arabidopsis - ps31
Arabidopsis_thaliana - ps32
Phylostratum

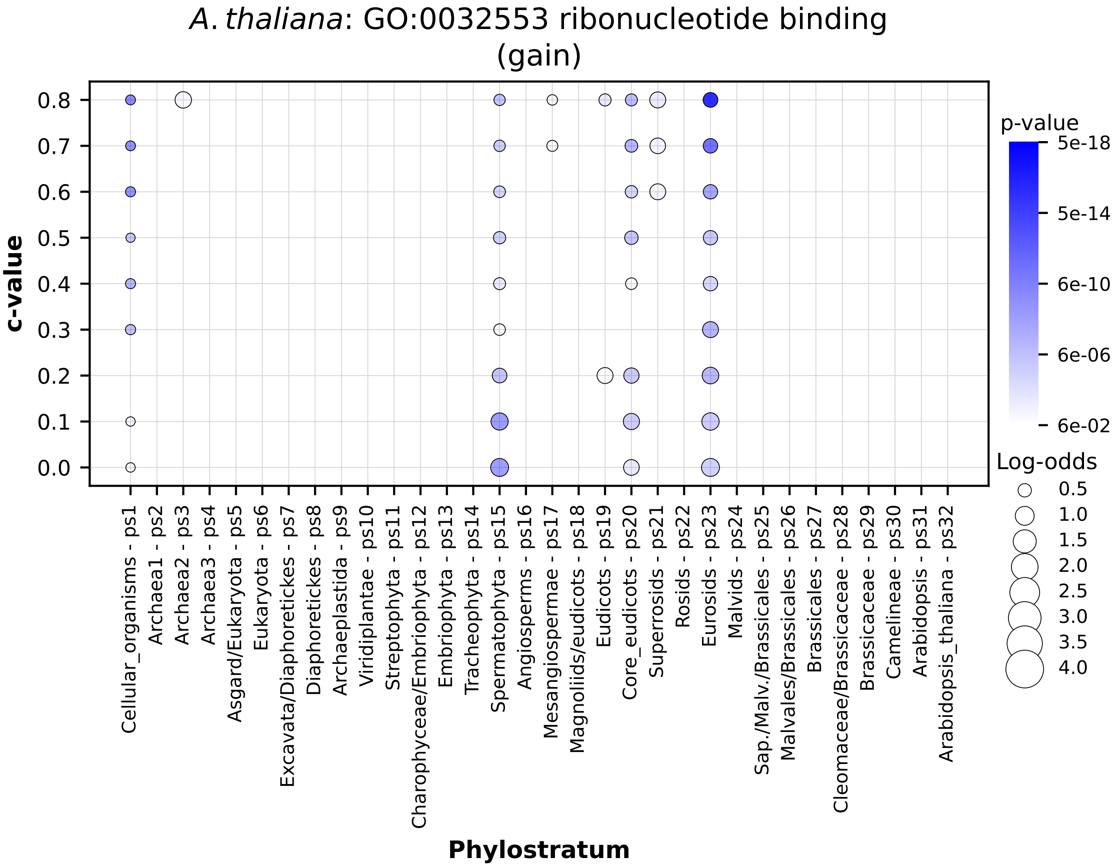

A. thaliana: GO:0032553 ribonucleotide binding (gain)
c-value
0.8
0.7
0.6
0.5
0.4
0.3
0.2
0.1
0.0
Phylostratum
Cellular_organisms - ps1
Archaea1 - ps2
Archaea2 - ps3
Archaea3 - ps4
Asgard/Eukaryota - ps5
Eukaryota - ps6
Excavata/Diaphoretickes - ps7
Diaphoretickes - ps8
Archaeplastida - ps9
Viridiplantae - ps10
Streptophyta - ps11
Charophyceae/Embriophyta - ps12
Embriophyta - ps13
Tracheophyta - ps14
Spermatophyta - ps15
Angiosperms - ps16
Mesangiospermae - ps17
Magnoliids/eudicots - ps18
Eudicots - ps19
Core_eudicots - ps20
Superrosids - ps21
Rosids - ps22
Eurosids - ps23
Malvids - ps24
Sap./Malv./Brassicales - ps25
Malvales/Brassicales - ps26
Brassicales - ps27
Cleomaceae/Brassicaceae - ps28
Brassicaceae - ps29
Camelineae - ps30
Arabidopsis - ps31
Arabidopsis_thaliana - ps32
p-value
5e-18
5e-14
6e-10
6e-06
6e-02
Log-odds
0.5
1.0
1.5
2.0
2.5
3.0
3.5
4.0

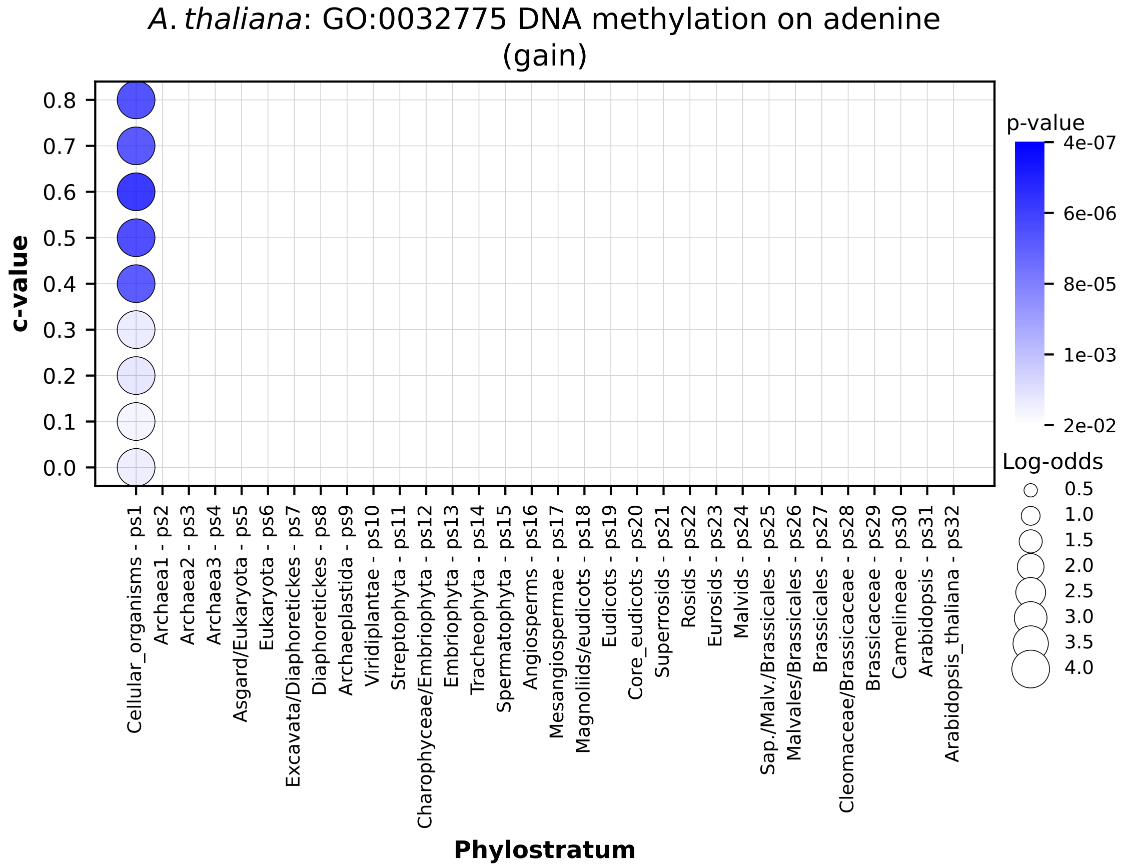

A. thaliana: GO:0032775 DNA methylation on adenine (gain)
c-value
0.8
0.7
0.6
0.5
0.4
0.3
0.2
0.1
0.0
p-value
4e-07
6e-06
8e-05
1e-03
2e-02
Log-odds
0.5
1.0
1.5
2.0
2.5
3.0
3.5
4.0
Cellular_organisms - ps1
Archaea1 - ps2
Archaea2 - ps3
Archaea3 - ps4
Asgard/Eukaryota - ps5
Eukaryota - ps6
Excavata/Diaphoretickes - ps7
Diaphoretickes - ps8
Archaeplastida - ps9
Viridiplantae - ps10
Streptophyta - ps11
Charophyceae/Embriophyta - ps12
Embriophyta - ps13
Tracheophyta - ps14
Spermatophyta - ps15
Angiosperms - ps16
Mesangiospermae - ps17
Magnoliids/eudicots - ps18
Eudicots - ps19
Core_eudicots - ps20
Superrosids - ps21
Rosids - ps22
Eurosids - ps23
Malvids - ps24
Sap./Malv./Brassicales - ps25
Malvales/Brassicales - ps26
Brassicales - ps27
Cleomaceae/Brassicaceae - ps28
Brassicaceae - ps29
Camelineae - ps30
Arabidopsis - ps31
Arabidopsis_thaliana - ps32
Phylostratum

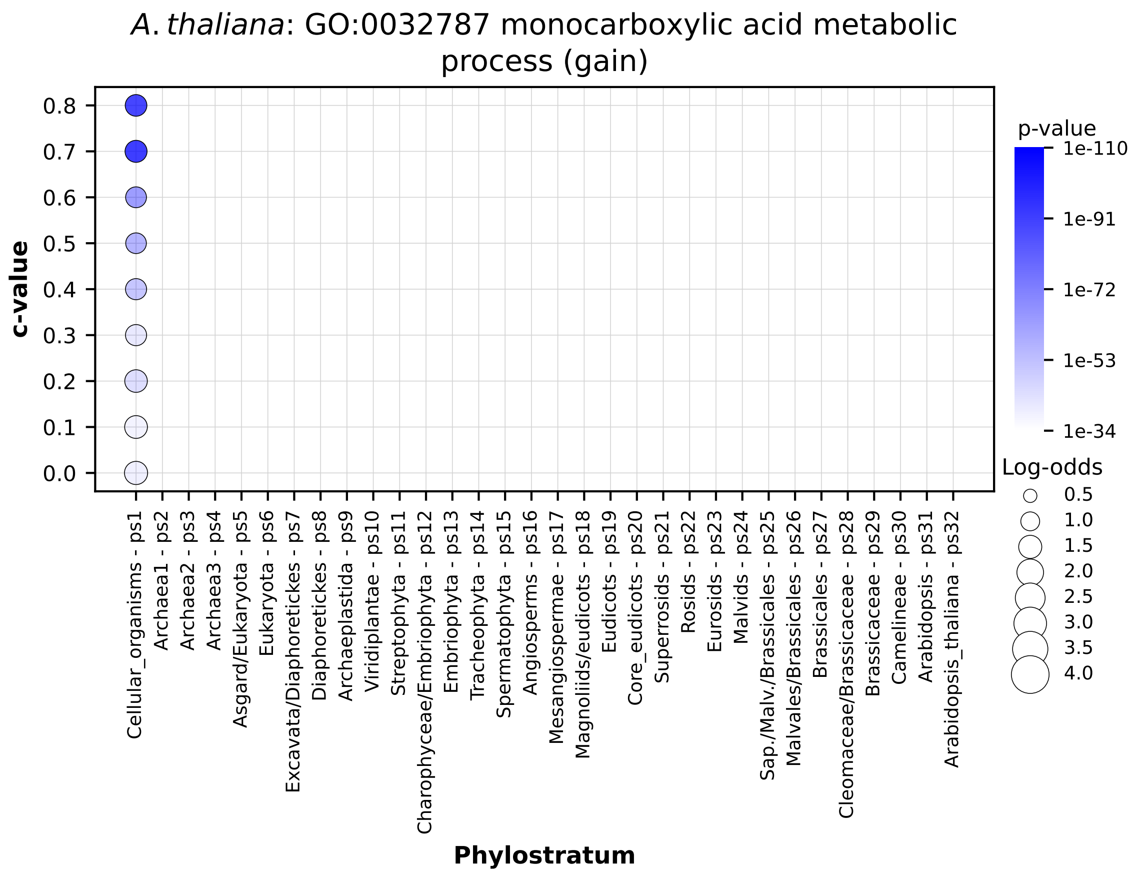

A. thaliana: GO:0032787 monocarboxylic acid metabolic process (gain)
c-value
0.8
0.7
0.6
0.5
0.4
0.3
0.2
0.1
0.0
p-value
1e-110
1e-91
1e-72
1e-53
1e-34
Log-odds
0.5
1.0
1.5
2.0
2.5
3.0
3.5
4.0
Cellular_organisms - ps1
Archaea1 - ps2
Archaea2 - ps3
Archaea3 - ps4
Asgard/Eukaryota - ps5
Eukaryota - ps6
Excavata/Diaphoretickes - ps7
Diaphoretickes - ps8
Archaeplastida - ps9
Viridiplantae - ps10
Streptophyta - ps11
Charophyceae/Embriophyta - ps12
Embriophyta - ps13
Tracheophyta - ps14
Spermatophyta - ps15
Angiosperms - ps16
Mesangiospermae - ps17
Magnoliids/eudicots - ps18
Eudicots - ps19
Core_eudicots - ps20
Superrosids - ps21
Rosids - ps22
Eurosids - ps23
Malvids - ps24
Sap./Malv./Brassicales - ps25
Malvales/Brassicales - ps26
Brassicales - ps27
Cleomaceae/Brassicaceae - ps28
Brassicaceae - ps29
Camelineae - ps30
Arabidopsis - ps31
Arabidopsis_thaliana - ps32
Phylostratum

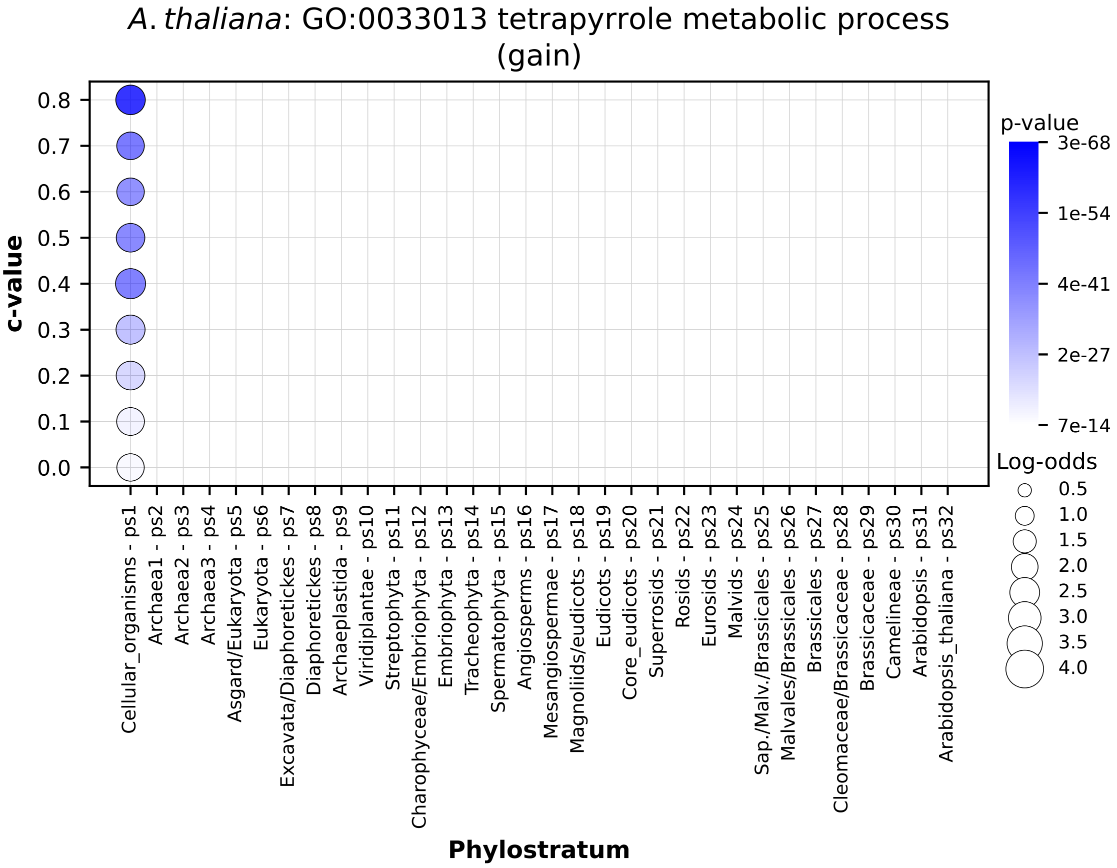

A. thaliana: GO:0033013 tetrapyrrole metabolic process
(gain)
c-value
0.8
0.7
0.6
0.5
0.4
0.3
0.2
0.1
0.0
p-value
3e-68
1e-54
4e-41
2e-27
7e-14
Log-odds
0.5
1.0
1.5
2.0
2.5
3.0
3.5
4.0
Cellular_organisms - ps1
Archaea1 - ps2
Archaea2 - ps3
Archaea3 - ps4
Asgard/Eukaryota - ps5
Eukaryota - ps6
Excavata/Diaphoretickes - ps7
Diaphoretickes - ps8
Archaeplastida - ps9
Viridiplantae - ps10
Streptophyta - ps11
Charophyceae/Embriophyta - ps12
Embriophyta - ps13
Tracheophyta - ps14
Spermatophyta - ps15
Angiosperms - ps16
Mesangiospermae - ps17
Magnoliids/eudicots - ps18
Eudicots - ps19
Core_eudicots - ps20
Superrosids - ps21
Rosids - ps22
Eurosids - ps23
Malvids - ps24
Sap./Malv./Brassicales - ps25
Malvales/Brassicales - ps26
Brassicales - ps27
Cleomaceae/Brassicaceae - ps28
Brassicaceae - ps29
Camelineae - ps30
Arabidopsis - ps31
Arabidopsis_thaliana - ps32
Phylostratum

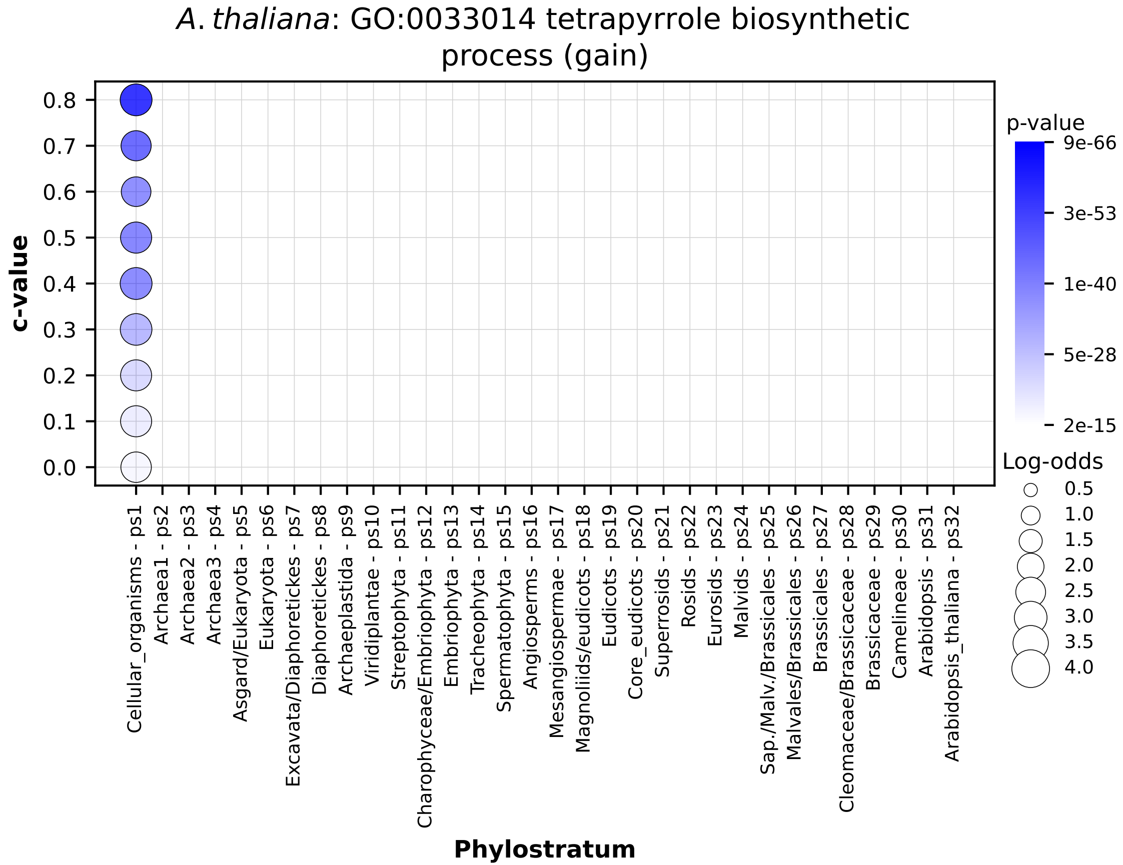
A. thaliana: GO:0033014 tetrapyrrole biosynthetic process (gain)
c-value
0.8
0.7
0.6
0.5
0.4
0.3
0.2
0.1
0.0
p-value
9e-66
3e-53
1e-40
5e-28
2e-15
Log-odds
0.5
1.0
1.5
2.0
2.5
3.0
3.5
4.0
Cellular_organisms - ps1
Archaea1 - ps2
Archaea2 - ps3
Archaea3 - ps4
Asgard/Eukaryota - ps5
Eukaryota - ps6
Excavata/Diaphoretickes - ps7
Diaphoretickes - ps8
Archaeplastida - ps9
Viridiplantae - ps10
Streptophyta - ps11
Charophyceae/Embriophyta - ps12
Embriophyta - ps13
Tracheophyta - ps14
Spermatophyta - ps15
Angiosperms - ps16
Mesangiospermae - ps17
Magnoliids/eudicots - ps18
Eudicots - ps19
Core_eudicots - ps20
Superrosids - ps21
Rosids - ps22
Eurosids - ps23
Malvids - ps24
Sap./Malv./Brassicales - ps25
Malvales/Brassicales - ps26
Brassicales - ps27
Cleomaceae/Brassicaceae - ps28
Brassicaceae - ps29
Camelineae - ps30
Arabidopsis - ps31
Arabidopsis_thaliana - ps32
Phylostratum

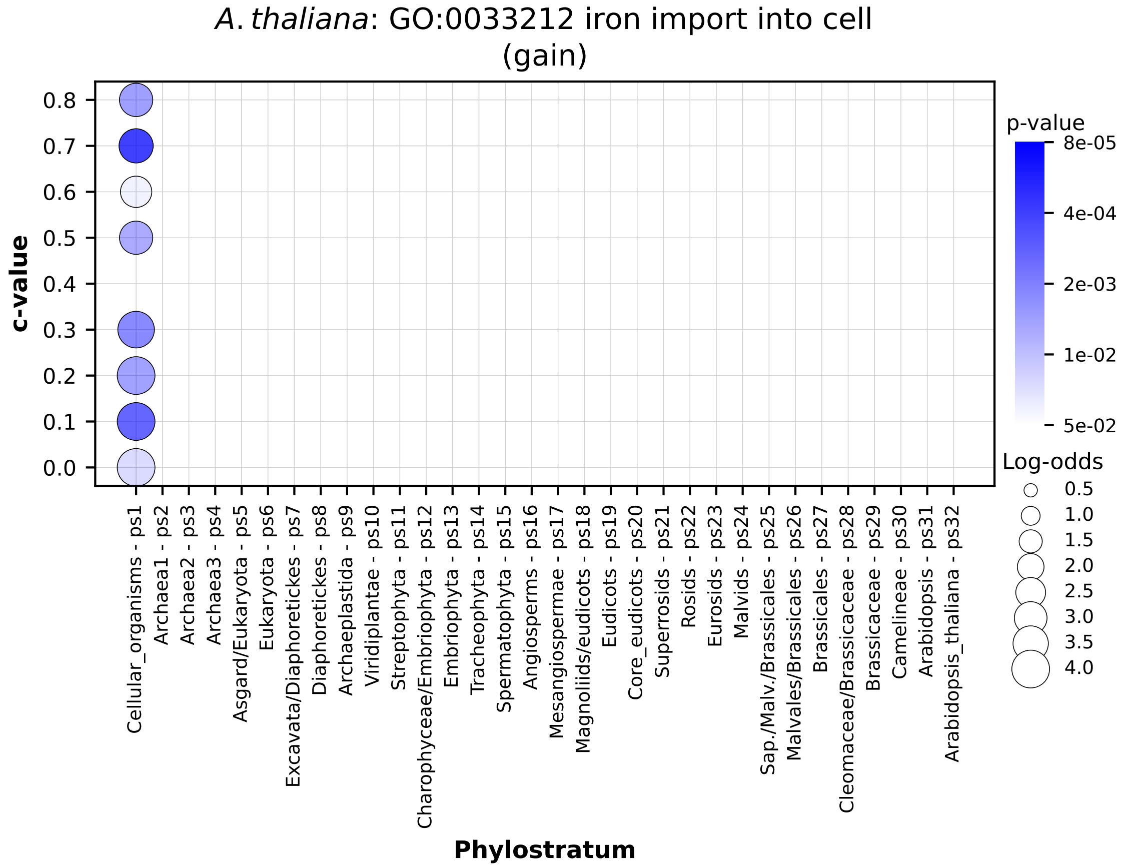

A. thaliana: GO:0033212 iron import into cell
(gain)
c-value
0.8
0.7
0.6
0.5
0.4
0.3
0.2
0.1
0.0
p-value
8e-05
4e-04
2e-03
1e-02
5e-02
Log-odds
0.5
1.0
1.5
2.0
2.5
3.0
3.5
4.0
Cellular_organisms - ps1
Archaea1 - ps2
Archaea2 - ps3
Archaea3 - ps4
Asgard/Eukaryota - ps5
Eukaryota - ps6
Excavata/Diaphoretickes - ps7
Diaphoretickes - ps8
Archaeplastida - ps9
Viridiplantae - ps10
Streptophyta - ps11
Charophyceae/Embriophyta - ps12
Embriophyta - ps13
Tracheophyta - ps14
Spermatophyta - ps15
Angiosperms - ps16
Mesangiospermae - ps17
Magnoliids/eudicots - ps18
Eudicots - ps19
Core_eudicots - ps20
Superrosids - ps21
Rosids - ps22
Eurosids - ps23
Malvids - ps24
Sap./Malv./Brassicales - ps25
Malvales/Brassicales - ps26
Brassicales - ps27
Cleomaceae/Brassicaceae - ps28
Brassicaceae - ps29
Camelineae - ps30
Arabidopsis - ps31
Arabidopsis_thaliana - ps32
Phylostratum

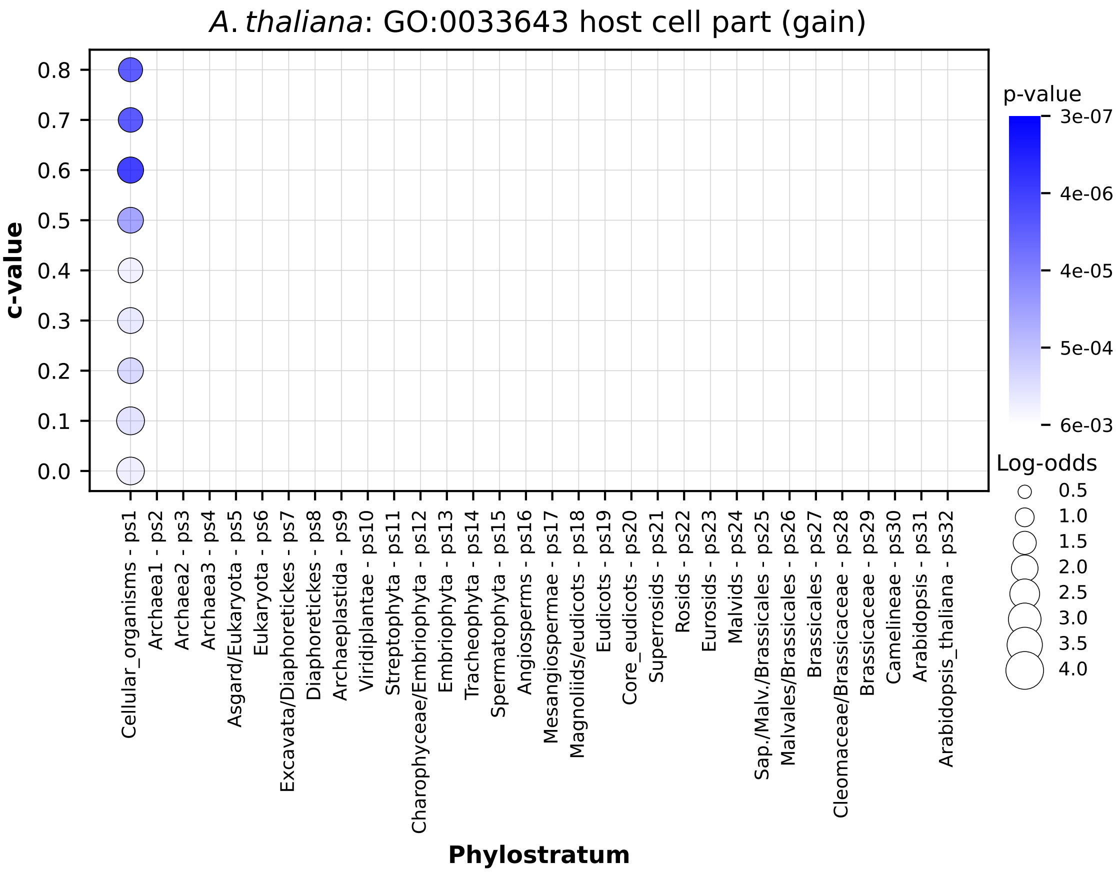

A. thaliana: GO:0033643 host cell part (gain)
c-value
0.0
0.1
0.2
0.3
0.4
0.5
0.6
0.7
0.8
Phylostratum
Cellular_organisms - ps1
Archaea1 - ps2
Archaea2 - ps3
Archaea3 - ps4
Asgard/Eukaryota - ps5
Eukaryota - ps6
Excavata/Diaphoretickes - ps7
Diaphoretickes - ps8
Archaeplastida - ps9
Viridiplantae - ps10
Streptophyta - ps11
Charophyceae/Embriophyta - ps12
Embriophyta - ps13
Tracheophyta - ps14
Spermatophyta - ps15
Angiosperms - ps16
Mesangiospermae - ps17
Magnoliids/eudicots - ps18
Eudicots - ps19
Core_eudicots - ps20
Superrosids - ps21
Rosids - ps22
Eurosids - ps23
Malvids - ps24
Sap./Malv./Brassicales - ps25
Malvales/Brassicales - ps26
Brassicales - ps27
Cleomaceae/Brassicaceae - ps28
Brassicaceae - ps29
Camelineae - ps30
Arabidopsis - ps31
Arabidopsis_thaliana - ps32
p-value
3e-07
4e-06
4e-05
5e-04
6e-03
Log-odds
0.5
1.0
1.5
2.0
2.5
3.0
3.5
4.0

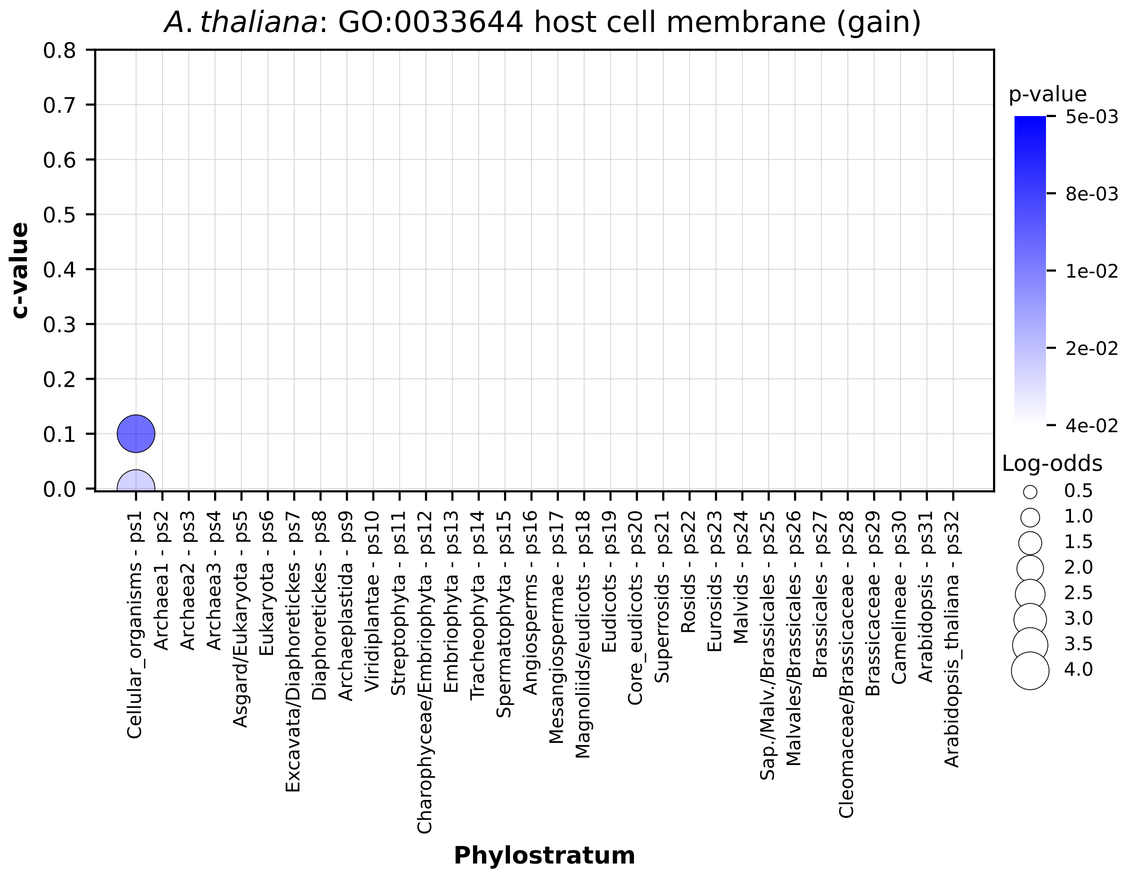

A. thaliana: GO:0033644 host cell membrane (gain)
c-value
0.8
0.7
0.6
0.5
0.4
0.3
0.2
0.1
0.0
Phylostratum
Cellular_organisms - ps1
Archaea1 - ps2
Archaea2 - ps3
Archaea3 - ps4
Asgard/Eukaryota - ps5
Eukaryota - ps6
Excavata/Diaphoretickes - ps7
Diaphoretickes - ps8
Archaeplastida - ps9
Viridiplantae - ps10
Streptophyta - ps11
Charophyceae/Embriophyta - ps12
Embriophyta - ps13
Tracheophyta - ps14
Spermatophyta - ps15
Angiosperms - ps16
Mesangiospermae - ps17
Magnoliids/eudicots - ps18
Eudicots - ps19
Core_eudicots - ps20
Superrosids - ps21
Rosids - ps22
Eurosids - ps23
Malvids - ps24
Sap./Malv./Brassicales - ps25
Malvales/Brassicales - ps26
Brassicales - ps27
Cleomaceae/Brassicaceae - ps28
Brassicaceae - ps29
Camelineae - ps30
Arabidopsis - ps31
Arabidopsis_thaliana - ps32
p-value
5e-03
8e-03
1e-02
2e-02
4e-02
Log-odds
0.5
1.0
1.5
2.0
2.5
3.0
3.5
4.0

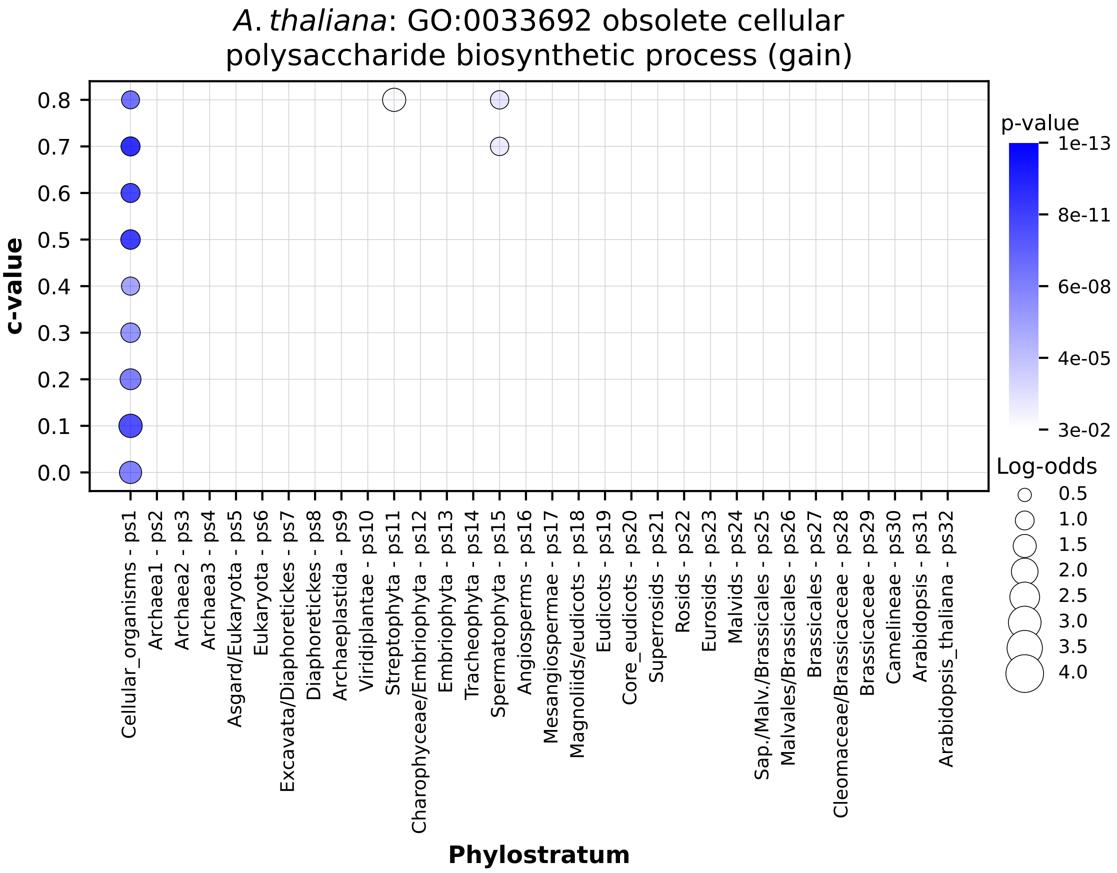
A. thaliana: GO:0033692 obsolete cellular polysaccharide biosynthetic process (gain)
c-value
0.0
0.1
0.2
0.3
0.4
0.5
0.6
0.7
0.8
Phylostratum
Cellular_organisms - ps1
Archaea1 - ps2
Archaea2 - ps3
Archaea3 - ps4
Asgard/Eukaryota - ps5
Eukaryota - ps6
Excavata/Diaphoretickes - ps7
Diaphoretickes - ps8
Archaeplastida - ps9
Viridiplantae - ps10
Streptophyta - ps11
Charophyceae/Embriophyta - ps12
Embriophyta - ps13
Tracheophyta - ps14
Spermatophyta - ps15
Angiosperms - ps16
Mesangiospermae - ps17
Magnoliids/eudicots - ps18
Eudicots - ps19
Core_eudicots - ps20
Superrosids - ps21
Rosids - ps22
Eurosids - ps23
Malvids - ps24
Sap./Malv./Brassicales - ps25
Malvales/Brassicales - ps26
Brassicales - ps27
Cleomaceae/Brassicaceae - ps28
Brassicaceae - ps29
Camelineae - ps30
Arabidopsis - ps31
Arabidopsis_thaliana - ps32
p-value
1e-13
8e-11
6e-08
4e-05
3e-02
Log-odds
0.5
1.0
1.5
2.0
2.5
3.0
3.5
4.0

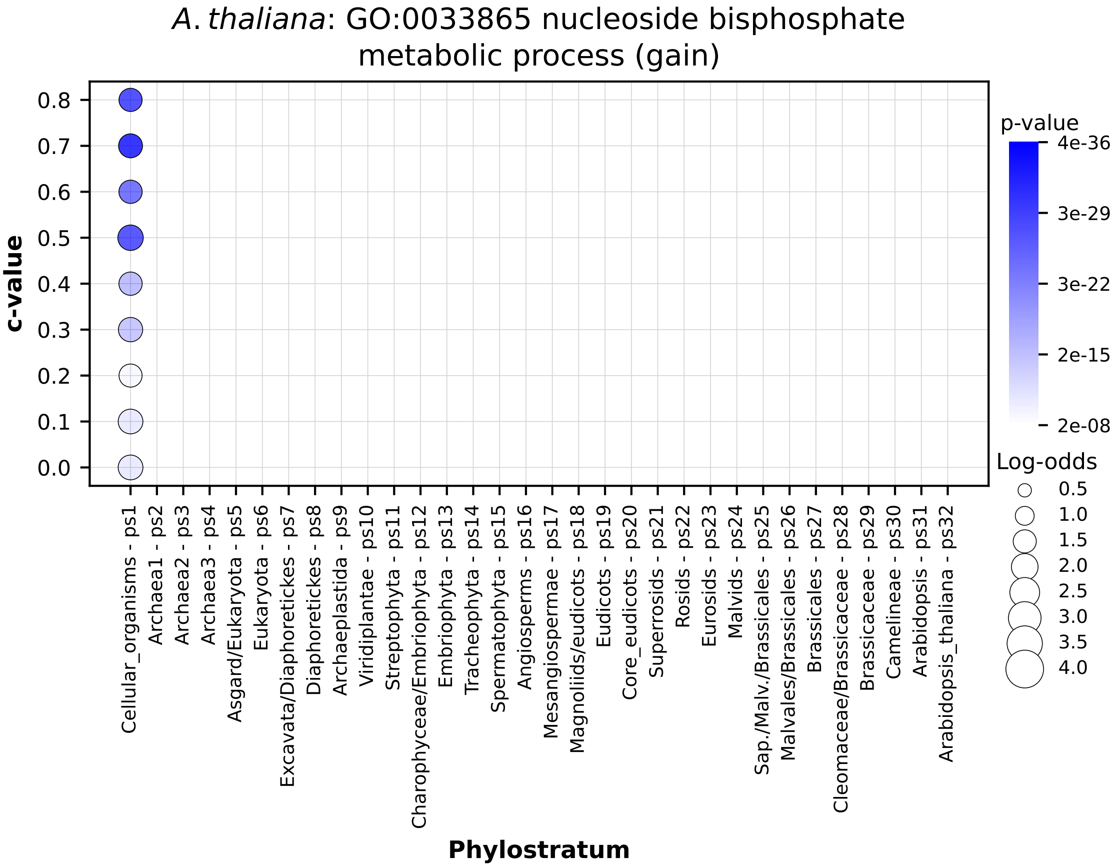

A. thaliana: GO:0033865 nucleoside bisphosphate metabolic process (gain)
c-value
0.0
0.1
0.2
0.3
0.4
0.5
0.6
0.7
0.8
Phylostratum
Cellular_organisms - ps1
Archaea1 - ps2
Archaea2 - ps3
Archaea3 - ps4
Asgard/Eukaryota - ps5
Eukaryota - ps6
Excavata/Diaphoretickes - ps7
Diaphoretickes - ps8
Archaeplastida - ps9
Viridiplantae - ps10
Streptophyta - ps11
Charophyceae/Embriophyta - ps12
Embriophyta - ps13
Tracheophyta - ps14
Spermatophyta - ps15
Angiosperms - ps16
Mesangiospermae - ps17
Magnoliids/eudicots - ps18
Eudicots - ps19
Core_eudicots - ps20
Superrosids - ps21
Rosids - ps22
Eurosids - ps23
Malvids - ps24
Sap./Malv./Brassicales - ps25
Malvales/Brassicales - ps26
Brassicales - ps27
Cleomaceae/Brassicaceae - ps28
Brassicaceae - ps29
Camelineae - ps30
Arabidopsis - ps31
Arabidopsis_thaliana - ps32
p-value
4e-36
3e-29
3e-22
2e-15
2e-08
Log-odds
0.5
1.0
1.5
2.0
2.5
3.0
3.5
4.0

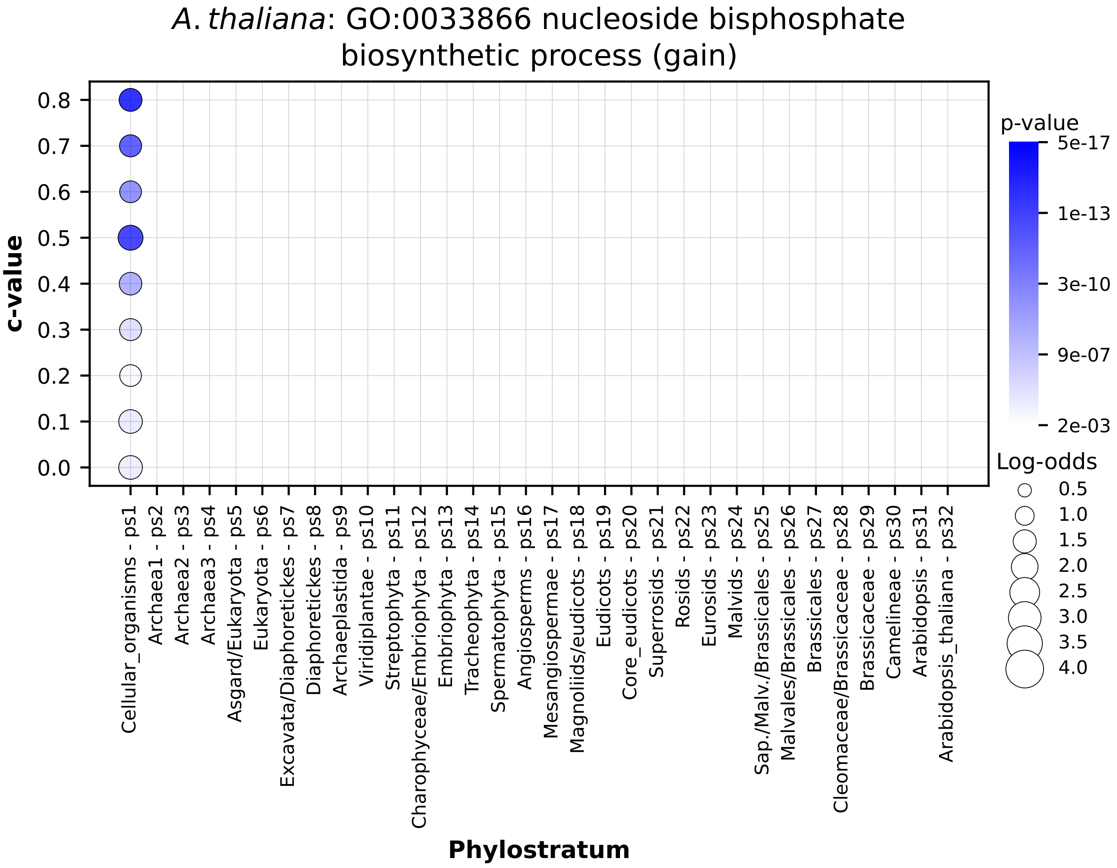

A. thaliana: GO:0033866 nucleoside bisphosphate biosynthetic process (gain)
c-value
0.0
0.1
0.2
0.3
0.4
0.5
0.6
0.7
0.8
Phylostratum
Cellular_organisms - ps1
Archaea1 - ps2
Archaea2 - ps3
Archaea3 - ps4
Asgard/Eukaryota - ps5
Eukaryota - ps6
Excavata/Diaphoretickes - ps7
Diaphoretickes - ps8
Archaeplastida - ps9
Viridiplantae - ps10
Streptophyta - ps11
Charophyceae/Embriophyta - ps12
Embriophyta - ps13
Tracheophyta - ps14
Spermatophyta - ps15
Angiosperms - ps16
Mesangiospermae - ps17
Magnoliids/eudicots - ps18
Eudicots - ps19
Core_eudicots - ps20
Superrosids - ps21
Rosids - ps22
Eurosids - ps23
Malvids - ps24
Sap./Malv./Brassicales - ps25
Malvales/Brassicales - ps26
Brassicales - ps27
Cleomaceae/Brassicaceae - ps28
Brassicaceae - ps29
Camelineae - ps30
Arabidopsis - ps31
Arabidopsis_thaliana - ps32
p-value
5e-17
1e-13
3e-10
9e-07
2e-03
Log-odds
0.5
1.0
1.5
2.0
2.5
3.0
3.5
4.0

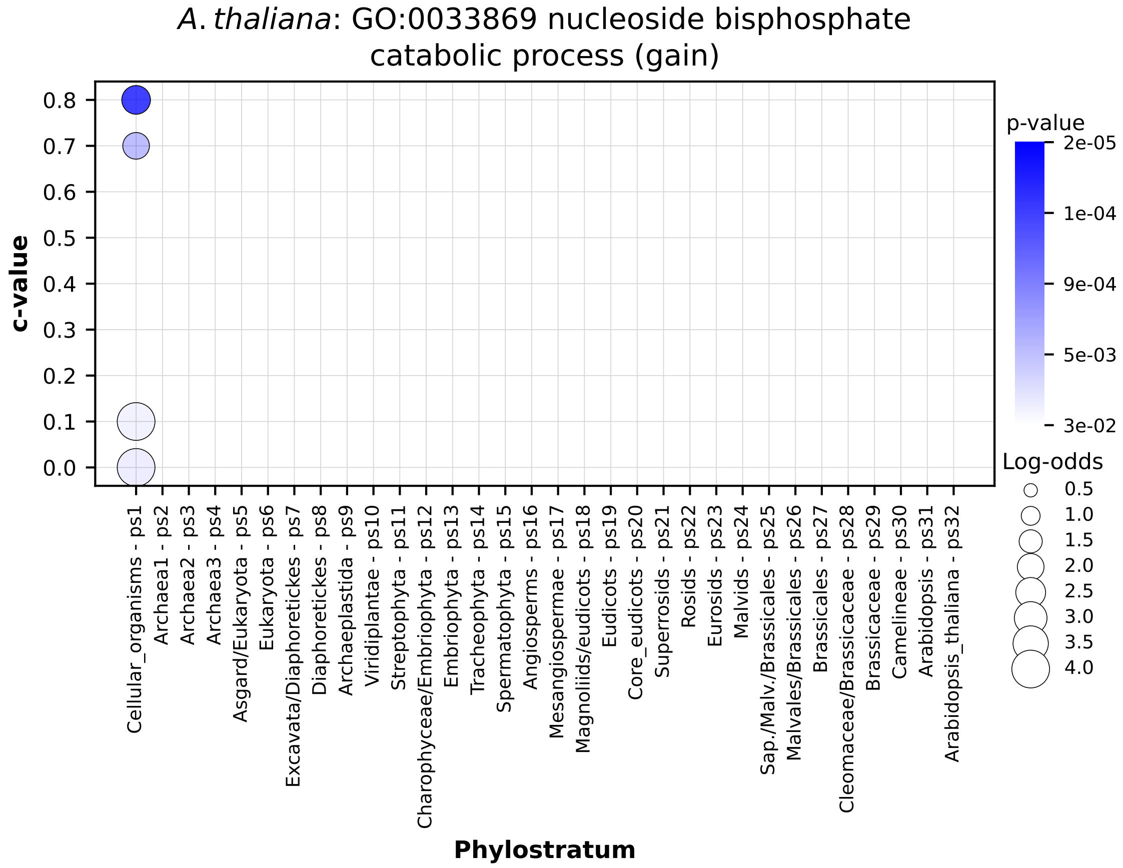

A. thaliana: GO:0033869 nucleoside bisphosphate catabolic process (gain)
c-value
0.8
0.7
0.6
0.5
0.4
0.3
0.2
0.1
0.0
p-value
2e-05
1e-04
9e-04
5e-03
3e-02
Log-odds
0.5
1.0
1.5
2.0
2.5
3.0
3.5
4.0
Cellular_organisms - ps1
Archaea1 - ps2
Archaea2 - ps3
Archaea3 - ps4
Asgard/Eukaryota - ps5
Eukaryota - ps6
Excavata/Diaphoretickes - ps7
Diaphoretickes - ps8
Archaeplastida - ps9
Viridiplantae - ps10
Streptophyta - ps11
Charophyceae/Embriophyta - ps12
Embriophyta - ps13
Tracheophyta - ps14
Spermatophyta - ps15
Angiosperms - ps16
Mesangiospermae - ps17
Magnoliids/eudicots - ps18
Eudicots - ps19
Core_eudicots - ps20
Superrosids - ps21
Rosids - ps22
Eurosids - ps23
Malvids - ps24
Sap./Malv./Brassicales - ps25
Malvales/Brassicales - ps26
Brassicales - ps27
Cleomaceae/Brassicaceae - ps28
Brassicaceae - ps29
Camelineae - ps30
Arabidopsis - ps31
Arabidopsis_thaliana - ps32
Phylostratum

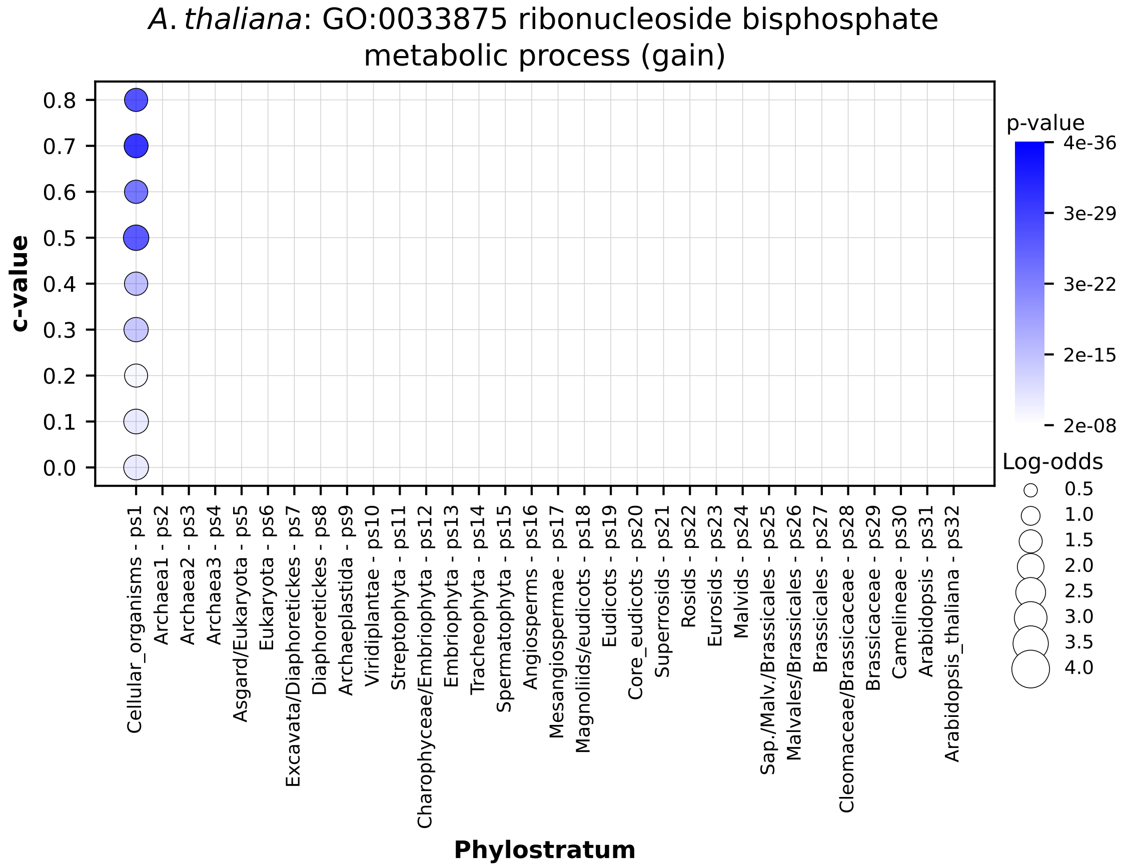

A. thaliana: GO:0033875 ribonucleoside bisphosphate metabolic process (gain)
c-value
0.8
0.7
0.6
0.5
0.4
0.3
0.2
0.1
0.0
p-value
4e-36
3e-29
3e-22
2e-15
2e-08
Log-odds
0.5
1.0
1.5
2.0
2.5
3.0
3.5
4.0
Cellular_organisms - ps1
Archaea1 - ps2
Archaea2 - ps3
Archaea3 - ps4
Asgard/Eukaryota - ps5
Eukaryota - ps6
Excavata/Diaphoretickes - ps7
Diaphoretickes - ps8
Archaeplastida - ps9
Viridiplantae - ps10
Streptophyta - ps11
Charophyceae/Embriophyta - ps12
Embriophyta - ps13
Tracheophyta - ps14
Spermatophyta - ps15
Angiosperms - ps16
Mesangiospermae - ps17
Magnoliids/eudicots - ps18
Eudicots - ps19
Core_eudicots - ps20
Superrosids - ps21
Rosids - ps22
Eurosids - ps23
Malvids - ps24
Sap./Malv./Brassicales - ps25
Malvales/Brassicales - ps26
Brassicales - ps27
Cleomaceae/Brassicaceae - ps28
Brassicaceae - ps29
Camelineae - ps30
Arabidopsis - ps31
Arabidopsis_thaliana - ps32
Phylostratum

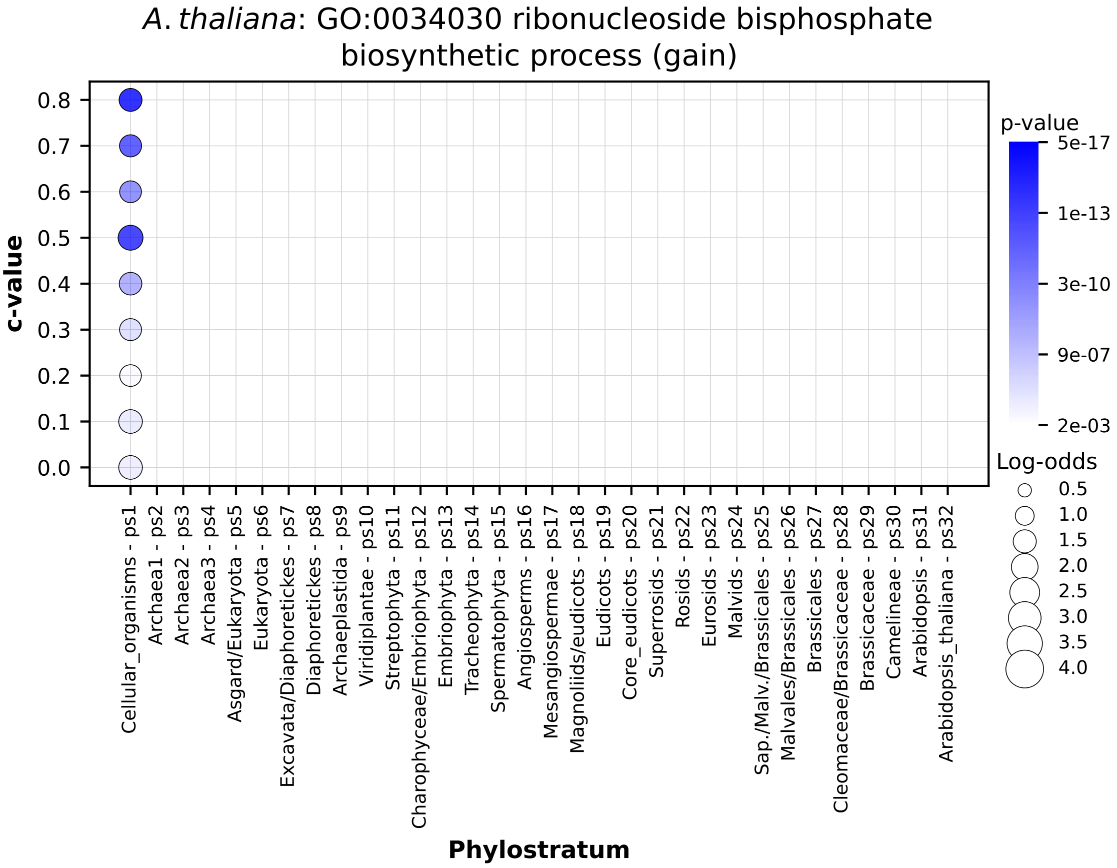
A. thaliana: GO:0034030 ribonucleoside bisphosphate biosynthetic process (gain)
c-value
0.8
0.7
0.6
0.5
0.4
0.3
0.2
0.1
0.0
Phylostratum
Cellular_organisms - ps1
Archaea1 - ps2
Archaea2 - ps3
Archaea3 - ps4
Asgard/Eukaryota - ps5
Eukaryota - ps6
Excavata/Diaphoretickes - ps7
Diaphoretickes - ps8
Archaeplastida - ps9
Viridiplantae - ps10
Streptophyta - ps11
Charophyceae/Embriophyta - ps12
Embriophyta - ps13
Tracheophyta - ps14
Spermatophyta - ps15
Angiosperms - ps16
Mesangiospermae - ps17
Magnoliids/eudicots - ps18
Eudicots - ps19
Core_eudicots - ps20
Superrosids - ps21
Rosids - ps22
Eurosids - ps23
Malvids - ps24
Sap./Malv./Brassicales - ps25
Malvales/Brassicales - ps26
Brassicales - ps27
Cleomaceae/Brassicaceae - ps28
Brassicaceae - ps29
Camelineae - ps30
Arabidopsis - ps31
Arabidopsis_thaliana - ps32
p-value
5e-17
1e-13
3e-10
9e-07
2e-03
Log-odds
0.5
1.0
1.5
2.0
2.5
3.0
3.5
4.0

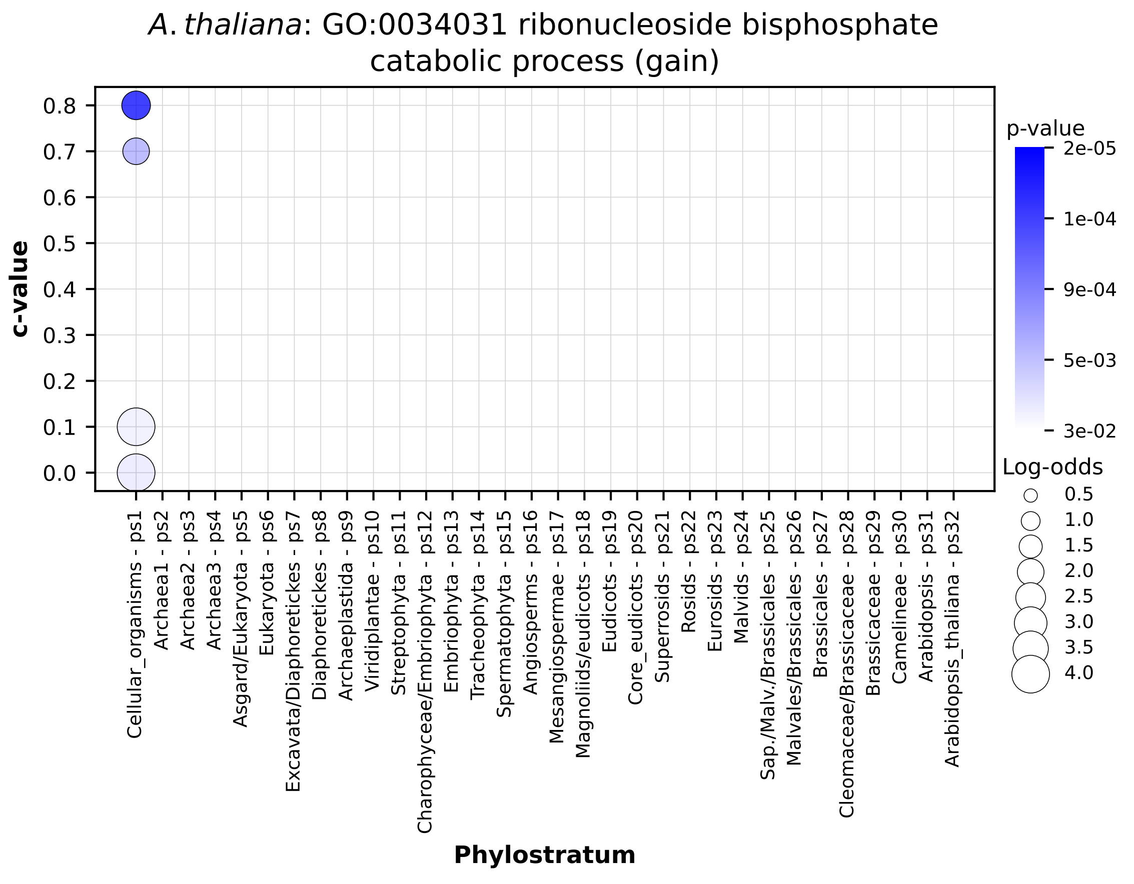

A. thaliana: GO:0034031 ribonucleoside bisphosphate catabolic process (gain)
c-value
0.8
0.7
0.6
0.5
0.4
0.3
0.2
0.1
0.0
p-value
2e-05
1e-04
9e-04
5e-03
3e-02
Log-odds
0.5
1.0
1.5
2.0
2.5
3.0
3.5
4.0
Cellular_organisms - ps1
Archaea1 - ps2
Archaea2 - ps3
Archaea3 - ps4
Asgard/Eukaryota - ps5
Eukaryota - ps6
Excavata/Diaphoretickes - ps7
Diaphoretickes - ps8
Archaeplastida - ps9
Viridiplantae - ps10
Streptophyta - ps11
Charophyceae/Embriophyta - ps12
Embriophyta - ps13
Tracheophyta - ps14
Spermatophyta - ps15
Angiosperms - ps16
Mesangiospermae - ps17
Magnoliids/eudicots - ps18
Eudicots - ps19
Core_eudicots - ps20
Superrosids - ps21
Rosids - ps22
Eurosids - ps23
Malvids - ps24
Sap./Malv./Brassicales - ps25
Malvales/Brassicales - ps26
Brassicales - ps27
Cleomaceae/Brassicaceae - ps28
Brassicaceae - ps29
Camelineae - ps30
Arabidopsis - ps31
Arabidopsis_thaliana - ps32
Phylostratum

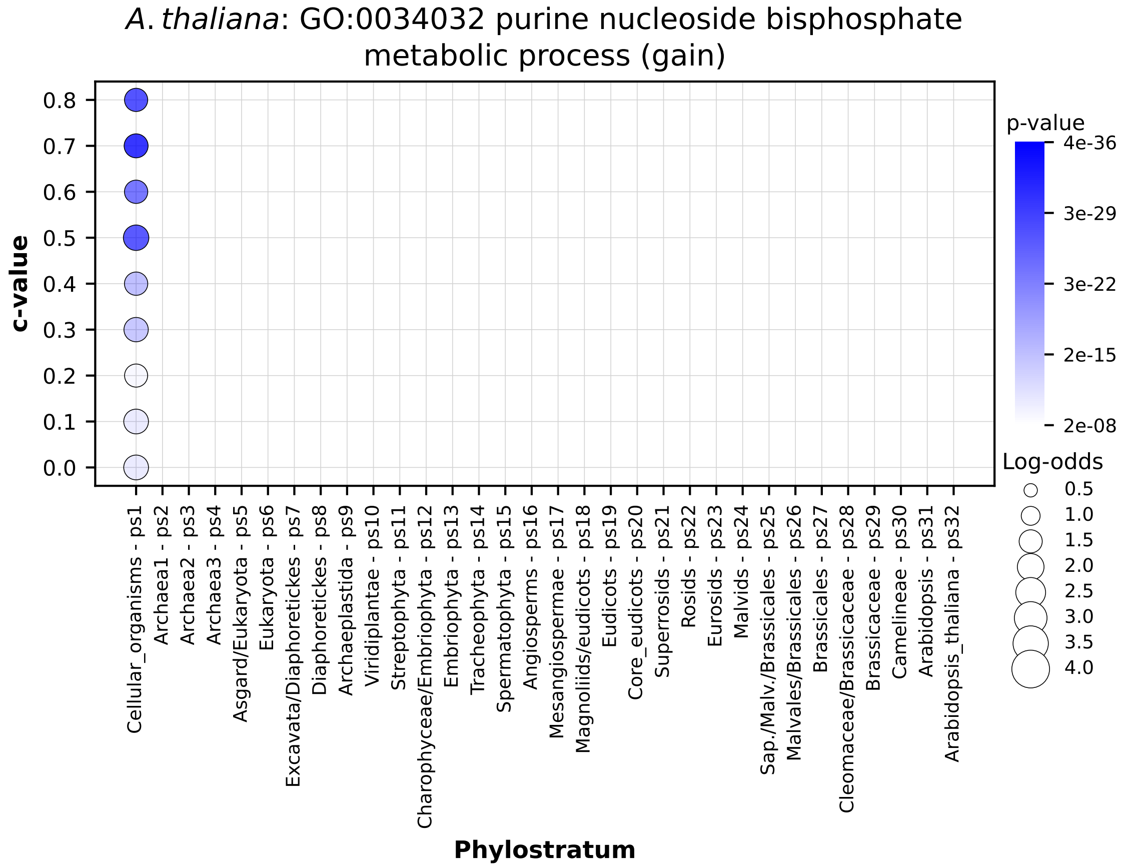
A. thaliana: GO:0034032 purine nucleoside bisphosphate metabolic process (gain)
c-value
0.0
0.1
0.2
0.3
0.4
0.5
0.6
0.7
0.8
Phylostratum
Cellular_organisms - ps1
Archaea1 - ps2
Archaea2 - ps3
Archaea3 - ps4
Asgard/Eukaryota - ps5
Eukaryota - ps6
Excavata/Diaphoretickes - ps7
Diaphoretickes - ps8
Archaeplastida - ps9
Viridiplantae - ps10
Streptophyta - ps11
Charophyceae/Embriophyta - ps12
Embriophyta - ps13
Tracheophyta - ps14
Spermatophyta - ps15
Angiosperms - ps16
Mesangiospermae - ps17
Magnoliids/eudicots - ps18
Eudicots - ps19
Core_eudicots - ps20
Superrosids - ps21
Rosids - ps22
Eurosids - ps23
Malvids - ps24
Sap./Malv./Brassicales - ps25
Malvales/Brassicales - ps26
Brassicales - ps27
Cleomaceae/Brassicaceae - ps28
Brassicaceae - ps29
Camelineae - ps30
Arabidopsis - ps31
Arabidopsis_thaliana - ps32
p-value
4e-36
3e-29
3e-22
2e-15
2e-08
Log-odds
0.5
1.0
1.5
2.0
2.5
3.0
3.5
4.0

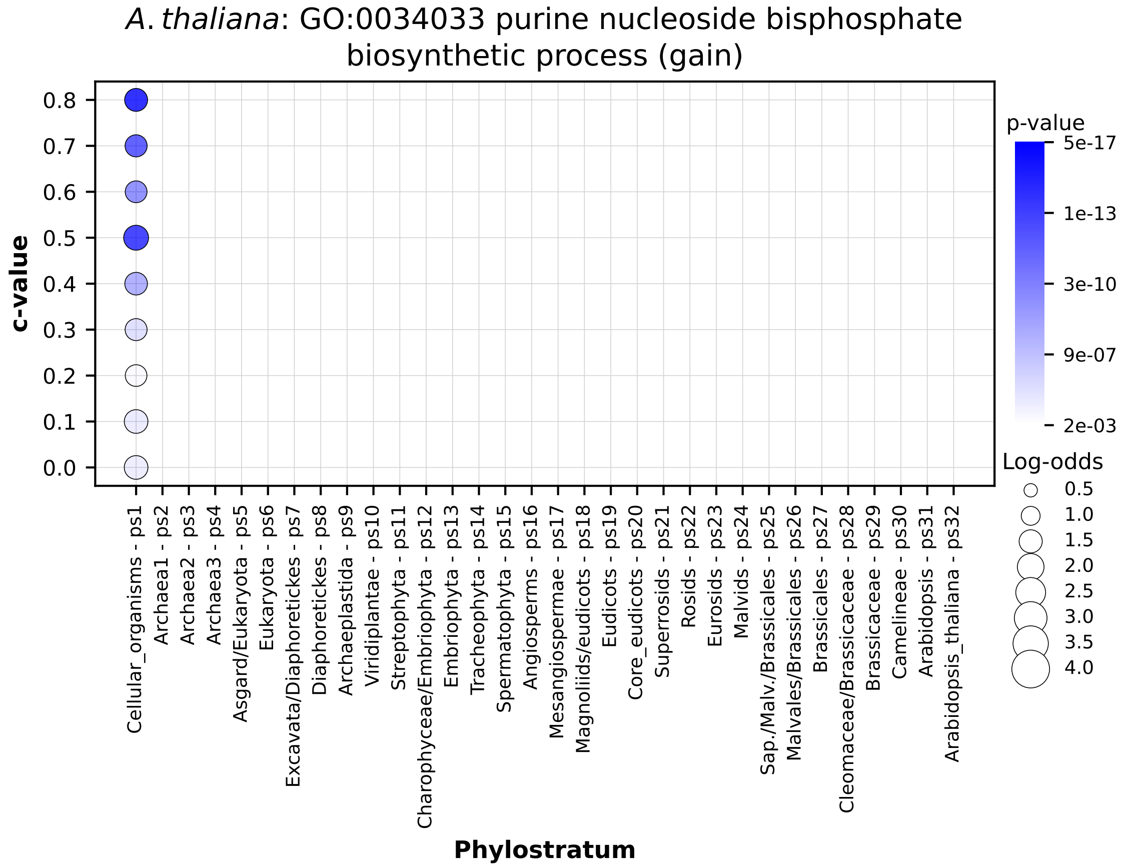

A. thaliana: GO:0034033 purine nucleoside bisphosphate biosynthetic process (gain)
c-value
0.8
0.7
0.6
0.5
0.4
0.3
0.2
0.1
0.0
p-value
5e-17
1e-13
3e-10
9e-07
2e-03
Log-odds
0.5
1.0
1.5
2.0
2.5
3.0
3.5
4.0
Cellular_organisms - ps1
Archaea1 - ps2
Archaea2 - ps3
Archaea3 - ps4
Asgard/Eukaryota - ps5
Eukaryota - ps6
Excavata/Diaphoretickes - ps7
Diaphoretickes - ps8
Archaeplastida - ps9
Viridiplantae - ps10
Streptophyta - ps11
Charophyceae/Embriophyta - ps12
Embriophyta - ps13
Tracheophyta - ps14
Spermatophyta - ps15
Angiosperms - ps16
Mesangiospermae - ps17
Magnoliids/eudicots - ps18
Eudicots - ps19
Core_eudicots - ps20
Superrosids - ps21
Rosids - ps22
Eurosids - ps23
Malvids - ps24
Sap./Malv./Brassicales - ps25
Malvales/Brassicales - ps26
Brassicales - ps27
Cleomaceae/Brassicaceae - ps28
Brassicaceae - ps29
Camelineae - ps30
Arabidopsis - ps31
Arabidopsis_thaliana - ps32
Phylostratum

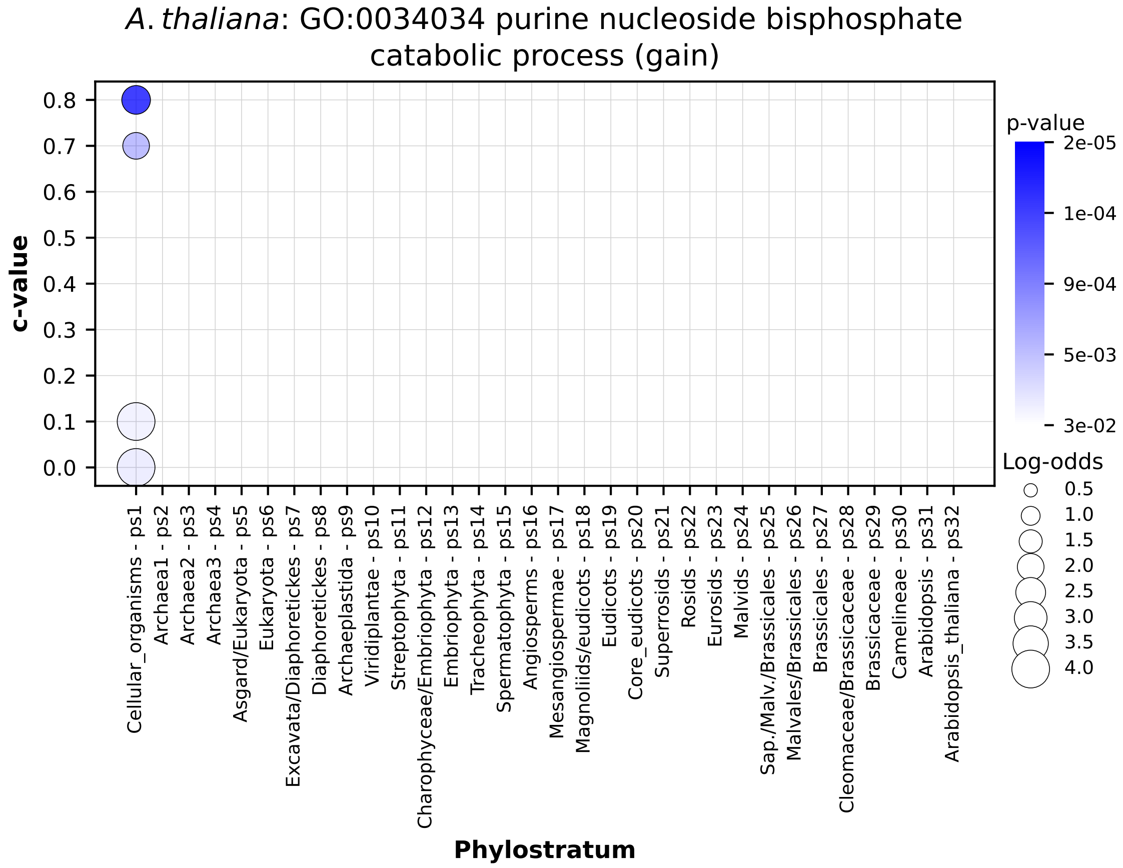

A. thaliana: GO:0034034 purine nucleoside bisphosphate catabolic process (gain)
c-value
0.8
0.7
0.6
0.5
0.4
0.3
0.2
0.1
0.0
p-value
2e-05
1e-04
9e-04
5e-03
3e-02
Log-odds
0.5
1.0
1.5
2.0
2.5
3.0
3.5
4.0
Cellular_organisms - ps1
Archaea1 - ps2
Archaea2 - ps3
Archaea3 - ps4
Asgard/Eukaryota - ps5
Eukaryota - ps6
Excavata/Diaphoretickes - ps7
Diaphoretickes - ps8
Archaeplastida - ps9
Viridiplantae - ps10
Streptophyta - ps11
Charophyceae/Embriophyta - ps12
Embriophyta - ps13
Tracheophyta - ps14
Spermatophyta - ps15
Angiosperms - ps16
Mesangiospermae - ps17
Magnoliids/eudicots - ps18
Eudicots - ps19
Core_eudicots - ps20
Superrosids - ps21
Rosids - ps22
Eurosids - ps23
Malvids - ps24
Sap./Malv./Brassicales - ps25
Malvales/Brassicales - ps26
Brassicales - ps27
Cleomaceae/Brassicaceae - ps28
Brassicaceae - ps29
Camelineae - ps30
Arabidopsis - ps31
Arabidopsis_thaliana - ps32
Phylostratum

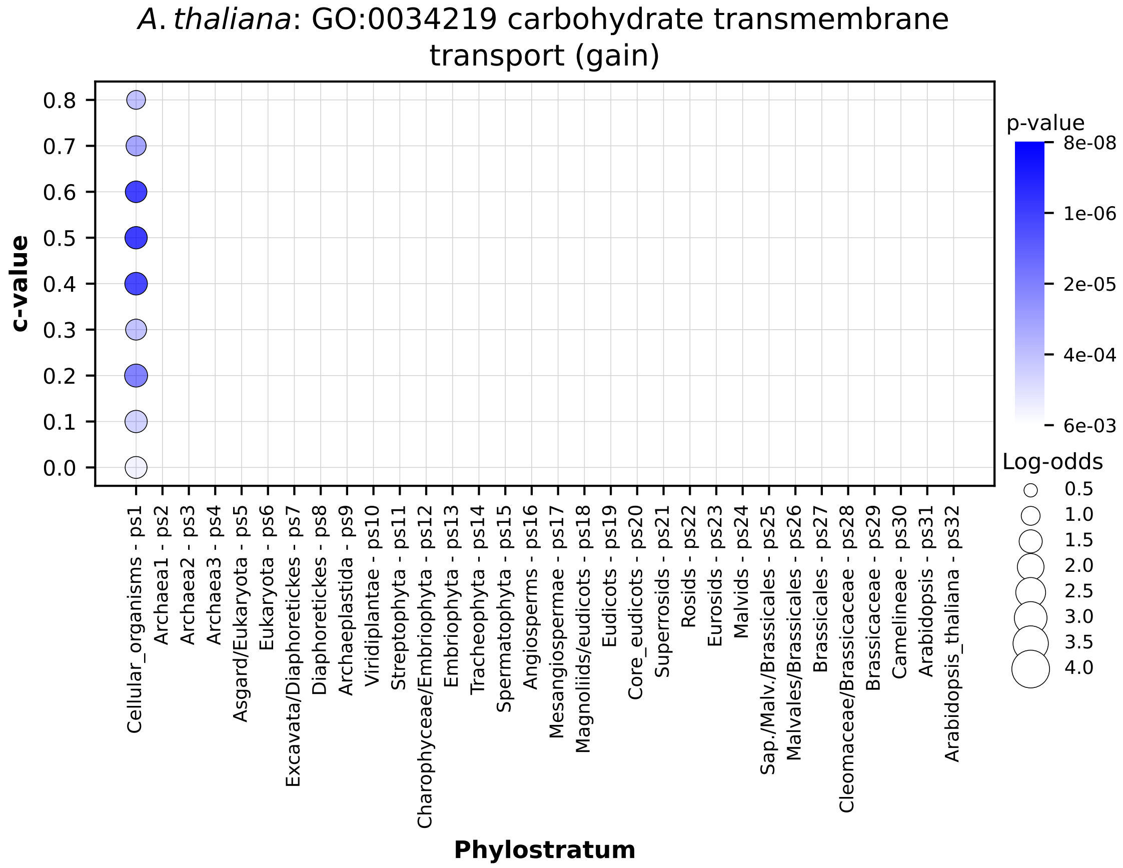

A. thaliana: GO:0034219 carbohydrate transmembrane transport (gain)
c-value
0.8
0.7
0.6
0.5
0.4
0.3
0.2
0.1
0.0
p-value
8e-08
1e-06
2e-05
4e-04
6e-03
Log-odds
0.5
1.0
1.5
2.0
2.5
3.0
3.5
4.0
Cellular_organisms - ps1
Archaea1 - ps2
Archaea2 - ps3
Archaea3 - ps4
Asgard/Eukaryota - ps5
Eukaryota - ps6
Excavata/Diaphoretickes - ps7
Diaphoretickes - ps8
Archaeplastida - ps9
Viridiplantae - ps10
Streptophyta - ps11
Charophyceae/Embriophyta - ps12
Embriophyta - ps13
Tracheophyta - ps14
Spermatophyta - ps15
Angiosperms - ps16
Mesangiospermae - ps17
Magnoliids/eudicots - ps18
Eudicots - ps19
Core_eudicots - ps20
Superrosids - ps21
Rosids - ps22
Eurosids - ps23
Malvids - ps24
Sap./Malv./Brassicales - ps25
Malvales/Brassicales - ps26
Brassicales - ps27
Cleomaceae/Brassicaceae - ps28
Brassicaceae - ps29
Camelineae - ps30
Arabidopsis - ps31
Arabidopsis_thaliana - ps32
Phylostratum

A. thaliana: GO:0034227 tRNA thio-modification (gain)

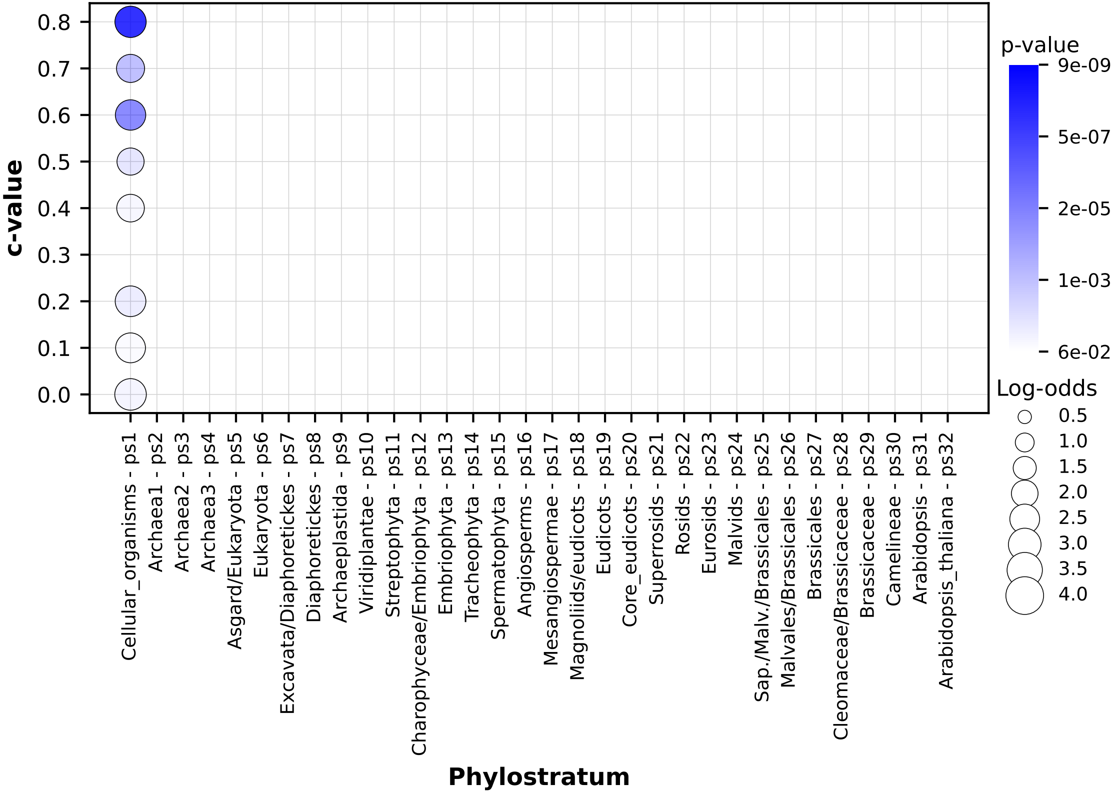

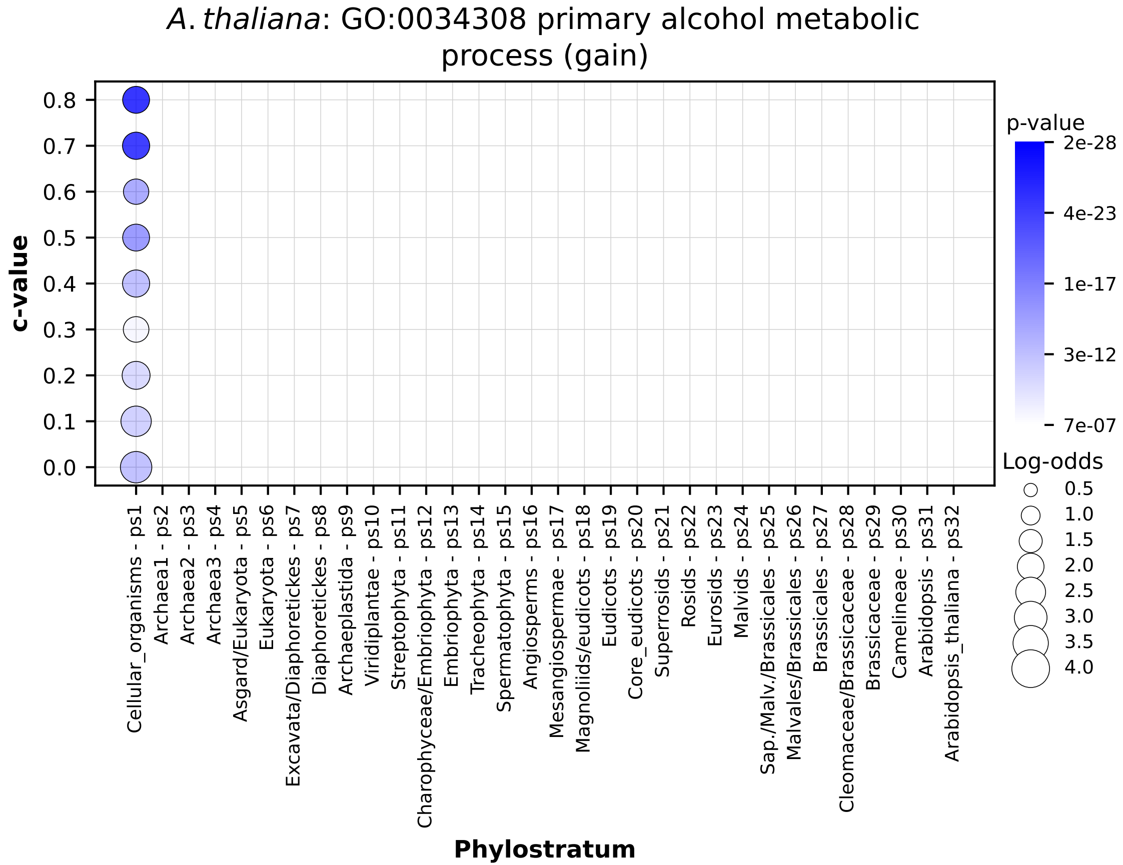

A. thaliana: GO:0034308 primary alcohol metabolic process (gain)
c-value
0.8
0.7
0.6
0.5
0.4
0.3
0.2
0.1
0.0
p-value
2e-28
4e-23
1e-17
3e-12
7e-07
Log-odds
0.5
1.0
1.5
2.0
2.5
3.0
3.5
4.0
Cellular_organisms - ps1
Archaea1 - ps2
Archaea2 - ps3
Archaea3 - ps4
Asgard/Eukaryota - ps5
Eukaryota - ps6
Excavata/Diaphoretickes - ps7
Diaphoretickes - ps8
Archaeplastida - ps9
Viridiplantae - ps10
Streptophyta - ps11
Charophyceae/Embriophyta - ps12
Embriophyta - ps13
Tracheophyta - ps14
Spermatophyta - ps15
Angiosperms - ps16
Mesangiospermae - ps17
Magnoliids/eudicots - ps18
Eudicots - ps19
Core_eudicots - ps20
Superrosids - ps21
Rosids - ps22
Eurosids - ps23
Malvids - ps24
Sap./Malv./Brassicales - ps25
Malvales/Brassicales - ps26
Brassicales - ps27
Cleomaceae/Brassicaceae - ps28
Brassicaceae - ps29
Camelineae - ps30
Arabidopsis - ps31
Arabidopsis_thaliana - ps32
Phylostratum

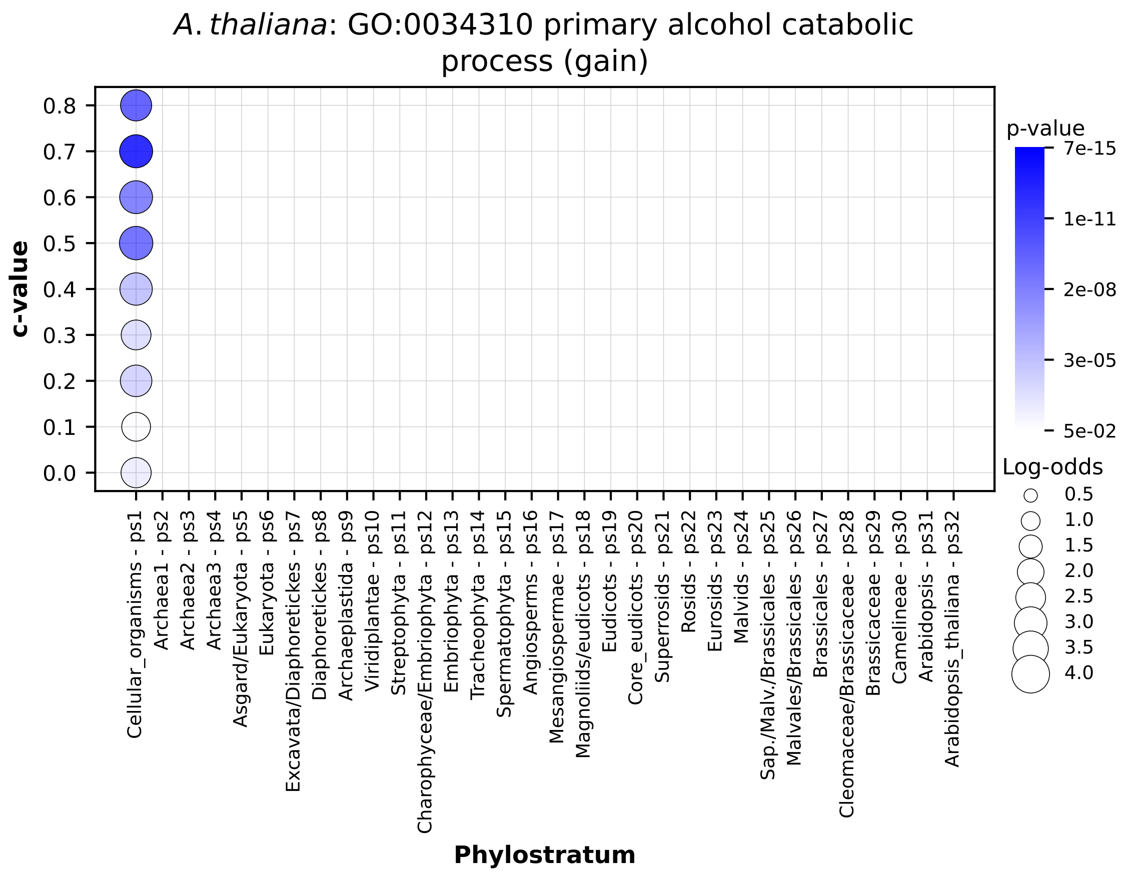

A. thaliana: GO:0034310 primary alcohol catabolic process (gain)
c-value
0.8
0.7
0.6
0.5
0.4
0.3
0.2
0.1
0.0
p-value
7e-15
1e-11
2e-08
3e-05
5e-02
Log-odds
0.5
1.0
1.5
2.0
2.5
3.0
3.5
4.0
Cellular_organisms - ps1
Archaea1 - ps2
Archaea2 - ps3
Archaea3 - ps4
Asgard/Eukaryota - ps5
Eukaryota - ps6
Excavata/Diaphoretickes - ps7
Diaphoretickes - ps8
Archaeplastida - ps9
Viridiplantae - ps10
Streptophyta - ps11
Charophyceae/Embriophyta - ps12
Embriophyta - ps13
Tracheophyta - ps14
Spermatophyta - ps15
Angiosperms - ps16
Mesangiospermae - ps17
Magnoliids/eudicots - ps18
Eudicots - ps19
Core_eudicots - ps20
Superrosids - ps21
Rosids - ps22
Eurosids - ps23
Malvids - ps24
Sap./Malv./Brassicales - ps25
Malvales/Brassicales - ps26
Brassicales - ps27
Cleomaceae/Brassicaceae - ps28
Brassicaceae - ps29
Camelineae - ps30
Arabidopsis - ps31
Arabidopsis_thaliana - ps32
Phylostratum

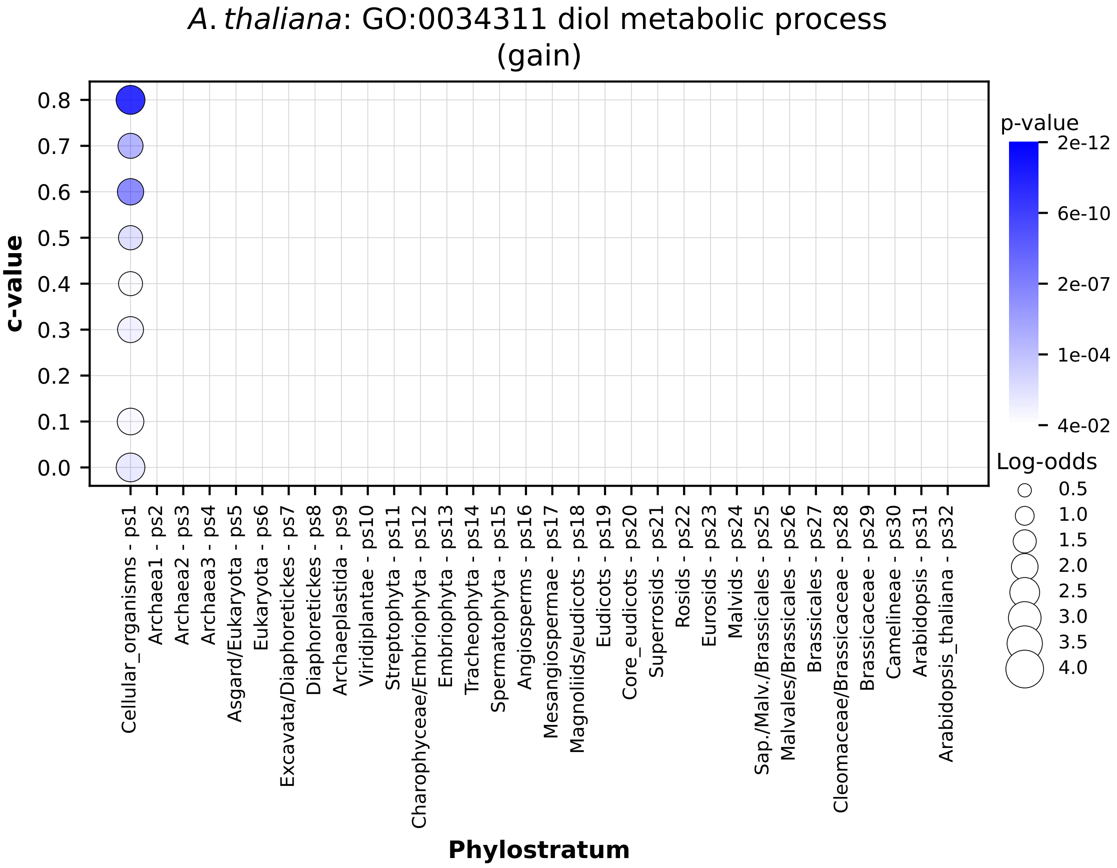
A. thaliana: GO:0034311 diol metabolic process
(gain)
c-value
0.8
0.7
0.6
0.5
0.4
0.3
0.2
0.1
0.0
p-value
2e-12
6e-10
2e-07
1e-04
4e-02
Log-odds
0.5
1.0
1.5
2.0
2.5
3.0
3.5
4.0
Cellular_organisms - ps1
Archaea1 - ps2
Archaea2 - ps3
Archaea3 - ps4
Asgard/Eukaryota - ps5
Eukaryota - ps6
Excavata/Diaphoretickes - ps7
Diaphoretickes - ps8
Archaeplastida - ps9
Viridiplantae - ps10
Streptophyta - ps11
Charophyceae/Embriophyta - ps12
Embriophyta - ps13
Tracheophyta - ps14
Spermatophyta - ps15
Angiosperms - ps16
Mesangiospermae - ps17
Magnoliids/eudicots - ps18
Eudicots - ps19
Core_eudicots - ps20
Superrosids - ps21
Rosids - ps22
Eurosids - ps23
Malvids - ps24
Sap./Malv./Brassicales - ps25
Malvales/Brassicales - ps26
Brassicales - ps27
Cleomaceae/Brassicaceae - ps28
Brassicaceae - ps29
Camelineae - ps30
Arabidopsis - ps31
Arabidopsis_thaliana - ps32
Phylostratum

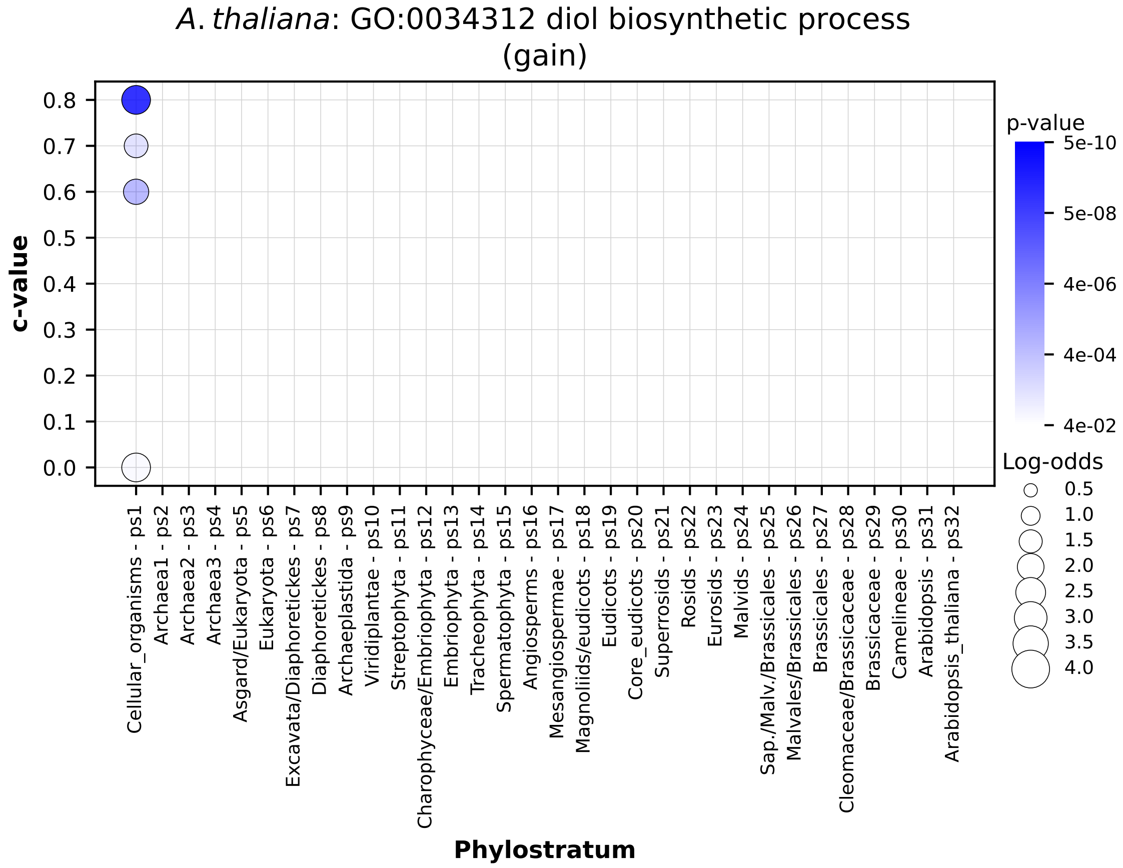

A. thaliana: GO:0034312 diol biosynthetic process
(gain)
c-value
0.8
0.7
0.6
0.5
0.4
0.3
0.2
0.1
0.0
p-value
5e-10
5e-08
4e-06
4e-04
4e-02
Log-odds
0.5
1.0
1.5
2.0
2.5
3.0
3.5
4.0
Cellular_organisms - ps1
Archaea1 - ps2
Archaea2 - ps3
Archaea3 - ps4
Asgard/Eukaryota - ps5
Eukaryota - ps6
Excavata/Diaphoretickes - ps7
Diaphoretickes - ps8
Archaeplastida - ps9
Viridiplantae - ps10
Streptophyta - ps11
Charophyceae/Embriophyta - ps12
Embriophyta - ps13
Tracheophyta - ps14
Spermatophyta - ps15
Angiosperms - ps16
Mesangiospermae - ps17
Magnoliids/eudicots - ps18
Eudicots - ps19
Core_eudicots - ps20
Superrosids - ps21
Rosids - ps22
Eurosids - ps23
Malvids - ps24
Sap./Malv./Brassicales - ps25
Malvales/Brassicales - ps26
Brassicales - ps27
Cleomaceae/Brassicaceae - ps28
Brassicaceae - ps29
Camelineae - ps30
Arabidopsis - ps31
Arabidopsis_thaliana - ps32
Phylostratum

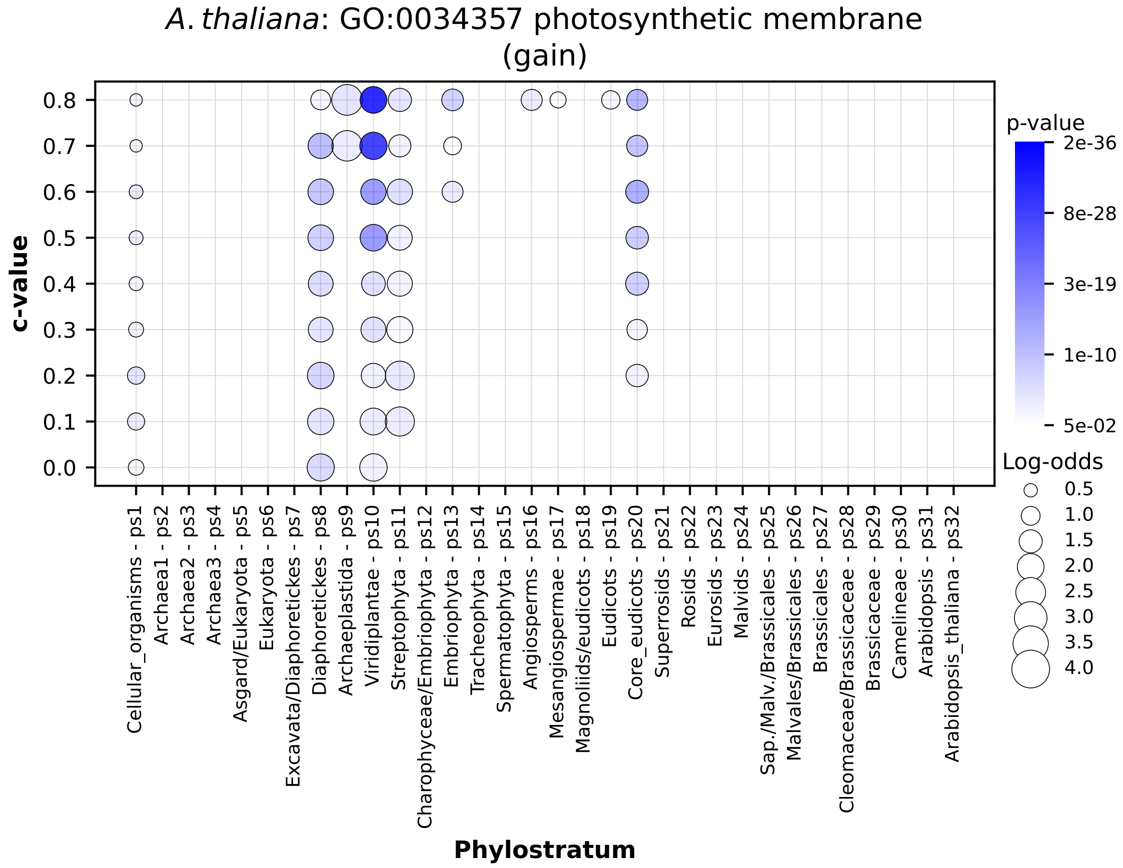

A. thaliana: GO:0034357 photosynthetic membrane
(gain)
c-value
0.8
0.7
0.6
0.5
0.4
0.3
0.2
0.1
0.0
Phylostratum
Cellular_organisms - ps1
Archaea1 - ps2
Archaea2 - ps3
Archaea3 - ps4
Asgard/Eukaryota - ps5
Eukaryota - ps6
Excavata/Diaphoretickes - ps7
Diaphoretickes - ps8
Archaeplastida - ps9
Viridiplantae - ps10
Streptophyta - ps11
Charophyceae/Embriophyta - ps12
Embriophyta - ps13
Tracheophyta - ps14
Spermatophyta - ps15
Angiosperms - ps16
Mesangiospermae - ps17
Magnoliids/eudicots - ps18
Eudicots - ps19
Core_eudicots - ps20
Superrosids - ps21
Rosids - ps22
Eurosids - ps23
Malvids - ps24
Sap./Malv./Brassicales - ps25
Malvales/Brassicales - ps26
Brassicales - ps27
Cleomaceae/Brassicaceae - ps28
Brassicaceae - ps29
Camelineae - ps30
Arabidopsis - ps31
Arabidopsis_thaliana - ps32
p-value
2e-36
8e-28
3e-19
1e-10
5e-02
Log-odds
0.5
1.0
1.5
2.0
2.5
3.0
3.5
4.0

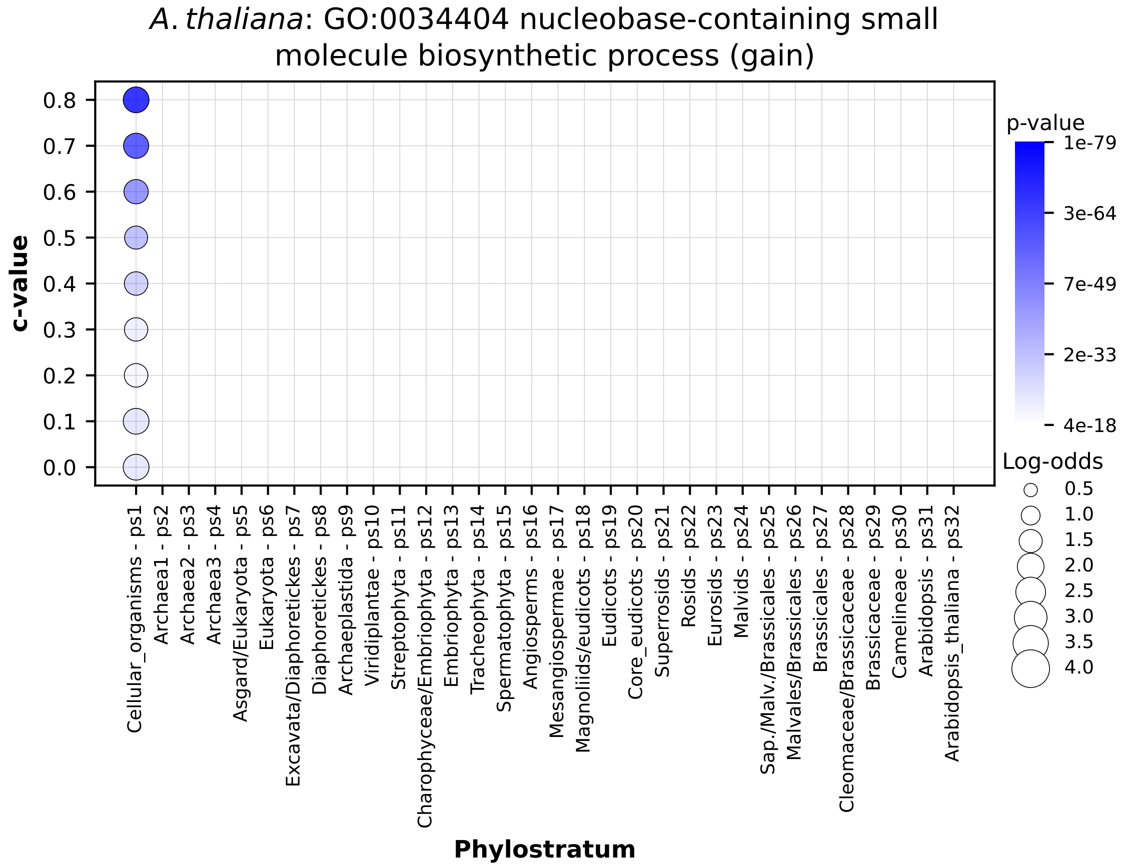
A. thaliana: GO:0034404 nucleobase-containing small molecule biosynthetic process (gain)
c-value
0.8
0.7
0.6
0.5
0.4
0.3
0.2
0.1
0.0
Phylostratum
Cellular_organisms - ps1
Archaea1 - ps2
Archaea2 - ps3
Archaea3 - ps4
Asgard/Eukaryota - ps5
Eukaryota - ps6
Excavata/Diaphoretickes - ps7
Diaphoretickes - ps8
Archaeplastida - ps9
Viridiplantae - ps10
Streptophyta - ps11
Charophyceae/Embriophyta - ps12
Embriophyta - ps13
Tracheophyta - ps14
Spermatophyta - ps15
Angiosperms - ps16
Mesangiospermae - ps17
Magnoliids/eudicots - ps18
Eudicots - ps19
Core_eudicots - ps20
Superrosids - ps21
Rosids - ps22
Eurosids - ps23
Malvids - ps24
Sap./Malv./Brassicales - ps25
Malvales/Brassicales - ps26
Brassicales - ps27
Cleomaceae/Brassicaceae - ps28
Brassicaceae - ps29
Camelineae - ps30
Arabidopsis - ps31
Arabidopsis_thaliana - ps32
p-value
1e-79
3e-64
7e-49
2e-33
4e-18
Log-odds
0.5
1.0
1.5
2.0
2.5
3.0
3.5
4.0

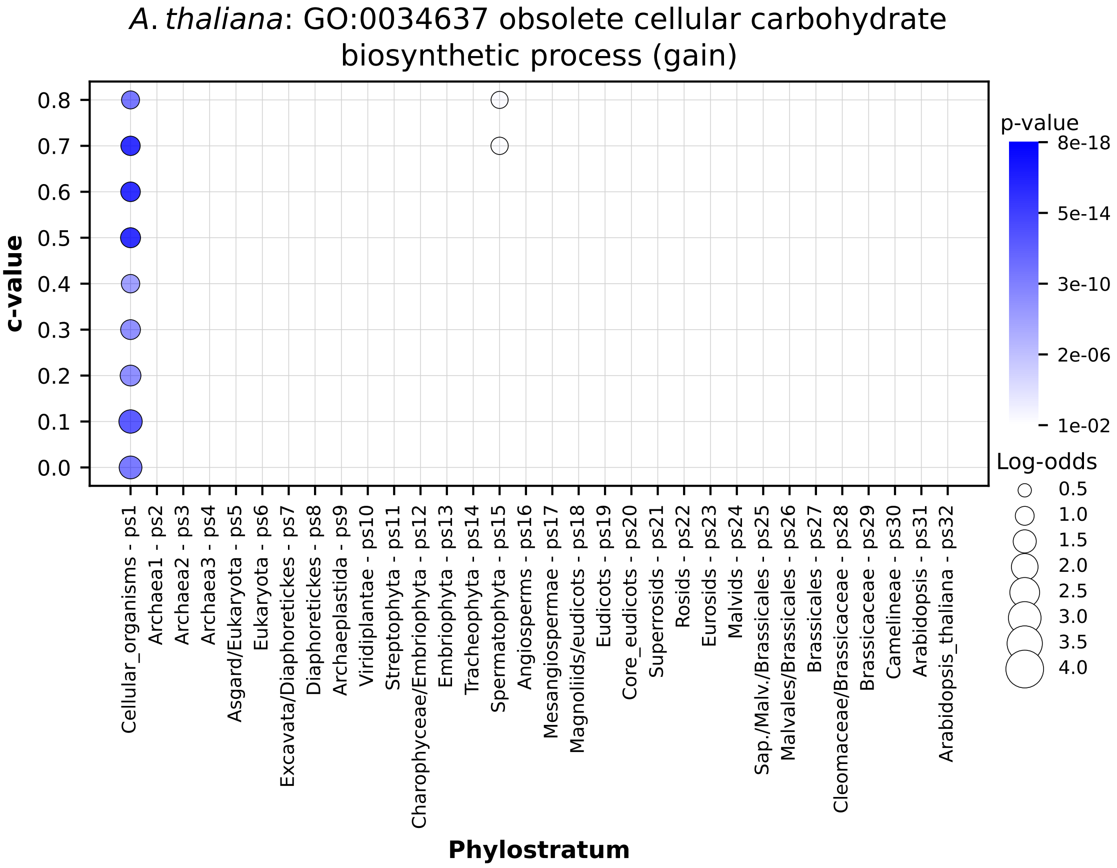
A. thaliana: GO:0034637 obsolete cellular carbohydrate biosynthetic process (gain)
c-value
0.8
0.7
0.6
0.5
0.4
0.3
0.2
0.1
0.0
Phylostratum
Cellular_organisms - ps1
Archaea1 - ps2
Archaea2 - ps3
Archaea3 - ps4
Asgard/Eukaryota - ps5
Eukaryota - ps6
Excavata/Diaphoretickes - ps7
Diaphoretickes - ps8
Archaeplastida - ps9
Viridiplantae - ps10
Streptophyta - ps11
Charophyceae/Embriophyta - ps12
Embriophyta - ps13
Tracheophyta - ps14
Spermatophyta - ps15
Angiosperms - ps16
Mesangiospermae - ps17
Magnoliids/eudicots - ps18
Eudicots - ps19
Core_eudicots - ps20
Superrosids - ps21
Rosids - ps22
Eurosids - ps23
Malvids - ps24
Sap./Malv./Brassicales - ps25
Malvales/Brassicales - ps26
Brassicales - ps27
Cleomaceae/Brassicaceae - ps28
Brassicaceae - ps29
Camelineae - ps30
Arabidopsis - ps31
Arabidopsis_thaliana - ps32
p-value
8e-18
5e-14
3e-10
2e-06
1e-02
Log-odds
0.5
1.0
1.5
2.0
2.5
3.0
3.5
4.0

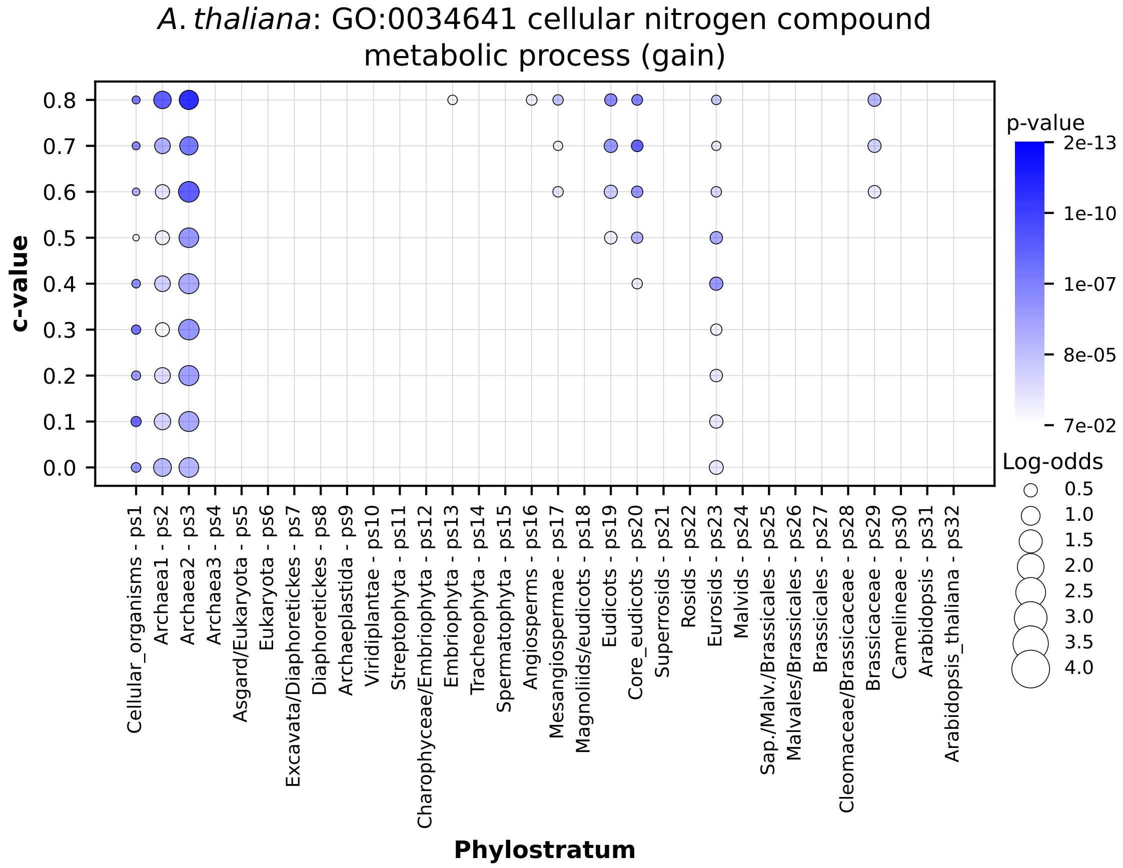

A. thaliana: GO:0034641 cellular nitrogen compound metabolic process (gain)
c-value
0.8
0.7
0.6
0.5
0.4
0.3
0.2
0.1
0.0
p-value
2e-13
1e-10
1e-07
8e-05
7e-02
Log-odds
0.5
1.0
1.5
2.0
2.5
3.0
3.5
4.0
Cellular_organisms - ps1
Archaea1 - ps2
Archaea2 - ps3
Archaea3 - ps4
Asgard/Eukaryota - ps5
Eukaryota - ps6
Excavata/Diaphoretickes - ps7
Diaphoretickes - ps8
Archaeplastida - ps9
Viridiplantae - ps10
Streptophyta - ps11
Charophyceae/Embriophyta - ps12
Embriophyta - ps13
Tracheophyta - ps14
Spermatophyta - ps15
Angiosperms - ps16
Mesangiospermae - ps17
Magnoliids/eudicots - ps18
Eudicots - ps19
Core_eudicots - ps20
Superrosids - ps21
Rosids - ps22
Eurosids - ps23
Malvids - ps24
Sap./Malv./Brassicales - ps25
Malvales/Brassicales - ps26
Brassicales - ps27
Cleomaceae/Brassicaceae - ps28
Brassicaceae - ps29
Camelineae - ps30
Arabidopsis - ps31
Arabidopsis_thaliana - ps32
Phylostratum

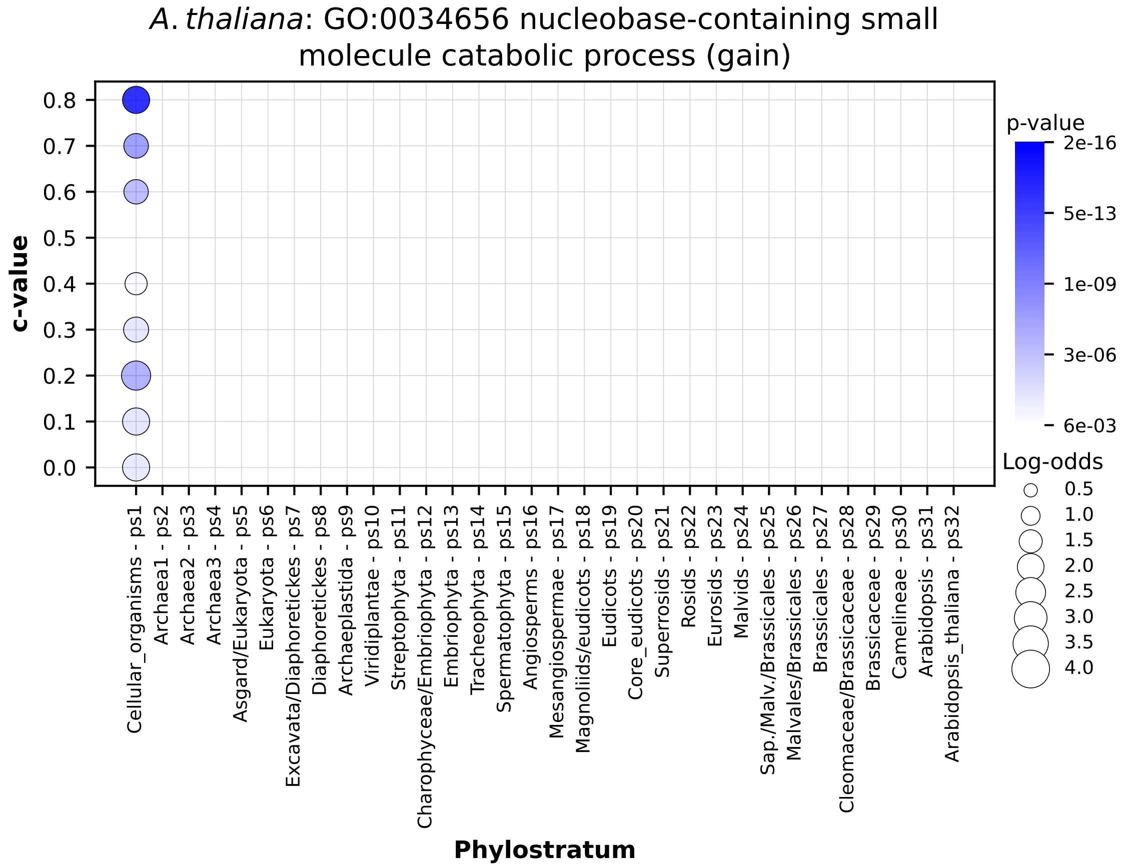

A. thaliana: GO:0034656 nucleobase-containing small molecule catabolic process (gain)
c-value
0.8
0.7
0.6
0.5
0.4
0.3
0.2
0.1
0.0
p-value
2e-16
5e-13
1e-09
3e-06
6e-03
Log-odds
0.5
1.0
1.5
2.0
2.5
3.0
3.5
4.0
Cellular_organisms - ps1
Archaea1 - ps2
Archaea2 - ps3
Archaea3 - ps4
Asgard/Eukaryota - ps5
Eukaryota - ps6
Excavata/Diaphoretickes - ps7
Diaphoretickes - ps8
Archaeplastida - ps9
Viridiplantae - ps10
Streptophyta - ps11
Charophyceae/Embriophyta - ps12
Embriophyta - ps13
Tracheophyta - ps14
Spermatophyta - ps15
Angiosperms - ps16
Mesangiospermae - ps17
Magnoliids/eudicots - ps18
Eudicots - ps19
Core_eudicots - ps20
Superrosids - ps21
Rosids - ps22
Eurosids - ps23
Malvids - ps24
Sap./Malv./Brassicales - ps25
Malvales/Brassicales - ps26
Brassicales - ps27
Cleomaceae/Brassicaceae - ps28
Brassicaceae - ps29
Camelineae - ps30
Arabidopsis - ps31
Arabidopsis_thaliana - ps32
Phylostratum

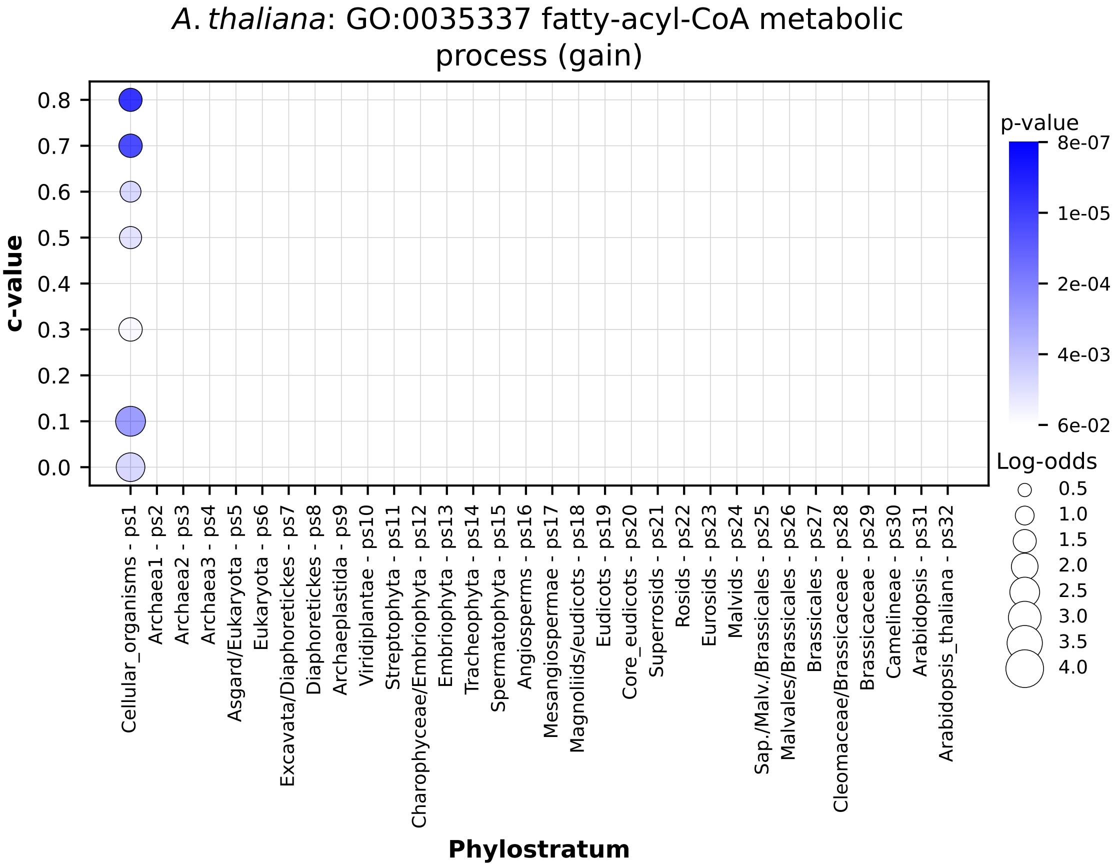

A. thaliana: GO:0035337 fatty-acyl-CoA metabolic process (gain)
c-value
0.8
0.7
0.6
0.5
0.4
0.3
0.2
0.1
0.0
p-value
8e-07
1e-05
2e-04
4e-03
6e-02
Log-odds
0.5
1.0
1.5
2.0
2.5
3.0
3.5
4.0
Cellular_organisms - ps1
Archaea1 - ps2
Archaea2 - ps3
Archaea3 - ps4
Asgard/Eukaryota - ps5
Eukaryota - ps6
Excavata/Diaphoretickes - ps7
Diaphoretickes - ps8
Archaeplastida - ps9
Viridiplantae - ps10
Streptophyta - ps11
Charophyceae/Embriophyta - ps12
Embriophyta - ps13
Tracheophyta - ps14
Spermatophyta - ps15
Angiosperms - ps16
Mesangiospermae - ps17
Magnoliids/eudicots - ps18
Eudicots - ps19
Core_eudicots - ps20
Superrosids - ps21
Rosids - ps22
Eurosids - ps23
Malvids - ps24
Sap./Malv./Brassicales - ps25
Malvales/Brassicales - ps26
Brassicales - ps27
Cleomaceae/Brassicaceae - ps28
Brassicaceae - ps29
Camelineae - ps30
Arabidopsis - ps31
Arabidopsis_thaliana - ps32
Phylostratum

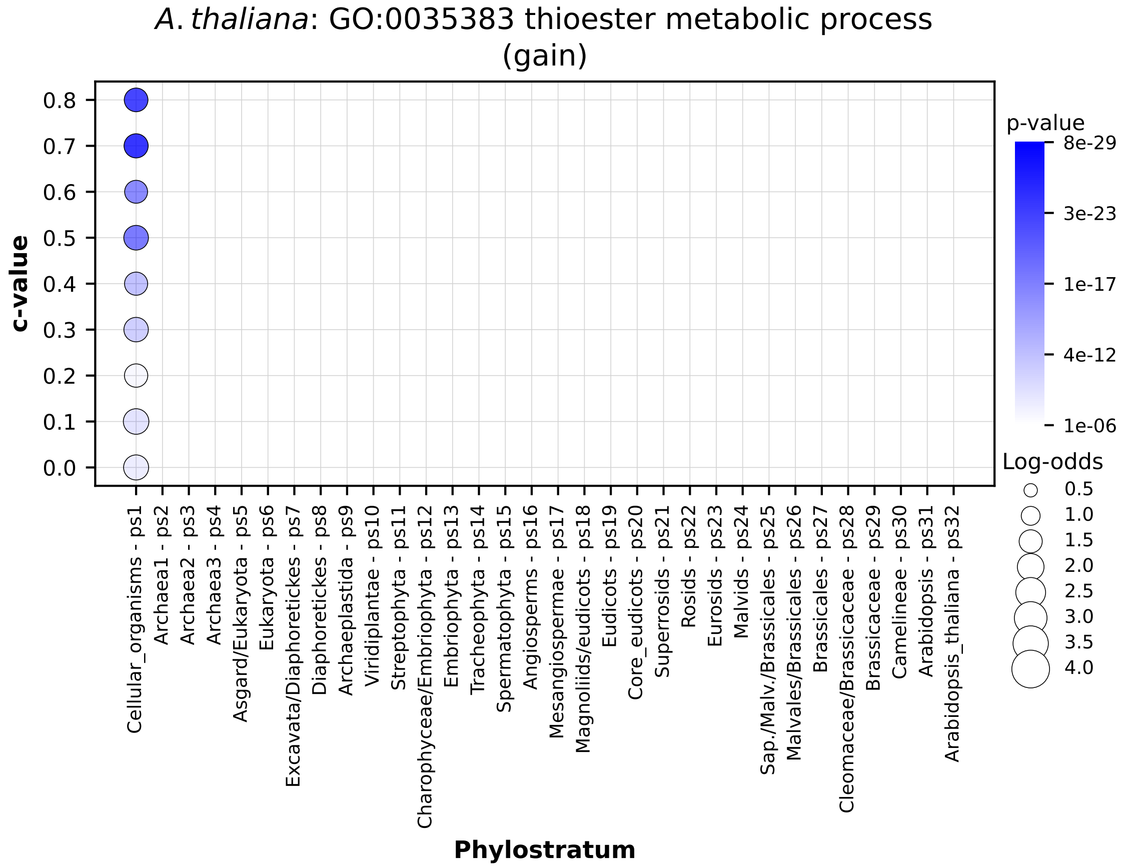

A. thaliana: GO:0035383 thioester metabolic process
(gain)
c-value
0.8
0.7
0.6
0.5
0.4
0.3
0.2
0.1
0.0
p-value
8e-29
3e-23
1e-17
4e-12
1e-06
Log-odds
0.5
1.0
1.5
2.0
2.5
3.0
3.5
4.0
Cellular_organisms - ps1
Archaea1 - ps2
Archaea2 - ps3
Archaea3 - ps4
Asgard/Eukaryota - ps5
Eukaryota - ps6
Excavata/Diaphoretickes - ps7
Diaphoretickes - ps8
Archaeplastida - ps9
Viridiplantae - ps10
Streptophyta - ps11
Charophyceae/Embriophyta - ps12
Embriophyta - ps13
Tracheophyta - ps14
Spermatophyta - ps15
Angiosperms - ps16
Mesangiospermae - ps17
Magnoliids/eudicots - ps18
Eudicots - ps19
Core_eudicots - ps20
Superrosids - ps21
Rosids - ps22
Eurosids - ps23
Malvids - ps24
Sap./Malv./Brassicales - ps25
Malvales/Brassicales - ps26
Brassicales - ps27
Cleomaceae/Brassicaceae - ps28
Brassicaceae - ps29
Camelineae - ps30
Arabidopsis - ps31
Arabidopsis_thaliana - ps32
Phylostratum

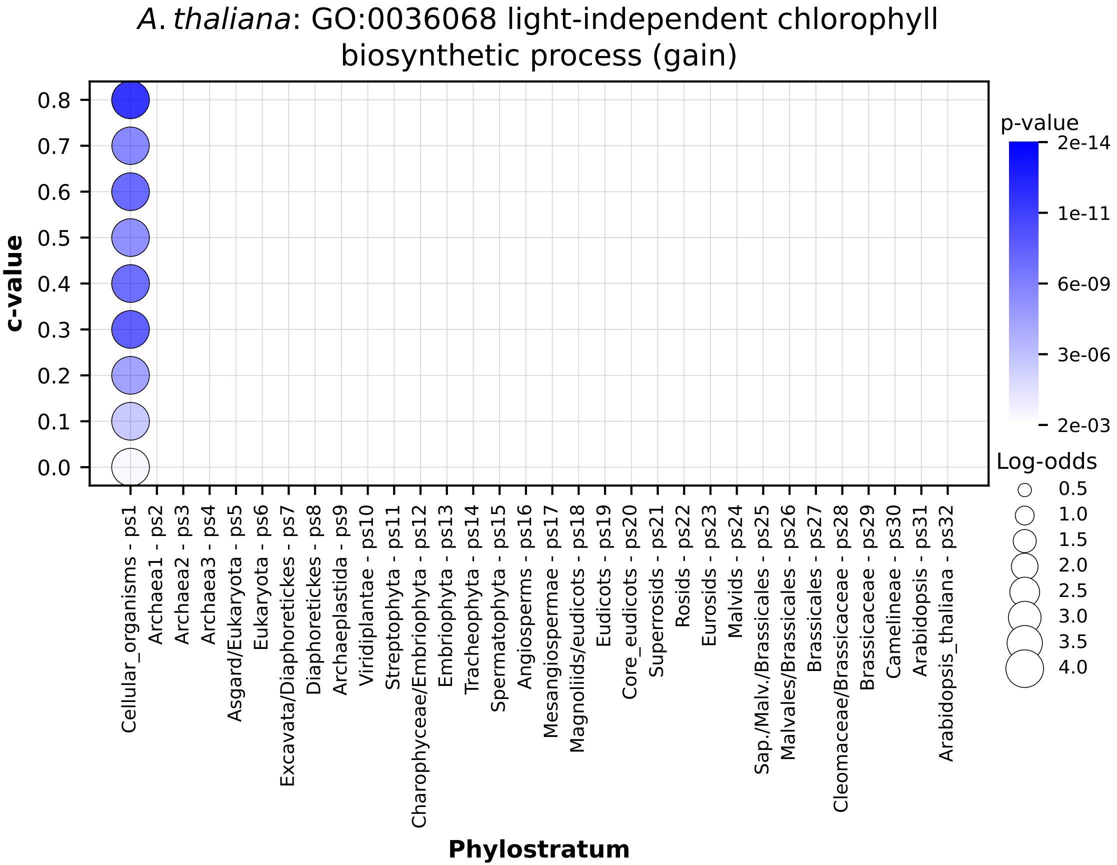

A. thaliana: GO:0036068 light-independent chlorophyll biosynthetic process (gain)
c-value
0.8
0.7
0.6
0.5
0.4
0.3
0.2
0.1
0.0
p-value
2e-14
1e-11
6e-09
3e-06
2e-03
Log-odds
0.5
1.0
1.5
2.0
2.5
3.0
3.5
4.0
Cellular_organisms - ps1
Archaea1 - ps2
Archaea2 - ps3
Archaea3 - ps4
Asgard/Eukaryota - ps5
Eukaryota - ps6
Excavata/Diaphoretickes - ps7
Diaphoretickes - ps8
Archaeplastida - ps9
Viridiplantae - ps10
Streptophyta - ps11
Charophyceae/Embriophyta - ps12
Embriophyta - ps13
Tracheophyta - ps14
Spermatophyta - ps15
Angiosperms - ps16
Mesangiospermae - ps17
Magnoliids/eudicots - ps18
Eudicots - ps19
Core_eudicots - ps20
Superrosids - ps21
Rosids - ps22
Eurosids - ps23
Malvids - ps24
Sap./Malv./Brassicales - ps25
Malvales/Brassicales - ps26
Brassicales - ps27
Cleomaceae/Brassicaceae - ps28
Brassicaceae - ps29
Camelineae - ps30
Arabidopsis - ps31
Arabidopsis_thaliana - ps32
Phylostratum

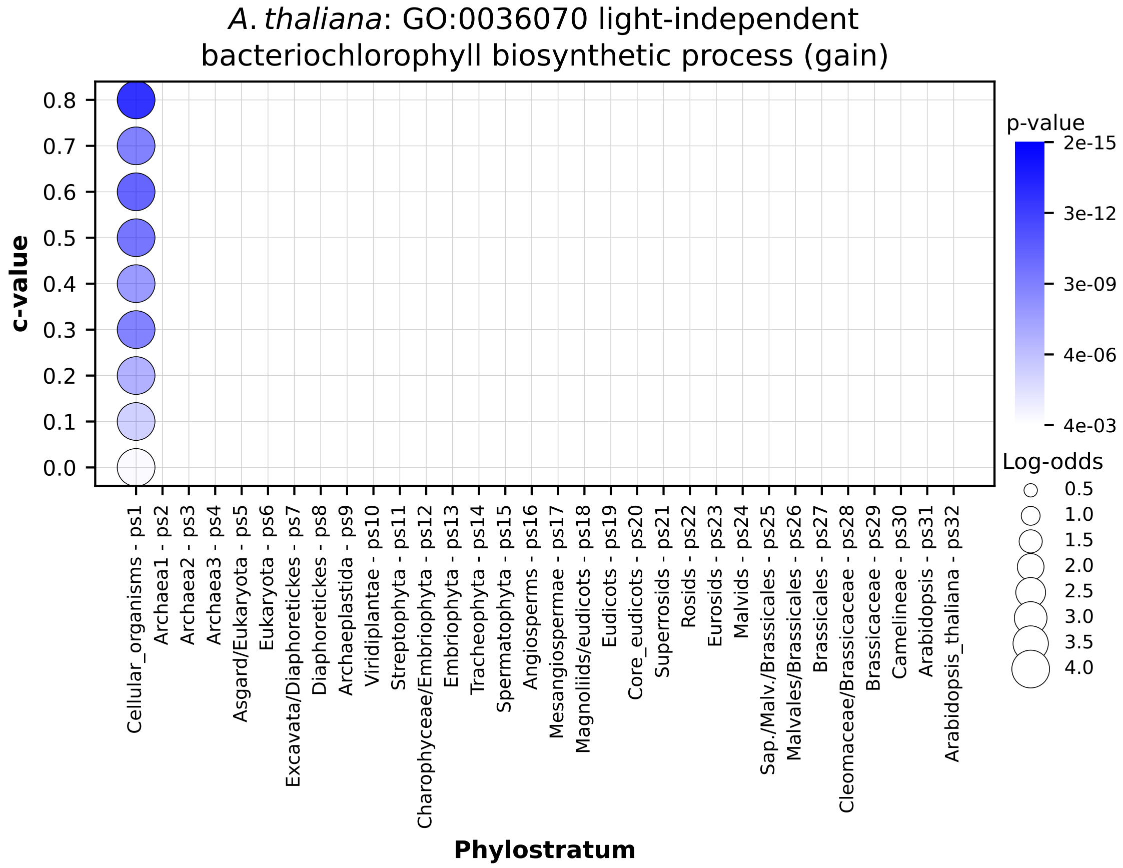

A. thaliana: GO:0036070 light-independent bacteriochlorophyll biosynthetic process (gain)
c-value
0.0
0.1
0.2
0.3
0.4
0.5
0.6
0.7
0.8
p-value
2e-15
3e-12
3e-09
4e-06
4e-03
Log-odds
0.5
1.0
1.5
2.0
2.5
3.0
3.5
4.0
Cellular_organisms - ps1
Archaea1 - ps2
Archaea2 - ps3
Archaea3 - ps4
Asgard/Eukaryota - ps5
Eukaryota - ps6
Excavata/Diaphoretickes - ps7
Diaphoretickes - ps8
Archaeplastida - ps9
Viridiplantae - ps10
Streptophyta - ps11
Charophyceae/Embriophyta - ps12
Embriophyta - ps13
Tracheophyta - ps14
Spermatophyta - ps15
Angiosperms - ps16
Mesangiospermae - ps17
Magnoliids/eudicots - ps18
Eudicots - ps19
Core_eudicots - ps20
Superrosids - ps21
Rosids - ps22
Eurosids - ps23
Malvids - ps24
Sap./Malv./Brassicales - ps25
Malvales/Brassicales - ps26
Brassicales - ps27
Cleomaceae/Brassicaceae - ps28
Brassicaceae - ps29
Camelineae - ps30
Arabidopsis - ps31
Arabidopsis_thaliana - ps32
Phylostratum

A. thaliana: GO:0036094 small molecule binding (gain)

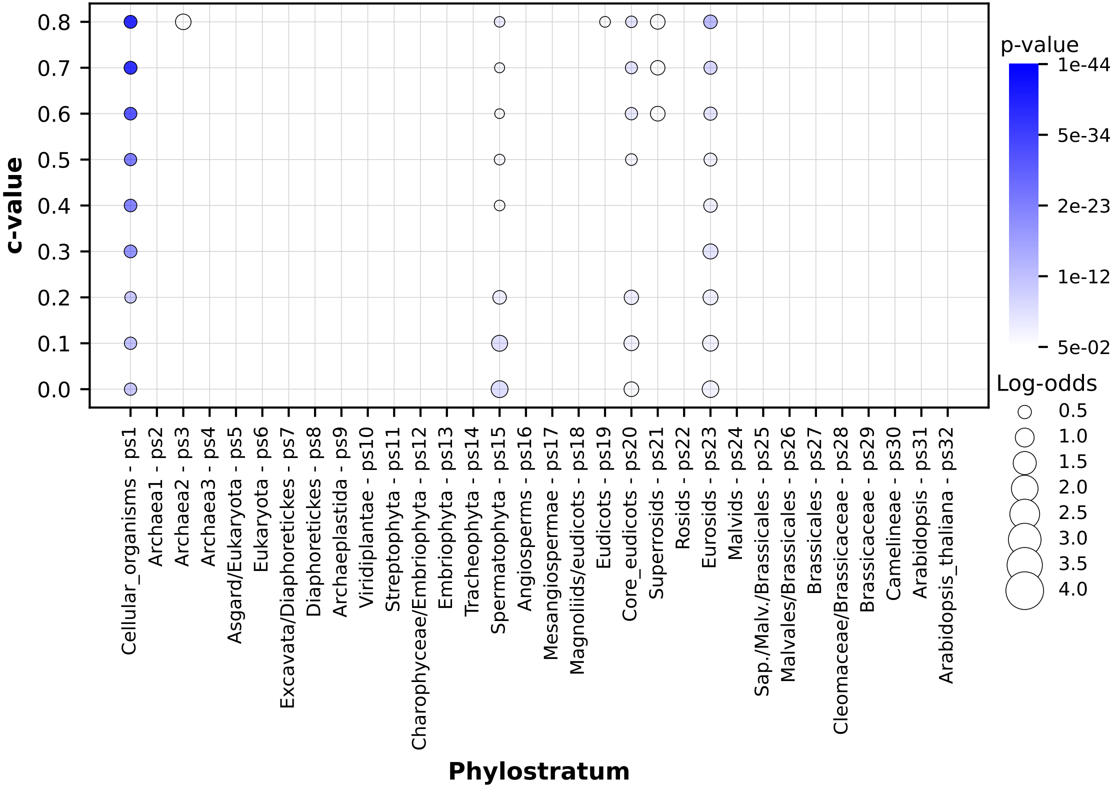

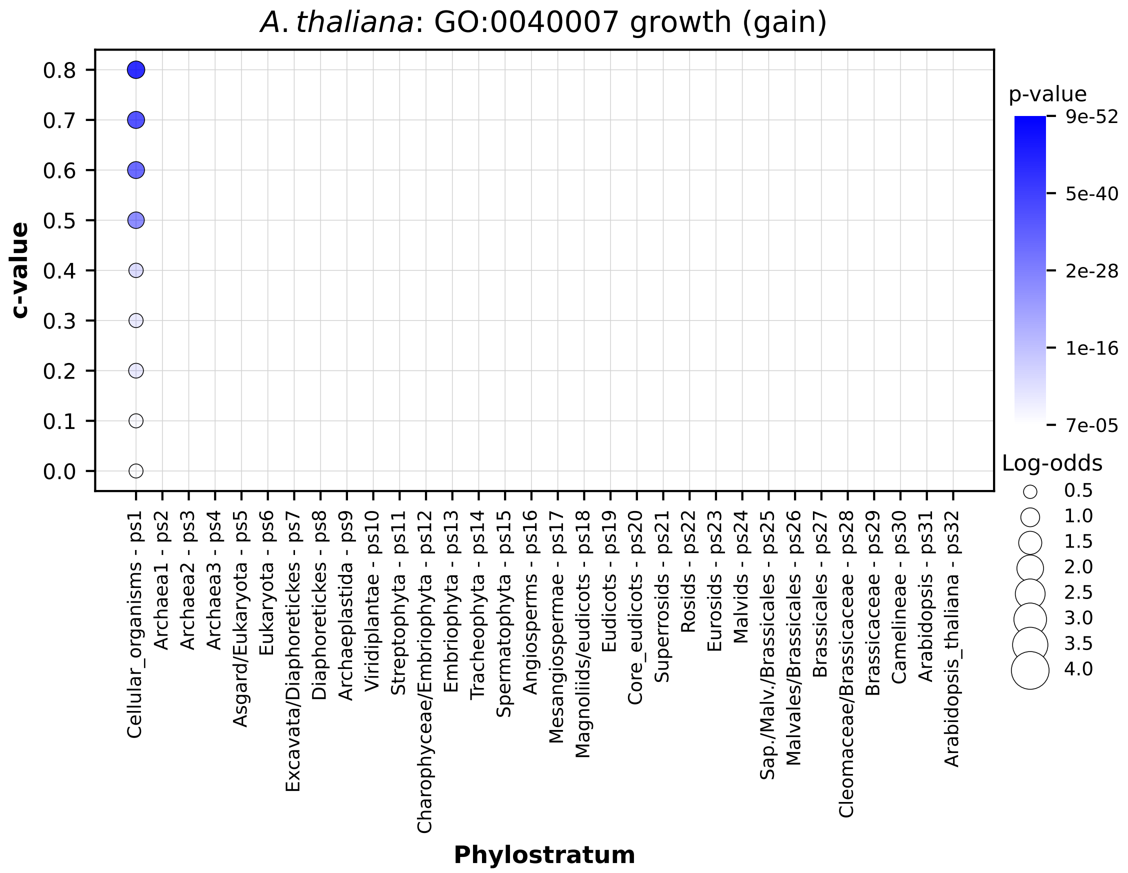

A. thaliana: GO:0040007 growth (gain)
c-value
0.0
0.1
0.2
0.3
0.4
0.5
0.6
0.7
0.8
Phylostratum
Cellular_organisms - ps1
Archaea1 - ps2
Archaea2 - ps3
Archaea3 - ps4
Asgard/Eukaryota - ps5
Eukaryota - ps6
Excavata/Diaphoretickes - ps7
Diaphoretickes - ps8
Archaeplastida - ps9
Viridiplantae - ps10
Streptophyta - ps11
Charophyceae/Embriophyta - ps12
Embriophyta - ps13
Tracheophyta - ps14
Spermatophyta - ps15
Angiosperms - ps16
Mesangiospermae - ps17
Magnoliids/eudicots - ps18
Eudicots - ps19
Core_eudicots - ps20
Superrosids - ps21
Rosids - ps22
Eurosids - ps23
Malvids - ps24
Sap./Malv./Brassicales - ps25
Malvales/Brassicales - ps26
Brassicales - ps27
Cleomaceae/Brassicaceae - ps28
Brassicaceae - ps29
Camelineae - ps30
Arabidopsis - ps31
Arabidopsis_thaliana - ps32
p-value
9e-52
5e-40
2e-28
1e-16
7e-05
Log-odds
0.5
1.0
1.5
2.0
2.5
3.0
3.5
4.0

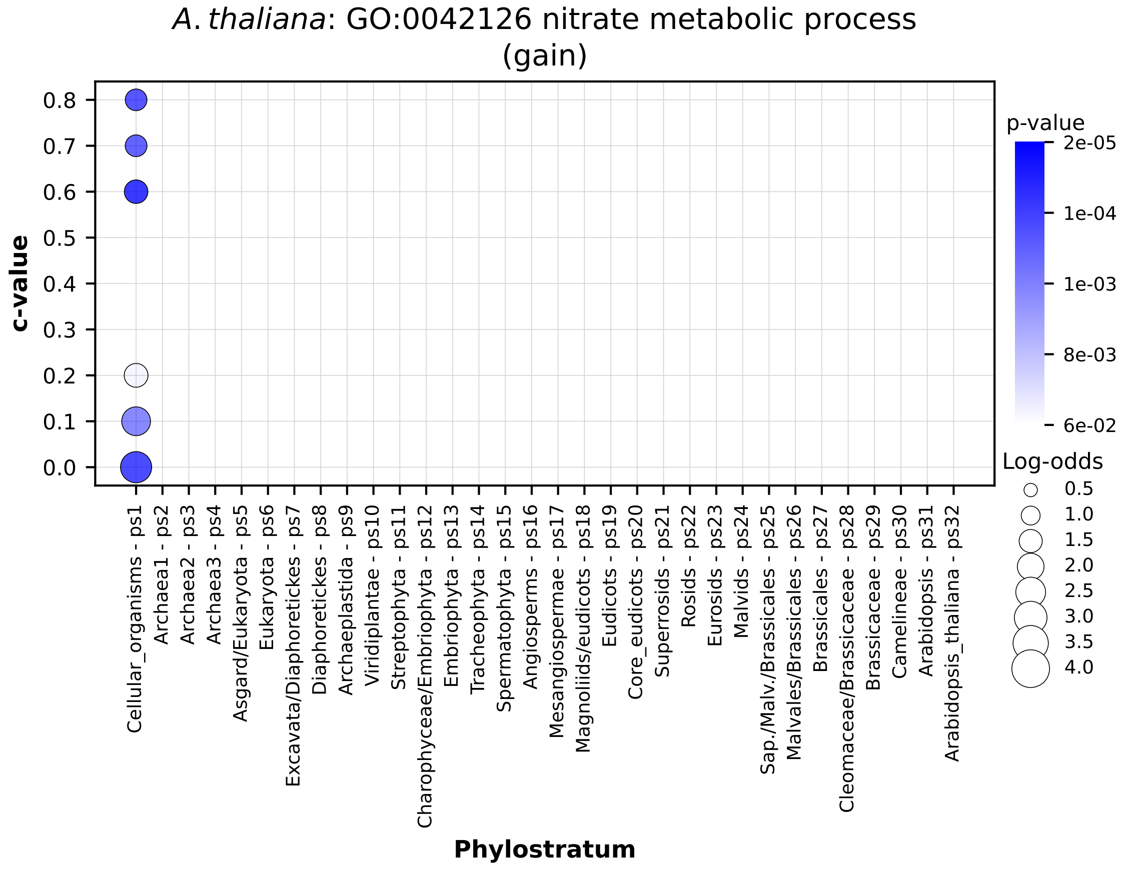

A. thaliana: GO:0042126 nitrate metabolic process (gain)
c-value
0.8
0.7
0.6
0.5
0.4
0.3
0.2
0.1
0.0
p-value
2e-05
1e-04
1e-03
8e-03
6e-02
Log-odds
0.5
1.0
1.5
2.0
2.5
3.0
3.5
4.0
Cellular_organisms - ps1
Archaea1 - ps2
Archaea2 - ps3
Archaea3 - ps4
Asgard/Eukaryota - ps5
Eukaryota - ps6
Excavata/Diaphoretickes - ps7
Diaphoretickes - ps8
Archaeplastida - ps9
Viridiplantae - ps10
Streptophyta - ps11
Charophyceae/Embriophyta - ps12
Embriophyta - ps13
Tracheophyta - ps14
Spermatophyta - ps15
Angiosperms - ps16
Mesangiospermae - ps17
Magnoliids/eudicots - ps18
Eudicots - ps19
Core_eudicots - ps20
Superrosids - ps21
Rosids - ps22
Eurosids - ps23
Malvids - ps24
Sap./Malv./Brassicales - ps25
Malvales/Brassicales - ps26
Brassicales - ps27
Cleomaceae/Brassicaceae - ps28
Brassicaceae - ps29
Camelineae - ps30
Arabidopsis - ps31
Arabidopsis_thaliana - ps32
Phylostratum

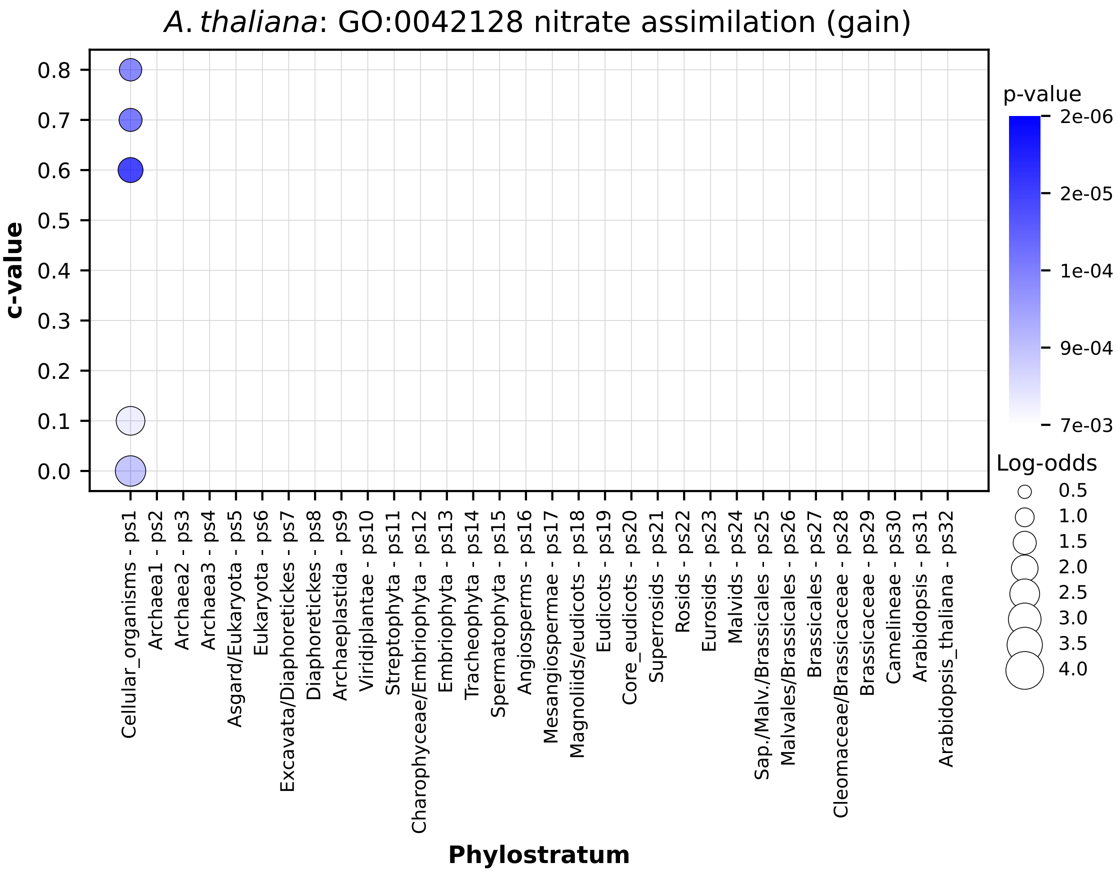
A. thaliana: GO:0042128 nitrate assimilation (gain)
c-value
0.8
0.7
0.6
0.5
0.4
0.3
0.2
0.1
0.0
Phylostratum
Cellular_organisms - ps1
Archaea1 - ps2
Archaea2 - ps3
Archaea3 - ps4
Asgard/Eukaryota - ps5
Eukaryota - ps6
Excavata/Diaphoretickes - ps7
Diaphoretickes - ps8
Archaeplastida - ps9
Viridiplantae - ps10
Streptophyta - ps11
Charophyceae/Embriophyta - ps12
Embriophyta - ps13
Tracheophyta - ps14
Spermatophyta - ps15
Angiosperms - ps16
Mesangiospermae - ps17
Magnoliids/eudicots - ps18
Eudicots - ps19
Core_eudicots - ps20
Superrosids - ps21
Rosids - ps22
Eurosids - ps23
Malvids - ps24
Sap./Malv./Brassicales - ps25
Malvales/Brassicales - ps26
Brassicales - ps27
Cleomaceae/Brassicaceae - ps28
Brassicaceae - ps29
Camelineae - ps30
Arabidopsis - ps31
Arabidopsis_thaliana - ps32
p-value
2e-06
2e-05
1e-04
9e-04
7e-03
Log-odds
0.5
1.0
1.5
2.0
2.5
3.0
3.5
4.0

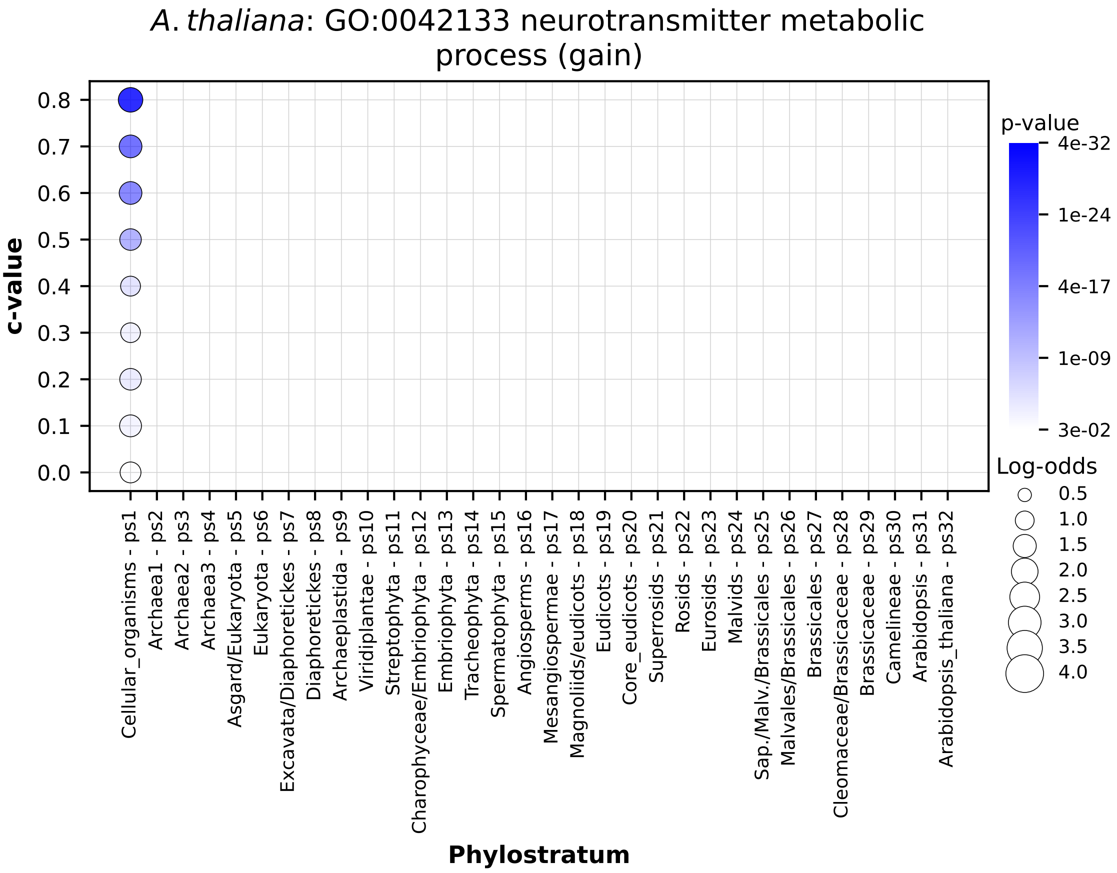

A. thaliana: GO:0042133 neurotransmitter metabolic process (gain)
c-value
0.8
0.7
0.6
0.5
0.4
0.3
0.2
0.1
0.0
p-value
4e-32
1e-24
4e-17
1e-09
3e-02
Log-odds
0.5
1.0
1.5
2.0
2.5
3.0
3.5
4.0
Cellular_organisms - ps1
Archaea1 - ps2
Archaea2 - ps3
Archaea3 - ps4
Asgard/Eukaryota - ps5
Eukaryota - ps6
Excavata/Diaphoretickes - ps7
Diaphoretickes - ps8
Archaeplastida - ps9
Viridiplantae - ps10
Streptophyta - ps11
Charophyceae/Embriophyta - ps12
Embriophyta - ps13
Tracheophyta - ps14
Spermatophyta - ps15
Angiosperms - ps16
Mesangiospermae - ps17
Magnoliids/eudicots - ps18
Eudicots - ps19
Core_eudicots - ps20
Superrosids - ps21
Rosids - ps22
Eurosids - ps23
Malvids - ps24
Sap./Malv./Brassicales - ps25
Malvales/Brassicales - ps26
Brassicales - ps27
Cleomaceae/Brassicaceae - ps28
Brassicaceae - ps29
Camelineae - ps30
Arabidopsis - ps31
Arabidopsis_thaliana - ps32
Phylostratum

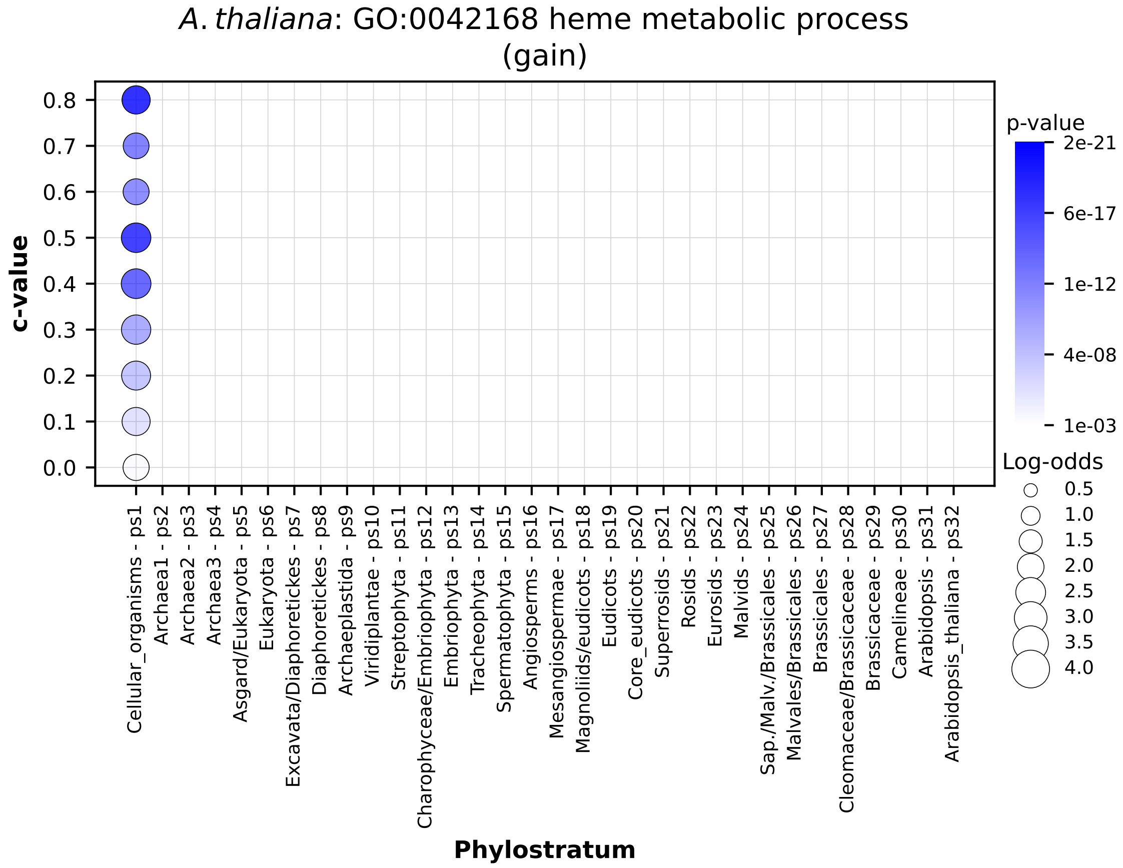
A. thaliana: GO:0042168 heme metabolic process (gain)
c-value
0.8
0.7
0.6
0.5
0.4
0.3
0.2
0.1
0.0
p-value
2e-21
6e-17
1e-12
4e-08
1e-03
Log-odds
0.5
1.0
1.5
2.0
2.5
3.0
3.5
4.0
Cellular_organisms - ps1
Archaea1 - ps2
Archaea2 - ps3
Archaea3 - ps4
Asgard/Eukaryota - ps5
Eukaryota - ps6
Excavata/Diaphoretickes - ps7
Diaphoretickes - ps8
Archaeplastida - ps9
Viridiplantae - ps10
Streptophyta - ps11
Charophyceae/Embriophyta - ps12
Embriophyta - ps13
Tracheophyta - ps14
Spermatophyta - ps15
Angiosperms - ps16
Mesangiospermae - ps17
Magnoliids/eudicots - ps18
Eudicots - ps19
Core_eudicots - ps20
Superrosids - ps21
Rosids - ps22
Eurosids - ps23
Malvids - ps24
Sap./Malv./Brassicales - ps25
Malvales/Brassicales - ps26
Brassicales - ps27
Cleomaceae/Brassicaceae - ps28
Brassicaceae - ps29
Camelineae - ps30
Arabidopsis - ps31
Arabidopsis_thaliana - ps32
Phylostratum

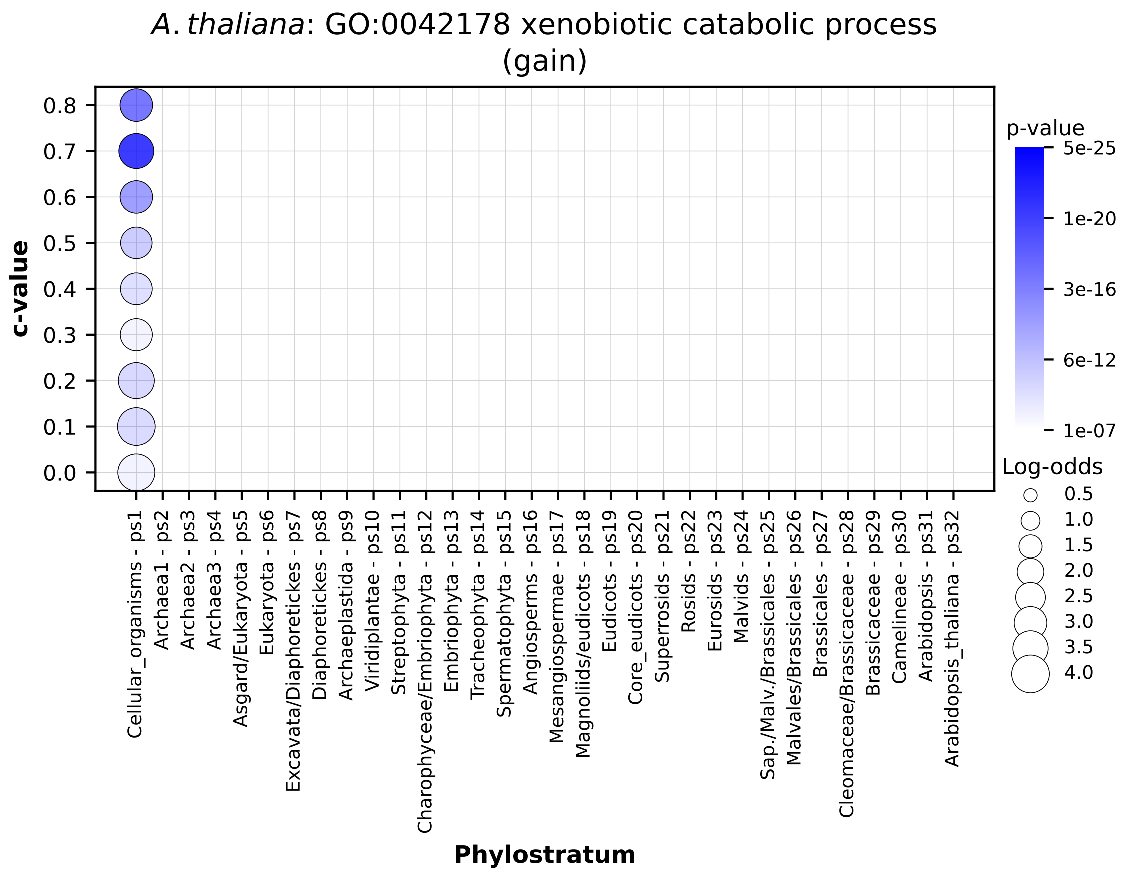

A. thaliana: GO:0042178 xenobiotic catabolic process
(gain)
c-value
0.8
0.7
0.6
0.5
0.4
0.3
0.2
0.1
0.0
p-value
5e-25
1e-20
3e-16
6e-12
1e-07
Log-odds
0.5
1.0
1.5
2.0
2.5
3.0
3.5
4.0
Cellular_organisms - ps1
Archaea1 - ps2
Archaea2 - ps3
Archaea3 - ps4
Asgard/Eukaryota - ps5
Eukaryota - ps6
Excavata/Diaphoretickes - ps7
Diaphoretickes - ps8
Archaeplastida - ps9
Viridiplantae - ps10
Streptophyta - ps11
Charophyceae/Embriophyta - ps12
Embriophyta - ps13
Tracheophyta - ps14
Spermatophyta - ps15
Angiosperms - ps16
Mesangiospermae - ps17
Magnoliids/eudicots - ps18
Eudicots - ps19
Core_eudicots - ps20
Superrosids - ps21
Rosids - ps22
Eurosids - ps23
Malvids - ps24
Sap./Malv./Brassicales - ps25
Malvales/Brassicales - ps26
Brassicales - ps27
Cleomaceae/Brassicaceae - ps28
Brassicaceae - ps29
Camelineae - ps30
Arabidopsis - ps31
Arabidopsis_thaliana - ps32
Phylostratum

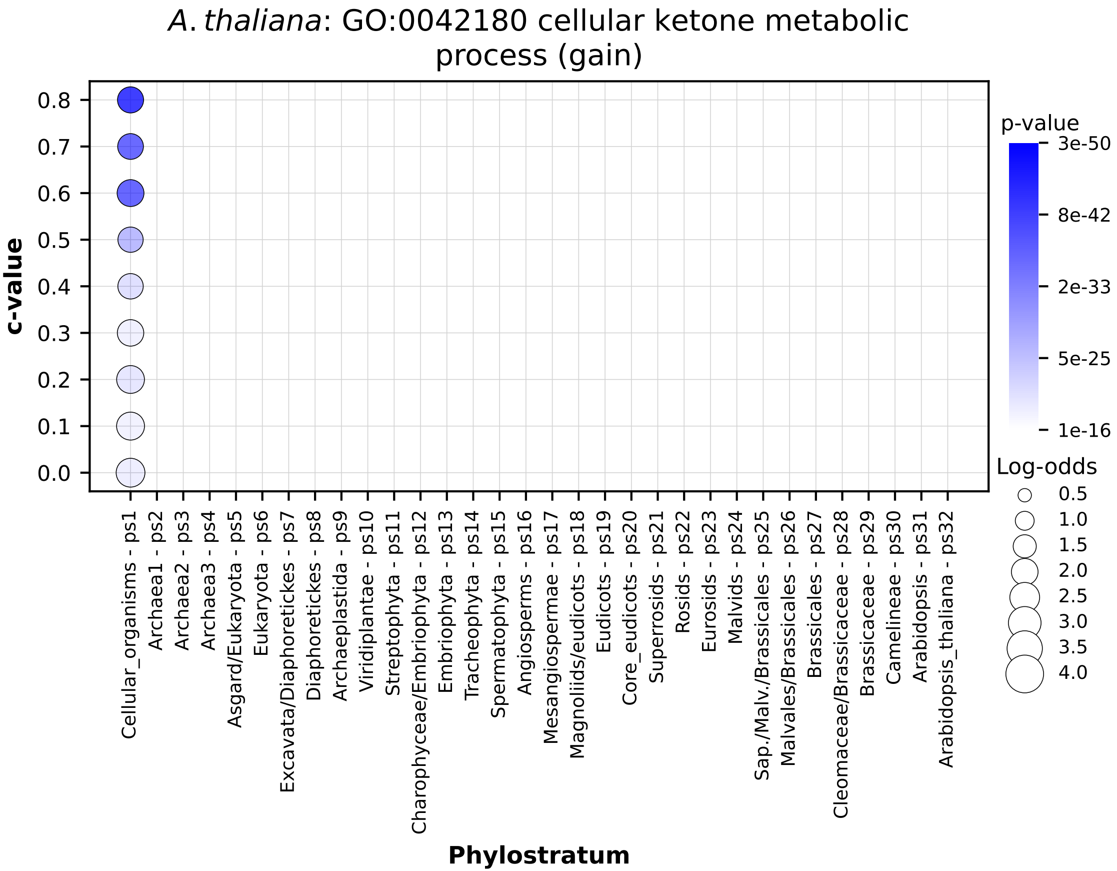

A. thaliana: GO:0042180 cellular ketone metabolic process (gain)
c-value
0.8
0.7
0.6
0.5
0.4
0.3
0.2
0.1
0.0
Phylostratum
Cellular_organisms - ps1
Archaea1 - ps2
Archaea2 - ps3
Archaea3 - ps4
Asgard/Eukaryota - ps5
Eukaryota - ps6
Excavata/Diaphoretickes - ps7
Diaphoretickes - ps8
Archaeplastida - ps9
Viridiplantae - ps10
Streptophyta - ps11
Charophyceae/Embriophyta - ps12
Embriophyta - ps13
Tracheophyta - ps14
Spermatophyta - ps15
Angiosperms - ps16
Mesangiospermae - ps17
Magnoliids/eudicots - ps18
Eudicots - ps19
Core_eudicots - ps20
Superrosids - ps21
Rosids - ps22
Eurosids - ps23
Malvids - ps24
Sap./Malv./Brassicales - ps25
Malvales/Brassicales - ps26
Brassicales - ps27
Cleomaceae/Brassicaceae - ps28
Brassicaceae - ps29
Camelineae - ps30
Arabidopsis - ps31
Arabidopsis_thaliana - ps32
p-value
3e-50
8e-42
2e-33
5e-25
1e-16
Log-odds
0.5
1.0
1.5
2.0
2.5
3.0
3.5
4.0

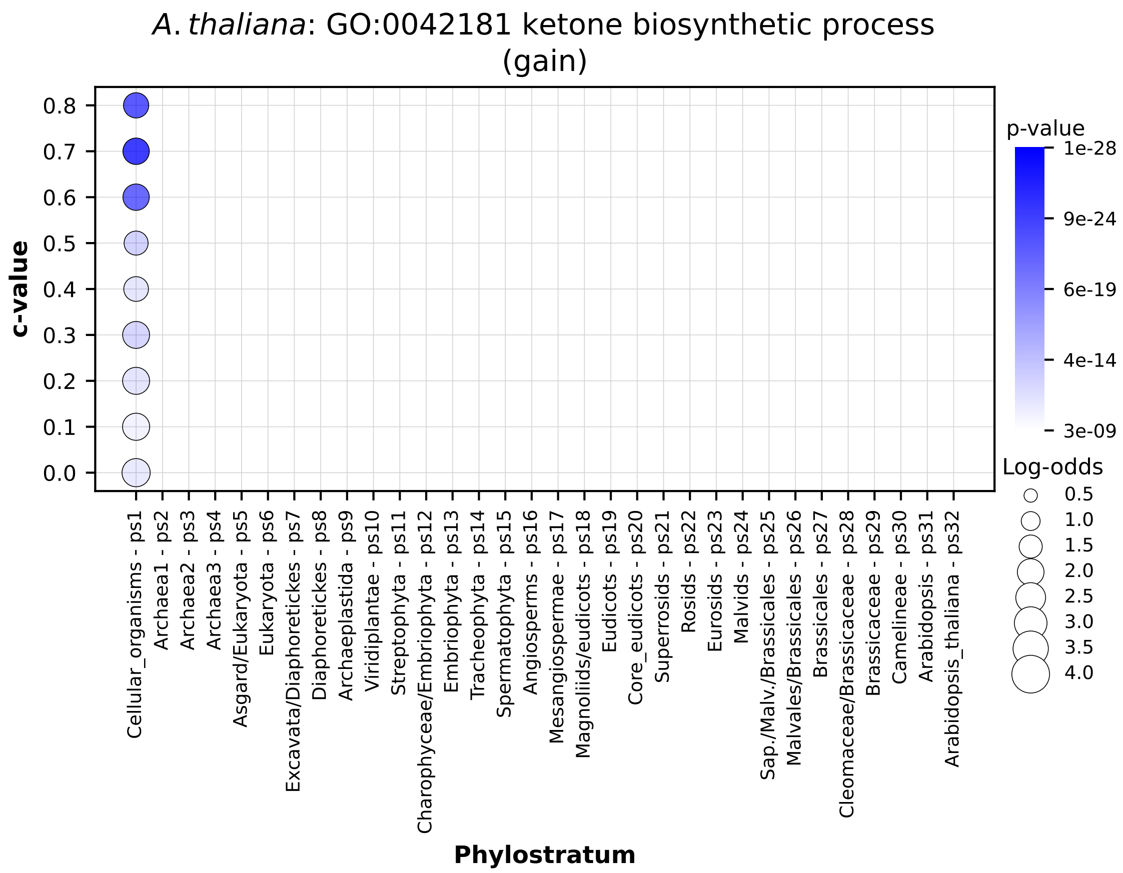

A. thaliana: GO:0042181 ketone biosynthetic process (gain)
c-value
0.0
0.1
0.2
0.3
0.4
0.5
0.6
0.7
0.8
p-value
1e-28
9e-24
6e-19
4e-14
3e-09
Log-odds
0.5
1.0
1.5
2.0
2.5
3.0
3.5
4.0
Cellular_organisms - ps1
Archaea1 - ps2
Archaea2 - ps3
Archaea3 - ps4
Asgard/Eukaryota - ps5
Eukaryota - ps6
Excavata/Diaphoretickes - ps7
Diaphoretickes - ps8
Archaeplastida - ps9
Viridiplantae - ps10
Streptophyta - ps11
Charophyceae/Embriophyta - ps12
Embriophyta - ps13
Tracheophyta - ps14
Spermatophyta - ps15
Angiosperms - ps16
Mesangiospermae - ps17
Magnoliids/eudicots - ps18
Eudicots - ps19
Core_eudicots - ps20
Superrosids - ps21
Rosids - ps22
Eurosids - ps23
Malvids - ps24
Sap./Malv./Brassicales - ps25
Malvales/Brassicales - ps26
Brassicales - ps27
Cleomaceae/Brassicaceae - ps28
Brassicaceae - ps29
Camelineae - ps30
Arabidopsis - ps31
Arabidopsis_thaliana - ps32
Phylostratum

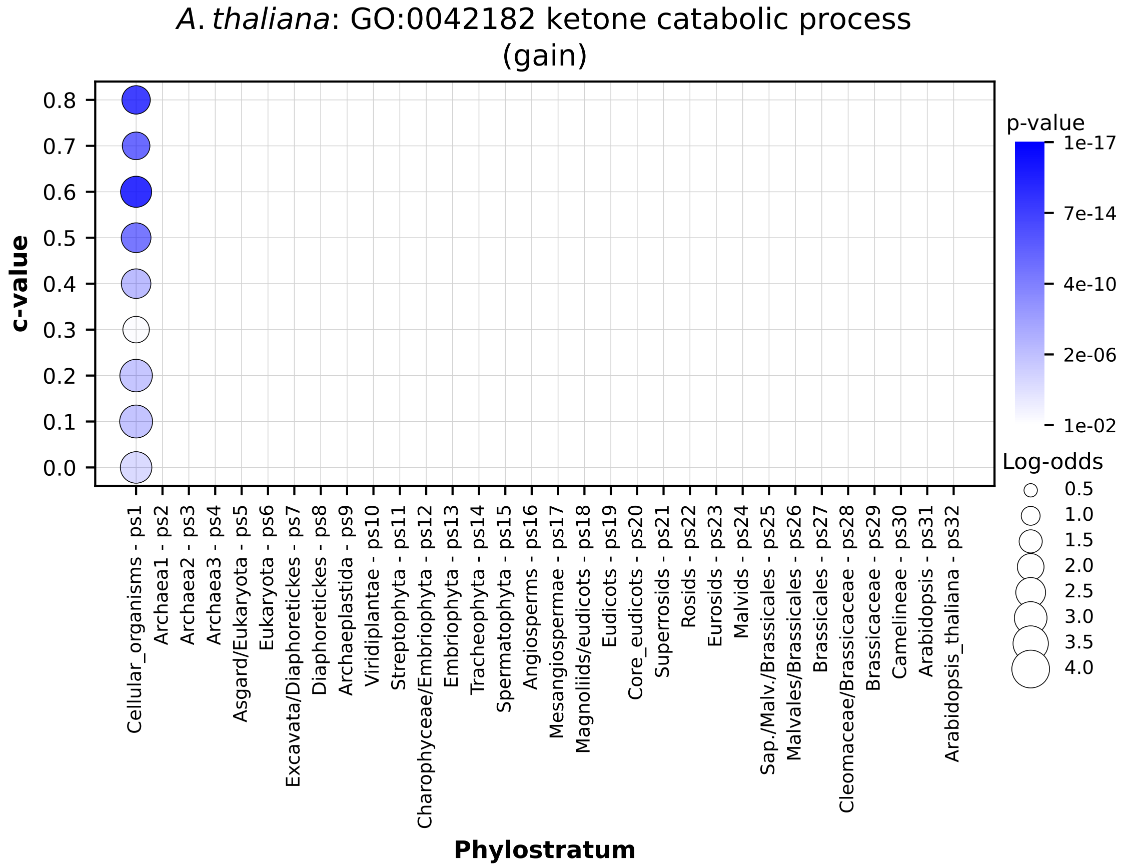
A. thaliana: GO:0042182 ketone catabolic process
(gain)
c-value
0.8
0.7
0.6
0.5
0.4
0.3
0.2
0.1
0.0
p-value
1e-17
7e-14
4e-10
2e-06
1e-02
Log-odds
0.5
1.0
1.5
2.0
2.5
3.0
3.5
4.0
Cellular_organisms - ps1
Archaea1 - ps2
Archaea2 - ps3
Archaea3 - ps4
Asgard/Eukaryota - ps5
Eukaryota - ps6
Excavata/Diaphoretickes - ps7
Diaphoretickes - ps8
Archaeplastida - ps9
Viridiplantae - ps10
Streptophyta - ps11
Charophyceae/Embriophyta - ps12
Embriophyta - ps13
Tracheophyta - ps14
Spermatophyta - ps15
Angiosperms - ps16
Mesangiospermae - ps17
Magnoliids/eudicots - ps18
Eudicots - ps19
Core_eudicots - ps20
Superrosids - ps21
Rosids - ps22
Eurosids - ps23
Malvids - ps24
Sap./Malv./Brassicales - ps25
Malvales/Brassicales - ps26
Brassicales - ps27
Cleomaceae/Brassicaceae - ps28
Brassicaceae - ps29
Camelineae - ps30
Arabidopsis - ps31
Arabidopsis_thaliana - ps32
Phylostratum

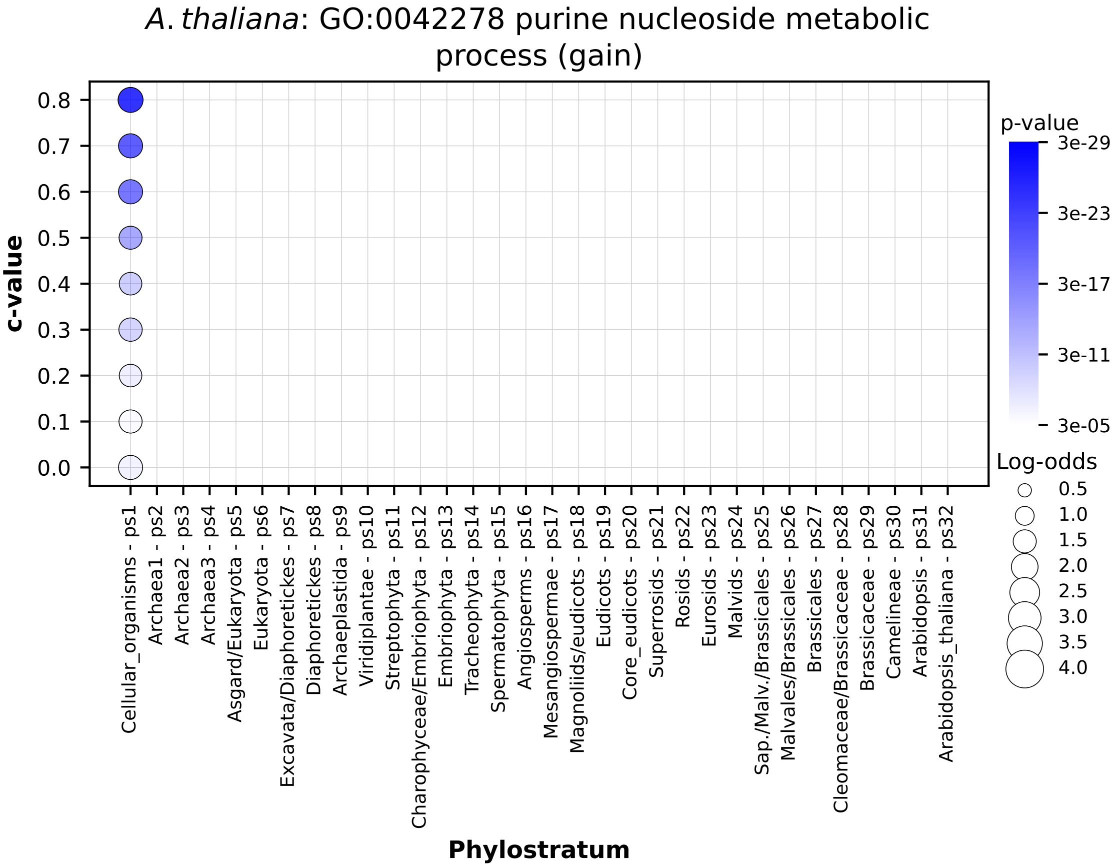

A. thaliana: GO:0042278 purine nucleoside metabolic process (gain)
c-value
0.8
0.7
0.6
0.5
0.4
0.3
0.2
0.1
0.0
Phylostratum
Cellular_organisms - ps1
Archaea1 - ps2
Archaea2 - ps3
Archaea3 - ps4
Asgard/Eukaryota - ps5
Eukaryota - ps6
Excavata/Diaphoretickes - ps7
Diaphoretickes - ps8
Archaeplastida - ps9
Viridiplantae - ps10
Streptophyta - ps11
Charophyceae/Embriophyta - ps12
Embriophyta - ps13
Tracheophyta - ps14
Spermatophyta - ps15
Angiosperms - ps16
Mesangiospermae - ps17
Magnoliids/eudicots - ps18
Eudicots - ps19
Core_eudicots - ps20
Superrosids - ps21
Rosids - ps22
Eurosids - ps23
Malvids - ps24
Sap./Malv./Brassicales - ps25
Malvales/Brassicales - ps26
Brassicales - ps27
Cleomaceae/Brassicaceae - ps28
Brassicaceae - ps29
Camelineae - ps30
Arabidopsis - ps31
Arabidopsis_thaliana - ps32
p-value
3e-29
3e-23
3e-17
3e-11
3e-05
Log-odds
0.5
1.0
1.5
2.0
2.5
3.0
3.5
4.0

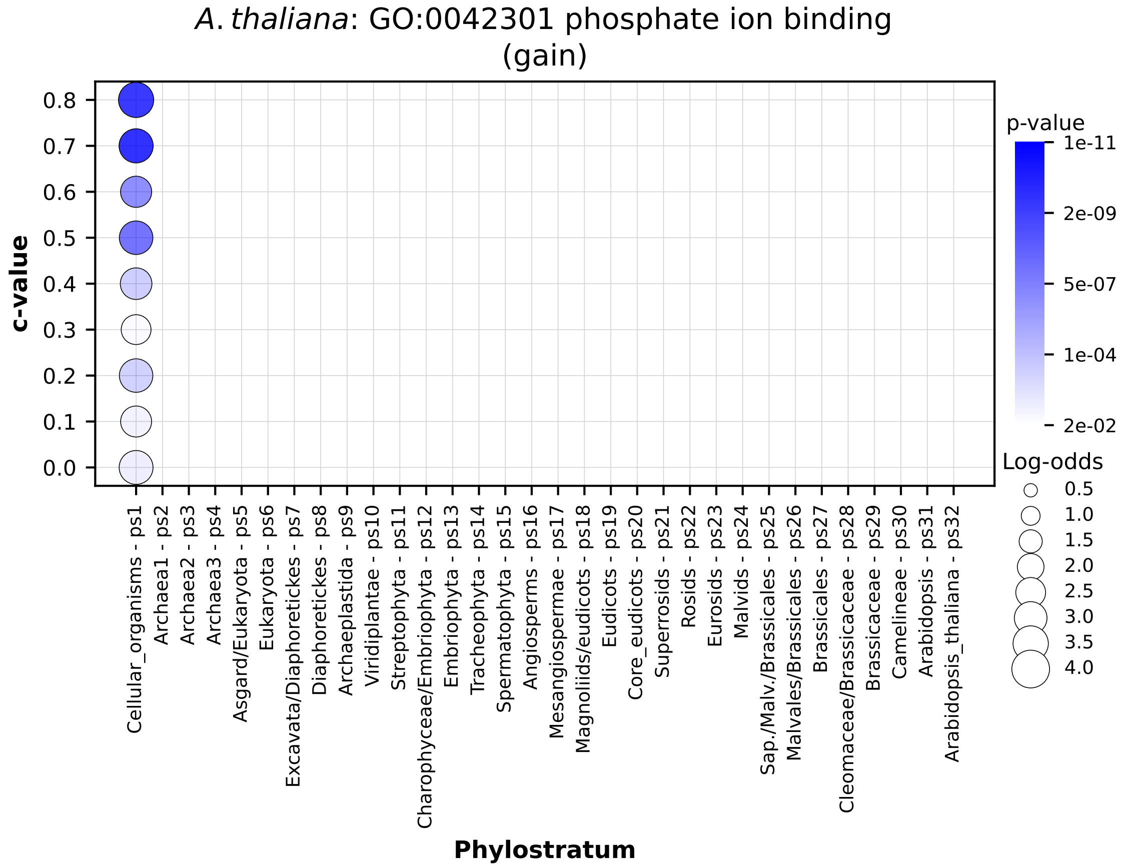

A. thaliana: GO:0042301 phosphate ion binding
(gain)
c-value
0.8
0.7
0.6
0.5
0.4
0.3
0.2
0.1
0.0
p-value
1e-11
2e-09
5e-07
1e-04
2e-02
Log-odds
0.5
1.0
1.5
2.0
2.5
3.0
3.5
4.0
Cellular_organisms - ps1
Archaea1 - ps2
Archaea2 - ps3
Archaea3 - ps4
Asgard/Eukaryota - ps5
Eukaryota - ps6
Excavata/Diaphoretickes - ps7
Diaphoretickes - ps8
Archaeplastida - ps9
Viridiplantae - ps10
Streptophyta - ps11
Charophyceae/Embriophyta - ps12
Embriophyta - ps13
Tracheophyta - ps14
Spermatophyta - ps15
Angiosperms - ps16
Mesangiospermae - ps17
Magnoliids/eudicots - ps18
Eudicots - ps19
Core_eudicots - ps20
Superrosids - ps21
Rosids - ps22
Eurosids - ps23
Malvids - ps24
Sap./Malv./Brassicales - ps25
Malvales/Brassicales - ps26
Brassicales - ps27
Cleomaceae/Brassicaceae - ps28
Brassicaceae - ps29
Camelineae - ps30
Arabidopsis - ps31
Arabidopsis_thaliana - ps32
Phylostratum

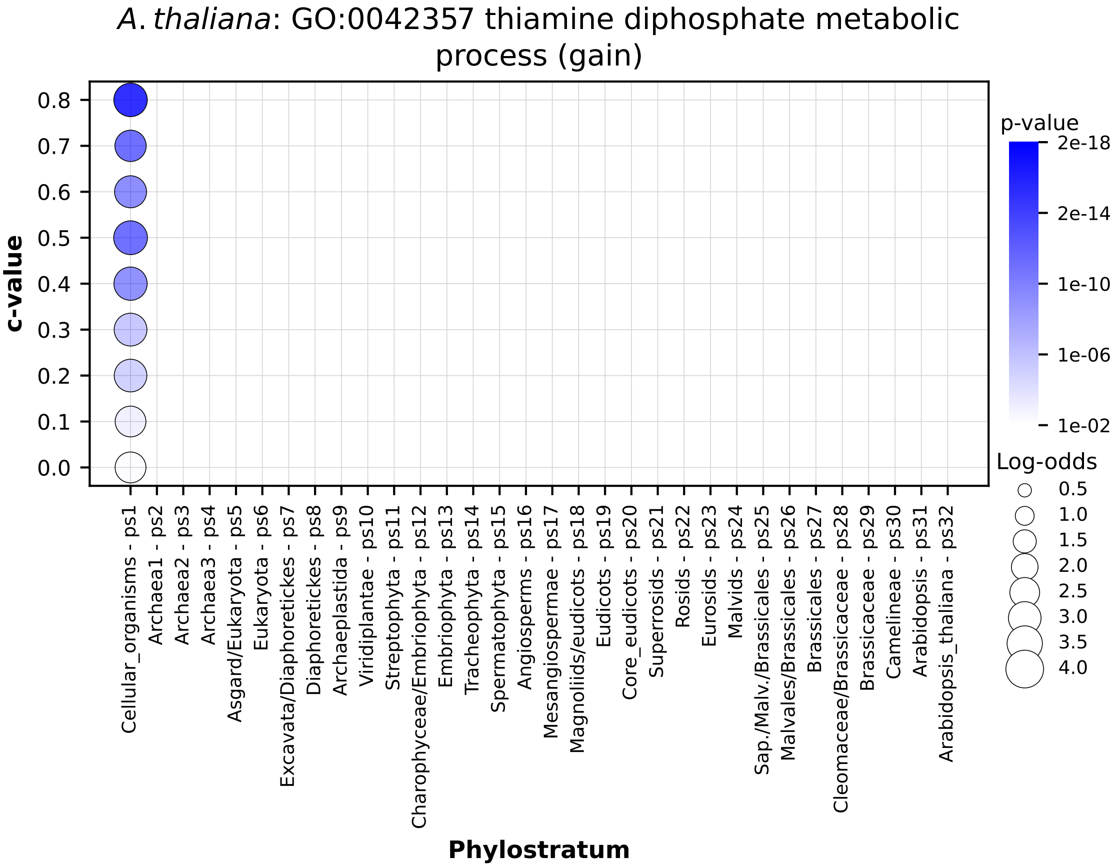
A. thaliana: GO:0042357 thiamine diphosphate metabolic process (gain)
c-value
0.8
0.7
0.6
0.5
0.4
0.3
0.2
0.1
0.0
p-value
2e-18
2e-14
1e-10
1e-06
1e-02
Log-odds
0.5
1.0
1.5
2.0
2.5
3.0
3.5
4.0
Cellular_organisms - ps1
Archaea1 - ps2
Archaea2 - ps3
Archaea3 - ps4
Asgard/Eukaryota - ps5
Eukaryota - ps6
Excavata/Diaphoretickes - ps7
Diaphoretickes - ps8
Archaeplastida - ps9
Viridiplantae - ps10
Streptophyta - ps11
Charophyceae/Embriophyta - ps12
Embriophyta - ps13
Tracheophyta - ps14
Spermatophyta - ps15
Angiosperms - ps16
Mesangiospermae - ps17
Magnoliids/eudicots - ps18
Eudicots - ps19
Core_eudicots - ps20
Superrosids - ps21
Rosids - ps22
Eurosids - ps23
Malvids - ps24
Sap./Malv./Brassicales - ps25
Malvales/Brassicales - ps26
Brassicales - ps27
Cleomaceae/Brassicaceae - ps28
Brassicaceae - ps29
Camelineae - ps30
Arabidopsis - ps31
Arabidopsis_thaliana - ps32
Phylostratum

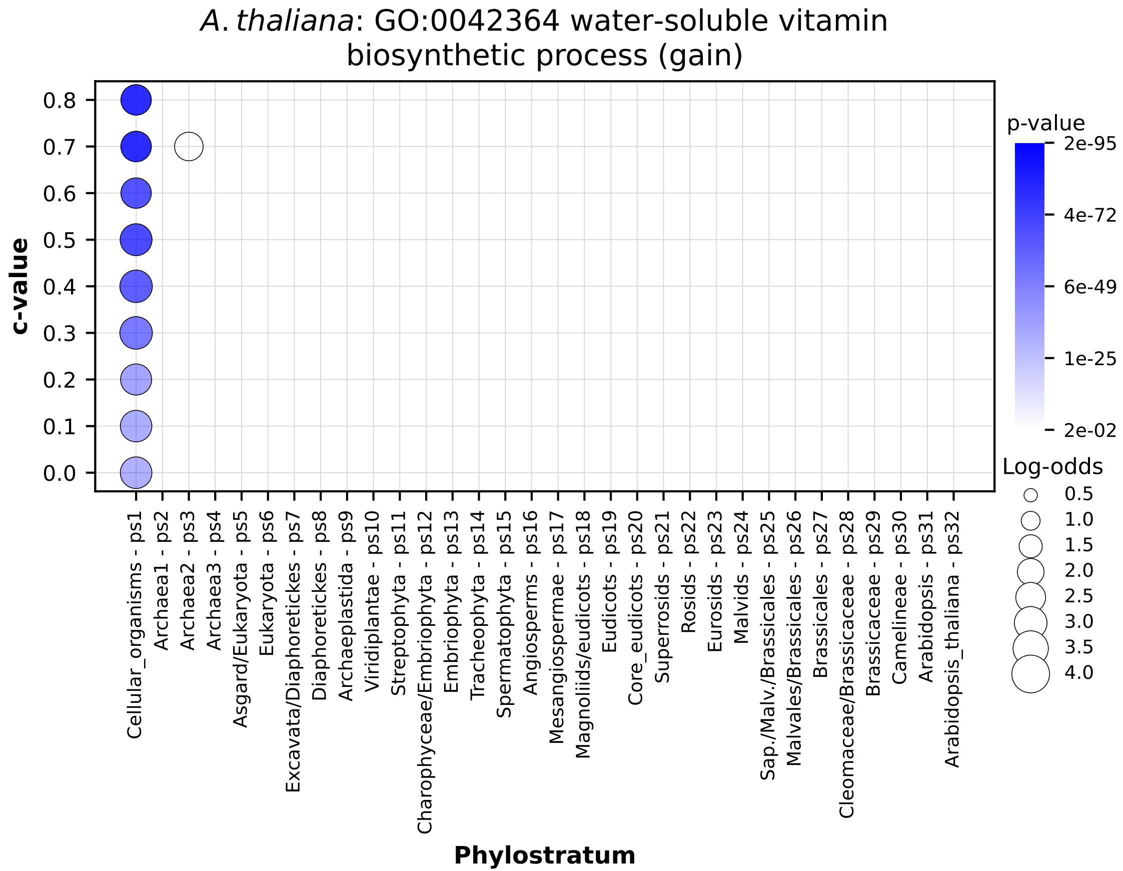
A. thaliana: GO:0042364 water-soluble vitamin biosynthetic process (gain)
c-value
0.8
0.7
0.6
0.5
0.4
0.3
0.2
0.1
0.0
Phylostratum
Cellular_organisms - ps1
Archaea1 - ps2
Archaea2 - ps3
Archaea3 - ps4
Asgard/Eukaryota - ps5
Eukaryota - ps6
Excavata/Diaphoretickes - ps7
Diaphoretickes - ps8
Archaeplastida - ps9
Viridiplantae - ps10
Streptophyta - ps11
Charophyceae/Embriophyta - ps12
Embriophyta - ps13
Tracheophyta - ps14
Spermatophyta - ps15
Angiosperms - ps16
Mesangiospermae - ps17
Magnoliids/eudicots - ps18
Eudicots - ps19
Core_eudicots - ps20
Superrosids - ps21
Rosids - ps22
Eurosids - ps23
Malvids - ps24
Sap./Malv./Brassicales - ps25
Malvales/Brassicales - ps26
Brassicales - ps27
Cleomaceae/Brassicaceae - ps28
Brassicaceae - ps29
Camelineae - ps30
Arabidopsis - ps31
Arabidopsis_thaliana - ps32
p-value
2e-95
4e-72
6e-49
1e-25
2e-02
Log-odds
0.5
1.0
1.5
2.0
2.5
3.0
3.5
4.0

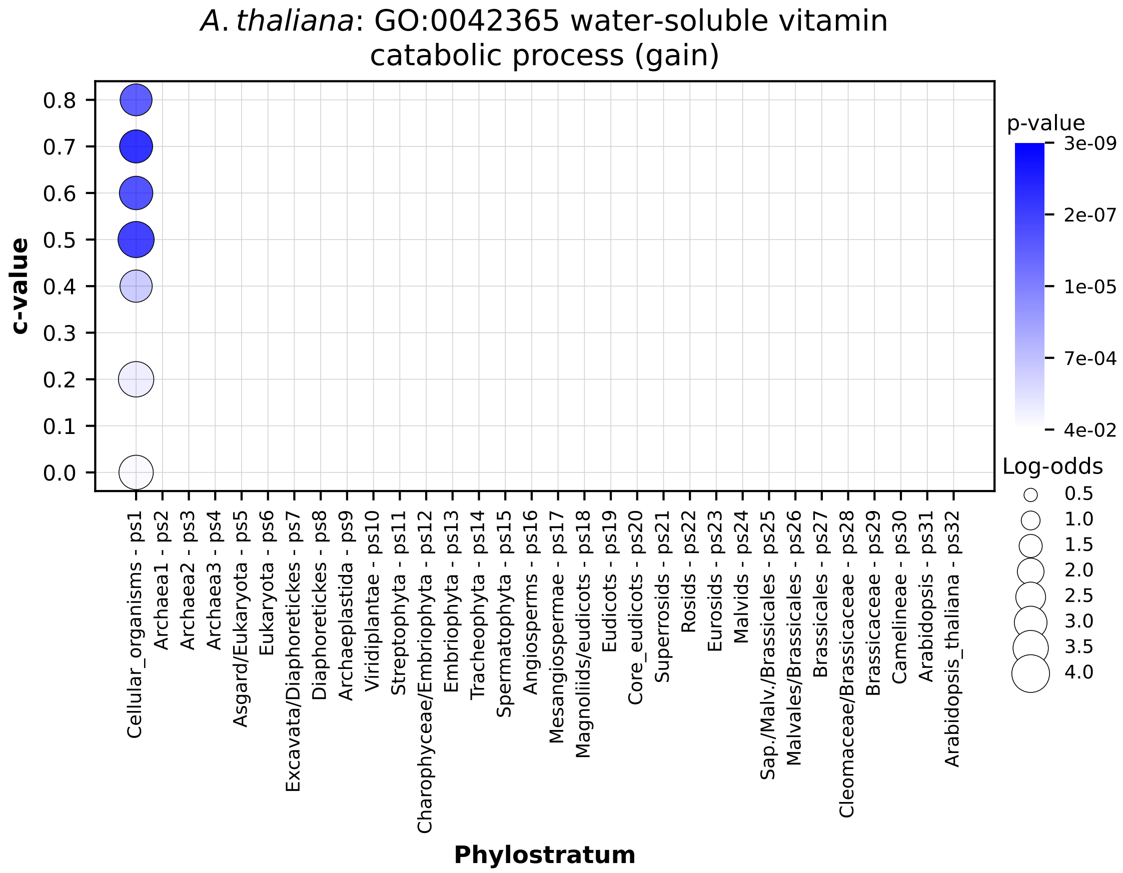

A. thaliana: GO:0042365 water-soluble vitamin catabolic process (gain)
c-value
0.8
0.7
0.6
0.5
0.4
0.3
0.2
0.1
0.0
p-value
3e-09
2e-07
1e-05
7e-04
4e-02
Log-odds
0.5
1.0
1.5
2.0
2.5
3.0
3.5
4.0
Cellular_organisms - ps1
Archaea1 - ps2
Archaea2 - ps3
Archaea3 - ps4
Asgard/Eukaryota - ps5
Eukaryota - ps6
Excavata/Diaphoretickes - ps7
Diaphoretickes - ps8
Archaeplastida - ps9
Viridiplantae - ps10
Streptophyta - ps11
Charophyceae/Embriophyta - ps12
Embriophyta - ps13
Tracheophyta - ps14
Spermatophyta - ps15
Angiosperms - ps16
Mesangiospermae - ps17
Magnoliids/eudicots - ps18
Eudicots - ps19
Core_eudicots - ps20
Superrosids - ps21
Rosids - ps22
Eurosids - ps23
Malvids - ps24
Sap./Malv./Brassicales - ps25
Malvales/Brassicales - ps26
Brassicales - ps27
Cleomaceae/Brassicaceae - ps28
Brassicaceae - ps29
Camelineae - ps30
Arabidopsis - ps31
Arabidopsis_thaliana - ps32
Phylostratum

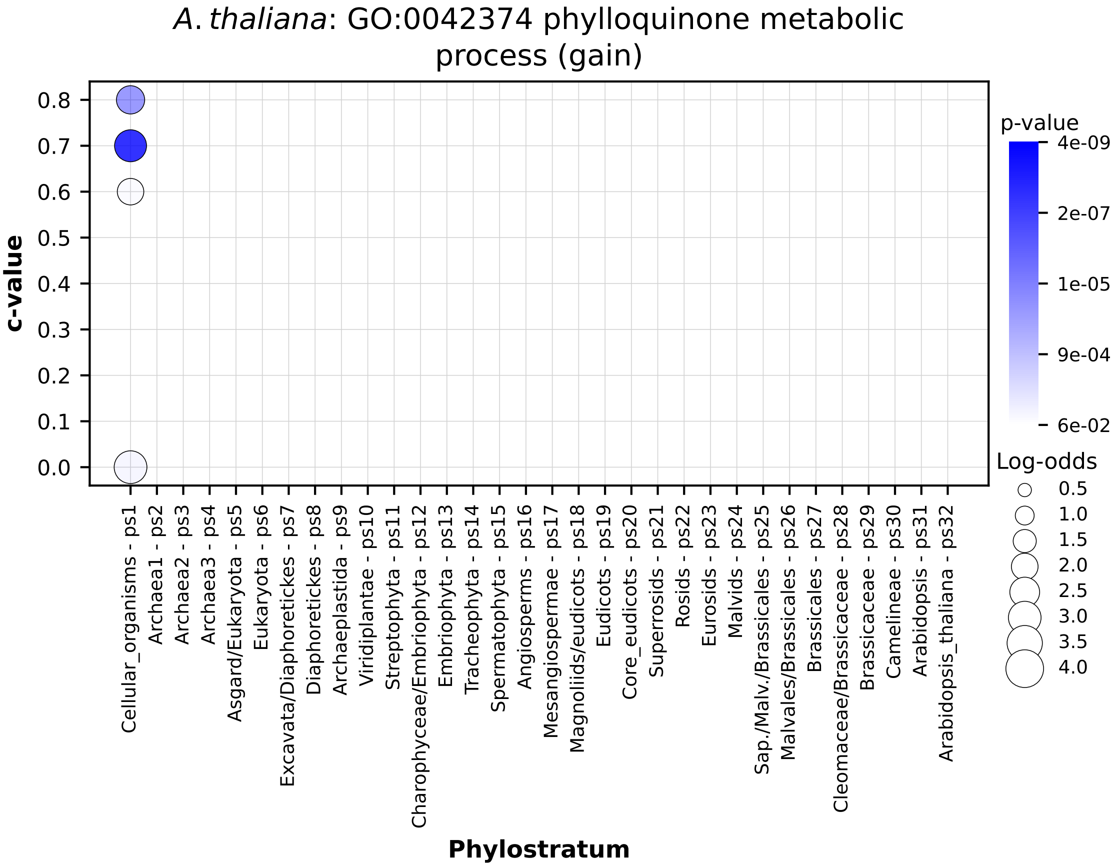

A. thaliana: GO:0042374 phylloquinone metabolic process (gain)
c-value
0.8
0.7
0.6
0.5
0.4
0.3
0.2
0.1
0.0
p-value
4e-09
2e-07
1e-05
9e-04
6e-02
Log-odds
0.5
1.0
1.5
2.0
2.5
3.0
3.5
4.0
Cellular_organisms - ps1
Archaea1 - ps2
Archaea2 - ps3
Archaea3 - ps4
Asgard/Eukaryota - ps5
Eukaryota - ps6
Excavata/Diaphoretickes - ps7
Diaphoretickes - ps8
Archaeplastida - ps9
Viridiplantae - ps10
Streptophyta - ps11
Charophyceae/Embriophyta - ps12
Embriophyta - ps13
Tracheophyta - ps14
Spermatophyta - ps15
Angiosperms - ps16
Mesangiospermae - ps17
Magnoliids/eudicots - ps18
Eudicots - ps19
Core_eudicots - ps20
Superrosids - ps21
Rosids - ps22
Eurosids - ps23
Malvids - ps24
Sap./Malv./Brassicales - ps25
Malvales/Brassicales - ps26
Brassicales - ps27
Cleomaceae/Brassicaceae - ps28
Brassicaceae - ps29
Camelineae - ps30
Arabidopsis - ps31
Arabidopsis_thaliana - ps32
Phylostratum

A. thaliana: GO:0042398 cellular modified amino acid biosynthetic process (gain)

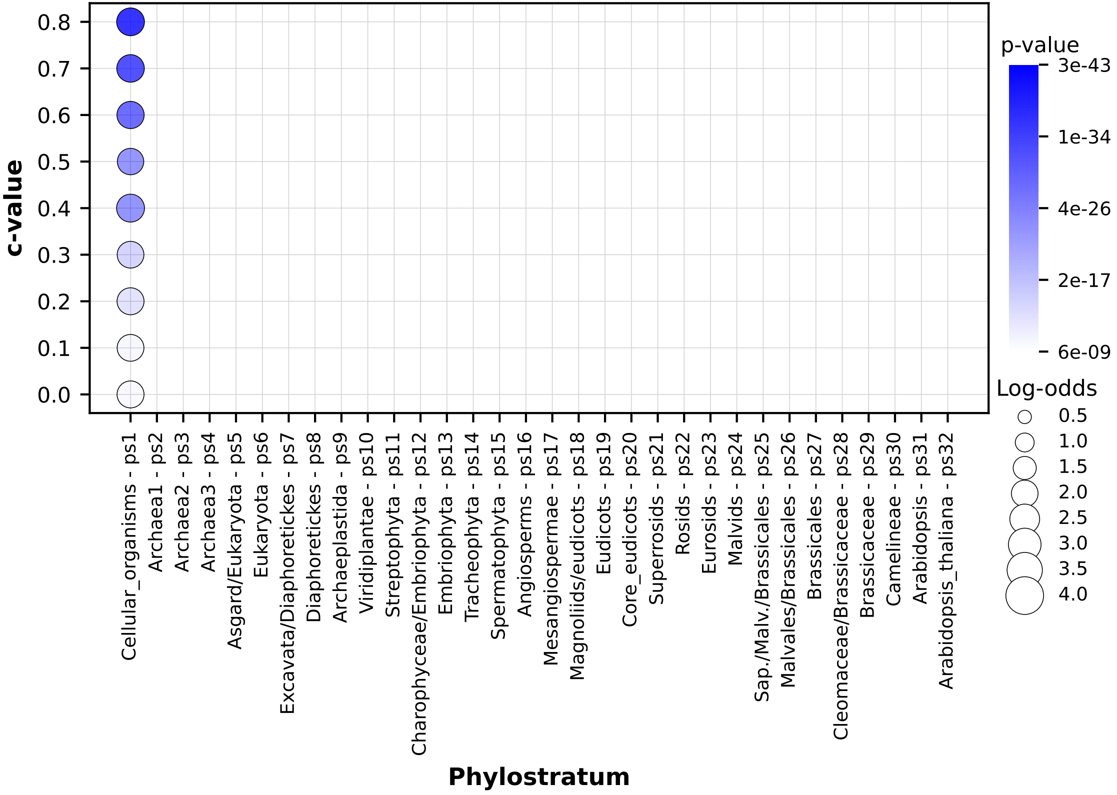

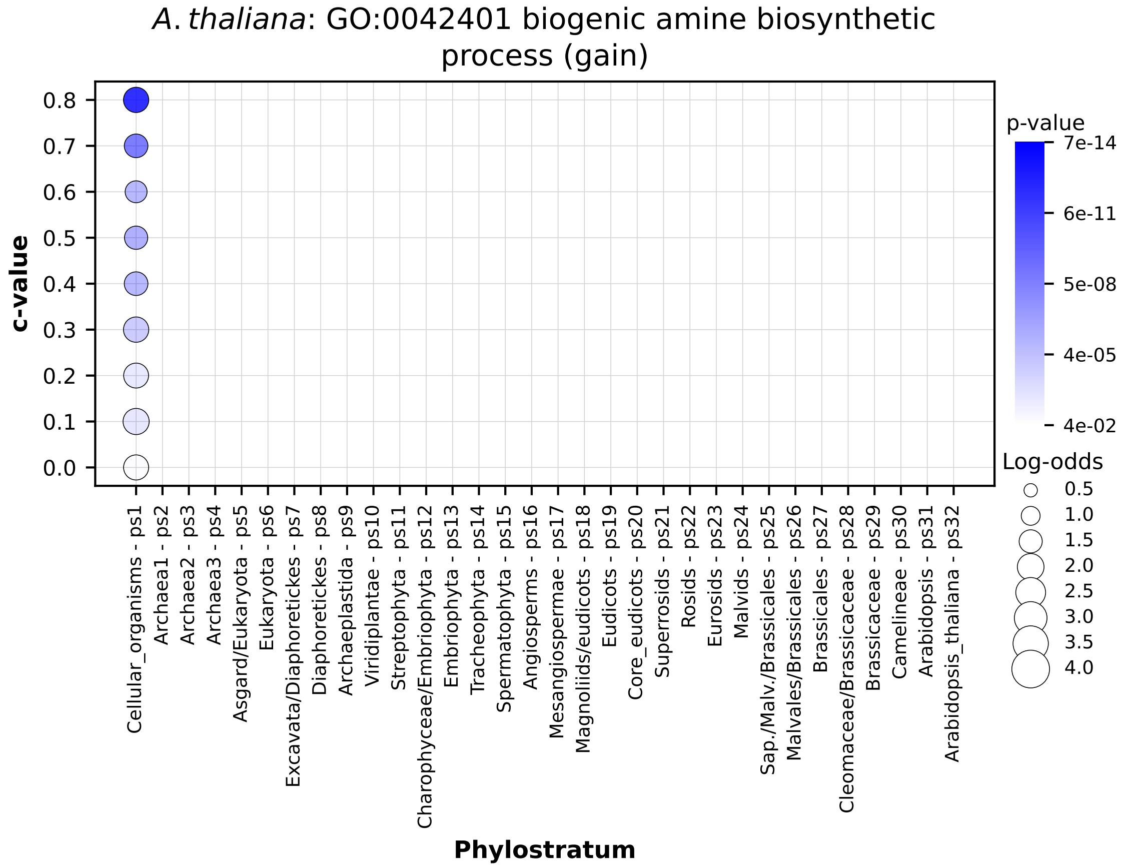

A. thaliana: GO:0042401 biogenic amine biosynthetic process (gain)
c-value
0.8
0.7
0.6
0.5
0.4
0.3
0.2
0.1
0.0
p-value
7e-14
6e-11
5e-08
4e-05
4e-02
Log-odds
0.5
1.0
1.5
2.0
2.5
3.0
3.5
4.0
Cellular_organisms - ps1
Archaea1 - ps2
Archaea2 - ps3
Archaea3 - ps4
Asgard/Eukaryota - ps5
Eukaryota - ps6
Excavata/Diaphoretickes - ps7
Diaphoretickes - ps8
Archaeplastida - ps9
Viridiplantae - ps10
Streptophyta - ps11
Charophyceae/Embriophyta - ps12
Embriophyta - ps13
Tracheophyta - ps14
Spermatophyta - ps15
Angiosperms - ps16
Mesangiospermae - ps17
Magnoliids/eudicots - ps18
Eudicots - ps19
Core_eudicots - ps20
Superrosids - ps21
Rosids - ps22
Eurosids - ps23
Malvids - ps24
Sap./Malv./Brassicales - ps25
Malvales/Brassicales - ps26
Brassicales - ps27
Cleomaceae/Brassicaceae - ps28
Brassicaceae - ps29
Camelineae - ps30
Arabidopsis - ps31
Arabidopsis_thaliana - ps32
Phylostratum

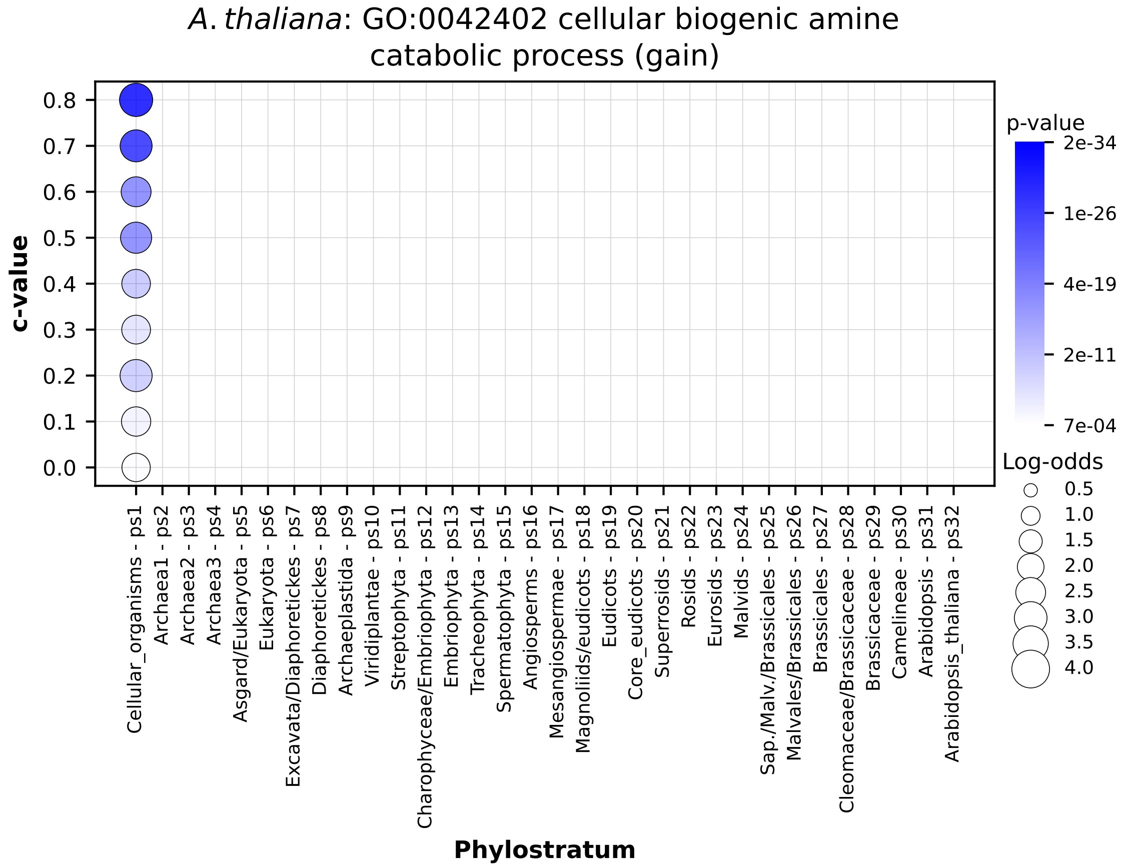

A. thaliana: GO:0042402 cellular biogenic amine catabolic process (gain)
c-value
0.8
0.7
0.6
0.5
0.4
0.3
0.2
0.1
0.0
p-value
2e-34
1e-26
4e-19
2e-11
7e-04
Log-odds
0.5
1.0
1.5
2.0
2.5
3.0
3.5
4.0
Cellular_organisms - ps1
Archaea1 - ps2
Archaea2 - ps3
Archaea3 - ps4
Asgard/Eukaryota - ps5
Eukaryota - ps6
Excavata/Diaphoretickes - ps7
Diaphoretickes - ps8
Archaeplastida - ps9
Viridiplantae - ps10
Streptophyta - ps11
Charophyceae/Embriophyta - ps12
Embriophyta - ps13
Tracheophyta - ps14
Spermatophyta - ps15
Angiosperms - ps16
Mesangiospermae - ps17
Magnoliids/eudicots - ps18
Eudicots - ps19
Core_eudicots - ps20
Superrosids - ps21
Rosids - ps22
Eurosids - ps23
Malvids - ps24
Sap./Malv./Brassicales - ps25
Malvales/Brassicales - ps26
Brassicales - ps27
Cleomaceae/Brassicaceae - ps28
Brassicaceae - ps29
Camelineae - ps30
Arabidopsis - ps31
Arabidopsis_thaliana - ps32
Phylostratum

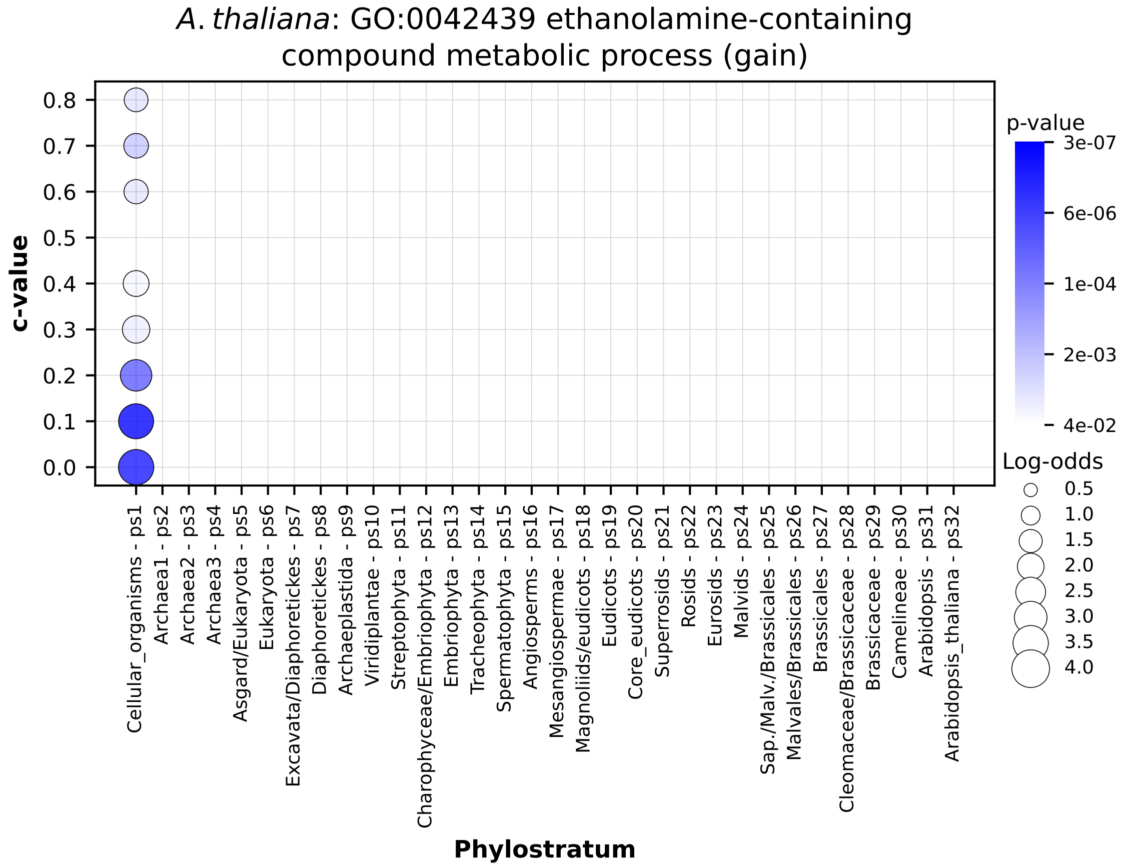

A. thaliana: GO:0042439 ethanolamine-containing compound metabolic process (gain)
c-value
0.8
0.7
0.6
0.5
0.4
0.3
0.2
0.1
0.0
p-value
3e-07
6e-06
1e-04
2e-03
4e-02
Log-odds
0.5
1.0
1.5
2.0
2.5
3.0
3.5
4.0
Cellular_organisms - ps1
Archaea1 - ps2
Archaea2 - ps3
Archaea3 - ps4
Asgard/Eukaryota - ps5
Eukaryota - ps6
Excavata/Diaphoretickes - ps7
Diaphoretickes - ps8
Archaeplastida - ps9
Viridiplantae - ps10
Streptophyta - ps11
Charophyceae/Embriophyta - ps12
Embriophyta - ps13
Tracheophyta - ps14
Spermatophyta - ps15
Angiosperms - ps16
Mesangiospermae - ps17
Magnoliids/eudicots - ps18
Eudicots - ps19
Core_eudicots - ps20
Superrosids - ps21
Rosids - ps22
Eurosids - ps23
Malvids - ps24
Sap./Malv./Brassicales - ps25
Malvales/Brassicales - ps26
Brassicales - ps27
Cleomaceae/Brassicaceae - ps28
Brassicaceae - ps29
Camelineae - ps30
Arabidopsis - ps31
Arabidopsis_thaliana - ps32
Phylostratum

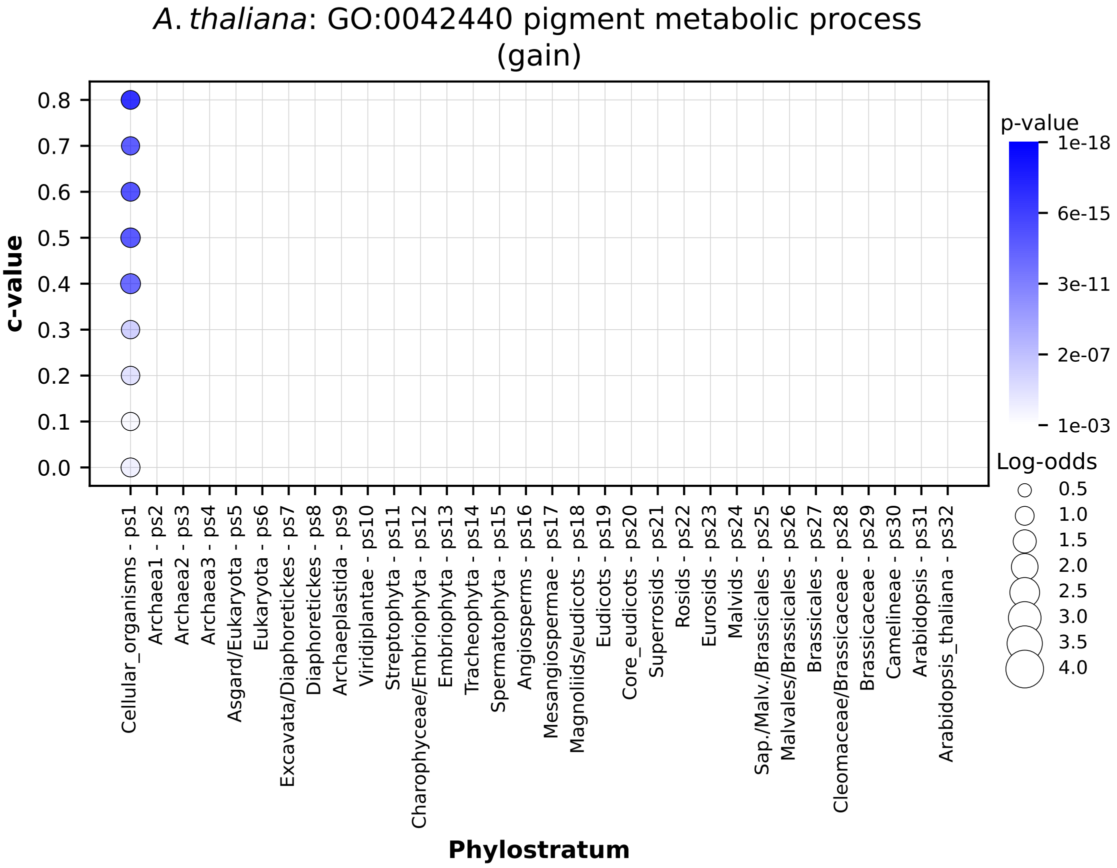

A. thaliana: GO:0042440 pigment metabolic process
(gain)
c-value
0.8
0.7
0.6
0.5
0.4
0.3
0.2
0.1
0.0
p-value
1e-18
6e-15
3e-11
2e-07
1e-03
Log-odds
0.5
1.0
1.5
2.0
2.5
3.0
3.5
4.0
Cellular_organisms - ps1
Archaea1 - ps2
Archaea2 - ps3
Archaea3 - ps4
Asgard/Eukaryota - ps5
Eukaryota - ps6
Excavata/Diaphoretickes - ps7
Diaphoretickes - ps8
Archaeplastida - ps9
Viridiplantae - ps10
Streptophyta - ps11
Charophyceae/Embriophyta - ps12
Embriophyta - ps13
Tracheophyta - ps14
Spermatophyta - ps15
Angiosperms - ps16
Mesangiospermae - ps17
Magnoliids/eudicots - ps18
Eudicots - ps19
Core_eudicots - ps20
Superrosids - ps21
Rosids - ps22
Eurosids - ps23
Malvids - ps24
Sap./Malv./Brassicales - ps25
Malvales/Brassicales - ps26
Brassicales - ps27
Cleomaceae/Brassicaceae - ps28
Brassicaceae - ps29
Camelineae - ps30
Arabidopsis - ps31
Arabidopsis_thaliana - ps32
Phylostratum

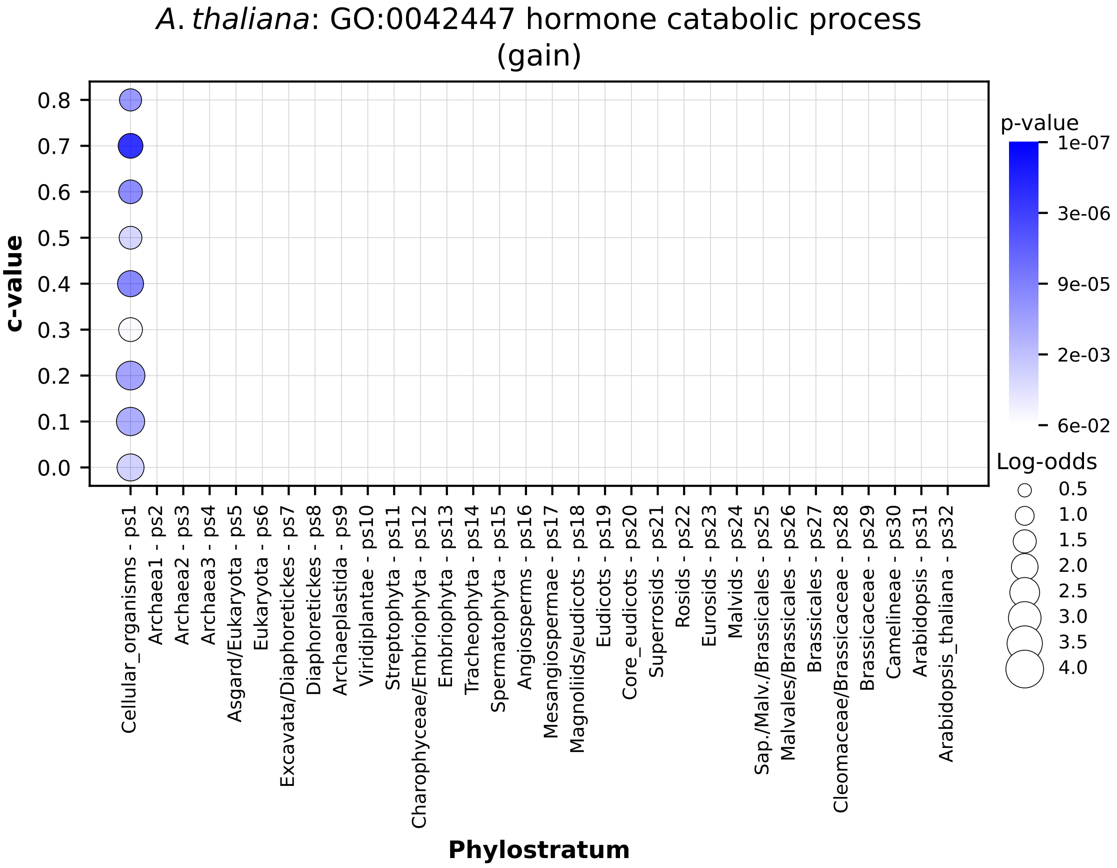

A. thaliana: GO:0042447 hormone catabolic process (gain)
c-value
0.0
0.1
0.2
0.3
0.4
0.5
0.6
0.7
0.8
Phylostratum
Cellular_organisms - ps1
Archaea1 - ps2
Archaea2 - ps3
Archaea3 - ps4
Asgard/Eukaryota - ps5
Eukaryota - ps6
Excavata/Diaphoretickes - ps7
Diaphoretickes - ps8
Archaeplastida - ps9
Viridiplantae - ps10
Streptophyta - ps11
Charophyceae/Embriophyta - ps12
Embriophyta - ps13
Tracheophyta - ps14
Spermatophyta - ps15
Angiosperms - ps16
Mesangiospermae - ps17
Magnoliids/eudicots - ps18
Eudicots - ps19
Core_eudicots - ps20
Superrosids - ps21
Rosids - ps22
Eurosids - ps23
Malvids - ps24
Sap./Malv./Brassicales - ps25
Malvales/Brassicales - ps26
Brassicales - ps27
Cleomaceae/Brassicaceae - ps28
Brassicaceae - ps29
Camelineae - ps30
Arabidopsis - ps31
Arabidopsis_thaliana - ps32
p-value
1e-07
3e-06
9e-05
2e-03
6e-02
Log-odds
0.5
1.0
1.5
2.0
2.5
3.0
3.5
4.0

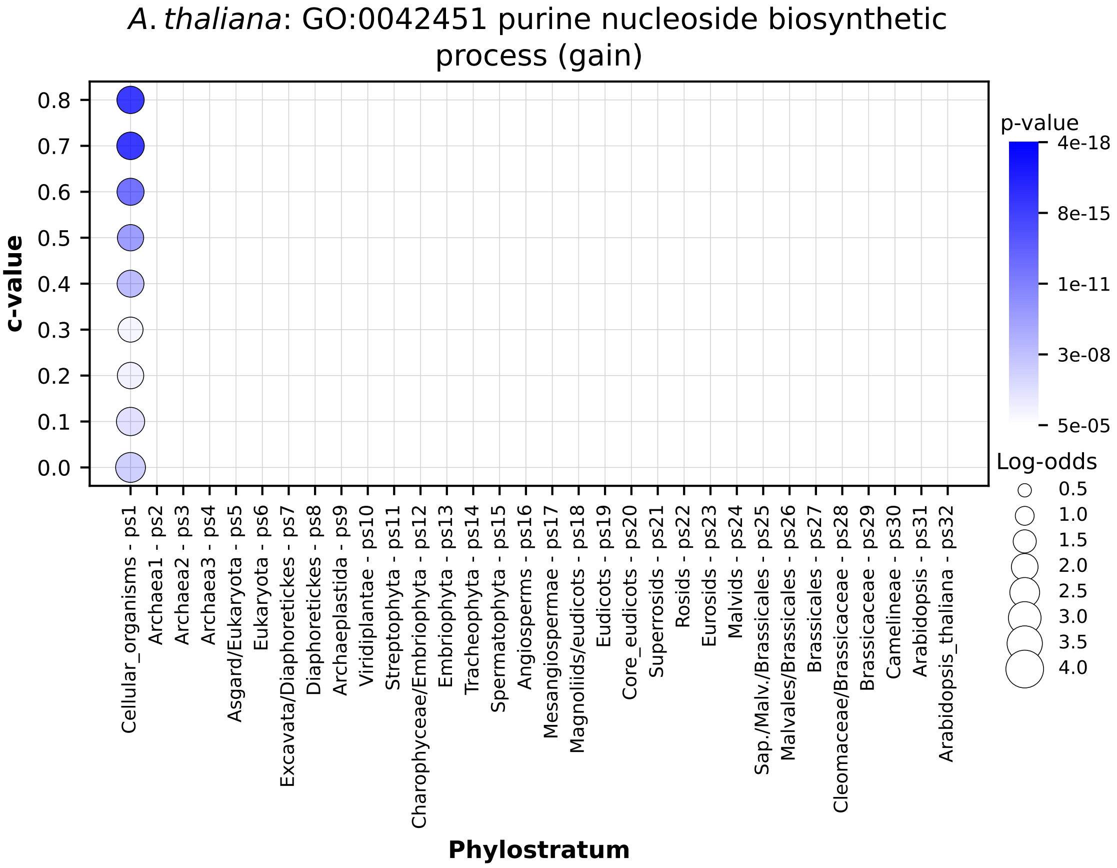
A. thaliana: GO:0042451 purine nucleoside biosynthetic process (gain)
c-value
0.8
0.7
0.6
0.5
0.4
0.3
0.2
0.1
0.0
p-value
4e-18
8e-15
1e-11
3e-08
5e-05
Log-odds
0.5
1.0
1.5
2.0
2.5
3.0
3.5
4.0
Cellular_organisms - ps1
Archaea1 - ps2
Archaea2 - ps3
Archaea3 - ps4
Asgard/Eukaryota - ps5
Eukaryota - ps6
Excavata/Diaphoretickes - ps7
Diaphoretickes - ps8
Archaeplastida - ps9
Viridiplantae - ps10
Streptophyta - ps11
Charophyceae/Embriophyta - ps12
Embriophyta - ps13
Tracheophyta - ps14
Spermatophyta - ps15
Angiosperms - ps16
Mesangiospermae - ps17
Magnoliids/eudicots - ps18
Eudicots - ps19
Core_eudicots - ps20
Superrosids - ps21
Rosids - ps22
Eurosids - ps23
Malvids - ps24
Sap./Malv./Brassicales - ps25
Malvales/Brassicales - ps26
Brassicales - ps27
Cleomaceae/Brassicaceae - ps28
Brassicaceae - ps29
Camelineae - ps30
Arabidopsis - ps31
Arabidopsis_thaliana - ps32
Phylostratum

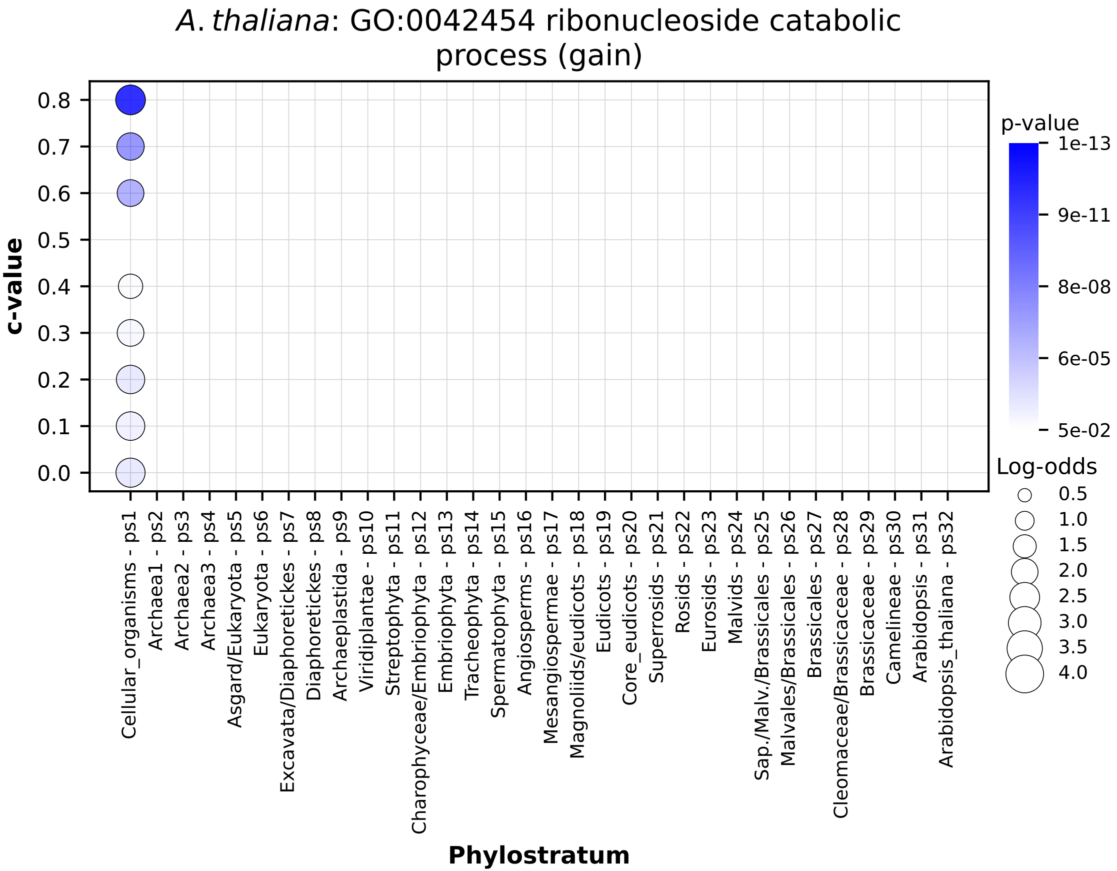

A. thaliana: GO:0042454 ribonucleoside catabolic process (gain)
c-value
0.8
0.7
0.6
0.5
0.4
0.3
0.2
0.1
0.0
p-value
1e-13
9e-11
8e-08
6e-05
5e-02
Log-odds
0.5
1.0
1.5
2.0
2.5
3.0
3.5
4.0
Cellular_organisms - ps1
Archaea1 - ps2
Archaea2 - ps3
Archaea3 - ps4
Asgard/Eukaryota - ps5
Eukaryota - ps6
Excavata/Diaphoretickes - ps7
Diaphoretickes - ps8
Archaeplastida - ps9
Viridiplantae - ps10
Streptophyta - ps11
Charophyceae/Embriophyta - ps12
Embriophyta - ps13
Tracheophyta - ps14
Spermatophyta - ps15
Angiosperms - ps16
Mesangiospermae - ps17
Magnoliids/eudicots - ps18
Eudicots - ps19
Core_eudicots - ps20
Superrosids - ps21
Rosids - ps22
Eurosids - ps23
Malvids - ps24
Sap./Malv./Brassicales - ps25
Malvales/Brassicales - ps26
Brassicales - ps27
Cleomaceae/Brassicaceae - ps28
Brassicaceae - ps29
Camelineae - ps30
Arabidopsis - ps31
Arabidopsis_thaliana - ps32
Phylostratum

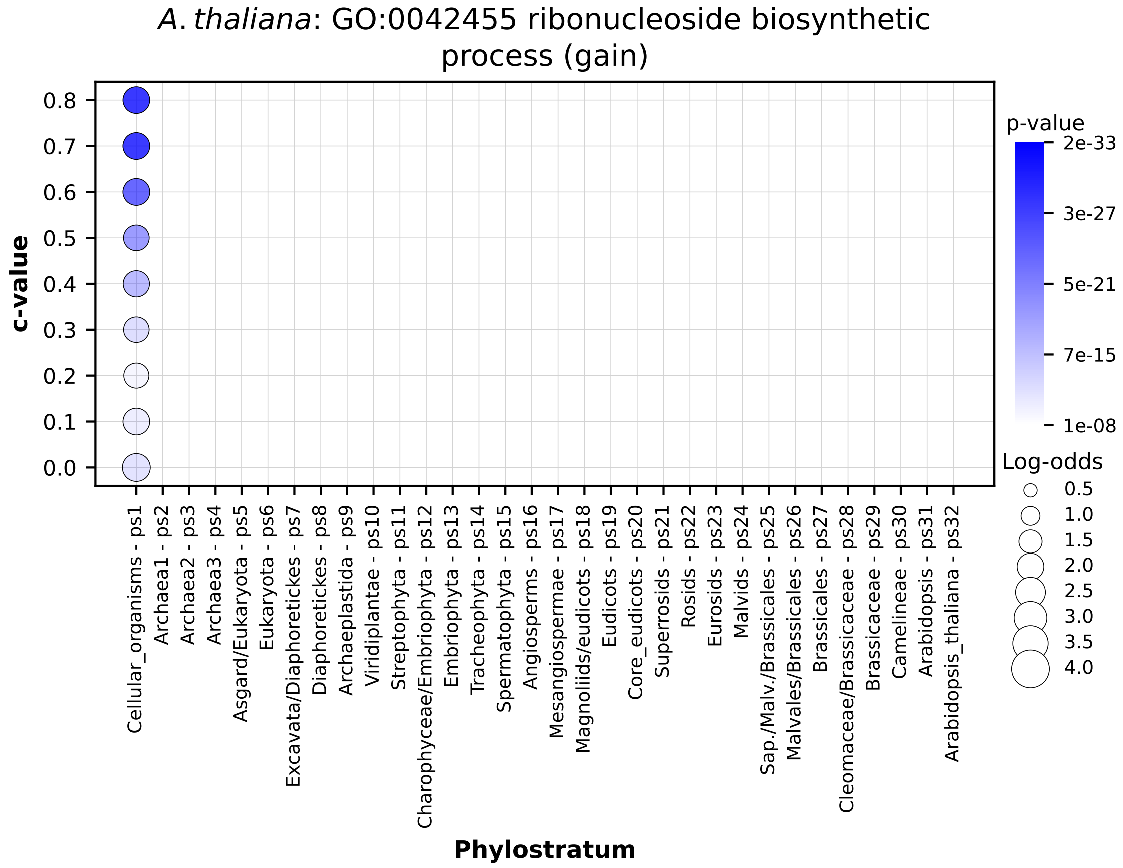
A. thaliana: GO:0042455 ribonucleoside biosynthetic process (gain)
c-value
0.8
0.7
0.6
0.5
0.4
0.3
0.2
0.1
0.0
p-value
2e-33
3e-27
5e-21
7e-15
1e-08
Log-odds
0.5
1.0
1.5
2.0
2.5
3.0
3.5
4.0
Cellular_organisms - ps1
Archaea1 - ps2
Archaea2 - ps3
Archaea3 - ps4
Asgard/Eukaryota - ps5
Eukaryota - ps6
Excavata/Diaphoretickes - ps7
Diaphoretickes - ps8
Archaeplastida - ps9
Viridiplantae - ps10
Streptophyta - ps11
Charophyceae/Embriophyta - ps12
Embriophyta - ps13
Tracheophyta - ps14
Spermatophyta - ps15
Angiosperms - ps16
Mesangiospermae - ps17
Magnoliids/eudicots - ps18
Eudicots - ps19
Core_eudicots - ps20
Superrosids - ps21
Rosids - ps22
Eurosids - ps23
Malvids - ps24
Sap./Malv./Brassicales - ps25
Malvales/Brassicales - ps26
Brassicales - ps27
Cleomaceae/Brassicaceae - ps28
Brassicaceae - ps29
Camelineae - ps30
Arabidopsis - ps31
Arabidopsis_thaliana - ps32
Phylostratum

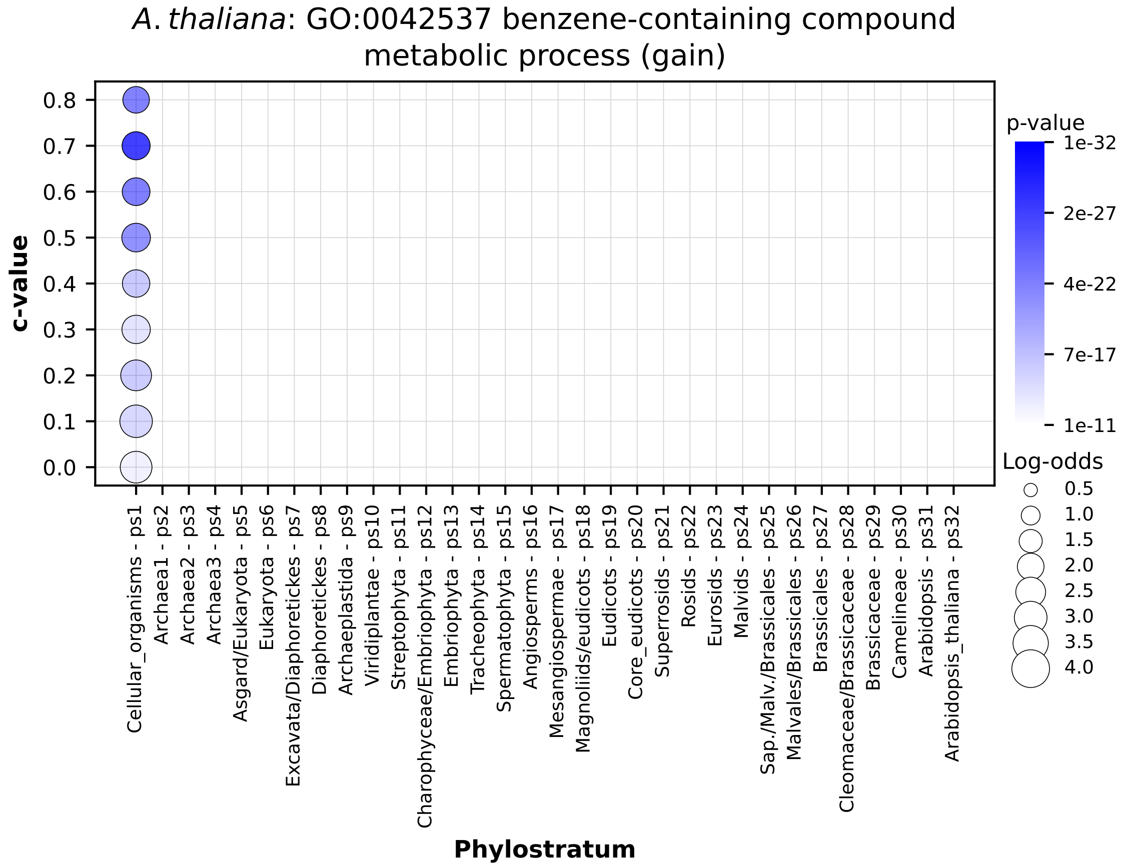

A. thaliana: GO:0042537 benzene-containing compound metabolic process (gain)
c-value
0.8
0.7
0.6
0.5
0.4
0.3
0.2
0.1
0.0
p-value
1e-32
2e-27
4e-22
7e-17
1e-11
Log-odds
0.5
1.0
1.5
2.0
2.5
3.0
3.5
4.0
Cellular_organisms - ps1
Archaea1 - ps2
Archaea2 - ps3
Archaea3 - ps4
Asgard/Eukaryota - ps5
Eukaryota - ps6
Excavata/Diaphoretickes - ps7
Diaphoretickes - ps8
Archaeplastida - ps9
Viridiplantae - ps10
Streptophyta - ps11
Charophyceae/Embriophyta - ps12
Embriophyta - ps13
Tracheophyta - ps14
Spermatophyta - ps15
Angiosperms - ps16
Mesangiospermae - ps17
Magnoliids/eudicots - ps18
Eudicots - ps19
Core_eudicots - ps20
Superrosids - ps21
Rosids - ps22
Eurosids - ps23
Malvids - ps24
Sap./Malv./Brassicales - ps25
Malvales/Brassicales - ps26
Brassicales - ps27
Cleomaceae/Brassicaceae - ps28
Brassicaceae - ps29
Camelineae - ps30
Arabidopsis - ps31
Arabidopsis_thaliana - ps32
Phylostratum

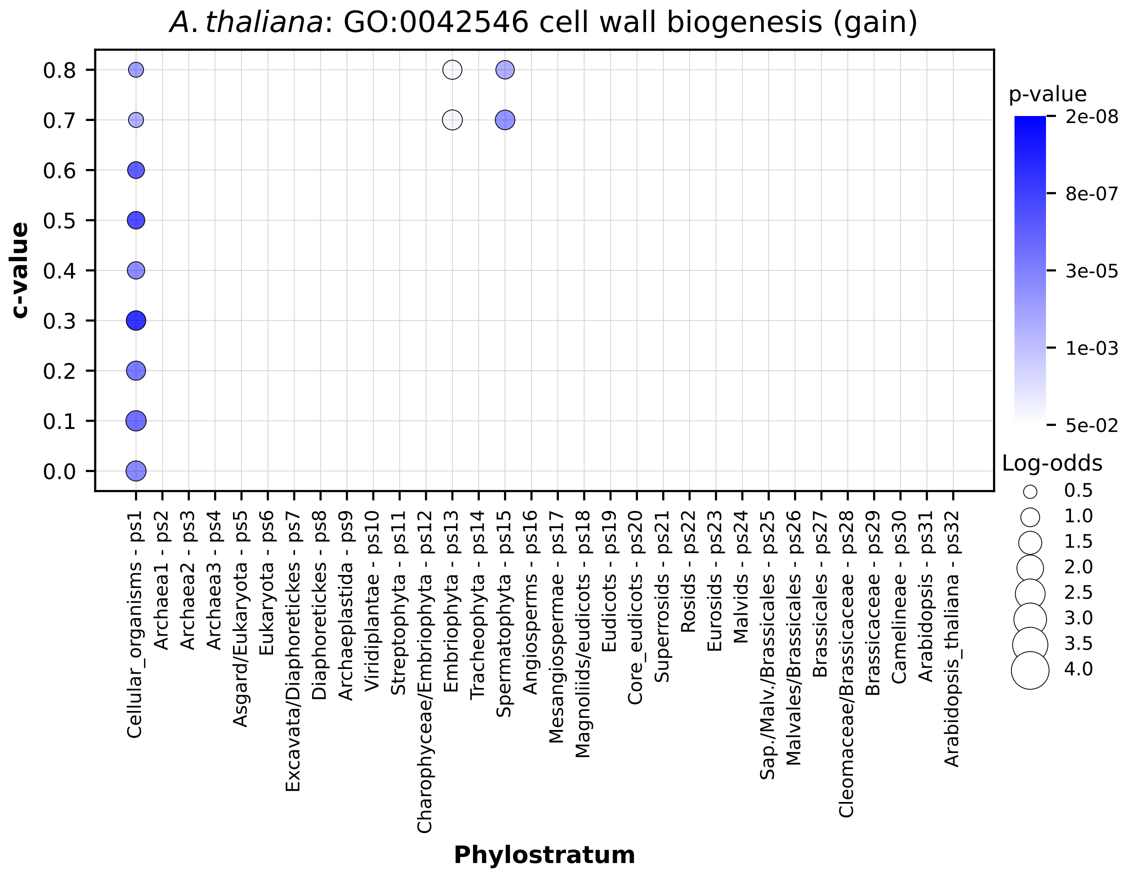

A. thaliana: GO:0042546 cell wall biogenesis (gain)
c-value
0.8
0.7
0.6
0.5
0.4
0.3
0.2
0.1
0.0
p-value
2e-08
8e-07
3e-05
1e-03
5e-02
Log-odds
0.5
1.0
1.5
2.0
2.5
3.0
3.5
4.0
Cellular_organisms - ps1
Archaea1 - ps2
Archaea2 - ps3
Archaea3 - ps4
Asgard/Eukaryota - ps5
Eukaryota - ps6
Excavata/Diaphoretickes - ps7
Diaphoretickes - ps8
Archaeplastida - ps9
Viridiplantae - ps10
Streptophyta - ps11
Charophyceae/Embriophyta - ps12
Embriophyta - ps13
Tracheophyta - ps14
Spermatophyta - ps15
Angiosperms - ps16
Mesangiospermae - ps17
Magnoliids/eudicots - ps18
Eudicots - ps19
Core_eudicots - ps20
Superrosids - ps21
Rosids - ps22
Eurosids - ps23
Malvids - ps24
Sap./Malv./Brassicales - ps25
Malvales/Brassicales - ps26
Brassicales - ps27
Cleomaceae/Brassicaceae - ps28
Brassicaceae - ps29
Camelineae - ps30
Arabidopsis - ps31
Arabidopsis_thaliana - ps32
Phylostratum

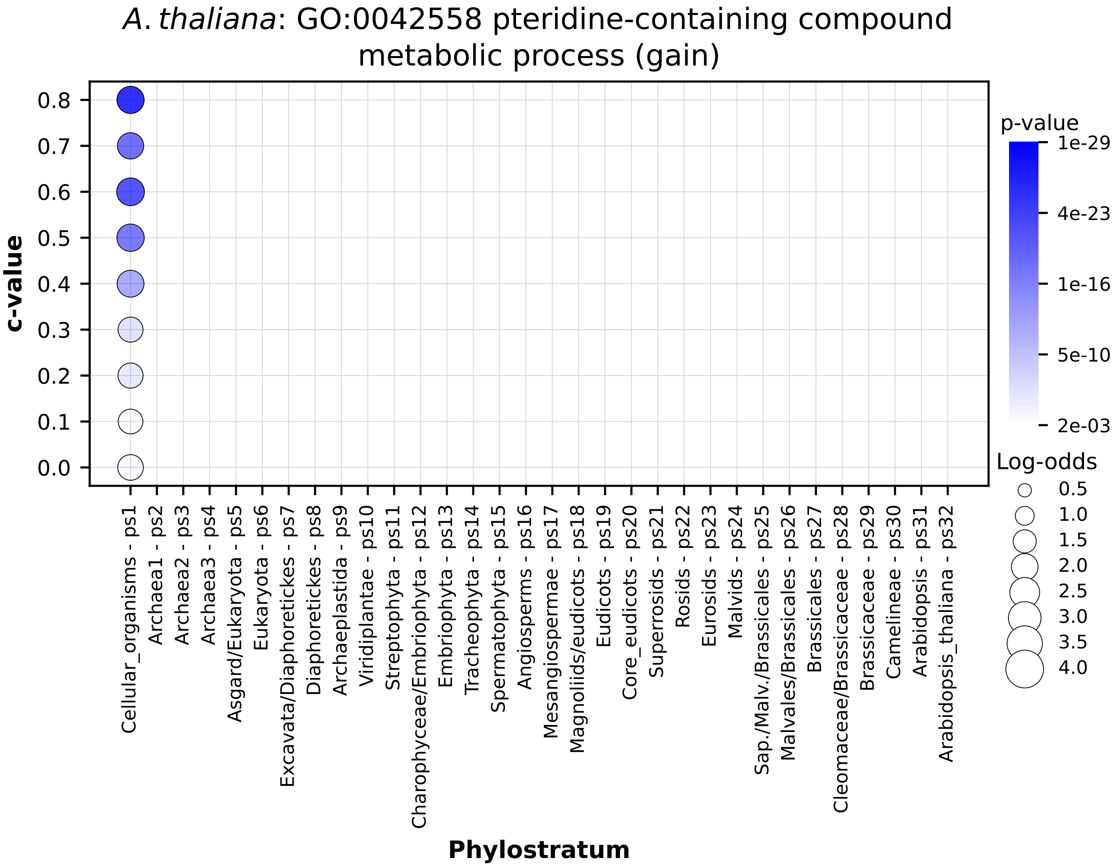
A. thaliana: GO:0042558 pteridine-containing compound metabolic process (gain)
c-value
0.8
0.7
0.6
0.5
0.4
0.3
0.2
0.1
0.0
p-value
1e-29
4e-23
1e-16
5e-10
2e-03
Log-odds
0.5
1.0
1.5
2.0
2.5
3.0
3.5
4.0
Cellular_organisms - ps1
Archaea1 - ps2
Archaea2 - ps3
Archaea3 - ps4
Asgard/Eukaryota - ps5
Eukaryota - ps6
Excavata/Diaphoretickes - ps7
Diaphoretickes - ps8
Archaeplastida - ps9
Viridiplantae - ps10
Streptophyta - ps11
Charophyceae/Embriophyta - ps12
Embriophyta - ps13
Tracheophyta - ps14
Spermatophyta - ps15
Angiosperms - ps16
Mesangiospermae - ps17
Magnoliids/eudicots - ps18
Eudicots - ps19
Core_eudicots - ps20
Superrosids - ps21
Rosids - ps22
Eurosids - ps23
Malvids - ps24
Sap./Malv./Brassicales - ps25
Malvales/Brassicales - ps26
Brassicales - ps27
Cleomaceae/Brassicaceae - ps28
Brassicaceae - ps29
Camelineae - ps30
Arabidopsis - ps31
Arabidopsis_thaliana - ps32
Phylostratum

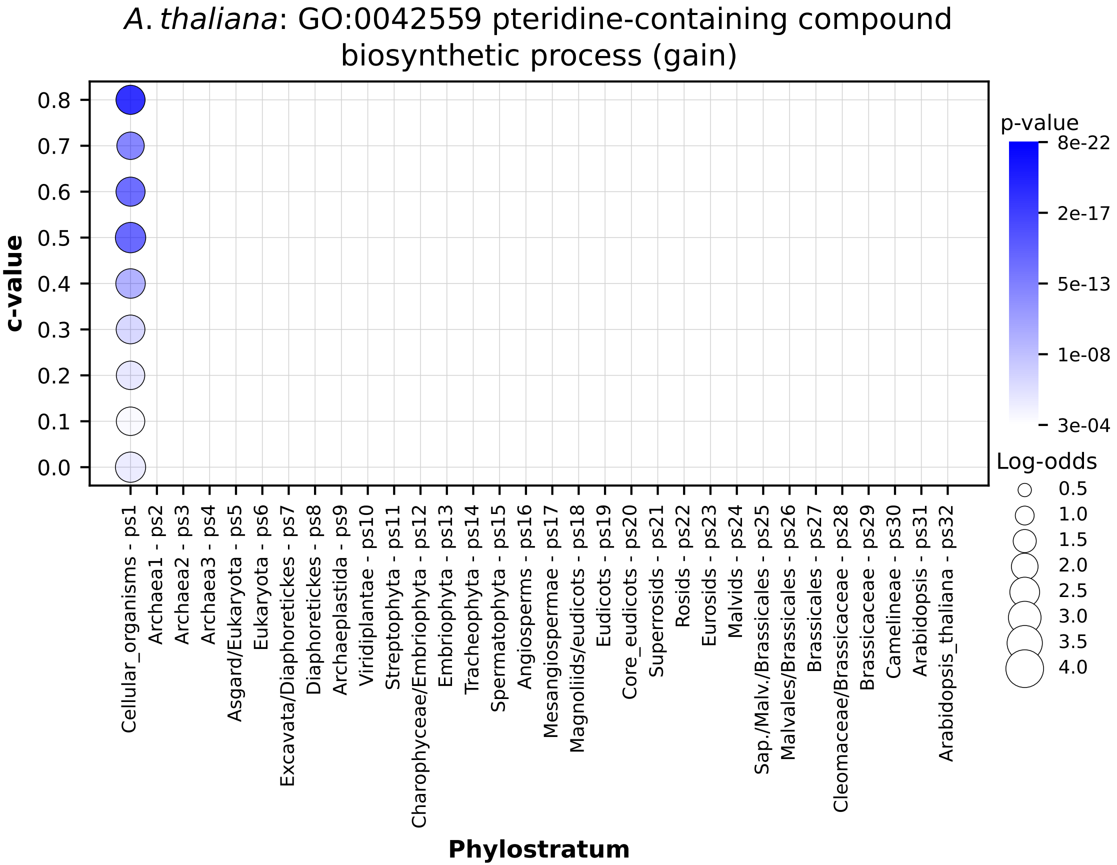

A. thaliana: GO:0042559 pteridine-containing compound biosynthetic process (gain)
c-value
0.8
0.7
0.6
0.5
0.4
0.3
0.2
0.1
0.0
p-value
8e-22
2e-17
5e-13
1e-08
3e-04
Log-odds
0.5
1.0
1.5
2.0
2.5
3.0
3.5
4.0
Cellular_organisms - ps1
Archaea1 - ps2
Archaea2 - ps3
Archaea3 - ps4
Asgard/Eukaryota - ps5
Eukaryota - ps6
Excavata/Diaphoretickes - ps7
Diaphoretickes - ps8
Archaeplastida - ps9
Viridiplantae - ps10
Streptophyta - ps11
Charophyceae/Embriophyta - ps12
Embriophyta - ps13
Tracheophyta - ps14
Spermatophyta - ps15
Angiosperms - ps16
Mesangiospermae - ps17
Magnoliids/eudicots - ps18
Eudicots - ps19
Core_eudicots - ps20
Superrosids - ps21
Rosids - ps22
Eurosids - ps23
Malvids - ps24
Sap./Malv./Brassicales - ps25
Malvales/Brassicales - ps26
Brassicales - ps27
Cleomaceae/Brassicaceae - ps28
Brassicaceae - ps29
Camelineae - ps30
Arabidopsis - ps31
Arabidopsis_thaliana - ps32
Phylostratum

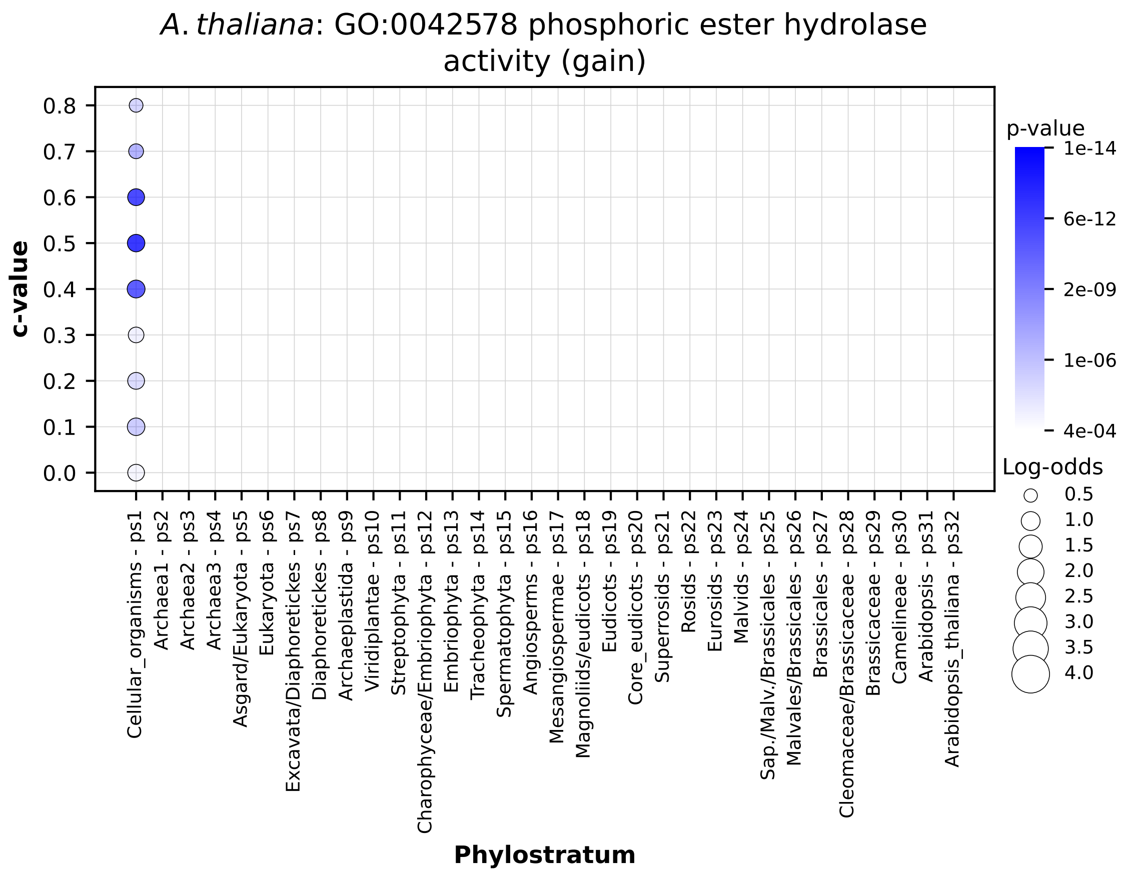

A. thaliana: GO:0042578 phosphoric ester hydrolase activity (gain)
c-value
0.8
0.7
0.6
0.5
0.4
0.3
0.2
0.1
0.0
p-value
1e-14
6e-12
2e-09
1e-06
4e-04
Log-odds
0.5
1.0
1.5
2.0
2.5
3.0
3.5
4.0
Cellular_organisms - ps1
Archaea1 - ps2
Archaea2 - ps3
Archaea3 - ps4
Asgard/Eukaryota - ps5
Eukaryota - ps6
Excavata/Diaphoretickes - ps7
Diaphoretickes - ps8
Archaeplastida - ps9
Viridiplantae - ps10
Streptophyta - ps11
Charophyceae/Embriophyta - ps12
Embriophyta - ps13
Tracheophyta - ps14
Spermatophyta - ps15
Angiosperms - ps16
Mesangiospermae - ps17
Magnoliids/eudicots - ps18
Eudicots - ps19
Core_eudicots - ps20
Superrosids - ps21
Rosids - ps22
Eurosids - ps23
Malvids - ps24
Sap./Malv./Brassicales - ps25
Malvales/Brassicales - ps26
Brassicales - ps27
Cleomaceae/Brassicaceae - ps28
Brassicaceae - ps29
Camelineae - ps30
Arabidopsis - ps31
Arabidopsis_thaliana - ps32
Phylostratum

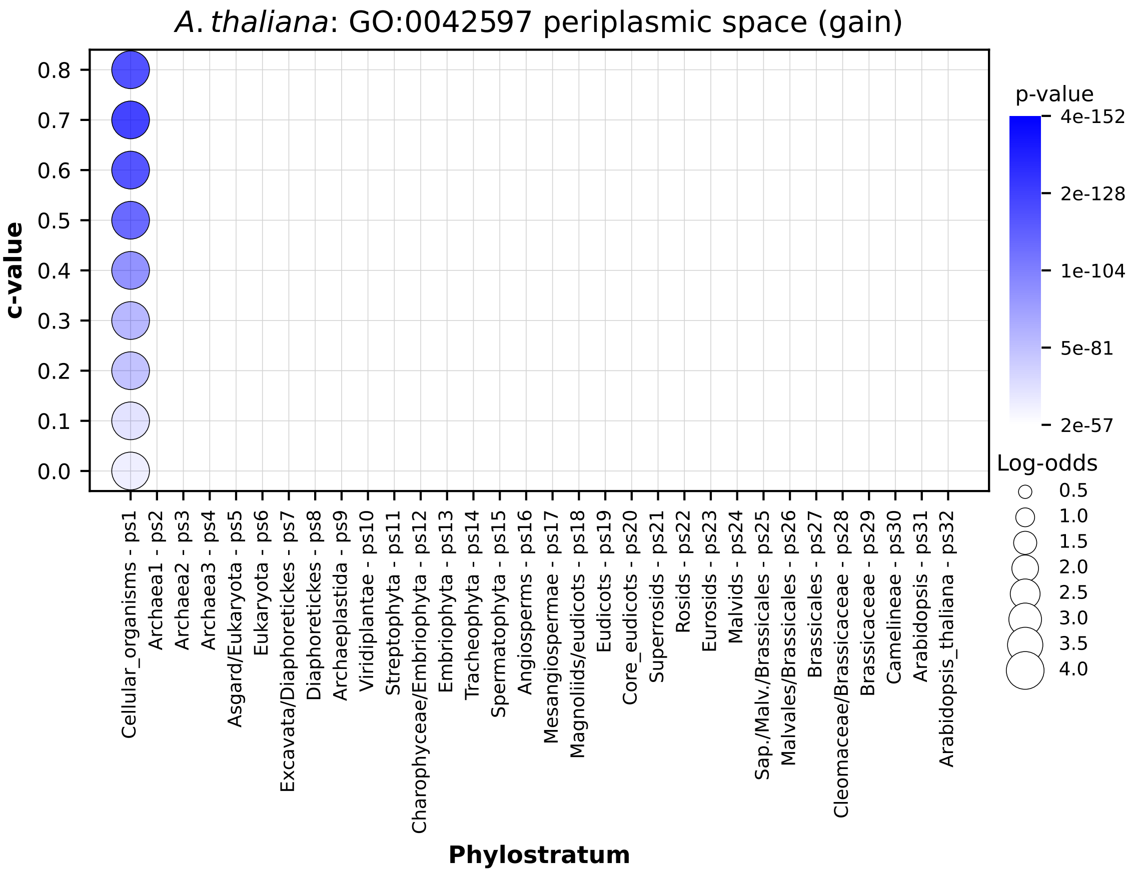

A. thaliana: GO:0042597 periplasmic space (gain)
c-value
0.0
0.1
0.2
0.3
0.4
0.5
0.6
0.7
0.8
p-value
4e-152
2e-128
1e-104
5e-81
2e-57
Log-odds
0.5
1.0
1.5
2.0
2.5
3.0
3.5
4.0
Cellular_organisms - ps1
Archaea1 - ps2
Archaea2 - ps3
Archaea3 - ps4
Asgard/Eukaryota - ps5
Eukaryota - ps6
Excavata/Diaphoretickes - ps7
Diaphoretickes - ps8
Archaeplastida - ps9
Viridiplantae - ps10
Streptophyta - ps11
Charophyceae/Embriophyta - ps12
Embriophyta - ps13
Tracheophyta - ps14
Spermatophyta - ps15
Angiosperms - ps16
Mesangiospermae - ps17
Magnoliids/eudicots - ps18
Eudicots - ps19
Core_eudicots - ps20
Superrosids - ps21
Rosids - ps22
Eurosids - ps23
Malvids - ps24
Sap./Malv./Brassicales - ps25
Malvales/Brassicales - ps26
Brassicales - ps27
Cleomaceae/Brassicaceae - ps28
Brassicaceae - ps29
Camelineae - ps30
Arabidopsis - ps31
Arabidopsis_thaliana - ps32
Phylostratum

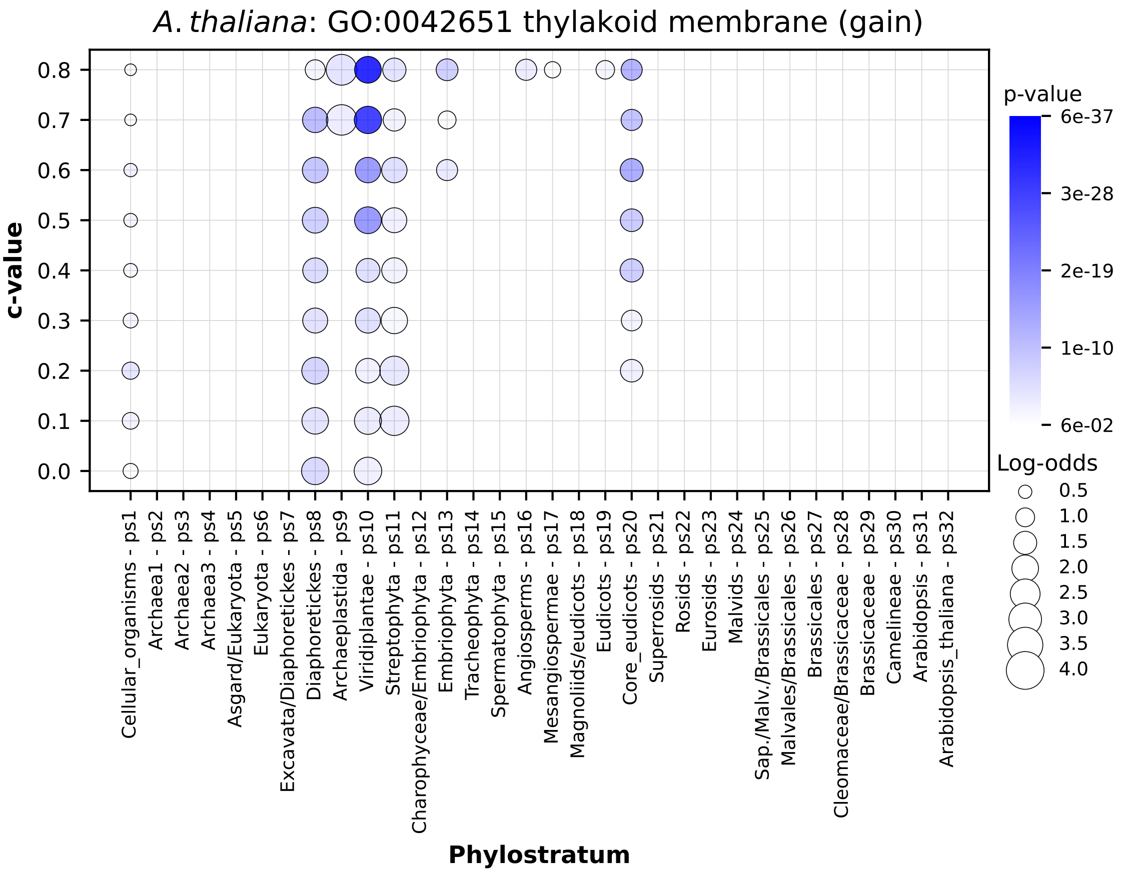
A. thaliana: GO:0042651 thylakoid membrane (gain)
c-value
0.8
0.7
0.6
0.5
0.4
0.3
0.2
0.1
0.0
Phylostratum
Cellular_organisms - ps1
Archaea1 - ps2
Archaea2 - ps3
Archaea3 - ps4
Asgard/Eukaryota - ps5
Eukaryota - ps6
Excavata/Diaphoretickes - ps7
Diaphoretickes - ps8
Archaeplastida - ps9
Viridiplantae - ps10
Streptophyta - ps11
Charophyceae/Embriophyta - ps12
Embriophyta - ps13
Tracheophyta - ps14
Spermatophyta - ps15
Angiosperms - ps16
Mesangiospermae - ps17
Magnoliids/eudicots - ps18
Eudicots - ps19
Core_eudicots - ps20
Superrosids - ps21
Rosids - ps22
Eurosids - ps23
Malvids - ps24
Sap./Malv./Brassicales - ps25
Malvales/Brassicales - ps26
Brassicales - ps27
Cleomaceae/Brassicaceae - ps28
Brassicaceae - ps29
Camelineae - ps30
Arabidopsis - ps31
Arabidopsis_thaliana - ps32
p-value
6e-37
3e-28
2e-19
1e-10
6e-02
Log-odds
0.5
1.0
1.5
2.0
2.5
3.0
3.5
4.0

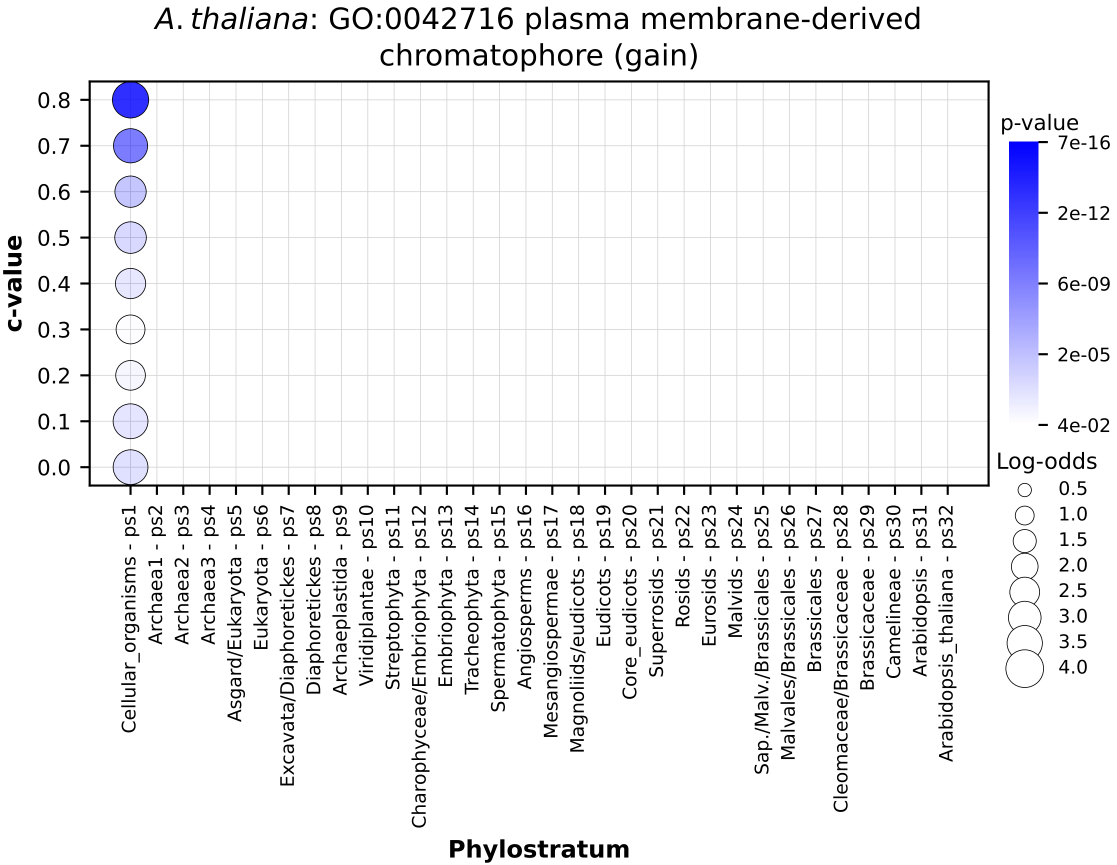
A. thaliana: GO:0042716 plasma membrane-derived chromatophore (gain)
c-value
0.0
0.1
0.2
0.3
0.4
0.5
0.6
0.7
0.8
p-value
7e-16
2e-12
6e-09
2e-05
4e-02
Log-odds
0.5
1.0
1.5
2.0
2.5
3.0
3.5
4.0
Cellular_organisms - ps1
Archaea1 - ps2
Archaea2 - ps3
Archaea3 - ps4
Asgard/Eukaryota - ps5
Eukaryota - ps6
Excavata/Diaphoretickes - ps7
Diaphoretickes - ps8
Archaeplastida - ps9
Viridiplantae - ps10
Streptophyta - ps11
Charophyceae/Embriophyta - ps12
Embriophyta - ps13
Tracheophyta - ps14
Spermatophyta - ps15
Angiosperms - ps16
Mesangiospermae - ps17
Magnoliids/eudicots - ps18
Eudicots - ps19
Core_eudicots - ps20
Superrosids - ps21
Rosids - ps22
Eurosids - ps23
Malvids - ps24
Sap./Malv./Brassicales - ps25
Malvales/Brassicales - ps26
Brassicales - ps27
Cleomaceae/Brassicaceae - ps28
Brassicaceae - ps29
Camelineae - ps30
Arabidopsis - ps31
Arabidopsis_thaliana - ps32
Phylostratum

A. thaliana: GO:0042717 plasma membrane-derived chromatophore membrane (gain)

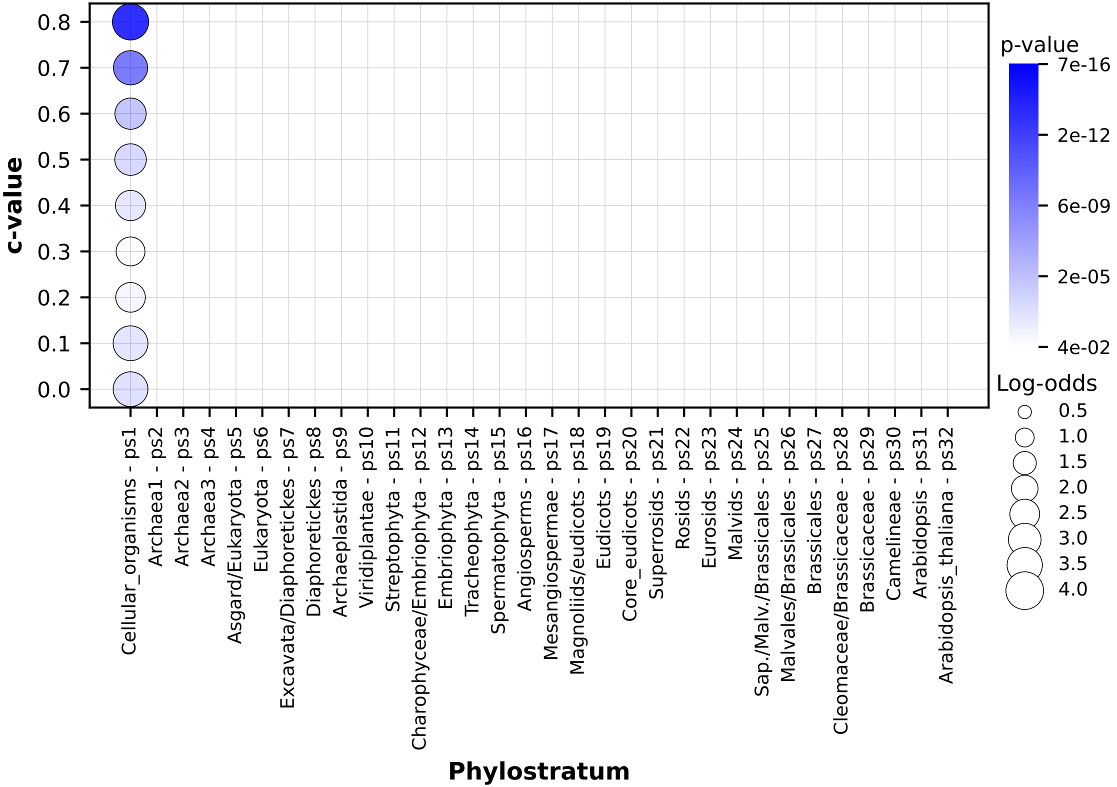

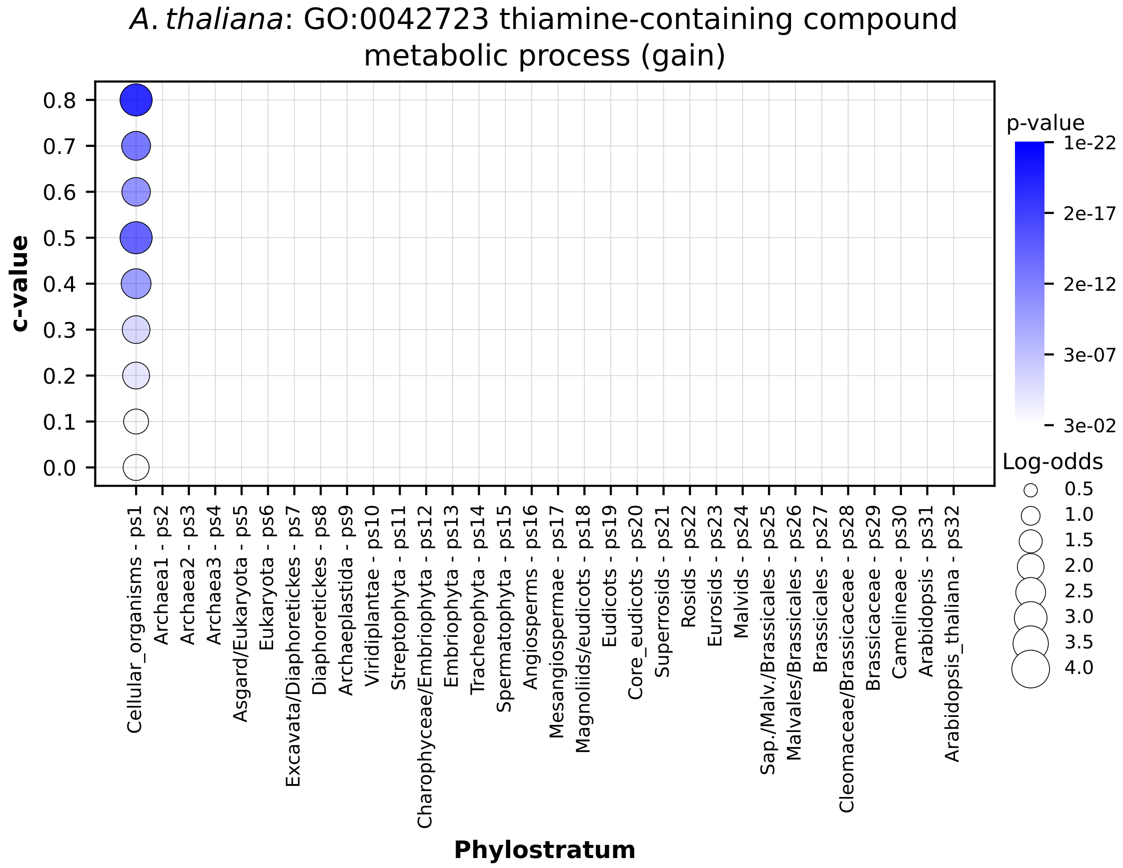
A. thaliana: GO:0042723 thiamine-containing compound metabolic process (gain)
c-value
0.8
0.7
0.6
0.5
0.4
0.3
0.2
0.1
0.0
p-value
1e-22
2e-17
2e-12
3e-07
3e-02
Log-odds
0.5
1.0
1.5
2.0
2.5
3.0
3.5
4.0
Cellular_organisms - ps1
Archaea1 - ps2
Archaea2 - ps3
Archaea3 - ps4
Asgard/Eukaryota - ps5
Eukaryota - ps6
Excavata/Diaphoretickes - ps7
Diaphoretickes - ps8
Archaeplastida - ps9
Viridiplantae - ps10
Streptophyta - ps11
Charophyceae/Embriophyta - ps12
Embriophyta - ps13
Tracheophyta - ps14
Spermatophyta - ps15
Angiosperms - ps16
Mesangiospermae - ps17
Magnoliids/eudicots - ps18
Eudicots - ps19
Core_eudicots - ps20
Superrosids - ps21
Rosids - ps22
Eurosids - ps23
Malvids - ps24
Sap./Malv./Brassicales - ps25
Malvales/Brassicales - ps26
Brassicales - ps27
Cleomaceae/Brassicaceae - ps28
Brassicaceae - ps29
Camelineae - ps30
Arabidopsis - ps31
Arabidopsis_thaliana - ps32
Phylostratum

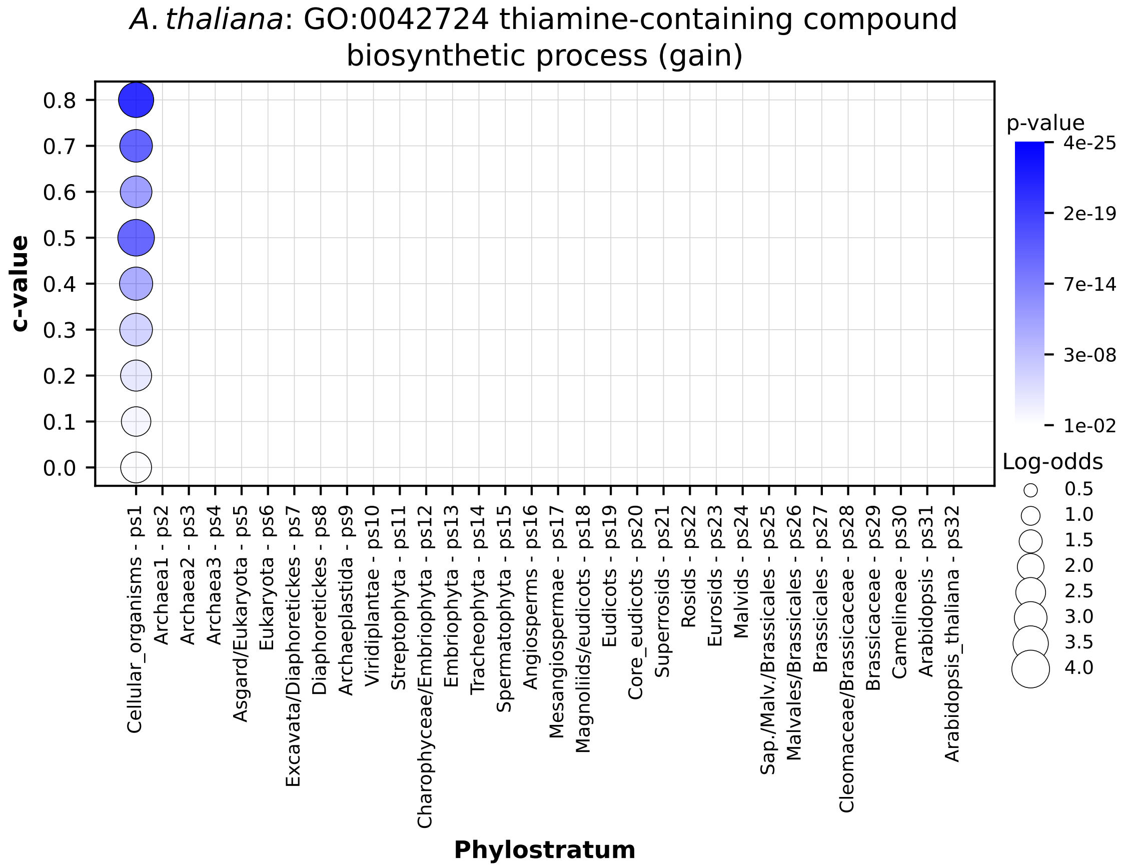

A. thaliana: GO:0042724 thiamine-containing compound biosynthetic process (gain)
c-value
0.8
0.7
0.6
0.5
0.4
0.3
0.2
0.1
0.0
Phylostratum
Cellular_organisms - ps1
Archaea1 - ps2
Archaea2 - ps3
Archaea3 - ps4
Asgard/Eukaryota - ps5
Eukaryota - ps6
Excavata/Diaphoretickes - ps7
Diaphoretickes - ps8
Archaeplastida - ps9
Viridiplantae - ps10
Streptophyta - ps11
Charophyceae/Embriophyta - ps12
Embriophyta - ps13
Tracheophyta - ps14
Spermatophyta - ps15
Angiosperms - ps16
Mesangiospermae - ps17
Magnoliids/eudicots - ps18
Eudicots - ps19
Core_eudicots - ps20
Superrosids - ps21
Rosids - ps22
Eurosids - ps23
Malvids - ps24
Sap./Malv./Brassicales - ps25
Malvales/Brassicales - ps26
Brassicales - ps27
Cleomaceae/Brassicaceae - ps28
Brassicaceae - ps29
Camelineae - ps30
Arabidopsis - ps31
Arabidopsis_thaliana - ps32
p-value
4e-25
2e-19
7e-14
3e-08
1e-02
Log-odds
0.5
1.0
1.5
2.0
2.5
3.0
3.5
4.0

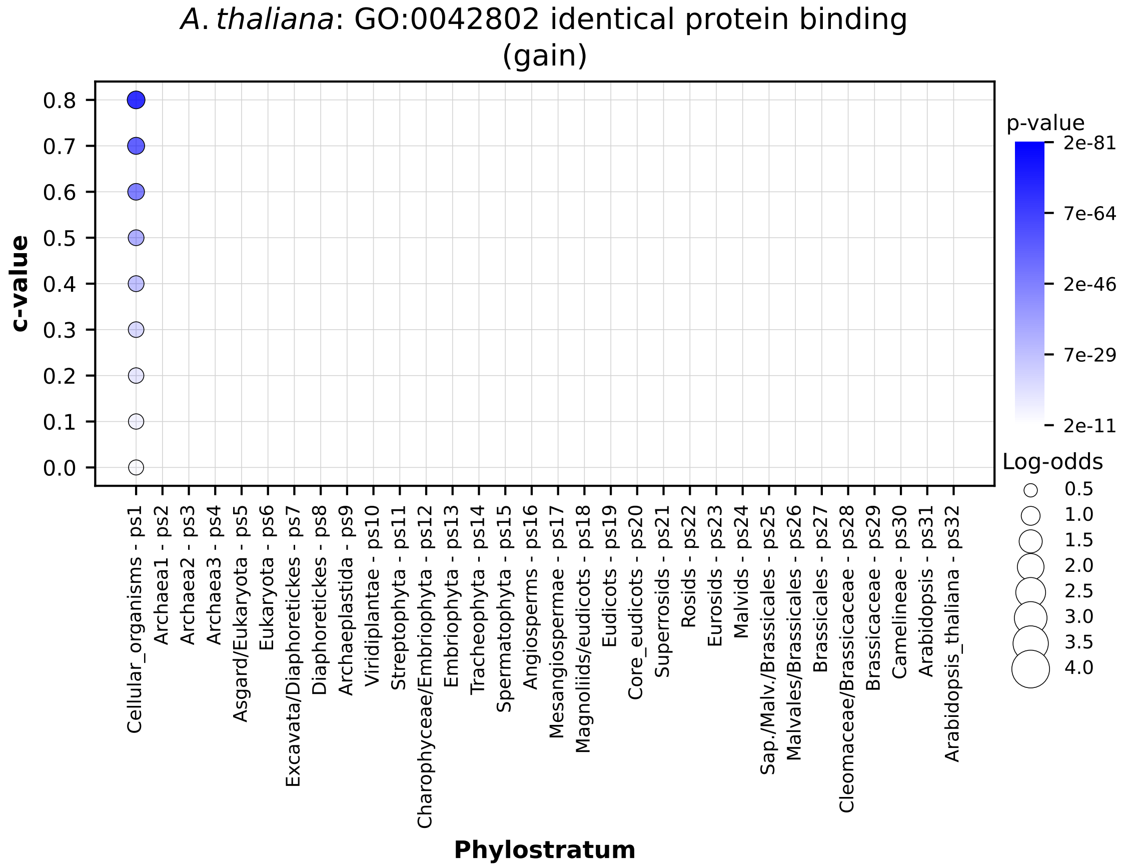

A. thaliana: GO:0042802 identical protein binding
(gain)
c-value
0.0
0.1
0.2
0.3
0.4
0.5
0.6
0.7
0.8
p-value
2e-81
7e-64
2e-46
7e-29
2e-11
Log-odds
0.5
1.0
1.5
2.0
2.5
3.0
3.5
4.0
Cellular_organisms - ps1
Archaea1 - ps2
Archaea2 - ps3
Archaea3 - ps4
Asgard/Eukaryota - ps5
Eukaryota - ps6
Excavata/Diaphoretickes - ps7
Diaphoretickes - ps8
Archaeplastida - ps9
Viridiplantae - ps10
Streptophyta - ps11
Charophyceae/Embriophyta - ps12
Embriophyta - ps13
Tracheophyta - ps14
Spermatophyta - ps15
Angiosperms - ps16
Mesangiospermae - ps17
Magnoliids/eudicots - ps18
Eudicots - ps19
Core_eudicots - ps20
Superrosids - ps21
Rosids - ps22
Eurosids - ps23
Malvids - ps24
Sap./Malv./Brassicales - ps25
Malvales/Brassicales - ps26
Brassicales - ps27
Cleomaceae/Brassicaceae - ps28
Brassicaceae - ps29
Camelineae - ps30
Arabidopsis - ps31
Arabidopsis_thaliana - ps32
Phylostratum

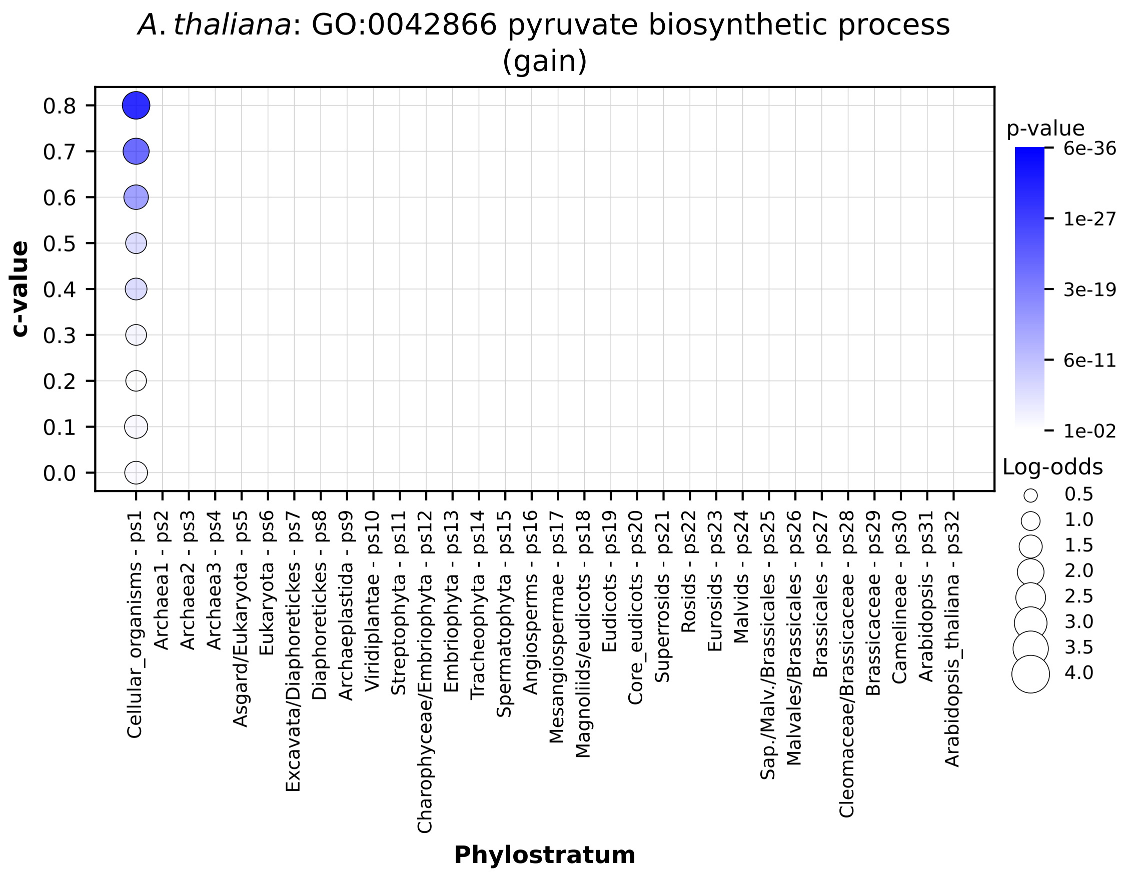

A. thaliana: GO:0042866 pyruvate biosynthetic process
(gain)
c-value
0.8
0.7
0.6
0.5
0.4
0.3
0.2
0.1
0.0
p-value
6e-36
1e-27
3e-19
6e-11
1e-02
Log-odds
0.5
1.0
1.5
2.0
2.5
3.0
3.5
4.0
Cellular_organisms - ps1
Archaea1 - ps2
Archaea2 - ps3
Archaea3 - ps4
Asgard/Eukaryota - ps5
Eukaryota - ps6
Excavata/Diaphoretickes - ps7
Diaphoretickes - ps8
Archaeplastida - ps9
Viridiplantae - ps10
Streptophyta - ps11
Charophyceae/Embriophyta - ps12
Embriophyta - ps13
Tracheophyta - ps14
Spermatophyta - ps15
Angiosperms - ps16
Mesangiospermae - ps17
Magnoliids/eudicots - ps18
Eudicots - ps19
Core_eudicots - ps20
Superrosids - ps21
Rosids - ps22
Eurosids - ps23
Malvids - ps24
Sap./Malv./Brassicales - ps25
Malvales/Brassicales - ps26
Brassicales - ps27
Cleomaceae/Brassicaceae - ps28
Brassicaceae - ps29
Camelineae - ps30
Arabidopsis - ps31
Arabidopsis_thaliana - ps32
Phylostratum

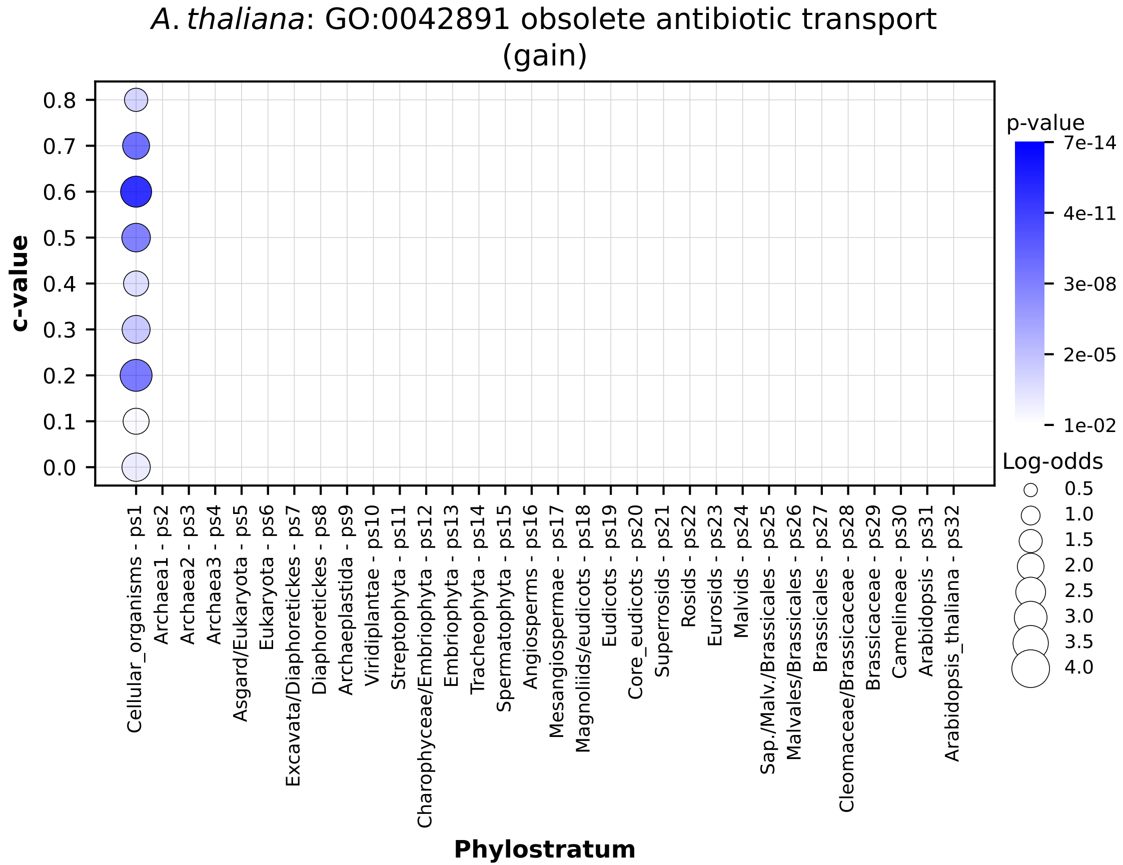
A. thaliana: GO:0042891 obsolete antibiotic transport (gain)
c-value
0.0
0.1
0.2
0.3
0.4
0.5
0.6
0.7
0.8
p-value
7e-14
4e-11
3e-08
2e-05
1e-02
Log-odds
0.5
1.0
1.5
2.0
2.5
3.0
3.5
4.0
Cellular_organisms - ps1
Archaea1 - ps2
Archaea2 - ps3
Archaea3 - ps4
Asgard/Eukaryota - ps5
Eukaryota - ps6
Excavata/Diaphoretickes - ps7
Diaphoretickes - ps8
Archaeplastida - ps9
Viridiplantae - ps10
Streptophyta - ps11
Charophyceae/Embriophyta - ps12
Embriophyta - ps13
Tracheophyta - ps14
Spermatophyta - ps15
Angiosperms - ps16
Mesangiospermae - ps17
Magnoliids/eudicots - ps18
Eudicots - ps19
Core_eudicots - ps20
Superrosids - ps21
Rosids - ps22
Eurosids - ps23
Malvids - ps24
Sap./Malv./Brassicales - ps25
Malvales/Brassicales - ps26
Brassicales - ps27
Cleomaceae/Brassicaceae - ps28
Brassicaceae - ps29
Camelineae - ps30
Arabidopsis - ps31
Arabidopsis_thaliana - ps32
Phylostratum

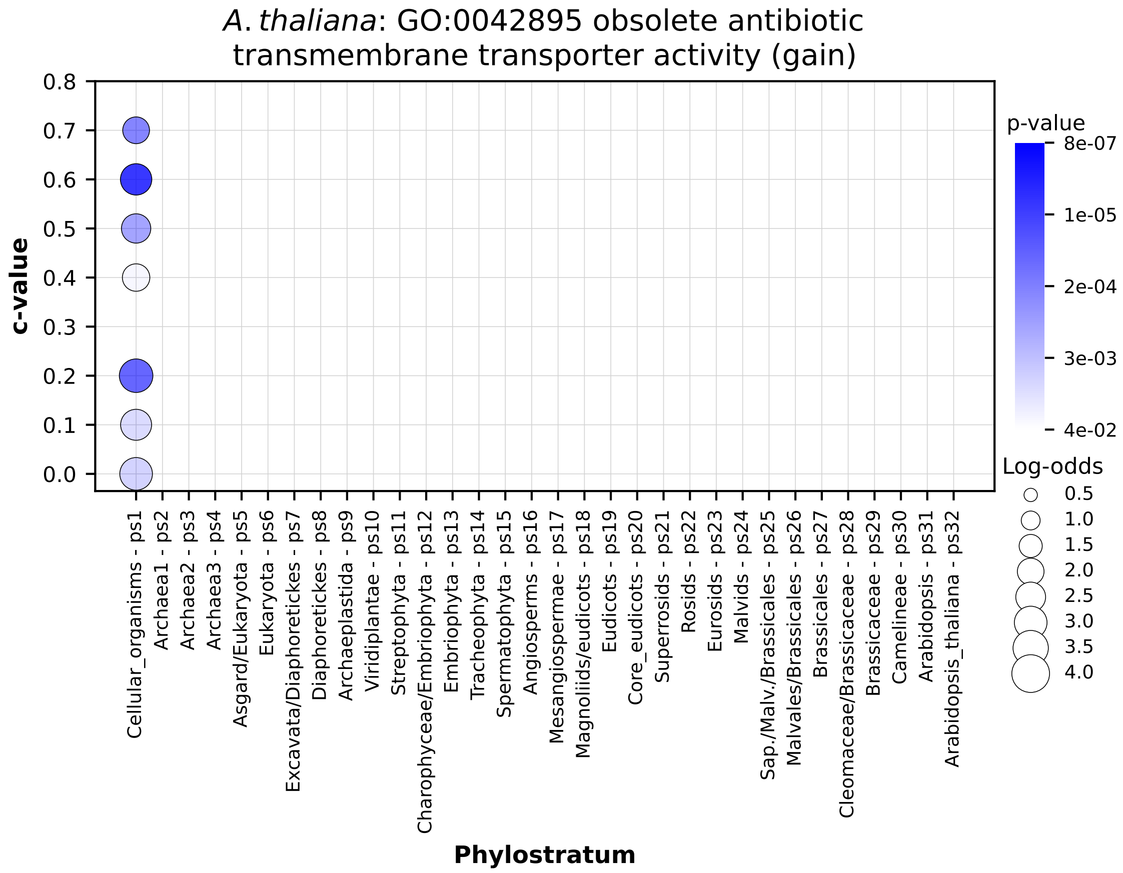

A. thaliana: GO:0042895 obsolete antibiotic transmembrane transporter activity (gain)
c-value
0.0
0.1
0.2
0.3
0.4
0.5
0.6
0.7
0.8
Phylostratum
Cellular_organisms - ps1
Archaea1 - ps2
Archaea2 - ps3
Archaea3 - ps4
Asgard/Eukaryota - ps5
Eukaryota - ps6
Excavata/Diaphoretickes - ps7
Diaphoretickes - ps8
Archaeplastida - ps9
Viridiplantae - ps10
Streptophyta - ps11
Charophyceae/Embriophyta - ps12
Embriophyta - ps13
Tracheophyta - ps14
Spermatophyta - ps15
Angiosperms - ps16
Mesangiospermae - ps17
Magnoliids/eudicots - ps18
Eudicots - ps19
Core_eudicots - ps20
Superrosids - ps21
Rosids - ps22
Eurosids - ps23
Malvids - ps24
Sap./Malv./Brassicales - ps25
Malvales/Brassicales - ps26
Brassicales - ps27
Cleomaceae/Brassicaceae - ps28
Brassicaceae - ps29
Camelineae - ps30
Arabidopsis - ps31
Arabidopsis_thaliana - ps32
p-value
8e-07
1e-05
2e-04
3e-03
4e-02
Log-odds
0.5
1.0
1.5
2.0
2.5
3.0
3.5
4.0

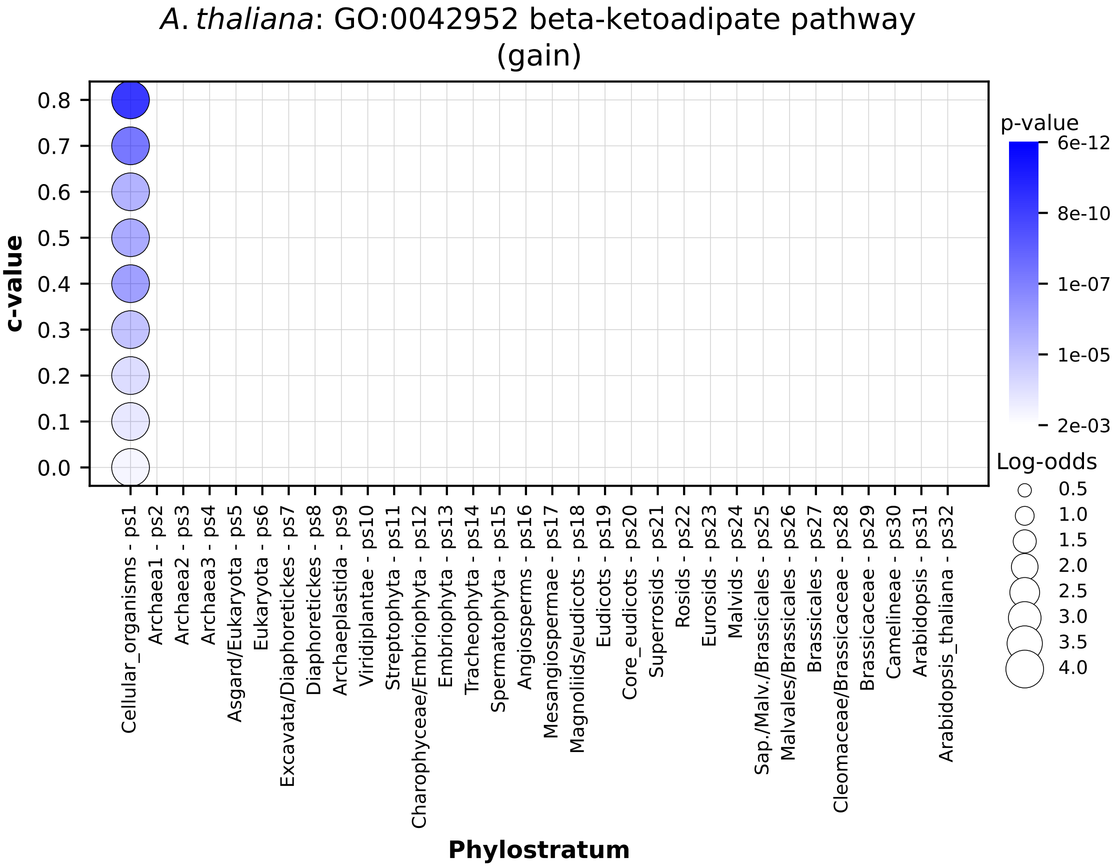

A. thaliana: GO:0042952 beta-ketoadipate pathway
(gain)
c-value
0.8
0.7
0.6
0.5
0.4
0.3
0.2
0.1
0.0
p-value
6e-12
8e-10
1e-07
1e-05
2e-03
Log-odds
0.5
1.0
1.5
2.0
2.5
3.0
3.5
4.0
Cellular_organisms - ps1
Archaea1 - ps2
Archaea2 - ps3
Archaea3 - ps4
Asgard/Eukaryota - ps5
Eukaryota - ps6
Excavata/Diaphoretickes - ps7
Diaphoretickes - ps8
Archaeplastida - ps9
Viridiplantae - ps10
Streptophyta - ps11
Charophyceae/Embriophyta - ps12
Embriophyta - ps13
Tracheophyta - ps14
Spermatophyta - ps15
Angiosperms - ps16
Mesangiospermae - ps17
Magnoliids/eudicots - ps18
Eudicots - ps19
Core_eudicots - ps20
Superrosids - ps21
Rosids - ps22
Eurosids - ps23
Malvids - ps24
Sap./Malv./Brassicales - ps25
Malvales/Brassicales - ps26
Brassicales - ps27
Cleomaceae/Brassicaceae - ps28
Brassicaceae - ps29
Camelineae - ps30
Arabidopsis - ps31
Arabidopsis_thaliana - ps32
Phylostratum

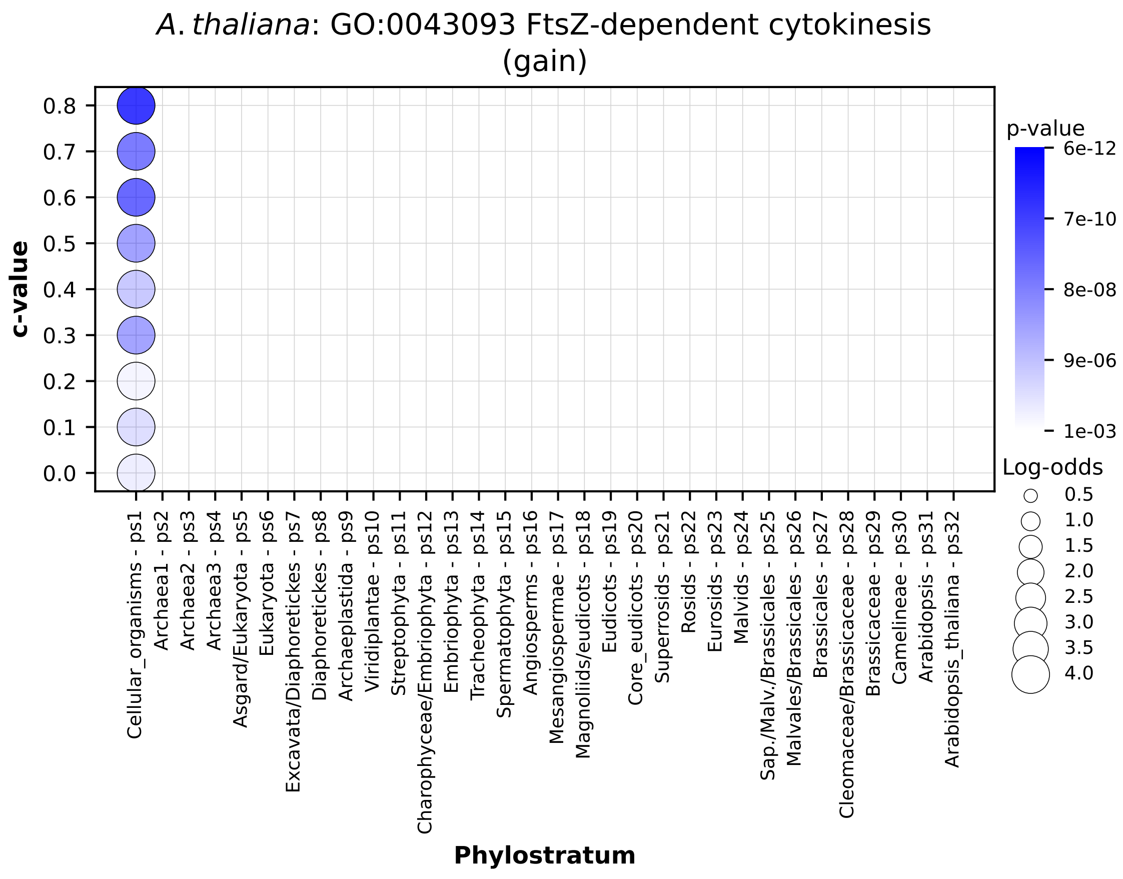

A. thaliana: GO:0043093 FtsZ-dependent cytokinesis
(gain)
c-value
0.0
0.1
0.2
0.3
0.4
0.5
0.6
0.7
0.8
p-value
6e-12
7e-10
8e-08
9e-06
1e-03
Log-odds
0.5
1.0
1.5
2.0
2.5
3.0
3.5
4.0
Cellular_organisms - ps1
Archaea1 - ps2
Archaea2 - ps3
Archaea3 - ps4
Asgard/Eukaryota - ps5
Eukaryota - ps6
Excavata/Diaphoretickes - ps7
Diaphoretickes - ps8
Archaeplastida - ps9
Viridiplantae - ps10
Streptophyta - ps11
Charophyceae/Embriophyta - ps12
Embriophyta - ps13
Tracheophyta - ps14
Spermatophyta - ps15
Angiosperms - ps16
Mesangiospermae - ps17
Magnoliids/eudicots - ps18
Eudicots - ps19
Core_eudicots - ps20
Superrosids - ps21
Rosids - ps22
Eurosids - ps23
Malvids - ps24
Sap./Malv./Brassicales - ps25
Malvales/Brassicales - ps26
Brassicales - ps27
Cleomaceae/Brassicaceae - ps28
Brassicaceae - ps29
Camelineae - ps30
Arabidopsis - ps31
Arabidopsis_thaliana - ps32
Phylostratum

A. thaliana: GO:0043094 cellular metabolic compound salvage (gain)

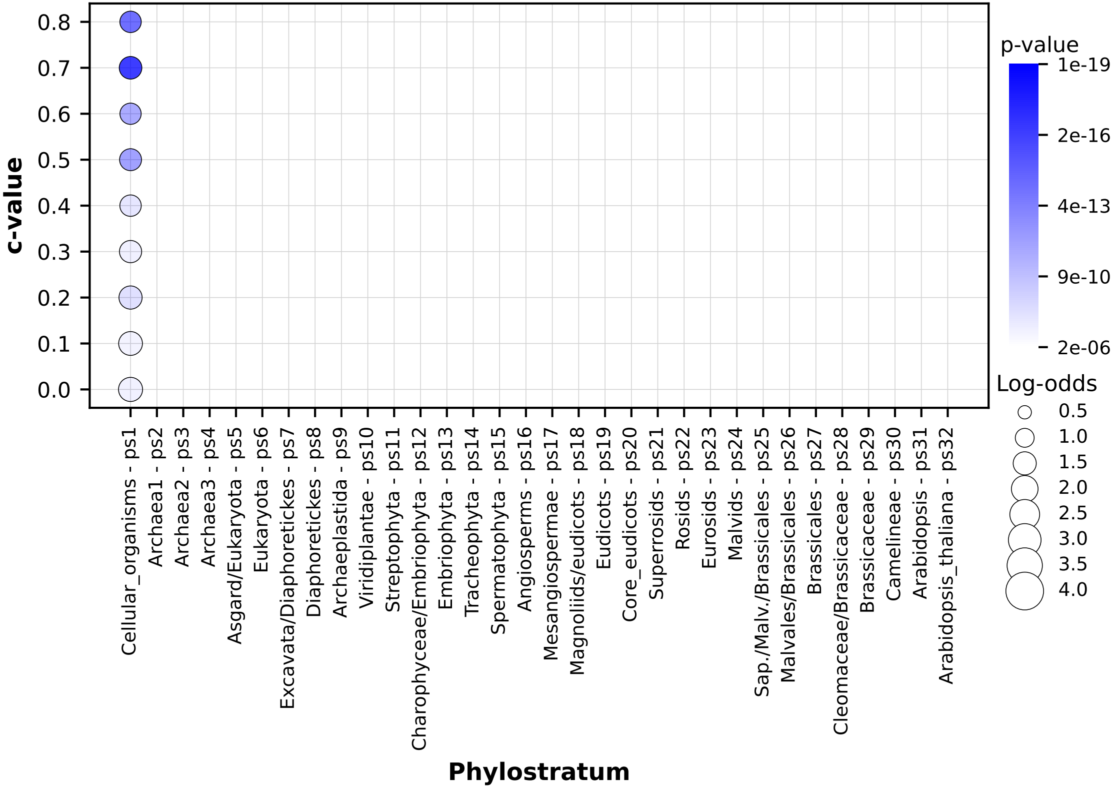

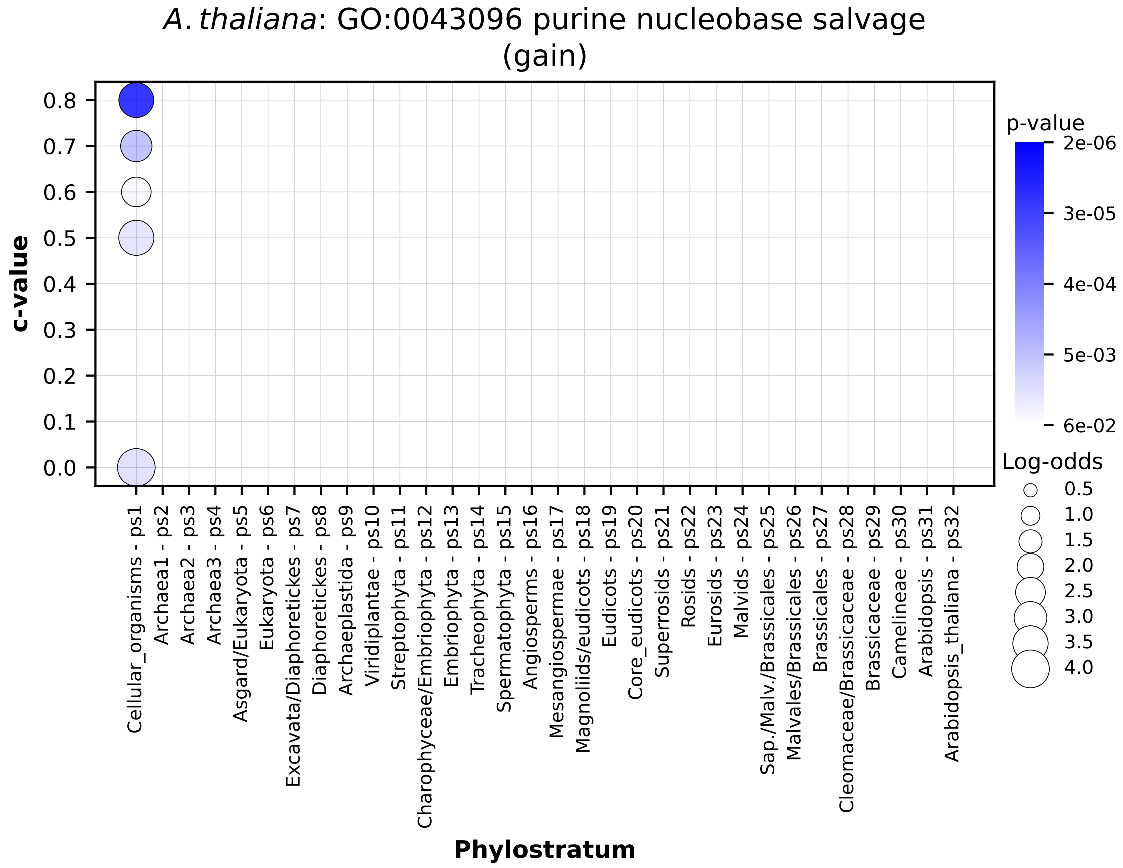

A. thaliana: GO:0043096 purine nucleobase salvage (gain)
c-value
0.8
0.7
0.6
0.5
0.4
0.3
0.2
0.1
0.0
p-value
2e-06
3e-05
4e-04
5e-03
6e-02
Log-odds
0.5
1.0
1.5
2.0
2.5
3.0
3.5
4.0
Cellular_organisms - ps1
Archaea1 - ps2
Archaea2 - ps3
Archaea3 - ps4
Asgard/Eukaryota - ps5
Eukaryota - ps6
Excavata/Diaphoretickes - ps7
Diaphoretickes - ps8
Archaeplastida - ps9
Viridiplantae - ps10
Streptophyta - ps11
Charophyceae/Embriophyta - ps12
Embriophyta - ps13
Tracheophyta - ps14
Spermatophyta - ps15
Angiosperms - ps16
Mesangiospermae - ps17
Magnoliids/eudicots - ps18
Eudicots - ps19
Core_eudicots - ps20
Superrosids - ps21
Rosids - ps22
Eurosids - ps23
Malvids - ps24
Sap./Malv./Brassicales - ps25
Malvales/Brassicales - ps26
Brassicales - ps27
Cleomaceae/Brassicaceae - ps28
Brassicaceae - ps29
Camelineae - ps30
Arabidopsis - ps31
Arabidopsis_thaliana - ps32
Phylostratum

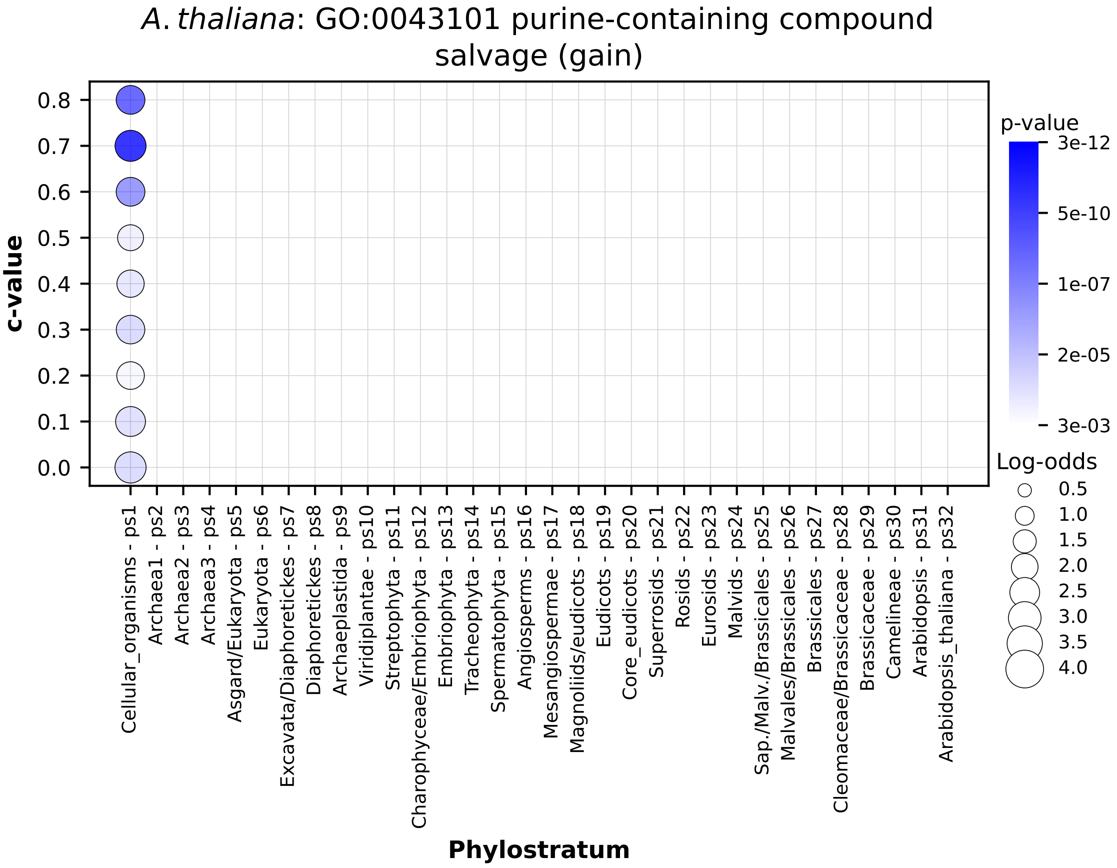

A. thaliana: GO:0043101 purine-containing compound salvage (gain)
c-value
0.8
0.7
0.6
0.5
0.4
0.3
0.2
0.1
0.0
Phylostratum
Cellular_organisms - ps1
Archaea1 - ps2
Archaea2 - ps3
Archaea3 - ps4
Asgard/Eukaryota - ps5
Eukaryota - ps6
Excavata/Diaphoretickes - ps7
Diaphoretickes - ps8
Archaeplastida - ps9
Viridiplantae - ps10
Streptophyta - ps11
Charophyceae/Embriophyta - ps12
Embriophyta - ps13
Tracheophyta - ps14
Spermatophyta - ps15
Angiosperms - ps16
Mesangiospermae - ps17
Magnoliids/eudicots - ps18
Eudicots - ps19
Core_eudicots - ps20
Superrosids - ps21
Rosids - ps22
Eurosids - ps23
Malvids - ps24
Sap./Malv./Brassicales - ps25
Malvales/Brassicales - ps26
Brassicales - ps27
Cleomaceae/Brassicaceae - ps28
Brassicaceae - ps29
Camelineae - ps30
Arabidopsis - ps31
Arabidopsis_thaliana - ps32
p-value
3e-12
5e-10
1e-07
2e-05
3e-03
Log-odds
0.5
1.0
1.5
2.0
2.5
3.0
3.5
4.0

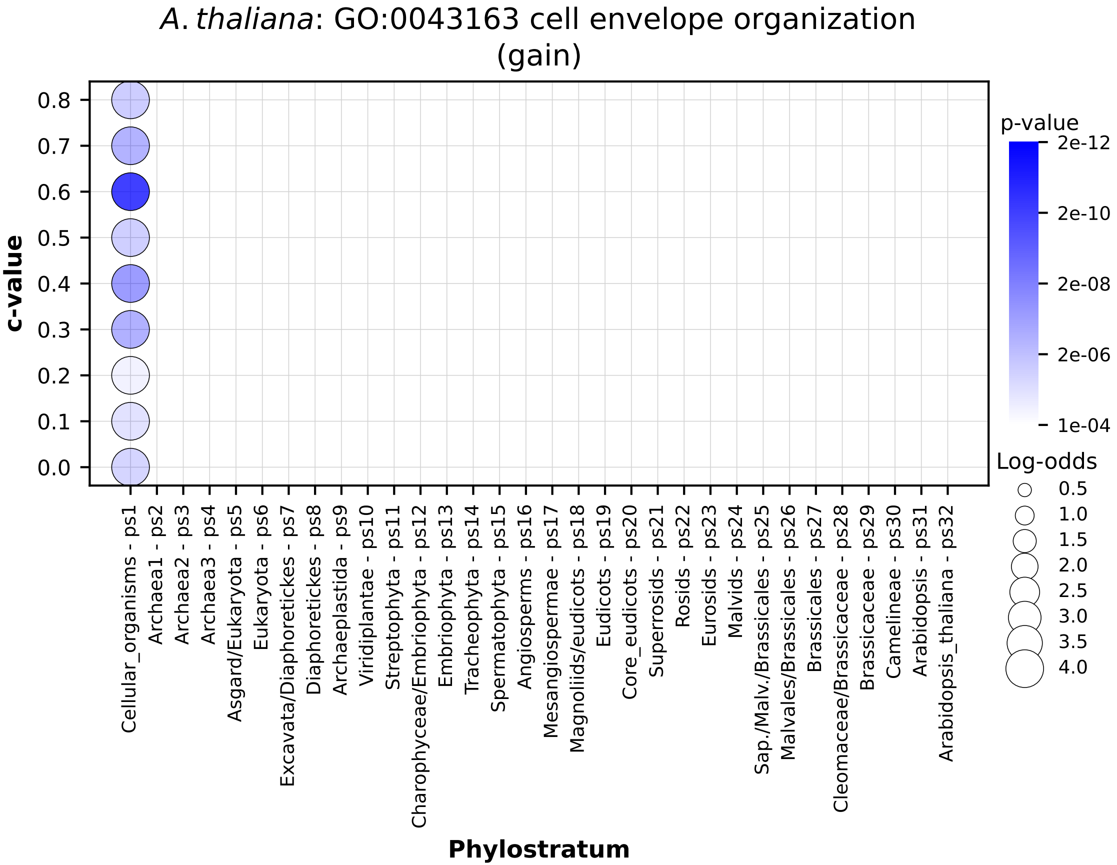

A. thaliana: GO:0043163 cell envelope organization (gain)
c-value
0.8
0.7
0.6
0.5
0.4
0.3
0.2
0.1
0.0
p-value
2e-12
2e-10
2e-08
2e-06
1e-04
Log-odds
0.5
1.0
1.5
2.0
2.5
3.0
3.5
4.0
Cellular_organisms - ps1
Archaea1 - ps2
Archaea2 - ps3
Archaea3 - ps4
Asgard/Eukaryota - ps5
Eukaryota - ps6
Excavata/Diaphoretickes - ps7
Diaphoretickes - ps8
Archaeplastida - ps9
Viridiplantae - ps10
Streptophyta - ps11
Charophyceae/Embriophyta - ps12
Embriophyta - ps13
Tracheophyta - ps14
Spermatophyta - ps15
Angiosperms - ps16
Mesangiospermae - ps17
Magnoliids/eudicots - ps18
Eudicots - ps19
Core_eudicots - ps20
Superrosids - ps21
Rosids - ps22
Eurosids - ps23
Malvids - ps24
Sap./Malv./Brassicales - ps25
Malvales/Brassicales - ps26
Brassicales - ps27
Cleomaceae/Brassicaceae - ps28
Brassicaceae - ps29
Camelineae - ps30
Arabidopsis - ps31
Arabidopsis_thaliana - ps32
Phylostratum

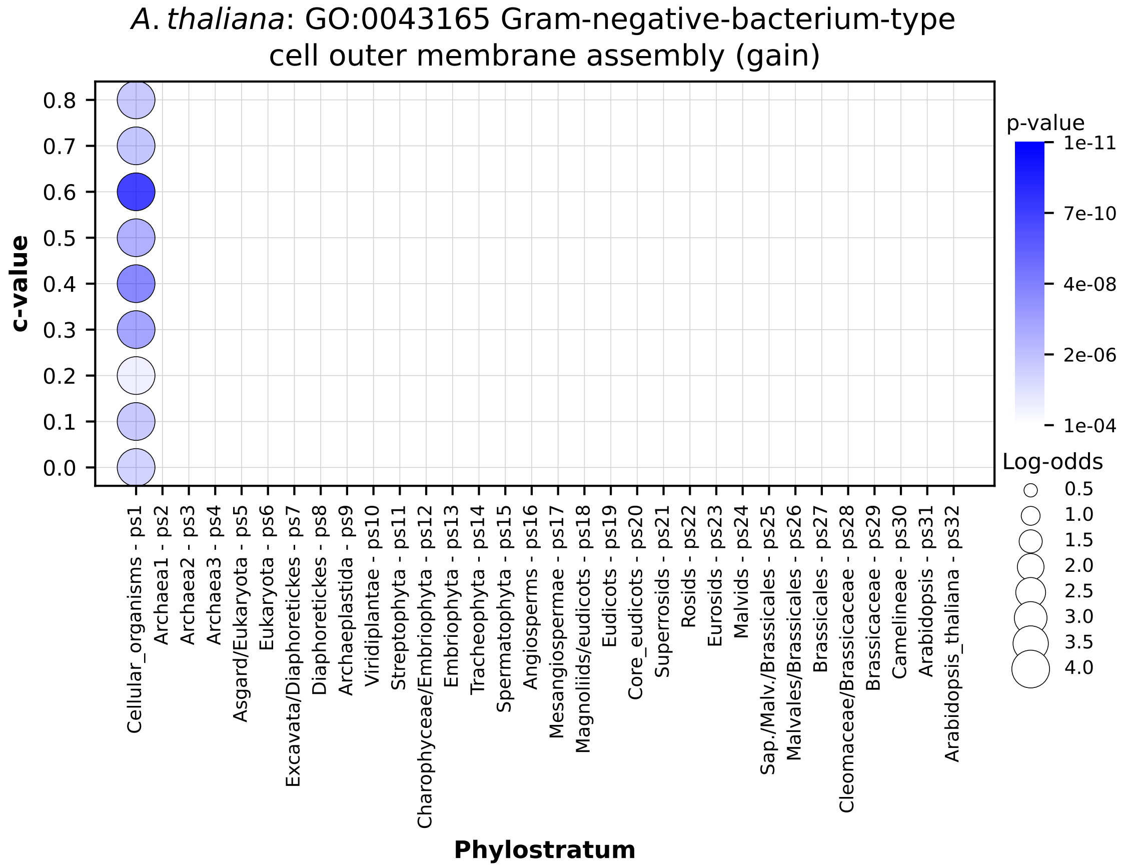

A. thaliana: GO:0043165 Gram-negative-bacterium-type cell outer membrane assembly (gain)
c-value
0.8
0.7
0.6
0.5
0.4
0.3
0.2
0.1
0.0
p-value
1e-11
7e-10
4e-08
2e-06
1e-04
Log-odds
0.5
1.0
1.5
2.0
2.5
3.0
3.5
4.0
Cellular_organisms - ps1
Archaea1 - ps2
Archaea2 - ps3
Archaea3 - ps4
Asgard/Eukaryota - ps5
Eukaryota - ps6
Excavata/Diaphoretickes - ps7
Diaphoretickes - ps8
Archaeplastida - ps9
Viridiplantae - ps10
Streptophyta - ps11
Charophyceae/Embriophyta - ps12
Embriophyta - ps13
Tracheophyta - ps14
Spermatophyta - ps15
Angiosperms - ps16
Mesangiospermae - ps17
Magnoliids/eudicots - ps18
Eudicots - ps19
Core_eudicots - ps20
Superrosids - ps21
Rosids - ps22
Eurosids - ps23
Malvids - ps24
Sap./Malv./Brassicales - ps25
Malvales/Brassicales - ps26
Brassicales - ps27
Cleomaceae/Brassicaceae - ps28
Brassicaceae - ps29
Camelineae - ps30
Arabidopsis - ps31
Arabidopsis_thaliana - ps32
Phylostratum

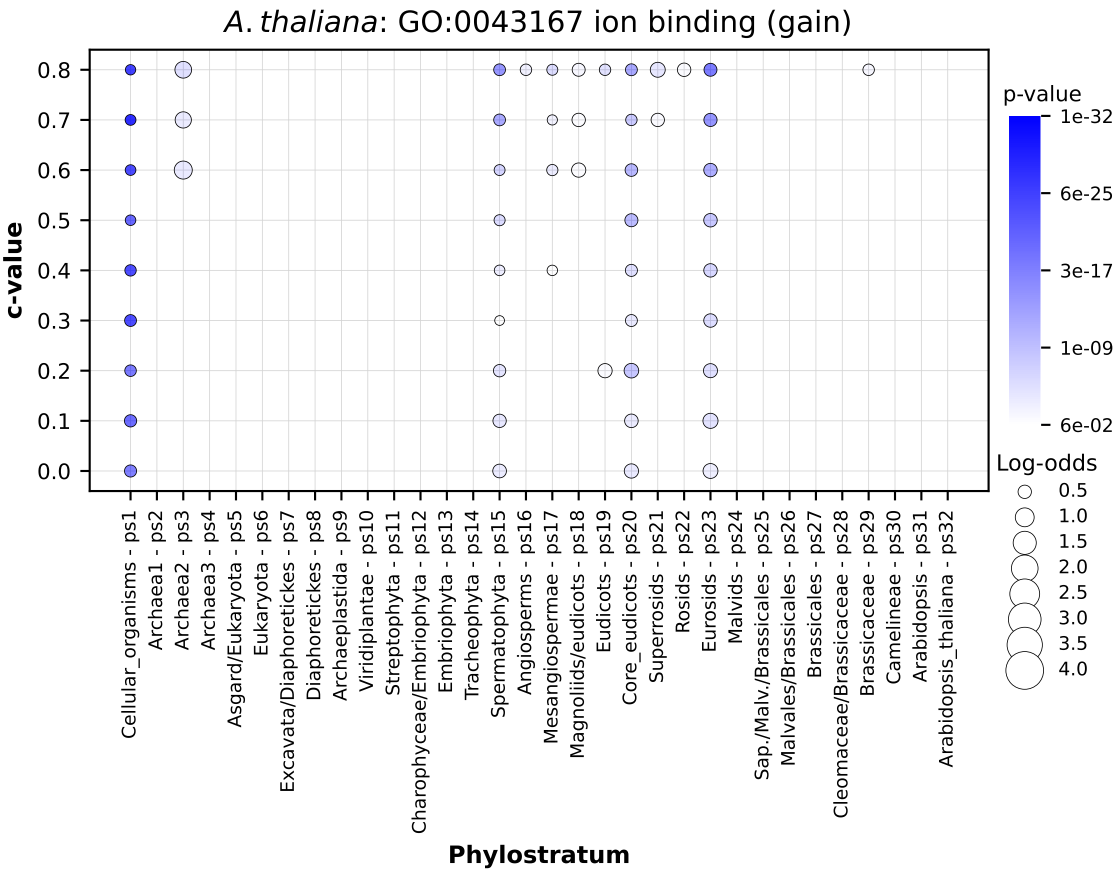

A. thaliana: GO:0043167 ion binding (gain)
c-value
0.8
0.7
0.6
0.5
0.4
0.3
0.2
0.1
0.0
p-value
1e-32
6e-25
3e-17
1e-09
6e-02
Log-odds
0.5
1.0
1.5
2.0
2.5
3.0
3.5
4.0
Cellular_organisms - ps1
Archaea1 - ps2
Archaea2 - ps3
Archaea3 - ps4
Asgard/Eukaryota - ps5
Eukaryota - ps6
Excavata/Diaphoretickes - ps7
Diaphoretickes - ps8
Archaeplastida - ps9
Viridiplantae - ps10
Streptophyta - ps11
Charophyceae/Embriophyta - ps12
Embriophyta - ps13
Tracheophyta - ps14
Spermatophyta - ps15
Angiosperms - ps16
Mesangiospermae - ps17
Magnoliids/eudicots - ps18
Eudicots - ps19
Core_eudicots - ps20
Superrosids - ps21
Rosids - ps22
Eurosids - ps23
Malvids - ps24
Sap./Malv./Brassicales - ps25
Malvales/Brassicales - ps26
Brassicales - ps27
Cleomaceae/Brassicaceae - ps28
Brassicaceae - ps29
Camelineae - ps30
Arabidopsis - ps31
Arabidopsis_thaliana - ps32
Phylostratum

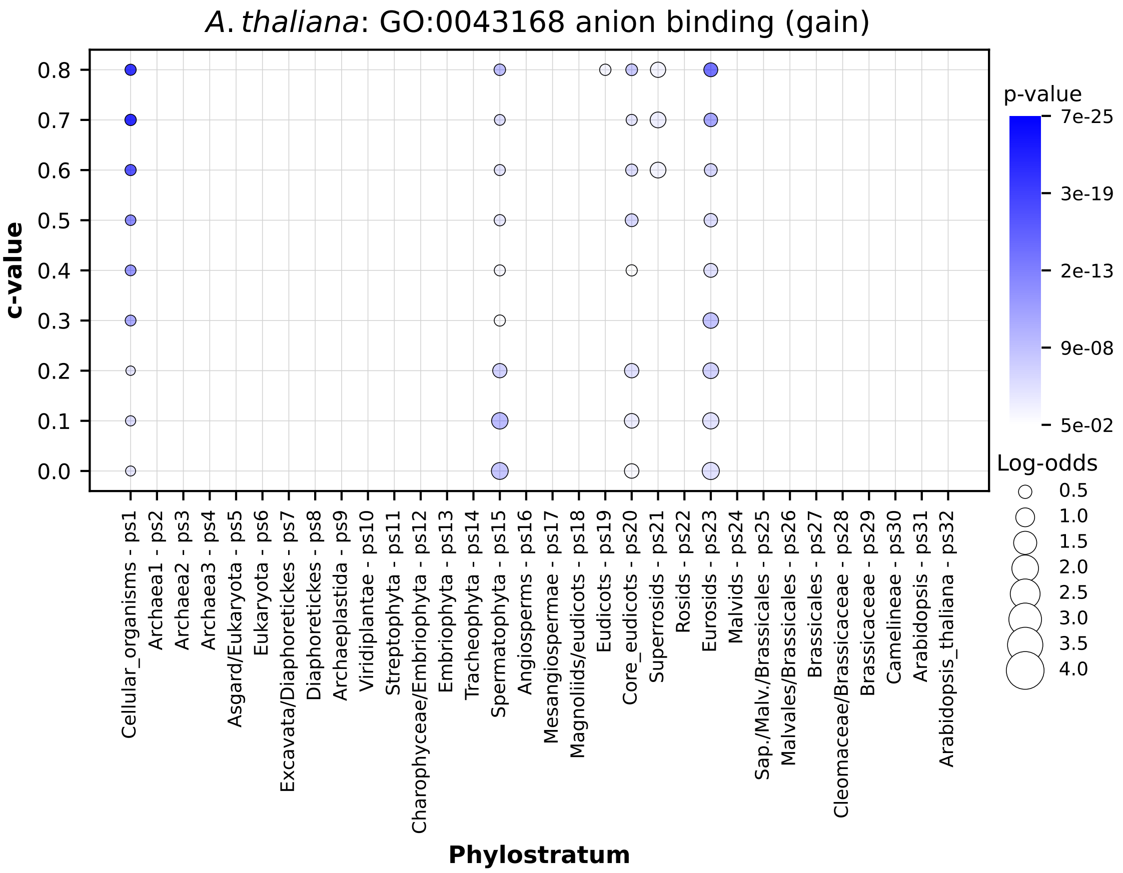

A. thaliana: GO:0043168 anion binding (gain)
c-value
0.0
0.1
0.2
0.3
0.4
0.5
0.6
0.7
0.8
p-value
7e-25
3e-19
2e-13
9e-08
5e-02
Log-odds
0.5
1.0
1.5
2.0
2.5
3.0
3.5
4.0
Cellular_organisms - ps1
Archaea1 - ps2
Archaea2 - ps3
Archaea3 - ps4
Asgard/Eukaryota - ps5
Eukaryota - ps6
Excavata/Diaphoretickes - ps7
Diaphoretickes - ps8
Archaeplastida - ps9
Viridiplantae - ps10
Streptophyta - ps11
Charophyceae/Embriophyta - ps12
Embriophyta - ps13
Tracheophyta - ps14
Spermatophyta - ps15
Angiosperms - ps16
Mesangiospermae - ps17
Magnoliids/eudicots - ps18
Eudicots - ps19
Core_eudicots - ps20
Superrosids - ps21
Rosids - ps22
Eurosids - ps23
Malvids - ps24
Sap./Malv./Brassicales - ps25
Malvales/Brassicales - ps26
Brassicales - ps27
Cleomaceae/Brassicaceae - ps28
Brassicaceae - ps29
Camelineae - ps30
Arabidopsis - ps31
Arabidopsis_thaliana - ps32
Phylostratum

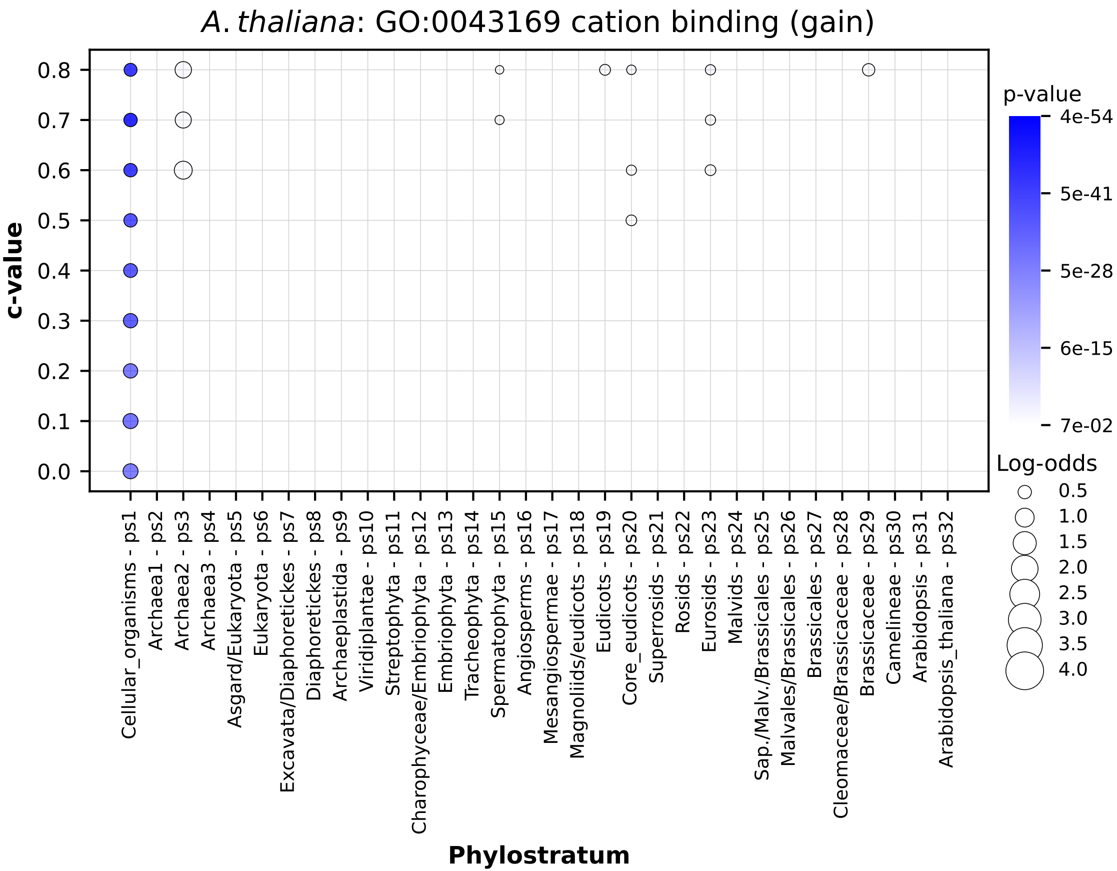

A. thaliana: GO:0043169 cation binding (gain)
c-value
0.0
0.1
0.2
0.3
0.4
0.5
0.6
0.7
0.8
p-value
4e-54
5e-41
5e-28
6e-15
7e-02
Log-odds
0.5
1.0
1.5
2.0
2.5
3.0
3.5
4.0
Cellular_organisms - ps1
Archaea1 - ps2
Archaea2 - ps3
Archaea3 - ps4
Asgard/Eukaryota - ps5
Eukaryota - ps6
Excavata/Diaphoretickes - ps7
Diaphoretickes - ps8
Archaeplastida - ps9
Viridiplantae - ps10
Streptophyta - ps11
Charophyceae/Embriophyta - ps12
Embriophyta - ps13
Tracheophyta - ps14
Spermatophyta - ps15
Angiosperms - ps16
Mesangiospermae - ps17
Magnoliids/eudicots - ps18
Eudicots - ps19
Core_eudicots - ps20
Superrosids - ps21
Rosids - ps22
Eurosids - ps23
Malvids - ps24
Sap./Malv./Brassicales - ps25
Malvales/Brassicales - ps26
Brassicales - ps27
Cleomaceae/Brassicaceae - ps28
Brassicaceae - ps29
Camelineae - ps30
Arabidopsis - ps31
Arabidopsis_thaliana - ps32
Phylostratum

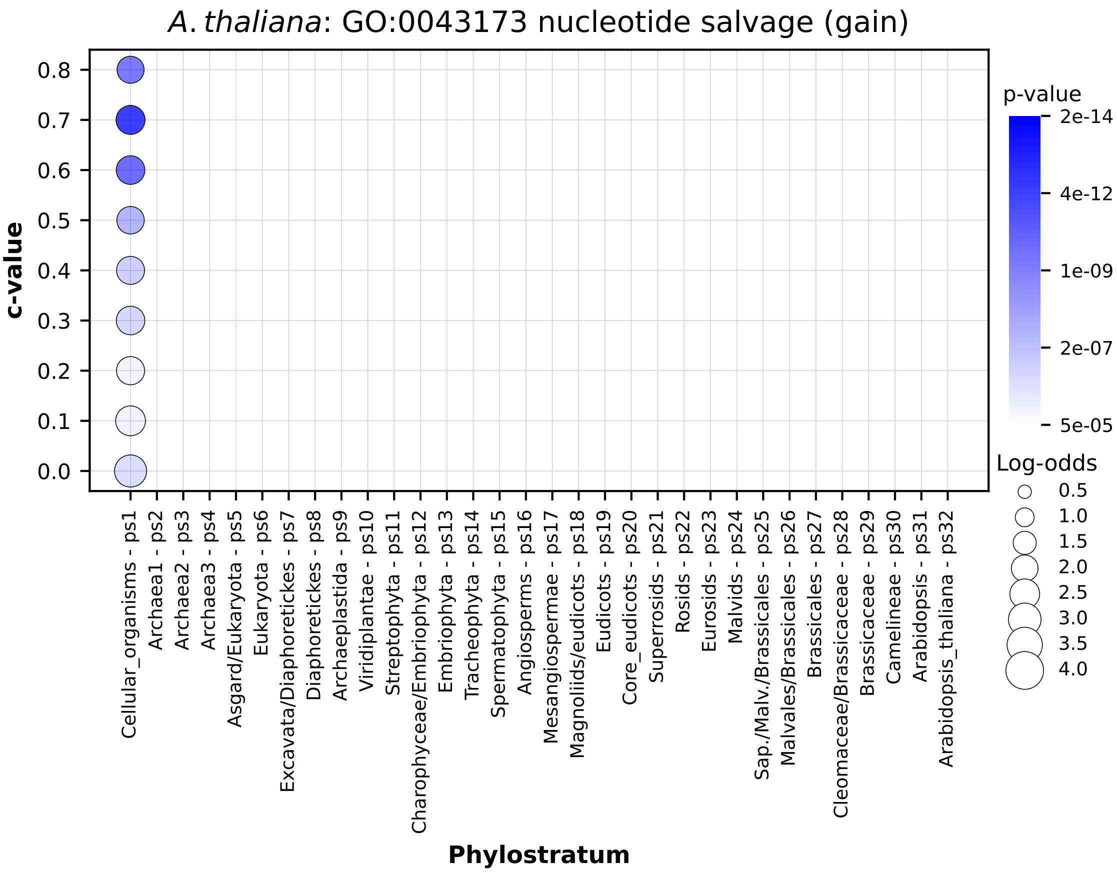

A. thaliana: GO:0043173 nucleotide salvage (gain)
c-value
0.0
0.1
0.2
0.3
0.4
0.5
0.6
0.7
0.8
Phylostratum
Cellular_organisms - ps1
Archaea1 - ps2
Archaea2 - ps3
Archaea3 - ps4
Asgard/Eukaryota - ps5
Eukaryota - ps6
Excavata/Diaphoretickes - ps7
Diaphoretickes - ps8
Archaeplastida - ps9
Viridiplantae - ps10
Streptophyta - ps11
Charophyceae/Embriophyta - ps12
Embriophyta - ps13
Tracheophyta - ps14
Spermatophyta - ps15
Angiosperms - ps16
Mesangiospermae - ps17
Magnoliids/eudicots - ps18
Eudicots - ps19
Core_eudicots - ps20
Superrosids - ps21
Rosids - ps22
Eurosids - ps23
Malvids - ps24
Sap./Malv./Brassicales - ps25
Malvales/Brassicales - ps26
Brassicales - ps27
Cleomaceae/Brassicaceae - ps28
Brassicaceae - ps29
Camelineae - ps30
Arabidopsis - ps31
Arabidopsis_thaliana - ps32
p-value
2e-14
4e-12
1e-09
2e-07
5e-05
Log-odds
0.5
1.0
1.5
2.0
2.5
3.0
3.5
4.0

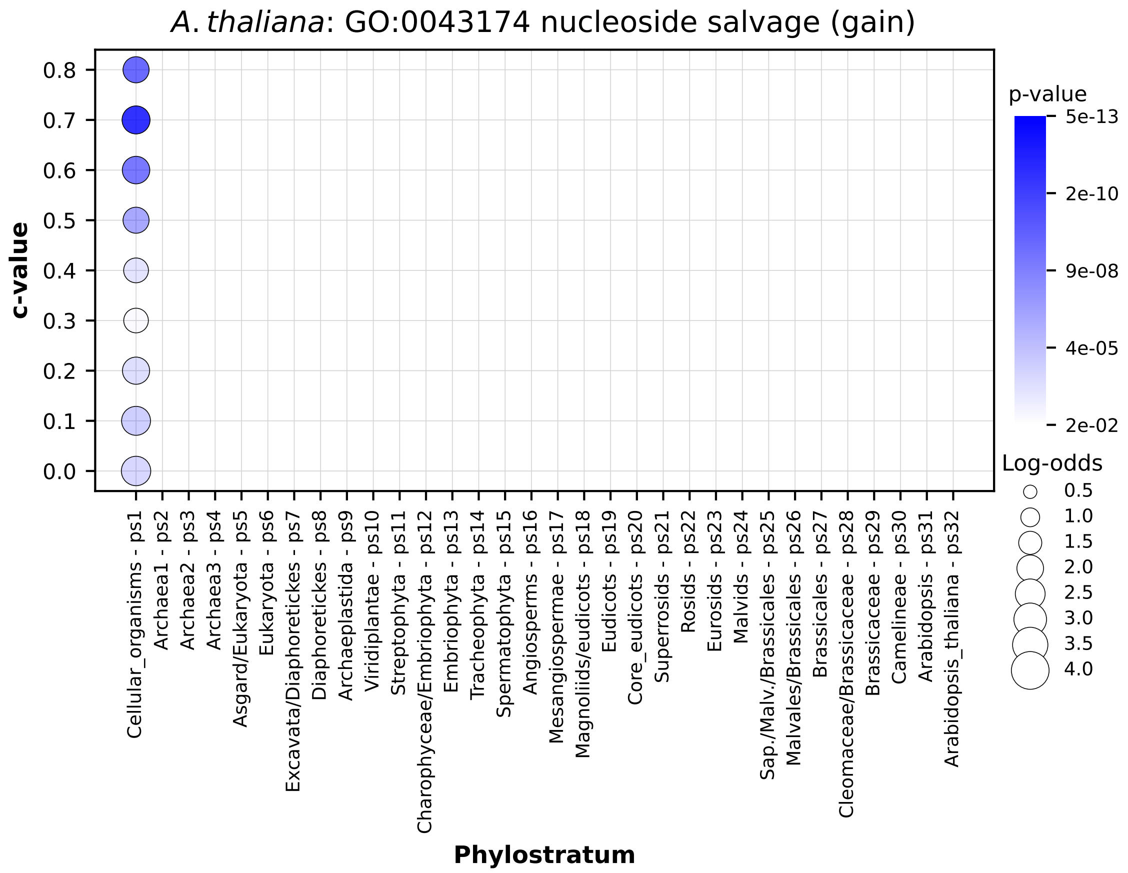
A. thaliana: GO:0043174 nucleoside salvage (gain)
c-value
0.8
0.7
0.6
0.5
0.4
0.3
0.2
0.1
0.0
p-value
5e-13
2e-10
9e-08
4e-05
2e-02
Log-odds
0.5
1.0
1.5
2.0
2.5
3.0
3.5
4.0
Cellular_organisms - ps1
Archaea1 - ps2
Archaea2 - ps3
Archaea3 - ps4
Asgard/Eukaryota - ps5
Eukaryota - ps6
Excavata/Diaphoretickes - ps7
Diaphoretickes - ps8
Archaeplastida - ps9
Viridiplantae - ps10
Streptophyta - ps11
Charophyceae/Embriophyta - ps12
Embriophyta - ps13
Tracheophyta - ps14
Spermatophyta - ps15
Angiosperms - ps16
Mesangiospermae - ps17
Magnoliids/eudicots - ps18
Eudicots - ps19
Core_eudicots - ps20
Superrosids - ps21
Rosids - ps22
Eurosids - ps23
Malvids - ps24
Sap./Malv./Brassicales - ps25
Malvales/Brassicales - ps26
Brassicales - ps27
Cleomaceae/Brassicaceae - ps28
Brassicaceae - ps29
Camelineae - ps30
Arabidopsis - ps31
Arabidopsis_thaliana - ps32
Phylostratum

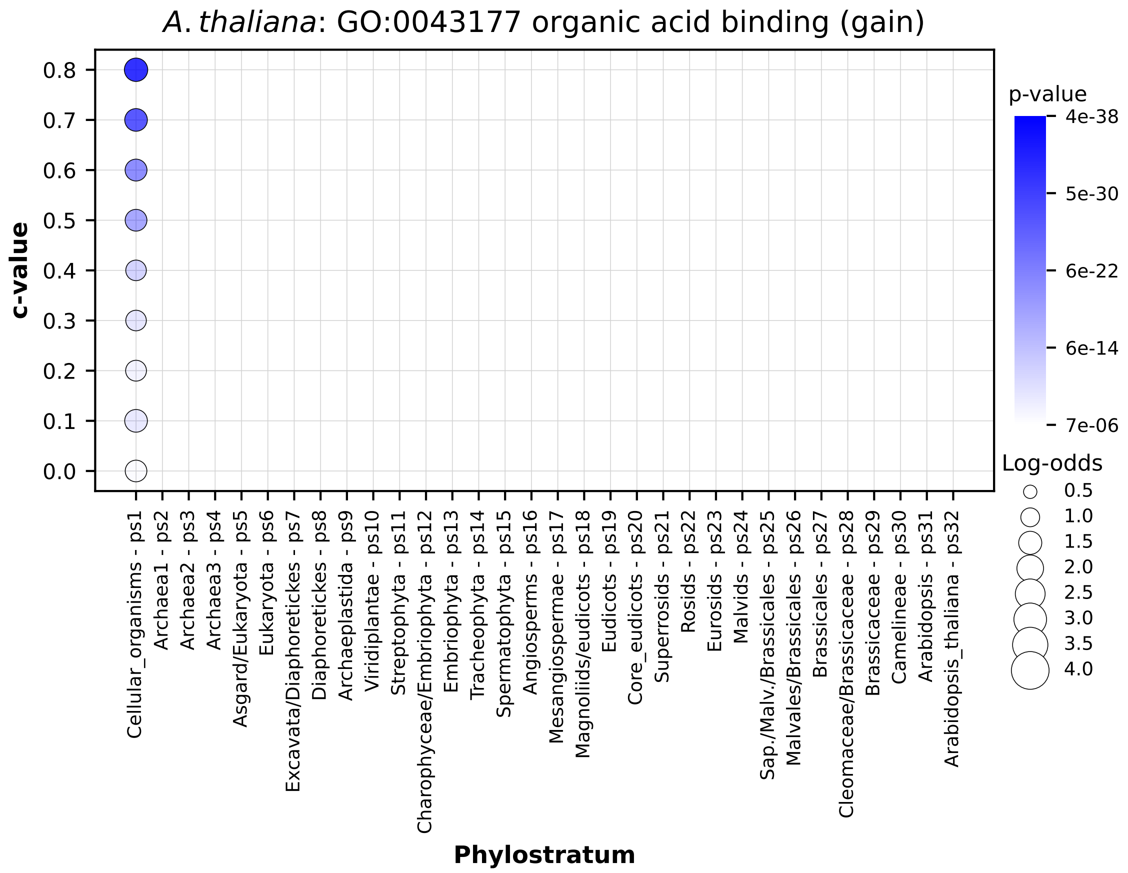

A. thaliana: GO:0043177 organic acid binding (gain)
c-value
0.0
0.1
0.2
0.3
0.4
0.5
0.6
0.7
0.8
Phylostratum
Cellular_organisms - ps1
Archaea1 - ps2
Archaea2 - ps3
Archaea3 - ps4
Asgard/Eukaryota - ps5
Eukaryota - ps6
Excavata/Diaphoretickes - ps7
Diaphoretickes - ps8
Archaeplastida - ps9
Viridiplantae - ps10
Streptophyta - ps11
Charophyceae/Embriophyta - ps12
Embriophyta - ps13
Tracheophyta - ps14
Spermatophyta - ps15
Angiosperms - ps16
Mesangiospermae - ps17
Magnoliids/eudicots - ps18
Eudicots - ps19
Core_eudicots - ps20
Superrosids - ps21
Rosids - ps22
Eurosids - ps23
Malvids - ps24
Sap./Malv./Brassicales - ps25
Malvales/Brassicales - ps26
Brassicales - ps27
Cleomaceae/Brassicaceae - ps28
Brassicaceae - ps29
Camelineae - ps30
Arabidopsis - ps31
Arabidopsis_thaliana - ps32
p-value
4e-38
5e-30
6e-22
6e-14
7e-06
Log-odds
0.5
1.0
1.5
2.0
2.5
3.0
3.5
4.0

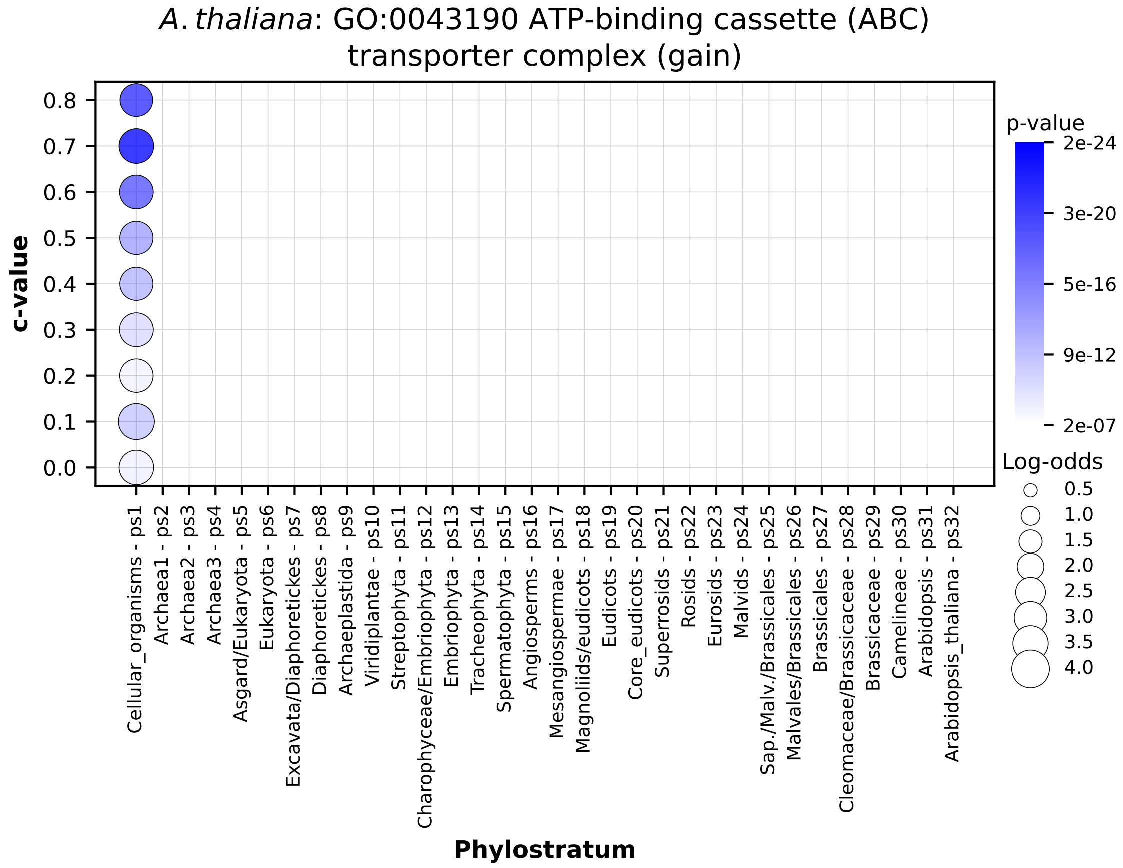
A. thaliana: GO:0043190 ATP-binding cassette (ABC) transporter complex (gain)
c-value
0.8
0.7
0.6
0.5
0.4
0.3
0.2
0.1
0.0
p-value
2e-24
3e-20
5e-16
9e-12
2e-07
Log-odds
0.5
1.0
1.5
2.0
2.5
3.0
3.5
4.0
Cellular_organisms - ps1
Archaea1 - ps2
Archaea2 - ps3
Archaea3 - ps4
Asgard/Eukaryota - ps5
Eukaryota - ps6
Excavata/Diaphoretickes - ps7
Diaphoretickes - ps8
Archaeplastida - ps9
Viridiplantae - ps10
Streptophyta - ps11
Charophyceae/Embriophyta - ps12
Embriophyta - ps13
Tracheophyta - ps14
Spermatophyta - ps15
Angiosperms - ps16
Mesangiospermae - ps17
Magnoliids/eudicots - ps18
Eudicots - ps19
Core_eudicots - ps20
Superrosids - ps21
Rosids - ps22
Eurosids - ps23
Malvids - ps24
Sap./Malv./Brassicales - ps25
Malvales/Brassicales - ps26
Brassicales - ps27
Cleomaceae/Brassicaceae - ps28
Brassicaceae - ps29
Camelineae - ps30
Arabidopsis - ps31
Arabidopsis_thaliana - ps32
Phylostratum

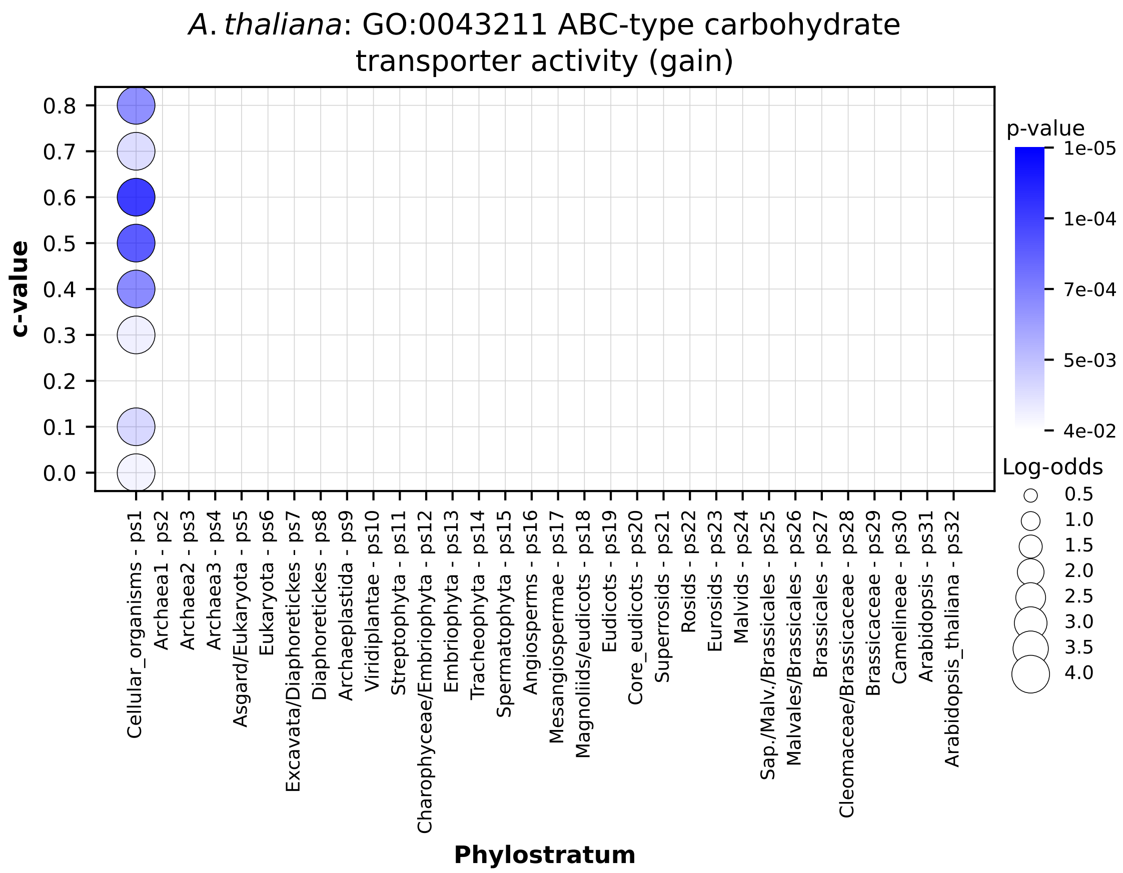

A. thaliana: GO:0043211 ABC-type carbohydrate transporter activity (gain)
c-value
0.0
0.1
0.2
0.3
0.4
0.5
0.6
0.7
0.8
p-value
1e-05
1e-04
7e-04
5e-03
4e-02
Log-odds
0.5
1.0
1.5
2.0
2.5
3.0
3.5
4.0
Cellular_organisms - ps1
Archaea1 - ps2
Archaea2 - ps3
Archaea3 - ps4
Asgard/Eukaryota - ps5
Eukaryota - ps6
Excavata/Diaphoretickes - ps7
Diaphoretickes - ps8
Archaeplastida - ps9
Viridiplantae - ps10
Streptophyta - ps11
Charophyceae/Embriophyta - ps12
Embriophyta - ps13
Tracheophyta - ps14
Spermatophyta - ps15
Angiosperms - ps16
Mesangiospermae - ps17
Magnoliids/eudicots - ps18
Eudicots - ps19
Core_eudicots - ps20
Superrosids - ps21
Rosids - ps22
Eurosids - ps23
Malvids - ps24
Sap./Malv./Brassicales - ps25
Malvales/Brassicales - ps26
Brassicales - ps27
Cleomaceae/Brassicaceae - ps28
Brassicaceae - ps29
Camelineae - ps30
Arabidopsis - ps31
Arabidopsis_thaliana - ps32
Phylostratum

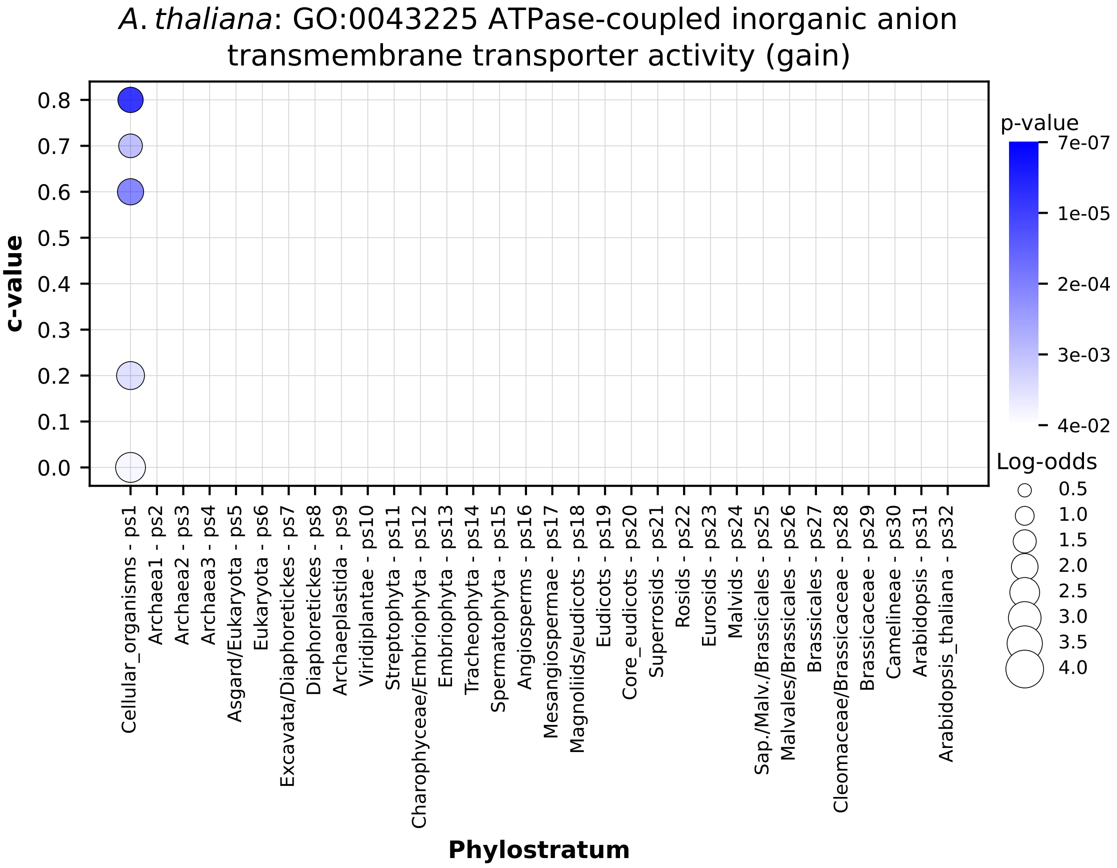

A. thaliana: GO:0043225 ATPase-coupled inorganic anion transmembrane transporter activity (gain)
c-value
0.8
0.7
0.6
0.5
0.4
0.3
0.2
0.1
0.0
p-value
7e-07
1e-05
2e-04
3e-03
4e-02
Log-odds
0.5
1.0
1.5
2.0
2.5
3.0
3.5
4.0
Cellular_organisms - ps1
Archaea1 - ps2
Archaea2 - ps3
Archaea3 - ps4
Asgard/Eukaryota - ps5
Eukaryota - ps6
Excavata/Diaphoretickes - ps7
Diaphoretickes - ps8
Archaeplastida - ps9
Viridiplantae - ps10
Streptophyta - ps11
Charophyceae/Embriophyta - ps12
Embriophyta - ps13
Tracheophyta - ps14
Spermatophyta - ps15
Angiosperms - ps16
Mesangiospermae - ps17
Magnoliids/eudicots - ps18
Eudicots - ps19
Core_eudicots - ps20
Superrosids - ps21
Rosids - ps22
Eurosids - ps23
Malvids - ps24
Sap./Malv./Brassicales - ps25
Malvales/Brassicales - ps26
Brassicales - ps27
Cleomaceae/Brassicaceae - ps28
Brassicaceae - ps29
Camelineae - ps30
Arabidopsis - ps31
Arabidopsis_thaliana - ps32
Phylostratum

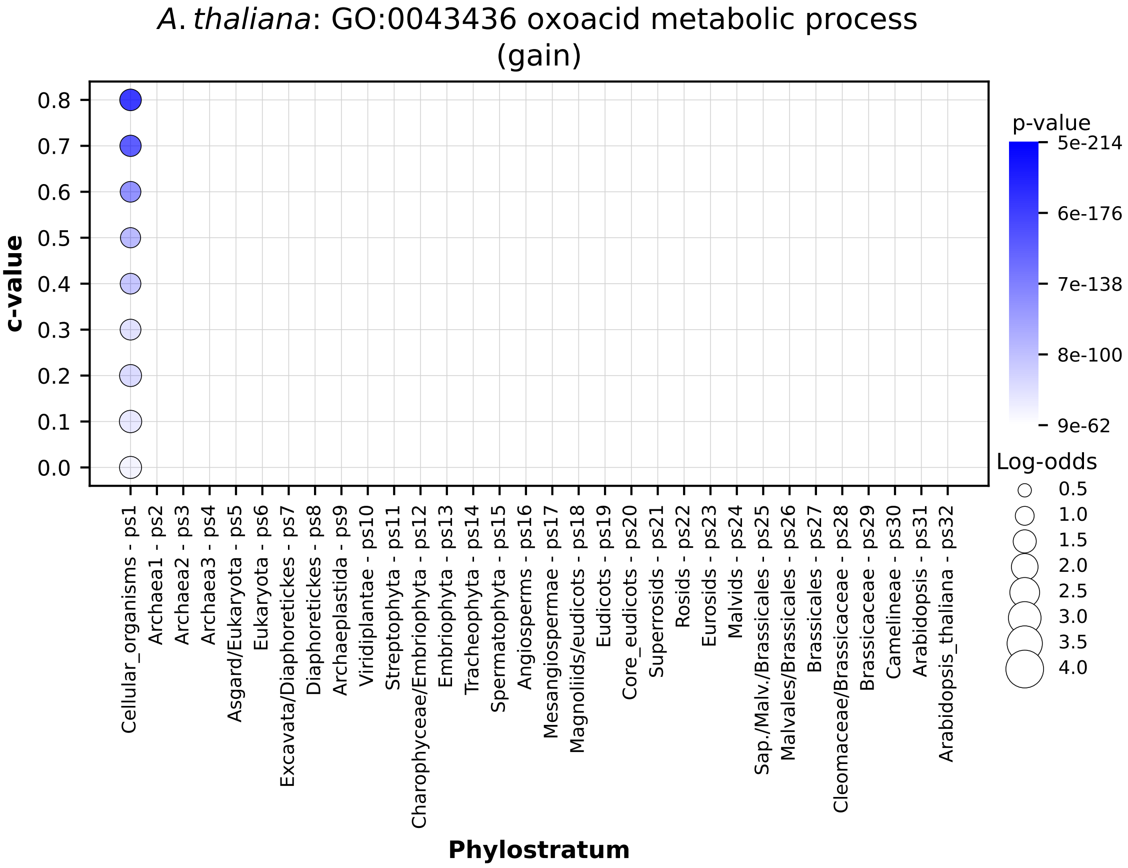

A. thaliana: GO:0043436 oxoacid metabolic process
(gain)
c-value
0.8
0.7
0.6
0.5
0.4
0.3
0.2
0.1
0.0
p-value
5e-214
6e-176
7e-138
8e-100
9e-62
Log-odds
0.5
1.0
1.5
2.0
2.5
3.0
3.5
4.0
Cellular_organisms - ps1
Archaea1 - ps2
Archaea2 - ps3
Archaea3 - ps4
Asgard/Eukaryota - ps5
Eukaryota - ps6
Excavata/Diaphoretickes - ps7
Diaphoretickes - ps8
Archaeplastida - ps9
Viridiplantae - ps10
Streptophyta - ps11
Charophyceae/Embriophyta - ps12
Embriophyta - ps13
Tracheophyta - ps14
Spermatophyta - ps15
Angiosperms - ps16
Mesangiospermae - ps17
Magnoliids/eudicots - ps18
Eudicots - ps19
Core_eudicots - ps20
Superrosids - ps21
Rosids - ps22
Eurosids - ps23
Malvids - ps24
Sap./Malv./Brassicales - ps25
Malvales/Brassicales - ps26
Brassicales - ps27
Cleomaceae/Brassicaceae - ps28
Brassicaceae - ps29
Camelineae - ps30
Arabidopsis - ps31
Arabidopsis_thaliana - ps32
Phylostratum

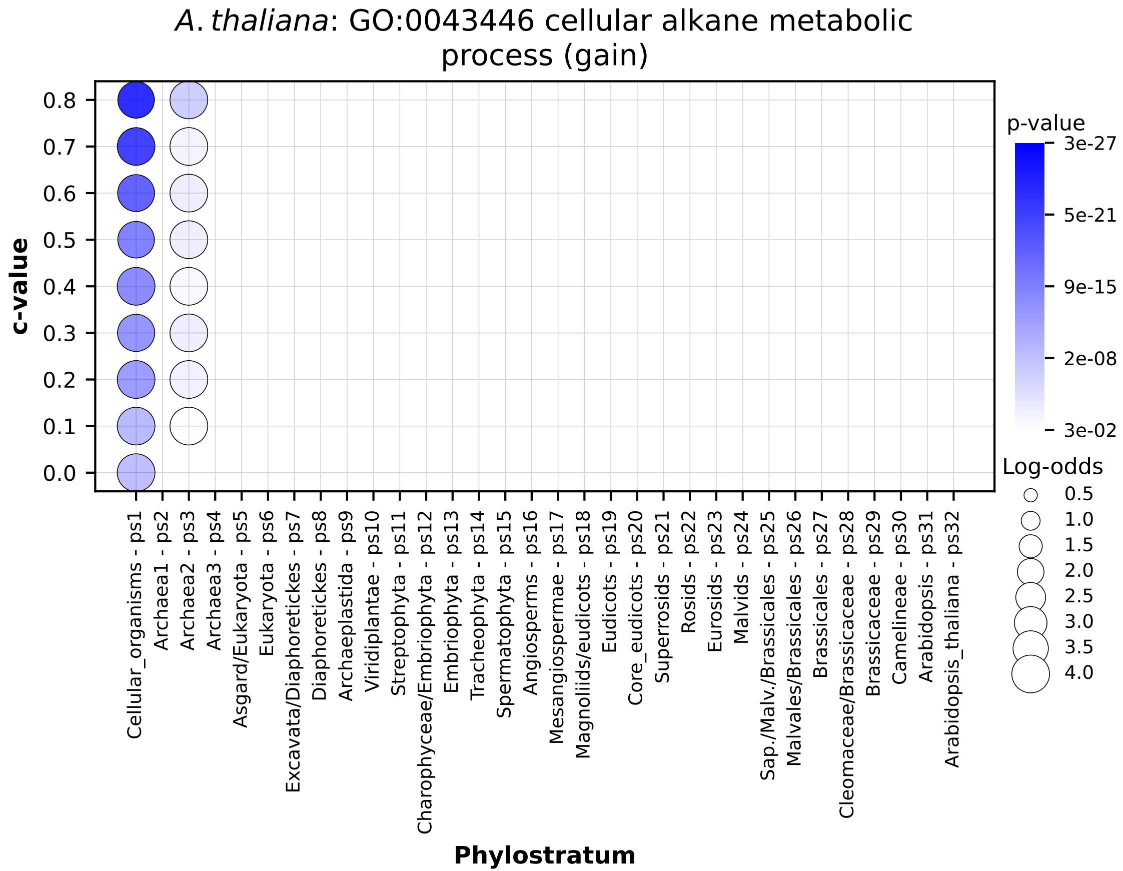

A. thaliana: GO:0043446 cellular alkane metabolic process (gain)
c-value
0.8
0.7
0.6
0.5
0.4
0.3
0.2
0.1
0.0
p-value
3e-27
5e-21
9e-15
2e-08
3e-02
Log-odds
0.5
1.0
1.5
2.0
2.5
3.0
3.5
4.0
Cellular_organisms - ps1
Archaea1 - ps2
Archaea2 - ps3
Archaea3 - ps4
Asgard/Eukaryota - ps5
Eukaryota - ps6
Excavata/Diaphoretickes - ps7
Diaphoretickes - ps8
Archaeplastida - ps9
Viridiplantae - ps10
Streptophyta - ps11
Charophyceae/Embriophyta - ps12
Embriophyta - ps13
Tracheophyta - ps14
Spermatophyta - ps15
Angiosperms - ps16
Mesangiospermae - ps17
Magnoliids/eudicots - ps18
Eudicots - ps19
Core_eudicots - ps20
Superrosids - ps21
Rosids - ps22
Eurosids - ps23
Malvids - ps24
Sap./Malv./Brassicales - ps25
Malvales/Brassicales - ps26
Brassicales - ps27
Cleomaceae/Brassicaceae - ps28
Brassicaceae - ps29
Camelineae - ps30
Arabidopsis - ps31
Arabidopsis_thaliana - ps32
Phylostratum

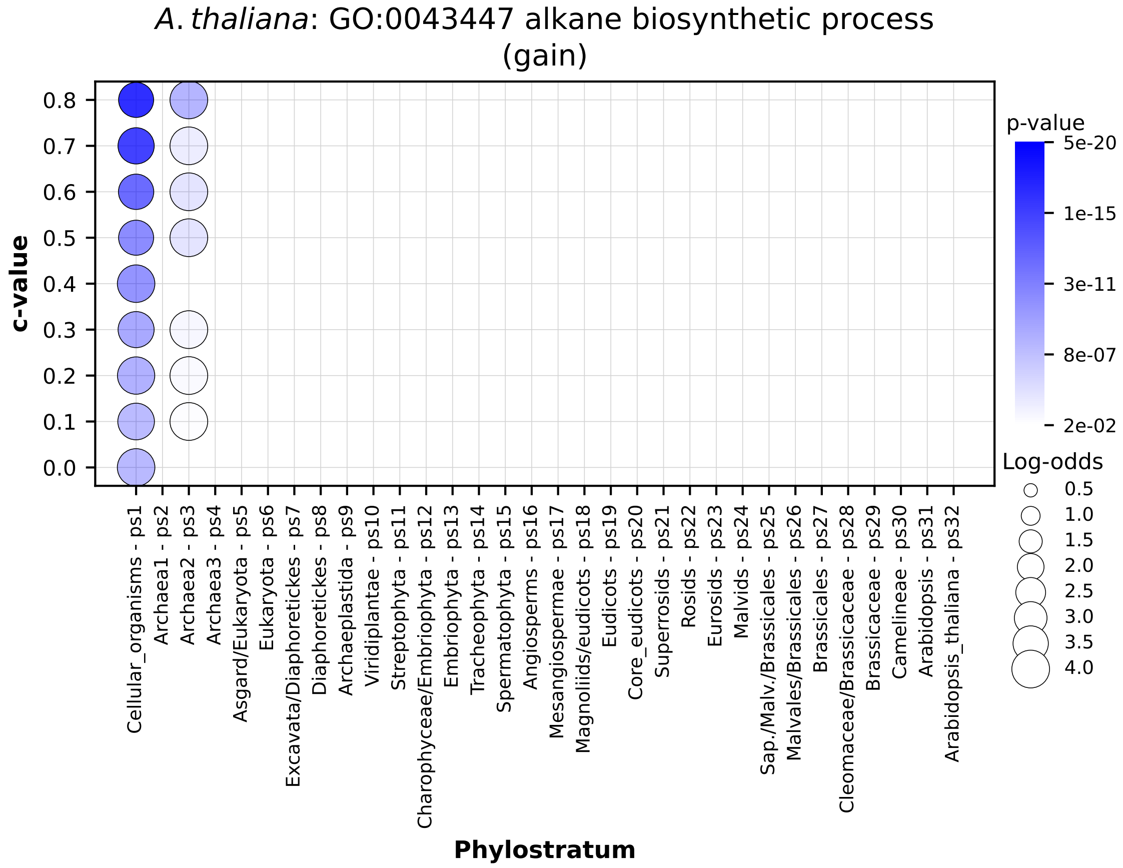

A. thaliana: GO:0043447 alkane biosynthetic process
(gain)
c-value
0.8
0.7
0.6
0.5
0.4
0.3
0.2
0.1
0.0
p-value
5e-20
1e-15
3e-11
8e-07
2e-02
Log-odds
0.5
1.0
1.5
2.0
2.5
3.0
3.5
4.0
Cellular_organisms - ps1
Archaea1 - ps2
Archaea2 - ps3
Archaea3 - ps4
Asgard/Eukaryota - ps5
Eukaryota - ps6
Excavata/Diaphoretickes - ps7
Diaphoretickes - ps8
Archaeplastida - ps9
Viridiplantae - ps10
Streptophyta - ps11
Charophyceae/Embriophyta - ps12
Embriophyta - ps13
Tracheophyta - ps14
Spermatophyta - ps15
Angiosperms - ps16
Mesangiospermae - ps17
Magnoliids/eudicots - ps18
Eudicots - ps19
Core_eudicots - ps20
Superrosids - ps21
Rosids - ps22
Eurosids - ps23
Malvids - ps24
Sap./Malv./Brassicales - ps25
Malvales/Brassicales - ps26
Brassicales - ps27
Cleomaceae/Brassicaceae - ps28
Brassicaceae - ps29
Camelineae - ps30
Arabidopsis - ps31
Arabidopsis_thaliana - ps32
Phylostratum

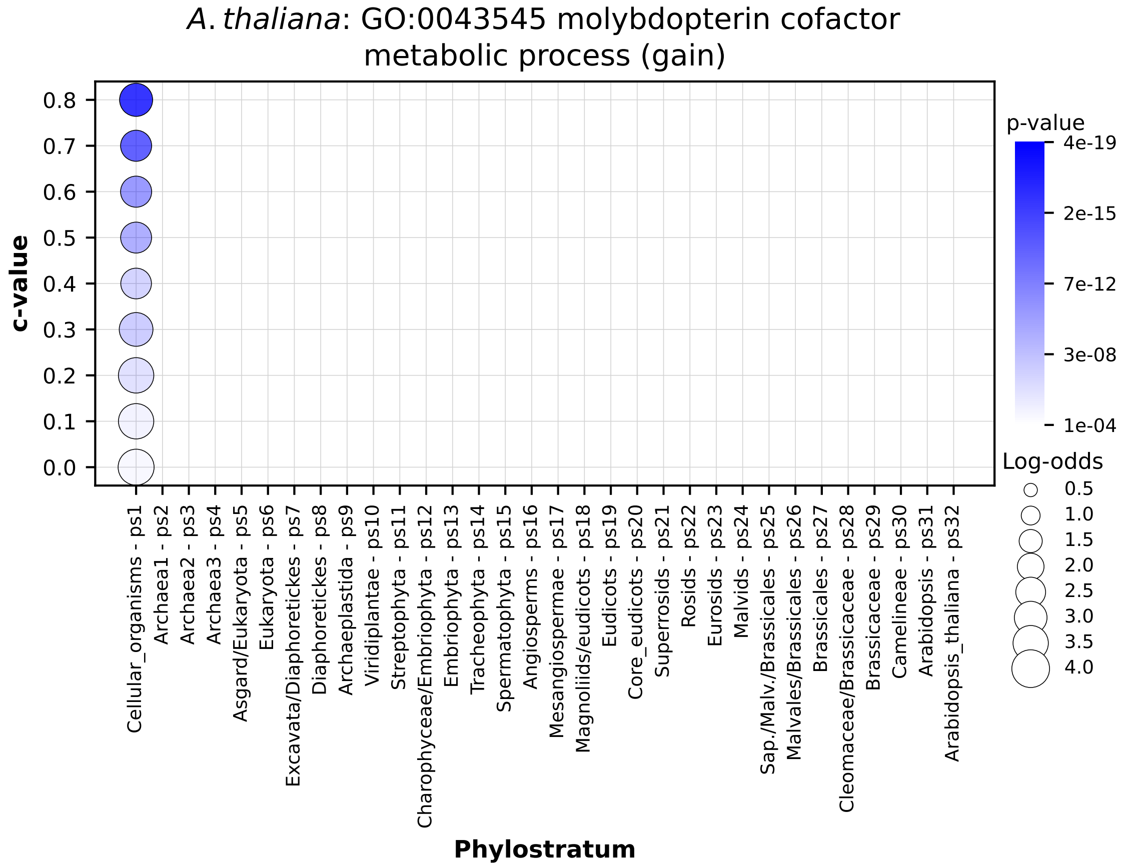

A. thaliana: GO:0043545 molybdopterin cofactor metabolic process (gain)
c-value
0.0
0.1
0.2
0.3
0.4
0.5
0.6
0.7
0.8
p-value
4e-19
2e-15
7e-12
3e-08
1e-04
Log-odds
0.5
1.0
1.5
2.0
2.5
3.0
3.5
4.0
Cellular_organisms - ps1
Archaea1 - ps2
Archaea2 - ps3
Archaea3 - ps4
Asgard/Eukaryota - ps5
Eukaryota - ps6
Excavata/Diaphoretickes - ps7
Diaphoretickes - ps8
Archaeplastida - ps9
Viridiplantae - ps10
Streptophyta - ps11
Charophyceae/Embriophyta - ps12
Embriophyta - ps13
Tracheophyta - ps14
Spermatophyta - ps15
Angiosperms - ps16
Mesangiospermae - ps17
Magnoliids/eudicots - ps18
Eudicots - ps19
Core_eudicots - ps20
Superrosids - ps21
Rosids - ps22
Eurosids - ps23
Malvids - ps24
Sap./Malv./Brassicales - ps25
Malvales/Brassicales - ps26
Brassicales - ps27
Cleomaceae/Brassicaceae - ps28
Brassicaceae - ps29
Camelineae - ps30
Arabidopsis - ps31
Arabidopsis_thaliana - ps32
Phylostratum

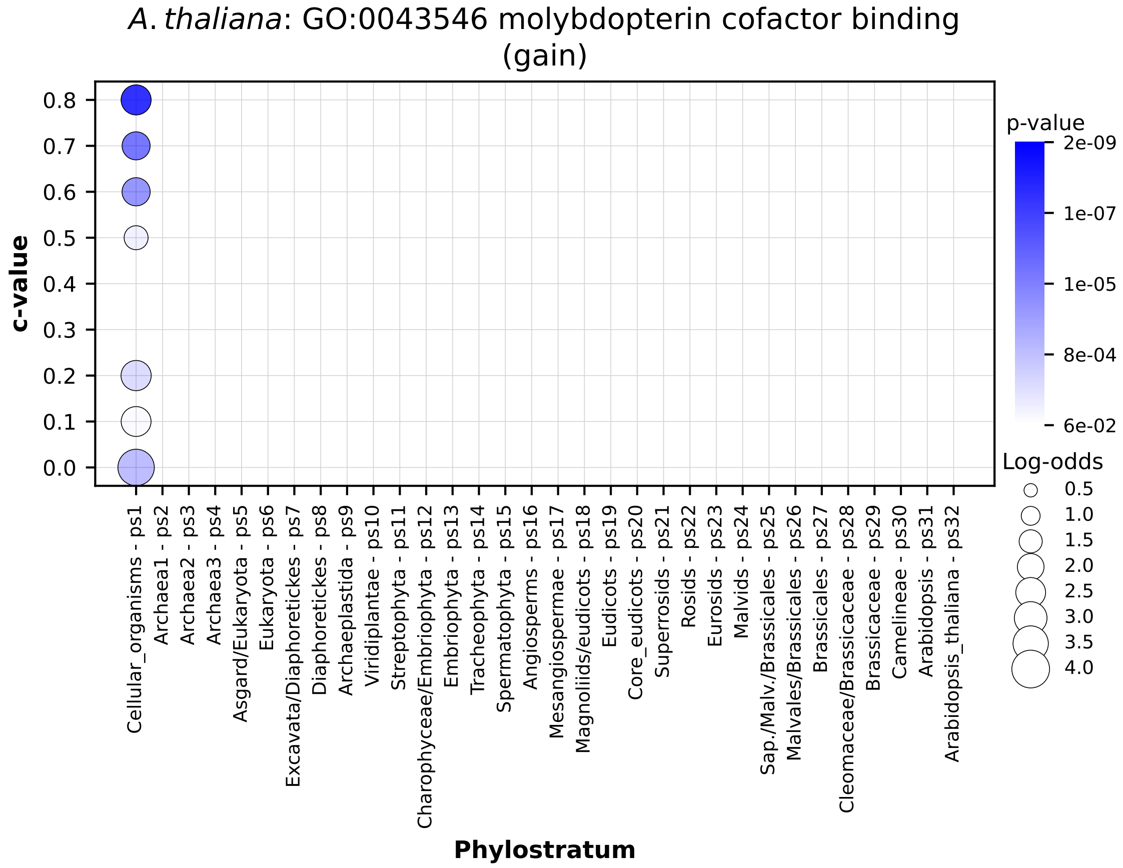

A. thaliana: GO:0043546 molybdopterin cofactor binding (gain)
c-value
0.8
0.7
0.6
0.5
0.4
0.3
0.2
0.1
0.0
p-value
2e-09
1e-07
1e-05
8e-04
6e-02
Log-odds
0.5
1.0
1.5
2.0
2.5
3.0
3.5
4.0
Cellular_organisms - ps1
Archaea1 - ps2
Archaea2 - ps3
Archaea3 - ps4
Asgard/Eukaryota - ps5
Eukaryota - ps6
Excavata/Diaphoretickes - ps7
Diaphoretickes - ps8
Archaeplastida - ps9
Viridiplantae - ps10
Streptophyta - ps11
Charophyceae/Embriophyta - ps12
Embriophyta - ps13
Tracheophyta - ps14
Spermatophyta - ps15
Angiosperms - ps16
Mesangiospermae - ps17
Magnoliids/eudicots - ps18
Eudicots - ps19
Core_eudicots - ps20
Superrosids - ps21
Rosids - ps22
Eurosids - ps23
Malvids - ps24
Sap./Malv./Brassicales - ps25
Malvales/Brassicales - ps26
Brassicales - ps27
Cleomaceae/Brassicaceae - ps28
Brassicaceae - ps29
Camelineae - ps30
Arabidopsis - ps31
Arabidopsis_thaliana - ps32
Phylostratum

A. thaliana: GO:0043571 maintenance of CRISPR repeat elements (gain)

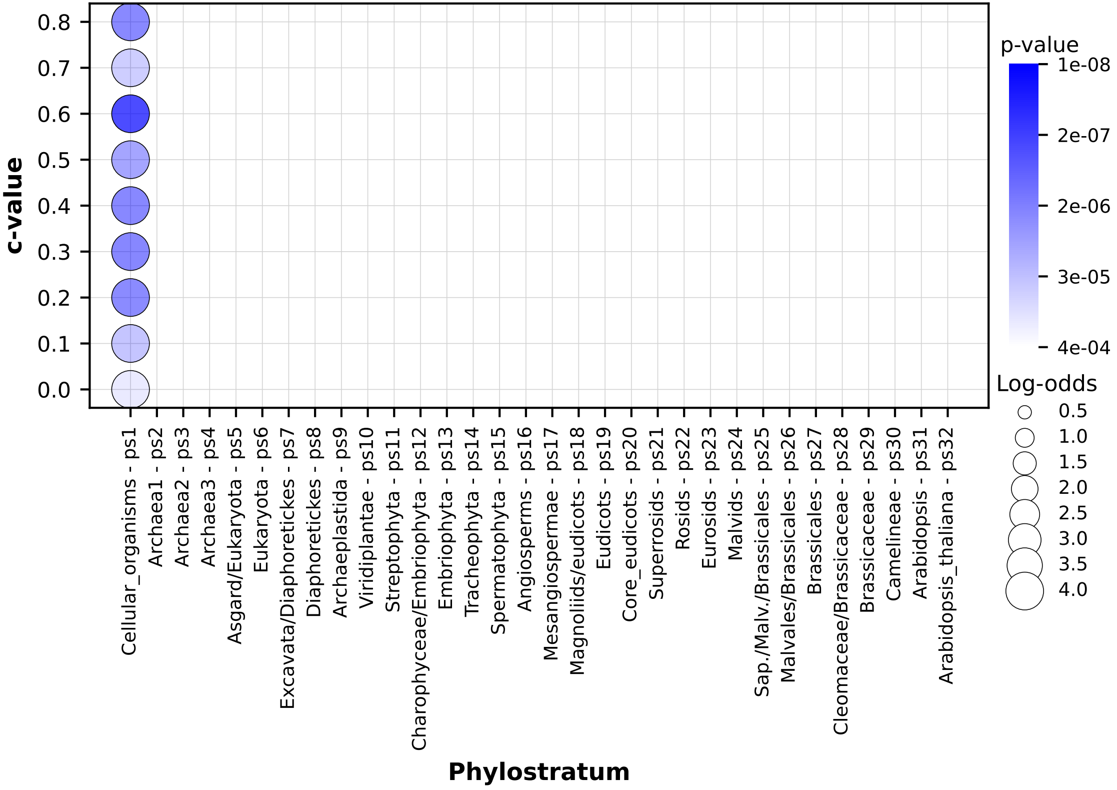

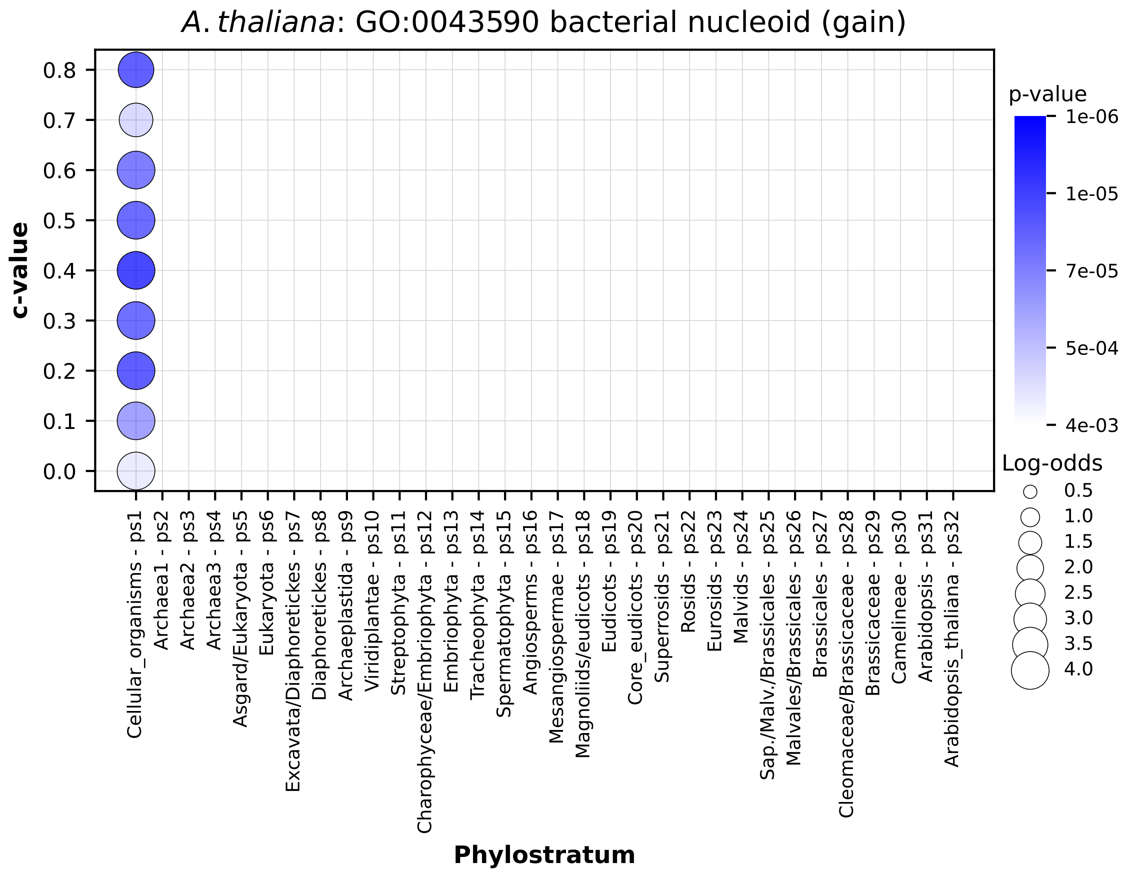

A. thaliana: GO:0043590 bacterial nucleoid (gain)
c-value
0.8
0.7
0.6
0.5
0.4
0.3
0.2
0.1
0.0
Phylostratum
Cellular_organisms - ps1
Archaea1 - ps2
Archaea2 - ps3
Archaea3 - ps4
Asgard/Eukaryota - ps5
Eukaryota - ps6
Excavata/Diaphoretickes - ps7
Diaphoretickes - ps8
Archaeplastida - ps9
Viridiplantae - ps10
Streptophyta - ps11
Charophyceae/Embriophyta - ps12
Embriophyta - ps13
Tracheophyta - ps14
Spermatophyta - ps15
Angiosperms - ps16
Mesangiospermae - ps17
Magnoliids/eudicots - ps18
Eudicots - ps19
Core_eudicots - ps20
Superrosids - ps21
Rosids - ps22
Eurosids - ps23
Malvids - ps24
Sap./Malv./Brassicales - ps25
Malvales/Brassicales - ps26
Brassicales - ps27
Cleomaceae/Brassicaceae - ps28
Brassicaceae - ps29
Camelineae - ps30
Arabidopsis - ps31
Arabidopsis_thaliana - ps32
p-value
1e-06
1e-05
7e-05
5e-04
4e-03
Log-odds
0.5
1.0
1.5
2.0
2.5
3.0
3.5
4.0

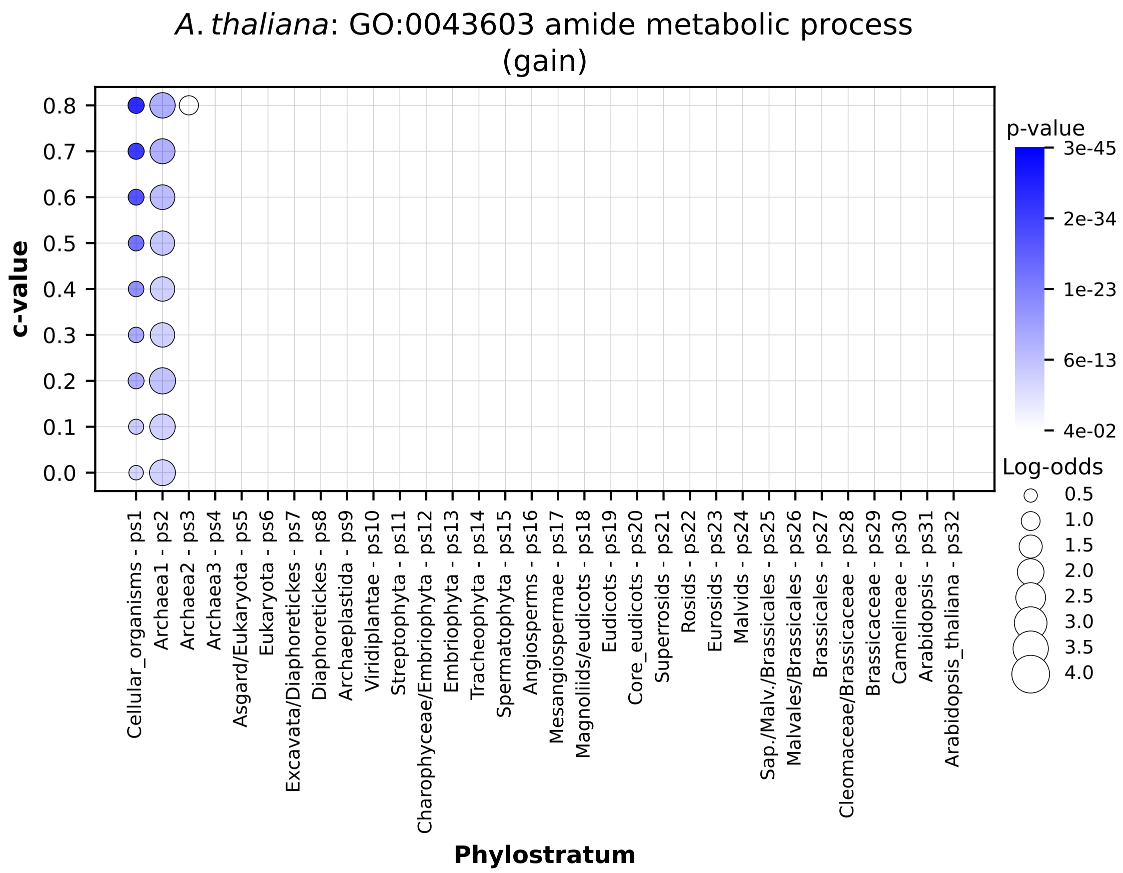

A. thaliana: GO:0043603 amide metabolic process
(gain)
c-value
0.8
0.7
0.6
0.5
0.4
0.3
0.2
0.1
0.0
p-value
3e-45
2e-34
1e-23
6e-13
4e-02
Log-odds
0.5
1.0
1.5
2.0
2.5
3.0
3.5
4.0
Cellular_organisms - ps1
Archaea1 - ps2
Archaea2 - ps3
Archaea3 - ps4
Asgard/Eukaryota - ps5
Eukaryota - ps6
Excavata/Diaphoretickes - ps7
Diaphoretickes - ps8
Archaeplastida - ps9
Viridiplantae - ps10
Streptophyta - ps11
Charophyceae/Embriophyta - ps12
Embriophyta - ps13
Tracheophyta - ps14
Spermatophyta - ps15
Angiosperms - ps16
Mesangiospermae - ps17
Magnoliids/eudicots - ps18
Eudicots - ps19
Core_eudicots - ps20
Superrosids - ps21
Rosids - ps22
Eurosids - ps23
Malvids - ps24
Sap./Malv./Brassicales - ps25
Malvales/Brassicales - ps26
Brassicales - ps27
Cleomaceae/Brassicaceae - ps28
Brassicaceae - ps29
Camelineae - ps30
Arabidopsis - ps31
Arabidopsis_thaliana - ps32
Phylostratum

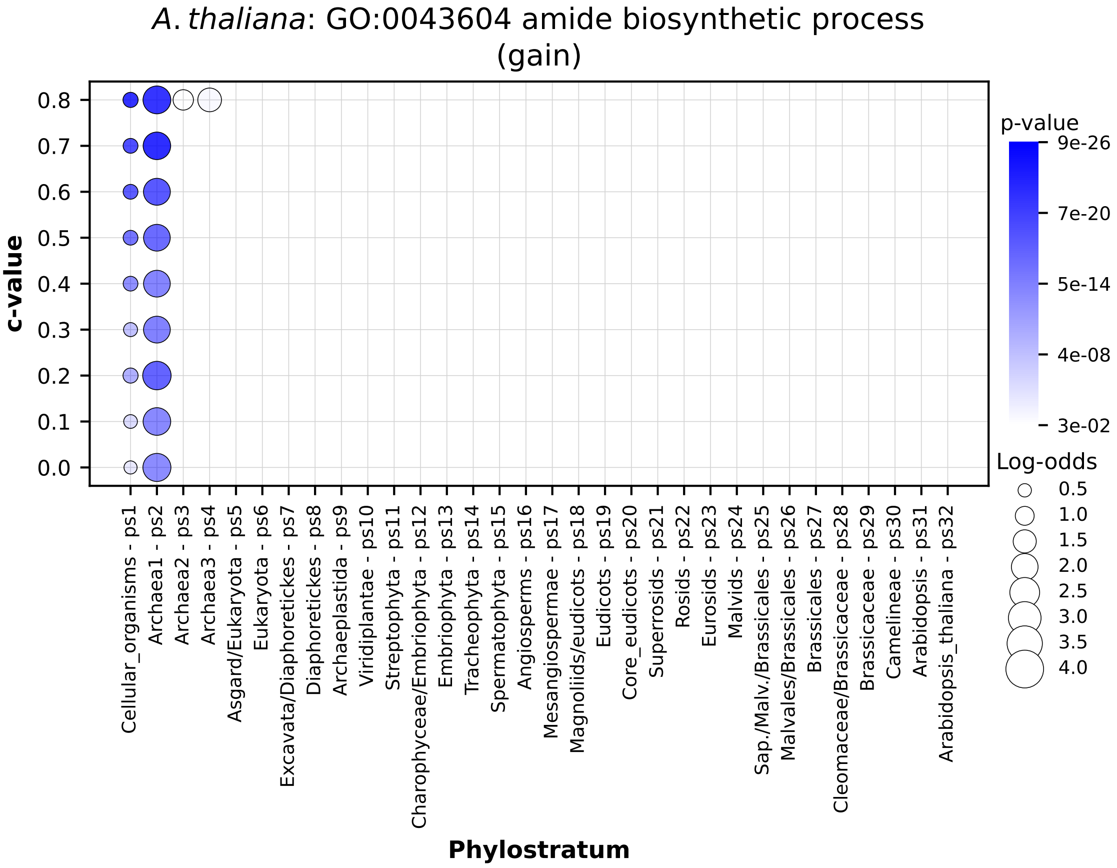

A. thaliana: GO:0043604 amide biosynthetic process (gain)
c-value
0.8
0.7
0.6
0.5
0.4
0.3
0.2
0.1
0.0
p-value
9e-26
7e-20
5e-14
4e-08
3e-02
Log-odds
0.5
1.0
1.5
2.0
2.5
3.0
3.5
4.0
Cellular_organisms - ps1
Archaea1 - ps2
Archaea2 - ps3
Archaea3 - ps4
Asgard/Eukaryota - ps5
Eukaryota - ps6
Excavata/Diaphoretickes - ps7
Diaphoretickes - ps8
Archaeplastida - ps9
Viridiplantae - ps10
Streptophyta - ps11
Charophyceae/Embriophyta - ps12
Embriophyta - ps13
Tracheophyta - ps14
Spermatophyta - ps15
Angiosperms - ps16
Mesangiospermae - ps17
Magnoliids/eudicots - ps18
Eudicots - ps19
Core_eudicots - ps20
Superrosids - ps21
Rosids - ps22
Eurosids - ps23
Malvids - ps24
Sap./Malv./Brassicales - ps25
Malvales/Brassicales - ps26
Brassicales - ps27
Cleomaceae/Brassicaceae - ps28
Brassicaceae - ps29
Camelineae - ps30
Arabidopsis - ps31
Arabidopsis_thaliana - ps32
Phylostratum

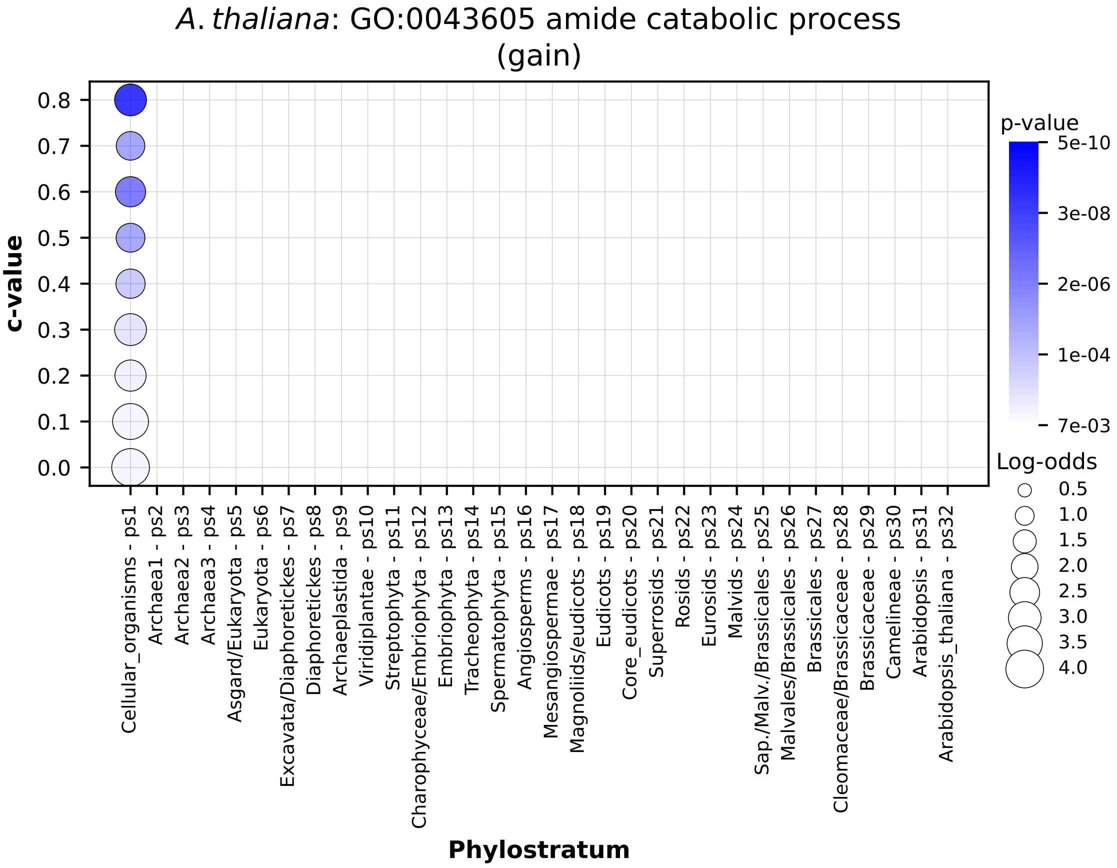
A. thaliana: GO:0043605 amide catabolic process
(gain)
c-value
0.8
0.7
0.6
0.5
0.4
0.3
0.2
0.1
0.0
p-value
5e-10
3e-08
2e-06
1e-04
7e-03
Log-odds
0.5
1.0
1.5
2.0
2.5
3.0
3.5
4.0
Cellular_organisms - ps1
Archaea1 - ps2
Archaea2 - ps3
Archaea3 - ps4
Asgard/Eukaryota - ps5
Eukaryota - ps6
Excavata/Diaphoretickes - ps7
Diaphoretickes - ps8
Archaeplastida - ps9
Viridiplantae - ps10
Streptophyta - ps11
Charophyceae/Embriophyta - ps12
Embriophyta - ps13
Tracheophyta - ps14
Spermatophyta - ps15
Angiosperms - ps16
Mesangiospermae - ps17
Magnoliids/eudicots - ps18
Eudicots - ps19
Core_eudicots - ps20
Superrosids - ps21
Rosids - ps22
Eurosids - ps23
Malvids - ps24
Sap./Malv./Brassicales - ps25
Malvales/Brassicales - ps26
Brassicales - ps27
Cleomaceae/Brassicaceae - ps28
Brassicaceae - ps29
Camelineae - ps30
Arabidopsis - ps31
Arabidopsis_thaliana - ps32
Phylostratum

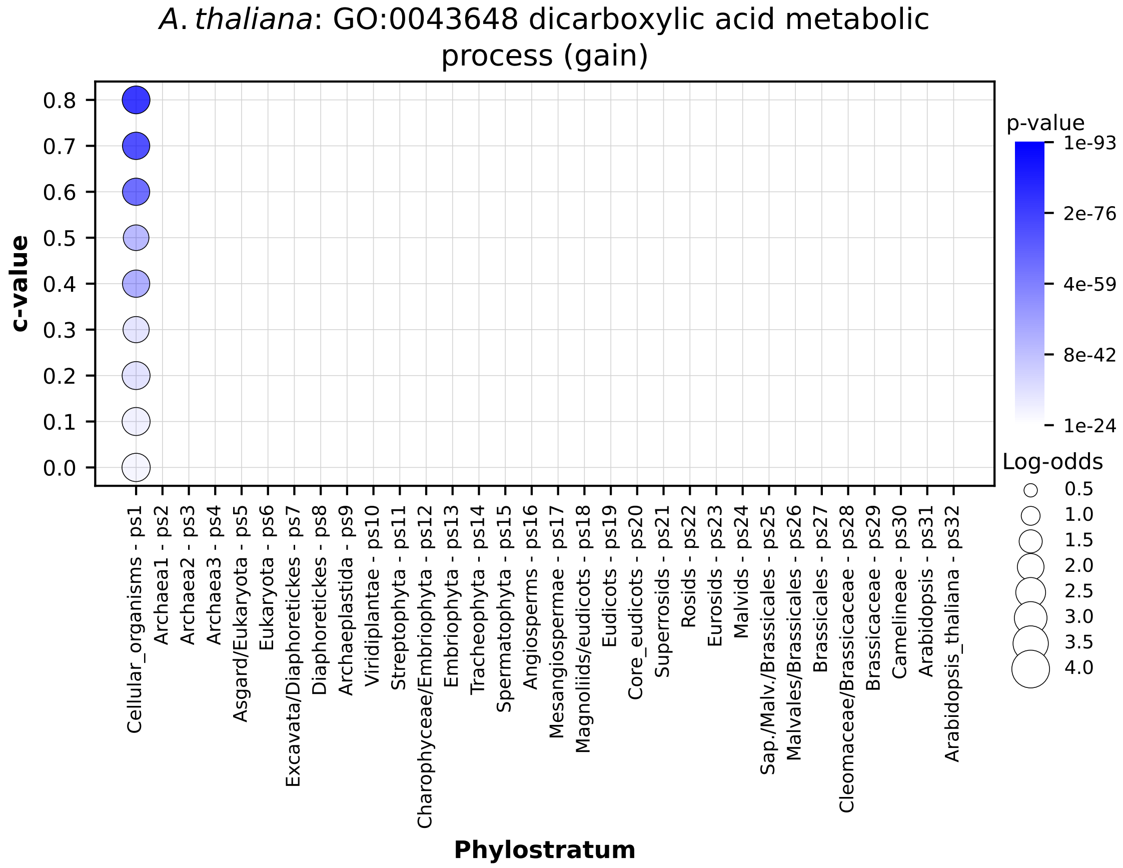

A. thaliana: GO:0043648 dicarboxylic acid metabolic process (gain)
c-value
0.8
0.7
0.6
0.5
0.4
0.3
0.2
0.1
0.0
p-value
1e-93
2e-76
4e-59
8e-42
1e-24
Log-odds
0.5
1.0
1.5
2.0
2.5
3.0
3.5
4.0
Cellular_organisms - ps1
Archaea1 - ps2
Archaea2 - ps3
Archaea3 - ps4
Asgard/Eukaryota - ps5
Eukaryota - ps6
Excavata/Diaphoretickes - ps7
Diaphoretickes - ps8
Archaeplastida - ps9
Viridiplantae - ps10
Streptophyta - ps11
Charophyceae/Embriophyta - ps12
Embriophyta - ps13
Tracheophyta - ps14
Spermatophyta - ps15
Angiosperms - ps16
Mesangiospermae - ps17
Magnoliids/eudicots - ps18
Eudicots - ps19
Core_eudicots - ps20
Superrosids - ps21
Rosids - ps22
Eurosids - ps23
Malvids - ps24
Sap./Malv./Brassicales - ps25
Malvales/Brassicales - ps26
Brassicales - ps27
Cleomaceae/Brassicaceae - ps28
Brassicaceae - ps29
Camelineae - ps30
Arabidopsis - ps31
Arabidopsis_thaliana - ps32
Phylostratum

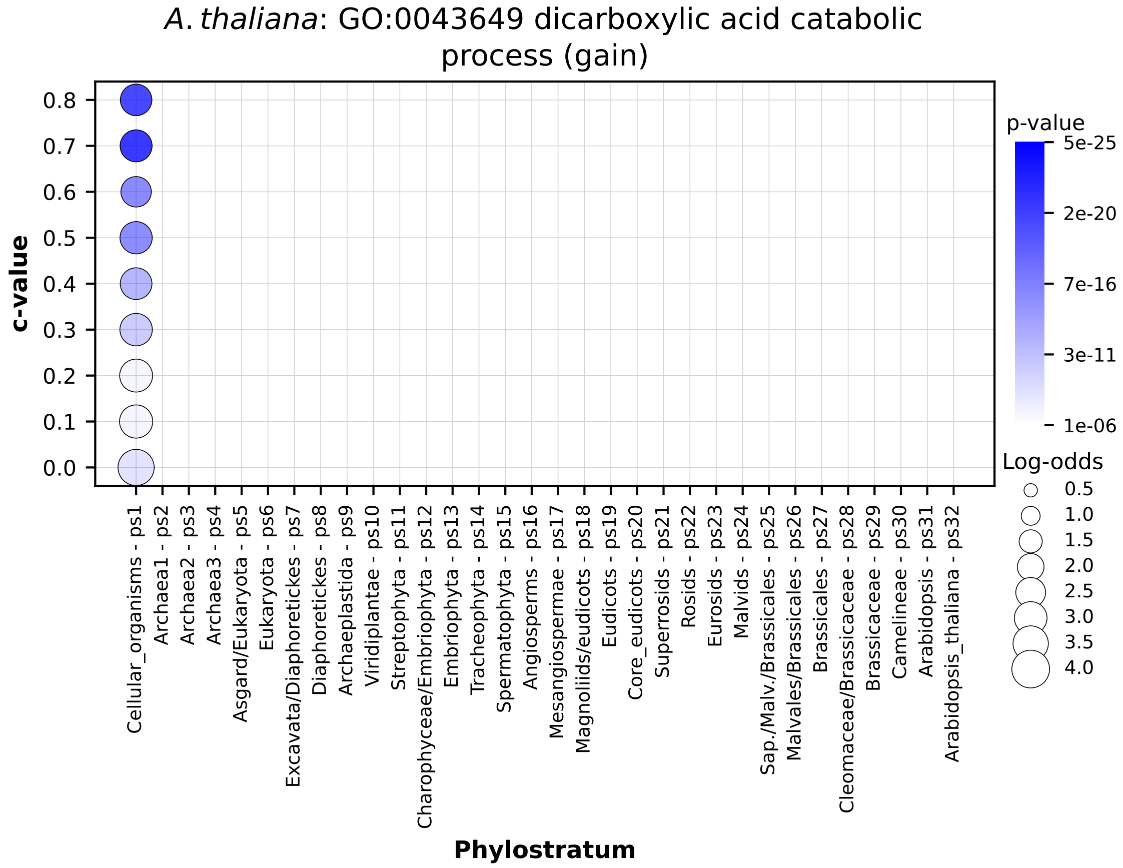

A. thaliana: GO:0043649 dicarboxylic acid catabolic process (gain)
c-value
0.8
0.7
0.6
0.5
0.4
0.3
0.2
0.1
0.0
p-value
5e-25
2e-20
7e-16
3e-11
1e-06
Log-odds
0.5
1.0
1.5
2.0
2.5
3.0
3.5
4.0
Cellular_organisms - ps1
Archaea1 - ps2
Archaea2 - ps3
Archaea3 - ps4
Asgard/Eukaryota - ps5
Eukaryota - ps6
Excavata/Diaphoretickes - ps7
Diaphoretickes - ps8
Archaeplastida - ps9
Viridiplantae - ps10
Streptophyta - ps11
Charophyceae/Embriophyta - ps12
Embriophyta - ps13
Tracheophyta - ps14
Spermatophyta - ps15
Angiosperms - ps16
Mesangiospermae - ps17
Magnoliids/eudicots - ps18
Eudicots - ps19
Core_eudicots - ps20
Superrosids - ps21
Rosids - ps22
Eurosids - ps23
Malvids - ps24
Sap./Malv./Brassicales - ps25
Malvales/Brassicales - ps26
Brassicales - ps27
Cleomaceae/Brassicaceae - ps28
Brassicaceae - ps29
Camelineae - ps30
Arabidopsis - ps31
Arabidopsis_thaliana - ps32
Phylostratum

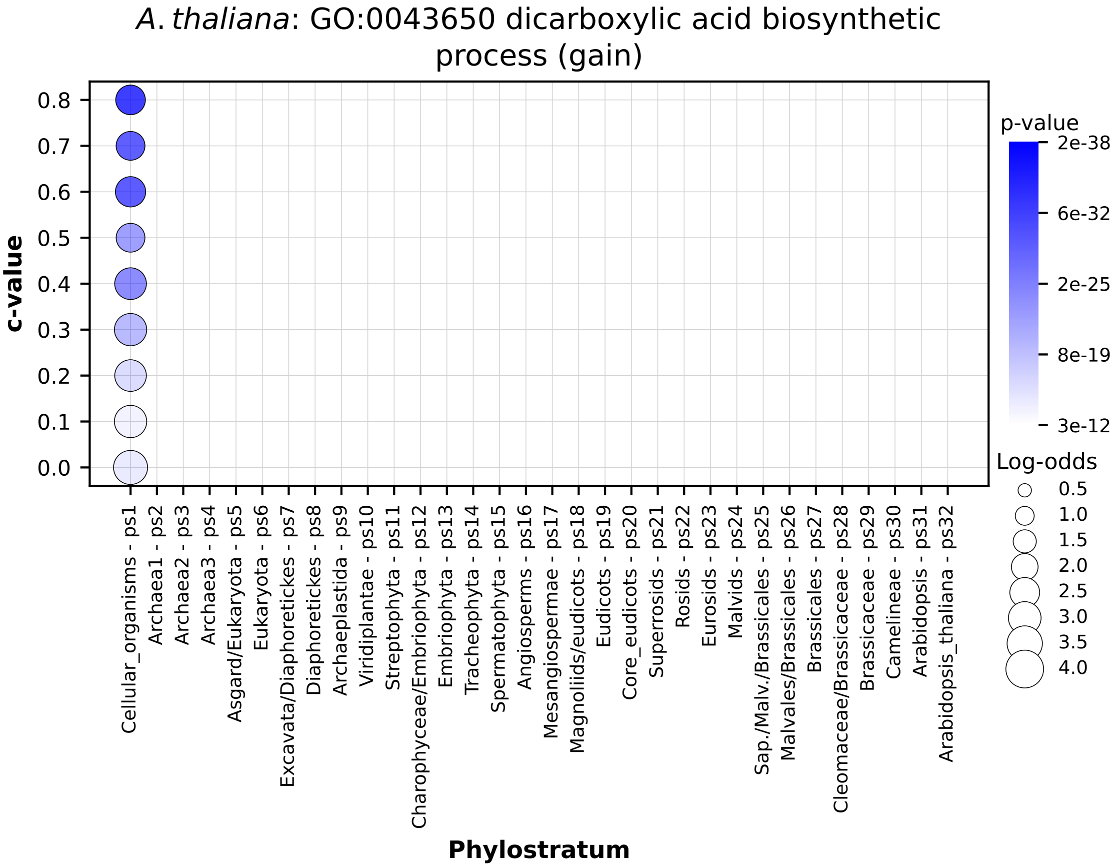

A. thaliana: GO:0043650 dicarboxylic acid biosynthetic process (gain)
c-value
0.8
0.7
0.6
0.5
0.4
0.3
0.2
0.1
0.0
Phylostratum
Cellular_organisms - ps1
Archaea1 - ps2
Archaea2 - ps3
Archaea3 - ps4
Asgard/Eukaryota - ps5
Eukaryota - ps6
Excavata/Diaphoretickes - ps7
Diaphoretickes - ps8
Archaeplastida - ps9
Viridiplantae - ps10
Streptophyta - ps11
Charophyceae/Embriophyta - ps12
Embriophyta - ps13
Tracheophyta - ps14
Spermatophyta - ps15
Angiosperms - ps16
Mesangiospermae - ps17
Magnoliids/eudicots - ps18
Eudicots - ps19
Core_eudicots - ps20
Superrosids - ps21
Rosids - ps22
Eurosids - ps23
Malvids - ps24
Sap./Malv./Brassicales - ps25
Malvales/Brassicales - ps26
Brassicales - ps27
Cleomaceae/Brassicaceae - ps28
Brassicaceae - ps29
Camelineae - ps30
Arabidopsis - ps31
Arabidopsis_thaliana - ps32
p-value
2e-38
6e-32
2e-25
8e-19
3e-12
Log-odds
0.5
1.0
1.5
2.0
2.5
3.0
3.5
4.0

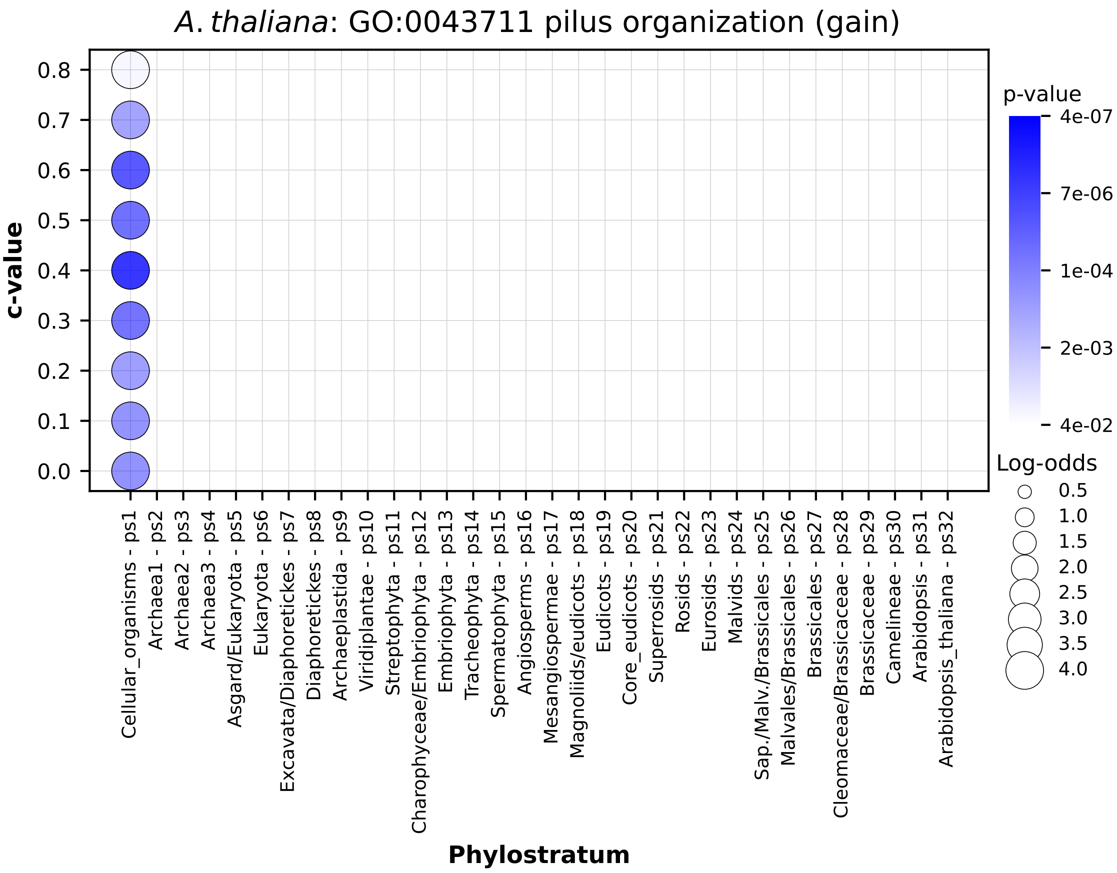

A. thaliana: GO:0043711 pilus organization (gain)
c-value
0.8
0.7
0.6
0.5
0.4
0.3
0.2
0.1
0.0
p-value
4e-07
7e-06
1e-04
2e-03
4e-02
Log-odds
0.5
1.0
1.5
2.0
2.5
3.0
3.5
4.0
Cellular_organisms - ps1
Archaea1 - ps2
Archaea2 - ps3
Archaea3 - ps4
Asgard/Eukaryota - ps5
Eukaryota - ps6
Excavata/Diaphoretickes - ps7
Diaphoretickes - ps8
Archaeplastida - ps9
Viridiplantae - ps10
Streptophyta - ps11
Charophyceae/Embriophyta - ps12
Embriophyta - ps13
Tracheophyta - ps14
Spermatophyta - ps15
Angiosperms - ps16
Mesangiospermae - ps17
Magnoliids/eudicots - ps18
Eudicots - ps19
Core_eudicots - ps20
Superrosids - ps21
Rosids - ps22
Eurosids - ps23
Malvids - ps24
Sap./Malv./Brassicales - ps25
Malvales/Brassicales - ps26
Brassicales - ps27
Cleomaceae/Brassicaceae - ps28
Brassicaceae - ps29
Camelineae - ps30
Arabidopsis - ps31
Arabidopsis_thaliana - ps32
Phylostratum

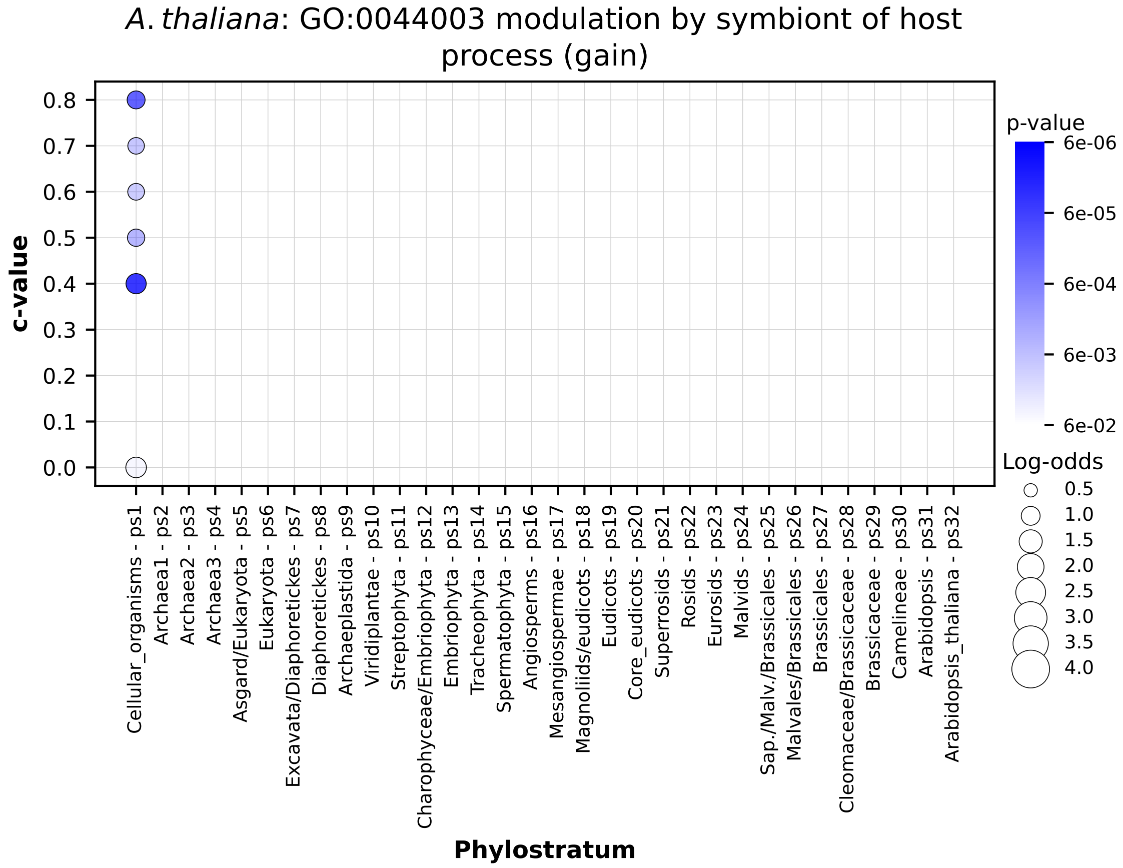
A. thaliana: GO:0044003 modulation by symbiont of host process (gain)
c-value
0.0
0.1
0.2
0.3
0.4
0.5
0.6
0.7
0.8
Phylostratum
Cellular_organisms - ps1
Archaea1 - ps2
Archaea2 - ps3
Archaea3 - ps4
Asgard/Eukaryota - ps5
Eukaryota - ps6
Excavata/Diaphoretickes - ps7
Diaphoretickes - ps8
Archaeplastida - ps9
Viridiplantae - ps10
Streptophyta - ps11
Charophyceae/Embriophyta - ps12
Embriophyta - ps13
Tracheophyta - ps14
Spermatophyta - ps15
Angiosperms - ps16
Mesangiospermae - ps17
Magnoliids/eudicots - ps18
Eudicots - ps19
Core_eudicots - ps20
Superrosids - ps21
Rosids - ps22
Eurosids - ps23
Malvids - ps24
Sap./Malv./Brassicales - ps25
Malvales/Brassicales - ps26
Brassicales - ps27
Cleomaceae/Brassicaceae - ps28
Brassicaceae - ps29
Camelineae - ps30
Arabidopsis - ps31
Arabidopsis_thaliana - ps32
p-value
6e-06
6e-05
6e-04
6e-03
6e-02
Log-odds
0.5
1.0
1.5
2.0
2.5
3.0
3.5
4.0

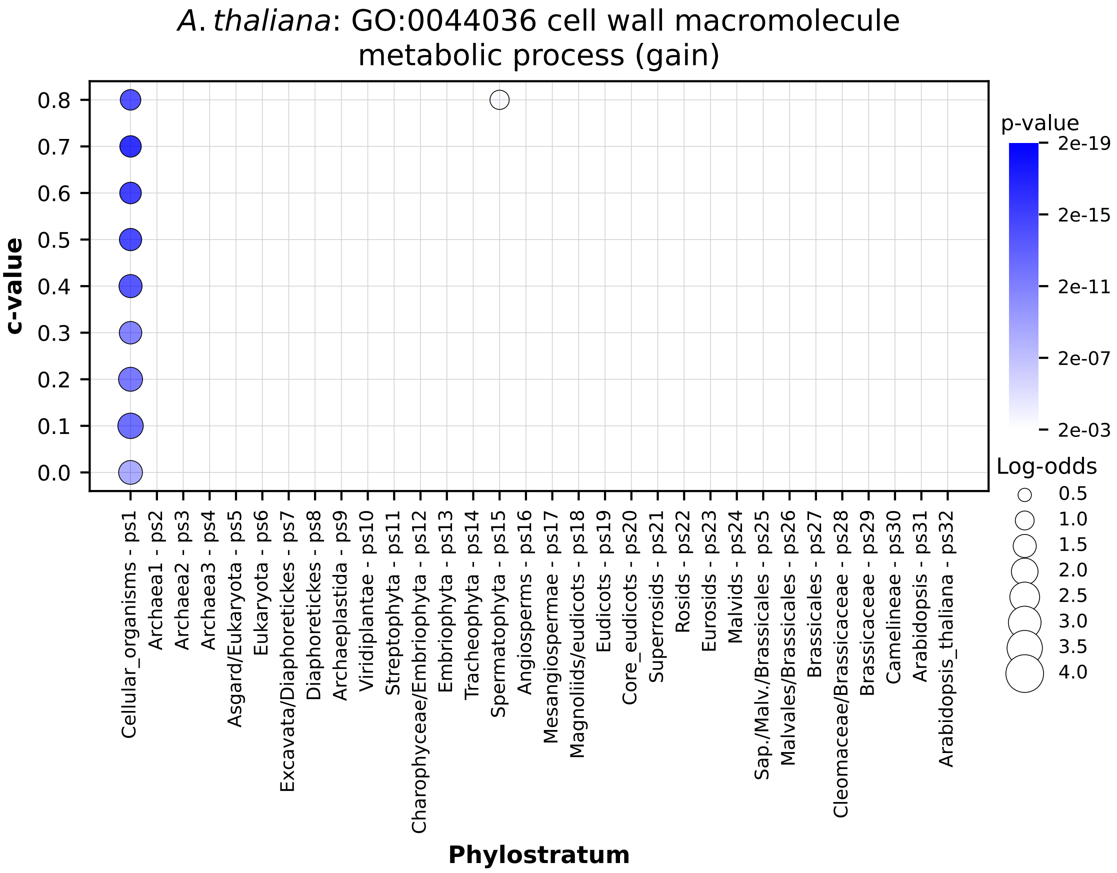

A. thaliana: GO:0044036 cell wall macromolecule metabolic process (gain)
c-value
0.8
0.7
0.6
0.5
0.4
0.3
0.2
0.1
0.0
p-value
2e-19
2e-15
2e-11
2e-07
2e-03
Log-odds
0.5
1.0
1.5
2.0
2.5
3.0
3.5
4.0
Cellular_organisms - ps1
Archaea1 - ps2
Archaea2 - ps3
Archaea3 - ps4
Asgard/Eukaryota - ps5
Eukaryota - ps6
Excavata/Diaphoretickes - ps7
Diaphoretickes - ps8
Archaeplastida - ps9
Viridiplantae - ps10
Streptophyta - ps11
Charophyceae/Embriophyta - ps12
Embriophyta - ps13
Tracheophyta - ps14
Spermatophyta - ps15
Angiosperms - ps16
Mesangiospermae - ps17
Magnoliids/eudicots - ps18
Eudicots - ps19
Core_eudicots - ps20
Superrosids - ps21
Rosids - ps22
Eurosids - ps23
Malvids - ps24
Sap./Malv./Brassicales - ps25
Malvales/Brassicales - ps26
Brassicales - ps27
Cleomaceae/Brassicaceae - ps28
Brassicaceae - ps29
Camelineae - ps30
Arabidopsis - ps31
Arabidopsis_thaliana - ps32
Phylostratum

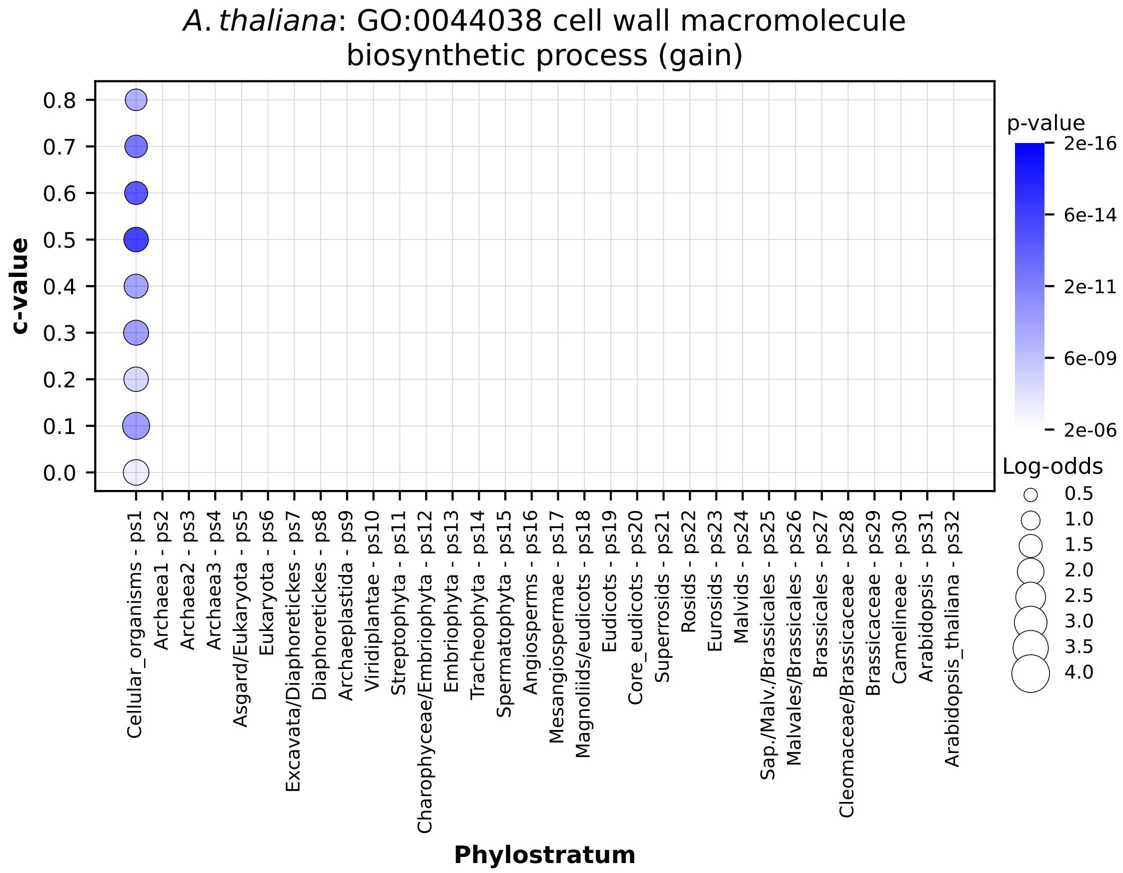

A. thaliana: GO:0044038 cell wall macromolecule biosynthetic process (gain)
c-value
0.8
0.7
0.6
0.5
0.4
0.3
0.2
0.1
0.0
p-value
2e-16
6e-14
2e-11
6e-09
2e-06
Log-odds
0.5
1.0
1.5
2.0
2.5
3.0
3.5
4.0
Cellular_organisms - ps1
Archaea1 - ps2
Archaea2 - ps3
Archaea3 - ps4
Asgard/Eukaryota - ps5
Eukaryota - ps6
Excavata/Diaphoretickes - ps7
Diaphoretickes - ps8
Archaeplastida - ps9
Viridiplantae - ps10
Streptophyta - ps11
Charophyceae/Embriophyta - ps12
Embriophyta - ps13
Tracheophyta - ps14
Spermatophyta - ps15
Angiosperms - ps16
Mesangiospermae - ps17
Magnoliids/eudicots - ps18
Eudicots - ps19
Core_eudicots - ps20
Superrosids - ps21
Rosids - ps22
Eurosids - ps23
Malvids - ps24
Sap./Malv./Brassicales - ps25
Malvales/Brassicales - ps26
Brassicales - ps27
Cleomaceae/Brassicaceae - ps28
Brassicaceae - ps29
Camelineae - ps30
Arabidopsis - ps31
Arabidopsis_thaliana - ps32
Phylostratum

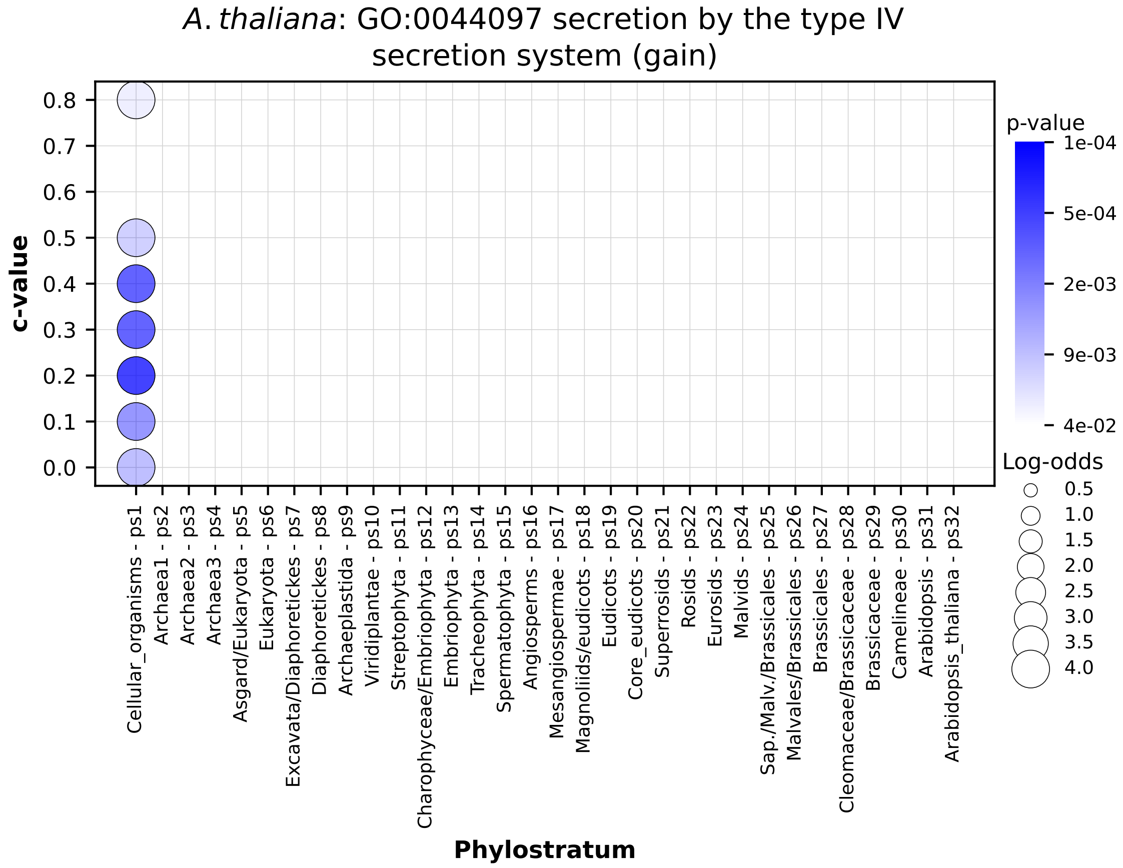

A. thaliana: GO:0044097 secretion by the type IV secretion system (gain)
c-value
0.8
0.7
0.6
0.5
0.4
0.3
0.2
0.1
0.0
p-value
1e-04
5e-04
2e-03
9e-03
4e-02
Log-odds
0.5
1.0
1.5
2.0
2.5
3.0
3.5
4.0
Cellular_organisms - ps1
Archaea1 - ps2
Archaea2 - ps3
Archaea3 - ps4
Asgard/Eukaryota - ps5
Eukaryota - ps6
Excavata/Diaphoretickes - ps7
Diaphoretickes - ps8
Archaeplastida - ps9
Viridiplantae - ps10
Streptophyta - ps11
Charophyceae/Embriophyta - ps12
Embriophyta - ps13
Tracheophyta - ps14
Spermatophyta - ps15
Angiosperms - ps16
Mesangiospermae - ps17
Magnoliids/eudicots - ps18
Eudicots - ps19
Core_eudicots - ps20
Superrosids - ps21
Rosids - ps22
Eurosids - ps23
Malvids - ps24
Sap./Malv./Brassicales - ps25
Malvales/Brassicales - ps26
Brassicales - ps27
Cleomaceae/Brassicaceae - ps28
Brassicaceae - ps29
Camelineae - ps30
Arabidopsis - ps31
Arabidopsis_thaliana - ps32
Phylostratum

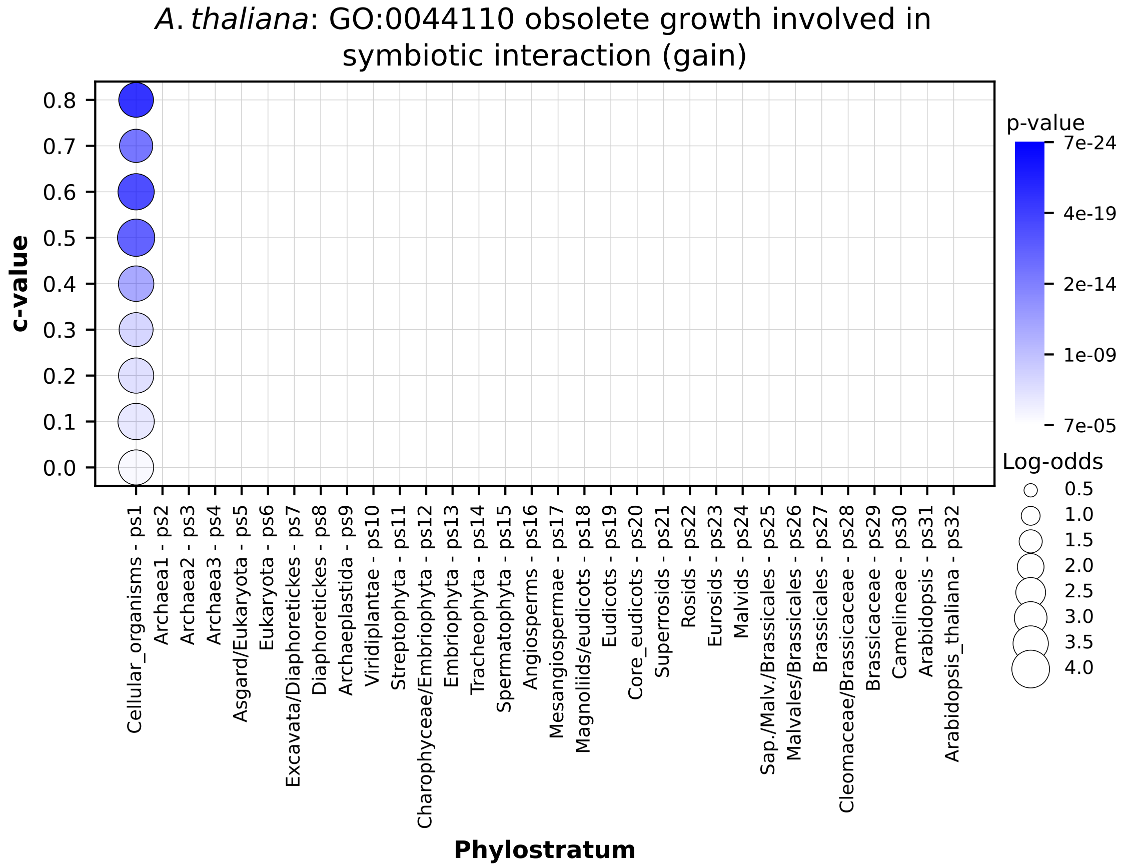

A. thaliana: GO:0044110 obsolete growth involved in symbiotic interaction (gain)
c-value
0.8
0.7
0.6
0.5
0.4
0.3
0.2
0.1
0.0
p-value
7e-24
4e-19
2e-14
1e-09
7e-05
Log-odds
0.5
1.0
1.5
2.0
2.5
3.0
3.5
4.0
Cellular_organisms - ps1
Archaea1 - ps2
Archaea2 - ps3
Archaea3 - ps4
Asgard/Eukaryota - ps5
Eukaryota - ps6
Excavata/Diaphoretickes - ps7
Diaphoretickes - ps8
Archaeplastida - ps9
Viridiplantae - ps10
Streptophyta - ps11
Charophyceae/Embriophyta - ps12
Embriophyta - ps13
Tracheophyta - ps14
Spermatophyta - ps15
Angiosperms - ps16
Mesangiospermae - ps17
Magnoliids/eudicots - ps18
Eudicots - ps19
Core_eudicots - ps20
Superrosids - ps21
Rosids - ps22
Eurosids - ps23
Malvids - ps24
Sap./Malv./Brassicales - ps25
Malvales/Brassicales - ps26
Brassicales - ps27
Cleomaceae/Brassicaceae - ps28
Brassicaceae - ps29
Camelineae - ps30
Arabidopsis - ps31
Arabidopsis_thaliana - ps32
Phylostratum

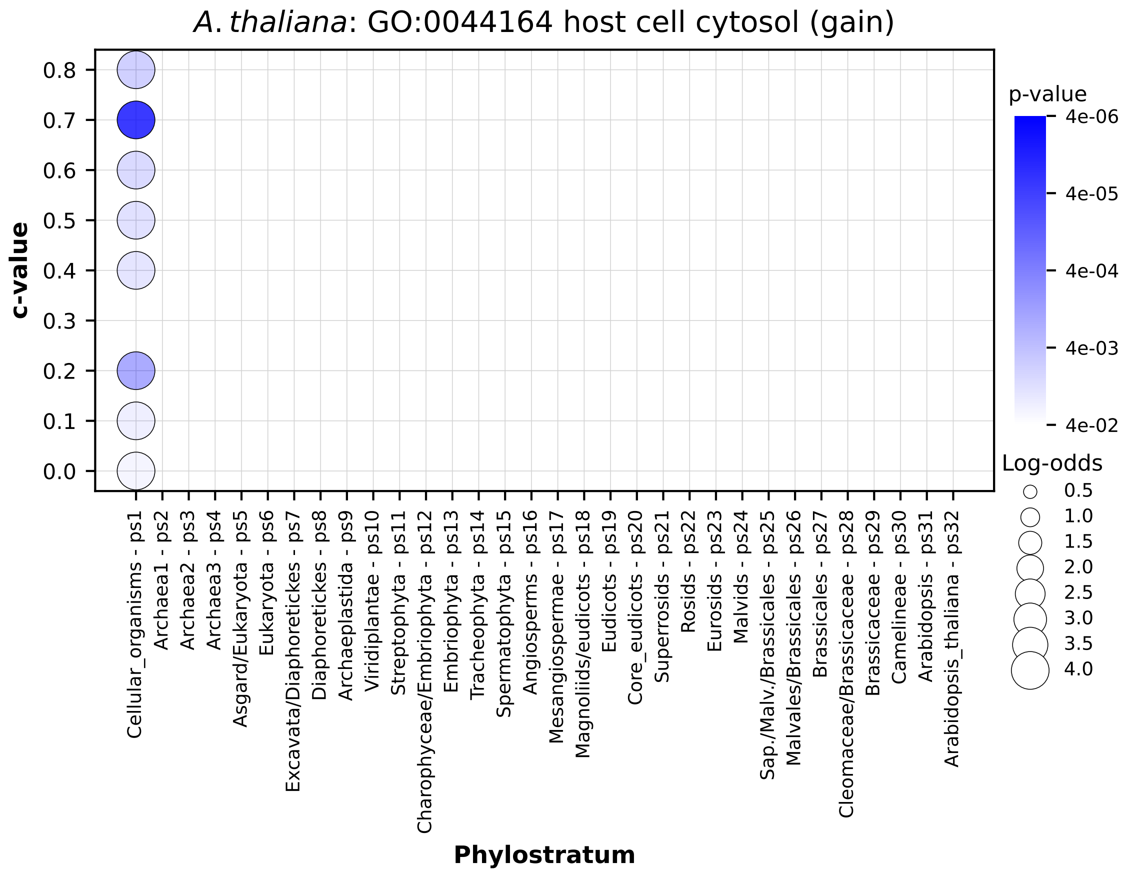

A. thaliana: GO:0044164 host cell cytosol (gain)
c-value
0.0
0.1
0.2
0.3
0.4
0.5
0.6
0.7
0.8
p-value
4e-06
4e-05
4e-04
4e-03
4e-02
Log-odds
0.5
1.0
1.5
2.0
2.5
3.0
3.5
4.0
Cellular_organisms - ps1
Archaea1 - ps2
Archaea2 - ps3
Archaea3 - ps4
Asgard/Eukaryota - ps5
Eukaryota - ps6
Excavata/Diaphoretickes - ps7
Diaphoretickes - ps8
Archaeplastida - ps9
Viridiplantae - ps10
Streptophyta - ps11
Charophyceae/Embriophyta - ps12
Embriophyta - ps13
Tracheophyta - ps14
Spermatophyta - ps15
Angiosperms - ps16
Mesangiospermae - ps17
Magnoliids/eudicots - ps18
Eudicots - ps19
Core_eudicots - ps20
Superrosids - ps21
Rosids - ps22
Eurosids - ps23
Malvids - ps24
Sap./Malv./Brassicales - ps25
Malvales/Brassicales - ps26
Brassicales - ps27
Cleomaceae/Brassicaceae - ps28
Brassicaceae - ps29
Camelineae - ps30
Arabidopsis - ps31
Arabidopsis_thaliana - ps32
Phylostratum

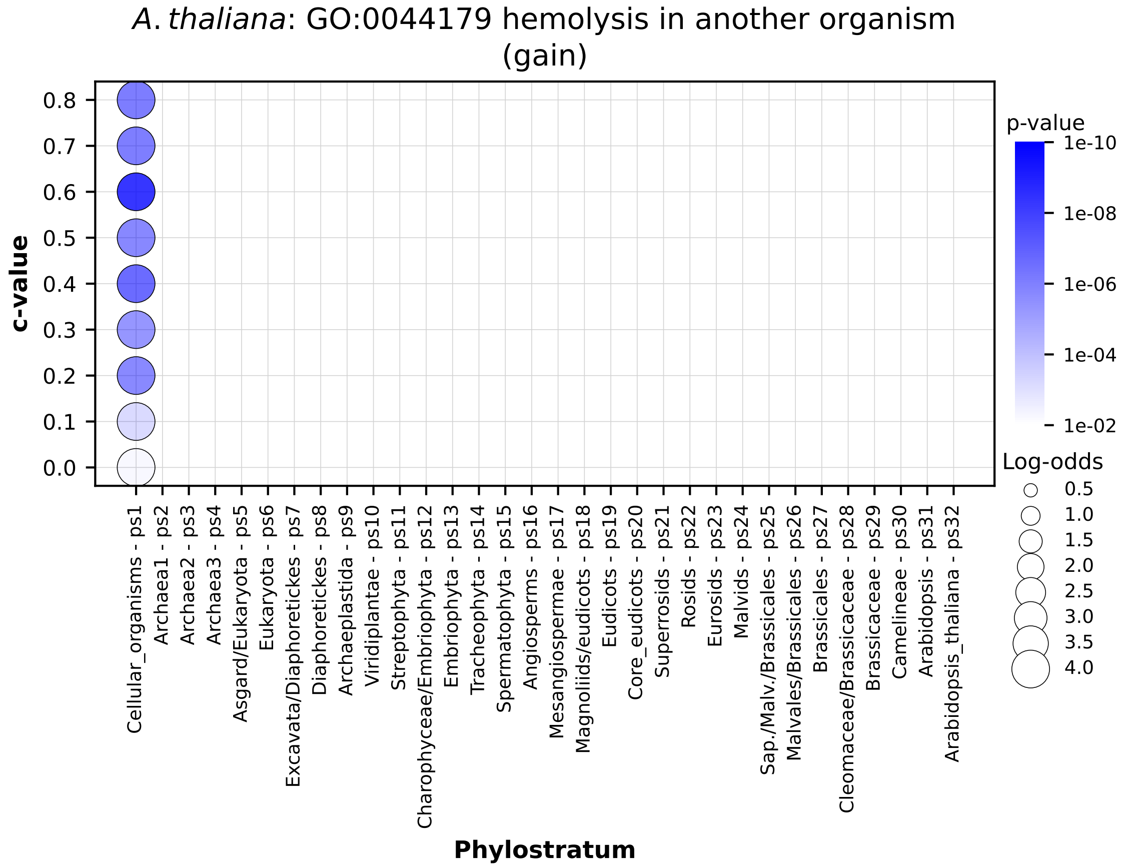
A. thaliana: GO:0044179 hemolysis in another organism (gain)
c-value
0.0
0.1
0.2
0.3
0.4
0.5
0.6
0.7
0.8
Phylostratum
Cellular_organisms - ps1
Archaea1 - ps2
Archaea2 - ps3
Archaea3 - ps4
Asgard/Eukaryota - ps5
Eukaryota - ps6
Excavata/Diaphoretickes - ps7
Diaphoretickes - ps8
Archaeplastida - ps9
Viridiplantae - ps10
Streptophyta - ps11
Charophyceae/Embriophyta - ps12
Embriophyta - ps13
Tracheophyta - ps14
Spermatophyta - ps15
Angiosperms - ps16
Mesangiospermae - ps17
Magnoliids/eudicots - ps18
Eudicots - ps19
Core_eudicots - ps20
Superrosids - ps21
Rosids - ps22
Eurosids - ps23
Malvids - ps24
Sap./Malv./Brassicales - ps25
Malvales/Brassicales - ps26
Brassicales - ps27
Cleomaceae/Brassicaceae - ps28
Brassicaceae - ps29
Camelineae - ps30
Arabidopsis - ps31
Arabidopsis_thaliana - ps32
p-value
1e-10
1e-08
1e-06
1e-04
1e-02
Log-odds
0.5
1.0
1.5
2.0
2.5
3.0
3.5
4.0

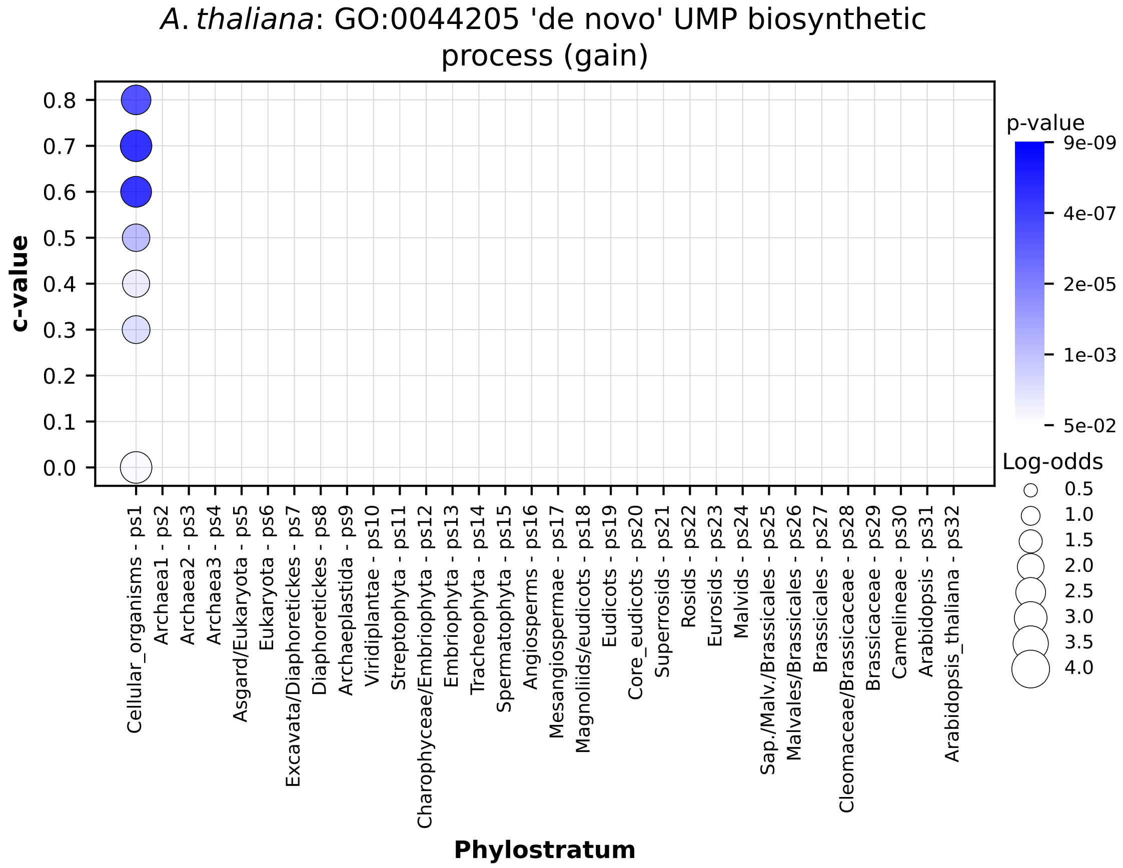

A. thaliana: GO:0044205 'de novo' UMP biosynthetic process (gain)
c-value
0.8
0.7
0.6
0.5
0.4
0.3
0.2
0.1
0.0
Phylostratum
Cellular_organisms - ps1
Archaea1 - ps2
Archaea2 - ps3
Archaea3 - ps4
Asgard/Eukaryota - ps5
Eukaryota - ps6
Excavata/Diaphoretickes - ps7
Diaphoretickes - ps8
Archaeplastida - ps9
Viridiplantae - ps10
Streptophyta - ps11
Charophyceae/Embriophyta - ps12
Embriophyta - ps13
Tracheophyta - ps14
Spermatophyta - ps15
Angiosperms - ps16
Mesangiospermae - ps17
Magnoliids/eudicots - ps18
Eudicots - ps19
Core_eudicots - ps20
Superrosids - ps21
Rosids - ps22
Eurosids - ps23
Malvids - ps24
Sap./Malv./Brassicales - ps25
Malvales/Brassicales - ps26
Brassicales - ps27
Cleomaceae/Brassicaceae - ps28
Brassicaceae - ps29
Camelineae - ps30
Arabidopsis - ps31
Arabidopsis_thaliana - ps32
p-value
9e-09
4e-07
2e-05
1e-03
5e-02
Log-odds
0.5
1.0
1.5
2.0
2.5
3.0
3.5
4.0

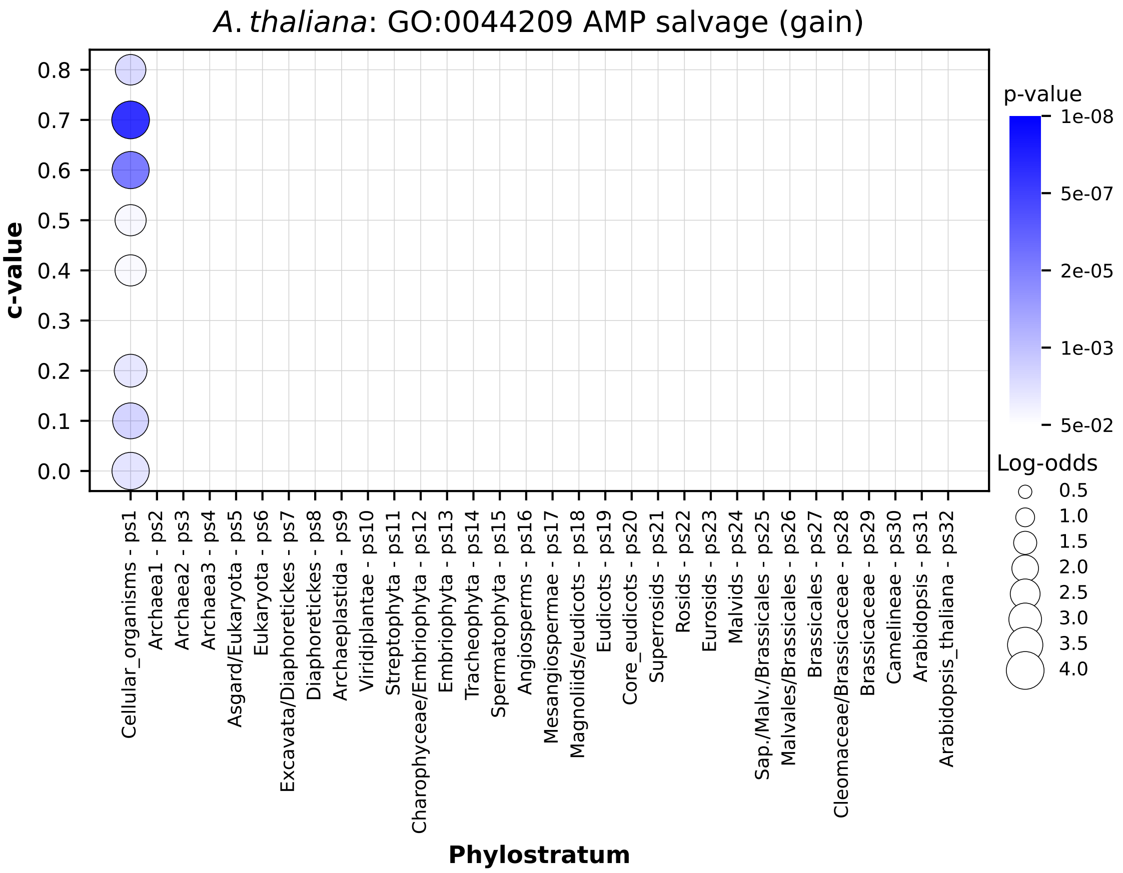
A. thaliana: GO:0044209 AMP salvage (gain)
c-value
0.0
0.1
0.2
0.3
0.4
0.5
0.6
0.7
0.8
Phylostratum
Cellular_organisms - ps1
Archaea1 - ps2
Archaea2 - ps3
Archaea3 - ps4
Asgard/Eukaryota - ps5
Eukaryota - ps6
Excavata/Diaphoretickes - ps7
Diaphoretickes - ps8
Archaeplastida - ps9
Viridiplantae - ps10
Streptophyta - ps11
Charophyceae/Embriophyta - ps12
Embriophyta - ps13
Tracheophyta - ps14
Spermatophyta - ps15
Angiosperms - ps16
Mesangiospermae - ps17
Magnoliids/eudicots - ps18
Eudicots - ps19
Core_eudicots - ps20
Superrosids - ps21
Rosids - ps22
Eurosids - ps23
Malvids - ps24
Sap./Malv./Brassicales - ps25
Malvales/Brassicales - ps26
Brassicales - ps27
Cleomaceae/Brassicaceae - ps28
Brassicaceae - ps29
Camelineae - ps30
Arabidopsis - ps31
Arabidopsis_thaliana - ps32
p-value
1e-08
5e-07
2e-05
1e-03
5e-02
Log-odds
0.5
1.0
1.5
2.0
2.5
3.0
3.5
4.0

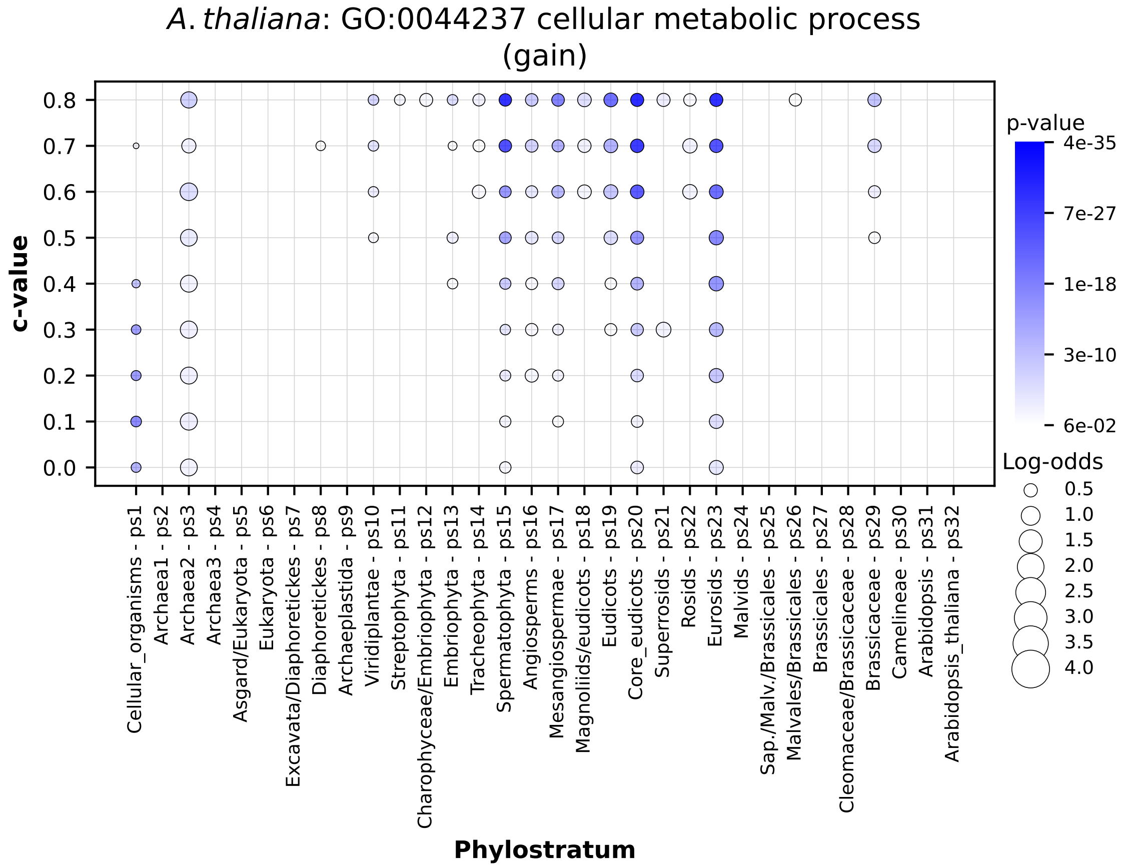
A. thaliana: GO:0044237 cellular metabolic process
(gain)
c-value
0.8
0.7
0.6
0.5
0.4
0.3
0.2
0.1
0.0
p-value
4e-35
7e-27
1e-18
3e-10
6e-02
Log-odds
0.5
1.0
1.5
2.0
2.5
3.0
3.5
4.0
Cellular_organisms - ps1
Archaea1 - ps2
Archaea2 - ps3
Archaea3 - ps4
Asgard/Eukaryota - ps5
Eukaryota - ps6
Excavata/Diaphoretickes - ps7
Diaphoretickes - ps8
Archaeplastida - ps9
Viridiplantae - ps10
Streptophyta - ps11
Charophyceae/Embriophyta - ps12
Embriophyta - ps13
Tracheophyta - ps14
Spermatophyta - ps15
Angiosperms - ps16
Mesangiospermae - ps17
Magnoliids/eudicots - ps18
Eudicots - ps19
Core_eudicots - ps20
Superrosids - ps21
Rosids - ps22
Eurosids - ps23
Malvids - ps24
Sap./Malv./Brassicales - ps25
Malvales/Brassicales - ps26
Brassicales - ps27
Cleomaceae/Brassicaceae - ps28
Brassicaceae - ps29
Camelineae - ps30
Arabidopsis - ps31
Arabidopsis_thaliana - ps32
Phylostratum

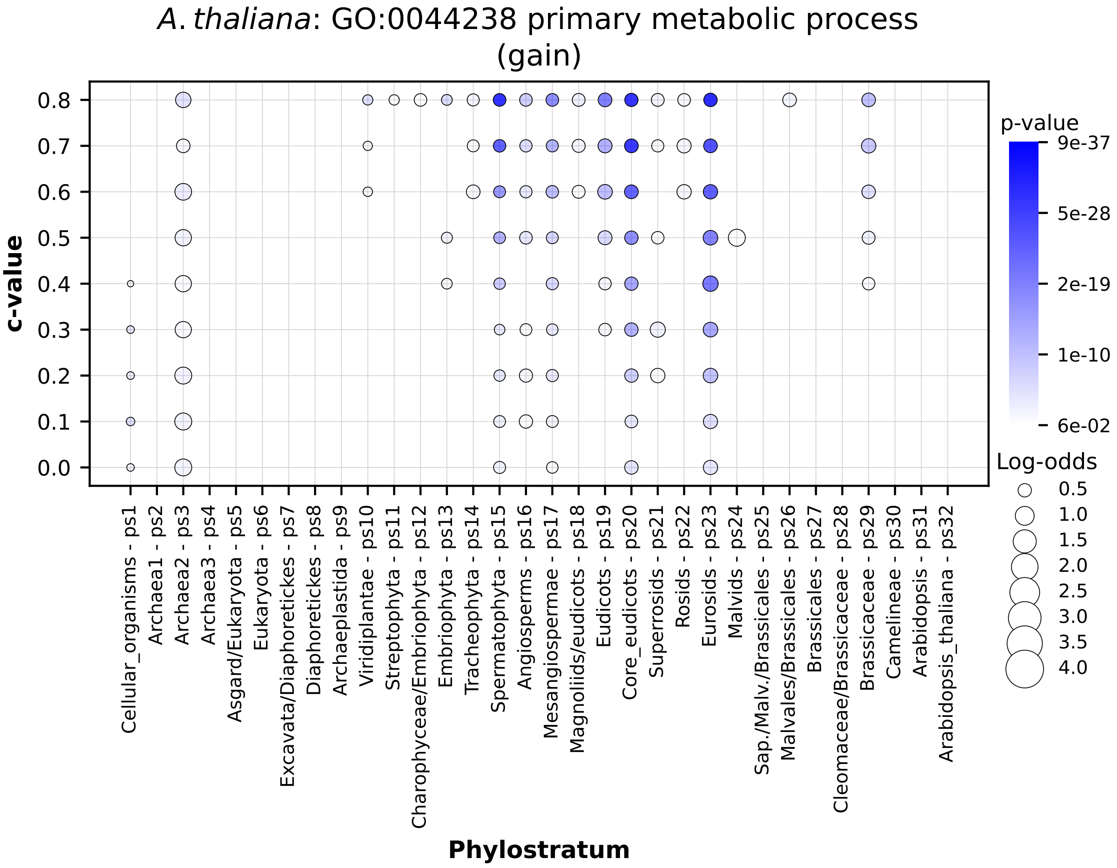
A. thaliana: GO:0044238 primary metabolic process
(gain)
c-value
0.8
0.7
0.6
0.5
0.4
0.3
0.2
0.1
0.0
Phylostratum
Cellular_organisms - ps1
Archaea1 - ps2
Archaea2 - ps3
Archaea3 - ps4
Asgard/Eukaryota - ps5
Eukaryota - ps6
Excavata/Diaphoretickes - ps7
Diaphoretickes - ps8
Archaeplastida - ps9
Viridiplantae - ps10
Streptophyta - ps11
Charophyceae/Embriophyta - ps12
Embriophyta - ps13
Tracheophyta - ps14
Spermatophyta - ps15
Angiosperms - ps16
Mesangiospermae - ps17
Magnoliids/eudicots - ps18
Eudicots - ps19
Core_eudicots - ps20
Superrosids - ps21
Rosids - ps22
Eurosids - ps23
Malvids - ps24
Sap./Malv./Brassicales - ps25
Malvales/Brassicales - ps26
Brassicales - ps27
Cleomaceae/Brassicaceae - ps28
Brassicaceae - ps29
Camelineae - ps30
Arabidopsis - ps31
Arabidopsis_thaliana - ps32
p-value
9e-37
5e-28
2e-19
1e-10
6e-02
Log-odds
0.5
1.0
1.5
2.0
2.5
3.0
3.5
4.0

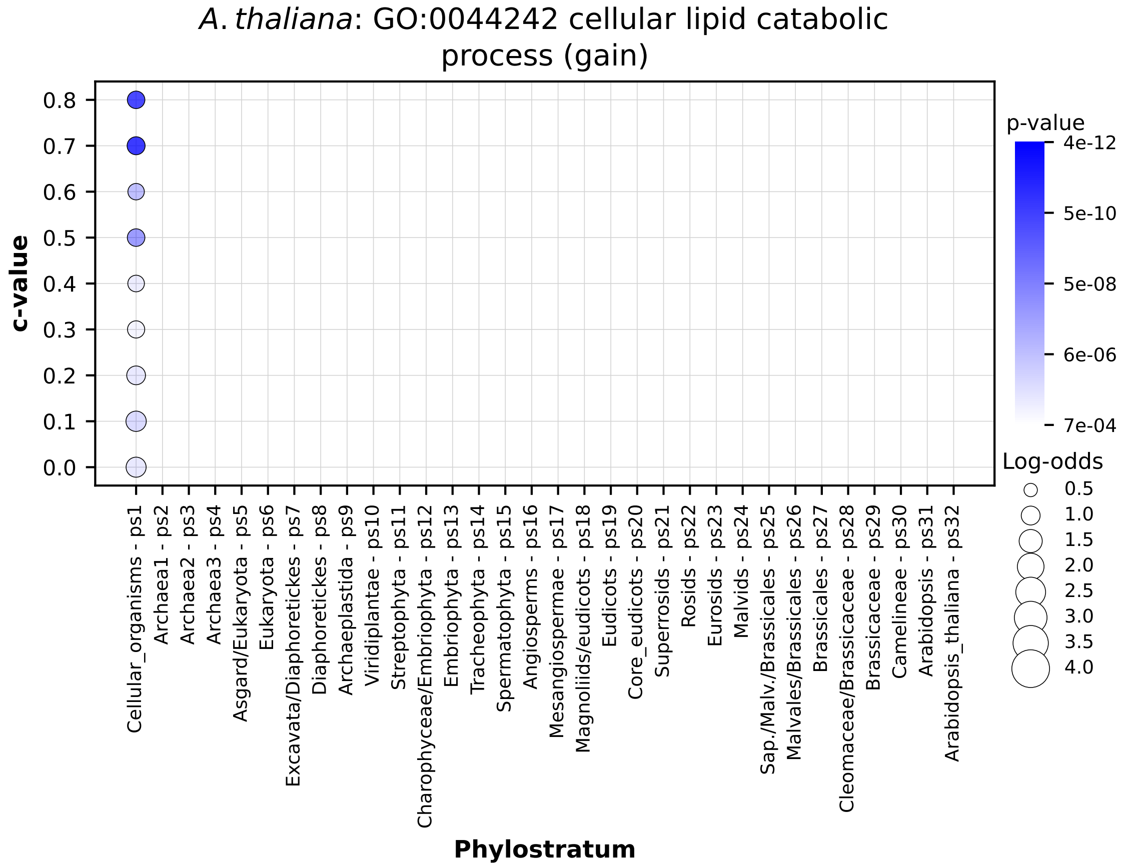
A. thaliana: GO:0044242 cellular lipid catabolic process (gain)
c-value
0.8
0.7
0.6
0.5
0.4
0.3
0.2
0.1
0.0
Phylostratum
Cellular_organisms - ps1
Archaea1 - ps2
Archaea2 - ps3
Archaea3 - ps4
Asgard/Eukaryota - ps5
Eukaryota - ps6
Excavata/Diaphoretickes - ps7
Diaphoretickes - ps8
Archaeplastida - ps9
Viridiplantae - ps10
Streptophyta - ps11
Charophyceae/Embriophyta - ps12
Embriophyta - ps13
Tracheophyta - ps14
Spermatophyta - ps15
Angiosperms - ps16
Mesangiospermae - ps17
Magnoliids/eudicots - ps18
Eudicots - ps19
Core_eudicots - ps20
Superrosids - ps21
Rosids - ps22
Eurosids - ps23
Malvids - ps24
Sap./Malv./Brassicales - ps25
Malvales/Brassicales - ps26
Brassicales - ps27
Cleomaceae/Brassicaceae - ps28
Brassicaceae - ps29
Camelineae - ps30
Arabidopsis - ps31
Arabidopsis_thaliana - ps32
p-value
4e-12
5e-10
5e-08
6e-06
7e-04
Log-odds
0.5
1.0
1.5
2.0
2.5
3.0
3.5
4.0

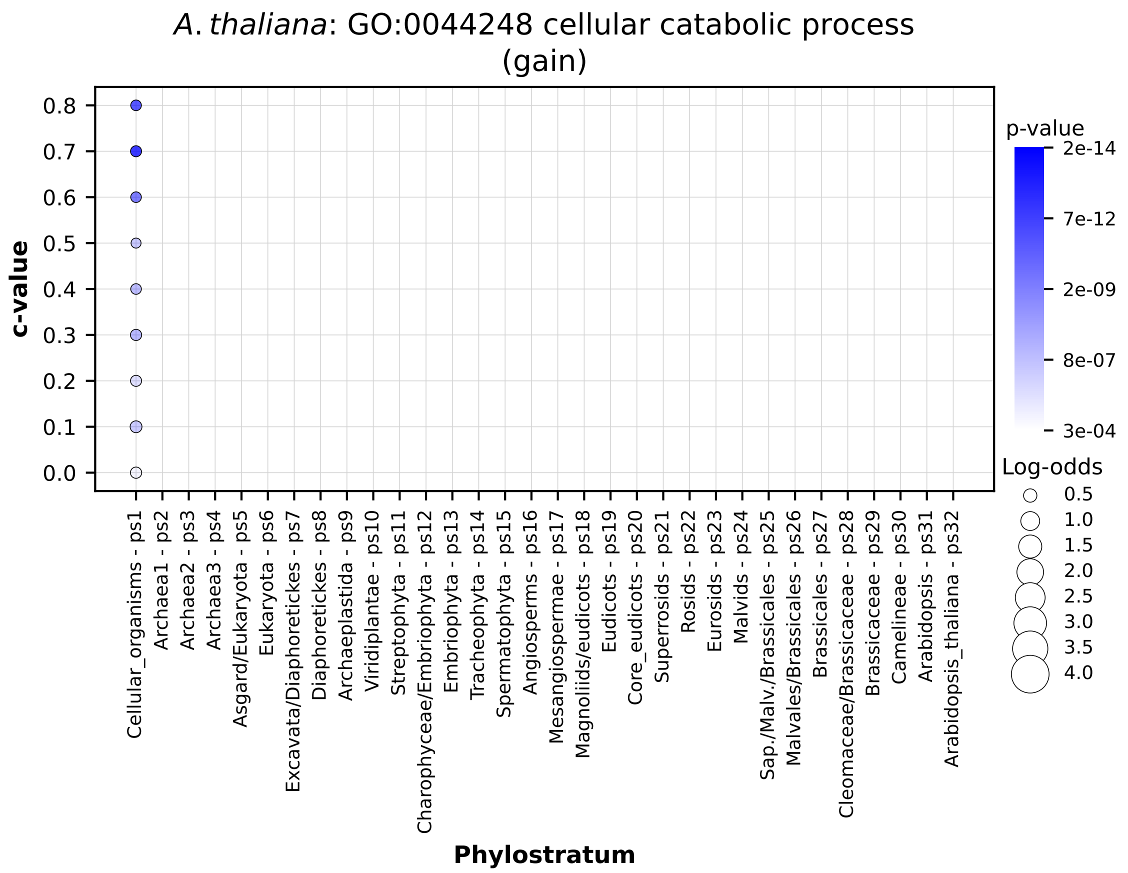
A. thaliana: GO:0044248 cellular catabolic process
(gain)
c-value
0.8
0.7
0.6
0.5
0.4
0.3
0.2
0.1
0.0
Phylostratum
Cellular_organisms - ps1
Archaea1 - ps2
Archaea2 - ps3
Archaea3 - ps4
Asgard/Eukaryota - ps5
Eukaryota - ps6
Excavata/Diaphoretickes - ps7
Diaphoretickes - ps8
Archaeplastida - ps9
Viridiplantae - ps10
Streptophyta - ps11
Charophyceae/Embriophyta - ps12
Embriophyta - ps13
Tracheophyta - ps14
Spermatophyta - ps15
Angiosperms - ps16
Mesangiospermae - ps17
Magnoliids/eudicots - ps18
Eudicots - ps19
Core_eudicots - ps20
Superrosids - ps21
Rosids - ps22
Eurosids - ps23
Malvids - ps24
Sap./Malv./Brassicales - ps25
Malvales/Brassicales - ps26
Brassicales - ps27
Cleomaceae/Brassicaceae - ps28
Brassicaceae - ps29
Camelineae - ps30
Arabidopsis - ps31
Arabidopsis_thaliana - ps32
p-value
2e-14
7e-12
2e-09
8e-07
3e-04
Log-odds
0.5
1.0
1.5
2.0
2.5
3.0
3.5
4.0

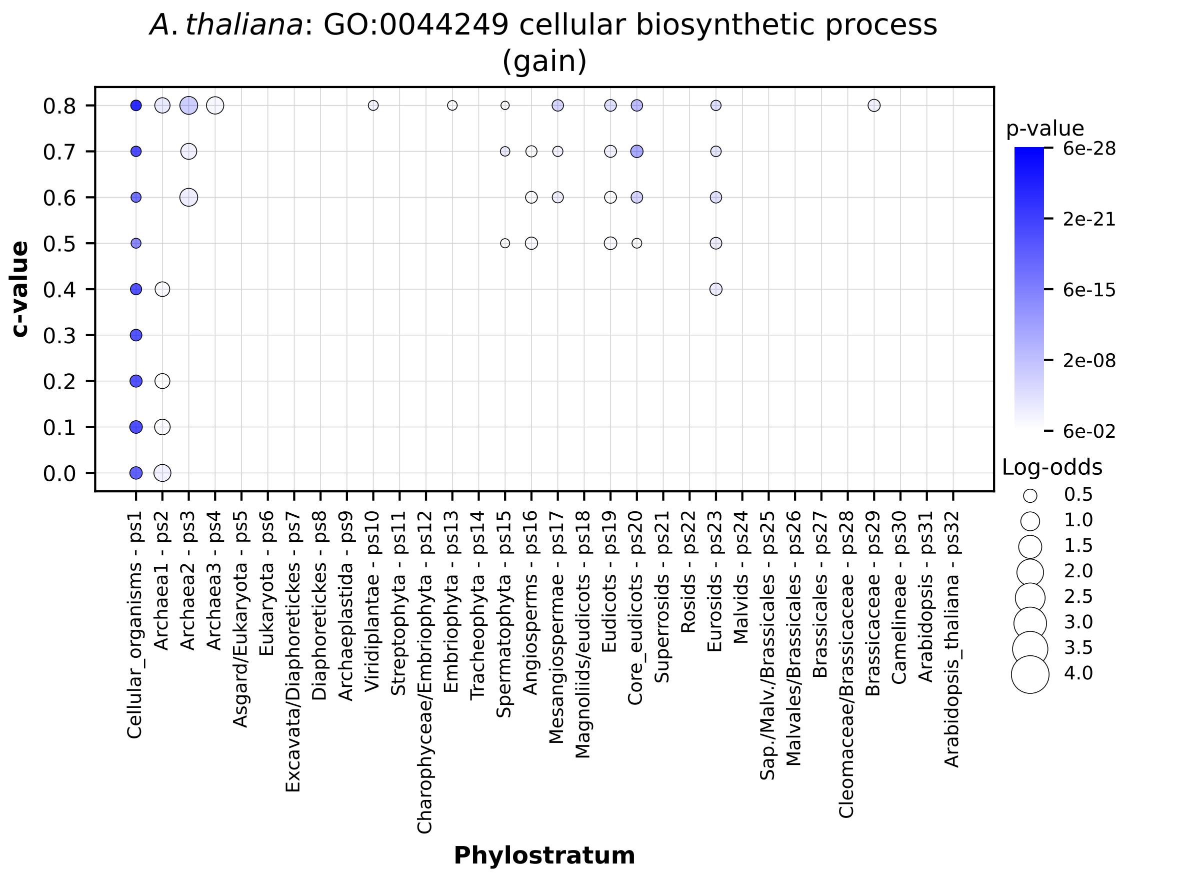

A. thaliana: GO:0044249 cellular biosynthetic process
(gain)
c-value
0.8
0.7
0.6
0.5
0.4
0.3
0.2
0.1
0.0
p-value
6e-28
2e-21
6e-15
2e-08
6e-02
Log-odds
0.5
1.0
1.5
2.0
2.5
3.0
3.5
4.0
Cellular_organisms - ps1
Archaea1 - ps2
Archaea2 - ps3
Archaea3 - ps4
Asgard/Eukaryota - ps5
Eukaryota - ps6
Excavata/Diaphoretickes - ps7
Diaphoretickes - ps8
Archaeplastida - ps9
Viridiplantae - ps10
Streptophyta - ps11
Charophyceae/Embriophyta - ps12
Embriophyta - ps13
Tracheophyta - ps14
Spermatophyta - ps15
Angiosperms - ps16
Mesangiospermae - ps17
Magnoliids/eudicots - ps18
Eudicots - ps19
Core_eudicots - ps20
Superrosids - ps21
Rosids - ps22
Eurosids - ps23
Malvids - ps24
Sap./Malv./Brassicales - ps25
Malvales/Brassicales - ps26
Brassicales - ps27
Cleomaceae/Brassicaceae - ps28
Brassicaceae - ps29
Camelineae - ps30
Arabidopsis - ps31
Arabidopsis_thaliana - ps32
Phylostratum

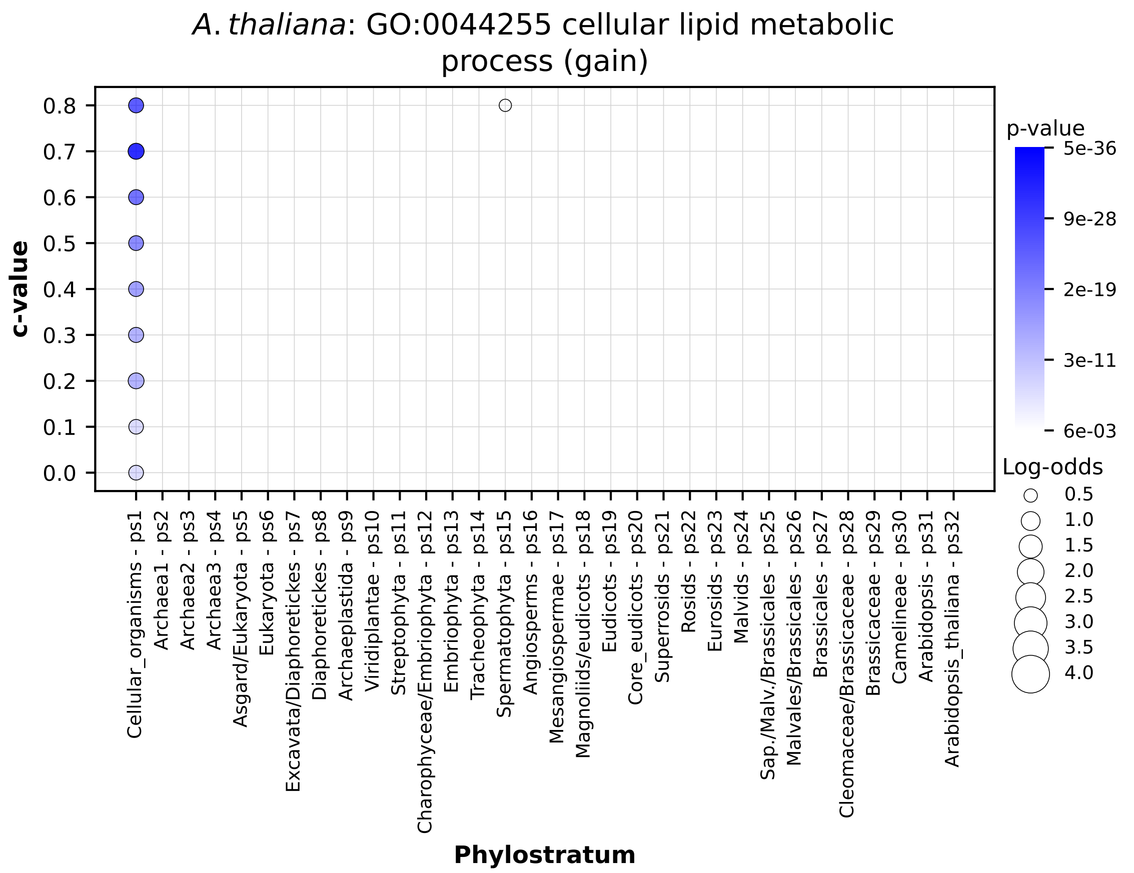

A. thaliana: GO:0044255 cellular lipid metabolic process (gain)
c-value
0.0
0.1
0.2
0.3
0.4
0.5
0.6
0.7
0.8
Phylostratum
Cellular_organisms - ps1
Archaea1 - ps2
Archaea2 - ps3
Archaea3 - ps4
Asgard/Eukaryota - ps5
Eukaryota - ps6
Excavata/Diaphoretickes - ps7
Diaphoretickes - ps8
Archaeplastida - ps9
Viridiplantae - ps10
Streptophyta - ps11
Charophyceae/Embriophyta - ps12
Embriophyta - ps13
Tracheophyta - ps14
Spermatophyta - ps15
Angiosperms - ps16
Mesangiospermae - ps17
Magnoliids/eudicots - ps18
Eudicots - ps19
Core_eudicots - ps20
Superrosids - ps21
Rosids - ps22
Eurosids - ps23
Malvids - ps24
Sap./Malv./Brassicales - ps25
Malvales/Brassicales - ps26
Brassicales - ps27
Cleomaceae/Brassicaceae - ps28
Brassicaceae - ps29
Camelineae - ps30
Arabidopsis - ps31
Arabidopsis_thaliana - ps32
p-value
5e-36
9e-28
2e-19
3e-11
6e-03
Log-odds
0.5
1.0
1.5
2.0
2.5
3.0
3.5
4.0

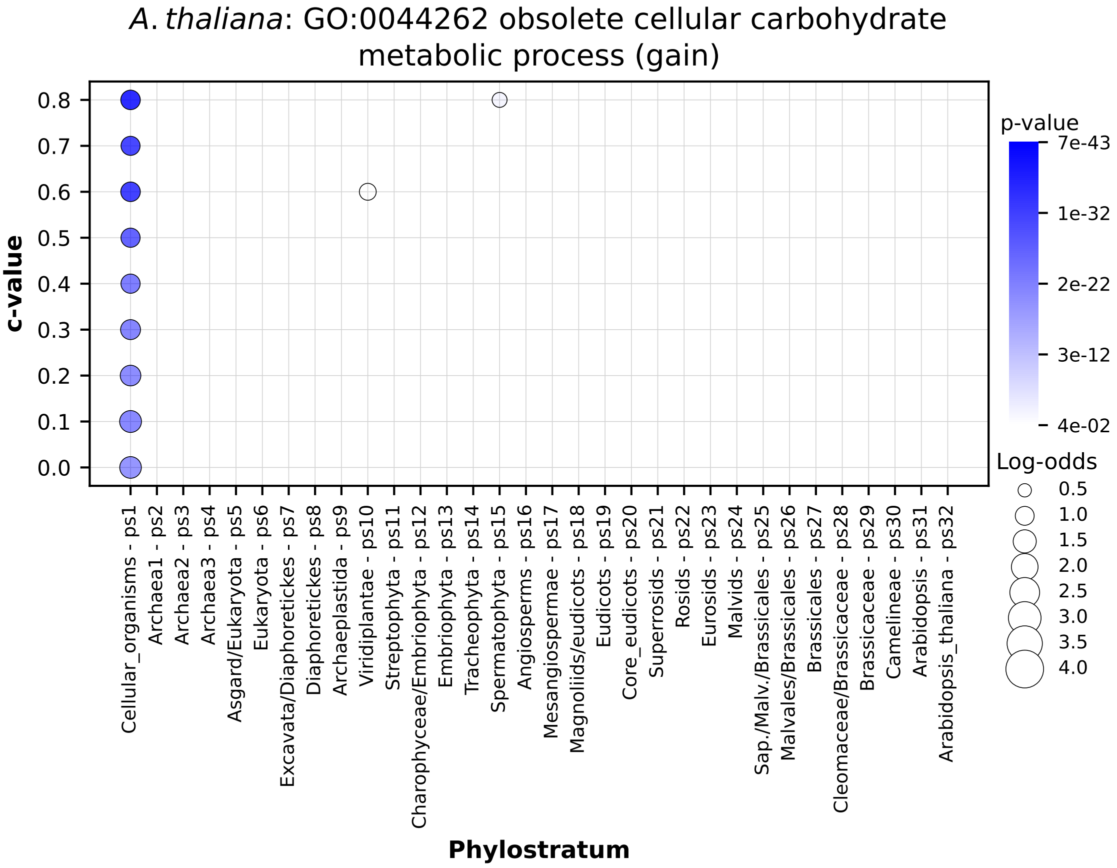

A. thaliana: GO:0044262 obsolete cellular carbohydrate metabolic process (gain)
c-value
0.8
0.7
0.6
0.5
0.4
0.3
0.2
0.1
0.0
p-value
7e-43
1e-32
2e-22
3e-12
4e-02
Log-odds
0.5
1.0
1.5
2.0
2.5
3.0
3.5
4.0
Cellular_organisms - ps1
Archaea1 - ps2
Archaea2 - ps3
Archaea3 - ps4
Asgard/Eukaryota - ps5
Eukaryota - ps6
Excavata/Diaphoretickes - ps7
Diaphoretickes - ps8
Archaeplastida - ps9
Viridiplantae - ps10
Streptophyta - ps11
Charophyceae/Embriophyta - ps12
Embriophyta - ps13
Tracheophyta - ps14
Spermatophyta - ps15
Angiosperms - ps16
Mesangiospermae - ps17
Magnoliids/eudicots - ps18
Eudicots - ps19
Core_eudicots - ps20
Superrosids - ps21
Rosids - ps22
Eurosids - ps23
Malvids - ps24
Sap./Malv./Brassicales - ps25
Malvales/Brassicales - ps26
Brassicales - ps27
Cleomaceae/Brassicaceae - ps28
Brassicaceae - ps29
Camelineae - ps30
Arabidopsis - ps31
Arabidopsis_thaliana - ps32
Phylostratum

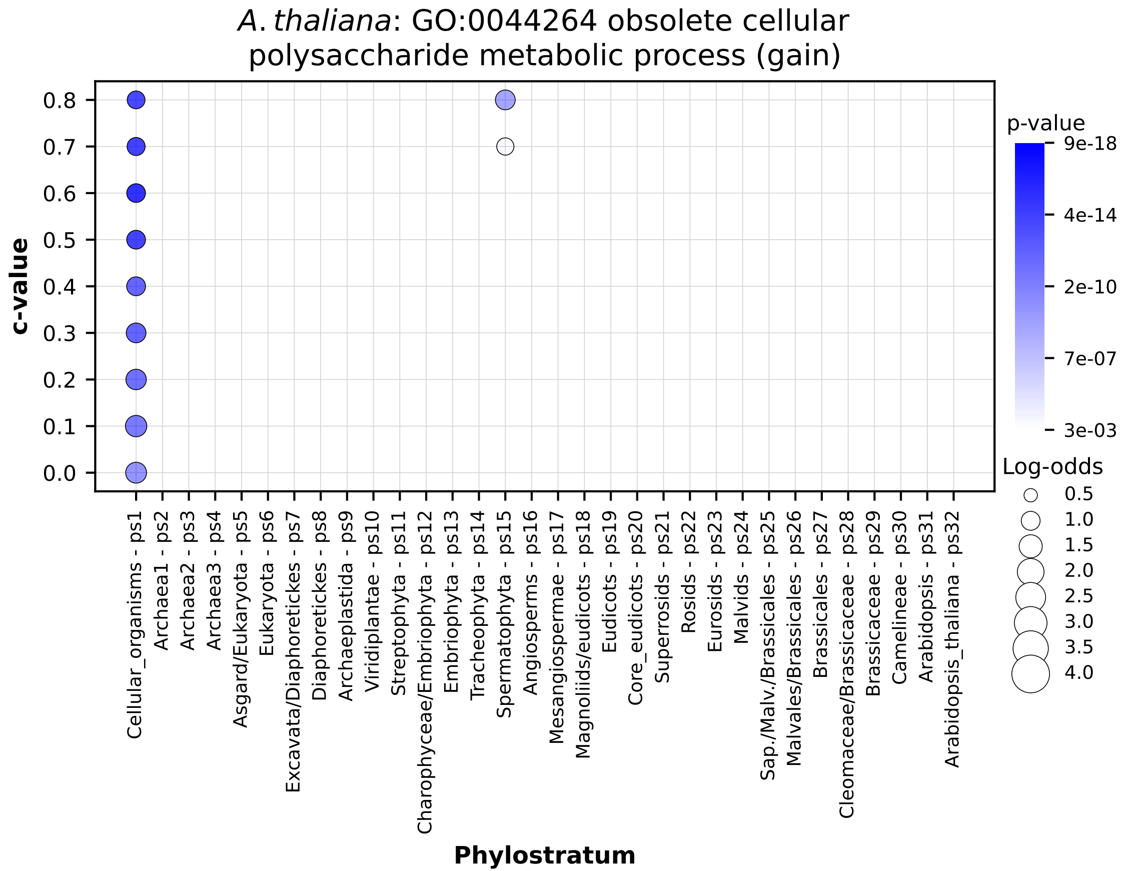

A. thaliana: GO:0044264 obsolete cellular polysaccharide metabolic process (gain)
c-value
0.8
0.7
0.6
0.5
0.4
0.3
0.2
0.1
0.0
p-value
9e-18
4e-14
2e-10
7e-07
3e-03
Log-odds
0.5
1.0
1.5
2.0
2.5
3.0
3.5
4.0
Cellular_organisms - ps1
Archaea1 - ps2
Archaea2 - ps3
Archaea3 - ps4
Asgard/Eukaryota - ps5
Eukaryota - ps6
Excavata/Diaphoretickes - ps7
Diaphoretickes - ps8
Archaeplastida - ps9
Viridiplantae - ps10
Streptophyta - ps11
Charophyceae/Embriophyta - ps12
Embriophyta - ps13
Tracheophyta - ps14
Spermatophyta - ps15
Angiosperms - ps16
Mesangiospermae - ps17
Magnoliids/eudicots - ps18
Eudicots - ps19
Core_eudicots - ps20
Superrosids - ps21
Rosids - ps22
Eurosids - ps23
Malvids - ps24
Sap./Malv./Brassicales - ps25
Malvales/Brassicales - ps26
Brassicales - ps27
Cleomaceae/Brassicaceae - ps28
Brassicaceae - ps29
Camelineae - ps30
Arabidopsis - ps31
Arabidopsis_thaliana - ps32
Phylostratum

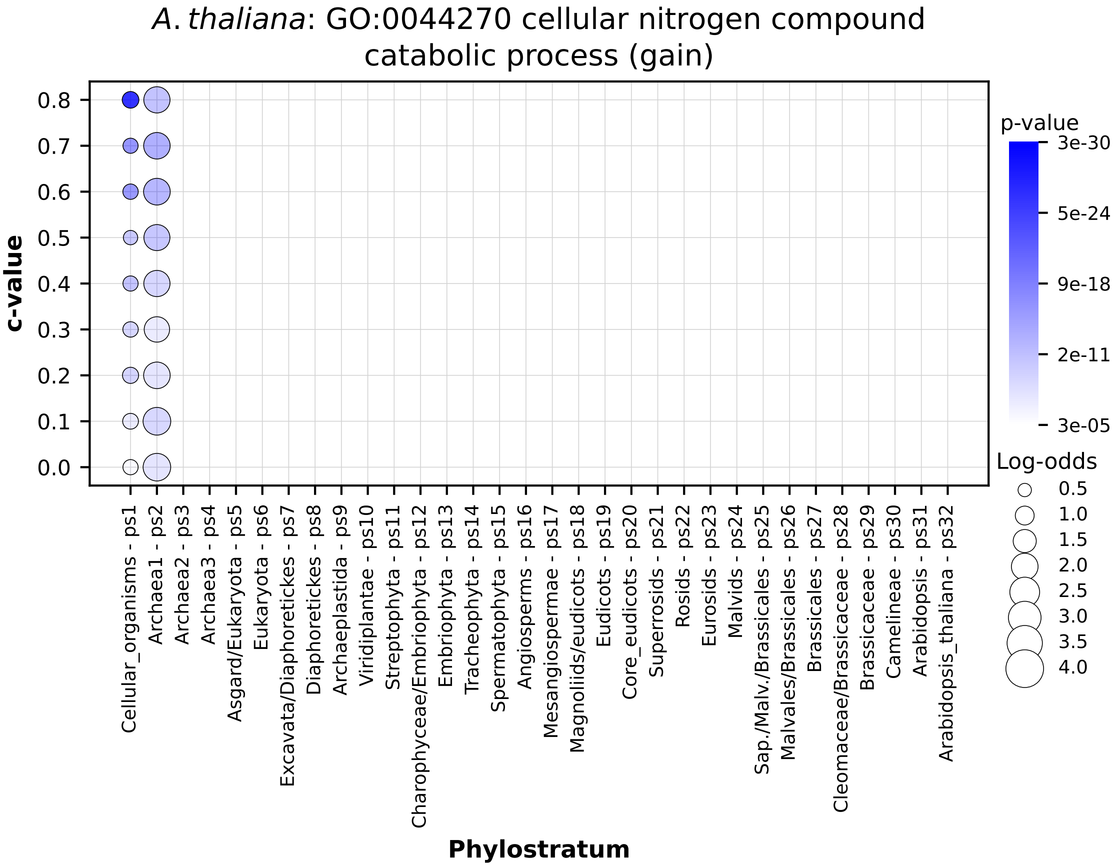
A. thaliana: GO:0044270 cellular nitrogen compound catabolic process (gain)
c-value
0.8
0.7
0.6
0.5
0.4
0.3
0.2
0.1
0.0
p-value
3e-30
5e-24
9e-18
2e-11
3e-05
Log-odds
0.5
1.0
1.5
2.0
2.5
3.0
3.5
4.0
Cellular_organisms - ps1
Archaea1 - ps2
Archaea2 - ps3
Archaea3 - ps4
Asgard/Eukaryota - ps5
Eukaryota - ps6
Excavata/Diaphoretickes - ps7
Diaphoretickes - ps8
Archaeplastida - ps9
Viridiplantae - ps10
Streptophyta - ps11
Charophyceae/Embriophyta - ps12
Embriophyta - ps13
Tracheophyta - ps14
Spermatophyta - ps15
Angiosperms - ps16
Mesangiospermae - ps17
Magnoliids/eudicots - ps18
Eudicots - ps19
Core_eudicots - ps20
Superrosids - ps21
Rosids - ps22
Eurosids - ps23
Malvids - ps24
Sap./Malv./Brassicales - ps25
Malvales/Brassicales - ps26
Brassicales - ps27
Cleomaceae/Brassicaceae - ps28
Brassicaceae - ps29
Camelineae - ps30
Arabidopsis - ps31
Arabidopsis_thaliana - ps32
Phylostratum

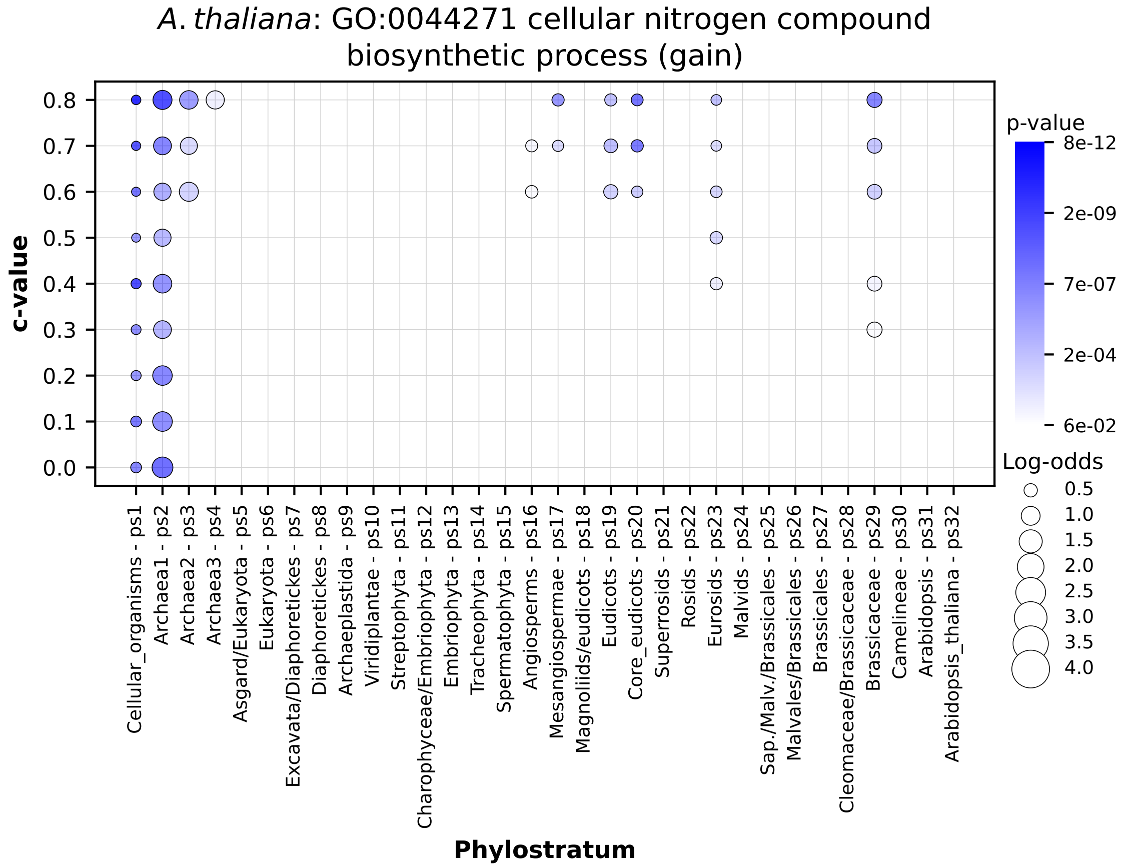

A. thaliana: GO:0044271 cellular nitrogen compound biosynthetic process (gain)
c-value
0.8
0.7
0.6
0.5
0.4
0.3
0.2
0.1
0.0
p-value
8e-12
2e-09
7e-07
2e-04
6e-02
Log-odds
0.5
1.0
1.5
2.0
2.5
3.0
3.5
4.0
Cellular_organisms - ps1
Archaea1 - ps2
Archaea2 - ps3
Archaea3 - ps4
Asgard/Eukaryota - ps5
Eukaryota - ps6
Excavata/Diaphoretickes - ps7
Diaphoretickes - ps8
Archaeplastida - ps9
Viridiplantae - ps10
Streptophyta - ps11
Charophyceae/Embriophyta - ps12
Embriophyta - ps13
Tracheophyta - ps14
Spermatophyta - ps15
Angiosperms - ps16
Mesangiospermae - ps17
Magnoliids/eudicots - ps18
Eudicots - ps19
Core_eudicots - ps20
Superrosids - ps21
Rosids - ps22
Eurosids - ps23
Malvids - ps24
Sap./Malv./Brassicales - ps25
Malvales/Brassicales - ps26
Brassicales - ps27
Cleomaceae/Brassicaceae - ps28
Brassicaceae - ps29
Camelineae - ps30
Arabidopsis - ps31
Arabidopsis_thaliana - ps32
Phylostratum

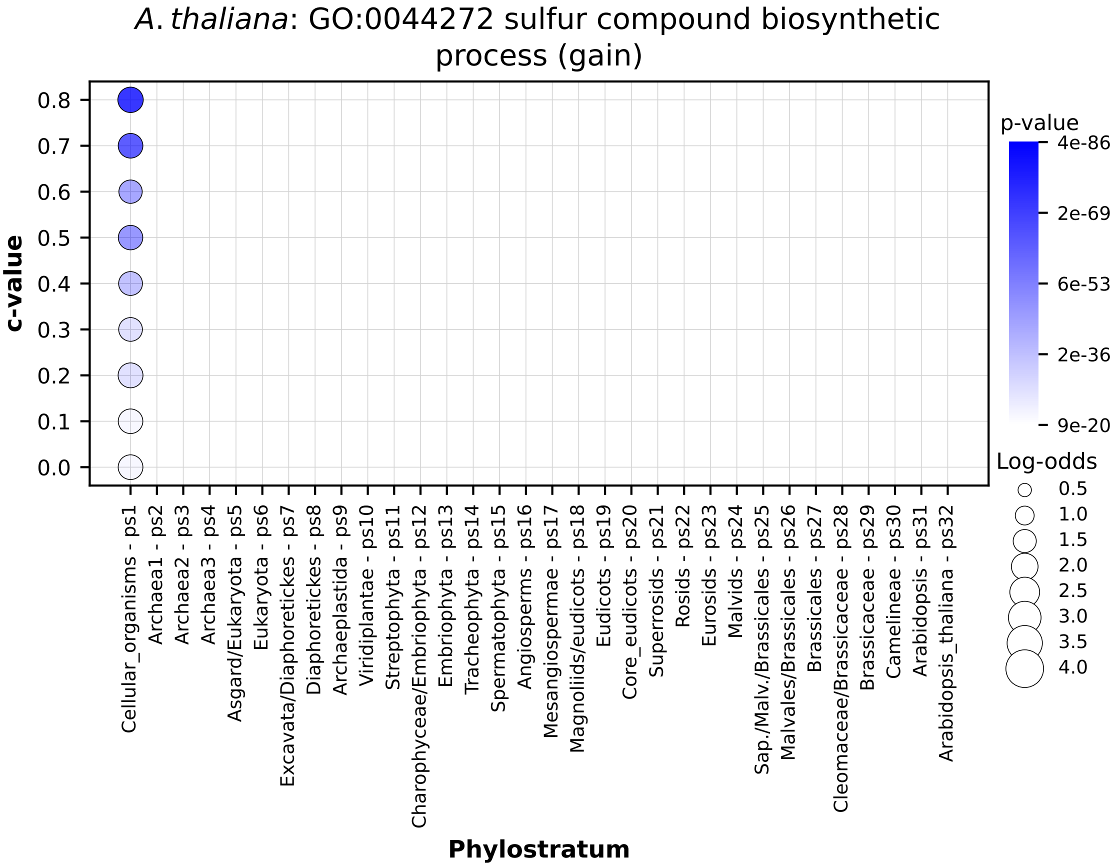

A. thaliana: GO:0044272 sulfur compound biosynthetic process (gain)
c-value
0.8
0.7
0.6
0.5
0.4
0.3
0.2
0.1
0.0
p-value
4e-86
2e-69
6e-53
2e-36
9e-20
Log-odds
0.5
1.0
1.5
2.0
2.5
3.0
3.5
4.0
Cellular_organisms - ps1
Archaea1 - ps2
Archaea2 - ps3
Archaea3 - ps4
Asgard/Eukaryota - ps5
Eukaryota - ps6
Excavata/Diaphoretickes - ps7
Diaphoretickes - ps8
Archaeplastida - ps9
Viridiplantae - ps10
Streptophyta - ps11
Charophyceae/Embriophyta - ps12
Embriophyta - ps13
Tracheophyta - ps14
Spermatophyta - ps15
Angiosperms - ps16
Mesangiospermae - ps17
Magnoliids/eudicots - ps18
Eudicots - ps19
Core_eudicots - ps20
Superrosids - ps21
Rosids - ps22
Eurosids - ps23
Malvids - ps24
Sap./Malv./Brassicales - ps25
Malvales/Brassicales - ps26
Brassicales - ps27
Cleomaceae/Brassicaceae - ps28
Brassicaceae - ps29
Camelineae - ps30
Arabidopsis - ps31
Arabidopsis_thaliana - ps32
Phylostratum

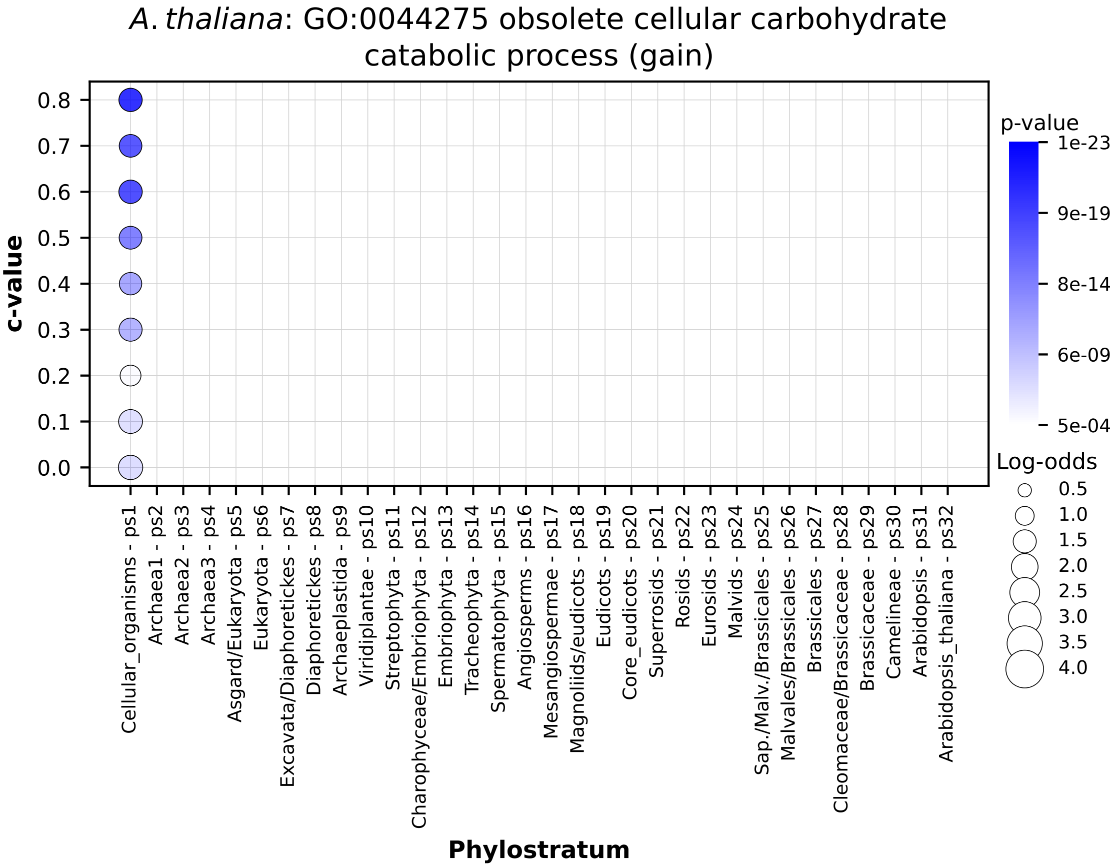
A. thaliana: GO:0044275 obsolete cellular carbohydrate catabolic process (gain)
c-value
0.8
0.7
0.6
0.5
0.4
0.3
0.2
0.1
0.0
p-value
1e-23
9e-19
8e-14
6e-09
5e-04
Log-odds
0.5
1.0
1.5
2.0
2.5
3.0
3.5
4.0
Cellular_organisms - ps1
Archaea1 - ps2
Archaea2 - ps3
Archaea3 - ps4
Asgard/Eukaryota - ps5
Eukaryota - ps6
Excavata/Diaphoretickes - ps7
Diaphoretickes - ps8
Archaeplastida - ps9
Viridiplantae - ps10
Streptophyta - ps11
Charophyceae/Embriophyta - ps12
Embriophyta - ps13
Tracheophyta - ps14
Spermatophyta - ps15
Angiosperms - ps16
Mesangiospermae - ps17
Magnoliids/eudicots - ps18
Eudicots - ps19
Core_eudicots - ps20
Superrosids - ps21
Rosids - ps22
Eurosids - ps23
Malvids - ps24
Sap./Malv./Brassicales - ps25
Malvales/Brassicales - ps26
Brassicales - ps27
Cleomaceae/Brassicaceae - ps28
Brassicaceae - ps29
Camelineae - ps30
Arabidopsis - ps31
Arabidopsis_thaliana - ps32
Phylostratum

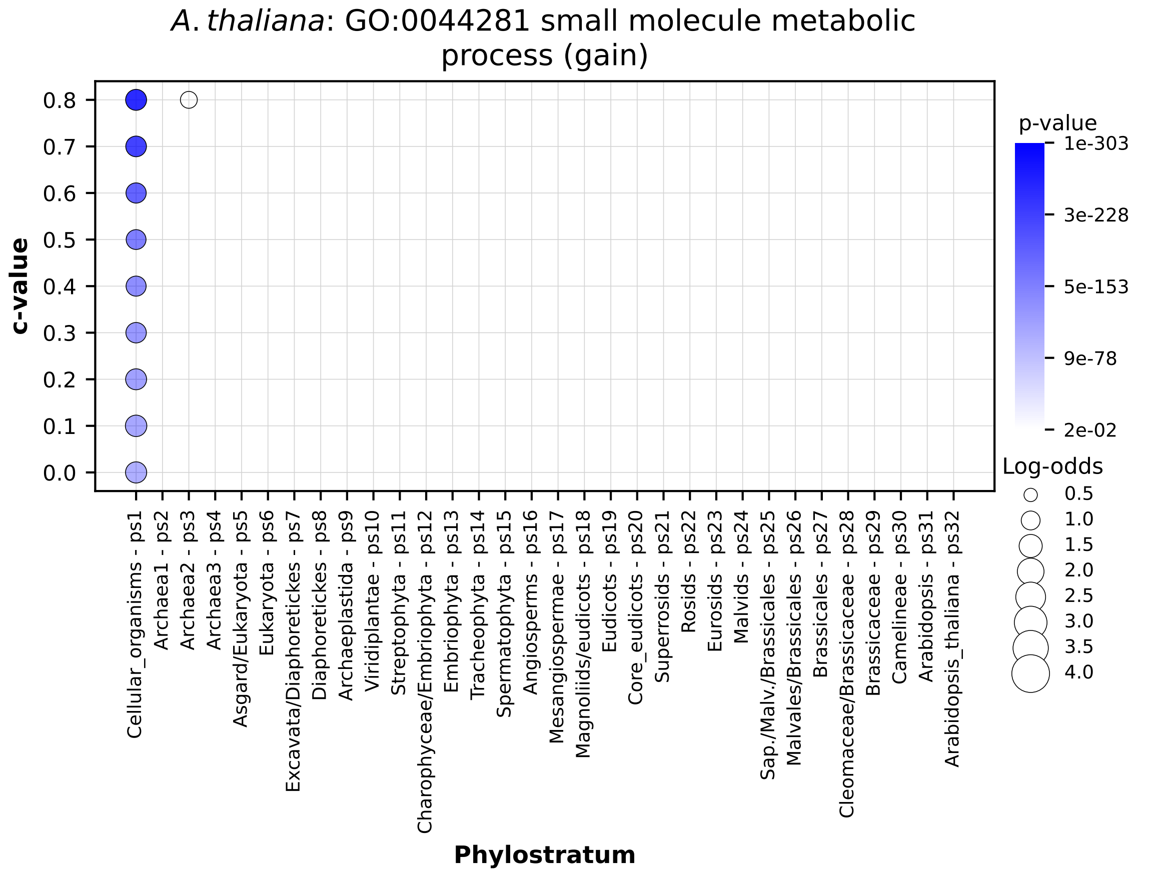

A. thaliana: GO:0044281 small molecule metabolic process (gain)
c-value
0.8
0.7
0.6
0.5
0.4
0.3
0.2
0.1
0.0
p-value
1e-303
3e-228
5e-153
9e-78
2e-02
Log-odds
0.5
1.0
1.5
2.0
2.5
3.0
3.5
4.0
Cellular_organisms - ps1
Archaea1 - ps2
Archaea2 - ps3
Archaea3 - ps4
Asgard/Eukaryota - ps5
Eukaryota - ps6
Excavata/Diaphoretickes - ps7
Diaphoretickes - ps8
Archaeplastida - ps9
Viridiplantae - ps10
Streptophyta - ps11
Charophyceae/Embriophyta - ps12
Embriophyta - ps13
Tracheophyta - ps14
Spermatophyta - ps15
Angiosperms - ps16
Mesangiospermae - ps17
Magnoliids/eudicots - ps18
Eudicots - ps19
Core_eudicots - ps20
Superrosids - ps21
Rosids - ps22
Eurosids - ps23
Malvids - ps24
Sap./Malv./Brassicales - ps25
Malvales/Brassicales - ps26
Brassicales - ps27
Cleomaceae/Brassicaceae - ps28
Brassicaceae - ps29
Camelineae - ps30
Arabidopsis - ps31
Arabidopsis_thaliana - ps32
Phylostratum

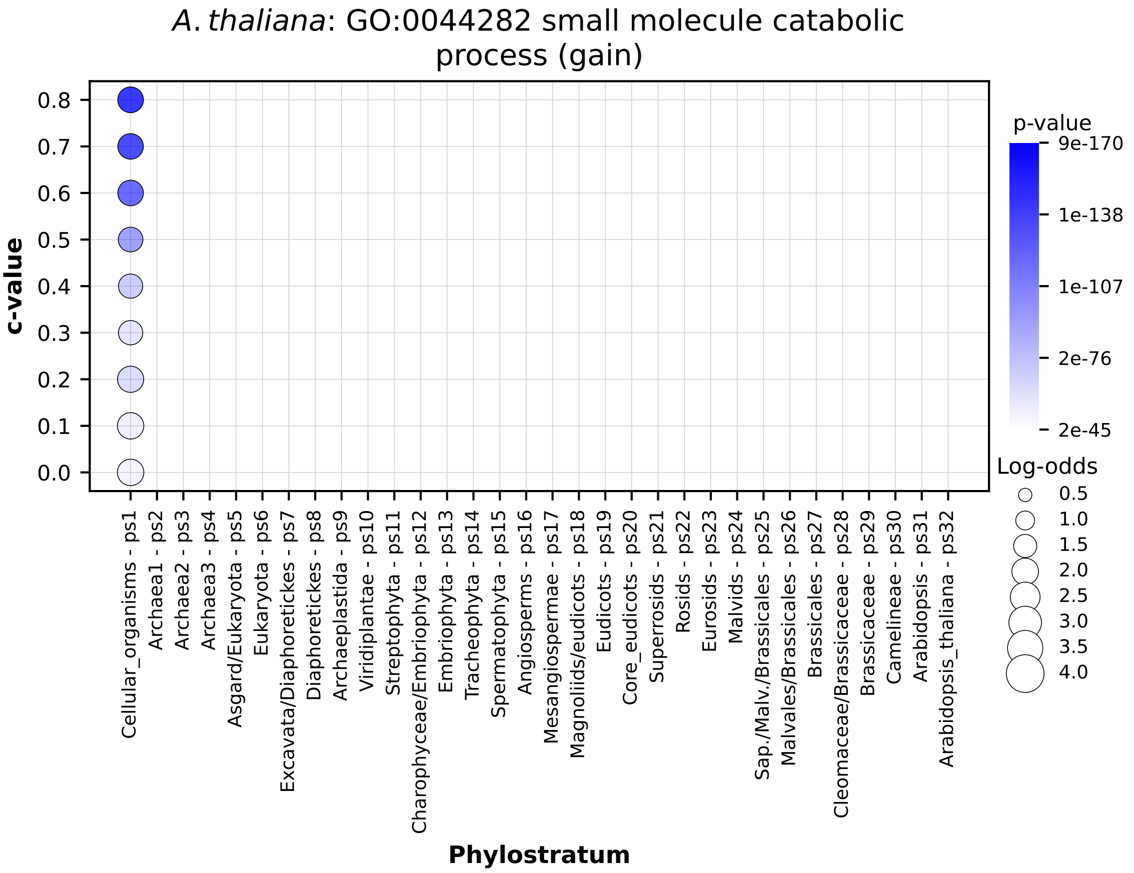

A. thaliana: GO:0044282 small molecule catabolic process (gain)
c-value
0.8
0.7
0.6
0.5
0.4
0.3
0.2
0.1
0.0
p-value
9e-170
1e-138
1e-107
2e-76
2e-45
Log-odds
0.5
1.0
1.5
2.0
2.5
3.0
3.5
4.0
Cellular_organisms - ps1
Archaea1 - ps2
Archaea2 - ps3
Archaea3 - ps4
Asgard/Eukaryota - ps5
Eukaryota - ps6
Excavata/Diaphoretickes - ps7
Diaphoretickes - ps8
Archaeplastida - ps9
Viridiplantae - ps10
Streptophyta - ps11
Charophyceae/Embriophyta - ps12
Embriophyta - ps13
Tracheophyta - ps14
Spermatophyta - ps15
Angiosperms - ps16
Mesangiospermae - ps17
Magnoliids/eudicots - ps18
Eudicots - ps19
Core_eudicots - ps20
Superrosids - ps21
Rosids - ps22
Eurosids - ps23
Malvids - ps24
Sap./Malv./Brassicales - ps25
Malvales/Brassicales - ps26
Brassicales - ps27
Cleomaceae/Brassicaceae - ps28
Brassicaceae - ps29
Camelineae - ps30
Arabidopsis - ps31
Arabidopsis_thaliana - ps32
Phylostratum

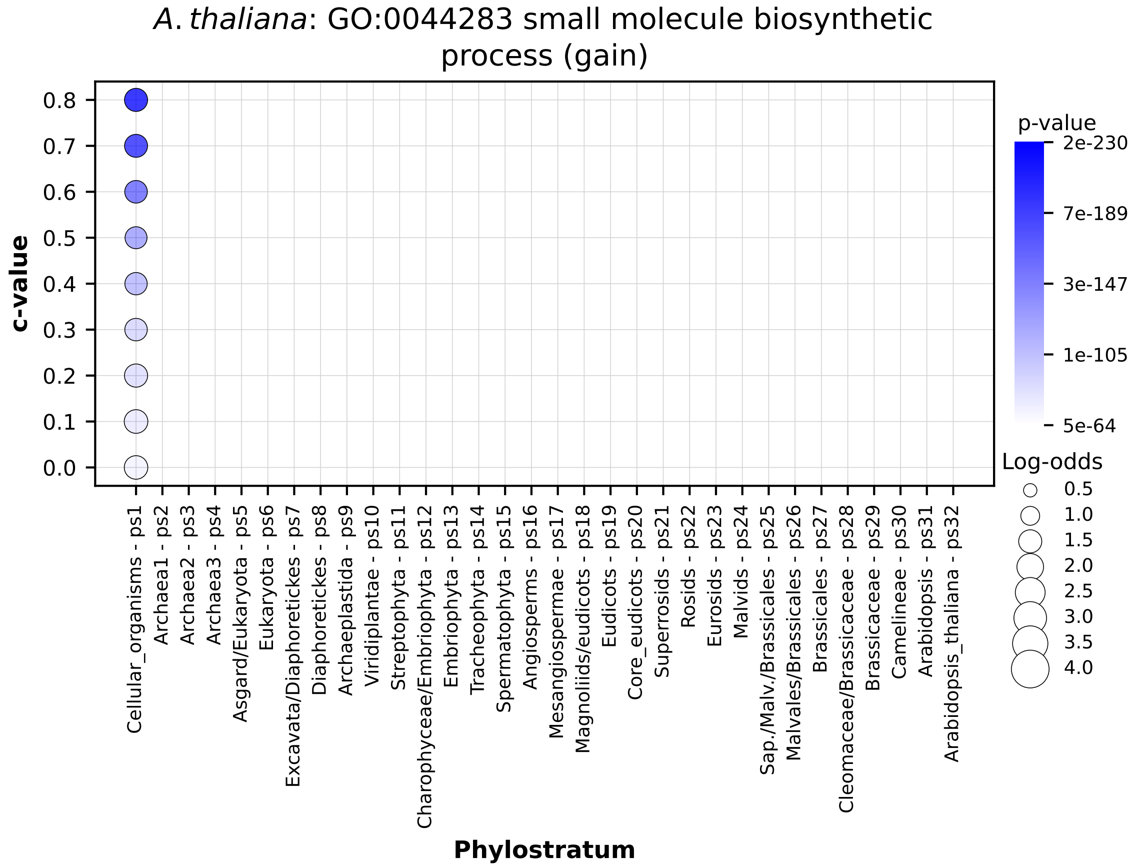

A. thaliana: GO:0044283 small molecule biosynthetic process (gain)
c-value
0.8
0.7
0.6
0.5
0.4
0.3
0.2
0.1
0.0
p-value
2e-230
7e-189
3e-147
1e-105
5e-64
Log-odds
0.5
1.0
1.5
2.0
2.5
3.0
3.5
4.0
Cellular_organisms - ps1
Archaea1 - ps2
Archaea2 - ps3
Archaea3 - ps4
Asgard/Eukaryota - ps5
Eukaryota - ps6
Excavata/Diaphoretickes - ps7
Diaphoretickes - ps8
Archaeplastida - ps9
Viridiplantae - ps10
Streptophyta - ps11
Charophyceae/Embriophyta - ps12
Embriophyta - ps13
Tracheophyta - ps14
Spermatophyta - ps15
Angiosperms - ps16
Mesangiospermae - ps17
Magnoliids/eudicots - ps18
Eudicots - ps19
Core_eudicots - ps20
Superrosids - ps21
Rosids - ps22
Eurosids - ps23
Malvids - ps24
Sap./Malv./Brassicales - ps25
Malvales/Brassicales - ps26
Brassicales - ps27
Cleomaceae/Brassicaceae - ps28
Brassicaceae - ps29
Camelineae - ps30
Arabidopsis - ps31
Arabidopsis_thaliana - ps32
Phylostratum

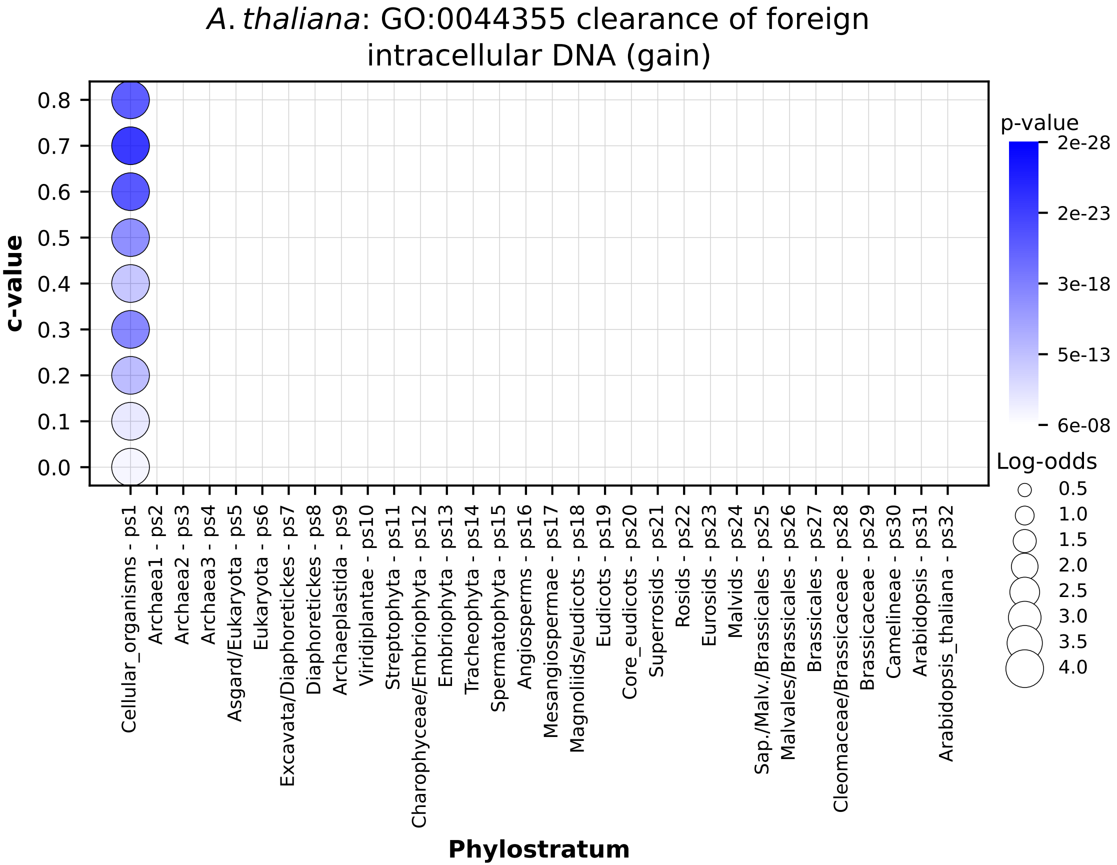

A. thaliana: GO:0044355 clearance of foreign intracellular DNA (gain)
c-value
0.8
0.7
0.6
0.5
0.4
0.3
0.2
0.1
0.0
p-value
2e-28
2e-23
3e-18
5e-13
6e-08
Log-odds
0.5
1.0
1.5
2.0
2.5
3.0
3.5
4.0
Cellular_organisms - ps1
Archaea1 - ps2
Archaea2 - ps3
Archaea3 - ps4
Asgard/Eukaryota - ps5
Eukaryota - ps6
Excavata/Diaphoretickes - ps7
Diaphoretickes - ps8
Archaeplastida - ps9
Viridiplantae - ps10
Streptophyta - ps11
Charophyceae/Embriophyta - ps12
Embriophyta - ps13
Tracheophyta - ps14
Spermatophyta - ps15
Angiosperms - ps16
Mesangiospermae - ps17
Magnoliids/eudicots - ps18
Eudicots - ps19
Core_eudicots - ps20
Superrosids - ps21
Rosids - ps22
Eurosids - ps23
Malvids - ps24
Sap./Malv./Brassicales - ps25
Malvales/Brassicales - ps26
Brassicales - ps27
Cleomaceae/Brassicaceae - ps28
Brassicaceae - ps29
Camelineae - ps30
Arabidopsis - ps31
Arabidopsis_thaliana - ps32
Phylostratum

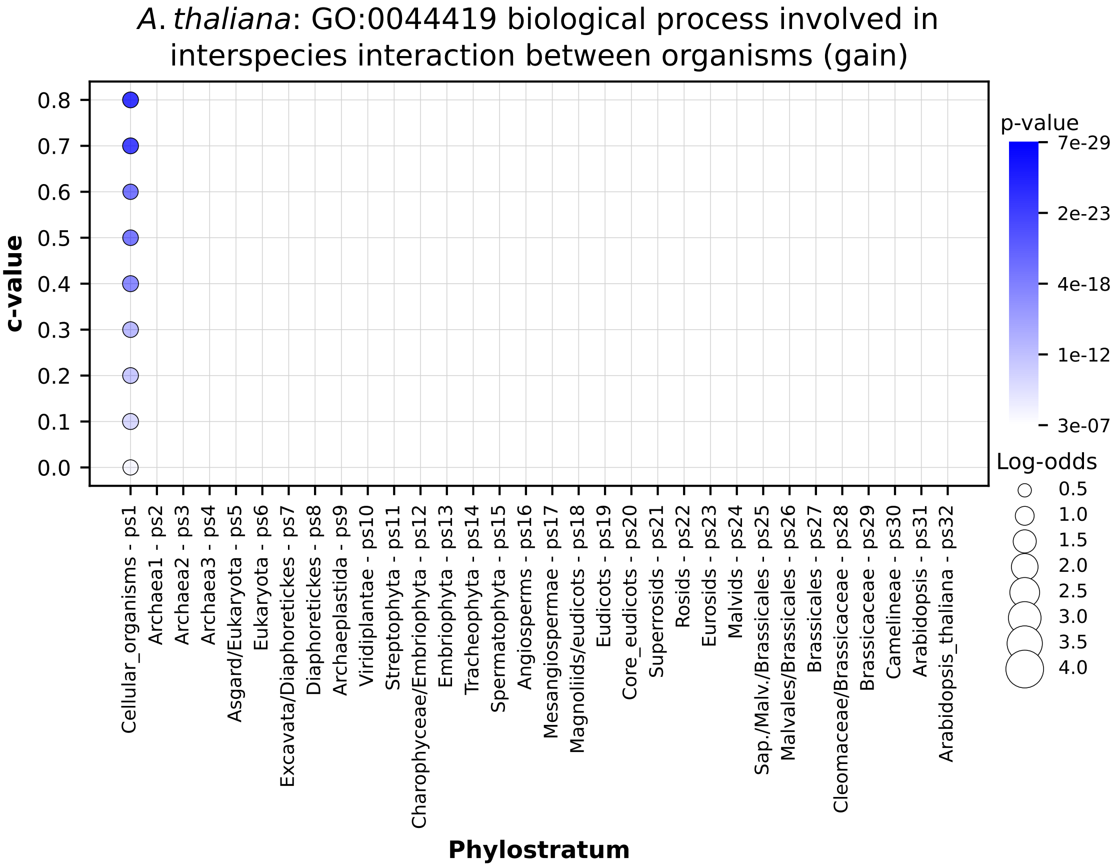
A. thaliana: GO:0044419 biological process involved in interspecies interaction between organisms (gain)
c-value
0.8
0.7
0.6
0.5
0.4
0.3
0.2
0.1
0.0
p-value
7e-29
2e-23
4e-18
1e-12
3e-07
Log-odds
0.5
1.0
1.5
2.0
2.5
3.0
3.5
4.0
Cellular_organisms - ps1
Archaea1 - ps2
Archaea2 - ps3
Archaea3 - ps4
Asgard/Eukaryota - ps5
Eukaryota - ps6
Excavata/Diaphoretickes - ps7
Diaphoretickes - ps8
Archaeplastida - ps9
Viridiplantae - ps10
Streptophyta - ps11
Charophyceae/Embriophyta - ps12
Embriophyta - ps13
Tracheophyta - ps14
Spermatophyta - ps15
Angiosperms - ps16
Mesangiospermae - ps17
Magnoliids/eudicots - ps18
Eudicots - ps19
Core_eudicots - ps20
Superrosids - ps21
Rosids - ps22
Eurosids - ps23
Malvids - ps24
Sap./Malv./Brassicales - ps25
Malvales/Brassicales - ps26
Brassicales - ps27
Cleomaceae/Brassicaceae - ps28
Brassicaceae - ps29
Camelineae - ps30
Arabidopsis - ps31
Arabidopsis_thaliana - ps32
Phylostratum

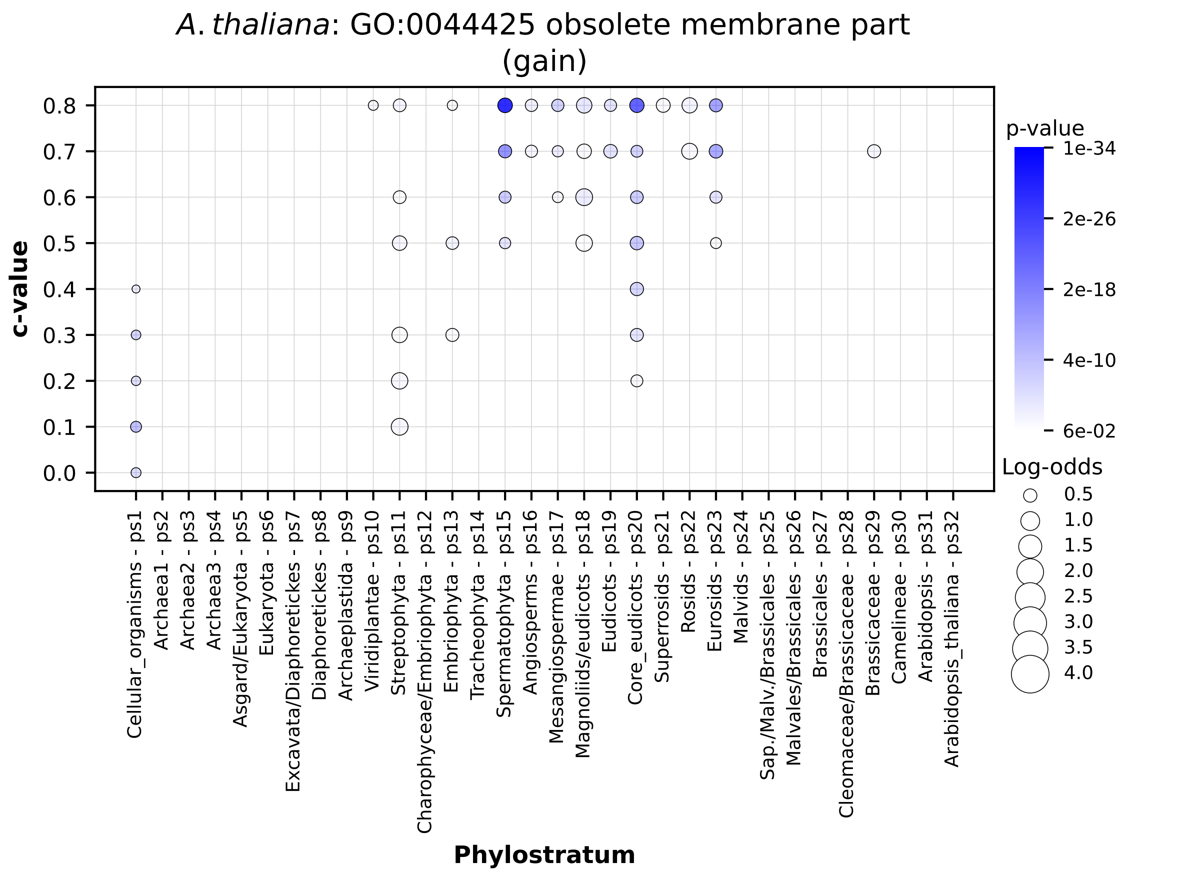

A. thaliana: GO:0044425 obsolete membrane part
(gain)
c-value
0.8
0.7
0.6
0.5
0.4
0.3
0.2
0.1
0.0
Phylostratum
Cellular_organisms - ps1
Archaea1 - ps2
Archaea2 - ps3
Archaea3 - ps4
Asgard/Eukaryota - ps5
Eukaryota - ps6
Excavata/Diaphoretickes - ps7
Diaphoretickes - ps8
Archaeplastida - ps9
Viridiplantae - ps10
Streptophyta - ps11
Charophyceae/Embriophyta - ps12
Embriophyta - ps13
Tracheophyta - ps14
Spermatophyta - ps15
Angiosperms - ps16
Mesangiospermae - ps17
Magnoliids/eudicots - ps18
Eudicots - ps19
Core_eudicots - ps20
Superrosids - ps21
Rosids - ps22
Eurosids - ps23
Malvids - ps24
Sap./Malv./Brassicales - ps25
Malvales/Brassicales - ps26
Brassicales - ps27
Cleomaceae/Brassicaceae - ps28
Brassicaceae - ps29
Camelineae - ps30
Arabidopsis - ps31
Arabidopsis_thaliana - ps32
p-value
1e-34
2e-26
2e-18
4e-10
6e-02
Log-odds
0.5
1.0
1.5
2.0
2.5
3.0
3.5
4.0

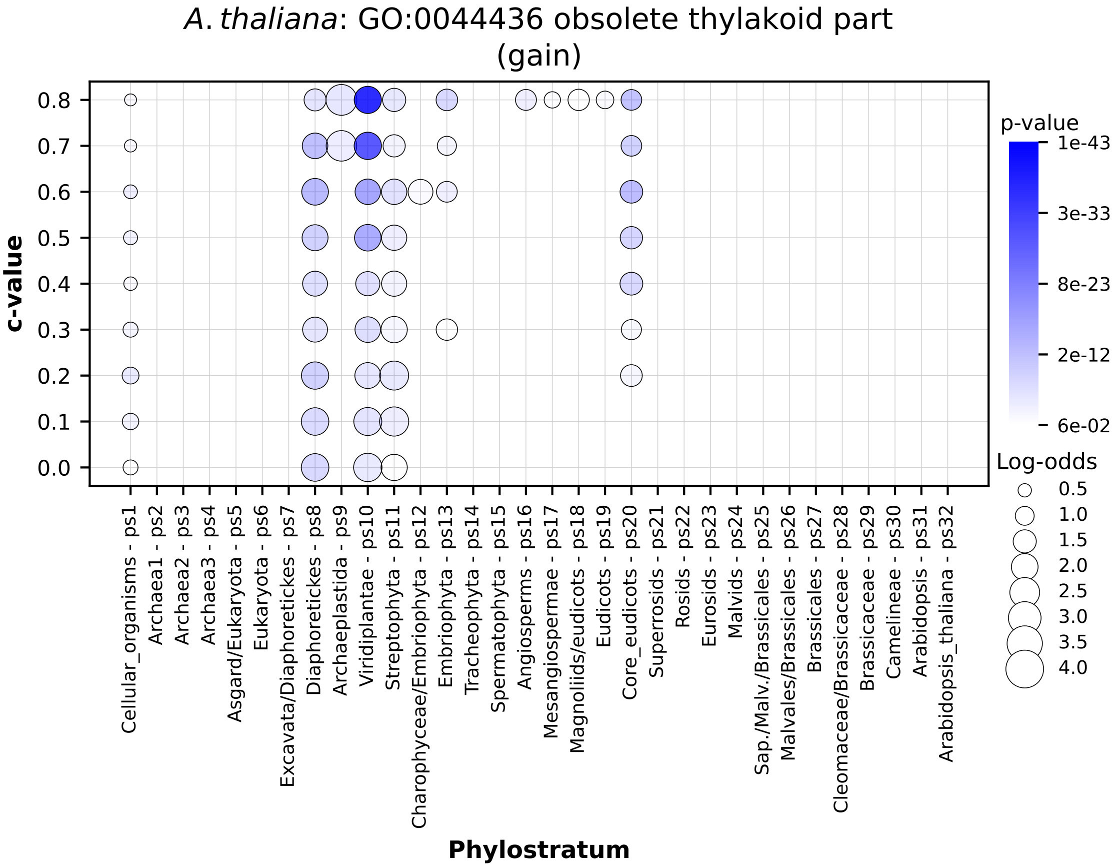

A. thaliana: GO:0044436 obsolete thylakoid part
(gain)
c-value
0.8
0.7
0.6
0.5
0.4
0.3
0.2
0.1
0.0
p-value
1e-43
3e-33
8e-23
2e-12
6e-02
Log-odds
0.5
1.0
1.5
2.0
2.5
3.0
3.5
4.0
Cellular_organisms - ps1
Archaea1 - ps2
Archaea2 - ps3
Archaea3 - ps4
Asgard/Eukaryota - ps5
Eukaryota - ps6
Excavata/Diaphoretickes - ps7
Diaphoretickes - ps8
Archaeplastida - ps9
Viridiplantae - ps10
Streptophyta - ps11
Charophyceae/Embriophyta - ps12
Embriophyta - ps13
Tracheophyta - ps14
Spermatophyta - ps15
Angiosperms - ps16
Mesangiospermae - ps17
Magnoliids/eudicots - ps18
Eudicots - ps19
Core_eudicots - ps20
Superrosids - ps21
Rosids - ps22
Eurosids - ps23
Malvids - ps24
Sap./Malv./Brassicales - ps25
Malvales/Brassicales - ps26
Brassicales - ps27
Cleomaceae/Brassicaceae - ps28
Brassicaceae - ps29
Camelineae - ps30
Arabidopsis - ps31
Arabidopsis_thaliana - ps32
Phylostratum

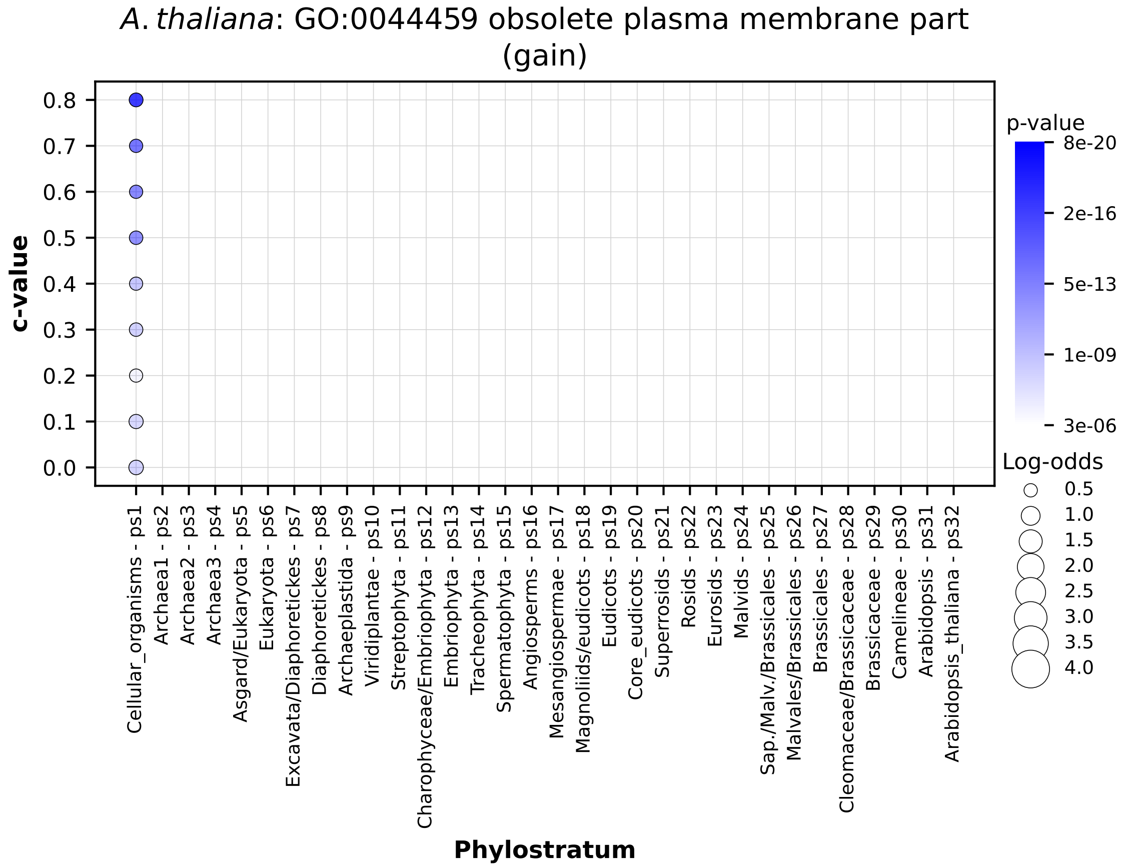

A. thaliana: GO:0044459 obsolete plasma membrane part (gain)
c-value
0.8
0.7
0.6
0.5
0.4
0.3
0.2
0.1
0.0
p-value
8e-20
2e-16
5e-13
1e-09
3e-06
Log-odds
0.5
1.0
1.5
2.0
2.5
3.0
3.5
4.0
Cellular_organisms - ps1
Archaea1 - ps2
Archaea2 - ps3
Archaea3 - ps4
Asgard/Eukaryota - ps5
Eukaryota - ps6
Excavata/Diaphoretickes - ps7
Diaphoretickes - ps8
Archaeplastida - ps9
Viridiplantae - ps10
Streptophyta - ps11
Charophyceae/Embriophyta - ps12
Embriophyta - ps13
Tracheophyta - ps14
Spermatophyta - ps15
Angiosperms - ps16
Mesangiospermae - ps17
Magnoliids/eudicots - ps18
Eudicots - ps19
Core_eudicots - ps20
Superrosids - ps21
Rosids - ps22
Eurosids - ps23
Malvids - ps24
Sap./Malv./Brassicales - ps25
Malvales/Brassicales - ps26
Brassicales - ps27
Cleomaceae/Brassicaceae - ps28
Brassicaceae - ps29
Camelineae - ps30
Arabidopsis - ps31
Arabidopsis_thaliana - ps32
Phylostratum

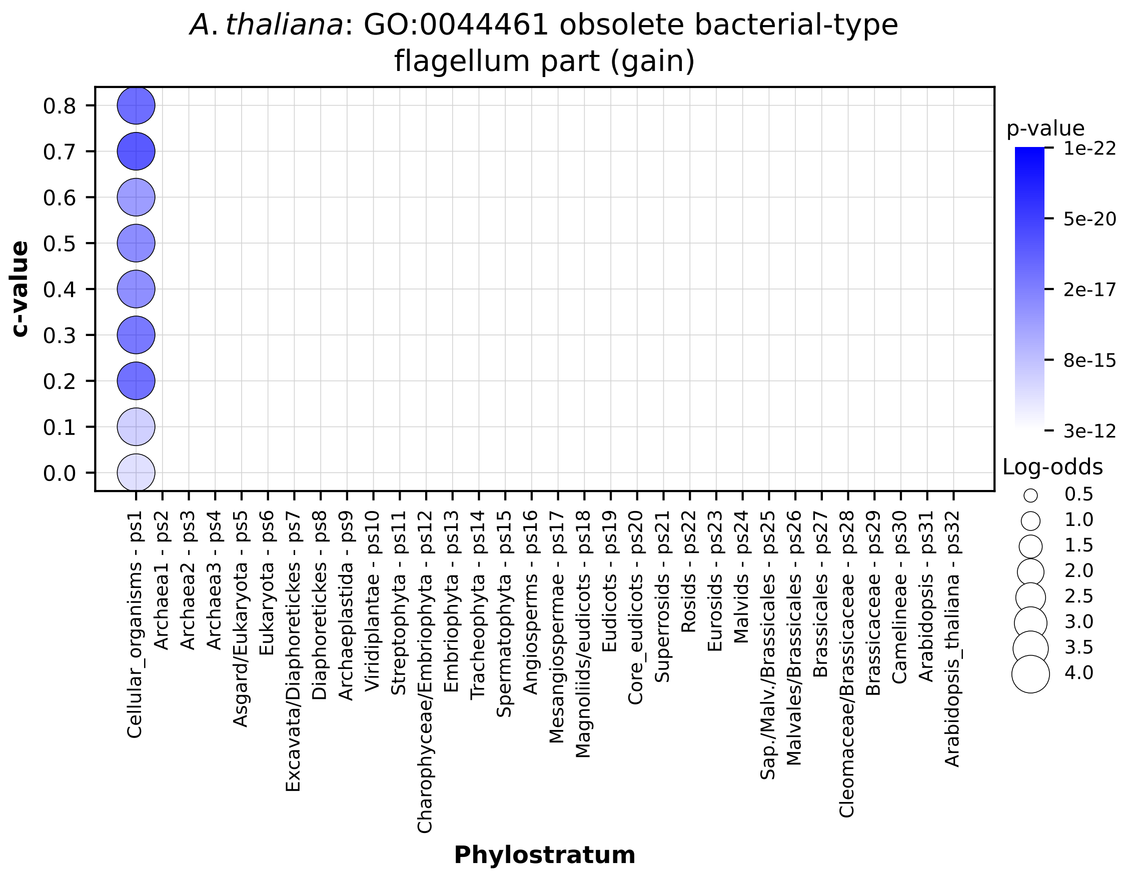
A. thaliana: GO:0044461 obsolete bacterial-type flagellum part (gain)
c-value
0.8
0.7
0.6
0.5
0.4
0.3
0.2
0.1
0.0
p-value
1e-22
5e-20
2e-17
8e-15
3e-12
Log-odds
0.5
1.0
1.5
2.0
2.5
3.0
3.5
4.0
Cellular_organisms - ps1
Archaea1 - ps2
Archaea2 - ps3
Archaea3 - ps4
Asgard/Eukaryota - ps5
Eukaryota - ps6
Excavata/Diaphoretickes - ps7
Diaphoretickes - ps8
Archaeplastida - ps9
Viridiplantae - ps10
Streptophyta - ps11
Charophyceae/Embriophyta - ps12
Embriophyta - ps13
Tracheophyta - ps14
Spermatophyta - ps15
Angiosperms - ps16
Mesangiospermae - ps17
Magnoliids/eudicots - ps18
Eudicots - ps19
Core_eudicots - ps20
Superrosids - ps21
Rosids - ps22
Eurosids - ps23
Malvids - ps24
Sap./Malv./Brassicales - ps25
Malvales/Brassicales - ps26
Brassicales - ps27
Cleomaceae/Brassicaceae - ps28
Brassicaceae - ps29
Camelineae - ps30
Arabidopsis - ps31
Arabidopsis_thaliana - ps32
Phylostratum

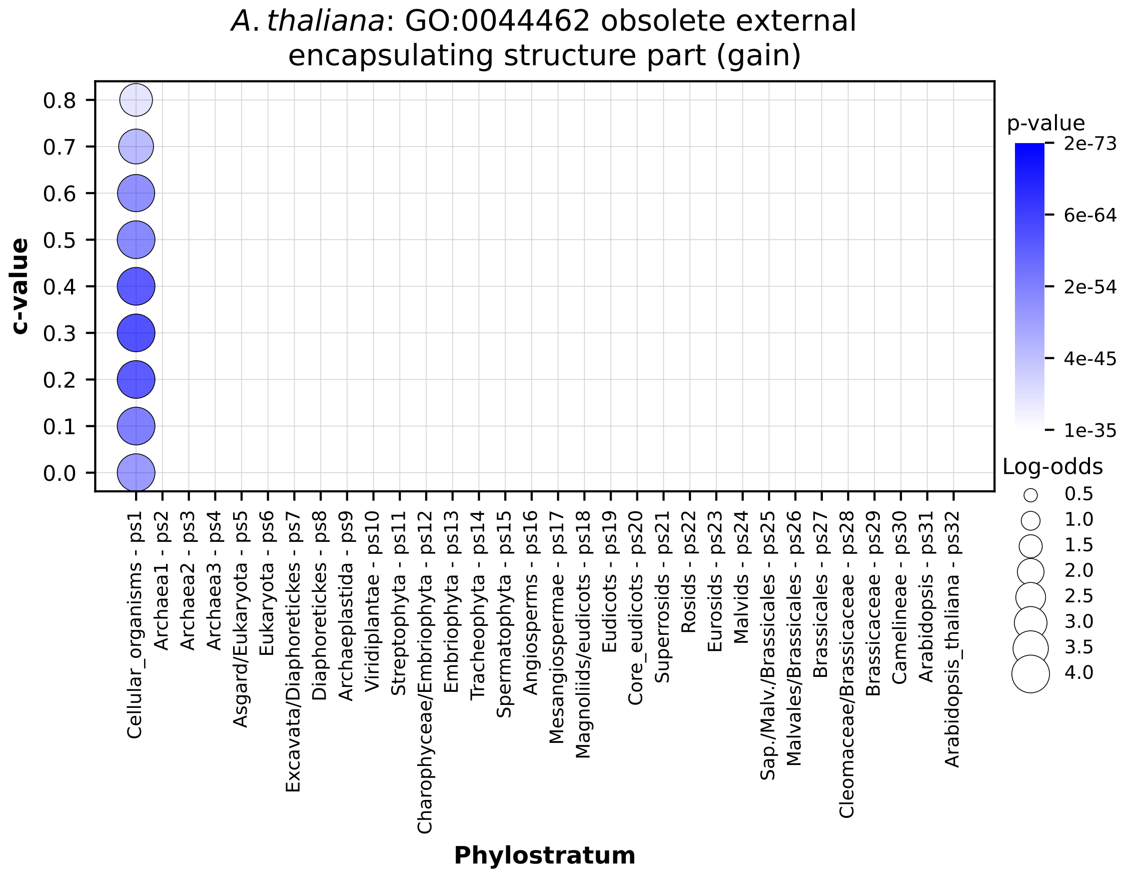

A. thaliana: GO:0044462 obsolete external encapsulating structure part (gain)
c-value
0.8
0.7
0.6
0.5
0.4
0.3
0.2
0.1
0.0
p-value
2e-73
6e-64
2e-54
4e-45
1e-35
Log-odds
0.5
1.0
1.5
2.0
2.5
3.0
3.5
4.0
Cellular_organisms - ps1
Archaea1 - ps2
Archaea2 - ps3
Archaea3 - ps4
Asgard/Eukaryota - ps5
Eukaryota - ps6
Excavata/Diaphoretickes - ps7
Diaphoretickes - ps8
Archaeplastida - ps9
Viridiplantae - ps10
Streptophyta - ps11
Charophyceae/Embriophyta - ps12
Embriophyta - ps13
Tracheophyta - ps14
Spermatophyta - ps15
Angiosperms - ps16
Mesangiospermae - ps17
Magnoliids/eudicots - ps18
Eudicots - ps19
Core_eudicots - ps20
Superrosids - ps21
Rosids - ps22
Eurosids - ps23
Malvids - ps24
Sap./Malv./Brassicales - ps25
Malvales/Brassicales - ps26
Brassicales - ps27
Cleomaceae/Brassicaceae - ps28
Brassicaceae - ps29
Camelineae - ps30
Arabidopsis - ps31
Arabidopsis_thaliana - ps32
Phylostratum

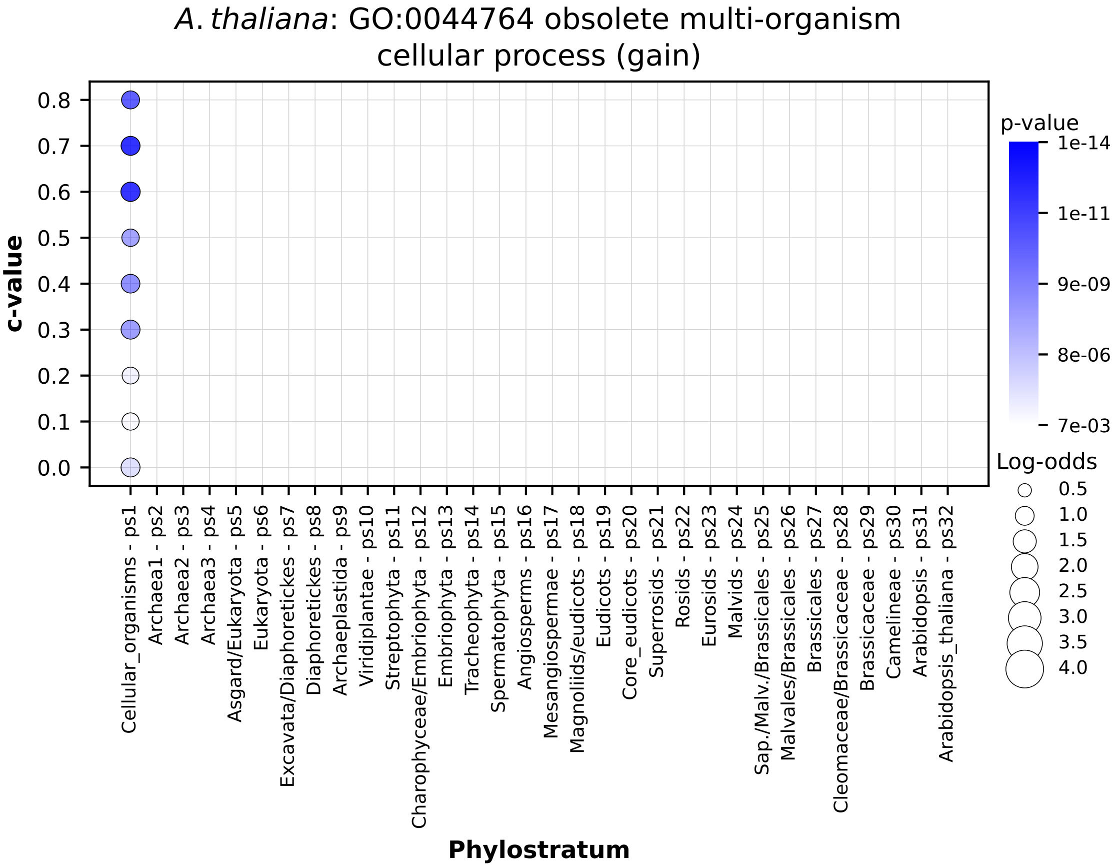

A. thaliana: GO:0044764 obsolete multi-organism cellular process (gain)
c-value
0.8
0.7
0.6
0.5
0.4
0.3
0.2
0.1
0.0
p-value
1e-14
1e-11
9e-09
8e-06
7e-03
Log-odds
0.5
1.0
1.5
2.0
2.5
3.0
3.5
4.0
Cellular_organisms - ps1
Archaea1 - ps2
Archaea2 - ps3
Archaea3 - ps4
Asgard/Eukaryota - ps5
Eukaryota - ps6
Excavata/Diaphoretickes - ps7
Diaphoretickes - ps8
Archaeplastida - ps9
Viridiplantae - ps10
Streptophyta - ps11
Charophyceae/Embriophyta - ps12
Embriophyta - ps13
Tracheophyta - ps14
Spermatophyta - ps15
Angiosperms - ps16
Mesangiospermae - ps17
Magnoliids/eudicots - ps18
Eudicots - ps19
Core_eudicots - ps20
Superrosids - ps21
Rosids - ps22
Eurosids - ps23
Malvids - ps24
Sap./Malv./Brassicales - ps25
Malvales/Brassicales - ps26
Brassicales - ps27
Cleomaceae/Brassicaceae - ps28
Brassicaceae - ps29
Camelineae - ps30
Arabidopsis - ps31
Arabidopsis_thaliana - ps32
Phylostratum

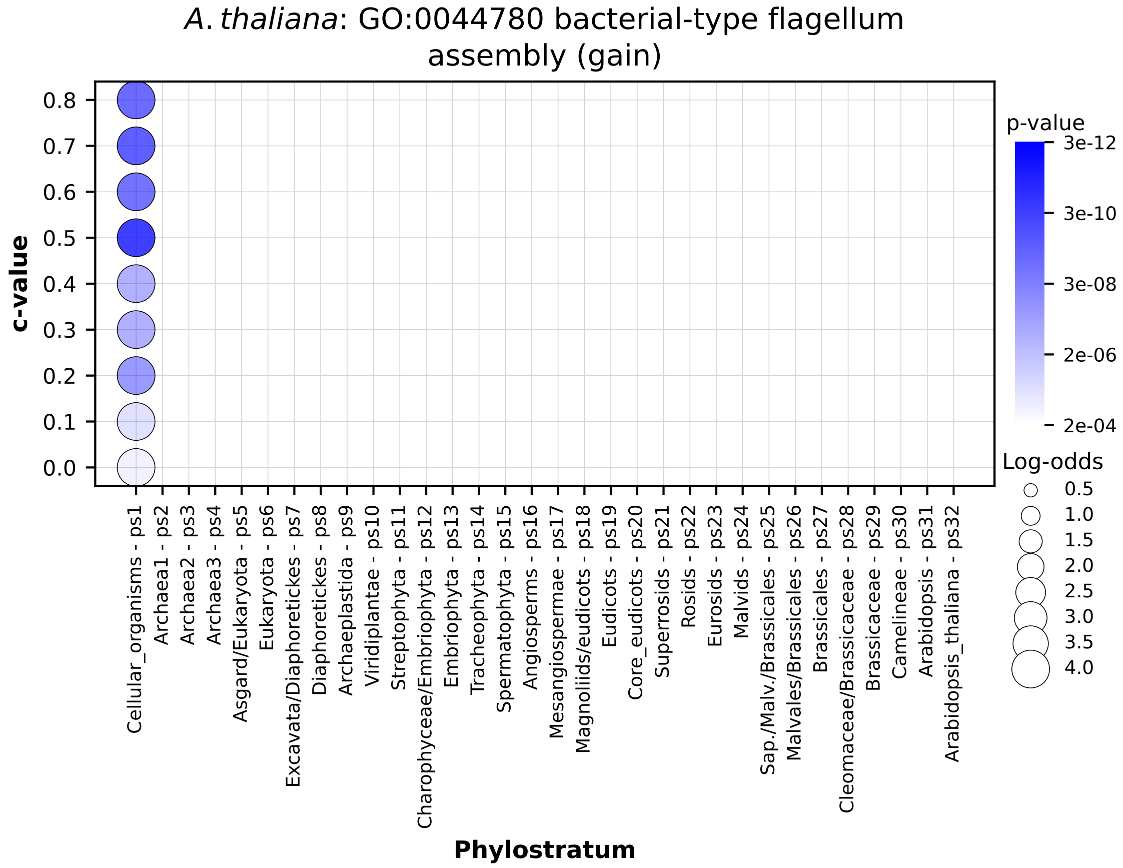

A. thaliana: GO:0044780 bacterial-type flagellum assembly (gain)
c-value
0.8
0.7
0.6
0.5
0.4
0.3
0.2
0.1
0.0
p-value
3e-12
3e-10
3e-08
2e-06
2e-04
Log-odds
0.5
1.0
1.5
2.0
2.5
3.0
3.5
4.0
Cellular_organisms - ps1
Archaea1 - ps2
Archaea2 - ps3
Archaea3 - ps4
Asgard/Eukaryota - ps5
Eukaryota - ps6
Excavata/Diaphoretickes - ps7
Diaphoretickes - ps8
Archaeplastida - ps9
Viridiplantae - ps10
Streptophyta - ps11
Charophyceae/Embriophyta - ps12
Embriophyta - ps13
Tracheophyta - ps14
Spermatophyta - ps15
Angiosperms - ps16
Mesangiospermae - ps17
Magnoliids/eudicots - ps18
Eudicots - ps19
Core_eudicots - ps20
Superrosids - ps21
Rosids - ps22
Eurosids - ps23
Malvids - ps24
Sap./Malv./Brassicales - ps25
Malvales/Brassicales - ps26
Brassicales - ps27
Cleomaceae/Brassicaceae - ps28
Brassicaceae - ps29
Camelineae - ps30
Arabidopsis - ps31
Arabidopsis_thaliana - ps32
Phylostratum

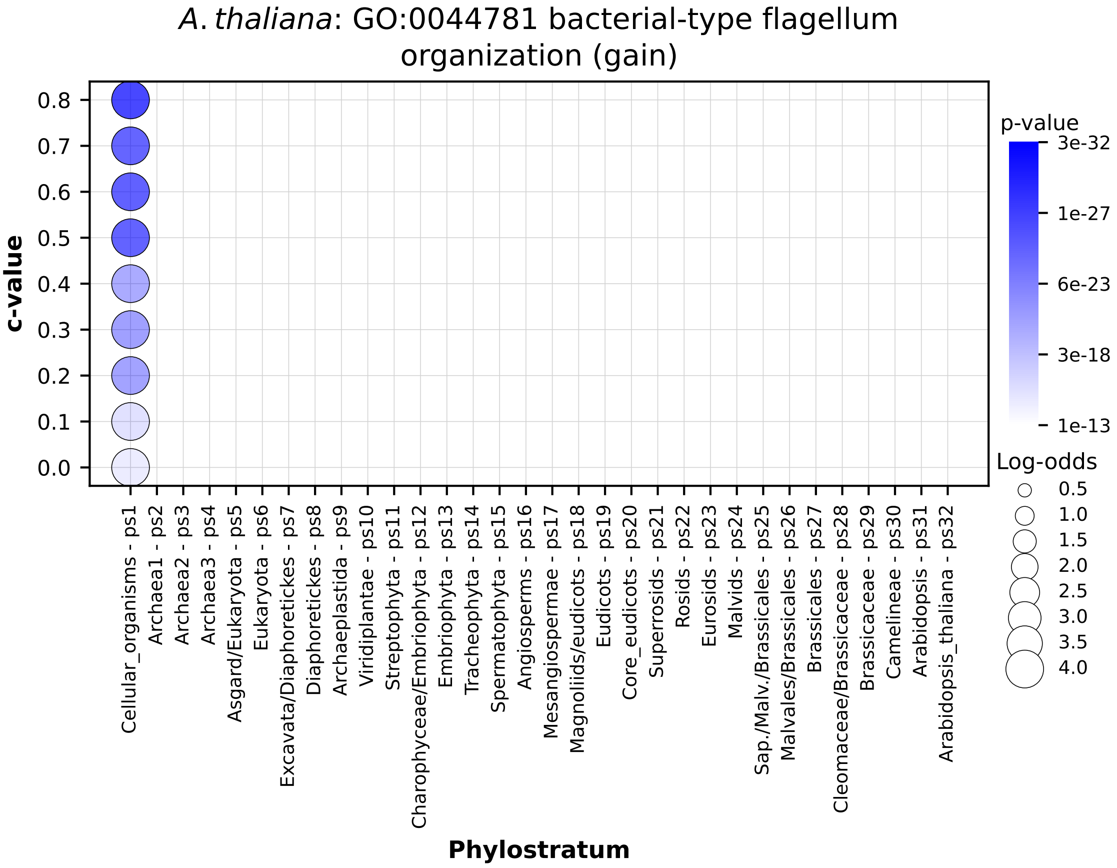

A. thaliana: GO:0044781 bacterial-type flagellum organization (gain)
c-value
0.8
0.7
0.6
0.5
0.4
0.3
0.2
0.1
0.0
p-value
3e-32
1e-27
6e-23
3e-18
1e-13
Log-odds
0.5
1.0
1.5
2.0
2.5
3.0
3.5
4.0
Cellular_organisms - ps1
Archaea1 - ps2
Archaea2 - ps3
Archaea3 - ps4
Asgard/Eukaryota - ps5
Eukaryota - ps6
Excavata/Diaphoretickes - ps7
Diaphoretickes - ps8
Archaeplastida - ps9
Viridiplantae - ps10
Streptophyta - ps11
Charophyceae/Embriophyta - ps12
Embriophyta - ps13
Tracheophyta - ps14
Spermatophyta - ps15
Angiosperms - ps16
Mesangiospermae - ps17
Magnoliids/eudicots - ps18
Eudicots - ps19
Core_eudicots - ps20
Superrosids - ps21
Rosids - ps22
Eurosids - ps23
Malvids - ps24
Sap./Malv./Brassicales - ps25
Malvales/Brassicales - ps26
Brassicales - ps27
Cleomaceae/Brassicaceae - ps28
Brassicaceae - ps29
Camelineae - ps30
Arabidopsis - ps31
Arabidopsis_thaliana - ps32
Phylostratum

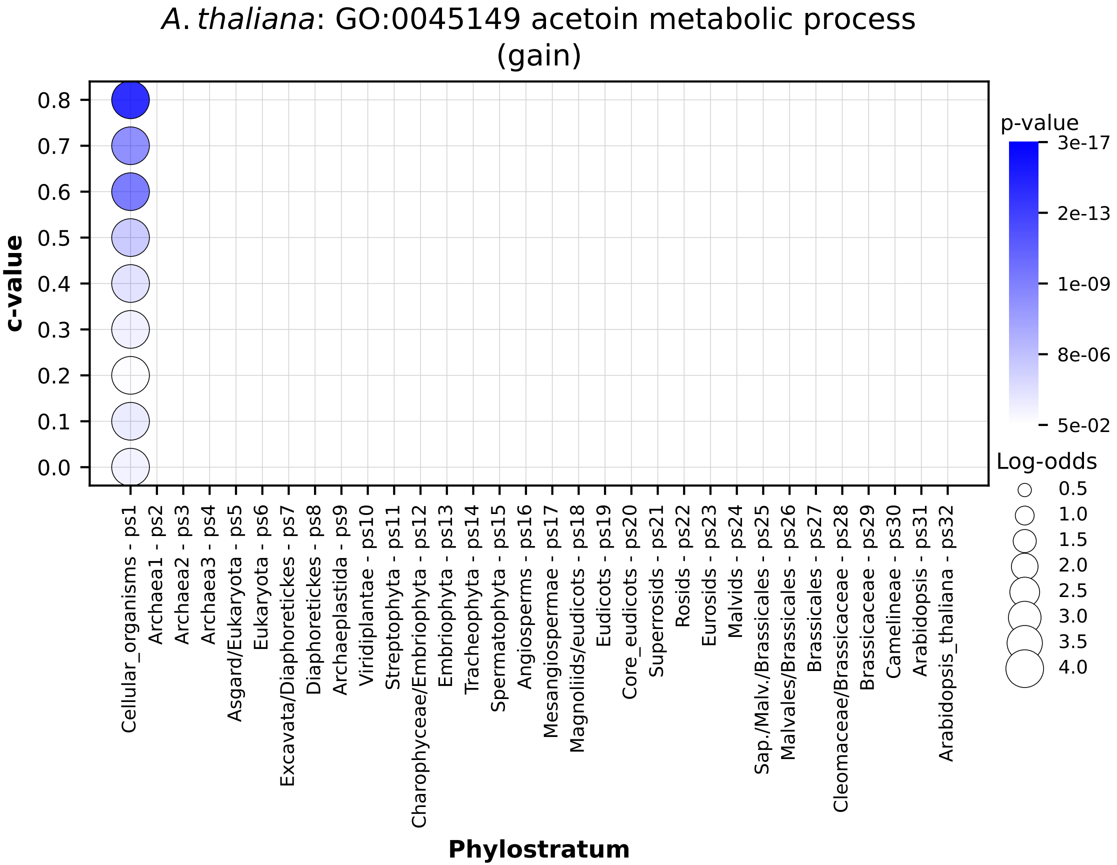

A. thaliana: GO:0045149 acetoin metabolic process (gain)
c-value
0.8
0.7
0.6
0.5
0.4
0.3
0.2
0.1
0.0
Phylostratum
Cellular_organisms - ps1
Archaea1 - ps2
Archaea2 - ps3
Archaea3 - ps4
Asgard/Eukaryota - ps5
Eukaryota - ps6
Excavata/Diaphoretickes - ps7
Diaphoretickes - ps8
Archaeplastida - ps9
Viridiplantae - ps10
Streptophyta - ps11
Charophyceae/Embriophyta - ps12
Embriophyta - ps13
Tracheophyta - ps14
Spermatophyta - ps15
Angiosperms - ps16
Mesangiospermae - ps17
Magnoliids/eudicots - ps18
Eudicots - ps19
Core_eudicots - ps20
Superrosids - ps21
Rosids - ps22
Eurosids - ps23
Malvids - ps24
Sap./Malv./Brassicales - ps25
Malvales/Brassicales - ps26
Brassicales - ps27
Cleomaceae/Brassicaceae - ps28
Brassicaceae - ps29
Camelineae - ps30
Arabidopsis - ps31
Arabidopsis_thaliana - ps32
p-value
3e-17
2e-13
1e-09
8e-06
5e-02
Log-odds
0.5
1.0
1.5
2.0
2.5
3.0
3.5
4.0

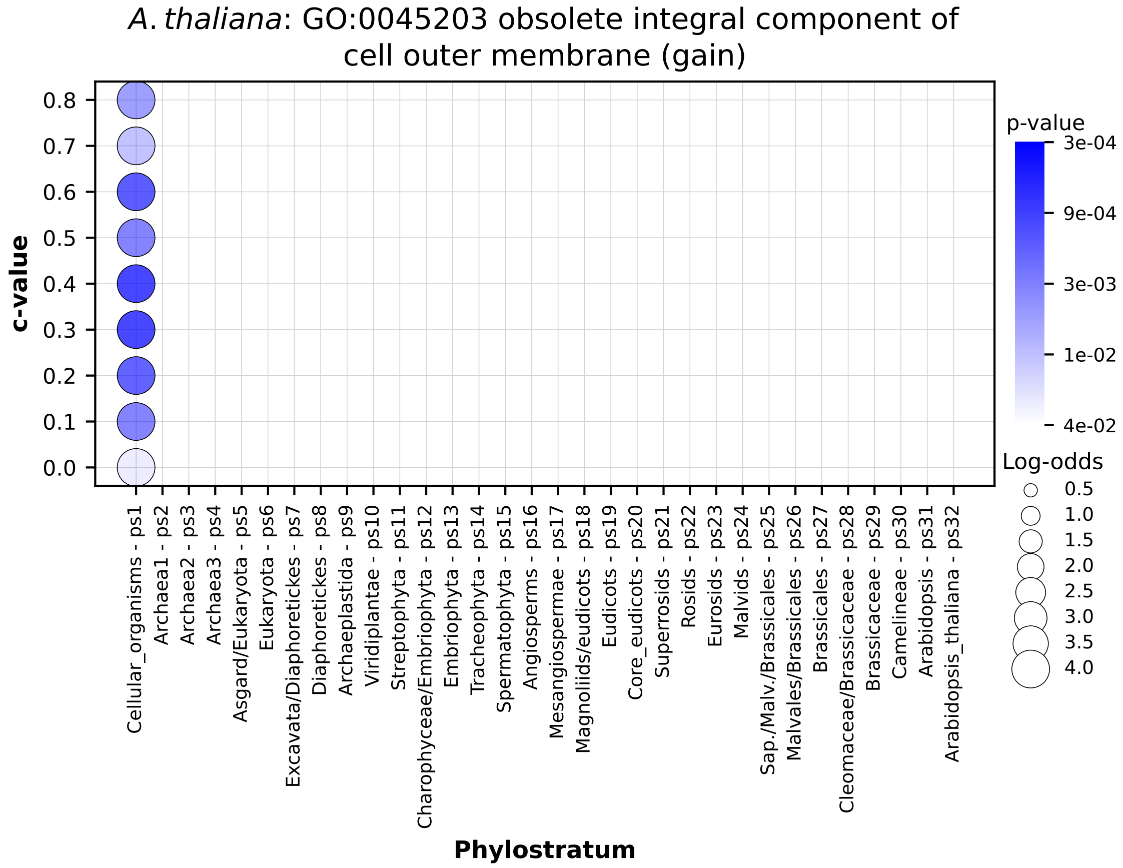

A. thaliana: GO:0045203 obsolete integral component of cell outer membrane (gain)
c-value
0.0
0.1
0.2
0.3
0.4
0.5
0.6
0.7
0.8
p-value
3e-04
9e-04
3e-03
1e-02
4e-02
Log-odds
0.5
1.0
1.5
2.0
2.5
3.0
3.5
4.0
Cellular_organisms - ps1
Archaea1 - ps2
Archaea2 - ps3
Archaea3 - ps4
Asgard/Eukaryota - ps5
Eukaryota - ps6
Excavata/Diaphoretickes - ps7
Diaphoretickes - ps8
Archaeplastida - ps9
Viridiplantae - ps10
Streptophyta - ps11
Charophyceae/Embriophyta - ps12
Embriophyta - ps13
Tracheophyta - ps14
Spermatophyta - ps15
Angiosperms - ps16
Mesangiospermae - ps17
Magnoliids/eudicots - ps18
Eudicots - ps19
Core_eudicots - ps20
Superrosids - ps21
Rosids - ps22
Eurosids - ps23
Malvids - ps24
Sap./Malv./Brassicales - ps25
Malvales/Brassicales - ps26
Brassicales - ps27
Cleomaceae/Brassicaceae - ps28
Brassicaceae - ps29
Camelineae - ps30
Arabidopsis - ps31
Arabidopsis_thaliana - ps32
Phylostratum

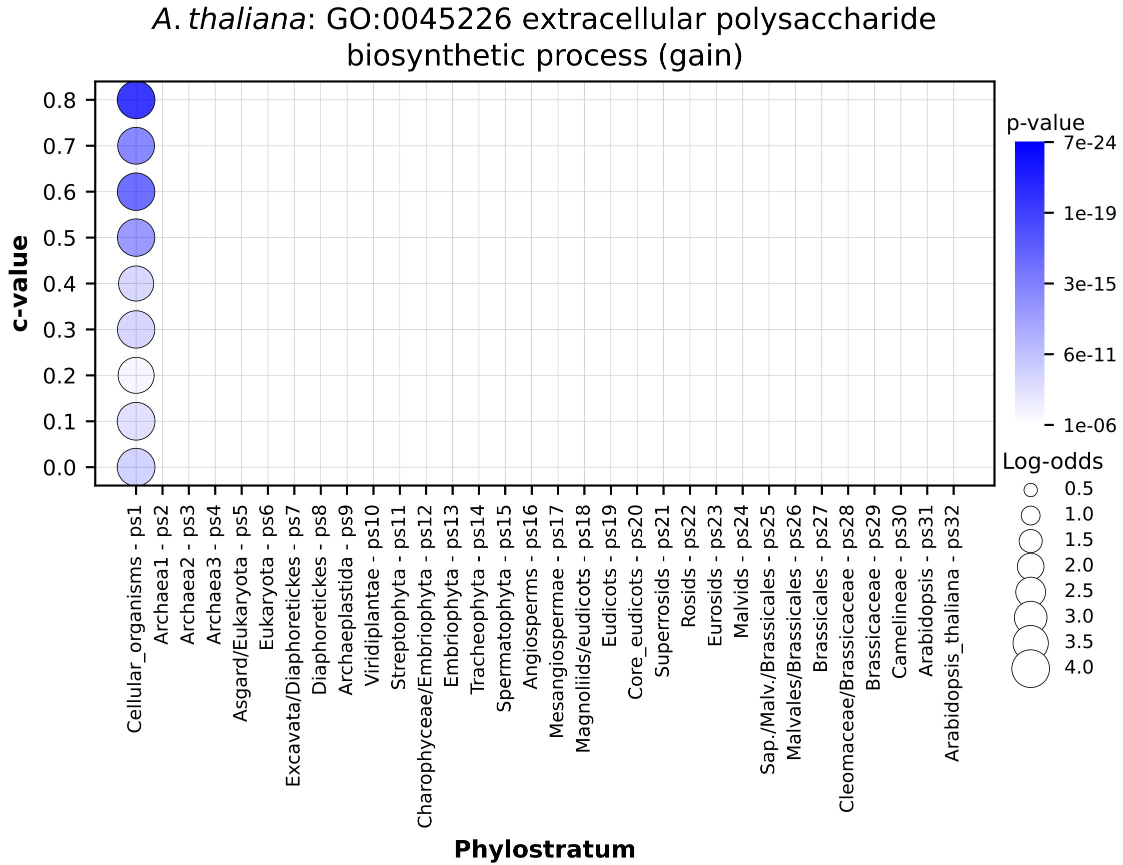
A. thaliana: GO:0045226 extracellular polysaccharide biosynthetic process (gain)
c-value
0.8
0.7
0.6
0.5
0.4
0.3
0.2
0.1
0.0
p-value
7e-24
1e-19
3e-15
6e-11
1e-06
Log-odds
0.5
1.0
1.5
2.0
2.5
3.0
3.5
4.0
Cellular_organisms - ps1
Archaea1 - ps2
Archaea2 - ps3
Archaea3 - ps4
Asgard/Eukaryota - ps5
Eukaryota - ps6
Excavata/Diaphoretickes - ps7
Diaphoretickes - ps8
Archaeplastida - ps9
Viridiplantae - ps10
Streptophyta - ps11
Charophyceae/Embriophyta - ps12
Embriophyta - ps13
Tracheophyta - ps14
Spermatophyta - ps15
Angiosperms - ps16
Mesangiospermae - ps17
Magnoliids/eudicots - ps18
Eudicots - ps19
Core_eudicots - ps20
Superrosids - ps21
Rosids - ps22
Eurosids - ps23
Malvids - ps24
Sap./Malv./Brassicales - ps25
Malvales/Brassicales - ps26
Brassicales - ps27
Cleomaceae/Brassicaceae - ps28
Brassicaceae - ps29
Camelineae - ps30
Arabidopsis - ps31
Arabidopsis_thaliana - ps32
Phylostratum

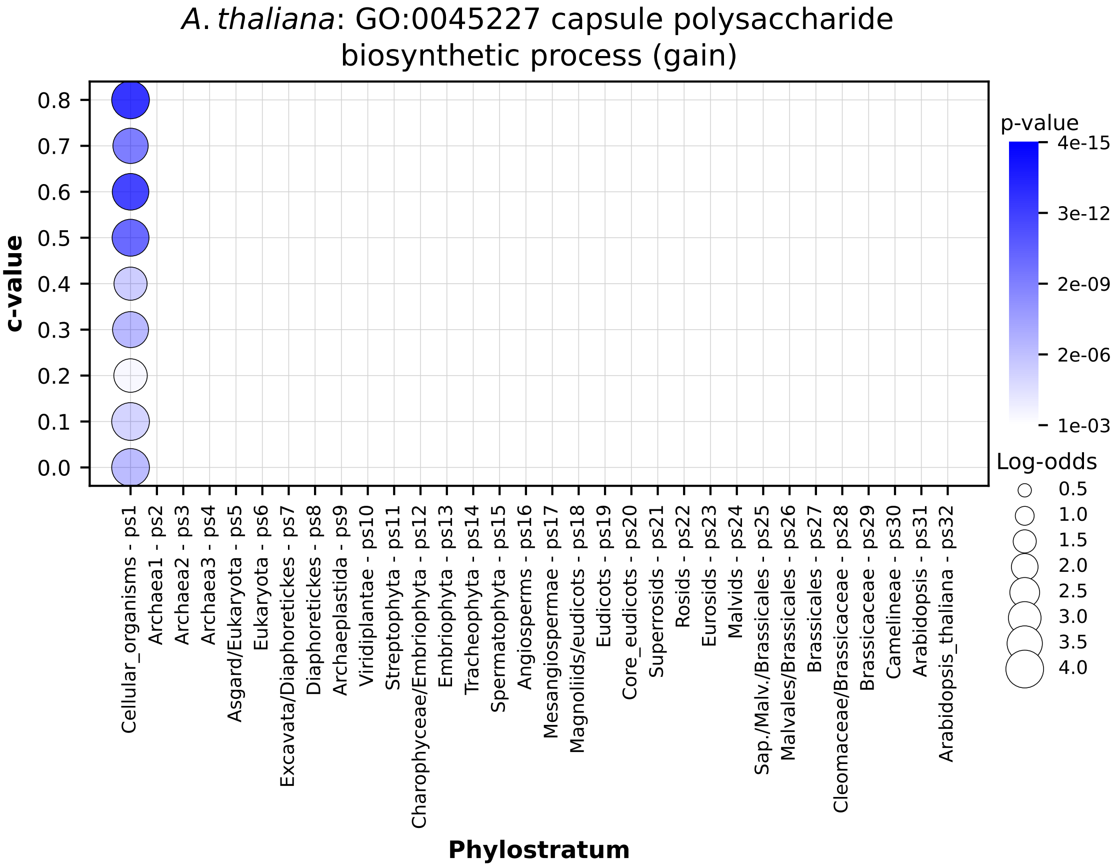

A. thaliana: GO:0045227 capsule polysaccharide biosynthetic process (gain)
c-value
0.8
0.7
0.6
0.5
0.4
0.3
0.2
0.1
0.0
p-value
4e-15
3e-12
2e-09
2e-06
1e-03
Log-odds
0.5
1.0
1.5
2.0
2.5
3.0
3.5
4.0
Cellular_organisms - ps1
Archaea1 - ps2
Archaea2 - ps3
Archaea3 - ps4
Asgard/Eukaryota - ps5
Eukaryota - ps6
Excavata/Diaphoretickes - ps7
Diaphoretickes - ps8
Archaeplastida - ps9
Viridiplantae - ps10
Streptophyta - ps11
Charophyceae/Embriophyta - ps12
Embriophyta - ps13
Tracheophyta - ps14
Spermatophyta - ps15
Angiosperms - ps16
Mesangiospermae - ps17
Magnoliids/eudicots - ps18
Eudicots - ps19
Core_eudicots - ps20
Superrosids - ps21
Rosids - ps22
Eurosids - ps23
Malvids - ps24
Sap./Malv./Brassicales - ps25
Malvales/Brassicales - ps26
Brassicales - ps27
Cleomaceae/Brassicaceae - ps28
Brassicaceae - ps29
Camelineae - ps30
Arabidopsis - ps31
Arabidopsis_thaliana - ps32
Phylostratum

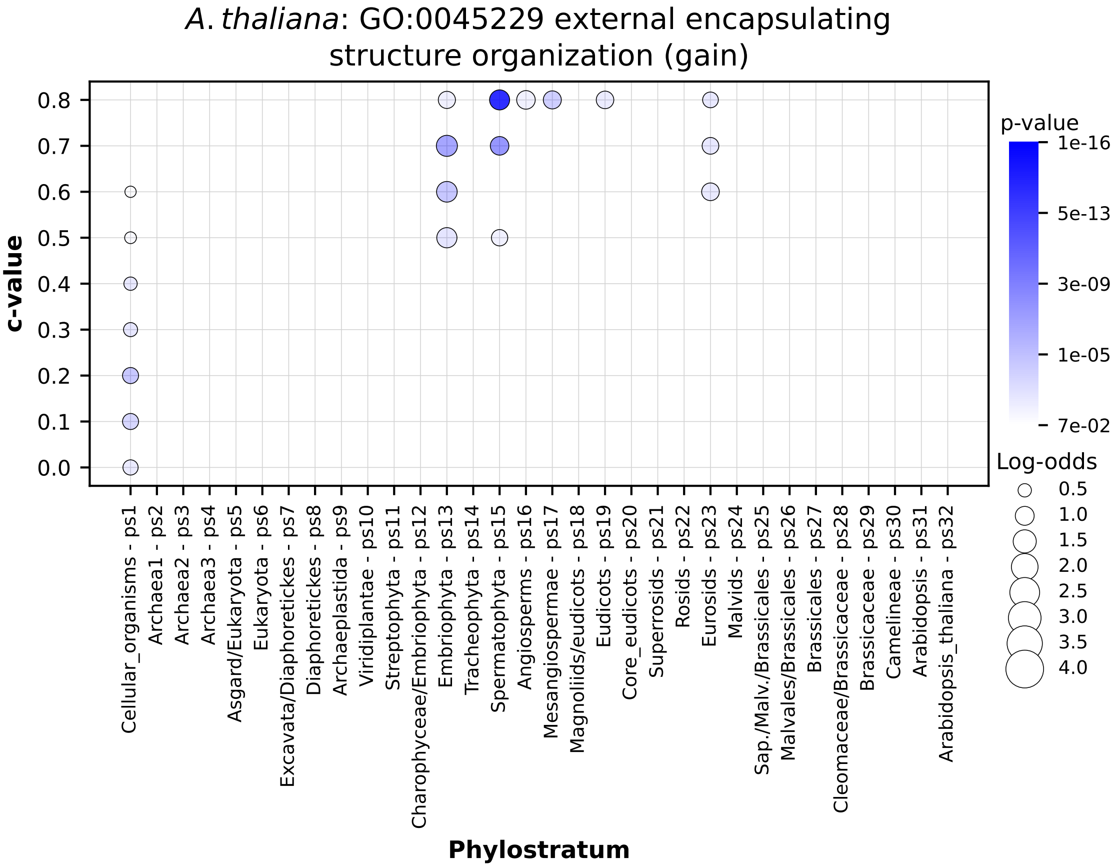
A. thaliana: GO:0045229 external encapsulating structure organization (gain)
c-value
0.0
0.1
0.2
0.3
0.4
0.5
0.6
0.7
0.8
Phylostratum
Cellular_organisms - ps1
Archaea1 - ps2
Archaea2 - ps3
Archaea3 - ps4
Asgard/Eukaryota - ps5
Eukaryota - ps6
Excavata/Diaphoretickes - ps7
Diaphoretickes - ps8
Archaeplastida - ps9
Viridiplantae - ps10
Streptophyta - ps11
Charophyceae/Embriophyta - ps12
Embriophyta - ps13
Tracheophyta - ps14
Spermatophyta - ps15
Angiosperms - ps16
Mesangiospermae - ps17
Magnoliids/eudicots - ps18
Eudicots - ps19
Core_eudicots - ps20
Superrosids - ps21
Rosids - ps22
Eurosids - ps23
Malvids - ps24
Sap./Malv./Brassicales - ps25
Malvales/Brassicales - ps26
Brassicales - ps27
Cleomaceae/Brassicaceae - ps28
Brassicaceae - ps29
Camelineae - ps30
Arabidopsis - ps31
Arabidopsis_thaliana - ps32
p-value
1e-16
5e-13
3e-09
1e-05
7e-02
Log-odds
0.5
1.0
1.5
2.0
2.5
3.0
3.5
4.0

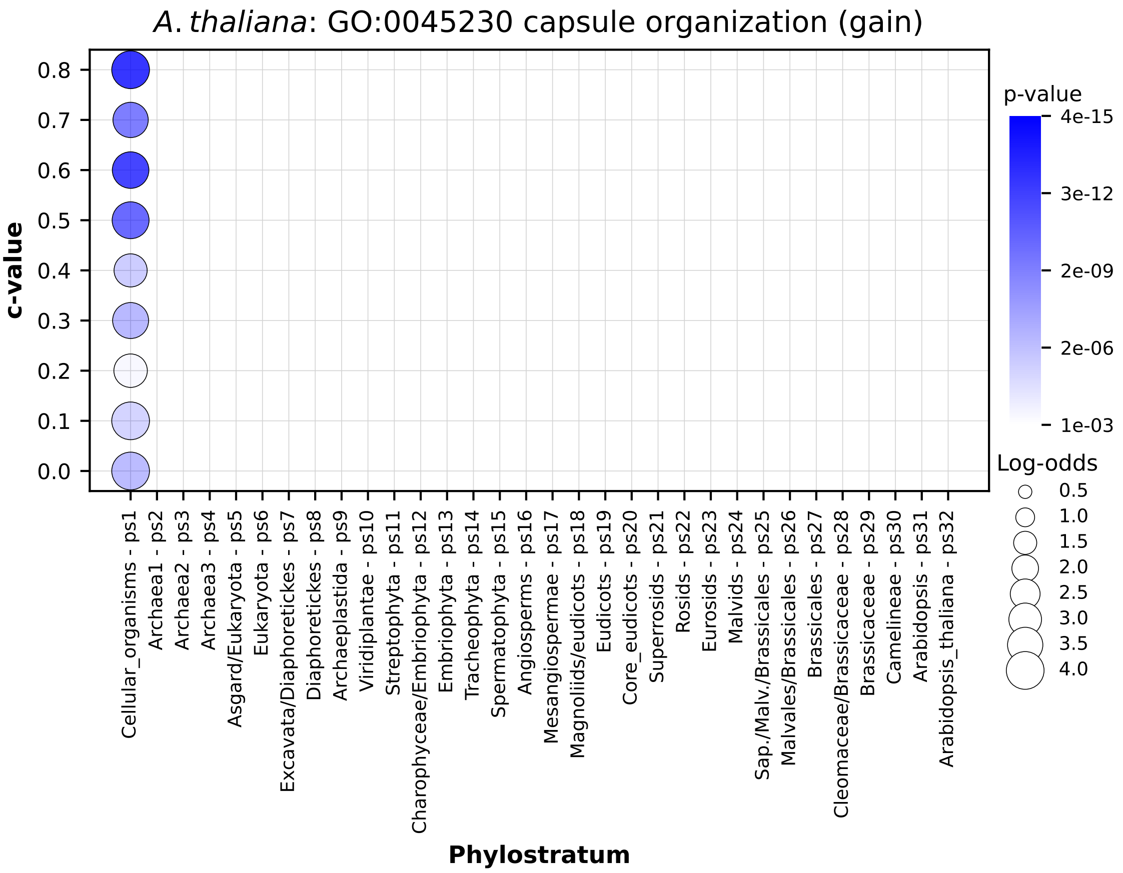

A. thaliana: GO:0045230 capsule organization (gain)
c-value
0.8
0.7
0.6
0.5
0.4
0.3
0.2
0.1
0.0
p-value
4e-15
3e-12
2e-09
2e-06
1e-03
Log-odds
0.5
1.0
1.5
2.0
2.5
3.0
3.5
4.0
Cellular_organisms - ps1
Archaea1 - ps2
Archaea2 - ps3
Archaea3 - ps4
Asgard/Eukaryota - ps5
Eukaryota - ps6
Excavata/Diaphoretickes - ps7
Diaphoretickes - ps8
Archaeplastida - ps9
Viridiplantae - ps10
Streptophyta - ps11
Charophyceae/Embriophyta - ps12
Embriophyta - ps13
Tracheophyta - ps14
Spermatophyta - ps15
Angiosperms - ps16
Mesangiospermae - ps17
Magnoliids/eudicots - ps18
Eudicots - ps19
Core_eudicots - ps20
Superrosids - ps21
Rosids - ps22
Eurosids - ps23
Malvids - ps24
Sap./Malv./Brassicales - ps25
Malvales/Brassicales - ps26
Brassicales - ps27
Cleomaceae/Brassicaceae - ps28
Brassicaceae - ps29
Camelineae - ps30
Arabidopsis - ps31
Arabidopsis_thaliana - ps32
Phylostratum

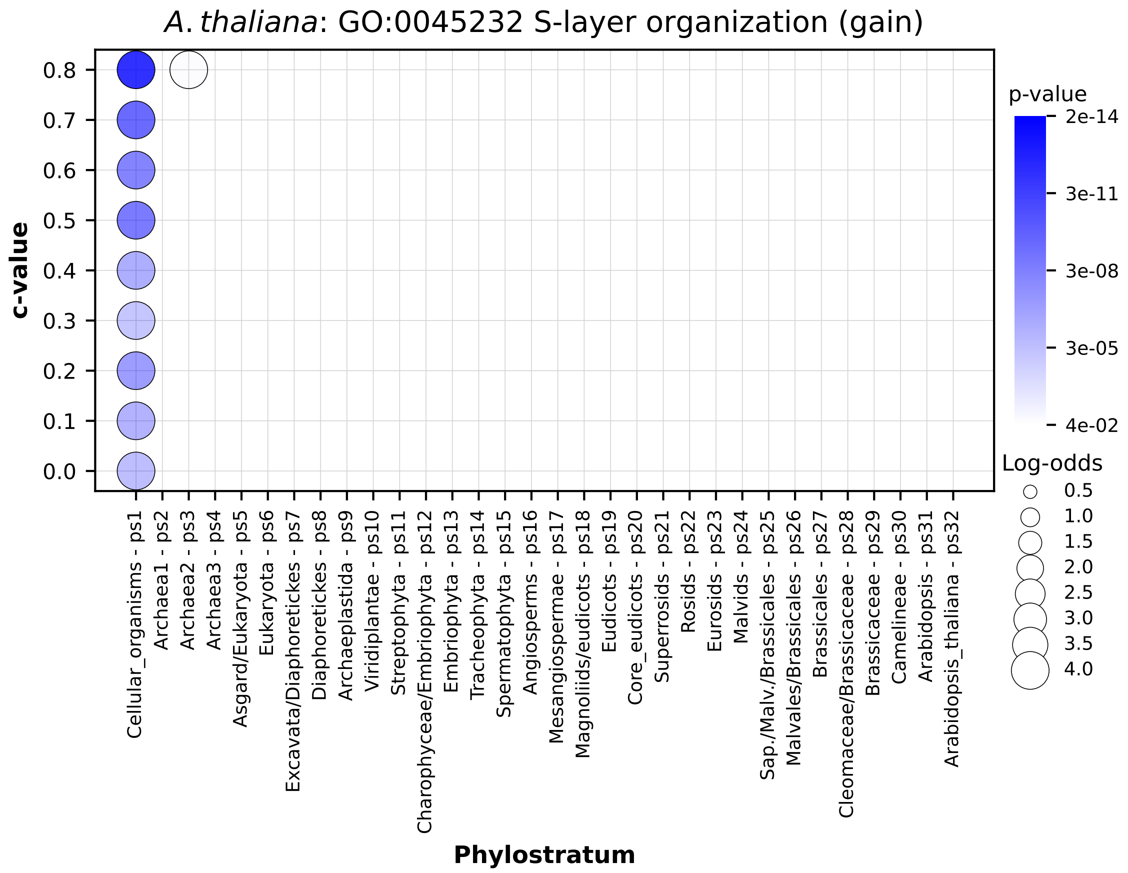

A. thaliana: GO:0045232 S-layer organization (gain)
c-value
0.0
0.1
0.2
0.3
0.4
0.5
0.6
0.7
0.8
p-value
2e-14
3e-11
3e-08
3e-05
4e-02
Log-odds
0.5
1.0
1.5
2.0
2.5
3.0
3.5
4.0
Cellular_organisms - ps1
Archaea1 - ps2
Archaea2 - ps3
Archaea3 - ps4
Asgard/Eukaryota - ps5
Eukaryota - ps6
Excavata/Diaphoretickes - ps7
Diaphoretickes - ps8
Archaeplastida - ps9
Viridiplantae - ps10
Streptophyta - ps11
Charophyceae/Embriophyta - ps12
Embriophyta - ps13
Tracheophyta - ps14
Spermatophyta - ps15
Angiosperms - ps16
Mesangiospermae - ps17
Magnoliids/eudicots - ps18
Eudicots - ps19
Core_eudicots - ps20
Superrosids - ps21
Rosids - ps22
Eurosids - ps23
Malvids - ps24
Sap./Malv./Brassicales - ps25
Malvales/Brassicales - ps26
Brassicales - ps27
Cleomaceae/Brassicaceae - ps28
Brassicaceae - ps29
Camelineae - ps30
Arabidopsis - ps31
Arabidopsis_thaliana - ps32
Phylostratum

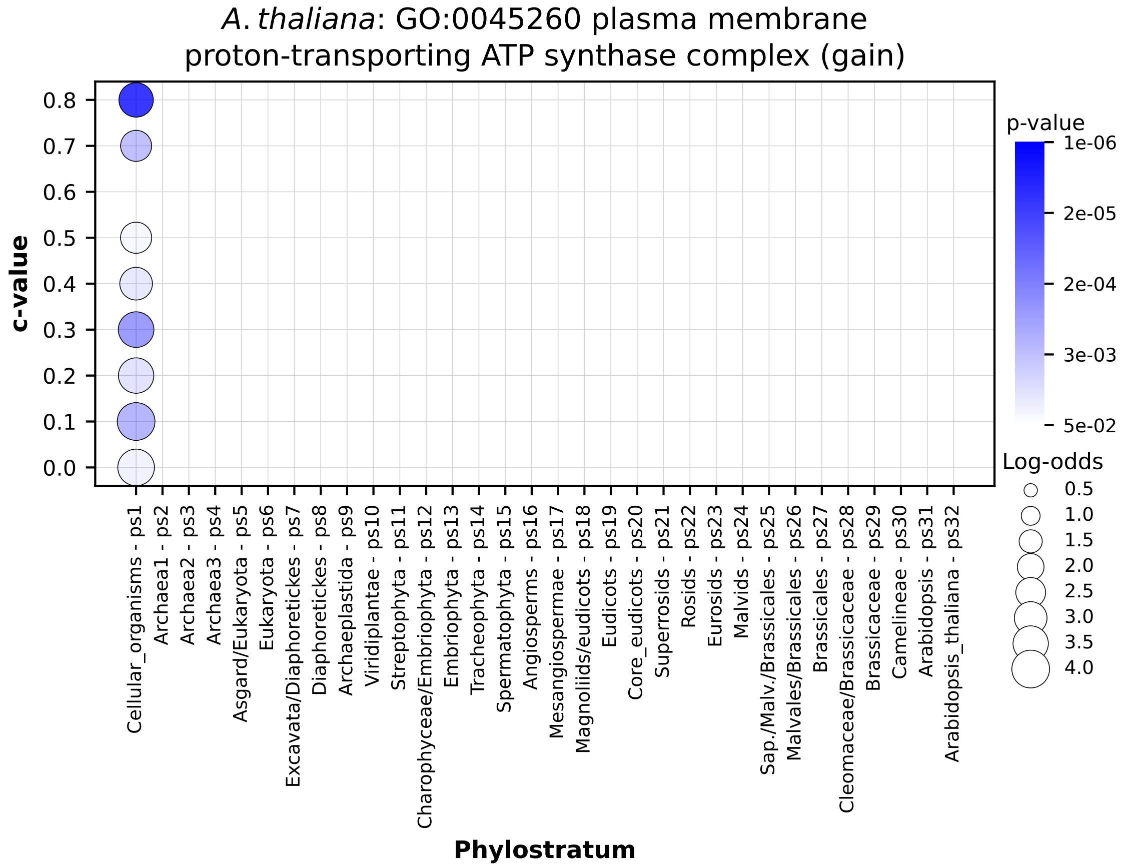

A. thaliana: GO:0045260 plasma membrane proton-transporting ATP synthase complex (gain)
c-value
0.8
0.7
0.6
0.5
0.4
0.3
0.2
0.1
0.0
Phylostratum
Cellular_organisms - ps1
Archaea1 - ps2
Archaea2 - ps3
Archaea3 - ps4
Asgard/Eukaryota - ps5
Eukaryota - ps6
Excavata/Diaphoretickes - ps7
Diaphoretickes - ps8
Archaeplastida - ps9
Viridiplantae - ps10
Streptophyta - ps11
Charophyceae/Embriophyta - ps12
Embriophyta - ps13
Tracheophyta - ps14
Spermatophyta - ps15
Angiosperms - ps16
Mesangiospermae - ps17
Magnoliids/eudicots - ps18
Eudicots - ps19
Core_eudicots - ps20
Superrosids - ps21
Rosids - ps22
Eurosids - ps23
Malvids - ps24
Sap./Malv./Brassicales - ps25
Malvales/Brassicales - ps26
Brassicales - ps27
Cleomaceae/Brassicaceae - ps28
Brassicaceae - ps29
Camelineae - ps30
Arabidopsis - ps31
Arabidopsis_thaliana - ps32
p-value
1e-06
2e-05
2e-04
3e-03
5e-02
Log-odds
0.5
1.0
1.5
2.0
2.5
3.0
3.5
4.0

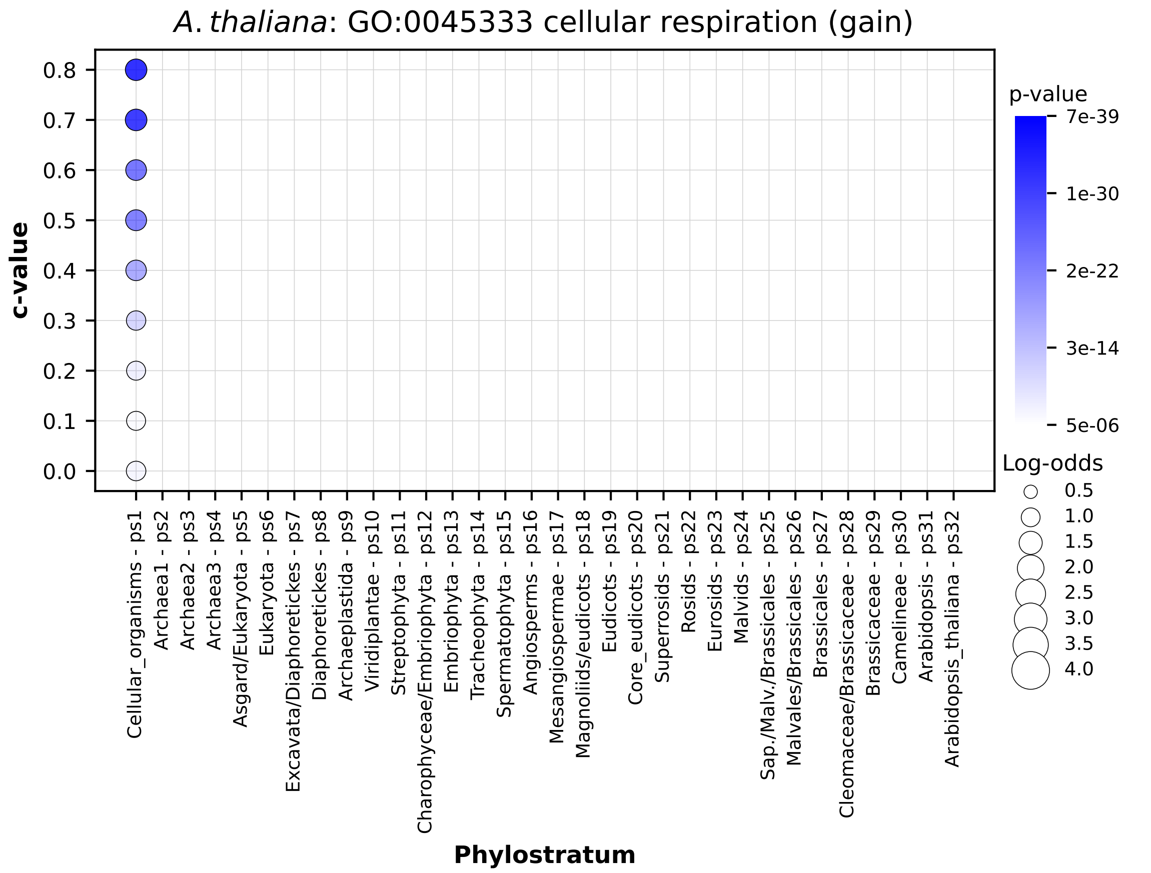

A. thaliana: GO:0045333 cellular respiration (gain)
c-value
0.8
0.7
0.6
0.5
0.4
0.3
0.2
0.1
0.0
p-value
7e-39
1e-30
2e-22
3e-14
5e-06
Log-odds
0.5
1.0
1.5
2.0
2.5
3.0
3.5
4.0
Cellular_organisms - ps1
Archaea1 - ps2
Archaea2 - ps3
Archaea3 - ps4
Asgard/Eukaryota - ps5
Eukaryota - ps6
Excavata/Diaphoretickes - ps7
Diaphoretickes - ps8
Archaeplastida - ps9
Viridiplantae - ps10
Streptophyta - ps11
Charophyceae/Embriophyta - ps12
Embriophyta - ps13
Tracheophyta - ps14
Spermatophyta - ps15
Angiosperms - ps16
Mesangiospermae - ps17
Magnoliids/eudicots - ps18
Eudicots - ps19
Core_eudicots - ps20
Superrosids - ps21
Rosids - ps22
Eurosids - ps23
Malvids - ps24
Sap./Malv./Brassicales - ps25
Malvales/Brassicales - ps26
Brassicales - ps27
Cleomaceae/Brassicaceae - ps28
Brassicaceae - ps29
Camelineae - ps30
Arabidopsis - ps31
Arabidopsis_thaliana - ps32
Phylostratum

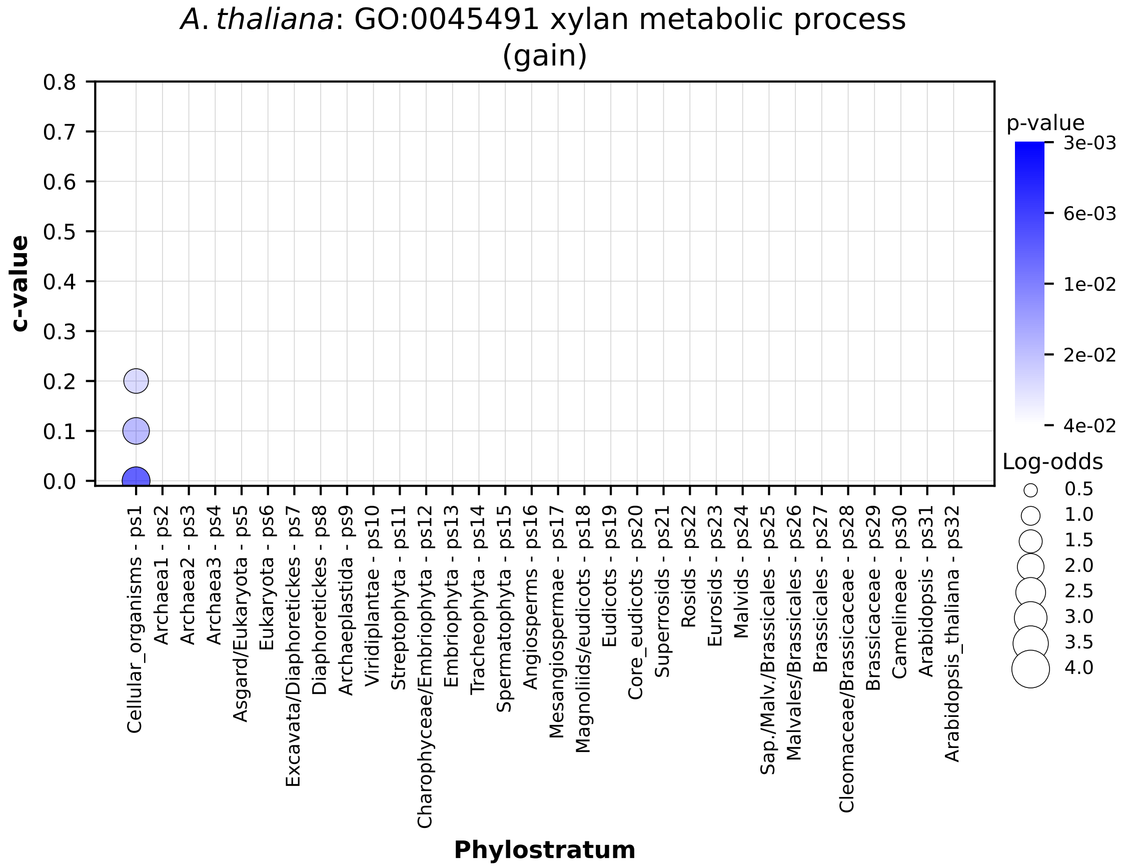

A. thaliana: GO:0045491 xylan metabolic process
(gain)
c-value
0.0
0.1
0.2
0.3
0.4
0.5
0.6
0.7
0.8
p-value
3e-03
6e-03
1e-02
2e-02
4e-02
Log-odds
0.5
1.0
1.5
2.0
2.5
3.0
3.5
4.0
Cellular_organisms - ps1
Archaea1 - ps2
Archaea2 - ps3
Archaea3 - ps4
Asgard/Eukaryota - ps5
Eukaryota - ps6
Excavata/Diaphoretickes - ps7
Diaphoretickes - ps8
Archaeplastida - ps9
Viridiplantae - ps10
Streptophyta - ps11
Charophyceae/Embriophyta - ps12
Embriophyta - ps13
Tracheophyta - ps14
Spermatophyta - ps15
Angiosperms - ps16
Mesangiospermae - ps17
Magnoliids/eudicots - ps18
Eudicots - ps19
Core_eudicots - ps20
Superrosids - ps21
Rosids - ps22
Eurosids - ps23
Malvids - ps24
Sap./Malv./Brassicales - ps25
Malvales/Brassicales - ps26
Brassicales - ps27
Cleomaceae/Brassicaceae - ps28
Brassicaceae - ps29
Camelineae - ps30
Arabidopsis - ps31
Arabidopsis_thaliana - ps32
Phylostratum

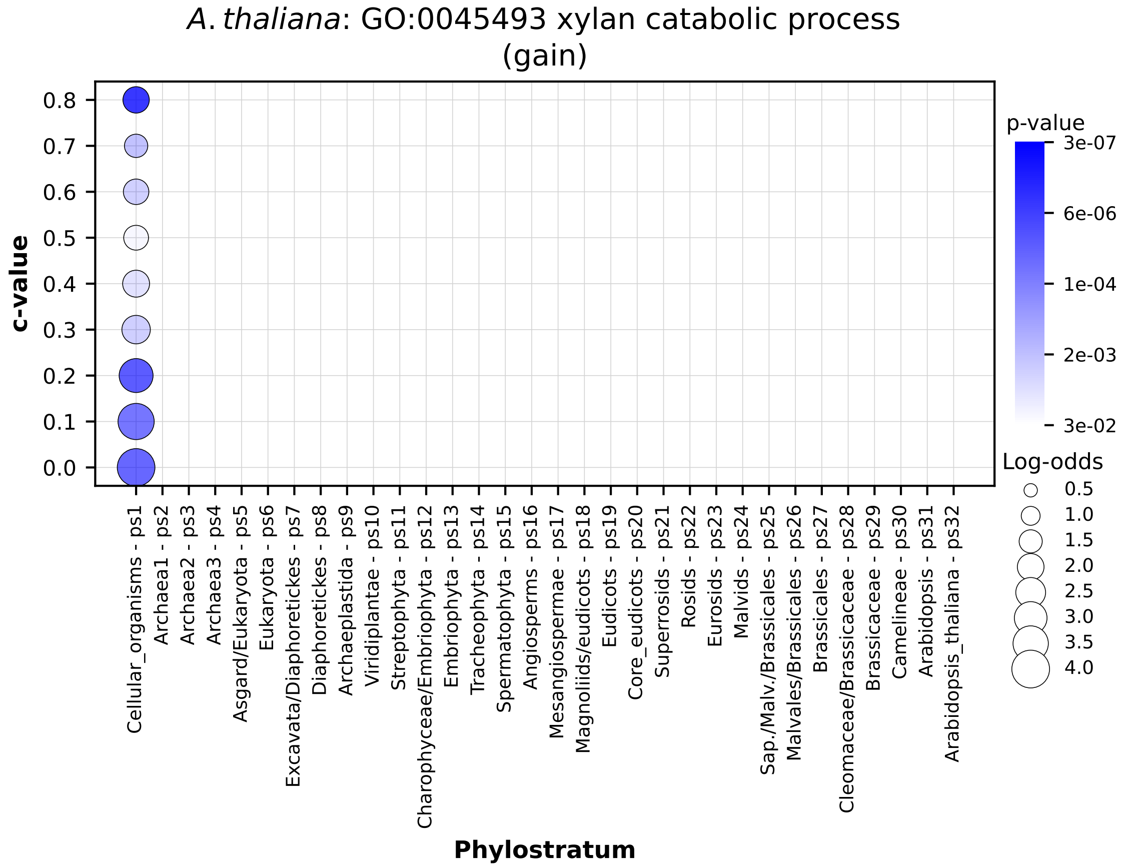

A. thaliana: GO:0045493 xylan catabolic process
(gain)
c-value
0.8
0.7
0.6
0.5
0.4
0.3
0.2
0.1
0.0
p-value
3e-07
6e-06
1e-04
2e-03
3e-02
Log-odds
0.5
1.0
1.5
2.0
2.5
3.0
3.5
4.0
Cellular_organisms - ps1
Archaea1 - ps2
Archaea2 - ps3
Archaea3 - ps4
Asgard/Eukaryota - ps5
Eukaryota - ps6
Excavata/Diaphoretickes - ps7
Diaphoretickes - ps8
Archaeplastida - ps9
Viridiplantae - ps10
Streptophyta - ps11
Charophyceae/Embriophyta - ps12
Embriophyta - ps13
Tracheophyta - ps14
Spermatophyta - ps15
Angiosperms - ps16
Mesangiospermae - ps17
Magnoliids/eudicots - ps18
Eudicots - ps19
Core_eudicots - ps20
Superrosids - ps21
Rosids - ps22
Eurosids - ps23
Malvids - ps24
Sap./Malv./Brassicales - ps25
Malvales/Brassicales - ps26
Brassicales - ps27
Cleomaceae/Brassicaceae - ps28
Brassicaceae - ps29
Camelineae - ps30
Arabidopsis - ps31
Arabidopsis_thaliana - ps32
Phylostratum

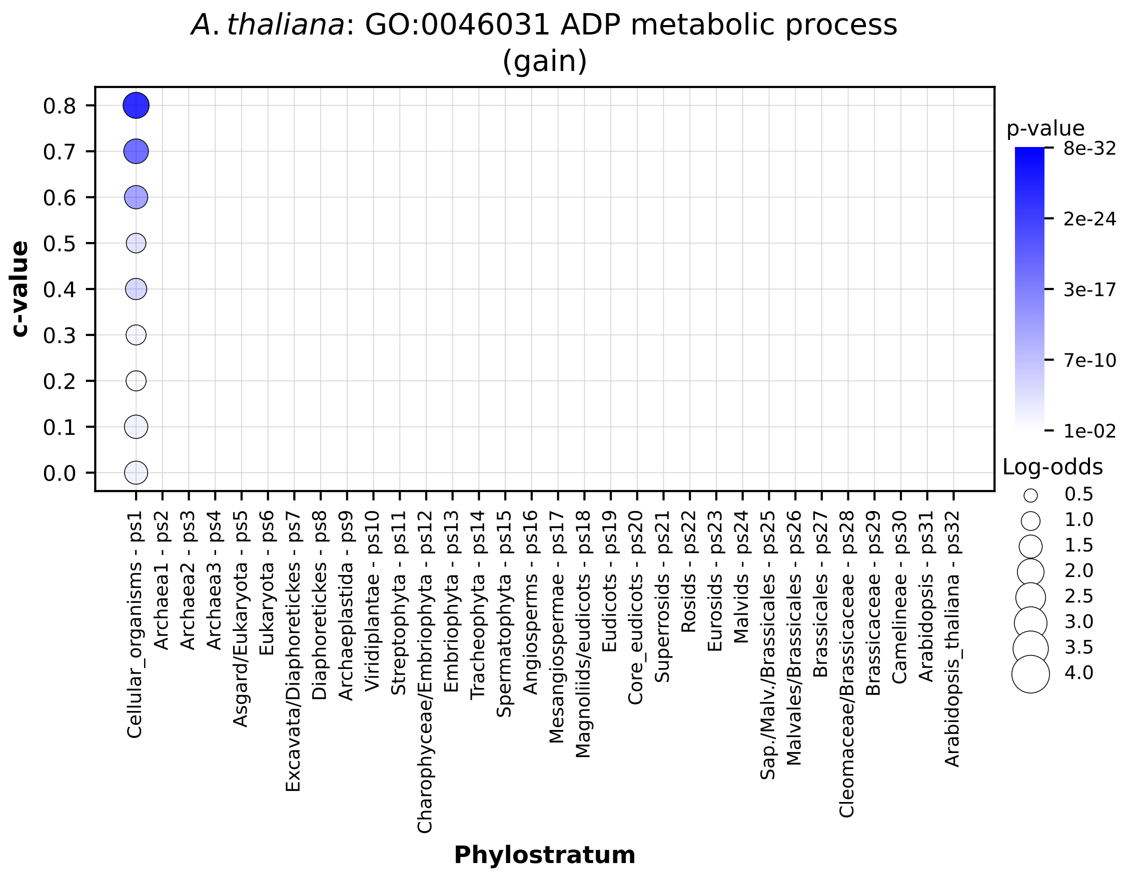
A. thaliana: GO:0046031 ADP metabolic process
(gain)
c-value
0.8
0.7
0.6
0.5
0.4
0.3
0.2
0.1
0.0
p-value
8e-32
2e-24
3e-17
7e-10
1e-02
Log-odds
0.5
1.0
1.5
2.0
2.5
3.0
3.5
4.0
Cellular_organisms - ps1
Archaea1 - ps2
Archaea2 - ps3
Archaea3 - ps4
Asgard/Eukaryota - ps5
Eukaryota - ps6
Excavata/Diaphoretickes - ps7
Diaphoretickes - ps8
Archaeplastida - ps9
Viridiplantae - ps10
Streptophyta - ps11
Charophyceae/Embriophyta - ps12
Embriophyta - ps13
Tracheophyta - ps14
Spermatophyta - ps15
Angiosperms - ps16
Mesangiospermae - ps17
Magnoliids/eudicots - ps18
Eudicots - ps19
Core_eudicots - ps20
Superrosids - ps21
Rosids - ps22
Eurosids - ps23
Malvids - ps24
Sap./Malv./Brassicales - ps25
Malvales/Brassicales - ps26
Brassicales - ps27
Cleomaceae/Brassicaceae - ps28
Brassicaceae - ps29
Camelineae - ps30
Arabidopsis - ps31
Arabidopsis_thaliana - ps32
Phylostratum

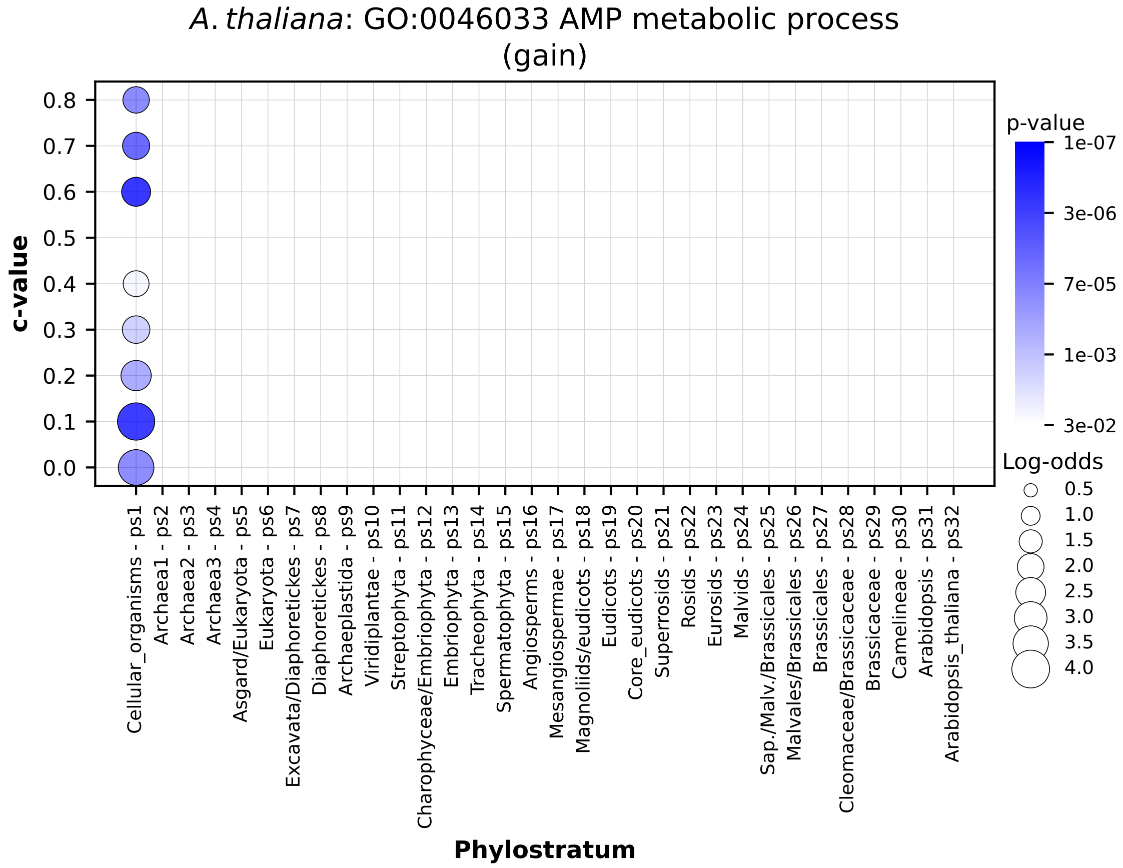

A. thaliana: GO:0046033 AMP metabolic process
(gain)
c-value
0.8
0.7
0.6
0.5
0.4
0.3
0.2
0.1
0.0
p-value
1e-07
3e-06
7e-05
1e-03
3e-02
Log-odds
0.5
1.0
1.5
2.0
2.5
3.0
3.5
4.0
Cellular_organisms - ps1
Archaea1 - ps2
Archaea2 - ps3
Archaea3 - ps4
Asgard/Eukaryota - ps5
Eukaryota - ps6
Excavata/Diaphoretickes - ps7
Diaphoretickes - ps8
Archaeplastida - ps9
Viridiplantae - ps10
Streptophyta - ps11
Charophyceae/Embriophyta - ps12
Embriophyta - ps13
Tracheophyta - ps14
Spermatophyta - ps15
Angiosperms - ps16
Mesangiospermae - ps17
Magnoliids/eudicots - ps18
Eudicots - ps19
Core_eudicots - ps20
Superrosids - ps21
Rosids - ps22
Eurosids - ps23
Malvids - ps24
Sap./Malv./Brassicales - ps25
Malvales/Brassicales - ps26
Brassicales - ps27
Cleomaceae/Brassicaceae - ps28
Brassicaceae - ps29
Camelineae - ps30
Arabidopsis - ps31
Arabidopsis_thaliana - ps32
Phylostratum

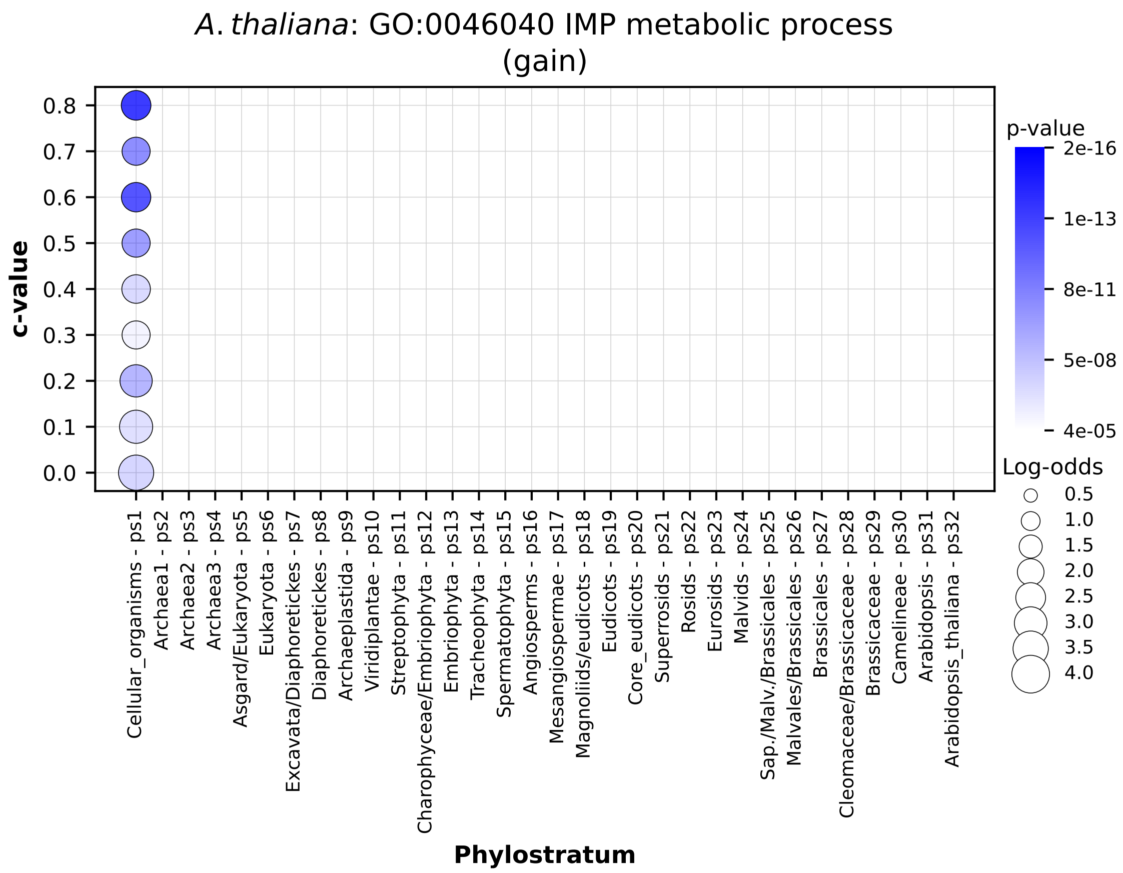
A. thaliana: GO:0046040 IMP metabolic process (gain)
c-value
0.0
0.1
0.2
0.3
0.4
0.5
0.6
0.7
0.8
Phylostratum
Cellular_organisms - ps1
Archaea1 - ps2
Archaea2 - ps3
Archaea3 - ps4
Asgard/Eukaryota - ps5
Eukaryota - ps6
Excavata/Diaphoretickes - ps7
Diaphoretickes - ps8
Archaeplastida - ps9
Viridiplantae - ps10
Streptophyta - ps11
Charophyceae/Embriophyta - ps12
Embriophyta - ps13
Tracheophyta - ps14
Spermatophyta - ps15
Angiosperms - ps16
Mesangiospermae - ps17
Magnoliids/eudicots - ps18
Eudicots - ps19
Core_eudicots - ps20
Superrosids - ps21
Rosids - ps22
Eurosids - ps23
Malvids - ps24
Sap./Malv./Brassicales - ps25
Malvales/Brassicales - ps26
Brassicales - ps27
Cleomaceae/Brassicaceae - ps28
Brassicaceae - ps29
Camelineae - ps30
Arabidopsis - ps31
Arabidopsis_thaliana - ps32
p-value
2e-16
1e-13
8e-11
5e-08
4e-05
Log-odds
0.5
1.0
1.5
2.0
2.5
3.0
3.5
4.0

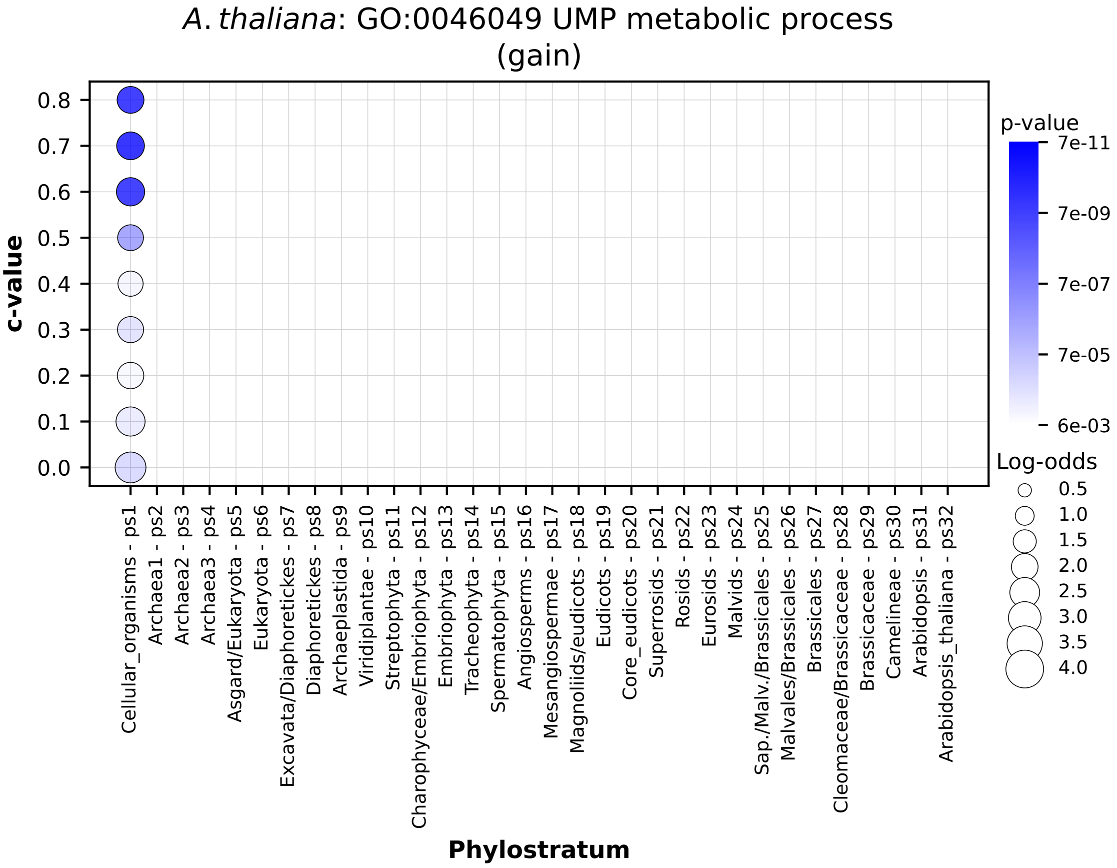

A. thaliana: GO:0046049 UMP metabolic process
(gain)
c-value
0.8
0.7
0.6
0.5
0.4
0.3
0.2
0.1
0.0
p-value
7e-11
7e-09
7e-07
7e-05
6e-03
Log-odds
0.5
1.0
1.5
2.0
2.5
3.0
3.5
4.0
Cellular_organisms - ps1
Archaea1 - ps2
Archaea2 - ps3
Archaea3 - ps4
Asgard/Eukaryota - ps5
Eukaryota - ps6
Excavata/Diaphoretickes - ps7
Diaphoretickes - ps8
Archaeplastida - ps9
Viridiplantae - ps10
Streptophyta - ps11
Charophyceae/Embriophyta - ps12
Embriophyta - ps13
Tracheophyta - ps14
Spermatophyta - ps15
Angiosperms - ps16
Mesangiospermae - ps17
Magnoliids/eudicots - ps18
Eudicots - ps19
Core_eudicots - ps20
Superrosids - ps21
Rosids - ps22
Eurosids - ps23
Malvids - ps24
Sap./Malv./Brassicales - ps25
Malvales/Brassicales - ps26
Brassicales - ps27
Cleomaceae/Brassicaceae - ps28
Brassicaceae - ps29
Camelineae - ps30
Arabidopsis - ps31
Arabidopsis_thaliana - ps32
Phylostratum

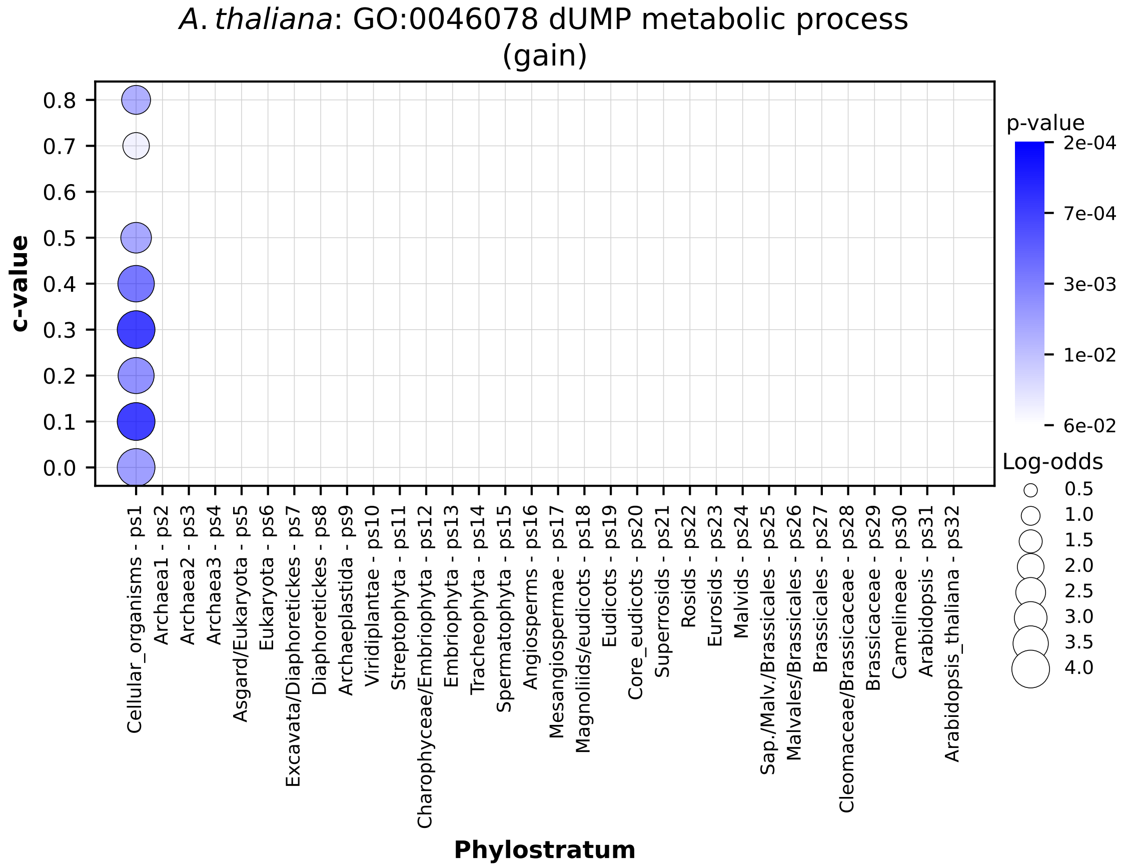

A. thaliana: GO:0046078 dUMP metabolic process
(gain)
c-value
0.8
0.7
0.6
0.5
0.4
0.3
0.2
0.1
0.0
p-value
2e-04
7e-04
3e-03
1e-02
6e-02
Log-odds
0.5
1.0
1.5
2.0
2.5
3.0
3.5
4.0
Cellular_organisms - ps1
Archaea1 - ps2
Archaea2 - ps3
Archaea3 - ps4
Asgard/Eukaryota - ps5
Eukaryota - ps6
Excavata/Diaphoretickes - ps7
Diaphoretickes - ps8
Archaeplastida - ps9
Viridiplantae - ps10
Streptophyta - ps11
Charophyceae/Embriophyta - ps12
Embriophyta - ps13
Tracheophyta - ps14
Spermatophyta - ps15
Angiosperms - ps16
Mesangiospermae - ps17
Magnoliids/eudicots - ps18
Eudicots - ps19
Core_eudicots - ps20
Superrosids - ps21
Rosids - ps22
Eurosids - ps23
Malvids - ps24
Sap./Malv./Brassicales - ps25
Malvales/Brassicales - ps26
Brassicales - ps27
Cleomaceae/Brassicaceae - ps28
Brassicaceae - ps29
Camelineae - ps30
Arabidopsis - ps31
Arabidopsis_thaliana - ps32
Phylostratum

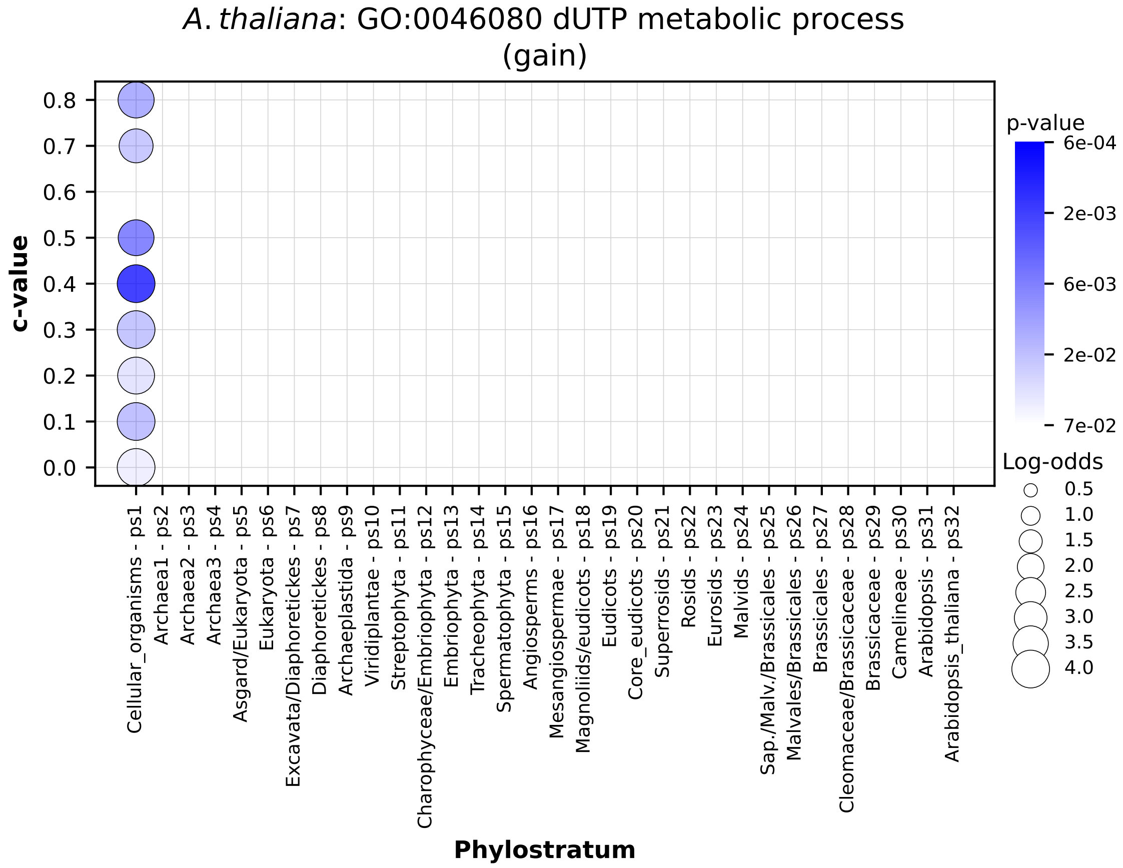
A. thaliana: GO:0046080 dUTP metabolic process
(gain)
c-value
0.8
0.7
0.6
0.5
0.4
0.3
0.2
0.1
0.0
Phylostratum
Cellular_organisms - ps1
Archaea1 - ps2
Archaea2 - ps3
Archaea3 - ps4
Asgard/Eukaryota - ps5
Eukaryota - ps6
Excavata/Diaphoretickes - ps7
Diaphoretickes - ps8
Archaeplastida - ps9
Viridiplantae - ps10
Streptophyta - ps11
Charophyceae/Embriophyta - ps12
Embriophyta - ps13
Tracheophyta - ps14
Spermatophyta - ps15
Angiosperms - ps16
Mesangiospermae - ps17
Magnoliids/eudicots - ps18
Eudicots - ps19
Core_eudicots - ps20
Superrosids - ps21
Rosids - ps22
Eurosids - ps23
Malvids - ps24
Sap./Malv./Brassicales - ps25
Malvales/Brassicales - ps26
Brassicales - ps27
Cleomaceae/Brassicaceae - ps28
Brassicaceae - ps29
Camelineae - ps30
Arabidopsis - ps31
Arabidopsis_thaliana - ps32
p-value
6e-04
2e-03
6e-03
2e-02
7e-02
Log-odds
0.5
1.0
1.5
2.0
2.5
3.0
3.5
4.0

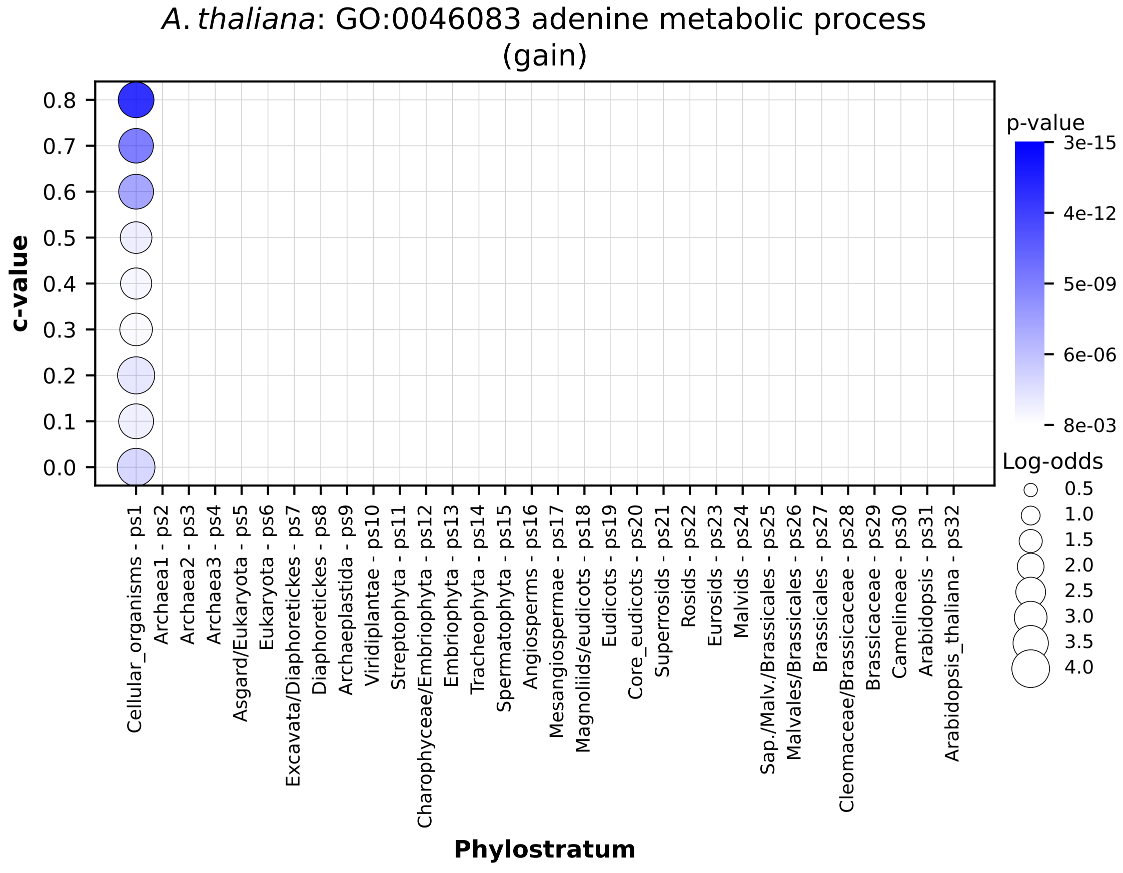

A. thaliana: GO:0046083 adenine metabolic process
(gain)
c-value
0.8
0.7
0.6
0.5
0.4
0.3
0.2
0.1
0.0
p-value
3e-15
4e-12
5e-09
6e-06
8e-03
Log-odds
0.5
1.0
1.5
2.0
2.5
3.0
3.5
4.0
Cellular_organisms - ps1
Archaea1 - ps2
Archaea2 - ps3
Archaea3 - ps4
Asgard/Eukaryota - ps5
Eukaryota - ps6
Excavata/Diaphoretickes - ps7
Diaphoretickes - ps8
Archaeplastida - ps9
Viridiplantae - ps10
Streptophyta - ps11
Charophyceae/Embriophyta - ps12
Embriophyta - ps13
Tracheophyta - ps14
Spermatophyta - ps15
Angiosperms - ps16
Mesangiospermae - ps17
Magnoliids/eudicots - ps18
Eudicots - ps19
Core_eudicots - ps20
Superrosids - ps21
Rosids - ps22
Eurosids - ps23
Malvids - ps24
Sap./Malv./Brassicales - ps25
Malvales/Brassicales - ps26
Brassicales - ps27
Cleomaceae/Brassicaceae - ps28
Brassicaceae - ps29
Camelineae - ps30
Arabidopsis - ps31
Arabidopsis_thaliana - ps32
Phylostratum

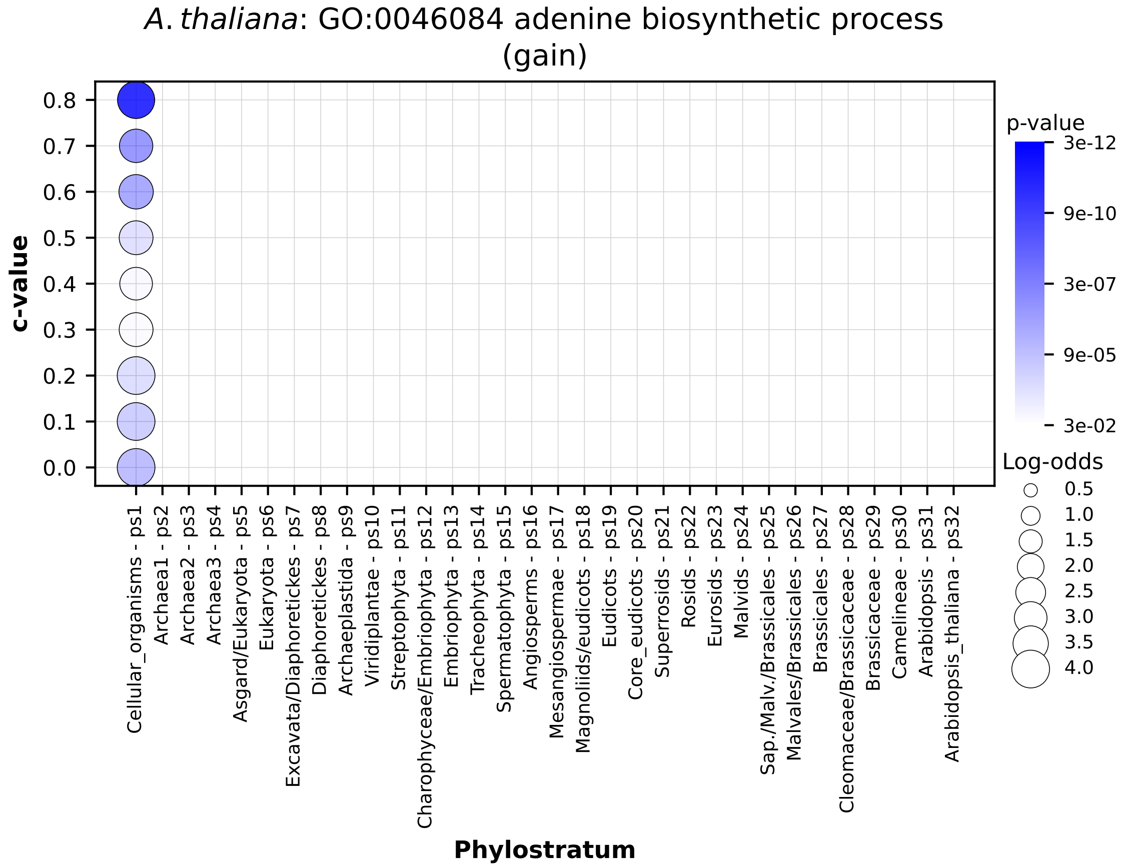

A. thaliana: GO:0046084 adenine biosynthetic process
(gain)
c-value
0.8
0.7
0.6
0.5
0.4
0.3
0.2
0.1
0.0
p-value
3e-12
9e-10
3e-07
9e-05
3e-02
Log-odds
0.5
1.0
1.5
2.0
2.5
3.0
3.5
4.0
Cellular_organisms - ps1
Archaea1 - ps2
Archaea2 - ps3
Archaea3 - ps4
Asgard/Eukaryota - ps5
Eukaryota - ps6
Excavata/Diaphoretickes - ps7
Diaphoretickes - ps8
Archaeplastida - ps9
Viridiplantae - ps10
Streptophyta - ps11
Charophyceae/Embriophyta - ps12
Embriophyta - ps13
Tracheophyta - ps14
Spermatophyta - ps15
Angiosperms - ps16
Mesangiospermae - ps17
Magnoliids/eudicots - ps18
Eudicots - ps19
Core_eudicots - ps20
Superrosids - ps21
Rosids - ps22
Eurosids - ps23
Malvids - ps24
Sap./Malv./Brassicales - ps25
Malvales/Brassicales - ps26
Brassicales - ps27
Cleomaceae/Brassicaceae - ps28
Brassicaceae - ps29
Camelineae - ps30
Arabidopsis - ps31
Arabidopsis_thaliana - ps32
Phylostratum

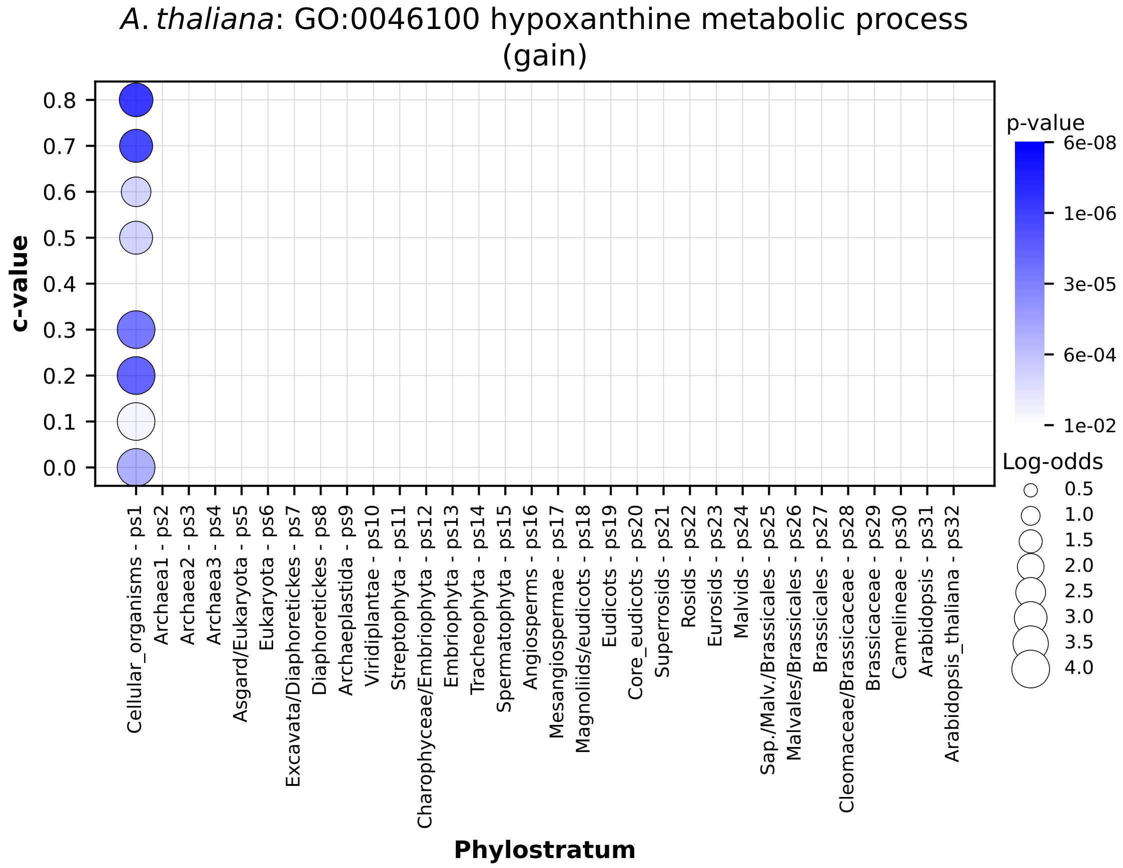

A. thaliana: GO:0046100 hypoxanthine metabolic process
(gain)
c-value
0.8
0.7
0.6
0.5
0.4
0.3
0.2
0.1
0.0
p-value
6e-08
1e-06
3e-05
6e-04
1e-02
Log-odds
0.5
1.0
1.5
2.0
2.5
3.0
3.5
4.0
Cellular_organisms - ps1
Archaea1 - ps2
Archaea2 - ps3
Archaea3 - ps4
Asgard/Eukaryota - ps5
Eukaryota - ps6
Excavata/Diaphoretickes - ps7
Diaphoretickes - ps8
Archaeplastida - ps9
Viridiplantae - ps10
Streptophyta - ps11
Charophyceae/Embriophyta - ps12
Embriophyta - ps13
Tracheophyta - ps14
Spermatophyta - ps15
Angiosperms - ps16
Mesangiospermae - ps17
Magnoliids/eudicots - ps18
Eudicots - ps19
Core_eudicots - ps20
Superrosids - ps21
Rosids - ps22
Eurosids - ps23
Malvids - ps24
Sap./Malv./Brassicales - ps25
Malvales/Brassicales - ps26
Brassicales - ps27
Cleomaceae/Brassicaceae - ps28
Brassicaceae - ps29
Camelineae - ps30
Arabidopsis - ps31
Arabidopsis_thaliana - ps32
Phylostratum

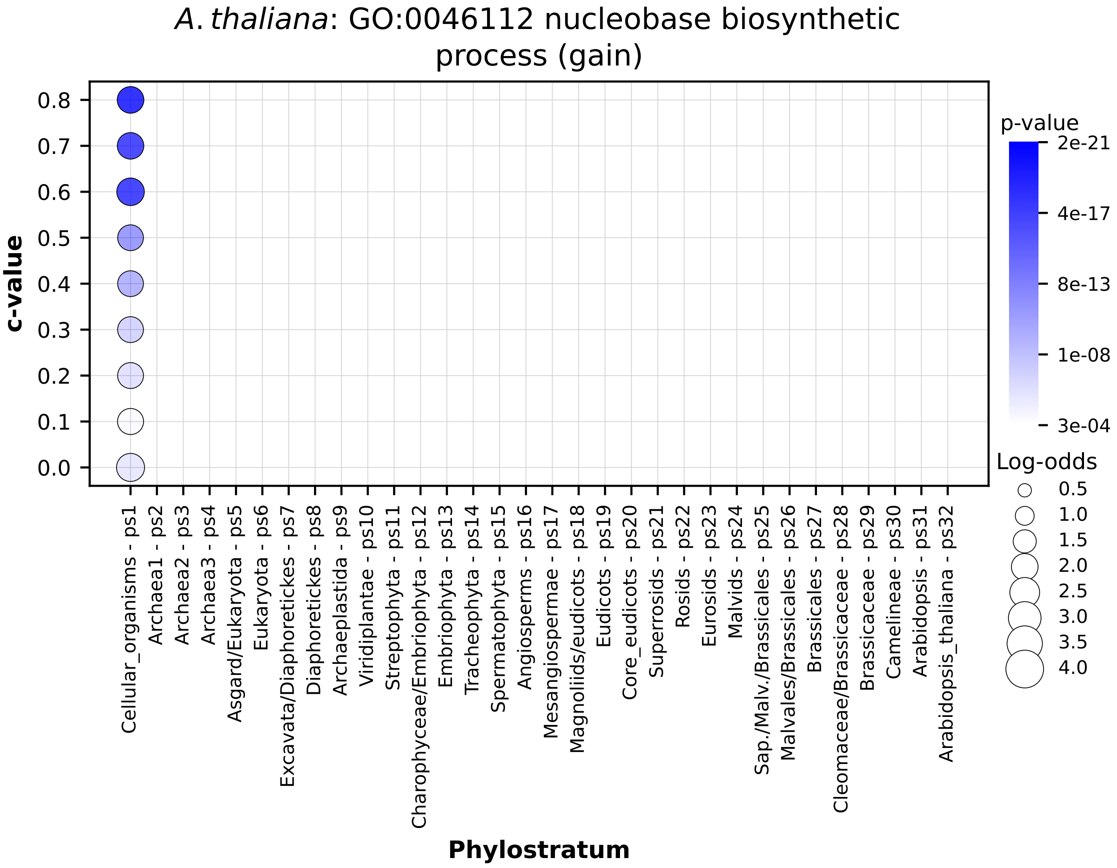

A. thaliana: GO:0046112 nucleobase biosynthetic process (gain)
c-value
0.8
0.7
0.6
0.5
0.4
0.3
0.2
0.1
0.0
p-value
2e-21
4e-17
8e-13
1e-08
3e-04
Log-odds
0.5
1.0
1.5
2.0
2.5
3.0
3.5
4.0
Cellular_organisms - ps1
Archaea1 - ps2
Archaea2 - ps3
Archaea3 - ps4
Asgard/Eukaryota - ps5
Eukaryota - ps6
Excavata/Diaphoretickes - ps7
Diaphoretickes - ps8
Archaeplastida - ps9
Viridiplantae - ps10
Streptophyta - ps11
Charophyceae/Embriophyta - ps12
Embriophyta - ps13
Tracheophyta - ps14
Spermatophyta - ps15
Angiosperms - ps16
Mesangiospermae - ps17
Magnoliids/eudicots - ps18
Eudicots - ps19
Core_eudicots - ps20
Superrosids - ps21
Rosids - ps22
Eurosids - ps23
Malvids - ps24
Sap./Malv./Brassicales - ps25
Malvales/Brassicales - ps26
Brassicales - ps27
Cleomaceae/Brassicaceae - ps28
Brassicaceae - ps29
Camelineae - ps30
Arabidopsis - ps31
Arabidopsis_thaliana - ps32
Phylostratum

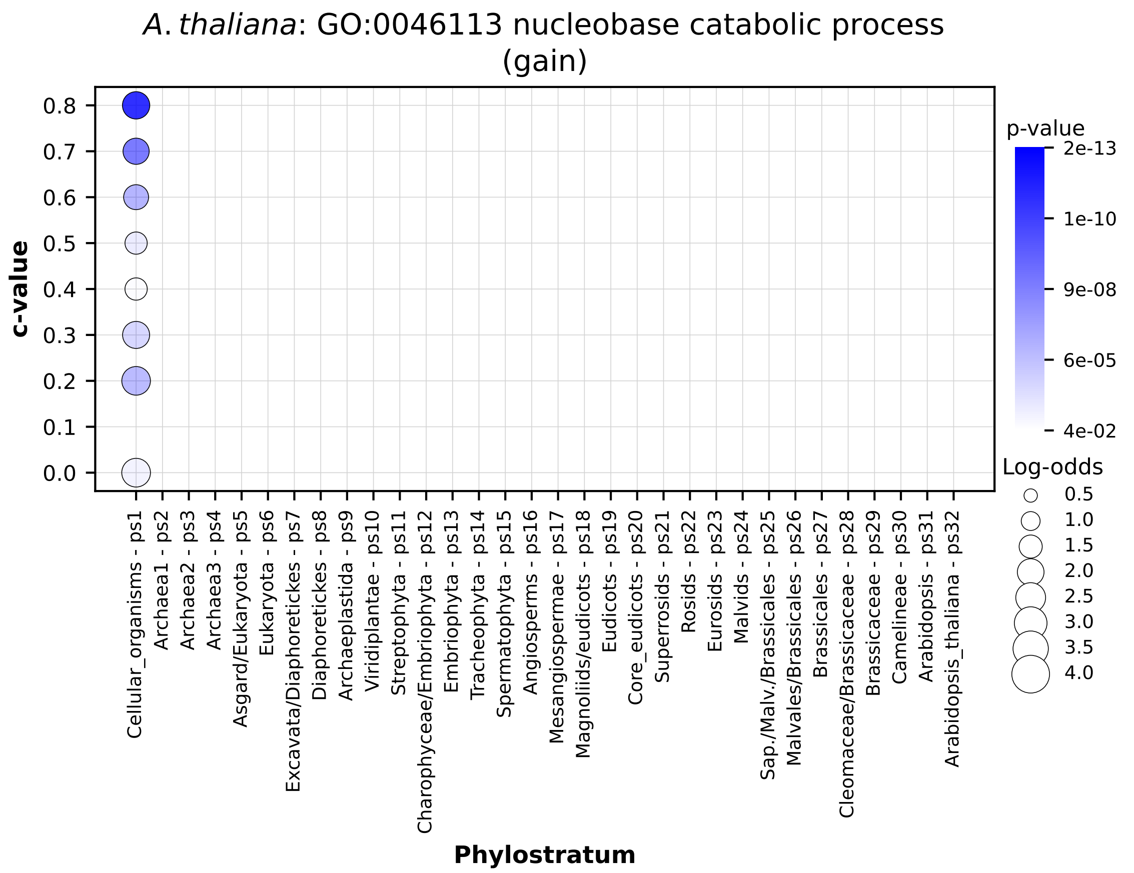

A. thaliana: GO:0046113 nucleobase catabolic process (gain)
c-value
0.0
0.1
0.2
0.3
0.4
0.5
0.6
0.7
0.8
p-value
2e-13
1e-10
9e-08
6e-05
4e-02
Log-odds
0.5
1.0
1.5
2.0
2.5
3.0
3.5
4.0
Cellular_organisms - ps1
Archaea1 - ps2
Archaea2 - ps3
Archaea3 - ps4
Asgard/Eukaryota - ps5
Eukaryota - ps6
Excavata/Diaphoretickes - ps7
Diaphoretickes - ps8
Archaeplastida - ps9
Viridiplantae - ps10
Streptophyta - ps11
Charophyceae/Embriophyta - ps12
Embriophyta - ps13
Tracheophyta - ps14
Spermatophyta - ps15
Angiosperms - ps16
Mesangiospermae - ps17
Magnoliids/eudicots - ps18
Eudicots - ps19
Core_eudicots - ps20
Superrosids - ps21
Rosids - ps22
Eurosids - ps23
Malvids - ps24
Sap./Malv./Brassicales - ps25
Malvales/Brassicales - ps26
Brassicales - ps27
Cleomaceae/Brassicaceae - ps28
Brassicaceae - ps29
Camelineae - ps30
Arabidopsis - ps31
Arabidopsis_thaliana - ps32
Phylostratum

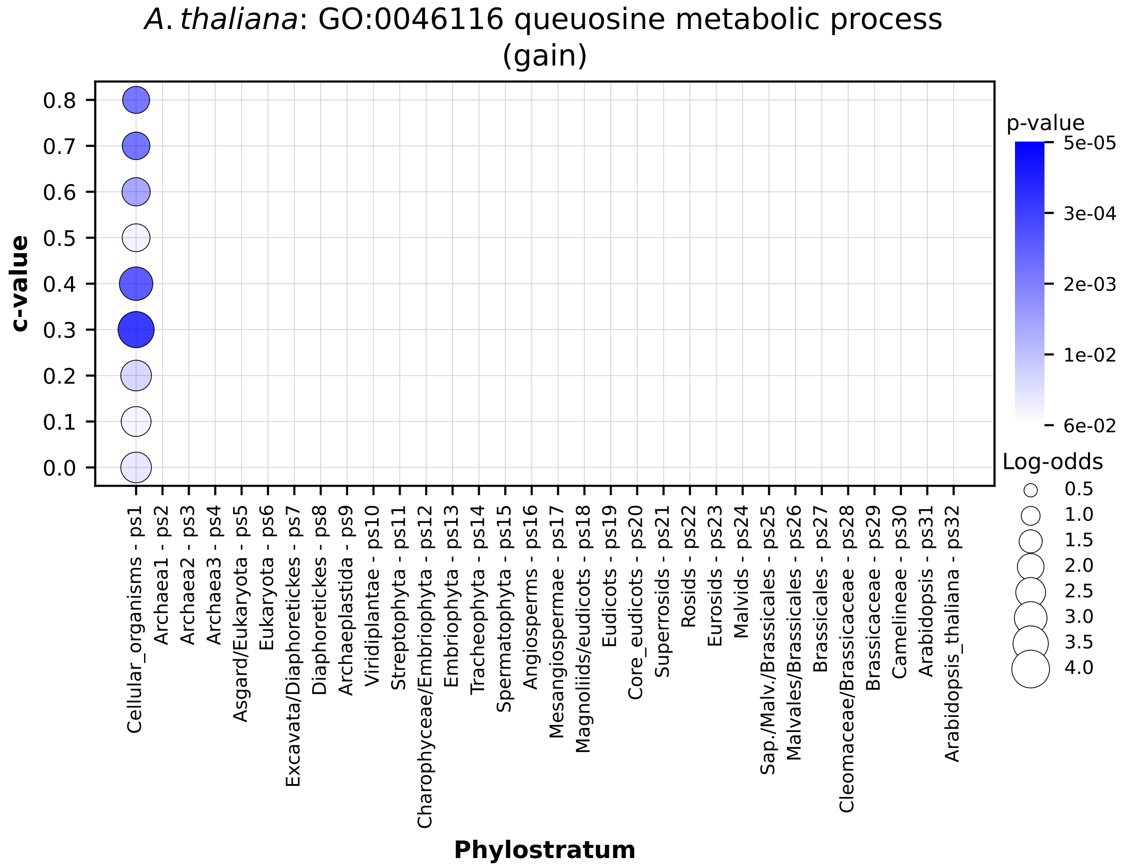

A. thaliana: GO:0046116 queuosine metabolic process (gain)
c-value
0.8
0.7
0.6
0.5
0.4
0.3
0.2
0.1
0.0
Phylostratum
Cellular_organisms - ps1
Archaea1 - ps2
Archaea2 - ps3
Archaea3 - ps4
Asgard/Eukaryota - ps5
Eukaryota - ps6
Excavata/Diaphoretickes - ps7
Diaphoretickes - ps8
Archaeplastida - ps9
Viridiplantae - ps10
Streptophyta - ps11
Charophyceae/Embriophyta - ps12
Embriophyta - ps13
Tracheophyta - ps14
Spermatophyta - ps15
Angiosperms - ps16
Mesangiospermae - ps17
Magnoliids/eudicots - ps18
Eudicots - ps19
Core_eudicots - ps20
Superrosids - ps21
Rosids - ps22
Eurosids - ps23
Malvids - ps24
Sap./Malv./Brassicales - ps25
Malvales/Brassicales - ps26
Brassicales - ps27
Cleomaceae/Brassicaceae - ps28
Brassicaceae - ps29
Camelineae - ps30
Arabidopsis - ps31
Arabidopsis_thaliana - ps32
p-value
5e-05
3e-04
2e-03
1e-02
6e-02
Log-odds
0.5
1.0
1.5
2.0
2.5
3.0
3.5
4.0

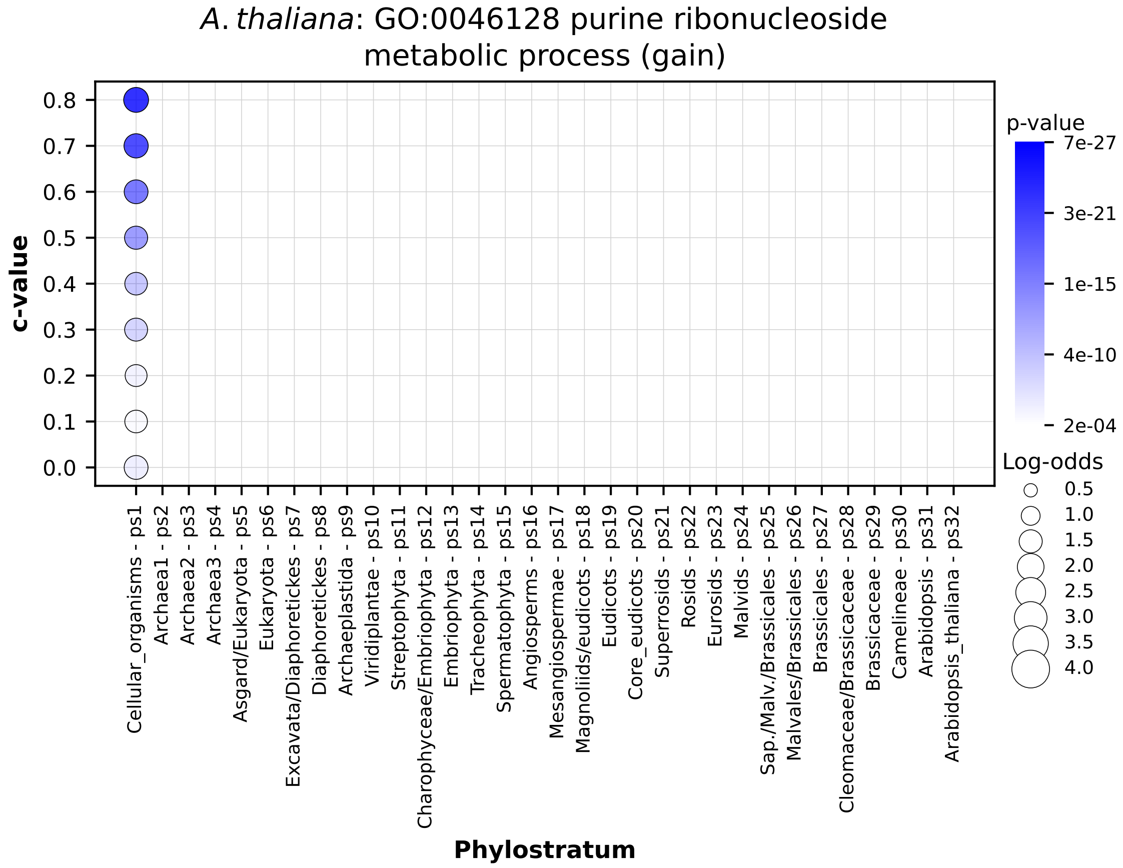

A. thaliana: GO:0046128 purine ribonucleoside metabolic process (gain)
c-value
0.8
0.7
0.6
0.5
0.4
0.3
0.2
0.1
0.0
p-value
7e-27
3e-21
1e-15
4e-10
2e-04
Log-odds
0.5
1.0
1.5
2.0
2.5
3.0
3.5
4.0
Cellular_organisms - ps1
Archaea1 - ps2
Archaea2 - ps3
Archaea3 - ps4
Asgard/Eukaryota - ps5
Eukaryota - ps6
Excavata/Diaphoretickes - ps7
Diaphoretickes - ps8
Archaeplastida - ps9
Viridiplantae - ps10
Streptophyta - ps11
Charophyceae/Embriophyta - ps12
Embriophyta - ps13
Tracheophyta - ps14
Spermatophyta - ps15
Angiosperms - ps16
Mesangiospermae - ps17
Magnoliids/eudicots - ps18
Eudicots - ps19
Core_eudicots - ps20
Superrosids - ps21
Rosids - ps22
Eurosids - ps23
Malvids - ps24
Sap./Malv./Brassicales - ps25
Malvales/Brassicales - ps26
Brassicales - ps27
Cleomaceae/Brassicaceae - ps28
Brassicaceae - ps29
Camelineae - ps30
Arabidopsis - ps31
Arabidopsis_thaliana - ps32
Phylostratum

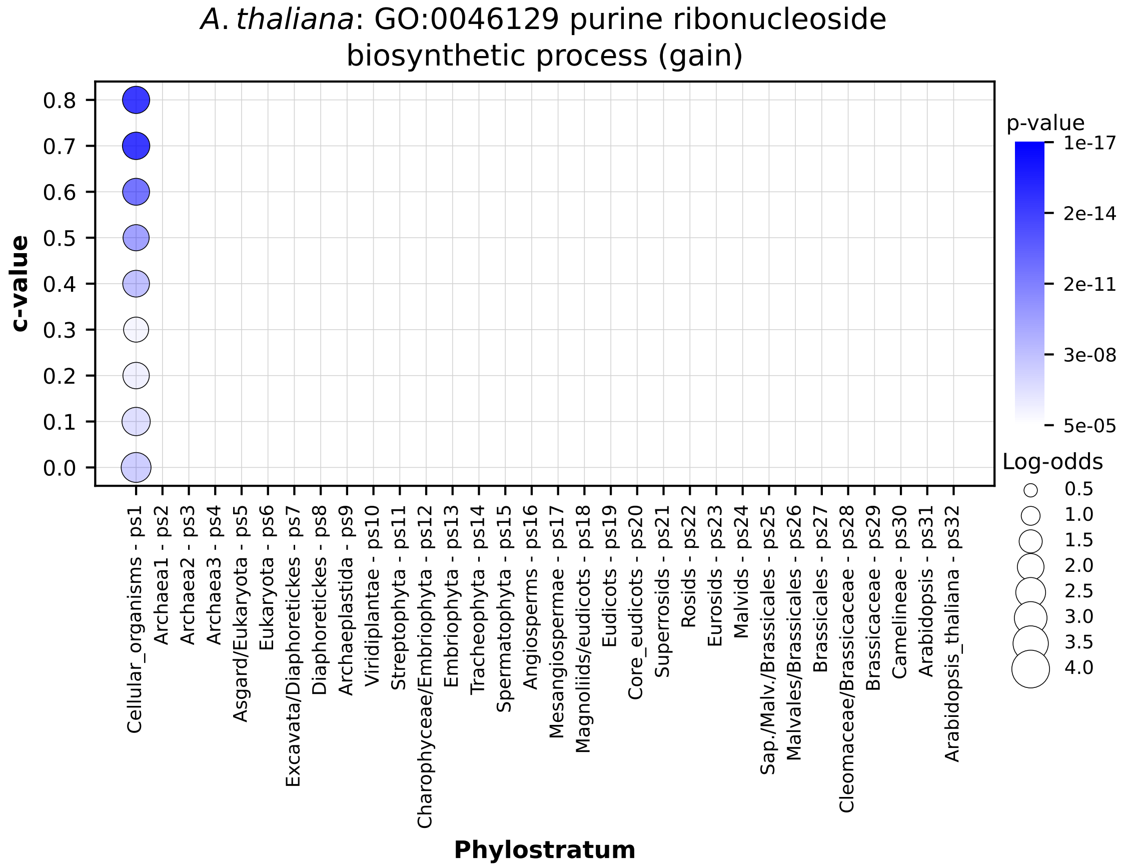

A. thaliana: GO:0046129 purine ribonucleoside biosynthetic process (gain)
c-value
0.8
0.7
0.6
0.5
0.4
0.3
0.2
0.1
0.0
p-value
1e-17
2e-14
2e-11
3e-08
5e-05
Log-odds
0.5
1.0
1.5
2.0
2.5
3.0
3.5
4.0
Cellular_organisms - ps1
Archaea1 - ps2
Archaea2 - ps3
Archaea3 - ps4
Asgard/Eukaryota - ps5
Eukaryota - ps6
Excavata/Diaphoretickes - ps7
Diaphoretickes - ps8
Archaeplastida - ps9
Viridiplantae - ps10
Streptophyta - ps11
Charophyceae/Embriophyta - ps12
Embriophyta - ps13
Tracheophyta - ps14
Spermatophyta - ps15
Angiosperms - ps16
Mesangiospermae - ps17
Magnoliids/eudicots - ps18
Eudicots - ps19
Core_eudicots - ps20
Superrosids - ps21
Rosids - ps22
Eurosids - ps23
Malvids - ps24
Sap./Malv./Brassicales - ps25
Malvales/Brassicales - ps26
Brassicales - ps27
Cleomaceae/Brassicaceae - ps28
Brassicaceae - ps29
Camelineae - ps30
Arabidopsis - ps31
Arabidopsis_thaliana - ps32
Phylostratum

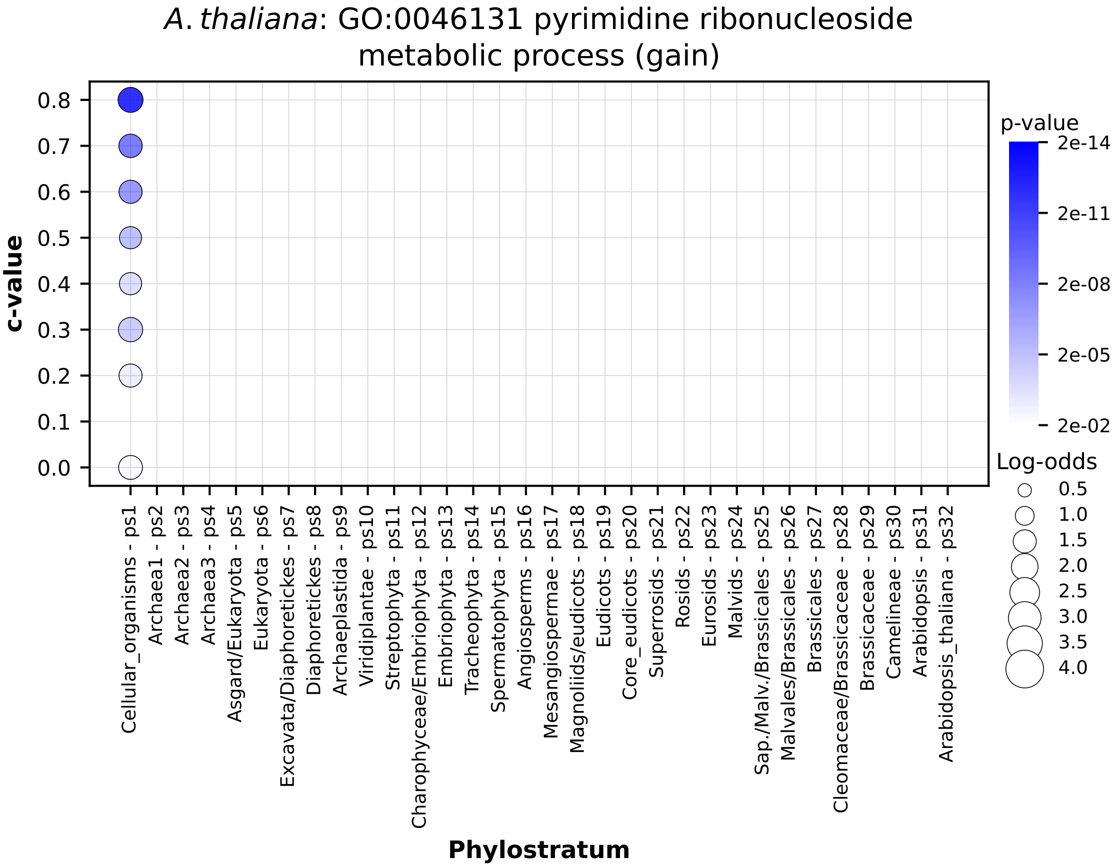
A. thaliana: GO:0046131 pyrimidine ribonucleoside metabolic process (gain)
c-value
0.8
0.7
0.6
0.5
0.4
0.3
0.2
0.1
0.0
p-value
2e-14
2e-11
2e-08
2e-05
2e-02
Log-odds
0.5
1.0
1.5
2.0
2.5
3.0
3.5
4.0
Cellular_organisms - ps1
Archaea1 - ps2
Archaea2 - ps3
Archaea3 - ps4
Asgard/Eukaryota - ps5
Eukaryota - ps6
Excavata/Diaphoretickes - ps7
Diaphoretickes - ps8
Archaeplastida - ps9
Viridiplantae - ps10
Streptophyta - ps11
Charophyceae/Embriophyta - ps12
Embriophyta - ps13
Tracheophyta - ps14
Spermatophyta - ps15
Angiosperms - ps16
Mesangiospermae - ps17
Magnoliids/eudicots - ps18
Eudicots - ps19
Core_eudicots - ps20
Superrosids - ps21
Rosids - ps22
Eurosids - ps23
Malvids - ps24
Sap./Malv./Brassicales - ps25
Malvales/Brassicales - ps26
Brassicales - ps27
Cleomaceae/Brassicaceae - ps28
Brassicaceae - ps29
Camelineae - ps30
Arabidopsis - ps31
Arabidopsis_thaliana - ps32
Phylostratum

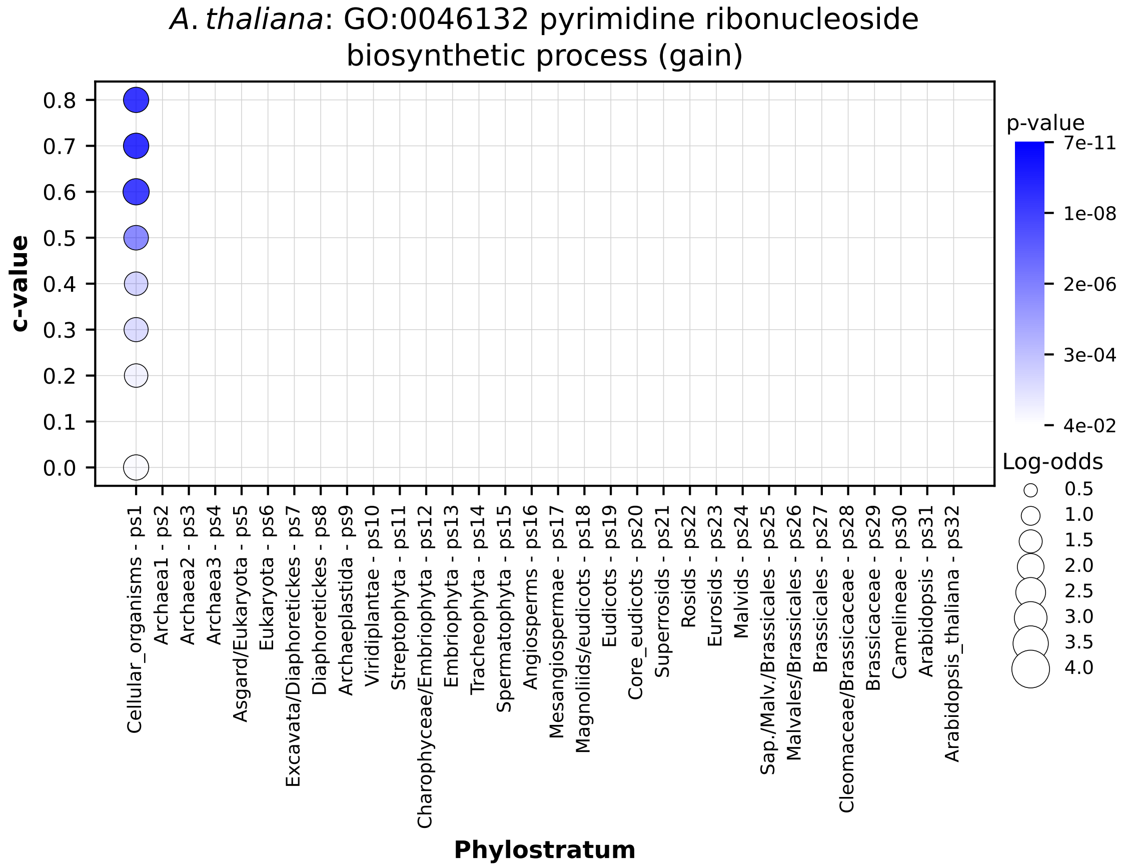

A. thaliana: GO:0046132 pyrimidine ribonucleoside biosynthetic process (gain)
c-value
0.8
0.7
0.6
0.5
0.4
0.3
0.2
0.1
0.0
p-value
7e-11
1e-08
2e-06
3e-04
4e-02
Log-odds
0.5
1.0
1.5
2.0
2.5
3.0
3.5
4.0
Cellular_organisms - ps1
Archaea1 - ps2
Archaea2 - ps3
Archaea3 - ps4
Asgard/Eukaryota - ps5
Eukaryota - ps6
Excavata/Diaphoretickes - ps7
Diaphoretickes - ps8
Archaeplastida - ps9
Viridiplantae - ps10
Streptophyta - ps11
Charophyceae/Embriophyta - ps12
Embriophyta - ps13
Tracheophyta - ps14
Spermatophyta - ps15
Angiosperms - ps16
Mesangiospermae - ps17
Magnoliids/eudicots - ps18
Eudicots - ps19
Core_eudicots - ps20
Superrosids - ps21
Rosids - ps22
Eurosids - ps23
Malvids - ps24
Sap./Malv./Brassicales - ps25
Malvales/Brassicales - ps26
Brassicales - ps27
Cleomaceae/Brassicaceae - ps28
Brassicaceae - ps29
Camelineae - ps30
Arabidopsis - ps31
Arabidopsis_thaliana - ps32
Phylostratum

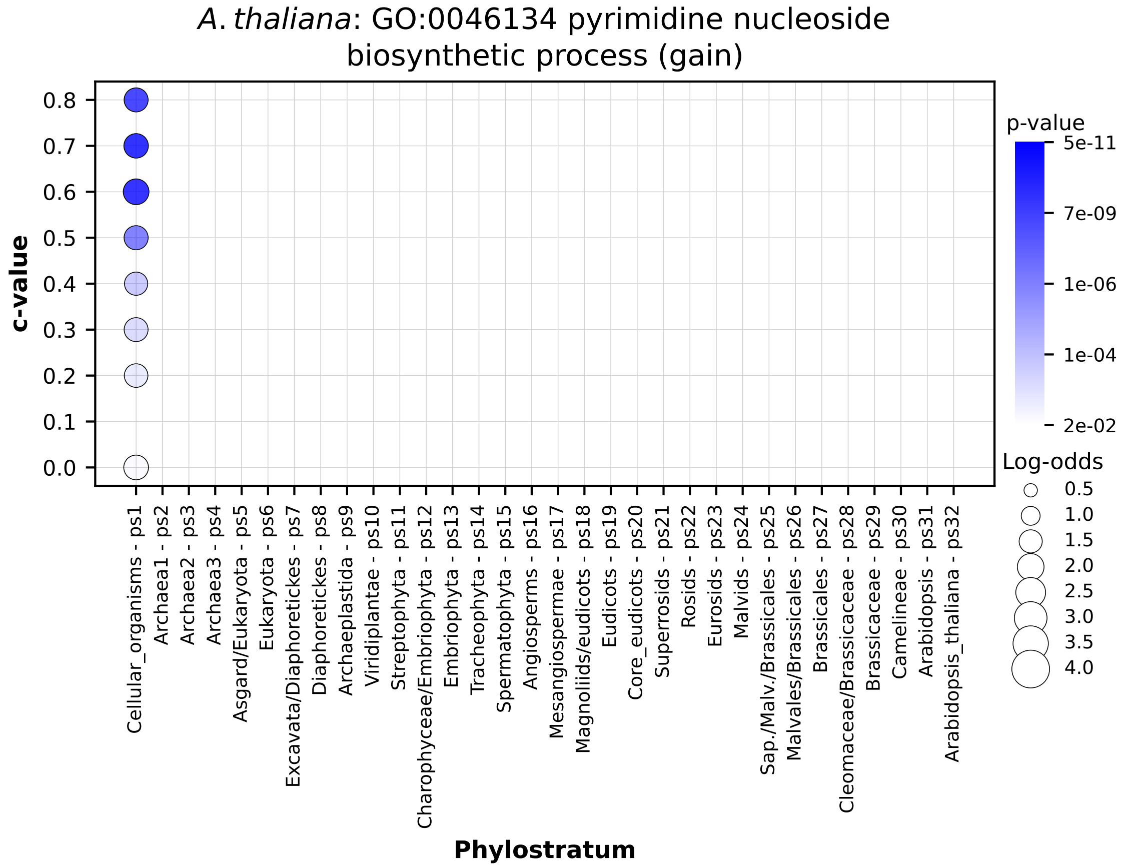
A. thaliana: GO:0046134 pyrimidine nucleoside biosynthetic process (gain)
c-value
0.0
0.1
0.2
0.3
0.4
0.5
0.6
0.7
0.8
Phylostratum
Cellular_organisms - ps1
Archaea1 - ps2
Archaea2 - ps3
Archaea3 - ps4
Asgard/Eukaryota - ps5
Eukaryota - ps6
Excavata/Diaphoretickes - ps7
Diaphoretickes - ps8
Archaeplastida - ps9
Viridiplantae - ps10
Streptophyta - ps11
Charophyceae/Embriophyta - ps12
Embriophyta - ps13
Tracheophyta - ps14
Spermatophyta - ps15
Angiosperms - ps16
Mesangiospermae - ps17
Magnoliids/eudicots - ps18
Eudicots - ps19
Core_eudicots - ps20
Superrosids - ps21
Rosids - ps22
Eurosids - ps23
Malvids - ps24
Sap./Malv./Brassicales - ps25
Malvales/Brassicales - ps26
Brassicales - ps27
Cleomaceae/Brassicaceae - ps28
Brassicaceae - ps29
Camelineae - ps30
Arabidopsis - ps31
Arabidopsis_thaliana - ps32
p-value
5e-11
7e-09
1e-06
1e-04
2e-02
Log-odds
0.5
1.0
1.5
2.0
2.5
3.0
3.5
4.0

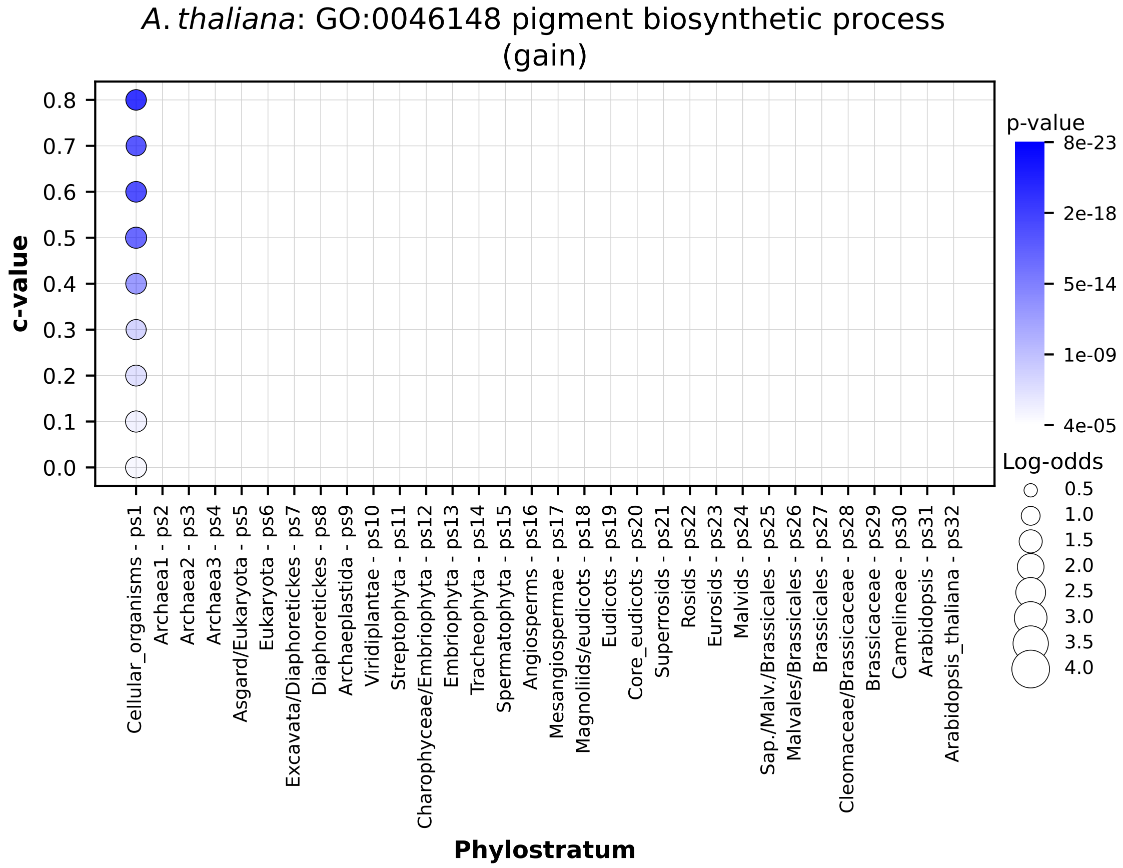
A. thaliana: GO:0046148 pigment biosynthetic process
(gain)
c-value
0.8
0.7
0.6
0.5
0.4
0.3
0.2
0.1
0.0
p-value
8e-23
2e-18
5e-14
1e-09
4e-05
Log-odds
0.5
1.0
1.5
2.0
2.5
3.0
3.5
4.0
Cellular_organisms - ps1
Archaea1 - ps2
Archaea2 - ps3
Archaea3 - ps4
Asgard/Eukaryota - ps5
Eukaryota - ps6
Excavata/Diaphoretickes - ps7
Diaphoretickes - ps8
Archaeplastida - ps9
Viridiplantae - ps10
Streptophyta - ps11
Charophyceae/Embriophyta - ps12
Embriophyta - ps13
Tracheophyta - ps14
Spermatophyta - ps15
Angiosperms - ps16
Mesangiospermae - ps17
Magnoliids/eudicots - ps18
Eudicots - ps19
Core_eudicots - ps20
Superrosids - ps21
Rosids - ps22
Eurosids - ps23
Malvids - ps24
Sap./Malv./Brassicales - ps25
Malvales/Brassicales - ps26
Brassicales - ps27
Cleomaceae/Brassicaceae - ps28
Brassicaceae - ps29
Camelineae - ps30
Arabidopsis - ps31
Arabidopsis_thaliana - ps32
Phylostratum

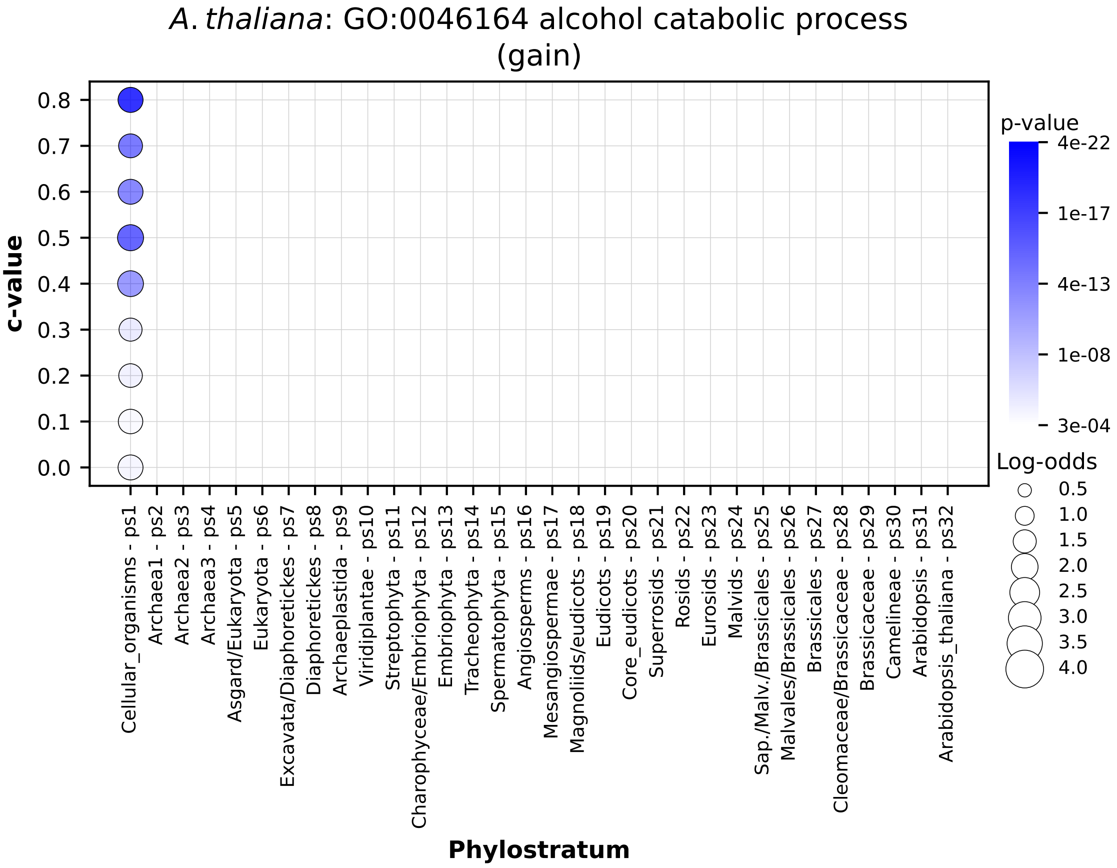

A. thaliana: GO:0046164 alcohol catabolic process
(gain)
c-value
0.8
0.7
0.6
0.5
0.4
0.3
0.2
0.1
0.0
p-value
4e-22
1e-17
4e-13
1e-08
3e-04
Log-odds
0.5
1.0
1.5
2.0
2.5
3.0
3.5
4.0
Cellular_organisms - ps1
Archaea1 - ps2
Archaea2 - ps3
Archaea3 - ps4
Asgard/Eukaryota - ps5
Eukaryota - ps6
Excavata/Diaphoretickes - ps7
Diaphoretickes - ps8
Archaeplastida - ps9
Viridiplantae - ps10
Streptophyta - ps11
Charophyceae/Embriophyta - ps12
Embriophyta - ps13
Tracheophyta - ps14
Spermatophyta - ps15
Angiosperms - ps16
Mesangiospermae - ps17
Magnoliids/eudicots - ps18
Eudicots - ps19
Core_eudicots - ps20
Superrosids - ps21
Rosids - ps22
Eurosids - ps23
Malvids - ps24
Sap./Malv./Brassicales - ps25
Malvales/Brassicales - ps26
Brassicales - ps27
Cleomaceae/Brassicaceae - ps28
Brassicaceae - ps29
Camelineae - ps30
Arabidopsis - ps31
Arabidopsis_thaliana - ps32
Phylostratum

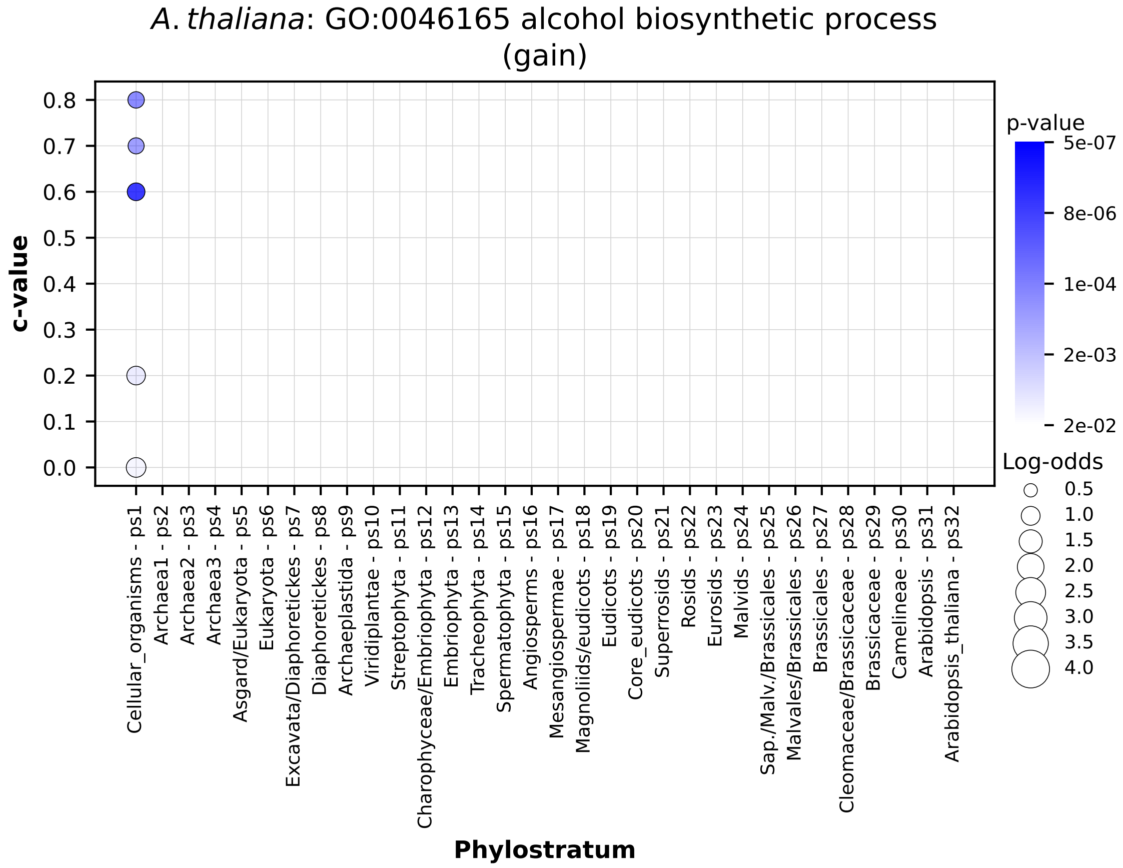
A. thaliana: GO:0046165 alcohol biosynthetic process
(gain)
c-value
0.8
0.7
0.6
0.5
0.4
0.3
0.2
0.1
0.0
Phylostratum
Cellular_organisms - ps1
Archaea1 - ps2
Archaea2 - ps3
Archaea3 - ps4
Asgard/Eukaryota - ps5
Eukaryota - ps6
Excavata/Diaphoretickes - ps7
Diaphoretickes - ps8
Archaeplastida - ps9
Viridiplantae - ps10
Streptophyta - ps11
Charophyceae/Embriophyta - ps12
Embriophyta - ps13
Tracheophyta - ps14
Spermatophyta - ps15
Angiosperms - ps16
Mesangiospermae - ps17
Magnoliids/eudicots - ps18
Eudicots - ps19
Core_eudicots - ps20
Superrosids - ps21
Rosids - ps22
Eurosids - ps23
Malvids - ps24
Sap./Malv./Brassicales - ps25
Malvales/Brassicales - ps26
Brassicales - ps27
Cleomaceae/Brassicaceae - ps28
Brassicaceae - ps29
Camelineae - ps30
Arabidopsis - ps31
Arabidopsis_thaliana - ps32
p-value
5e-07
8e-06
1e-04
2e-03
2e-02
Log-odds
0.5
1.0
1.5
2.0
2.5
3.0
3.5
4.0

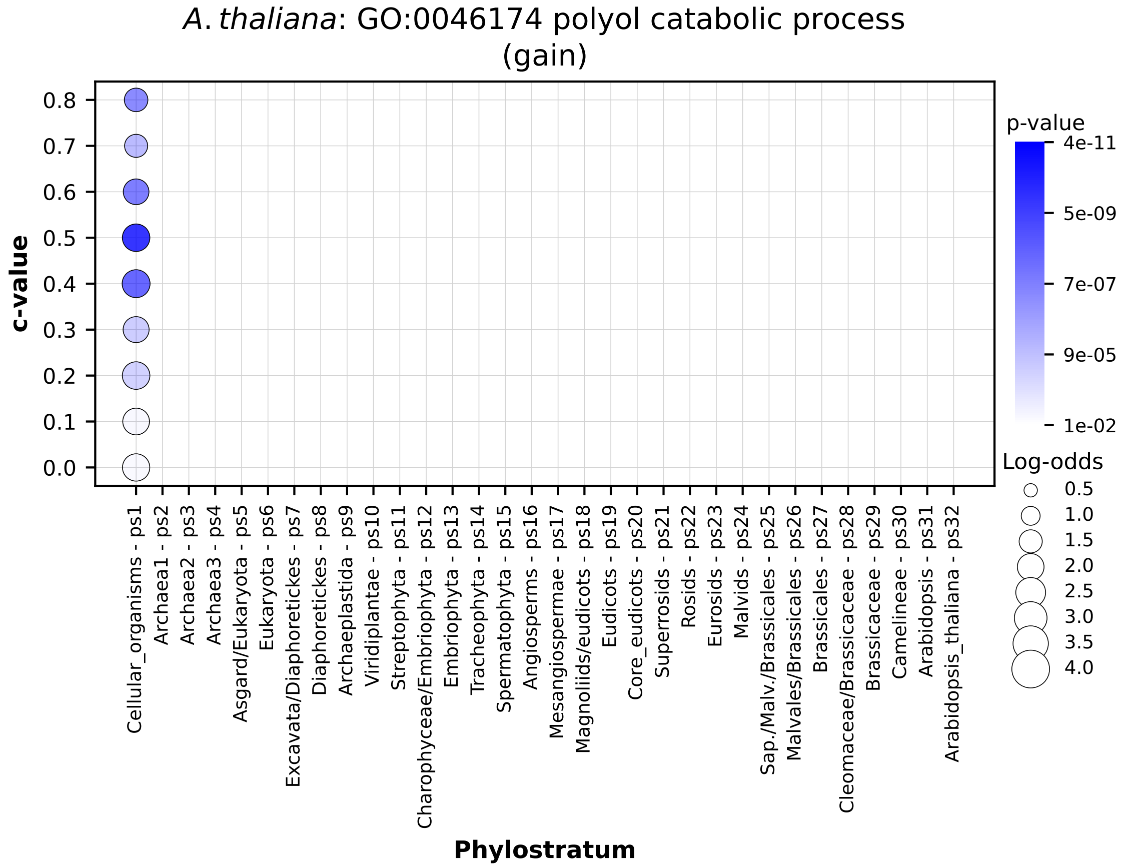

A. thaliana: GO:0046174 polyol catabolic process
(gain)
c-value
0.0
0.1
0.2
0.3
0.4
0.5
0.6
0.7
0.8
p-value
4e-11
5e-09
7e-07
9e-05
1e-02
Log-odds
0.5
1.0
1.5
2.0
2.5
3.0
3.5
4.0
Cellular_organisms - ps1
Archaea1 - ps2
Archaea2 - ps3
Archaea3 - ps4
Asgard/Eukaryota - ps5
Eukaryota - ps6
Excavata/Diaphoretickes - ps7
Diaphoretickes - ps8
Archaeplastida - ps9
Viridiplantae - ps10
Streptophyta - ps11
Charophyceae/Embriophyta - ps12
Embriophyta - ps13
Tracheophyta - ps14
Spermatophyta - ps15
Angiosperms - ps16
Mesangiospermae - ps17
Magnoliids/eudicots - ps18
Eudicots - ps19
Core_eudicots - ps20
Superrosids - ps21
Rosids - ps22
Eurosids - ps23
Malvids - ps24
Sap./Malv./Brassicales - ps25
Malvales/Brassicales - ps26
Brassicales - ps27
Cleomaceae/Brassicaceae - ps28
Brassicaceae - ps29
Camelineae - ps30
Arabidopsis - ps31
Arabidopsis_thaliana - ps32
Phylostratum

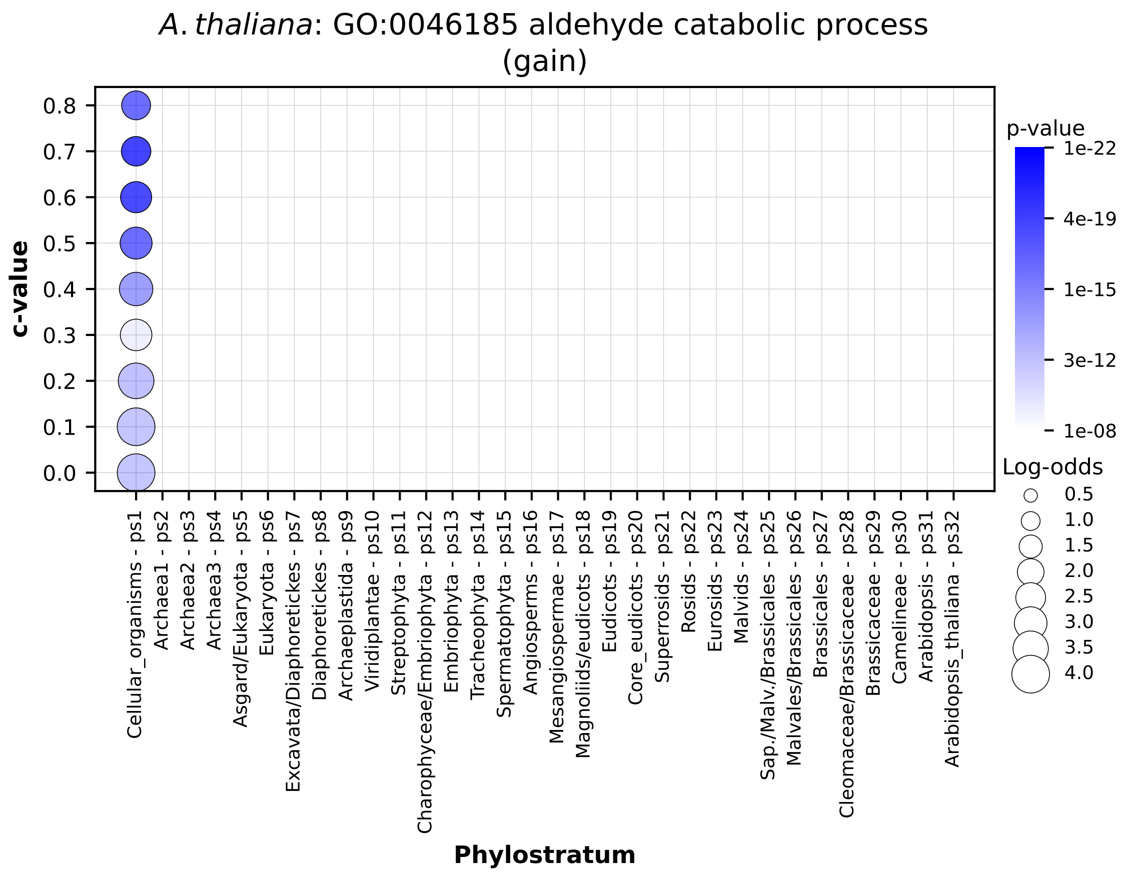

A. thaliana: GO:0046185 aldehyde catabolic process (gain)
c-value
0.0
0.1
0.2
0.3
0.4
0.5
0.6
0.7
0.8
p-value
1e-22
4e-19
1e-15
3e-12
1e-08
Log-odds
0.5
1.0
1.5
2.0
2.5
3.0
3.5
4.0
Cellular_organisms - ps1
Archaea1 - ps2
Archaea2 - ps3
Archaea3 - ps4
Asgard/Eukaryota - ps5
Eukaryota - ps6
Excavata/Diaphoretickes - ps7
Diaphoretickes - ps8
Archaeplastida - ps9
Viridiplantae - ps10
Streptophyta - ps11
Charophyceae/Embriophyta - ps12
Embriophyta - ps13
Tracheophyta - ps14
Spermatophyta - ps15
Angiosperms - ps16
Mesangiospermae - ps17
Magnoliids/eudicots - ps18
Eudicots - ps19
Core_eudicots - ps20
Superrosids - ps21
Rosids - ps22
Eurosids - ps23
Malvids - ps24
Sap./Malv./Brassicales - ps25
Malvales/Brassicales - ps26
Brassicales - ps27
Cleomaceae/Brassicaceae - ps28
Brassicaceae - ps29
Camelineae - ps30
Arabidopsis - ps31
Arabidopsis_thaliana - ps32
Phylostratum

A. thaliana: GO:0046189 phenol-containing compound biosynthetic process (gain)

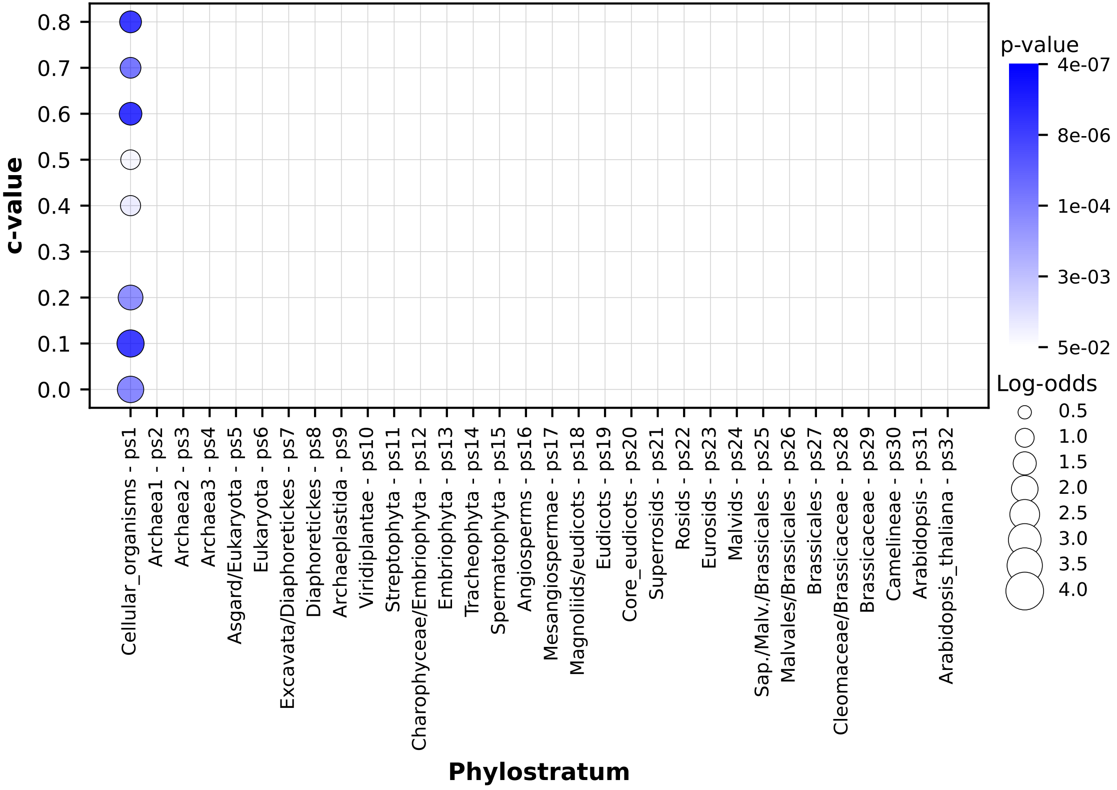

A. thaliana: GO:0046224 bacteriocin metabolic process (gain)

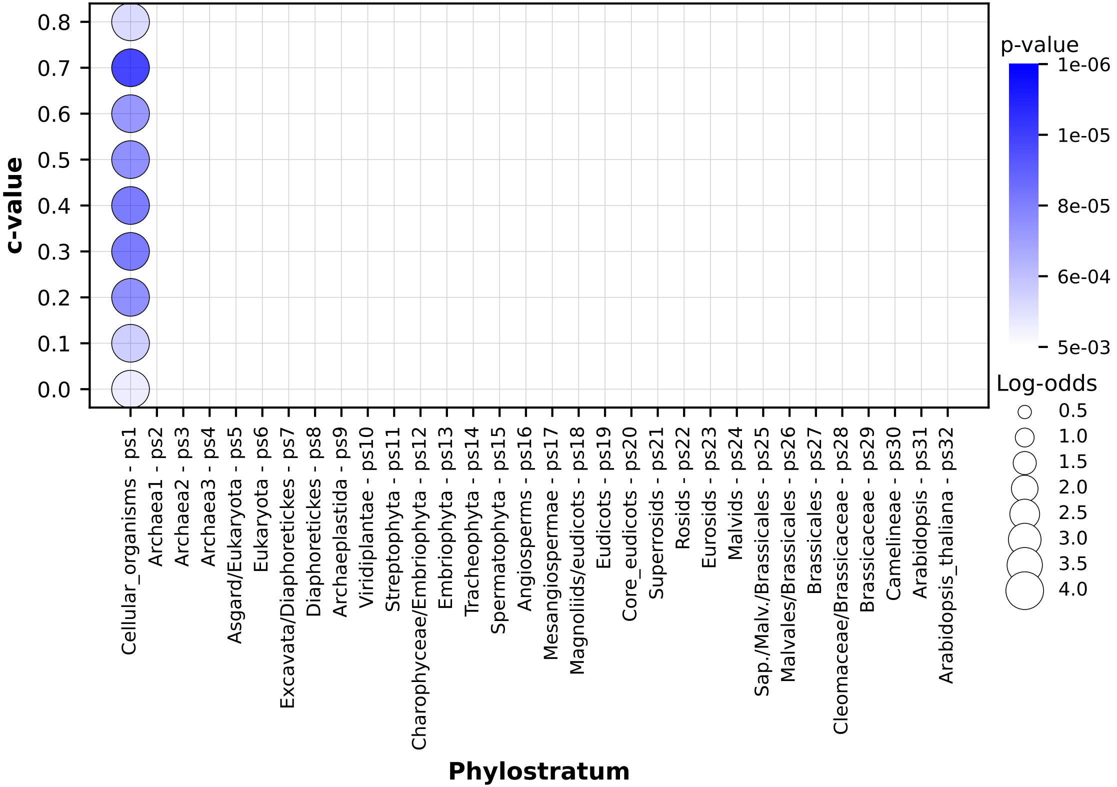

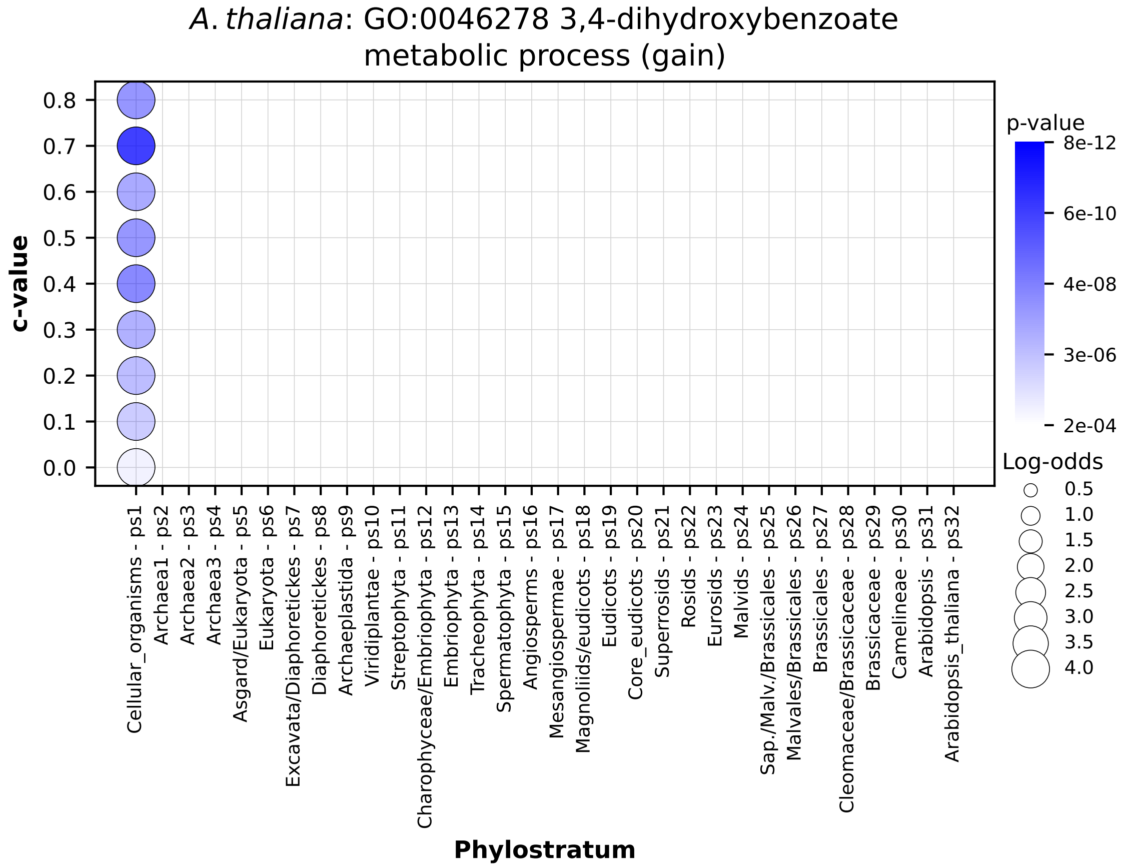
A. thaliana: GO:0046278 3,4-dihydroxybenzoate metabolic process (gain)
c-value
0.8
0.7
0.6
0.5
0.4
0.3
0.2
0.1
0.0
p-value
8e-12
6e-10
4e-08
3e-06
2e-04
Log-odds
0.5
1.0
1.5
2.0
2.5
3.0
3.5
4.0
Cellular_organisms - ps1
Archaea1 - ps2
Archaea2 - ps3
Archaea3 - ps4
Asgard/Eukaryota - ps5
Eukaryota - ps6
Excavata/Diaphoretickes - ps7
Diaphoretickes - ps8
Archaeplastida - ps9
Viridiplantae - ps10
Streptophyta - ps11
Charophyceae/Embriophyta - ps12
Embriophyta - ps13
Tracheophyta - ps14
Spermatophyta - ps15
Angiosperms - ps16
Mesangiospermae - ps17
Magnoliids/eudicots - ps18
Eudicots - ps19
Core_eudicots - ps20
Superrosids - ps21
Rosids - ps22
Eurosids - ps23
Malvids - ps24
Sap./Malv./Brassicales - ps25
Malvales/Brassicales - ps26
Brassicales - ps27
Cleomaceae/Brassicaceae - ps28
Brassicaceae - ps29
Camelineae - ps30
Arabidopsis - ps31
Arabidopsis_thaliana - ps32
Phylostratum

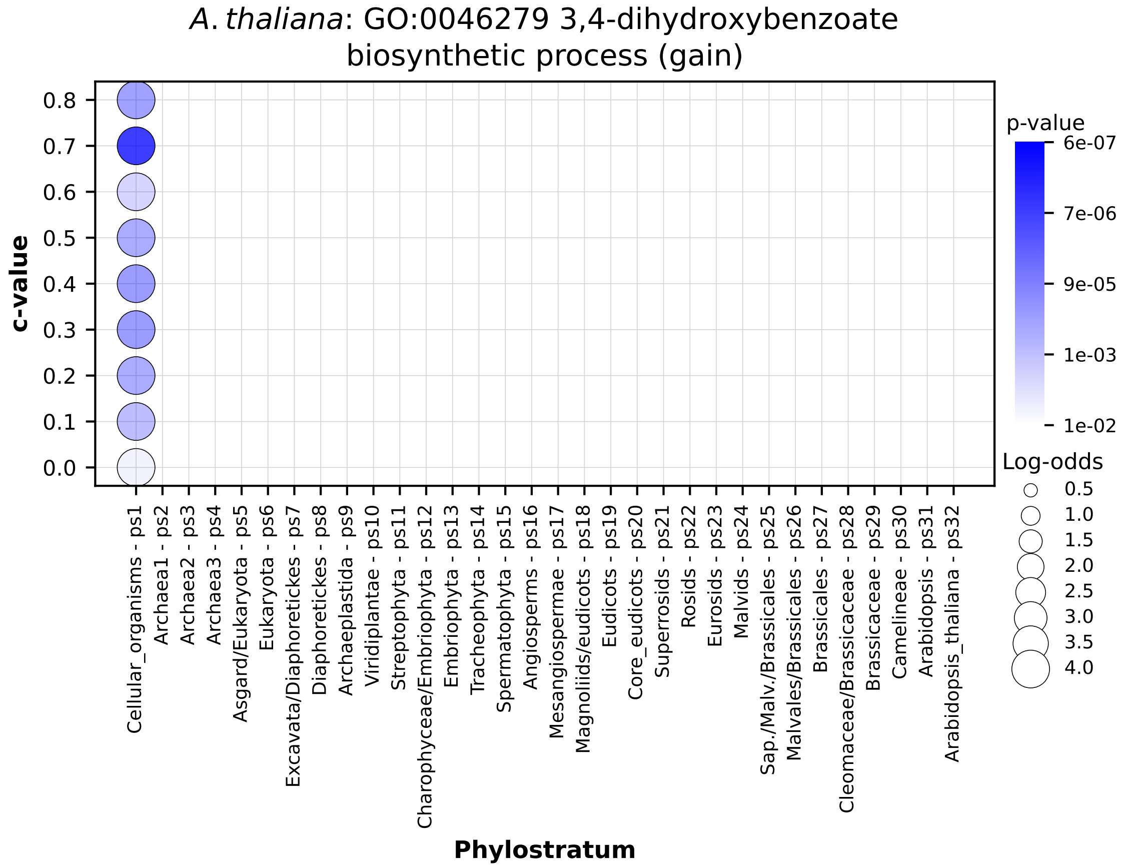
A. thaliana: GO:0046279 3,4-dihydroxybenzoate biosynthetic process (gain)
c-value
0.8
0.7
0.6
0.5
0.4
0.3
0.2
0.1
0.0
p-value
6e-07
7e-06
9e-05
1e-03
1e-02
Log-odds
0.5
1.0
1.5
2.0
2.5
3.0
3.5
4.0
Cellular_organisms - ps1
Archaea1 - ps2
Archaea2 - ps3
Archaea3 - ps4
Asgard/Eukaryota - ps5
Eukaryota - ps6
Excavata/Diaphoretickes - ps7
Diaphoretickes - ps8
Archaeplastida - ps9
Viridiplantae - ps10
Streptophyta - ps11
Charophyceae/Embriophyta - ps12
Embriophyta - ps13
Tracheophyta - ps14
Spermatophyta - ps15
Angiosperms - ps16
Mesangiospermae - ps17
Magnoliids/eudicots - ps18
Eudicots - ps19
Core_eudicots - ps20
Superrosids - ps21
Rosids - ps22
Eurosids - ps23
Malvids - ps24
Sap./Malv./Brassicales - ps25
Malvales/Brassicales - ps26
Brassicales - ps27
Cleomaceae/Brassicaceae - ps28
Brassicaceae - ps29
Camelineae - ps30
Arabidopsis - ps31
Arabidopsis_thaliana - ps32
Phylostratum

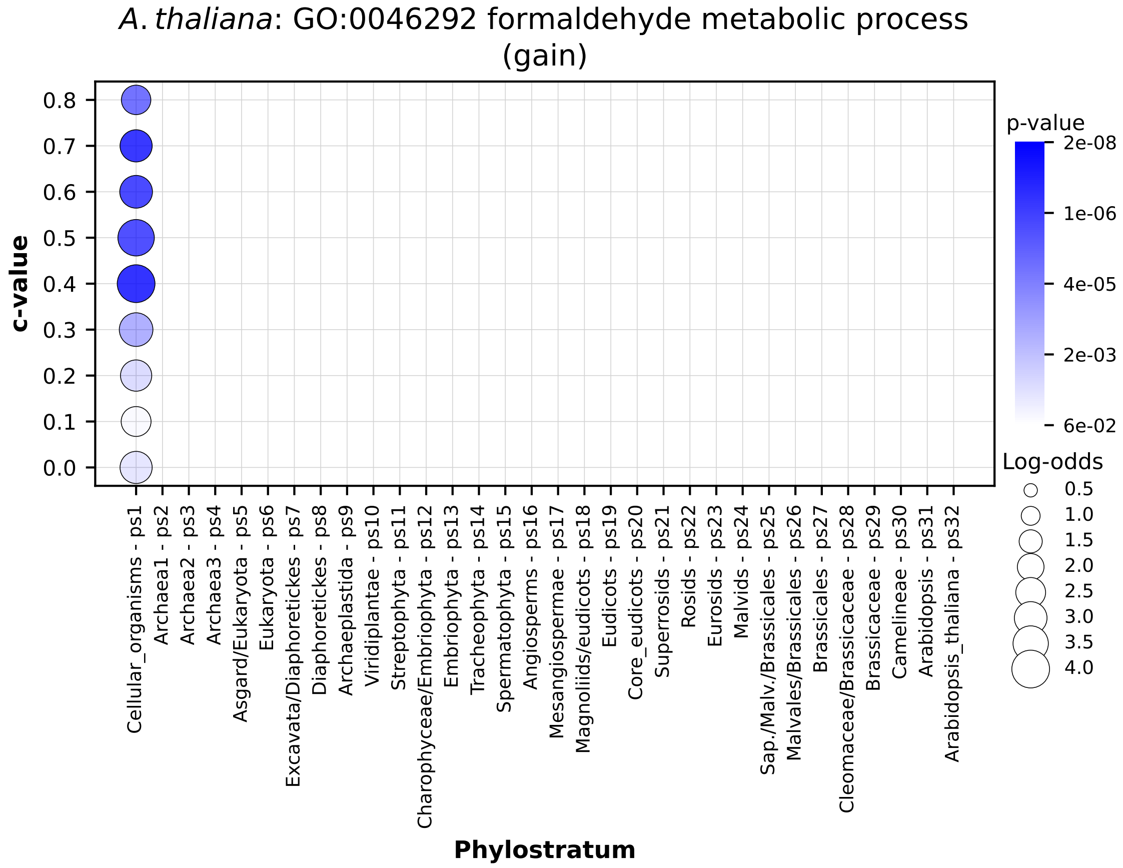

A. thaliana: GO:0046292 formaldehyde metabolic process (gain)
c-value
0.8
0.7
0.6
0.5
0.4
0.3
0.2
0.1
0.0
p-value
2e-08
1e-06
4e-05
2e-03
6e-02
Log-odds
0.5
1.0
1.5
2.0
2.5
3.0
3.5
4.0
Cellular_organisms - ps1
Archaea1 - ps2
Archaea2 - ps3
Archaea3 - ps4
Asgard/Eukaryota - ps5
Eukaryota - ps6
Excavata/Diaphoretickes - ps7
Diaphoretickes - ps8
Archaeplastida - ps9
Viridiplantae - ps10
Streptophyta - ps11
Charophyceae/Embriophyta - ps12
Embriophyta - ps13
Tracheophyta - ps14
Spermatophyta - ps15
Angiosperms - ps16
Mesangiospermae - ps17
Magnoliids/eudicots - ps18
Eudicots - ps19
Core_eudicots - ps20
Superrosids - ps21
Rosids - ps22
Eurosids - ps23
Malvids - ps24
Sap./Malv./Brassicales - ps25
Malvales/Brassicales - ps26
Brassicales - ps27
Cleomaceae/Brassicaceae - ps28
Brassicaceae - ps29
Camelineae - ps30
Arabidopsis - ps31
Arabidopsis_thaliana - ps32
Phylostratum

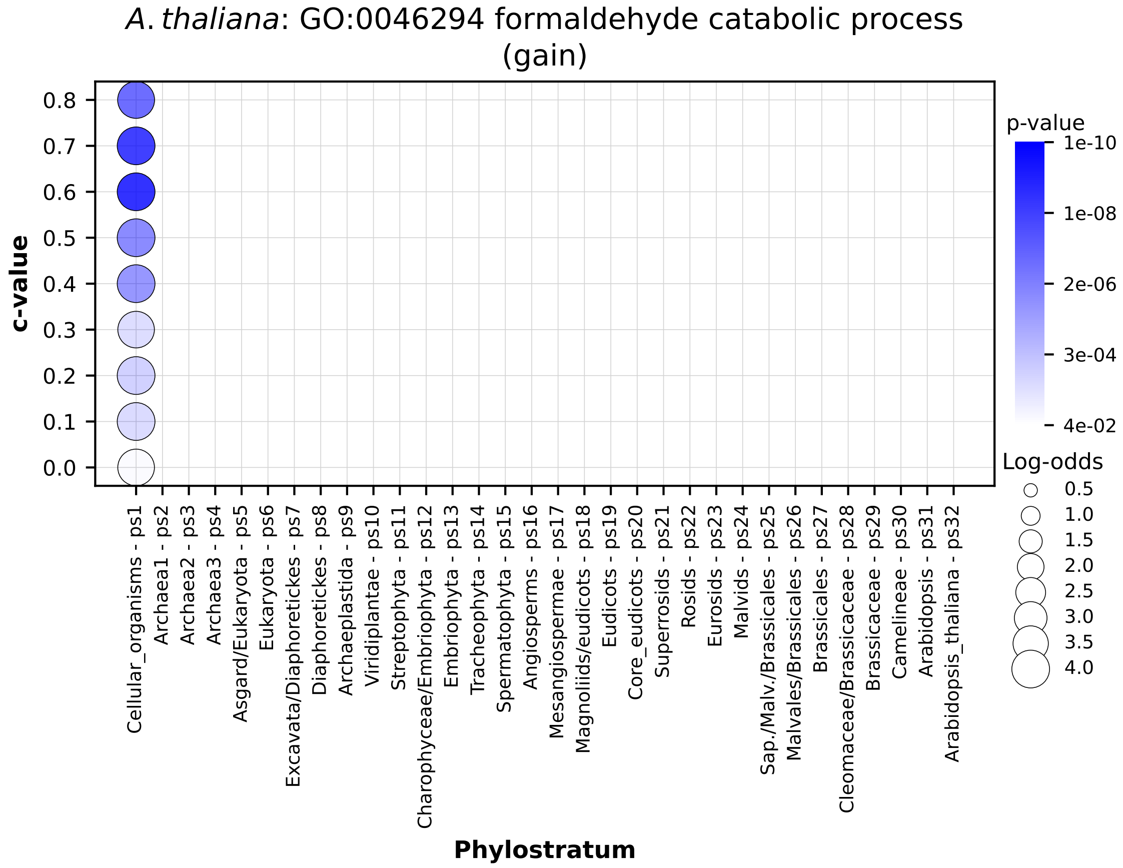

A. thaliana: GO:0046294 formaldehyde catabolic process (gain)
c-value
0.0
0.1
0.2
0.3
0.4
0.5
0.6
0.7
0.8
p-value
1e-10
1e-08
2e-06
3e-04
4e-02
Log-odds
0.5
1.0
1.5
2.0
2.5
3.0
3.5
4.0
Cellular_organisms - ps1
Archaea1 - ps2
Archaea2 - ps3
Archaea3 - ps4
Asgard/Eukaryota - ps5
Eukaryota - ps6
Excavata/Diaphoretickes - ps7
Diaphoretickes - ps8
Archaeplastida - ps9
Viridiplantae - ps10
Streptophyta - ps11
Charophyceae/Embriophyta - ps12
Embriophyta - ps13
Tracheophyta - ps14
Spermatophyta - ps15
Angiosperms - ps16
Mesangiospermae - ps17
Magnoliids/eudicots - ps18
Eudicots - ps19
Core_eudicots - ps20
Superrosids - ps21
Rosids - ps22
Eurosids - ps23
Malvids - ps24
Sap./Malv./Brassicales - ps25
Malvales/Brassicales - ps26
Brassicales - ps27
Cleomaceae/Brassicaceae - ps28
Brassicaceae - ps29
Camelineae - ps30
Arabidopsis - ps31
Arabidopsis_thaliana - ps32
Phylostratum

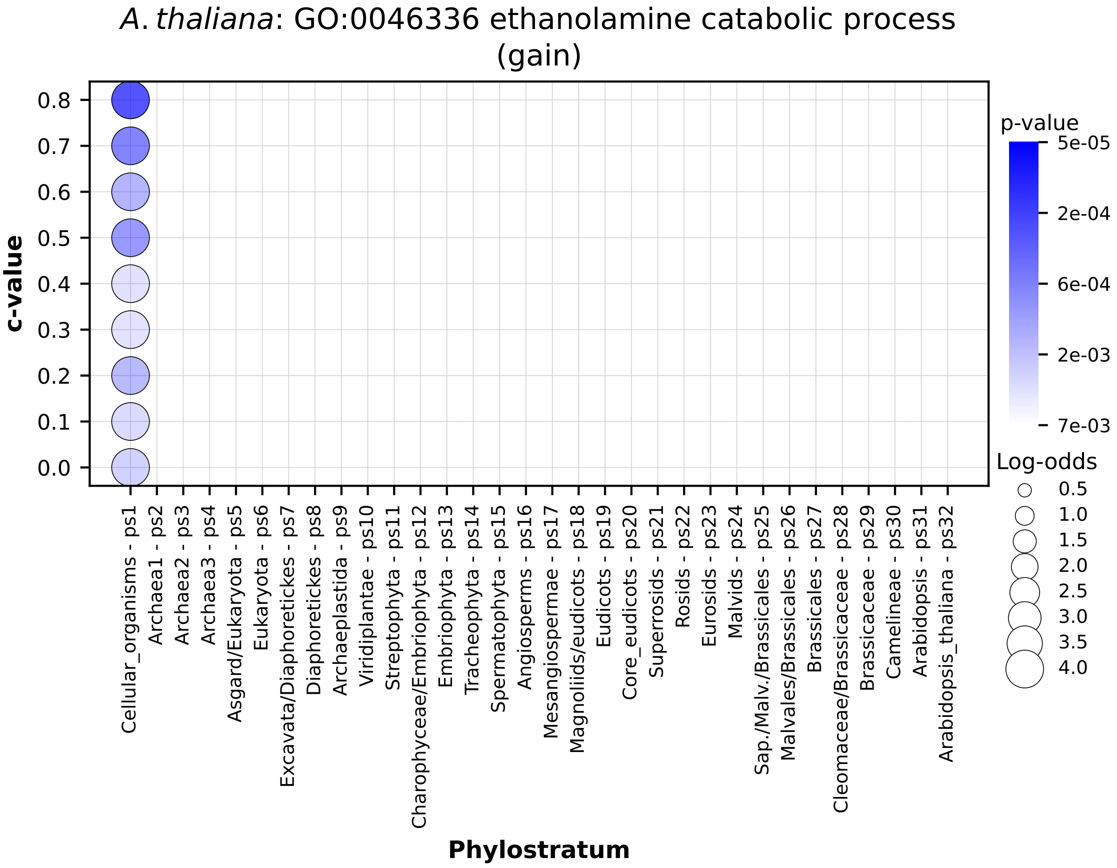

A. thaliana: GO:0046336 ethanolamine catabolic process (gain)
c-value
0.8
0.7
0.6
0.5
0.4
0.3
0.2
0.1
0.0
p-value
5e-05
2e-04
6e-04
2e-03
7e-03
Log-odds
0.5
1.0
1.5
2.0
2.5
3.0
3.5
4.0
Cellular_organisms - ps1
Archaea1 - ps2
Archaea2 - ps3
Archaea3 - ps4
Asgard/Eukaryota - ps5
Eukaryota - ps6
Excavata/Diaphoretickes - ps7
Diaphoretickes - ps8
Archaeplastida - ps9
Viridiplantae - ps10
Streptophyta - ps11
Charophyceae/Embriophyta - ps12
Embriophyta - ps13
Tracheophyta - ps14
Spermatophyta - ps15
Angiosperms - ps16
Mesangiospermae - ps17
Magnoliids/eudicots - ps18
Eudicots - ps19
Core_eudicots - ps20
Superrosids - ps21
Rosids - ps22
Eurosids - ps23
Malvids - ps24
Sap./Malv./Brassicales - ps25
Malvales/Brassicales - ps26
Brassicales - ps27
Cleomaceae/Brassicaceae - ps28
Brassicaceae - ps29
Camelineae - ps30
Arabidopsis - ps31
Arabidopsis_thaliana - ps32
Phylostratum

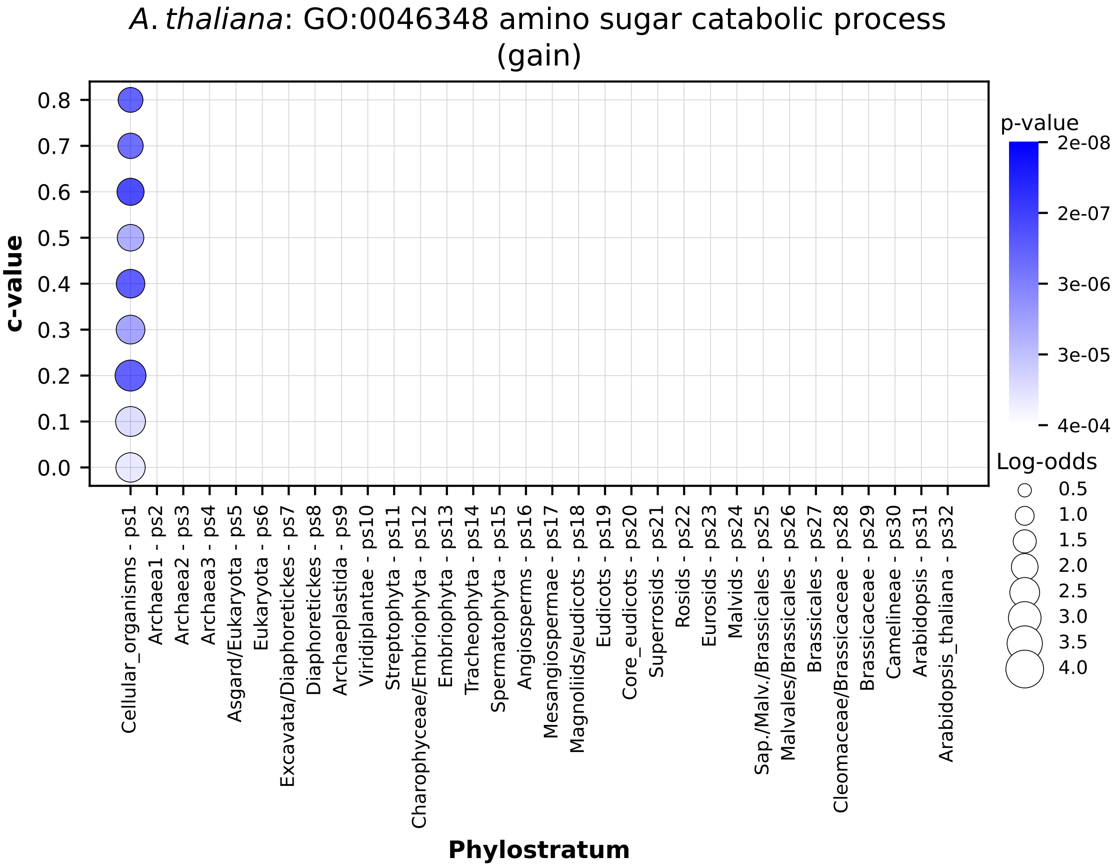

A. thaliana: GO:0046348 amino sugar catabolic process
(gain)
c-value
0.8
0.7
0.6
0.5
0.4
0.3
0.2
0.1
0.0
p-value
2e-08
2e-07
3e-06
3e-05
4e-04
Log-odds
0.5
1.0
1.5
2.0
2.5
3.0
3.5
4.0
Cellular_organisms - ps1
Archaea1 - ps2
Archaea2 - ps3
Archaea3 - ps4
Asgard/Eukaryota - ps5
Eukaryota - ps6
Excavata/Diaphoretickes - ps7
Diaphoretickes - ps8
Archaeplastida - ps9
Viridiplantae - ps10
Streptophyta - ps11
Charophyceae/Embriophyta - ps12
Embriophyta - ps13
Tracheophyta - ps14
Spermatophyta - ps15
Angiosperms - ps16
Mesangiospermae - ps17
Magnoliids/eudicots - ps18
Eudicots - ps19
Core_eudicots - ps20
Superrosids - ps21
Rosids - ps22
Eurosids - ps23
Malvids - ps24
Sap./Malv./Brassicales - ps25
Malvales/Brassicales - ps26
Brassicales - ps27
Cleomaceae/Brassicaceae - ps28
Brassicaceae - ps29
Camelineae - ps30
Arabidopsis - ps31
Arabidopsis_thaliana - ps32
Phylostratum

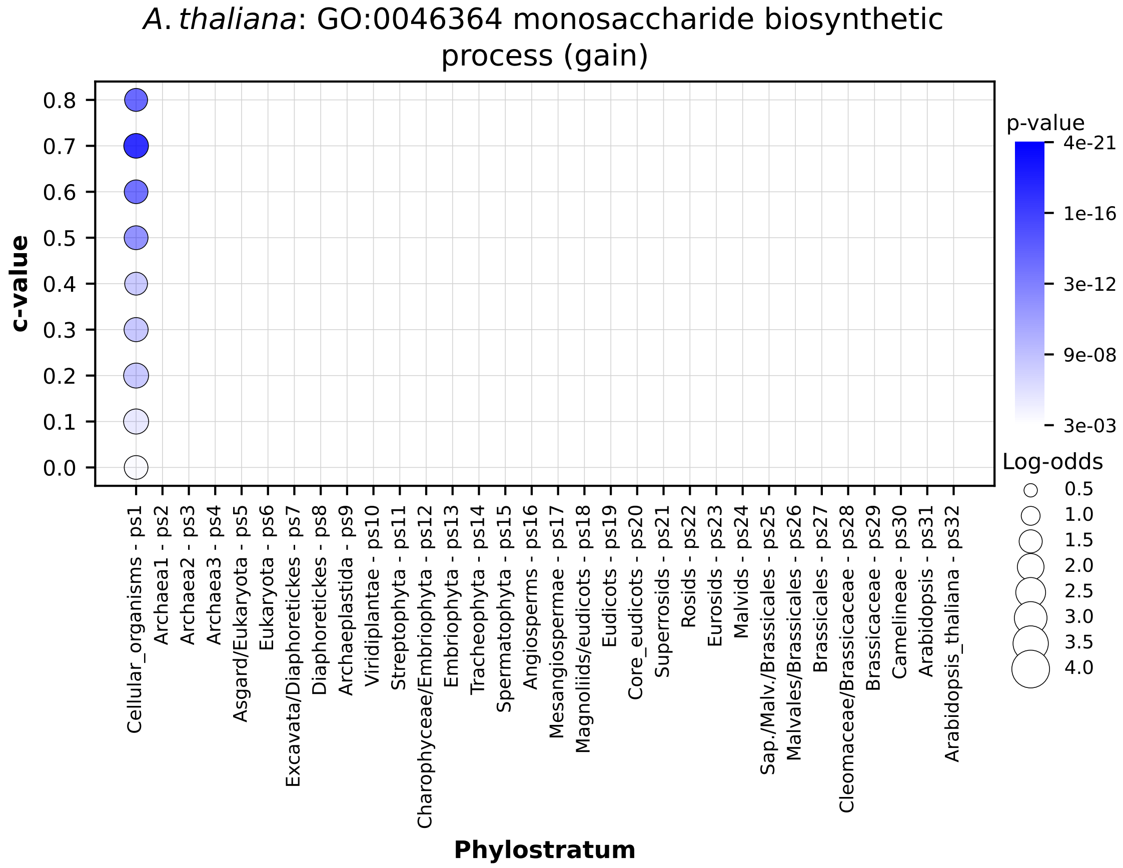

A. thaliana: GO:0046364 monosaccharide biosynthetic process (gain)
c-value
0.8
0.7
0.6
0.5
0.4
0.3
0.2
0.1
0.0
p-value
4e-21
1e-16
3e-12
9e-08
3e-03
Log-odds
0.5
1.0
1.5
2.0
2.5
3.0
3.5
4.0
Cellular_organisms - ps1
Archaea1 - ps2
Archaea2 - ps3
Archaea3 - ps4
Asgard/Eukaryota - ps5
Eukaryota - ps6
Excavata/Diaphoretickes - ps7
Diaphoretickes - ps8
Archaeplastida - ps9
Viridiplantae - ps10
Streptophyta - ps11
Charophyceae/Embriophyta - ps12
Embriophyta - ps13
Tracheophyta - ps14
Spermatophyta - ps15
Angiosperms - ps16
Mesangiospermae - ps17
Magnoliids/eudicots - ps18
Eudicots - ps19
Core_eudicots - ps20
Superrosids - ps21
Rosids - ps22
Eurosids - ps23
Malvids - ps24
Sap./Malv./Brassicales - ps25
Malvales/Brassicales - ps26
Brassicales - ps27
Cleomaceae/Brassicaceae - ps28
Brassicaceae - ps29
Camelineae - ps30
Arabidopsis - ps31
Arabidopsis_thaliana - ps32
Phylostratum

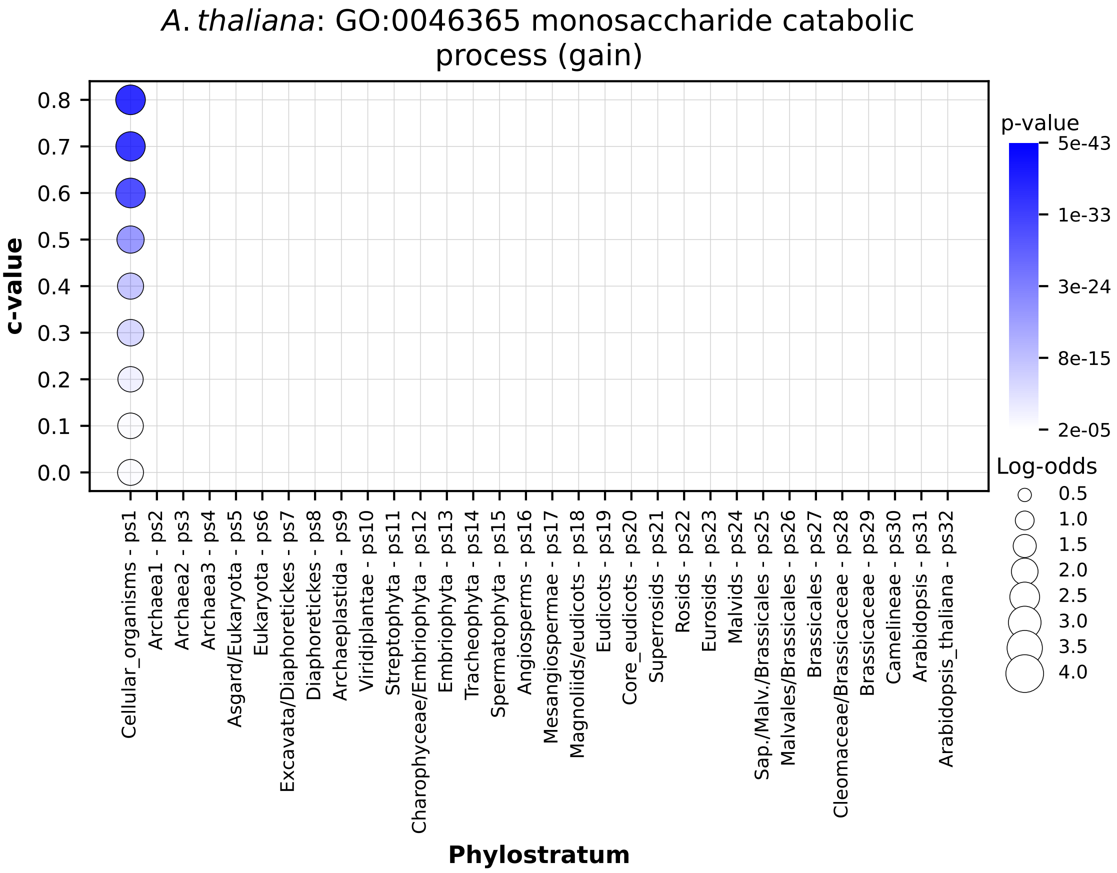
A. thaliana: GO:0046365 monosaccharide catabolic process (gain)
c-value
0.8
0.7
0.6
0.5
0.4
0.3
0.2
0.1
0.0
p-value
5e-43
1e-33
3e-24
8e-15
2e-05
Log-odds
0.5
1.0
1.5
2.0
2.5
3.0
3.5
4.0
Cellular_organisms - ps1
Archaea1 - ps2
Archaea2 - ps3
Archaea3 - ps4
Asgard/Eukaryota - ps5
Eukaryota - ps6
Excavata/Diaphoretickes - ps7
Diaphoretickes - ps8
Archaeplastida - ps9
Viridiplantae - ps10
Streptophyta - ps11
Charophyceae/Embriophyta - ps12
Embriophyta - ps13
Tracheophyta - ps14
Spermatophyta - ps15
Angiosperms - ps16
Mesangiospermae - ps17
Magnoliids/eudicots - ps18
Eudicots - ps19
Core_eudicots - ps20
Superrosids - ps21
Rosids - ps22
Eurosids - ps23
Malvids - ps24
Sap./Malv./Brassicales - ps25
Malvales/Brassicales - ps26
Brassicales - ps27
Cleomaceae/Brassicaceae - ps28
Brassicaceae - ps29
Camelineae - ps30
Arabidopsis - ps31
Arabidopsis_thaliana - ps32
Phylostratum

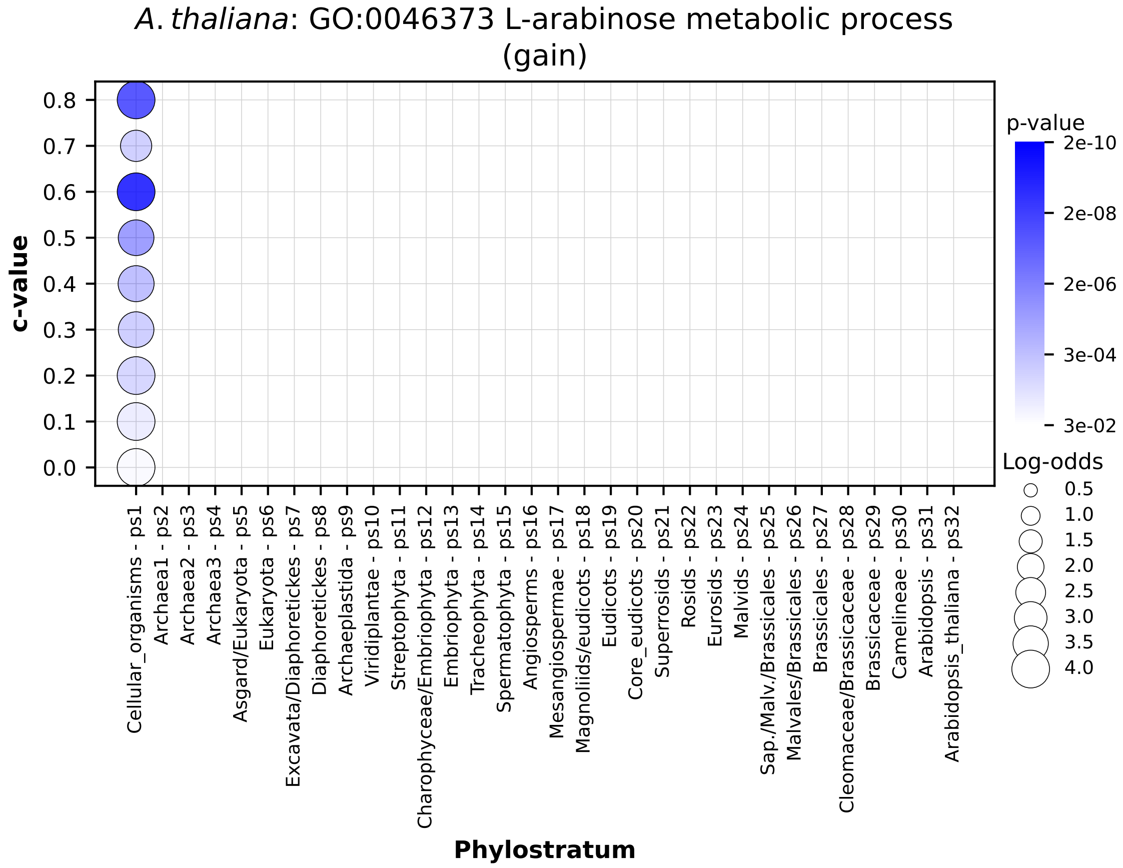

A. thaliana: GO:0046373 L-arabinose metabolic process (gain)
c-value
0.8
0.7
0.6
0.5
0.4
0.3
0.2
0.1
0.0
p-value
2e-10
2e-08
2e-06
3e-04
3e-02
Log-odds
0.5
1.0
1.5
2.0
2.5
3.0
3.5
4.0
Cellular_organisms - ps1
Archaea1 - ps2
Archaea2 - ps3
Archaea3 - ps4
Asgard/Eukaryota - ps5
Eukaryota - ps6
Excavata/Diaphoretickes - ps7
Diaphoretickes - ps8
Archaeplastida - ps9
Viridiplantae - ps10
Streptophyta - ps11
Charophyceae/Embriophyta - ps12
Embriophyta - ps13
Tracheophyta - ps14
Spermatophyta - ps15
Angiosperms - ps16
Mesangiospermae - ps17
Magnoliids/eudicots - ps18
Eudicots - ps19
Core_eudicots - ps20
Superrosids - ps21
Rosids - ps22
Eurosids - ps23
Malvids - ps24
Sap./Malv./Brassicales - ps25
Malvales/Brassicales - ps26
Brassicales - ps27
Cleomaceae/Brassicaceae - ps28
Brassicaceae - ps29
Camelineae - ps30
Arabidopsis - ps31
Arabidopsis_thaliana - ps32
Phylostratum

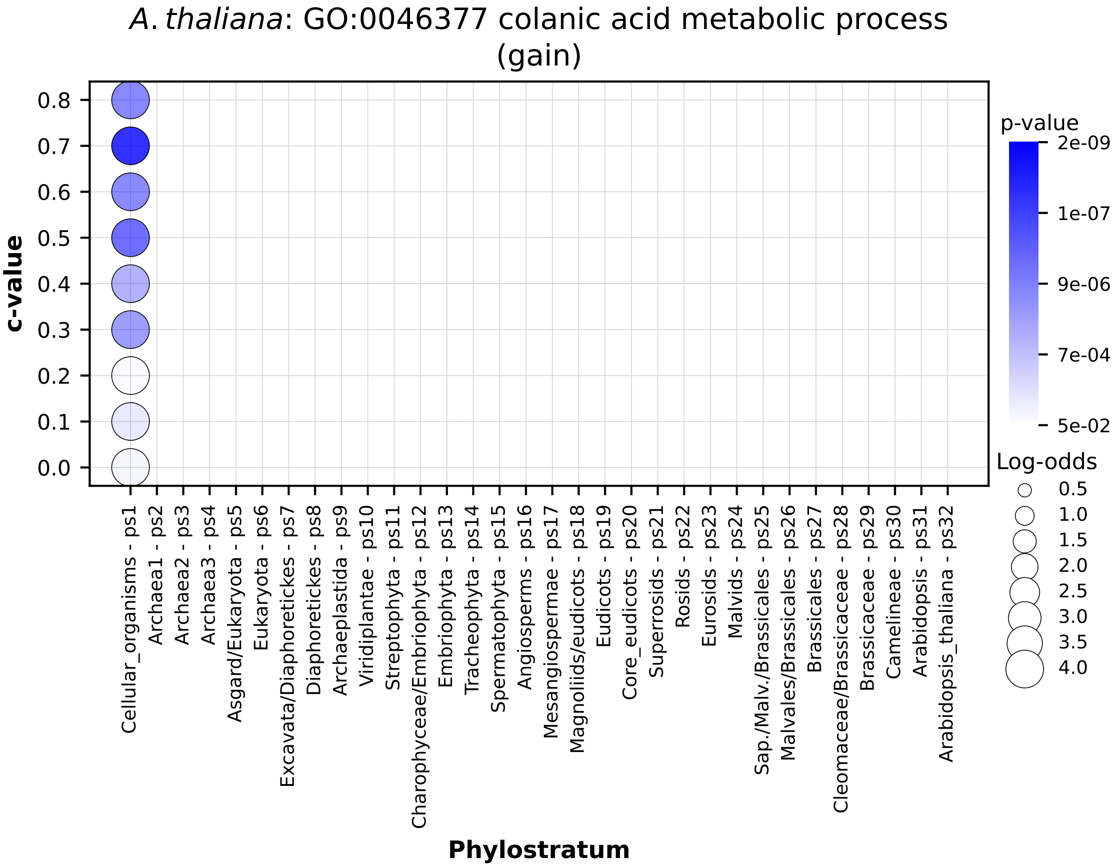

A. thaliana: GO:0046377 colanic acid metabolic process
(gain)
c-value
0.0
0.1
0.2
0.3
0.4
0.5
0.6
0.7
0.8
p-value
2e-09
1e-07
9e-06
7e-04
5e-02
Log-odds
0.5
1.0
1.5
2.0
2.5
3.0
3.5
4.0
Cellular_organisms - ps1
Archaea1 - ps2
Archaea2 - ps3
Archaea3 - ps4
Asgard/Eukaryota - ps5
Eukaryota - ps6
Excavata/Diaphoretickes - ps7
Diaphoretickes - ps8
Archaeplastida - ps9
Viridiplantae - ps10
Streptophyta - ps11
Charophyceae/Embriophyta - ps12
Embriophyta - ps13
Tracheophyta - ps14
Spermatophyta - ps15
Angiosperms - ps16
Mesangiospermae - ps17
Magnoliids/eudicots - ps18
Eudicots - ps19
Core_eudicots - ps20
Superrosids - ps21
Rosids - ps22
Eurosids - ps23
Malvids - ps24
Sap./Malv./Brassicales - ps25
Malvales/Brassicales - ps26
Brassicales - ps27
Cleomaceae/Brassicaceae - ps28
Brassicaceae - ps29
Camelineae - ps30
Arabidopsis - ps31
Arabidopsis_thaliana - ps32
Phylostratum

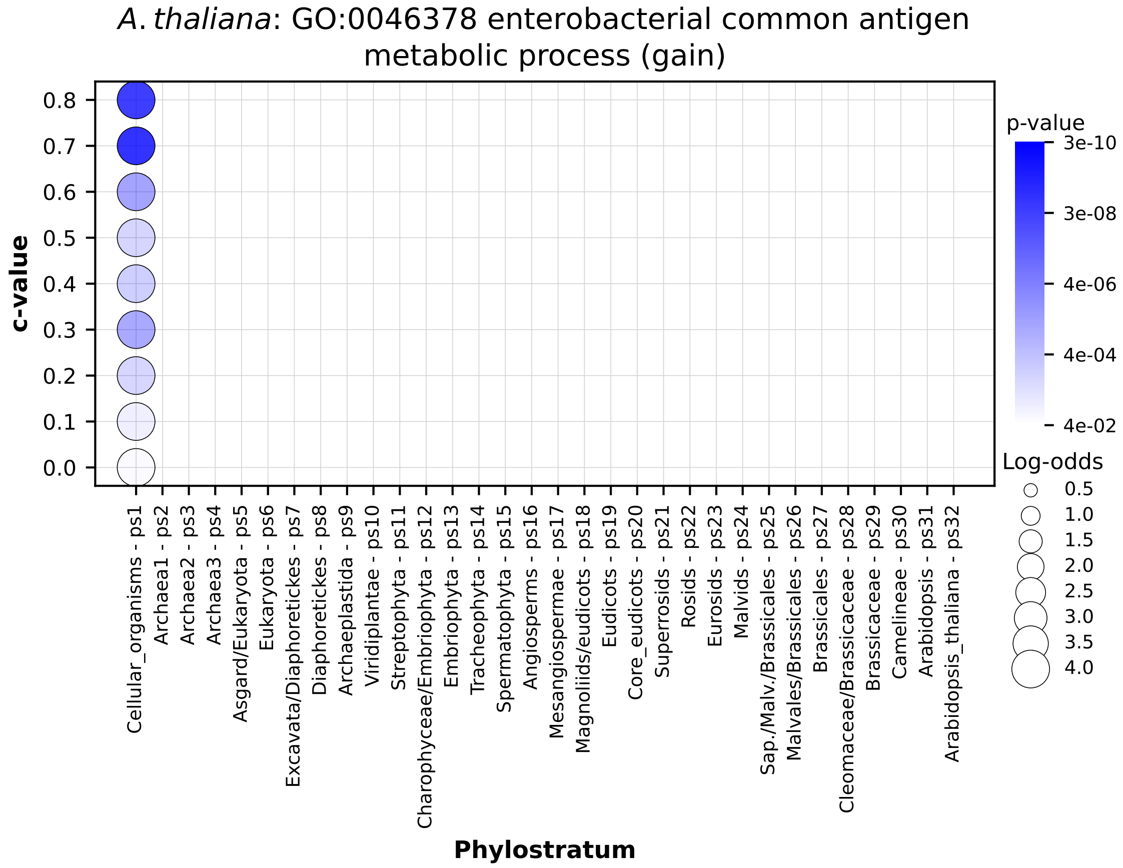

A. thaliana: GO:0046378 enterobacterial common antigen metabolic process (gain)
c-value
0.8
0.7
0.6
0.5
0.4
0.3
0.2
0.1
0.0
p-value
3e-10
3e-08
4e-06
4e-04
4e-02
Log-odds
0.5
1.0
1.5
2.0
2.5
3.0
3.5
4.0
Cellular_organisms - ps1
Archaea1 - ps2
Archaea2 - ps3
Archaea3 - ps4
Asgard/Eukaryota - ps5
Eukaryota - ps6
Excavata/Diaphoretickes - ps7
Diaphoretickes - ps8
Archaeplastida - ps9
Viridiplantae - ps10
Streptophyta - ps11
Charophyceae/Embriophyta - ps12
Embriophyta - ps13
Tracheophyta - ps14
Spermatophyta - ps15
Angiosperms - ps16
Mesangiospermae - ps17
Magnoliids/eudicots - ps18
Eudicots - ps19
Core_eudicots - ps20
Superrosids - ps21
Rosids - ps22
Eurosids - ps23
Malvids - ps24
Sap./Malv./Brassicales - ps25
Malvales/Brassicales - ps26
Brassicales - ps27
Cleomaceae/Brassicaceae - ps28
Brassicaceae - ps29
Camelineae - ps30
Arabidopsis - ps31
Arabidopsis_thaliana - ps32
Phylostratum

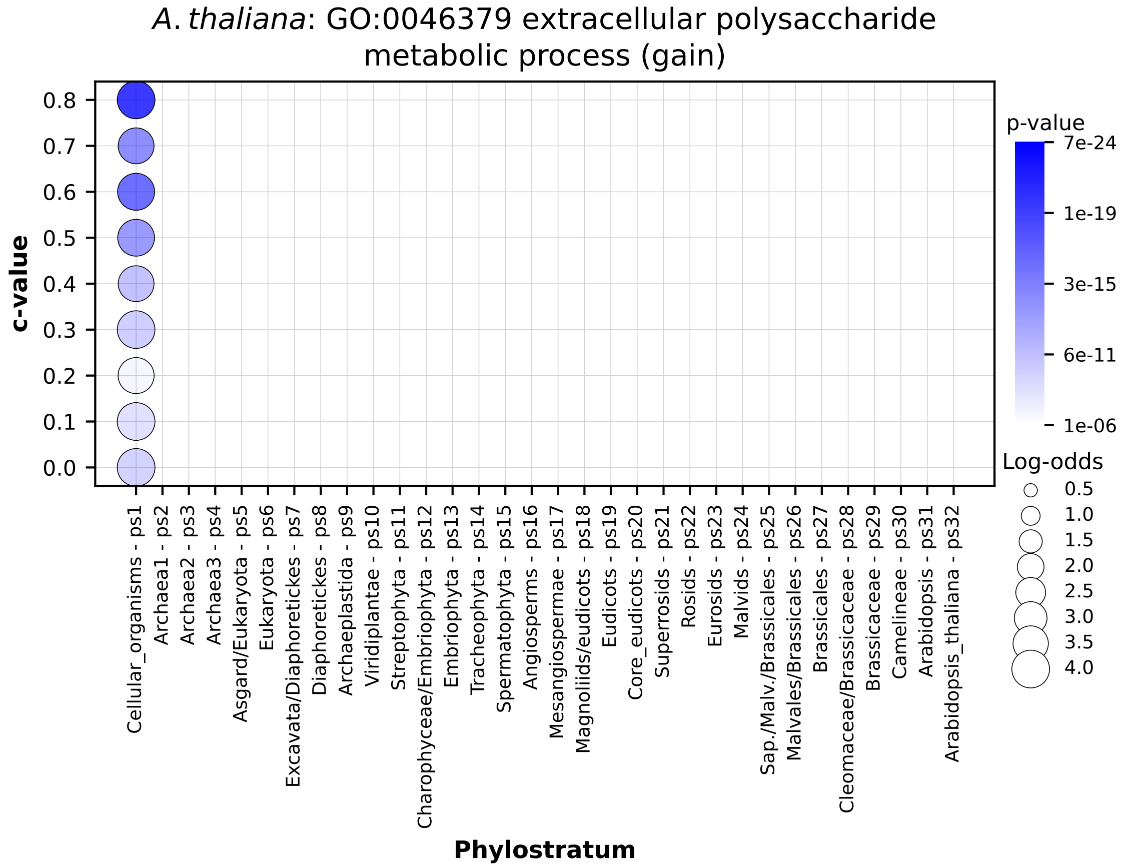

A. thaliana: GO:0046379 extracellular polysaccharide metabolic process (gain)
c-value
0.8
0.7
0.6
0.5
0.4
0.3
0.2
0.1
0.0
p-value
7e-24
1e-19
3e-15
6e-11
1e-06
Log-odds
0.5
1.0
1.5
2.0
2.5
3.0
3.5
4.0
Cellular_organisms - ps1
Archaea1 - ps2
Archaea2 - ps3
Archaea3 - ps4
Asgard/Eukaryota - ps5
Eukaryota - ps6
Excavata/Diaphoretickes - ps7
Diaphoretickes - ps8
Archaeplastida - ps9
Viridiplantae - ps10
Streptophyta - ps11
Charophyceae/Embriophyta - ps12
Embriophyta - ps13
Tracheophyta - ps14
Spermatophyta - ps15
Angiosperms - ps16
Mesangiospermae - ps17
Magnoliids/eudicots - ps18
Eudicots - ps19
Core_eudicots - ps20
Superrosids - ps21
Rosids - ps22
Eurosids - ps23
Malvids - ps24
Sap./Malv./Brassicales - ps25
Malvales/Brassicales - ps26
Brassicales - ps27
Cleomaceae/Brassicaceae - ps28
Brassicaceae - ps29
Camelineae - ps30
Arabidopsis - ps31
Arabidopsis_thaliana - ps32
Phylostratum

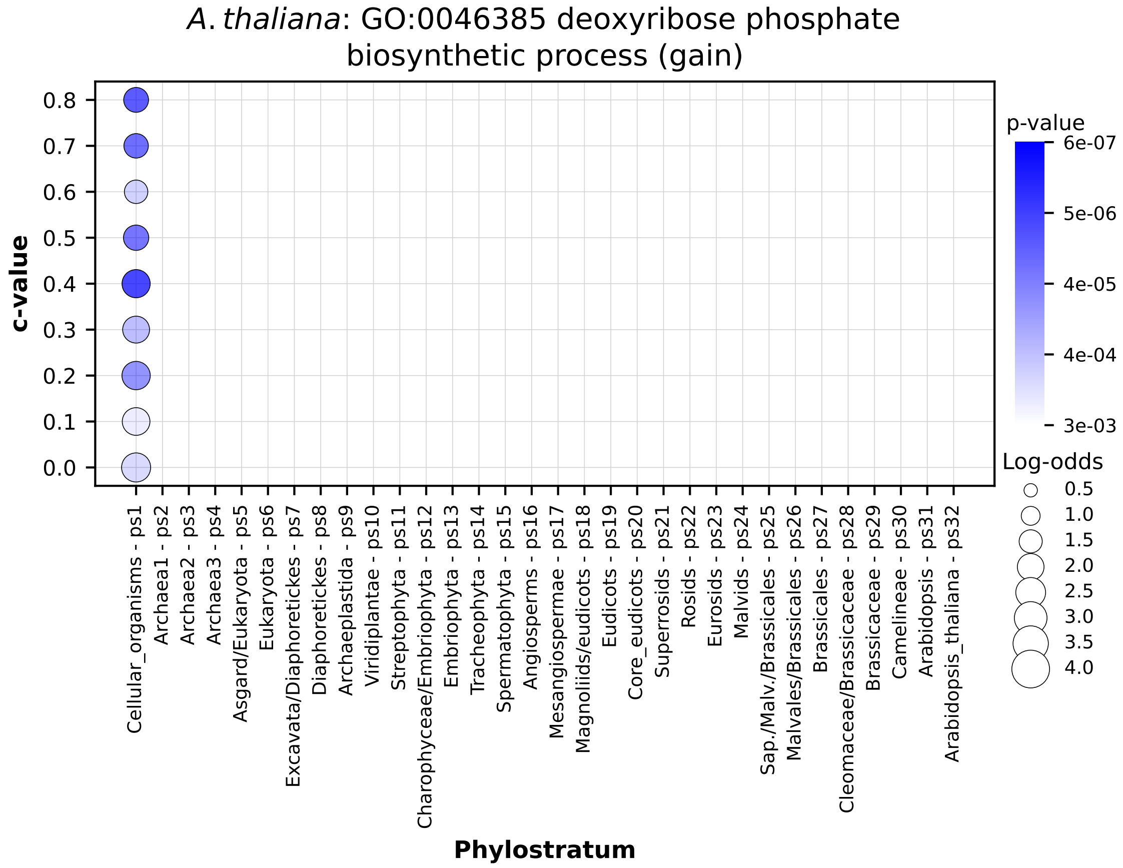

A. thaliana: GO:0046385 deoxyribose phosphate biosynthetic process (gain)
c-value
0.8
0.7
0.6
0.5
0.4
0.3
0.2
0.1
0.0
p-value
6e-07
5e-06
4e-05
4e-04
3e-03
Log-odds
0.5
1.0
1.5
2.0
2.5
3.0
3.5
4.0
Cellular_organisms - ps1
Archaea1 - ps2
Archaea2 - ps3
Archaea3 - ps4
Asgard/Eukaryota - ps5
Eukaryota - ps6
Excavata/Diaphoretickes - ps7
Diaphoretickes - ps8
Archaeplastida - ps9
Viridiplantae - ps10
Streptophyta - ps11
Charophyceae/Embriophyta - ps12
Embriophyta - ps13
Tracheophyta - ps14
Spermatophyta - ps15
Angiosperms - ps16
Mesangiospermae - ps17
Magnoliids/eudicots - ps18
Eudicots - ps19
Core_eudicots - ps20
Superrosids - ps21
Rosids - ps22
Eurosids - ps23
Malvids - ps24
Sap./Malv./Brassicales - ps25
Malvales/Brassicales - ps26
Brassicales - ps27
Cleomaceae/Brassicaceae - ps28
Brassicaceae - ps29
Camelineae - ps30
Arabidopsis - ps31
Arabidopsis_thaliana - ps32
Phylostratum

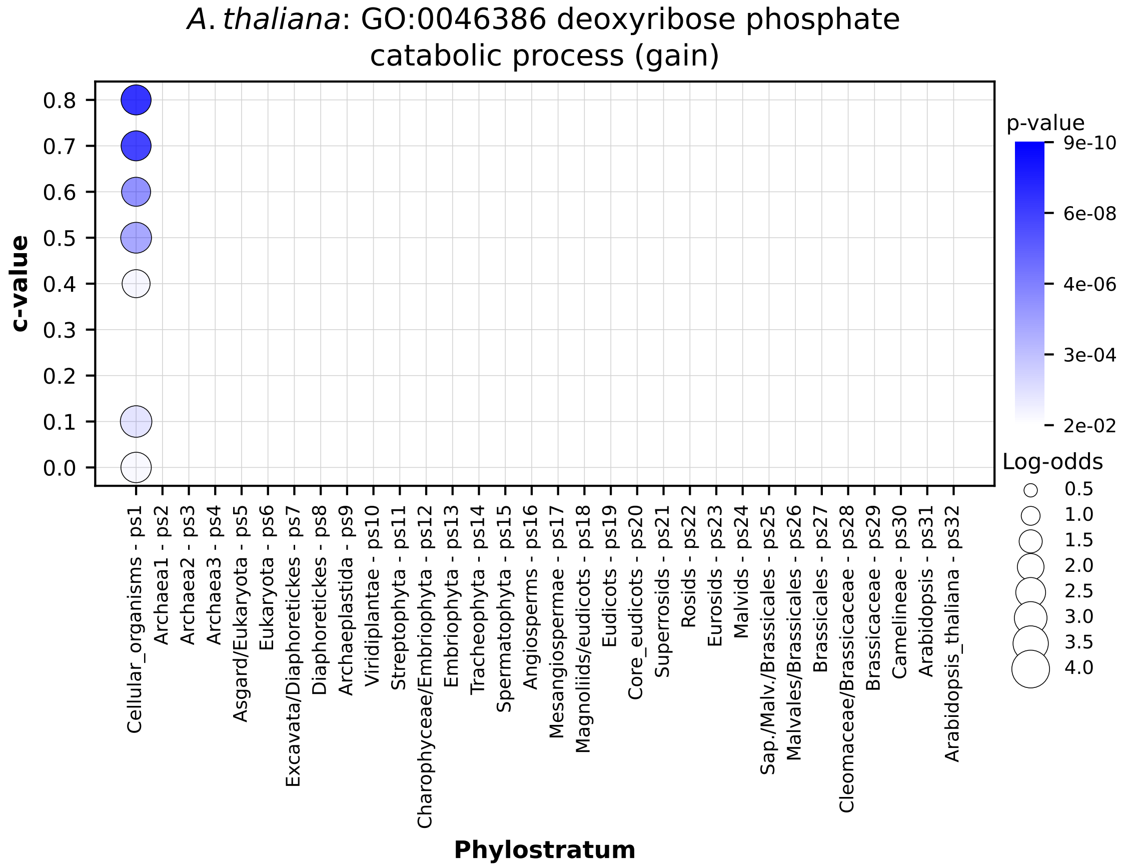

A. thaliana: GO:0046386 deoxyribose phosphate catabolic process (gain)
c-value
0.8
0.7
0.6
0.5
0.4
0.3
0.2
0.1
0.0
p-value
9e-10
6e-08
4e-06
3e-04
2e-02
Log-odds
0.5
1.0
1.5
2.0
2.5
3.0
3.5
4.0
Cellular_organisms - ps1
Archaea1 - ps2
Archaea2 - ps3
Archaea3 - ps4
Asgard/Eukaryota - ps5
Eukaryota - ps6
Excavata/Diaphoretickes - ps7
Diaphoretickes - ps8
Archaeplastida - ps9
Viridiplantae - ps10
Streptophyta - ps11
Charophyceae/Embriophyta - ps12
Embriophyta - ps13
Tracheophyta - ps14
Spermatophyta - ps15
Angiosperms - ps16
Mesangiospermae - ps17
Magnoliids/eudicots - ps18
Eudicots - ps19
Core_eudicots - ps20
Superrosids - ps21
Rosids - ps22
Eurosids - ps23
Malvids - ps24
Sap./Malv./Brassicales - ps25
Malvales/Brassicales - ps26
Brassicales - ps27
Cleomaceae/Brassicaceae - ps28
Brassicaceae - ps29
Camelineae - ps30
Arabidopsis - ps31
Arabidopsis_thaliana - ps32
Phylostratum

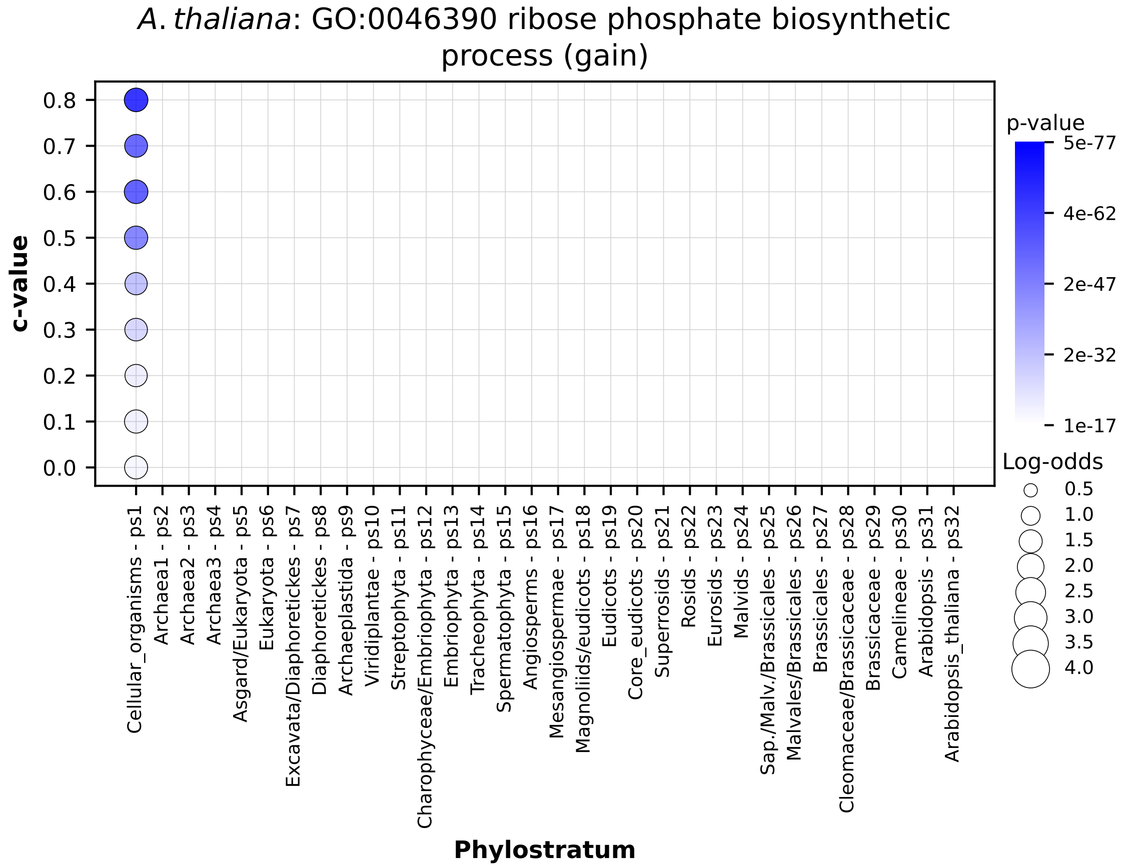
A. thaliana: GO:0046390 ribose phosphate biosynthetic process (gain)
c-value
0.8
0.7
0.6
0.5
0.4
0.3
0.2
0.1
0.0
p-value
5e-77
4e-62
2e-47
2e-32
1e-17
Log-odds
0.5
1.0
1.5
2.0
2.5
3.0
3.5
4.0
Cellular_organisms - ps1
Archaea1 - ps2
Archaea2 - ps3
Archaea3 - ps4
Asgard/Eukaryota - ps5
Eukaryota - ps6
Excavata/Diaphoretickes - ps7
Diaphoretickes - ps8
Archaeplastida - ps9
Viridiplantae - ps10
Streptophyta - ps11
Charophyceae/Embriophyta - ps12
Embriophyta - ps13
Tracheophyta - ps14
Spermatophyta - ps15
Angiosperms - ps16
Mesangiospermae - ps17
Magnoliids/eudicots - ps18
Eudicots - ps19
Core_eudicots - ps20
Superrosids - ps21
Rosids - ps22
Eurosids - ps23
Malvids - ps24
Sap./Malv./Brassicales - ps25
Malvales/Brassicales - ps26
Brassicales - ps27
Cleomaceae/Brassicaceae - ps28
Brassicaceae - ps29
Camelineae - ps30
Arabidopsis - ps31
Arabidopsis_thaliana - ps32
Phylostratum

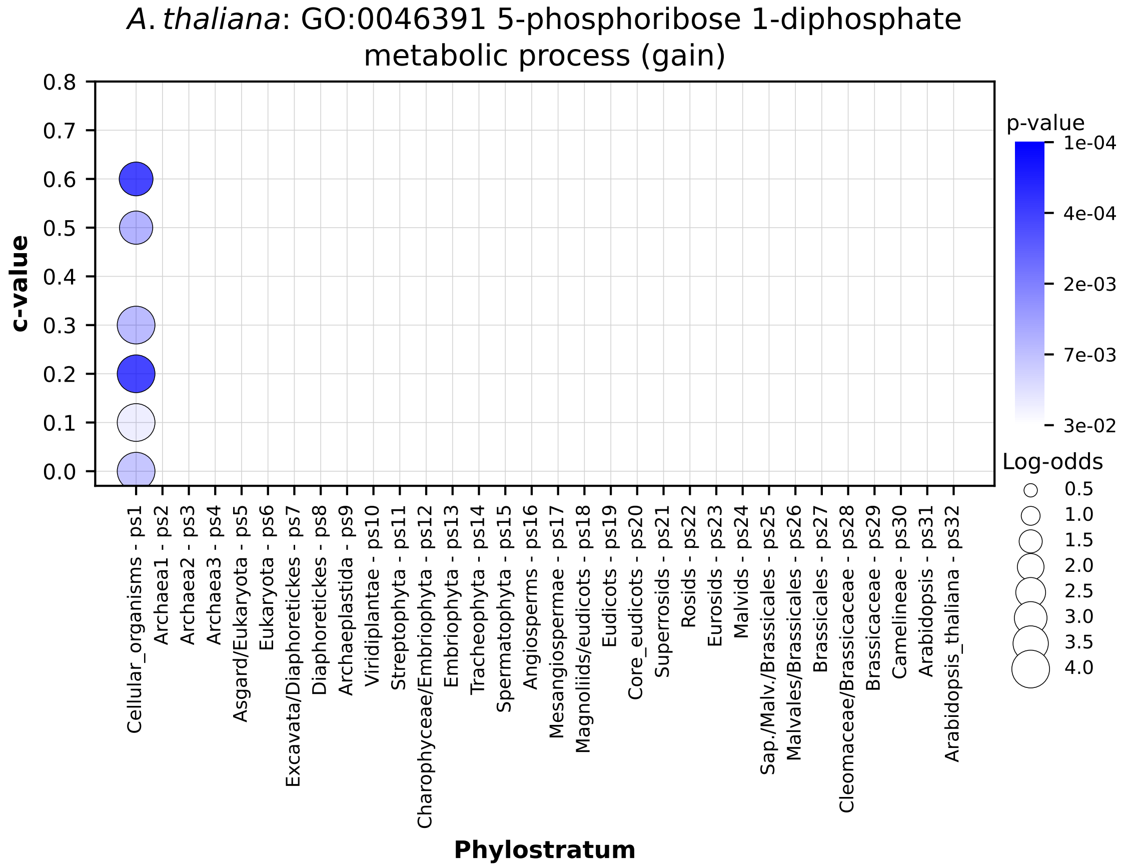

A. thaliana: GO:0046391 5-phosphoribose 1-diphosphate metabolic process (gain)
c-value
0.8
0.7
0.6
0.5
0.4
0.3
0.2
0.1
0.0
Phylostratum
Cellular_organisms - ps1
Archaea1 - ps2
Archaea2 - ps3
Archaea3 - ps4
Asgard/Eukaryota - ps5
Eukaryota - ps6
Excavata/Diaphoretickes - ps7
Diaphoretickes - ps8
Archaeplastida - ps9
Viridiplantae - ps10
Streptophyta - ps11
Charophyceae/Embriophyta - ps12
Embriophyta - ps13
Tracheophyta - ps14
Spermatophyta - ps15
Angiosperms - ps16
Mesangiospermae - ps17
Magnoliids/eudicots - ps18
Eudicots - ps19
Core_eudicots - ps20
Superrosids - ps21
Rosids - ps22
Eurosids - ps23
Malvids - ps24
Sap./Malv./Brassicales - ps25
Malvales/Brassicales - ps26
Brassicales - ps27
Cleomaceae/Brassicaceae - ps28
Brassicaceae - ps29
Camelineae - ps30
Arabidopsis - ps31
Arabidopsis_thaliana - ps32
p-value
1e-04
4e-04
2e-03
7e-03
3e-02
Log-odds
0.5
1.0
1.5
2.0
2.5
3.0
3.5
4.0

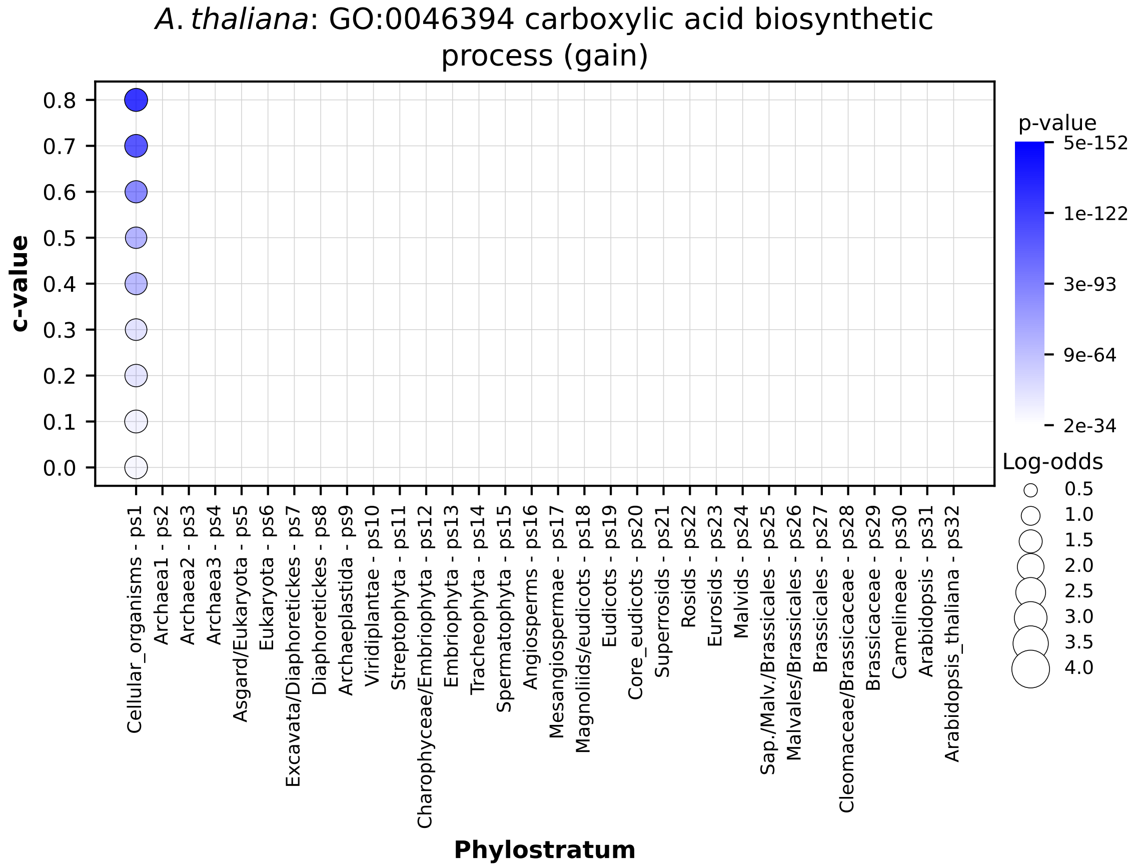
A. thaliana: GO:0046394 carboxylic acid biosynthetic process (gain)
c-value
0.8
0.7
0.6
0.5
0.4
0.3
0.2
0.1
0.0
Phylostratum
Cellular_organisms - ps1
Archaea1 - ps2
Archaea2 - ps3
Archaea3 - ps4
Asgard/Eukaryota - ps5
Eukaryota - ps6
Excavata/Diaphoretickes - ps7
Diaphoretickes - ps8
Archaeplastida - ps9
Viridiplantae - ps10
Streptophyta - ps11
Charophyceae/Embriophyta - ps12
Embriophyta - ps13
Tracheophyta - ps14
Spermatophyta - ps15
Angiosperms - ps16
Mesangiospermae - ps17
Magnoliids/eudicots - ps18
Eudicots - ps19
Core_eudicots - ps20
Superrosids - ps21
Rosids - ps22
Eurosids - ps23
Malvids - ps24
Sap./Malv./Brassicales - ps25
Malvales/Brassicales - ps26
Brassicales - ps27
Cleomaceae/Brassicaceae - ps28
Brassicaceae - ps29
Camelineae - ps30
Arabidopsis - ps31
Arabidopsis_thaliana - ps32
p-value
5e-152
1e-122
3e-93
9e-64
2e-34
Log-odds
0.5
1.0
1.5
2.0
2.5
3.0
3.5
4.0

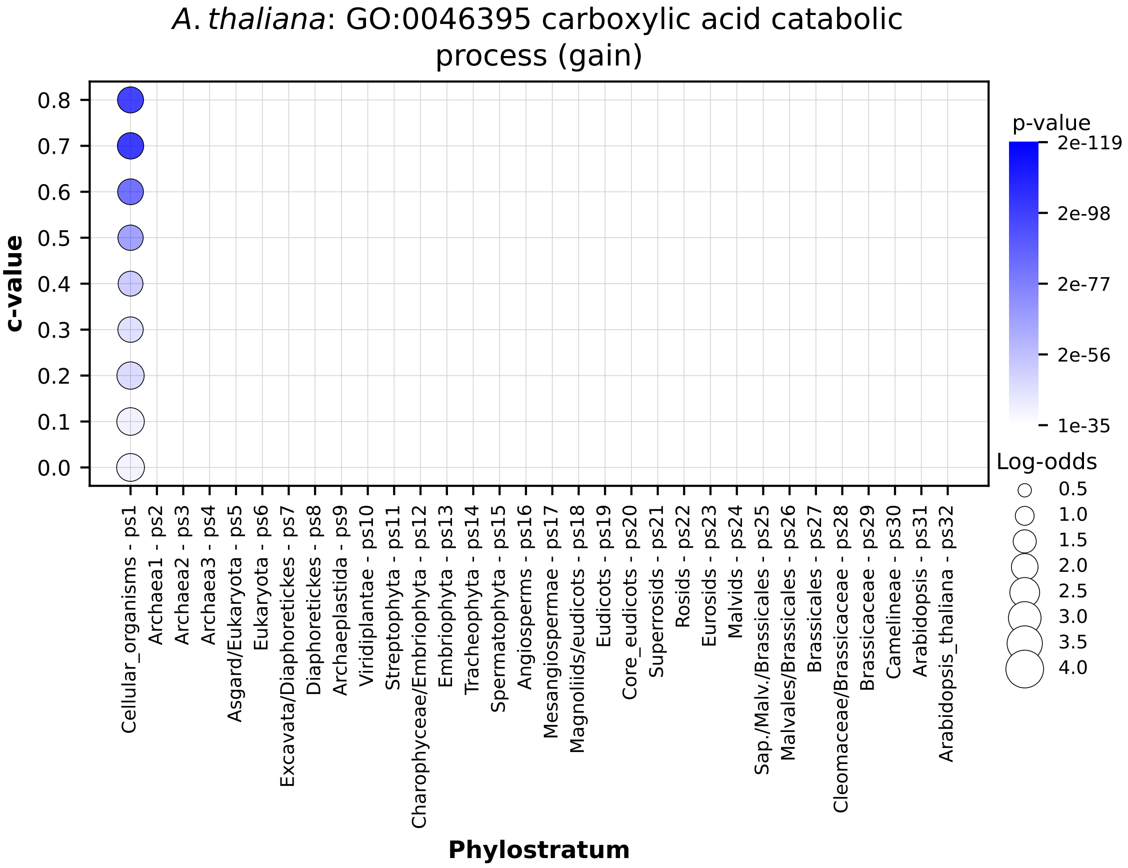

A. thaliana: GO:0046395 carboxylic acid catabolic process (gain)
c-value
0.8
0.7
0.6
0.5
0.4
0.3
0.2
0.1
0.0
p-value
2e-119
2e-98
2e-77
2e-56
1e-35
Log-odds
0.5
1.0
1.5
2.0
2.5
3.0
3.5
4.0
Cellular_organisms - ps1
Archaea1 - ps2
Archaea2 - ps3
Archaea3 - ps4
Asgard/Eukaryota - ps5
Eukaryota - ps6
Excavata/Diaphoretickes - ps7
Diaphoretickes - ps8
Archaeplastida - ps9
Viridiplantae - ps10
Streptophyta - ps11
Charophyceae/Embriophyta - ps12
Embriophyta - ps13
Tracheophyta - ps14
Spermatophyta - ps15
Angiosperms - ps16
Mesangiospermae - ps17
Magnoliids/eudicots - ps18
Eudicots - ps19
Core_eudicots - ps20
Superrosids - ps21
Rosids - ps22
Eurosids - ps23
Malvids - ps24
Sap./Malv./Brassicales - ps25
Malvales/Brassicales - ps26
Brassicales - ps27
Cleomaceae/Brassicaceae - ps28
Brassicaceae - ps29
Camelineae - ps30
Arabidopsis - ps31
Arabidopsis_thaliana - ps32
Phylostratum

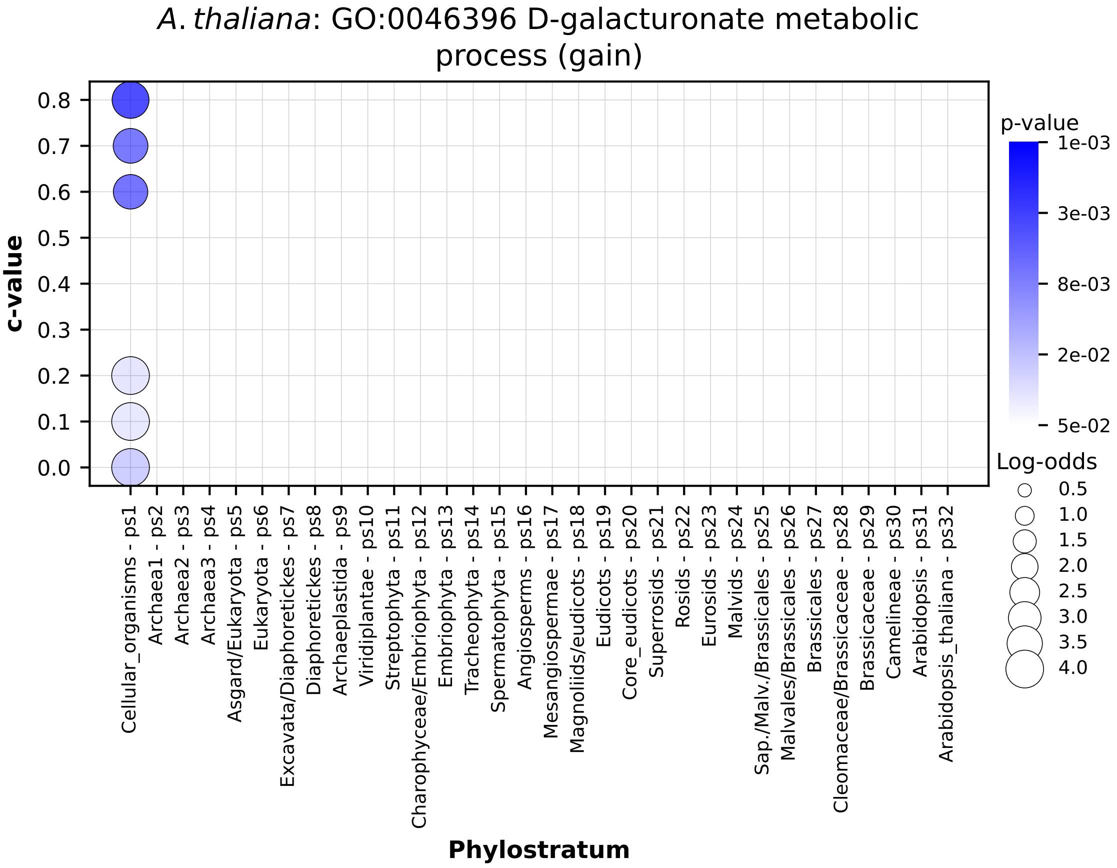

A. thaliana: GO:0046396 D-galacturonate metabolic process (gain)
c-value
0.0
0.1
0.2
0.3
0.4
0.5
0.6
0.7
0.8
Phylostratum
Cellular_organisms - ps1
Archaea1 - ps2
Archaea2 - ps3
Archaea3 - ps4
Asgard/Eukaryota - ps5
Eukaryota - ps6
Excavata/Diaphoretickes - ps7
Diaphoretickes - ps8
Archaeplastida - ps9
Viridiplantae - ps10
Streptophyta - ps11
Charophyceae/Embriophyta - ps12
Embriophyta - ps13
Tracheophyta - ps14
Spermatophyta - ps15
Angiosperms - ps16
Mesangiospermae - ps17
Magnoliids/eudicots - ps18
Eudicots - ps19
Core_eudicots - ps20
Superrosids - ps21
Rosids - ps22
Eurosids - ps23
Malvids - ps24
Sap./Malv./Brassicales - ps25
Malvales/Brassicales - ps26
Brassicales - ps27
Cleomaceae/Brassicaceae - ps28
Brassicaceae - ps29
Camelineae - ps30
Arabidopsis - ps31
Arabidopsis_thaliana - ps32
p-value
1e-03
3e-03
8e-03
2e-02
5e-02
Log-odds
0.5
1.0
1.5
2.0
2.5
3.0
3.5
4.0

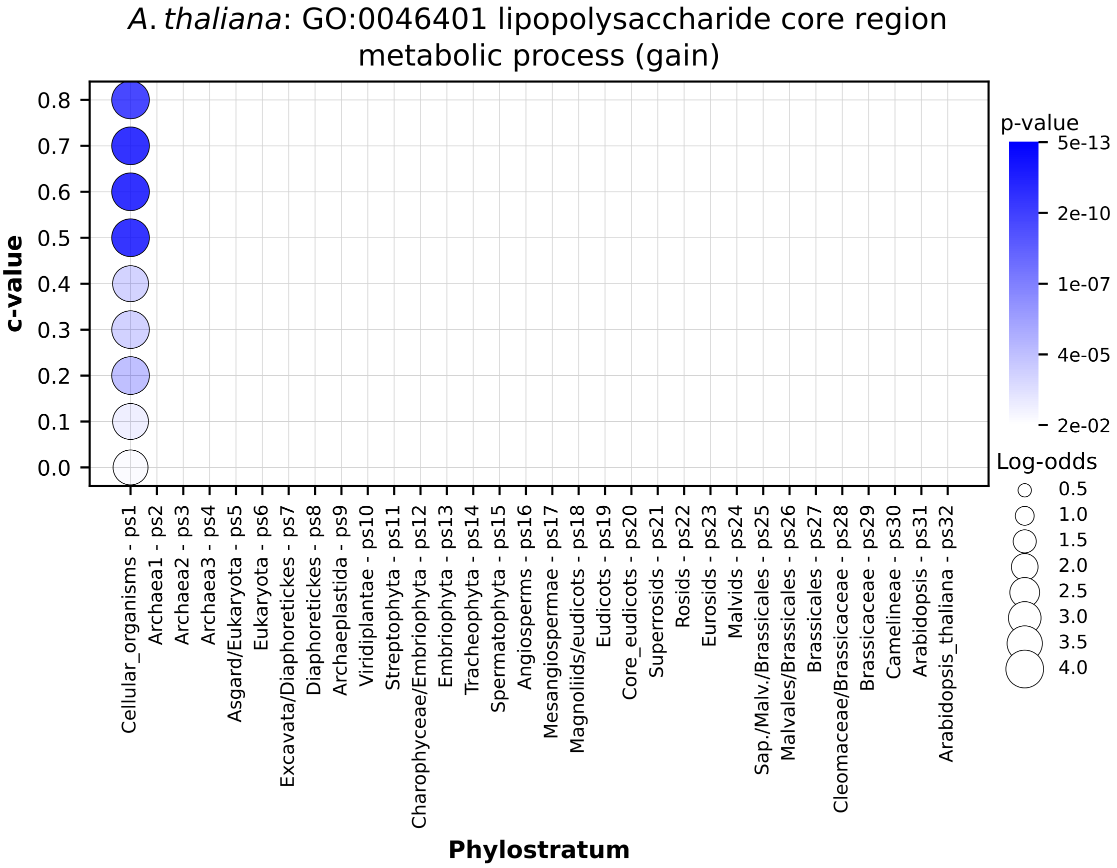
A. thaliana: GO:0046401 lipopolysaccharide core region metabolic process (gain)
c-value
0.0
0.1
0.2
0.3
0.4
0.5
0.6
0.7
0.8
p-value
5e-13
2e-10
1e-07
4e-05
2e-02
Log-odds
0.5
1.0
1.5
2.0
2.5
3.0
3.5
4.0
Cellular_organisms - ps1
Archaea1 - ps2
Archaea2 - ps3
Archaea3 - ps4
Asgard/Eukaryota - ps5
Eukaryota - ps6
Excavata/Diaphoretickes - ps7
Diaphoretickes - ps8
Archaeplastida - ps9
Viridiplantae - ps10
Streptophyta - ps11
Charophyceae/Embriophyta - ps12
Embriophyta - ps13
Tracheophyta - ps14
Spermatophyta - ps15
Angiosperms - ps16
Mesangiospermae - ps17
Magnoliids/eudicots - ps18
Eudicots - ps19
Core_eudicots - ps20
Superrosids - ps21
Rosids - ps22
Eurosids - ps23
Malvids - ps24
Sap./Malv./Brassicales - ps25
Malvales/Brassicales - ps26
Brassicales - ps27
Cleomaceae/Brassicaceae - ps28
Brassicaceae - ps29
Camelineae - ps30
Arabidopsis - ps31
Arabidopsis_thaliana - ps32
Phylostratum

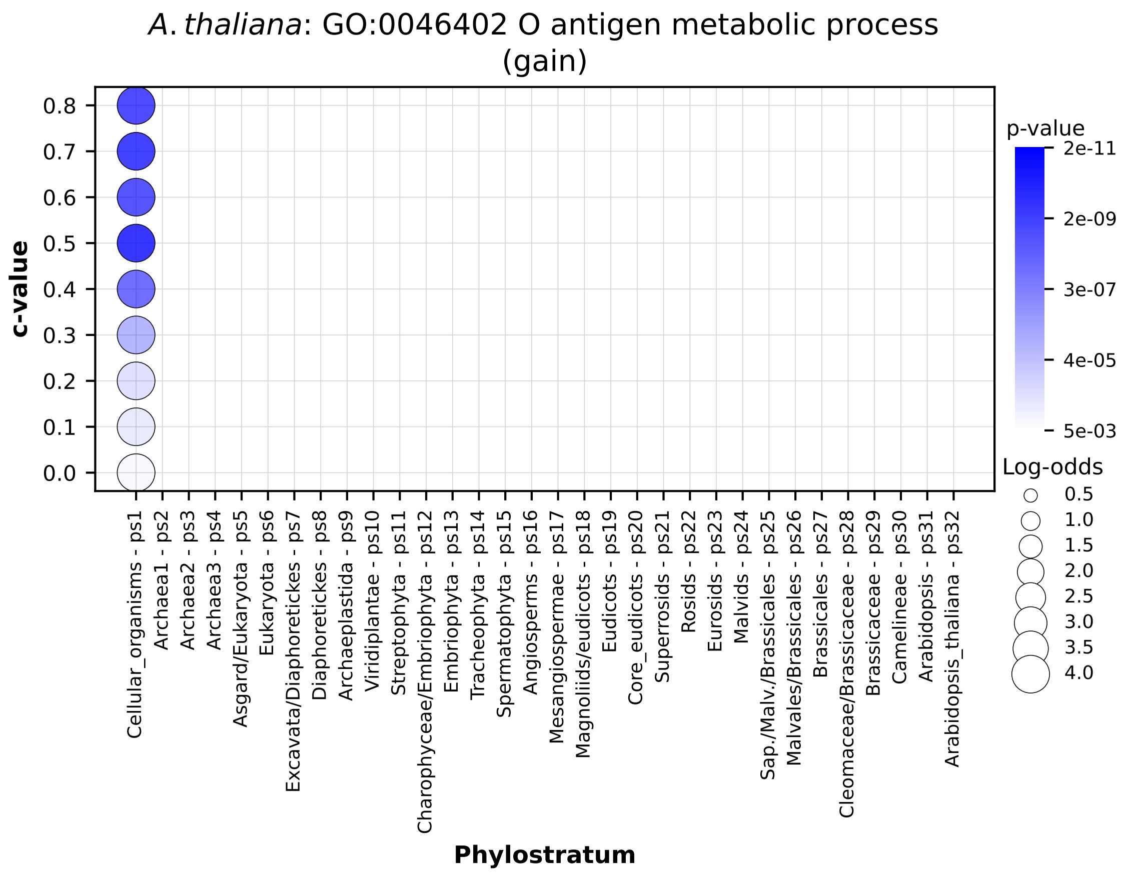

A. thaliana: GO:0046402 O antigen metabolic process
(gain)
c-value
0.8
0.7
0.6
0.5
0.4
0.3
0.2
0.1
0.0
p-value
2e-11
2e-09
3e-07
4e-05
5e-03
Log-odds
0.5
1.0
1.5
2.0
2.5
3.0
3.5
4.0
Cellular_organisms - ps1
Archaea1 - ps2
Archaea2 - ps3
Archaea3 - ps4
Asgard/Eukaryota - ps5
Eukaryota - ps6
Excavata/Diaphoretickes - ps7
Diaphoretickes - ps8
Archaeplastida - ps9
Viridiplantae - ps10
Streptophyta - ps11
Charophyceae/Embriophyta - ps12
Embriophyta - ps13
Tracheophyta - ps14
Spermatophyta - ps15
Angiosperms - ps16
Mesangiospermae - ps17
Magnoliids/eudicots - ps18
Eudicots - ps19
Core_eudicots - ps20
Superrosids - ps21
Rosids - ps22
Eurosids - ps23
Malvids - ps24
Sap./Malv./Brassicales - ps25
Malvales/Brassicales - ps26
Brassicales - ps27
Cleomaceae/Brassicaceae - ps28
Brassicaceae - ps29
Camelineae - ps30
Arabidopsis - ps31
Arabidopsis_thaliana - ps32
Phylostratum

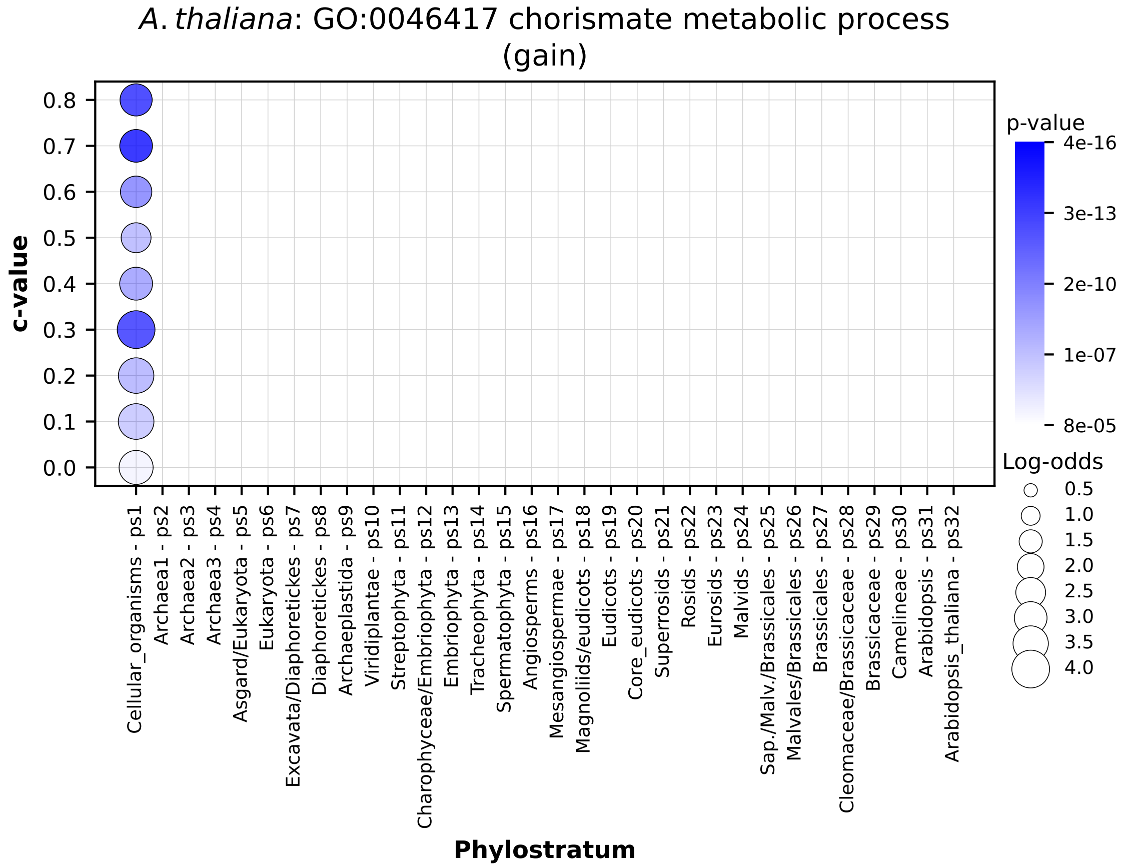
A. thaliana: GO:0046417 chorismate metabolic process
(gain)
c-value
0.8
0.7
0.6
0.5
0.4
0.3
0.2
0.1
0.0
p-value
4e-16
3e-13
2e-10
1e-07
8e-05
Log-odds
0.5
1.0
1.5
2.0
2.5
3.0
3.5
4.0
Cellular_organisms - ps1
Archaea1 - ps2
Archaea2 - ps3
Archaea3 - ps4
Asgard/Eukaryota - ps5
Eukaryota - ps6
Excavata/Diaphoretickes - ps7
Diaphoretickes - ps8
Archaeplastida - ps9
Viridiplantae - ps10
Streptophyta - ps11
Charophyceae/Embriophyta - ps12
Embriophyta - ps13
Tracheophyta - ps14
Spermatophyta - ps15
Angiosperms - ps16
Mesangiospermae - ps17
Magnoliids/eudicots - ps18
Eudicots - ps19
Core_eudicots - ps20
Superrosids - ps21
Rosids - ps22
Eurosids - ps23
Malvids - ps24
Sap./Malv./Brassicales - ps25
Malvales/Brassicales - ps26
Brassicales - ps27
Cleomaceae/Brassicaceae - ps28
Brassicaceae - ps29
Camelineae - ps30
Arabidopsis - ps31
Arabidopsis_thaliana - ps32
Phylostratum

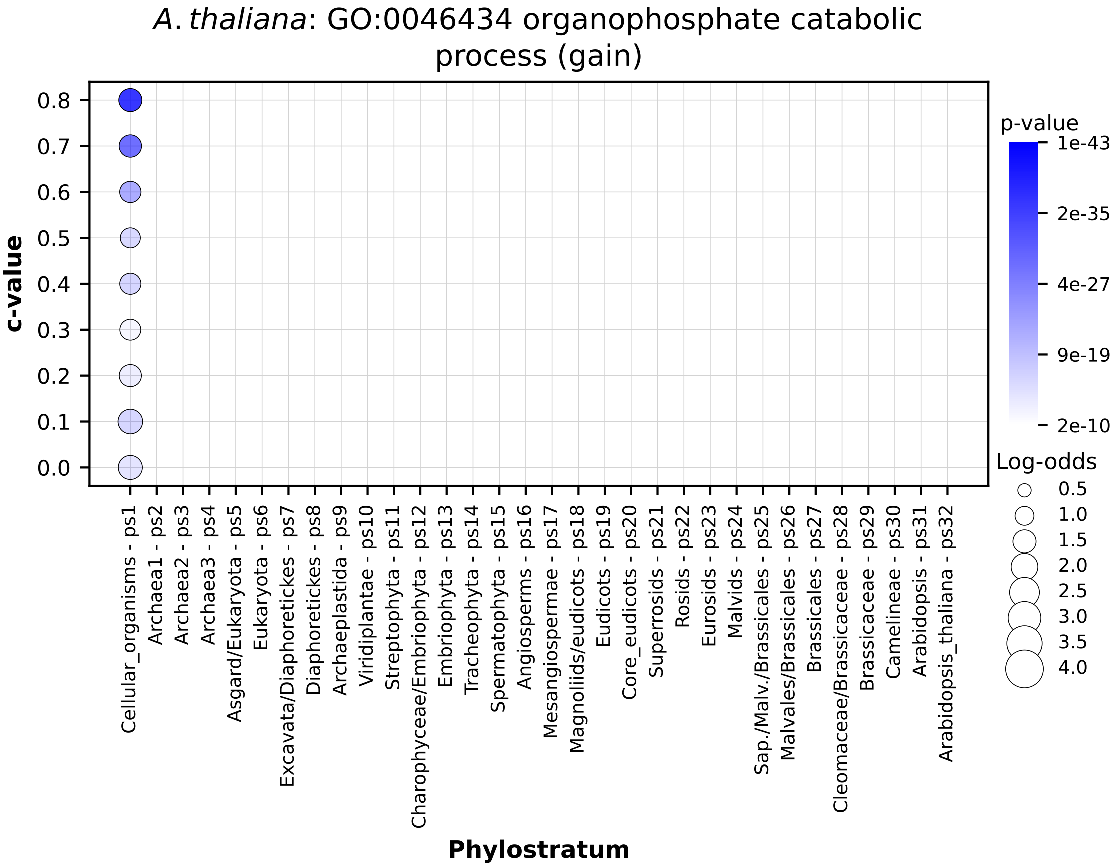
A. thaliana: GO:0046434 organophosphate catabolic process (gain)
c-value
0.0
0.1
0.2
0.3
0.4
0.5
0.6
0.7
0.8
p-value
1e-43
2e-35
4e-27
9e-19
2e-10
Log-odds
0.5
1.0
1.5
2.0
2.5
3.0
3.5
4.0
Cellular_organisms - ps1
Archaea1 - ps2
Archaea2 - ps3
Archaea3 - ps4
Asgard/Eukaryota - ps5
Eukaryota - ps6
Excavata/Diaphoretickes - ps7
Diaphoretickes - ps8
Archaeplastida - ps9
Viridiplantae - ps10
Streptophyta - ps11
Charophyceae/Embriophyta - ps12
Embriophyta - ps13
Tracheophyta - ps14
Spermatophyta - ps15
Angiosperms - ps16
Mesangiospermae - ps17
Magnoliids/eudicots - ps18
Eudicots - ps19
Core_eudicots - ps20
Superrosids - ps21
Rosids - ps22
Eurosids - ps23
Malvids - ps24
Sap./Malv./Brassicales - ps25
Malvales/Brassicales - ps26
Brassicales - ps27
Cleomaceae/Brassicaceae - ps28
Brassicaceae - ps29
Camelineae - ps30
Arabidopsis - ps31
Arabidopsis_thaliana - ps32
Phylostratum

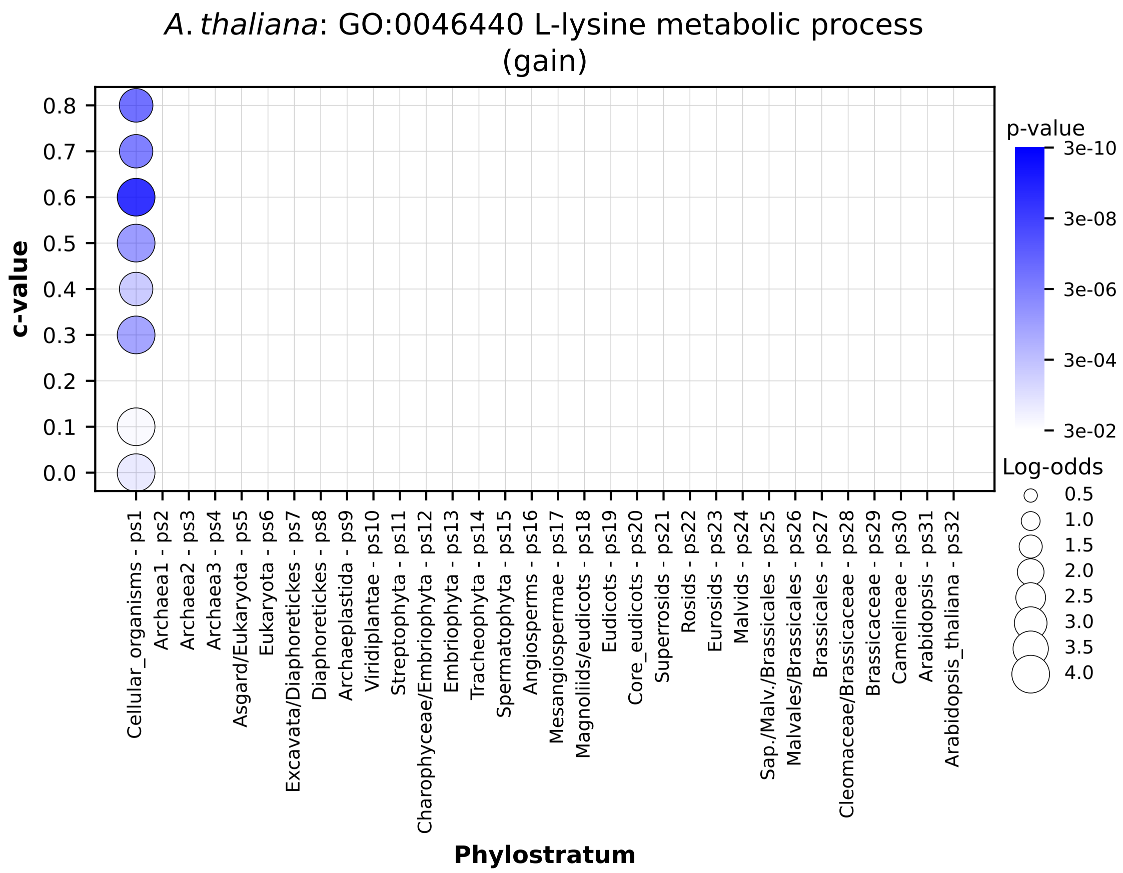

A. thaliana: GO:0046440 L-lysine metabolic process (gain)
c-value
0.8
0.7
0.6
0.5
0.4
0.3
0.2
0.1
0.0
p-value
3e-10
3e-08
3e-06
3e-04
3e-02
Log-odds
0.5
1.0
1.5
2.0
2.5
3.0
3.5
4.0
Cellular_organisms - ps1
Archaea1 - ps2
Archaea2 - ps3
Archaea3 - ps4
Asgard/Eukaryota - ps5
Eukaryota - ps6
Excavata/Diaphoretickes - ps7
Diaphoretickes - ps8
Archaeplastida - ps9
Viridiplantae - ps10
Streptophyta - ps11
Charophyceae/Embriophyta - ps12
Embriophyta - ps13
Tracheophyta - ps14
Spermatophyta - ps15
Angiosperms - ps16
Mesangiospermae - ps17
Magnoliids/eudicots - ps18
Eudicots - ps19
Core_eudicots - ps20
Superrosids - ps21
Rosids - ps22
Eurosids - ps23
Malvids - ps24
Sap./Malv./Brassicales - ps25
Malvales/Brassicales - ps26
Brassicales - ps27
Cleomaceae/Brassicaceae - ps28
Brassicaceae - ps29
Camelineae - ps30
Arabidopsis - ps31
Arabidopsis_thaliana - ps32
Phylostratum

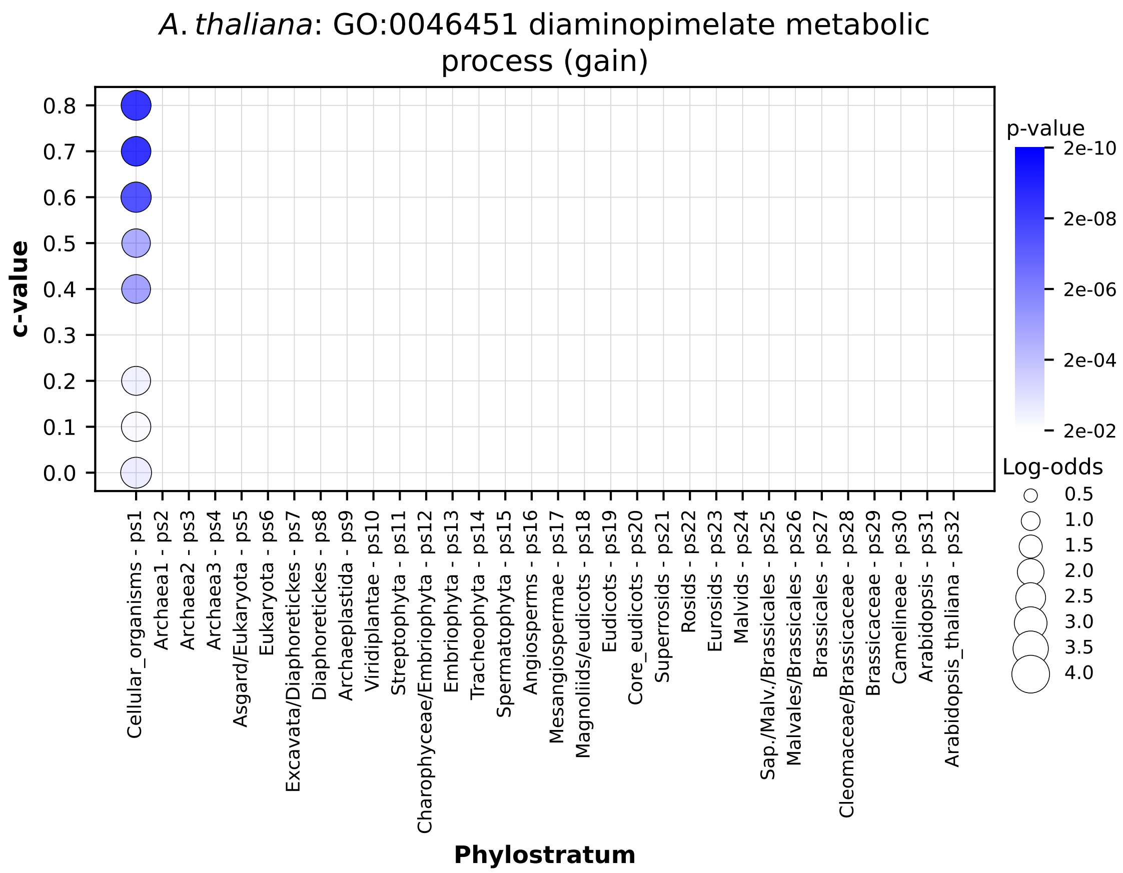

A. thaliana: GO:0046451 diaminopimelate metabolic process (gain)
c-value
0.8
0.7
0.6
0.5
0.4
0.3
0.2
0.1
0.0
p-value
2e-10
2e-08
2e-06
2e-04
2e-02
Log-odds
0.5
1.0
1.5
2.0
2.5
3.0
3.5
4.0
Cellular_organisms - ps1
Archaea1 - ps2
Archaea2 - ps3
Archaea3 - ps4
Asgard/Eukaryota - ps5
Eukaryota - ps6
Excavata/Diaphoretickes - ps7
Diaphoretickes - ps8
Archaeplastida - ps9
Viridiplantae - ps10
Streptophyta - ps11
Charophyceae/Embriophyta - ps12
Embriophyta - ps13
Tracheophyta - ps14
Spermatophyta - ps15
Angiosperms - ps16
Mesangiospermae - ps17
Magnoliids/eudicots - ps18
Eudicots - ps19
Core_eudicots - ps20
Superrosids - ps21
Rosids - ps22
Eurosids - ps23
Malvids - ps24
Sap./Malv./Brassicales - ps25
Malvales/Brassicales - ps26
Brassicales - ps27
Cleomaceae/Brassicaceae - ps28
Brassicaceae - ps29
Camelineae - ps30
Arabidopsis - ps31
Arabidopsis_thaliana - ps32
Phylostratum

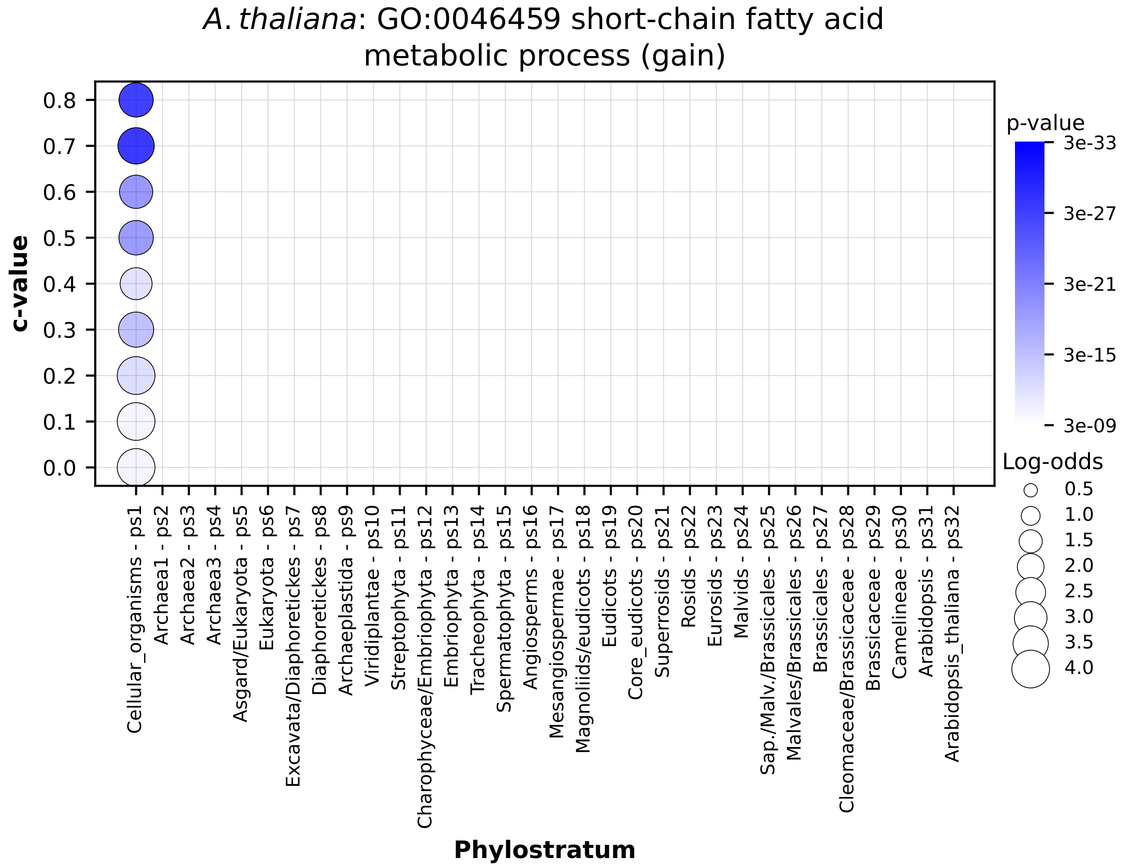

$A. thaliana$: GO:0046459 short-chain fatty acid metabolic process (gain)
c-value
0.0
0.1
0.2
0.3
0.4
0.5
0.6
0.7
0.8
p-value
3e-33
3e-27
3e-21
3e-15
3e-09
Log-odds
0.5
1.0
1.5
2.0
2.5
3.0
3.5
4.0
Cellular_organisms - ps1
Archaea1 - ps2
Archaea2 - ps3
Archaea3 - ps4
Asgard/Eukaryota - ps5
Eukaryota - ps6
Excavata/Diaphoretickes - ps7
Diaphoretickes - ps8
Archaeplastida - ps9
Viridiplantae - ps10
Streptophyta - ps11
Charophyceae/Embriophyta - ps12
Embriophyta - ps13
Tracheophyta - ps14
Spermatophyta - ps15
Angiosperms - ps16
Mesangiospermae - ps17
Magnoliids/eudicots - ps18
Eudicots - ps19
Core_eudicots - ps20
Superrosids - ps21
Rosids - ps22
Eurosids - ps23
Malvids - ps24
Sap./Malv./Brassicales - ps25
Malvales/Brassicales - ps26
Brassicales - ps27
Cleomaceae/Brassicaceae - ps28
Brassicaceae - ps29
Camelineae - ps30
Arabidopsis - ps31
Arabidopsis_thaliana - ps32
Phylostratum

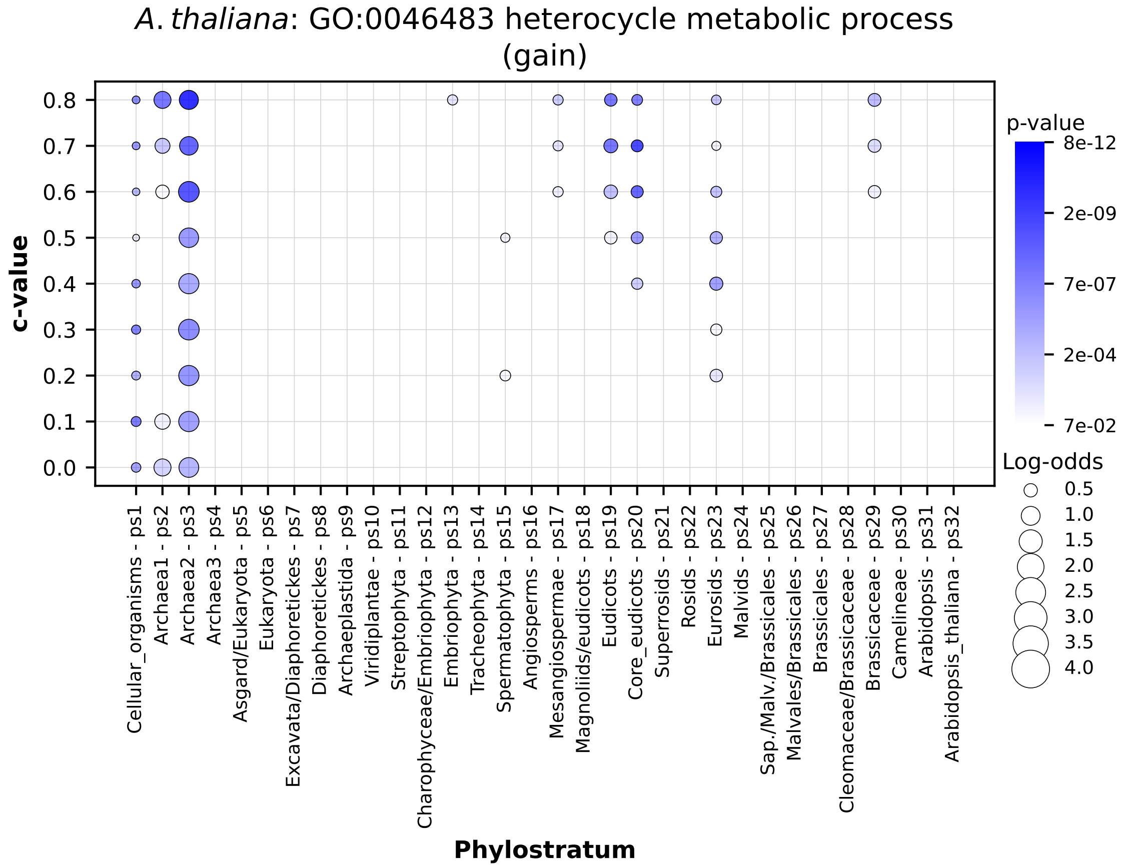

A. thaliana: GO:0046483 heterocycle metabolic process (gain)
c-value
0.8
0.7
0.6
0.5
0.4
0.3
0.2
0.1
0.0
Phylostratum
Cellular_organisms - ps1
Archaea1 - ps2
Archaea2 - ps3
Archaea3 - ps4
Asgard/Eukaryota - ps5
Eukaryota - ps6
Excavata/Diaphoretickes - ps7
Diaphoretickes - ps8
Archaeplastida - ps9
Viridiplantae - ps10
Streptophyta - ps11
Charophyceae/Embriophyta - ps12
Embriophyta - ps13
Tracheophyta - ps14
Spermatophyta - ps15
Angiosperms - ps16
Mesangiospermae - ps17
Magnoliids/eudicots - ps18
Eudicots - ps19
Core_eudicots - ps20
Superrosids - ps21
Rosids - ps22
Eurosids - ps23
Malvids - ps24
Sap./Malv./Brassicales - ps25
Malvales/Brassicales - ps26
Brassicales - ps27
Cleomaceae/Brassicaceae - ps28
Brassicaceae - ps29
Camelineae - ps30
Arabidopsis - ps31
Arabidopsis_thaliana - ps32
p-value
8e-12
2e-09
7e-07
2e-04
7e-02
Log-odds
0.5
1.0
1.5
2.0
2.5
3.0
3.5
4.0

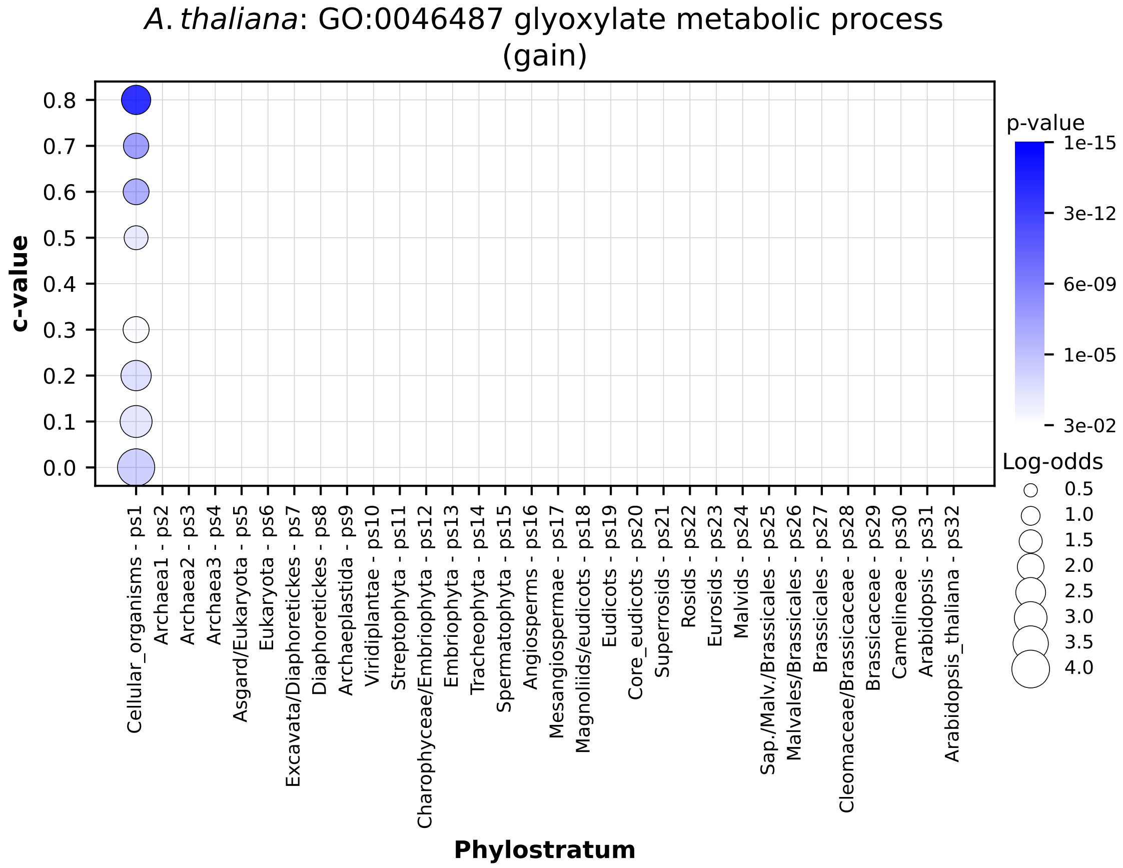

A. thaliana: GO:0046487 glyoxylate metabolic process
(gain)
c-value
0.8
0.7
0.6
0.5
0.4
0.3
0.2
0.1
0.0
p-value
1e-15
3e-12
6e-09
1e-05
3e-02
Log-odds
0.5
1.0
1.5
2.0
2.5
3.0
3.5
4.0
Cellular_organisms - ps1
Archaea1 - ps2
Archaea2 - ps3
Archaea3 - ps4
Asgard/Eukaryota - ps5
Eukaryota - ps6
Excavata/Diaphoretickes - ps7
Diaphoretickes - ps8
Archaeplastida - ps9
Viridiplantae - ps10
Streptophyta - ps11
Charophyceae/Embriophyta - ps12
Embriophyta - ps13
Tracheophyta - ps14
Spermatophyta - ps15
Angiosperms - ps16
Mesangiospermae - ps17
Magnoliids/eudicots - ps18
Eudicots - ps19
Core_eudicots - ps20
Superrosids - ps21
Rosids - ps22
Eurosids - ps23
Malvids - ps24
Sap./Malv./Brassicales - ps25
Malvales/Brassicales - ps26
Brassicales - ps27
Cleomaceae/Brassicaceae - ps28
Brassicaceae - ps29
Camelineae - ps30
Arabidopsis - ps31
Arabidopsis_thaliana - ps32
Phylostratum

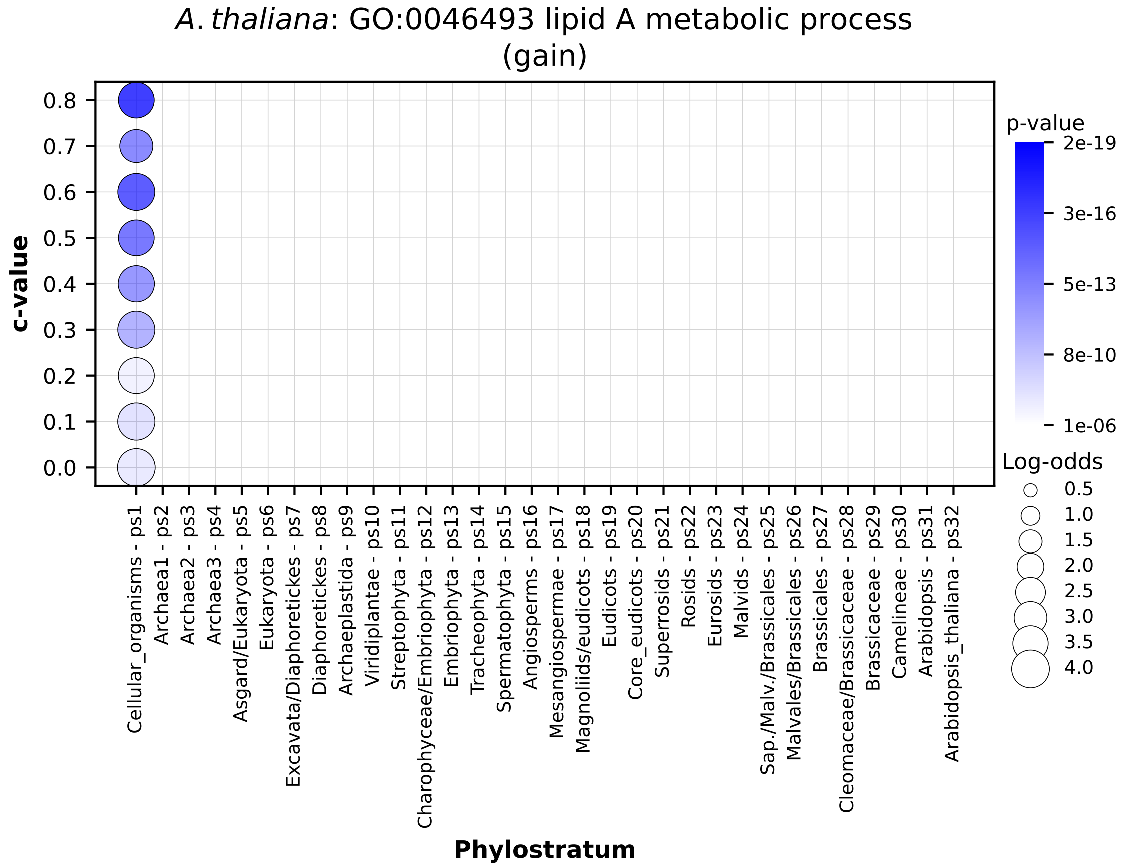

$A. thaliana$: GO:0046493 lipid A metabolic process
(gain)
c-value
0.8
0.7
0.6
0.5
0.4
0.3
0.2
0.1
0.0
p-value
2e-19
3e-16
5e-13
8e-10
1e-06
Log-odds
0.5
1.0
1.5
2.0
2.5
3.0
3.5
4.0
Cellular_organisms - ps1
Archaea1 - ps2
Archaea2 - ps3
Archaea3 - ps4
Asgard/Eukaryota - ps5
Eukaryota - ps6
Excavata/Diaphoretickes - ps7
Diaphoretickes - ps8
Archaeplastida - ps9
Viridiplantae - ps10
Streptophyta - ps11
Charophyceae/Embriophyta - ps12
Embriophyta - ps13
Tracheophyta - ps14
Spermatophyta - ps15
Angiosperms - ps16
Mesangiospermae - ps17
Magnoliids/eudicots - ps18
Eudicots - ps19
Core_eudicots - ps20
Superrosids - ps21
Rosids - ps22
Eurosids - ps23
Malvids - ps24
Sap./Malv./Brassicales - ps25
Malvales/Brassicales - ps26
Brassicales - ps27
Cleomaceae/Brassicaceae - ps28
Brassicaceae - ps29
Camelineae - ps30
Arabidopsis - ps31
Arabidopsis_thaliana - ps32
Phylostratum

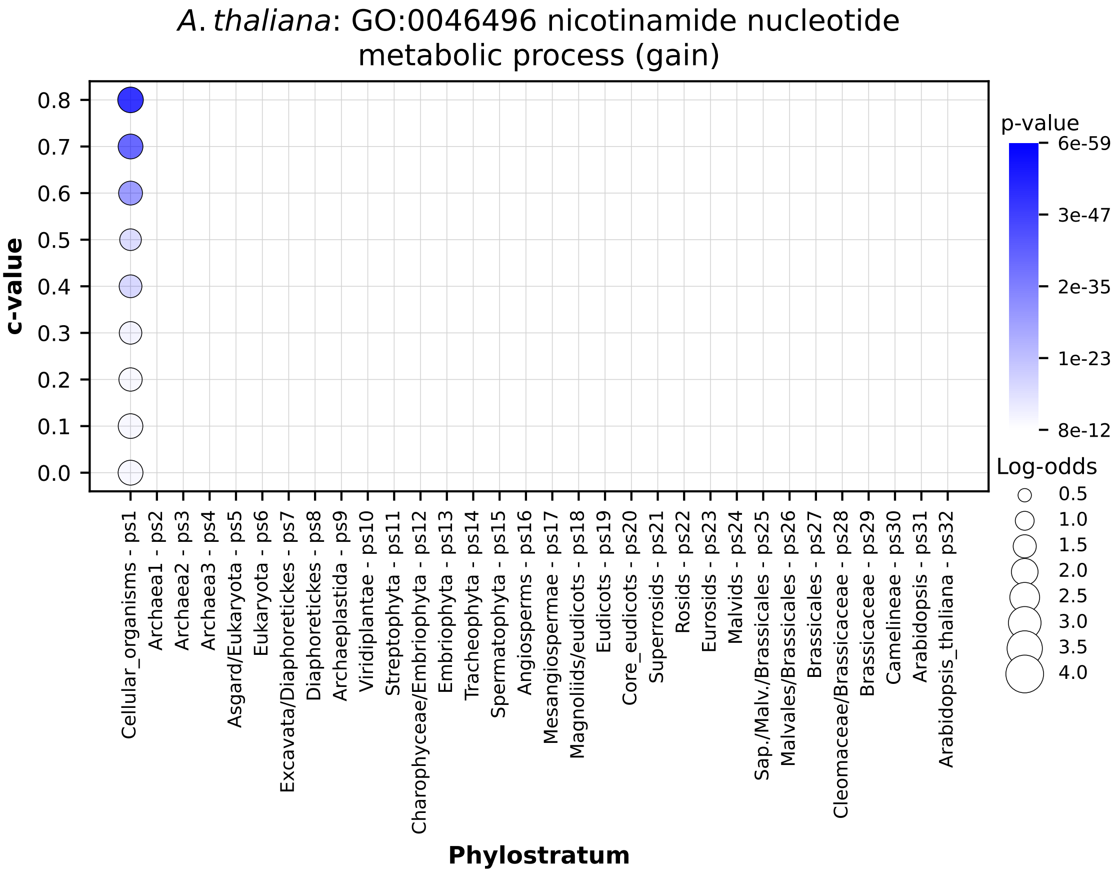

A. thaliana: GO:0046496 nicotinamide nucleotide metabolic process (gain)
c-value
0.8
0.7
0.6
0.5
0.4
0.3
0.2
0.1
0.0
Phylostratum
Cellular_organisms - ps1
Archaea1 - ps2
Archaea2 - ps3
Archaea3 - ps4
Asgard/Eukaryota - ps5
Eukaryota - ps6
Excavata/Diaphoretickes - ps7
Diaphoretickes - ps8
Archaeplastida - ps9
Viridiplantae - ps10
Streptophyta - ps11
Charophyceae/Embriophyta - ps12
Embriophyta - ps13
Tracheophyta - ps14
Spermatophyta - ps15
Angiosperms - ps16
Mesangiospermae - ps17
Magnoliids/eudicots - ps18
Eudicots - ps19
Core_eudicots - ps20
Superrosids - ps21
Rosids - ps22
Eurosids - ps23
Malvids - ps24
Sap./Malv./Brassicales - ps25
Malvales/Brassicales - ps26
Brassicales - ps27
Cleomaceae/Brassicaceae - ps28
Brassicaceae - ps29
Camelineae - ps30
Arabidopsis - ps31
Arabidopsis_thaliana - ps32
p-value
6e-59
3e-47
2e-35
1e-23
8e-12
Log-odds
0.5
1.0
1.5
2.0
2.5
3.0
3.5
4.0

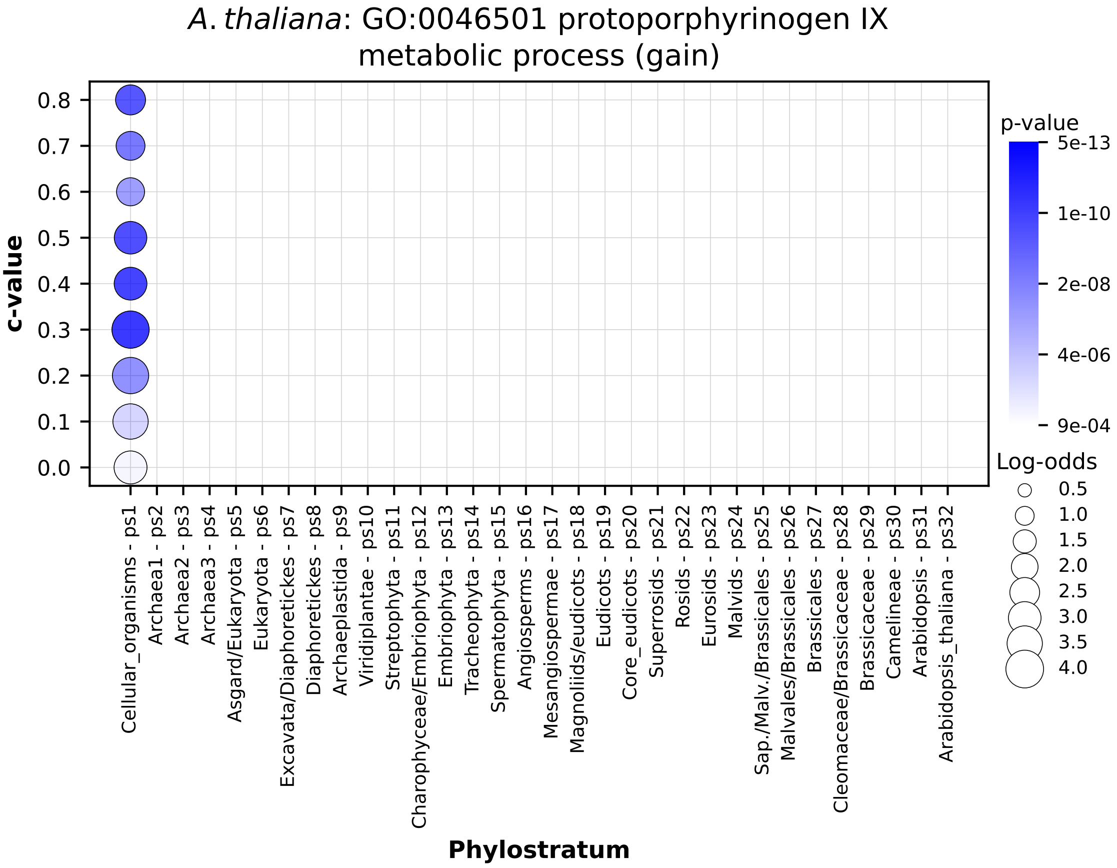

A. thaliana: GO:0046501 protoporphyrinogen IX metabolic process (gain)
c-value
0.8
0.7
0.6
0.5
0.4
0.3
0.2
0.1
0.0
p-value
5e-13
1e-10
2e-08
4e-06
9e-04
Log-odds
0.5
1.0
1.5
2.0
2.5
3.0
3.5
4.0
Cellular_organisms - ps1
Archaea1 - ps2
Archaea2 - ps3
Archaea3 - ps4
Asgard/Eukaryota - ps5
Eukaryota - ps6
Excavata/Diaphoretickes - ps7
Diaphoretickes - ps8
Archaeplastida - ps9
Viridiplantae - ps10
Streptophyta - ps11
Charophyceae/Embriophyta - ps12
Embriophyta - ps13
Tracheophyta - ps14
Spermatophyta - ps15
Angiosperms - ps16
Mesangiospermae - ps17
Magnoliids/eudicots - ps18
Eudicots - ps19
Core_eudicots - ps20
Superrosids - ps21
Rosids - ps22
Eurosids - ps23
Malvids - ps24
Sap./Malv./Brassicales - ps25
Malvales/Brassicales - ps26
Brassicales - ps27
Cleomaceae/Brassicaceae - ps28
Brassicaceae - ps29
Camelineae - ps30
Arabidopsis - ps31
Arabidopsis_thaliana - ps32
Phylostratum

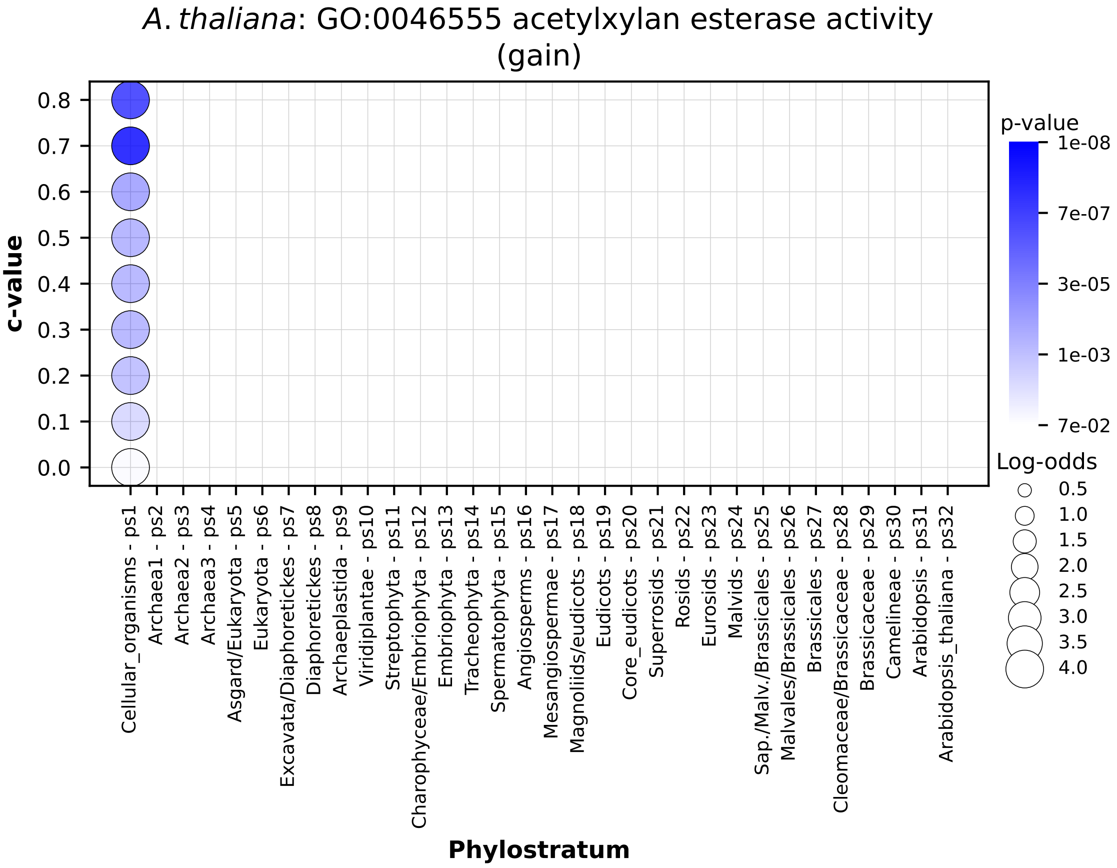

A. thaliana: GO:0046555 acetylxylan esterase activity (gain)
c-value
0.0
0.1
0.2
0.3
0.4
0.5
0.6
0.7
0.8
p-value
1e-08
7e-07
3e-05
1e-03
7e-02
Log-odds
0.5
1.0
1.5
2.0
2.5
3.0
3.5
4.0
Cellular_organisms - ps1
Archaea1 - ps2
Archaea2 - ps3
Archaea3 - ps4
Asgard/Eukaryota - ps5
Eukaryota - ps6
Excavata/Diaphoretickes - ps7
Diaphoretickes - ps8
Archaeplastida - ps9
Viridiplantae - ps10
Streptophyta - ps11
Charophyceae/Embriophyta - ps12
Embriophyta - ps13
Tracheophyta - ps14
Spermatophyta - ps15
Angiosperms - ps16
Mesangiospermae - ps17
Magnoliids/eudicots - ps18
Eudicots - ps19
Core_eudicots - ps20
Superrosids - ps21
Rosids - ps22
Eurosids - ps23
Malvids - ps24
Sap./Malv./Brassicales - ps25
Malvales/Brassicales - ps26
Brassicales - ps27
Cleomaceae/Brassicaceae - ps28
Brassicaceae - ps29
Camelineae - ps30
Arabidopsis - ps31
Arabidopsis_thaliana - ps32
Phylostratum

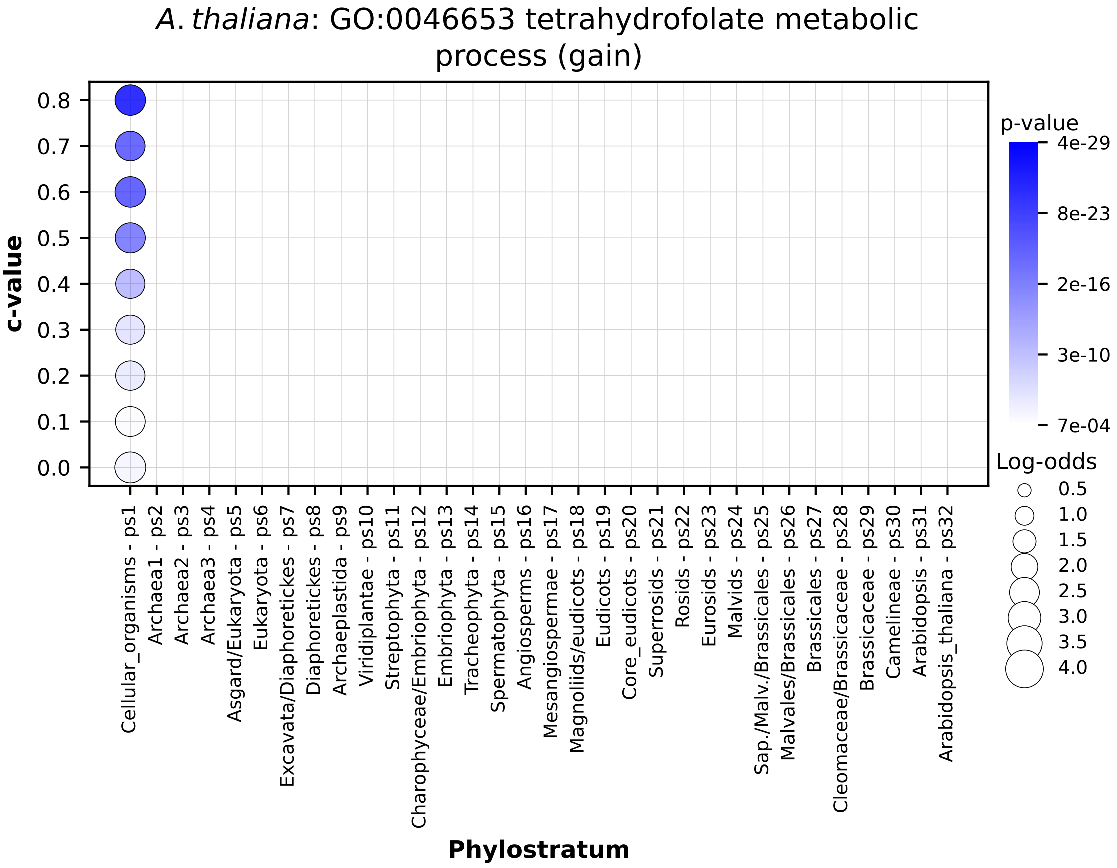

A. thaliana: GO:0046653 tetrahydrofolate metabolic process (gain)
c-value
0.8
0.7
0.6
0.5
0.4
0.3
0.2
0.1
0.0
p-value
4e-29
8e-23
2e-16
3e-10
7e-04
Log-odds
0.5
1.0
1.5
2.0
2.5
3.0
3.5
4.0
Cellular_organisms - ps1
Archaea1 - ps2
Archaea2 - ps3
Archaea3 - ps4
Asgard/Eukaryota - ps5
Eukaryota - ps6
Excavata/Diaphoretickes - ps7
Diaphoretickes - ps8
Archaeplastida - ps9
Viridiplantae - ps10
Streptophyta - ps11
Charophyceae/Embriophyta - ps12
Embriophyta - ps13
Tracheophyta - ps14
Spermatophyta - ps15
Angiosperms - ps16
Mesangiospermae - ps17
Magnoliids/eudicots - ps18
Eudicots - ps19
Core_eudicots - ps20
Superrosids - ps21
Rosids - ps22
Eurosids - ps23
Malvids - ps24
Sap./Malv./Brassicales - ps25
Malvales/Brassicales - ps26
Brassicales - ps27
Cleomaceae/Brassicaceae - ps28
Brassicaceae - ps29
Camelineae - ps30
Arabidopsis - ps31
Arabidopsis_thaliana - ps32
Phylostratum

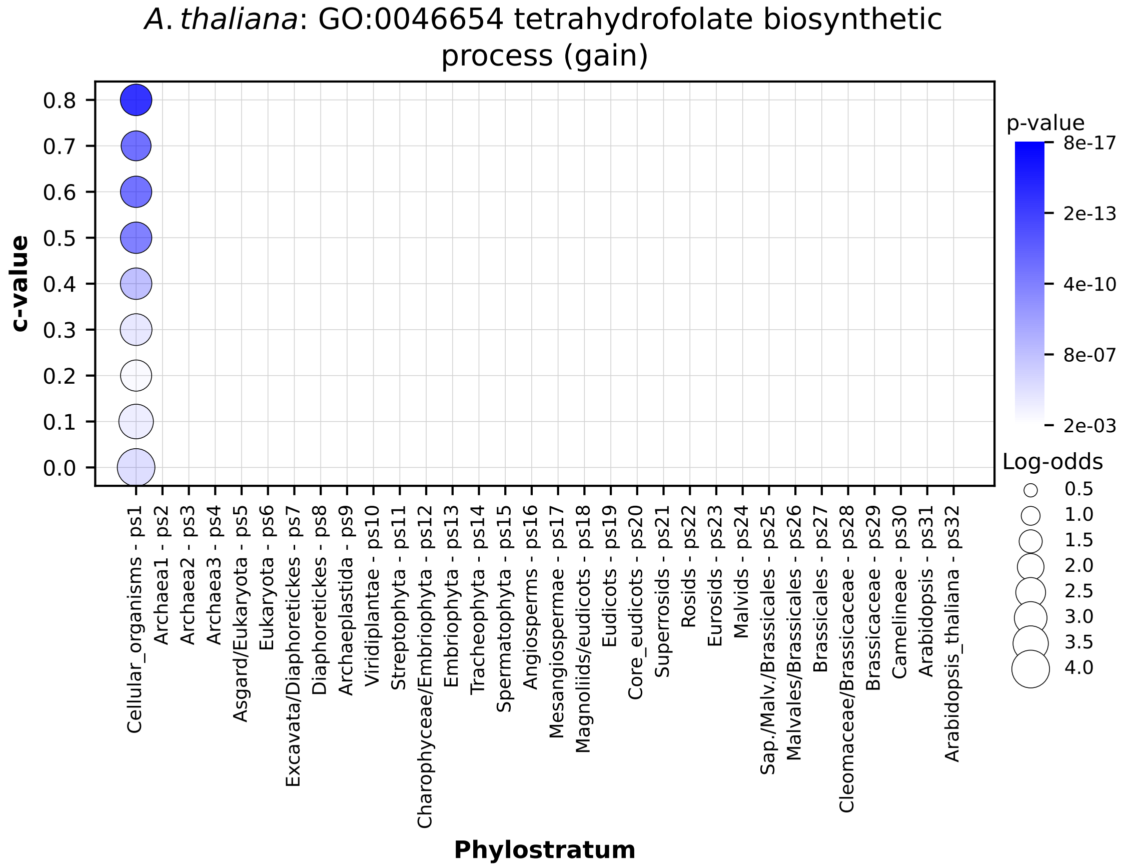
A. thaliana: GO:0046654 tetrahydrofolate biosynthetic process (gain)
c-value
0.8
0.7
0.6
0.5
0.4
0.3
0.2
0.1
0.0
p-value
8e-17
2e-13
4e-10
8e-07
2e-03
Log-odds
0.5
1.0
1.5
2.0
2.5
3.0
3.5
4.0
Cellular_organisms - ps1
Archaea1 - ps2
Archaea2 - ps3
Archaea3 - ps4
Asgard/Eukaryota - ps5
Eukaryota - ps6
Excavata/Diaphoretickes - ps7
Diaphoretickes - ps8
Archaeplastida - ps9
Viridiplantae - ps10
Streptophyta - ps11
Charophyceae/Embriophyta - ps12
Embriophyta - ps13
Tracheophyta - ps14
Spermatophyta - ps15
Angiosperms - ps16
Mesangiospermae - ps17
Magnoliids/eudicots - ps18
Eudicots - ps19
Core_eudicots - ps20
Superrosids - ps21
Rosids - ps22
Eurosids - ps23
Malvids - ps24
Sap./Malv./Brassicales - ps25
Malvales/Brassicales - ps26
Brassicales - ps27
Cleomaceae/Brassicaceae - ps28
Brassicaceae - ps29
Camelineae - ps30
Arabidopsis - ps31
Arabidopsis_thaliana - ps32
Phylostratum

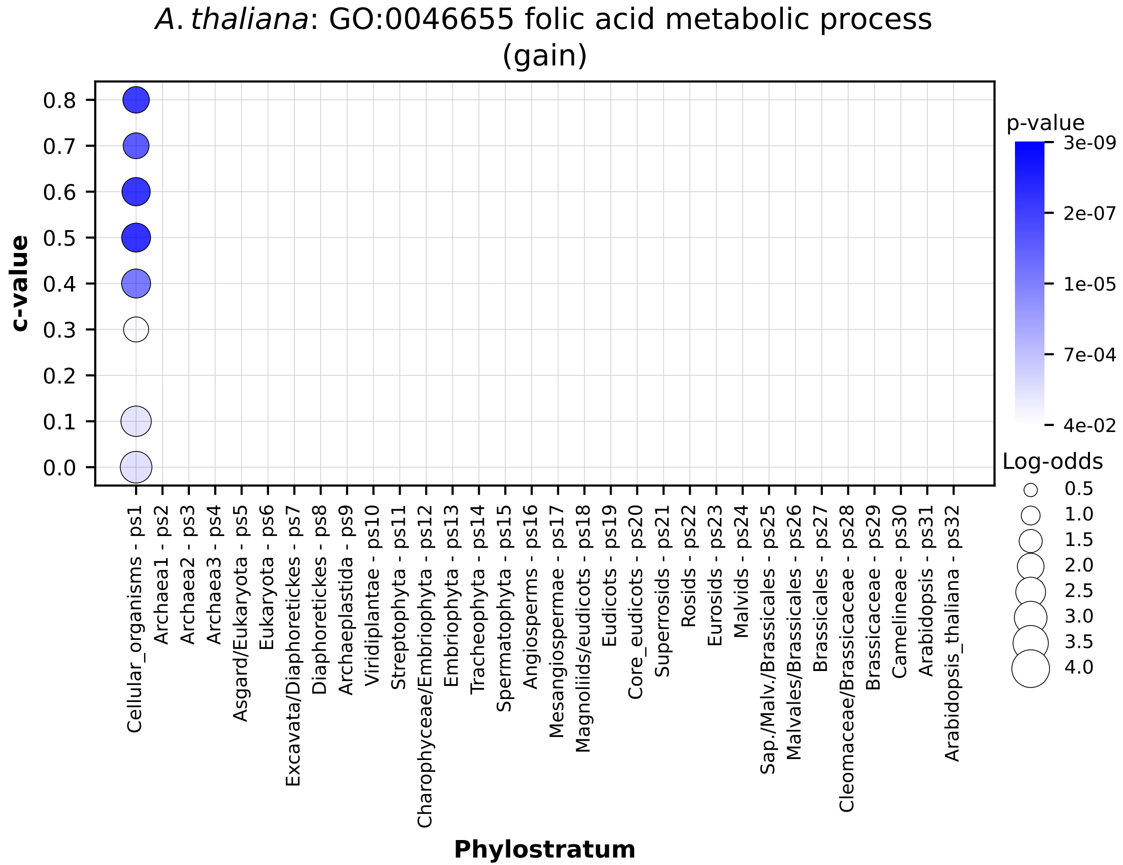

A. thaliana: GO:0046655 folic acid metabolic process (gain)
c-value
0.8
0.7
0.6
0.5
0.4
0.3
0.2
0.1
0.0
p-value
3e-09
2e-07
1e-05
7e-04
4e-02
Log-odds
0.5
1.0
1.5
2.0
2.5
3.0
3.5
4.0
Cellular_organisms - ps1
Archaea1 - ps2
Archaea2 - ps3
Archaea3 - ps4
Asgard/Eukaryota - ps5
Eukaryota - ps6
Excavata/Diaphoretickes - ps7
Diaphoretickes - ps8
Archaeplastida - ps9
Viridiplantae - ps10
Streptophyta - ps11
Charophyceae/Embriophyta - ps12
Embriophyta - ps13
Tracheophyta - ps14
Spermatophyta - ps15
Angiosperms - ps16
Mesangiospermae - ps17
Magnoliids/eudicots - ps18
Eudicots - ps19
Core_eudicots - ps20
Superrosids - ps21
Rosids - ps22
Eurosids - ps23
Malvids - ps24
Sap./Malv./Brassicales - ps25
Malvales/Brassicales - ps26
Brassicales - ps27
Cleomaceae/Brassicaceae - ps28
Brassicaceae - ps29
Camelineae - ps30
Arabidopsis - ps31
Arabidopsis_thaliana - ps32
Phylostratum

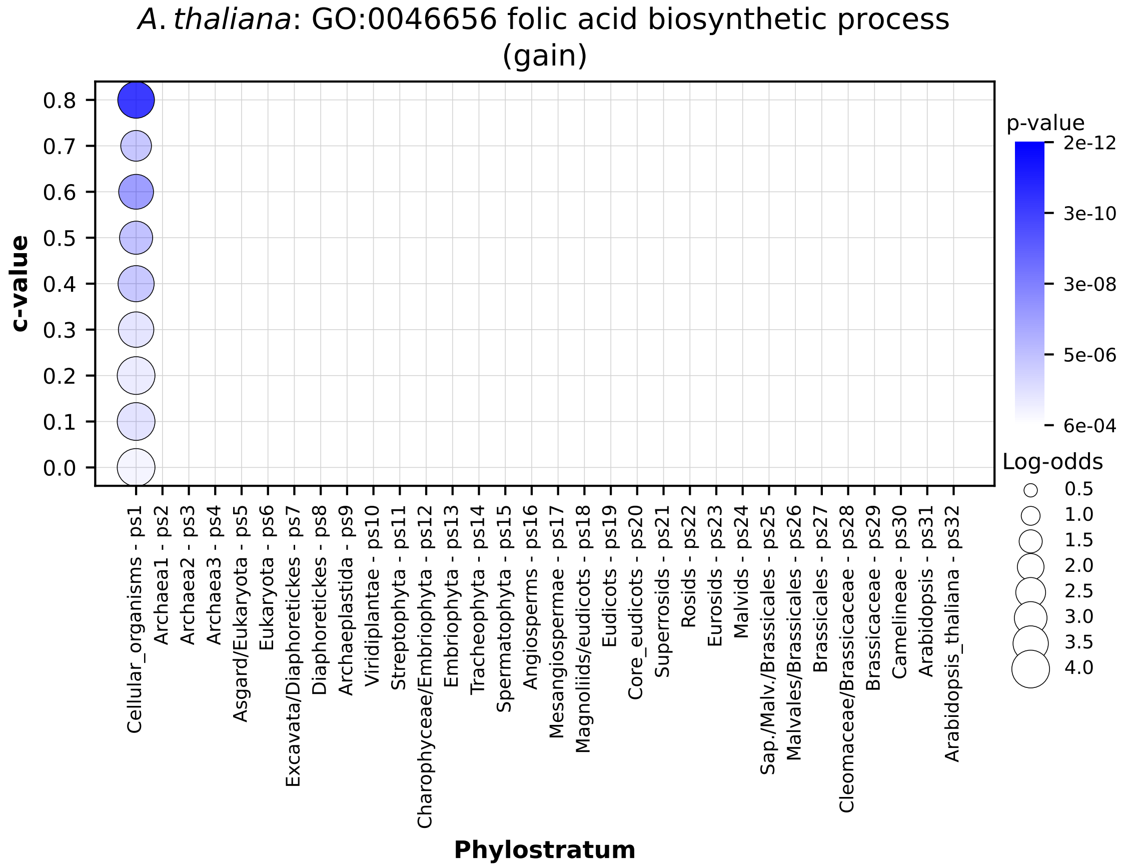

A. thaliana: GO:0046656 folic acid biosynthetic process
(gain)
c-value
0.8
0.7
0.6
0.5
0.4
0.3
0.2
0.1
0.0
p-value
2e-12
3e-10
3e-08
5e-06
6e-04
Log-odds
0.5
1.0
1.5
2.0
2.5
3.0
3.5
4.0
Cellular_organisms - ps1
Archaea1 - ps2
Archaea2 - ps3
Archaea3 - ps4
Asgard/Eukaryota - ps5
Eukaryota - ps6
Excavata/Diaphoretickes - ps7
Diaphoretickes - ps8
Archaeplastida - ps9
Viridiplantae - ps10
Streptophyta - ps11
Charophyceae/Embriophyta - ps12
Embriophyta - ps13
Tracheophyta - ps14
Spermatophyta - ps15
Angiosperms - ps16
Mesangiospermae - ps17
Magnoliids/eudicots - ps18
Eudicots - ps19
Core_eudicots - ps20
Superrosids - ps21
Rosids - ps22
Eurosids - ps23
Malvids - ps24
Sap./Malv./Brassicales - ps25
Malvales/Brassicales - ps26
Brassicales - ps27
Cleomaceae/Brassicaceae - ps28
Brassicaceae - ps29
Camelineae - ps30
Arabidopsis - ps31
Arabidopsis_thaliana - ps32
Phylostratum

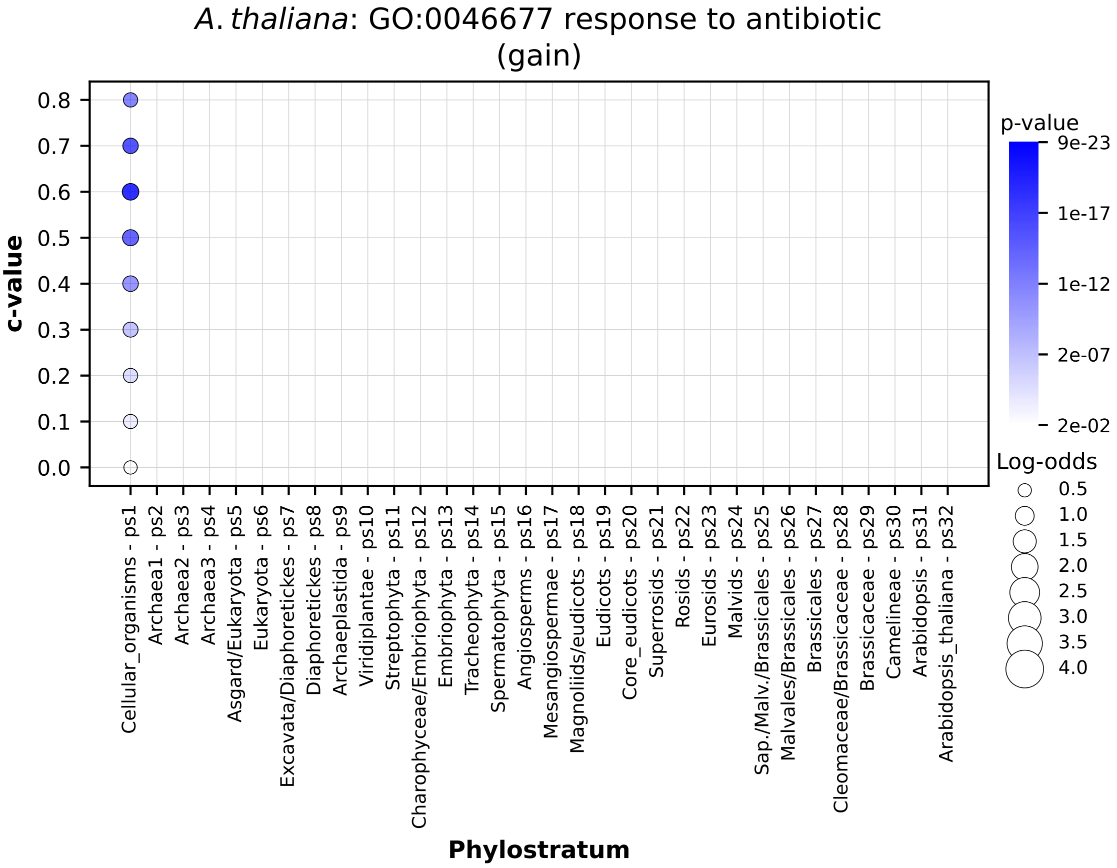

A. thaliana: GO:0046677 response to antibiotic
(gain)
c-value
0.8
0.7
0.6
0.5
0.4
0.3
0.2
0.1
0.0
p-value
9e-23
1e-17
1e-12
2e-07
2e-02
Log-odds
0.5
1.0
1.5
2.0
2.5
3.0
3.5
4.0
Cellular_organisms - ps1
Archaea1 - ps2
Archaea2 - ps3
Archaea3 - ps4
Asgard/Eukaryota - ps5
Eukaryota - ps6
Excavata/Diaphoretickes - ps7
Diaphoretickes - ps8
Archaeplastida - ps9
Viridiplantae - ps10
Streptophyta - ps11
Charophyceae/Embriophyta - ps12
Embriophyta - ps13
Tracheophyta - ps14
Spermatophyta - ps15
Angiosperms - ps16
Mesangiospermae - ps17
Magnoliids/eudicots - ps18
Eudicots - ps19
Core_eudicots - ps20
Superrosids - ps21
Rosids - ps22
Eurosids - ps23
Malvids - ps24
Sap./Malv./Brassicales - ps25
Malvales/Brassicales - ps26
Brassicales - ps27
Cleomaceae/Brassicaceae - ps28
Brassicaceae - ps29
Camelineae - ps30
Arabidopsis - ps31
Arabidopsis_thaliana - ps32
Phylostratum

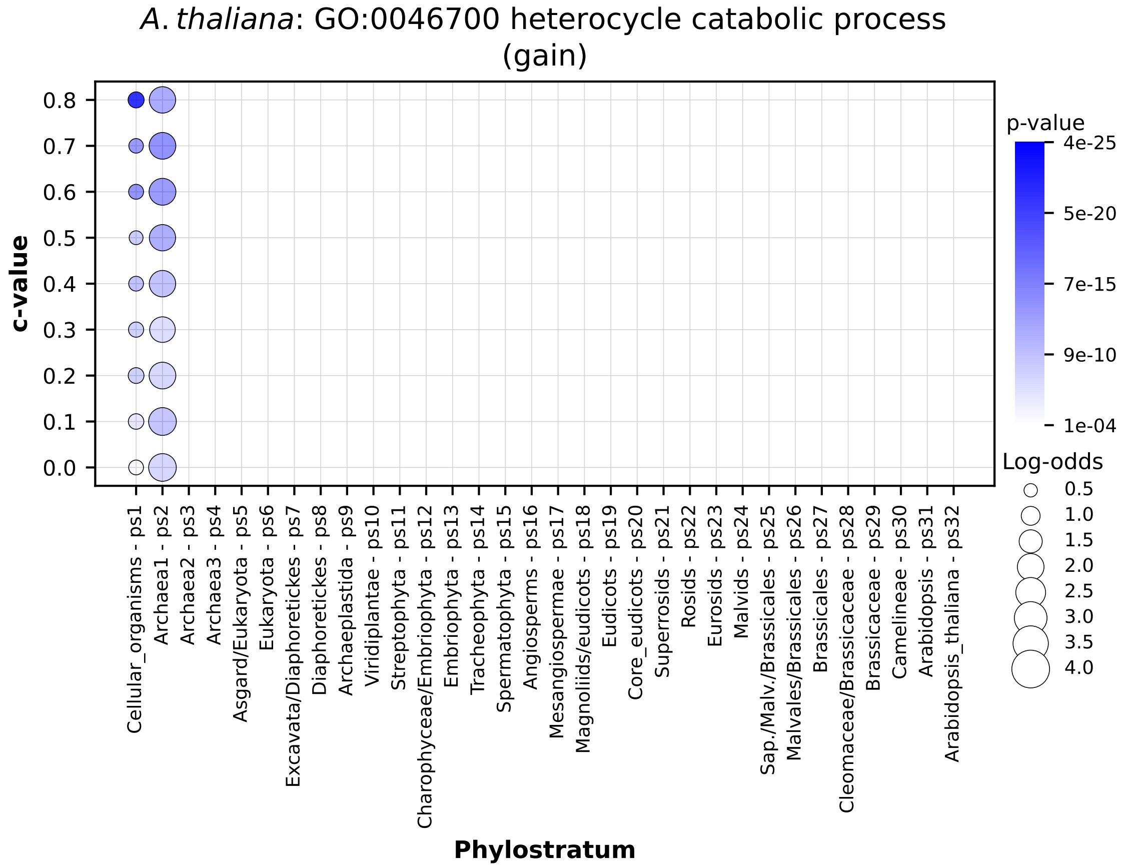

A. thaliana: GO:0046700 heterocycle catabolic process
(gain)
c-value
0.8
0.7
0.6
0.5
0.4
0.3
0.2
0.1
0.0
p-value
4e-25
5e-20
7e-15
9e-10
1e-04
Log-odds
0.5
1.0
1.5
2.0
2.5
3.0
3.5
4.0
Cellular_organisms - ps1
Archaea1 - ps2
Archaea2 - ps3
Archaea3 - ps4
Asgard/Eukaryota - ps5
Eukaryota - ps6
Excavata/Diaphoretickes - ps7
Diaphoretickes - ps8
Archaeplastida - ps9
Viridiplantae - ps10
Streptophyta - ps11
Charophyceae/Embriophyta - ps12
Embriophyta - ps13
Tracheophyta - ps14
Spermatophyta - ps15
Angiosperms - ps16
Mesangiospermae - ps17
Magnoliids/eudicots - ps18
Eudicots - ps19
Core_eudicots - ps20
Superrosids - ps21
Rosids - ps22
Eurosids - ps23
Malvids - ps24
Sap./Malv./Brassicales - ps25
Malvales/Brassicales - ps26
Brassicales - ps27
Cleomaceae/Brassicaceae - ps28
Brassicaceae - ps29
Camelineae - ps30
Arabidopsis - ps31
Arabidopsis_thaliana - ps32
Phylostratum

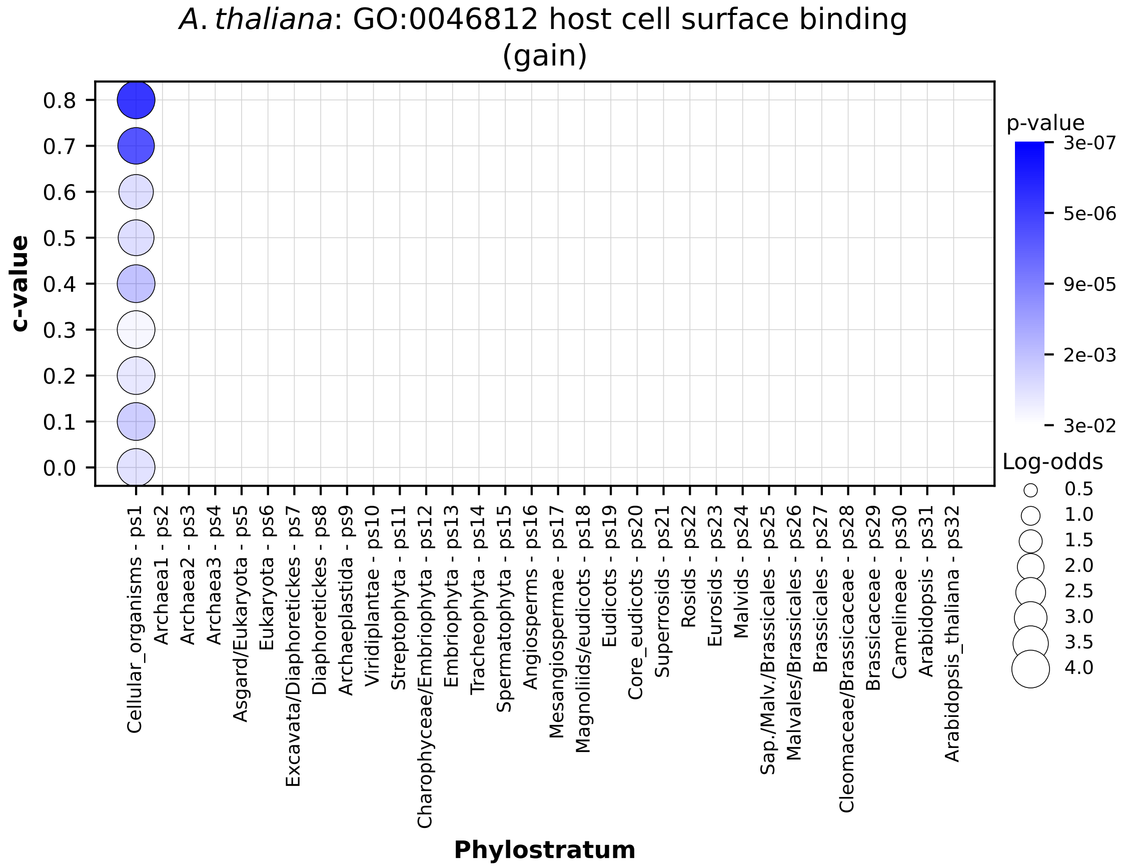

A. thaliana: GO:0046812 host cell surface binding (gain)
c-value
0.0
0.1
0.2
0.3
0.4
0.5
0.6
0.7
0.8
p-value
3e-07
5e-06
9e-05
2e-03
3e-02
Log-odds
0.5
1.0
1.5
2.0
2.5
3.0
3.5
4.0
Cellular_organisms - ps1
Archaea1 - ps2
Archaea2 - ps3
Archaea3 - ps4
Asgard/Eukaryota - ps5
Eukaryota - ps6
Excavata/Diaphoretickes - ps7
Diaphoretickes - ps8
Archaeplastida - ps9
Viridiplantae - ps10
Streptophyta - ps11
Charophyceae/Embriophyta - ps12
Embriophyta - ps13
Tracheophyta - ps14
Spermatophyta - ps15
Angiosperms - ps16
Mesangiospermae - ps17
Magnoliids/eudicots - ps18
Eudicots - ps19
Core_eudicots - ps20
Superrosids - ps21
Rosids - ps22
Eurosids - ps23
Malvids - ps24
Sap./Malv./Brassicales - ps25
Malvales/Brassicales - ps26
Brassicales - ps27
Cleomaceae/Brassicaceae - ps28
Brassicaceae - ps29
Camelineae - ps30
Arabidopsis - ps31
Arabidopsis_thaliana - ps32
Phylostratum

A. thaliana: GO:0046835 carbohydrate phosphorylation (gain)

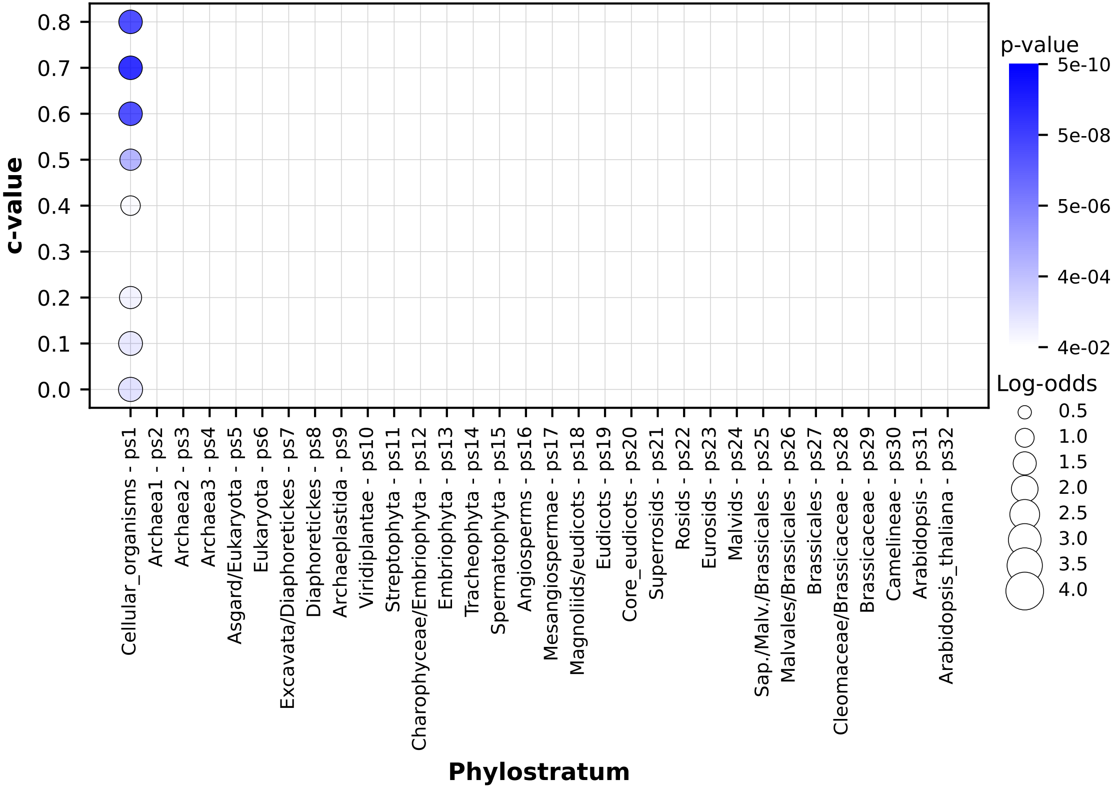

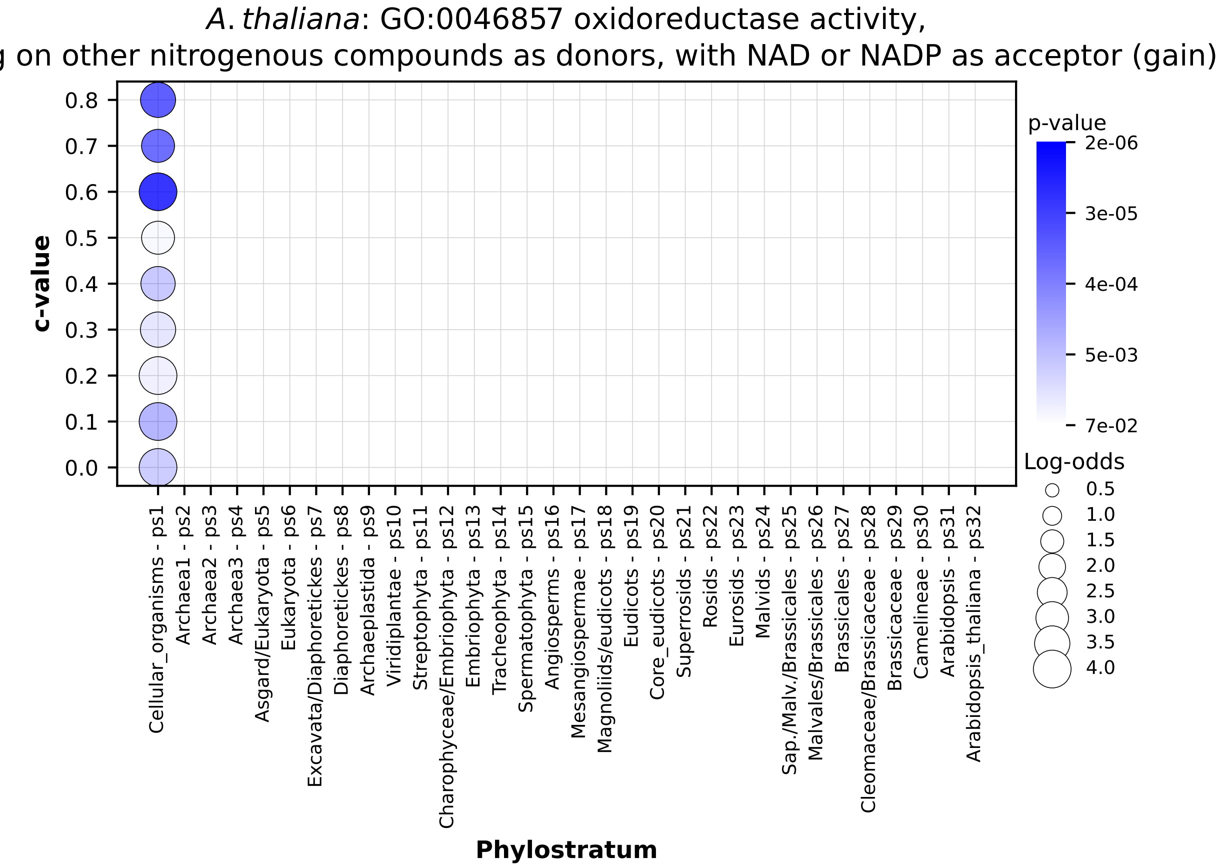

A. thaliana: GO:0046857 oxidoreductase activity,
g on other nitrogenous compounds as donors, with NAD or NADP as acceptor (gain)
c-value
0.8
0.7
0.6
0.5
0.4
0.3
0.2
0.1
0.0
p-value
2e-06
3e-05
4e-04
5e-03
7e-02
Log-odds
0.5
1.0
1.5
2.0
2.5
3.0
3.5
4.0
Cellular_organisms - ps1
Archaea1 - ps2
Archaea2 - ps3
Archaea3 - ps4
Asgard/Eukaryota - ps5
Eukaryota - ps6
Excavata/Diaphoretickes - ps7
Diaphoretickes - ps8
Archaeplastida - ps9
Viridiplantae - ps10
Streptophyta - ps11
Charophyceae/Embriophyta - ps12
Embriophyta - ps13
Tracheophyta - ps14
Spermatophyta - ps15
Angiosperms - ps16
Mesangiospermae - ps17
Magnoliids/eudicots - ps18
Eudicots - ps19
Core_eudicots - ps20
Superrosids - ps21
Rosids - ps22
Eurosids - ps23
Malvids - ps24
Sap./Malv./Brassicales - ps25
Malvales/Brassicales - ps26
Brassicales - ps27
Cleomaceae/Brassicaceae - ps28
Brassicaceae - ps29
Camelineae - ps30
Arabidopsis - ps31
Arabidopsis_thaliana - ps32
Phylostratum

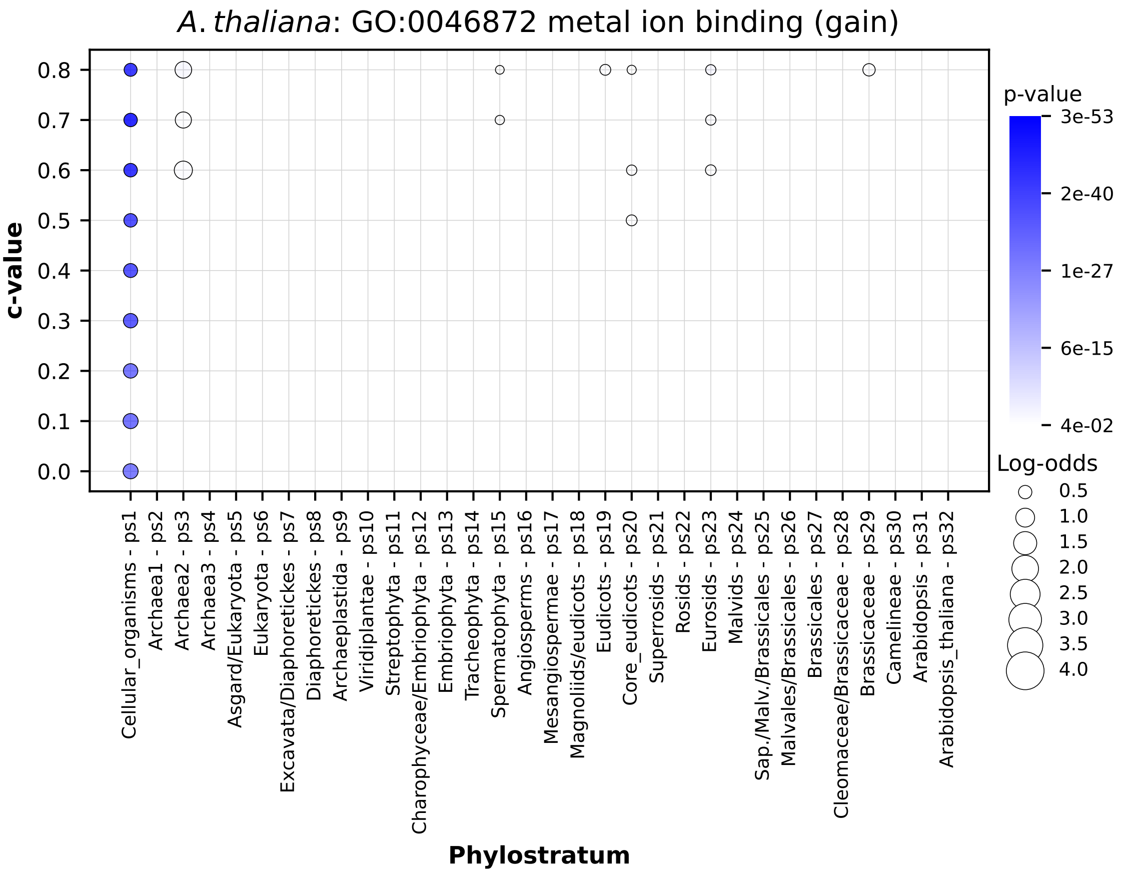

A. thaliana: GO:0046872 metal ion binding (gain)
c-value
0.8
0.7
0.6
0.5
0.4
0.3
0.2
0.1
0.0
p-value
3e-53
2e-40
1e-27
6e-15
4e-02
Log-odds
0.5
1.0
1.5
2.0
2.5
3.0
3.5
4.0
Cellular_organisms - ps1
Archaea1 - ps2
Archaea2 - ps3
Archaea3 - ps4
Asgard/Eukaryota - ps5
Eukaryota - ps6
Excavata/Diaphoretickes - ps7
Diaphoretickes - ps8
Archaeplastida - ps9
Viridiplantae - ps10
Streptophyta - ps11
Charophyceae/Embriophyta - ps12
Embriophyta - ps13
Tracheophyta - ps14
Spermatophyta - ps15
Angiosperms - ps16
Mesangiospermae - ps17
Magnoliids/eudicots - ps18
Eudicots - ps19
Core_eudicots - ps20
Superrosids - ps21
Rosids - ps22
Eurosids - ps23
Malvids - ps24
Sap./Malv./Brassicales - ps25
Malvales/Brassicales - ps26
Brassicales - ps27
Cleomaceae/Brassicaceae - ps28
Brassicaceae - ps29
Camelineae - ps30
Arabidopsis - ps31
Arabidopsis_thaliana - ps32
Phylostratum

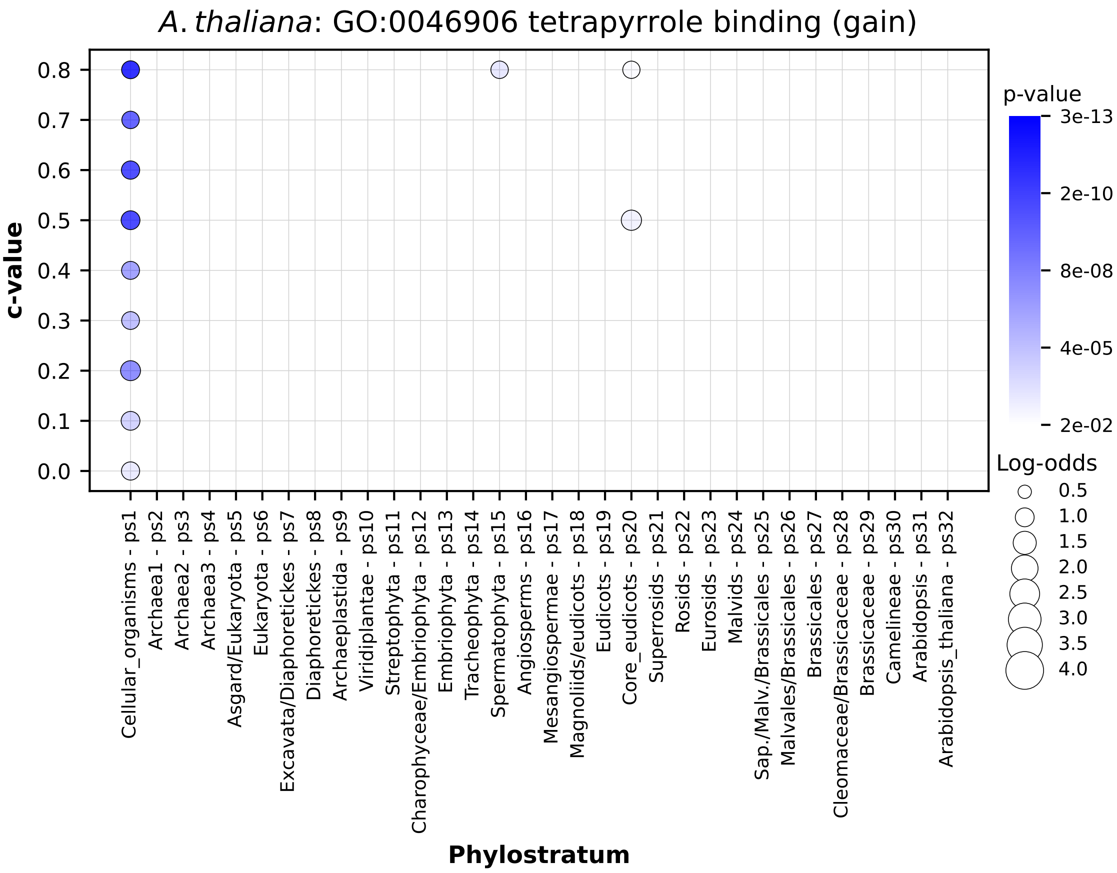
A. thaliana: GO:0046906 tetrapyrrole binding (gain)
c-value
0.8
0.7
0.6
0.5
0.4
0.3
0.2
0.1
0.0
p-value
3e-13
2e-10
8e-08
4e-05
2e-02
Log-odds
0.5
1.0
1.5
2.0
2.5
3.0
3.5
4.0
Cellular_organisms - ps1
Archaea1 - ps2
Archaea2 - ps3
Archaea3 - ps4
Asgard/Eukaryota - ps5
Eukaryota - ps6
Excavata/Diaphoretickes - ps7
Diaphoretickes - ps8
Archaeplastida - ps9
Viridiplantae - ps10
Streptophyta - ps11
Charophyceae/Embriophyta - ps12
Embriophyta - ps13
Tracheophyta - ps14
Spermatophyta - ps15
Angiosperms - ps16
Mesangiospermae - ps17
Magnoliids/eudicots - ps18
Eudicots - ps19
Core_eudicots - ps20
Superrosids - ps21
Rosids - ps22
Eurosids - ps23
Malvids - ps24
Sap./Malv./Brassicales - ps25
Malvales/Brassicales - ps26
Brassicales - ps27
Cleomaceae/Brassicaceae - ps28
Brassicaceae - ps29
Camelineae - ps30
Arabidopsis - ps31
Arabidopsis_thaliana - ps32
Phylostratum

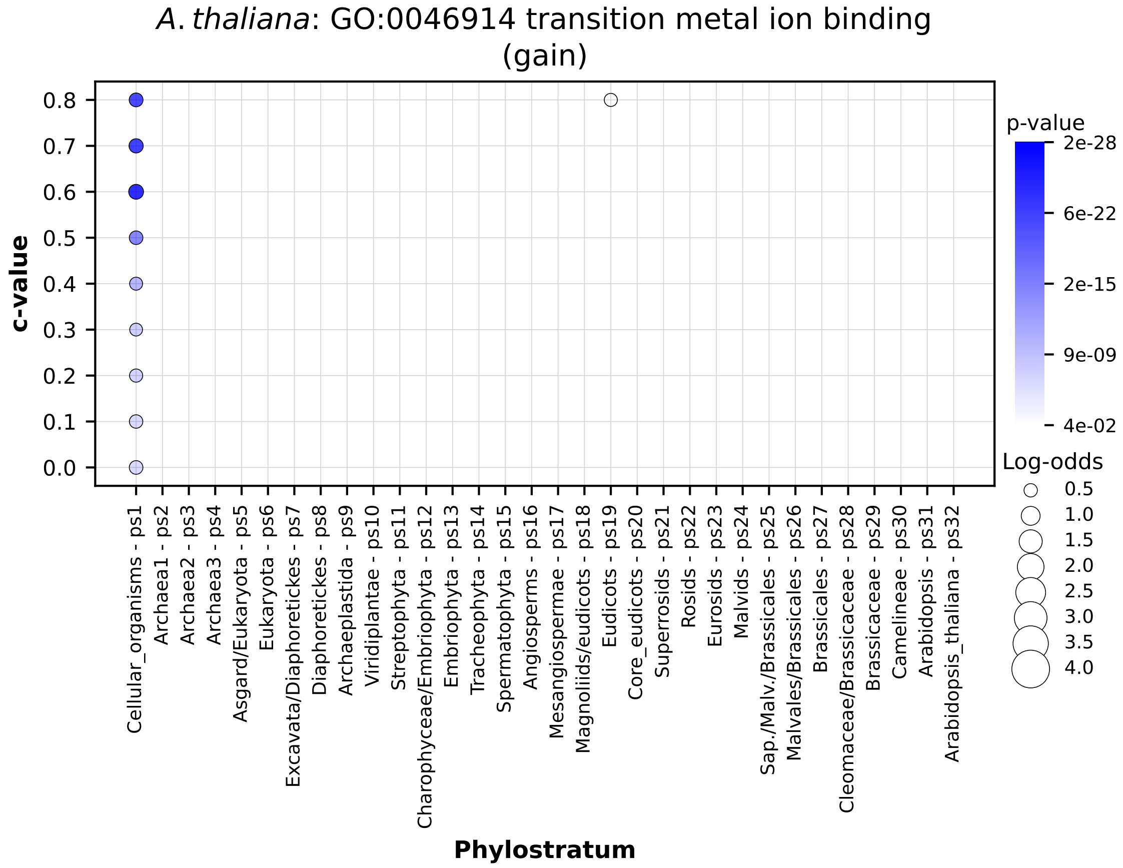
A. thaliana: GO:0046914 transition metal ion binding
(gain)
c-value
0.8
0.7
0.6
0.5
0.4
0.3
0.2
0.1
0.0
p-value
2e-28
6e-22
2e-15
9e-09
4e-02
Log-odds
0.5
1.0
1.5
2.0
2.5
3.0
3.5
4.0
Cellular_organisms - ps1
Archaea1 - ps2
Archaea2 - ps3
Archaea3 - ps4
Asgard/Eukaryota - ps5
Eukaryota - ps6
Excavata/Diaphoretickes - ps7
Diaphoretickes - ps8
Archaeplastida - ps9
Viridiplantae - ps10
Streptophyta - ps11
Charophyceae/Embriophyta - ps12
Embriophyta - ps13
Tracheophyta - ps14
Spermatophyta - ps15
Angiosperms - ps16
Mesangiospermae - ps17
Magnoliids/eudicots - ps18
Eudicots - ps19
Core_eudicots - ps20
Superrosids - ps21
Rosids - ps22
Eurosids - ps23
Malvids - ps24
Sap./Malv./Brassicales - ps25
Malvales/Brassicales - ps26
Brassicales - ps27
Cleomaceae/Brassicaceae - ps28
Brassicaceae - ps29
Camelineae - ps30
Arabidopsis - ps31
Arabidopsis_thaliana - ps32
Phylostratum

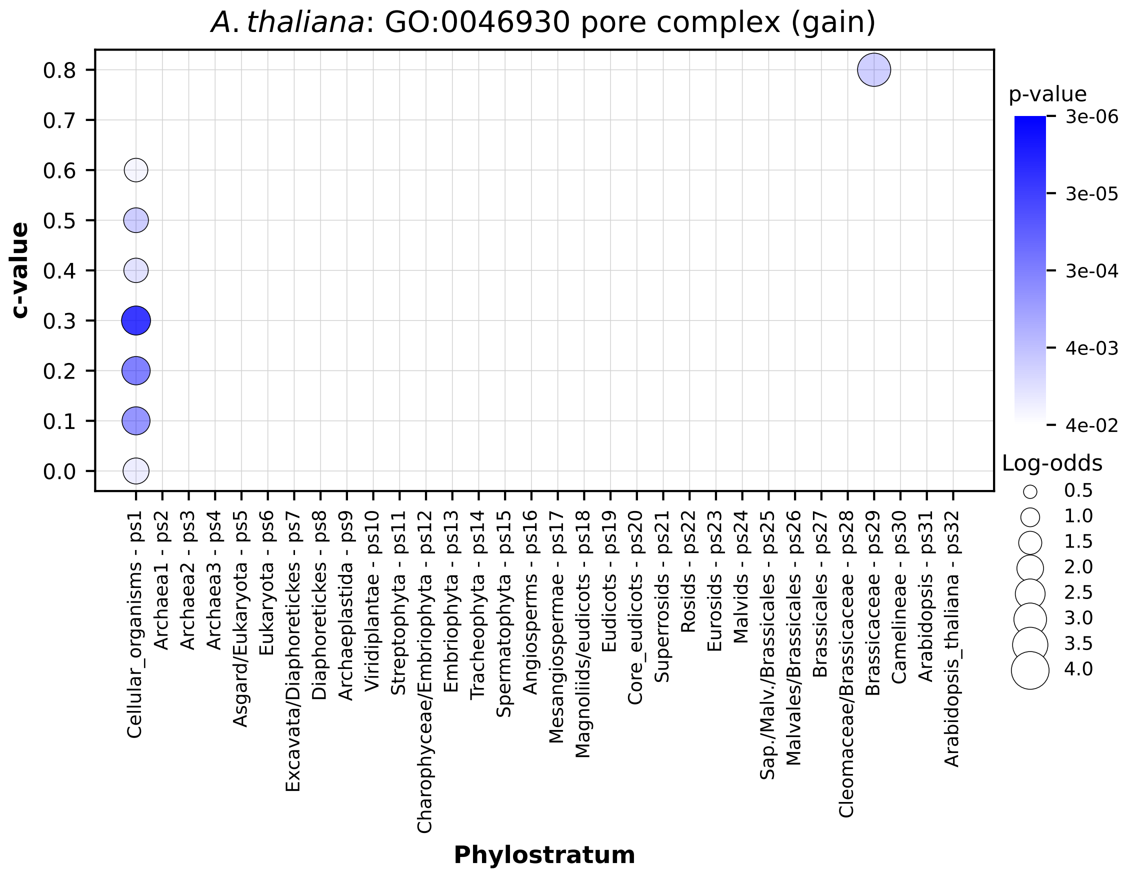

A. thaliana: GO:0046930 pore complex (gain)
c-value
0.0
0.1
0.2
0.3
0.4
0.5
0.6
0.7
0.8
Phylostratum
Cellular_organisms - ps1
Archaea1 - ps2
Archaea2 - ps3
Archaea3 - ps4
Asgard/Eukaryota - ps5
Eukaryota - ps6
Excavata/Diaphoretickes - ps7
Diaphoretickes - ps8
Archaeplastida - ps9
Viridiplantae - ps10
Streptophyta - ps11
Charophyceae/Embriophyta - ps12
Embriophyta - ps13
Tracheophyta - ps14
Spermatophyta - ps15
Angiosperms - ps16
Mesangiospermae - ps17
Magnoliids/eudicots - ps18
Eudicots - ps19
Core_eudicots - ps20
Superrosids - ps21
Rosids - ps22
Eurosids - ps23
Malvids - ps24
Sap./Malv./Brassicales - ps25
Malvales/Brassicales - ps26
Brassicales - ps27
Cleomaceae/Brassicaceae - ps28
Brassicaceae - ps29
Camelineae - ps30
Arabidopsis - ps31
Arabidopsis_thaliana - ps32
p-value
3e-06
3e-05
3e-04
4e-03
4e-02
Log-odds
0.5
1.0
1.5
2.0
2.5
3.0
3.5
4.0

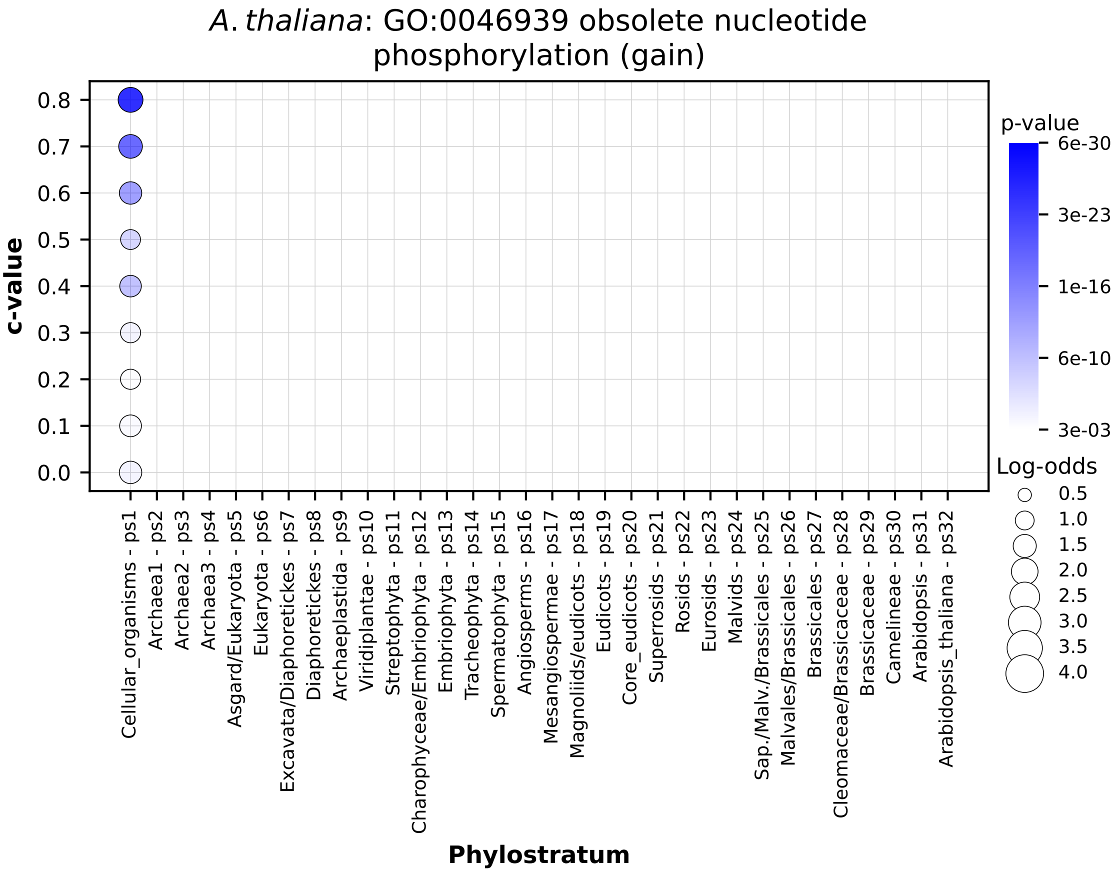

A. thaliana: GO:0046939 obsolete nucleotide phosphorylation (gain)
c-value
0.8
0.7
0.6
0.5
0.4
0.3
0.2
0.1
0.0
p-value
6e-30
3e-23
1e-16
6e-10
3e-03
Log-odds
0.5
1.0
1.5
2.0
2.5
3.0
3.5
4.0
Cellular_organisms - ps1
Archaea1 - ps2
Archaea2 - ps3
Archaea3 - ps4
Asgard/Eukaryota - ps5
Eukaryota - ps6
Excavata/Diaphoretickes - ps7
Diaphoretickes - ps8
Archaeplastida - ps9
Viridiplantae - ps10
Streptophyta - ps11
Charophyceae/Embriophyta - ps12
Embriophyta - ps13
Tracheophyta - ps14
Spermatophyta - ps15
Angiosperms - ps16
Mesangiospermae - ps17
Magnoliids/eudicots - ps18
Eudicots - ps19
Core_eudicots - ps20
Superrosids - ps21
Rosids - ps22
Eurosids - ps23
Malvids - ps24
Sap./Malv./Brassicales - ps25
Malvales/Brassicales - ps26
Brassicales - ps27
Cleomaceae/Brassicaceae - ps28
Brassicaceae - ps29
Camelineae - ps30
Arabidopsis - ps31
Arabidopsis_thaliana - ps32
Phylostratum

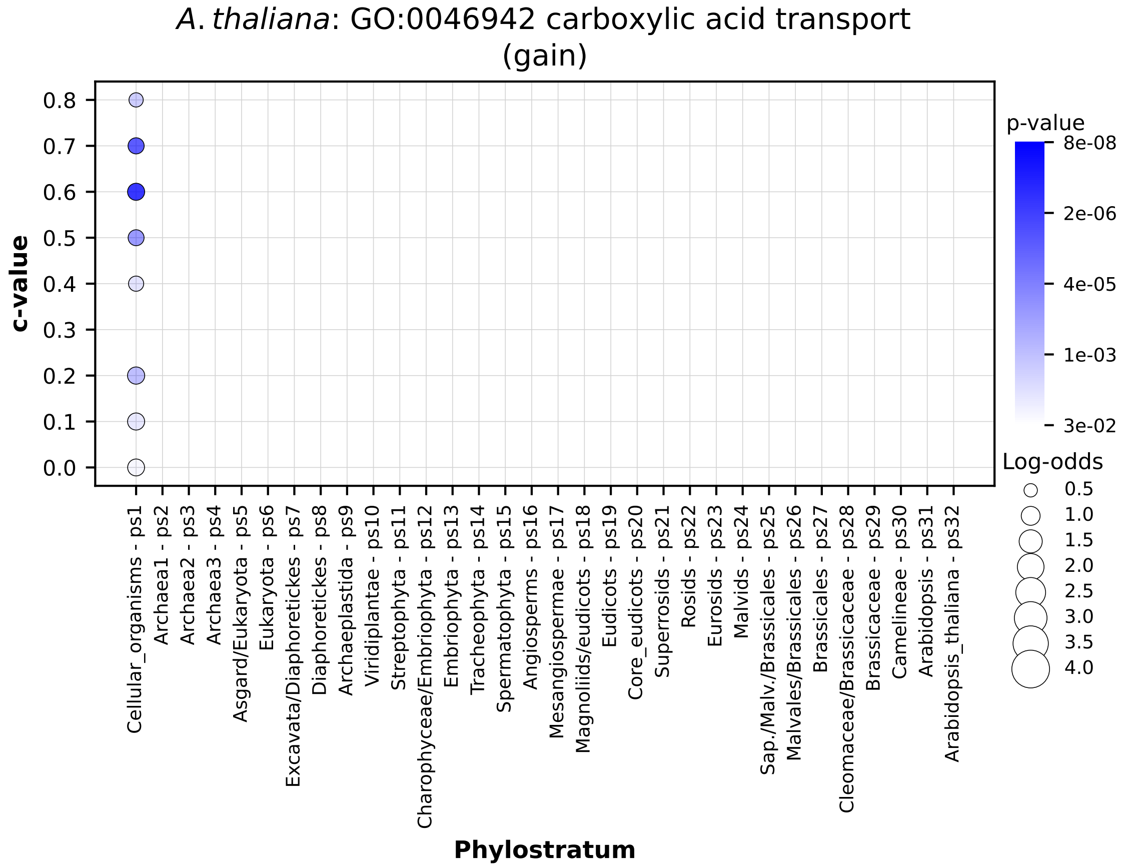

A. thaliana: GO:0046942 carboxylic acid transport
(gain)
c-value
0.8
0.7
0.6
0.5
0.4
0.3
0.2
0.1
0.0
p-value
8e-08
2e-06
4e-05
1e-03
3e-02
Log-odds
0.5
1.0
1.5
2.0
2.5
3.0
3.5
4.0
Cellular_organisms - ps1
Archaea1 - ps2
Archaea2 - ps3
Archaea3 - ps4
Asgard/Eukaryota - ps5
Eukaryota - ps6
Excavata/Diaphoretickes - ps7
Diaphoretickes - ps8
Archaeplastida - ps9
Viridiplantae - ps10
Streptophyta - ps11
Charophyceae/Embriophyta - ps12
Embriophyta - ps13
Tracheophyta - ps14
Spermatophyta - ps15
Angiosperms - ps16
Mesangiospermae - ps17
Magnoliids/eudicots - ps18
Eudicots - ps19
Core_eudicots - ps20
Superrosids - ps21
Rosids - ps22
Eurosids - ps23
Malvids - ps24
Sap./Malv./Brassicales - ps25
Malvales/Brassicales - ps26
Brassicales - ps27
Cleomaceae/Brassicaceae - ps28
Brassicaceae - ps29
Camelineae - ps30
Arabidopsis - ps31
Arabidopsis_thaliana - ps32
Phylostratum

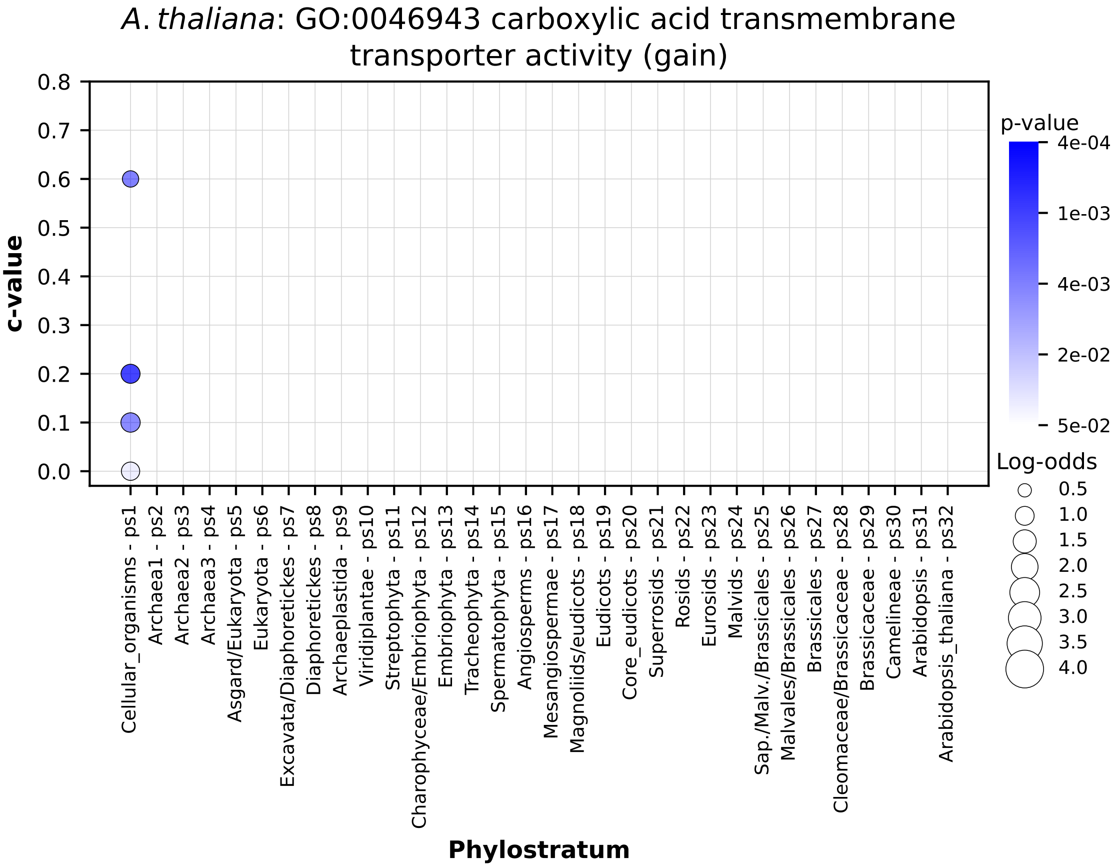

A. thaliana: GO:0046943 carboxylic acid transmembrane transporter activity (gain)
c-value
0.8
0.7
0.6
0.5
0.4
0.3
0.2
0.1
0.0
p-value
4e-04
1e-03
4e-03
2e-02
5e-02
Log-odds
0.5
1.0
1.5
2.0
2.5
3.0
3.5
4.0
Cellular_organisms - ps1
Archaea1 - ps2
Archaea2 - ps3
Archaea3 - ps4
Asgard/Eukaryota - ps5
Eukaryota - ps6
Excavata/Diaphoretickes - ps7
Diaphoretickes - ps8
Archaeplastida - ps9
Viridiplantae - ps10
Streptophyta - ps11
Charophyceae/Embriophyta - ps12
Embriophyta - ps13
Tracheophyta - ps14
Spermatophyta - ps15
Angiosperms - ps16
Mesangiospermae - ps17
Magnoliids/eudicots - ps18
Eudicots - ps19
Core_eudicots - ps20
Superrosids - ps21
Rosids - ps22
Eurosids - ps23
Malvids - ps24
Sap./Malv./Brassicales - ps25
Malvales/Brassicales - ps26
Brassicales - ps27
Cleomaceae/Brassicaceae - ps28
Brassicaceae - ps29
Camelineae - ps30
Arabidopsis - ps31
Arabidopsis_thaliana - ps32
Phylostratum

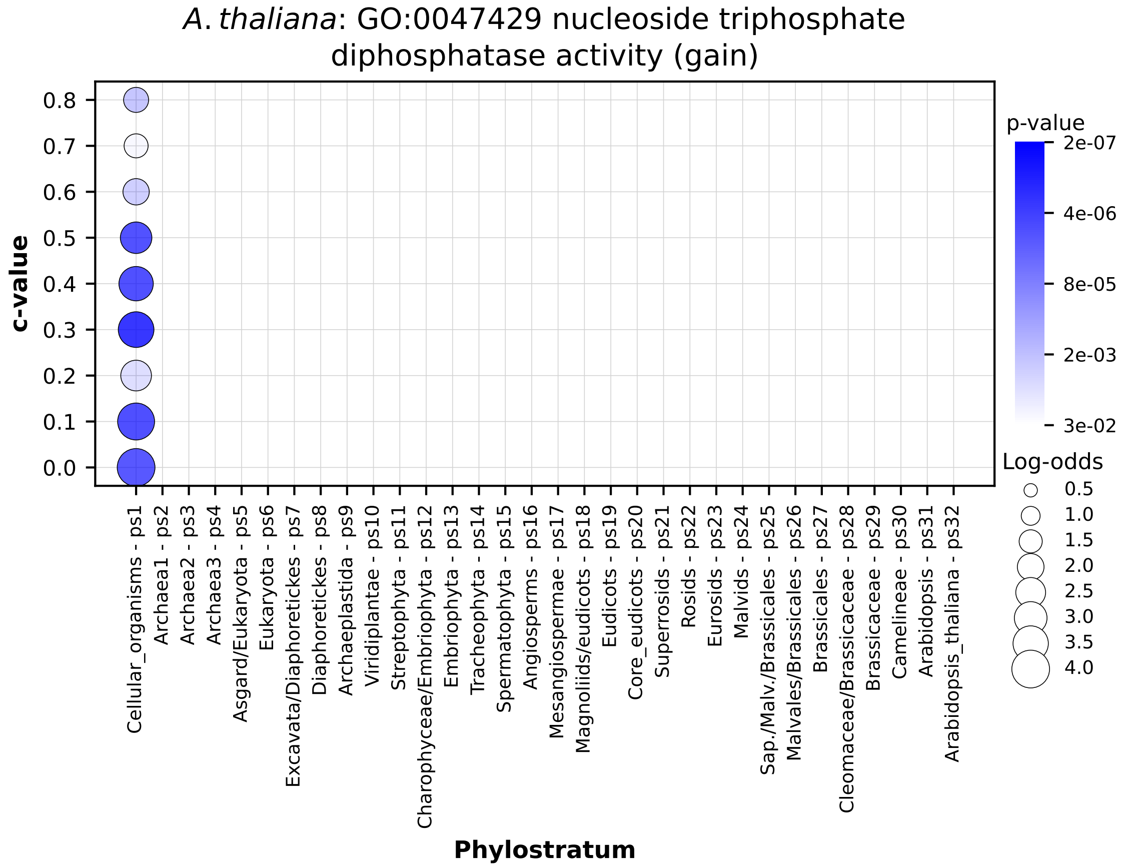
A. thaliana: GO:0047429 nucleoside triphosphate diphosphatase activity (gain)
c-value
0.8
0.7
0.6
0.5
0.4
0.3
0.2
0.1
0.0
p-value
2e-07
4e-06
8e-05
2e-03
3e-02
Log-odds
0.5
1.0
1.5
2.0
2.5
3.0
3.5
4.0
Cellular_organisms - ps1
Archaea1 - ps2
Archaea2 - ps3
Archaea3 - ps4
Asgard/Eukaryota - ps5
Eukaryota - ps6
Excavata/Diaphoretickes - ps7
Diaphoretickes - ps8
Archaeplastida - ps9
Viridiplantae - ps10
Streptophyta - ps11
Charophyceae/Embriophyta - ps12
Embriophyta - ps13
Tracheophyta - ps14
Spermatophyta - ps15
Angiosperms - ps16
Mesangiospermae - ps17
Magnoliids/eudicots - ps18
Eudicots - ps19
Core_eudicots - ps20
Superrosids - ps21
Rosids - ps22
Eurosids - ps23
Malvids - ps24
Sap./Malv./Brassicales - ps25
Malvales/Brassicales - ps26
Brassicales - ps27
Cleomaceae/Brassicaceae - ps28
Brassicaceae - ps29
Camelineae - ps30
Arabidopsis - ps31
Arabidopsis_thaliana - ps32
Phylostratum

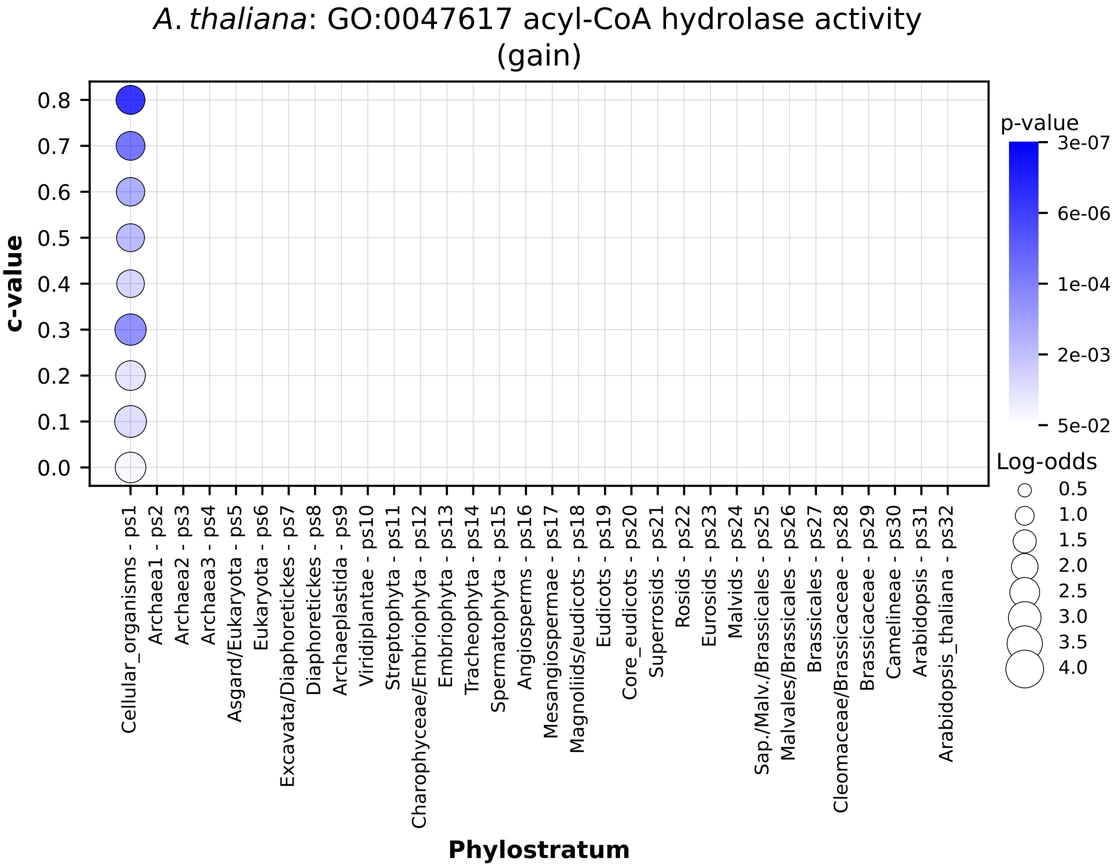

A. thaliana: GO:0047617 acyl-CoA hydrolase activity (gain)
c-value
0.8
0.7
0.6
0.5
0.4
0.3
0.2
0.1
0.0
p-value
3e-07
6e-06
1e-04
2e-03
5e-02
Log-odds
0.5
1.0
1.5
2.0
2.5
3.0
3.5
4.0
Cellular_organisms - ps1
Archaea1 - ps2
Archaea2 - ps3
Archaea3 - ps4
Asgard/Eukaryota - ps5
Eukaryota - ps6
Excavata/Diaphoretickes - ps7
Diaphoretickes - ps8
Archaeplastida - ps9
Viridiplantae - ps10
Streptophyta - ps11
Charophyceae/Embriophyta - ps12
Embriophyta - ps13
Tracheophyta - ps14
Spermatophyta - ps15
Angiosperms - ps16
Mesangiospermae - ps17
Magnoliids/eudicots - ps18
Eudicots - ps19
Core_eudicots - ps20
Superrosids - ps21
Rosids - ps22
Eurosids - ps23
Malvids - ps24
Sap./Malv./Brassicales - ps25
Malvales/Brassicales - ps26
Brassicales - ps27
Cleomaceae/Brassicaceae - ps28
Brassicaceae - ps29
Camelineae - ps30
Arabidopsis - ps31
Arabidopsis_thaliana - ps32
Phylostratum

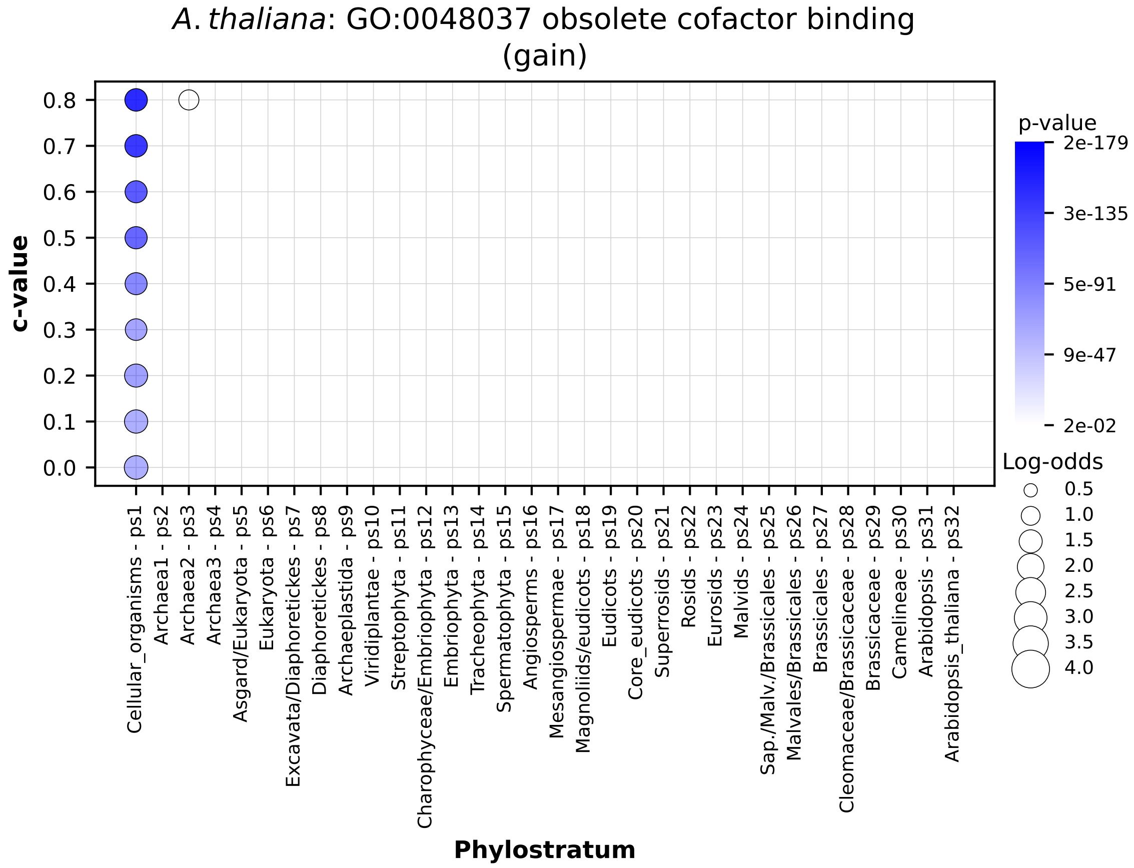

A. thaliana: GO:0048037 obsolete cofactor binding (gain)
c-value
0.8
0.7
0.6
0.5
0.4
0.3
0.2
0.1
0.0
p-value
2e-179
3e-135
5e-91
9e-47
2e-02
Log-odds
0.5
1.0
1.5
2.0
2.5
3.0
3.5
4.0
Cellular_organisms - ps1
Archaea1 - ps2
Archaea2 - ps3
Archaea3 - ps4
Asgard/Eukaryota - ps5
Eukaryota - ps6
Excavata/Diaphoretickes - ps7
Diaphoretickes - ps8
Archaeplastida - ps9
Viridiplantae - ps10
Streptophyta - ps11
Charophyceae/Embriophyta - ps12
Embriophyta - ps13
Tracheophyta - ps14
Spermatophyta - ps15
Angiosperms - ps16
Mesangiospermae - ps17
Magnoliids/eudicots - ps18
Eudicots - ps19
Core_eudicots - ps20
Superrosids - ps21
Rosids - ps22
Eurosids - ps23
Malvids - ps24
Sap./Malv./Brassicales - ps25
Malvales/Brassicales - ps26
Brassicales - ps27
Cleomaceae/Brassicaceae - ps28
Brassicaceae - ps29
Camelineae - ps30
Arabidopsis - ps31
Arabidopsis_thaliana - ps32
Phylostratum

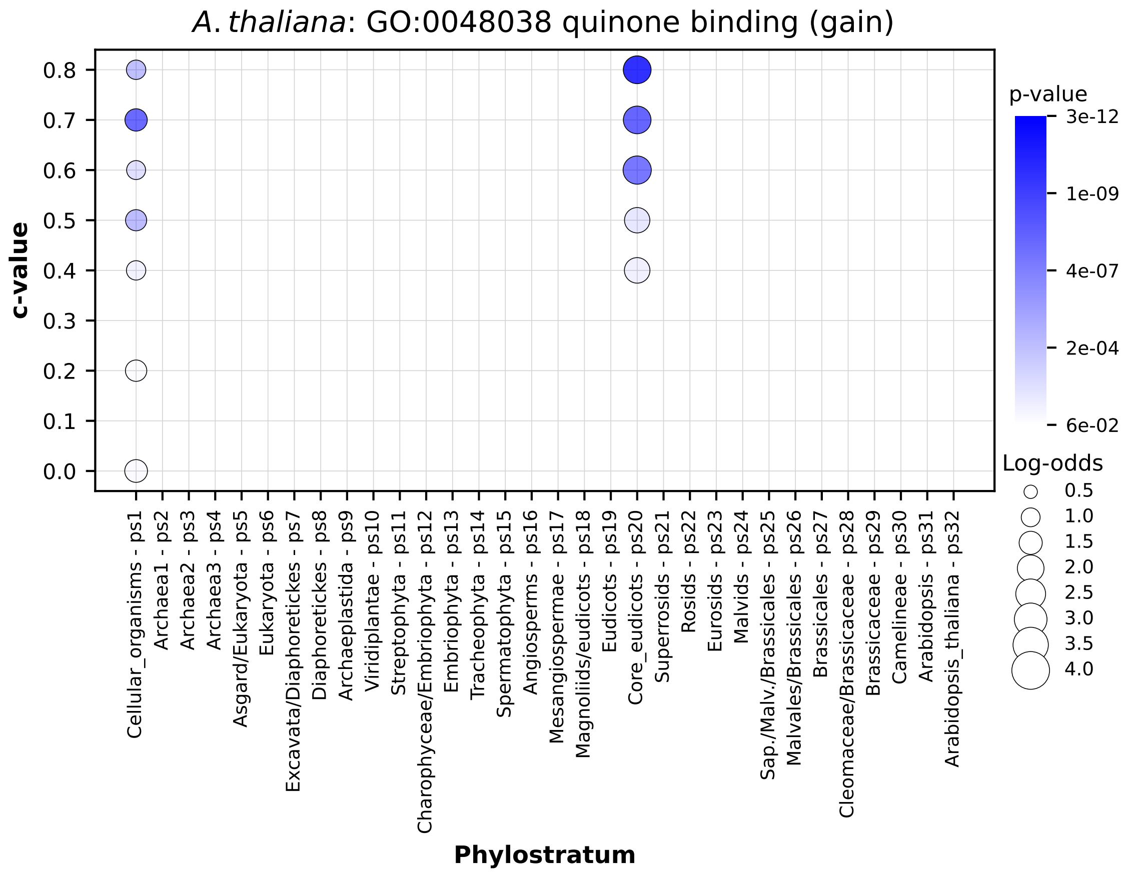
A. thaliana: GO:0048038 quinone binding (gain)
c-value
0.8
0.7
0.6
0.5
0.4
0.3
0.2
0.1
0.0
p-value
3e-12
1e-09
4e-07
2e-04
6e-02
Log-odds
0.5
1.0
1.5
2.0
2.5
3.0
3.5
4.0
Cellular_organisms - ps1
Archaea1 - ps2
Archaea2 - ps3
Archaea3 - ps4
Asgard/Eukaryota - ps5
Eukaryota - ps6
Excavata/Diaphoretickes - ps7
Diaphoretickes - ps8
Archaeplastida - ps9
Viridiplantae - ps10
Streptophyta - ps11
Charophyceae/Embriophyta - ps12
Embriophyta - ps13
Tracheophyta - ps14
Spermatophyta - ps15
Angiosperms - ps16
Mesangiospermae - ps17
Magnoliids/eudicots - ps18
Eudicots - ps19
Core_eudicots - ps20
Superrosids - ps21
Rosids - ps22
Eurosids - ps23
Malvids - ps24
Sap./Malv./Brassicales - ps25
Malvales/Brassicales - ps26
Brassicales - ps27
Cleomaceae/Brassicaceae - ps28
Brassicaceae - ps29
Camelineae - ps30
Arabidopsis - ps31
Arabidopsis_thaliana - ps32
Phylostratum

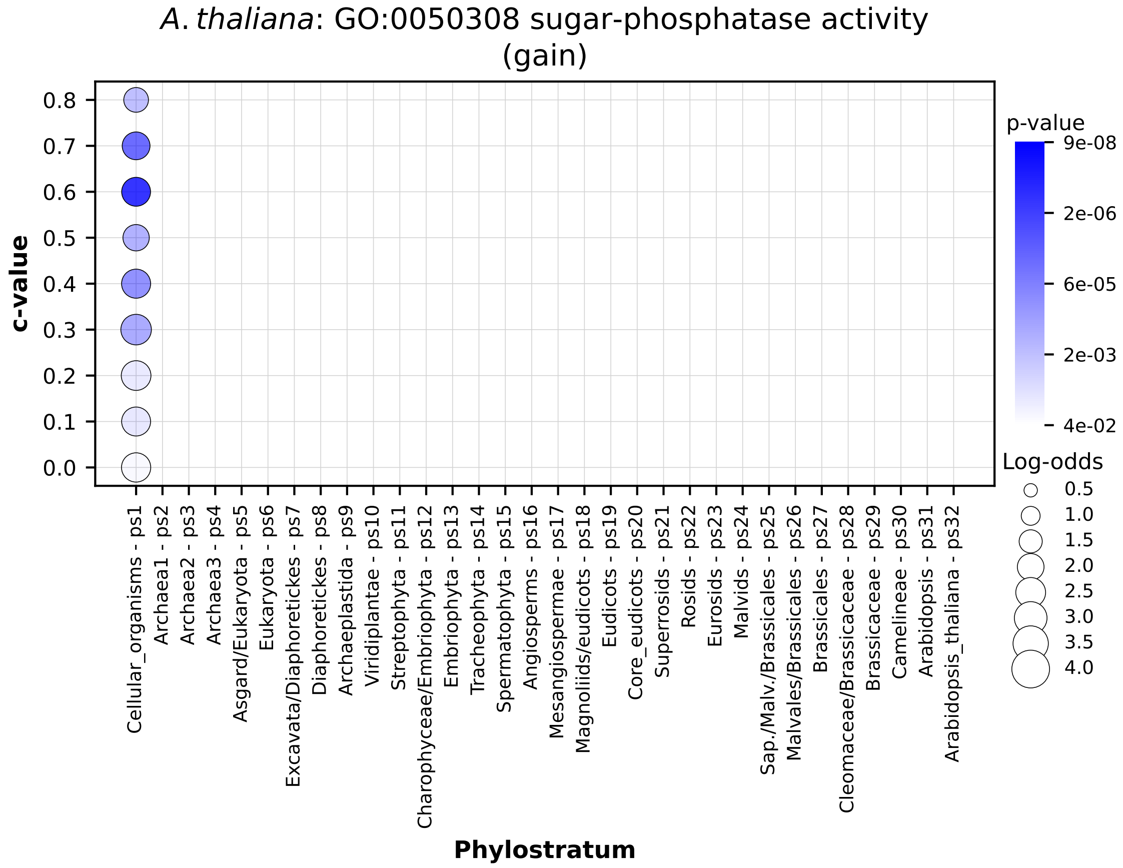

A. thaliana: GO:0050308 sugar-phosphatase activity
(gain)
c-value
0.8
0.7
0.6
0.5
0.4
0.3
0.2
0.1
0.0
p-value
9e-08
2e-06
6e-05
2e-03
4e-02
Log-odds
0.5
1.0
1.5
2.0
2.5
3.0
3.5
4.0
Cellular_organisms - ps1
Archaea1 - ps2
Archaea2 - ps3
Archaea3 - ps4
Asgard/Eukaryota - ps5
Eukaryota - ps6
Excavata/Diaphoretickes - ps7
Diaphoretickes - ps8
Archaeplastida - ps9
Viridiplantae - ps10
Streptophyta - ps11
Charophyceae/Embriophyta - ps12
Embriophyta - ps13
Tracheophyta - ps14
Spermatophyta - ps15
Angiosperms - ps16
Mesangiospermae - ps17
Magnoliids/eudicots - ps18
Eudicots - ps19
Core_eudicots - ps20
Superrosids - ps21
Rosids - ps22
Eurosids - ps23
Malvids - ps24
Sap./Malv./Brassicales - ps25
Malvales/Brassicales - ps26
Brassicales - ps27
Cleomaceae/Brassicaceae - ps28
Brassicaceae - ps29
Camelineae - ps30
Arabidopsis - ps31
Arabidopsis_thaliana - ps32
Phylostratum

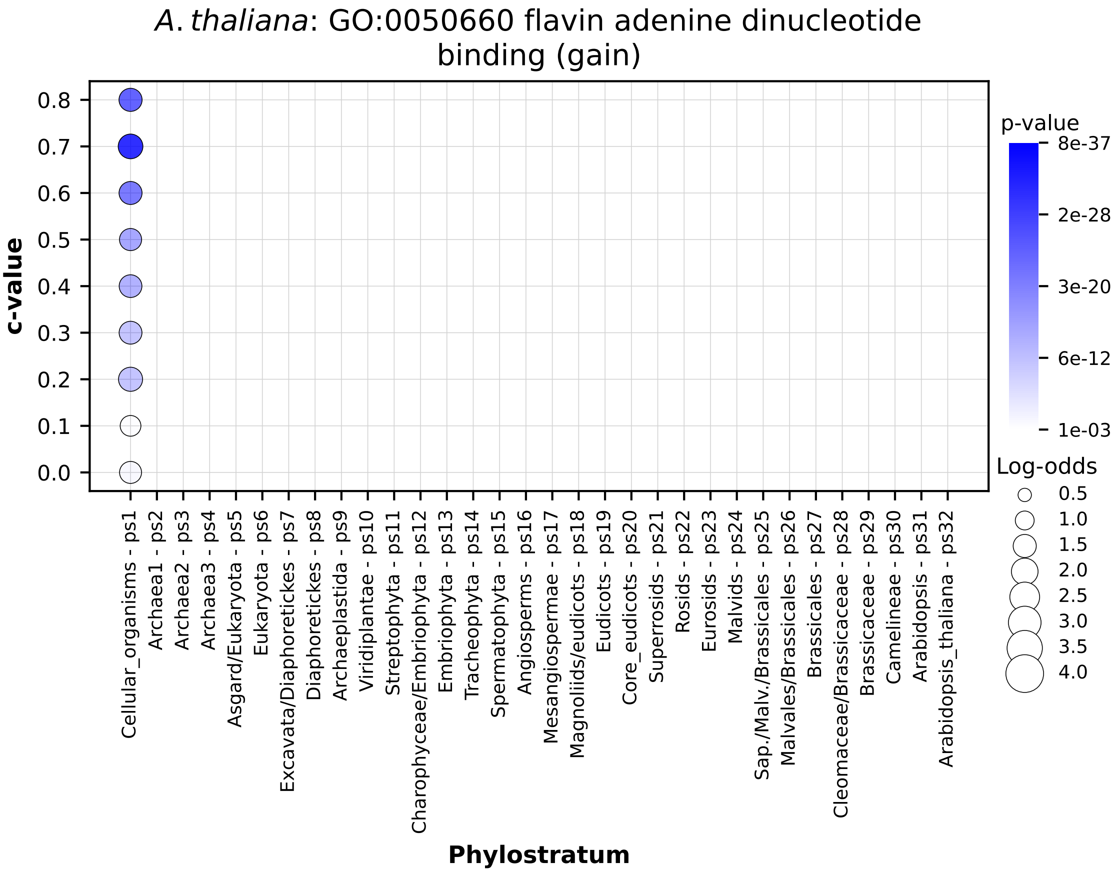

A. thaliana: GO:0050660 flavin adenine dinucleotide binding (gain)
c-value
0.8
0.7
0.6
0.5
0.4
0.3
0.2
0.1
0.0
p-value
8e-37
2e-28
3e-20
6e-12
1e-03
Log-odds
0.5
1.0
1.5
2.0
2.5
3.0
3.5
4.0
Cellular_organisms - ps1
Archaea1 - ps2
Archaea2 - ps3
Archaea3 - ps4
Asgard/Eukaryota - ps5
Eukaryota - ps6
Excavata/Diaphoretickes - ps7
Diaphoretickes - ps8
Archaeplastida - ps9
Viridiplantae - ps10
Streptophyta - ps11
Charophyceae/Embriophyta - ps12
Embriophyta - ps13
Tracheophyta - ps14
Spermatophyta - ps15
Angiosperms - ps16
Mesangiospermae - ps17
Magnoliids/eudicots - ps18
Eudicots - ps19
Core_eudicots - ps20
Superrosids - ps21
Rosids - ps22
Eurosids - ps23
Malvids - ps24
Sap./Malv./Brassicales - ps25
Malvales/Brassicales - ps26
Brassicales - ps27
Cleomaceae/Brassicaceae - ps28
Brassicaceae - ps29
Camelineae - ps30
Arabidopsis - ps31
Arabidopsis_thaliana - ps32
Phylostratum

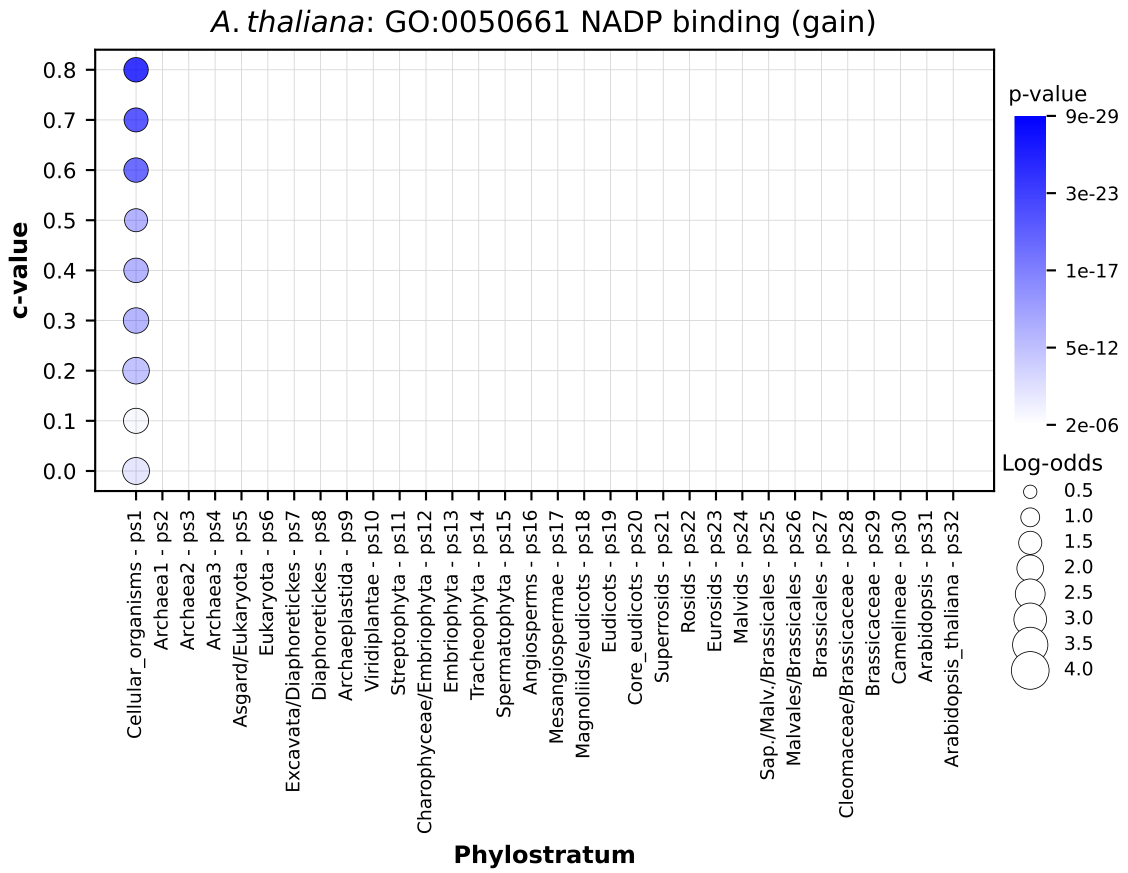
A. thaliana: GO:0050661 NADP binding (gain)
c-value
0.0
0.1
0.2
0.3
0.4
0.5
0.6
0.7
0.8
p-value
9e-29
3e-23
1e-17
5e-12
2e-06
Log-odds
0.5
1.0
1.5
2.0
2.5
3.0
3.5
4.0
Cellular_organisms - ps1
Archaea1 - ps2
Archaea2 - ps3
Archaea3 - ps4
Asgard/Eukaryota - ps5
Eukaryota - ps6
Excavata/Diaphoretickes - ps7
Diaphoretickes - ps8
Archaeplastida - ps9
Viridiplantae - ps10
Streptophyta - ps11
Charophyceae/Embriophyta - ps12
Embriophyta - ps13
Tracheophyta - ps14
Spermatophyta - ps15
Angiosperms - ps16
Mesangiospermae - ps17
Magnoliids/eudicots - ps18
Eudicots - ps19
Core_eudicots - ps20
Superrosids - ps21
Rosids - ps22
Eurosids - ps23
Malvids - ps24
Sap./Malv./Brassicales - ps25
Malvales/Brassicales - ps26
Brassicales - ps27
Cleomaceae/Brassicaceae - ps28
Brassicaceae - ps29
Camelineae - ps30
Arabidopsis - ps31
Arabidopsis_thaliana - ps32
Phylostratum

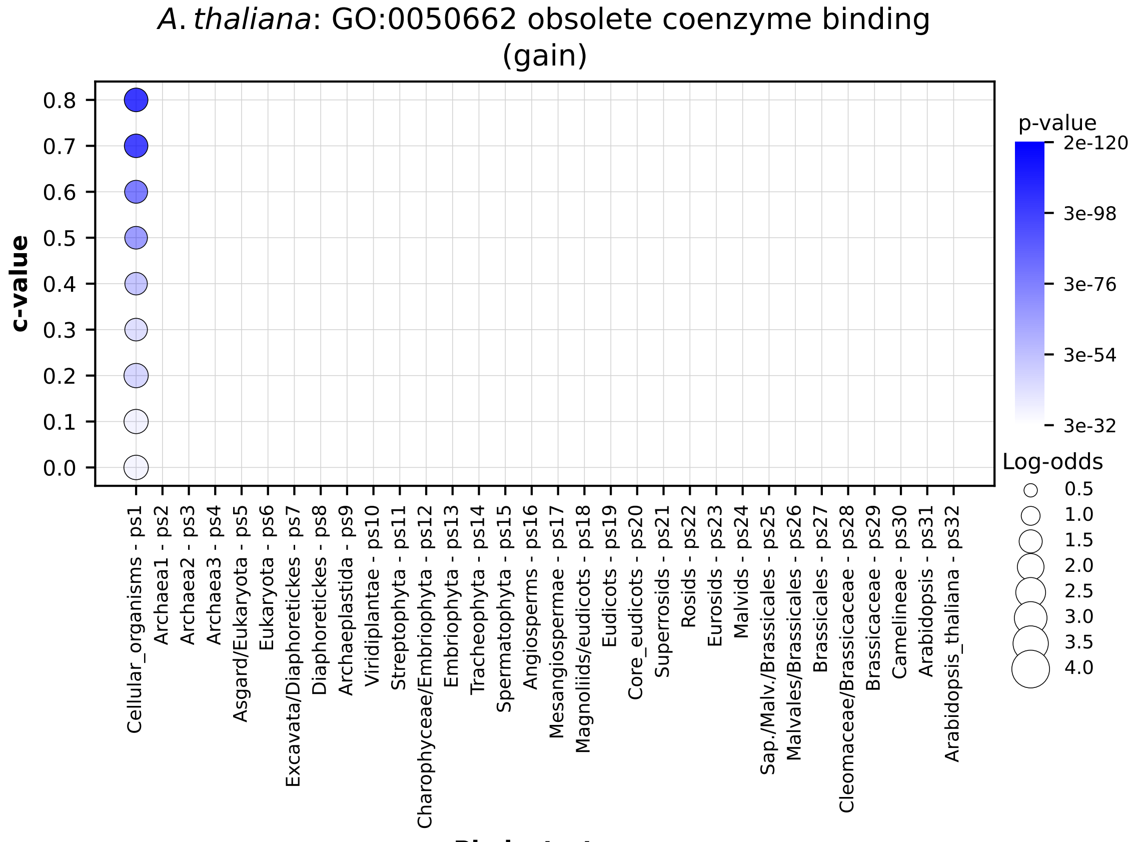
A. thaliana: GO:0050662 obsolete coenzyme binding
(gain)
c-value
0.8
0.7
0.6
0.5
0.4
0.3
0.2
0.1
0.0
Phylostratum
Cellular_organisms - ps1
Archaea1 - ps2
Archaea2 - ps3
Archaea3 - ps4
Asgard/Eukaryota - ps5
Eukaryota - ps6
Excavata/Diaphoretickes - ps7
Diaphoretickes - ps8
Archaeplastida - ps9
Viridiplantae - ps10
Streptophyta - ps11
Charophyceae/Embriophyta - ps12
Embriophyta - ps13
Tracheophyta - ps14
Spermatophyta - ps15
Angiosperms - ps16
Mesangiospermae - ps17
Magnoliids/eudicots - ps18
Eudicots - ps19
Core_eudicots - ps20
Superrosids - ps21
Rosids - ps22
Eurosids - ps23
Malvids - ps24
Sap./Malv./Brassicales - ps25
Malvales/Brassicales - ps26
Brassicales - ps27
Cleomaceae/Brassicaceae - ps28
Brassicaceae - ps29
Camelineae - ps30
Arabidopsis - ps31
Arabidopsis_thaliana - ps32
p-value
2e-120
3e-98
3e-76
3e-54
3e-32
Log-odds
0.5
1.0
1.5
2.0
2.5
3.0
3.5
4.0

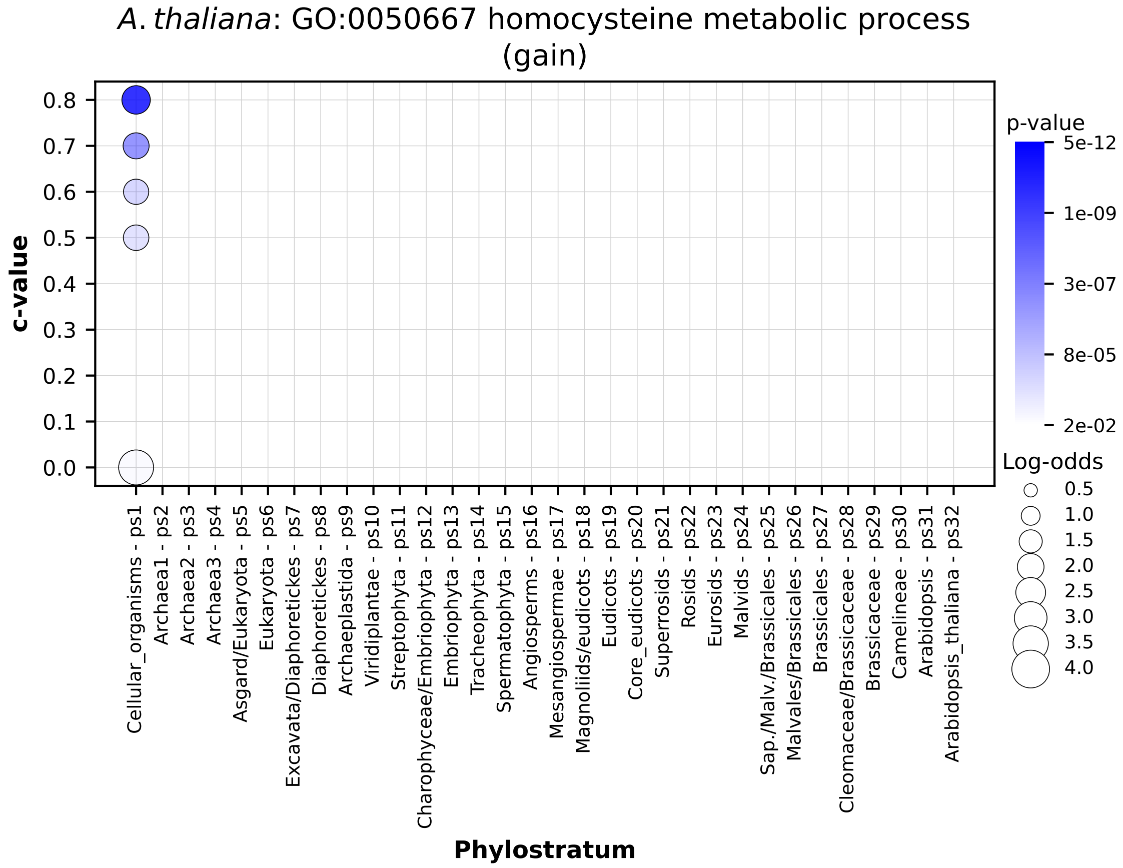

A. thaliana: GO:0050667 homocysteine metabolic process
(gain)
c-value
0.8
0.7
0.6
0.5
0.4
0.3
0.2
0.1
0.0
p-value
5e-12
1e-09
3e-07
8e-05
2e-02
Log-odds
0.5
1.0
1.5
2.0
2.5
3.0
3.5
4.0
Cellular_organisms - ps1
Archaea1 - ps2
Archaea2 - ps3
Archaea3 - ps4
Asgard/Eukaryota - ps5
Eukaryota - ps6
Excavata/Diaphoretickes - ps7
Diaphoretickes - ps8
Archaeplastida - ps9
Viridiplantae - ps10
Streptophyta - ps11
Charophyceae/Embriophyta - ps12
Embriophyta - ps13
Tracheophyta - ps14
Spermatophyta - ps15
Angiosperms - ps16
Mesangiospermae - ps17
Magnoliids/eudicots - ps18
Eudicots - ps19
Core_eudicots - ps20
Superrosids - ps21
Rosids - ps22
Eurosids - ps23
Malvids - ps24
Sap./Malv./Brassicales - ps25
Malvales/Brassicales - ps26
Brassicales - ps27
Cleomaceae/Brassicaceae - ps28
Brassicaceae - ps29
Camelineae - ps30
Arabidopsis - ps31
Arabidopsis_thaliana - ps32
Phylostratum

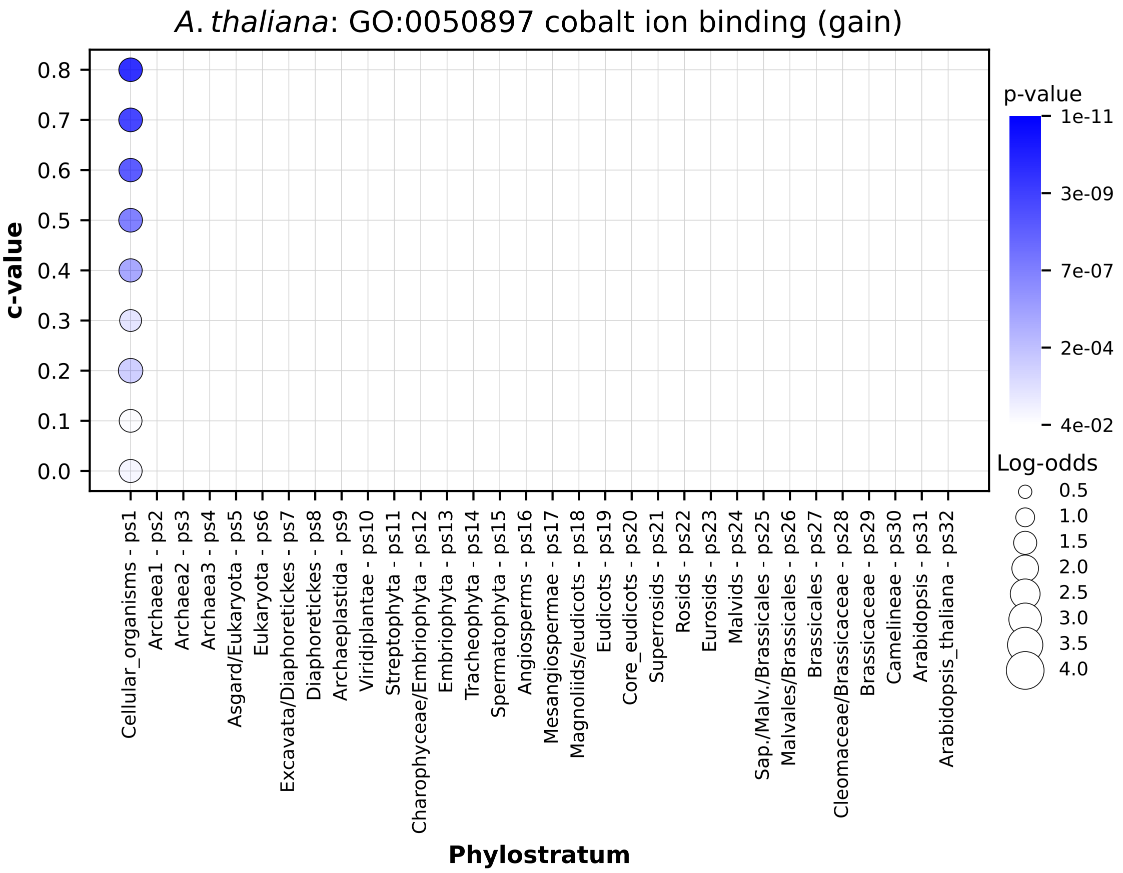

A. thaliana: GO:0050897 cobalt ion binding (gain)
c-value
0.0
0.1
0.2
0.3
0.4
0.5
0.6
0.7
0.8
p-value
1e-11
3e-09
7e-07
2e-04
4e-02
Log-odds
0.5
1.0
1.5
2.0
2.5
3.0
3.5
4.0
Cellular_organisms - ps1
Archaea1 - ps2
Archaea2 - ps3
Archaea3 - ps4
Asgard/Eukaryota - ps5
Eukaryota - ps6
Excavata/Diaphoretickes - ps7
Diaphoretickes - ps8
Archaeplastida - ps9
Viridiplantae - ps10
Streptophyta - ps11
Charophyceae/Embriophyta - ps12
Embriophyta - ps13
Tracheophyta - ps14
Spermatophyta - ps15
Angiosperms - ps16
Mesangiospermae - ps17
Magnoliids/eudicots - ps18
Eudicots - ps19
Core_eudicots - ps20
Superrosids - ps21
Rosids - ps22
Eurosids - ps23
Malvids - ps24
Sap./Malv./Brassicales - ps25
Malvales/Brassicales - ps26
Brassicales - ps27
Cleomaceae/Brassicaceae - ps28
Brassicaceae - ps29
Camelineae - ps30
Arabidopsis - ps31
Arabidopsis_thaliana - ps32
Phylostratum

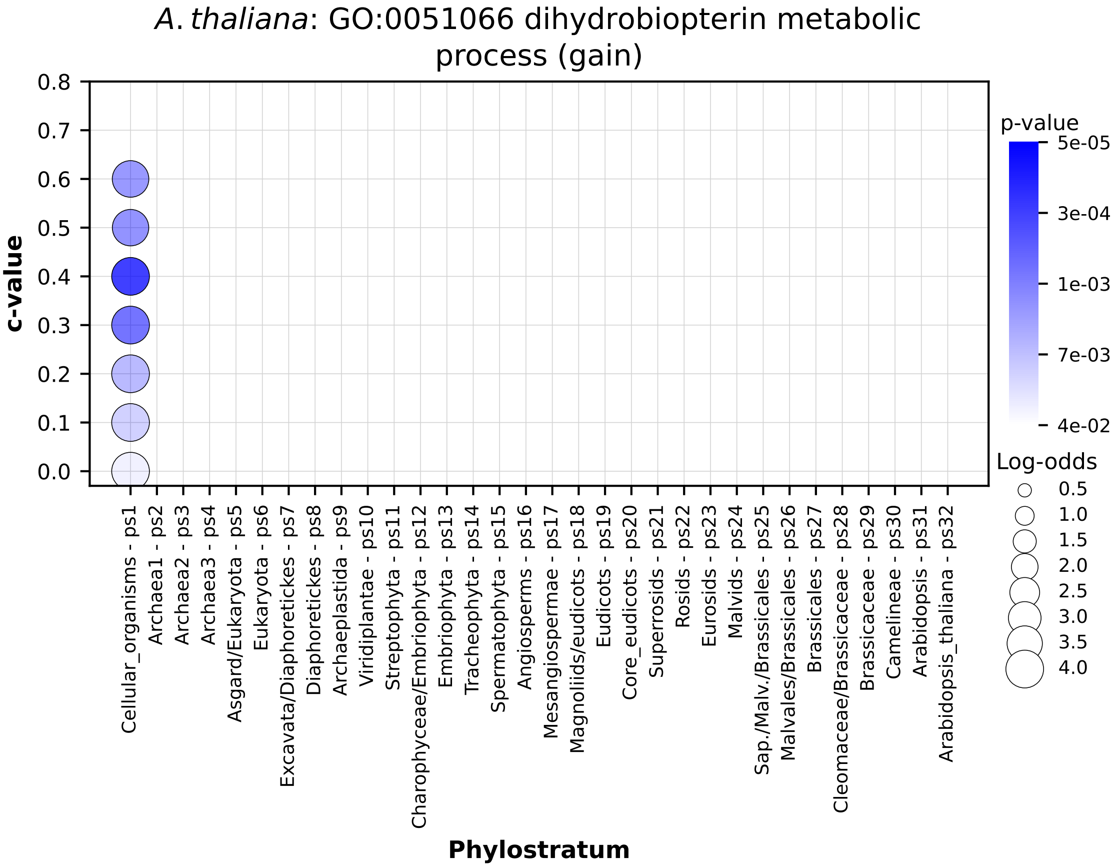
A. thaliana: GO:0051066 dihydrobiopterin metabolic process (gain)
c-value
0.8
0.7
0.6
0.5
0.4
0.3
0.2
0.1
0.0
p-value
5e-05
3e-04
1e-03
7e-03
4e-02
Log-odds
0.5
1.0
1.5
2.0
2.5
3.0
3.5
4.0
Cellular_organisms - ps1
Archaea1 - ps2
Archaea2 - ps3
Archaea3 - ps4
Asgard/Eukaryota - ps5
Eukaryota - ps6
Excavata/Diaphoretickes - ps7
Diaphoretickes - ps8
Archaeplastida - ps9
Viridiplantae - ps10
Streptophyta - ps11
Charophyceae/Embriophyta - ps12
Embriophyta - ps13
Tracheophyta - ps14
Spermatophyta - ps15
Angiosperms - ps16
Mesangiospermae - ps17
Magnoliids/eudicots - ps18
Eudicots - ps19
Core_eudicots - ps20
Superrosids - ps21
Rosids - ps22
Eurosids - ps23
Malvids - ps24
Sap./Malv./Brassicales - ps25
Malvales/Brassicales - ps26
Brassicales - ps27
Cleomaceae/Brassicaceae - ps28
Brassicaceae - ps29
Camelineae - ps30
Arabidopsis - ps31
Arabidopsis_thaliana - ps32
Phylostratum

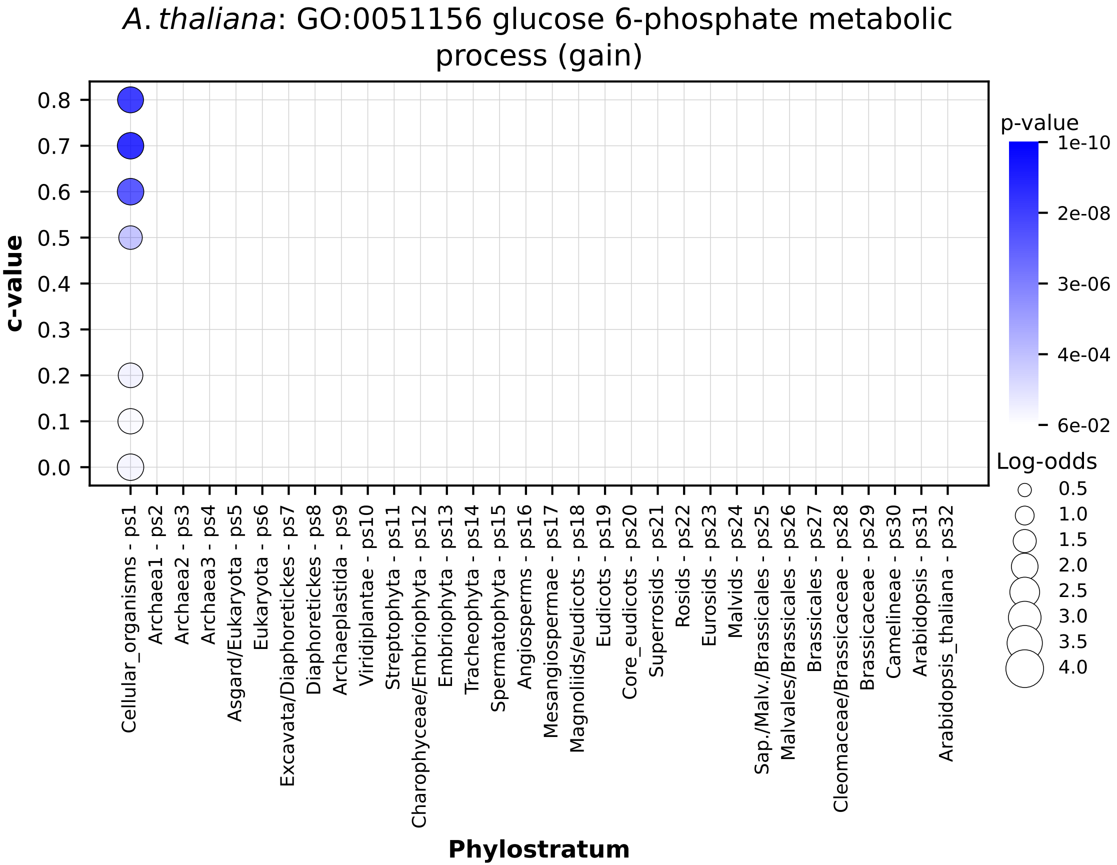
A. thaliana: GO:0051156 glucose 6-phosphate metabolic process (gain)
c-value
0.8
0.7
0.6
0.5
0.4
0.3
0.2
0.1
0.0
p-value
1e-10
2e-08
3e-06
4e-04
6e-02
Log-odds
0.5
1.0
1.5
2.0
2.5
3.0
3.5
4.0
Cellular_organisms - ps1
Archaea1 - ps2
Archaea2 - ps3
Archaea3 - ps4
Asgard/Eukaryota - ps5
Eukaryota - ps6
Excavata/Diaphoretickes - ps7
Diaphoretickes - ps8
Archaeplastida - ps9
Viridiplantae - ps10
Streptophyta - ps11
Charophyceae/Embriophyta - ps12
Embriophyta - ps13
Tracheophyta - ps14
Spermatophyta - ps15
Angiosperms - ps16
Mesangiospermae - ps17
Magnoliids/eudicots - ps18
Eudicots - ps19
Core_eudicots - ps20
Superrosids - ps21
Rosids - ps22
Eurosids - ps23
Malvids - ps24
Sap./Malv./Brassicales - ps25
Malvales/Brassicales - ps26
Brassicales - ps27
Cleomaceae/Brassicaceae - ps28
Brassicaceae - ps29
Camelineae - ps30
Arabidopsis - ps31
Arabidopsis_thaliana - ps32
Phylostratum

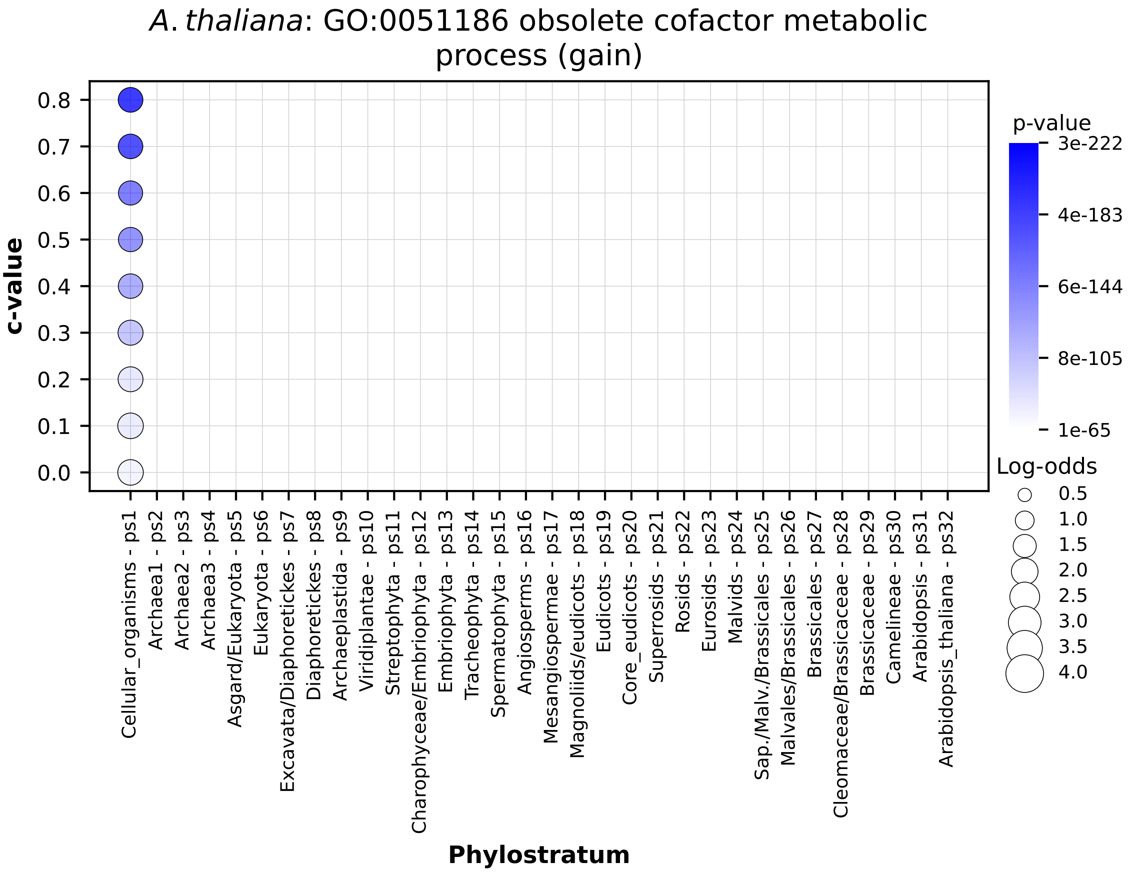

A. thaliana: GO:0051186 obsolete cofactor metabolic process (gain)
c-value
0.8
0.7
0.6
0.5
0.4
0.3
0.2
0.1
0.0
p-value
3e-222
4e-183
6e-144
8e-105
1e-65
Log-odds
0.5
1.0
1.5
2.0
2.5
3.0
3.5
4.0
Cellular_organisms - ps1
Archaea1 - ps2
Archaea2 - ps3
Archaea3 - ps4
Asgard/Eukaryota - ps5
Eukaryota - ps6
Excavata/Diaphoretickes - ps7
Diaphoretickes - ps8
Archaeplastida - ps9
Viridiplantae - ps10
Streptophyta - ps11
Charophyceae/Embriophyta - ps12
Embriophyta - ps13
Tracheophyta - ps14
Spermatophyta - ps15
Angiosperms - ps16
Mesangiospermae - ps17
Magnoliids/eudicots - ps18
Eudicots - ps19
Core_eudicots - ps20
Superrosids - ps21
Rosids - ps22
Eurosids - ps23
Malvids - ps24
Sap./Malv./Brassicales - ps25
Malvales/Brassicales - ps26
Brassicales - ps27
Cleomaceae/Brassicaceae - ps28
Brassicaceae - ps29
Camelineae - ps30
Arabidopsis - ps31
Arabidopsis_thaliana - ps32
Phylostratum

A. thaliana: GO:0051188 obsolete cofactor biosynthetic process (gain)

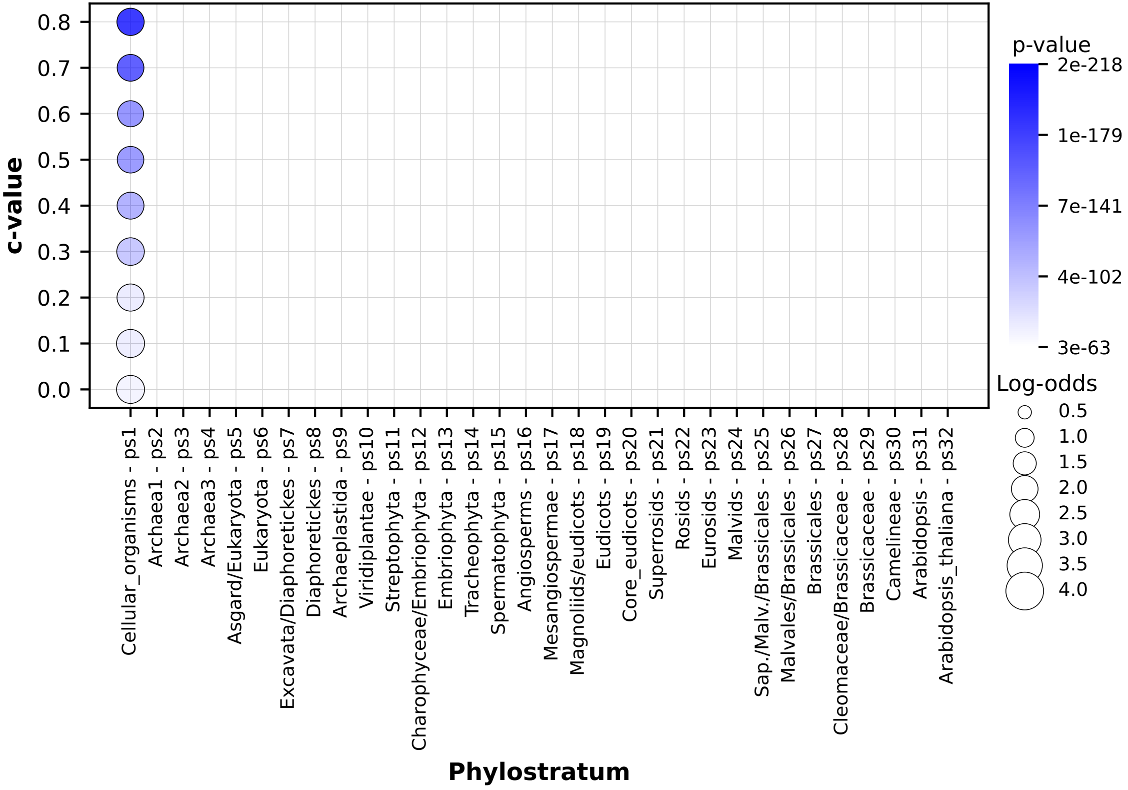

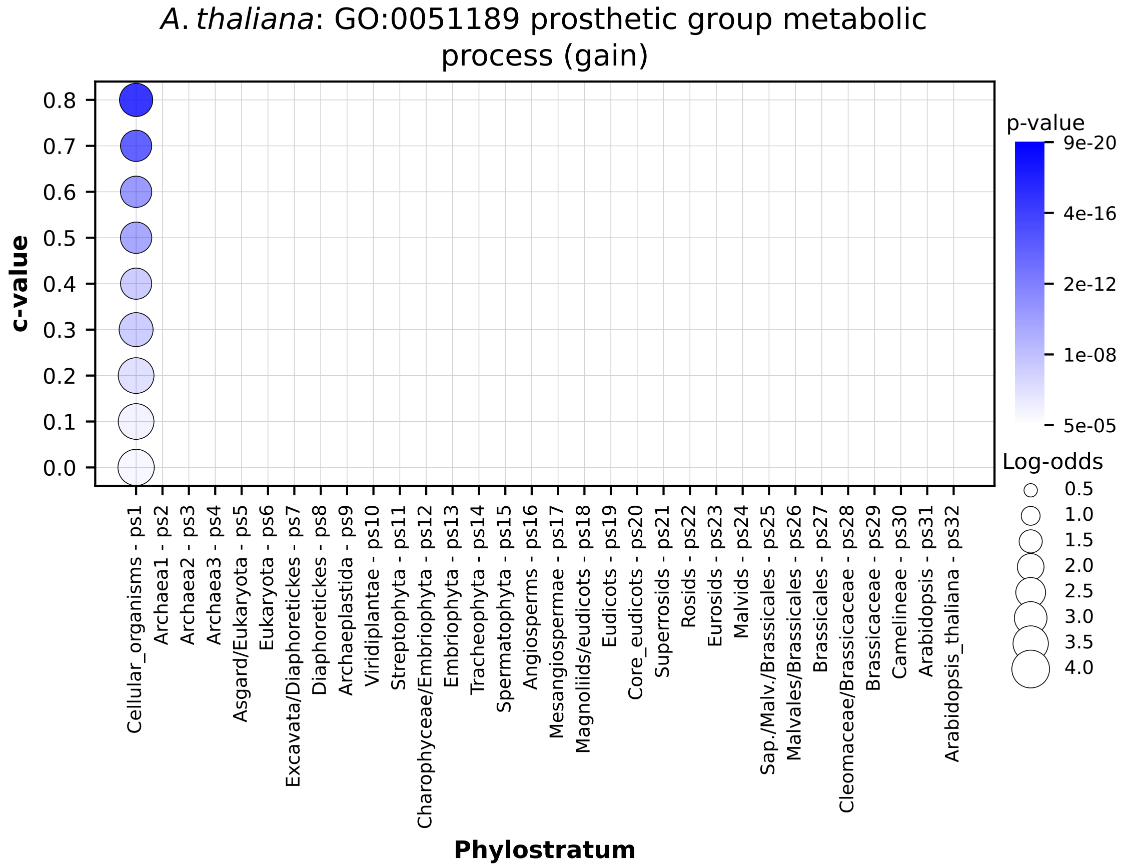

A. thaliana: GO:0051189 prosthetic group metabolic process (gain)
c-value
0.8
0.7
0.6
0.5
0.4
0.3
0.2
0.1
0.0
p-value
9e-20
4e-16
2e-12
1e-08
5e-05
Log-odds
0.5
1.0
1.5
2.0
2.5
3.0
3.5
4.0
Cellular_organisms - ps1
Archaea1 - ps2
Archaea2 - ps3
Archaea3 - ps4
Asgard/Eukaryota - ps5
Eukaryota - ps6
Excavata/Diaphoretickes - ps7
Diaphoretickes - ps8
Archaeplastida - ps9
Viridiplantae - ps10
Streptophyta - ps11
Charophyceae/Embriophyta - ps12
Embriophyta - ps13
Tracheophyta - ps14
Spermatophyta - ps15
Angiosperms - ps16
Mesangiospermae - ps17
Magnoliids/eudicots - ps18
Eudicots - ps19
Core_eudicots - ps20
Superrosids - ps21
Rosids - ps22
Eurosids - ps23
Malvids - ps24
Sap./Malv./Brassicales - ps25
Malvales/Brassicales - ps26
Brassicales - ps27
Cleomaceae/Brassicaceae - ps28
Brassicaceae - ps29
Camelineae - ps30
Arabidopsis - ps31
Arabidopsis_thaliana - ps32
Phylostratum

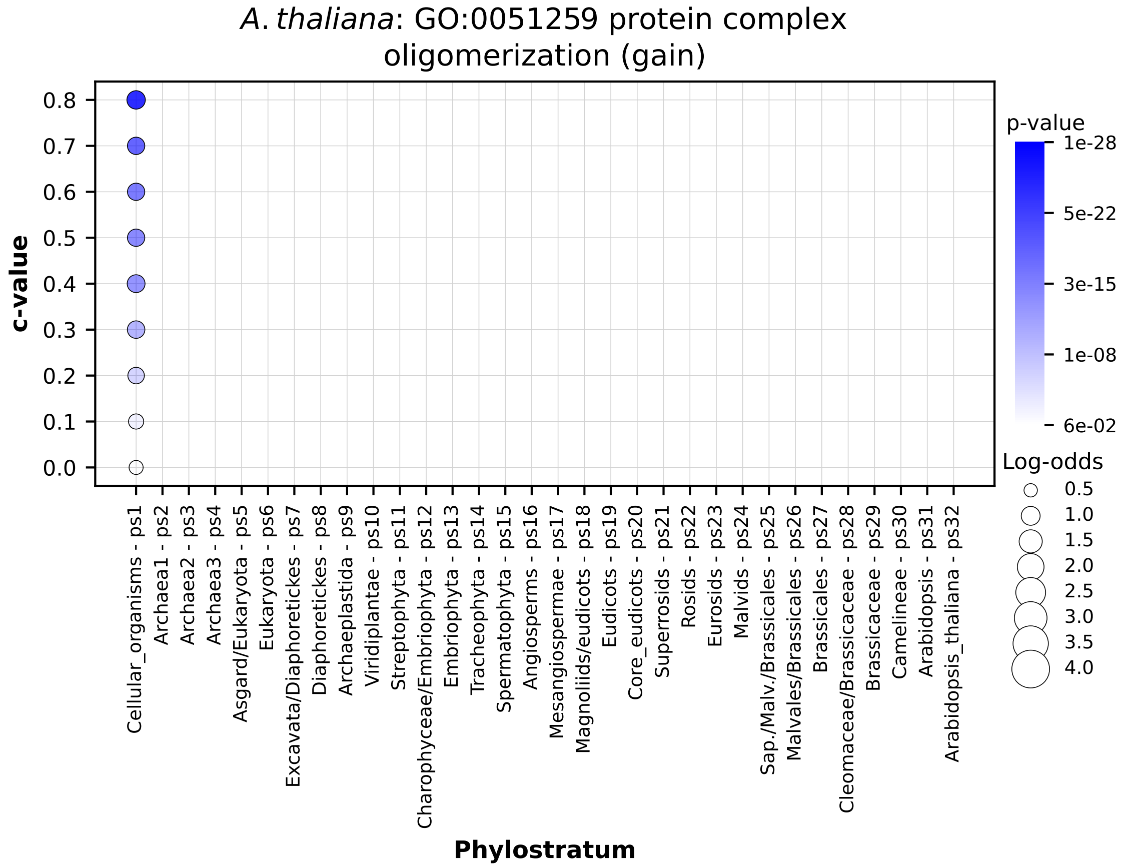
A. thaliana: GO:0051259 protein complex oligomerization (gain)
c-value
0.8
0.7
0.6
0.5
0.4
0.3
0.2
0.1
0.0
p-value
1e-28
5e-22
3e-15
1e-08
6e-02
Log-odds
0.5
1.0
1.5
2.0
2.5
3.0
3.5
4.0
Cellular_organisms - ps1
Archaea1 - ps2
Archaea2 - ps3
Archaea3 - ps4
Asgard/Eukaryota - ps5
Eukaryota - ps6
Excavata/Diaphoretickes - ps7
Diaphoretickes - ps8
Archaeplastida - ps9
Viridiplantae - ps10
Streptophyta - ps11
Charophyceae/Embriophyta - ps12
Embriophyta - ps13
Tracheophyta - ps14
Spermatophyta - ps15
Angiosperms - ps16
Mesangiospermae - ps17
Magnoliids/eudicots - ps18
Eudicots - ps19
Core_eudicots - ps20
Superrosids - ps21
Rosids - ps22
Eurosids - ps23
Malvids - ps24
Sap./Malv./Brassicales - ps25
Malvales/Brassicales - ps26
Brassicales - ps27
Cleomaceae/Brassicaceae - ps28
Brassicaceae - ps29
Camelineae - ps30
Arabidopsis - ps31
Arabidopsis_thaliana - ps32
Phylostratum

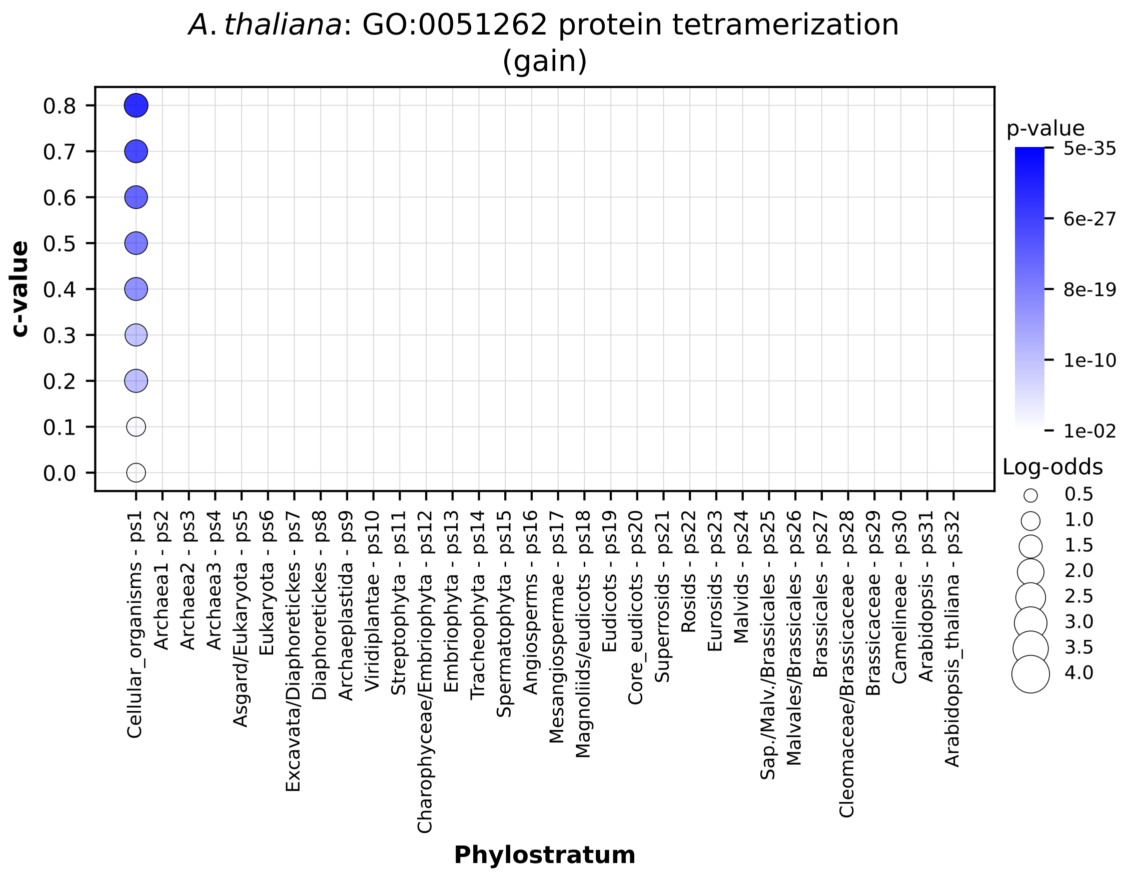

A. thaliana: GO:0051262 protein tetramerization (gain)
c-value
0.8
0.7
0.6
0.5
0.4
0.3
0.2
0.1
0.0
p-value
5e-35
6e-27
8e-19
1e-10
1e-02
Log-odds
0.5
1.0
1.5
2.0
2.5
3.0
3.5
4.0
Cellular_organisms - ps1
Archaea1 - ps2
Archaea2 - ps3
Archaea3 - ps4
Asgard/Eukaryota - ps5
Eukaryota - ps6
Excavata/Diaphoretickes - ps7
Diaphoretickes - ps8
Archaeplastida - ps9
Viridiplantae - ps10
Streptophyta - ps11
Charophyceae/Embriophyta - ps12
Embriophyta - ps13
Tracheophyta - ps14
Spermatophyta - ps15
Angiosperms - ps16
Mesangiospermae - ps17
Magnoliids/eudicots - ps18
Eudicots - ps19
Core_eudicots - ps20
Superrosids - ps21
Rosids - ps22
Eurosids - ps23
Malvids - ps24
Sap./Malv./Brassicales - ps25
Malvales/Brassicales - ps26
Brassicales - ps27
Cleomaceae/Brassicaceae - ps28
Brassicaceae - ps29
Camelineae - ps30
Arabidopsis - ps31
Arabidopsis_thaliana - ps32
Phylostratum

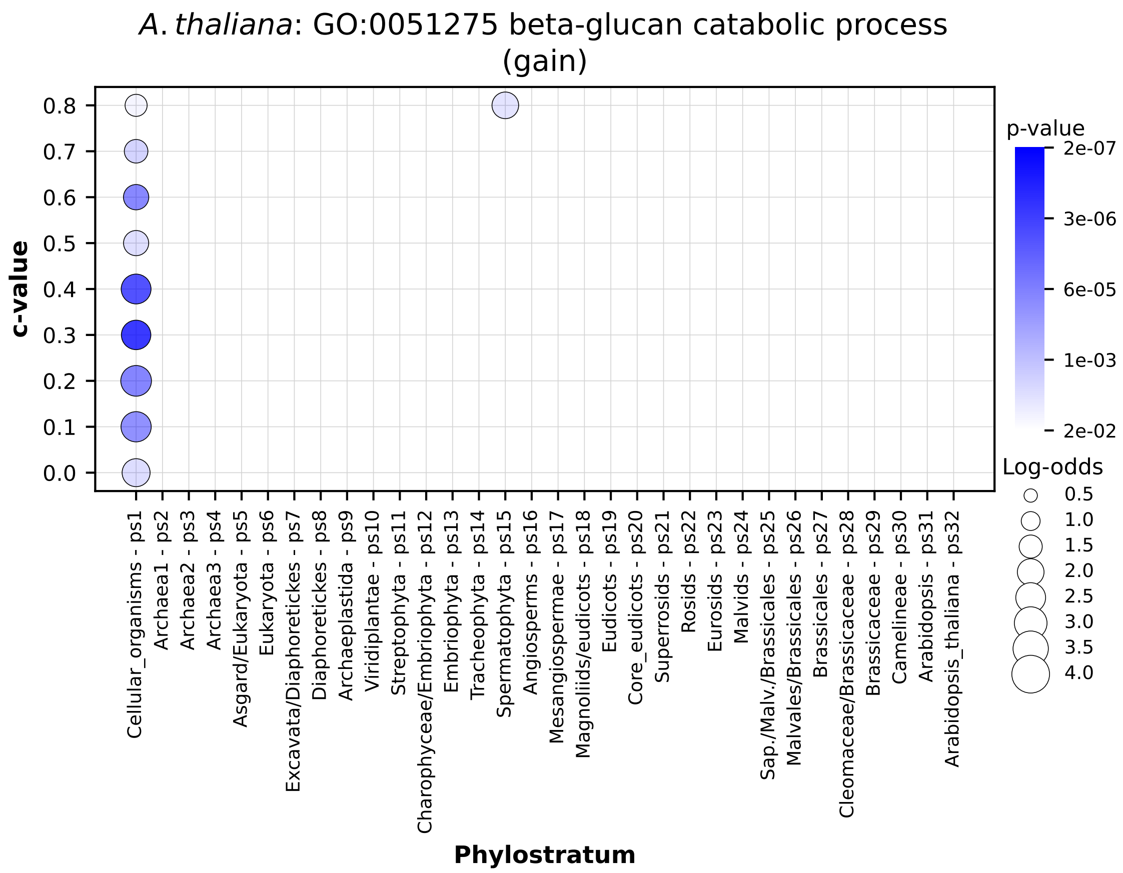

A. thaliana: GO:0051275 beta-glucan catabolic process (gain)
c-value
0.0
0.1
0.2
0.3
0.4
0.5
0.6
0.7
0.8
Phylostratum
Cellular_organisms - ps1
Archaea1 - ps2
Archaea2 - ps3
Archaea3 - ps4
Asgard/Eukaryota - ps5
Eukaryota - ps6
Excavata/Diaphoretickes - ps7
Diaphoretickes - ps8
Archaeplastida - ps9
Viridiplantae - ps10
Streptophyta - ps11
Charophyceae/Embriophyta - ps12
Embriophyta - ps13
Tracheophyta - ps14
Spermatophyta - ps15
Angiosperms - ps16
Mesangiospermae - ps17
Magnoliids/eudicots - ps18
Eudicots - ps19
Core_eudicots - ps20
Superrosids - ps21
Rosids - ps22
Eurosids - ps23
Malvids - ps24
Sap./Malv./Brassicales - ps25
Malvales/Brassicales - ps26
Brassicales - ps27
Cleomaceae/Brassicaceae - ps28
Brassicaceae - ps29
Camelineae - ps30
Arabidopsis - ps31
Arabidopsis_thaliana - ps32
p-value
2e-07
3e-06
6e-05
1e-03
2e-02
Log-odds
0.5
1.0
1.5
2.0
2.5
3.0
3.5
4.0

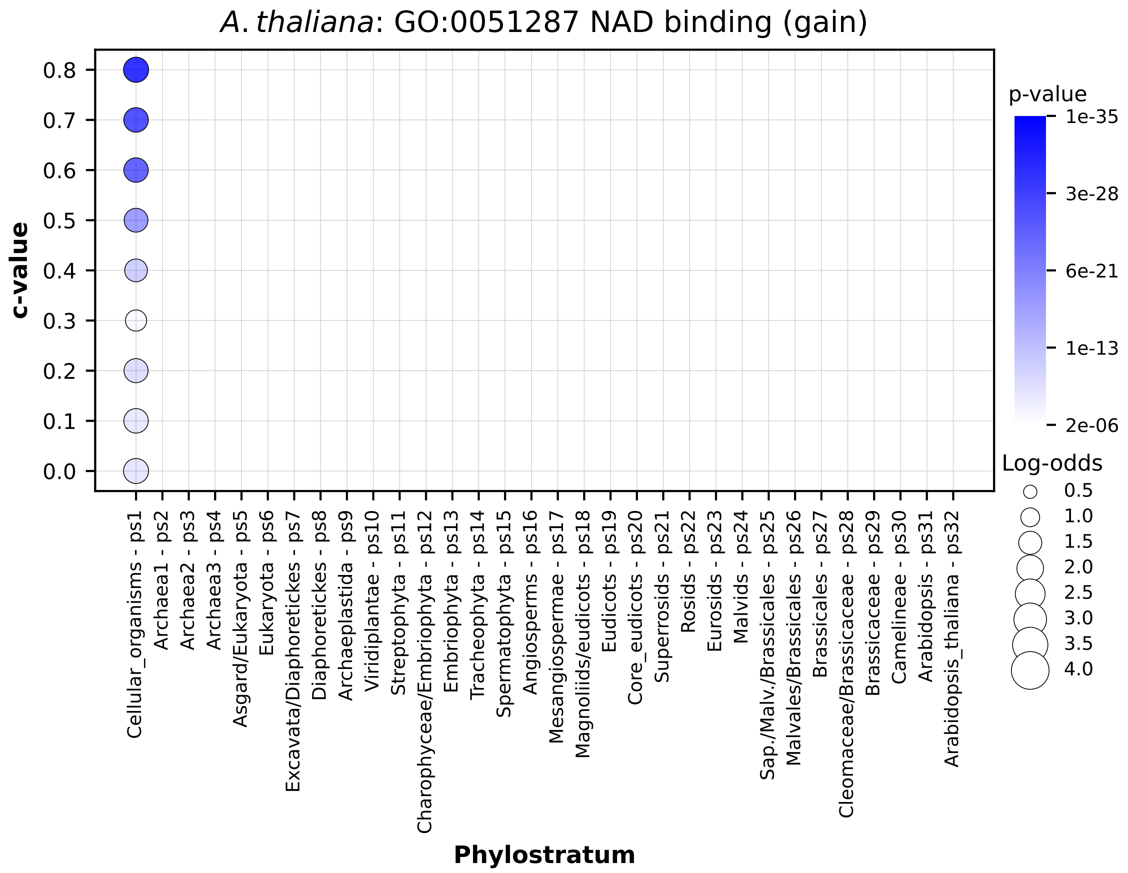

A. thaliana: GO:0051287 NAD binding (gain)
c-value
0.0
0.1
0.2
0.3
0.4
0.5
0.6
0.7
0.8
p-value
1e-35
3e-28
6e-21
1e-13
2e-06
Log-odds
0.5
1.0
1.5
2.0
2.5
3.0
3.5
4.0
Cellular_organisms - ps1
Archaea1 - ps2
Archaea2 - ps3
Archaea3 - ps4
Asgard/Eukaryota - ps5
Eukaryota - ps6
Excavata/Diaphoretickes - ps7
Diaphoretickes - ps8
Archaeplastida - ps9
Viridiplantae - ps10
Streptophyta - ps11
Charophyceae/Embriophyta - ps12
Embriophyta - ps13
Tracheophyta - ps14
Spermatophyta - ps15
Angiosperms - ps16
Mesangiospermae - ps17
Magnoliids/eudicots - ps18
Eudicots - ps19
Core_eudicots - ps20
Superrosids - ps21
Rosids - ps22
Eurosids - ps23
Malvids - ps24
Sap./Malv./Brassicales - ps25
Malvales/Brassicales - ps26
Brassicales - ps27
Cleomaceae/Brassicaceae - ps28
Brassicaceae - ps29
Camelineae - ps30
Arabidopsis - ps31
Arabidopsis_thaliana - ps32
Phylostratum

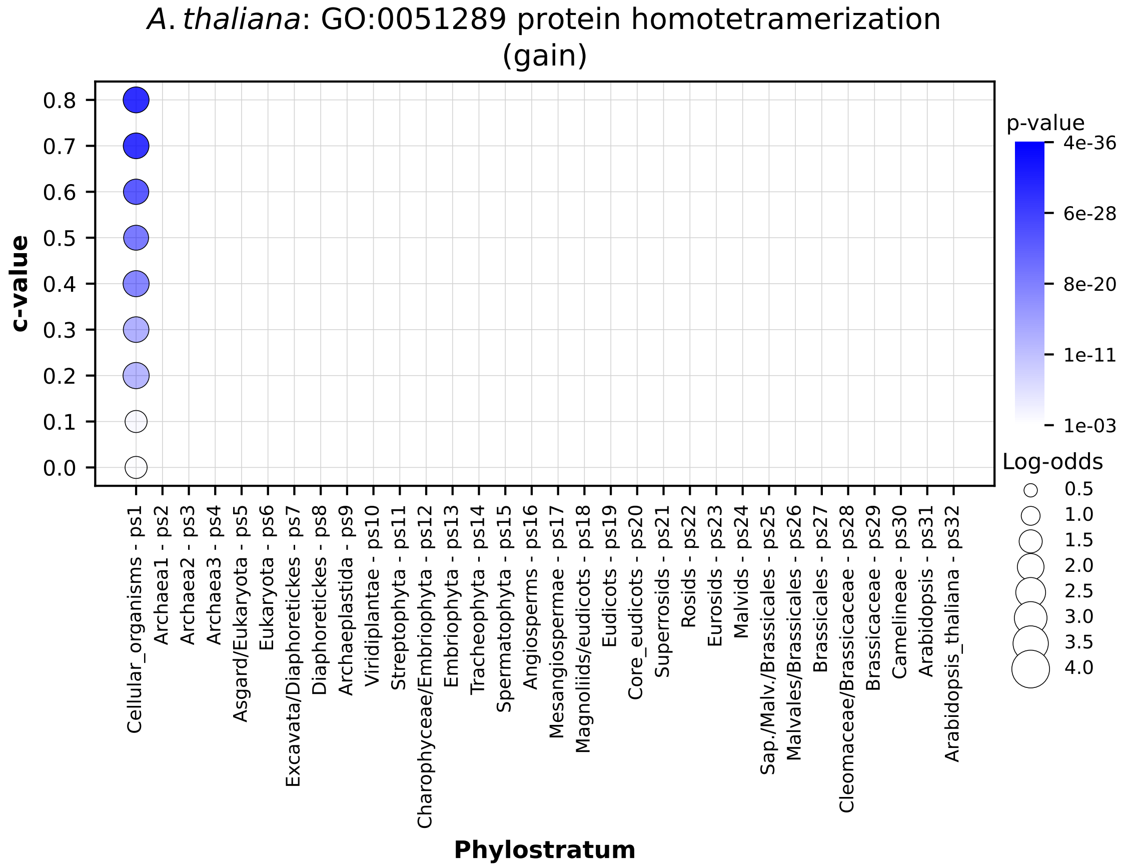

A. thaliana: GO:0051289 protein homotetramerization
(gain)
c-value
0.8
0.7
0.6
0.5
0.4
0.3
0.2
0.1
0.0
p-value
4e-36
6e-28
8e-20
1e-11
1e-03
Log-odds
0.5
1.0
1.5
2.0
2.5
3.0
3.5
4.0
Cellular_organisms - ps1
Archaea1 - ps2
Archaea2 - ps3
Archaea3 - ps4
Asgard/Eukaryota - ps5
Eukaryota - ps6
Excavata/Diaphoretickes - ps7
Diaphoretickes - ps8
Archaeplastida - ps9
Viridiplantae - ps10
Streptophyta - ps11
Charophyceae/Embriophyta - ps12
Embriophyta - ps13
Tracheophyta - ps14
Spermatophyta - ps15
Angiosperms - ps16
Mesangiospermae - ps17
Magnoliids/eudicots - ps18
Eudicots - ps19
Core_eudicots - ps20
Superrosids - ps21
Rosids - ps22
Eurosids - ps23
Malvids - ps24
Sap./Malv./Brassicales - ps25
Malvales/Brassicales - ps26
Brassicales - ps27
Cleomaceae/Brassicaceae - ps28
Brassicaceae - ps29
Camelineae - ps30
Arabidopsis - ps31
Arabidopsis_thaliana - ps32
Phylostratum

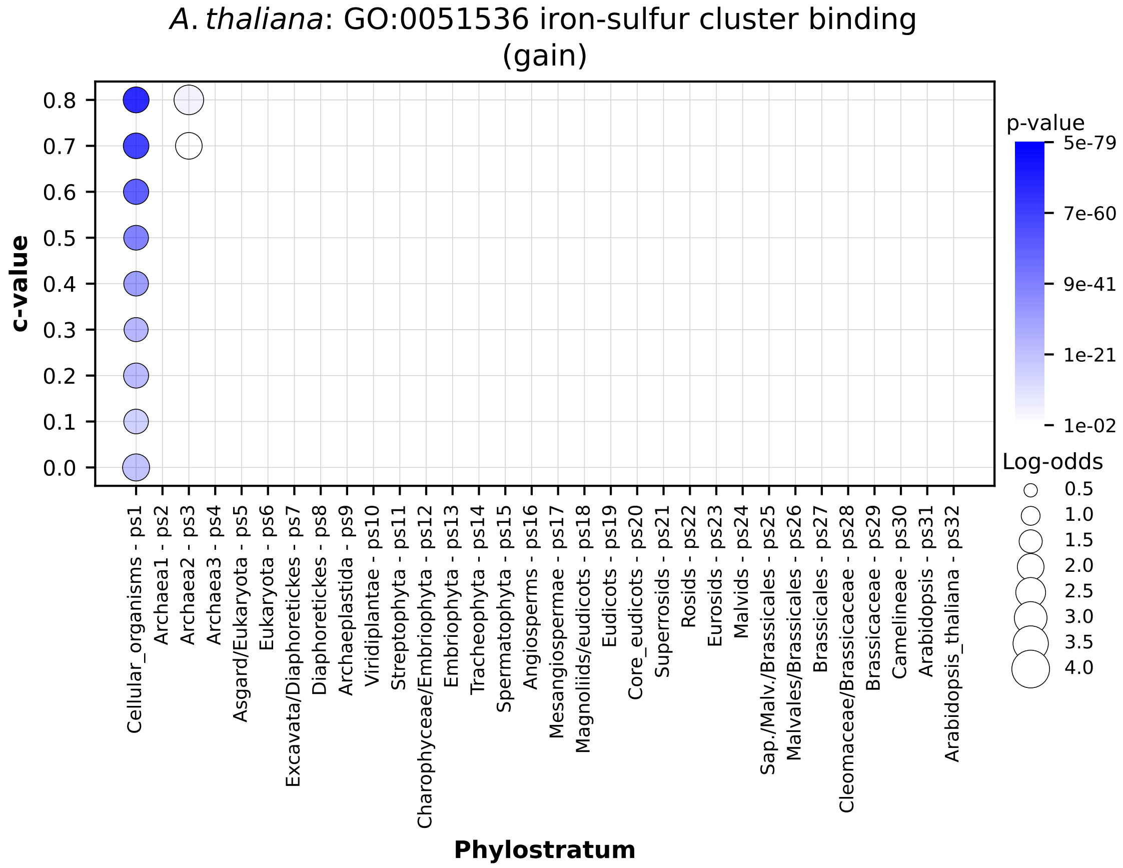

A. thaliana: GO:0051536 iron-sulfur cluster binding (gain)
c-value
0.8
0.7
0.6
0.5
0.4
0.3
0.2
0.1
0.0
p-value
5e-79
7e-60
9e-41
1e-21
1e-02
Log-odds
0.5
1.0
1.5
2.0
2.5
3.0
3.5
4.0
Cellular_organisms - ps1
Archaea1 - ps2
Archaea2 - ps3
Archaea3 - ps4
Asgard/Eukaryota - ps5
Eukaryota - ps6
Excavata/Diaphoretickes - ps7
Diaphoretickes - ps8
Archaeplastida - ps9
Viridiplantae - ps10
Streptophyta - ps11
Charophyceae/Embriophyta - ps12
Embriophyta - ps13
Tracheophyta - ps14
Spermatophyta - ps15
Angiosperms - ps16
Mesangiospermae - ps17
Magnoliids/eudicots - ps18
Eudicots - ps19
Core_eudicots - ps20
Superrosids - ps21
Rosids - ps22
Eurosids - ps23
Malvids - ps24
Sap./Malv./Brassicales - ps25
Malvales/Brassicales - ps26
Brassicales - ps27
Cleomaceae/Brassicaceae - ps28
Brassicaceae - ps29
Camelineae - ps30
Arabidopsis - ps31
Arabidopsis_thaliana - ps32
Phylostratum

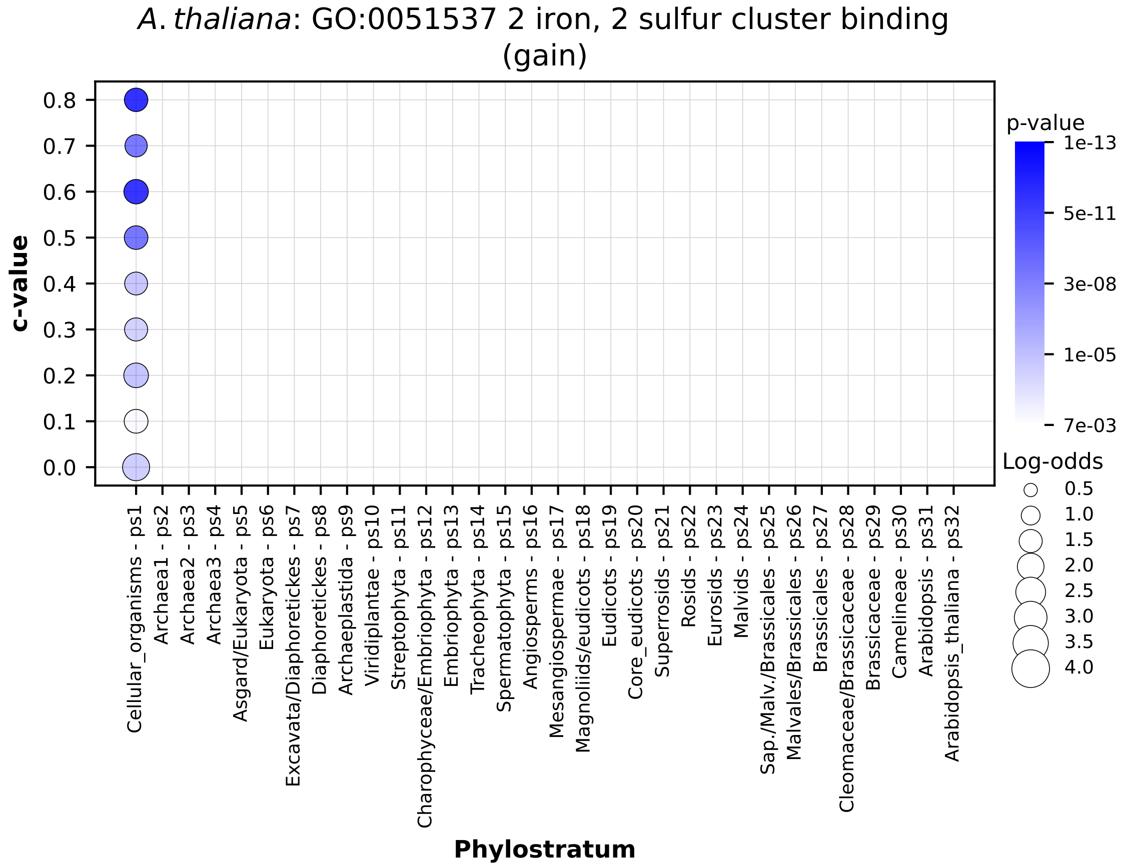
A. thaliana: GO:0051537 2 iron, 2 sulfur cluster binding
(gain)
c-value
0.8
0.7
0.6
0.5
0.4
0.3
0.2
0.1
0.0
p-value
1e-13
5e-11
3e-08
1e-05
7e-03
Log-odds
0.5
1.0
1.5
2.0
2.5
3.0
3.5
4.0
Cellular_organisms - ps1
Archaea1 - ps2
Archaea2 - ps3
Archaea3 - ps4
Asgard/Eukaryota - ps5
Eukaryota - ps6
Excavata/Diaphoretickes - ps7
Diaphoretickes - ps8
Archaeplastida - ps9
Viridiplantae - ps10
Streptophyta - ps11
Charophyceae/Embriophyta - ps12
Embriophyta - ps13
Tracheophyta - ps14
Spermatophyta - ps15
Angiosperms - ps16
Mesangiospermae - ps17
Magnoliids/eudicots - ps18
Eudicots - ps19
Core_eudicots - ps20
Superrosids - ps21
Rosids - ps22
Eurosids - ps23
Malvids - ps24
Sap./Malv./Brassicales - ps25
Malvales/Brassicales - ps26
Brassicales - ps27
Cleomaceae/Brassicaceae - ps28
Brassicaceae - ps29
Camelineae - ps30
Arabidopsis - ps31
Arabidopsis_thaliana - ps32
Phylostratum

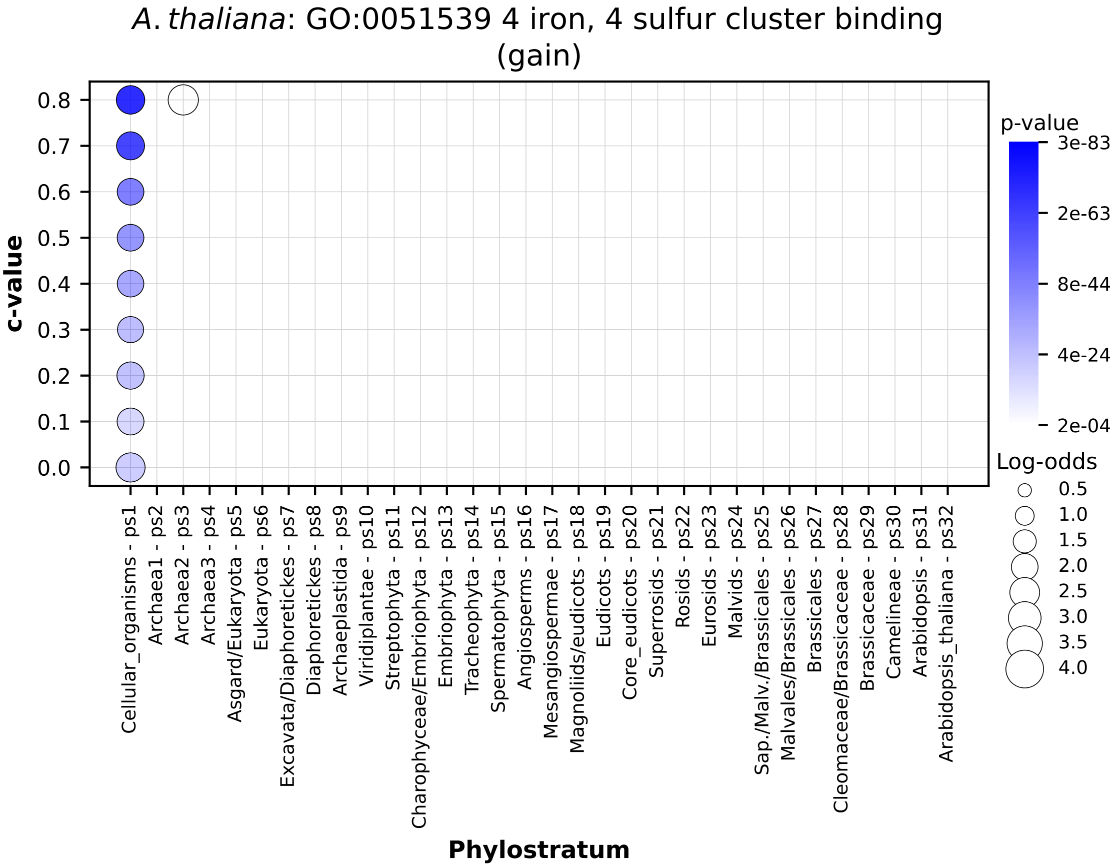

A. thaliana: GO:0051539 4 iron, 4 sulfur cluster binding
(gain)
c-value
0.8
0.7
0.6
0.5
0.4
0.3
0.2
0.1
0.0
p-value
3e-83
2e-63
8e-44
4e-24
2e-04
Log-odds
0.5
1.0
1.5
2.0
2.5
3.0
3.5
4.0
Cellular_organisms - ps1
Archaea1 - ps2
Archaea2 - ps3
Archaea3 - ps4
Asgard/Eukaryota - ps5
Eukaryota - ps6
Excavata/Diaphoretickes - ps7
Diaphoretickes - ps8
Archaeplastida - ps9
Viridiplantae - ps10
Streptophyta - ps11
Charophyceae/Embriophyta - ps12
Embriophyta - ps13
Tracheophyta - ps14
Spermatophyta - ps15
Angiosperms - ps16
Mesangiospermae - ps17
Magnoliids/eudicots - ps18
Eudicots - ps19
Core_eudicots - ps20
Superrosids - ps21
Rosids - ps22
Eurosids - ps23
Malvids - ps24
Sap./Malv./Brassicales - ps25
Malvales/Brassicales - ps26
Brassicales - ps27
Cleomaceae/Brassicaceae - ps28
Brassicaceae - ps29
Camelineae - ps30
Arabidopsis - ps31
Arabidopsis_thaliana - ps32
Phylostratum

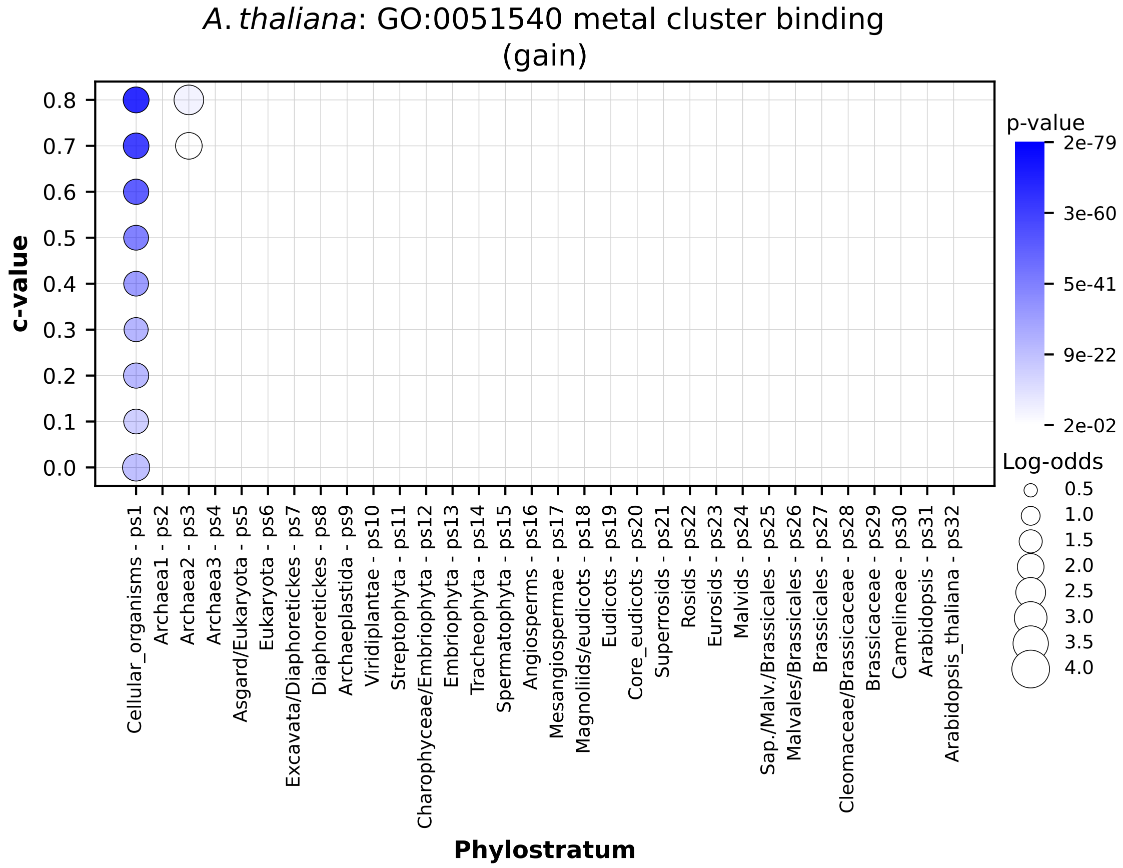
A. thaliana: GO:0051540 metal cluster binding
(gain)
c-value
0.8
0.7
0.6
0.5
0.4
0.3
0.2
0.1
0.0
p-value
2e-79
3e-60
5e-41
9e-22
2e-02
Log-odds
0.5
1.0
1.5
2.0
2.5
3.0
3.5
4.0
Cellular_organisms - ps1
Archaea1 - ps2
Archaea2 - ps3
Archaea3 - ps4
Asgard/Eukaryota - ps5
Eukaryota - ps6
Excavata/Diaphoretickes - ps7
Diaphoretickes - ps8
Archaeplastida - ps9
Viridiplantae - ps10
Streptophyta - ps11
Charophyceae/Embriophyta - ps12
Embriophyta - ps13
Tracheophyta - ps14
Spermatophyta - ps15
Angiosperms - ps16
Mesangiospermae - ps17
Magnoliids/eudicots - ps18
Eudicots - ps19
Core_eudicots - ps20
Superrosids - ps21
Rosids - ps22
Eurosids - ps23
Malvids - ps24
Sap./Malv./Brassicales - ps25
Malvales/Brassicales - ps26
Brassicales - ps27
Cleomaceae/Brassicaceae - ps28
Brassicaceae - ps29
Camelineae - ps30
Arabidopsis - ps31
Arabidopsis_thaliana - ps32
Phylostratum

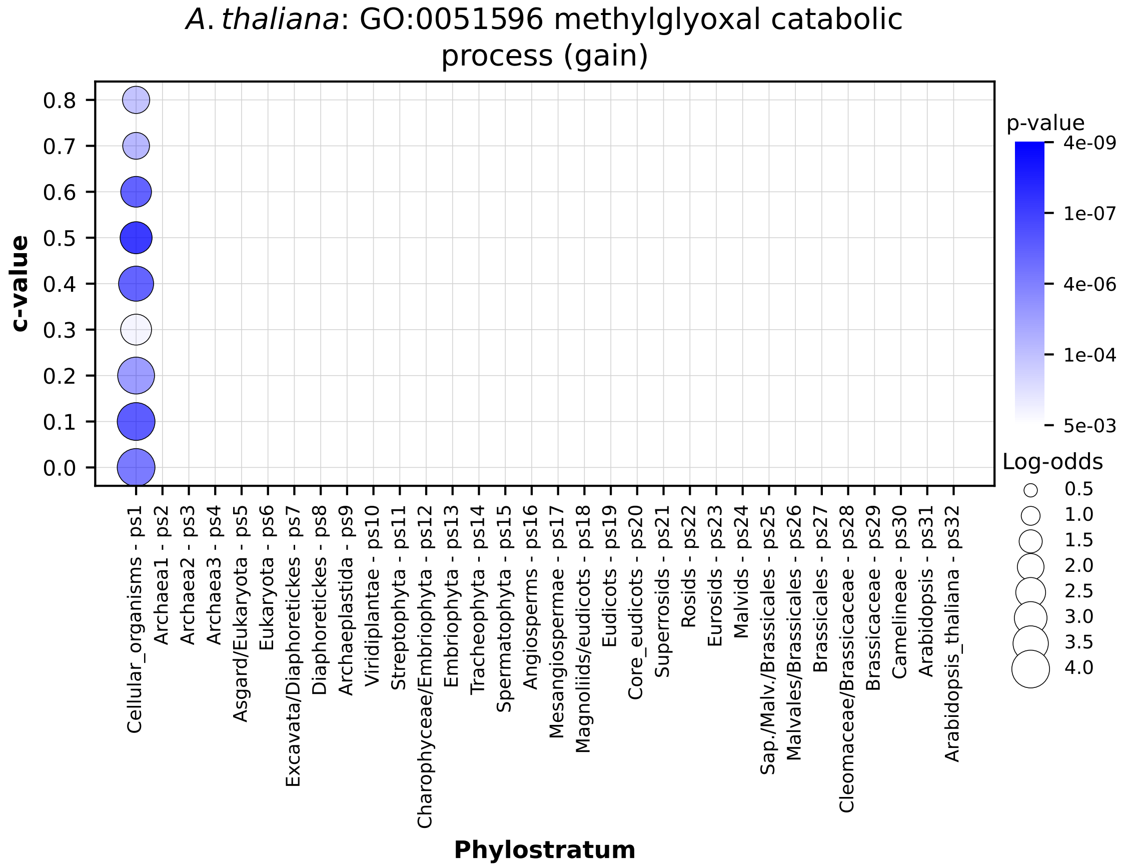
A. thaliana: GO:0051596 methylglyoxal catabolic process (gain)
c-value
0.8
0.7
0.6
0.5
0.4
0.3
0.2
0.1
0.0
p-value
4e-09
1e-07
4e-06
1e-04
5e-03
Log-odds
0.5
1.0
1.5
2.0
2.5
3.0
3.5
4.0
Cellular_organisms - ps1
Archaea1 - ps2
Archaea2 - ps3
Archaea3 - ps4
Asgard/Eukaryota - ps5
Eukaryota - ps6
Excavata/Diaphoretickes - ps7
Diaphoretickes - ps8
Archaeplastida - ps9
Viridiplantae - ps10
Streptophyta - ps11
Charophyceae/Embriophyta - ps12
Embriophyta - ps13
Tracheophyta - ps14
Spermatophyta - ps15
Angiosperms - ps16
Mesangiospermae - ps17
Magnoliids/eudicots - ps18
Eudicots - ps19
Core_eudicots - ps20
Superrosids - ps21
Rosids - ps22
Eurosids - ps23
Malvids - ps24
Sap./Malv./Brassicales - ps25
Malvales/Brassicales - ps26
Brassicales - ps27
Cleomaceae/Brassicaceae - ps28
Brassicaceae - ps29
Camelineae - ps30
Arabidopsis - ps31
Arabidopsis_thaliana - ps32
Phylostratum

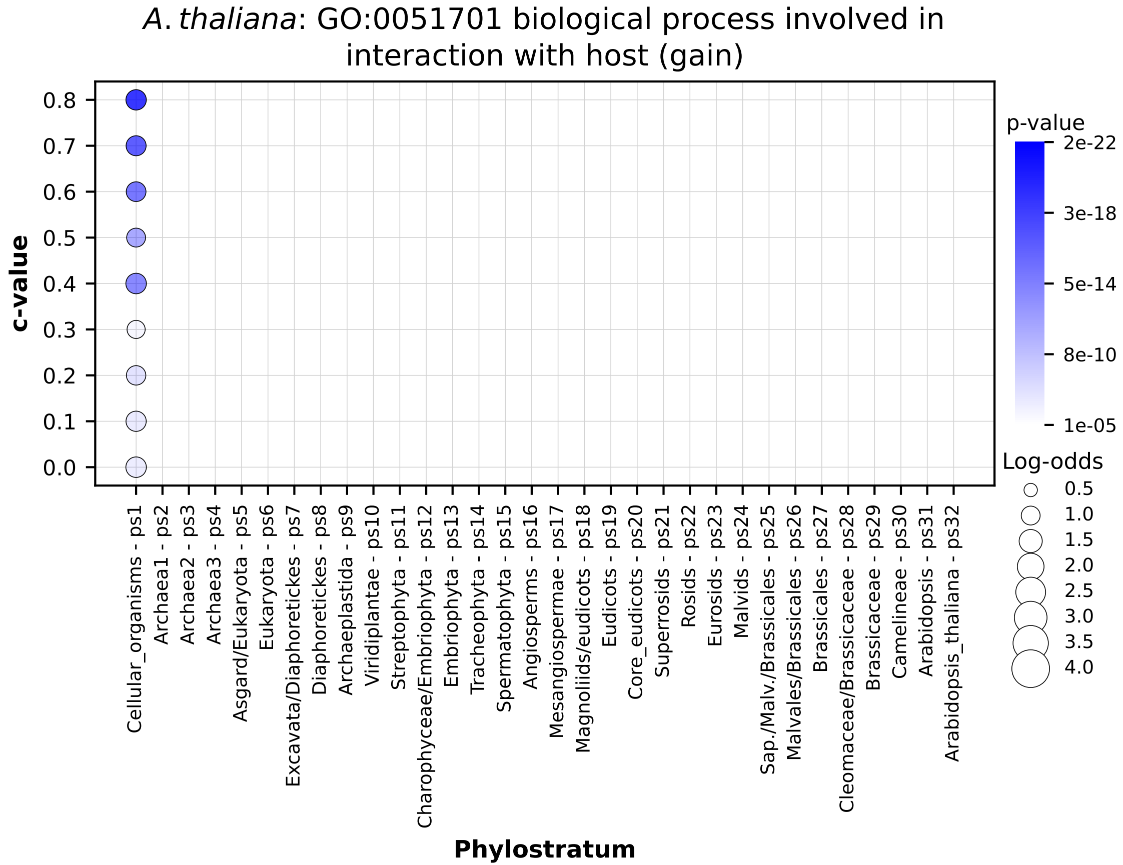

A. thaliana: GO:0051701 biological process involved in interaction with host (gain)
c-value
0.8
0.7
0.6
0.5
0.4
0.3
0.2
0.1
0.0
p-value
2e-22
3e-18
5e-14
8e-10
1e-05
Log-odds
0.5
1.0
1.5
2.0
2.5
3.0
3.5
4.0
Cellular_organisms - ps1
Archaea1 - ps2
Archaea2 - ps3
Archaea3 - ps4
Asgard/Eukaryota - ps5
Eukaryota - ps6
Excavata/Diaphoretickes - ps7
Diaphoretickes - ps8
Archaeplastida - ps9
Viridiplantae - ps10
Streptophyta - ps11
Charophyceae/Embriophyta - ps12
Embriophyta - ps13
Tracheophyta - ps14
Spermatophyta - ps15
Angiosperms - ps16
Mesangiospermae - ps17
Magnoliids/eudicots - ps18
Eudicots - ps19
Core_eudicots - ps20
Superrosids - ps21
Rosids - ps22
Eurosids - ps23
Malvids - ps24
Sap./Malv./Brassicales - ps25
Malvales/Brassicales - ps26
Brassicales - ps27
Cleomaceae/Brassicaceae - ps28
Brassicaceae - ps29
Camelineae - ps30
Arabidopsis - ps31
Arabidopsis_thaliana - ps32
Phylostratum

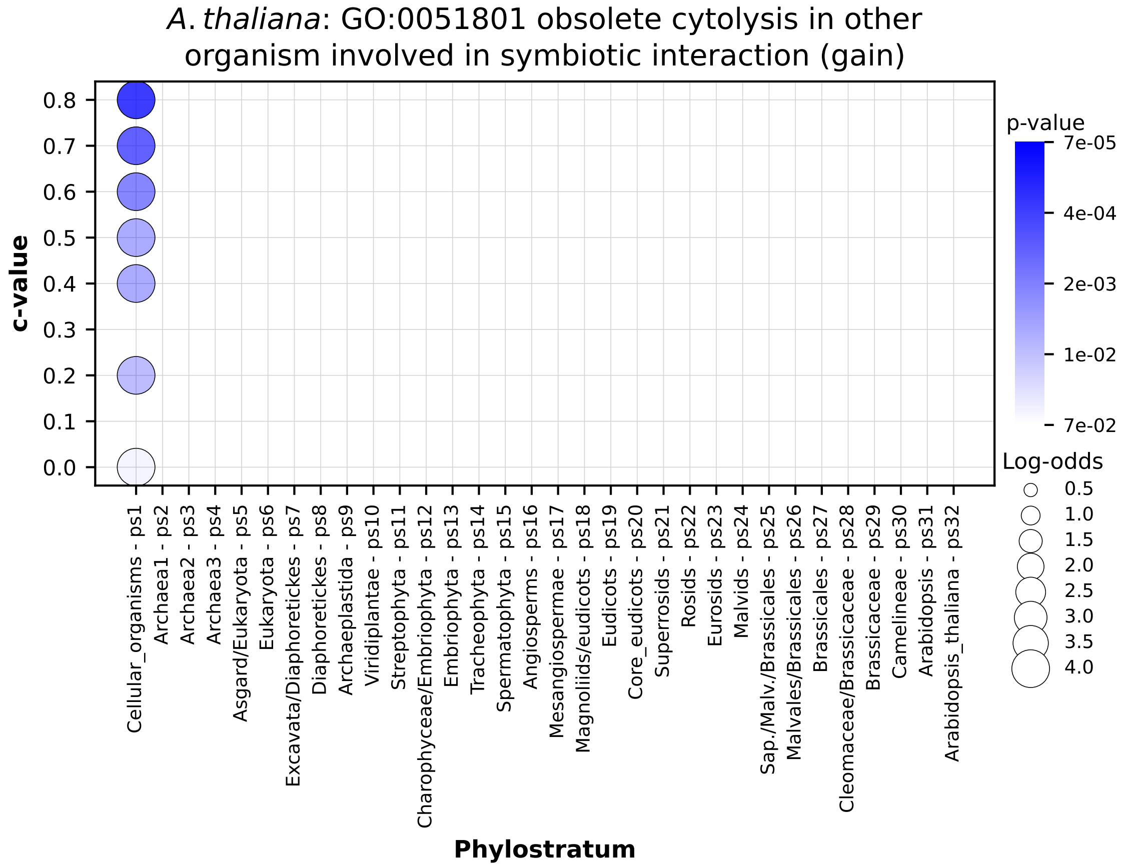

A. thaliana: GO:0051801 obsolete cytolysis in other organism involved in symbiotic interaction (gain)
c-value
0.8
0.7
0.6
0.5
0.4
0.3
0.2
0.1
0.0
p-value
7e-05
4e-04
2e-03
1e-02
7e-02
Log-odds
0.5
1.0
1.5
2.0
2.5
3.0
3.5
4.0
Cellular_organisms - ps1
Archaea1 - ps2
Archaea2 - ps3
Archaea3 - ps4
Asgard/Eukaryota - ps5
Eukaryota - ps6
Excavata/Diaphoretickes - ps7
Diaphoretickes - ps8
Archaeplastida - ps9
Viridiplantae - ps10
Streptophyta - ps11
Charophyceae/Embriophyta - ps12
Embriophyta - ps13
Tracheophyta - ps14
Spermatophyta - ps15
Angiosperms - ps16
Mesangiospermae - ps17
Magnoliids/eudicots - ps18
Eudicots - ps19
Core_eudicots - ps20
Superrosids - ps21
Rosids - ps22
Eurosids - ps23
Malvids - ps24
Sap./Malv./Brassicales - ps25
Malvales/Brassicales - ps26
Brassicales - ps27
Cleomaceae/Brassicaceae - ps28
Brassicaceae - ps29
Camelineae - ps30
Arabidopsis - ps31
Arabidopsis_thaliana - ps32
Phylostratum

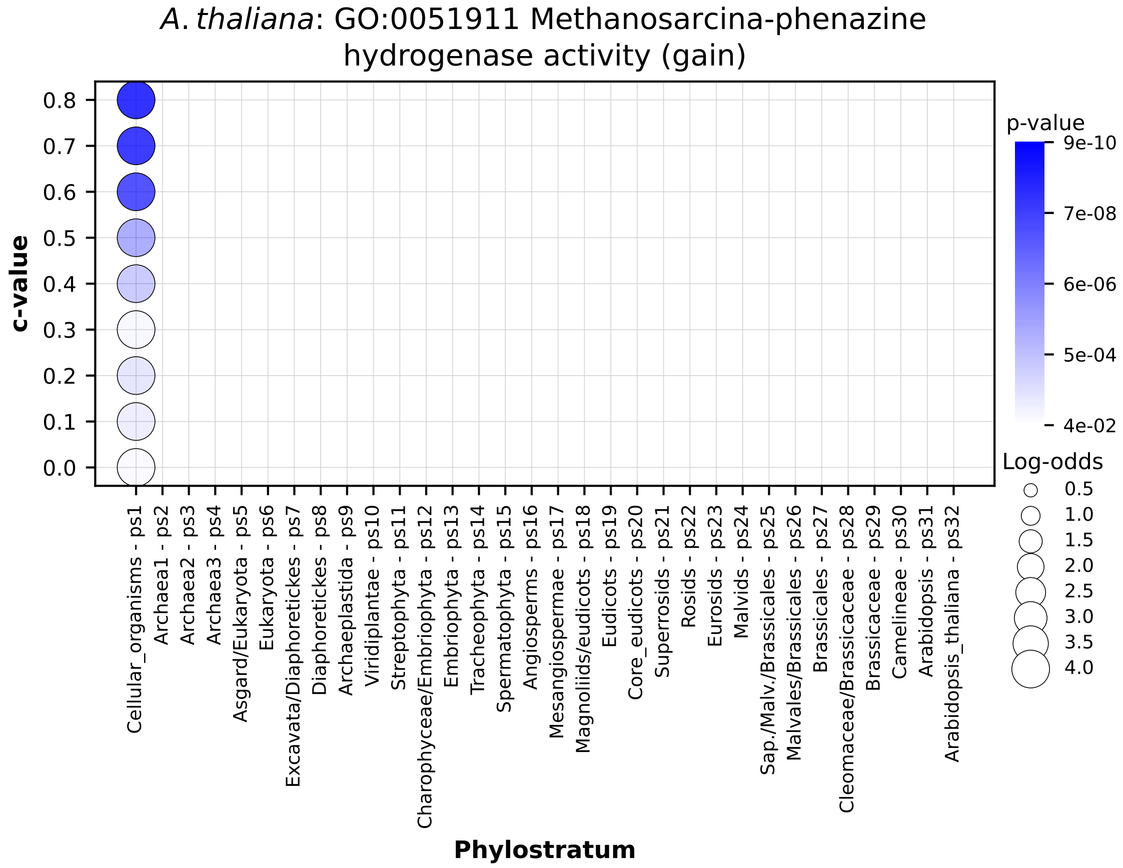

A. thaliana: GO:0051911 Methanosarcina-phenazine hydrogenase activity (gain)
c-value
0.8
0.7
0.6
0.5
0.4
0.3
0.2
0.1
0.0
p-value
9e-10
7e-08
6e-06
5e-04
4e-02
Log-odds
0.5
1.0
1.5
2.0
2.5
3.0
3.5
4.0
Cellular_organisms - ps1
Archaea1 - ps2
Archaea2 - ps3
Archaea3 - ps4
Asgard/Eukaryota - ps5
Eukaryota - ps6
Excavata/Diaphoretickes - ps7
Diaphoretickes - ps8
Archaeplastida - ps9
Viridiplantae - ps10
Streptophyta - ps11
Charophyceae/Embriophyta - ps12
Embriophyta - ps13
Tracheophyta - ps14
Spermatophyta - ps15
Angiosperms - ps16
Mesangiospermae - ps17
Magnoliids/eudicots - ps18
Eudicots - ps19
Core_eudicots - ps20
Superrosids - ps21
Rosids - ps22
Eurosids - ps23
Malvids - ps24
Sap./Malv./Brassicales - ps25
Malvales/Brassicales - ps26
Brassicales - ps27
Cleomaceae/Brassicaceae - ps28
Brassicaceae - ps29
Camelineae - ps30
Arabidopsis - ps31
Arabidopsis_thaliana - ps32
Phylostratum

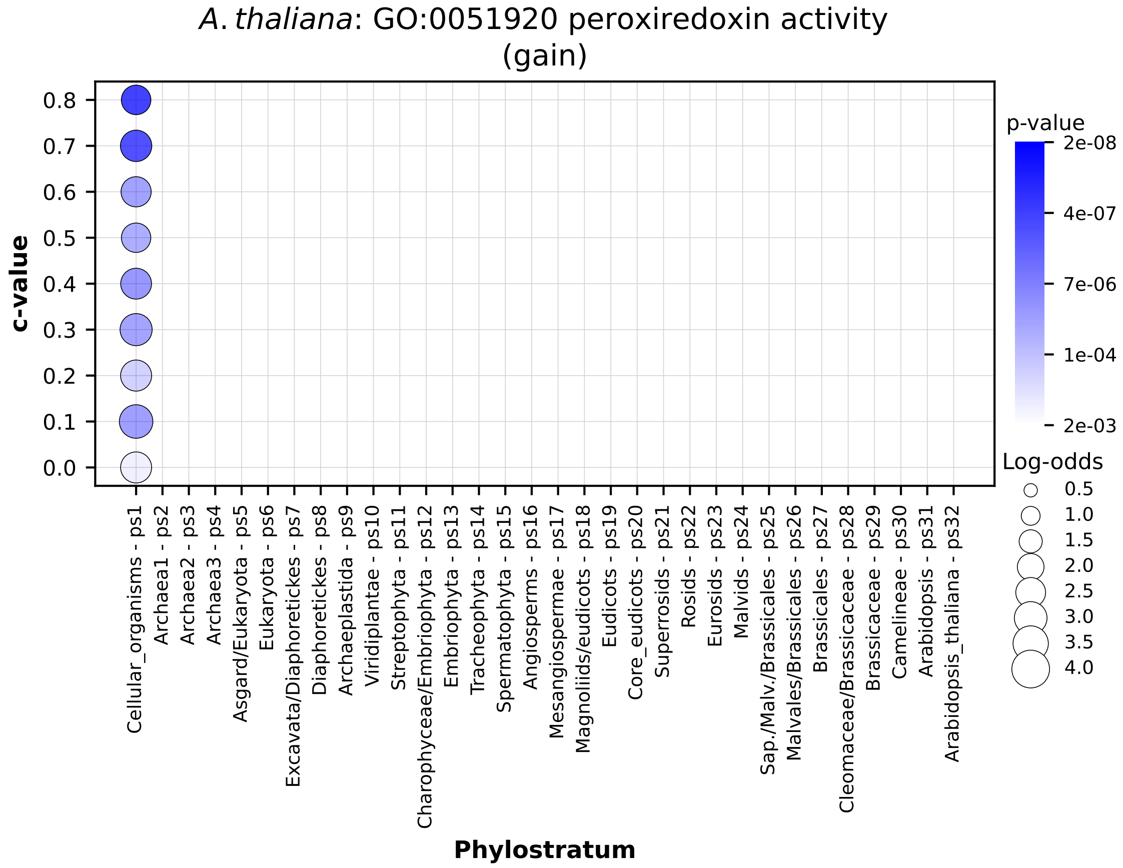

A. thaliana: GO:0051920 peroxiredoxin activity
(gain)
c-value
0.8
0.7
0.6
0.5
0.4
0.3
0.2
0.1
0.0
p-value
2e-08
4e-07
7e-06
1e-04
2e-03
Log-odds
0.5
1.0
1.5
2.0
2.5
3.0
3.5
4.0
Cellular_organisms - ps1
Archaea1 - ps2
Archaea2 - ps3
Archaea3 - ps4
Asgard/Eukaryota - ps5
Eukaryota - ps6
Excavata/Diaphoretickes - ps7
Diaphoretickes - ps8
Archaeplastida - ps9
Viridiplantae - ps10
Streptophyta - ps11
Charophyceae/Embriophyta - ps12
Embriophyta - ps13
Tracheophyta - ps14
Spermatophyta - ps15
Angiosperms - ps16
Mesangiospermae - ps17
Magnoliids/eudicots - ps18
Eudicots - ps19
Core_eudicots - ps20
Superrosids - ps21
Rosids - ps22
Eurosids - ps23
Malvids - ps24
Sap./Malv./Brassicales - ps25
Malvales/Brassicales - ps26
Brassicales - ps27
Cleomaceae/Brassicaceae - ps28
Brassicaceae - ps29
Camelineae - ps30
Arabidopsis - ps31
Arabidopsis_thaliana - ps32
Phylostratum

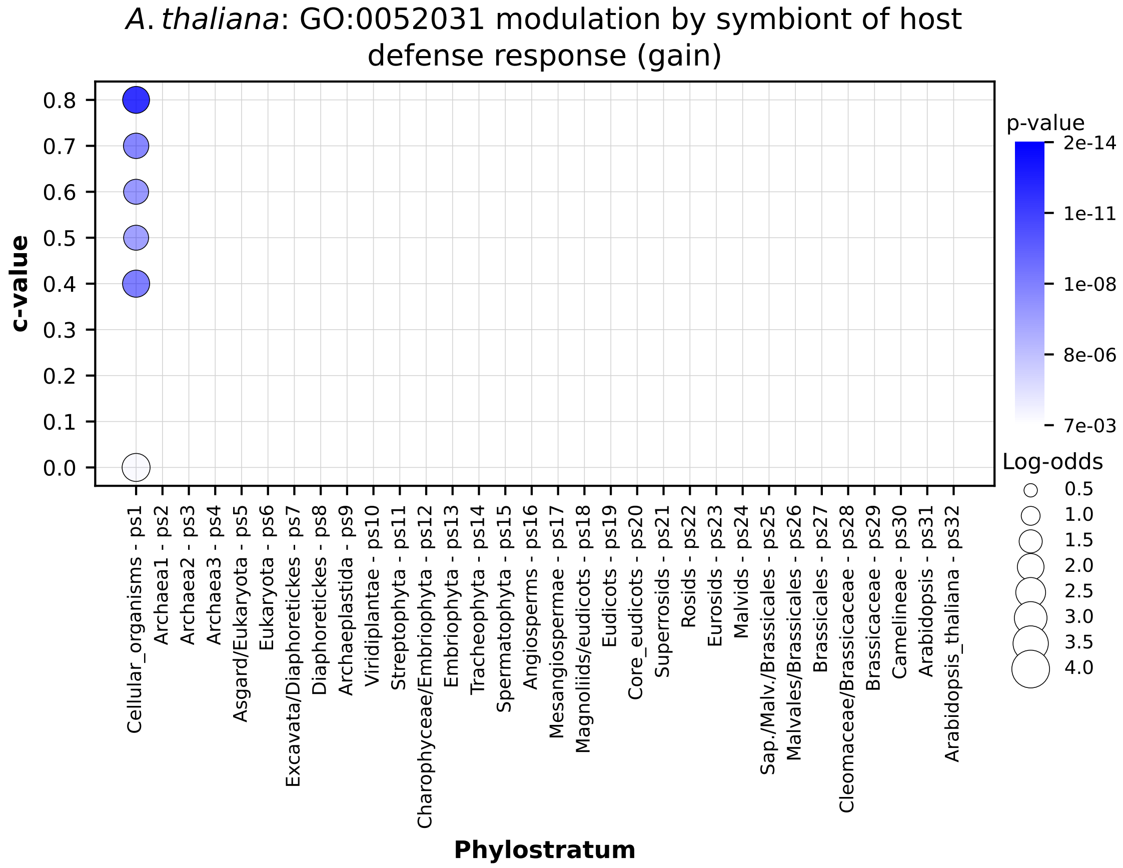

A. thaliana: GO:0052031 modulation by symbiont of host defense response (gain)
c-value
0.8
0.7
0.6
0.5
0.4
0.3
0.2
0.1
0.0
Phylostratum
Cellular_organisms - ps1
Archaea1 - ps2
Archaea2 - ps3
Archaea3 - ps4
Asgard/Eukaryota - ps5
Eukaryota - ps6
Excavata/Diaphoretickes - ps7
Diaphoretickes - ps8
Archaeplastida - ps9
Viridiplantae - ps10
Streptophyta - ps11
Charophyceae/Embriophyta - ps12
Embriophyta - ps13
Tracheophyta - ps14
Spermatophyta - ps15
Angiosperms - ps16
Mesangiospermae - ps17
Magnoliids/eudicots - ps18
Eudicots - ps19
Core_eudicots - ps20
Superrosids - ps21
Rosids - ps22
Eurosids - ps23
Malvids - ps24
Sap./Malv./Brassicales - ps25
Malvales/Brassicales - ps26
Brassicales - ps27
Cleomaceae/Brassicaceae - ps28
Brassicaceae - ps29
Camelineae - ps30
Arabidopsis - ps31
Arabidopsis_thaliana - ps32
p-value
2e-14
1e-11
1e-08
8e-06
7e-03
Log-odds
0.5
1.0
1.5
2.0
2.5
3.0
3.5
4.0

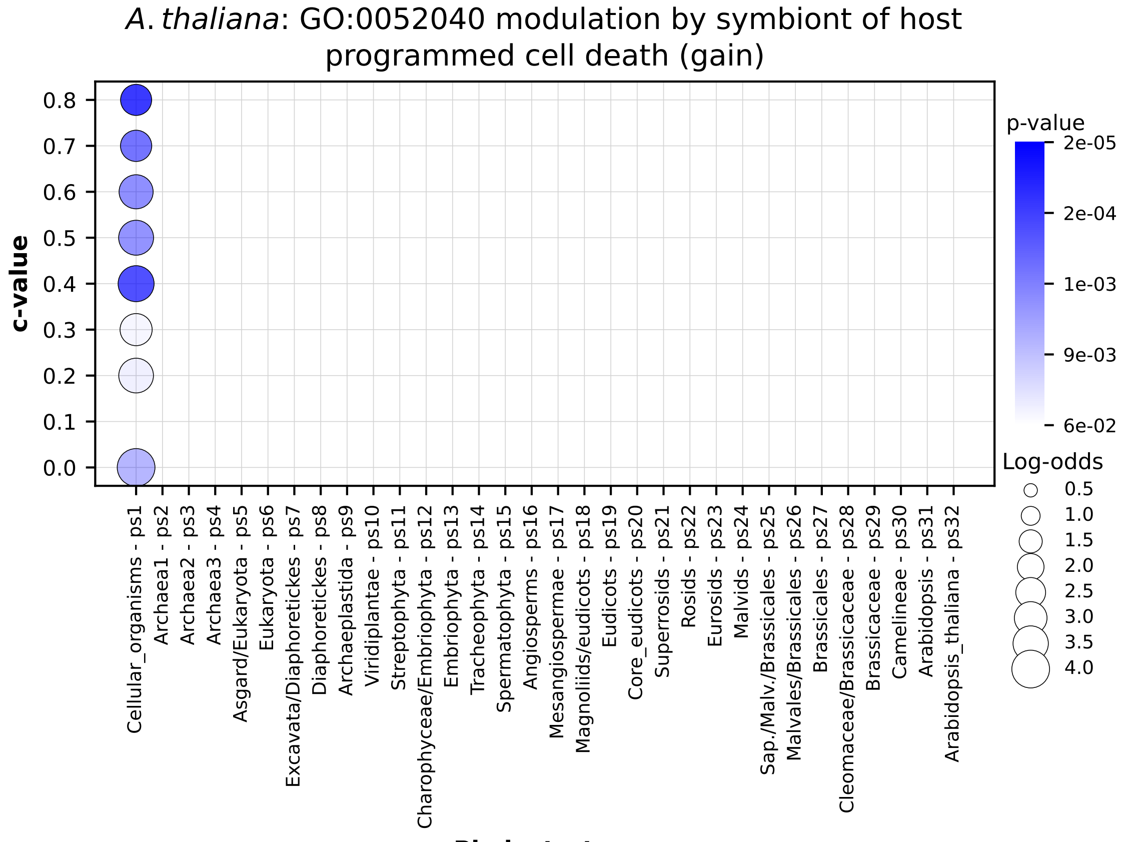

A. thaliana: GO:0052040 modulation by symbiont of host programmed cell death (gain)
c-value
0.0
0.1
0.2
0.3
0.4
0.5
0.6
0.7
0.8
Phylostratum
Cellular_organisms - ps1
Archaea1 - ps2
Archaea2 - ps3
Archaea3 - ps4
Asgard/Eukaryota - ps5
Eukaryota - ps6
Excavata/Diaphoretickes - ps7
Diaphoretickes - ps8
Archaeplastida - ps9
Viridiplantae - ps10
Streptophyta - ps11
Charophyceae/Embriophyta - ps12
Embriophyta - ps13
Tracheophyta - ps14
Spermatophyta - ps15
Angiosperms - ps16
Mesangiospermae - ps17
Magnoliids/eudicots - ps18
Eudicots - ps19
Core_eudicots - ps20
Superrosids - ps21
Rosids - ps22
Eurosids - ps23
Malvids - ps24
Sap./Malv./Brassicales - ps25
Malvales/Brassicales - ps26
Brassicales - ps27
Cleomaceae/Brassicaceae - ps28
Brassicaceae - ps29
Camelineae - ps30
Arabidopsis - ps31
Arabidopsis_thaliana - ps32
p-value
2e-05
2e-04
1e-03
9e-03
6e-02
Log-odds
0.5
1.0
1.5
2.0
2.5
3.0
3.5
4.0

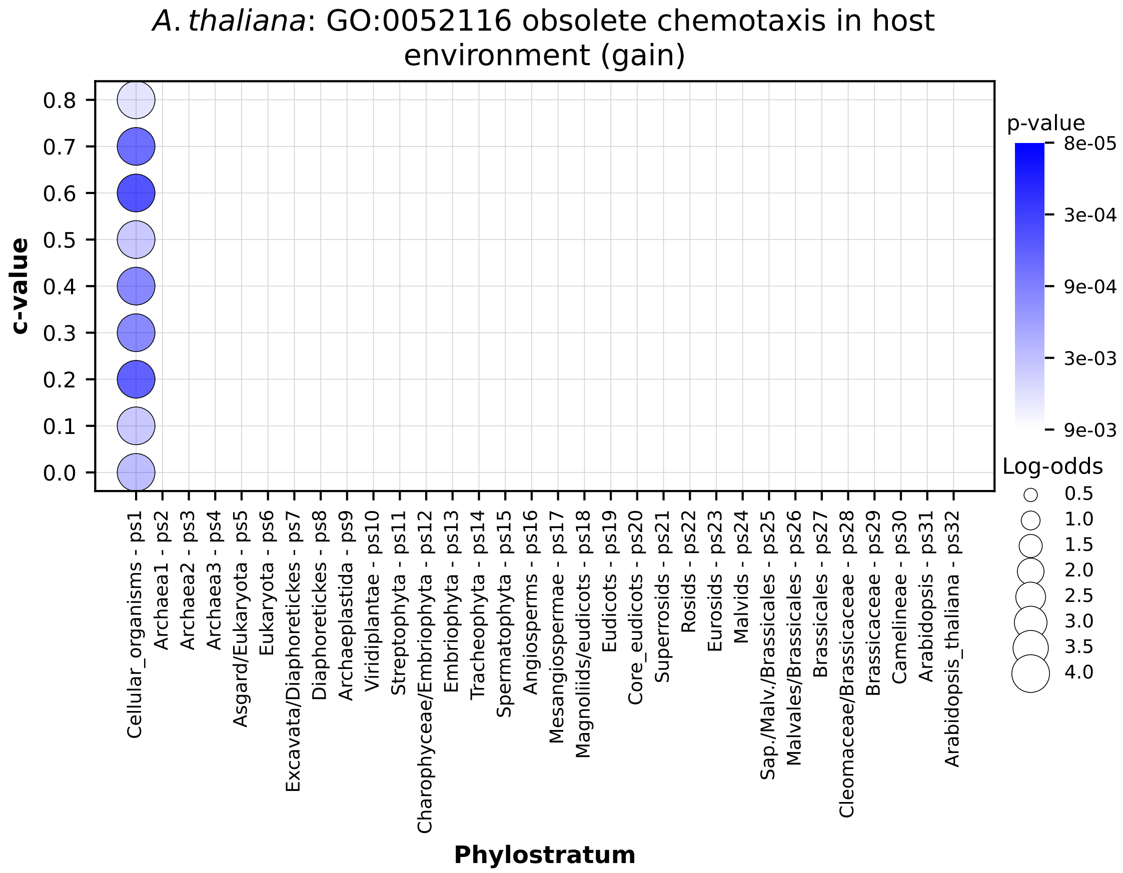
A. thaliana: GO:0052116 obsolete chemotaxis in host environment (gain)
c-value
0.0
0.1
0.2
0.3
0.4
0.5
0.6
0.7
0.8
Phylostratum
Cellular_organisms - ps1
Archaea1 - ps2
Archaea2 - ps3
Archaea3 - ps4
Asgard/Eukaryota - ps5
Eukaryota - ps6
Excavata/Diaphoretickes - ps7
Diaphoretickes - ps8
Archaeplastida - ps9
Viridiplantae - ps10
Streptophyta - ps11
Charophyceae/Embriophyta - ps12
Embriophyta - ps13
Tracheophyta - ps14
Spermatophyta - ps15
Angiosperms - ps16
Mesangiospermae - ps17
Magnoliids/eudicots - ps18
Eudicots - ps19
Core_eudicots - ps20
Superrosids - ps21
Rosids - ps22
Eurosids - ps23
Malvids - ps24
Sap./Malv./Brassicales - ps25
Malvales/Brassicales - ps26
Brassicales - ps27
Cleomaceae/Brassicaceae - ps28
Brassicaceae - ps29
Camelineae - ps30
Arabidopsis - ps31
Arabidopsis_thaliana - ps32
p-value
8e-05
3e-04
9e-04
3e-03
9e-03
Log-odds
0.5
1.0
1.5
2.0
2.5
3.0
3.5
4.0

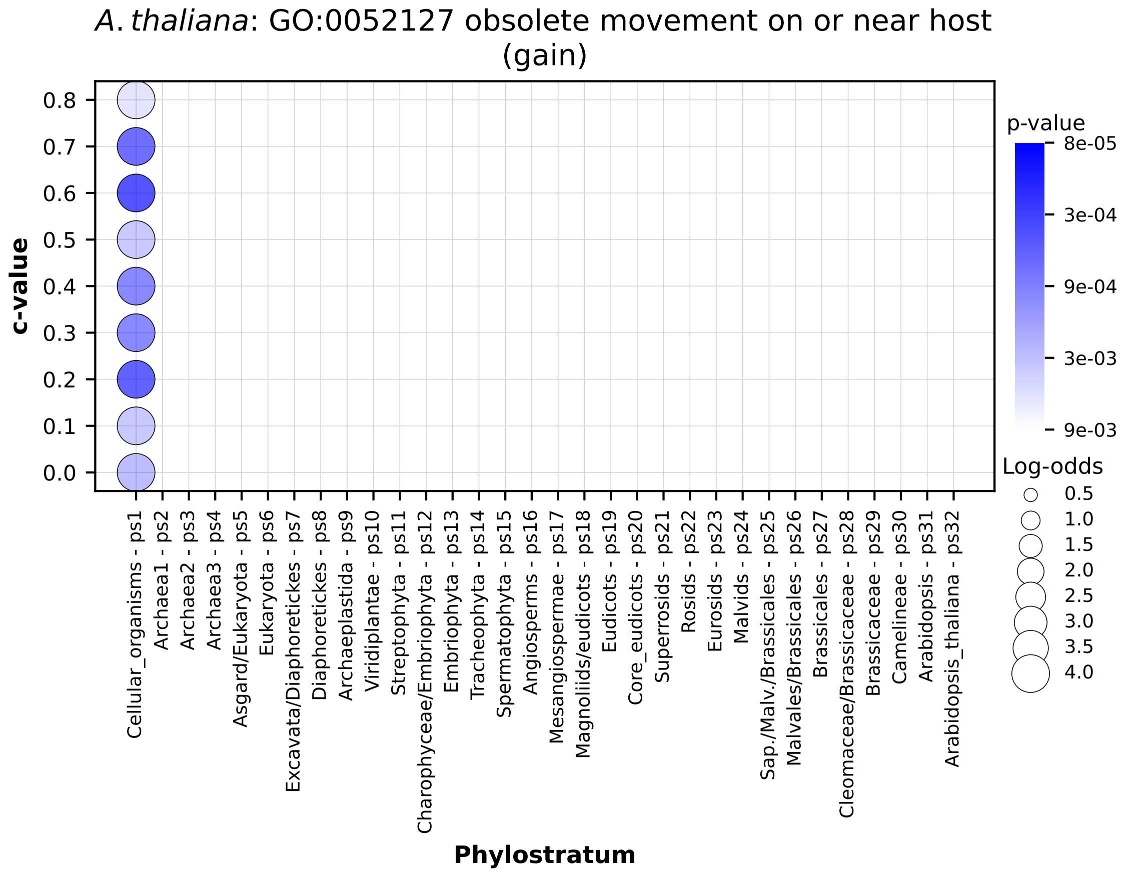

A. thaliana: GO:0052127 obsolete movement on or near host (gain)
c-value
0.8
0.7
0.6
0.5
0.4
0.3
0.2
0.1
0.0
p-value
8e-05
3e-04
9e-04
3e-03
9e-03
Log-odds
0.5
1.0
1.5
2.0
2.5
3.0
3.5
4.0
Cellular_organisms - ps1
Archaea1 - ps2
Archaea2 - ps3
Archaea3 - ps4
Asgard/Eukaryota - ps5
Eukaryota - ps6
Excavata/Diaphoretickes - ps7
Diaphoretickes - ps8
Archaeplastida - ps9
Viridiplantae - ps10
Streptophyta - ps11
Charophyceae/Embriophyta - ps12
Embriophyta - ps13
Tracheophyta - ps14
Spermatophyta - ps15
Angiosperms - ps16
Mesangiospermae - ps17
Magnoliids/eudicots - ps18
Eudicots - ps19
Core_eudicots - ps20
Superrosids - ps21
Rosids - ps22
Eurosids - ps23
Malvids - ps24
Sap./Malv./Brassicales - ps25
Malvales/Brassicales - ps26
Brassicales - ps27
Cleomaceae/Brassicaceae - ps28
Brassicaceae - ps29
Camelineae - ps30
Arabidopsis - ps31
Arabidopsis_thaliana - ps32
Phylostratum

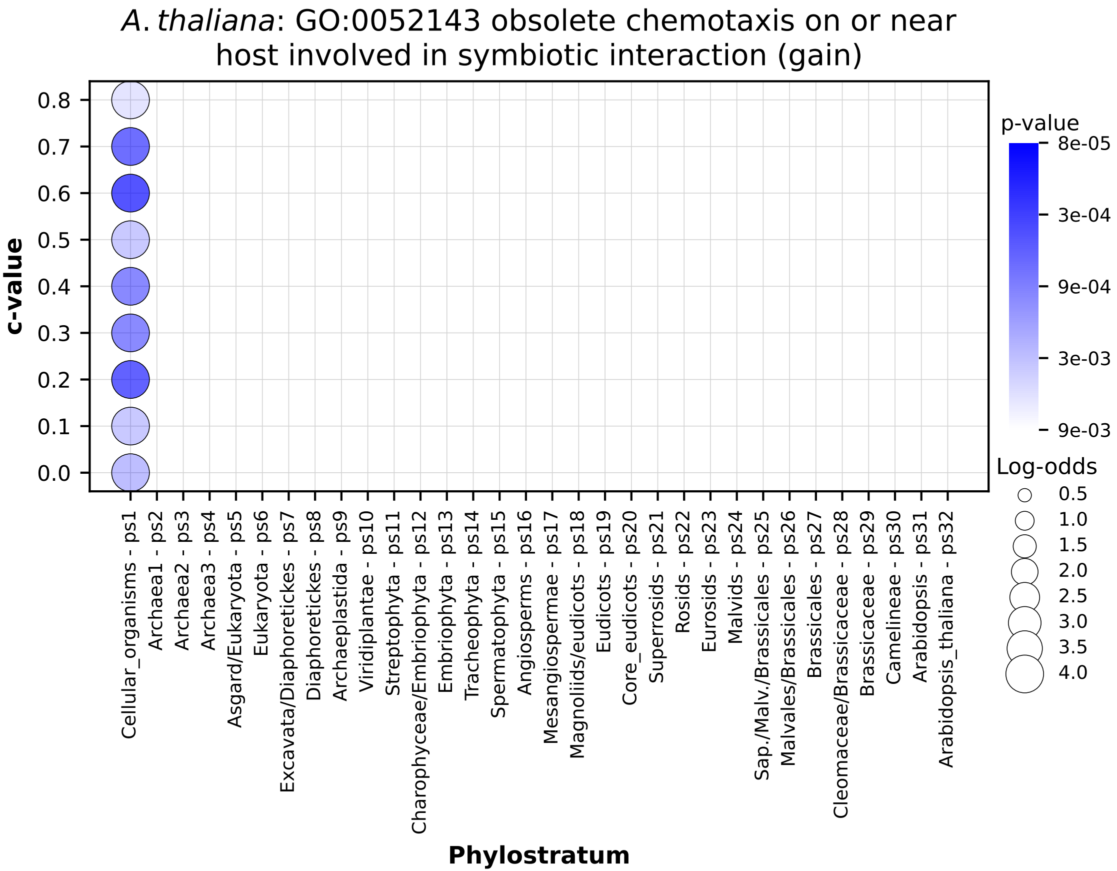

A. thaliana: GO:0052143 obsolete chemotaxis on or near host involved in symbiotic interaction (gain)
c-value
0.8
0.7
0.6
0.5
0.4
0.3
0.2
0.1
0.0
p-value
8e-05
3e-04
9e-04
3e-03
9e-03
Log-odds
0.5
1.0
1.5
2.0
2.5
3.0
3.5
4.0
Cellular_organisms - ps1
Archaea1 - ps2
Archaea2 - ps3
Archaea3 - ps4
Asgard/Eukaryota - ps5
Eukaryota - ps6
Excavata/Diaphoretickes - ps7
Diaphoretickes - ps8
Archaeplastida - ps9
Viridiplantae - ps10
Streptophyta - ps11
Charophyceae/Embriophyta - ps12
Embriophyta - ps13
Tracheophyta - ps14
Spermatophyta - ps15
Angiosperms - ps16
Mesangiospermae - ps17
Magnoliids/eudicots - ps18
Eudicots - ps19
Core_eudicots - ps20
Superrosids - ps21
Rosids - ps22
Eurosids - ps23
Malvids - ps24
Sap./Malv./Brassicales - ps25
Malvales/Brassicales - ps26
Brassicales - ps27
Cleomaceae/Brassicaceae - ps28
Brassicaceae - ps29
Camelineae - ps30
Arabidopsis - ps31
Arabidopsis_thaliana - ps32
Phylostratum

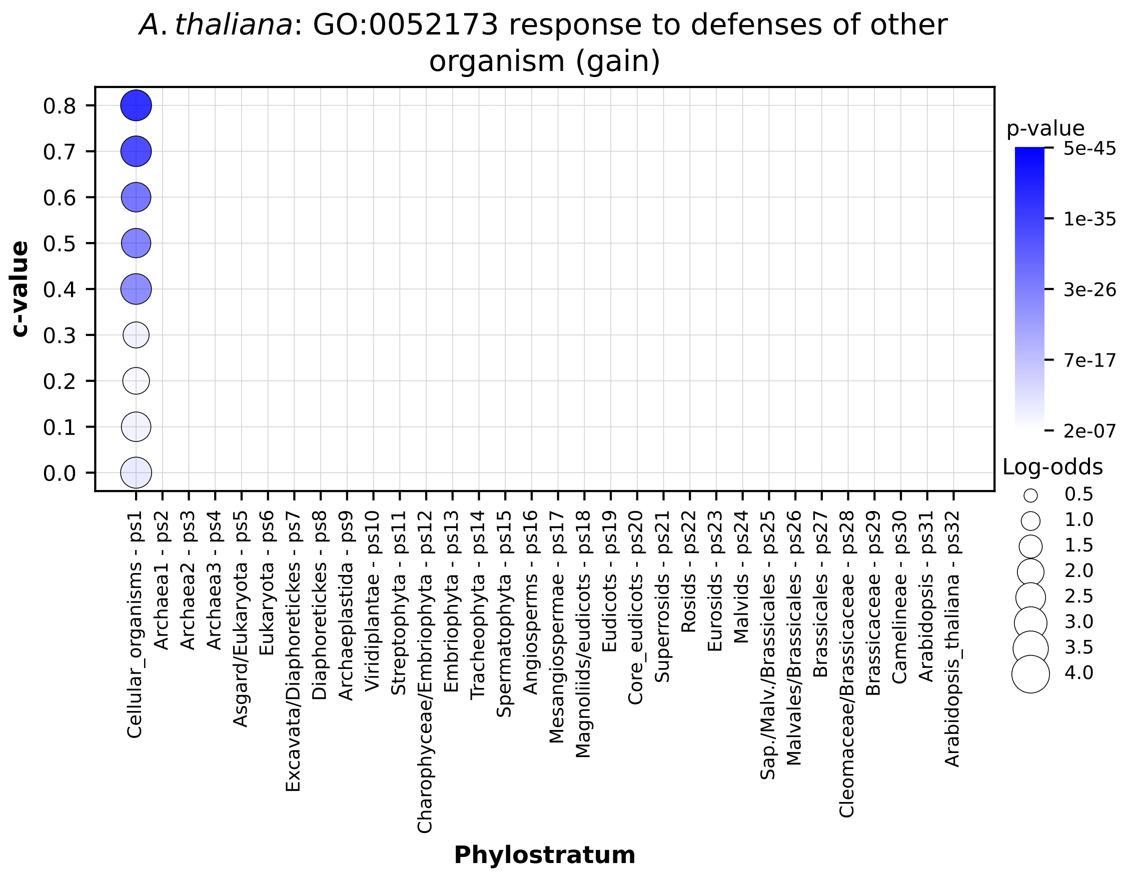

A. thaliana: GO:0052173 response to defenses of other organism (gain)
c-value
0.0
0.1
0.2
0.3
0.4
0.5
0.6
0.7
0.8
p-value
5e-45
1e-35
3e-26
7e-17
2e-07
Log-odds
0.5
1.0
1.5
2.0
2.5
3.0
3.5
4.0
Cellular_organisms - ps1
Archaea1 - ps2
Archaea2 - ps3
Archaea3 - ps4
Asgard/Eukaryota - ps5
Eukaryota - ps6
Excavata/Diaphoretickes - ps7
Diaphoretickes - ps8
Archaeplastida - ps9
Viridiplantae - ps10
Streptophyta - ps11
Charophyceae/Embriophyta - ps12
Embriophyta - ps13
Tracheophyta - ps14
Spermatophyta - ps15
Angiosperms - ps16
Mesangiospermae - ps17
Magnoliids/eudicots - ps18
Eudicots - ps19
Core_eudicots - ps20
Superrosids - ps21
Rosids - ps22
Eurosids - ps23
Malvids - ps24
Sap./Malv./Brassicales - ps25
Malvales/Brassicales - ps26
Brassicales - ps27
Cleomaceae/Brassicaceae - ps28
Brassicaceae - ps29
Camelineae - ps30
Arabidopsis - ps31
Arabidopsis_thaliana - ps32
Phylostratum

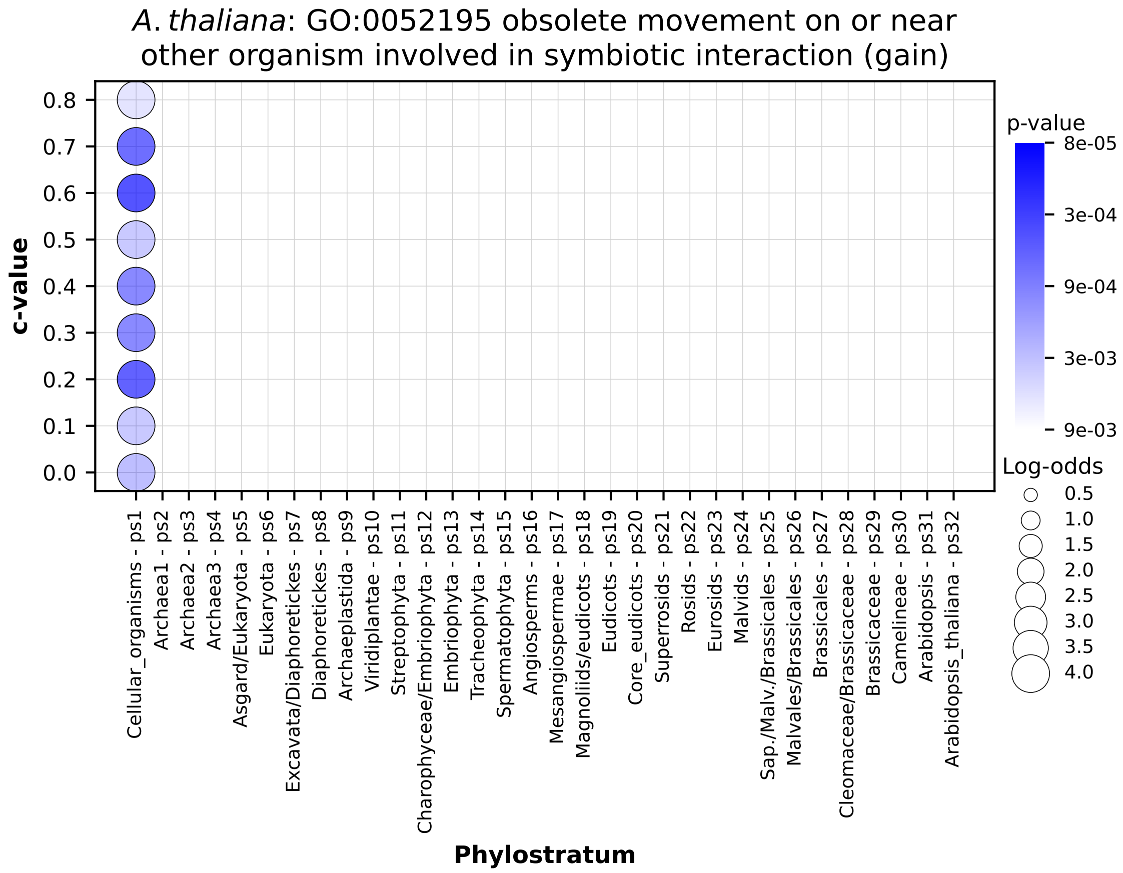
A. thaliana: GO:0052195 obsolete movement on or near other organism involved in symbiotic interaction (gain)
c-value
0.8
0.7
0.6
0.5
0.4
0.3
0.2
0.1
0.0
Phylostratum
Cellular_organisms - ps1
Archaea1 - ps2
Archaea2 - ps3
Archaea3 - ps4
Asgard/Eukaryota - ps5
Eukaryota - ps6
Excavata/Diaphoretickes - ps7
Diaphoretickes - ps8
Archaeplastida - ps9
Viridiplantae - ps10
Streptophyta - ps11
Charophyceae/Embriophyta - ps12
Embriophyta - ps13
Tracheophyta - ps14
Spermatophyta - ps15
Angiosperms - ps16
Mesangiospermae - ps17
Magnoliids/eudicots - ps18
Eudicots - ps19
Core_eudicots - ps20
Superrosids - ps21
Rosids - ps22
Eurosids - ps23
Malvids - ps24
Sap./Malv./Brassicales - ps25
Malvales/Brassicales - ps26
Brassicales - ps27
Cleomaceae/Brassicaceae - ps28
Brassicaceae - ps29
Camelineae - ps30
Arabidopsis - ps31
Arabidopsis_thaliana - ps32
p-value
8e-05
3e-04
9e-04
3e-03
9e-03
Log-odds
0.5
1.0
1.5
2.0
2.5
3.0
3.5
4.0

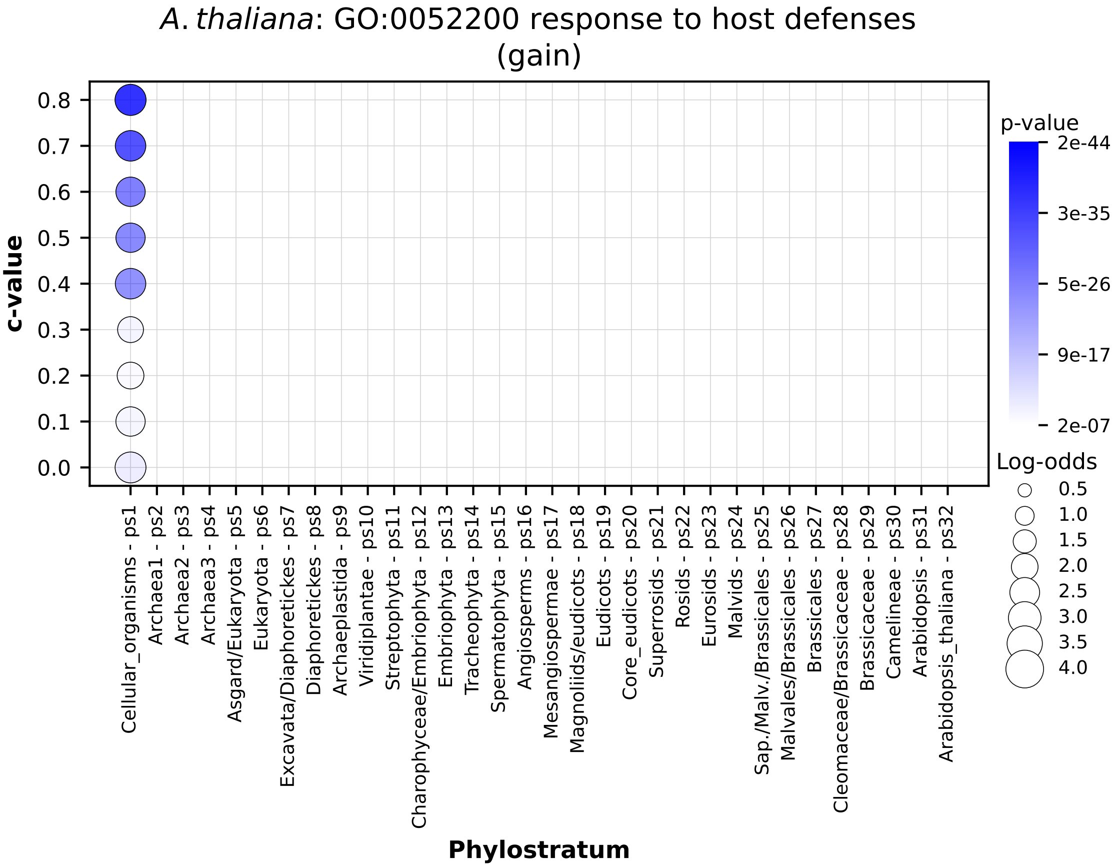

A. thaliana: GO:0052200 response to host defenses
(gain)
c-value
0.8
0.7
0.6
0.5
0.4
0.3
0.2
0.1
0.0
p-value
2e-44
3e-35
5e-26
9e-17
2e-07
Log-odds
0.5
1.0
1.5
2.0
2.5
3.0
3.5
4.0
Cellular_organisms - ps1
Archaea1 - ps2
Archaea2 - ps3
Archaea3 - ps4
Asgard/Eukaryota - ps5
Eukaryota - ps6
Excavata/Diaphoretickes - ps7
Diaphoretickes - ps8
Archaeplastida - ps9
Viridiplantae - ps10
Streptophyta - ps11
Charophyceae/Embriophyta - ps12
Embriophyta - ps13
Tracheophyta - ps14
Spermatophyta - ps15
Angiosperms - ps16
Mesangiospermae - ps17
Magnoliids/eudicots - ps18
Eudicots - ps19
Core_eudicots - ps20
Superrosids - ps21
Rosids - ps22
Eurosids - ps23
Malvids - ps24
Sap./Malv./Brassicales - ps25
Malvales/Brassicales - ps26
Brassicales - ps27
Cleomaceae/Brassicaceae - ps28
Brassicaceae - ps29
Camelineae - ps30
Arabidopsis - ps31
Arabidopsis_thaliana - ps32
Phylostratum

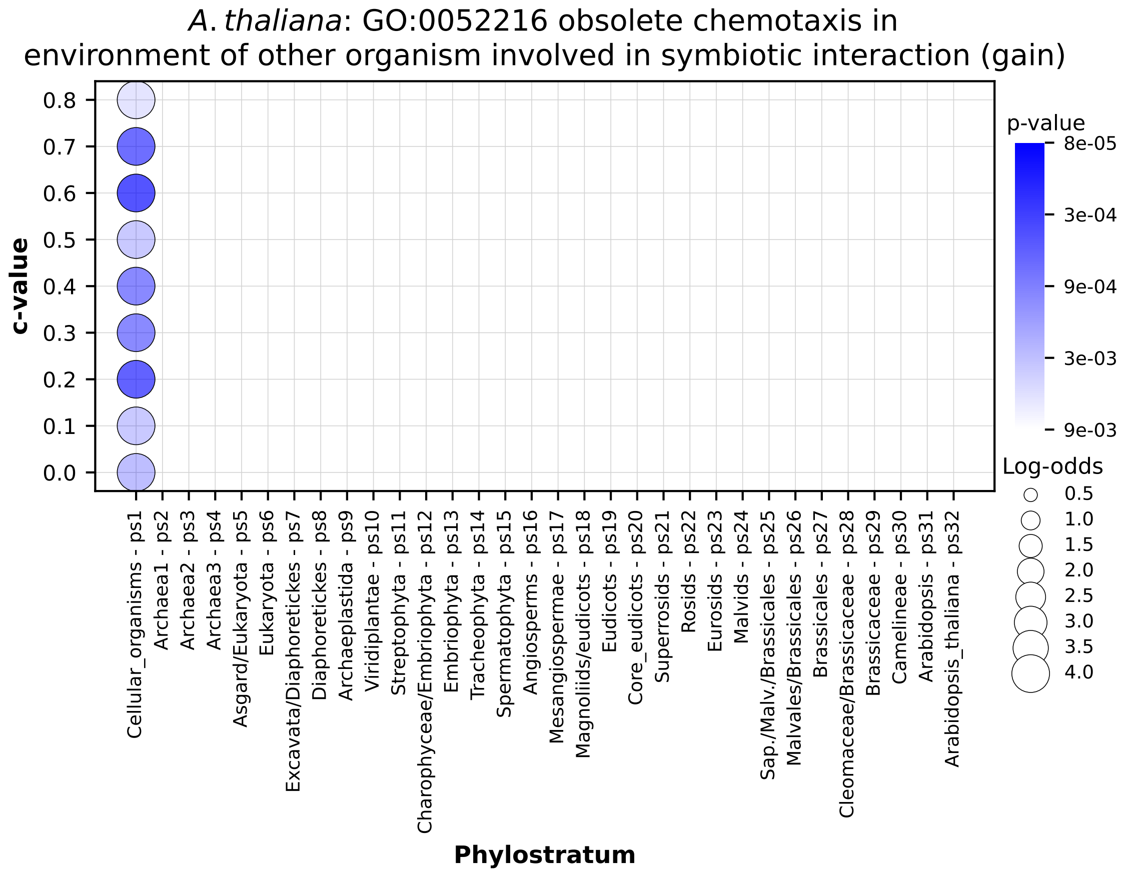
A. thaliana: GO:0052216 obsolete chemotaxis in environment of other organism involved in symbiotic interaction (gain)
c-value
0.8
0.7
0.6
0.5
0.4
0.3
0.2
0.1
0.0
p-value
8e-05
3e-04
9e-04
3e-03
9e-03
Log-odds
0.5
1.0
1.5
2.0
2.5
3.0
3.5
4.0
Cellular_organisms - ps1
Archaea1 - ps2
Archaea2 - ps3
Archaea3 - ps4
Asgard/Eukaryota - ps5
Eukaryota - ps6
Excavata/Diaphoretickes - ps7
Diaphoretickes - ps8
Archaeplastida - ps9
Viridiplantae - ps10
Streptophyta - ps11
Charophyceae/Embriophyta - ps12
Embriophyta - ps13
Tracheophyta - ps14
Spermatophyta - ps15
Angiosperms - ps16
Mesangiospermae - ps17
Magnoliids/eudicots - ps18
Eudicots - ps19
Core_eudicots - ps20
Superrosids - ps21
Rosids - ps22
Eurosids - ps23
Malvids - ps24
Sap./Malv./Brassicales - ps25
Malvales/Brassicales - ps26
Brassicales - ps27
Cleomaceae/Brassicaceae - ps28
Brassicaceae - ps29
Camelineae - ps30
Arabidopsis - ps31
Arabidopsis_thaliana - ps32
Phylostratum

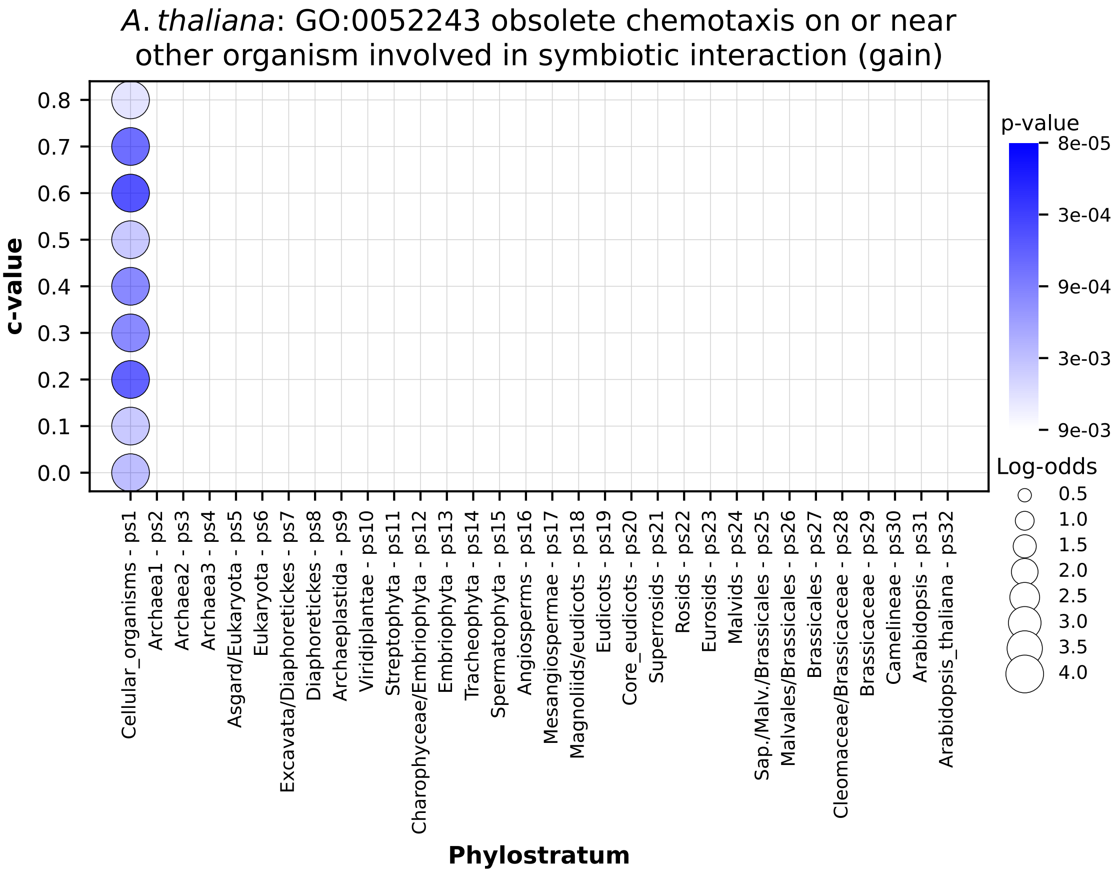

A. thaliana: GO:0052243 obsolete chemotaxis on or near other organism involved in symbiotic interaction (gain)
c-value
0.8
0.7
0.6
0.5
0.4
0.3
0.2
0.1
0.0
p-value
8e-05
3e-04
9e-04
3e-03
9e-03
Log-odds
0.5
1.0
1.5
2.0
2.5
3.0
3.5
4.0
Cellular_organisms - ps1
Archaea1 - ps2
Archaea2 - ps3
Archaea3 - ps4
Asgard/Eukaryota - ps5
Eukaryota - ps6
Excavata/Diaphoretickes - ps7
Diaphoretickes - ps8
Archaeplastida - ps9
Viridiplantae - ps10
Streptophyta - ps11
Charophyceae/Embriophyta - ps12
Embriophyta - ps13
Tracheophyta - ps14
Spermatophyta - ps15
Angiosperms - ps16
Mesangiospermae - ps17
Magnoliids/eudicots - ps18
Eudicots - ps19
Core_eudicots - ps20
Superrosids - ps21
Rosids - ps22
Eurosids - ps23
Malvids - ps24
Sap./Malv./Brassicales - ps25
Malvales/Brassicales - ps26
Brassicales - ps27
Cleomaceae/Brassicaceae - ps28
Brassicaceae - ps29
Camelineae - ps30
Arabidopsis - ps31
Arabidopsis_thaliana - ps32
Phylostratum

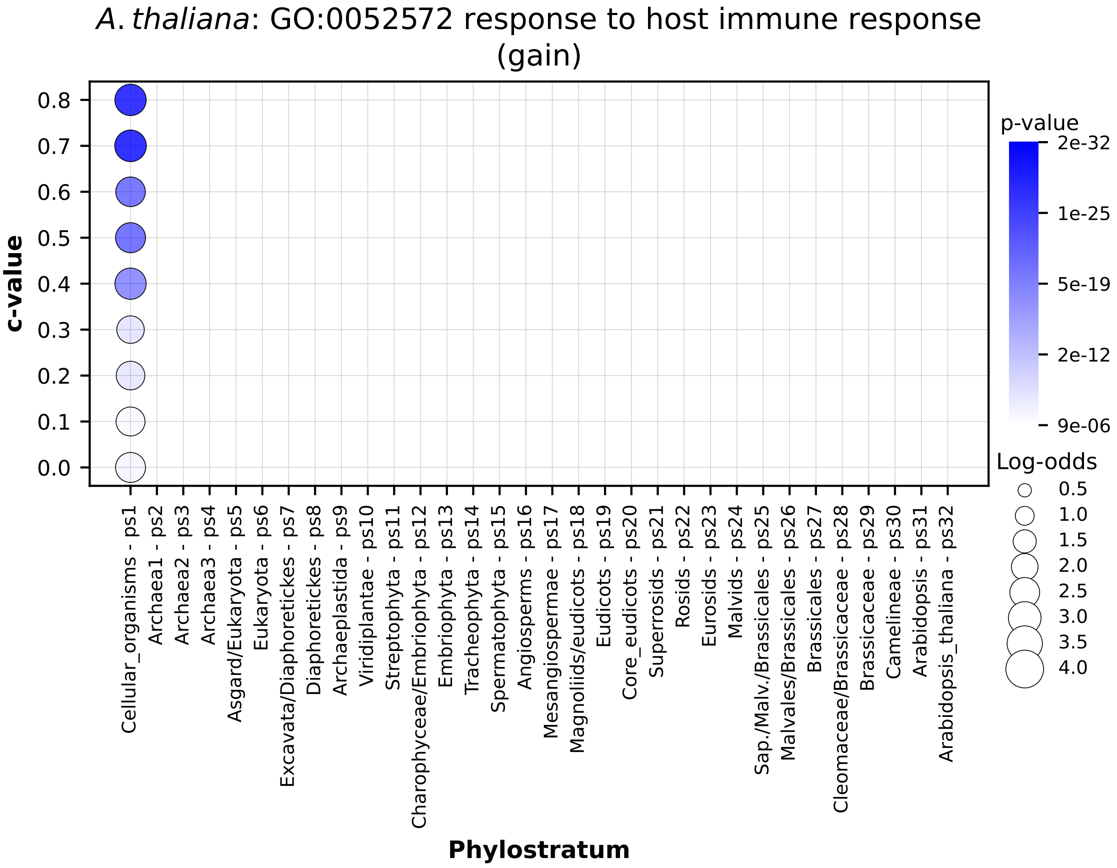
A. thaliana: GO:0052572 response to host immune response (gain)
c-value
0.8
0.7
0.6
0.5
0.4
0.3
0.2
0.1
0.0
Phylostratum
Cellular_organisms - ps1
Archaea1 - ps2
Archaea2 - ps3
Archaea3 - ps4
Asgard/Eukaryota - ps5
Eukaryota - ps6
Excavata/Diaphoretickes - ps7
Diaphoretickes - ps8
Archaeplastida - ps9
Viridiplantae - ps10
Streptophyta - ps11
Charophyceae/Embriophyta - ps12
Embriophyta - ps13
Tracheophyta - ps14
Spermatophyta - ps15
Angiosperms - ps16
Mesangiospermae - ps17
Magnoliids/eudicots - ps18
Eudicots - ps19
Core_eudicots - ps20
Superrosids - ps21
Rosids - ps22
Eurosids - ps23
Malvids - ps24
Sap./Malv./Brassicales - ps25
Malvales/Brassicales - ps26
Brassicales - ps27
Cleomaceae/Brassicaceae - ps28
Brassicaceae - ps29
Camelineae - ps30
Arabidopsis - ps31
Arabidopsis_thaliana - ps32
p-value
2e-32
1e-25
5e-19
2e-12
9e-06
Log-odds
0.5
1.0
1.5
2.0
2.5
3.0
3.5
4.0

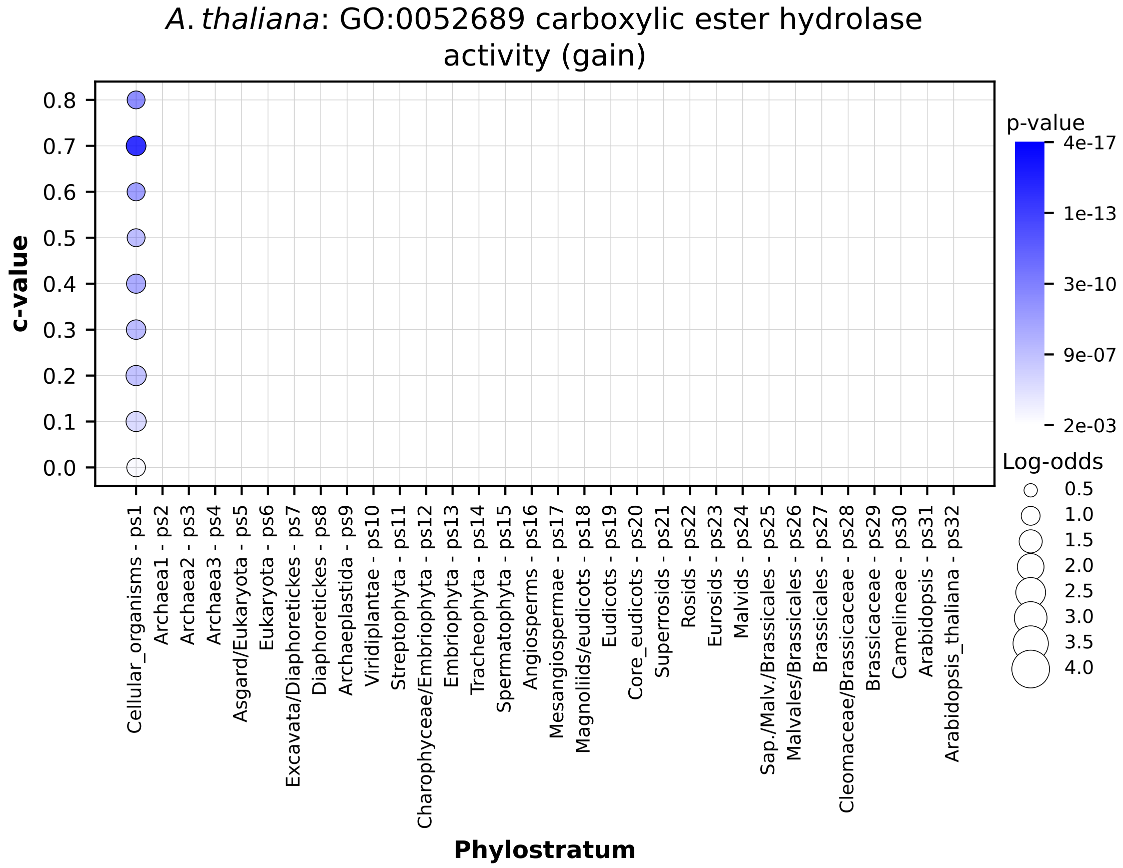

A. thaliana: GO:0052689 carboxylic ester hydrolase activity (gain)
c-value
0.8
0.7
0.6
0.5
0.4
0.3
0.2
0.1
0.0
p-value
4e-17
1e-13
3e-10
9e-07
2e-03
Log-odds
0.5
1.0
1.5
2.0
2.5
3.0
3.5
4.0
Cellular_organisms - ps1
Archaea1 - ps2
Archaea2 - ps3
Archaea3 - ps4
Asgard/Eukaryota - ps5
Eukaryota - ps6
Excavata/Diaphoretickes - ps7
Diaphoretickes - ps8
Archaeplastida - ps9
Viridiplantae - ps10
Streptophyta - ps11
Charophyceae/Embriophyta - ps12
Embriophyta - ps13
Tracheophyta - ps14
Spermatophyta - ps15
Angiosperms - ps16
Mesangiospermae - ps17
Magnoliids/eudicots - ps18
Eudicots - ps19
Core_eudicots - ps20
Superrosids - ps21
Rosids - ps22
Eurosids - ps23
Malvids - ps24
Sap./Malv./Brassicales - ps25
Malvales/Brassicales - ps26
Brassicales - ps27
Cleomaceae/Brassicaceae - ps28
Brassicaceae - ps29
Camelineae - ps30
Arabidopsis - ps31
Arabidopsis_thaliana - ps32
Phylostratum

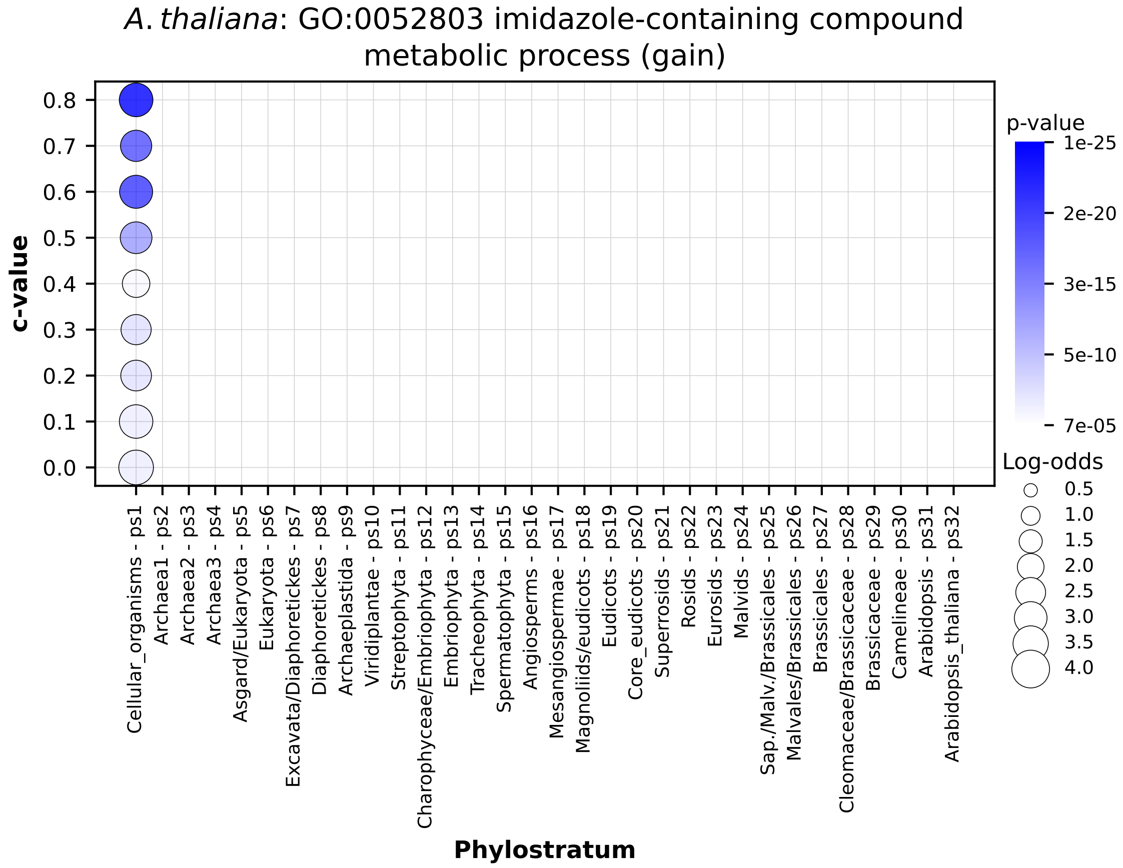

A. thaliana: GO:0052803 imidazole-containing compound metabolic process (gain)
c-value
0.8
0.7
0.6
0.5
0.4
0.3
0.2
0.1
0.0
p-value
1e-25
2e-20
3e-15
5e-10
7e-05
Log-odds
0.5
1.0
1.5
2.0
2.5
3.0
3.5
4.0
Cellular_organisms - ps1
Archaea1 - ps2
Archaea2 - ps3
Archaea3 - ps4
Asgard/Eukaryota - ps5
Eukaryota - ps6
Excavata/Diaphoretickes - ps7
Diaphoretickes - ps8
Archaeplastida - ps9
Viridiplantae - ps10
Streptophyta - ps11
Charophyceae/Embriophyta - ps12
Embriophyta - ps13
Tracheophyta - ps14
Spermatophyta - ps15
Angiosperms - ps16
Mesangiospermae - ps17
Magnoliids/eudicots - ps18
Eudicots - ps19
Core_eudicots - ps20
Superrosids - ps21
Rosids - ps22
Eurosids - ps23
Malvids - ps24
Sap./Malv./Brassicales - ps25
Malvales/Brassicales - ps26
Brassicales - ps27
Cleomaceae/Brassicaceae - ps28
Brassicaceae - ps29
Camelineae - ps30
Arabidopsis - ps31
Arabidopsis_thaliana - ps32
Phylostratum

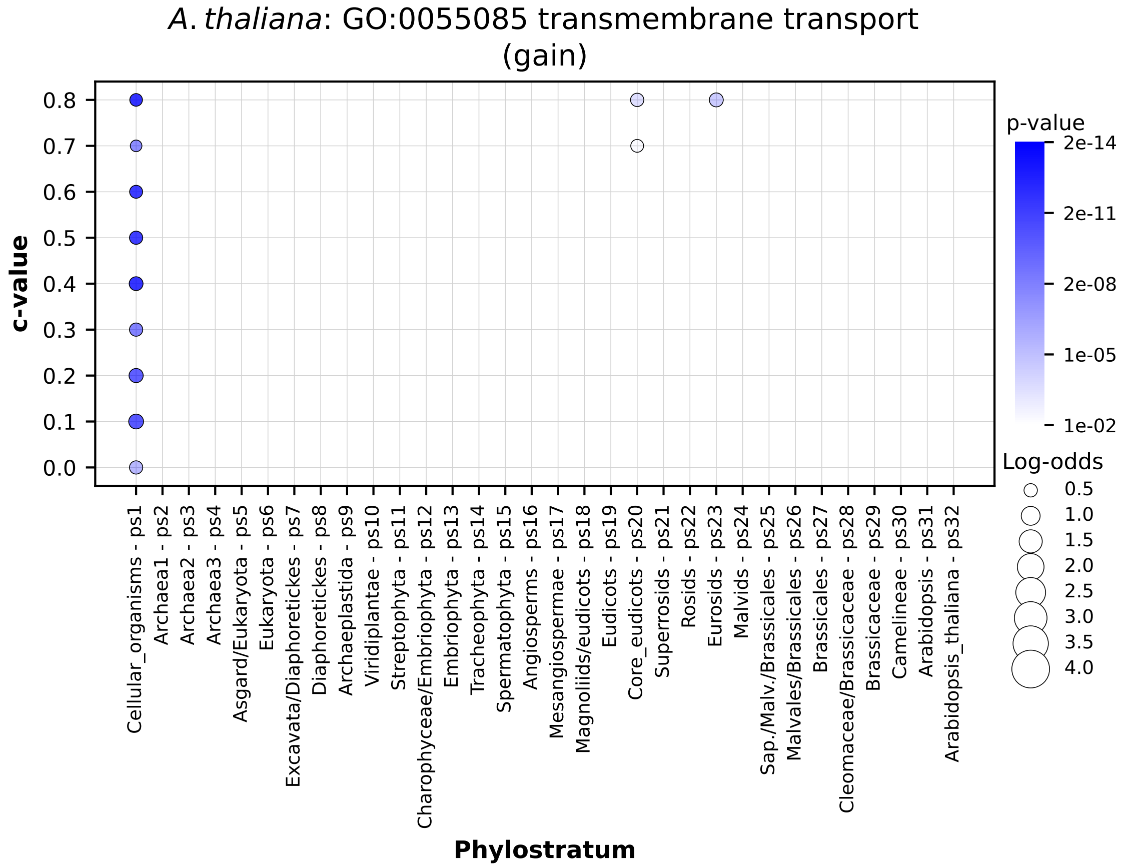

A. thaliana: GO:0055085 transmembrane transport
(gain)
c-value
0.8
0.7
0.6
0.5
0.4
0.3
0.2
0.1
0.0
p-value
2e-14
2e-11
2e-08
1e-05
1e-02
Log-odds
0.5
1.0
1.5
2.0
2.5
3.0
3.5
4.0
Cellular_organisms - ps1
Archaea1 - ps2
Archaea2 - ps3
Archaea3 - ps4
Asgard/Eukaryota - ps5
Eukaryota - ps6
Excavata/Diaphoretickes - ps7
Diaphoretickes - ps8
Archaeplastida - ps9
Viridiplantae - ps10
Streptophyta - ps11
Charophyceae/Embriophyta - ps12
Embriophyta - ps13
Tracheophyta - ps14
Spermatophyta - ps15
Angiosperms - ps16
Mesangiospermae - ps17
Magnoliids/eudicots - ps18
Eudicots - ps19
Core_eudicots - ps20
Superrosids - ps21
Rosids - ps22
Eurosids - ps23
Malvids - ps24
Sap./Malv./Brassicales - ps25
Malvales/Brassicales - ps26
Brassicales - ps27
Cleomaceae/Brassicaceae - ps28
Brassicaceae - ps29
Camelineae - ps30
Arabidopsis - ps31
Arabidopsis_thaliana - ps32
Phylostratum

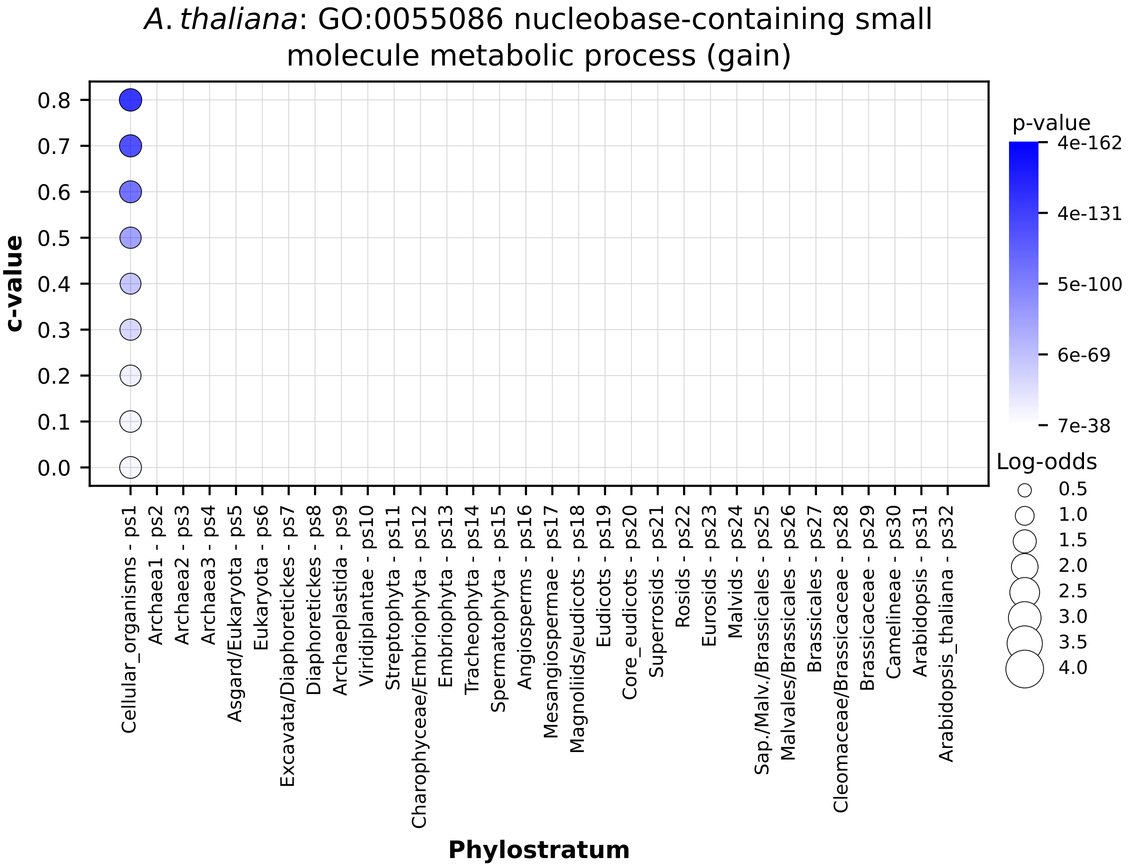
A. thaliana: GO:0055086 nucleobase-containing small molecule metabolic process (gain)
c-value
0.0
0.1
0.2
0.3
0.4
0.5
0.6
0.7
0.8
p-value
4e-162
4e-131
5e-100
6e-69
7e-38
Log-odds
0.5
1.0
1.5
2.0
2.5
3.0
3.5
4.0
Cellular_organisms - ps1
Archaea1 - ps2
Archaea2 - ps3
Archaea3 - ps4
Asgard/Eukaryota - ps5
Eukaryota - ps6
Excavata/Diaphoretickes - ps7
Diaphoretickes - ps8
Archaeplastida - ps9
Viridiplantae - ps10
Streptophyta - ps11
Charophyceae/Embriophyta - ps12
Embriophyta - ps13
Tracheophyta - ps14
Spermatophyta - ps15
Angiosperms - ps16
Mesangiospermae - ps17
Magnoliids/eudicots - ps18
Eudicots - ps19
Core_eudicots - ps20
Superrosids - ps21
Rosids - ps22
Eurosids - ps23
Malvids - ps24
Sap./Malv./Brassicales - ps25
Malvales/Brassicales - ps26
Brassicales - ps27
Cleomaceae/Brassicaceae - ps28
Brassicaceae - ps29
Camelineae - ps30
Arabidopsis - ps31
Arabidopsis_thaliana - ps32
Phylostratum

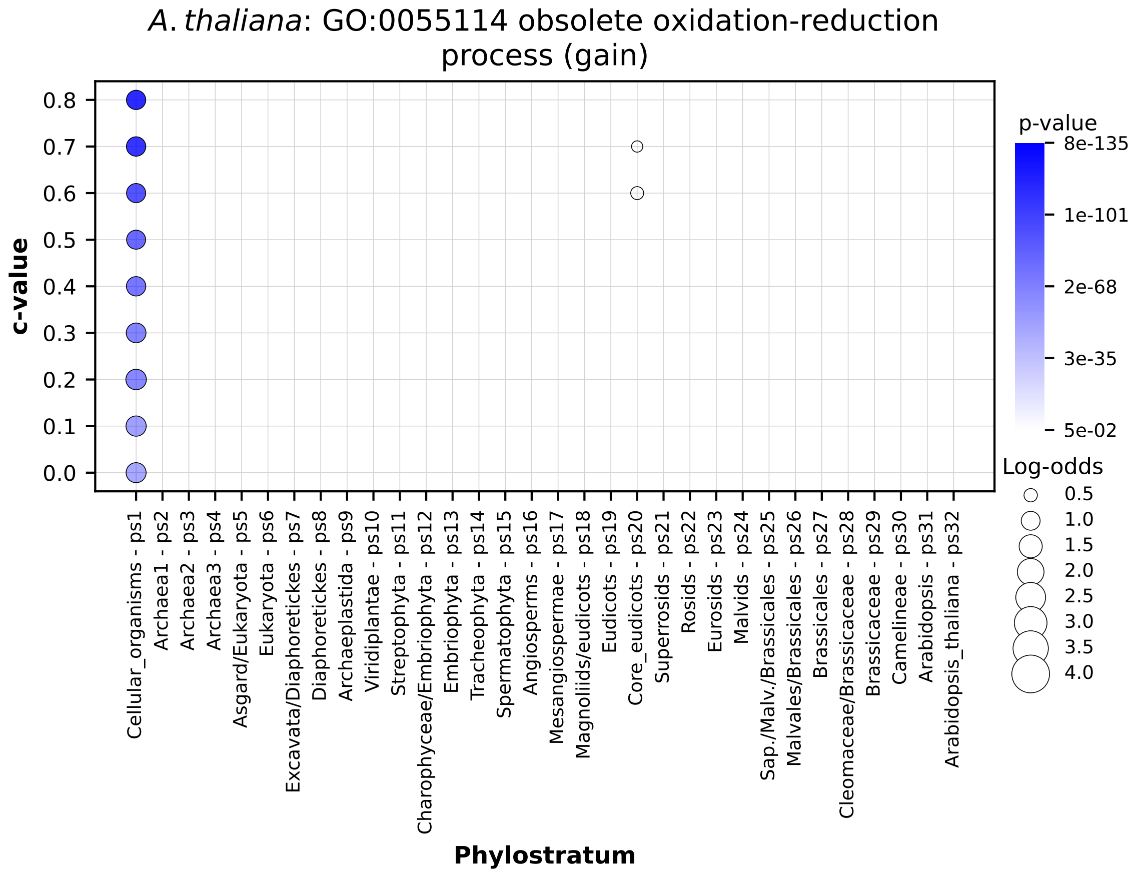
A. thaliana: GO:0055114 obsolete oxidation-reduction process (gain)
c-value
0.8
0.7
0.6
0.5
0.4
0.3
0.2
0.1
0.0
p-value
8e-135
1e-101
2e-68
3e-35
5e-02
Log-odds
0.5
1.0
1.5
2.0
2.5
3.0
3.5
4.0
Cellular_organisms - ps1
Archaea1 - ps2
Archaea2 - ps3
Archaea3 - ps4
Asgard/Eukaryota - ps5
Eukaryota - ps6
Excavata/Diaphoretickes - ps7
Diaphoretickes - ps8
Archaeplastida - ps9
Viridiplantae - ps10
Streptophyta - ps11
Charophyceae/Embriophyta - ps12
Embriophyta - ps13
Tracheophyta - ps14
Spermatophyta - ps15
Angiosperms - ps16
Mesangiospermae - ps17
Magnoliids/eudicots - ps18
Eudicots - ps19
Core_eudicots - ps20
Superrosids - ps21
Rosids - ps22
Eurosids - ps23
Malvids - ps24
Sap./Malv./Brassicales - ps25
Malvales/Brassicales - ps26
Brassicales - ps27
Cleomaceae/Brassicaceae - ps28
Brassicaceae - ps29
Camelineae - ps30
Arabidopsis - ps31
Arabidopsis_thaliana - ps32
Phylostratum

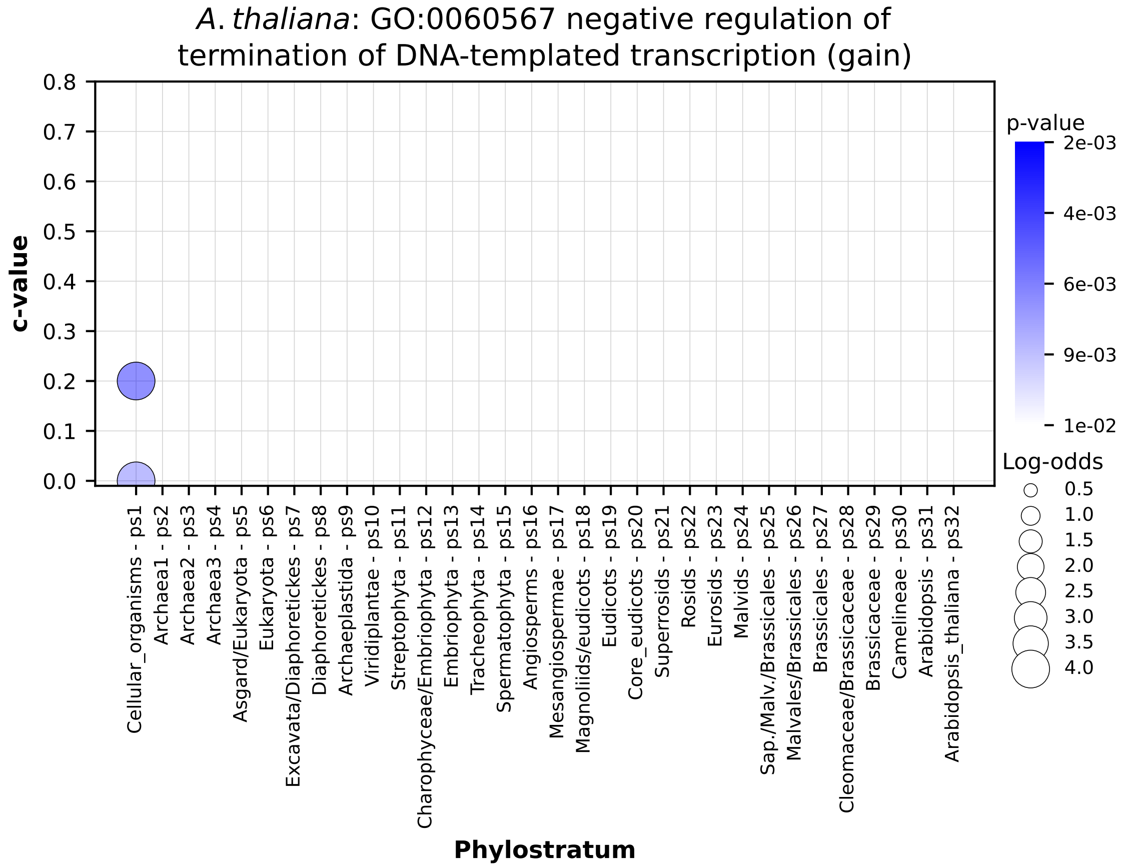

A. thaliana: GO:0060567 negative regulation of termination of DNA-templated transcription (gain)
c-value
0.8
0.7
0.6
0.5
0.4
0.3
0.2
0.1
0.0
p-value
2e-03
4e-03
6e-03
9e-03
1e-02
Log-odds
0.5
1.0
1.5
2.0
2.5
3.0
3.5
4.0
Cellular_organisms - ps1
Archaea1 - ps2
Archaea2 - ps3
Archaea3 - ps4
Asgard/Eukaryota - ps5
Eukaryota - ps6
Excavata/Diaphoretickes - ps7
Diaphoretickes - ps8
Archaeplastida - ps9
Viridiplantae - ps10
Streptophyta - ps11
Charophyceae/Embriophyta - ps12
Embriophyta - ps13
Tracheophyta - ps14
Spermatophyta - ps15
Angiosperms - ps16
Mesangiospermae - ps17
Magnoliids/eudicots - ps18
Eudicots - ps19
Core_eudicots - ps20
Superrosids - ps21
Rosids - ps22
Eurosids - ps23
Malvids - ps24
Sap./Malv./Brassicales - ps25
Malvales/Brassicales - ps26
Brassicales - ps27
Cleomaceae/Brassicaceae - ps28
Brassicaceae - ps29
Camelineae - ps30
Arabidopsis - ps31
Arabidopsis_thaliana - ps32
Phylostratum

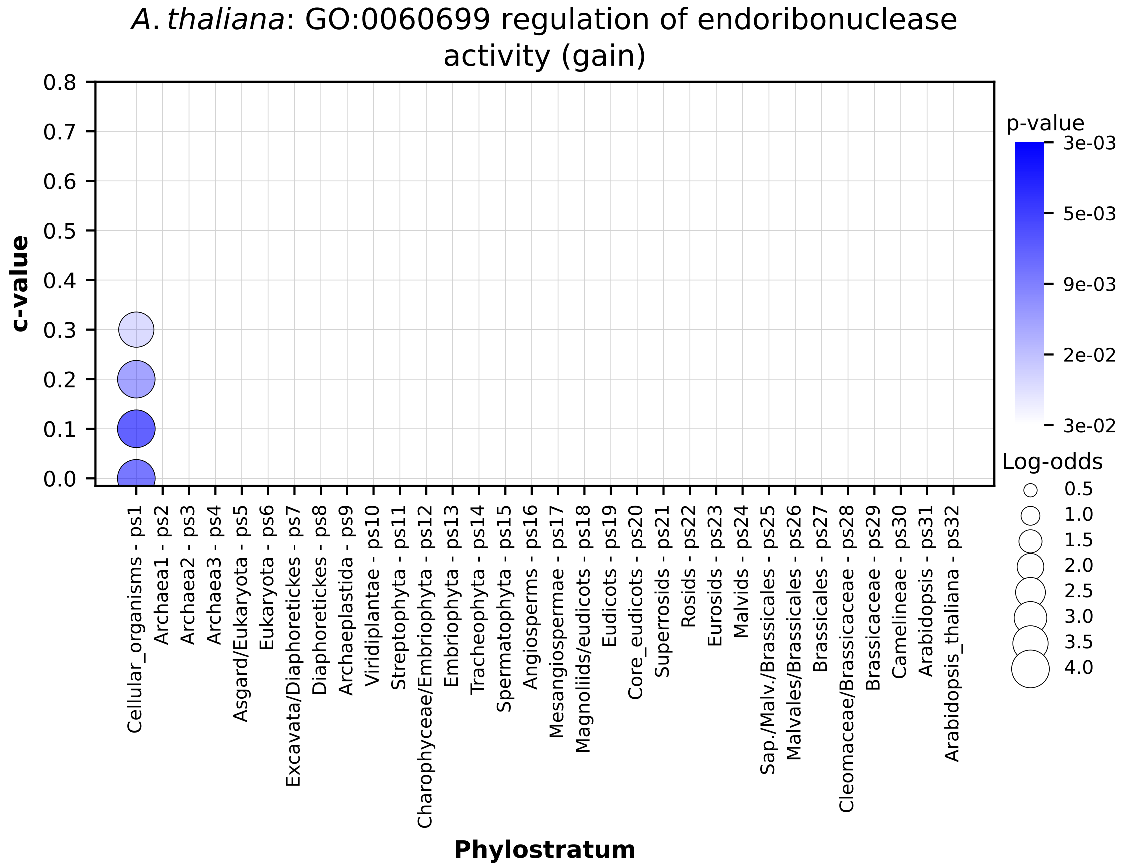

$A. thaliana$: GO:0060699 regulation of endoribonuclease activity (gain)
c-value
0.8
0.7
0.6
0.5
0.4
0.3
0.2
0.1
0.0
p-value
3e-03
5e-03
9e-03
2e-02
3e-02
Log-odds
0.5
1.0
1.5
2.0
2.5
3.0
3.5
4.0
Cellular_organisms - ps1
Archaea1 - ps2
Archaea2 - ps3
Archaea3 - ps4
Asgard/Eukaryota - ps5
Eukaryota - ps6
Excavata/Diaphoretickes - ps7
Diaphoretickes - ps8
Archaeplastida - ps9
Viridiplantae - ps10
Streptophyta - ps11
Charophyceae/Embriophyta - ps12
Embriophyta - ps13
Tracheophyta - ps14
Spermatophyta - ps15
Angiosperms - ps16
Mesangiospermae - ps17
Magnoliids/eudicots - ps18
Eudicots - ps19
Core_eudicots - ps20
Superrosids - ps21
Rosids - ps22
Eurosids - ps23
Malvids - ps24
Sap./Malv./Brassicales - ps25
Malvales/Brassicales - ps26
Brassicales - ps27
Cleomaceae/Brassicaceae - ps28
Brassicaceae - ps29
Camelineae - ps30
Arabidopsis - ps31
Arabidopsis_thaliana - ps32
Phylostratum

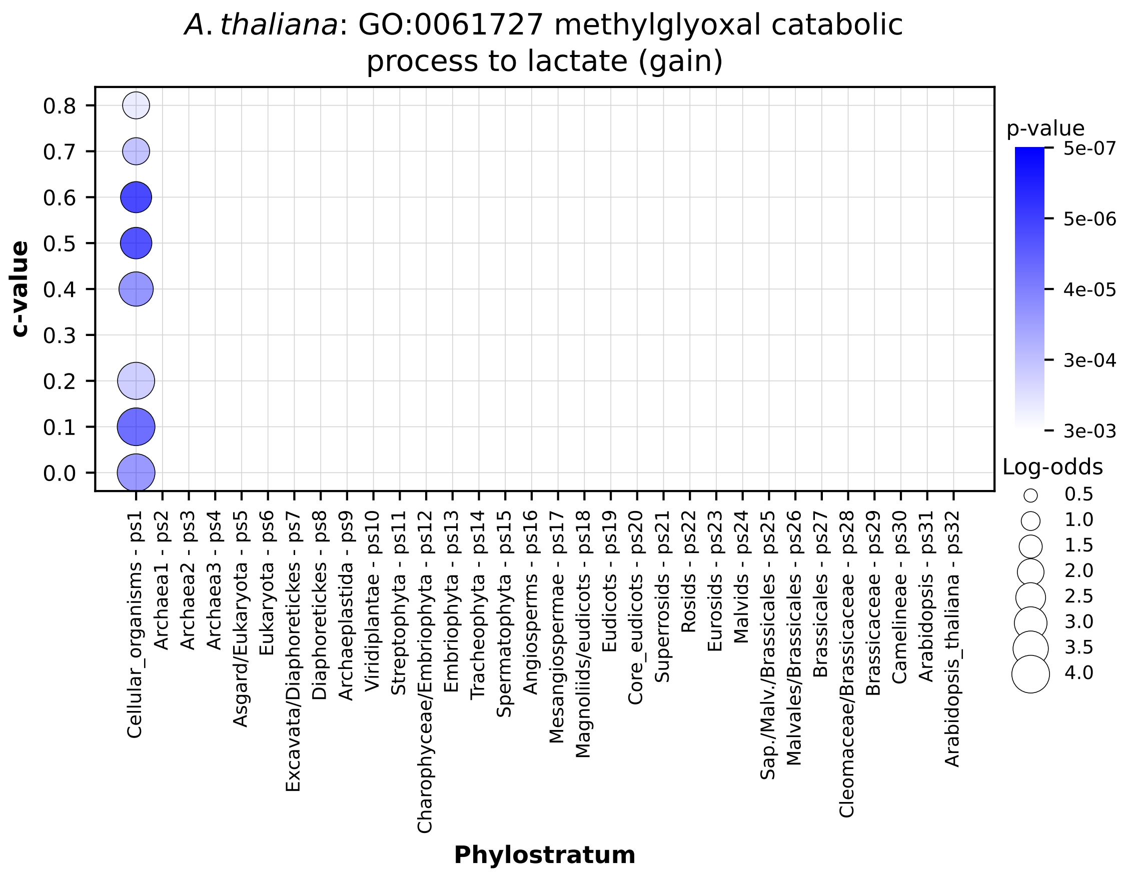

A. thaliana: GO:0061727 methylglyoxal catabolic process to lactate (gain)
c-value
0.8
0.7
0.6
0.5
0.4
0.3
0.2
0.1
0.0
Phylostratum
Cellular_organisms - ps1
Archaea1 - ps2
Archaea2 - ps3
Archaea3 - ps4
Asgard/Eukaryota - ps5
Eukaryota - ps6
Excavata/Diaphoretickes - ps7
Diaphoretickes - ps8
Archaeplastida - ps9
Viridiplantae - ps10
Streptophyta - ps11
Charophyceae/Embriophyta - ps12
Embriophyta - ps13
Tracheophyta - ps14
Spermatophyta - ps15
Angiosperms - ps16
Mesangiospermae - ps17
Magnoliids/eudicots - ps18
Eudicots - ps19
Core_eudicots - ps20
Superrosids - ps21
Rosids - ps22
Eurosids - ps23
Malvids - ps24
Sap./Malv./Brassicales - ps25
Malvales/Brassicales - ps26
Brassicales - ps27
Cleomaceae/Brassicaceae - ps28
Brassicaceae - ps29
Camelineae - ps30
Arabidopsis - ps31
Arabidopsis_thaliana - ps32
p-value
5e-07
5e-06
4e-05
3e-04
3e-03
Log-odds
0.5
1.0
1.5
2.0
2.5
3.0
3.5
4.0

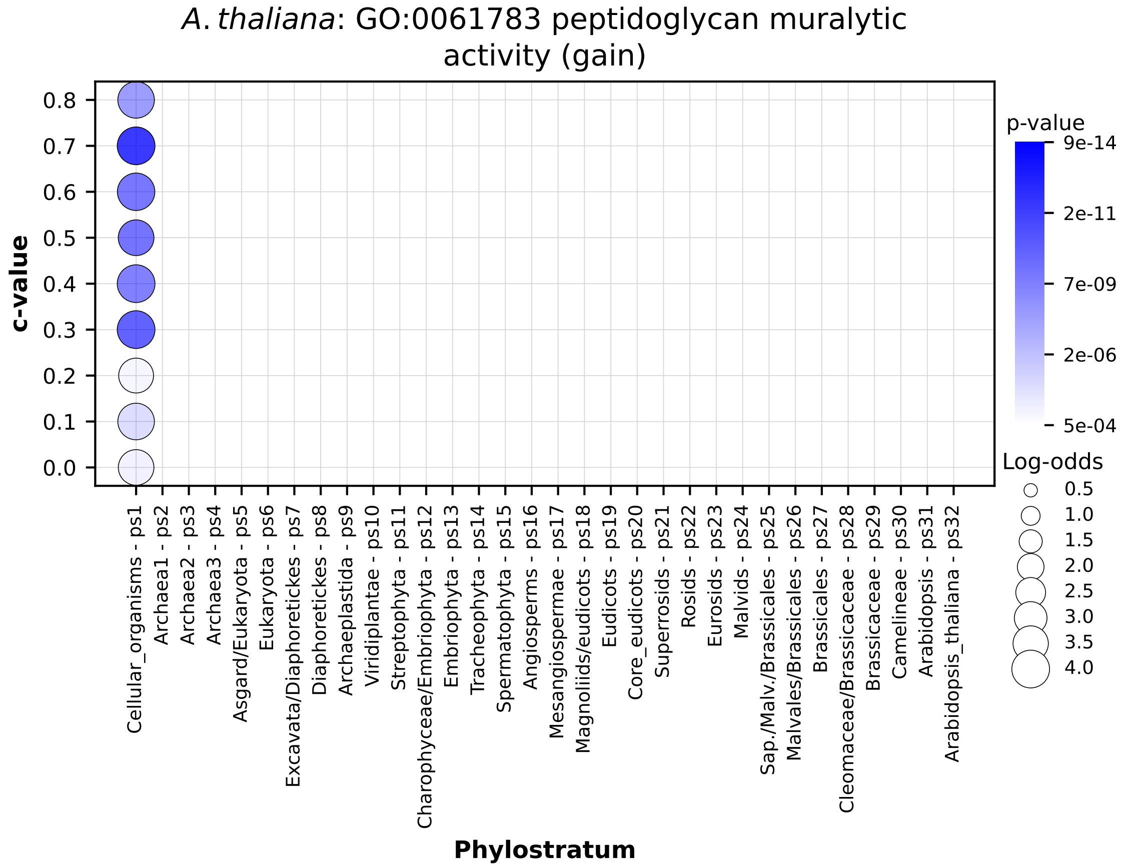
A. thaliana: GO:0061783 peptidoglycan muralytic activity (gain)
c-value
0.8
0.7
0.6
0.5
0.4
0.3
0.2
0.1
0.0
p-value
9e-14
2e-11
7e-09
2e-06
5e-04
Log-odds
0.5
1.0
1.5
2.0
2.5
3.0
3.5
4.0
Cellular_organisms - ps1
Archaea1 - ps2
Archaea2 - ps3
Archaea3 - ps4
Asgard/Eukaryota - ps5
Eukaryota - ps6
Excavata/Diaphoretickes - ps7
Diaphoretickes - ps8
Archaeplastida - ps9
Viridiplantae - ps10
Streptophyta - ps11
Charophyceae/Embriophyta - ps12
Embriophyta - ps13
Tracheophyta - ps14
Spermatophyta - ps15
Angiosperms - ps16
Mesangiospermae - ps17
Magnoliids/eudicots - ps18
Eudicots - ps19
Core_eudicots - ps20
Superrosids - ps21
Rosids - ps22
Eurosids - ps23
Malvids - ps24
Sap./Malv./Brassicales - ps25
Malvales/Brassicales - ps26
Brassicales - ps27
Cleomaceae/Brassicaceae - ps28
Brassicaceae - ps29
Camelineae - ps30
Arabidopsis - ps31
Arabidopsis_thaliana - ps32
Phylostratum

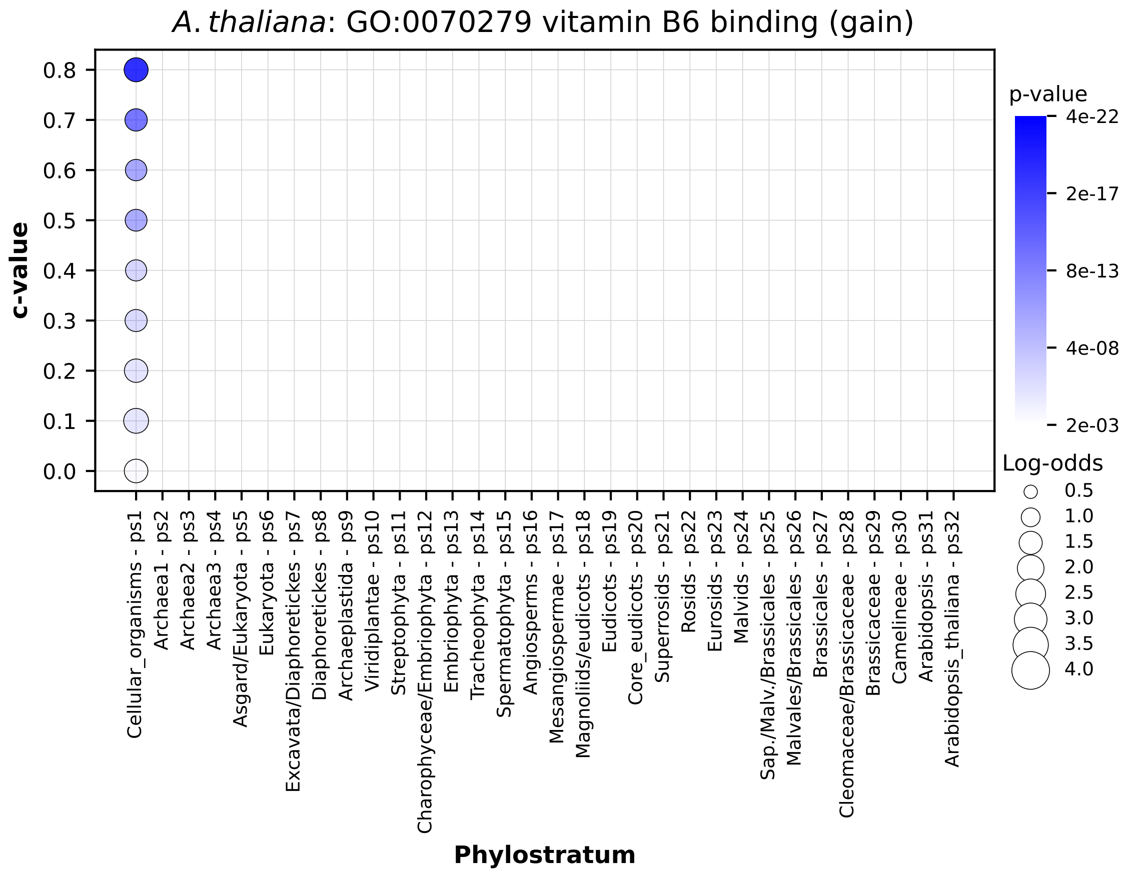

A. thaliana: GO:0070279 vitamin B6 binding (gain)
c-value
0.8
0.7
0.6
0.5
0.4
0.3
0.2
0.1
0.0
p-value
4e-22
2e-17
8e-13
4e-08
2e-03
Log-odds
0.5
1.0
1.5
2.0
2.5
3.0
3.5
4.0
Cellular_organisms - ps1
Archaea1 - ps2
Archaea2 - ps3
Archaea3 - ps4
Asgard/Eukaryota - ps5
Eukaryota - ps6
Excavata/Diaphoretickes - ps7
Diaphoretickes - ps8
Archaeplastida - ps9
Viridiplantae - ps10
Streptophyta - ps11
Charophyceae/Embriophyta - ps12
Embriophyta - ps13
Tracheophyta - ps14
Spermatophyta - ps15
Angiosperms - ps16
Mesangiospermae - ps17
Magnoliids/eudicots - ps18
Eudicots - ps19
Core_eudicots - ps20
Superrosids - ps21
Rosids - ps22
Eurosids - ps23
Malvids - ps24
Sap./Malv./Brassicales - ps25
Malvales/Brassicales - ps26
Brassicales - ps27
Cleomaceae/Brassicaceae - ps28
Brassicaceae - ps29
Camelineae - ps30
Arabidopsis - ps31
Arabidopsis_thaliana - ps32
Phylostratum

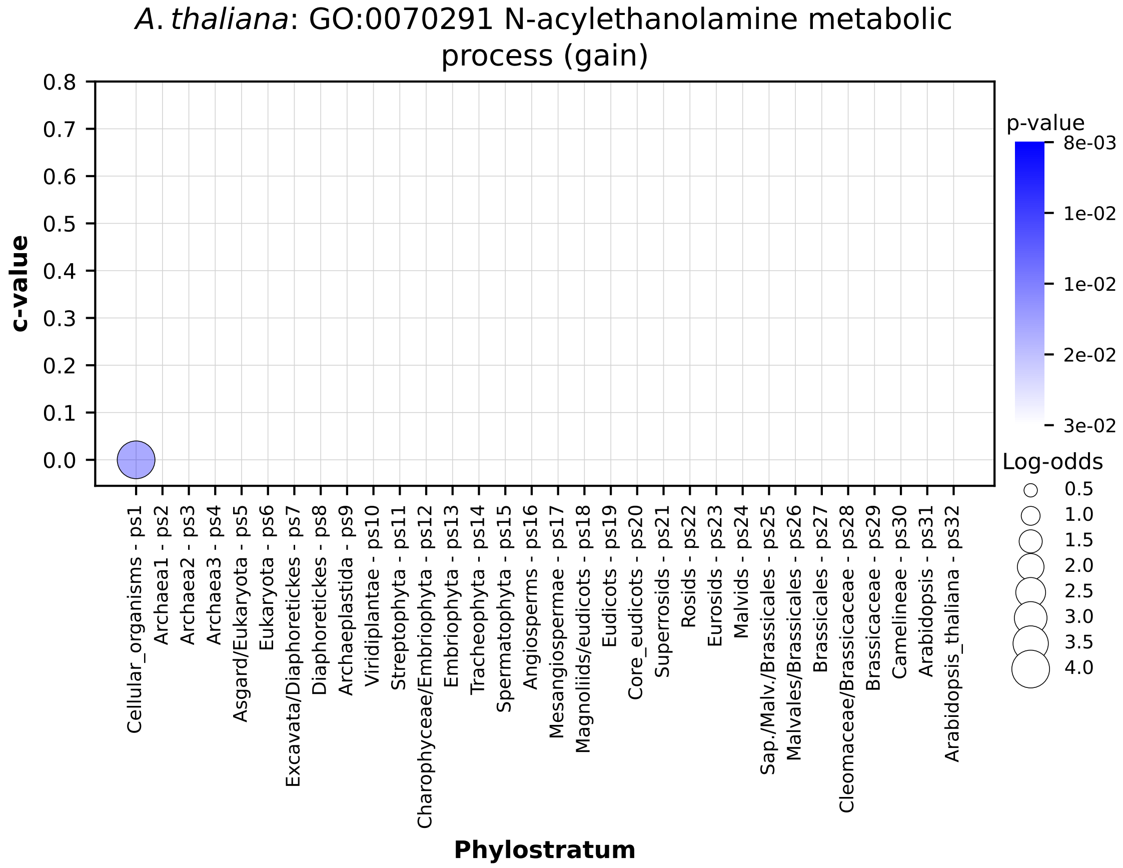
A. thaliana: GO:0070291 N-acylethanolamine metabolic process (gain)
c-value
0.8
0.7
0.6
0.5
0.4
0.3
0.2
0.1
0.0
p-value
8e-03
1e-02
1e-02
2e-02
3e-02
Log-odds
0.5
1.0
1.5
2.0
2.5
3.0
3.5
4.0
Cellular_organisms - ps1
Archaea1 - ps2
Archaea2 - ps3
Archaea3 - ps4
Asgard/Eukaryota - ps5
Eukaryota - ps6
Excavata/Diaphoretickes - ps7
Diaphoretickes - ps8
Archaeplastida - ps9
Viridiplantae - ps10
Streptophyta - ps11
Charophyceae/Embriophyta - ps12
Embriophyta - ps13
Tracheophyta - ps14
Spermatophyta - ps15
Angiosperms - ps16
Mesangiospermae - ps17
Magnoliids/eudicots - ps18
Eudicots - ps19
Core_eudicots - ps20
Superrosids - ps21
Rosids - ps22
Eurosids - ps23
Malvids - ps24
Sap./Malv./Brassicales - ps25
Malvales/Brassicales - ps26
Brassicales - ps27
Cleomaceae/Brassicaceae - ps28
Brassicaceae - ps29
Camelineae - ps30
Arabidopsis - ps31
Arabidopsis_thaliana - ps32
Phylostratum

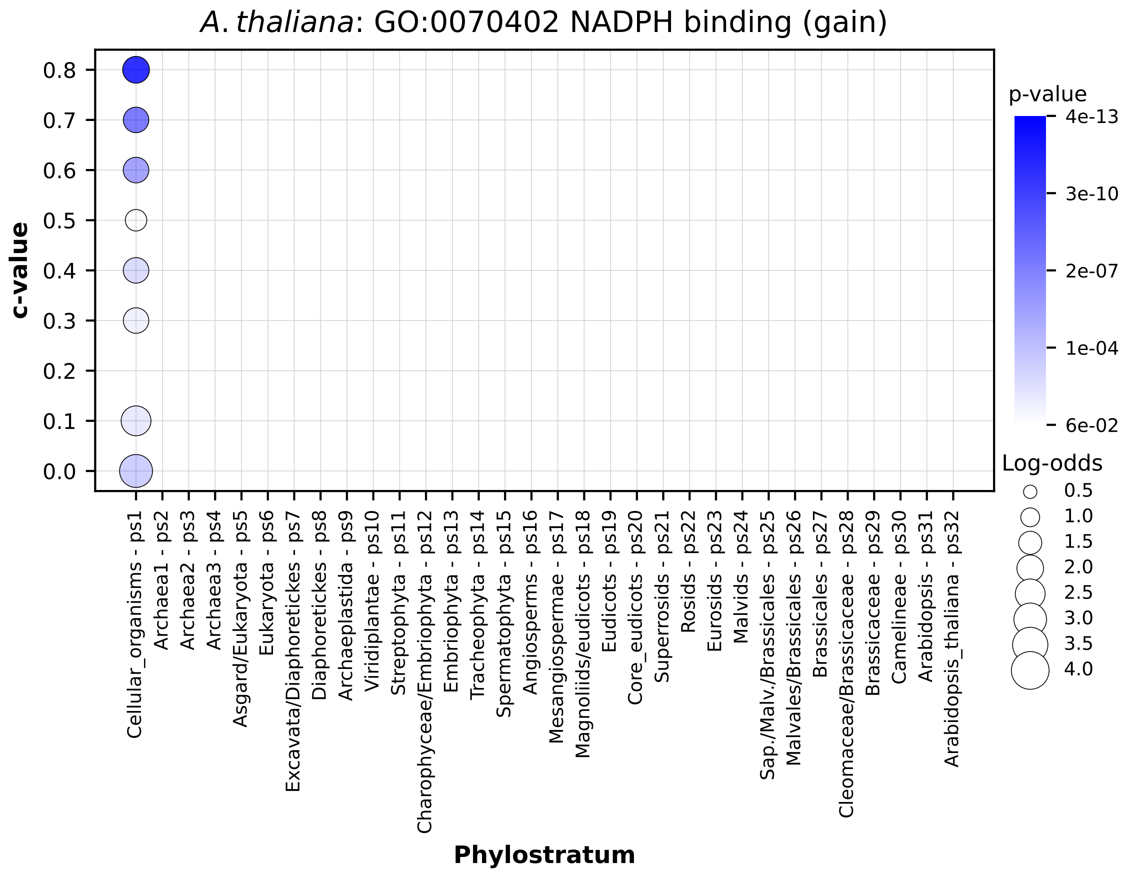

A. thaliana: GO:0070402 NADPH binding (gain)
c-value
0.8
0.7
0.6
0.5
0.4
0.3
0.2
0.1
0.0
p-value
4e-13
3e-10
2e-07
1e-04
6e-02
Log-odds
0.5
1.0
1.5
2.0
2.5
3.0
3.5
4.0
Cellular_organisms - ps1
Archaea1 - ps2
Archaea2 - ps3
Archaea3 - ps4
Asgard/Eukaryota - ps5
Eukaryota - ps6
Excavata/Diaphoretickes - ps7
Diaphoretickes - ps8
Archaeplastida - ps9
Viridiplantae - ps10
Streptophyta - ps11
Charophyceae/Embriophyta - ps12
Embriophyta - ps13
Tracheophyta - ps14
Spermatophyta - ps15
Angiosperms - ps16
Mesangiospermae - ps17
Magnoliids/eudicots - ps18
Eudicots - ps19
Core_eudicots - ps20
Superrosids - ps21
Rosids - ps22
Eurosids - ps23
Malvids - ps24
Sap./Malv./Brassicales - ps25
Malvales/Brassicales - ps26
Brassicales - ps27
Cleomaceae/Brassicaceae - ps28
Brassicaceae - ps29
Camelineae - ps30
Arabidopsis - ps31
Arabidopsis_thaliana - ps32
Phylostratum

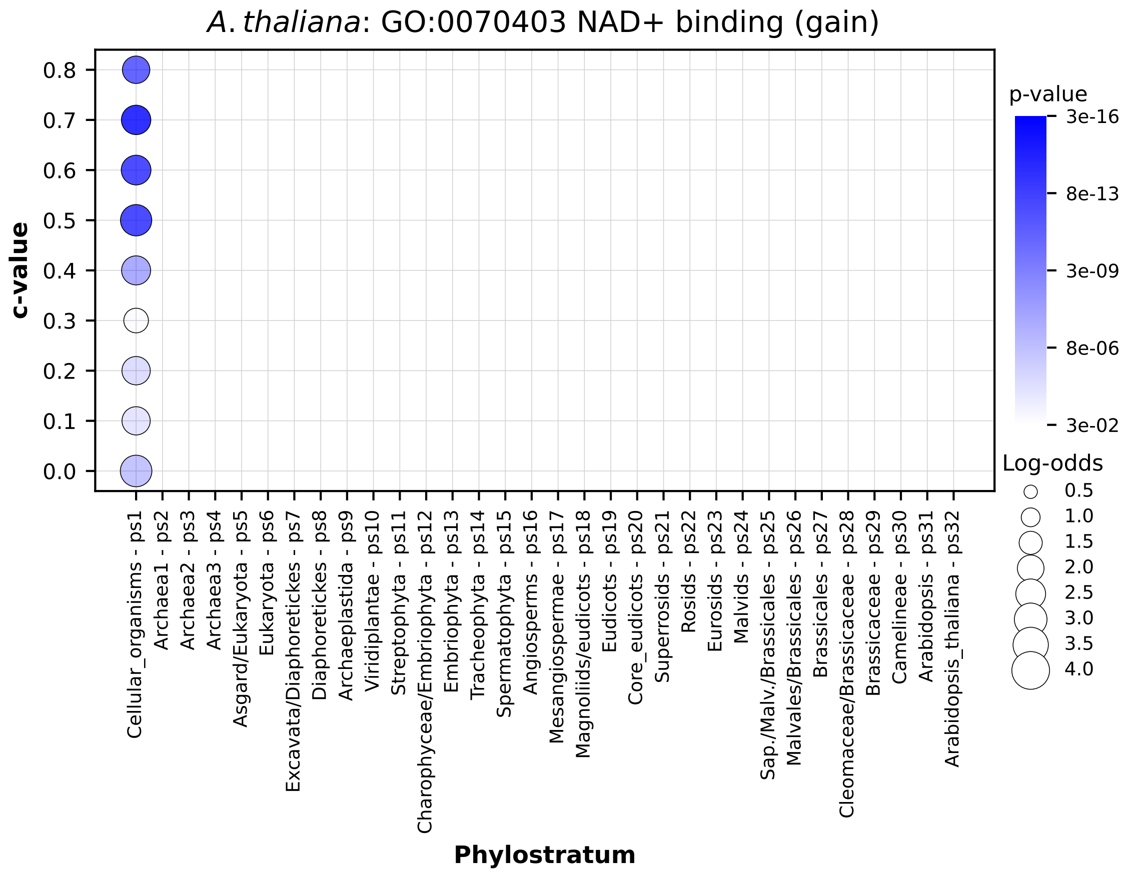

A. thaliana: GO:0070403 NAD+ binding (gain)
c-value
0.8
0.7
0.6
0.5
0.4
0.3
0.2
0.1
0.0
p-value
3e-16
8e-13
3e-09
8e-06
3e-02
Log-odds
0.5
1.0
1.5
2.0
2.5
3.0
3.5
4.0
Cellular_organisms - ps1
Archaea1 - ps2
Archaea2 - ps3
Archaea3 - ps4
Asgard/Eukaryota - ps5
Eukaryota - ps6
Excavata/Diaphoretickes - ps7
Diaphoretickes - ps8
Archaeplastida - ps9
Viridiplantae - ps10
Streptophyta - ps11
Charophyceae/Embriophyta - ps12
Embriophyta - ps13
Tracheophyta - ps14
Spermatophyta - ps15
Angiosperms - ps16
Mesangiospermae - ps17
Magnoliids/eudicots - ps18
Eudicots - ps19
Core_eudicots - ps20
Superrosids - ps21
Rosids - ps22
Eurosids - ps23
Malvids - ps24
Sap./Malv./Brassicales - ps25
Malvales/Brassicales - ps26
Brassicales - ps27
Cleomaceae/Brassicaceae - ps28
Brassicaceae - ps29
Camelineae - ps30
Arabidopsis - ps31
Arabidopsis_thaliana - ps32
Phylostratum

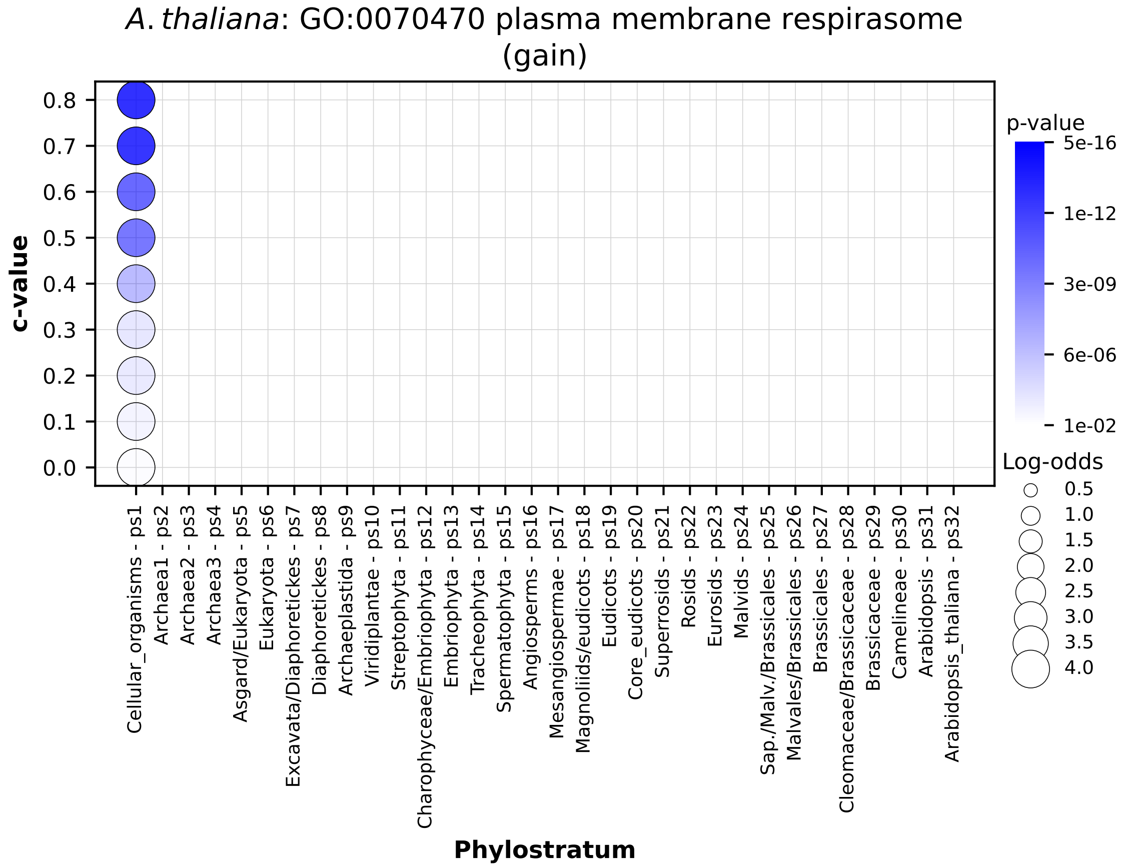
A. thaliana: GO:0070470 plasma membrane respirasome (gain)
c-value
0.8
0.7
0.6
0.5
0.4
0.3
0.2
0.1
0.0
p-value
5e-16
1e-12
3e-09
6e-06
1e-02
Log-odds
0.5
1.0
1.5
2.0
2.5
3.0
3.5
4.0
Cellular_organisms - ps1
Archaea1 - ps2
Archaea2 - ps3
Archaea3 - ps4
Asgard/Eukaryota - ps5
Eukaryota - ps6
Excavata/Diaphoretickes - ps7
Diaphoretickes - ps8
Archaeplastida - ps9
Viridiplantae - ps10
Streptophyta - ps11
Charophyceae/Embriophyta - ps12
Embriophyta - ps13
Tracheophyta - ps14
Spermatophyta - ps15
Angiosperms - ps16
Mesangiospermae - ps17
Magnoliids/eudicots - ps18
Eudicots - ps19
Core_eudicots - ps20
Superrosids - ps21
Rosids - ps22
Eurosids - ps23
Malvids - ps24
Sap./Malv./Brassicales - ps25
Malvales/Brassicales - ps26
Brassicales - ps27
Cleomaceae/Brassicaceae - ps28
Brassicaceae - ps29
Camelineae - ps30
Arabidopsis - ps31
Arabidopsis_thaliana - ps32
Phylostratum

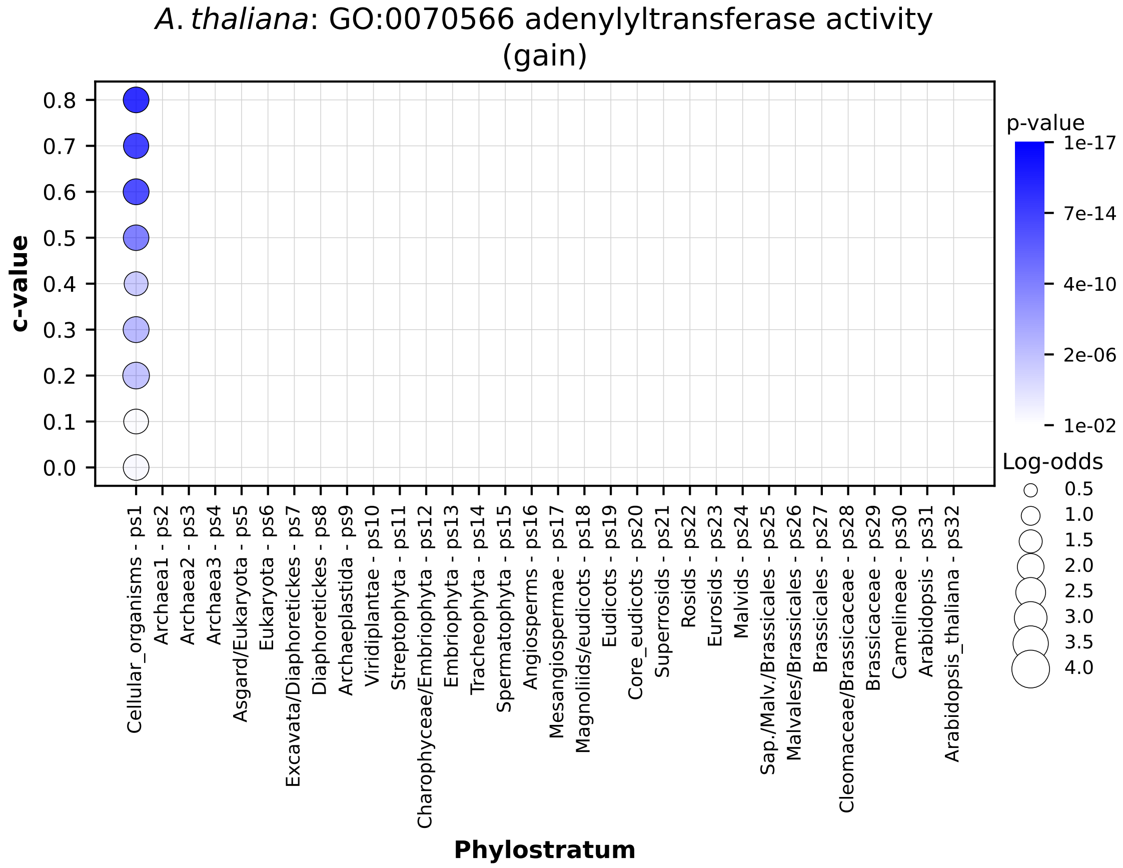

A. thaliana: GO:0070566 adenylyltransferase activity (gain)
c-value
0.8
0.7
0.6
0.5
0.4
0.3
0.2
0.1
0.0
p-value
1e-17
7e-14
4e-10
2e-06
1e-02
Log-odds
0.5
1.0
1.5
2.0
2.5
3.0
3.5
4.0
Cellular_organisms - ps1
Archaea1 - ps2
Archaea2 - ps3
Archaea3 - ps4
Asgard/Eukaryota - ps5
Eukaryota - ps6
Excavata/Diaphoretickes - ps7
Diaphoretickes - ps8
Archaeplastida - ps9
Viridiplantae - ps10
Streptophyta - ps11
Charophyceae/Embriophyta - ps12
Embriophyta - ps13
Tracheophyta - ps14
Spermatophyta - ps15
Angiosperms - ps16
Mesangiospermae - ps17
Magnoliids/eudicots - ps18
Eudicots - ps19
Core_eudicots - ps20
Superrosids - ps21
Rosids - ps22
Eurosids - ps23
Malvids - ps24
Sap./Malv./Brassicales - ps25
Malvales/Brassicales - ps26
Brassicales - ps27
Cleomaceae/Brassicaceae - ps28
Brassicaceae - ps29
Camelineae - ps30
Arabidopsis - ps31
Arabidopsis_thaliana - ps32
Phylostratum

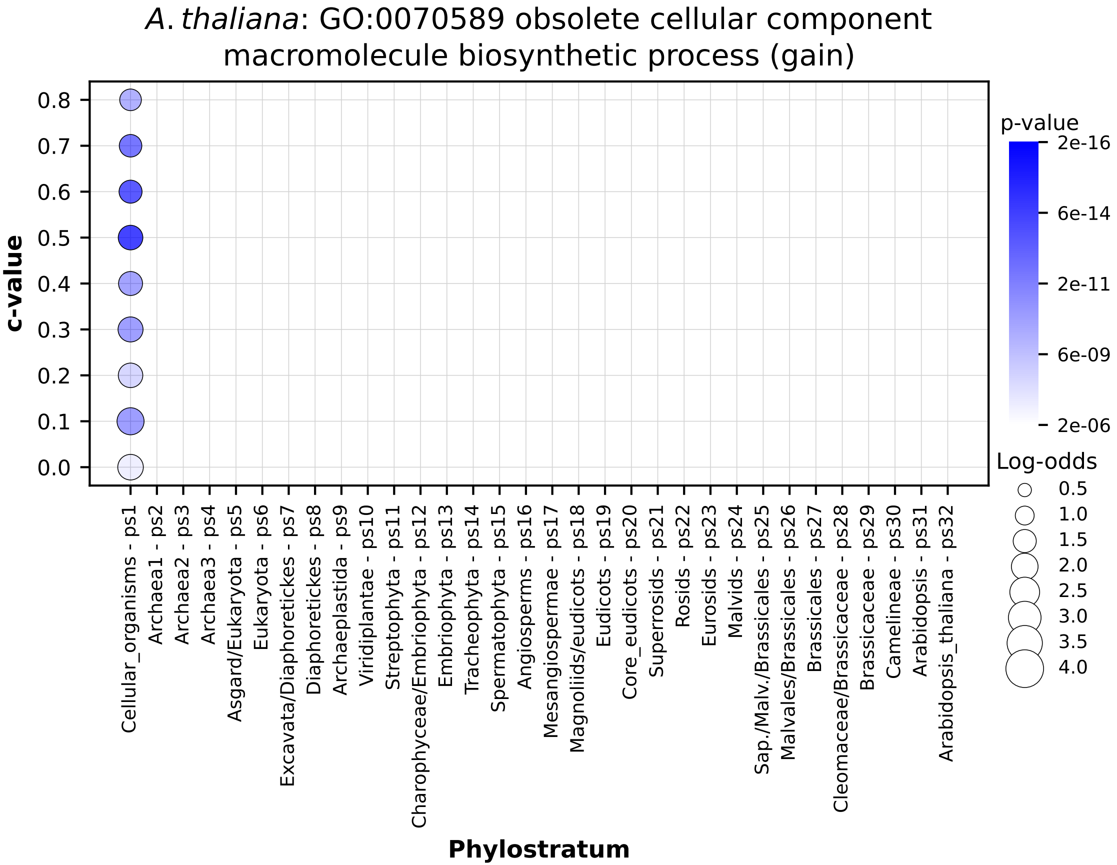

A. thaliana: GO:0070589 obsolete cellular component macromolecule biosynthetic process (gain)
c-value
0.0
0.1
0.2
0.3
0.4
0.5
0.6
0.7
0.8
p-value
2e-16
6e-14
2e-11
6e-09
2e-06
Log-odds
0.5
1.0
1.5
2.0
2.5
3.0
3.5
4.0
Cellular_organisms - ps1
Archaea1 - ps2
Archaea2 - ps3
Archaea3 - ps4
Asgard/Eukaryota - ps5
Eukaryota - ps6
Excavata/Diaphoretickes - ps7
Diaphoretickes - ps8
Archaeplastida - ps9
Viridiplantae - ps10
Streptophyta - ps11
Charophyceae/Embriophyta - ps12
Embriophyta - ps13
Tracheophyta - ps14
Spermatophyta - ps15
Angiosperms - ps16
Mesangiospermae - ps17
Magnoliids/eudicots - ps18
Eudicots - ps19
Core_eudicots - ps20
Superrosids - ps21
Rosids - ps22
Eurosids - ps23
Malvids - ps24
Sap./Malv./Brassicales - ps25
Malvales/Brassicales - ps26
Brassicales - ps27
Cleomaceae/Brassicaceae - ps28
Brassicaceae - ps29
Camelineae - ps30
Arabidopsis - ps31
Arabidopsis_thaliana - ps32
Phylostratum

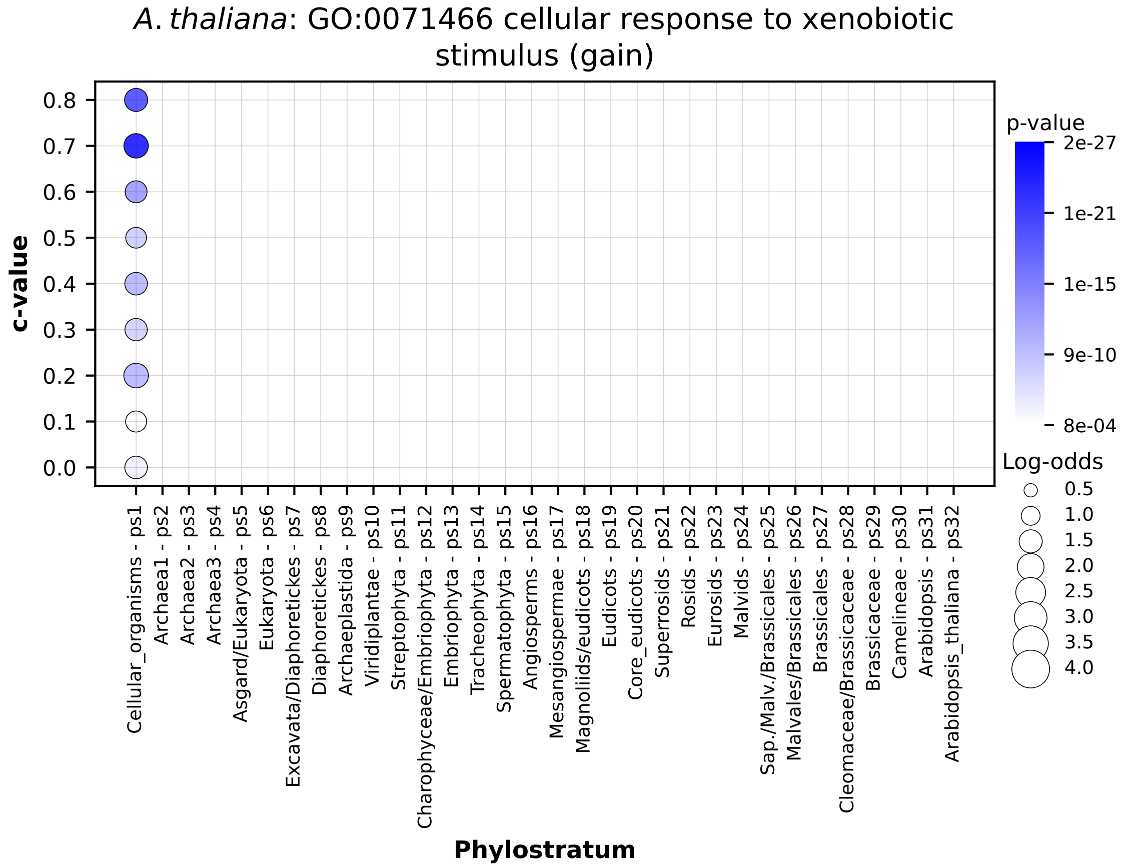
A. thaliana: GO:0071466 cellular response to xenobiotic stimulus (gain)
c-value
0.8
0.7
0.6
0.5
0.4
0.3
0.2
0.1
0.0
Phylostratum
Cellular_organisms - ps1
Archaea1 - ps2
Archaea2 - ps3
Archaea3 - ps4
Asgard/Eukaryota - ps5
Eukaryota - ps6
Excavata/Diaphoretickes - ps7
Diaphoretickes - ps8
Archaeplastida - ps9
Viridiplantae - ps10
Streptophyta - ps11
Charophyceae/Embriophyta - ps12
Embriophyta - ps13
Tracheophyta - ps14
Spermatophyta - ps15
Angiosperms - ps16
Mesangiospermae - ps17
Magnoliids/eudicots - ps18
Eudicots - ps19
Core_eudicots - ps20
Superrosids - ps21
Rosids - ps22
Eurosids - ps23
Malvids - ps24
Sap./Malv./Brassicales - ps25
Malvales/Brassicales - ps26
Brassicales - ps27
Cleomaceae/Brassicaceae - ps28
Brassicaceae - ps29
Camelineae - ps30
Arabidopsis - ps31
Arabidopsis_thaliana - ps32
p-value
2e-27
1e-21
1e-15
9e-10
8e-04
Log-odds
0.5
1.0
1.5
2.0
2.5
3.0
3.5
4.0

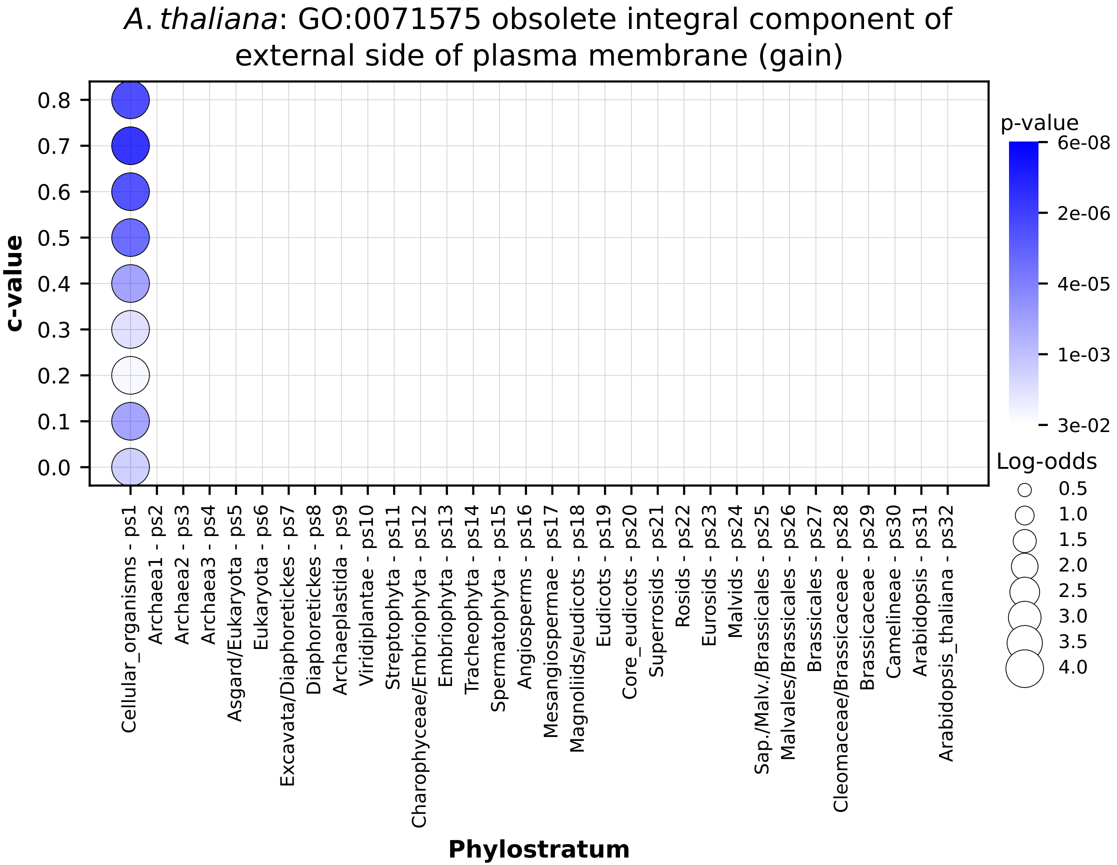

A. thaliana: GO:0071575 obsolete integral component of external side of plasma membrane (gain)
c-value
0.8
0.7
0.6
0.5
0.4
0.3
0.2
0.1
0.0
p-value
6e-08
2e-06
4e-05
1e-03
3e-02
Log-odds
0.5
1.0
1.5
2.0
2.5
3.0
3.5
4.0
Cellular_organisms - ps1
Archaea1 - ps2
Archaea2 - ps3
Archaea3 - ps4
Asgard/Eukaryota - ps5
Eukaryota - ps6
Excavata/Diaphoretickes - ps7
Diaphoretickes - ps8
Archaeplastida - ps9
Viridiplantae - ps10
Streptophyta - ps11
Charophyceae/Embriophyta - ps12
Embriophyta - ps13
Tracheophyta - ps14
Spermatophyta - ps15
Angiosperms - ps16
Mesangiospermae - ps17
Magnoliids/eudicots - ps18
Eudicots - ps19
Core_eudicots - ps20
Superrosids - ps21
Rosids - ps22
Eurosids - ps23
Malvids - ps24
Sap./Malv./Brassicales - ps25
Malvales/Brassicales - ps26
Brassicales - ps27
Cleomaceae/Brassicaceae - ps28
Brassicaceae - ps29
Camelineae - ps30
Arabidopsis - ps31
Arabidopsis_thaliana - ps32
Phylostratum

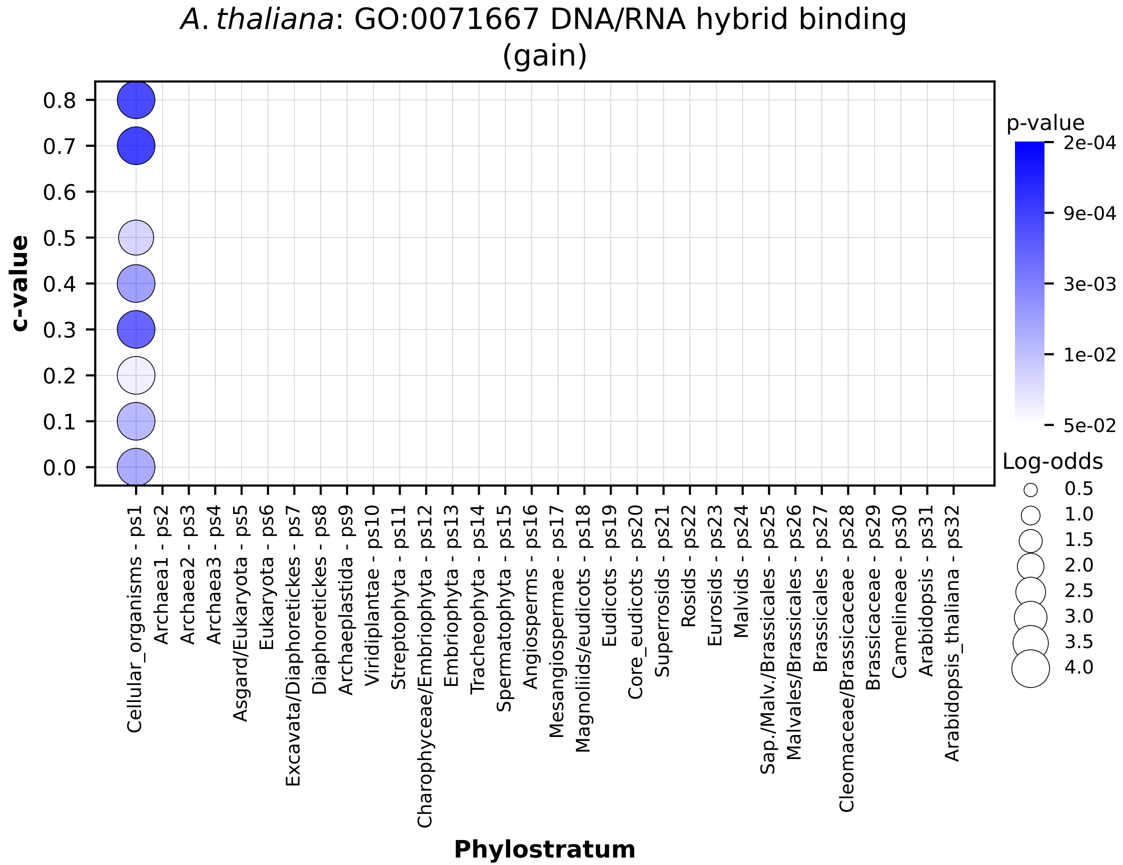

A. thaliana: GO:0071667 DNA/RNA hybrid binding
(gain)
c-value
0.8
0.7
0.6
0.5
0.4
0.3
0.2
0.1
0.0
Phylostratum
Cellular_organisms - ps1
Archaea1 - ps2
Archaea2 - ps3
Archaea3 - ps4
Asgard/Eukaryota - ps5
Eukaryota - ps6
Excavata/Diaphoretickes - ps7
Diaphoretickes - ps8
Archaeplastida - ps9
Viridiplantae - ps10
Streptophyta - ps11
Charophyceae/Embriophyta - ps12
Embriophyta - ps13
Tracheophyta - ps14
Spermatophyta - ps15
Angiosperms - ps16
Mesangiospermae - ps17
Magnoliids/eudicots - ps18
Eudicots - ps19
Core_eudicots - ps20
Superrosids - ps21
Rosids - ps22
Eurosids - ps23
Malvids - ps24
Sap./Malv./Brassicales - ps25
Malvales/Brassicales - ps26
Brassicales - ps27
Cleomaceae/Brassicaceae - ps28
Brassicaceae - ps29
Camelineae - ps30
Arabidopsis - ps31
Arabidopsis_thaliana - ps32
p-value
2e-04
9e-04
3e-03
1e-02
5e-02
Log-odds
0.5
1.0
1.5
2.0
2.5
3.0
3.5
4.0

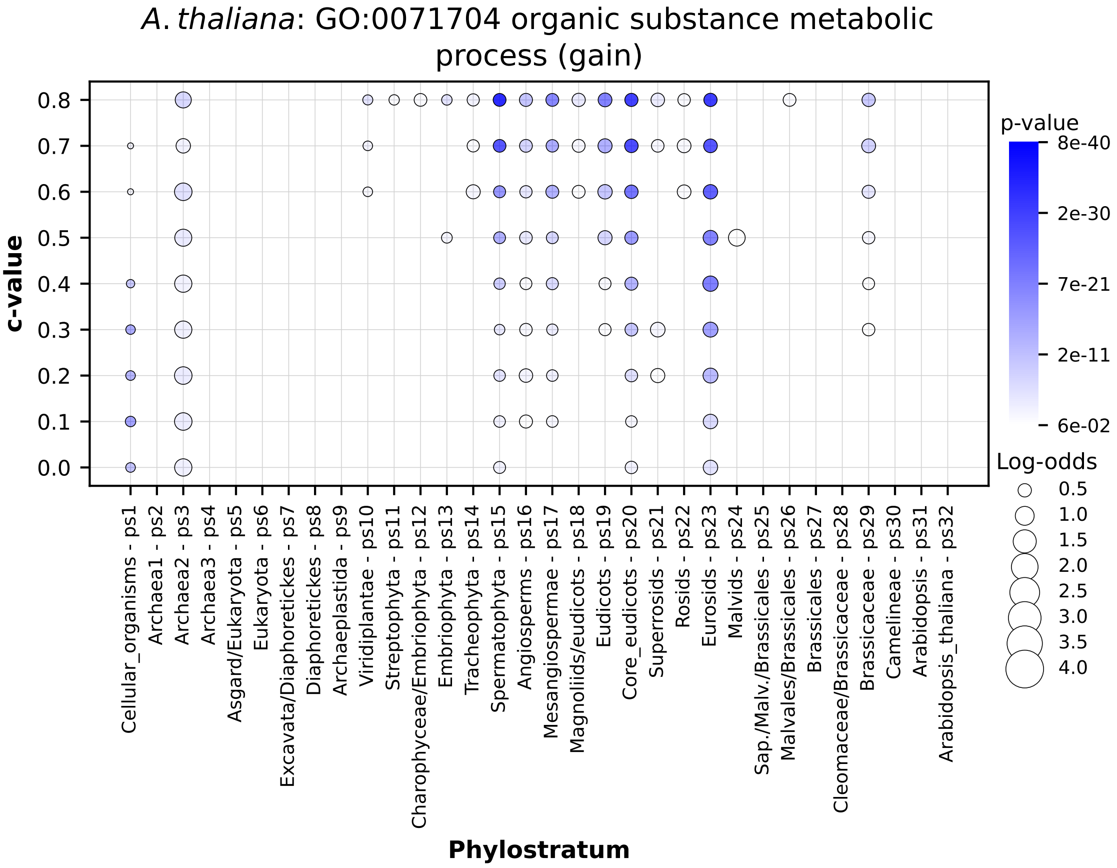

A. thaliana: GO:0071704 organic substance metabolic process (gain)
c-value
0.8
0.7
0.6
0.5
0.4
0.3
0.2
0.1
0.0
Phylostratum
Cellular_organisms - ps1
Archaea1 - ps2
Archaea2 - ps3
Archaea3 - ps4
Asgard/Eukaryota - ps5
Eukaryota - ps6
Excavata/Diaphoretickes - ps7
Diaphoretickes - ps8
Archaeplastida - ps9
Viridiplantae - ps10
Streptophyta - ps11
Charophyceae/Embriophyta - ps12
Embriophyta - ps13
Tracheophyta - ps14
Spermatophyta - ps15
Angiosperms - ps16
Mesangiospermae - ps17
Magnoliids/eudicots - ps18
Eudicots - ps19
Core_eudicots - ps20
Superrosids - ps21
Rosids - ps22
Eurosids - ps23
Malvids - ps24
Sap./Malv./Brassicales - ps25
Malvales/Brassicales - ps26
Brassicales - ps27
Cleomaceae/Brassicaceae - ps28
Brassicaceae - ps29
Camelineae - ps30
Arabidopsis - ps31
Arabidopsis_thaliana - ps32
p-value
8e-40
2e-30
7e-21
2e-11
6e-02
Log-odds
0.5
1.0
1.5
2.0
2.5
3.0
3.5
4.0

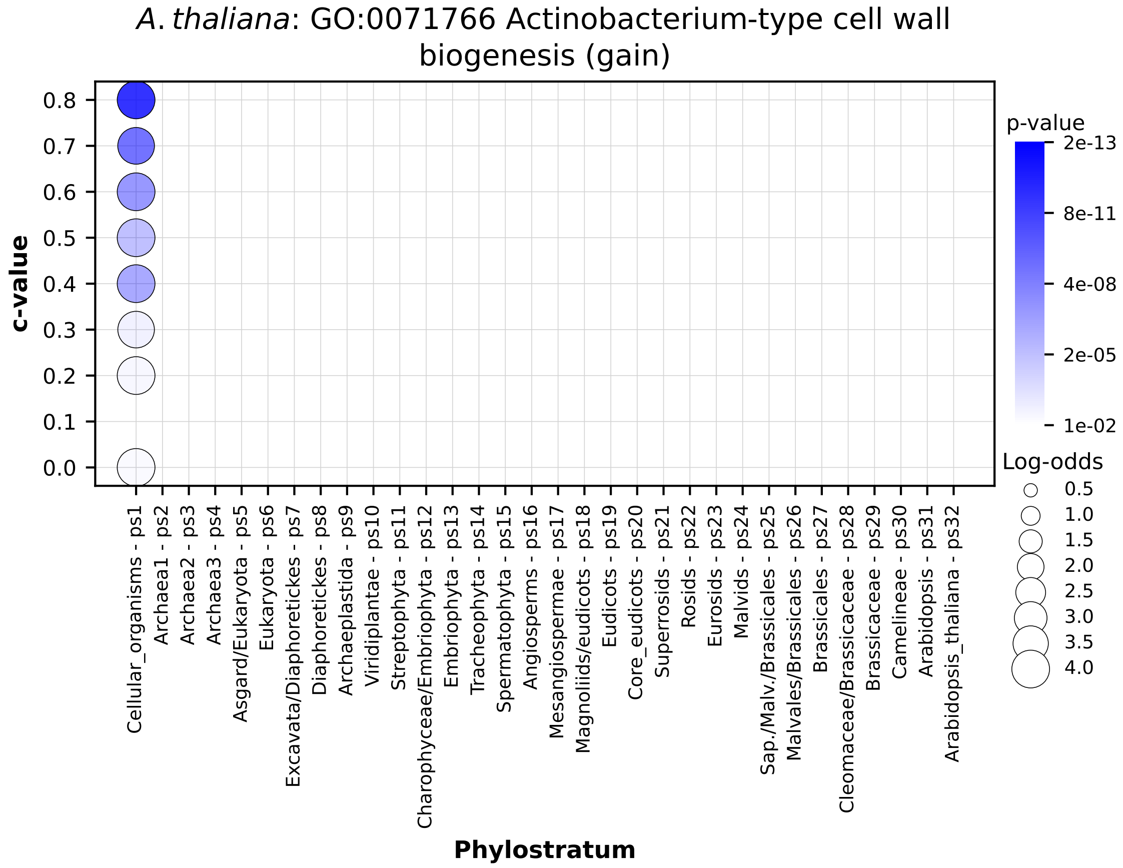

A. thaliana: GO:0071766 Actinobacterium-type cell wall biogenesis (gain)
c-value
0.8
0.7
0.6
0.5
0.4
0.3
0.2
0.1
0.0
p-value
2e-13
8e-11
4e-08
2e-05
1e-02
Log-odds
0.5
1.0
1.5
2.0
2.5
3.0
3.5
4.0
Cellular_organisms - ps1
Archaea1 - ps2
Archaea2 - ps3
Archaea3 - ps4
Asgard/Eukaryota - ps5
Eukaryota - ps6
Excavata/Diaphoretickes - ps7
Diaphoretickes - ps8
Archaeplastida - ps9
Viridiplantae - ps10
Streptophyta - ps11
Charophyceae/Embriophyta - ps12
Embriophyta - ps13
Tracheophyta - ps14
Spermatophyta - ps15
Angiosperms - ps16
Mesangiospermae - ps17
Magnoliids/eudicots - ps18
Eudicots - ps19
Core_eudicots - ps20
Superrosids - ps21
Rosids - ps22
Eurosids - ps23
Malvids - ps24
Sap./Malv./Brassicales - ps25
Malvales/Brassicales - ps26
Brassicales - ps27
Cleomaceae/Brassicaceae - ps28
Brassicaceae - ps29
Camelineae - ps30
Arabidopsis - ps31
Arabidopsis_thaliana - ps32
Phylostratum

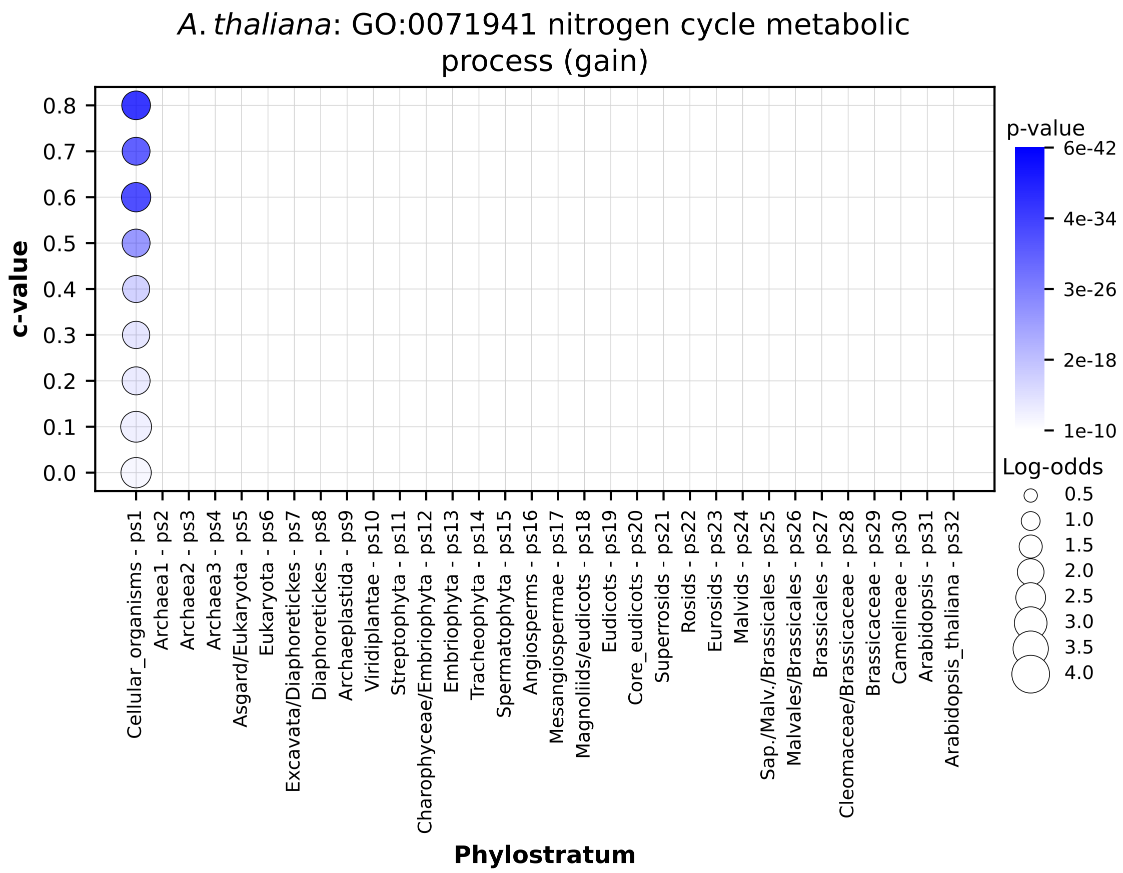

A. thaliana: GO:0071941 nitrogen cycle metabolic process (gain)
c-value
0.8
0.7
0.6
0.5
0.4
0.3
0.2
0.1
0.0
p-value
6e-42
4e-34
3e-26
2e-18
1e-10
Log-odds
0.5
1.0
1.5
2.0
2.5
3.0
3.5
4.0
Cellular_organisms - ps1
Archaea1 - ps2
Archaea2 - ps3
Archaea3 - ps4
Asgard/Eukaryota - ps5
Eukaryota - ps6
Excavata/Diaphoretickes - ps7
Diaphoretickes - ps8
Archaeplastida - ps9
Viridiplantae - ps10
Streptophyta - ps11
Charophyceae/Embriophyta - ps12
Embriophyta - ps13
Tracheophyta - ps14
Spermatophyta - ps15
Angiosperms - ps16
Mesangiospermae - ps17
Magnoliids/eudicots - ps18
Eudicots - ps19
Core_eudicots - ps20
Superrosids - ps21
Rosids - ps22
Eurosids - ps23
Malvids - ps24
Sap./Malv./Brassicales - ps25
Malvales/Brassicales - ps26
Brassicales - ps27
Cleomaceae/Brassicaceae - ps28
Brassicaceae - ps29
Camelineae - ps30
Arabidopsis - ps31
Arabidopsis_thaliana - ps32
Phylostratum

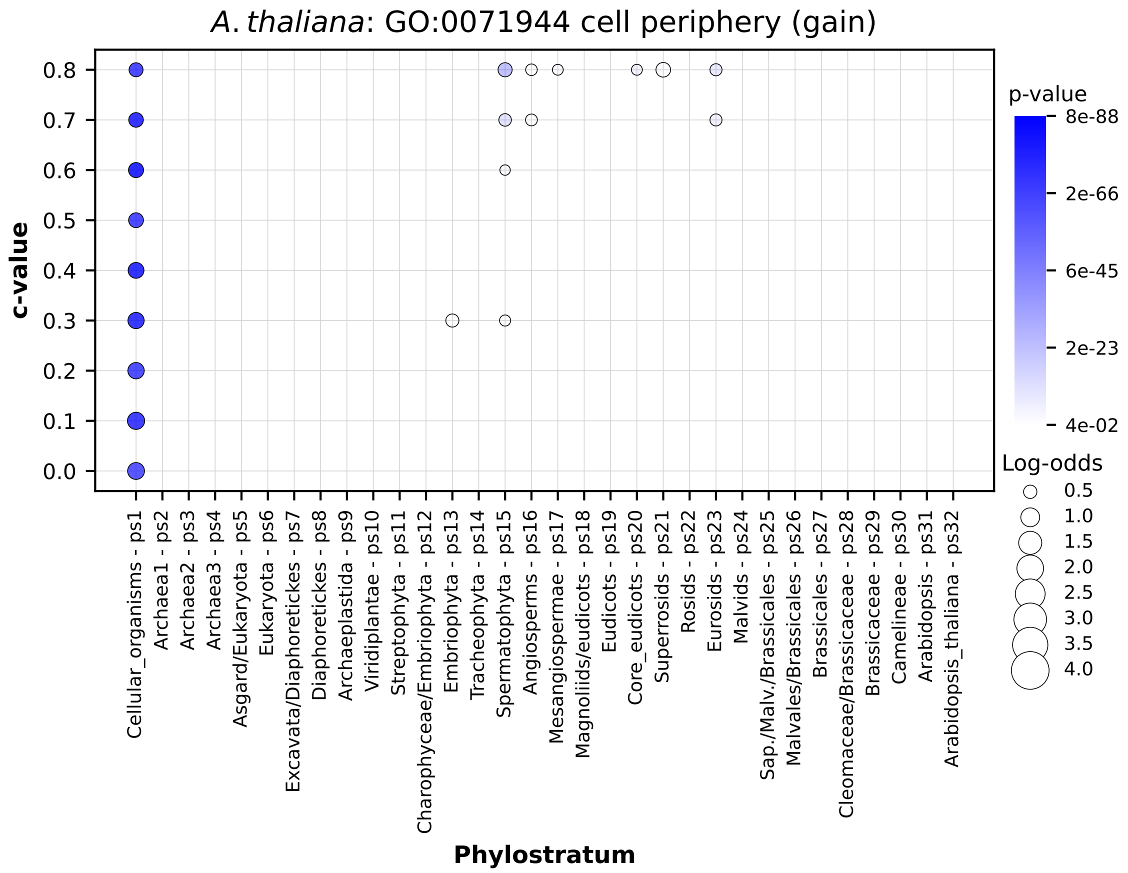

A. thaliana: GO:0071944 cell periphery (gain)
c-value
0.8
0.7
0.6
0.5
0.4
0.3
0.2
0.1
0.0
p-value
8e-88
2e-66
6e-45
2e-23
4e-02
Log-odds
0.5
1.0
1.5
2.0
2.5
3.0
3.5
4.0
Cellular_organisms - ps1
Archaea1 - ps2
Archaea2 - ps3
Archaea3 - ps4
Asgard/Eukaryota - ps5
Eukaryota - ps6
Excavata/Diaphoretickes - ps7
Diaphoretickes - ps8
Archaeplastida - ps9
Viridiplantae - ps10
Streptophyta - ps11
Charophyceae/Embriophyta - ps12
Embriophyta - ps13
Tracheophyta - ps14
Spermatophyta - ps15
Angiosperms - ps16
Mesangiospermae - ps17
Magnoliids/eudicots - ps18
Eudicots - ps19
Core_eudicots - ps20
Superrosids - ps21
Rosids - ps22
Eurosids - ps23
Malvids - ps24
Sap./Malv./Brassicales - ps25
Malvales/Brassicales - ps26
Brassicales - ps27
Cleomaceae/Brassicaceae - ps28
Brassicaceae - ps29
Camelineae - ps30
Arabidopsis - ps31
Arabidopsis_thaliana - ps32
Phylostratum

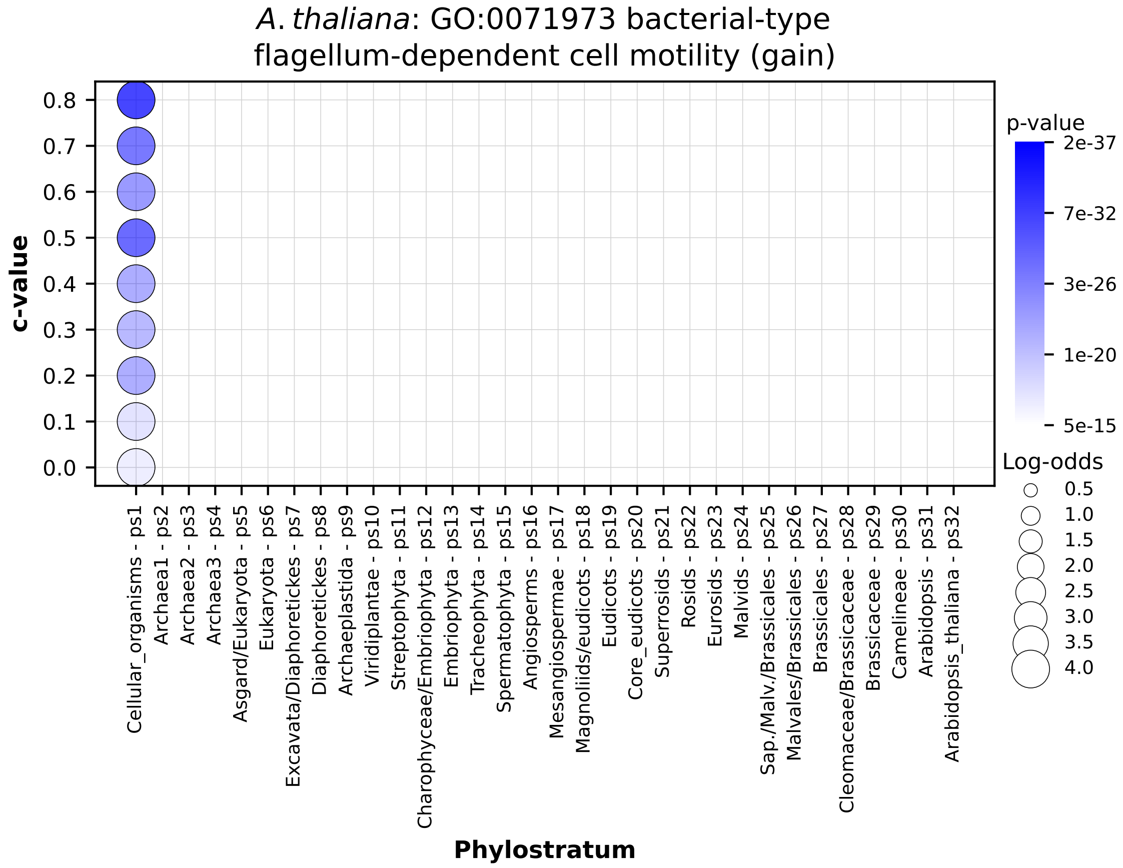

A. thaliana: GO:0071973 bacterial-type flagellum-dependent cell motility (gain)
c-value
0.8
0.7
0.6
0.5
0.4
0.3
0.2
0.1
0.0
p-value
2e-37
7e-32
3e-26
1e-20
5e-15
Log-odds
0.5
1.0
1.5
2.0
2.5
3.0
3.5
4.0
Cellular_organisms - ps1
Archaea1 - ps2
Archaea2 - ps3
Archaea3 - ps4
Asgard/Eukaryota - ps5
Eukaryota - ps6
Excavata/Diaphoretickes - ps7
Diaphoretickes - ps8
Archaeplastida - ps9
Viridiplantae - ps10
Streptophyta - ps11
Charophyceae/Embriophyta - ps12
Embriophyta - ps13
Tracheophyta - ps14
Spermatophyta - ps15
Angiosperms - ps16
Mesangiospermae - ps17
Magnoliids/eudicots - ps18
Eudicots - ps19
Core_eudicots - ps20
Superrosids - ps21
Rosids - ps22
Eurosids - ps23
Malvids - ps24
Sap./Malv./Brassicales - ps25
Malvales/Brassicales - ps26
Brassicales - ps27
Cleomaceae/Brassicaceae - ps28
Brassicaceae - ps29
Camelineae - ps30
Arabidopsis - ps31
Arabidopsis_thaliana - ps32
Phylostratum

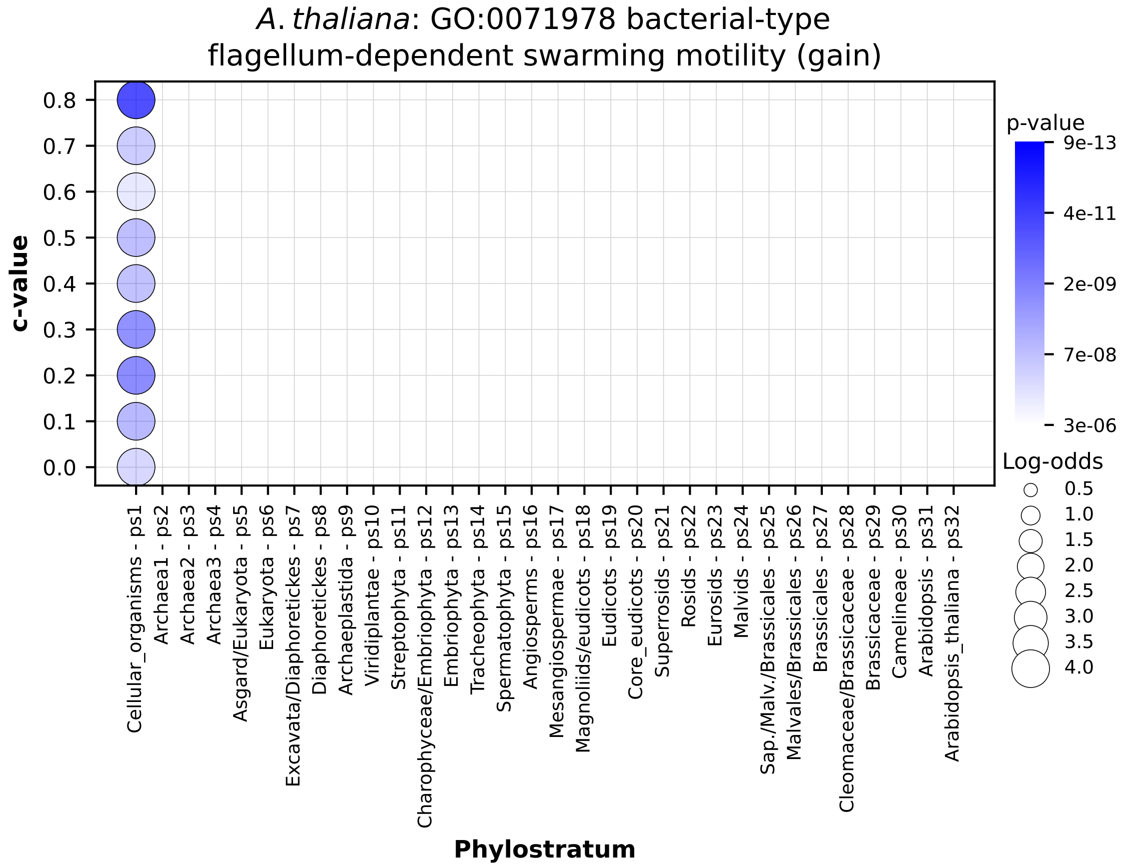
A. thaliana: GO:0071978 bacterial-type flagellum-dependent swarming motility (gain)
c-value
0.0
0.1
0.2
0.3
0.4
0.5
0.6
0.7
0.8
p-value
9e-13
4e-11
2e-09
7e-08
3e-06
Log-odds
0.5
1.0
1.5
2.0
2.5
3.0
3.5
4.0
Cellular_organisms - ps1
Archaea1 - ps2
Archaea2 - ps3
Archaea3 - ps4
Asgard/Eukaryota - ps5
Eukaryota - ps6
Excavata/Diaphoretickes - ps7
Diaphoretickes - ps8
Archaeplastida - ps9
Viridiplantae - ps10
Streptophyta - ps11
Charophyceae/Embriophyta - ps12
Embriophyta - ps13
Tracheophyta - ps14
Spermatophyta - ps15
Angiosperms - ps16
Mesangiospermae - ps17
Magnoliids/eudicots - ps18
Eudicots - ps19
Core_eudicots - ps20
Superrosids - ps21
Rosids - ps22
Eurosids - ps23
Malvids - ps24
Sap./Malv./Brassicales - ps25
Malvales/Brassicales - ps26
Brassicales - ps27
Cleomaceae/Brassicaceae - ps28
Brassicaceae - ps29
Camelineae - ps30
Arabidopsis - ps31
Arabidopsis_thaliana - ps32
Phylostratum

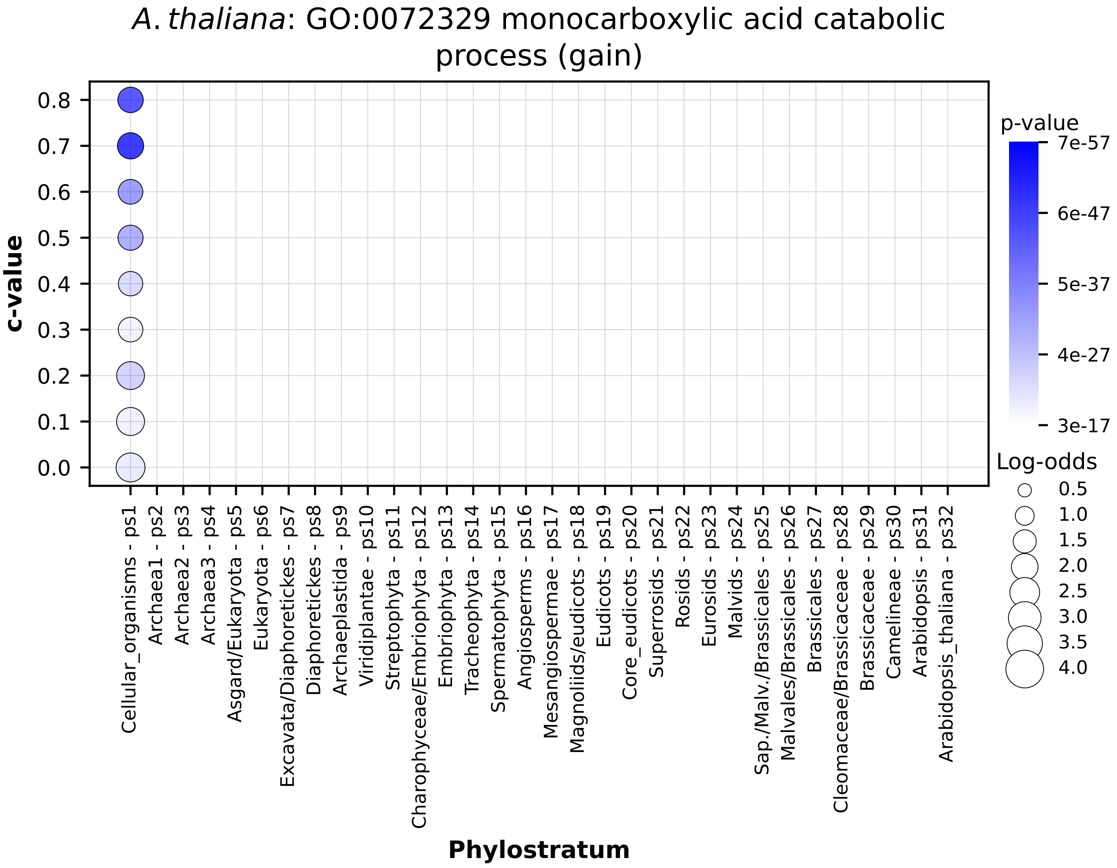

A. thaliana: GO:0072329 monocarboxylic acid catabolic process (gain)
c-value
0.0
0.1
0.2
0.3
0.4
0.5
0.6
0.7
0.8
Phylostratum
Cellular_organisms - ps1
Archaea1 - ps2
Archaea2 - ps3
Archaea3 - ps4
Asgard/Eukaryota - ps5
Eukaryota - ps6
Excavata/Diaphoretickes - ps7
Diaphoretickes - ps8
Archaeplastida - ps9
Viridiplantae - ps10
Streptophyta - ps11
Charophyceae/Embriophyta - ps12
Embriophyta - ps13
Tracheophyta - ps14
Spermatophyta - ps15
Angiosperms - ps16
Mesangiospermae - ps17
Magnoliids/eudicots - ps18
Eudicots - ps19
Core_eudicots - ps20
Superrosids - ps21
Rosids - ps22
Eurosids - ps23
Malvids - ps24
Sap./Malv./Brassicales - ps25
Malvales/Brassicales - ps26
Brassicales - ps27
Cleomaceae/Brassicaceae - ps28
Brassicaceae - ps29
Camelineae - ps30
Arabidopsis - ps31
Arabidopsis_thaliana - ps32
p-value
7e-57
6e-47
5e-37
4e-27
3e-17
Log-odds
0.5
1.0
1.5
2.0
2.5
3.0
3.5
4.0

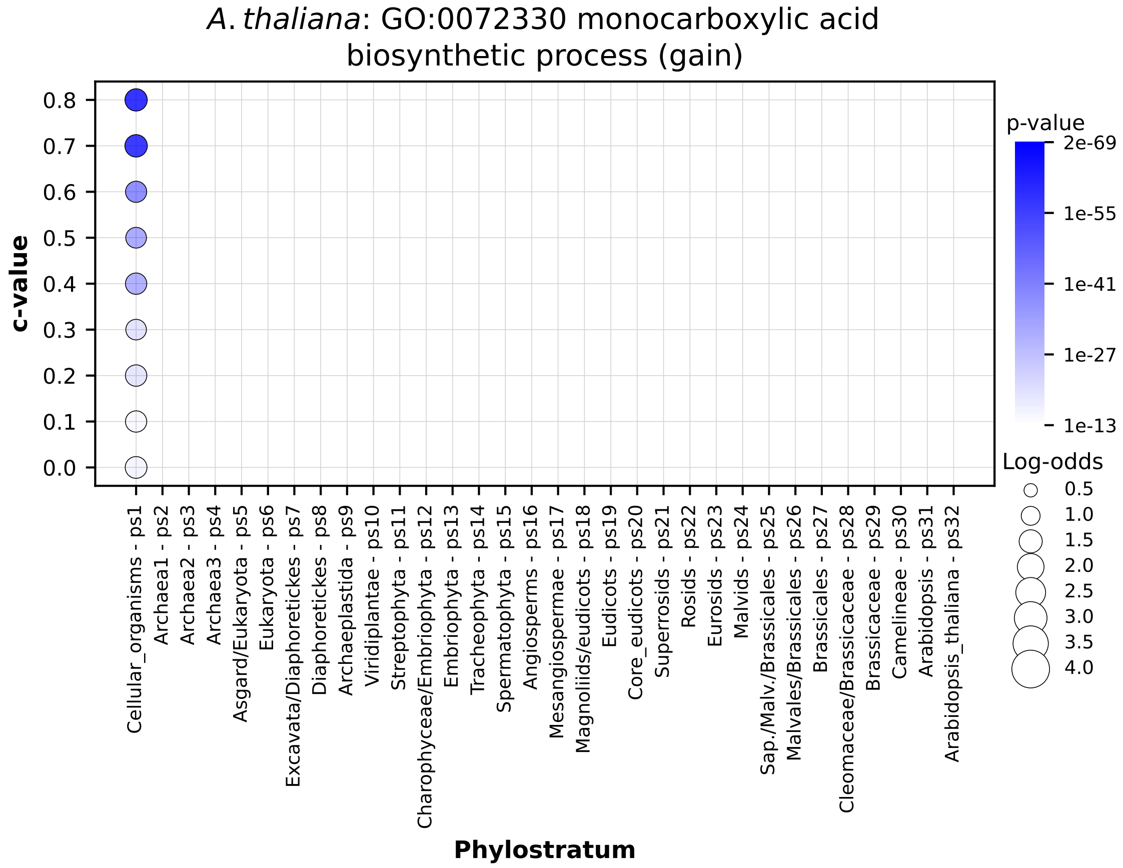

A. thaliana: GO:0072330 monocarboxylic acid biosynthetic process (gain)
c-value
0.0
0.1
0.2
0.3
0.4
0.5
0.6
0.7
0.8
p-value
2e-69
1e-55
1e-41
1e-27
1e-13
Log-odds
0.5
1.0
1.5
2.0
2.5
3.0
3.5
4.0
Cellular_organisms - ps1
Archaea1 - ps2
Archaea2 - ps3
Archaea3 - ps4
Asgard/Eukaryota - ps5
Eukaryota - ps6
Excavata/Diaphoretickes - ps7
Diaphoretickes - ps8
Archaeplastida - ps9
Viridiplantae - ps10
Streptophyta - ps11
Charophyceae/Embriophyta - ps12
Embriophyta - ps13
Tracheophyta - ps14
Spermatophyta - ps15
Angiosperms - ps16
Mesangiospermae - ps17
Magnoliids/eudicots - ps18
Eudicots - ps19
Core_eudicots - ps20
Superrosids - ps21
Rosids - ps22
Eurosids - ps23
Malvids - ps24
Sap./Malv./Brassicales - ps25
Malvales/Brassicales - ps26
Brassicales - ps27
Cleomaceae/Brassicaceae - ps28
Brassicaceae - ps29
Camelineae - ps30
Arabidopsis - ps31
Arabidopsis_thaliana - ps32
Phylostratum

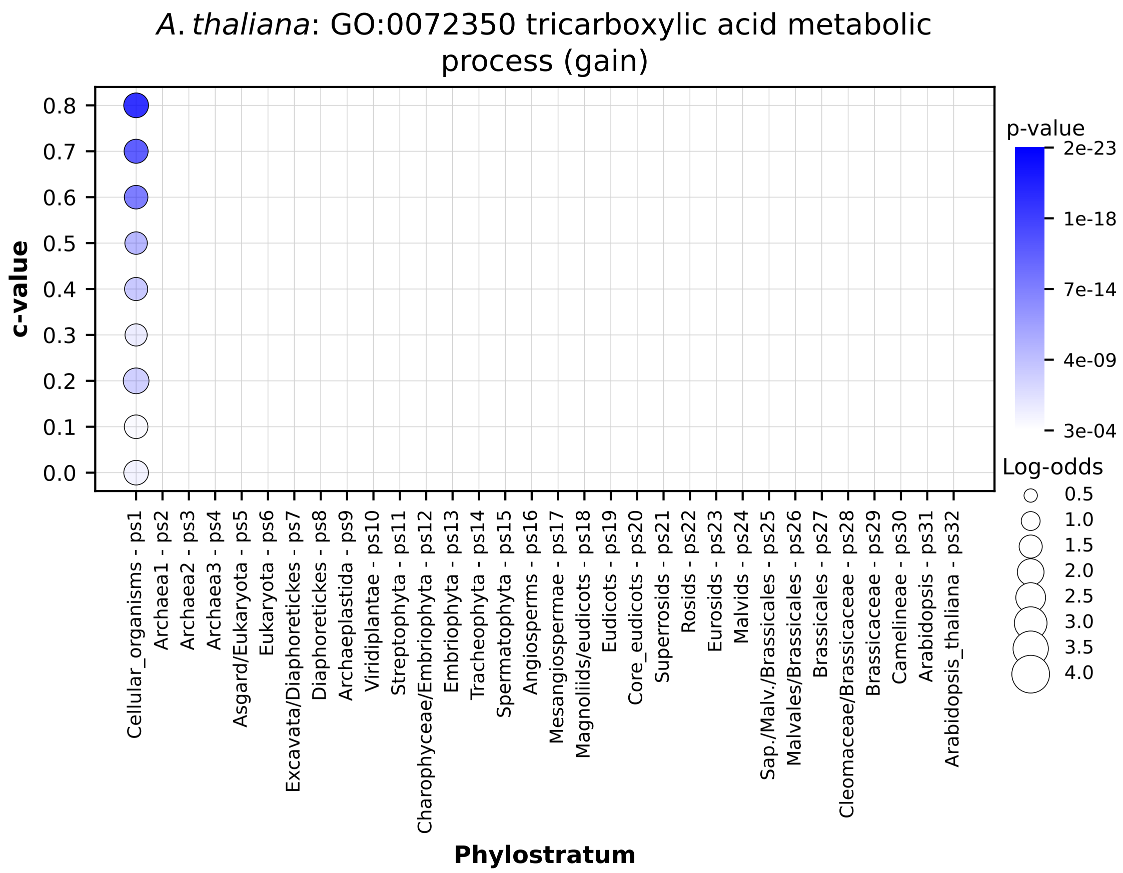
A. thaliana: GO:0072350 tricarboxylic acid metabolic process (gain)
c-value
0.8
0.7
0.6
0.5
0.4
0.3
0.2
0.1
0.0
p-value
2e-23
1e-18
7e-14
4e-09
3e-04
Log-odds
0.5
1.0
1.5
2.0
2.5
3.0
3.5
4.0
Cellular_organisms - ps1
Archaea1 - ps2
Archaea2 - ps3
Archaea3 - ps4
Asgard/Eukaryota - ps5
Eukaryota - ps6
Excavata/Diaphoretickes - ps7
Diaphoretickes - ps8
Archaeplastida - ps9
Viridiplantae - ps10
Streptophyta - ps11
Charophyceae/Embriophyta - ps12
Embriophyta - ps13
Tracheophyta - ps14
Spermatophyta - ps15
Angiosperms - ps16
Mesangiospermae - ps17
Magnoliids/eudicots - ps18
Eudicots - ps19
Core_eudicots - ps20
Superrosids - ps21
Rosids - ps22
Eurosids - ps23
Malvids - ps24
Sap./Malv./Brassicales - ps25
Malvales/Brassicales - ps26
Brassicales - ps27
Cleomaceae/Brassicaceae - ps28
Brassicaceae - ps29
Camelineae - ps30
Arabidopsis - ps31
Arabidopsis_thaliana - ps32
Phylostratum

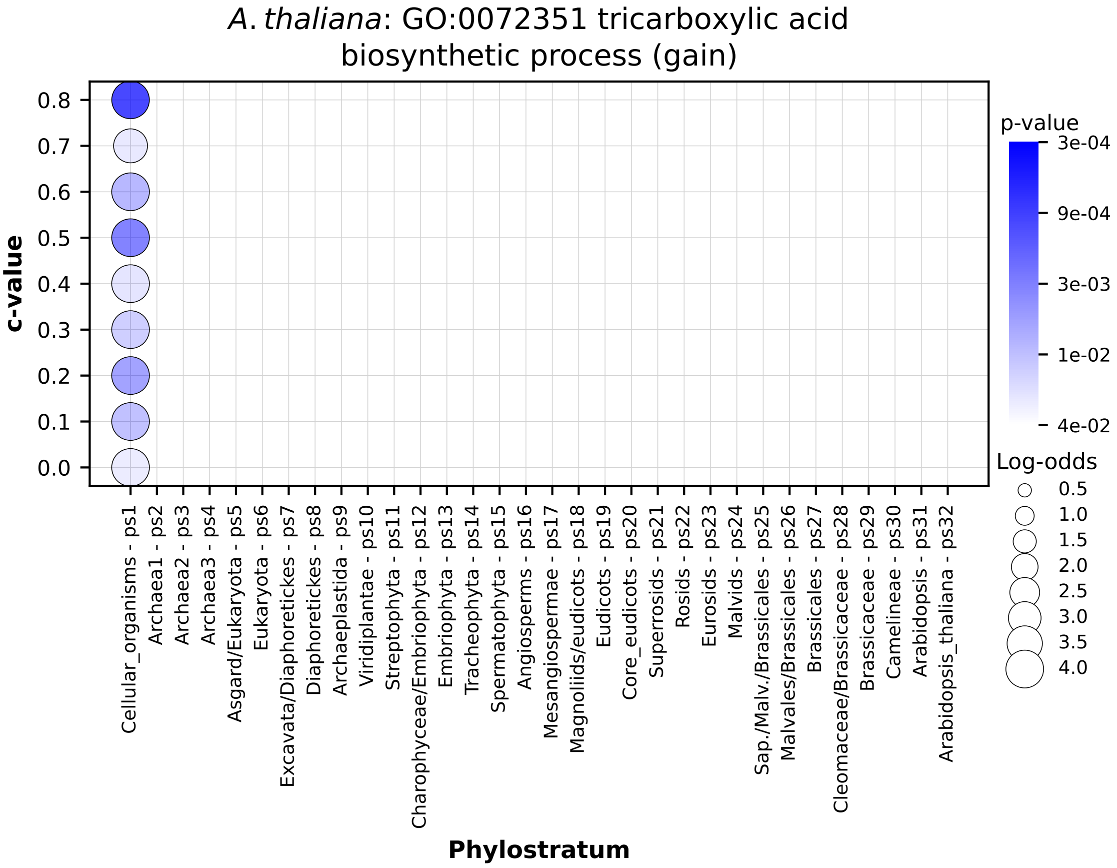

A. thaliana: GO:0072351 tricarboxylic acid biosynthetic process (gain)
c-value
0.0
0.1
0.2
0.3
0.4
0.5
0.6
0.7
0.8
p-value
3e-04
9e-04
3e-03
1e-02
4e-02
Log-odds
0.5
1.0
1.5
2.0
2.5
3.0
3.5
4.0
Cellular_organisms - ps1
Archaea1 - ps2
Archaea2 - ps3
Archaea3 - ps4
Asgard/Eukaryota - ps5
Eukaryota - ps6
Excavata/Diaphoretickes - ps7
Diaphoretickes - ps8
Archaeplastida - ps9
Viridiplantae - ps10
Streptophyta - ps11
Charophyceae/Embriophyta - ps12
Embriophyta - ps13
Tracheophyta - ps14
Spermatophyta - ps15
Angiosperms - ps16
Mesangiospermae - ps17
Magnoliids/eudicots - ps18
Eudicots - ps19
Core_eudicots - ps20
Superrosids - ps21
Rosids - ps22
Eurosids - ps23
Malvids - ps24
Sap./Malv./Brassicales - ps25
Malvales/Brassicales - ps26
Brassicales - ps27
Cleomaceae/Brassicaceae - ps28
Brassicaceae - ps29
Camelineae - ps30
Arabidopsis - ps31
Arabidopsis_thaliana - ps32
Phylostratum

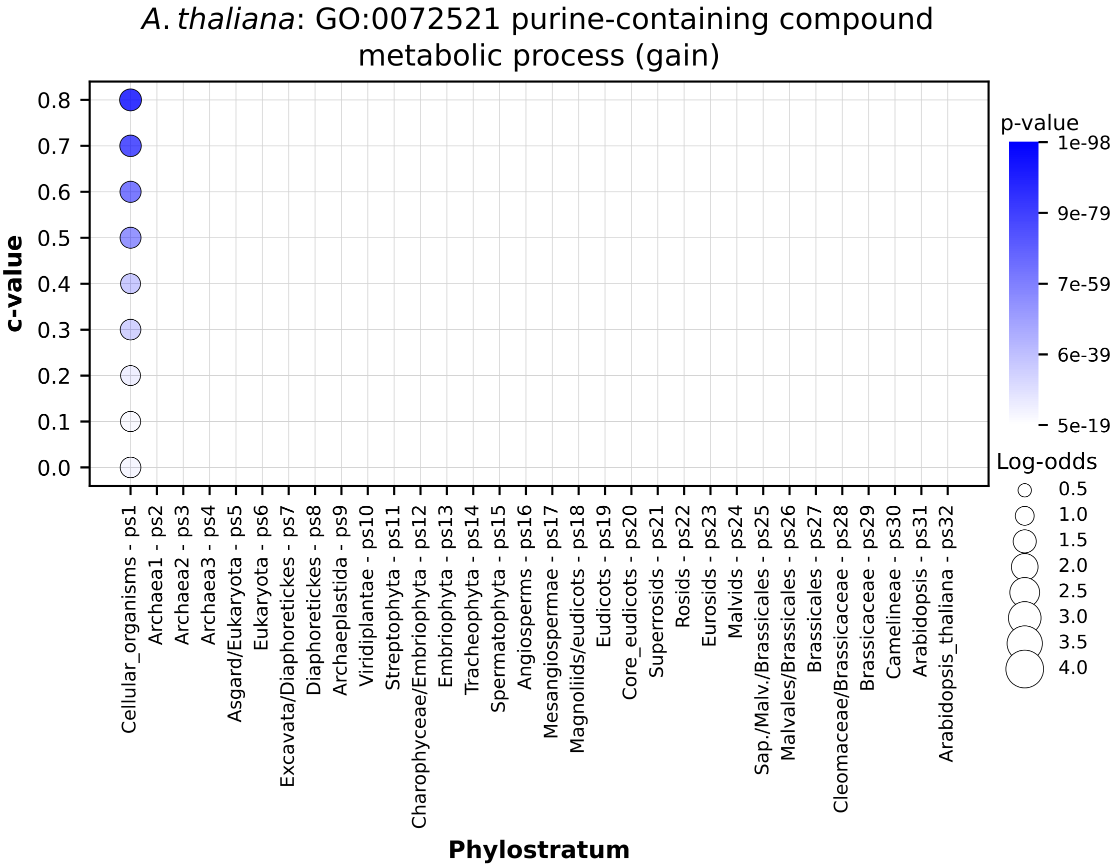
A. thaliana: GO:0072521 purine-containing compound metabolic process (gain)
c-value
0.8
0.7
0.6
0.5
0.4
0.3
0.2
0.1
0.0
p-value
1e-98
9e-79
7e-59
6e-39
5e-19
Log-odds
0.5
1.0
1.5
2.0
2.5
3.0
3.5
4.0
Cellular_organisms - ps1
Archaea1 - ps2
Archaea2 - ps3
Archaea3 - ps4
Asgard/Eukaryota - ps5
Eukaryota - ps6
Excavata/Diaphoretickes - ps7
Diaphoretickes - ps8
Archaeplastida - ps9
Viridiplantae - ps10
Streptophyta - ps11
Charophyceae/Embriophyta - ps12
Embriophyta - ps13
Tracheophyta - ps14
Spermatophyta - ps15
Angiosperms - ps16
Mesangiospermae - ps17
Magnoliids/eudicots - ps18
Eudicots - ps19
Core_eudicots - ps20
Superrosids - ps21
Rosids - ps22
Eurosids - ps23
Malvids - ps24
Sap./Malv./Brassicales - ps25
Malvales/Brassicales - ps26
Brassicales - ps27
Cleomaceae/Brassicaceae - ps28
Brassicaceae - ps29
Camelineae - ps30
Arabidopsis - ps31
Arabidopsis_thaliana - ps32
Phylostratum

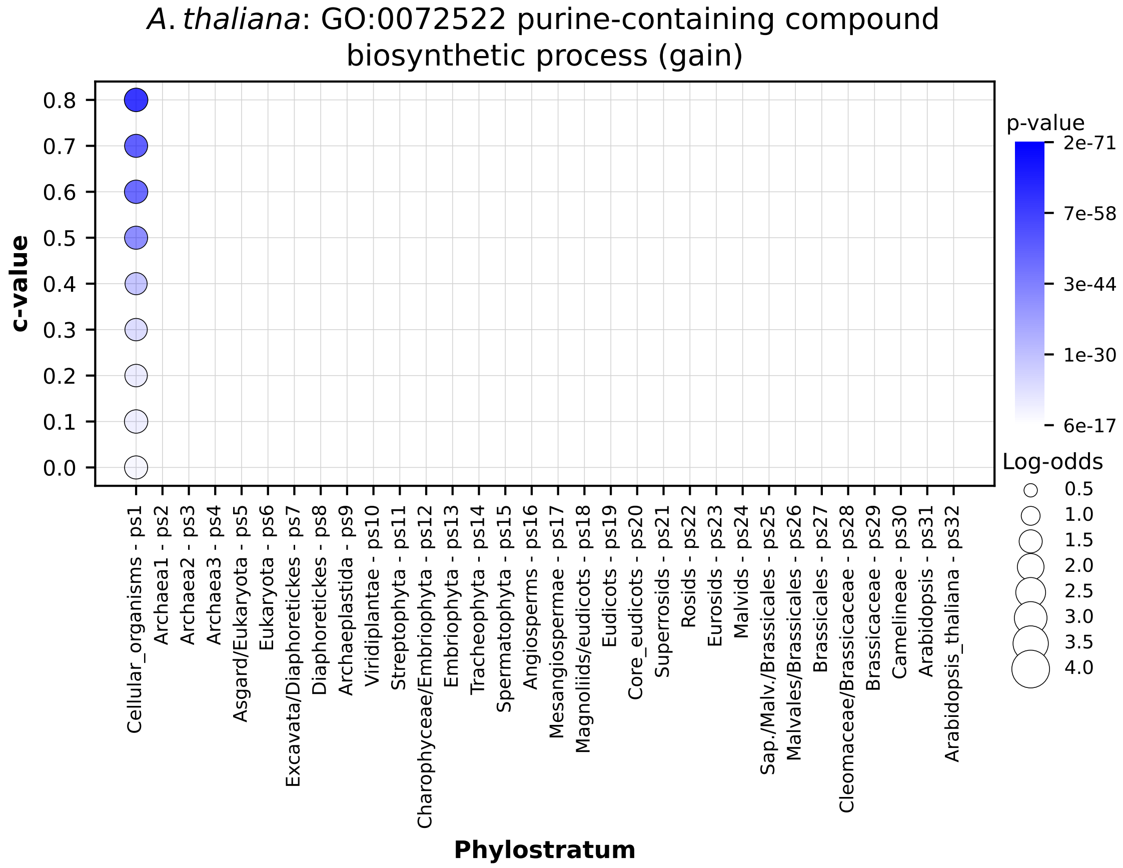

A. thaliana: GO:0072522 purine-containing compound biosynthetic process (gain)
c-value
0.8
0.7
0.6
0.5
0.4
0.3
0.2
0.1
0.0
p-value
2e-71
7e-58
3e-44
1e-30
6e-17
Log-odds
0.5
1.0
1.5
2.0
2.5
3.0
3.5
4.0
Cellular_organisms - ps1
Archaea1 - ps2
Archaea2 - ps3
Archaea3 - ps4
Asgard/Eukaryota - ps5
Eukaryota - ps6
Excavata/Diaphoretickes - ps7
Diaphoretickes - ps8
Archaeplastida - ps9
Viridiplantae - ps10
Streptophyta - ps11
Charophyceae/Embriophyta - ps12
Embriophyta - ps13
Tracheophyta - ps14
Spermatophyta - ps15
Angiosperms - ps16
Mesangiospermae - ps17
Magnoliids/eudicots - ps18
Eudicots - ps19
Core_eudicots - ps20
Superrosids - ps21
Rosids - ps22
Eurosids - ps23
Malvids - ps24
Sap./Malv./Brassicales - ps25
Malvales/Brassicales - ps26
Brassicales - ps27
Cleomaceae/Brassicaceae - ps28
Brassicaceae - ps29
Camelineae - ps30
Arabidopsis - ps31
Arabidopsis_thaliana - ps32
Phylostratum

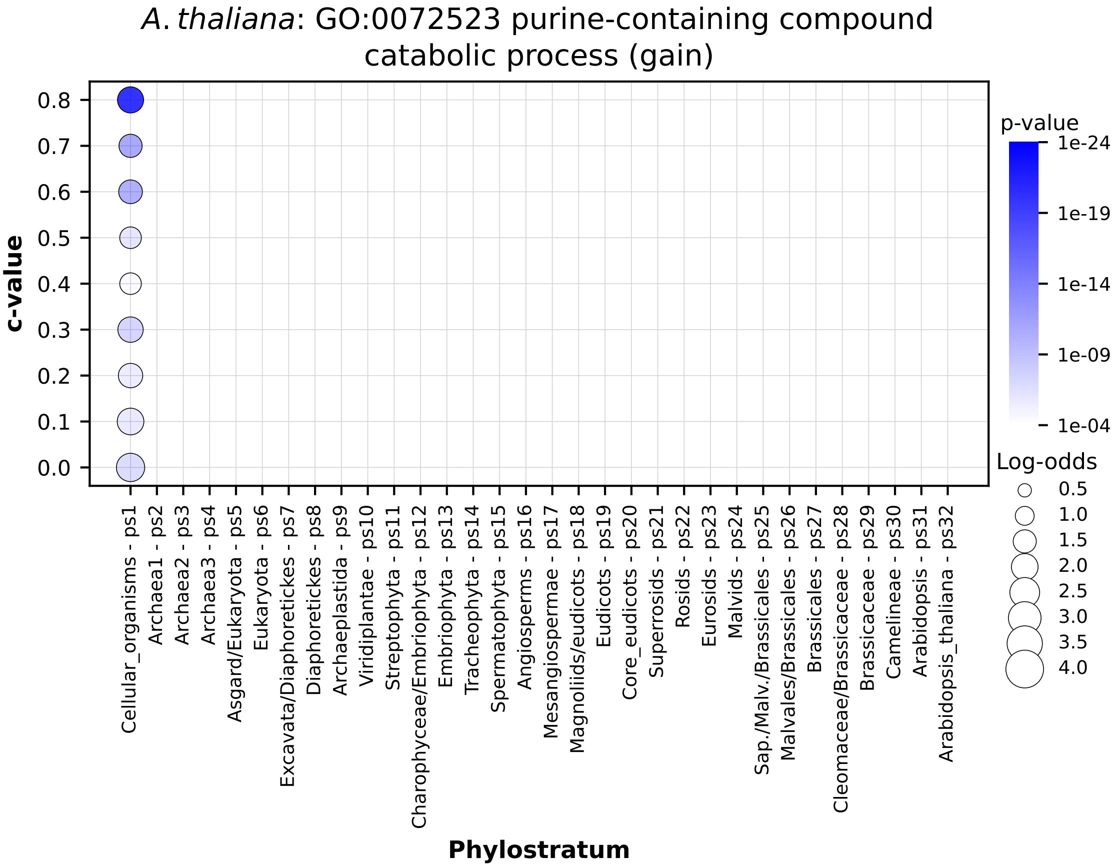
A. thaliana: GO:0072523 purine-containing compound catabolic process (gain)
c-value
0.8
0.7
0.6
0.5
0.4
0.3
0.2
0.1
0.0
p-value
1e-24
1e-19
1e-14
1e-09
1e-04
Log-odds
0.5
1.0
1.5
2.0
2.5
3.0
3.5
4.0
Cellular_organisms - ps1
Archaea1 - ps2
Archaea2 - ps3
Archaea3 - ps4
Asgard/Eukaryota - ps5
Eukaryota - ps6
Excavata/Diaphoretickes - ps7
Diaphoretickes - ps8
Archaeplastida - ps9
Viridiplantae - ps10
Streptophyta - ps11
Charophyceae/Embriophyta - ps12
Embriophyta - ps13
Tracheophyta - ps14
Spermatophyta - ps15
Angiosperms - ps16
Mesangiospermae - ps17
Magnoliids/eudicots - ps18
Eudicots - ps19
Core_eudicots - ps20
Superrosids - ps21
Rosids - ps22
Eurosids - ps23
Malvids - ps24
Sap./Malv./Brassicales - ps25
Malvales/Brassicales - ps26
Brassicales - ps27
Cleomaceae/Brassicaceae - ps28
Brassicaceae - ps29
Camelineae - ps30
Arabidopsis - ps31
Arabidopsis_thaliana - ps32
Phylostratum

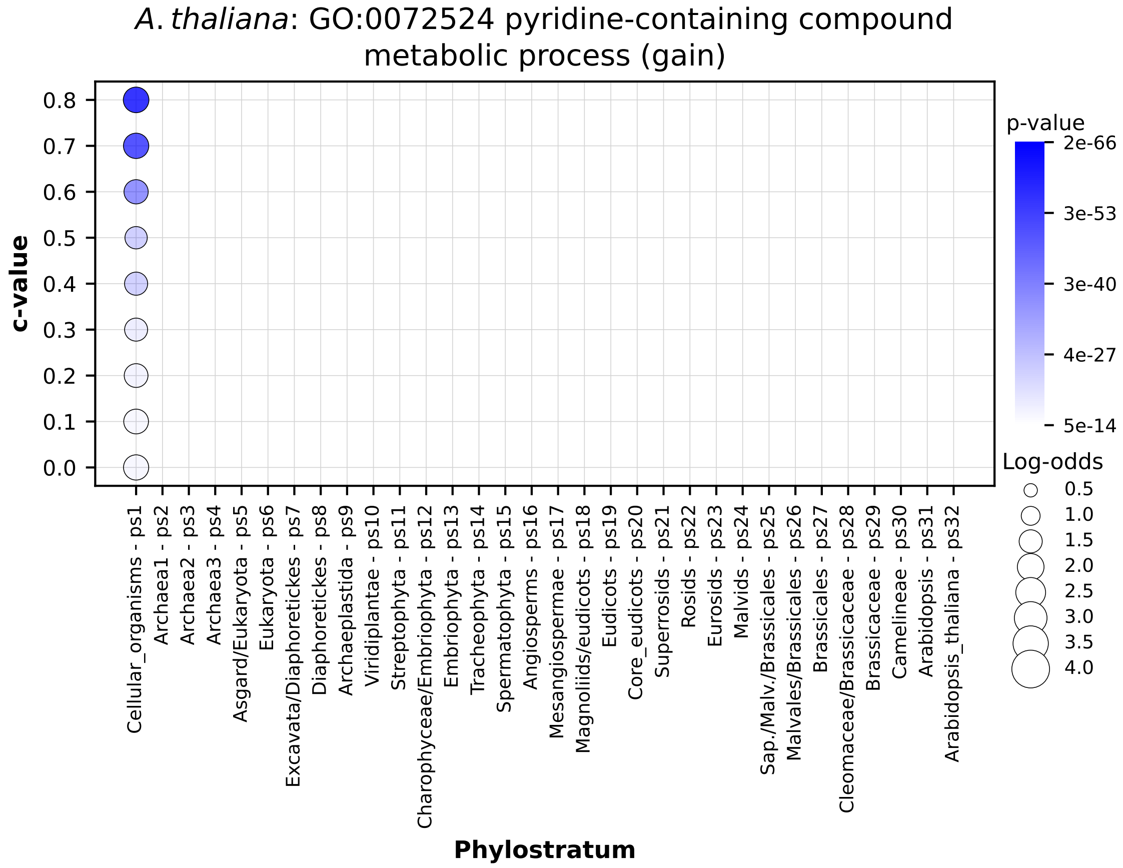
A. thaliana: GO:0072524 pyridine-containing compound metabolic process (gain)
c-value
0.8
0.7
0.6
0.5
0.4
0.3
0.2
0.1
0.0
p-value
2e-66
3e-53
3e-40
4e-27
5e-14
Log-odds
0.5
1.0
1.5
2.0
2.5
3.0
3.5
4.0
Cellular_organisms - ps1
Archaea1 - ps2
Archaea2 - ps3
Archaea3 - ps4
Asgard/Eukaryota - ps5
Eukaryota - ps6
Excavata/Diaphoretickes - ps7
Diaphoretickes - ps8
Archaeplastida - ps9
Viridiplantae - ps10
Streptophyta - ps11
Charophyceae/Embriophyta - ps12
Embriophyta - ps13
Tracheophyta - ps14
Spermatophyta - ps15
Angiosperms - ps16
Mesangiospermae - ps17
Magnoliids/eudicots - ps18
Eudicots - ps19
Core_eudicots - ps20
Superrosids - ps21
Rosids - ps22
Eurosids - ps23
Malvids - ps24
Sap./Malv./Brassicales - ps25
Malvales/Brassicales - ps26
Brassicales - ps27
Cleomaceae/Brassicaceae - ps28
Brassicaceae - ps29
Camelineae - ps30
Arabidopsis - ps31
Arabidopsis_thaliana - ps32
Phylostratum

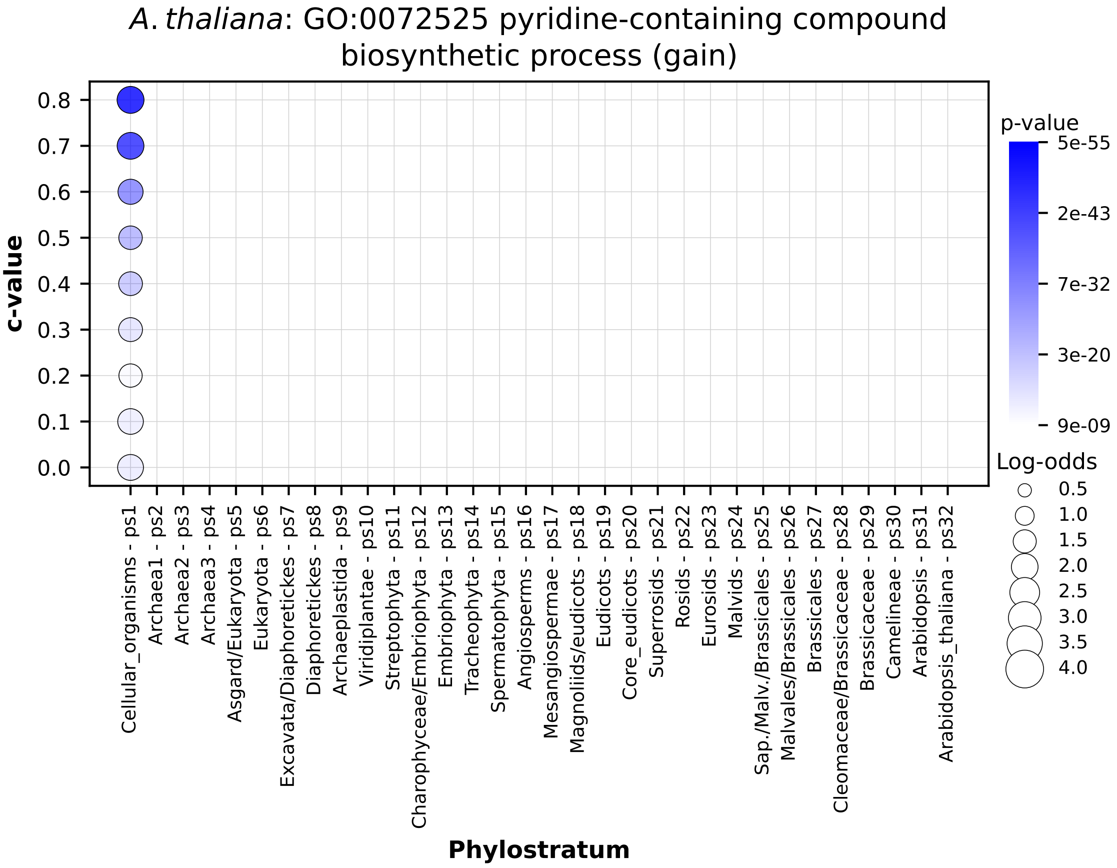

A. thaliana: GO:0072525 pyridine-containing compound biosynthetic process (gain)
c-value
0.0
0.1
0.2
0.3
0.4
0.5
0.6
0.7
0.8
Phylostratum
Cellular_organisms - ps1
Archaea1 - ps2
Archaea2 - ps3
Archaea3 - ps4
Asgard/Eukaryota - ps5
Eukaryota - ps6
Excavata/Diaphoretickes - ps7
Diaphoretickes - ps8
Archaeplastida - ps9
Viridiplantae - ps10
Streptophyta - ps11
Charophyceae/Embriophyta - ps12
Embriophyta - ps13
Tracheophyta - ps14
Spermatophyta - ps15
Angiosperms - ps16
Mesangiospermae - ps17
Magnoliids/eudicots - ps18
Eudicots - ps19
Core_eudicots - ps20
Superrosids - ps21
Rosids - ps22
Eurosids - ps23
Malvids - ps24
Sap./Malv./Brassicales - ps25
Malvales/Brassicales - ps26
Brassicales - ps27
Cleomaceae/Brassicaceae - ps28
Brassicaceae - ps29
Camelineae - ps30
Arabidopsis - ps31
Arabidopsis_thaliana - ps32
p-value
5e-55
2e-43
7e-32
3e-20
9e-09
Log-odds
0.5
1.0
1.5
2.0
2.5
3.0
3.5
4.0

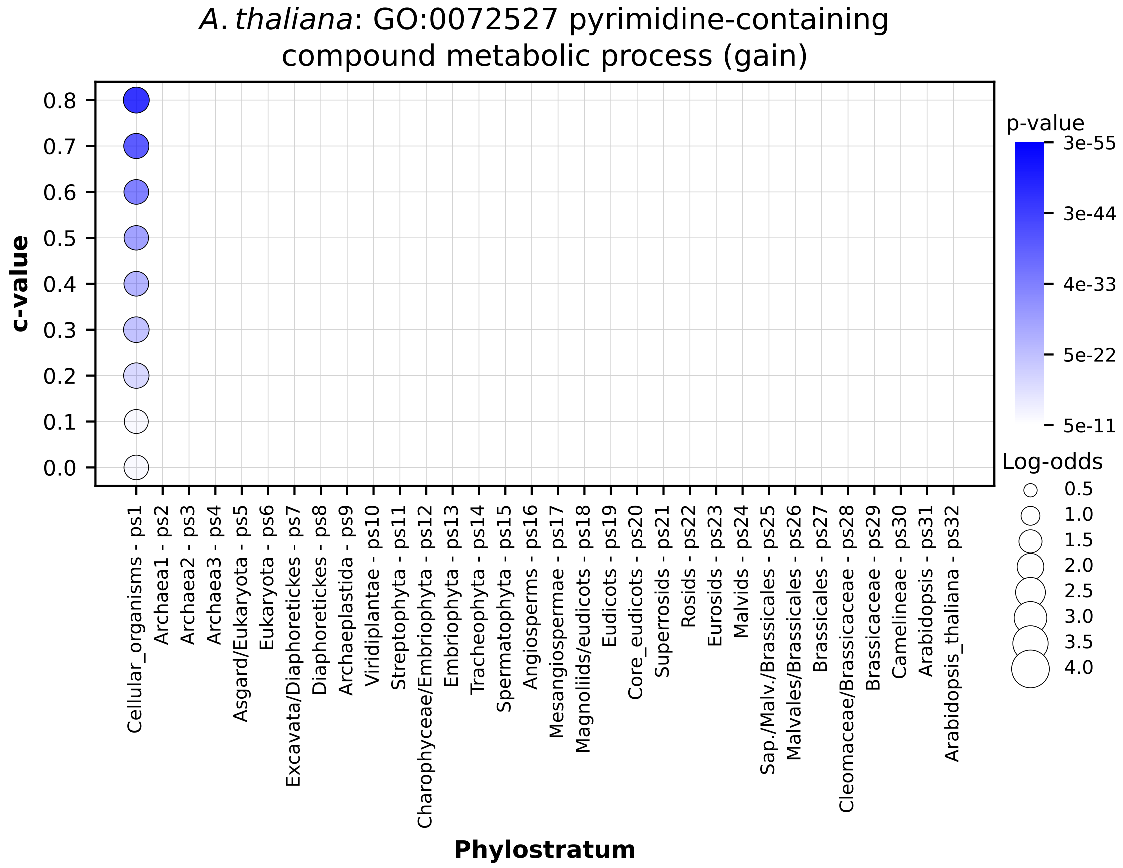
A. thaliana: GO:0072527 pyrimidine-containing compound metabolic process (gain)
c-value
0.0
0.1
0.2
0.3
0.4
0.5
0.6
0.7
0.8
p-value
3e-55
3e-44
4e-33
5e-22
5e-11
Log-odds
0.5
1.0
1.5
2.0
2.5
3.0
3.5
4.0
Phylostratum
Cellular_organisms - ps1
Archaea1 - ps2
Archaea2 - ps3
Archaea3 - ps4
Asgard/Eukaryota - ps5
Eukaryota - ps6
Excavata/Diaphoretickes - ps7
Diaphoretickes - ps8
Archaeplastida - ps9
Viridiplantae - ps10
Streptophyta - ps11
Charophyceae/Embriophyta - ps12
Embriophyta - ps13
Tracheophyta - ps14
Spermatophyta - ps15
Angiosperms - ps16
Mesangiospermae - ps17
Magnoliids/eudicots - ps18
Eudicots - ps19
Core_eudicots - ps20
Superrosids - ps21
Rosids - ps22
Eurosids - ps23
Malvids - ps24
Sap./Malv./Brassicales - ps25
Malvales/Brassicales - ps26
Brassicales - ps27
Cleomaceae/Brassicaceae - ps28
Brassicaceae - ps29
Camelineae - ps30
Arabidopsis - ps31
Arabidopsis_thaliana - ps32

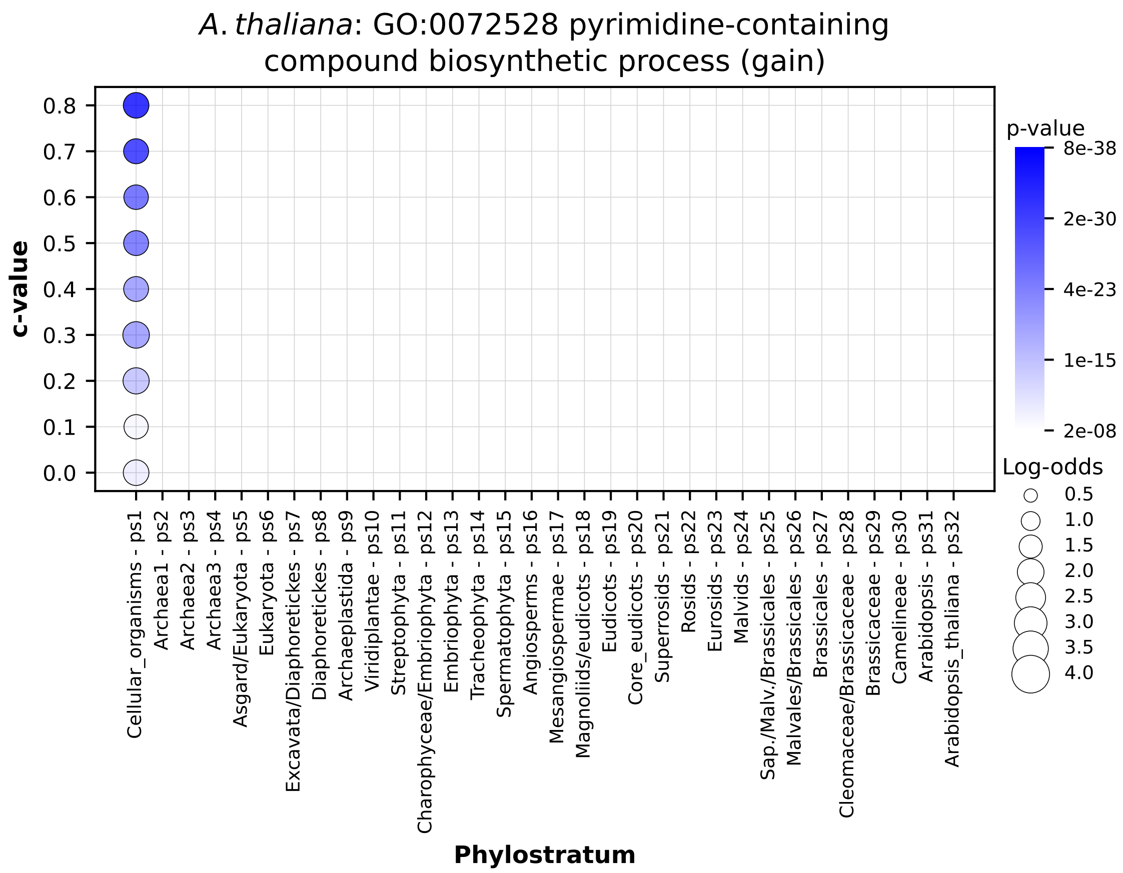

A. thaliana: GO:0072528 pyrimidine-containing compound biosynthetic process (gain)
c-value
0.8
0.7
0.6
0.5
0.4
0.3
0.2
0.1
0.0
p-value
8e-38
2e-30
4e-23
1e-15
2e-08
Log-odds
0.5
1.0
1.5
2.0
2.5
3.0
3.5
4.0
Cellular_organisms - ps1
Archaea1 - ps2
Archaea2 - ps3
Archaea3 - ps4
Asgard/Eukaryota - ps5
Eukaryota - ps6
Excavata/Diaphoretickes - ps7
Diaphoretickes - ps8
Archaeplastida - ps9
Viridiplantae - ps10
Streptophyta - ps11
Charophyceae/Embriophyta - ps12
Embriophyta - ps13
Tracheophyta - ps14
Spermatophyta - ps15
Angiosperms - ps16
Mesangiospermae - ps17
Magnoliids/eudicots - ps18
Eudicots - ps19
Core_eudicots - ps20
Superrosids - ps21
Rosids - ps22
Eurosids - ps23
Malvids - ps24
Sap./Malv./Brassicales - ps25
Malvales/Brassicales - ps26
Brassicales - ps27
Cleomaceae/Brassicaceae - ps28
Brassicaceae - ps29
Camelineae - ps30
Arabidopsis - ps31
Arabidopsis_thaliana - ps32
Phylostratum

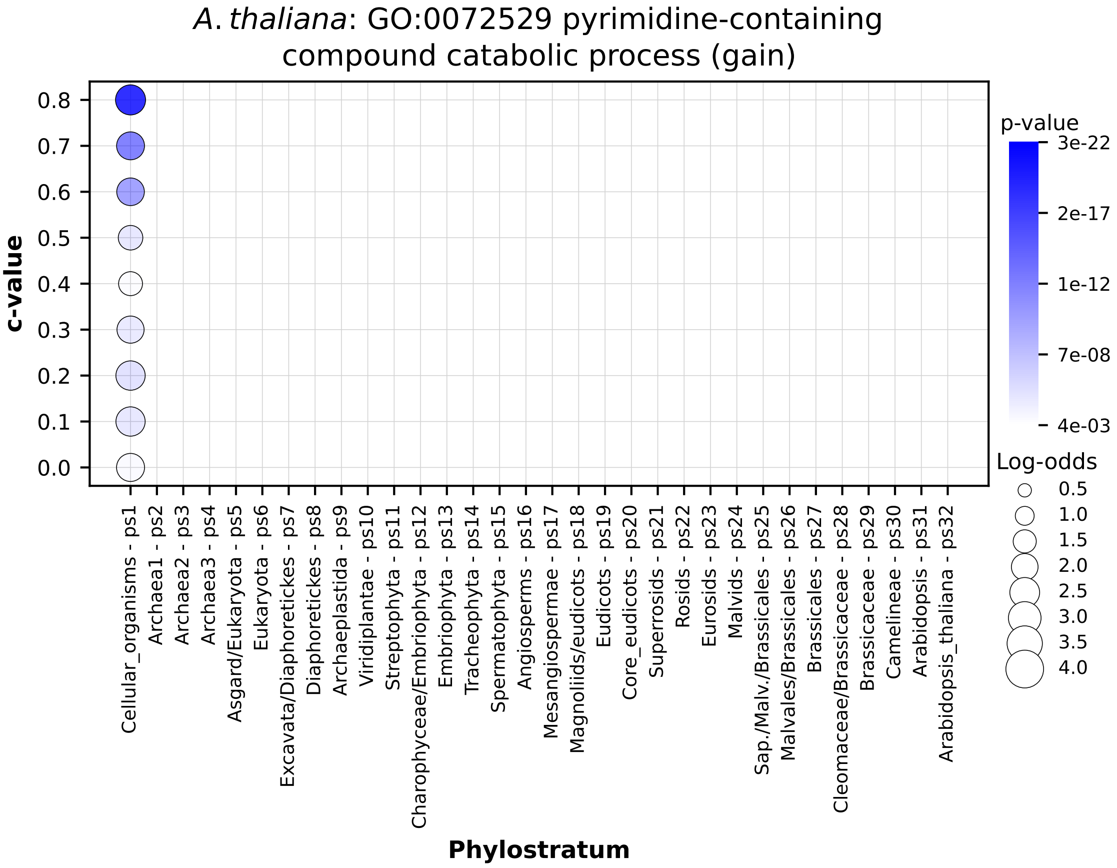

A. thaliana: GO:0072529 pyrimidine-containing compound catabolic process (gain)
c-value
0.8
0.7
0.6
0.5
0.4
0.3
0.2
0.1
0.0
p-value
3e-22
2e-17
1e-12
7e-08
4e-03
Log-odds
0.5
1.0
1.5
2.0
2.5
3.0
3.5
4.0
Cellular_organisms - ps1
Archaea1 - ps2
Archaea2 - ps3
Archaea3 - ps4
Asgard/Eukaryota - ps5
Eukaryota - ps6
Excavata/Diaphoretickes - ps7
Diaphoretickes - ps8
Archaeplastida - ps9
Viridiplantae - ps10
Streptophyta - ps11
Charophyceae/Embriophyta - ps12
Embriophyta - ps13
Tracheophyta - ps14
Spermatophyta - ps15
Angiosperms - ps16
Mesangiospermae - ps17
Magnoliids/eudicots - ps18
Eudicots - ps19
Core_eudicots - ps20
Superrosids - ps21
Rosids - ps22
Eurosids - ps23
Malvids - ps24
Sap./Malv./Brassicales - ps25
Malvales/Brassicales - ps26
Brassicales - ps27
Cleomaceae/Brassicaceae - ps28
Brassicaceae - ps29
Camelineae - ps30
Arabidopsis - ps31
Arabidopsis_thaliana - ps32
Phylostratum

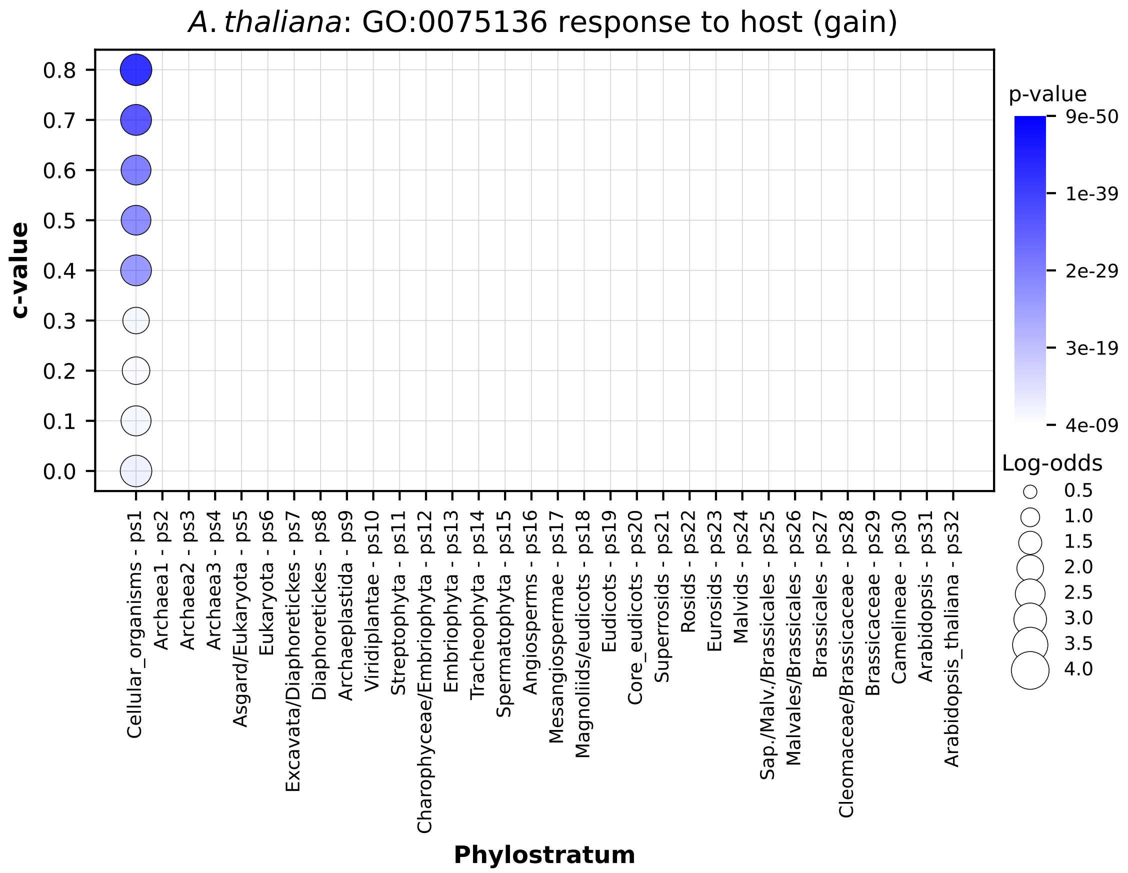
A. thaliana: GO:0075136 response to host (gain)
c-value
0.8
0.7
0.6
0.5
0.4
0.3
0.2
0.1
0.0
p-value
9e-50
1e-39
2e-29
3e-19
4e-09
Log-odds
0.5
1.0
1.5
2.0
2.5
3.0
3.5
4.0
Cellular_organisms - ps1
Archaea1 - ps2
Archaea2 - ps3
Archaea3 - ps4
Asgard/Eukaryota - ps5
Eukaryota - ps6
Excavata/Diaphoretickes - ps7
Diaphoretickes - ps8
Archaeplastida - ps9
Viridiplantae - ps10
Streptophyta - ps11
Charophyceae/Embriophyta - ps12
Embriophyta - ps13
Tracheophyta - ps14
Spermatophyta - ps15
Angiosperms - ps16
Mesangiospermae - ps17
Magnoliids/eudicots - ps18
Eudicots - ps19
Core_eudicots - ps20
Superrosids - ps21
Rosids - ps22
Eurosids - ps23
Malvids - ps24
Sap./Malv./Brassicales - ps25
Malvales/Brassicales - ps26
Brassicales - ps27
Cleomaceae/Brassicaceae - ps28
Brassicaceae - ps29
Camelineae - ps30
Arabidopsis - ps31
Arabidopsis_thaliana - ps32
Phylostratum

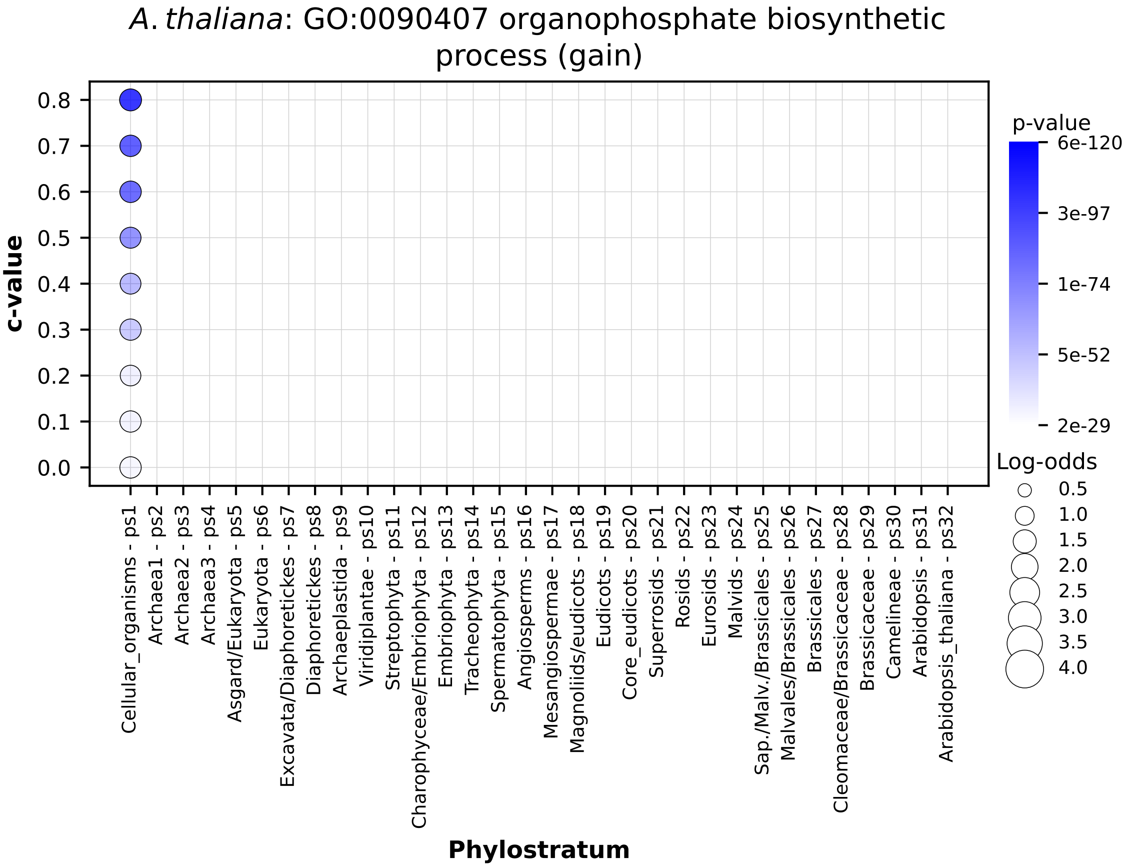
A. thaliana: GO:0090407 organophosphate biosynthetic process (gain)
c-value
0.8
0.7
0.6
0.5
0.4
0.3
0.2
0.1
0.0
p-value
6e-120
3e-97
1e-74
5e-52
2e-29
Log-odds
0.5
1.0
1.5
2.0
2.5
3.0
3.5
4.0
Cellular_organisms - ps1
Archaea1 - ps2
Archaea2 - ps3
Archaea3 - ps4
Asgard/Eukaryota - ps5
Eukaryota - ps6
Excavata/Diaphoretickes - ps7
Diaphoretickes - ps8
Archaeplastida - ps9
Viridiplantae - ps10
Streptophyta - ps11
Charophyceae/Embriophyta - ps12
Embriophyta - ps13
Tracheophyta - ps14
Spermatophyta - ps15
Angiosperms - ps16
Mesangiospermae - ps17
Magnoliids/eudicots - ps18
Eudicots - ps19
Core_eudicots - ps20
Superrosids - ps21
Rosids - ps22
Eurosids - ps23
Malvids - ps24
Sap./Malv./Brassicales - ps25
Malvales/Brassicales - ps26
Brassicales - ps27
Cleomaceae/Brassicaceae - ps28
Brassicaceae - ps29
Camelineae - ps30
Arabidopsis - ps31
Arabidopsis_thaliana - ps32
Phylostratum

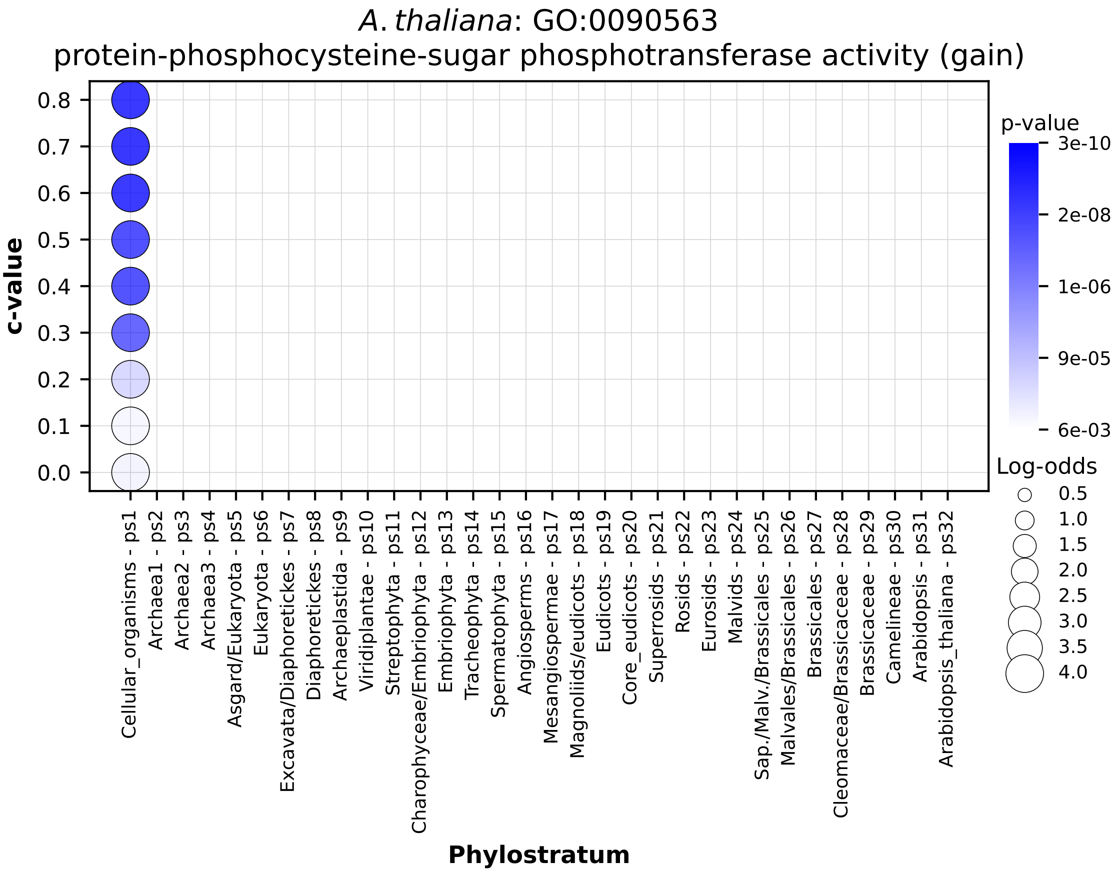

A. thaliana: GO:0090563
protein-phosphocysteine-sugar phosphotransferase activity (gain)
c-value
0.8
0.7
0.6
0.5
0.4
0.3
0.2
0.1
0.0
p-value
3e-10
2e-08
1e-06
9e-05
6e-03
Log-odds
0.5
1.0
1.5
2.0
2.5
3.0
3.5
4.0
Cellular_organisms - ps1
Archaea1 - ps2
Archaea2 - ps3
Archaea3 - ps4
Asgard/Eukaryota - ps5
Eukaryota - ps6
Excavata/Diaphoretickes - ps7
Diaphoretickes - ps8
Archaeplastida - ps9
Viridiplantae - ps10
Streptophyta - ps11
Charophyceae/Embriophyta - ps12
Embriophyta - ps13
Tracheophyta - ps14
Spermatophyta - ps15
Angiosperms - ps16
Mesangiospermae - ps17
Magnoliids/eudicots - ps18
Eudicots - ps19
Core_eudicots - ps20
Superrosids - ps21
Rosids - ps22
Eurosids - ps23
Malvids - ps24
Sap./Malv./Brassicales - ps25
Malvales/Brassicales - ps26
Brassicales - ps27
Cleomaceae/Brassicaceae - ps28
Brassicaceae - ps29
Camelineae - ps30
Arabidopsis - ps31
Arabidopsis_thaliana - ps32
Phylostratum

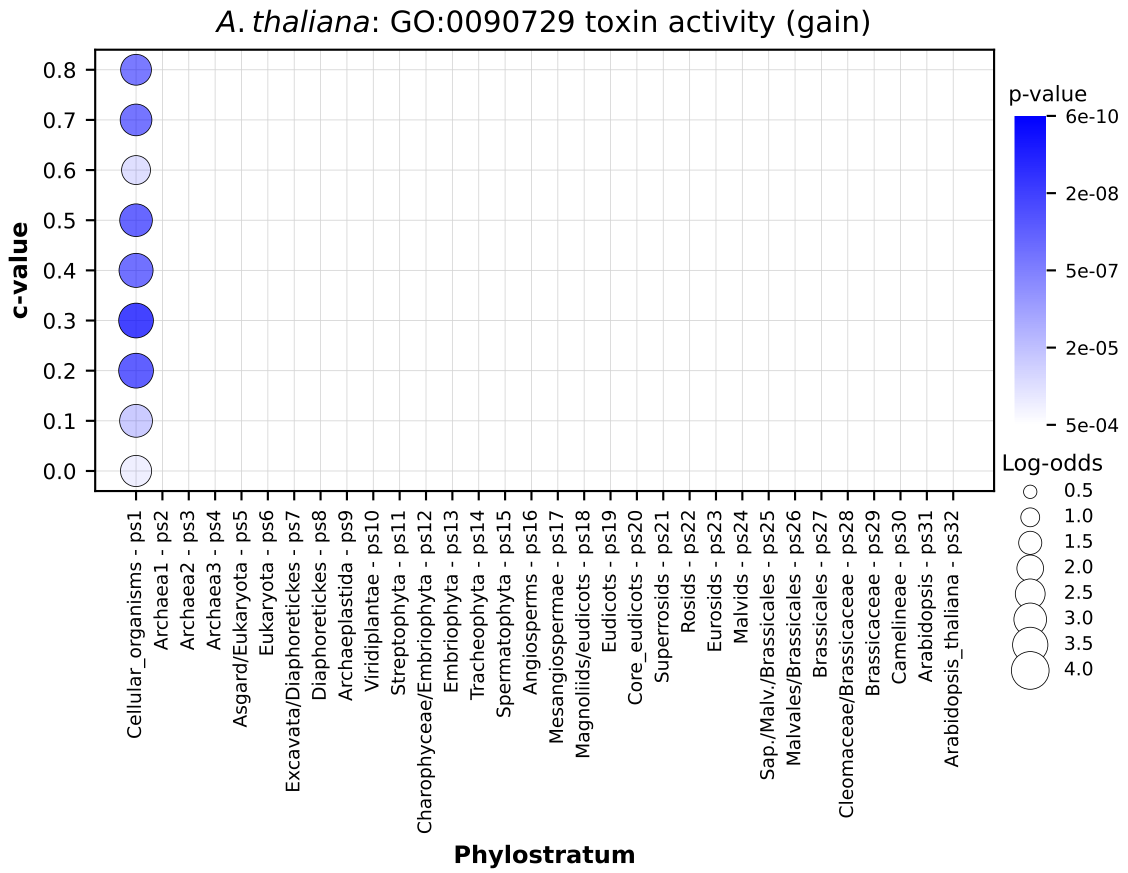

A. thaliana: GO:0090729 toxin activity (gain)
c-value
0.0
0.1
0.2
0.3
0.4
0.5
0.6
0.7
0.8
Phylostratum
Cellular_organisms - ps1
Archaea1 - ps2
Archaea2 - ps3
Archaea3 - ps4
Asgard/Eukaryota - ps5
Eukaryota - ps6
Excavata/Diaphoretickes - ps7
Diaphoretickes - ps8
Archaeplastida - ps9
Viridiplantae - ps10
Streptophyta - ps11
Charophyceae/Embriophyta - ps12
Embriophyta - ps13
Tracheophyta - ps14
Spermatophyta - ps15
Angiosperms - ps16
Mesangiospermae - ps17
Magnoliids/eudicots - ps18
Eudicots - ps19
Core_eudicots - ps20
Superrosids - ps21
Rosids - ps22
Eurosids - ps23
Malvids - ps24
Sap./Malv./Brassicales - ps25
Malvales/Brassicales - ps26
Brassicales - ps27
Cleomaceae/Brassicaceae - ps28
Brassicaceae - ps29
Camelineae - ps30
Arabidopsis - ps31
Arabidopsis_thaliana - ps32
p-value
6e-10
2e-08
5e-07
2e-05
5e-04
Log-odds
0.5
1.0
1.5
2.0
2.5
3.0
3.5
4.0

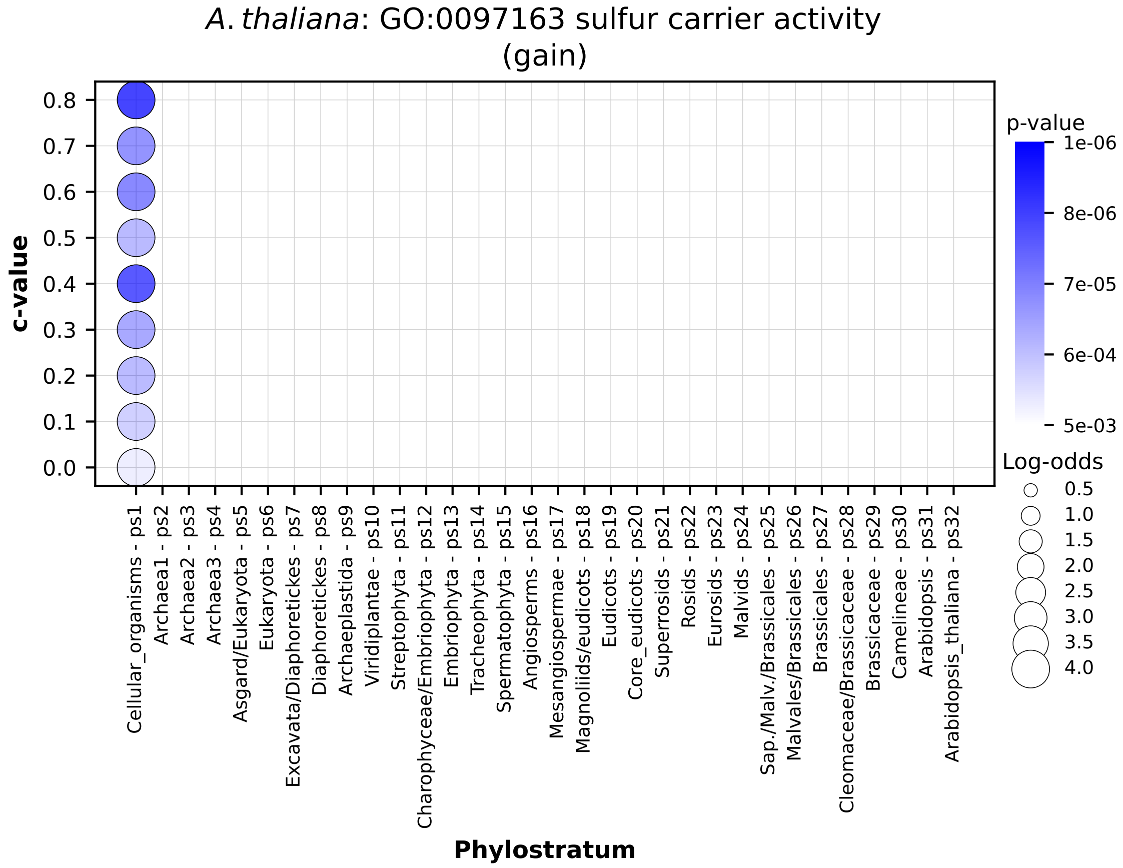
A. thaliana: GO:0097163 sulfur carrier activity
(gain)
c-value
0.8
0.7
0.6
0.5
0.4
0.3
0.2
0.1
0.0
p-value
1e-06
8e-06
7e-05
6e-04
5e-03
Log-odds
0.5
1.0
1.5
2.0
2.5
3.0
3.5
4.0
Cellular_organisms - ps1
Archaea1 - ps2
Archaea2 - ps3
Archaea3 - ps4
Asgard/Eukaryota - ps5
Eukaryota - ps6
Excavata/Diaphoretickes - ps7
Diaphoretickes - ps8
Archaeplastida - ps9
Viridiplantae - ps10
Streptophyta - ps11
Charophyceae/Embriophyta - ps12
Embriophyta - ps13
Tracheophyta - ps14
Spermatophyta - ps15
Angiosperms - ps16
Mesangiospermae - ps17
Magnoliids/eudicots - ps18
Eudicots - ps19
Core_eudicots - ps20
Superrosids - ps21
Rosids - ps22
Eurosids - ps23
Malvids - ps24
Sap./Malv./Brassicales - ps25
Malvales/Brassicales - ps26
Brassicales - ps27
Cleomaceae/Brassicaceae - ps28
Brassicaceae - ps29
Camelineae - ps30
Arabidopsis - ps31
Arabidopsis_thaliana - ps32
Phylostratum

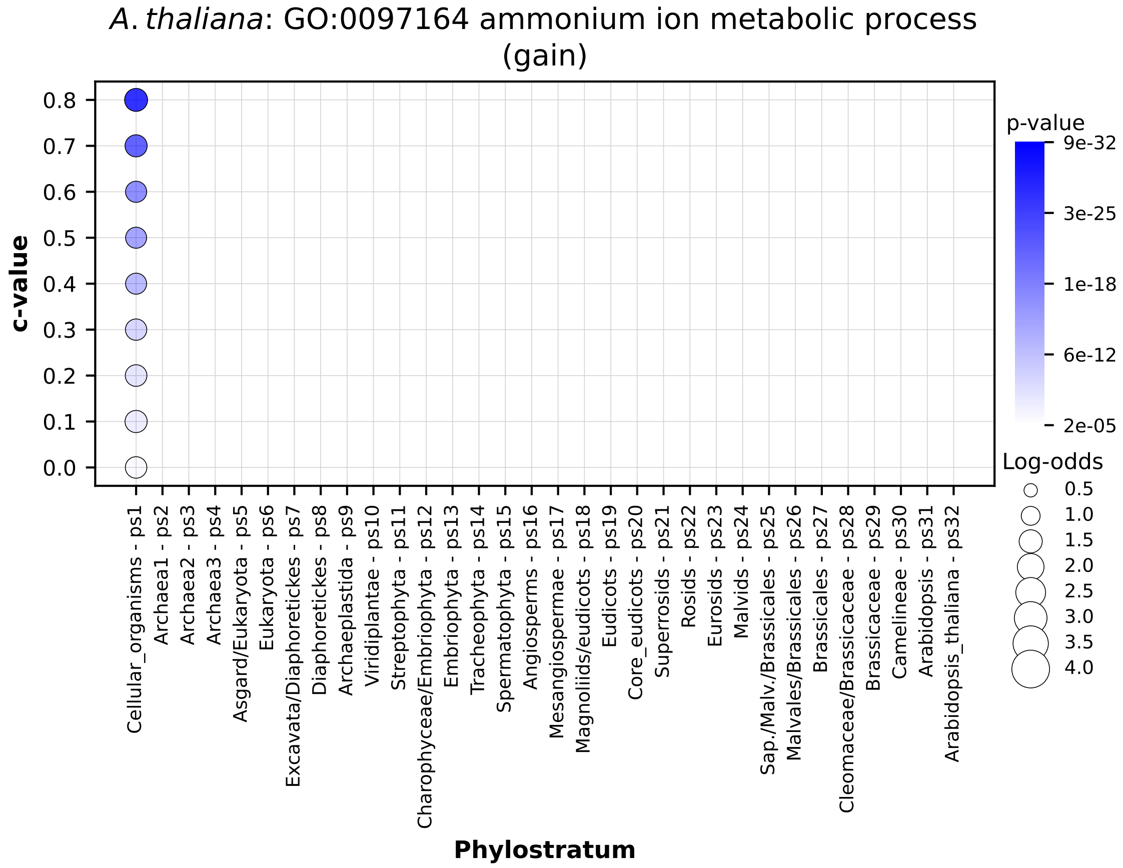

A. thaliana: GO:0097164 ammonium ion metabolic process (gain)
c-value
0.8
0.7
0.6
0.5
0.4
0.3
0.2
0.1
0.0
p-value
9e-32
3e-25
1e-18
6e-12
2e-05
Log-odds
0.5
1.0
1.5
2.0
2.5
3.0
3.5
4.0
Cellular_organisms - ps1
Archaea1 - ps2
Archaea2 - ps3
Archaea3 - ps4
Asgard/Eukaryota - ps5
Eukaryota - ps6
Excavata/Diaphoretickes - ps7
Diaphoretickes - ps8
Archaeplastida - ps9
Viridiplantae - ps10
Streptophyta - ps11
Charophyceae/Embriophyta - ps12
Embriophyta - ps13
Tracheophyta - ps14
Spermatophyta - ps15
Angiosperms - ps16
Mesangiospermae - ps17
Magnoliids/eudicots - ps18
Eudicots - ps19
Core_eudicots - ps20
Superrosids - ps21
Rosids - ps22
Eurosids - ps23
Malvids - ps24
Sap./Malv./Brassicales - ps25
Malvales/Brassicales - ps26
Brassicales - ps27
Cleomaceae/Brassicaceae - ps28
Brassicaceae - ps29
Camelineae - ps30
Arabidopsis - ps31
Arabidopsis_thaliana - ps32
Phylostratum

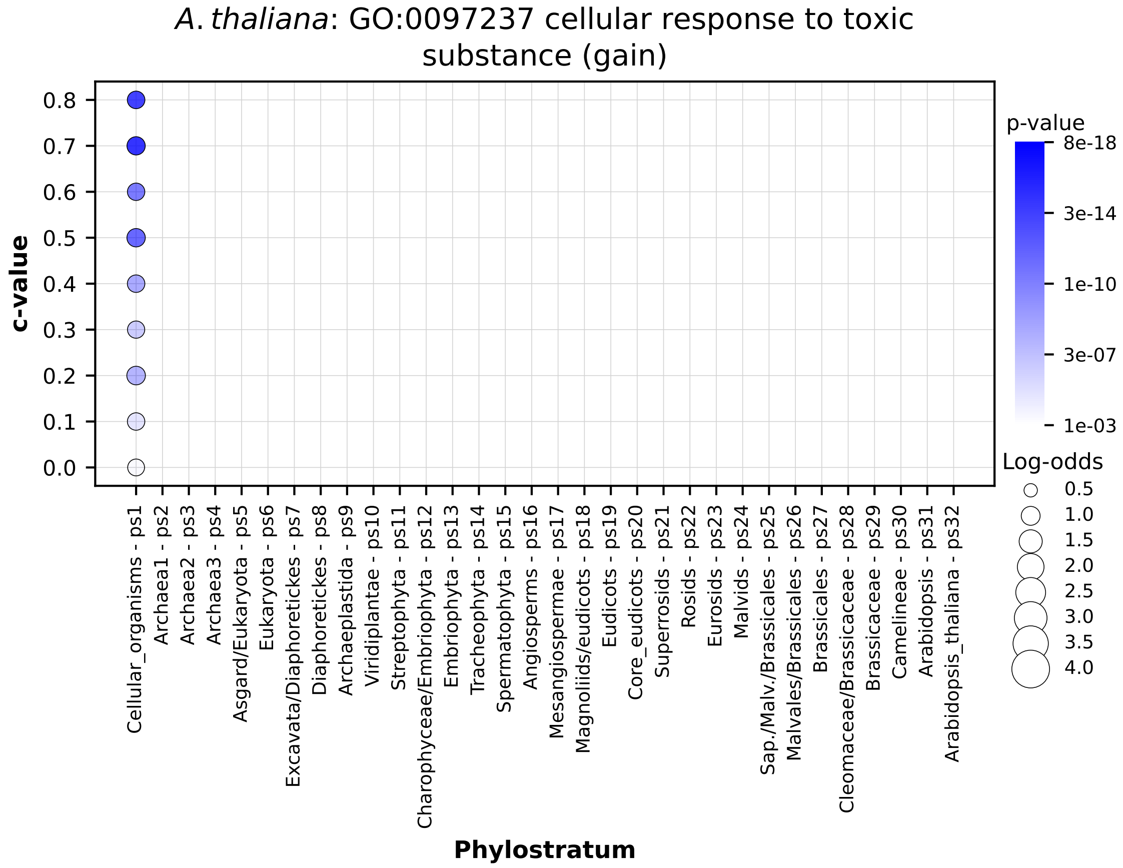

A. thaliana: GO:0097237 cellular response to toxic substance (gain)
c-value
0.0
0.1
0.2
0.3
0.4
0.5
0.6
0.7
0.8
Phylostratum
Cellular_organisms - ps1
Archaea1 - ps2
Archaea2 - ps3
Archaea3 - ps4
Asgard/Eukaryota - ps5
Eukaryota - ps6
Excavata/Diaphoretickes - ps7
Diaphoretickes - ps8
Archaeplastida - ps9
Viridiplantae - ps10
Streptophyta - ps11
Charophyceae/Embriophyta - ps12
Embriophyta - ps13
Tracheophyta - ps14
Spermatophyta - ps15
Angiosperms - ps16
Mesangiospermae - ps17
Magnoliids/eudicots - ps18
Eudicots - ps19
Core_eudicots - ps20
Superrosids - ps21
Rosids - ps22
Eurosids - ps23
Malvids - ps24
Sap./Malv./Brassicales - ps25
Malvales/Brassicales - ps26
Brassicales - ps27
Cleomaceae/Brassicaceae - ps28
Brassicaceae - ps29
Camelineae - ps30
Arabidopsis - ps31
Arabidopsis_thaliana - ps32
p-value
8e-18
3e-14
1e-10
3e-07
1e-03
Log-odds
0.5
1.0
1.5
2.0
2.5
3.0
3.5
4.0

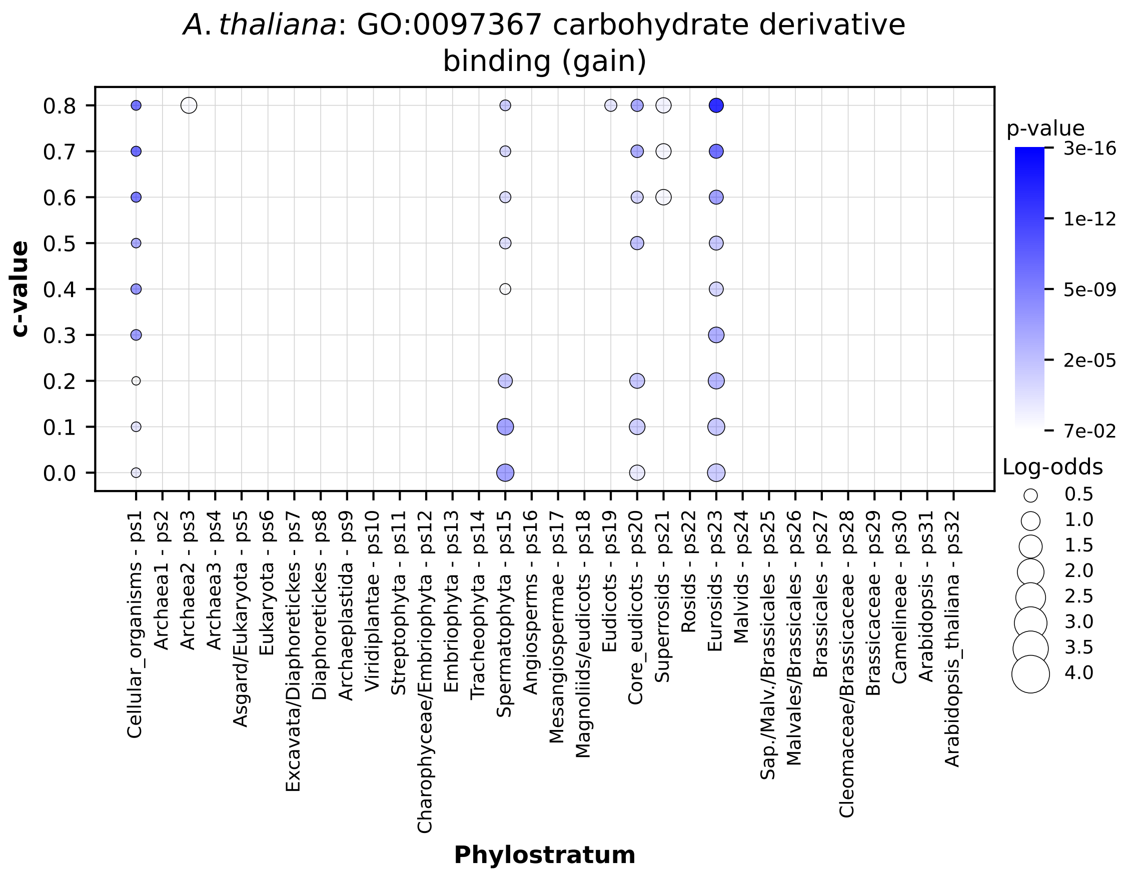
A. thaliana: GO:0097367 carbohydrate derivative binding (gain)
c-value
0.8
0.7
0.6
0.5
0.4
0.3
0.2
0.1
0.0
Phylostratum
Cellular_organisms - ps1
Archaea1 - ps2
Archaea2 - ps3
Archaea3 - ps4
Asgard/Eukaryota - ps5
Eukaryota - ps6
Excavata/Diaphoretickes - ps7
Diaphoretickes - ps8
Archaeplastida - ps9
Viridiplantae - ps10
Streptophyta - ps11
Charophyceae/Embriophyta - ps12
Embriophyta - ps13
Tracheophyta - ps14
Spermatophyta - ps15
Angiosperms - ps16
Mesangiospermae - ps17
Magnoliids/eudicots - ps18
Eudicots - ps19
Core_eudicots - ps20
Superrosids - ps21
Rosids - ps22
Eurosids - ps23
Malvids - ps24
Sap./Malv./Brassicales - ps25
Malvales/Brassicales - ps26
Brassicales - ps27
Cleomaceae/Brassicaceae - ps28
Brassicaceae - ps29
Camelineae - ps30
Arabidopsis - ps31
Arabidopsis_thaliana - ps32
p-value
3e-16
1e-12
5e-09
2e-05
7e-02
Log-odds
0.5
1.0
1.5
2.0
2.5
3.0
3.5
4.0

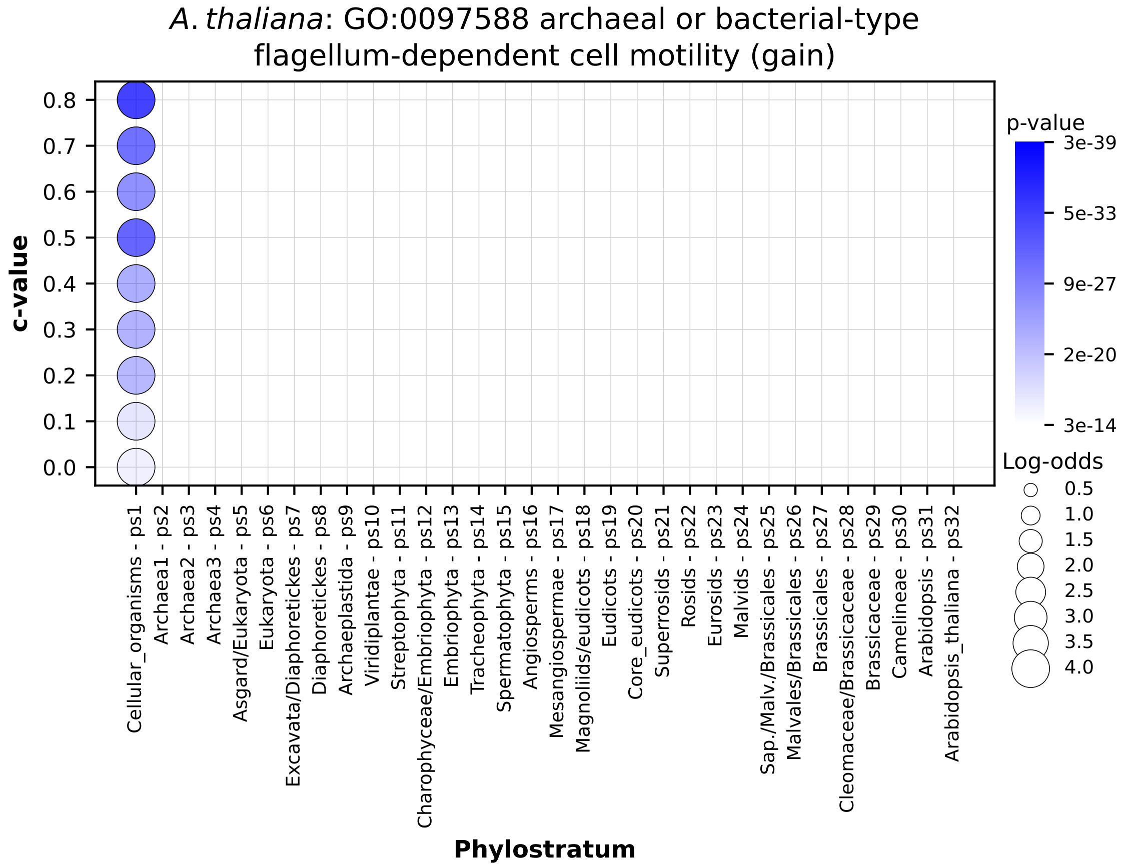
A. thaliana: GO:0097588 archaeal or bacterial-type flagellum-dependent cell motility (gain)
c-value
0.8
0.7
0.6
0.5
0.4
0.3
0.2
0.1
0.0
p-value
3e-39
5e-33
9e-27
2e-20
3e-14
Log-odds
0.5
1.0
1.5
2.0
2.5
3.0
3.5
4.0
Cellular_organisms - ps1
Archaea1 - ps2
Archaea2 - ps3
Archaea3 - ps4
Asgard/Eukaryota - ps5
Eukaryota - ps6
Excavata/Diaphoretickes - ps7
Diaphoretickes - ps8
Archaeplastida - ps9
Viridiplantae - ps10
Streptophyta - ps11
Charophyceae/Embriophyta - ps12
Embriophyta - ps13
Tracheophyta - ps14
Spermatophyta - ps15
Angiosperms - ps16
Mesangiospermae - ps17
Magnoliids/eudicots - ps18
Eudicots - ps19
Core_eudicots - ps20
Superrosids - ps21
Rosids - ps22
Eurosids - ps23
Malvids - ps24
Sap./Malv./Brassicales - ps25
Malvales/Brassicales - ps26
Brassicales - ps27
Cleomaceae/Brassicaceae - ps28
Brassicaceae - ps29
Camelineae - ps30
Arabidopsis - ps31
Arabidopsis_thaliana - ps32
Phylostratum

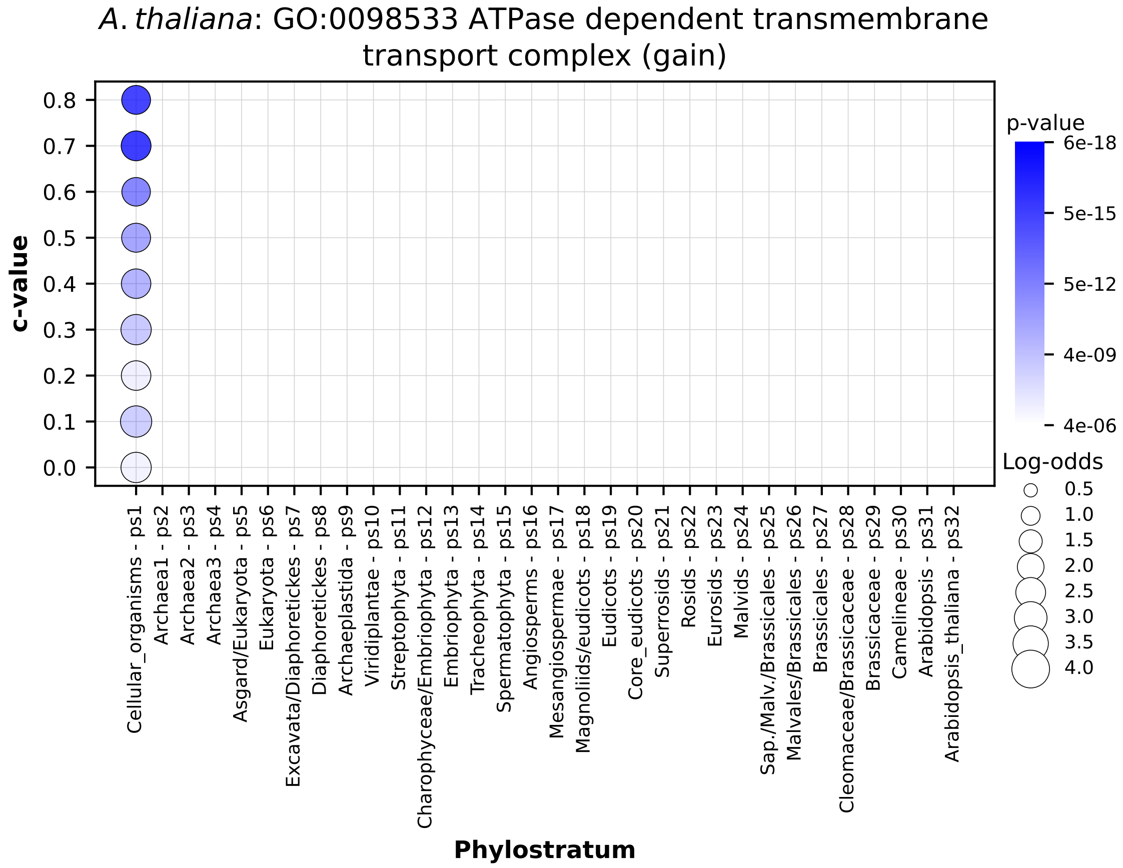

A. thaliana: GO:0098533 ATPase dependent transmembrane transport complex (gain)
c-value
0.8
0.7
0.6
0.5
0.4
0.3
0.2
0.1
0.0
p-value
6e-18
5e-15
5e-12
4e-09
4e-06
Log-odds
0.5
1.0
1.5
2.0
2.5
3.0
3.5
4.0
Cellular_organisms - ps1
Archaea1 - ps2
Archaea2 - ps3
Archaea3 - ps4
Asgard/Eukaryota - ps5
Eukaryota - ps6
Excavata/Diaphoretickes - ps7
Diaphoretickes - ps8
Archaeplastida - ps9
Viridiplantae - ps10
Streptophyta - ps11
Charophyceae/Embriophyta - ps12
Embriophyta - ps13
Tracheophyta - ps14
Spermatophyta - ps15
Angiosperms - ps16
Mesangiospermae - ps17
Magnoliids/eudicots - ps18
Eudicots - ps19
Core_eudicots - ps20
Superrosids - ps21
Rosids - ps22
Eurosids - ps23
Malvids - ps24
Sap./Malv./Brassicales - ps25
Malvales/Brassicales - ps26
Brassicales - ps27
Cleomaceae/Brassicaceae - ps28
Brassicaceae - ps29
Camelineae - ps30
Arabidopsis - ps31
Arabidopsis_thaliana - ps32
Phylostratum

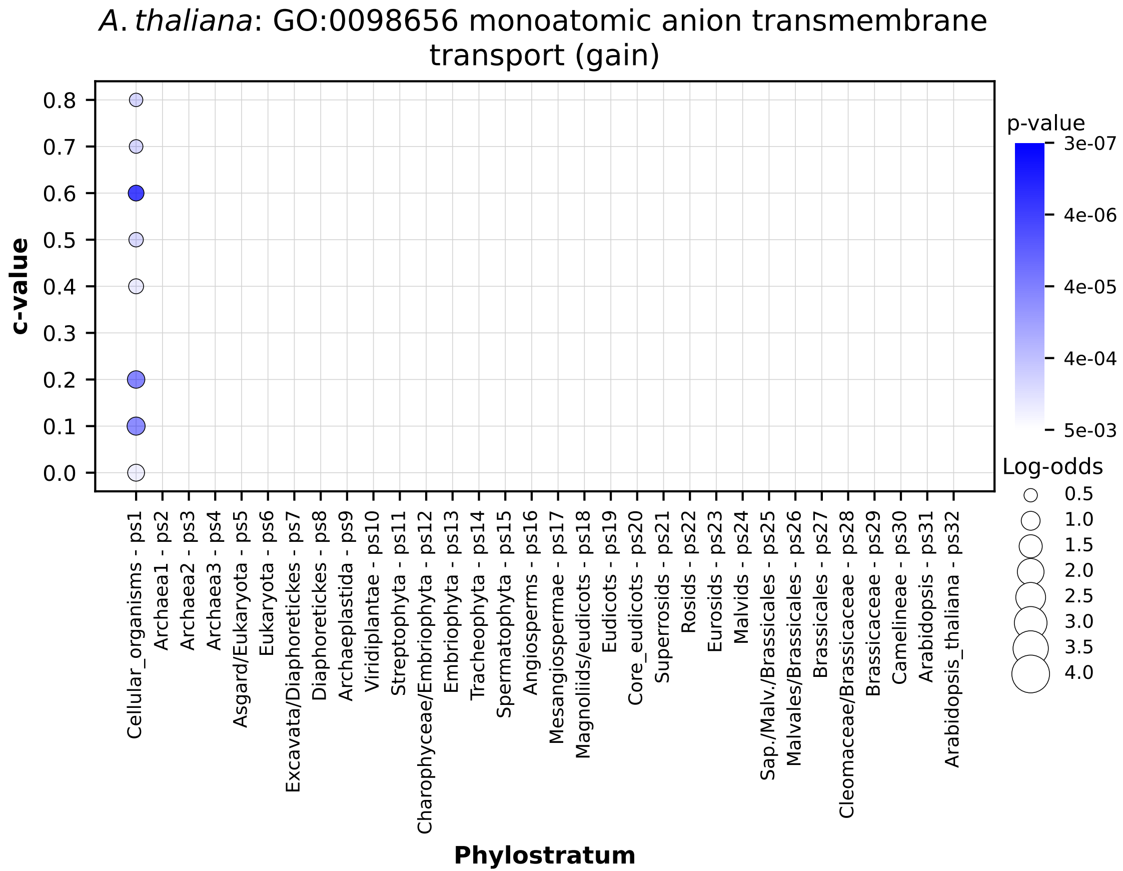

A. thaliana: GO:0098656 monoatomic anion transmembrane transport (gain)
c-value
0.8
0.7
0.6
0.5
0.4
0.3
0.2
0.1
0.0
p-value
3e-07
4e-06
4e-05
4e-04
5e-03
Log-odds
0.5
1.0
1.5
2.0
2.5
3.0
3.5
4.0
Cellular_organisms - ps1
Archaea1 - ps2
Archaea2 - ps3
Archaea3 - ps4
Asgard/Eukaryota - ps5
Eukaryota - ps6
Excavata/Diaphoretickes - ps7
Diaphoretickes - ps8
Archaeplastida - ps9
Viridiplantae - ps10
Streptophyta - ps11
Charophyceae/Embriophyta - ps12
Embriophyta - ps13
Tracheophyta - ps14
Spermatophyta - ps15
Angiosperms - ps16
Mesangiospermae - ps17
Magnoliids/eudicots - ps18
Eudicots - ps19
Core_eudicots - ps20
Superrosids - ps21
Rosids - ps22
Eurosids - ps23
Malvids - ps24
Sap./Malv./Brassicales - ps25
Malvales/Brassicales - ps26
Brassicales - ps27
Cleomaceae/Brassicaceae - ps28
Brassicaceae - ps29
Camelineae - ps30
Arabidopsis - ps31
Arabidopsis_thaliana - ps32
Phylostratum

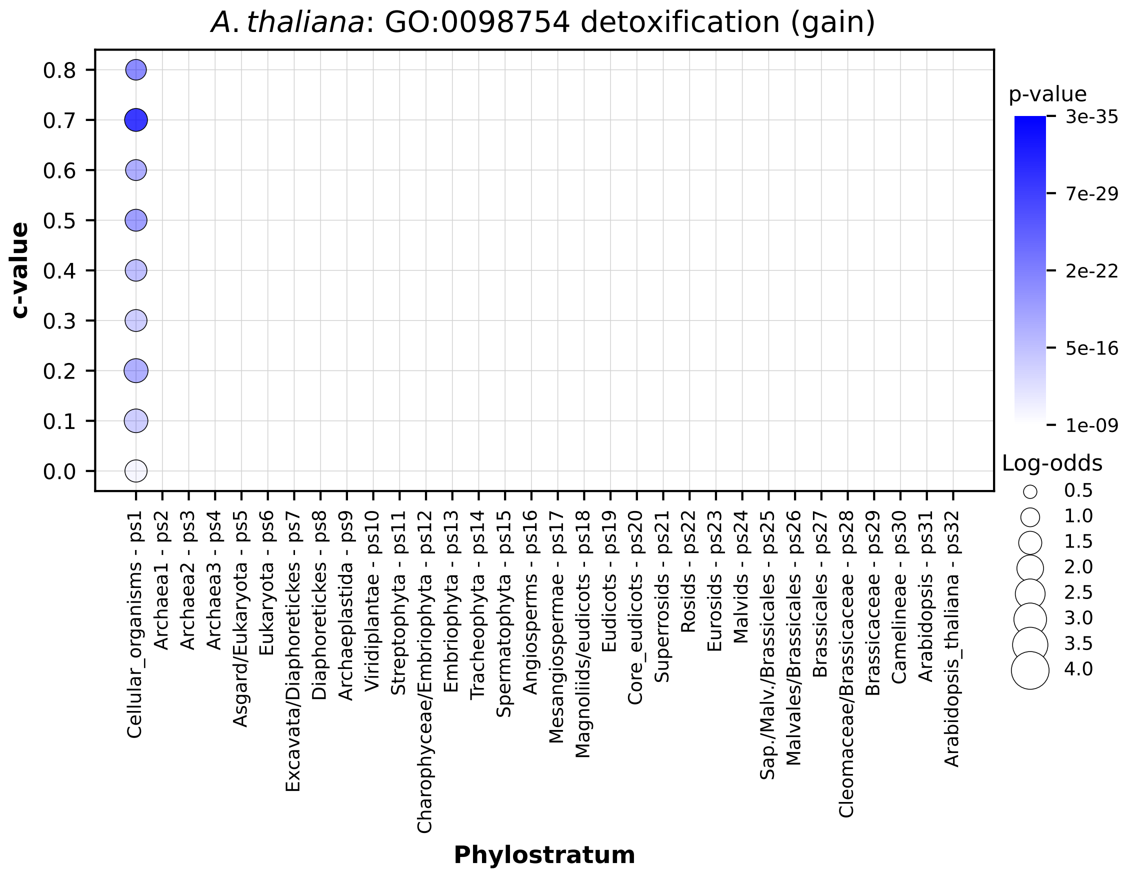

A. thaliana: GO:0098754 detoxification (gain)
c-value
0.8
0.7
0.6
0.5
0.4
0.3
0.2
0.1
0.0
p-value
3e-35
7e-29
2e-22
5e-16
1e-09
Log-odds
0.5
1.0
1.5
2.0
2.5
3.0
3.5
4.0
Cellular_organisms - ps1
Archaea1 - ps2
Archaea2 - ps3
Archaea3 - ps4
Asgard/Eukaryota - ps5
Eukaryota - ps6
Excavata/Diaphoretickes - ps7
Diaphoretickes - ps8
Archaeplastida - ps9
Viridiplantae - ps10
Streptophyta - ps11
Charophyceae/Embriophyta - ps12
Embriophyta - ps13
Tracheophyta - ps14
Spermatophyta - ps15
Angiosperms - ps16
Mesangiospermae - ps17
Magnoliids/eudicots - ps18
Eudicots - ps19
Core_eudicots - ps20
Superrosids - ps21
Rosids - ps22
Eurosids - ps23
Malvids - ps24
Sap./Malv./Brassicales - ps25
Malvales/Brassicales - ps26
Brassicales - ps27
Cleomaceae/Brassicaceae - ps28
Brassicaceae - ps29
Camelineae - ps30
Arabidopsis - ps31
Arabidopsis_thaliana - ps32
Phylostratum

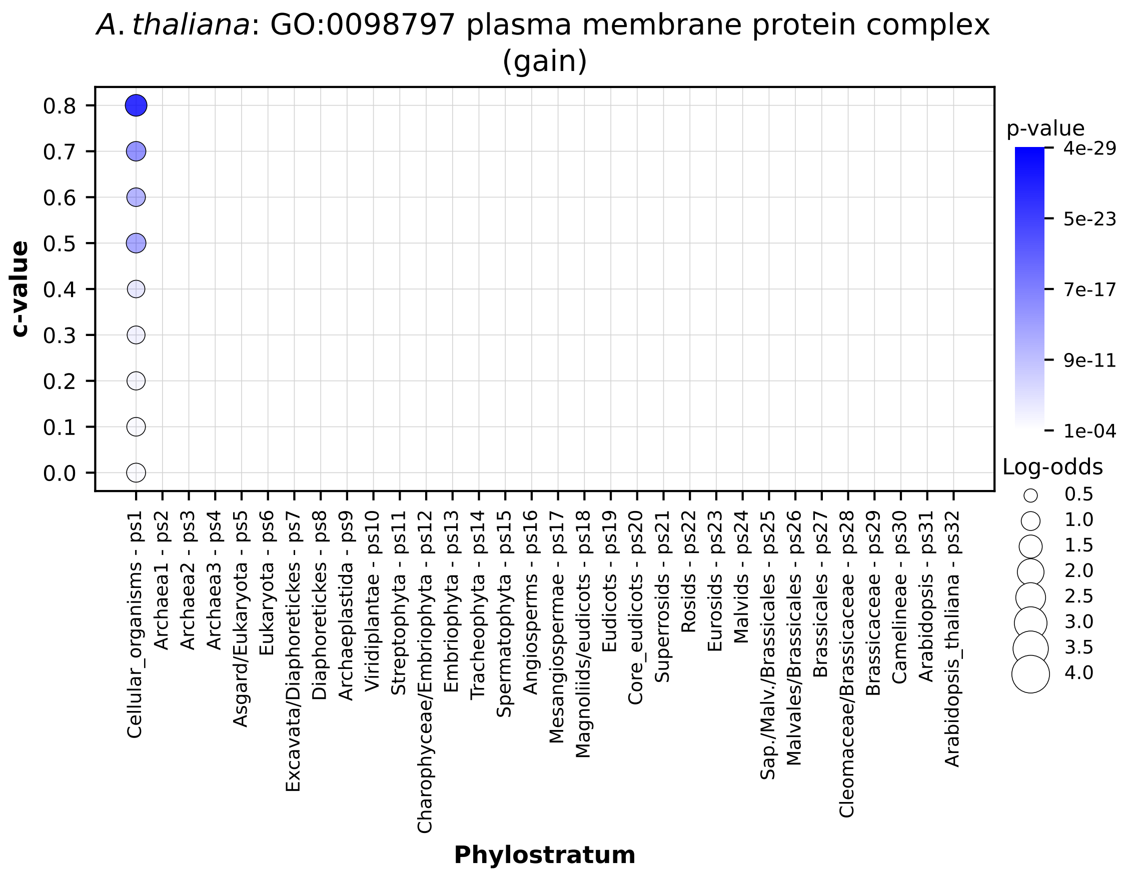

A. thaliana: GO:0098797 plasma membrane protein complex
(gain)
c-value
0.8
0.7
0.6
0.5
0.4
0.3
0.2
0.1
0.0
p-value
4e-29
5e-23
7e-17
9e-11
1e-04
Log-odds
0.5
1.0
1.5
2.0
2.5
3.0
3.5
4.0
Cellular_organisms - ps1
Archaea1 - ps2
Archaea2 - ps3
Archaea3 - ps4
Asgard/Eukaryota - ps5
Eukaryota - ps6
Excavata/Diaphoretickes - ps7
Diaphoretickes - ps8
Archaeplastida - ps9
Viridiplantae - ps10
Streptophyta - ps11
Charophyceae/Embriophyta - ps12
Embriophyta - ps13
Tracheophyta - ps14
Spermatophyta - ps15
Angiosperms - ps16
Mesangiospermae - ps17
Magnoliids/eudicots - ps18
Eudicots - ps19
Core_eudicots - ps20
Superrosids - ps21
Rosids - ps22
Eurosids - ps23
Malvids - ps24
Sap./Malv./Brassicales - ps25
Malvales/Brassicales - ps26
Brassicales - ps27
Cleomaceae/Brassicaceae - ps28
Brassicaceae - ps29
Camelineae - ps30
Arabidopsis - ps31
Arabidopsis_thaliana - ps32
Phylostratum

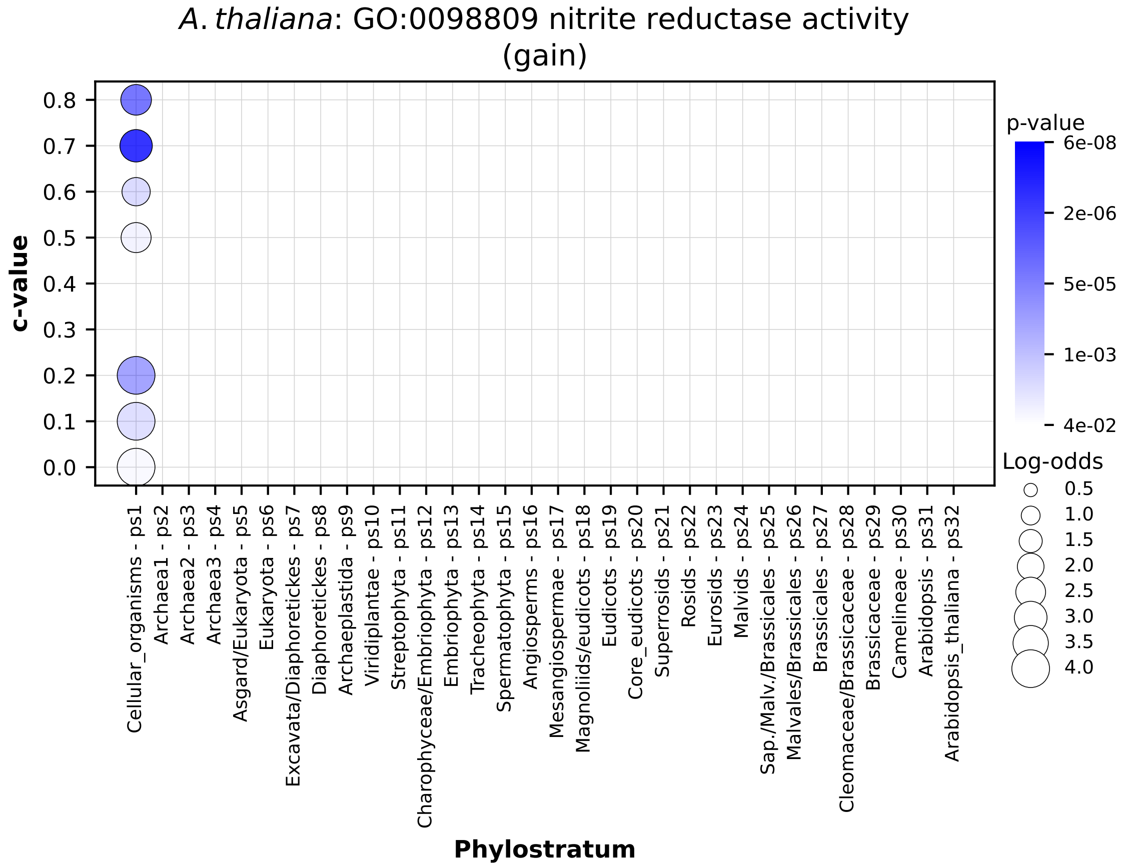

A. thaliana: GO:0098809 nitrite reductase activity
(gain)
c-value
0.8
0.7
0.6
0.5
0.4
0.3
0.2
0.1
0.0
p-value
6e-08
2e-06
5e-05
1e-03
4e-02
Log-odds
0.5
1.0
1.5
2.0
2.5
3.0
3.5
4.0
Cellular_organisms - ps1
Archaea1 - ps2
Archaea2 - ps3
Archaea3 - ps4
Asgard/Eukaryota - ps5
Eukaryota - ps6
Excavata/Diaphoretickes - ps7
Diaphoretickes - ps8
Archaeplastida - ps9
Viridiplantae - ps10
Streptophyta - ps11
Charophyceae/Embriophyta - ps12
Embriophyta - ps13
Tracheophyta - ps14
Spermatophyta - ps15
Angiosperms - ps16
Mesangiospermae - ps17
Magnoliids/eudicots - ps18
Eudicots - ps19
Core_eudicots - ps20
Superrosids - ps21
Rosids - ps22
Eurosids - ps23
Malvids - ps24
Sap./Malv./Brassicales - ps25
Malvales/Brassicales - ps26
Brassicales - ps27
Cleomaceae/Brassicaceae - ps28
Brassicaceae - ps29
Camelineae - ps30
Arabidopsis - ps31
Arabidopsis_thaliana - ps32
Phylostratum

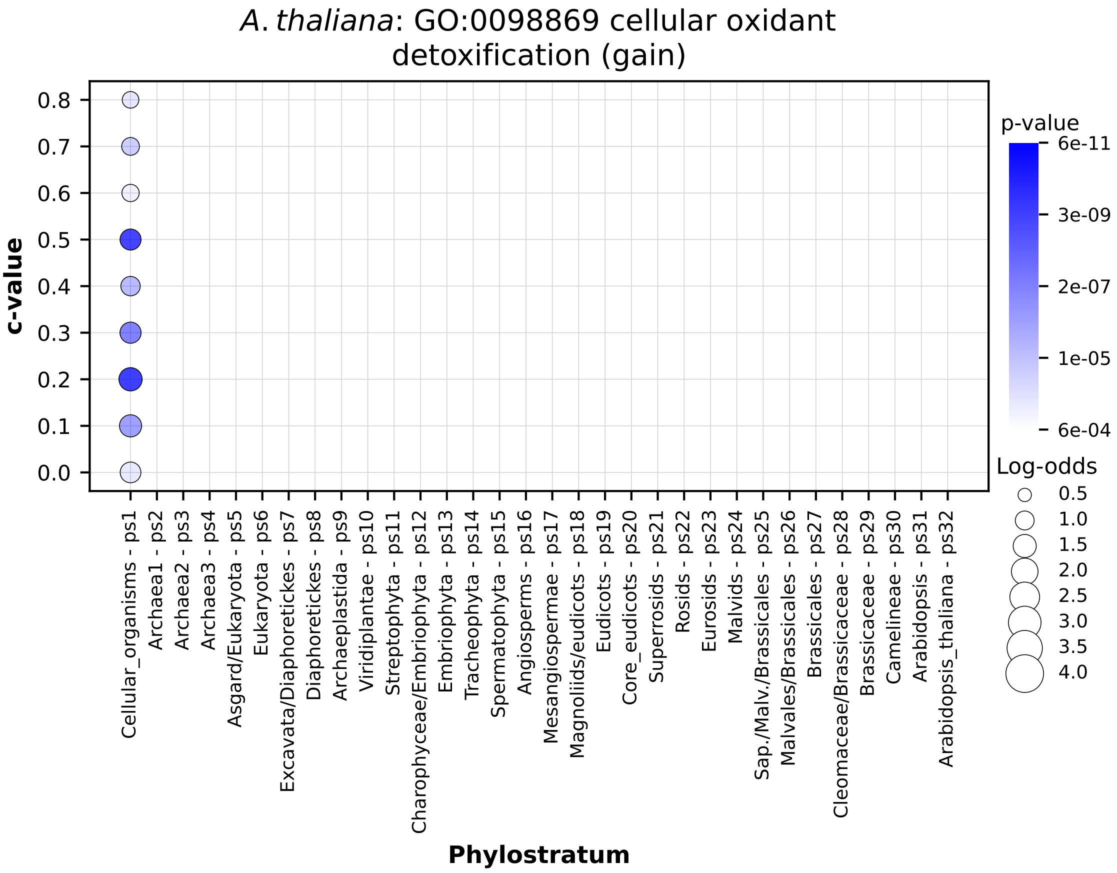

A. thaliana: GO:0098869 cellular oxidant detoxification (gain)
c-value
0.8
0.7
0.6
0.5
0.4
0.3
0.2
0.1
0.0
Phylostratum
Cellular_organisms - ps1
Archaea1 - ps2
Archaea2 - ps3
Archaea3 - ps4
Asgard/Eukaryota - ps5
Eukaryota - ps6
Excavata/Diaphoretickes - ps7
Diaphoretickes - ps8
Archaeplastida - ps9
Viridiplantae - ps10
Streptophyta - ps11
Charophyceae/Embriophyta - ps12
Embriophyta - ps13
Tracheophyta - ps14
Spermatophyta - ps15
Angiosperms - ps16
Mesangiospermae - ps17
Magnoliids/eudicots - ps18
Eudicots - ps19
Core_eudicots - ps20
Superrosids - ps21
Rosids - ps22
Eurosids - ps23
Malvids - ps24
Sap./Malv./Brassicales - ps25
Malvales/Brassicales - ps26
Brassicales - ps27
Cleomaceae/Brassicaceae - ps28
Brassicaceae - ps29
Camelineae - ps30
Arabidopsis - ps31
Arabidopsis_thaliana - ps32
p-value
6e-11
3e-09
2e-07
1e-05
6e-04
Log-odds
0.5
1.0
1.5
2.0
2.5
3.0
3.5
4.0

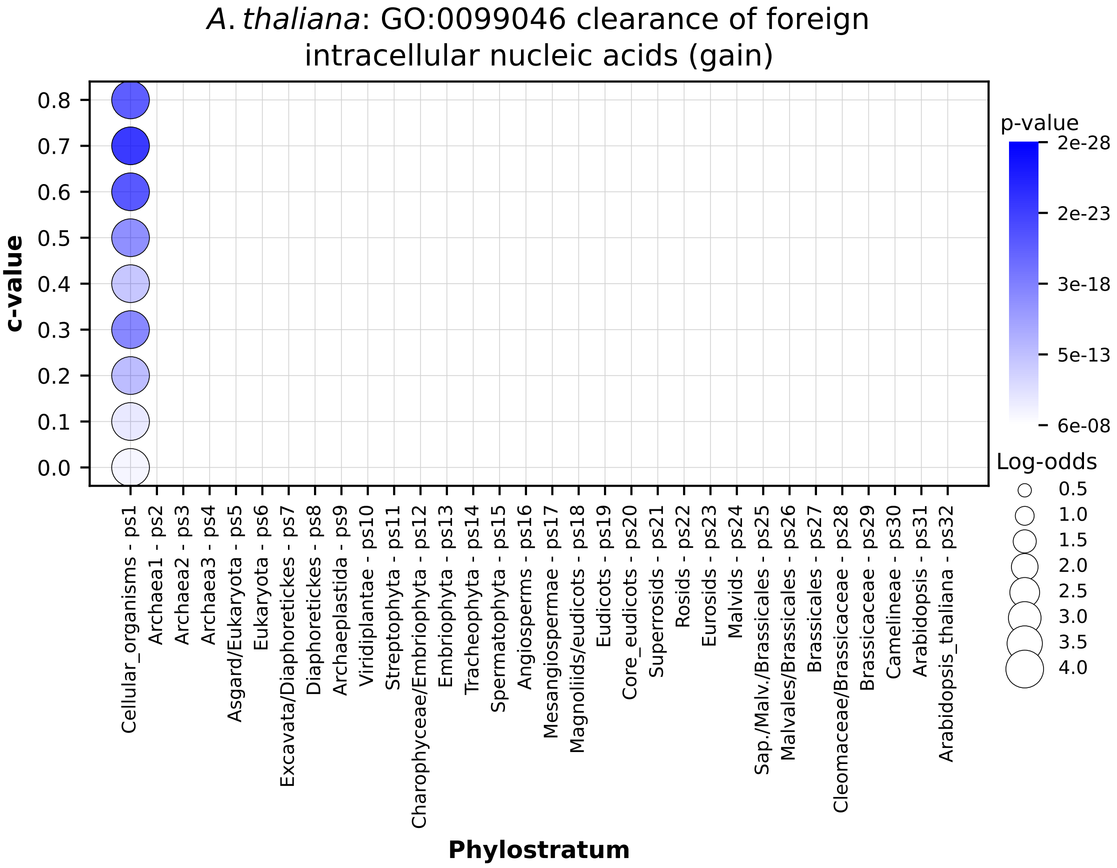
A. thaliana: GO:0099046 clearance of foreign intracellular nucleic acids (gain)
c-value
0.0
0.1
0.2
0.3
0.4
0.5
0.6
0.7
0.8
p-value
2e-28
2e-23
3e-18
5e-13
6e-08
Log-odds
0.5
1.0
1.5
2.0
2.5
3.0
3.5
4.0
Cellular_organisms - ps1
Archaea1 - ps2
Archaea2 - ps3
Archaea3 - ps4
Asgard/Eukaryota - ps5
Eukaryota - ps6
Excavata/Diaphoretickes - ps7
Diaphoretickes - ps8
Archaeplastida - ps9
Viridiplantae - ps10
Streptophyta - ps11
Charophyceae/Embriophyta - ps12
Embriophyta - ps13
Tracheophyta - ps14
Spermatophyta - ps15
Angiosperms - ps16
Mesangiospermae - ps17
Magnoliids/eudicots - ps18
Eudicots - ps19
Core_eudicots - ps20
Superrosids - ps21
Rosids - ps22
Eurosids - ps23
Malvids - ps24
Sap./Malv./Brassicales - ps25
Malvales/Brassicales - ps26
Brassicales - ps27
Cleomaceae/Brassicaceae - ps28
Brassicaceae - ps29
Camelineae - ps30
Arabidopsis - ps31
Arabidopsis_thaliana - ps32
Phylostratum

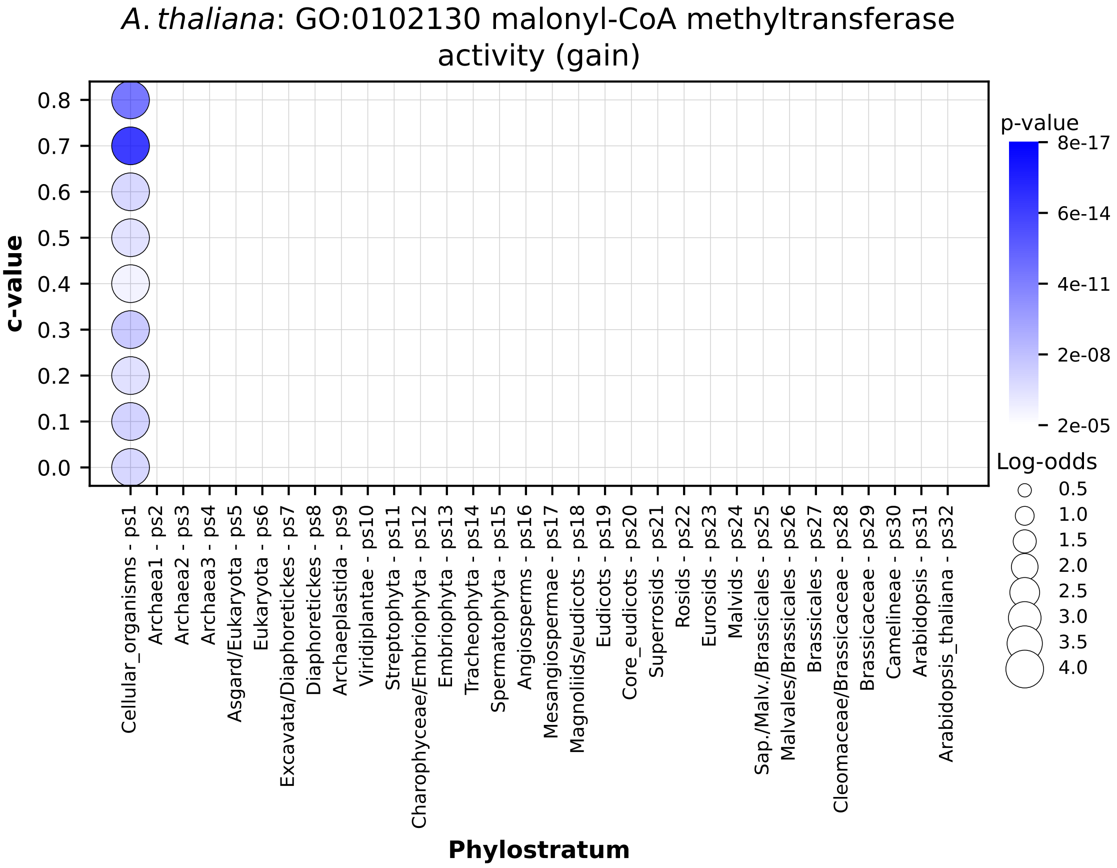

A. thaliana: GO:0102130 malonyl-CoA methyltransferase activity (gain)
c-value
0.0
0.1
0.2
0.3
0.4
0.5
0.6
0.7
0.8
Phylostratum
Cellular_organisms - ps1
Archaea1 - ps2
Archaea2 - ps3
Archaea3 - ps4
Asgard/Eukaryota - ps5
Eukaryota - ps6
Excavata/Diaphoretickes - ps7
Diaphoretickes - ps8
Archaeplastida - ps9
Viridiplantae - ps10
Streptophyta - ps11
Charophyceae/Embriophyta - ps12
Embriophyta - ps13
Tracheophyta - ps14
Spermatophyta - ps15
Angiosperms - ps16
Mesangiospermae - ps17
Magnoliids/eudicots - ps18
Eudicots - ps19
Core_eudicots - ps20
Superrosids - ps21
Rosids - ps22
Eurosids - ps23
Malvids - ps24
Sap./Malv./Brassicales - ps25
Malvales/Brassicales - ps26
Brassicales - ps27
Cleomaceae/Brassicaceae - ps28
Brassicaceae - ps29
Camelineae - ps30
Arabidopsis - ps31
Arabidopsis_thaliana - ps32
p-value
8e-17
6e-14
4e-11
2e-08
2e-05
Log-odds
0.5
1.0
1.5
2.0
2.5
3.0
3.5
4.0

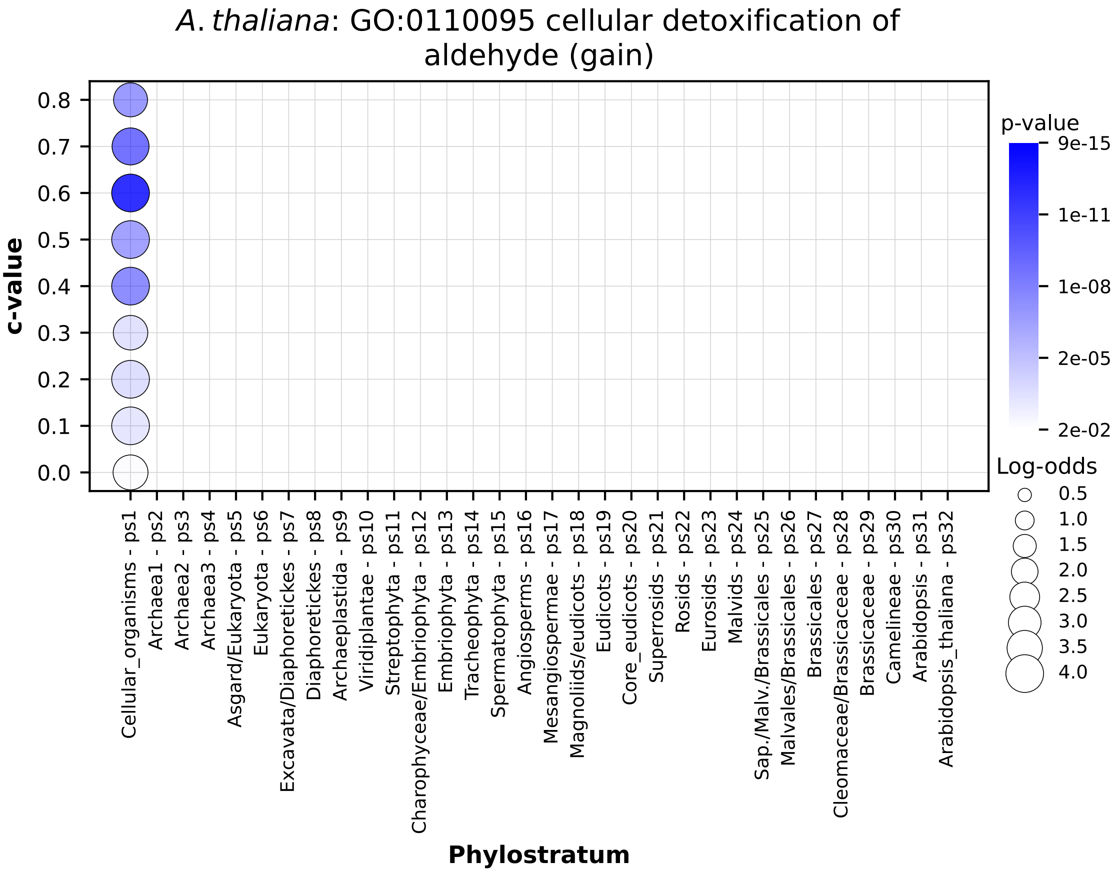

A. thaliana: GO:0110095 cellular detoxification of aldehyde (gain)
c-value
0.8
0.7
0.6
0.5
0.4
0.3
0.2
0.1
0.0
p-value
9e-15
1e-11
1e-08
2e-05
2e-02
Log-odds
0.5
1.0
1.5
2.0
2.5
3.0
3.5
4.0
Cellular_organisms - ps1
Archaea1 - ps2
Archaea2 - ps3
Archaea3 - ps4
Asgard/Eukaryota - ps5
Eukaryota - ps6
Excavata/Diaphoretickes - ps7
Diaphoretickes - ps8
Archaeplastida - ps9
Viridiplantae - ps10
Streptophyta - ps11
Charophyceae/Embriophyta - ps12
Embriophyta - ps13
Tracheophyta - ps14
Spermatophyta - ps15
Angiosperms - ps16
Mesangiospermae - ps17
Magnoliids/eudicots - ps18
Eudicots - ps19
Core_eudicots - ps20
Superrosids - ps21
Rosids - ps22
Eurosids - ps23
Malvids - ps24
Sap./Malv./Brassicales - ps25
Malvales/Brassicales - ps26
Brassicales - ps27
Cleomaceae/Brassicaceae - ps28
Brassicaceae - ps29
Camelineae - ps30
Arabidopsis - ps31
Arabidopsis_thaliana - ps32
Phylostratum

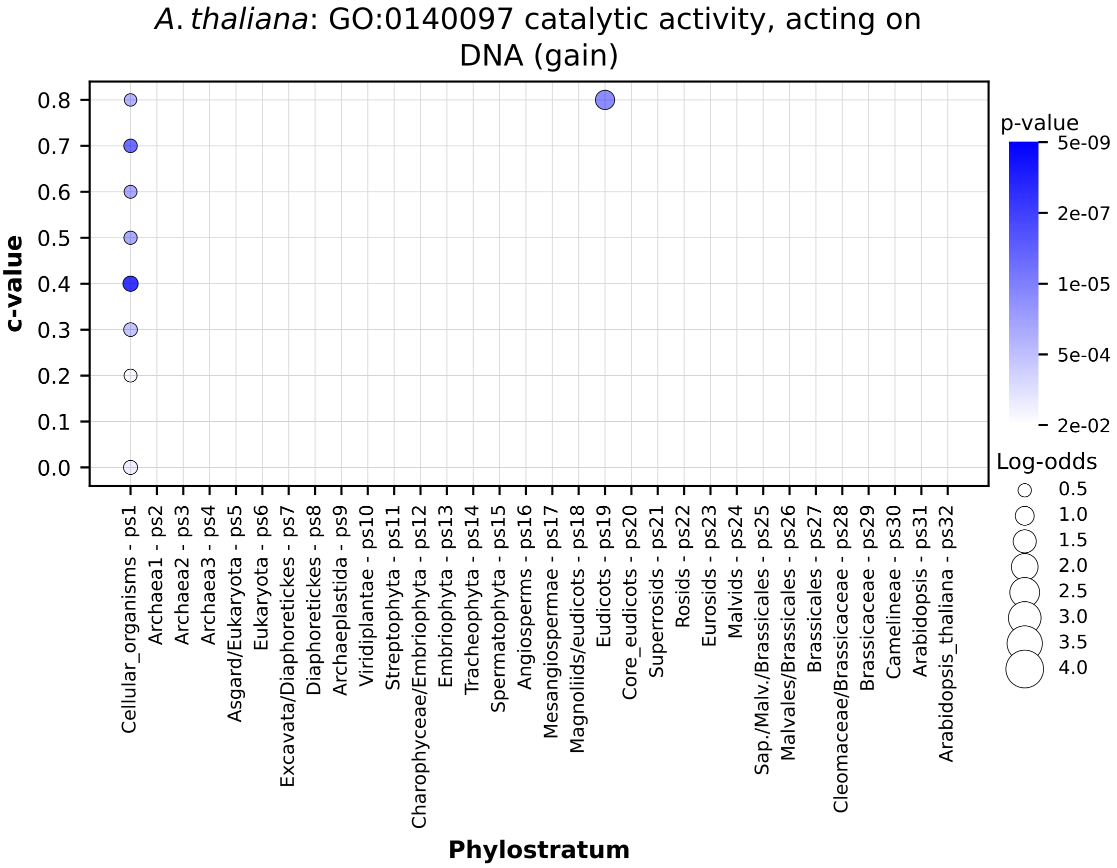

A. thaliana: GO:0140097 catalytic activity, acting on DNA (gain)
c-value
0.8
0.7
0.6
0.5
0.4
0.3
0.2
0.1
0.0
p-value
5e-09
2e-07
1e-05
5e-04
2e-02
Log-odds
0.5
1.0
1.5
2.0
2.5
3.0
3.5
4.0
Cellular_organisms - ps1
Archaea1 - ps2
Archaea2 - ps3
Archaea3 - ps4
Asgard/Eukaryota - ps5
Eukaryota - ps6
Excavata/Diaphoretickes - ps7
Diaphoretickes - ps8
Archaeplastida - ps9
Viridiplantae - ps10
Streptophyta - ps11
Charophyceae/Embriophyta - ps12
Embriophyta - ps13
Tracheophyta - ps14
Spermatophyta - ps15
Angiosperms - ps16
Mesangiospermae - ps17
Magnoliids/eudicots - ps18
Eudicots - ps19
Core_eudicots - ps20
Superrosids - ps21
Rosids - ps22
Eurosids - ps23
Malvids - ps24
Sap./Malv./Brassicales - ps25
Malvales/Brassicales - ps26
Brassicales - ps27
Cleomaceae/Brassicaceae - ps28
Brassicaceae - ps29
Camelineae - ps30
Arabidopsis - ps31
Arabidopsis_thaliana - ps32
Phylostratum

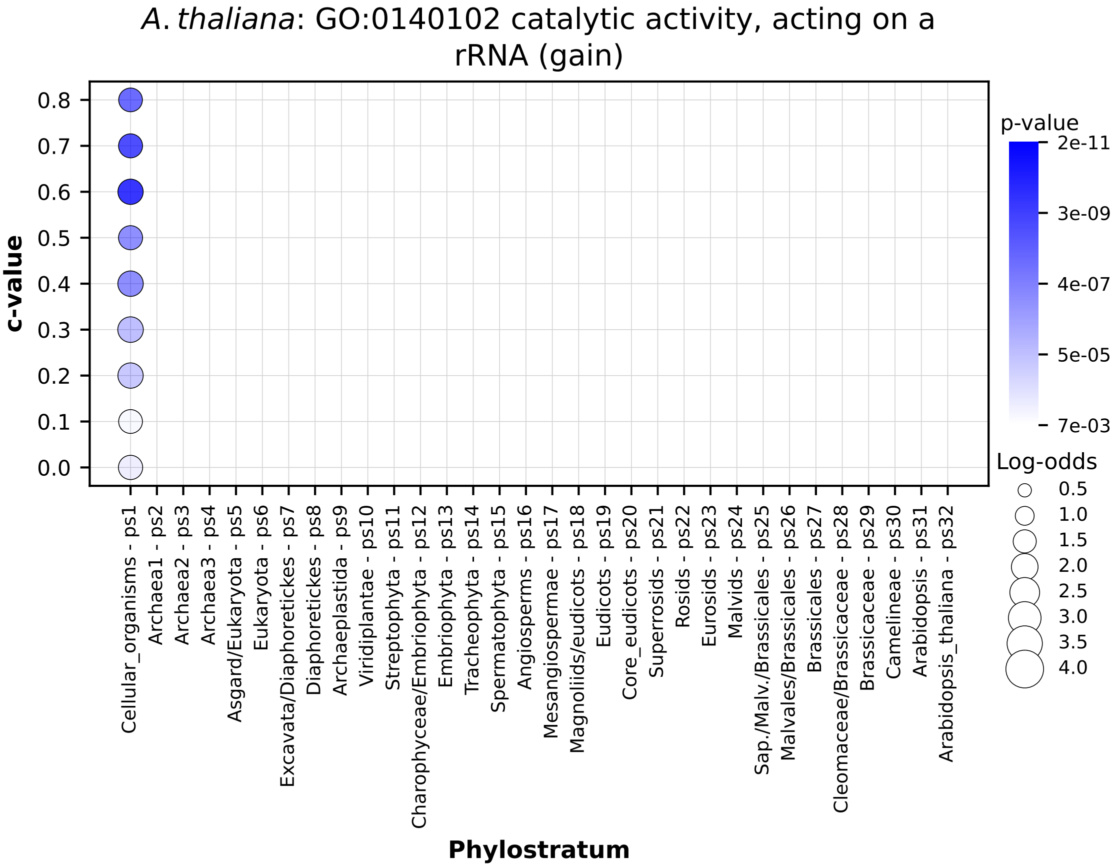

A. thaliana: GO:0140102 catalytic activity, acting on a rRNA (gain)
c-value
0.8
0.7
0.6
0.5
0.4
0.3
0.2
0.1
0.0
p-value
2e-11
3e-09
4e-07
5e-05
7e-03
Log-odds
0.5
1.0
1.5
2.0
2.5
3.0
3.5
4.0
Cellular_organisms - ps1
Archaea1 - ps2
Archaea2 - ps3
Archaea3 - ps4
Asgard/Eukaryota - ps5
Eukaryota - ps6
Excavata/Diaphoretickes - ps7
Diaphoretickes - ps8
Archaeplastida - ps9
Viridiplantae - ps10
Streptophyta - ps11
Charophyceae/Embriophyta - ps12
Embriophyta - ps13
Tracheophyta - ps14
Spermatophyta - ps15
Angiosperms - ps16
Mesangiospermae - ps17
Magnoliids/eudicots - ps18
Eudicots - ps19
Core_eudicots - ps20
Superrosids - ps21
Rosids - ps22
Eurosids - ps23
Malvids - ps24
Sap./Malv./Brassicales - ps25
Malvales/Brassicales - ps26
Brassicales - ps27
Cleomaceae/Brassicaceae - ps28
Brassicaceae - ps29
Camelineae - ps30
Arabidopsis - ps31
Arabidopsis_thaliana - ps32
Phylostratum

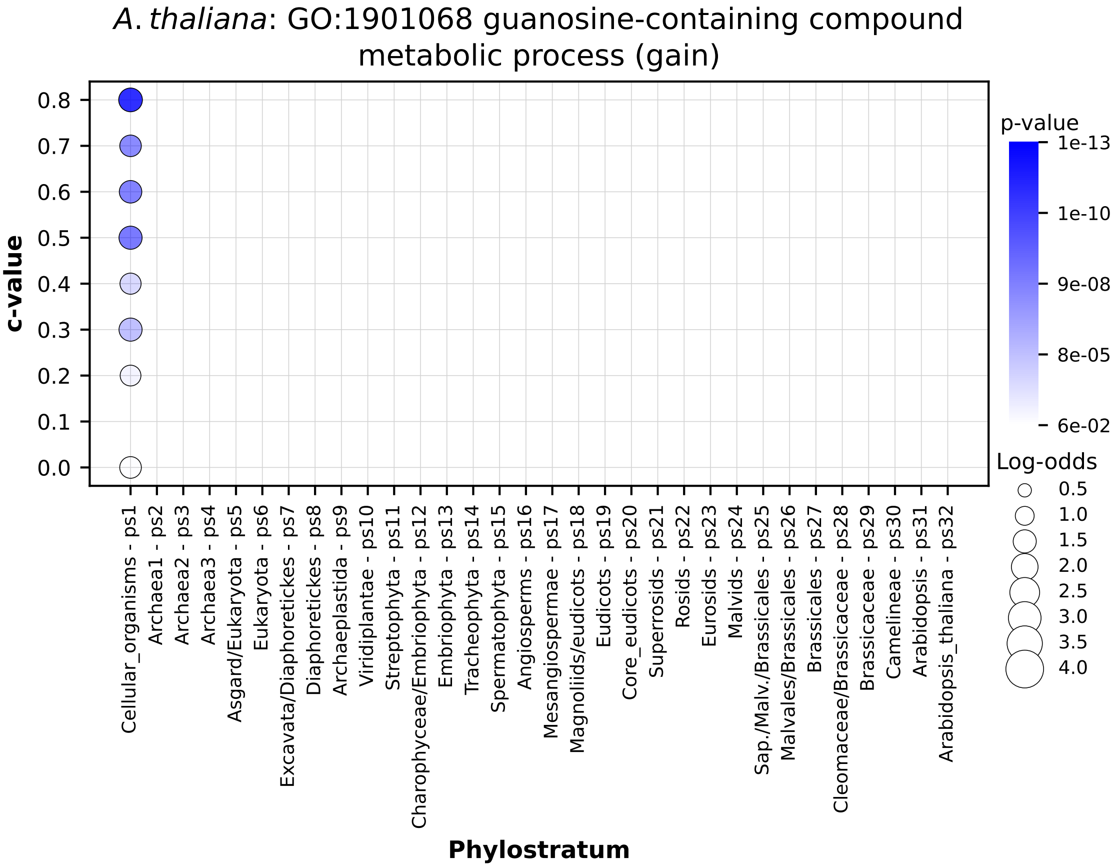

A. thaliana: GO:1901068 guanosine-containing compound metabolic process (gain)
c-value
0.8
0.7
0.6
0.5
0.4
0.3
0.2
0.1
0.0
p-value
1e-13
1e-10
9e-08
8e-05
6e-02
Log-odds
0.5
1.0
1.5
2.0
2.5
3.0
3.5
4.0
Cellular_organisms - ps1
Archaea1 - ps2
Archaea2 - ps3
Archaea3 - ps4
Asgard/Eukaryota - ps5
Eukaryota - ps6
Excavata/Diaphoretickes - ps7
Diaphoretickes - ps8
Archaeplastida - ps9
Viridiplantae - ps10
Streptophyta - ps11
Charophyceae/Embriophyta - ps12
Embriophyta - ps13
Tracheophyta - ps14
Spermatophyta - ps15
Angiosperms - ps16
Mesangiospermae - ps17
Magnoliids/eudicots - ps18
Eudicots - ps19
Core_eudicots - ps20
Superrosids - ps21
Rosids - ps22
Eurosids - ps23
Malvids - ps24
Sap./Malv./Brassicales - ps25
Malvales/Brassicales - ps26
Brassicales - ps27
Cleomaceae/Brassicaceae - ps28
Brassicaceae - ps29
Camelineae - ps30
Arabidopsis - ps31
Arabidopsis_thaliana - ps32
Phylostratum

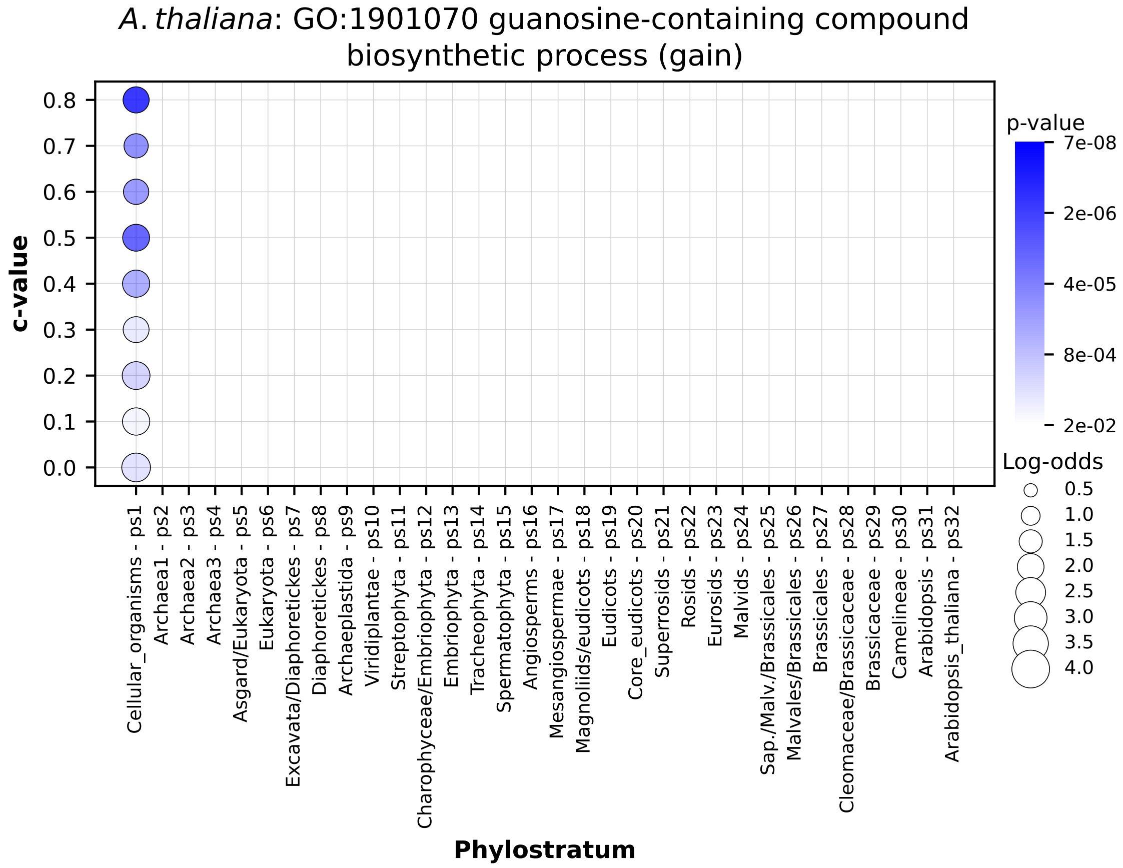

A. thaliana: GO:1901070 guanosine-containing compound biosynthetic process (gain)
c-value
0.8
0.7
0.6
0.5
0.4
0.3
0.2
0.1
0.0
p-value
7e-08
2e-06
4e-05
8e-04
2e-02
Log-odds
0.5
1.0
1.5
2.0
2.5
3.0
3.5
4.0
Cellular_organisms - ps1
Archaea1 - ps2
Archaea2 - ps3
Archaea3 - ps4
Asgard/Eukaryota - ps5
Eukaryota - ps6
Excavata/Diaphoretickes - ps7
Diaphoretickes - ps8
Archaeplastida - ps9
Viridiplantae - ps10
Streptophyta - ps11
Charophyceae/Embriophyta - ps12
Embriophyta - ps13
Tracheophyta - ps14
Spermatophyta - ps15
Angiosperms - ps16
Mesangiospermae - ps17
Magnoliids/eudicots - ps18
Eudicots - ps19
Core_eudicots - ps20
Superrosids - ps21
Rosids - ps22
Eurosids - ps23
Malvids - ps24
Sap./Malv./Brassicales - ps25
Malvales/Brassicales - ps26
Brassicales - ps27
Cleomaceae/Brassicaceae - ps28
Brassicaceae - ps29
Camelineae - ps30
Arabidopsis - ps31
Arabidopsis_thaliana - ps32
Phylostratum

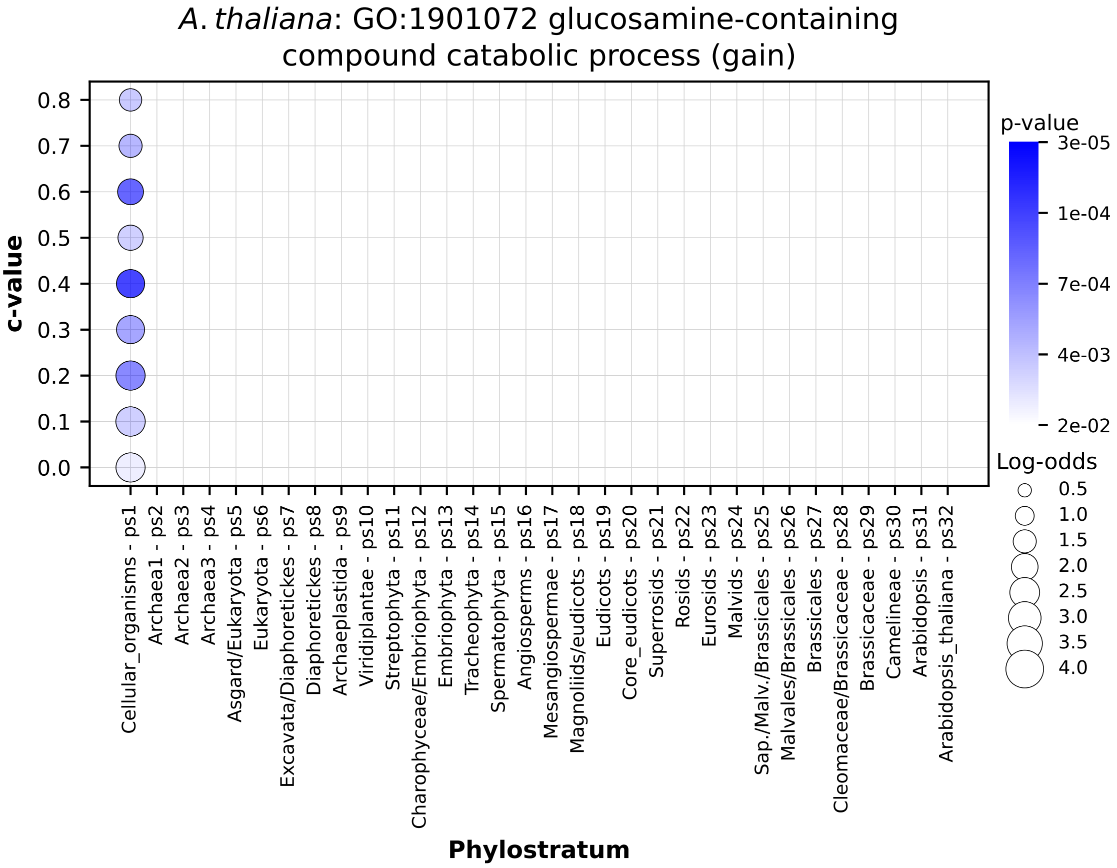

A. thaliana: GO:1901072 glucosamine-containing compound catabolic process (gain)
c-value
0.0
0.1
0.2
0.3
0.4
0.5
0.6
0.7
0.8
p-value
3e-05
1e-04
7e-04
4e-03
2e-02
Log-odds
0.5
1.0
1.5
2.0
2.5
3.0
3.5
4.0
Cellular_organisms - ps1
Archaea1 - ps2
Archaea2 - ps3
Archaea3 - ps4
Asgard/Eukaryota - ps5
Eukaryota - ps6
Excavata/Diaphoretickes - ps7
Diaphoretickes - ps8
Archaeplastida - ps9
Viridiplantae - ps10
Streptophyta - ps11
Charophyceae/Embriophyta - ps12
Embriophyta - ps13
Tracheophyta - ps14
Spermatophyta - ps15
Angiosperms - ps16
Mesangiospermae - ps17
Magnoliids/eudicots - ps18
Eudicots - ps19
Core_eudicots - ps20
Superrosids - ps21
Rosids - ps22
Eurosids - ps23
Malvids - ps24
Sap./Malv./Brassicales - ps25
Malvales/Brassicales - ps26
Brassicales - ps27
Cleomaceae/Brassicaceae - ps28
Brassicaceae - ps29
Camelineae - ps30
Arabidopsis - ps31
Arabidopsis_thaliana - ps32
Phylostratum

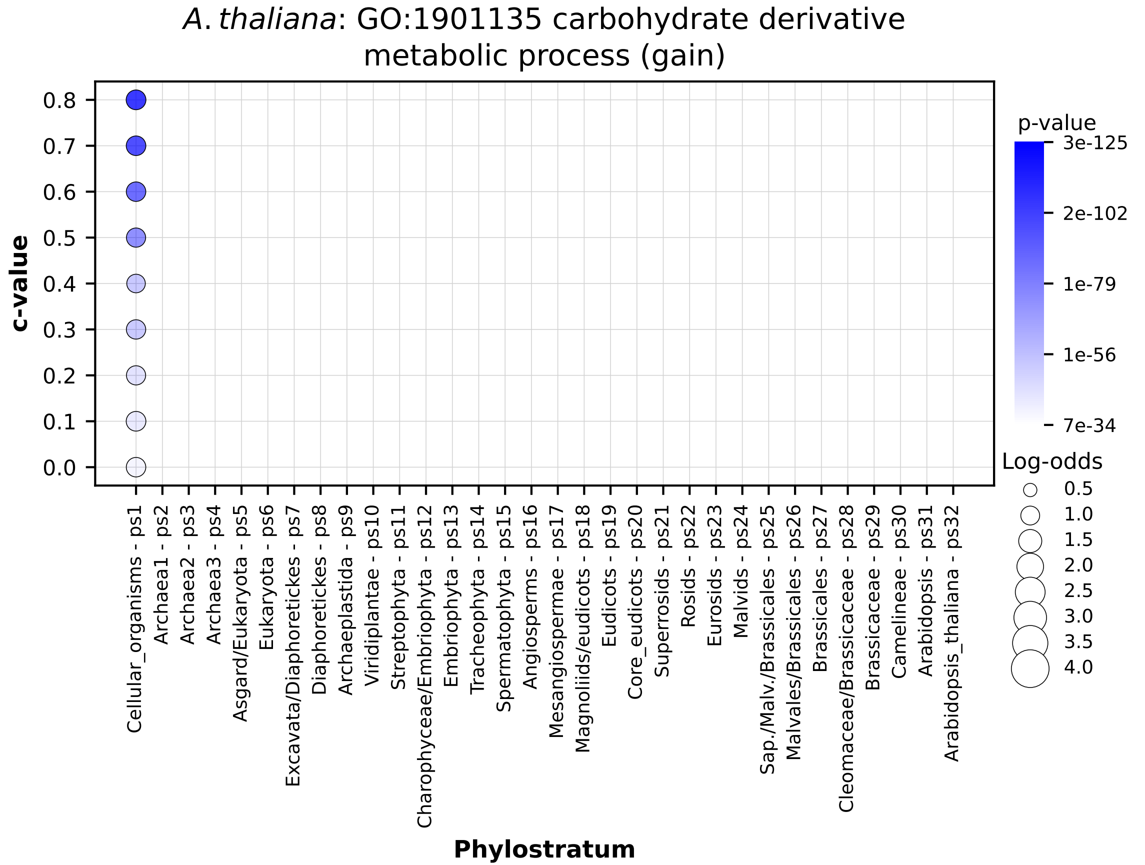

A. thaliana: GO:1901135 carbohydrate derivative metabolic process (gain)
c-value
0.8
0.7
0.6
0.5
0.4
0.3
0.2
0.1
0.0
p-value
3e-125
2e-102
1e-79
1e-56
7e-34
Log-odds
0.5
1.0
1.5
2.0
2.5
3.0
3.5
4.0
Cellular_organisms - ps1
Archaea1 - ps2
Archaea2 - ps3
Archaea3 - ps4
Asgard/Eukaryota - ps5
Eukaryota - ps6
Excavata/Diaphoretickes - ps7
Diaphoretickes - ps8
Archaeplastida - ps9
Viridiplantae - ps10
Streptophyta - ps11
Charophyceae/Embriophyta - ps12
Embriophyta - ps13
Tracheophyta - ps14
Spermatophyta - ps15
Angiosperms - ps16
Mesangiospermae - ps17
Magnoliids/eudicots - ps18
Eudicots - ps19
Core_eudicots - ps20
Superrosids - ps21
Rosids - ps22
Eurosids - ps23
Malvids - ps24
Sap./Malv./Brassicales - ps25
Malvales/Brassicales - ps26
Brassicales - ps27
Cleomaceae/Brassicaceae - ps28
Brassicaceae - ps29
Camelineae - ps30
Arabidopsis - ps31
Arabidopsis_thaliana - ps32
Phylostratum

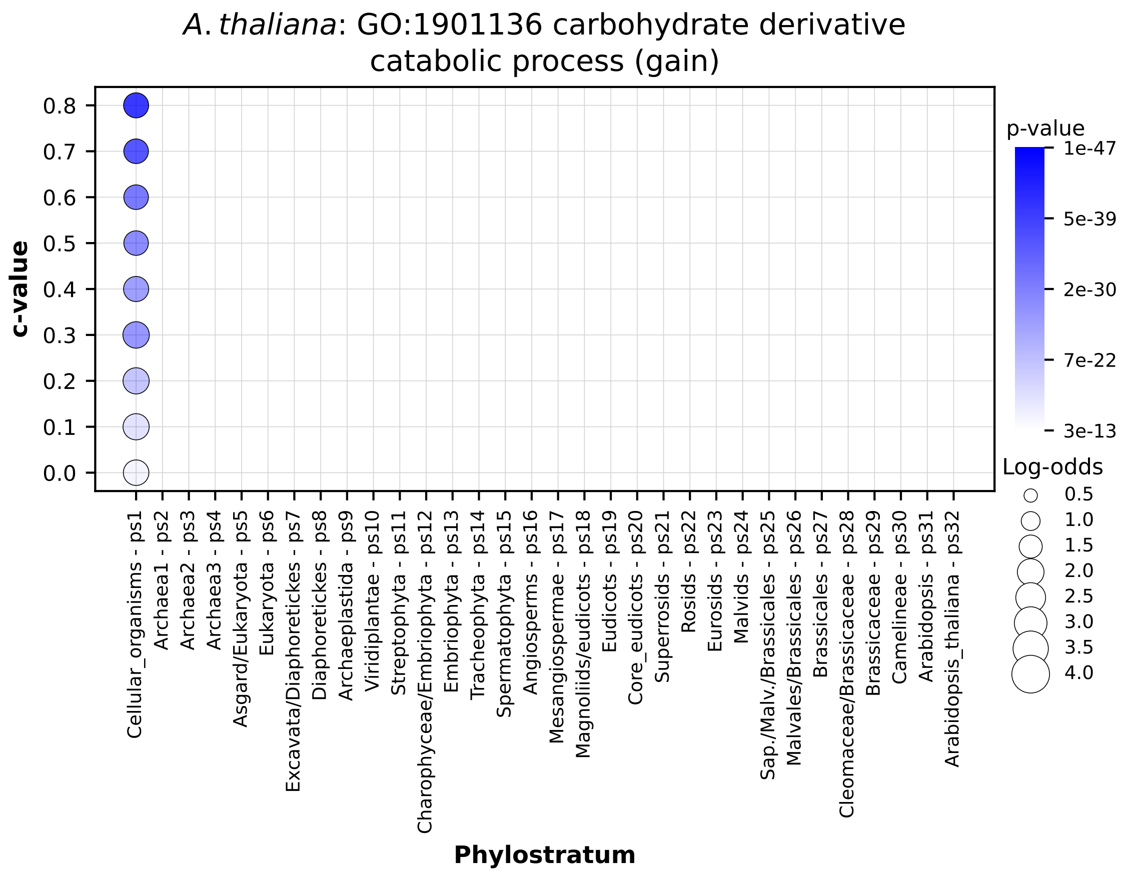

A. thaliana: GO:1901136 carbohydrate derivative catabolic process (gain)
c-value
0.8
0.7
0.6
0.5
0.4
0.3
0.2
0.1
0.0
p-value
1e-47
5e-39
2e-30
7e-22
3e-13
Log-odds
0.5
1.0
1.5
2.0
2.5
3.0
3.5
4.0
Cellular_organisms - ps1
Archaea1 - ps2
Archaea2 - ps3
Archaea3 - ps4
Asgard/Eukaryota - ps5
Eukaryota - ps6
Excavata/Diaphoretickes - ps7
Diaphoretickes - ps8
Archaeplastida - ps9
Viridiplantae - ps10
Streptophyta - ps11
Charophyceae/Embriophyta - ps12
Embriophyta - ps13
Tracheophyta - ps14
Spermatophyta - ps15
Angiosperms - ps16
Mesangiospermae - ps17
Magnoliids/eudicots - ps18
Eudicots - ps19
Core_eudicots - ps20
Superrosids - ps21
Rosids - ps22
Eurosids - ps23
Malvids - ps24
Sap./Malv./Brassicales - ps25
Malvales/Brassicales - ps26
Brassicales - ps27
Cleomaceae/Brassicaceae - ps28
Brassicaceae - ps29
Camelineae - ps30
Arabidopsis - ps31
Arabidopsis_thaliana - ps32
Phylostratum

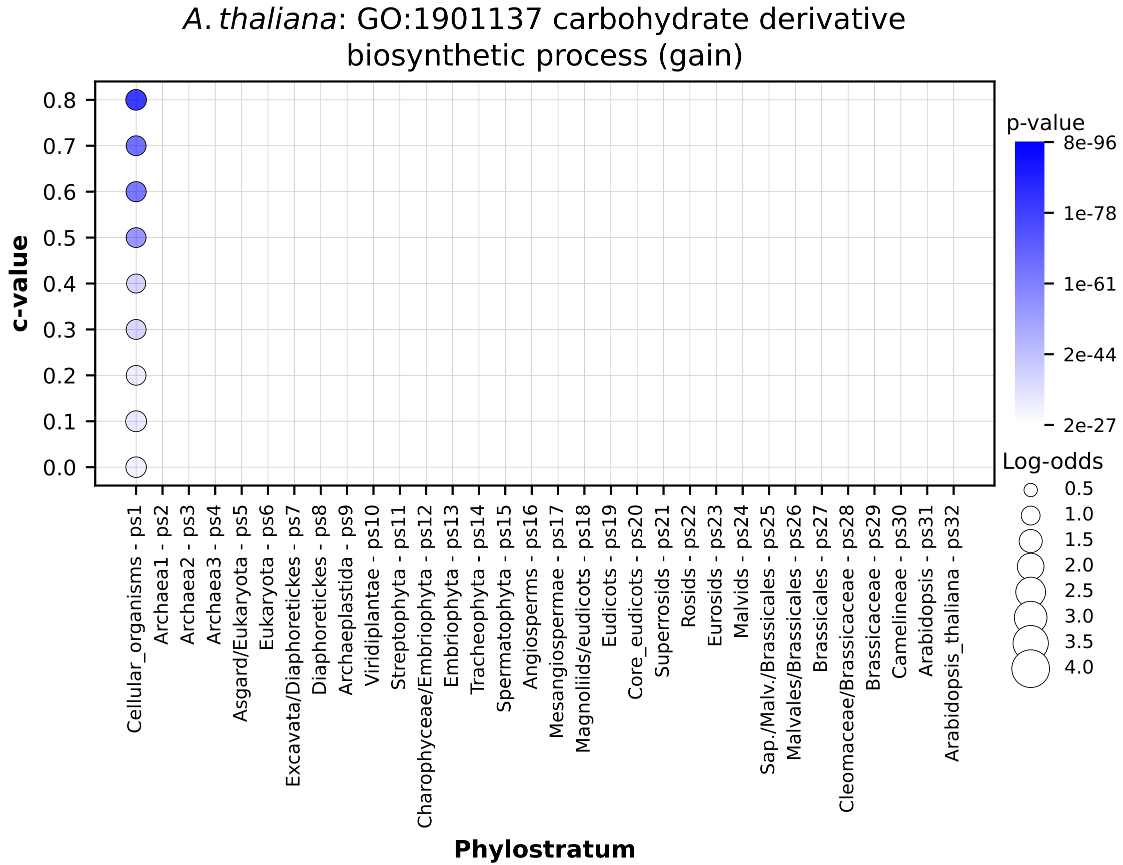

A. thaliana: GO:1901137 carbohydrate derivative biosynthetic process (gain)
c-value
0.8
0.7
0.6
0.5
0.4
0.3
0.2
0.1
0.0
p-value
8e-96
1e-78
1e-61
2e-44
2e-27
Log-odds
0.5
1.0
1.5
2.0
2.5
3.0
3.5
4.0
Cellular_organisms - ps1
Archaea1 - ps2
Archaea2 - ps3
Archaea3 - ps4
Asgard/Eukaryota - ps5
Eukaryota - ps6
Excavata/Diaphoretickes - ps7
Diaphoretickes - ps8
Archaeplastida - ps9
Viridiplantae - ps10
Streptophyta - ps11
Charophyceae/Embriophyta - ps12
Embriophyta - ps13
Tracheophyta - ps14
Spermatophyta - ps15
Angiosperms - ps16
Mesangiospermae - ps17
Magnoliids/eudicots - ps18
Eudicots - ps19
Core_eudicots - ps20
Superrosids - ps21
Rosids - ps22
Eurosids - ps23
Malvids - ps24
Sap./Malv./Brassicales - ps25
Malvales/Brassicales - ps26
Brassicales - ps27
Cleomaceae/Brassicaceae - ps28
Brassicaceae - ps29
Camelineae - ps30
Arabidopsis - ps31
Arabidopsis_thaliana - ps32
Phylostratum

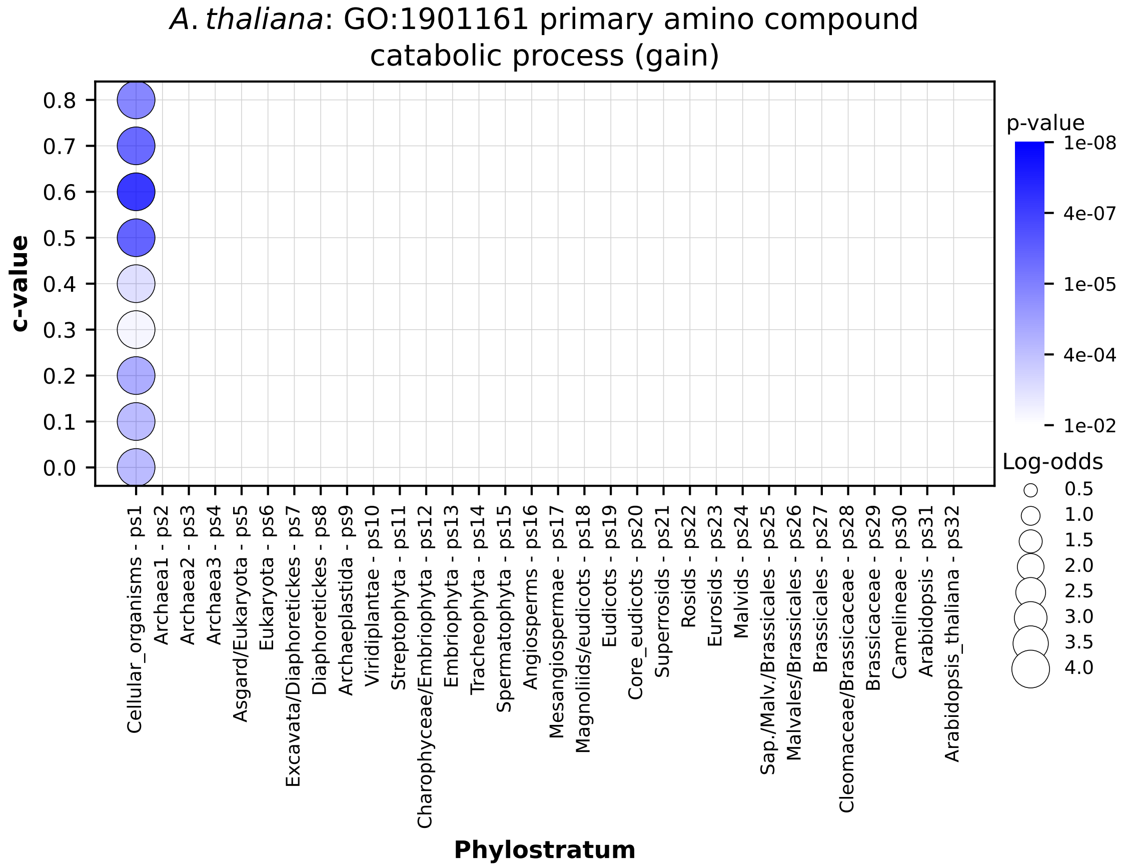
A. thaliana: GO:1901161 primary amino compound catabolic process (gain)
c-value
0.8
0.7
0.6
0.5
0.4
0.3
0.2
0.1
0.0
p-value
1e-08
4e-07
1e-05
4e-04
1e-02
Log-odds
0.5
1.0
1.5
2.0
2.5
3.0
3.5
4.0
Cellular_organisms - ps1
Archaea1 - ps2
Archaea2 - ps3
Archaea3 - ps4
Asgard/Eukaryota - ps5
Eukaryota - ps6
Excavata/Diaphoretickes - ps7
Diaphoretickes - ps8
Archaeplastida - ps9
Viridiplantae - ps10
Streptophyta - ps11
Charophyceae/Embriophyta - ps12
Embriophyta - ps13
Tracheophyta - ps14
Spermatophyta - ps15
Angiosperms - ps16
Mesangiospermae - ps17
Magnoliids/eudicots - ps18
Eudicots - ps19
Core_eudicots - ps20
Superrosids - ps21
Rosids - ps22
Eurosids - ps23
Malvids - ps24
Sap./Malv./Brassicales - ps25
Malvales/Brassicales - ps26
Brassicales - ps27
Cleomaceae/Brassicaceae - ps28
Brassicaceae - ps29
Camelineae - ps30
Arabidopsis - ps31
Arabidopsis_thaliana - ps32
Phylostratum

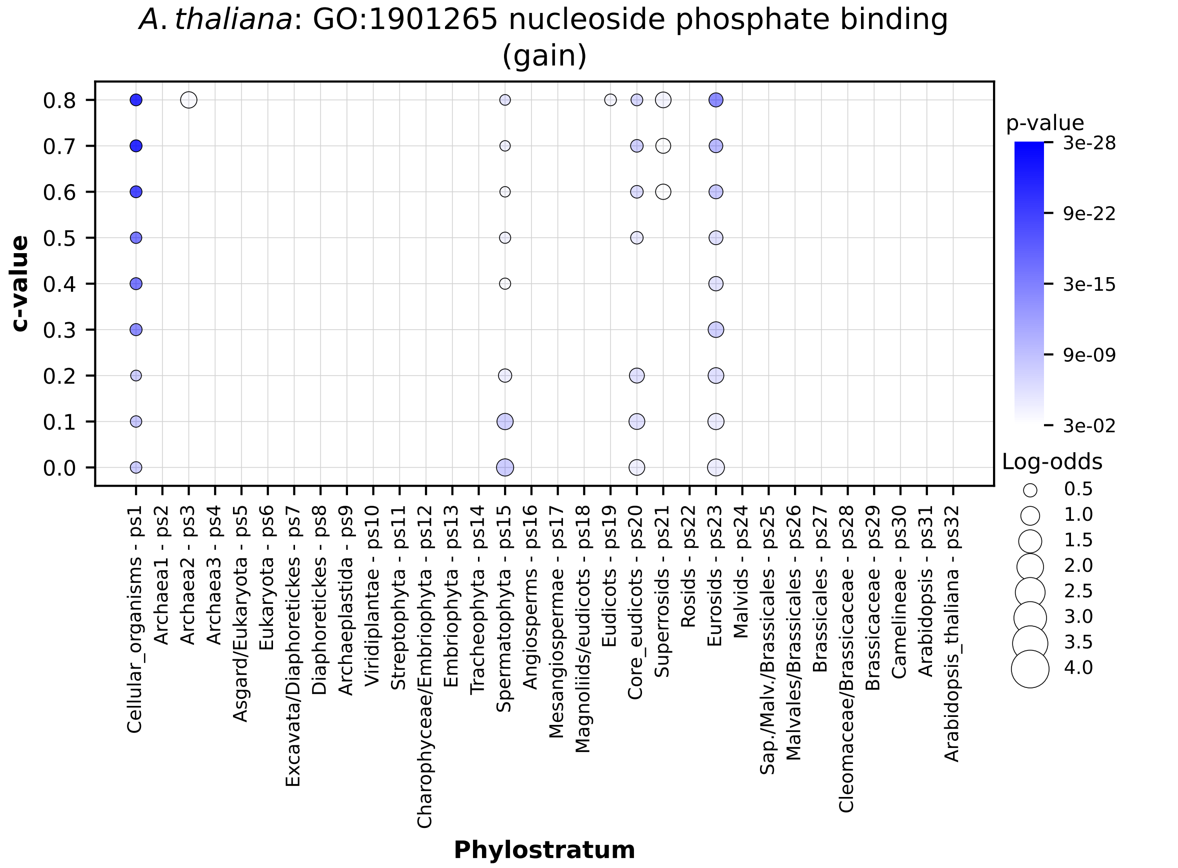

A. thaliana: GO:1901265 nucleoside phosphate binding (gain)
c-value
0.8
0.7
0.6
0.5
0.4
0.3
0.2
0.1
0.0
p-value
3e-28
9e-22
3e-15
9e-09
3e-02
Log-odds
0.5
1.0
1.5
2.0
2.5
3.0
3.5
4.0
Cellular_organisms - ps1
Archaea1 - ps2
Archaea2 - ps3
Archaea3 - ps4
Asgard/Eukaryota - ps5
Eukaryota - ps6
Excavata/Diaphoretickes - ps7
Diaphoretickes - ps8
Archaeplastida - ps9
Viridiplantae - ps10
Streptophyta - ps11
Charophyceae/Embriophyta - ps12
Embriophyta - ps13
Tracheophyta - ps14
Spermatophyta - ps15
Angiosperms - ps16
Mesangiospermae - ps17
Magnoliids/eudicots - ps18
Eudicots - ps19
Core_eudicots - ps20
Superrosids - ps21
Rosids - ps22
Eurosids - ps23
Malvids - ps24
Sap./Malv./Brassicales - ps25
Malvales/Brassicales - ps26
Brassicales - ps27
Cleomaceae/Brassicaceae - ps28
Brassicaceae - ps29
Camelineae - ps30
Arabidopsis - ps31
Arabidopsis_thaliana - ps32
Phylostratum

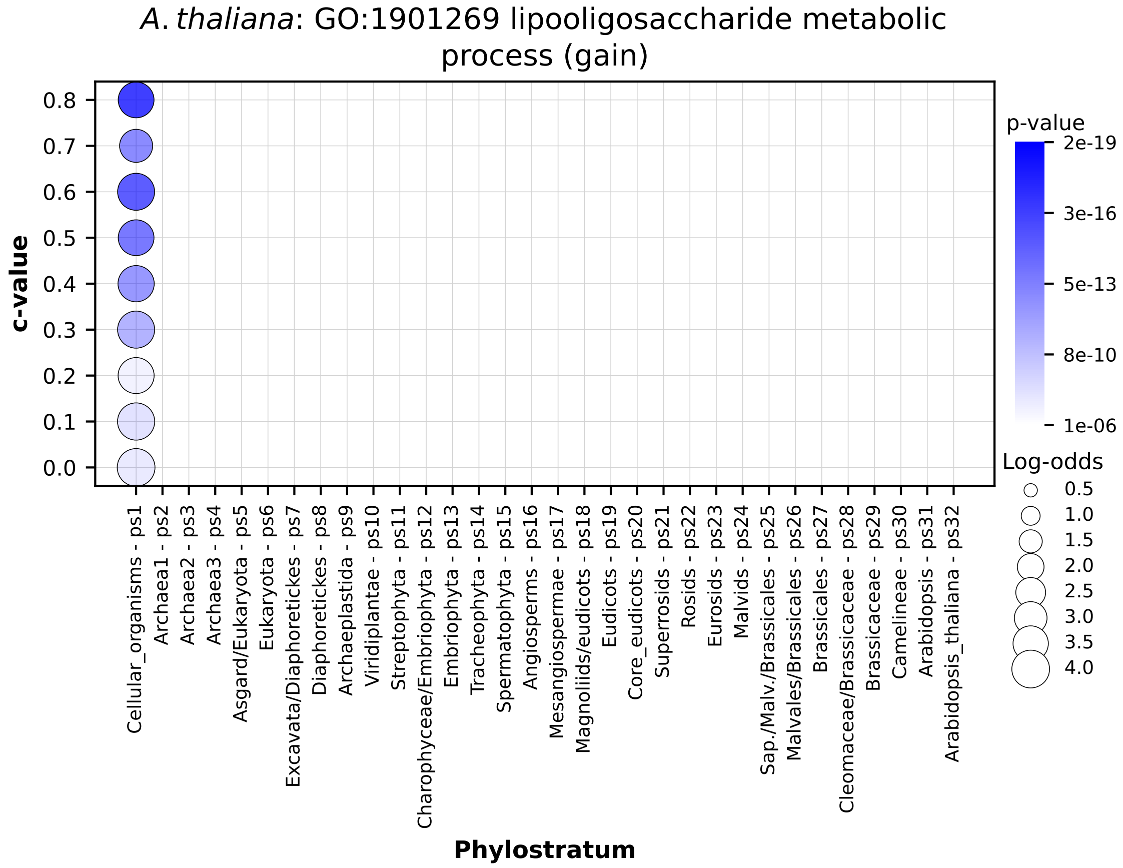

A. thaliana: GO:1901269 lipooligosaccharide metabolic process (gain)
c-value
0.8
0.7
0.6
0.5
0.4
0.3
0.2
0.1
0.0
p-value
2e-19
3e-16
5e-13
8e-10
1e-06
Log-odds
0.5
1.0
1.5
2.0
2.5
3.0
3.5
4.0
Cellular_organisms - ps1
Archaea1 - ps2
Archaea2 - ps3
Archaea3 - ps4
Asgard/Eukaryota - ps5
Eukaryota - ps6
Excavata/Diaphoretickes - ps7
Diaphoretickes - ps8
Archaeplastida - ps9
Viridiplantae - ps10
Streptophyta - ps11
Charophyceae/Embriophyta - ps12
Embriophyta - ps13
Tracheophyta - ps14
Spermatophyta - ps15
Angiosperms - ps16
Mesangiospermae - ps17
Magnoliids/eudicots - ps18
Eudicots - ps19
Core_eudicots - ps20
Superrosids - ps21
Rosids - ps22
Eurosids - ps23
Malvids - ps24
Sap./Malv./Brassicales - ps25
Malvales/Brassicales - ps26
Brassicales - ps27
Cleomaceae/Brassicaceae - ps28
Brassicaceae - ps29
Camelineae - ps30
Arabidopsis - ps31
Arabidopsis_thaliana - ps32
Phylostratum

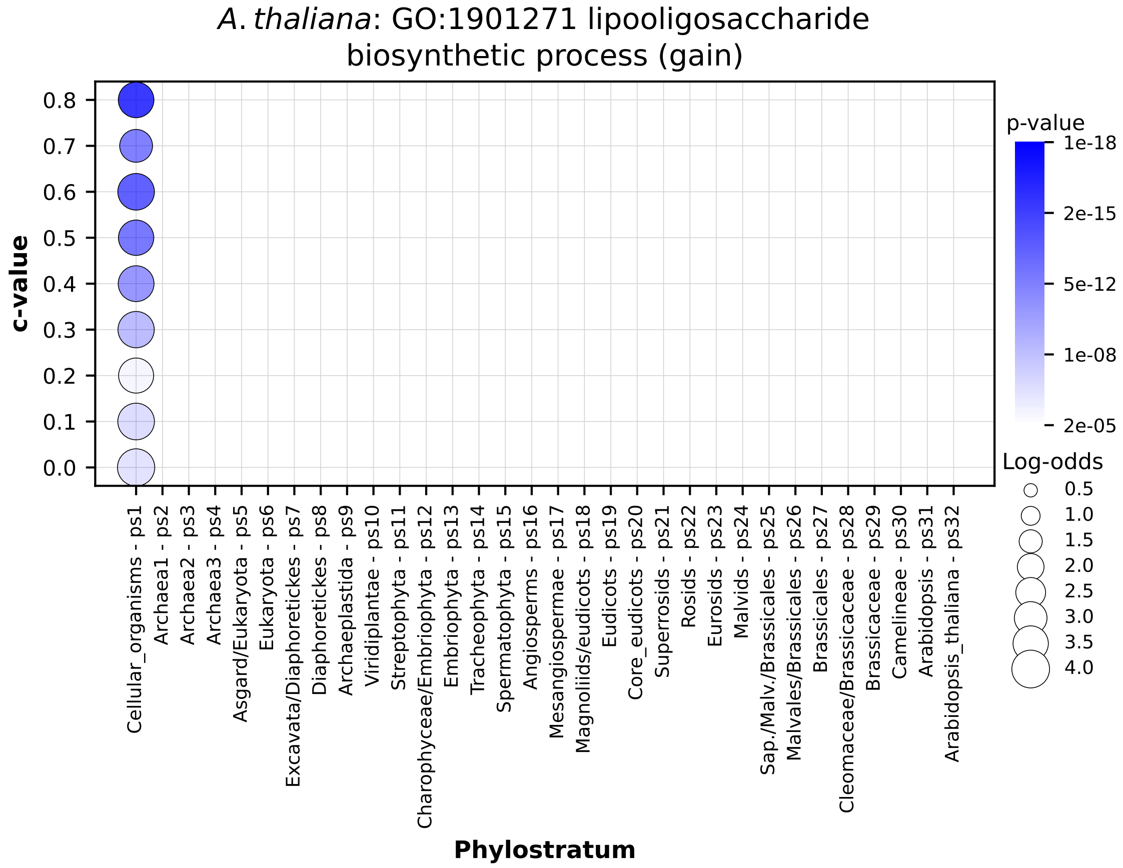

A. thaliana: GO:1901271 lipooligosaccharide biosynthetic process (gain)
c-value
0.8
0.7
0.6
0.5
0.4
0.3
0.2
0.1
0.0
p-value
1e-18
2e-15
5e-12
1e-08
2e-05
Log-odds
0.5
1.0
1.5
2.0
2.5
3.0
3.5
4.0
Cellular_organisms - ps1
Archaea1 - ps2
Archaea2 - ps3
Archaea3 - ps4
Asgard/Eukaryota - ps5
Eukaryota - ps6
Excavata/Diaphoretickes - ps7
Diaphoretickes - ps8
Archaeplastida - ps9
Viridiplantae - ps10
Streptophyta - ps11
Charophyceae/Embriophyta - ps12
Embriophyta - ps13
Tracheophyta - ps14
Spermatophyta - ps15
Angiosperms - ps16
Mesangiospermae - ps17
Magnoliids/eudicots - ps18
Eudicots - ps19
Core_eudicots - ps20
Superrosids - ps21
Rosids - ps22
Eurosids - ps23
Malvids - ps24
Sap./Malv./Brassicales - ps25
Malvales/Brassicales - ps26
Brassicales - ps27
Cleomaceae/Brassicaceae - ps28
Brassicaceae - ps29
Camelineae - ps30
Arabidopsis - ps31
Arabidopsis_thaliana - ps32
Phylostratum

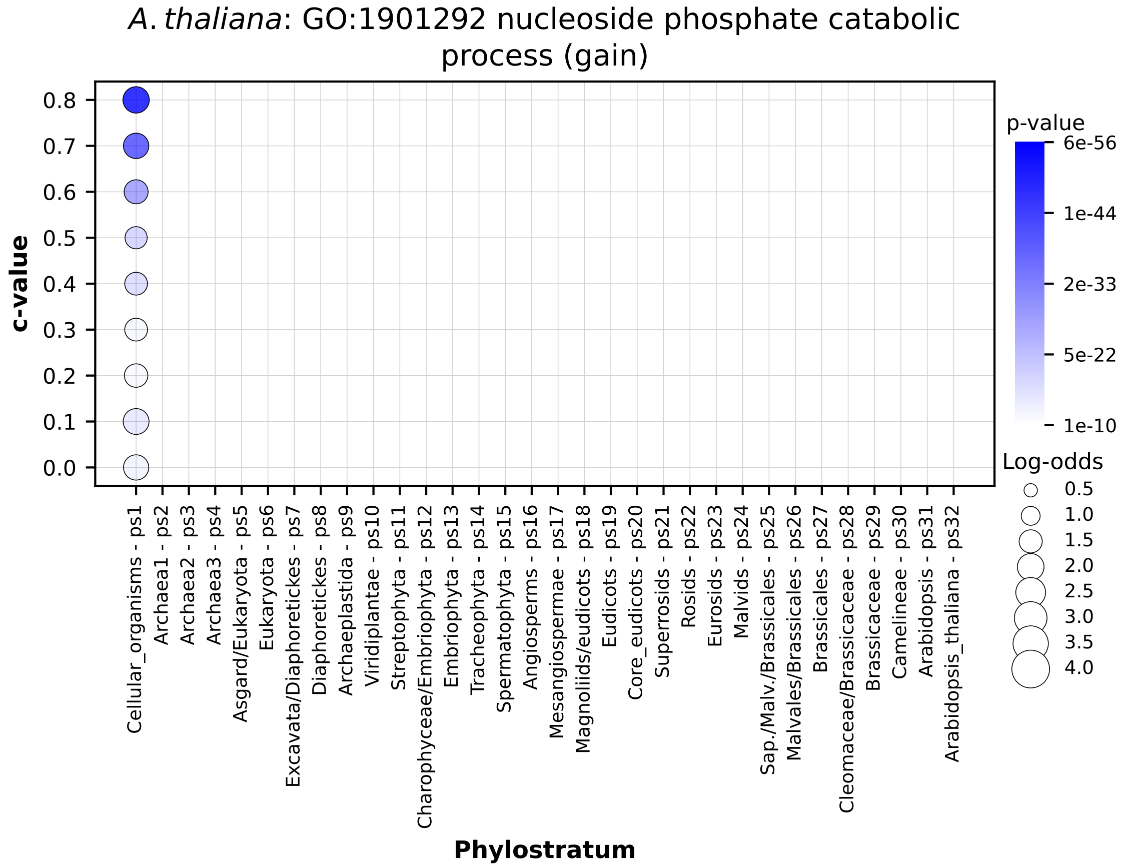
A. thaliana: GO:1901292 nucleoside phosphate catabolic process (gain)
c-value
0.8
0.7
0.6
0.5
0.4
0.3
0.2
0.1
0.0
p-value
6e-56
1e-44
2e-33
5e-22
1e-10
Log-odds
0.5
1.0
1.5
2.0
2.5
3.0
3.5
4.0
Cellular_organisms - ps1
Archaea1 - ps2
Archaea2 - ps3
Archaea3 - ps4
Asgard/Eukaryota - ps5
Eukaryota - ps6
Excavata/Diaphoretickes - ps7
Diaphoretickes - ps8
Archaeplastida - ps9
Viridiplantae - ps10
Streptophyta - ps11
Charophyceae/Embriophyta - ps12
Embriophyta - ps13
Tracheophyta - ps14
Spermatophyta - ps15
Angiosperms - ps16
Mesangiospermae - ps17
Magnoliids/eudicots - ps18
Eudicots - ps19
Core_eudicots - ps20
Superrosids - ps21
Rosids - ps22
Eurosids - ps23
Malvids - ps24
Sap./Malv./Brassicales - ps25
Malvales/Brassicales - ps26
Brassicales - ps27
Cleomaceae/Brassicaceae - ps28
Brassicaceae - ps29
Camelineae - ps30
Arabidopsis - ps31
Arabidopsis_thaliana - ps32
Phylostratum

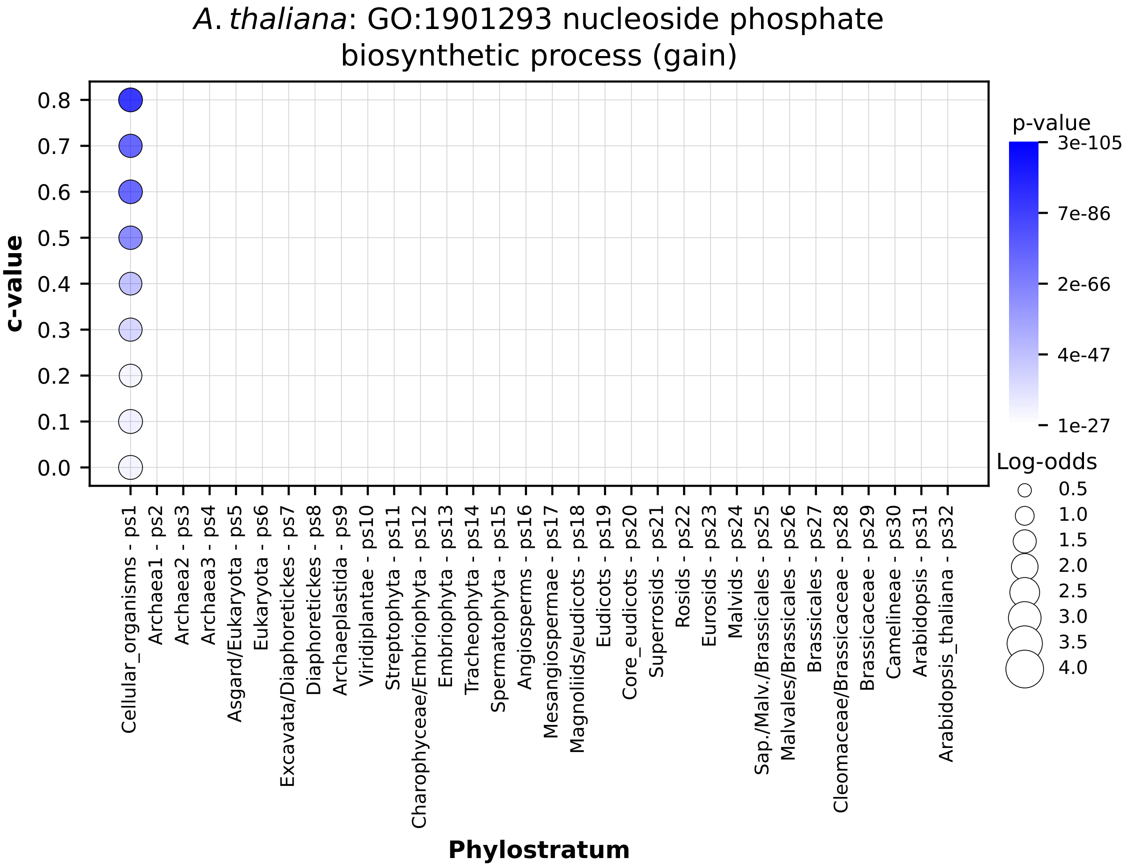
A. thaliana: GO:1901293 nucleoside phosphate biosynthetic process (gain)
c-value
0.8
0.7
0.6
0.5
0.4
0.3
0.2
0.1
0.0
p-value
3e-105
7e-86
2e-66
4e-47
1e-27
Log-odds
0.5
1.0
1.5
2.0
2.5
3.0
3.5
4.0
Cellular_organisms - ps1
Archaea1 - ps2
Archaea2 - ps3
Archaea3 - ps4
Asgard/Eukaryota - ps5
Eukaryota - ps6
Excavata/Diaphoretickes - ps7
Diaphoretickes - ps8
Archaeplastida - ps9
Viridiplantae - ps10
Streptophyta - ps11
Charophyceae/Embriophyta - ps12
Embriophyta - ps13
Tracheophyta - ps14
Spermatophyta - ps15
Angiosperms - ps16
Mesangiospermae - ps17
Magnoliids/eudicots - ps18
Eudicots - ps19
Core_eudicots - ps20
Superrosids - ps21
Rosids - ps22
Eurosids - ps23
Malvids - ps24
Sap./Malv./Brassicales - ps25
Malvales/Brassicales - ps26
Brassicales - ps27
Cleomaceae/Brassicaceae - ps28
Brassicaceae - ps29
Camelineae - ps30
Arabidopsis - ps31
Arabidopsis_thaliana - ps32
Phylostratum

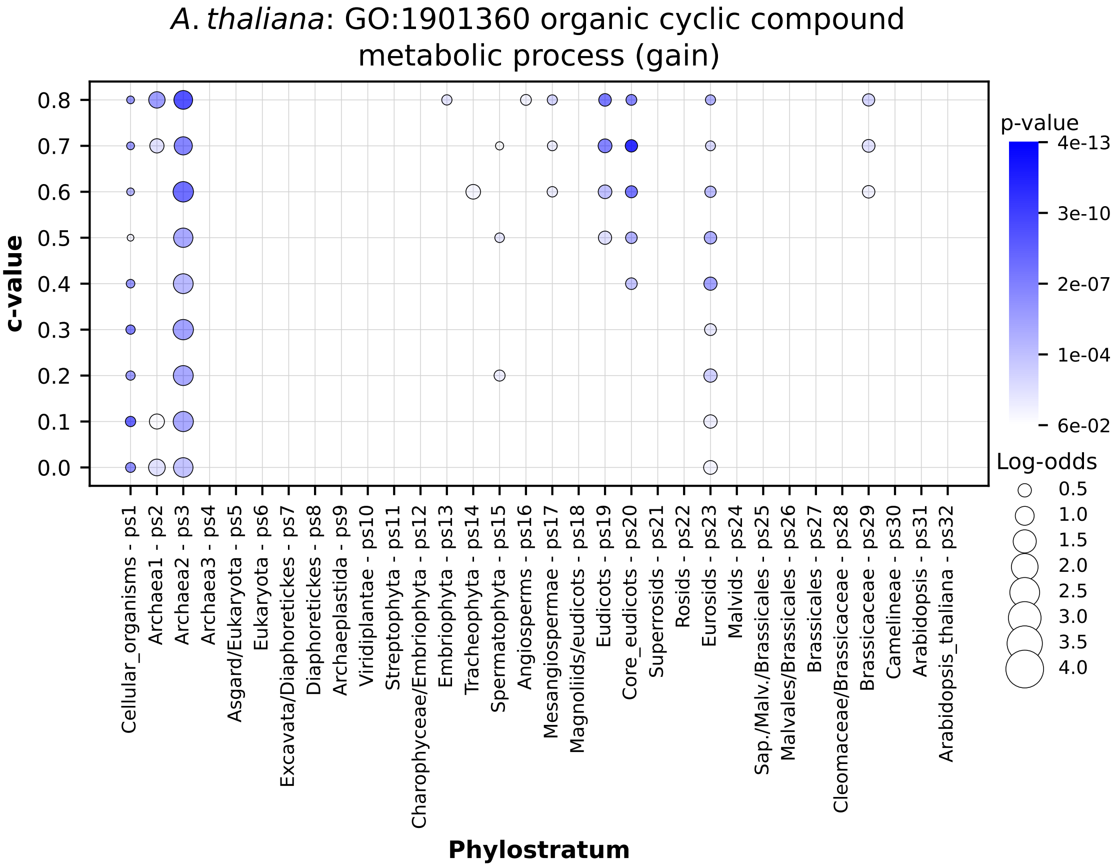
A. thaliana: GO:1901360 organic cyclic compound metabolic process (gain)
c-value
0.8
0.7
0.6
0.5
0.4
0.3
0.2
0.1
0.0
p-value
4e-13
3e-10
2e-07
1e-04
6e-02
Log-odds
0.5
1.0
1.5
2.0
2.5
3.0
3.5
4.0
Cellular_organisms - ps1
Archaea1 - ps2
Archaea2 - ps3
Archaea3 - ps4
Asgard/Eukaryota - ps5
Eukaryota - ps6
Excavata/Diaphoretickes - ps7
Diaphoretickes - ps8
Archaeplastida - ps9
Viridiplantae - ps10
Streptophyta - ps11
Charophyceae/Embriophyta - ps12
Embriophyta - ps13
Tracheophyta - ps14
Spermatophyta - ps15
Angiosperms - ps16
Mesangiospermae - ps17
Magnoliids/eudicots - ps18
Eudicots - ps19
Core_eudicots - ps20
Superrosids - ps21
Rosids - ps22
Eurosids - ps23
Malvids - ps24
Sap./Malv./Brassicales - ps25
Malvales/Brassicales - ps26
Brassicales - ps27
Cleomaceae/Brassicaceae - ps28
Brassicaceae - ps29
Camelineae - ps30
Arabidopsis - ps31
Arabidopsis_thaliana - ps32
Phylostratum

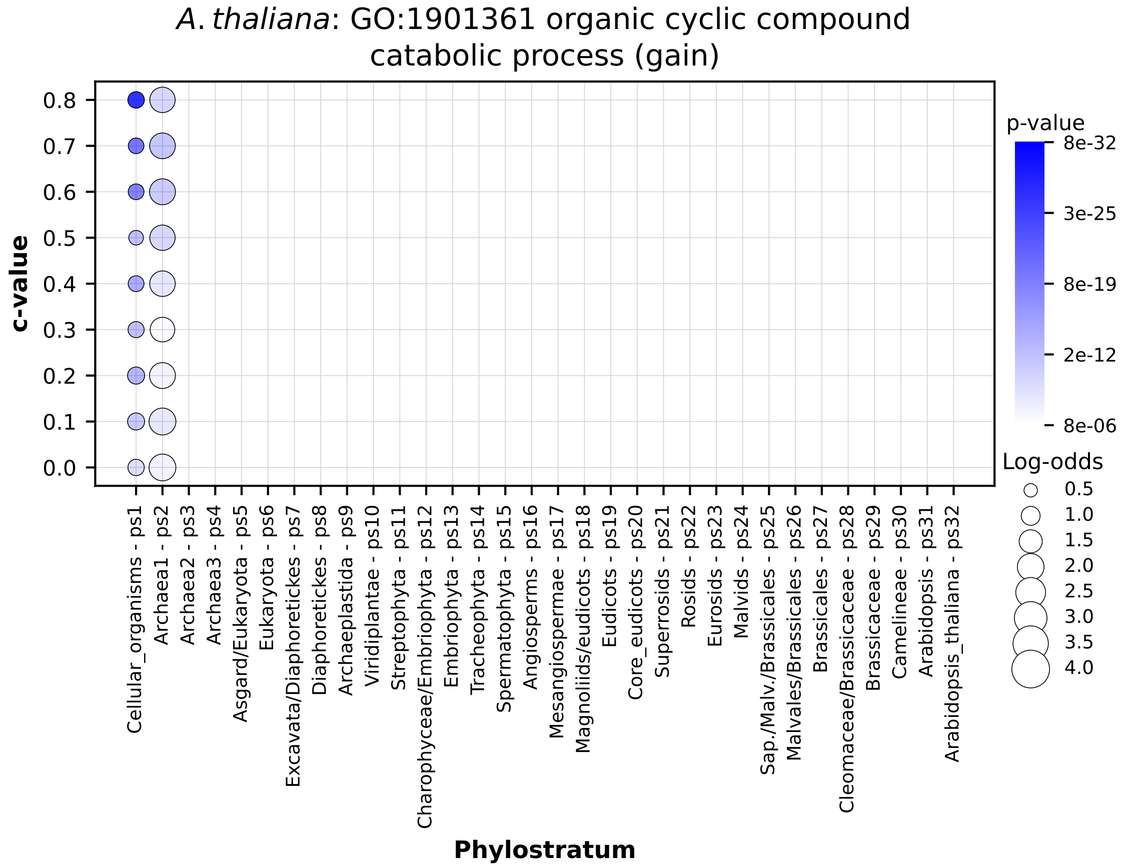

A. thaliana: GO:1901361 organic cyclic compound catabolic process (gain)
c-value
0.8
0.7
0.6
0.5
0.4
0.3
0.2
0.1
0.0
p-value
8e-32
3e-25
8e-19
2e-12
8e-06
Log-odds
0.5
1.0
1.5
2.0
2.5
3.0
3.5
4.0
Cellular_organisms - ps1
Archaea1 - ps2
Archaea2 - ps3
Archaea3 - ps4
Asgard/Eukaryota - ps5
Eukaryota - ps6
Excavata/Diaphoretickes - ps7
Diaphoretickes - ps8
Archaeplastida - ps9
Viridiplantae - ps10
Streptophyta - ps11
Charophyceae/Embriophyta - ps12
Embriophyta - ps13
Tracheophyta - ps14
Spermatophyta - ps15
Angiosperms - ps16
Mesangiospermae - ps17
Magnoliids/eudicots - ps18
Eudicots - ps19
Core_eudicots - ps20
Superrosids - ps21
Rosids - ps22
Eurosids - ps23
Malvids - ps24
Sap./Malv./Brassicales - ps25
Malvales/Brassicales - ps26
Brassicales - ps27
Cleomaceae/Brassicaceae - ps28
Brassicaceae - ps29
Camelineae - ps30
Arabidopsis - ps31
Arabidopsis_thaliana - ps32
Phylostratum

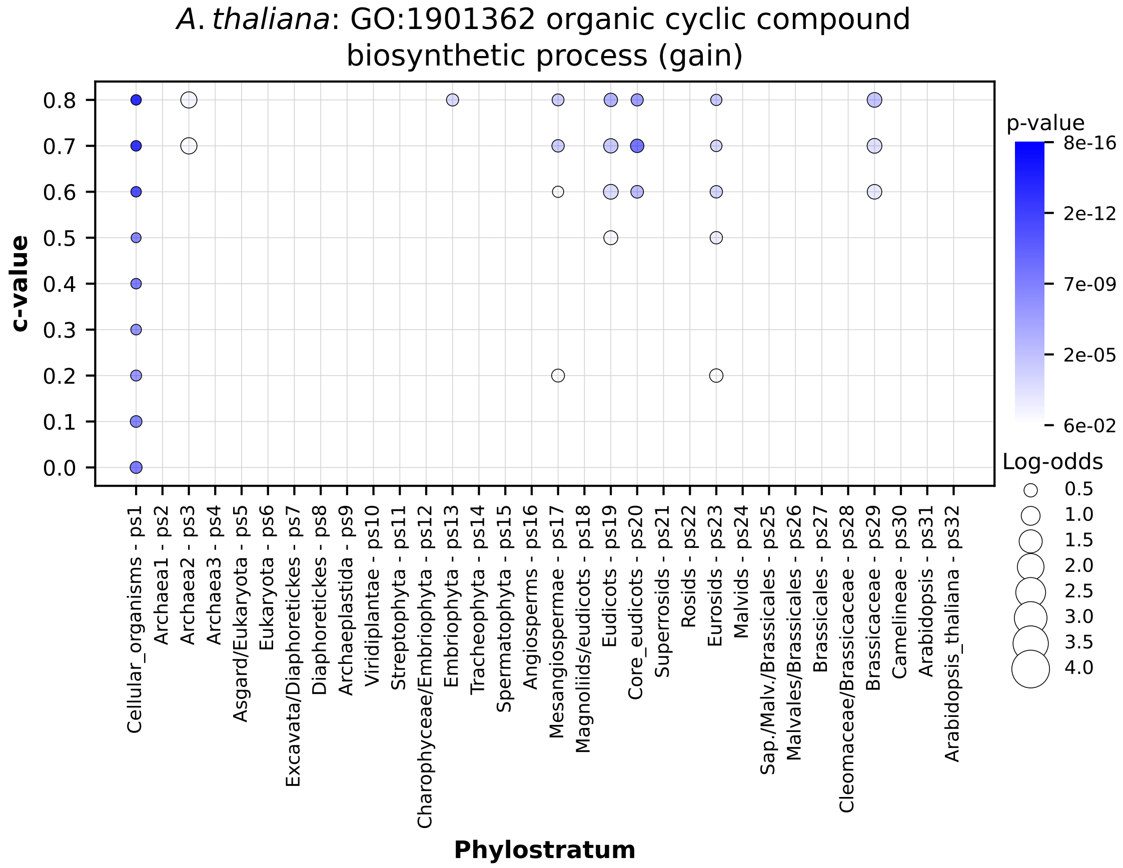
A. thaliana: GO:1901362 organic cyclic compound biosynthetic process (gain)
c-value
0.8
0.7
0.6
0.5
0.4
0.3
0.2
0.1
0.0
p-value
8e-16
2e-12
7e-09
2e-05
6e-02
Log-odds
0.5
1.0
1.5
2.0
2.5
3.0
3.5
4.0
Cellular_organisms - ps1
Archaea1 - ps2
Archaea2 - ps3
Archaea3 - ps4
Asgard/Eukaryota - ps5
Eukaryota - ps6
Excavata/Diaphoretickes - ps7
Diaphoretickes - ps8
Archaeplastida - ps9
Viridiplantae - ps10
Streptophyta - ps11
Charophyceae/Embriophyta - ps12
Embriophyta - ps13
Tracheophyta - ps14
Spermatophyta - ps15
Angiosperms - ps16
Mesangiospermae - ps17
Magnoliids/eudicots - ps18
Eudicots - ps19
Core_eudicots - ps20
Superrosids - ps21
Rosids - ps22
Eurosids - ps23
Malvids - ps24
Sap./Malv./Brassicales - ps25
Malvales/Brassicales - ps26
Brassicales - ps27
Cleomaceae/Brassicaceae - ps28
Brassicaceae - ps29
Camelineae - ps30
Arabidopsis - ps31
Arabidopsis_thaliana - ps32
Phylostratum

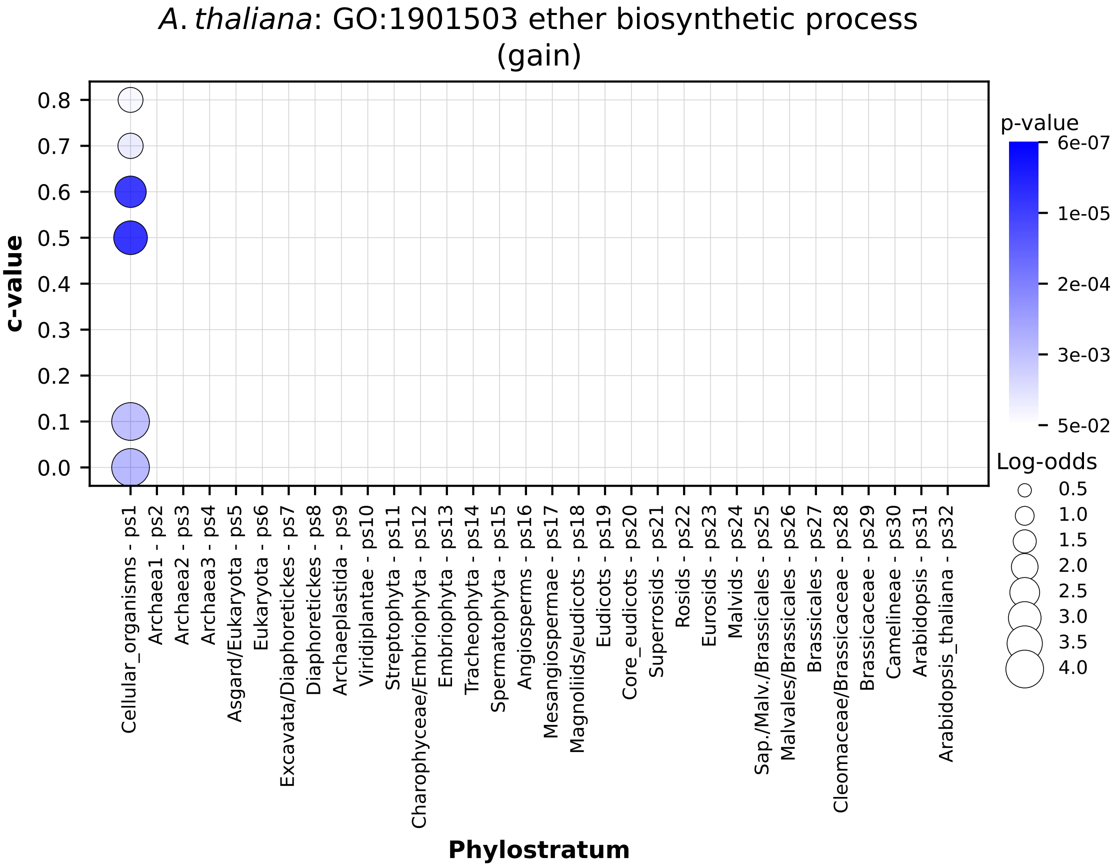

A. thaliana: GO:1901503 ether biosynthetic process
(gain)
c-value
0.8
0.7
0.6
0.5
0.4
0.3
0.2
0.1
0.0
p-value
6e-07
1e-05
2e-04
3e-03
5e-02
Log-odds
0.5
1.0
1.5
2.0
2.5
3.0
3.5
4.0
Cellular_organisms - ps1
Archaea1 - ps2
Archaea2 - ps3
Archaea3 - ps4
Asgard/Eukaryota - ps5
Eukaryota - ps6
Excavata/Diaphoretickes - ps7
Diaphoretickes - ps8
Archaeplastida - ps9
Viridiplantae - ps10
Streptophyta - ps11
Charophyceae/Embriophyta - ps12
Embriophyta - ps13
Tracheophyta - ps14
Spermatophyta - ps15
Angiosperms - ps16
Mesangiospermae - ps17
Magnoliids/eudicots - ps18
Eudicots - ps19
Core_eudicots - ps20
Superrosids - ps21
Rosids - ps22
Eurosids - ps23
Malvids - ps24
Sap./Malv./Brassicales - ps25
Malvales/Brassicales - ps26
Brassicales - ps27
Cleomaceae/Brassicaceae - ps28
Brassicaceae - ps29
Camelineae - ps30
Arabidopsis - ps31
Arabidopsis_thaliana - ps32
Phylostratum

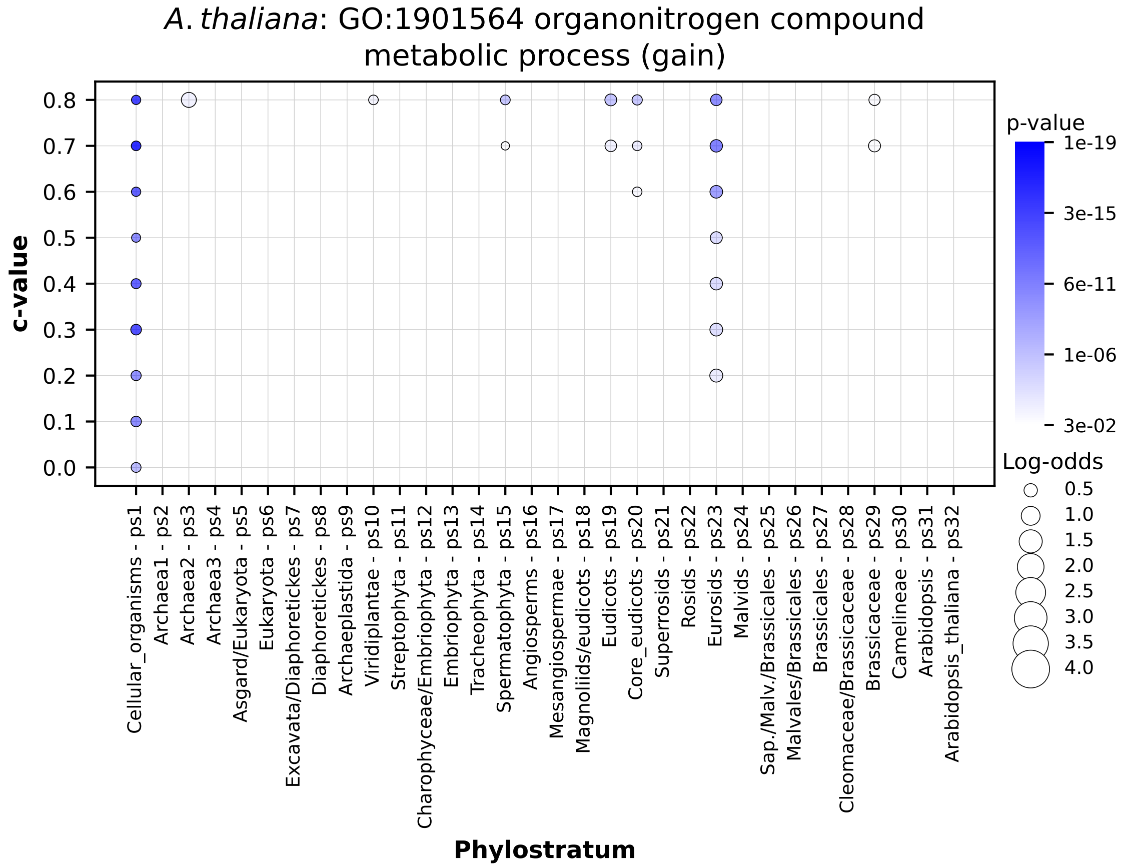

A. thaliana: GO:1901564 organonitrogen compound metabolic process (gain)
c-value
0.8
0.7
0.6
0.5
0.4
0.3
0.2
0.1
0.0
p-value
1e-19
3e-15
6e-11
1e-06
3e-02
Log-odds
0.5
1.0
1.5
2.0
2.5
3.0
3.5
4.0
Cellular_organisms - ps1
Archaea1 - ps2
Archaea2 - ps3
Archaea3 - ps4
Asgard/Eukaryota - ps5
Eukaryota - ps6
Excavata/Diaphoretickes - ps7
Diaphoretickes - ps8
Archaeplastida - ps9
Viridiplantae - ps10
Streptophyta - ps11
Charophyceae/Embriophyta - ps12
Embriophyta - ps13
Tracheophyta - ps14
Spermatophyta - ps15
Angiosperms - ps16
Mesangiospermae - ps17
Magnoliids/eudicots - ps18
Eudicots - ps19
Core_eudicots - ps20
Superrosids - ps21
Rosids - ps22
Eurosids - ps23
Malvids - ps24
Sap./Malv./Brassicales - ps25
Malvales/Brassicales - ps26
Brassicales - ps27
Cleomaceae/Brassicaceae - ps28
Brassicaceae - ps29
Camelineae - ps30
Arabidopsis - ps31
Arabidopsis_thaliana - ps32
Phylostratum

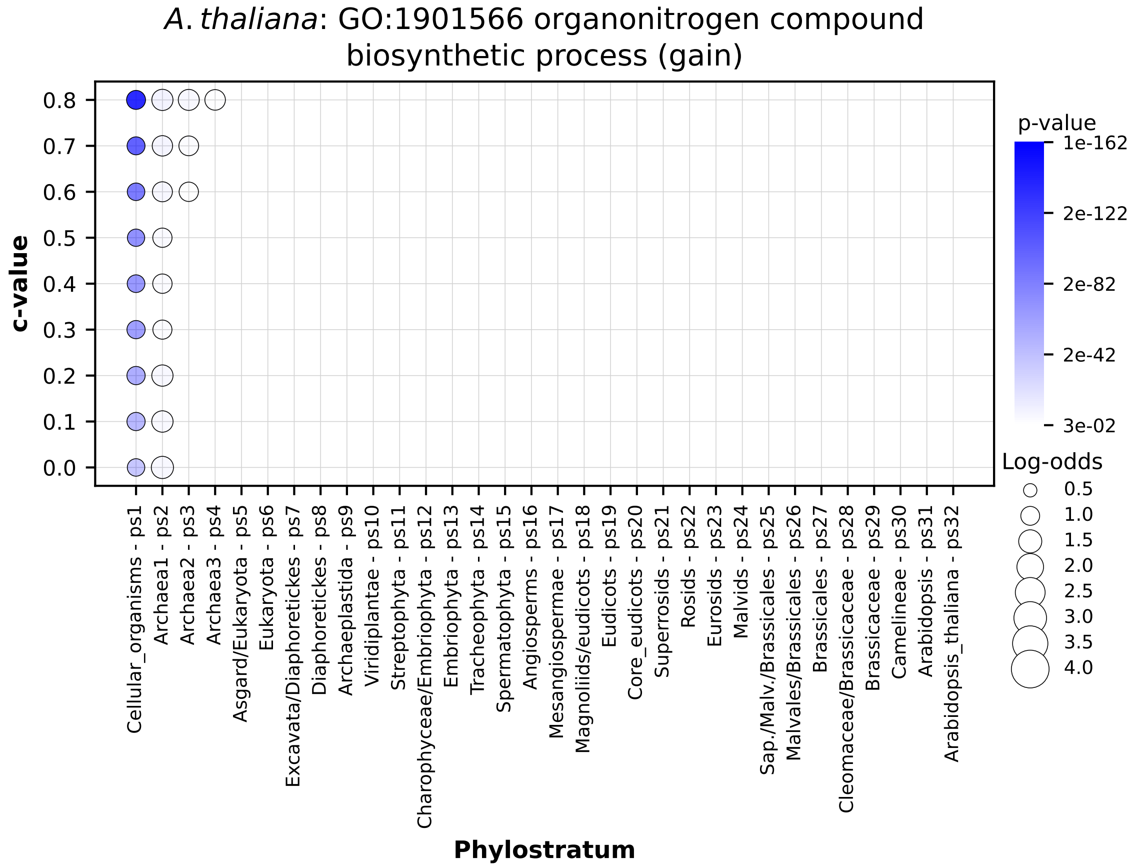
A. thaliana: GO:1901566 organonitrogen compound biosynthetic process (gain)
c-value
0.8
0.7
0.6
0.5
0.4
0.3
0.2
0.1
0.0
p-value
1e-162
2e-122
2e-82
2e-42
3e-02
Log-odds
0.5
1.0
1.5
2.0
2.5
3.0
3.5
4.0
Cellular_organisms - ps1
Archaea1 - ps2
Archaea2 - ps3
Archaea3 - ps4
Asgard/Eukaryota - ps5
Eukaryota - ps6
Excavata/Diaphoretickes - ps7
Diaphoretickes - ps8
Archaeplastida - ps9
Viridiplantae - ps10
Streptophyta - ps11
Charophyceae/Embriophyta - ps12
Embriophyta - ps13
Tracheophyta - ps14
Spermatophyta - ps15
Angiosperms - ps16
Mesangiospermae - ps17
Magnoliids/eudicots - ps18
Eudicots - ps19
Core_eudicots - ps20
Superrosids - ps21
Rosids - ps22
Eurosids - ps23
Malvids - ps24
Sap./Malv./Brassicales - ps25
Malvales/Brassicales - ps26
Brassicales - ps27
Cleomaceae/Brassicaceae - ps28
Brassicaceae - ps29
Camelineae - ps30
Arabidopsis - ps31
Arabidopsis_thaliana - ps32
Phylostratum

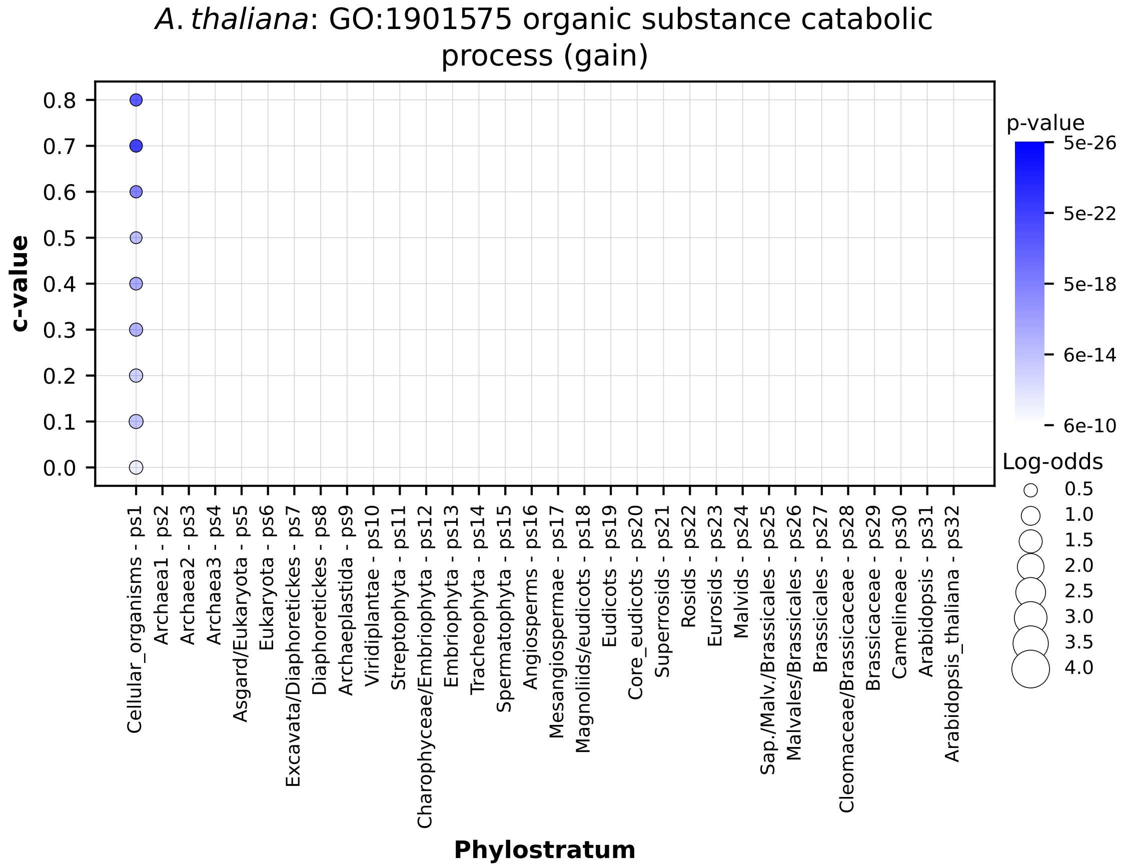
A. thaliana: GO:1901575 organic substance catabolic process (gain)
c-value
0.0
0.1
0.2
0.3
0.4
0.5
0.6
0.7
0.8
Phylostratum
Cellular_organisms - ps1
Archaea1 - ps2
Archaea2 - ps3
Archaea3 - ps4
Asgard/Eukaryota - ps5
Eukaryota - ps6
Excavata/Diaphoretickes - ps7
Diaphoretickes - ps8
Archaeplastida - ps9
Viridiplantae - ps10
Streptophyta - ps11
Charophyceae/Embriophyta - ps12
Embriophyta - ps13
Tracheophyta - ps14
Spermatophyta - ps15
Angiosperms - ps16
Mesangiospermae - ps17
Magnoliids/eudicots - ps18
Eudicots - ps19
Core_eudicots - ps20
Superrosids - ps21
Rosids - ps22
Eurosids - ps23
Malvids - ps24
Sap./Malv./Brassicales - ps25
Malvales/Brassicales - ps26
Brassicales - ps27
Cleomaceae/Brassicaceae - ps28
Brassicaceae - ps29
Camelineae - ps30
Arabidopsis - ps31
Arabidopsis_thaliana - ps32
p-value
5e-26
5e-22
5e-18
6e-14
6e-10
Log-odds
0.5
1.0
1.5
2.0
2.5
3.0
3.5
4.0

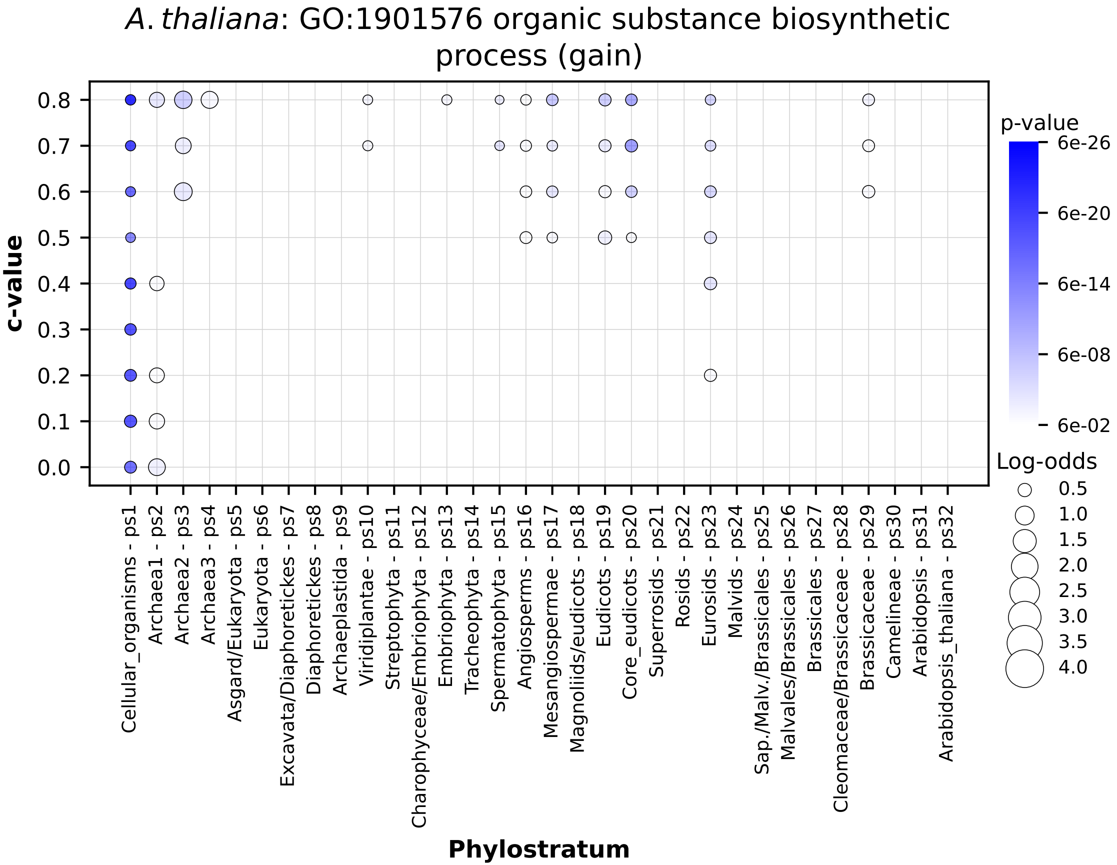

A. thaliana: GO:1901576 organic substance biosynthetic process (gain)
c-value
0.8
0.7
0.6
0.5
0.4
0.3
0.2
0.1
0.0
Phylostratum
Cellular_organisms - ps1
Archaea1 - ps2
Archaea2 - ps3
Archaea3 - ps4
Asgard/Eukaryota - ps5
Eukaryota - ps6
Excavata/Diaphoretickes - ps7
Diaphoretickes - ps8
Archaeplastida - ps9
Viridiplantae - ps10
Streptophyta - ps11
Charophyceae/Embriophyta - ps12
Embriophyta - ps13
Tracheophyta - ps14
Spermatophyta - ps15
Angiosperms - ps16
Mesangiospermae - ps17
Magnoliids/eudicots - ps18
Eudicots - ps19
Core_eudicots - ps20
Superrosids - ps21
Rosids - ps22
Eurosids - ps23
Malvids - ps24
Sap./Malv./Brassicales - ps25
Malvales/Brassicales - ps26
Brassicales - ps27
Cleomaceae/Brassicaceae - ps28
Brassicaceae - ps29
Camelineae - ps30
Arabidopsis - ps31
Arabidopsis_thaliana - ps32
p-value
6e-26
6e-20
6e-14
6e-08
6e-02
Log-odds
0.5
1.0
1.5
2.0
2.5
3.0
3.5
4.0

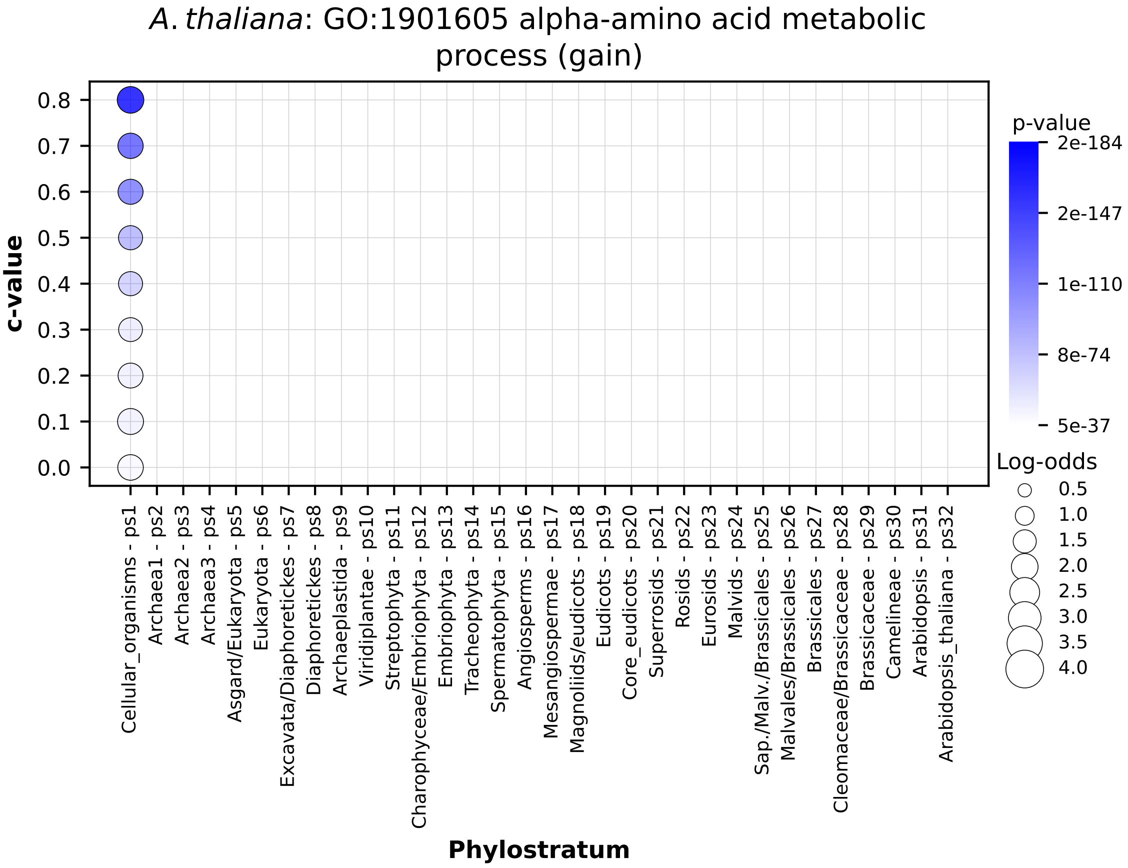
A. thaliana: GO:1901605 alpha-amino acid metabolic process (gain)
c-value
0.8
0.7
0.6
0.5
0.4
0.3
0.2
0.1
0.0
p-value
2e-184
2e-147
1e-110
8e-74
5e-37
Log-odds
0.5
1.0
1.5
2.0
2.5
3.0
3.5
4.0
Cellular_organisms - ps1
Archaea1 - ps2
Archaea2 - ps3
Archaea3 - ps4
Asgard/Eukaryota - ps5
Eukaryota - ps6
Excavata/Diaphoretickes - ps7
Diaphoretickes - ps8
Archaeplastida - ps9
Viridiplantae - ps10
Streptophyta - ps11
Charophyceae/Embriophyta - ps12
Embriophyta - ps13
Tracheophyta - ps14
Spermatophyta - ps15
Angiosperms - ps16
Mesangiospermae - ps17
Magnoliids/eudicots - ps18
Eudicots - ps19
Core_eudicots - ps20
Superrosids - ps21
Rosids - ps22
Eurosids - ps23
Malvids - ps24
Sap./Malv./Brassicales - ps25
Malvales/Brassicales - ps26
Brassicales - ps27
Cleomaceae/Brassicaceae - ps28
Brassicaceae - ps29
Camelineae - ps30
Arabidopsis - ps31
Arabidopsis_thaliana - ps32
Phylostratum

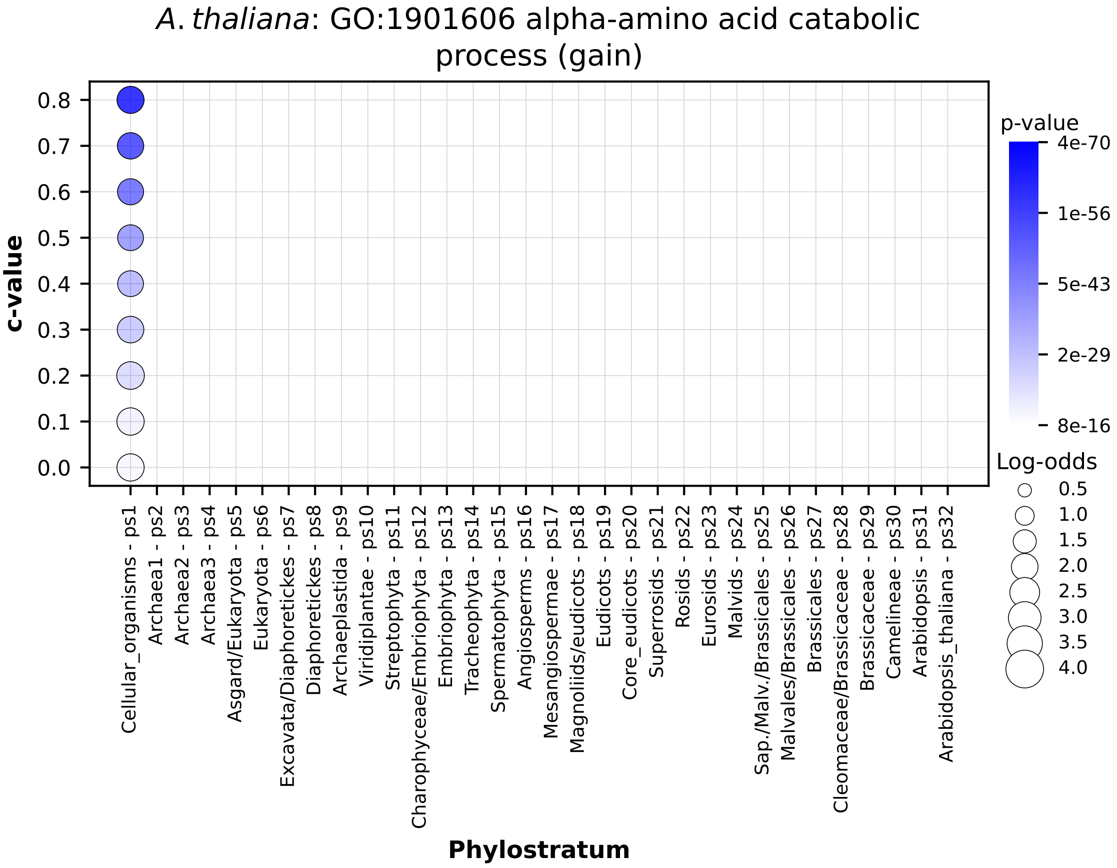

A. thaliana: GO:1901606 alpha-amino acid catabolic process (gain)
c-value
0.8
0.7
0.6
0.5
0.4
0.3
0.2
0.1
0.0
p-value
4e-70
1e-56
5e-43
2e-29
8e-16
Log-odds
0.5
1.0
1.5
2.0
2.5
3.0
3.5
4.0
Cellular_organisms - ps1
Archaea1 - ps2
Archaea2 - ps3
Archaea3 - ps4
Asgard/Eukaryota - ps5
Eukaryota - ps6
Excavata/Diaphoretickes - ps7
Diaphoretickes - ps8
Archaeplastida - ps9
Viridiplantae - ps10
Streptophyta - ps11
Charophyceae/Embriophyta - ps12
Embriophyta - ps13
Tracheophyta - ps14
Spermatophyta - ps15
Angiosperms - ps16
Mesangiospermae - ps17
Magnoliids/eudicots - ps18
Eudicots - ps19
Core_eudicots - ps20
Superrosids - ps21
Rosids - ps22
Eurosids - ps23
Malvids - ps24
Sap./Malv./Brassicales - ps25
Malvales/Brassicales - ps26
Brassicales - ps27
Cleomaceae/Brassicaceae - ps28
Brassicaceae - ps29
Camelineae - ps30
Arabidopsis - ps31
Arabidopsis_thaliana - ps32
Phylostratum

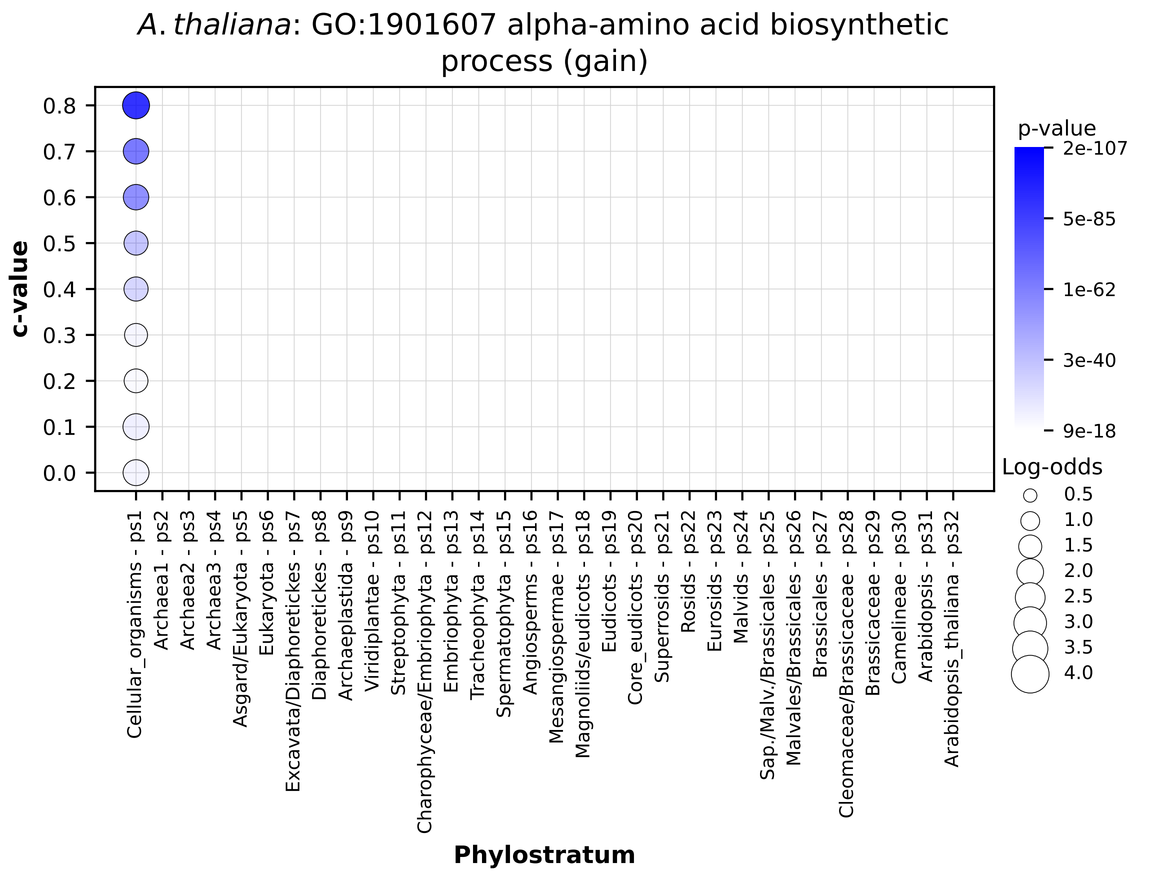

A. thaliana: GO:1901607 alpha-amino acid biosynthetic process (gain)
c-value
0.8
0.7
0.6
0.5
0.4
0.3
0.2
0.1
0.0
p-value
2e-107
5e-85
1e-62
3e-40
9e-18
Log-odds
0.5
1.0
1.5
2.0
2.5
3.0
3.5
4.0
Cellular_organisms - ps1
Archaea1 - ps2
Archaea2 - ps3
Archaea3 - ps4
Asgard/Eukaryota - ps5
Eukaryota - ps6
Excavata/Diaphoretickes - ps7
Diaphoretickes - ps8
Archaeplastida - ps9
Viridiplantae - ps10
Streptophyta - ps11
Charophyceae/Embriophyta - ps12
Embriophyta - ps13
Tracheophyta - ps14
Spermatophyta - ps15
Angiosperms - ps16
Mesangiospermae - ps17
Magnoliids/eudicots - ps18
Eudicots - ps19
Core_eudicots - ps20
Superrosids - ps21
Rosids - ps22
Eurosids - ps23
Malvids - ps24
Sap./Malv./Brassicales - ps25
Malvales/Brassicales - ps26
Brassicales - ps27
Cleomaceae/Brassicaceae - ps28
Brassicaceae - ps29
Camelineae - ps30
Arabidopsis - ps31
Arabidopsis_thaliana - ps32
Phylostratum

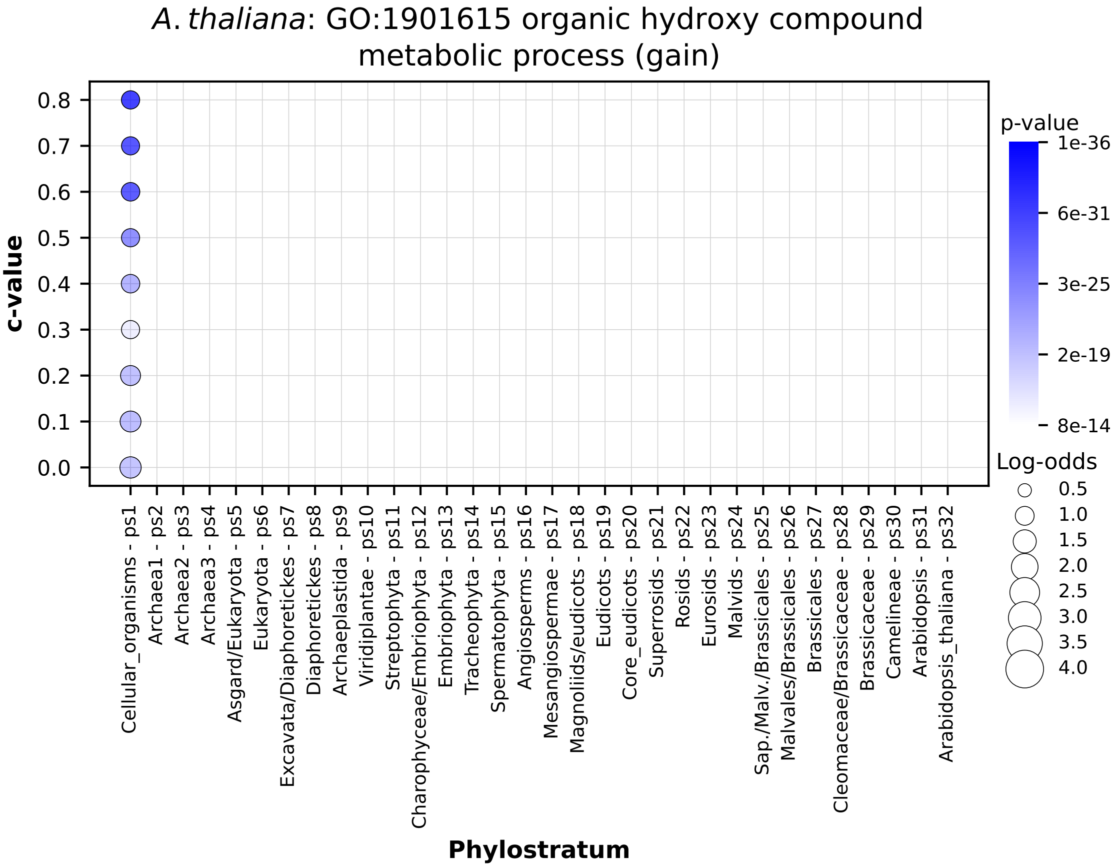
A. thaliana: GO:1901615 organic hydroxy compound metabolic process (gain)
c-value
0.8
0.7
0.6
0.5
0.4
0.3
0.2
0.1
0.0
p-value
1e-36
6e-31
3e-25
2e-19
8e-14
Log-odds
0.5
1.0
1.5
2.0
2.5
3.0
3.5
4.0
Cellular_organisms - ps1
Archaea1 - ps2
Archaea2 - ps3
Archaea3 - ps4
Asgard/Eukaryota - ps5
Eukaryota - ps6
Excavata/Diaphoretickes - ps7
Diaphoretickes - ps8
Archaeplastida - ps9
Viridiplantae - ps10
Streptophyta - ps11
Charophyceae/Embriophyta - ps12
Embriophyta - ps13
Tracheophyta - ps14
Spermatophyta - ps15
Angiosperms - ps16
Mesangiospermae - ps17
Magnoliids/eudicots - ps18
Eudicots - ps19
Core_eudicots - ps20
Superrosids - ps21
Rosids - ps22
Eurosids - ps23
Malvids - ps24
Sap./Malv./Brassicales - ps25
Malvales/Brassicales - ps26
Brassicales - ps27
Cleomaceae/Brassicaceae - ps28
Brassicaceae - ps29
Camelineae - ps30
Arabidopsis - ps31
Arabidopsis_thaliana - ps32
Phylostratum

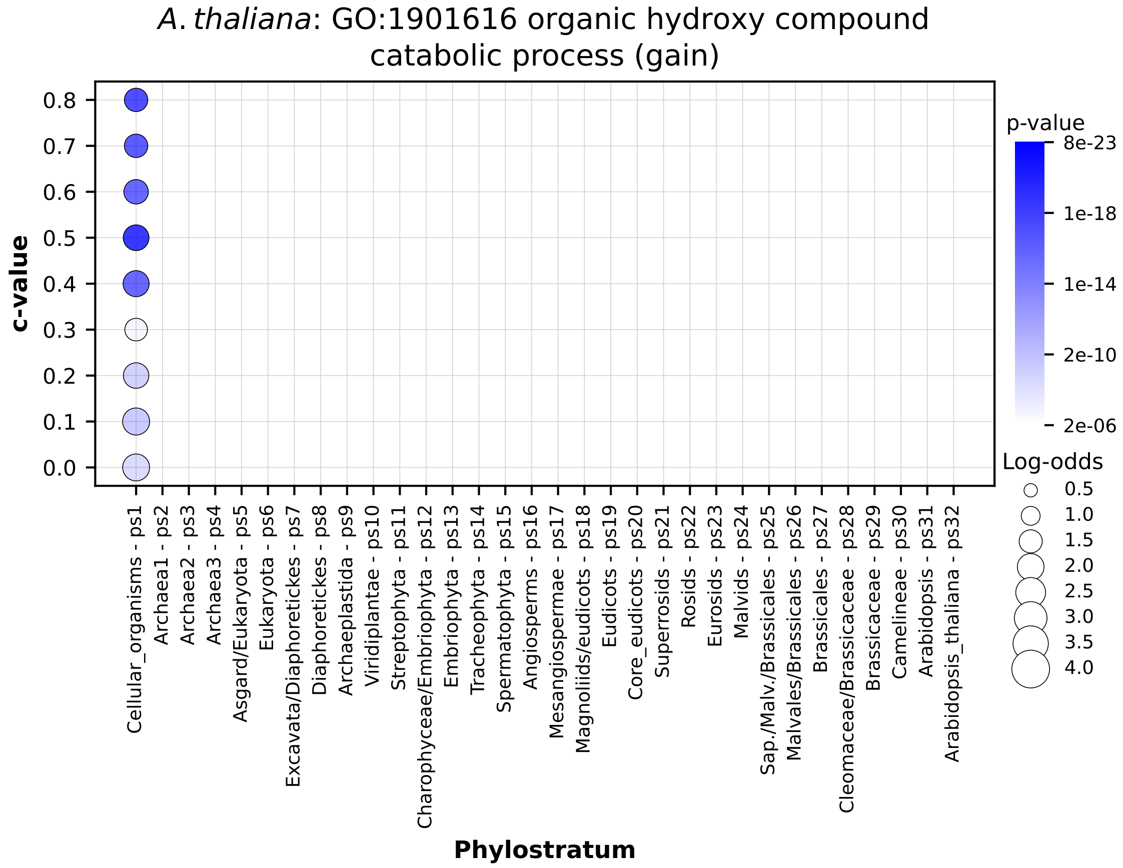
A. thaliana: GO:1901616 organic hydroxy compound catabolic process (gain)
c-value
0.8
0.7
0.6
0.5
0.4
0.3
0.2
0.1
0.0
p-value
8e-23
1e-18
1e-14
2e-10
2e-06
Log-odds
0.5
1.0
1.5
2.0
2.5
3.0
3.5
4.0
Cellular_organisms - ps1
Archaea1 - ps2
Archaea2 - ps3
Archaea3 - ps4
Asgard/Eukaryota - ps5
Eukaryota - ps6
Excavata/Diaphoretickes - ps7
Diaphoretickes - ps8
Archaeplastida - ps9
Viridiplantae - ps10
Streptophyta - ps11
Charophyceae/Embriophyta - ps12
Embriophyta - ps13
Tracheophyta - ps14
Spermatophyta - ps15
Angiosperms - ps16
Mesangiospermae - ps17
Magnoliids/eudicots - ps18
Eudicots - ps19
Core_eudicots - ps20
Superrosids - ps21
Rosids - ps22
Eurosids - ps23
Malvids - ps24
Sap./Malv./Brassicales - ps25
Malvales/Brassicales - ps26
Brassicales - ps27
Cleomaceae/Brassicaceae - ps28
Brassicaceae - ps29
Camelineae - ps30
Arabidopsis - ps31
Arabidopsis_thaliana - ps32
Phylostratum

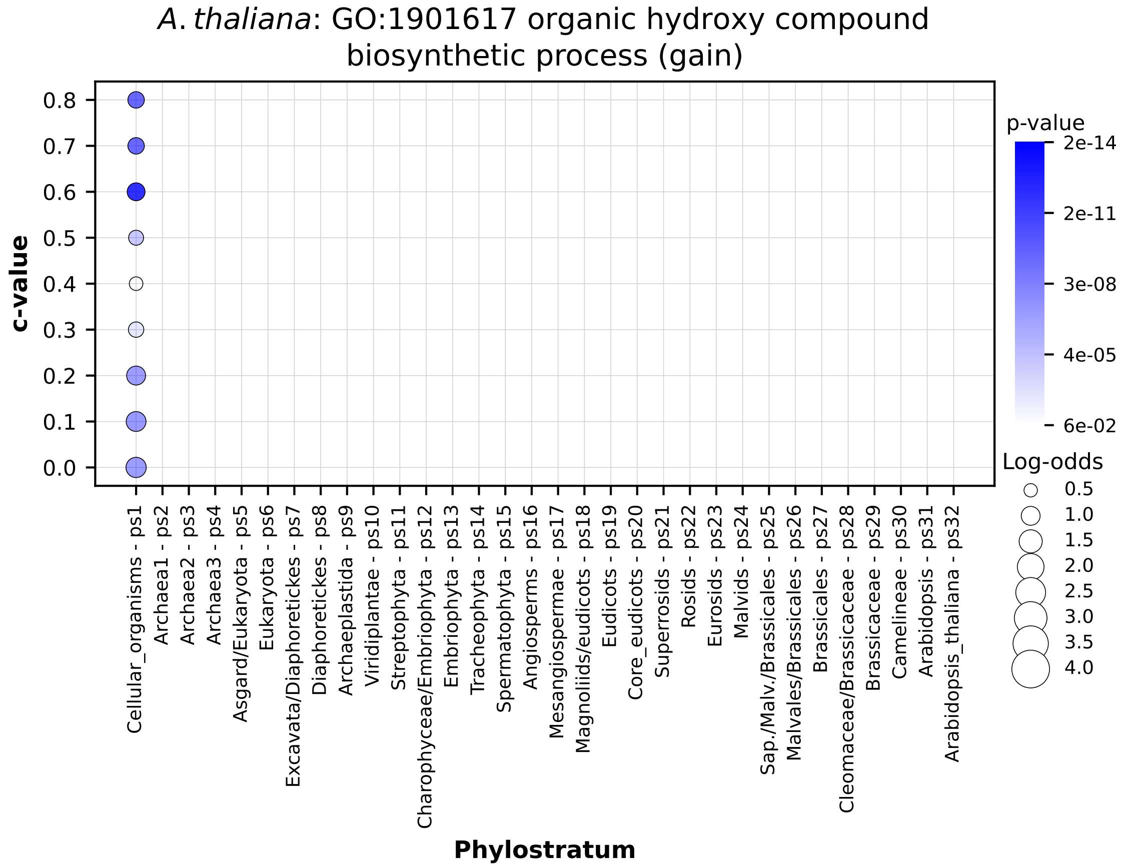

A. thaliana: GO:1901617 organic hydroxy compound biosynthetic process (gain)
c-value
0.0
0.1
0.2
0.3
0.4
0.5
0.6
0.7
0.8
Phylostratum
Cellular_organisms - ps1
Archaea1 - ps2
Archaea2 - ps3
Archaea3 - ps4
Asgard/Eukaryota - ps5
Eukaryota - ps6
Excavata/Diaphoretickes - ps7
Diaphoretickes - ps8
Archaeplastida - ps9
Viridiplantae - ps10
Streptophyta - ps11
Charophyceae/Embriophyta - ps12
Embriophyta - ps13
Tracheophyta - ps14
Spermatophyta - ps15
Angiosperms - ps16
Mesangiospermae - ps17
Magnoliids/eudicots - ps18
Eudicots - ps19
Core_eudicots - ps20
Superrosids - ps21
Rosids - ps22
Eurosids - ps23
Malvids - ps24
Sap./Malv./Brassicales - ps25
Malvales/Brassicales - ps26
Brassicales - ps27
Cleomaceae/Brassicaceae - ps28
Brassicaceae - ps29
Camelineae - ps30
Arabidopsis - ps31
Arabidopsis_thaliana - ps32
p-value
2e-14
2e-11
3e-08
4e-05
6e-02
Log-odds
0.5
1.0
1.5
2.0
2.5
3.0
3.5
4.0

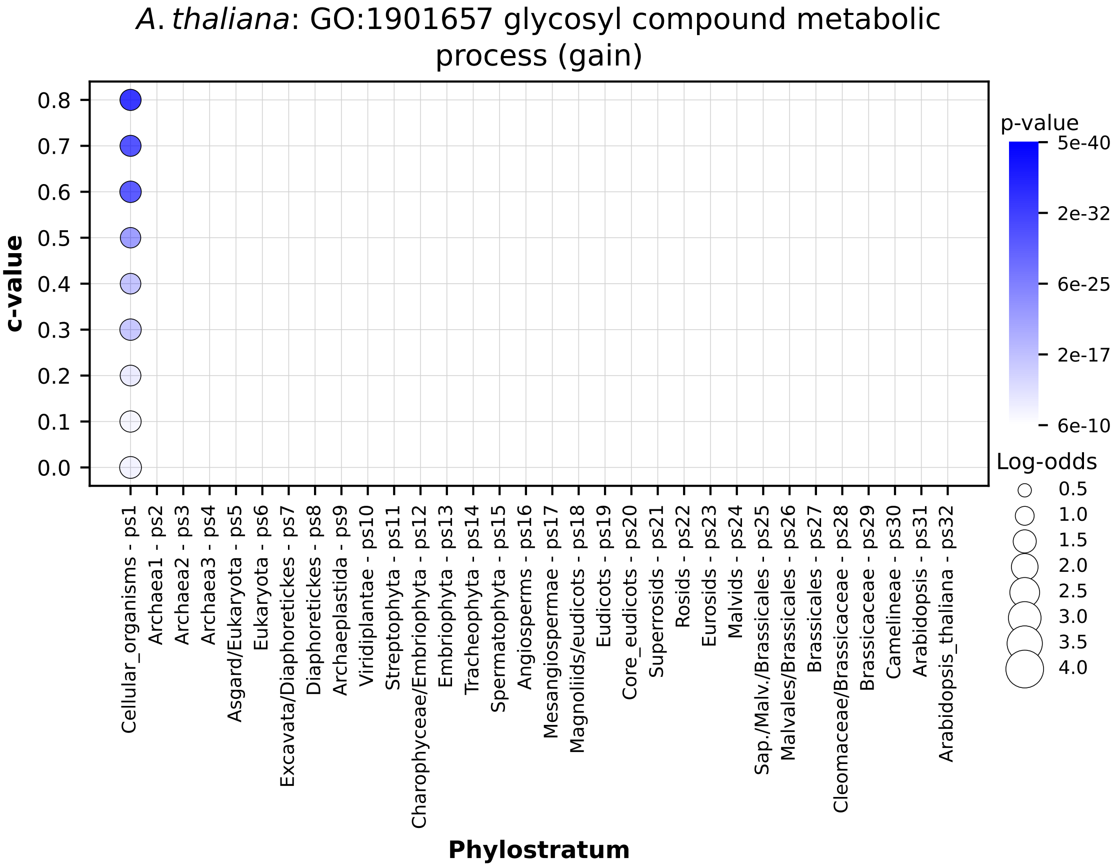

A. thaliana: GO:1901657 glycosyl compound metabolic process (gain)
c-value
0.8
0.7
0.6
0.5
0.4
0.3
0.2
0.1
0.0
p-value
5e-40
2e-32
6e-25
2e-17
6e-10
Log-odds
0.5
1.0
1.5
2.0
2.5
3.0
3.5
4.0
Cellular_organisms - ps1
Archaea1 - ps2
Archaea2 - ps3
Archaea3 - ps4
Asgard/Eukaryota - ps5
Eukaryota - ps6
Excavata/Diaphoretickes - ps7
Diaphoretickes - ps8
Archaeplastida - ps9
Viridiplantae - ps10
Streptophyta - ps11
Charophyceae/Embriophyta - ps12
Embriophyta - ps13
Tracheophyta - ps14
Spermatophyta - ps15
Angiosperms - ps16
Mesangiospermae - ps17
Magnoliids/eudicots - ps18
Eudicots - ps19
Core_eudicots - ps20
Superrosids - ps21
Rosids - ps22
Eurosids - ps23
Malvids - ps24
Sap./Malv./Brassicales - ps25
Malvales/Brassicales - ps26
Brassicales - ps27
Cleomaceae/Brassicaceae - ps28
Brassicaceae - ps29
Camelineae - ps30
Arabidopsis - ps31
Arabidopsis_thaliana - ps32
Phylostratum

A. thaliana: GO:1901658 glycosyl compound catabolic process (gain)

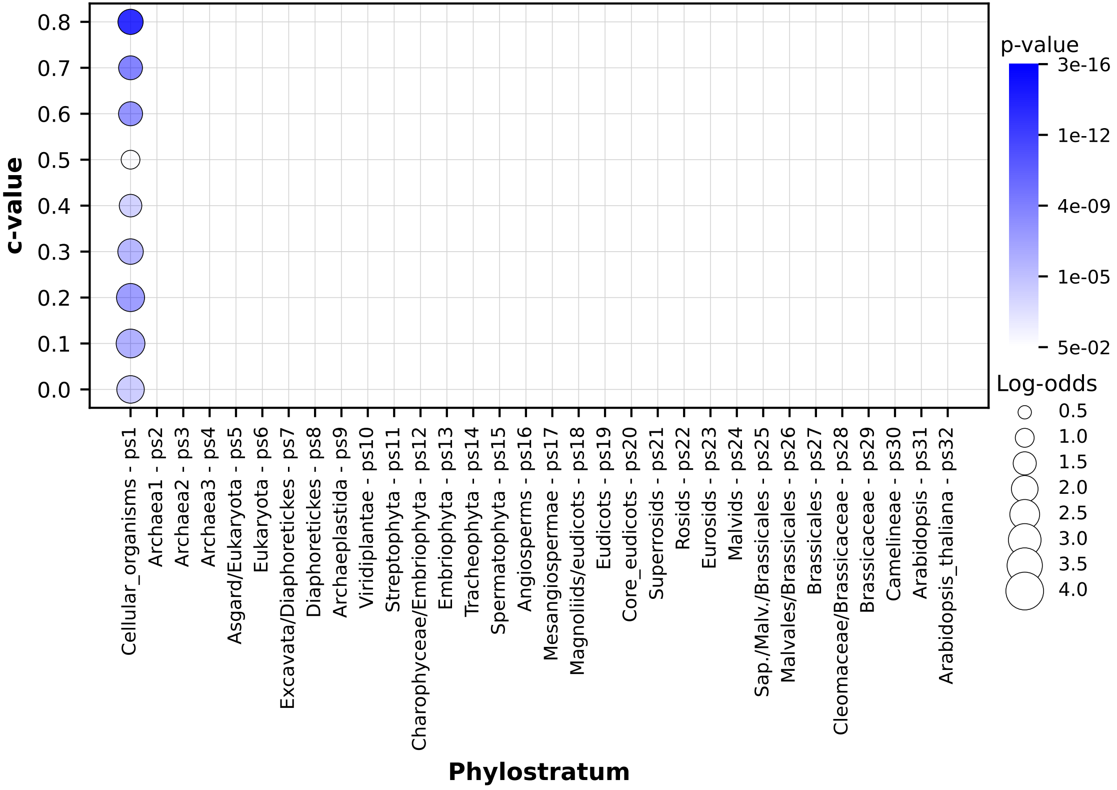

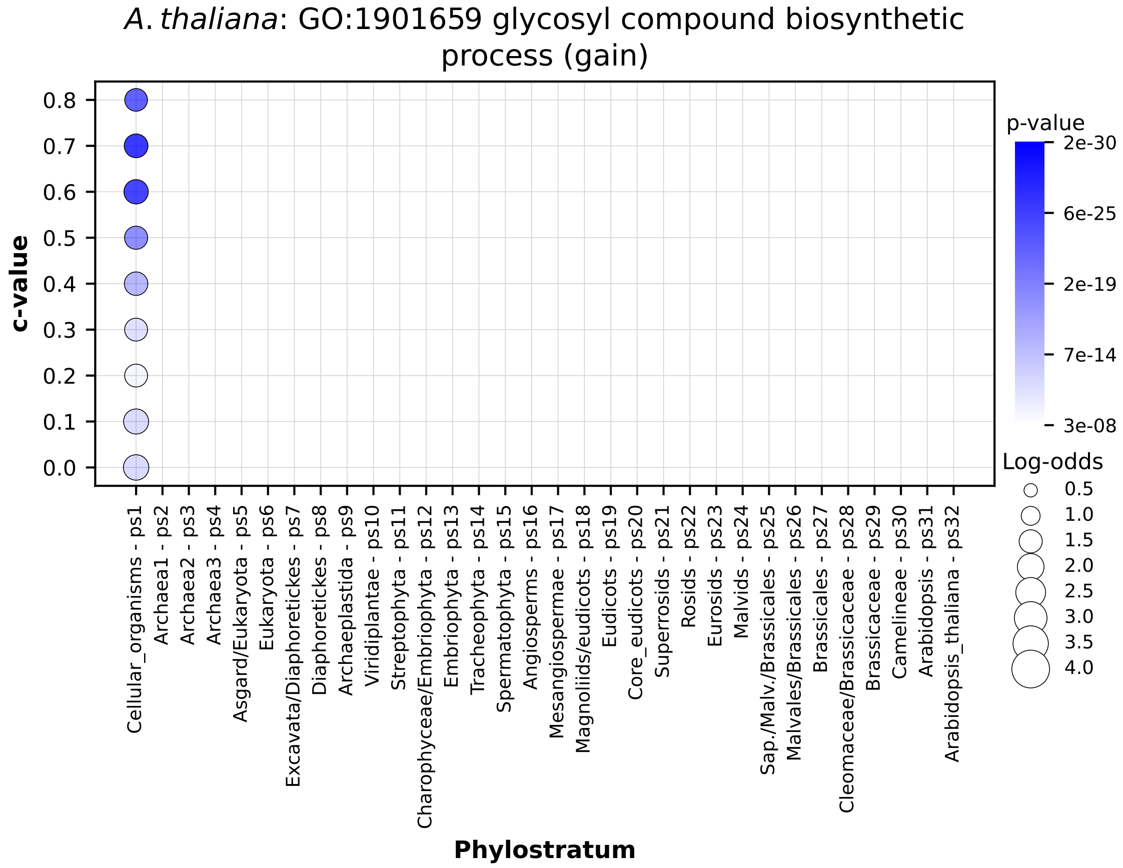

A. thaliana: GO:1901659 glycosyl compound biosynthetic process (gain)
c-value
0.8
0.7
0.6
0.5
0.4
0.3
0.2
0.1
0.0
p-value
2e-30
6e-25
2e-19
7e-14
3e-08
Log-odds
0.5
1.0
1.5
2.0
2.5
3.0
3.5
4.0
Cellular_organisms - ps1
Archaea1 - ps2
Archaea2 - ps3
Archaea3 - ps4
Asgard/Eukaryota - ps5
Eukaryota - ps6
Excavata/Diaphoretickes - ps7
Diaphoretickes - ps8
Archaeplastida - ps9
Viridiplantae - ps10
Streptophyta - ps11
Charophyceae/Embriophyta - ps12
Embriophyta - ps13
Tracheophyta - ps14
Spermatophyta - ps15
Angiosperms - ps16
Mesangiospermae - ps17
Magnoliids/eudicots - ps18
Eudicots - ps19
Core_eudicots - ps20
Superrosids - ps21
Rosids - ps22
Eurosids - ps23
Malvids - ps24
Sap./Malv./Brassicales - ps25
Malvales/Brassicales - ps26
Brassicales - ps27
Cleomaceae/Brassicaceae - ps28
Brassicaceae - ps29
Camelineae - ps30
Arabidopsis - ps31
Arabidopsis_thaliana - ps32
Phylostratum

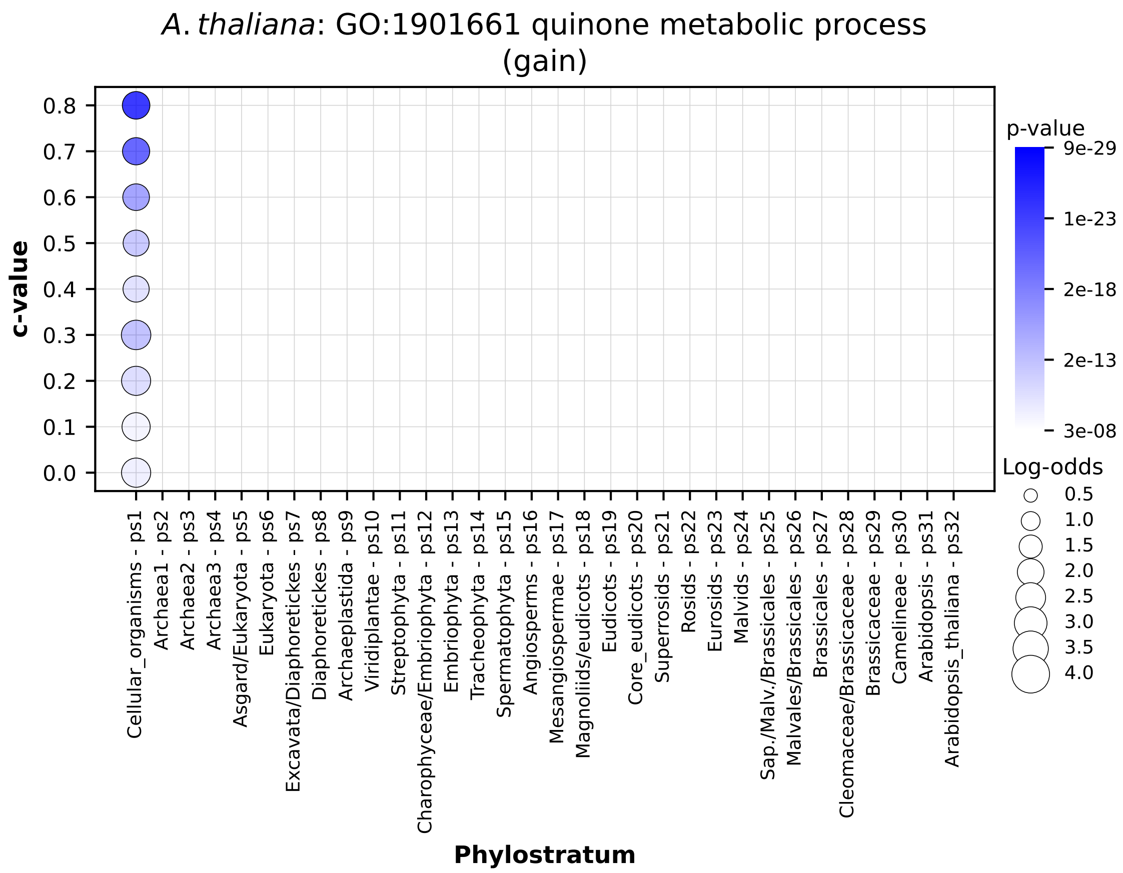

$A. thaliana$: GO:1901661 quinone metabolic process
(gain)
c-value
0.8
0.7
0.6
0.5
0.4
0.3
0.2
0.1
0.0
p-value
9e-29
1e-23
2e-18
2e-13
3e-08
Log-odds
0.5
1.0
1.5
2.0
2.5
3.0
3.5
4.0
Cellular_organisms - ps1
Archaea1 - ps2
Archaea2 - ps3
Archaea3 - ps4
Asgard/Eukaryota - ps5
Eukaryota - ps6
Excavata/Diaphoretickes - ps7
Diaphoretickes - ps8
Archaeplastida - ps9
Viridiplantae - ps10
Streptophyta - ps11
Charophyceae/Embriophyta - ps12
Embriophyta - ps13
Tracheophyta - ps14
Spermatophyta - ps15
Angiosperms - ps16
Mesangiospermae - ps17
Magnoliids/eudicots - ps18
Eudicots - ps19
Core_eudicots - ps20
Superrosids - ps21
Rosids - ps22
Eurosids - ps23
Malvids - ps24
Sap./Malv./Brassicales - ps25
Malvales/Brassicales - ps26
Brassicales - ps27
Cleomaceae/Brassicaceae - ps28
Brassicaceae - ps29
Camelineae - ps30
Arabidopsis - ps31
Arabidopsis_thaliana - ps32
Phylostratum

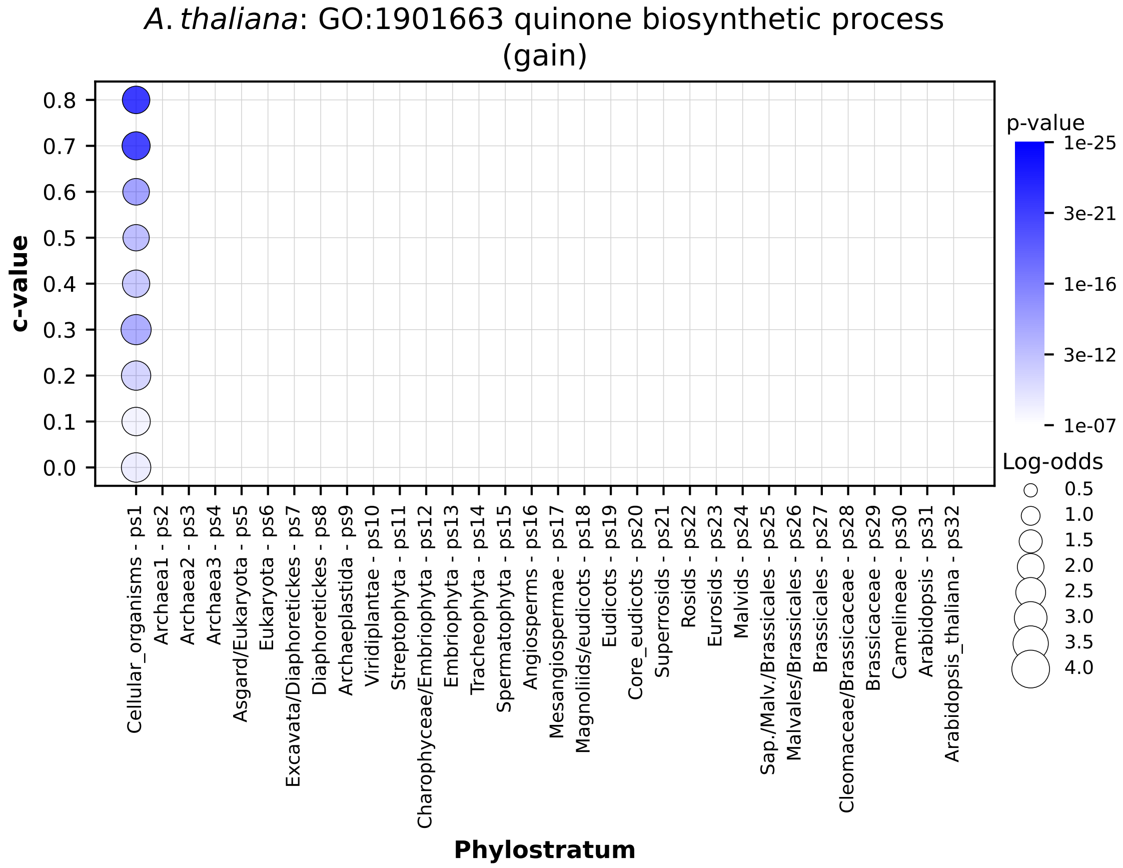

A. thaliana: GO:1901663 quinone biosynthetic process (gain)
c-value
0.8
0.7
0.6
0.5
0.4
0.3
0.2
0.1
0.0
p-value
1e-25
3e-21
1e-16
3e-12
1e-07
Log-odds
0.5
1.0
1.5
2.0
2.5
3.0
3.5
4.0
Cellular_organisms - ps1
Archaea1 - ps2
Archaea2 - ps3
Archaea3 - ps4
Asgard/Eukaryota - ps5
Eukaryota - ps6
Excavata/Diaphoretickes - ps7
Diaphoretickes - ps8
Archaeplastida - ps9
Viridiplantae - ps10
Streptophyta - ps11
Charophyceae/Embriophyta - ps12
Embriophyta - ps13
Tracheophyta - ps14
Spermatophyta - ps15
Angiosperms - ps16
Mesangiospermae - ps17
Magnoliids/eudicots - ps18
Eudicots - ps19
Core_eudicots - ps20
Superrosids - ps21
Rosids - ps22
Eurosids - ps23
Malvids - ps24
Sap./Malv./Brassicales - ps25
Malvales/Brassicales - ps26
Brassicales - ps27
Cleomaceae/Brassicaceae - ps28
Brassicaceae - ps29
Camelineae - ps30
Arabidopsis - ps31
Arabidopsis_thaliana - ps32
Phylostratum

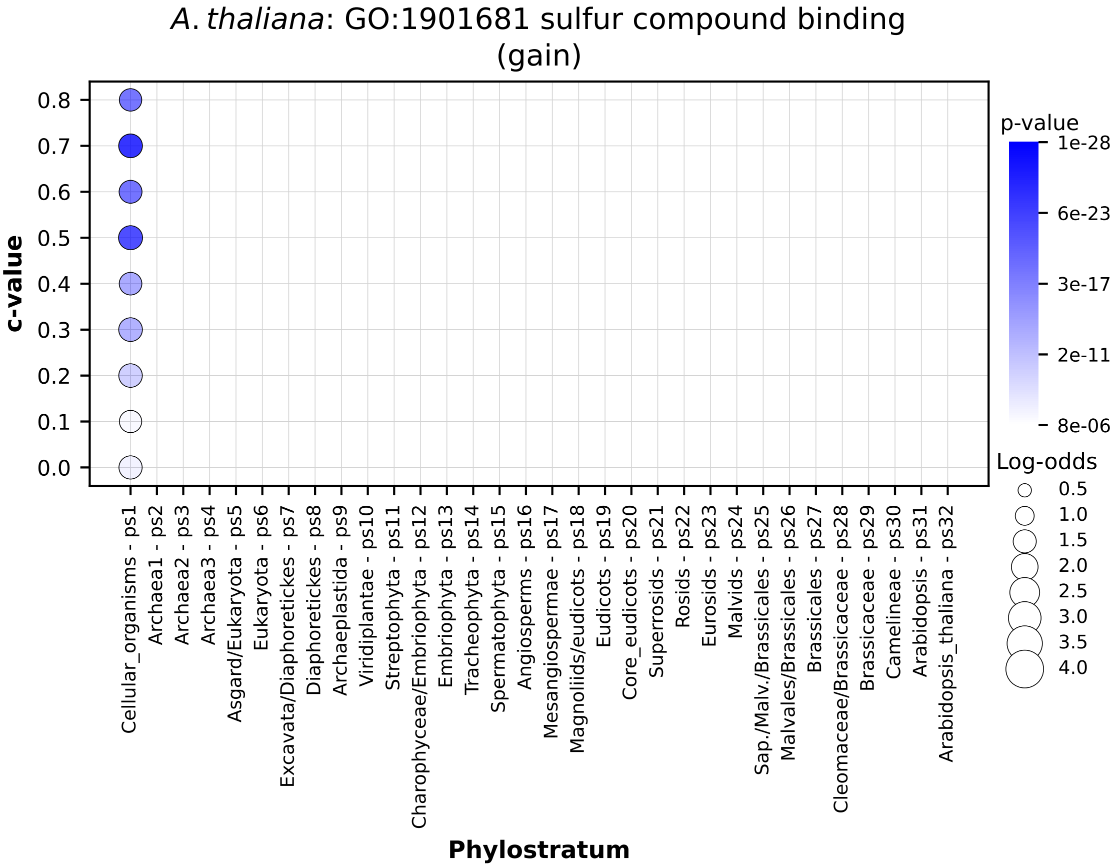

A. thaliana: GO:1901681 sulfur compound binding
(gain)
c-value
0.8
0.7
0.6
0.5
0.4
0.3
0.2
0.1
0.0
Phylostratum
Cellular_organisms - ps1
Archaea1 - ps2
Archaea2 - ps3
Archaea3 - ps4
Asgard/Eukaryota - ps5
Eukaryota - ps6
Excavata/Diaphoretickes - ps7
Diaphoretickes - ps8
Archaeplastida - ps9
Viridiplantae - ps10
Streptophyta - ps11
Charophyceae/Embriophyta - ps12
Embriophyta - ps13
Tracheophyta - ps14
Spermatophyta - ps15
Angiosperms - ps16
Mesangiospermae - ps17
Magnoliids/eudicots - ps18
Eudicots - ps19
Core_eudicots - ps20
Superrosids - ps21
Rosids - ps22
Eurosids - ps23
Malvids - ps24
Sap./Malv./Brassicales - ps25
Malvales/Brassicales - ps26
Brassicales - ps27
Cleomaceae/Brassicaceae - ps28
Brassicaceae - ps29
Camelineae - ps30
Arabidopsis - ps31
Arabidopsis_thaliana - ps32
p-value
1e-28
6e-23
3e-17
2e-11
8e-06
Log-odds
0.5
1.0
1.5
2.0
2.5
3.0
3.5
4.0

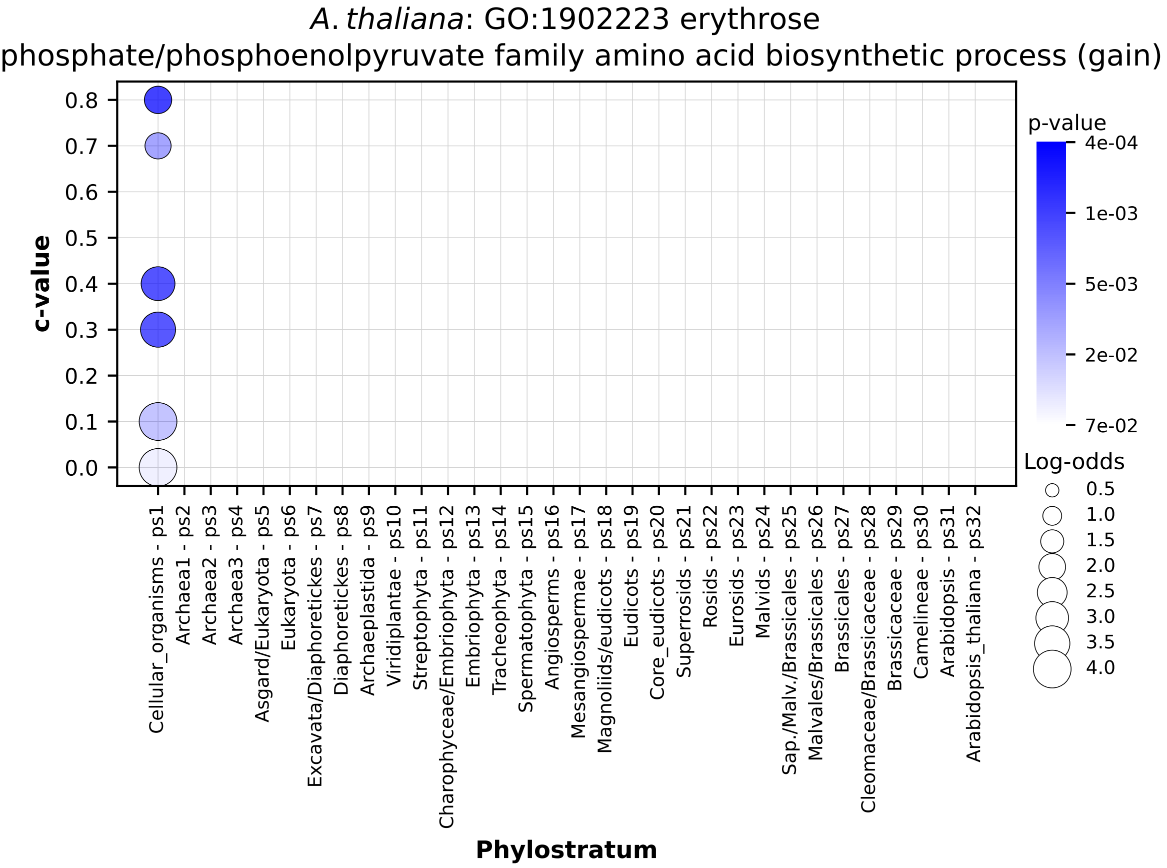

A. thaliana: GO:1902223 erythrose phosphate/phosphoenolpyruvate family amino acid biosynthetic process (gain)
c-value
0.8
0.7
0.6
0.5
0.4
0.3
0.2
0.1
0.0
p-value
4e-04
1e-03
5e-03
2e-02
7e-02
Log-odds
0.5
1.0
1.5
2.0
2.5
3.0
3.5
4.0
Cellular_organisms - ps1
Archaea1 - ps2
Archaea2 - ps3
Archaea3 - ps4
Asgard/Eukaryota - ps5
Eukaryota - ps6
Excavata/Diaphoretickes - ps7
Diaphoretickes - ps8
Archaeplastida - ps9
Viridiplantae - ps10
Streptophyta - ps11
Charophyceae/Embriophyta - ps12
Embriophyta - ps13
Tracheophyta - ps14
Spermatophyta - ps15
Angiosperms - ps16
Mesangiospermae - ps17
Magnoliids/eudicots - ps18
Eudicots - ps19
Core_eudicots - ps20
Superrosids - ps21
Rosids - ps22
Eurosids - ps23
Malvids - ps24
Sap./Malv./Brassicales - ps25
Malvales/Brassicales - ps26
Brassicales - ps27
Cleomaceae/Brassicaceae - ps28
Brassicaceae - ps29
Camelineae - ps30
Arabidopsis - ps31
Arabidopsis_thaliana - ps32
Phylostratum

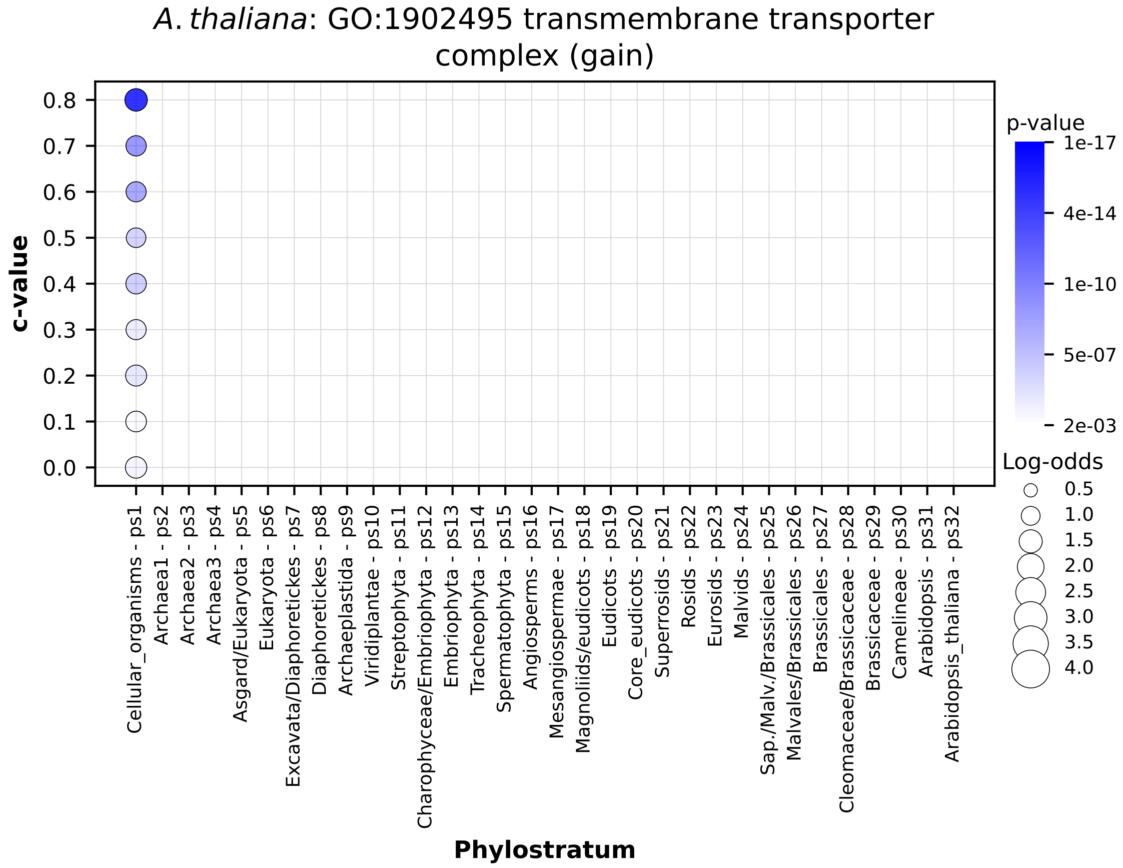
A. thaliana: GO:1902495 transmembrane transporter complex (gain)
c-value
0.0
0.1
0.2
0.3
0.4
0.5
0.6
0.7
0.8
Phylostratum
Cellular_organisms - ps1
Archaea1 - ps2
Archaea2 - ps3
Archaea3 - ps4
Asgard/Eukaryota - ps5
Eukaryota - ps6
Excavata/Diaphoretickes - ps7
Diaphoretickes - ps8
Archaeplastida - ps9
Viridiplantae - ps10
Streptophyta - ps11
Charophyceae/Embriophyta - ps12
Embriophyta - ps13
Tracheophyta - ps14
Spermatophyta - ps15
Angiosperms - ps16
Mesangiospermae - ps17
Magnoliids/eudicots - ps18
Eudicots - ps19
Core_eudicots - ps20
Superrosids - ps21
Rosids - ps22
Eurosids - ps23
Malvids - ps24
Sap./Malv./Brassicales - ps25
Malvales/Brassicales - ps26
Brassicales - ps27
Cleomaceae/Brassicaceae - ps28
Brassicaceae - ps29
Camelineae - ps30
Arabidopsis - ps31
Arabidopsis_thaliana - ps32
p-value
1e-17
4e-14
1e-10
5e-07
2e-03
Log-odds
0.5
1.0
1.5
2.0
2.5
3.0
3.5
4.0

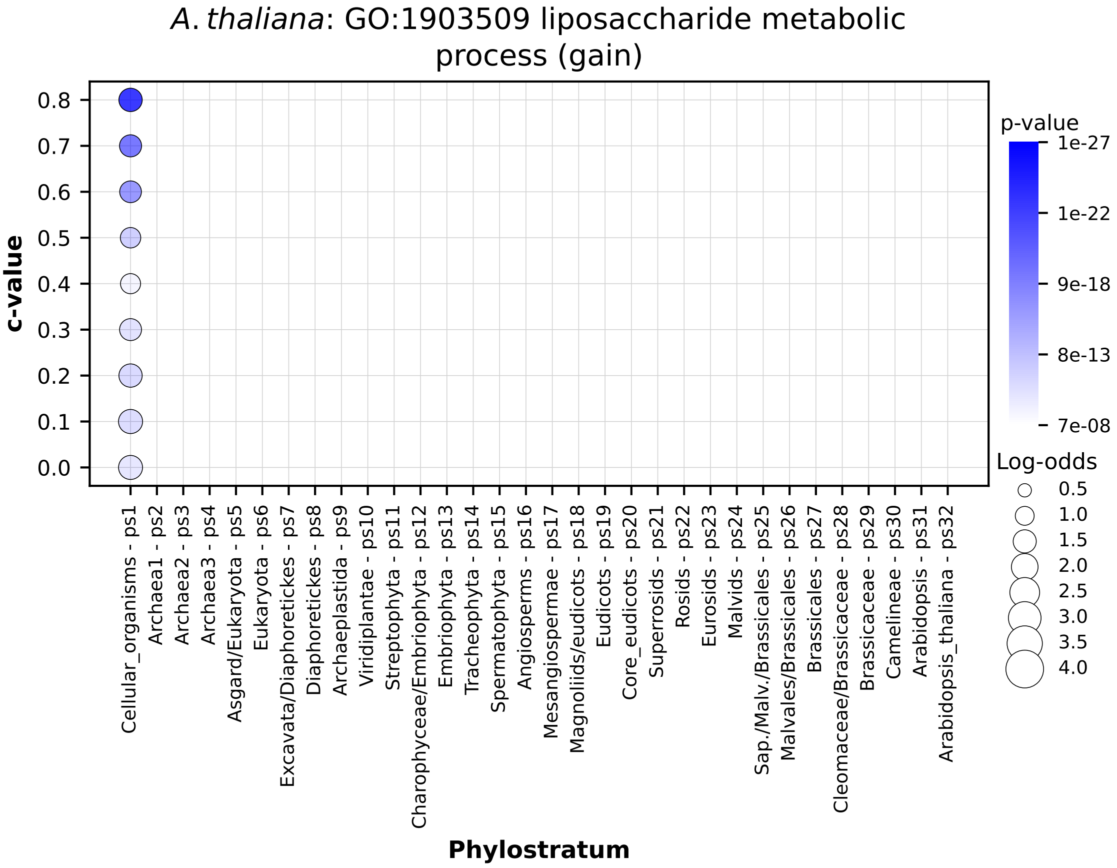

A. thaliana: GO:1903509 liposaccharide metabolic process (gain)
c-value
0.8
0.7
0.6
0.5
0.4
0.3
0.2
0.1
0.0
p-value
1e-27
1e-22
9e-18
8e-13
7e-08
Log-odds
0.5
1.0
1.5
2.0
2.5
3.0
3.5
4.0
Cellular_organisms - ps1
Archaea1 - ps2
Archaea2 - ps3
Archaea3 - ps4
Asgard/Eukaryota - ps5
Eukaryota - ps6
Excavata/Diaphoretickes - ps7
Diaphoretickes - ps8
Archaeplastida - ps9
Viridiplantae - ps10
Streptophyta - ps11
Charophyceae/Embriophyta - ps12
Embriophyta - ps13
Tracheophyta - ps14
Spermatophyta - ps15
Angiosperms - ps16
Mesangiospermae - ps17
Magnoliids/eudicots - ps18
Eudicots - ps19
Core_eudicots - ps20
Superrosids - ps21
Rosids - ps22
Eurosids - ps23
Malvids - ps24
Sap./Malv./Brassicales - ps25
Malvales/Brassicales - ps26
Brassicales - ps27
Cleomaceae/Brassicaceae - ps28
Brassicaceae - ps29
Camelineae - ps30
Arabidopsis - ps31
Arabidopsis_thaliana - ps32
Phylostratum

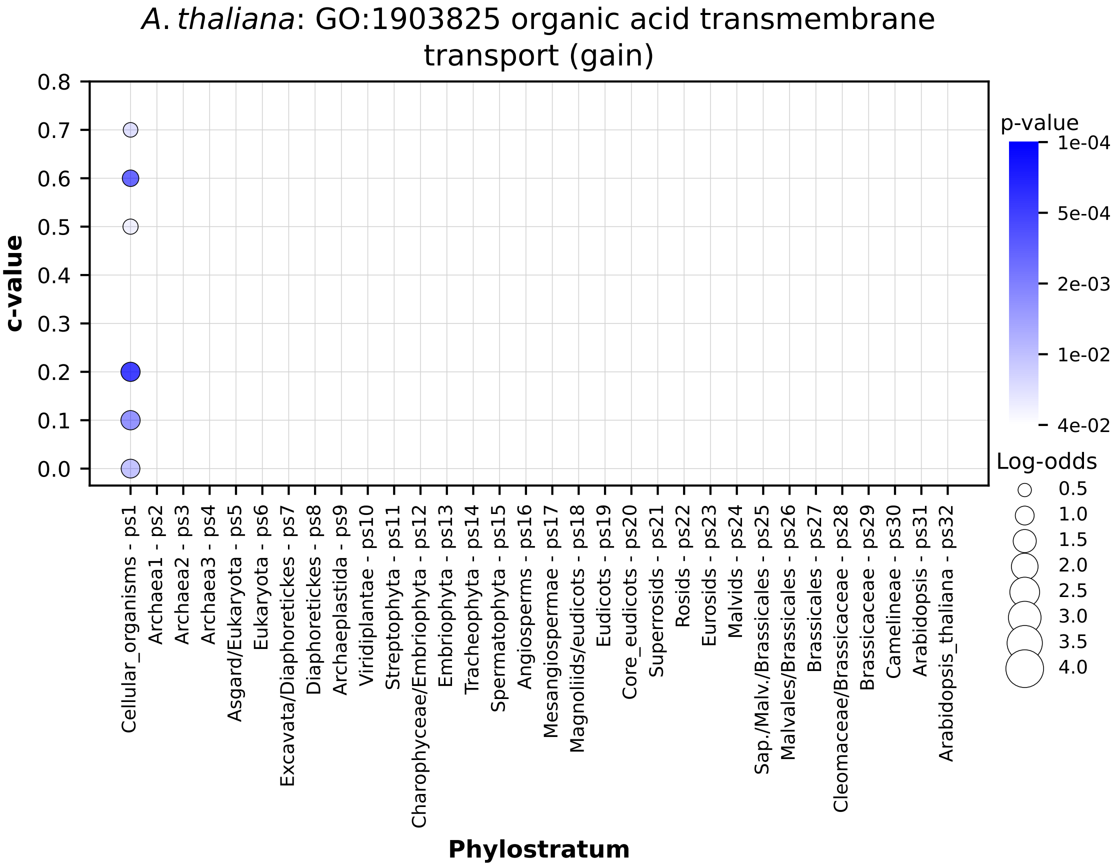

A. thaliana: GO:1903825 organic acid transmembrane transport (gain)
c-value
0.0
0.1
0.2
0.3
0.4
0.5
0.6
0.7
0.8
Phylostratum
Cellular_organisms - ps1
Archaea1 - ps2
Archaea2 - ps3
Archaea3 - ps4
Asgard/Eukaryota - ps5
Eukaryota - ps6
Excavata/Diaphoretickes - ps7
Diaphoretickes - ps8
Archaeplastida - ps9
Viridiplantae - ps10
Streptophyta - ps11
Charophyceae/Embriophyta - ps12
Embriophyta - ps13
Tracheophyta - ps14
Spermatophyta - ps15
Angiosperms - ps16
Mesangiospermae - ps17
Magnoliids/eudicots - ps18
Eudicots - ps19
Core_eudicots - ps20
Superrosids - ps21
Rosids - ps22
Eurosids - ps23
Malvids - ps24
Sap./Malv./Brassicales - ps25
Malvales/Brassicales - ps26
Brassicales - ps27
Cleomaceae/Brassicaceae - ps28
Brassicaceae - ps29
Camelineae - ps30
Arabidopsis - ps31
Arabidopsis_thaliana - ps32
p-value
1e-04
5e-04
2e-03
1e-02
4e-02
Log-odds
0.5
1.0
1.5
2.0
2.5
3.0
3.5
4.0

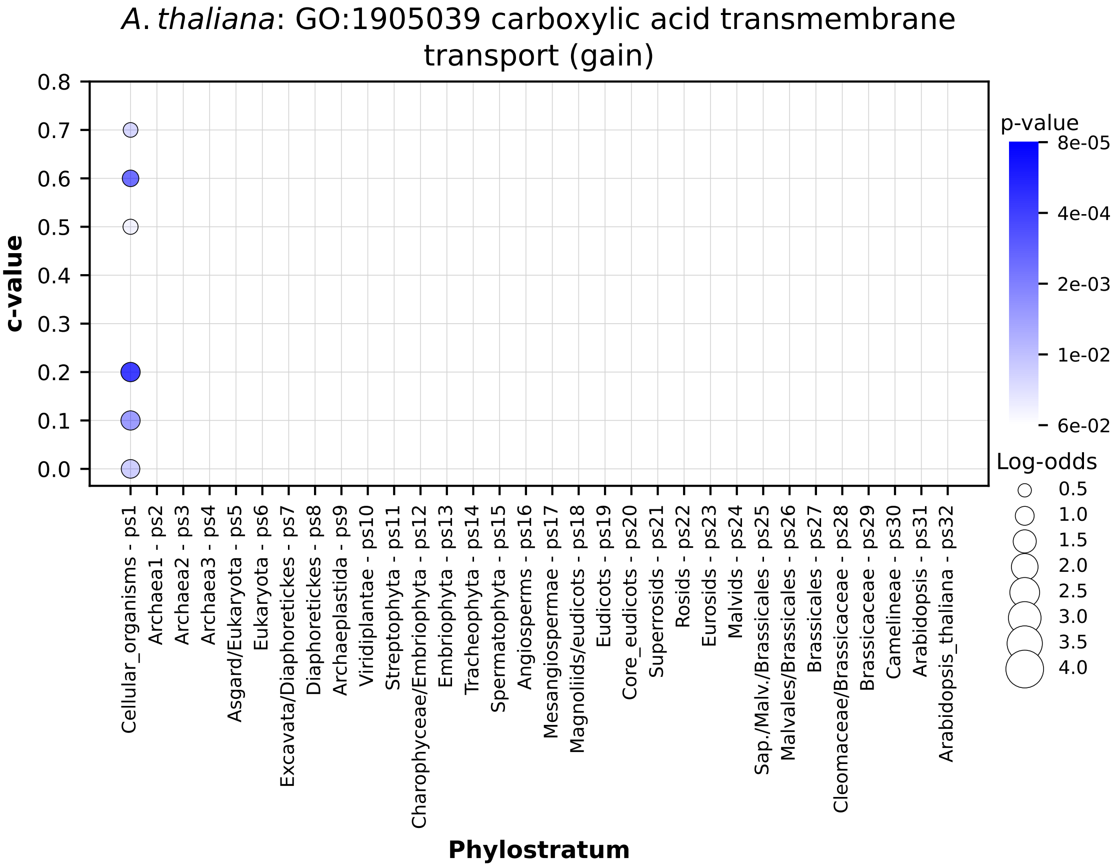
A. thaliana: GO:1905039 carboxylic acid transmembrane transport (gain)
c-value
Phylostratum
p-value
8e-05
4e-04
2e-03
1e-02
6e-02
Log-odds
0.5
1.0
1.5
2.0
2.5
3.0
3.5
4.0
0.0
0.1
0.2
0.3
0.4
0.5
0.6
0.7
0.8
Cellular_organisms - ps1
Archaea1 - ps2
Archaea2 - ps3
Archaea3 - ps4
Asgard/Eukaryota - ps5
Eukaryota - ps6
Excavata/Diaphoretickes - ps7
Diaphoretickes - ps8
Archaeplastida - ps9
Viridiplantae - ps10
Streptophyta - ps11
Charophyceae/Embriophyta - ps12
Embriophyta - ps13
Tracheophyta - ps14
Spermatophyta - ps15
Angiosperms - ps16
Mesangiospermae - ps17
Magnoliids/eudicots - ps18
Eudicots - ps19
Core_eudicots - ps20
Superrosids - ps21
Rosids - ps22
Eurosids - ps23
Malvids - ps24
Sap./Malv./Brassicales - ps25
Malvales/Brassicales - ps26
Brassicales - ps27
Cleomaceae/Brassicaceae - ps28
Brassicaceae - ps29
Camelineae - ps30
Arabidopsis - ps31
Arabidopsis_thaliana - ps32

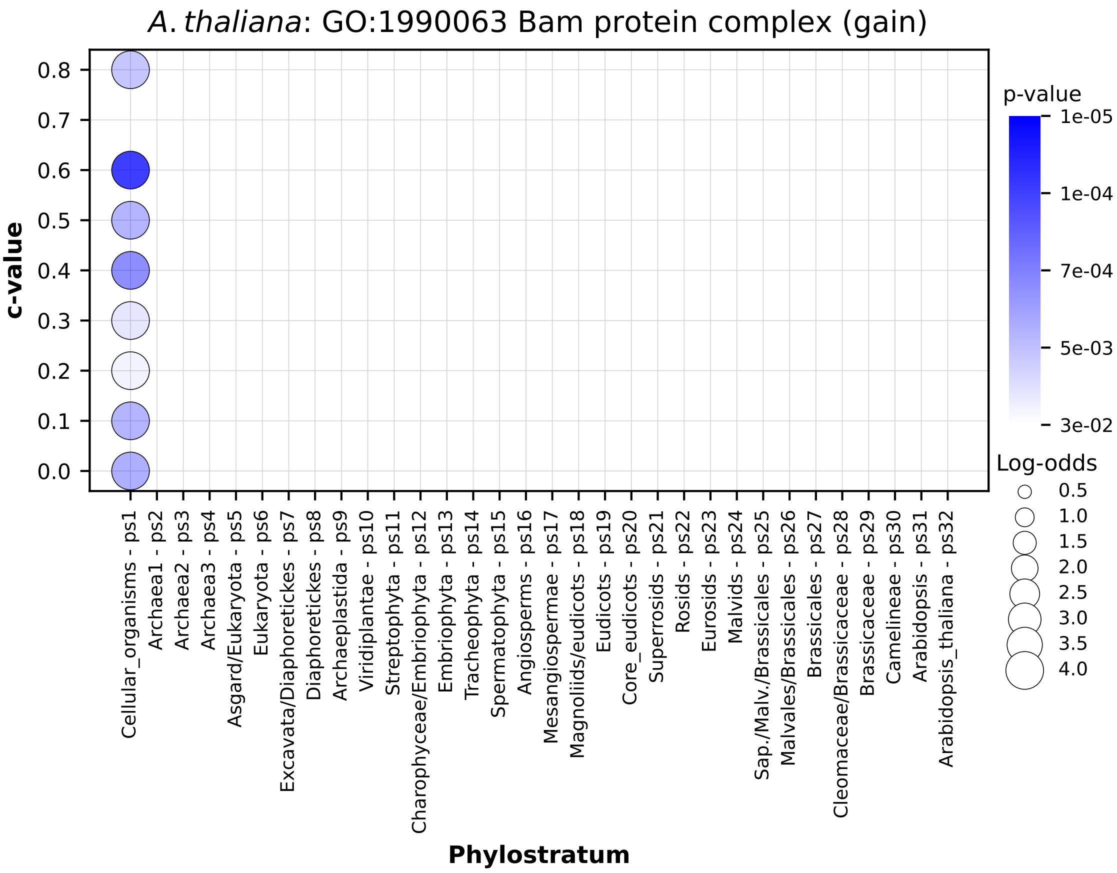
A. thaliana: GO:1990063 Bam protein complex (gain)
c-value
0.0
0.1
0.2
0.3
0.4
0.5
0.6
0.7
0.8
Phylostratum
Cellular_organisms - ps1
Archaea1 - ps2
Archaea2 - ps3
Archaea3 - ps4
Asgard/Eukaryota - ps5
Eukaryota - ps6
Excavata/Diaphoretickes - ps7
Diaphoretickes - ps8
Archaeplastida - ps9
Viridiplantae - ps10
Streptophyta - ps11
Charophyceae/Embriophyta - ps12
Embriophyta - ps13
Tracheophyta - ps14
Spermatophyta - ps15
Angiosperms - ps16
Mesangiospermae - ps17
Magnoliids/eudicots - ps18
Eudicots - ps19
Core_eudicots - ps20
Superrosids - ps21
Rosids - ps22
Eurosids - ps23
Malvids - ps24
Sap./Malv./Brassicales - ps25
Malvales/Brassicales - ps26
Brassicales - ps27
Cleomaceae/Brassicaceae - ps28
Brassicaceae - ps29
Camelineae - ps30
Arabidopsis - ps31
Arabidopsis_thaliana - ps32
p-value
1e-05
1e-04
7e-04
5e-03
3e-02
Log-odds
0.5
1.0
1.5
2.0
2.5
3.0
3.5
4.0

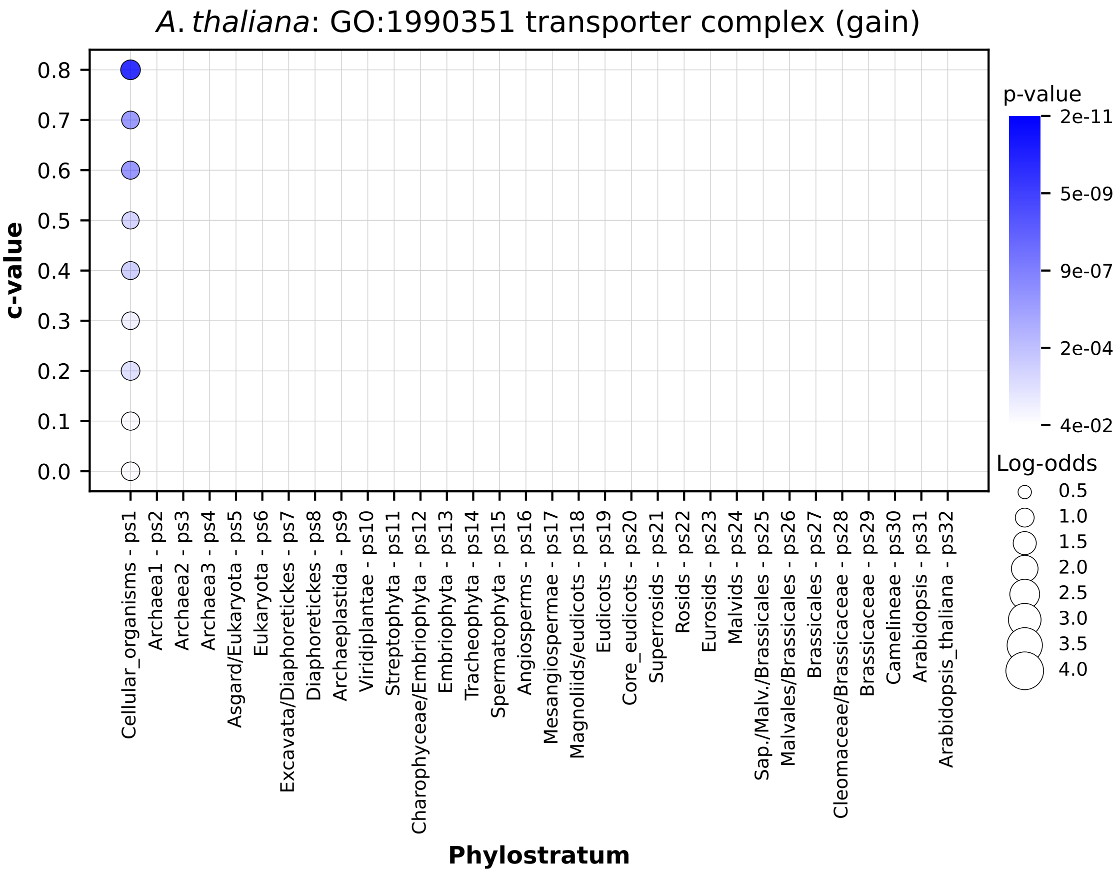
A. thaliana: GO:1990351 transporter complex (gain)
c-value
0.0
0.1
0.2
0.3
0.4
0.5
0.6
0.7
0.8
Phylostratum
Cellular_organisms - ps1
Archaea1 - ps2
Archaea2 - ps3
Archaea3 - ps4
Asgard/Eukaryota - ps5
Eukaryota - ps6
Excavata/Diaphoretickes - ps7
Diaphoretickes - ps8
Archaeplastida - ps9
Viridiplantae - ps10
Streptophyta - ps11
Charophyceae/Embriophyta - ps12
Embriophyta - ps13
Tracheophyta - ps14
Spermatophyta - ps15
Angiosperms - ps16
Mesangiospermae - ps17
Magnoliids/eudicots - ps18
Eudicots - ps19
Core_eudicots - ps20
Superrosids - ps21
Rosids - ps22
Eurosids - ps23
Malvids - ps24
Sap./Malv./Brassicales - ps25
Malvales/Brassicales - ps26
Brassicales - ps27
Cleomaceae/Brassicaceae - ps28
Brassicaceae - ps29
Camelineae - ps30
Arabidopsis - ps31
Arabidopsis_thaliana - ps32
p-value
2e-11
5e-09
9e-07
2e-04
4e-02
Log-odds
0.5
1.0
1.5
2.0
2.5
3.0
3.5
4.0

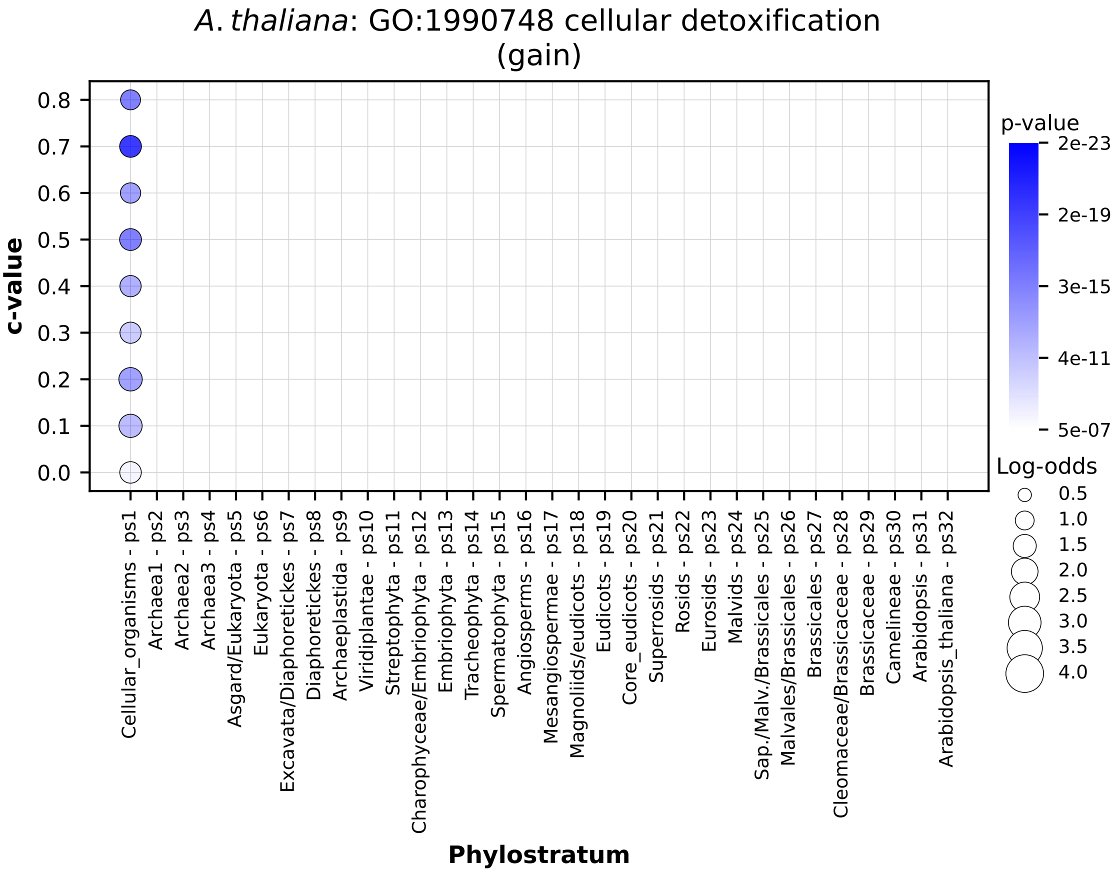
A. thaliana: GO:1990748 cellular detoxification
(gain)
c-value
0.8
0.7
0.6
0.5
0.4
0.3
0.2
0.1
0.0
p-value
2e-23
2e-19
3e-15
4e-11
5e-07
Log-odds
0.5
1.0
1.5
2.0
2.5
3.0
3.5
4.0
Cellular_organisms - ps1
Archaea1 - ps2
Archaea2 - ps3
Archaea3 - ps4
Asgard/Eukaryota - ps5
Eukaryota - ps6
Excavata/Diaphoretickes - ps7
Diaphoretickes - ps8
Archaeplastida - ps9
Viridiplantae - ps10
Streptophyta - ps11
Charophyceae/Embriophyta - ps12
Embriophyta - ps13
Tracheophyta - ps14
Spermatophyta - ps15
Angiosperms - ps16
Mesangiospermae - ps17
Magnoliids/eudicots - ps18
Eudicots - ps19
Core_eudicots - ps20
Superrosids - ps21
Rosids - ps22
Eurosids - ps23
Malvids - ps24
Sap./Malv./Brassicales - ps25
Malvales/Brassicales - ps26
Brassicales - ps27
Cleomaceae/Brassicaceae - ps28
Brassicaceae - ps29
Camelineae - ps30
Arabidopsis - ps31
Arabidopsis_thaliana - ps32
Phylostratum

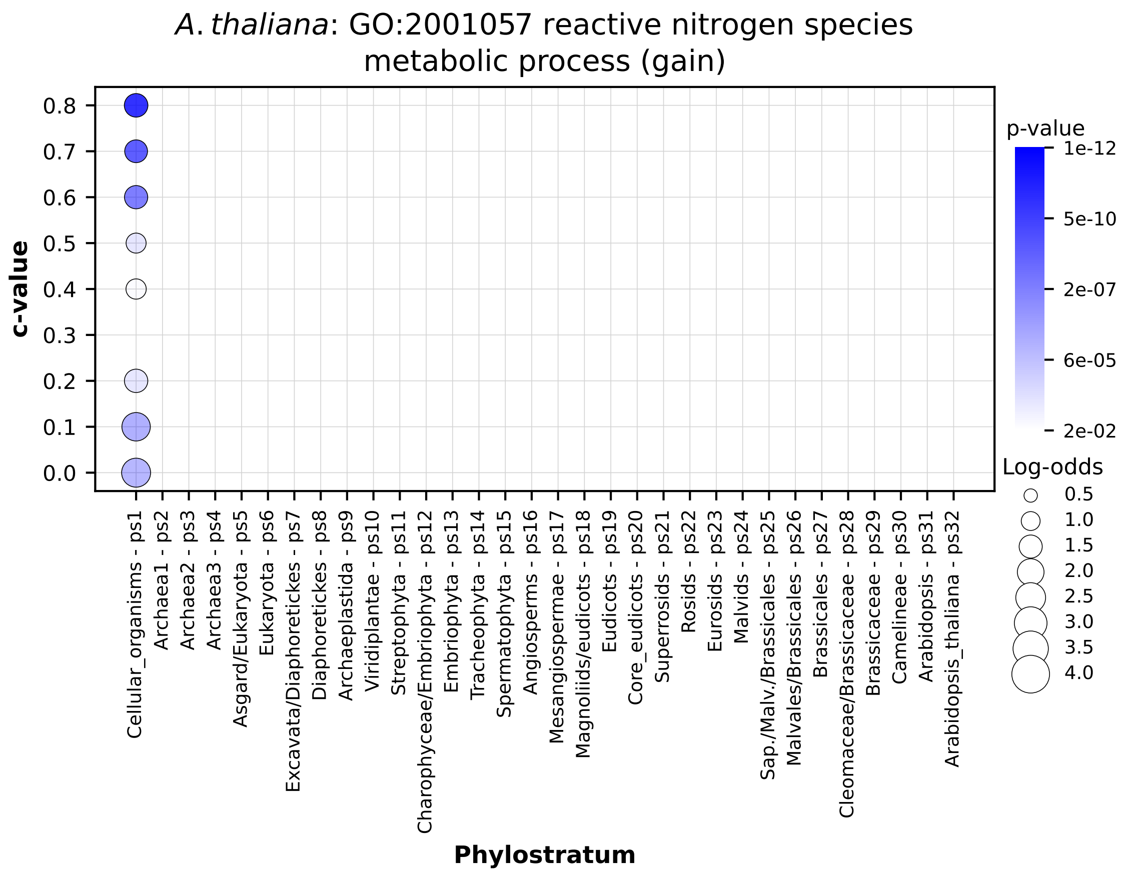

A. thaliana: GO:2001057 reactive nitrogen species metabolic process (gain)
c-value
0.8
0.7
0.6
0.5
0.4
0.3
0.2
0.1
0.0
p-value
1e-12
5e-10
2e-07
6e-05
2e-02
Log-odds
0.5
1.0
1.5
2.0
2.5
3.0
3.5
4.0
Cellular_organisms - ps1
Archaea1 - ps2
Archaea2 - ps3
Archaea3 - ps4
Asgard/Eukaryota - ps5
Eukaryota - ps6
Excavata/Diaphoretickes - ps7
Diaphoretickes - ps8
Archaeplastida - ps9
Viridiplantae - ps10
Streptophyta - ps11
Charophyceae/Embriophyta - ps12
Embriophyta - ps13
Tracheophyta - ps14
Spermatophyta - ps15
Angiosperms - ps16
Mesangiospermae - ps17
Magnoliids/eudicots - ps18
Eudicots - ps19
Core_eudicots - ps20
Superrosids - ps21
Rosids - ps22
Eurosids - ps23
Malvids - ps24
Sap./Malv./Brassicales - ps25
Malvales/Brassicales - ps26
Brassicales - ps27
Cleomaceae/Brassicaceae - ps28
Brassicaceae - ps29
Camelineae - ps30
Arabidopsis - ps31
Arabidopsis_thaliana - ps32
Phylostratum

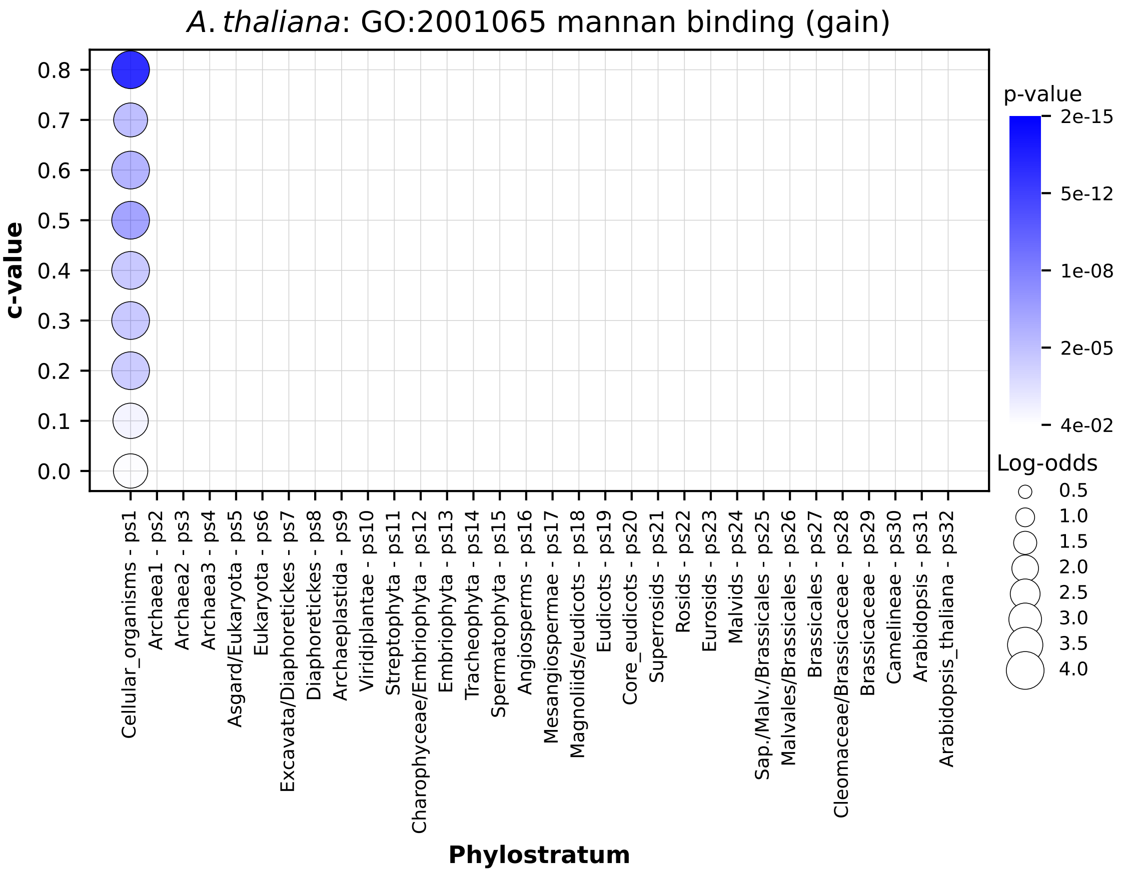

A. thaliana: GO:2001065 mannan binding (gain)
c-value
0.8
0.7
0.6
0.5
0.4
0.3
0.2
0.1
0.0
Phylostratum
Cellular_organisms - ps1
Archaea1 - ps2
Archaea2 - ps3
Archaea3 - ps4
Asgard/Eukaryota - ps5
Eukaryota - ps6
Excavata/Diaphoretickes - ps7
Diaphoretickes - ps8
Archaeplastida - ps9
Viridiplantae - ps10
Streptophyta - ps11
Charophyceae/Embriophyta - ps12
Embriophyta - ps13
Tracheophyta - ps14
Spermatophyta - ps15
Angiosperms - ps16
Mesangiospermae - ps17
Magnoliids/eudicots - ps18
Eudicots - ps19
Core_eudicots - ps20
Superrosids - ps21
Rosids - ps22
Eurosids - ps23
Malvids - ps24
Sap./Malv./Brassicales - ps25
Malvales/Brassicales - ps26
Brassicales - ps27
Cleomaceae/Brassicaceae - ps28
Brassicaceae - ps29
Camelineae - ps30
Arabidopsis - ps31
Arabidopsis_thaliana - ps32
p-value
2e-15
5e-12
1e-08
2e-05
4e-02
Log-odds
0.5
1.0
1.5
2.0
2.5
3.0
3.5
4.0

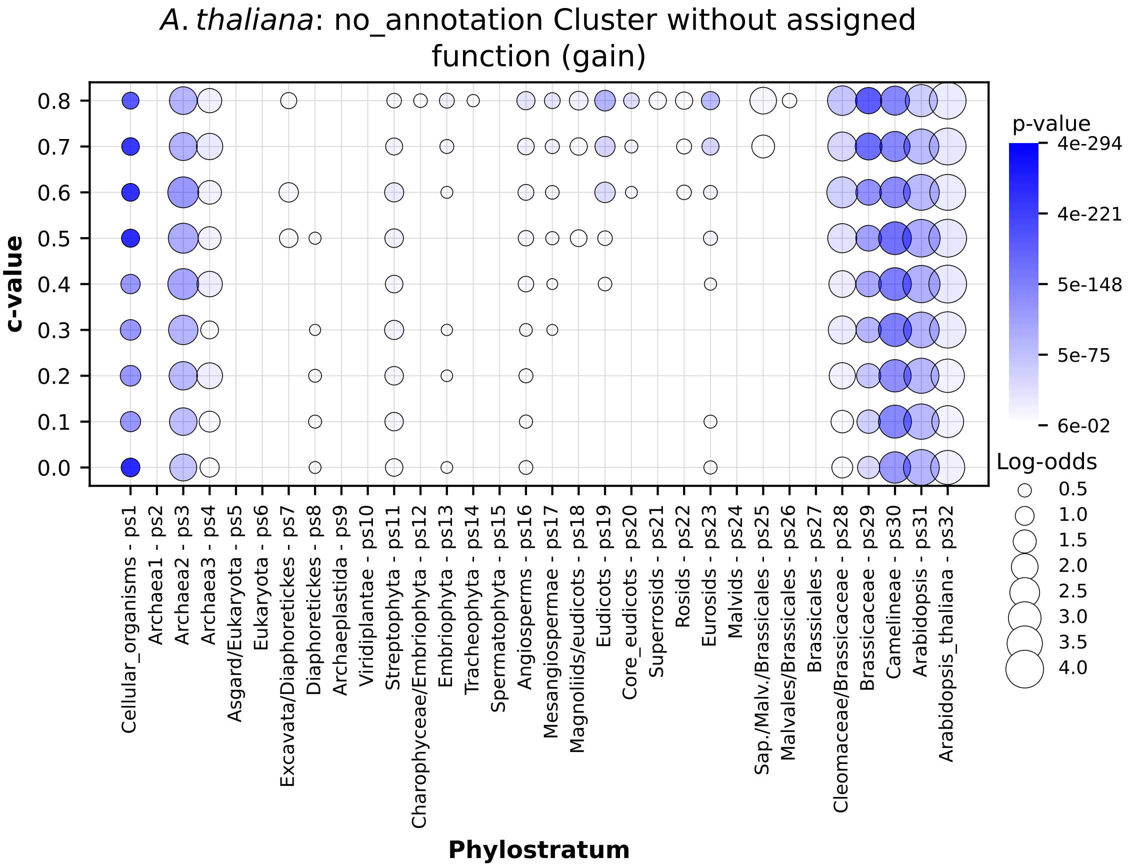

A. thaliana: no_annotation Cluster without assigned function (gain)
c-value
0.8
0.7
0.6
0.5
0.4
0.3
0.2
0.1
0.0
Phylostratum
Cellular_organisms - ps1
Archaea1 - ps2
Archaea2 - ps3
Archaea3 - ps4
Asgard/Eukaryota - ps5
Eukaryota - ps6
Excavata/Diaphoretickes - ps7
Diaphoretickes - ps8
Archaeplastida - ps9
Viridiplantae - ps10
Streptophyta - ps11
Charophyceae/Embriophyta - ps12
Embriophyta - ps13
Tracheophyta - ps14
Spermatophyta - ps15
Angiospermae - ps16
Mesangiospermae - ps17
Magnoliids/eudicots - ps18
Eudicots - ps19
Core_eudicots - ps20
Superrosids - ps21
Rosids - ps22
Eurosids - ps23
Malvids - ps24
Sap./Malv./Brassicales - ps25
Malvales/Brassicales - ps26
Brassicales - ps27
Cleomaceae/Brassicaceae - ps28
Brassicaceae - ps29
Camelineae - ps30
Arabidopsis - ps31
Arabidopsis_thaliana - ps32
p-value
4e-294
4e-221
5e-148
5e-75
6e-02
Log-odds
0.5
1.0
1.5
2.0
2.5
3.0
3.5
4.0

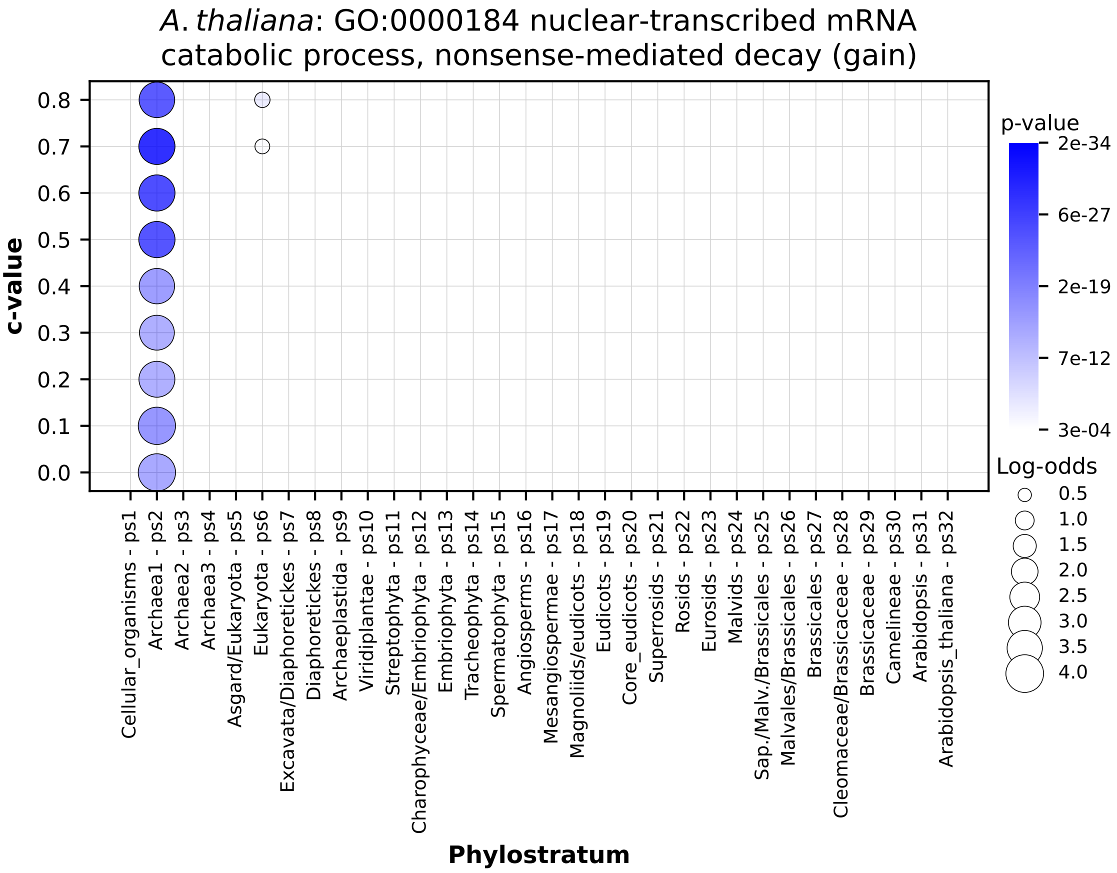

A. thaliana: GO:0000184 nuclear-transcribed mRNA catabolic process, nonsense-mediated decay (gain)
c-value
0.8
0.7
0.6
0.5
0.4
0.3
0.2
0.1
0.0
p-value
2e-34
6e-27
2e-19
7e-12
3e-04
Log-odds
0.5
1.0
1.5
2.0
2.5
3.0
3.5
4.0
Cellular_organisms - ps1
Archaea1 - ps2
Archaea2 - ps3
Archaea3 - ps4
Asgard/Eukaryota - ps5
Eukaryota - ps6
Excavata/Diaphoretickes - ps7
Diaphoretickes - ps8
Archaeplastida - ps9
Viridiplantae - ps10
Streptophyta - ps11
Charophyceae/Embriophyta - ps12
Embriophyta - ps13
Tracheophyta - ps14
Spermatophyta - ps15
Angiosperms - ps16
Mesangiospermae - ps17
Magnoliids/eudicots - ps18
Eudicots - ps19
Core_eudicots - ps20
Superrosids - ps21
Rosids - ps22
Eurosids - ps23
Malvids - ps24
Sap./Malv./Brassicales - ps25
Malvales/Brassicales - ps26
Brassicales - ps27
Cleomaceae/Brassicaceae - ps28
Brassicaceae - ps29
Camelineae - ps30
Arabidopsis - ps31
Arabidopsis_thaliana - ps32
Phylostratum

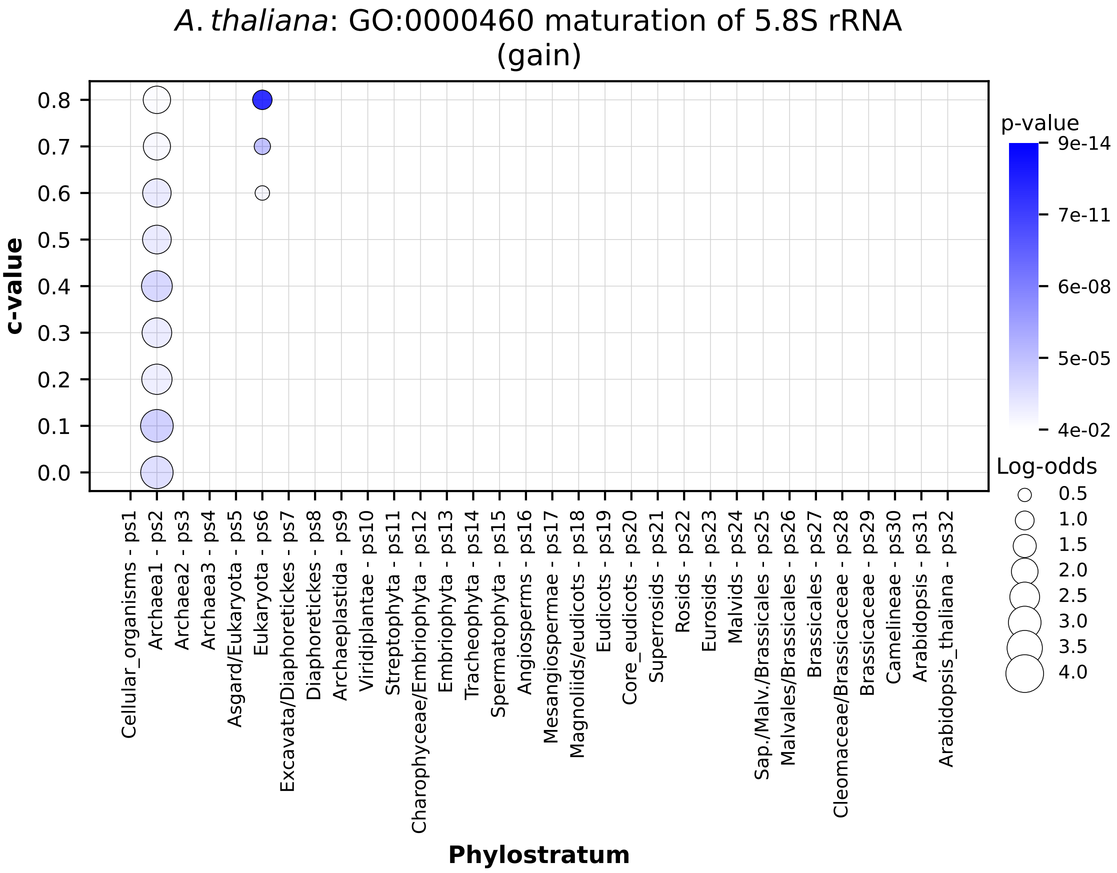
A. thaliana: GO:0000460 maturation of 5.8S rRNA
(gain)
c-value
0.8
0.7
0.6
0.5
0.4
0.3
0.2
0.1
0.0
p-value
9e-14
7e-11
6e-08
5e-05
4e-02
Log-odds
0.5
1.0
1.5
2.0
2.5
3.0
3.5
4.0
Cellular_organisms - ps1
Archaea1 - ps2
Archaea2 - ps3
Archaea3 - ps4
Asgard/Eukaryota - ps5
Eukaryota - ps6
Excavata/Diaphoretickes - ps7
Diaphoretickes - ps8
Archaeplastida - ps9
Viridiplantae - ps10
Streptophyta - ps11
Charophyceae/Embriophyta - ps12
Embriophyta - ps13
Tracheophyta - ps14
Spermatophyta - ps15
Angiosperms - ps16
Mesangiospermae - ps17
Magnoliids/eudicots - ps18
Eudicots - ps19
Core_eudicots - ps20
Superrosids - ps21
Rosids - ps22
Eurosids - ps23
Malvids - ps24
Sap./Malv./Brassicales - ps25
Malvales/Brassicales - ps26
Brassicales - ps27
Cleomaceae/Brassicaceae - ps28
Brassicaceae - ps29
Camelineae - ps30
Arabidopsis - ps31
Arabidopsis_thaliana - ps32
Phylostratum

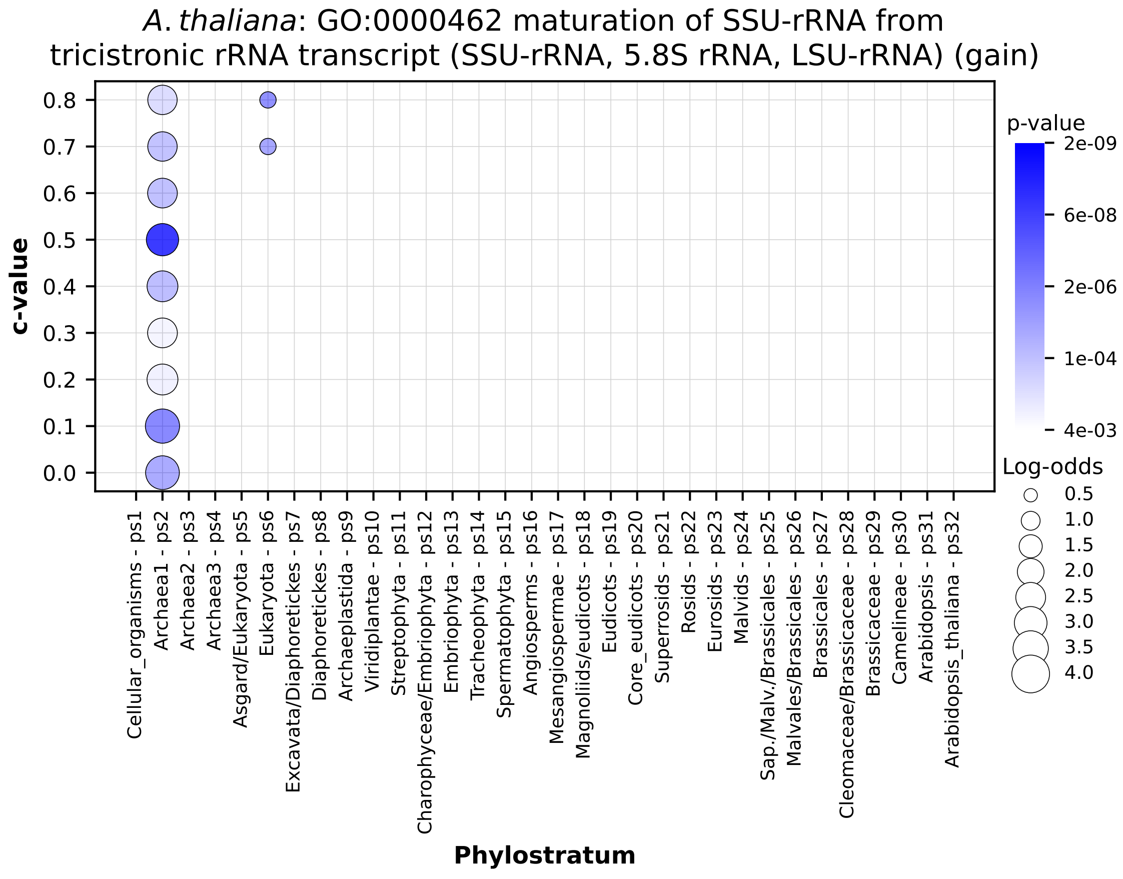

A. thaliana: GO:0000462 maturation of SSU-rRNA from tricistronic rRNA transcript (SSU-rRNA, 5.8S rRNA, LSU-rRNA) (gain)
c-value
0.8
0.7
0.6
0.5
0.4
0.3
0.2
0.1
0.0
p-value
2e-09
6e-08
2e-06
1e-04
4e-03
Log-odds
0.5
1.0
1.5
2.0
2.5
3.0
3.5
4.0
Cellular_organisms - ps1
Archaea1 - ps2
Archaea2 - ps3
Archaea3 - ps4
Asgard/Eukaryota - ps5
Eukaryota - ps6
Excavata/Diaphoretickes - ps7
Diaphoretickes - ps8
Archaeplastida - ps9
Viridiplantae - ps10
Streptophyta - ps11
Charophyceae/Embriophyta - ps12
Embriophyta - ps13
Tracheophyta - ps14
Spermatophyta - ps15
Angiosperms - ps16
Mesangiospermae - ps17
Magnoliids/eudicots - ps18
Eudicots - ps19
Core_eudicots - ps20
Superrosids - ps21
Rosids - ps22
Eurosids - ps23
Malvids - ps24
Sap./Malv./Brassicales - ps25
Malvales/Brassicales - ps26
Brassicales - ps27
Cleomaceae/Brassicaceae - ps28
Brassicaceae - ps29
Camelineae - ps30
Arabidopsis - ps31
Arabidopsis_thaliana - ps32
Phylostratum

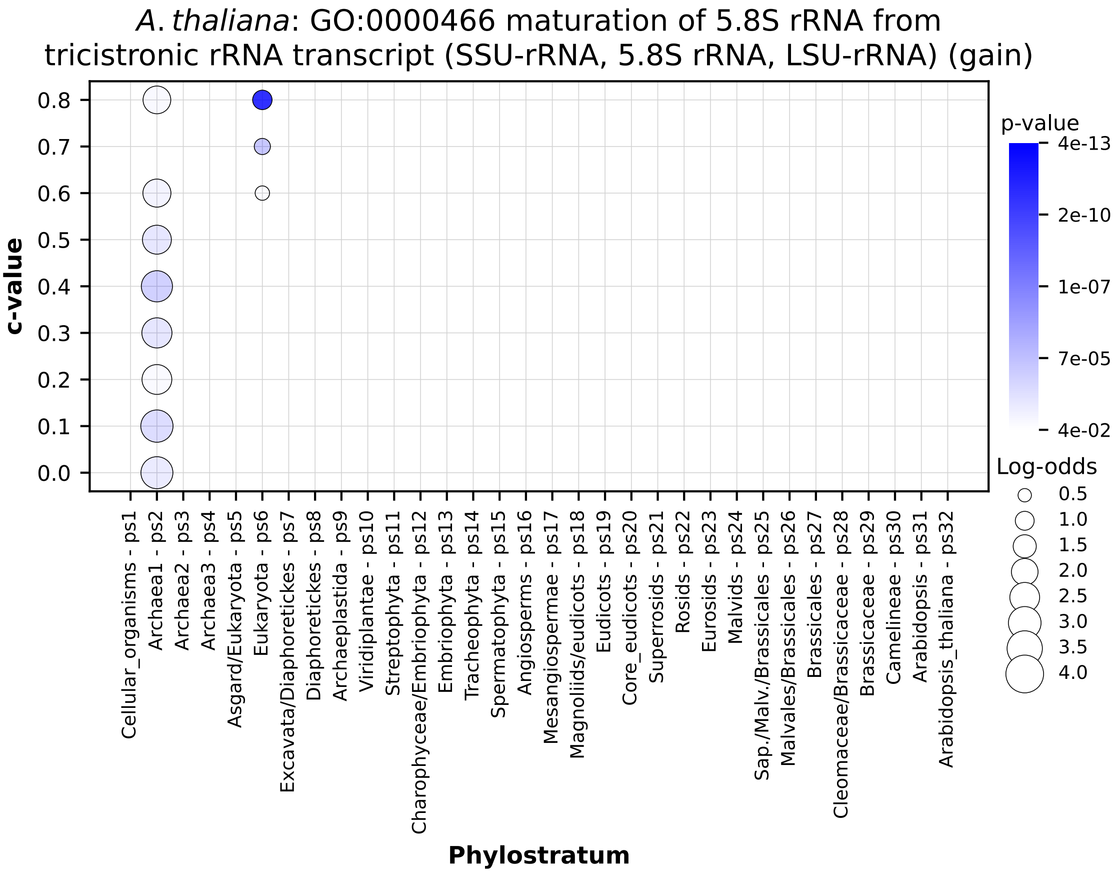

A. thaliana: GO:0000466 maturation of 5.8S rRNA from tricistronic rRNA transcript (SSU-rRNA, 5.8S rRNA, LSU-rRNA) (gain)
c-value
0.8
0.7
0.6
0.5
0.4
0.3
0.2
0.1
0.0
p-value
4e-13
2e-10
1e-07
7e-05
4e-02
Log-odds
0.5
1.0
1.5
2.0
2.5
3.0
3.5
4.0
Cellular_organisms - ps1
Archaea1 - ps2
Archaea2 - ps3
Archaea3 - ps4
Asgard/Eukaryota - ps5
Eukaryota - ps6
Excavata/Diaphoretickes - ps7
Diaphoretickes - ps8
Archaeplastida - ps9
Viridiplantae - ps10
Streptophyta - ps11
Charophyceae/Embriophyta - ps12
Embriophyta - ps13
Tracheophyta - ps14
Spermatophyta - ps15
Angiosperms - ps16
Mesangiospermae - ps17
Magnoliids/eudicots - ps18
Eudicots - ps19
Core_eudicots - ps20
Superrosids - ps21
Rosids - ps22
Eurosids - ps23
Malvids - ps24
Sap./Malv./Brassicales - ps25
Malvales/Brassicales - ps26
Brassicales - ps27
Cleomaceae/Brassicaceae - ps28
Brassicaceae - ps29
Camelineae - ps30
Arabidopsis - ps31
Arabidopsis_thaliana - ps32
Phylostratum

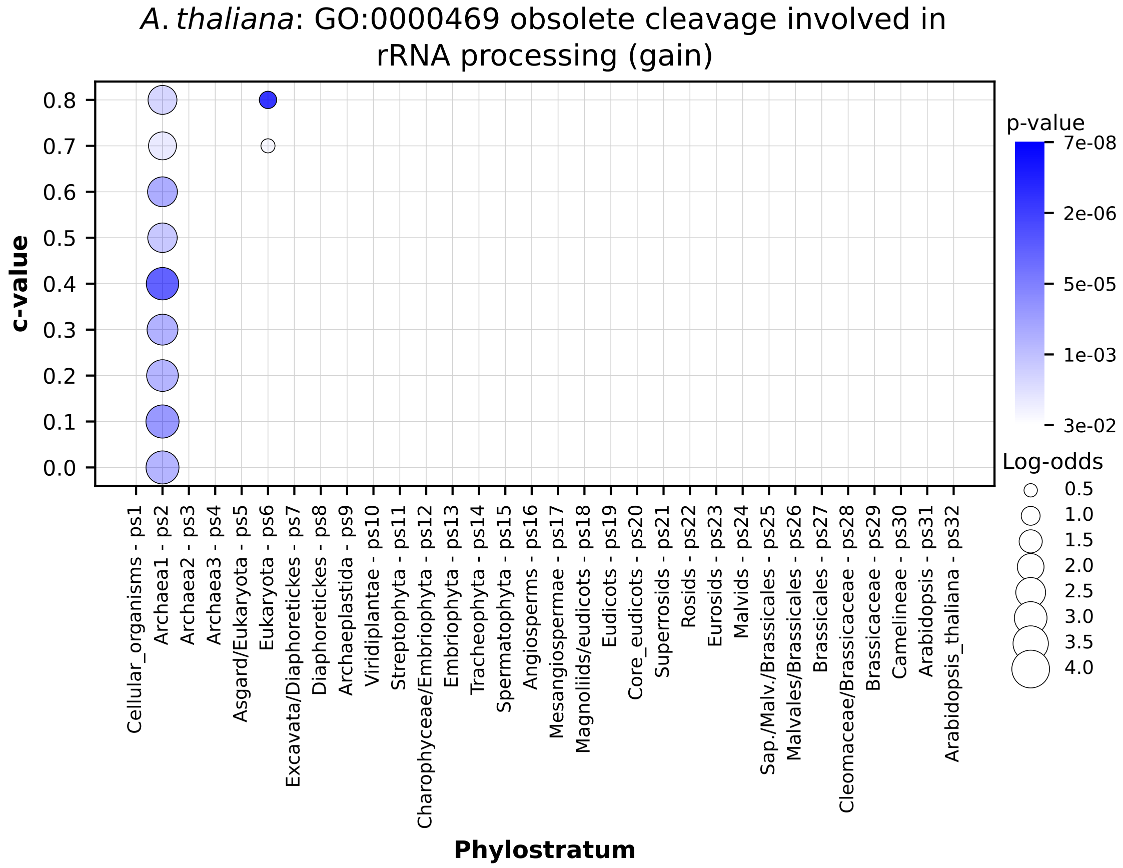

A. thaliana: GO:0000469 obsolete cleavage involved in rRNA processing (gain)
c-value
0.8
0.7
0.6
0.5
0.4
0.3
0.2
0.1
0.0
Phylostratum
Cellular_organisms - ps1
Archaea1 - ps2
Archaea2 - ps3
Archaea3 - ps4
Asgard/Eukaryota - ps5
Eukaryota - ps6
Excavata/Diaphoretickes - ps7
Diaphoretickes - ps8
Archaeplastida - ps9
Viridiplantae - ps10
Streptophyta - ps11
Charophyceae/Embriophyta - ps12
Embriophyta - ps13
Tracheophyta - ps14
Spermatophyta - ps15
Angiosperms - ps16
Mesangiospermae - ps17
Magnoliids/eudicots - ps18
Eudicots - ps19
Core_eudicots - ps20
Superrosids - ps21
Rosids - ps22
Eurosids - ps23
Malvids - ps24
Sap./Malv./Brassicales - ps25
Malvales/Brassicales - ps26
Brassicales - ps27
Cleomaceae/Brassicaceae - ps28
Brassicaceae - ps29
Camelineae - ps30
Arabidopsis - ps31
Arabidopsis_thaliana - ps32
p-value
7e-08
2e-06
5e-05
1e-03
3e-02
Log-odds
0.5
1.0
1.5
2.0
2.5
3.0
3.5
4.0

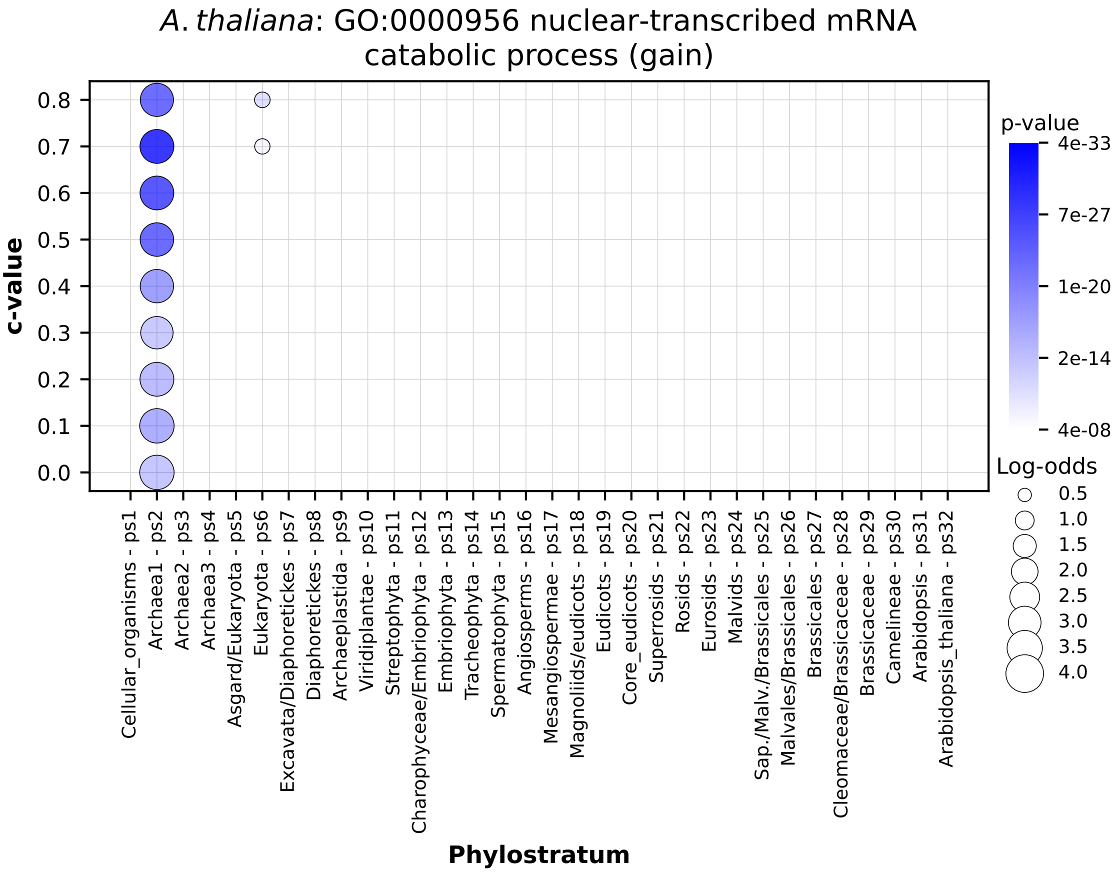
A. thaliana: GO:0000956 nuclear-transcribed mRNA catabolic process (gain)
c-value
0.0
0.1
0.2
0.3
0.4
0.5
0.6
0.7
0.8
Phylostratum
Cellular_organisms - ps1
Archaea1 - ps2
Archaea2 - ps3
Archaea3 - ps4
Asgard/Eukaryota - ps5
Eukaryota - ps6
Excavata/Diaphoretickes - ps7
Diaphoretickes - ps8
Archaeplastida - ps9
Viridiplantae - ps10
Streptophyta - ps11
Charophyceae/Embriophyta - ps12
Embriophyta - ps13
Tracheophyta - ps14
Spermatophyta - ps15
Angiosperms - ps16
Mesangiospermae - ps17
Magnoliids/eudicots - ps18
Eudicots - ps19
Core_eudicots - ps20
Superrosids - ps21
Rosids - ps22
Eurosids - ps23
Malvids - ps24
Sap./Malv./Brassicales - ps25
Malvales/Brassicales - ps26
Brassicales - ps27
Cleomaceae/Brassicaceae - ps28
Brassicaceae - ps29
Camelineae - ps30
Arabidopsis - ps31
Arabidopsis_thaliana - ps32
p-value
4e-33
7e-27
1e-20
2e-14
4e-08
Log-odds
0.5
1.0
1.5
2.0
2.5
3.0
3.5
4.0

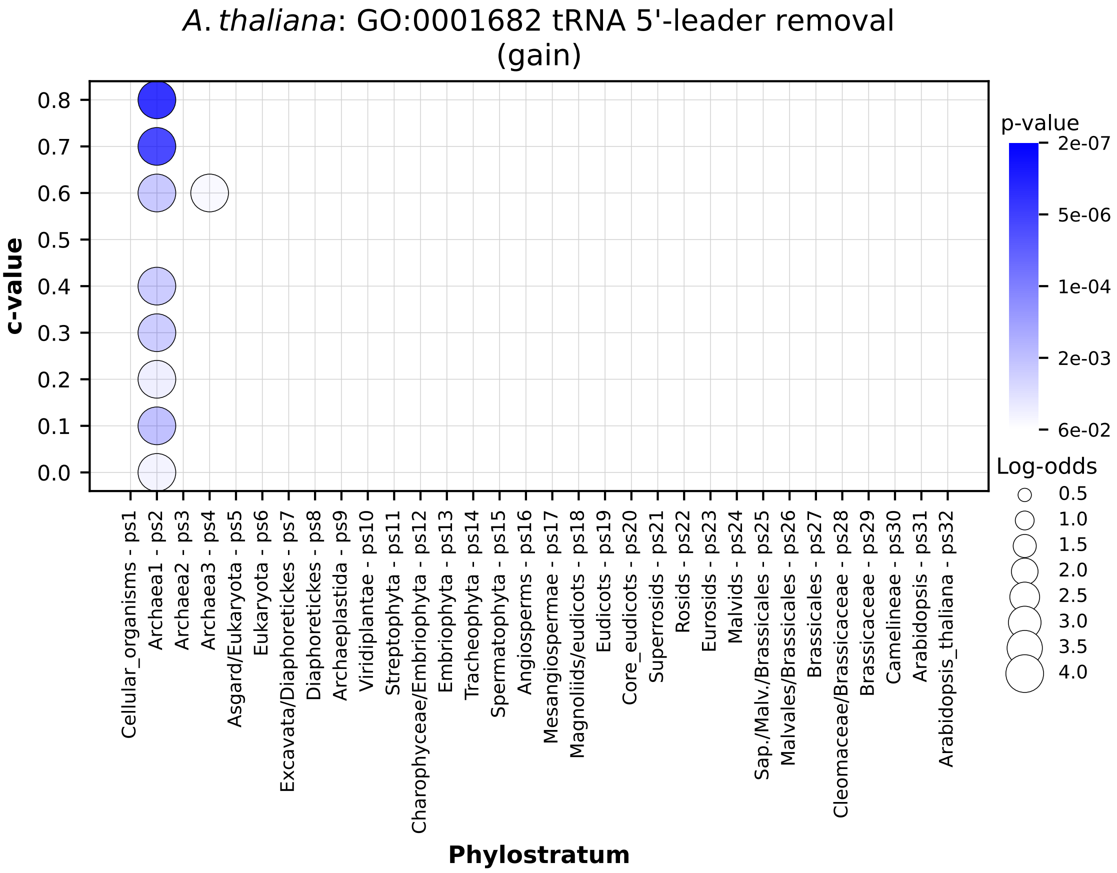
A. thaliana: GO:0001682 tRNA 5'-leader removal
(gain)
c-value
0.0
0.1
0.2
0.3
0.4
0.5
0.6
0.7
0.8
p-value
2e-07
5e-06
1e-04
2e-03
6e-02
Log-odds
0.5
1.0
1.5
2.0
2.5
3.0
3.5
4.0
Cellular_organisms - ps1
Archaea1 - ps2
Archaea2 - ps3
Archaea3 - ps4
Asgard/Eukaryota - ps5
Eukaryota - ps6
Excavata/Diaphoretickes - ps7
Diaphoretickes - ps8
Archaeplastida - ps9
Viridiplantae - ps10
Streptophyta - ps11
Charophyceae/Embriophyta - ps12
Embriophyta - ps13
Tracheophyta - ps14
Spermatophyta - ps15
Angiosperms - ps16
Mesangiospermae - ps17
Magnoliids/eudicots - ps18
Eudicots - ps19
Core_eudicots - ps20
Superrosids - ps21
Rosids - ps22
Eurosids - ps23
Malvids - ps24
Sap./Malv./Brassicales - ps25
Malvales/Brassicales - ps26
Brassicales - ps27
Cleomaceae/Brassicaceae - ps28
Brassicaceae - ps29
Camelineae - ps30
Arabidopsis - ps31
Arabidopsis_thaliana - ps32
Phylostratum

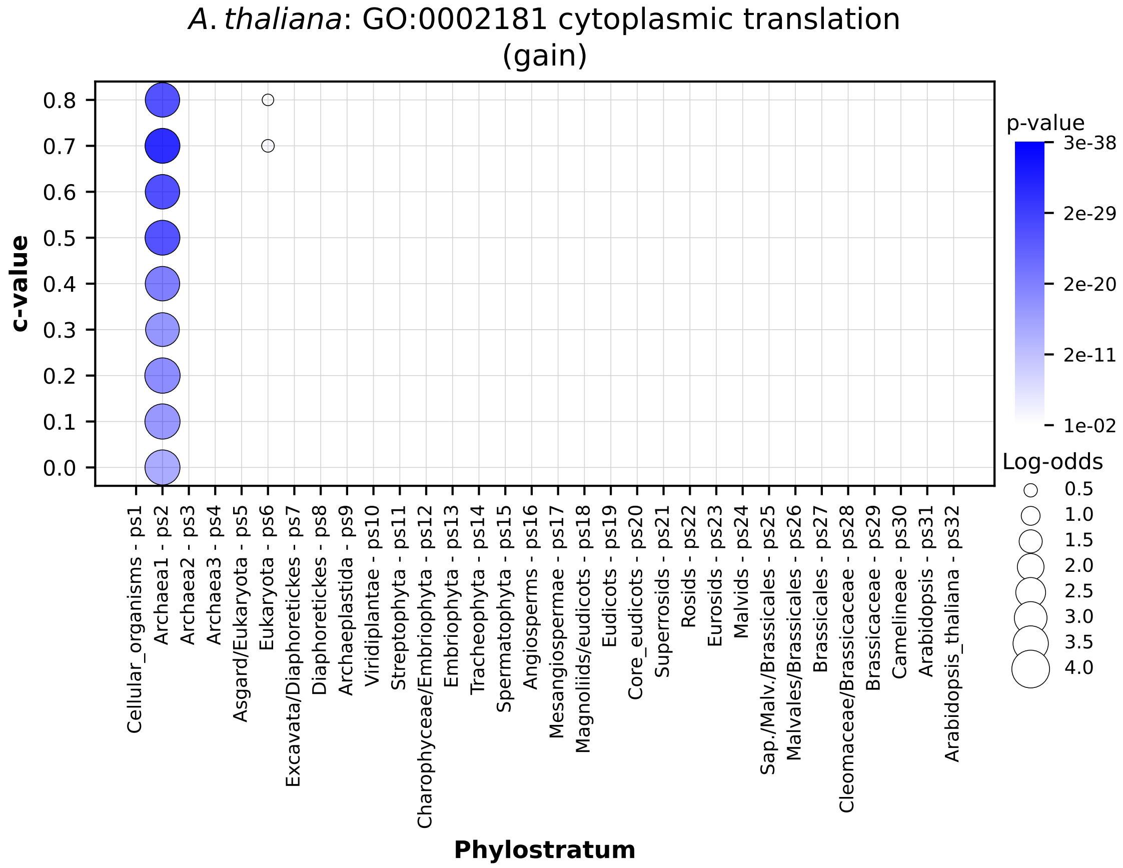
A. thaliana: GO:0002181 cytoplasmic translation
(gain)
c-value
0.8
0.7
0.6
0.5
0.4
0.3
0.2
0.1
0.0
p-value
3e-38
2e-29
2e-20
2e-11
1e-02
Log-odds
0.5
1.0
1.5
2.0
2.5
3.0
3.5
4.0
Cellular_organisms - ps1
Archaea1 - ps2
Archaea2 - ps3
Archaea3 - ps4
Asgard/Eukaryota - ps5
Eukaryota - ps6
Excavata/Diaphoretickes - ps7
Diaphoretickes - ps8
Archaeplastida - ps9
Viridiplantae - ps10
Streptophyta - ps11
Charophyceae/Embriophyta - ps12
Embriophyta - ps13
Tracheophyta - ps14
Spermatophyta - ps15
Angiosperms - ps16
Mesangiospermae - ps17
Magnoliids/eudicots - ps18
Eudicots - ps19
Core_eudicots - ps20
Superrosids - ps21
Rosids - ps22
Eurosids - ps23
Malvids - ps24
Sap./Malv./Brassicales - ps25
Malvales/Brassicales - ps26
Brassicales - ps27
Cleomaceae/Brassicaceae - ps28
Brassicaceae - ps29
Camelineae - ps30
Arabidopsis - ps31
Arabidopsis_thaliana - ps32
Phylostratum

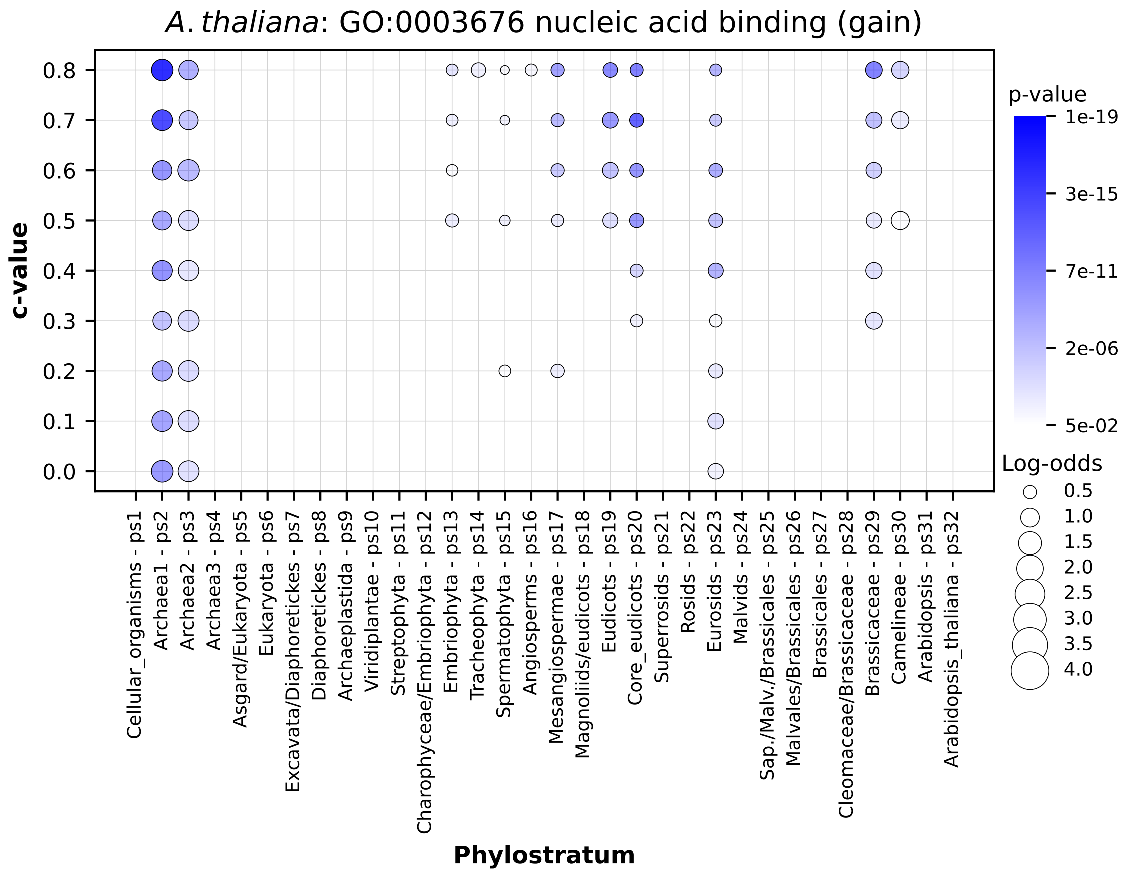

A. thaliana: GO:0003676 nucleic acid binding (gain)
c-value
0.8
0.7
0.6
0.5
0.4
0.3
0.2
0.1
0.0
p-value
1e-19
3e-15
7e-11
2e-06
5e-02
Log-odds
0.5
1.0
1.5
2.0
2.5
3.0
3.5
4.0
Cellular_organisms - ps1
Archaea1 - ps2
Archaea2 - ps3
Archaea3 - ps4
Asgard/Eukaryota - ps5
Eukaryota - ps6
Excavata/Diaphoretickes - ps7
Diaphoretickes - ps8
Archaeplastida - ps9
Viridiplantae - ps10
Streptophyta - ps11
Charophyceae/Embriophyta - ps12
Embriophyta - ps13
Tracheophyta - ps14
Spermatophyta - ps15
Angiosperms - ps16
Mesangiospermae - ps17
Magnoliids/eudicots - ps18
Eudicots - ps19
Core_eudicots - ps20
Superrosids - ps21
Rosids - ps22
Eurosids - ps23
Malvids - ps24
Sap./Malv./Brassicales - ps25
Malvales/Brassicales - ps26
Brassicales - ps27
Cleomaceae/Brassicaceae - ps28
Brassicaceae - ps29
Camelineae - ps30
Arabidopsis - ps31
Arabidopsis_thaliana - ps32
Phylostratum

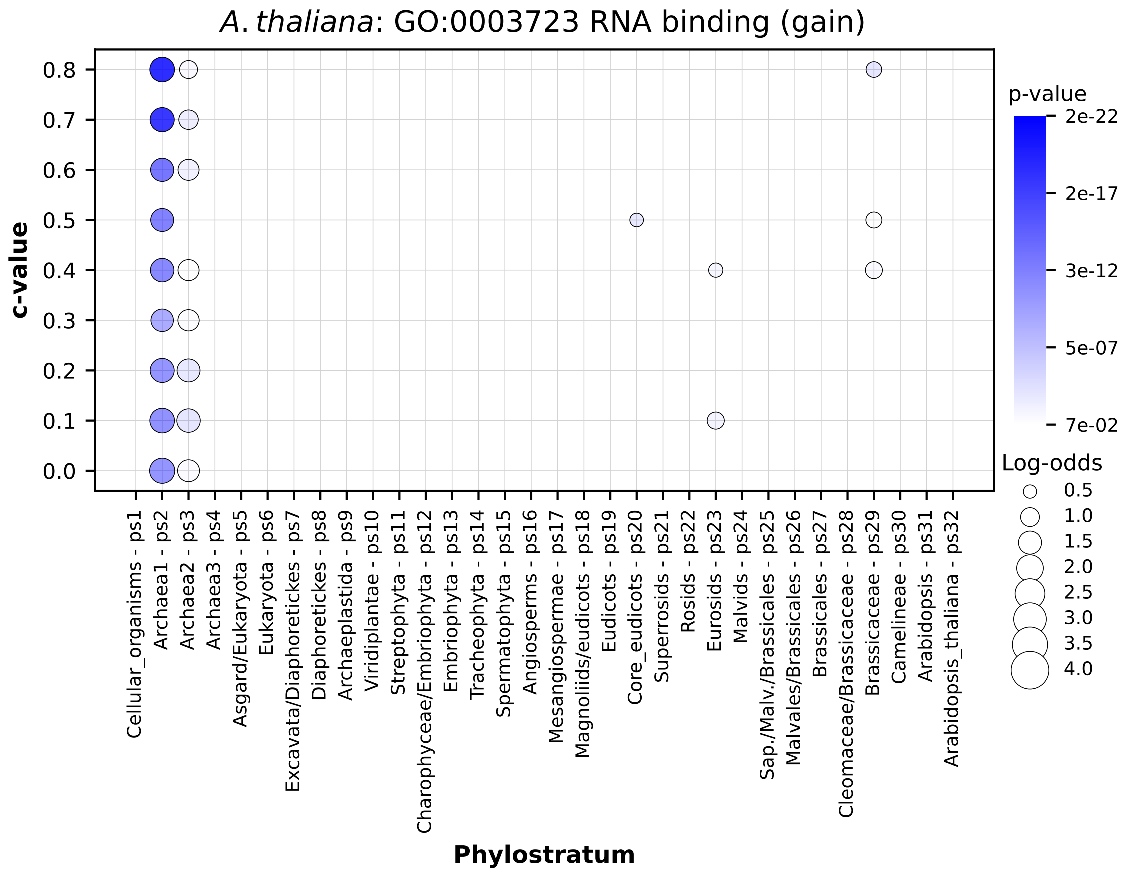

A. thaliana: GO:0003723 RNA binding (gain)
c-value
0.8
0.7
0.6
0.5
0.4
0.3
0.2
0.1
0.0
Phylostratum
Cellular_organisms - ps1
Archaea1 - ps2
Archaea2 - ps3
Archaea3 - ps4
Asgard/Eukaryota - ps5
Eukaryota - ps6
Excavata/Diaphoretickes - ps7
Diaphoretickes - ps8
Archaeplastida - ps9
Viridiplantae - ps10
Streptophyta - ps11
Charophyceae/Embriophyta - ps12
Embriophyta - ps13
Tracheophyta - ps14
Spermatophyta - ps15
Angiosperms - ps16
Mesangiospermae - ps17
Magnoliids/eudicots - ps18
Eudicots - ps19
Core_eudicots - ps20
Superrosids - ps21
Rosids - ps22
Eurosids - ps23
Malvids - ps24
Sap./Malv./Brassicales - ps25
Malvales/Brassicales - ps26
Brassicales - ps27
Cleomaceae/Brassicaceae - ps28
Brassicaceae - ps29
Camelineae - ps30
Arabidopsis - ps31
Arabidopsis_thaliana - ps32
p-value
2e-22
2e-17
3e-12
5e-07
7e-02
Log-odds
0.5
1.0
1.5
2.0
2.5
3.0
3.5
4.0

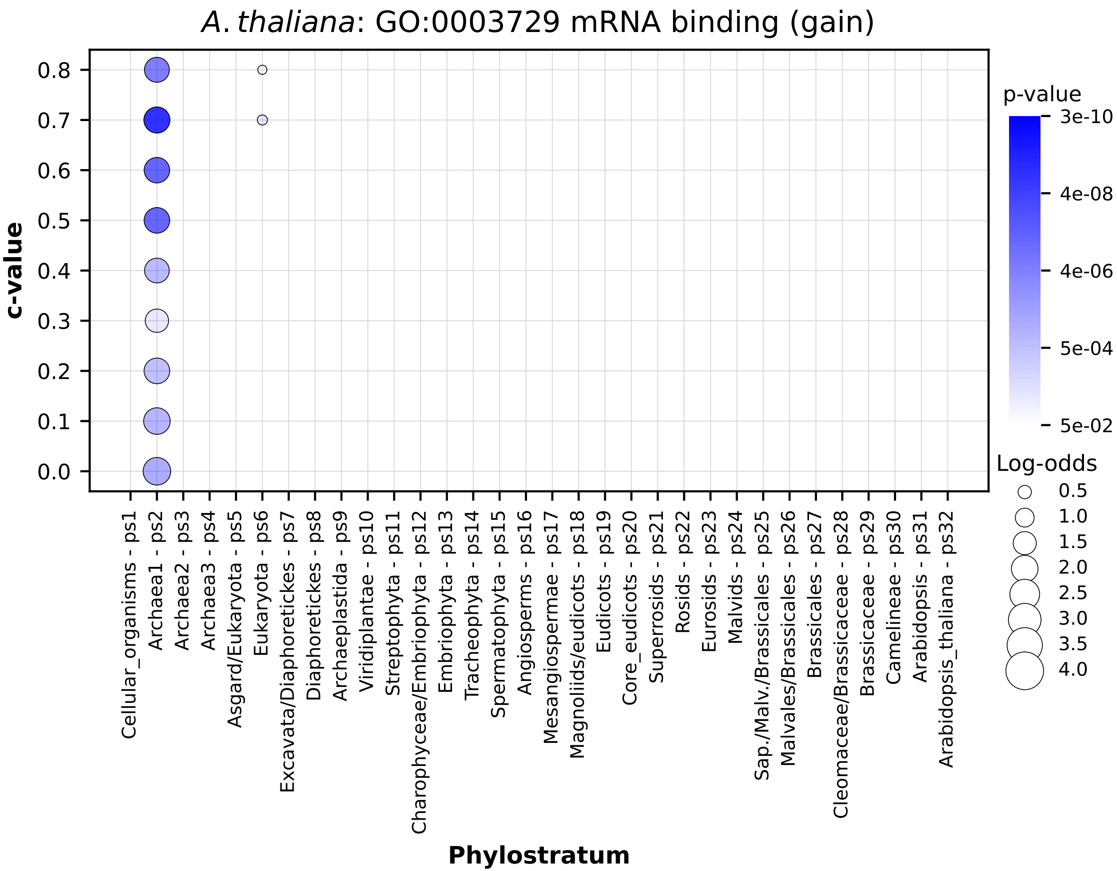

A. thaliana: GO:0003729 mRNA binding (gain)
c-value
0.0
0.1
0.2
0.3
0.4
0.5
0.6
0.7
0.8
Phylostratum
Cellular_organisms - ps1
Archaea1 - ps2
Archaea2 - ps3
Archaea3 - ps4
Asgard/Eukaryota - ps5
Eukaryota - ps6
Excavata/Diaphoretickes - ps7
Diaphoretickes - ps8
Archaeplastida - ps9
Viridiplantae - ps10
Streptophyta - ps11
Charophyceae/Embriophyta - ps12
Embriophyta - ps13
Tracheophyta - ps14
Spermatophyta - ps15
Angiosperms - ps16
Mesangiospermae - ps17
Magnoliids/eudicots - ps18
Eudicots - ps19
Core_eudicots - ps20
Superrosids - ps21
Rosids - ps22
Eurosids - ps23
Malvids - ps24
Sap./Malv./Brassicales - ps25
Malvales/Brassicales - ps26
Brassicales - ps27
Cleomaceae/Brassicaceae - ps28
Brassicaceae - ps29
Camelineae - ps30
Arabidopsis - ps31
Arabidopsis_thaliana - ps32
p-value
3e-10
4e-08
4e-06
5e-04
5e-02
Log-odds
0.5
1.0
1.5
2.0
2.5
3.0
3.5
4.0

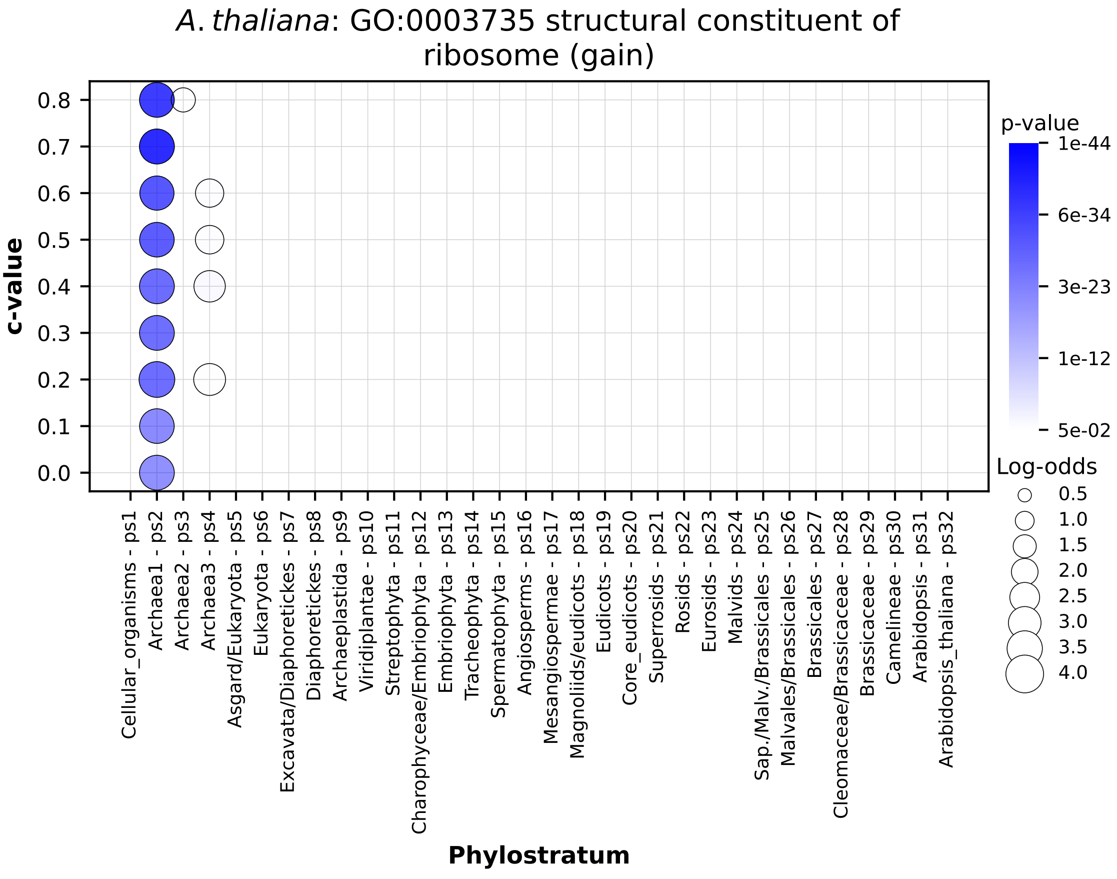

A. thaliana: GO:0003735 structural constituent of ribosome (gain)
c-value
0.8
0.7
0.6
0.5
0.4
0.3
0.2
0.1
0.0
p-value
1e-44
6e-34
3e-23
1e-12
5e-02
Log-odds
0.5
1.0
1.5
2.0
2.5
3.0
3.5
4.0
Cellular_organisms - ps1
Archaea1 - ps2
Archaea2 - ps3
Archaea3 - ps4
Asgard/Eukaryota - ps5
Eukaryota - ps6
Excavata/Diaphoretickes - ps7
Diaphoretickes - ps8
Archaeplastida - ps9
Viridiplantae - ps10
Streptophyta - ps11
Charophyceae/Embriophyta - ps12
Embriophyta - ps13
Tracheophyta - ps14
Spermatophyta - ps15
Angiosperms - ps16
Mesangiospermae - ps17
Magnoliids/eudicots - ps18
Eudicots - ps19
Core_eudicots - ps20
Superrosids - ps21
Rosids - ps22
Eurosids - ps23
Malvids - ps24
Sap./Malv./Brassicales - ps25
Malvales/Brassicales - ps26
Brassicales - ps27
Cleomaceae/Brassicaceae - ps28
Brassicaceae - ps29
Camelineae - ps30
Arabidopsis - ps31
Arabidopsis_thaliana - ps32
Phylostratum

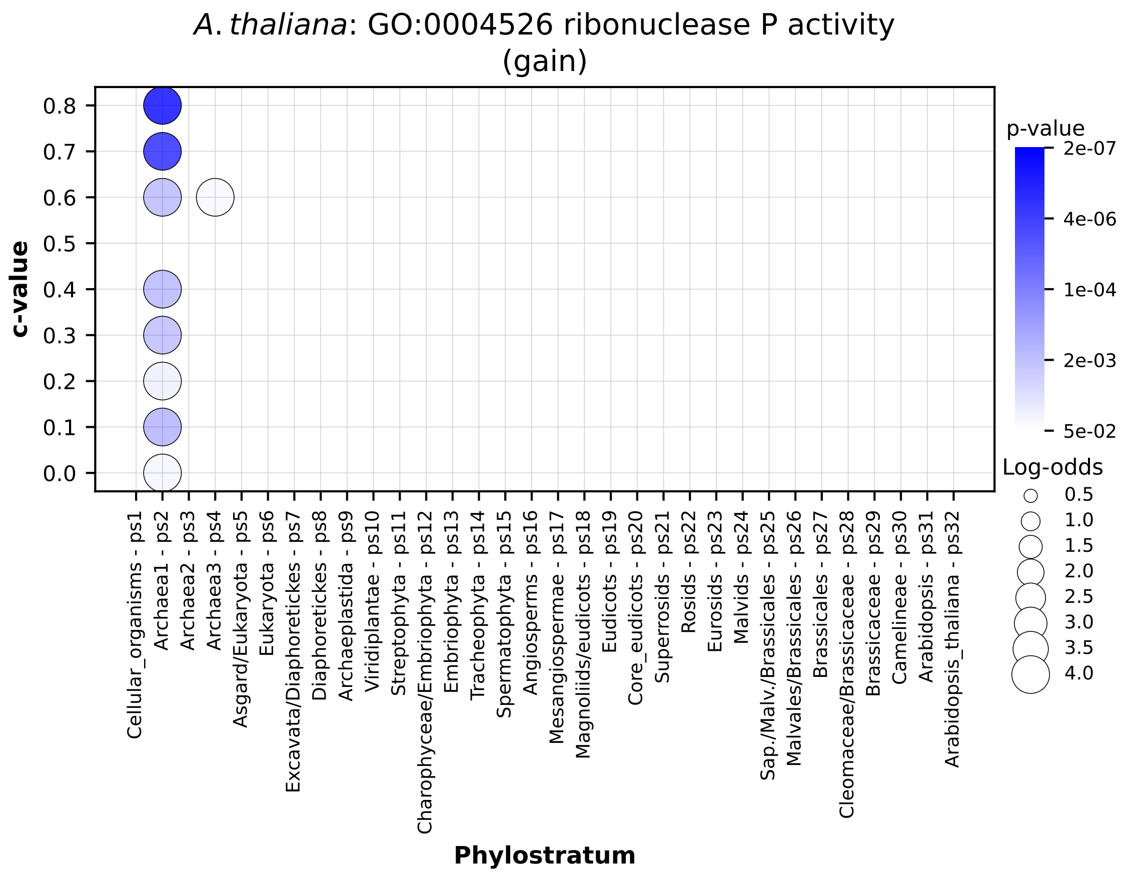

A. thaliana: GO:0004526 ribonuclease P activity
(gain)
c-value
0.8
0.7
0.6
0.5
0.4
0.3
0.2
0.1
0.0
p-value
2e-07
4e-06
1e-04
2e-03
5e-02
Log-odds
0.5
1.0
1.5
2.0
2.5
3.0
3.5
4.0
Cellular_organisms - ps1
Archaea1 - ps2
Archaea2 - ps3
Archaea3 - ps4
Asgard/Eukaryota - ps5
Eukaryota - ps6
Excavata/Diaphoretickes - ps7
Diaphoretickes - ps8
Archaeplastida - ps9
Viridiplantae - ps10
Streptophyta - ps11
Charophyceae/Embriophyta - ps12
Embriophyta - ps13
Tracheophyta - ps14
Spermatophyta - ps15
Angiosperms - ps16
Mesangiospermae - ps17
Magnoliids/eudicots - ps18
Eudicots - ps19
Core_eudicots - ps20
Superrosids - ps21
Rosids - ps22
Eurosids - ps23
Malvids - ps24
Sap./Malv./Brassicales - ps25
Malvales/Brassicales - ps26
Brassicales - ps27
Cleomaceae/Brassicaceae - ps28
Brassicaceae - ps29
Camelineae - ps30
Arabidopsis - ps31
Arabidopsis_thaliana - ps32
Phylostratum

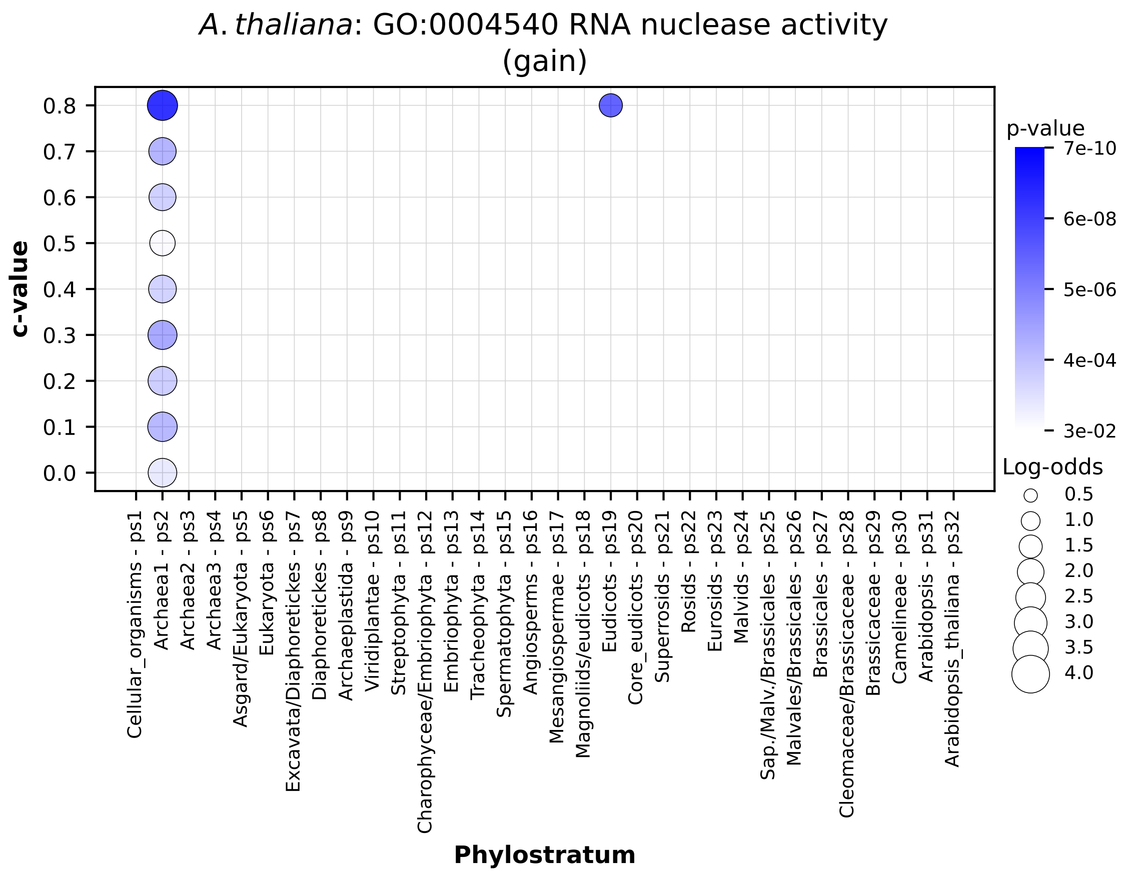

A. thaliana: GO:0004540 RNA nuclease activity
(gain)
c-value
0.8
0.7
0.6
0.5
0.4
0.3
0.2
0.1
0.0
p-value
7e-10
6e-08
5e-06
4e-04
3e-02
Log-odds
0.5
1.0
1.5
2.0
2.5
3.0
3.5
4.0
Cellular_organisms - ps1
Archaea1 - ps2
Archaea2 - ps3
Archaea3 - ps4
Asgard/Eukaryota - ps5
Eukaryota - ps6
Excavata/Diaphoretickes - ps7
Diaphoretickes - ps8
Archaeplastida - ps9
Viridiplantae - ps10
Streptophyta - ps11
Charophyceae/Embriophyta - ps12
Embriophyta - ps13
Tracheophyta - ps14
Spermatophyta - ps15
Angiosperms - ps16
Mesangiospermae - ps17
Magnoliids/eudicots - ps18
Eudicots - ps19
Core_eudicots - ps20
Superrosids - ps21
Rosids - ps22
Eurosids - ps23
Malvids - ps24
Sap./Malv./Brassicales - ps25
Malvales/Brassicales - ps26
Brassicales - ps27
Cleomaceae/Brassicaceae - ps28
Brassicaceae - ps29
Camelineae - ps30
Arabidopsis - ps31
Arabidopsis_thaliana - ps32
Phylostratum

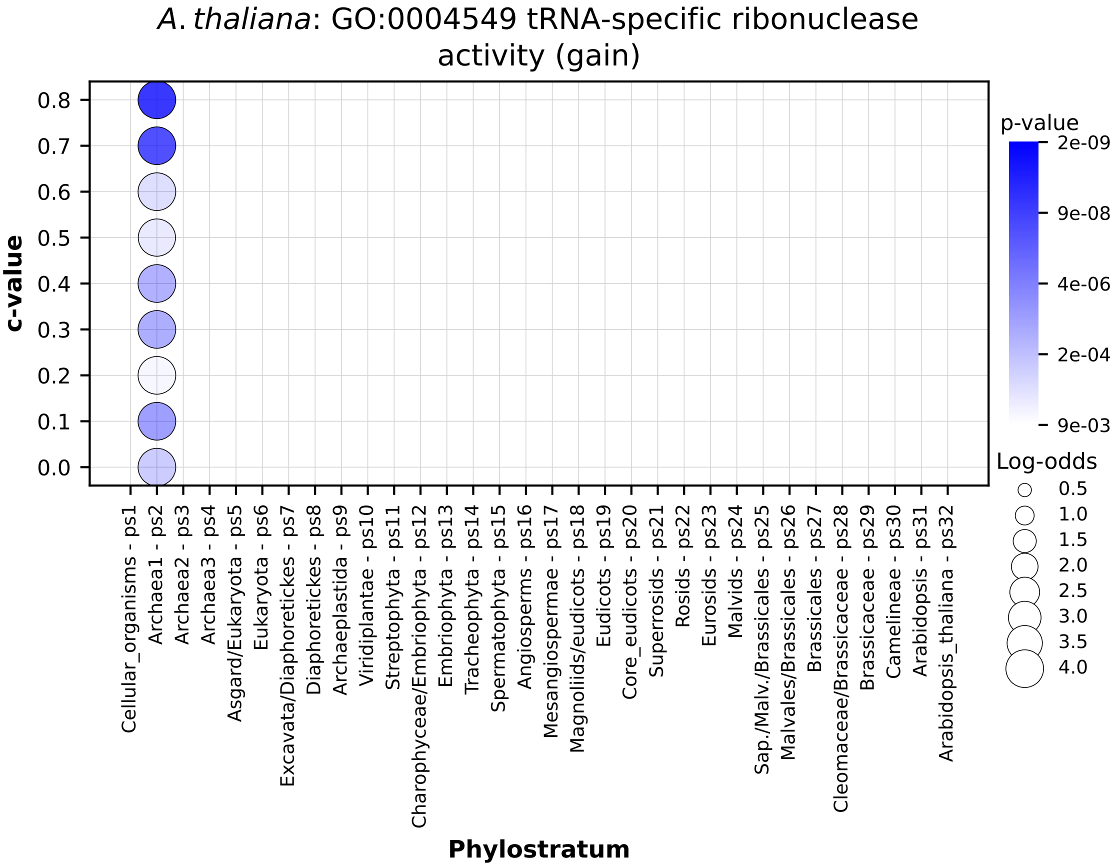

A. thaliana: GO:0004549 tRNA-specific ribonuclease activity (gain)
c-value
0.0
0.1
0.2
0.3
0.4
0.5
0.6
0.7
0.8
Phylostratum
Cellular_organisms - ps1
Archaea1 - ps2
Archaea2 - ps3
Archaea3 - ps4
Asgard/Eukaryota - ps5
Eukaryota - ps6
Excavata/Diaphoretickes - ps7
Diaphoretickes - ps8
Archaeplastida - ps9
Viridiplantae - ps10
Streptophyta - ps11
Charophyceae/Embriophyta - ps12
Embriophyta - ps13
Tracheophyta - ps14
Spermatophyta - ps15
Angiosperms - ps16
Mesangiospermae - ps17
Magnoliids/eudicots - ps18
Eudicots - ps19
Core_eudicots - ps20
Superrosids - ps21
Rosids - ps22
Eurosids - ps23
Malvids - ps24
Sap./Malv./Brassicales - ps25
Malvales/Brassicales - ps26
Brassicales - ps27
Cleomaceae/Brassicaceae - ps28
Brassicaceae - ps29
Camelineae - ps30
Arabidopsis - ps31
Arabidopsis_thaliana - ps32
p-value
2e-09
9e-08
4e-06
2e-04
9e-03
Log-odds
0.5
1.0
1.5
2.0
2.5
3.0
3.5
4.0

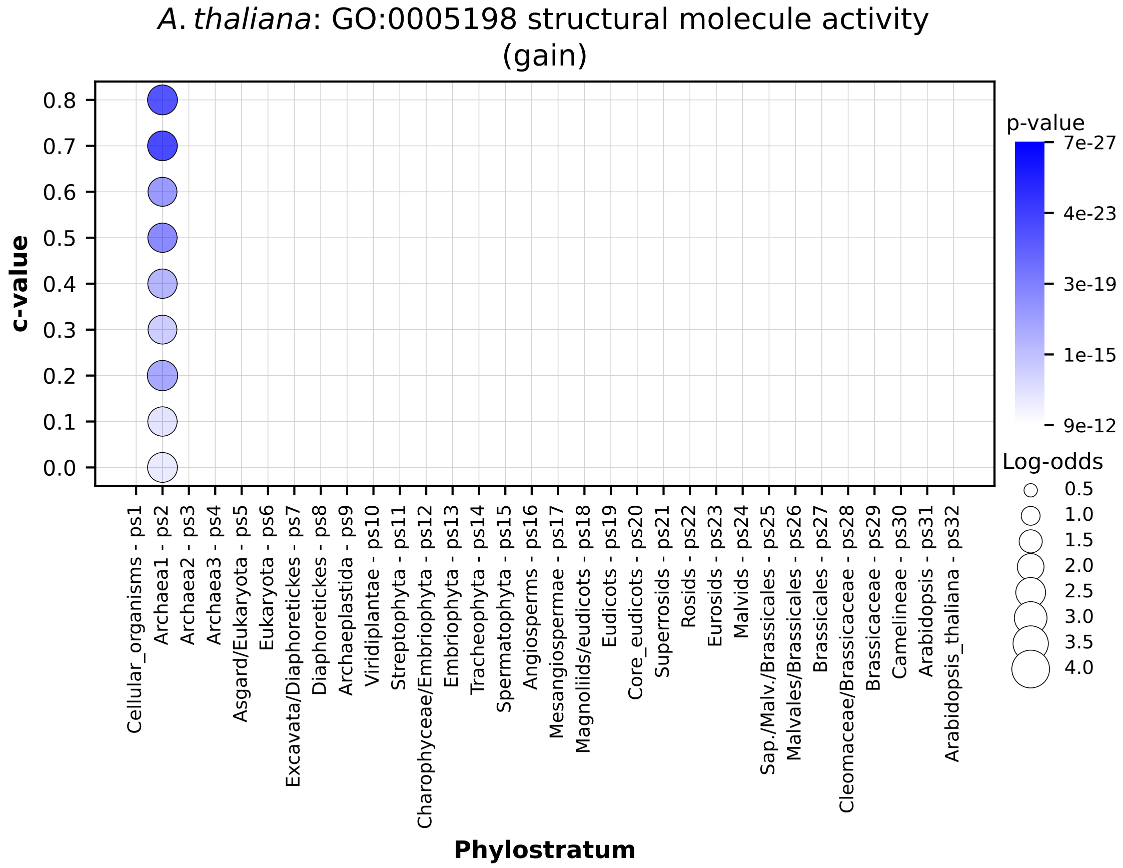
A. thaliana: GO:0005198 structural molecule activity
(gain)
c-value
0.8
0.7
0.6
0.5
0.4
0.3
0.2
0.1
0.0
p-value
7e-27
4e-23
3e-19
1e-15
9e-12
Log-odds
0.5
1.0
1.5
2.0
2.5
3.0
3.5
4.0
Cellular_organisms - ps1
Archaea1 - ps2
Archaea2 - ps3
Archaea3 - ps4
Asgard/Eukaryota - ps5
Eukaryota - ps6
Excavata/Diaphoretickes - ps7
Diaphoretickes - ps8
Archaeplastida - ps9
Viridiplantae - ps10
Streptophyta - ps11
Charophyceae/Embriophyta - ps12
Embriophyta - ps13
Tracheophyta - ps14
Spermatophyta - ps15
Angiosperms - ps16
Mesangiospermae - ps17
Magnoliids/eudicots - ps18
Eudicots - ps19
Core_eudicots - ps20
Superrosids - ps21
Rosids - ps22
Eurosids - ps23
Malvids - ps24
Sap./Malv./Brassicales - ps25
Malvales/Brassicales - ps26
Brassicales - ps27
Cleomaceae/Brassicaceae - ps28
Brassicaceae - ps29
Camelineae - ps30
Arabidopsis - ps31
Arabidopsis_thaliana - ps32
Phylostratum

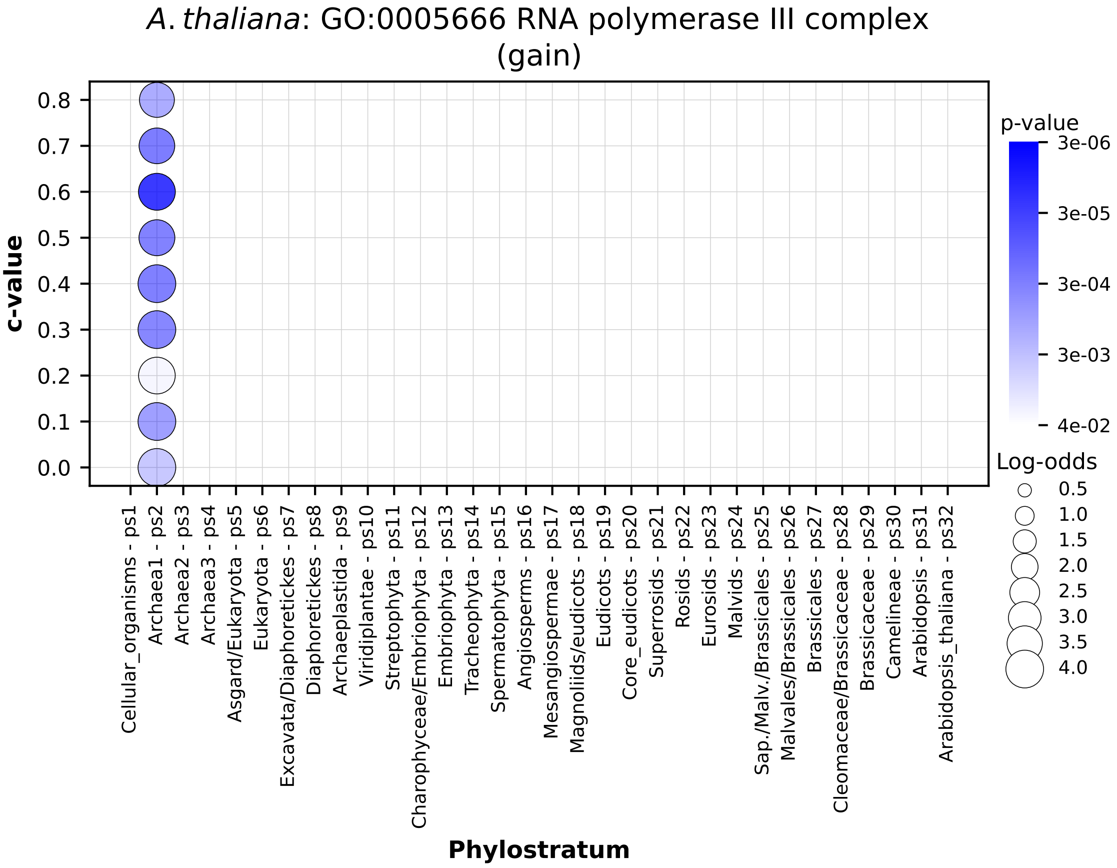

A. thaliana: GO:0005666 RNA polymerase III complex
(gain)
c-value
0.8
0.7
0.6
0.5
0.4
0.3
0.2
0.1
0.0
p-value
3e-06
3e-05
3e-04
3e-03
4e-02
Log-odds
0.5
1.0
1.5
2.0
2.5
3.0
3.5
4.0
Cellular_organisms - ps1
Archaea1 - ps2
Archaea2 - ps3
Archaea3 - ps4
Asgard/Eukaryota - ps5
Eukaryota - ps6
Excavata/Diaphoretickes - ps7
Diaphoretickes - ps8
Archaeplastida - ps9
Viridiplantae - ps10
Streptophyta - ps11
Charophyceae/Embriophyta - ps12
Embriophyta - ps13
Tracheophyta - ps14
Spermatophyta - ps15
Angiosperms - ps16
Mesangiospermae - ps17
Magnoliids/eudicots - ps18
Eudicots - ps19
Core_eudicots - ps20
Superrosids - ps21
Rosids - ps22
Eurosids - ps23
Malvids - ps24
Sap./Malv./Brassicales - ps25
Malvales/Brassicales - ps26
Brassicales - ps27
Cleomaceae/Brassicaceae - ps28
Brassicaceae - ps29
Camelineae - ps30
Arabidopsis - ps31
Arabidopsis_thaliana - ps32
Phylostratum

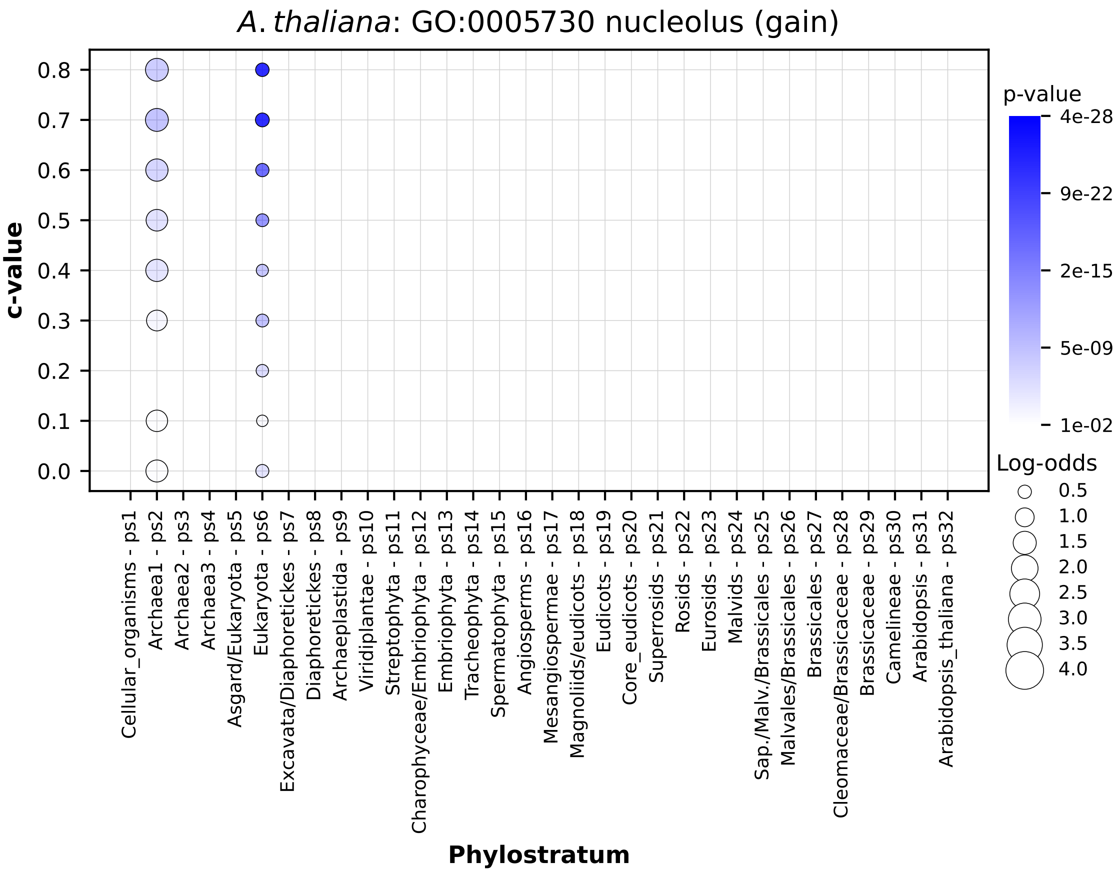

A. thaliana: GO:0005730 nucleolus (gain)
c-value
0.8
0.7
0.6
0.5
0.4
0.3
0.2
0.1
0.0
p-value
4e-28
9e-22
2e-15
5e-09
1e-02
Log-odds
0.5
1.0
1.5
2.0
2.5
3.0
3.5
4.0
Cellular_organisms - ps1
Archaea1 - ps2
Archaea2 - ps3
Archaea3 - ps4
Asgard/Eukaryota - ps5
Eukaryota - ps6
Excavata/Diaphoretickes - ps7
Diaphoretickes - ps8
Archaeplastida - ps9
Viridiplantae - ps10
Streptophyta - ps11
Charophyceae/Embriophyta - ps12
Embriophyta - ps13
Tracheophyta - ps14
Spermatophyta - ps15
Angiosperms - ps16
Mesangiospermae - ps17
Magnoliids/eudicots - ps18
Eudicots - ps19
Core_eudicots - ps20
Superrosids - ps21
Rosids - ps22
Eurosids - ps23
Malvids - ps24
Sap./Malv./Brassicales - ps25
Malvales/Brassicales - ps26
Brassicales - ps27
Cleomaceae/Brassicaceae - ps28
Brassicaceae - ps29
Camelineae - ps30
Arabidopsis - ps31
Arabidopsis_thaliana - ps32
Phylostratum

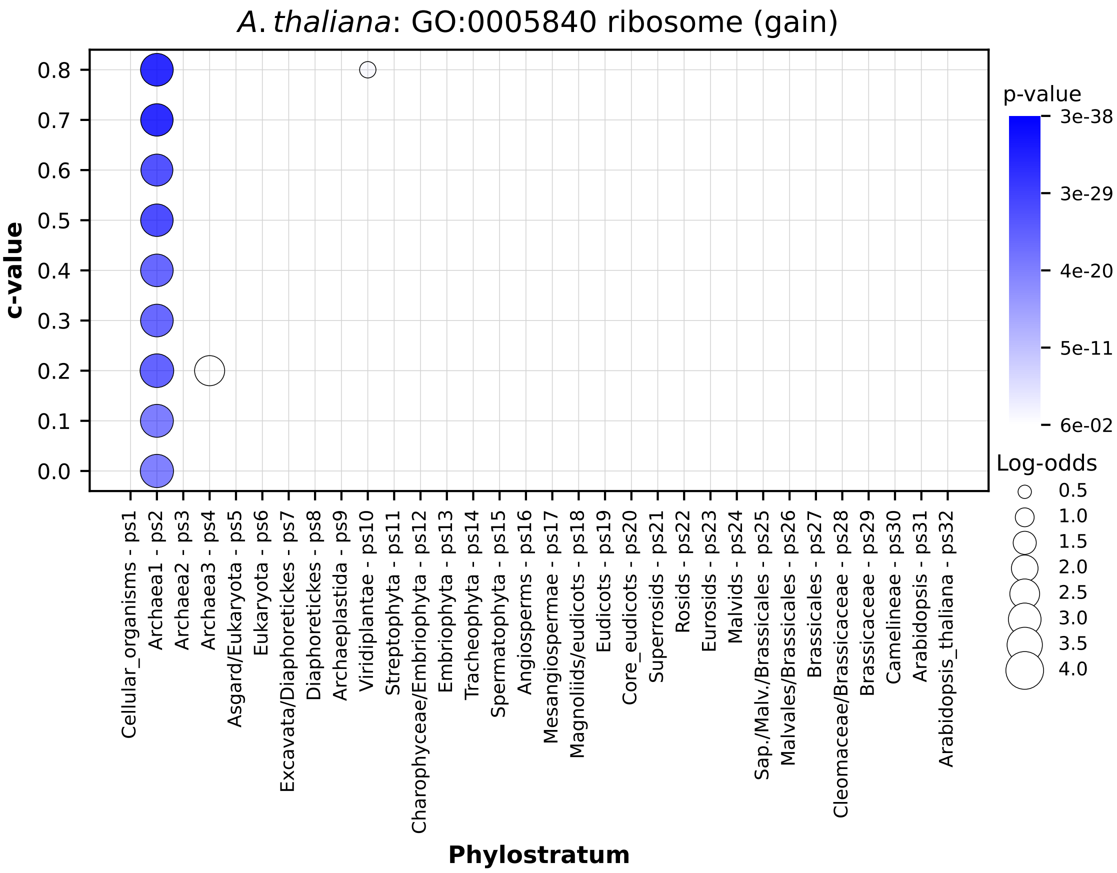

A. thaliana: GO:0005840 ribosome (gain)
c-value
0.8
0.7
0.6
0.5
0.4
0.3
0.2
0.1
0.0
p-value
3e-38
3e-29
4e-20
5e-11
6e-02
Log-odds
0.5
1.0
1.5
2.0
2.5
3.0
3.5
4.0
Cellular_organisms - ps1
Archaea1 - ps2
Archaea2 - ps3
Archaea3 - ps4
Asgard/Eukaryota - ps5
Eukaryota - ps6
Excavata/Diaphoretickes - ps7
Diaphoretickes - ps8
Archaeplastida - ps9
Viridiplantae - ps10
Streptophyta - ps11
Charophyceae/Embriophyta - ps12
Embriophyta - ps13
Tracheophyta - ps14
Spermatophyta - ps15
Angiosperms - ps16
Mesangiospermae - ps17
Magnoliids/eudicots - ps18
Eudicots - ps19
Core_eudicots - ps20
Superrosids - ps21
Rosids - ps22
Eurosids - ps23
Malvids - ps24
Sap./Malv./Brassicales - ps25
Malvales/Brassicales - ps26
Brassicales - ps27
Cleomaceae/Brassicaceae - ps28
Brassicaceae - ps29
Camelineae - ps30
Arabidopsis - ps31
Arabidopsis_thaliana - ps32
Phylostratum

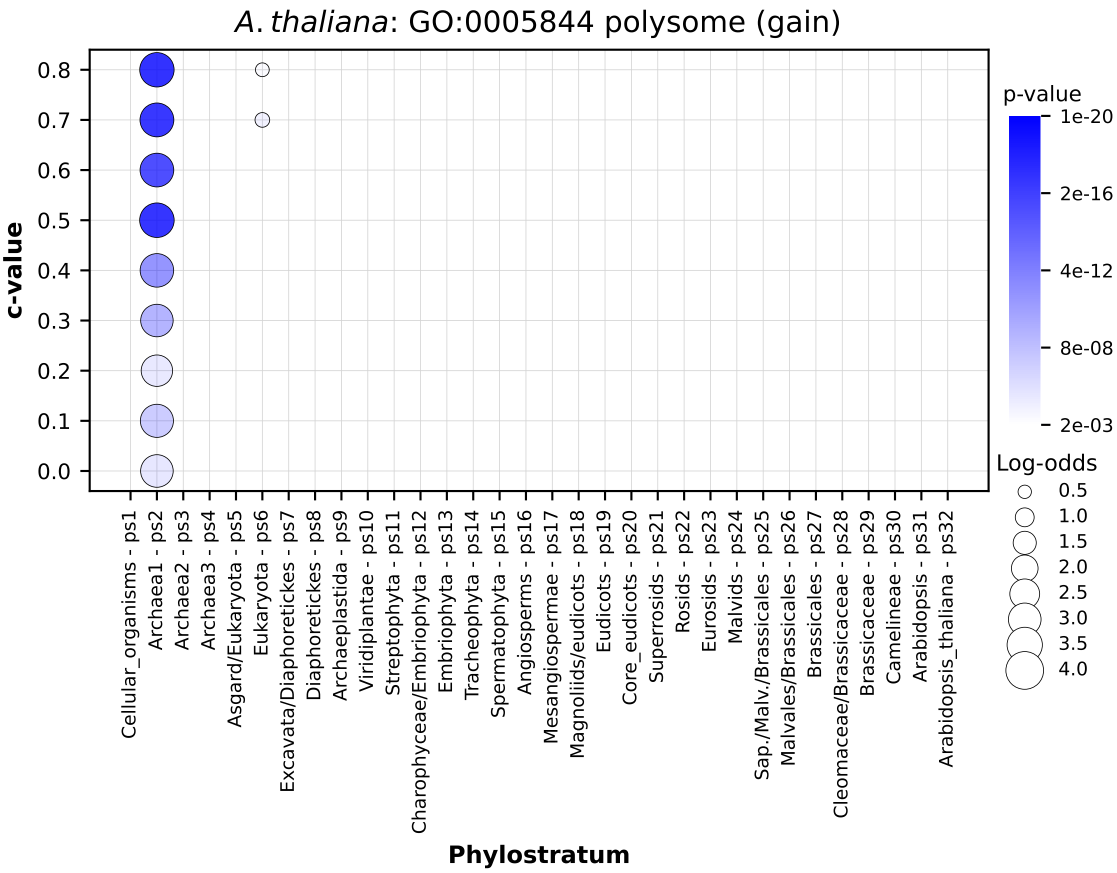

A. thaliana: GO:0005844 polysome (gain)
c-value
0.8
0.7
0.6
0.5
0.4
0.3
0.2
0.1
0.0
p-value
1e-20
2e-16
4e-12
8e-08
2e-03
Log-odds
0.5
1.0
1.5
2.0
2.5
3.0
3.5
4.0
Cellular_organisms - ps1
Archaea1 - ps2
Archaea2 - ps3
Archaea3 - ps4
Asgard/Eukaryota - ps5
Eukaryota - ps6
Excavata/Diaphoretickes - ps7
Diaphoretickes - ps8
Archaeplastida - ps9
Viridiplantae - ps10
Streptophyta - ps11
Charophyceae/Embriophyta - ps12
Embriophyta - ps13
Tracheophyta - ps14
Spermatophyta - ps15
Angiosperms - ps16
Mesangiospermae - ps17
Magnoliids/eudicots - ps18
Eudicots - ps19
Core_eudicots - ps20
Superrosids - ps21
Rosids - ps22
Eurosids - ps23
Malvids - ps24
Sap./Malv./Brassicales - ps25
Malvales/Brassicales - ps26
Brassicales - ps27
Cleomaceae/Brassicaceae - ps28
Brassicaceae - ps29
Camelineae - ps30
Arabidopsis - ps31
Arabidopsis_thaliana - ps32
Phylostratum

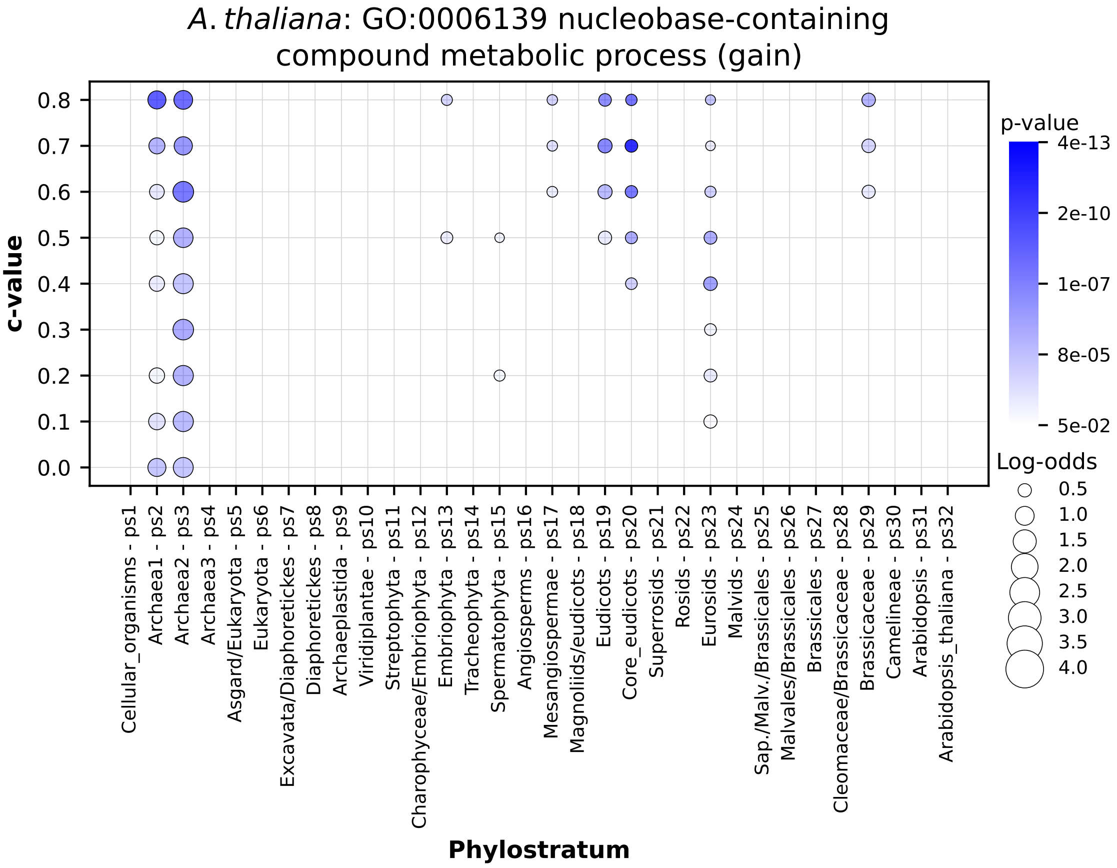

A. thaliana: GO:0006139 nucleobase-containing compound metabolic process (gain)
c-value
0.8
0.7
0.6
0.5
0.4
0.3
0.2
0.1
0.0
Phylostratum
Cellular_organisms - ps1
Archaea1 - ps2
Archaea2 - ps3
Archaea3 - ps4
Asgard/Eukaryota - ps5
Eukaryota - ps6
Excavata/Diaphoretickes - ps7
Diaphoretickes - ps8
Archaeplastida - ps9
Viridiplantae - ps10
Streptophyta - ps11
Charophyceae/Embriophyta - ps12
Embriophyta - ps13
Tracheophyta - ps14
Spermatophyta - ps15
Angiosperms - ps16
Mesangiospermae - ps17
Magnoliids/eudicots - ps18
Eudicots - ps19
Core_eudicots - ps20
Superrosids - ps21
Rosids - ps22
Eurosids - ps23
Malvids - ps24
Sap./Malv./Brassicales - ps25
Malvales/Brassicales - ps26
Brassicales - ps27
Cleomaceae/Brassicaceae - ps28
Brassicaceae - ps29
Camelineae - ps30
Arabidopsis - ps31
Arabidopsis_thaliana - ps32
p-value
4e-13
2e-10
1e-07
8e-05
5e-02
Log-odds
0.5
1.0
1.5
2.0
2.5
3.0
3.5
4.0

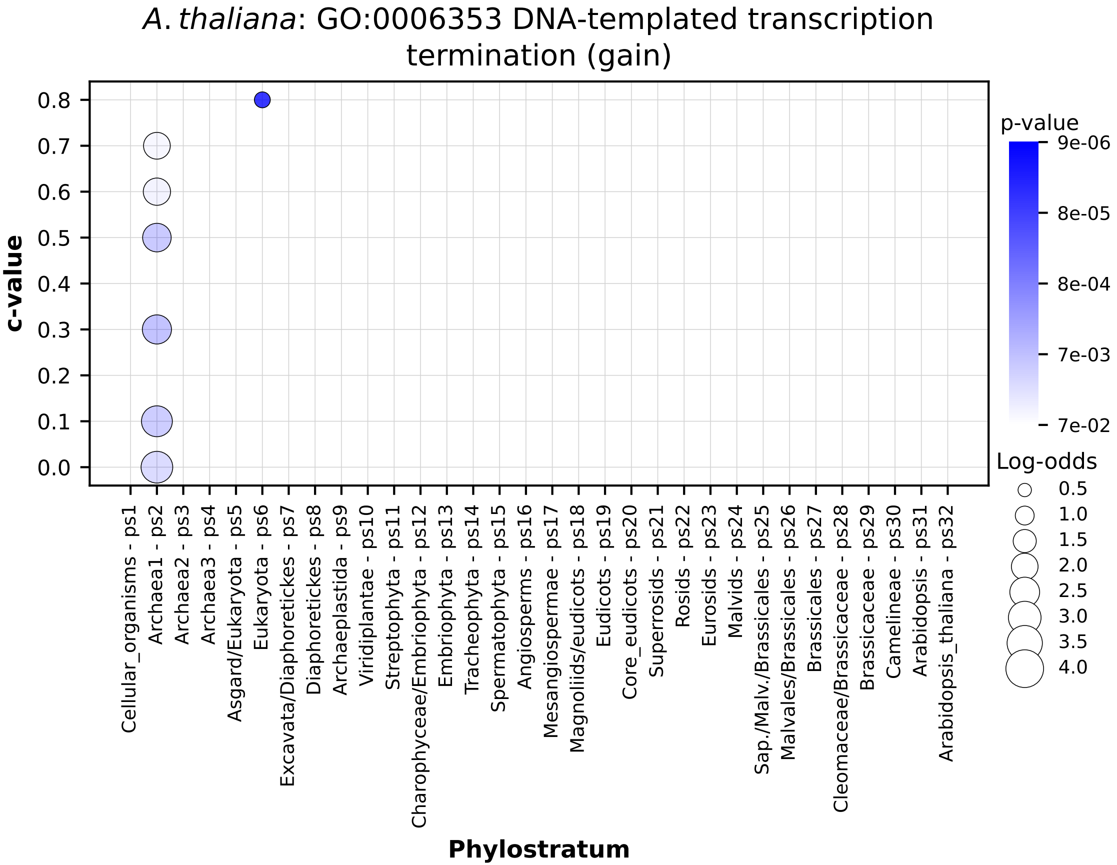

A. thaliana: GO:0006353 DNA-templated transcription termination (gain)
c-value
0.8
0.7
0.6
0.5
0.4
0.3
0.2
0.1
0.0
p-value
9e-06
8e-05
8e-04
7e-03
7e-02
Log-odds
0.5
1.0
1.5
2.0
2.5
3.0
3.5
4.0
Cellular_organisms - ps1
Archaea1 - ps2
Archaea2 - ps3
Archaea3 - ps4
Asgard/Eukaryota - ps5
Eukaryota - ps6
Excavata/Diaphoretickes - ps7
Diaphoretickes - ps8
Archaeplastida - ps9
Viridiplantae - ps10
Streptophyta - ps11
Charophyceae/Embriophyta - ps12
Embriophyta - ps13
Tracheophyta - ps14
Spermatophyta - ps15
Angiosperms - ps16
Mesangiospermae - ps17
Magnoliids/eudicots - ps18
Eudicots - ps19
Core_eudicots - ps20
Superrosids - ps21
Rosids - ps22
Eurosids - ps23
Malvids - ps24
Sap./Malv./Brassicales - ps25
Malvales/Brassicales - ps26
Brassicales - ps27
Cleomaceae/Brassicaceae - ps28
Brassicaceae - ps29
Camelineae - ps30
Arabidopsis - ps31
Arabidopsis_thaliana - ps32
Phylostratum

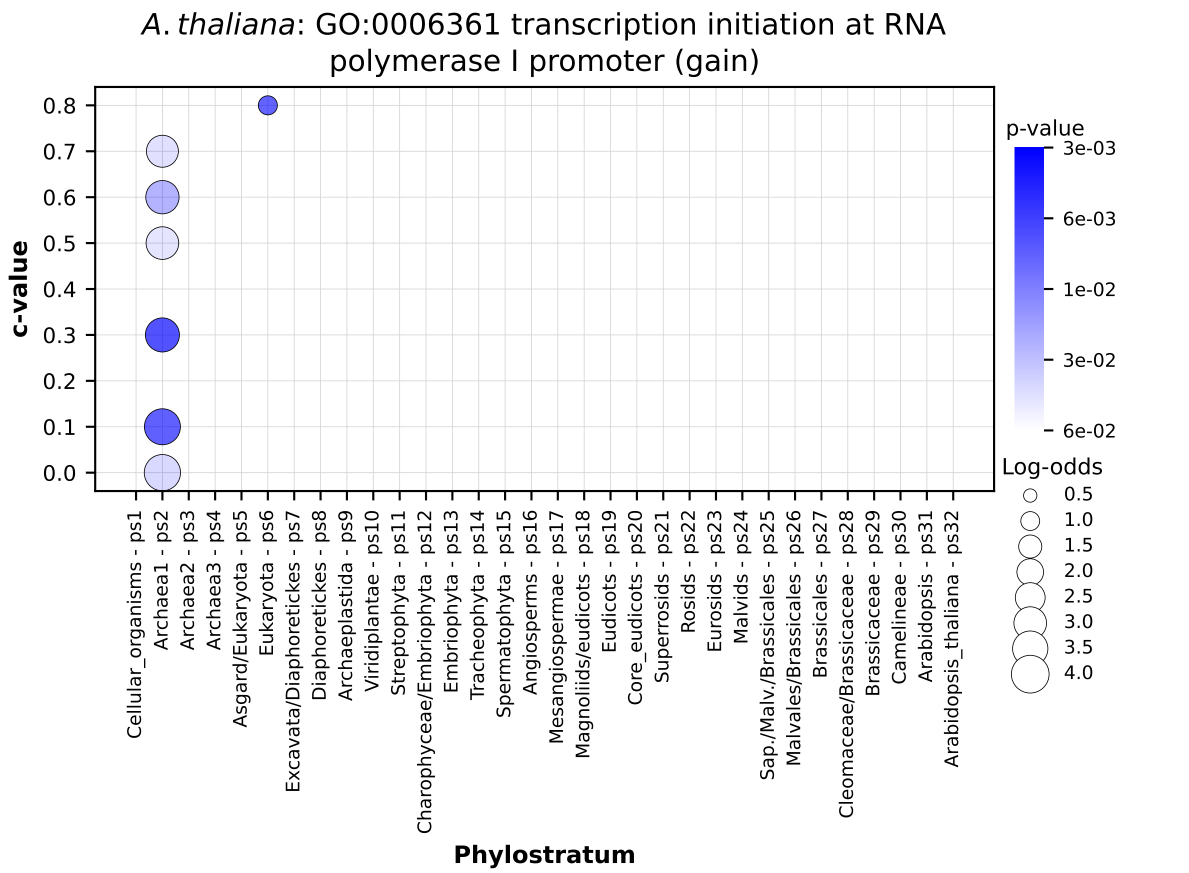

A. thaliana: GO:0006361 transcription initiation at RNA polymerase I promoter (gain)
c-value
0.0
0.1
0.2
0.3
0.4
0.5
0.6
0.7
0.8
Phylostratum
Cellular_organisms - ps1
Archaea1 - ps2
Archaea2 - ps3
Archaea3 - ps4
Asgard/Eukaryota - ps5
Eukaryota - ps6
Excavata/Diaphoretickes - ps7
Diaphoretickes - ps8
Archaeplastida - ps9
Viridiplantae - ps10
Streptophyta - ps11
Charophyceae/Embriophyta - ps12
Embriophyta - ps13
Tracheophyta - ps14
Spermatophyta - ps15
Angiosperms - ps16
Mesangiospermae - ps17
Magnoliids/eudicots - ps18
Eudicots - ps19
Core_eudicots - ps20
Superrosids - ps21
Rosids - ps22
Eurosids - ps23
Malvids - ps24
Sap./Malv./Brassicales - ps25
Malvales/Brassicales - ps26
Brassicales - ps27
Cleomaceae/Brassicaceae - ps28
Brassicaceae - ps29
Camelineae - ps30
Arabidopsis - ps31
Arabidopsis_thaliana - ps32
p-value
3e-03
6e-03
1e-02
3e-02
6e-02
Log-odds
0.5
1.0
1.5
2.0
2.5
3.0
3.5
4.0

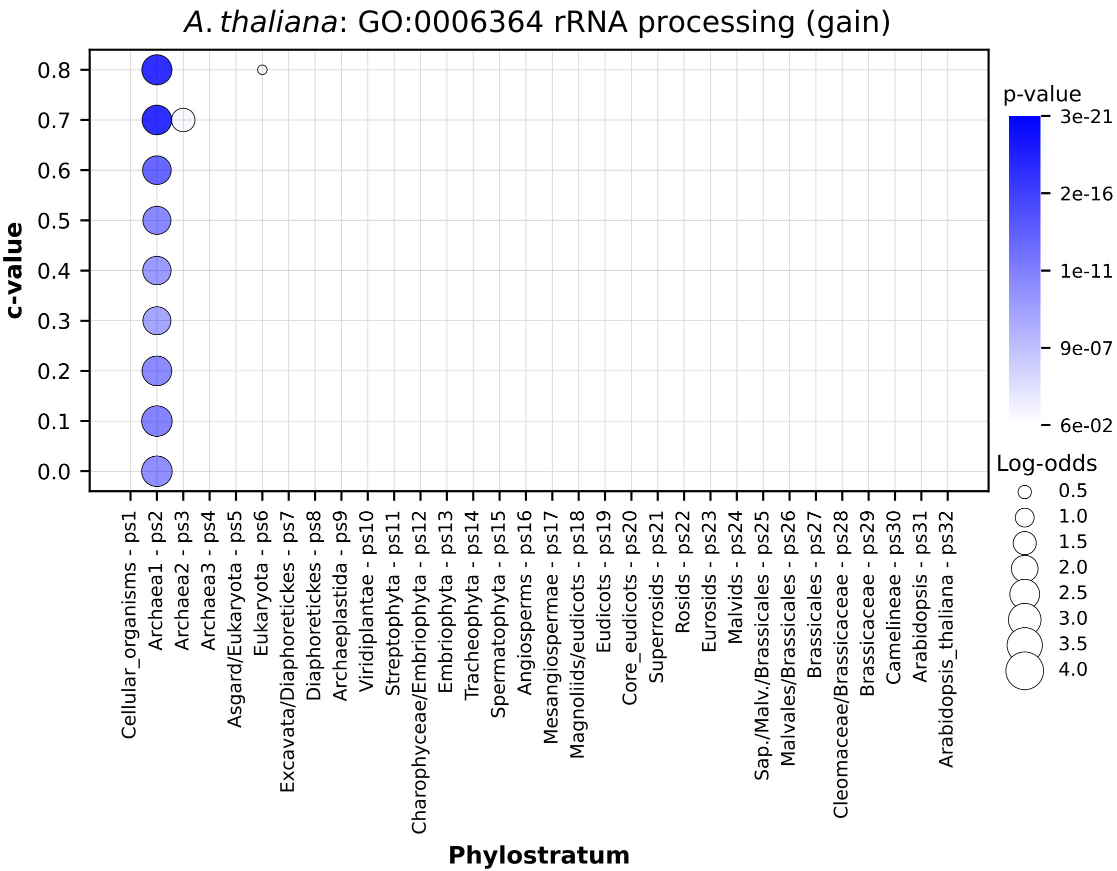

A. thaliana: GO:0006364 rRNA processing (gain)
c-value
0.8
0.7
0.6
0.5
0.4
0.3
0.2
0.1
0.0
p-value
3e-21
2e-16
1e-11
9e-07
6e-02
Log-odds
0.5
1.0
1.5
2.0
2.5
3.0
3.5
4.0
Cellular_organisms - ps1
Archaea1 - ps2
Archaea2 - ps3
Archaea3 - ps4
Asgard/Eukaryota - ps5
Eukaryota - ps6
Excavata/Diaphoretickes - ps7
Diaphoretickes - ps8
Archaeplastida - ps9
Viridiplantae - ps10
Streptophyta - ps11
Charophyceae/Embriophyta - ps12
Embriophyta - ps13
Tracheophyta - ps14
Spermatophyta - ps15
Angiosperms - ps16
Mesangiospermae - ps17
Magnoliids/eudicots - ps18
Eudicots - ps19
Core_eudicots - ps20
Superrosids - ps21
Rosids - ps22
Eurosids - ps23
Malvids - ps24
Sap./Malv./Brassicales - ps25
Malvales/Brassicales - ps26
Brassicales - ps27
Cleomaceae/Brassicaceae - ps28
Brassicaceae - ps29
Camelineae - ps30
Arabidopsis - ps31
Arabidopsis_thaliana - ps32
Phylostratum

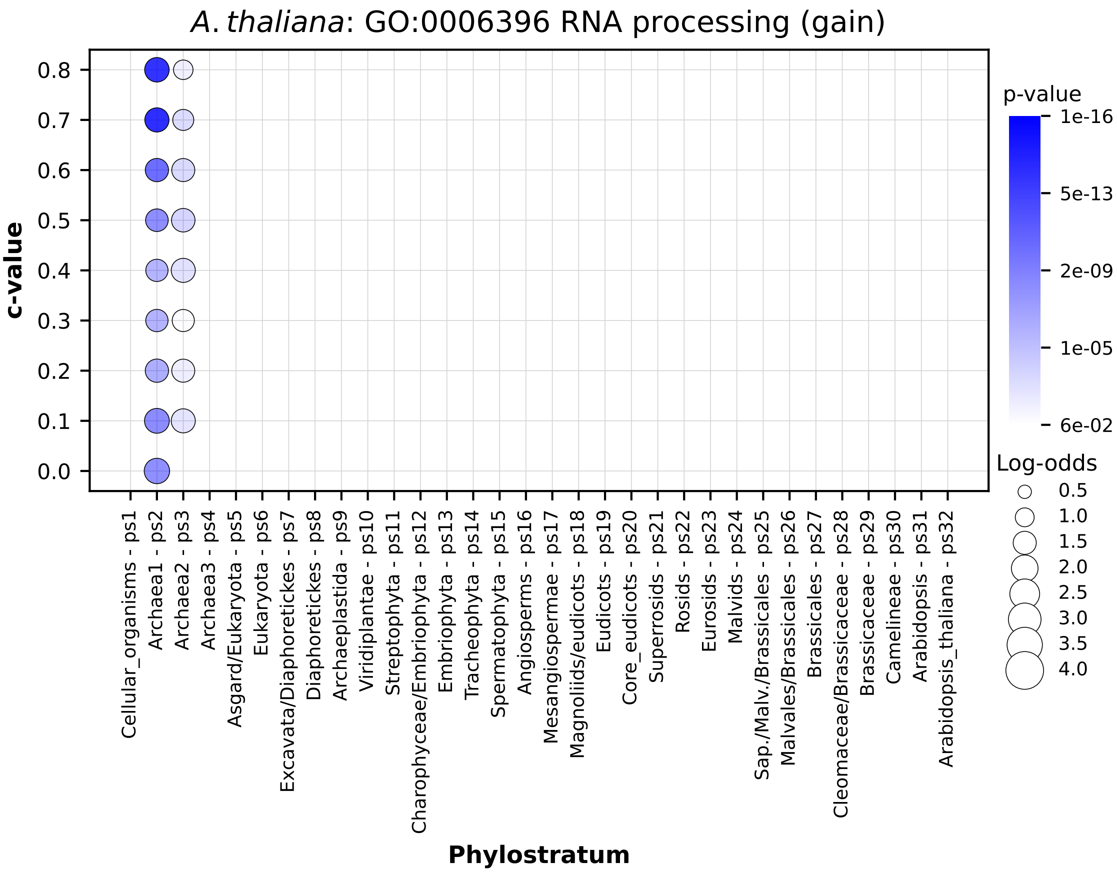
A. thaliana: GO:0006396 RNA processing (gain)
c-value
0.8
0.7
0.6
0.5
0.4
0.3
0.2
0.1
0.0
p-value
1e-16
5e-13
2e-09
1e-05
6e-02
Log-odds
0.5
1.0
1.5
2.0
2.5
3.0
3.5
4.0
Cellular_organisms - ps1
Archaea1 - ps2
Archaea2 - ps3
Archaea3 - ps4
Asgard/Eukaryota - ps5
Eukaryota - ps6
Excavata/Diaphoretickes - ps7
Diaphoretickes - ps8
Archaeplastida - ps9
Viridiplantae - ps10
Streptophyta - ps11
Charophyceae/Embriophyta - ps12
Embriophyta - ps13
Tracheophyta - ps14
Spermatophyta - ps15
Angiosperms - ps16
Mesangiospermae - ps17
Magnoliids/eudicots - ps18
Eudicots - ps19
Core_eudicots - ps20
Superrosids - ps21
Rosids - ps22
Eurosids - ps23
Malvids - ps24
Sap./Malv./Brassicales - ps25
Malvales/Brassicales - ps26
Brassicales - ps27
Cleomaceae/Brassicaceae - ps28
Brassicaceae - ps29
Camelineae - ps30
Arabidopsis - ps31
Arabidopsis_thaliana - ps32
Phylostratum

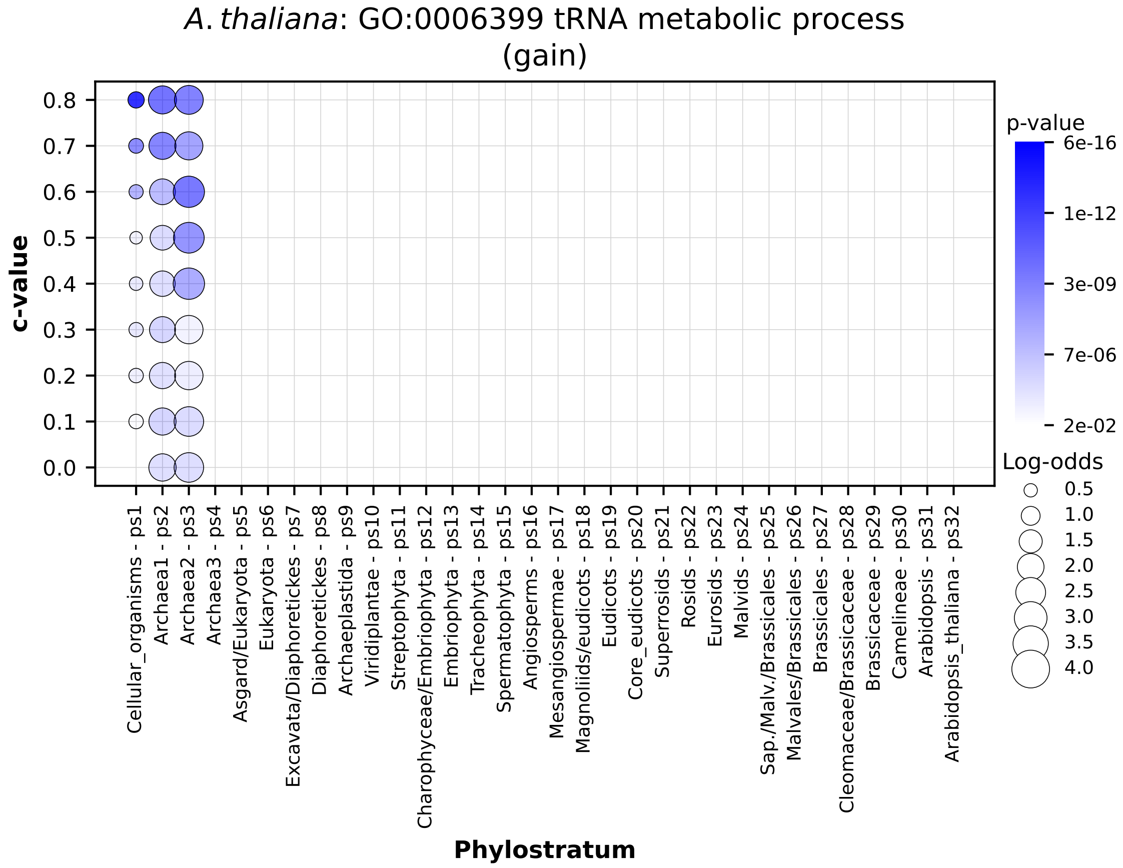

A. thaliana: GO:0006399 tRNA metabolic process
(gain)
c-value
0.8
0.7
0.6
0.5
0.4
0.3
0.2
0.1
0.0
p-value
6e-16
1e-12
3e-09
7e-06
2e-02
Log-odds
0.5
1.0
1.5
2.0
2.5
3.0
3.5
4.0
Cellular_organisms - ps1
Archaea1 - ps2
Archaea2 - ps3
Archaea3 - ps4
Asgard/Eukaryota - ps5
Eukaryota - ps6
Excavata/Diaphoretickes - ps7
Diaphoretickes - ps8
Archaeplastida - ps9
Viridiplantae - ps10
Streptophyta - ps11
Charophyceae/Embriophyta - ps12
Embriophyta - ps13
Tracheophyta - ps14
Spermatophyta - ps15
Angiosperms - ps16
Mesangiospermae - ps17
Magnoliids/eudicots - ps18
Eudicots - ps19
Core_eudicots - ps20
Superrosids - ps21
Rosids - ps22
Eurosids - ps23
Malvids - ps24
Sap./Malv./Brassicales - ps25
Malvales/Brassicales - ps26
Brassicales - ps27
Cleomaceae/Brassicaceae - ps28
Brassicaceae - ps29
Camelineae - ps30
Arabidopsis - ps31
Arabidopsis_thaliana - ps32
Phylostratum

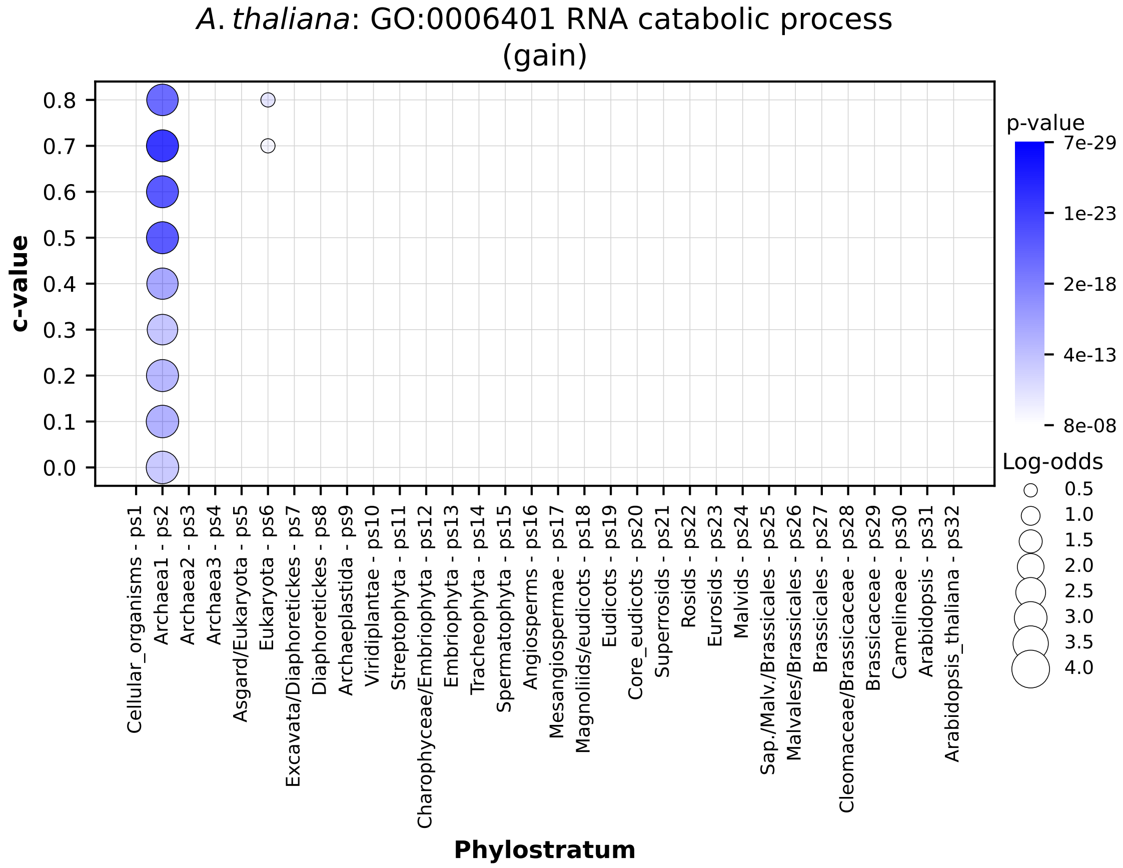

A. thaliana: GO:0006401 RNA catabolic process
(gain)
c-value
0.8
0.7
0.6
0.5
0.4
0.3
0.2
0.1
0.0
Phylostratum
Cellular_organisms - ps1
Archaea1 - ps2
Archaea2 - ps3
Archaea3 - ps4
Asgard/Eukaryota - ps5
Eukaryota - ps6
Excavata/Diaphoretickes - ps7
Diaphoretickes - ps8
Archaeplastida - ps9
Viridiplantae - ps10
Streptophyta - ps11
Charophyceae/Embriophyta - ps12
Embriophyta - ps13
Tracheophyta - ps14
Spermatophyta - ps15
Angiosperms - ps16
Mesangiospermae - ps17
Magnoliids/eudicots - ps18
Eudicots - ps19
Core_eudicots - ps20
Superrosids - ps21
Rosids - ps22
Eurosids - ps23
Malvids - ps24
Sap./Malv./Brassicales - ps25
Malvales/Brassicales - ps26
Brassicales - ps27
Cleomaceae/Brassicaceae - ps28
Brassicaceae - ps29
Camelineae - ps30
Arabidopsis - ps31
Arabidopsis_thaliana - ps32
p-value
7e-29
1e-23
2e-18
4e-13
8e-08
Log-odds
0.5
1.0
1.5
2.0
2.5
3.0
3.5
4.0

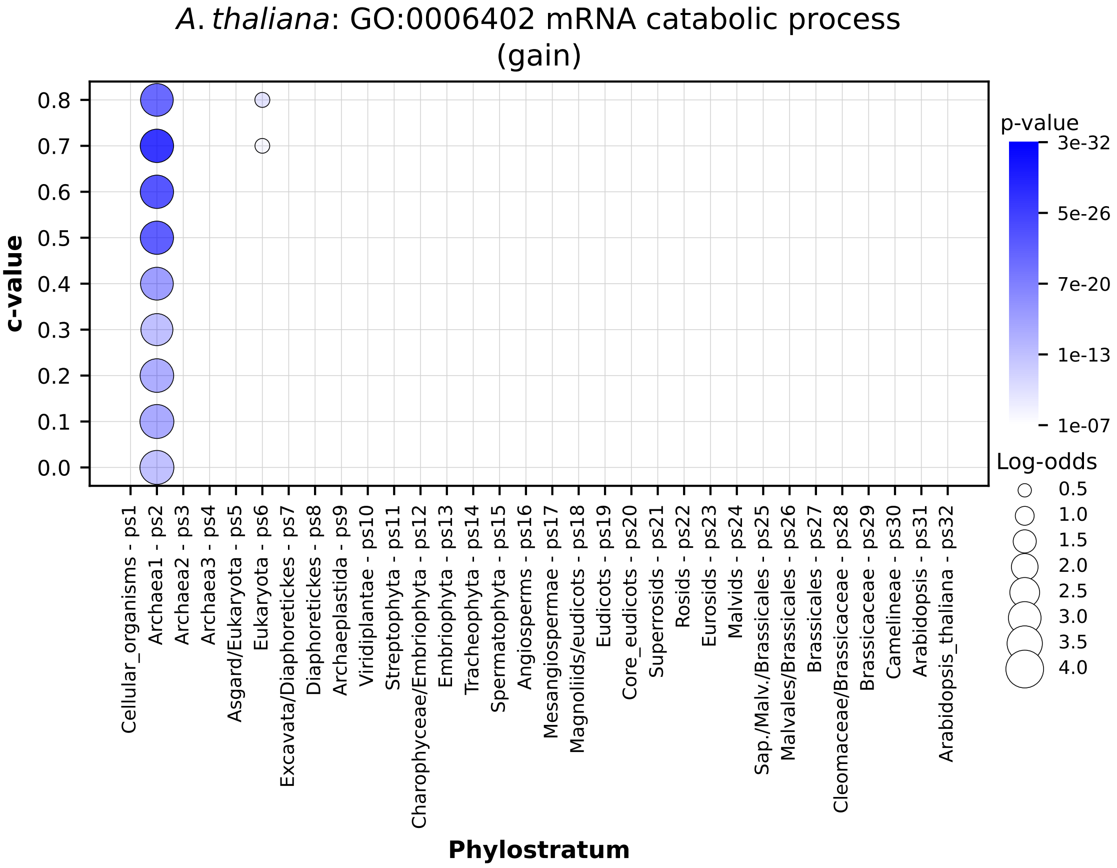

A. thaliana: GO:0006402 mRNA catabolic process
(gain)
c-value
0.8
0.7
0.6
0.5
0.4
0.3
0.2
0.1
0.0
p-value
3e-32
5e-26
7e-20
1e-13
1e-07
Log-odds
0.5
1.0
1.5
2.0
2.5
3.0
3.5
4.0
Cellular_organisms - ps1
Archaea1 - ps2
Archaea2 - ps3
Archaea3 - ps4
Asgard/Eukaryota - ps5
Eukaryota - ps6
Excavata/Diaphoretickes - ps7
Diaphoretickes - ps8
Archaeplastida - ps9
Viridiplantae - ps10
Streptophyta - ps11
Charophyceae/Embriophyta - ps12
Embriophyta - ps13
Tracheophyta - ps14
Spermatophyta - ps15
Angiosperms - ps16
Mesangiospermae - ps17
Magnoliids/eudicots - ps18
Eudicots - ps19
Core_eudicots - ps20
Superrosids - ps21
Rosids - ps22
Eurosids - ps23
Malvids - ps24
Sap./Malv./Brassicales - ps25
Malvales/Brassicales - ps26
Brassicales - ps27
Cleomaceae/Brassicaceae - ps28
Brassicaceae - ps29
Camelineae - ps30
Arabidopsis - ps31
Arabidopsis_thaliana - ps32
Phylostratum

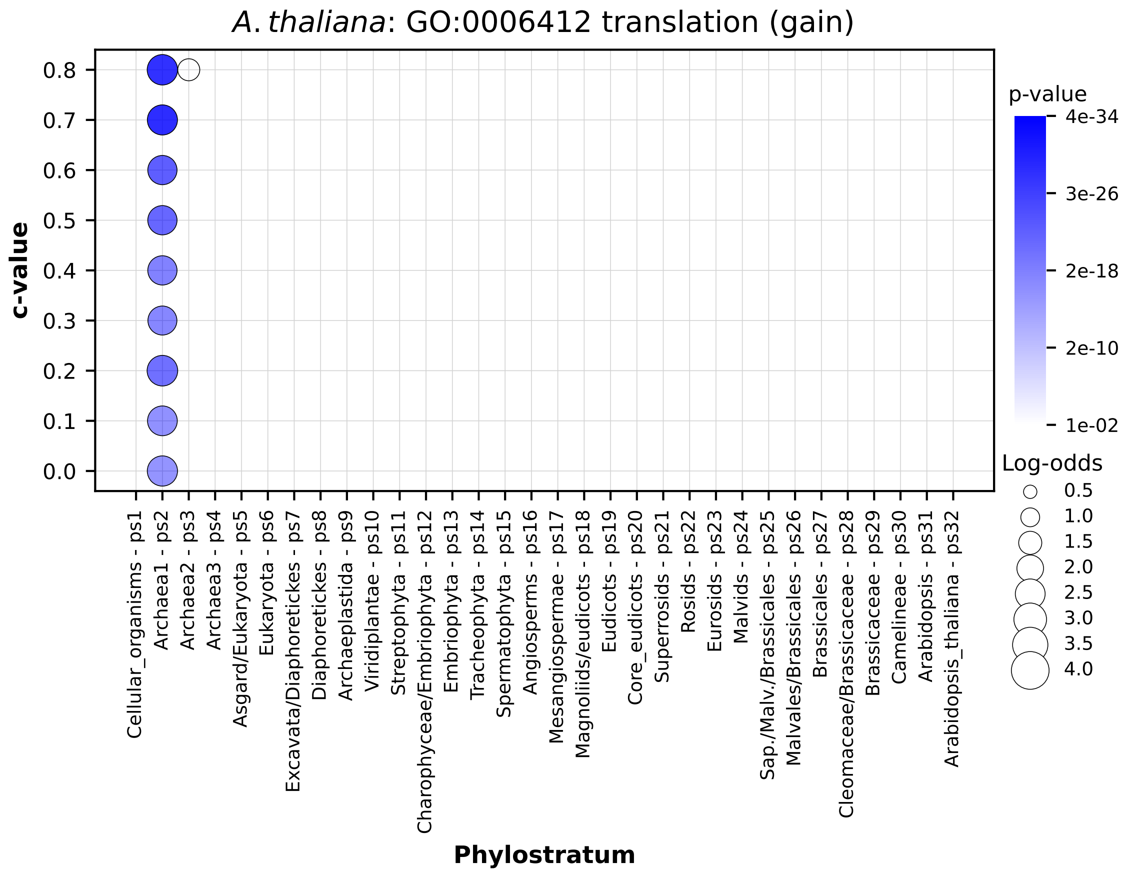

A. thaliana: GO:0006412 translation (gain)
c-value
0.8
0.7
0.6
0.5
0.4
0.3
0.2
0.1
0.0
p-value
4e-34
3e-26
2e-18
2e-10
1e-02
Log-odds
0.5
1.0
1.5
2.0
2.5
3.0
3.5
4.0
Cellular_organisms - ps1
Archaea1 - ps2
Archaea2 - ps3
Archaea3 - ps4
Asgard/Eukaryota - ps5
Eukaryota - ps6
Excavata/Diaphoretickes - ps7
Diaphoretickes - ps8
Archaeplastida - ps9
Viridiplantae - ps10
Streptophyta - ps11
Charophyceae/Embriophyta - ps12
Embriophyta - ps13
Tracheophyta - ps14
Spermatophyta - ps15
Angiosperms - ps16
Mesangiospermae - ps17
Magnoliids/eudicots - ps18
Eudicots - ps19
Core_eudicots - ps20
Superrosids - ps21
Rosids - ps22
Eurosids - ps23
Malvids - ps24
Sap./Malv./Brassicales - ps25
Malvales/Brassicales - ps26
Brassicales - ps27
Cleomaceae/Brassicaceae - ps28
Brassicaceae - ps29
Camelineae - ps30
Arabidopsis - ps31
Arabidopsis_thaliana - ps32
Phylostratum

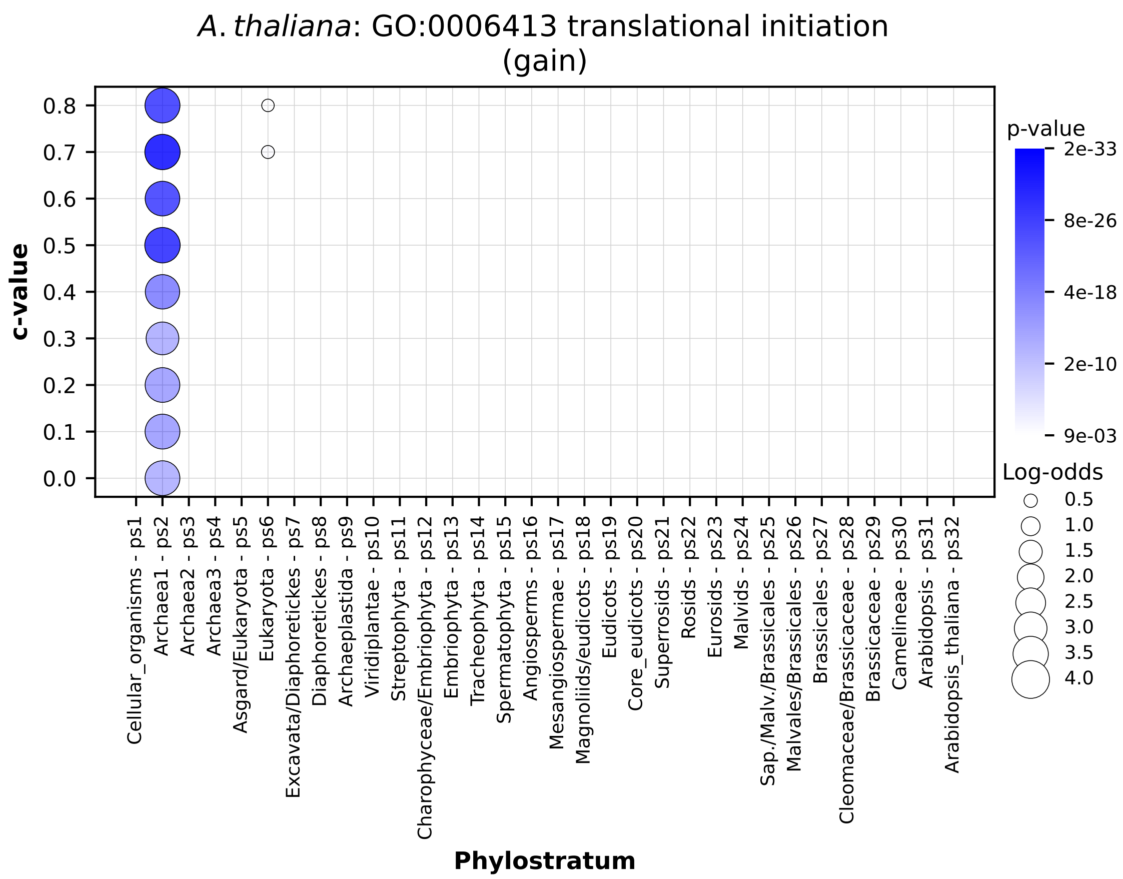

A. thaliana: GO:0006413 translational initiation
(gain)
c-value
0.8
0.7
0.6
0.5
0.4
0.3
0.2
0.1
0.0
p-value
2e-33
8e-26
4e-18
2e-10
9e-03
Log-odds
0.5
1.0
1.5
2.0
2.5
3.0
3.5
4.0
Cellular_organisms - ps1
Archaea1 - ps2
Archaea2 - ps3
Archaea3 - ps4
Asgard/Eukaryota - ps5
Eukaryota - ps6
Excavata/Diaphoretickes - ps7
Diaphoretickes - ps8
Archaeplastida - ps9
Viridiplantae - ps10
Streptophyta - ps11
Charophyceae/Embriophyta - ps12
Embriophyta - ps13
Tracheophyta - ps14
Spermatophyta - ps15
Angiosperms - ps16
Mesangiospermae - ps17
Magnoliids/eudicots - ps18
Eudicots - ps19
Core_eudicots - ps20
Superrosids - ps21
Rosids - ps22
Eurosids - ps23
Malvids - ps24
Sap./Malv./Brassicales - ps25
Malvales/Brassicales - ps26
Brassicales - ps27
Cleomaceae/Brassicaceae - ps28
Brassicaceae - ps29
Camelineae - ps30
Arabidopsis - ps31
Arabidopsis_thaliana - ps32
Phylostratum

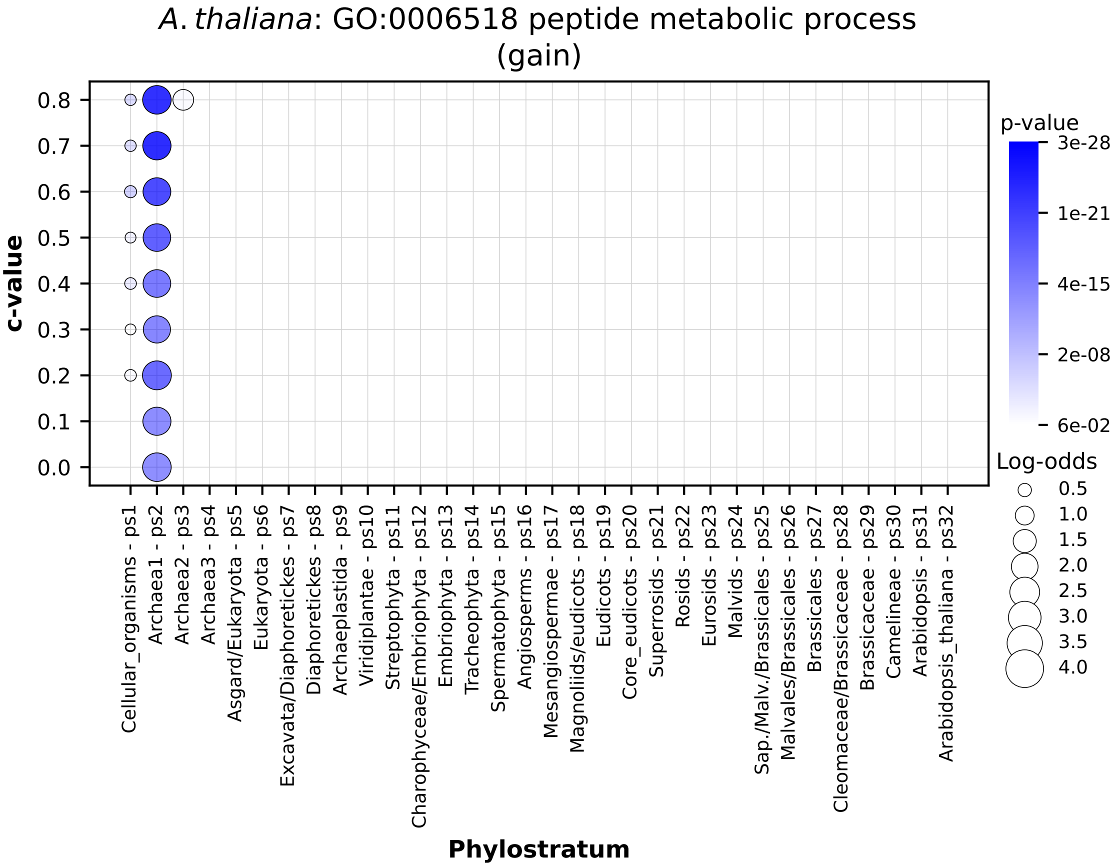

A. thaliana: GO:0006518 peptide metabolic process
(gain)
c-value
0.8
0.7
0.6
0.5
0.4
0.3
0.2
0.1
0.0
p-value
3e-28
1e-21
4e-15
2e-08
6e-02
Log-odds
0.5
1.0
1.5
2.0
2.5
3.0
3.5
4.0
Cellular_organisms - ps1
Archaea1 - ps2
Archaea2 - ps3
Archaea3 - ps4
Asgard/Eukaryota - ps5
Eukaryota - ps6
Excavata/Diaphoretickes - ps7
Diaphoretickes - ps8
Archaeplastida - ps9
Viridiplantae - ps10
Streptophyta - ps11
Charophyceae/Embriophyta - ps12
Embriophyta - ps13
Tracheophyta - ps14
Spermatophyta - ps15
Angiosperms - ps16
Mesangiospermae - ps17
Magnoliids/eudicots - ps18
Eudicots - ps19
Core_eudicots - ps20
Superrosids - ps21
Rosids - ps22
Eurosids - ps23
Malvids - ps24
Sap./Malv./Brassicales - ps25
Malvales/Brassicales - ps26
Brassicales - ps27
Cleomaceae/Brassicaceae - ps28
Brassicaceae - ps29
Camelineae - ps30
Arabidopsis - ps31
Arabidopsis_thaliana - ps32
Phylostratum

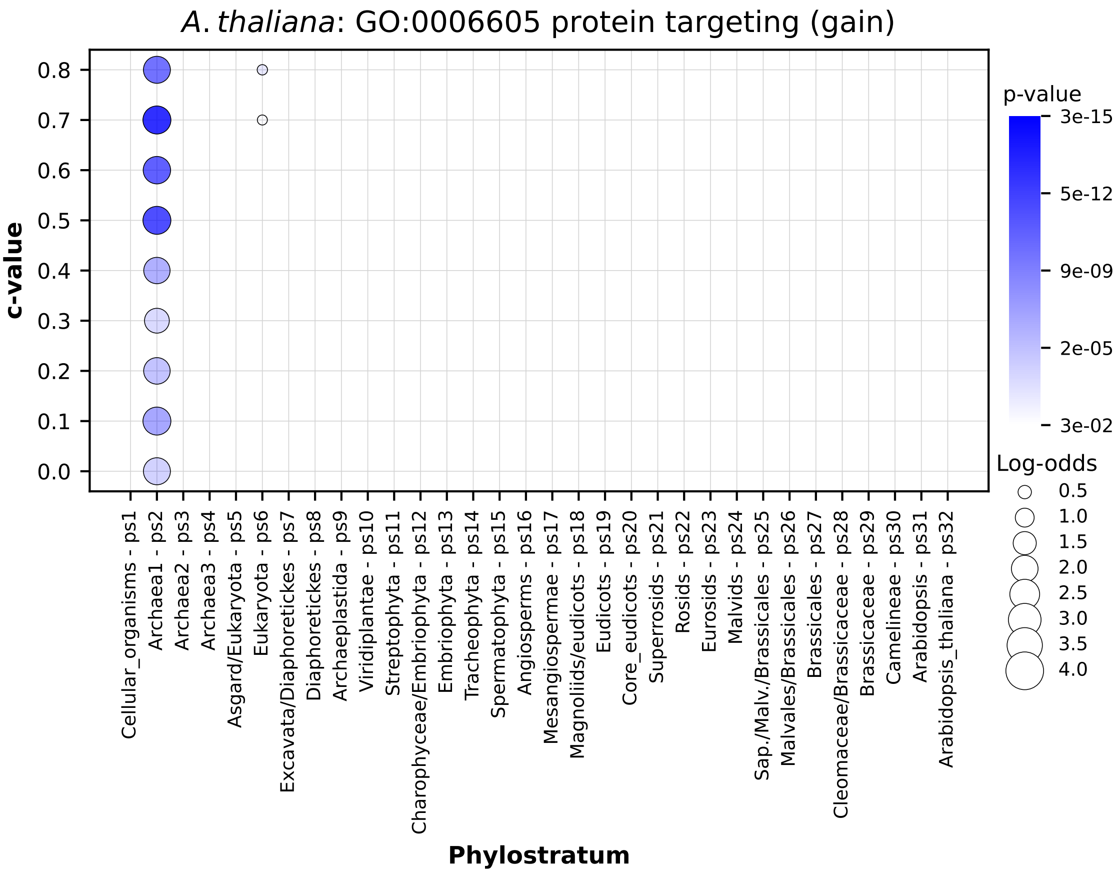

A. thaliana: GO:0006605 protein targeting (gain)
c-value
0.8
0.7
0.6
0.5
0.4
0.3
0.2
0.1
0.0
Phylostratum
Cellular_organisms - ps1
Archaea1 - ps2
Archaea2 - ps3
Archaea3 - ps4
Asgard/Eukaryota - ps5
Eukaryota - ps6
Excavata/Diaphoretickes - ps7
Diaphoretickes - ps8
Archaeplastida - ps9
Viridiplantae - ps10
Streptophyta - ps11
Charophyceae/Embriophyta - ps12
Embriophyta - ps13
Tracheophyta - ps14
Spermatophyta - ps15
Angiosperms - ps16
Mesangiospermae - ps17
Magnoliids/eudicots - ps18
Eudicots - ps19
Core_eudicots - ps20
Superrosids - ps21
Rosids - ps22
Eurosids - ps23
Malvids - ps24
Sap./Malv./Brassicales - ps25
Malvales/Brassicales - ps26
Brassicales - ps27
Cleomaceae/Brassicaceae - ps28
Brassicaceae - ps29
Camelineae - ps30
Arabidopsis - ps31
Arabidopsis_thaliana - ps32
p-value
3e-15
5e-12
9e-09
2e-05
3e-02
Log-odds
0.5
1.0
1.5
2.0
2.5
3.0
3.5
4.0

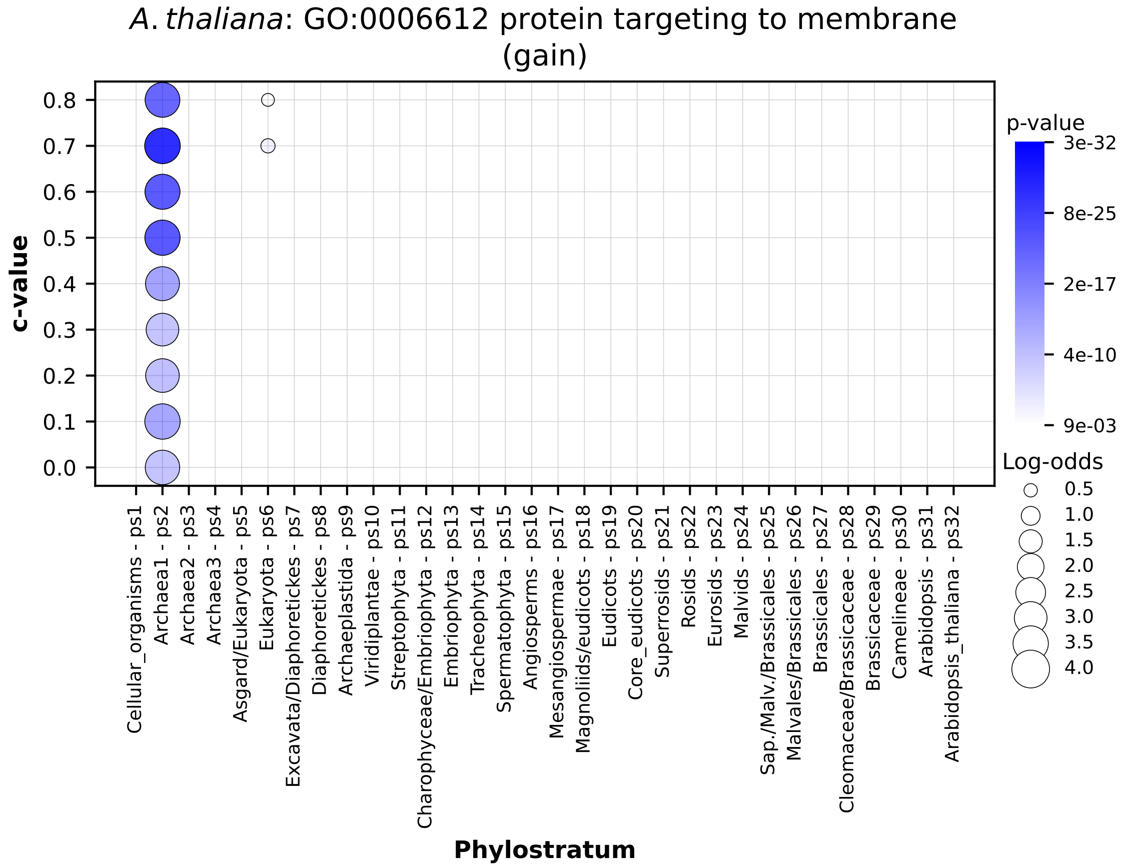

A. thaliana: GO:0006612 protein targeting to membrane (gain)
c-value
0.8
0.7
0.6
0.5
0.4
0.3
0.2
0.1
0.0
p-value
3e-32
8e-25
2e-17
4e-10
9e-03
Log-odds
0.5
1.0
1.5
2.0
2.5
3.0
3.5
4.0
Cellular_organisms - ps1
Archaea1 - ps2
Archaea2 - ps3
Archaea3 - ps4
Asgard/Eukaryota - ps5
Eukaryota - ps6
Excavata/Diaphoretickes - ps7
Diaphoretickes - ps8
Archaeplastida - ps9
Viridiplantae - ps10
Streptophyta - ps11
Charophyceae/Embriophyta - ps12
Embriophyta - ps13
Tracheophyta - ps14
Spermatophyta - ps15
Angiosperms - ps16
Mesangiospermae - ps17
Magnoliids/eudicots - ps18
Eudicots - ps19
Core_eudicots - ps20
Superrosids - ps21
Rosids - ps22
Eurosids - ps23
Malvids - ps24
Sap./Malv./Brassicales - ps25
Malvales/Brassicales - ps26
Brassicales - ps27
Cleomaceae/Brassicaceae - ps28
Brassicaceae - ps29
Camelineae - ps30
Arabidopsis - ps31
Arabidopsis_thaliana - ps32
Phylostratum

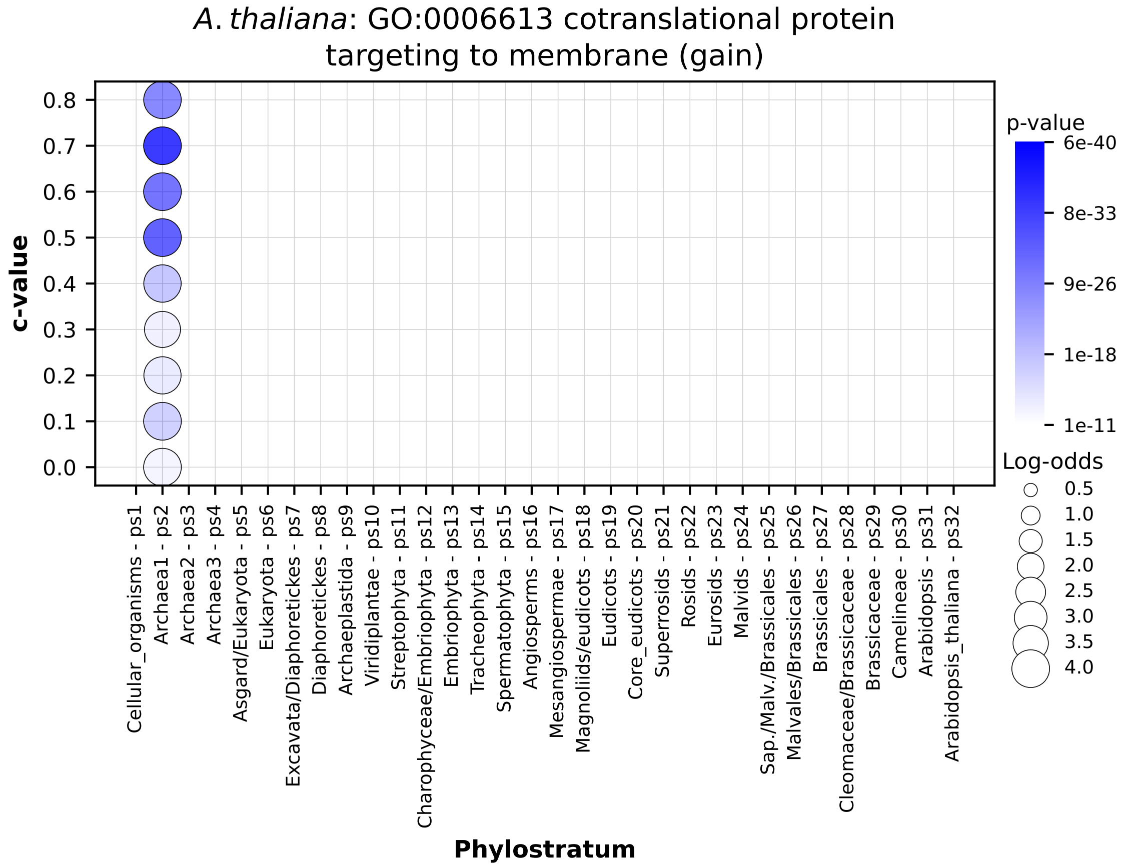
A. thaliana: GO:0006613 cotranslational protein targeting to membrane (gain)
c-value
0.8
0.7
0.6
0.5
0.4
0.3
0.2
0.1
0.0
p-value
6e-40
8e-33
9e-26
1e-18
1e-11
Log-odds
0.5
1.0
1.5
2.0
2.5
3.0
3.5
4.0
Cellular_organisms - ps1
Archaea1 - ps2
Archaea2 - ps3
Archaea3 - ps4
Asgard/Eukaryota - ps5
Eukaryota - ps6
Excavata/Diaphoretickes - ps7
Diaphoretickes - ps8
Archaeplastida - ps9
Viridiplantae - ps10
Streptophyta - ps11
Charophyceae/Embriophyta - ps12
Embriophyta - ps13
Tracheophyta - ps14
Spermatophyta - ps15
Angiosperms - ps16
Mesangiospermae - ps17
Magnoliids/eudicots - ps18
Eudicots - ps19
Core_eudicots - ps20
Superrosids - ps21
Rosids - ps22
Eurosids - ps23
Malvids - ps24
Sap./Malv./Brassicales - ps25
Malvales/Brassicales - ps26
Brassicales - ps27
Cleomaceae/Brassicaceae - ps28
Brassicaceae - ps29
Camelineae - ps30
Arabidopsis - ps31
Arabidopsis_thaliana - ps32
Phylostratum

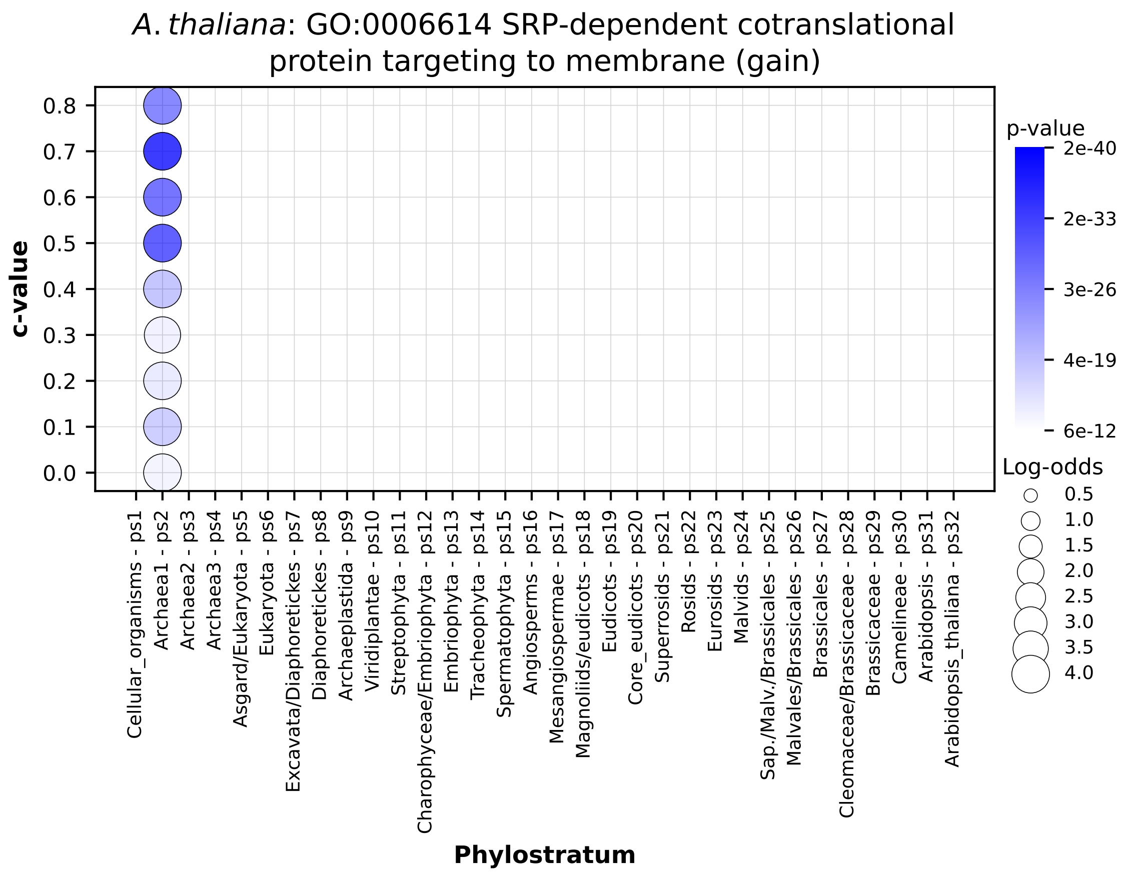

A. thaliana: GO:0006614 SRP-dependent cotranslational protein targeting to membrane (gain)
c-value
0.8
0.7
0.6
0.5
0.4
0.3
0.2
0.1
0.0
p-value
2e-40
2e-33
3e-26
4e-19
6e-12
Log-odds
0.5
1.0
1.5
2.0
2.5
3.0
3.5
4.0
Cellular_organisms - ps1
Archaea1 - ps2
Archaea2 - ps3
Archaea3 - ps4
Asgard/Eukaryota - ps5
Eukaryota - ps6
Excavata/Diaphoretickes - ps7
Diaphoretickes - ps8
Archaeplastida - ps9
Viridiplantae - ps10
Streptophyta - ps11
Charophyceae/Embriophyta - ps12
Embriophyta - ps13
Tracheophyta - ps14
Spermatophyta - ps15
Angiosperms - ps16
Mesangiospermae - ps17
Magnoliids/eudicots - ps18
Eudicots - ps19
Core_eudicots - ps20
Superrosids - ps21
Rosids - ps22
Eurosids - ps23
Malvids - ps24
Sap./Malv./Brassicales - ps25
Malvales/Brassicales - ps26
Brassicales - ps27
Cleomaceae/Brassicaceae - ps28
Brassicaceae - ps29
Camelineae - ps30
Arabidopsis - ps31
Arabidopsis_thaliana - ps32
Phylostratum

A. thaliana: GO:0009057 macromolecule catabolic process (gain)

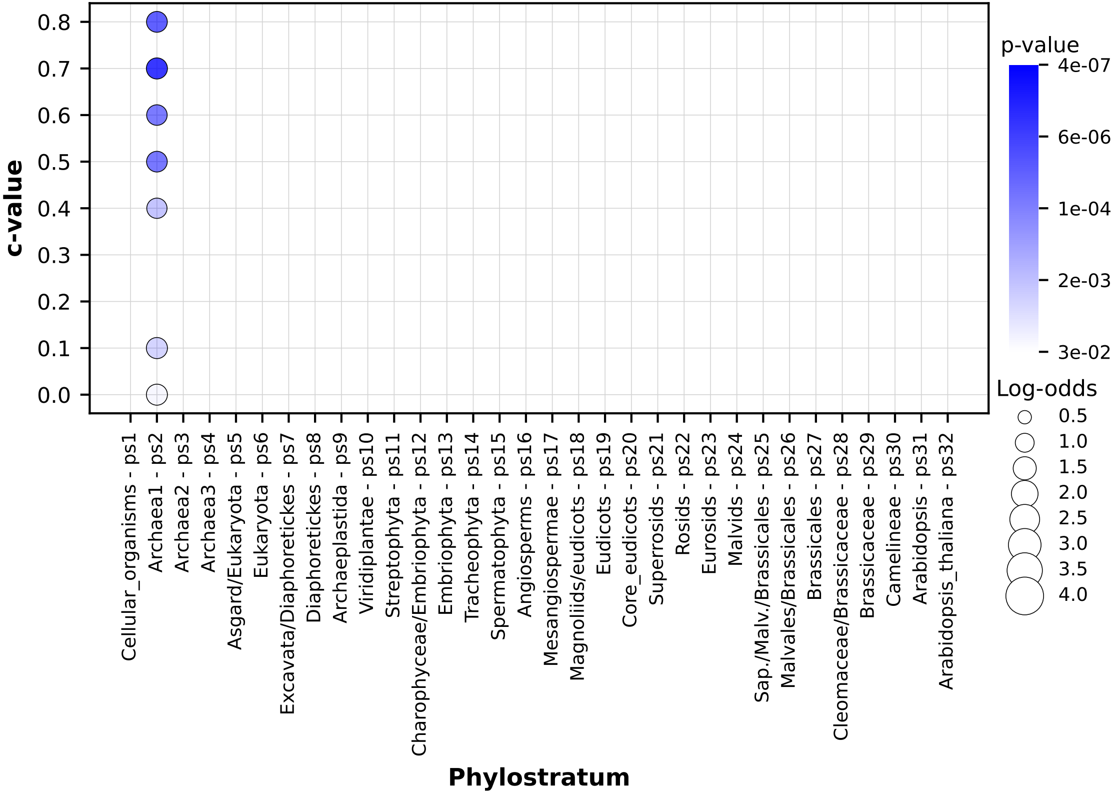

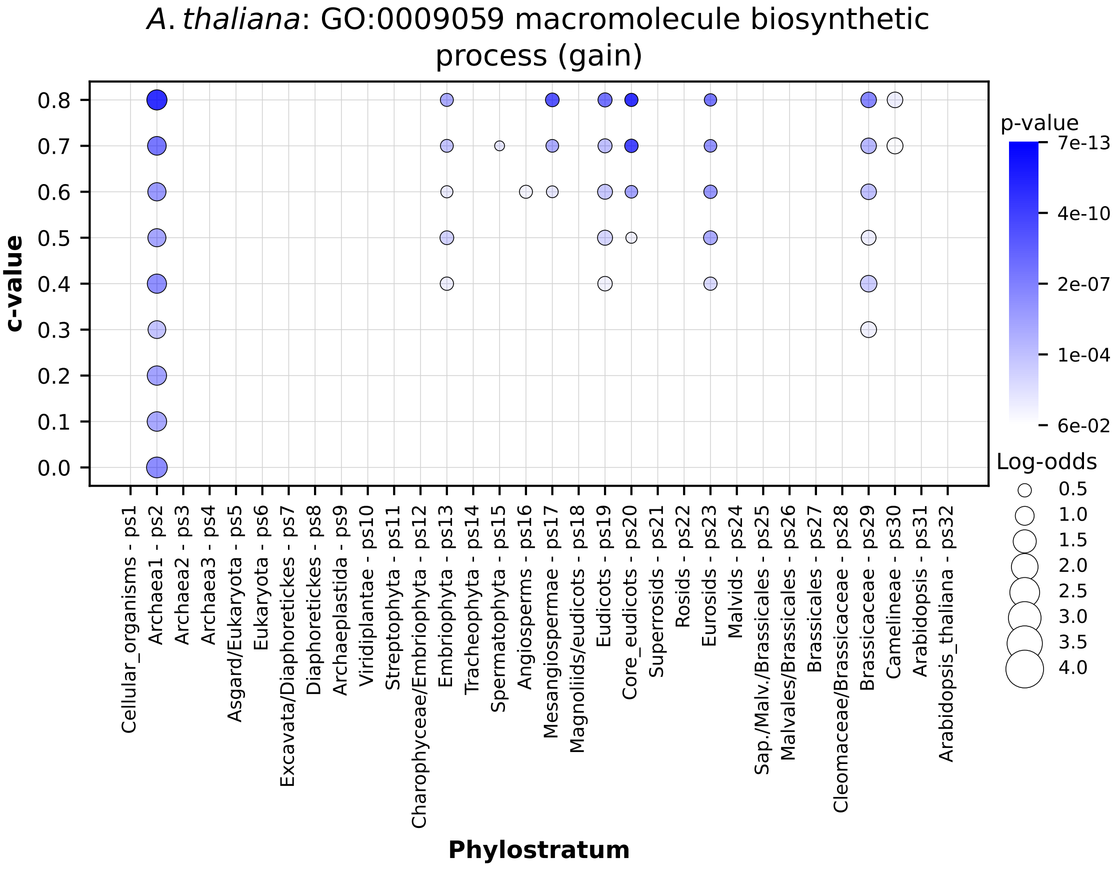

A. thaliana: GO:0009059 macromolecule biosynthetic process (gain)
c-value
0.8
0.7
0.6
0.5
0.4
0.3
0.2
0.1
0.0
p-value
7e-13
4e-10
2e-07
1e-04
6e-02
Log-odds
0.5
1.0
1.5
2.0
2.5
3.0
3.5
4.0
Cellular_organisms - ps1
Archaea1 - ps2
Archaea2 - ps3
Archaea3 - ps4
Asgard/Eukaryota - ps5
Eukaryota - ps6
Excavata/Diaphoretickes - ps7
Diaphoretickes - ps8
Archaeplastida - ps9
Viridiplantae - ps10
Streptophyta - ps11
Charophyceae/Embriophyta - ps12
Embriophyta - ps13
Tracheophyta - ps14
Spermatophyta - ps15
Angiosperms - ps16
Mesangiospermae - ps17
Magnoliids/eudicots - ps18
Eudicots - ps19
Core_eudicots - ps20
Superrosids - ps21
Rosids - ps22
Eurosids - ps23
Malvids - ps24
Sap./Malv./Brassicales - ps25
Malvales/Brassicales - ps26
Brassicales - ps27
Cleomaceae/Brassicaceae - ps28
Brassicaceae - ps29
Camelineae - ps30
Arabidopsis - ps31
Arabidopsis_thaliana - ps32
Phylostratum

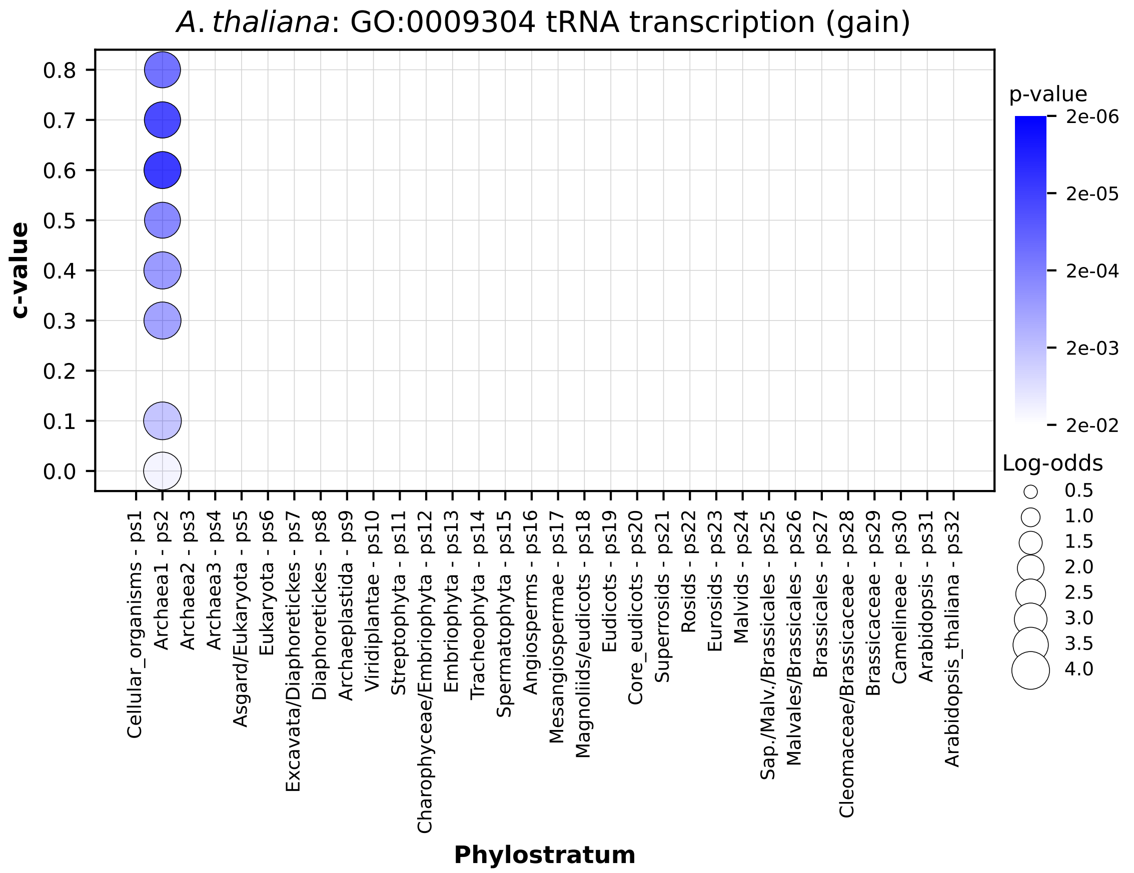

A. thaliana: GO:0009304 tRNA transcription (gain)
c-value
0.8
0.7
0.6
0.5
0.4
0.3
0.2
0.1
0.0
p-value
2e-06
2e-05
2e-04
2e-03
2e-02
Log-odds
0.5
1.0
1.5
2.0
2.5
3.0
3.5
4.0
Cellular_organisms - ps1
Archaea1 - ps2
Archaea2 - ps3
Archaea3 - ps4
Asgard/Eukaryota - ps5
Eukaryota - ps6
Excavata/Diaphoretickes - ps7
Diaphoretickes - ps8
Archaeplastida - ps9
Viridiplantae - ps10
Streptophyta - ps11
Charophyceae/Embriophyta - ps12
Embriophyta - ps13
Tracheophyta - ps14
Spermatophyta - ps15
Angiosperms - ps16
Mesangiospermae - ps17
Magnoliids/eudicots - ps18
Eudicots - ps19
Core_eudicots - ps20
Superrosids - ps21
Rosids - ps22
Eurosids - ps23
Malvids - ps24
Sap./Malv./Brassicales - ps25
Malvales/Brassicales - ps26
Brassicales - ps27
Cleomaceae/Brassicaceae - ps28
Brassicaceae - ps29
Camelineae - ps30
Arabidopsis - ps31
Arabidopsis_thaliana - ps32
Phylostratum

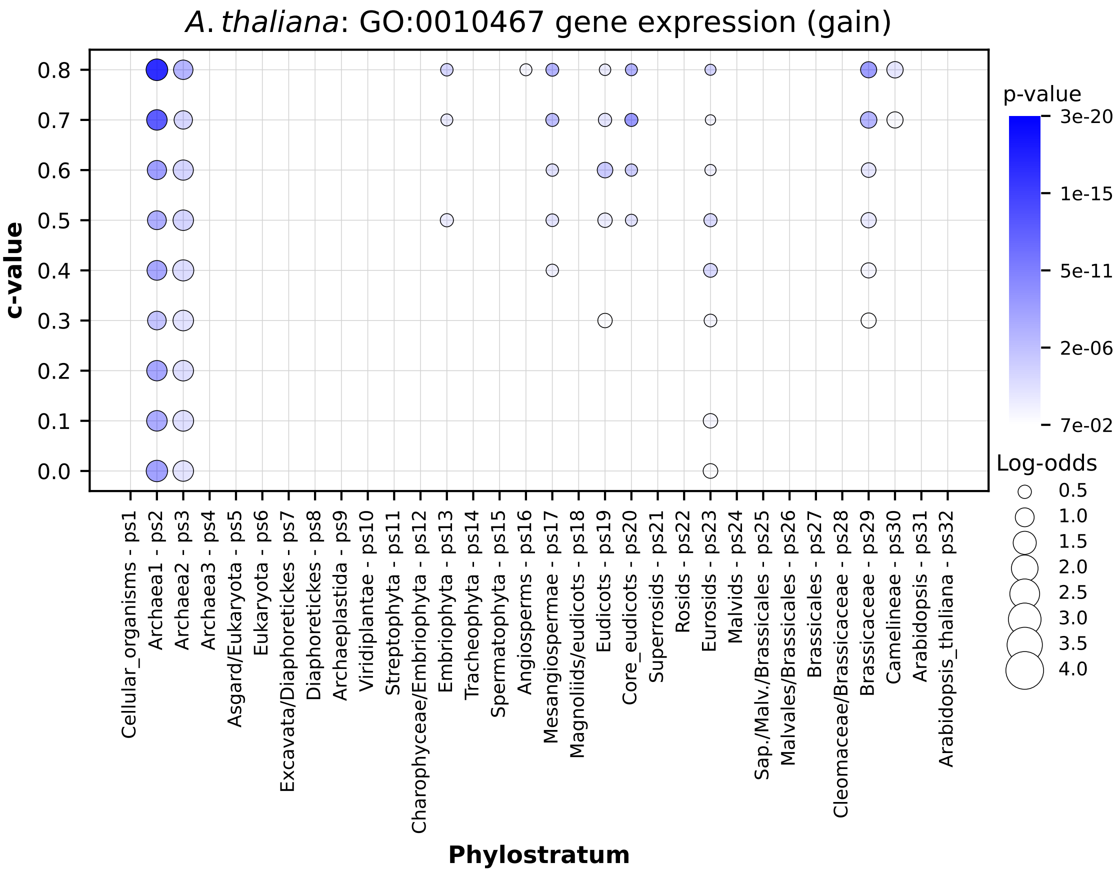

A. thaliana: GO:0010467 gene expression (gain)
c-value
0.8
0.7
0.6
0.5
0.4
0.3
0.2
0.1
0.0
p-value
3e-20
1e-15
5e-11
2e-06
7e-02
Log-odds
0.5
1.0
1.5
2.0
2.5
3.0
3.5
4.0
Cellular_organisms - ps1
Archaea1 - ps2
Archaea2 - ps3
Archaea3 - ps4
Asgard/Eukaryota - ps5
Eukaryota - ps6
Excavata/Diaphoretickes - ps7
Diaphoretickes - ps8
Archaeplastida - ps9
Viridiplantae - ps10
Streptophyta - ps11
Charophyceae/Embriophyta - ps12
Embriophyta - ps13
Tracheophyta - ps14
Spermatophyta - ps15
Angiosperms - ps16
Mesangiospermae - ps17
Magnoliids/eudicots - ps18
Eudicots - ps19
Core_eudicots - ps20
Superrosids - ps21
Rosids - ps22
Eurosids - ps23
Malvids - ps24
Sap./Malv./Brassicales - ps25
Malvales/Brassicales - ps26
Brassicales - ps27
Cleomaceae/Brassicaceae - ps28
Brassicaceae - ps29
Camelineae - ps30
Arabidopsis - ps31
Arabidopsis_thaliana - ps32
Phylostratum

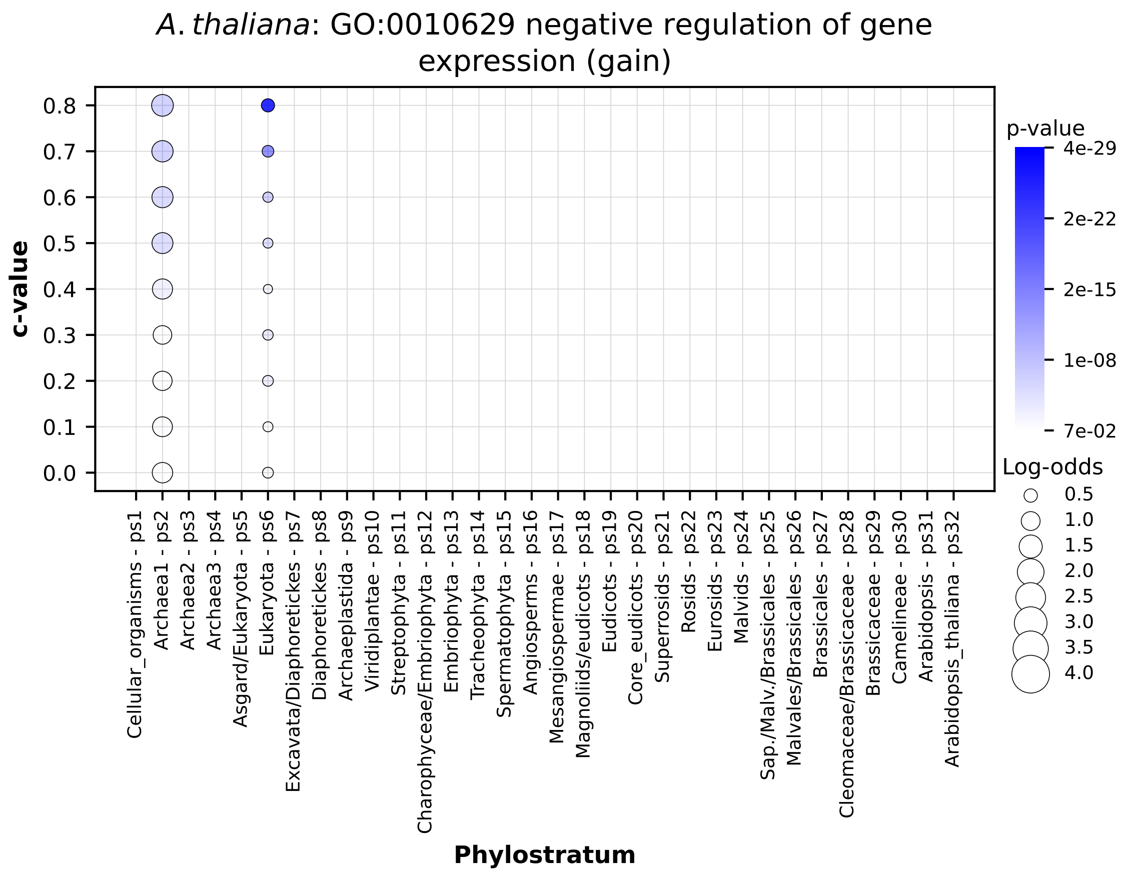

A. thaliana: GO:0010629 negative regulation of gene expression (gain)
c-value
0.8
0.7
0.6
0.5
0.4
0.3
0.2
0.1
0.0
p-value
4e-29
2e-22
2e-15
1e-08
7e-02
Log-odds
0.5
1.0
1.5
2.0
2.5
3.0
3.5
4.0
Cellular_organisms - ps1
Archaea1 - ps2
Archaea2 - ps3
Archaea3 - ps4
Asgard/Eukaryota - ps5
Eukaryota - ps6
Excavata/Diaphoretickes - ps7
Diaphoretickes - ps8
Archaeplastida - ps9
Viridiplantae - ps10
Streptophyta - ps11
Charophyceae/Embriophyta - ps12
Embriophyta - ps13
Tracheophyta - ps14
Spermatophyta - ps15
Angiosperms - ps16
Mesangiospermae - ps17
Magnoliids/eudicots - ps18
Eudicots - ps19
Core_eudicots - ps20
Superrosids - ps21
Rosids - ps22
Eurosids - ps23
Malvids - ps24
Sap./Malv./Brassicales - ps25
Malvales/Brassicales - ps26
Brassicales - ps27
Cleomaceae/Brassicaceae - ps28
Brassicaceae - ps29
Camelineae - ps30
Arabidopsis - ps31
Arabidopsis_thaliana - ps32
Phylostratum

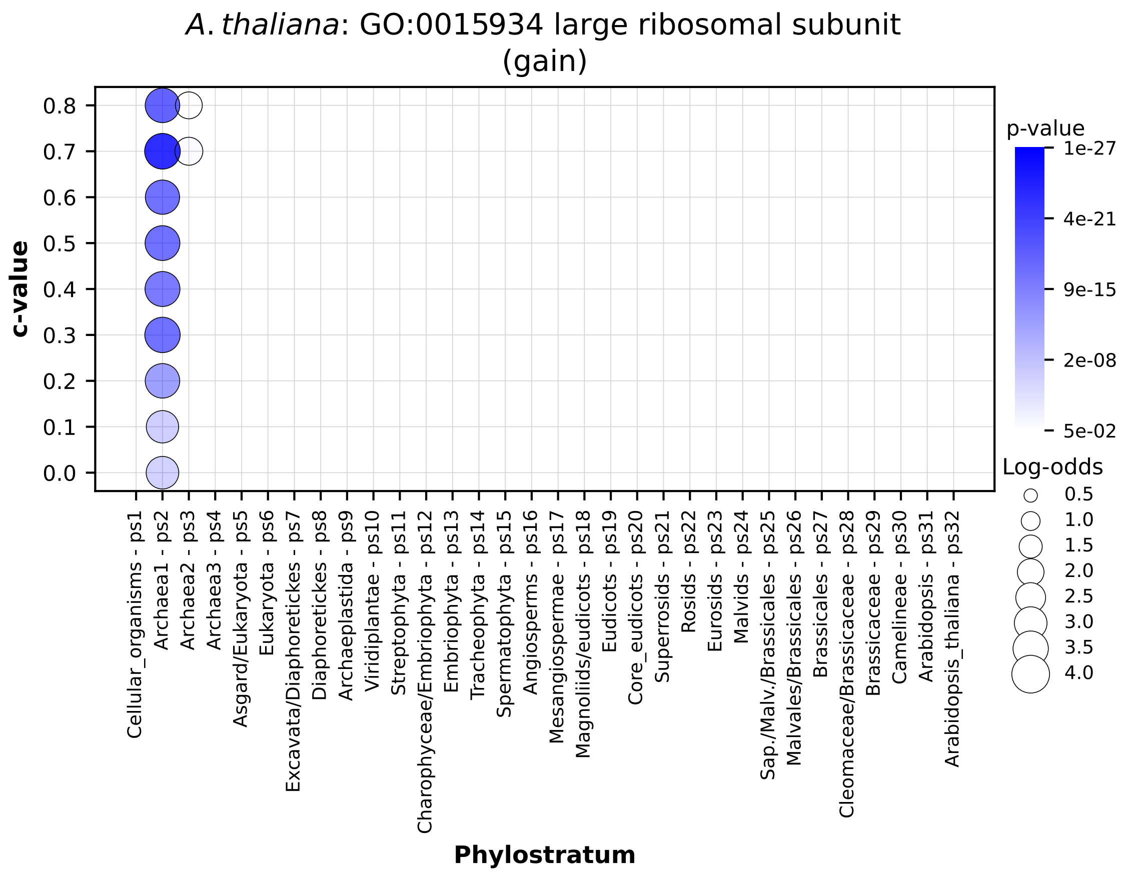

A. thaliana: GO:0015934 large ribosomal subunit (gain)
c-value
0.8
0.7
0.6
0.5
0.4
0.3
0.2
0.1
0.0
Phylostratum
Cellular_organisms - ps1
Archaea1 - ps2
Archaea2 - ps3
Archaea3 - ps4
Asgard/Eukaryota - ps5
Eukaryota - ps6
Excavata/Diaphoretickes - ps7
Diaphoretickes - ps8
Archaeplastida - ps9
Viridiplantae - ps10
Streptophyta - ps11
Charophyceae/Embriophyta - ps12
Embriophyta - ps13
Tracheophyta - ps14
Spermatophyta - ps15
Angiosperms - ps16
Mesangiospermae - ps17
Magnoliids/eudicots - ps18
Eudicots - ps19
Core_eudicots - ps20
Superrosids - ps21
Rosids - ps22
Eurosids - ps23
Malvids - ps24
Sap./Malv./Brassicales - ps25
Malvales/Brassicales - ps26
Brassicales - ps27
Cleomaceae/Brassicaceae - ps28
Brassicaceae - ps29
Camelineae - ps30
Arabidopsis - ps31
Arabidopsis_thaliana - ps32
p-value
1e-27
4e-21
9e-15
2e-08
5e-02
Log-odds
0.5
1.0
1.5
2.0
2.5
3.0
3.5
4.0

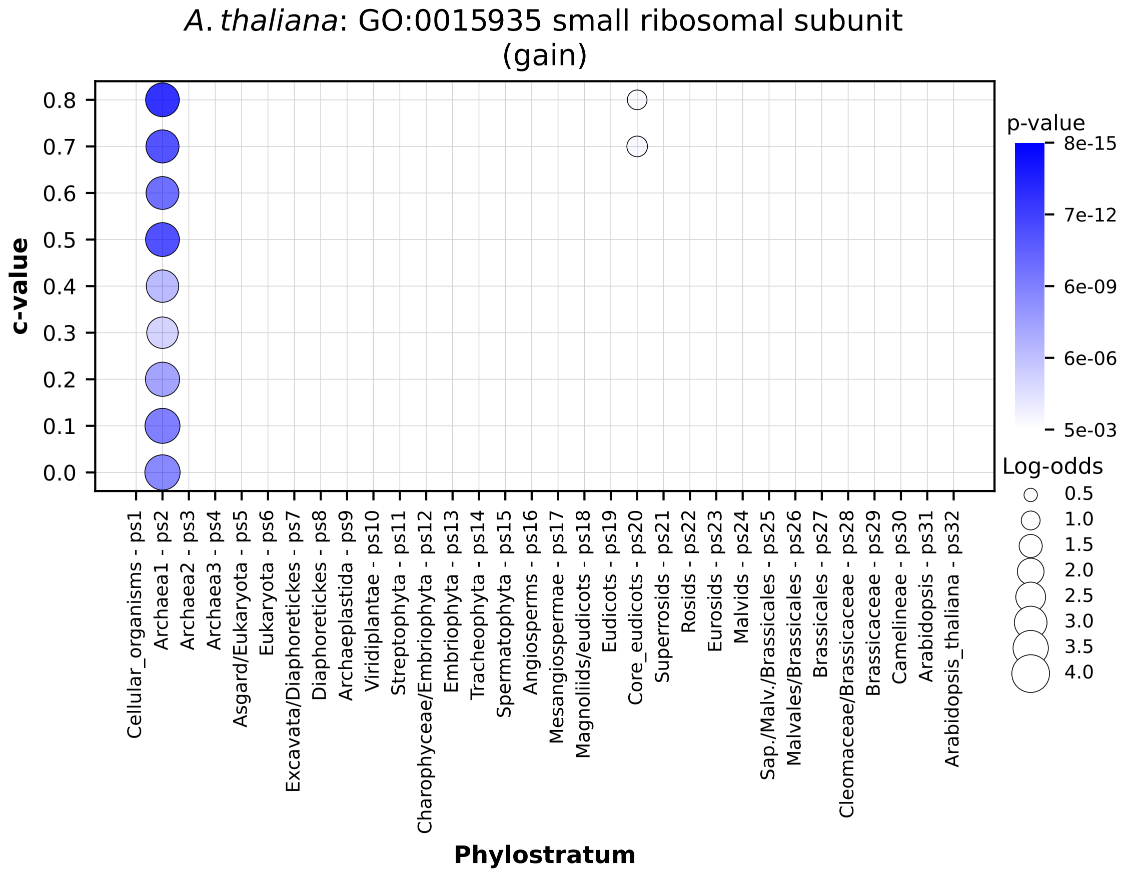
A. thaliana: GO:0015935 small ribosomal subunit
(gain)
c-value
0.8
0.7
0.6
0.5
0.4
0.3
0.2
0.1
0.0
p-value
8e-15
7e-12
6e-09
6e-06
5e-03
Log-odds
0.5
1.0
1.5
2.0
2.5
3.0
3.5
4.0
Cellular_organisms - ps1
Archaea1 - ps2
Archaea2 - ps3
Archaea3 - ps4
Asgard/Eukaryota - ps5
Eukaryota - ps6
Excavata/Diaphoretickes - ps7
Diaphoretickes - ps8
Archaeplastida - ps9
Viridiplantae - ps10
Streptophyta - ps11
Charophyceae/Embriophyta - ps12
Embriophyta - ps13
Tracheophyta - ps14
Spermatophyta - ps15
Angiosperms - ps16
Mesangiospermae - ps17
Magnoliids/eudicots - ps18
Eudicots - ps19
Core_eudicots - ps20
Superrosids - ps21
Rosids - ps22
Eurosids - ps23
Malvids - ps24
Sap./Malv./Brassicales - ps25
Malvales/Brassicales - ps26
Brassicales - ps27
Cleomaceae/Brassicaceae - ps28
Brassicaceae - ps29
Camelineae - ps30
Arabidopsis - ps31
Arabidopsis_thaliana - ps32
Phylostratum

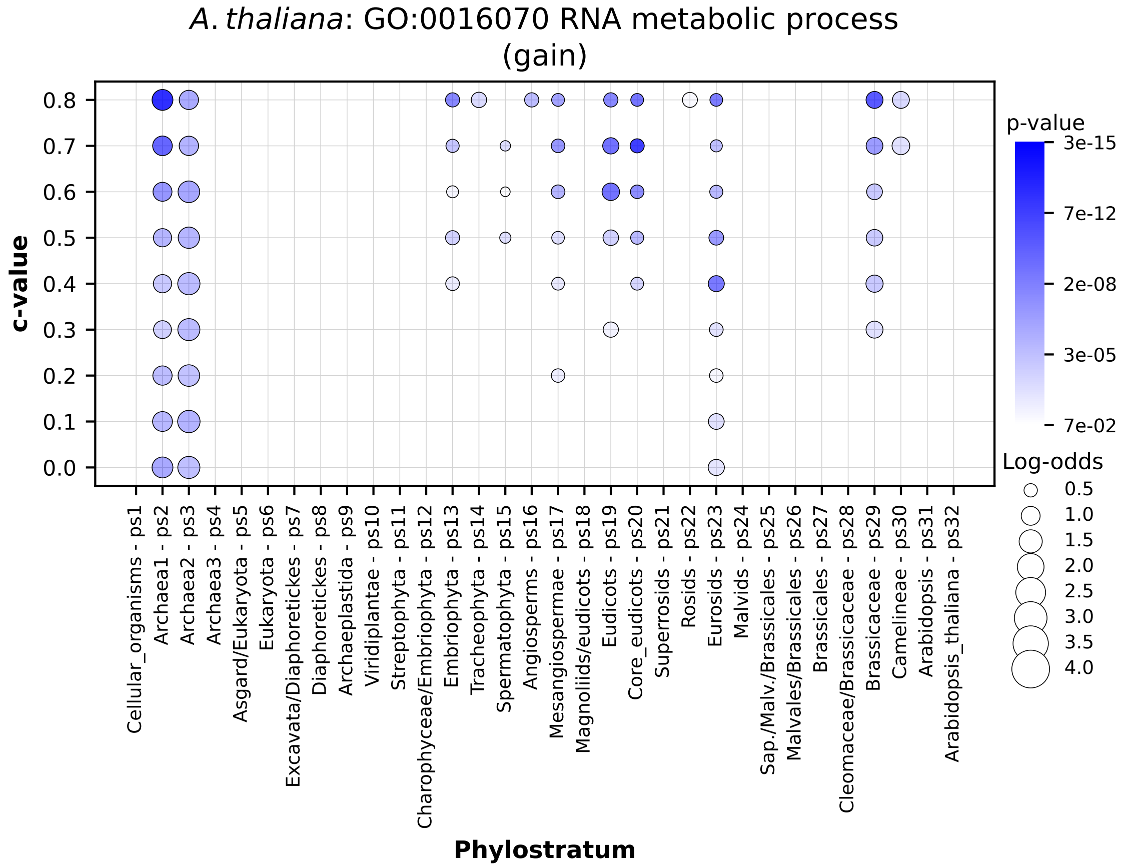

A. thaliana: GO:0016070 RNA metabolic process
(gain)
c-value
0.8
0.7
0.6
0.5
0.4
0.3
0.2
0.1
0.0
Phylostratum
Cellular_organisms - ps1
Archaea1 - ps2
Archaea2 - ps3
Archaea3 - ps4
Asgard/Eukaryota - ps5
Eukaryota - ps6
Excavata/Diaphoretickes - ps7
Diaphoretickes - ps8
Archaeplastida - ps9
Viridiplantae - ps10
Streptophyta - ps11
Charophyceae/Embriophyta - ps12
Embriophyta - ps13
Tracheophyta - ps14
Spermatophyta - ps15
Angiosperms - ps16
Mesangiospermae - ps17
Magnoliids/eudicots - ps18
Eudicots - ps19
Core_eudicots - ps20
Superrosids - ps21
Rosids - ps22
Eurosids - ps23
Malvids - ps24
Sap./Malv./Brassicales - ps25
Malvales/Brassicales - ps26
Brassicales - ps27
Cleomaceae/Brassicaceae - ps28
Brassicaceae - ps29
Camelineae - ps30
Arabidopsis - ps31
Arabidopsis_thaliana - ps32
p-value
3e-15
7e-12
2e-08
3e-05
7e-02
Log-odds
0.5
1.0
1.5
2.0
2.5
3.0
3.5
4.0

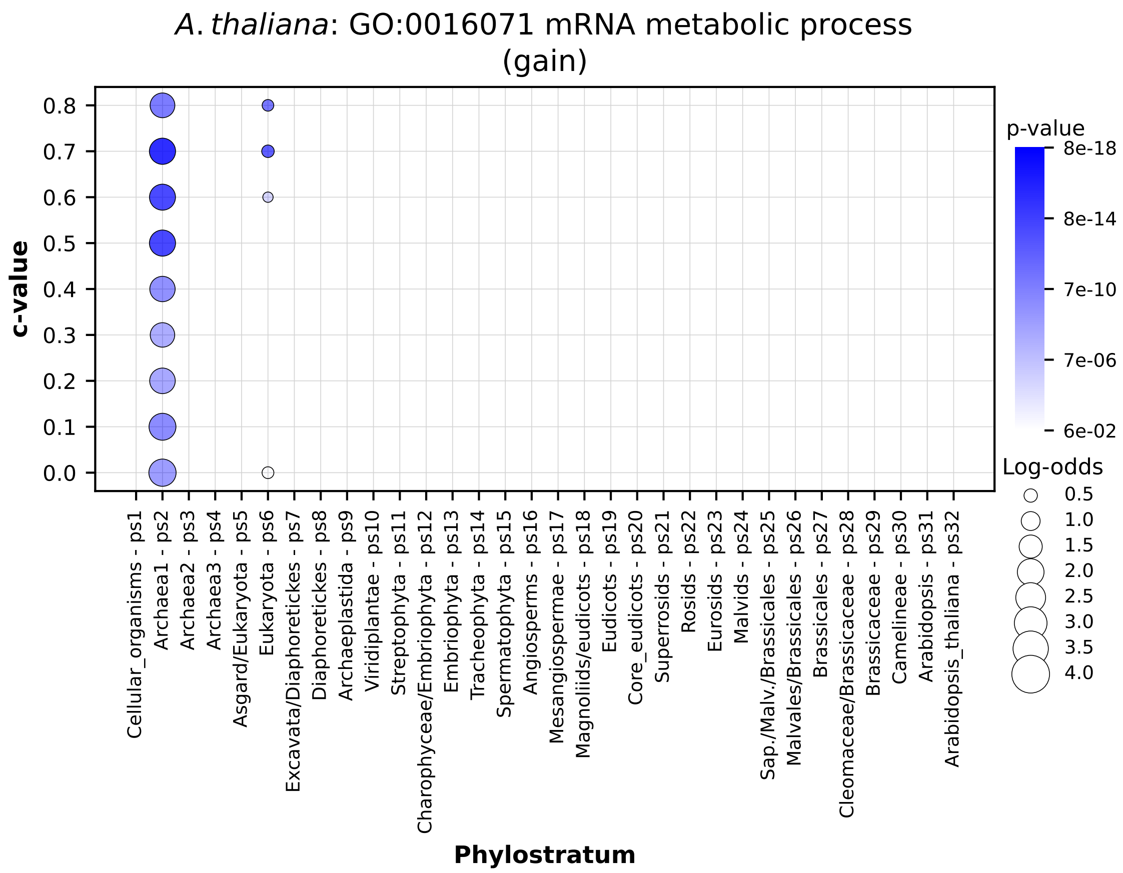

A. thaliana: GO:0016071 mRNA metabolic process
(gain)
c-value
0.8
0.7
0.6
0.5
0.4
0.3
0.2
0.1
0.0
p-value
8e-18
8e-14
7e-10
7e-06
6e-02
Log-odds
0.5
1.0
1.5
2.0
2.5
3.0
3.5
4.0
Cellular_organisms - ps1
Archaea1 - ps2
Archaea2 - ps3
Archaea3 - ps4
Asgard/Eukaryota - ps5
Eukaryota - ps6
Excavata/Diaphoretickes - ps7
Diaphoretickes - ps8
Archaeplastida - ps9
Viridiplantae - ps10
Streptophyta - ps11
Charophyceae/Embriophyta - ps12
Embriophyta - ps13
Tracheophyta - ps14
Spermatophyta - ps15
Angiosperms - ps16
Mesangiospermae - ps17
Magnoliids/eudicots - ps18
Eudicots - ps19
Core_eudicots - ps20
Superrosids - ps21
Rosids - ps22
Eurosids - ps23
Malvids - ps24
Sap./Malv./Brassicales - ps25
Malvales/Brassicales - ps26
Brassicales - ps27
Cleomaceae/Brassicaceae - ps28
Brassicaceae - ps29
Camelineae - ps30
Arabidopsis - ps31
Arabidopsis_thaliana - ps32
Phylostratum

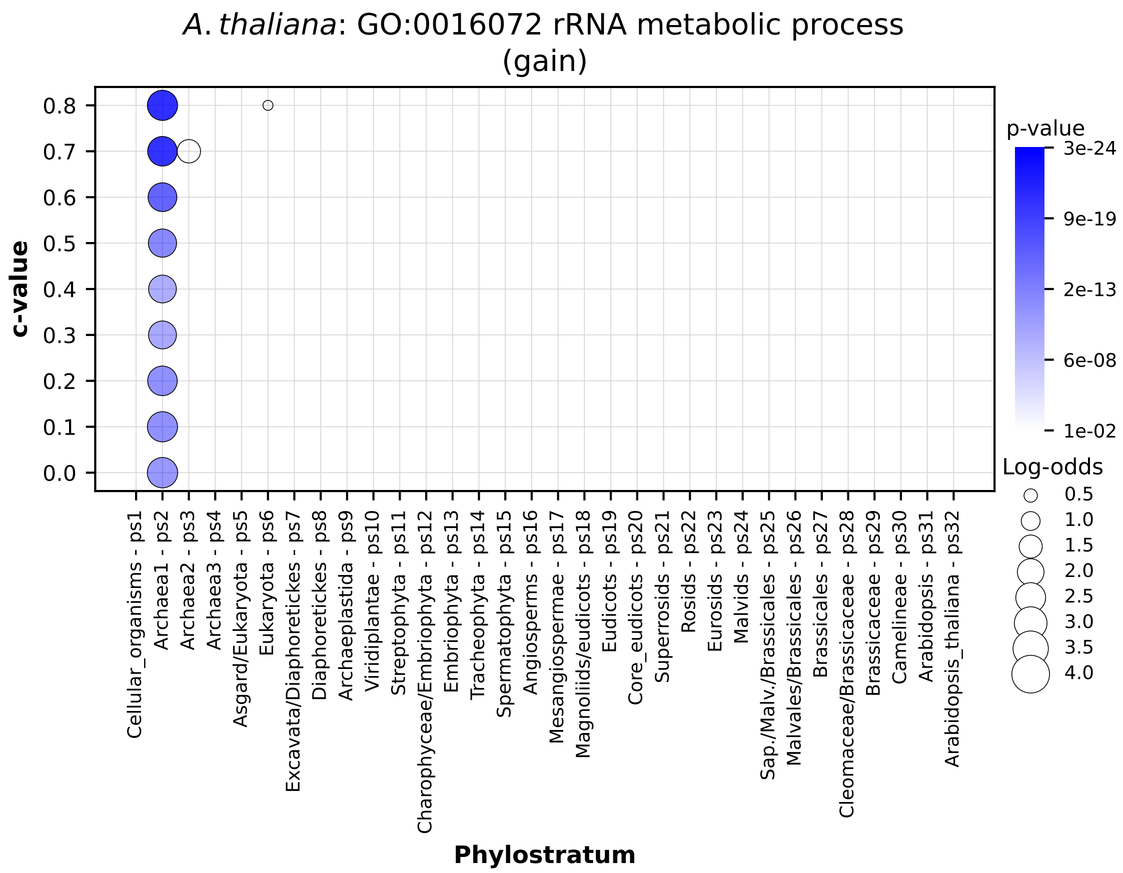

A. thaliana: GO:0016072 rRNA metabolic process (gain)
c-value
0.8
0.7
0.6
0.5
0.4
0.3
0.2
0.1
0.0
p-value
3e-24
9e-19
2e-13
6e-08
1e-02
Log-odds
0.5
1.0
1.5
2.0
2.5
3.0
3.5
4.0
Cellular_organisms - ps1
Archaea1 - ps2
Archaea2 - ps3
Archaea3 - ps4
Asgard/Eukaryota - ps5
Eukaryota - ps6
Excavata/Diaphoretickes - ps7
Diaphoretickes - ps8
Archaeplastida - ps9
Viridiplantae - ps10
Streptophyta - ps11
Charophyceae/Embriophyta - ps12
Embriophyta - ps13
Tracheophyta - ps14
Spermatophyta - ps15
Angiosperms - ps16
Mesangiospermae - ps17
Magnoliids/eudicots - ps18
Eudicots - ps19
Core_eudicots - ps20
Superrosids - ps21
Rosids - ps22
Eurosids - ps23
Malvids - ps24
Sap./Malv./Brassicales - ps25
Malvales/Brassicales - ps26
Brassicales - ps27
Cleomaceae/Brassicaceae - ps28
Brassicaceae - ps29
Camelineae - ps30
Arabidopsis - ps31
Arabidopsis_thaliana - ps32
Phylostratum

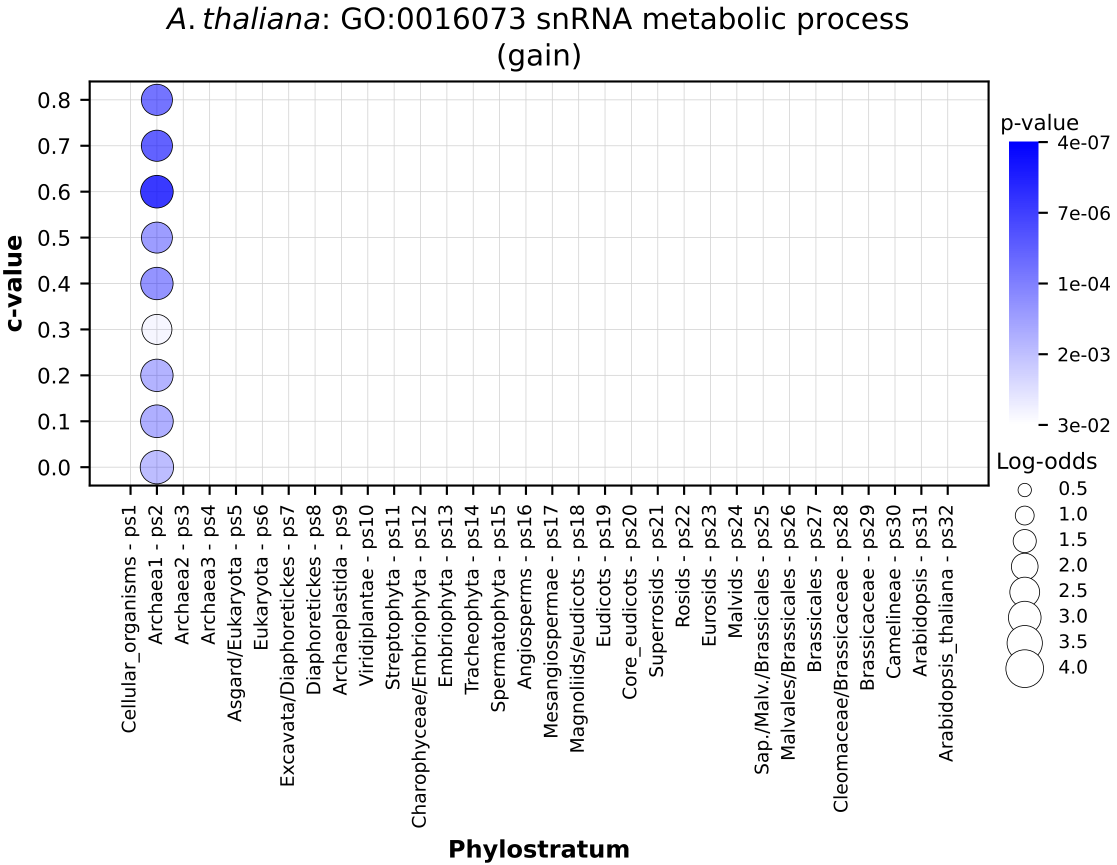

A. thaliana: GO:0016073 snRNA metabolic process
(gain)
c-value
0.8
0.7
0.6
0.5
0.4
0.3
0.2
0.1
0.0
p-value
4e-07
7e-06
1e-04
2e-03
3e-02
Log-odds
0.5
1.0
1.5
2.0
2.5
3.0
3.5
4.0
Cellular_organisms - ps1
Archaea1 - ps2
Archaea2 - ps3
Archaea3 - ps4
Asgard/Eukaryota - ps5
Eukaryota - ps6
Excavata/Diaphoretickes - ps7
Diaphoretickes - ps8
Archaeplastida - ps9
Viridiplantae - ps10
Streptophyta - ps11
Charophyceae/Embriophyta - ps12
Embriophyta - ps13
Tracheophyta - ps14
Spermatophyta - ps15
Angiosperms - ps16
Mesangiospermae - ps17
Magnoliids/eudicots - ps18
Eudicots - ps19
Core_eudicots - ps20
Superrosids - ps21
Rosids - ps22
Eurosids - ps23
Malvids - ps24
Sap./Malv./Brassicales - ps25
Malvales/Brassicales - ps26
Brassicales - ps27
Cleomaceae/Brassicaceae - ps28
Brassicaceae - ps29
Camelineae - ps30
Arabidopsis - ps31
Arabidopsis_thaliana - ps32
Phylostratum

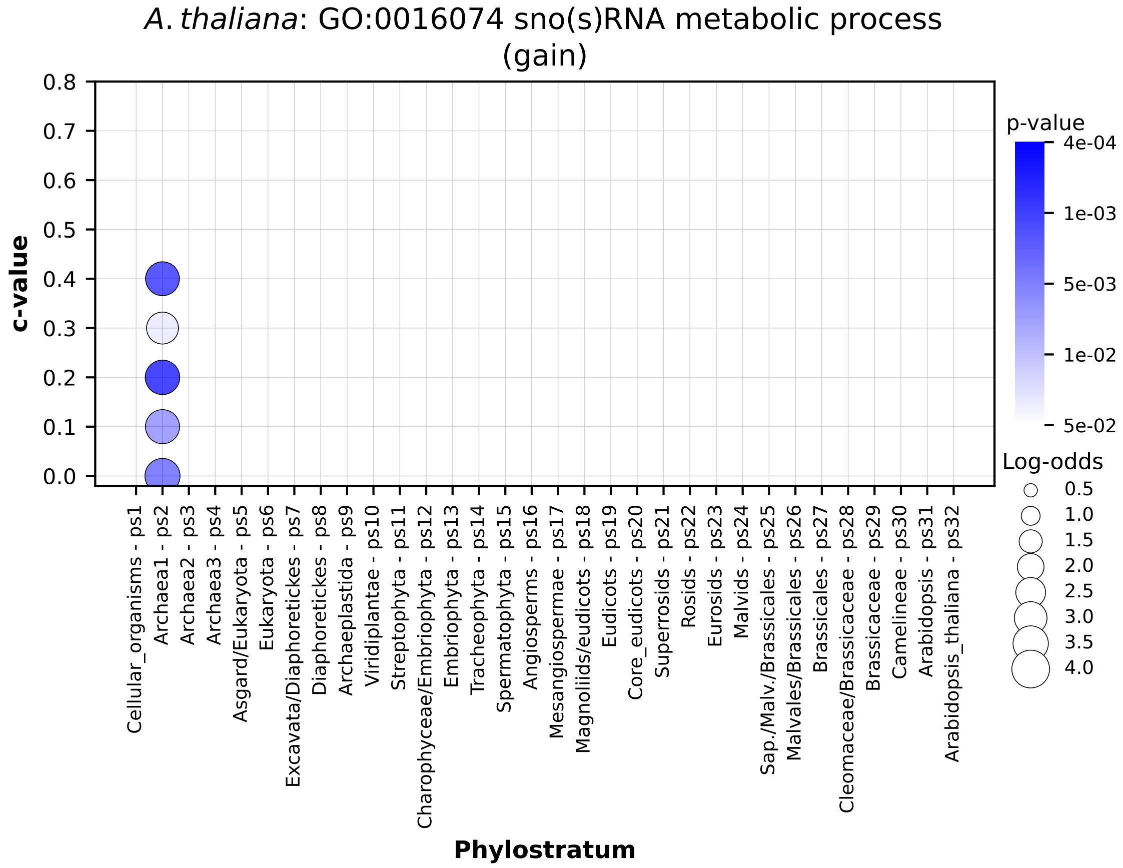
A. thaliana: GO:0016074 sno(s)RNA metabolic process (gain)
c-value
0.0
0.1
0.2
0.3
0.4
0.5
0.6
0.7
0.8
p-value
4e-04
1e-03
5e-03
1e-02
5e-02
Log-odds
0.5
1.0
1.5
2.0
2.5
3.0
3.5
4.0
Cellular_organisms - ps1
Archaea1 - ps2
Archaea2 - ps3
Archaea3 - ps4
Asgard/Eukaryota - ps5
Eukaryota - ps6
Excavata/Diaphoretickes - ps7
Diaphoretickes - ps8
Archaeplastida - ps9
Viridiplantae - ps10
Streptophyta - ps11
Charophyceae/Embriophyta - ps12
Embriophyta - ps13
Tracheophyta - ps14
Spermatophyta - ps15
Angiosperms - ps16
Mesangiospermae - ps17
Magnoliids/eudicots - ps18
Eudicots - ps19
Core_eudicots - ps20
Superrosids - ps21
Rosids - ps22
Eurosids - ps23
Malvids - ps24
Sap./Malv./Brassicales - ps25
Malvales/Brassicales - ps26
Brassicales - ps27
Cleomaceae/Brassicaceae - ps28
Brassicaceae - ps29
Camelineae - ps30
Arabidopsis - ps31
Arabidopsis_thaliana - ps32
Phylostratum

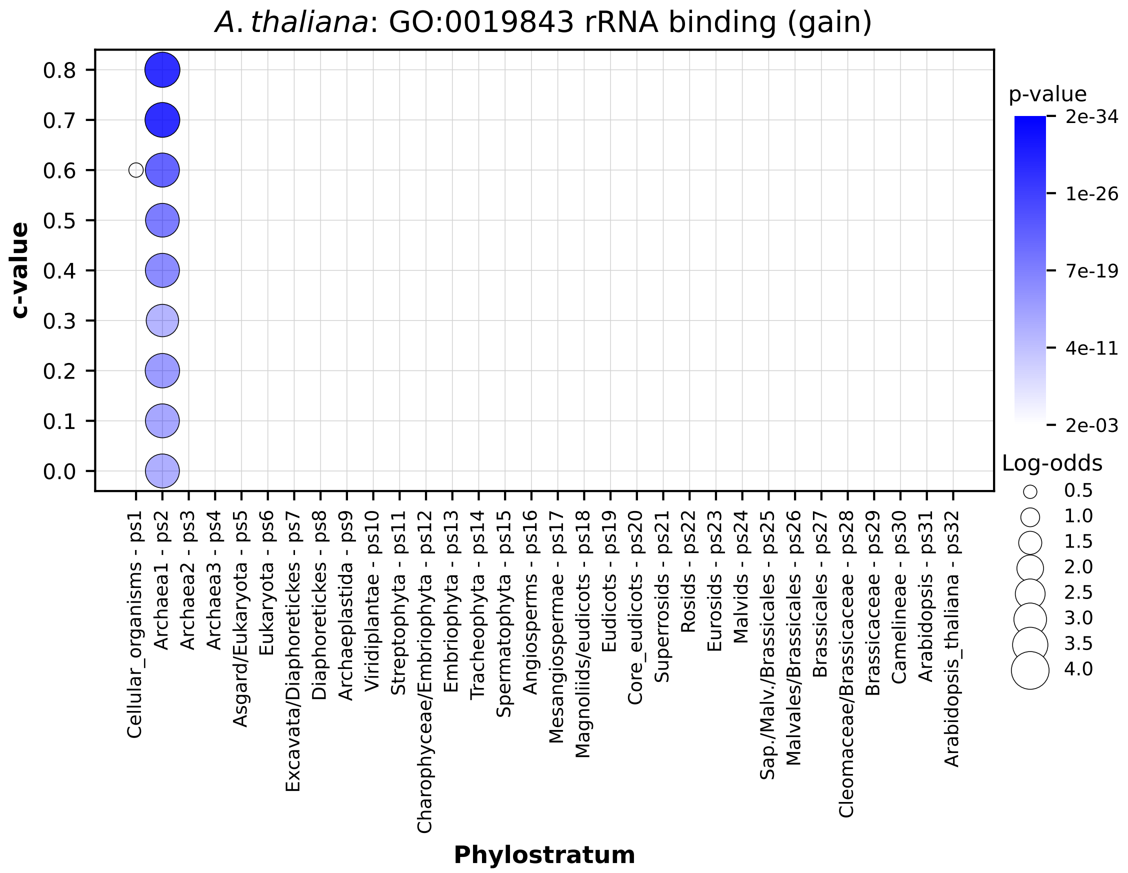

A. thaliana: GO:0019843 rRNA binding (gain)
c-value
0.8
0.7
0.6
0.5
0.4
0.3
0.2
0.1
0.0
p-value
2e-34
1e-26
7e-19
4e-11
2e-03
Log-odds
0.5
1.0
1.5
2.0
2.5
3.0
3.5
4.0
Cellular_organisms - ps1
Archaea1 - ps2
Archaea2 - ps3
Archaea3 - ps4
Asgard/Eukaryota - ps5
Eukaryota - ps6
Excavata/Diaphoretickes - ps7
Diaphoretickes - ps8
Archaeplastida - ps9
Viridiplantae - ps10
Streptophyta - ps11
Charophyceae/Embriophyta - ps12
Embriophyta - ps13
Tracheophyta - ps14
Spermatophyta - ps15
Angiosperms - ps16
Mesangiospermae - ps17
Magnoliids/eudicots - ps18
Eudicots - ps19
Core_eudicots - ps20
Superrosids - ps21
Rosids - ps22
Eurosids - ps23
Malvids - ps24
Sap./Malv./Brassicales - ps25
Malvales/Brassicales - ps26
Brassicales - ps27
Cleomaceae/Brassicaceae - ps28
Brassicaceae - ps29
Camelineae - ps30
Arabidopsis - ps31
Arabidopsis_thaliana - ps32
Phylostratum

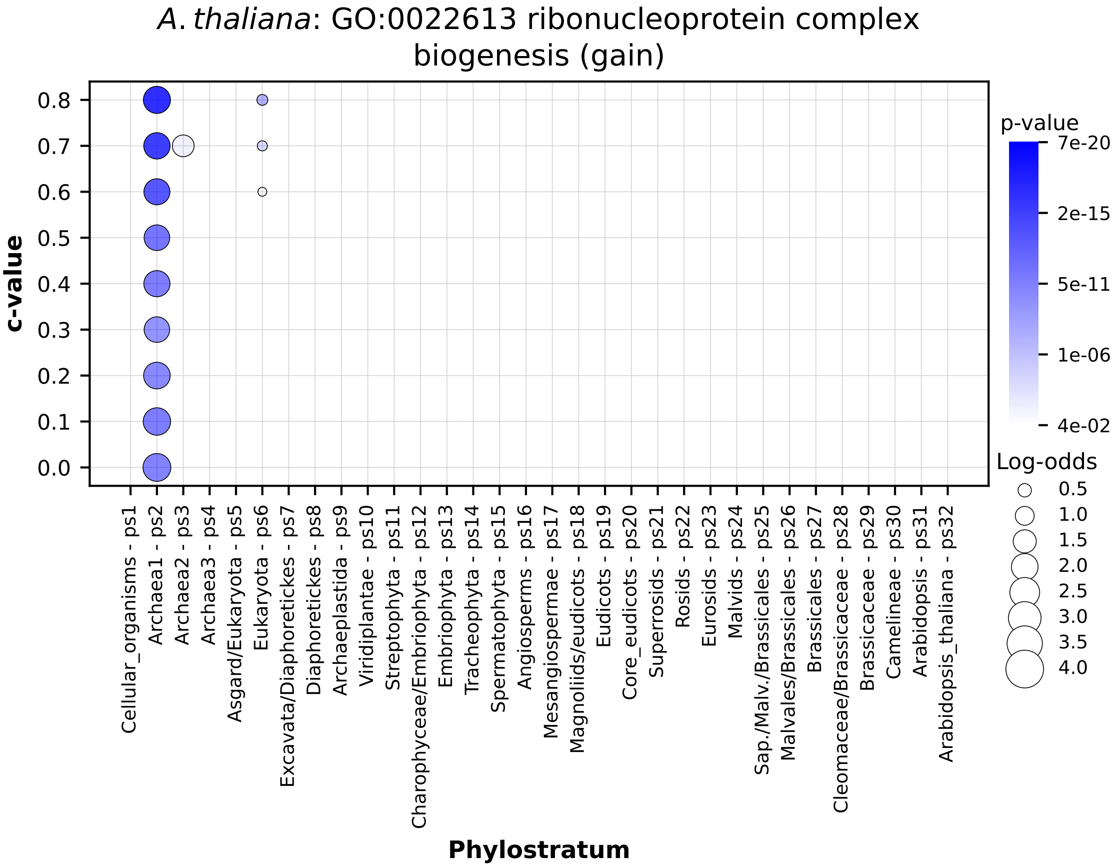
A. thaliana: GO:0022613 ribonucleoprotein complex biogenesis (gain)
c-value
0.8
0.7
0.6
0.5
0.4
0.3
0.2
0.1
0.0
Phylostratum
Cellular_organisms - ps1
Archaea1 - ps2
Archaea2 - ps3
Archaea3 - ps4
Asgard/Eukaryota - ps5
Eukaryota - ps6
Excavata/Diaphoretickes - ps7
Diaphoretickes - ps8
Archaeplastida - ps9
Viridiplantae - ps10
Streptophyta - ps11
Charophyceae/Embriophyta - ps12
Embriophyta - ps13
Tracheophyta - ps14
Spermatophyta - ps15
Angiosperms - ps16
Mesangiospermae - ps17
Magnoliids/eudicots - ps18
Eudicots - ps19
Core_eudicots - ps20
Superrosids - ps21
Rosids - ps22
Eurosids - ps23
Malvids - ps24
Sap./Malv./Brassicales - ps25
Malvales/Brassicales - ps26
Brassicales - ps27
Cleomaceae/Brassicaceae - ps28
Brassicaceae - ps29
Camelineae - ps30
Arabidopsis - ps31
Arabidopsis_thaliana - ps32
p-value
7e-20
2e-15
5e-11
1e-06
4e-02
Log-odds
0.5
1.0
1.5
2.0
2.5
3.0
3.5
4.0

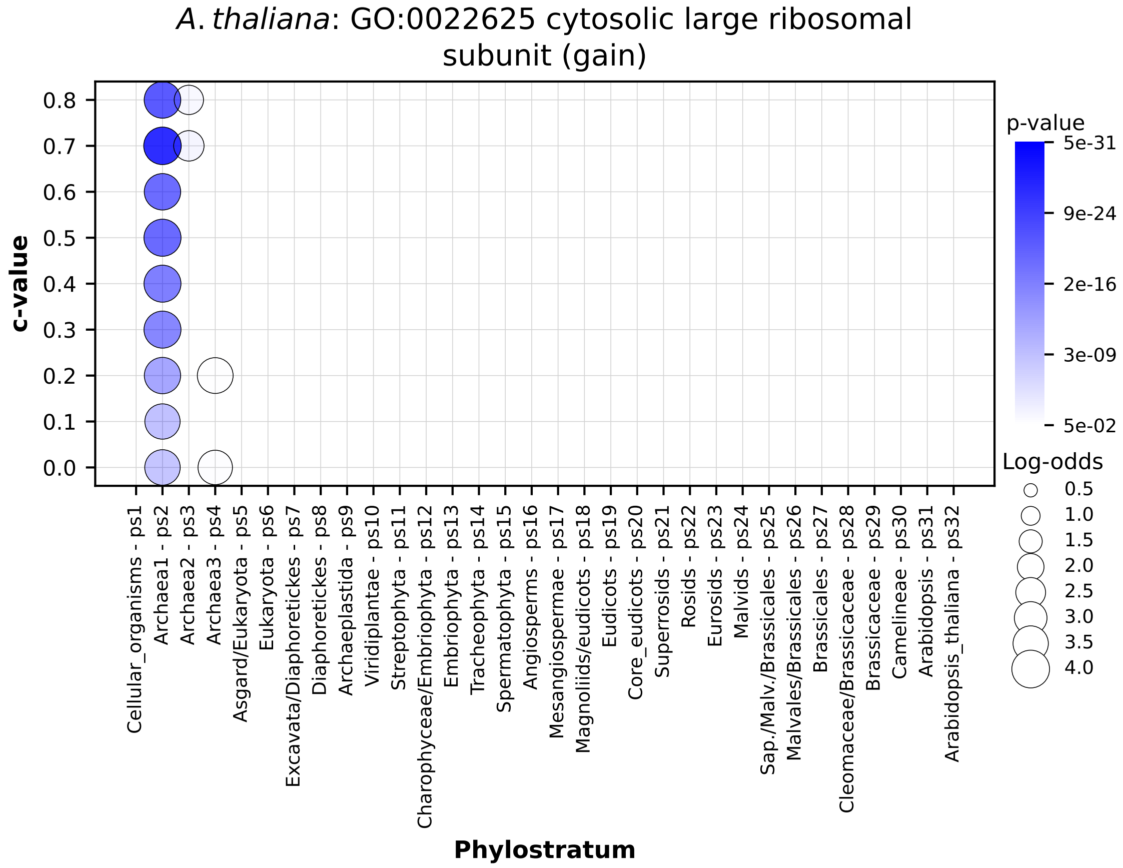
A. thaliana: GO:0022625 cytosolic large ribosomal subunit (gain)
c-value
0.8
0.7
0.6
0.5
0.4
0.3
0.2
0.1
0.0
Phylostratum
Cellular_organisms - ps1
Archaea1 - ps2
Archaea2 - ps3
Archaea3 - ps4
Asgard/Eukaryota - ps5
Eukaryota - ps6
Excavata/Diaphoretickes - ps7
Diaphoretickes - ps8
Archaeplastida - ps9
Viridiplantae - ps10
Streptophyta - ps11
Charophyceae/Embriophyta - ps12
Embriophyta - ps13
Tracheophyta - ps14
Spermatophyta - ps15
Angiosperms - ps16
Mesangiospermae - ps17
Magnoliids/eudicots - ps18
Eudicots - ps19
Core_eudicots - ps20
Superrosids - ps21
Rosids - ps22
Eurosids - ps23
Malvids - ps24
Sap./Malv./Brassicales - ps25
Malvales/Brassicales - ps26
Brassicales - ps27
Cleomaceae/Brassicaceae - ps28
Brassicaceae - ps29
Camelineae - ps30
Arabidopsis - ps31
Arabidopsis_thaliana - ps32
p-value
5e-31
9e-24
2e-16
3e-09
5e-02
Log-odds
0.5
1.0
1.5
2.0
2.5
3.0
3.5
4.0

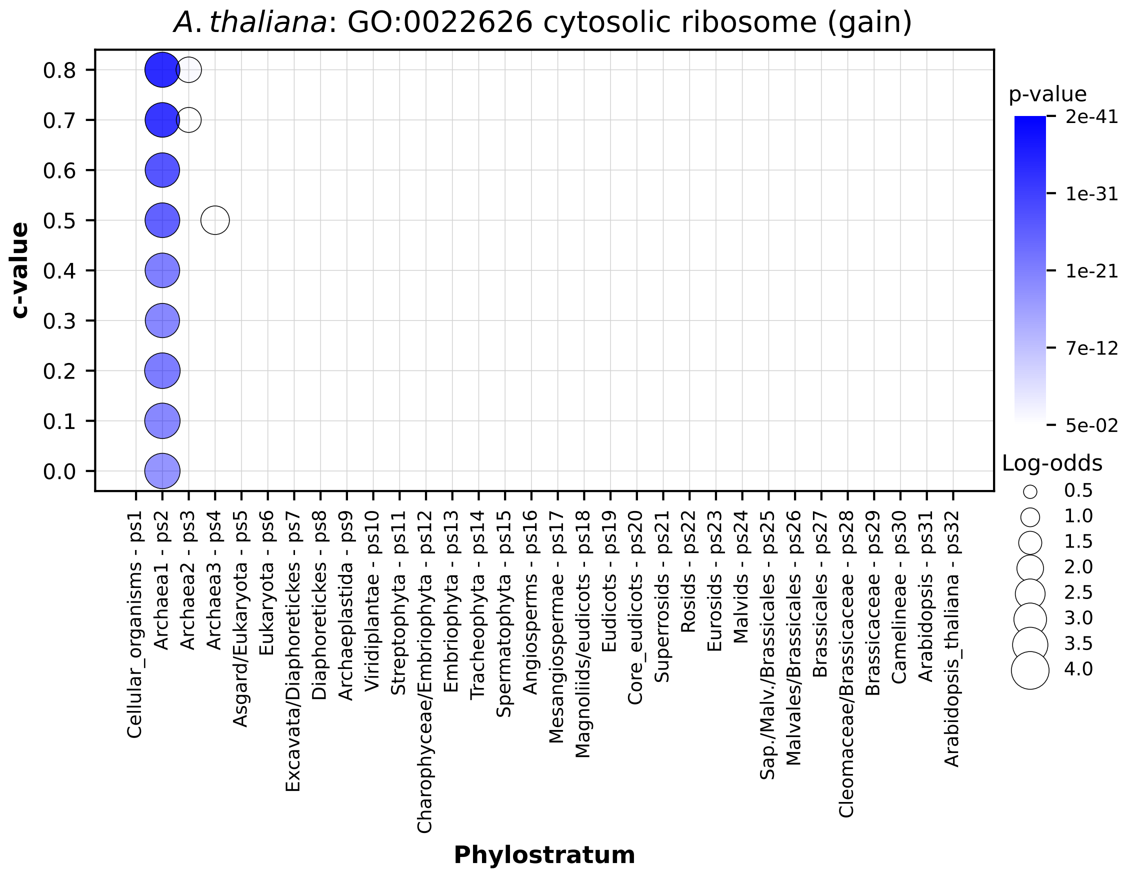

A. thaliana: GO:0022626 cytosolic ribosome (gain)
c-value
0.8
0.7
0.6
0.5
0.4
0.3
0.2
0.1
0.0
p-value
2e-41
1e-31
1e-21
7e-12
5e-02
Log-odds
0.5
1.0
1.5
2.0
2.5
3.0
3.5
4.0
Cellular_organisms - ps1
Archaea1 - ps2
Archaea2 - ps3
Archaea3 - ps4
Asgard/Eukaryota - ps5
Eukaryota - ps6
Excavata/Diaphoretickes - ps7
Diaphoretickes - ps8
Archaeplastida - ps9
Viridiplantae - ps10
Streptophyta - ps11
Charophyceae/Embriophyta - ps12
Embriophyta - ps13
Tracheophyta - ps14
Spermatophyta - ps15
Angiosperms - ps16
Mesangiospermae - ps17
Magnoliids/eudicots - ps18
Eudicots - ps19
Core_eudicots - ps20
Superrosids - ps21
Rosids - ps22
Eurosids - ps23
Malvids - ps24
Sap./Malv./Brassicales - ps25
Malvales/Brassicales - ps26
Brassicales - ps27
Cleomaceae/Brassicaceae - ps28
Brassicaceae - ps29
Camelineae - ps30
Arabidopsis - ps31
Arabidopsis_thaliana - ps32
Phylostratum

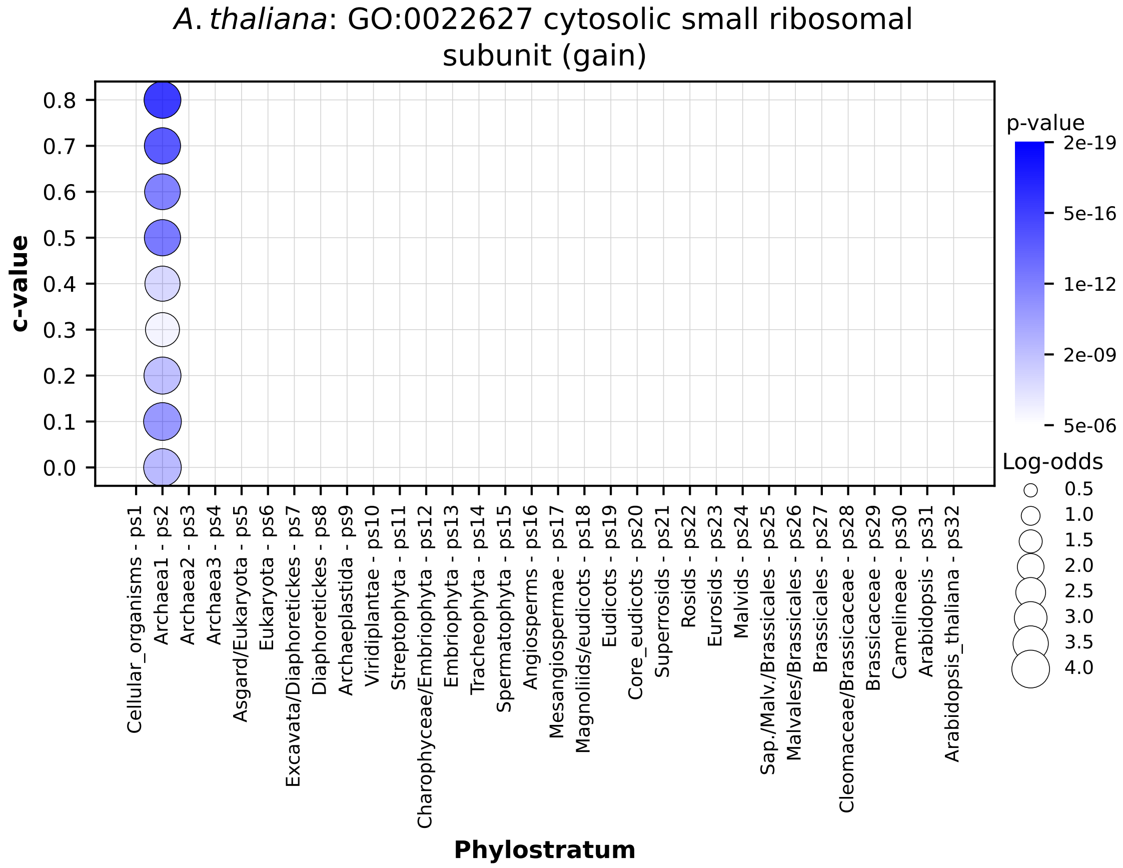
A. thaliana: GO:0022627 cytosolic small ribosomal subunit (gain)
c-value
0.8
0.7
0.6
0.5
0.4
0.3
0.2
0.1
0.0
p-value
2e-19
5e-16
1e-12
2e-09
5e-06
Log-odds
0.5
1.0
1.5
2.0
2.5
3.0
3.5
4.0
Cellular_organisms - ps1
Archaea1 - ps2
Archaea2 - ps3
Archaea3 - ps4
Asgard/Eukaryota - ps5
Eukaryota - ps6
Excavata/Diaphoretickes - ps7
Diaphoretickes - ps8
Archaeplastida - ps9
Viridiplantae - ps10
Streptophyta - ps11
Charophyceae/Embriophyta - ps12
Embriophyta - ps13
Tracheophyta - ps14
Spermatophyta - ps15
Angiosperms - ps16
Mesangiospermae - ps17
Magnoliids/eudicots - ps18
Eudicots - ps19
Core_eudicots - ps20
Superrosids - ps21
Rosids - ps22
Eurosids - ps23
Malvids - ps24
Sap./Malv./Brassicales - ps25
Malvales/Brassicales - ps26
Brassicales - ps27
Cleomaceae/Brassicaceae - ps28
Brassicaceae - ps29
Camelineae - ps30
Arabidopsis - ps31
Arabidopsis_thaliana - ps32
Phylostratum

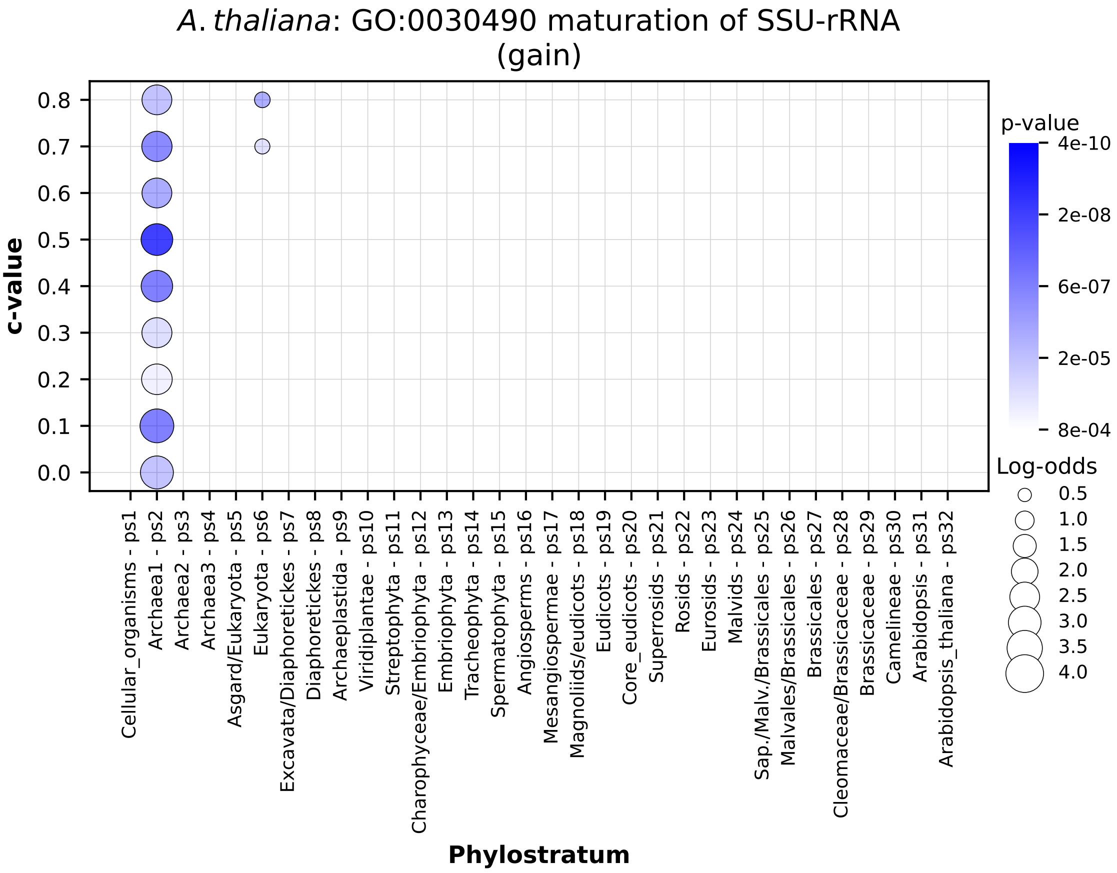

A. thaliana: GO:0030490 maturation of SSU-rRNA (gain)
c-value
0.8
0.7
0.6
0.5
0.4
0.3
0.2
0.1
0.0
p-value
4e-10
2e-08
6e-07
2e-05
8e-04
Log-odds
0.5
1.0
1.5
2.0
2.5
3.0
3.5
4.0
Cellular_organisms - ps1
Archaea1 - ps2
Archaea2 - ps3
Archaea3 - ps4
Asgard/Eukaryota - ps5
Eukaryota - ps6
Excavata/Diaphoretickes - ps7
Diaphoretickes - ps8
Archaeplastida - ps9
Viridiplantae - ps10
Streptophyta - ps11
Charophyceae/Embriophyta - ps12
Embriophyta - ps13
Tracheophyta - ps14
Spermatophyta - ps15
Angiosperms - ps16
Mesangiospermae - ps17
Magnoliids/eudicots - ps18
Eudicots - ps19
Core_eudicots - ps20
Superrosids - ps21
Rosids - ps22
Eurosids - ps23
Malvids - ps24
Sap./Malv./Brassicales - ps25
Malvales/Brassicales - ps26
Brassicales - ps27
Cleomaceae/Brassicaceae - ps28
Brassicaceae - ps29
Camelineae - ps30
Arabidopsis - ps31
Arabidopsis_thaliana - ps32
Phylostratum

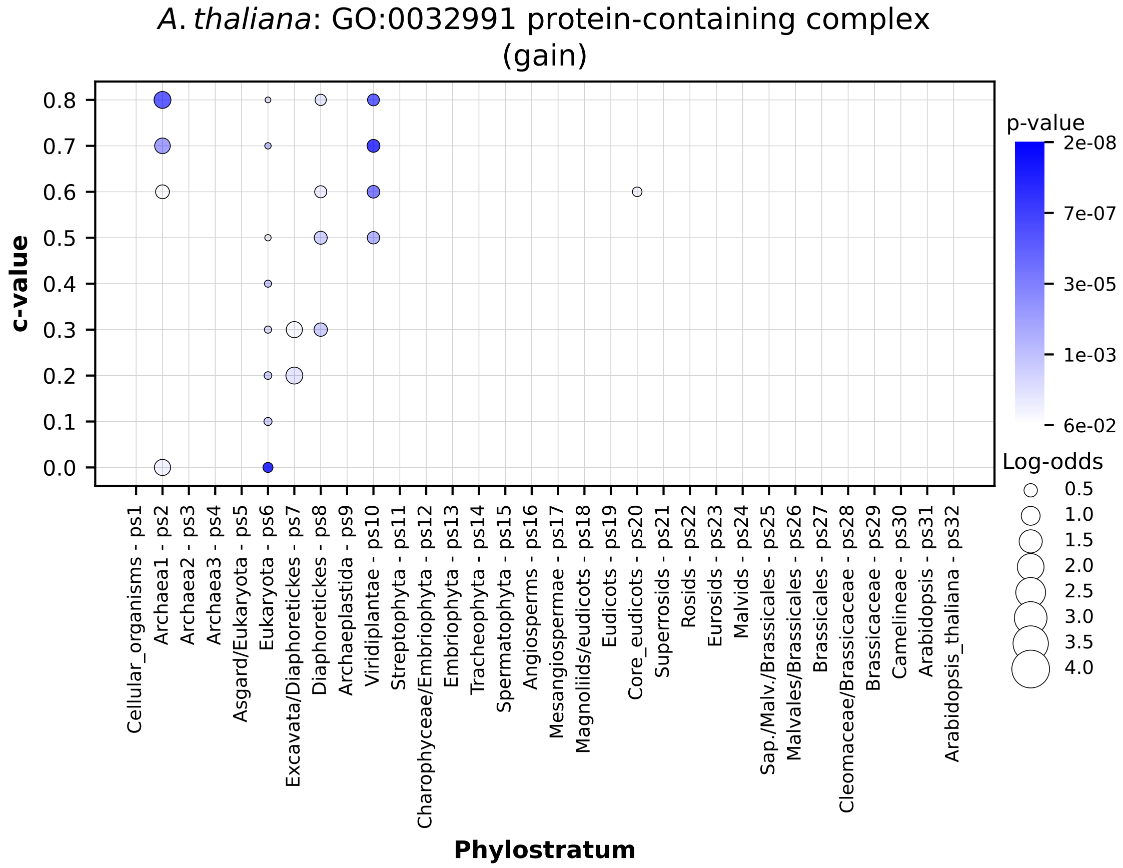

A. thaliana: GO:0032991 protein-containing complex
(gain)
c-value
0.8
0.7
0.6
0.5
0.4
0.3
0.2
0.1
0.0
p-value
2e-08
7e-07
3e-05
1e-03
6e-02
Log-odds
0.5
1.0
1.5
2.0
2.5
3.0
3.5
4.0
Cellular_organisms - ps1
Archaea1 - ps2
Archaea2 - ps3
Archaea3 - ps4
Asgard/Eukaryota - ps5
Eukaryota - ps6
Excavata/Diaphoretickes - ps7
Diaphoretickes - ps8
Archaeplastida - ps9
Viridiplantae - ps10
Streptophyta - ps11
Charophyceae/Embriophyta - ps12
Embriophyta - ps13
Tracheophyta - ps14
Spermatophyta - ps15
Angiosperms - ps16
Mesangiospermae - ps17
Magnoliids/eudicots - ps18
Eudicots - ps19
Core_eudicots - ps20
Superrosids - ps21
Rosids - ps22
Eurosids - ps23
Malvids - ps24
Sap./Malv./Brassicales - ps25
Malvales/Brassicales - ps26
Brassicales - ps27
Cleomaceae/Brassicaceae - ps28
Brassicaceae - ps29
Camelineae - ps30
Arabidopsis - ps31
Arabidopsis_thaliana - ps32
Phylostratum

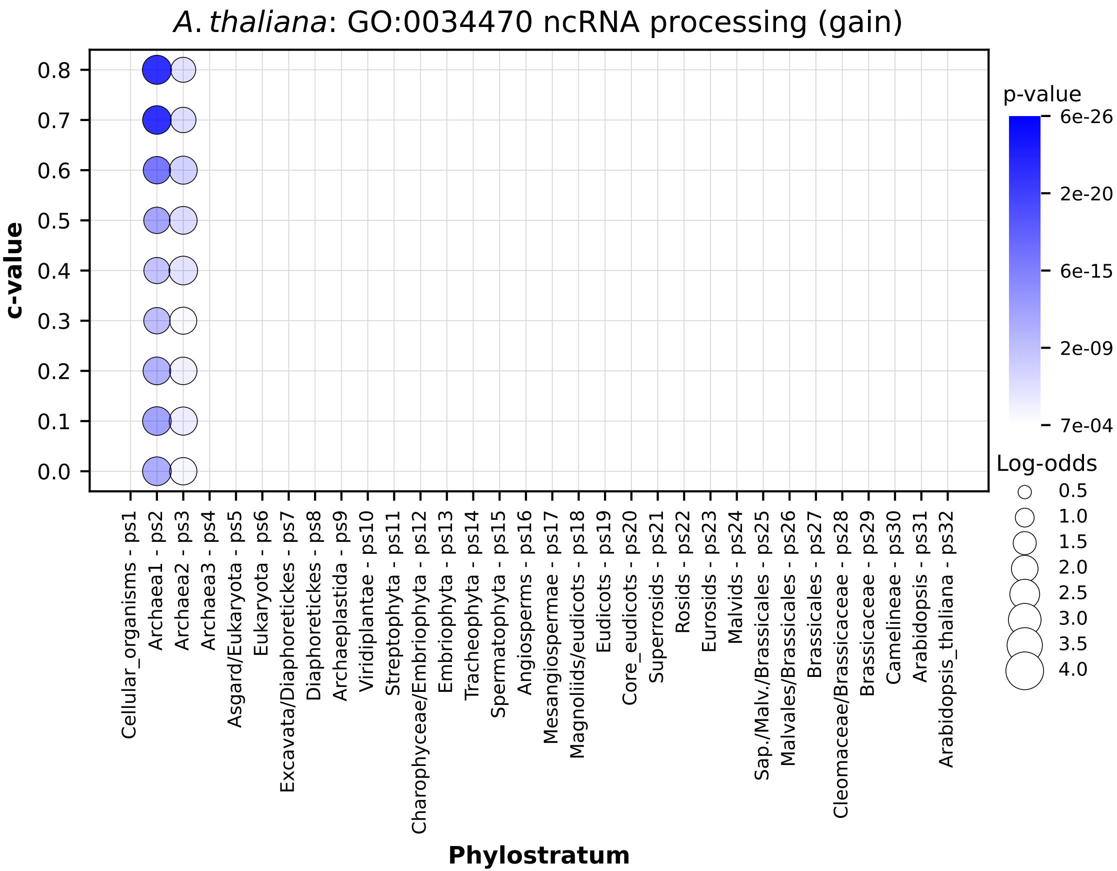

A. thaliana: GO:0034470 ncRNA processing (gain)
c-value
0.8
0.7
0.6
0.5
0.4
0.3
0.2
0.1
0.0
p-value
6e-26
2e-20
6e-15
2e-09
7e-04
Log-odds
0.5
1.0
1.5
2.0
2.5
3.0
3.5
4.0
Cellular_organisms - ps1
Archaea1 - ps2
Archaea2 - ps3
Archaea3 - ps4
Asgard/Eukaryota - ps5
Eukaryota - ps6
Excavata/Diaphoretickes - ps7
Diaphoretickes - ps8
Archaeplastida - ps9
Viridiplantae - ps10
Streptophyta - ps11
Charophyceae/Embriophyta - ps12
Embriophyta - ps13
Tracheophyta - ps14
Spermatophyta - ps15
Angiosperms - ps16
Mesangiospermae - ps17
Magnoliids/eudicots - ps18
Eudicots - ps19
Core_eudicots - ps20
Superrosids - ps21
Rosids - ps22
Eurosids - ps23
Malvids - ps24
Sap./Malv./Brassicales - ps25
Malvales/Brassicales - ps26
Brassicales - ps27
Cleomaceae/Brassicaceae - ps28
Brassicaceae - ps29
Camelineae - ps30
Arabidopsis - ps31
Arabidopsis_thaliana - ps32
Phylostratum

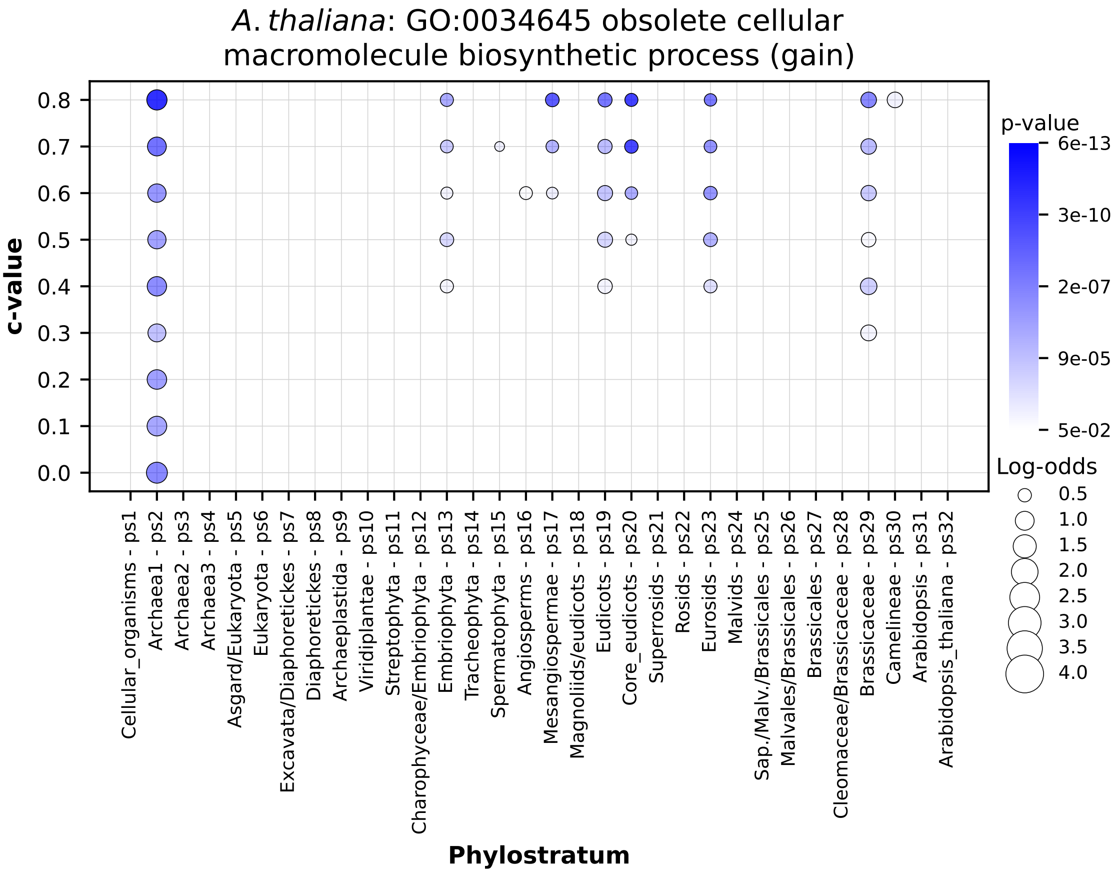

A. thaliana: GO:0034645 obsolete cellular macromolecule biosynthetic process (gain)
c-value
0.8
0.7
0.6
0.5
0.4
0.3
0.2
0.1
0.0
p-value
6e-13
3e-10
2e-07
9e-05
5e-02
Log-odds
0.5
1.0
1.5
2.0
2.5
3.0
3.5
4.0
Cellular_organisms - ps1
Archaea1 - ps2
Archaea2 - ps3
Archaea3 - ps4
Asgard/Eukaryota - ps5
Eukaryota - ps6
Excavata/Diaphoretickes - ps7
Diaphoretickes - ps8
Archaeplastida - ps9
Viridiplantae - ps10
Streptophyta - ps11
Charophyceae/Embriophyta - ps12
Embriophyta - ps13
Tracheophyta - ps14
Spermatophyta - ps15
Angiosperms - ps16
Mesangiospermae - ps17
Magnoliids/eudicots - ps18
Eudicots - ps19
Core_eudicots - ps20
Superrosids - ps21
Rosids - ps22
Eurosids - ps23
Malvids - ps24
Sap./Malv./Brassicales - ps25
Malvales/Brassicales - ps26
Brassicales - ps27
Cleomaceae/Brassicaceae - ps28
Brassicaceae - ps29
Camelineae - ps30
Arabidopsis - ps31
Arabidopsis_thaliana - ps32
Phylostratum

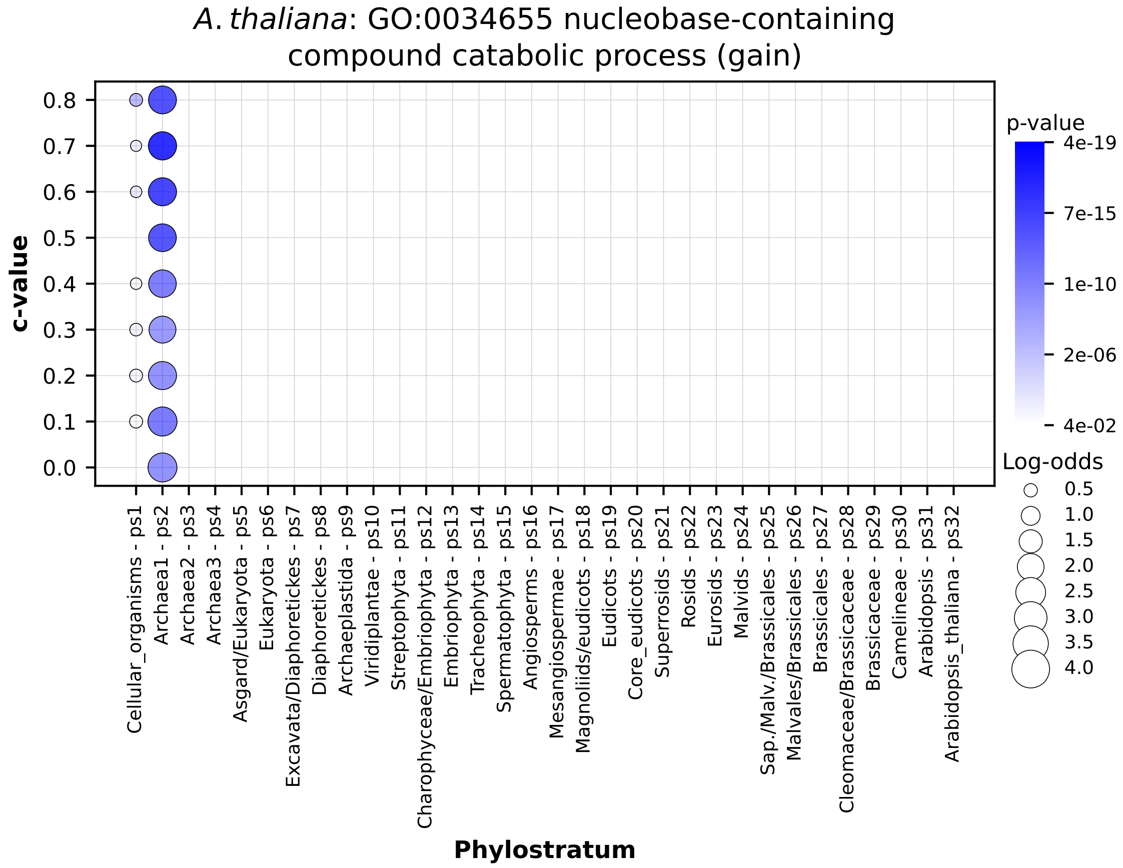

A. thaliana: GO:0034655 nucleobase-containing compound catabolic process (gain)
c-value
0.8
0.7
0.6
0.5
0.4
0.3
0.2
0.1
0.0
p-value
4e-19
7e-15
1e-10
2e-06
4e-02
Log-odds
0.5
1.0
1.5
2.0
2.5
3.0
3.5
4.0
Cellular_organisms - ps1
Archaea1 - ps2
Archaea2 - ps3
Archaea3 - ps4
Asgard/Eukaryota - ps5
Eukaryota - ps6
Excavata/Diaphoretickes - ps7
Diaphoretickes - ps8
Archaeplastida - ps9
Viridiplantae - ps10
Streptophyta - ps11
Charophyceae/Embriophyta - ps12
Embriophyta - ps13
Tracheophyta - ps14
Spermatophyta - ps15
Angiosperms - ps16
Mesangiospermae - ps17
Magnoliids/eudicots - ps18
Eudicots - ps19
Core_eudicots - ps20
Superrosids - ps21
Rosids - ps22
Eurosids - ps23
Malvids - ps24
Sap./Malv./Brassicales - ps25
Malvales/Brassicales - ps26
Brassicales - ps27
Cleomaceae/Brassicaceae - ps28
Brassicaceae - ps29
Camelineae - ps30
Arabidopsis - ps31
Arabidopsis_thaliana - ps32
Phylostratum

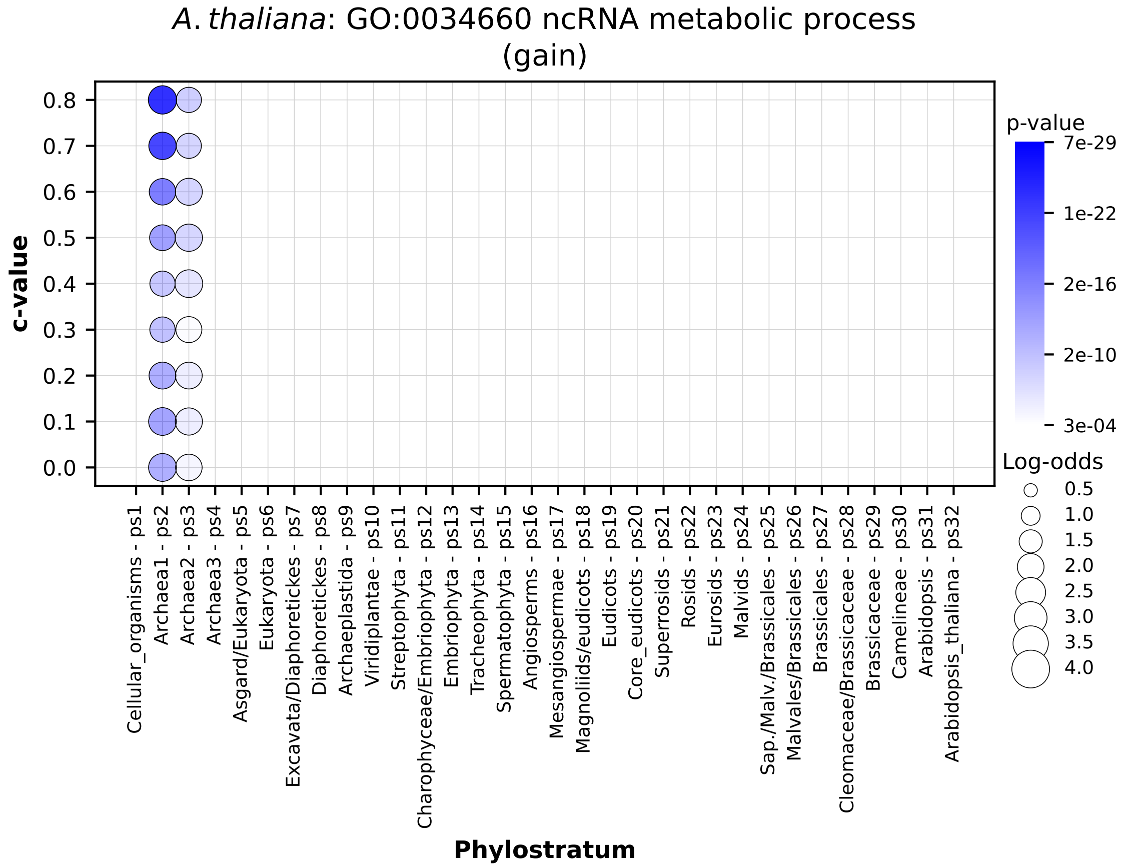

A. thaliana: GO:0034660 ncRNA metabolic process
(gain)
c-value
0.8
0.7
0.6
0.5
0.4
0.3
0.2
0.1
0.0
p-value
7e-29
1e-22
2e-16
2e-10
3e-04
Log-odds
0.5
1.0
1.5
2.0
2.5
3.0
3.5
4.0
Cellular_organisms - ps1
Archaea1 - ps2
Archaea2 - ps3
Archaea3 - ps4
Asgard/Eukaryota - ps5
Eukaryota - ps6
Excavata/Diaphoretickes - ps7
Diaphoretickes - ps8
Archaeplastida - ps9
Viridiplantae - ps10
Streptophyta - ps11
Charophyceae/Embriophyta - ps12
Embriophyta - ps13
Tracheophyta - ps14
Spermatophyta - ps15
Angiosperms - ps16
Mesangiospermae - ps17
Magnoliids/eudicots - ps18
Eudicots - ps19
Core_eudicots - ps20
Superrosids - ps21
Rosids - ps22
Eurosids - ps23
Malvids - ps24
Sap./Malv./Brassicales - ps25
Malvales/Brassicales - ps26
Brassicales - ps27
Cleomaceae/Brassicaceae - ps28
Brassicaceae - ps29
Camelineae - ps30
Arabidopsis - ps31
Arabidopsis_thaliana - ps32
Phylostratum

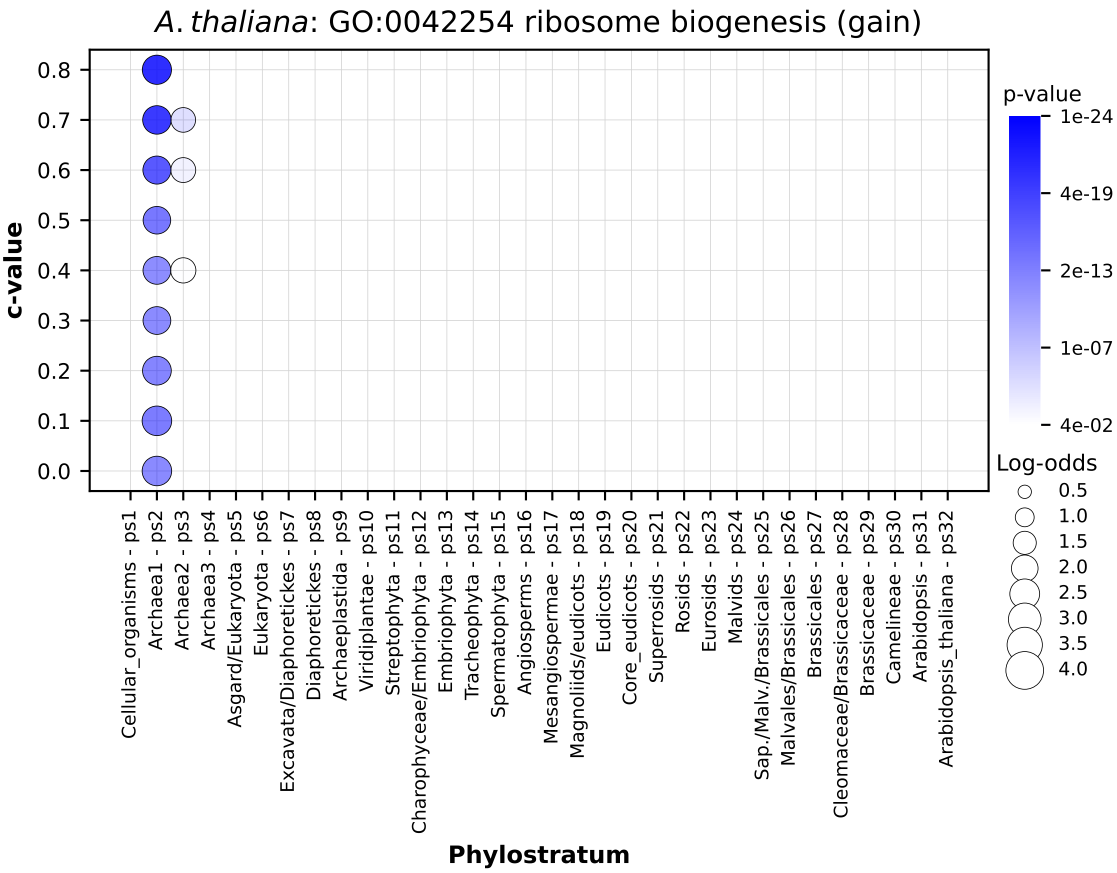

A. thaliana: GO:0042254 ribosome biogenesis (gain)
c-value
0.0
0.1
0.2
0.3
0.4
0.5
0.6
0.7
0.8
Phylostratum
Cellular_organisms - ps1
Archaea1 - ps2
Archaea2 - ps3
Archaea3 - ps4
Asgard/Eukaryota - ps5
Eukaryota - ps6
Excavata/Diaphoretickes - ps7
Diaphoretickes - ps8
Archaeplastida - ps9
Viridiplantae - ps10
Streptophyta - ps11
Charophyceae/Embriophyta - ps12
Embriophyta - ps13
Tracheophyta - ps14
Spermatophyta - ps15
Angiosperms - ps16
Mesangiospermae - ps17
Magnoliids/eudicots - ps18
Eudicots - ps19
Core_eudicots - ps20
Superrosids - ps21
Rosids - ps22
Eurosids - ps23
Malvids - ps24
Sap./Malv./Brassicales - ps25
Malvales/Brassicales - ps26
Brassicales - ps27
Cleomaceae/Brassicaceae - ps28
Brassicaceae - ps29
Camelineae - ps30
Arabidopsis - ps31
Arabidopsis_thaliana - ps32
p-value
1e-24
4e-19
2e-13
1e-07
4e-02
Log-odds
0.5
1.0
1.5
2.0
2.5
3.0
3.5
4.0

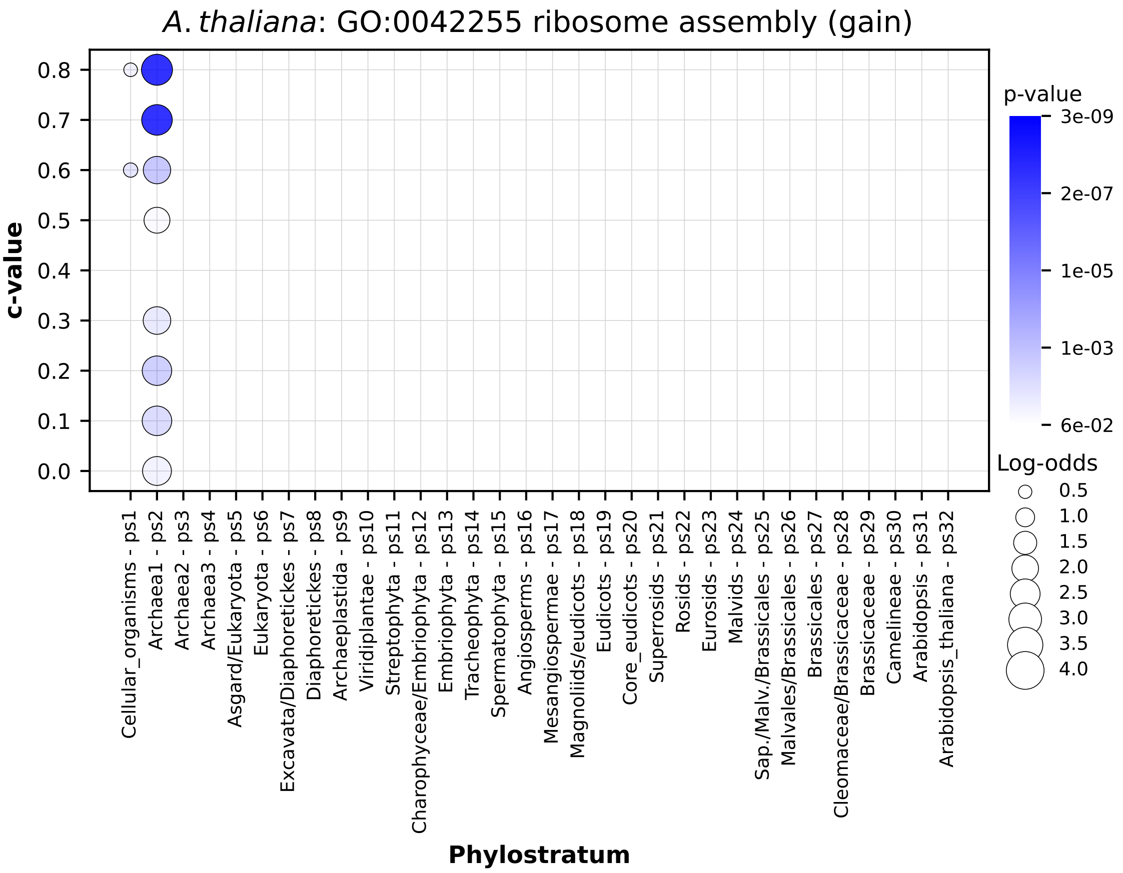

A. thaliana: GO:0042255 ribosome assembly (gain)
c-value
0.8
0.7
0.6
0.5
0.4
0.3
0.2
0.1
0.0
p-value
3e-09
2e-07
1e-05
1e-03
6e-02
Log-odds
0.5
1.0
1.5
2.0
2.5
3.0
3.5
4.0
Cellular_organisms - ps1
Archaea1 - ps2
Archaea2 - ps3
Archaea3 - ps4
Asgard/Eukaryota - ps5
Eukaryota - ps6
Excavata/Diaphoretickes - ps7
Diaphoretickes - ps8
Archaeplastida - ps9
Viridiplantae - ps10
Streptophyta - ps11
Charophyceae/Embriophyta - ps12
Embriophyta - ps13
Tracheophyta - ps14
Spermatophyta - ps15
Angiosperms - ps16
Mesangiospermae - ps17
Magnoliids/eudicots - ps18
Eudicots - ps19
Core_eudicots - ps20
Superrosids - ps21
Rosids - ps22
Eurosids - ps23
Malvids - ps24
Sap./Malv./Brassicales - ps25
Malvales/Brassicales - ps26
Brassicales - ps27
Cleomaceae/Brassicaceae - ps28
Brassicaceae - ps29
Camelineae - ps30
Arabidopsis - ps31
Arabidopsis_thaliana - ps32
Phylostratum

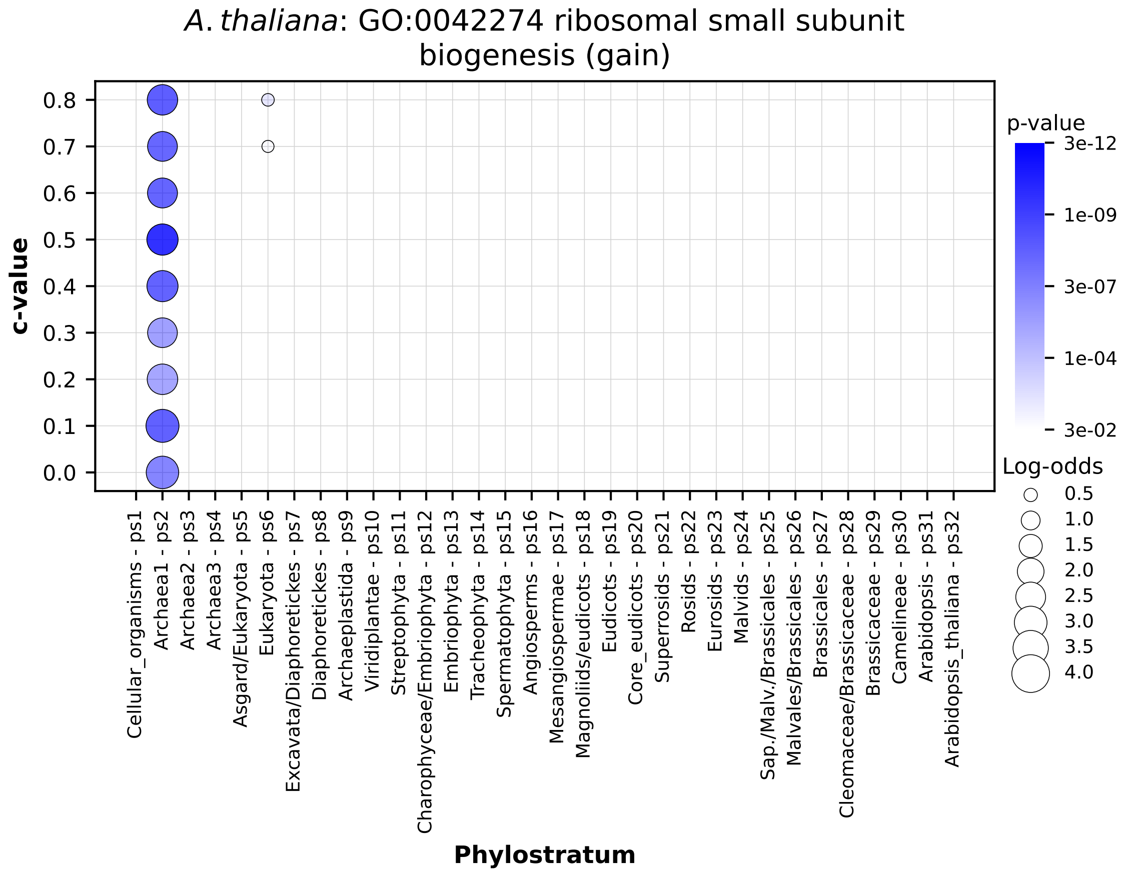
A. thaliana: GO:0042274 ribosomal small subunit biogenesis (gain)
c-value
0.8
0.7
0.6
0.5
0.4
0.3
0.2
0.1
0.0
p-value
3e-12
1e-09
3e-07
1e-04
3e-02
Log-odds
0.5
1.0
1.5
2.0
2.5
3.0
3.5
4.0
Cellular_organisms - ps1
Archaea1 - ps2
Archaea2 - ps3
Archaea3 - ps4
Asgard/Eukaryota - ps5
Eukaryota - ps6
Excavata/Diaphoretickes - ps7
Diaphoretickes - ps8
Archaeplastida - ps9
Viridiplantae - ps10
Streptophyta - ps11
Charophyceae/Embriophyta - ps12
Embriophyta - ps13
Tracheophyta - ps14
Spermatophyta - ps15
Angiosperms - ps16
Mesangiospermae - ps17
Magnoliids/eudicots - ps18
Eudicots - ps19
Core_eudicots - ps20
Superrosids - ps21
Rosids - ps22
Eurosids - ps23
Malvids - ps24
Sap./Malv./Brassicales - ps25
Malvales/Brassicales - ps26
Brassicales - ps27
Cleomaceae/Brassicaceae - ps28
Brassicaceae - ps29
Camelineae - ps30
Arabidopsis - ps31
Arabidopsis_thaliana - ps32
Phylostratum

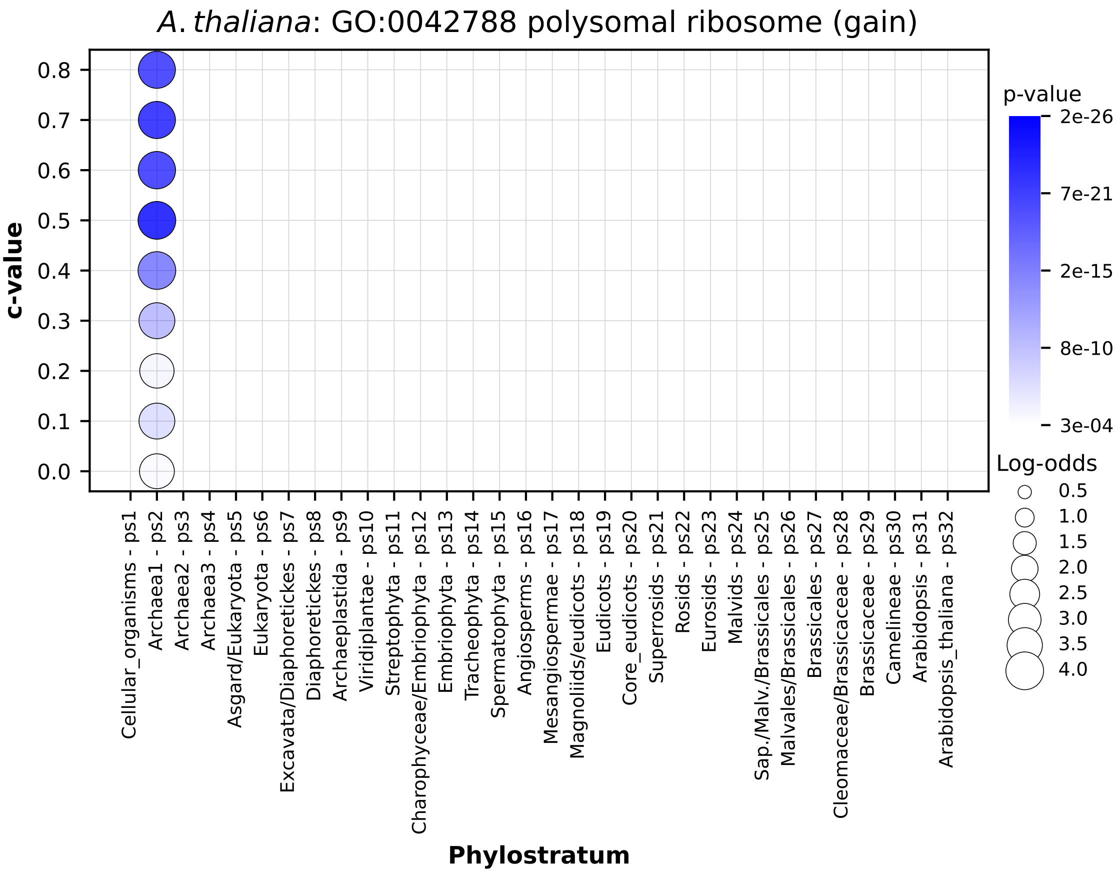

A. thaliana: GO:0042788 polysomal ribosome (gain)
c-value
0.0
0.1
0.2
0.3
0.4
0.5
0.6
0.7
0.8
Phylostratum
Cellular_organisms - ps1
Archaea1 - ps2
Archaea2 - ps3
Archaea3 - ps4
Asgard/Eukaryota - ps5
Eukaryota - ps6
Excavata/Diaphoretickes - ps7
Diaphoretickes - ps8
Archaeplastida - ps9
Viridiplantae - ps10
Streptophyta - ps11
Charophyceae/Embriophyta - ps12
Embriophyta - ps13
Tracheophyta - ps14
Spermatophyta - ps15
Angiosperms - ps16
Mesangiospermae - ps17
Magnoliids/eudicots - ps18
Eudicots - ps19
Core_eudicots - ps20
Superrosids - ps21
Rosids - ps22
Eurosids - ps23
Malvids - ps24
Sap./Malv./Brassicales - ps25
Malvales/Brassicales - ps26
Brassicales - ps27
Cleomaceae/Brassicaceae - ps28
Brassicaceae - ps29
Camelineae - ps30
Arabidopsis - ps31
Arabidopsis_thaliana - ps32
p-value
2e-26
7e-21
2e-15
8e-10
3e-04
Log-odds
0.5
1.0
1.5
2.0
2.5
3.0
3.5
4.0

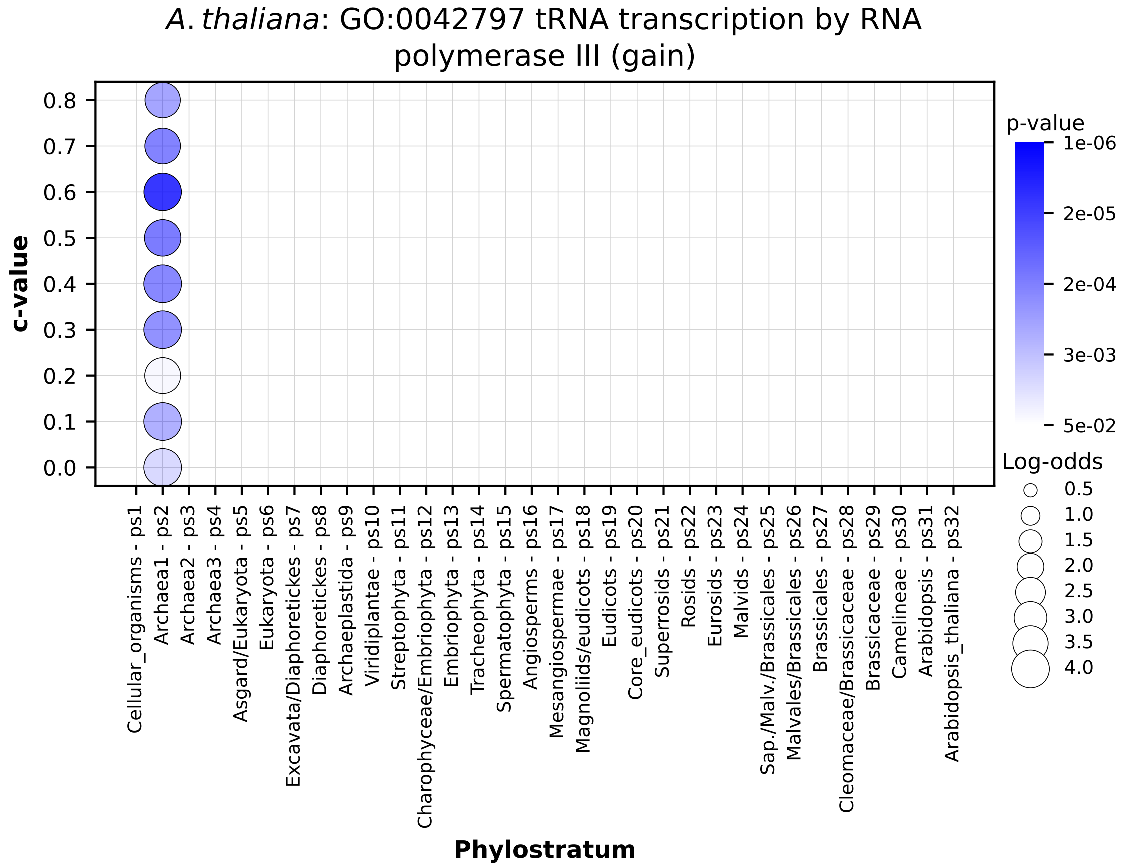
A. thaliana: GO:0042797 tRNA transcription by RNA polymerase III (gain)
c-value
0.0
0.1
0.2
0.3
0.4
0.5
0.6
0.7
0.8
p-value
1e-06
2e-05
2e-04
3e-03
5e-02
Log-odds
0.5
1.0
1.5
2.0
2.5
3.0
3.5
4.0
Cellular_organisms - ps1
Archaea1 - ps2
Archaea2 - ps3
Archaea3 - ps4
Asgard/Eukaryota - ps5
Eukaryota - ps6
Excavata/Diaphoretickes - ps7
Diaphoretickes - ps8
Archaeplastida - ps9
Viridiplantae - ps10
Streptophyta - ps11
Charophyceae/Embriophyta - ps12
Embriophyta - ps13
Tracheophyta - ps14
Spermatophyta - ps15
Angiosperms - ps16
Mesangiospermae - ps17
Magnoliids/eudicots - ps18
Eudicots - ps19
Core_eudicots - ps20
Superrosids - ps21
Rosids - ps22
Eurosids - ps23
Malvids - ps24
Sap./Malv./Brassicales - ps25
Malvales/Brassicales - ps26
Brassicales - ps27
Cleomaceae/Brassicaceae - ps28
Brassicaceae - ps29
Camelineae - ps30
Arabidopsis - ps31
Arabidopsis_thaliana - ps32
Phylostratum

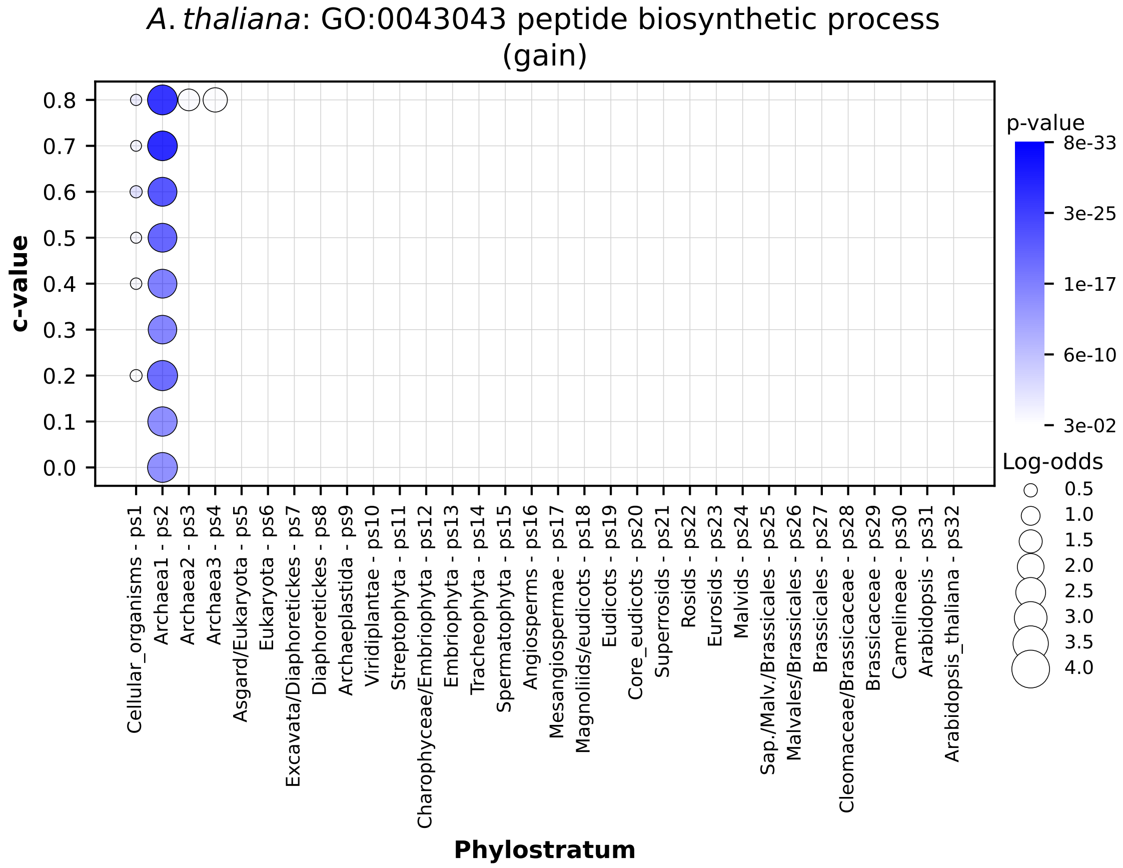

A. thaliana: GO:0043043 peptide biosynthetic process
(gain)
c-value
0.8
0.7
0.6
0.5
0.4
0.3
0.2
0.1
0.0
p-value
8e-33
3e-25
1e-17
6e-10
3e-02
Log-odds
0.5
1.0
1.5
2.0
2.5
3.0
3.5
4.0
Cellular_organisms - ps1
Archaea1 - ps2
Archaea2 - ps3
Archaea3 - ps4
Asgard/Eukaryota - ps5
Eukaryota - ps6
Excavata/Diaphoretickes - ps7
Diaphoretickes - ps8
Archaeplastida - ps9
Viridiplantae - ps10
Streptophyta - ps11
Charophyceae/Embriophyta - ps12
Embriophyta - ps13
Tracheophyta - ps14
Spermatophyta - ps15
Angiosperms - ps16
Mesangiospermae - ps17
Magnoliids/eudicots - ps18
Eudicots - ps19
Core_eudicots - ps20
Superrosids - ps21
Rosids - ps22
Eurosids - ps23
Malvids - ps24
Sap./Malv./Brassicales - ps25
Malvales/Brassicales - ps26
Brassicales - ps27
Cleomaceae/Brassicaceae - ps28
Brassicaceae - ps29
Camelineae - ps30
Arabidopsis - ps31
Arabidopsis_thaliana - ps32
Phylostratum

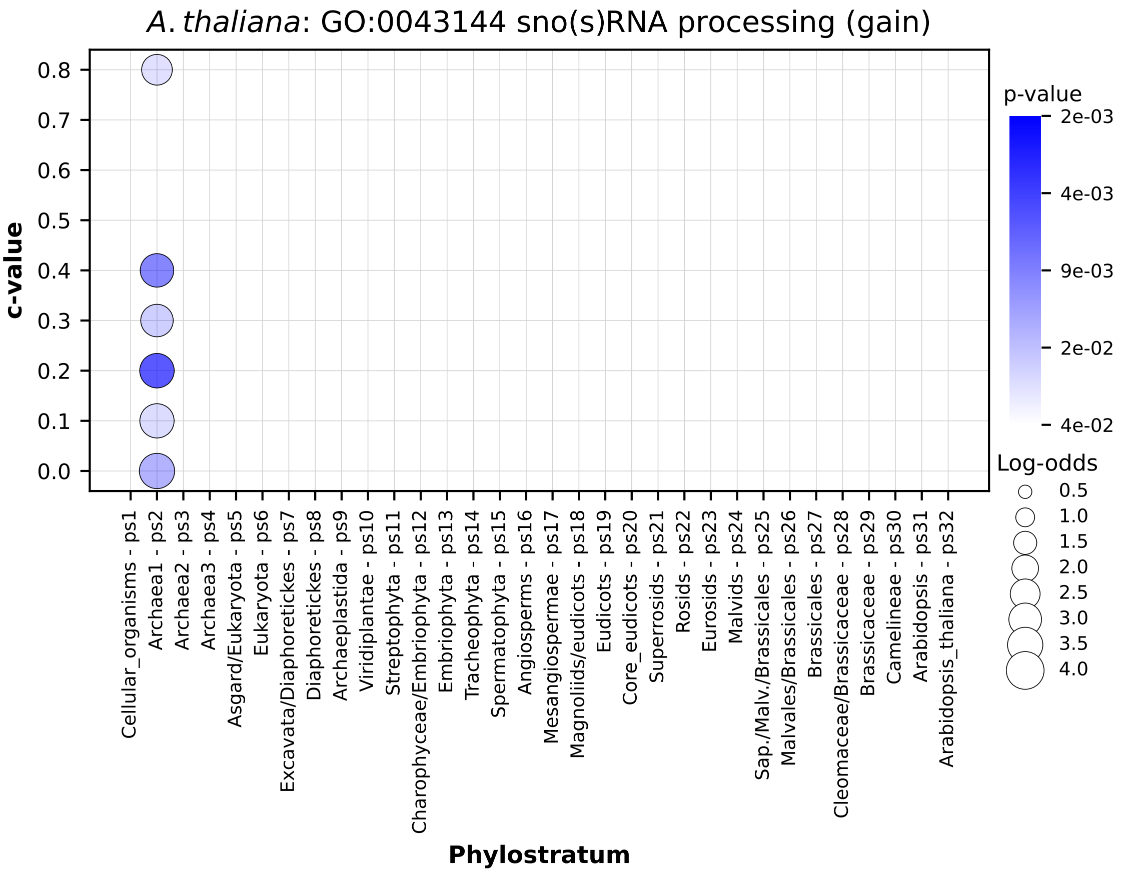

A. thaliana: GO:0043144 sno(s)RNA processing (gain)
c-value
0.8
0.7
0.6
0.5
0.4
0.3
0.2
0.1
0.0
p-value
2e-03
4e-03
9e-03
2e-02
4e-02
Log-odds
0.5
1.0
1.5
2.0
2.5
3.0
3.5
4.0
Cellular_organisms - ps1
Archaea1 - ps2
Archaea2 - ps3
Archaea3 - ps4
Asgard/Eukaryota - ps5
Eukaryota - ps6
Excavata/Diaphoretickes - ps7
Diaphoretickes - ps8
Archaeplastida - ps9
Viridiplantae - ps10
Streptophyta - ps11
Charophyceae/Embriophyta - ps12
Embriophyta - ps13
Tracheophyta - ps14
Spermatophyta - ps15
Angiosperms - ps16
Mesangiospermae - ps17
Magnoliids/eudicots - ps18
Eudicots - ps19
Core_eudicots - ps20
Superrosids - ps21
Rosids - ps22
Eurosids - ps23
Malvids - ps24
Sap./Malv./Brassicales - ps25
Malvales/Brassicales - ps26
Brassicales - ps27
Cleomaceae/Brassicaceae - ps28
Brassicaceae - ps29
Camelineae - ps30
Arabidopsis - ps31
Arabidopsis_thaliana - ps32
Phylostratum

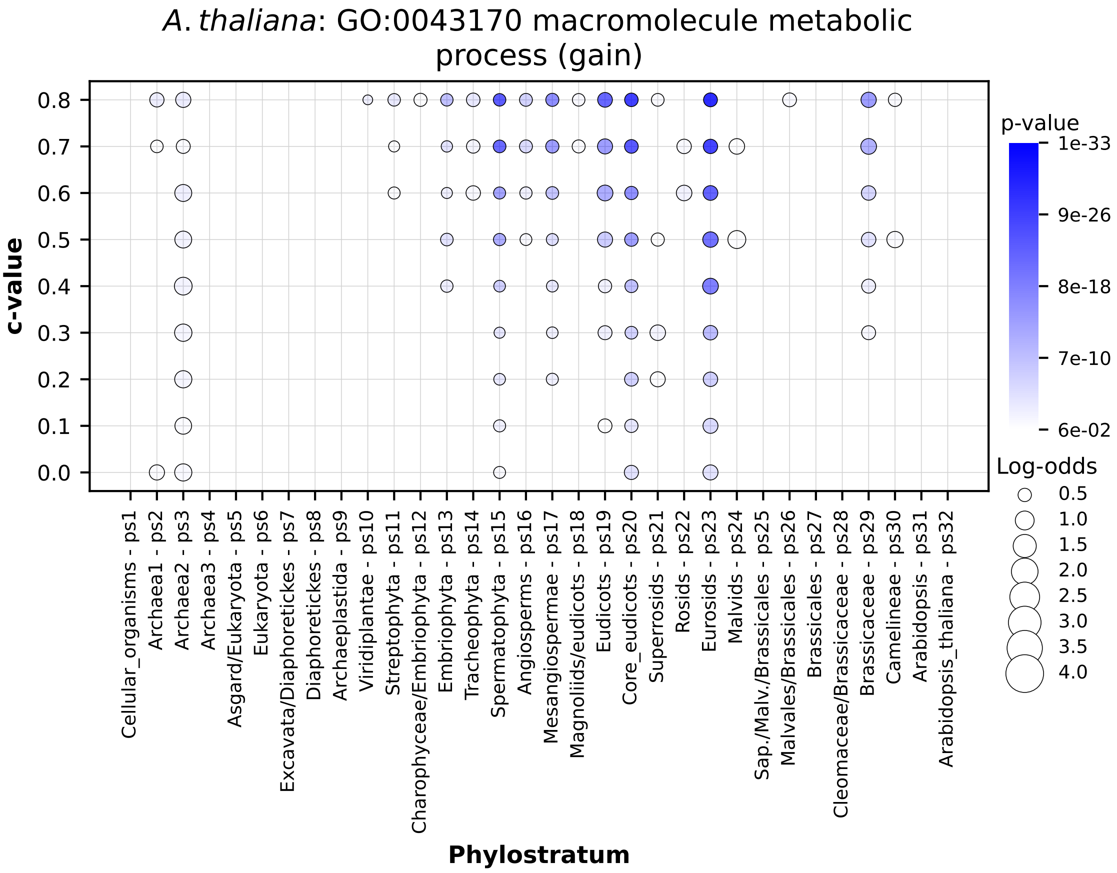

A. thaliana: GO:0043170 macromolecule metabolic process (gain)
c-value
0.8
0.7
0.6
0.5
0.4
0.3
0.2
0.1
0.0
p-value
1e-33
9e-26
8e-18
7e-10
6e-02
Log-odds
0.5
1.0
1.5
2.0
2.5
3.0
3.5
4.0
Cellular_organisms - ps1
Archaea1 - ps2
Archaea2 - ps3
Archaea3 - ps4
Asgard/Eukaryota - ps5
Eukaryota - ps6
Excavata/Diaphoretickes - ps7
Diaphoretickes - ps8
Archaeplastida - ps9
Viridiplantae - ps10
Streptophyta - ps11
Charophyceae/Embriophyta - ps12
Embriophyta - ps13
Tracheophyta - ps14
Spermatophyta - ps15
Angiosperms - ps16
Mesangiospermae - ps17
Magnoliids/eudicots - ps18
Eudicots - ps19
Core_eudicots - ps20
Superrosids - ps21
Rosids - ps22
Eurosids - ps23
Malvids - ps24
Sap./Malv./Brassicales - ps25
Malvales/Brassicales - ps26
Brassicales - ps27
Cleomaceae/Brassicaceae - ps28
Brassicaceae - ps29
Camelineae - ps30
Arabidopsis - ps31
Arabidopsis_thaliana - ps32
Phylostratum

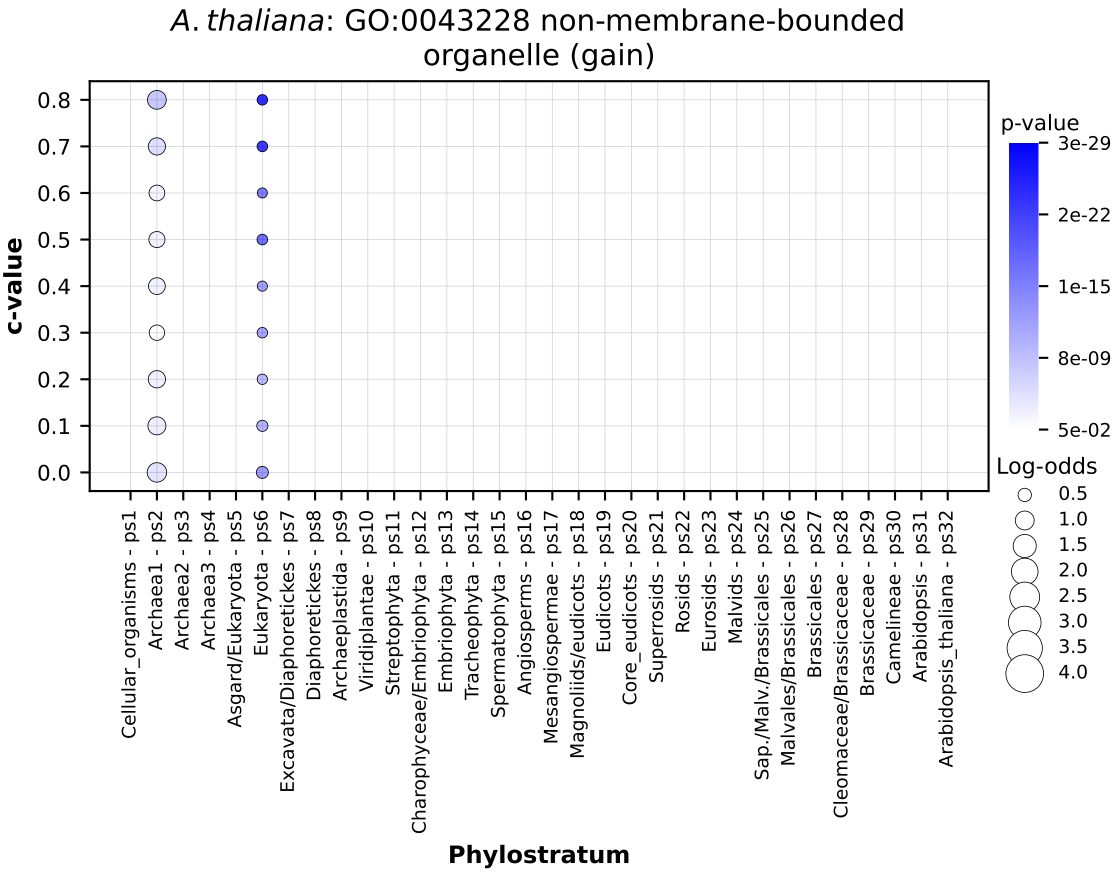
A. thaliana: GO:0043228 non-membrane-bounded organelle (gain)
c-value
0.8
0.7
0.6
0.5
0.4
0.3
0.2
0.1
0.0
p-value
3e-29
2e-22
1e-15
8e-09
5e-02
Log-odds
0.5
1.0
1.5
2.0
2.5
3.0
3.5
4.0
Cellular_organisms - ps1
Archaea1 - ps2
Archaea2 - ps3
Archaea3 - ps4
Asgard/Eukaryota - ps5
Eukaryota - ps6
Excavata/Diaphoretickes - ps7
Diaphoretickes - ps8
Archaeplastida - ps9
Viridiplantae - ps10
Streptophyta - ps11
Charophyceae/Embriophyta - ps12
Embriophyta - ps13
Tracheophyta - ps14
Spermatophyta - ps15
Angiosperms - ps16
Mesangiospermae - ps17
Magnoliids/eudicots - ps18
Eudicots - ps19
Core_eudicots - ps20
Superrosids - ps21
Rosids - ps22
Eurosids - ps23
Malvids - ps24
Sap./Malv./Brassicales - ps25
Malvales/Brassicales - ps26
Brassicales - ps27
Cleomaceae/Brassicaceae - ps28
Brassicaceae - ps29
Camelineae - ps30
Arabidopsis - ps31
Arabidopsis_thaliana - ps32
Phylostratum

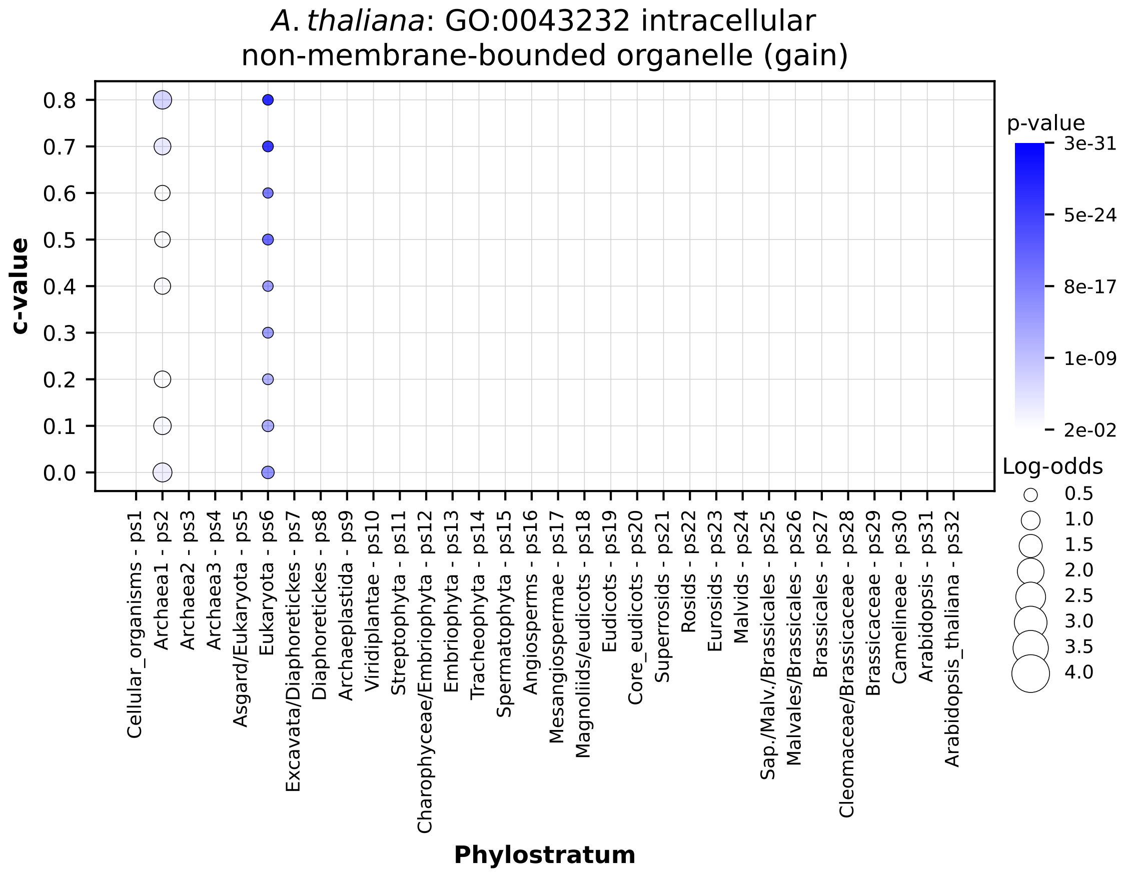

A. thaliana: GO:0043232 intracellular non-membrane-bounded organelle (gain)
c-value
0.8
0.7
0.6
0.5
0.4
0.3
0.2
0.1
0.0
p-value
3e-31
5e-24
8e-17
1e-09
2e-02
Log-odds
0.5
1.0
1.5
2.0
2.5
3.0
3.5
4.0
Cellular_organisms - ps1
Archaea1 - ps2
Archaea2 - ps3
Archaea3 - ps4
Asgard/Eukaryota - ps5
Eukaryota - ps6
Excavata/Diaphoretickes - ps7
Diaphoretickes - ps8
Archaeplastida - ps9
Viridiplantae - ps10
Streptophyta - ps11
Charophyceae/Embriophyta - ps12
Embriophyta - ps13
Tracheophyta - ps14
Spermatophyta - ps15
Angiosperms - ps16
Mesangiospermae - ps17
Magnoliids/eudicots - ps18
Eudicots - ps19
Core_eudicots - ps20
Superrosids - ps21
Rosids - ps22
Eurosids - ps23
Malvids - ps24
Sap./Malv./Brassicales - ps25
Malvales/Brassicales - ps26
Brassicales - ps27
Cleomaceae/Brassicaceae - ps28
Brassicaceae - ps29
Camelineae - ps30
Arabidopsis - ps31
Arabidopsis_thaliana - ps32
Phylostratum

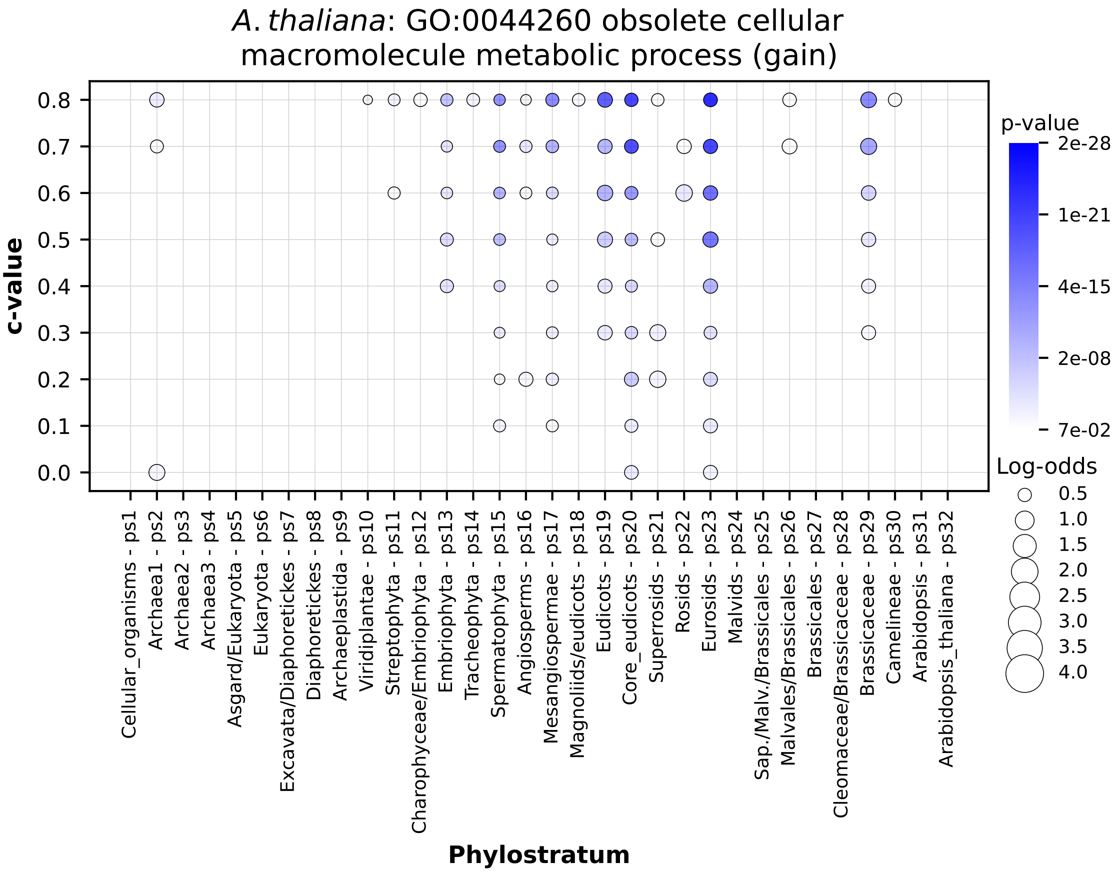

A. thaliana: GO:0044260 obsolete cellular macromolecule metabolic process (gain)
c-value
0.8
0.7
0.6
0.5
0.4
0.3
0.2
0.1
0.0
p-value
2e-28
1e-21
4e-15
2e-08
7e-02
Log-odds
0.5
1.0
1.5
2.0
2.5
3.0
3.5
4.0
Cellular_organisms - ps1
Archaea1 - ps2
Archaea2 - ps3
Archaea3 - ps4
Asgard/Eukaryota - ps5
Eukaryota - ps6
Excavata/Diaphoretickes - ps7
Diaphoretickes - ps8
Archaeplastida - ps9
Viridiplantae - ps10
Streptophyta - ps11
Charophyceae/Embriophyta - ps12
Embriophyta - ps13
Tracheophyta - ps14
Spermatophyta - ps15
Angiosperms - ps16
Mesangiospermae - ps17
Magnoliids/eudicots - ps18
Eudicots - ps19
Core_eudicots - ps20
Superrosids - ps21
Rosids - ps22
Eurosids - ps23
Malvids - ps24
Sap./Malv./Brassicales - ps25
Malvales/Brassicales - ps26
Brassicales - ps27
Cleomaceae/Brassicaceae - ps28
Brassicaceae - ps29
Camelineae - ps30
Arabidopsis - ps31
Arabidopsis_thaliana - ps32
Phylostratum

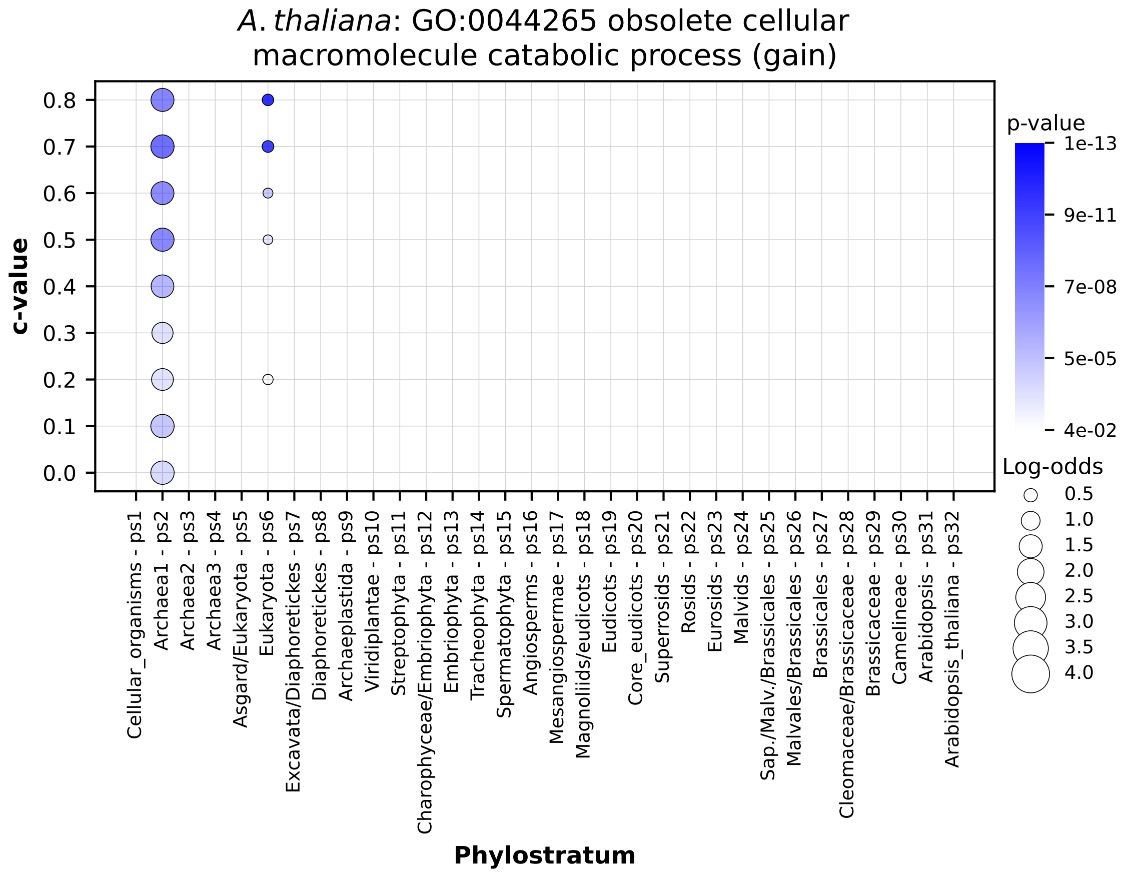

A. thaliana: GO:0044265 obsolete cellular macromolecule catabolic process (gain)
c-value
0.8
0.7
0.6
0.5
0.4
0.3
0.2
0.1
0.0
p-value
1e-13
9e-11
7e-08
5e-05
4e-02
Log-odds
0.5
1.0
1.5
2.0
2.5
3.0
3.5
4.0
Cellular_organisms - ps1
Archaea1 - ps2
Archaea2 - ps3
Archaea3 - ps4
Asgard/Eukaryota - ps5
Eukaryota - ps6
Excavata/Diaphoretickes - ps7
Diaphoretickes - ps8
Archaeplastida - ps9
Viridiplantae - ps10
Streptophyta - ps11
Charophyceae/Embriophyta - ps12
Embriophyta - ps13
Tracheophyta - ps14
Spermatophyta - ps15
Angiosperms - ps16
Mesangiospermae - ps17
Magnoliids/eudicots - ps18
Eudicots - ps19
Core_eudicots - ps20
Superrosids - ps21
Rosids - ps22
Eurosids - ps23
Malvids - ps24
Sap./Malv./Brassicales - ps25
Malvales/Brassicales - ps26
Brassicales - ps27
Cleomaceae/Brassicaceae - ps28
Brassicaceae - ps29
Camelineae - ps30
Arabidopsis - ps31
Arabidopsis_thaliana - ps32
Phylostratum

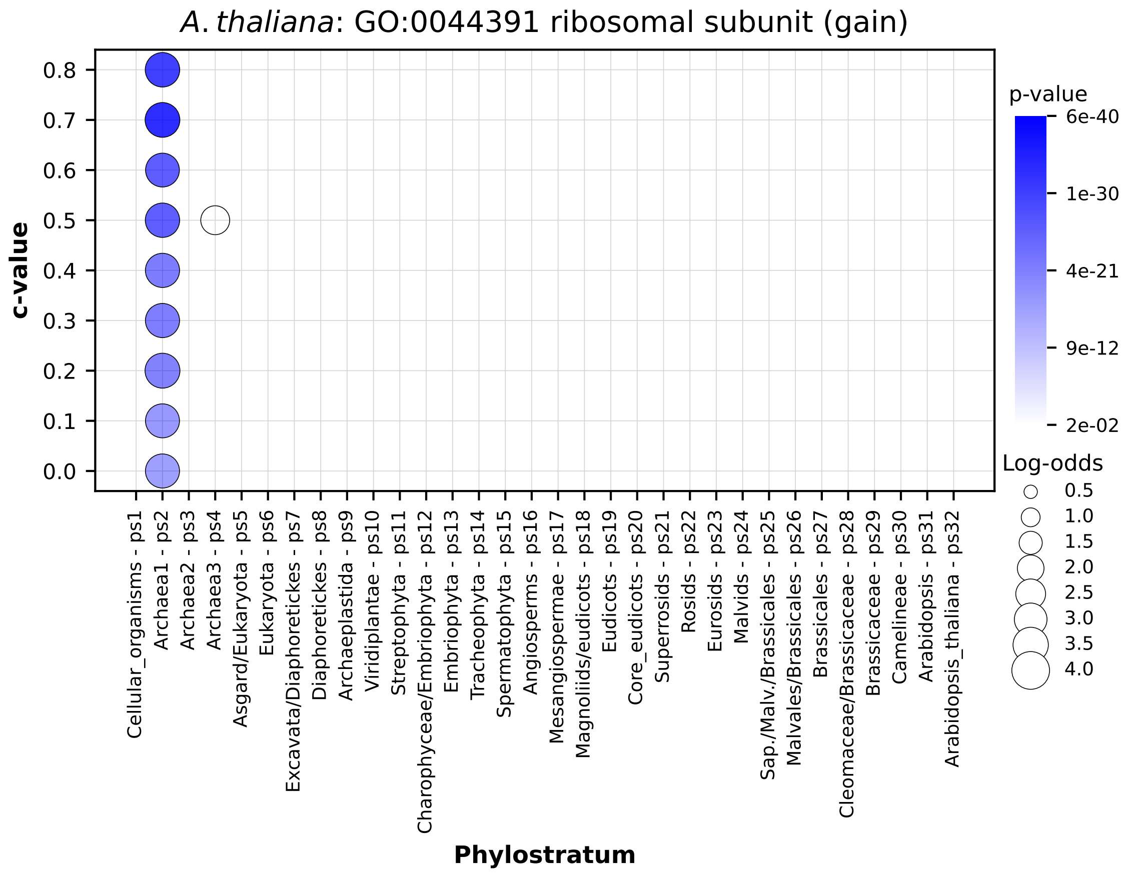
A. thaliana: GO:0044391 ribosomal subunit (gain)
c-value
0.0
0.1
0.2
0.3
0.4
0.5
0.6
0.7
0.8
p-value
6e-40
1e-30
4e-21
9e-12
2e-02
Log-odds
0.5
1.0
1.5
2.0
2.5
3.0
3.5
4.0
Cellular_organisms - ps1
Archaea1 - ps2
Archaea2 - ps3
Archaea3 - ps4
Asgard/Eukaryota - ps5
Eukaryota - ps6
Excavata/Diaphoretickes - ps7
Diaphoretickes - ps8
Archaeplastida - ps9
Viridiplantae - ps10
Streptophyta - ps11
Charophyceae/Embriophyta - ps12
Embriophyta - ps13
Tracheophyta - ps14
Spermatophyta - ps15
Angiosperms - ps16
Mesangiospermae - ps17
Magnoliids/eudicots - ps18
Eudicots - ps19
Core_eudicots - ps20
Superrosids - ps21
Rosids - ps22
Eurosids - ps23
Malvids - ps24
Sap./Malv./Brassicales - ps25
Malvales/Brassicales - ps26
Brassicales - ps27
Cleomaceae/Brassicaceae - ps28
Brassicaceae - ps29
Camelineae - ps30
Arabidopsis - ps31
Arabidopsis_thaliana - ps32
Phylostratum

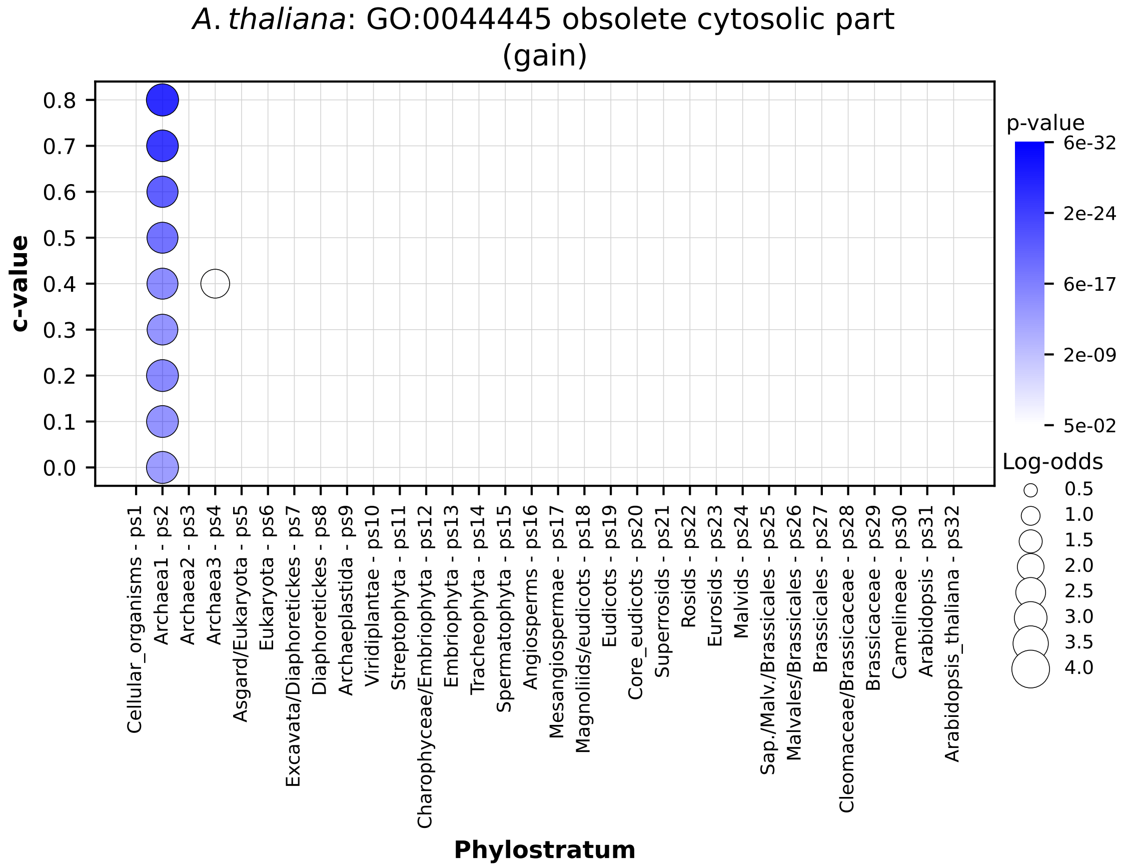

A. thaliana: GO:0044445 obsolete cytosolic part (gain)
c-value
0.0
0.1
0.2
0.3
0.4
0.5
0.6
0.7
0.8
p-value
6e-32
2e-24
6e-17
2e-09
5e-02
Log-odds
0.5
1.0
1.5
2.0
2.5
3.0
3.5
4.0
Cellular_organisms - ps1
Archaea1 - ps2
Archaea2 - ps3
Archaea3 - ps4
Asgard/Eukaryota - ps5
Eukaryota - ps6
Excavata/Diaphoretickes - ps7
Diaphoretickes - ps8
Archaeplastida - ps9
Viridiplantae - ps10
Streptophyta - ps11
Charophyceae/Embriophyta - ps12
Embriophyta - ps13
Tracheophyta - ps14
Spermatophyta - ps15
Angiosperms - ps16
Mesangiospermae - ps17
Magnoliids/eudicots - ps18
Eudicots - ps19
Core_eudicots - ps20
Superrosids - ps21
Rosids - ps22
Eurosids - ps23
Malvids - ps24
Sap./Malv./Brassicales - ps25
Malvales/Brassicales - ps26
Brassicales - ps27
Cleomaceae/Brassicaceae - ps28
Brassicaceae - ps29
Camelineae - ps30
Arabidopsis - ps31
Arabidopsis_thaliana - ps32
Phylostratum

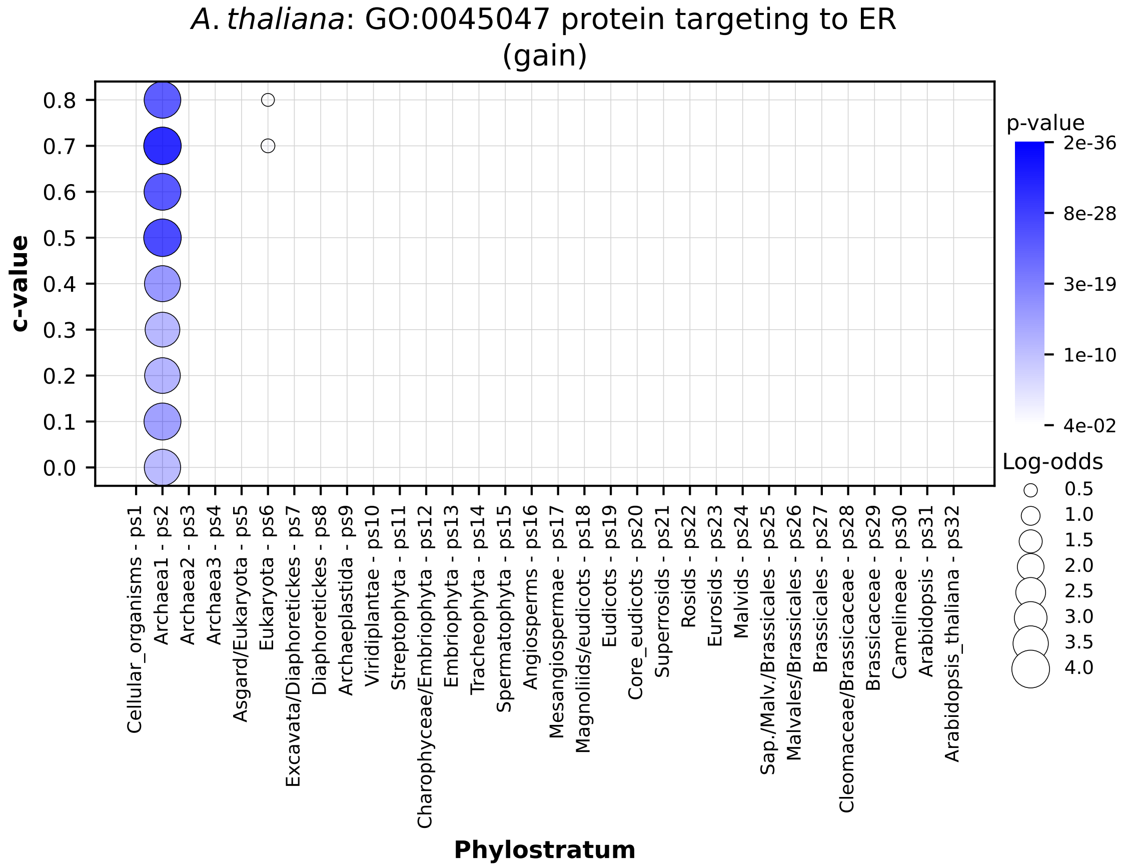

A. thaliana: GO:0045047 protein targeting to ER
(gain)
c-value
0.8
0.7
0.6
0.5
0.4
0.3
0.2
0.1
0.0
p-value
2e-36
8e-28
3e-19
1e-10
4e-02
Log-odds
0.5
1.0
1.5
2.0
2.5
3.0
3.5
4.0
Cellular_organisms - ps1
Archaea1 - ps2
Archaea2 - ps3
Archaea3 - ps4
Asgard/Eukaryota - ps5
Eukaryota - ps6
Excavata/Diaphoretickes - ps7
Diaphoretickes - ps8
Archaeplastida - ps9
Viridiplantae - ps10
Streptophyta - ps11
Charophyceae/Embriophyta - ps12
Embriophyta - ps13
Tracheophyta - ps14
Spermatophyta - ps15
Angiosperms - ps16
Mesangiospermae - ps17
Magnoliids/eudicots - ps18
Eudicots - ps19
Core_eudicots - ps20
Superrosids - ps21
Rosids - ps22
Eurosids - ps23
Malvids - ps24
Sap./Malv./Brassicales - ps25
Malvales/Brassicales - ps26
Brassicales - ps27
Cleomaceae/Brassicaceae - ps28
Brassicaceae - ps29
Camelineae - ps30
Arabidopsis - ps31
Arabidopsis_thaliana - ps32
Phylostratum

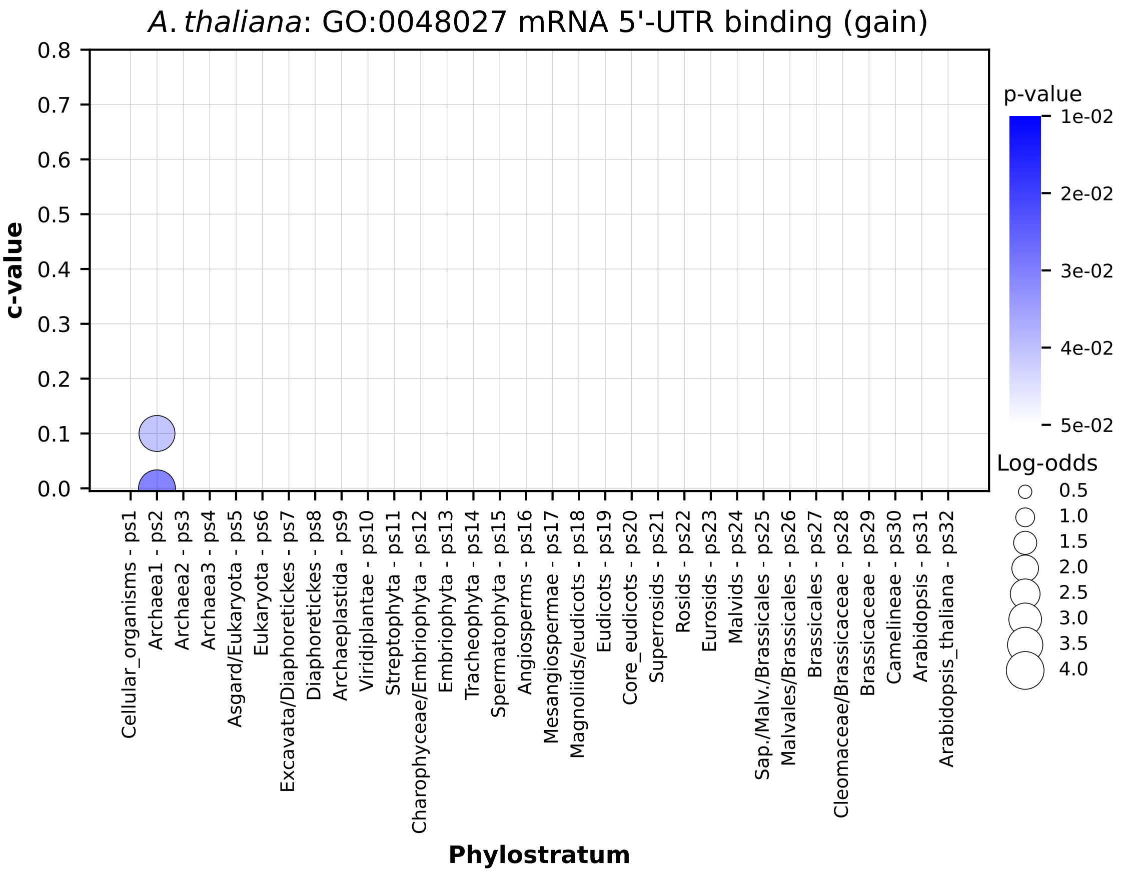

A. thaliana: GO:0048027 mRNA 5'-UTR binding (gain)
c-value
0.0
0.1
0.2
0.3
0.4
0.5
0.6
0.7
0.8
p-value
1e-02
2e-02
3e-02
4e-02
5e-02
Log-odds
0.5
1.0
1.5
2.0
2.5
3.0
3.5
4.0
Cellular_organisms - ps1
Archaea1 - ps2
Archaea2 - ps3
Archaea3 - ps4
Asgard/Eukaryota - ps5
Eukaryota - ps6
Excavata/Diaphoretickes - ps7
Diaphoretickes - ps8
Archaeplastida - ps9
Viridiplantae - ps10
Streptophyta - ps11
Charophyceae/Embriophyta - ps12
Embriophyta - ps13
Tracheophyta - ps14
Spermatophyta - ps15
Angiosperms - ps16
Mesangiospermae - ps17
Magnoliids/eudicots - ps18
Eudicots - ps19
Core_eudicots - ps20
Superrosids - ps21
Rosids - ps22
Eurosids - ps23
Malvids - ps24
Sap./Malv./Brassicales - ps25
Malvales/Brassicales - ps26
Brassicales - ps27
Cleomaceae/Brassicaceae - ps28
Brassicaceae - ps29
Camelineae - ps30
Arabidopsis - ps31
Arabidopsis_thaliana - ps32
Phylostratum

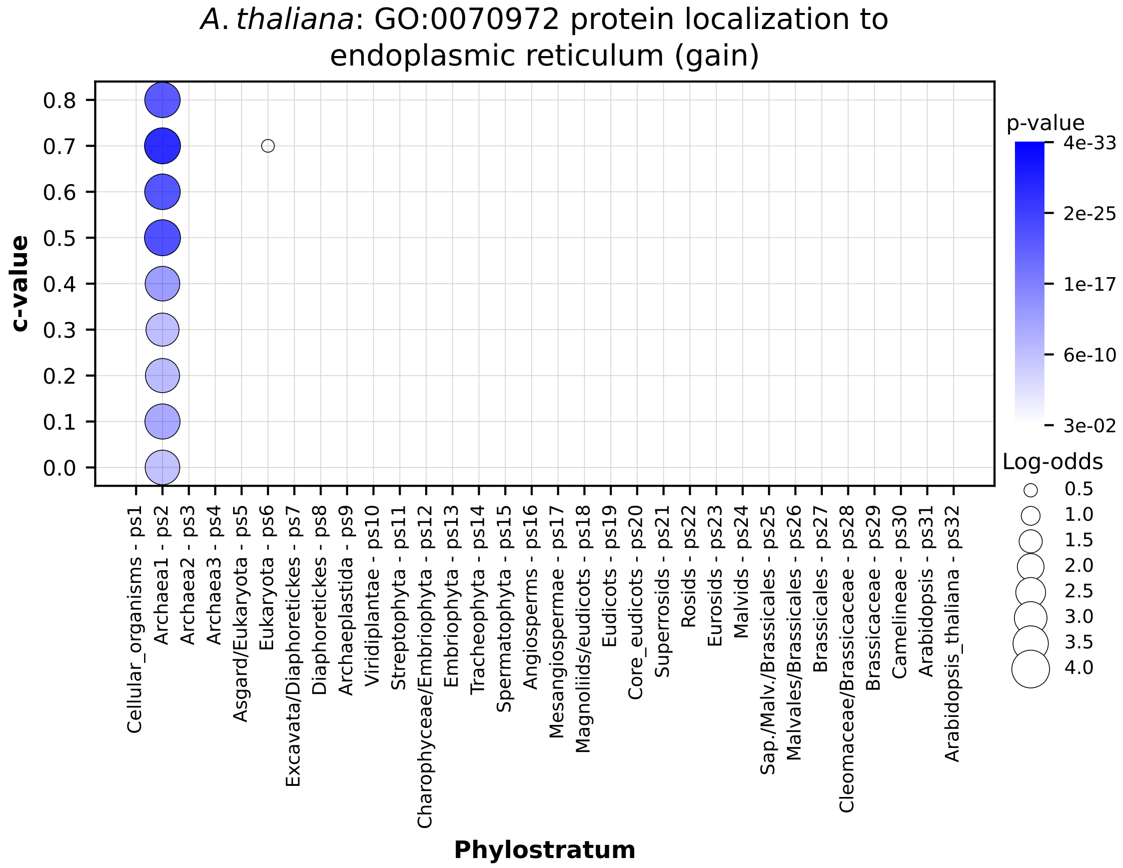

A. thaliana: GO:0070972 protein localization to endoplasmic reticulum (gain)
c-value
0.8
0.7
0.6
0.5
0.4
0.3
0.2
0.1
0.0
p-value
4e-33
2e-25
1e-17
6e-10
3e-02
Log-odds
0.5
1.0
1.5
2.0
2.5
3.0
3.5
4.0
Cellular_organisms - ps1
Archaea1 - ps2
Archaea2 - ps3
Archaea3 - ps4
Asgard/Eukaryota - ps5
Eukaryota - ps6
Excavata/Diaphoretickes - ps7
Diaphoretickes - ps8
Archaeplastida - ps9
Viridiplantae - ps10
Streptophyta - ps11
Charophyceae/Embriophyta - ps12
Embriophyta - ps13
Tracheophyta - ps14
Spermatophyta - ps15
Angiosperms - ps16
Mesangiospermae - ps17
Magnoliids/eudicots - ps18
Eudicots - ps19
Core_eudicots - ps20
Superrosids - ps21
Rosids - ps22
Eurosids - ps23
Malvids - ps24
Sap./Malv./Brassicales - ps25
Malvales/Brassicales - ps26
Brassicales - ps27
Cleomaceae/Brassicaceae - ps28
Brassicaceae - ps29
Camelineae - ps30
Arabidopsis - ps31
Arabidopsis_thaliana - ps32
Phylostratum

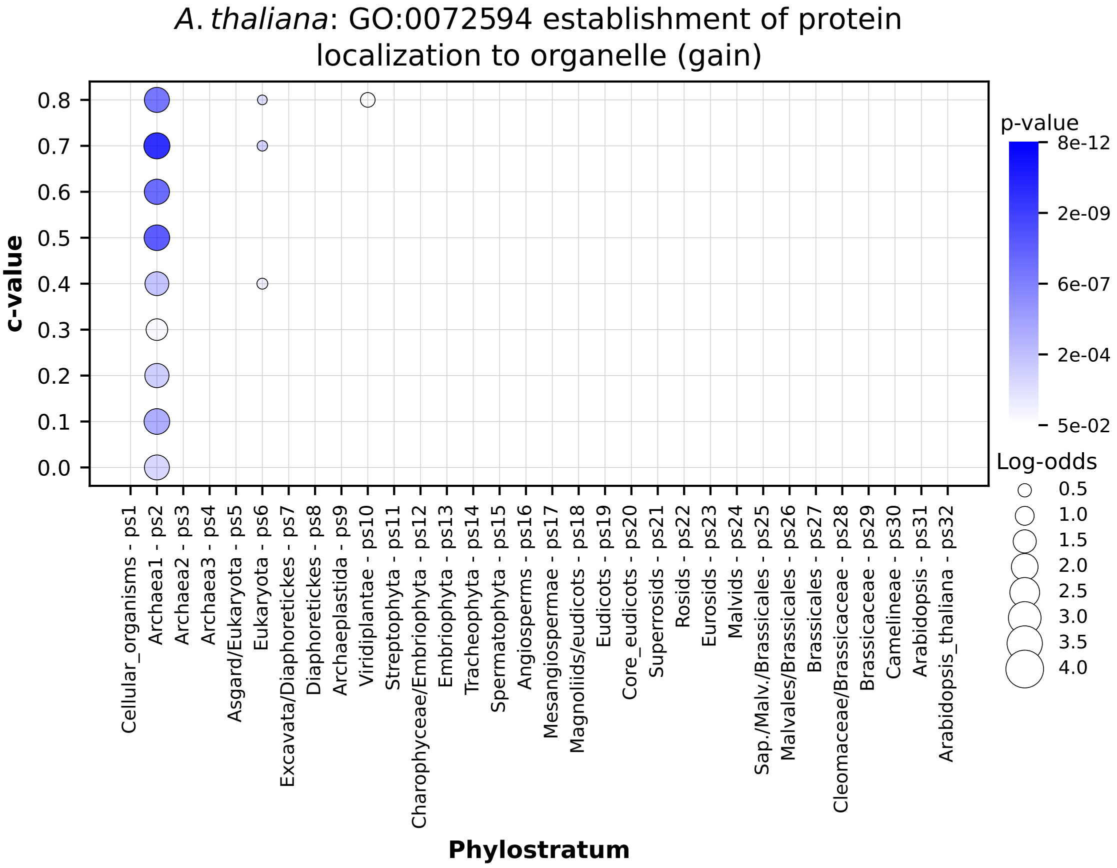

A. thaliana: GO:0072594 establishment of protein localization to organelle (gain)
c-value
0.8
0.7
0.6
0.5
0.4
0.3
0.2
0.1
0.0
p-value
8e-12
2e-09
6e-07
2e-04
5e-02
Log-odds
0.5
1.0
1.5
2.0
2.5
3.0
3.5
4.0
Cellular_organisms - ps1
Archaea1 - ps2
Archaea2 - ps3
Archaea3 - ps4
Asgard/Eukaryota - ps5
Eukaryota - ps6
Excavata/Diaphoretickes - ps7
Diaphoretickes - ps8
Archaeplastida - ps9
Viridiplantae - ps10
Streptophyta - ps11
Charophyceae/Embriophyta - ps12
Embriophyta - ps13
Tracheophyta - ps14
Spermatophyta - ps15
Angiosperms - ps16
Mesangiospermae - ps17
Magnoliids/eudicots - ps18
Eudicots - ps19
Core_eudicots - ps20
Superrosids - ps21
Rosids - ps22
Eurosids - ps23
Malvids - ps24
Sap./Malv./Brassicales - ps25
Malvales/Brassicales - ps26
Brassicales - ps27
Cleomaceae/Brassicaceae - ps28
Brassicaceae - ps29
Camelineae - ps30
Arabidopsis - ps31
Arabidopsis_thaliana - ps32
Phylostratum

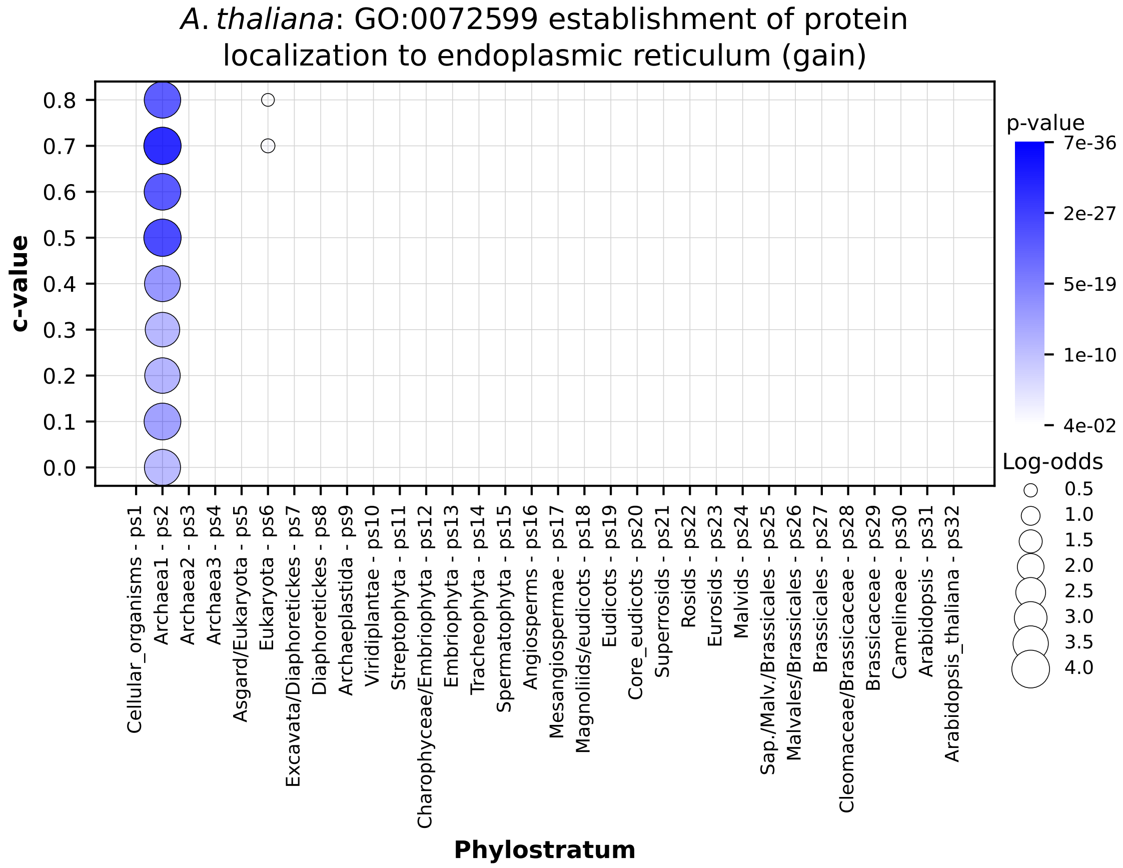
A. thaliana: GO:0072599 establishment of protein localization to endoplasmic reticulum (gain)
c-value
0.8
0.7
0.6
0.5
0.4
0.3
0.2
0.1
0.0
Phylostratum
Cellular_organisms - ps1
Archaea1 - ps2
Archaea2 - ps3
Archaea3 - ps4
Asgard/Eukaryota - ps5
Eukaryota - ps6
Excavata/Diaphoretickes - ps7
Diaphoretickes - ps8
Archaeplastida - ps9
Viridiplantae - ps10
Streptophyta - ps11
Charophyceae/Embriophyta - ps12
Embriophyta - ps13
Tracheophyta - ps14
Spermatophyta - ps15
Angiosperms - ps16
Mesangiospermae - ps17
Magnoliids/eudicots - ps18
Eudicots - ps19
Core_eudicots - ps20
Superrosids - ps21
Rosids - ps22
Eurosids - ps23
Malvids - ps24
Sap./Malv./Brassicales - ps25
Malvales/Brassicales - ps26
Brassicales - ps27
Cleomaceae/Brassicaceae - ps28
Brassicaceae - ps29
Camelineae - ps30
Arabidopsis - ps31
Arabidopsis_thaliana - ps32
p-value
7e-36
2e-27
5e-19
1e-10
4e-02
Log-odds
0.5
1.0
1.5
2.0
2.5
3.0
3.5
4.0

A. thaliana: GO:0072657 protein localization to membrane (gain)

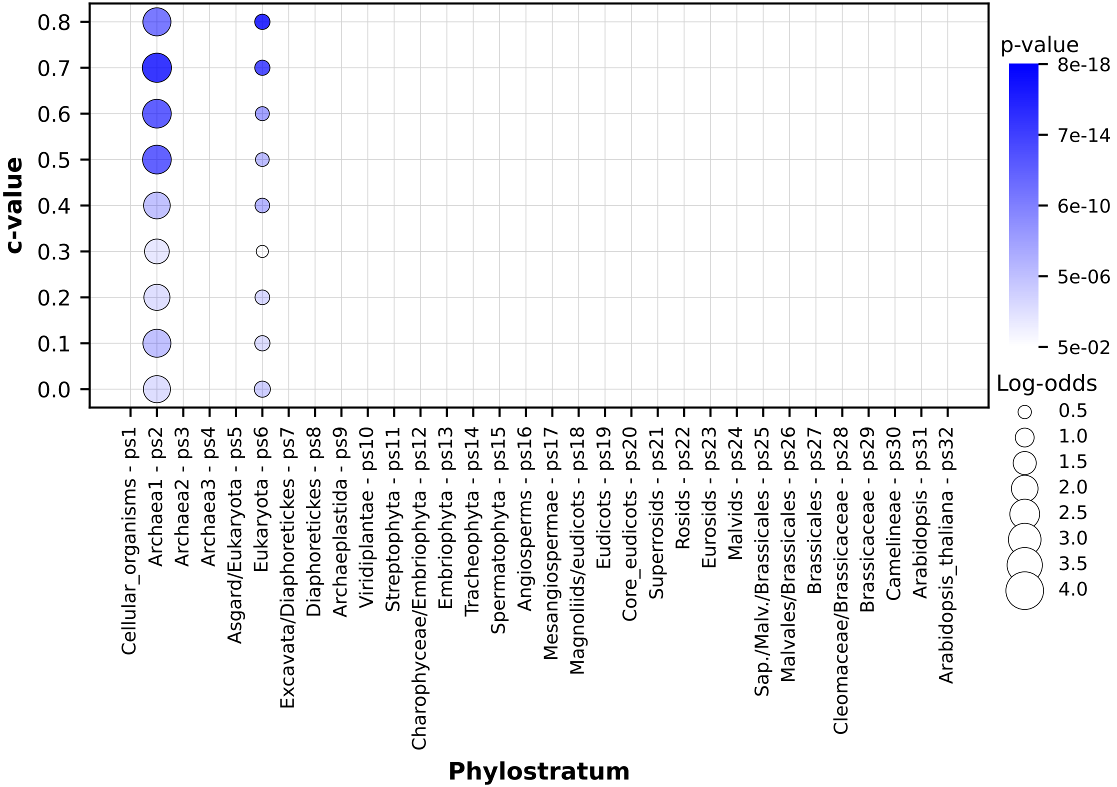

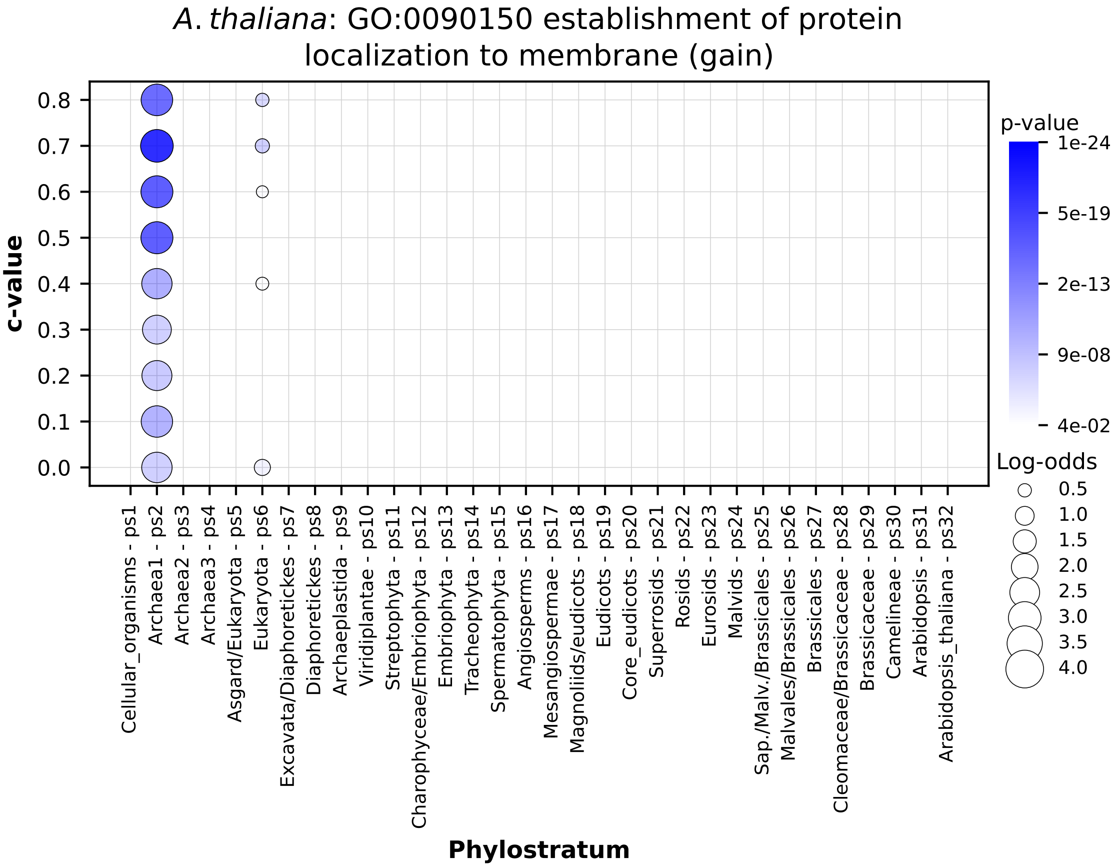

A. thaliana: GO:0090150 establishment of protein localization to membrane (gain)
c-value
0.8
0.7
0.6
0.5
0.4
0.3
0.2
0.1
0.0
Phylostratum
Cellular_organisms - ps1
Archaea1 - ps2
Archaea2 - ps3
Archaea3 - ps4
Asgard/Eukaryota - ps5
Eukaryota - ps6
Excavata/Diaphoretickes - ps7
Diaphoretickes - ps8
Archaeplastida - ps9
Viridiplantae - ps10
Streptophyta - ps11
Charophyceae/Embriophyta - ps12
Embriophyta - ps13
Tracheophyta - ps14
Spermatophyta - ps15
Angiosperms - ps16
Mesangiospermae - ps17
Magnoliids/eudicots - ps18
Eudicots - ps19
Core_eudicots - ps20
Superrosids - ps21
Rosids - ps22
Eurosids - ps23
Malvids - ps24
Sap./Malv./Brassicales - ps25
Malvales/Brassicales - ps26
Brassicales - ps27
Cleomaceae/Brassicaceae - ps28
Brassicaceae - ps29
Camelineae - ps30
Arabidopsis - ps31
Arabidopsis_thaliana - ps32
p-value
1e-24
5e-19
2e-13
9e-08
4e-02
Log-odds
0.5
1.0
1.5
2.0
2.5
3.0
3.5
4.0

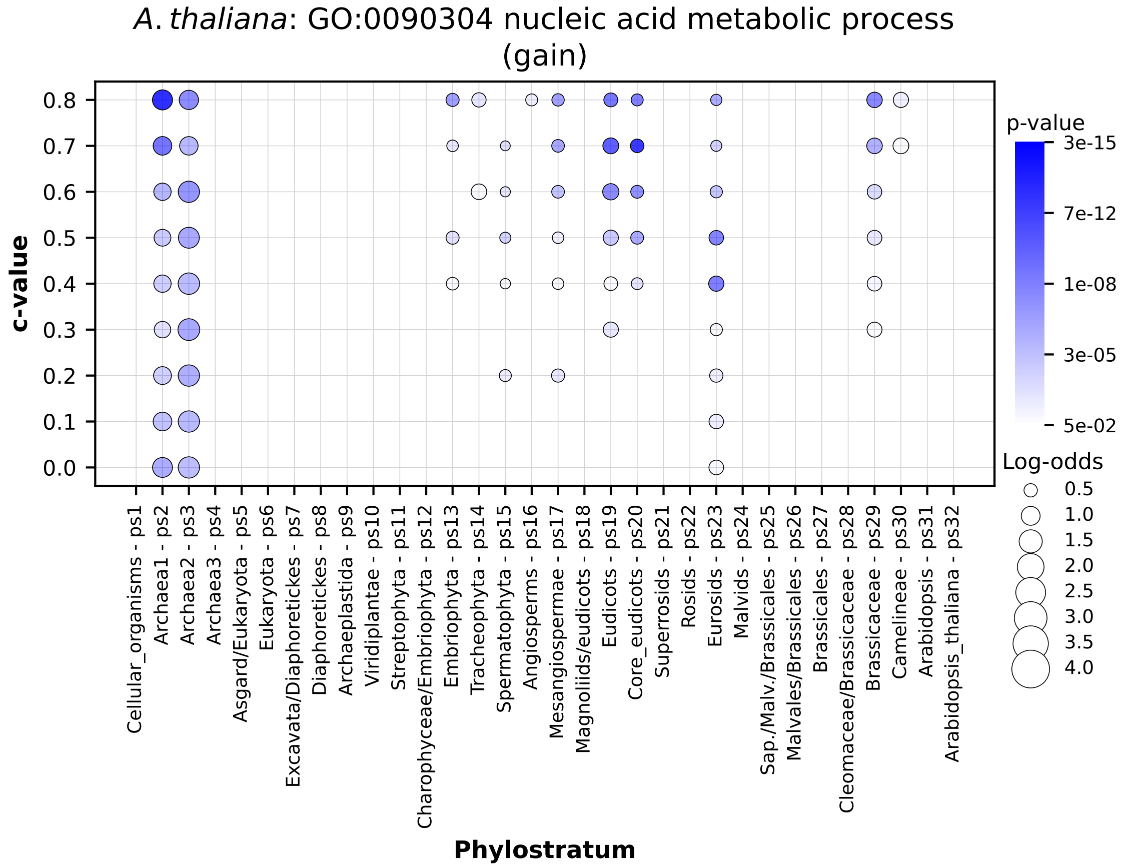

A. thaliana: GO:0090304 nucleic acid metabolic process (gain)
c-value
0.8
0.7
0.6
0.5
0.4
0.3
0.2
0.1
0.0
p-value
3e-15
7e-12
1e-08
3e-05
5e-02
Log-odds
0.5
1.0
1.5
2.0
2.5
3.0
3.5
4.0
Cellular_organisms - ps1
Archaea1 - ps2
Archaea2 - ps3
Archaea3 - ps4
Asgard/Eukaryota - ps5
Eukaryota - ps6
Excavata/Diaphoretickes - ps7
Diaphoretickes - ps8
Archaeplastida - ps9
Viridiplantae - ps10
Streptophyta - ps11
Charophyceae/Embriophyta - ps12
Embriophyta - ps13
Tracheophyta - ps14
Spermatophyta - ps15
Angiosperms - ps16
Mesangiospermae - ps17
Magnoliids/eudicots - ps18
Eudicots - ps19
Core_eudicots - ps20
Superrosids - ps21
Rosids - ps22
Eurosids - ps23
Malvids - ps24
Sap./Malv./Brassicales - ps25
Malvales/Brassicales - ps26
Brassicales - ps27
Cleomaceae/Brassicaceae - ps28
Brassicaceae - ps29
Camelineae - ps30
Arabidopsis - ps31
Arabidopsis_thaliana - ps32
Phylostratum

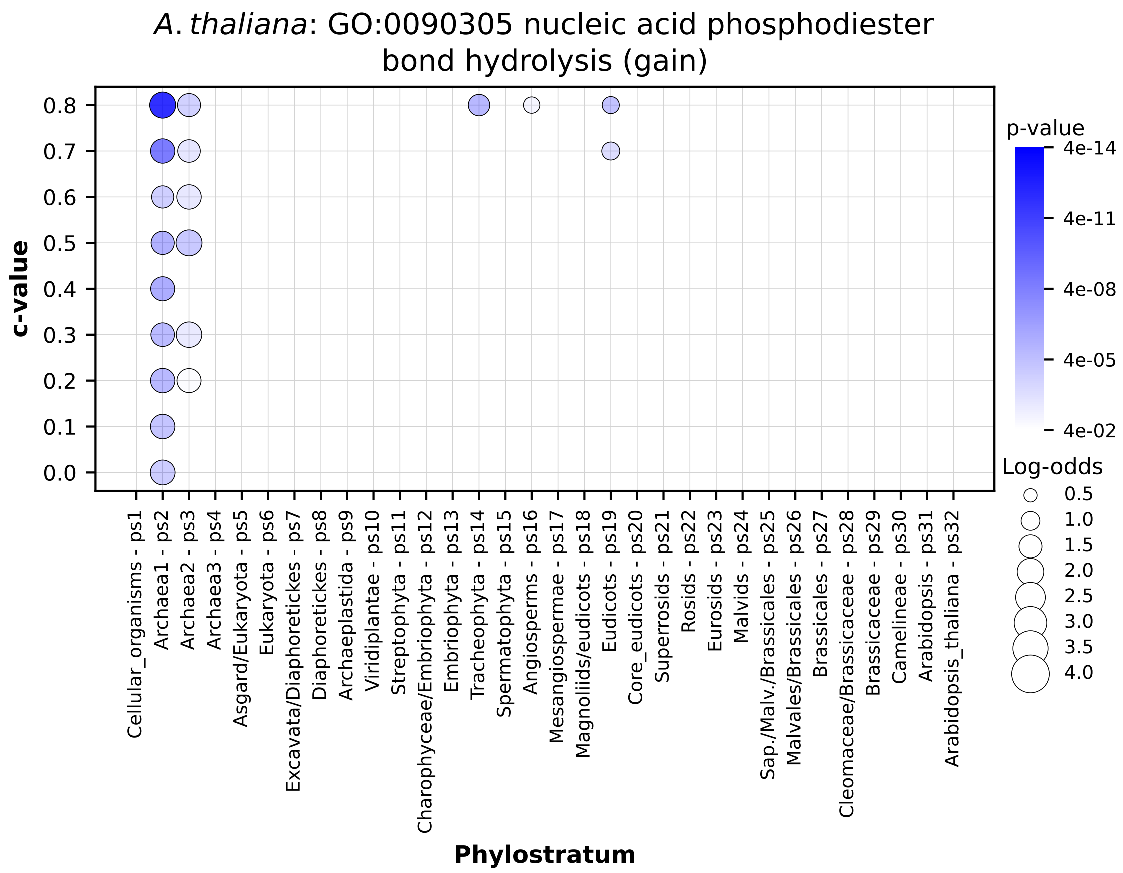
A. thaliana: GO:0090305 nucleic acid phosphodiester bond hydrolysis (gain)
c-value
0.8
0.7
0.6
0.5
0.4
0.3
0.2
0.1
0.0
p-value
4e-14
4e-11
4e-08
4e-05
4e-02
Log-odds
0.5
1.0
1.5
2.0
2.5
3.0
3.5
4.0
Cellular_organisms - ps1
Archaea1 - ps2
Archaea2 - ps3
Archaea3 - ps4
Asgard/Eukaryota - ps5
Eukaryota - ps6
Excavata/Diaphoretickes - ps7
Diaphoretickes - ps8
Archaeplastida - ps9
Viridiplantae - ps10
Streptophyta - ps11
Charophyceae/Embriophyta - ps12
Embriophyta - ps13
Tracheophyta - ps14
Spermatophyta - ps15
Angiosperms - ps16
Mesangiospermae - ps17
Magnoliids/eudicots - ps18
Eudicots - ps19
Core_eudicots - ps20
Superrosids - ps21
Rosids - ps22
Eurosids - ps23
Malvids - ps24
Sap./Malv./Brassicales - ps25
Malvales/Brassicales - ps26
Brassicales - ps27
Cleomaceae/Brassicaceae - ps28
Brassicaceae - ps29
Camelineae - ps30
Arabidopsis - ps31
Arabidopsis_thaliana - ps32
Phylostratum

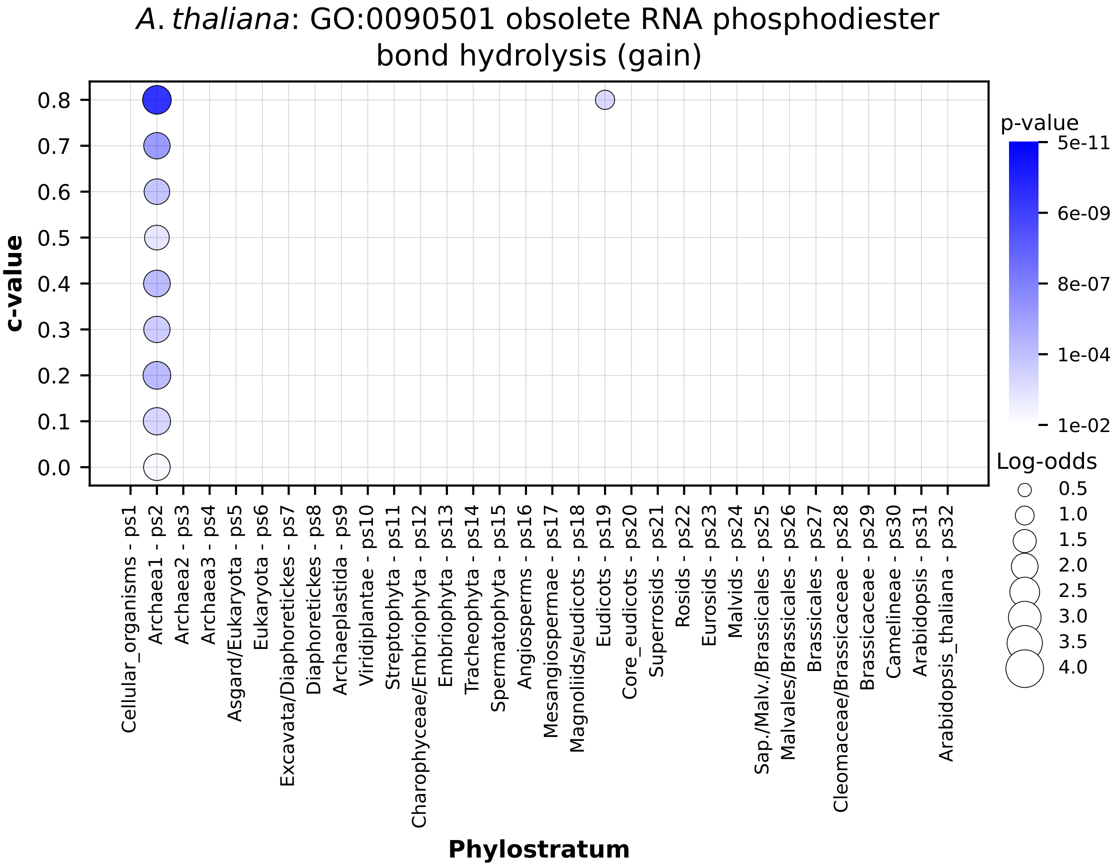

A. thaliana: GO:0090501 obsolete RNA phosphodiester bond hydrolysis (gain)
c-value
0.8
0.7
0.6
0.5
0.4
0.3
0.2
0.1
0.0
p-value
5e-11
6e-09
8e-07
1e-04
1e-02
Log-odds
0.5
1.0
1.5
2.0
2.5
3.0
3.5
4.0
Cellular_organisms - ps1
Archaea1 - ps2
Archaea2 - ps3
Archaea3 - ps4
Asgard/Eukaryota - ps5
Eukaryota - ps6
Excavata/Diaphoretickes - ps7
Diaphoretickes - ps8
Archaeplastida - ps9
Viridiplantae - ps10
Streptophyta - ps11
Charophyceae/Embriophyta - ps12
Embriophyta - ps13
Tracheophyta - ps14
Spermatophyta - ps15
Angiosperms - ps16
Mesangiospermae - ps17
Magnoliids/eudicots - ps18
Eudicots - ps19
Core_eudicots - ps20
Superrosids - ps21
Rosids - ps22
Eurosids - ps23
Malvids - ps24
Sap./Malv./Brassicales - ps25
Malvales/Brassicales - ps26
Brassicales - ps27
Cleomaceae/Brassicaceae - ps28
Brassicaceae - ps29
Camelineae - ps30
Arabidopsis - ps31
Arabidopsis_thaliana - ps32
Phylostratum

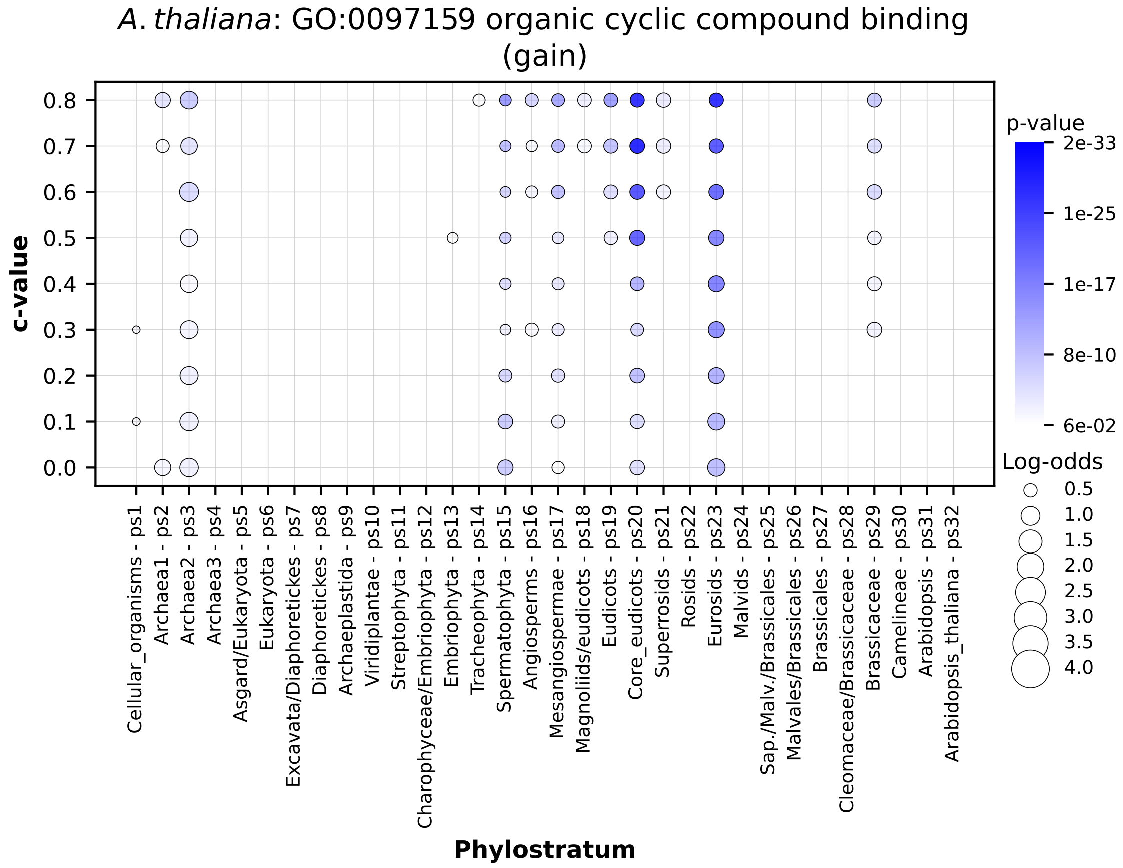

A. thaliana: GO:0097159 organic cyclic compound binding (gain)
c-value
0.8
0.7
0.6
0.5
0.4
0.3
0.2
0.1
0.0
p-value
2e-33
1e-25
1e-17
8e-10
6e-02
Log-odds
0.5
1.0
1.5
2.0
2.5
3.0
3.5
4.0
Cellular_organisms - ps1
Archaea1 - ps2
Archaea2 - ps3
Archaea3 - ps4
Asgard/Eukaryota - ps5
Eukaryota - ps6
Excavata/Diaphoretickes - ps7
Diaphoretickes - ps8
Archaeplastida - ps9
Viridiplantae - ps10
Streptophyta - ps11
Charophyceae/Embriophyta - ps12
Embriophyta - ps13
Tracheophyta - ps14
Spermatophyta - ps15
Angiosperms - ps16
Mesangiospermae - ps17
Magnoliids/eudicots - ps18
Eudicots - ps19
Core_eudicots - ps20
Superrosids - ps21
Rosids - ps22
Eurosids - ps23
Malvids - ps24
Sap./Malv./Brassicales - ps25
Malvales/Brassicales - ps26
Brassicales - ps27
Cleomaceae/Brassicaceae - ps28
Brassicaceae - ps29
Camelineae - ps30
Arabidopsis - ps31
Arabidopsis_thaliana - ps32
Phylostratum

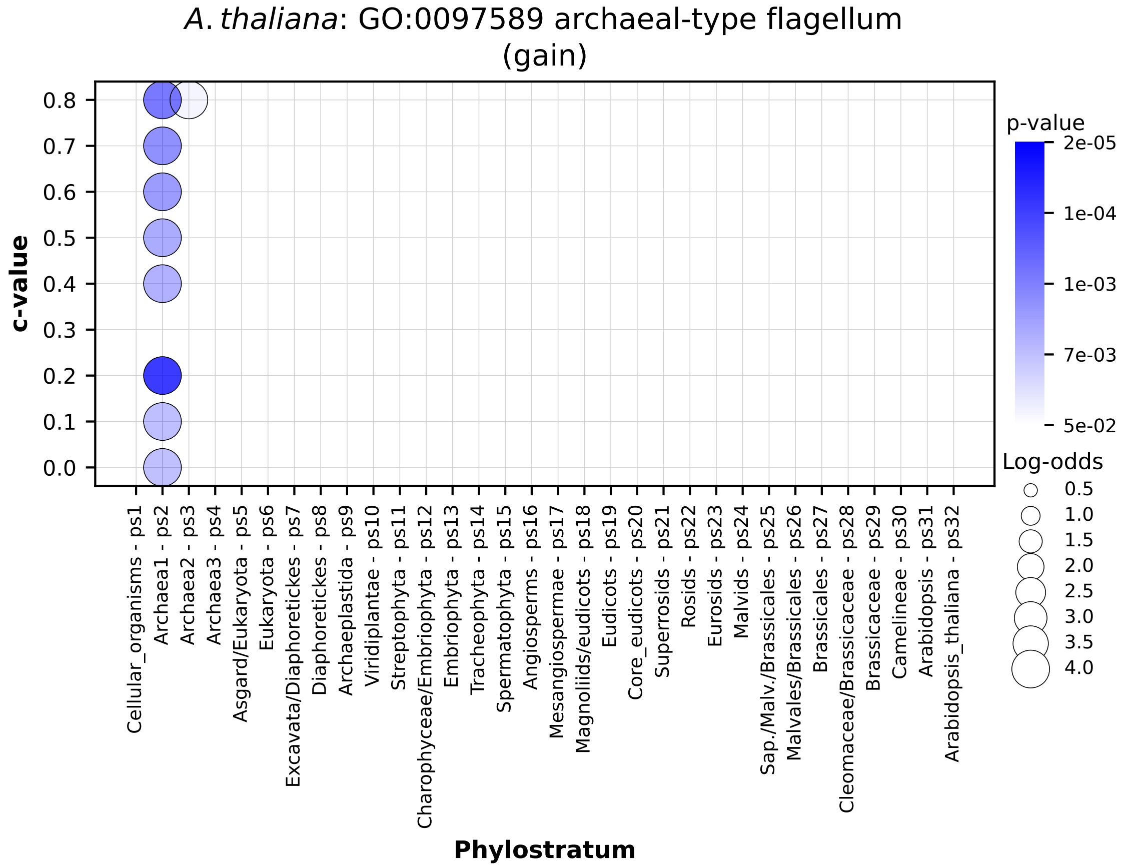
A. thaliana: GO:0097589 archaeal-type flagellum
(gain)
c-value
0.8
0.7
0.6
0.5
0.4
0.3
0.2
0.1
0.0
p-value
2e-05
1e-04
1e-03
7e-03
5e-02
Log-odds
0.5
1.0
1.5
2.0
2.5
3.0
3.5
4.0
Cellular_organisms - ps1
Archaea1 - ps2
Archaea2 - ps3
Archaea3 - ps4
Asgard/Eukaryota - ps5
Eukaryota - ps6
Excavata/Diaphoretickes - ps7
Diaphoretickes - ps8
Archaeplastida - ps9
Viridiplantae - ps10
Streptophyta - ps11
Charophyceae/Embriophyta - ps12
Embriophyta - ps13
Tracheophyta - ps14
Spermatophyta - ps15
Angiosperms - ps16
Mesangiospermae - ps17
Magnoliids/eudicots - ps18
Eudicots - ps19
Core_eudicots - ps20
Superrosids - ps21
Rosids - ps22
Eurosids - ps23
Malvids - ps24
Sap./Malv./Brassicales - ps25
Malvales/Brassicales - ps26
Brassicales - ps27
Cleomaceae/Brassicaceae - ps28
Brassicaceae - ps29
Camelineae - ps30
Arabidopsis - ps31
Arabidopsis_thaliana - ps32
Phylostratum

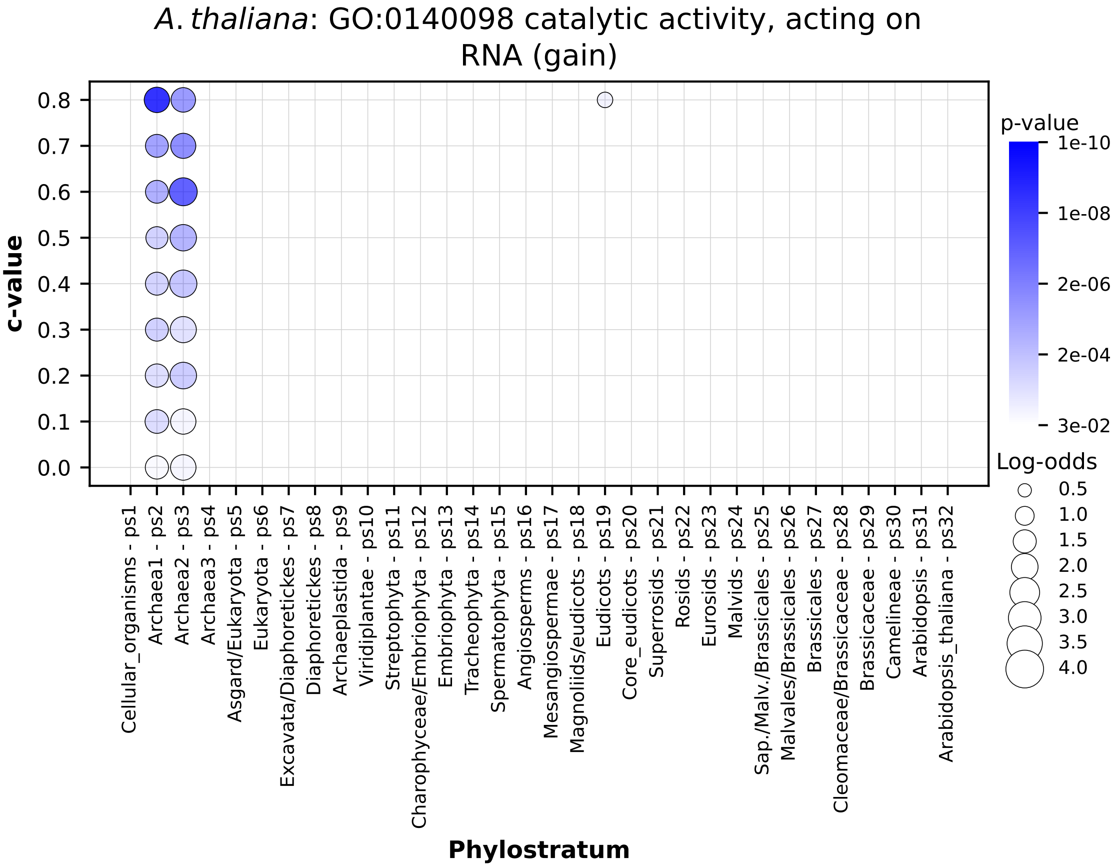

A. thaliana: GO:0140098 catalytic activity, acting on RNA (gain)
c-value
0.8
0.7
0.6
0.5
0.4
0.3
0.2
0.1
0.0
p-value
1e-10
1e-08
2e-06
2e-04
3e-02
Log-odds
0.5
1.0
1.5
2.0
2.5
3.0
3.5
4.0
Cellular_organisms - ps1
Archaea1 - ps2
Archaea2 - ps3
Archaea3 - ps4
Asgard/Eukaryota - ps5
Eukaryota - ps6
Excavata/Diaphoretickes - ps7
Diaphoretickes - ps8
Archaeplastida - ps9
Viridiplantae - ps10
Streptophyta - ps11
Charophyceae/Embriophyta - ps12
Embriophyta - ps13
Tracheophyta - ps14
Spermatophyta - ps15
Angiosperms - ps16
Mesangiospermae - ps17
Magnoliids/eudicots - ps18
Eudicots - ps19
Core_eudicots - ps20
Superrosids - ps21
Rosids - ps22
Eurosids - ps23
Malvids - ps24
Sap./Malv./Brassicales - ps25
Malvales/Brassicales - ps26
Brassicales - ps27
Cleomaceae/Brassicaceae - ps28
Brassicaceae - ps29
Camelineae - ps30
Arabidopsis - ps31
Arabidopsis_thaliana - ps32
Phylostratum

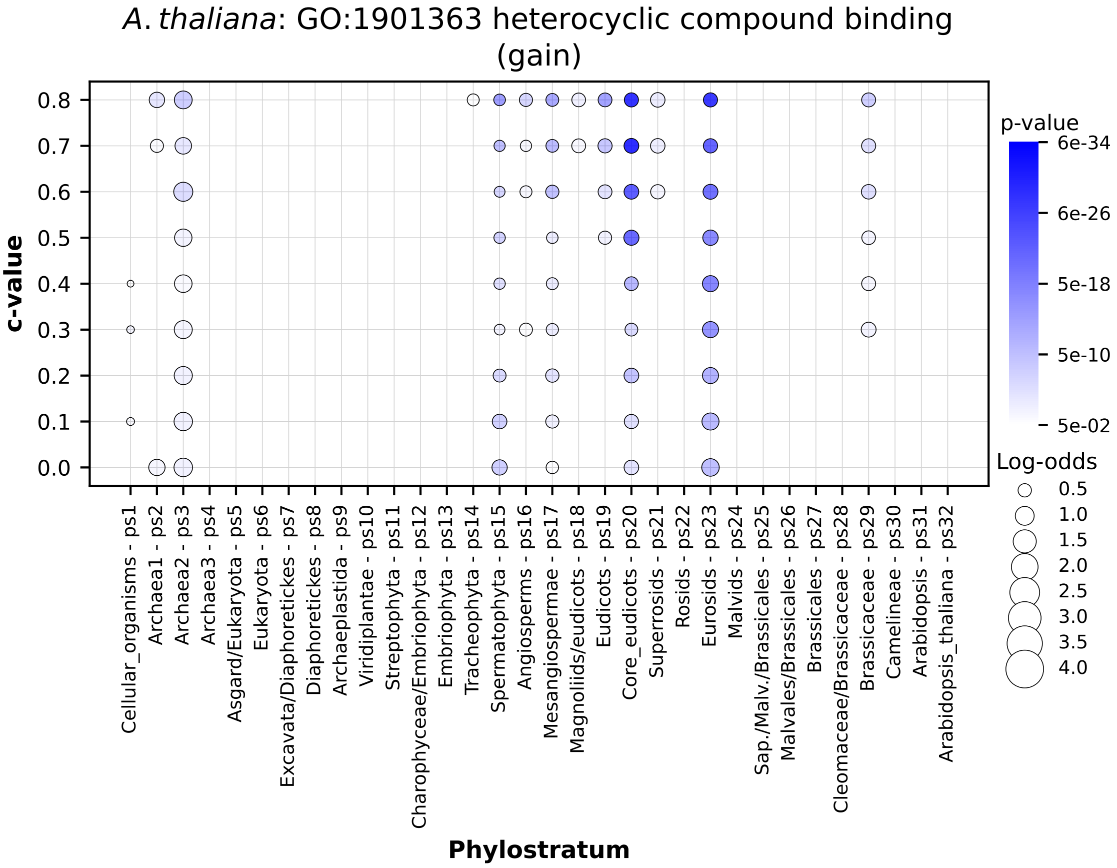
A. thaliana: GO:1901363 heterocyclic compound binding
(gain)
c-value
0.8
0.7
0.6
0.5
0.4
0.3
0.2
0.1
0.0
p-value
6e-34
6e-26
5e-18
5e-10
5e-02
Log-odds
0.5
1.0
1.5
2.0
2.5
3.0
3.5
4.0
Cellular_organisms - ps1
Archaea1 - ps2
Archaea2 - ps3
Archaea3 - ps4
Asgard/Eukaryota - ps5
Eukaryota - ps6
Excavata/Diaphoretickes - ps7
Diaphoretickes - ps8
Archaeplastida - ps9
Viridiplantae - ps10
Streptophyta - ps11
Charophyceae/Embriophyta - ps12
Embriophyta - ps13
Tracheophyta - ps14
Spermatophyta - ps15
Angiosperms - ps16
Mesangiospermae - ps17
Magnoliids/eudicots - ps18
Eudicots - ps19
Core_eudicots - ps20
Superrosids - ps21
Rosids - ps22
Eurosids - ps23
Malvids - ps24
Sap./Malv./Brassicales - ps25
Malvales/Brassicales - ps26
Brassicales - ps27
Cleomaceae/Brassicaceae - ps28
Brassicaceae - ps29
Camelineae - ps30
Arabidopsis - ps31
Arabidopsis_thaliana - ps32
Phylostratum

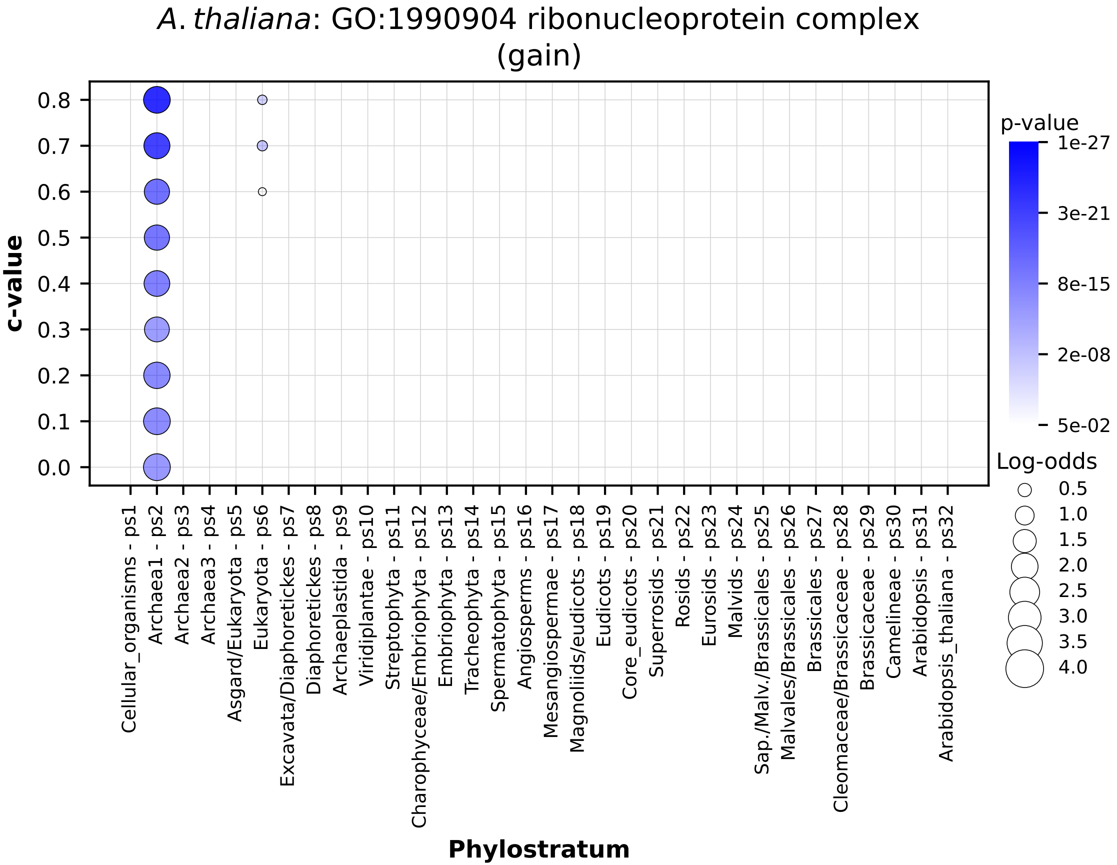

A. thaliana: GO:1990904 ribonucleoprotein complex (gain)
c-value
0.8
0.7
0.6
0.5
0.4
0.3
0.2
0.1
0.0
p-value
1e-27
3e-21
8e-15
2e-08
5e-02
Log-odds
0.5
1.0
1.5
2.0
2.5
3.0
3.5
4.0
Cellular_organisms - ps1
Archaea1 - ps2
Archaea2 - ps3
Archaea3 - ps4
Asgard/Eukaryota - ps5
Eukaryota - ps6
Excavata/Diaphoretickes - ps7
Diaphoretickes - ps8
Archaeplastida - ps9
Viridiplantae - ps10
Streptophyta - ps11
Charophyceae/Embriophyta - ps12
Embriophyta - ps13
Tracheophyta - ps14
Spermatophyta - ps15
Angiosperms - ps16
Mesangiospermae - ps17
Magnoliids/eudicots - ps18
Eudicots - ps19
Core_eudicots - ps20
Superrosids - ps21
Rosids - ps22
Eurosids - ps23
Malvids - ps24
Sap./Malv./Brassicales - ps25
Malvales/Brassicales - ps26
Brassicales - ps27
Cleomaceae/Brassicaceae - ps28
Brassicaceae - ps29
Camelineae - ps30
Arabidopsis - ps31
Arabidopsis_thaliana - ps32
Phylostratum

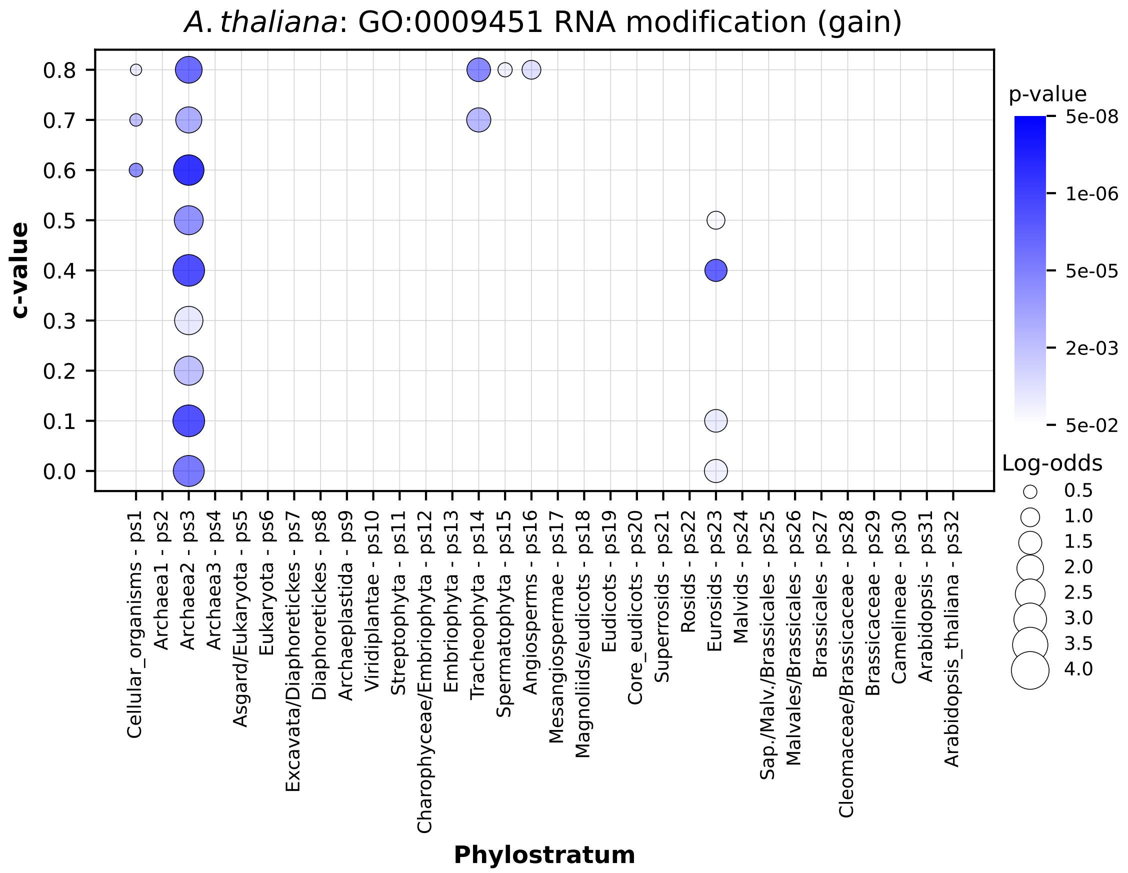

A. thaliana: GO:0009451 RNA modification (gain)
c-value
0.8
0.7
0.6
0.5
0.4
0.3
0.2
0.1
0.0
Phylostratum
Cellular_organisms - ps1
Archaea1 - ps2
Archaea2 - ps3
Archaea3 - ps4
Asgard/Eukaryota - ps5
Eukaryota - ps6
Excavata/Diaphoretickes - ps7
Diaphoretickes - ps8
Archaeplastida - ps9
Viridiplantae - ps10
Streptophyta - ps11
Charophyceae/Embriophyta - ps12
Embriophyta - ps13
Tracheophyta - ps14
Spermatophyta - ps15
Angiosperms - ps16
Mesangiospermae - ps17
Magnoliids/eudicots - ps18
Eudicots - ps19
Core_eudicots - ps20
Superrosids - ps21
Rosids - ps22
Eurosids - ps23
Malvids - ps24
Sap./Malv./Brassicales - ps25
Malvales/Brassicales - ps26
Brassicales - ps27
Cleomaceae/Brassicaceae - ps28
Brassicaceae - ps29
Camelineae - ps30
Arabidopsis - ps31
Arabidopsis_thaliana - ps32
p-value
5e-08
1e-06
5e-05
2e-03
5e-02
Log-odds
0.5
1.0
1.5
2.0
2.5
3.0
3.5
4.0

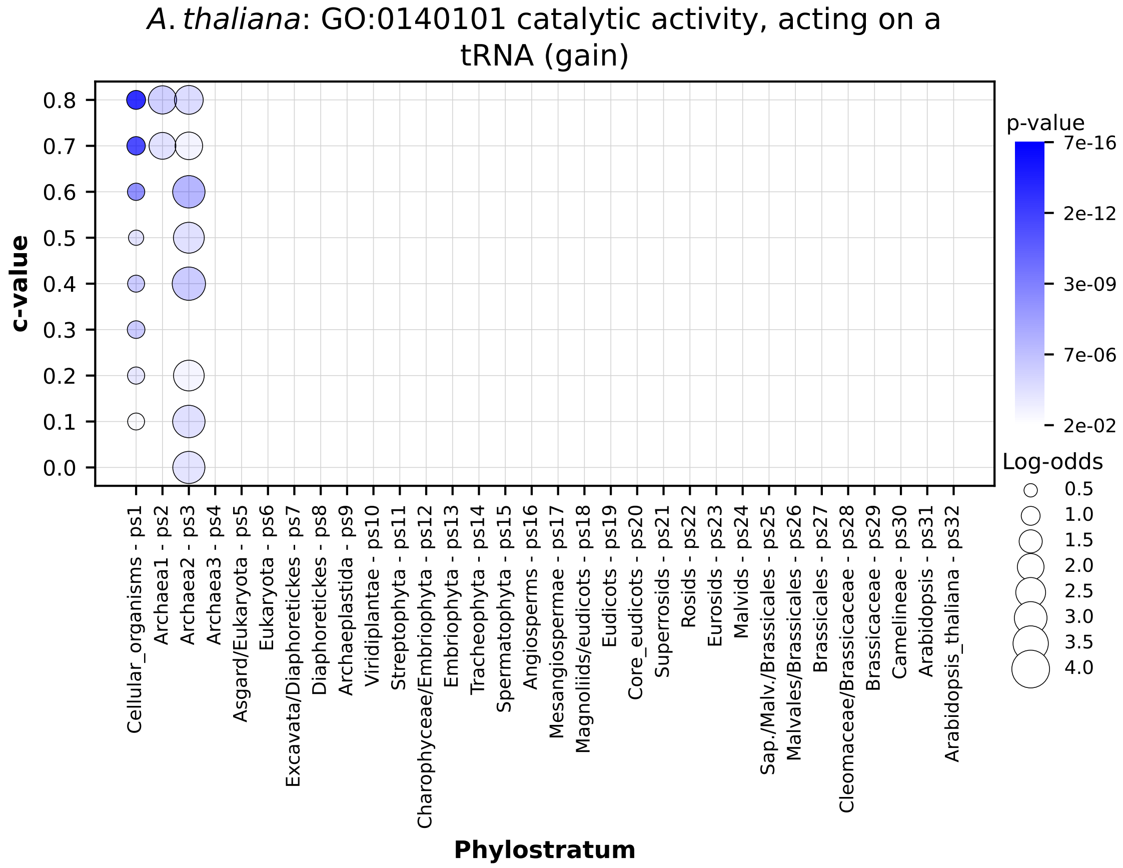

A. thaliana: GO:0140101 catalytic activity, acting on a tRNA (gain)
c-value
0.8
0.7
0.6
0.5
0.4
0.3
0.2
0.1
0.0
p-value
7e-16
2e-12
3e-09
7e-06
2e-02
Log-odds
0.5
1.0
1.5
2.0
2.5
3.0
3.5
4.0
Cellular_organisms - ps1
Archaea1 - ps2
Archaea2 - ps3
Archaea3 - ps4
Asgard/Eukaryota - ps5
Eukaryota - ps6
Excavata/Diaphoretickes - ps7
Diaphoretickes - ps8
Archaeplastida - ps9
Viridiplantae - ps10
Streptophyta - ps11
Charophyceae/Embriophyta - ps12
Embriophyta - ps13
Tracheophyta - ps14
Spermatophyta - ps15
Angiosperms - ps16
Mesangiospermae - ps17
Magnoliids/eudicots - ps18
Eudicots - ps19
Core_eudicots - ps20
Superrosids - ps21
Rosids - ps22
Eurosids - ps23
Malvids - ps24
Sap./Malv./Brassicales - ps25
Malvales/Brassicales - ps26
Brassicales - ps27
Cleomaceae/Brassicaceae - ps28
Brassicaceae - ps29
Camelineae - ps30
Arabidopsis - ps31
Arabidopsis_thaliana - ps32
Phylostratum

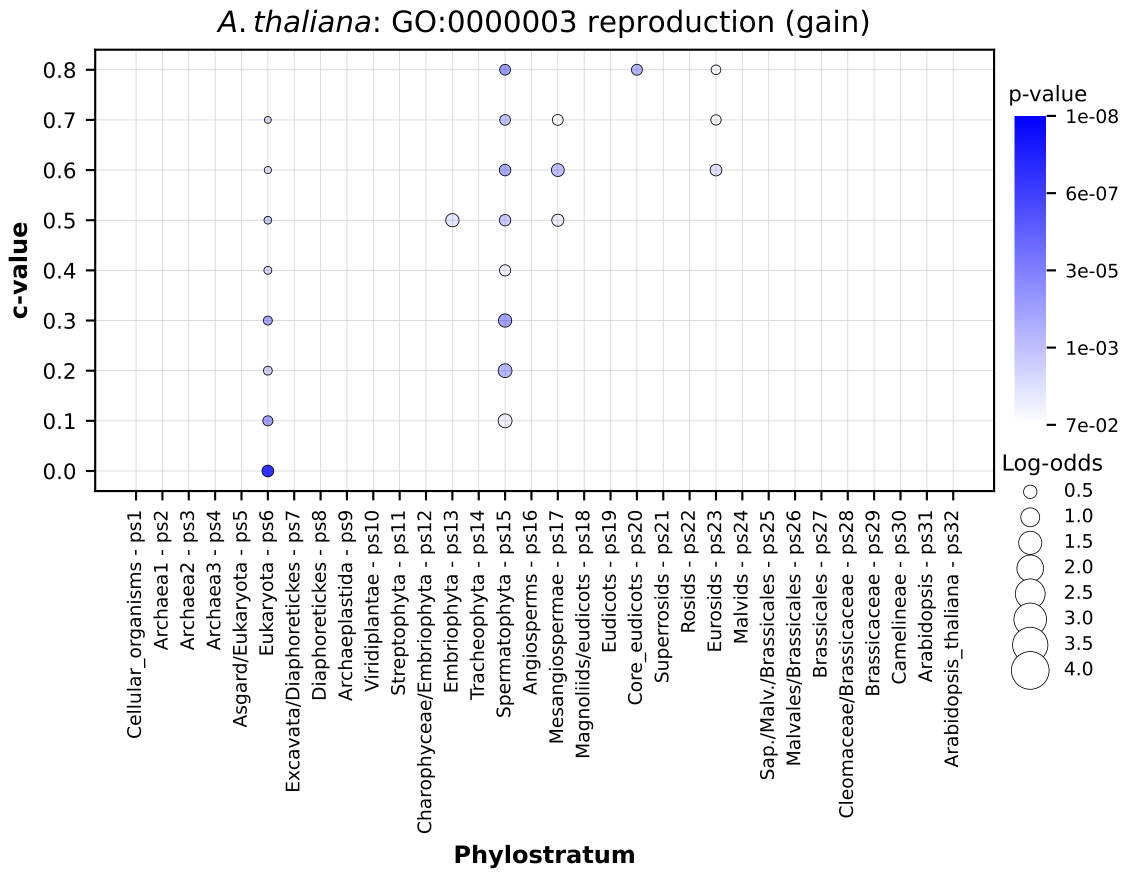

A. thaliana: GO:0000003 reproduction (gain)
c-value
Phylostratum
p-value
1e-08
6e-07
3e-05
1e-03
7e-02
Log-odds
0.5
1.0
1.5
2.0
2.5
3.0
3.5
4.0
0.0
0.1
0.2
0.3
0.4
0.5
0.6
0.7
0.8
Cellular_organisms - ps1
Archaea1 - ps2
Archaea2 - ps3
Archaea3 - ps4
Asgard/Eukaryota - ps5
Eukaryota - ps6
Excavata/Diaphoretickes - ps7
Diaphoretickes - ps8
Archaeplastida - ps9
Viridiplantae - ps10
Streptophyta - ps11
Charophyceae/Embriophyta - ps12
Embriophyta - ps13
Tracheophyta - ps14
Spermatophyta - ps15
Angiosperms - ps16
Mesangiospermae - ps17
Magnoliids/eudicots - ps18
Eudicots - ps19
Core_eudicots - ps20
Superrosids - ps21
Rosids - ps22
Eurosids - ps23
Malvids - ps24
Sap./Malv./Brassicales - ps25
Malvales/Brassicales - ps26
Brassicales - ps27
Cleomaceae/Brassicaceae - ps28
Brassicaceae - ps29
Camelineae - ps30
Arabidopsis - ps31
Arabidopsis_thaliana - ps32

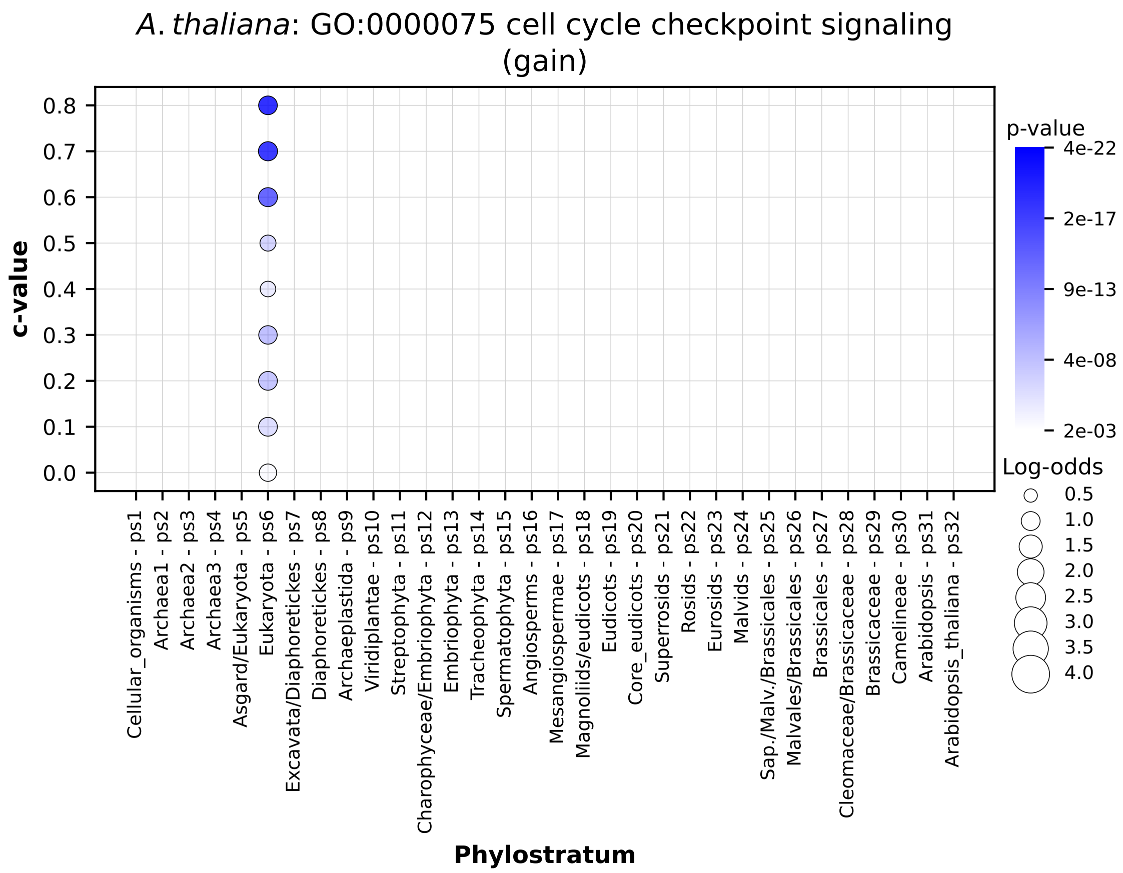
A. thaliana: GO:0000075 cell cycle checkpoint signaling
(gain)
c-value
0.8
0.7
0.6
0.5
0.4
0.3
0.2
0.1
0.0
p-value
4e-22
2e-17
9e-13
4e-08
2e-03
Log-odds
0.5
1.0
1.5
2.0
2.5
3.0
3.5
4.0
Cellular_organisms - ps1
Archaea1 - ps2
Archaea2 - ps3
Archaea3 - ps4
Asgard/Eukaryota - ps5
Eukaryota - ps6
Excavata/Diaphoretickes - ps7
Diaphoretickes - ps8
Archaeplastida - ps9
Viridiplantae - ps10
Streptophyta - ps11
Charophyceae/Embriophyta - ps12
Embriophyta - ps13
Tracheophyta - ps14
Spermatophyta - ps15
Angiosperms - ps16
Mesangiospermae - ps17
Magnoliids/eudicots - ps18
Eudicots - ps19
Core_eudicots - ps20
Superrosids - ps21
Rosids - ps22
Eurosids - ps23
Malvids - ps24
Sap./Malv./Brassicales - ps25
Malvales/Brassicales - ps26
Brassicales - ps27
Cleomaceae/Brassicaceae - ps28
Brassicaceae - ps29
Camelineae - ps30
Arabidopsis - ps31
Arabidopsis_thaliana - ps32
Phylostratum

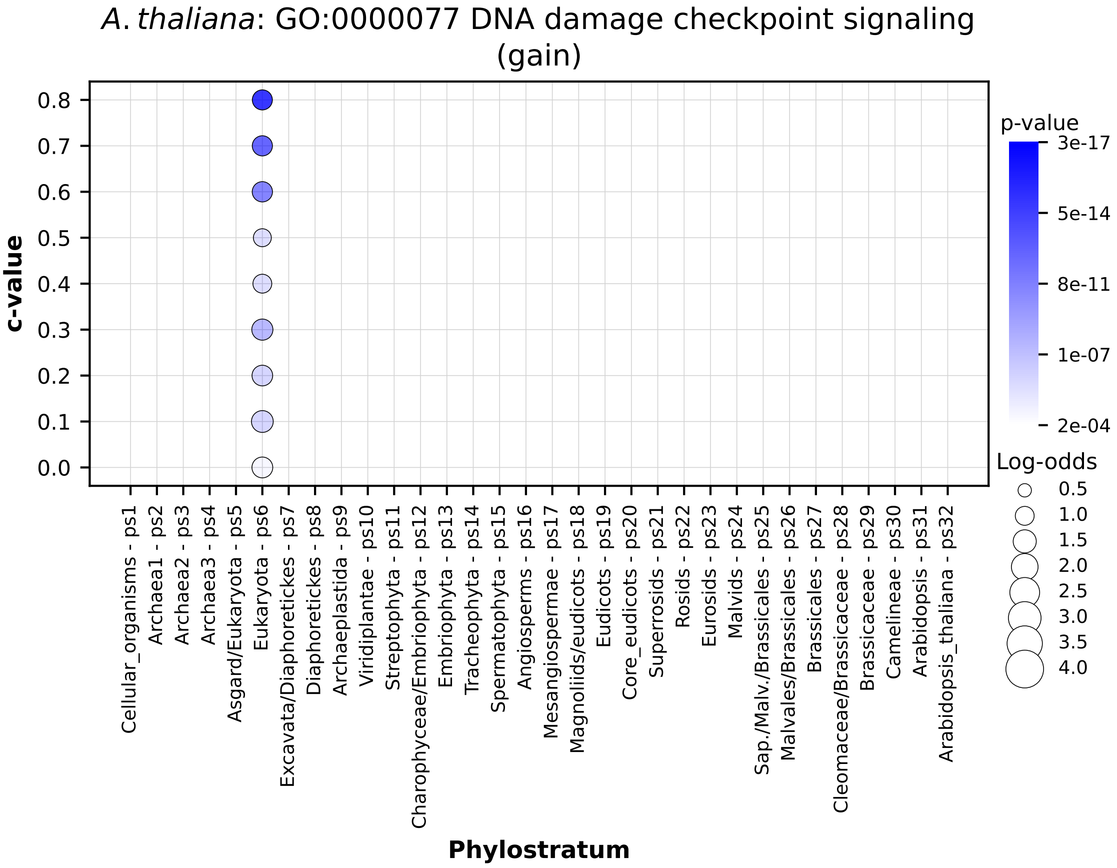

A. thaliana: GO:0000077 DNA damage checkpoint signaling (gain)
c-value
0.0
0.1
0.2
0.3
0.4
0.5
0.6
0.7
0.8
p-value
3e-17
5e-14
8e-11
1e-07
2e-04
Log-odds
0.5
1.0
1.5
2.0
2.5
3.0
3.5
4.0
Cellular_organisms - ps1
Archaea1 - ps2
Archaea2 - ps3
Archaea3 - ps4
Asgard/Eukaryota - ps5
Eukaryota - ps6
Excavata/Diaphoretickes - ps7
Diaphoretickes - ps8
Archaeplastida - ps9
Viridiplantae - ps10
Streptophyta - ps11
Charophyceae/Embriophyta - ps12
Embriophyta - ps13
Tracheophyta - ps14
Spermatophyta - ps15
Angiosperms - ps16
Mesangiospermae - ps17
Magnoliids/eudicots - ps18
Eudicots - ps19
Core_eudicots - ps20
Superrosids - ps21
Rosids - ps22
Eurosids - ps23
Malvids - ps24
Sap./Malv./Brassicales - ps25
Malvales/Brassicales - ps26
Brassicales - ps27
Cleomaceae/Brassicaceae - ps28
Brassicaceae - ps29
Camelineae - ps30
Arabidopsis - ps31
Arabidopsis_thaliana - ps32
Phylostratum

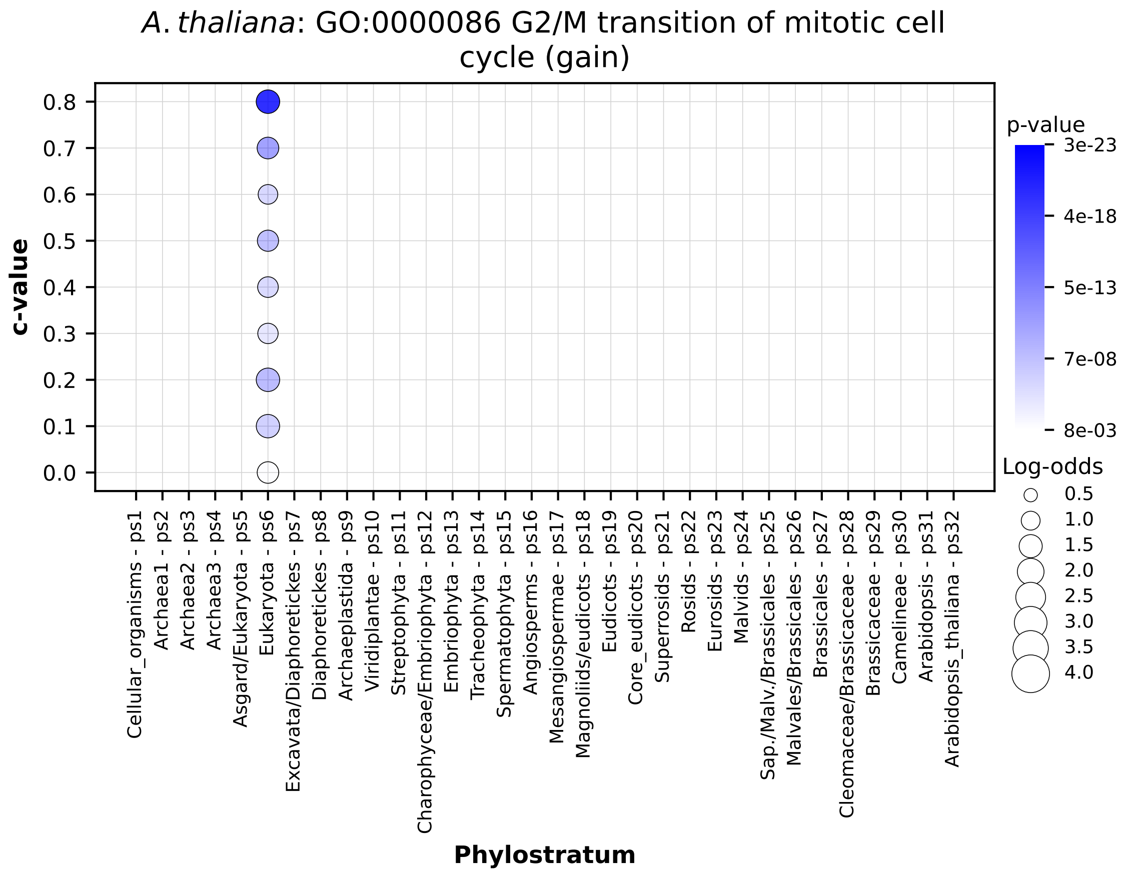

A. thaliana: GO:0000086 G2/M transition of mitotic cell cycle (gain)
c-value
0.8
0.7
0.6
0.5
0.4
0.3
0.2
0.1
0.0
p-value
3e-23
4e-18
5e-13
7e-08
8e-03
Log-odds
0.5
1.0
1.5
2.0
2.5
3.0
3.5
4.0
Cellular_organisms - ps1
Archaea1 - ps2
Archaea2 - ps3
Archaea3 - ps4
Asgard/Eukaryota - ps5
Eukaryota - ps6
Excavata/Diaphoretickes - ps7
Diaphoretickes - ps8
Archaeplastida - ps9
Viridiplantae - ps10
Streptophyta - ps11
Charophyceae/Embriophyta - ps12
Embriophyta - ps13
Tracheophyta - ps14
Spermatophyta - ps15
Angiosperms - ps16
Mesangiospermae - ps17
Magnoliids/eudicots - ps18
Eudicots - ps19
Core_eudicots - ps20
Superrosids - ps21
Rosids - ps22
Eurosids - ps23
Malvids - ps24
Sap./Malv./Brassicales - ps25
Malvales/Brassicales - ps26
Brassicales - ps27
Cleomaceae/Brassicaceae - ps28
Brassicaceae - ps29
Camelineae - ps30
Arabidopsis - ps31
Arabidopsis_thaliana - ps32
Phylostratum

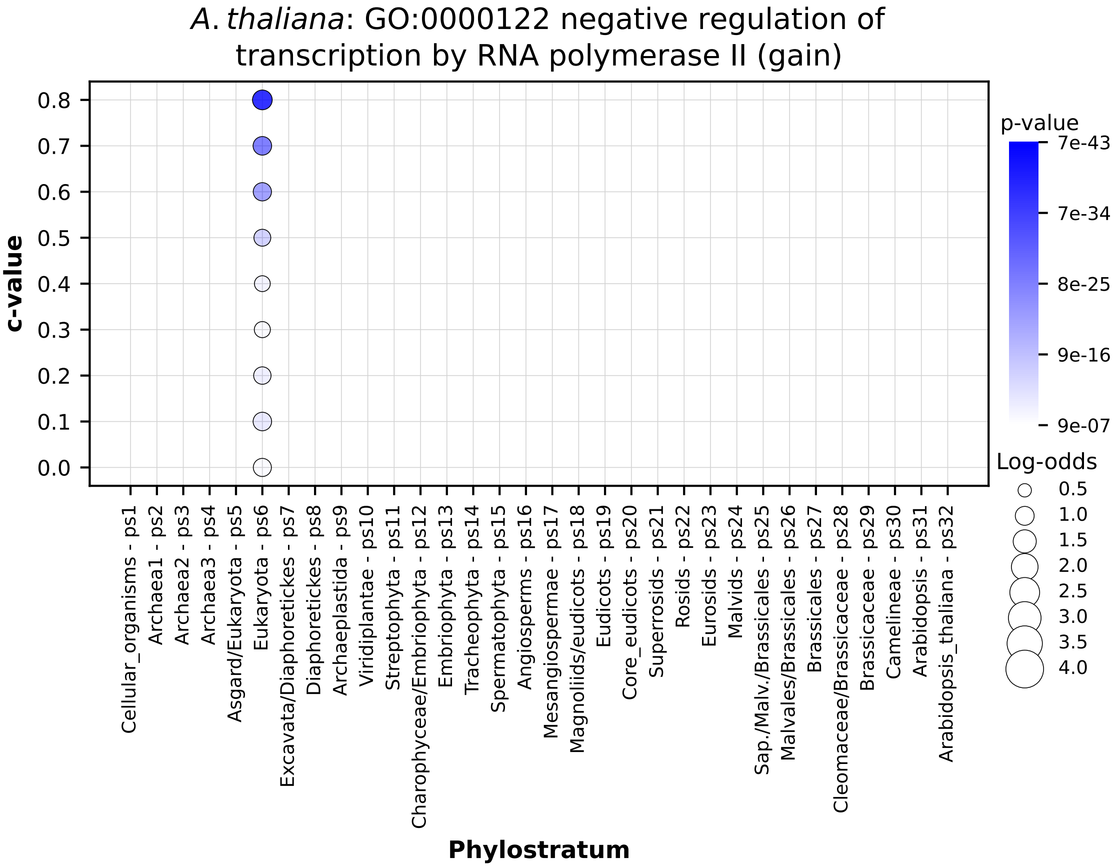
A. thaliana: GO:0000122 negative regulation of
transcription by RNA polymerase II (gain)
c-value
0.8
0.7
0.6
0.5
0.4
0.3
0.2
0.1
0.0
Phylostratum
Cellular_organisms - ps1
Archaea1 - ps2
Archaea2 - ps3
Archaea3 - ps4
Asgard/Eukaryota - ps5
Eukaryota - ps6
Excavata/Diaphoretickes - ps7
Diaphoretickes - ps8
Archaeplastida - ps9
Viridiplantae - ps10
Streptophyta - ps11
Charophyceae/Embriophyta - ps12
Embriophyta - ps13
Tracheophyta - ps14
Spermatophyta - ps15
Angiosperms - ps16
Mesangiospermae - ps17
Magnoliids/eudicots - ps18
Eudicots - ps19
Core_eudicots - ps20
Superrosids - ps21
Rosids - ps22
Eurosids - ps23
Malvids - ps24
Sap./Malv./Brassicales - ps25
Malvales/Brassicales - ps26
Brassicales - ps27
Cleomaceae/Brassicaceae - ps28
Brassicaceae - ps29
Camelineae - ps30
Arabidopsis - ps31
Arabidopsis_thaliana - ps32
p-value
7e-43
7e-34
8e-25
9e-16
9e-07
Log-odds
0.5
1.0
1.5
2.0
2.5
3.0
3.5
4.0

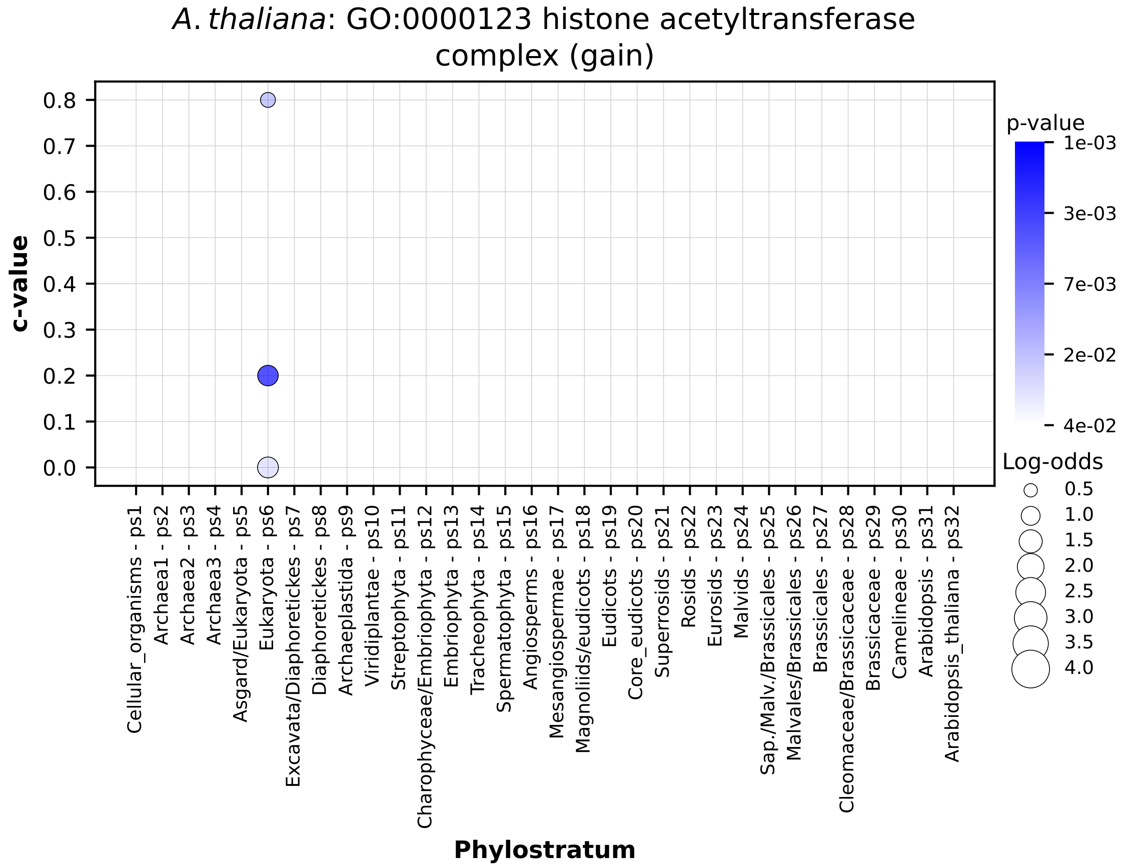

A. thaliana: GO:0000123 histone acetyltransferase complex (gain)
c-value
0.0
0.1
0.2
0.3
0.4
0.5
0.6
0.7
0.8
Phylostratum
Cellular_organisms - ps1
Archaea1 - ps2
Archaea2 - ps3
Archaea3 - ps4
Asgard/Eukaryota - ps5
Eukaryota - ps6
Excavata/Diaphoretickes - ps7
Diaphoretickes - ps8
Archaeplastida - ps9
Viridiplantae - ps10
Streptophyta - ps11
Charophyceae/Embriophyta - ps12
Embriophyta - ps13
Tracheophyta - ps14
Spermatophyta - ps15
Angiosperms - ps16
Mesangiospermae - ps17
Magnoliids/eudicots - ps18
Eudicots - ps19
Core_eudicots - ps20
Superrosids - ps21
Rosids - ps22
Eurosids - ps23
Malvids - ps24
Sap./Malv./Brassicales - ps25
Malvales/Brassicales - ps26
Brassicales - ps27
Cleomaceae/Brassicaceae - ps28
Brassicaceae - ps29
Camelineae - ps30
Arabidopsis - ps31
Arabidopsis_thaliana - ps32
p-value
1e-03
3e-03
7e-03
2e-02
4e-02
Log-odds
0.5
1.0
1.5
2.0
2.5
3.0
3.5
4.0

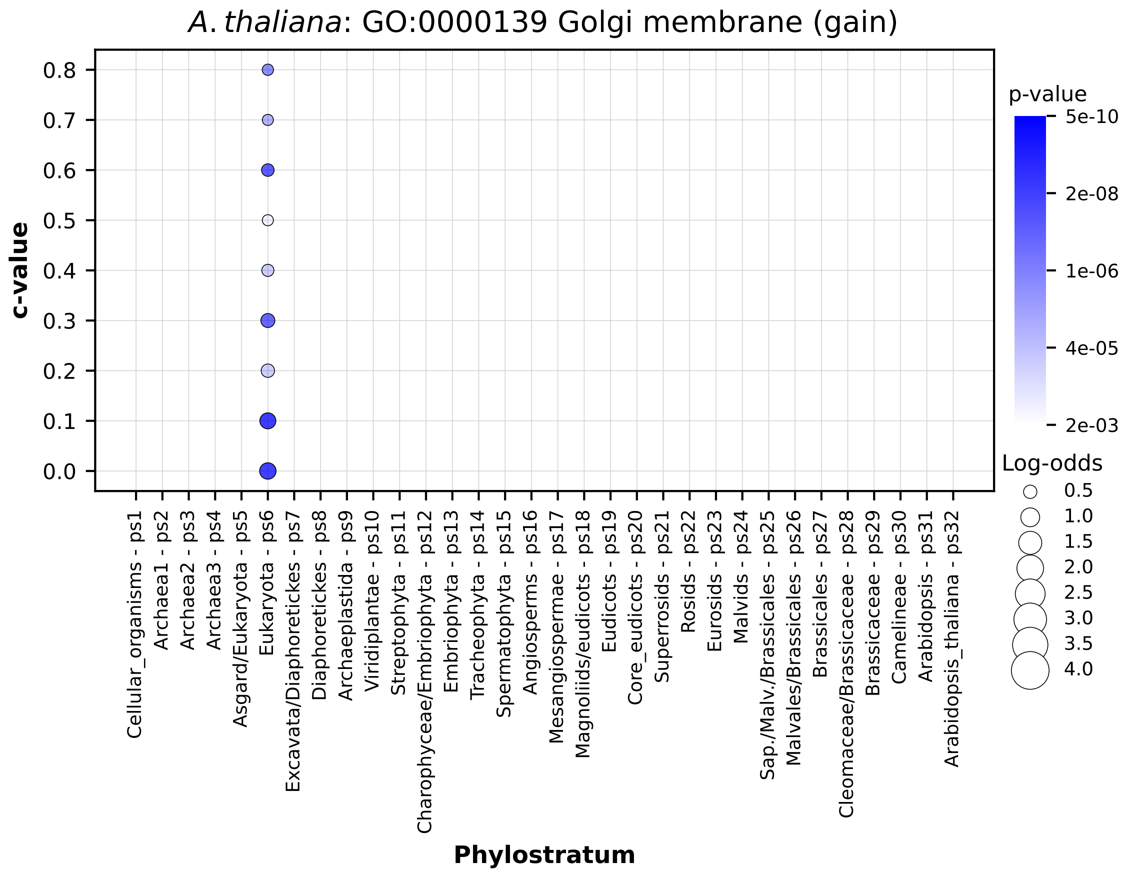
A. thaliana: GO:0000139 Golgi membrane (gain)
c-value
0.8
0.7
0.6
0.5
0.4
0.3
0.2
0.1
0.0
Phylostratum
Cellular_organisms - ps1
Archaea1 - ps2
Archaea2 - ps3
Archaea3 - ps4
Asgard/Eukaryota - ps5
Eukaryota - ps6
Excavata/Diaphoretickes - ps7
Diaphoretickes - ps8
Archaeplastida - ps9
Viridiplantae - ps10
Streptophyta - ps11
Charophyceae/Embriophyta - ps12
Embriophyta - ps13
Tracheophyta - ps14
Spermatophyta - ps15
Angiosperms - ps16
Mesangiospermae - ps17
Magnoliids/eudicots - ps18
Eudicots - ps19
Core_eudicots - ps20
Superrosids - ps21
Rosids - ps22
Eurosids - ps23
Malvids - ps24
Sap./Malv./Brassicales - ps25
Malvales/Brassicales - ps26
Brassicales - ps27
Cleomaceae/Brassicaceae - ps28
Brassicaceae - ps29
Camelineae - ps30
Arabidopsis - ps31
Arabidopsis_thaliana - ps32
p-value
5e-10
2e-08
1e-06
4e-05
2e-03
Log-odds
0.5
1.0
1.5
2.0
2.5
3.0
3.5
4.0

A. thaliana: GO:0000149 SNARE binding (gain)

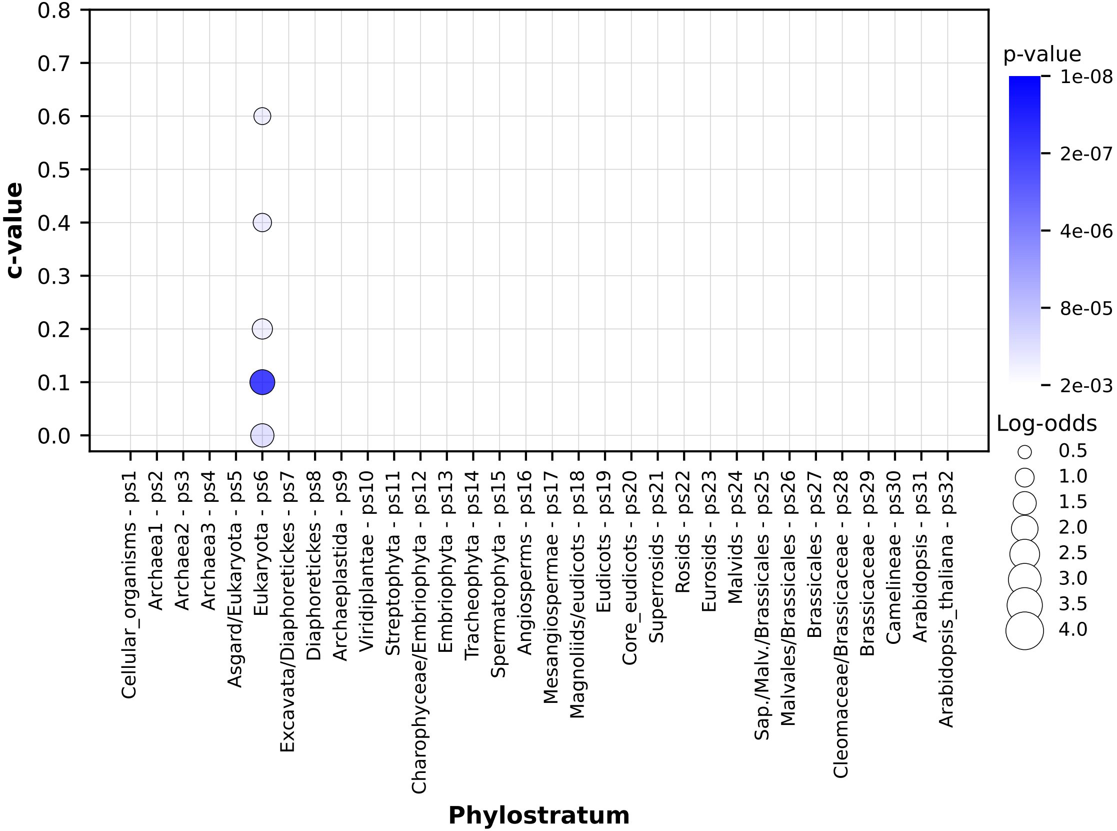

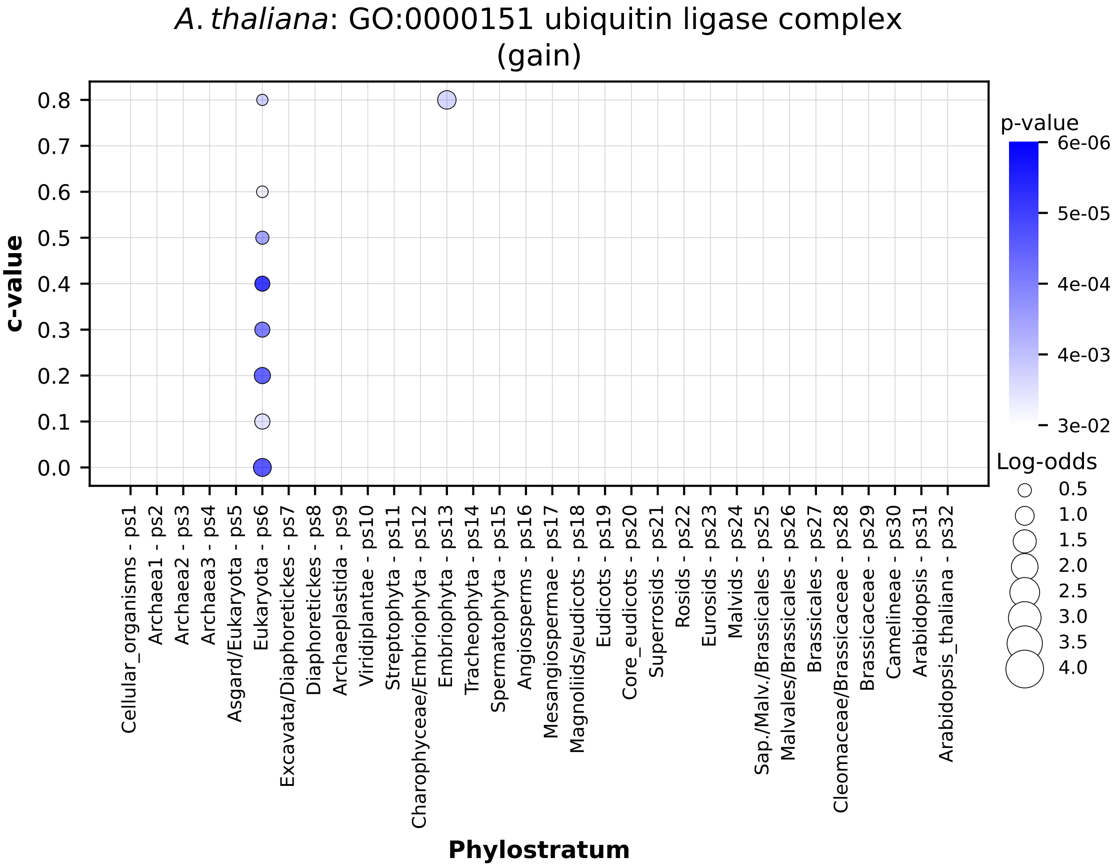

A. thaliana: GO:0000151 ubiquitin ligase complex (gain)
c-value
0.8
0.7
0.6
0.5
0.4
0.3
0.2
0.1
0.0
p-value
6e-06
5e-05
4e-04
4e-03
3e-02
Log-odds
0.5
1.0
1.5
2.0
2.5
3.0
3.5
4.0
Cellular_organisms - ps1
Archaea1 - ps2
Archaea2 - ps3
Archaea3 - ps4
Asgard/Eukaryota - ps5
Eukaryota - ps6
Excavata/Diaphoretickes - ps7
Diaphoretickes - ps8
Archaeplastida - ps9
Viridiplantae - ps10
Streptophyta - ps11
Charophyceae/Embriophyta - ps12
Embriophyta - ps13
Tracheophyta - ps14
Spermatophyta - ps15
Angiosperms - ps16
Mesangiospermae - ps17
Magnoliids/eudicots - ps18
Eudicots - ps19
Core_eudicots - ps20
Superrosids - ps21
Rosids - ps22
Eurosids - ps23
Malvids - ps24
Sap./Malv./Brassicales - ps25
Malvales/Brassicales - ps26
Brassicales - ps27
Cleomaceae/Brassicaceae - ps28
Brassicaceae - ps29
Camelineae - ps30
Arabidopsis - ps31
Arabidopsis_thaliana - ps32
Phylostratum

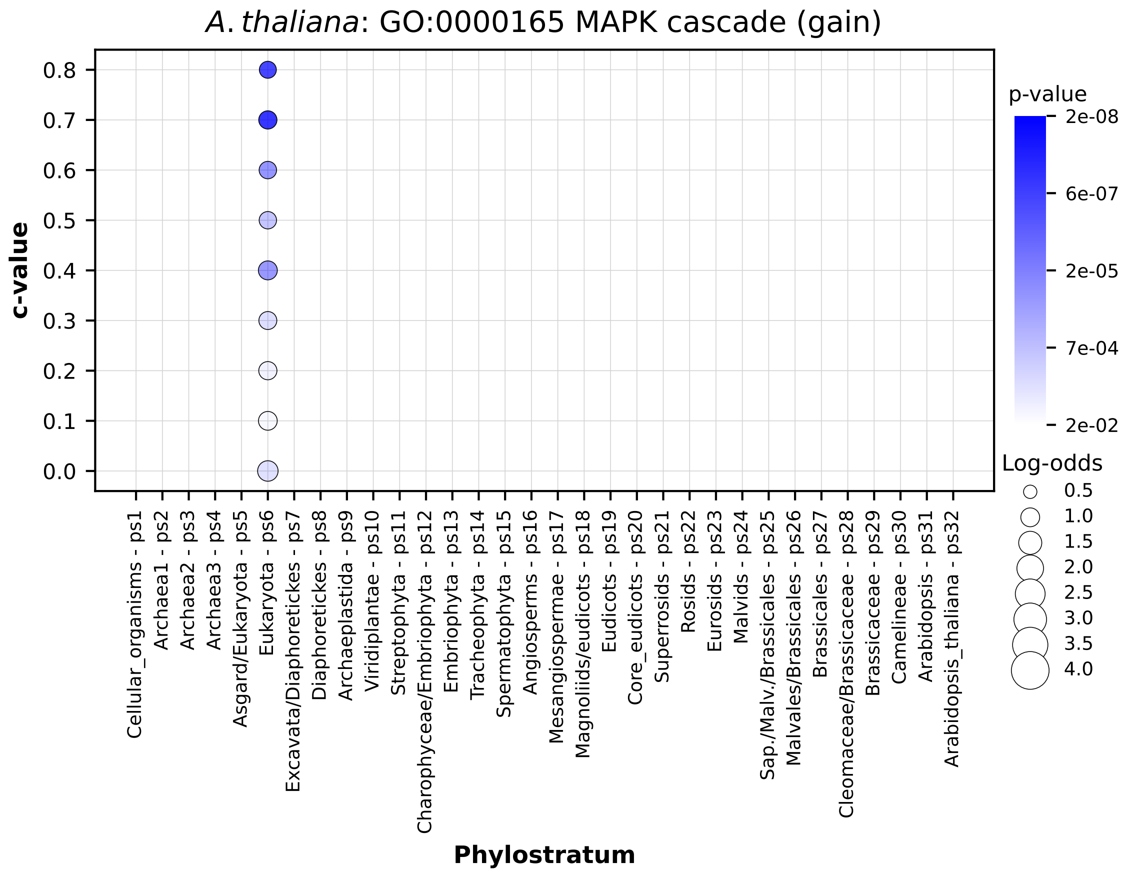
A. thaliana: GO:0000165 MAPK cascade (gain)
c-value
0.0
0.1
0.2
0.3
0.4
0.5
0.6
0.7
0.8
Phylostratum
Cellular_organisms - ps1
Archaea1 - ps2
Archaea2 - ps3
Archaea3 - ps4
Asgard/Eukaryota - ps5
Eukaryota - ps6
Excavata/Diaphoretickes - ps7
Diaphoretickes - ps8
Archaeplastida - ps9
Viridiplantae - ps10
Streptophyta - ps11
Charophyceae/Embriophyta - ps12
Embriophyta - ps13
Tracheophyta - ps14
Spermatophyta - ps15
Angiosperms - ps16
Mesangiospermae - ps17
Magnoliids/eudicots - ps18
Eudicots - ps19
Core_eudicots - ps20
Superrosids - ps21
Rosids - ps22
Eurosids - ps23
Malvids - ps24
Sap./Malv./Brassicales - ps25
Malvales/Brassicales - ps26
Brassicales - ps27
Cleomaceae/Brassicaceae - ps28
Brassicaceae - ps29
Camelineae - ps30
Arabidopsis - ps31
Arabidopsis_thaliana - ps32
p-value
2e-08
6e-07
2e-05
7e-04
2e-02
Log-odds
0.5
1.0
1.5
2.0
2.5
3.0
3.5
4.0

A. thaliana: GO:0000226 microtubule cytoskeleton organization (gain)

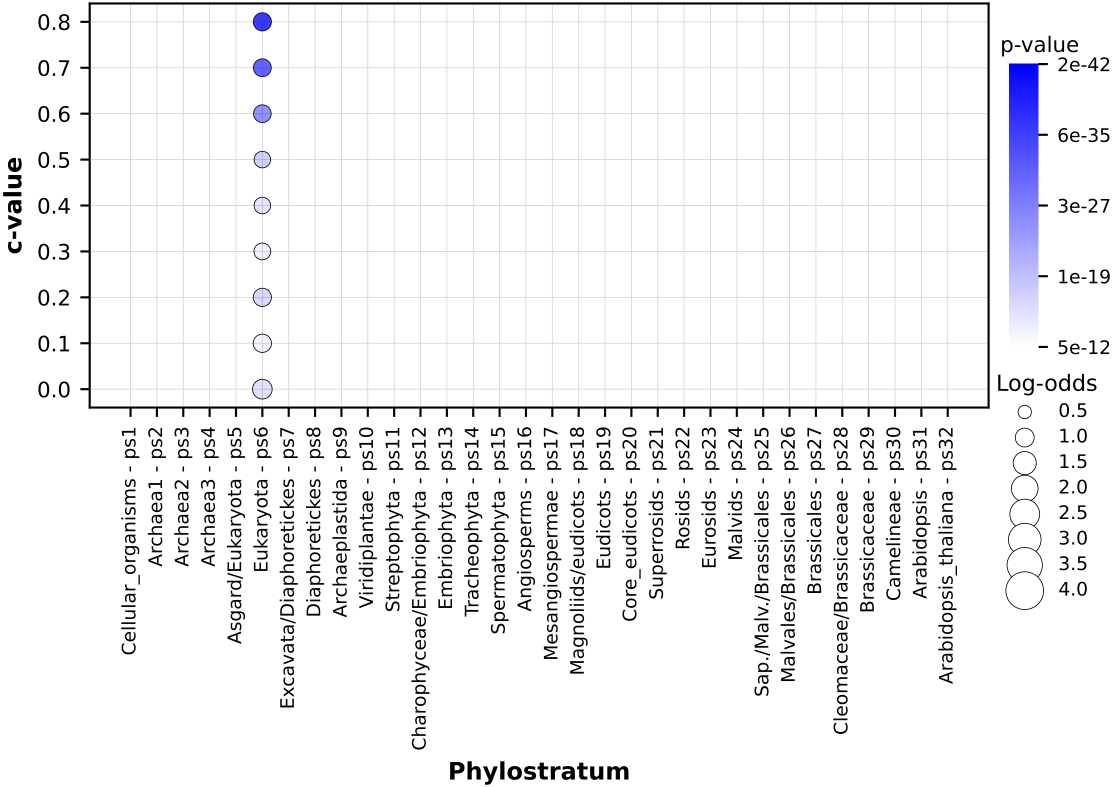

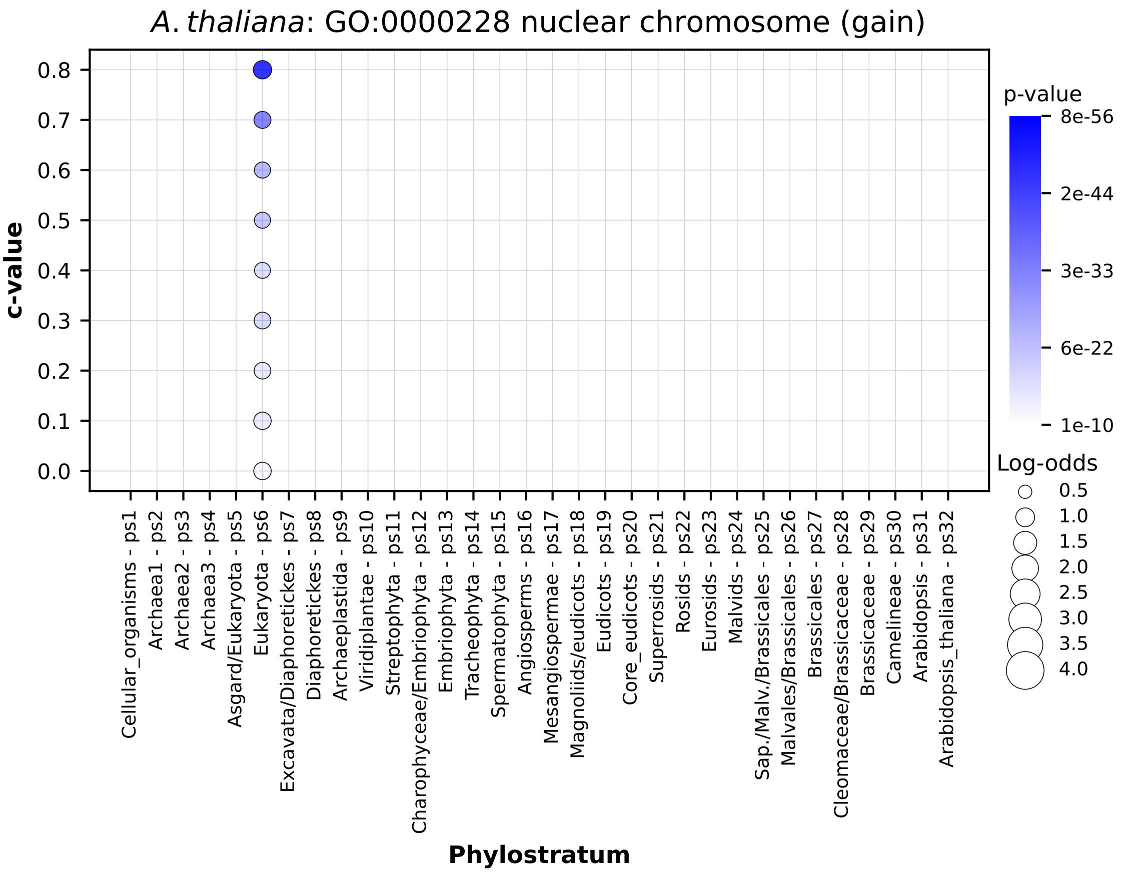

A. thaliana: GO:0000228 nuclear chromosome (gain)
c-value
0.8
0.7
0.6
0.5
0.4
0.3
0.2
0.1
0.0
p-value
8e-56
2e-44
3e-33
6e-22
1e-10
Log-odds
0.5
1.0
1.5
2.0
2.5
3.0
3.5
4.0
Cellular_organisms - ps1
Archaea1 - ps2
Archaea2 - ps3
Archaea3 - ps4
Asgard/Eukaryota - ps5
Eukaryota - ps6
Excavata/Diaphoretickes - ps7
Diaphoretickes - ps8
Archaeplastida - ps9
Viridiplantae - ps10
Streptophyta - ps11
Charophyceae/Embriophyta - ps12
Embriophyta - ps13
Tracheophyta - ps14
Spermatophyta - ps15
Angiosperms - ps16
Mesangiospermae - ps17
Magnoliids/eudicots - ps18
Eudicots - ps19
Core_eudicots - ps20
Superrosids - ps21
Rosids - ps22
Eurosids - ps23
Malvids - ps24
Sap./Malv./Brassicales - ps25
Malvales/Brassicales - ps26
Brassicales - ps27
Cleomaceae/Brassicaceae - ps28
Brassicaceae - ps29
Camelineae - ps30
Arabidopsis - ps31
Arabidopsis_thaliana - ps32
Phylostratum

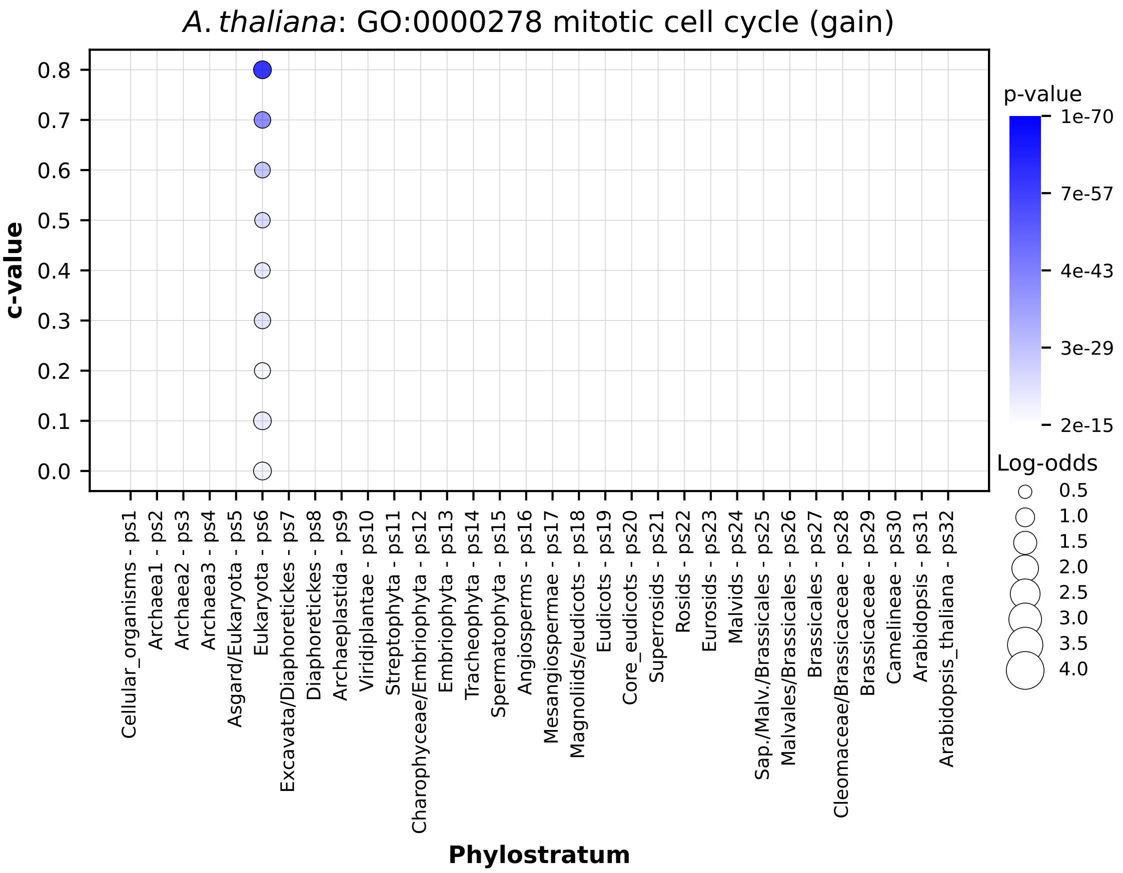
A. thaliana: GO:0000278 mitotic cell cycle (gain)
c-value
0.8
0.7
0.6
0.5
0.4
0.3
0.2
0.1
0.0
p-value
1e-70
7e-57
4e-43
3e-29
2e-15
Log-odds
0.5
1.0
1.5
2.0
2.5
3.0
3.5
4.0
Cellular_organisms - ps1
Archaea1 - ps2
Archaea2 - ps3
Archaea3 - ps4
Asgard/Eukaryota - ps5
Eukaryota - ps6
Excavata/Diaphoretickes - ps7
Diaphoretickes - ps8
Archaeplastida - ps9
Viridiplantae - ps10
Streptophyta - ps11
Charophyceae/Embriophyta - ps12
Embriophyta - ps13
Tracheophyta - ps14
Spermatophyta - ps15
Angiosperms - ps16
Mesangiospermae - ps17
Magnoliids/eudicots - ps18
Eudicots - ps19
Core_eudicots - ps20
Superrosids - ps21
Rosids - ps22
Eurosids - ps23
Malvids - ps24
Sap./Malv./Brassicales - ps25
Malvales/Brassicales - ps26
Brassicales - ps27
Cleomaceae/Brassicaceae - ps28
Brassicaceae - ps29
Camelineae - ps30
Arabidopsis - ps31
Arabidopsis_thaliana - ps32
Phylostratum

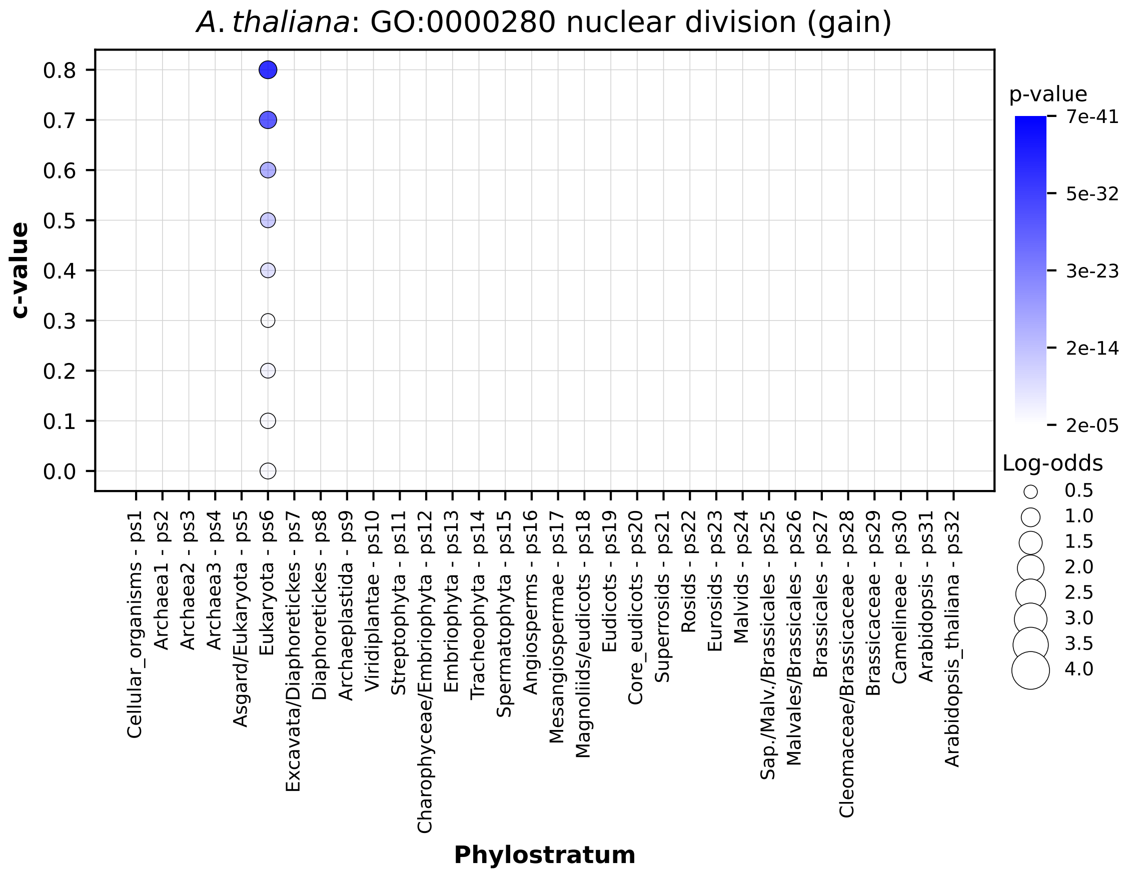

A. thaliana: GO:0000280 nuclear division (gain)
c-value
0.0
0.1
0.2
0.3
0.4
0.5
0.6
0.7
0.8
Phylostratum
Cellular_organisms - ps1
Archaea1 - ps2
Archaea2 - ps3
Archaea3 - ps4
Asgard/Eukaryota - ps5
Eukaryota - ps6
Excavata/Diaphoretickes - ps7
Diaphoretickes - ps8
Archaeplastida - ps9
Viridiplantae - ps10
Streptophyta - ps11
Charophyceae/Embriophyta - ps12
Embriophyta - ps13
Tracheophyta - ps14
Spermatophyta - ps15
Angiosperms - ps16
Mesangiospermae - ps17
Magnoliids/eudicots - ps18
Eudicots - ps19
Core_eudicots - ps20
Superrosids - ps21
Rosids - ps22
Eurosids - ps23
Malvids - ps24
Sap./Malv./Brassicales - ps25
Malvales/Brassicales - ps26
Brassicales - ps27
Cleomaceae/Brassicaceae - ps28
Brassicaceae - ps29
Camelineae - ps30
Arabidopsis - ps31
Arabidopsis_thaliana - ps32
p-value
7e-41
5e-32
3e-23
2e-14
2e-05
Log-odds
0.5
1.0
1.5
2.0
2.5
3.0
3.5
4.0

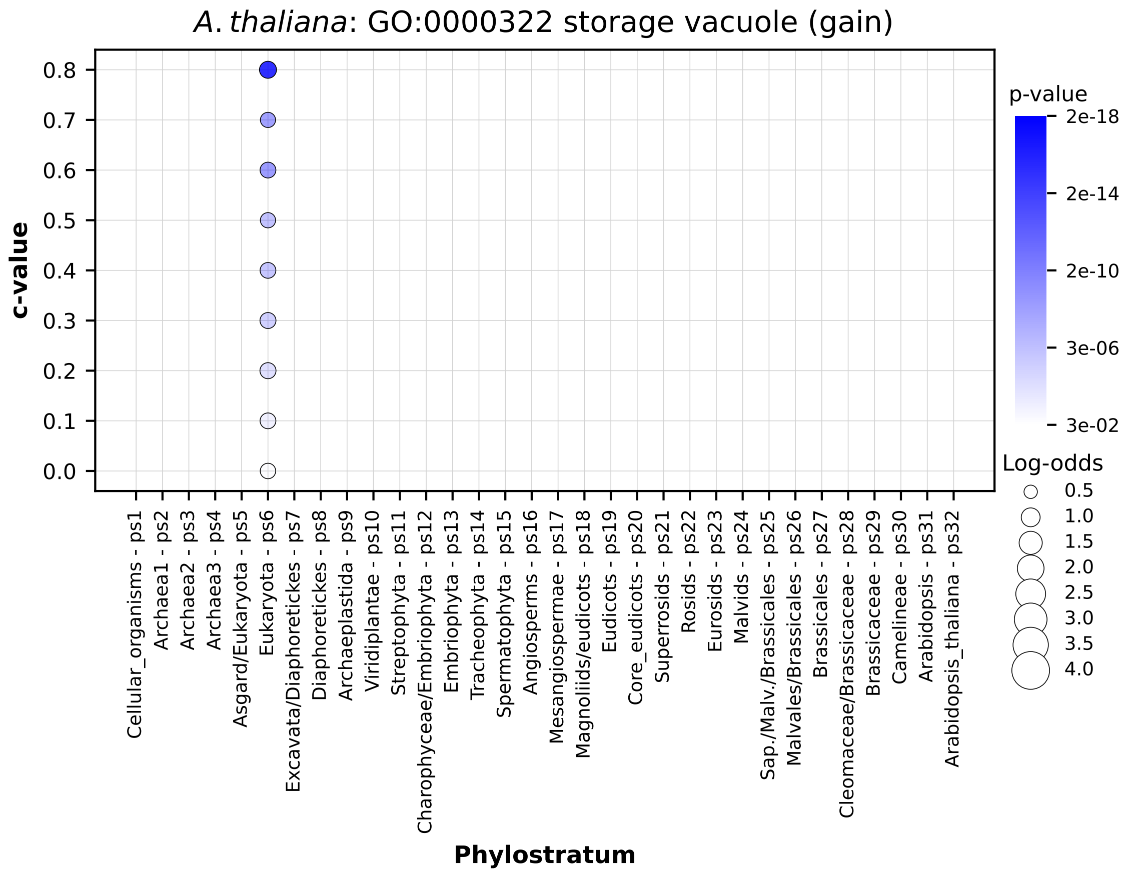

A. thaliana: GO:0000322 storage vacuole (gain)
c-value
0.8
0.7
0.6
0.5
0.4
0.3
0.2
0.1
0.0
p-value
2e-18
2e-14
2e-10
3e-06
3e-02
Log-odds
0.5
1.0
1.5
2.0
2.5
3.0
3.5
4.0
Cellular_organisms - ps1
Archaea1 - ps2
Archaea2 - ps3
Archaea3 - ps4
Asgard/Eukaryota - ps5
Eukaryota - ps6
Excavata/Diaphoretickes - ps7
Diaphoretickes - ps8
Archaeplastida - ps9
Viridiplantae - ps10
Streptophyta - ps11
Charophyceae/Embriophyta - ps12
Embriophyta - ps13
Tracheophyta - ps14
Spermatophyta - ps15
Angiosperms - ps16
Mesangiospermae - ps17
Magnoliids/eudicots - ps18
Eudicots - ps19
Core_eudicots - ps20
Superrosids - ps21
Rosids - ps22
Eurosids - ps23
Malvids - ps24
Sap./Malv./Brassicales - ps25
Malvales/Brassicales - ps26
Brassicales - ps27
Cleomaceae/Brassicaceae - ps28
Brassicaceae - ps29
Camelineae - ps30
Arabidopsis - ps31
Arabidopsis_thaliana - ps32
Phylostratum

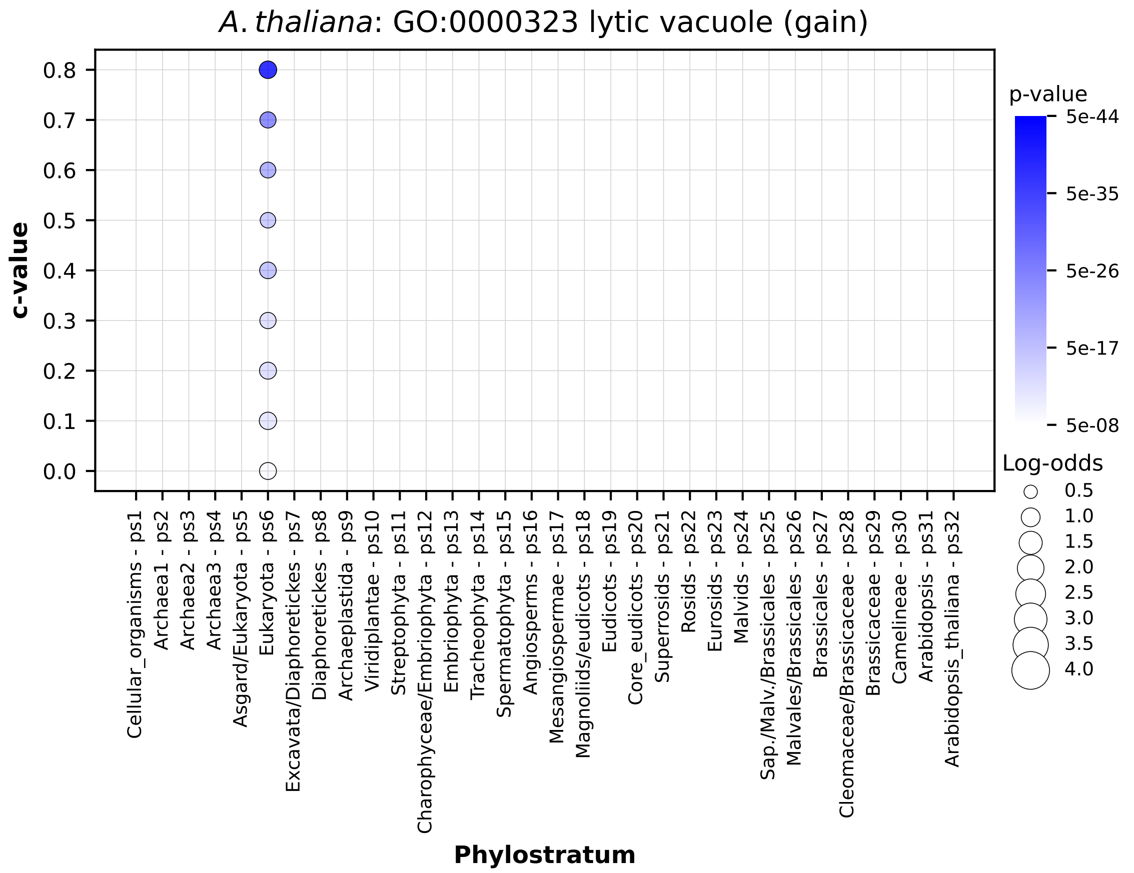

A. thaliana: GO:0000323 lytic vacuole (gain)
c-value
0.8
0.7
0.6
0.5
0.4
0.3
0.2
0.1
0.0
p-value
5e-44
5e-35
5e-26
5e-17
5e-08
Log-odds
0.5
1.0
1.5
2.0
2.5
3.0
3.5
4.0
Cellular_organisms - ps1
Archaea1 - ps2
Archaea2 - ps3
Archaea3 - ps4
Asgard/Eukaryota - ps5
Eukaryota - ps6
Excavata/Diaphoretickes - ps7
Diaphoretickes - ps8
Archaeplastida - ps9
Viridiplantae - ps10
Streptophyta - ps11
Charophyceae/Embriophyta - ps12
Embriophyta - ps13
Tracheophyta - ps14
Spermatophyta - ps15
Angiosperms - ps16
Mesangiospermae - ps17
Magnoliids/eudicots - ps18
Eudicots - ps19
Core_eudicots - ps20
Superrosids - ps21
Rosids - ps22
Eurosids - ps23
Malvids - ps24
Sap./Malv./Brassicales - ps25
Malvales/Brassicales - ps26
Brassicales - ps27
Cleomaceae/Brassicaceae - ps28
Brassicaceae - ps29
Camelineae - ps30
Arabidopsis - ps31
Arabidopsis_thaliana - ps32
Phylostratum

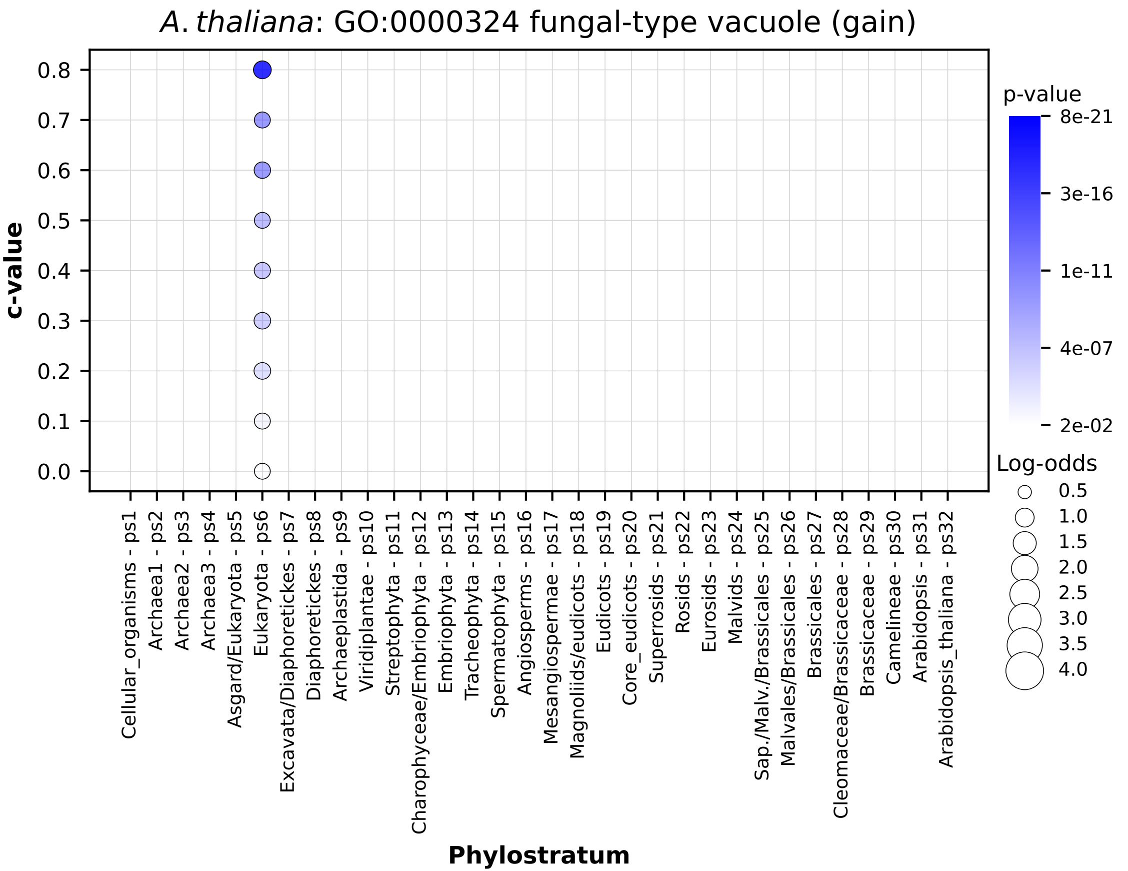

A. thaliana: GO:0000324 fungal-type vacuole (gain)
c-value
0.0
0.1
0.2
0.3
0.4
0.5
0.6
0.7
0.8
Phylostratum
Cellular_organisms - ps1
Archaea1 - ps2
Archaea2 - ps3
Archaea3 - ps4
Asgard/Eukaryota - ps5
Eukaryota - ps6
Excavata/Diaphoretickes - ps7
Diaphoretickes - ps8
Archaeplastida - ps9
Viridiplantae - ps10
Streptophyta - ps11
Charophyceae/Embriophyta - ps12
Embriophyta - ps13
Tracheophyta - ps14
Spermatophyta - ps15
Angiosperms - ps16
Mesangiospermae - ps17
Magnoliids/eudicots - ps18
Eudicots - ps19
Core_eudicots - ps20
Superrosids - ps21
Rosids - ps22
Eurosids - ps23
Malvids - ps24
Sap./Malv./Brassicales - ps25
Malvales/Brassicales - ps26
Brassicales - ps27
Cleomaceae/Brassicaceae - ps28
Brassicaceae - ps29
Camelineae - ps30
Arabidopsis - ps31
Arabidopsis_thaliana - ps32
p-value
8e-21
3e-16
1e-11
4e-07
2e-02
Log-odds
0.5
1.0
1.5
2.0
2.5
3.0
3.5
4.0

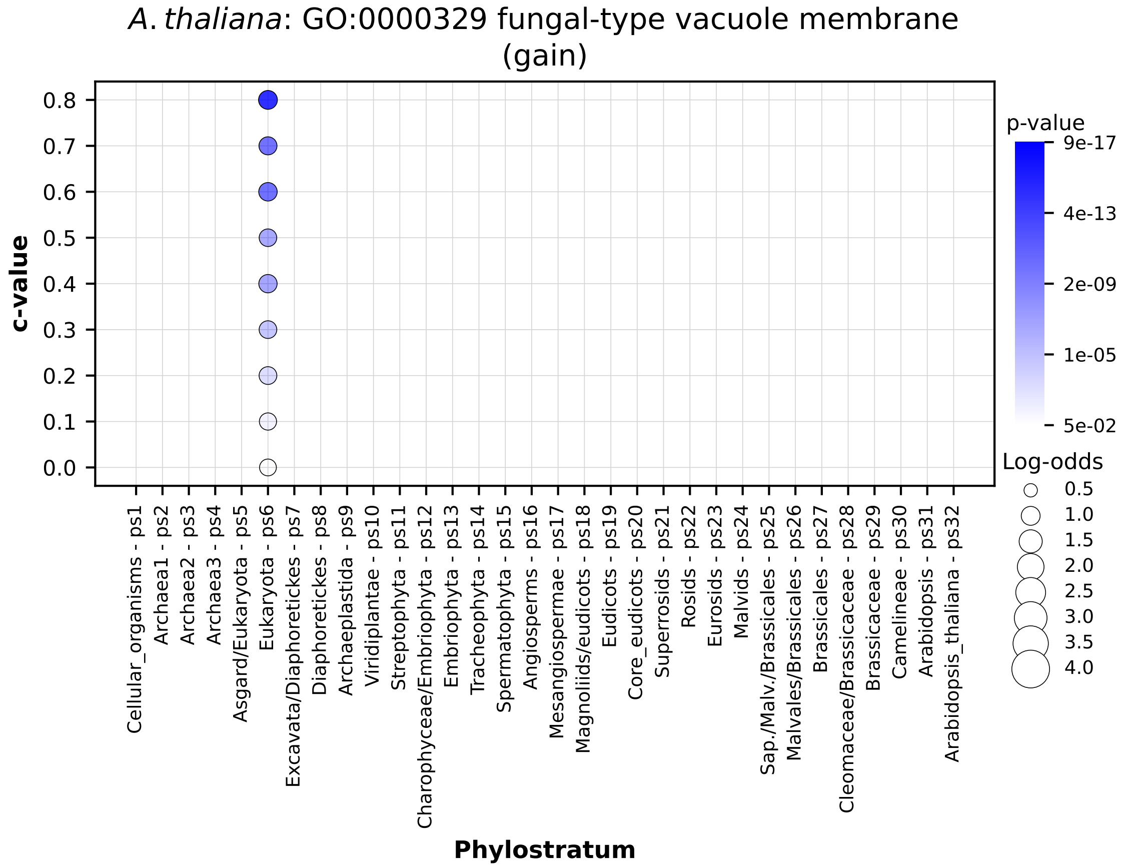

A. thaliana: GO:0000329 fungal-type vacuole membrane (gain)
c-value
0.8
0.7
0.6
0.5
0.4
0.3
0.2
0.1
0.0
p-value
9e-17
4e-13
2e-09
1e-05
5e-02
Log-odds
0.5
1.0
1.5
2.0
2.5
3.0
3.5
4.0
Cellular_organisms - ps1
Archaea1 - ps2
Archaea2 - ps3
Archaea3 - ps4
Asgard/Eukaryota - ps5
Eukaryota - ps6
Excavata/Diaphoretickes - ps7
Diaphoretickes - ps8
Archaeplastida - ps9
Viridiplantae - ps10
Streptophyta - ps11
Charophyceae/Embriophyta - ps12
Embriophyta - ps13
Tracheophyta - ps14
Spermatophyta - ps15
Angiosperms - ps16
Mesangiospermae - ps17
Magnoliids/eudicots - ps18
Eudicots - ps19
Core_eudicots - ps20
Superrosids - ps21
Rosids - ps22
Eurosids - ps23
Malvids - ps24
Sap./Malv./Brassicales - ps25
Malvales/Brassicales - ps26
Brassicales - ps27
Cleomaceae/Brassicaceae - ps28
Brassicaceae - ps29
Camelineae - ps30
Arabidopsis - ps31
Arabidopsis_thaliana - ps32
Phylostratum

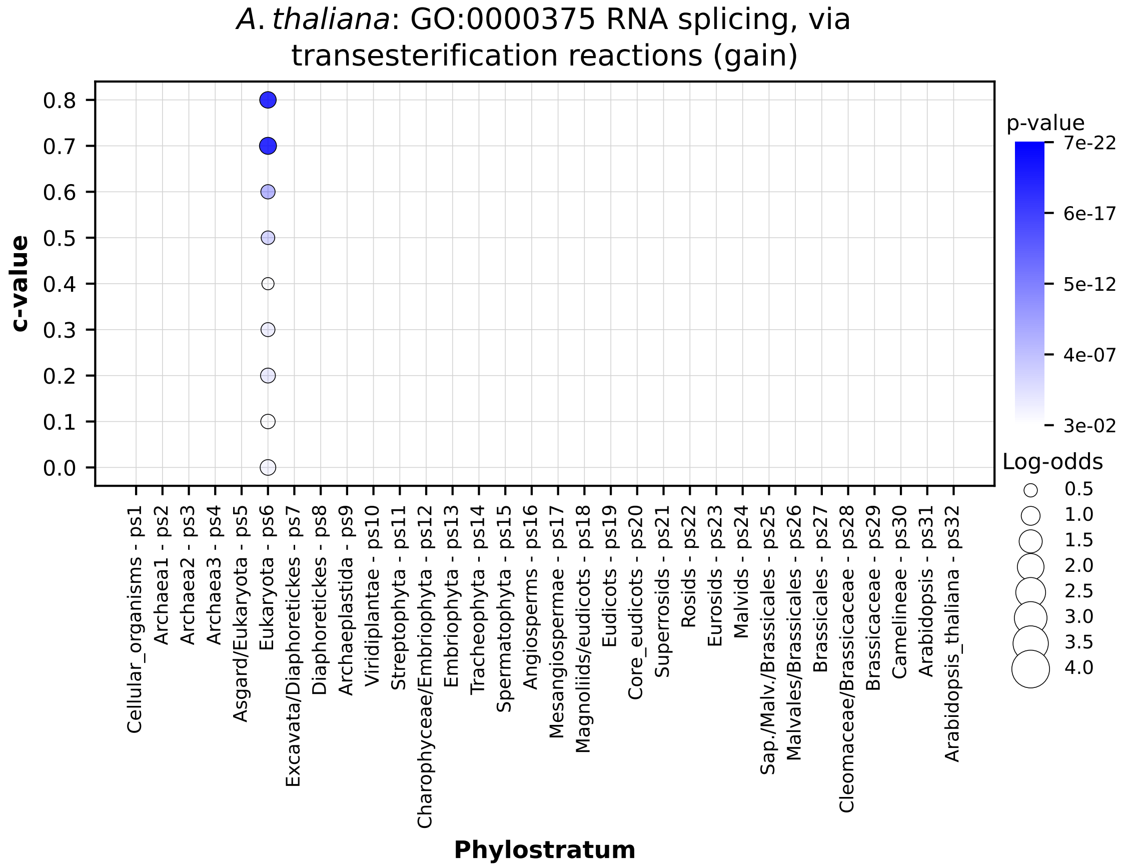
A. thaliana: GO:0000375 RNA splicing, via transesterification reactions (gain)
c-value
0.8
0.7
0.6
0.5
0.4
0.3
0.2
0.1
0.0
p-value
7e-22
6e-17
5e-12
4e-07
3e-02
Log-odds
0.5
1.0
1.5
2.0
2.5
3.0
3.5
4.0
Cellular_organisms - ps1
Archaea1 - ps2
Archaea2 - ps3
Archaea3 - ps4
Asgard/Eukaryota - ps5
Eukaryota - ps6
Excavata/Diaphoretickes - ps7
Diaphoretickes - ps8
Archaeplastida - ps9
Viridiplantae - ps10
Streptophyta - ps11
Charophyceae/Embriophyta - ps12
Embriophyta - ps13
Tracheophyta - ps14
Spermatophyta - ps15
Angiosperms - ps16
Mesangiospermae - ps17
Magnoliids/eudicots - ps18
Eudicots - ps19
Core_eudicots - ps20
Superrosids - ps21
Rosids - ps22
Eurosids - ps23
Malvids - ps24
Sap./Malv./Brassicales - ps25
Malvales/Brassicales - ps26
Brassicales - ps27
Cleomaceae/Brassicaceae - ps28
Brassicaceae - ps29
Camelineae - ps30
Arabidopsis - ps31
Arabidopsis_thaliana - ps32
Phylostratum

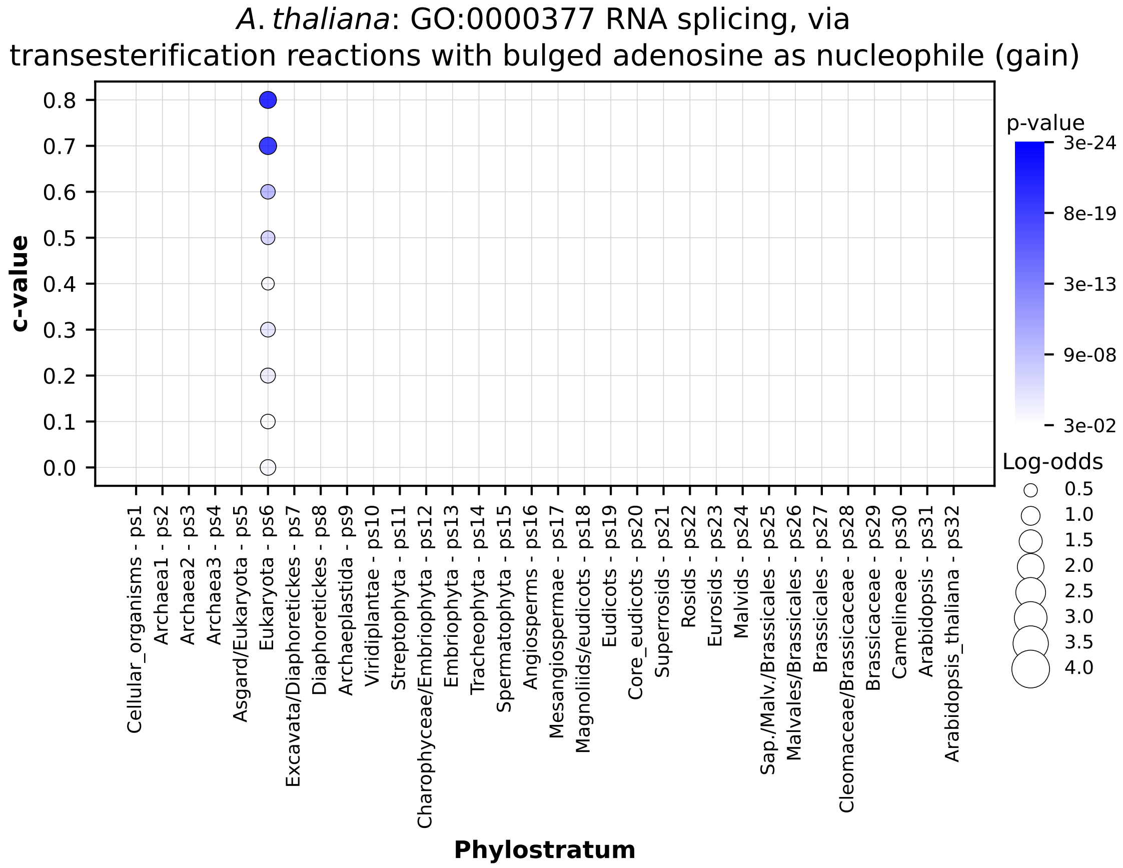

A. thaliana: GO:0000377 RNA splicing, via transesterification reactions with bulged adenosine as nucleophile (gain)
c-value
0.8
0.7
0.6
0.5
0.4
0.3
0.2
0.1
0.0
p-value
3e-24
8e-19
3e-13
9e-08
3e-02
Log-odds
0.5
1.0
1.5
2.0
2.5
3.0
3.5
4.0
Cellular_organisms - ps1
Archaea1 - ps2
Archaea2 - ps3
Archaea3 - ps4
Asgard/Eukaryota - ps5
Eukaryota - ps6
Excavata/Diaphoretickes - ps7
Diaphoretickes - ps8
Archaeplastida - ps9
Viridiplantae - ps10
Streptophyta - ps11
Charophyceae/Embriophyta - ps12
Embriophyta - ps13
Tracheophyta - ps14
Spermatophyta - ps15
Angiosperms - ps16
Mesangiospermae - ps17
Magnoliids/eudicots - ps18
Eudicots - ps19
Core_eudicots - ps20
Superrosids - ps21
Rosids - ps22
Eurosids - ps23
Malvids - ps24
Sap./Malv./Brassicales - ps25
Malvales/Brassicales - ps26
Brassicales - ps27
Cleomaceae/Brassicaceae - ps28
Brassicaceae - ps29
Camelineae - ps30
Arabidopsis - ps31
Arabidopsis_thaliana - ps32
Phylostratum

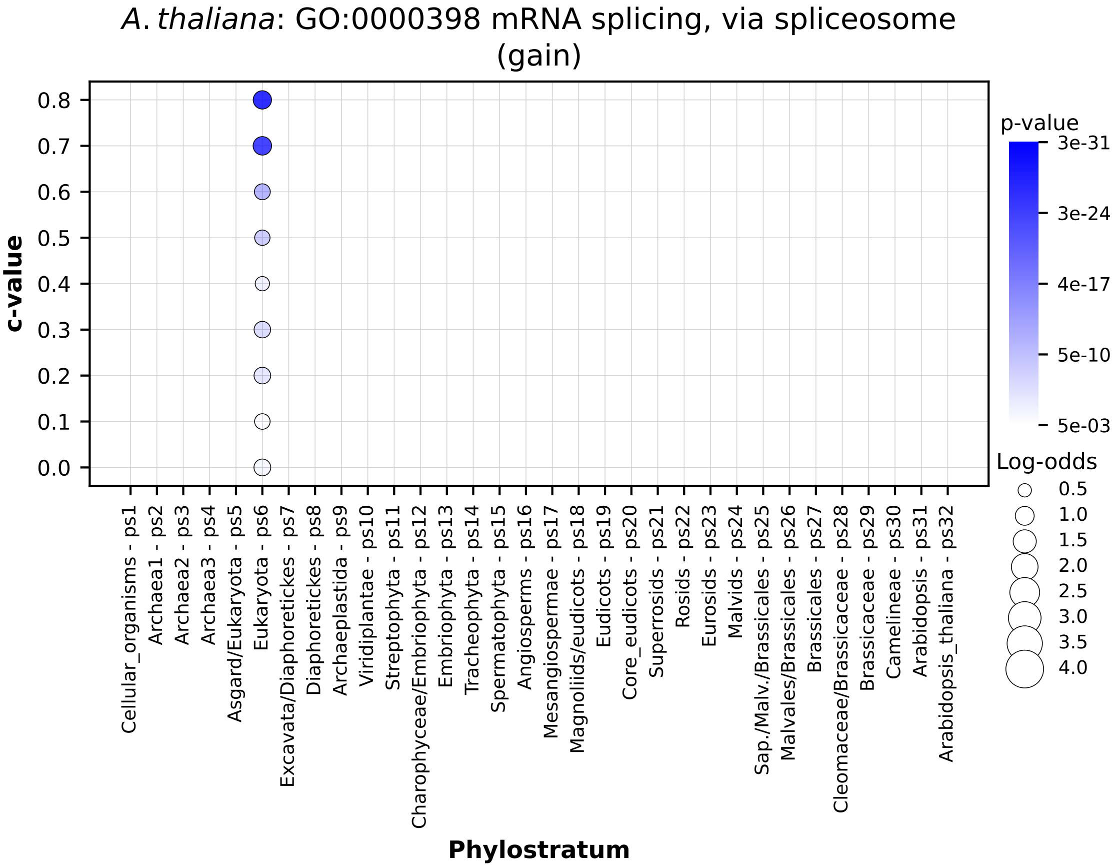

A. thaliana: GO:0000398 mRNA splicing, via spliceosome
(gain)
c-value
0.8
0.7
0.6
0.5
0.4
0.3
0.2
0.1
0.0
p-value
3e-31
3e-24
4e-17
5e-10
5e-03
Log-odds
0.5
1.0
1.5
2.0
2.5
3.0
3.5
4.0
Cellular_organisms - ps1
Archaea1 - ps2
Archaea2 - ps3
Archaea3 - ps4
Asgard/Eukaryota - ps5
Eukaryota - ps6
Excavata/Diaphoretickes - ps7
Diaphoretickes - ps8
Archaeplastida - ps9
Viridiplantae - ps10
Streptophyta - ps11
Charophyceae/Embriophyta - ps12
Embriophyta - ps13
Tracheophyta - ps14
Spermatophyta - ps15
Angiosperms - ps16
Mesangiospermae - ps17
Magnoliids/eudicots - ps18
Eudicots - ps19
Core_eudicots - ps20
Superrosids - ps21
Rosids - ps22
Eurosids - ps23
Malvids - ps24
Sap./Malv./Brassicales - ps25
Malvales/Brassicales - ps26
Brassicales - ps27
Cleomaceae/Brassicaceae - ps28
Brassicaceae - ps29
Camelineae - ps30
Arabidopsis - ps31
Arabidopsis_thaliana - ps32
Phylostratum

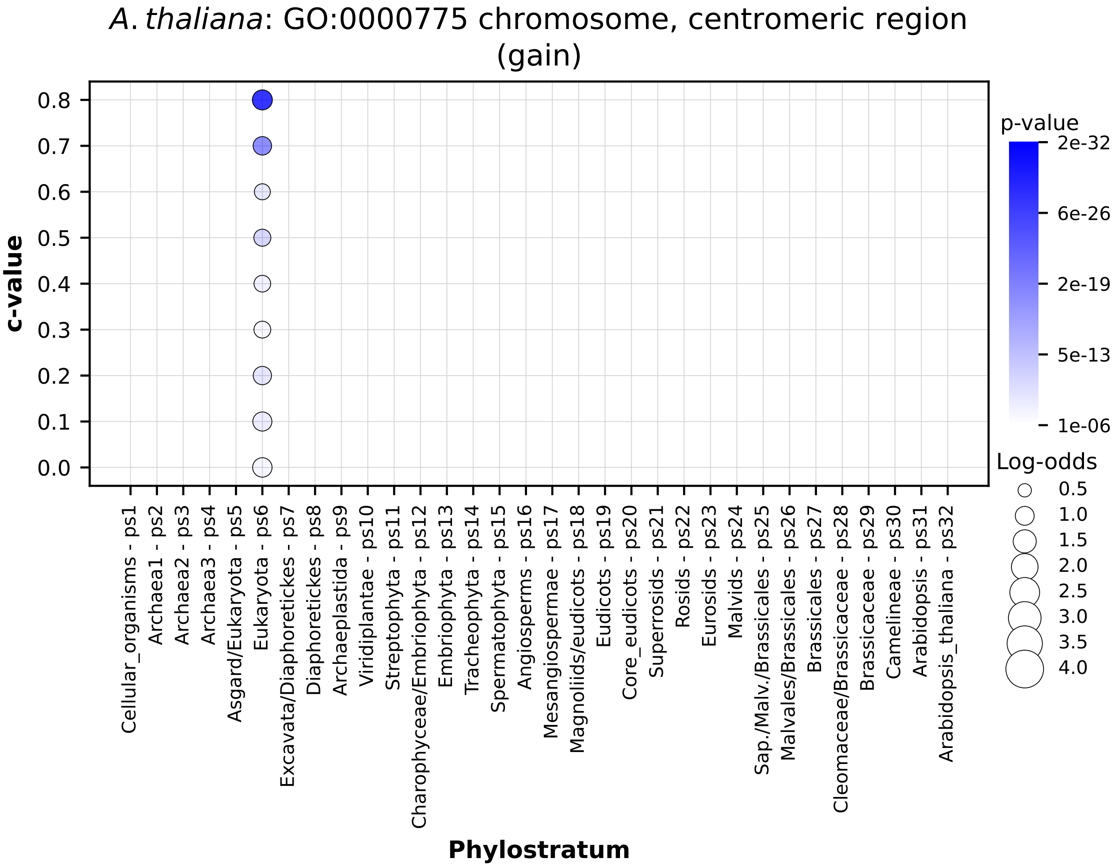

A. thaliana: GO:0000775 chromosome, centromeric region (gain)
c-value
0.0
0.1
0.2
0.3
0.4
0.5
0.6
0.7
0.8
Phylostratum
Cellular_organisms - ps1
Archaea1 - ps2
Archaea2 - ps3
Archaea3 - ps4
Asgard/Eukaryota - ps5
Eukaryota - ps6
Excavata/Diaphoretickes - ps7
Diaphoretickes - ps8
Archaeplastida - ps9
Viridiplantae - ps10
Streptophyta - ps11
Charophyceae/Embriophyta - ps12
Embriophyta - ps13
Tracheophyta - ps14
Spermatophyta - ps15
Angiosperms - ps16
Mesangiospermae - ps17
Magnoliids/eudicots - ps18
Eudicots - ps19
Core_eudicots - ps20
Superrosids - ps21
Rosids - ps22
Eurosids - ps23
Malvids - ps24
Sap./Malv./Brassicales - ps25
Malvales/Brassicales - ps26
Brassicales - ps27
Cleomaceae/Brassicaceae - ps28
Brassicaceae - ps29
Camelineae - ps30
Arabidopsis - ps31
Arabidopsis_thaliana - ps32
p-value
2e-32
6e-26
2e-19
5e-13
1e-06
Log-odds
0.5
1.0
1.5
2.0
2.5
3.0
3.5
4.0

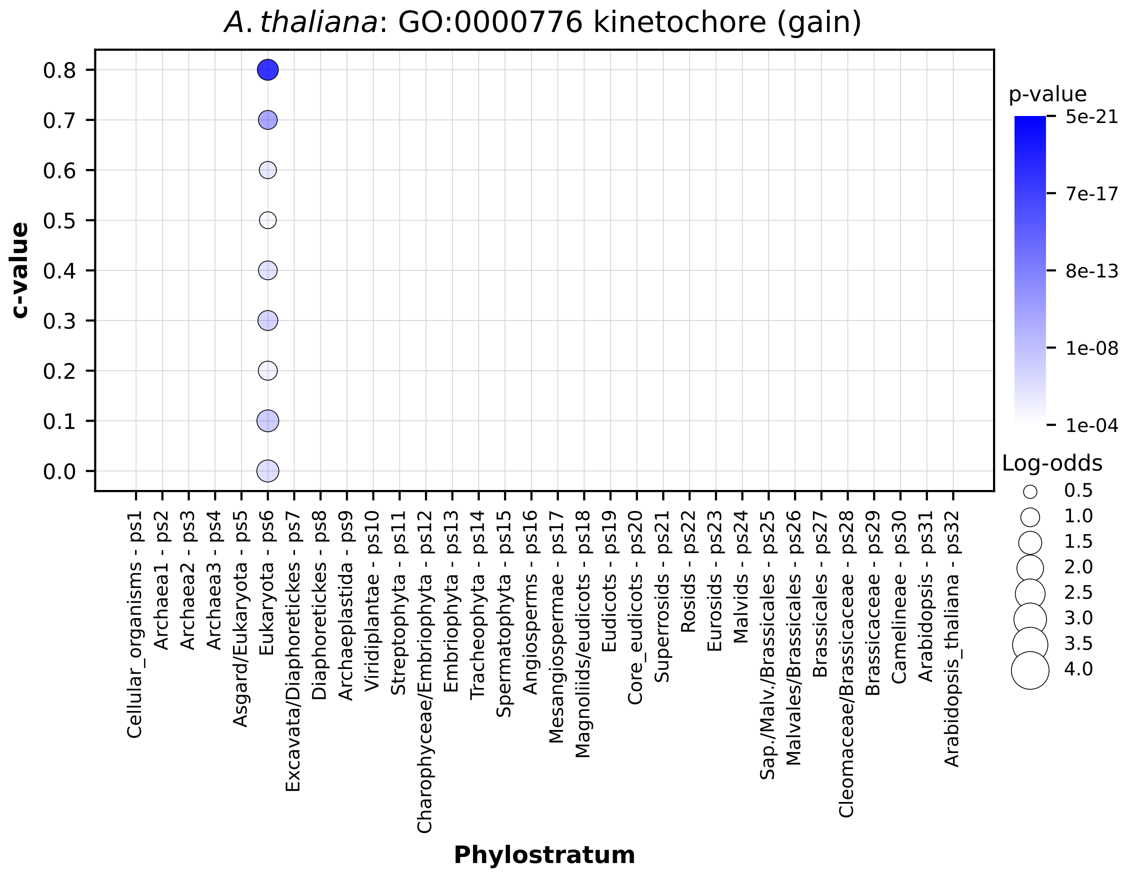
A. thaliana: GO:0000776 kinetochore (gain)
c-value
0.8
0.7
0.6
0.5
0.4
0.3
0.2
0.1
0.0
p-value
5e-21
7e-17
8e-13
1e-08
1e-04
Log-odds
0.5
1.0
1.5
2.0
2.5
3.0
3.5
4.0
Cellular_organisms - ps1
Archaea1 - ps2
Archaea2 - ps3
Archaea3 - ps4
Asgard/Eukaryota - ps5
Eukaryota - ps6
Excavata/Diaphoretickes - ps7
Diaphoretickes - ps8
Archaeplastida - ps9
Viridiplantae - ps10
Streptophyta - ps11
Charophyceae/Embriophyta - ps12
Embriophyta - ps13
Tracheophyta - ps14
Spermatophyta - ps15
Angiosperms - ps16
Mesangiospermae - ps17
Magnoliids/eudicots - ps18
Eudicots - ps19
Core_eudicots - ps20
Superrosids - ps21
Rosids - ps22
Eurosids - ps23
Malvids - ps24
Sap./Malv./Brassicales - ps25
Malvales/Brassicales - ps26
Brassicales - ps27
Cleomaceae/Brassicaceae - ps28
Brassicaceae - ps29
Camelineae - ps30
Arabidopsis - ps31
Arabidopsis_thaliana - ps32
Phylostratum

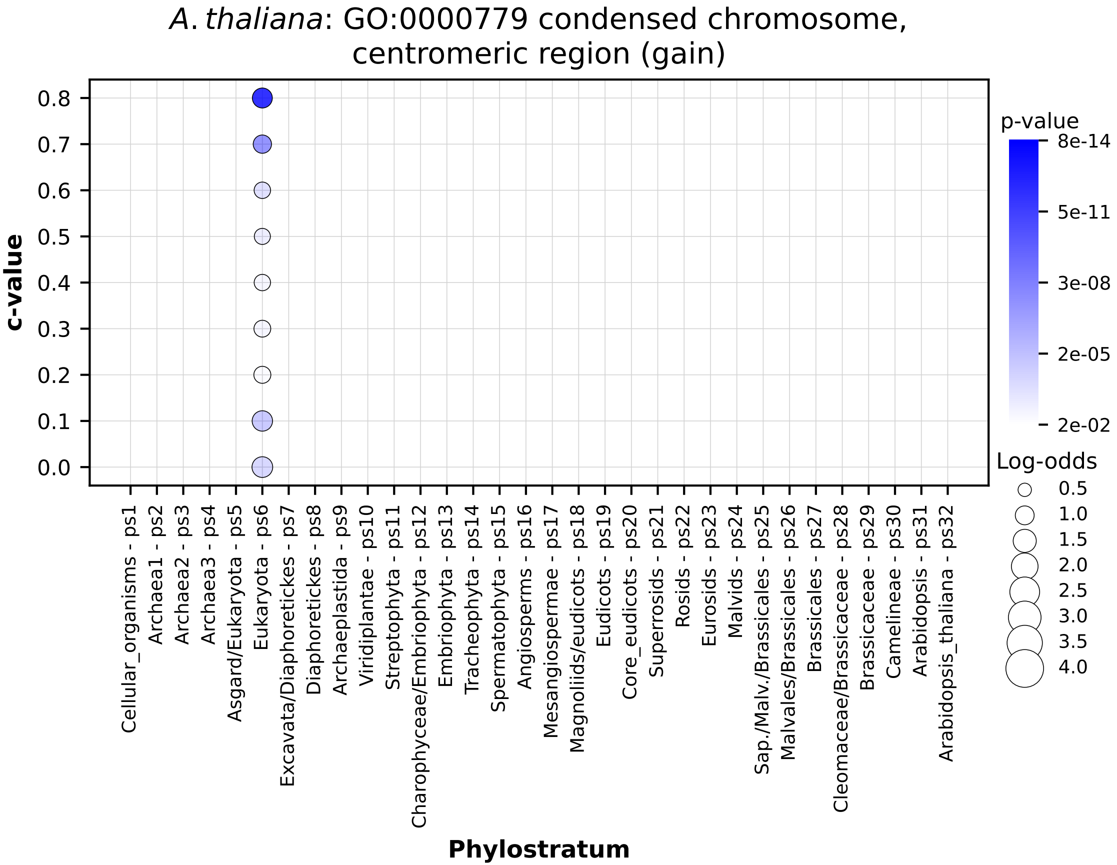

A. thaliana: GO:0000779 condensed chromosome, centromeric region (gain)
c-value
0.8
0.7
0.6
0.5
0.4
0.3
0.2
0.1
0.0
p-value
8e-14
5e-11
3e-08
2e-05
2e-02
Log-odds
0.5
1.0
1.5
2.0
2.5
3.0
3.5
4.0
Cellular_organisms - ps1
Archaea1 - ps2
Archaea2 - ps3
Archaea3 - ps4
Asgard/Eukaryota - ps5
Eukaryota - ps6
Excavata/Diaphoretickes - ps7
Diaphoretickes - ps8
Archaeplastida - ps9
Viridiplantae - ps10
Streptophyta - ps11
Charophyceae/Embriophyta - ps12
Embriophyta - ps13
Tracheophyta - ps14
Spermatophyta - ps15
Angiosperms - ps16
Mesangiospermae - ps17
Magnoliids/eudicots - ps18
Eudicots - ps19
Core_eudicots - ps20
Superrosids - ps21
Rosids - ps22
Eurosids - ps23
Malvids - ps24
Sap./Malv./Brassicales - ps25
Malvales/Brassicales - ps26
Brassicales - ps27
Cleomaceae/Brassicaceae - ps28
Brassicaceae - ps29
Camelineae - ps30
Arabidopsis - ps31
Arabidopsis_thaliana - ps32
Phylostratum

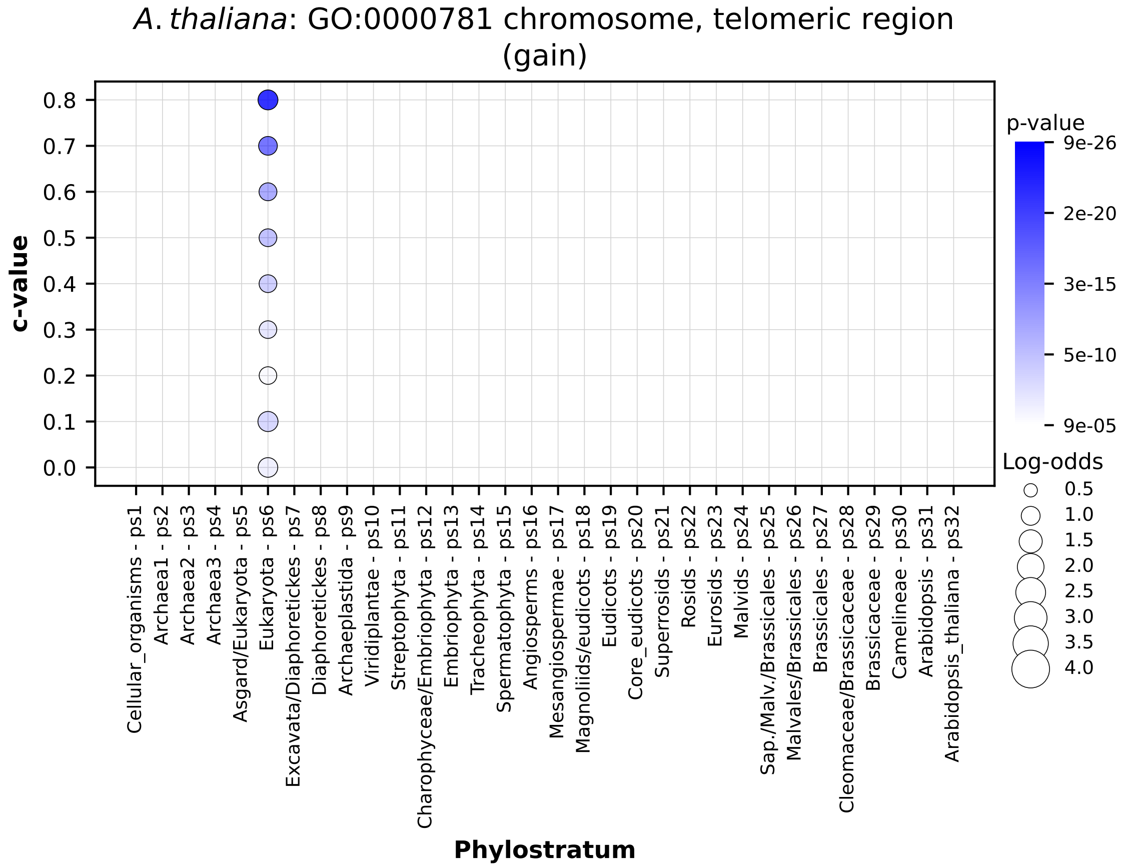

A. thaliana: GO:0000781 chromosome, telomeric region
(gain)
c-value
0.8
0.7
0.6
0.5
0.4
0.3
0.2
0.1
0.0
p-value
9e-26
2e-20
3e-15
5e-10
9e-05
Log-odds
0.5
1.0
1.5
2.0
2.5
3.0
3.5
4.0
Cellular_organisms - ps1
Archaea1 - ps2
Archaea2 - ps3
Archaea3 - ps4
Asgard/Eukaryota - ps5
Eukaryota - ps6
Excavata/Diaphoretickes - ps7
Diaphoretickes - ps8
Archaeplastida - ps9
Viridiplantae - ps10
Streptophyta - ps11
Charophyceae/Embriophyta - ps12
Embriophyta - ps13
Tracheophyta - ps14
Spermatophyta - ps15
Angiosperms - ps16
Mesangiospermae - ps17
Magnoliids/eudicots - ps18
Eudicots - ps19
Core_eudicots - ps20
Superrosids - ps21
Rosids - ps22
Eurosids - ps23
Malvids - ps24
Sap./Malv./Brassicales - ps25
Malvales/Brassicales - ps26
Brassicales - ps27
Cleomaceae/Brassicaceae - ps28
Brassicaceae - ps29
Camelineae - ps30
Arabidopsis - ps31
Arabidopsis_thaliana - ps32
Phylostratum

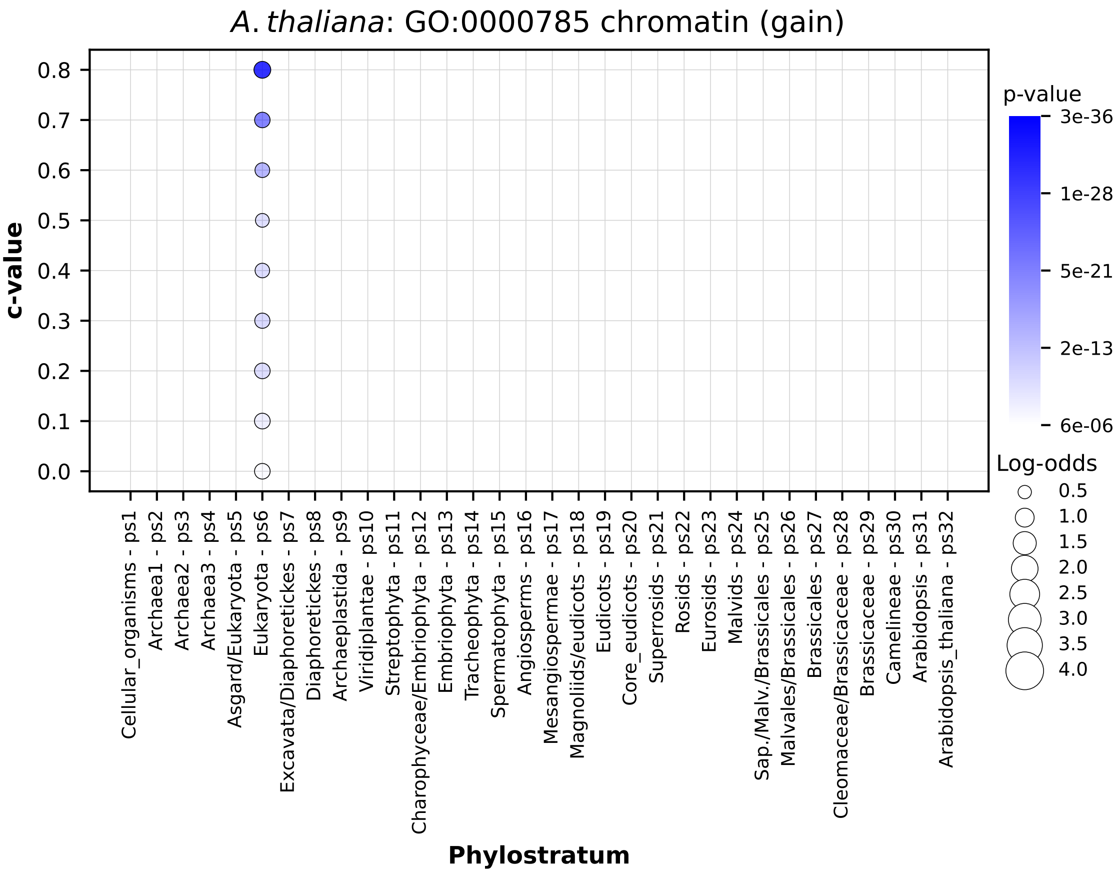
A. thaliana: GO:0000785 chromatin (gain)
c-value
0.8
0.7
0.6
0.5
0.4
0.3
0.2
0.1
0.0
p-value
3e-36
1e-28
5e-21
2e-13
6e-06
Log-odds
0.5
1.0
1.5
2.0
2.5
3.0
3.5
4.0
Cellular_organisms - ps1
Archaea1 - ps2
Archaea2 - ps3
Archaea3 - ps4
Asgard/Eukaryota - ps5
Eukaryota - ps6
Excavata/Diaphoretickes - ps7
Diaphoretickes - ps8
Archaeplastida - ps9
Viridiplantae - ps10
Streptophyta - ps11
Charophyceae/Embriophyta - ps12
Embriophyta - ps13
Tracheophyta - ps14
Spermatophyta - ps15
Angiosperms - ps16
Mesangiospermae - ps17
Magnoliids/eudicots - ps18
Eudicots - ps19
Core_eudicots - ps20
Superrosids - ps21
Rosids - ps22
Eurosids - ps23
Malvids - ps24
Sap./Malv./Brassicales - ps25
Malvales/Brassicales - ps26
Brassicales - ps27
Cleomaceae/Brassicaceae - ps28
Brassicaceae - ps29
Camelineae - ps30
Arabidopsis - ps31
Arabidopsis_thaliana - ps32
Phylostratum

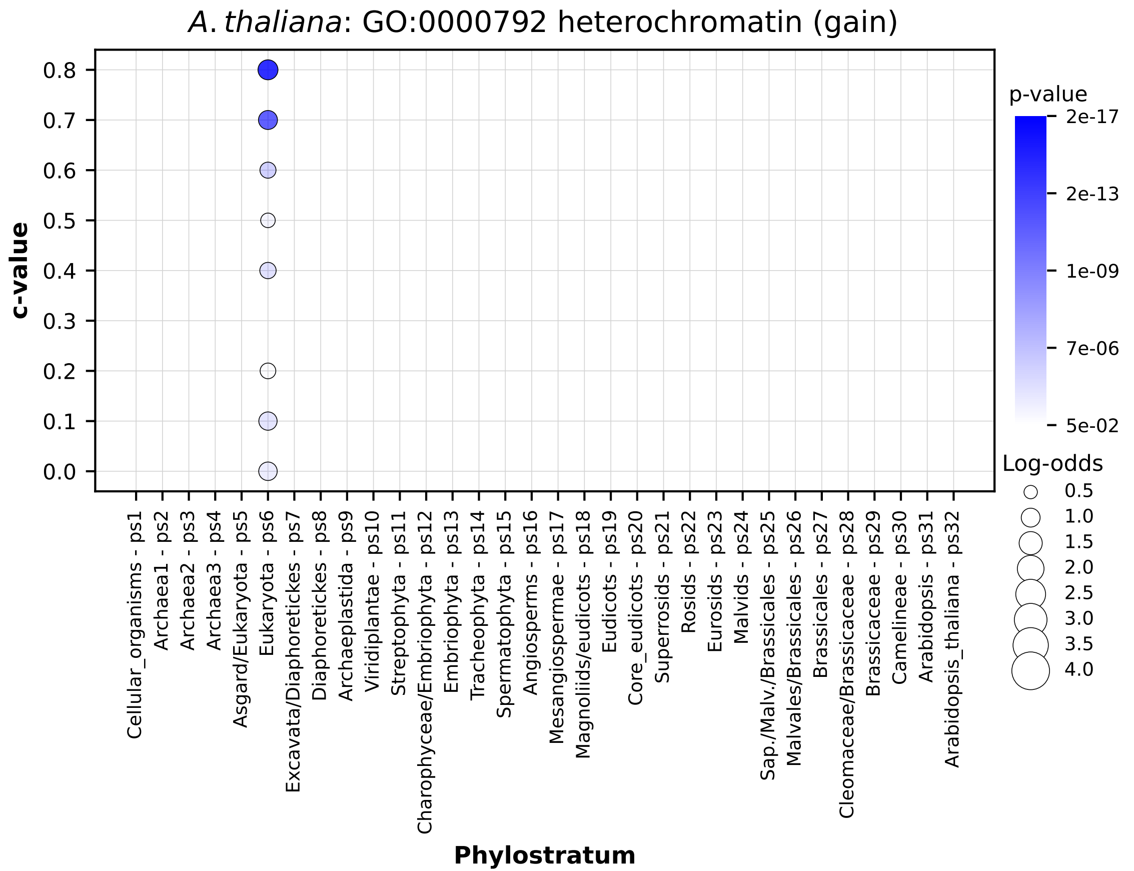
A. thaliana: GO:0000792 heterochromatin (gain)
c-value
0.0
0.1
0.2
0.3
0.4
0.5
0.6
0.7
0.8
p-value
2e-17
2e-13
1e-09
7e-06
5e-02
Log-odds
0.5
1.0
1.5
2.0
2.5
3.0
3.5
4.0
Cellular_organisms - ps1
Archaea1 - ps2
Archaea2 - ps3
Archaea3 - ps4
Asgard/Eukaryota - ps5
Eukaryota - ps6
Excavata/Diaphoretickes - ps7
Diaphoretickes - ps8
Archaeplastida - ps9
Viridiplantae - ps10
Streptophyta - ps11
Charophyceae/Embriophyta - ps12
Embriophyta - ps13
Tracheophyta - ps14
Spermatophyta - ps15
Angiosperms - ps16
Mesangiospermae - ps17
Magnoliids/eudicots - ps18
Eudicots - ps19
Core_eudicots - ps20
Superrosids - ps21
Rosids - ps22
Eurosids - ps23
Malvids - ps24
Sap./Malv./Brassicales - ps25
Malvales/Brassicales - ps26
Brassicales - ps27
Cleomaceae/Brassicaceae - ps28
Brassicaceae - ps29
Camelineae - ps30
Arabidopsis - ps31
Arabidopsis_thaliana - ps32
Phylostratum

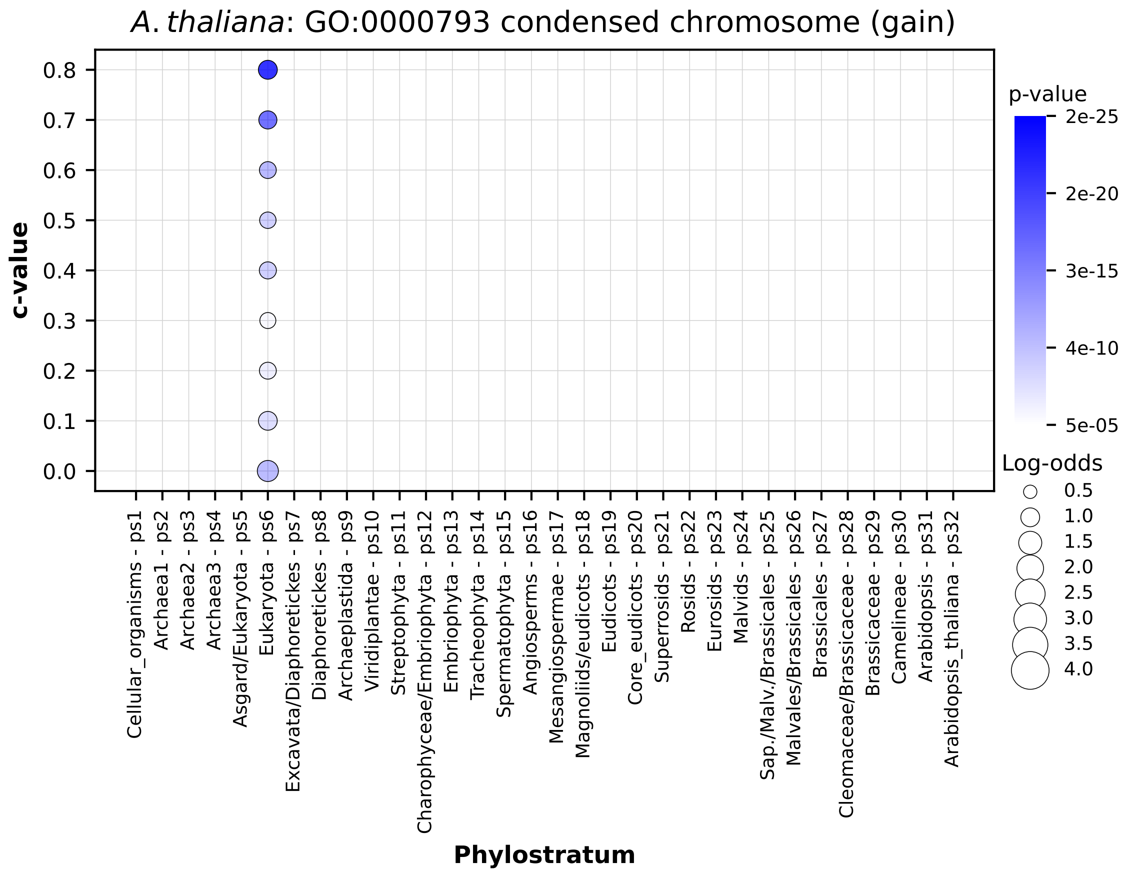

A. thaliana: GO:0000793 condensed chromosome (gain)
c-value
0.8
0.7
0.6
0.5
0.4
0.3
0.2
0.1
0.0
p-value
2e-25
2e-20
3e-15
4e-10
5e-05
Log-odds
0.5
1.0
1.5
2.0
2.5
3.0
3.5
4.0
Cellular_organisms - ps1
Archaea1 - ps2
Archaea2 - ps3
Archaea3 - ps4
Asgard/Eukaryota - ps5
Eukaryota - ps6
Excavata/Diaphoretickes - ps7
Diaphoretickes - ps8
Archaeplastida - ps9
Viridiplantae - ps10
Streptophyta - ps11
Charophyceae/Embriophyta - ps12
Embriophyta - ps13
Tracheophyta - ps14
Spermatophyta - ps15
Angiosperms - ps16
Mesangiospermae - ps17
Magnoliids/eudicots - ps18
Eudicots - ps19
Core_eudicots - ps20
Superrosids - ps21
Rosids - ps22
Eurosids - ps23
Malvids - ps24
Sap./Malv./Brassicales - ps25
Malvales/Brassicales - ps26
Brassicales - ps27
Cleomaceae/Brassicaceae - ps28
Brassicaceae - ps29
Camelineae - ps30
Arabidopsis - ps31
Arabidopsis_thaliana - ps32
Phylostratum

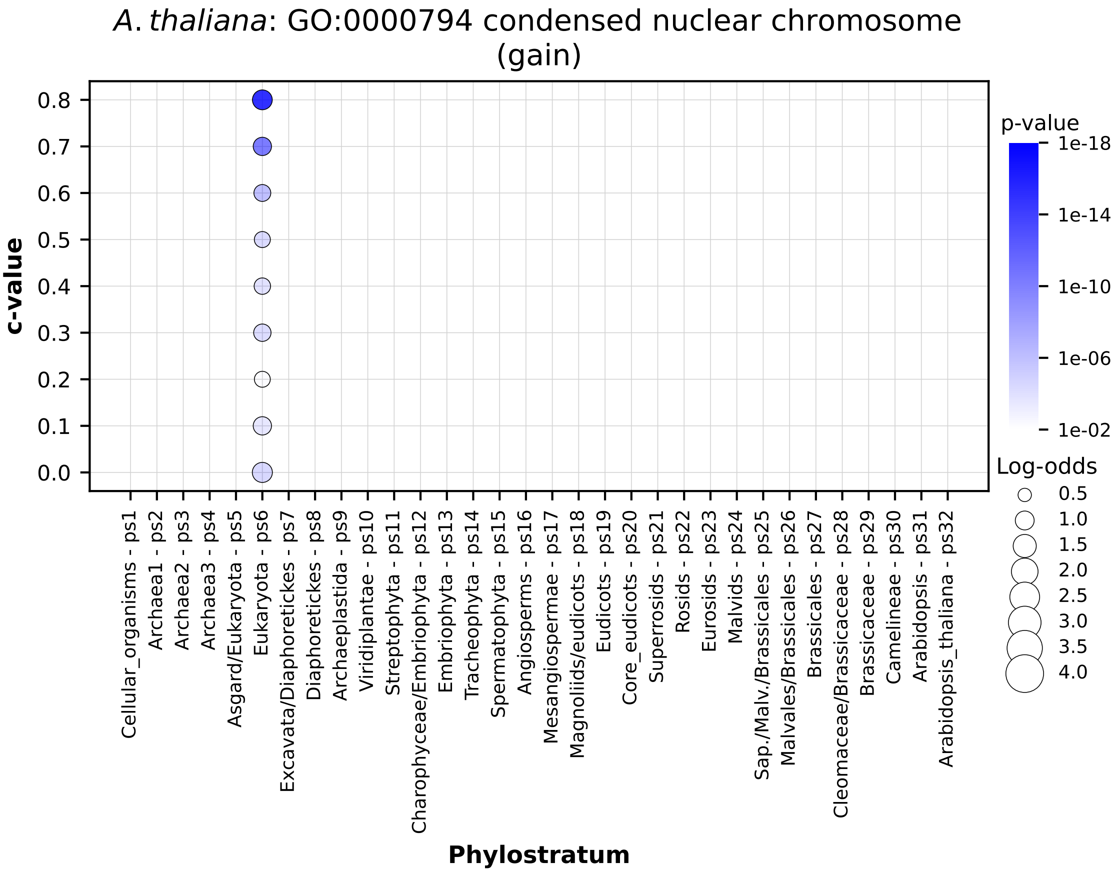
A. thaliana: GO:0000794 condensed nuclear chromosome (gain)
c-value
0.0
0.1
0.2
0.3
0.4
0.5
0.6
0.7
0.8
p-value
1e-18
1e-14
1e-10
1e-06
1e-02
Log-odds
0.5
1.0
1.5
2.0
2.5
3.0
3.5
4.0
Cellular_organisms - ps1
Archaea1 - ps2
Archaea2 - ps3
Archaea3 - ps4
Asgard/Eukaryota - ps5
Eukaryota - ps6
Excavata/Diaphoretickes - ps7
Diaphoretickes - ps8
Archaeplastida - ps9
Viridiplantae - ps10
Streptophyta - ps11
Charophyceae/Embriophyta - ps12
Embriophyta - ps13
Tracheophyta - ps14
Spermatophyta - ps15
Angiosperms - ps16
Mesangiospermae - ps17
Magnoliids/eudicots - ps18
Eudicots - ps19
Core_eudicots - ps20
Superrosids - ps21
Rosids - ps22
Eurosids - ps23
Malvids - ps24
Sap./Malv./Brassicales - ps25
Malvales/Brassicales - ps26
Brassicales - ps27
Cleomaceae/Brassicaceae - ps28
Brassicaceae - ps29
Camelineae - ps30
Arabidopsis - ps31
Arabidopsis_thaliana - ps32
Phylostratum

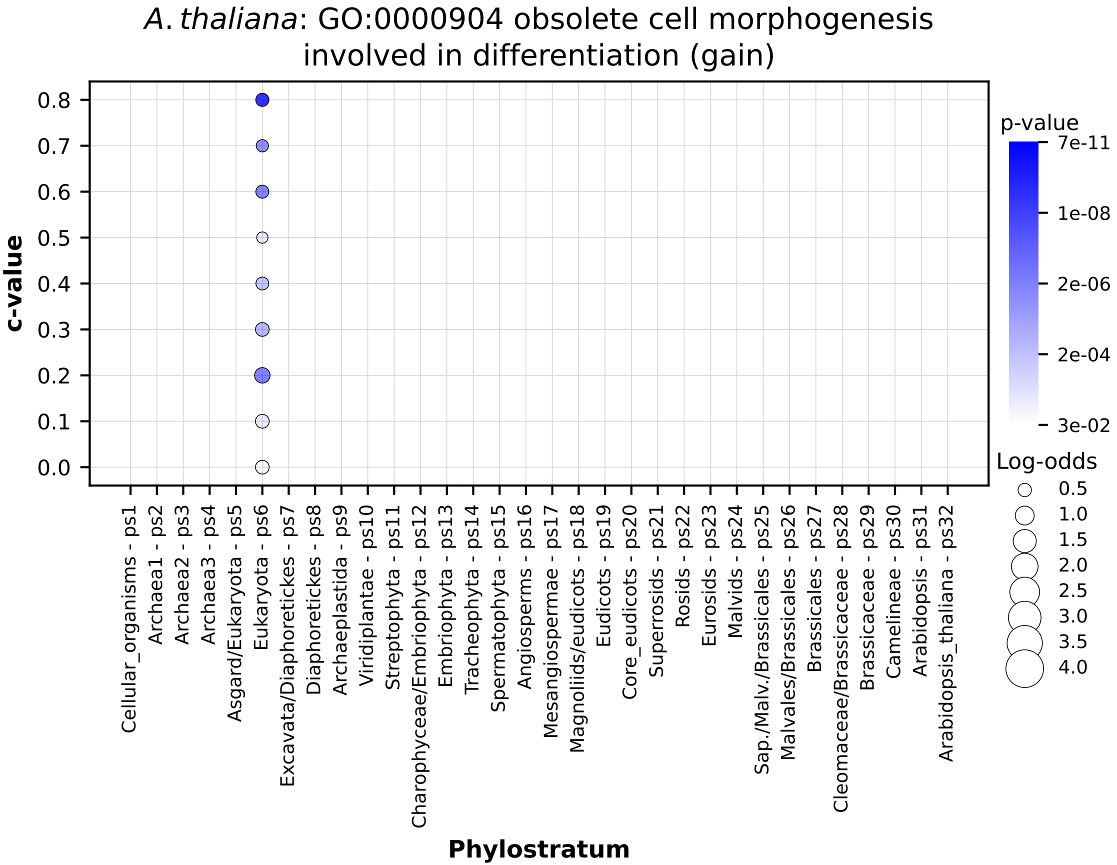
A. thaliana: GO:0000904 obsolete cell morphogenesis involved in differentiation (gain)
c-value
0.0
0.1
0.2
0.3
0.4
0.5
0.6
0.7
0.8
Phylostratum
Cellular_organisms - ps1
Archaea1 - ps2
Archaea2 - ps3
Archaea3 - ps4
Asgard/Eukaryota - ps5
Eukaryota - ps6
Excavata/Diaphoretickes - ps7
Diaphoretickes - ps8
Archaeplastida - ps9
Viridiplantae - ps10
Streptophyta - ps11
Charophyceae/Embriophyta - ps12
Embriophyta - ps13
Tracheophyta - ps14
Spermatophyta - ps15
Angiosperms - ps16
Mesangiospermae - ps17
Magnoliids/eudicots - ps18
Eudicots - ps19
Core_eudicots - ps20
Superrosids - ps21
Rosids - ps22
Eurosids - ps23
Malvids - ps24
Sap./Malv./Brassicales - ps25
Malvales/Brassicales - ps26
Brassicales - ps27
Cleomaceae/Brassicaceae - ps28
Brassicaceae - ps29
Camelineae - ps30
Arabidopsis - ps31
Arabidopsis_thaliana - ps32
p-value
7e-11
1e-08
2e-06
2e-04
3e-02
Log-odds
0.5
1.0
1.5
2.0
2.5
3.0
3.5
4.0

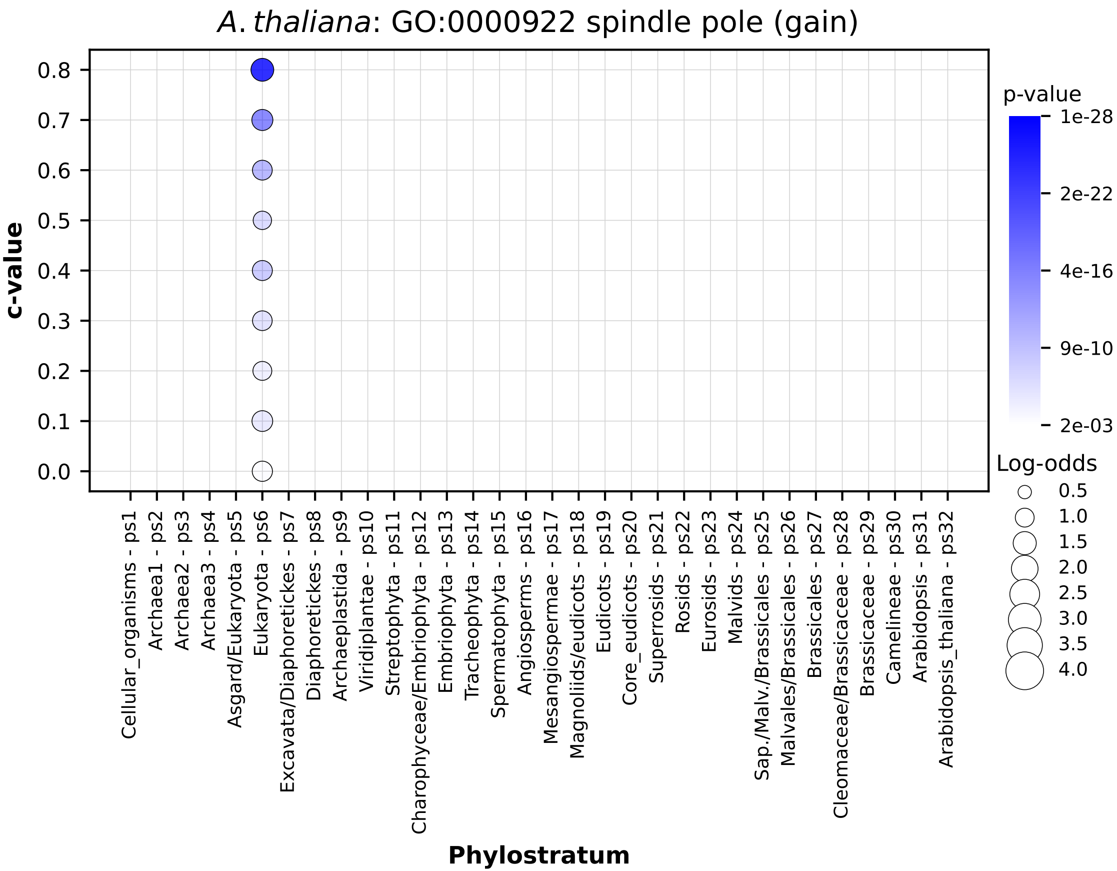

A. thaliana: GO:0000922 spindle pole (gain)
c-value
0.8
0.7
0.6
0.5
0.4
0.3
0.2
0.1
0.0
p-value
1e-28
2e-22
4e-16
9e-10
2e-03
Log-odds
0.5
1.0
1.5
2.0
2.5
3.0
3.5
4.0
Cellular_organisms - ps1
Archaea1 - ps2
Archaea2 - ps3
Archaea3 - ps4
Asgard/Eukaryota - ps5
Eukaryota - ps6
Excavata/Diaphoretickes - ps7
Diaphoretickes - ps8
Archaeplastida - ps9
Viridiplantae - ps10
Streptophyta - ps11
Charophyceae/Embriophyta - ps12
Embriophyta - ps13
Tracheophyta - ps14
Spermatophyta - ps15
Angiosperms - ps16
Mesangiospermae - ps17
Magnoliids/eudicots - ps18
Eudicots - ps19
Core_eudicots - ps20
Superrosids - ps21
Rosids - ps22
Eurosids - ps23
Malvids - ps24
Sap./Malv./Brassicales - ps25
Malvales/Brassicales - ps26
Brassicales - ps27
Cleomaceae/Brassicaceae - ps28
Brassicaceae - ps29
Camelineae - ps30
Arabidopsis - ps31
Arabidopsis_thaliana - ps32
Phylostratum

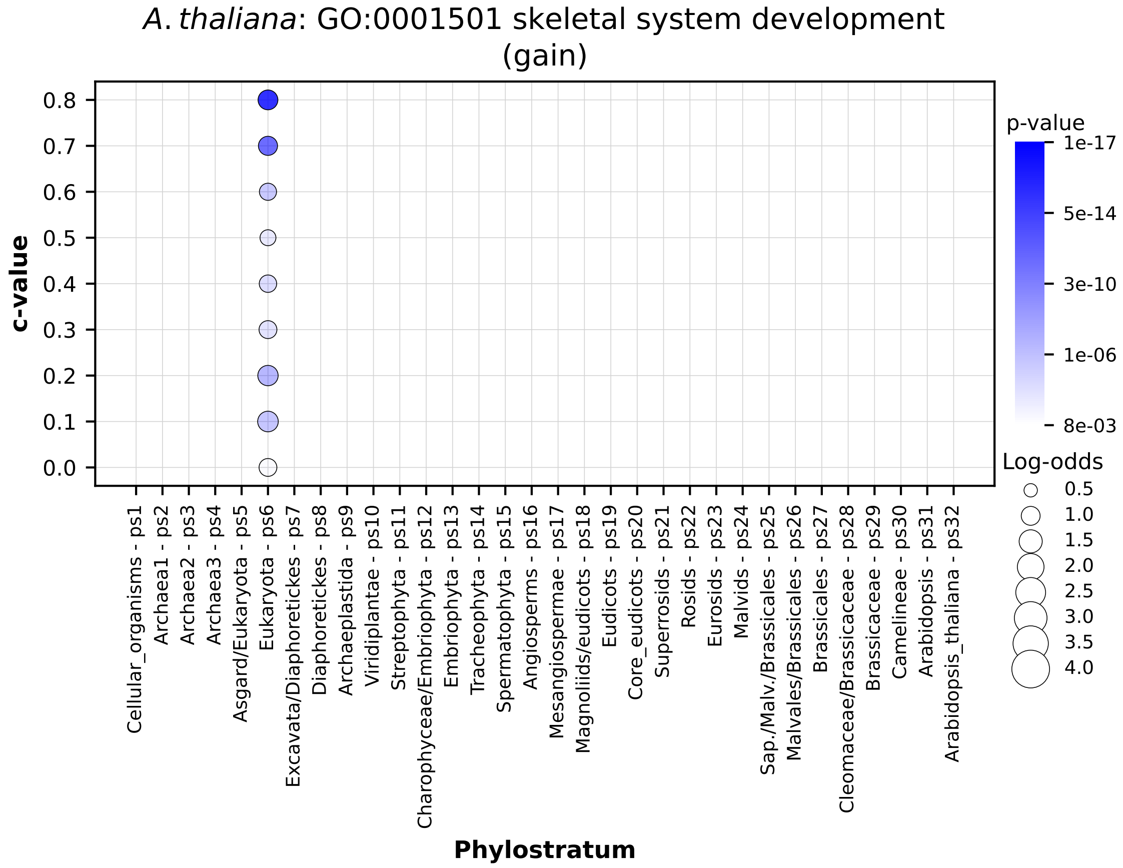

A. thaliana: GO:0001501 skeletal system development
(gain)
c-value
0.0
0.1
0.2
0.3
0.4
0.5
0.6
0.7
0.8
Phylostratum
Cellular_organisms - ps1
Archaea1 - ps2
Archaea2 - ps3
Archaea3 - ps4
Asgard/Eukaryota - ps5
Eukaryota - ps6
Excavata/Diaphoretickes - ps7
Diaphoretickes - ps8
Archaeplastida - ps9
Viridiplantae - ps10
Streptophyta - ps11
Charophyceae/Embriophyta - ps12
Embriophyta - ps13
Tracheophyta - ps14
Spermatophyta - ps15
Angiosperms - ps16
Mesangiospermae - ps17
Magnoliids/eudicots - ps18
Eudicots - ps19
Core_eudicots - ps20
Superrosids - ps21
Rosids - ps22
Eurosids - ps23
Malvids - ps24
Sap./Malv./Brassicales - ps25
Malvales/Brassicales - ps26
Brassicales - ps27
Cleomaceae/Brassicaceae - ps28
Brassicaceae - ps29
Camelineae - ps30
Arabidopsis - ps31
Arabidopsis_thaliana - ps32
p-value
1e-17
5e-14
3e-10
1e-06
8e-03
Log-odds
0.5
1.0
1.5
2.0
2.5
3.0
3.5
4.0

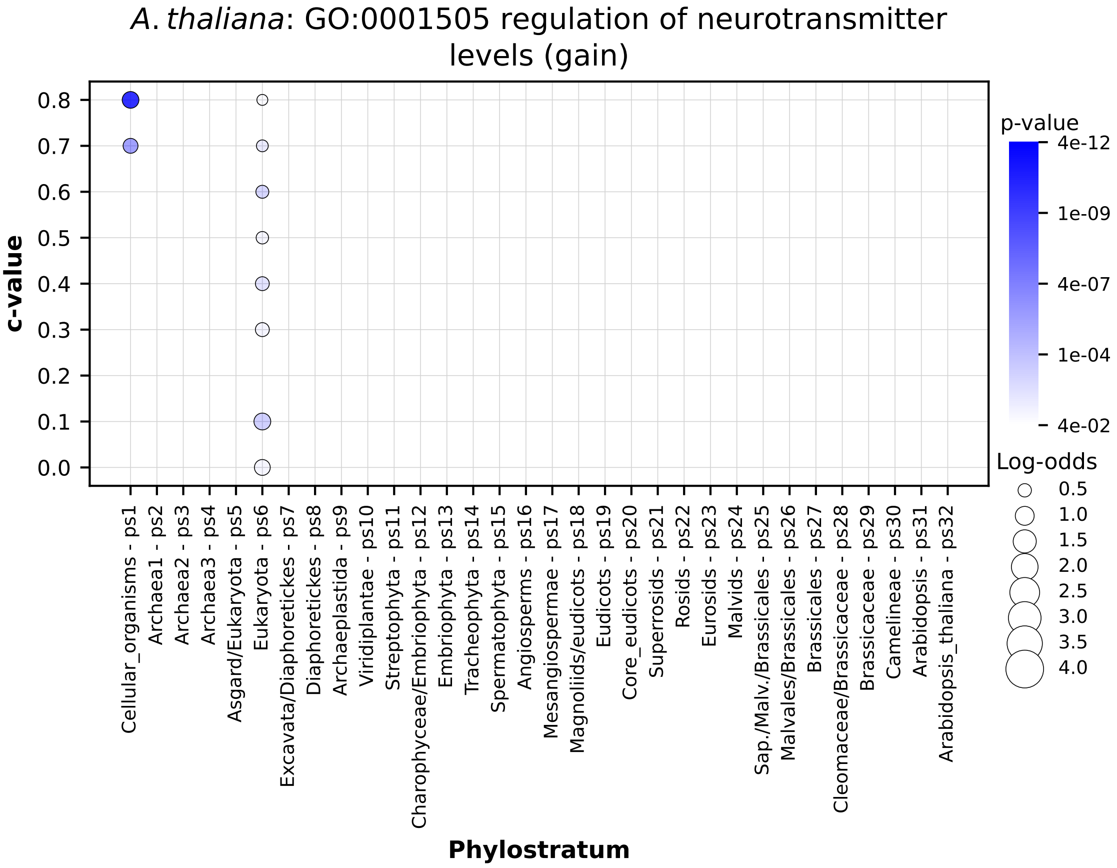
A. thaliana: GO:0001505 regulation of neurotransmitter levels (gain)
c-value
0.8
0.7
0.6
0.5
0.4
0.3
0.2
0.1
0.0
p-value
4e-12
1e-09
4e-07
1e-04
4e-02
Log-odds
0.5
1.0
1.5
2.0
2.5
3.0
3.5
4.0
Cellular_organisms - ps1
Archaea1 - ps2
Archaea2 - ps3
Archaea3 - ps4
Asgard/Eukaryota - ps5
Eukaryota - ps6
Excavata/Diaphoretickes - ps7
Diaphoretickes - ps8
Archaeplastida - ps9
Viridiplantae - ps10
Streptophyta - ps11
Charophyceae/Embriophyta - ps12
Embriophyta - ps13
Tracheophyta - ps14
Spermatophyta - ps15
Angiosperms - ps16
Mesangiospermae - ps17
Magnoliids/eudicots - ps18
Eudicots - ps19
Core_eudicots - ps20
Superrosids - ps21
Rosids - ps22
Eurosids - ps23
Malvids - ps24
Sap./Malv./Brassicales - ps25
Malvales/Brassicales - ps26
Brassicales - ps27
Cleomaceae/Brassicaceae - ps28
Brassicaceae - ps29
Camelineae - ps30
Arabidopsis - ps31
Arabidopsis_thaliana - ps32
Phylostratum

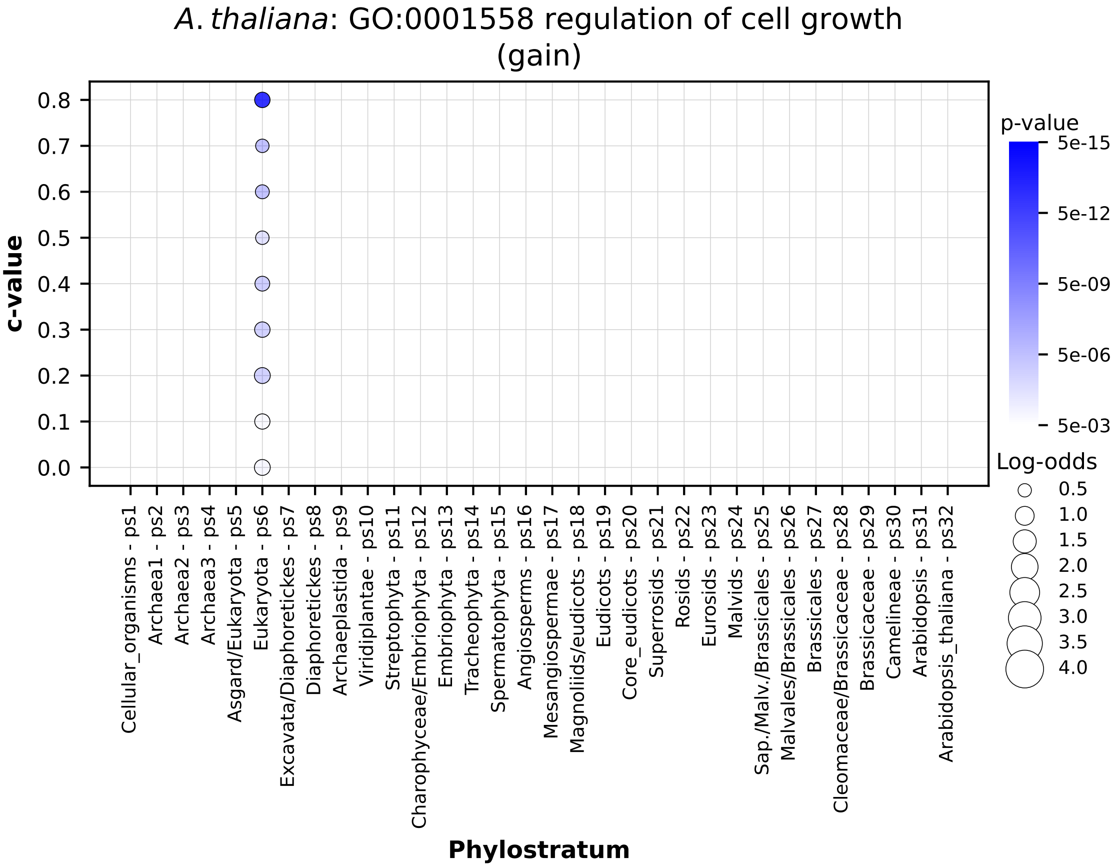

A. thaliana: GO:0001558 regulation of cell growth
(gain)
c-value
0.8
0.7
0.6
0.5
0.4
0.3
0.2
0.1
0.0
p-value
5e-15
5e-12
5e-09
5e-06
5e-03
Log-odds
0.5
1.0
1.5
2.0
2.5
3.0
3.5
4.0
Cellular_organisms - ps1
Archaea1 - ps2
Archaea2 - ps3
Archaea3 - ps4
Asgard/Eukaryota - ps5
Eukaryota - ps6
Excavata/Diaphoretickes - ps7
Diaphoretickes - ps8
Archaeplastida - ps9
Viridiplantae - ps10
Streptophyta - ps11
Charophyceae/Embriophyta - ps12
Embriophyta - ps13
Tracheophyta - ps14
Spermatophyta - ps15
Angiosperms - ps16
Mesangiospermae - ps17
Magnoliids/eudicots - ps18
Eudicots - ps19
Core_eudicots - ps20
Superrosids - ps21
Rosids - ps22
Eurosids - ps23
Malvids - ps24
Sap./Malv./Brassicales - ps25
Malvales/Brassicales - ps26
Brassicales - ps27
Cleomaceae/Brassicaceae - ps28
Brassicaceae - ps29
Camelineae - ps30
Arabidopsis - ps31
Arabidopsis_thaliana - ps32
Phylostratum

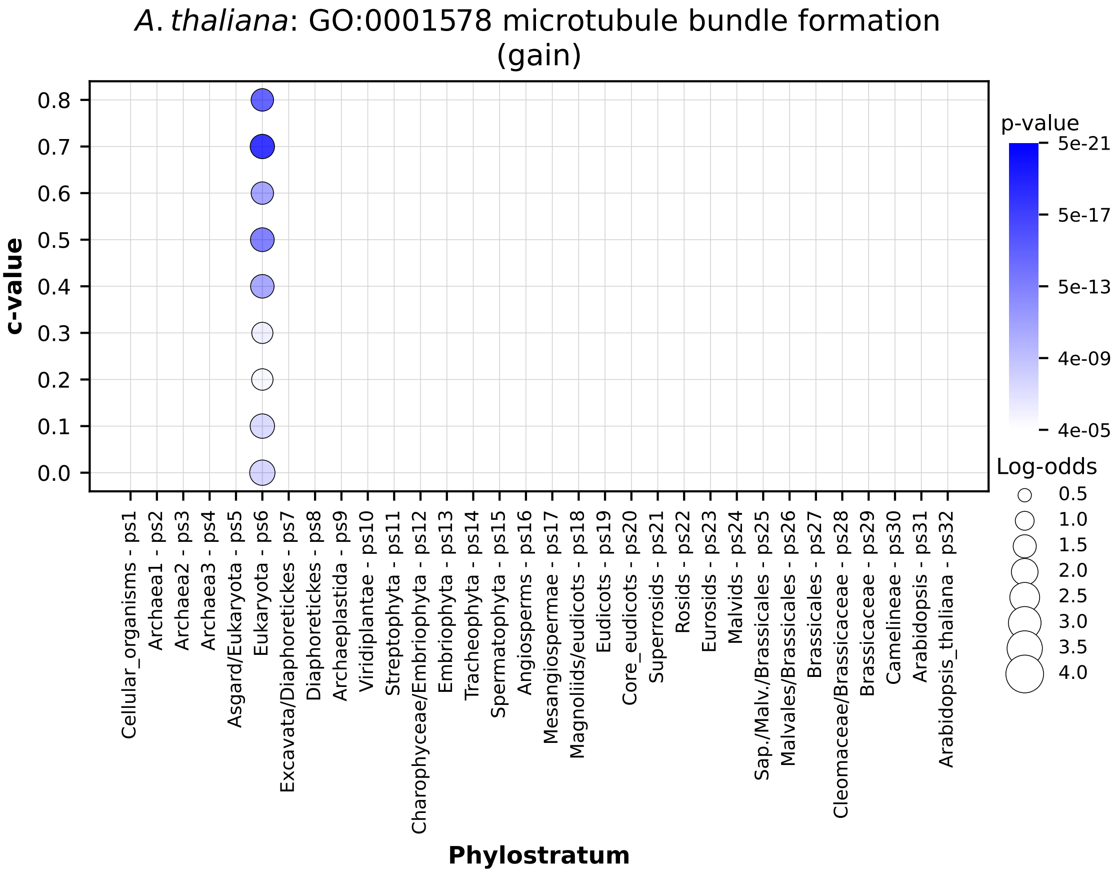
A. thaliana: GO:0001578 microtubule bundle formation (gain)
c-value
0.8
0.7
0.6
0.5
0.4
0.3
0.2
0.1
0.0
p-value
5e-21
5e-17
5e-13
4e-09
4e-05
Log-odds
0.5
1.0
1.5
2.0
2.5
3.0
3.5
4.0
Cellular_organisms - ps1
Archaea1 - ps2
Archaea2 - ps3
Archaea3 - ps4
Asgard/Eukaryota - ps5
Eukaryota - ps6
Excavata/Diaphoretickes - ps7
Diaphoretickes - ps8
Archaeplastida - ps9
Viridiplantae - ps10
Streptophyta - ps11
Charophyceae/Embriophyta - ps12
Embriophyta - ps13
Tracheophyta - ps14
Spermatophyta - ps15
Angiosperms - ps16
Mesangiospermae - ps17
Magnoliids/eudicots - ps18
Eudicots - ps19
Core_eudicots - ps20
Superrosids - ps21
Rosids - ps22
Eurosids - ps23
Malvids - ps24
Sap./Malv./Brassicales - ps25
Malvales/Brassicales - ps26
Brassicales - ps27
Cleomaceae/Brassicaceae - ps28
Brassicaceae - ps29
Camelineae - ps30
Arabidopsis - ps31
Arabidopsis_thaliana - ps32
Phylostratum

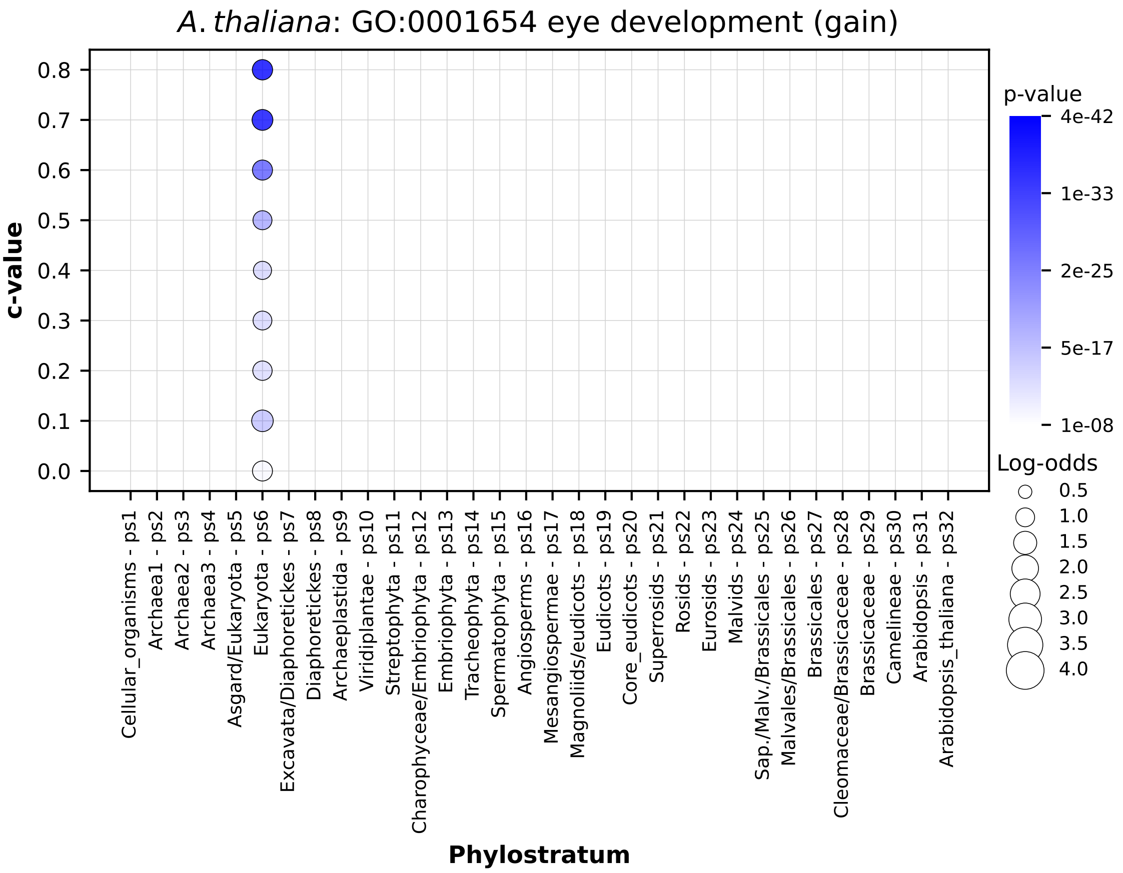
A. thaliana: GO:0001654 eye development (gain)
c-value
0.8
0.7
0.6
0.5
0.4
0.3
0.2
0.1
0.0
Phylostratum
Cellular_organisms - ps1
Archaea1 - ps2
Archaea2 - ps3
Archaea3 - ps4
Asgard/Eukaryota - ps5
Eukaryota - ps6
Excavata/Diaphoretickes - ps7
Diaphoretickes - ps8
Archaeplastida - ps9
Viridiplantae - ps10
Streptophyta - ps11
Charophyceae/Embriophyta - ps12
Embriophyta - ps13
Tracheophyta - ps14
Spermatophyta - ps15
Angiosperms - ps16
Mesangiospermae - ps17
Magnoliids/eudicots - ps18
Eudicots - ps19
Core_eudicots - ps20
Superrosids - ps21
Rosids - ps22
Eurosids - ps23
Malvids - ps24
Sap./Malv./Brassicales - ps25
Malvales/Brassicales - ps26
Brassicales - ps27
Cleomaceae/Brassicaceae - ps28
Brassicaceae - ps29
Camelineae - ps30
Arabidopsis - ps31
Arabidopsis_thaliana - ps32
p-value
4e-42
1e-33
2e-25
5e-17
1e-08
Log-odds
0.5
1.0
1.5
2.0
2.5
3.0
3.5
4.0

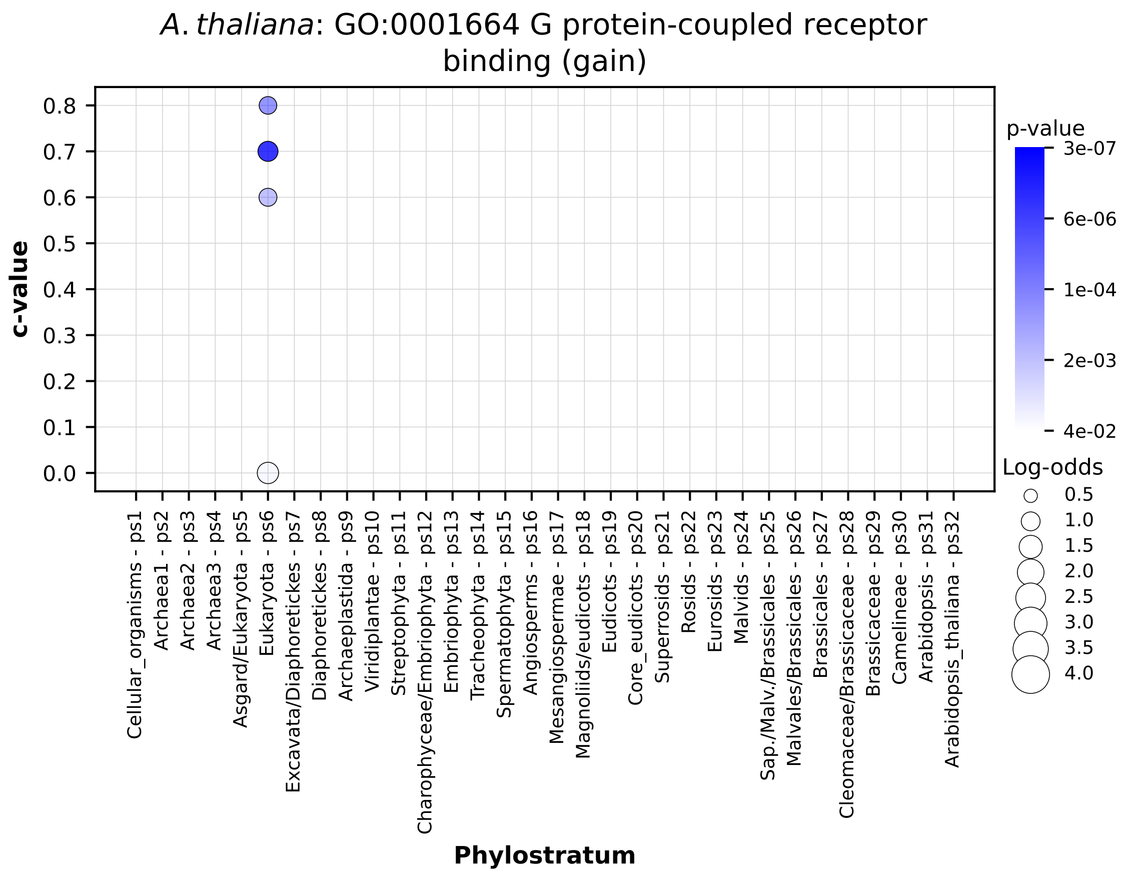

A. thaliana: GO:0001664 G protein-coupled receptor binding (gain)
c-value
0.8
0.7
0.6
0.5
0.4
0.3
0.2
0.1
0.0
p-value
3e-07
6e-06
1e-04
2e-03
4e-02
Log-odds
0.5
1.0
1.5
2.0
2.5
3.0
3.5
4.0
Cellular_organisms - ps1
Archaea1 - ps2
Archaea2 - ps3
Archaea3 - ps4
Asgard/Eukaryota - ps5
Eukaryota - ps6
Excavata/Diaphoretickes - ps7
Diaphoretickes - ps8
Archaeplastida - ps9
Viridiplantae - ps10
Streptophyta - ps11
Charophyceae/Embriophyta - ps12
Embriophyta - ps13
Tracheophyta - ps14
Spermatophyta - ps15
Angiosperms - ps16
Mesangiospermae - ps17
Magnoliids/eudicots - ps18
Eudicots - ps19
Core_eudicots - ps20
Superrosids - ps21
Rosids - ps22
Eurosids - ps23
Malvids - ps24
Sap./Malv./Brassicales - ps25
Malvales/Brassicales - ps26
Brassicales - ps27
Cleomaceae/Brassicaceae - ps28
Brassicaceae - ps29
Camelineae - ps30
Arabidopsis - ps31
Arabidopsis_thaliana - ps32
Phylostratum

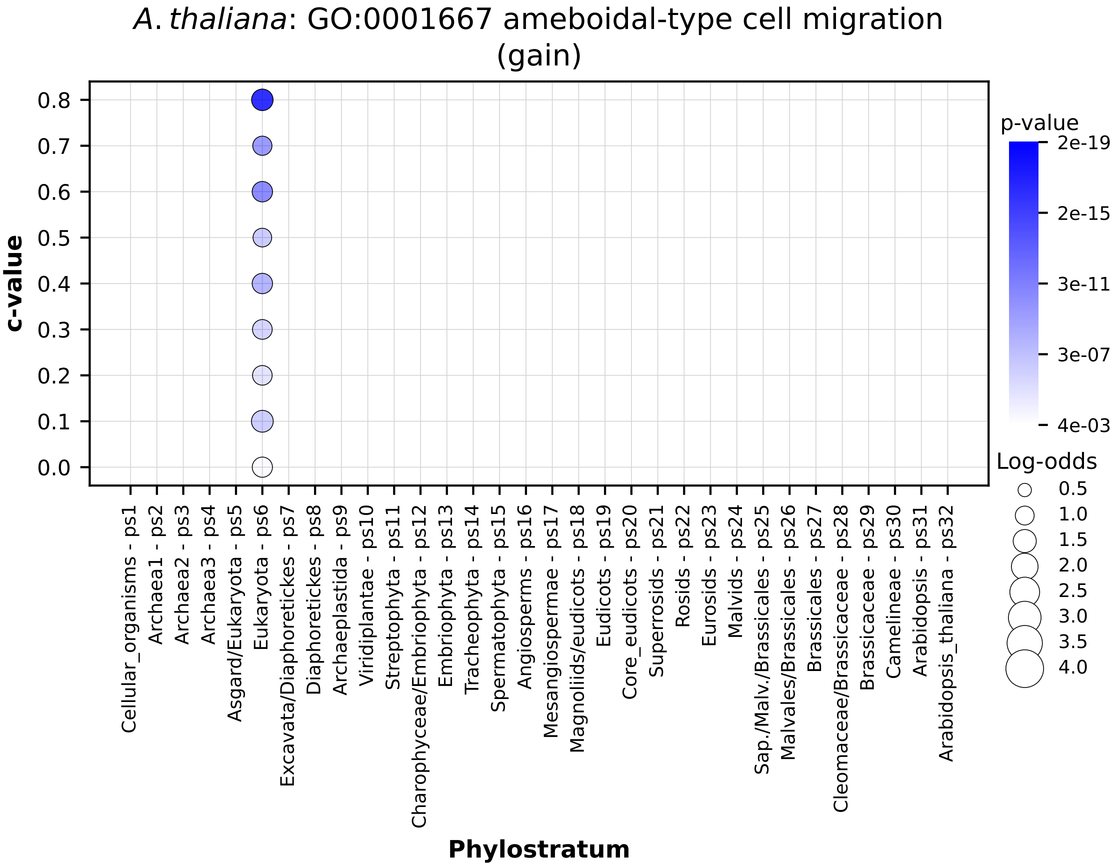

A. thaliana: GO:0001667 ameboidal-type cell migration
(gain)
c-value
0.8
0.7
0.6
0.5
0.4
0.3
0.2
0.1
0.0
p-value
2e-19
2e-15
3e-11
3e-07
4e-03
Log-odds
0.5
1.0
1.5
2.0
2.5
3.0
3.5
4.0
Cellular_organisms - ps1
Archaea1 - ps2
Archaea2 - ps3
Archaea3 - ps4
Asgard/Eukaryota - ps5
Eukaryota - ps6
Excavata/Diaphoretickes - ps7
Diaphoretickes - ps8
Archaeplastida - ps9
Viridiplantae - ps10
Streptophyta - ps11
Charophyceae/Embriophyta - ps12
Embriophyta - ps13
Tracheophyta - ps14
Spermatophyta - ps15
Angiosperms - ps16
Mesangiospermae - ps17
Magnoliids/eudicots - ps18
Eudicots - ps19
Core_eudicots - ps20
Superrosids - ps21
Rosids - ps22
Eurosids - ps23
Malvids - ps24
Sap./Malv./Brassicales - ps25
Malvales/Brassicales - ps26
Brassicales - ps27
Cleomaceae/Brassicaceae - ps28
Brassicaceae - ps29
Camelineae - ps30
Arabidopsis - ps31
Arabidopsis_thaliana - ps32
Phylostratum

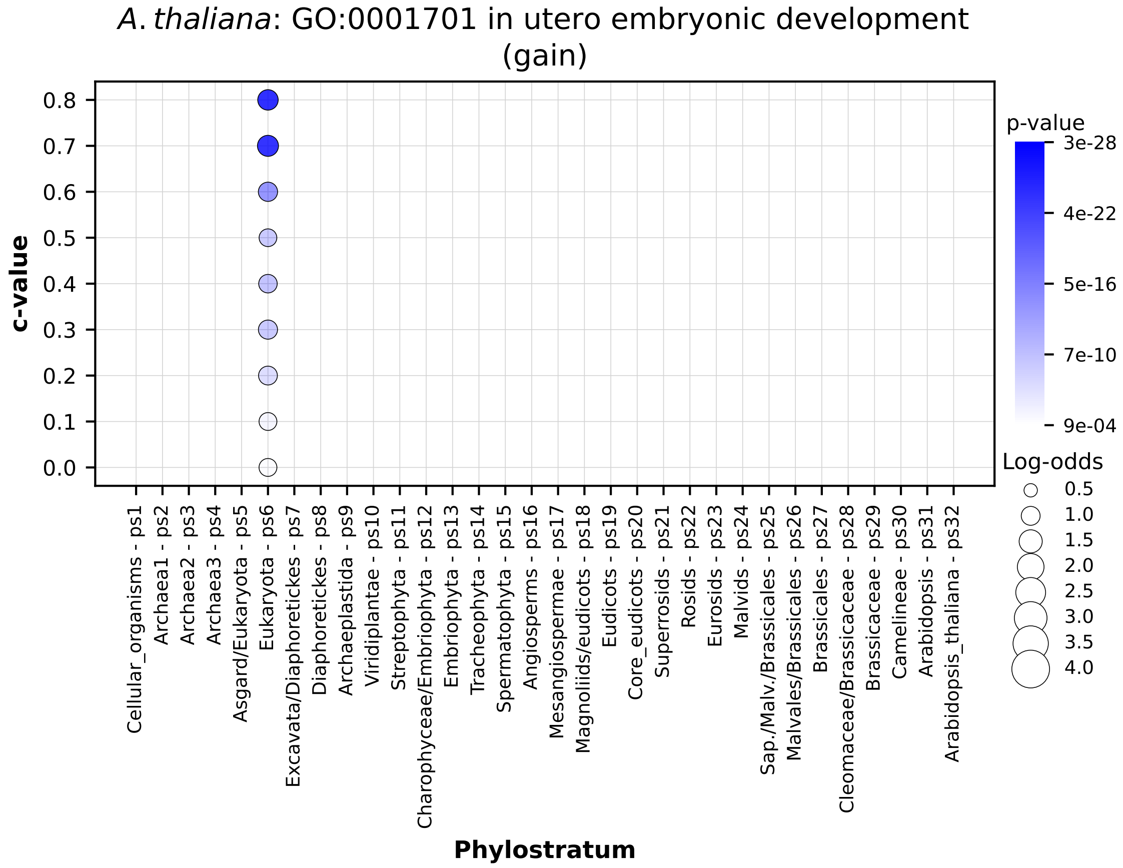

A. thaliana: GO:0001701 in utero embryonic development
(gain)
c-value
0.8
0.7
0.6
0.5
0.4
0.3
0.2
0.1
0.0
p-value
3e-28
4e-22
5e-16
7e-10
9e-04
Log-odds
0.5
1.0
1.5
2.0
2.5
3.0
3.5
4.0
Cellular_organisms - ps1
Archaea1 - ps2
Archaea2 - ps3
Archaea3 - ps4
Asgard/Eukaryota - ps5
Eukaryota - ps6
Excavata/Diaphoretickes - ps7
Diaphoretickes - ps8
Archaeplastida - ps9
Viridiplantae - ps10
Streptophyta - ps11
Charophyceae/Embriophyta - ps12
Embriophyta - ps13
Tracheophyta - ps14
Spermatophyta - ps15
Angiosperms - ps16
Mesangiospermae - ps17
Magnoliids/eudicots - ps18
Eudicots - ps19
Core_eudicots - ps20
Superrosids - ps21
Rosids - ps22
Eurosids - ps23
Malvids - ps24
Sap./Malv./Brassicales - ps25
Malvales/Brassicales - ps26
Brassicales - ps27
Cleomaceae/Brassicaceae - ps28
Brassicaceae - ps29
Camelineae - ps30
Arabidopsis - ps31
Arabidopsis_thaliana - ps32
Phylostratum

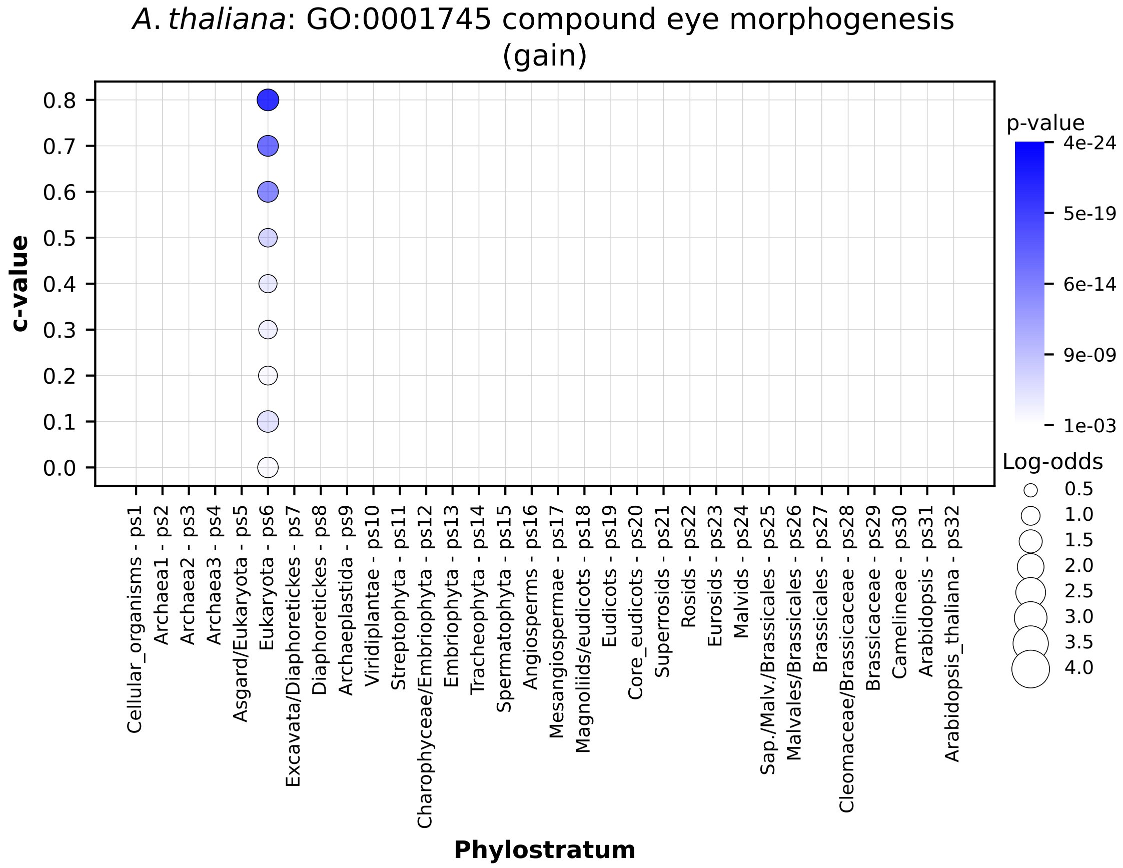

A. thaliana: GO:0001745 compound eye morphogenesis
(gain)
c-value
0.8
0.7
0.6
0.5
0.4
0.3
0.2
0.1
0.0
p-value
4e-24
5e-19
6e-14
9e-09
1e-03
Log-odds
0.5
1.0
1.5
2.0
2.5
3.0
3.5
4.0
Cellular_organisms - ps1
Archaea1 - ps2
Archaea2 - ps3
Archaea3 - ps4
Asgard/Eukaryota - ps5
Eukaryota - ps6
Excavata/Diaphoretickes - ps7
Diaphoretickes - ps8
Archaeplastida - ps9
Viridiplantae - ps10
Streptophyta - ps11
Charophyceae/Embriophyta - ps12
Embriophyta - ps13
Tracheophyta - ps14
Spermatophyta - ps15
Angiosperms - ps16
Mesangiospermae - ps17
Magnoliids/eudicots - ps18
Eudicots - ps19
Core_eudicots - ps20
Superrosids - ps21
Rosids - ps22
Eurosids - ps23
Malvids - ps24
Sap./Malv./Brassicales - ps25
Malvales/Brassicales - ps26
Brassicales - ps27
Cleomaceae/Brassicaceae - ps28
Brassicaceae - ps29
Camelineae - ps30
Arabidopsis - ps31
Arabidopsis_thaliana - ps32
Phylostratum

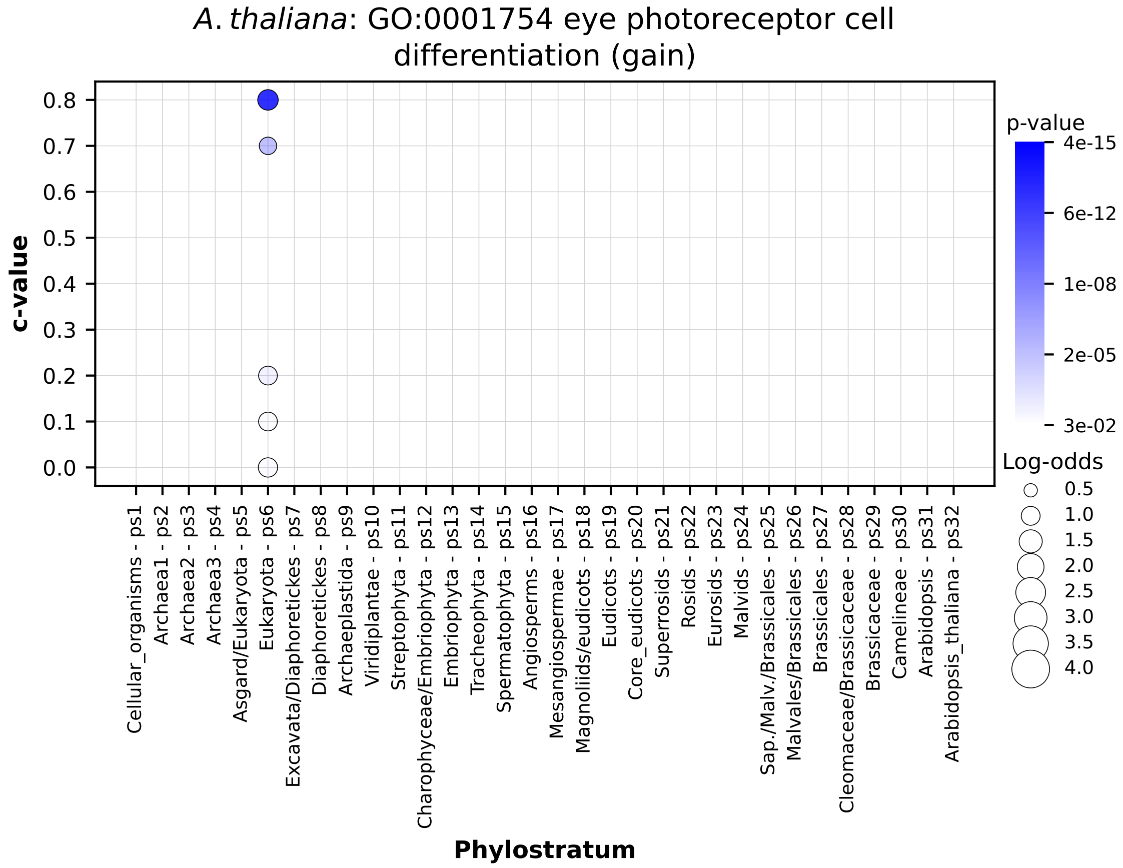

A. thaliana: GO:0001754 eye photoreceptor cell differentiation (gain)
c-value
0.8
0.7
0.6
0.5
0.4
0.3
0.2
0.1
0.0
p-value
4e-15
6e-12
1e-08
2e-05
3e-02
Log-odds
0.5
1.0
1.5
2.0
2.5
3.0
3.5
4.0
Cellular_organisms - ps1
Archaea1 - ps2
Archaea2 - ps3
Archaea3 - ps4
Asgard/Eukaryota - ps5
Eukaryota - ps6
Excavata/Diaphoretickes - ps7
Diaphoretickes - ps8
Archaeplastida - ps9
Viridiplantae - ps10
Streptophyta - ps11
Charophyceae/Embriophyta - ps12
Embriophyta - ps13
Tracheophyta - ps14
Spermatophyta - ps15
Angiosperms - ps16
Mesangiospermae - ps17
Magnoliids/eudicots - ps18
Eudicots - ps19
Core_eudicots - ps20
Superrosids - ps21
Rosids - ps22
Eurosids - ps23
Malvids - ps24
Sap./Malv./Brassicales - ps25
Malvales/Brassicales - ps26
Brassicales - ps27
Cleomaceae/Brassicaceae - ps28
Brassicaceae - ps29
Camelineae - ps30
Arabidopsis - ps31
Arabidopsis_thaliana - ps32
Phylostratum

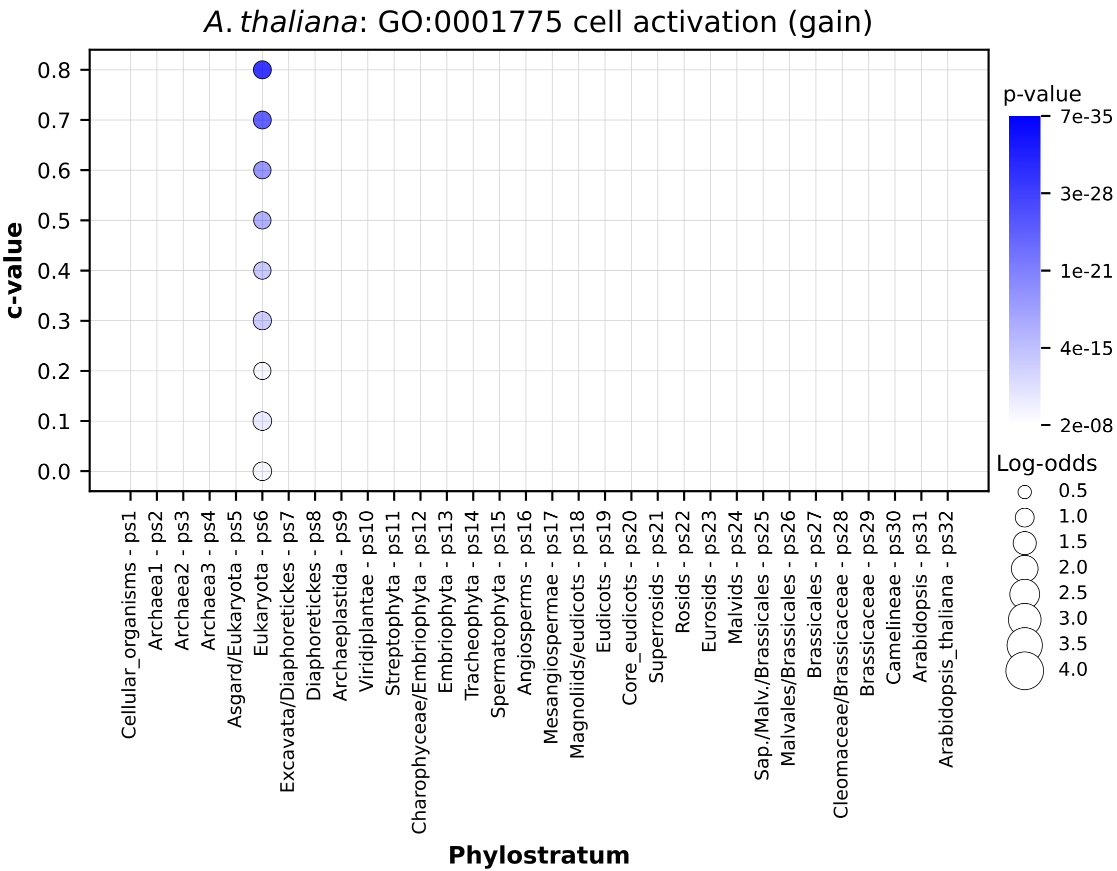

A. thaliana: GO:0001775 cell activation (gain)
c-value
0.8
0.7
0.6
0.5
0.4
0.3
0.2
0.1
0.0
p-value
7e-35
3e-28
1e-21
4e-15
2e-08
Log-odds
0.5
1.0
1.5
2.0
2.5
3.0
3.5
4.0
Cellular_organisms - ps1
Archaea1 - ps2
Archaea2 - ps3
Archaea3 - ps4
Asgard/Eukaryota - ps5
Eukaryota - ps6
Excavata/Diaphoretickes - ps7
Diaphoretickes - ps8
Archaeplastida - ps9
Viridiplantae - ps10
Streptophyta - ps11
Charophyceae/Embriophyta - ps12
Embriophyta - ps13
Tracheophyta - ps14
Spermatophyta - ps15
Angiosperms - ps16
Mesangiospermae - ps17
Magnoliids/eudicots - ps18
Eudicots - ps19
Core_eudicots - ps20
Superrosids - ps21
Rosids - ps22
Eurosids - ps23
Malvids - ps24
Sap./Malv./Brassicales - ps25
Malvales/Brassicales - ps26
Brassicales - ps27
Cleomaceae/Brassicaceae - ps28
Brassicaceae - ps29
Camelineae - ps30
Arabidopsis - ps31
Arabidopsis_thaliana - ps32
Phylostratum

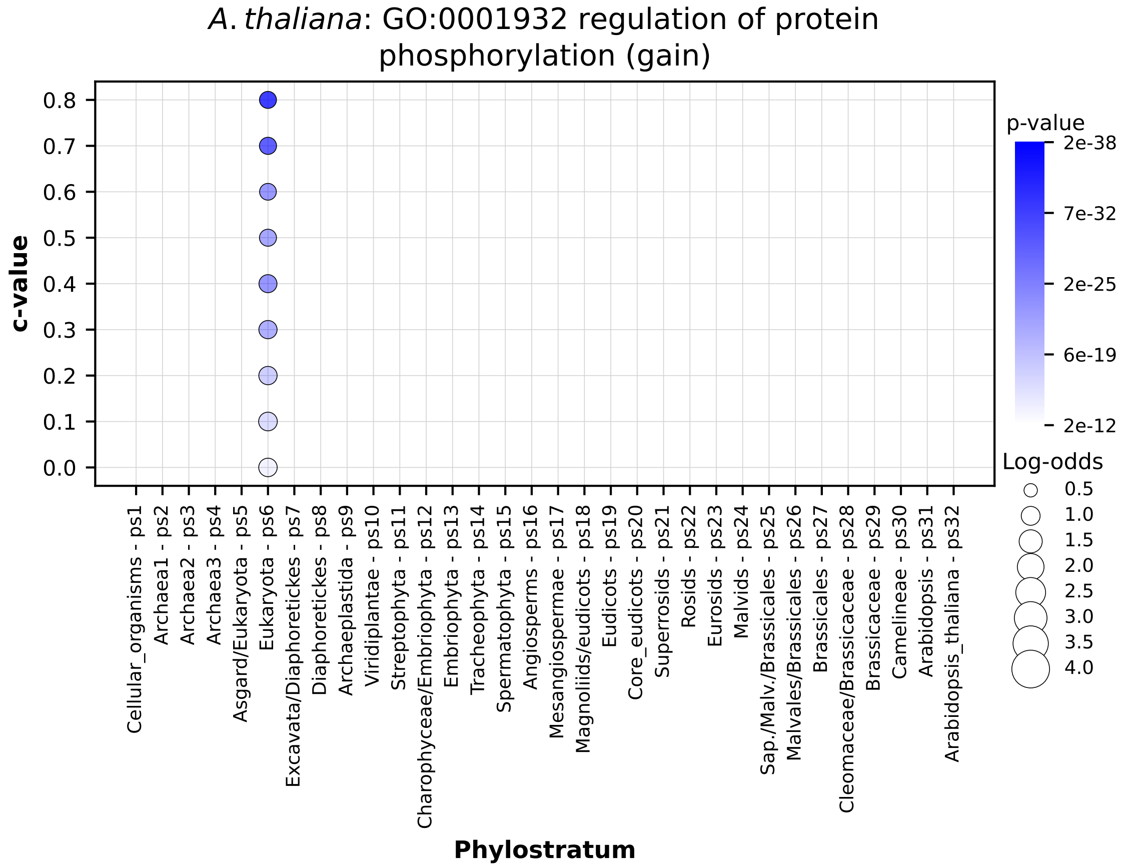

A. thaliana: GO:0001932 regulation of protein phosphorylation (gain)
c-value
0.8
0.7
0.6
0.5
0.4
0.3
0.2
0.1
0.0
p-value
2e-38
7e-32
2e-25
6e-19
2e-12
Log-odds
0.5
1.0
1.5
2.0
2.5
3.0
3.5
4.0
Cellular_organisms - ps1
Archaea1 - ps2
Archaea2 - ps3
Archaea3 - ps4
Asgard/Eukaryota - ps5
Eukaryota - ps6
Excavata/Diaphoretickes - ps7
Diaphoretickes - ps8
Archaeplastida - ps9
Viridiplantae - ps10
Streptophyta - ps11
Charophyceae/Embriophyta - ps12
Embriophyta - ps13
Tracheophyta - ps14
Spermatophyta - ps15
Angiosperms - ps16
Mesangiospermae - ps17
Magnoliids/eudicots - ps18
Eudicots - ps19
Core_eudicots - ps20
Superrosids - ps21
Rosids - ps22
Eurosids - ps23
Malvids - ps24
Sap./Malv./Brassicales - ps25
Malvales/Brassicales - ps26
Brassicales - ps27
Cleomaceae/Brassicaceae - ps28
Brassicaceae - ps29
Camelineae - ps30
Arabidopsis - ps31
Arabidopsis_thaliana - ps32
Phylostratum

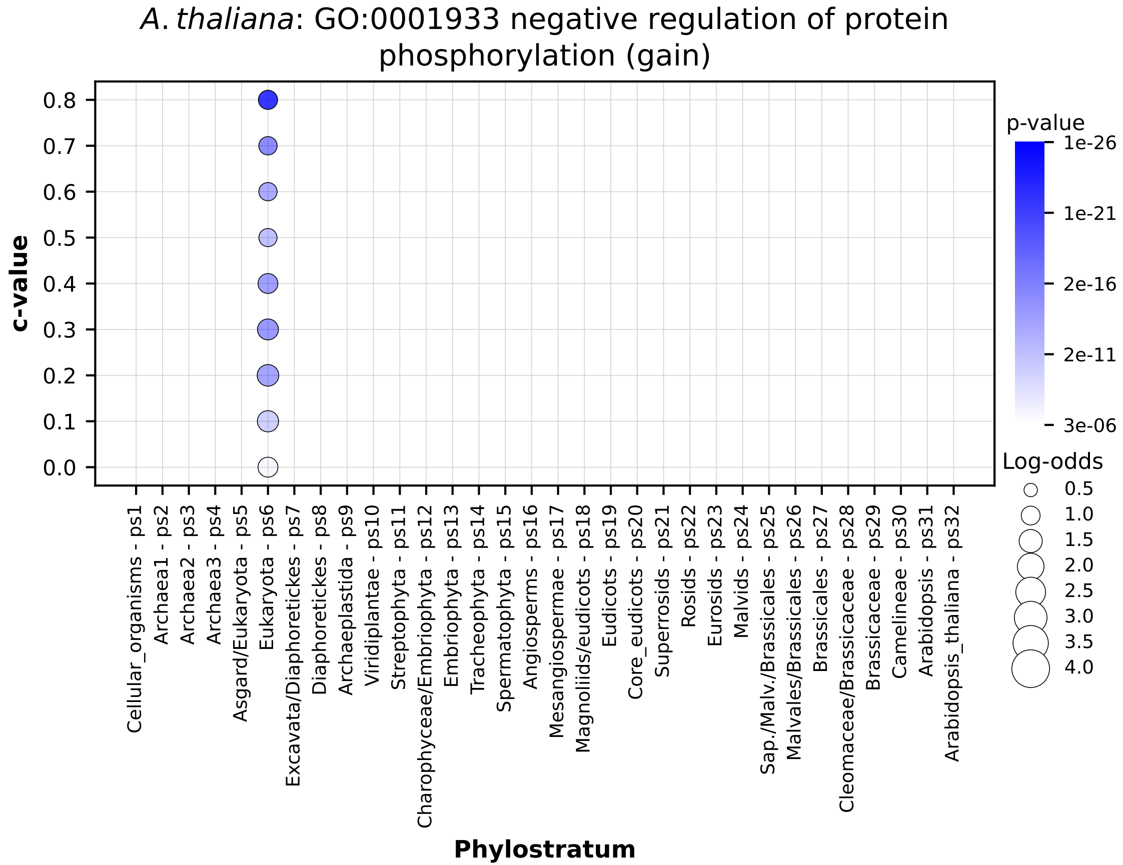
A. thaliana: GO:0001933 negative regulation of protein phosphorylation (gain)
c-value
0.8
0.7
0.6
0.5
0.4
0.3
0.2
0.1
0.0
p-value
1e-26
1e-21
2e-16
2e-11
3e-06
Log-odds
0.5
1.0
1.5
2.0
2.5
3.0
3.5
4.0
Cellular_organisms - ps1
Archaea1 - ps2
Archaea2 - ps3
Archaea3 - ps4
Asgard/Eukaryota - ps5
Eukaryota - ps6
Excavata/Diaphoretickes - ps7
Diaphoretickes - ps8
Archaeplastida - ps9
Viridiplantae - ps10
Streptophyta - ps11
Charophyceae/Embriophyta - ps12
Embriophyta - ps13
Tracheophyta - ps14
Spermatophyta - ps15
Angiosperms - ps16
Mesangiospermae - ps17
Magnoliids/eudicots - ps18
Eudicots - ps19
Core_eudicots - ps20
Superrosids - ps21
Rosids - ps22
Eurosids - ps23
Malvids - ps24
Sap./Malv./Brassicales - ps25
Malvales/Brassicales - ps26
Brassicales - ps27
Cleomaceae/Brassicaceae - ps28
Brassicaceae - ps29
Camelineae - ps30
Arabidopsis - ps31
Arabidopsis_thaliana - ps32
Phylostratum

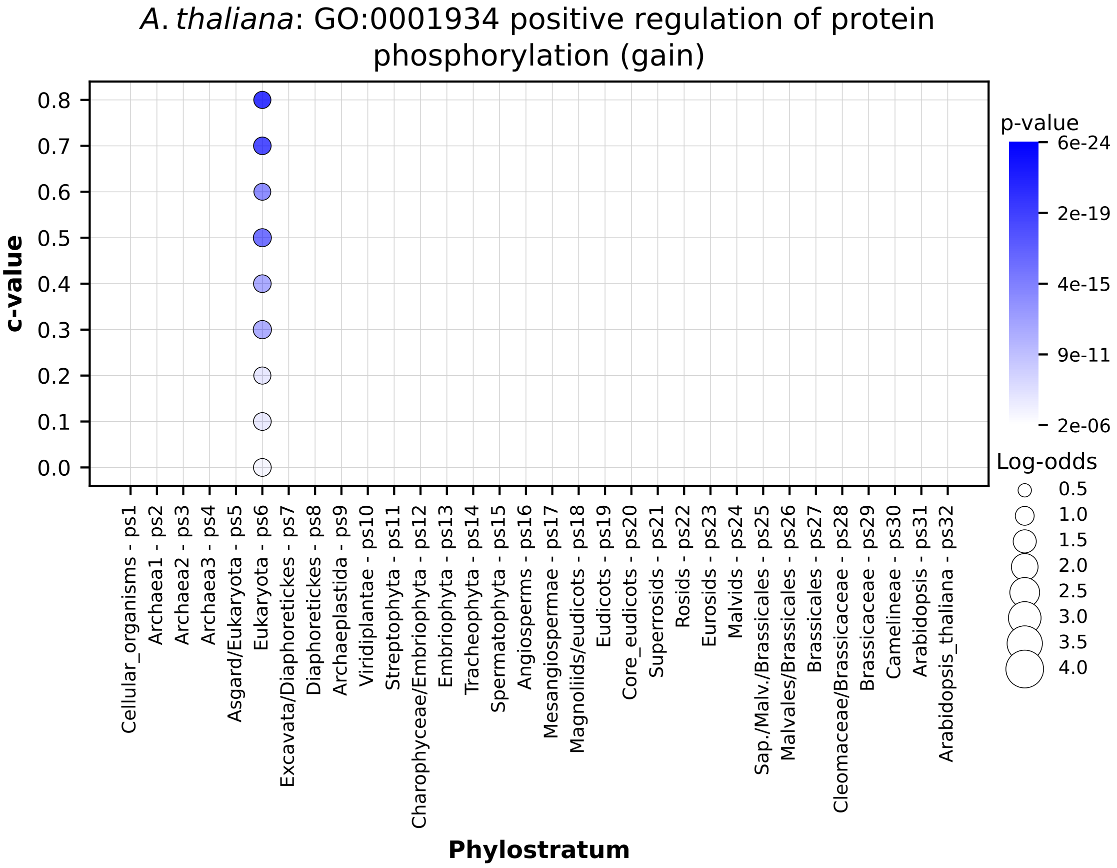

A. thaliana: GO:0001934 positive regulation of protein phosphorylation (gain)
c-value
0.8
0.7
0.6
0.5
0.4
0.3
0.2
0.1
0.0
p-value
6e-24
2e-19
4e-15
9e-11
2e-06
Log-odds
0.5
1.0
1.5
2.0
2.5
3.0
3.5
4.0
Cellular_organisms - ps1
Archaea1 - ps2
Archaea2 - ps3
Archaea3 - ps4
Asgard/Eukaryota - ps5
Eukaryota - ps6
Excavata/Diaphoretickes - ps7
Diaphoretickes - ps8
Archaeplastida - ps9
Viridiplantae - ps10
Streptophyta - ps11
Charophyceae/Embriophyta - ps12
Embriophyta - ps13
Tracheophyta - ps14
Spermatophyta - ps15
Angiosperms - ps16
Mesangiospermae - ps17
Magnoliids/eudicots - ps18
Eudicots - ps19
Core_eudicots - ps20
Superrosids - ps21
Rosids - ps22
Eurosids - ps23
Malvids - ps24
Sap./Malv./Brassicales - ps25
Malvales/Brassicales - ps26
Brassicales - ps27
Cleomaceae/Brassicaceae - ps28
Brassicaceae - ps29
Camelineae - ps30
Arabidopsis - ps31
Arabidopsis_thaliana - ps32
Phylostratum

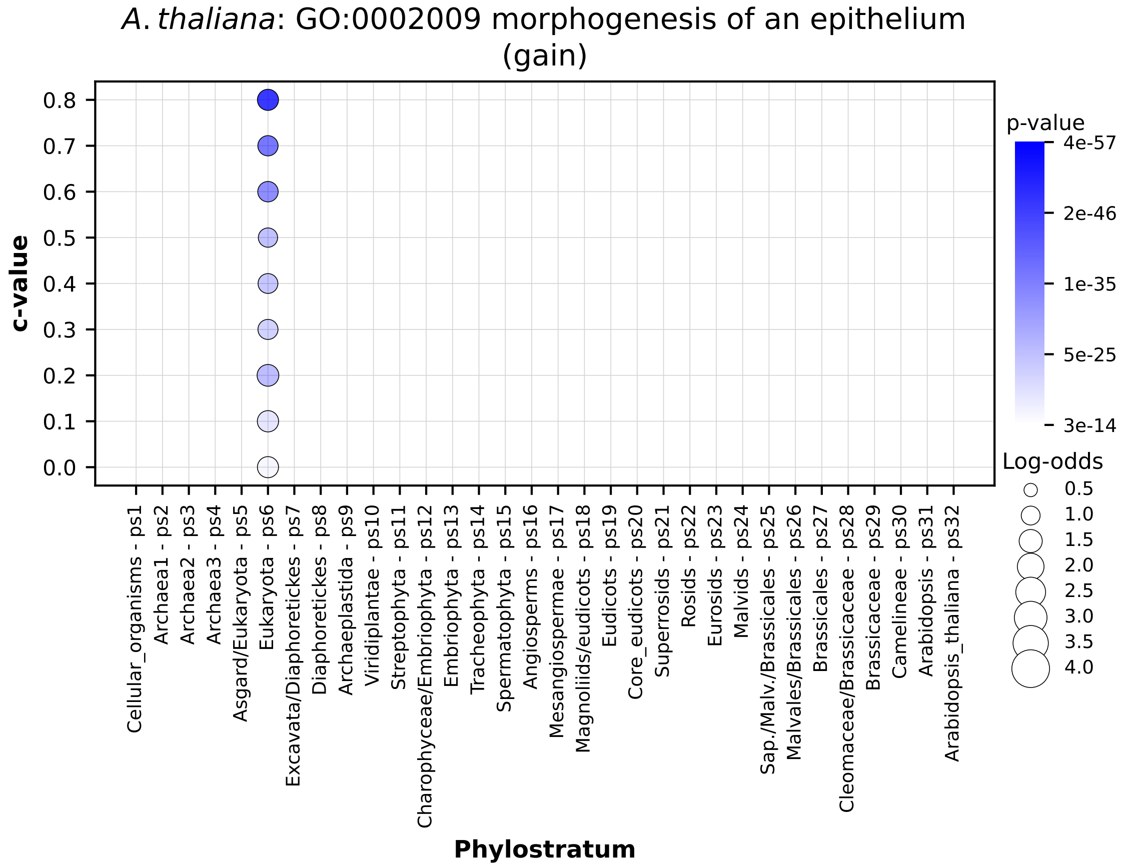
A. thaliana: GO:0002009 morphogenesis of an epithelium
(gain)
c-value
0.8
0.7
0.6
0.5
0.4
0.3
0.2
0.1
0.0
p-value
4e-57
2e-46
1e-35
5e-25
3e-14
Log-odds
0.5
1.0
1.5
2.0
2.5
3.0
3.5
4.0
Cellular_organisms - ps1
Archaea1 - ps2
Archaea2 - ps3
Archaea3 - ps4
Asgard/Eukaryota - ps5
Eukaryota - ps6
Excavata/Diaphoretickes - ps7
Diaphoretickes - ps8
Archaeplastida - ps9
Viridiplantae - ps10
Streptophyta - ps11
Charophyceae/Embriophyta - ps12
Embriophyta - ps13
Tracheophyta - ps14
Spermatophyta - ps15
Angiosperms - ps16
Mesangiospermae - ps17
Magnoliids/eudicots - ps18
Eudicots - ps19
Core_eudicots - ps20
Superrosids - ps21
Rosids - ps22
Eurosids - ps23
Malvids - ps24
Sap./Malv./Brassicales - ps25
Malvales/Brassicales - ps26
Brassicales - ps27
Cleomaceae/Brassicaceae - ps28
Brassicaceae - ps29
Camelineae - ps30
Arabidopsis - ps31
Arabidopsis_thaliana - ps32
Phylostratum

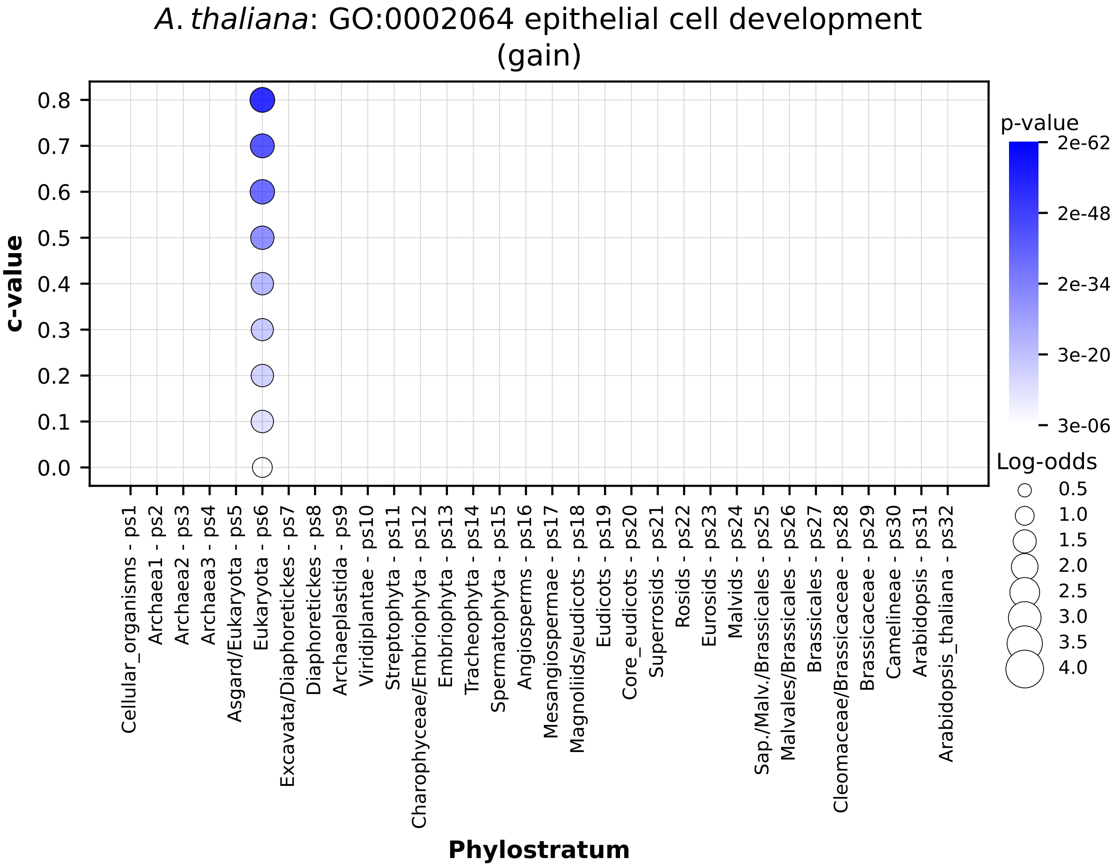

A. thaliana: GO:0002064 epithelial cell development (gain)
c-value
0.8
0.7
0.6
0.5
0.4
0.3
0.2
0.1
0.0
p-value
2e-62
2e-48
2e-34
3e-20
3e-06
Log-odds
0.5
1.0
1.5
2.0
2.5
3.0
3.5
4.0
Cellular_organisms - ps1
Archaea1 - ps2
Archaea2 - ps3
Archaea3 - ps4
Asgard/Eukaryota - ps5
Eukaryota - ps6
Excavata/Diaphoretickes - ps7
Diaphoretickes - ps8
Archaeplastida - ps9
Viridiplantae - ps10
Streptophyta - ps11
Charophyceae/Embriophyta - ps12
Embriophyta - ps13
Tracheophyta - ps14
Spermatophyta - ps15
Angiosperms - ps16
Mesangiospermae - ps17
Magnoliids/eudicots - ps18
Eudicots - ps19
Core_eudicots - ps20
Superrosids - ps21
Rosids - ps22
Eurosids - ps23
Malvids - ps24
Sap./Malv./Brassicales - ps25
Malvales/Brassicales - ps26
Brassicales - ps27
Cleomaceae/Brassicaceae - ps28
Brassicaceae - ps29
Camelineae - ps30
Arabidopsis - ps31
Arabidopsis_thaliana - ps32
Phylostratum

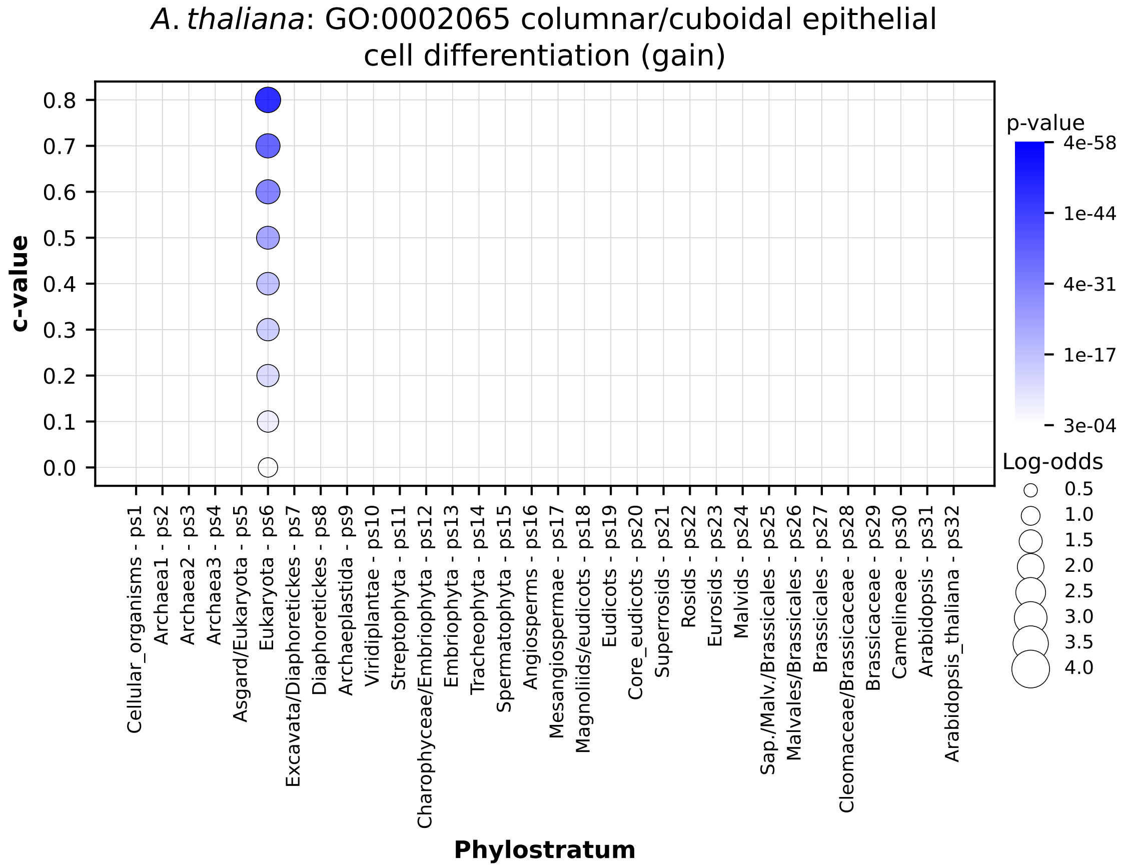

A. thaliana: GO:0002065 columnar/cuboidal epithelial cell differentiation (gain)
c-value
0.8
0.7
0.6
0.5
0.4
0.3
0.2
0.1
0.0
p-value
4e-58
1e-44
4e-31
1e-17
3e-04
Log-odds
0.5
1.0
1.5
2.0
2.5
3.0
3.5
4.0
Cellular_organisms - ps1
Archaea1 - ps2
Archaea2 - ps3
Archaea3 - ps4
Asgard/Eukaryota - ps5
Eukaryota - ps6
Excavata/Diaphoretickes - ps7
Diaphoretickes - ps8
Archaeplastida - ps9
Viridiplantae - ps10
Streptophyta - ps11
Charophyceae/Embriophyta - ps12
Embriophyta - ps13
Tracheophyta - ps14
Spermatophyta - ps15
Angiosperms - ps16
Mesangiospermae - ps17
Magnoliids/eudicots - ps18
Eudicots - ps19
Core_eudicots - ps20
Superrosids - ps21
Rosids - ps22
Eurosids - ps23
Malvids - ps24
Sap./Malv./Brassicales - ps25
Malvales/Brassicales - ps26
Brassicales - ps27
Cleomaceae/Brassicaceae - ps28
Brassicaceae - ps29
Camelineae - ps30
Arabidopsis - ps31
Arabidopsis_thaliana - ps32
Phylostratum

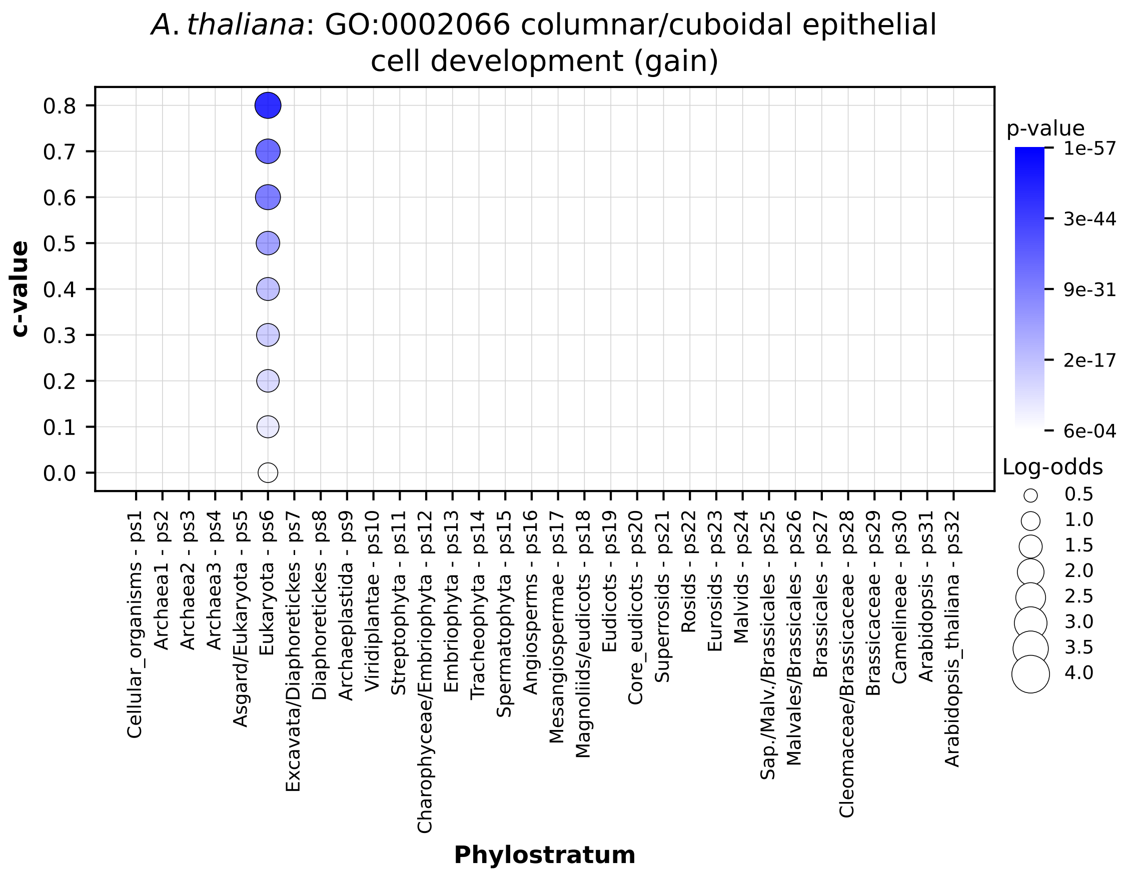

A. thaliana: GO:0002066 columnar/cuboidal epithelial cell development (gain)
c-value
0.8
0.7
0.6
0.5
0.4
0.3
0.2
0.1
0.0
p-value
1e-57
3e-44
9e-31
2e-17
6e-04
Log-odds
0.5
1.0
1.5
2.0
2.5
3.0
3.5
4.0
Cellular_organisms - ps1
Archaea1 - ps2
Archaea2 - ps3
Archaea3 - ps4
Asgard/Eukaryota - ps5
Eukaryota - ps6
Excavata/Diaphoretickes - ps7
Diaphoretickes - ps8
Archaeplastida - ps9
Viridiplantae - ps10
Streptophyta - ps11
Charophyceae/Embriophyta - ps12
Embriophyta - ps13
Tracheophyta - ps14
Spermatophyta - ps15
Angiosperms - ps16
Mesangiospermae - ps17
Magnoliids/eudicots - ps18
Eudicots - ps19
Core_eudicots - ps20
Superrosids - ps21
Rosids - ps22
Eurosids - ps23
Malvids - ps24
Sap./Malv./Brassicales - ps25
Malvales/Brassicales - ps26
Brassicales - ps27
Cleomaceae/Brassicaceae - ps28
Brassicaceae - ps29
Camelineae - ps30
Arabidopsis - ps31
Arabidopsis_thaliana - ps32
Phylostratum

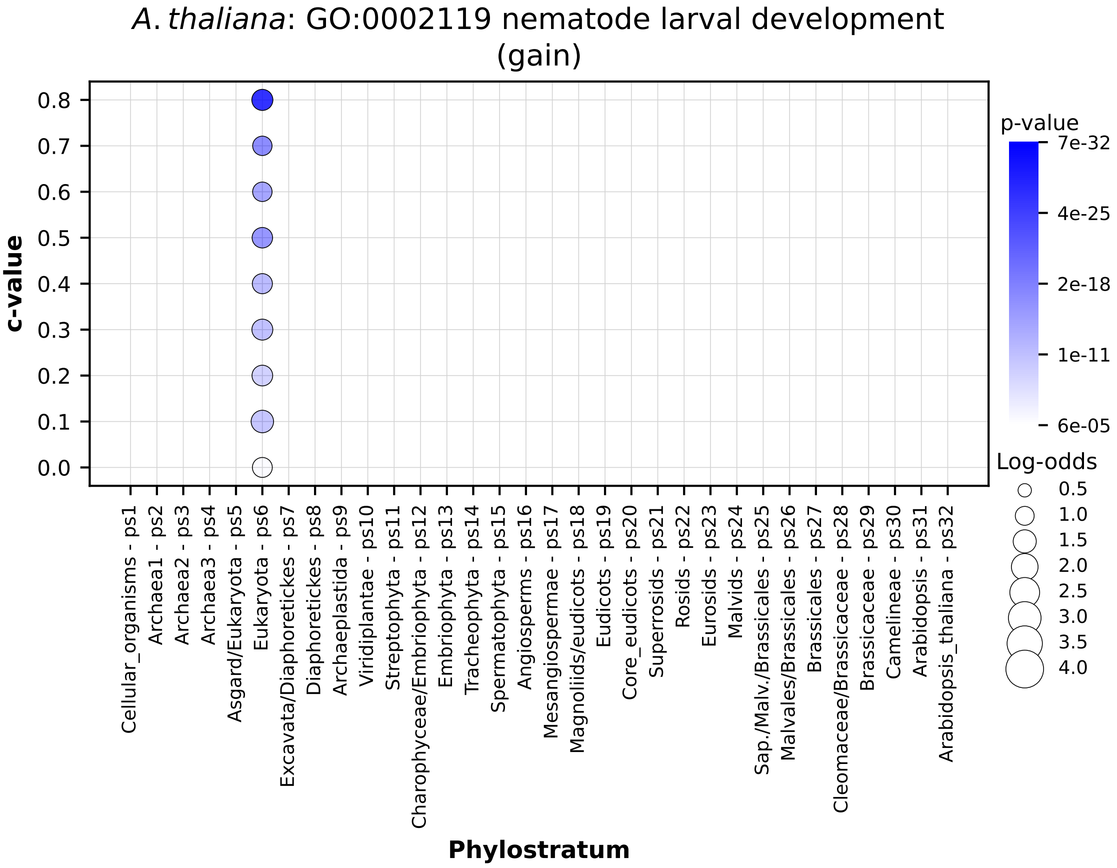
A. thaliana: GO:0002119 nematode larval development
(gain)
c-value
0.8
0.7
0.6
0.5
0.4
0.3
0.2
0.1
0.0
Phylostratum
Cellular_organisms - ps1
Archaea1 - ps2
Archaea2 - ps3
Archaea3 - ps4
Asgard/Eukaryota - ps5
Eukaryota - ps6
Excavata/Diaphoretickes - ps7
Diaphoretickes - ps8
Archaeplastida - ps9
Viridiplantae - ps10
Streptophyta - ps11
Charophyceae/Embriophyta - ps12
Embriophyta - ps13
Tracheophyta - ps14
Spermatophyta - ps15
Angiosperms - ps16
Mesangiospermae - ps17
Magnoliids/eudicots - ps18
Eudicots - ps19
Core_eudicots - ps20
Superrosids - ps21
Rosids - ps22
Eurosids - ps23
Malvids - ps24
Sap./Malv./Brassicales - ps25
Malvales/Brassicales - ps26
Brassicales - ps27
Cleomaceae/Brassicaceae - ps28
Brassicaceae - ps29
Camelineae - ps30
Arabidopsis - ps31
Arabidopsis_thaliana - ps32
p-value
7e-32
4e-25
2e-18
1e-11
6e-05
Log-odds
0.5
1.0
1.5
2.0
2.5
3.0
3.5
4.0

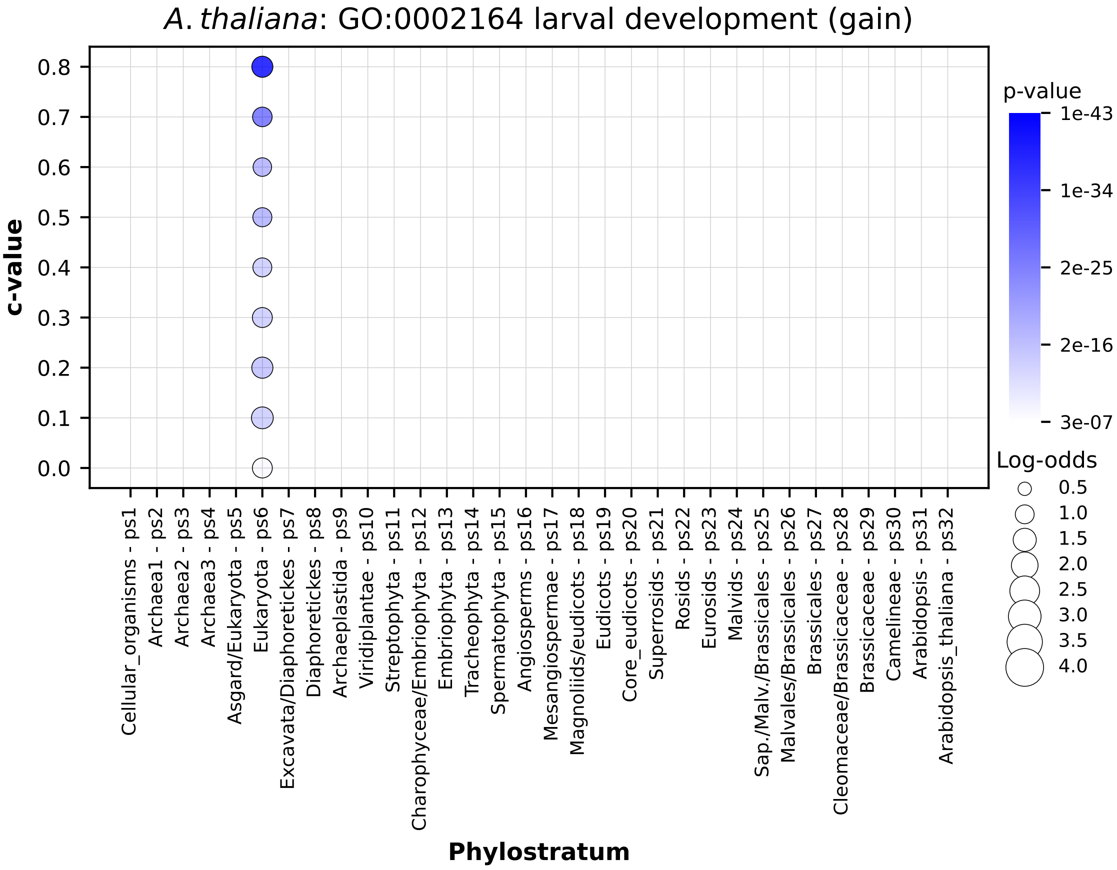

A. thaliana: GO:0002164 larval development (gain)
c-value
0.8
0.7
0.6
0.5
0.4
0.3
0.2
0.1
0.0
p-value
1e-43
1e-34
2e-25
2e-16
3e-07
Log-odds
0.5
1.0
1.5
2.0
2.5
3.0
3.5
4.0
Cellular_organisms - ps1
Archaea1 - ps2
Archaea2 - ps3
Archaea3 - ps4
Asgard/Eukaryota - ps5
Eukaryota - ps6
Excavata/Diaphoretickes - ps7
Diaphoretickes - ps8
Archaeplastida - ps9
Viridiplantae - ps10
Streptophyta - ps11
Charophyceae/Embriophyta - ps12
Embriophyta - ps13
Tracheophyta - ps14
Spermatophyta - ps15
Angiosperms - ps16
Mesangiospermae - ps17
Magnoliids/eudicots - ps18
Eudicots - ps19
Core_eudicots - ps20
Superrosids - ps21
Rosids - ps22
Eurosids - ps23
Malvids - ps24
Sap./Malv./Brassicales - ps25
Malvales/Brassicales - ps26
Brassicales - ps27
Cleomaceae/Brassicaceae - ps28
Brassicaceae - ps29
Camelineae - ps30
Arabidopsis - ps31
Arabidopsis_thaliana - ps32
Phylostratum

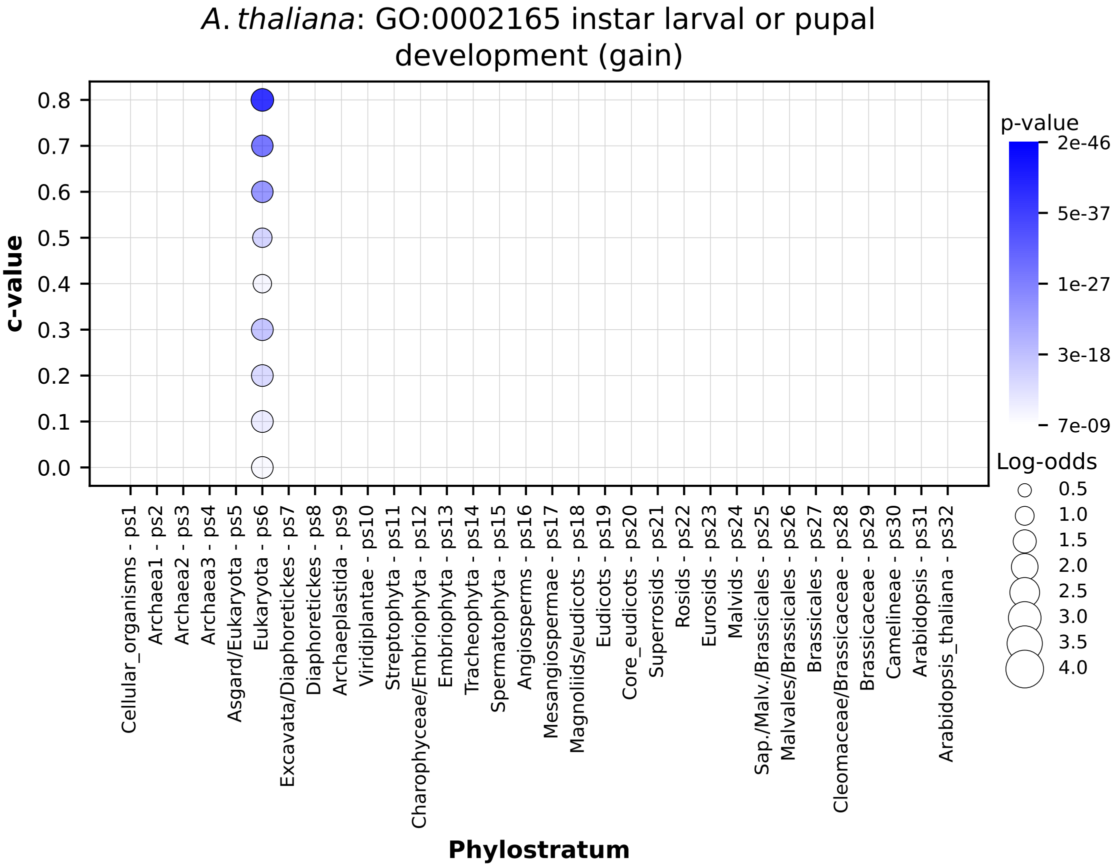

A. thaliana: GO:0002165 instar larval or pupal development (gain)
c-value
0.8
0.7
0.6
0.5
0.4
0.3
0.2
0.1
0.0
p-value
2e-46
5e-37
1e-27
3e-18
7e-09
Log-odds
0.5
1.0
1.5
2.0
2.5
3.0
3.5
4.0
Cellular_organisms - ps1
Archaea1 - ps2
Archaea2 - ps3
Archaea3 - ps4
Asgard/Eukaryota - ps5
Eukaryota - ps6
Excavata/Diaphoretickes - ps7
Diaphoretickes - ps8
Archaeplastida - ps9
Viridiplantae - ps10
Streptophyta - ps11
Charophyceae/Embriophyta - ps12
Embriophyta - ps13
Tracheophyta - ps14
Spermatophyta - ps15
Angiosperms - ps16
Mesangiospermae - ps17
Magnoliids/eudicots - ps18
Eudicots - ps19
Core_eudicots - ps20
Superrosids - ps21
Rosids - ps22
Eurosids - ps23
Malvids - ps24
Sap./Malv./Brassicales - ps25
Malvales/Brassicales - ps26
Brassicales - ps27
Cleomaceae/Brassicaceae - ps28
Brassicaceae - ps29
Camelineae - ps30
Arabidopsis - ps31
Arabidopsis_thaliana - ps32
Phylostratum

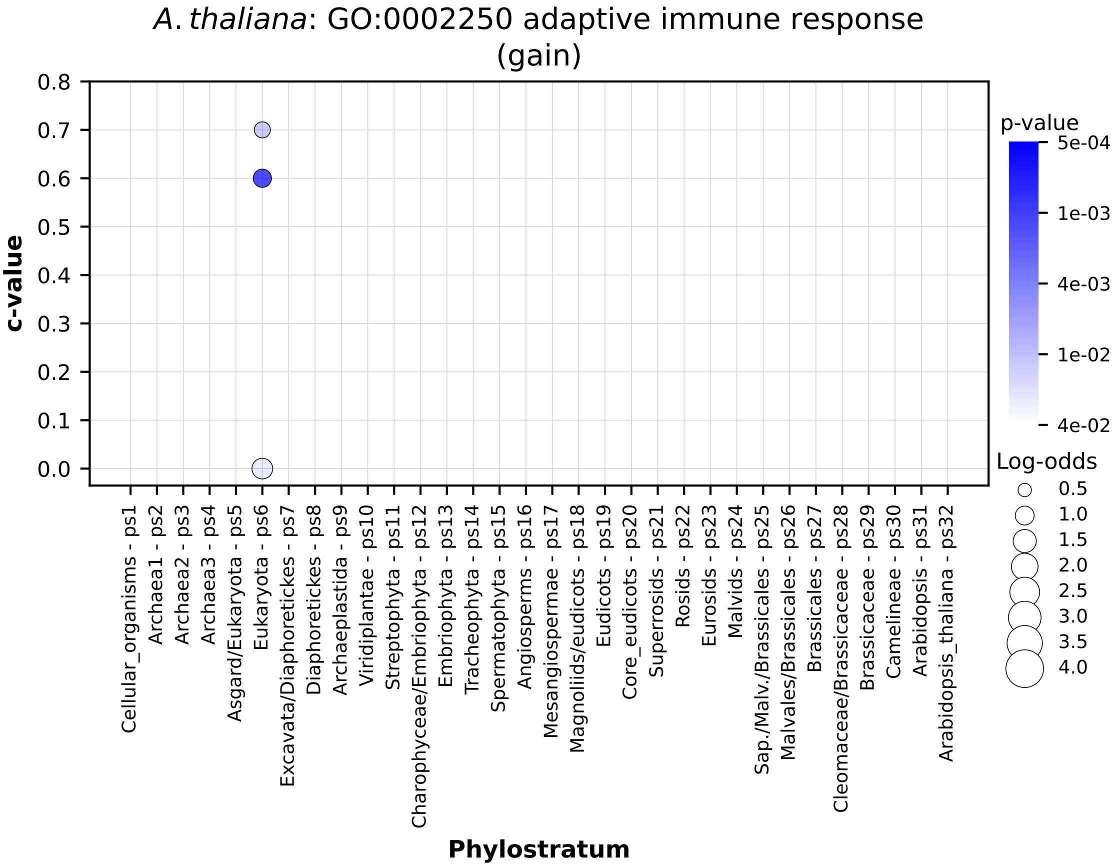
A. thaliana: GO:0002250 adaptive immune response
(gain)
c-value
0.0
0.1
0.2
0.3
0.4
0.5
0.6
0.7
0.8
Phylostratum
Cellular_organisms - ps1
Archaea1 - ps2
Archaea2 - ps3
Archaea3 - ps4
Asgard/Eukaryota - ps5
Eukaryota - ps6
Excavata/Diaphoretickes - ps7
Diaphoretickes - ps8
Archaeplastida - ps9
Viridiplantae - ps10
Streptophyta - ps11
Charophyceae/Embriophyta - ps12
Embriophyta - ps13
Tracheophyta - ps14
Spermatophyta - ps15
Angiosperms - ps16
Mesangiospermae - ps17
Magnoliids/eudicots - ps18
Eudicots - ps19
Core_eudicots - ps20
Superrosids - ps21
Rosids - ps22
Eurosids - ps23
Malvids - ps24
Sap./Malv./Brassicales - ps25
Malvales/Brassicales - ps26
Brassicales - ps27
Cleomaceae/Brassicaceae - ps28
Brassicaceae - ps29
Camelineae - ps30
Arabidopsis - ps31
Arabidopsis_thaliana - ps32
p-value
5e-04
1e-03
4e-03
1e-02
4e-02
Log-odds
0.5
1.0
1.5
2.0
2.5
3.0
3.5
4.0

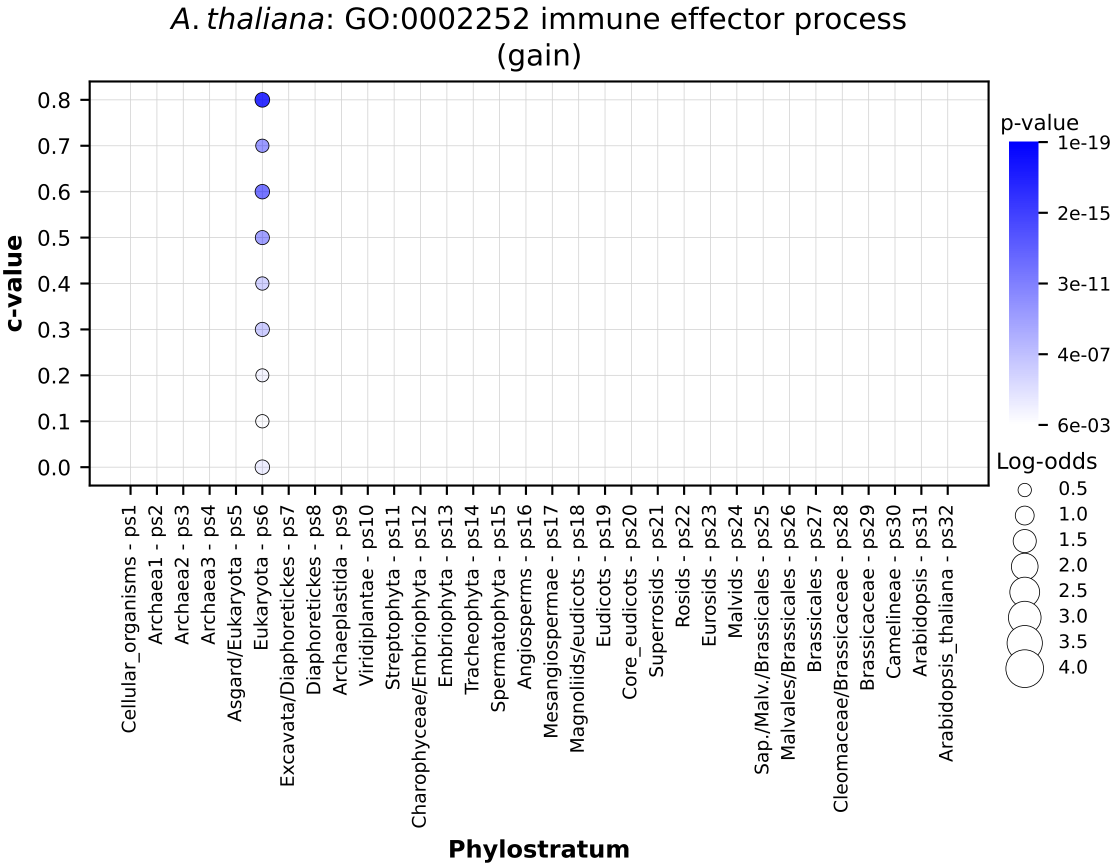

A. thaliana: GO:0002252 immune effector process
(gain)
c-value
0.8
0.7
0.6
0.5
0.4
0.3
0.2
0.1
0.0
p-value
1e-19
2e-15
3e-11
4e-07
6e-03
Log-odds
0.5
1.0
1.5
2.0
2.5
3.0
3.5
4.0
Cellular_organisms - ps1
Archaea1 - ps2
Archaea2 - ps3
Archaea3 - ps4
Asgard/Eukaryota - ps5
Eukaryota - ps6
Excavata/Diaphoretickes - ps7
Diaphoretickes - ps8
Archaeplastida - ps9
Viridiplantae - ps10
Streptophyta - ps11
Charophyceae/Embriophyta - ps12
Embriophyta - ps13
Tracheophyta - ps14
Spermatophyta - ps15
Angiosperms - ps16
Mesangiospermae - ps17
Magnoliids/eudicots - ps18
Eudicots - ps19
Core_eudicots - ps20
Superrosids - ps21
Rosids - ps22
Eurosids - ps23
Malvids - ps24
Sap./Malv./Brassicales - ps25
Malvales/Brassicales - ps26
Brassicales - ps27
Cleomaceae/Brassicaceae - ps28
Brassicaceae - ps29
Camelineae - ps30
Arabidopsis - ps31
Arabidopsis_thaliana - ps32
Phylostratum

A. thaliana: GO:0002253 activation of immune response (gain)

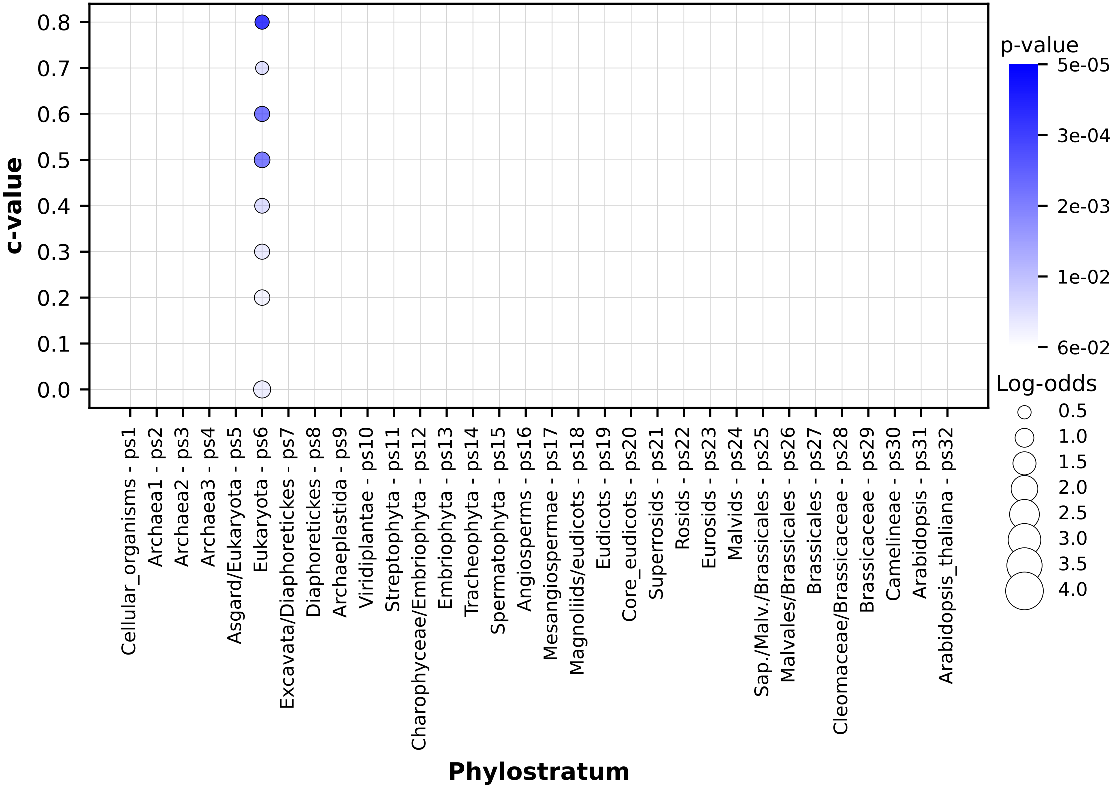

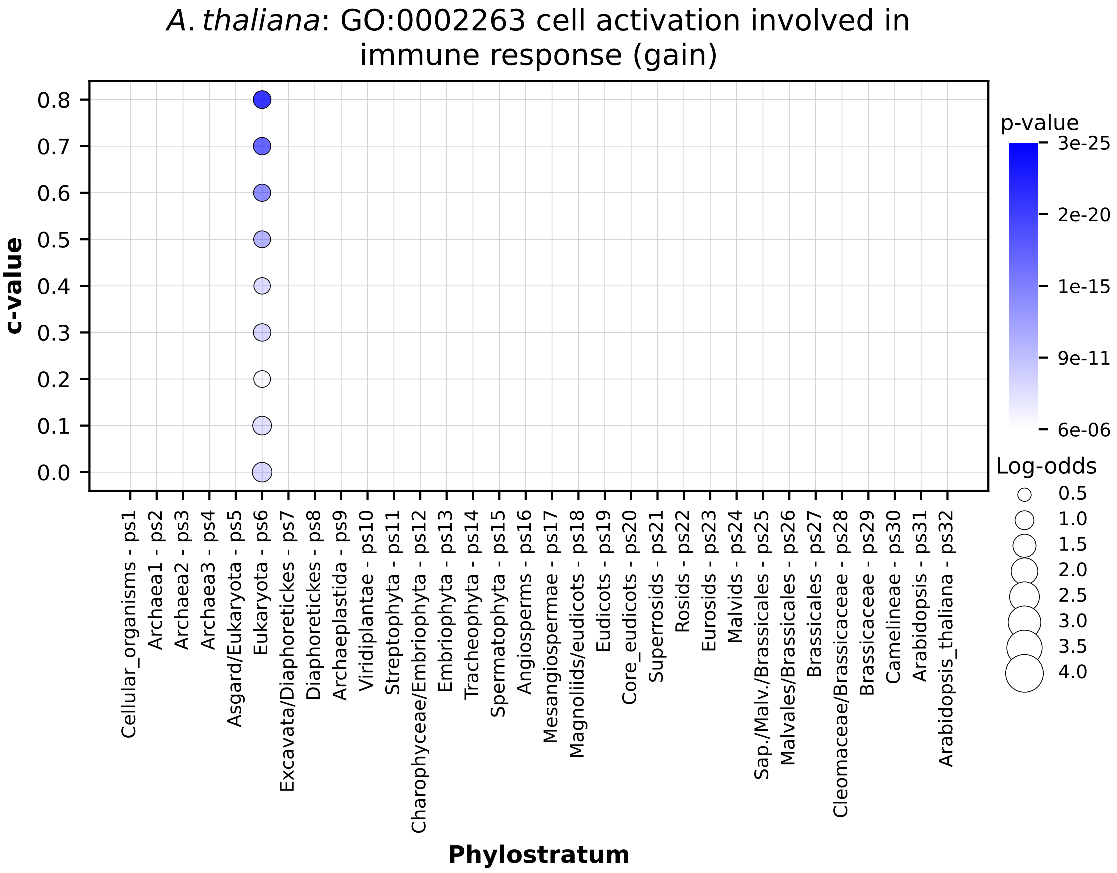

A. thaliana: GO:0002263 cell activation involved in immune response (gain)
c-value
0.8
0.7
0.6
0.5
0.4
0.3
0.2
0.1
0.0
p-value
3e-25
2e-20
1e-15
9e-11
6e-06
Log-odds
0.5
1.0
1.5
2.0
2.5
3.0
3.5
4.0
Cellular_organisms - ps1
Archaea1 - ps2
Archaea2 - ps3
Archaea3 - ps4
Asgard/Eukaryota - ps5
Eukaryota - ps6
Excavata/Diaphoretickes - ps7
Diaphoretickes - ps8
Archaeplastida - ps9
Viridiplantae - ps10
Streptophyta - ps11
Charophyceae/Embriophyta - ps12
Embriophyta - ps13
Tracheophyta - ps14
Spermatophyta - ps15
Angiosperms - ps16
Mesangiospermae - ps17
Magnoliids/eudicots - ps18
Eudicots - ps19
Core_eudicots - ps20
Superrosids - ps21
Rosids - ps22
Eurosids - ps23
Malvids - ps24
Sap./Malv./Brassicales - ps25
Malvales/Brassicales - ps26
Brassicales - ps27
Cleomaceae/Brassicaceae - ps28
Brassicaceae - ps29
Camelineae - ps30
Arabidopsis - ps31
Arabidopsis_thaliana - ps32
Phylostratum

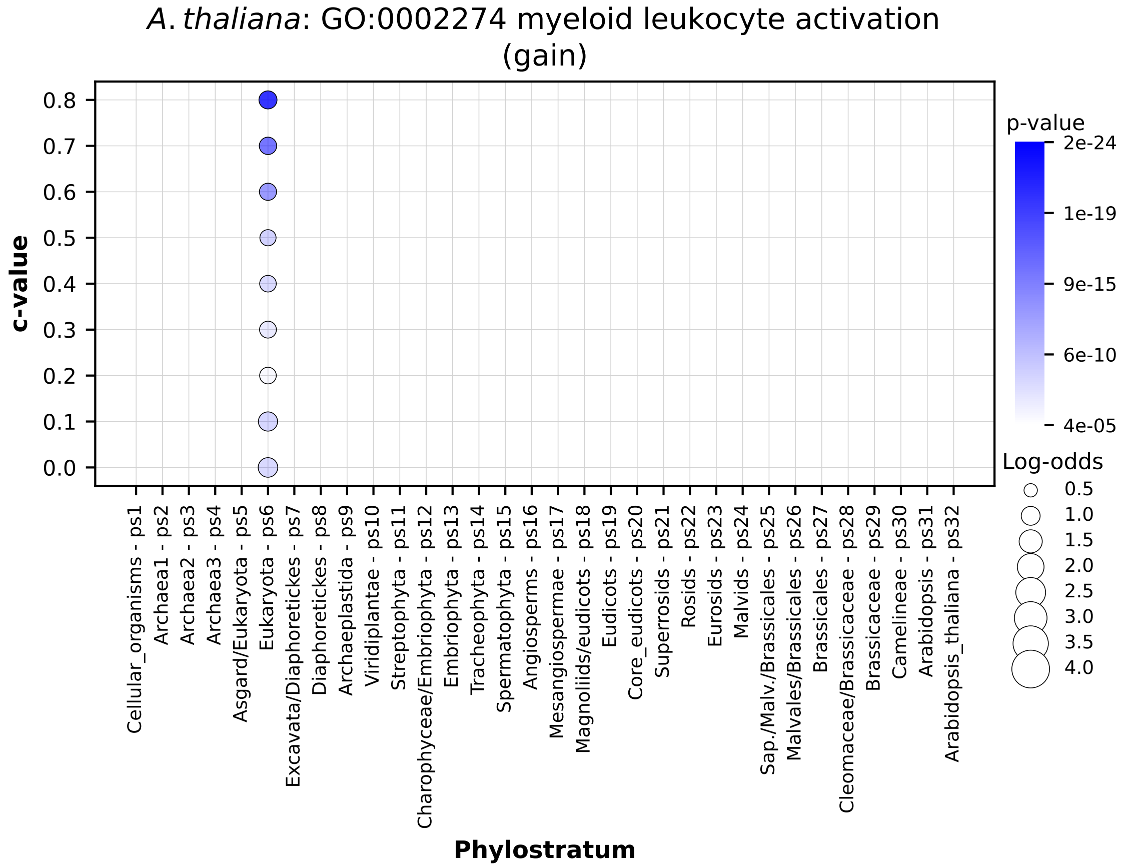

A. thaliana: GO:0002274 myeloid leukocyte activation
(gain)
c-value
0.8
0.7
0.6
0.5
0.4
0.3
0.2
0.1
0.0
p-value
2e-24
1e-19
9e-15
6e-10
4e-05
Log-odds
0.5
1.0
1.5
2.0
2.5
3.0
3.5
4.0
Cellular_organisms - ps1
Archaea1 - ps2
Archaea2 - ps3
Archaea3 - ps4
Asgard/Eukaryota - ps5
Eukaryota - ps6
Excavata/Diaphoretickes - ps7
Diaphoretickes - ps8
Archaeplastida - ps9
Viridiplantae - ps10
Streptophyta - ps11
Charophyceae/Embriophyta - ps12
Embriophyta - ps13
Tracheophyta - ps14
Spermatophyta - ps15
Angiosperms - ps16
Mesangiospermae - ps17
Magnoliids/eudicots - ps18
Eudicots - ps19
Core_eudicots - ps20
Superrosids - ps21
Rosids - ps22
Eurosids - ps23
Malvids - ps24
Sap./Malv./Brassicales - ps25
Malvales/Brassicales - ps26
Brassicales - ps27
Cleomaceae/Brassicaceae - ps28
Brassicaceae - ps29
Camelineae - ps30
Arabidopsis - ps31
Arabidopsis_thaliana - ps32
Phylostratum

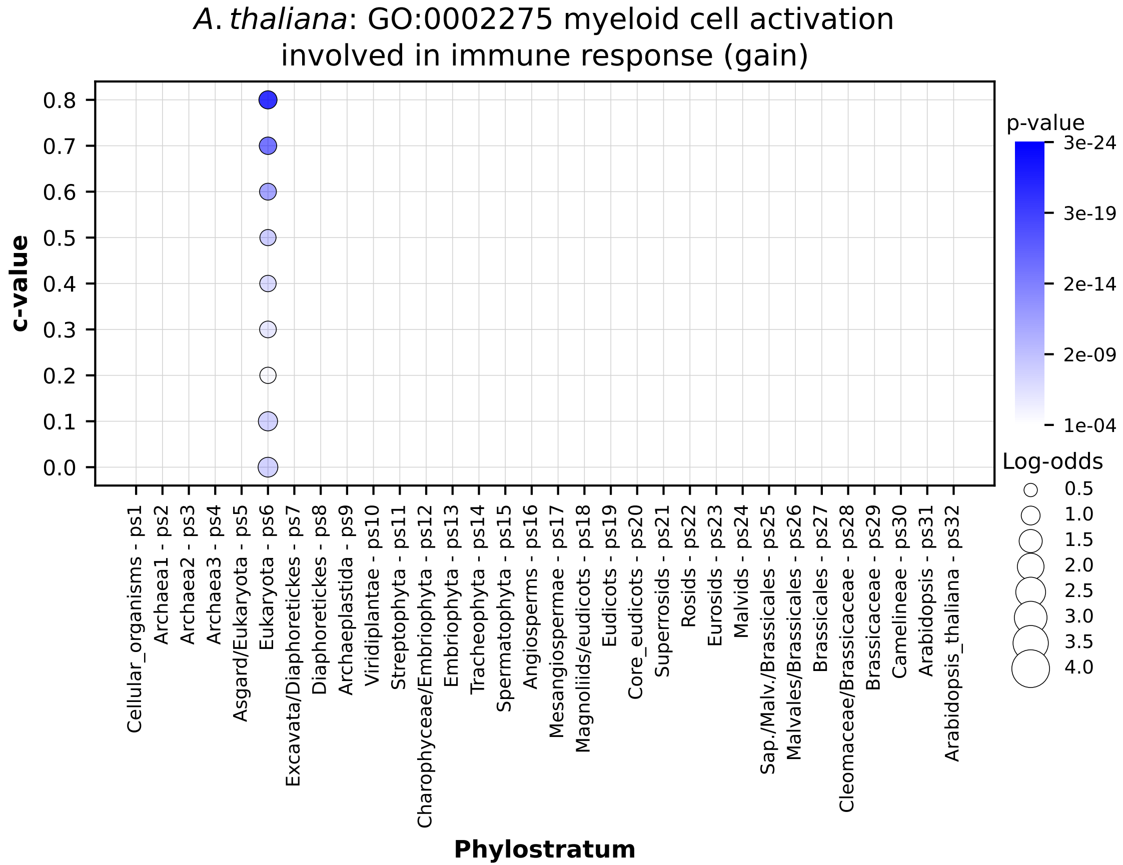

A. thaliana: GO:0002275 myeloid cell activation involved in immune response (gain)
c-value
0.0
0.1
0.2
0.3
0.4
0.5
0.6
0.7
0.8
Phylostratum
Cellular_organisms - ps1
Archaea1 - ps2
Archaea2 - ps3
Archaea3 - ps4
Asgard/Eukaryota - ps5
Eukaryota - ps6
Excavata/Diaphoretickes - ps7
Diaphoretickes - ps8
Archaeplastida - ps9
Viridiplantae - ps10
Streptophyta - ps11
Charophyceae/Embriophyta - ps12
Embriophyta - ps13
Tracheophyta - ps14
Spermatophyta - ps15
Angiosperms - ps16
Mesangiospermae - ps17
Magnoliids/eudicots - ps18
Eudicots - ps19
Core_eudicots - ps20
Superrosids - ps21
Rosids - ps22
Eurosids - ps23
Malvids - ps24
Sap./Malv./Brassicales - ps25
Malvales/Brassicales - ps26
Brassicales - ps27
Cleomaceae/Brassicaceae - ps28
Brassicaceae - ps29
Camelineae - ps30
Arabidopsis - ps31
Arabidopsis_thaliana - ps32
p-value
3e-24
3e-19
2e-14
2e-09
1e-04
Log-odds
0.5
1.0
1.5
2.0
2.5
3.0
3.5
4.0

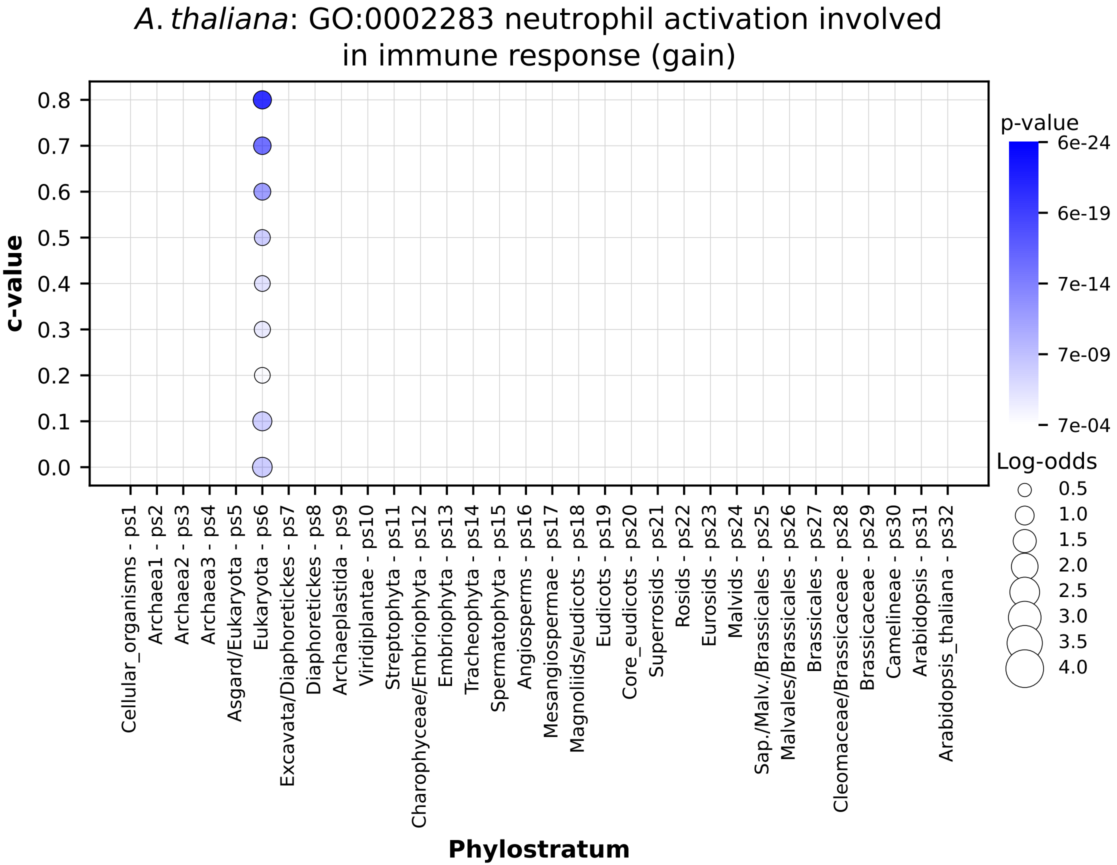

A. thaliana: GO:0002283 neutrophil activation involved in immune response (gain)
c-value
0.8
0.7
0.6
0.5
0.4
0.3
0.2
0.1
0.0
p-value
6e-24
6e-19
7e-14
7e-09
7e-04
Log-odds
0.5
1.0
1.5
2.0
2.5
3.0
3.5
4.0
Cellular_organisms - ps1
Archaea1 - ps2
Archaea2 - ps3
Archaea3 - ps4
Asgard/Eukaryota - ps5
Eukaryota - ps6
Excavata/Diaphoretickes - ps7
Diaphoretickes - ps8
Archaeplastida - ps9
Viridiplantae - ps10
Streptophyta - ps11
Charophyceae/Embriophyta - ps12
Embriophyta - ps13
Tracheophyta - ps14
Spermatophyta - ps15
Angiosperms - ps16
Mesangiospermae - ps17
Magnoliids/eudicots - ps18
Eudicots - ps19
Core_eudicots - ps20
Superrosids - ps21
Rosids - ps22
Eurosids - ps23
Malvids - ps24
Sap./Malv./Brassicales - ps25
Malvales/Brassicales - ps26
Brassicales - ps27
Cleomaceae/Brassicaceae - ps28
Brassicaceae - ps29
Camelineae - ps30
Arabidopsis - ps31
Arabidopsis_thaliana - ps32
Phylostratum

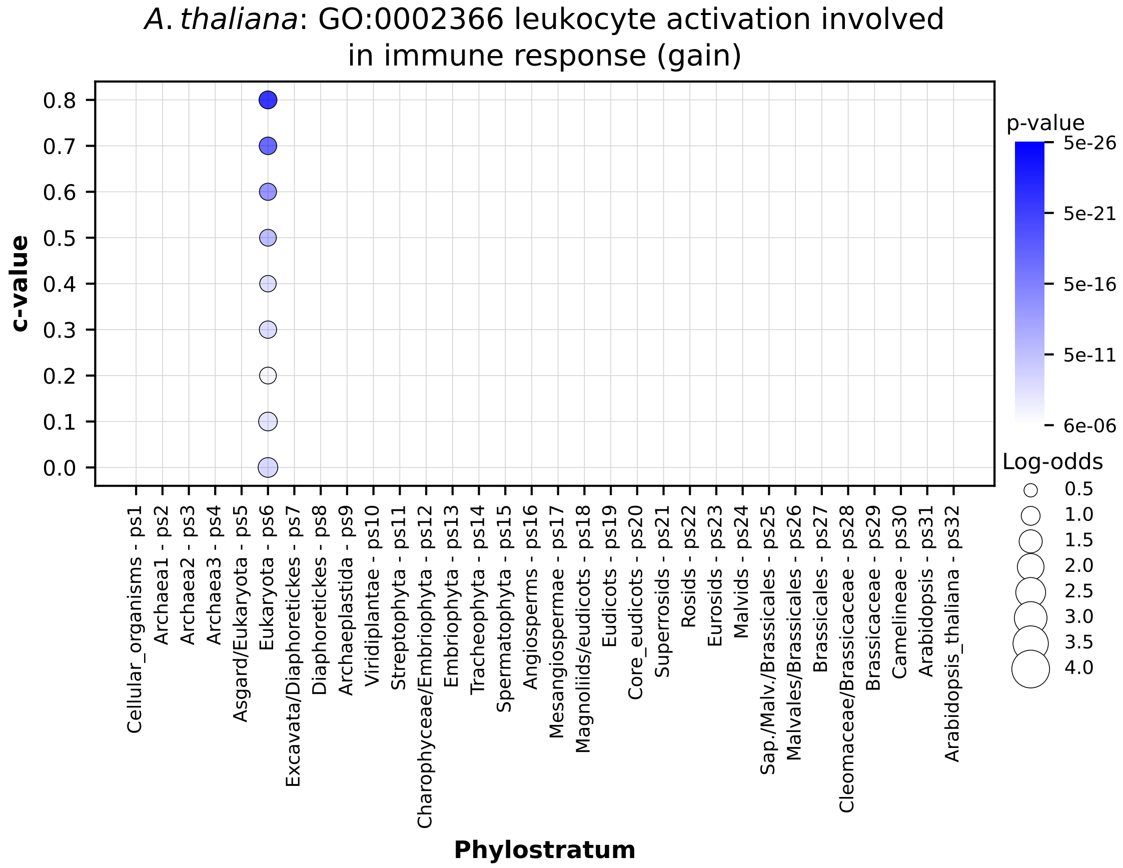
A. thaliana: GO:0002366 leukocyte activation involved in immune response (gain)
c-value
0.0
0.1
0.2
0.3
0.4
0.5
0.6
0.7
0.8
p-value
5e-26
5e-21
5e-16
5e-11
6e-06
Log-odds
0.5
1.0
1.5
2.0
2.5
3.0
3.5
4.0
Cellular_organisms - ps1
Archaea1 - ps2
Archaea2 - ps3
Archaea3 - ps4
Asgard/Eukaryota - ps5
Eukaryota - ps6
Excavata/Diaphoretickes - ps7
Diaphoretickes - ps8
Archaeplastida - ps9
Viridiplantae - ps10
Streptophyta - ps11
Charophyceae/Embriophyta - ps12
Embriophyta - ps13
Tracheophyta - ps14
Spermatophyta - ps15
Angiosperms - ps16
Mesangiospermae - ps17
Magnoliids/eudicots - ps18
Eudicots - ps19
Core_eudicots - ps20
Superrosids - ps21
Rosids - ps22
Eurosids - ps23
Malvids - ps24
Sap./Malv./Brassicales - ps25
Malvales/Brassicales - ps26
Brassicales - ps27
Cleomaceae/Brassicaceae - ps28
Brassicaceae - ps29
Camelineae - ps30
Arabidopsis - ps31
Arabidopsis_thaliana - ps32
Phylostratum

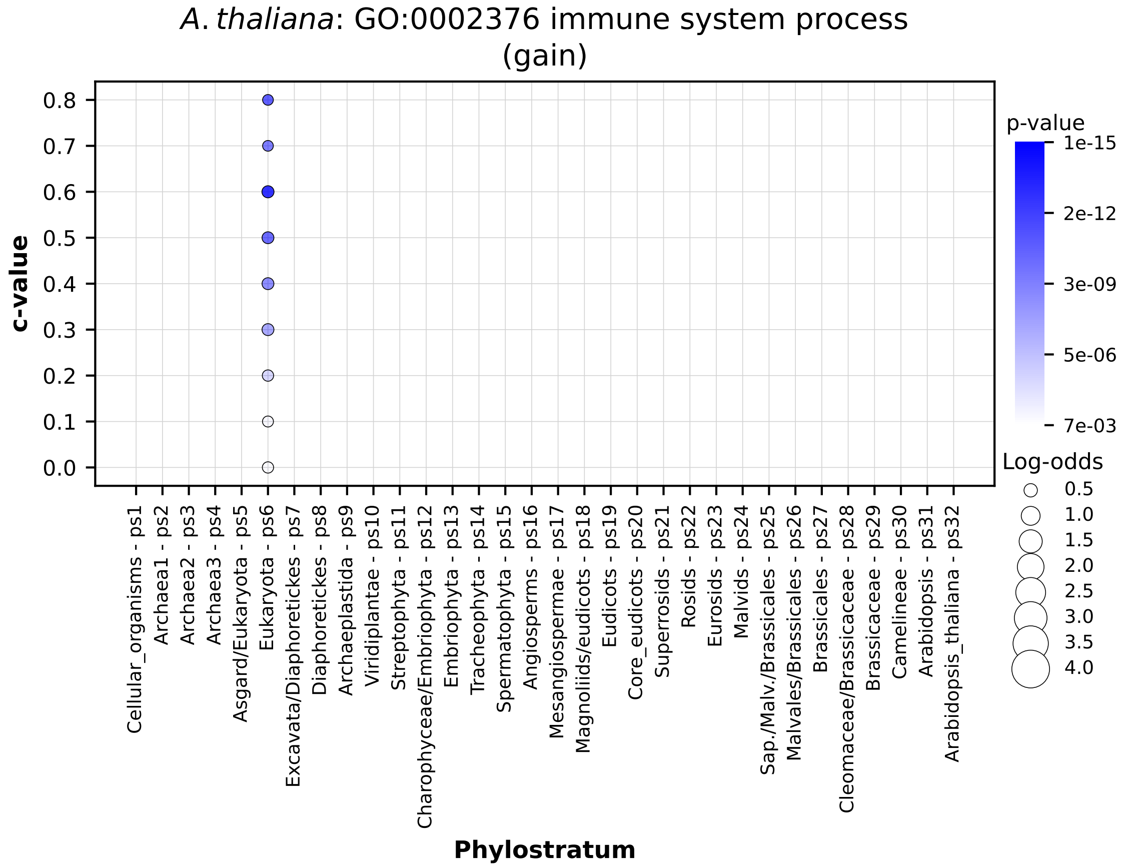

A. thaliana: GO:0002376 immune system process
(gain)
c-value
0.8
0.7
0.6
0.5
0.4
0.3
0.2
0.1
0.0
p-value
1e-15
2e-12
3e-09
5e-06
7e-03
Log-odds
0.5
1.0
1.5
2.0
2.5
3.0
3.5
4.0
Cellular_organisms - ps1
Archaea1 - ps2
Archaea2 - ps3
Archaea3 - ps4
Asgard/Eukaryota - ps5
Eukaryota - ps6
Excavata/Diaphoretickes - ps7
Diaphoretickes - ps8
Archaeplastida - ps9
Viridiplantae - ps10
Streptophyta - ps11
Charophyceae/Embriophyta - ps12
Embriophyta - ps13
Tracheophyta - ps14
Spermatophyta - ps15
Angiosperms - ps16
Mesangiospermae - ps17
Magnoliids/eudicots - ps18
Eudicots - ps19
Core_eudicots - ps20
Superrosids - ps21
Rosids - ps22
Eurosids - ps23
Malvids - ps24
Sap./Malv./Brassicales - ps25
Malvales/Brassicales - ps26
Brassicales - ps27
Cleomaceae/Brassicaceae - ps28
Brassicaceae - ps29
Camelineae - ps30
Arabidopsis - ps31
Arabidopsis_thaliana - ps32
Phylostratum

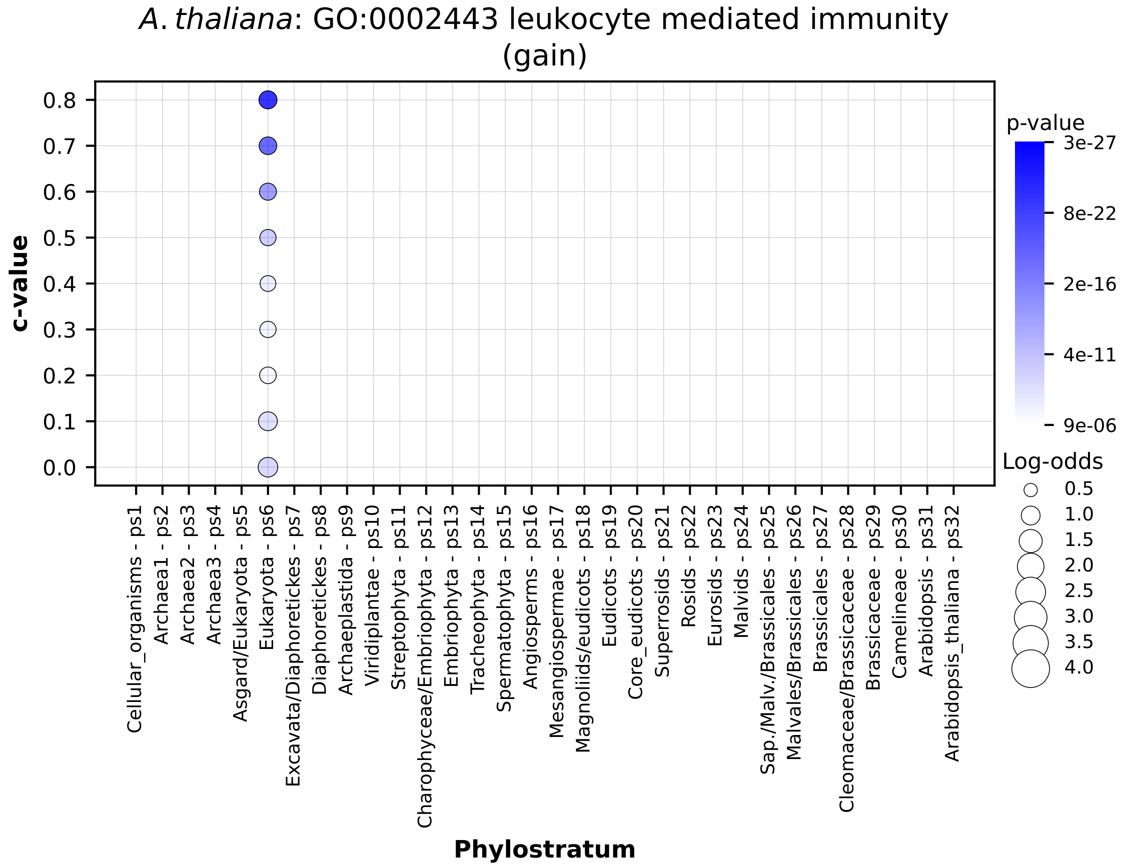
A. thaliana: GO:0002443 leukocyte mediated immunity
(gain)
c-value
0.8
0.7
0.6
0.5
0.4
0.3
0.2
0.1
0.0
p-value
3e-27
8e-22
2e-16
4e-11
9e-06
Log-odds
0.5
1.0
1.5
2.0
2.5
3.0
3.5
4.0
Cellular_organisms - ps1
Archaea1 - ps2
Archaea2 - ps3
Archaea3 - ps4
Asgard/Eukaryota - ps5
Eukaryota - ps6
Excavata/Diaphoretickes - ps7
Diaphoretickes - ps8
Archaeplastida - ps9
Viridiplantae - ps10
Streptophyta - ps11
Charophyceae/Embriophyta - ps12
Embriophyta - ps13
Tracheophyta - ps14
Spermatophyta - ps15
Angiosperms - ps16
Mesangiospermae - ps17
Magnoliids/eudicots - ps18
Eudicots - ps19
Core_eudicots - ps20
Superrosids - ps21
Rosids - ps22
Eurosids - ps23
Malvids - ps24
Sap./Malv./Brassicales - ps25
Malvales/Brassicales - ps26
Brassicales - ps27
Cleomaceae/Brassicaceae - ps28
Brassicaceae - ps29
Camelineae - ps30
Arabidopsis - ps31
Arabidopsis_thaliana - ps32
Phylostratum

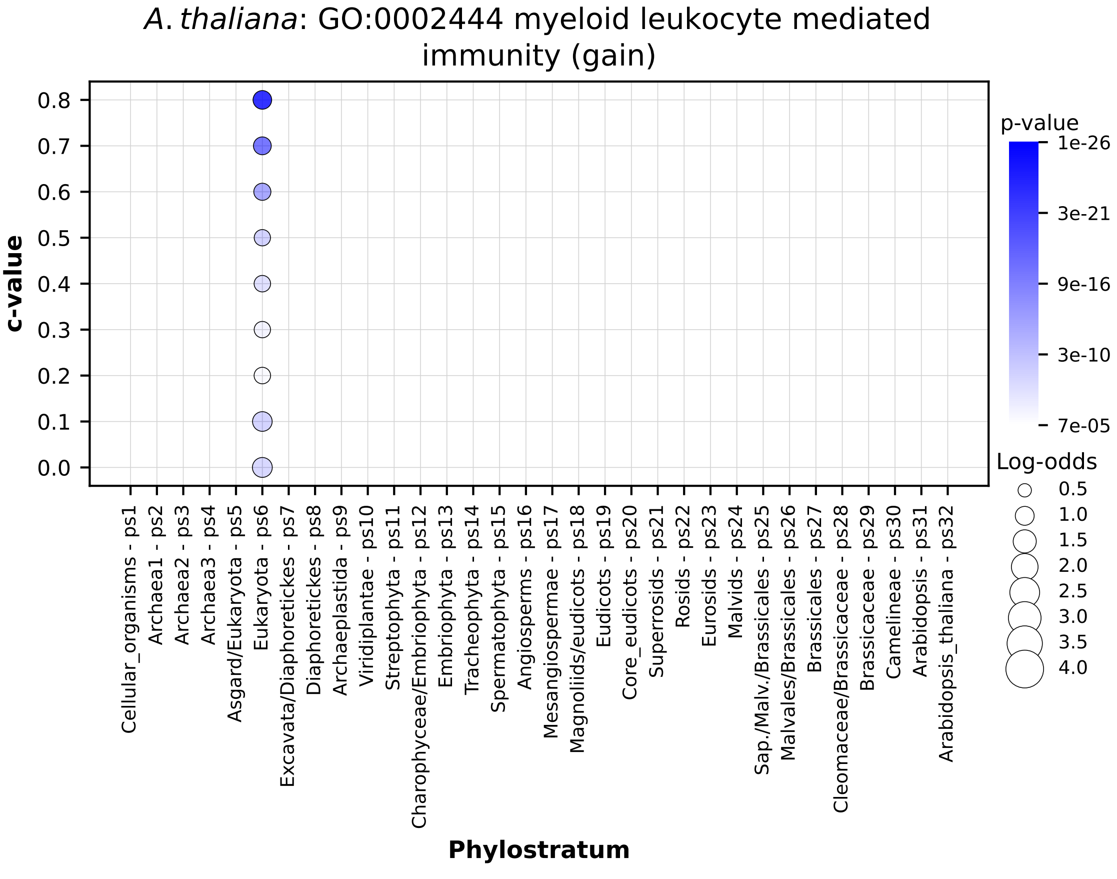

A. thaliana: GO:0002444 myeloid leukocyte mediated immunity (gain)
c-value
0.8
0.7
0.6
0.5
0.4
0.3
0.2
0.1
0.0
p-value
1e-26
3e-21
9e-16
3e-10
7e-05
Log-odds
0.5
1.0
1.5
2.0
2.5
3.0
3.5
4.0
Cellular_organisms - ps1
Archaea1 - ps2
Archaea2 - ps3
Archaea3 - ps4
Asgard/Eukaryota - ps5
Eukaryota - ps6
Excavata/Diaphoretickes - ps7
Diaphoretickes - ps8
Archaeplastida - ps9
Viridiplantae - ps10
Streptophyta - ps11
Charophyceae/Embriophyta - ps12
Embriophyta - ps13
Tracheophyta - ps14
Spermatophyta - ps15
Angiosperms - ps16
Mesangiospermae - ps17
Magnoliids/eudicots - ps18
Eudicots - ps19
Core_eudicots - ps20
Superrosids - ps21
Rosids - ps22
Eurosids - ps23
Malvids - ps24
Sap./Malv./Brassicales - ps25
Malvales/Brassicales - ps26
Brassicales - ps27
Cleomaceae/Brassicaceae - ps28
Brassicaceae - ps29
Camelineae - ps30
Arabidopsis - ps31
Arabidopsis_thaliana - ps32
Phylostratum

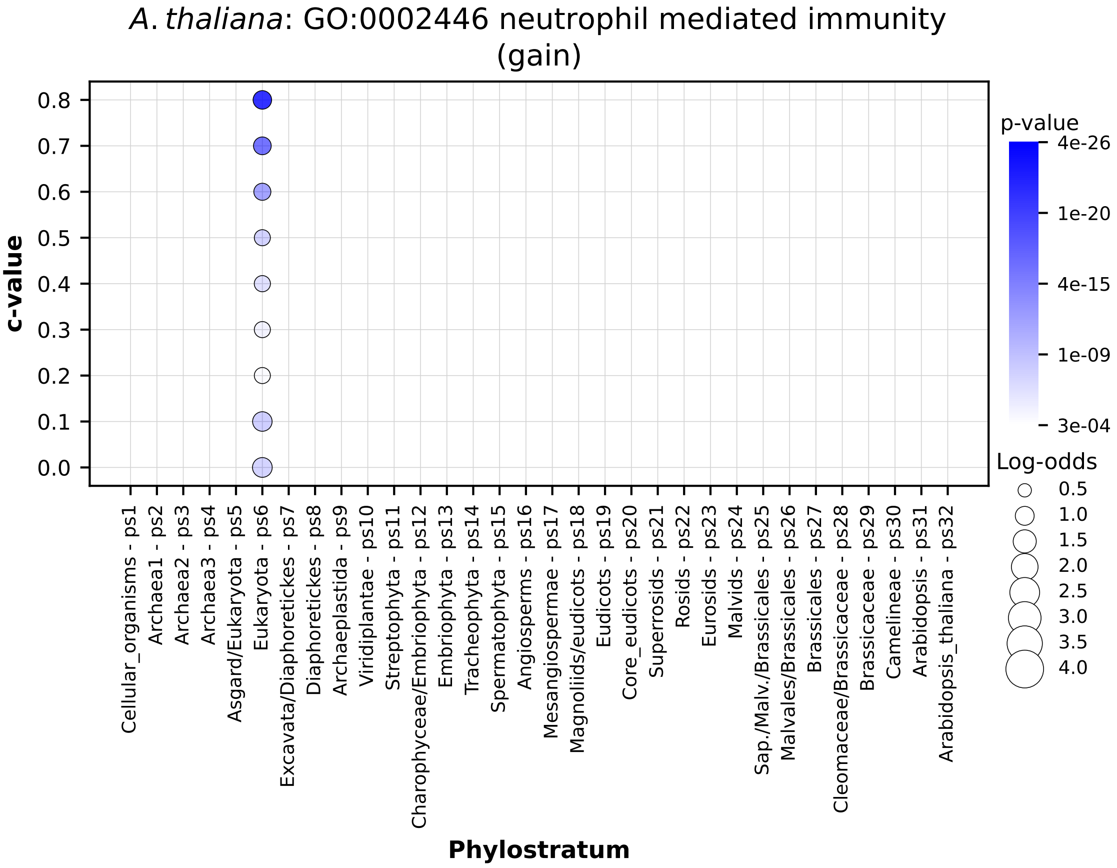

A. thaliana: GO:0002446 neutrophil mediated immunity
(gain)
c-value
0.8
0.7
0.6
0.5
0.4
0.3
0.2
0.1
0.0
p-value
4e-26
1e-20
4e-15
1e-09
3e-04
Log-odds
0.5
1.0
1.5
2.0
2.5
3.0
3.5
4.0
Cellular_organisms - ps1
Archaea1 - ps2
Archaea2 - ps3
Archaea3 - ps4
Asgard/Eukaryota - ps5
Eukaryota - ps6
Excavata/Diaphoretickes - ps7
Diaphoretickes - ps8
Archaeplastida - ps9
Viridiplantae - ps10
Streptophyta - ps11
Charophyceae/Embriophyta - ps12
Embriophyta - ps13
Tracheophyta - ps14
Spermatophyta - ps15
Angiosperms - ps16
Mesangiospermae - ps17
Magnoliids/eudicots - ps18
Eudicots - ps19
Core_eudicots - ps20
Superrosids - ps21
Rosids - ps22
Eurosids - ps23
Malvids - ps24
Sap./Malv./Brassicales - ps25
Malvales/Brassicales - ps26
Brassicales - ps27
Cleomaceae/Brassicaceae - ps28
Brassicaceae - ps29
Camelineae - ps30
Arabidopsis - ps31
Arabidopsis_thaliana - ps32
Phylostratum

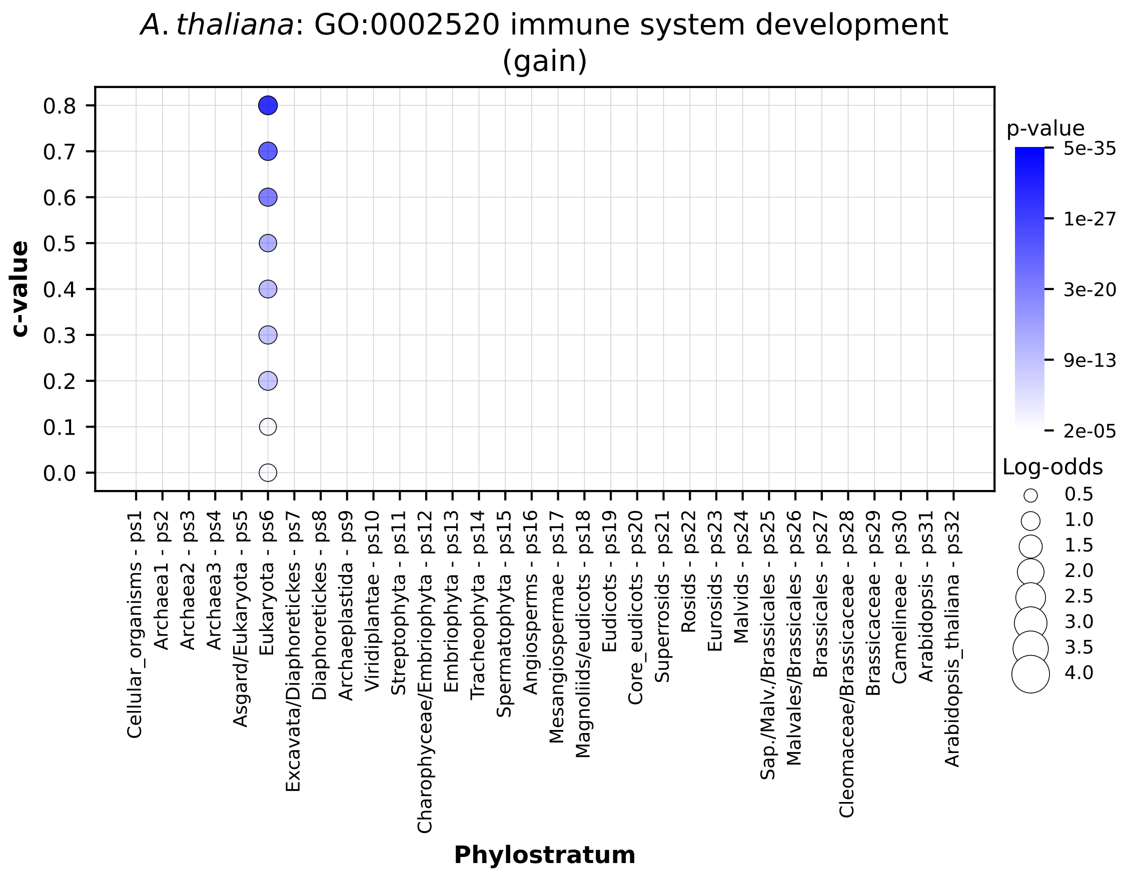

A. thaliana: GO:0002520 immune system development (gain)
c-value
0.8
0.7
0.6
0.5
0.4
0.3
0.2
0.1
0.0
p-value
5e-35
1e-27
3e-20
9e-13
2e-05
Log-odds
0.5
1.0
1.5
2.0
2.5
3.0
3.5
4.0
Cellular_organisms - ps1
Archaea1 - ps2
Archaea2 - ps3
Archaea3 - ps4
Asgard/Eukaryota - ps5
Eukaryota - ps6
Excavata/Diaphoretickes - ps7
Diaphoretickes - ps8
Archaeplastida - ps9
Viridiplantae - ps10
Streptophyta - ps11
Charophyceae/Embriophyta - ps12
Embriophyta - ps13
Tracheophyta - ps14
Spermatophyta - ps15
Angiosperms - ps16
Mesangiospermae - ps17
Magnoliids/eudicots - ps18
Eudicots - ps19
Core_eudicots - ps20
Superrosids - ps21
Rosids - ps22
Eurosids - ps23
Malvids - ps24
Sap./Malv./Brassicales - ps25
Malvales/Brassicales - ps26
Brassicales - ps27
Cleomaceae/Brassicaceae - ps28
Brassicaceae - ps29
Camelineae - ps30
Arabidopsis - ps31
Arabidopsis_thaliana - ps32
Phylostratum

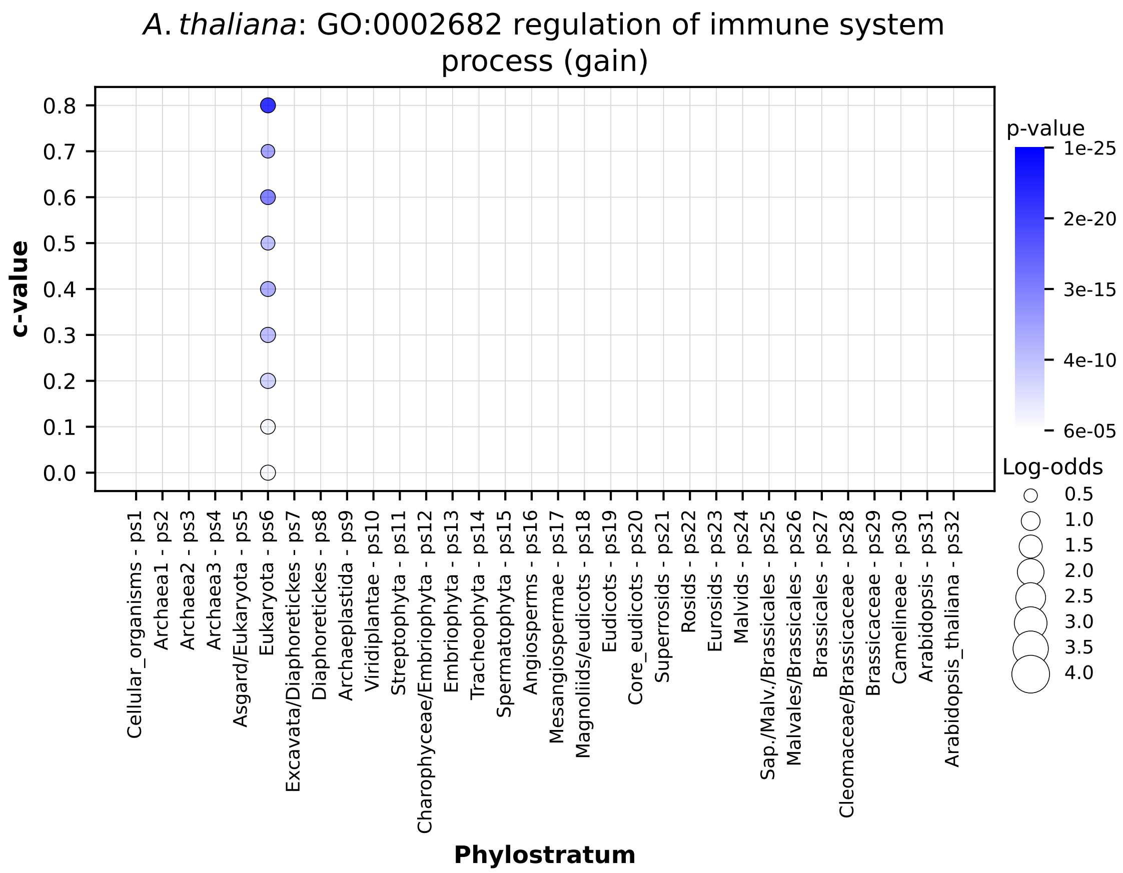

A. thaliana: GO:0002682 regulation of immune system process (gain)
c-value
0.8
0.7
0.6
0.5
0.4
0.3
0.2
0.1
0.0
p-value
1e-25
2e-20
3e-15
4e-10
6e-05
Log-odds
0.5
1.0
1.5
2.0
2.5
3.0
3.5
4.0
Cellular_organisms - ps1
Archaea1 - ps2
Archaea2 - ps3
Archaea3 - ps4
Asgard/Eukaryota - ps5
Eukaryota - ps6
Excavata/Diaphoretickes - ps7
Diaphoretickes - ps8
Archaeplastida - ps9
Viridiplantae - ps10
Streptophyta - ps11
Charophyceae/Embriophyta - ps12
Embriophyta - ps13
Tracheophyta - ps14
Spermatophyta - ps15
Angiosperms - ps16
Mesangiospermae - ps17
Magnoliids/eudicots - ps18
Eudicots - ps19
Core_eudicots - ps20
Superrosids - ps21
Rosids - ps22
Eurosids - ps23
Malvids - ps24
Sap./Malv./Brassicales - ps25
Malvales/Brassicales - ps26
Brassicales - ps27
Cleomaceae/Brassicaceae - ps28
Brassicaceae - ps29
Camelineae - ps30
Arabidopsis - ps31
Arabidopsis_thaliana - ps32
Phylostratum

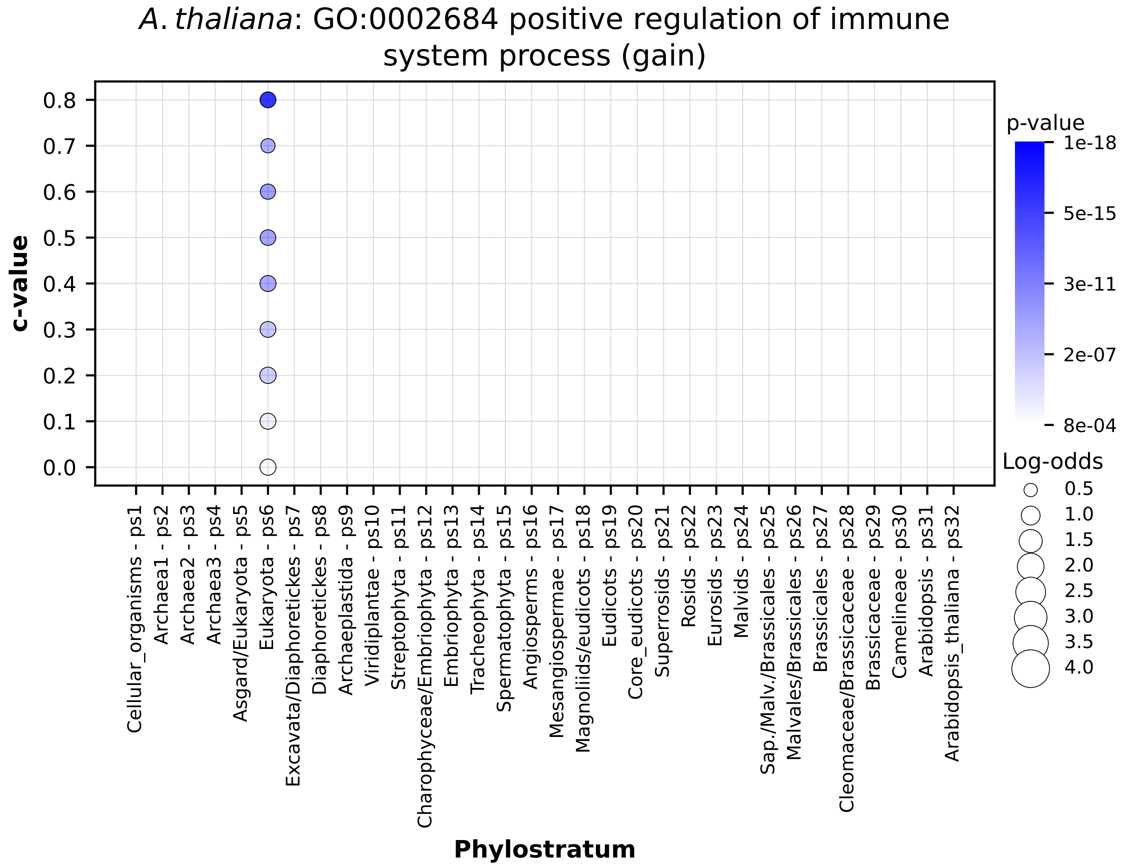

A. thaliana: GO:0002684 positive regulation of immune system process (gain)
c-value
0.8
0.7
0.6
0.5
0.4
0.3
0.2
0.1
0.0
p-value
1e-18
5e-15
3e-11
2e-07
8e-04
Log-odds
0.5
1.0
1.5
2.0
2.5
3.0
3.5
4.0
Cellular_organisms - ps1
Archaea1 - ps2
Archaea2 - ps3
Archaea3 - ps4
Asgard/Eukaryota - ps5
Eukaryota - ps6
Excavata/Diaphoretickes - ps7
Diaphoretickes - ps8
Archaeplastida - ps9
Viridiplantae - ps10
Streptophyta - ps11
Charophyceae/Embriophyta - ps12
Embriophyta - ps13
Tracheophyta - ps14
Spermatophyta - ps15
Angiosperms - ps16
Mesangiospermae - ps17
Magnoliids/eudicots - ps18
Eudicots - ps19
Core_eudicots - ps20
Superrosids - ps21
Rosids - ps22
Eurosids - ps23
Malvids - ps24
Sap./Malv./Brassicales - ps25
Malvales/Brassicales - ps26
Brassicales - ps27
Cleomaceae/Brassicaceae - ps28
Brassicaceae - ps29
Camelineae - ps30
Arabidopsis - ps31
Arabidopsis_thaliana - ps32
Phylostratum

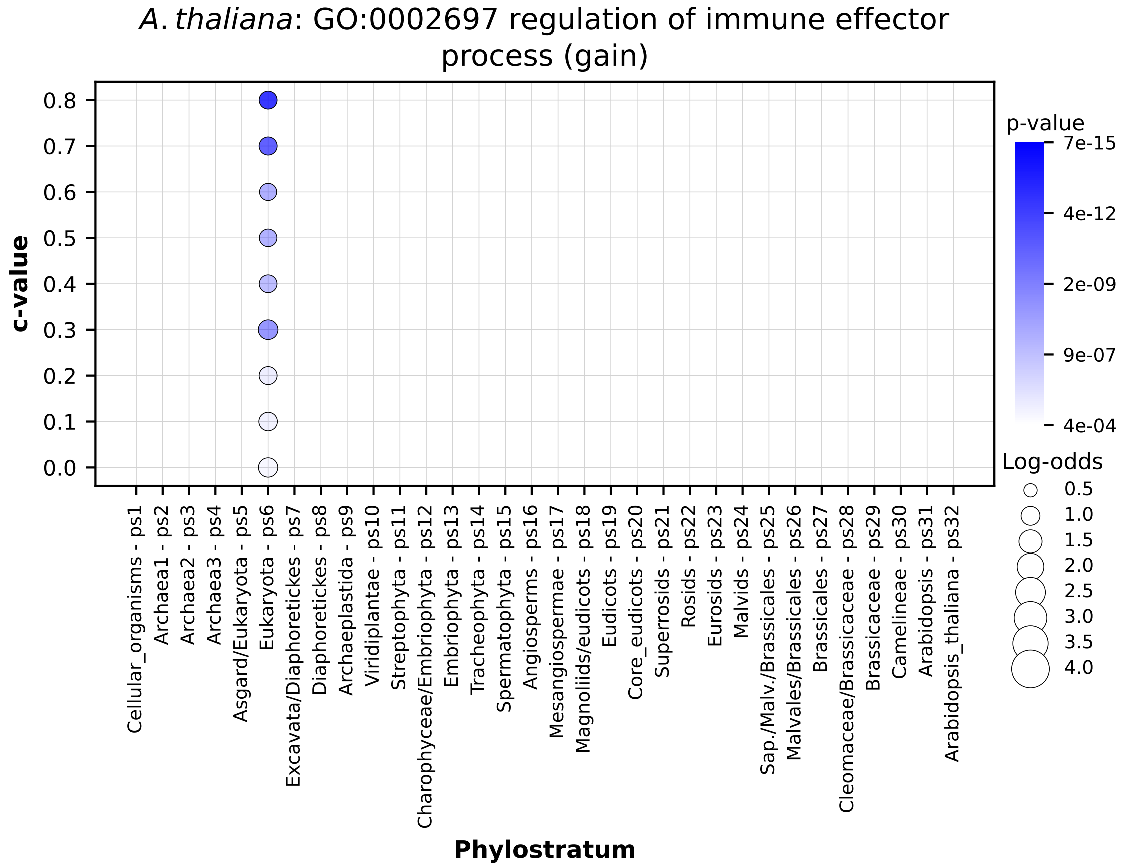

A. thaliana: GO:0002697 regulation of immune effector process (gain)
c-value
0.8
0.7
0.6
0.5
0.4
0.3
0.2
0.1
0.0
p-value
7e-15
4e-12
2e-09
9e-07
4e-04
Log-odds
0.5
1.0
1.5
2.0
2.5
3.0
3.5
4.0
Cellular_organisms - ps1
Archaea1 - ps2
Archaea2 - ps3
Archaea3 - ps4
Asgard/Eukaryota - ps5
Eukaryota - ps6
Excavata/Diaphoretickes - ps7
Diaphoretickes - ps8
Archaeplastida - ps9
Viridiplantae - ps10
Streptophyta - ps11
Charophyceae/Embriophyta - ps12
Embriophyta - ps13
Tracheophyta - ps14
Spermatophyta - ps15
Angiosperms - ps16
Mesangiospermae - ps17
Magnoliids/eudicots - ps18
Eudicots - ps19
Core_eudicots - ps20
Superrosids - ps21
Rosids - ps22
Eurosids - ps23
Malvids - ps24
Sap./Malv./Brassicales - ps25
Malvales/Brassicales - ps26
Brassicales - ps27
Cleomaceae/Brassicaceae - ps28
Brassicaceae - ps29
Camelineae - ps30
Arabidopsis - ps31
Arabidopsis_thaliana - ps32
Phylostratum

A. thaliana: GO:0002699 positive regulation of immune effector process (gain)

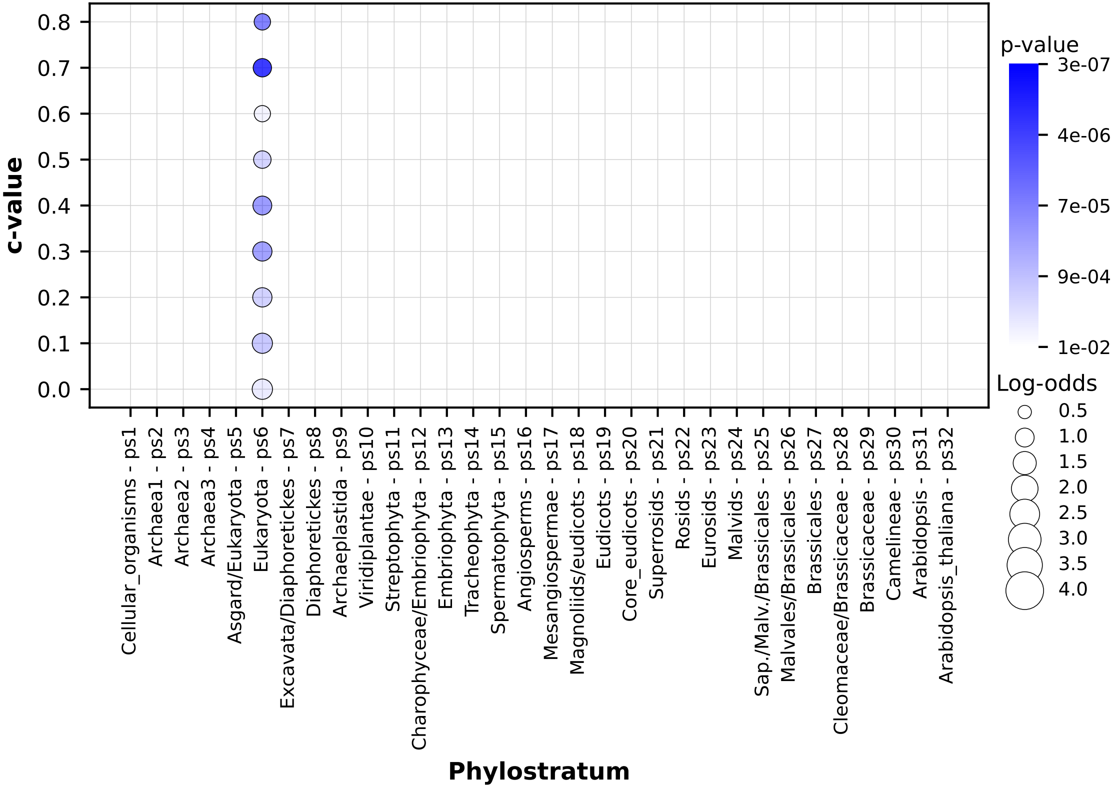

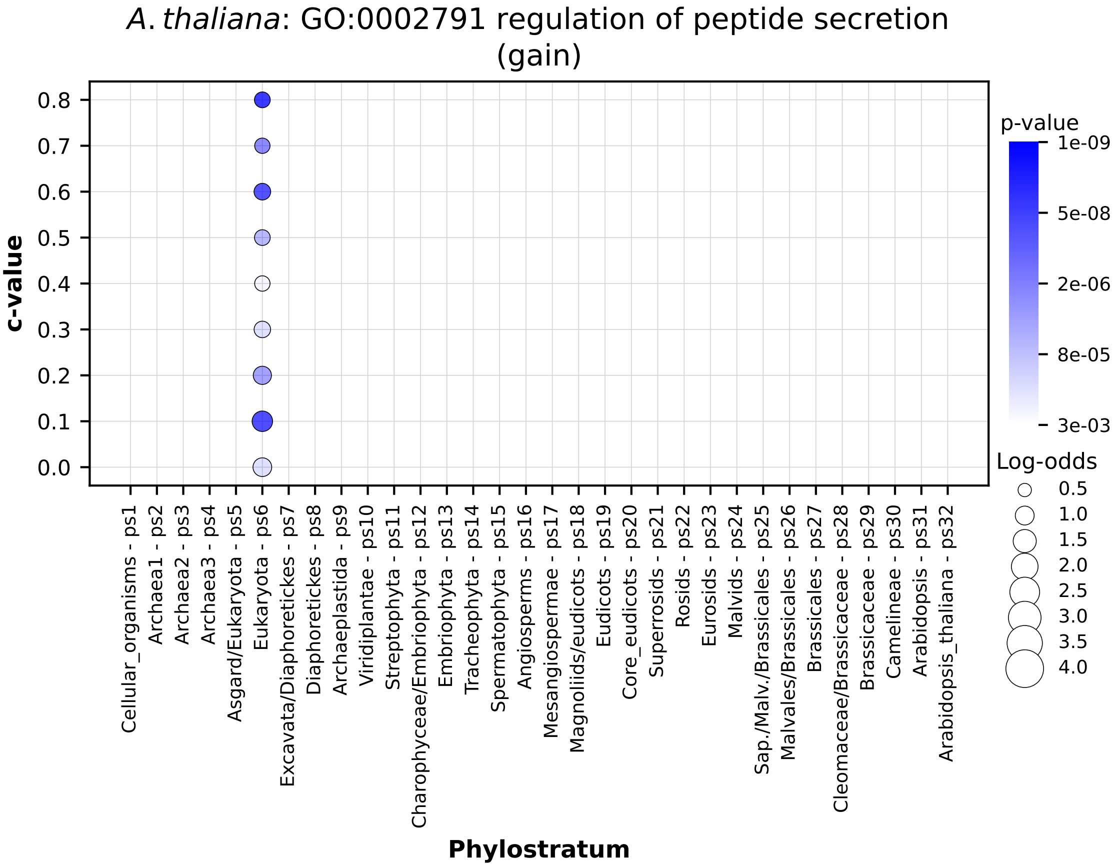

$A. thaliana$: GO:0002791 regulation of peptide secretion (gain)
c-value
0.8
0.7
0.6
0.5
0.4
0.3
0.2
0.1
0.0
Phylostratum
Cellular_organisms - ps1
Archaea1 - ps2
Archaea2 - ps3
Archaea3 - ps4
Asgard/Eukaryota - ps5
Eukaryota - ps6
Excavata/Diaphoretickes - ps7
Diaphoretickes - ps8
Archaeplastida - ps9
Viridiplantae - ps10
Streptophyta - ps11
Charophyceae/Embriophyta - ps12
Embriophyta - ps13
Tracheophyta - ps14
Spermatophyta - ps15
Angiosperms - ps16
Mesangiospermae - ps17
Magnoliids/eudicots - ps18
Eudicots - ps19
Core_eudicots - ps20
Superrosids - ps21
Rosids - ps22
Eurosids - ps23
Malvids - ps24
Sap./Malv./Brassicales - ps25
Malvales/Brassicales - ps26
Brassicales - ps27
Cleomaceae/Brassicaceae - ps28
Brassicaceae - ps29
Camelineae - ps30
Arabidopsis - ps31
Arabidopsis_thaliana - ps32
p-value
1e-09
5e-08
2e-06
8e-05
3e-03
Log-odds
0.5
1.0
1.5
2.0
2.5
3.0
3.5
4.0

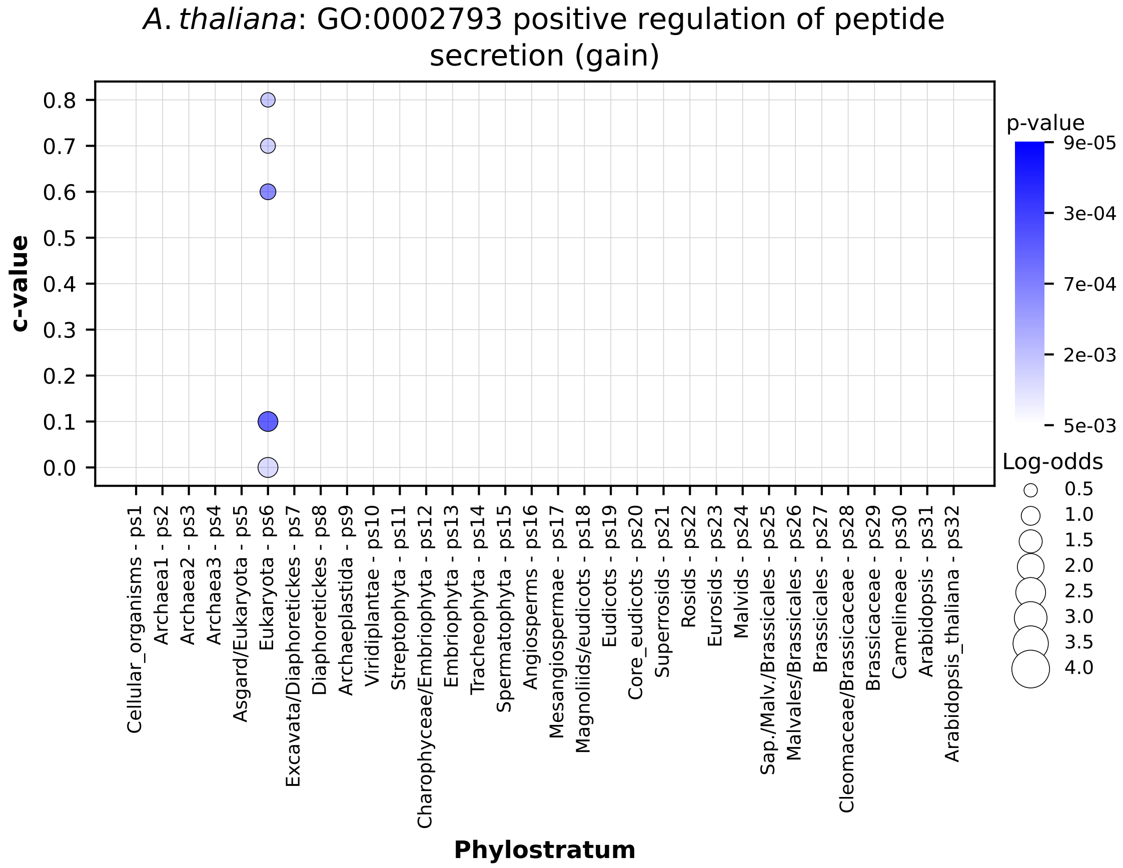

A. thaliana: GO:0002793 positive regulation of peptide secretion (gain)
c-value
0.8
0.7
0.6
0.5
0.4
0.3
0.2
0.1
0.0
Phylostratum
Cellular_organisms - ps1
Archaea1 - ps2
Archaea2 - ps3
Archaea3 - ps4
Asgard/Eukaryota - ps5
Eukaryota - ps6
Excavata/Diaphoretickes - ps7
Diaphoretickes - ps8
Archaeplastida - ps9
Viridiplantae - ps10
Streptophyta - ps11
Charophyceae/Embriophyta - ps12
Embriophyta - ps13
Tracheophyta - ps14
Spermatophyta - ps15
Angiosperms - ps16
Mesangiospermae - ps17
Magnoliids/eudicots - ps18
Eudicots - ps19
Core_eudicots - ps20
Superrosids - ps21
Rosids - ps22
Eurosids - ps23
Malvids - ps24
Sap./Malv./Brassicales - ps25
Malvales/Brassicales - ps26
Brassicales - ps27
Cleomaceae/Brassicaceae - ps28
Brassicaceae - ps29
Camelineae - ps30
Arabidopsis - ps31
Arabidopsis_thaliana - ps32
p-value
9e-05
3e-04
7e-04
2e-03
5e-03
Log-odds
0.5
1.0
1.5
2.0
2.5
3.0
3.5
4.0

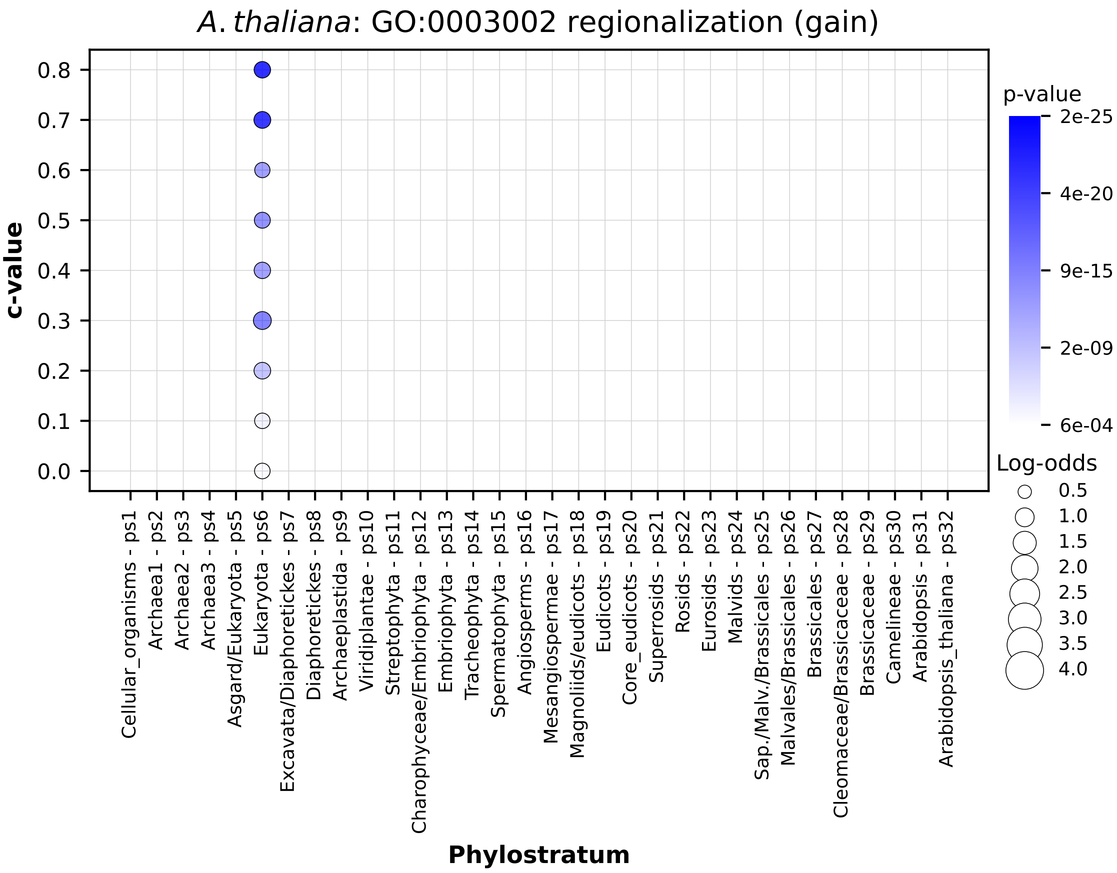

A. thaliana: GO:0003002 regionalization (gain)
c-value
0.8
0.7
0.6
0.5
0.4
0.3
0.2
0.1
0.0
Cellular_organisms - ps1
Archaea1 - ps2
Archaea2 - ps3
Archaea3 - ps4
Asgard/Eukaryota - ps5
Eukaryota - ps6
Excavata/Diaphoretickes - ps7
Diaphoretickes - ps8
Archaeplastida - ps9
Viridiplantae - ps10
Streptophyta - ps11
Charophyceae/Embriophyta - ps12
Embriophyta - ps13
Tracheophyta - ps14
Spermatophyta - ps15
Angiosperms - ps16
Mesangiospermae - ps17
Magnoliids/eudicots - ps18
Eudicots - ps19
Core_eudicots - ps20
Superrosids - ps21
Rosids - ps22
Eurosids - ps23
Malvids - ps24
Sap./Malv./Brassicales - ps25
Malvales/Brassicales - ps26
Brassicales - ps27
Cleomaceae/Brassicaceae - ps28
Brassicaceae - ps29
Camelineae - ps30
Arabidopsis - ps31
Arabidopsis_thaliana - ps32
Phylostratum
p-value
2e-25
4e-20
9e-15
2e-09
6e-04
Log-odds
0.5
1.0
1.5
2.0
2.5
3.0
3.5
4.0

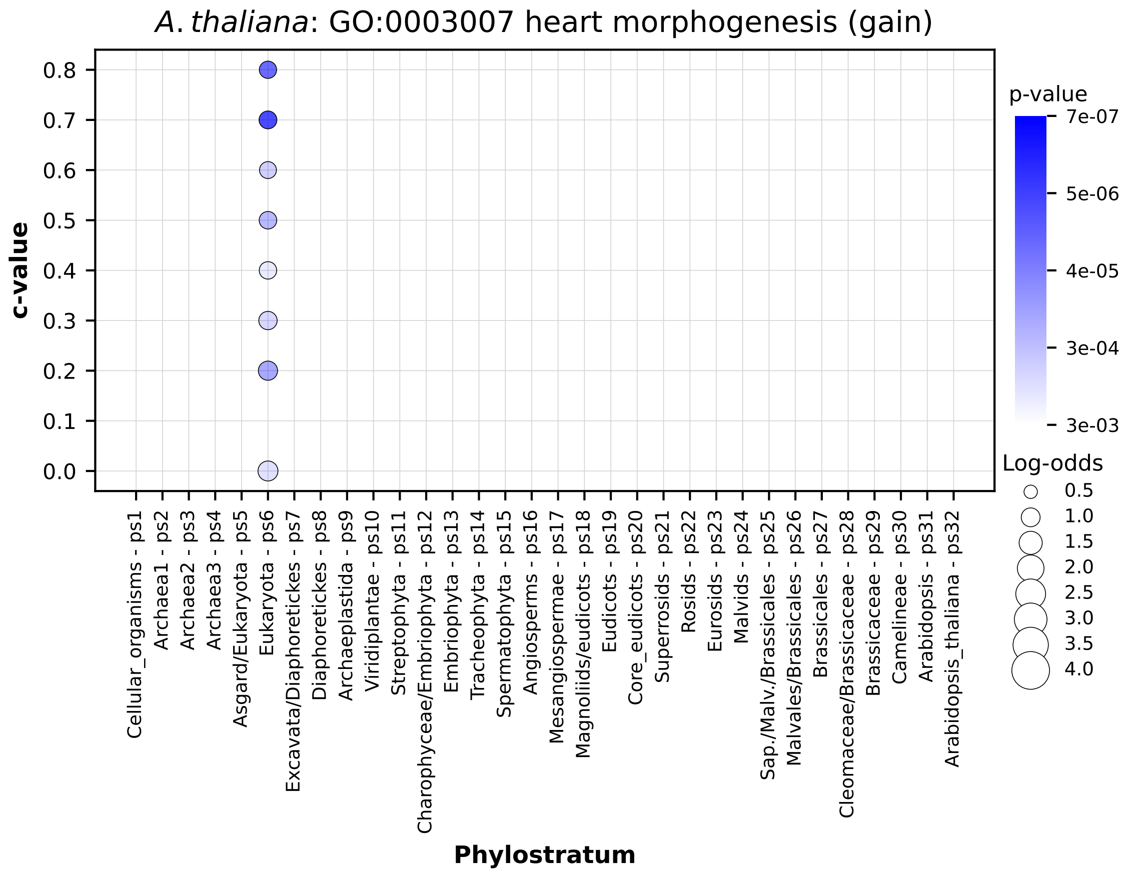
A. thaliana: GO:0003007 heart morphogenesis (gain)
c-value
0.8
0.7
0.6
0.5
0.4
0.3
0.2
0.1
0.0
Phylostratum
Cellular_organisms - ps1
Archaea1 - ps2
Archaea2 - ps3
Archaea3 - ps4
Asgard/Eukaryota - ps5
Eukaryota - ps6
Excavata/Diaphoretickes - ps7
Diaphoretickes - ps8
Archaeplastida - ps9
Viridiplantae - ps10
Streptophyta - ps11
Charophyceae/Embriophyta - ps12
Embriophyta - ps13
Tracheophyta - ps14
Spermatophyta - ps15
Angiosperms - ps16
Mesangiospermae - ps17
Magnoliids/eudicots - ps18
Eudicots - ps19
Core_eudicots - ps20
Superrosids - ps21
Rosids - ps22
Eurosids - ps23
Malvids - ps24
Sap./Malv./Brassicales - ps25
Malvales/Brassicales - ps26
Brassicales - ps27
Cleomaceae/Brassicaceae - ps28
Brassicaceae - ps29
Camelineae - ps30
Arabidopsis - ps31
Arabidopsis_thaliana - ps32
p-value
7e-07
5e-06
4e-05
3e-04
3e-03
Log-odds
0.5
1.0
1.5
2.0
2.5
3.0
3.5
4.0

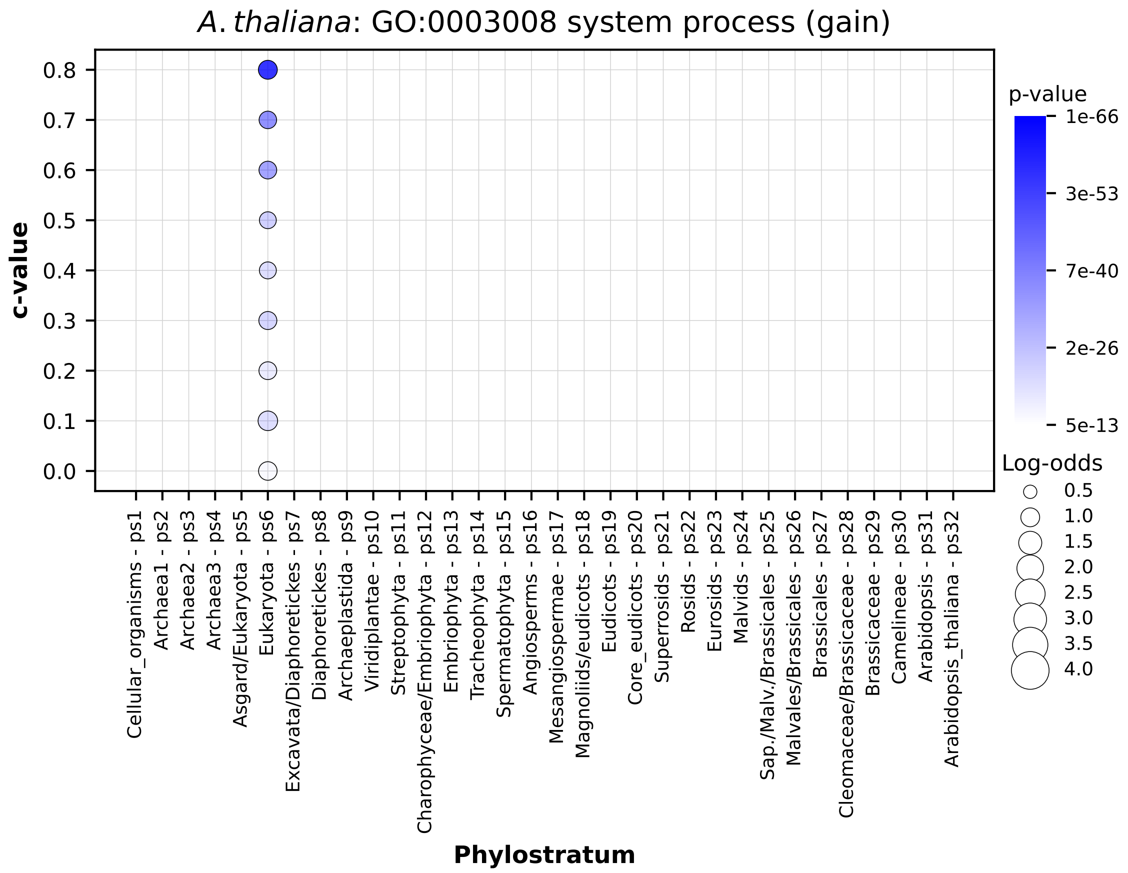
A. thaliana: GO:0003008 system process (gain)
c-value
0.8
0.7
0.6
0.5
0.4
0.3
0.2
0.1
0.0
p-value
1e-66
3e-53
7e-40
2e-26
5e-13
Log-odds
0.5
1.0
1.5
2.0
2.5
3.0
3.5
4.0
Cellular_organisms - ps1
Archaea1 - ps2
Archaea2 - ps3
Archaea3 - ps4
Asgard/Eukaryota - ps5
Eukaryota - ps6
Excavata/Diaphoretickes - ps7
Diaphoretickes - ps8
Archaeplastida - ps9
Viridiplantae - ps10
Streptophyta - ps11
Charophyceae/Embriophyta - ps12
Embriophyta - ps13
Tracheophyta - ps14
Spermatophyta - ps15
Angiosperms - ps16
Mesangiospermae - ps17
Magnoliids/eudicots - ps18
Eudicots - ps19
Core_eudicots - ps20
Superrosids - ps21
Rosids - ps22
Eurosids - ps23
Malvids - ps24
Sap./Malv./Brassicales - ps25
Malvales/Brassicales - ps26
Brassicales - ps27
Cleomaceae/Brassicaceae - ps28
Brassicaceae - ps29
Camelineae - ps30
Arabidopsis - ps31
Arabidopsis_thaliana - ps32
Phylostratum

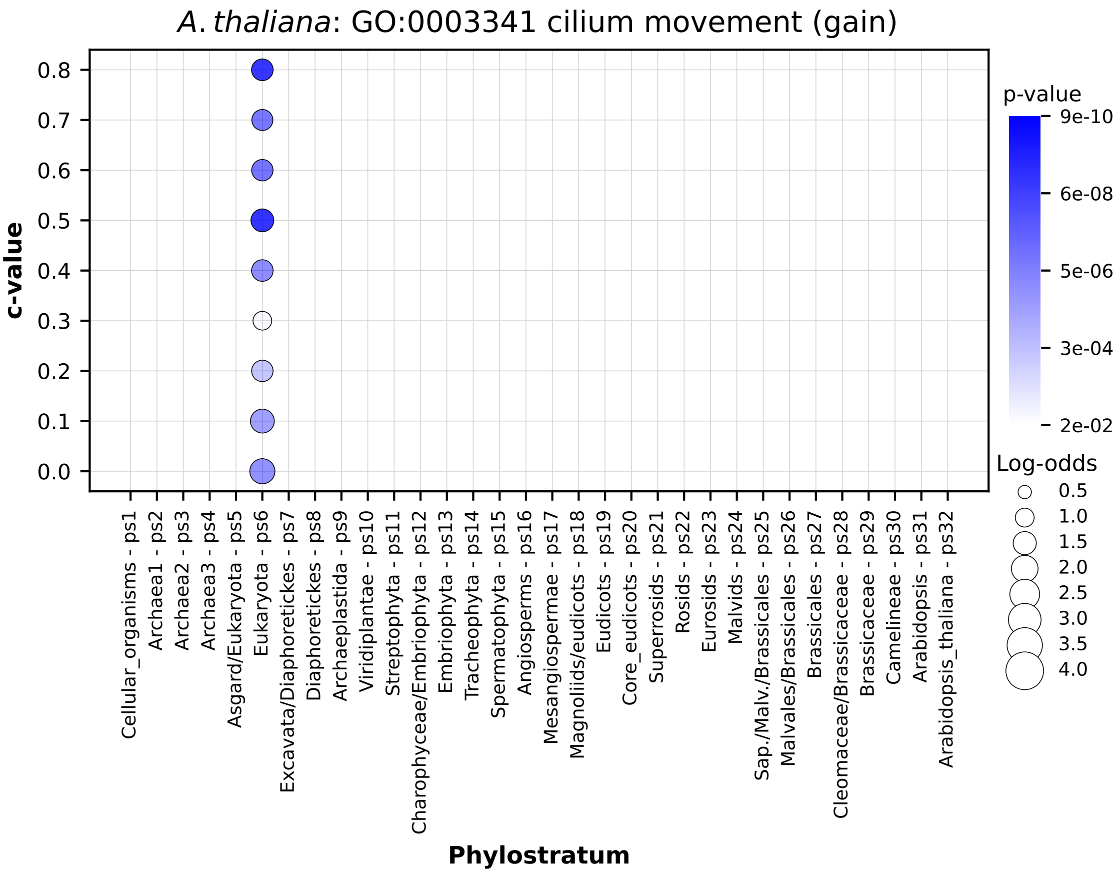

A. thaliana: GO:0003341 cilium movement (gain)
c-value
0.0
0.1
0.2
0.3
0.4
0.5
0.6
0.7
0.8
Phylostratum
Cellular_organisms - ps1
Archaea1 - ps2
Archaea2 - ps3
Archaea3 - ps4
Asgard/Eukaryota - ps5
Eukaryota - ps6
Excavata/Diaphoretickes - ps7
Diaphoretickes - ps8
Archaeplastida - ps9
Viridiplantae - ps10
Streptophyta - ps11
Charophyceae/Embriophyta - ps12
Embriophyta - ps13
Tracheophyta - ps14
Spermatophyta - ps15
Angiosperms - ps16
Mesangiospermae - ps17
Magnoliids/eudicots - ps18
Eudicots - ps19
Core_eudicots - ps20
Superrosids - ps21
Rosids - ps22
Eurosids - ps23
Malvids - ps24
Sap./Malv./Brassicales - ps25
Malvales/Brassicales - ps26
Brassicales - ps27
Cleomaceae/Brassicaceae - ps28
Brassicaceae - ps29
Camelineae - ps30
Arabidopsis - ps31
Arabidopsis_thaliana - ps32
p-value
9e-10
6e-08
5e-06
3e-04
2e-02
Log-odds
0.5
1.0
1.5
2.0
2.5
3.0
3.5
4.0

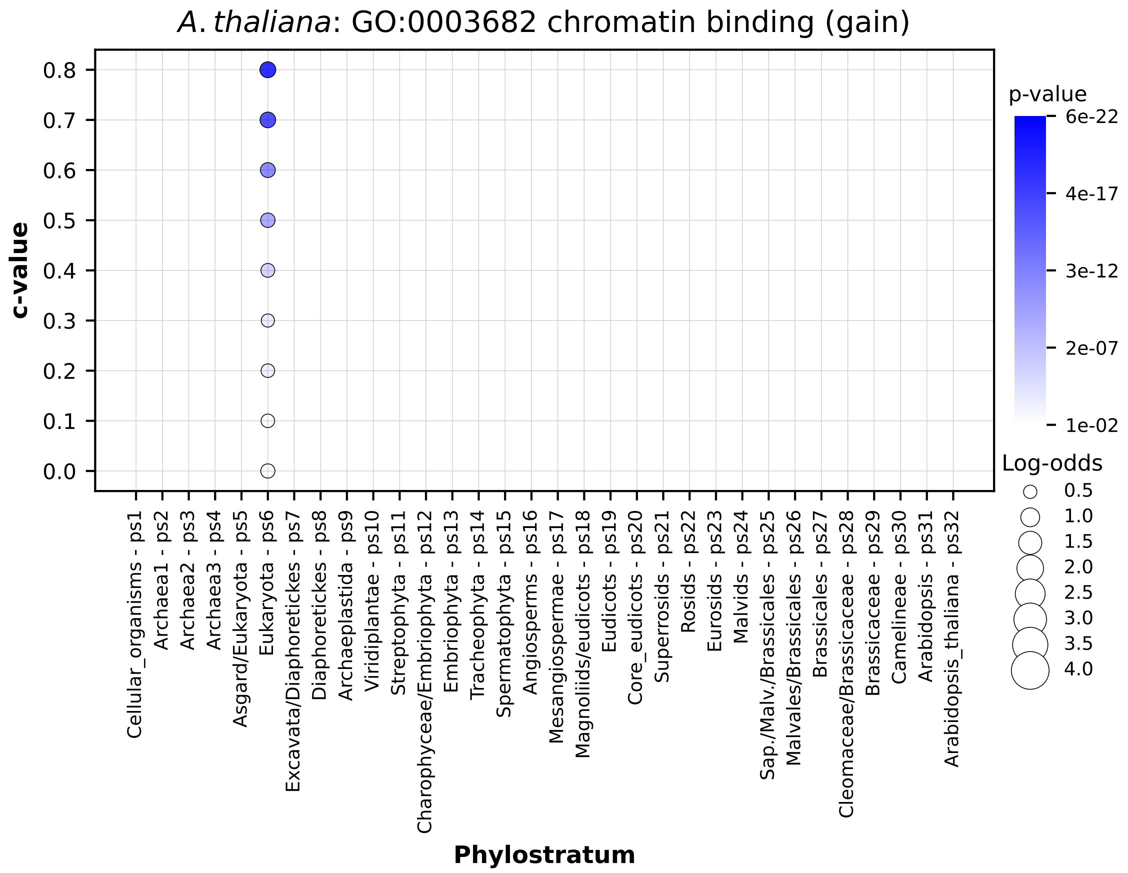
A. thaliana: GO:0003682 chromatin binding (gain)
c-value
0.8
0.7
0.6
0.5
0.4
0.3
0.2
0.1
0.0
p-value
6e-22
4e-17
3e-12
2e-07
1e-02
Log-odds
0.5
1.0
1.5
2.0
2.5
3.0
3.5
4.0
Cellular_organisms - ps1
Archaea1 - ps2
Archaea2 - ps3
Archaea3 - ps4
Asgard/Eukaryota - ps5
Eukaryota - ps6
Excavata/Diaphoretickes - ps7
Diaphoretickes - ps8
Archaeplastida - ps9
Viridiplantae - ps10
Streptophyta - ps11
Charophyceae/Embriophyta - ps12
Embriophyta - ps13
Tracheophyta - ps14
Spermatophyta - ps15
Angiosperms - ps16
Mesangiospermae - ps17
Magnoliids/eudicots - ps18
Eudicots - ps19
Core_eudicots - ps20
Superrosids - ps21
Rosids - ps22
Eurosids - ps23
Malvids - ps24
Sap./Malv./Brassicales - ps25
Malvales/Brassicales - ps26
Brassicales - ps27
Cleomaceae/Brassicaceae - ps28
Brassicaceae - ps29
Camelineae - ps30
Arabidopsis - ps31
Arabidopsis_thaliana - ps32
Phylostratum

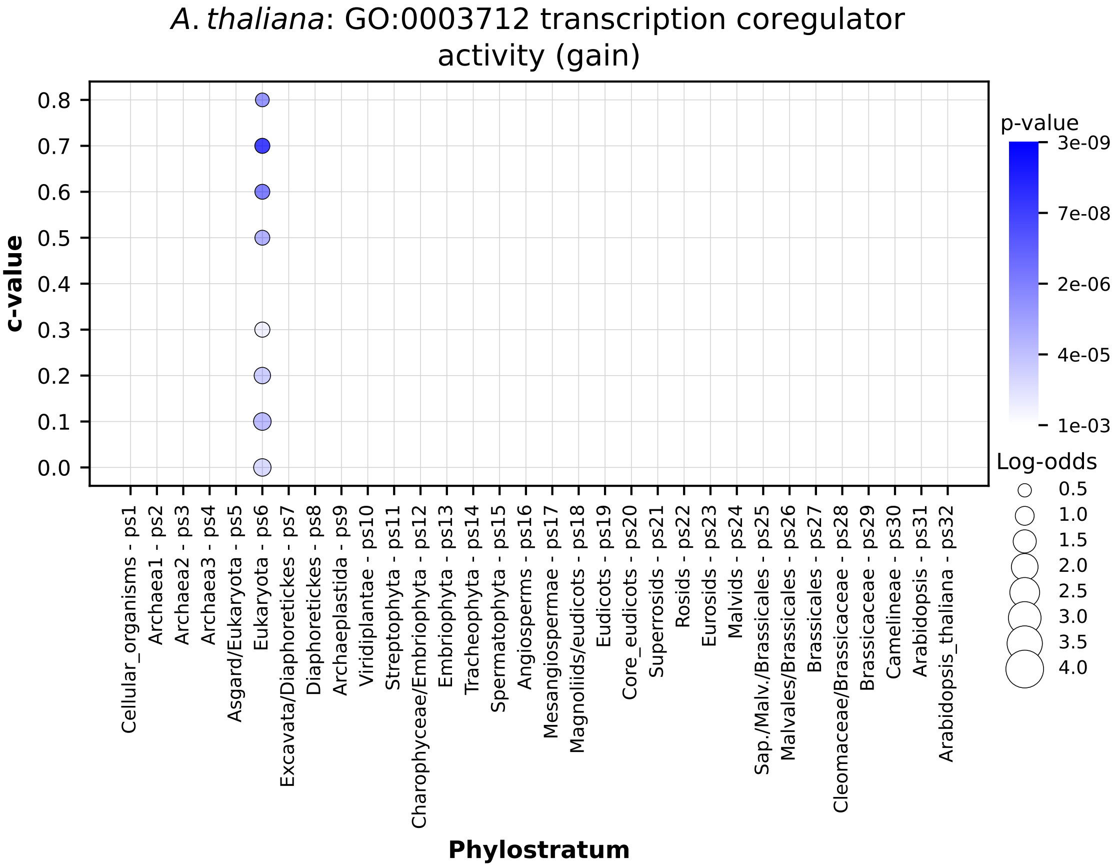
A. thaliana: GO:0003712 transcription coregulator activity (gain)
c-value
0.8
0.7
0.6
0.5
0.4
0.3
0.2
0.1
0.0
p-value
3e-09
7e-08
2e-06
4e-05
1e-03
Log-odds
0.5
1.0
1.5
2.0
2.5
3.0
3.5
4.0
Cellular_organisms - ps1
Archaea1 - ps2
Archaea2 - ps3
Archaea3 - ps4
Asgard/Eukaryota - ps5
Eukaryota - ps6
Excavata/Diaphoretickes - ps7
Diaphoretickes - ps8
Archaeplastida - ps9
Viridiplantae - ps10
Streptophyta - ps11
Charophyceae/Embriophyta - ps12
Embriophyta - ps13
Tracheophyta - ps14
Spermatophyta - ps15
Angiosperms - ps16
Mesangiospermae - ps17
Magnoliids/eudicots - ps18
Eudicots - ps19
Core_eudicots - ps20
Superrosids - ps21
Rosids - ps22
Eurosids - ps23
Malvids - ps24
Sap./Malv./Brassicales - ps25
Malvales/Brassicales - ps26
Brassicales - ps27
Cleomaceae/Brassicaceae - ps28
Brassicaceae - ps29
Camelineae - ps30
Arabidopsis - ps31
Arabidopsis_thaliana - ps32
Phylostratum

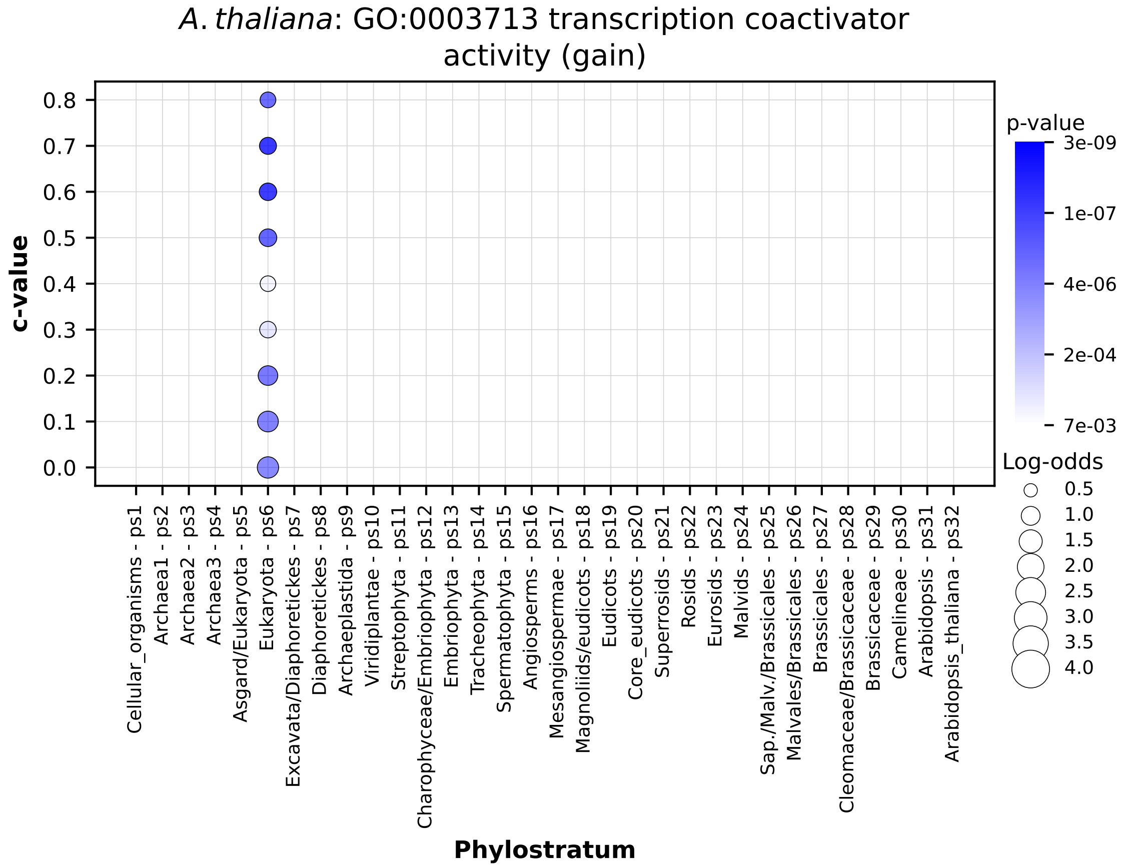

A. thaliana: GO:0003713 transcription coactivator activity (gain)
c-value
0.8
0.7
0.6
0.5
0.4
0.3
0.2
0.1
0.0
Phylostratum
Cellular_organisms - ps1
Archaea1 - ps2
Archaea2 - ps3
Archaea3 - ps4
Asgard/Eukaryota - ps5
Eukaryota - ps6
Excavata/Diaphoretickes - ps7
Diaphoretickes - ps8
Archaeplastida - ps9
Viridiplantae - ps10
Streptophyta - ps11
Charophyceae/Embriophyta - ps12
Embriophyta - ps13
Tracheophyta - ps14
Spermatophyta - ps15
Angiosperms - ps16
Mesangiospermae - ps17
Magnoliids/eudicots - ps18
Eudicots - ps19
Core_eudicots - ps20
Superrosids - ps21
Rosids - ps22
Eurosids - ps23
Malvids - ps24
Sap./Malv./Brassicales - ps25
Malvales/Brassicales - ps26
Brassicales - ps27
Cleomaceae/Brassicaceae - ps28
Brassicaceae - ps29
Camelineae - ps30
Arabidopsis - ps31
Arabidopsis_thaliana - ps32
p-value
3e-09
1e-07
4e-06
2e-04
7e-03
Log-odds
0.5
1.0
1.5
2.0
2.5
3.0
3.5
4.0

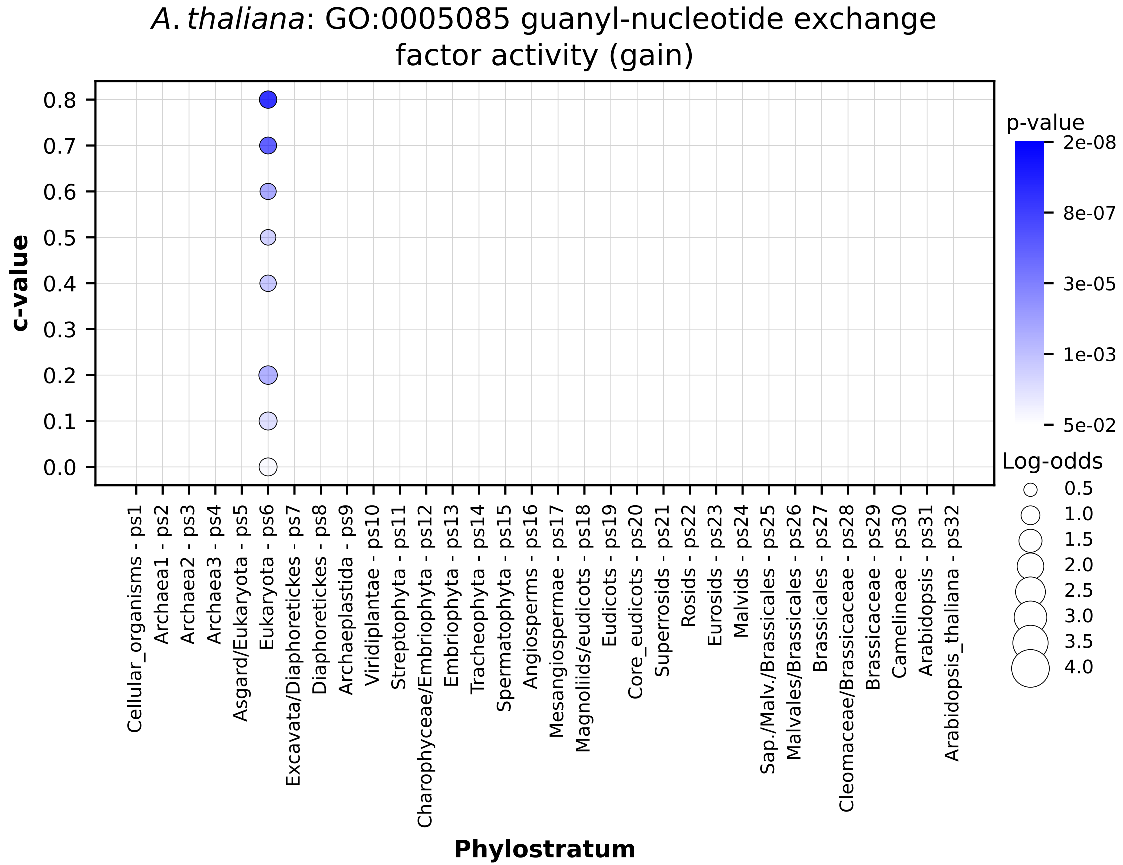
A. thaliana: GO:0005085 guanyl-nucleotide exchange factor activity (gain)
c-value
0.8
0.7
0.6
0.5
0.4
0.3
0.2
0.1
0.0
Phylostratum
Cellular_organisms - ps1
Archaea1 - ps2
Archaea2 - ps3
Archaea3 - ps4
Asgard/Eukaryota - ps5
Eukaryota - ps6
Excavata/Diaphoretickes - ps7
Diaphoretickes - ps8
Archaeplastida - ps9
Viridiplantae - ps10
Streptophyta - ps11
Charophyceae/Embriophyta - ps12
Embriophyta - ps13
Tracheophyta - ps14
Spermatophyta - ps15
Angiosperms - ps16
Mesangiospermae - ps17
Magnoliids/eudicots - ps18
Eudicots - ps19
Core_eudicots - ps20
Superrosids - ps21
Rosids - ps22
Eurosids - ps23
Malvids - ps24
Sap./Malv./Brassicales - ps25
Malvales/Brassicales - ps26
Brassicales - ps27
Cleomaceae/Brassicaceae - ps28
Brassicaceae - ps29
Camelineae - ps30
Arabidopsis - ps31
Arabidopsis_thaliana - ps32
p-value
2e-08
8e-07
3e-05
1e-03
5e-02
Log-odds
0.5
1.0
1.5
2.0
2.5
3.0
3.5
4.0

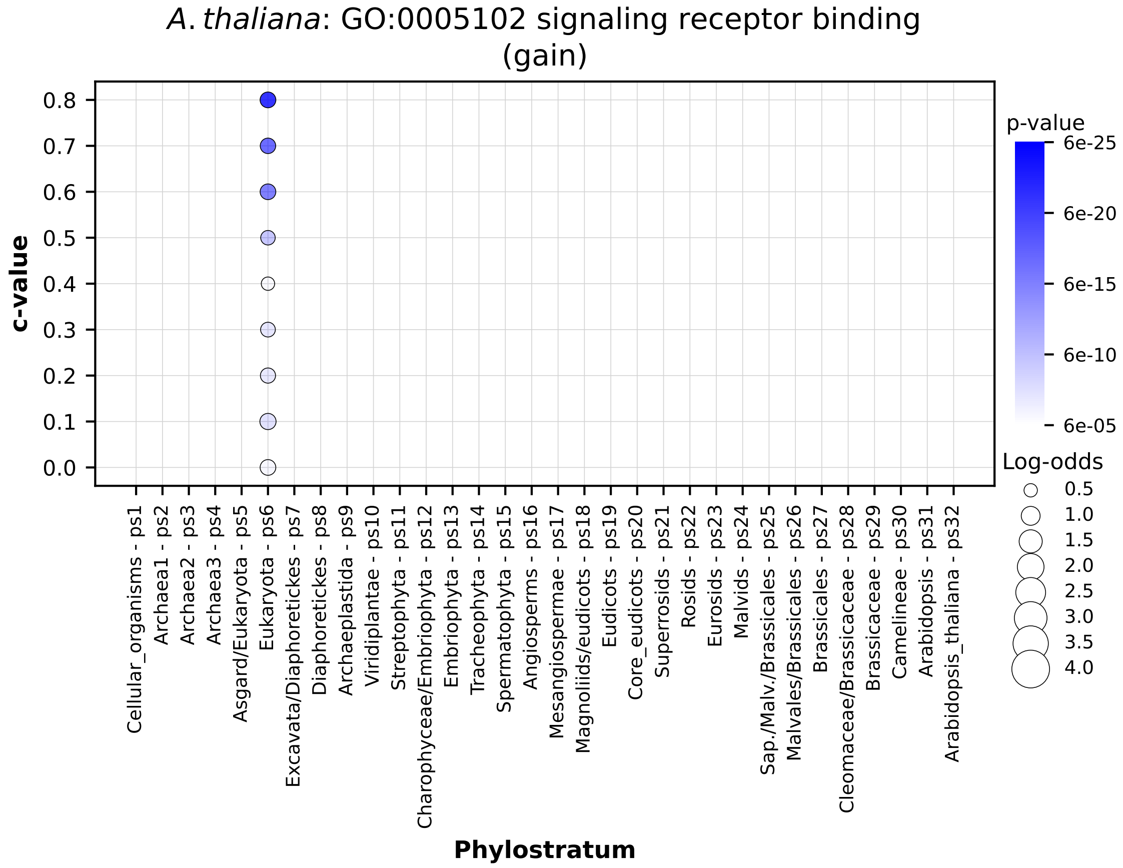

A. thaliana: GO:0005102 signaling receptor binding
(gain)
c-value
0.8
0.7
0.6
0.5
0.4
0.3
0.2
0.1
0.0
p-value
6e-25
6e-20
6e-15
6e-10
6e-05
Log-odds
0.5
1.0
1.5
2.0
2.5
3.0
3.5
4.0
Cellular_organisms - ps1
Archaea1 - ps2
Archaea2 - ps3
Archaea3 - ps4
Asgard/Eukaryota - ps5
Eukaryota - ps6
Excavata/Diaphoretickes - ps7
Diaphoretickes - ps8
Archaeplastida - ps9
Viridiplantae - ps10
Streptophyta - ps11
Charophyceae/Embriophyta - ps12
Embriophyta - ps13
Tracheophyta - ps14
Spermatophyta - ps15
Angiosperms - ps16
Mesangiospermae - ps17
Magnoliids/eudicots - ps18
Eudicots - ps19
Core_eudicots - ps20
Superrosids - ps21
Rosids - ps22
Eurosids - ps23
Malvids - ps24
Sap./Malv./Brassicales - ps25
Malvales/Brassicales - ps26
Brassicales - ps27
Cleomaceae/Brassicaceae - ps28
Brassicaceae - ps29
Camelineae - ps30
Arabidopsis - ps31
Arabidopsis_thaliana - ps32
Phylostratum

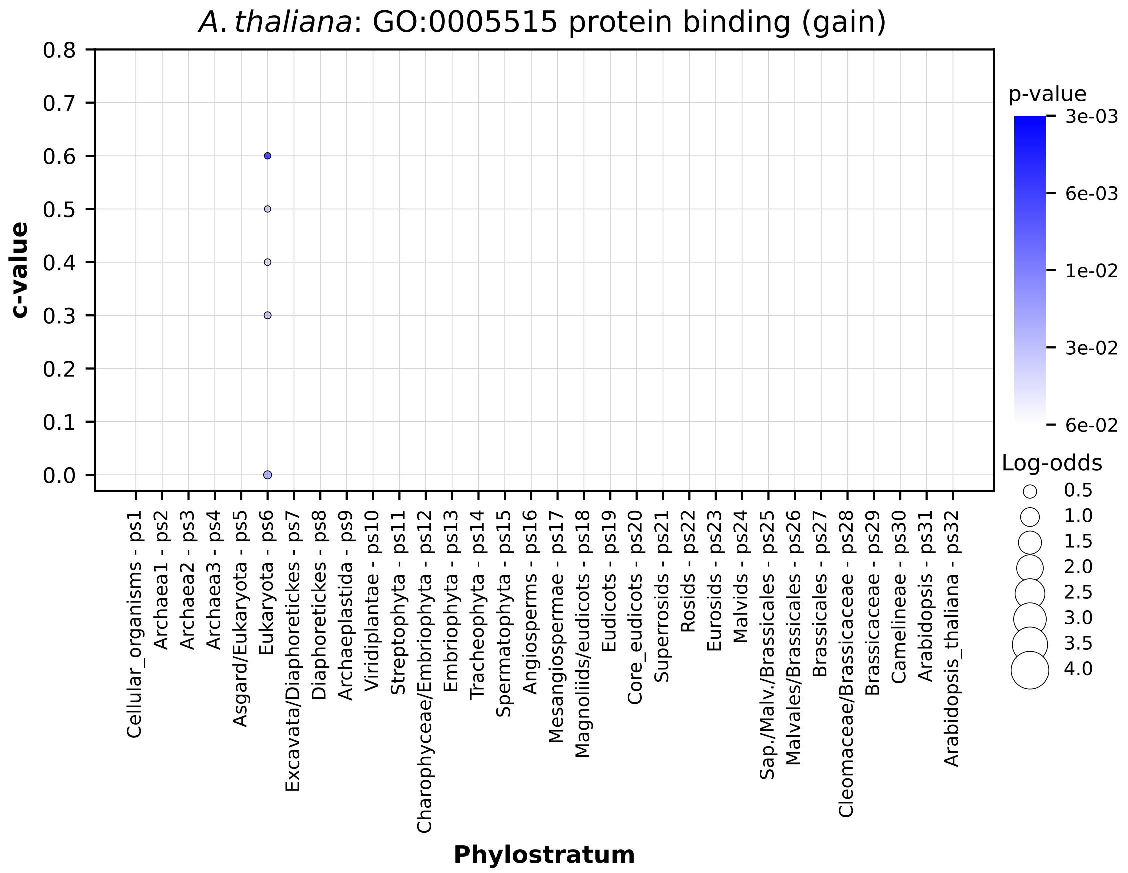

A. thaliana: GO:0005515 protein binding (gain)
c-value
0.0
0.1
0.2
0.3
0.4
0.5
0.6
0.7
0.8
Phylostratum
Cellular_organisms - ps1
Archaea1 - ps2
Archaea2 - ps3
Archaea3 - ps4
Asgard/Eukaryota - ps5
Eukaryota - ps6
Excavata/Diaphoretickes - ps7
Diaphoretickes - ps8
Archaeplastida - ps9
Viridiplantae - ps10
Streptophyta - ps11
Charophyceae/Embriophyta - ps12
Embriophyta - ps13
Tracheophyta - ps14
Spermatophyta - ps15
Angiosperms - ps16
Mesangiospermae - ps17
Magnoliids/eudicots - ps18
Eudicots - ps19
Core_eudicots - ps20
Superrosids - ps21
Rosids - ps22
Eurosids - ps23
Malvids - ps24
Sap./Malv./Brassicales - ps25
Malvales/Brassicales - ps26
Brassicales - ps27
Cleomaceae/Brassicaceae - ps28
Brassicaceae - ps29
Camelineae - ps30
Arabidopsis - ps31
Arabidopsis_thaliana - ps32
p-value
3e-03
6e-03
1e-02
3e-02
6e-02
Log-odds
0.5
1.0
1.5
2.0
2.5
3.0
3.5
4.0

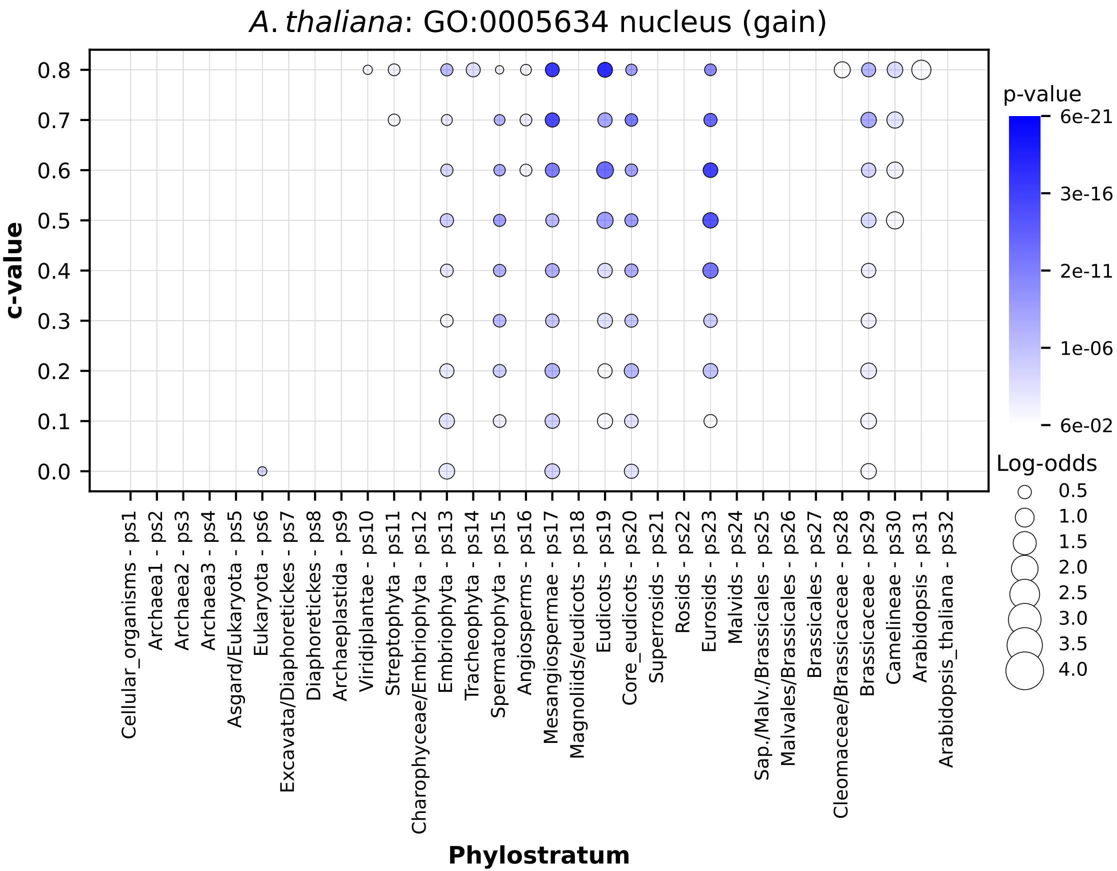

A. thaliana: GO:0005634 nucleus (gain)
c-value
0.8
0.7
0.6
0.5
0.4
0.3
0.2
0.1
0.0
Phylostratum
Cellular_organisms - ps1
Archaea1 - ps2
Archaea2 - ps3
Archaea3 - ps4
Asgard/Eukaryota - ps5
Eukaryota - ps6
Excavata/Diaphoretickes - ps7
Diaphoretickes - ps8
Archaeplastida - ps9
Viridiplantae - ps10
Streptophyta - ps11
Charophyceae/Embriophyta - ps12
Embriophyta - ps13
Tracheophyta - ps14
Spermatophyta - ps15
Angiosperms - ps16
Mesangiospermae - ps17
Magnoliids/eudicots - ps18
Eudicots - ps19
Core_eudicots - ps20
Superrosids - ps21
Rosids - ps22
Eurosids - ps23
Malvids - ps24
Sap./Malv./Brassicales - ps25
Malvales/Brassicales - ps26
Brassicales - ps27
Cleomaceae/Brassicaceae - ps28
Brassicaceae - ps29
Camelineae - ps30
Arabidopsis - ps31
Arabidopsis_thaliana - ps32
p-value
6e-21
3e-16
2e-11
1e-06
6e-02
Log-odds
0.5
1.0
1.5
2.0
2.5
3.0
3.5
4.0

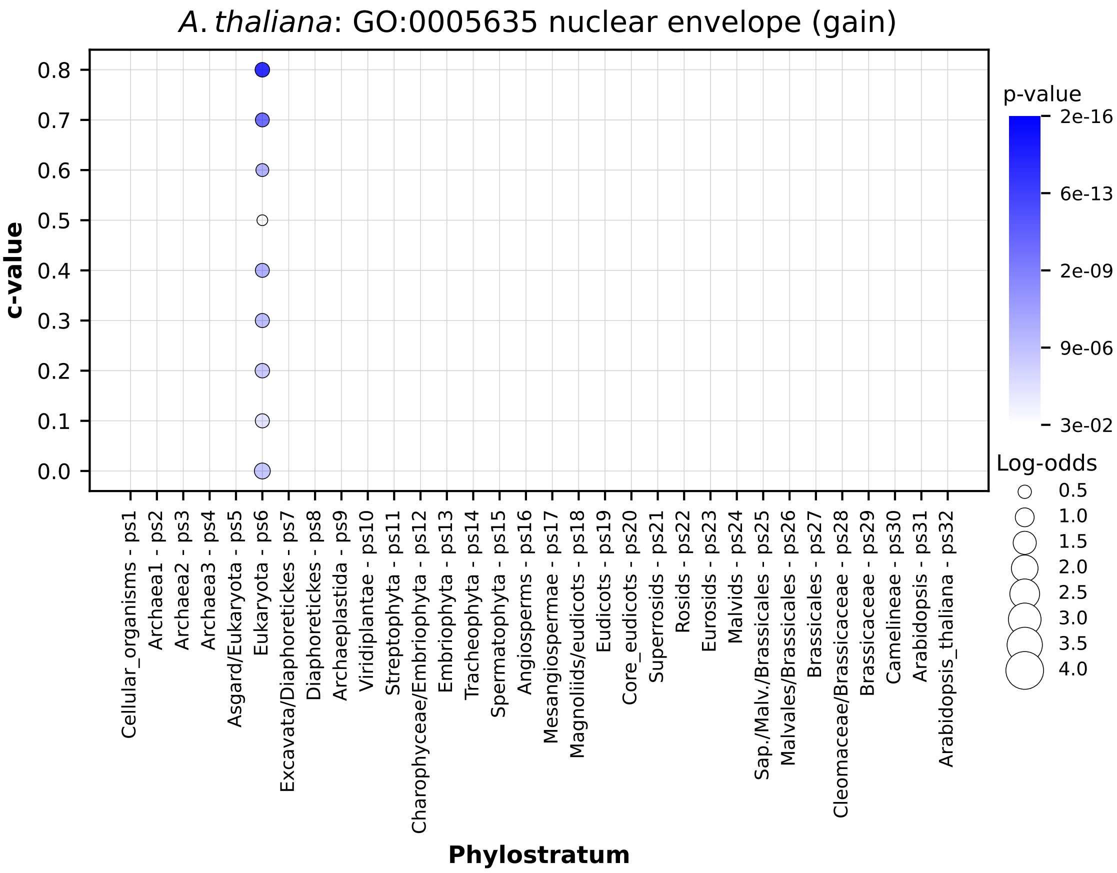

A. thaliana: GO:0005635 nuclear envelope (gain)
c-value
0.8
0.7
0.6
0.5
0.4
0.3
0.2
0.1
0.0
Phylostratum
Cellular_organisms - ps1
Archaea1 - ps2
Archaea2 - ps3
Archaea3 - ps4
Asgard/Eukaryota - ps5
Eukaryota - ps6
Excavata/Diaphoretickes - ps7
Diaphoretickes - ps8
Archaeplastida - ps9
Viridiplantae - ps10
Streptophyta - ps11
Charophyceae/Embriophyta - ps12
Embriophyta - ps13
Tracheophyta - ps14
Spermatophyta - ps15
Angiosperms - ps16
Mesangiospermae - ps17
Magnoliids/eudicots - ps18
Eudicots - ps19
Core_eudicots - ps20
Superrosids - ps21
Rosids - ps22
Eurosids - ps23
Malvids - ps24
Sap./Malv./Brassicales - ps25
Malvales/Brassicales - ps26
Brassicales - ps27
Cleomaceae/Brassicaceae - ps28
Brassicaceae - ps29
Camelineae - ps30
Arabidopsis - ps31
Arabidopsis_thaliana - ps32
p-value
2e-16
6e-13
2e-09
9e-06
3e-02
Log-odds
0.5
1.0
1.5
2.0
2.5
3.0
3.5
4.0

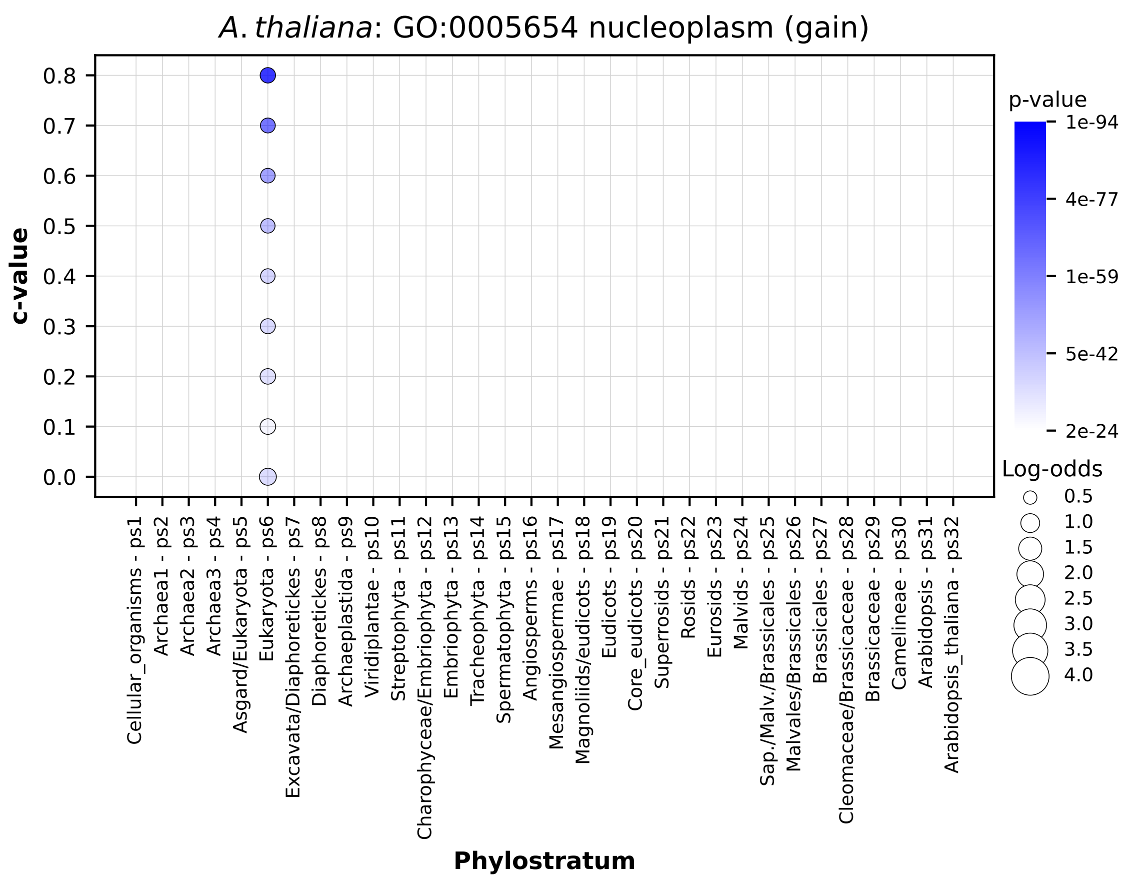
A. thaliana: GO:0005654 nucleoplasm (gain)
c-value
0.0
0.1
0.2
0.3
0.4
0.5
0.6
0.7
0.8
Phylostratum
Cellular_organisms - ps1
Archaea1 - ps2
Archaea2 - ps3
Archaea3 - ps4
Asgard/Eukaryota - ps5
Eukaryota - ps6
Excavata/Diaphoretickes - ps7
Diaphoretickes - ps8
Archaeplastida - ps9
Viridiplantae - ps10
Streptophyta - ps11
Charophyceae/Embriophyta - ps12
Embriophyta - ps13
Tracheophyta - ps14
Spermatophyta - ps15
Angiosperms - ps16
Mesangiospermae - ps17
Magnoliids/eudicots - ps18
Eudicots - ps19
Core_eudicots - ps20
Superrosids - ps21
Rosids - ps22
Eurosids - ps23
Malvids - ps24
Sap./Malv./Brassicales - ps25
Malvales/Brassicales - ps26
Brassicales - ps27
Cleomaceae/Brassicaceae - ps28
Brassicaceae - ps29
Camelineae - ps30
Arabidopsis - ps31
Arabidopsis_thaliana - ps32
p-value
1e-94
4e-77
1e-59
5e-42
2e-24
Log-odds
0.5
1.0
1.5
2.0
2.5
3.0
3.5
4.0

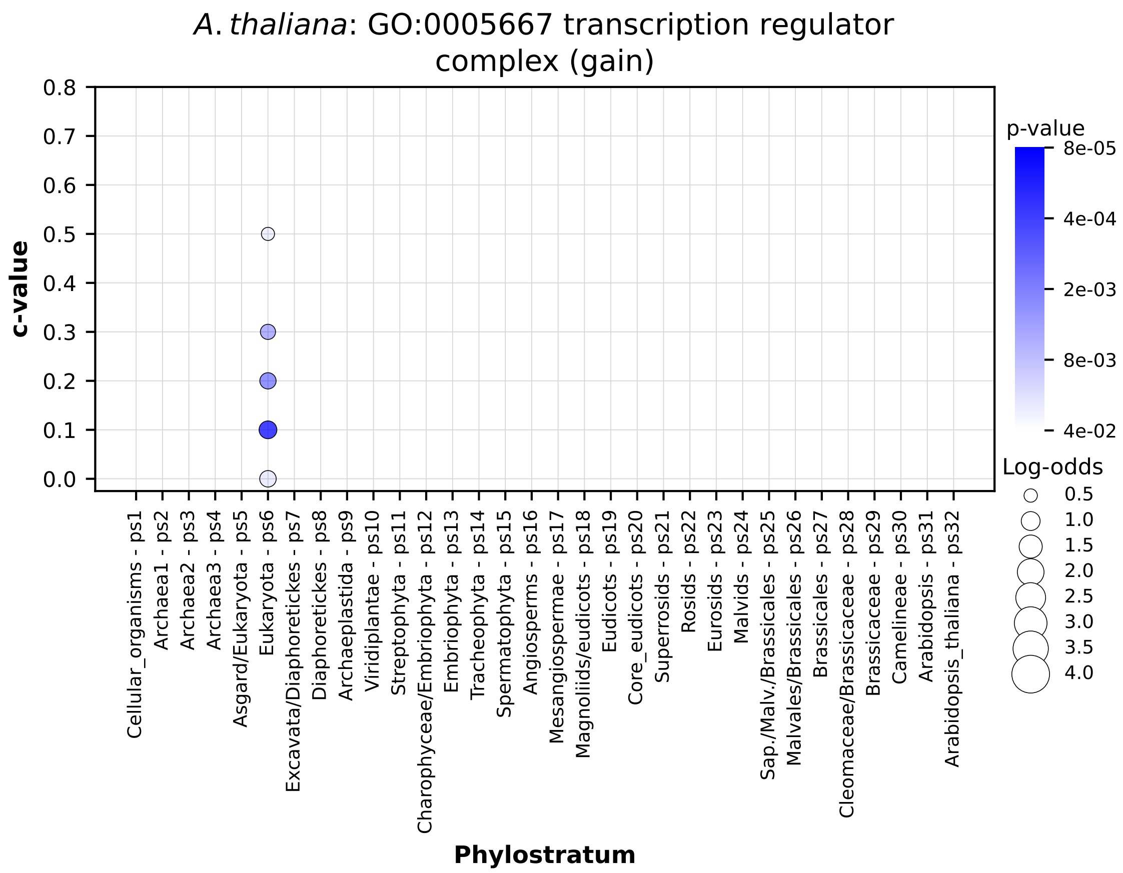
A. thaliana: GO:0005667 transcription regulator complex (gain)
c-value
0.8
0.7
0.6
0.5
0.4
0.3
0.2
0.1
0.0
Phylostratum
Cellular_organisms - ps1
Archaea1 - ps2
Archaea2 - ps3
Archaea3 - ps4
Asgard/Eukaryota - ps5
Eukaryota - ps6
Excavata/Diaphoretickes - ps7
Diaphoretickes - ps8
Archaeplastida - ps9
Viridiplantae - ps10
Streptophyta - ps11
Charophyceae/Embriophyta - ps12
Embriophyta - ps13
Tracheophyta - ps14
Spermatophyta - ps15
Angiosperms - ps16
Mesangiospermae - ps17
Magnoliids/eudicots - ps18
Eudicots - ps19
Core_eudicots - ps20
Superrosids - ps21
Rosids - ps22
Eurosids - ps23
Malvids - ps24
Sap./Malv./Brassicales - ps25
Malvales/Brassicales - ps26
Brassicales - ps27
Cleomaceae/Brassicaceae - ps28
Brassicaceae - ps29
Camelineae - ps30
Arabidopsis - ps31
Arabidopsis_thaliana - ps32
p-value
8e-05
4e-04
2e-03
8e-03
4e-02
Log-odds
0.5
1.0
1.5
2.0
2.5
3.0
3.5
4.0

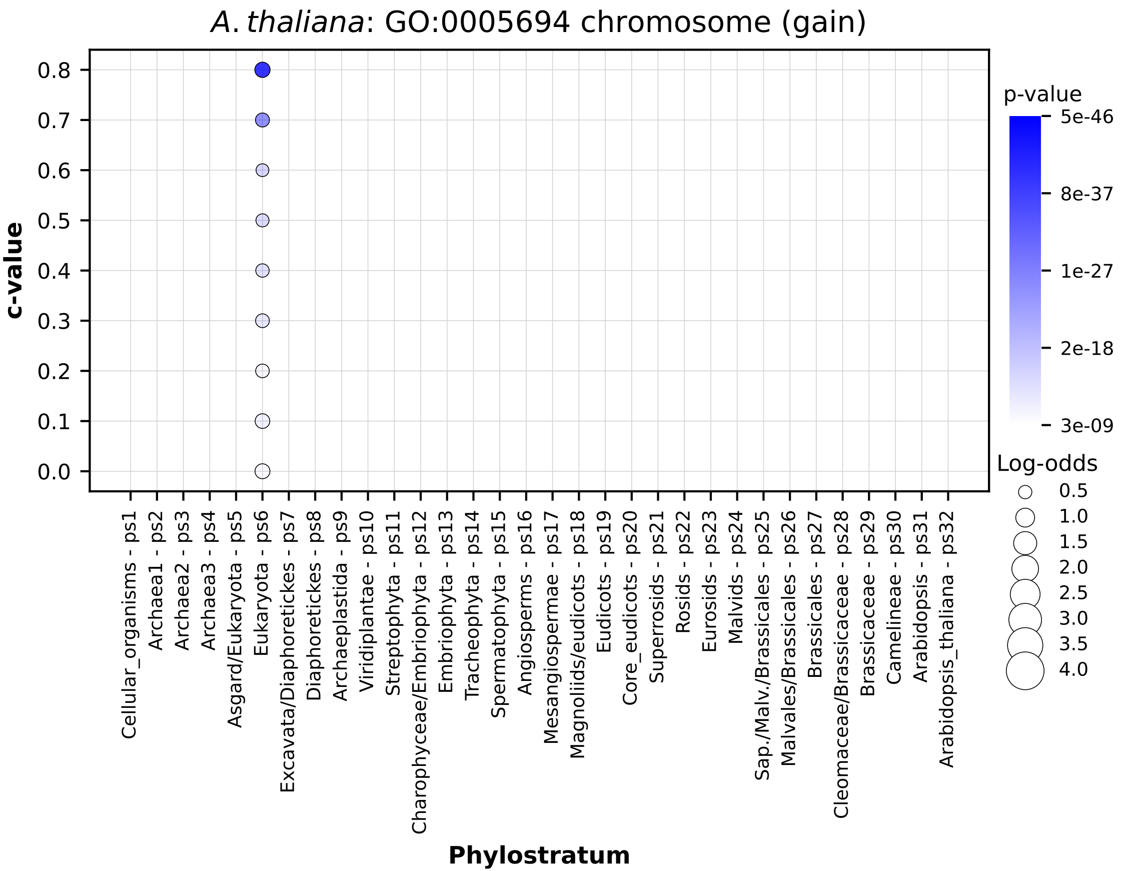

A. thaliana: GO:0005694 chromosome (gain)
c-value
0.8
0.7
0.6
0.5
0.4
0.3
0.2
0.1
0.0
p-value
5e-46
8e-37
1e-27
2e-18
3e-09
Log-odds
0.5
1.0
1.5
2.0
2.5
3.0
3.5
4.0
Cellular_organisms - ps1
Archaea1 - ps2
Archaea2 - ps3
Archaea3 - ps4
Asgard/Eukaryota - ps5
Eukaryota - ps6
Excavata/Diaphoretickes - ps7
Diaphoretickes - ps8
Archaeplastida - ps9
Viridiplantae - ps10
Streptophyta - ps11
Charophyceae/Embriophyta - ps12
Embriophyta - ps13
Tracheophyta - ps14
Spermatophyta - ps15
Angiosperms - ps16
Mesangiospermae - ps17
Magnoliids/eudicots - ps18
Eudicots - ps19
Core_eudicots - ps20
Superrosids - ps21
Rosids - ps22
Eurosids - ps23
Malvids - ps24
Sap./Malv./Brassicales - ps25
Malvales/Brassicales - ps26
Brassicales - ps27
Cleomaceae/Brassicaceae - ps28
Brassicaceae - ps29
Camelineae - ps30
Arabidopsis - ps31
Arabidopsis_thaliana - ps32
Phylostratum

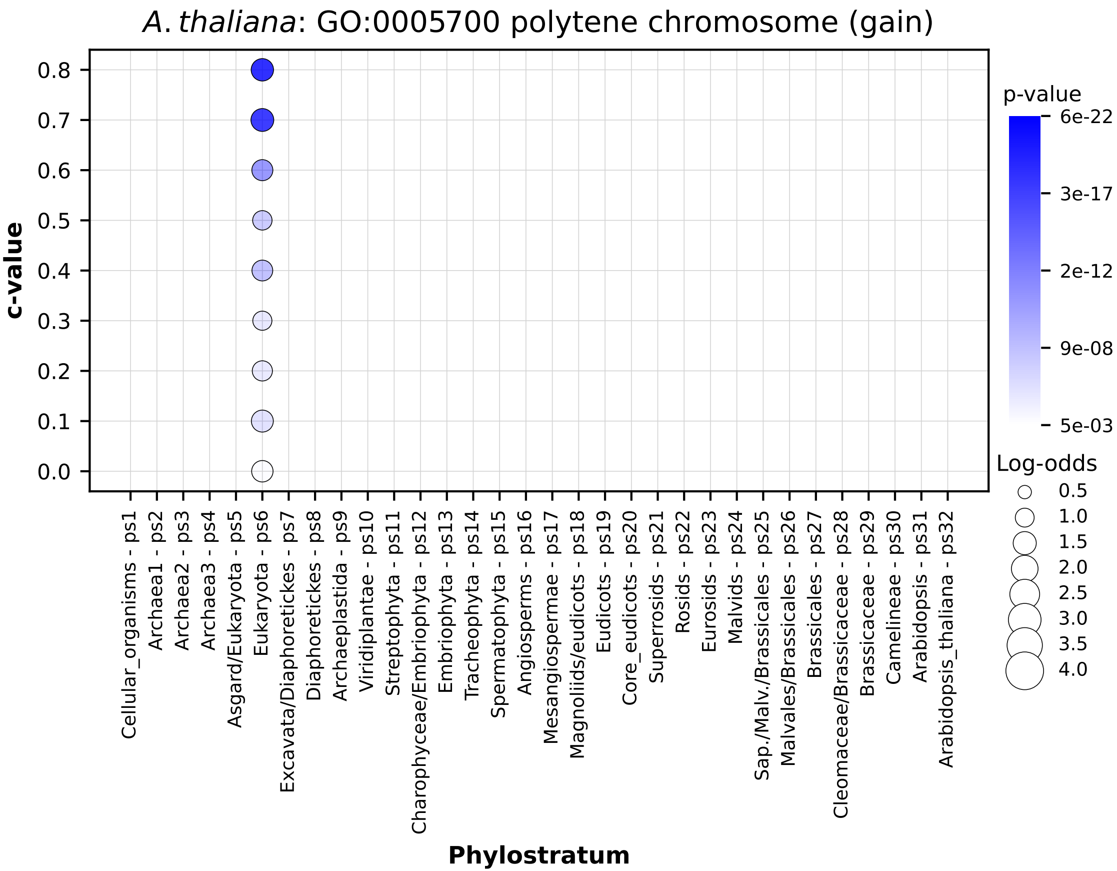
A. thaliana: GO:0005700 polytene chromosome (gain)
c-value
0.0
0.1
0.2
0.3
0.4
0.5
0.6
0.7
0.8
Phylostratum
Cellular_organisms - ps1
Archaea1 - ps2
Archaea2 - ps3
Archaea3 - ps4
Asgard/Eukaryota - ps5
Eukaryota - ps6
Excavata/Diaphoretickes - ps7
Diaphoretickes - ps8
Archaeplastida - ps9
Viridiplantae - ps10
Streptophyta - ps11
Charophyceae/Embriophyta - ps12
Embriophyta - ps13
Tracheophyta - ps14
Spermatophyta - ps15
Angiosperms - ps16
Mesangiospermae - ps17
Magnoliids/eudicots - ps18
Eudicots - ps19
Core_eudicots - ps20
Superrosids - ps21
Rosids - ps22
Eurosids - ps23
Malvids - ps24
Sap./Malv./Brassicales - ps25
Malvales/Brassicales - ps26
Brassicales - ps27
Cleomaceae/Brassicaceae - ps28
Brassicaceae - ps29
Camelineae - ps30
Arabidopsis - ps31
Arabidopsis_thaliana - ps32
p-value
6e-22
3e-17
2e-12
9e-08
5e-03
Log-odds
0.5
1.0
1.5
2.0
2.5
3.0
3.5
4.0

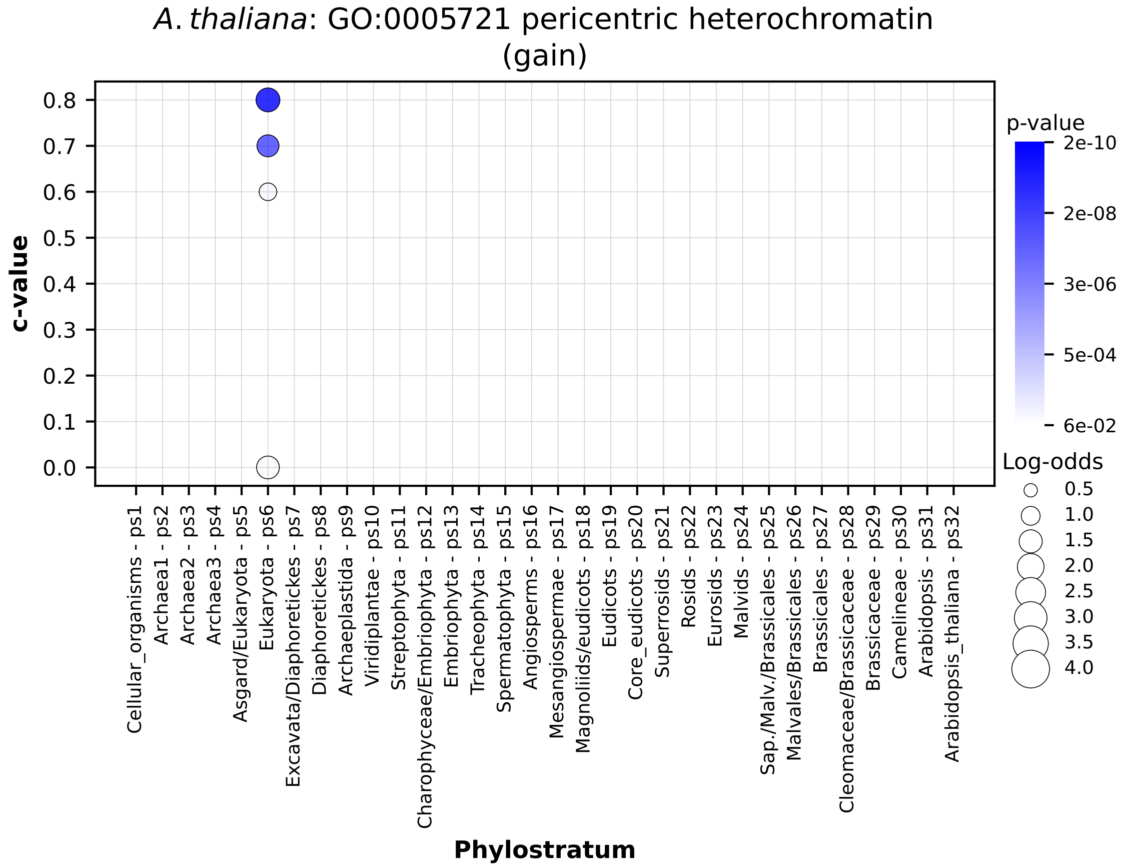

A. thaliana: GO:0005721 pericentric heterochromatin
(gain)
c-value
0.8
0.7
0.6
0.5
0.4
0.3
0.2
0.1
0.0
p-value
2e-10
2e-08
3e-06
5e-04
6e-02
Log-odds
0.5
1.0
1.5
2.0
2.5
3.0
3.5
4.0
Cellular_organisms - ps1
Archaea1 - ps2
Archaea2 - ps3
Archaea3 - ps4
Asgard/Eukaryota - ps5
Eukaryota - ps6
Excavata/Diaphoretickes - ps7
Diaphoretickes - ps8
Archaeplastida - ps9
Viridiplantae - ps10
Streptophyta - ps11
Charophyceae/Embriophyta - ps12
Embriophyta - ps13
Tracheophyta - ps14
Spermatophyta - ps15
Angiosperms - ps16
Mesangiospermae - ps17
Magnoliids/eudicots - ps18
Eudicots - ps19
Core_eudicots - ps20
Superrosids - ps21
Rosids - ps22
Eurosids - ps23
Malvids - ps24
Sap./Malv./Brassicales - ps25
Malvales/Brassicales - ps26
Brassicales - ps27
Cleomaceae/Brassicaceae - ps28
Brassicaceae - ps29
Camelineae - ps30
Arabidopsis - ps31
Arabidopsis_thaliana - ps32
Phylostratum

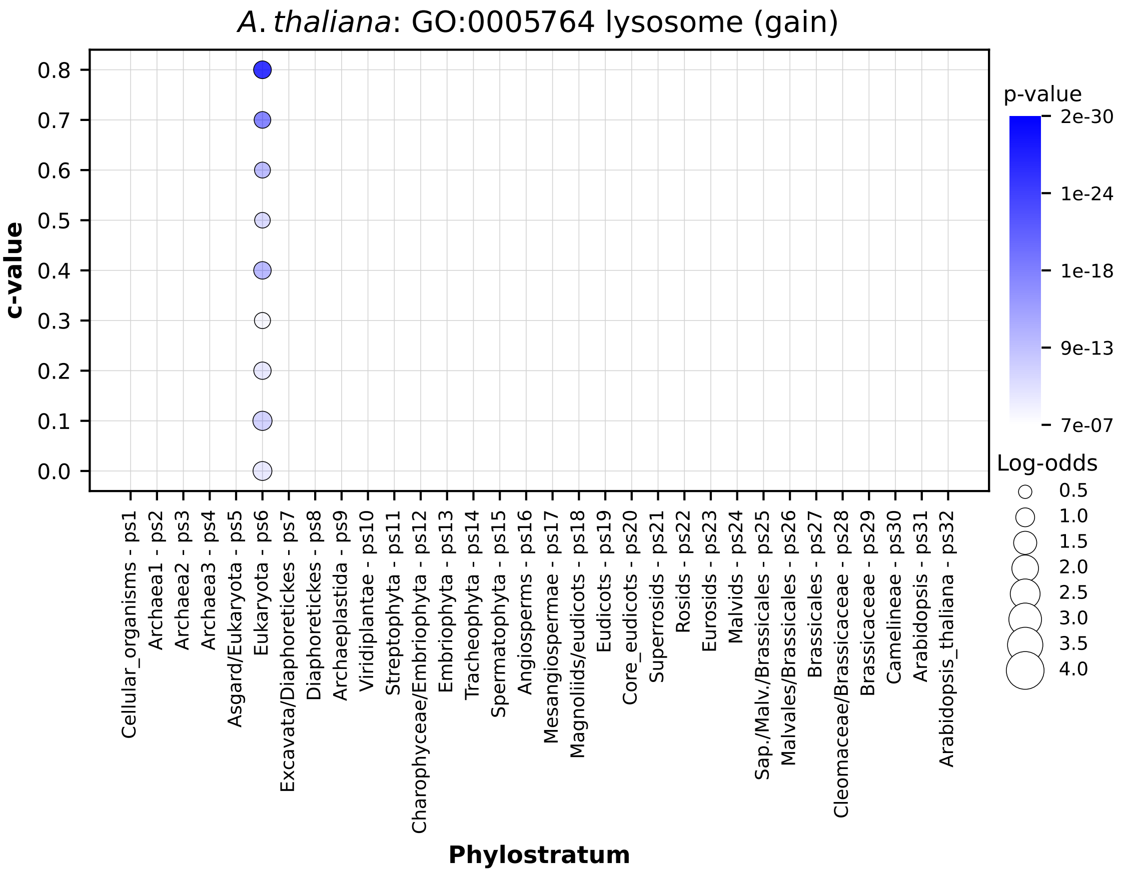

A. thaliana: GO:0005764 lysosome (gain)
c-value
0.8
0.7
0.6
0.5
0.4
0.3
0.2
0.1
0.0
p-value
2e-30
1e-24
1e-18
9e-13
7e-07
Log-odds
0.5
1.0
1.5
2.0
2.5
3.0
3.5
4.0
Cellular_organisms - ps1
Archaea1 - ps2
Archaea2 - ps3
Archaea3 - ps4
Asgard/Eukaryota - ps5
Eukaryota - ps6
Excavata/Diaphoretickes - ps7
Diaphoretickes - ps8
Archaeplastida - ps9
Viridiplantae - ps10
Streptophyta - ps11
Charophyceae/Embriophyta - ps12
Embriophyta - ps13
Tracheophyta - ps14
Spermatophyta - ps15
Angiosperms - ps16
Mesangiospermae - ps17
Magnoliids/eudicots - ps18
Eudicots - ps19
Core_eudicots - ps20
Superrosids - ps21
Rosids - ps22
Eurosids - ps23
Malvids - ps24
Sap./Malv./Brassicales - ps25
Malvales/Brassicales - ps26
Brassicales - ps27
Cleomaceae/Brassicaceae - ps28
Brassicaceae - ps29
Camelineae - ps30
Arabidopsis - ps31
Arabidopsis_thaliana - ps32
Phylostratum

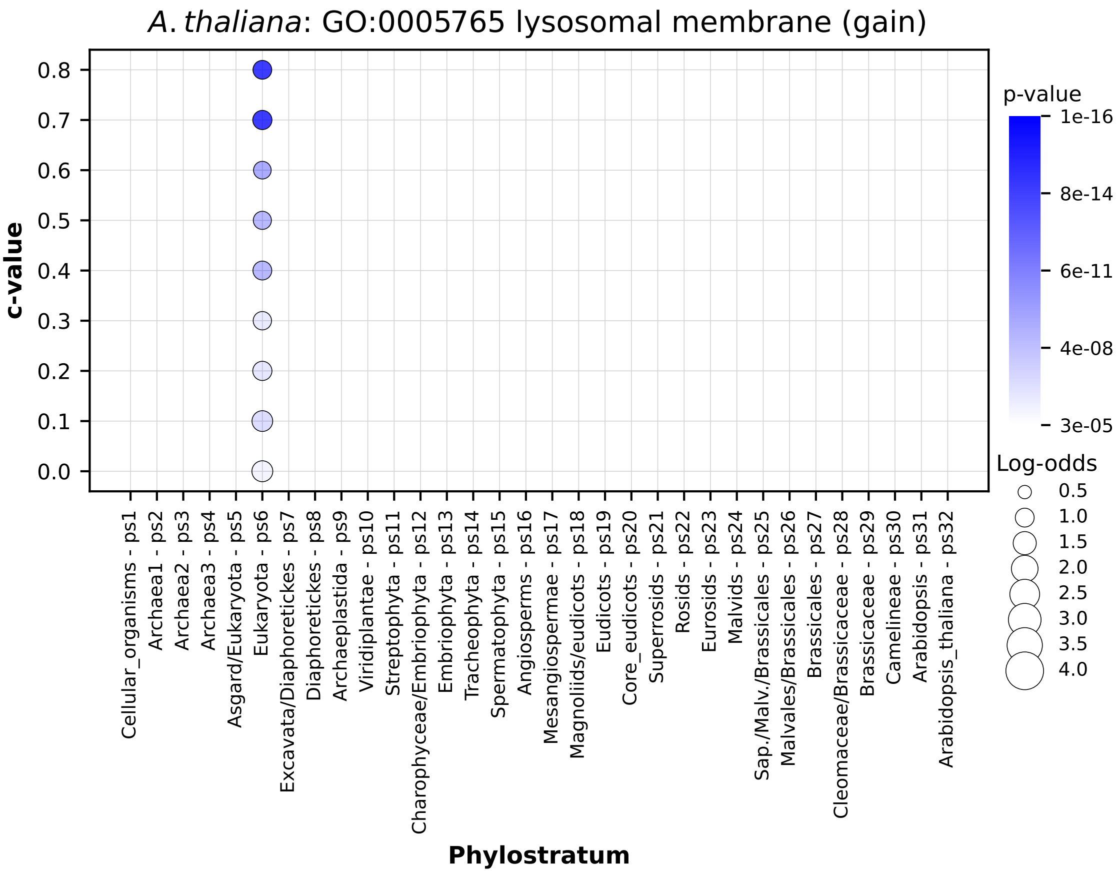

A. thaliana: GO:0005765 lysosomal membrane (gain)
c-value
0.8
0.7
0.6
0.5
0.4
0.3
0.2
0.1
0.0
Phylostratum
Cellular_organisms - ps1
Archaea1 - ps2
Archaea2 - ps3
Archaea3 - ps4
Asgard/Eukaryota - ps5
Eukaryota - ps6
Excavata/Diaphoretickes - ps7
Diaphoretickes - ps8
Archaeplastida - ps9
Viridiplantae - ps10
Streptophyta - ps11
Charophyceae/Embriophyta - ps12
Embriophyta - ps13
Tracheophyta - ps14
Spermatophyta - ps15
Angiosperms - ps16
Mesangiospermae - ps17
Magnoliids/eudicots - ps18
Eudicots - ps19
Core_eudicots - ps20
Superrosids - ps21
Rosids - ps22
Eurosids - ps23
Malvids - ps24
Sap./Malv./Brassicales - ps25
Malvales/Brassicales - ps26
Brassicales - ps27
Cleomaceae/Brassicaceae - ps28
Brassicaceae - ps29
Camelineae - ps30
Arabidopsis - ps31
Arabidopsis_thaliana - ps32
p-value
1e-16
8e-14
6e-11
4e-08
3e-05
Log-odds
0.5
1.0
1.5
2.0
2.5
3.0
3.5
4.0

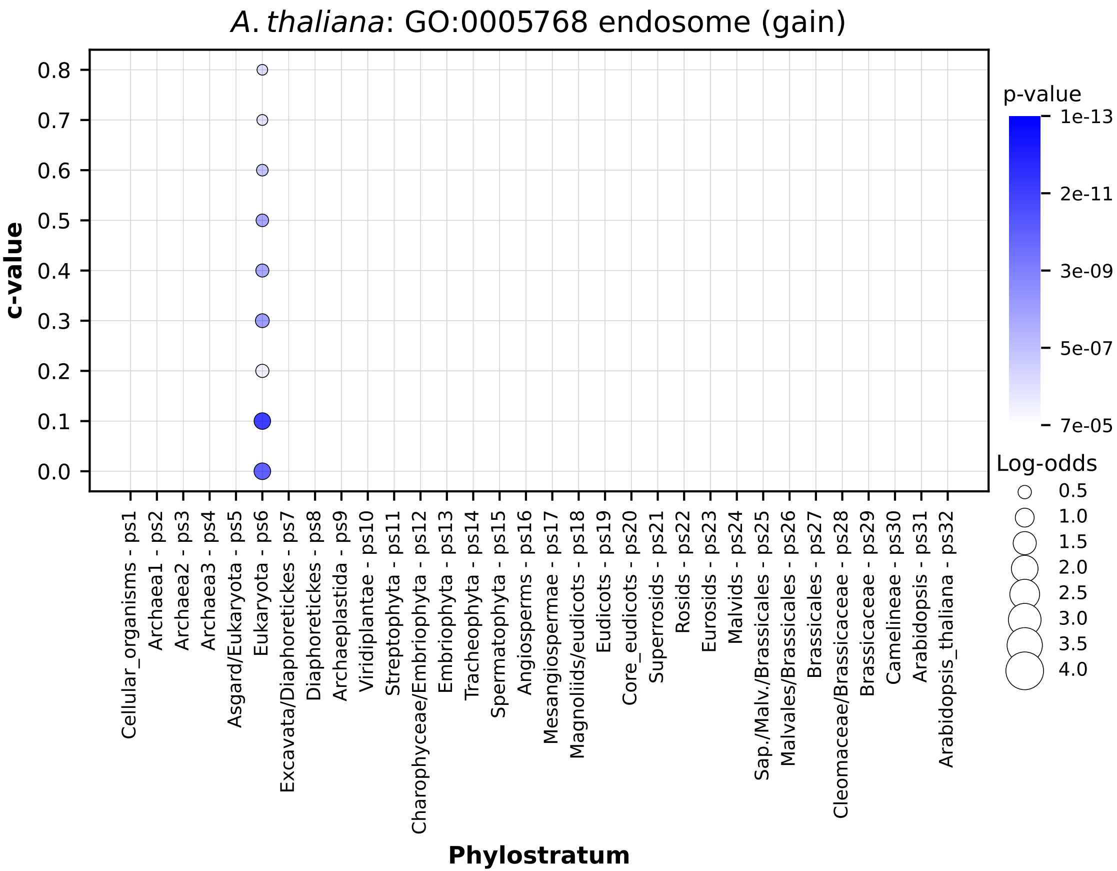

A. thaliana: GO:0005768 endosome (gain)
c-value
0.0
0.1
0.2
0.3
0.4
0.5
0.6
0.7
0.8
Phylostratum
Cellular_organisms - ps1
Archaea1 - ps2
Archaea2 - ps3
Archaea3 - ps4
Asgard/Eukaryota - ps5
Eukaryota - ps6
Excavata/Diaphoretickes - ps7
Diaphoretickes - ps8
Archaeplastida - ps9
Viridiplantae - ps10
Streptophyta - ps11
Charophyceae/Embriophyta - ps12
Embriophyta - ps13
Tracheophyta - ps14
Spermatophyta - ps15
Angiosperms - ps16
Mesangiospermae - ps17
Magnoliids/eudicots - ps18
Eudicots - ps19
Core_eudicots - ps20
Superrosids - ps21
Rosids - ps22
Eurosids - ps23
Malvids - ps24
Sap./Malv./Brassicales - ps25
Malvales/Brassicales - ps26
Brassicales - ps27
Cleomaceae/Brassicaceae - ps28
Brassicaceae - ps29
Camelineae - ps30
Arabidopsis - ps31
Arabidopsis_thaliana - ps32
p-value
1e-13
2e-11
3e-09
5e-07
7e-05
Log-odds
0.5
1.0
1.5
2.0
2.5
3.0
3.5
4.0

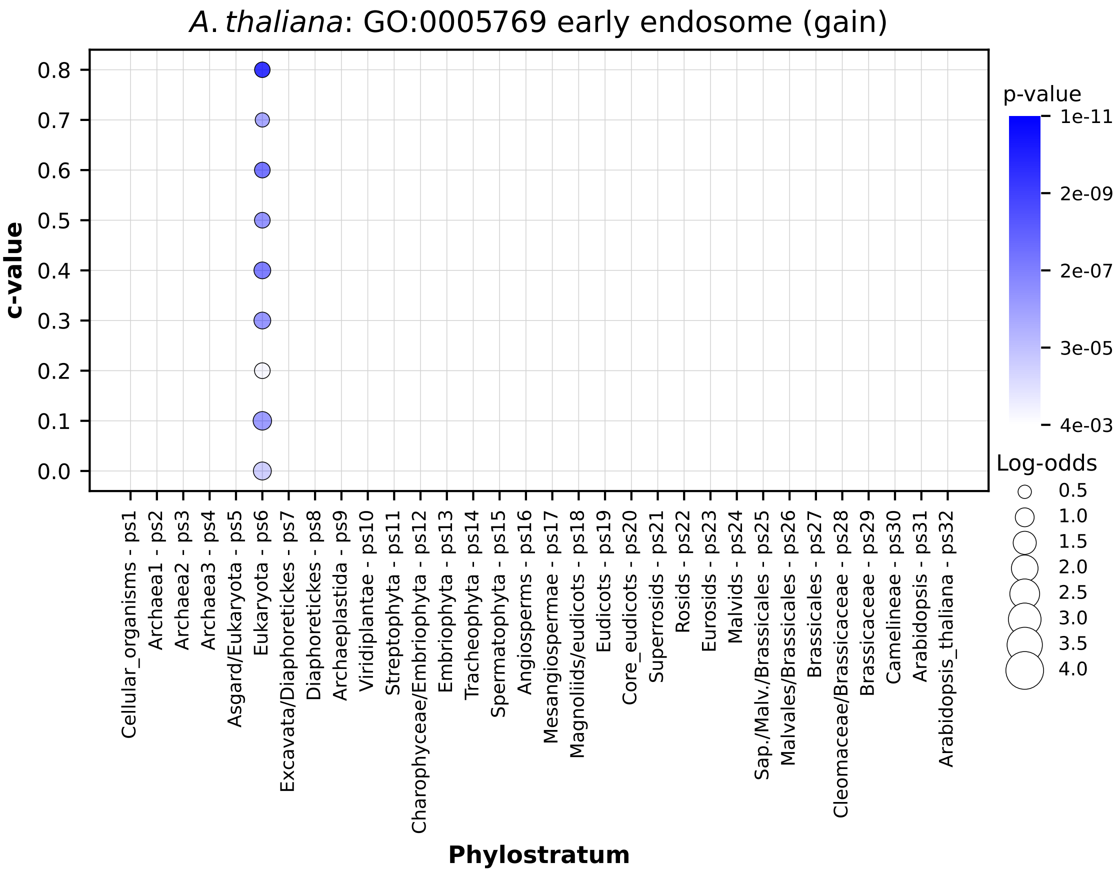
A. thaliana: GO:0005769 early endosome (gain)
c-value
0.8
0.7
0.6
0.5
0.4
0.3
0.2
0.1
0.0
p-value
1e-11
2e-09
2e-07
3e-05
4e-03
Log-odds
0.5
1.0
1.5
2.0
2.5
3.0
3.5
4.0
Cellular_organisms - ps1
Archaea1 - ps2
Archaea2 - ps3
Archaea3 - ps4
Asgard/Eukaryota - ps5
Eukaryota - ps6
Excavata/Diaphoretickes - ps7
Diaphoretickes - ps8
Archaeplastida - ps9
Viridiplantae - ps10
Streptophyta - ps11
Charophyceae/Embriophyta - ps12
Embriophyta - ps13
Tracheophyta - ps14
Spermatophyta - ps15
Angiosperms - ps16
Mesangiospermae - ps17
Magnoliids/eudicots - ps18
Eudicots - ps19
Core_eudicots - ps20
Superrosids - ps21
Rosids - ps22
Eurosids - ps23
Malvids - ps24
Sap./Malv./Brassicales - ps25
Malvales/Brassicales - ps26
Brassicales - ps27
Cleomaceae/Brassicaceae - ps28
Brassicaceae - ps29
Camelineae - ps30
Arabidopsis - ps31
Arabidopsis_thaliana - ps32
Phylostratum

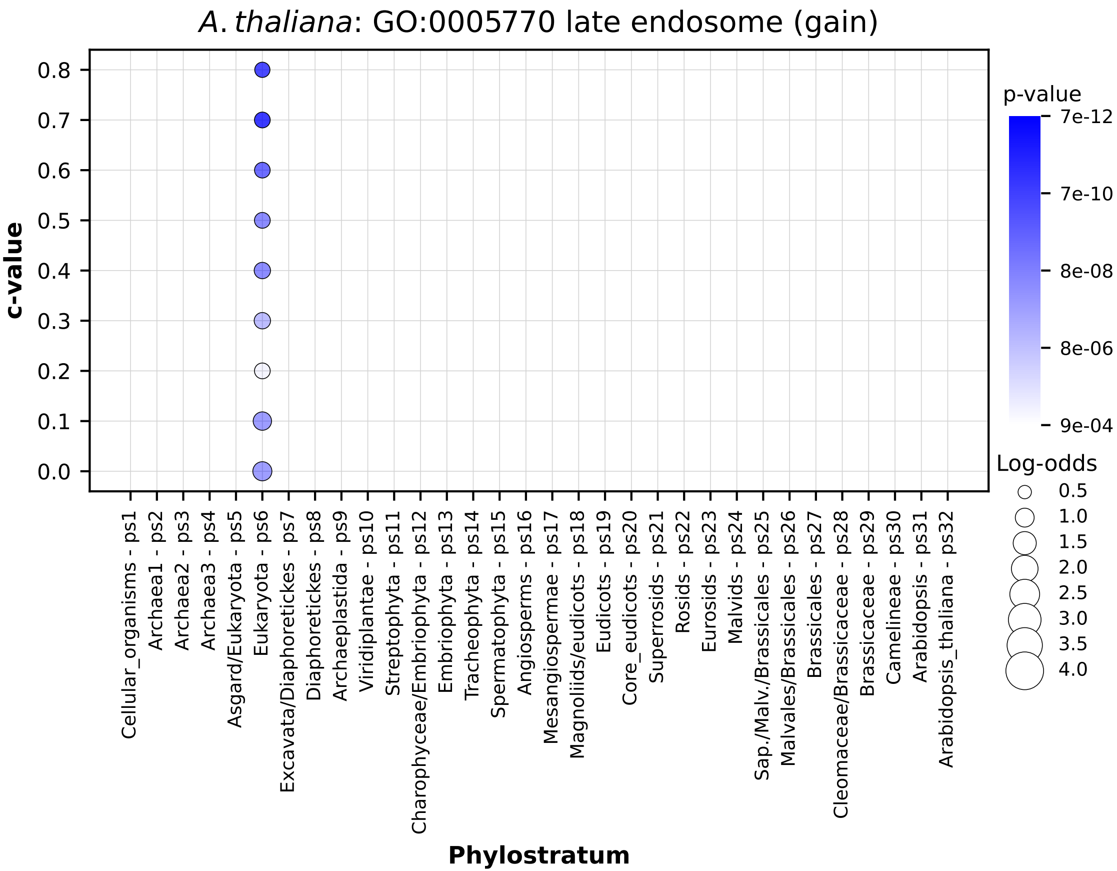
A. thaliana: GO:0005770 late endosome (gain)
c-value
0.8
0.7
0.6
0.5
0.4
0.3
0.2
0.1
0.0
p-value
7e-12
7e-10
8e-08
8e-06
9e-04
Log-odds
0.5
1.0
1.5
2.0
2.5
3.0
3.5
4.0
Cellular_organisms - ps1
Archaea1 - ps2
Archaea2 - ps3
Archaea3 - ps4
Asgard/Eukaryota - ps5
Eukaryota - ps6
Excavata/Diaphoretickes - ps7
Diaphoretickes - ps8
Archaeplastida - ps9
Viridiplantae - ps10
Streptophyta - ps11
Charophyceae/Embriophyta - ps12
Embriophyta - ps13
Tracheophyta - ps14
Spermatophyta - ps15
Angiosperms - ps16
Mesangiospermae - ps17
Magnoliids/eudicots - ps18
Eudicots - ps19
Core_eudicots - ps20
Superrosids - ps21
Rosids - ps22
Eurosids - ps23
Malvids - ps24
Sap./Malv./Brassicales - ps25
Malvales/Brassicales - ps26
Brassicales - ps27
Cleomaceae/Brassicaceae - ps28
Brassicaceae - ps29
Camelineae - ps30
Arabidopsis - ps31
Arabidopsis_thaliana - ps32
Phylostratum

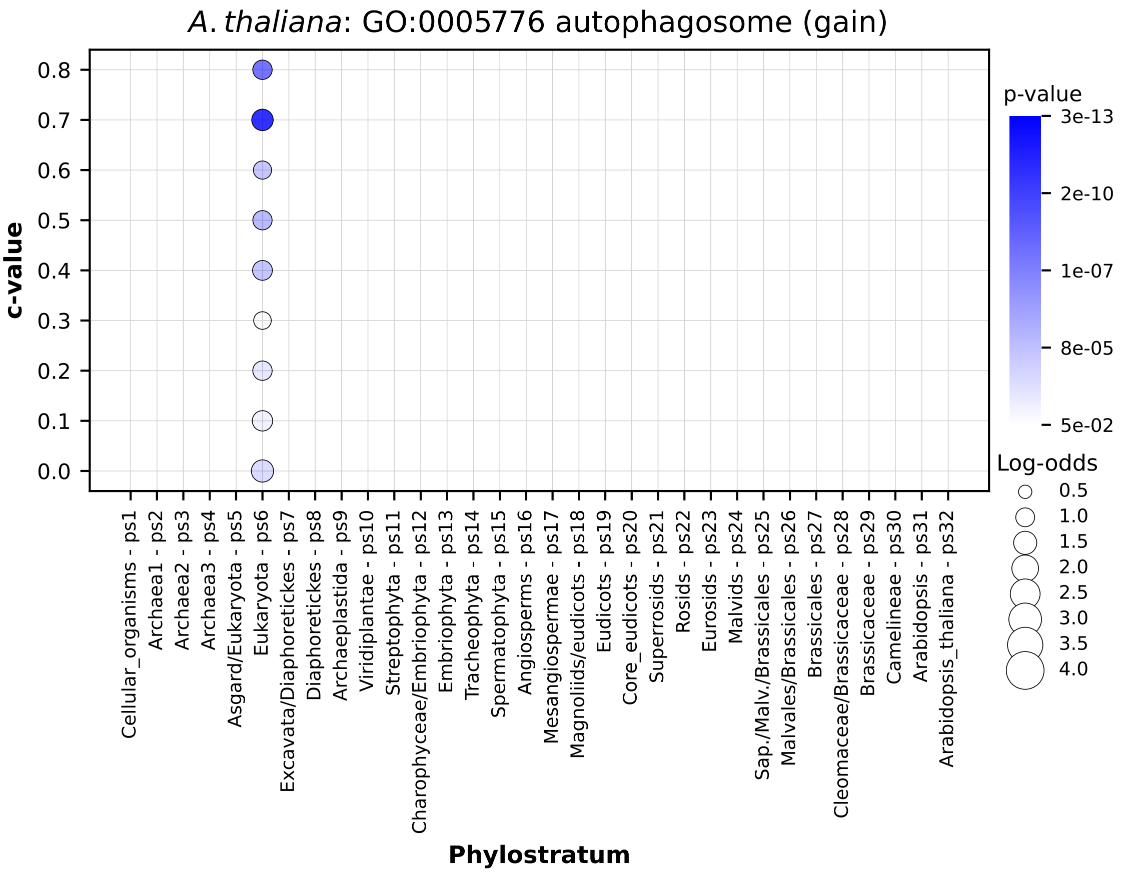

A. thaliana: GO:0005776 autophagosome (gain)
c-value
0.0
0.1
0.2
0.3
0.4
0.5
0.6
0.7
0.8
Phylostratum
Cellular_organisms - ps1
Archaea1 - ps2
Archaea2 - ps3
Archaea3 - ps4
Asgard/Eukaryota - ps5
Eukaryota - ps6
Excavata/Diaphoretickes - ps7
Diaphoretickes - ps8
Archaeplastida - ps9
Viridiplantae - ps10
Streptophyta - ps11
Charophyceae/Embriophyta - ps12
Embriophyta - ps13
Tracheophyta - ps14
Spermatophyta - ps15
Angiosperms - ps16
Mesangiospermae - ps17
Magnoliids/eudicots - ps18
Eudicots - ps19
Core_eudicots - ps20
Superrosids - ps21
Rosids - ps22
Eurosids - ps23
Malvids - ps24
Sap./Malv./Brassicales - ps25
Malvales/Brassicales - ps26
Brassicales - ps27
Cleomaceae/Brassicaceae - ps28
Brassicaceae - ps29
Camelineae - ps30
Arabidopsis - ps31
Arabidopsis_thaliana - ps32
p-value
3e-13
2e-10
1e-07
8e-05
5e-02
Log-odds
0.5
1.0
1.5
2.0
2.5
3.0
3.5
4.0

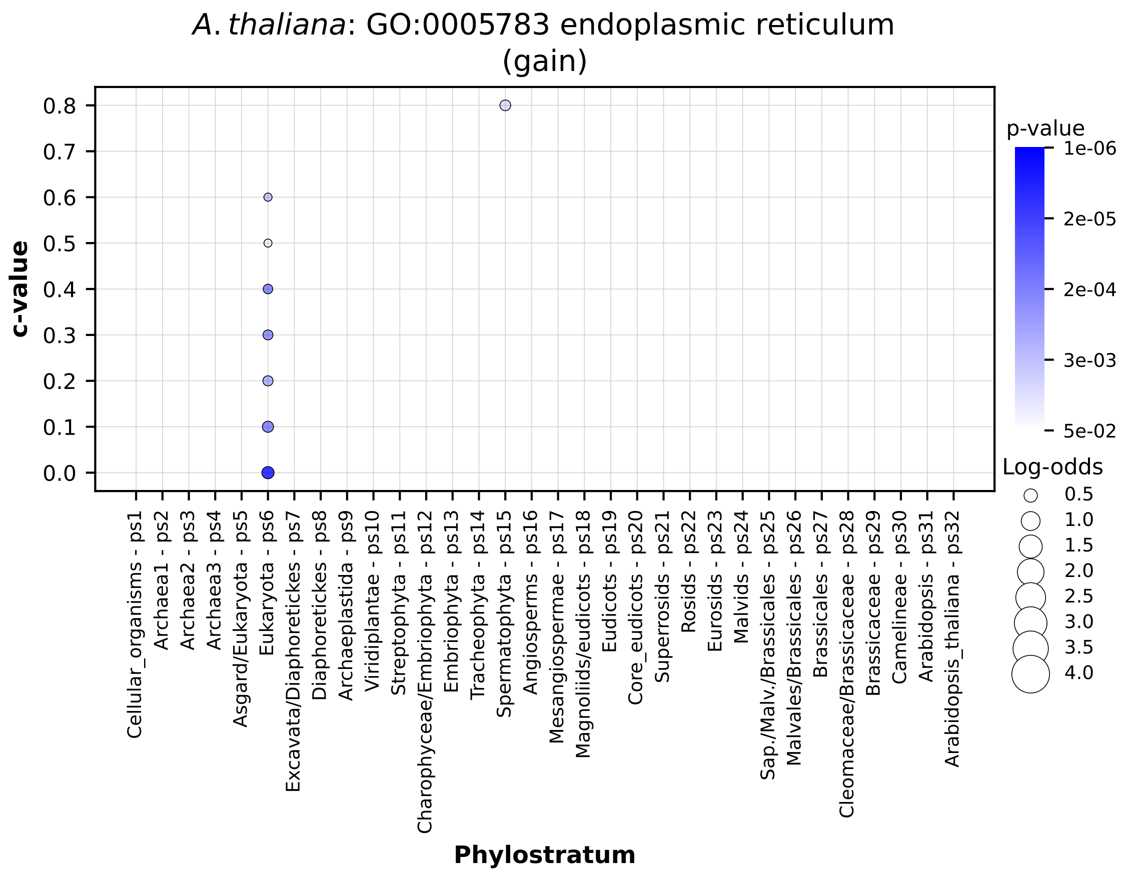

A. thaliana: GO:0005783 endoplasmic reticulum
(gain)
c-value
0.8
0.7
0.6
0.5
0.4
0.3
0.2
0.1
0.0
p-value
1e-06
2e-05
2e-04
3e-03
5e-02
Log-odds
0.5
1.0
1.5
2.0
2.5
3.0
3.5
4.0
Cellular_organisms - ps1
Archaea1 - ps2
Archaea2 - ps3
Archaea3 - ps4
Asgard/Eukaryota - ps5
Eukaryota - ps6
Excavata/Diaphoretickes - ps7
Diaphoretickes - ps8
Archaeplastida - ps9
Viridiplantae - ps10
Streptophyta - ps11
Charophyceae/Embriophyta - ps12
Embriophyta - ps13
Tracheophyta - ps14
Spermatophyta - ps15
Angiosperms - ps16
Mesangiospermae - ps17
Magnoliids/eudicots - ps18
Eudicots - ps19
Core_eudicots - ps20
Superrosids - ps21
Rosids - ps22
Eurosids - ps23
Malvids - ps24
Sap./Malv./Brassicales - ps25
Malvales/Brassicales - ps26
Brassicales - ps27
Cleomaceae/Brassicaceae - ps28
Brassicaceae - ps29
Camelineae - ps30
Arabidopsis - ps31
Arabidopsis_thaliana - ps32
Phylostratum

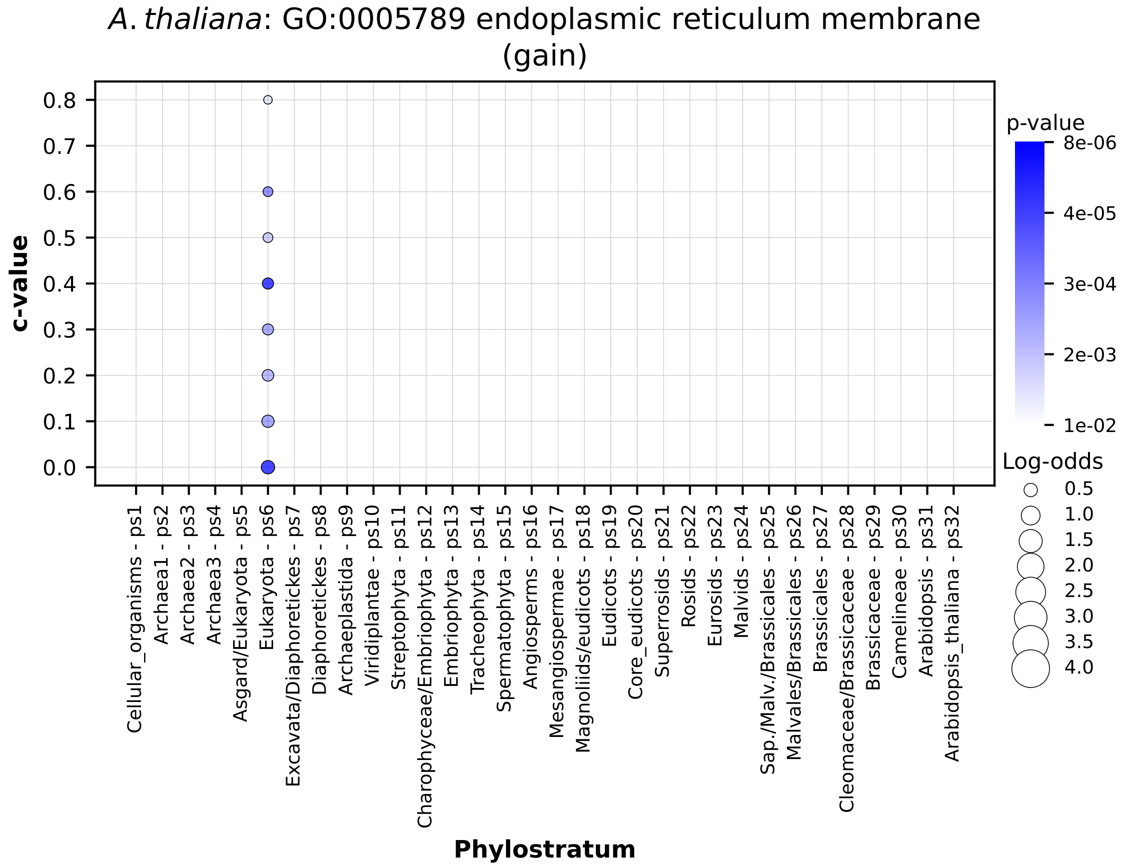

A. thaliana: GO:0005789 endoplasmic reticulum membrane
(gain)
c-value
0.8
0.7
0.6
0.5
0.4
0.3
0.2
0.1
0.0
p-value
8e-06
4e-05
3e-04
2e-03
1e-02
Log-odds
0.5
1.0
1.5
2.0
2.5
3.0
3.5
4.0
Cellular_organisms - ps1
Archaea1 - ps2
Archaea2 - ps3
Archaea3 - ps4
Asgard/Eukaryota - ps5
Eukaryota - ps6
Excavata/Diaphoretickes - ps7
Diaphoretickes - ps8
Archaeplastida - ps9
Viridiplantae - ps10
Streptophyta - ps11
Charophyceae/Embriophyta - ps12
Embriophyta - ps13
Tracheophyta - ps14
Spermatophyta - ps15
Angiosperms - ps16
Mesangiospermae - ps17
Magnoliids/eudicots - ps18
Eudicots - ps19
Core_eudicots - ps20
Superrosids - ps21
Rosids - ps22
Eurosids - ps23
Malvids - ps24
Sap./Malv./Brassicales - ps25
Malvales/Brassicales - ps26
Brassicales - ps27
Cleomaceae/Brassicaceae - ps28
Brassicaceae - ps29
Camelineae - ps30
Arabidopsis - ps31
Arabidopsis_thaliana - ps32
Phylostratum

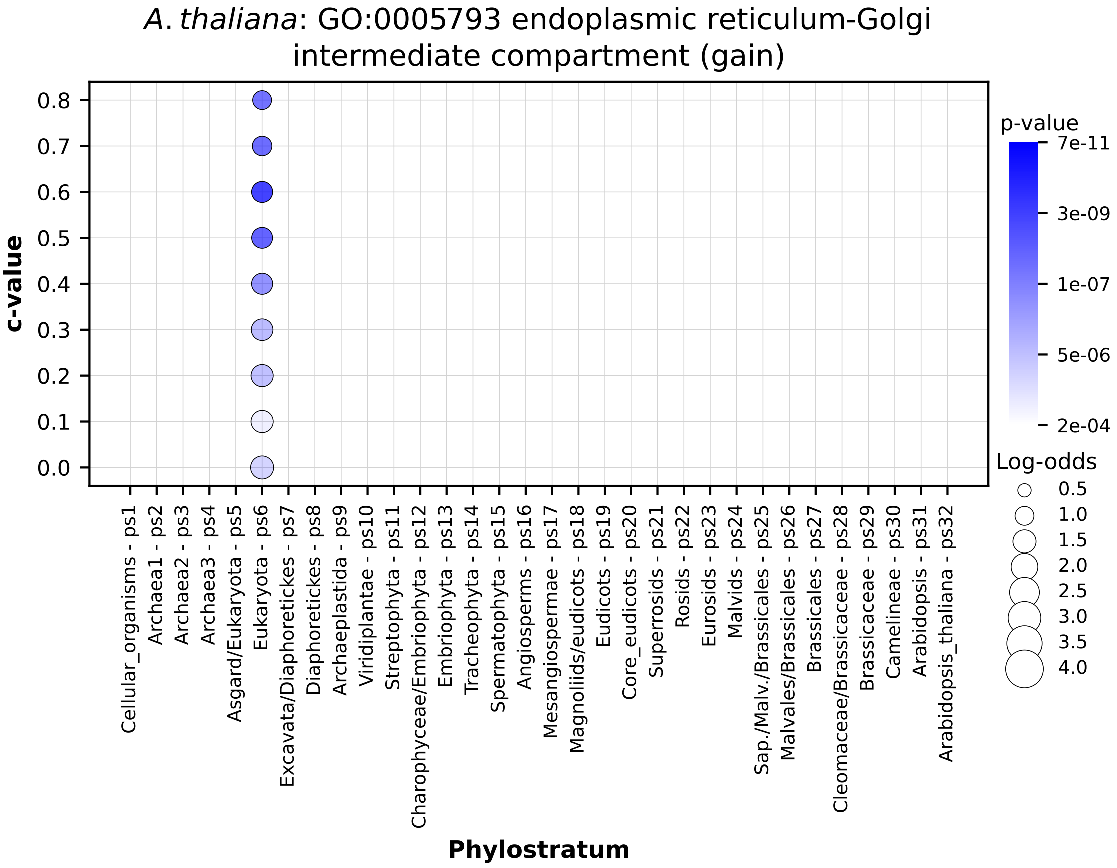
A. thaliana: GO:0005793 endoplasmic reticulum-Golgi intermediate compartment (gain)
c-value
0.8
0.7
0.6
0.5
0.4
0.3
0.2
0.1
0.0
p-value
7e-11
3e-09
1e-07
5e-06
2e-04
Log-odds
0.5
1.0
1.5
2.0
2.5
3.0
3.5
4.0
Cellular_organisms - ps1
Archaea1 - ps2
Archaea2 - ps3
Archaea3 - ps4
Asgard/Eukaryota - ps5
Eukaryota - ps6
Excavata/Diaphoretickes - ps7
Diaphoretickes - ps8
Archaeplastida - ps9
Viridiplantae - ps10
Streptophyta - ps11
Charophyceae/Embriophyta - ps12
Embriophyta - ps13
Tracheophyta - ps14
Spermatophyta - ps15
Angiosperms - ps16
Mesangiospermae - ps17
Magnoliids/eudicots - ps18
Eudicots - ps19
Core_eudicots - ps20
Superrosids - ps21
Rosids - ps22
Eurosids - ps23
Malvids - ps24
Sap./Malv./Brassicales - ps25
Malvales/Brassicales - ps26
Brassicales - ps27
Cleomaceae/Brassicaceae - ps28
Brassicaceae - ps29
Camelineae - ps30
Arabidopsis - ps31
Arabidopsis_thaliana - ps32
Phylostratum

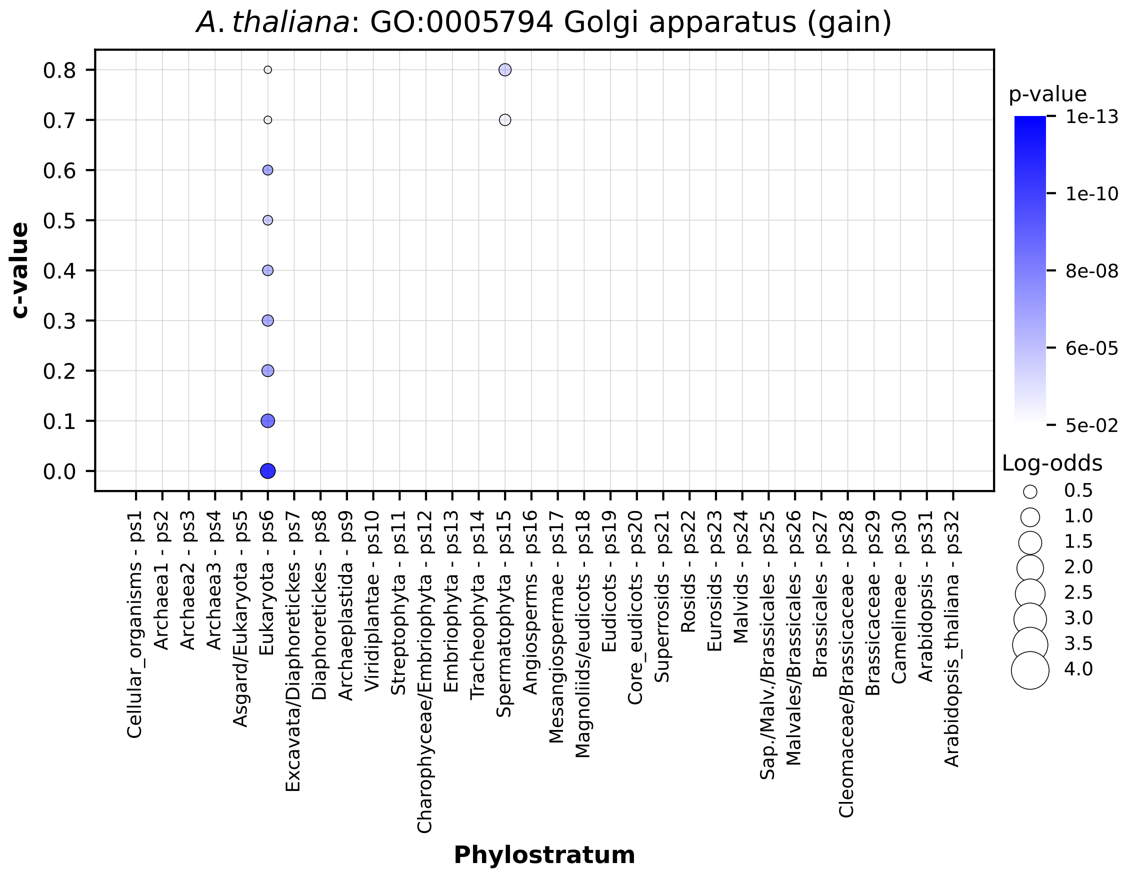
A. thaliana: GO:0005794 Golgi apparatus (gain)
c-value
0.8
0.7
0.6
0.5
0.4
0.3
0.2
0.1
0.0
Phylostratum
Cellular_organisms - ps1
Archaea1 - ps2
Archaea2 - ps3
Archaea3 - ps4
Asgard/Eukaryota - ps5
Eukaryota - ps6
Excavata/Diaphoretickes - ps7
Diaphoretickes - ps8
Archaeplastida - ps9
Viridiplantae - ps10
Streptophyta - ps11
Charophyceae/Embriophyta - ps12
Embriophyta - ps13
Tracheophyta - ps14
Spermatophyta - ps15
Angiosperms - ps16
Mesangiospermae - ps17
Magnoliids/eudicots - ps18
Eudicots - ps19
Core_eudicots - ps20
Superrosids - ps21
Rosids - ps22
Eurosids - ps23
Malvids - ps24
Sap./Malv./Brassicales - ps25
Malvales/Brassicales - ps26
Brassicales - ps27
Cleomaceae/Brassicaceae - ps28
Brassicaceae - ps29
Camelineae - ps30
Arabidopsis - ps31
Arabidopsis_thaliana - ps32
p-value
1e-13
1e-10
8e-08
6e-05
5e-02
Log-odds
0.5
1.0
1.5
2.0
2.5
3.0
3.5
4.0

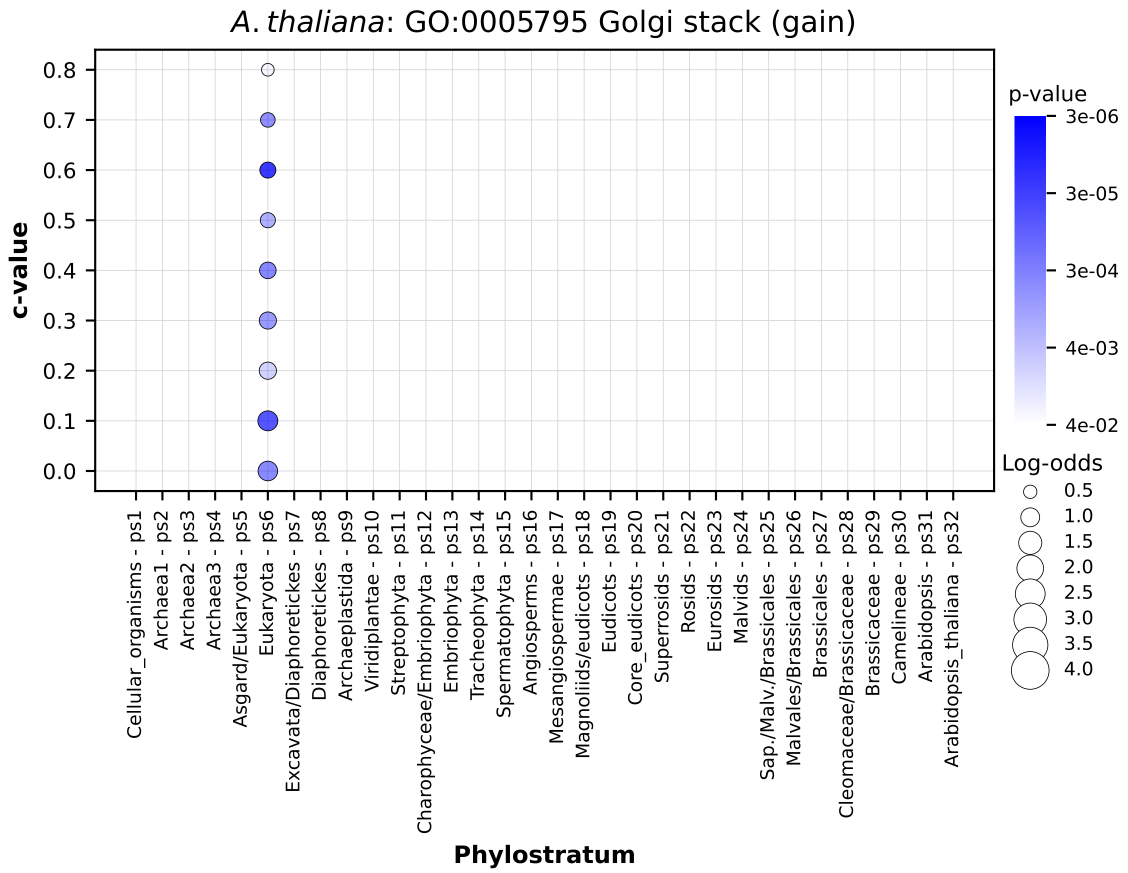

A. thaliana: GO:0005795 Golgi stack (gain)
c-value
0.8
0.7
0.6
0.5
0.4
0.3
0.2
0.1
0.0
p-value
3e-06
3e-05
3e-04
4e-03
4e-02
Log-odds
0.5
1.0
1.5
2.0
2.5
3.0
3.5
4.0
Cellular_organisms - ps1
Archaea1 - ps2
Archaea2 - ps3
Archaea3 - ps4
Asgard/Eukaryota - ps5
Eukaryota - ps6
Excavata/Diaphoretickes - ps7
Diaphoretickes - ps8
Archaeplastida - ps9
Viridiplantae - ps10
Streptophyta - ps11
Charophyceae/Embriophyta - ps12
Embriophyta - ps13
Tracheophyta - ps14
Spermatophyta - ps15
Angiosperms - ps16
Mesangiospermae - ps17
Magnoliids/eudicots - ps18
Eudicots - ps19
Core_eudicots - ps20
Superrosids - ps21
Rosids - ps22
Eurosids - ps23
Malvids - ps24
Sap./Malv./Brassicales - ps25
Malvales/Brassicales - ps26
Brassicales - ps27
Cleomaceae/Brassicaceae - ps28
Brassicaceae - ps29
Camelineae - ps30
Arabidopsis - ps31
Arabidopsis_thaliana - ps32
Phylostratum

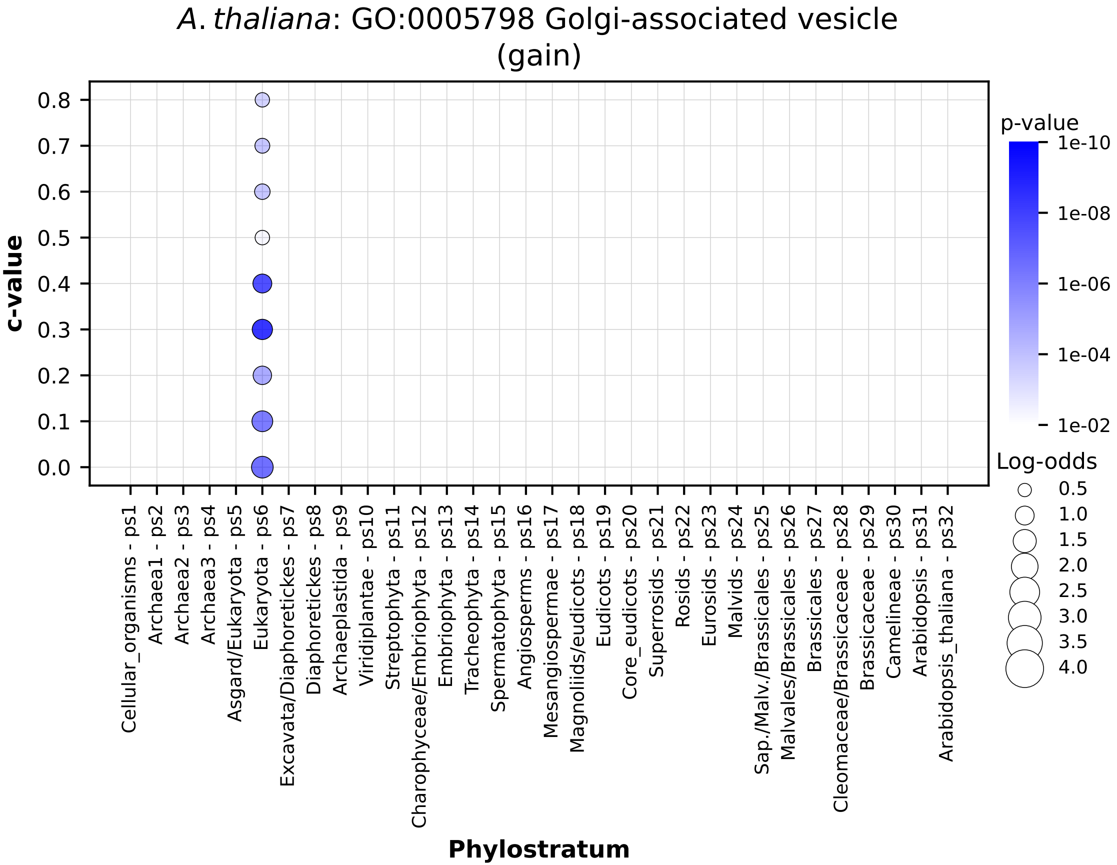
A. thaliana: GO:0005798 Golgi-associated vesicle
(gain)
c-value
0.8
0.7
0.6
0.5
0.4
0.3
0.2
0.1
0.0
p-value
1e-10
1e-08
1e-06
1e-04
1e-02
Log-odds
0.5
1.0
1.5
2.0
2.5
3.0
3.5
4.0
Cellular_organisms - ps1
Archaea1 - ps2
Archaea2 - ps3
Archaea3 - ps4
Asgard/Eukaryota - ps5
Eukaryota - ps6
Excavata/Diaphoretickes - ps7
Diaphoretickes - ps8
Archaeplastida - ps9
Viridiplantae - ps10
Streptophyta - ps11
Charophyceae/Embriophyta - ps12
Embriophyta - ps13
Tracheophyta - ps14
Spermatophyta - ps15
Angiosperms - ps16
Mesangiospermae - ps17
Magnoliids/eudicots - ps18
Eudicots - ps19
Core_eudicots - ps20
Superrosids - ps21
Rosids - ps22
Eurosids - ps23
Malvids - ps24
Sap./Malv./Brassicales - ps25
Malvales/Brassicales - ps26
Brassicales - ps27
Cleomaceae/Brassicaceae - ps28
Brassicaceae - ps29
Camelineae - ps30
Arabidopsis - ps31
Arabidopsis_thaliana - ps32
Phylostratum

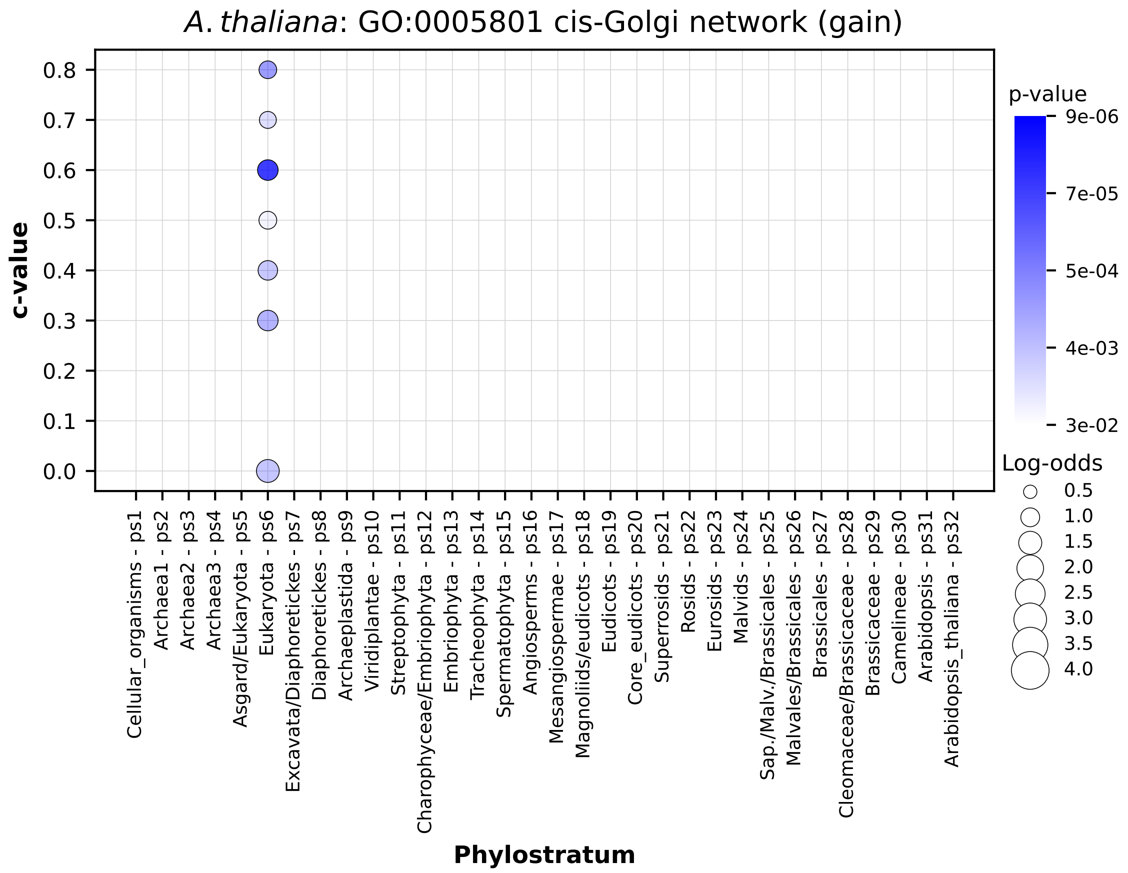
A. thaliana: GO:0005801 cis-Golgi network (gain)
c-value
0.8
0.7
0.6
0.5
0.4
0.3
0.2
0.1
0.0
p-value
9e-06
7e-05
5e-04
4e-03
3e-02
Log-odds
0.5
1.0
1.5
2.0
2.5
3.0
3.5
4.0
Cellular_organisms - ps1
Archaea1 - ps2
Archaea2 - ps3
Archaea3 - ps4
Asgard/Eukaryota - ps5
Eukaryota - ps6
Excavata/Diaphoretickes - ps7
Diaphoretickes - ps8
Archaeplastida - ps9
Viridiplantae - ps10
Streptophyta - ps11
Charophyceae/Embriophyta - ps12
Embriophyta - ps13
Tracheophyta - ps14
Spermatophyta - ps15
Angiosperms - ps16
Mesangiospermae - ps17
Magnoliids/eudicots - ps18
Eudicots - ps19
Core_eudicots - ps20
Superrosids - ps21
Rosids - ps22
Eurosids - ps23
Malvids - ps24
Sap./Malv./Brassicales - ps25
Malvales/Brassicales - ps26
Brassicales - ps27
Cleomaceae/Brassicaceae - ps28
Brassicaceae - ps29
Camelineae - ps30
Arabidopsis - ps31
Arabidopsis_thaliana - ps32
Phylostratum

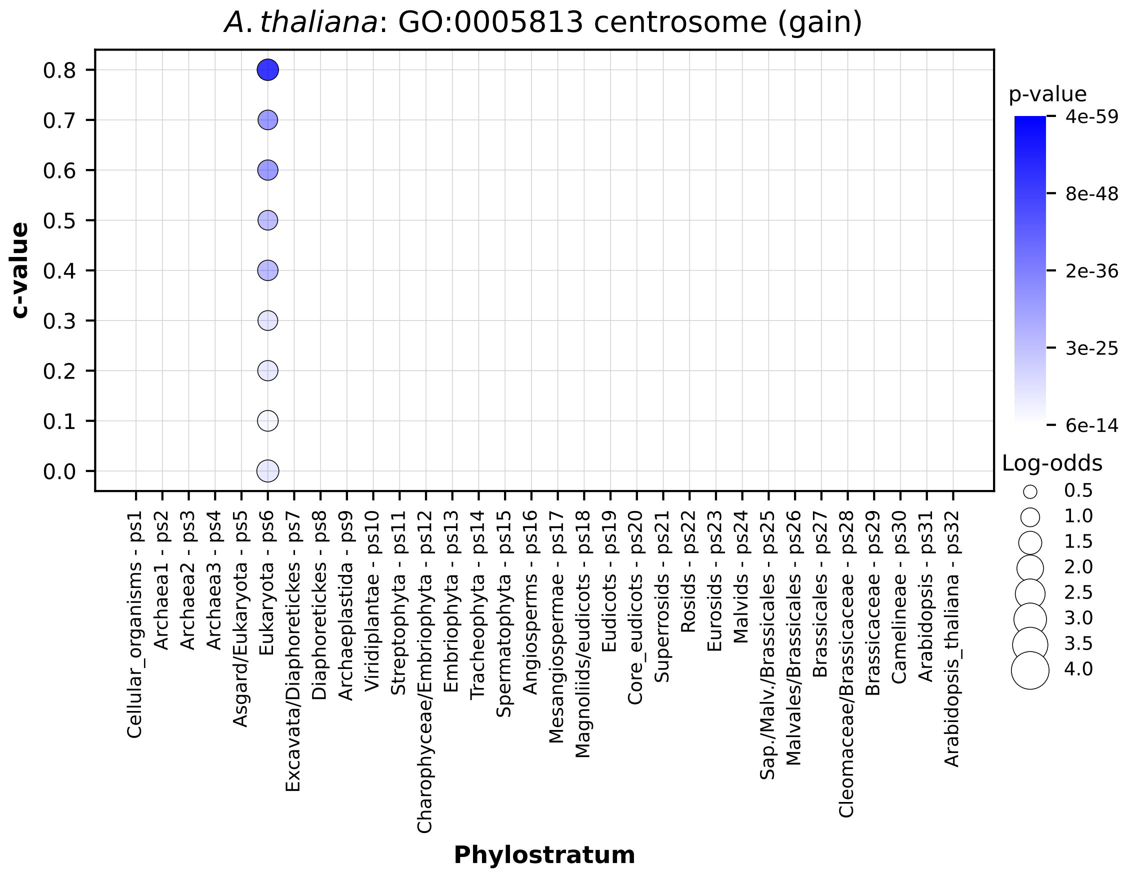

A. thaliana: GO:0005813 centrosome (gain)
c-value
0.8
0.7
0.6
0.5
0.4
0.3
0.2
0.1
0.0
p-value
4e-59
8e-48
2e-36
3e-25
6e-14
Log-odds
0.5
1.0
1.5
2.0
2.5
3.0
3.5
4.0
Cellular_organisms - ps1
Archaea1 - ps2
Archaea2 - ps3
Archaea3 - ps4
Asgard/Eukaryota - ps5
Eukaryota - ps6
Excavata/Diaphoretickes - ps7
Diaphoretickes - ps8
Archaeplastida - ps9
Viridiplantae - ps10
Streptophyta - ps11
Charophyceae/Embriophyta - ps12
Embriophyta - ps13
Tracheophyta - ps14
Spermatophyta - ps15
Angiosperms - ps16
Mesangiospermae - ps17
Magnoliids/eudicots - ps18
Eudicots - ps19
Core_eudicots - ps20
Superrosids - ps21
Rosids - ps22
Eurosids - ps23
Malvids - ps24
Sap./Malv./Brassicales - ps25
Malvales/Brassicales - ps26
Brassicales - ps27
Cleomaceae/Brassicaceae - ps28
Brassicaceae - ps29
Camelineae - ps30
Arabidopsis - ps31
Arabidopsis_thaliana - ps32
Phylostratum

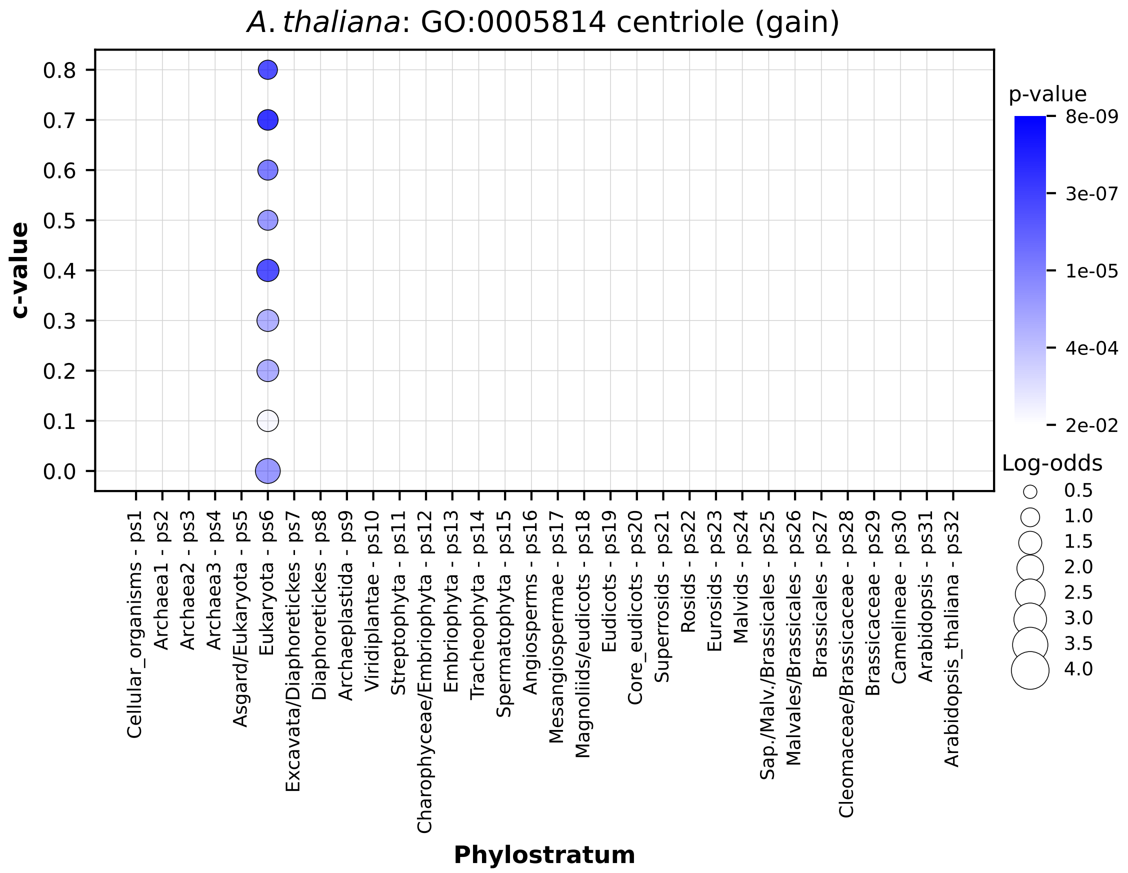

A. thaliana: GO:0005814 centriole (gain)
c-value
0.8
0.7
0.6
0.5
0.4
0.3
0.2
0.1
0.0
p-value
8e-09
3e-07
1e-05
4e-04
2e-02
Log-odds
0.5
1.0
1.5
2.0
2.5
3.0
3.5
4.0
Cellular_organisms - ps1
Archaea1 - ps2
Archaea2 - ps3
Archaea3 - ps4
Asgard/Eukaryota - ps5
Eukaryota - ps6
Excavata/Diaphoretickes - ps7
Diaphoretickes - ps8
Archaeplastida - ps9
Viridiplantae - ps10
Streptophyta - ps11
Charophyceae/Embriophyta - ps12
Embriophyta - ps13
Tracheophyta - ps14
Spermatophyta - ps15
Angiosperms - ps16
Mesangiospermae - ps17
Magnoliids/eudicots - ps18
Eudicots - ps19
Core_eudicots - ps20
Superrosids - ps21
Rosids - ps22
Eurosids - ps23
Malvids - ps24
Sap./Malv./Brassicales - ps25
Malvales/Brassicales - ps26
Brassicales - ps27
Cleomaceae/Brassicaceae - ps28
Brassicaceae - ps29
Camelineae - ps30
Arabidopsis - ps31
Arabidopsis_thaliana - ps32
Phylostratum

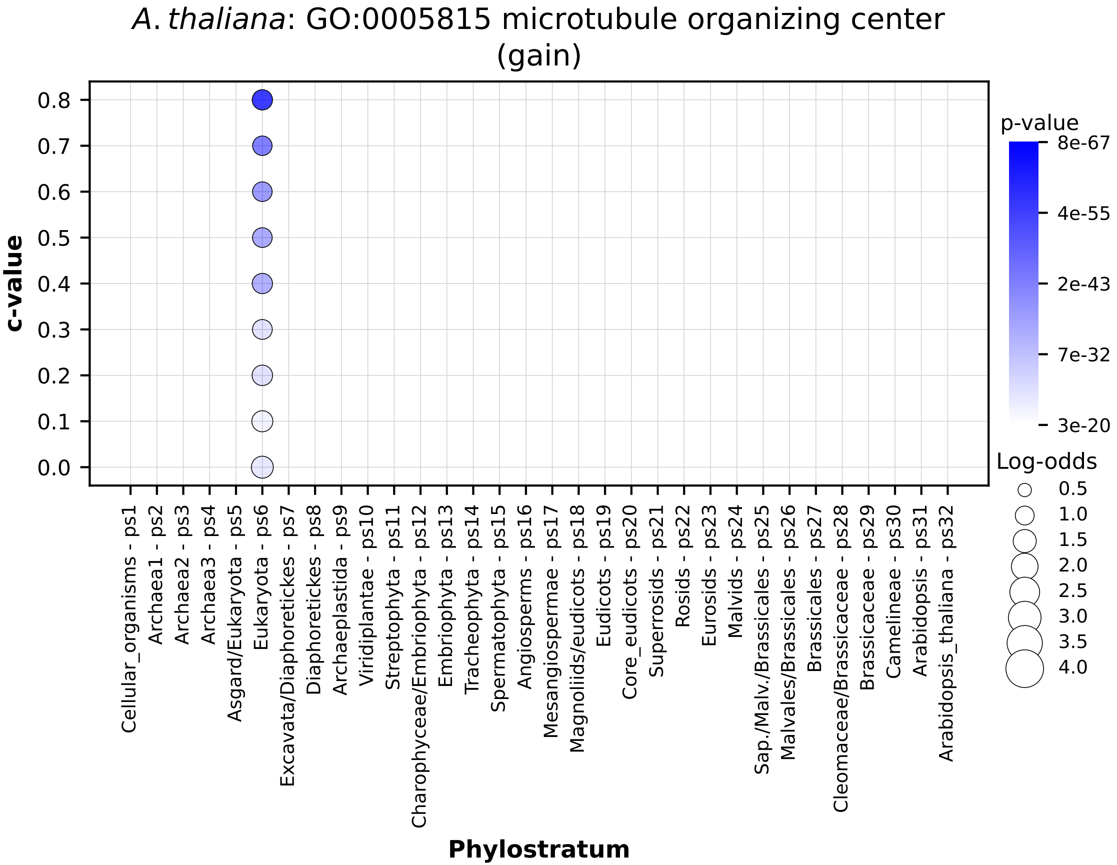

A. thaliana: GO:0005815 microtubule organizing center
(gain)
c-value
0.8
0.7
0.6
0.5
0.4
0.3
0.2
0.1
0.0
p-value
8e-67
4e-55
2e-43
7e-32
3e-20
Log-odds
0.5
1.0
1.5
2.0
2.5
3.0
3.5
4.0
Cellular_organisms - ps1
Archaea1 - ps2
Archaea2 - ps3
Archaea3 - ps4
Asgard/Eukaryota - ps5
Eukaryota - ps6
Excavata/Diaphoretickes - ps7
Diaphoretickes - ps8
Archaeplastida - ps9
Viridiplantae - ps10
Streptophyta - ps11
Charophyceae/Embriophyta - ps12
Embriophyta - ps13
Tracheophyta - ps14
Spermatophyta - ps15
Angiosperms - ps16
Mesangiospermae - ps17
Magnoliids/eudicots - ps18
Eudicots - ps19
Core_eudicots - ps20
Superrosids - ps21
Rosids - ps22
Eurosids - ps23
Malvids - ps24
Sap./Malv./Brassicales - ps25
Malvales/Brassicales - ps26
Brassicales - ps27
Cleomaceae/Brassicaceae - ps28
Brassicaceae - ps29
Camelineae - ps30
Arabidopsis - ps31
Arabidopsis_thaliana - ps32
Phylostratum

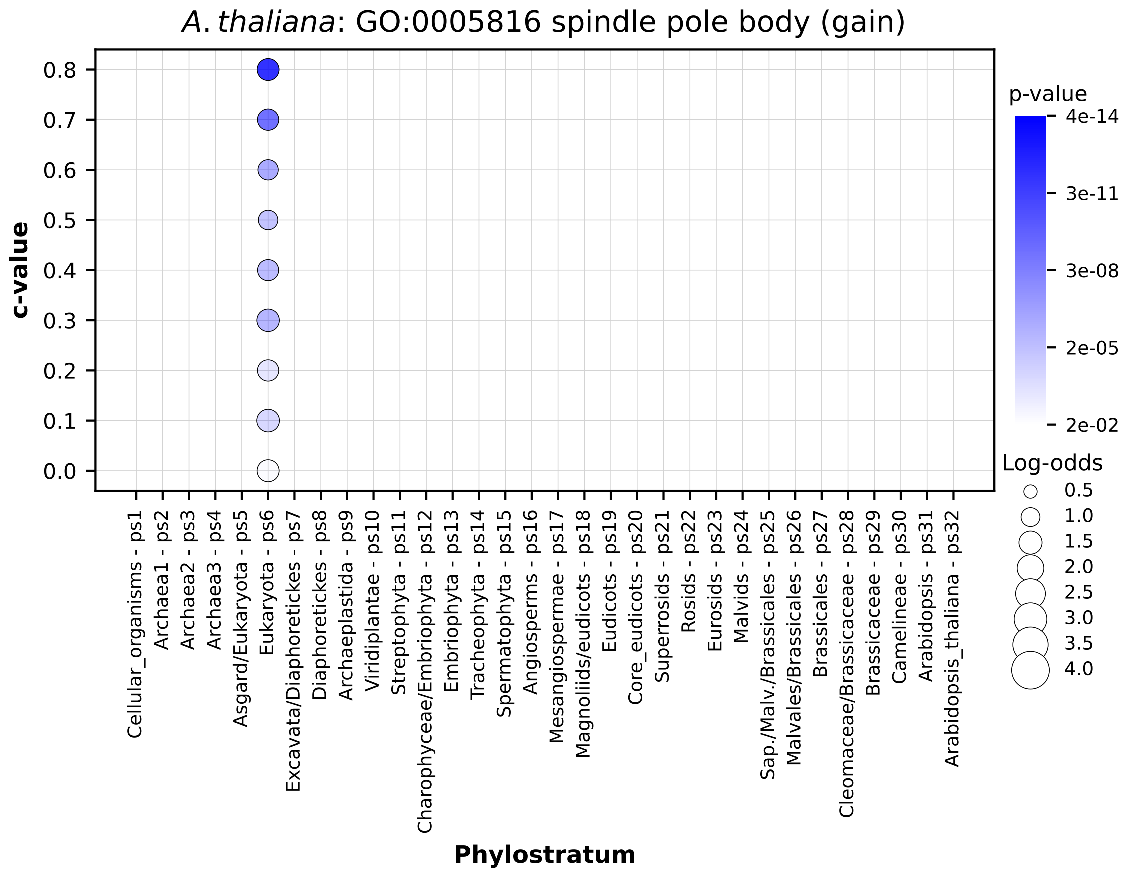

A. thaliana: GO:0005816 spindle pole body (gain)
c-value
0.8
0.7
0.6
0.5
0.4
0.3
0.2
0.1
0.0
p-value
4e-14
3e-11
3e-08
2e-05
2e-02
Log-odds
0.5
1.0
1.5
2.0
2.5
3.0
3.5
4.0
Cellular_organisms - ps1
Archaea1 - ps2
Archaea2 - ps3
Archaea3 - ps4
Asgard/Eukaryota - ps5
Eukaryota - ps6
Excavata/Diaphoretickes - ps7
Diaphoretickes - ps8
Archaeplastida - ps9
Viridiplantae - ps10
Streptophyta - ps11
Charophyceae/Embriophyta - ps12
Embriophyta - ps13
Tracheophyta - ps14
Spermatophyta - ps15
Angiosperms - ps16
Mesangiospermae - ps17
Magnoliids/eudicots - ps18
Eudicots - ps19
Core_eudicots - ps20
Superrosids - ps21
Rosids - ps22
Eurosids - ps23
Malvids - ps24
Sap./Malv./Brassicales - ps25
Malvales/Brassicales - ps26
Brassicales - ps27
Cleomaceae/Brassicaceae - ps28
Brassicaceae - ps29
Camelineae - ps30
Arabidopsis - ps31
Arabidopsis_thaliana - ps32
Phylostratum

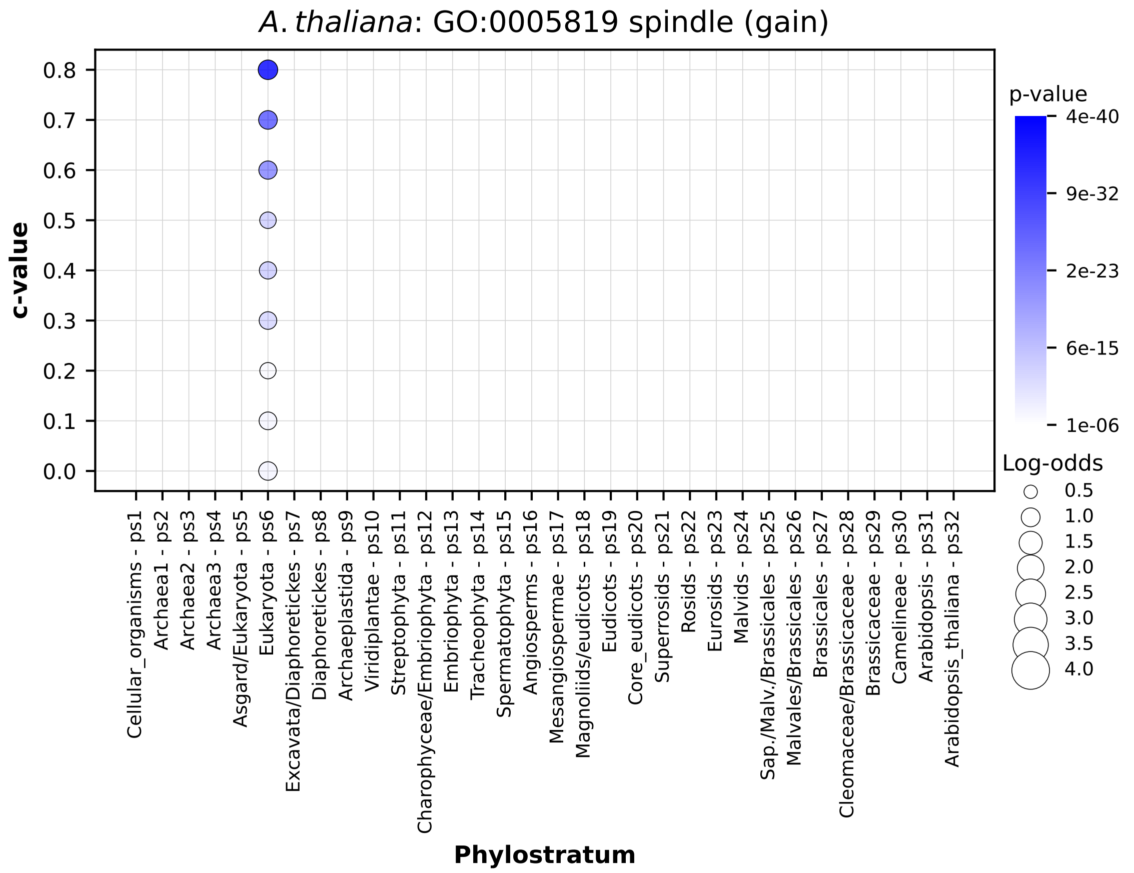
A. thaliana: GO:0005819 spindle (gain)
c-value
0.8
0.7
0.6
0.5
0.4
0.3
0.2
0.1
0.0
p-value
4e-40
9e-32
2e-23
6e-15
1e-06
Log-odds
0.5
1.0
1.5
2.0
2.5
3.0
3.5
4.0
Cellular_organisms - ps1
Archaea1 - ps2
Archaea2 - ps3
Archaea3 - ps4
Asgard/Eukaryota - ps5
Eukaryota - ps6
Excavata/Diaphoretickes - ps7
Diaphoretickes - ps8
Archaeplastida - ps9
Viridiplantae - ps10
Streptophyta - ps11
Charophyceae/Embriophyta - ps12
Embriophyta - ps13
Tracheophyta - ps14
Spermatophyta - ps15
Angiosperms - ps16
Mesangiospermae - ps17
Magnoliids/eudicots - ps18
Eudicots - ps19
Core_eudicots - ps20
Superrosids - ps21
Rosids - ps22
Eurosids - ps23
Malvids - ps24
Sap./Malv./Brassicales - ps25
Malvales/Brassicales - ps26
Brassicales - ps27
Cleomaceae/Brassicaceae - ps28
Brassicaceae - ps29
Camelineae - ps30
Arabidopsis - ps31
Arabidopsis_thaliana - ps32
Phylostratum

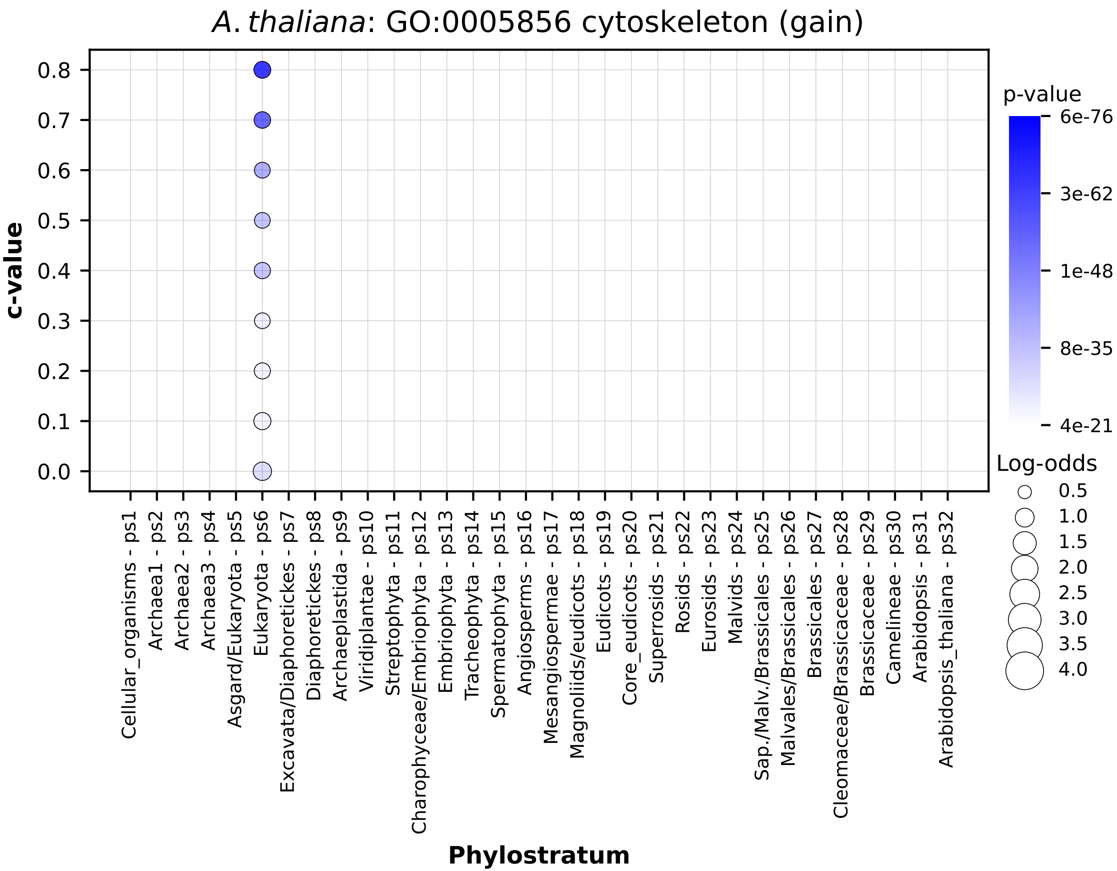
A. thaliana: GO:0005856 cytoskeleton (gain)
c-value
0.8
0.7
0.6
0.5
0.4
0.3
0.2
0.1
0.0
p-value
6e-76
3e-62
1e-48
8e-35
4e-21
Log-odds
0.5
1.0
1.5
2.0
2.5
3.0
3.5
4.0
Cellular_organisms - ps1
Archaea1 - ps2
Archaea2 - ps3
Archaea3 - ps4
Asgard/Eukaryota - ps5
Eukaryota - ps6
Excavata/Diaphoretickes - ps7
Diaphoretickes - ps8
Archaeplastida - ps9
Viridiplantae - ps10
Streptophyta - ps11
Charophyceae/Embriophyta - ps12
Embriophyta - ps13
Tracheophyta - ps14
Spermatophyta - ps15
Angiosperms - ps16
Mesangiospermae - ps17
Magnoliids/eudicots - ps18
Eudicots - ps19
Core_eudicots - ps20
Superrosids - ps21
Rosids - ps22
Eurosids - ps23
Malvids - ps24
Sap./Malv./Brassicales - ps25
Malvales/Brassicales - ps26
Brassicales - ps27
Cleomaceae/Brassicaceae - ps28
Brassicaceae - ps29
Camelineae - ps30
Arabidopsis - ps31
Arabidopsis_thaliana - ps32
Phylostratum

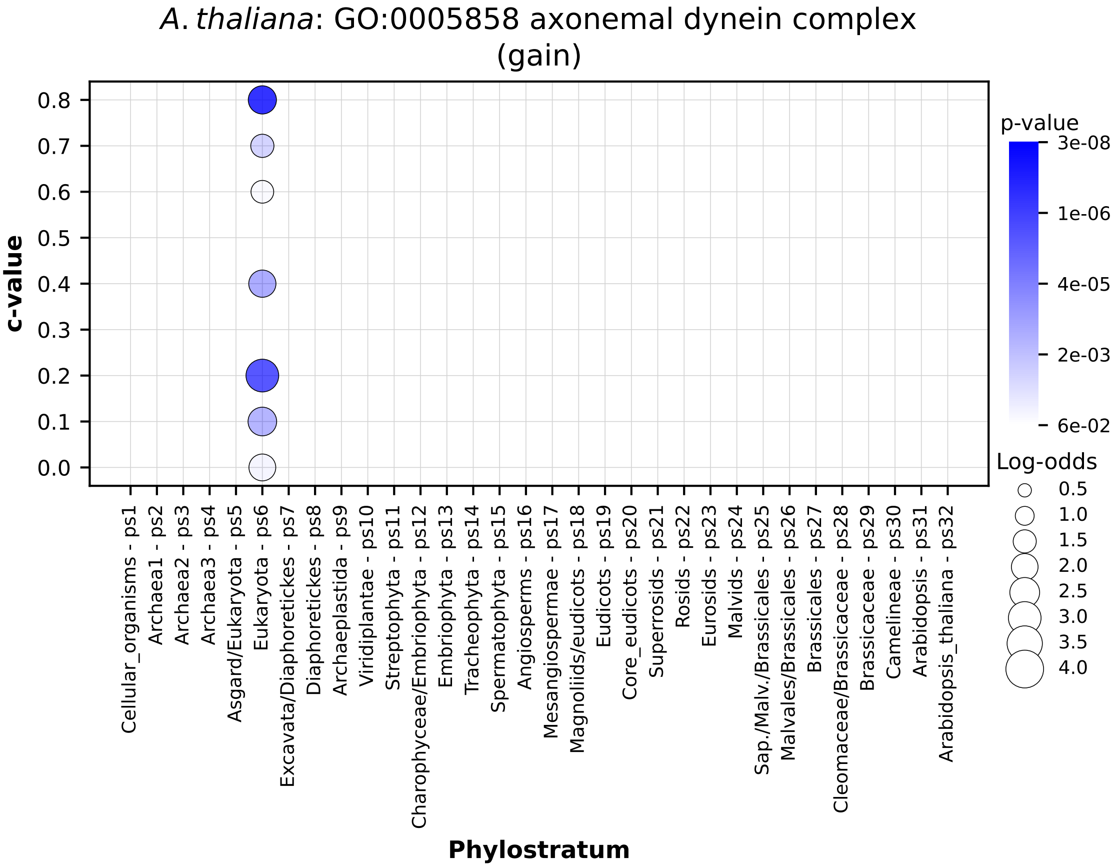

A. thaliana: GO:0005858 axonemal dynein complex
(gain)
c-value
0.8
0.7
0.6
0.5
0.4
0.3
0.2
0.1
0.0
p-value
3e-08
1e-06
4e-05
2e-03
6e-02
Log-odds
0.5
1.0
1.5
2.0
2.5
3.0
3.5
4.0
Cellular_organisms - ps1
Archaea1 - ps2
Archaea2 - ps3
Archaea3 - ps4
Asgard/Eukaryota - ps5
Eukaryota - ps6
Excavata/Diaphoretickes - ps7
Diaphoretickes - ps8
Archaeplastida - ps9
Viridiplantae - ps10
Streptophyta - ps11
Charophyceae/Embriophyta - ps12
Embriophyta - ps13
Tracheophyta - ps14
Spermatophyta - ps15
Angiosperms - ps16
Mesangiospermae - ps17
Magnoliids/eudicots - ps18
Eudicots - ps19
Core_eudicots - ps20
Superrosids - ps21
Rosids - ps22
Eurosids - ps23
Malvids - ps24
Sap./Malv./Brassicales - ps25
Malvales/Brassicales - ps26
Brassicales - ps27
Cleomaceae/Brassicaceae - ps28
Brassicaceae - ps29
Camelineae - ps30
Arabidopsis - ps31
Arabidopsis_thaliana - ps32
Phylostratum

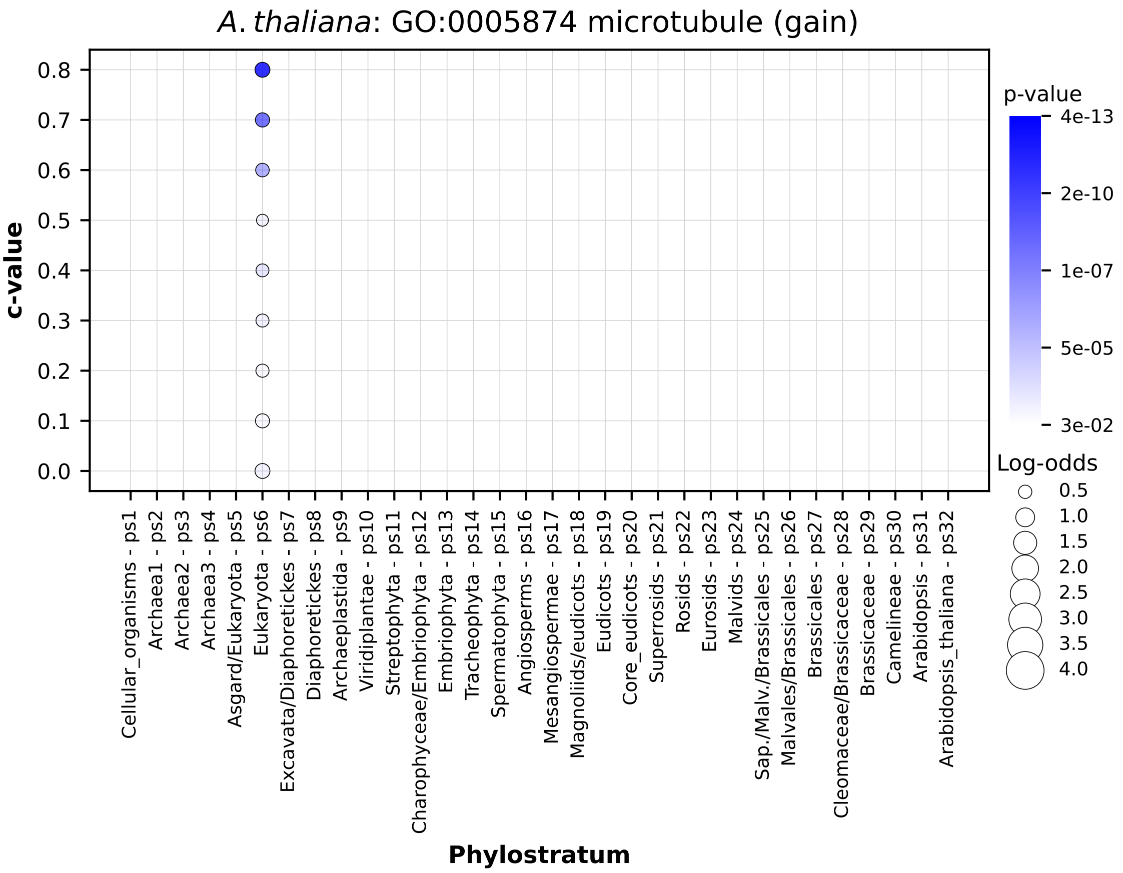

A. thaliana: GO:0005874 microtubule (gain)
c-value
0.8
0.7
0.6
0.5
0.4
0.3
0.2
0.1
0.0
Phylostratum
Cellular_organisms - ps1
Archaea1 - ps2
Archaea2 - ps3
Archaea3 - ps4
Asgard/Eukaryota - ps5
Eukaryota - ps6
Excavata/Diaphoretickes - ps7
Diaphoretickes - ps8
Archaeplastida - ps9
Viridiplantae - ps10
Streptophyta - ps11
Charophyceae/Embriophyta - ps12
Embriophyta - ps13
Tracheophyta - ps14
Spermatophyta - ps15
Angiosperms - ps16
Mesangiospermae - ps17
Magnoliids/eudicots - ps18
Eudicots - ps19
Core_eudicots - ps20
Superrosids - ps21
Rosids - ps22
Eurosids - ps23
Malvids - ps24
Sap./Malv./Brassicales - ps25
Malvales/Brassicales - ps26
Brassicales - ps27
Cleomaceae/Brassicaceae - ps28
Brassicaceae - ps29
Camelineae - ps30
Arabidopsis - ps31
Arabidopsis_thaliana - ps32
p-value
4e-13
2e-10
1e-07
5e-05
3e-02
Log-odds
0.5
1.0
1.5
2.0
2.5
3.0
3.5
4.0

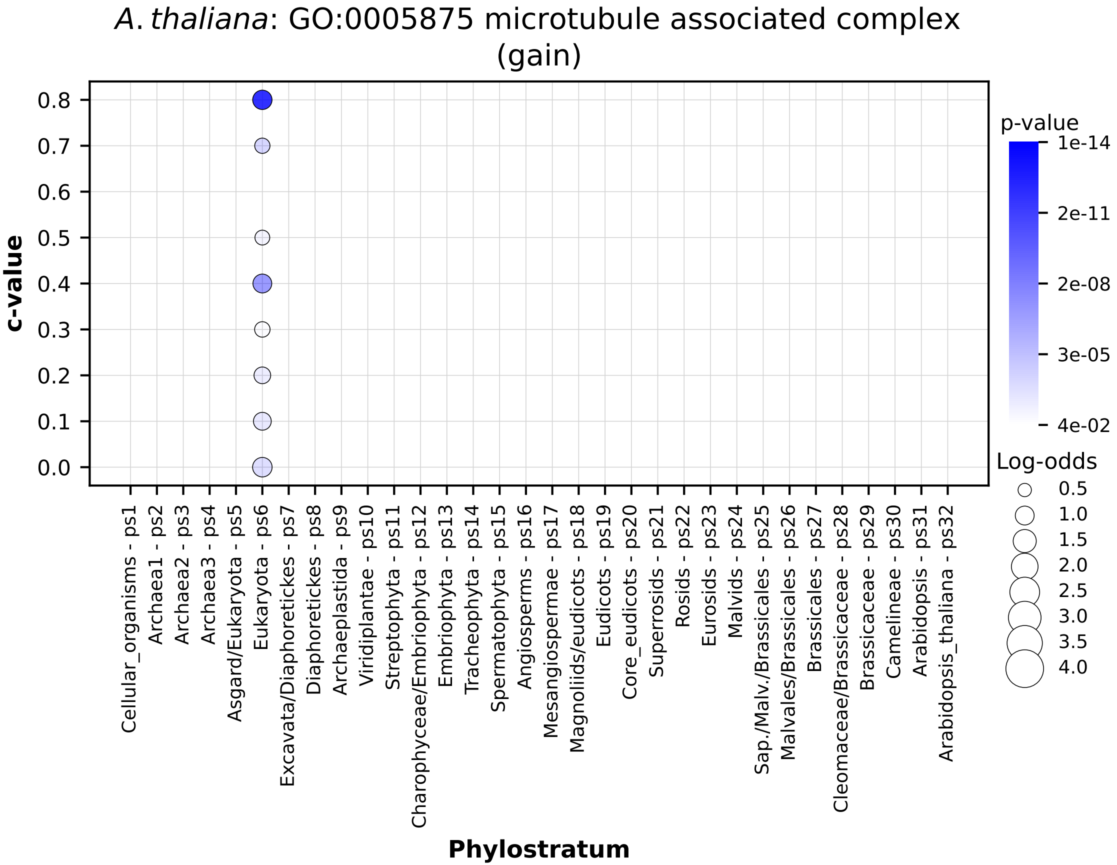

A. thaliana: GO:0005875 microtubule associated complex (gain)
c-value
0.8
0.7
0.6
0.5
0.4
0.3
0.2
0.1
0.0
p-value
1e-14
2e-11
2e-08
3e-05
4e-02
Log-odds
0.5
1.0
1.5
2.0
2.5
3.0
3.5
4.0
Cellular_organisms - ps1
Archaea1 - ps2
Archaea2 - ps3
Archaea3 - ps4
Asgard/Eukaryota - ps5
Eukaryota - ps6
Excavata/Diaphoretickes - ps7
Diaphoretickes - ps8
Archaeplastida - ps9
Viridiplantae - ps10
Streptophyta - ps11
Charophyceae/Embriophyta - ps12
Embriophyta - ps13
Tracheophyta - ps14
Spermatophyta - ps15
Angiosperms - ps16
Mesangiospermae - ps17
Magnoliids/eudicots - ps18
Eudicots - ps19
Core_eudicots - ps20
Superrosids - ps21
Rosids - ps22
Eurosids - ps23
Malvids - ps24
Sap./Malv./Brassicales - ps25
Malvales/Brassicales - ps26
Brassicales - ps27
Cleomaceae/Brassicaceae - ps28
Brassicaceae - ps29
Camelineae - ps30
Arabidopsis - ps31
Arabidopsis_thaliana - ps32
Phylostratum

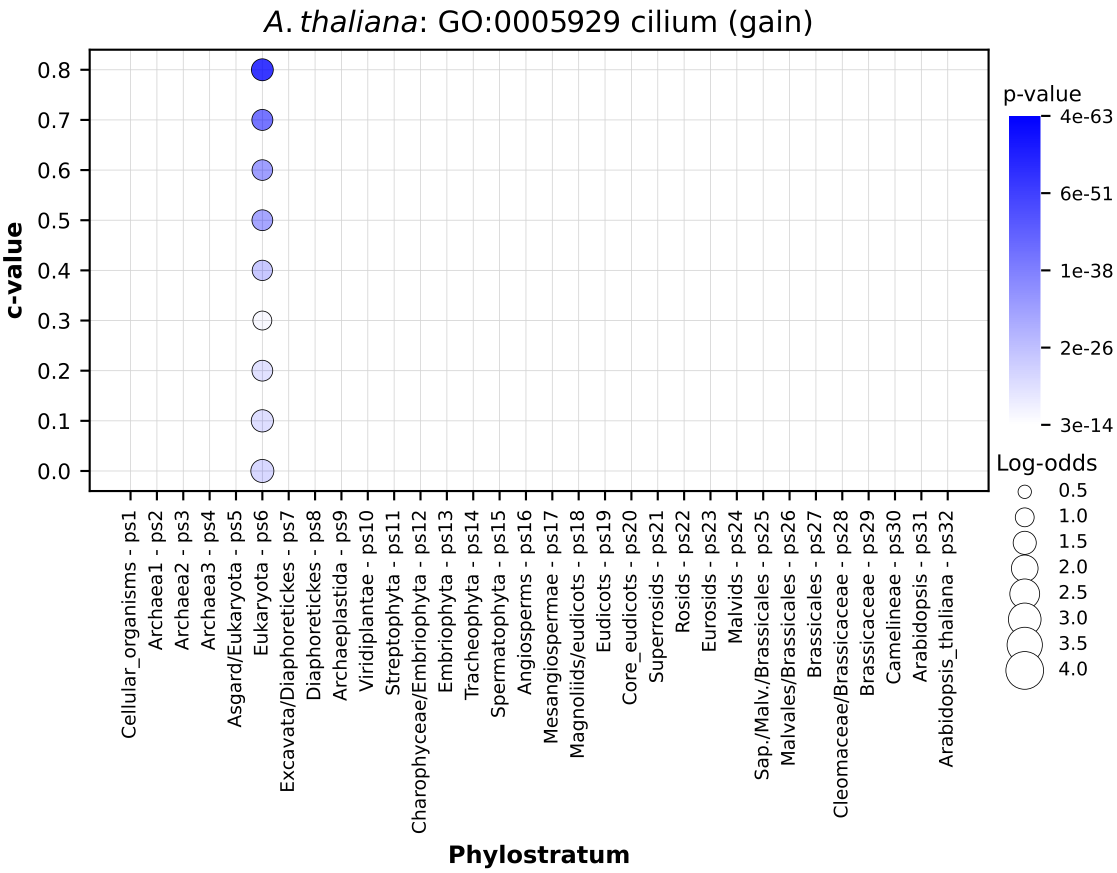

A. thaliana: GO:0005929 cilium (gain)
c-value
0.8
0.7
0.6
0.5
0.4
0.3
0.2
0.1
0.0
p-value
4e-63
6e-51
1e-38
2e-26
3e-14
Log-odds
0.5
1.0
1.5
2.0
2.5
3.0
3.5
4.0
Cellular_organisms - ps1
Archaea1 - ps2
Archaea2 - ps3
Archaea3 - ps4
Asgard/Eukaryota - ps5
Eukaryota - ps6
Excavata/Diaphoretickes - ps7
Diaphoretickes - ps8
Archaeplastida - ps9
Viridiplantae - ps10
Streptophyta - ps11
Charophyceae/Embriophyta - ps12
Embriophyta - ps13
Tracheophyta - ps14
Spermatophyta - ps15
Angiosperms - ps16
Mesangiospermae - ps17
Magnoliids/eudicots - ps18
Eudicots - ps19
Core_eudicots - ps20
Superrosids - ps21
Rosids - ps22
Eurosids - ps23
Malvids - ps24
Sap./Malv./Brassicales - ps25
Malvales/Brassicales - ps26
Brassicales - ps27
Cleomaceae/Brassicaceae - ps28
Brassicaceae - ps29
Camelineae - ps30
Arabidopsis - ps31
Arabidopsis_thaliana - ps32
Phylostratum

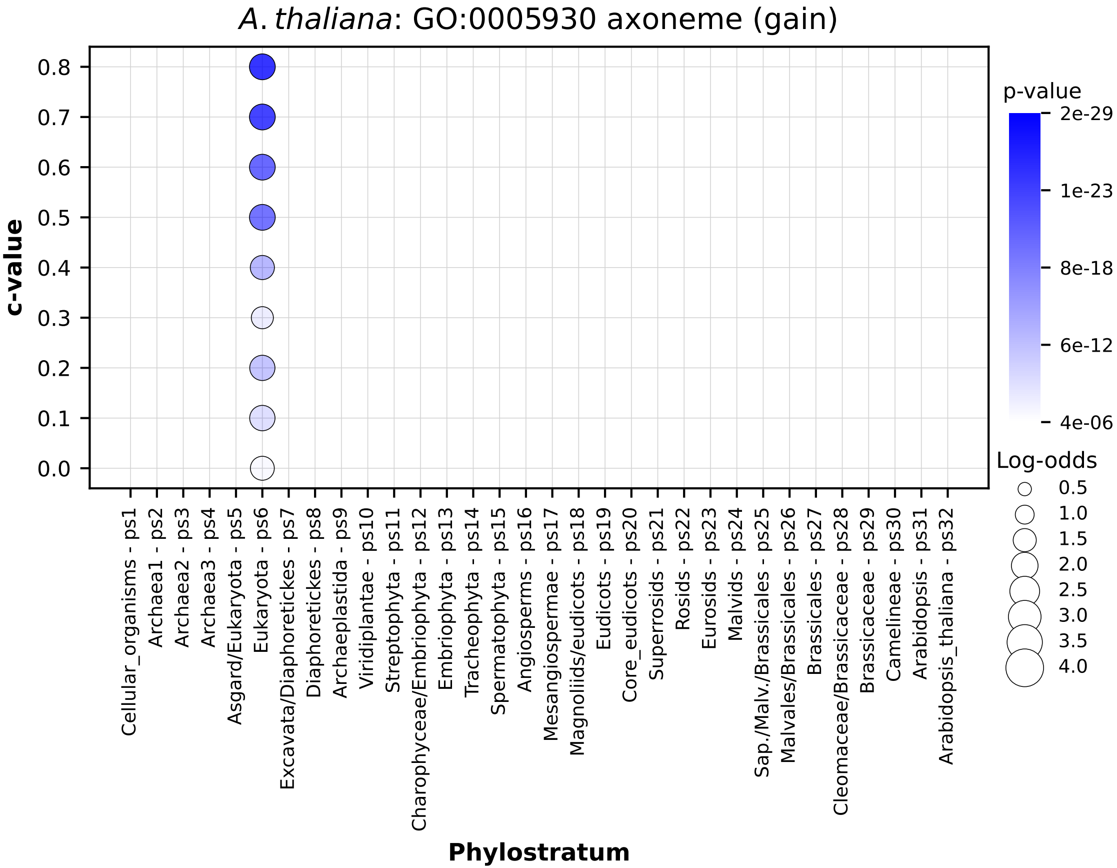

A. thaliana: GO:0005930 axoneme (gain)
c-value
0.8
0.7
0.6
0.5
0.4
0.3
0.2
0.1
0.0
p-value
2e-29
1e-23
8e-18
6e-12
4e-06
Log-odds
0.5
1.0
1.5
2.0
2.5
3.0
3.5
4.0
Cellular_organisms - ps1
Archaea1 - ps2
Archaea2 - ps3
Archaea3 - ps4
Asgard/Eukaryota - ps5
Eukaryota - ps6
Excavata/Diaphoretickes - ps7
Diaphoretickes - ps8
Archaeplastida - ps9
Viridiplantae - ps10
Streptophyta - ps11
Charophyceae/Embriophyta - ps12
Embriophyta - ps13
Tracheophyta - ps14
Spermatophyta - ps15
Angiosperms - ps16
Mesangiospermae - ps17
Magnoliids/eudicots - ps18
Eudicots - ps19
Core_eudicots - ps20
Superrosids - ps21
Rosids - ps22
Eurosids - ps23
Malvids - ps24
Sap./Malv./Brassicales - ps25
Malvales/Brassicales - ps26
Brassicales - ps27
Cleomaceae/Brassicaceae - ps28
Brassicaceae - ps29
Camelineae - ps30
Arabidopsis - ps31
Arabidopsis_thaliana - ps32
Phylostratum

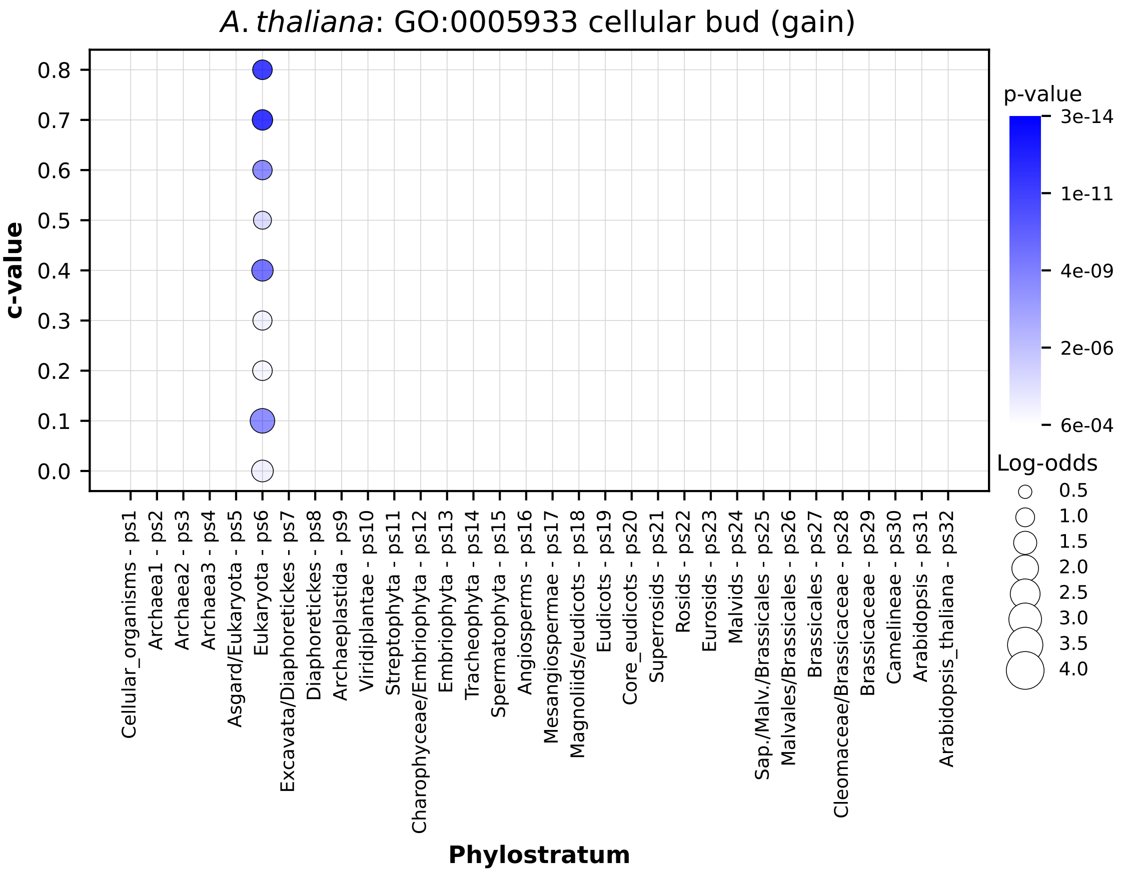

A. thaliana: GO:0005933 cellular bud (gain)
c-value
0.8
0.7
0.6
0.5
0.4
0.3
0.2
0.1
0.0
p-value
3e-14
1e-11
4e-09
2e-06
6e-04
Log-odds
0.5
1.0
1.5
2.0
2.5
3.0
3.5
4.0
Cellular_organisms - ps1
Archaea1 - ps2
Archaea2 - ps3
Archaea3 - ps4
Asgard/Eukaryota - ps5
Eukaryota - ps6
Excavata/Diaphoretickes - ps7
Diaphoretickes - ps8
Archaeplastida - ps9
Viridiplantae - ps10
Streptophyta - ps11
Charophyceae/Embriophyta - ps12
Embriophyta - ps13
Tracheophyta - ps14
Spermatophyta - ps15
Angiosperms - ps16
Mesangiospermae - ps17
Magnoliids/eudicots - ps18
Eudicots - ps19
Core_eudicots - ps20
Superrosids - ps21
Rosids - ps22
Eurosids - ps23
Malvids - ps24
Sap./Malv./Brassicales - ps25
Malvales/Brassicales - ps26
Brassicales - ps27
Cleomaceae/Brassicaceae - ps28
Brassicaceae - ps29
Camelineae - ps30
Arabidopsis - ps31
Arabidopsis_thaliana - ps32
Phylostratum

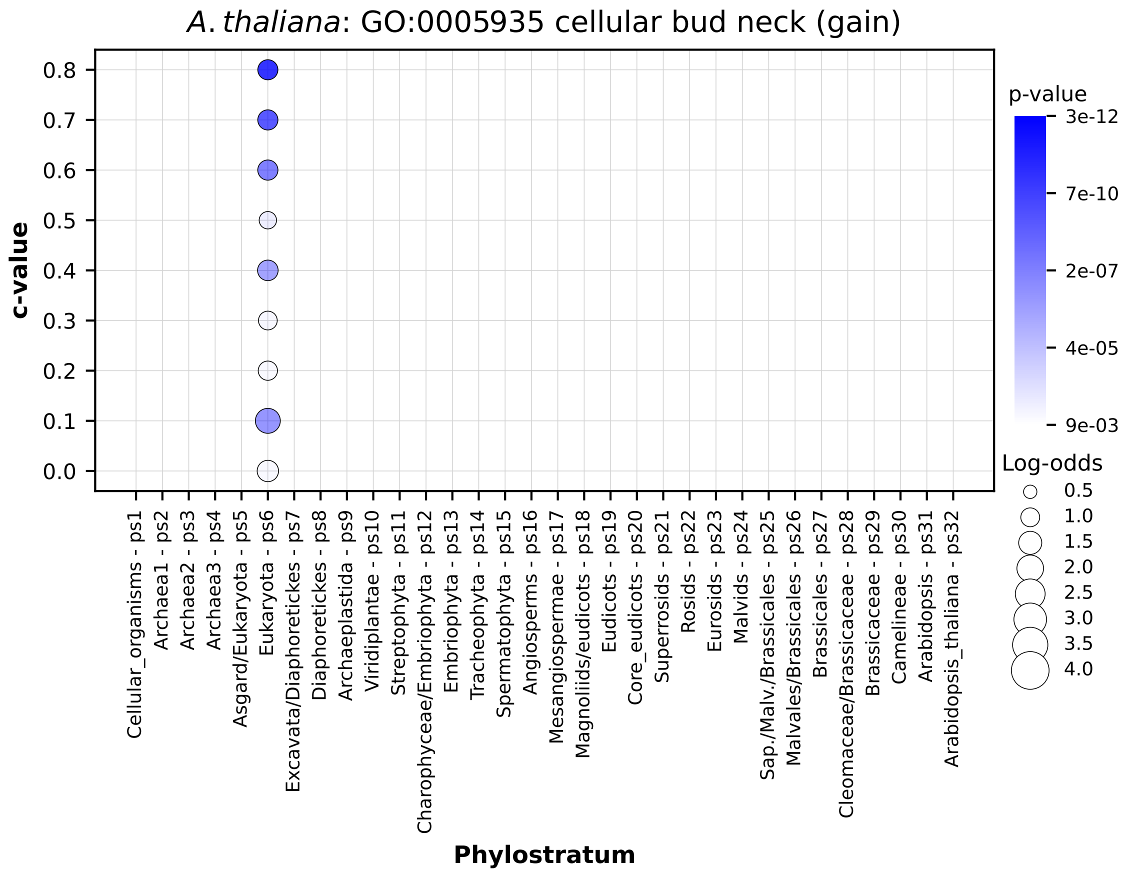
A. thaliana: GO:0005935 cellular bud neck (gain)
c-value
0.0
0.1
0.2
0.3
0.4
0.5
0.6
0.7
0.8
p-value
3e-12
7e-10
2e-07
4e-05
9e-03
Log-odds
0.5
1.0
1.5
2.0
2.5
3.0
3.5
4.0
Cellular_organisms - ps1
Archaea1 - ps2
Archaea2 - ps3
Archaea3 - ps4
Asgard/Eukaryota - ps5
Eukaryota - ps6
Excavata/Diaphoretickes - ps7
Diaphoretickes - ps8
Archaeplastida - ps9
Viridiplantae - ps10
Streptophyta - ps11
Charophyceae/Embriophyta - ps12
Embriophyta - ps13
Tracheophyta - ps14
Spermatophyta - ps15
Angiosperms - ps16
Mesangiospermae - ps17
Magnoliids/eudicots - ps18
Eudicots - ps19
Core_eudicots - ps20
Superrosids - ps21
Rosids - ps22
Eurosids - ps23
Malvids - ps24
Sap./Malv./Brassicales - ps25
Malvales/Brassicales - ps26
Brassicales - ps27
Cleomaceae/Brassicaceae - ps28
Brassicaceae - ps29
Camelineae - ps30
Arabidopsis - ps31
Arabidopsis_thaliana - ps32
Phylostratum

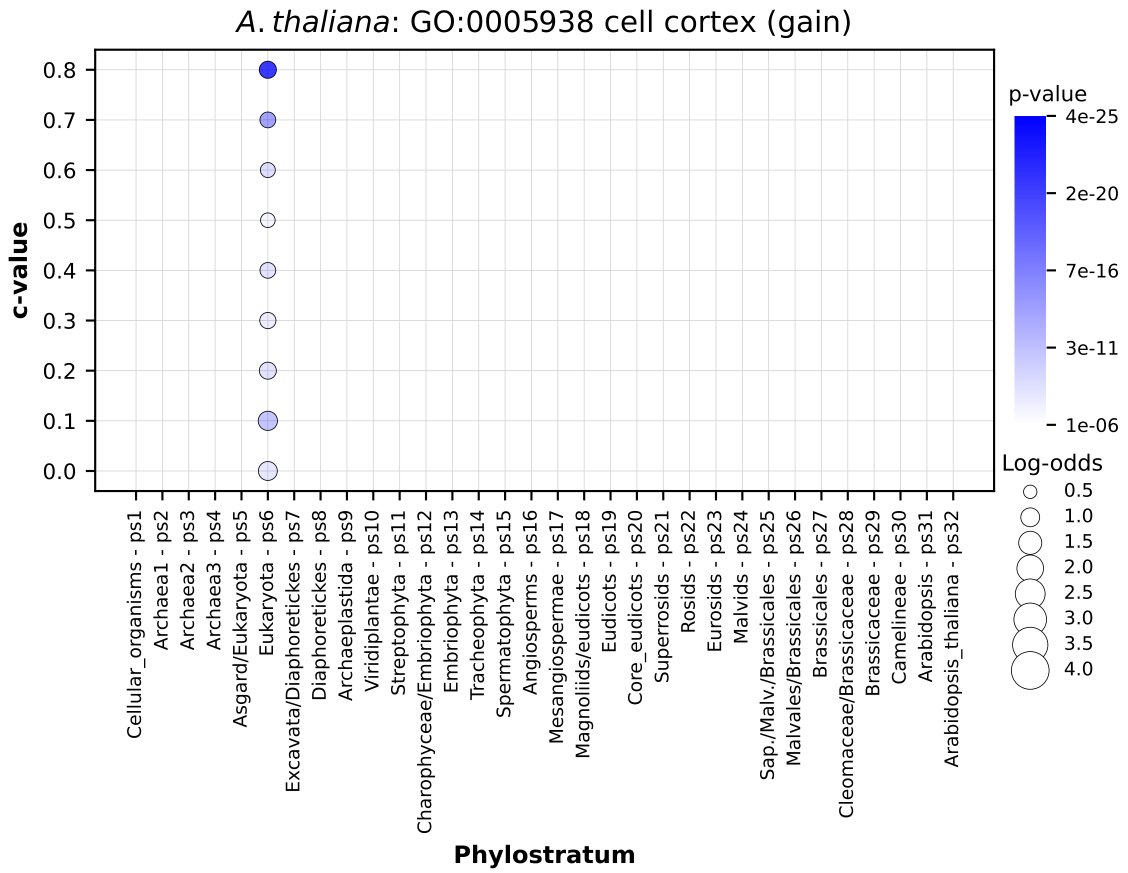
A. thaliana: GO:0005938 cell cortex (gain)
c-value
0.8
0.7
0.6
0.5
0.4
0.3
0.2
0.1
0.0
Phylostratum
Cellular_organisms - ps1
Archaea1 - ps2
Archaea2 - ps3
Archaea3 - ps4
Asgard/Eukaryota - ps5
Eukaryota - ps6
Excavata/Diaphoretickes - ps7
Diaphoretickes - ps8
Archaeplastida - ps9
Viridiplantae - ps10
Streptophyta - ps11
Charophyceae/Embriophyta - ps12
Embriophyta - ps13
Tracheophyta - ps14
Spermatophyta - ps15
Angiosperms - ps16
Mesangiospermae - ps17
Magnoliids/eudicots - ps18
Eudicots - ps19
Core_eudicots - ps20
Superrosids - ps21
Rosids - ps22
Eurosids - ps23
Malvids - ps24
Sap./Malv./Brassicales - ps25
Malvales/Brassicales - ps26
Brassicales - ps27
Cleomaceae/Brassicaceae - ps28
Brassicaceae - ps29
Camelineae - ps30
Arabidopsis - ps31
Arabidopsis_thaliana - ps32
p-value
4e-25
2e-20
7e-16
3e-11
1e-06
Log-odds
0.5
1.0
1.5
2.0
2.5
3.0
3.5
4.0

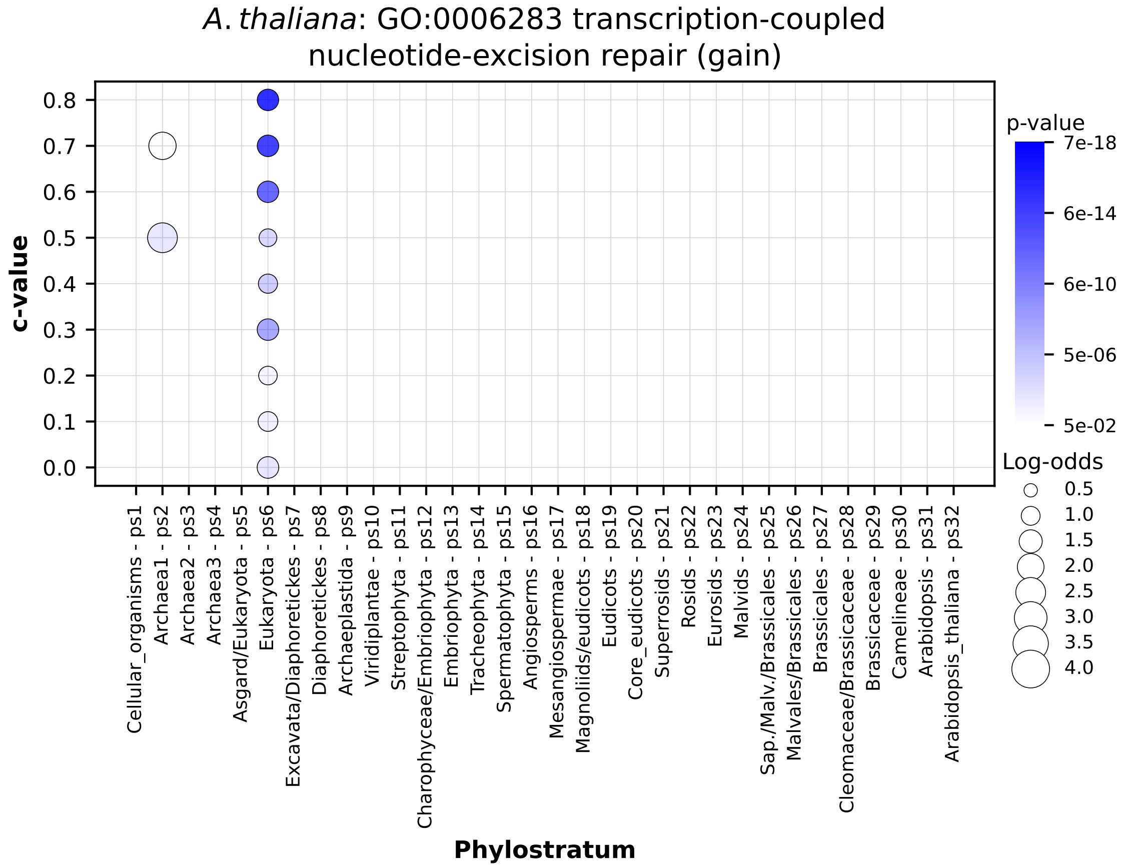
A. thaliana: GO:0006283 transcription-coupled nucleotide-excision repair (gain)
c-value
0.8
0.7
0.6
0.5
0.4
0.3
0.2
0.1
0.0
p-value
7e-18
6e-14
6e-10
5e-06
5e-02
Log-odds
0.5
1.0
1.5
2.0
2.5
3.0
3.5
4.0
Cellular_organisms - ps1
Archaea1 - ps2
Archaea2 - ps3
Archaea3 - ps4
Asgard/Eukaryota - ps5
Eukaryota - ps6
Excavata/Diaphoretickes - ps7
Diaphoretickes - ps8
Archaeplastida - ps9
Viridiplantae - ps10
Streptophyta - ps11
Charophyceae/Embriophyta - ps12
Embriophyta - ps13
Tracheophyta - ps14
Spermatophyta - ps15
Angiosperms - ps16
Mesangiospermae - ps17
Magnoliids/eudicots - ps18
Eudicots - ps19
Core_eudicots - ps20
Superrosids - ps21
Rosids - ps22
Eurosids - ps23
Malvids - ps24
Sap./Malv./Brassicales - ps25
Malvales/Brassicales - ps26
Brassicales - ps27
Cleomaceae/Brassicaceae - ps28
Brassicaceae - ps29
Camelineae - ps30
Arabidopsis - ps31
Arabidopsis_thaliana - ps32
Phylostratum

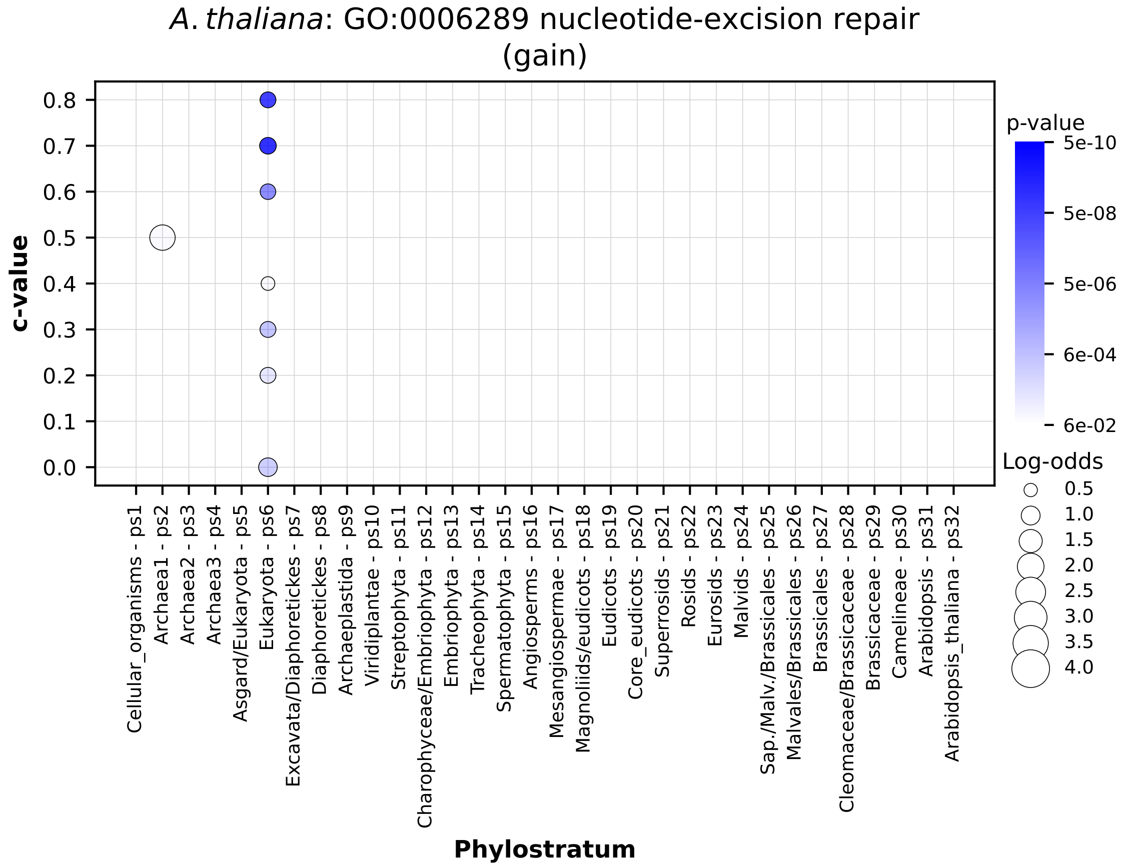

A. thaliana: GO:0006289 nucleotide-excision repair
(gain)
c-value
0.8
0.7
0.6
0.5
0.4
0.3
0.2
0.1
0.0
Phylostratum
Cellular_organisms - ps1
Archaea1 - ps2
Archaea2 - ps3
Archaea3 - ps4
Asgard/Eukaryota - ps5
Eukaryota - ps6
Excavata/Diaphoretickes - ps7
Diaphoretickes - ps8
Archaeplastida - ps9
Viridiplantae - ps10
Streptophyta - ps11
Charophyceae/Embriophyta - ps12
Embriophyta - ps13
Tracheophyta - ps14
Spermatophyta - ps15
Angiosperms - ps16
Mesangiospermae - ps17
Magnoliids/eudicots - ps18
Eudicots - ps19
Core_eudicots - ps20
Superrosids - ps21
Rosids - ps22
Eurosids - ps23
Malvids - ps24
Sap./Malv./Brassicales - ps25
Malvales/Brassicales - ps26
Brassicales - ps27
Cleomaceae/Brassicaceae - ps28
Brassicaceae - ps29
Camelineae - ps30
Arabidopsis - ps31
Arabidopsis_thaliana - ps32
p-value
5e-10
5e-08
5e-06
6e-04
6e-02
Log-odds
0.5
1.0
1.5
2.0
2.5
3.0
3.5
4.0

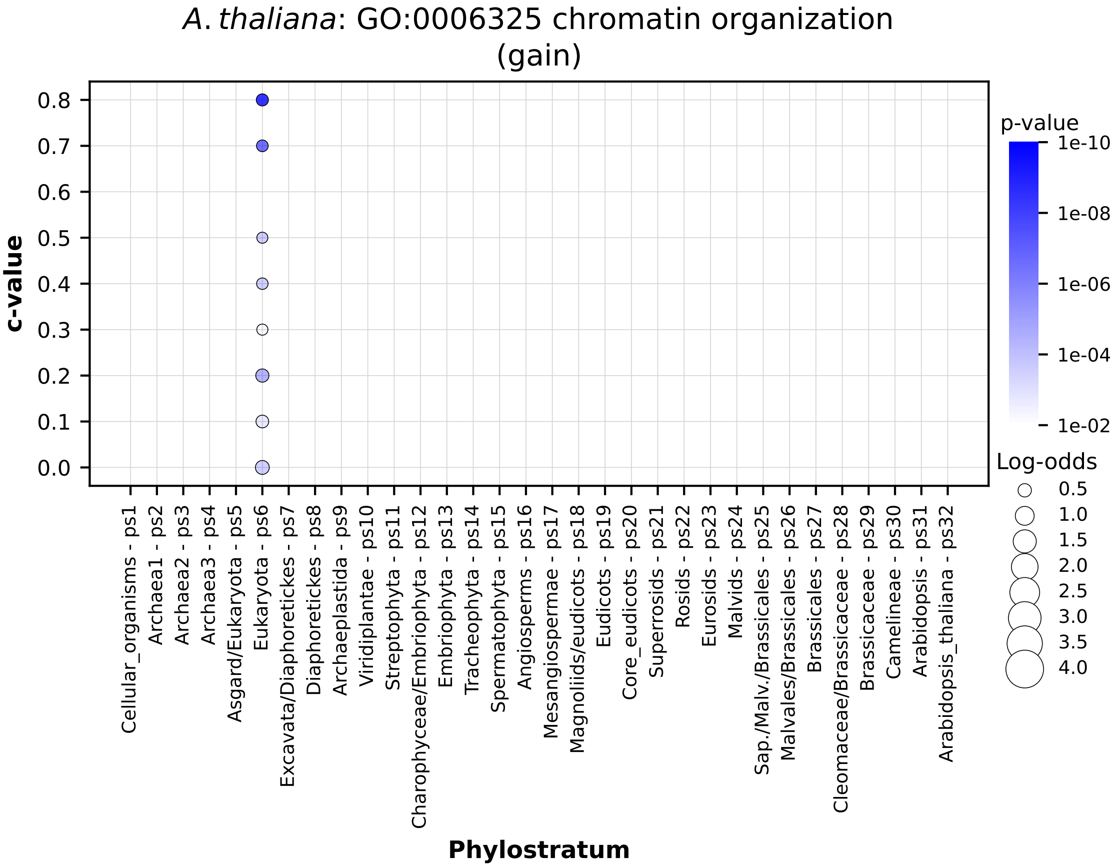

A. thaliana: GO:0006325 chromatin organization (gain)
c-value
0.8
0.7
0.6
0.5
0.4
0.3
0.2
0.1
0.0
p-value
1e-10
1e-08
1e-06
1e-04
1e-02
Log-odds
0.5
1.0
1.5
2.0
2.5
3.0
3.5
4.0
Cellular_organisms - ps1
Archaea1 - ps2
Archaea2 - ps3
Archaea3 - ps4
Asgard/Eukaryota - ps5
Eukaryota - ps6
Excavata/Diaphoretickes - ps7
Diaphoretickes - ps8
Archaeplastida - ps9
Viridiplantae - ps10
Streptophyta - ps11
Charophyceae/Embriophyta - ps12
Embriophyta - ps13
Tracheophyta - ps14
Spermatophyta - ps15
Angiosperms - ps16
Mesangiospermae - ps17
Magnoliids/eudicots - ps18
Eudicots - ps19
Core_eudicots - ps20
Superrosids - ps21
Rosids - ps22
Eurosids - ps23
Malvids - ps24
Sap./Malv./Brassicales - ps25
Malvales/Brassicales - ps26
Brassicales - ps27
Cleomaceae/Brassicaceae - ps28
Brassicaceae - ps29
Camelineae - ps30
Arabidopsis - ps31
Arabidopsis_thaliana - ps32
Phylostratum

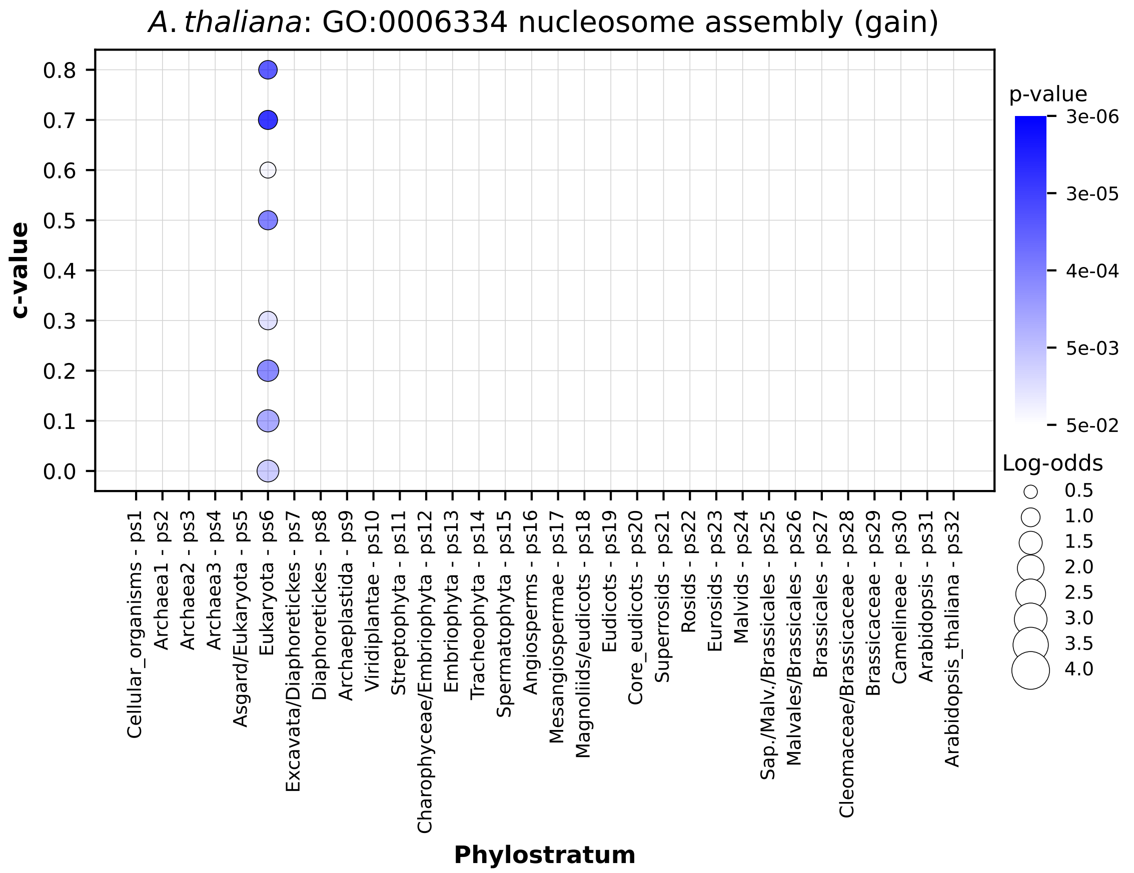

A. thaliana: GO:0006334 nucleosome assembly (gain)
c-value
0.8
0.7
0.6
0.5
0.4
0.3
0.2
0.1
0.0
Phylostratum
Cellular_organisms - ps1
Archaea1 - ps2
Archaea2 - ps3
Archaea3 - ps4
Asgard/Eukaryota - ps5
Eukaryota - ps6
Excavata/Diaphoretickes - ps7
Diaphoretickes - ps8
Archaeplastida - ps9
Viridiplantae - ps10
Streptophyta - ps11
Charophyceae/Embriophyta - ps12
Embriophyta - ps13
Tracheophyta - ps14
Spermatophyta - ps15
Angiosperms - ps16
Mesangiospermae - ps17
Magnoliids/eudicots - ps18
Eudicots - ps19
Core_eudicots - ps20
Superrosids - ps21
Rosids - ps22
Eurosids - ps23
Malvids - ps24
Sap./Malv./Brassicales - ps25
Malvales/Brassicales - ps26
Brassicales - ps27
Cleomaceae/Brassicaceae - ps28
Brassicaceae - ps29
Camelineae - ps30
Arabidopsis - ps31
Arabidopsis_thaliana - ps32
p-value
3e-06
3e-05
4e-04
5e-03
5e-02
Log-odds
0.5
1.0
1.5
2.0
2.5
3.0
3.5
4.0

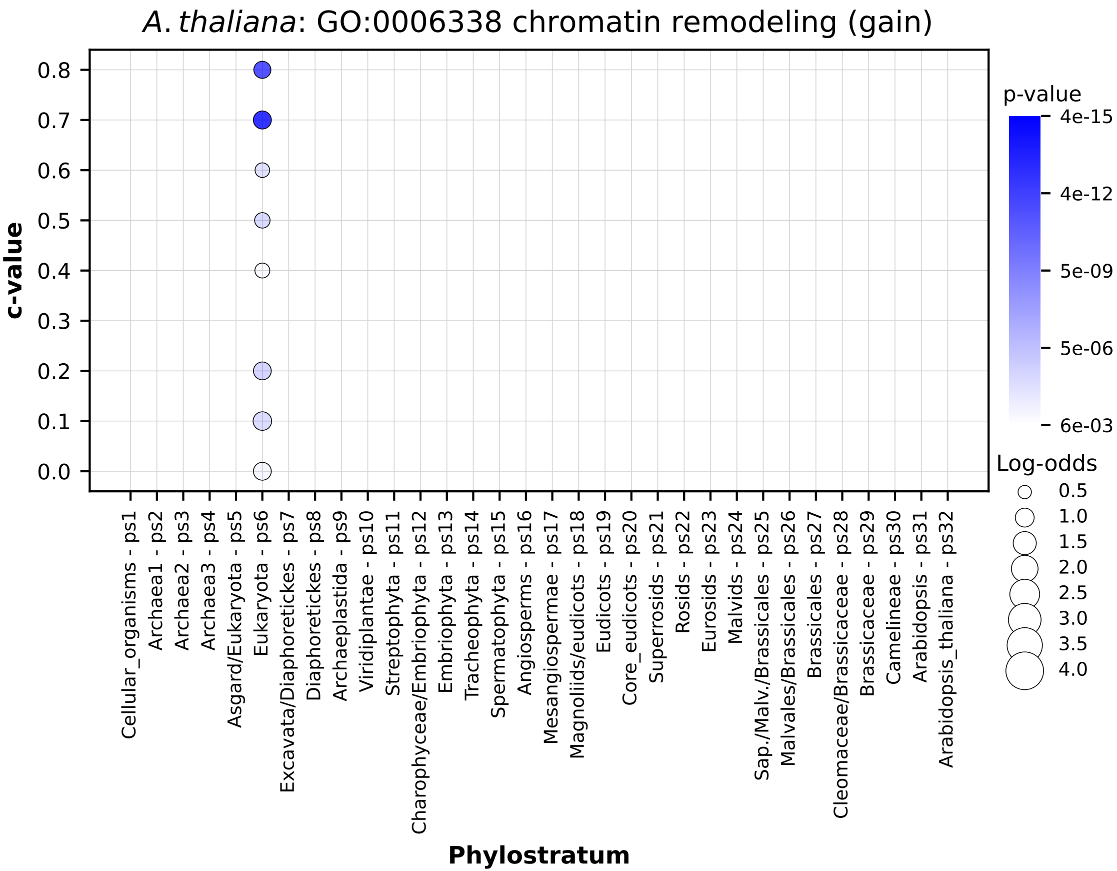

A. thaliana: GO:0006338 chromatin remodeling (gain)
c-value
0.8
0.7
0.6
0.5
0.4
0.3
0.2
0.1
0.0
p-value
4e-15
4e-12
5e-09
5e-06
6e-03
Log-odds
0.5
1.0
1.5
2.0
2.5
3.0
3.5
4.0
Cellular_organisms - ps1
Archaea1 - ps2
Archaea2 - ps3
Archaea3 - ps4
Asgard/Eukaryota - ps5
Eukaryota - ps6
Excavata/Diaphoretickes - ps7
Diaphoretickes - ps8
Archaeplastida - ps9
Viridiplantae - ps10
Streptophyta - ps11
Charophyceae/Embriophyta - ps12
Embriophyta - ps13
Tracheophyta - ps14
Spermatophyta - ps15
Angiosperms - ps16
Mesangiospermae - ps17
Magnoliids/eudicots - ps18
Eudicots - ps19
Core_eudicots - ps20
Superrosids - ps21
Rosids - ps22
Eurosids - ps23
Malvids - ps24
Sap./Malv./Brassicales - ps25
Malvales/Brassicales - ps26
Brassicales - ps27
Cleomaceae/Brassicaceae - ps28
Brassicaceae - ps29
Camelineae - ps30
Arabidopsis - ps31
Arabidopsis_thaliana - ps32
Phylostratum

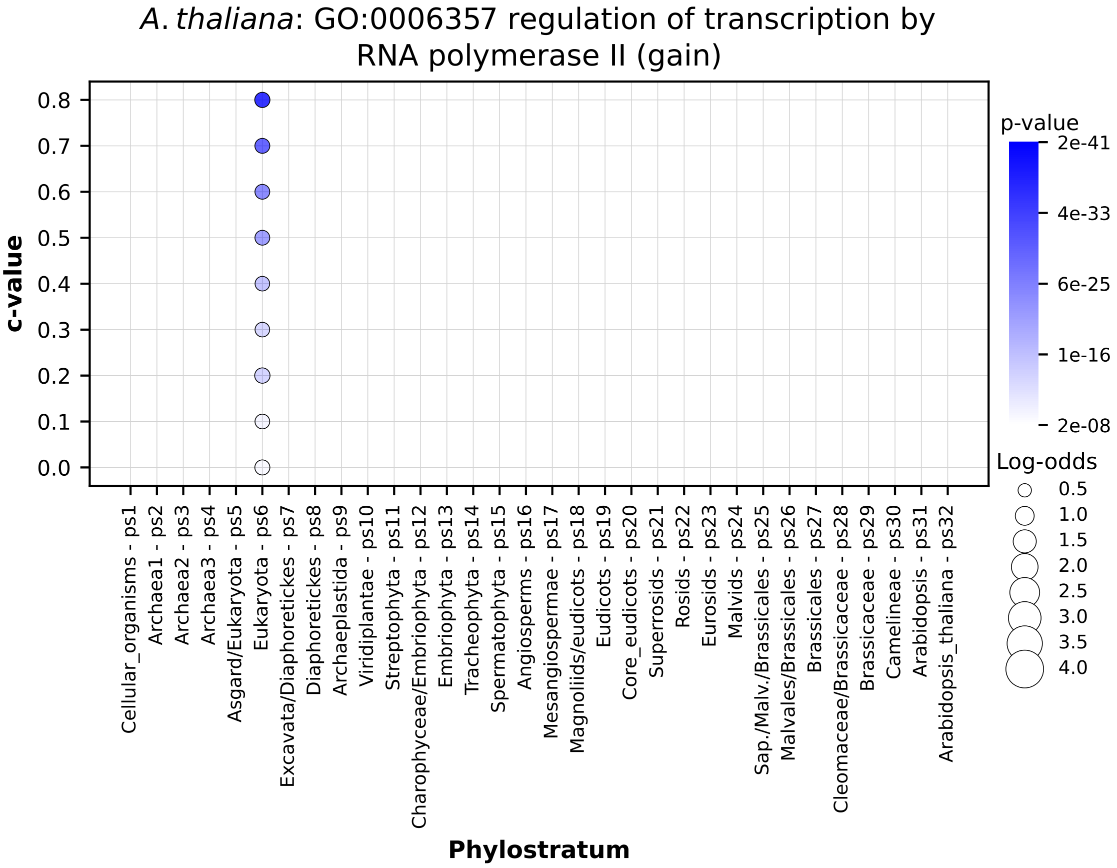
A. thaliana: GO:0006357 regulation of transcription by RNA polymerase II (gain)
c-value
0.8
0.7
0.6
0.5
0.4
0.3
0.2
0.1
0.0
p-value
2e-41
4e-33
6e-25
1e-16
2e-08
Log-odds
0.5
1.0
1.5
2.0
2.5
3.0
3.5
4.0
Cellular_organisms - ps1
Archaea1 - ps2
Archaea2 - ps3
Archaea3 - ps4
Asgard/Eukaryota - ps5
Eukaryota - ps6
Excavata/Diaphoretickes - ps7
Diaphoretickes - ps8
Archaeplastida - ps9
Viridiplantae - ps10
Streptophyta - ps11
Charophyceae/Embriophyta - ps12
Embriophyta - ps13
Tracheophyta - ps14
Spermatophyta - ps15
Angiosperms - ps16
Mesangiospermae - ps17
Magnoliids/eudicots - ps18
Eudicots - ps19
Core_eudicots - ps20
Superrosids - ps21
Rosids - ps22
Eurosids - ps23
Malvids - ps24
Sap./Malv./Brassicales - ps25
Malvales/Brassicales - ps26
Brassicales - ps27
Cleomaceae/Brassicaceae - ps28
Brassicaceae - ps29
Camelineae - ps30
Arabidopsis - ps31
Arabidopsis_thaliana - ps32
Phylostratum

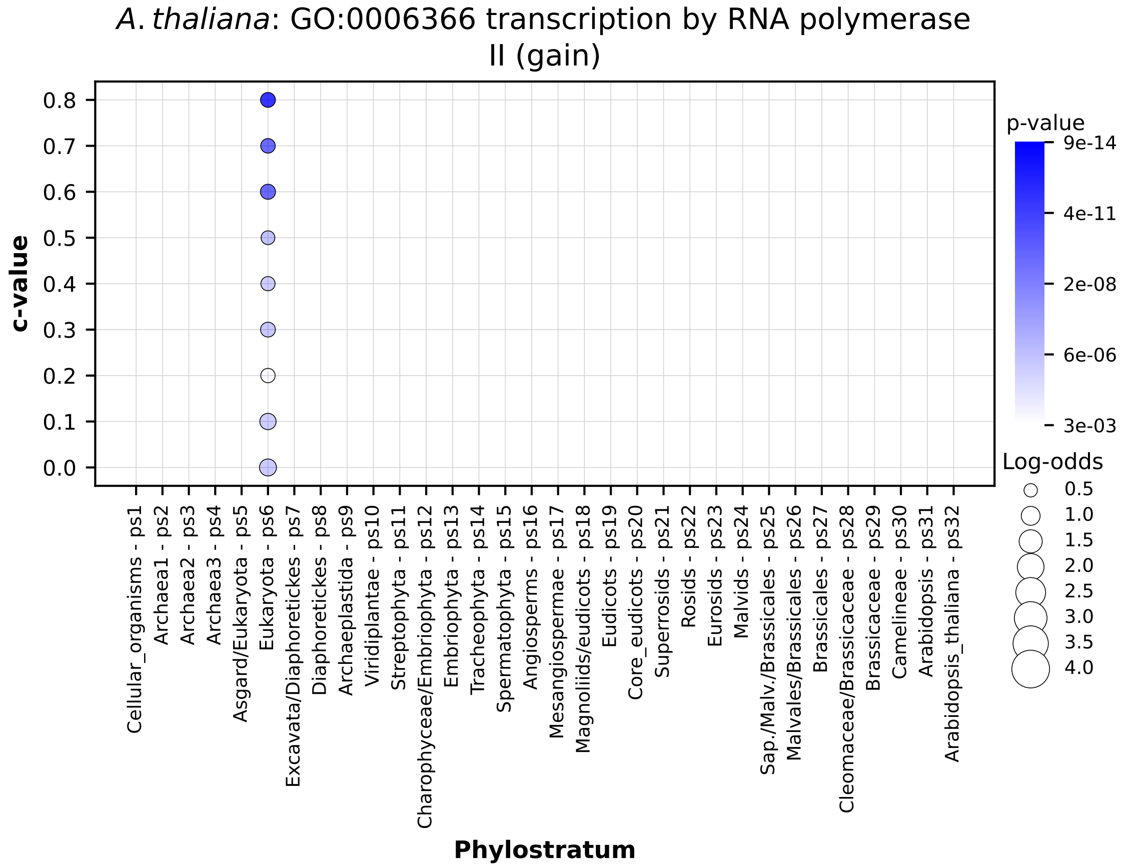

A. thaliana: GO:0006366 transcription by RNA polymerase II (gain)
c-value
0.8
0.7
0.6
0.5
0.4
0.3
0.2
0.1
0.0
Phylostratum
Cellular_organisms - ps1
Archaea1 - ps2
Archaea2 - ps3
Archaea3 - ps4
Asgard/Eukaryota - ps5
Eukaryota - ps6
Excavata/Diaphoretickes - ps7
Diaphoretickes - ps8
Archaeplastida - ps9
Viridiplantae - ps10
Streptophyta - ps11
Charophyceae/Embriophyta - ps12
Embriophyta - ps13
Tracheophyta - ps14
Spermatophyta - ps15
Angiosperms - ps16
Mesangiospermae - ps17
Magnoliids/eudicots - ps18
Eudicots - ps19
Core_eudicots - ps20
Superrosids - ps21
Rosids - ps22
Eurosids - ps23
Malvids - ps24
Sap./Malv./Brassicales - ps25
Malvales/Brassicales - ps26
Brassicales - ps27
Cleomaceae/Brassicaceae - ps28
Brassicaceae - ps29
Camelineae - ps30
Arabidopsis - ps31
Arabidopsis_thaliana - ps32
p-value
9e-14
4e-11
2e-08
6e-06
3e-03
Log-odds
0.5
1.0
1.5
2.0
2.5
3.0
3.5
4.0

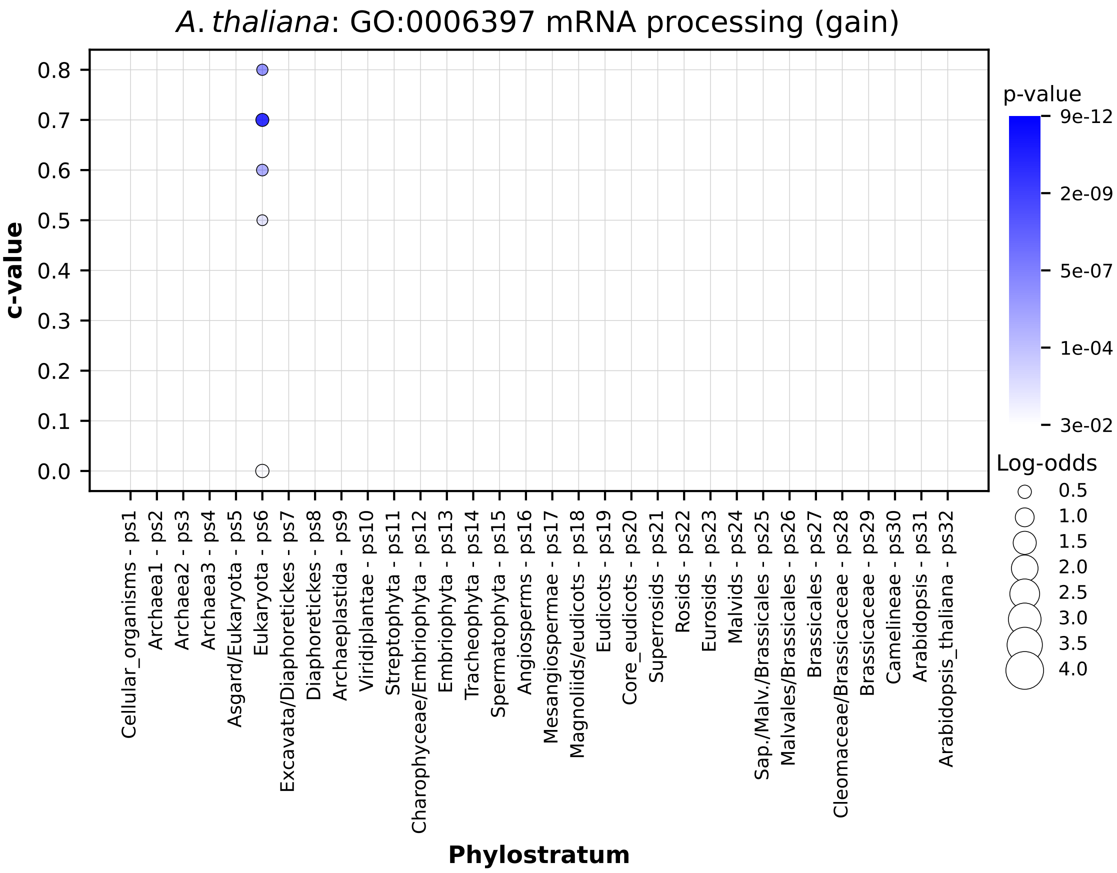
A. thaliana: GO:0006397 mRNA processing (gain)
c-value
0.8
0.7
0.6
0.5
0.4
0.3
0.2
0.1
0.0
Phylostratum
Cellular_organisms - ps1
Archaea1 - ps2
Archaea2 - ps3
Archaea3 - ps4
Asgard/Eukaryota - ps5
Eukaryota - ps6
Excavata/Diaphoretickes - ps7
Diaphoretickes - ps8
Archaeplastida - ps9
Viridiplantae - ps10
Streptophyta - ps11
Charophyceae/Embriophyta - ps12
Embriophyta - ps13
Tracheophyta - ps14
Spermatophyta - ps15
Angiosperms - ps16
Mesangiospermae - ps17
Magnoliids/eudicots - ps18
Eudicots - ps19
Core_eudicots - ps20
Superrosids - ps21
Rosids - ps22
Eurosids - ps23
Malvids - ps24
Sap./Malv./Brassicales - ps25
Malvales/Brassicales - ps26
Brassicales - ps27
Cleomaceae/Brassicaceae - ps28
Brassicaceae - ps29
Camelineae - ps30
Arabidopsis - ps31
Arabidopsis_thaliana - ps32
p-value
9e-12
2e-09
5e-07
1e-04
3e-02
Log-odds
0.5
1.0
1.5
2.0
2.5
3.0
3.5
4.0

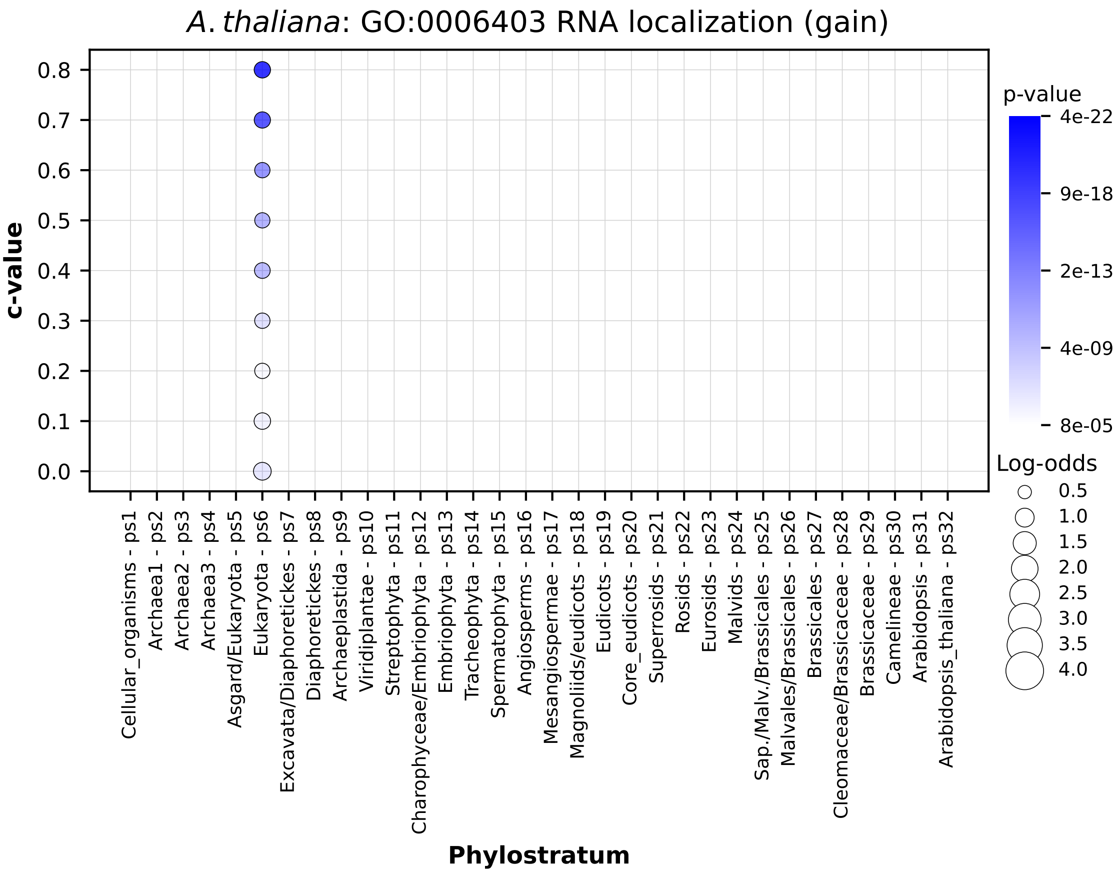

A. thaliana: GO:0006403 RNA localization (gain)
c-value
0.8
0.7
0.6
0.5
0.4
0.3
0.2
0.1
0.0
p-value
4e-22
9e-18
2e-13
4e-09
8e-05
Log-odds
0.5
1.0
1.5
2.0
2.5
3.0
3.5
4.0
Cellular_organisms - ps1
Archaea1 - ps2
Archaea2 - ps3
Archaea3 - ps4
Asgard/Eukaryota - ps5
Eukaryota - ps6
Excavata/Diaphoretickes - ps7
Diaphoretickes - ps8
Archaeplastida - ps9
Viridiplantae - ps10
Streptophyta - ps11
Charophyceae/Embriophyta - ps12
Embriophyta - ps13
Tracheophyta - ps14
Spermatophyta - ps15
Angiosperms - ps16
Mesangiospermae - ps17
Magnoliids/eudicots - ps18
Eudicots - ps19
Core_eudicots - ps20
Superrosids - ps21
Rosids - ps22
Eurosids - ps23
Malvids - ps24
Sap./Malv./Brassicales - ps25
Malvales/Brassicales - ps26
Brassicales - ps27
Cleomaceae/Brassicaceae - ps28
Brassicaceae - ps29
Camelineae - ps30
Arabidopsis - ps31
Arabidopsis_thaliana - ps32
Phylostratum

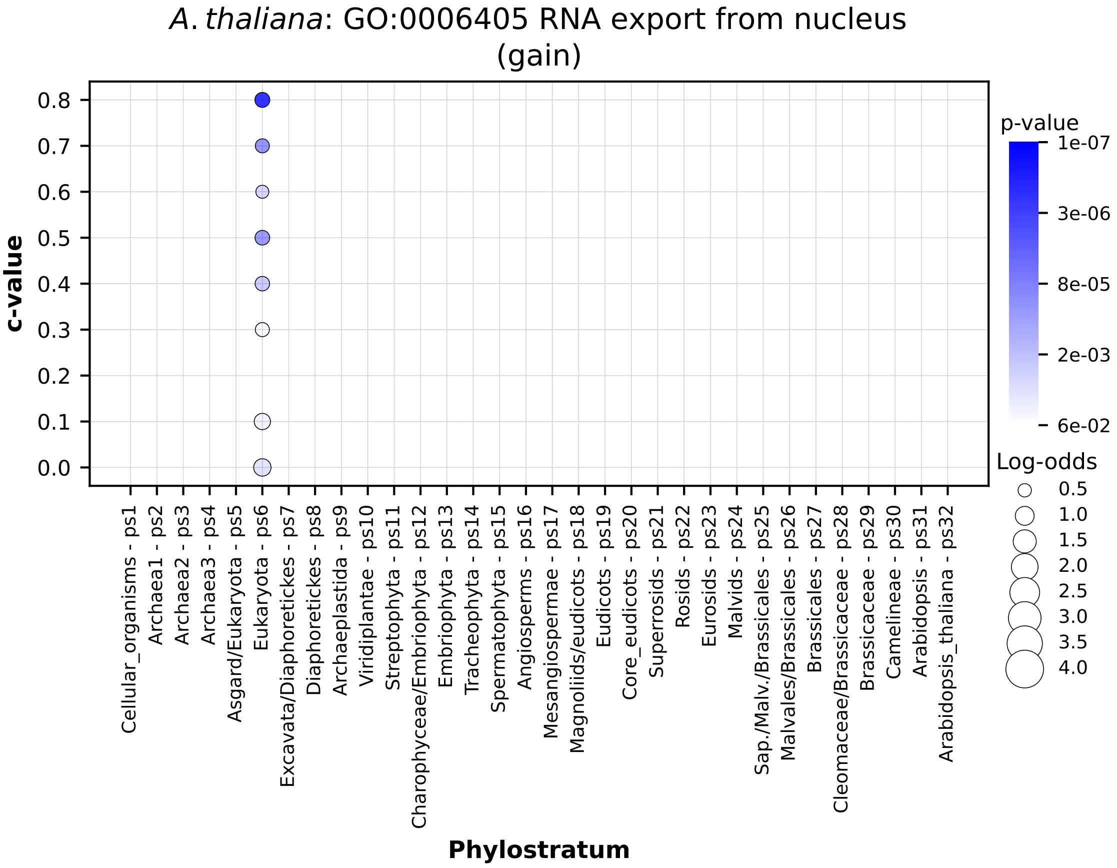
A. thaliana: GO:0006405 RNA export from nucleus
(gain)
c-value
0.8
0.7
0.6
0.5
0.4
0.3
0.2
0.1
0.0
Phylostratum
Cellular_organisms - ps1
Archaea1 - ps2
Archaea2 - ps3
Archaea3 - ps4
Asgard/Eukaryota - ps5
Eukaryota - ps6
Excavata/Diaphoretickes - ps7
Diaphoretickes - ps8
Archaeplastida - ps9
Viridiplantae - ps10
Streptophyta - ps11
Charophyceae/Embriophyta - ps12
Embriophyta - ps13
Tracheophyta - ps14
Spermatophyta - ps15
Angiosperms - ps16
Mesangiospermae - ps17
Magnoliids/eudicots - ps18
Eudicots - ps19
Core_eudicots - ps20
Superrosids - ps21
Rosids - ps22
Eurosids - ps23
Malvids - ps24
Sap./Malv./Brassicales - ps25
Malvales/Brassicales - ps26
Brassicales - ps27
Cleomaceae/Brassicaceae - ps28
Brassicaceae - ps29
Camelineae - ps30
Arabidopsis - ps31
Arabidopsis_thaliana - ps32
p-value
1e-07
3e-06
8e-05
2e-03
6e-02
Log-odds
0.5
1.0
1.5
2.0
2.5
3.0
3.5
4.0

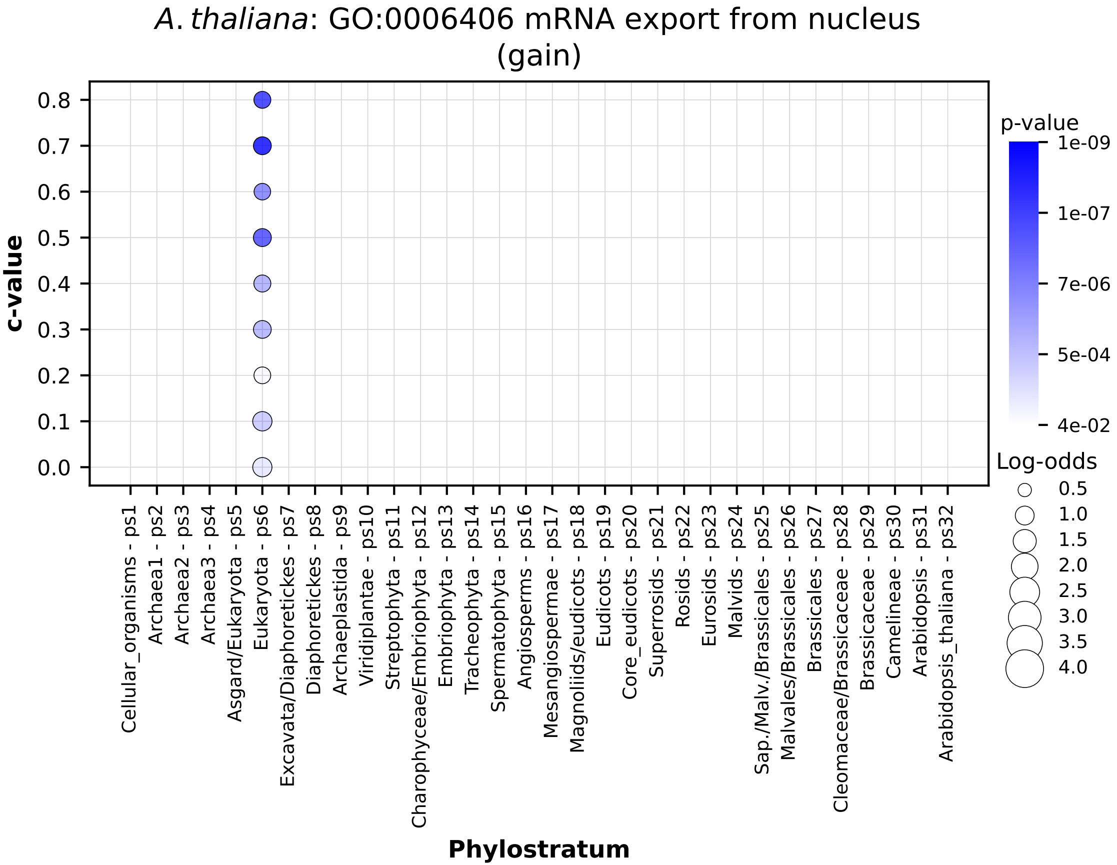

A. thaliana: GO:0006406 mRNA export from nucleus
(gain)
c-value
0.8
0.7
0.6
0.5
0.4
0.3
0.2
0.1
0.0
p-value
1e-09
1e-07
7e-06
5e-04
4e-02
Log-odds
0.5
1.0
1.5
2.0
2.5
3.0
3.5
4.0
Cellular_organisms - ps1
Archaea1 - ps2
Archaea2 - ps3
Archaea3 - ps4
Asgard/Eukaryota - ps5
Eukaryota - ps6
Excavata/Diaphoretickes - ps7
Diaphoretickes - ps8
Archaeplastida - ps9
Viridiplantae - ps10
Streptophyta - ps11
Charophyceae/Embriophyta - ps12
Embriophyta - ps13
Tracheophyta - ps14
Spermatophyta - ps15
Angiosperms - ps16
Mesangiospermae - ps17
Magnoliids/eudicots - ps18
Eudicots - ps19
Core_eudicots - ps20
Superrosids - ps21
Rosids - ps22
Eurosids - ps23
Malvids - ps24
Sap./Malv./Brassicales - ps25
Malvales/Brassicales - ps26
Brassicales - ps27
Cleomaceae/Brassicaceae - ps28
Brassicaceae - ps29
Camelineae - ps30
Arabidopsis - ps31
Arabidopsis_thaliana - ps32
Phylostratum

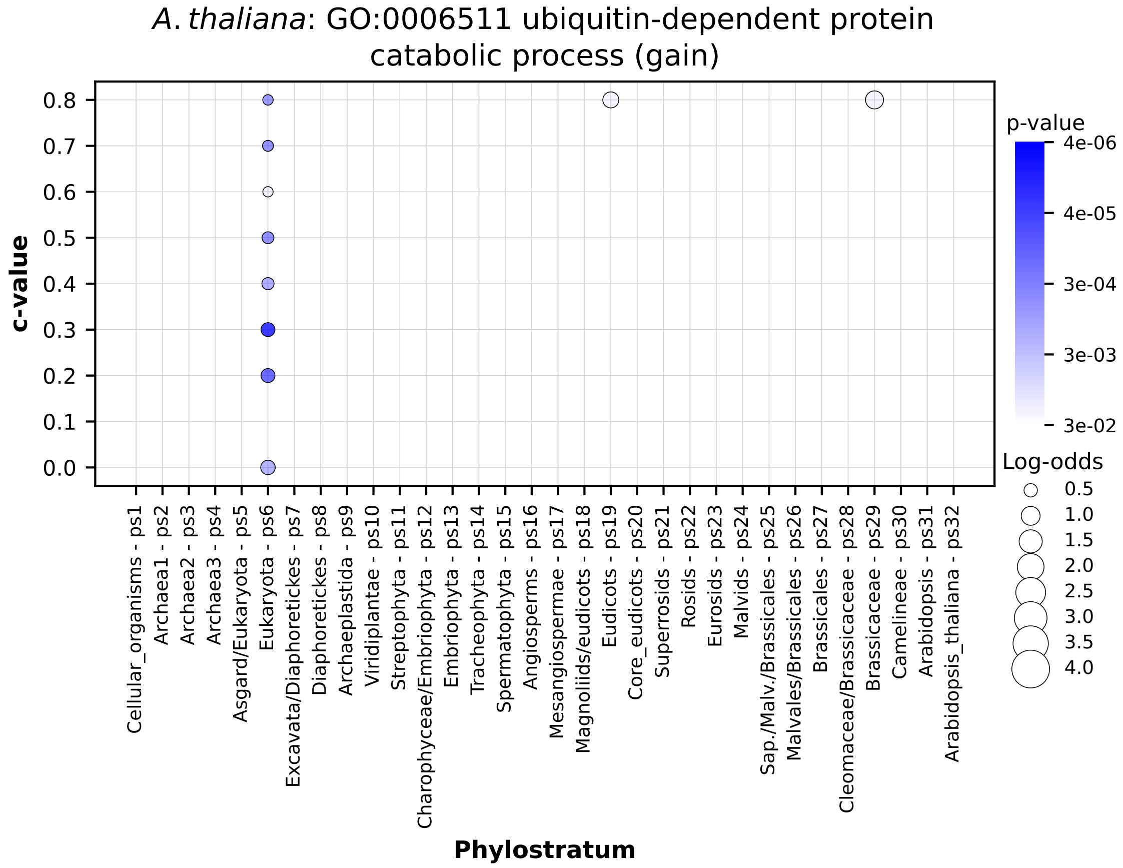

A. thaliana: GO:0006511 ubiquitin-dependent protein catabolic process (gain)
c-value
0.8
0.7
0.6
0.5
0.4
0.3
0.2
0.1
0.0
p-value
4e-06
4e-05
3e-04
3e-03
3e-02
Log-odds
0.5
1.0
1.5
2.0
2.5
3.0
3.5
4.0
Cellular_organisms - ps1
Archaea1 - ps2
Archaea2 - ps3
Archaea3 - ps4
Asgard/Eukaryota - ps5
Eukaryota - ps6
Excavata/Diaphoretickes - ps7
Diaphoretickes - ps8
Archaeplastida - ps9
Viridiplantae - ps10
Streptophyta - ps11
Charophyceae/Embriophyta - ps12
Embriophyta - ps13
Tracheophyta - ps14
Spermatophyta - ps15
Angiosperms - ps16
Mesangiospermae - ps17
Magnoliids/eudicots - ps18
Eudicots - ps19
Core_eudicots - ps20
Superrosids - ps21
Rosids - ps22
Eurosids - ps23
Malvids - ps24
Sap./Malv./Brassicales - ps25
Malvales/Brassicales - ps26
Brassicales - ps27
Cleomaceae/Brassicaceae - ps28
Brassicaceae - ps29
Camelineae - ps30
Arabidopsis - ps31
Arabidopsis_thaliana - ps32
Phylostratum

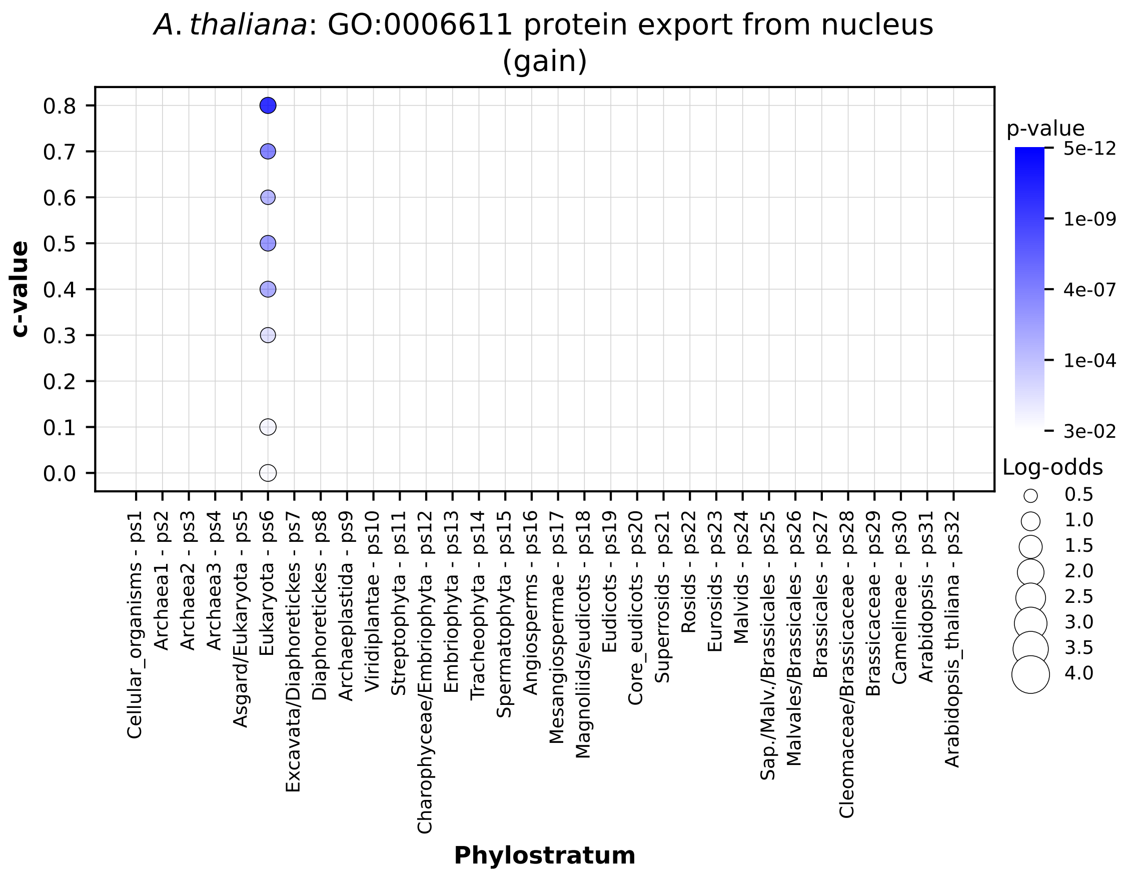

A. thaliana: GO:0006611 protein export from nucleus
(gain)
c-value
0.8
0.7
0.6
0.5
0.4
0.3
0.2
0.1
0.0
p-value
5e-12
1e-09
4e-07
1e-04
3e-02
Log-odds
0.5
1.0
1.5
2.0
2.5
3.0
3.5
4.0
Cellular_organisms - ps1
Archaea1 - ps2
Archaea2 - ps3
Archaea3 - ps4
Asgard/Eukaryota - ps5
Eukaryota - ps6
Excavata/Diaphoretickes - ps7
Diaphoretickes - ps8
Archaeplastida - ps9
Viridiplantae - ps10
Streptophyta - ps11
Charophyceae/Embriophyta - ps12
Embriophyta - ps13
Tracheophyta - ps14
Spermatophyta - ps15
Angiosperms - ps16
Mesangiospermae - ps17
Magnoliids/eudicots - ps18
Eudicots - ps19
Core_eudicots - ps20
Superrosids - ps21
Rosids - ps22
Eurosids - ps23
Malvids - ps24
Sap./Malv./Brassicales - ps25
Malvales/Brassicales - ps26
Brassicales - ps27
Cleomaceae/Brassicaceae - ps28
Brassicaceae - ps29
Camelineae - ps30
Arabidopsis - ps31
Arabidopsis_thaliana - ps32
Phylostratum

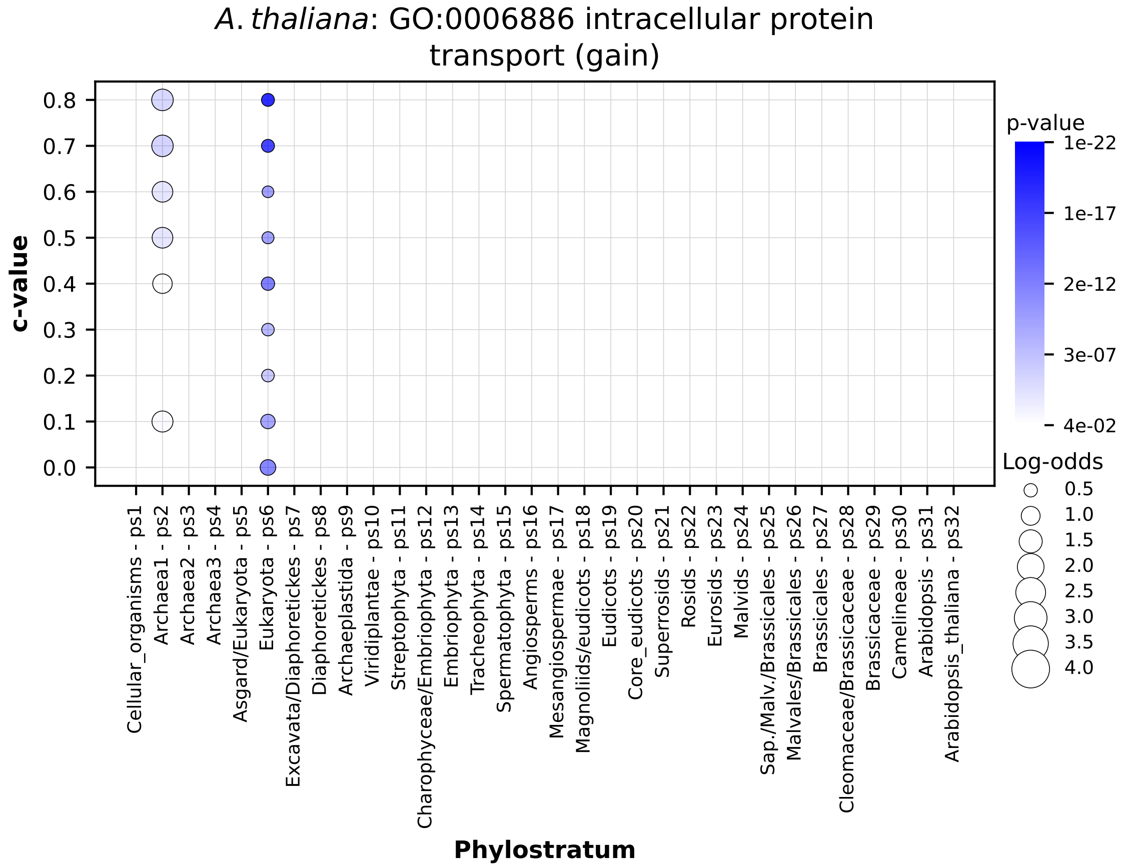

A. thaliana: GO:0006886 intracellular protein transport (gain)
c-value
0.0
0.1
0.2
0.3
0.4
0.5
0.6
0.7
0.8
Phylostratum
Cellular_organisms - ps1
Archaea1 - ps2
Archaea2 - ps3
Archaea3 - ps4
Asgard/Eukaryota - ps5
Eukaryota - ps6
Excavata/Diaphoretickes - ps7
Diaphoretickes - ps8
Archaeplastida - ps9
Viridiplantae - ps10
Streptophyta - ps11
Charophyceae/Embriophyta - ps12
Embriophyta - ps13
Tracheophyta - ps14
Spermatophyta - ps15
Angiosperms - ps16
Mesangiospermae - ps17
Magnoliids/eudicots - ps18
Eudicots - ps19
Core_eudicots - ps20
Superrosids - ps21
Rosids - ps22
Eurosids - ps23
Malvids - ps24
Sap./Malv./Brassicales - ps25
Malvales/Brassicales - ps26
Brassicales - ps27
Cleomaceae/Brassicaceae - ps28
Brassicaceae - ps29
Camelineae - ps30
Arabidopsis - ps31
Arabidopsis_thaliana - ps32
p-value
1e-22
1e-17
2e-12
3e-07
4e-02
Log-odds
0.5
1.0
1.5
2.0
2.5
3.0
3.5
4.0

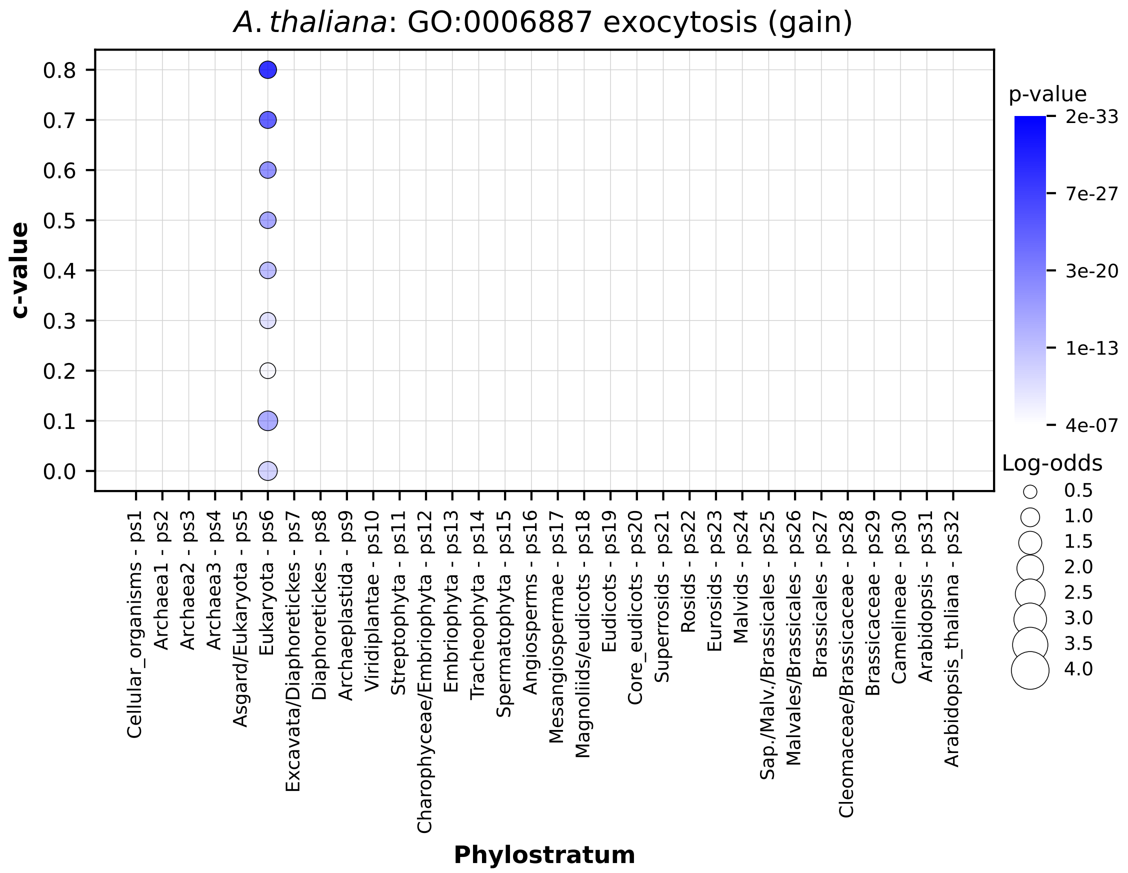

A. thaliana: GO:0006887 exocytosis (gain)
c-value
0.8
0.7
0.6
0.5
0.4
0.3
0.2
0.1
0.0
p-value
2e-33
7e-27
3e-20
1e-13
4e-07
Log-odds
0.5
1.0
1.5
2.0
2.5
3.0
3.5
4.0
Cellular_organisms - ps1
Archaea1 - ps2
Archaea2 - ps3
Archaea3 - ps4
Asgard/Eukaryota - ps5
Eukaryota - ps6
Excavata/Diaphoretickes - ps7
Diaphoretickes - ps8
Archaeplastida - ps9
Viridiplantae - ps10
Streptophyta - ps11
Charophyceae/Embriophyta - ps12
Embriophyta - ps13
Tracheophyta - ps14
Spermatophyta - ps15
Angiosperms - ps16
Mesangiospermae - ps17
Magnoliids/eudicots - ps18
Eudicots - ps19
Core_eudicots - ps20
Superrosids - ps21
Rosids - ps22
Eurosids - ps23
Malvids - ps24
Sap./Malv./Brassicales - ps25
Malvales/Brassicales - ps26
Brassicales - ps27
Cleomaceae/Brassicaceae - ps28
Brassicaceae - ps29
Camelineae - ps30
Arabidopsis - ps31
Arabidopsis_thaliana - ps32
Phylostratum

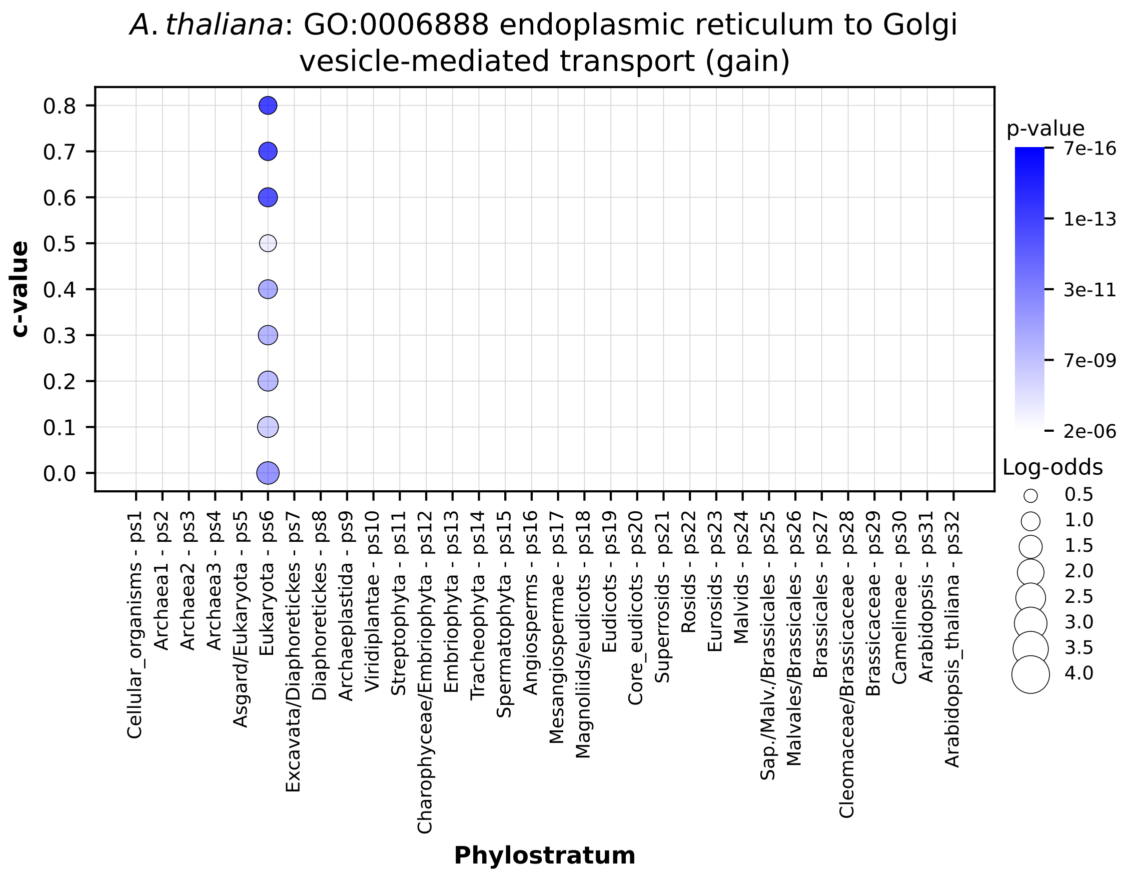

A. thaliana: GO:0006888 endoplasmic reticulum to Golgi vesicle-mediated transport (gain)
c-value
0.8
0.7
0.6
0.5
0.4
0.3
0.2
0.1
0.0
p-value
7e-16
1e-13
3e-11
7e-09
2e-06
Log-odds
0.5
1.0
1.5
2.0
2.5
3.0
3.5
4.0
Cellular_organisms - ps1
Archaea1 - ps2
Archaea2 - ps3
Archaea3 - ps4
Asgard/Eukaryota - ps5
Eukaryota - ps6
Excavata/Diaphoretickes - ps7
Diaphoretickes - ps8
Archaeplastida - ps9
Viridiplantae - ps10
Streptophyta - ps11
Charophyceae/Embriophyta - ps12
Embriophyta - ps13
Tracheophyta - ps14
Spermatophyta - ps15
Angiosperms - ps16
Mesangiospermae - ps17
Magnoliids/eudicots - ps18
Eudicots - ps19
Core_eudicots - ps20
Superrosids - ps21
Rosids - ps22
Eurosids - ps23
Malvids - ps24
Sap./Malv./Brassicales - ps25
Malvales/Brassicales - ps26
Brassicales - ps27
Cleomaceae/Brassicaceae - ps28
Brassicaceae - ps29
Camelineae - ps30
Arabidopsis - ps31
Arabidopsis_thaliana - ps32
Phylostratum

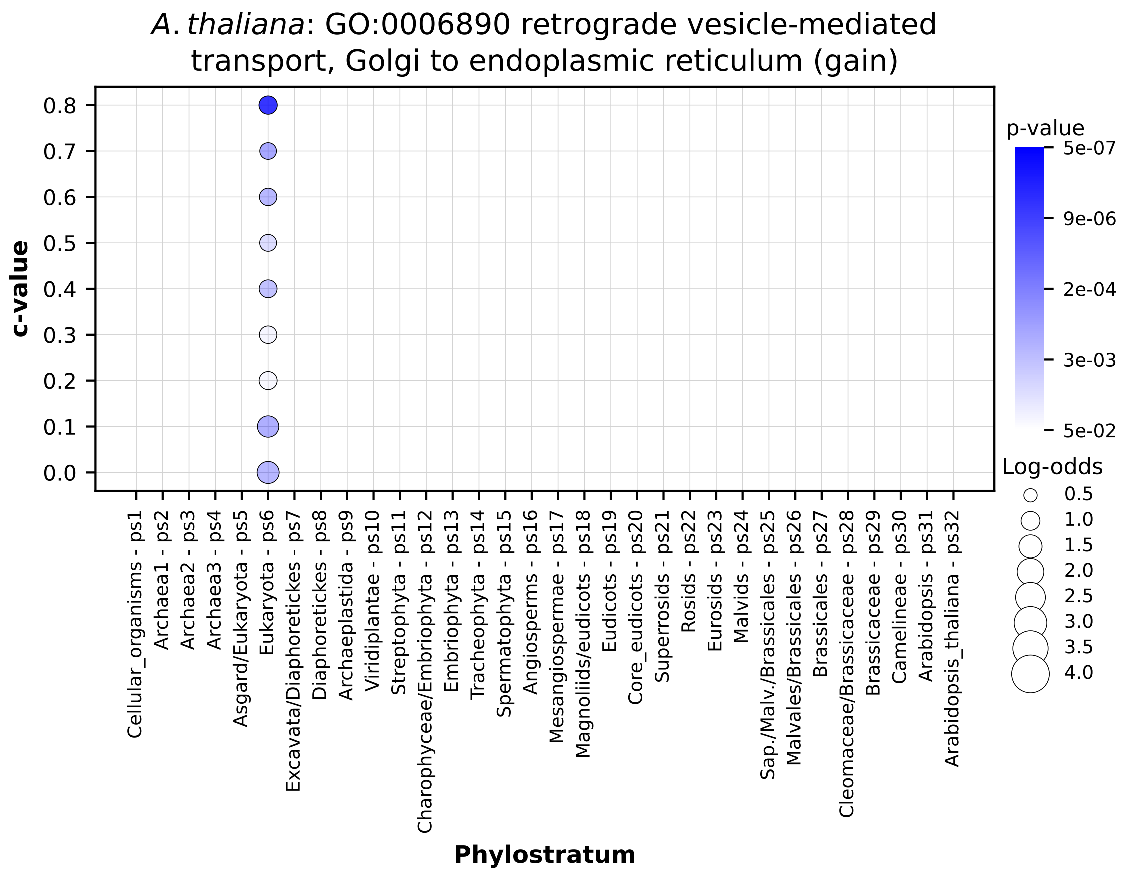
A. thaliana: GO:0006890 retrograde vesicle-mediated transport, Golgi to endoplasmic reticulum (gain)
c-value
0.8
0.7
0.6
0.5
0.4
0.3
0.2
0.1
0.0
p-value
5e-07
9e-06
2e-04
3e-03
5e-02
Log-odds
0.5
1.0
1.5
2.0
2.5
3.0
3.5
4.0
Cellular_organisms - ps1
Archaea1 - ps2
Archaea2 - ps3
Archaea3 - ps4
Asgard/Eukaryota - ps5
Eukaryota - ps6
Excavata/Diaphoretickes - ps7
Diaphoretickes - ps8
Archaeplastida - ps9
Viridiplantae - ps10
Streptophyta - ps11
Charophyceae/Embriophyta - ps12
Embriophyta - ps13
Tracheophyta - ps14
Spermatophyta - ps15
Angiosperms - ps16
Mesangiospermae - ps17
Magnoliids/eudicots - ps18
Eudicots - ps19
Core_eudicots - ps20
Superrosids - ps21
Rosids - ps22
Eurosids - ps23
Malvids - ps24
Sap./Malv./Brassicales - ps25
Malvales/Brassicales - ps26
Brassicales - ps27
Cleomaceae/Brassicaceae - ps28
Brassicaceae - ps29
Camelineae - ps30
Arabidopsis - ps31
Arabidopsis_thaliana - ps32
Phylostratum

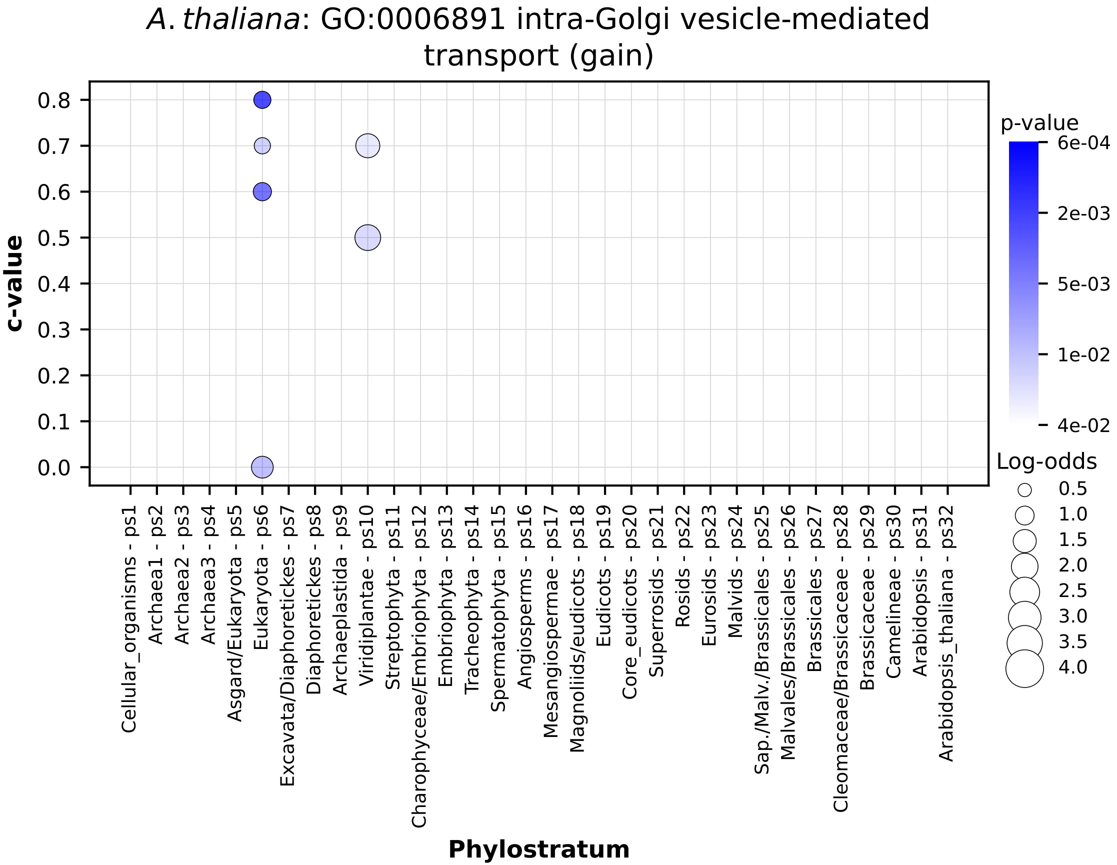
A. thaliana: GO:0006891 intra-Golgi vesicle-mediated transport (gain)
c-value
0.8
0.7
0.6
0.5
0.4
0.3
0.2
0.1
0.0
Phylostratum
Cellular_organisms - ps1
Archaea1 - ps2
Archaea2 - ps3
Archaea3 - ps4
Asgard/Eukaryota - ps5
Eukaryota - ps6
Excavata/Diaphoretickes - ps7
Diaphoretickes - ps8
Archaeplastida - ps9
Viridiplantae - ps10
Streptophyta - ps11
Charophyceae/Embriophyta - ps12
Embriophyta - ps13
Tracheophyta - ps14
Spermatophyta - ps15
Angiosperms - ps16
Mesangiospermae - ps17
Magnoliids/eudicots - ps18
Eudicots - ps19
Core_eudicots - ps20
Superrosids - ps21
Rosids - ps22
Eurosids - ps23
Malvids - ps24
Sap./Malv./Brassicales - ps25
Malvales/Brassicales - ps26
Brassicales - ps27
Cleomaceae/Brassicaceae - ps28
Brassicaceae - ps29
Camelineae - ps30
Arabidopsis - ps31
Arabidopsis_thaliana - ps32
p-value
6e-04
2e-03
5e-03
1e-02
4e-02
Log-odds
0.5
1.0
1.5
2.0
2.5
3.0
3.5
4.0

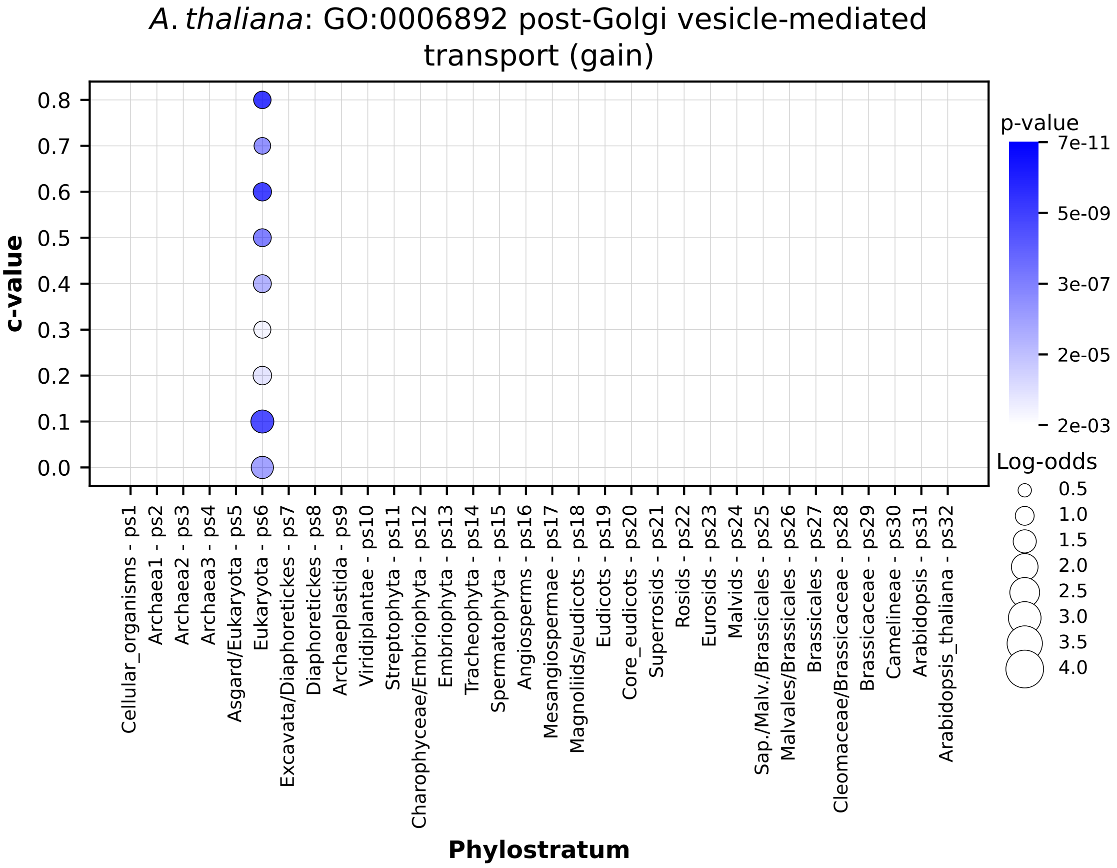

A. thaliana: GO:0006892 post-Golgi vesicle-mediated transport (gain)
c-value
0.0
0.1
0.2
0.3
0.4
0.5
0.6
0.7
0.8
Phylostratum
Cellular_organisms - ps1
Archaea1 - ps2
Archaea2 - ps3
Archaea3 - ps4
Asgard/Eukaryota - ps5
Eukaryota - ps6
Excavata/Diaphoretickes - ps7
Diaphoretickes - ps8
Archaeplastida - ps9
Viridiplantae - ps10
Streptophyta - ps11
Charophyceae/Embriophyta - ps12
Embriophyta - ps13
Tracheophyta - ps14
Spermatophyta - ps15
Angiosperms - ps16
Mesangiospermae - ps17
Magnoliids/eudicots - ps18
Eudicots - ps19
Core_eudicots - ps20
Superrosids - ps21
Rosids - ps22
Eurosids - ps23
Malvids - ps24
Sap./Malv./Brassicales - ps25
Malvales/Brassicales - ps26
Brassicales - ps27
Cleomaceae/Brassicaceae - ps28
Brassicaceae - ps29
Camelineae - ps30
Arabidopsis - ps31
Arabidopsis_thaliana - ps32
p-value
7e-11
5e-09
3e-07
2e-05
2e-03
Log-odds
0.5
1.0
1.5
2.0
2.5
3.0
3.5
4.0

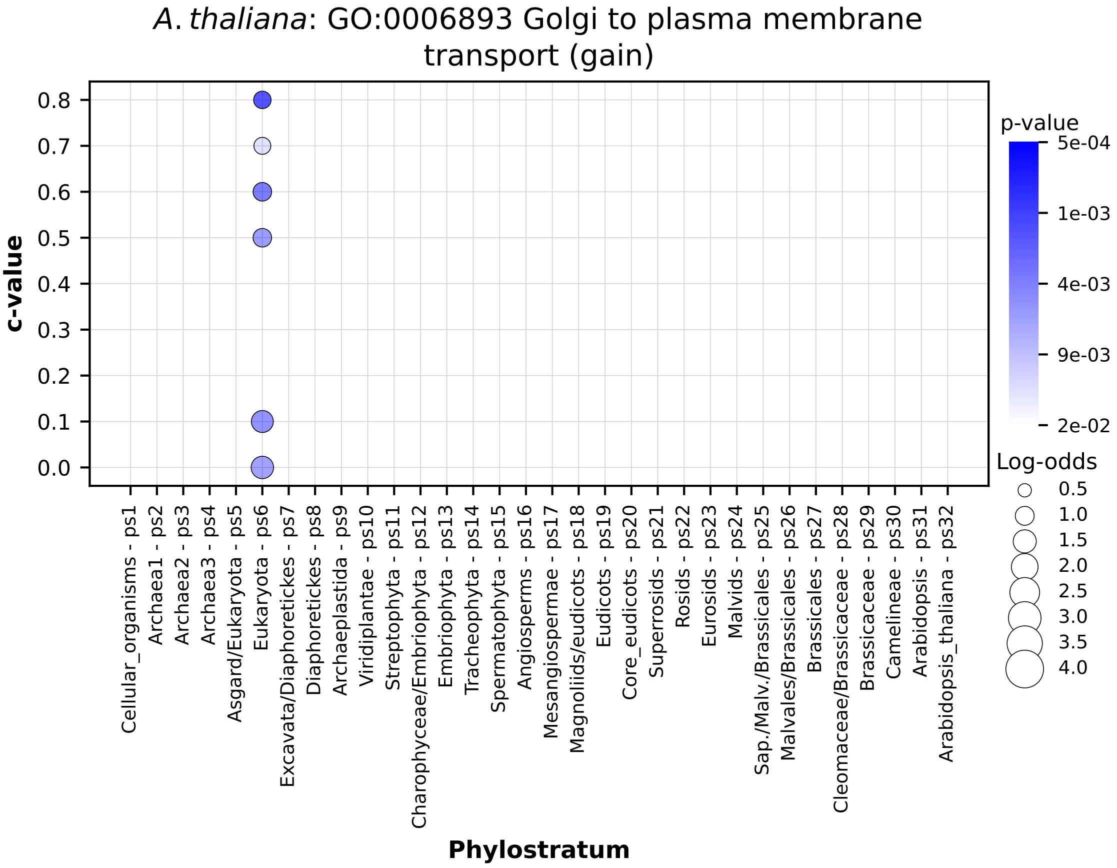

A. thaliana: GO:0006893 Golgi to plasma membrane transport (gain)
c-value
0.8
0.7
0.6
0.5
0.4
0.3
0.2
0.1
0.0
p-value
5e-04
1e-03
4e-03
9e-03
2e-02
Log-odds
0.5
1.0
1.5
2.0
2.5
3.0
3.5
4.0
Cellular_organisms - ps1
Archaea1 - ps2
Archaea2 - ps3
Archaea3 - ps4
Asgard/Eukaryota - ps5
Eukaryota - ps6
Excavata/Diaphoretickes - ps7
Diaphoretickes - ps8
Archaeplastida - ps9
Viridiplantae - ps10
Streptophyta - ps11
Charophyceae/Embriophyta - ps12
Embriophyta - ps13
Tracheophyta - ps14
Spermatophyta - ps15
Angiosperms - ps16
Mesangiospermae - ps17
Magnoliids/eudicots - ps18
Eudicots - ps19
Core_eudicots - ps20
Superrosids - ps21
Rosids - ps22
Eurosids - ps23
Malvids - ps24
Sap./Malv./Brassicales - ps25
Malvales/Brassicales - ps26
Brassicales - ps27
Cleomaceae/Brassicaceae - ps28
Brassicaceae - ps29
Camelineae - ps30
Arabidopsis - ps31
Arabidopsis_thaliana - ps32
Phylostratum

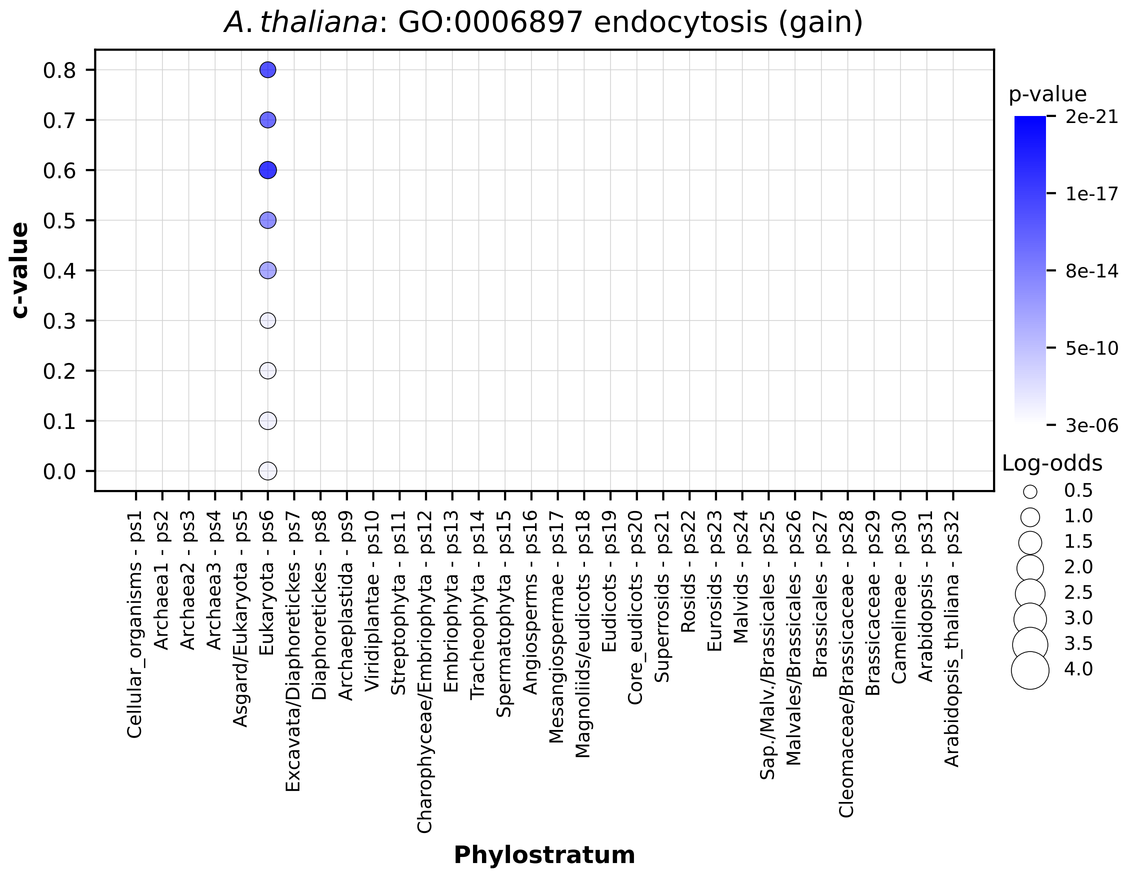

A. thaliana: GO:0006897 endocytosis (gain)
c-value
0.0
0.1
0.2
0.3
0.4
0.5
0.6
0.7
0.8
Phylostratum
Cellular_organisms - ps1
Archaea1 - ps2
Archaea2 - ps3
Archaea3 - ps4
Asgard/Eukaryota - ps5
Eukaryota - ps6
Excavata/Diaphoretickes - ps7
Diaphoretickes - ps8
Archaeplastida - ps9
Viridiplantae - ps10
Streptophyta - ps11
Charophyceae/Embriophyta - ps12
Embriophyta - ps13
Tracheophyta - ps14
Spermatophyta - ps15
Angiosperms - ps16
Mesangiospermae - ps17
Magnoliids/eudicots - ps18
Eudicots - ps19
Core_eudicots - ps20
Superrosids - ps21
Rosids - ps22
Eurosids - ps23
Malvids - ps24
Sap./Malv./Brassicales - ps25
Malvales/Brassicales - ps26
Brassicales - ps27
Cleomaceae/Brassicaceae - ps28
Brassicaceae - ps29
Camelineae - ps30
Arabidopsis - ps31
Arabidopsis_thaliana - ps32
p-value
2e-21
1e-17
8e-14
5e-10
3e-06
Log-odds
0.5
1.0
1.5
2.0
2.5
3.0
3.5
4.0

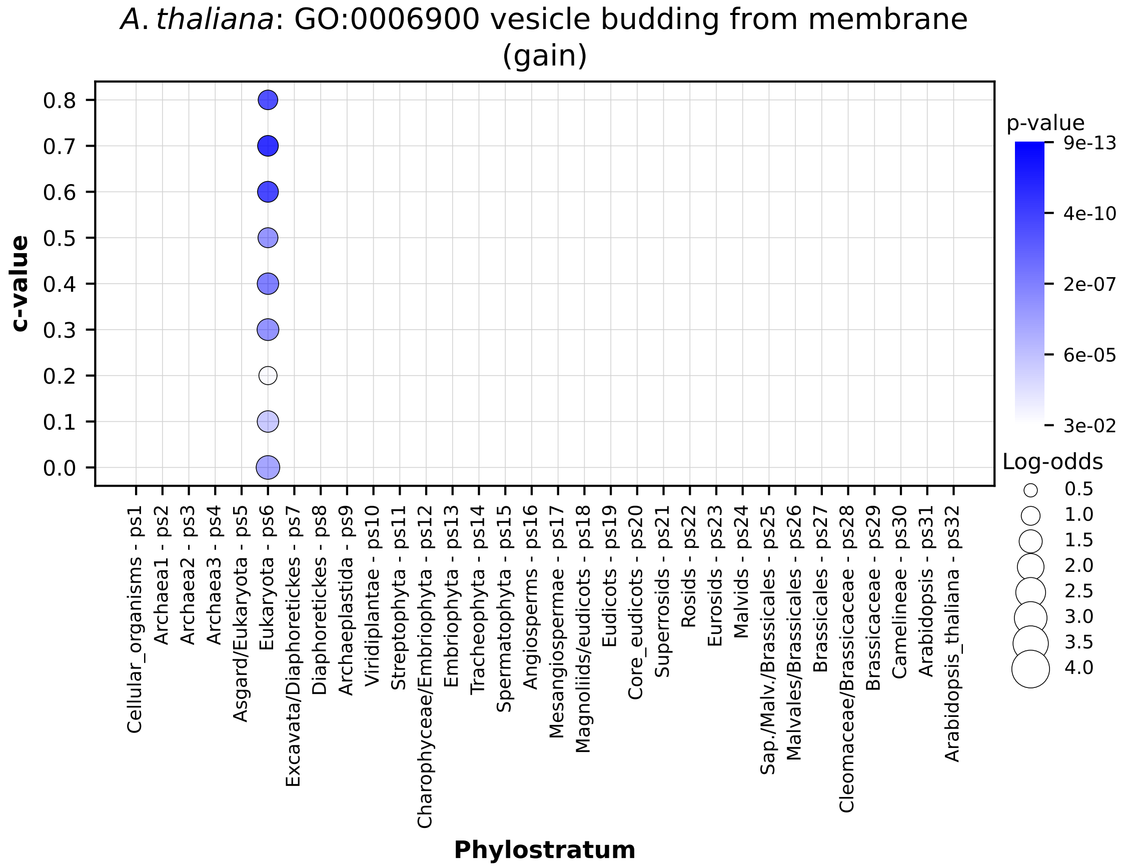

A. thaliana: GO:0006900 vesicle budding from membrane (gain)
c-value
0.8
0.7
0.6
0.5
0.4
0.3
0.2
0.1
0.0
p-value
9e-13
4e-10
2e-07
6e-05
3e-02
Log-odds
0.5
1.0
1.5
2.0
2.5
3.0
3.5
4.0
Cellular_organisms - ps1
Archaea1 - ps2
Archaea2 - ps3
Archaea3 - ps4
Asgard/Eukaryota - ps5
Eukaryota - ps6
Excavata/Diaphoretickes - ps7
Diaphoretickes - ps8
Archaeplastida - ps9
Viridiplantae - ps10
Streptophyta - ps11
Charophyceae/Embriophyta - ps12
Embriophyta - ps13
Tracheophyta - ps14
Spermatophyta - ps15
Angiosperms - ps16
Mesangiospermae - ps17
Magnoliids/eudicots - ps18
Eudicots - ps19
Core_eudicots - ps20
Superrosids - ps21
Rosids - ps22
Eurosids - ps23
Malvids - ps24
Sap./Malv./Brassicales - ps25
Malvales/Brassicales - ps26
Brassicales - ps27
Cleomaceae/Brassicaceae - ps28
Brassicaceae - ps29
Camelineae - ps30
Arabidopsis - ps31
Arabidopsis_thaliana - ps32
Phylostratum

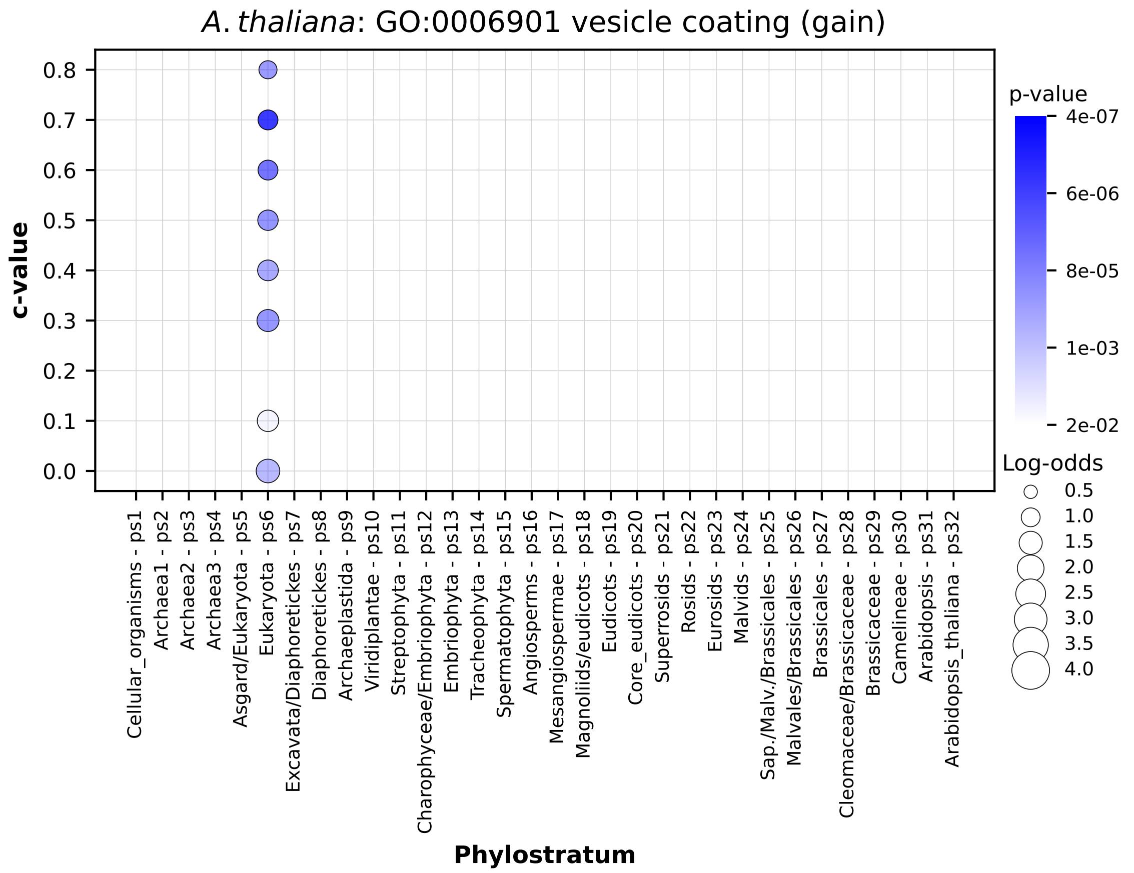

A. thaliana: GO:0006901 vesicle coating (gain)
c-value
0.0
0.1
0.2
0.3
0.4
0.5
0.6
0.7
0.8
p-value
4e-07
6e-06
8e-05
1e-03
2e-02
Log-odds
0.5
1.0
1.5
2.0
2.5
3.0
3.5
4.0
Cellular_organisms - ps1
Archaea1 - ps2
Archaea2 - ps3
Archaea3 - ps4
Asgard/Eukaryota - ps5
Eukaryota - ps6
Excavata/Diaphoretickes - ps7
Diaphoretickes - ps8
Archaeplastida - ps9
Viridiplantae - ps10
Streptophyta - ps11
Charophyceae/Embriophyta - ps12
Embriophyta - ps13
Tracheophyta - ps14
Spermatophyta - ps15
Angiosperms - ps16
Mesangiospermae - ps17
Magnoliids/eudicots - ps18
Eudicots - ps19
Core_eudicots - ps20
Superrosids - ps21
Rosids - ps22
Eurosids - ps23
Malvids - ps24
Sap./Malv./Brassicales - ps25
Malvales/Brassicales - ps26
Brassicales - ps27
Cleomaceae/Brassicaceae - ps28
Brassicaceae - ps29
Camelineae - ps30
Arabidopsis - ps31
Arabidopsis_thaliana - ps32
Phylostratum

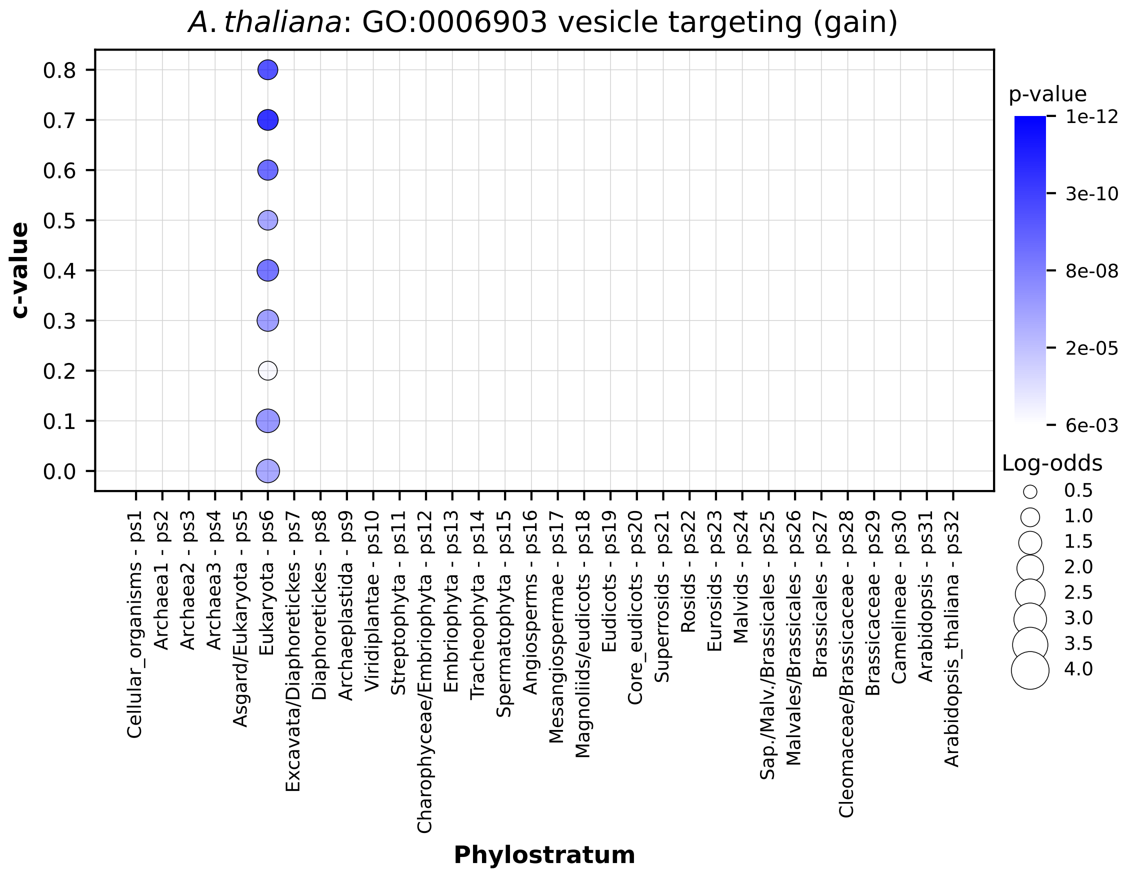

A. thaliana: GO:0006903 vesicle targeting (gain)
c-value
0.8
0.7
0.6
0.5
0.4
0.3
0.2
0.1
0.0
p-value
1e-12
3e-10
8e-08
2e-05
6e-03
Log-odds
0.5
1.0
1.5
2.0
2.5
3.0
3.5
4.0
Cellular_organisms - ps1
Archaea1 - ps2
Archaea2 - ps3
Archaea3 - ps4
Asgard/Eukaryota - ps5
Eukaryota - ps6
Excavata/Diaphoretickes - ps7
Diaphoretickes - ps8
Archaeplastida - ps9
Viridiplantae - ps10
Streptophyta - ps11
Charophyceae/Embriophyta - ps12
Embriophyta - ps13
Tracheophyta - ps14
Spermatophyta - ps15
Angiosperms - ps16
Mesangiospermae - ps17
Magnoliids/eudicots - ps18
Eudicots - ps19
Core_eudicots - ps20
Superrosids - ps21
Rosids - ps22
Eurosids - ps23
Malvids - ps24
Sap./Malv./Brassicales - ps25
Malvales/Brassicales - ps26
Brassicales - ps27
Cleomaceae/Brassicaceae - ps28
Brassicaceae - ps29
Camelineae - ps30
Arabidopsis - ps31
Arabidopsis_thaliana - ps32
Phylostratum

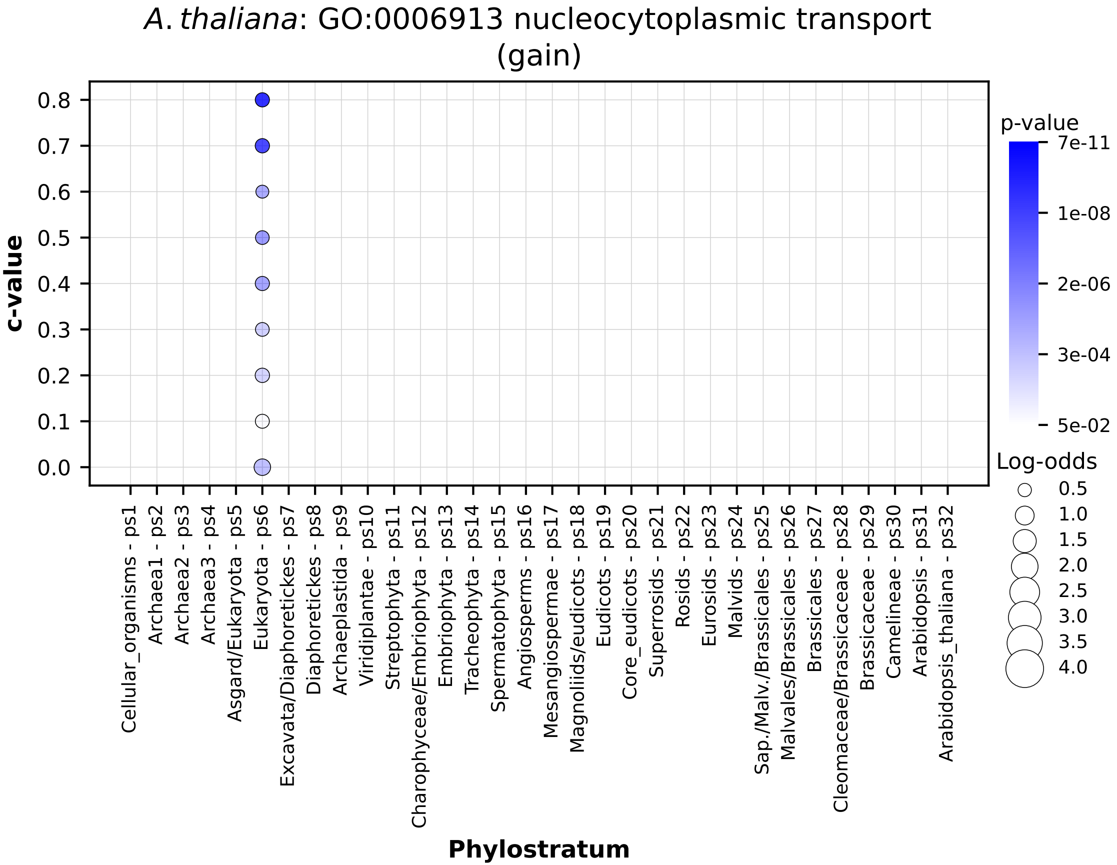
A. thaliana: GO:0006913 nucleocytoplasmic transport
(gain)
c-value
0.8
0.7
0.6
0.5
0.4
0.3
0.2
0.1
0.0
p-value
7e-11
1e-08
2e-06
3e-04
5e-02
Log-odds
0.5
1.0
1.5
2.0
2.5
3.0
3.5
4.0
Cellular_organisms - ps1
Archaea1 - ps2
Archaea2 - ps3
Archaea3 - ps4
Asgard/Eukaryota - ps5
Eukaryota - ps6
Excavata/Diaphoretickes - ps7
Diaphoretickes - ps8
Archaeplastida - ps9
Viridiplantae - ps10
Streptophyta - ps11
Charophyceae/Embriophyta - ps12
Embriophyta - ps13
Tracheophyta - ps14
Spermatophyta - ps15
Angiosperms - ps16
Mesangiospermae - ps17
Magnoliids/eudicots - ps18
Eudicots - ps19
Core_eudicots - ps20
Superrosids - ps21
Rosids - ps22
Eurosids - ps23
Malvids - ps24
Sap./Malv./Brassicales - ps25
Malvales/Brassicales - ps26
Brassicales - ps27
Cleomaceae/Brassicaceae - ps28
Brassicaceae - ps29
Camelineae - ps30
Arabidopsis - ps31
Arabidopsis_thaliana - ps32
Phylostratum

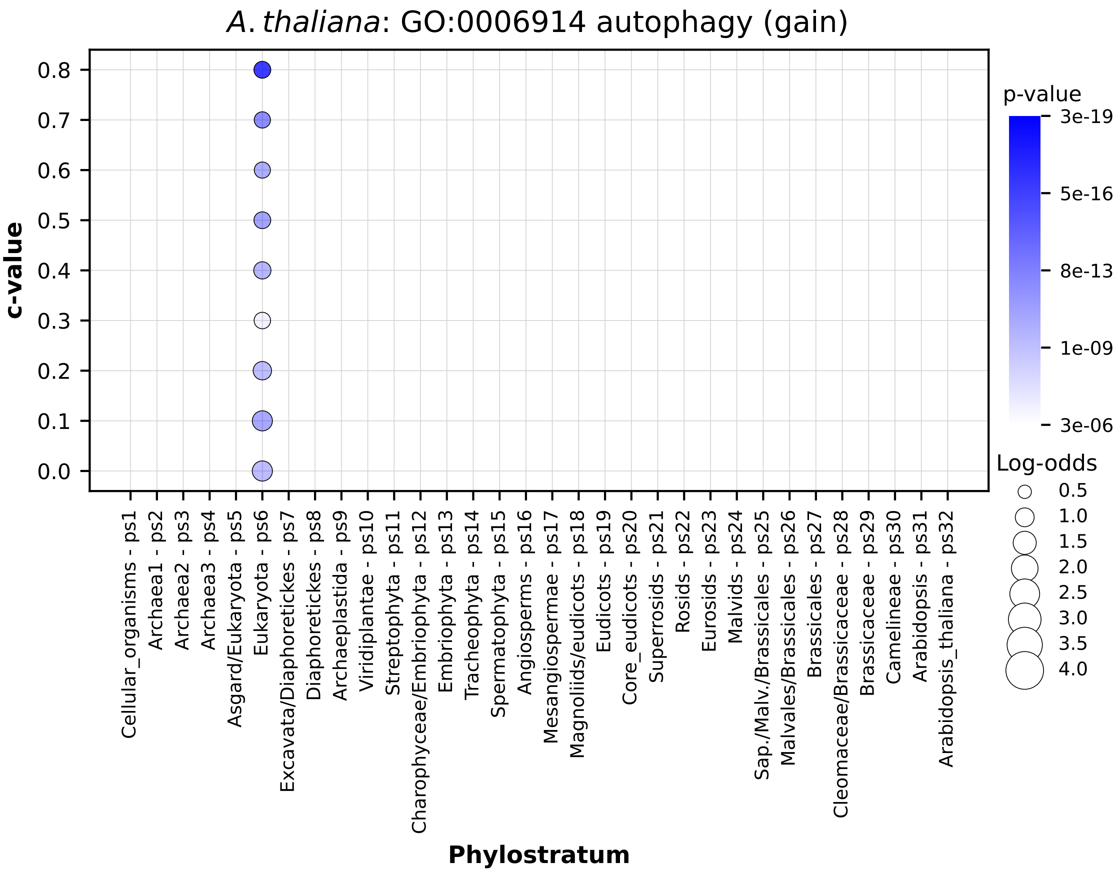
A. thaliana: GO:0006914 autophagy (gain)
c-value
0.8
0.7
0.6
0.5
0.4
0.3
0.2
0.1
0.0
p-value
3e-19
5e-16
8e-13
1e-09
3e-06
Log-odds
0.5
1.0
1.5
2.0
2.5
3.0
3.5
4.0
Cellular_organisms - ps1
Archaea1 - ps2
Archaea2 - ps3
Archaea3 - ps4
Asgard/Eukaryota - ps5
Eukaryota - ps6
Excavata/Diaphoretickes - ps7
Diaphoretickes - ps8
Archaeplastida - ps9
Viridiplantae - ps10
Streptophyta - ps11
Charophyceae/Embriophyta - ps12
Embriophyta - ps13
Tracheophyta - ps14
Spermatophyta - ps15
Angiosperms - ps16
Mesangiospermae - ps17
Magnoliids/eudicots - ps18
Eudicots - ps19
Core_eudicots - ps20
Superrosids - ps21
Rosids - ps22
Eurosids - ps23
Malvids - ps24
Sap./Malv./Brassicales - ps25
Malvales/Brassicales - ps26
Brassicales - ps27
Cleomaceae/Brassicaceae - ps28
Brassicaceae - ps29
Camelineae - ps30
Arabidopsis - ps31
Arabidopsis_thaliana - ps32
Phylostratum

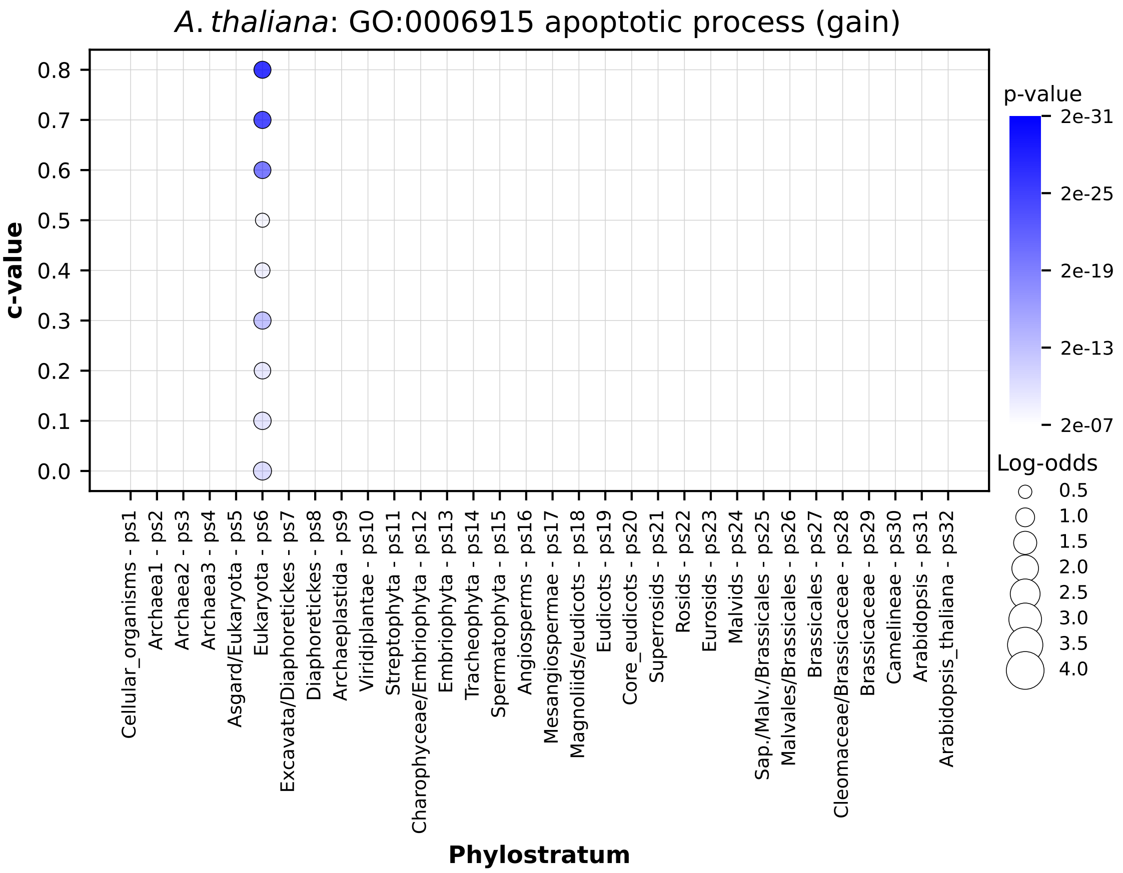

A. thaliana: GO:0006915 apoptotic process (gain)
c-value
0.0
0.1
0.2
0.3
0.4
0.5
0.6
0.7
0.8
Phylostratum
Cellular_organisms - ps1
Archaea1 - ps2
Archaea2 - ps3
Archaea3 - ps4
Asgard/Eukaryota - ps5
Eukaryota - ps6
Excavata/Diaphoretickes - ps7
Diaphoretickes - ps8
Archaeplastida - ps9
Viridiplantae - ps10
Streptophyta - ps11
Charophyceae/Embriophyta - ps12
Embriophyta - ps13
Tracheophyta - ps14
Spermatophyta - ps15
Angiosperms - ps16
Mesangiospermae - ps17
Magnoliids/eudicots - ps18
Eudicots - ps19
Core_eudicots - ps20
Superrosids - ps21
Rosids - ps22
Eurosids - ps23
Malvids - ps24
Sap./Malv./Brassicales - ps25
Malvales/Brassicales - ps26
Brassicales - ps27
Cleomaceae/Brassicaceae - ps28
Brassicaceae - ps29
Camelineae - ps30
Arabidopsis - ps31
Arabidopsis_thaliana - ps32
p-value
2e-31
2e-25
2e-19
2e-13
2e-07
Log-odds
0.5
1.0
1.5
2.0
2.5
3.0
3.5
4.0

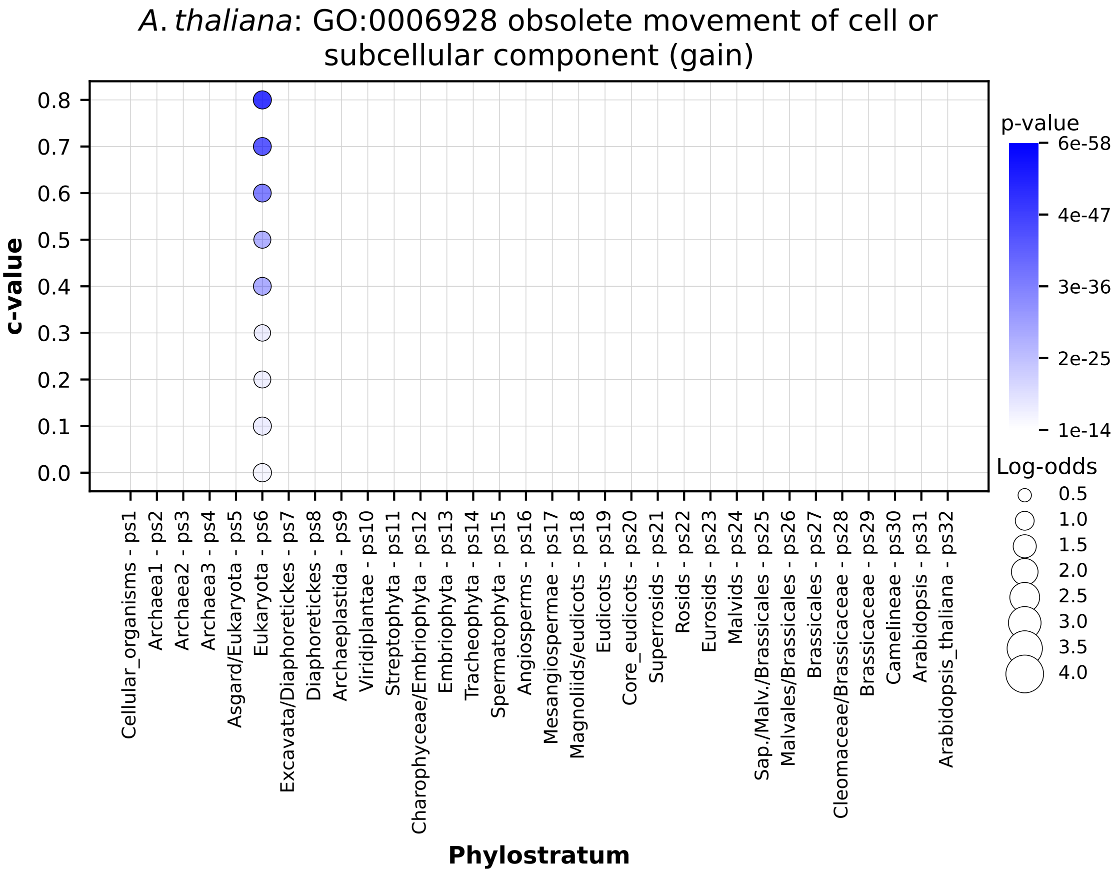

A. thaliana: GO:0006928 obsolete movement of cell or subcellular component (gain)
c-value
0.8
0.7
0.6
0.5
0.4
0.3
0.2
0.1
0.0
p-value
6e-58
4e-47
3e-36
2e-25
1e-14
Log-odds
0.5
1.0
1.5
2.0
2.5
3.0
3.5
4.0
Cellular_organisms - ps1
Archaea1 - ps2
Archaea2 - ps3
Archaea3 - ps4
Asgard/Eukaryota - ps5
Eukaryota - ps6
Excavata/Diaphoretickes - ps7
Diaphoretickes - ps8
Archaeplastida - ps9
Viridiplantae - ps10
Streptophyta - ps11
Charophyceae/Embriophyta - ps12
Embriophyta - ps13
Tracheophyta - ps14
Spermatophyta - ps15
Angiosperms - ps16
Mesangiospermae - ps17
Magnoliids/eudicots - ps18
Eudicots - ps19
Core_eudicots - ps20
Superrosids - ps21
Rosids - ps22
Eurosids - ps23
Malvids - ps24
Sap./Malv./Brassicales - ps25
Malvales/Brassicales - ps26
Brassicales - ps27
Cleomaceae/Brassicaceae - ps28
Brassicaceae - ps29
Camelineae - ps30
Arabidopsis - ps31
Arabidopsis_thaliana - ps32
Phylostratum

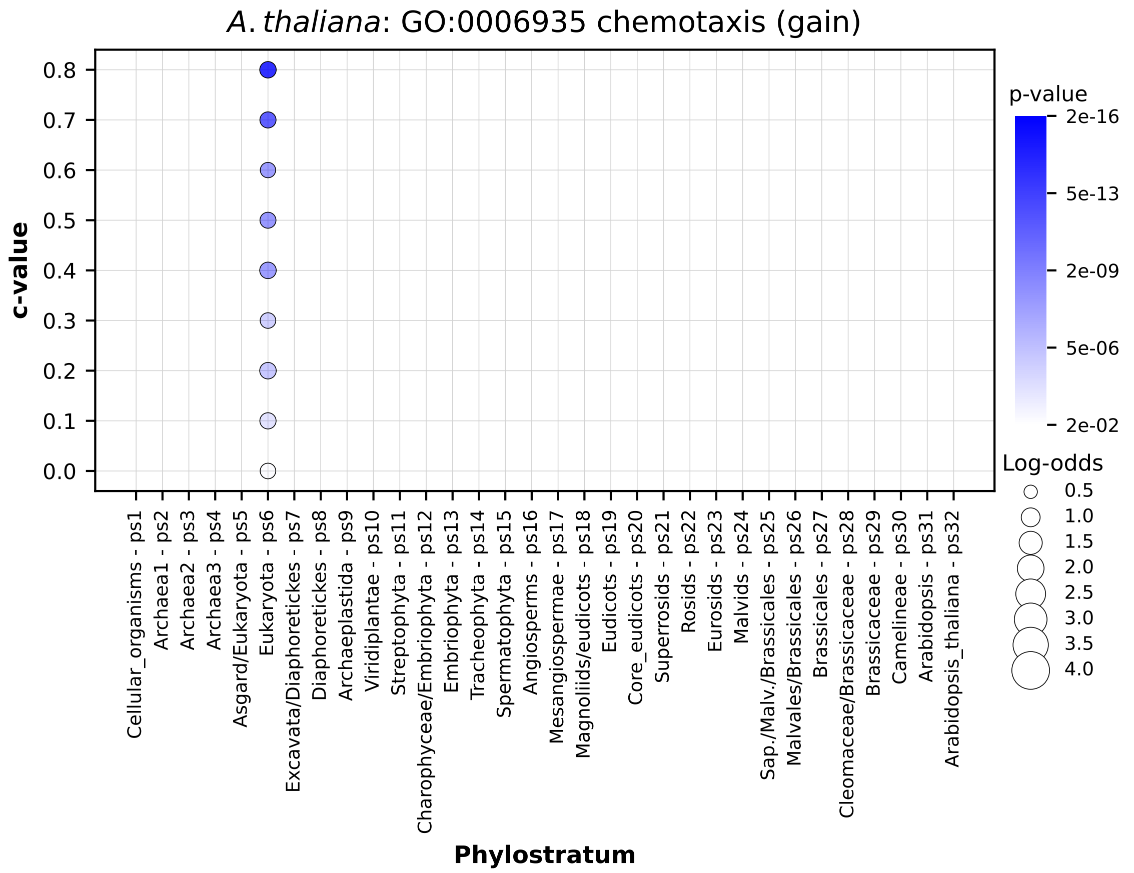

A. thaliana: GO:0006935 chemotaxis (gain)
c-value
0.8
0.7
0.6
0.5
0.4
0.3
0.2
0.1
0.0
Phylostratum
Cellular_organisms - ps1
Archaea1 - ps2
Archaea2 - ps3
Archaea3 - ps4
Asgard/Eukaryota - ps5
Eukaryota - ps6
Excavata/Diaphoretickes - ps7
Diaphoretickes - ps8
Archaeplastida - ps9
Viridiplantae - ps10
Streptophyta - ps11
Charophyceae/Embriophyta - ps12
Embriophyta - ps13
Tracheophyta - ps14
Spermatophyta - ps15
Angiosperms - ps16
Mesangiospermae - ps17
Magnoliids/eudicots - ps18
Eudicots - ps19
Core_eudicots - ps20
Superrosids - ps21
Rosids - ps22
Eurosids - ps23
Malvids - ps24
Sap./Malv./Brassicales - ps25
Malvales/Brassicales - ps26
Brassicales - ps27
Cleomaceae/Brassicaceae - ps28
Brassicaceae - ps29
Camelineae - ps30
Arabidopsis - ps31
Arabidopsis_thaliana - ps32
p-value
2e-16
5e-13
2e-09
5e-06
2e-02
Log-odds
0.5
1.0
1.5
2.0
2.5
3.0
3.5
4.0

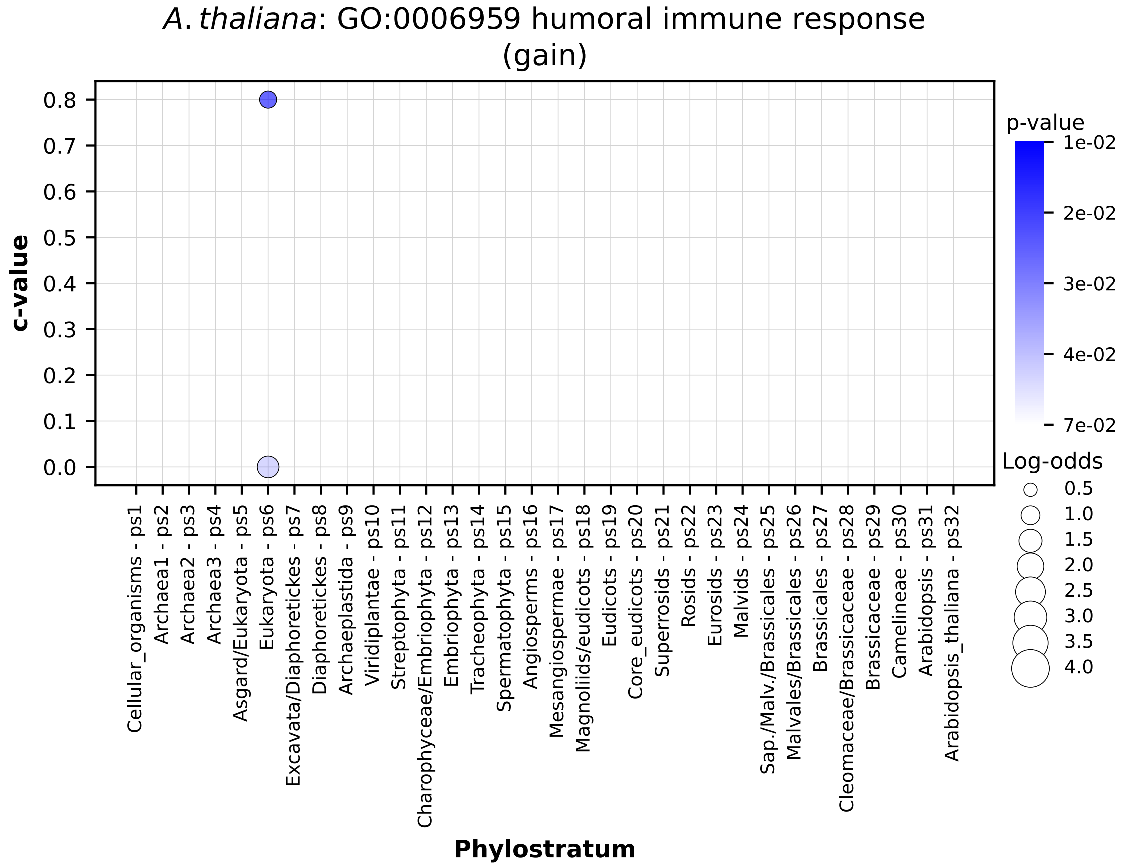

A. thaliana: GO:0006959 humoral immune response
(gain)
c-value
0.8
0.7
0.6
0.5
0.4
0.3
0.2
0.1
0.0
p-value
1e-02
2e-02
3e-02
4e-02
7e-02
Log-odds
0.5
1.0
1.5
2.0
2.5
3.0
3.5
4.0
Cellular_organisms - ps1
Archaea1 - ps2
Archaea2 - ps3
Archaea3 - ps4
Asgard/Eukaryota - ps5
Eukaryota - ps6
Excavata/Diaphoretickes - ps7
Diaphoretickes - ps8
Archaeplastida - ps9
Viridiplantae - ps10
Streptophyta - ps11
Charophyceae/Embriophyta - ps12
Embriophyta - ps13
Tracheophyta - ps14
Spermatophyta - ps15
Angiosperms - ps16
Mesangiospermae - ps17
Magnoliids/eudicots - ps18
Eudicots - ps19
Core_eudicots - ps20
Superrosids - ps21
Rosids - ps22
Eurosids - ps23
Malvids - ps24
Sap./Malv./Brassicales - ps25
Malvales/Brassicales - ps26
Brassicales - ps27
Cleomaceae/Brassicaceae - ps28
Brassicaceae - ps29
Camelineae - ps30
Arabidopsis - ps31
Arabidopsis_thaliana - ps32
Phylostratum

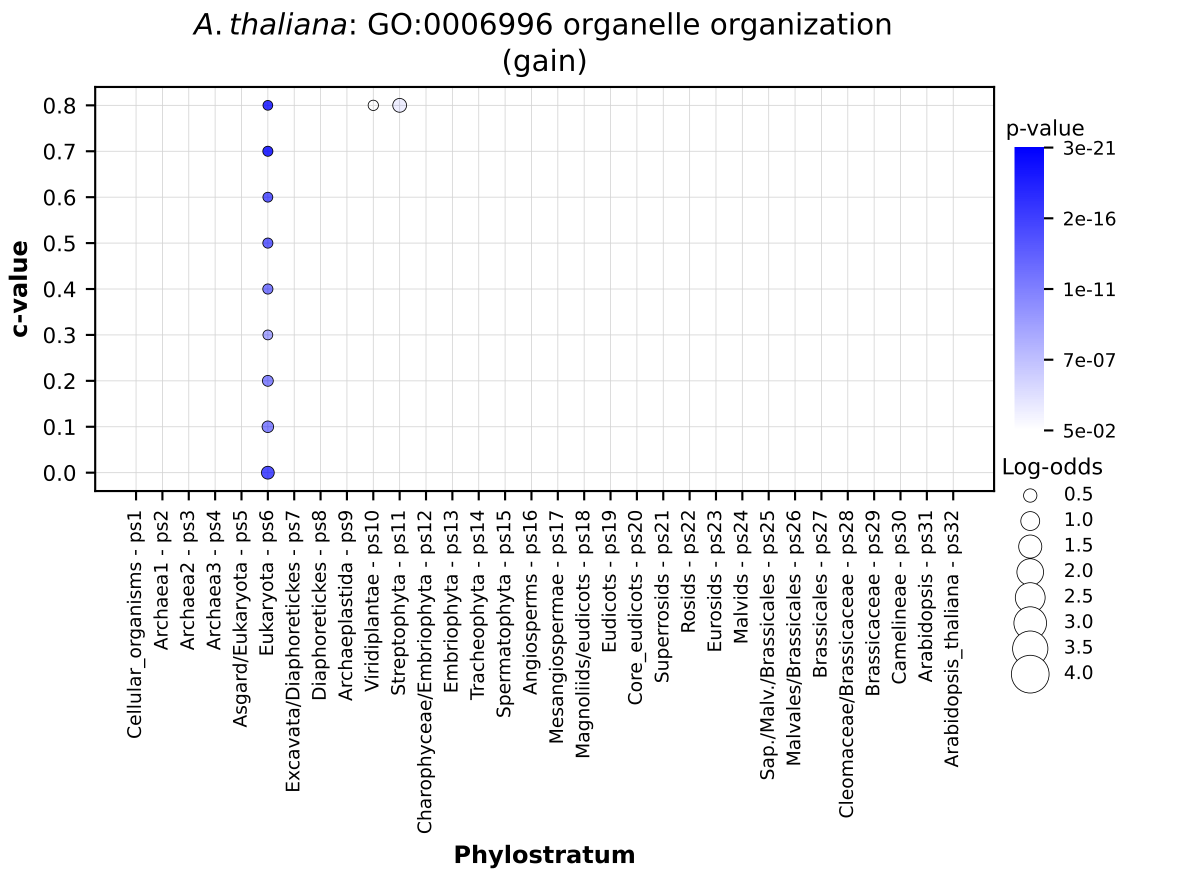

A. thaliana: GO:0006996 organelle organization
(gain)
c-value
0.8
0.7
0.6
0.5
0.4
0.3
0.2
0.1
0.0
p-value
3e-21
2e-16
1e-11
7e-07
5e-02
Log-odds
0.5
1.0
1.5
2.0
2.5
3.0
3.5
4.0
Cellular_organisms - ps1
Archaea1 - ps2
Archaea2 - ps3
Archaea3 - ps4
Asgard/Eukaryota - ps5
Eukaryota - ps6
Excavata/Diaphoretickes - ps7
Diaphoretickes - ps8
Archaeplastida - ps9
Viridiplantae - ps10
Streptophyta - ps11
Charophyceae/Embriophyta - ps12
Embriophyta - ps13
Tracheophyta - ps14
Spermatophyta - ps15
Angiosperms - ps16
Mesangiospermae - ps17
Magnoliids/eudicots - ps18
Eudicots - ps19
Core_eudicots - ps20
Superrosids - ps21
Rosids - ps22
Eurosids - ps23
Malvids - ps24
Sap./Malv./Brassicales - ps25
Malvales/Brassicales - ps26
Brassicales - ps27
Cleomaceae/Brassicaceae - ps28
Brassicaceae - ps29
Camelineae - ps30
Arabidopsis - ps31
Arabidopsis_thaliana - ps32
Phylostratum

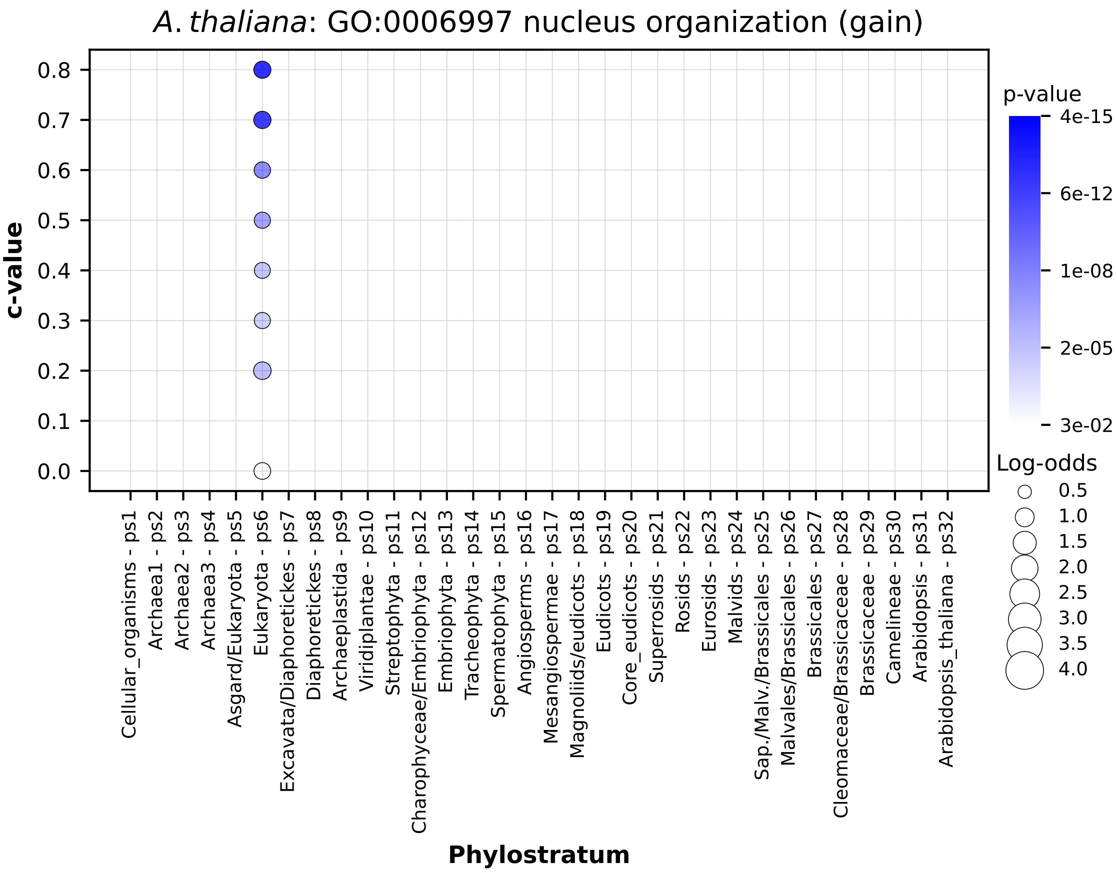
A. thaliana: GO:0006997 nucleus organization (gain)
c-value
0.8
0.7
0.6
0.5
0.4
0.3
0.2
0.1
0.0
p-value
4e-15
6e-12
1e-08
2e-05
3e-02
Log-odds
0.5
1.0
1.5
2.0
2.5
3.0
3.5
4.0
Cellular_organisms - ps1
Archaea1 - ps2
Archaea2 - ps3
Archaea3 - ps4
Asgard/Eukaryota - ps5
Eukaryota - ps6
Excavata/Diaphoretickes - ps7
Diaphoretickes - ps8
Archaeplastida - ps9
Viridiplantae - ps10
Streptophyta - ps11
Charophyceae/Embriophyta - ps12
Embriophyta - ps13
Tracheophyta - ps14
Spermatophyta - ps15
Angiosperms - ps16
Mesangiospermae - ps17
Magnoliids/eudicots - ps18
Eudicots - ps19
Core_eudicots - ps20
Superrosids - ps21
Rosids - ps22
Eurosids - ps23
Malvids - ps24
Sap./Malv./Brassicales - ps25
Malvales/Brassicales - ps26
Brassicales - ps27
Cleomaceae/Brassicaceae - ps28
Brassicaceae - ps29
Camelineae - ps30
Arabidopsis - ps31
Arabidopsis_thaliana - ps32
Phylostratum

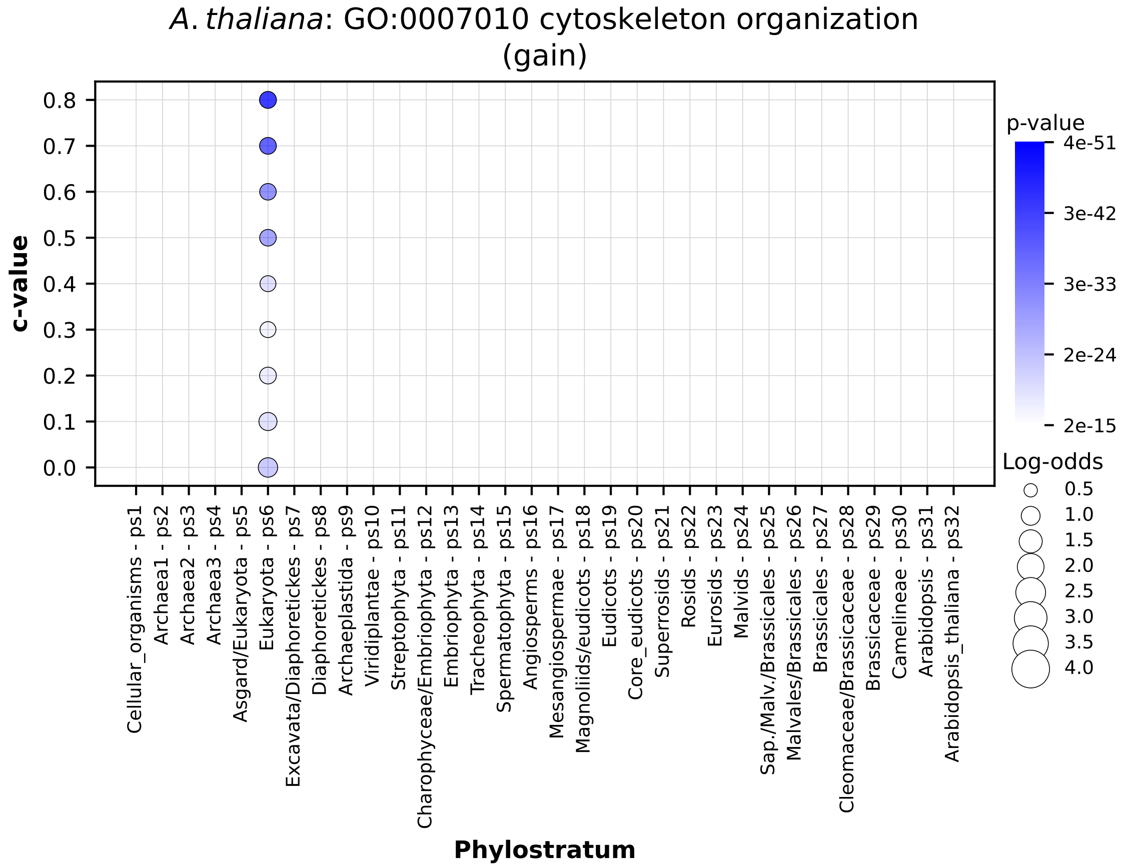

A. thaliana: GO:0007010 cytoskeleton organization
(gain)
c-value
0.8
0.7
0.6
0.5
0.4
0.3
0.2
0.1
0.0
p-value
4e-51
3e-42
3e-33
2e-24
2e-15
Log-odds
0.5
1.0
1.5
2.0
2.5
3.0
3.5
4.0
Cellular_organisms - ps1
Archaea1 - ps2
Archaea2 - ps3
Archaea3 - ps4
Asgard/Eukaryota - ps5
Eukaryota - ps6
Excavata/Diaphoretickes - ps7
Diaphoretickes - ps8
Archaeplastida - ps9
Viridiplantae - ps10
Streptophyta - ps11
Charophyceae/Embriophyta - ps12
Embriophyta - ps13
Tracheophyta - ps14
Spermatophyta - ps15
Angiosperms - ps16
Mesangiospermae - ps17
Magnoliids/eudicots - ps18
Eudicots - ps19
Core_eudicots - ps20
Superrosids - ps21
Rosids - ps22
Eurosids - ps23
Malvids - ps24
Sap./Malv./Brassicales - ps25
Malvales/Brassicales - ps26
Brassicales - ps27
Cleomaceae/Brassicaceae - ps28
Brassicaceae - ps29
Camelineae - ps30
Arabidopsis - ps31
Arabidopsis_thaliana - ps32
Phylostratum

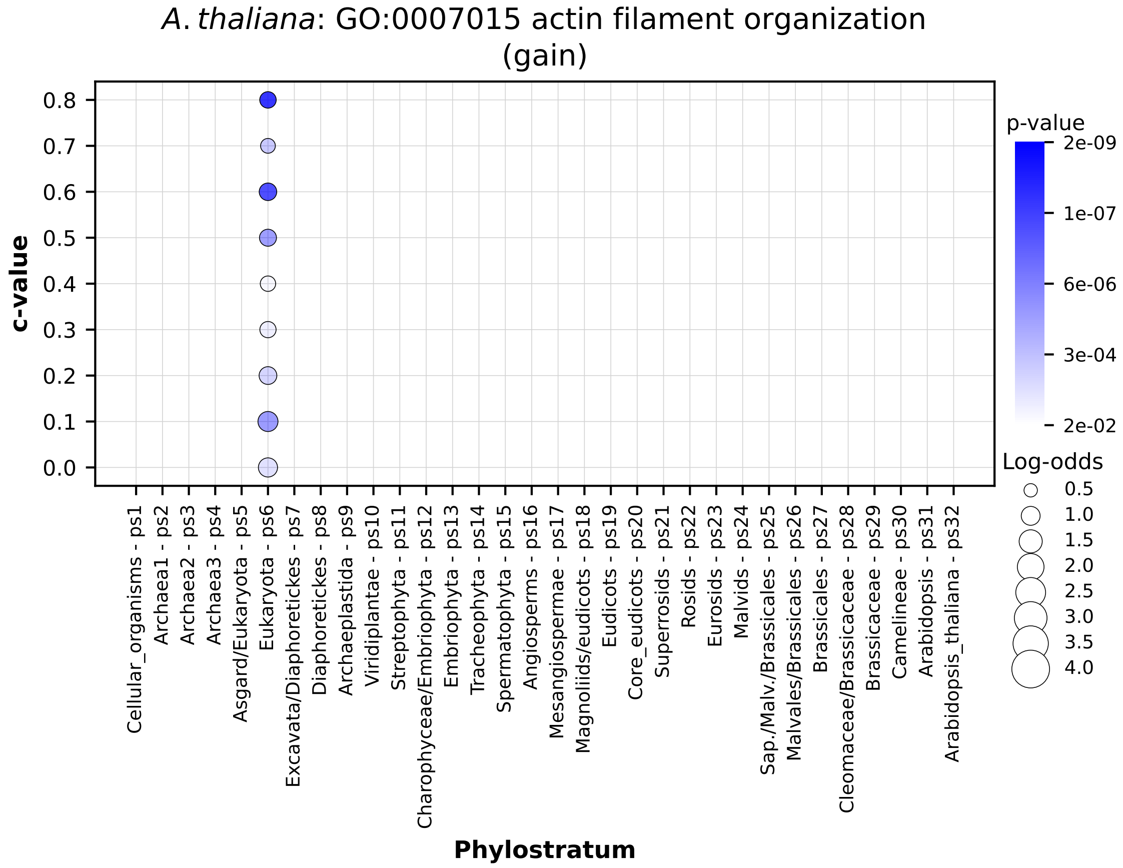

$A. thaliana$: GO:0007015 actin filament organization (gain)
c-value
0.0
0.1
0.2
0.3
0.4
0.5
0.6
0.7
0.8
p-value
2e-09
1e-07
6e-06
3e-04
2e-02
Log-odds
0.5
1.0
1.5
2.0
2.5
3.0
3.5
4.0
Cellular_organisms - ps1
Archaea1 - ps2
Archaea2 - ps3
Archaea3 - ps4
Asgard/Eukaryota - ps5
Eukaryota - ps6
Excavata/Diaphoretickes - ps7
Diaphoretickes - ps8
Archaeplastida - ps9
Viridiplantae - ps10
Streptophyta - ps11
Charophyceae/Embriophyta - ps12
Embriophyta - ps13
Tracheophyta - ps14
Spermatophyta - ps15
Angiosperms - ps16
Mesangiospermae - ps17
Magnoliids/eudicots - ps18
Eudicots - ps19
Core_eudicots - ps20
Superrosids - ps21
Rosids - ps22
Eurosids - ps23
Malvids - ps24
Sap./Malv./Brassicales - ps25
Malvales/Brassicales - ps26
Brassicales - ps27
Cleomaceae/Brassicaceae - ps28
Brassicaceae - ps29
Camelineae - ps30
Arabidopsis - ps31
Arabidopsis_thaliana - ps32
Phylostratum

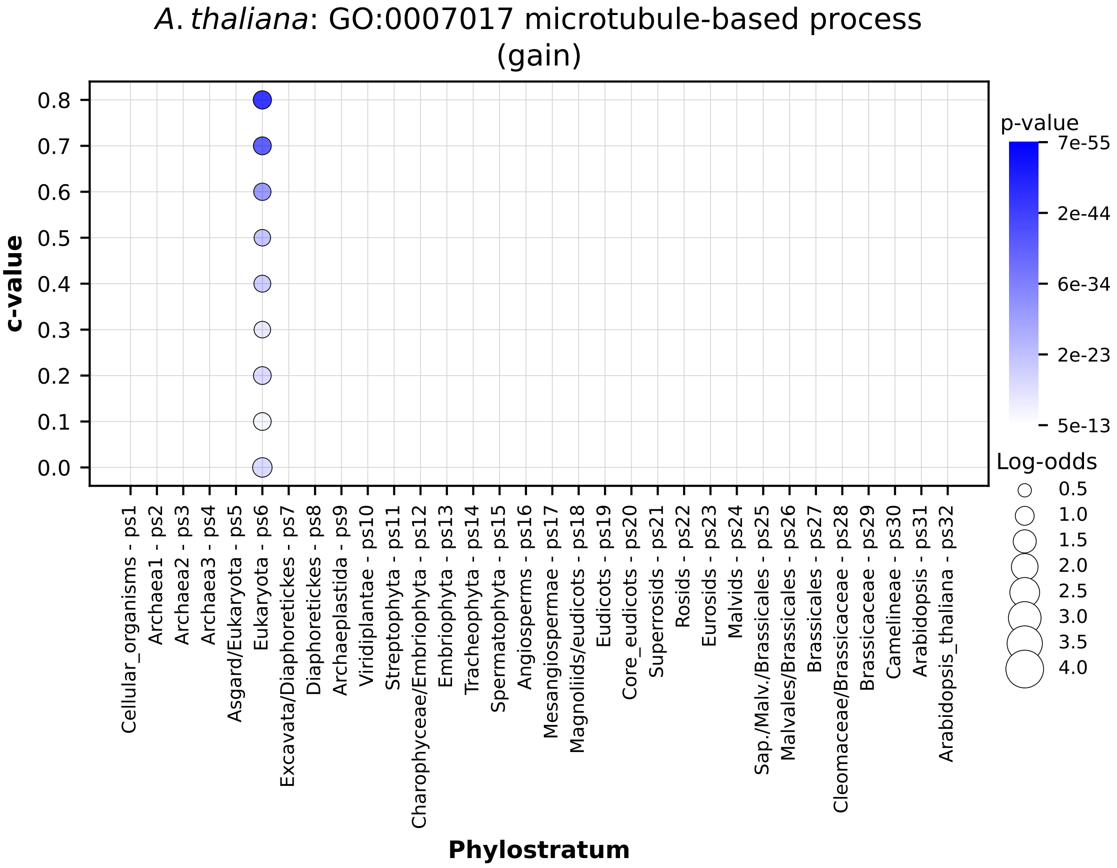

A. thaliana: GO:0007017 microtubule-based process
(gain)
c-value
0.8
0.7
0.6
0.5
0.4
0.3
0.2
0.1
0.0
Phylostratum
Cellular_organisms - ps1
Archaea1 - ps2
Archaea2 - ps3
Archaea3 - ps4
Asgard/Eukaryota - ps5
Eukaryota - ps6
Excavata/Diaphoretickes - ps7
Diaphoretickes - ps8
Archaeplastida - ps9
Viridiplantae - ps10
Streptophyta - ps11
Charophyceae/Embriophyta - ps12
Embriophyta - ps13
Tracheophyta - ps14
Spermatophyta - ps15
Angiosperms - ps16
Mesangiospermae - ps17
Magnoliids/eudicots - ps18
Eudicots - ps19
Core_eudicots - ps20
Superrosids - ps21
Rosids - ps22
Eurosids - ps23
Malvids - ps24
Sap./Malv./Brassicales - ps25
Malvales/Brassicales - ps26
Brassicales - ps27
Cleomaceae/Brassicaceae - ps28
Brassicaceae - ps29
Camelineae - ps30
Arabidopsis - ps31
Arabidopsis_thaliana - ps32
p-value
7e-55
2e-44
6e-34
2e-23
5e-13
Log-odds
0.5
1.0
1.5
2.0
2.5
3.0
3.5
4.0

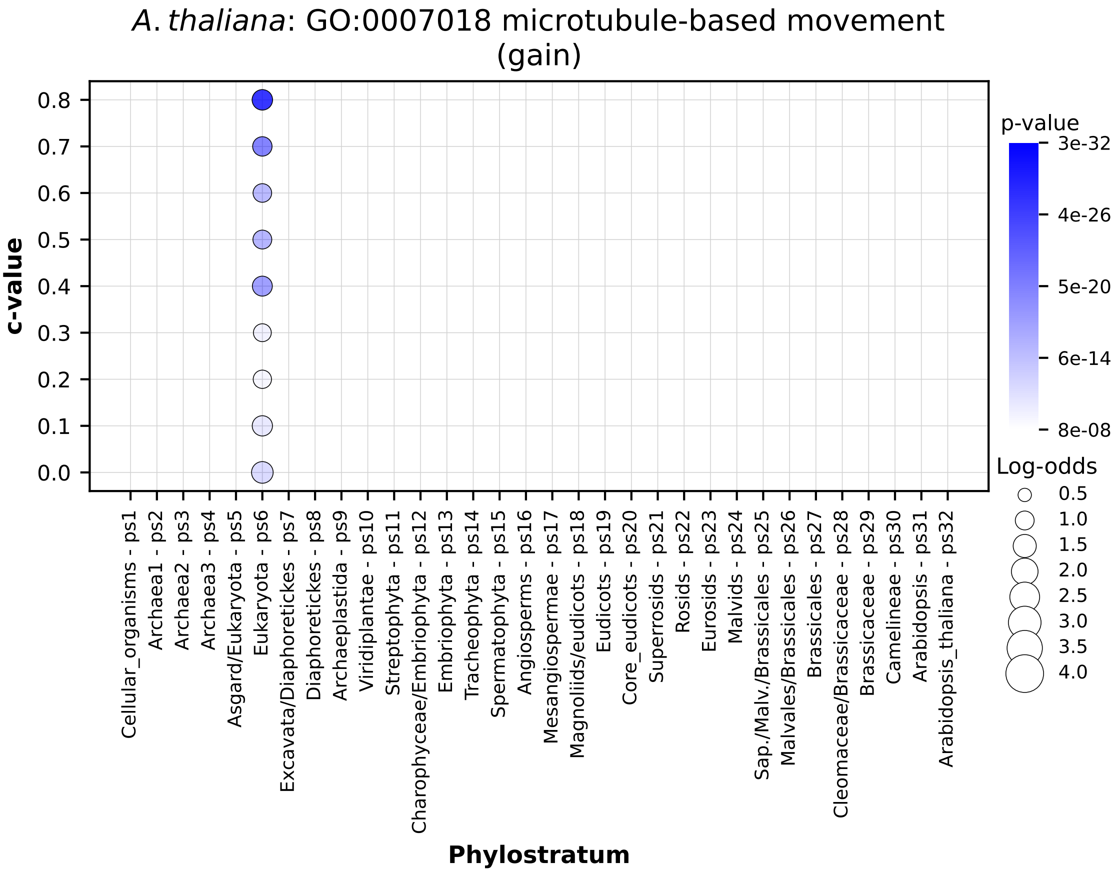

A. thaliana: GO:0007018 microtubule-based movement (gain)
c-value
0.8
0.7
0.6
0.5
0.4
0.3
0.2
0.1
0.0
p-value
3e-32
4e-26
5e-20
6e-14
8e-08
Log-odds
0.5
1.0
1.5
2.0
2.5
3.0
3.5
4.0
Cellular_organisms - ps1
Archaea1 - ps2
Archaea2 - ps3
Archaea3 - ps4
Asgard/Eukaryota - ps5
Eukaryota - ps6
Excavata/Diaphoretickes - ps7
Diaphoretickes - ps8
Archaeplastida - ps9
Viridiplantae - ps10
Streptophyta - ps11
Charophyceae/Embriophyta - ps12
Embriophyta - ps13
Tracheophyta - ps14
Spermatophyta - ps15
Angiosperms - ps16
Mesangiospermae - ps17
Magnoliids/eudicots - ps18
Eudicots - ps19
Core_eudicots - ps20
Superrosids - ps21
Rosids - ps22
Eurosids - ps23
Malvids - ps24
Sap./Malv./Brassicales - ps25
Malvales/Brassicales - ps26
Brassicales - ps27
Cleomaceae/Brassicaceae - ps28
Brassicaceae - ps29
Camelineae - ps30
Arabidopsis - ps31
Arabidopsis_thaliana - ps32
Phylostratum

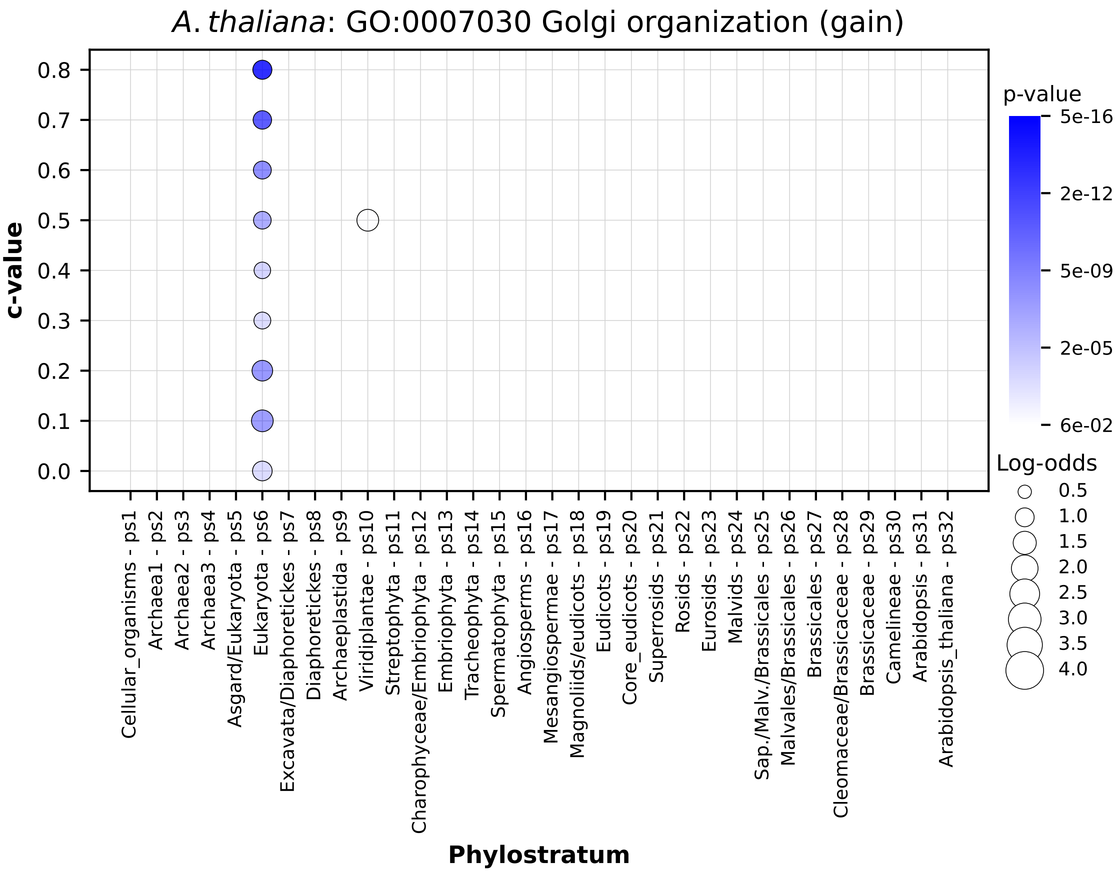

A. thaliana: GO:0007030 Golgi organization (gain)
c-value
0.8
0.7
0.6
0.5
0.4
0.3
0.2
0.1
0.0
p-value
5e-16
2e-12
5e-09
2e-05
6e-02
Log-odds
0.5
1.0
1.5
2.0
2.5
3.0
3.5
4.0
Cellular_organisms - ps1
Archaea1 - ps2
Archaea2 - ps3
Archaea3 - ps4
Asgard/Eukaryota - ps5
Eukaryota - ps6
Excavata/Diaphoretickes - ps7
Diaphoretickes - ps8
Archaeplastida - ps9
Viridiplantae - ps10
Streptophyta - ps11
Charophyceae/Embriophyta - ps12
Embriophyta - ps13
Tracheophyta - ps14
Spermatophyta - ps15
Angiosperms - ps16
Mesangiospermae - ps17
Magnoliids/eudicots - ps18
Eudicots - ps19
Core_eudicots - ps20
Superrosids - ps21
Rosids - ps22
Eurosids - ps23
Malvids - ps24
Sap./Malv./Brassicales - ps25
Malvales/Brassicales - ps26
Brassicales - ps27
Cleomaceae/Brassicaceae - ps28
Brassicaceae - ps29
Camelineae - ps30
Arabidopsis - ps31
Arabidopsis_thaliana - ps32
Phylostratum

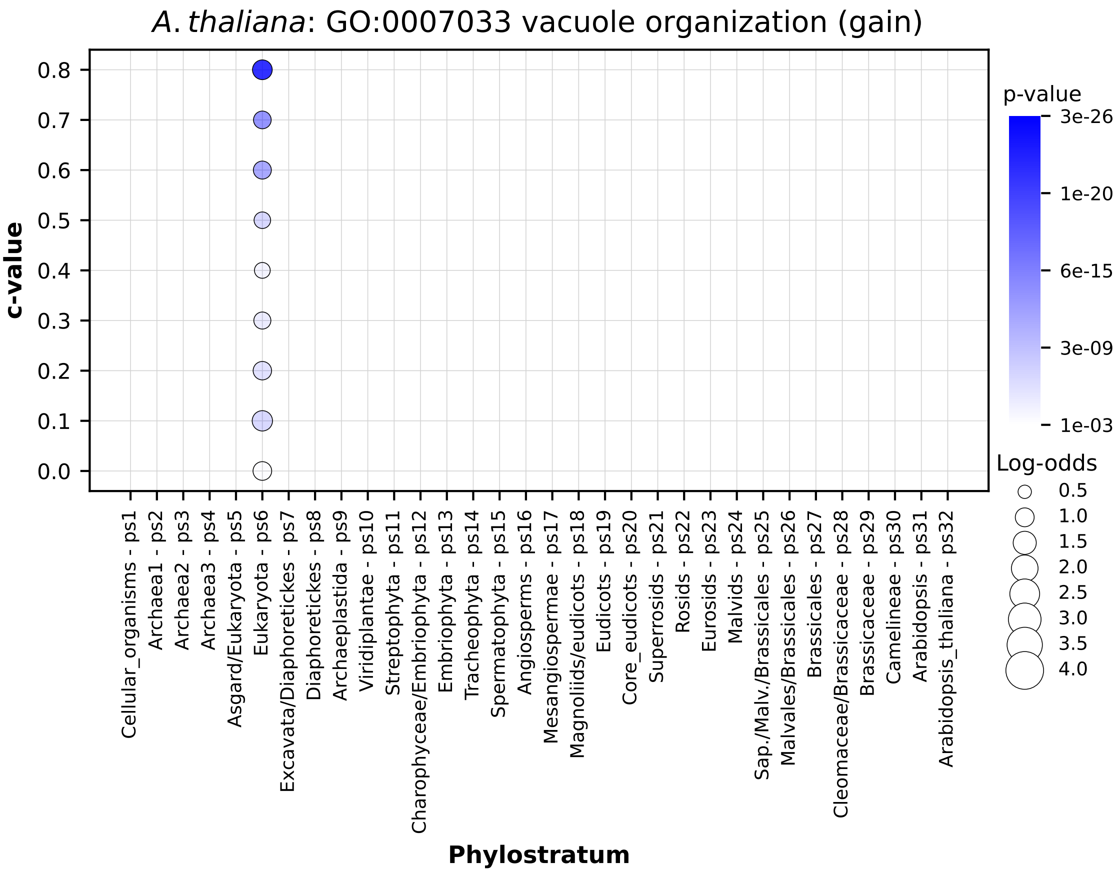
A. thaliana: GO:0007033 vacuole organization (gain)
c-value
0.0
0.1
0.2
0.3
0.4
0.5
0.6
0.7
0.8
Phylostratum
Cellular_organisms - ps1
Archaea1 - ps2
Archaea2 - ps3
Archaea3 - ps4
Asgard/Eukaryota - ps5
Eukaryota - ps6
Excavata/Diaphoretickes - ps7
Diaphoretickes - ps8
Archaeplastida - ps9
Viridiplantae - ps10
Streptophyta - ps11
Charophyceae/Embriophyta - ps12
Embriophyta - ps13
Tracheophyta - ps14
Spermatophyta - ps15
Angiosperms - ps16
Mesangiospermae - ps17
Magnoliids/eudicots - ps18
Eudicots - ps19
Core_eudicots - ps20
Superrosids - ps21
Rosids - ps22
Eurosids - ps23
Malvids - ps24
Sap./Malv./Brassicales - ps25
Malvales/Brassicales - ps26
Brassicales - ps27
Cleomaceae/Brassicaceae - ps28
Brassicaceae - ps29
Camelineae - ps30
Arabidopsis - ps31
Arabidopsis_thaliana - ps32
p-value
3e-26
1e-20
6e-15
3e-09
1e-03
Log-odds
0.5
1.0
1.5
2.0
2.5
3.0
3.5
4.0

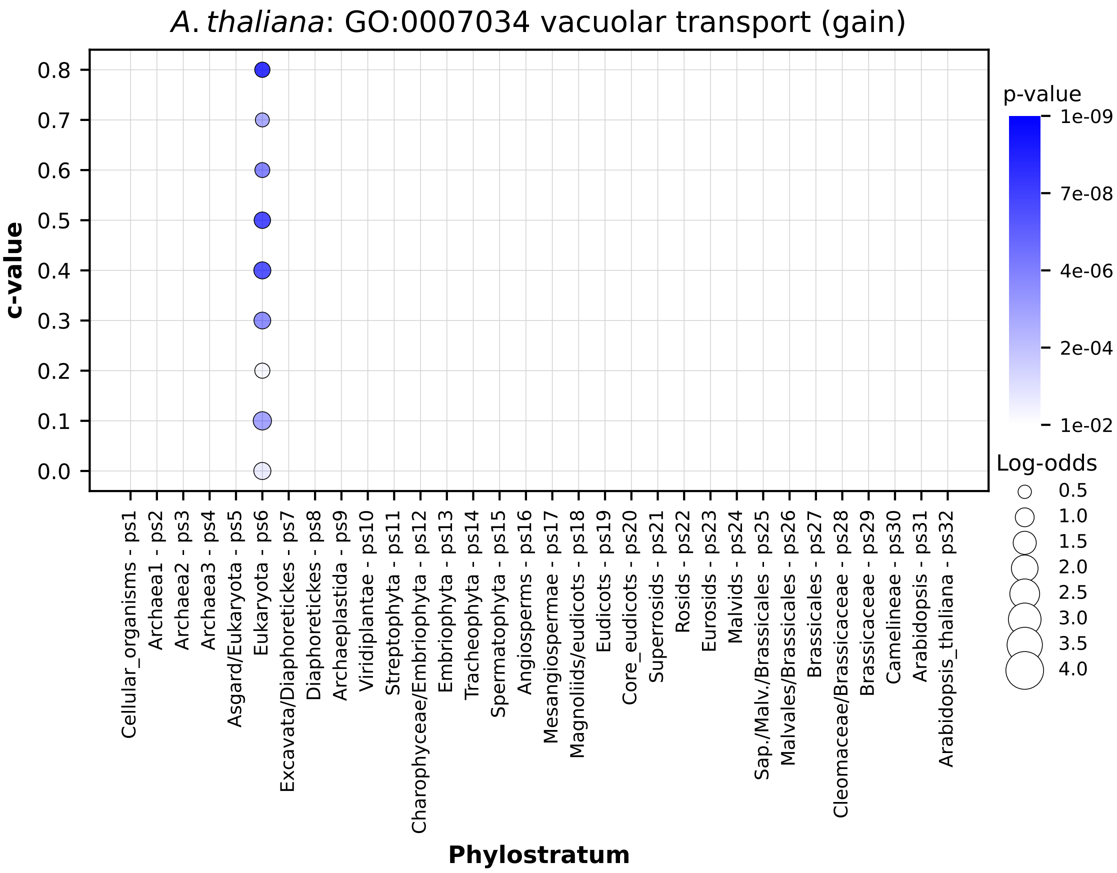

A. thaliana: GO:0007034 vacuolar transport (gain)
c-value
0.8
0.7
0.6
0.5
0.4
0.3
0.2
0.1
0.0
Phylostratum
Cellular_organisms - ps1
Archaea1 - ps2
Archaea2 - ps3
Archaea3 - ps4
Asgard/Eukaryota - ps5
Eukaryota - ps6
Excavata/Diaphoretickes - ps7
Diaphoretickes - ps8
Archaeplastida - ps9
Viridiplantae - ps10
Streptophyta - ps11
Charophyceae/Embriophyta - ps12
Embriophyta - ps13
Tracheophyta - ps14
Spermatophyta - ps15
Angiosperms - ps16
Mesangiospermae - ps17
Magnoliids/eudicots - ps18
Eudicots - ps19
Core_eudicots - ps20
Superrosids - ps21
Rosids - ps22
Eurosids - ps23
Malvids - ps24
Sap./Malv./Brassicales - ps25
Malvales/Brassicales - ps26
Brassicales - ps27
Cleomaceae/Brassicaceae - ps28
Brassicaceae - ps29
Camelineae - ps30
Arabidopsis - ps31
Arabidopsis_thaliana - ps32
p-value
1e-09
7e-08
4e-06
2e-04
1e-02
Log-odds
0.5
1.0
1.5
2.0
2.5
3.0
3.5
4.0

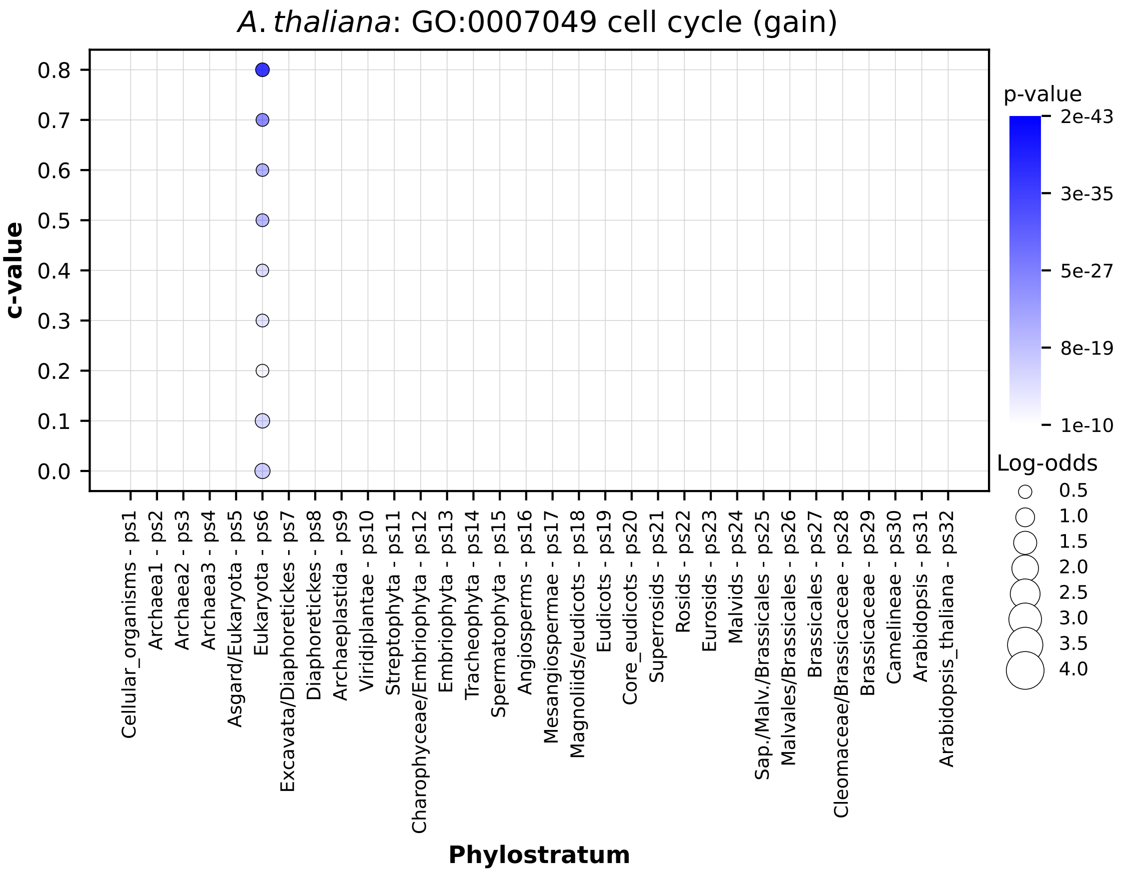
A. thaliana: GO:0007049 cell cycle (gain)
c-value
0.8
0.7
0.6
0.5
0.4
0.3
0.2
0.1
0.0
Phylostratum
Cellular_organisms - ps1
Archaea1 - ps2
Archaea2 - ps3
Archaea3 - ps4
Asgard/Eukaryota - ps5
Eukaryota - ps6
Excavata/Diaphoretickes - ps7
Diaphoretickes - ps8
Archaeplastida - ps9
Viridiplantae - ps10
Streptophyta - ps11
Charophyceae/Embriophyta - ps12
Embriophyta - ps13
Tracheophyta - ps14
Spermatophyta - ps15
Angiosperms - ps16
Mesangiospermae - ps17
Magnoliids/eudicots - ps18
Eudicots - ps19
Core_eudicots - ps20
Superrosids - ps21
Rosids - ps22
Eurosids - ps23
Malvids - ps24
Sap./Malv./Brassicales - ps25
Malvales/Brassicales - ps26
Brassicales - ps27
Cleomaceae/Brassicaceae - ps28
Brassicaceae - ps29
Camelineae - ps30
Arabidopsis - ps31
Arabidopsis_thaliana - ps32
p-value
2e-43
3e-35
5e-27
8e-19
1e-10
Log-odds
0.5
1.0
1.5
2.0
2.5
3.0
3.5
4.0

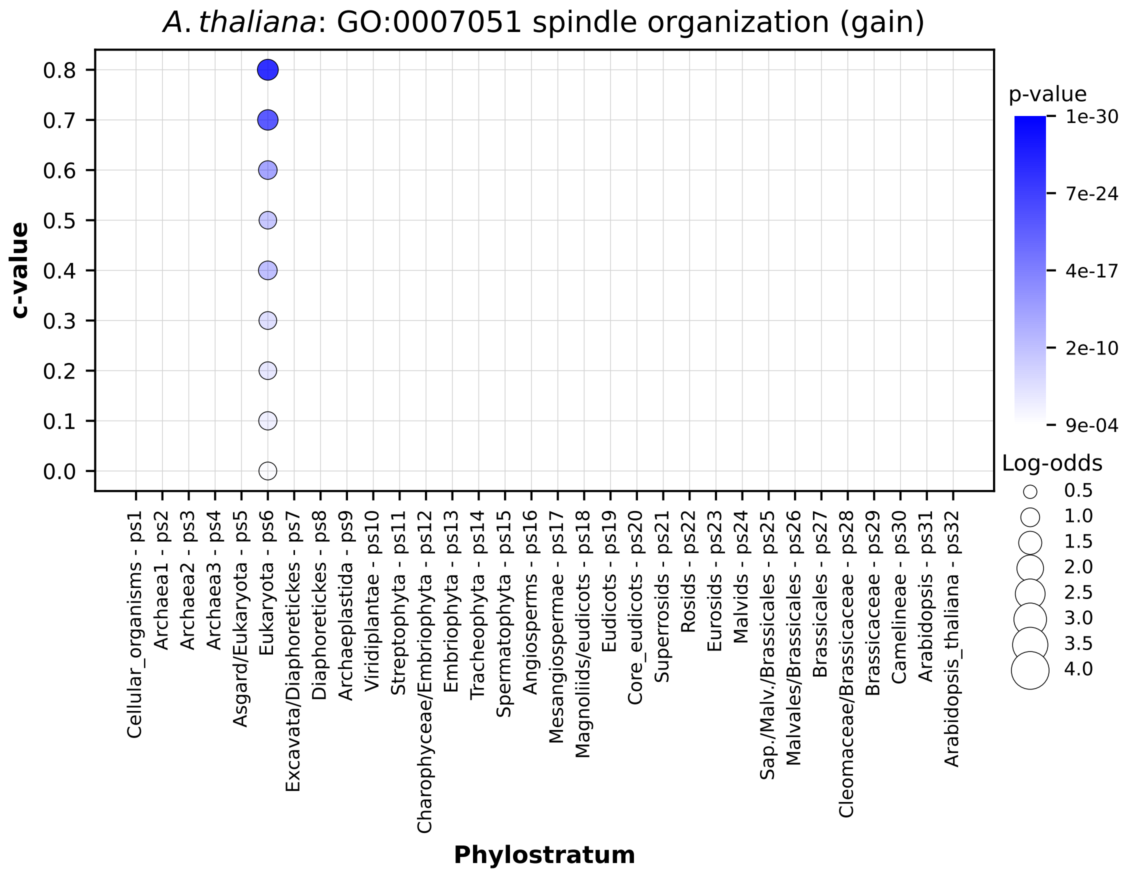
A. thaliana: GO:0007051 spindle organization (gain)
c-value
0.8
0.7
0.6
0.5
0.4
0.3
0.2
0.1
0.0
Phylostratum
Cellular_organisms - ps1
Archaea1 - ps2
Archaea2 - ps3
Archaea3 - ps4
Asgard/Eukaryota - ps5
Eukaryota - ps6
Excavata/Diaphoretickes - ps7
Diaphoretickes - ps8
Archaeplastida - ps9
Viridiplantae - ps10
Streptophyta - ps11
Charophyceae/Embriophyta - ps12
Embriophyta - ps13
Tracheophyta - ps14
Spermatophyta - ps15
Angiosperms - ps16
Mesangiospermae - ps17
Magnoliids/eudicots - ps18
Eudicots - ps19
Core_eudicots - ps20
Superrosids - ps21
Rosids - ps22
Eurosids - ps23
Malvids - ps24
Sap./Malv./Brassicales - ps25
Malvales/Brassicales - ps26
Brassicales - ps27
Cleomaceae/Brassicaceae - ps28
Brassicaceae - ps29
Camelineae - ps30
Arabidopsis - ps31
Arabidopsis_thaliana - ps32
p-value
1e-30
7e-24
4e-17
2e-10
9e-04
Log-odds
0.5
1.0
1.5
2.0
2.5
3.0
3.5
4.0

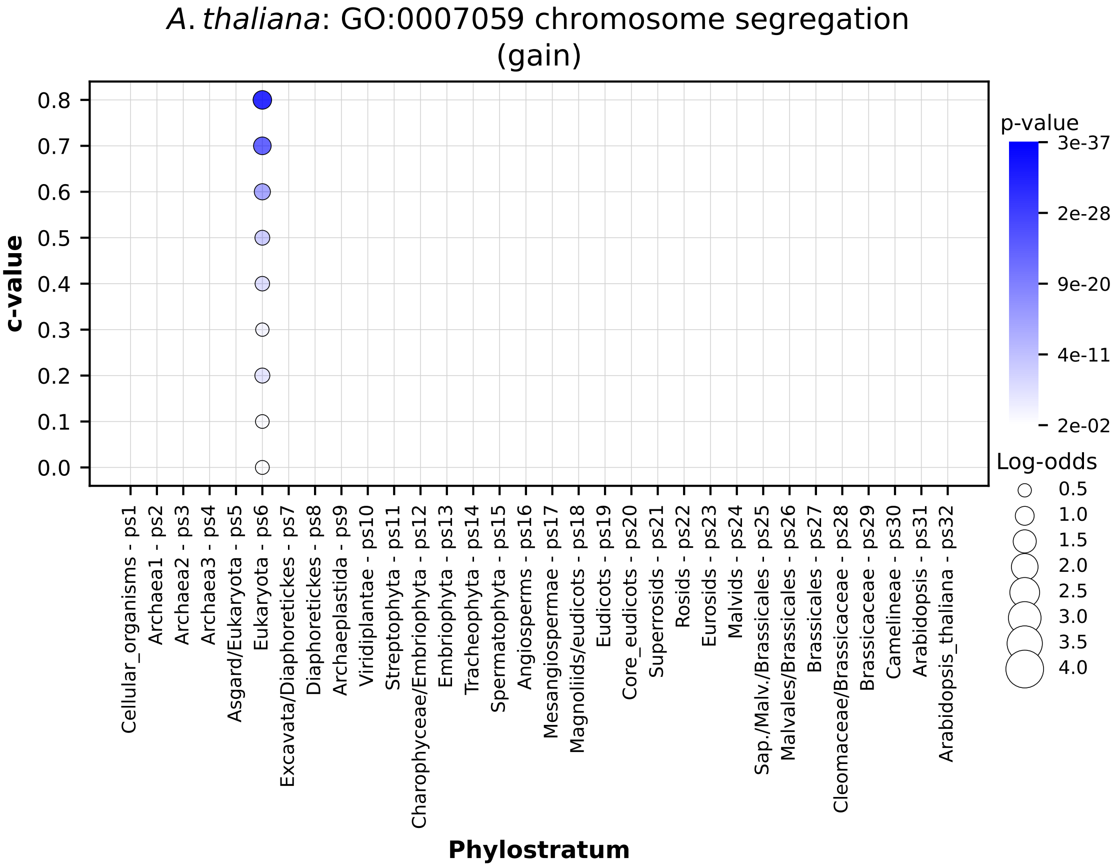
A. thaliana: GO:0007059 chromosome segregation (gain)
c-value
0.8
0.7
0.6
0.5
0.4
0.3
0.2
0.1
0.0
p-value
3e-37
2e-28
9e-20
4e-11
2e-02
Log-odds
0.5
1.0
1.5
2.0
2.5
3.0
3.5
4.0
Cellular_organisms - ps1
Archaea1 - ps2
Archaea2 - ps3
Archaea3 - ps4
Asgard/Eukaryota - ps5
Eukaryota - ps6
Excavata/Diaphoretickes - ps7
Diaphoretickes - ps8
Archaeplastida - ps9
Viridiplantae - ps10
Streptophyta - ps11
Charophyceae/Embriophyta - ps12
Embriophyta - ps13
Tracheophyta - ps14
Spermatophyta - ps15
Angiosperms - ps16
Mesangiospermae - ps17
Magnoliids/eudicots - ps18
Eudicots - ps19
Core_eudicots - ps20
Superrosids - ps21
Rosids - ps22
Eurosids - ps23
Malvids - ps24
Sap./Malv./Brassicales - ps25
Malvales/Brassicales - ps26
Brassicales - ps27
Cleomaceae/Brassicaceae - ps28
Brassicaceae - ps29
Camelineae - ps30
Arabidopsis - ps31
Arabidopsis_thaliana - ps32
Phylostratum

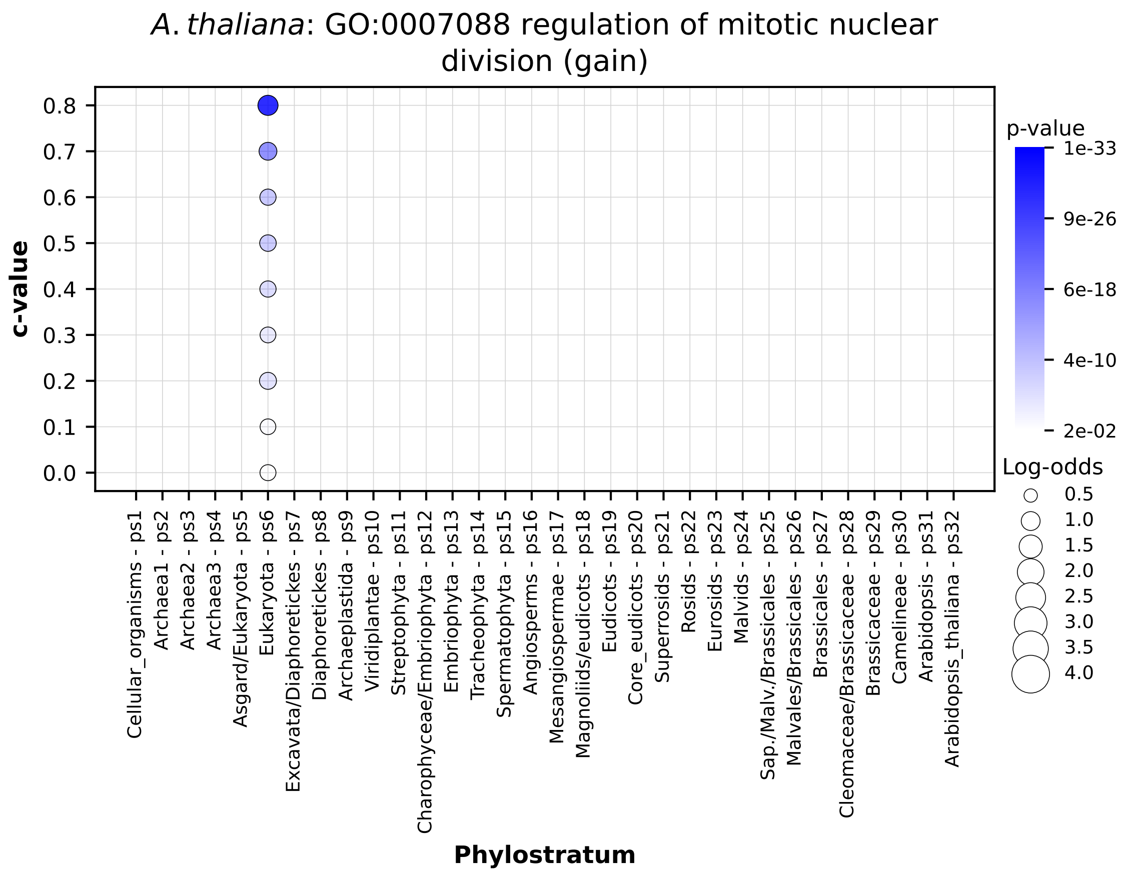

A. thaliana: GO:0007088 regulation of mitotic nuclear division (gain)
c-value
0.8
0.7
0.6
0.5
0.4
0.3
0.2
0.1
0.0
p-value
1e-33
9e-26
6e-18
4e-10
2e-02
Log-odds
0.5
1.0
1.5
2.0
2.5
3.0
3.5
4.0
Cellular_organisms - ps1
Archaea1 - ps2
Archaea2 - ps3
Archaea3 - ps4
Asgard/Eukaryota - ps5
Eukaryota - ps6
Excavata/Diaphoretickes - ps7
Diaphoretickes - ps8
Archaeplastida - ps9
Viridiplantae - ps10
Streptophyta - ps11
Charophyceae/Embriophyta - ps12
Embriophyta - ps13
Tracheophyta - ps14
Spermatophyta - ps15
Angiosperms - ps16
Mesangiospermae - ps17
Magnoliids/eudicots - ps18
Eudicots - ps19
Core_eudicots - ps20
Superrosids - ps21
Rosids - ps22
Eurosids - ps23
Malvids - ps24
Sap./Malv./Brassicales - ps25
Malvales/Brassicales - ps26
Brassicales - ps27
Cleomaceae/Brassicaceae - ps28
Brassicaceae - ps29
Camelineae - ps30
Arabidopsis - ps31
Arabidopsis_thaliana - ps32
Phylostratum

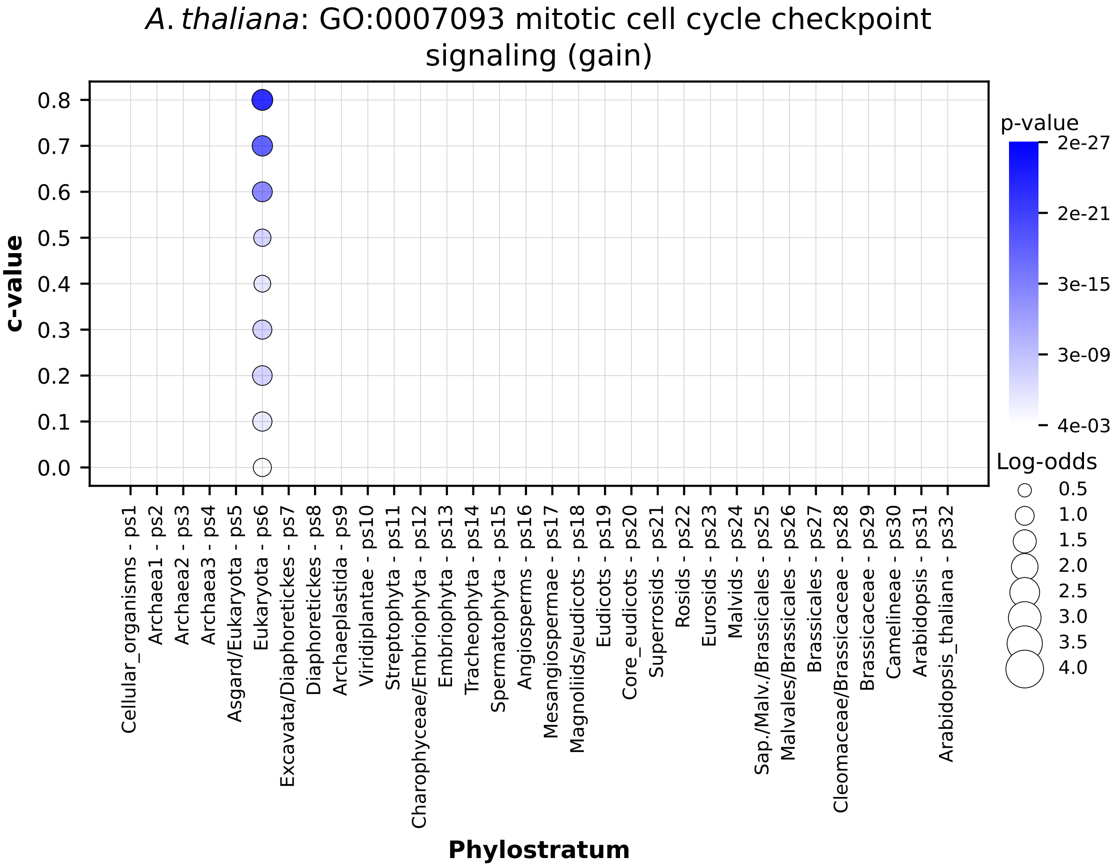
A. thaliana: GO:0007093 mitotic cell cycle checkpoint signaling (gain)
c-value
0.8
0.7
0.6
0.5
0.4
0.3
0.2
0.1
0.0
p-value
2e-27
2e-21
3e-15
3e-09
4e-03
Log-odds
0.5
1.0
1.5
2.0
2.5
3.0
3.5
4.0
Cellular_organisms - ps1
Archaea1 - ps2
Archaea2 - ps3
Archaea3 - ps4
Asgard/Eukaryota - ps5
Eukaryota - ps6
Excavata/Diaphoretickes - ps7
Diaphoretickes - ps8
Archaeplastida - ps9
Viridiplantae - ps10
Streptophyta - ps11
Charophyceae/Embriophyta - ps12
Embriophyta - ps13
Tracheophyta - ps14
Spermatophyta - ps15
Angiosperms - ps16
Mesangiospermae - ps17
Magnoliids/eudicots - ps18
Eudicots - ps19
Core_eudicots - ps20
Superrosids - ps21
Rosids - ps22
Eurosids - ps23
Malvids - ps24
Sap./Malv./Brassicales - ps25
Malvales/Brassicales - ps26
Brassicales - ps27
Cleomaceae/Brassicaceae - ps28
Brassicaceae - ps29
Camelineae - ps30
Arabidopsis - ps31
Arabidopsis_thaliana - ps32
Phylostratum

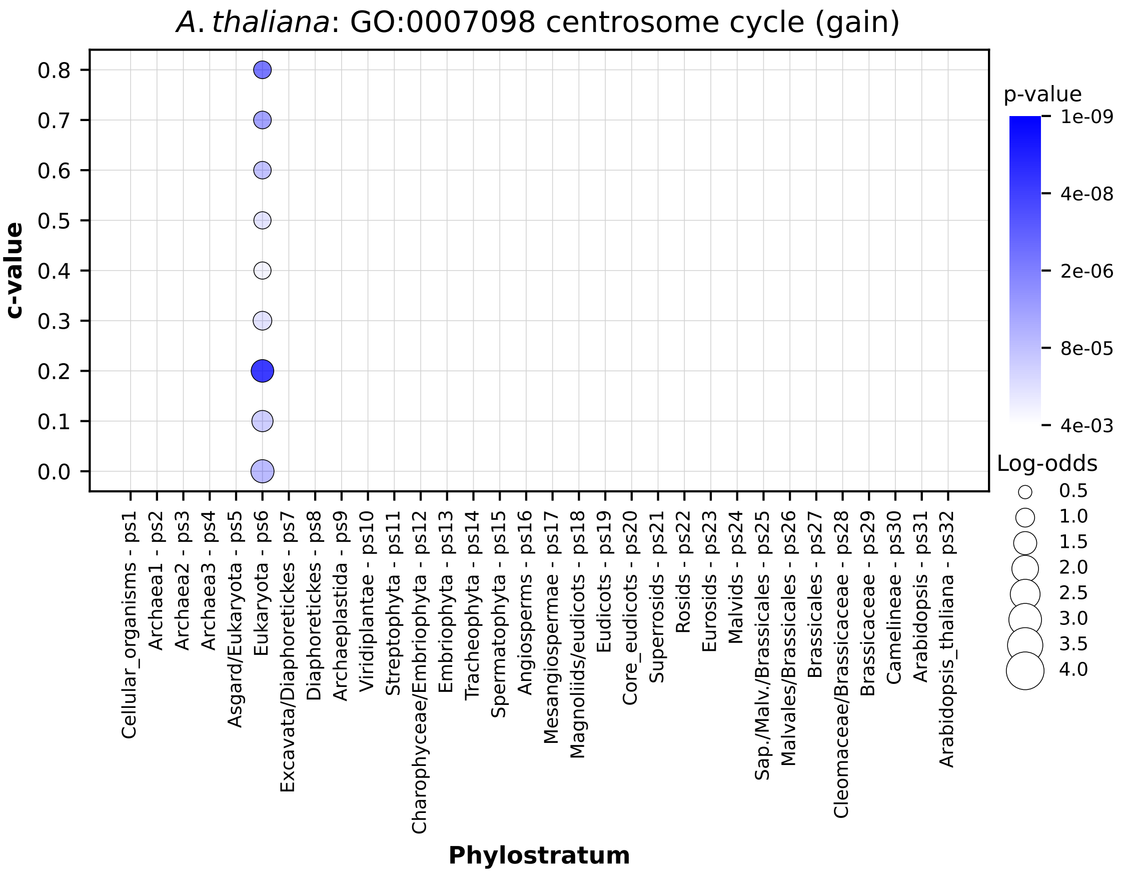

A. thaliana: GO:0007098 centrosome cycle (gain)
c-value
0.8
0.7
0.6
0.5
0.4
0.3
0.2
0.1
0.0
Phylostratum
Cellular_organisms - ps1
Archaea1 - ps2
Archaea2 - ps3
Archaea3 - ps4
Asgard/Eukaryota - ps5
Eukaryota - ps6
Excavata/Diaphoretickes - ps7
Diaphoretickes - ps8
Archaeplastida - ps9
Viridiplantae - ps10
Streptophyta - ps11
Charophyceae/Embriophyta - ps12
Embriophyta - ps13
Tracheophyta - ps14
Spermatophyta - ps15
Angiosperms - ps16
Mesangiospermae - ps17
Magnoliids/eudicots - ps18
Eudicots - ps19
Core_eudicots - ps20
Superrosids - ps21
Rosids - ps22
Eurosids - ps23
Malvids - ps24
Sap./Malv./Brassicales - ps25
Malvales/Brassicales - ps26
Brassicales - ps27
Cleomaceae/Brassicaceae - ps28
Brassicaceae - ps29
Camelineae - ps30
Arabidopsis - ps31
Arabidopsis_thaliana - ps32
p-value
1e-09
4e-08
2e-06
8e-05
4e-03
Log-odds
0.5
1.0
1.5
2.0
2.5
3.0
3.5
4.0

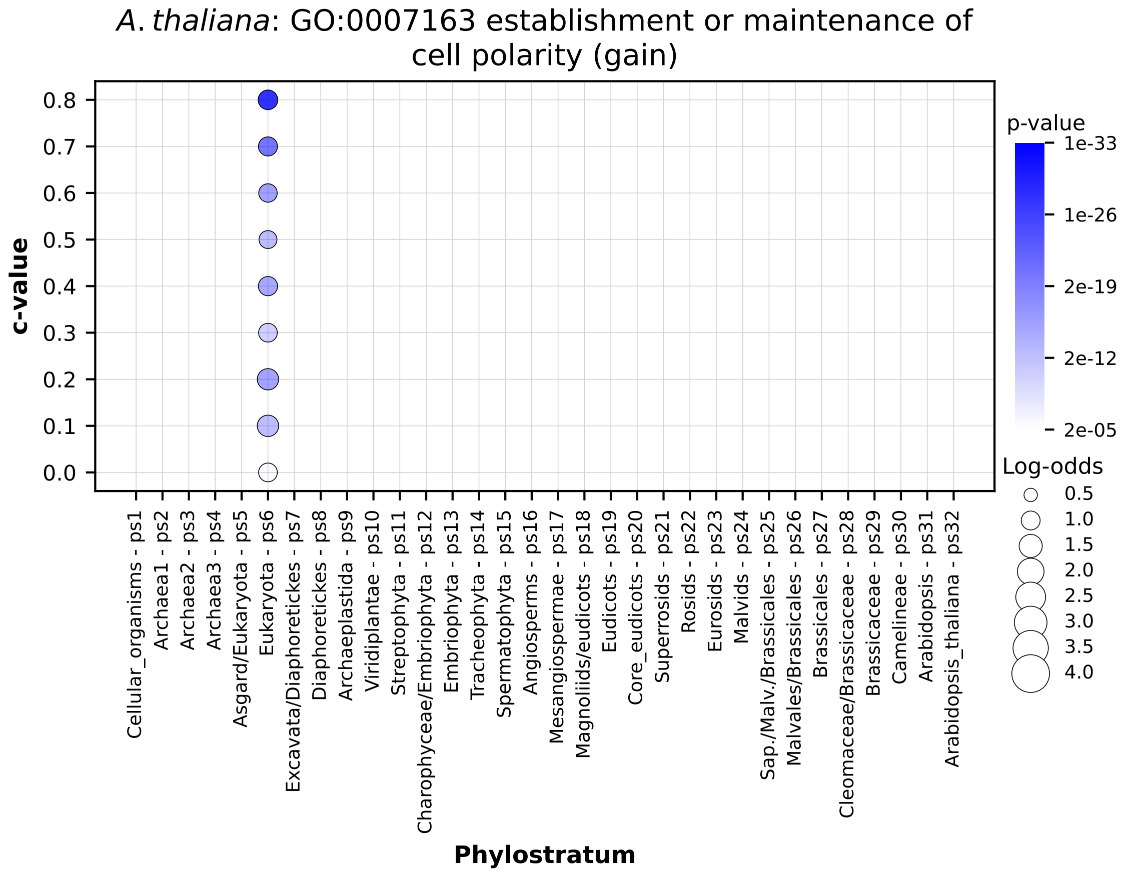
A. thaliana: GO:0007163 establishment or maintenance of cell polarity (gain)
c-value
0.8
0.7
0.6
0.5
0.4
0.3
0.2
0.1
0.0
Phylostratum
Cellular_organisms - ps1
Archaea1 - ps2
Archaea2 - ps3
Archaea3 - ps4
Asgard/Eukaryota - ps5
Eukaryota - ps6
Excavata/Diaphoretickes - ps7
Diaphoretickes - ps8
Archaeplastida - ps9
Viridiplantae - ps10
Streptophyta - ps11
Charophyceae/Embriophyta - ps12
Embriophyta - ps13
Tracheophyta - ps14
Spermatophyta - ps15
Angiosperms - ps16
Mesangiospermae - ps17
Magnoliids/eudicots - ps18
Eudicots - ps19
Core_eudicots - ps20
Superrosids - ps21
Rosids - ps22
Eurosids - ps23
Malvids - ps24
Sap./Malv./Brassicales - ps25
Malvales/Brassicales - ps26
Brassicales - ps27
Cleomaceae/Brassicaceae - ps28
Brassicaceae - ps29
Camelineae - ps30
Arabidopsis - ps31
Arabidopsis_thaliana - ps32
p-value
1e-33
1e-26
2e-19
2e-12
2e-05
Log-odds
0.5
1.0
1.5
2.0
2.5
3.0
3.5
4.0

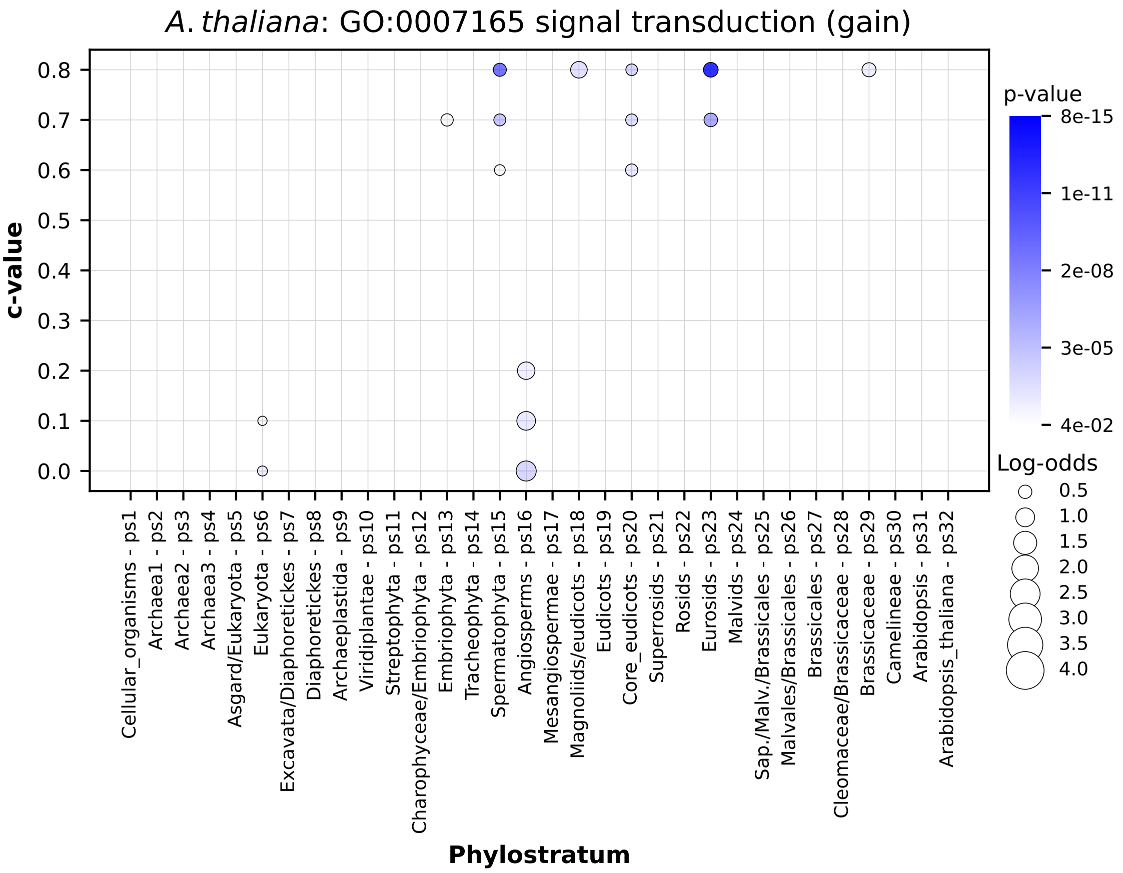

A. thaliana: GO:0007165 signal transduction (gain)
c-value
0.8
0.7
0.6
0.5
0.4
0.3
0.2
0.1
0.0
p-value
8e-15
1e-11
2e-08
3e-05
4e-02
Log-odds
0.5
1.0
1.5
2.0
2.5
3.0
3.5
4.0
Cellular_organisms - ps1
Archaea1 - ps2
Archaea2 - ps3
Archaea3 - ps4
Asgard/Eukaryota - ps5
Eukaryota - ps6
Excavata/Diaphoretickes - ps7
Diaphoretickes - ps8
Archaeplastida - ps9
Viridiplantae - ps10
Streptophyta - ps11
Charophyceae/Embriophyta - ps12
Embriophyta - ps13
Tracheophyta - ps14
Spermatophyta - ps15
Angiosperms - ps16
Mesangiospermae - ps17
Magnoliids/eudicots - ps18
Eudicots - ps19
Core_eudicots - ps20
Superrosids - ps21
Rosids - ps22
Eurosids - ps23
Malvids - ps24
Sap./Malv./Brassicales - ps25
Malvales/Brassicales - ps26
Brassicales - ps27
Cleomaceae/Brassicaceae - ps28
Brassicaceae - ps29
Camelineae - ps30
Arabidopsis - ps31
Arabidopsis_thaliana - ps32
Phylostratum

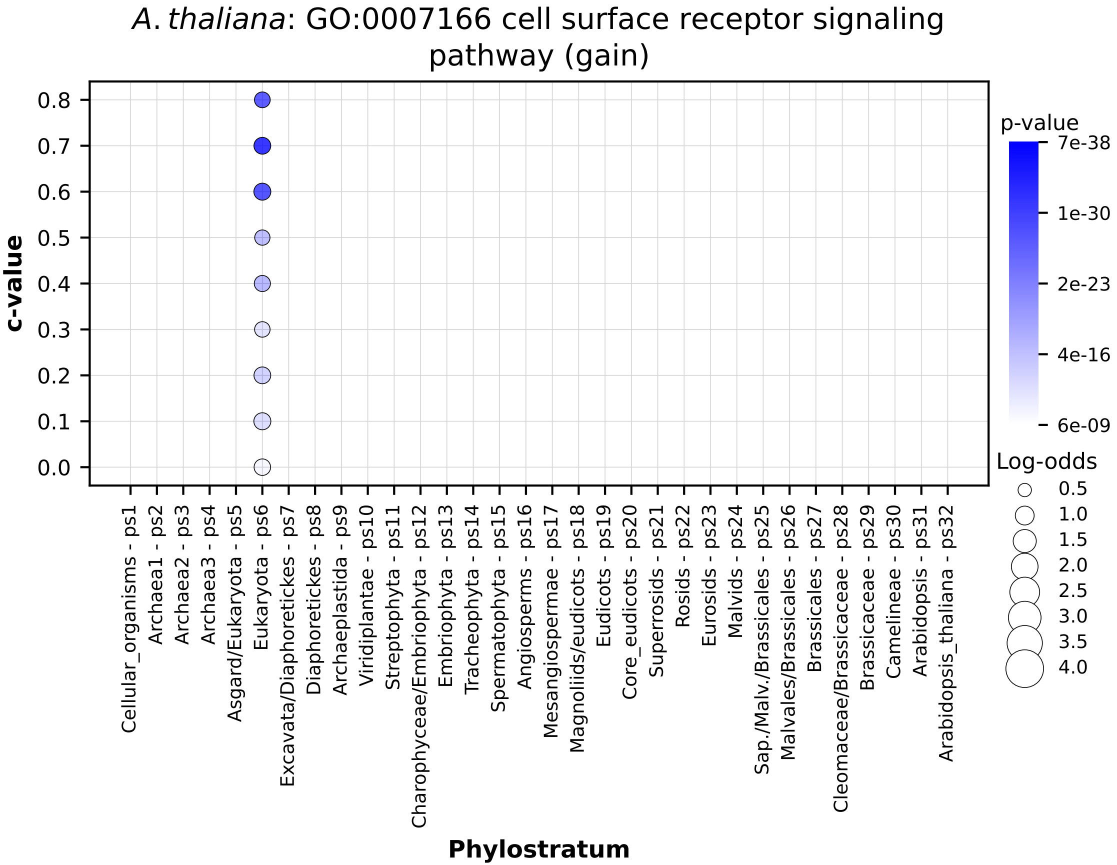

A. thaliana: GO:0007166 cell surface receptor signaling pathway (gain)
c-value
0.8
0.7
0.6
0.5
0.4
0.3
0.2
0.1
0.0
p-value
7e-38
1e-30
2e-23
4e-16
6e-09
Log-odds
0.5
1.0
1.5
2.0
2.5
3.0
3.5
4.0
Cellular_organisms - ps1
Archaea1 - ps2
Archaea2 - ps3
Archaea3 - ps4
Asgard/Eukaryota - ps5
Eukaryota - ps6
Excavata/Diaphoretickes - ps7
Diaphoretickes - ps8
Archaeplastida - ps9
Viridiplantae - ps10
Streptophyta - ps11
Charophyceae/Embriophyta - ps12
Embriophyta - ps13
Tracheophyta - ps14
Spermatophyta - ps15
Angiosperms - ps16
Mesangiospermae - ps17
Magnoliids/eudicots - ps18
Eudicots - ps19
Core_eudicots - ps20
Superrosids - ps21
Rosids - ps22
Eurosids - ps23
Malvids - ps24
Sap./Malv./Brassicales - ps25
Malvales/Brassicales - ps26
Brassicales - ps27
Cleomaceae/Brassicaceae - ps28
Brassicaceae - ps29
Camelineae - ps30
Arabidopsis - ps31
Arabidopsis_thaliana - ps32
Phylostratum

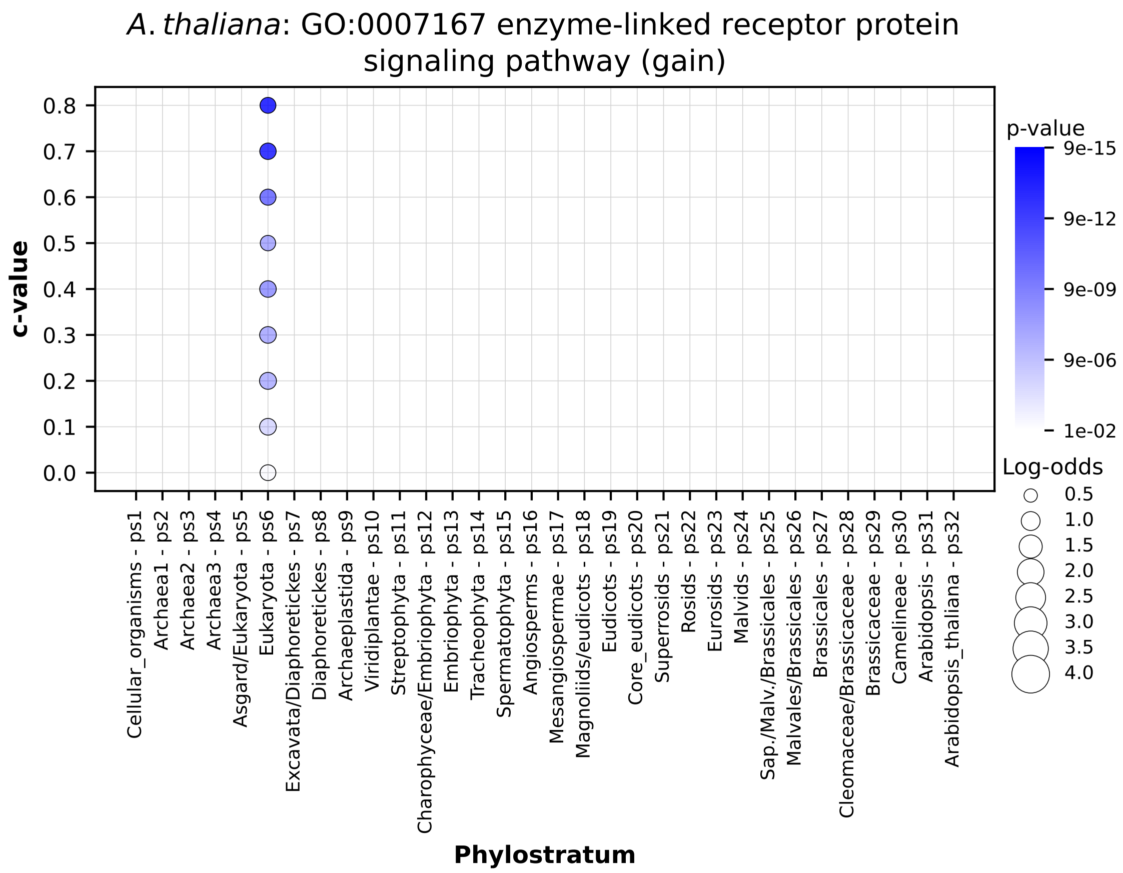
A. thaliana: GO:0007167 enzyme-linked receptor protein signaling pathway (gain)
c-value
0.0
0.1
0.2
0.3
0.4
0.5
0.6
0.7
0.8
Phylostratum
Cellular_organisms - ps1
Archaea1 - ps2
Archaea2 - ps3
Archaea3 - ps4
Asgard/Eukaryota - ps5
Eukaryota - ps6
Excavata/Diaphoretickes - ps7
Diaphoretickes - ps8
Archaeplastida - ps9
Viridiplantae - ps10
Streptophyta - ps11
Charophyceae/Embriophyta - ps12
Embriophyta - ps13
Tracheophyta - ps14
Spermatophyta - ps15
Angiosperms - ps16
Mesangiospermae - ps17
Magnoliids/eudicots - ps18
Eudicots - ps19
Core_eudicots - ps20
Superrosids - ps21
Rosids - ps22
Eurosids - ps23
Malvids - ps24
Sap./Malv./Brassicales - ps25
Malvales/Brassicales - ps26
Brassicales - ps27
Cleomaceae/Brassicaceae - ps28
Brassicaceae - ps29
Camelineae - ps30
Arabidopsis - ps31
Arabidopsis_thaliana - ps32
p-value
9e-15
9e-12
9e-09
9e-06
1e-02
Log-odds
0.5
1.0
1.5
2.0
2.5
3.0
3.5
4.0

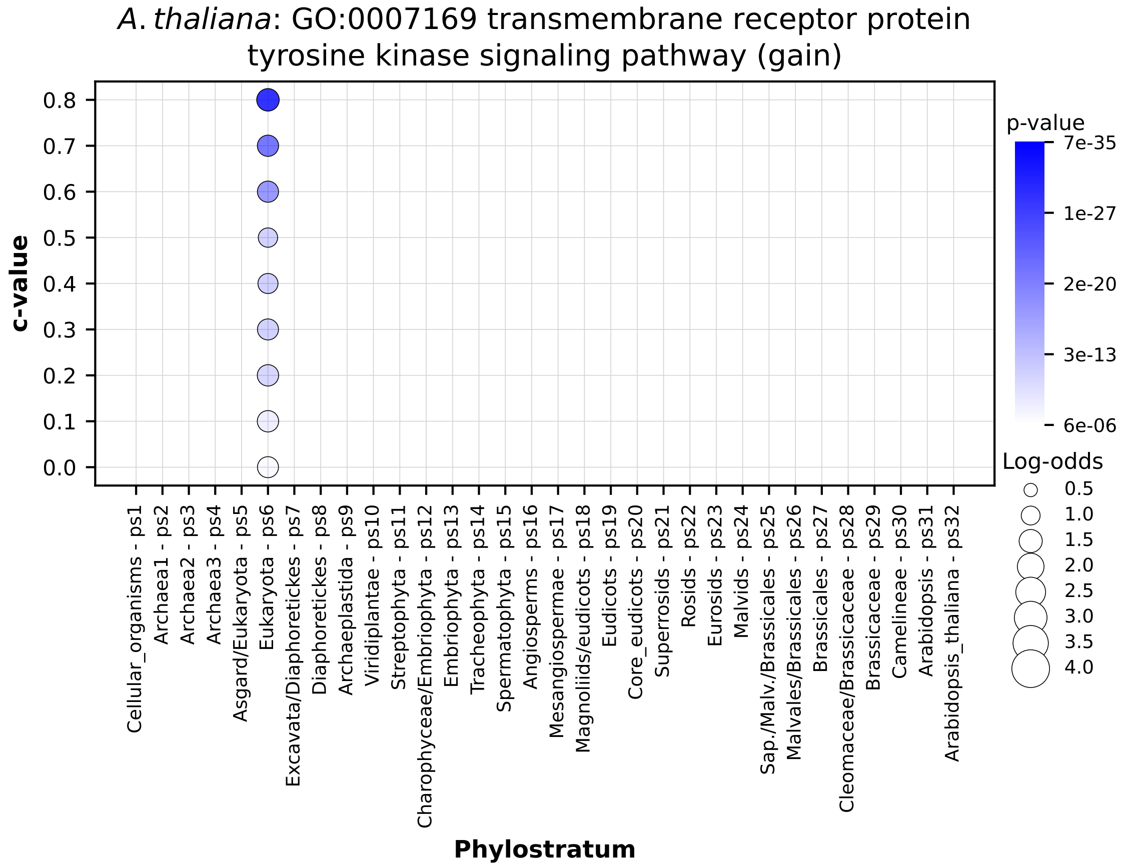

A. thaliana: GO:0007169 transmembrane receptor protein tyrosine kinase signaling pathway (gain)
c-value
0.0
0.1
0.2
0.3
0.4
0.5
0.6
0.7
0.8
p-value
7e-35
1e-27
2e-20
3e-13
6e-06
Log-odds
0.5
1.0
1.5
2.0
2.5
3.0
3.5
4.0
Cellular_organisms - ps1
Archaea1 - ps2
Archaea2 - ps3
Archaea3 - ps4
Asgard/Eukaryota - ps5
Eukaryota - ps6
Excavata/Diaphoretickes - ps7
Diaphoretickes - ps8
Archaeplastida - ps9
Viridiplantae - ps10
Streptophyta - ps11
Charophyceae/Embriophyta - ps12
Embriophyta - ps13
Tracheophyta - ps14
Spermatophyta - ps15
Angiosperms - ps16
Mesangiospermae - ps17
Magnoliids/eudicots - ps18
Eudicots - ps19
Core_eudicots - ps20
Superrosids - ps21
Rosids - ps22
Eurosids - ps23
Malvids - ps24
Sap./Malv./Brassicales - ps25
Malvales/Brassicales - ps26
Brassicales - ps27
Cleomaceae/Brassicaceae - ps28
Brassicaceae - ps29
Camelineae - ps30
Arabidopsis - ps31
Arabidopsis_thaliana - ps32
Phylostratum

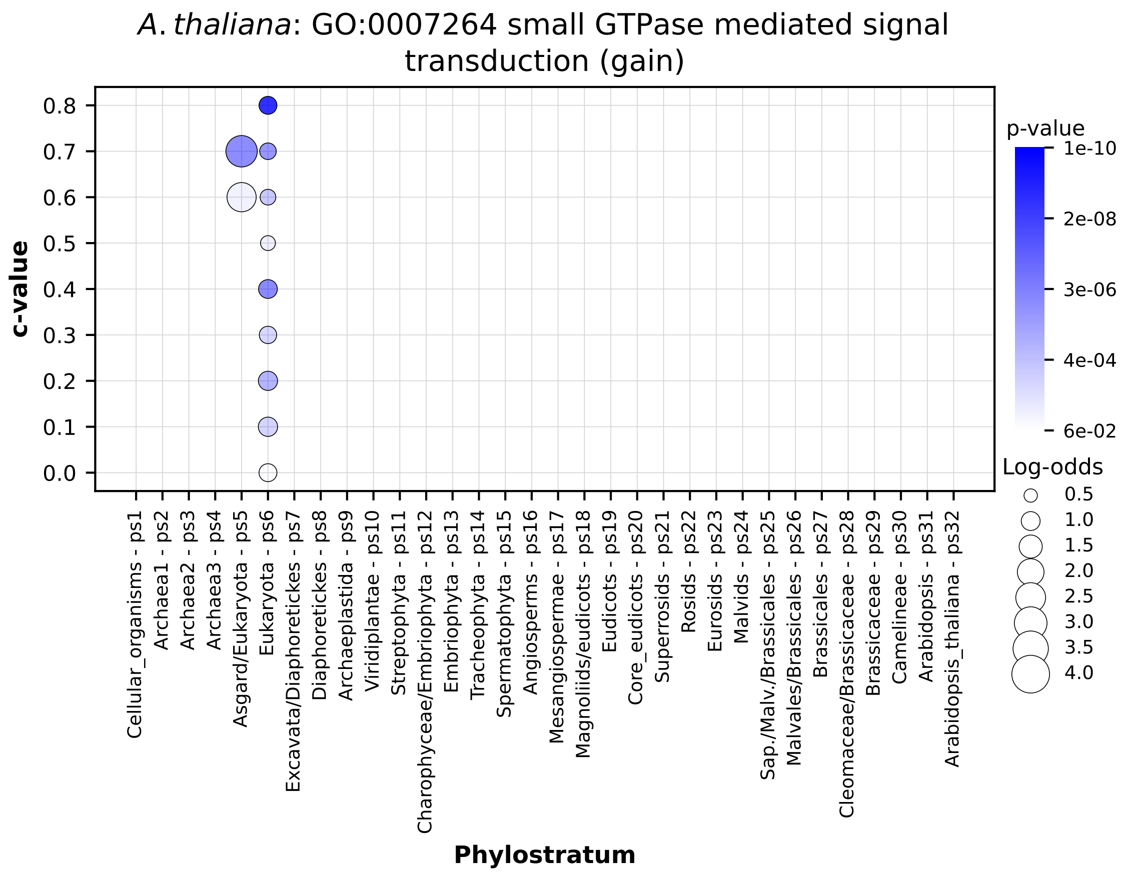
A. thaliana: GO:0007264 small GTPase mediated signal transduction (gain)
c-value
0.8
0.7
0.6
0.5
0.4
0.3
0.2
0.1
0.0
p-value
1e-10
2e-08
3e-06
4e-04
6e-02
Log-odds
0.5
1.0
1.5
2.0
2.5
3.0
3.5
4.0
Cellular_organisms - ps1
Archaea1 - ps2
Archaea2 - ps3
Archaea3 - ps4
Asgard/Eukaryota - ps5
Eukaryota - ps6
Excavata/Diaphoretickes - ps7
Diaphoretickes - ps8
Archaeplastida - ps9
Viridiplantae - ps10
Streptophyta - ps11
Charophyceae/Embriophyta - ps12
Embriophyta - ps13
Tracheophyta - ps14
Spermatophyta - ps15
Angiosperms - ps16
Mesangiospermae - ps17
Magnoliids/eudicots - ps18
Eudicots - ps19
Core_eudicots - ps20
Superrosids - ps21
Rosids - ps22
Eurosids - ps23
Malvids - ps24
Sap./Malv./Brassicales - ps25
Malvales/Brassicales - ps26
Brassicales - ps27
Cleomaceae/Brassicaceae - ps28
Brassicaceae - ps29
Camelineae - ps30
Arabidopsis - ps31
Arabidopsis_thaliana - ps32
Phylostratum

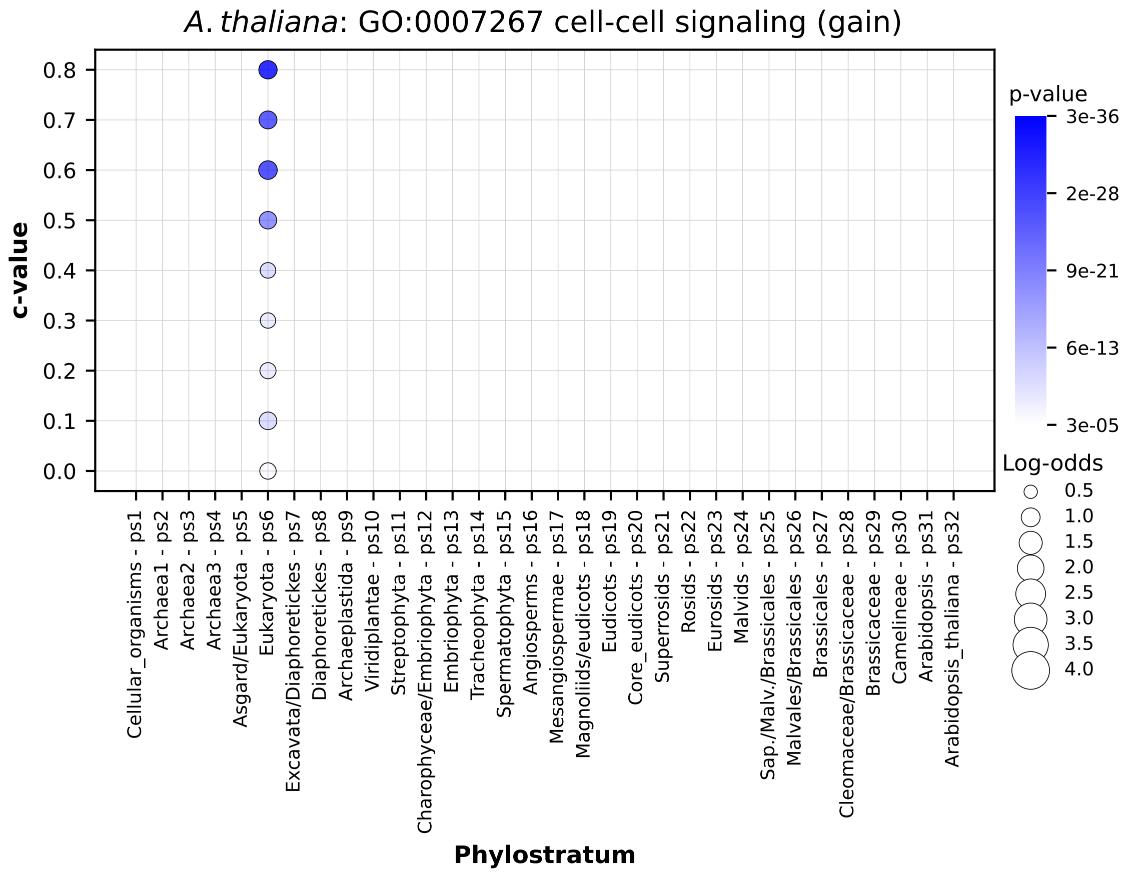

A. thaliana: GO:0007267 cell-cell signaling (gain)
c-value
0.8
0.7
0.6
0.5
0.4
0.3
0.2
0.1
0.0
Phylostratum
Cellular_organisms - ps1
Archaea1 - ps2
Archaea2 - ps3
Archaea3 - ps4
Asgard/Eukaryota - ps5
Eukaryota - ps6
Excavata/Diaphoretickes - ps7
Diaphoretickes - ps8
Archaeplastida - ps9
Viridiplantae - ps10
Streptophyta - ps11
Charophyceae/Embriophyta - ps12
Embriophyta - ps13
Tracheophyta - ps14
Spermatophyta - ps15
Angiosperms - ps16
Mesangiospermae - ps17
Magnoliids/eudicots - ps18
Eudicots - ps19
Core_eudicots - ps20
Superrosids - ps21
Rosids - ps22
Eurosids - ps23
Malvids - ps24
Sap./Malv./Brassicales - ps25
Malvales/Brassicales - ps26
Brassicales - ps27
Cleomaceae/Brassicaceae - ps28
Brassicaceae - ps29
Camelineae - ps30
Arabidopsis - ps31
Arabidopsis_thaliana - ps32
p-value
3e-36
2e-28
9e-21
6e-13
3e-05
Log-odds
0.5
1.0
1.5
2.0
2.5
3.0
3.5
4.0

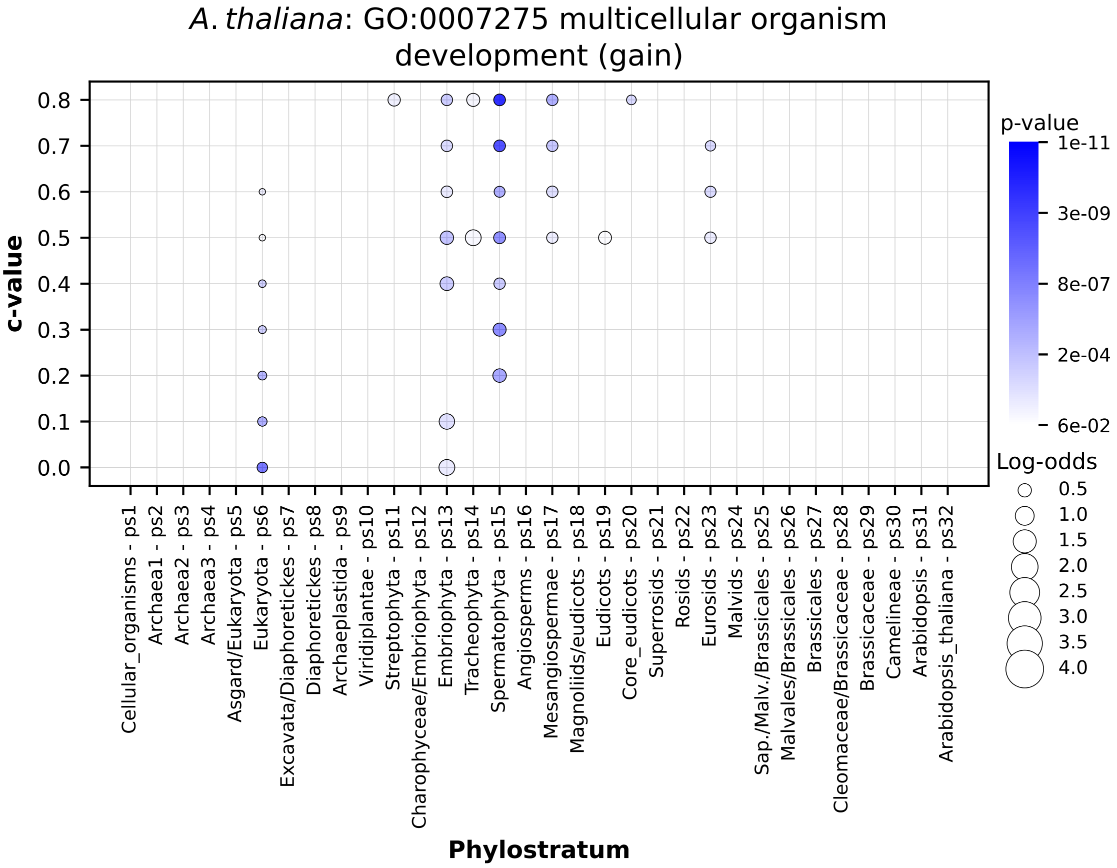

A. thaliana: GO:0007275 multicellular organism development (gain)
c-value
0.8
0.7
0.6
0.5
0.4
0.3
0.2
0.1
0.0
p-value
1e-11
3e-09
8e-07
2e-04
6e-02
Log-odds
0.5
1.0
1.5
2.0
2.5
3.0
3.5
4.0
Cellular_organisms - ps1
Archaea1 - ps2
Archaea2 - ps3
Archaea3 - ps4
Asgard/Eukaryota - ps5
Eukaryota - ps6
Excavata/Diaphoretickes - ps7
Diaphoretickes - ps8
Archaeplastida - ps9
Viridiplantae - ps10
Streptophyta - ps11
Charophyceae/Embriophyta - ps12
Embriophyta - ps13
Tracheophyta - ps14
Spermatophyta - ps15
Angiosperms - ps16
Mesangiospermae - ps17
Magnoliids/eudicots - ps18
Eudicots - ps19
Core_eudicots - ps20
Superrosids - ps21
Rosids - ps22
Eurosids - ps23
Malvids - ps24
Sap./Malv./Brassicales - ps25
Malvales/Brassicales - ps26
Brassicales - ps27
Cleomaceae/Brassicaceae - ps28
Brassicaceae - ps29
Camelineae - ps30
Arabidopsis - ps31
Arabidopsis_thaliana - ps32
Phylostratum

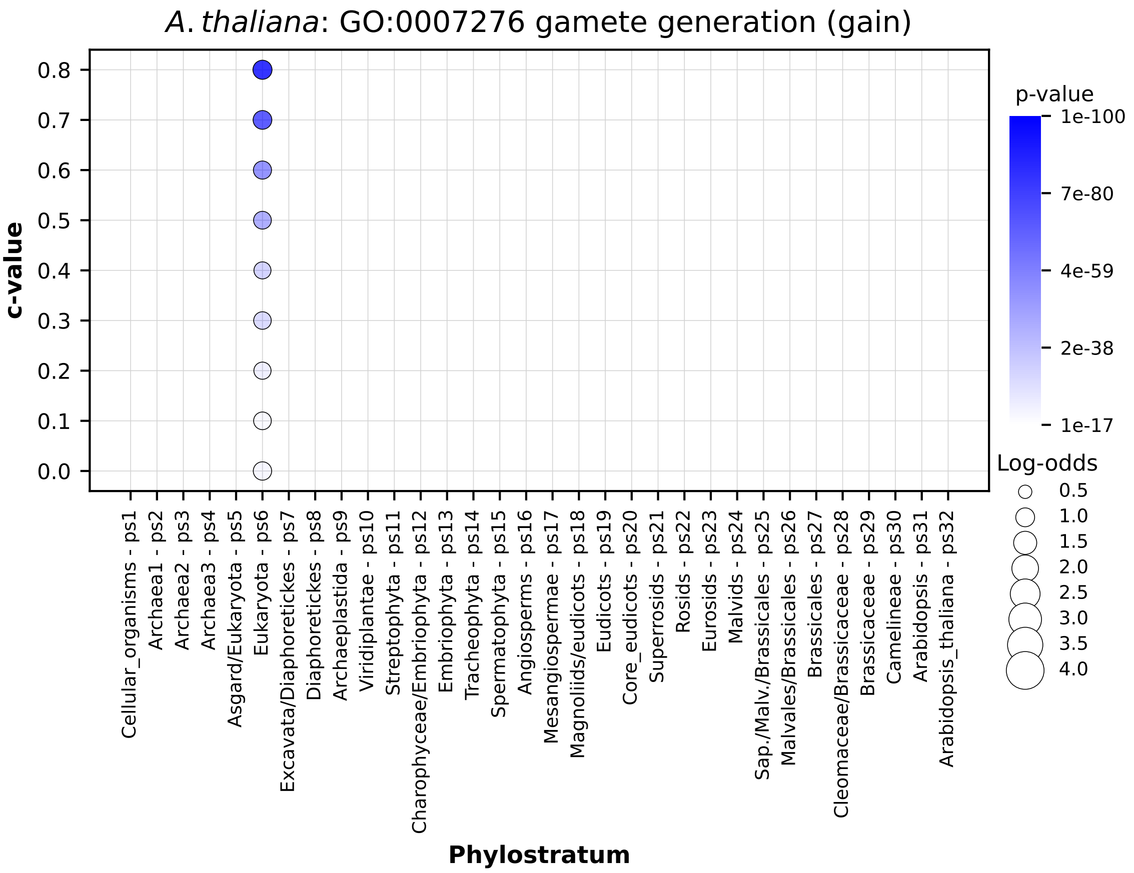

A. thaliana: GO:0007276 gamete generation (gain)
c-value
0.8
0.7
0.6
0.5
0.4
0.3
0.2
0.1
0.0
p-value
1e-100
7e-80
4e-59
2e-38
1e-17
Log-odds
0.5
1.0
1.5
2.0
2.5
3.0
3.5
4.0
Cellular_organisms - ps1
Archaea1 - ps2
Archaea2 - ps3
Archaea3 - ps4
Asgard/Eukaryota - ps5
Eukaryota - ps6
Excavata/Diaphoretickes - ps7
Diaphoretickes - ps8
Archaeplastida - ps9
Viridiplantae - ps10
Streptophyta - ps11
Charophyceae/Embriophyta - ps12
Embriophyta - ps13
Tracheophyta - ps14
Spermatophyta - ps15
Angiosperms - ps16
Mesangiospermae - ps17
Magnoliids/eudicots - ps18
Eudicots - ps19
Core_eudicots - ps20
Superrosids - ps21
Rosids - ps22
Eurosids - ps23
Malvids - ps24
Sap./Malv./Brassicales - ps25
Malvales/Brassicales - ps26
Brassicales - ps27
Cleomaceae/Brassicaceae - ps28
Brassicaceae - ps29
Camelineae - ps30
Arabidopsis - ps31
Arabidopsis_thaliana - ps32
Phylostratum

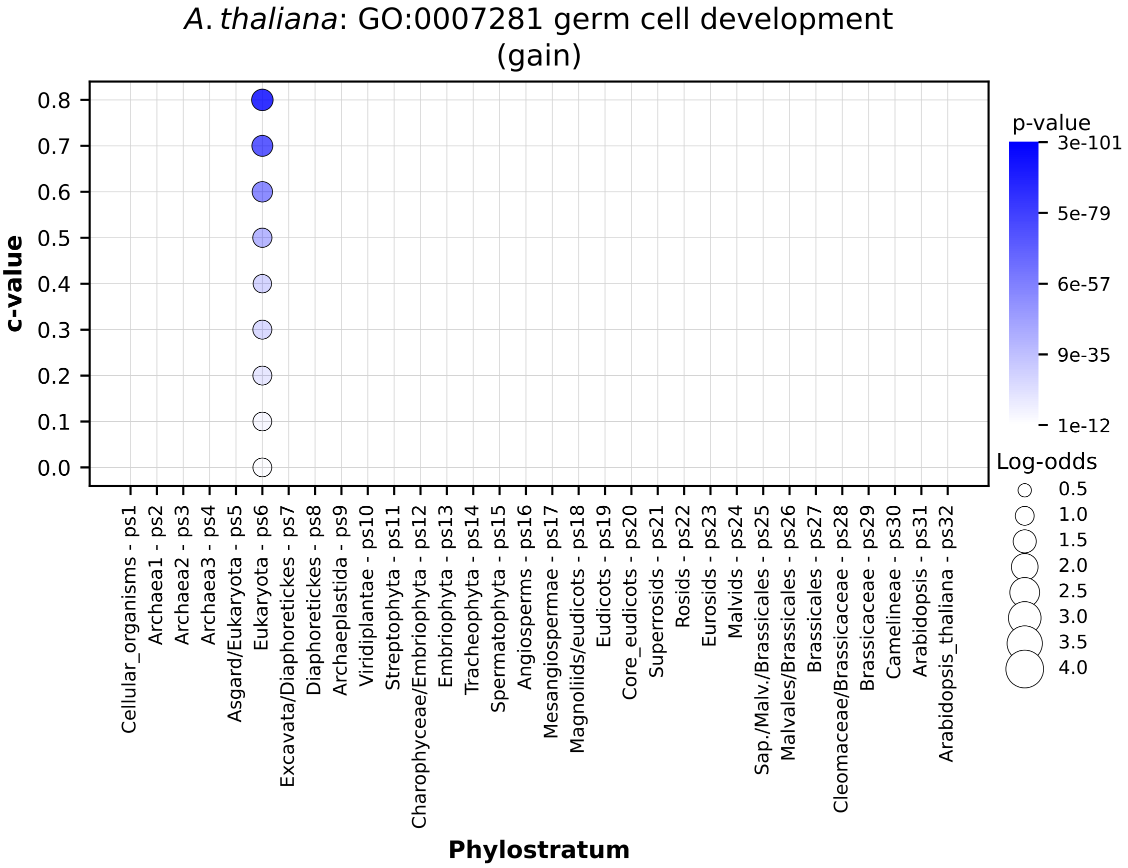

A. thaliana: GO:0007281 germ cell development
(gain)
c-value
0.0
0.1
0.2
0.3
0.4
0.5
0.6
0.7
0.8
Phylostratum
Cellular_organisms - ps1
Archaea1 - ps2
Archaea2 - ps3
Archaea3 - ps4
Asgard/Eukaryota - ps5
Eukaryota - ps6
Excavata/Diaphoretickes - ps7
Diaphoretickes - ps8
Archaeplastida - ps9
Viridiplantae - ps10
Streptophyta - ps11
Charophyceae/Embriophyta - ps12
Embriophyta - ps13
Tracheophyta - ps14
Spermatophyta - ps15
Angiosperms - ps16
Mesangiospermae - ps17
Magnoliids/eudicots - ps18
Eudicots - ps19
Core_eudicots - ps20
Superrosids - ps21
Rosids - ps22
Eurosids - ps23
Malvids - ps24
Sap./Malv./Brassicales - ps25
Malvales/Brassicales - ps26
Brassicales - ps27
Cleomaceae/Brassicaceae - ps28
Brassicaceae - ps29
Camelineae - ps30
Arabidopsis - ps31
Arabidopsis_thaliana - ps32
p-value
3e-101
5e-79
6e-57
9e-35
1e-12
Log-odds
0.5
1.0
1.5
2.0
2.5
3.0
3.5
4.0

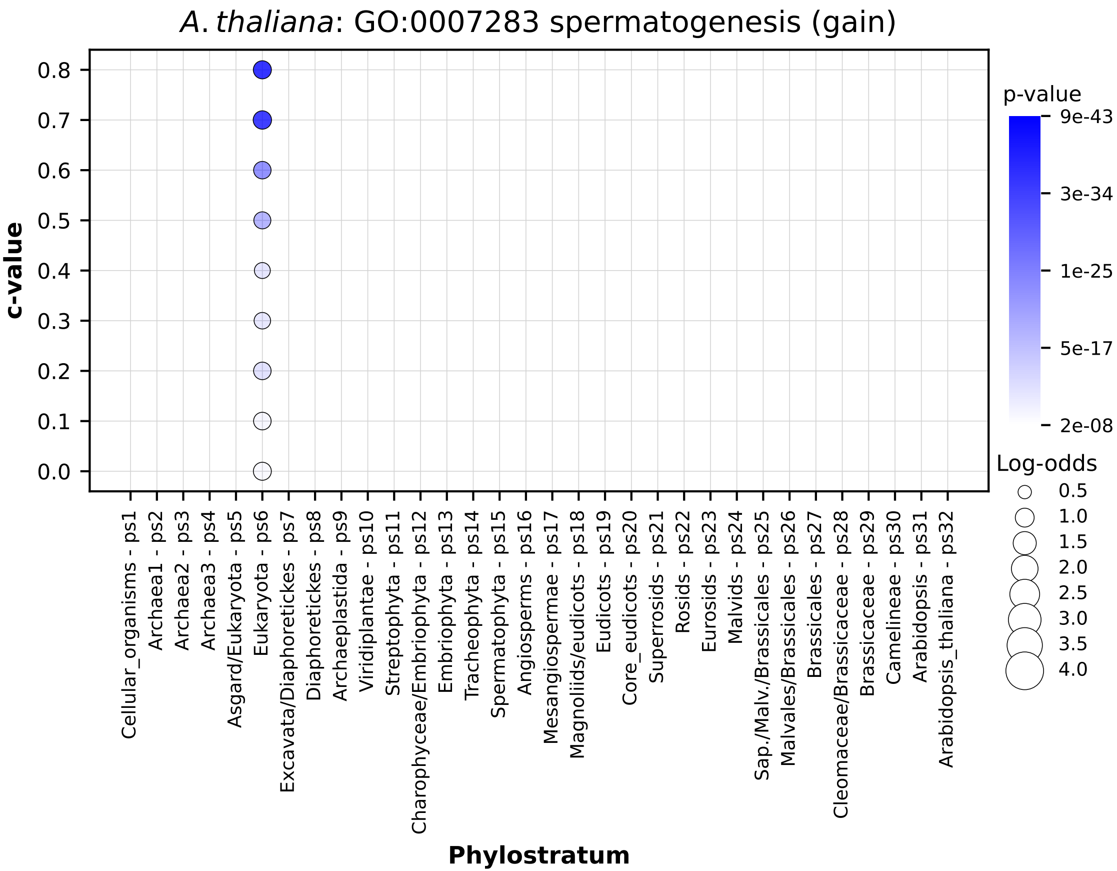

A. thaliana: GO:0007283 spermatogenesis (gain)
c-value
0.8
0.7
0.6
0.5
0.4
0.3
0.2
0.1
0.0
p-value
9e-43
3e-34
1e-25
5e-17
2e-08
Log-odds
0.5
1.0
1.5
2.0
2.5
3.0
3.5
4.0
Cellular_organisms - ps1
Archaea1 - ps2
Archaea2 - ps3
Archaea3 - ps4
Asgard/Eukaryota - ps5
Eukaryota - ps6
Excavata/Diaphoretickes - ps7
Diaphoretickes - ps8
Archaeplastida - ps9
Viridiplantae - ps10
Streptophyta - ps11
Charophyceae/Embriophyta - ps12
Embriophyta - ps13
Tracheophyta - ps14
Spermatophyta - ps15
Angiosperms - ps16
Mesangiospermae - ps17
Magnoliids/eudicots - ps18
Eudicots - ps19
Core_eudicots - ps20
Superrosids - ps21
Rosids - ps22
Eurosids - ps23
Malvids - ps24
Sap./Malv./Brassicales - ps25
Malvales/Brassicales - ps26
Brassicales - ps27
Cleomaceae/Brassicaceae - ps28
Brassicaceae - ps29
Camelineae - ps30
Arabidopsis - ps31
Arabidopsis_thaliana - ps32
Phylostratum

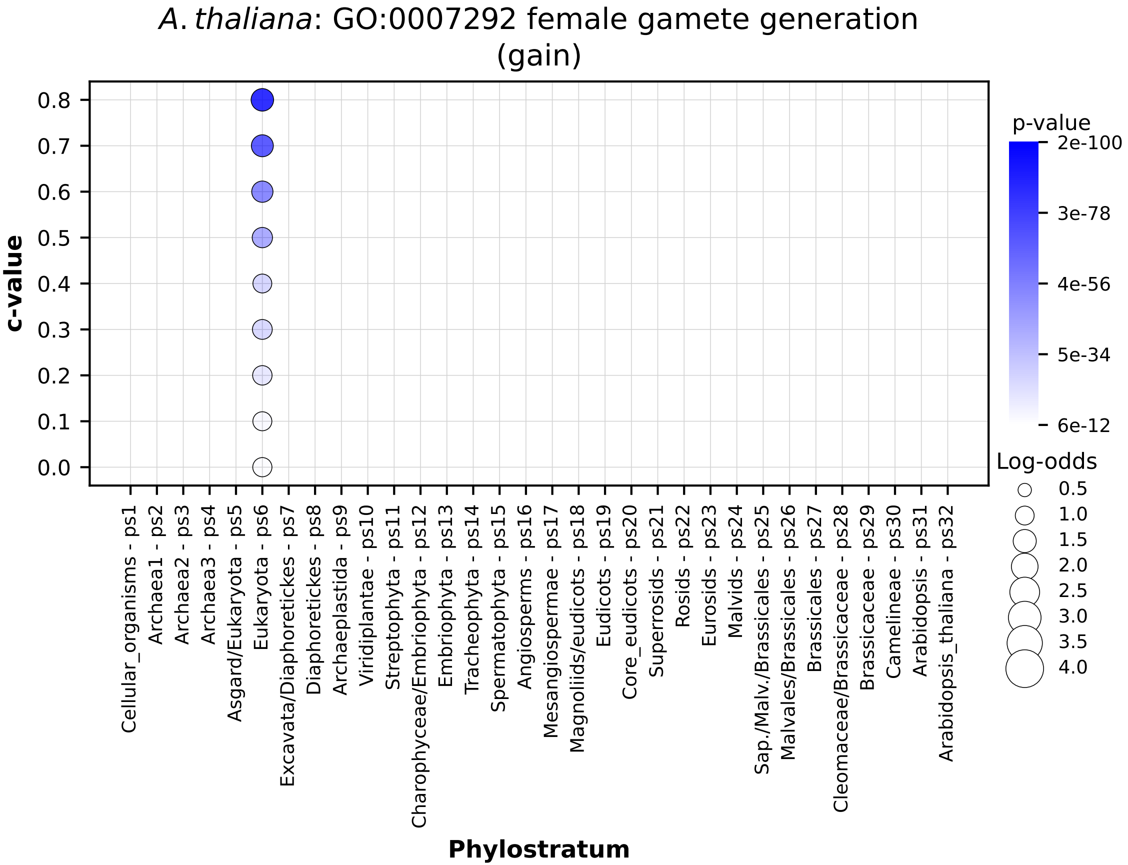
A. thaliana: GO:0007292 female gamete generation
(gain)
c-value
0.8
0.7
0.6
0.5
0.4
0.3
0.2
0.1
0.0
p-value
2e-100
3e-78
4e-56
5e-34
6e-12
Log-odds
0.5
1.0
1.5
2.0
2.5
3.0
3.5
4.0
Cellular_organisms - ps1
Archaea1 - ps2
Archaea2 - ps3
Archaea3 - ps4
Asgard/Eukaryota - ps5
Eukaryota - ps6
Excavata/Diaphoretickes - ps7
Diaphoretickes - ps8
Archaeplastida - ps9
Viridiplantae - ps10
Streptophyta - ps11
Charophyceae/Embriophyta - ps12
Embriophyta - ps13
Tracheophyta - ps14
Spermatophyta - ps15
Angiosperms - ps16
Mesangiospermae - ps17
Magnoliids/eudicots - ps18
Eudicots - ps19
Core_eudicots - ps20
Superrosids - ps21
Rosids - ps22
Eurosids - ps23
Malvids - ps24
Sap./Malv./Brassicales - ps25
Malvales/Brassicales - ps26
Brassicales - ps27
Cleomaceae/Brassicaceae - ps28
Brassicaceae - ps29
Camelineae - ps30
Arabidopsis - ps31
Arabidopsis_thaliana - ps32
Phylostratum

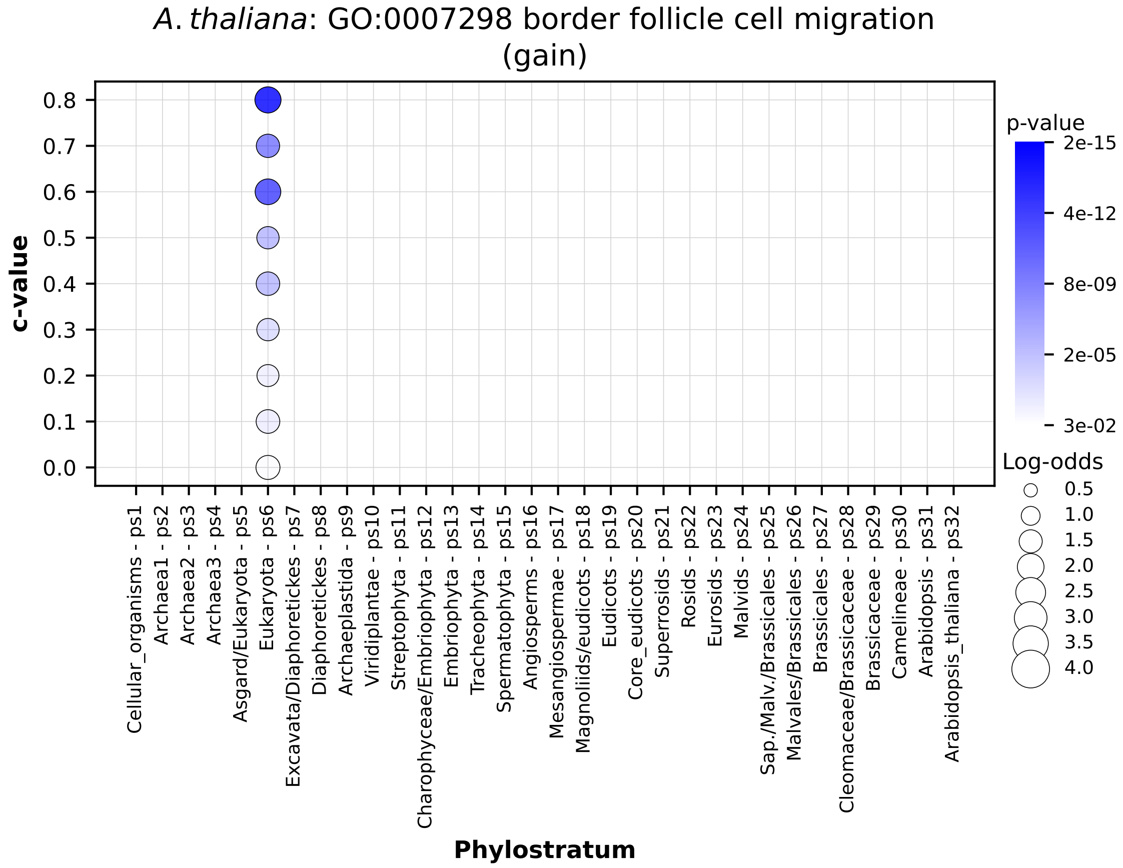

A. thaliana: GO:0007298 border follicle cell migration (gain)
c-value
0.0
0.1
0.2
0.3
0.4
0.5
0.6
0.7
0.8
Phylostratum
Cellular_organisms - ps1
Archaea1 - ps2
Archaea2 - ps3
Archaea3 - ps4
Asgard/Eukaryota - ps5
Eukaryota - ps6
Excavata/Diaphoretickes - ps7
Diaphoretickes - ps8
Archaeplastida - ps9
Viridiplantae - ps10
Streptophyta - ps11
Charophyceae/Embriophyta - ps12
Embriophyta - ps13
Tracheophyta - ps14
Spermatophyta - ps15
Angiosperms - ps16
Mesangiospermae - ps17
Magnoliids/eudicots - ps18
Eudicots - ps19
Core_eudicots - ps20
Superrosids - ps21
Rosids - ps22
Eurosids - ps23
Malvids - ps24
Sap./Malv./Brassicales - ps25
Malvales/Brassicales - ps26
Brassicales - ps27
Cleomaceae/Brassicaceae - ps28
Brassicaceae - ps29
Camelineae - ps30
Arabidopsis - ps31
Arabidopsis_thaliana - ps32
p-value
2e-15
4e-12
8e-09
2e-05
3e-02
Log-odds
0.5
1.0
1.5
2.0
2.5
3.0
3.5
4.0

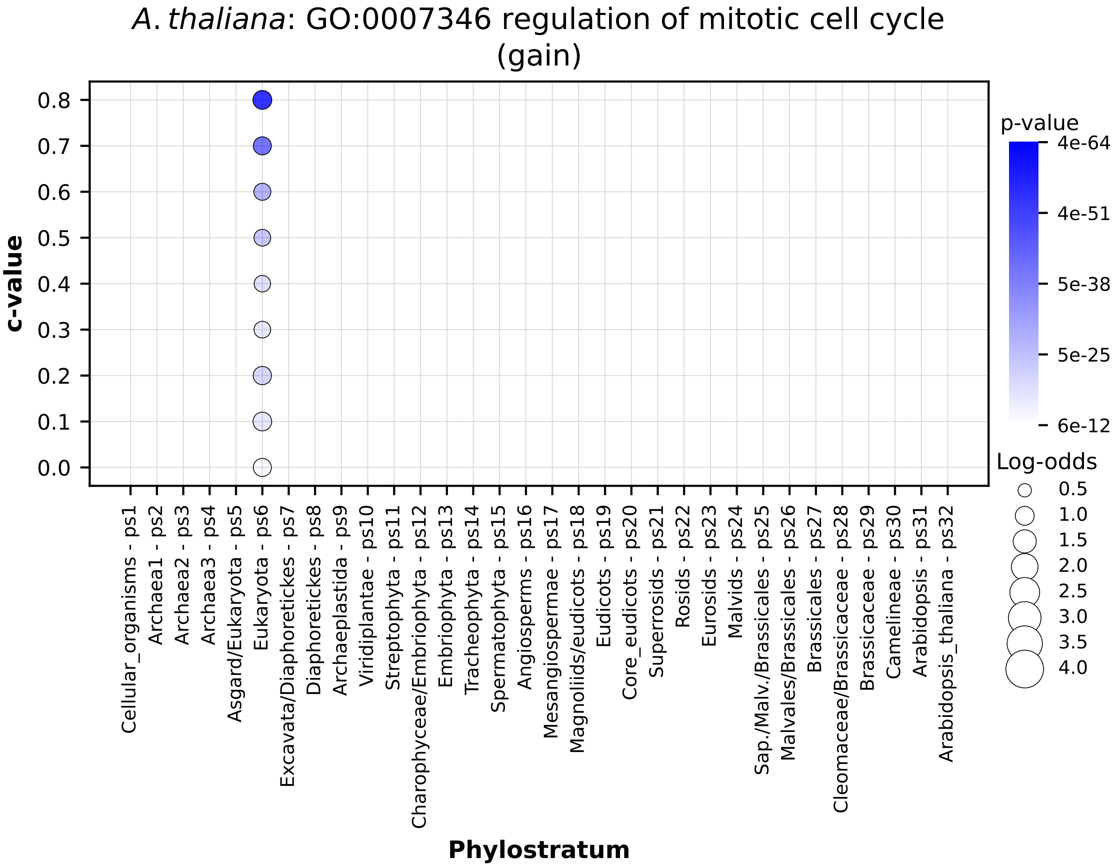

A. thaliana: GO:0007346 regulation of mitotic cell cycle
(gain)
c-value
0.8
0.7
0.6
0.5
0.4
0.3
0.2
0.1
0.0
p-value
4e-64
4e-51
5e-38
5e-25
6e-12
Log-odds
0.5
1.0
1.5
2.0
2.5
3.0
3.5
4.0
Cellular_organisms - ps1
Archaea1 - ps2
Archaea2 - ps3
Archaea3 - ps4
Asgard/Eukaryota - ps5
Eukaryota - ps6
Excavata/Diaphoretickes - ps7
Diaphoretickes - ps8
Archaeplastida - ps9
Viridiplantae - ps10
Streptophyta - ps11
Charophyceae/Embriophyta - ps12
Embriophyta - ps13
Tracheophyta - ps14
Spermatophyta - ps15
Angiosperms - ps16
Mesangiospermae - ps17
Magnoliids/eudicots - ps18
Eudicots - ps19
Core_eudicots - ps20
Superrosids - ps21
Rosids - ps22
Eurosids - ps23
Malvids - ps24
Sap./Malv./Brassicales - ps25
Malvales/Brassicales - ps26
Brassicales - ps27
Cleomaceae/Brassicaceae - ps28
Brassicaceae - ps29
Camelineae - ps30
Arabidopsis - ps31
Arabidopsis_thaliana - ps32
Phylostratum

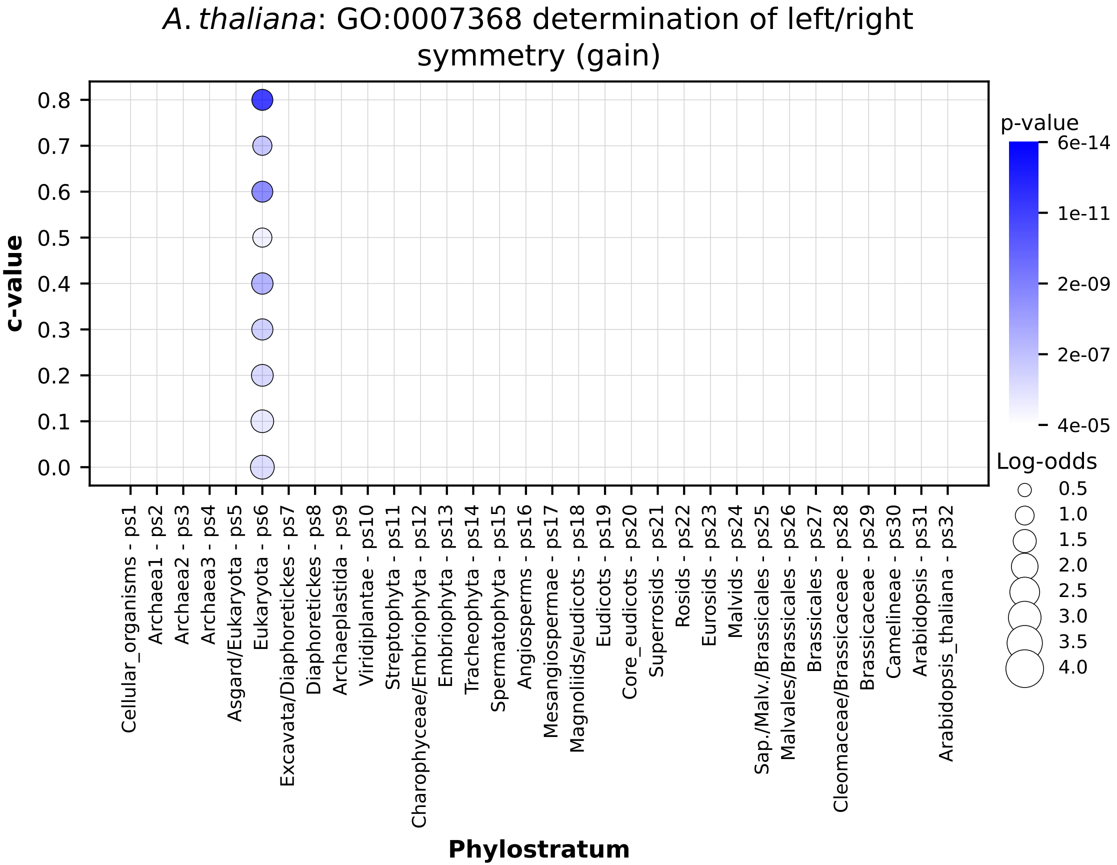
A. thaliana: GO:0007368 determination of left/right symmetry (gain)
c-value
0.8
0.7
0.6
0.5
0.4
0.3
0.2
0.1
0.0
p-value
6e-14
1e-11
2e-09
2e-07
4e-05
Log-odds
0.5
1.0
1.5
2.0
2.5
3.0
3.5
4.0
Cellular_organisms - ps1
Archaea1 - ps2
Archaea2 - ps3
Archaea3 - ps4
Asgard/Eukaryota - ps5
Eukaryota - ps6
Excavata/Diaphoretickes - ps7
Diaphoretickes - ps8
Archaeplastida - ps9
Viridiplantae - ps10
Streptophyta - ps11
Charophyceae/Embriophyta - ps12
Embriophyta - ps13
Tracheophyta - ps14
Spermatophyta - ps15
Angiosperms - ps16
Mesangiospermae - ps17
Magnoliids/eudicots - ps18
Eudicots - ps19
Core_eudicots - ps20
Superrosids - ps21
Rosids - ps22
Eurosids - ps23
Malvids - ps24
Sap./Malv./Brassicales - ps25
Malvales/Brassicales - ps26
Brassicales - ps27
Cleomaceae/Brassicaceae - ps28
Brassicaceae - ps29
Camelineae - ps30
Arabidopsis - ps31
Arabidopsis_thaliana - ps32
Phylostratum

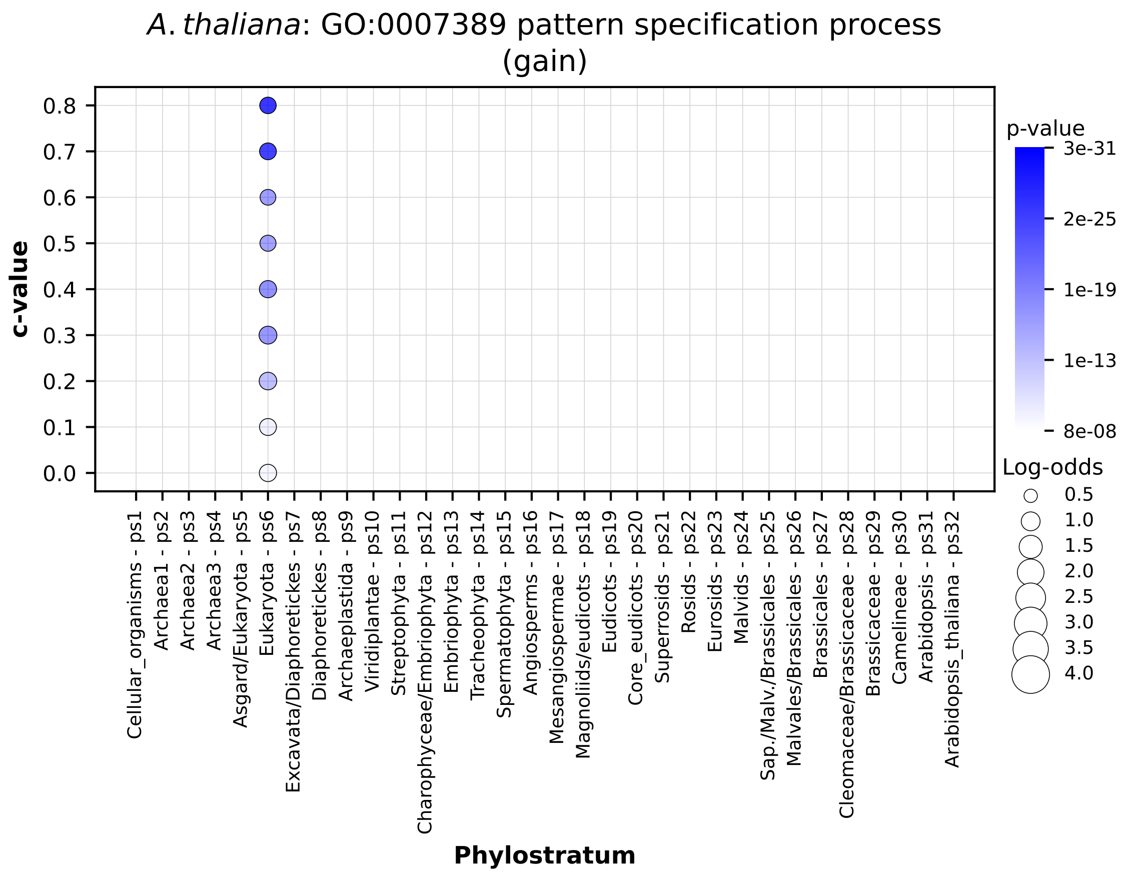

A. thaliana: GO:0007389 pattern specification process
(gain)
c-value
0.8
0.7
0.6
0.5
0.4
0.3
0.2
0.1
0.0
p-value
3e-31
2e-25
1e-19
1e-13
8e-08
Log-odds
0.5
1.0
1.5
2.0
2.5
3.0
3.5
4.0
Cellular_organisms - ps1
Archaea1 - ps2
Archaea2 - ps3
Archaea3 - ps4
Asgard/Eukaryota - ps5
Eukaryota - ps6
Excavata/Diaphoretickes - ps7
Diaphoretickes - ps8
Archaeplastida - ps9
Viridiplantae - ps10
Streptophyta - ps11
Charophyceae/Embriophyta - ps12
Embriophyta - ps13
Tracheophyta - ps14
Spermatophyta - ps15
Angiosperms - ps16
Mesangiospermae - ps17
Magnoliids/eudicots - ps18
Eudicots - ps19
Core_eudicots - ps20
Superrosids - ps21
Rosids - ps22
Eurosids - ps23
Malvids - ps24
Sap./Malv./Brassicales - ps25
Malvales/Brassicales - ps26
Brassicales - ps27
Cleomaceae/Brassicaceae - ps28
Brassicaceae - ps29
Camelineae - ps30
Arabidopsis - ps31
Arabidopsis_thaliana - ps32
Phylostratum

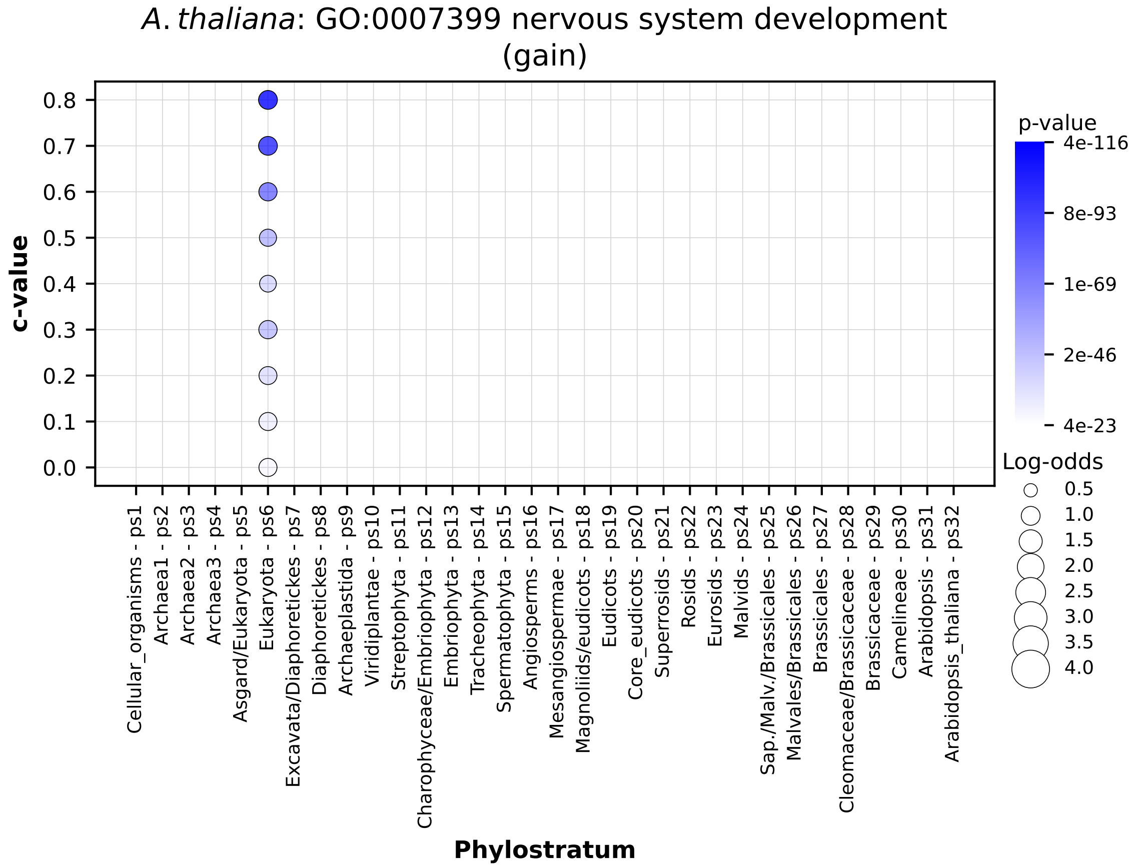

A. thaliana: GO:0007399 nervous system development
(gain)
c-value
0.8
0.7
0.6
0.5
0.4
0.3
0.2
0.1
0.0
p-value
4e-116
8e-93
1e-69
2e-46
4e-23
Log-odds
0.5
1.0
1.5
2.0
2.5
3.0
3.5
4.0
Cellular_organisms - ps1
Archaea1 - ps2
Archaea2 - ps3
Archaea3 - ps4
Asgard/Eukaryota - ps5
Eukaryota - ps6
Excavata/Diaphoretickes - ps7
Diaphoretickes - ps8
Archaeplastida - ps9
Viridiplantae - ps10
Streptophyta - ps11
Charophyceae/Embriophyta - ps12
Embriophyta - ps13
Tracheophyta - ps14
Spermatophyta - ps15
Angiosperms - ps16
Mesangiospermae - ps17
Magnoliids/eudicots - ps18
Eudicots - ps19
Core_eudicots - ps20
Superrosids - ps21
Rosids - ps22
Eurosids - ps23
Malvids - ps24
Sap./Malv./Brassicales - ps25
Malvales/Brassicales - ps26
Brassicales - ps27
Cleomaceae/Brassicaceae - ps28
Brassicaceae - ps29
Camelineae - ps30
Arabidopsis - ps31
Arabidopsis_thaliana - ps32
Phylostratum

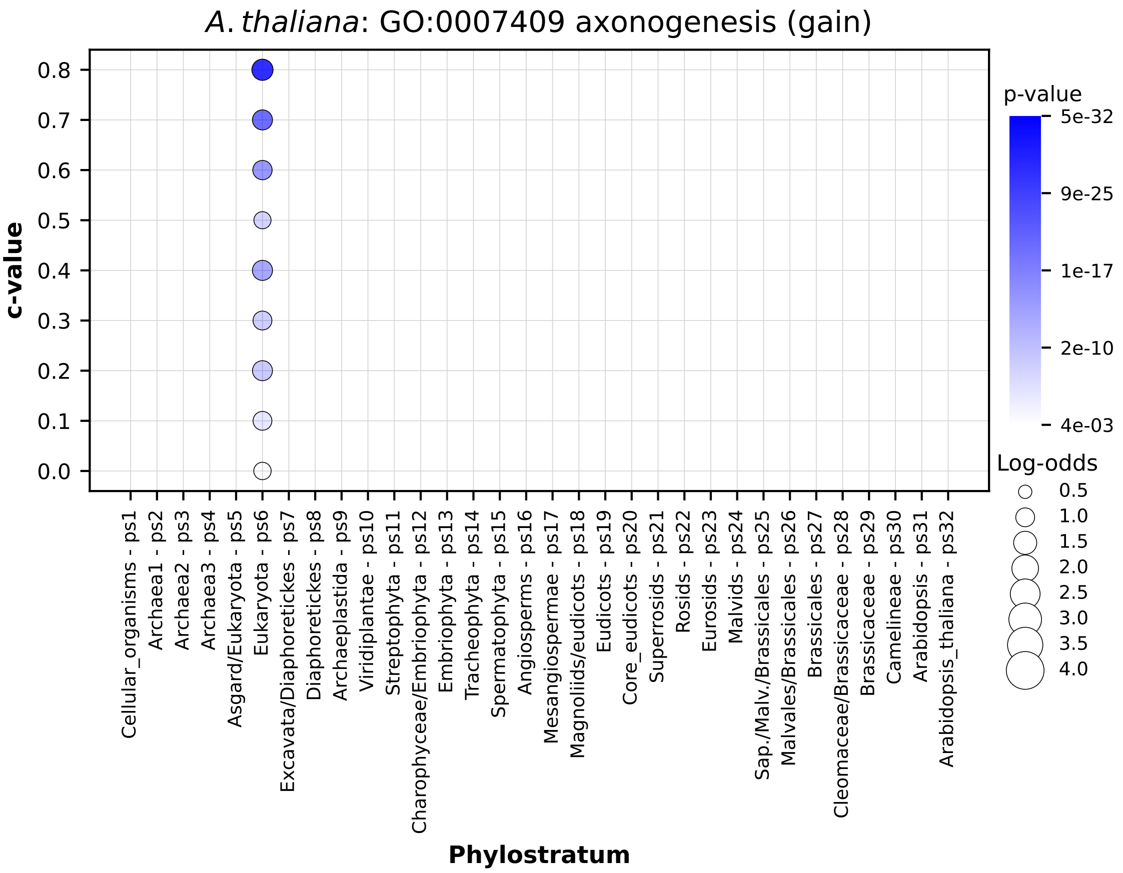
A. thaliana: GO:0007409 axonogenesis (gain)
c-value
0.0
0.1
0.2
0.3
0.4
0.5
0.6
0.7
0.8
p-value
5e-32
9e-25
1e-17
2e-10
4e-03
Log-odds
0.5
1.0
1.5
2.0
2.5
3.0
3.5
4.0
Cellular_organisms - ps1
Archaea1 - ps2
Archaea2 - ps3
Archaea3 - ps4
Asgard/Eukaryota - ps5
Eukaryota - ps6
Excavata/Diaphoretickes - ps7
Diaphoretickes - ps8
Archaeplastida - ps9
Viridiplantae - ps10
Streptophyta - ps11
Charophyceae/Embriophyta - ps12
Embriophyta - ps13
Tracheophyta - ps14
Spermatophyta - ps15
Angiosperms - ps16
Mesangiospermae - ps17
Magnoliids/eudicots - ps18
Eudicots - ps19
Core_eudicots - ps20
Superrosids - ps21
Rosids - ps22
Eurosids - ps23
Malvids - ps24
Sap./Malv./Brassicales - ps25
Malvales/Brassicales - ps26
Brassicales - ps27
Cleomaceae/Brassicaceae - ps28
Brassicaceae - ps29
Camelineae - ps30
Arabidopsis - ps31
Arabidopsis_thaliana - ps32
Phylostratum

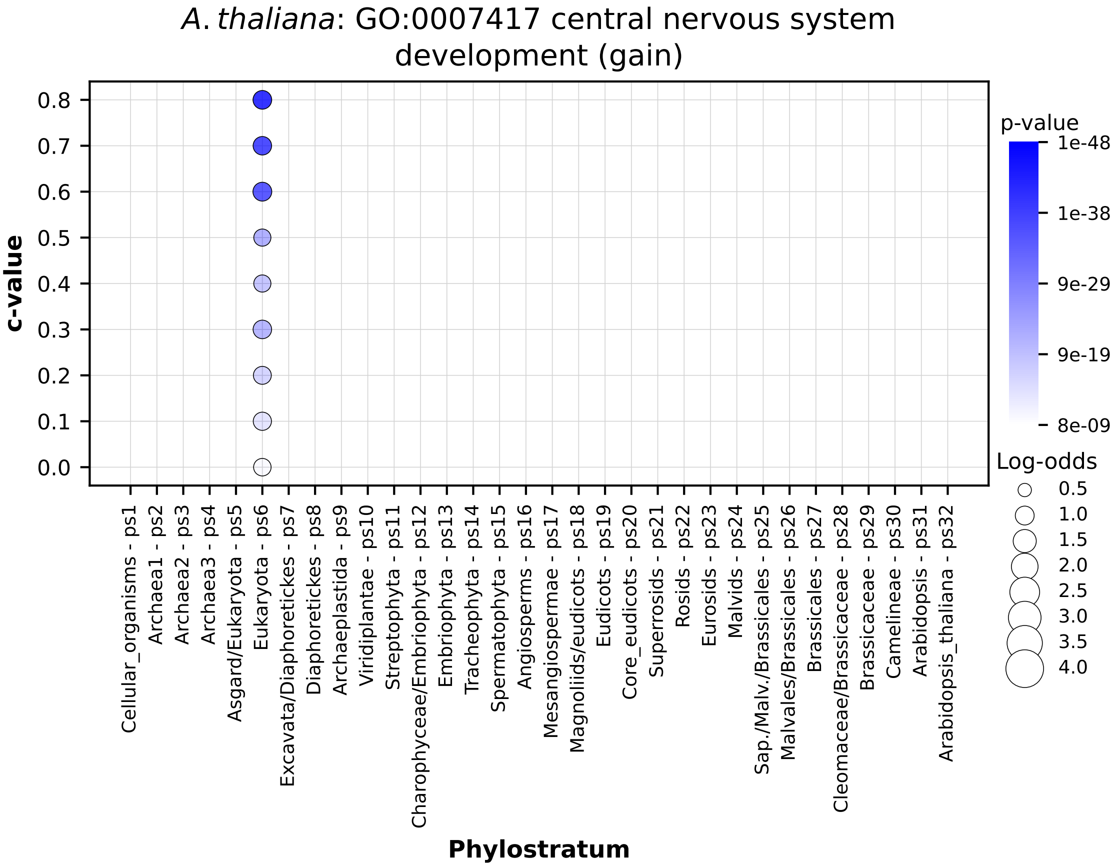

A. thaliana: GO:0007417 central nervous system development (gain)
c-value
0.8
0.7
0.6
0.5
0.4
0.3
0.2
0.1
0.0
p-value
1e-48
1e-38
9e-29
9e-19
8e-09
Log-odds
0.5
1.0
1.5
2.0
2.5
3.0
3.5
4.0
Cellular_organisms - ps1
Archaea1 - ps2
Archaea2 - ps3
Archaea3 - ps4
Asgard/Eukaryota - ps5
Eukaryota - ps6
Excavata/Diaphoretickes - ps7
Diaphoretickes - ps8
Archaeplastida - ps9
Viridiplantae - ps10
Streptophyta - ps11
Charophyceae/Embriophyta - ps12
Embriophyta - ps13
Tracheophyta - ps14
Spermatophyta - ps15
Angiosperms - ps16
Mesangiospermae - ps17
Magnoliids/eudicots - ps18
Eudicots - ps19
Core_eudicots - ps20
Superrosids - ps21
Rosids - ps22
Eurosids - ps23
Malvids - ps24
Sap./Malv./Brassicales - ps25
Malvales/Brassicales - ps26
Brassicales - ps27
Cleomaceae/Brassicaceae - ps28
Brassicaceae - ps29
Camelineae - ps30
Arabidopsis - ps31
Arabidopsis_thaliana - ps32
Phylostratum

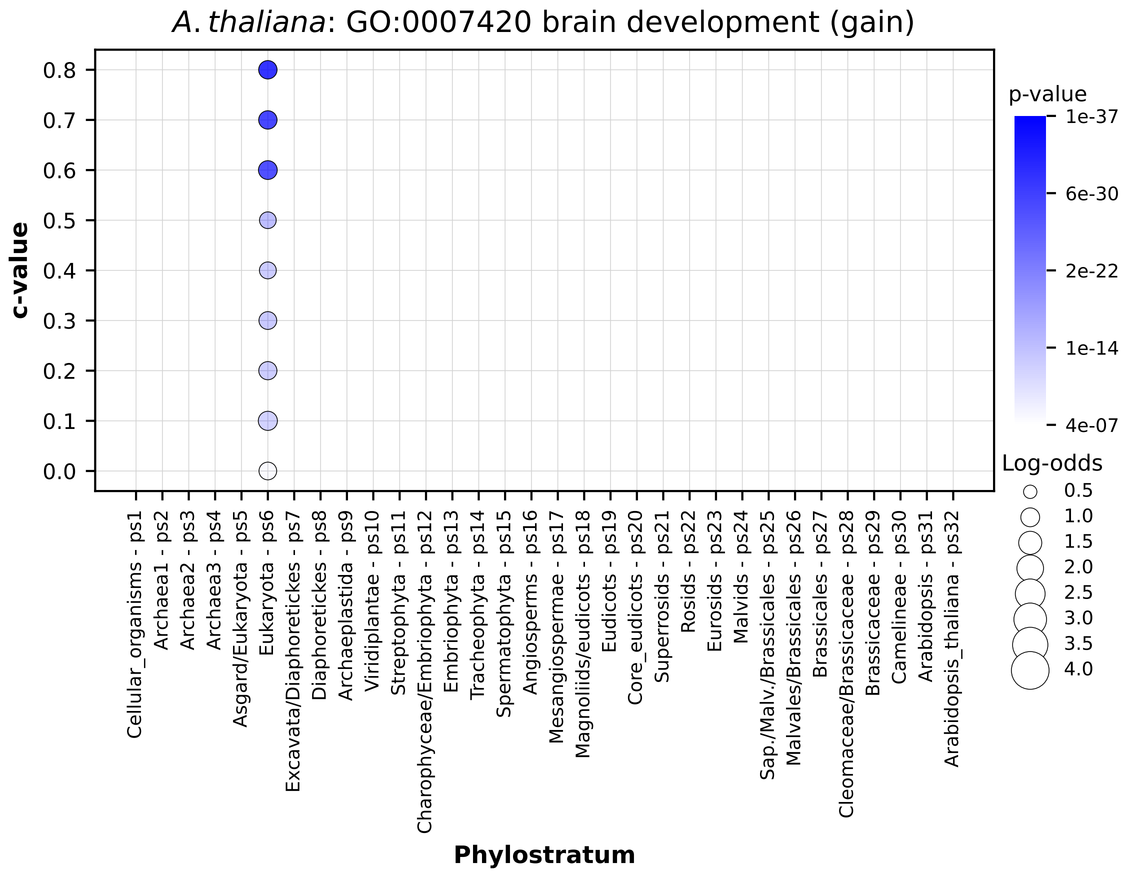
A. thaliana: GO:0007420 brain development (gain)
c-value
0.8
0.7
0.6
0.5
0.4
0.3
0.2
0.1
0.0
p-value
1e-37
6e-30
2e-22
1e-14
4e-07
Log-odds
0.5
1.0
1.5
2.0
2.5
3.0
3.5
4.0
Cellular_organisms - ps1
Archaea1 - ps2
Archaea2 - ps3
Archaea3 - ps4
Asgard/Eukaryota - ps5
Eukaryota - ps6
Excavata/Diaphoretickes - ps7
Diaphoretickes - ps8
Archaeplastida - ps9
Viridiplantae - ps10
Streptophyta - ps11
Charophyceae/Embriophyta - ps12
Embriophyta - ps13
Tracheophyta - ps14
Spermatophyta - ps15
Angiosperms - ps16
Mesangiospermae - ps17
Magnoliids/eudicots - ps18
Eudicots - ps19
Core_eudicots - ps20
Superrosids - ps21
Rosids - ps22
Eurosids - ps23
Malvids - ps24
Sap./Malv./Brassicales - ps25
Malvales/Brassicales - ps26
Brassicales - ps27
Cleomaceae/Brassicaceae - ps28
Brassicaceae - ps29
Camelineae - ps30
Arabidopsis - ps31
Arabidopsis_thaliana - ps32
Phylostratum

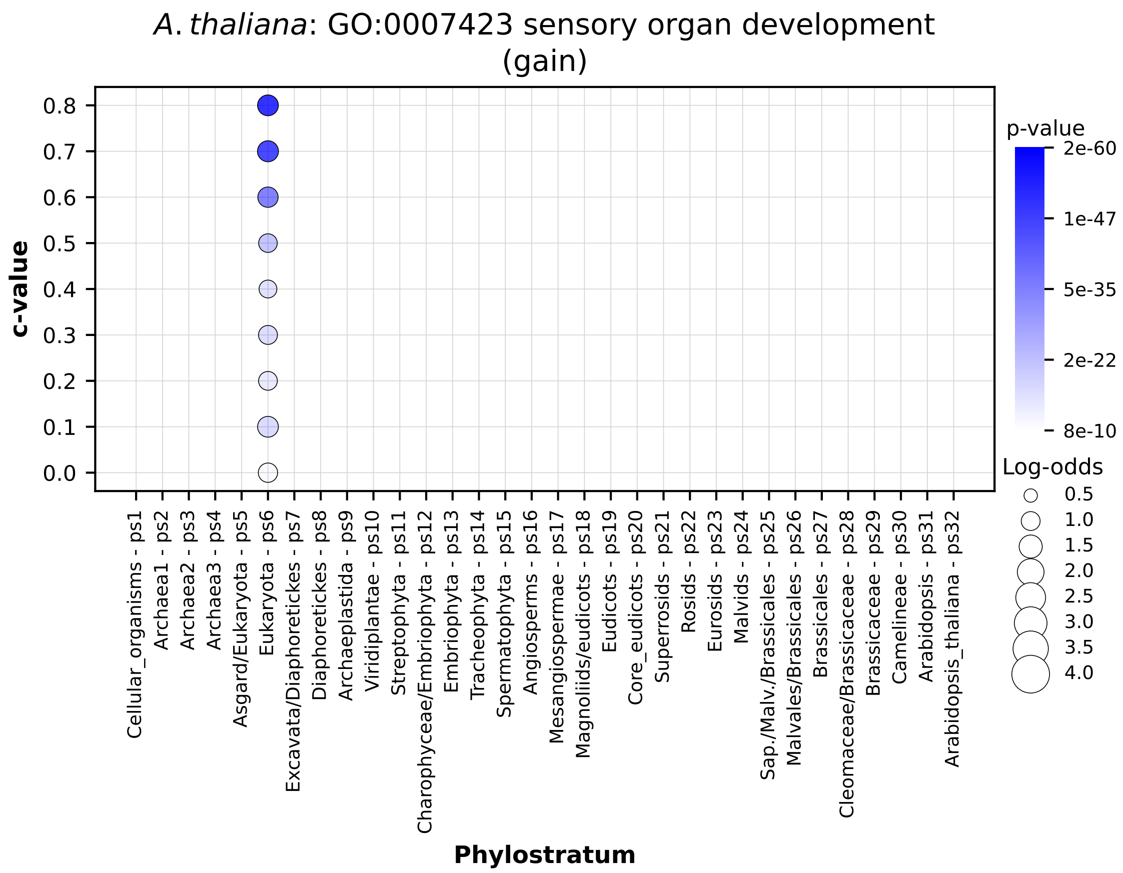

A. thaliana: GO:0007423 sensory organ development (gain)
c-value
0.8
0.7
0.6
0.5
0.4
0.3
0.2
0.1
0.0
p-value
2e-60
1e-47
5e-35
2e-22
8e-10
Log-odds
0.5
1.0
1.5
2.0
2.5
3.0
3.5
4.0
Cellular_organisms - ps1
Archaea1 - ps2
Archaea2 - ps3
Archaea3 - ps4
Asgard/Eukaryota - ps5
Eukaryota - ps6
Excavata/Diaphoretickes - ps7
Diaphoretickes - ps8
Archaeplastida - ps9
Viridiplantae - ps10
Streptophyta - ps11
Charophyceae/Embriophyta - ps12
Embriophyta - ps13
Tracheophyta - ps14
Spermatophyta - ps15
Angiosperms - ps16
Mesangiospermae - ps17
Magnoliids/eudicots - ps18
Eudicots - ps19
Core_eudicots - ps20
Superrosids - ps21
Rosids - ps22
Eurosids - ps23
Malvids - ps24
Sap./Malv./Brassicales - ps25
Malvales/Brassicales - ps26
Brassicales - ps27
Cleomaceae/Brassicaceae - ps28
Brassicaceae - ps29
Camelineae - ps30
Arabidopsis - ps31
Arabidopsis_thaliana - ps32
Phylostratum

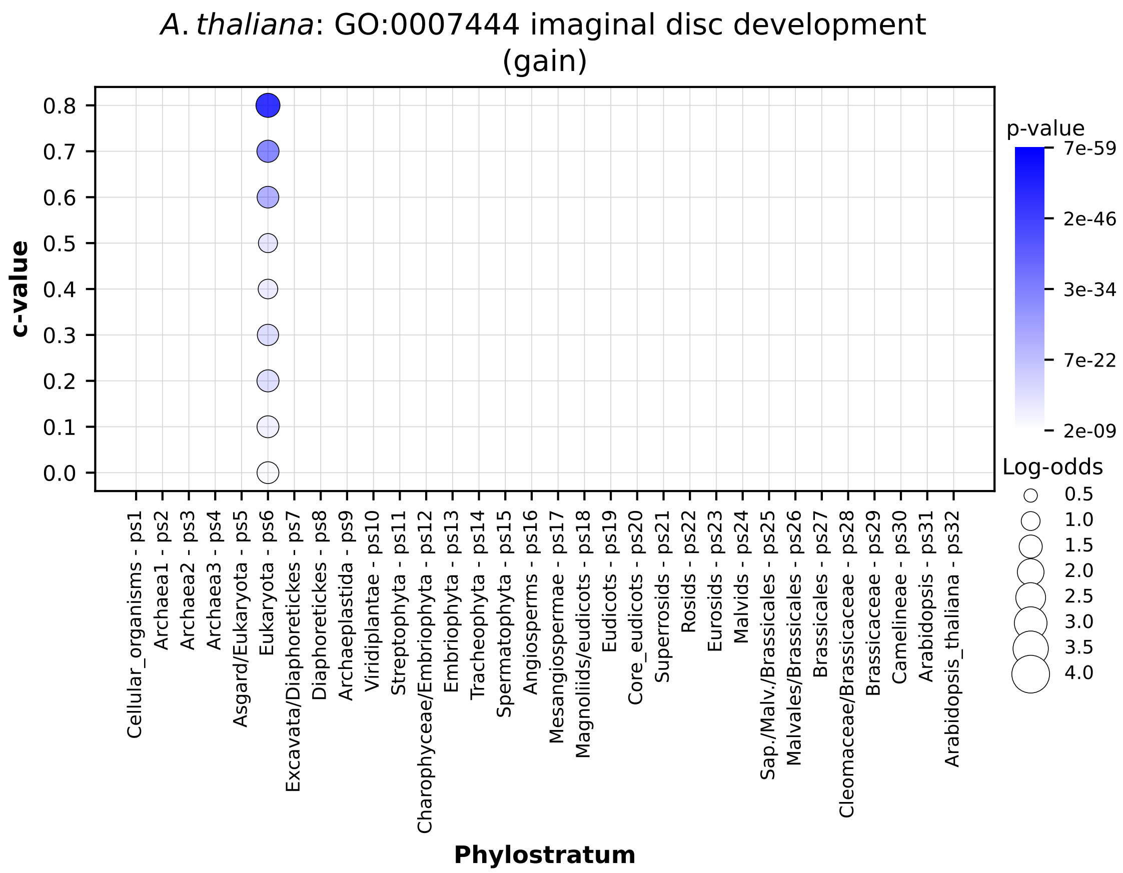

A. thaliana: GO:0007444 imaginal disc development
(gain)
c-value
0.8
0.7
0.6
0.5
0.4
0.3
0.2
0.1
0.0
p-value
7e-59
2e-46
3e-34
7e-22
2e-09
Log-odds
0.5
1.0
1.5
2.0
2.5
3.0
3.5
4.0
Cellular_organisms - ps1
Archaea1 - ps2
Archaea2 - ps3
Archaea3 - ps4
Asgard/Eukaryota - ps5
Eukaryota - ps6
Excavata/Diaphoretickes - ps7
Diaphoretickes - ps8
Archaeplastida - ps9
Viridiplantae - ps10
Streptophyta - ps11
Charophyceae/Embriophyta - ps12
Embriophyta - ps13
Tracheophyta - ps14
Spermatophyta - ps15
Angiosperms - ps16
Mesangiospermae - ps17
Magnoliids/eudicots - ps18
Eudicots - ps19
Core_eudicots - ps20
Superrosids - ps21
Rosids - ps22
Eurosids - ps23
Malvids - ps24
Sap./Malv./Brassicales - ps25
Malvales/Brassicales - ps26
Brassicales - ps27
Cleomaceae/Brassicaceae - ps28
Brassicaceae - ps29
Camelineae - ps30
Arabidopsis - ps31
Arabidopsis_thaliana - ps32
Phylostratum

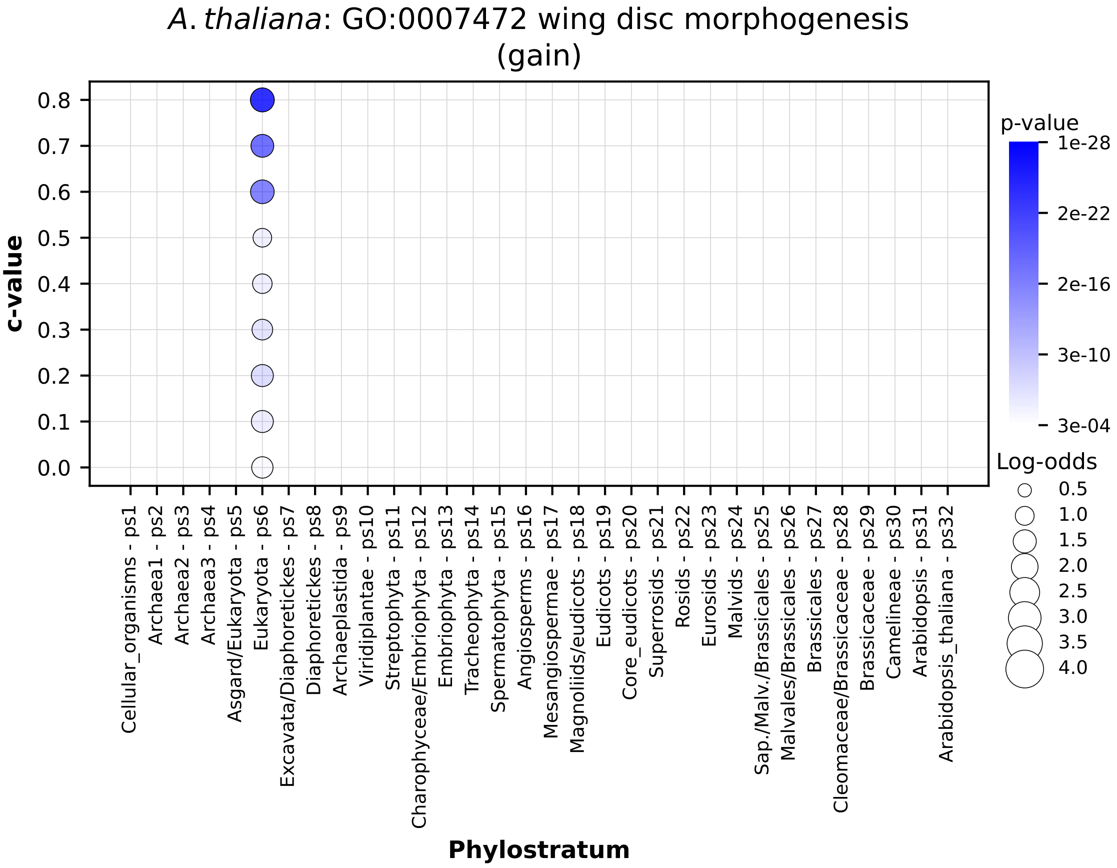
A. thaliana: GO:0007472 wing disc morphogenesis
(gain)
c-value
0.8
0.7
0.6
0.5
0.4
0.3
0.2
0.1
0.0
Phylostratum
Cellular_organisms - ps1
Archaea1 - ps2
Archaea2 - ps3
Archaea3 - ps4
Asgard/Eukaryota - ps5
Eukaryota - ps6
Excavata/Diaphoretickes - ps7
Diaphoretickes - ps8
Archaeplastida - ps9
Viridiplantae - ps10
Streptophyta - ps11
Charophyceae/Embriophyta - ps12
Embriophyta - ps13
Tracheophyta - ps14
Spermatophyta - ps15
Angiosperms - ps16
Mesangiospermae - ps17
Magnoliids/eudicots - ps18
Eudicots - ps19
Core_eudicots - ps20
Superrosids - ps21
Rosids - ps22
Eurosids - ps23
Malvids - ps24
Sap./Malv./Brassicales - ps25
Malvales/Brassicales - ps26
Brassicales - ps27
Cleomaceae/Brassicaceae - ps28
Brassicaceae - ps29
Camelineae - ps30
Arabidopsis - ps31
Arabidopsis_thaliana - ps32
p-value
1e-28
2e-22
2e-16
3e-10
3e-04
Log-odds
0.5
1.0
1.5
2.0
2.5
3.0
3.5
4.0

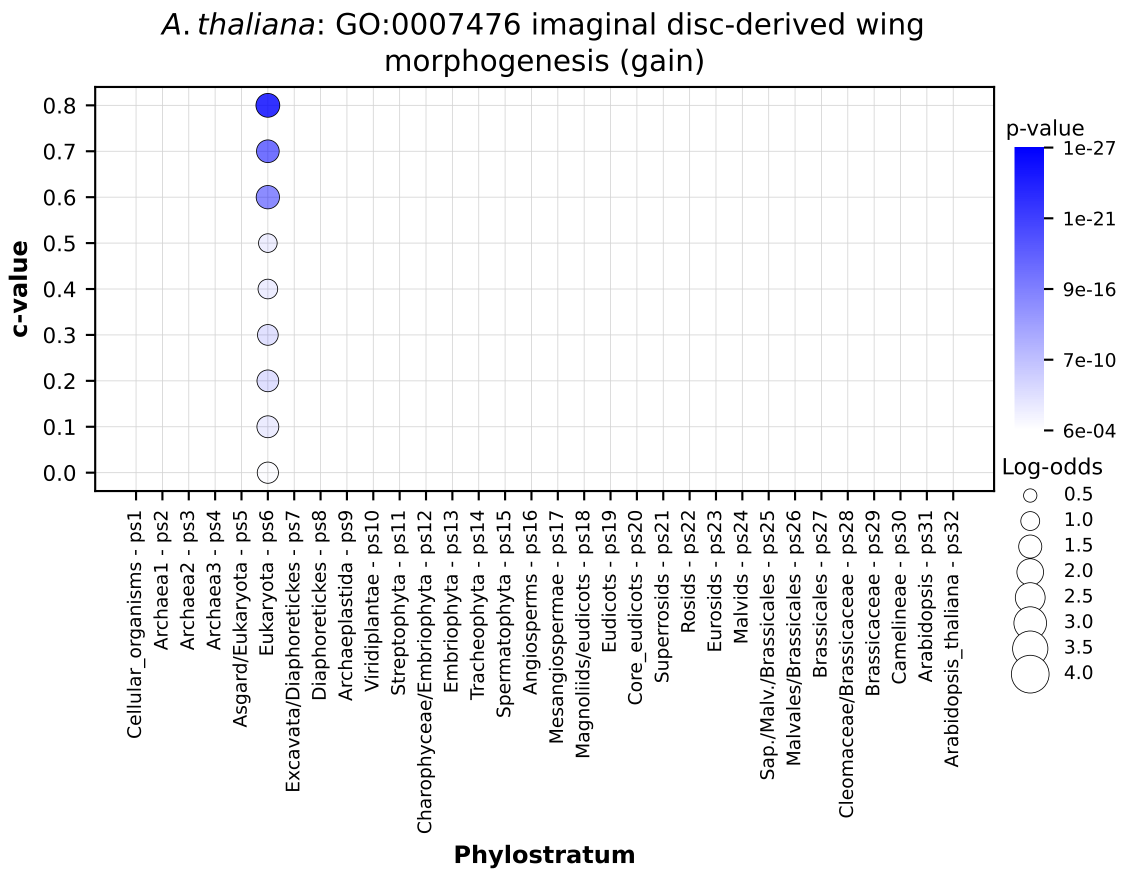

A. thaliana: GO:0007476 imaginal disc-derived wing morphogenesis (gain)
c-value
0.8
0.7
0.6
0.5
0.4
0.3
0.2
0.1
0.0
p-value
1e-27
1e-21
9e-16
7e-10
6e-04
Log-odds
0.5
1.0
1.5
2.0
2.5
3.0
3.5
4.0
Cellular_organisms - ps1
Archaea1 - ps2
Archaea2 - ps3
Archaea3 - ps4
Asgard/Eukaryota - ps5
Eukaryota - ps6
Excavata/Diaphoretickes - ps7
Diaphoretickes - ps8
Archaeplastida - ps9
Viridiplantae - ps10
Streptophyta - ps11
Charophyceae/Embriophyta - ps12
Embriophyta - ps13
Tracheophyta - ps14
Spermatophyta - ps15
Angiosperms - ps16
Mesangiospermae - ps17
Magnoliids/eudicots - ps18
Eudicots - ps19
Core_eudicots - ps20
Superrosids - ps21
Rosids - ps22
Eurosids - ps23
Malvids - ps24
Sap./Malv./Brassicales - ps25
Malvales/Brassicales - ps26
Brassicales - ps27
Cleomaceae/Brassicaceae - ps28
Brassicaceae - ps29
Camelineae - ps30
Arabidopsis - ps31
Arabidopsis_thaliana - ps32
Phylostratum

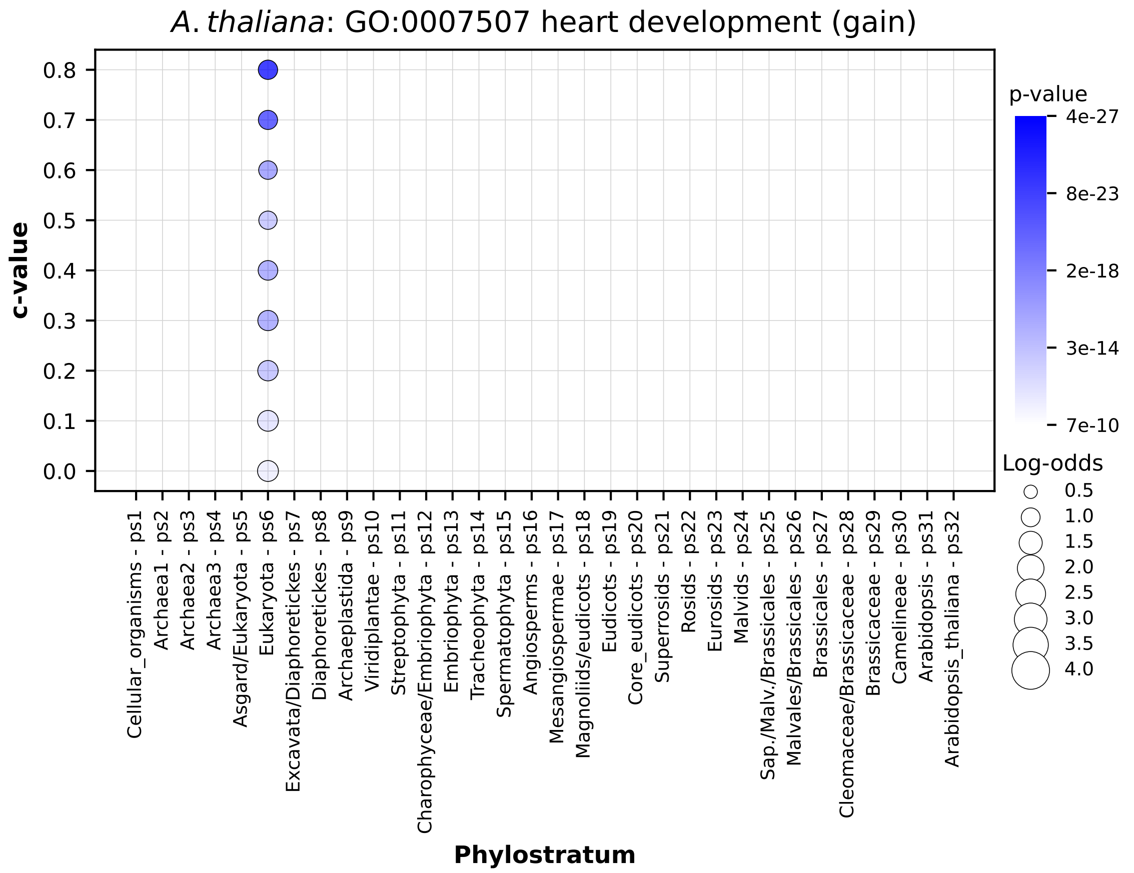
A. thaliana: GO:0007507 heart development (gain)
c-value
0.8
0.7
0.6
0.5
0.4
0.3
0.2
0.1
0.0
p-value
4e-27
8e-23
2e-18
3e-14
7e-10
Log-odds
0.5
1.0
1.5
2.0
2.5
3.0
3.5
4.0
Cellular_organisms - ps1
Archaea1 - ps2
Archaea2 - ps3
Archaea3 - ps4
Asgard/Eukaryota - ps5
Eukaryota - ps6
Excavata/Diaphoretickes - ps7
Diaphoretickes - ps8
Archaeplastida - ps9
Viridiplantae - ps10
Streptophyta - ps11
Charophyceae/Embriophyta - ps12
Embriophyta - ps13
Tracheophyta - ps14
Spermatophyta - ps15
Angiosperms - ps16
Mesangiospermae - ps17
Magnoliids/eudicots - ps18
Eudicots - ps19
Core_eudicots - ps20
Superrosids - ps21
Rosids - ps22
Eurosids - ps23
Malvids - ps24
Sap./Malv./Brassicales - ps25
Malvales/Brassicales - ps26
Brassicales - ps27
Cleomaceae/Brassicaceae - ps28
Brassicaceae - ps29
Camelineae - ps30
Arabidopsis - ps31
Arabidopsis_thaliana - ps32
Phylostratum

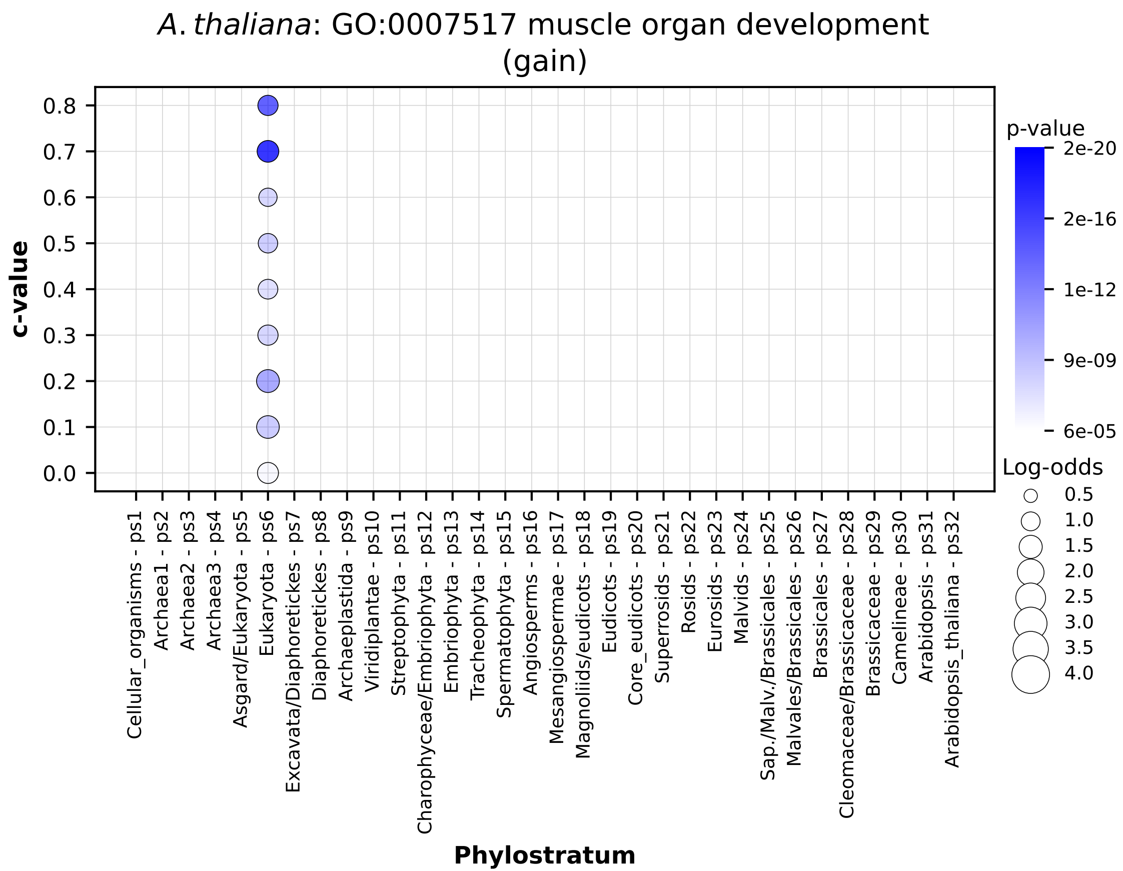

A. thaliana: GO:0007517 muscle organ development
(gain)
c-value
0.8
0.7
0.6
0.5
0.4
0.3
0.2
0.1
0.0
p-value
2e-20
2e-16
1e-12
9e-09
6e-05
Log-odds
0.5
1.0
1.5
2.0
2.5
3.0
3.5
4.0
Cellular_organisms - ps1
Archaea1 - ps2
Archaea2 - ps3
Archaea3 - ps4
Asgard/Eukaryota - ps5
Eukaryota - ps6
Excavata/Diaphoretickes - ps7
Diaphoretickes - ps8
Archaeplastida - ps9
Viridiplantae - ps10
Streptophyta - ps11
Charophyceae/Embriophyta - ps12
Embriophyta - ps13
Tracheophyta - ps14
Spermatophyta - ps15
Angiosperms - ps16
Mesangiospermae - ps17
Magnoliids/eudicots - ps18
Eudicots - ps19
Core_eudicots - ps20
Superrosids - ps21
Rosids - ps22
Eurosids - ps23
Malvids - ps24
Sap./Malv./Brassicales - ps25
Malvales/Brassicales - ps26
Brassicales - ps27
Cleomaceae/Brassicaceae - ps28
Brassicaceae - ps29
Camelineae - ps30
Arabidopsis - ps31
Arabidopsis_thaliana - ps32
Phylostratum

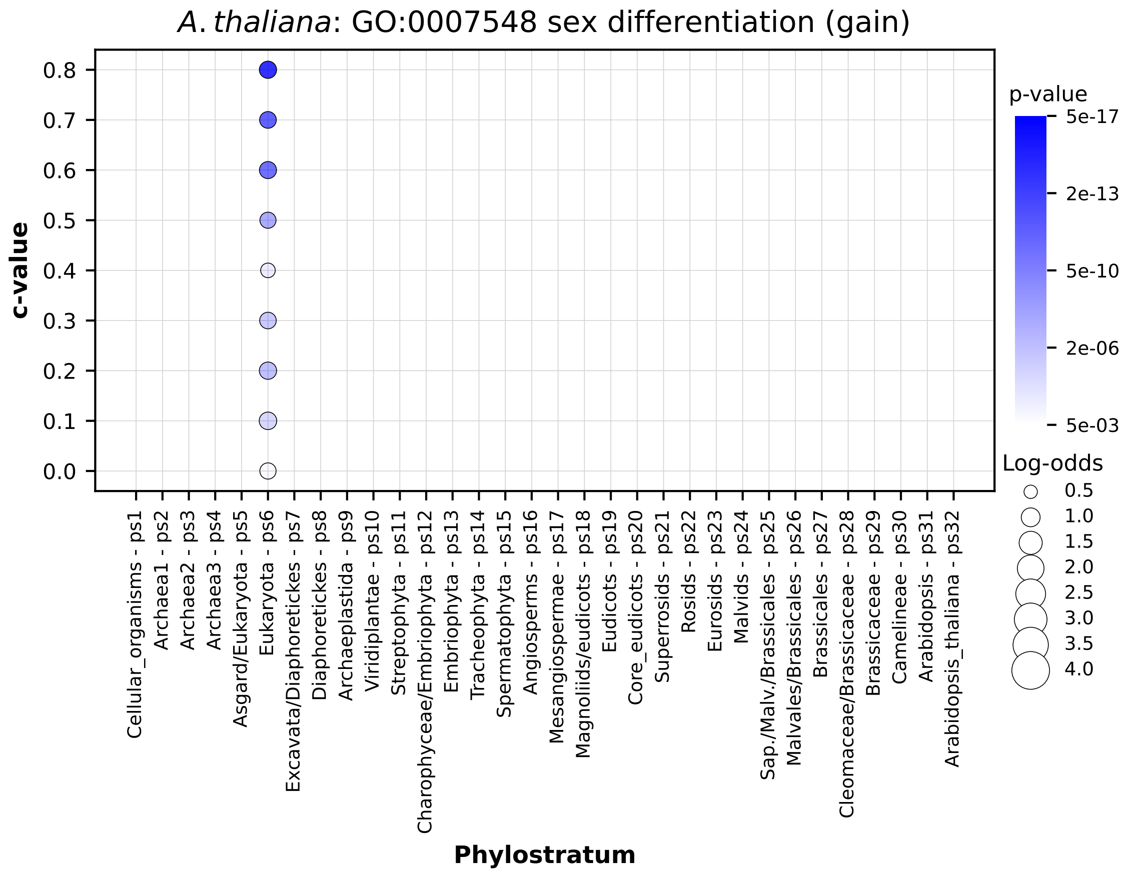
A. thaliana: GO:0007548 sex differentiation (gain)
c-value
0.8
0.7
0.6
0.5
0.4
0.3
0.2
0.1
0.0
p-value
5e-17
2e-13
5e-10
2e-06
5e-03
Log-odds
0.5
1.0
1.5
2.0
2.5
3.0
3.5
4.0
Cellular_organisms - ps1
Archaea1 - ps2
Archaea2 - ps3
Archaea3 - ps4
Asgard/Eukaryota - ps5
Eukaryota - ps6
Excavata/Diaphoretickes - ps7
Diaphoretickes - ps8
Archaeplastida - ps9
Viridiplantae - ps10
Streptophyta - ps11
Charophyceae/Embriophyta - ps12
Embriophyta - ps13
Tracheophyta - ps14
Spermatophyta - ps15
Angiosperms - ps16
Mesangiospermae - ps17
Magnoliids/eudicots - ps18
Eudicots - ps19
Core_eudicots - ps20
Superrosids - ps21
Rosids - ps22
Eurosids - ps23
Malvids - ps24
Sap./Malv./Brassicales - ps25
Malvales/Brassicales - ps26
Brassicales - ps27
Cleomaceae/Brassicaceae - ps28
Brassicaceae - ps29
Camelineae - ps30
Arabidopsis - ps31
Arabidopsis_thaliana - ps32
Phylostratum

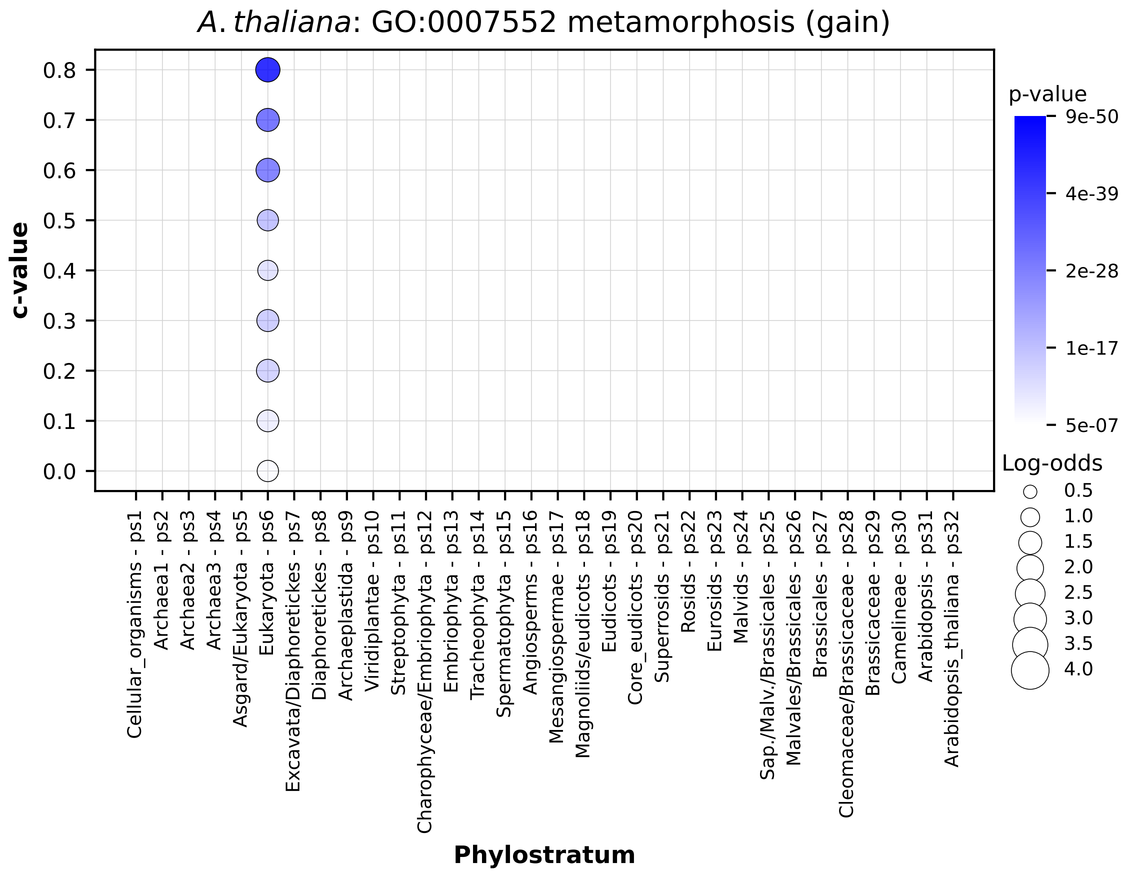

A. thaliana: GO:0007552 metamorphosis (gain)
c-value
0.0
0.1
0.2
0.3
0.4
0.5
0.6
0.7
0.8
Phylostratum
Cellular_organisms - ps1
Archaea1 - ps2
Archaea2 - ps3
Archaea3 - ps4
Asgard/Eukaryota - ps5
Eukaryota - ps6
Excavata/Diaphoretickes - ps7
Diaphoretickes - ps8
Archaeplastida - ps9
Viridiplantae - ps10
Streptophyta - ps11
Charophyceae/Embriophyta - ps12
Embriophyta - ps13
Tracheophyta - ps14
Spermatophyta - ps15
Angiosperms - ps16
Mesangiospermae - ps17
Magnoliids/eudicots - ps18
Eudicots - ps19
Core_eudicots - ps20
Superrosids - ps21
Rosids - ps22
Eurosids - ps23
Malvids - ps24
Sap./Malv./Brassicales - ps25
Malvales/Brassicales - ps26
Brassicales - ps27
Cleomaceae/Brassicaceae - ps28
Brassicaceae - ps29
Camelineae - ps30
Arabidopsis - ps31
Arabidopsis_thaliana - ps32
p-value
9e-50
4e-39
2e-28
1e-17
5e-07
Log-odds
0.5
1.0
1.5
2.0
2.5
3.0
3.5
4.0

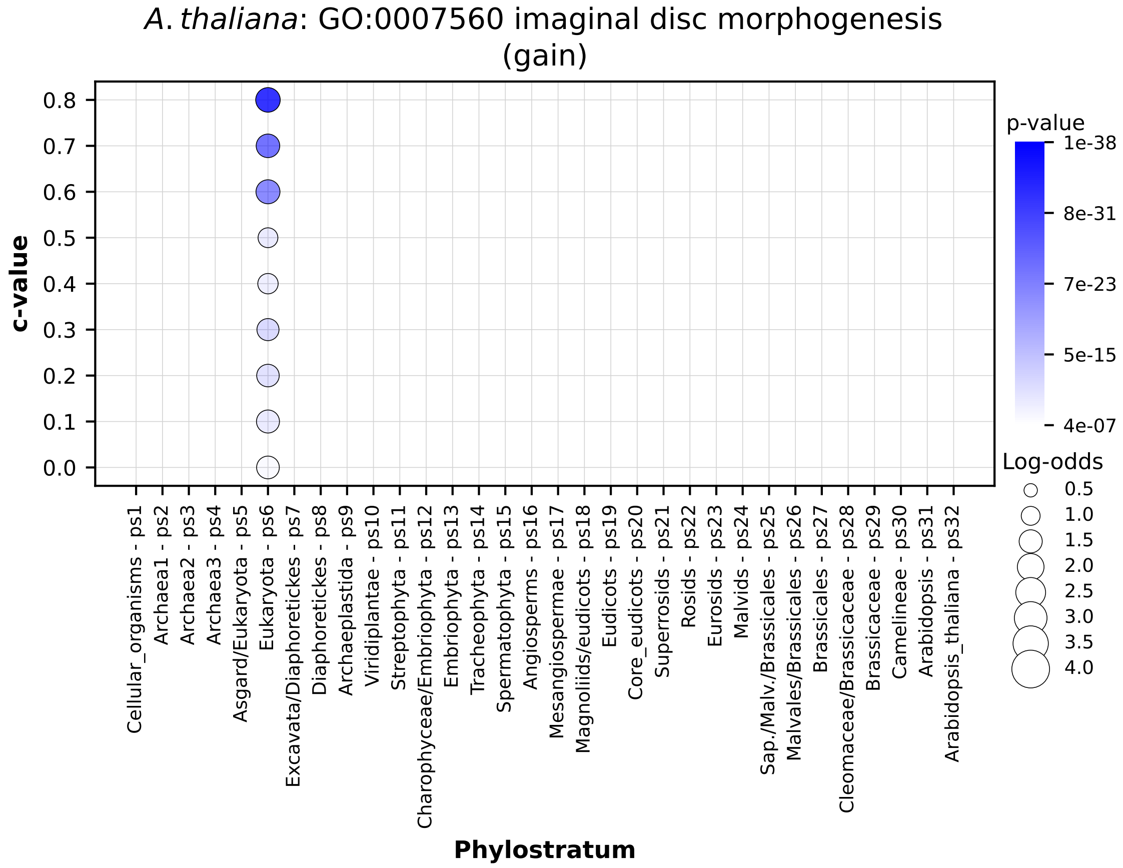

A. thaliana: GO:0007560 imaginal disc morphogenesis
(gain)
c-value
0.0
0.1
0.2
0.3
0.4
0.5
0.6
0.7
0.8
p-value
1e-38
8e-31
7e-23
5e-15
4e-07
Log-odds
0.5
1.0
1.5
2.0
2.5
3.0
3.5
4.0
Cellular_organisms - ps1
Archaea1 - ps2
Archaea2 - ps3
Archaea3 - ps4
Asgard/Eukaryota - ps5
Eukaryota - ps6
Excavata/Diaphoretickes - ps7
Diaphoretickes - ps8
Archaeplastida - ps9
Viridiplantae - ps10
Streptophyta - ps11
Charophyceae/Embriophyta - ps12
Embriophyta - ps13
Tracheophyta - ps14
Spermatophyta - ps15
Angiosperms - ps16
Mesangiospermae - ps17
Magnoliids/eudicots - ps18
Eudicots - ps19
Core_eudicots - ps20
Superrosids - ps21
Rosids - ps22
Eurosids - ps23
Malvids - ps24
Sap./Malv./Brassicales - ps25
Malvales/Brassicales - ps26
Brassicales - ps27
Cleomaceae/Brassicaceae - ps28
Brassicaceae - ps29
Camelineae - ps30
Arabidopsis - ps31
Arabidopsis_thaliana - ps32
Phylostratum

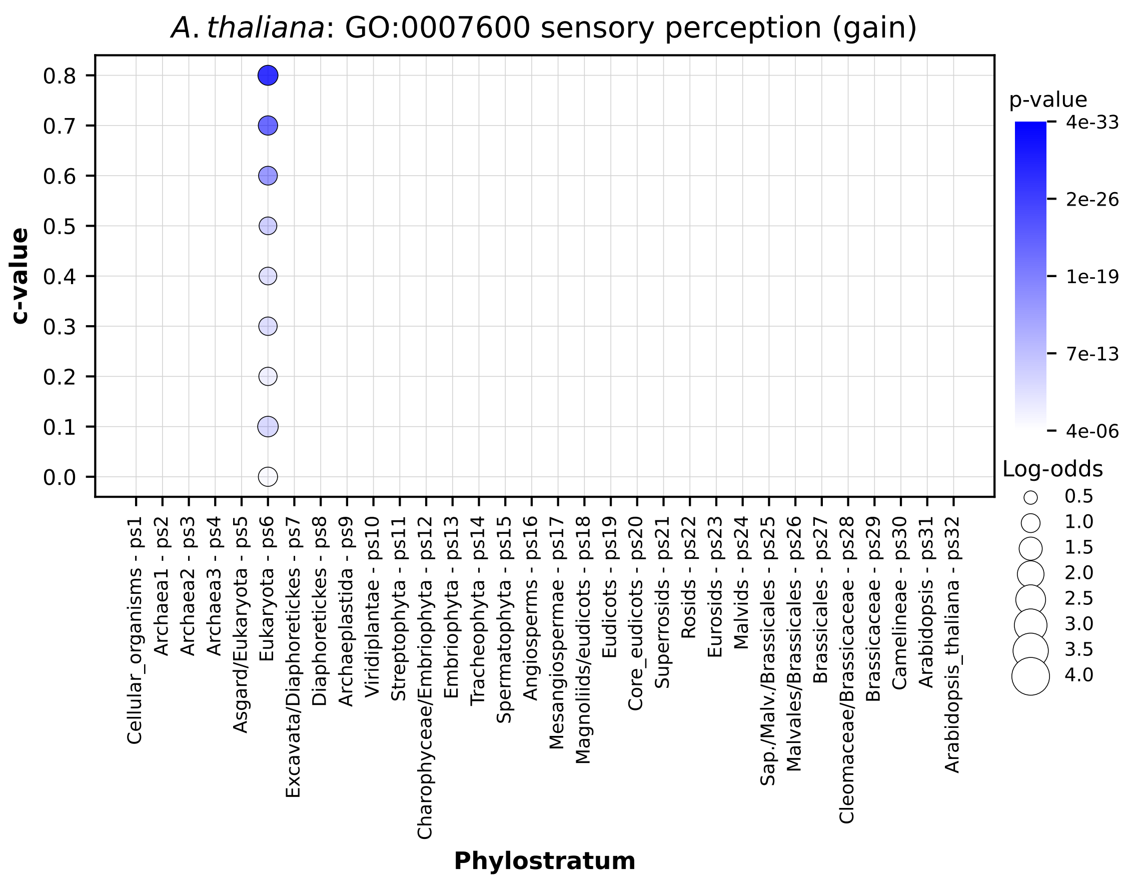
A. thaliana: GO:0007600 sensory perception (gain)
c-value
0.8
0.7
0.6
0.5
0.4
0.3
0.2
0.1
0.0
p-value
4e-33
2e-26
1e-19
7e-13
4e-06
Log-odds
0.5
1.0
1.5
2.0
2.5
3.0
3.5
4.0
Cellular_organisms - ps1
Archaea1 - ps2
Archaea2 - ps3
Archaea3 - ps4
Asgard/Eukaryota - ps5
Eukaryota - ps6
Excavata/Diaphoretickes - ps7
Diaphoretickes - ps8
Archaeplastida - ps9
Viridiplantae - ps10
Streptophyta - ps11
Charophyceae/Embriophyta - ps12
Embriophyta - ps13
Tracheophyta - ps14
Spermatophyta - ps15
Angiosperms - ps16
Mesangiospermae - ps17
Magnoliids/eudicots - ps18
Eudicots - ps19
Core_eudicots - ps20
Superrosids - ps21
Rosids - ps22
Eurosids - ps23
Malvids - ps24
Sap./Malv./Brassicales - ps25
Malvales/Brassicales - ps26
Brassicales - ps27
Cleomaceae/Brassicaceae - ps28
Brassicaceae - ps29
Camelineae - ps30
Arabidopsis - ps31
Arabidopsis_thaliana - ps32
Phylostratum

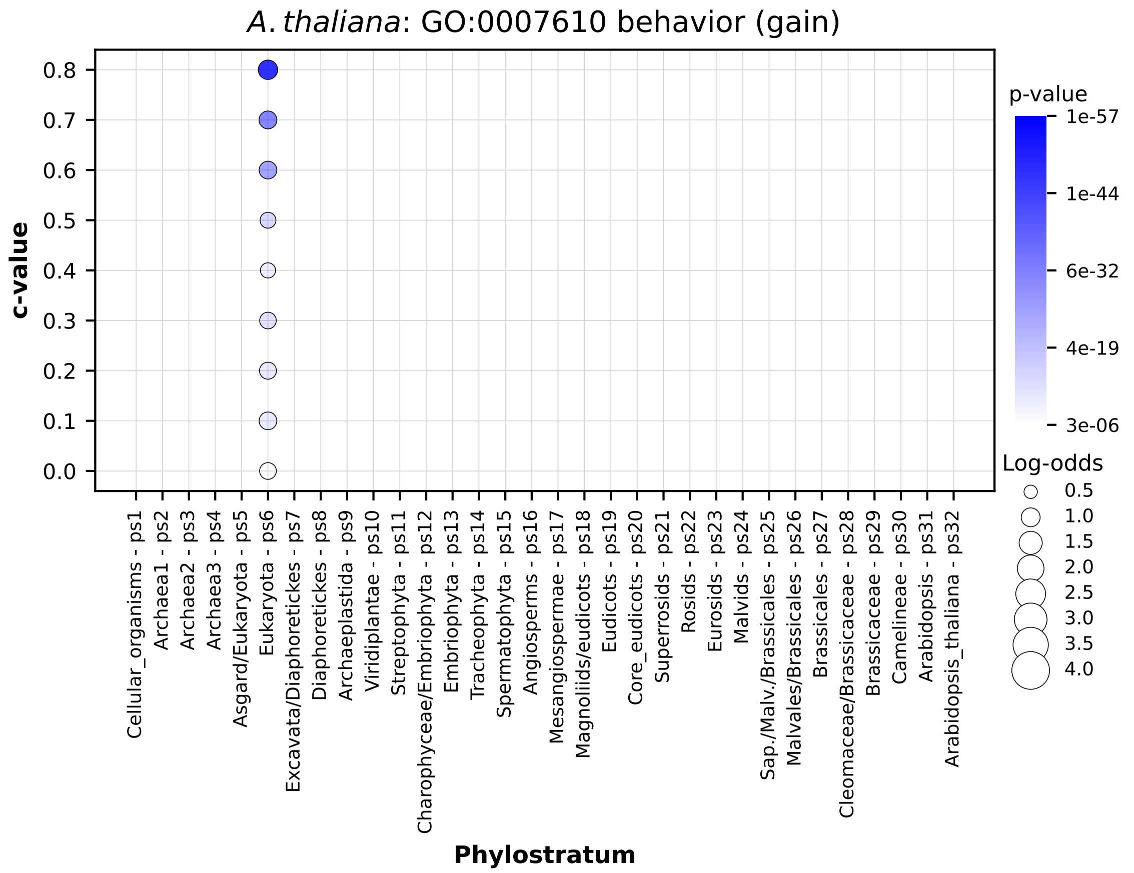

A. thaliana: GO:0007610 behavior (gain)
c-value
0.0
0.1
0.2
0.3
0.4
0.5
0.6
0.7
0.8
p-value
1e-57
1e-44
6e-32
4e-19
3e-06
Log-odds
0.5
1.0
1.5
2.0
2.5
3.0
3.5
4.0
Cellular_organisms - ps1
Archaea1 - ps2
Archaea2 - ps3
Archaea3 - ps4
Asgard/Eukaryota - ps5
Eukaryota - ps6
Excavata/Diaphoretickes - ps7
Diaphoretickes - ps8
Archaeplastida - ps9
Viridiplantae - ps10
Streptophyta - ps11
Charophyceae/Embriophyta - ps12
Embriophyta - ps13
Tracheophyta - ps14
Spermatophyta - ps15
Angiosperms - ps16
Mesangiospermae - ps17
Magnoliids/eudicots - ps18
Eudicots - ps19
Core_eudicots - ps20
Superrosids - ps21
Rosids - ps22
Eurosids - ps23
Malvids - ps24
Sap./Malv./Brassicales - ps25
Malvales/Brassicales - ps26
Brassicales - ps27
Cleomaceae/Brassicaceae - ps28
Brassicaceae - ps29
Camelineae - ps30
Arabidopsis - ps31
Arabidopsis_thaliana - ps32
Phylostratum

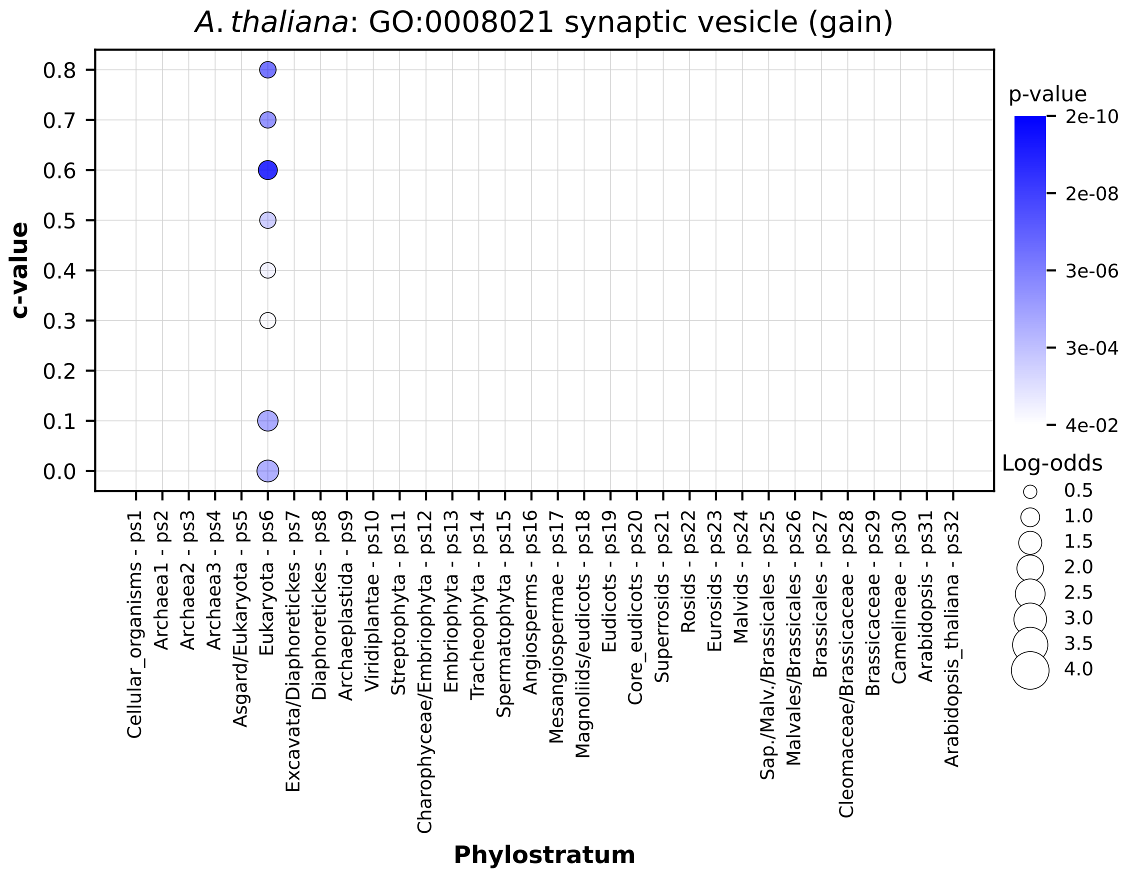

A. thaliana: GO:0008021 synaptic vesicle (gain)
c-value
0.8
0.7
0.6
0.5
0.4
0.3
0.2
0.1
0.0
p-value
2e-10
2e-08
3e-06
3e-04
4e-02
Log-odds
0.5
1.0
1.5
2.0
2.5
3.0
3.5
4.0
Cellular_organisms - ps1
Archaea1 - ps2
Archaea2 - ps3
Archaea3 - ps4
Asgard/Eukaryota - ps5
Eukaryota - ps6
Excavata/Diaphoretickes - ps7
Diaphoretickes - ps8
Archaeplastida - ps9
Viridiplantae - ps10
Streptophyta - ps11
Charophyceae/Embriophyta - ps12
Embriophyta - ps13
Tracheophyta - ps14
Spermatophyta - ps15
Angiosperms - ps16
Mesangiospermae - ps17
Magnoliids/eudicots - ps18
Eudicots - ps19
Core_eudicots - ps20
Superrosids - ps21
Rosids - ps22
Eurosids - ps23
Malvids - ps24
Sap./Malv./Brassicales - ps25
Malvales/Brassicales - ps26
Brassicales - ps27
Cleomaceae/Brassicaceae - ps28
Brassicaceae - ps29
Camelineae - ps30
Arabidopsis - ps31
Arabidopsis_thaliana - ps32
Phylostratum

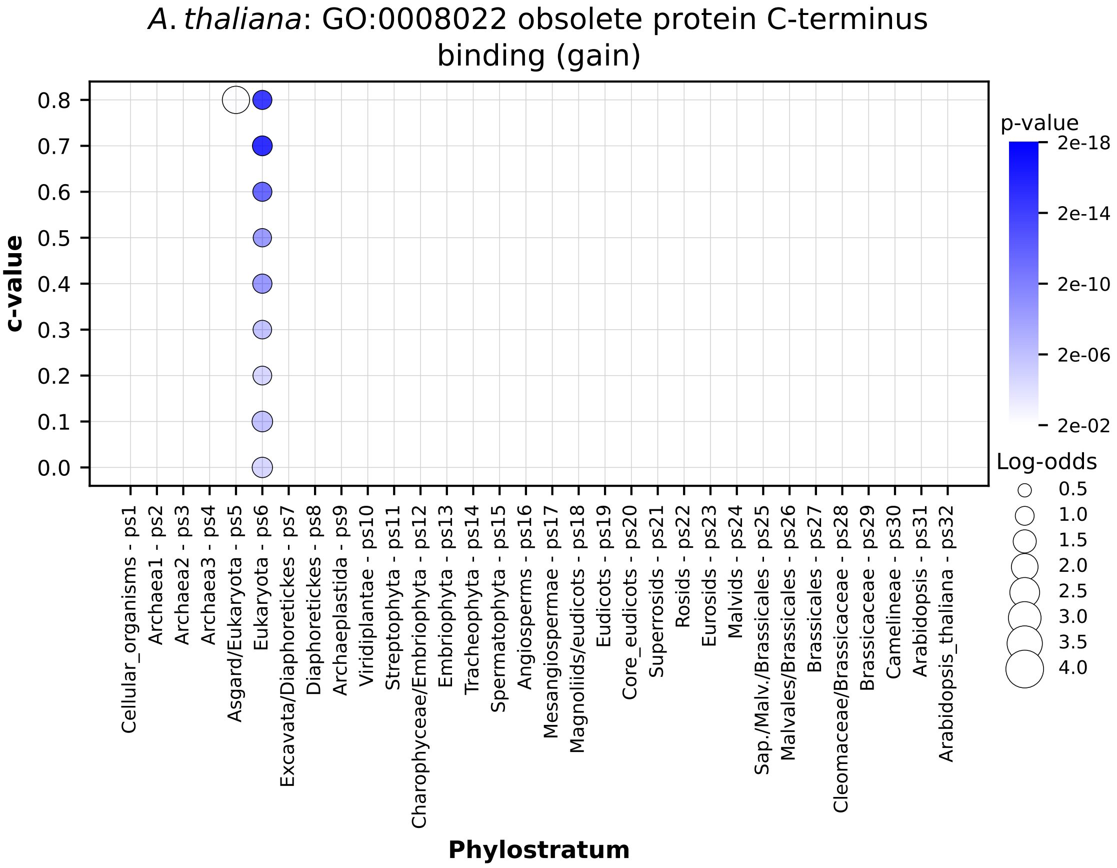

A. thaliana: GO:0008022 obsolete protein C-terminus binding (gain)
c-value
0.8
0.7
0.6
0.5
0.4
0.3
0.2
0.1
0.0
p-value
2e-18
2e-14
2e-10
2e-06
2e-02
Log-odds
0.5
1.0
1.5
2.0
2.5
3.0
3.5
4.0
Cellular_organisms - ps1
Archaea1 - ps2
Archaea2 - ps3
Archaea3 - ps4
Asgard/Eukaryota - ps5
Eukaryota - ps6
Excavata/Diaphoretickes - ps7
Diaphoretickes - ps8
Archaeplastida - ps9
Viridiplantae - ps10
Streptophyta - ps11
Charophyceae/Embriophyta - ps12
Embriophyta - ps13
Tracheophyta - ps14
Spermatophyta - ps15
Angiosperms - ps16
Mesangiospermae - ps17
Magnoliids/eudicots - ps18
Eudicots - ps19
Core_eudicots - ps20
Superrosids - ps21
Rosids - ps22
Eurosids - ps23
Malvids - ps24
Sap./Malv./Brassicales - ps25
Malvales/Brassicales - ps26
Brassicales - ps27
Cleomaceae/Brassicaceae - ps28
Brassicaceae - ps29
Camelineae - ps30
Arabidopsis - ps31
Arabidopsis_thaliana - ps32
Phylostratum

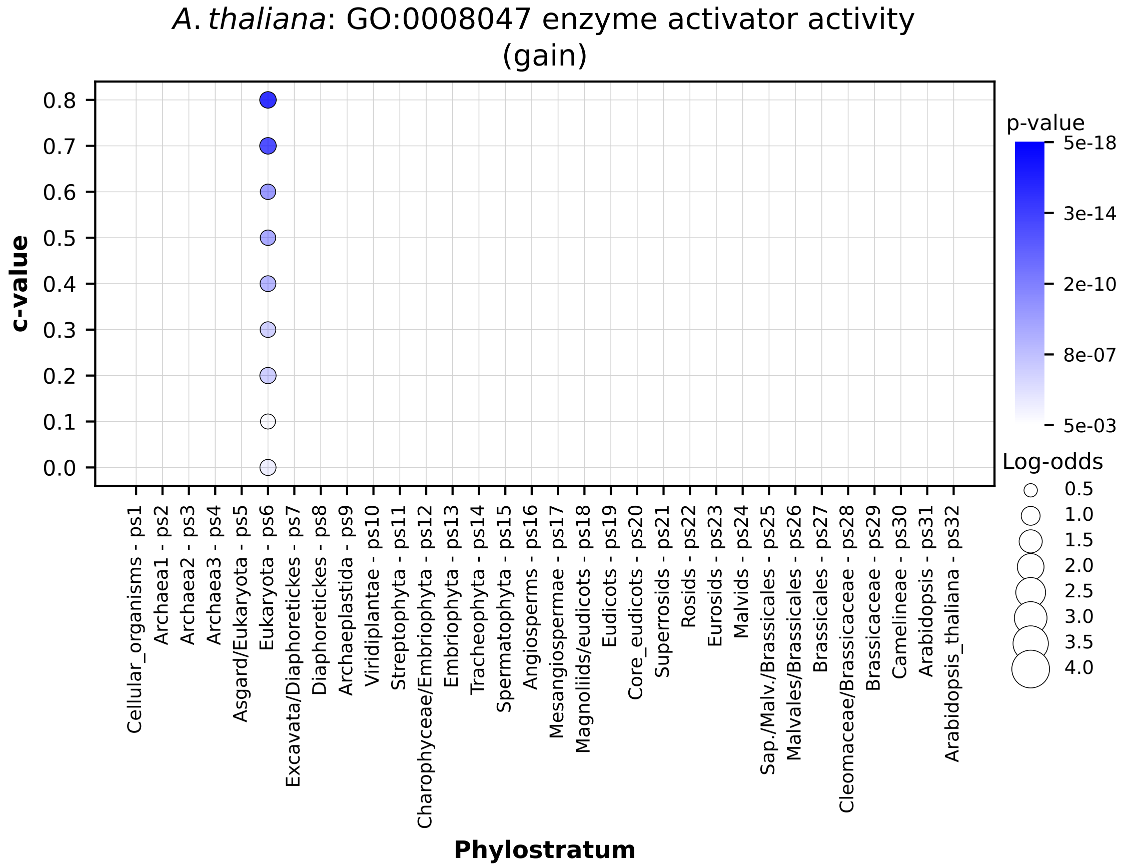

A. thaliana: GO:0008047 enzyme activator activity
(gain)
c-value
0.8
0.7
0.6
0.5
0.4
0.3
0.2
0.1
0.0
p-value
5e-18
3e-14
2e-10
8e-07
5e-03
Log-odds
0.5
1.0
1.5
2.0
2.5
3.0
3.5
4.0
Cellular_organisms - ps1
Archaea1 - ps2
Archaea2 - ps3
Archaea3 - ps4
Asgard/Eukaryota - ps5
Eukaryota - ps6
Excavata/Diaphoretickes - ps7
Diaphoretickes - ps8
Archaeplastida - ps9
Viridiplantae - ps10
Streptophyta - ps11
Charophyceae/Embriophyta - ps12
Embriophyta - ps13
Tracheophyta - ps14
Spermatophyta - ps15
Angiosperms - ps16
Mesangiospermae - ps17
Magnoliids/eudicots - ps18
Eudicots - ps19
Core_eudicots - ps20
Superrosids - ps21
Rosids - ps22
Eurosids - ps23
Malvids - ps24
Sap./Malv./Brassicales - ps25
Malvales/Brassicales - ps26
Brassicales - ps27
Cleomaceae/Brassicaceae - ps28
Brassicaceae - ps29
Camelineae - ps30
Arabidopsis - ps31
Arabidopsis_thaliana - ps32
Phylostratum

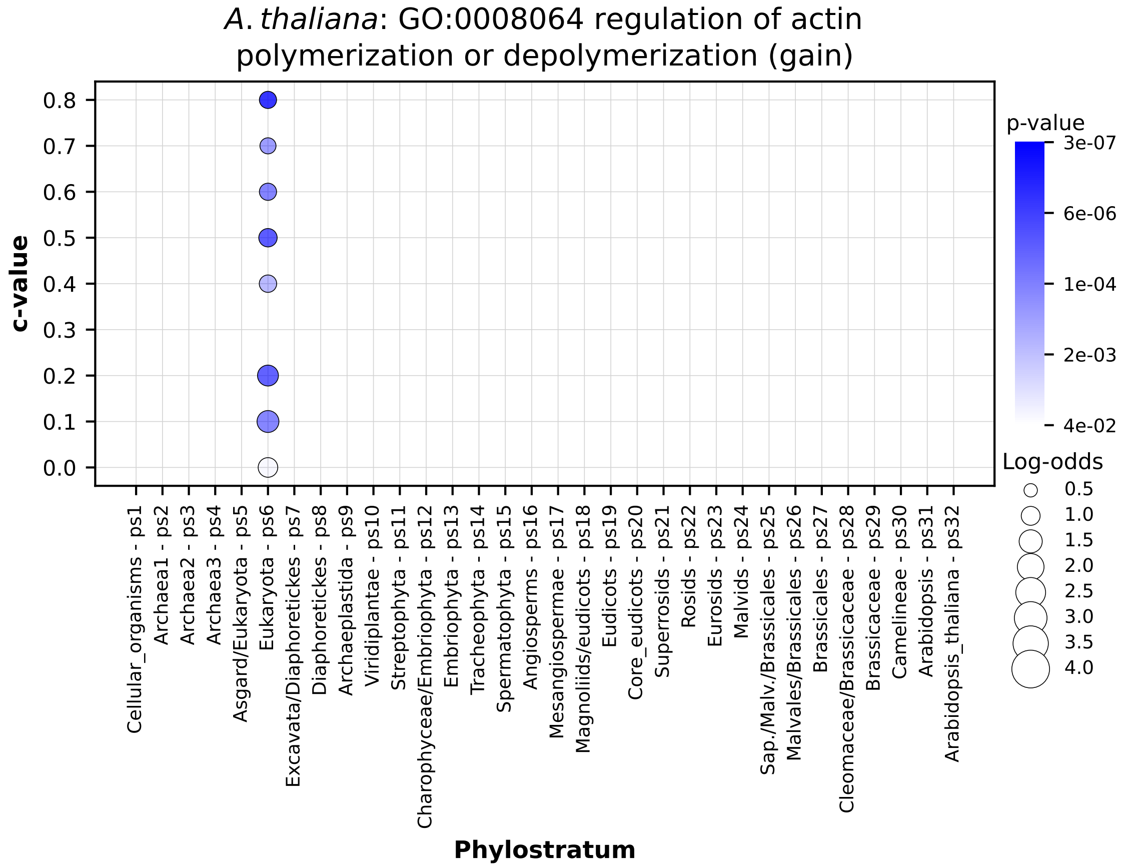

A. thaliana: GO:0008064 regulation of actin polymerization or depolymerization (gain)
c-value
0.8
0.7
0.6
0.5
0.4
0.3
0.2
0.1
0.0
p-value
3e-07
6e-06
1e-04
2e-03
4e-02
Log-odds
0.5
1.0
1.5
2.0
2.5
3.0
3.5
4.0
Cellular_organisms - ps1
Archaea1 - ps2
Archaea2 - ps3
Archaea3 - ps4
Asgard/Eukaryota - ps5
Eukaryota - ps6
Excavata/Diaphoretickes - ps7
Diaphoretickes - ps8
Archaeplastida - ps9
Viridiplantae - ps10
Streptophyta - ps11
Charophyceae/Embriophyta - ps12
Embriophyta - ps13
Tracheophyta - ps14
Spermatophyta - ps15
Angiosperms - ps16
Mesangiospermae - ps17
Magnoliids/eudicots - ps18
Eudicots - ps19
Core_eudicots - ps20
Superrosids - ps21
Rosids - ps22
Eurosids - ps23
Malvids - ps24
Sap./Malv./Brassicales - ps25
Malvales/Brassicales - ps26
Brassicales - ps27
Cleomaceae/Brassicaceae - ps28
Brassicaceae - ps29
Camelineae - ps30
Arabidopsis - ps31
Arabidopsis_thaliana - ps32
Phylostratum

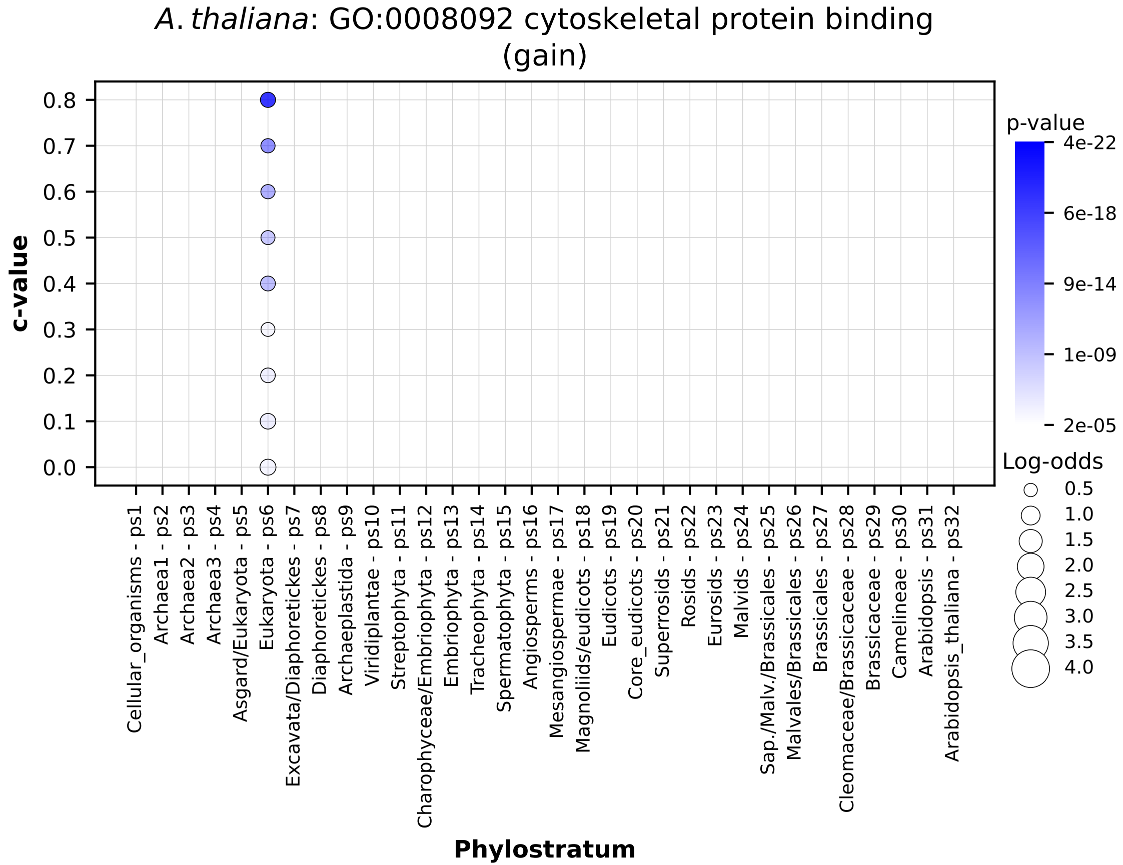

A. thaliana: GO:0008092 cytoskeletal protein binding (gain)
c-value
0.8
0.7
0.6
0.5
0.4
0.3
0.2
0.1
0.0
p-value
4e-22
6e-18
9e-14
1e-09
2e-05
Log-odds
0.5
1.0
1.5
2.0
2.5
3.0
3.5
4.0
Cellular_organisms - ps1
Archaea1 - ps2
Archaea2 - ps3
Archaea3 - ps4
Asgard/Eukaryota - ps5
Eukaryota - ps6
Excavata/Diaphoretickes - ps7
Diaphoretickes - ps8
Archaeplastida - ps9
Viridiplantae - ps10
Streptophyta - ps11
Charophyceae/Embriophyta - ps12
Embriophyta - ps13
Tracheophyta - ps14
Spermatophyta - ps15
Angiosperms - ps16
Mesangiospermae - ps17
Magnoliids/eudicots - ps18
Eudicots - ps19
Core_eudicots - ps20
Superrosids - ps21
Rosids - ps22
Eurosids - ps23
Malvids - ps24
Sap./Malv./Brassicales - ps25
Malvales/Brassicales - ps26
Brassicales - ps27
Cleomaceae/Brassicaceae - ps28
Brassicaceae - ps29
Camelineae - ps30
Arabidopsis - ps31
Arabidopsis_thaliana - ps32
Phylostratum

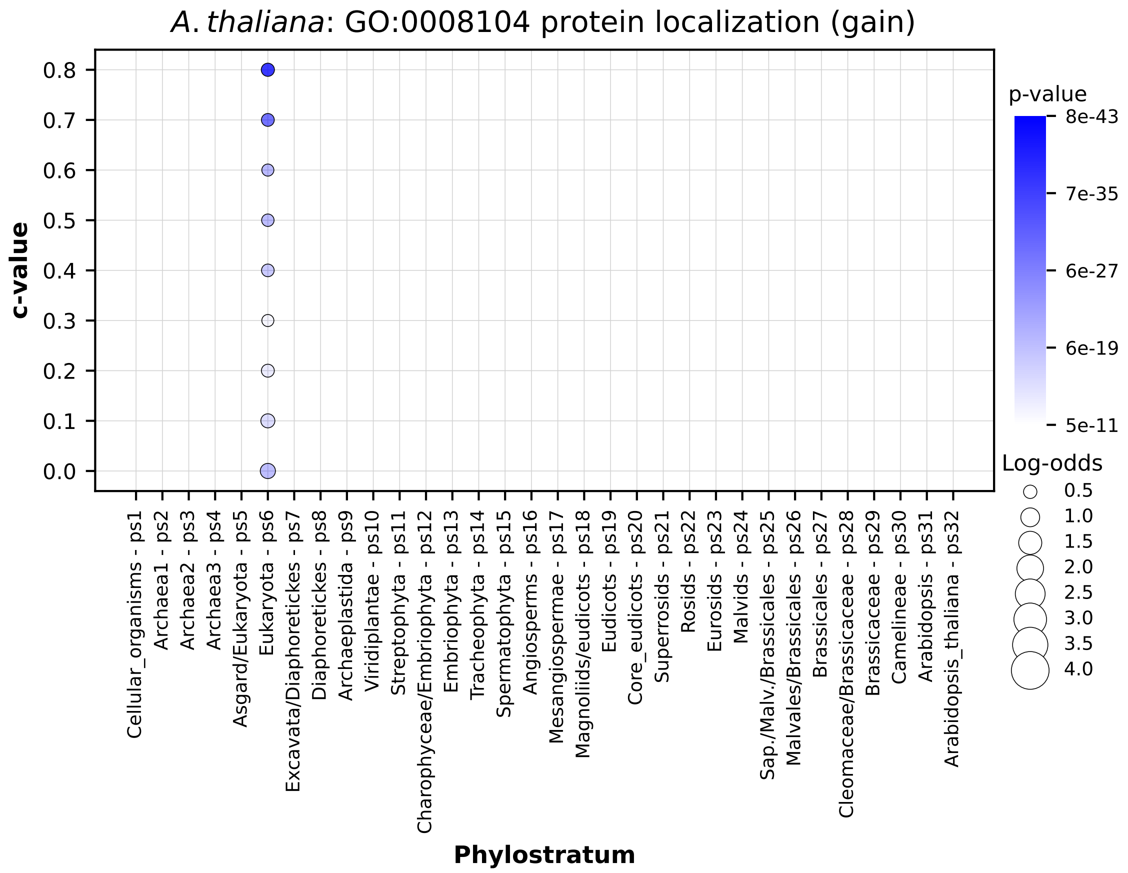

A. thaliana: GO:0008104 protein localization (gain)
c-value
0.0
0.1
0.2
0.3
0.4
0.5
0.6
0.7
0.8
Phylostratum
Cellular_organisms - ps1
Archaea1 - ps2
Archaea2 - ps3
Archaea3 - ps4
Asgard/Eukaryota - ps5
Eukaryota - ps6
Excavata/Diaphoretickes - ps7
Diaphoretickes - ps8
Archaeplastida - ps9
Viridiplantae - ps10
Streptophyta - ps11
Charophyceae/Embriophyta - ps12
Embriophyta - ps13
Tracheophyta - ps14
Spermatophyta - ps15
Angiosperms - ps16
Mesangiospermae - ps17
Magnoliids/eudicots - ps18
Eudicots - ps19
Core_eudicots - ps20
Superrosids - ps21
Rosids - ps22
Eurosids - ps23
Malvids - ps24
Sap./Malv./Brassicales - ps25
Malvales/Brassicales - ps26
Brassicales - ps27
Cleomaceae/Brassicaceae - ps28
Brassicaceae - ps29
Camelineae - ps30
Arabidopsis - ps31
Arabidopsis_thaliana - ps32
p-value
8e-43
7e-35
6e-27
6e-19
5e-11
Log-odds
0.5
1.0
1.5
2.0
2.5
3.0
3.5
4.0

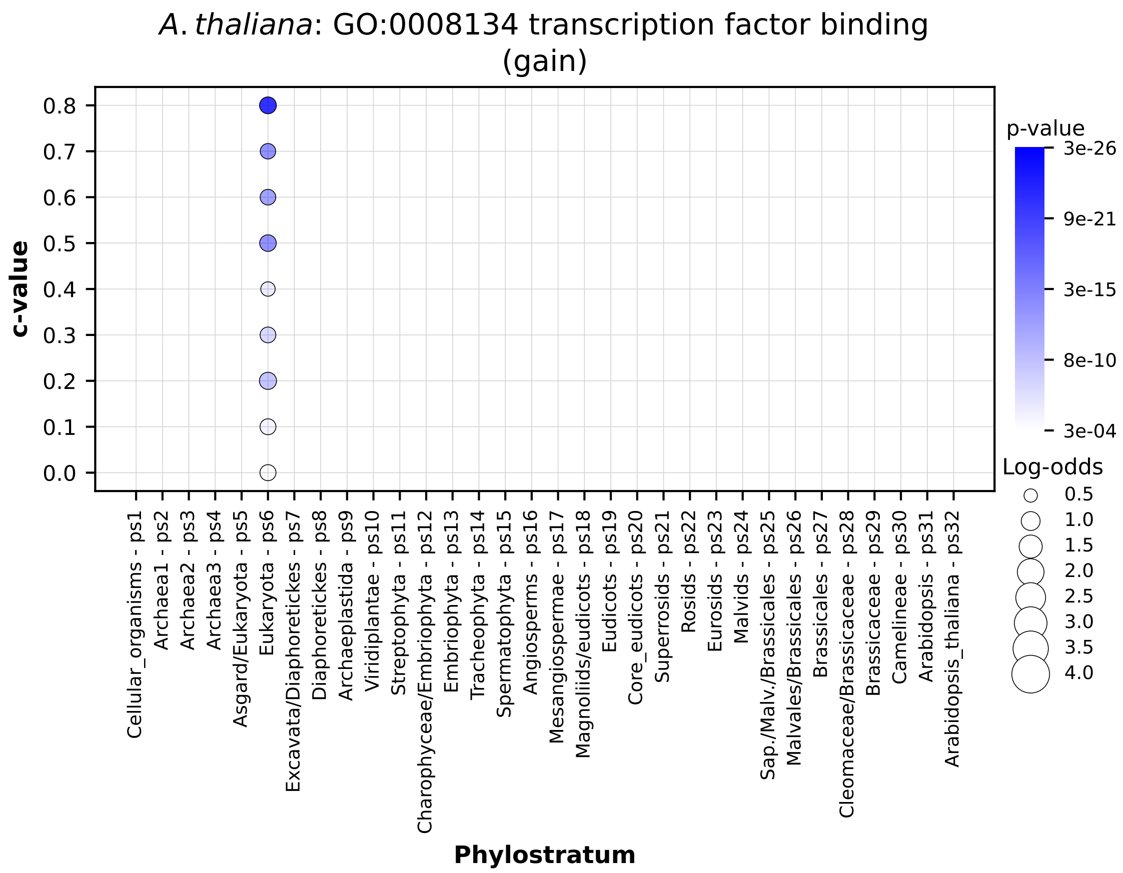
A. thaliana: GO:0008134 transcription factor binding
(gain)
c-value
0.0
0.1
0.2
0.3
0.4
0.5
0.6
0.7
0.8
p-value
3e-26
9e-21
3e-15
8e-10
3e-04
Log-odds
0.5
1.0
1.5
2.0
2.5
3.0
3.5
4.0
Cellular_organisms - ps1
Archaea1 - ps2
Archaea2 - ps3
Archaea3 - ps4
Asgard/Eukaryota - ps5
Eukaryota - ps6
Excavata/Diaphoretickes - ps7
Diaphoretickes - ps8
Archaeplastida - ps9
Viridiplantae - ps10
Streptophyta - ps11
Charophyceae/Embriophyta - ps12
Embriophyta - ps13
Tracheophyta - ps14
Spermatophyta - ps15
Angiosperms - ps16
Mesangiospermae - ps17
Magnoliids/eudicots - ps18
Eudicots - ps19
Core_eudicots - ps20
Superrosids - ps21
Rosids - ps22
Eurosids - ps23
Malvids - ps24
Sap./Malv./Brassicales - ps25
Malvales/Brassicales - ps26
Brassicales - ps27
Cleomaceae/Brassicaceae - ps28
Brassicaceae - ps29
Camelineae - ps30
Arabidopsis - ps31
Arabidopsis_thaliana - ps32
Phylostratum

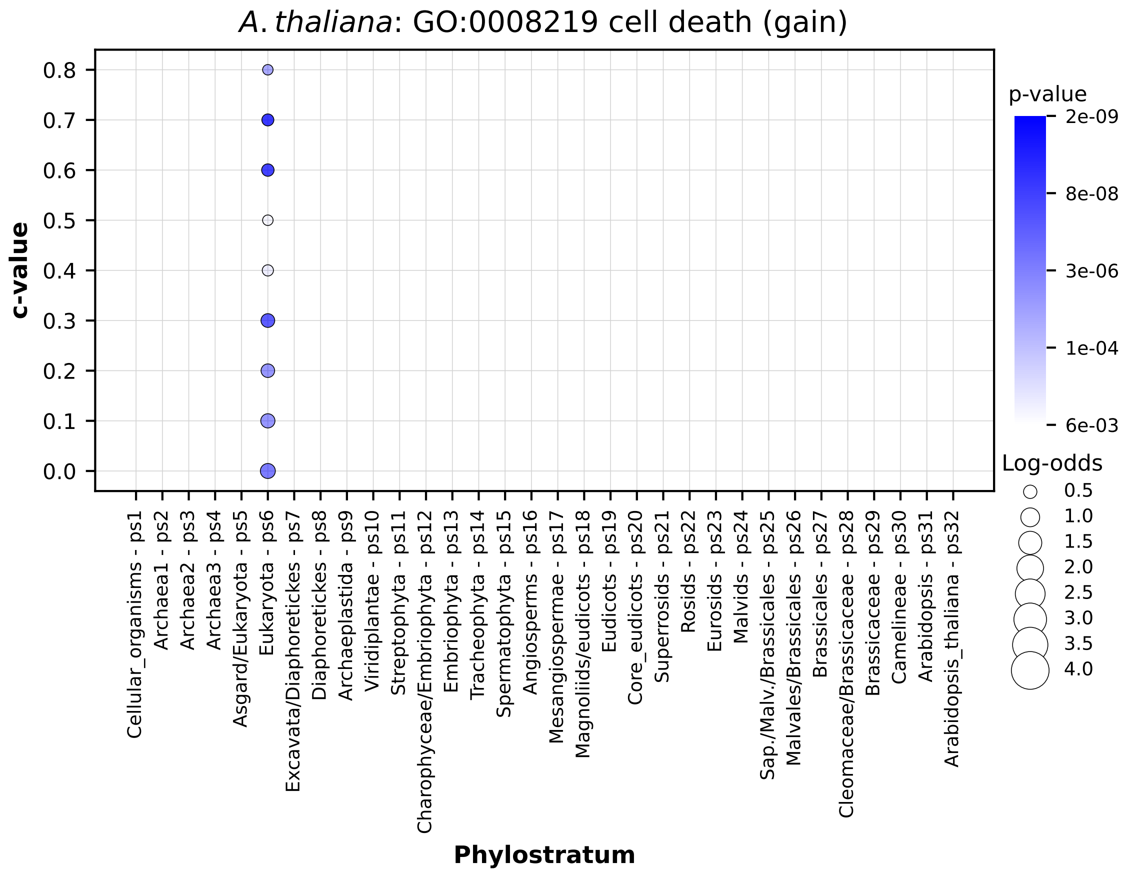

A. thaliana: GO:0008219 cell death (gain)
c-value
0.8
0.7
0.6
0.5
0.4
0.3
0.2
0.1
0.0
p-value
2e-09
8e-08
3e-06
1e-04
6e-03
Log-odds
0.5
1.0
1.5
2.0
2.5
3.0
3.5
4.0
Cellular_organisms - ps1
Archaea1 - ps2
Archaea2 - ps3
Archaea3 - ps4
Asgard/Eukaryota - ps5
Eukaryota - ps6
Excavata/Diaphoretickes - ps7
Diaphoretickes - ps8
Archaeplastida - ps9
Viridiplantae - ps10
Streptophyta - ps11
Charophyceae/Embriophyta - ps12
Embriophyta - ps13
Tracheophyta - ps14
Spermatophyta - ps15
Angiosperms - ps16
Mesangiospermae - ps17
Magnoliids/eudicots - ps18
Eudicots - ps19
Core_eudicots - ps20
Superrosids - ps21
Rosids - ps22
Eurosids - ps23
Malvids - ps24
Sap./Malv./Brassicales - ps25
Malvales/Brassicales - ps26
Brassicales - ps27
Cleomaceae/Brassicaceae - ps28
Brassicaceae - ps29
Camelineae - ps30
Arabidopsis - ps31
Arabidopsis_thaliana - ps32
Phylostratum

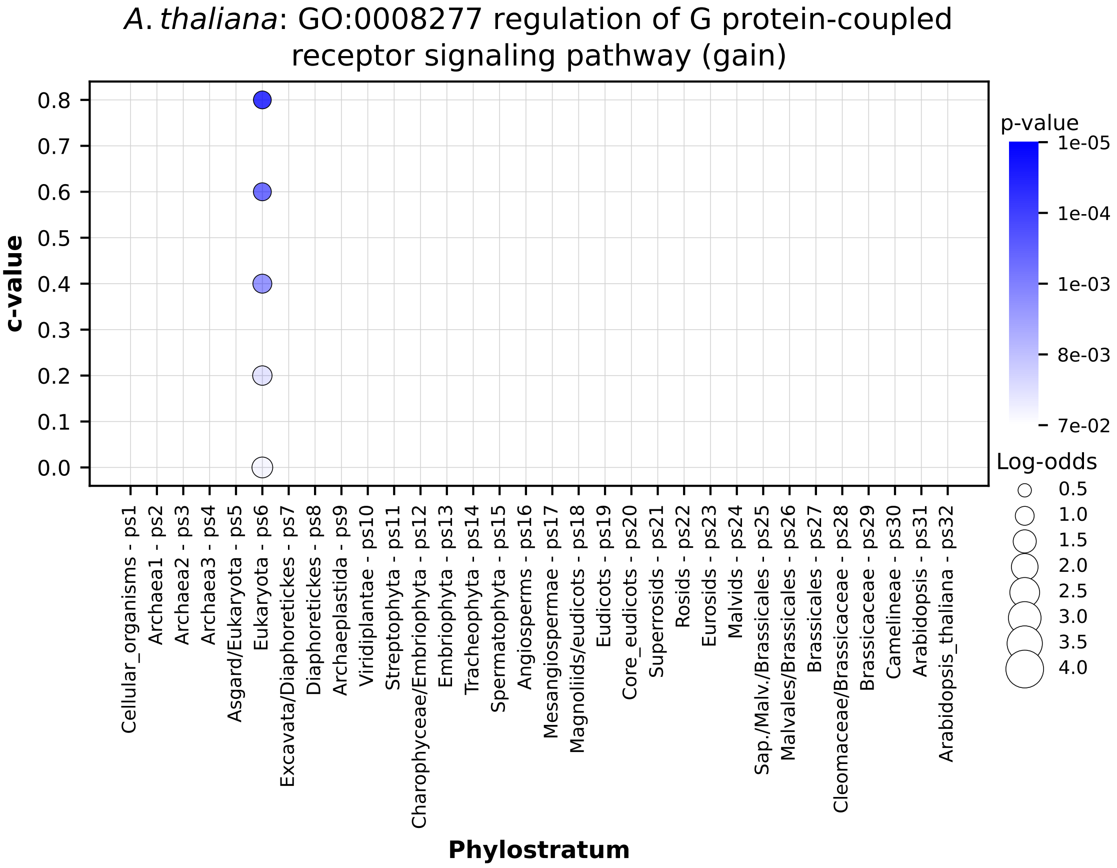

A. thaliana: GO:0008277 regulation of G protein-coupled receptor signaling pathway (gain)
c-value
0.8
0.7
0.6
0.5
0.4
0.3
0.2
0.1
0.0
p-value
1e-05
1e-04
1e-03
8e-03
7e-02
Log-odds
0.5
1.0
1.5
2.0
2.5
3.0
3.5
4.0
Cellular_organisms - ps1
Archaea1 - ps2
Archaea2 - ps3
Archaea3 - ps4
Asgard/Eukaryota - ps5
Eukaryota - ps6
Excavata/Diaphoretickes - ps7
Diaphoretickes - ps8
Archaeplastida - ps9
Viridiplantae - ps10
Streptophyta - ps11
Charophyceae/Embriophyta - ps12
Embriophyta - ps13
Tracheophyta - ps14
Spermatophyta - ps15
Angiosperms - ps16
Mesangiospermae - ps17
Magnoliids/eudicots - ps18
Eudicots - ps19
Core_eudicots - ps20
Superrosids - ps21
Rosids - ps22
Eurosids - ps23
Malvids - ps24
Sap./Malv./Brassicales - ps25
Malvales/Brassicales - ps26
Brassicales - ps27
Cleomaceae/Brassicaceae - ps28
Brassicaceae - ps29
Camelineae - ps30
Arabidopsis - ps31
Arabidopsis_thaliana - ps32
Phylostratum

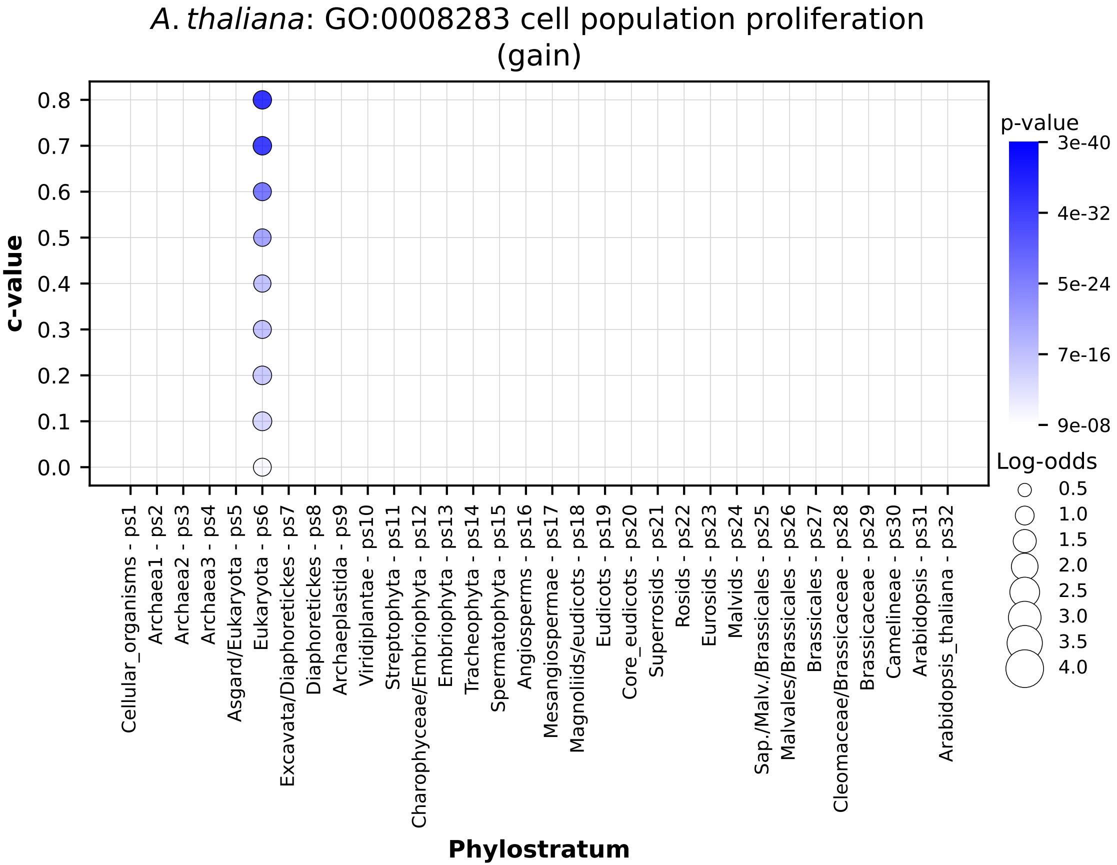
A. thaliana: GO:0008283 cell population proliferation
(gain)
c-value
0.8
0.7
0.6
0.5
0.4
0.3
0.2
0.1
0.0
Phylostratum
Cellular_organisms - ps1
Archaea1 - ps2
Archaea2 - ps3
Archaea3 - ps4
Asgard/Eukaryota - ps5
Eukaryota - ps6
Excavata/Diaphoretickes - ps7
Diaphoretickes - ps8
Archaeplastida - ps9
Viridiplantae - ps10
Streptophyta - ps11
Charophyceae/Embriophyta - ps12
Embriophyta - ps13
Tracheophyta - ps14
Spermatophyta - ps15
Angiosperms - ps16
Mesangiospermae - ps17
Magnoliids/eudicots - ps18
Eudicots - ps19
Core_eudicots - ps20
Superrosids - ps21
Rosids - ps22
Eurosids - ps23
Malvids - ps24
Sap./Malv./Brassicales - ps25
Malvales/Brassicales - ps26
Brassicales - ps27
Cleomaceae/Brassicaceae - ps28
Brassicaceae - ps29
Camelineae - ps30
Arabidopsis - ps31
Arabidopsis_thaliana - ps32
p-value
3e-40
4e-32
5e-24
7e-16
9e-08
Log-odds
0.5
1.0
1.5
2.0
2.5
3.0
3.5
4.0

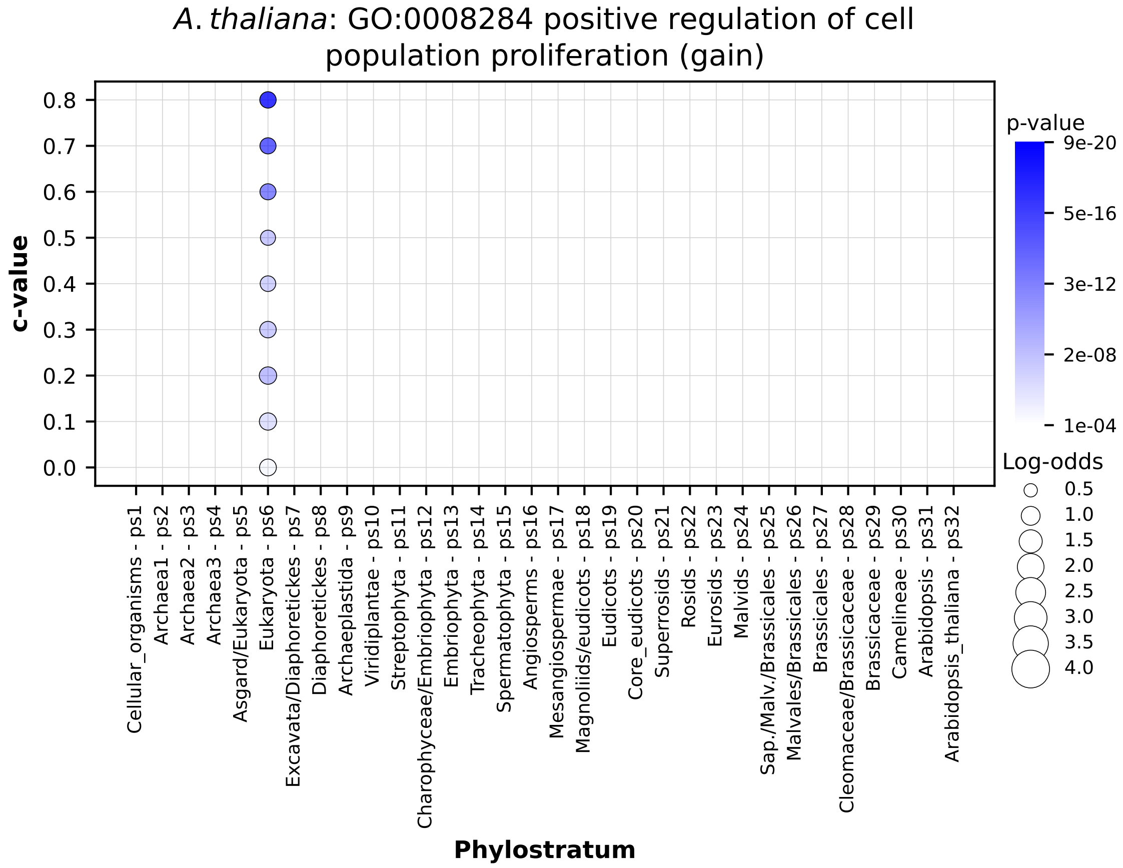
A. thaliana: GO:0008284 positive regulation of cell population proliferation (gain)
c-value
0.8
0.7
0.6
0.5
0.4
0.3
0.2
0.1
0.0
Phylostratum
Cellular_organisms - ps1
Archaea1 - ps2
Archaea2 - ps3
Archaea3 - ps4
Asgard/Eukaryota - ps5
Eukaryota - ps6
Excavata/Diaphoretickes - ps7
Diaphoretickes - ps8
Archaeplastida - ps9
Viridiplantae - ps10
Streptophyta - ps11
Charophyceae/Embriophyta - ps12
Embriophyta - ps13
Tracheophyta - ps14
Spermatophyta - ps15
Angiosperms - ps16
Mesangiospermae - ps17
Magnoliids/eudicots - ps18
Eudicots - ps19
Core_eudicots - ps20
Superrosids - ps21
Rosids - ps22
Eurosids - ps23
Malvids - ps24
Sap./Malv./Brassicales - ps25
Malvales/Brassicales - ps26
Brassicales - ps27
Cleomaceae/Brassicaceae - ps28
Brassicaceae - ps29
Camelineae - ps30
Arabidopsis - ps31
Arabidopsis_thaliana - ps32
p-value
9e-20
5e-16
3e-12
2e-08
1e-04
Log-odds
0.5
1.0
1.5
2.0
2.5
3.0
3.5
4.0

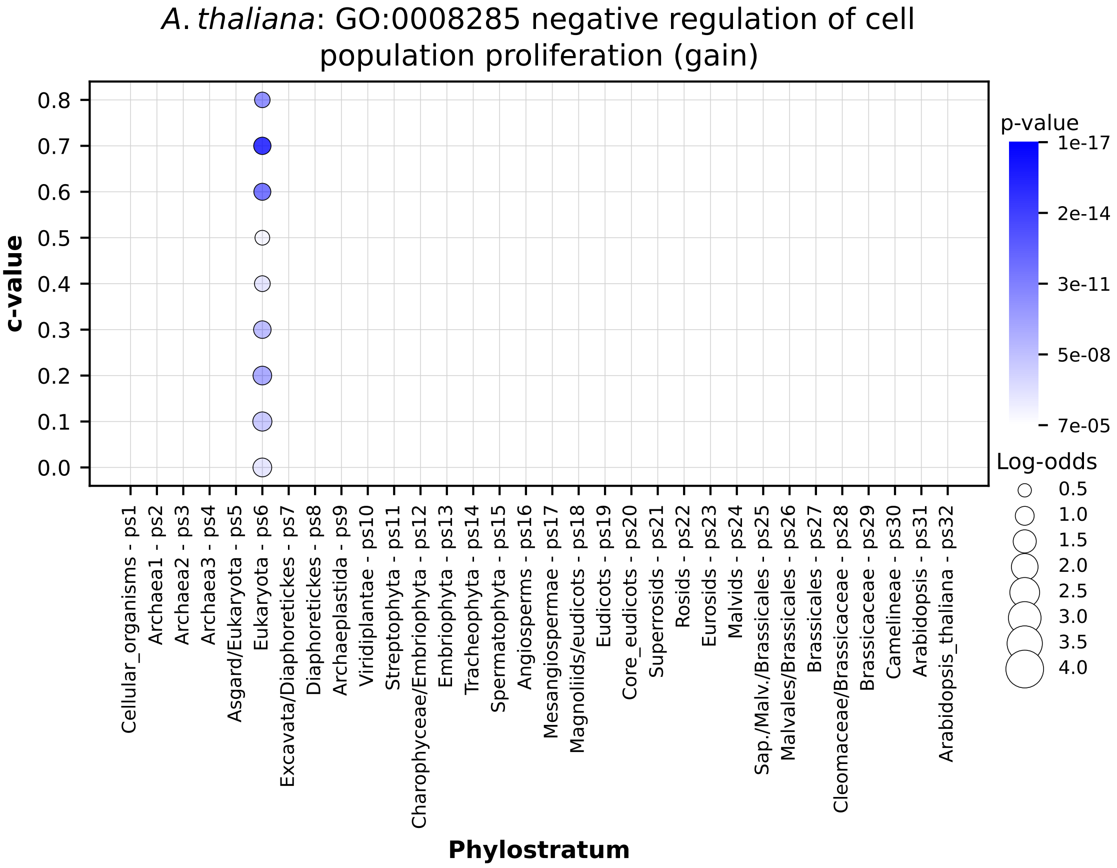
A. thaliana: GO:0008285 negative regulation of cell population proliferation (gain)
c-value
0.8
0.7
0.6
0.5
0.4
0.3
0.2
0.1
0.0
p-value
1e-17
2e-14
3e-11
5e-08
7e-05
Log-odds
0.5
1.0
1.5
2.0
2.5
3.0
3.5
4.0
Cellular_organisms - ps1
Archaea1 - ps2
Archaea2 - ps3
Archaea3 - ps4
Asgard/Eukaryota - ps5
Eukaryota - ps6
Excavata/Diaphoretickes - ps7
Diaphoretickes - ps8
Archaeplastida - ps9
Viridiplantae - ps10
Streptophyta - ps11
Charophyceae/Embriophyta - ps12
Embriophyta - ps13
Tracheophyta - ps14
Spermatophyta - ps15
Angiosperms - ps16
Mesangiospermae - ps17
Magnoliids/eudicots - ps18
Eudicots - ps19
Core_eudicots - ps20
Superrosids - ps21
Rosids - ps22
Eurosids - ps23
Malvids - ps24
Sap./Malv./Brassicales - ps25
Malvales/Brassicales - ps26
Brassicales - ps27
Cleomaceae/Brassicaceae - ps28
Brassicaceae - ps29
Camelineae - ps30
Arabidopsis - ps31
Arabidopsis_thaliana - ps32
Phylostratum

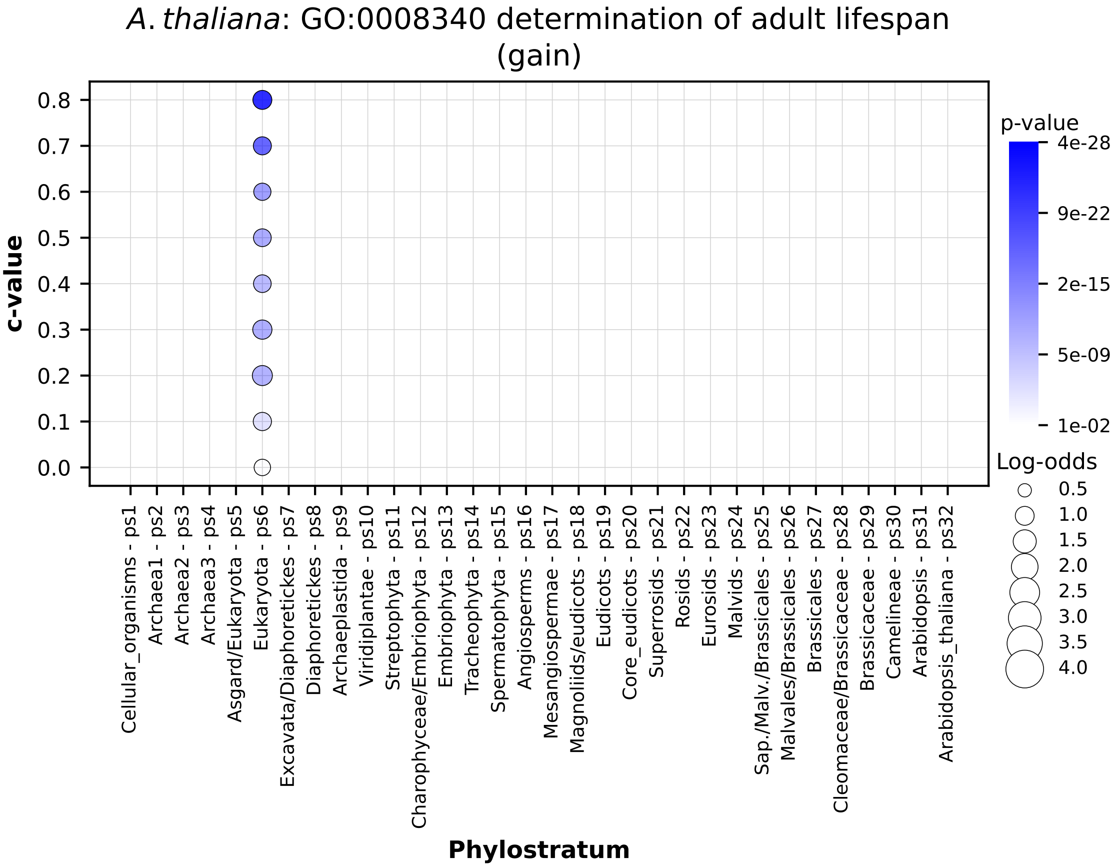

A. thaliana: GO:0008340 determination of adult lifespan
(gain)
c-value
0.8
0.7
0.6
0.5
0.4
0.3
0.2
0.1
0.0
p-value
4e-28
9e-22
2e-15
5e-09
1e-02
Log-odds
0.5
1.0
1.5
2.0
2.5
3.0
3.5
4.0
Cellular_organisms - ps1
Archaea1 - ps2
Archaea2 - ps3
Archaea3 - ps4
Asgard/Eukaryota - ps5
Eukaryota - ps6
Excavata/Diaphoretickes - ps7
Diaphoretickes - ps8
Archaeplastida - ps9
Viridiplantae - ps10
Streptophyta - ps11
Charophyceae/Embriophyta - ps12
Embriophyta - ps13
Tracheophyta - ps14
Spermatophyta - ps15
Angiosperms - ps16
Mesangiospermae - ps17
Magnoliids/eudicots - ps18
Eudicots - ps19
Core_eudicots - ps20
Superrosids - ps21
Rosids - ps22
Eurosids - ps23
Malvids - ps24
Sap./Malv./Brassicales - ps25
Malvales/Brassicales - ps26
Brassicales - ps27
Cleomaceae/Brassicaceae - ps28
Brassicaceae - ps29
Camelineae - ps30
Arabidopsis - ps31
Arabidopsis_thaliana - ps32
Phylostratum

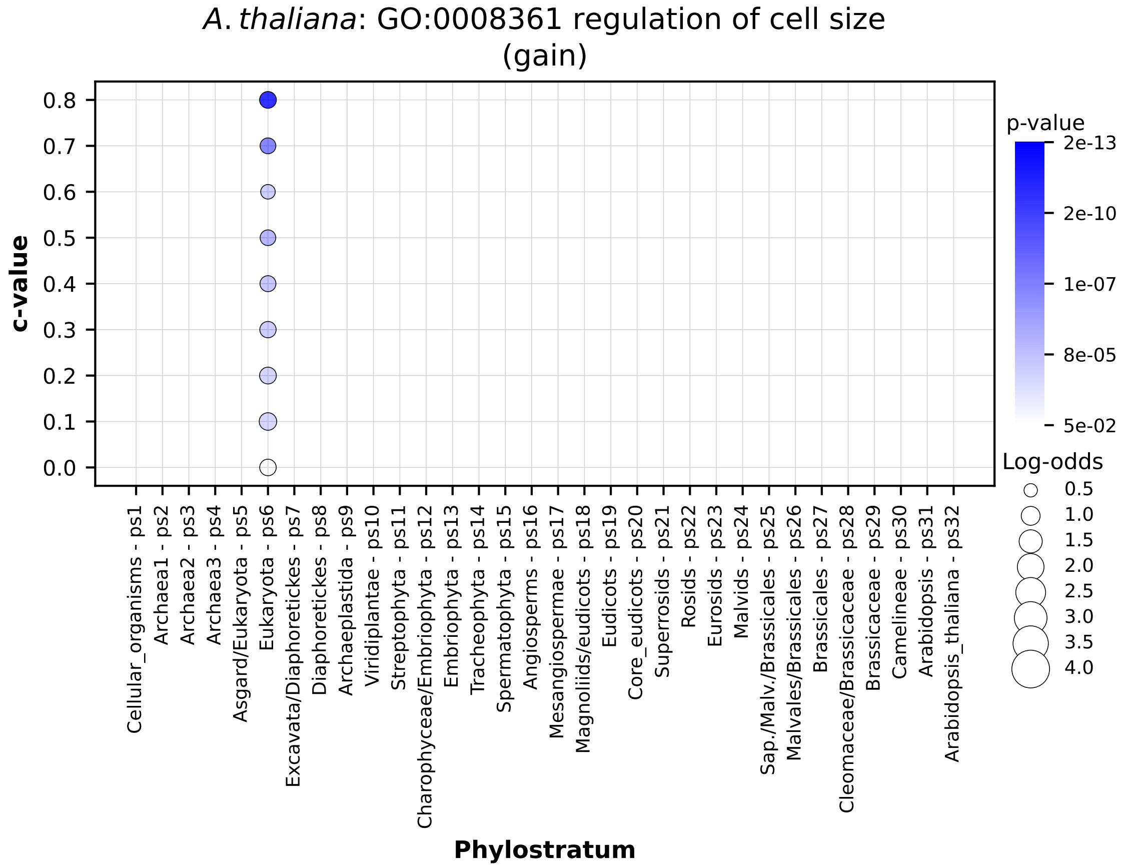
A. thaliana: GO:0008361 regulation of cell size
(gain)
c-value
0.8
0.7
0.6
0.5
0.4
0.3
0.2
0.1
0.0
p-value
2e-13
2e-10
1e-07
8e-05
5e-02
Log-odds
0.5
1.0
1.5
2.0
2.5
3.0
3.5
4.0
Cellular_organisms - ps1
Archaea1 - ps2
Archaea2 - ps3
Archaea3 - ps4
Asgard/Eukaryota - ps5
Eukaryota - ps6
Excavata/Diaphoretickes - ps7
Diaphoretickes - ps8
Archaeplastida - ps9
Viridiplantae - ps10
Streptophyta - ps11
Charophyceae/Embriophyta - ps12
Embriophyta - ps13
Tracheophyta - ps14
Spermatophyta - ps15
Angiosperms - ps16
Mesangiospermae - ps17
Magnoliids/eudicots - ps18
Eudicots - ps19
Core_eudicots - ps20
Superrosids - ps21
Rosids - ps22
Eurosids - ps23
Malvids - ps24
Sap./Malv./Brassicales - ps25
Malvales/Brassicales - ps26
Brassicales - ps27
Cleomaceae/Brassicaceae - ps28
Brassicaceae - ps29
Camelineae - ps30
Arabidopsis - ps31
Arabidopsis_thaliana - ps32
Phylostratum

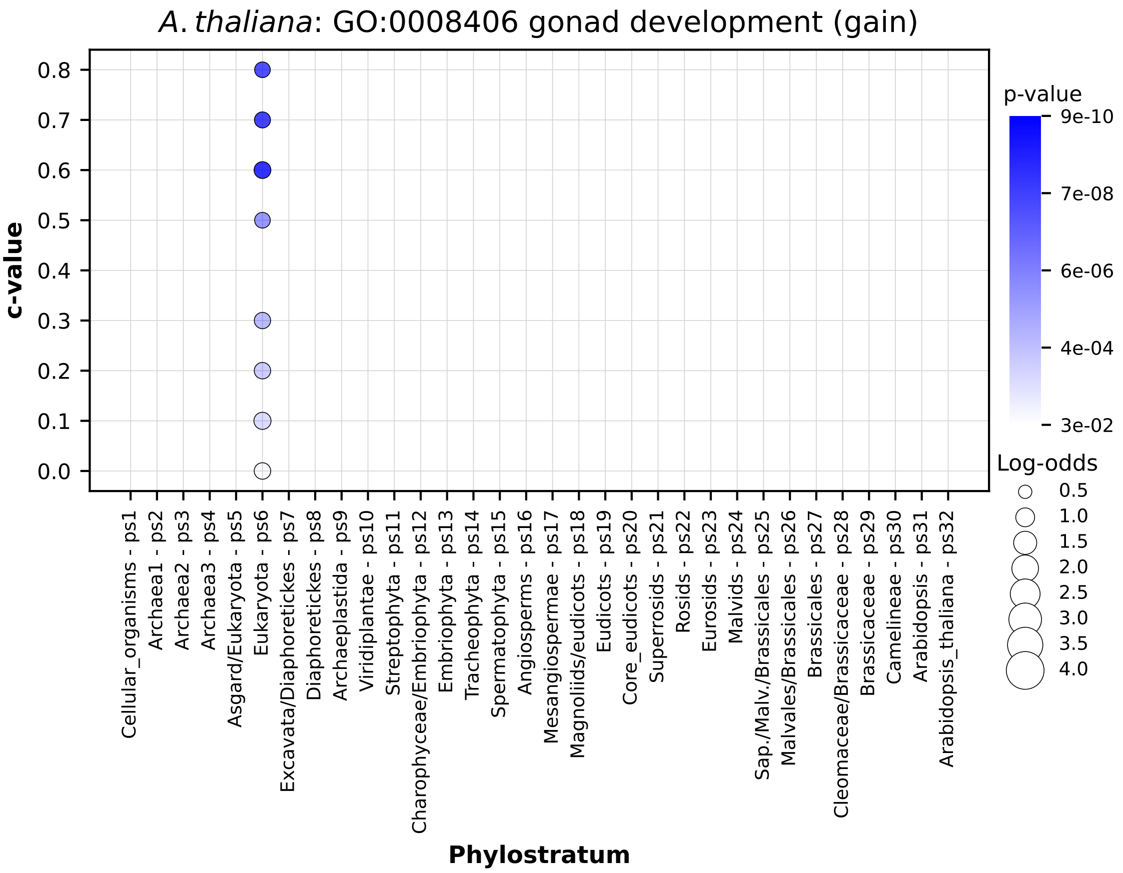

A. thaliana: GO:0008406 gonad development (gain)
c-value
0.8
0.7
0.6
0.5
0.4
0.3
0.2
0.1
0.0
p-value
9e-10
7e-08
6e-06
4e-04
3e-02
Log-odds
0.5
1.0
1.5
2.0
2.5
3.0
3.5
4.0
Cellular_organisms - ps1
Archaea1 - ps2
Archaea2 - ps3
Archaea3 - ps4
Asgard/Eukaryota - ps5
Eukaryota - ps6
Excavata/Diaphoretickes - ps7
Diaphoretickes - ps8
Archaeplastida - ps9
Viridiplantae - ps10
Streptophyta - ps11
Charophyceae/Embriophyta - ps12
Embriophyta - ps13
Tracheophyta - ps14
Spermatophyta - ps15
Angiosperms - ps16
Mesangiospermae - ps17
Magnoliids/eudicots - ps18
Eudicots - ps19
Core_eudicots - ps20
Superrosids - ps21
Rosids - ps22
Eurosids - ps23
Malvids - ps24
Sap./Malv./Brassicales - ps25
Malvales/Brassicales - ps26
Brassicales - ps27
Cleomaceae/Brassicaceae - ps28
Brassicaceae - ps29
Camelineae - ps30
Arabidopsis - ps31
Arabidopsis_thaliana - ps32
Phylostratum

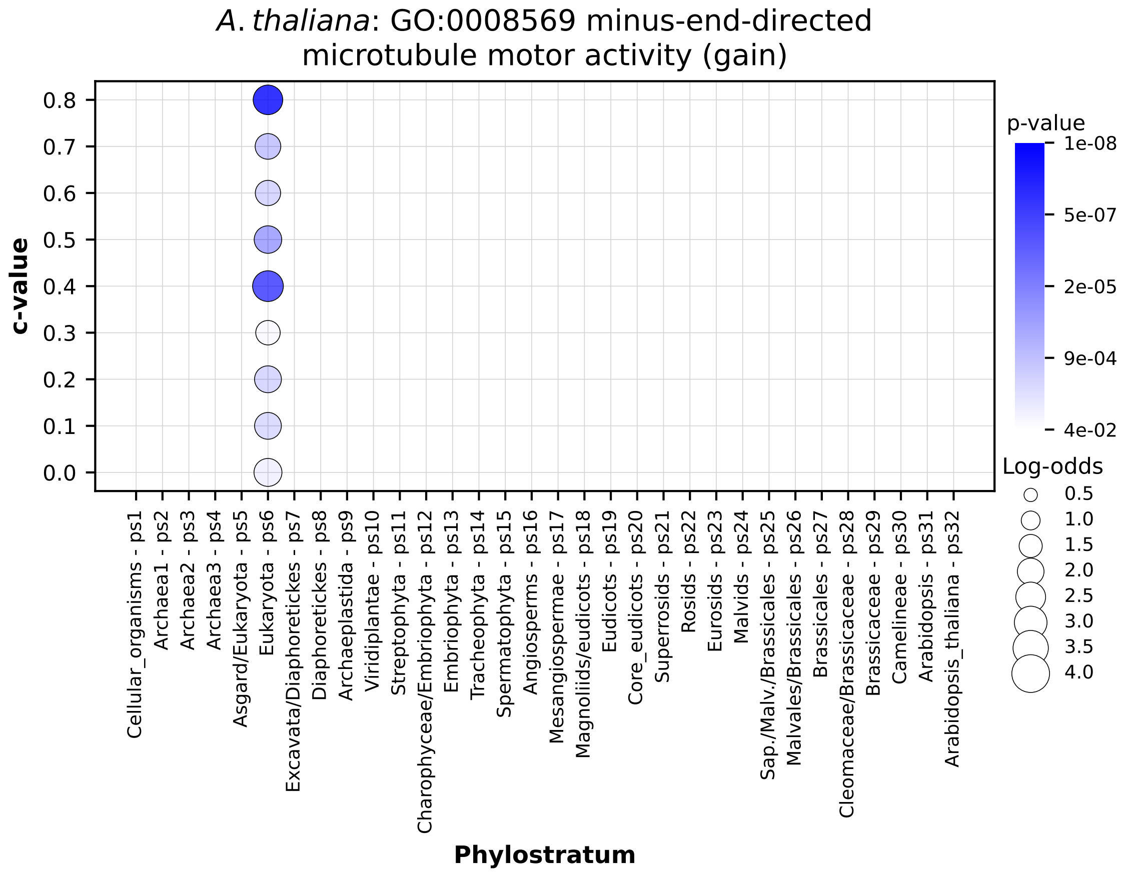

A. thaliana: GO:0008569 minus-end-directed microtubule motor activity (gain)
c-value
0.8
0.7
0.6
0.5
0.4
0.3
0.2
0.1
0.0
p-value
1e-08
5e-07
2e-05
9e-04
4e-02
Log-odds
0.5
1.0
1.5
2.0
2.5
3.0
3.5
4.0
Cellular_organisms - ps1
Archaea1 - ps2
Archaea2 - ps3
Archaea3 - ps4
Asgard/Eukaryota - ps5
Eukaryota - ps6
Excavata/Diaphoretickes - ps7
Diaphoretickes - ps8
Archaeplastida - ps9
Viridiplantae - ps10
Streptophyta - ps11
Charophyceae/Embriophyta - ps12
Embriophyta - ps13
Tracheophyta - ps14
Spermatophyta - ps15
Angiosperms - ps16
Mesangiospermae - ps17
Magnoliids/eudicots - ps18
Eudicots - ps19
Core_eudicots - ps20
Superrosids - ps21
Rosids - ps22
Eurosids - ps23
Malvids - ps24
Sap./Malv./Brassicales - ps25
Malvales/Brassicales - ps26
Brassicales - ps27
Cleomaceae/Brassicaceae - ps28
Brassicaceae - ps29
Camelineae - ps30
Arabidopsis - ps31
Arabidopsis_thaliana - ps32
Phylostratum

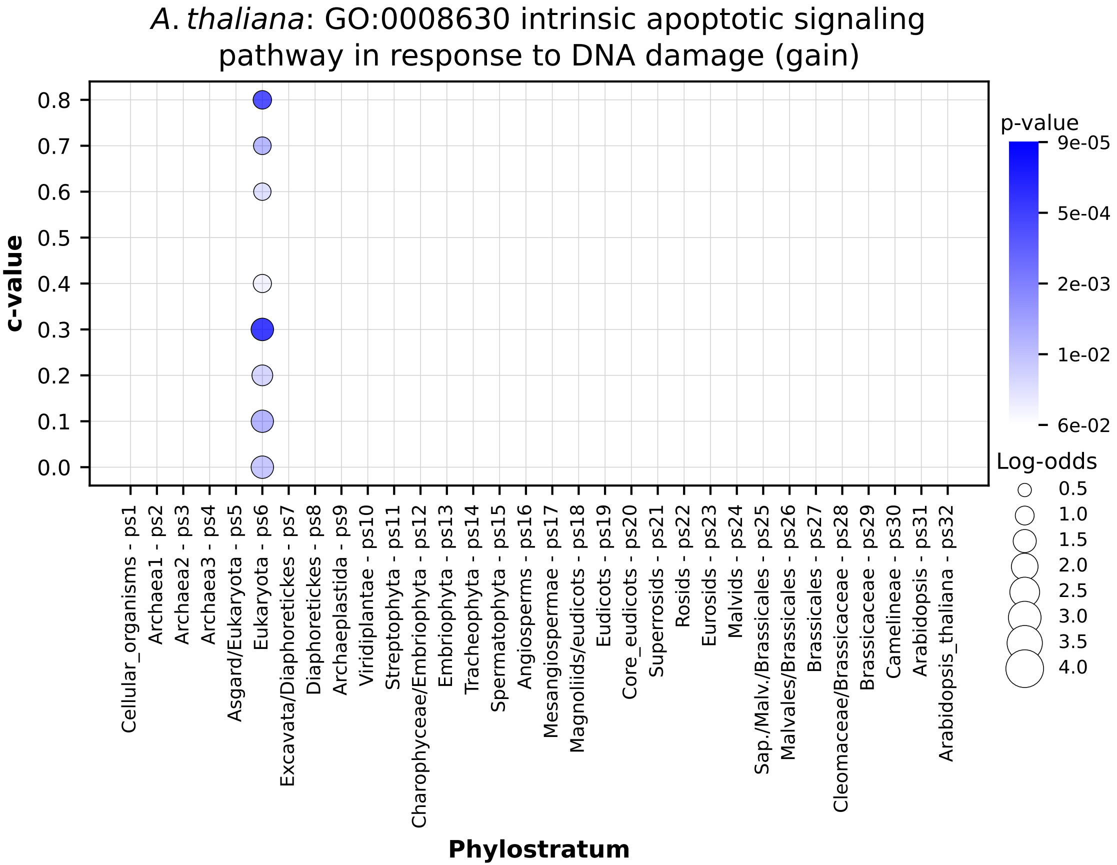

A. thaliana: GO:0008630 intrinsic apoptotic signaling pathway in response to DNA damage (gain)
c-value
0.8
0.7
0.6
0.5
0.4
0.3
0.2
0.1
0.0
Phylostratum
Cellular_organisms - ps1
Archaea1 - ps2
Archaea2 - ps3
Archaea3 - ps4
Asgard/Eukaryota - ps5
Eukaryota - ps6
Excavata/Diaphoretickes - ps7
Diaphoretickes - ps8
Archaeplastida - ps9
Viridiplantae - ps10
Streptophyta - ps11
Charophyceae/Embriophyta - ps12
Embriophyta - ps13
Tracheophyta - ps14
Spermatophyta - ps15
Angiosperms - ps16
Mesangiospermae - ps17
Magnoliids/eudicots - ps18
Eudicots - ps19
Core_eudicots - ps20
Superrosids - ps21
Rosids - ps22
Eurosids - ps23
Malvids - ps24
Sap./Malv./Brassicales - ps25
Malvales/Brassicales - ps26
Brassicales - ps27
Cleomaceae/Brassicaceae - ps28
Brassicaceae - ps29
Camelineae - ps30
Arabidopsis - ps31
Arabidopsis_thaliana - ps32
p-value
9e-05
5e-04
2e-03
1e-02
6e-02
Log-odds
0.5
1.0
1.5
2.0
2.5
3.0
3.5
4.0

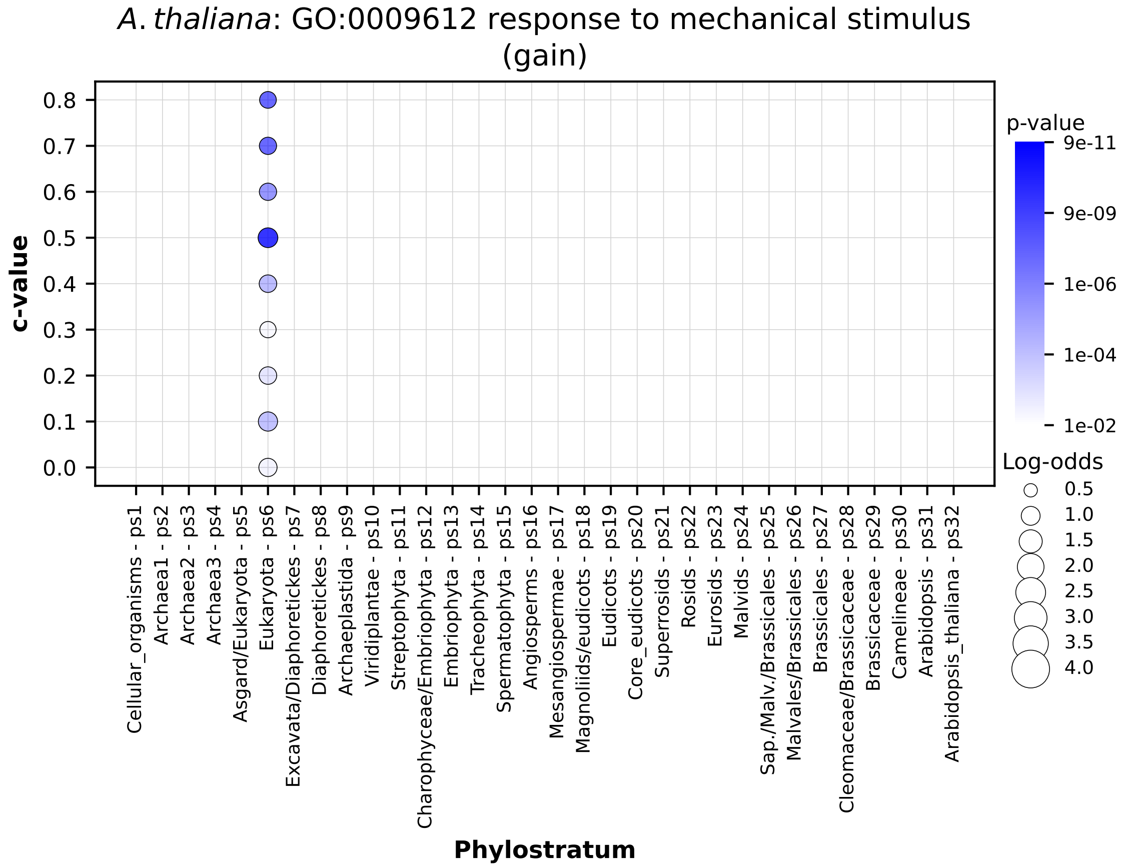

A. thaliana: GO:0009612 response to mechanical stimulus (gain)
c-value
0.8
0.7
0.6
0.5
0.4
0.3
0.2
0.1
0.0
p-value
9e-11
9e-09
1e-06
1e-04
1e-02
Log-odds
0.5
1.0
1.5
2.0
2.5
3.0
3.5
4.0
Cellular_organisms - ps1
Archaea1 - ps2
Archaea2 - ps3
Archaea3 - ps4
Asgard/Eukaryota - ps5
Eukaryota - ps6
Excavata/Diaphoretickes - ps7
Diaphoretickes - ps8
Archaeplastida - ps9
Viridiplantae - ps10
Streptophyta - ps11
Charophyceae/Embriophyta - ps12
Embriophyta - ps13
Tracheophyta - ps14
Spermatophyta - ps15
Angiosperms - ps16
Mesangiospermae - ps17
Magnoliids/eudicots - ps18
Eudicots - ps19
Core_eudicots - ps20
Superrosids - ps21
Rosids - ps22
Eurosids - ps23
Malvids - ps24
Sap./Malv./Brassicales - ps25
Malvales/Brassicales - ps26
Brassicales - ps27
Cleomaceae/Brassicaceae - ps28
Brassicaceae - ps29
Camelineae - ps30
Arabidopsis - ps31
Arabidopsis_thaliana - ps32
Phylostratum

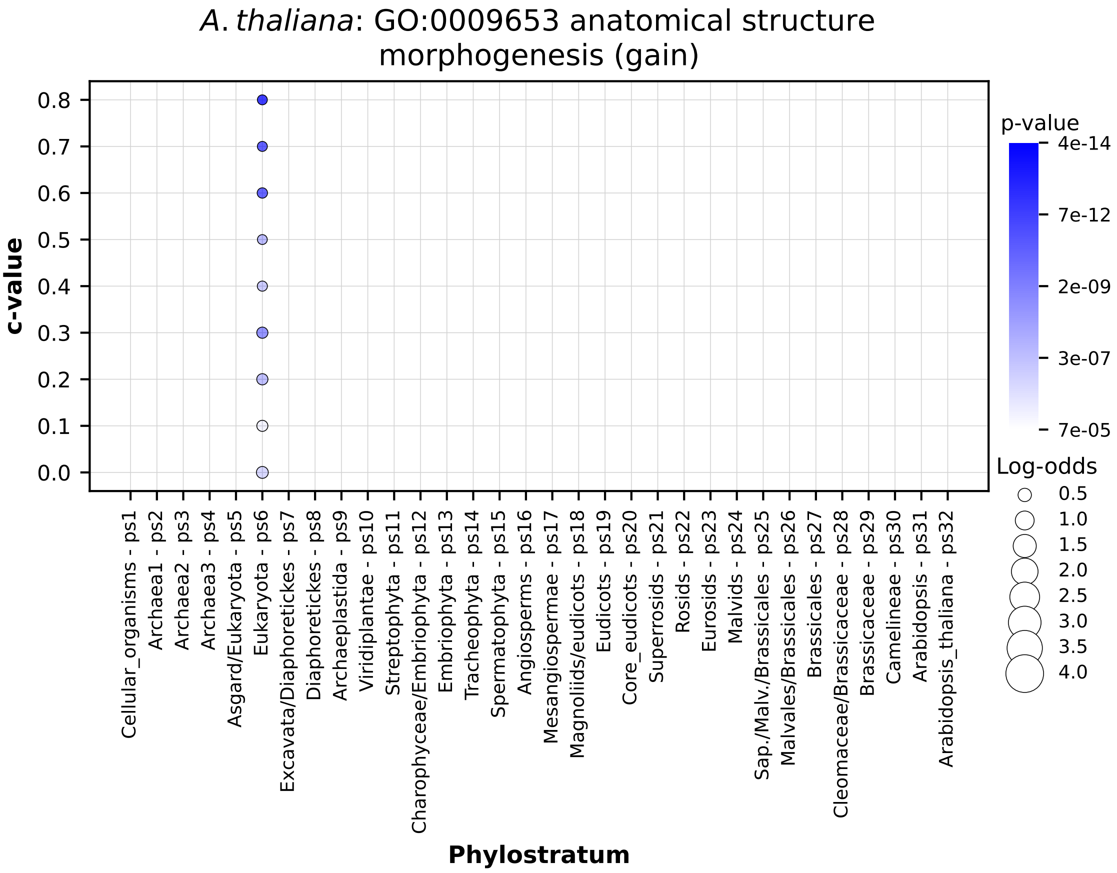

A. thaliana: GO:0009653 anatomical structure morphogenesis (gain)
c-value
0.8
0.7
0.6
0.5
0.4
0.3
0.2
0.1
0.0
p-value
4e-14
7e-12
2e-09
3e-07
7e-05
Log-odds
0.5
1.0
1.5
2.0
2.5
3.0
3.5
4.0
Cellular_organisms - ps1
Archaea1 - ps2
Archaea2 - ps3
Archaea3 - ps4
Asgard/Eukaryota - ps5
Eukaryota - ps6
Excavata/Diaphoretickes - ps7
Diaphoretickes - ps8
Archaeplastida - ps9
Viridiplantae - ps10
Streptophyta - ps11
Charophyceae/Embriophyta - ps12
Embriophyta - ps13
Tracheophyta - ps14
Spermatophyta - ps15
Angiosperms - ps16
Mesangiospermae - ps17
Magnoliids/eudicots - ps18
Eudicots - ps19
Core_eudicots - ps20
Superrosids - ps21
Rosids - ps22
Eurosids - ps23
Malvids - ps24
Sap./Malv./Brassicales - ps25
Malvales/Brassicales - ps26
Brassicales - ps27
Cleomaceae/Brassicaceae - ps28
Brassicaceae - ps29
Camelineae - ps30
Arabidopsis - ps31
Arabidopsis_thaliana - ps32
Phylostratum

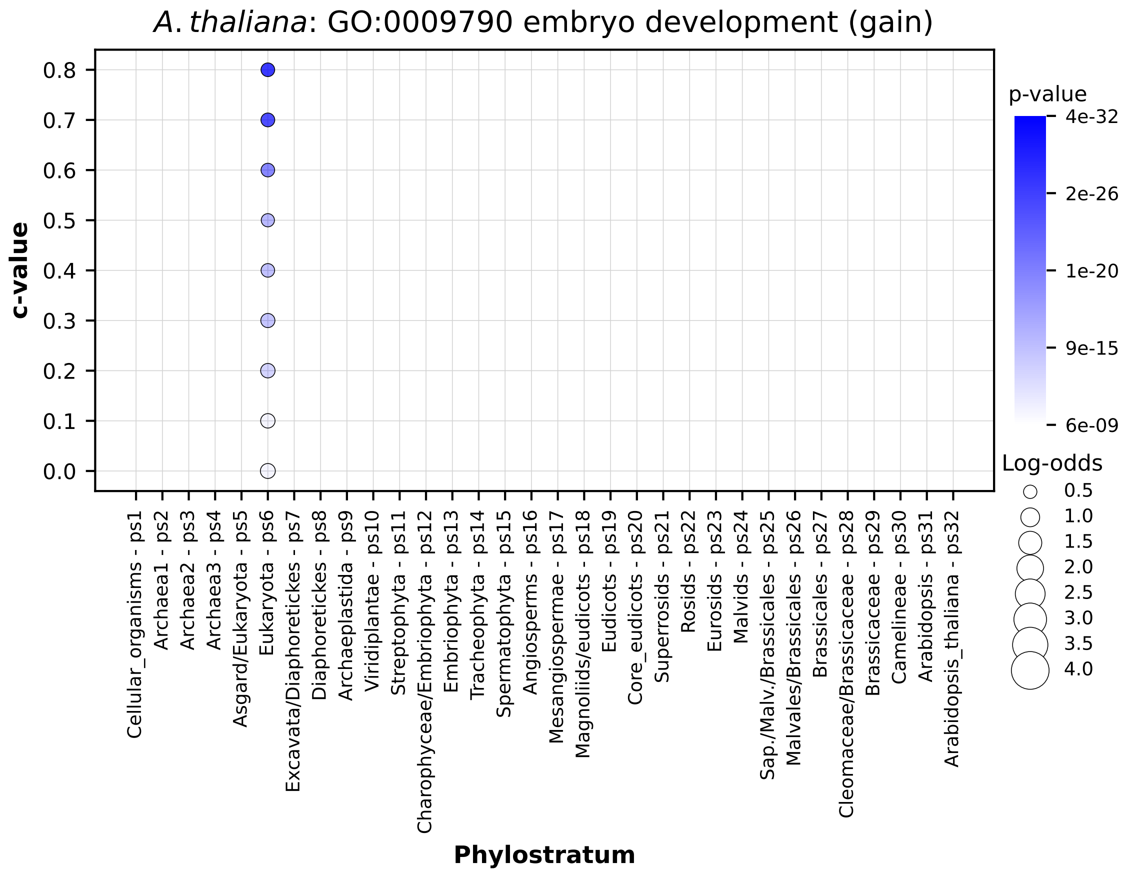

A. thaliana: GO:0009790 embryo development (gain)
c-value
0.8
0.7
0.6
0.5
0.4
0.3
0.2
0.1
0.0
Phylostratum
Cellular_organisms - ps1
Archaea1 - ps2
Archaea2 - ps3
Archaea3 - ps4
Asgard/Eukaryota - ps5
Eukaryota - ps6
Excavata/Diaphoretickes - ps7
Diaphoretickes - ps8
Archaeplastida - ps9
Viridiplantae - ps10
Streptophyta - ps11
Charophyceae/Embriophyta - ps12
Embriophyta - ps13
Tracheophyta - ps14
Spermatophyta - ps15
Angiosperms - ps16
Mesangiospermae - ps17
Magnoliids/eudicots - ps18
Eudicots - ps19
Core_eudicots - ps20
Superrosids - ps21
Rosids - ps22
Eurosids - ps23
Malvids - ps24
Sap./Malv./Brassicales - ps25
Malvales/Brassicales - ps26
Brassicales - ps27
Cleomaceae/Brassicaceae - ps28
Brassicaceae - ps29
Camelineae - ps30
Arabidopsis - ps31
Arabidopsis_thaliana - ps32
p-value
4e-32
2e-26
1e-20
9e-15
6e-09
Log-odds
0.5
1.0
1.5
2.0
2.5
3.0
3.5
4.0

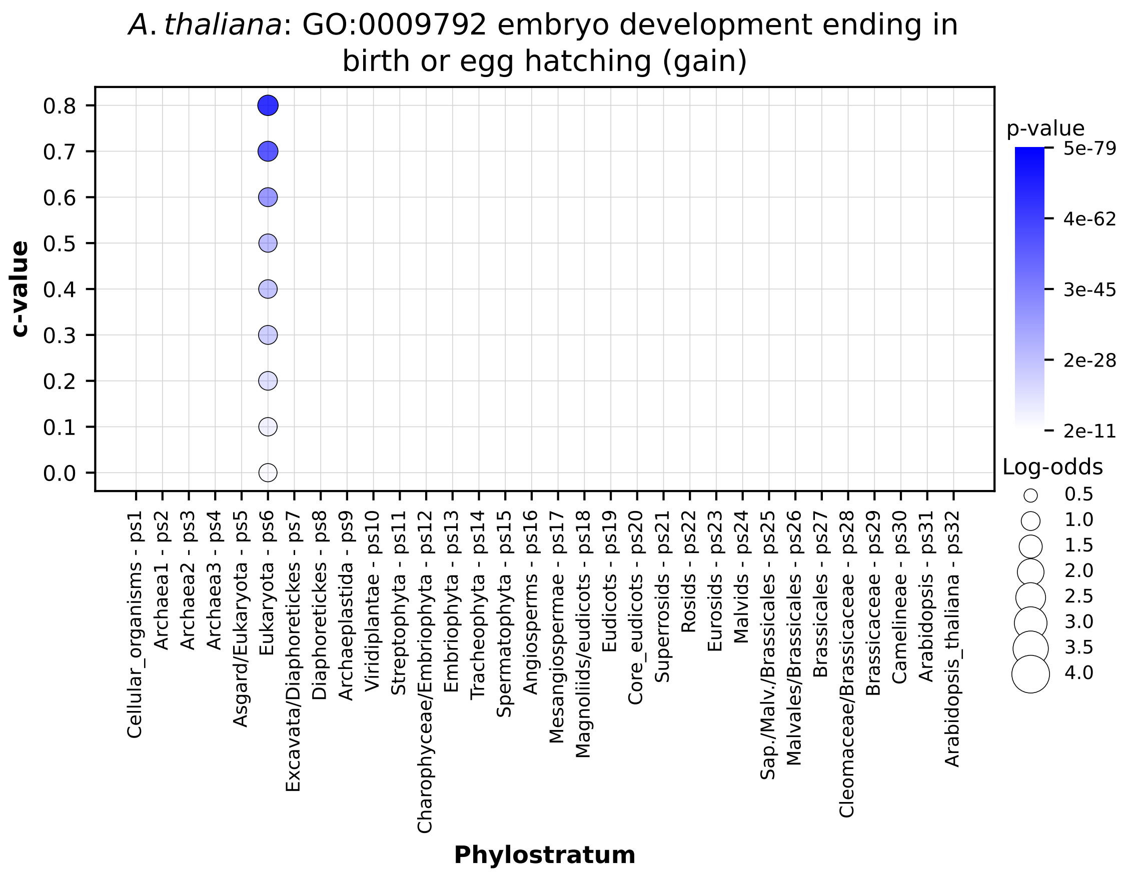
A. thaliana: GO:0009792 embryo development ending in birth or egg hatching (gain)
c-value
0.8
0.7
0.6
0.5
0.4
0.3
0.2
0.1
0.0
p-value
5e-79
4e-62
3e-45
2e-28
2e-11
Log-odds
0.5
1.0
1.5
2.0
2.5
3.0
3.5
4.0
Cellular_organisms - ps1
Archaea1 - ps2
Archaea2 - ps3
Archaea3 - ps4
Asgard/Eukaryota - ps5
Eukaryota - ps6
Excavata/Diaphoretickes - ps7
Diaphoretickes - ps8
Archaeplastida - ps9
Viridiplantae - ps10
Streptophyta - ps11
Charophyceae/Embriophyta - ps12
Embriophyta - ps13
Tracheophyta - ps14
Spermatophyta - ps15
Angiosperms - ps16
Mesangiospermae - ps17
Magnoliids/eudicots - ps18
Eudicots - ps19
Core_eudicots - ps20
Superrosids - ps21
Rosids - ps22
Eurosids - ps23
Malvids - ps24
Sap./Malv./Brassicales - ps25
Malvales/Brassicales - ps26
Brassicales - ps27
Cleomaceae/Brassicaceae - ps28
Brassicaceae - ps29
Camelineae - ps30
Arabidopsis - ps31
Arabidopsis_thaliana - ps32
Phylostratum

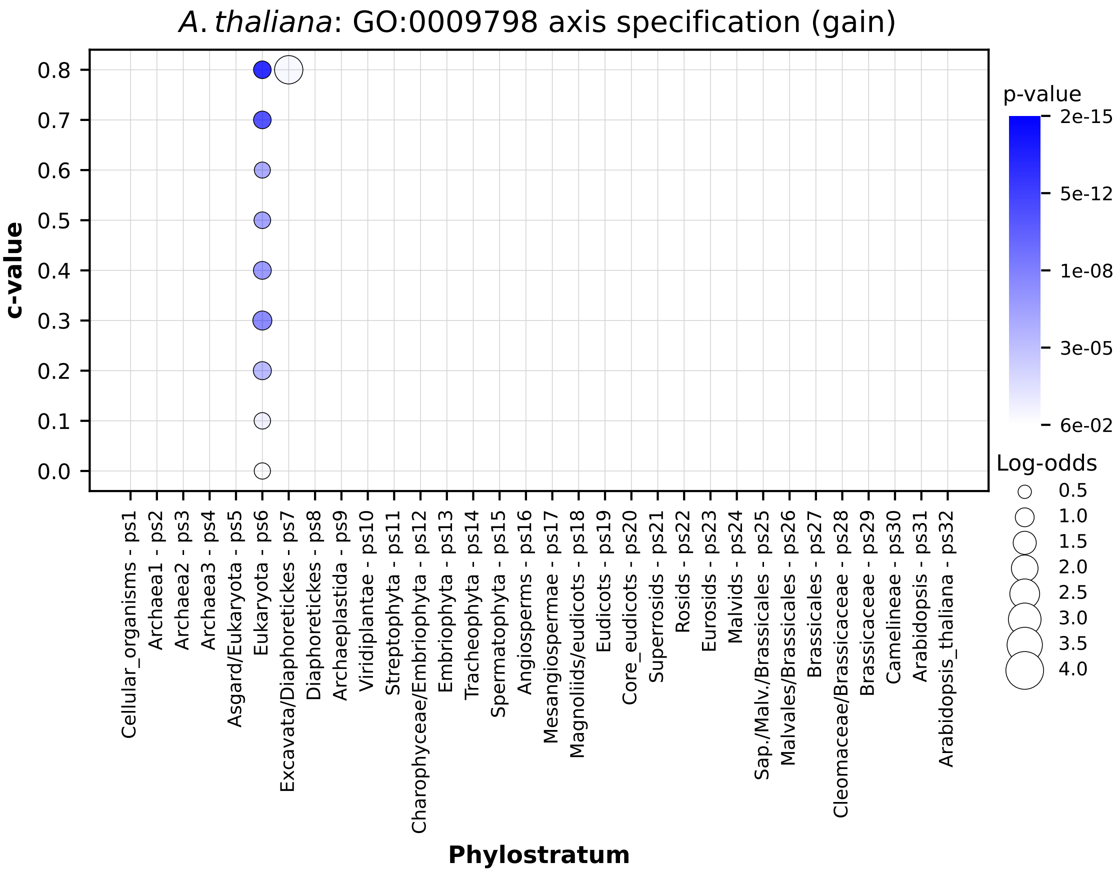
A. thaliana: GO:0009798 axis specification (gain)
c-value
0.0
0.1
0.2
0.3
0.4
0.5
0.6
0.7
0.8
p-value
2e-15
5e-12
1e-08
3e-05
6e-02
Log-odds
0.5
1.0
1.5
2.0
2.5
3.0
3.5
4.0
Cellular_organisms - ps1
Archaea1 - ps2
Archaea2 - ps3
Archaea3 - ps4
Asgard/Eukaryota - ps5
Eukaryota - ps6
Excavata/Diaphoretickes - ps7
Diaphoretickes - ps8
Archaeplastida - ps9
Viridiplantae - ps10
Streptophyta - ps11
Charophyceae/Embriophyta - ps12
Embriophyta - ps13
Tracheophyta - ps14
Spermatophyta - ps15
Angiosperms - ps16
Mesangiospermae - ps17
Magnoliids/eudicots - ps18
Eudicots - ps19
Core_eudicots - ps20
Superrosids - ps21
Rosids - ps22
Eurosids - ps23
Malvids - ps24
Sap./Malv./Brassicales - ps25
Malvales/Brassicales - ps26
Brassicales - ps27
Cleomaceae/Brassicaceae - ps28
Brassicaceae - ps29
Camelineae - ps30
Arabidopsis - ps31
Arabidopsis_thaliana - ps32
Phylostratum

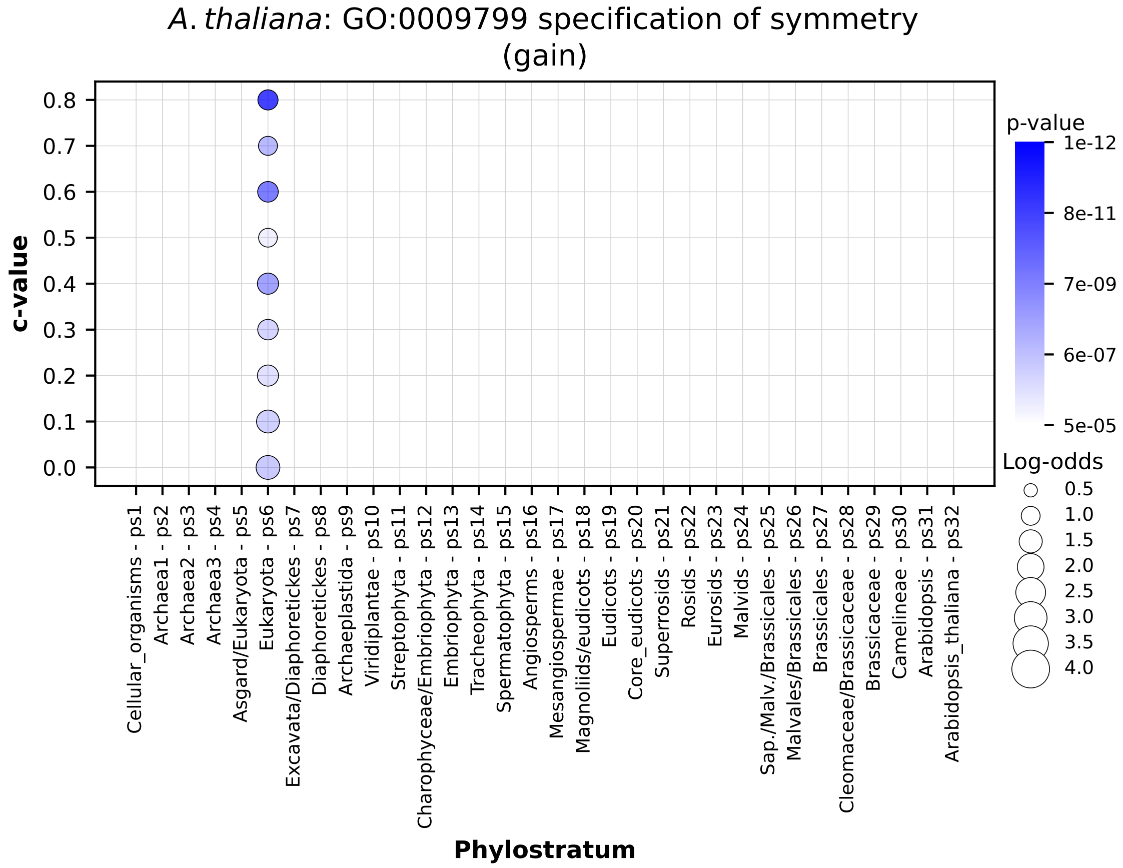

A. thaliana: GO:0009799 specification of symmetry
(gain)
c-value
0.0
0.1
0.2
0.3
0.4
0.5
0.6
0.7
0.8
Phylostratum
Cellular_organisms - ps1
Archaea1 - ps2
Archaea2 - ps3
Archaea3 - ps4
Asgard/Eukaryota - ps5
Eukaryota - ps6
Excavata/Diaphoretickes - ps7
Diaphoretickes - ps8
Archaeplastida - ps9
Viridiplantae - ps10
Streptophyta - ps11
Charophyceae/Embriophyta - ps12
Embriophyta - ps13
Tracheophyta - ps14
Spermatophyta - ps15
Angiosperms - ps16
Mesangiospermae - ps17
Magnoliids/eudicots - ps18
Eudicots - ps19
Core_eudicots - ps20
Superrosids - ps21
Rosids - ps22
Eurosids - ps23
Malvids - ps24
Sap./Malv./Brassicales - ps25
Malvales/Brassicales - ps26
Brassicales - ps27
Cleomaceae/Brassicaceae - ps28
Brassicaceae - ps29
Camelineae - ps30
Arabidopsis - ps31
Arabidopsis_thaliana - ps32
p-value
1e-12
8e-11
7e-09
6e-07
5e-05
Log-odds
0.5
1.0
1.5
2.0
2.5
3.0
3.5
4.0

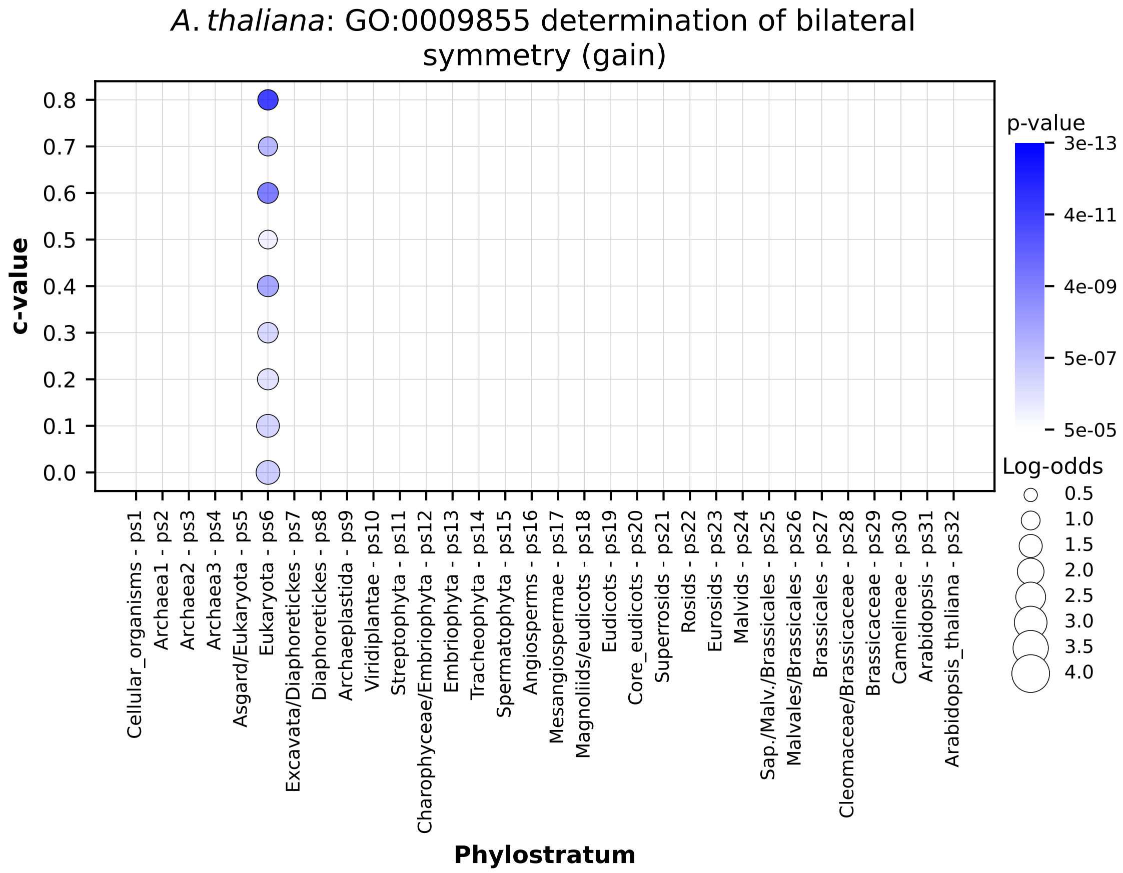

A. thaliana: GO:0009855 determination of bilateral symmetry (gain)
c-value
0.0
0.1
0.2
0.3
0.4
0.5
0.6
0.7
0.8
p-value
3e-13
4e-11
4e-09
5e-07
5e-05
Log-odds
0.5
1.0
1.5
2.0
2.5
3.0
3.5
4.0
Cellular_organisms - ps1
Archaea1 - ps2
Archaea2 - ps3
Archaea3 - ps4
Asgard/Eukaryota - ps5
Eukaryota - ps6
Excavata/Diaphoretickes - ps7
Diaphoretickes - ps8
Archaeplastida - ps9
Viridiplantae - ps10
Streptophyta - ps11
Charophyceae/Embriophyta - ps12
Embriophyta - ps13
Tracheophyta - ps14
Spermatophyta - ps15
Angiosperms - ps16
Mesangiospermae - ps17
Magnoliids/eudicots - ps18
Eudicots - ps19
Core_eudicots - ps20
Superrosids - ps21
Rosids - ps22
Eurosids - ps23
Malvids - ps24
Sap./Malv./Brassicales - ps25
Malvales/Brassicales - ps26
Brassicales - ps27
Cleomaceae/Brassicaceae - ps28
Brassicaceae - ps29
Camelineae - ps30
Arabidopsis - ps31
Arabidopsis_thaliana - ps32
Phylostratum

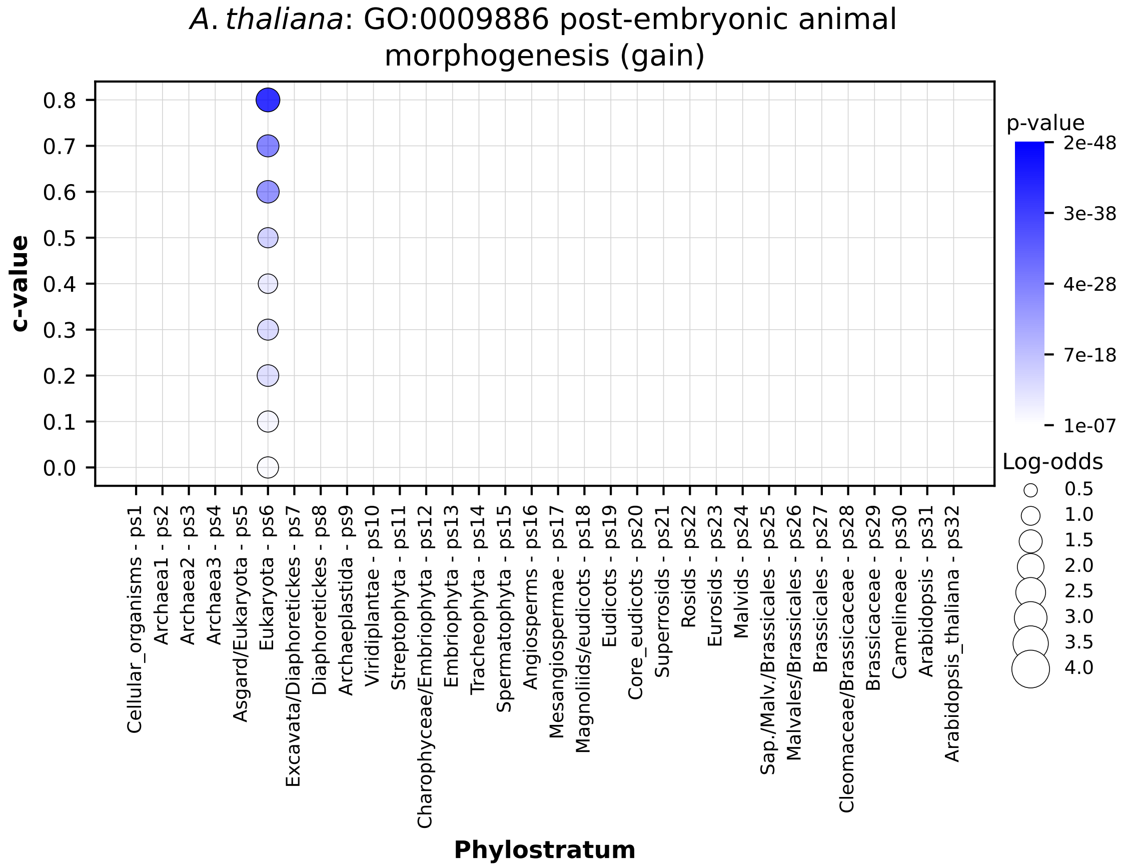

A. thaliana: GO:0009886 post-embryonic animal morphogenesis (gain)
c-value
0.8
0.7
0.6
0.5
0.4
0.3
0.2
0.1
0.0
p-value
2e-48
3e-38
4e-28
7e-18
1e-07
Log-odds
0.5
1.0
1.5
2.0
2.5
3.0
3.5
4.0
Cellular_organisms - ps1
Archaea1 - ps2
Archaea2 - ps3
Archaea3 - ps4
Asgard/Eukaryota - ps5
Eukaryota - ps6
Excavata/Diaphoretickes - ps7
Diaphoretickes - ps8
Archaeplastida - ps9
Viridiplantae - ps10
Streptophyta - ps11
Charophyceae/Embriophyta - ps12
Embriophyta - ps13
Tracheophyta - ps14
Spermatophyta - ps15
Angiosperms - ps16
Mesangiospermae - ps17
Magnoliids/eudicots - ps18
Eudicots - ps19
Core_eudicots - ps20
Superrosids - ps21
Rosids - ps22
Eurosids - ps23
Malvids - ps24
Sap./Malv./Brassicales - ps25
Malvales/Brassicales - ps26
Brassicales - ps27
Cleomaceae/Brassicaceae - ps28
Brassicaceae - ps29
Camelineae - ps30
Arabidopsis - ps31
Arabidopsis_thaliana - ps32
Phylostratum

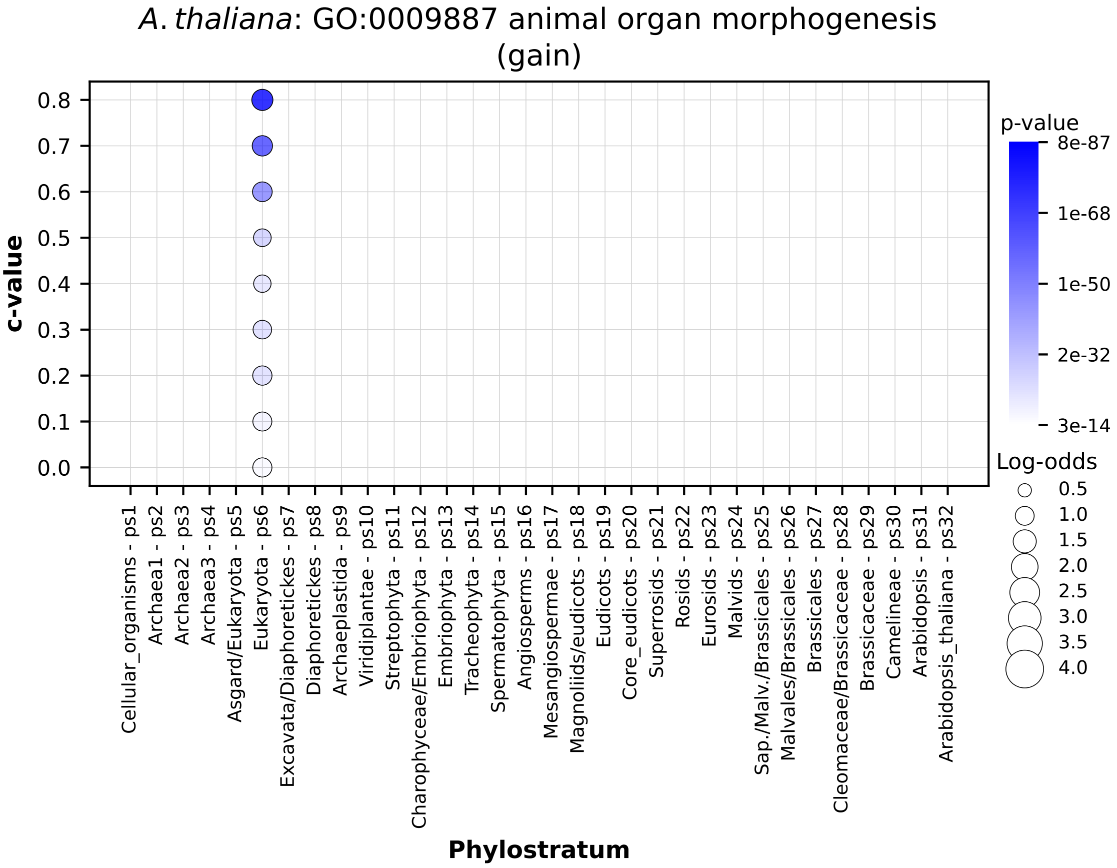
A. thaliana: GO:0009887 animal organ morphogenesis
(gain)
c-value
0.8
0.7
0.6
0.5
0.4
0.3
0.2
0.1
0.0
Phylostratum
Cellular_organisms - ps1
Archaea1 - ps2
Archaea2 - ps3
Archaea3 - ps4
Asgard/Eukaryota - ps5
Eukaryota - ps6
Excavata/Diaphoretickes - ps7
Diaphoretickes - ps8
Archaeplastida - ps9
Viridiplantae - ps10
Streptophyta - ps11
Charophyceae/Embriophyta - ps12
Embriophyta - ps13
Tracheophyta - ps14
Spermatophyta - ps15
Angiosperms - ps16
Mesangiospermae - ps17
Magnoliids/eudicots - ps18
Eudicots - ps19
Core_eudicots - ps20
Superrosids - ps21
Rosids - ps22
Eurosids - ps23
Malvids - ps24
Sap./Malv./Brassicales - ps25
Malvales/Brassicales - ps26
Brassicales - ps27
Cleomaceae/Brassicaceae - ps28
Brassicaceae - ps29
Camelineae - ps30
Arabidopsis - ps31
Arabidopsis_thaliana - ps32
p-value
8e-87
1e-68
1e-50
2e-32
3e-14
Log-odds
0.5
1.0
1.5
2.0
2.5
3.0
3.5
4.0

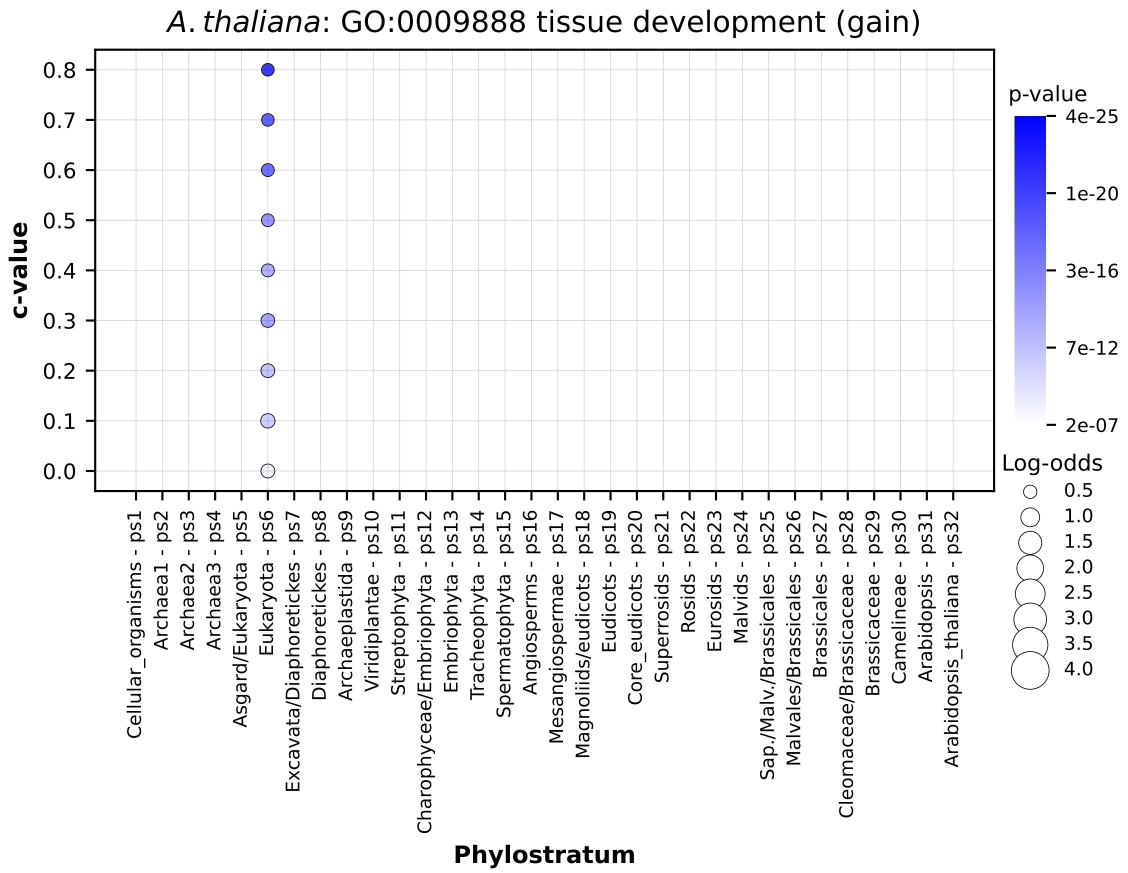

A. thaliana: GO:0009888 tissue development (gain)
c-value
0.8
0.7
0.6
0.5
0.4
0.3
0.2
0.1
0.0
p-value
4e-25
1e-20
3e-16
7e-12
2e-07
Log-odds
0.5
1.0
1.5
2.0
2.5
3.0
3.5
4.0
Cellular_organisms - ps1
Archaea1 - ps2
Archaea2 - ps3
Archaea3 - ps4
Asgard/Eukaryota - ps5
Eukaryota - ps6
Excavata/Diaphoretickes - ps7
Diaphoretickes - ps8
Archaeplastida - ps9
Viridiplantae - ps10
Streptophyta - ps11
Charophyceae/Embriophyta - ps12
Embriophyta - ps13
Tracheophyta - ps14
Spermatophyta - ps15
Angiosperms - ps16
Mesangiospermae - ps17
Magnoliids/eudicots - ps18
Eudicots - ps19
Core_eudicots - ps20
Superrosids - ps21
Rosids - ps22
Eurosids - ps23
Malvids - ps24
Sap./Malv./Brassicales - ps25
Malvales/Brassicales - ps26
Brassicales - ps27
Cleomaceae/Brassicaceae - ps28
Brassicaceae - ps29
Camelineae - ps30
Arabidopsis - ps31
Arabidopsis_thaliana - ps32
Phylostratum

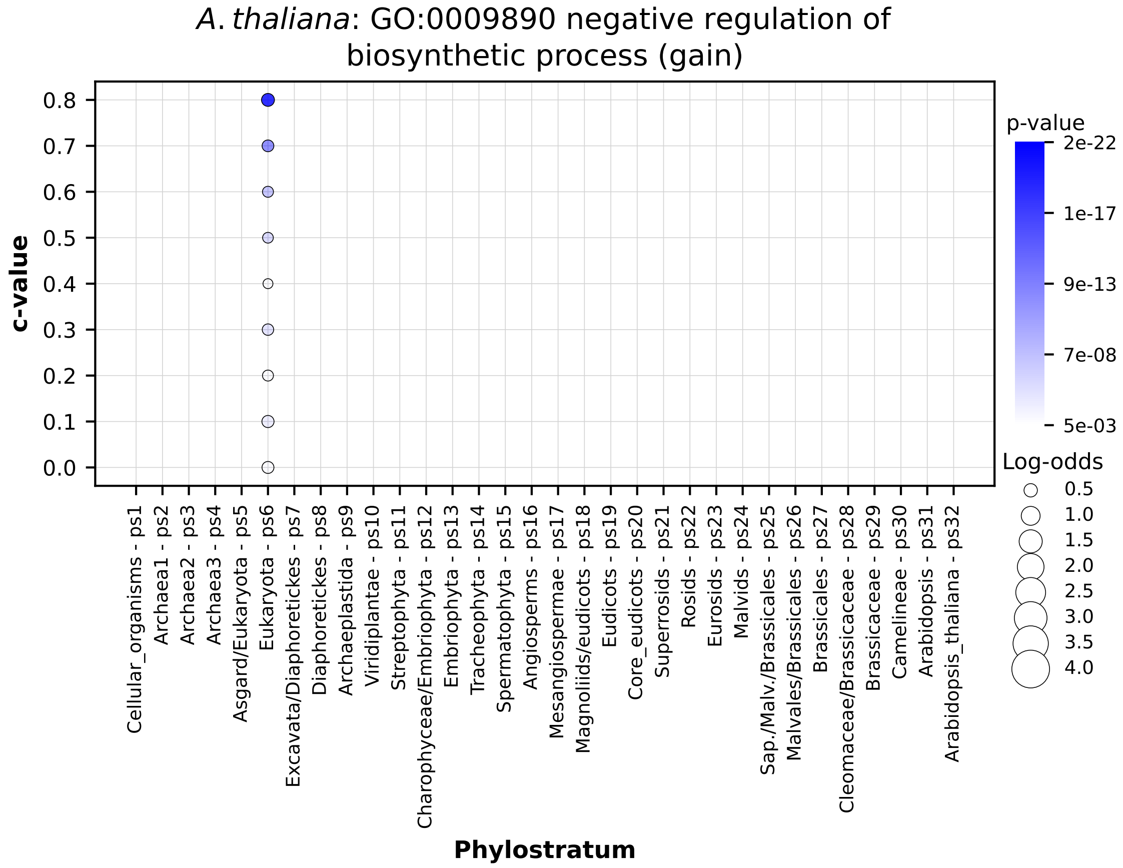
A. thaliana: GO:0009890 negative regulation of biosynthetic process (gain)
c-value
0.8
0.7
0.6
0.5
0.4
0.3
0.2
0.1
0.0
p-value
2e-22
1e-17
9e-13
7e-08
5e-03
Log-odds
0.5
1.0
1.5
2.0
2.5
3.0
3.5
4.0
Cellular_organisms - ps1
Archaea1 - ps2
Archaea2 - ps3
Archaea3 - ps4
Asgard/Eukaryota - ps5
Eukaryota - ps6
Excavata/Diaphoretickes - ps7
Diaphoretickes - ps8
Archaeplastida - ps9
Viridiplantae - ps10
Streptophyta - ps11
Charophyceae/Embriophyta - ps12
Embriophyta - ps13
Tracheophyta - ps14
Spermatophyta - ps15
Angiosperms - ps16
Mesangiospermae - ps17
Magnoliids/eudicots - ps18
Eudicots - ps19
Core_eudicots - ps20
Superrosids - ps21
Rosids - ps22
Eurosids - ps23
Malvids - ps24
Sap./Malv./Brassicales - ps25
Malvales/Brassicales - ps26
Brassicales - ps27
Cleomaceae/Brassicaceae - ps28
Brassicaceae - ps29
Camelineae - ps30
Arabidopsis - ps31
Arabidopsis_thaliana - ps32
Phylostratum

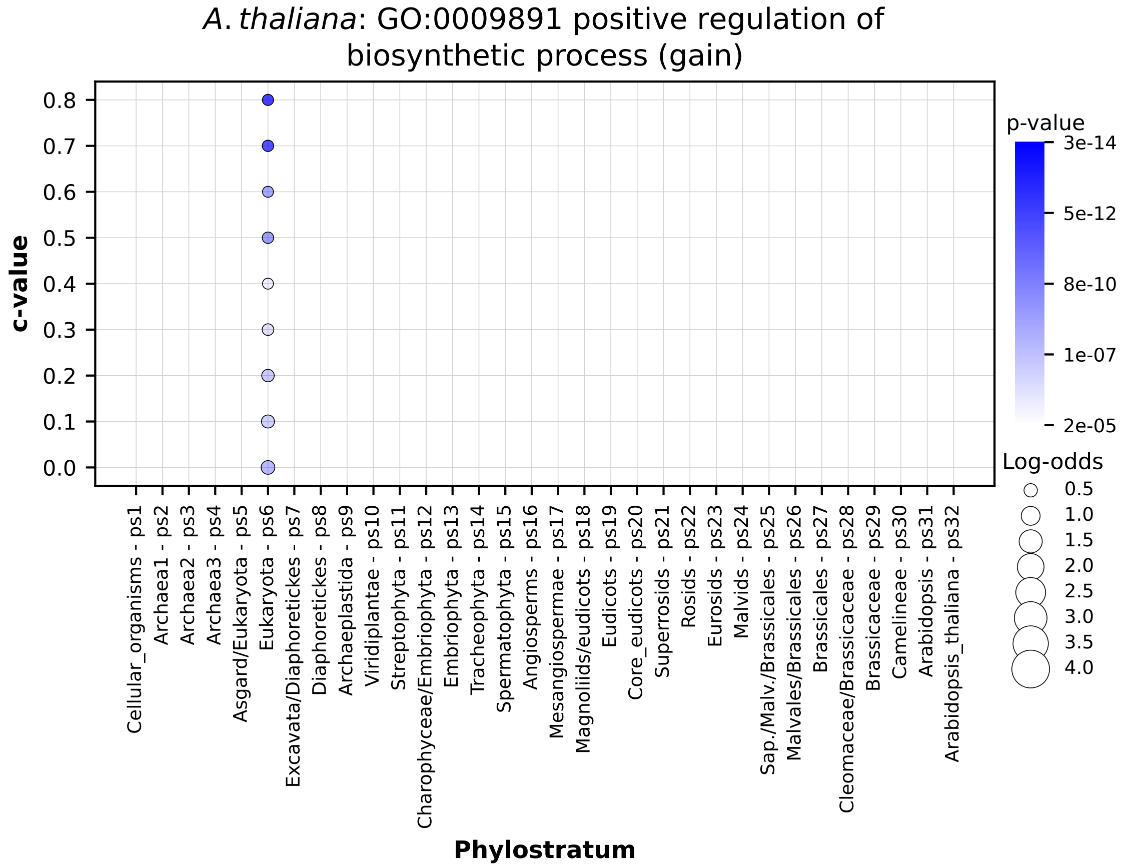
A. thaliana: GO:0009891 positive regulation of biosynthetic process (gain)
c-value
0.0
0.1
0.2
0.3
0.4
0.5
0.6
0.7
0.8
Phylostratum
Cellular_organisms - ps1
Archaea1 - ps2
Archaea2 - ps3
Archaea3 - ps4
Asgard/Eukaryota - ps5
Eukaryota - ps6
Excavata/Diaphoretickes - ps7
Diaphoretickes - ps8
Archaeplastida - ps9
Viridiplantae - ps10
Streptophyta - ps11
Charophyceae/Embriophyta - ps12
Embriophyta - ps13
Tracheophyta - ps14
Spermatophyta - ps15
Angiosperms - ps16
Mesangiospermae - ps17
Magnoliids/eudicots - ps18
Eudicots - ps19
Core_eudicots - ps20
Superrosids - ps21
Rosids - ps22
Eurosids - ps23
Malvids - ps24
Sap./Malv./Brassicales - ps25
Malvales/Brassicales - ps26
Brassicales - ps27
Cleomaceae/Brassicaceae - ps28
Brassicaceae - ps29
Camelineae - ps30
Arabidopsis - ps31
Arabidopsis_thaliana - ps32
p-value
3e-14
5e-12
8e-10
1e-07
2e-05
Log-odds
0.5
1.0
1.5
2.0
2.5
3.0
3.5
4.0

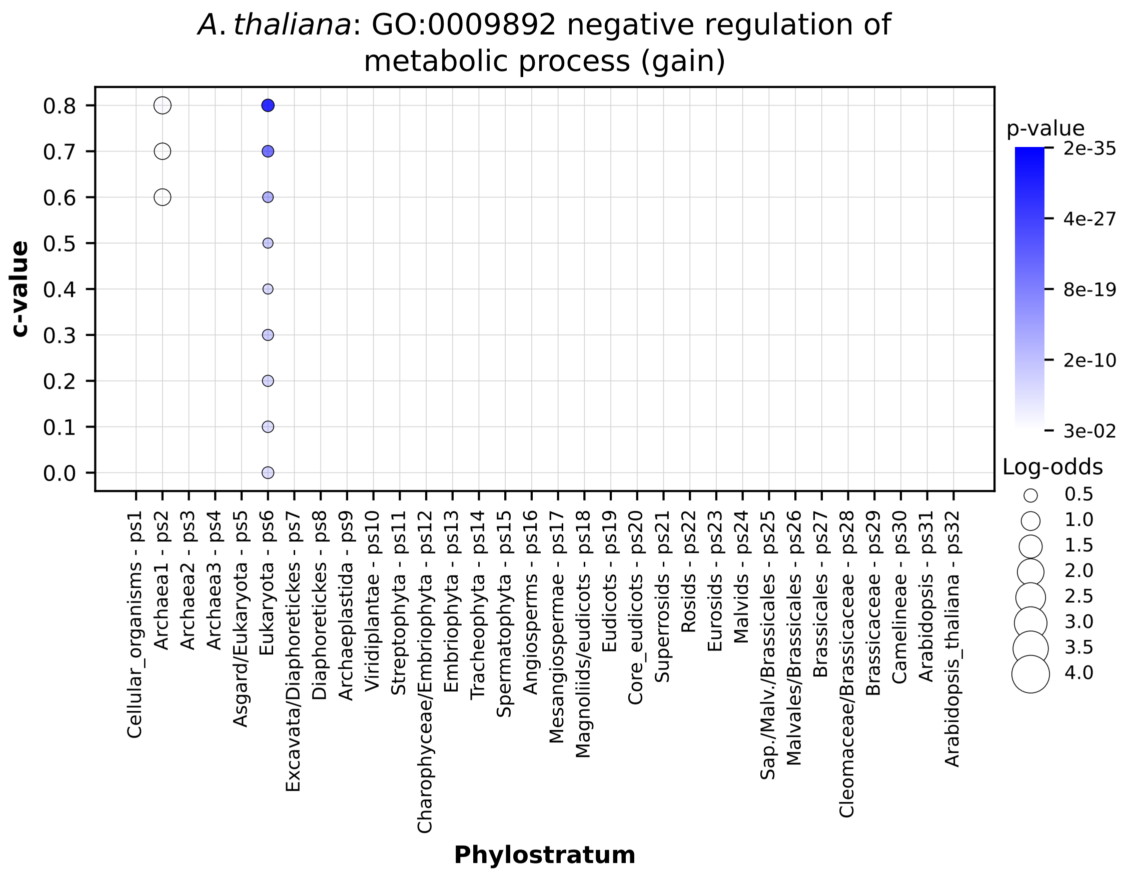
A. thaliana: GO:0009892 negative regulation of metabolic process (gain)
c-value
0.8
0.7
0.6
0.5
0.4
0.3
0.2
0.1
0.0
p-value
2e-35
4e-27
8e-19
2e-10
3e-02
Log-odds
0.5
1.0
1.5
2.0
2.5
3.0
3.5
4.0
Cellular_organisms - ps1
Archaea1 - ps2
Archaea2 - ps3
Archaea3 - ps4
Asgard/Eukaryota - ps5
Eukaryota - ps6
Excavata/Diaphoretickes - ps7
Diaphoretickes - ps8
Archaeplastida - ps9
Viridiplantae - ps10
Streptophyta - ps11
Charophyceae/Embriophyta - ps12
Embriophyta - ps13
Tracheophyta - ps14
Spermatophyta - ps15
Angiosperms - ps16
Mesangiospermae - ps17
Magnoliids/eudicots - ps18
Eudicots - ps19
Core_eudicots - ps20
Superrosids - ps21
Rosids - ps22
Eurosids - ps23
Malvids - ps24
Sap./Malv./Brassicales - ps25
Malvales/Brassicales - ps26
Brassicales - ps27
Cleomaceae/Brassicaceae - ps28
Brassicaceae - ps29
Camelineae - ps30
Arabidopsis - ps31
Arabidopsis_thaliana - ps32
Phylostratum

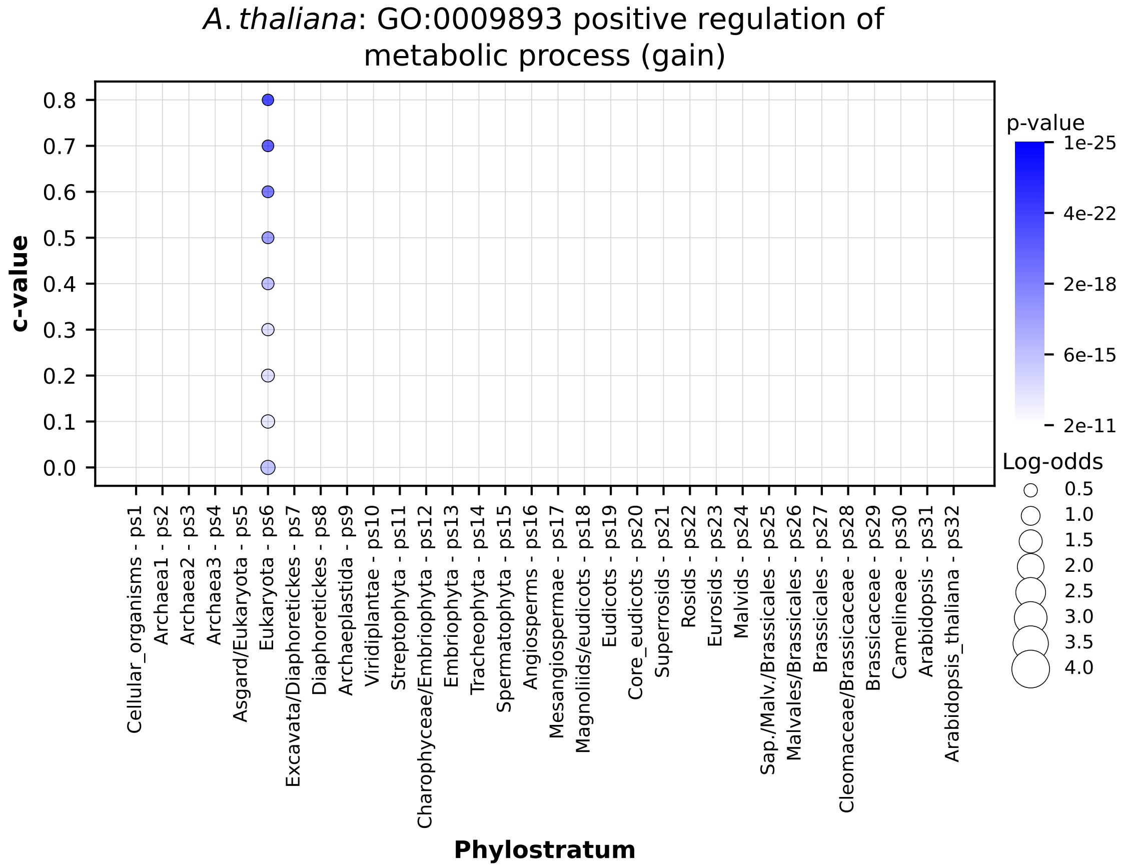

A. thaliana: GO:0009893 positive regulation of metabolic process (gain)
c-value
0.8
0.7
0.6
0.5
0.4
0.3
0.2
0.1
0.0
p-value
1e-25
4e-22
2e-18
6e-15
2e-11
Log-odds
0.5
1.0
1.5
2.0
2.5
3.0
3.5
4.0
Cellular_organisms - ps1
Archaea1 - ps2
Archaea2 - ps3
Archaea3 - ps4
Asgard/Eukaryota - ps5
Eukaryota - ps6
Excavata/Diaphoretickes - ps7
Diaphoretickes - ps8
Archaeplastida - ps9
Viridiplantae - ps10
Streptophyta - ps11
Charophyceae/Embriophyta - ps12
Embriophyta - ps13
Tracheophyta - ps14
Spermatophyta - ps15
Angiosperms - ps16
Mesangiospermae - ps17
Magnoliids/eudicots - ps18
Eudicots - ps19
Core_eudicots - ps20
Superrosids - ps21
Rosids - ps22
Eurosids - ps23
Malvids - ps24
Sap./Malv./Brassicales - ps25
Malvales/Brassicales - ps26
Brassicales - ps27
Cleomaceae/Brassicaceae - ps28
Brassicaceae - ps29
Camelineae - ps30
Arabidopsis - ps31
Arabidopsis_thaliana - ps32
Phylostratum

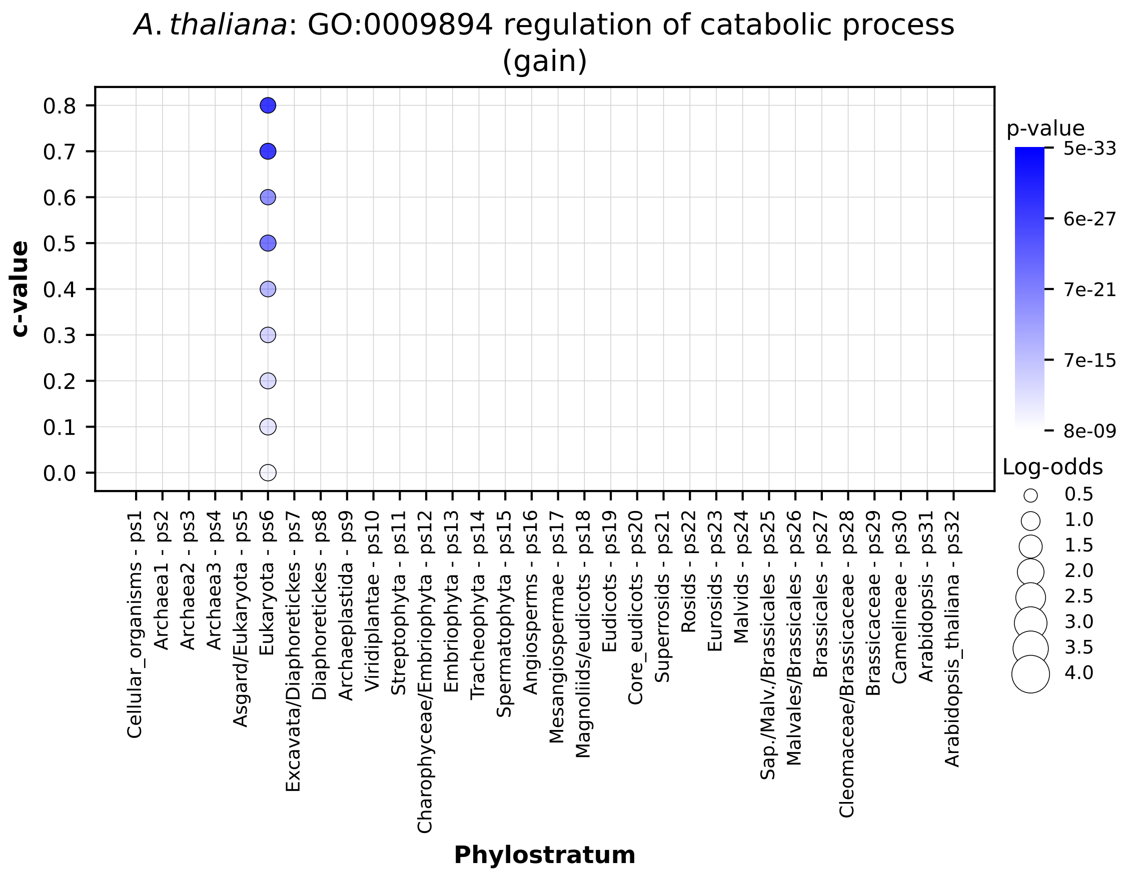

A. thaliana: GO:0009894 regulation of catabolic process (gain)
c-value
0.8
0.7
0.6
0.5
0.4
0.3
0.2
0.1
0.0
p-value
5e-33
6e-27
7e-21
7e-15
8e-09
Log-odds
0.5
1.0
1.5
2.0
2.5
3.0
3.5
4.0
Cellular_organisms - ps1
Archaea1 - ps2
Archaea2 - ps3
Archaea3 - ps4
Asgard/Eukaryota - ps5
Eukaryota - ps6
Excavata/Diaphoretickes - ps7
Diaphoretickes - ps8
Archaeplastida - ps9
Viridiplantae - ps10
Streptophyta - ps11
Charophyceae/Embriophyta - ps12
Embriophyta - ps13
Tracheophyta - ps14
Spermatophyta - ps15
Angiosperms - ps16
Mesangiospermae - ps17
Magnoliids/eudicots - ps18
Eudicots - ps19
Core_eudicots - ps20
Superrosids - ps21
Rosids - ps22
Eurosids - ps23
Malvids - ps24
Sap./Malv./Brassicales - ps25
Malvales/Brassicales - ps26
Brassicales - ps27
Cleomaceae/Brassicaceae - ps28
Brassicaceae - ps29
Camelineae - ps30
Arabidopsis - ps31
Arabidopsis_thaliana - ps32
Phylostratum

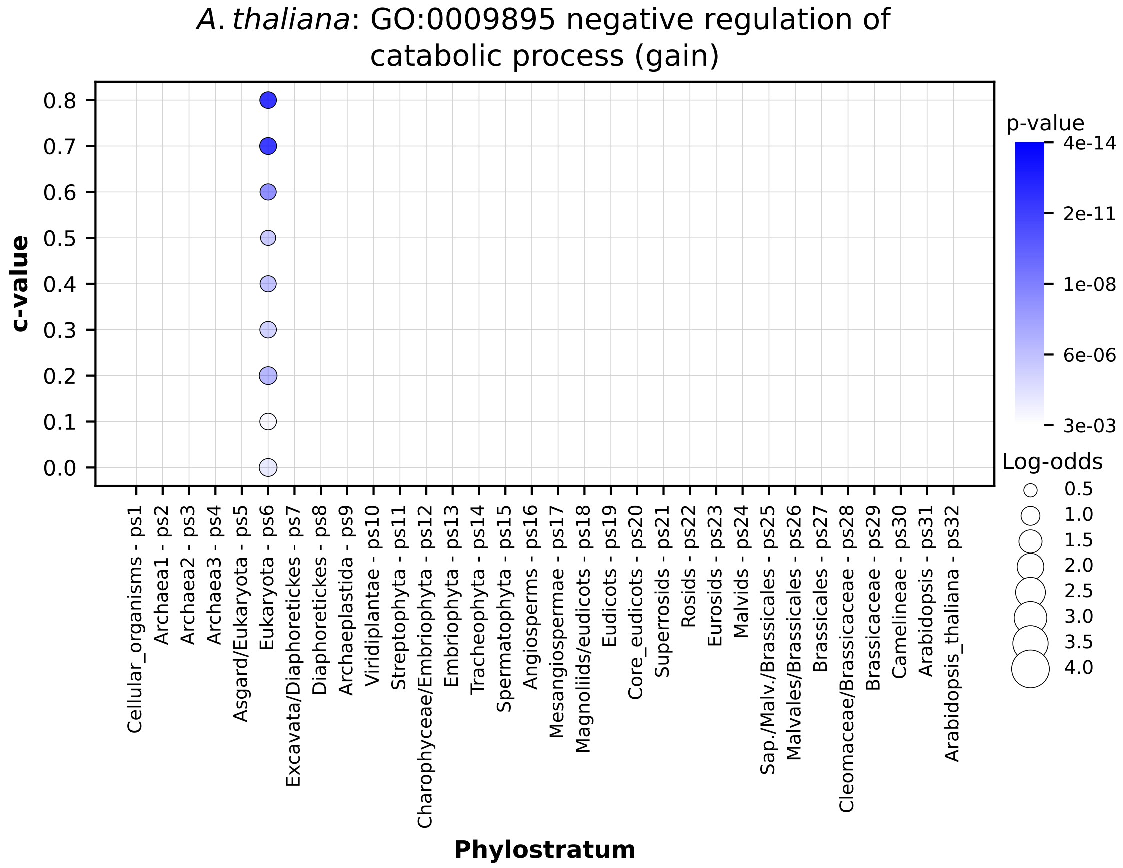

A. thaliana: GO:0009895 negative regulation of catabolic process (gain)
c-value
0.0
0.1
0.2
0.3
0.4
0.5
0.6
0.7
0.8
p-value
4e-14
2e-11
1e-08
6e-06
3e-03
Log-odds
0.5
1.0
1.5
2.0
2.5
3.0
3.5
4.0
Cellular_organisms - ps1
Archaea1 - ps2
Archaea2 - ps3
Archaea3 - ps4
Asgard/Eukaryota - ps5
Eukaryota - ps6
Excavata/Diaphoretickes - ps7
Diaphoretickes - ps8
Archaeplastida - ps9
Viridiplantae - ps10
Streptophyta - ps11
Charophyceae/Embriophyta - ps12
Embriophyta - ps13
Tracheophyta - ps14
Spermatophyta - ps15
Angiosperms - ps16
Mesangiospermae - ps17
Magnoliids/eudicots - ps18
Eudicots - ps19
Core_eudicots - ps20
Superrosids - ps21
Rosids - ps22
Eurosids - ps23
Malvids - ps24
Sap./Malv./Brassicales - ps25
Malvales/Brassicales - ps26
Brassicales - ps27
Cleomaceae/Brassicaceae - ps28
Brassicaceae - ps29
Camelineae - ps30
Arabidopsis - ps31
Arabidopsis_thaliana - ps32
Phylostratum

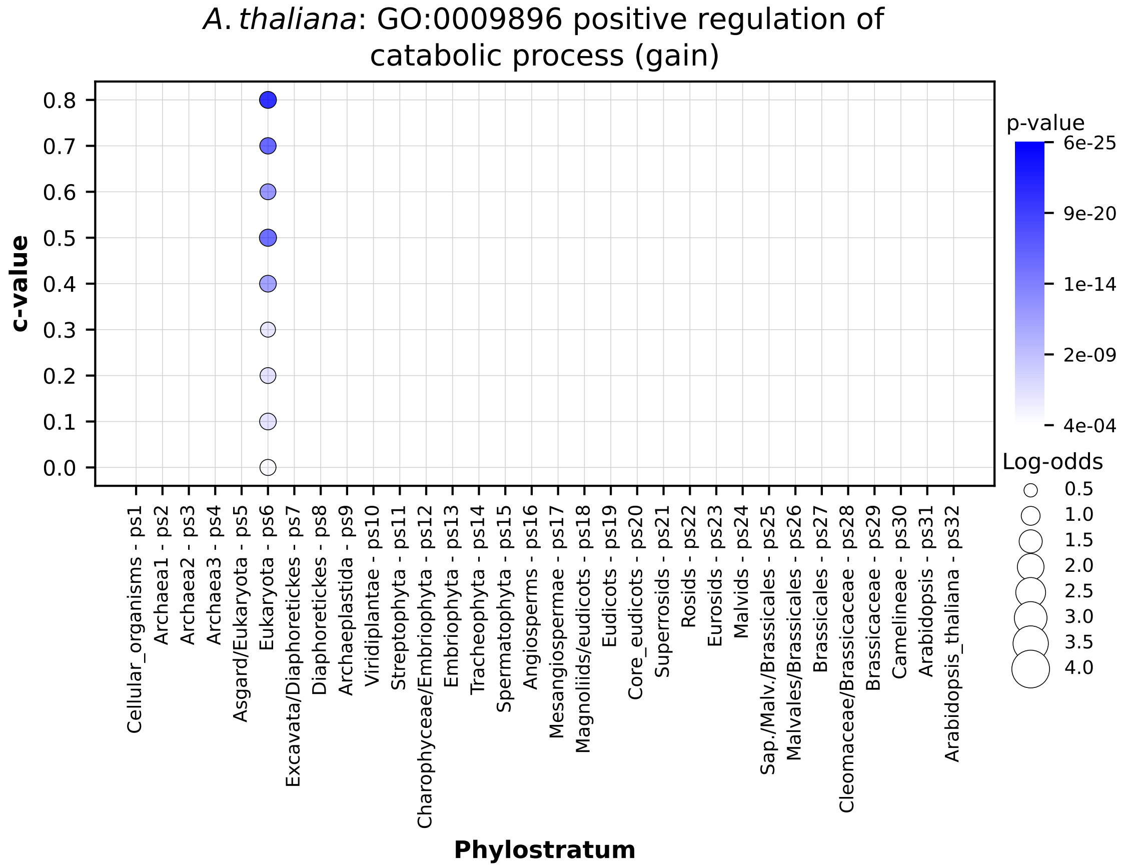

A. thaliana: GO:0009896 positive regulation of catabolic process (gain)
c-value
0.8
0.7
0.6
0.5
0.4
0.3
0.2
0.1
0.0
p-value
6e-25
9e-20
1e-14
2e-09
4e-04
Log-odds
0.5
1.0
1.5
2.0
2.5
3.0
3.5
4.0
Cellular_organisms - ps1
Archaea1 - ps2
Archaea2 - ps3
Archaea3 - ps4
Asgard/Eukaryota - ps5
Eukaryota - ps6
Excavata/Diaphoretickes - ps7
Diaphoretickes - ps8
Archaeplastida - ps9
Viridiplantae - ps10
Streptophyta - ps11
Charophyceae/Embriophyta - ps12
Embriophyta - ps13
Tracheophyta - ps14
Spermatophyta - ps15
Angiosperms - ps16
Mesangiospermae - ps17
Magnoliids/eudicots - ps18
Eudicots - ps19
Core_eudicots - ps20
Superrosids - ps21
Rosids - ps22
Eurosids - ps23
Malvids - ps24
Sap./Malv./Brassicales - ps25
Malvales/Brassicales - ps26
Brassicales - ps27
Cleomaceae/Brassicaceae - ps28
Brassicaceae - ps29
Camelineae - ps30
Arabidopsis - ps31
Arabidopsis_thaliana - ps32
Phylostratum

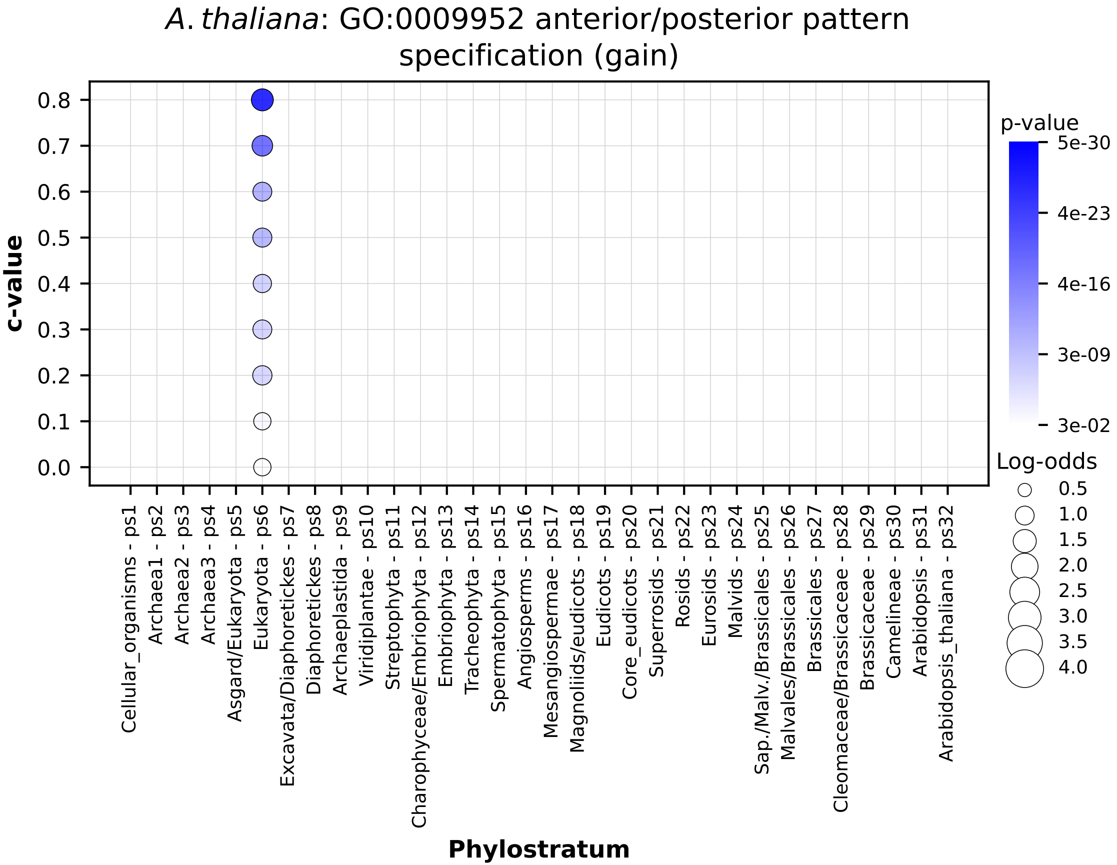

A. thaliana: GO:0009952 anterior/posterior pattern specification (gain)
c-value
0.8
0.7
0.6
0.5
0.4
0.3
0.2
0.1
0.0
p-value
5e-30
4e-23
4e-16
3e-09
3e-02
Log-odds
0.5
1.0
1.5
2.0
2.5
3.0
3.5
4.0
Cellular_organisms - ps1
Archaea1 - ps2
Archaea2 - ps3
Archaea3 - ps4
Asgard/Eukaryota - ps5
Eukaryota - ps6
Excavata/Diaphoretickes - ps7
Diaphoretickes - ps8
Archaeplastida - ps9
Viridiplantae - ps10
Streptophyta - ps11
Charophyceae/Embriophyta - ps12
Embriophyta - ps13
Tracheophyta - ps14
Spermatophyta - ps15
Angiosperms - ps16
Mesangiospermae - ps17
Magnoliids/eudicots - ps18
Eudicots - ps19
Core_eudicots - ps20
Superrosids - ps21
Rosids - ps22
Eurosids - ps23
Malvids - ps24
Sap./Malv./Brassicales - ps25
Malvales/Brassicales - ps26
Brassicales - ps27
Cleomaceae/Brassicaceae - ps28
Brassicaceae - ps29
Camelineae - ps30
Arabidopsis - ps31
Arabidopsis_thaliana - ps32
Phylostratum

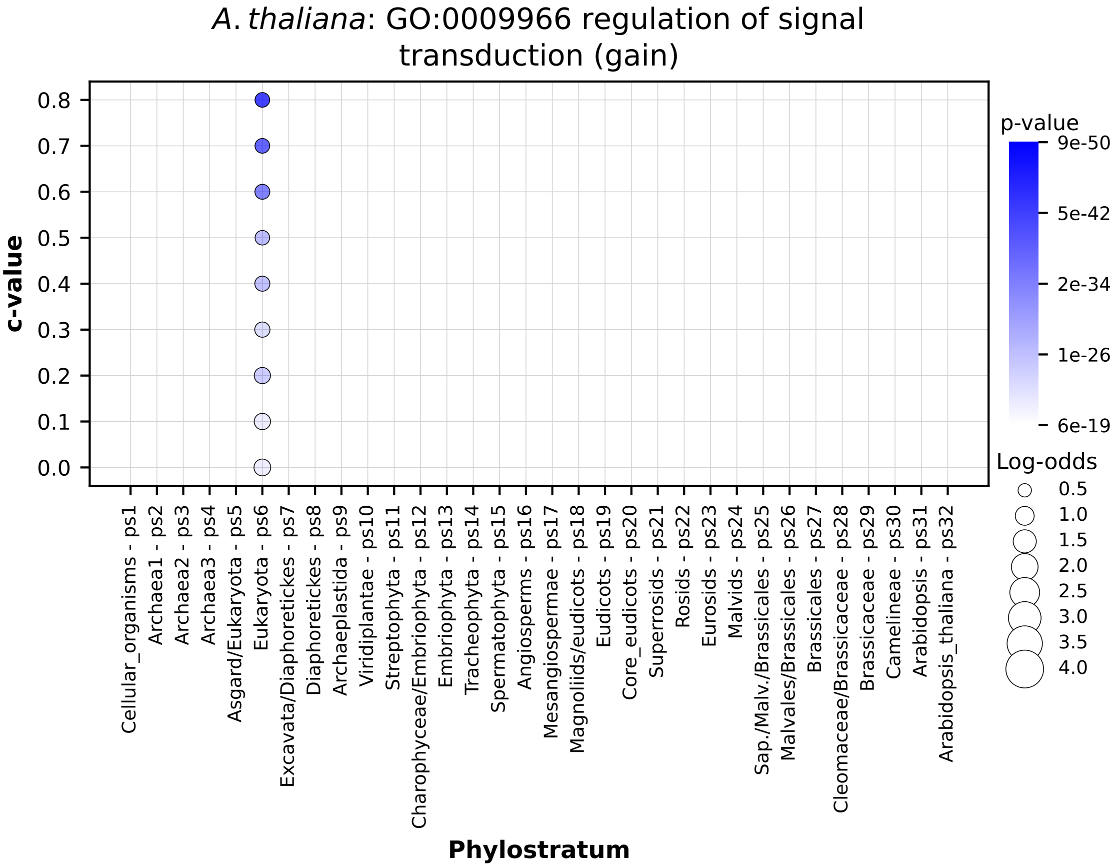

A. thaliana: GO:0009966 regulation of signal transduction (gain)
c-value
0.8
0.7
0.6
0.5
0.4
0.3
0.2
0.1
0.0
p-value
9e-50
5e-42
2e-34
1e-26
6e-19
Log-odds
0.5
1.0
1.5
2.0
2.5
3.0
3.5
4.0
Cellular_organisms - ps1
Archaea1 - ps2
Archaea2 - ps3
Archaea3 - ps4
Asgard/Eukaryota - ps5
Eukaryota - ps6
Excavata/Diaphoretickes - ps7
Diaphoretickes - ps8
Archaeplastida - ps9
Viridiplantae - ps10
Streptophyta - ps11
Charophyceae/Embriophyta - ps12
Embriophyta - ps13
Tracheophyta - ps14
Spermatophyta - ps15
Angiosperms - ps16
Mesangiospermae - ps17
Magnoliids/eudicots - ps18
Eudicots - ps19
Core_eudicots - ps20
Superrosids - ps21
Rosids - ps22
Eurosids - ps23
Malvids - ps24
Sap./Malv./Brassicales - ps25
Malvales/Brassicales - ps26
Brassicales - ps27
Cleomaceae/Brassicaceae - ps28
Brassicaceae - ps29
Camelineae - ps30
Arabidopsis - ps31
Arabidopsis_thaliana - ps32
Phylostratum

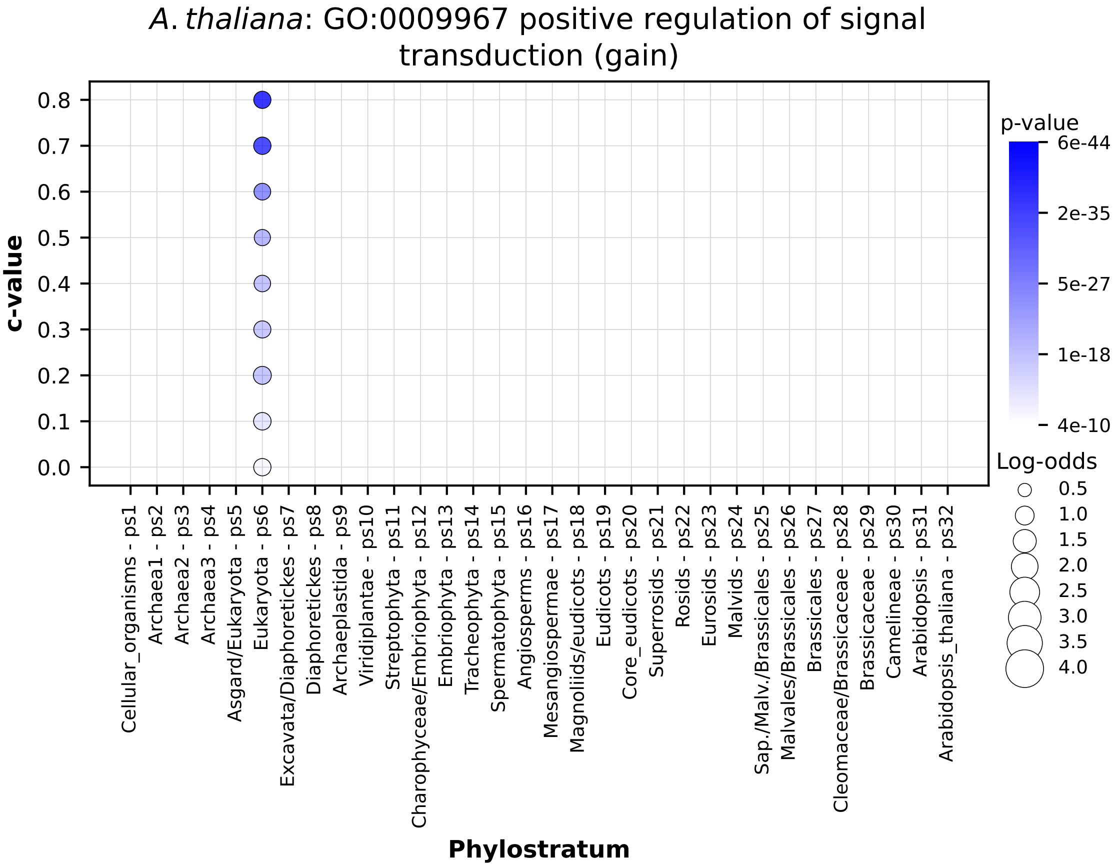

A. thaliana: GO:0009967 positive regulation of signal transduction (gain)
c-value
0.0
0.1
0.2
0.3
0.4
0.5
0.6
0.7
0.8
Phylostratum
Cellular_organisms - ps1
Archaea1 - ps2
Archaea2 - ps3
Archaea3 - ps4
Asgard/Eukaryota - ps5
Eukaryota - ps6
Excavata/Diaphoretickes - ps7
Diaphoretickes - ps8
Archaeplastida - ps9
Viridiplantae - ps10
Streptophyta - ps11
Charophyceae/Embriophyta - ps12
Embriophyta - ps13
Tracheophyta - ps14
Spermatophyta - ps15
Angiosperms - ps16
Mesangiospermae - ps17
Magnoliids/eudicots - ps18
Eudicots - ps19
Core_eudicots - ps20
Superrosids - ps21
Rosids - ps22
Eurosids - ps23
Malvids - ps24
Sap./Malv./Brassicales - ps25
Malvales/Brassicales - ps26
Brassicales - ps27
Cleomaceae/Brassicaceae - ps28
Brassicaceae - ps29
Camelineae - ps30
Arabidopsis - ps31
Arabidopsis_thaliana - ps32
p-value
6e-44
2e-35
5e-27
1e-18
4e-10
Log-odds
0.5
1.0
1.5
2.0
2.5
3.0
3.5
4.0

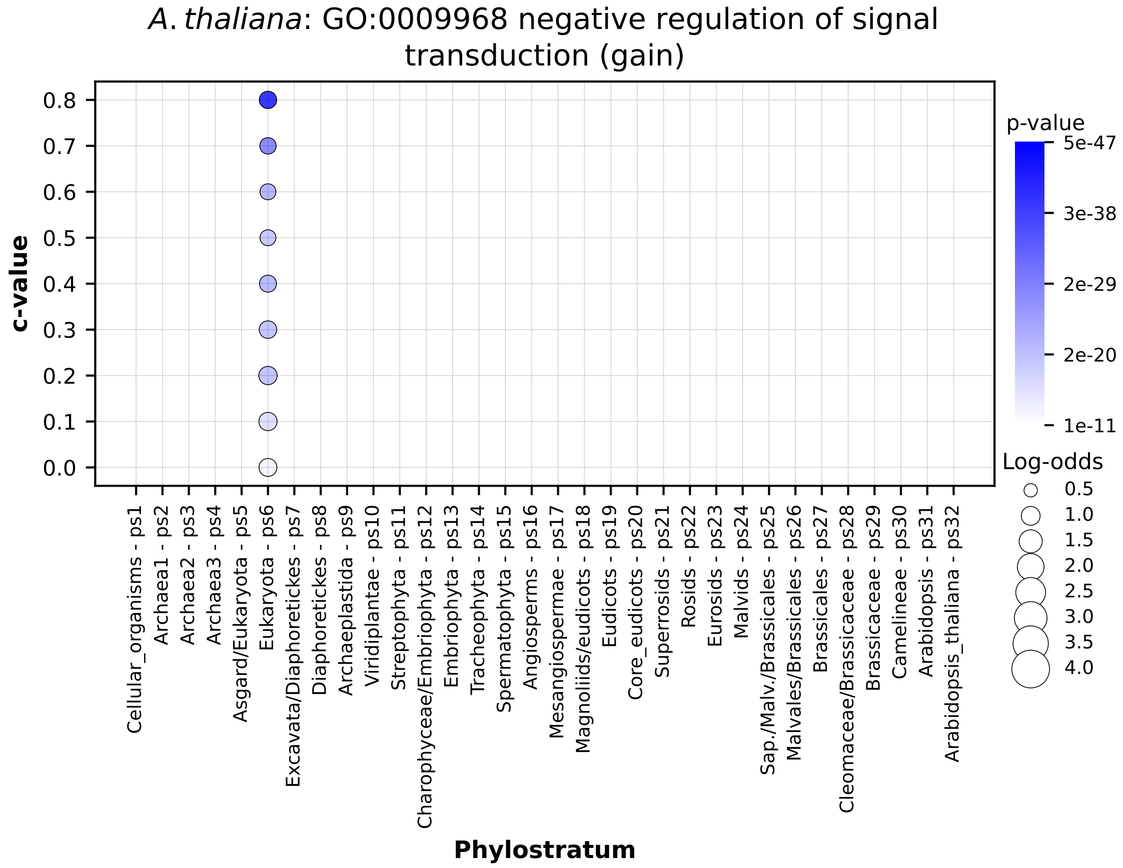
A. thaliana: GO:0009968 negative regulation of signal transduction (gain)
c-value
0.0
0.1
0.2
0.3
0.4
0.5
0.6
0.7
0.8
p-value
5e-47
3e-38
2e-29
2e-20
1e-11
Log-odds
0.5
1.0
1.5
2.0
2.5
3.0
3.5
4.0
Cellular_organisms - ps1
Archaea1 - ps2
Archaea2 - ps3
Archaea3 - ps4
Asgard/Eukaryota - ps5
Eukaryota - ps6
Excavata/Diaphoretickes - ps7
Diaphoretickes - ps8
Archaeplastida - ps9
Viridiplantae - ps10
Streptophyta - ps11
Charophyceae/Embriophyta - ps12
Embriophyta - ps13
Tracheophyta - ps14
Spermatophyta - ps15
Angiosperms - ps16
Mesangiospermae - ps17
Magnoliids/eudicots - ps18
Eudicots - ps19
Core_eudicots - ps20
Superrosids - ps21
Rosids - ps22
Eurosids - ps23
Malvids - ps24
Sap./Malv./Brassicales - ps25
Malvales/Brassicales - ps26
Brassicales - ps27
Cleomaceae/Brassicaceae - ps28
Brassicaceae - ps29
Camelineae - ps30
Arabidopsis - ps31
Arabidopsis_thaliana - ps32
Phylostratum

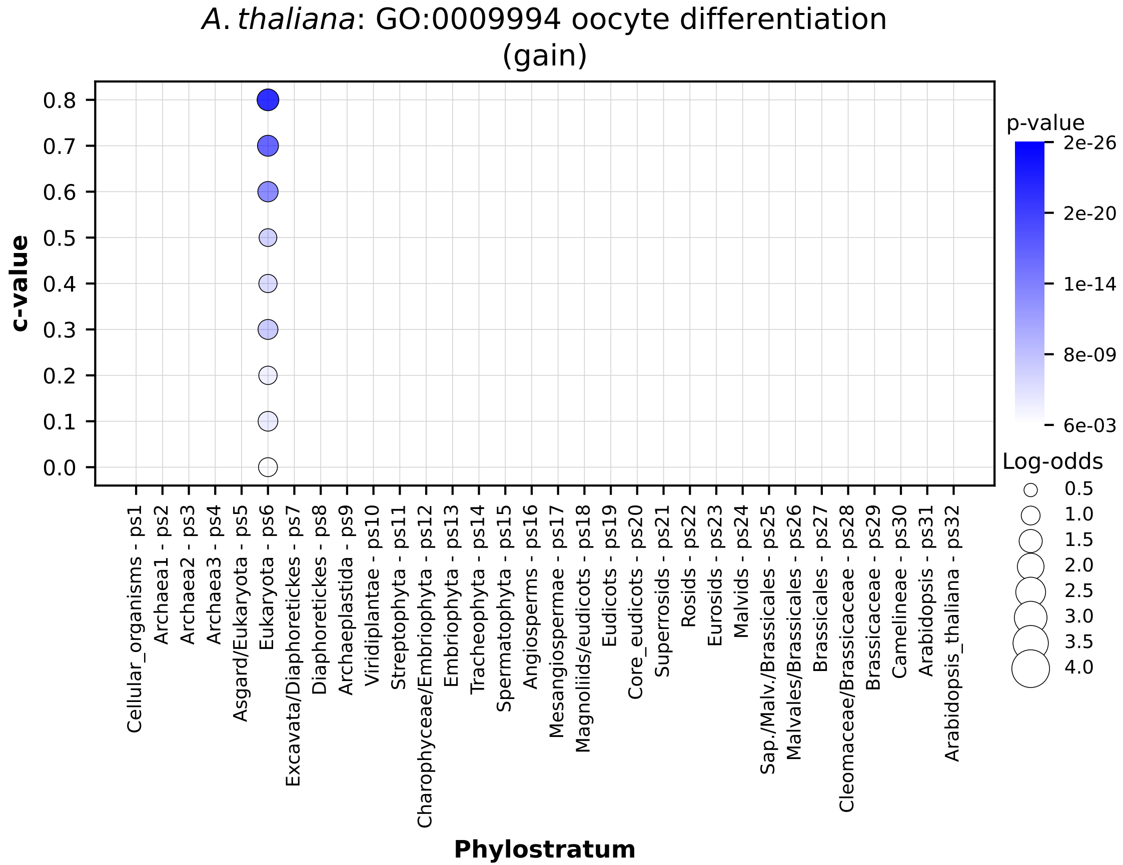

A. thaliana: GO:0009994 oocyte differentiation
(gain)
c-value
0.8
0.7
0.6
0.5
0.4
0.3
0.2
0.1
0.0
p-value
2e-26
2e-20
1e-14
8e-09
6e-03
Log-odds
0.5
1.0
1.5
2.0
2.5
3.0
3.5
4.0
Cellular_organisms - ps1
Archaea1 - ps2
Archaea2 - ps3
Archaea3 - ps4
Asgard/Eukaryota - ps5
Eukaryota - ps6
Excavata/Diaphoretickes - ps7
Diaphoretickes - ps8
Archaeplastida - ps9
Viridiplantae - ps10
Streptophyta - ps11
Charophyceae/Embriophyta - ps12
Embriophyta - ps13
Tracheophyta - ps14
Spermatophyta - ps15
Angiosperms - ps16
Mesangiospermae - ps17
Magnoliids/eudicots - ps18
Eudicots - ps19
Core_eudicots - ps20
Superrosids - ps21
Rosids - ps22
Eurosids - ps23
Malvids - ps24
Sap./Malv./Brassicales - ps25
Malvales/Brassicales - ps26
Brassicales - ps27
Cleomaceae/Brassicaceae - ps28
Brassicaceae - ps29
Camelineae - ps30
Arabidopsis - ps31
Arabidopsis_thaliana - ps32
Phylostratum

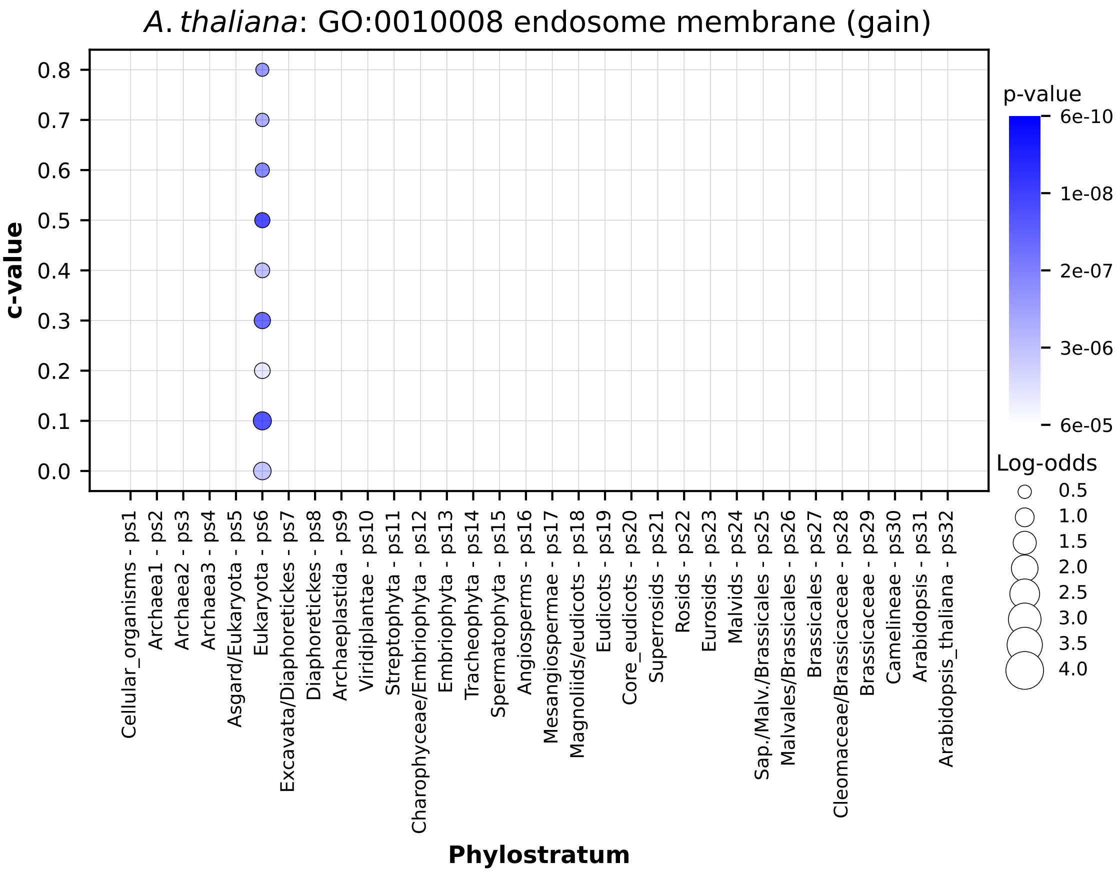

A. thaliana: GO:0010008 endosome membrane (gain)
c-value
0.0
0.1
0.2
0.3
0.4
0.5
0.6
0.7
0.8
p-value
6e-10
1e-08
2e-07
3e-06
6e-05
Log-odds
0.5
1.0
1.5
2.0
2.5
3.0
3.5
4.0
Cellular_organisms - ps1
Archaea1 - ps2
Archaea2 - ps3
Archaea3 - ps4
Asgard/Eukaryota - ps5
Eukaryota - ps6
Excavata/Diaphoretickes - ps7
Diaphoretickes - ps8
Archaeplastida - ps9
Viridiplantae - ps10
Streptophyta - ps11
Charophyceae/Embriophyta - ps12
Embriophyta - ps13
Tracheophyta - ps14
Spermatophyta - ps15
Angiosperms - ps16
Mesangiospermae - ps17
Magnoliids/eudicots - ps18
Eudicots - ps19
Core_eudicots - ps20
Superrosids - ps21
Rosids - ps22
Eurosids - ps23
Malvids - ps24
Sap./Malv./Brassicales - ps25
Malvales/Brassicales - ps26
Brassicales - ps27
Cleomaceae/Brassicaceae - ps28
Brassicaceae - ps29
Camelineae - ps30
Arabidopsis - ps31
Arabidopsis_thaliana - ps32
Phylostratum

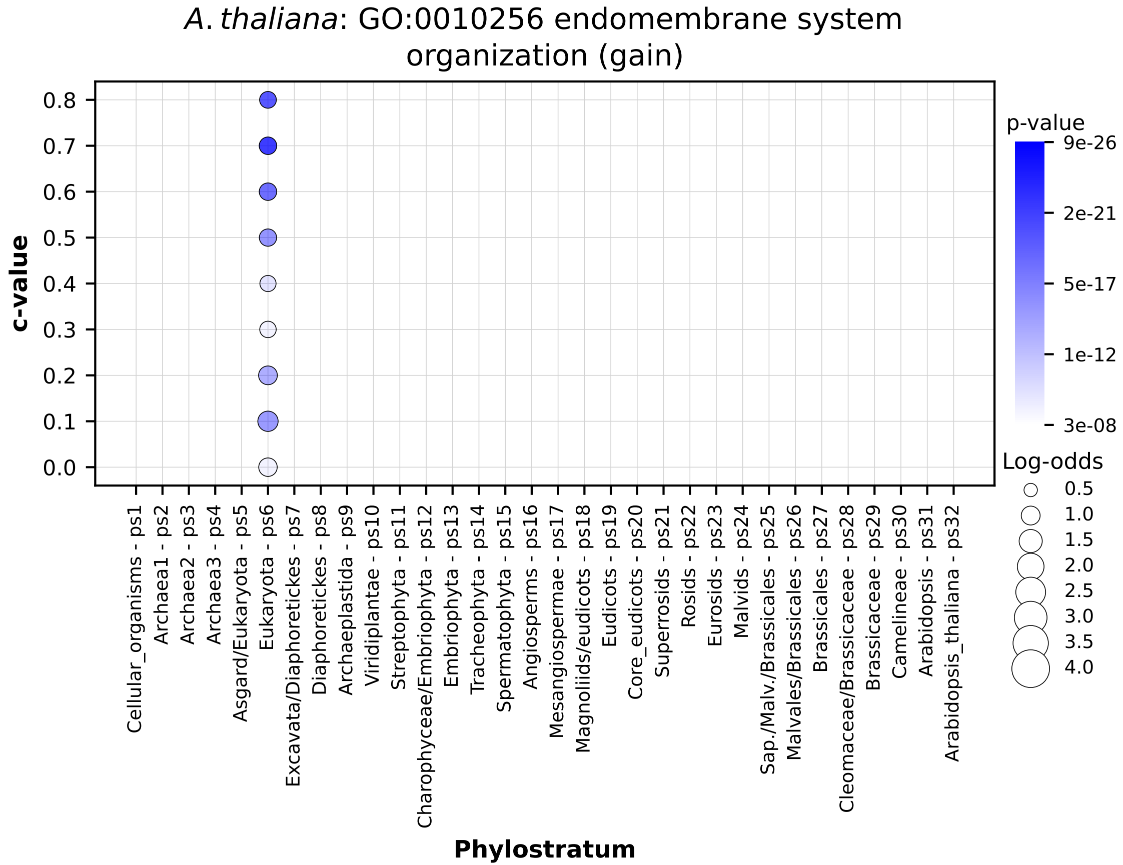

A. thaliana: GO:0010256 endomembrane system organization (gain)
c-value
0.8
0.7
0.6
0.5
0.4
0.3
0.2
0.1
0.0
p-value
9e-26
2e-21
5e-17
1e-12
3e-08
Log-odds
0.5
1.0
1.5
2.0
2.5
3.0
3.5
4.0
Cellular_organisms - ps1
Archaea1 - ps2
Archaea2 - ps3
Archaea3 - ps4
Asgard/Eukaryota - ps5
Eukaryota - ps6
Excavata/Diaphoretickes - ps7
Diaphoretickes - ps8
Archaeplastida - ps9
Viridiplantae - ps10
Streptophyta - ps11
Charophyceae/Embriophyta - ps12
Embriophyta - ps13
Tracheophyta - ps14
Spermatophyta - ps15
Angiosperms - ps16
Mesangiospermae - ps17
Magnoliids/eudicots - ps18
Eudicots - ps19
Core_eudicots - ps20
Superrosids - ps21
Rosids - ps22
Eurosids - ps23
Malvids - ps24
Sap./Malv./Brassicales - ps25
Malvales/Brassicales - ps26
Brassicales - ps27
Cleomaceae/Brassicaceae - ps28
Brassicaceae - ps29
Camelineae - ps30
Arabidopsis - ps31
Arabidopsis_thaliana - ps32
Phylostratum

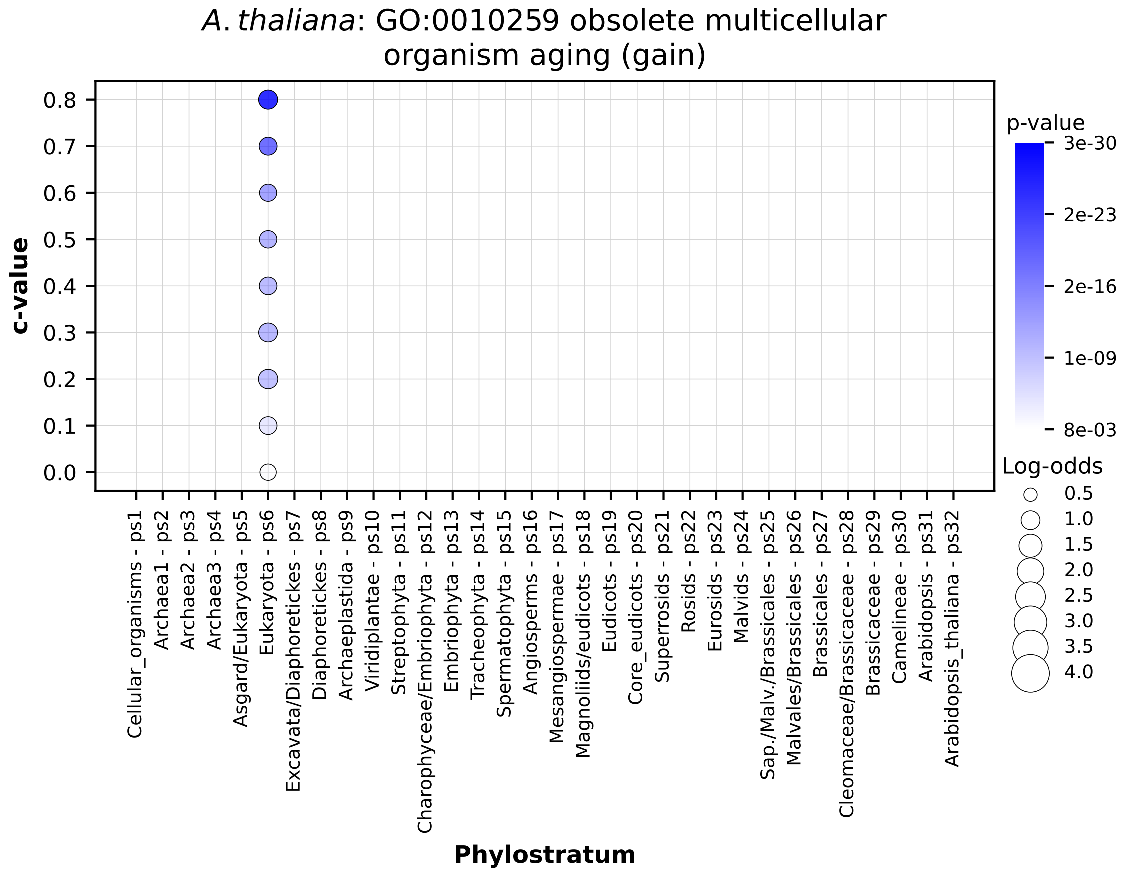
A. thaliana: GO:0010259 obsolete multicellular organism aging (gain)
c-value
0.0
0.1
0.2
0.3
0.4
0.5
0.6
0.7
0.8
p-value
3e-30
2e-23
2e-16
1e-09
8e-03
Log-odds
0.5
1.0
1.5
2.0
2.5
3.0
3.5
4.0
Cellular_organisms - ps1
Archaea1 - ps2
Archaea2 - ps3
Archaea3 - ps4
Asgard/Eukaryota - ps5
Eukaryota - ps6
Excavata/Diaphoretickes - ps7
Diaphoretickes - ps8
Archaeplastida - ps9
Viridiplantae - ps10
Streptophyta - ps11
Charophyceae/Embriophyta - ps12
Embriophyta - ps13
Tracheophyta - ps14
Spermatophyta - ps15
Angiosperms - ps16
Mesangiospermae - ps17
Magnoliids/eudicots - ps18
Eudicots - ps19
Core_eudicots - ps20
Superrosids - ps21
Rosids - ps22
Eurosids - ps23
Malvids - ps24
Sap./Malv./Brassicales - ps25
Malvales/Brassicales - ps26
Brassicales - ps27
Cleomaceae/Brassicaceae - ps28
Brassicaceae - ps29
Camelineae - ps30
Arabidopsis - ps31
Arabidopsis_thaliana - ps32
Phylostratum

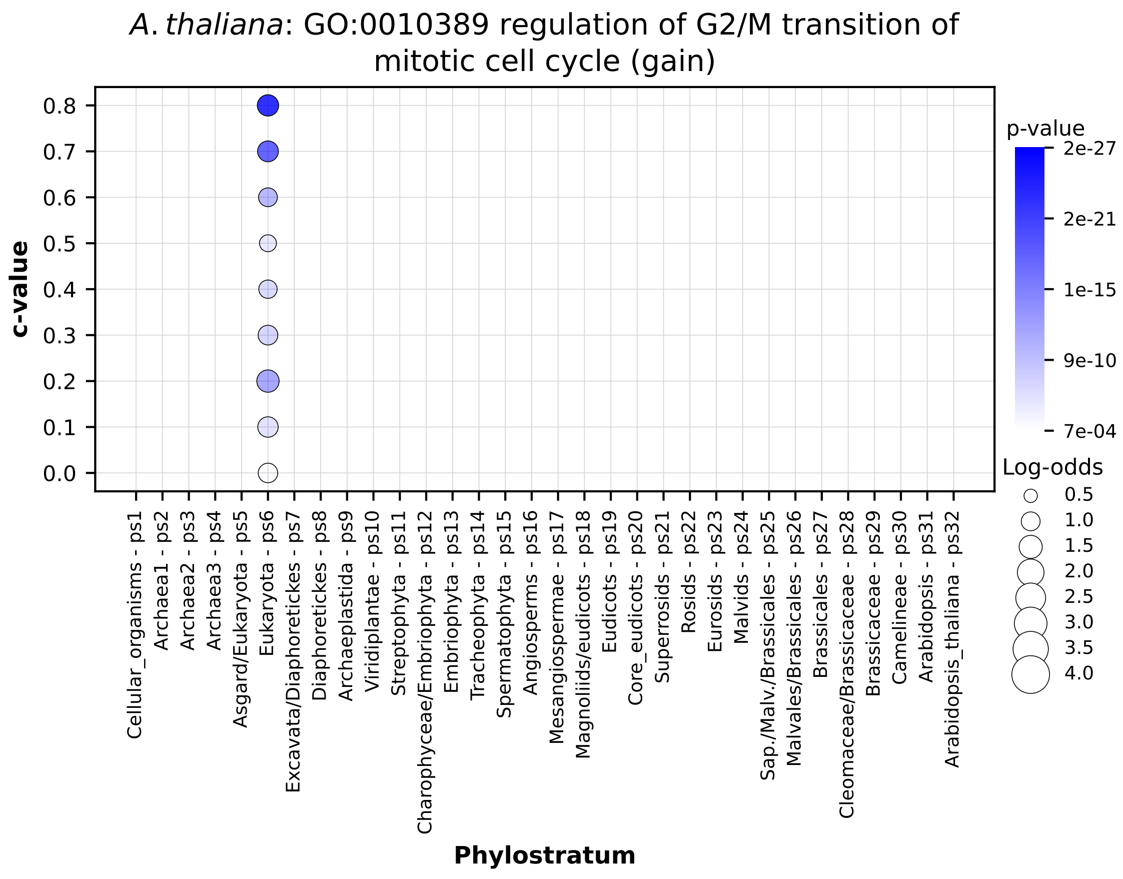
A. thaliana: GO:0010389 regulation of G2/M transition of mitotic cell cycle (gain)
c-value
0.0
0.1
0.2
0.3
0.4
0.5
0.6
0.7
0.8
Phylostratum
Cellular_organisms - ps1
Archaea1 - ps2
Archaea2 - ps3
Archaea3 - ps4
Asgard/Eukaryota - ps5
Eukaryota - ps6
Excavata/Diaphoretickes - ps7
Diaphoretickes - ps8
Archaeplastida - ps9
Viridiplantae - ps10
Streptophyta - ps11
Charophyceae/Embriophyta - ps12
Embriophyta - ps13
Tracheophyta - ps14
Spermatophyta - ps15
Angiosperms - ps16
Mesangiospermae - ps17
Magnoliids/eudicots - ps18
Eudicots - ps19
Core_eudicots - ps20
Superrosids - ps21
Rosids - ps22
Eurosids - ps23
Malvids - ps24
Sap./Malv./Brassicales - ps25
Malvales/Brassicales - ps26
Brassicales - ps27
Cleomaceae/Brassicaceae - ps28
Brassicaceae - ps29
Camelineae - ps30
Arabidopsis - ps31
Arabidopsis_thaliana - ps32
p-value
2e-27
2e-21
1e-15
9e-10
7e-04
Log-odds
0.5
1.0
1.5
2.0
2.5
3.0
3.5
4.0

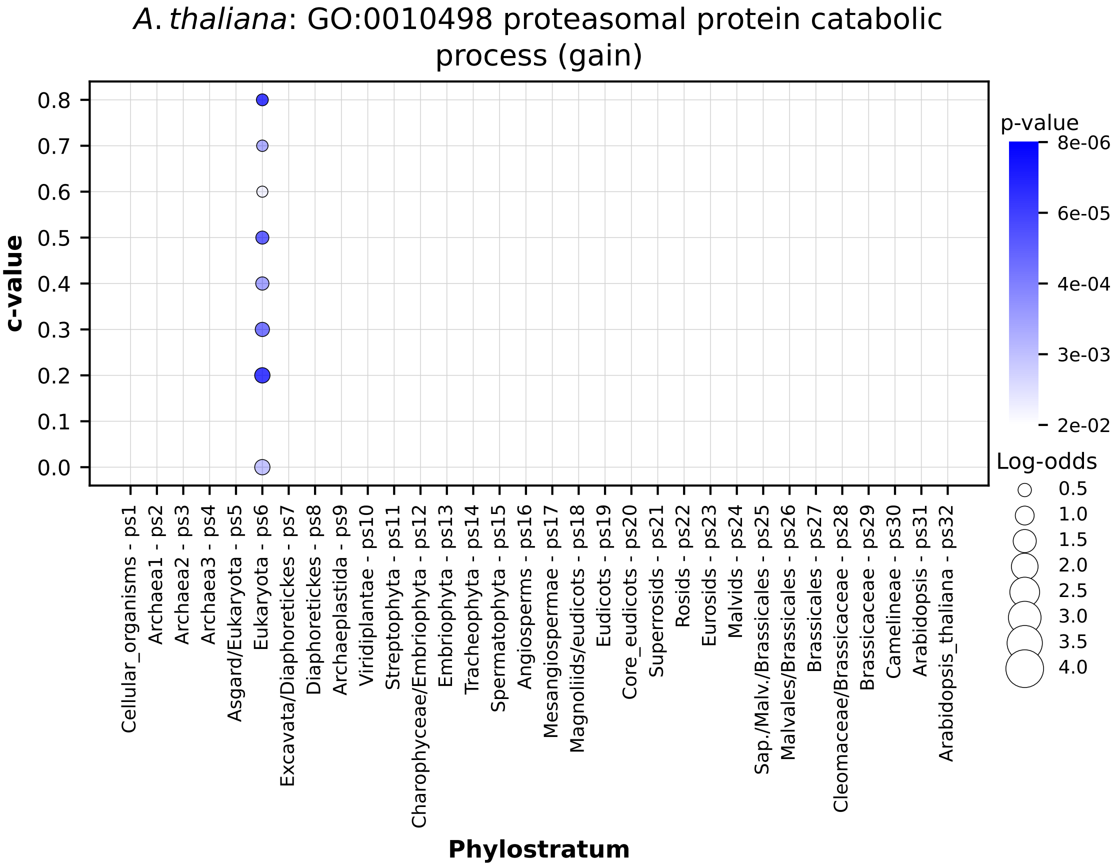

A. thaliana: GO:0010498 proteasomal protein catabolic process (gain)
c-value
0.8
0.7
0.6
0.5
0.4
0.3
0.2
0.1
0.0
Phylostratum
Cellular_organisms - ps1
Archaea1 - ps2
Archaea2 - ps3
Archaea3 - ps4
Asgard/Eukaryota - ps5
Eukaryota - ps6
Excavata/Diaphoretickes - ps7
Diaphoretickes - ps8
Archaeplastida - ps9
Viridiplantae - ps10
Streptophyta - ps11
Charophyceae/Embriophyta - ps12
Embriophyta - ps13
Tracheophyta - ps14
Spermatophyta - ps15
Angiosperms - ps16
Mesangiospermae - ps17
Magnoliids/eudicots - ps18
Eudicots - ps19
Core_eudicots - ps20
Superrosids - ps21
Rosids - ps22
Eurosids - ps23
Malvids - ps24
Sap./Malv./Brassicales - ps25
Malvales/Brassicales - ps26
Brassicales - ps27
Cleomaceae/Brassicaceae - ps28
Brassicaceae - ps29
Camelineae - ps30
Arabidopsis - ps31
Arabidopsis_thaliana - ps32
p-value
8e-06
6e-05
4e-04
3e-03
2e-02
Log-odds
0.5
1.0
1.5
2.0
2.5
3.0
3.5
4.0

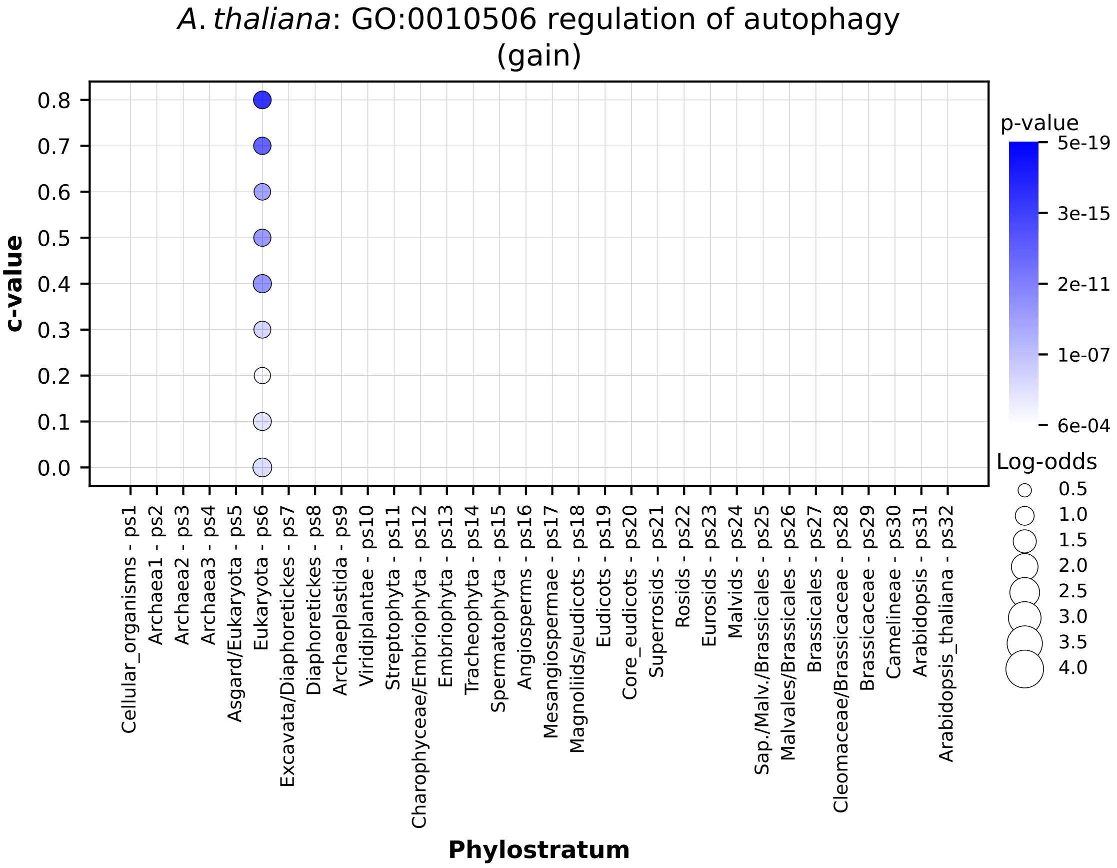

A. thaliana: GO:0010506 regulation of autophagy
(gain)
c-value
0.8
0.7
0.6
0.5
0.4
0.3
0.2
0.1
0.0
p-value
5e-19
3e-15
2e-11
1e-07
6e-04
Log-odds
0.5
1.0
1.5
2.0
2.5
3.0
3.5
4.0
Cellular_organisms - ps1
Archaea1 - ps2
Archaea2 - ps3
Archaea3 - ps4
Asgard/Eukaryota - ps5
Eukaryota - ps6
Excavata/Diaphoretickes - ps7
Diaphoretickes - ps8
Archaeplastida - ps9
Viridiplantae - ps10
Streptophyta - ps11
Charophyceae/Embriophyta - ps12
Embriophyta - ps13
Tracheophyta - ps14
Spermatophyta - ps15
Angiosperms - ps16
Mesangiospermae - ps17
Magnoliids/eudicots - ps18
Eudicots - ps19
Core_eudicots - ps20
Superrosids - ps21
Rosids - ps22
Eurosids - ps23
Malvids - ps24
Sap./Malv./Brassicales - ps25
Malvales/Brassicales - ps26
Brassicales - ps27
Cleomaceae/Brassicaceae - ps28
Brassicaceae - ps29
Camelineae - ps30
Arabidopsis - ps31
Arabidopsis_thaliana - ps32
Phylostratum

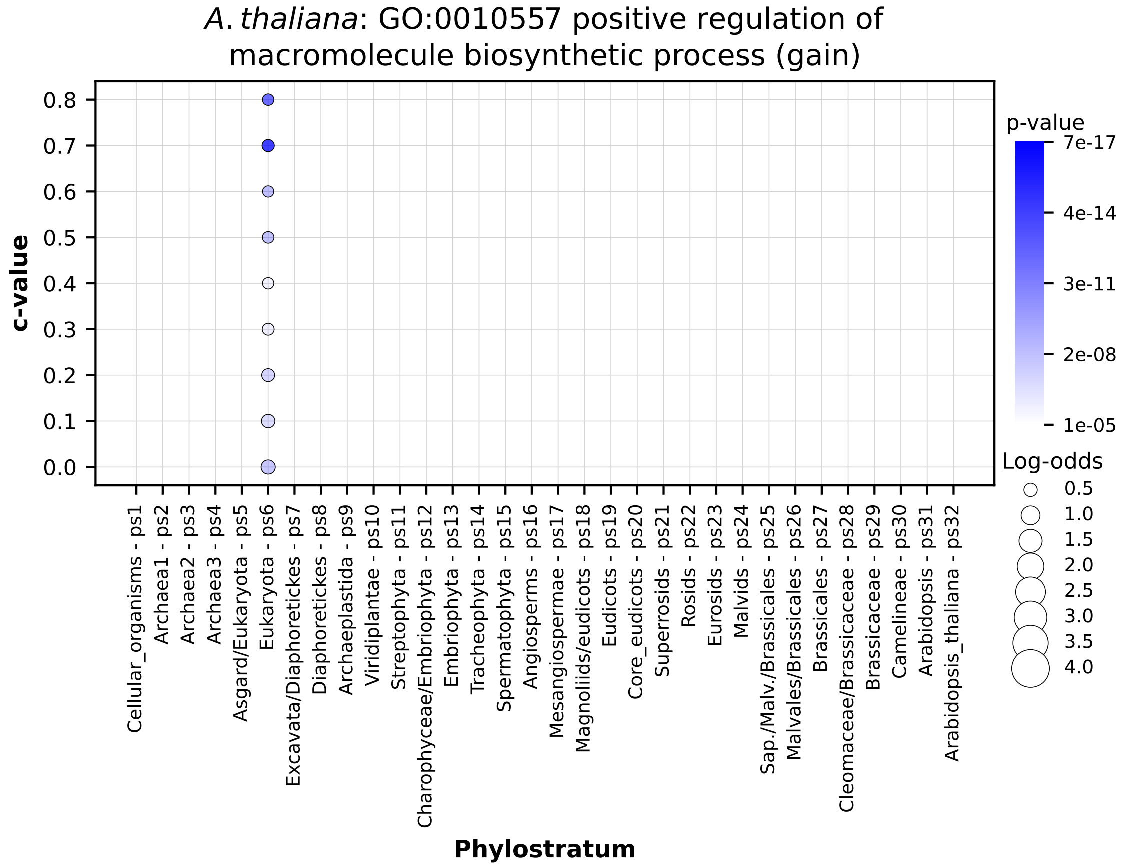

A. thaliana: GO:0010557 positive regulation of macromolecule biosynthetic process (gain)
c-value
0.8
0.7
0.6
0.5
0.4
0.3
0.2
0.1
0.0
p-value
7e-17
4e-14
3e-11
2e-08
1e-05
Log-odds
0.5
1.0
1.5
2.0
2.5
3.0
3.5
4.0
Cellular_organisms - ps1
Archaea1 - ps2
Archaea2 - ps3
Archaea3 - ps4
Asgard/Eukaryota - ps5
Eukaryota - ps6
Excavata/Diaphoretickes - ps7
Diaphoretickes - ps8
Archaeplastida - ps9
Viridiplantae - ps10
Streptophyta - ps11
Charophyceae/Embriophyta - ps12
Embriophyta - ps13
Tracheophyta - ps14
Spermatophyta - ps15
Angiosperms - ps16
Mesangiospermae - ps17
Magnoliids/eudicots - ps18
Eudicots - ps19
Core_eudicots - ps20
Superrosids - ps21
Rosids - ps22
Eurosids - ps23
Malvids - ps24
Sap./Malv./Brassicales - ps25
Malvales/Brassicales - ps26
Brassicales - ps27
Cleomaceae/Brassicaceae - ps28
Brassicaceae - ps29
Camelineae - ps30
Arabidopsis - ps31
Arabidopsis_thaliana - ps32
Phylostratum

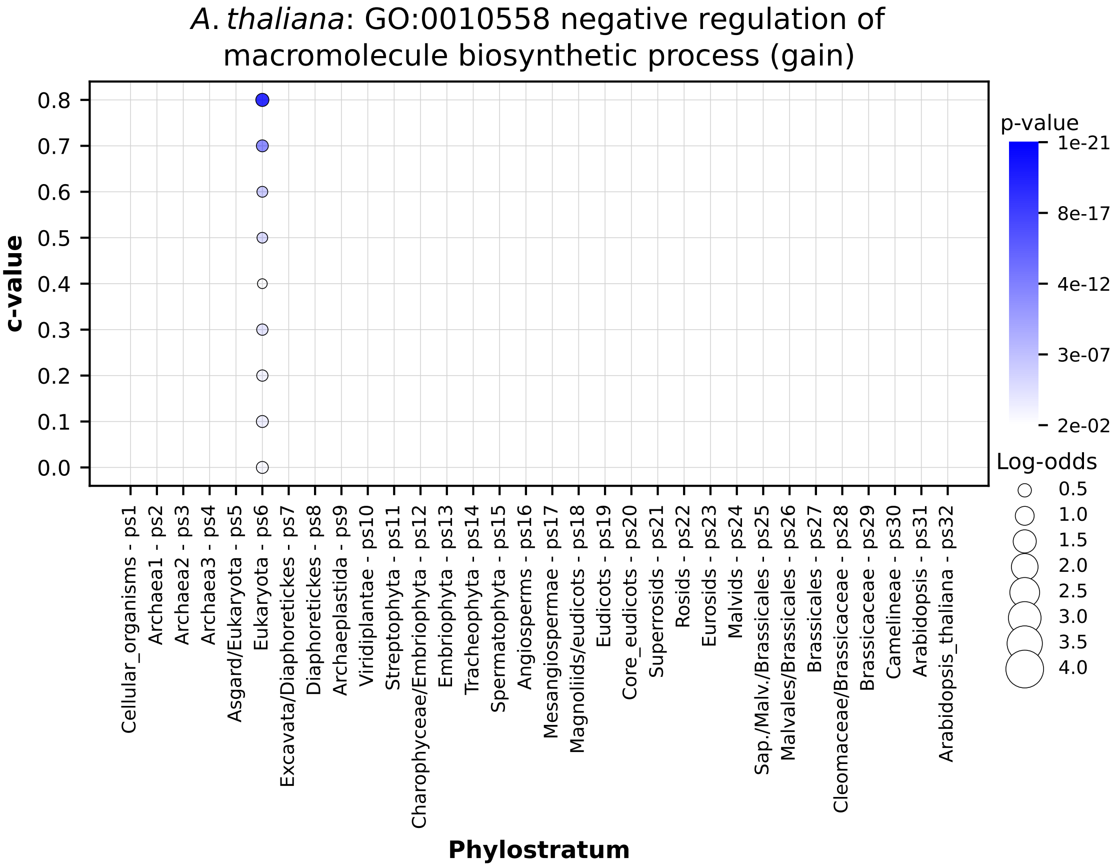

A. thaliana: GO:0010558 negative regulation of macromolecule biosynthetic process (gain)
c-value
0.0
0.1
0.2
0.3
0.4
0.5
0.6
0.7
0.8
p-value
1e-21
8e-17
4e-12
3e-07
2e-02
Log-odds
0.5
1.0
1.5
2.0
2.5
3.0
3.5
4.0
Cellular_organisms - ps1
Archaea1 - ps2
Archaea2 - ps3
Archaea3 - ps4
Asgard/Eukaryota - ps5
Eukaryota - ps6
Excavata/Diaphoretickes - ps7
Diaphoretickes - ps8
Archaeplastida - ps9
Viridiplantae - ps10
Streptophyta - ps11
Charophyceae/Embriophyta - ps12
Embriophyta - ps13
Tracheophyta - ps14
Spermatophyta - ps15
Angiosperms - ps16
Mesangiospermae - ps17
Magnoliids/eudicots - ps18
Eudicots - ps19
Core_eudicots - ps20
Superrosids - ps21
Rosids - ps22
Eurosids - ps23
Malvids - ps24
Sap./Malv./Brassicales - ps25
Malvales/Brassicales - ps26
Brassicales - ps27
Cleomaceae/Brassicaceae - ps28
Brassicaceae - ps29
Camelineae - ps30
Arabidopsis - ps31
Arabidopsis_thaliana - ps32
Phylostratum

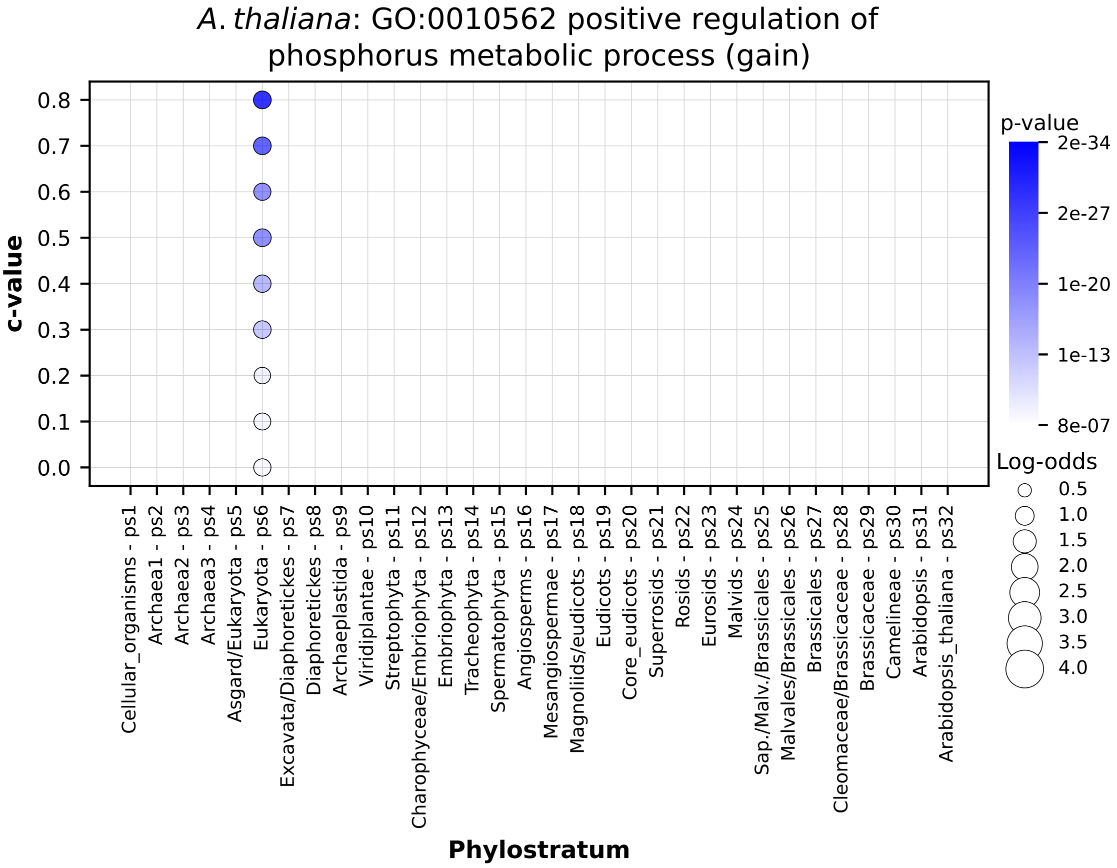

A. thaliana: GO:0010562 positive regulation of phosphorus metabolic process (gain)
c-value
0.0
0.1
0.2
0.3
0.4
0.5
0.6
0.7
0.8
Phylostratum
Cellular_organisms - ps1
Archaea1 - ps2
Archaea2 - ps3
Archaea3 - ps4
Asgard/Eukaryota - ps5
Eukaryota - ps6
Excavata/Diaphoretickes - ps7
Diaphoretickes - ps8
Archaeplastida - ps9
Viridiplantae - ps10
Streptophyta - ps11
Charophyceae/Embriophyta - ps12
Embriophyta - ps13
Tracheophyta - ps14
Spermatophyta - ps15
Angiosperms - ps16
Mesangiospermae - ps17
Magnoliids/eudicots - ps18
Eudicots - ps19
Core_eudicots - ps20
Superrosids - ps21
Rosids - ps22
Eurosids - ps23
Malvids - ps24
Sap./Malv./Brassicales - ps25
Malvales/Brassicales - ps26
Brassicales - ps27
Cleomaceae/Brassicaceae - ps28
Brassicaceae - ps29
Camelineae - ps30
Arabidopsis - ps31
Arabidopsis_thaliana - ps32
p-value
2e-34
2e-27
1e-20
1e-13
8e-07
Log-odds
0.5
1.0
1.5
2.0
2.5
3.0
3.5
4.0

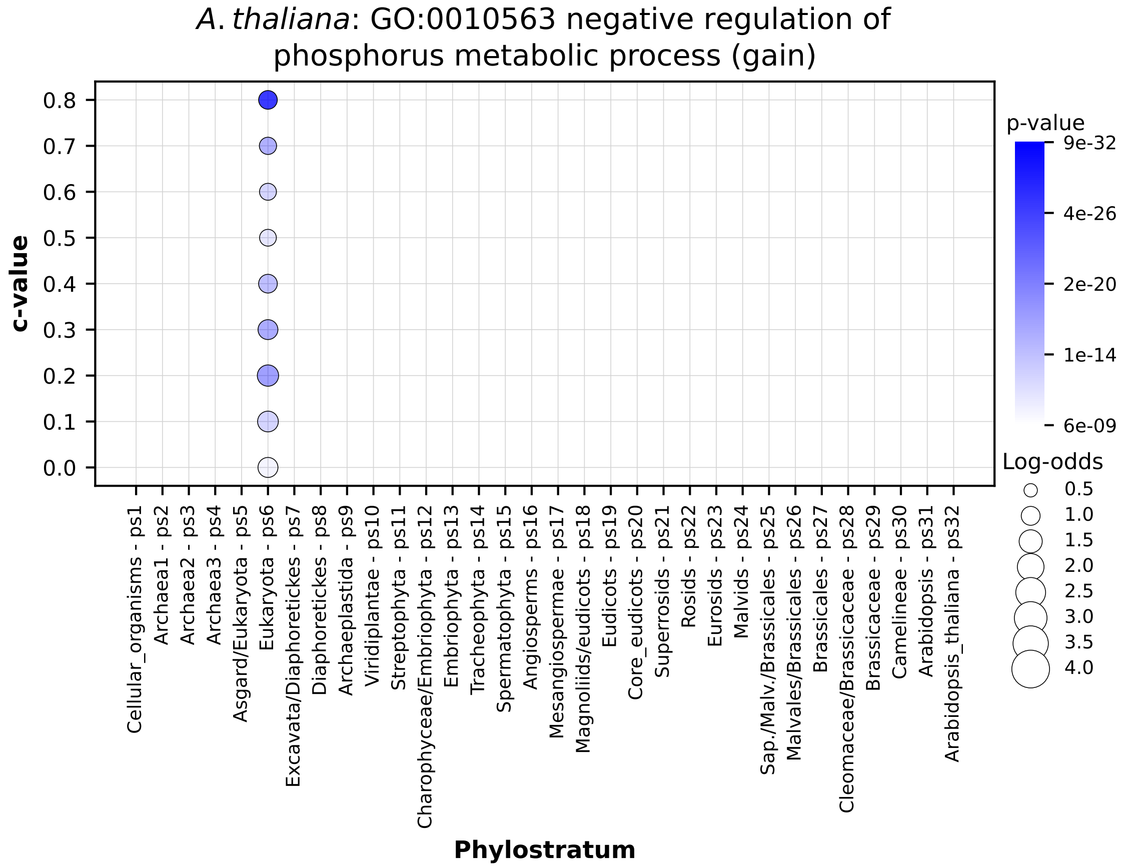
A. thaliana: GO:0010563 negative regulation of phosphorus metabolic process (gain)
c-value
0.0
0.1
0.2
0.3
0.4
0.5
0.6
0.7
0.8
p-value
9e-32
4e-26
2e-20
1e-14
6e-09
Log-odds
0.5
1.0
1.5
2.0
2.5
3.0
3.5
4.0
Cellular_organisms - ps1
Archaea1 - ps2
Archaea2 - ps3
Archaea3 - ps4
Asgard/Eukaryota - ps5
Eukaryota - ps6
Excavata/Diaphoretickes - ps7
Diaphoretickes - ps8
Archaeplastida - ps9
Viridiplantae - ps10
Streptophyta - ps11
Charophyceae/Embriophyta - ps12
Embriophyta - ps13
Tracheophyta - ps14
Spermatophyta - ps15
Angiosperms - ps16
Mesangiospermae - ps17
Magnoliids/eudicots - ps18
Eudicots - ps19
Core_eudicots - ps20
Superrosids - ps21
Rosids - ps22
Eurosids - ps23
Malvids - ps24
Sap./Malv./Brassicales - ps25
Malvales/Brassicales - ps26
Brassicales - ps27
Cleomaceae/Brassicaceae - ps28
Brassicaceae - ps29
Camelineae - ps30
Arabidopsis - ps31
Arabidopsis_thaliana - ps32
Phylostratum

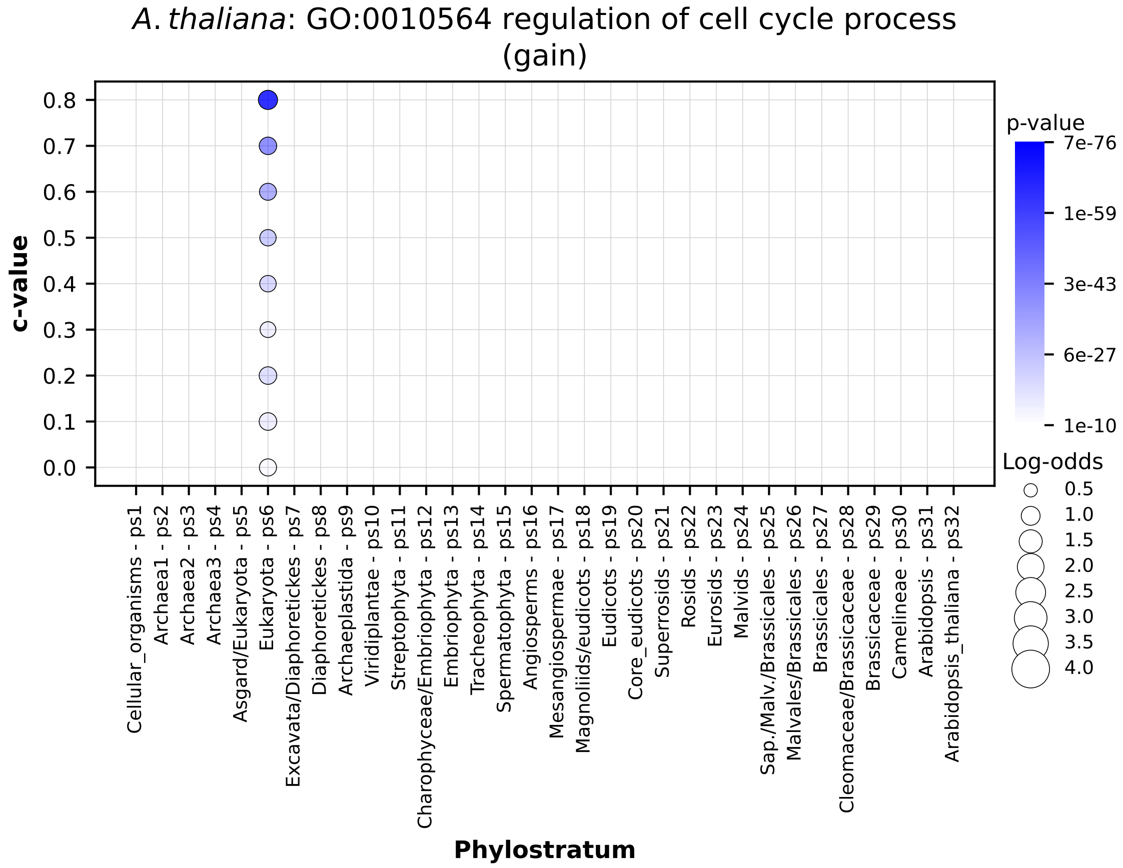

A. thaliana: GO:0010564 regulation of cell cycle process (gain)
c-value
0.8
0.7
0.6
0.5
0.4
0.3
0.2
0.1
0.0
p-value
7e-76
1e-59
3e-43
6e-27
1e-10
Log-odds
0.5
1.0
1.5
2.0
2.5
3.0
3.5
4.0
Cellular_organisms - ps1
Archaea1 - ps2
Archaea2 - ps3
Archaea3 - ps4
Asgard/Eukaryota - ps5
Eukaryota - ps6
Excavata/Diaphoretickes - ps7
Diaphoretickes - ps8
Archaeplastida - ps9
Viridiplantae - ps10
Streptophyta - ps11
Charophyceae/Embriophyta - ps12
Embriophyta - ps13
Tracheophyta - ps14
Spermatophyta - ps15
Angiosperms - ps16
Mesangiospermae - ps17
Magnoliids/eudicots - ps18
Eudicots - ps19
Core_eudicots - ps20
Superrosids - ps21
Rosids - ps22
Eurosids - ps23
Malvids - ps24
Sap./Malv./Brassicales - ps25
Malvales/Brassicales - ps26
Brassicales - ps27
Cleomaceae/Brassicaceae - ps28
Brassicaceae - ps29
Camelineae - ps30
Arabidopsis - ps31
Arabidopsis_thaliana - ps32
Phylostratum

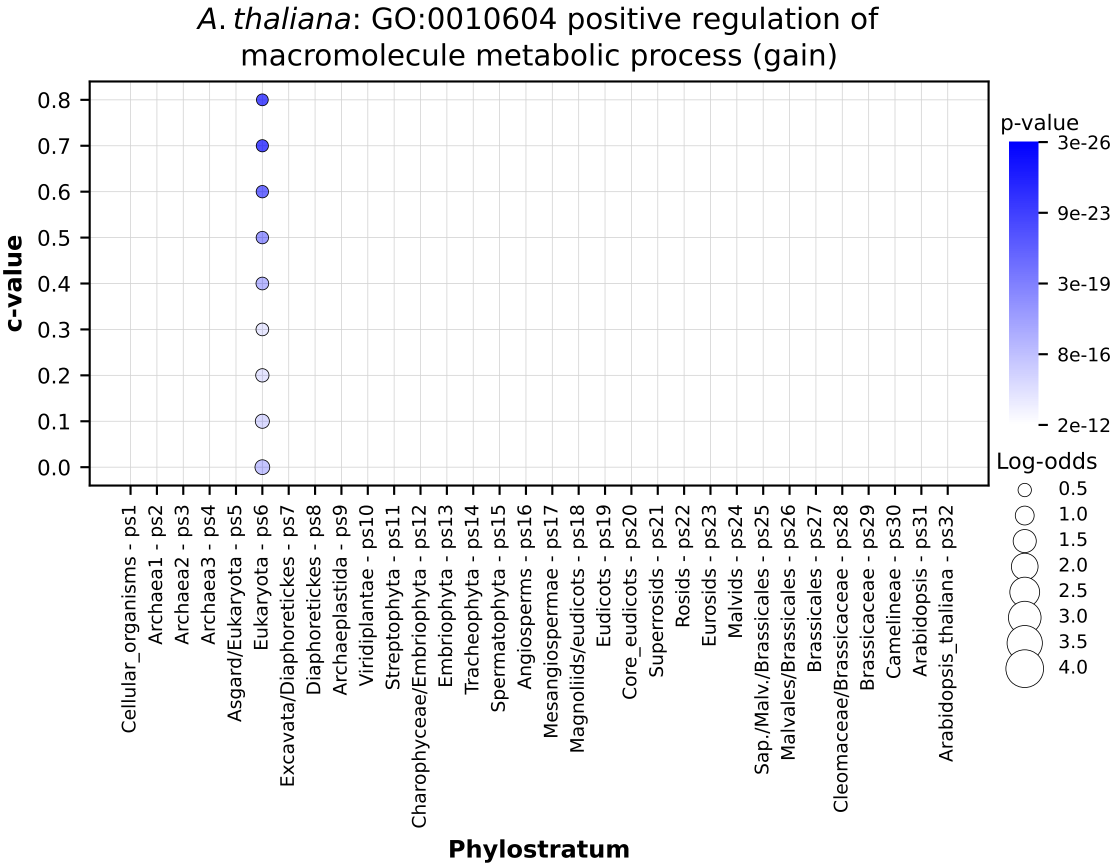

A. thaliana: GO:0010604 positive regulation of macromolecule metabolic process (gain)
c-value
0.8
0.7
0.6
0.5
0.4
0.3
0.2
0.1
0.0
p-value
3e-26
9e-23
3e-19
8e-16
2e-12
Log-odds
0.5
1.0
1.5
2.0
2.5
3.0
3.5
4.0
Cellular_organisms - ps1
Archaea1 - ps2
Archaea2 - ps3
Archaea3 - ps4
Asgard/Eukaryota - ps5
Eukaryota - ps6
Excavata/Diaphoretickes - ps7
Diaphoretickes - ps8
Archaeplastida - ps9
Viridiplantae - ps10
Streptophyta - ps11
Charophyceae/Embriophyta - ps12
Embriophyta - ps13
Tracheophyta - ps14
Spermatophyta - ps15
Angiosperms - ps16
Mesangiospermae - ps17
Magnoliids/eudicots - ps18
Eudicots - ps19
Core_eudicots - ps20
Superrosids - ps21
Rosids - ps22
Eurosids - ps23
Malvids - ps24
Sap./Malv./Brassicales - ps25
Malvales/Brassicales - ps26
Brassicales - ps27
Cleomaceae/Brassicaceae - ps28
Brassicaceae - ps29
Camelineae - ps30
Arabidopsis - ps31
Arabidopsis_thaliana - ps32
Phylostratum

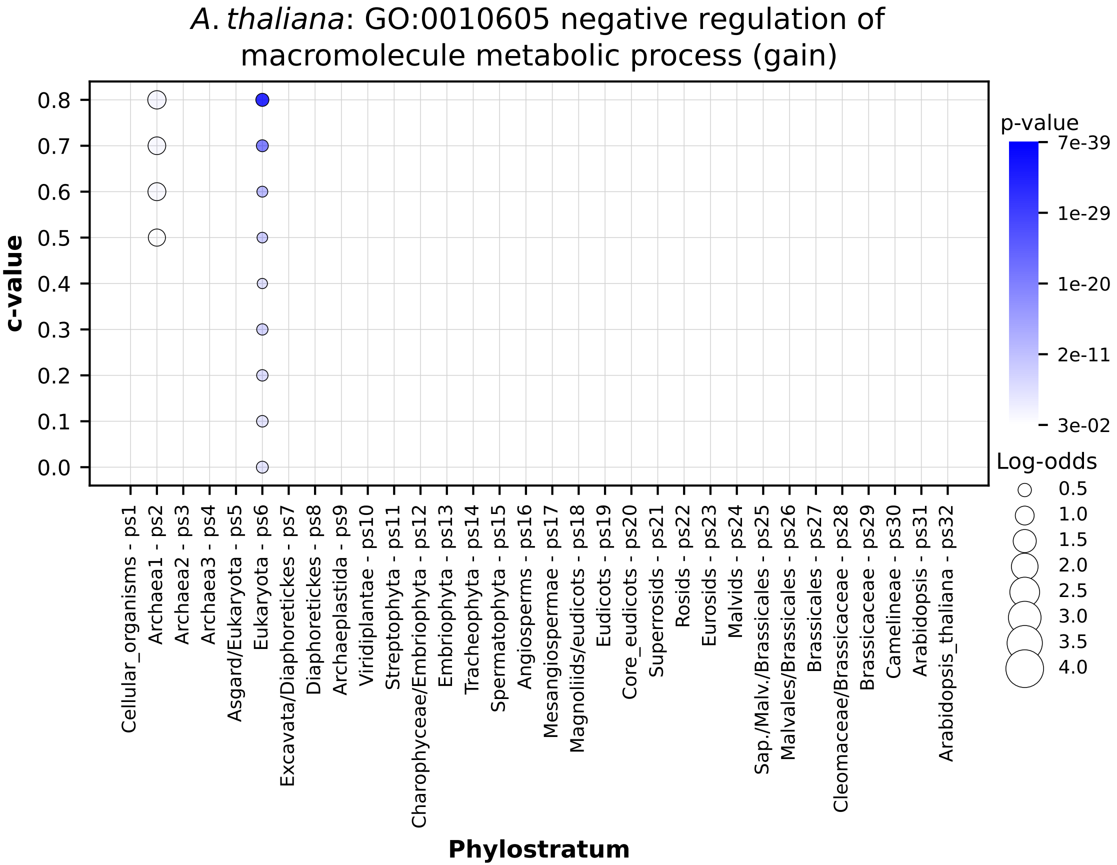
A. thaliana: GO:0010605 negative regulation of macromolecule metabolic process (gain)
c-value
0.8
0.7
0.6
0.5
0.4
0.3
0.2
0.1
0.0
Phylostratum
Cellular_organisms - ps1
Archaea1 - ps2
Archaea2 - ps3
Archaea3 - ps4
Asgard/Eukaryota - ps5
Eukaryota - ps6
Excavata/Diaphoretickes - ps7
Diaphoretickes - ps8
Archaeplastida - ps9
Viridiplantae - ps10
Streptophyta - ps11
Charophyceae/Embriophyta - ps12
Embriophyta - ps13
Tracheophyta - ps14
Spermatophyta - ps15
Angiosperms - ps16
Mesangiospermae - ps17
Magnoliids/eudicots - ps18
Eudicots - ps19
Core_eudicots - ps20
Superrosids - ps21
Rosids - ps22
Eurosids - ps23
Malvids - ps24
Sap./Malv./Brassicales - ps25
Malvales/Brassicales - ps26
Brassicales - ps27
Cleomaceae/Brassicaceae - ps28
Brassicaceae - ps29
Camelineae - ps30
Arabidopsis - ps31
Arabidopsis_thaliana - ps32
p-value
7e-39
1e-29
1e-20
2e-11
3e-02
Log-odds
0.5
1.0
1.5
2.0
2.5
3.0
3.5
4.0

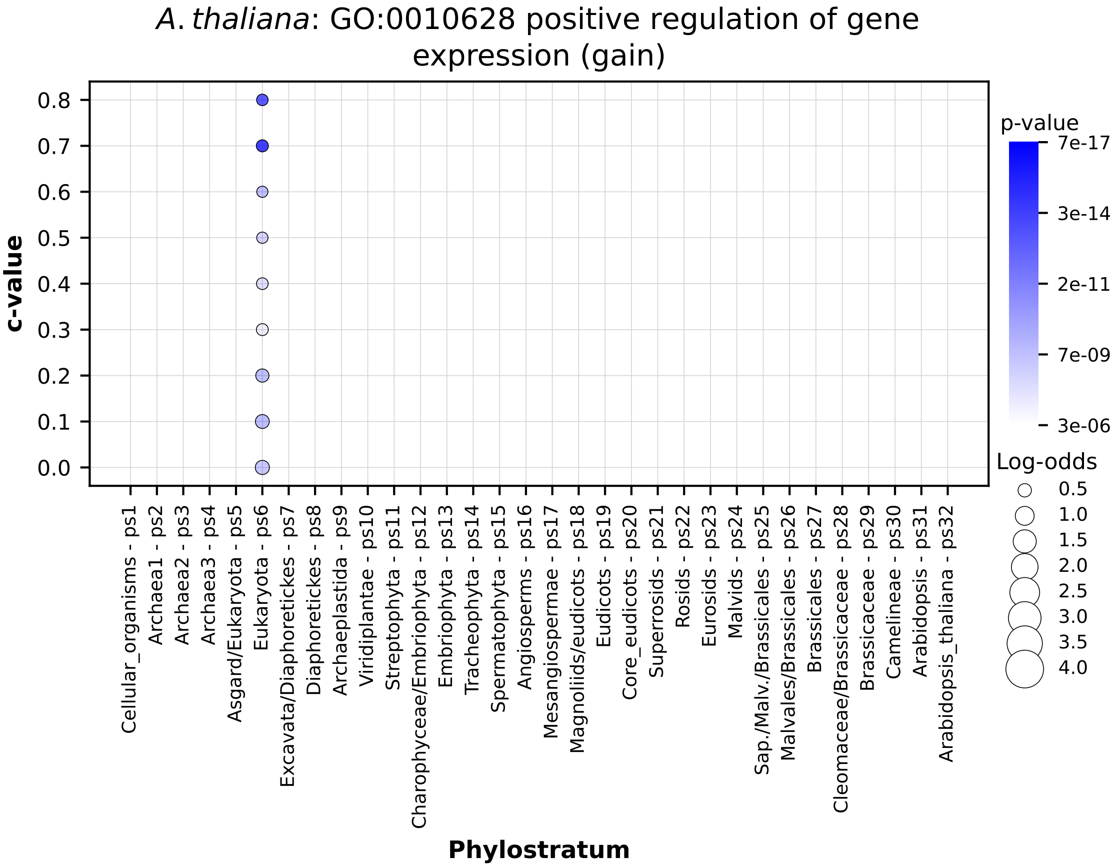

A. thaliana: GO:0010628 positive regulation of gene expression (gain)
c-value
0.8
0.7
0.6
0.5
0.4
0.3
0.2
0.1
0.0
p-value
7e-17
3e-14
2e-11
7e-09
3e-06
Log-odds
0.5
1.0
1.5
2.0
2.5
3.0
3.5
4.0
Cellular_organisms - ps1
Archaea1 - ps2
Archaea2 - ps3
Archaea3 - ps4
Asgard/Eukaryota - ps5
Eukaryota - ps6
Excavata/Diaphoretickes - ps7
Diaphoretickes - ps8
Archaeplastida - ps9
Viridiplantae - ps10
Streptophyta - ps11
Charophyceae/Embriophyta - ps12
Embriophyta - ps13
Tracheophyta - ps14
Spermatophyta - ps15
Angiosperms - ps16
Mesangiospermae - ps17
Magnoliids/eudicots - ps18
Eudicots - ps19
Core_eudicots - ps20
Superrosids - ps21
Rosids - ps22
Eurosids - ps23
Malvids - ps24
Sap./Malv./Brassicales - ps25
Malvales/Brassicales - ps26
Brassicales - ps27
Cleomaceae/Brassicaceae - ps28
Brassicaceae - ps29
Camelineae - ps30
Arabidopsis - ps31
Arabidopsis_thaliana - ps32
Phylostratum

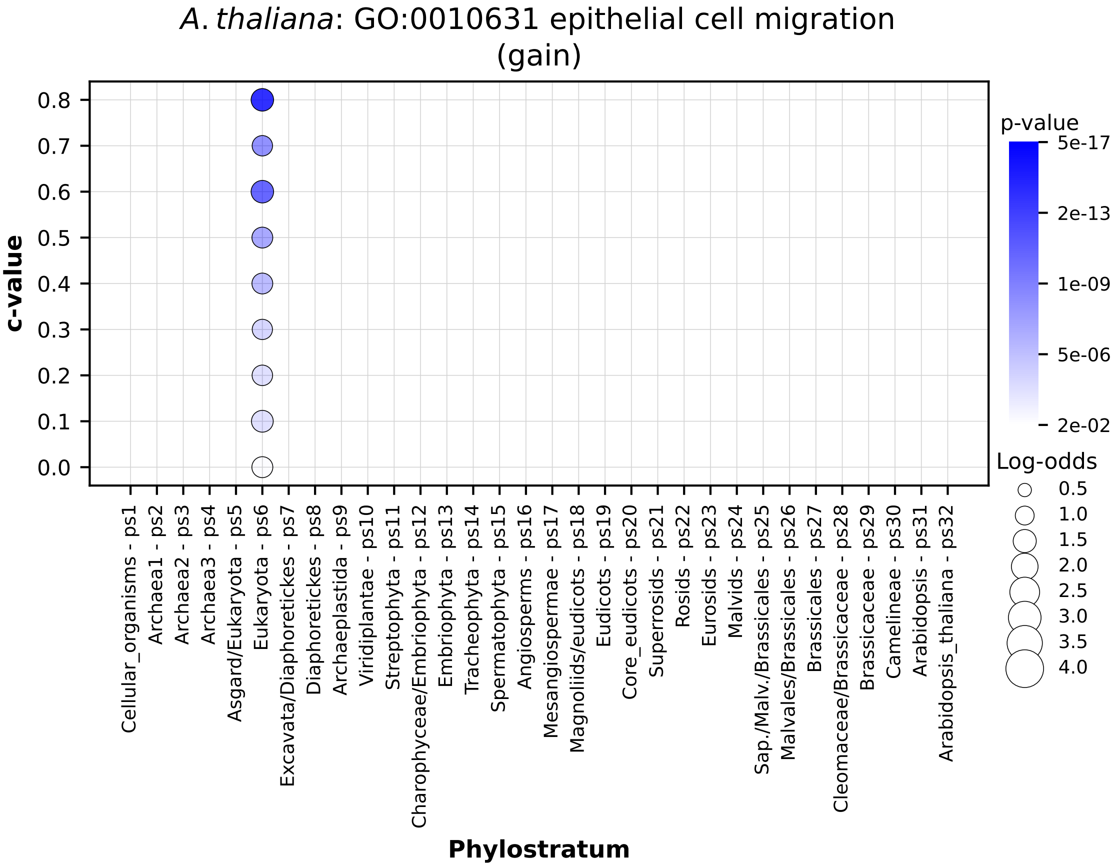
A. thaliana: GO:0010631 epithelial cell migration
(gain)
c-value
0.0
0.1
0.2
0.3
0.4
0.5
0.6
0.7
0.8
p-value
5e-17
2e-13
1e-09
5e-06
2e-02
Log-odds
0.5
1.0
1.5
2.0
2.5
3.0
3.5
4.0
Cellular_organisms - ps1
Archaea1 - ps2
Archaea2 - ps3
Archaea3 - ps4
Asgard/Eukaryota - ps5
Eukaryota - ps6
Excavata/Diaphoretickes - ps7
Diaphoretickes - ps8
Archaeplastida - ps9
Viridiplantae - ps10
Streptophyta - ps11
Charophyceae/Embriophyta - ps12
Embriophyta - ps13
Tracheophyta - ps14
Spermatophyta - ps15
Angiosperms - ps16
Mesangiospermae - ps17
Magnoliids/eudicots - ps18
Eudicots - ps19
Core_eudicots - ps20
Superrosids - ps21
Rosids - ps22
Eurosids - ps23
Malvids - ps24
Sap./Malv./Brassicales - ps25
Malvales/Brassicales - ps26
Brassicales - ps27
Cleomaceae/Brassicaceae - ps28
Brassicaceae - ps29
Camelineae - ps30
Arabidopsis - ps31
Arabidopsis_thaliana - ps32
Phylostratum

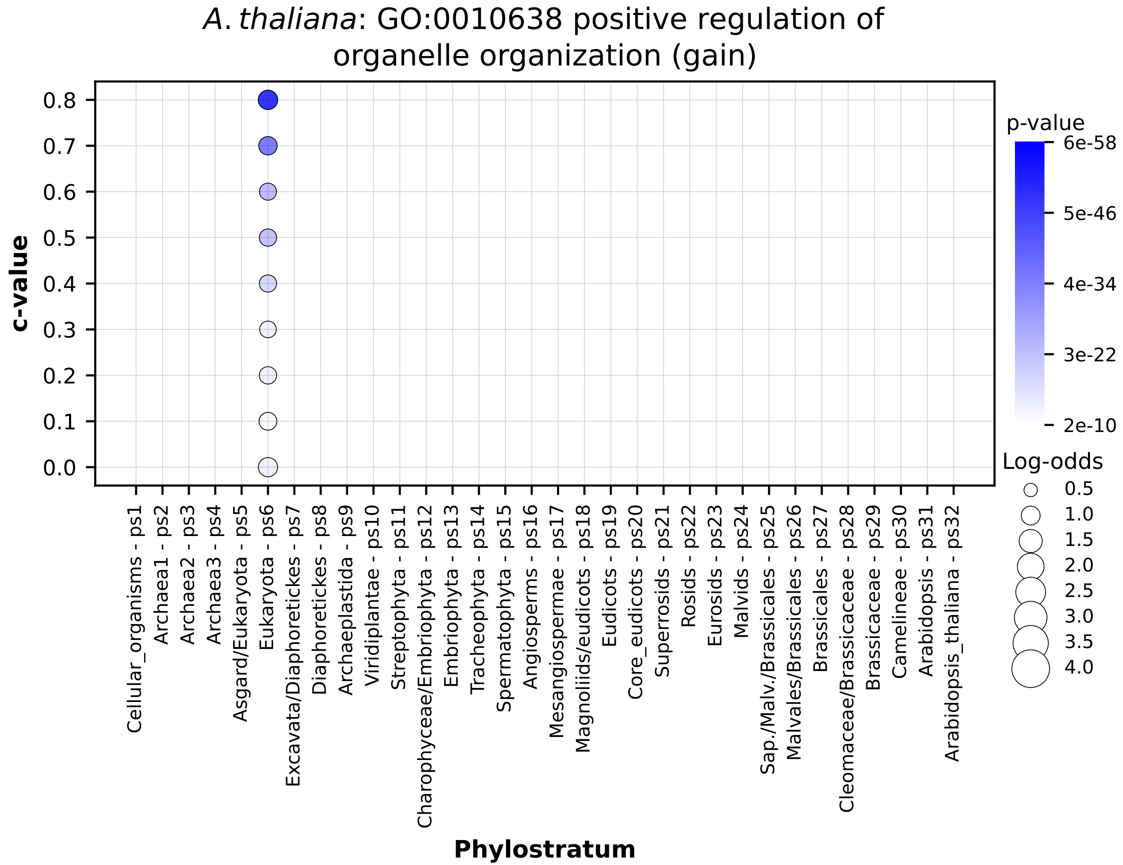

A. thaliana: GO:0010638 positive regulation of organelle organization (gain)
c-value
0.8
0.7
0.6
0.5
0.4
0.3
0.2
0.1
0.0
p-value
6e-58
5e-46
4e-34
3e-22
2e-10
Log-odds
0.5
1.0
1.5
2.0
2.5
3.0
3.5
4.0
Cellular_organisms - ps1
Archaea1 - ps2
Archaea2 - ps3
Archaea3 - ps4
Asgard/Eukaryota - ps5
Eukaryota - ps6
Excavata/Diaphoretickes - ps7
Diaphoretickes - ps8
Archaeplastida - ps9
Viridiplantae - ps10
Streptophyta - ps11
Charophyceae/Embriophyta - ps12
Embriophyta - ps13
Tracheophyta - ps14
Spermatophyta - ps15
Angiosperms - ps16
Mesangiospermae - ps17
Magnoliids/eudicots - ps18
Eudicots - ps19
Core_eudicots - ps20
Superrosids - ps21
Rosids - ps22
Eurosids - ps23
Malvids - ps24
Sap./Malv./Brassicales - ps25
Malvales/Brassicales - ps26
Brassicales - ps27
Cleomaceae/Brassicaceae - ps28
Brassicaceae - ps29
Camelineae - ps30
Arabidopsis - ps31
Arabidopsis_thaliana - ps32
Phylostratum

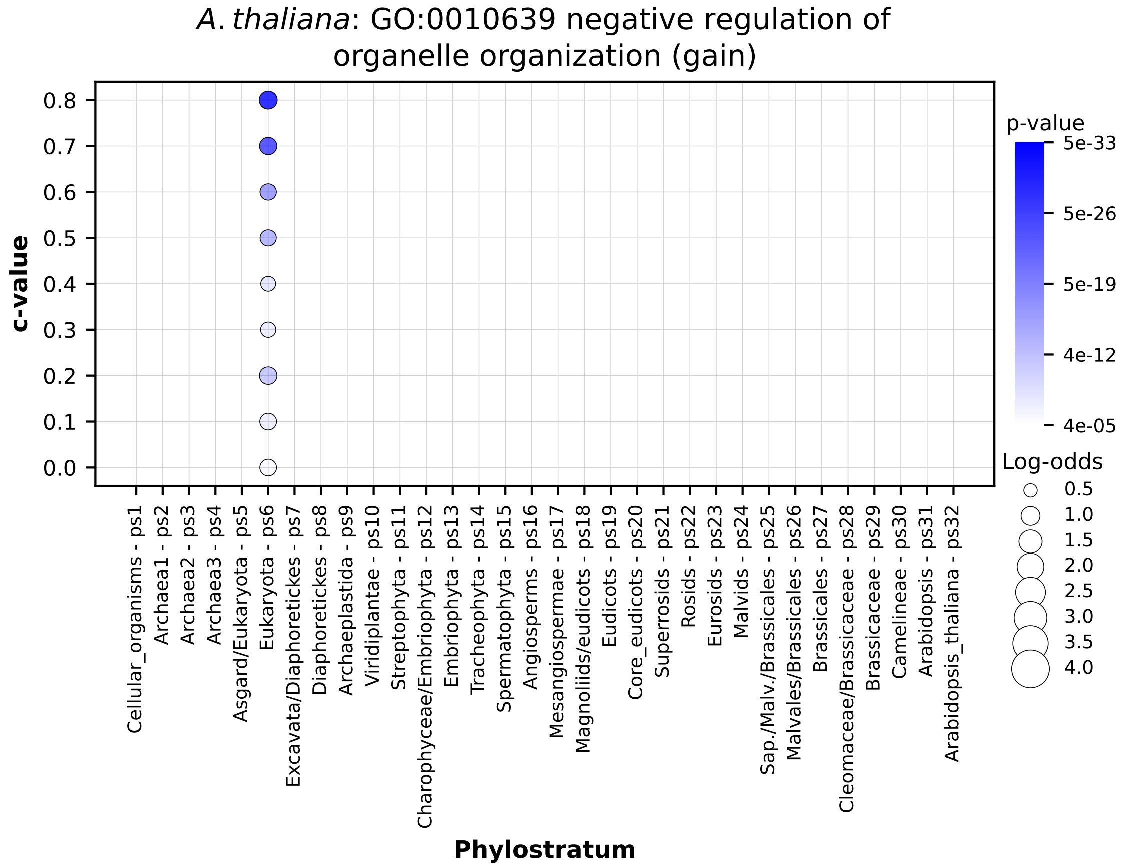
A. thaliana: GO:0010639 negative regulation of organelle organization (gain)
c-value
0.0
0.1
0.2
0.3
0.4
0.5
0.6
0.7
0.8
p-value
5e-33
5e-26
5e-19
4e-12
4e-05
Log-odds
0.5
1.0
1.5
2.0
2.5
3.0
3.5
4.0
Cellular_organisms - ps1
Archaea1 - ps2
Archaea2 - ps3
Archaea3 - ps4
Asgard/Eukaryota - ps5
Eukaryota - ps6
Excavata/Diaphoretickes - ps7
Diaphoretickes - ps8
Archaeplastida - ps9
Viridiplantae - ps10
Streptophyta - ps11
Charophyceae/Embriophyta - ps12
Embriophyta - ps13
Tracheophyta - ps14
Spermatophyta - ps15
Angiosperms - ps16
Mesangiospermae - ps17
Magnoliids/eudicots - ps18
Eudicots - ps19
Core_eudicots - ps20
Superrosids - ps21
Rosids - ps22
Eurosids - ps23
Malvids - ps24
Sap./Malv./Brassicales - ps25
Malvales/Brassicales - ps26
Brassicales - ps27
Cleomaceae/Brassicaceae - ps28
Brassicaceae - ps29
Camelineae - ps30
Arabidopsis - ps31
Arabidopsis_thaliana - ps32
Phylostratum

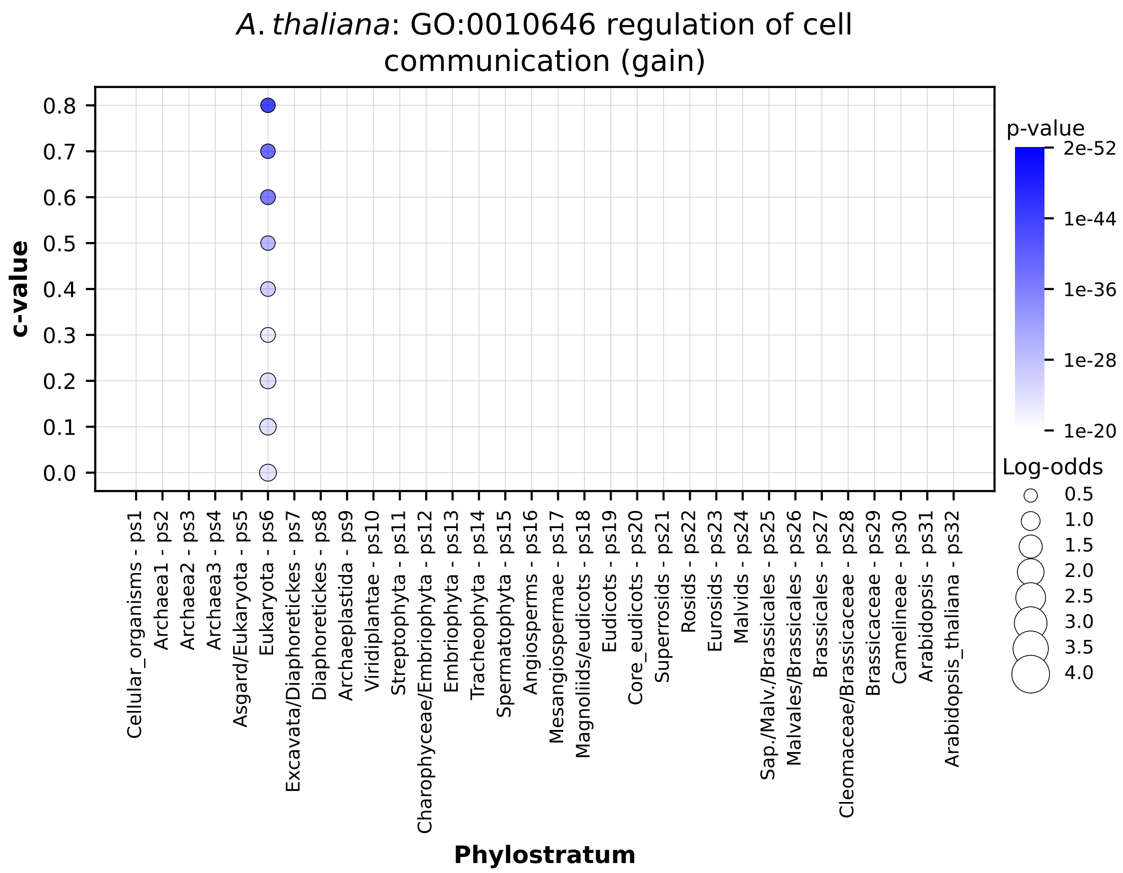

A. thaliana: GO:0010646 regulation of cell communication (gain)
c-value
0.8
0.7
0.6
0.5
0.4
0.3
0.2
0.1
0.0
p-value
2e-52
1e-44
1e-36
1e-28
1e-20
Log-odds
0.5
1.0
1.5
2.0
2.5
3.0
3.5
4.0
Cellular_organisms - ps1
Archaea1 - ps2
Archaea2 - ps3
Archaea3 - ps4
Asgard/Eukaryota - ps5
Eukaryota - ps6
Excavata/Diaphoretickes - ps7
Diaphoretickes - ps8
Archaeplastida - ps9
Viridiplantae - ps10
Streptophyta - ps11
Charophyceae/Embriophyta - ps12
Embriophyta - ps13
Tracheophyta - ps14
Spermatophyta - ps15
Angiosperms - ps16
Mesangiospermae - ps17
Magnoliids/eudicots - ps18
Eudicots - ps19
Core_eudicots - ps20
Superrosids - ps21
Rosids - ps22
Eurosids - ps23
Malvids - ps24
Sap./Malv./Brassicales - ps25
Malvales/Brassicales - ps26
Brassicales - ps27
Cleomaceae/Brassicaceae - ps28
Brassicaceae - ps29
Camelineae - ps30
Arabidopsis - ps31
Arabidopsis_thaliana - ps32
Phylostratum

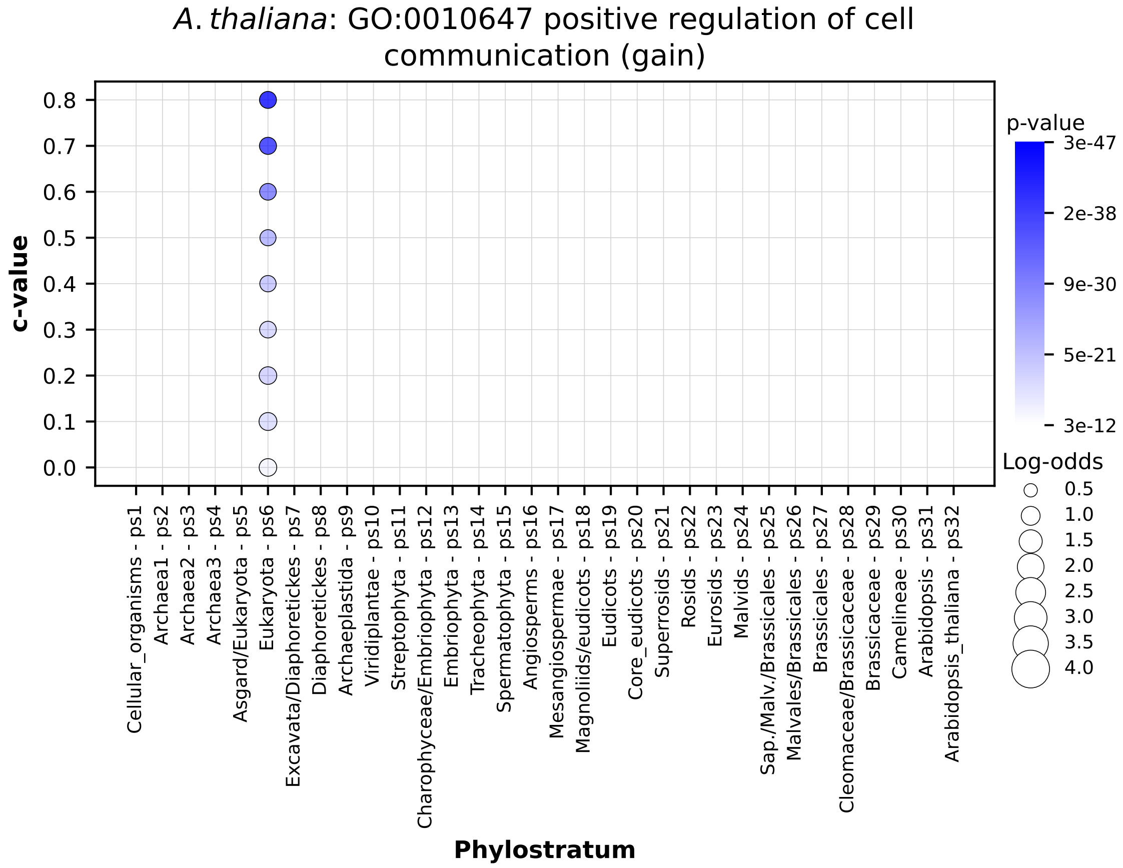

A. thaliana: GO:0010647 positive regulation of cell communication (gain)
c-value
0.8
0.7
0.6
0.5
0.4
0.3
0.2
0.1
0.0
p-value
3e-47
2e-38
9e-30
5e-21
3e-12
Log-odds
0.5
1.0
1.5
2.0
2.5
3.0
3.5
4.0
Cellular_organisms - ps1
Archaea1 - ps2
Archaea2 - ps3
Archaea3 - ps4
Asgard/Eukaryota - ps5
Eukaryota - ps6
Excavata/Diaphoretickes - ps7
Diaphoretickes - ps8
Archaeplastida - ps9
Viridiplantae - ps10
Streptophyta - ps11
Charophyceae/Embriophyta - ps12
Embriophyta - ps13
Tracheophyta - ps14
Spermatophyta - ps15
Angiosperms - ps16
Mesangiospermae - ps17
Magnoliids/eudicots - ps18
Eudicots - ps19
Core_eudicots - ps20
Superrosids - ps21
Rosids - ps22
Eurosids - ps23
Malvids - ps24
Sap./Malv./Brassicales - ps25
Malvales/Brassicales - ps26
Brassicales - ps27
Cleomaceae/Brassicaceae - ps28
Brassicaceae - ps29
Camelineae - ps30
Arabidopsis - ps31
Arabidopsis_thaliana - ps32
Phylostratum

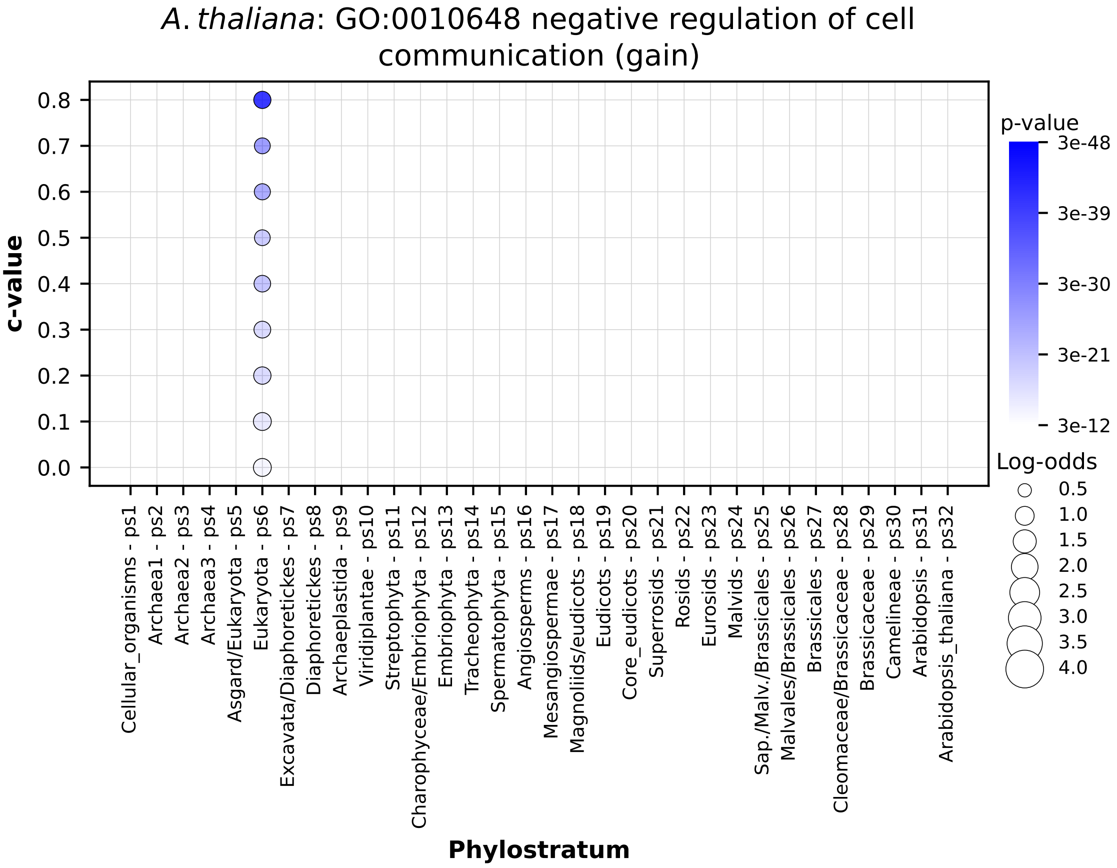
A. thaliana: GO:0010648 negative regulation of cell communication (gain)
c-value
0.8
0.7
0.6
0.5
0.4
0.3
0.2
0.1
0.0
p-value
3e-48
3e-39
3e-30
3e-21
3e-12
Log-odds
0.5
1.0
1.5
2.0
2.5
3.0
3.5
4.0
Cellular_organisms - ps1
Archaea1 - ps2
Archaea2 - ps3
Archaea3 - ps4
Asgard/Eukaryota - ps5
Eukaryota - ps6
Excavata/Diaphoretickes - ps7
Diaphoretickes - ps8
Archaeplastida - ps9
Viridiplantae - ps10
Streptophyta - ps11
Charophyceae/Embriophyta - ps12
Embriophyta - ps13
Tracheophyta - ps14
Spermatophyta - ps15
Angiosperms - ps16
Mesangiospermae - ps17
Magnoliids/eudicots - ps18
Eudicots - ps19
Core_eudicots - ps20
Superrosids - ps21
Rosids - ps22
Eurosids - ps23
Malvids - ps24
Sap./Malv./Brassicales - ps25
Malvales/Brassicales - ps26
Brassicales - ps27
Cleomaceae/Brassicaceae - ps28
Brassicaceae - ps29
Camelineae - ps30
Arabidopsis - ps31
Arabidopsis_thaliana - ps32
Phylostratum

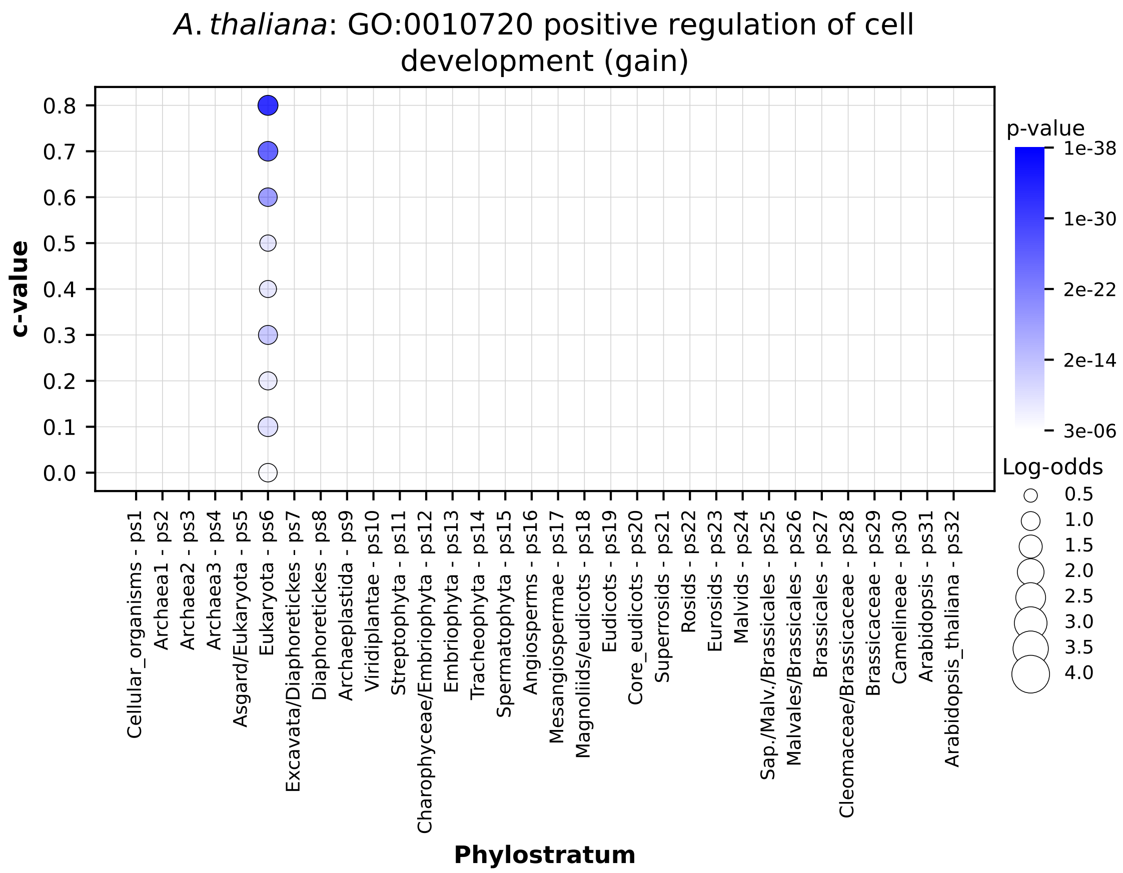

A. thaliana: GO:0010720 positive regulation of cell development (gain)
c-value
0.8
0.7
0.6
0.5
0.4
0.3
0.2
0.1
0.0
p-value
1e-38
1e-30
2e-22
2e-14
3e-06
Log-odds
0.5
1.0
1.5
2.0
2.5
3.0
3.5
4.0
Cellular_organisms - ps1
Archaea1 - ps2
Archaea2 - ps3
Archaea3 - ps4
Asgard/Eukaryota - ps5
Eukaryota - ps6
Excavata/Diaphoretickes - ps7
Diaphoretickes - ps8
Archaeplastida - ps9
Viridiplantae - ps10
Streptophyta - ps11
Charophyceae/Embriophyta - ps12
Embriophyta - ps13
Tracheophyta - ps14
Spermatophyta - ps15
Angiosperms - ps16
Mesangiospermae - ps17
Magnoliids/eudicots - ps18
Eudicots - ps19
Core_eudicots - ps20
Superrosids - ps21
Rosids - ps22
Eurosids - ps23
Malvids - ps24
Sap./Malv./Brassicales - ps25
Malvales/Brassicales - ps26
Brassicales - ps27
Cleomaceae/Brassicaceae - ps28
Brassicaceae - ps29
Camelineae - ps30
Arabidopsis - ps31
Arabidopsis_thaliana - ps32
Phylostratum

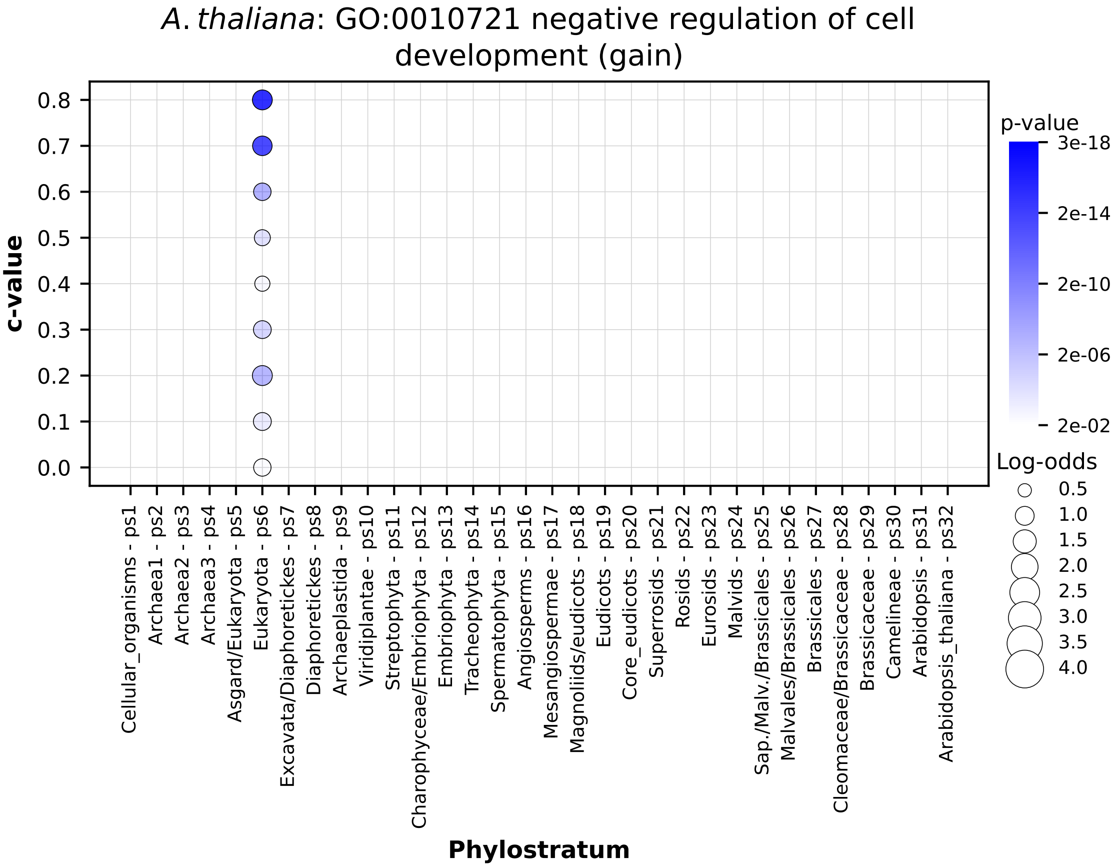

A. thaliana: GO:0010721 negative regulation of cell development (gain)
c-value
0.0
0.1
0.2
0.3
0.4
0.5
0.6
0.7
0.8
Phylostratum
Cellular_organisms - ps1
Archaea1 - ps2
Archaea2 - ps3
Archaea3 - ps4
Asgard/Eukaryota - ps5
Eukaryota - ps6
Excavata/Diaphoretickes - ps7
Diaphoretickes - ps8
Archaeplastida - ps9
Viridiplantae - ps10
Streptophyta - ps11
Charophyceae/Embriophyta - ps12
Embriophyta - ps13
Tracheophyta - ps14
Spermatophyta - ps15
Angiosperms - ps16
Mesangiospermae - ps17
Magnoliids/eudicots - ps18
Eudicots - ps19
Core_eudicots - ps20
Superrosids - ps21
Rosids - ps22
Eurosids - ps23
Malvids - ps24
Sap./Malv./Brassicales - ps25
Malvales/Brassicales - ps26
Brassicales - ps27
Cleomaceae/Brassicaceae - ps28
Brassicaceae - ps29
Camelineae - ps30
Arabidopsis - ps31
Arabidopsis_thaliana - ps32
p-value
3e-18
2e-14
2e-10
2e-06
2e-02
Log-odds
0.5
1.0
1.5
2.0
2.5
3.0
3.5
4.0

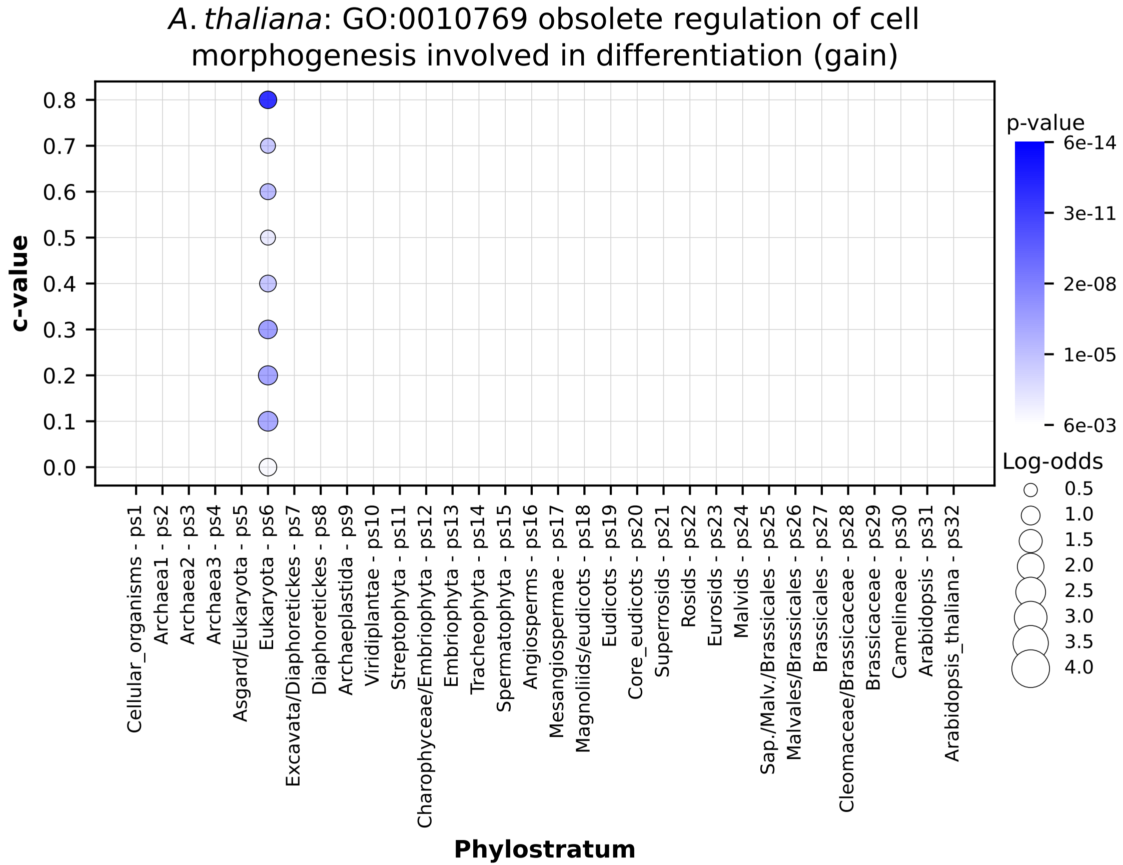

A. thaliana: GO:0010769 obsolete regulation of cell morphogenesis involved in differentiation (gain)
c-value
0.8
0.7
0.6
0.5
0.4
0.3
0.2
0.1
0.0
p-value
6e-14
3e-11
2e-08
1e-05
6e-03
Log-odds
0.5
1.0
1.5
2.0
2.5
3.0
3.5
4.0
Cellular_organisms - ps1
Archaea1 - ps2
Archaea2 - ps3
Archaea3 - ps4
Asgard/Eukaryota - ps5
Eukaryota - ps6
Excavata/Diaphoretickes - ps7
Diaphoretickes - ps8
Archaeplastida - ps9
Viridiplantae - ps10
Streptophyta - ps11
Charophyceae/Embriophyta - ps12
Embriophyta - ps13
Tracheophyta - ps14
Spermatophyta - ps15
Angiosperms - ps16
Mesangiospermae - ps17
Magnoliids/eudicots - ps18
Eudicots - ps19
Core_eudicots - ps20
Superrosids - ps21
Rosids - ps22
Eurosids - ps23
Malvids - ps24
Sap./Malv./Brassicales - ps25
Malvales/Brassicales - ps26
Brassicales - ps27
Cleomaceae/Brassicaceae - ps28
Brassicaceae - ps29
Camelineae - ps30
Arabidopsis - ps31
Arabidopsis_thaliana - ps32
Phylostratum

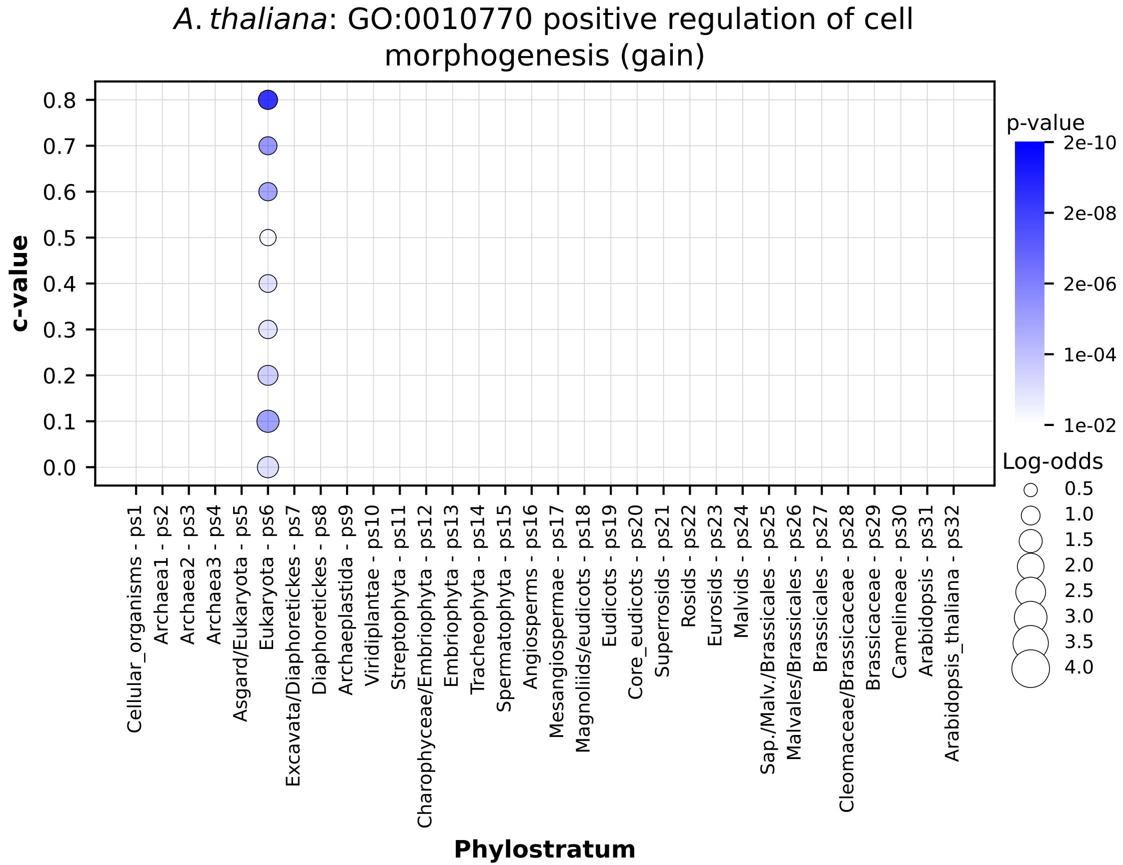
A. thaliana: GO:0010770 positive regulation of cell morphogenesis (gain)
c-value
0.8
0.7
0.6
0.5
0.4
0.3
0.2
0.1
0.0
p-value
2e-10
2e-08
2e-06
1e-04
1e-02
Log-odds
0.5
1.0
1.5
2.0
2.5
3.0
3.5
4.0
Cellular_organisms - ps1
Archaea1 - ps2
Archaea2 - ps3
Archaea3 - ps4
Asgard/Eukaryota - ps5
Eukaryota - ps6
Excavata/Diaphoretickes - ps7
Diaphoretickes - ps8
Archaeplastida - ps9
Viridiplantae - ps10
Streptophyta - ps11
Charophyceae/Embriophyta - ps12
Embriophyta - ps13
Tracheophyta - ps14
Spermatophyta - ps15
Angiosperms - ps16
Mesangiospermae - ps17
Magnoliids/eudicots - ps18
Eudicots - ps19
Core_eudicots - ps20
Superrosids - ps21
Rosids - ps22
Eurosids - ps23
Malvids - ps24
Sap./Malv./Brassicales - ps25
Malvales/Brassicales - ps26
Brassicales - ps27
Cleomaceae/Brassicaceae - ps28
Brassicaceae - ps29
Camelineae - ps30
Arabidopsis - ps31
Arabidopsis_thaliana - ps32
Phylostratum

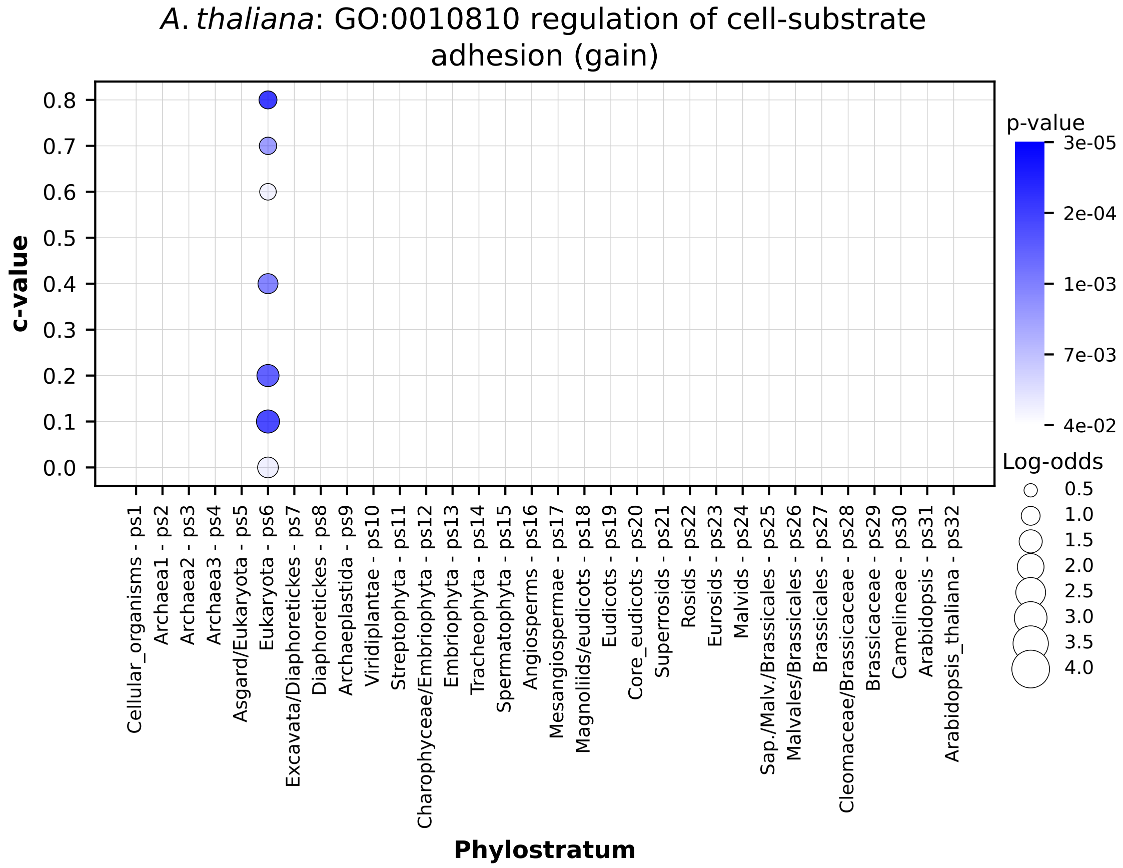
A. thaliana: GO:0010810 regulation of cell-substrate adhesion (gain)
c-value
0.8
0.7
0.6
0.5
0.4
0.3
0.2
0.1
0.0
Phylostratum
Cellular_organisms - ps1
Archaea1 - ps2
Archaea2 - ps3
Archaea3 - ps4
Asgard/Eukaryota - ps5
Eukaryota - ps6
Excavata/Diaphoretickes - ps7
Diaphoretickes - ps8
Archaeplastida - ps9
Viridiplantae - ps10
Streptophyta - ps11
Charophyceae/Embriophyta - ps12
Embriophyta - ps13
Tracheophyta - ps14
Spermatophyta - ps15
Angiosperms - ps16
Mesangiospermae - ps17
Magnoliids/eudicots - ps18
Eudicots - ps19
Core_eudicots - ps20
Superrosids - ps21
Rosids - ps22
Eurosids - ps23
Malvids - ps24
Sap./Malv./Brassicales - ps25
Malvales/Brassicales - ps26
Brassicales - ps27
Cleomaceae/Brassicaceae - ps28
Brassicaceae - ps29
Camelineae - ps30
Arabidopsis - ps31
Arabidopsis_thaliana - ps32
p-value
3e-05
2e-04
1e-03
7e-03
4e-02
Log-odds
0.5
1.0
1.5
2.0
2.5
3.0
3.5
4.0

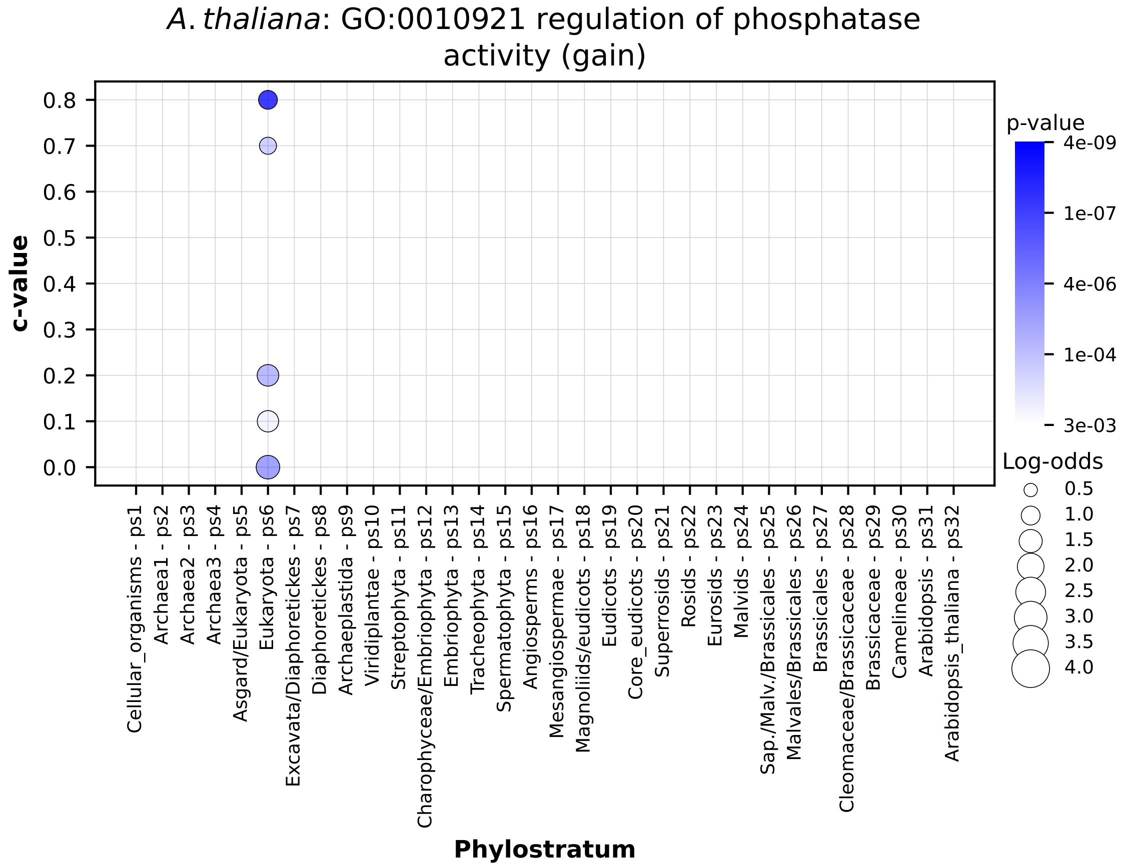

A. thaliana: GO:0010921 regulation of phosphatase activity (gain)
c-value
0.8
0.7
0.6
0.5
0.4
0.3
0.2
0.1
0.0
Phylostratum
Cellular_organisms - ps1
Archaea1 - ps2
Archaea2 - ps3
Archaea3 - ps4
Asgard/Eukaryota - ps5
Eukaryota - ps6
Excavata/Diaphoretickes - ps7
Diaphoretickes - ps8
Archaeplastida - ps9
Viridiplantae - ps10
Streptophyta - ps11
Charophyceae/Embriophyta - ps12
Embriophyta - ps13
Tracheophyta - ps14
Spermatophyta - ps15
Angiosperms - ps16
Mesangiospermae - ps17
Magnoliids/eudicots - ps18
Eudicots - ps19
Core_eudicots - ps20
Superrosids - ps21
Rosids - ps22
Eurosids - ps23
Malvids - ps24
Sap./Malv./Brassicales - ps25
Malvales/Brassicales - ps26
Brassicales - ps27
Cleomaceae/Brassicaceae - ps28
Brassicaceae - ps29
Camelineae - ps30
Arabidopsis - ps31
Arabidopsis_thaliana - ps32
p-value
4e-09
1e-07
4e-06
1e-04
3e-03
Log-odds
0.5
1.0
1.5
2.0
2.5
3.0
3.5
4.0

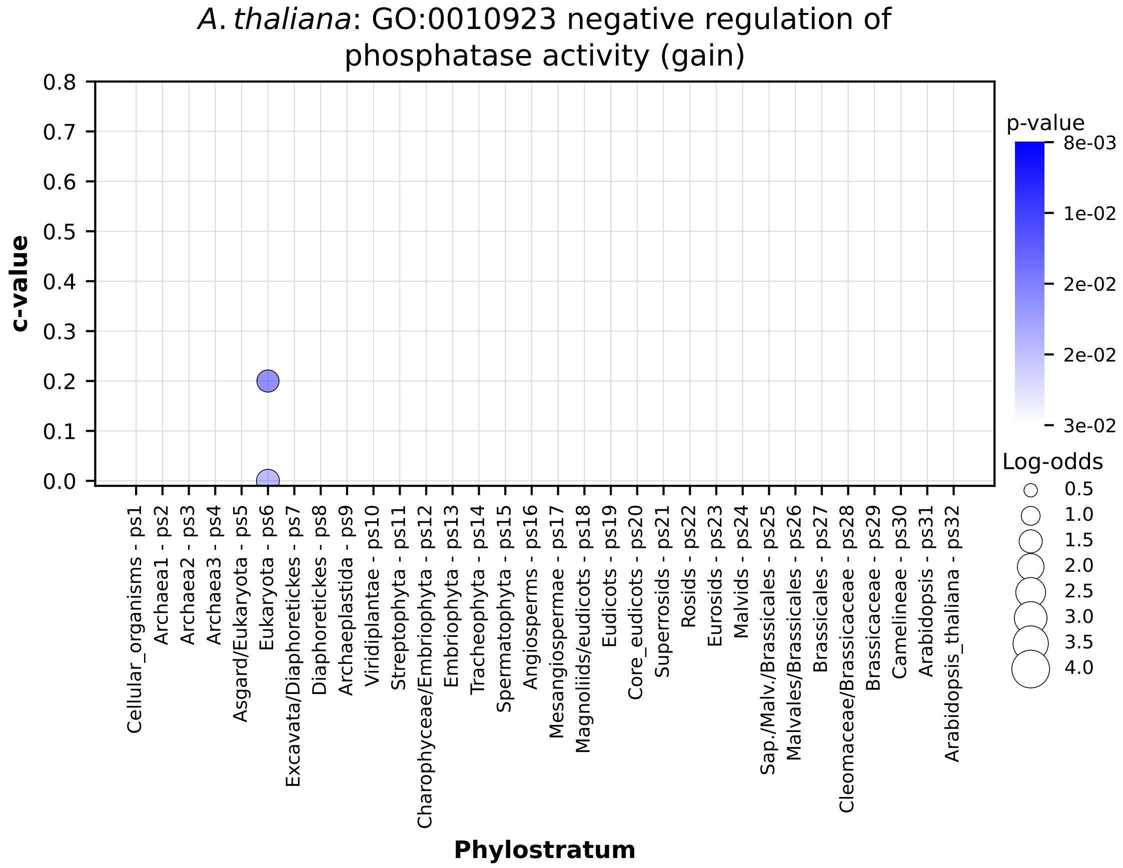

A. thaliana: GO:0010923 negative regulation of phosphatase activity (gain)
c-value
0.8
0.7
0.6
0.5
0.4
0.3
0.2
0.1
0.0
p-value
8e-03
1e-02
2e-02
2e-02
3e-02
Log-odds
0.5
1.0
1.5
2.0
2.5
3.0
3.5
4.0
Cellular_organisms - ps1
Archaea1 - ps2
Archaea2 - ps3
Archaea3 - ps4
Asgard/Eukaryota - ps5
Eukaryota - ps6
Excavata/Diaphoretickes - ps7
Diaphoretickes - ps8
Archaeplastida - ps9
Viridiplantae - ps10
Streptophyta - ps11
Charophyceae/Embriophyta - ps12
Embriophyta - ps13
Tracheophyta - ps14
Spermatophyta - ps15
Angiosperms - ps16
Mesangiospermae - ps17
Magnoliids/eudicots - ps18
Eudicots - ps19
Core_eudicots - ps20
Superrosids - ps21
Rosids - ps22
Eurosids - ps23
Malvids - ps24
Sap./Malv./Brassicales - ps25
Malvales/Brassicales - ps26
Brassicales - ps27
Cleomaceae/Brassicaceae - ps28
Brassicaceae - ps29
Camelineae - ps30
Arabidopsis - ps31
Arabidopsis_thaliana - ps32
Phylostratum

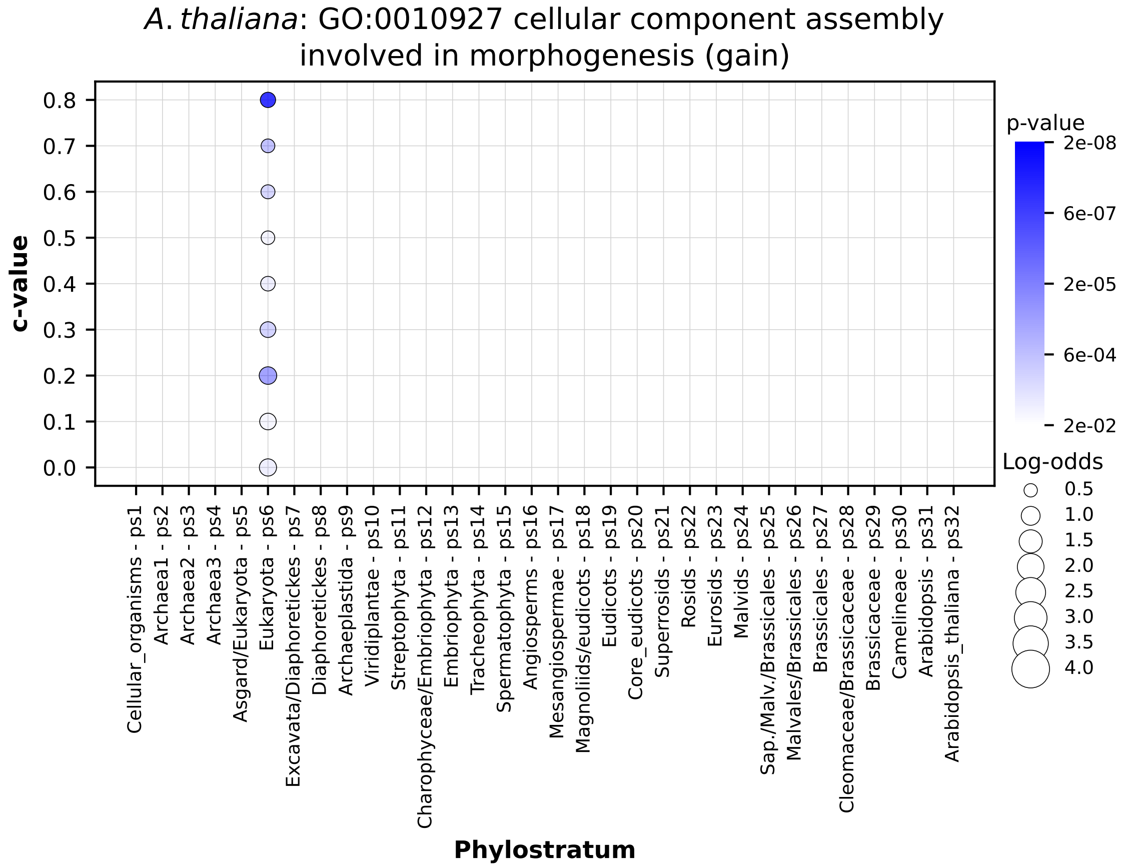

A. thaliana: GO:0010927 cellular component assembly involved in morphogenesis (gain)
c-value
0.8
0.7
0.6
0.5
0.4
0.3
0.2
0.1
0.0
p-value
2e-08
6e-07
2e-05
6e-04
2e-02
Log-odds
0.5
1.0
1.5
2.0
2.5
3.0
3.5
4.0
Cellular_organisms - ps1
Archaea1 - ps2
Archaea2 - ps3
Archaea3 - ps4
Asgard/Eukaryota - ps5
Eukaryota - ps6
Excavata/Diaphoretickes - ps7
Diaphoretickes - ps8
Archaeplastida - ps9
Viridiplantae - ps10
Streptophyta - ps11
Charophyceae/Embriophyta - ps12
Embriophyta - ps13
Tracheophyta - ps14
Spermatophyta - ps15
Angiosperms - ps16
Mesangiospermae - ps17
Magnoliids/eudicots - ps18
Eudicots - ps19
Core_eudicots - ps20
Superrosids - ps21
Rosids - ps22
Eurosids - ps23
Malvids - ps24
Sap./Malv./Brassicales - ps25
Malvales/Brassicales - ps26
Brassicales - ps27
Cleomaceae/Brassicaceae - ps28
Brassicaceae - ps29
Camelineae - ps30
Arabidopsis - ps31
Arabidopsis_thaliana - ps32
Phylostratum

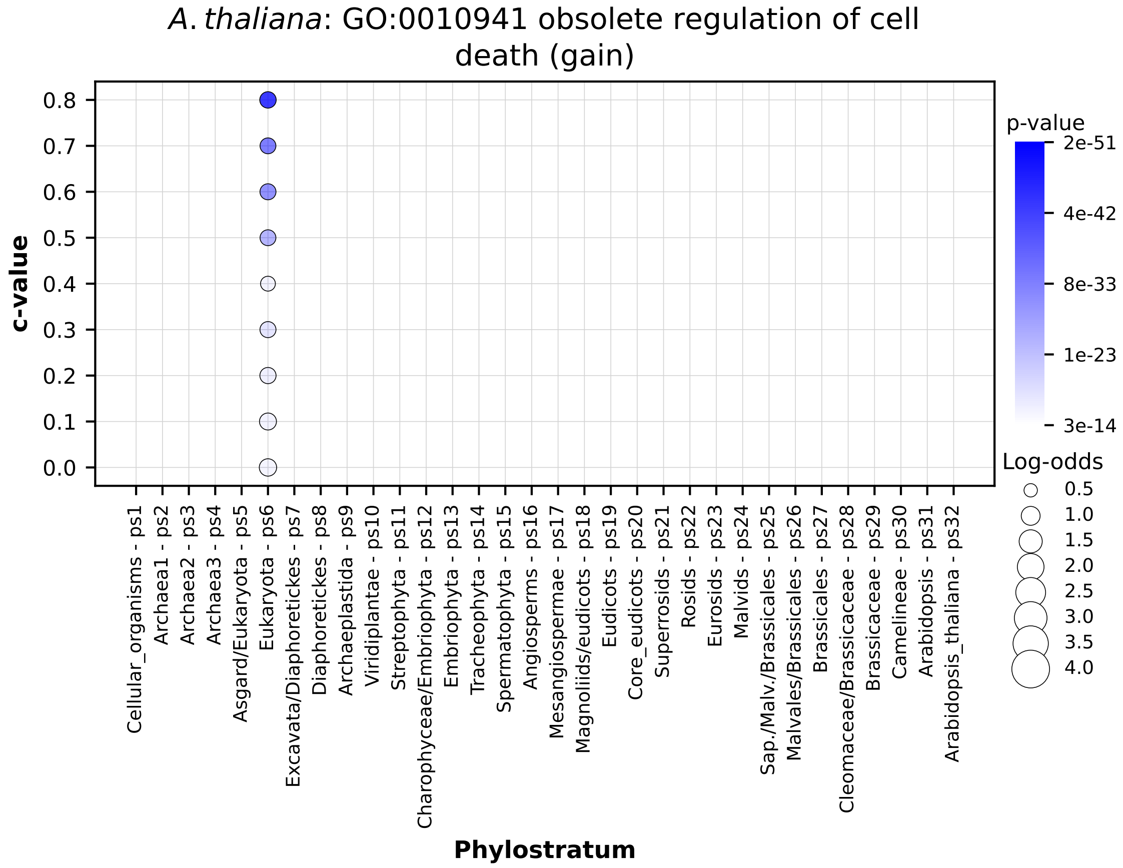

A. thaliana: GO:0010941 obsolete regulation of cell death (gain)
c-value
0.8
0.7
0.6
0.5
0.4
0.3
0.2
0.1
0.0
p-value
2e-51
4e-42
8e-33
1e-23
3e-14
Log-odds
0.5
1.0
1.5
2.0
2.5
3.0
3.5
4.0
Cellular_organisms - ps1
Archaea1 - ps2
Archaea2 - ps3
Archaea3 - ps4
Asgard/Eukaryota - ps5
Eukaryota - ps6
Excavata/Diaphoretickes - ps7
Diaphoretickes - ps8
Archaeplastida - ps9
Viridiplantae - ps10
Streptophyta - ps11
Charophyceae/Embriophyta - ps12
Embriophyta - ps13
Tracheophyta - ps14
Spermatophyta - ps15
Angiosperms - ps16
Mesangiospermae - ps17
Magnoliids/eudicots - ps18
Eudicots - ps19
Core_eudicots - ps20
Superrosids - ps21
Rosids - ps22
Eurosids - ps23
Malvids - ps24
Sap./Malv./Brassicales - ps25
Malvales/Brassicales - ps26
Brassicales - ps27
Cleomaceae/Brassicaceae - ps28
Brassicaceae - ps29
Camelineae - ps30
Arabidopsis - ps31
Arabidopsis_thaliana - ps32
Phylostratum

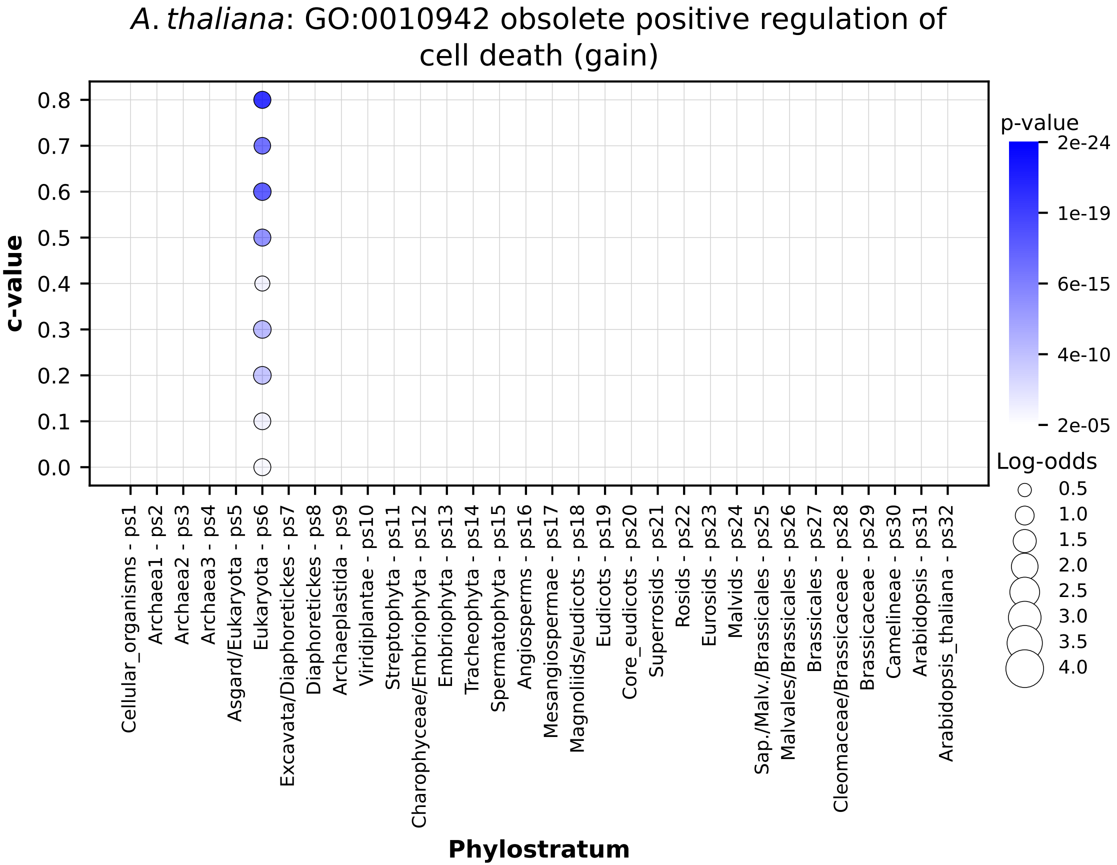

A. thaliana: GO:0010942 obsolete positive regulation of cell death (gain)
c-value
0.8
0.7
0.6
0.5
0.4
0.3
0.2
0.1
0.0
p-value
2e-24
1e-19
6e-15
4e-10
2e-05
Log-odds
0.5
1.0
1.5
2.0
2.5
3.0
3.5
4.0
Cellular_organisms - ps1
Archaea1 - ps2
Archaea2 - ps3
Archaea3 - ps4
Asgard/Eukaryota - ps5
Eukaryota - ps6
Excavata/Diaphoretickes - ps7
Diaphoretickes - ps8
Archaeplastida - ps9
Viridiplantae - ps10
Streptophyta - ps11
Charophyceae/Embriophyta - ps12
Embriophyta - ps13
Tracheophyta - ps14
Spermatophyta - ps15
Angiosperms - ps16
Mesangiospermae - ps17
Magnoliids/eudicots - ps18
Eudicots - ps19
Core_eudicots - ps20
Superrosids - ps21
Rosids - ps22
Eurosids - ps23
Malvids - ps24
Sap./Malv./Brassicales - ps25
Malvales/Brassicales - ps26
Brassicales - ps27
Cleomaceae/Brassicaceae - ps28
Brassicaceae - ps29
Camelineae - ps30
Arabidopsis - ps31
Arabidopsis_thaliana - ps32
Phylostratum

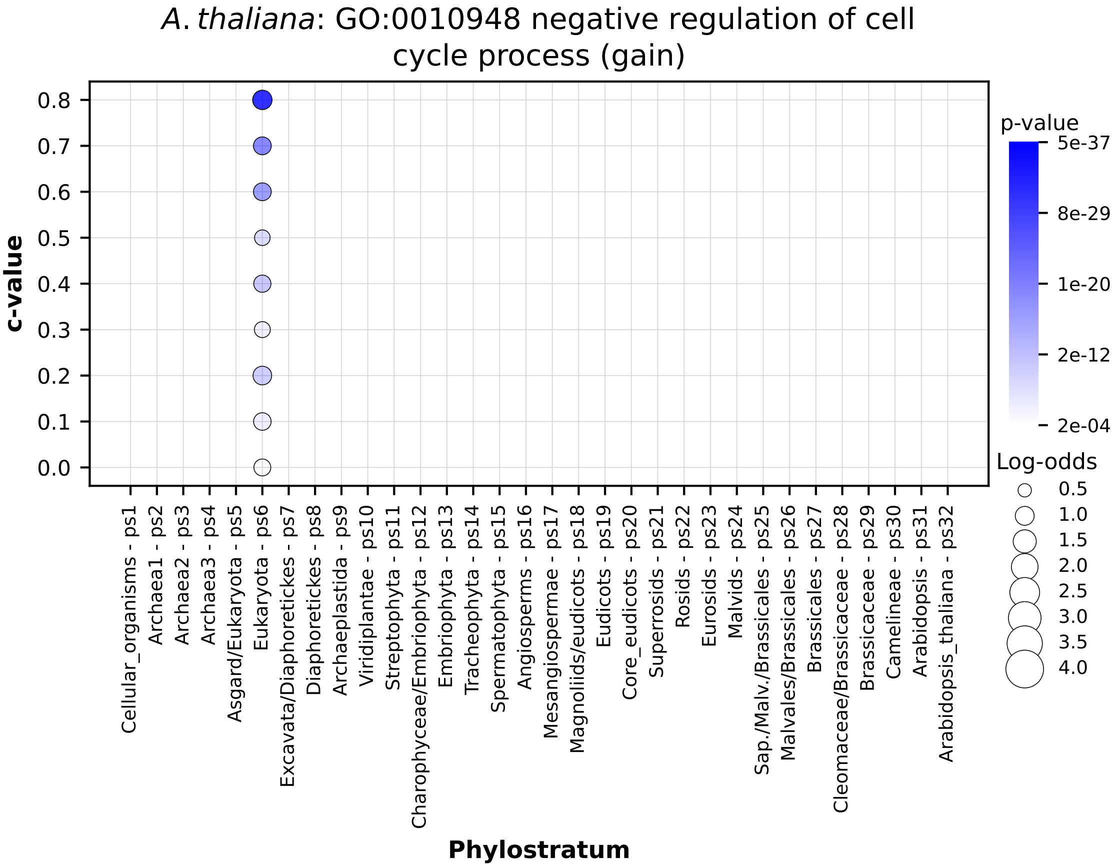

A. thaliana: GO:0010948 negative regulation of cell cycle process (gain)
c-value
0.8
0.7
0.6
0.5
0.4
0.3
0.2
0.1
0.0
p-value
5e-37
8e-29
1e-20
2e-12
2e-04
Log-odds
0.5
1.0
1.5
2.0
2.5
3.0
3.5
4.0
Cellular_organisms - ps1
Archaea1 - ps2
Archaea2 - ps3
Archaea3 - ps4
Asgard/Eukaryota - ps5
Eukaryota - ps6
Excavata/Diaphoretickes - ps7
Diaphoretickes - ps8
Archaeplastida - ps9
Viridiplantae - ps10
Streptophyta - ps11
Charophyceae/Embriophyta - ps12
Embriophyta - ps13
Tracheophyta - ps14
Spermatophyta - ps15
Angiosperms - ps16
Mesangiospermae - ps17
Magnoliids/eudicots - ps18
Eudicots - ps19
Core_eudicots - ps20
Superrosids - ps21
Rosids - ps22
Eurosids - ps23
Malvids - ps24
Sap./Malv./Brassicales - ps25
Malvales/Brassicales - ps26
Brassicales - ps27
Cleomaceae/Brassicaceae - ps28
Brassicaceae - ps29
Camelineae - ps30
Arabidopsis - ps31
Arabidopsis_thaliana - ps32
Phylostratum

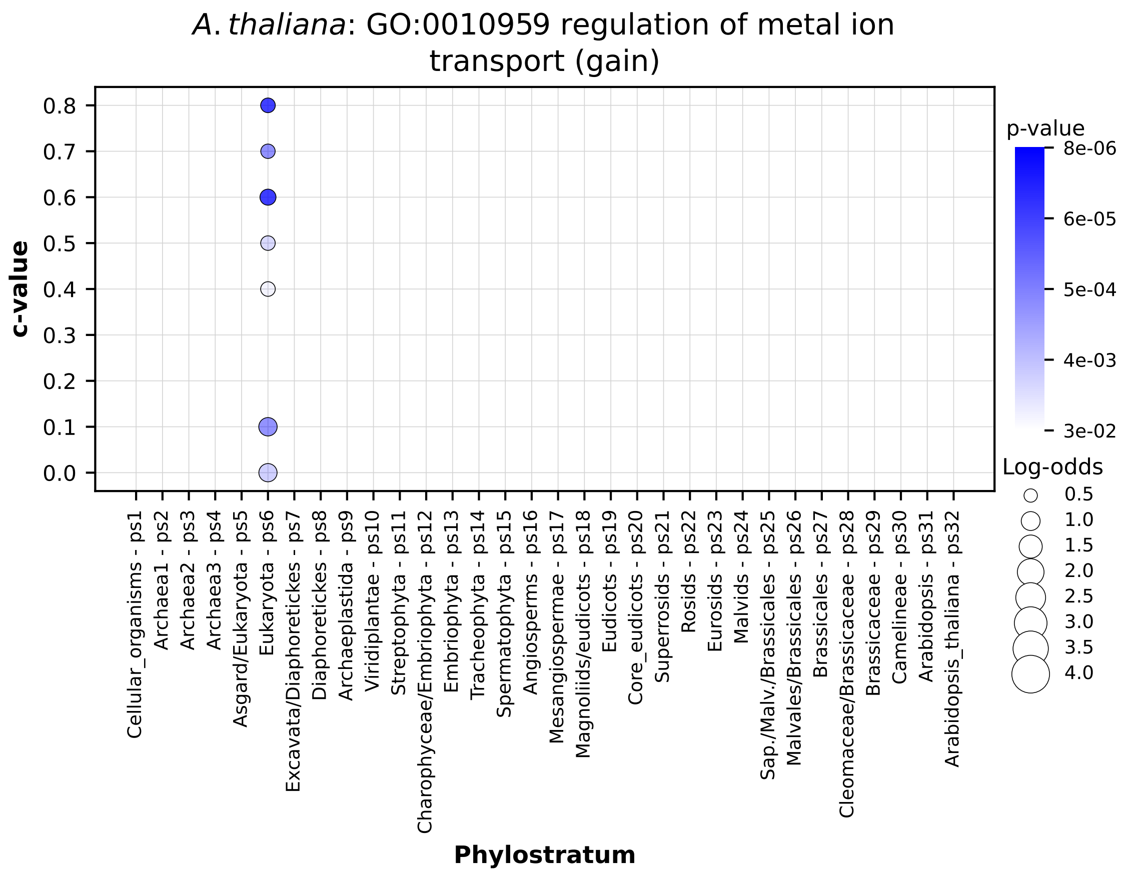

A. thaliana: GO:0010959 regulation of metal ion transport (gain)
c-value
0.8
0.7
0.6
0.5
0.4
0.3
0.2
0.1
0.0
p-value
8e-06
6e-05
5e-04
4e-03
3e-02
Log-odds
0.5
1.0
1.5
2.0
2.5
3.0
3.5
4.0
Cellular_organisms - ps1
Archaea1 - ps2
Archaea2 - ps3
Archaea3 - ps4
Asgard/Eukaryota - ps5
Eukaryota - ps6
Excavata/Diaphoretickes - ps7
Diaphoretickes - ps8
Archaeplastida - ps9
Viridiplantae - ps10
Streptophyta - ps11
Charophyceae/Embriophyta - ps12
Embriophyta - ps13
Tracheophyta - ps14
Spermatophyta - ps15
Angiosperms - ps16
Mesangiospermae - ps17
Magnoliids/eudicots - ps18
Eudicots - ps19
Core_eudicots - ps20
Superrosids - ps21
Rosids - ps22
Eurosids - ps23
Malvids - ps24
Sap./Malv./Brassicales - ps25
Malvales/Brassicales - ps26
Brassicales - ps27
Cleomaceae/Brassicaceae - ps28
Brassicaceae - ps29
Camelineae - ps30
Arabidopsis - ps31
Arabidopsis_thaliana - ps32
Phylostratum

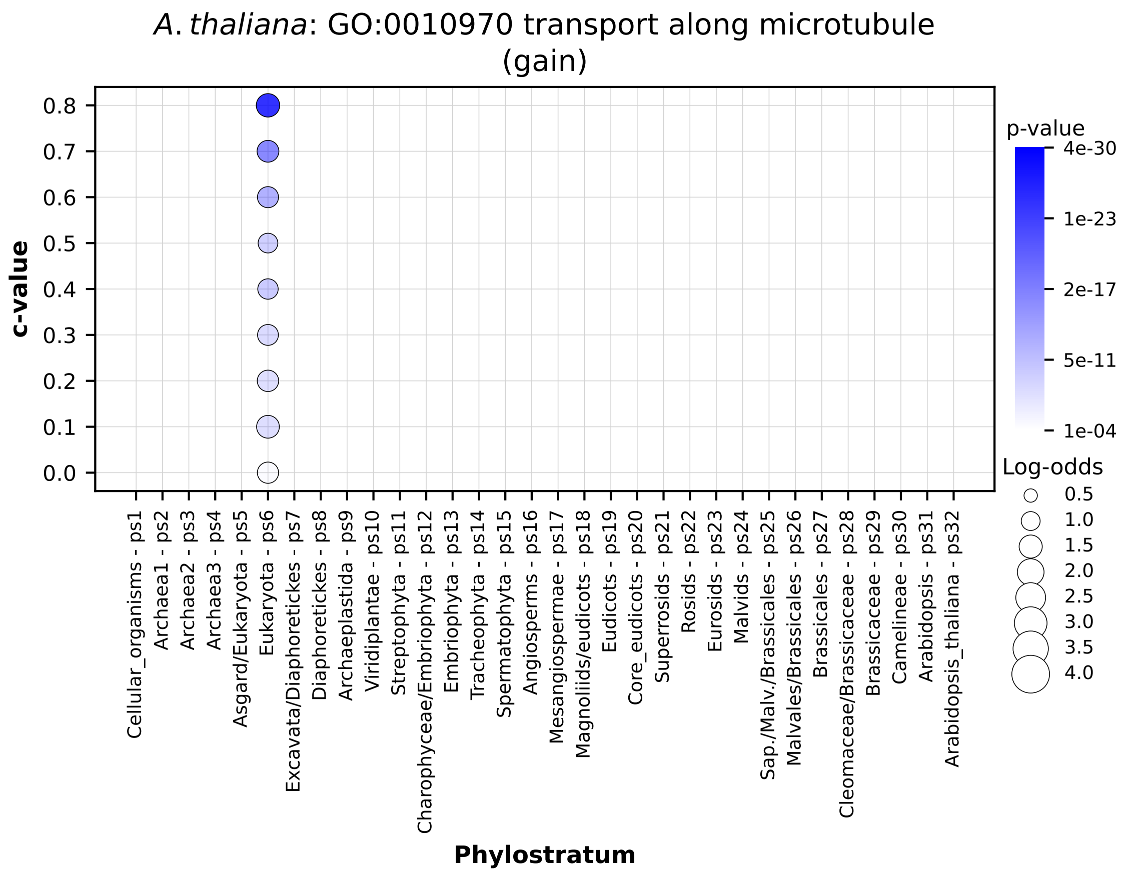
A. thaliana: GO:0010970 transport along microtubule
(gain)
c-value
0.8
0.7
0.6
0.5
0.4
0.3
0.2
0.1
0.0
p-value
4e-30
1e-23
2e-17
5e-11
1e-04
Log-odds
0.5
1.0
1.5
2.0
2.5
3.0
3.5
4.0
Cellular_organisms - ps1
Archaea1 - ps2
Archaea2 - ps3
Archaea3 - ps4
Asgard/Eukaryota - ps5
Eukaryota - ps6
Excavata/Diaphoretickes - ps7
Diaphoretickes - ps8
Archaeplastida - ps9
Viridiplantae - ps10
Streptophyta - ps11
Charophyceae/Embriophyta - ps12
Embriophyta - ps13
Tracheophyta - ps14
Spermatophyta - ps15
Angiosperms - ps16
Mesangiospermae - ps17
Magnoliids/eudicots - ps18
Eudicots - ps19
Core_eudicots - ps20
Superrosids - ps21
Rosids - ps22
Eurosids - ps23
Malvids - ps24
Sap./Malv./Brassicales - ps25
Malvales/Brassicales - ps26
Brassicales - ps27
Cleomaceae/Brassicaceae - ps28
Brassicaceae - ps29
Camelineae - ps30
Arabidopsis - ps31
Arabidopsis_thaliana - ps32
Phylostratum

A. thaliana: GO:0010975 regulation of neuron projection development (gain)

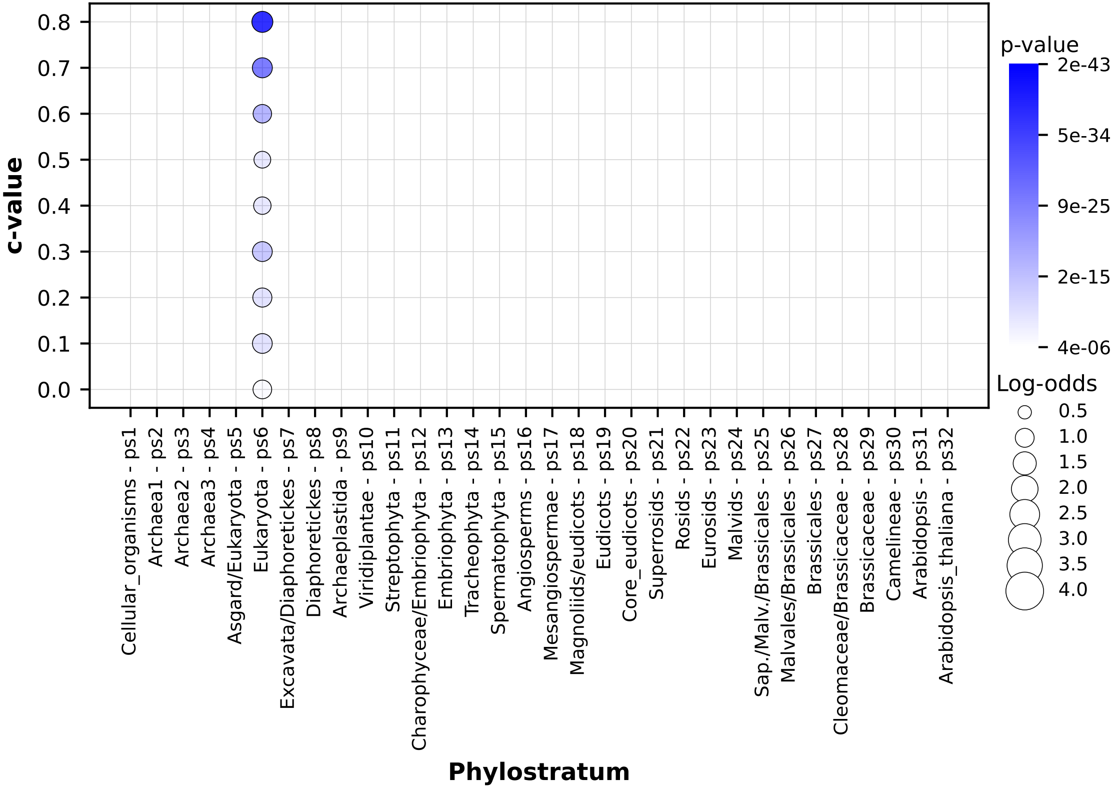

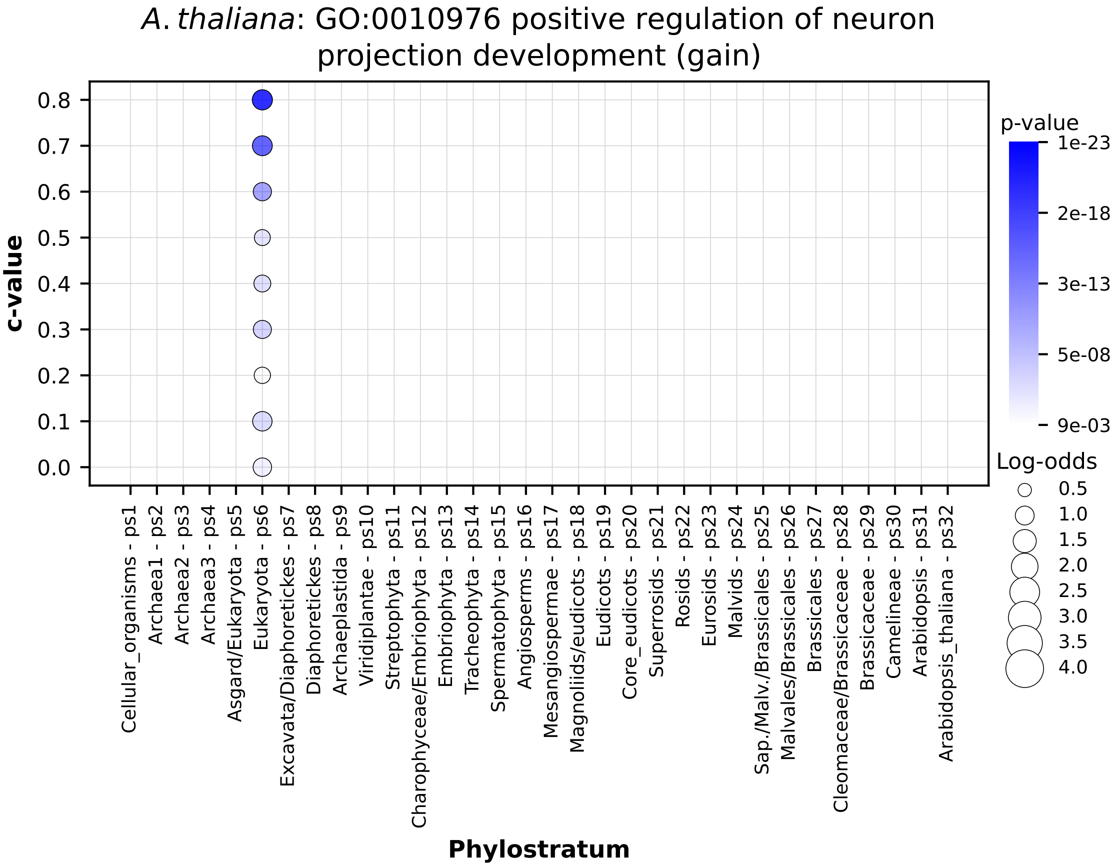

A. thaliana: GO:0010976 positive regulation of neuron projection development (gain)
c-value
0.8
0.7
0.6
0.5
0.4
0.3
0.2
0.1
0.0
p-value
1e-23
2e-18
3e-13
5e-08
9e-03
Log-odds
0.5
1.0
1.5
2.0
2.5
3.0
3.5
4.0
Cellular_organisms - ps1
Archaea1 - ps2
Archaea2 - ps3
Archaea3 - ps4
Asgard/Eukaryota - ps5
Eukaryota - ps6
Excavata/Diaphoretickes - ps7
Diaphoretickes - ps8
Archaeplastida - ps9
Viridiplantae - ps10
Streptophyta - ps11
Charophyceae/Embriophyta - ps12
Embriophyta - ps13
Tracheophyta - ps14
Spermatophyta - ps15
Angiosperms - ps16
Mesangiospermae - ps17
Magnoliids/eudicots - ps18
Eudicots - ps19
Core_eudicots - ps20
Superrosids - ps21
Rosids - ps22
Eurosids - ps23
Malvids - ps24
Sap./Malv./Brassicales - ps25
Malvales/Brassicales - ps26
Brassicales - ps27
Cleomaceae/Brassicaceae - ps28
Brassicaceae - ps29
Camelineae - ps30
Arabidopsis - ps31
Arabidopsis_thaliana - ps32
Phylostratum

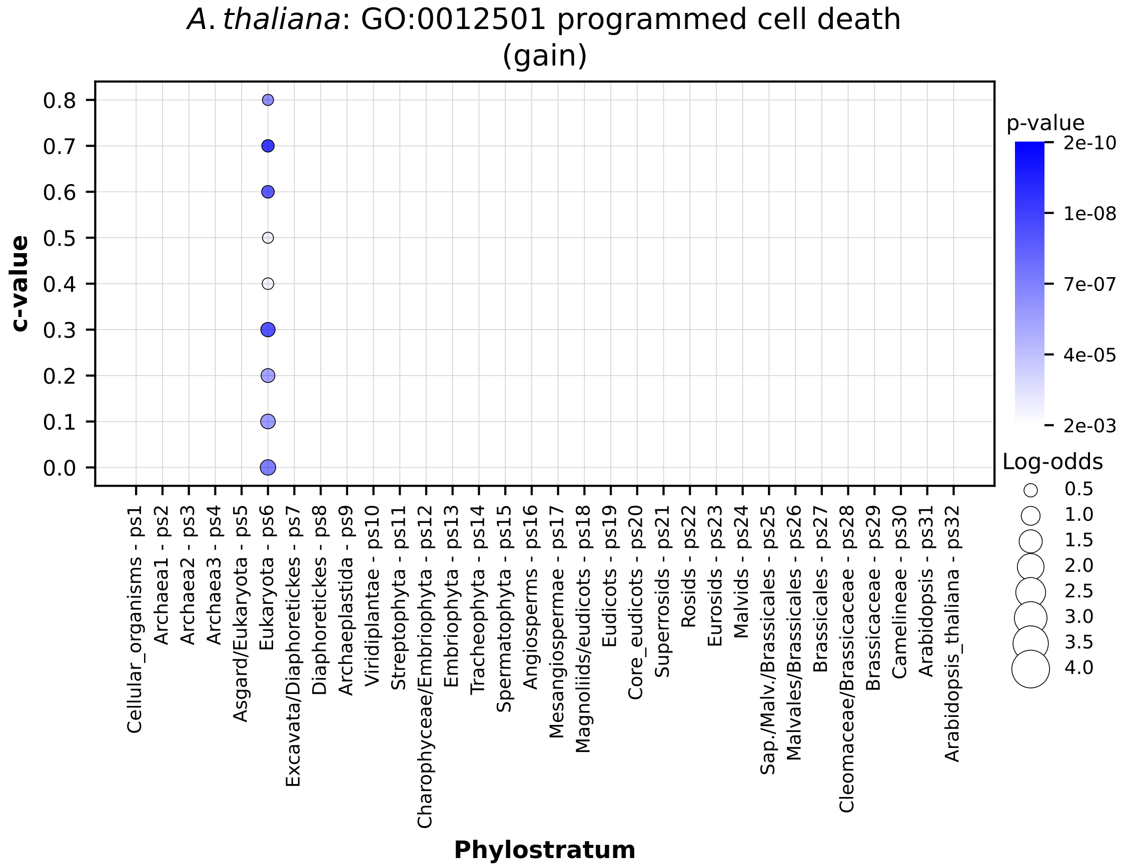

A. thaliana: GO:0012501 programmed cell death
(gain)
c-value
0.8
0.7
0.6
0.5
0.4
0.3
0.2
0.1
0.0
p-value
2e-10
1e-08
7e-07
4e-05
2e-03
Log-odds
0.5
1.0
1.5
2.0
2.5
3.0
3.5
4.0
Cellular_organisms - ps1
Archaea1 - ps2
Archaea2 - ps3
Archaea3 - ps4
Asgard/Eukaryota - ps5
Eukaryota - ps6
Excavata/Diaphoretickes - ps7
Diaphoretickes - ps8
Archaeplastida - ps9
Viridiplantae - ps10
Streptophyta - ps11
Charophyceae/Embriophyta - ps12
Embriophyta - ps13
Tracheophyta - ps14
Spermatophyta - ps15
Angiosperms - ps16
Mesangiospermae - ps17
Magnoliids/eudicots - ps18
Eudicots - ps19
Core_eudicots - ps20
Superrosids - ps21
Rosids - ps22
Eurosids - ps23
Malvids - ps24
Sap./Malv./Brassicales - ps25
Malvales/Brassicales - ps26
Brassicales - ps27
Cleomaceae/Brassicaceae - ps28
Brassicaceae - ps29
Camelineae - ps30
Arabidopsis - ps31
Arabidopsis_thaliana - ps32
Phylostratum

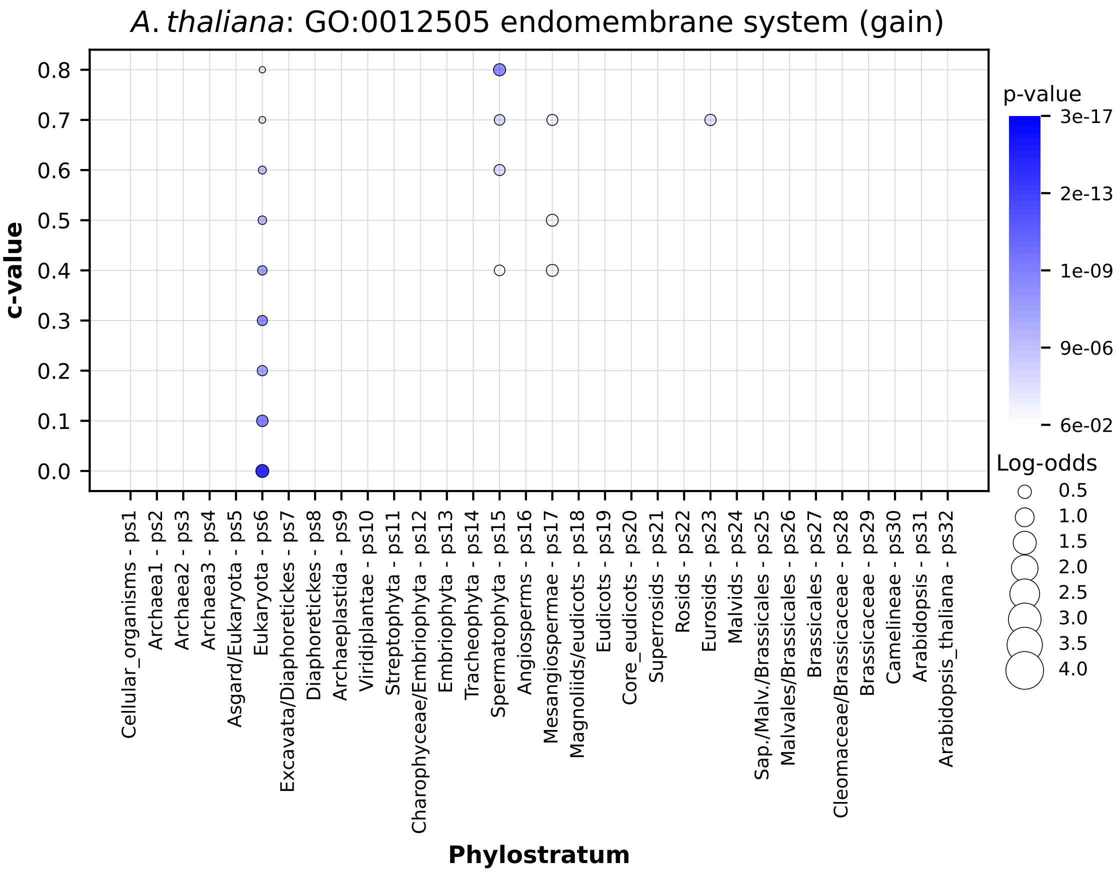
A. thaliana: GO:0012505 endomembrane system (gain)
c-value
0.0
0.1
0.2
0.3
0.4
0.5
0.6
0.7
0.8
Phylostratum
Cellular_organisms - ps1
Archaea1 - ps2
Archaea2 - ps3
Archaea3 - ps4
Asgard/Eukaryota - ps5
Eukaryota - ps6
Excavata/Diaphoretickes - ps7
Diaphoretickes - ps8
Archaeplastida - ps9
Viridiplantae - ps10
Streptophyta - ps11
Charophyceae/Embriophyta - ps12
Embriophyta - ps13
Tracheophyta - ps14
Spermatophyta - ps15
Angiosperms - ps16
Mesangiospermae - ps17
Magnoliids/eudicots - ps18
Eudicots - ps19
Core_eudicots - ps20
Superrosids - ps21
Rosids - ps22
Eurosids - ps23
Malvids - ps24
Sap./Malv./Brassicales - ps25
Malvales/Brassicales - ps26
Brassicales - ps27
Cleomaceae/Brassicaceae - ps28
Brassicaceae - ps29
Camelineae - ps30
Arabidopsis - ps31
Arabidopsis_thaliana - ps32
p-value
3e-17
2e-13
1e-09
9e-06
6e-02
Log-odds
0.5
1.0
1.5
2.0
2.5
3.0
3.5
4.0

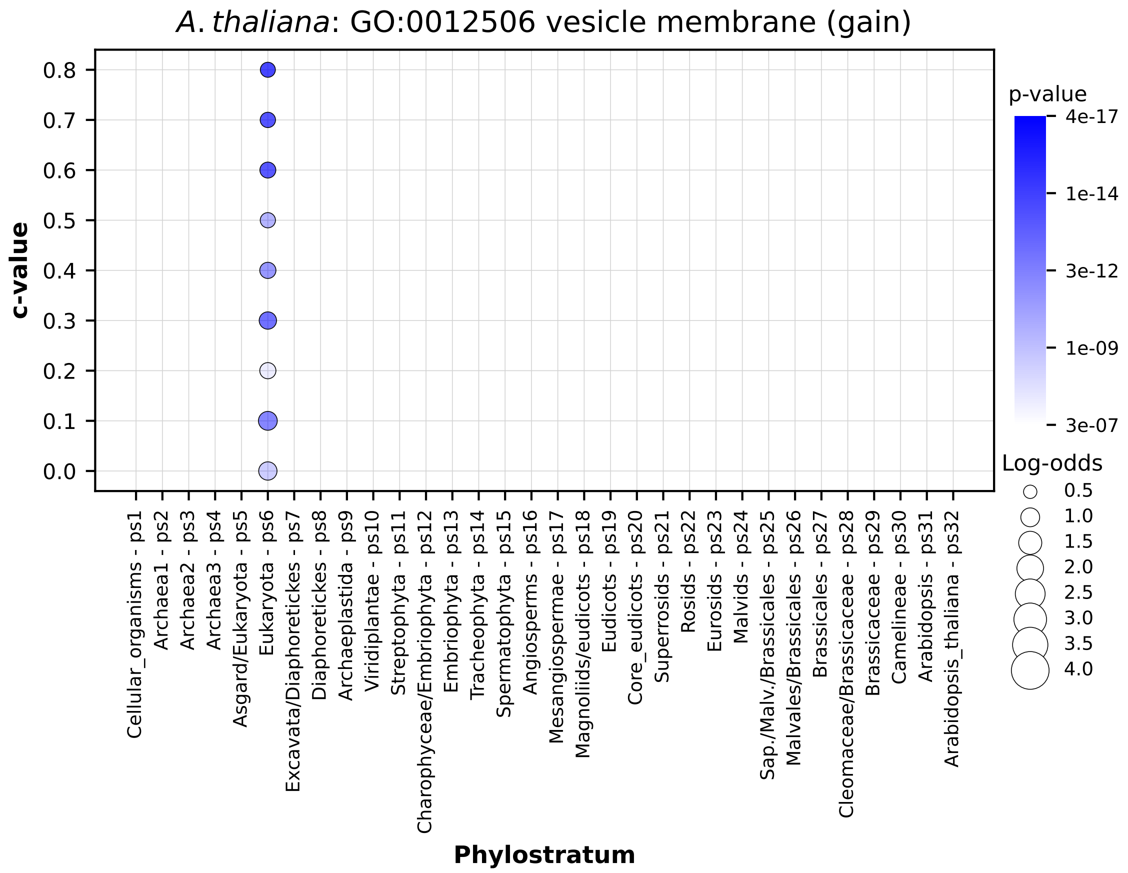

A. thaliana: GO:0012506 vesicle membrane (gain)
c-value
0.8
0.7
0.6
0.5
0.4
0.3
0.2
0.1
0.0
p-value
4e-17
1e-14
3e-12
1e-09
3e-07
Log-odds
0.5
1.0
1.5
2.0
2.5
3.0
3.5
4.0
Cellular_organisms - ps1
Archaea1 - ps2
Archaea2 - ps3
Archaea3 - ps4
Asgard/Eukaryota - ps5
Eukaryota - ps6
Excavata/Diaphoretickes - ps7
Diaphoretickes - ps8
Archaeplastida - ps9
Viridiplantae - ps10
Streptophyta - ps11
Charophyceae/Embriophyta - ps12
Embriophyta - ps13
Tracheophyta - ps14
Spermatophyta - ps15
Angiosperms - ps16
Mesangiospermae - ps17
Magnoliids/eudicots - ps18
Eudicots - ps19
Core_eudicots - ps20
Superrosids - ps21
Rosids - ps22
Eurosids - ps23
Malvids - ps24
Sap./Malv./Brassicales - ps25
Malvales/Brassicales - ps26
Brassicales - ps27
Cleomaceae/Brassicaceae - ps28
Brassicaceae - ps29
Camelineae - ps30
Arabidopsis - ps31
Arabidopsis_thaliana - ps32
Phylostratum

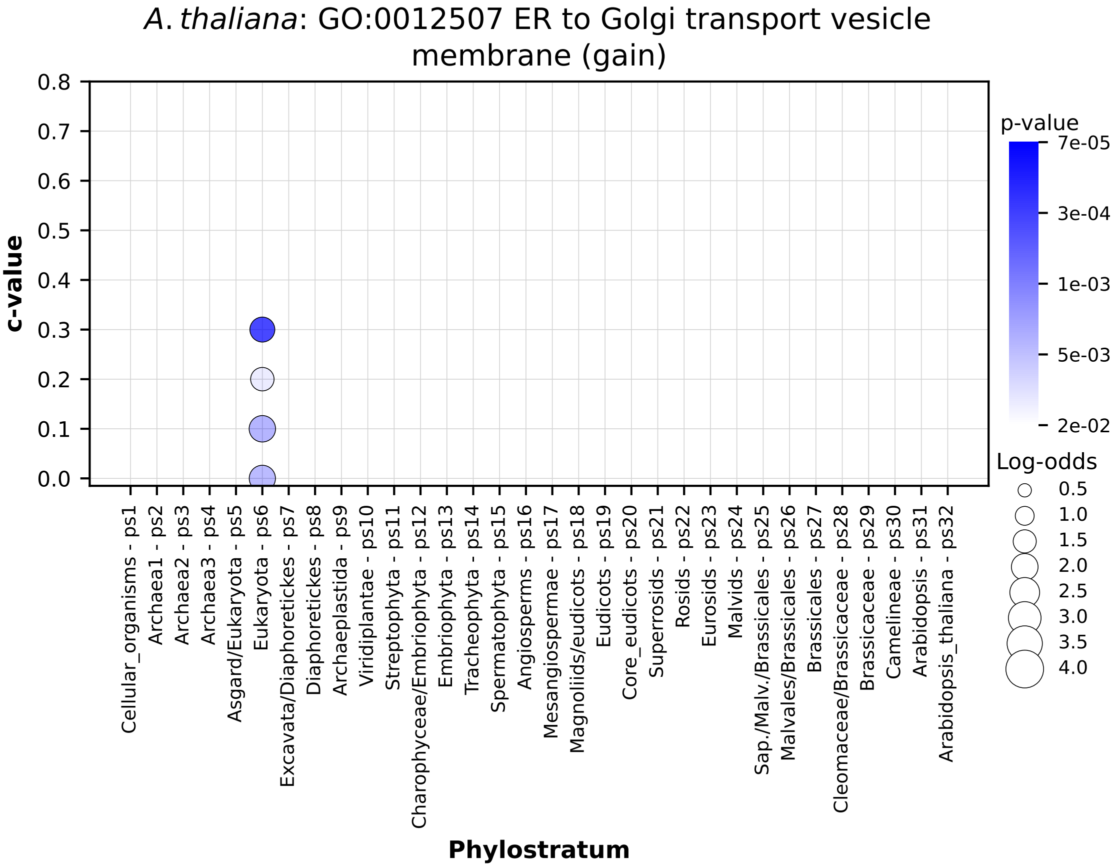

A. thaliana: GO:0012507 ER to Golgi transport vesicle membrane (gain)
c-value
0.8
0.7
0.6
0.5
0.4
0.3
0.2
0.1
0.0
p-value
7e-05
3e-04
1e-03
5e-03
2e-02
Log-odds
0.5
1.0
1.5
2.0
2.5
3.0
3.5
4.0
Cellular_organisms - ps1
Archaea1 - ps2
Archaea2 - ps3
Archaea3 - ps4
Asgard/Eukaryota - ps5
Eukaryota - ps6
Excavata/Diaphoretickes - ps7
Diaphoretickes - ps8
Archaeplastida - ps9
Viridiplantae - ps10
Streptophyta - ps11
Charophyceae/Embriophyta - ps12
Embriophyta - ps13
Tracheophyta - ps14
Spermatophyta - ps15
Angiosperms - ps16
Mesangiospermae - ps17
Magnoliids/eudicots - ps18
Eudicots - ps19
Core_eudicots - ps20
Superrosids - ps21
Rosids - ps22
Eurosids - ps23
Malvids - ps24
Sap./Malv./Brassicales - ps25
Malvales/Brassicales - ps26
Brassicales - ps27
Cleomaceae/Brassicaceae - ps28
Brassicaceae - ps29
Camelineae - ps30
Arabidopsis - ps31
Arabidopsis_thaliana - ps32
Phylostratum

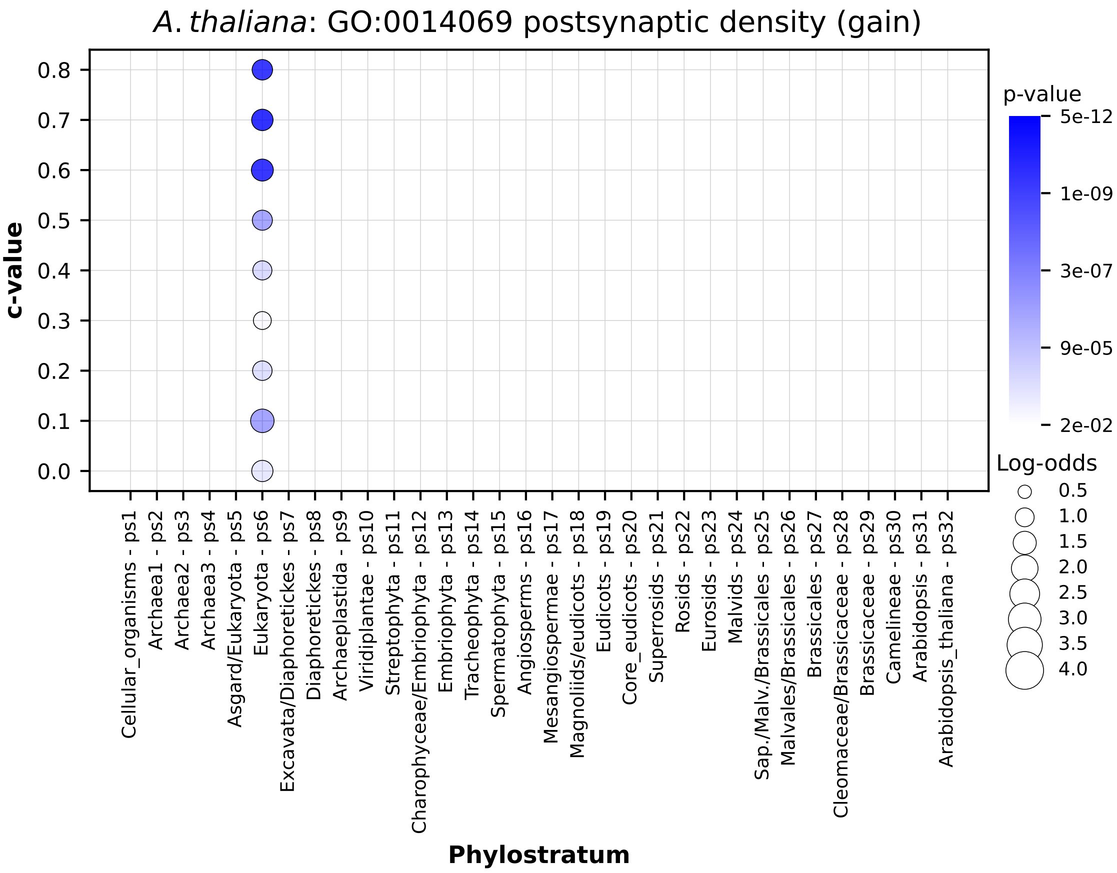

A. thaliana: GO:0014069 postsynaptic density (gain)
c-value
0.8
0.7
0.6
0.5
0.4
0.3
0.2
0.1
0.0
p-value
5e-12
1e-09
3e-07
9e-05
2e-02
Log-odds
0.5
1.0
1.5
2.0
2.5
3.0
3.5
4.0
Cellular_organisms - ps1
Archaea1 - ps2
Archaea2 - ps3
Archaea3 - ps4
Asgard/Eukaryota - ps5
Eukaryota - ps6
Excavata/Diaphoretickes - ps7
Diaphoretickes - ps8
Archaeplastida - ps9
Viridiplantae - ps10
Streptophyta - ps11
Charophyceae/Embriophyta - ps12
Embriophyta - ps13
Tracheophyta - ps14
Spermatophyta - ps15
Angiosperms - ps16
Mesangiospermae - ps17
Magnoliids/eudicots - ps18
Eudicots - ps19
Core_eudicots - ps20
Superrosids - ps21
Rosids - ps22
Eurosids - ps23
Malvids - ps24
Sap./Malv./Brassicales - ps25
Malvales/Brassicales - ps26
Brassicales - ps27
Cleomaceae/Brassicaceae - ps28
Brassicaceae - ps29
Camelineae - ps30
Arabidopsis - ps31
Arabidopsis_thaliana - ps32
Phylostratum

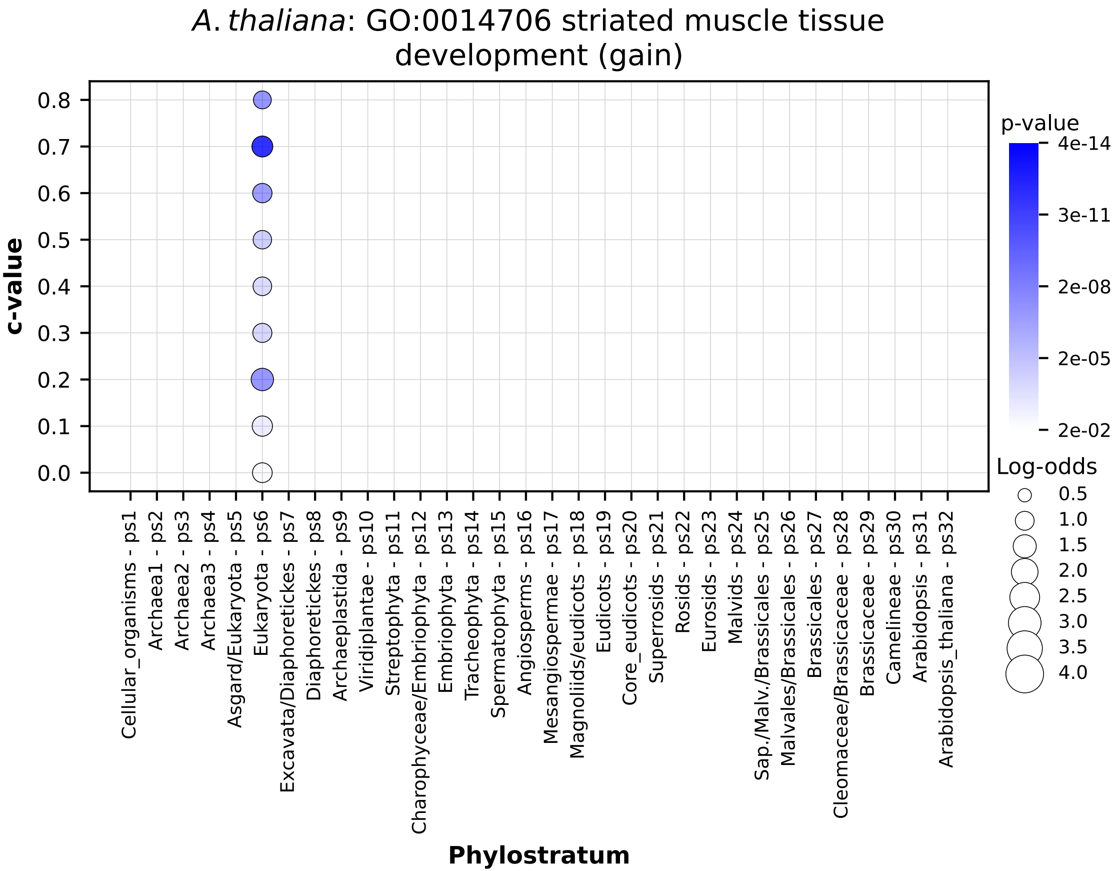
A. thaliana: GO:0014706 striated muscle tissue development (gain)
c-value
0.8
0.7
0.6
0.5
0.4
0.3
0.2
0.1
0.0
p-value
4e-14
3e-11
2e-08
2e-05
2e-02
Log-odds
0.5
1.0
1.5
2.0
2.5
3.0
3.5
4.0
Cellular_organisms - ps1
Archaea1 - ps2
Archaea2 - ps3
Archaea3 - ps4
Asgard/Eukaryota - ps5
Eukaryota - ps6
Excavata/Diaphoretickes - ps7
Diaphoretickes - ps8
Archaeplastida - ps9
Viridiplantae - ps10
Streptophyta - ps11
Charophyceae/Embriophyta - ps12
Embriophyta - ps13
Tracheophyta - ps14
Spermatophyta - ps15
Angiosperms - ps16
Mesangiospermae - ps17
Magnoliids/eudicots - ps18
Eudicots - ps19
Core_eudicots - ps20
Superrosids - ps21
Rosids - ps22
Eurosids - ps23
Malvids - ps24
Sap./Malv./Brassicales - ps25
Malvales/Brassicales - ps26
Brassicales - ps27
Cleomaceae/Brassicaceae - ps28
Brassicaceae - ps29
Camelineae - ps30
Arabidopsis - ps31
Arabidopsis_thaliana - ps32
Phylostratum

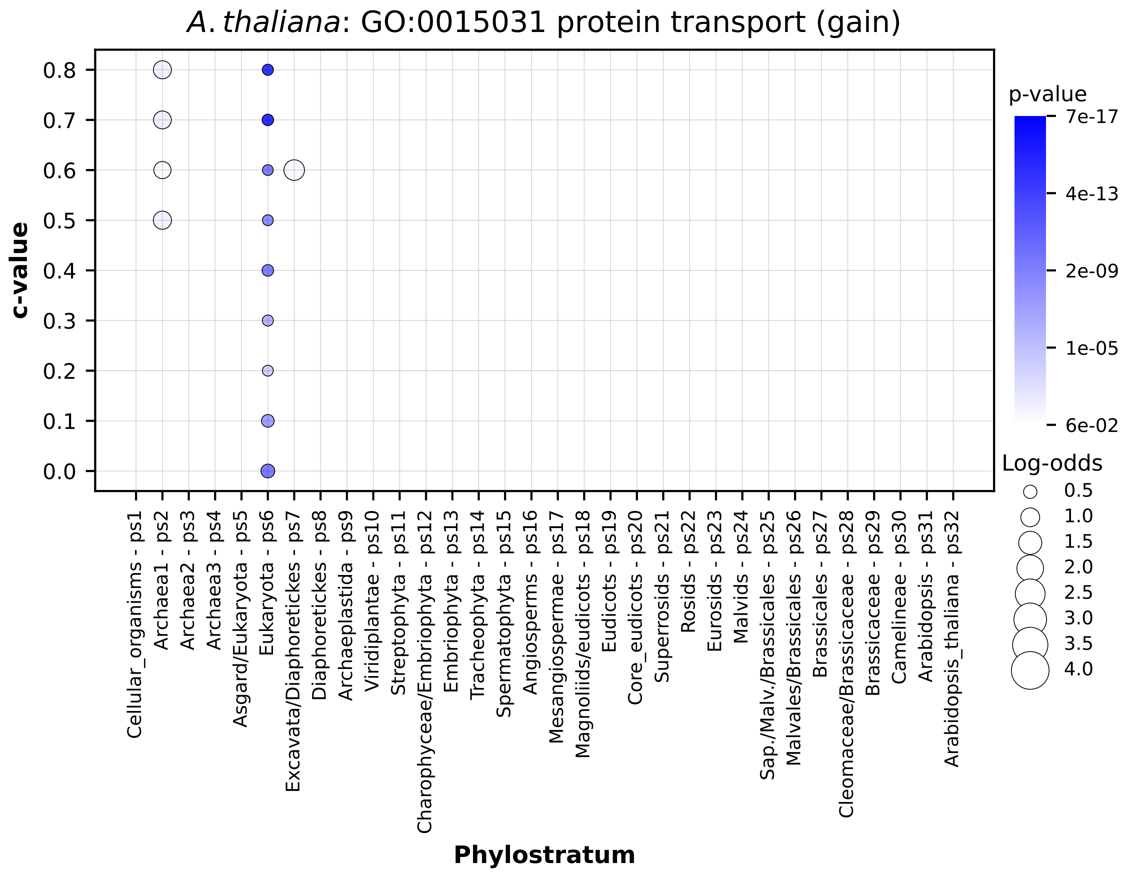

A. thaliana: GO:0015031 protein transport (gain)
c-value
0.8
0.7
0.6
0.5
0.4
0.3
0.2
0.1
0.0
Phylostratum
Cellular_organisms - ps1
Archaea1 - ps2
Archaea2 - ps3
Archaea3 - ps4
Asgard/Eukaryota - ps5
Eukaryota - ps6
Excavata/Diaphoretickes - ps7
Diaphoretickes - ps8
Archaeplastida - ps9
Viridiplantae - ps10
Streptophyta - ps11
Charophyceae/Embriophyta - ps12
Embriophyta - ps13
Tracheophyta - ps14
Spermatophyta - ps15
Angiosperms - ps16
Mesangiospermae - ps17
Magnoliids/eudicots - ps18
Eudicots - ps19
Core_eudicots - ps20
Superrosids - ps21
Rosids - ps22
Eurosids - ps23
Malvids - ps24
Sap./Malv./Brassicales - ps25
Malvales/Brassicales - ps26
Brassicales - ps27
Cleomaceae/Brassicaceae - ps28
Brassicaceae - ps29
Camelineae - ps30
Arabidopsis - ps31
Arabidopsis_thaliana - ps32
p-value
7e-17
4e-13
2e-09
1e-05
6e-02
Log-odds
0.5
1.0
1.5
2.0
2.5
3.0
3.5
4.0

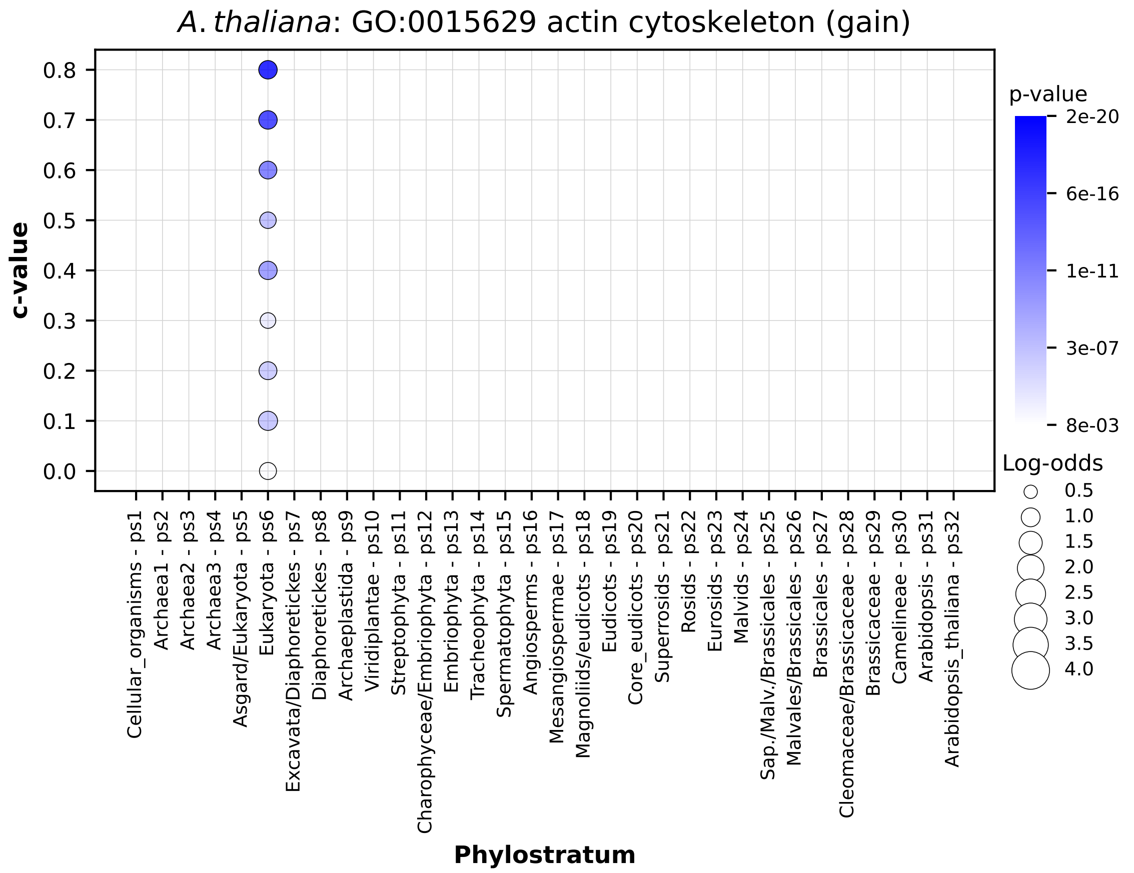

A. thaliana: GO:0015629 actin cytoskeleton (gain)
c-value
0.0
0.1
0.2
0.3
0.4
0.5
0.6
0.7
0.8
p-value
2e-20
6e-16
1e-11
3e-07
8e-03
Log-odds
0.5
1.0
1.5
2.0
2.5
3.0
3.5
4.0
Cellular_organisms - ps1
Archaea1 - ps2
Archaea2 - ps3
Archaea3 - ps4
Asgard/Eukaryota - ps5
Eukaryota - ps6
Excavata/Diaphoretickes - ps7
Diaphoretickes - ps8
Archaeplastida - ps9
Viridiplantae - ps10
Streptophyta - ps11
Charophyceae/Embriophyta - ps12
Embriophyta - ps13
Tracheophyta - ps14
Spermatophyta - ps15
Angiosperms - ps16
Mesangiospermae - ps17
Magnoliids/eudicots - ps18
Eudicots - ps19
Core_eudicots - ps20
Superrosids - ps21
Rosids - ps22
Eurosids - ps23
Malvids - ps24
Sap./Malv./Brassicales - ps25
Malvales/Brassicales - ps26
Brassicales - ps27
Cleomaceae/Brassicaceae - ps28
Brassicaceae - ps29
Camelineae - ps30
Arabidopsis - ps31
Arabidopsis_thaliana - ps32
Phylostratum

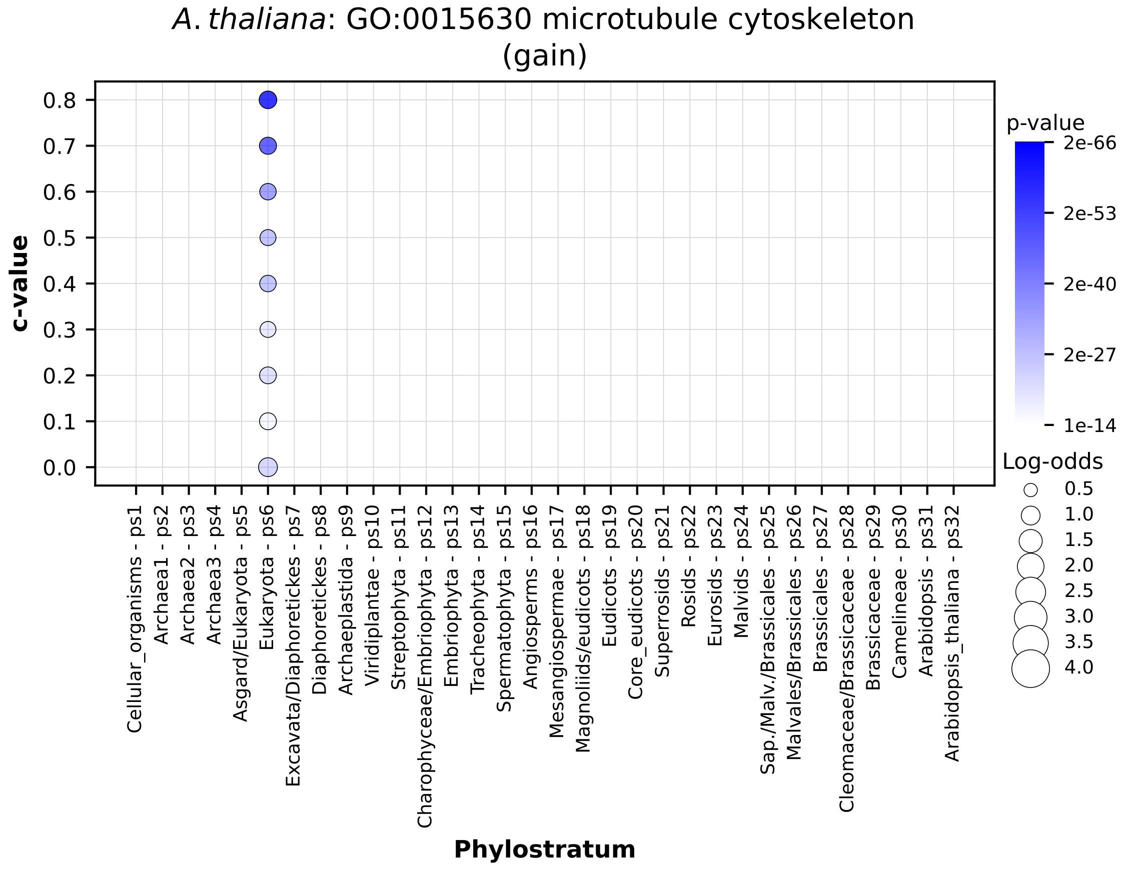
A. thaliana: GO:0015630 microtubule cytoskeleton
(gain)
c-value
0.8
0.7
0.6
0.5
0.4
0.3
0.2
0.1
0.0
p-value
2e-66
2e-53
2e-40
2e-27
1e-14
Log-odds
0.5
1.0
1.5
2.0
2.5
3.0
3.5
4.0
Cellular_organisms - ps1
Archaea1 - ps2
Archaea2 - ps3
Archaea3 - ps4
Asgard/Eukaryota - ps5
Eukaryota - ps6
Excavata/Diaphoretickes - ps7
Diaphoretickes - ps8
Archaeplastida - ps9
Viridiplantae - ps10
Streptophyta - ps11
Charophyceae/Embriophyta - ps12
Embriophyta - ps13
Tracheophyta - ps14
Spermatophyta - ps15
Angiosperms - ps16
Mesangiospermae - ps17
Magnoliids/eudicots - ps18
Eudicots - ps19
Core_eudicots - ps20
Superrosids - ps21
Rosids - ps22
Eurosids - ps23
Malvids - ps24
Sap./Malv./Brassicales - ps25
Malvales/Brassicales - ps26
Brassicales - ps27
Cleomaceae/Brassicaceae - ps28
Brassicaceae - ps29
Camelineae - ps30
Arabidopsis - ps31
Arabidopsis_thaliana - ps32
Phylostratum

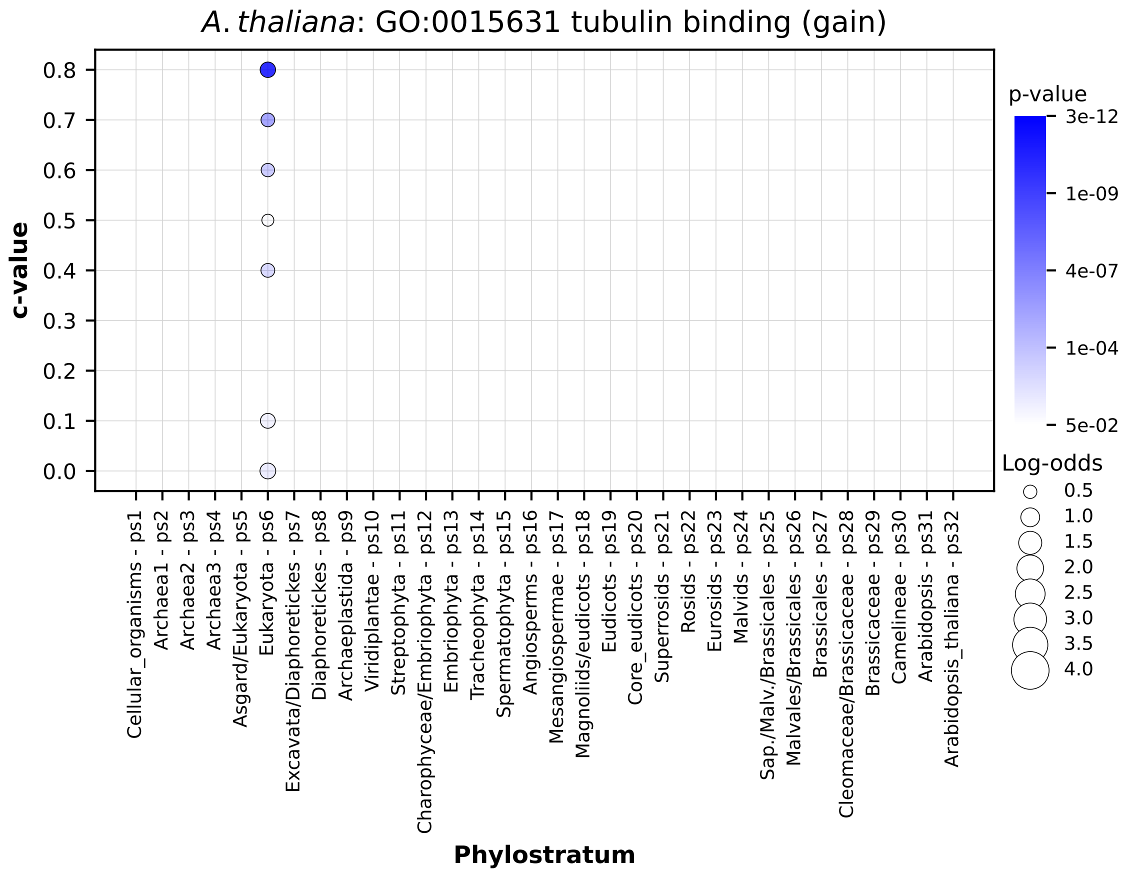

A. thaliana: GO:0015631 tubulin binding (gain)
c-value
0.8
0.7
0.6
0.5
0.4
0.3
0.2
0.1
0.0
p-value
3e-12
1e-09
4e-07
1e-04
5e-02
Log-odds
0.5
1.0
1.5
2.0
2.5
3.0
3.5
4.0
Cellular_organisms - ps1
Archaea1 - ps2
Archaea2 - ps3
Archaea3 - ps4
Asgard/Eukaryota - ps5
Eukaryota - ps6
Excavata/Diaphoretickes - ps7
Diaphoretickes - ps8
Archaeplastida - ps9
Viridiplantae - ps10
Streptophyta - ps11
Charophyceae/Embriophyta - ps12
Embriophyta - ps13
Tracheophyta - ps14
Spermatophyta - ps15
Angiosperms - ps16
Mesangiospermae - ps17
Magnoliids/eudicots - ps18
Eudicots - ps19
Core_eudicots - ps20
Superrosids - ps21
Rosids - ps22
Eurosids - ps23
Malvids - ps24
Sap./Malv./Brassicales - ps25
Malvales/Brassicales - ps26
Brassicales - ps27
Cleomaceae/Brassicaceae - ps28
Brassicaceae - ps29
Camelineae - ps30
Arabidopsis - ps31
Arabidopsis_thaliana - ps32
Phylostratum

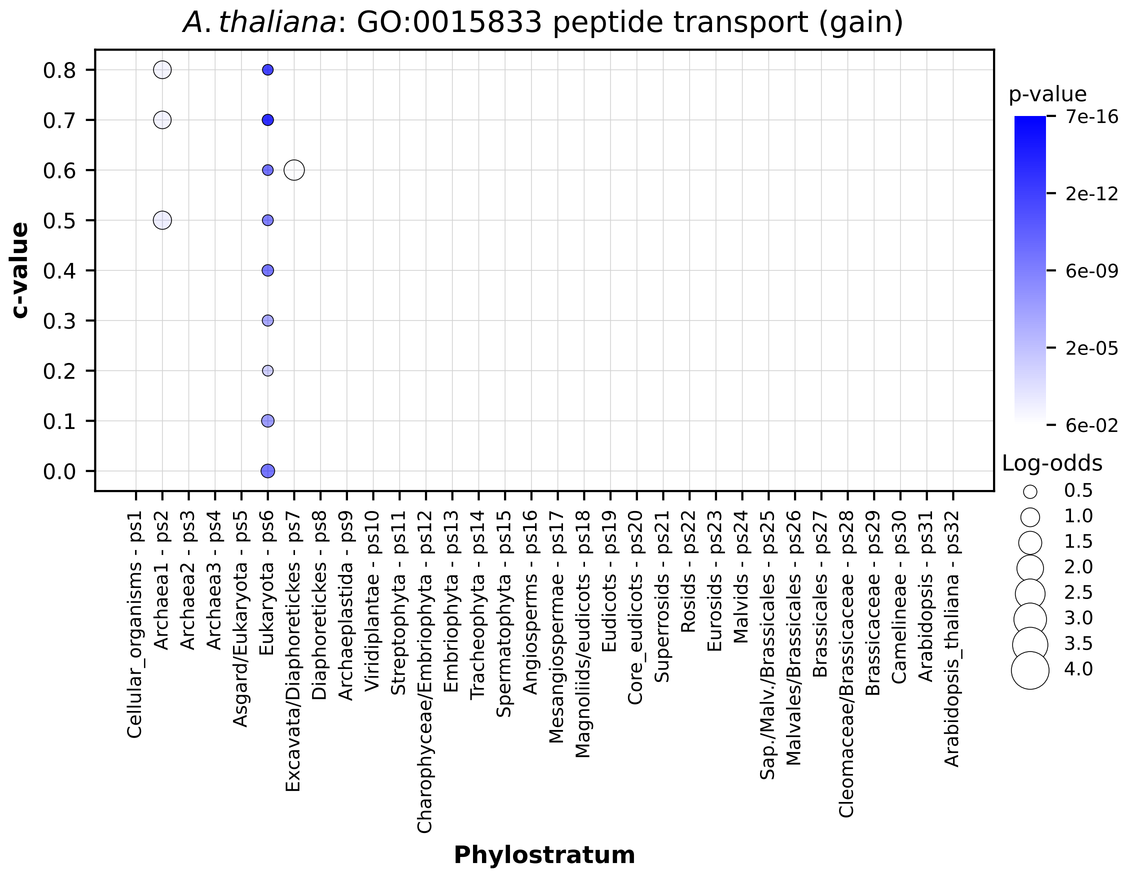
A. thaliana: GO:0015833 peptide transport (gain)
c-value
Phylostratum
p-value
7e-16
2e-12
6e-09
2e-05
6e-02
Log-odds
0.5
1.0
1.5
2.0
2.5
3.0
3.5
4.0
Cellular_organisms - ps1
Archaea1 - ps2
Archaea2 - ps3
Archaea3 - ps4
Asgard/Eukaryota - ps5
Eukaryota - ps6
Excavata/Diaphoretickes - ps7
Diaphoretickes - ps8
Archaeplastida - ps9
Viridiplantae - ps10
Streptophyta - ps11
Charophyceae/Embriophyta - ps12
Embriophyta - ps13
Tracheophyta - ps14
Spermatophyta - ps15
Angiosperms - ps16
Mesangiospermae - ps17
Magnoliids/eudicots - ps18
Eudicots - ps19
Core_eudicots - ps20
Superrosids - ps21
Rosids - ps22
Eurosids - ps23
Malvids - ps24
Sap./Malv./Brassicales - ps25
Malvales/Brassicales - ps26
Brassicales - ps27
Cleomaceae/Brassicaceae - ps28
Brassicaceae - ps29
Camelineae - ps30
Arabidopsis - ps31
Arabidopsis_thaliana - ps32

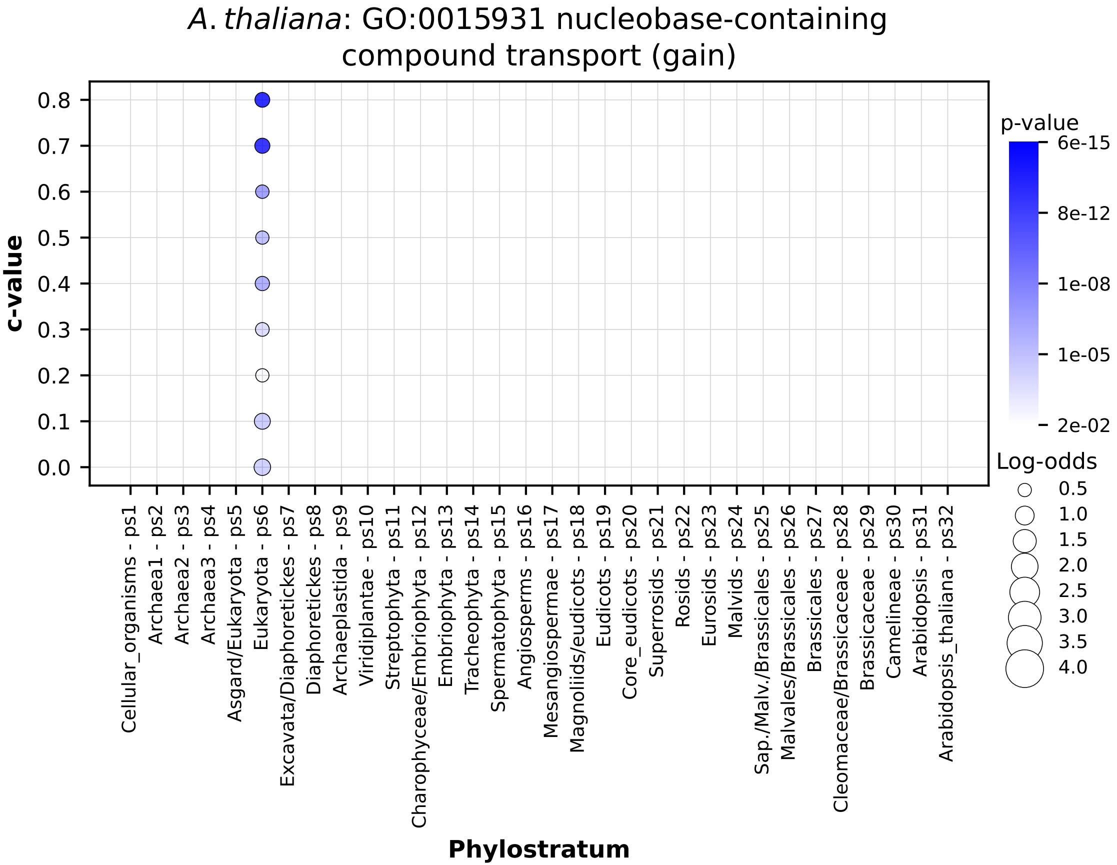

A. thaliana: GO:0015931 nucleobase-containing compound transport (gain)
c-value
0.8
0.7
0.6
0.5
0.4
0.3
0.2
0.1
0.0
p-value
6e-15
8e-12
1e-08
1e-05
2e-02
Log-odds
0.5
1.0
1.5
2.0
2.5
3.0
3.5
4.0
Cellular_organisms - ps1
Archaea1 - ps2
Archaea2 - ps3
Archaea3 - ps4
Asgard/Eukaryota - ps5
Eukaryota - ps6
Excavata/Diaphoretickes - ps7
Diaphoretickes - ps8
Archaeplastida - ps9
Viridiplantae - ps10
Streptophyta - ps11
Charophyceae/Embriophyta - ps12
Embriophyta - ps13
Tracheophyta - ps14
Spermatophyta - ps15
Angiosperms - ps16
Mesangiospermae - ps17
Magnoliids/eudicots - ps18
Eudicots - ps19
Core_eudicots - ps20
Superrosids - ps21
Rosids - ps22
Eurosids - ps23
Malvids - ps24
Sap./Malv./Brassicales - ps25
Malvales/Brassicales - ps26
Brassicales - ps27
Cleomaceae/Brassicaceae - ps28
Brassicaceae - ps29
Camelineae - ps30
Arabidopsis - ps31
Arabidopsis_thaliana - ps32
Phylostratum

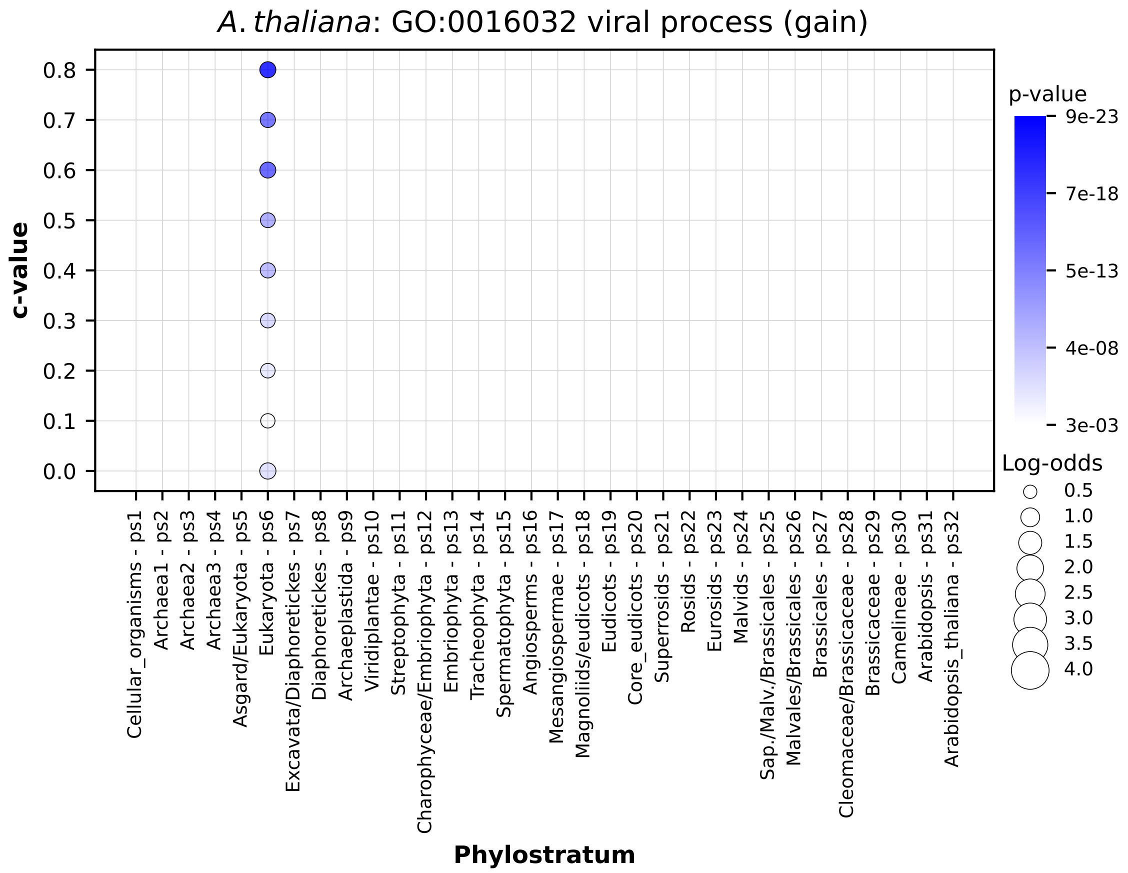

A. thaliana: GO:0016032 viral process (gain)
c-value
0.8
0.7
0.6
0.5
0.4
0.3
0.2
0.1
0.0
p-value
9e-23
7e-18
5e-13
4e-08
3e-03
Log-odds
0.5
1.0
1.5
2.0
2.5
3.0
3.5
4.0
Cellular_organisms - ps1
Archaea1 - ps2
Archaea2 - ps3
Archaea3 - ps4
Asgard/Eukaryota - ps5
Eukaryota - ps6
Excavata/Diaphoretickes - ps7
Diaphoretickes - ps8
Archaeplastida - ps9
Viridiplantae - ps10
Streptophyta - ps11
Charophyceae/Embriophyta - ps12
Embriophyta - ps13
Tracheophyta - ps14
Spermatophyta - ps15
Angiosperms - ps16
Mesangiospermae - ps17
Magnoliids/eudicots - ps18
Eudicots - ps19
Core_eudicots - ps20
Superrosids - ps21
Rosids - ps22
Eurosids - ps23
Malvids - ps24
Sap./Malv./Brassicales - ps25
Malvales/Brassicales - ps26
Brassicales - ps27
Cleomaceae/Brassicaceae - ps28
Brassicaceae - ps29
Camelineae - ps30
Arabidopsis - ps31
Arabidopsis_thaliana - ps32
Phylostratum

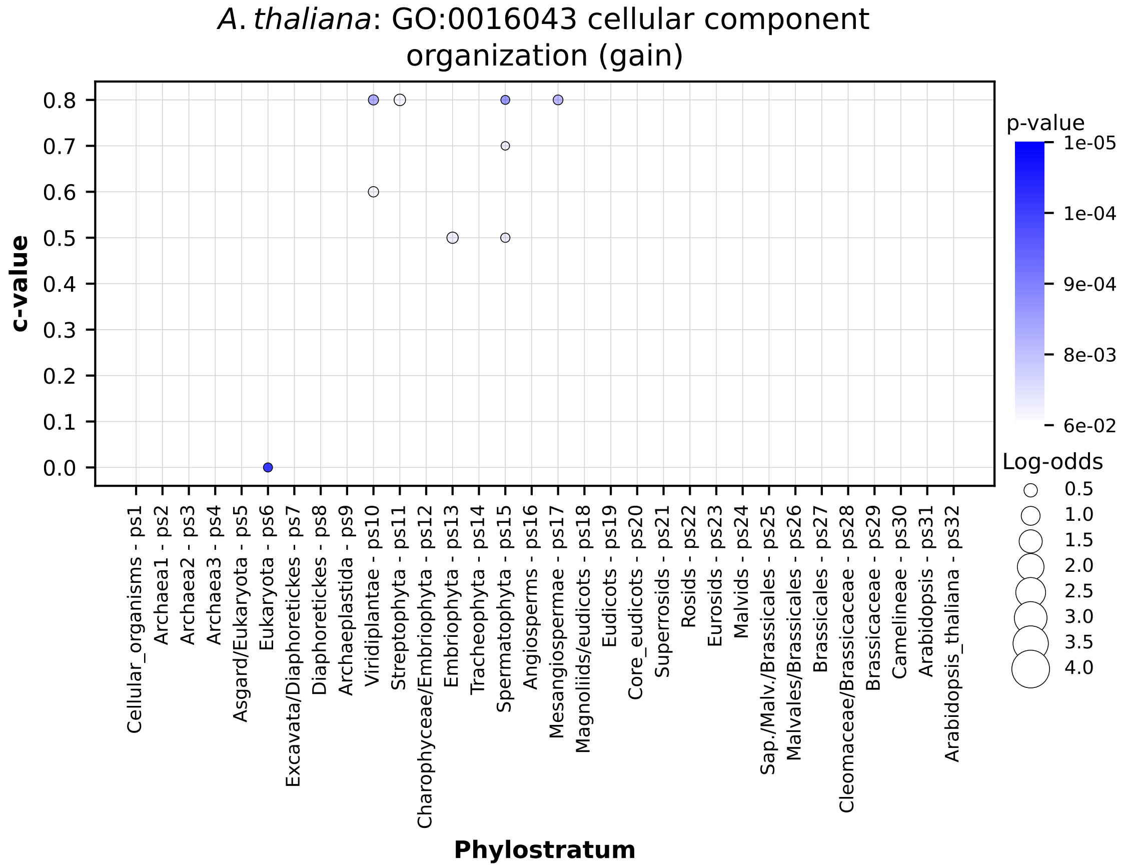
A. thaliana: GO:0016043 cellular component organization (gain)
c-value
0.8
0.7
0.6
0.5
0.4
0.3
0.2
0.1
0.0
Phylostratum
Cellular_organisms - ps1
Archaea1 - ps2
Archaea2 - ps3
Archaea3 - ps4
Asgard/Eukaryota - ps5
Eukaryota - ps6
Excavata/Diaphoretickes - ps7
Diaphoretickes - ps8
Archaeplastida - ps9
Viridiplantae - ps10
Streptophyta - ps11
Charophyceae/Embriophyta - ps12
Embriophyta - ps13
Tracheophyta - ps14
Spermatophyta - ps15
Angiosperms - ps16
Mesangiospermae - ps17
Magnoliids/eudicots - ps18
Eudicots - ps19
Core_eudicots - ps20
Superrosids - ps21
Rosids - ps22
Eurosids - ps23
Malvids - ps24
Sap./Malv./Brassicales - ps25
Malvales/Brassicales - ps26
Brassicales - ps27
Cleomaceae/Brassicaceae - ps28
Brassicaceae - ps29
Camelineae - ps30
Arabidopsis - ps31
Arabidopsis_thaliana - ps32
p-value
1e-05
1e-04
9e-04
8e-03
6e-02
Log-odds
0.5
1.0
1.5
2.0
2.5
3.0
3.5
4.0

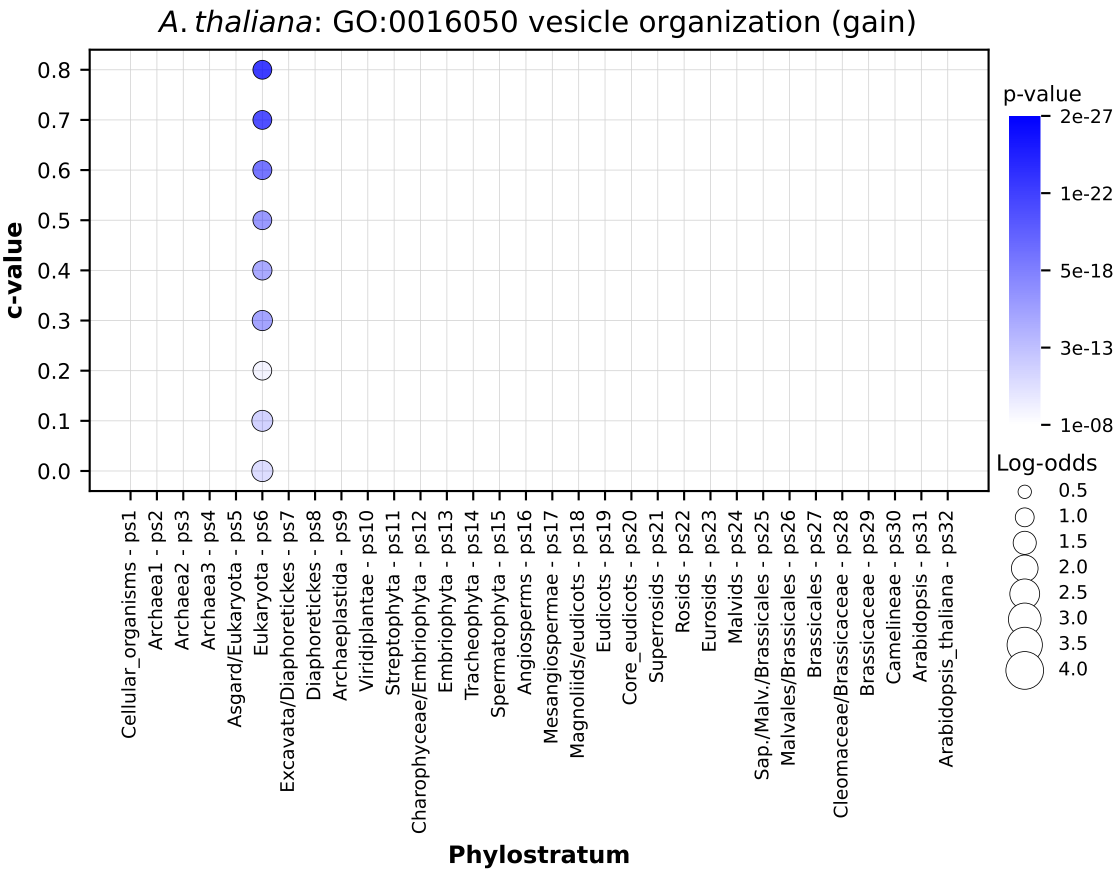

A. thaliana: GO:0016050 vesicle organization (gain)
c-value
0.8
0.7
0.6
0.5
0.4
0.3
0.2
0.1
0.0
p-value
2e-27
1e-22
5e-18
3e-13
1e-08
Log-odds
0.5
1.0
1.5
2.0
2.5
3.0
3.5
4.0
Cellular_organisms - ps1
Archaea1 - ps2
Archaea2 - ps3
Archaea3 - ps4
Asgard/Eukaryota - ps5
Eukaryota - ps6
Excavata/Diaphoretickes - ps7
Diaphoretickes - ps8
Archaeplastida - ps9
Viridiplantae - ps10
Streptophyta - ps11
Charophyceae/Embriophyta - ps12
Embriophyta - ps13
Tracheophyta - ps14
Spermatophyta - ps15
Angiosperms - ps16
Mesangiospermae - ps17
Magnoliids/eudicots - ps18
Eudicots - ps19
Core_eudicots - ps20
Superrosids - ps21
Rosids - ps22
Eurosids - ps23
Malvids - ps24
Sap./Malv./Brassicales - ps25
Malvales/Brassicales - ps26
Brassicales - ps27
Cleomaceae/Brassicaceae - ps28
Brassicaceae - ps29
Camelineae - ps30
Arabidopsis - ps31
Arabidopsis_thaliana - ps32
Phylostratum

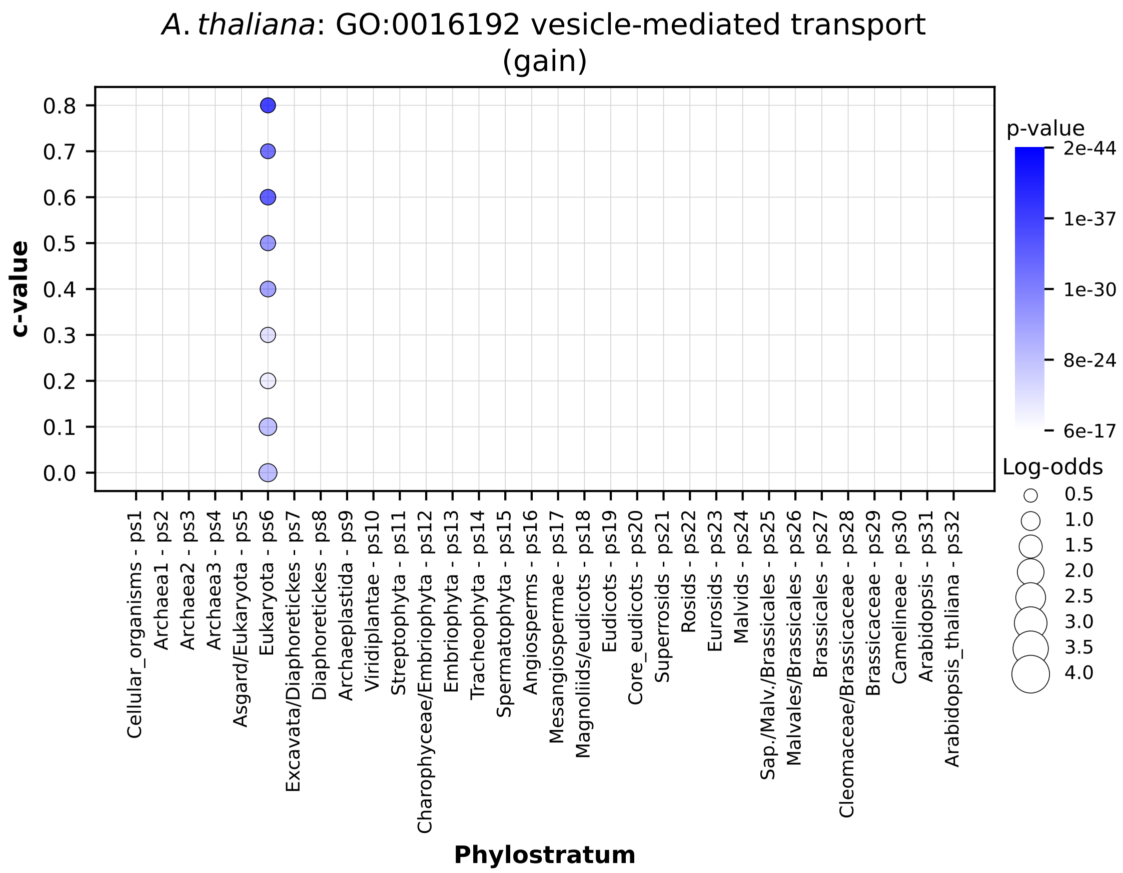

A. thaliana: GO:0016192 vesicle-mediated transport (gain)
c-value
0.8
0.7
0.6
0.5
0.4
0.3
0.2
0.1
0.0
p-value
2e-44
1e-37
1e-30
8e-24
6e-17
Log-odds
0.5
1.0
1.5
2.0
2.5
3.0
3.5
4.0
Cellular_organisms - ps1
Archaea1 - ps2
Archaea2 - ps3
Archaea3 - ps4
Asgard/Eukaryota - ps5
Eukaryota - ps6
Excavata/Diaphoretickes - ps7
Diaphoretickes - ps8
Archaeplastida - ps9
Viridiplantae - ps10
Streptophyta - ps11
Charophyceae/Embriophyta - ps12
Embriophyta - ps13
Tracheophyta - ps14
Spermatophyta - ps15
Angiosperms - ps16
Mesangiospermae - ps17
Magnoliids/eudicots - ps18
Eudicots - ps19
Core_eudicots - ps20
Superrosids - ps21
Rosids - ps22
Eurosids - ps23
Malvids - ps24
Sap./Malv./Brassicales - ps25
Malvales/Brassicales - ps26
Brassicales - ps27
Cleomaceae/Brassicaceae - ps28
Brassicaceae - ps29
Camelineae - ps30
Arabidopsis - ps31
Arabidopsis_thaliana - ps32
Phylostratum

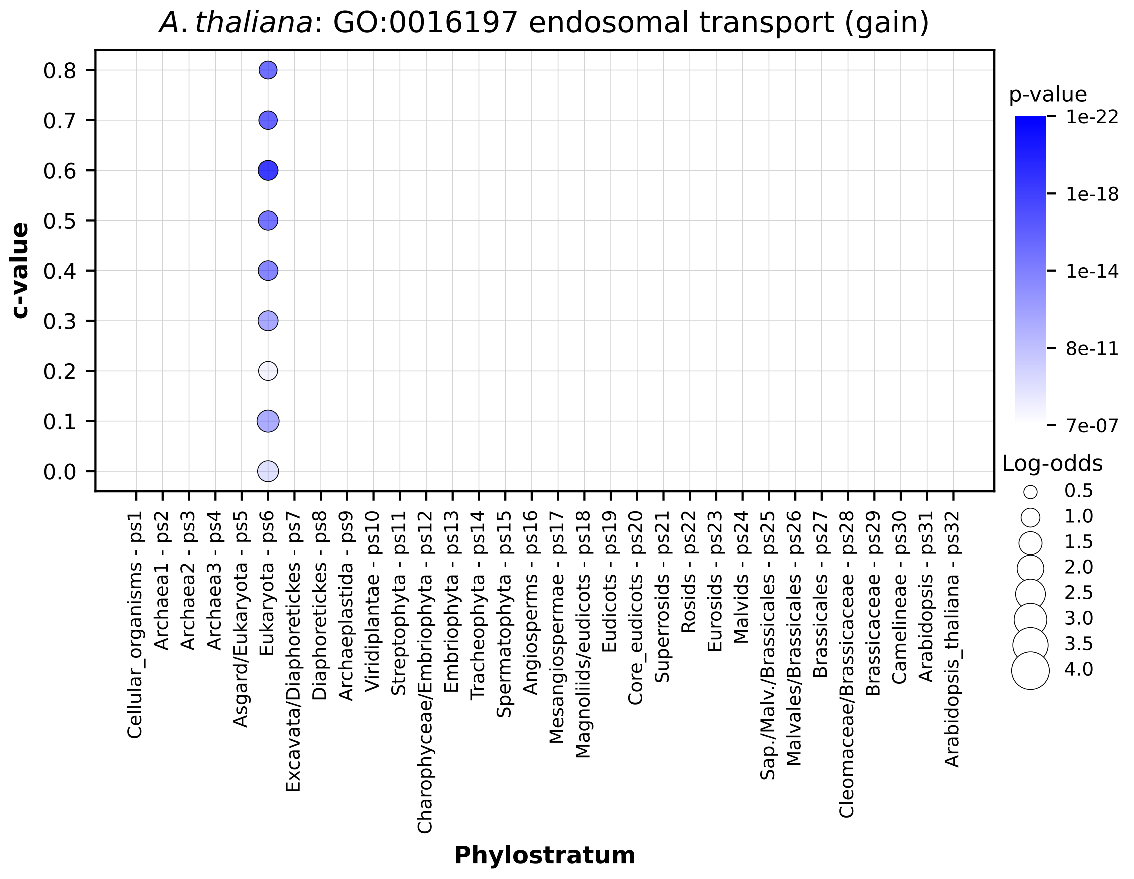

A. thaliana: GO:0016197 endosomal transport (gain)
c-value
0.8
0.7
0.6
0.5
0.4
0.3
0.2
0.1
0.0
p-value
1e-22
1e-18
1e-14
8e-11
7e-07
Log-odds
0.5
1.0
1.5
2.0
2.5
3.0
3.5
4.0
Cellular_organisms - ps1
Archaea1 - ps2
Archaea2 - ps3
Archaea3 - ps4
Asgard/Eukaryota - ps5
Eukaryota - ps6
Excavata/Diaphoretickes - ps7
Diaphoretickes - ps8
Archaeplastida - ps9
Viridiplantae - ps10
Streptophyta - ps11
Charophyceae/Embriophyta - ps12
Embriophyta - ps13
Tracheophyta - ps14
Spermatophyta - ps15
Angiosperms - ps16
Mesangiospermae - ps17
Magnoliids/eudicots - ps18
Eudicots - ps19
Core_eudicots - ps20
Superrosids - ps21
Rosids - ps22
Eurosids - ps23
Malvids - ps24
Sap./Malv./Brassicales - ps25
Malvales/Brassicales - ps26
Brassicales - ps27
Cleomaceae/Brassicaceae - ps28
Brassicaceae - ps29
Camelineae - ps30
Arabidopsis - ps31
Arabidopsis_thaliana - ps32
Phylostratum

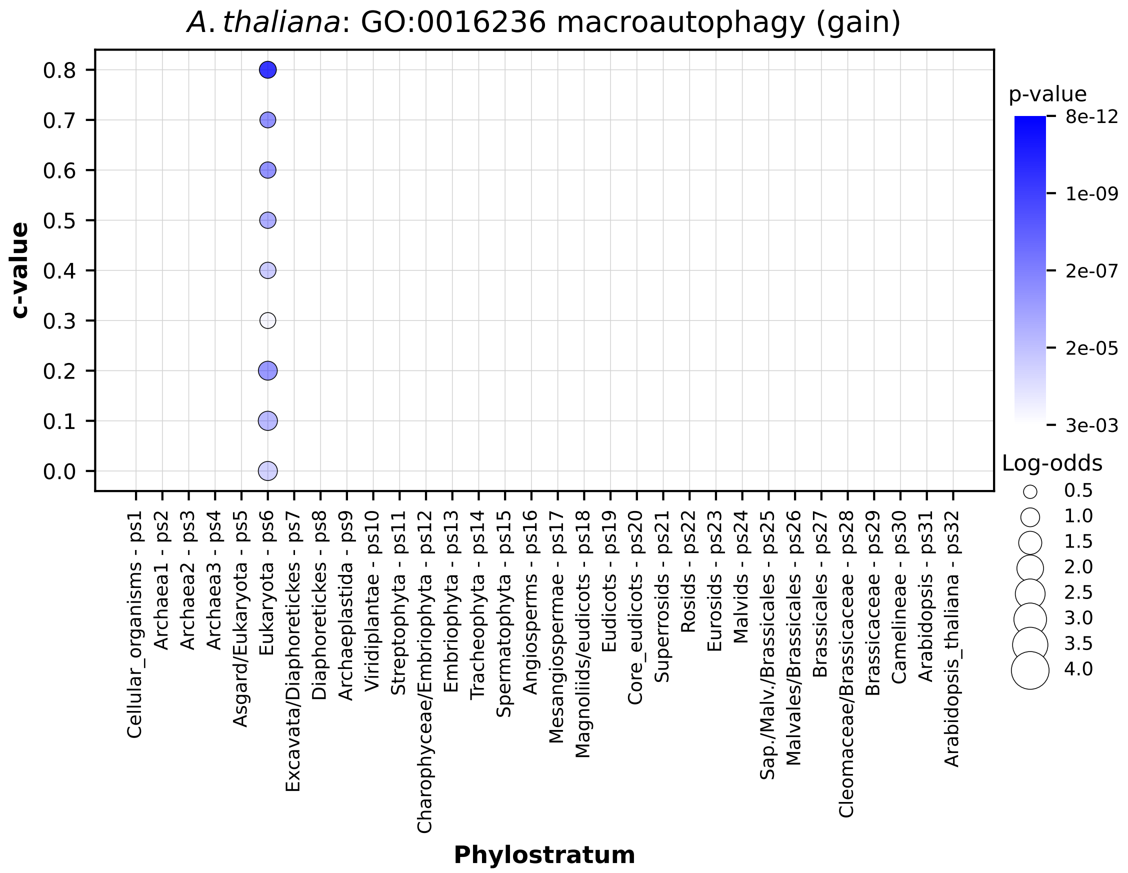

A. thaliana: GO:0016236 macroautophagy (gain)
c-value
0.8
0.7
0.6
0.5
0.4
0.3
0.2
0.1
0.0
p-value
8e-12
1e-09
2e-07
2e-05
3e-03
Log-odds
0.5
1.0
1.5
2.0
2.5
3.0
3.5
4.0
Cellular_organisms - ps1
Archaea1 - ps2
Archaea2 - ps3
Archaea3 - ps4
Asgard/Eukaryota - ps5
Eukaryota - ps6
Excavata/Diaphoretickes - ps7
Diaphoretickes - ps8
Archaeplastida - ps9
Viridiplantae - ps10
Streptophyta - ps11
Charophyceae/Embriophyta - ps12
Embriophyta - ps13
Tracheophyta - ps14
Spermatophyta - ps15
Angiosperms - ps16
Mesangiospermae - ps17
Magnoliids/eudicots - ps18
Eudicots - ps19
Core_eudicots - ps20
Superrosids - ps21
Rosids - ps22
Eurosids - ps23
Malvids - ps24
Sap./Malv./Brassicales - ps25
Malvales/Brassicales - ps26
Brassicales - ps27
Cleomaceae/Brassicaceae - ps28
Brassicaceae - ps29
Camelineae - ps30
Arabidopsis - ps31
Arabidopsis_thaliana - ps32
Phylostratum

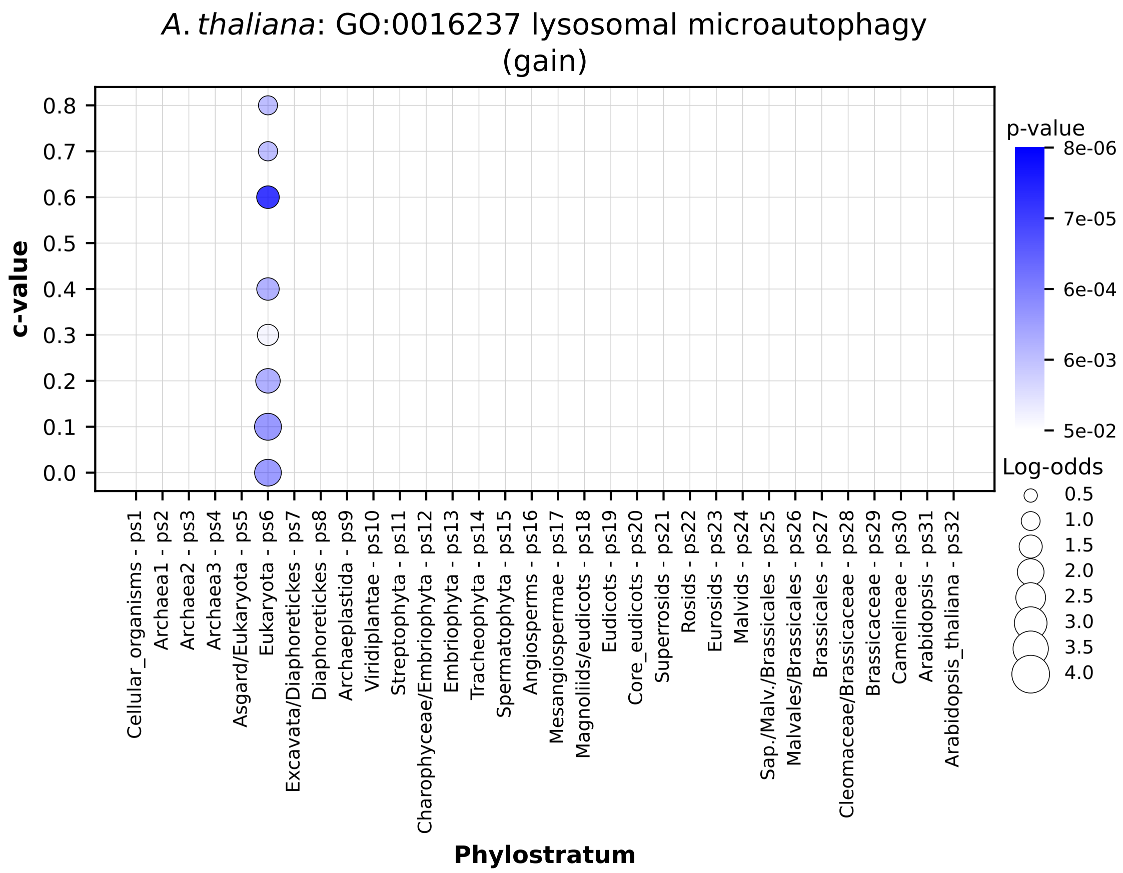

A. thaliana: GO:0016237 lysosomal microautophagy
(gain)
c-value
0.8
0.7
0.6
0.5
0.4
0.3
0.2
0.1
0.0
p-value
8e-06
7e-05
6e-04
6e-03
5e-02
Log-odds
0.5
1.0
1.5
2.0
2.5
3.0
3.5
4.0
Cellular_organisms - ps1
Archaea1 - ps2
Archaea2 - ps3
Archaea3 - ps4
Asgard/Eukaryota - ps5
Eukaryota - ps6
Excavata/Diaphoretickes - ps7
Diaphoretickes - ps8
Archaeplastida - ps9
Viridiplantae - ps10
Streptophyta - ps11
Charophyceae/Embriophyta - ps12
Embriophyta - ps13
Tracheophyta - ps14
Spermatophyta - ps15
Angiosperms - ps16
Mesangiospermae - ps17
Magnoliids/eudicots - ps18
Eudicots - ps19
Core_eudicots - ps20
Superrosids - ps21
Rosids - ps22
Eurosids - ps23
Malvids - ps24
Sap./Malv./Brassicales - ps25
Malvales/Brassicales - ps26
Brassicales - ps27
Cleomaceae/Brassicaceae - ps28
Brassicaceae - ps29
Camelineae - ps30
Arabidopsis - ps31
Arabidopsis_thaliana - ps32
Phylostratum

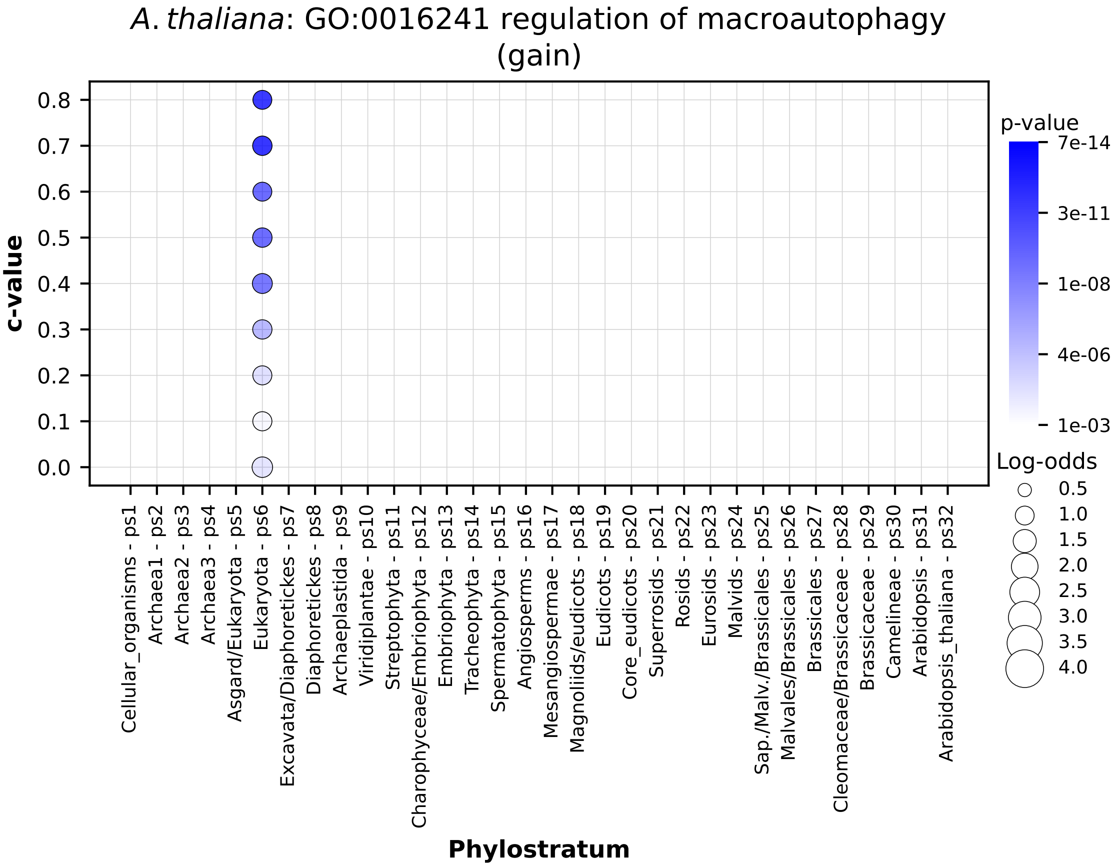

A. thaliana: GO:0016241 regulation of macroautophagy
(gain)
c-value
0.8
0.7
0.6
0.5
0.4
0.3
0.2
0.1
0.0
p-value
7e-14
3e-11
1e-08
4e-06
1e-03
Log-odds
0.5
1.0
1.5
2.0
2.5
3.0
3.5
4.0
Cellular_organisms - ps1
Archaea1 - ps2
Archaea2 - ps3
Archaea3 - ps4
Asgard/Eukaryota - ps5
Eukaryota - ps6
Excavata/Diaphoretickes - ps7
Diaphoretickes - ps8
Archaeplastida - ps9
Viridiplantae - ps10
Streptophyta - ps11
Charophyceae/Embriophyta - ps12
Embriophyta - ps13
Tracheophyta - ps14
Spermatophyta - ps15
Angiosperms - ps16
Mesangiospermae - ps17
Magnoliids/eudicots - ps18
Eudicots - ps19
Core_eudicots - ps20
Superrosids - ps21
Rosids - ps22
Eurosids - ps23
Malvids - ps24
Sap./Malv./Brassicales - ps25
Malvales/Brassicales - ps26
Brassicales - ps27
Cleomaceae/Brassicaceae - ps28
Brassicaceae - ps29
Camelineae - ps30
Arabidopsis - ps31
Arabidopsis_thaliana - ps32
Phylostratum

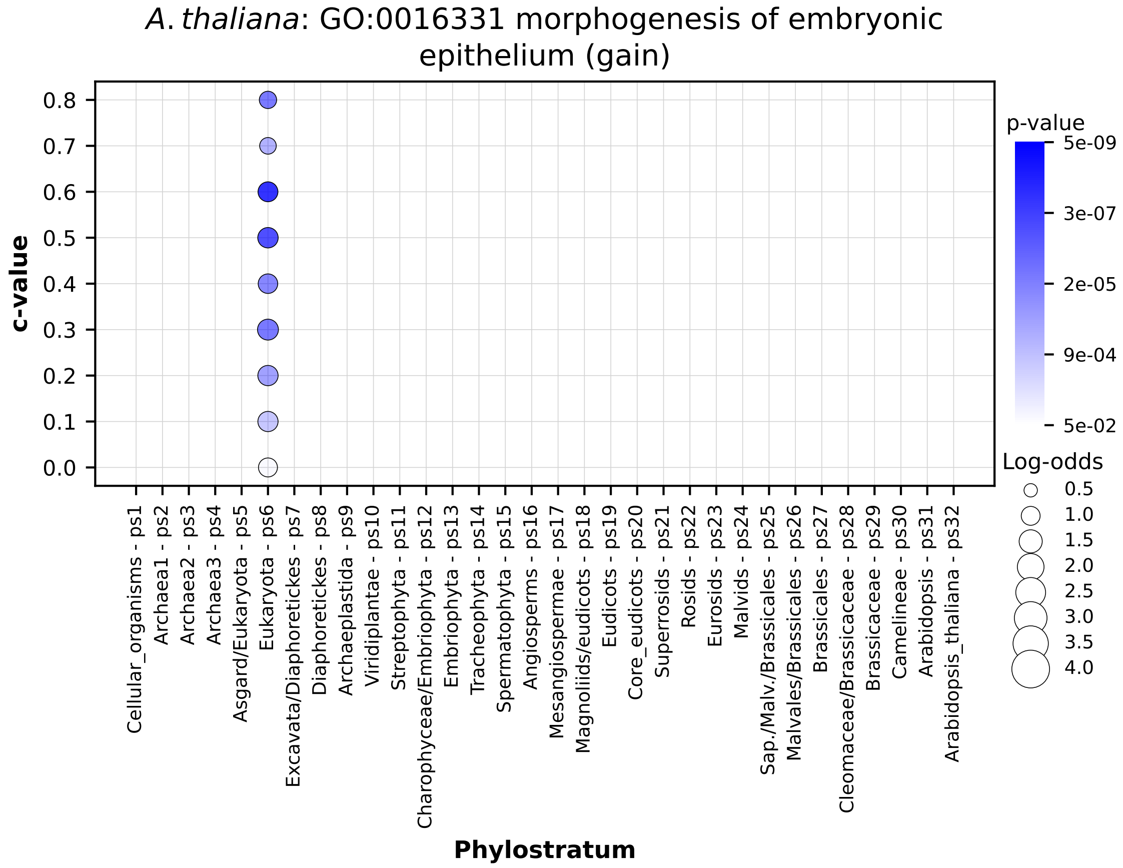

A. thaliana: GO:0016331 morphogenesis of embryonic epithelium (gain)
c-value
0.8
0.7
0.6
0.5
0.4
0.3
0.2
0.1
0.0
p-value
5e-09
3e-07
2e-05
9e-04
5e-02
Log-odds
0.5
1.0
1.5
2.0
2.5
3.0
3.5
4.0
Cellular_organisms - ps1
Archaea1 - ps2
Archaea2 - ps3
Archaea3 - ps4
Asgard/Eukaryota - ps5
Eukaryota - ps6
Excavata/Diaphoretickes - ps7
Diaphoretickes - ps8
Archaeplastida - ps9
Viridiplantae - ps10
Streptophyta - ps11
Charophyceae/Embriophyta - ps12
Embriophyta - ps13
Tracheophyta - ps14
Spermatophyta - ps15
Angiosperms - ps16
Mesangiospermae - ps17
Magnoliids/eudicots - ps18
Eudicots - ps19
Core_eudicots - ps20
Superrosids - ps21
Rosids - ps22
Eurosids - ps23
Malvids - ps24
Sap./Malv./Brassicales - ps25
Malvales/Brassicales - ps26
Brassicales - ps27
Cleomaceae/Brassicaceae - ps28
Brassicaceae - ps29
Camelineae - ps30
Arabidopsis - ps31
Arabidopsis_thaliana - ps32
Phylostratum

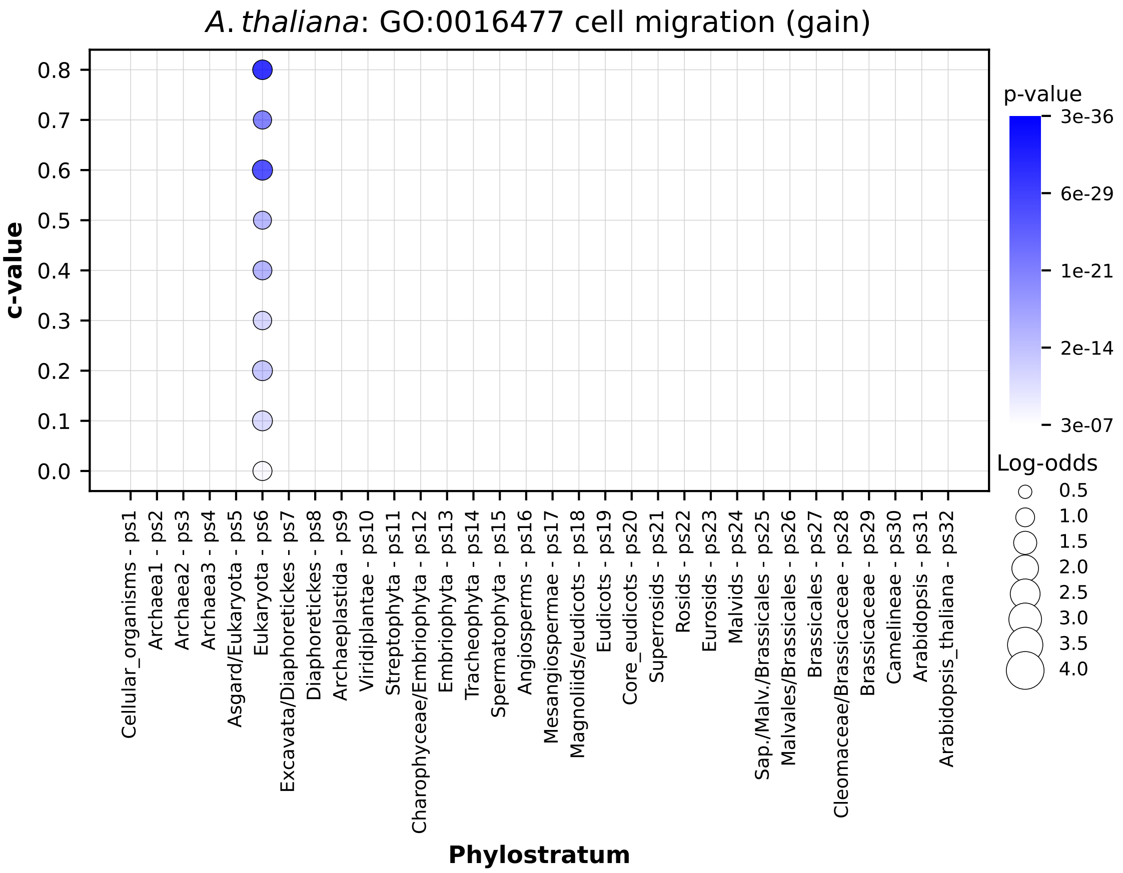

A. thaliana: GO:0016477 cell migration (gain)
c-value
0.8
0.7
0.6
0.5
0.4
0.3
0.2
0.1
0.0
Phylostratum
Cellular_organisms - ps1
Archaea1 - ps2
Archaea2 - ps3
Archaea3 - ps4
Asgard/Eukaryota - ps5
Eukaryota - ps6
Excavata/Diaphoretickes - ps7
Diaphoretickes - ps8
Archaeplastida - ps9
Viridiplantae - ps10
Streptophyta - ps11
Charophyceae/Embriophyta - ps12
Embriophyta - ps13
Tracheophyta - ps14
Spermatophyta - ps15
Angiosperms - ps16
Mesangiospermae - ps17
Magnoliids/eudicots - ps18
Eudicots - ps19
Core_eudicots - ps20
Superrosids - ps21
Rosids - ps22
Eurosids - ps23
Malvids - ps24
Sap./Malv./Brassicales - ps25
Malvales/Brassicales - ps26
Brassicales - ps27
Cleomaceae/Brassicaceae - ps28
Brassicaceae - ps29
Camelineae - ps30
Arabidopsis - ps31
Arabidopsis_thaliana - ps32
p-value
3e-36
6e-29
1e-21
2e-14
3e-07
Log-odds
0.5
1.0
1.5
2.0
2.5
3.0
3.5
4.0

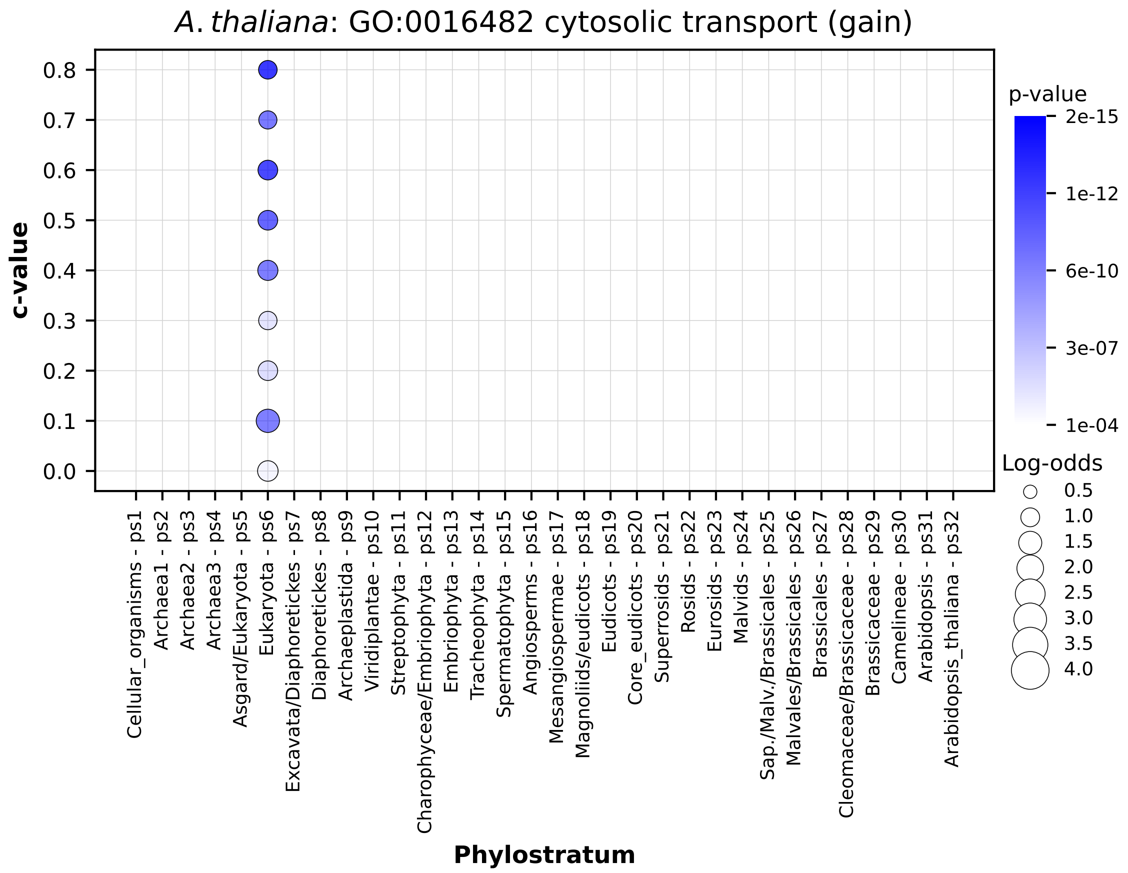
A. thaliana: GO:0016482 cytosolic transport (gain)
c-value
0.0
0.1
0.2
0.3
0.4
0.5
0.6
0.7
0.8
p-value
2e-15
1e-12
6e-10
3e-07
1e-04
Log-odds
0.5
1.0
1.5
2.0
2.5
3.0
3.5
4.0
Cellular_organisms - ps1
Archaea1 - ps2
Archaea2 - ps3
Archaea3 - ps4
Asgard/Eukaryota - ps5
Eukaryota - ps6
Excavata/Diaphoretickes - ps7
Diaphoretickes - ps8
Archaeplastida - ps9
Viridiplantae - ps10
Streptophyta - ps11
Charophyceae/Embriophyta - ps12
Embriophyta - ps13
Tracheophyta - ps14
Spermatophyta - ps15
Angiosperms - ps16
Mesangiospermae - ps17
Magnoliids/eudicots - ps18
Eudicots - ps19
Core_eudicots - ps20
Superrosids - ps21
Rosids - ps22
Eurosids - ps23
Malvids - ps24
Sap./Malv./Brassicales - ps25
Malvales/Brassicales - ps26
Brassicales - ps27
Cleomaceae/Brassicaceae - ps28
Brassicaceae - ps29
Camelineae - ps30
Arabidopsis - ps31
Arabidopsis_thaliana - ps32
Phylostratum

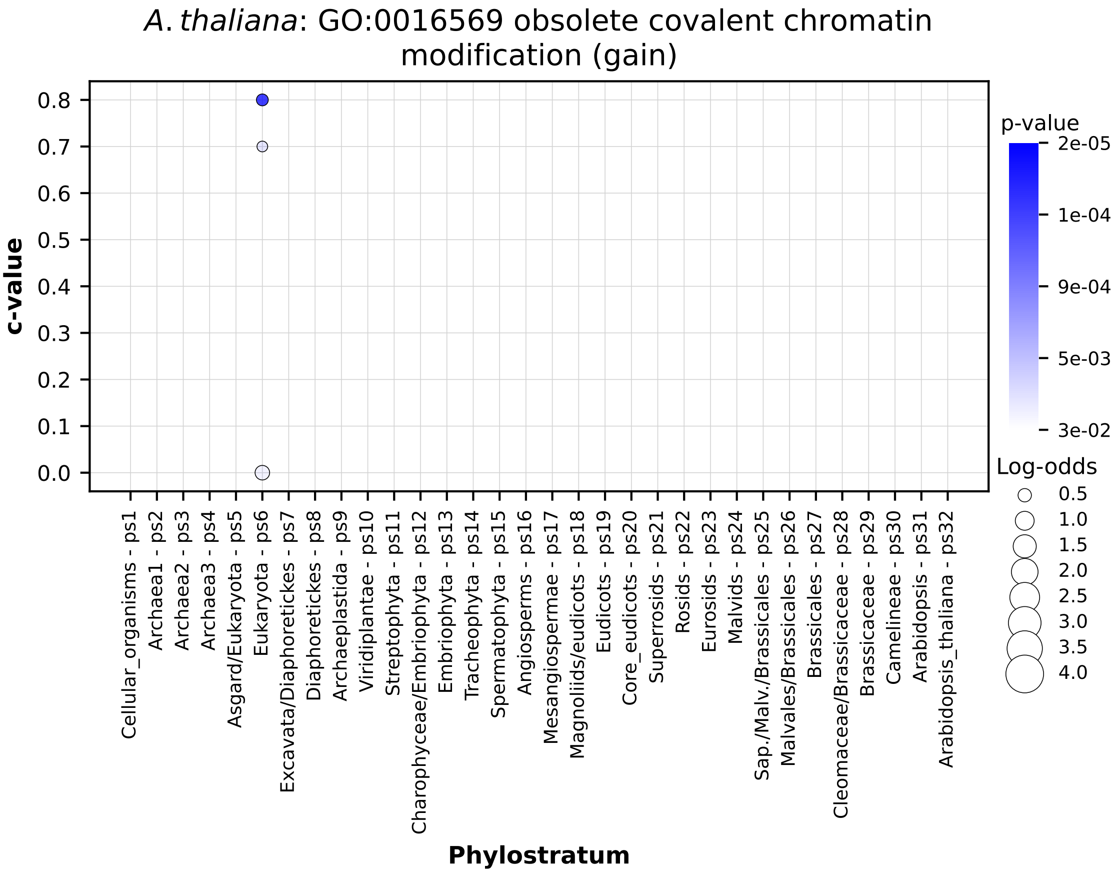
A. thaliana: GO:0016569 obsolete covalent chromatin modification (gain)
c-value
0.8
0.7
0.6
0.5
0.4
0.3
0.2
0.1
0.0
p-value
2e-05
1e-04
9e-04
5e-03
3e-02
Log-odds
0.5
1.0
1.5
2.0
2.5
3.0
3.5
4.0
Cellular_organisms - ps1
Archaea1 - ps2
Archaea2 - ps3
Archaea3 - ps4
Asgard/Eukaryota - ps5
Eukaryota - ps6
Excavata/Diaphoretickes - ps7
Diaphoretickes - ps8
Archaeplastida - ps9
Viridiplantae - ps10
Streptophyta - ps11
Charophyceae/Embriophyta - ps12
Embriophyta - ps13
Tracheophyta - ps14
Spermatophyta - ps15
Angiosperms - ps16
Mesangiospermae - ps17
Magnoliids/eudicots - ps18
Eudicots - ps19
Core_eudicots - ps20
Superrosids - ps21
Rosids - ps22
Eurosids - ps23
Malvids - ps24
Sap./Malv./Brassicales - ps25
Malvales/Brassicales - ps26
Brassicales - ps27
Cleomaceae/Brassicaceae - ps28
Brassicaceae - ps29
Camelineae - ps30
Arabidopsis - ps31
Arabidopsis_thaliana - ps32
Phylostratum

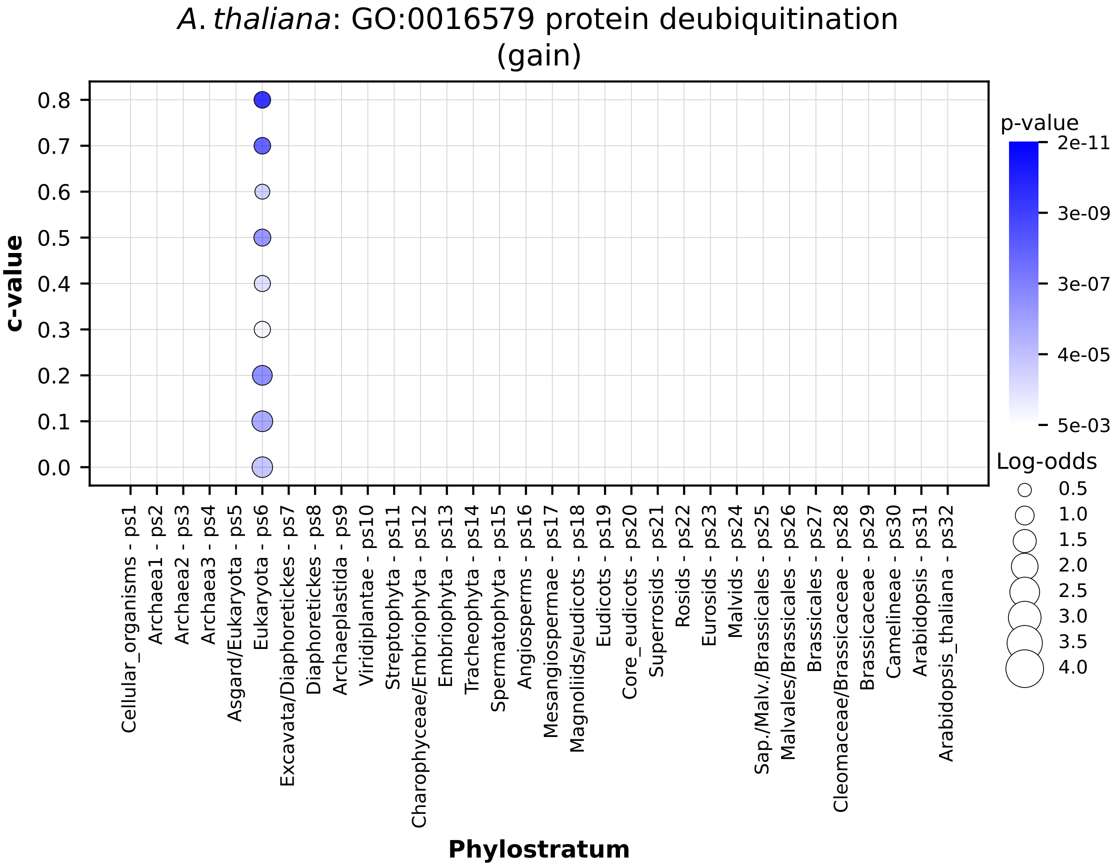

A. thaliana: GO:0016579 protein deubiquitination
(gain)
c-value
0.8
0.7
0.6
0.5
0.4
0.3
0.2
0.1
0.0
p-value
2e-11
3e-09
3e-07
4e-05
5e-03
Log-odds
0.5
1.0
1.5
2.0
2.5
3.0
3.5
4.0
Cellular_organisms - ps1
Archaea1 - ps2
Archaea2 - ps3
Archaea3 - ps4
Asgard/Eukaryota - ps5
Eukaryota - ps6
Excavata/Diaphoretickes - ps7
Diaphoretickes - ps8
Archaeplastida - ps9
Viridiplantae - ps10
Streptophyta - ps11
Charophyceae/Embriophyta - ps12
Embriophyta - ps13
Tracheophyta - ps14
Spermatophyta - ps15
Angiosperms - ps16
Mesangiospermae - ps17
Magnoliids/eudicots - ps18
Eudicots - ps19
Core_eudicots - ps20
Superrosids - ps21
Rosids - ps22
Eurosids - ps23
Malvids - ps24
Sap./Malv./Brassicales - ps25
Malvales/Brassicales - ps26
Brassicales - ps27
Cleomaceae/Brassicaceae - ps28
Brassicaceae - ps29
Camelineae - ps30
Arabidopsis - ps31
Arabidopsis_thaliana - ps32
Phylostratum

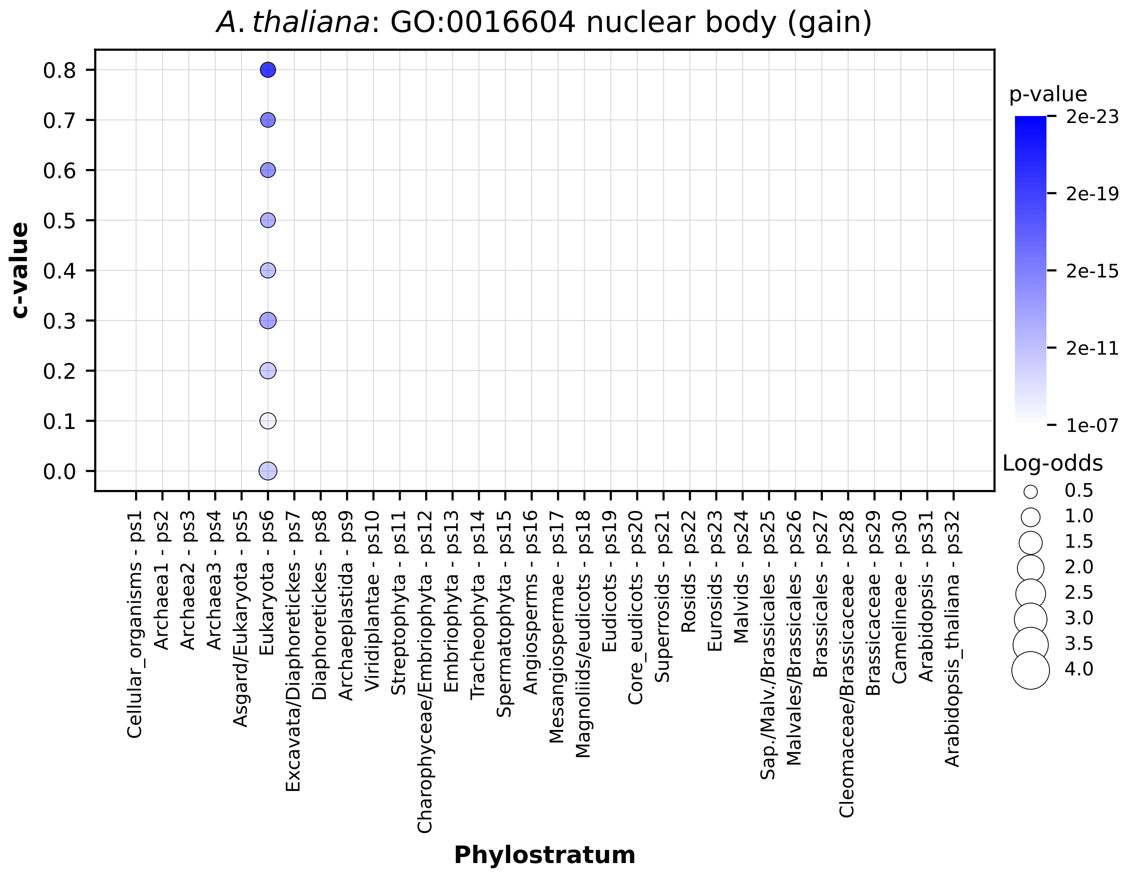
A. thaliana: GO:0016604 nuclear body (gain)
c-value
0.8
0.7
0.6
0.5
0.4
0.3
0.2
0.1
0.0
p-value
2e-23
2e-19
2e-15
2e-11
1e-07
Log-odds
0.5
1.0
1.5
2.0
2.5
3.0
3.5
4.0
Cellular_organisms - ps1
Archaea1 - ps2
Archaea2 - ps3
Archaea3 - ps4
Asgard/Eukaryota - ps5
Eukaryota - ps6
Excavata/Diaphoretickes - ps7
Diaphoretickes - ps8
Archaeplastida - ps9
Viridiplantae - ps10
Streptophyta - ps11
Charophyceae/Embriophyta - ps12
Embriophyta - ps13
Tracheophyta - ps14
Spermatophyta - ps15
Angiosperms - ps16
Mesangiospermae - ps17
Magnoliids/eudicots - ps18
Eudicots - ps19
Core_eudicots - ps20
Superrosids - ps21
Rosids - ps22
Eurosids - ps23
Malvids - ps24
Sap./Malv./Brassicales - ps25
Malvales/Brassicales - ps26
Brassicales - ps27
Cleomaceae/Brassicaceae - ps28
Brassicaceae - ps29
Camelineae - ps30
Arabidopsis - ps31
Arabidopsis_thaliana - ps32
Phylostratum

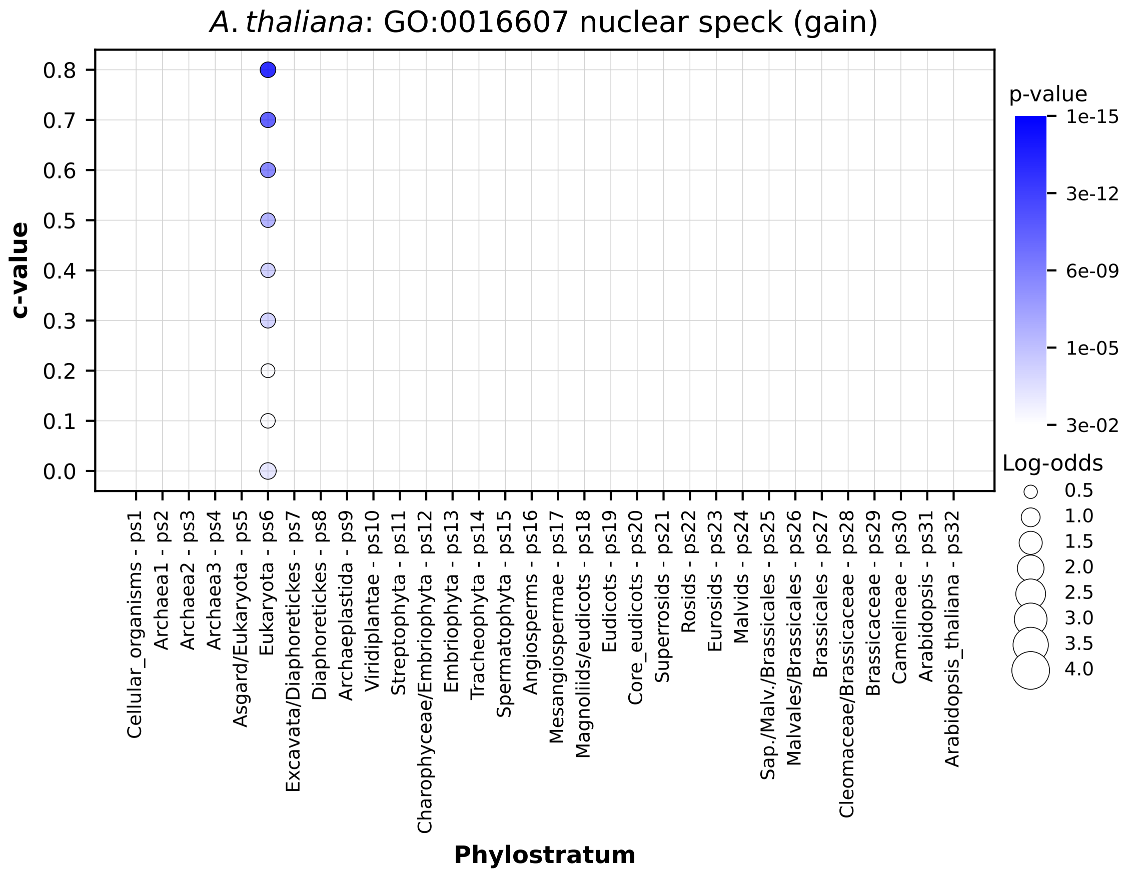
A. thaliana: GO:0016607 nuclear speck (gain)
c-value
0.8
0.7
0.6
0.5
0.4
0.3
0.2
0.1
0.0
Phylostratum
Cellular_organisms - ps1
Archaea1 - ps2
Archaea2 - ps3
Archaea3 - ps4
Asgard/Eukaryota - ps5
Eukaryota - ps6
Excavata/Diaphoretickes - ps7
Diaphoretickes - ps8
Archaeplastida - ps9
Viridiplantae - ps10
Streptophyta - ps11
Charophyceae/Embriophyta - ps12
Embriophyta - ps13
Tracheophyta - ps14
Spermatophyta - ps15
Angiosperms - ps16
Mesangiospermae - ps17
Magnoliids/eudicots - ps18
Eudicots - ps19
Core_eudicots - ps20
Superrosids - ps21
Rosids - ps22
Eurosids - ps23
Malvids - ps24
Sap./Malv./Brassicales - ps25
Malvales/Brassicales - ps26
Brassicales - ps27
Cleomaceae/Brassicaceae - ps28
Brassicaceae - ps29
Camelineae - ps30
Arabidopsis - ps31
Arabidopsis_thaliana - ps32
p-value
1e-15
3e-12
6e-09
1e-05
3e-02
Log-odds
0.5
1.0
1.5
2.0
2.5
3.0
3.5
4.0

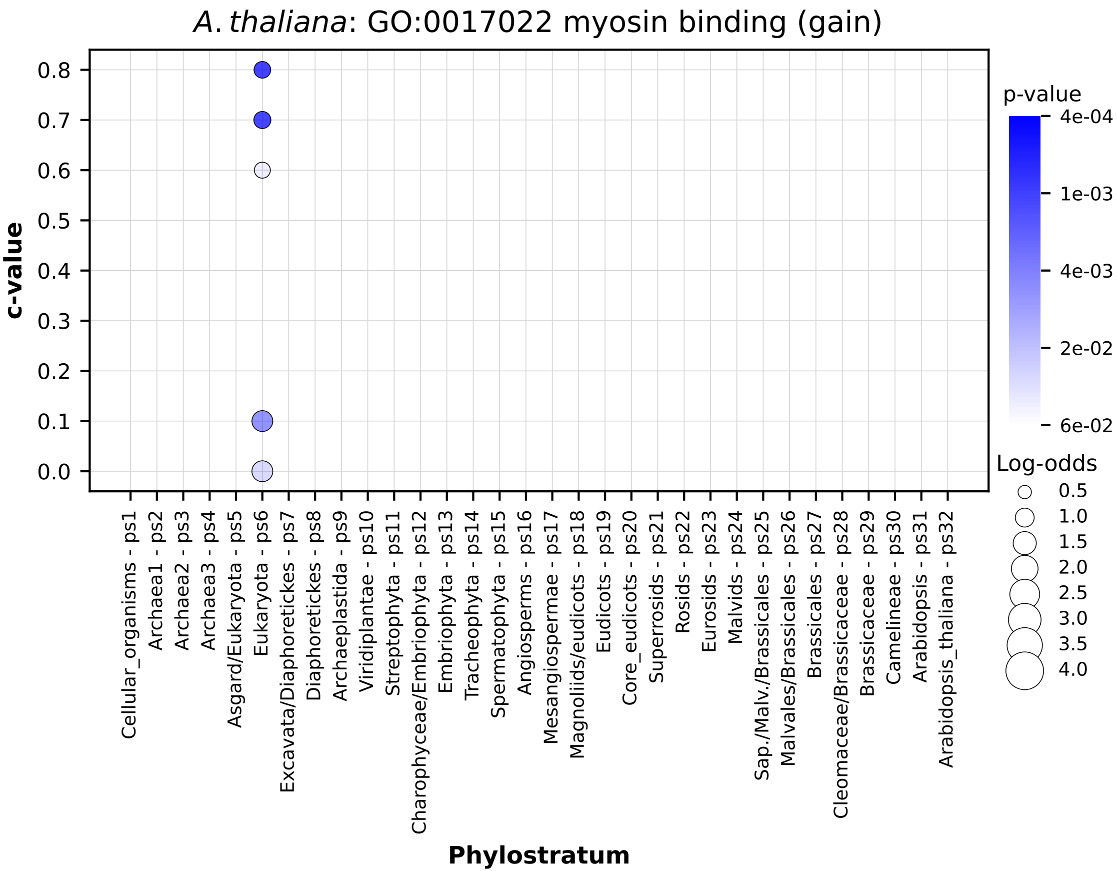

A. thaliana: GO:0017022 myosin binding (gain)
c-value
0.8
0.7
0.6
0.5
0.4
0.3
0.2
0.1
0.0
p-value
4e-04
1e-03
4e-03
2e-02
6e-02
Log-odds
0.5
1.0
1.5
2.0
2.5
3.0
3.5
4.0
Cellular_organisms - ps1
Archaea1 - ps2
Archaea2 - ps3
Archaea3 - ps4
Asgard/Eukaryota - ps5
Eukaryota - ps6
Excavata/Diaphoretickes - ps7
Diaphoretickes - ps8
Archaeplastida - ps9
Viridiplantae - ps10
Streptophyta - ps11
Charophyceae/Embriophyta - ps12
Embriophyta - ps13
Tracheophyta - ps14
Spermatophyta - ps15
Angiosperms - ps16
Mesangiospermae - ps17
Magnoliids/eudicots - ps18
Eudicots - ps19
Core_eudicots - ps20
Superrosids - ps21
Rosids - ps22
Eurosids - ps23
Malvids - ps24
Sap./Malv./Brassicales - ps25
Malvales/Brassicales - ps26
Brassicales - ps27
Cleomaceae/Brassicaceae - ps28
Brassicaceae - ps29
Camelineae - ps30
Arabidopsis - ps31
Arabidopsis_thaliana - ps32
Phylostratum

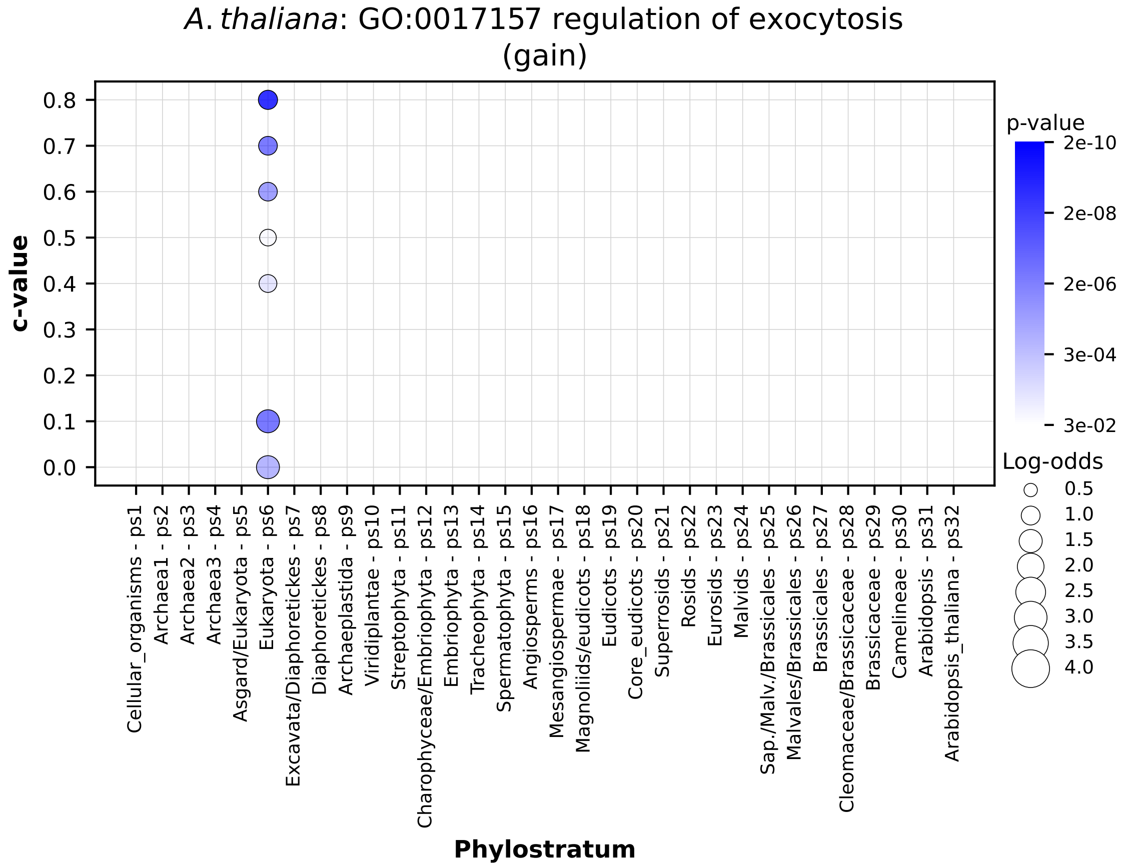

A. thaliana: GO:0017157 regulation of exocytosis (gain)
c-value
0.0
0.1
0.2
0.3
0.4
0.5
0.6
0.7
0.8
Phylostratum
Cellular_organisms - ps1
Archaea1 - ps2
Archaea2 - ps3
Archaea3 - ps4
Asgard/Eukaryota - ps5
Eukaryota - ps6
Excavata/Diaphoretickes - ps7
Diaphoretickes - ps8
Archaeplastida - ps9
Viridiplantae - ps10
Streptophyta - ps11
Charophyceae/Embriophyta - ps12
Embriophyta - ps13
Tracheophyta - ps14
Spermatophyta - ps15
Angiosperms - ps16
Mesangiospermae - ps17
Magnoliids/eudicots - ps18
Eudicots - ps19
Core_eudicots - ps20
Superrosids - ps21
Rosids - ps22
Eurosids - ps23
Malvids - ps24
Sap./Malv./Brassicales - ps25
Malvales/Brassicales - ps26
Brassicales - ps27
Cleomaceae/Brassicaceae - ps28
Brassicaceae - ps29
Camelineae - ps30
Arabidopsis - ps31
Arabidopsis_thaliana - ps32
p-value
2e-10
2e-08
2e-06
3e-04
3e-02
Log-odds
0.5
1.0
1.5
2.0
2.5
3.0
3.5
4.0

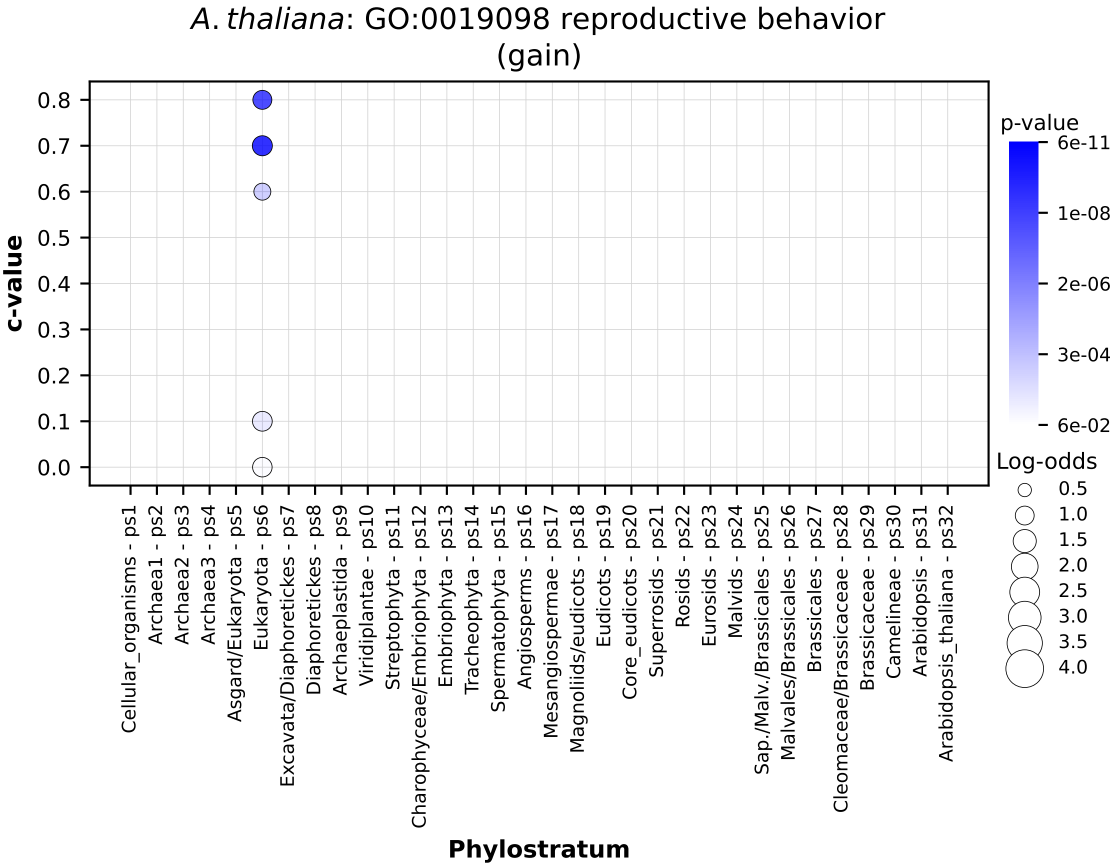

A. thaliana: GO:0019098 reproductive behavior
(gain)
c-value
0.0
0.1
0.2
0.3
0.4
0.5
0.6
0.7
0.8
p-value
6e-11
1e-08
2e-06
3e-04
6e-02
Log-odds
0.5
1.0
1.5
2.0
2.5
3.0
3.5
4.0
Cellular_organisms - ps1
Archaea1 - ps2
Archaea2 - ps3
Archaea3 - ps4
Asgard/Eukaryota - ps5
Eukaryota - ps6
Excavata/Diaphoretickes - ps7
Diaphoretickes - ps8
Archaeplastida - ps9
Viridiplantae - ps10
Streptophyta - ps11
Charophyceae/Embriophyta - ps12
Embriophyta - ps13
Tracheophyta - ps14
Spermatophyta - ps15
Angiosperms - ps16
Mesangiospermae - ps17
Magnoliids/eudicots - ps18
Eudicots - ps19
Core_eudicots - ps20
Superrosids - ps21
Rosids - ps22
Eurosids - ps23
Malvids - ps24
Sap./Malv./Brassicales - ps25
Malvales/Brassicales - ps26
Brassicales - ps27
Cleomaceae/Brassicaceae - ps28
Brassicaceae - ps29
Camelineae - ps30
Arabidopsis - ps31
Arabidopsis_thaliana - ps32
Phylostratum

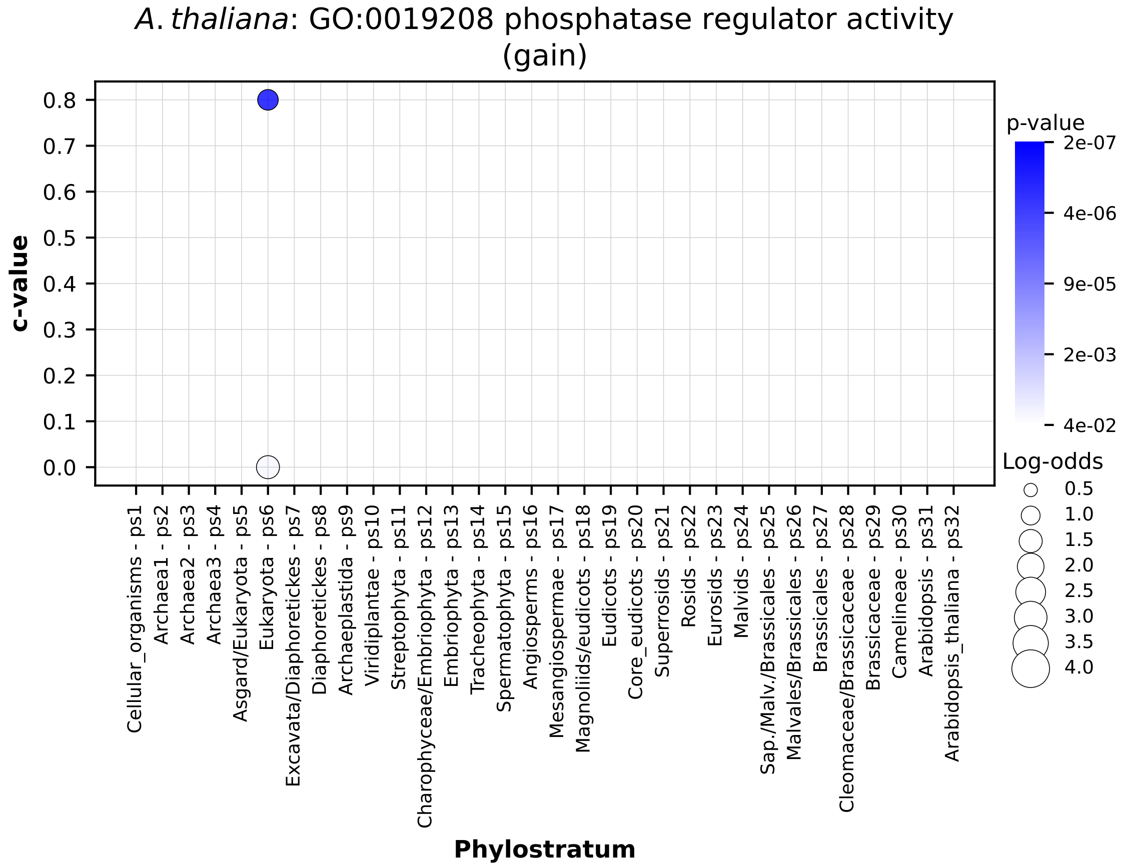
A. thaliana: GO:0019208 phosphatase regulator activity (gain)
c-value
0.0
0.1
0.2
0.3
0.4
0.5
0.6
0.7
0.8
p-value
2e-07
4e-06
9e-05
2e-03
4e-02
Log-odds
0.5
1.0
1.5
2.0
2.5
3.0
3.5
4.0
Cellular_organisms - ps1
Archaea1 - ps2
Archaea2 - ps3
Archaea3 - ps4
Asgard/Eukaryota - ps5
Eukaryota - ps6
Excavata/Diaphoretickes - ps7
Diaphoretickes - ps8
Archaeplastida - ps9
Viridiplantae - ps10
Streptophyta - ps11
Charophyceae/Embriophyta - ps12
Embriophyta - ps13
Tracheophyta - ps14
Spermatophyta - ps15
Angiosperms - ps16
Mesangiospermae - ps17
Magnoliids/eudicots - ps18
Eudicots - ps19
Core_eudicots - ps20
Superrosids - ps21
Rosids - ps22
Eurosids - ps23
Malvids - ps24
Sap./Malv./Brassicales - ps25
Malvales/Brassicales - ps26
Brassicales - ps27
Cleomaceae/Brassicaceae - ps28
Brassicaceae - ps29
Camelineae - ps30
Arabidopsis - ps31
Arabidopsis_thaliana - ps32
Phylostratum

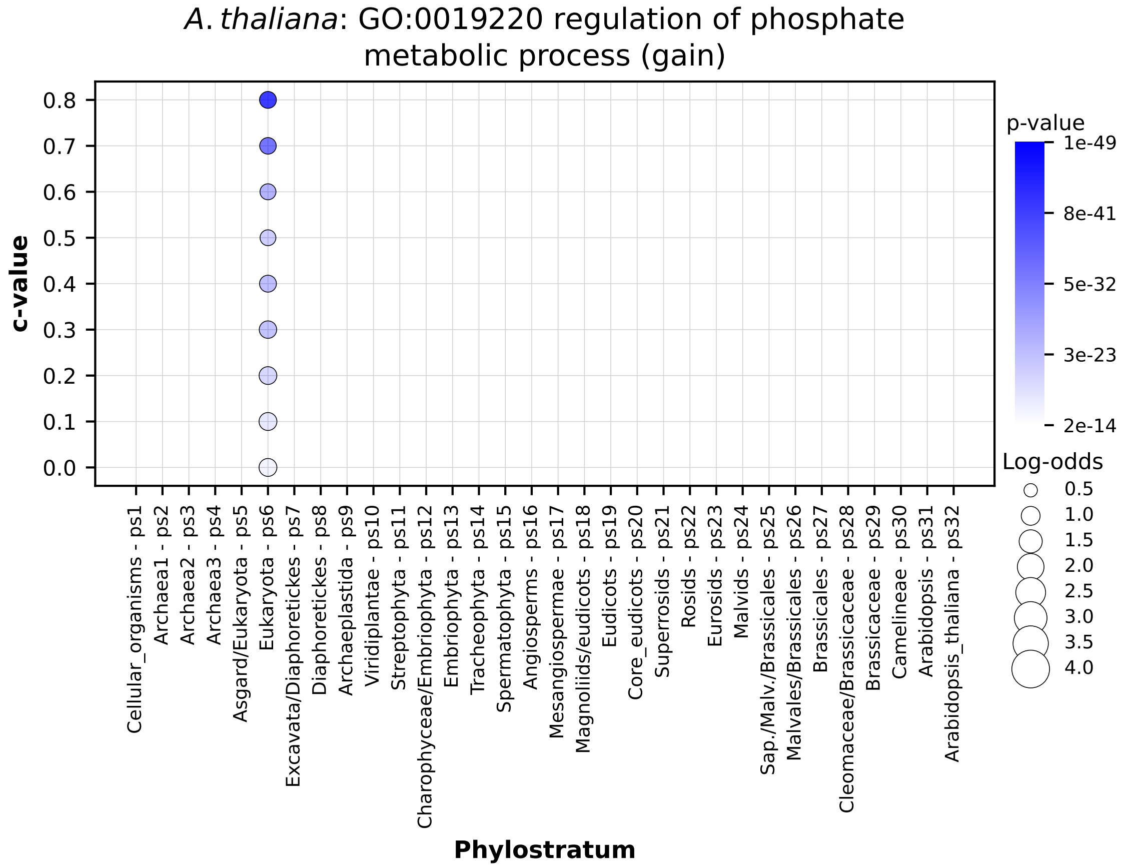

A. thaliana: GO:0019220 regulation of phosphate metabolic process (gain)
c-value
0.8
0.7
0.6
0.5
0.4
0.3
0.2
0.1
0.0
p-value
1e-49
8e-41
5e-32
3e-23
2e-14
Log-odds
0.5
1.0
1.5
2.0
2.5
3.0
3.5
4.0
Cellular_organisms - ps1
Archaea1 - ps2
Archaea2 - ps3
Archaea3 - ps4
Asgard/Eukaryota - ps5
Eukaryota - ps6
Excavata/Diaphoretickes - ps7
Diaphoretickes - ps8
Archaeplastida - ps9
Viridiplantae - ps10
Streptophyta - ps11
Charophyceae/Embriophyta - ps12
Embriophyta - ps13
Tracheophyta - ps14
Spermatophyta - ps15
Angiosperms - ps16
Mesangiospermae - ps17
Magnoliids/eudicots - ps18
Eudicots - ps19
Core_eudicots - ps20
Superrosids - ps21
Rosids - ps22
Eurosids - ps23
Malvids - ps24
Sap./Malv./Brassicales - ps25
Malvales/Brassicales - ps26
Brassicales - ps27
Cleomaceae/Brassicaceae - ps28
Brassicaceae - ps29
Camelineae - ps30
Arabidopsis - ps31
Arabidopsis_thaliana - ps32
Phylostratum

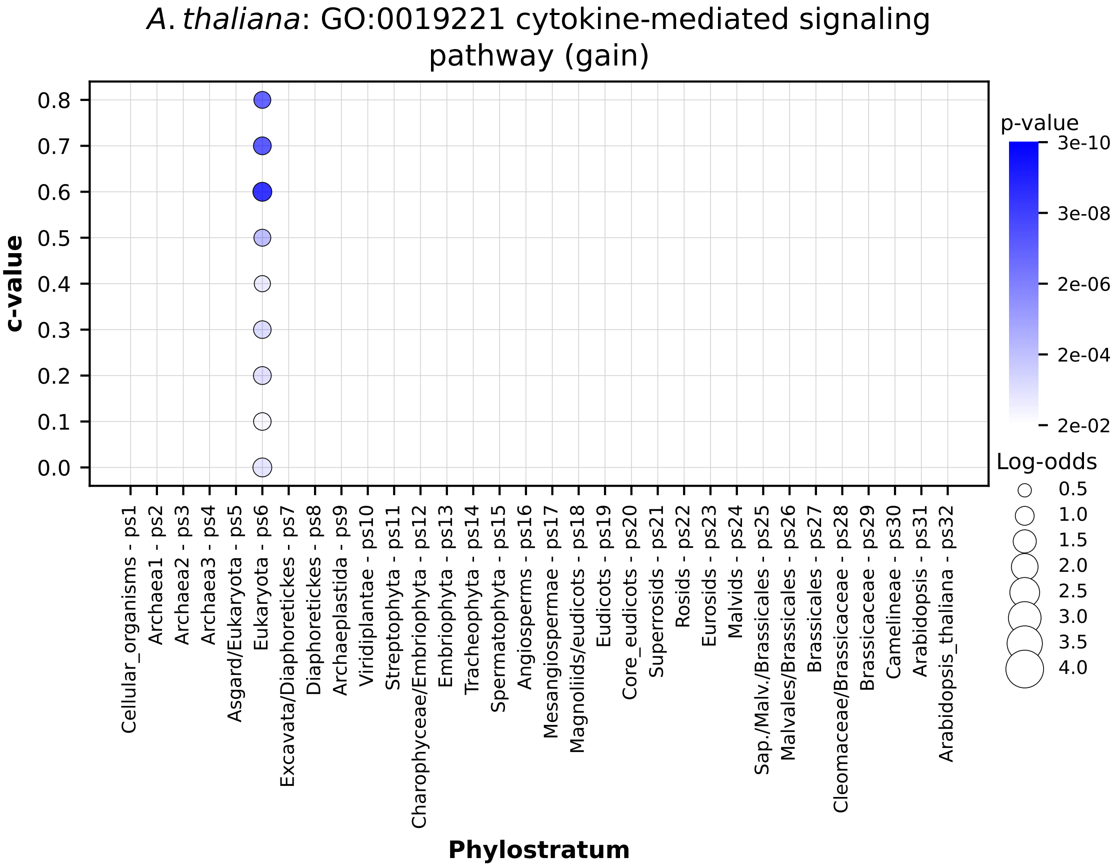

A. thaliana: GO:0019221 cytokine-mediated signaling pathway (gain)
c-value
0.8
0.7
0.6
0.5
0.4
0.3
0.2
0.1
0.0
p-value
3e-10
3e-08
2e-06
2e-04
2e-02
Log-odds
0.5
1.0
1.5
2.0
2.5
3.0
3.5
4.0
Cellular_organisms - ps1
Archaea1 - ps2
Archaea2 - ps3
Archaea3 - ps4
Asgard/Eukaryota - ps5
Eukaryota - ps6
Excavata/Diaphoretickes - ps7
Diaphoretickes - ps8
Archaeplastida - ps9
Viridiplantae - ps10
Streptophyta - ps11
Charophyceae/Embriophyta - ps12
Embriophyta - ps13
Tracheophyta - ps14
Spermatophyta - ps15
Angiosperms - ps16
Mesangiospermae - ps17
Magnoliids/eudicots - ps18
Eudicots - ps19
Core_eudicots - ps20
Superrosids - ps21
Rosids - ps22
Eurosids - ps23
Malvids - ps24
Sap./Malv./Brassicales - ps25
Malvales/Brassicales - ps26
Brassicales - ps27
Cleomaceae/Brassicaceae - ps28
Brassicaceae - ps29
Camelineae - ps30
Arabidopsis - ps31
Arabidopsis_thaliana - ps32
Phylostratum

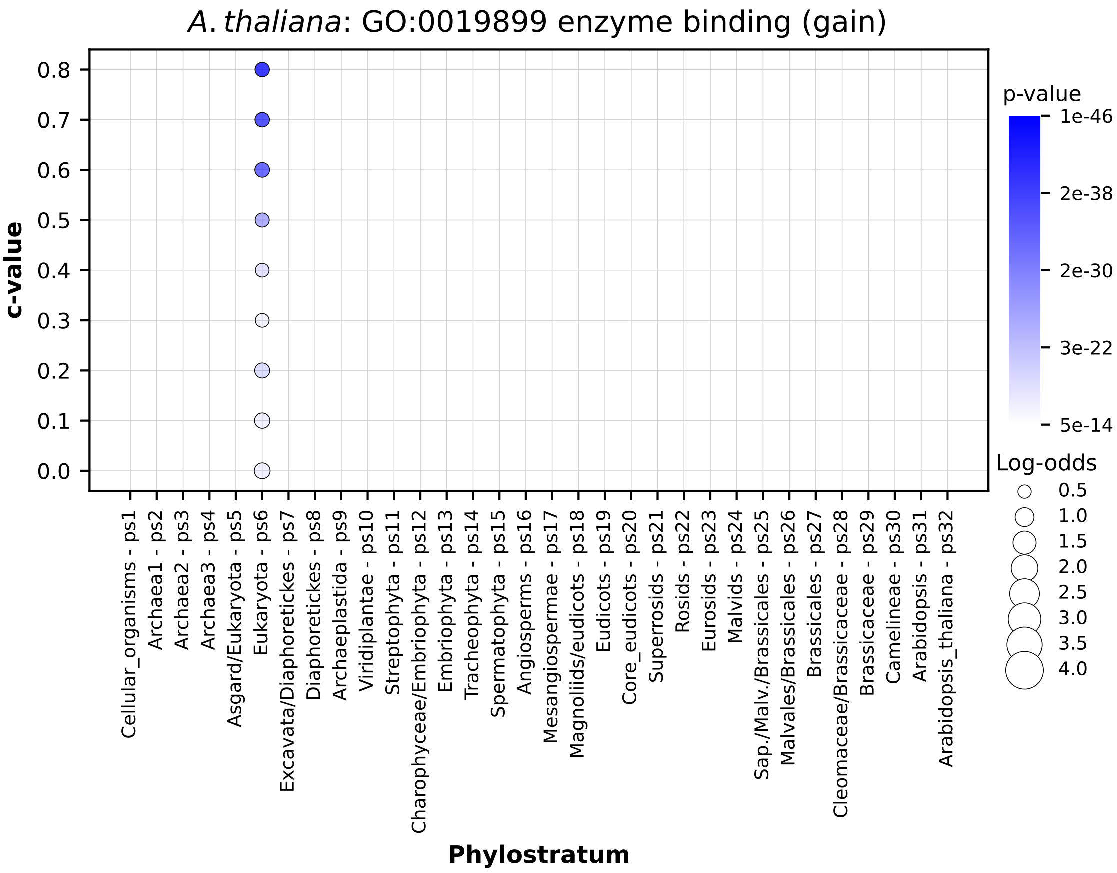
A. thaliana: GO:0019899 enzyme binding (gain)
c-value
0.8
0.7
0.6
0.5
0.4
0.3
0.2
0.1
0.0
Phylostratum
Cellular_organisms - ps1
Archaea1 - ps2
Archaea2 - ps3
Archaea3 - ps4
Asgard/Eukaryota - ps5
Eukaryota - ps6
Excavata/Diaphoretickes - ps7
Diaphoretickes - ps8
Archaeplastida - ps9
Viridiplantae - ps10
Streptophyta - ps11
Charophyceae/Embriophyta - ps12
Embriophyta - ps13
Tracheophyta - ps14
Spermatophyta - ps15
Angiosperms - ps16
Mesangiospermae - ps17
Magnoliids/eudicots - ps18
Eudicots - ps19
Core_eudicots - ps20
Superrosids - ps21
Rosids - ps22
Eurosids - ps23
Malvids - ps24
Sap./Malv./Brassicales - ps25
Malvales/Brassicales - ps26
Brassicales - ps27
Cleomaceae/Brassicaceae - ps28
Brassicaceae - ps29
Camelineae - ps30
Arabidopsis - ps31
Arabidopsis_thaliana - ps32
p-value
1e-46
2e-38
2e-30
3e-22
5e-14
Log-odds
0.5
1.0
1.5
2.0
2.5
3.0
3.5
4.0

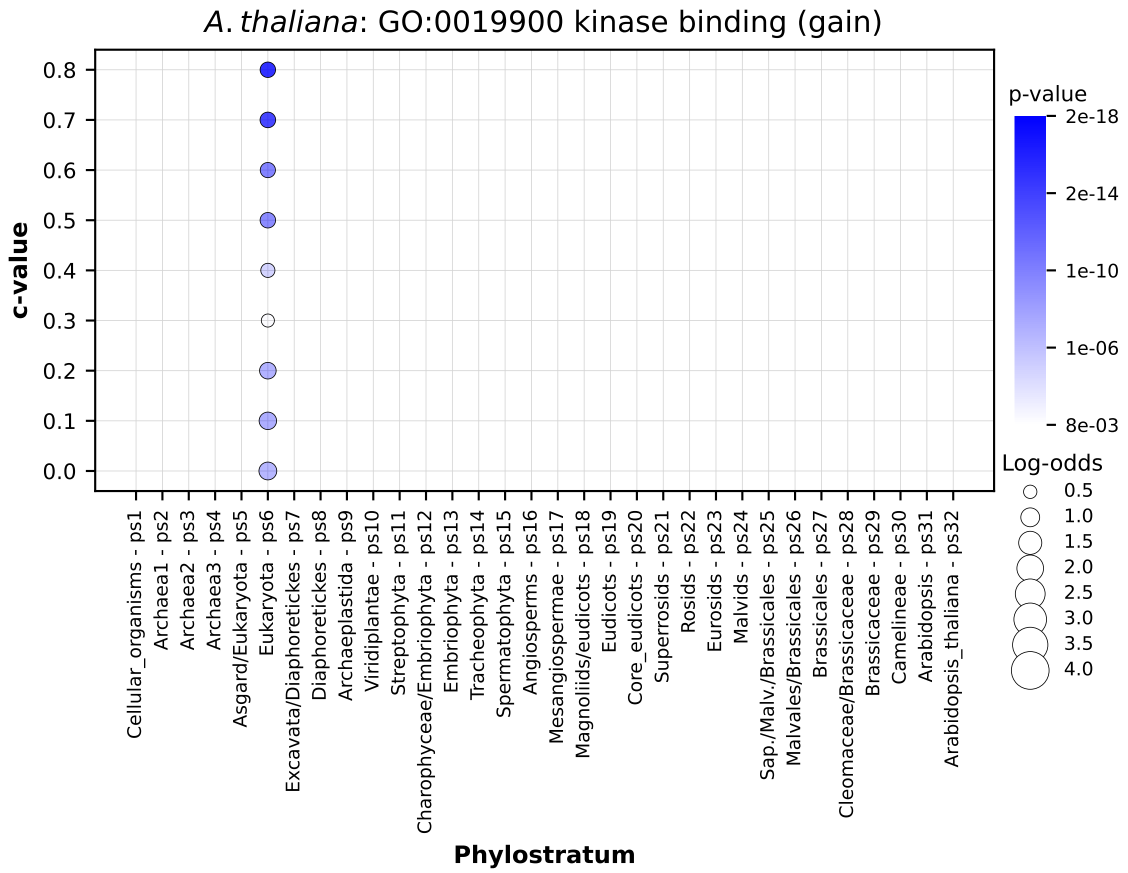

A. thaliana: GO:0019900 kinase binding (gain)
c-value
0.8
0.7
0.6
0.5
0.4
0.3
0.2
0.1
0.0
p-value
2e-18
2e-14
1e-10
1e-06
8e-03
Log-odds
0.5
1.0
1.5
2.0
2.5
3.0
3.5
4.0
Cellular_organisms - ps1
Archaea1 - ps2
Archaea2 - ps3
Archaea3 - ps4
Asgard/Eukaryota - ps5
Eukaryota - ps6
Excavata/Diaphoretickes - ps7
Diaphoretickes - ps8
Archaeplastida - ps9
Viridiplantae - ps10
Streptophyta - ps11
Charophyceae/Embriophyta - ps12
Embriophyta - ps13
Tracheophyta - ps14
Spermatophyta - ps15
Angiosperms - ps16
Mesangiospermae - ps17
Magnoliids/eudicots - ps18
Eudicots - ps19
Core_eudicots - ps20
Superrosids - ps21
Rosids - ps22
Eurosids - ps23
Malvids - ps24
Sap./Malv./Brassicales - ps25
Malvales/Brassicales - ps26
Brassicales - ps27
Cleomaceae/Brassicaceae - ps28
Brassicaceae - ps29
Camelineae - ps30
Arabidopsis - ps31
Arabidopsis_thaliana - ps32
Phylostratum

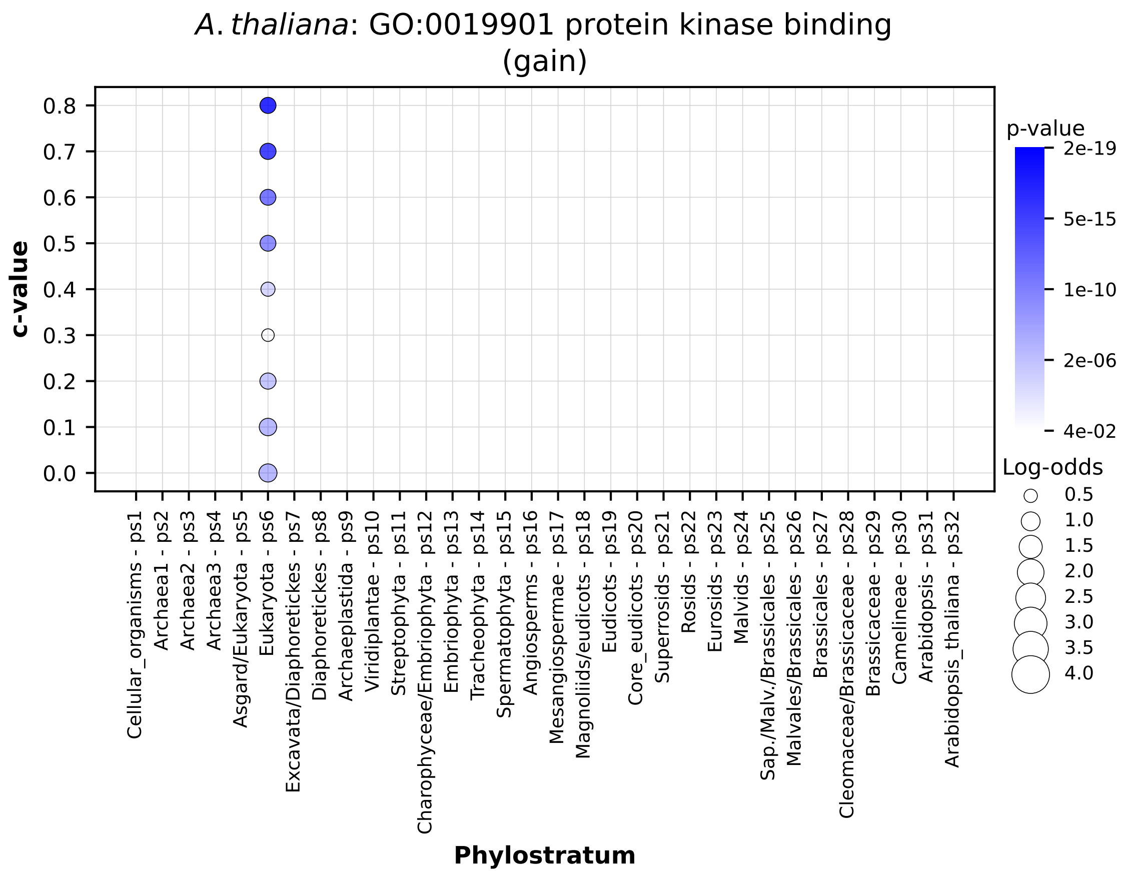

A. thaliana: GO:0019901 protein kinase binding
(gain)
c-value
0.8
0.7
0.6
0.5
0.4
0.3
0.2
0.1
0.0
p-value
2e-19
5e-15
1e-10
2e-06
4e-02
Log-odds
0.5
1.0
1.5
2.0
2.5
3.0
3.5
4.0
Cellular_organisms - ps1
Archaea1 - ps2
Archaea2 - ps3
Archaea3 - ps4
Asgard/Eukaryota - ps5
Eukaryota - ps6
Excavata/Diaphoretickes - ps7
Diaphoretickes - ps8
Archaeplastida - ps9
Viridiplantae - ps10
Streptophyta - ps11
Charophyceae/Embriophyta - ps12
Embriophyta - ps13
Tracheophyta - ps14
Spermatophyta - ps15
Angiosperms - ps16
Mesangiospermae - ps17
Magnoliids/eudicots - ps18
Eudicots - ps19
Core_eudicots - ps20
Superrosids - ps21
Rosids - ps22
Eurosids - ps23
Malvids - ps24
Sap./Malv./Brassicales - ps25
Malvales/Brassicales - ps26
Brassicales - ps27
Cleomaceae/Brassicaceae - ps28
Brassicaceae - ps29
Camelineae - ps30
Arabidopsis - ps31
Arabidopsis_thaliana - ps32
Phylostratum

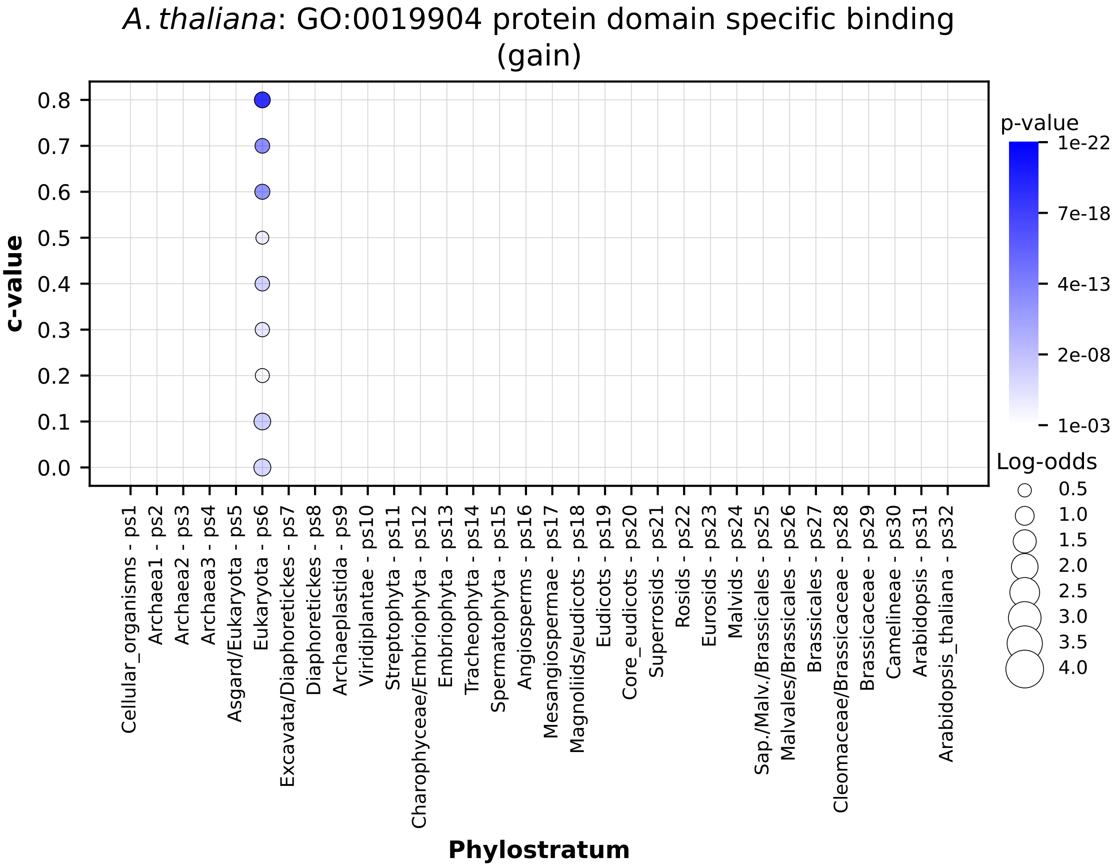

A. thaliana: GO:0019904 protein domain specific binding (gain)
c-value
0.8
0.7
0.6
0.5
0.4
0.3
0.2
0.1
0.0
p-value
1e-22
7e-18
4e-13
2e-08
1e-03
Log-odds
0.5
1.0
1.5
2.0
2.5
3.0
3.5
4.0
Cellular_organisms - ps1
Archaea1 - ps2
Archaea2 - ps3
Archaea3 - ps4
Asgard/Eukaryota - ps5
Eukaryota - ps6
Excavata/Diaphoretickes - ps7
Diaphoretickes - ps8
Archaeplastida - ps9
Viridiplantae - ps10
Streptophyta - ps11
Charophyceae/Embriophyta - ps12
Embriophyta - ps13
Tracheophyta - ps14
Spermatophyta - ps15
Angiosperms - ps16
Mesangiospermae - ps17
Magnoliids/eudicots - ps18
Eudicots - ps19
Core_eudicots - ps20
Superrosids - ps21
Rosids - ps22
Eurosids - ps23
Malvids - ps24
Sap./Malv./Brassicales - ps25
Malvales/Brassicales - ps26
Brassicales - ps27
Cleomaceae/Brassicaceae - ps28
Brassicaceae - ps29
Camelineae - ps30
Arabidopsis - ps31
Arabidopsis_thaliana - ps32
Phylostratum

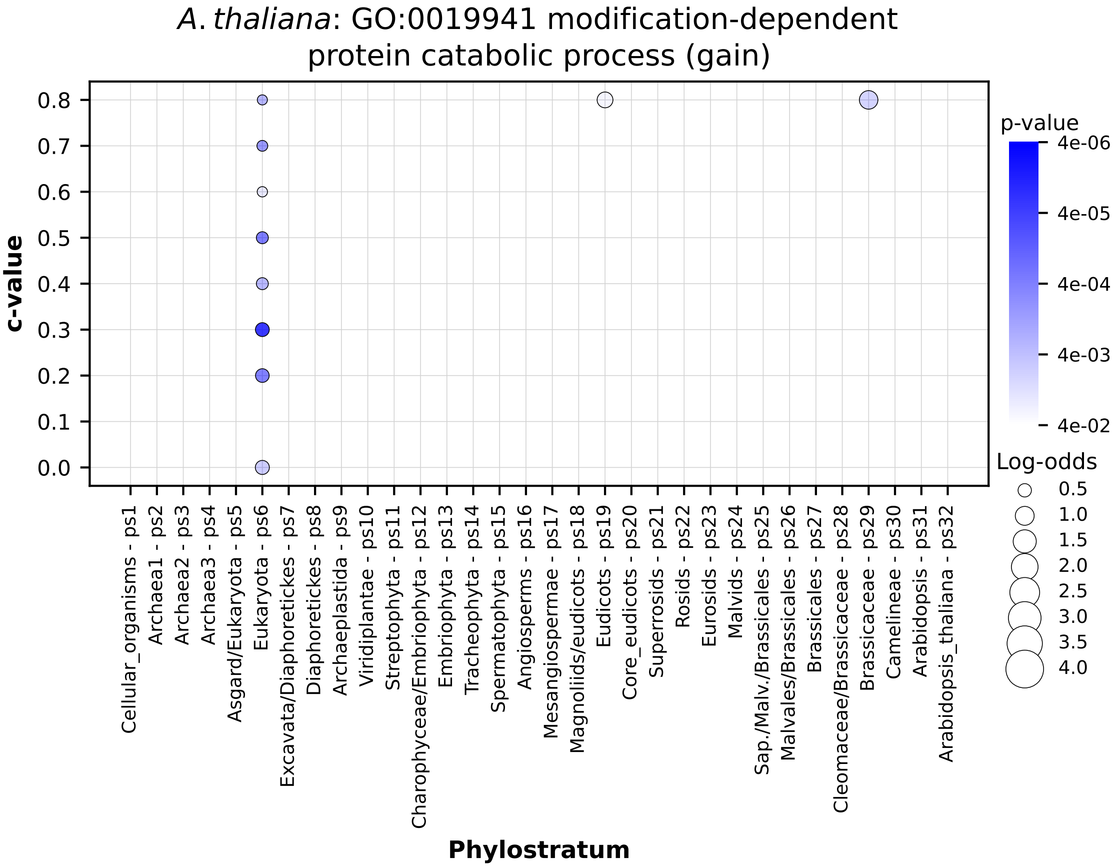

A. thaliana: GO:0019941 modification-dependent protein catabolic process (gain)
c-value
0.8
0.7
0.6
0.5
0.4
0.3
0.2
0.1
0.0
p-value
4e-06
4e-05
4e-04
4e-03
4e-02
Log-odds
0.5
1.0
1.5
2.0
2.5
3.0
3.5
4.0
Cellular_organisms - ps1
Archaea1 - ps2
Archaea2 - ps3
Archaea3 - ps4
Asgard/Eukaryota - ps5
Eukaryota - ps6
Excavata/Diaphoretickes - ps7
Diaphoretickes - ps8
Archaeplastida - ps9
Viridiplantae - ps10
Streptophyta - ps11
Charophyceae/Embriophyta - ps12
Embriophyta - ps13
Tracheophyta - ps14
Spermatophyta - ps15
Angiosperms - ps16
Mesangiospermae - ps17
Magnoliids/eudicots - ps18
Eudicots - ps19
Core_eudicots - ps20
Superrosids - ps21
Rosids - ps22
Eurosids - ps23
Malvids - ps24
Sap./Malv./Brassicales - ps25
Malvales/Brassicales - ps26
Brassicales - ps27
Cleomaceae/Brassicaceae - ps28
Brassicaceae - ps29
Camelineae - ps30
Arabidopsis - ps31
Arabidopsis_thaliana - ps32
Phylostratum

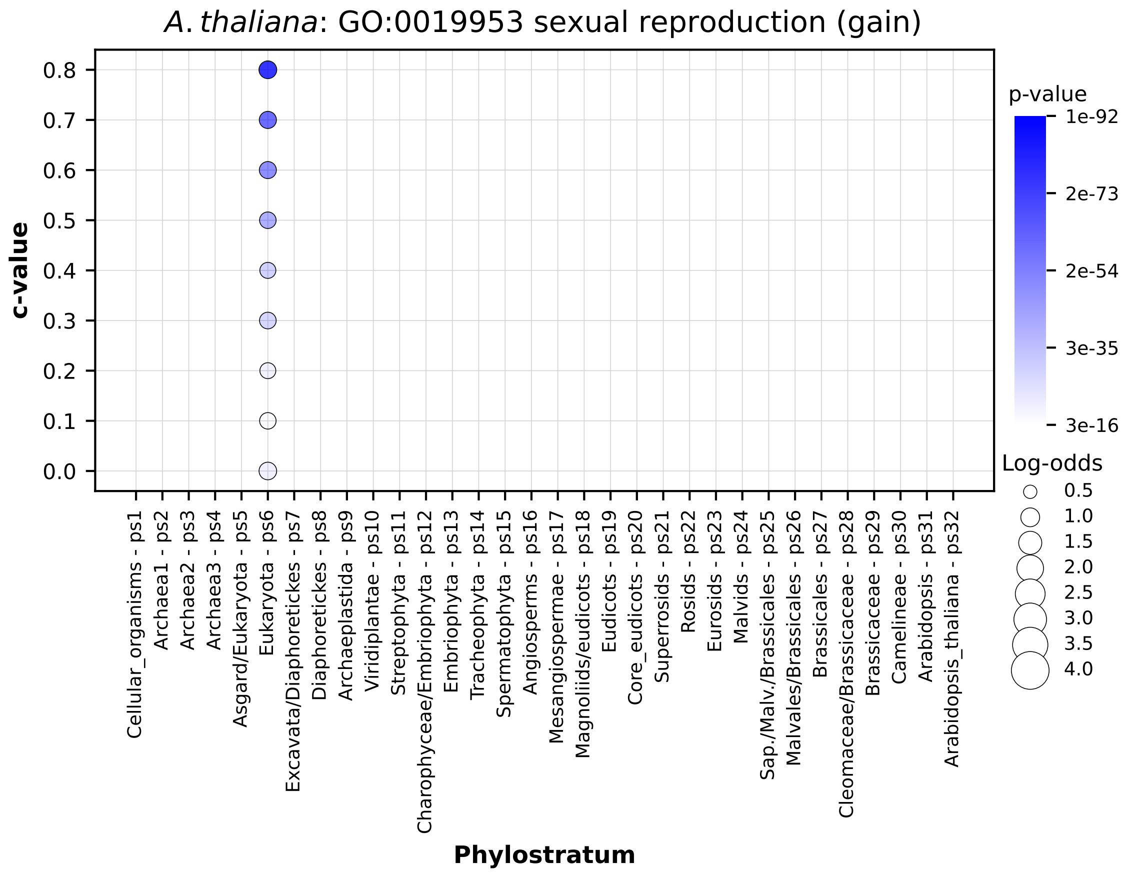

A. thaliana: GO:0019953 sexual reproduction (gain)
c-value
0.8
0.7
0.6
0.5
0.4
0.3
0.2
0.1
0.0
Phylostratum
Cellular_organisms - ps1
Archaea1 - ps2
Archaea2 - ps3
Archaea3 - ps4
Asgard/Eukaryota - ps5
Eukaryota - ps6
Excavata/Diaphoretickes - ps7
Diaphoretickes - ps8
Archaeplastida - ps9
Viridiplantae - ps10
Streptophyta - ps11
Charophyceae/Embriophyta - ps12
Embriophyta - ps13
Tracheophyta - ps14
Spermatophyta - ps15
Angiosperms - ps16
Mesangiospermae - ps17
Magnoliids/eudicots - ps18
Eudicots - ps19
Core_eudicots - ps20
Superrosids - ps21
Rosids - ps22
Eurosids - ps23
Malvids - ps24
Sap./Malv./Brassicales - ps25
Malvales/Brassicales - ps26
Brassicales - ps27
Cleomaceae/Brassicaceae - ps28
Brassicaceae - ps29
Camelineae - ps30
Arabidopsis - ps31
Arabidopsis_thaliana - ps32
p-value
1e-92
2e-73
2e-54
3e-35
3e-16
Log-odds
0.5
1.0
1.5
2.0
2.5
3.0
3.5
4.0

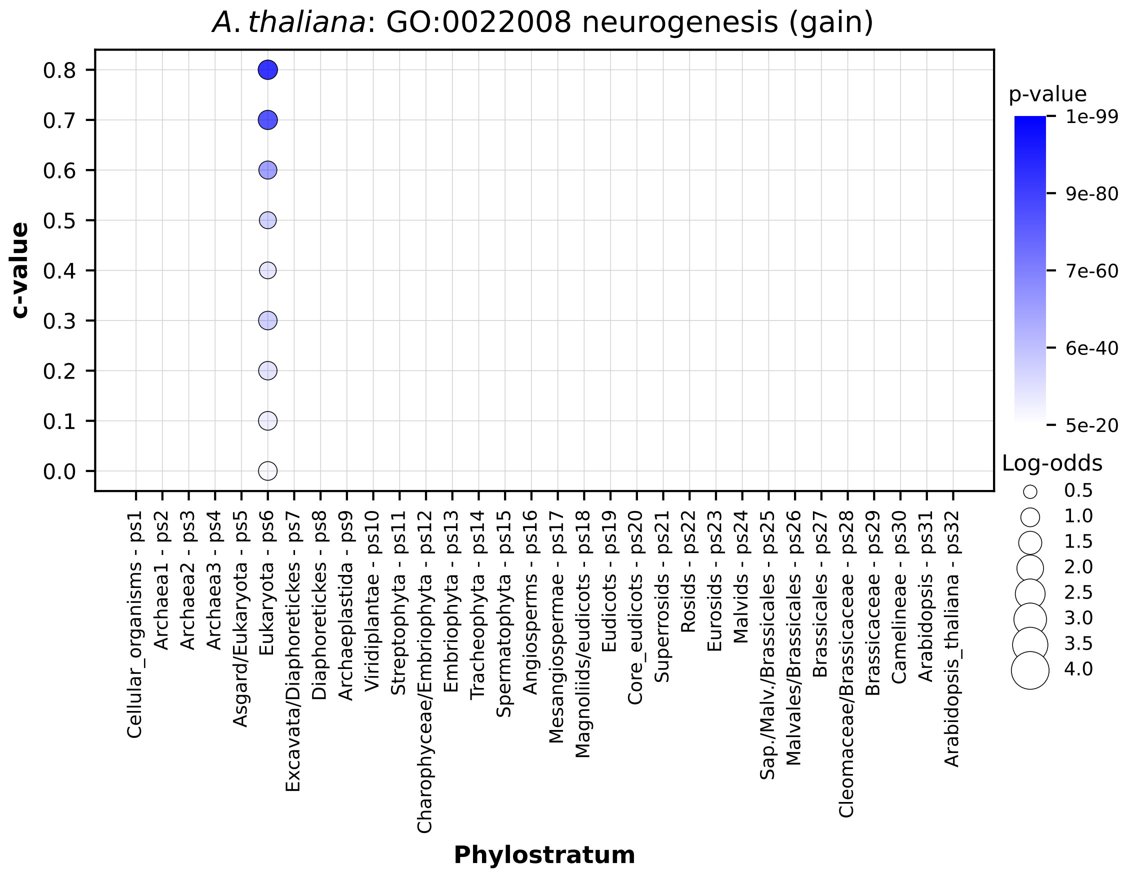
A. thaliana: GO:0022008 neurogenesis (gain)
c-value
0.0
0.1
0.2
0.3
0.4
0.5
0.6
0.7
0.8
p-value
1e-99
9e-80
7e-60
6e-40
5e-20
Log-odds
0.5
1.0
1.5
2.0
2.5
3.0
3.5
4.0
Cellular_organisms - ps1
Archaea1 - ps2
Archaea2 - ps3
Archaea3 - ps4
Asgard/Eukaryota - ps5
Eukaryota - ps6
Excavata/Diaphoretickes - ps7
Diaphoretickes - ps8
Archaeplastida - ps9
Viridiplantae - ps10
Streptophyta - ps11
Charophyceae/Embriophyta - ps12
Embriophyta - ps13
Tracheophyta - ps14
Spermatophyta - ps15
Angiosperms - ps16
Mesangiospermae - ps17
Magnoliids/eudicots - ps18
Eudicots - ps19
Core_eudicots - ps20
Superrosids - ps21
Rosids - ps22
Eurosids - ps23
Malvids - ps24
Sap./Malv./Brassicales - ps25
Malvales/Brassicales - ps26
Brassicales - ps27
Cleomaceae/Brassicaceae - ps28
Brassicaceae - ps29
Camelineae - ps30
Arabidopsis - ps31
Arabidopsis_thaliana - ps32
Phylostratum

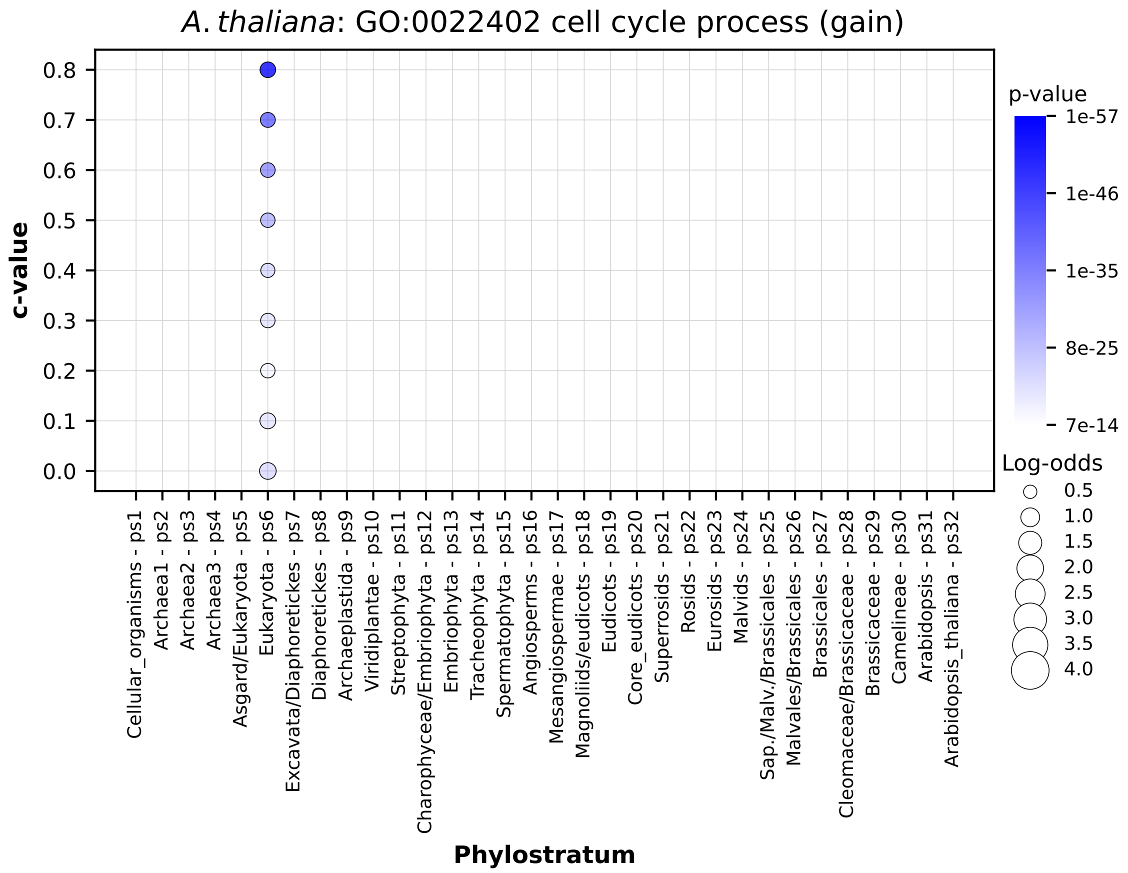
A. thaliana: GO:0022402 cell cycle process (gain)
c-value
0.8
0.7
0.6
0.5
0.4
0.3
0.2
0.1
0.0
Phylostratum
Cellular_organisms - ps1
Archaea1 - ps2
Archaea2 - ps3
Archaea3 - ps4
Asgard/Eukaryota - ps5
Eukaryota - ps6
Excavata/Diaphoretickes - ps7
Diaphoretickes - ps8
Archaeplastida - ps9
Viridiplantae - ps10
Streptophyta - ps11
Charophyceae/Embriophyta - ps12
Embriophyta - ps13
Tracheophyta - ps14
Spermatophyta - ps15
Angiosperms - ps16
Mesangiospermae - ps17
Magnoliids/eudicots - ps18
Eudicots - ps19
Core_eudicots - ps20
Superrosids - ps21
Rosids - ps22
Eurosids - ps23
Malvids - ps24
Sap./Malv./Brassicales - ps25
Malvales/Brassicales - ps26
Brassicales - ps27
Cleomaceae/Brassicaceae - ps28
Brassicaceae - ps29
Camelineae - ps30
Arabidopsis - ps31
Arabidopsis_thaliana - ps32
p-value
1e-57
1e-46
1e-35
8e-25
7e-14
Log-odds
0.5
1.0
1.5
2.0
2.5
3.0
3.5
4.0

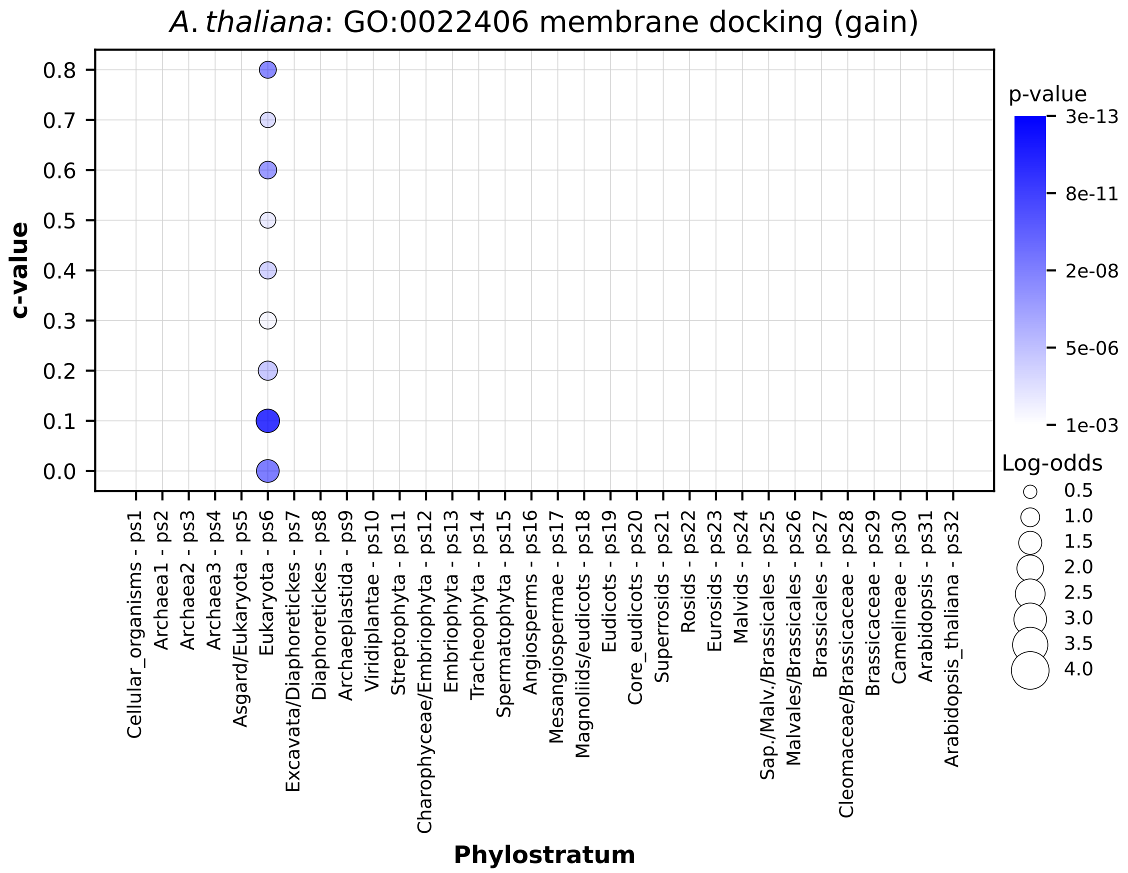

A. thaliana: GO:0022406 membrane docking (gain)
c-value
0.8
0.7
0.6
0.5
0.4
0.3
0.2
0.1
0.0
p-value
3e-13
8e-11
2e-08
5e-06
1e-03
Log-odds
0.5
1.0
1.5
2.0
2.5
3.0
3.5
4.0
Cellular_organisms - ps1
Archaea1 - ps2
Archaea2 - ps3
Archaea3 - ps4
Asgard/Eukaryota - ps5
Eukaryota - ps6
Excavata/Diaphoretickes - ps7
Diaphoretickes - ps8
Archaeplastida - ps9
Viridiplantae - ps10
Streptophyta - ps11
Charophyceae/Embriophyta - ps12
Embriophyta - ps13
Tracheophyta - ps14
Spermatophyta - ps15
Angiosperms - ps16
Mesangiospermae - ps17
Magnoliids/eudicots - ps18
Eudicots - ps19
Core_eudicots - ps20
Superrosids - ps21
Rosids - ps22
Eurosids - ps23
Malvids - ps24
Sap./Malv./Brassicales - ps25
Malvales/Brassicales - ps26
Brassicales - ps27
Cleomaceae/Brassicaceae - ps28
Brassicaceae - ps29
Camelineae - ps30
Arabidopsis - ps31
Arabidopsis_thaliana - ps32
Phylostratum

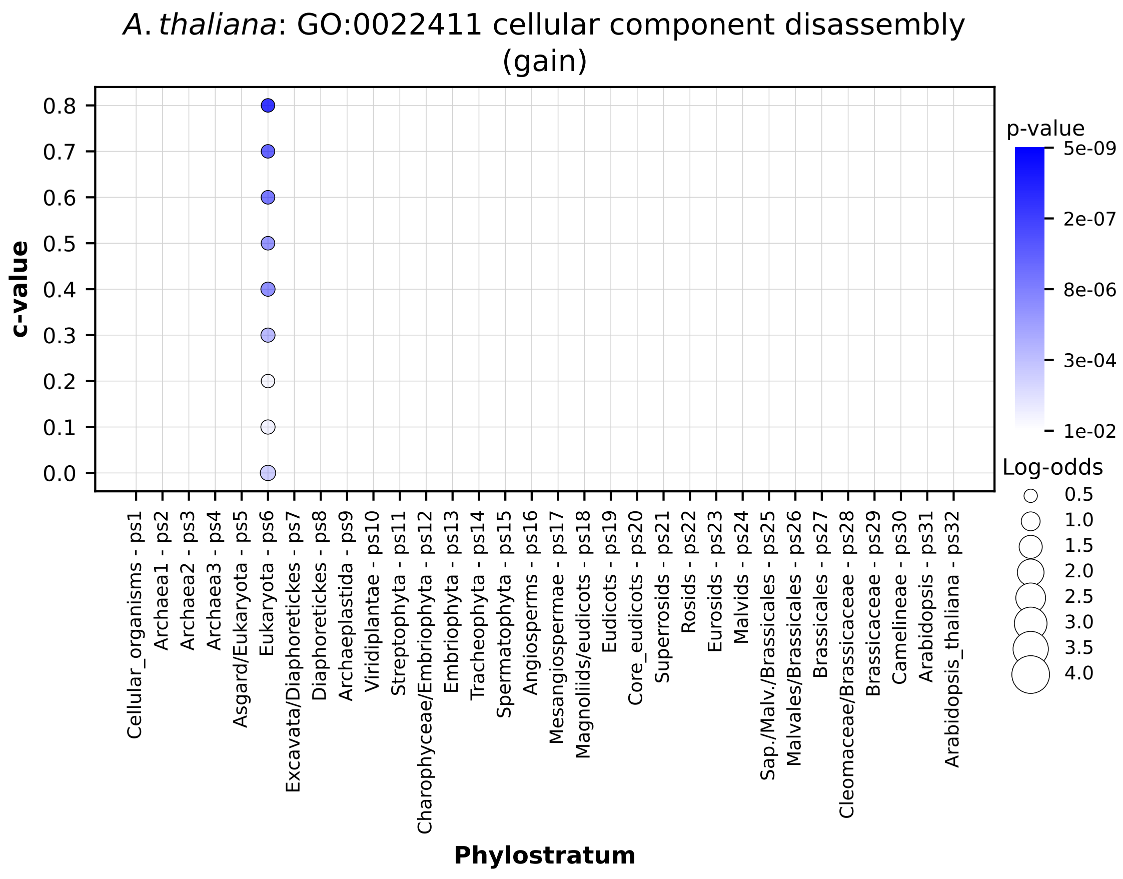
A. thaliana: GO:0022411 cellular component disassembly
(gain)
c-value
0.8
0.7
0.6
0.5
0.4
0.3
0.2
0.1
0.0
Phylostratum
Cellular_organisms - ps1
Archaea1 - ps2
Archaea2 - ps3
Archaea3 - ps4
Asgard/Eukaryota - ps5
Eukaryota - ps6
Excavata/Diaphoretickes - ps7
Diaphoretickes - ps8
Archaeplastida - ps9
Viridiplantae - ps10
Streptophyta - ps11
Charophyceae/Embriophyta - ps12
Embriophyta - ps13
Tracheophyta - ps14
Spermatophyta - ps15
Angiosperms - ps16
Mesangiospermae - ps17
Magnoliids/eudicots - ps18
Eudicots - ps19
Core_eudicots - ps20
Superrosids - ps21
Rosids - ps22
Eurosids - ps23
Malvids - ps24
Sap./Malv./Brassicales - ps25
Malvales/Brassicales - ps26
Brassicales - ps27
Cleomaceae/Brassicaceae - ps28
Brassicaceae - ps29
Camelineae - ps30
Arabidopsis - ps31
Arabidopsis_thaliana - ps32
p-value
5e-09
2e-07
8e-06
3e-04
1e-02
Log-odds
0.5
1.0
1.5
2.0
2.5
3.0
3.5
4.0

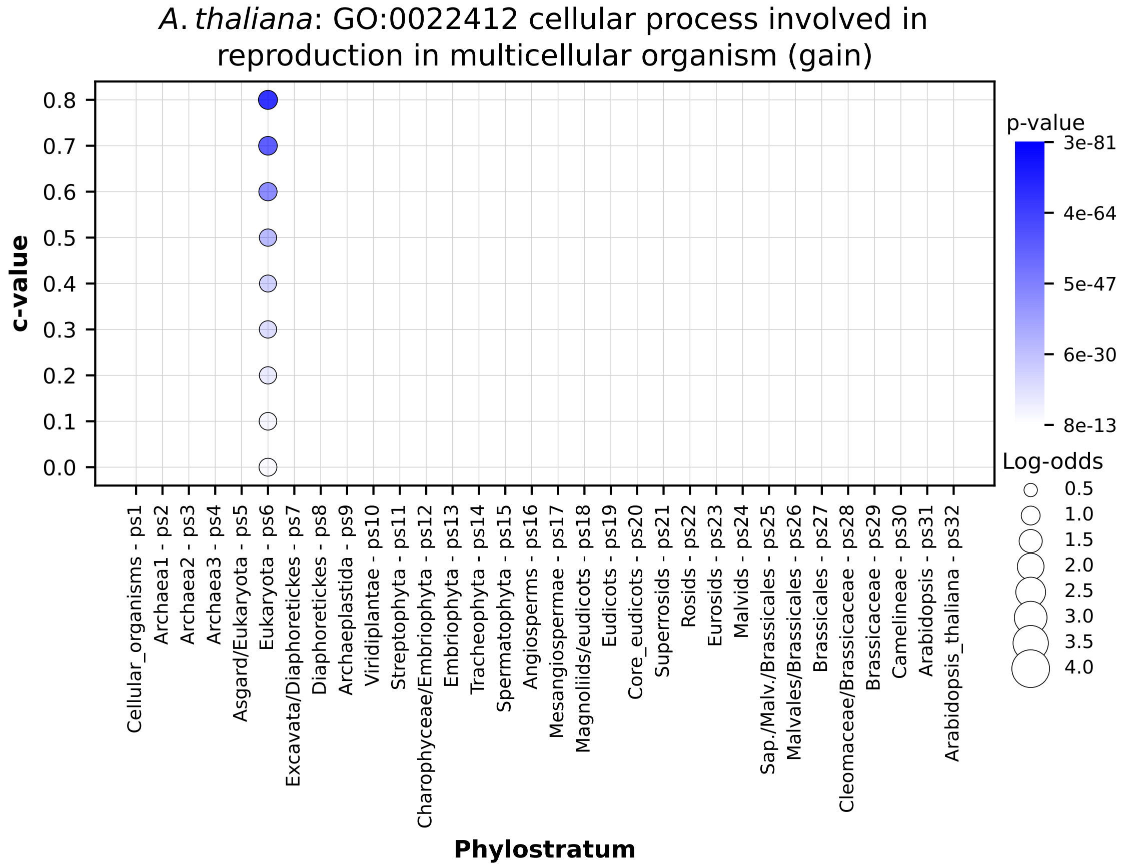
A. thaliana: GO:0022412 cellular process involved in reproduction in multicellular organism (gain)
c-value
0.8
0.7
0.6
0.5
0.4
0.3
0.2
0.1
0.0
p-value
3e-81
4e-64
5e-47
6e-30
8e-13
Log-odds
0.5
1.0
1.5
2.0
2.5
3.0
3.5
4.0
Cellular_organisms - ps1
Archaea1 - ps2
Archaea2 - ps3
Archaea3 - ps4
Asgard/Eukaryota - ps5
Eukaryota - ps6
Excavata/Diaphoretickes - ps7
Diaphoretickes - ps8
Archaeplastida - ps9
Viridiplantae - ps10
Streptophyta - ps11
Charophyceae/Embriophyta - ps12
Embriophyta - ps13
Tracheophyta - ps14
Spermatophyta - ps15
Angiosperms - ps16
Mesangiospermae - ps17
Magnoliids/eudicots - ps18
Eudicots - ps19
Core_eudicots - ps20
Superrosids - ps21
Rosids - ps22
Eurosids - ps23
Malvids - ps24
Sap./Malv./Brassicales - ps25
Malvales/Brassicales - ps26
Brassicales - ps27
Cleomaceae/Brassicaceae - ps28
Brassicaceae - ps29
Camelineae - ps30
Arabidopsis - ps31
Arabidopsis_thaliana - ps32
Phylostratum

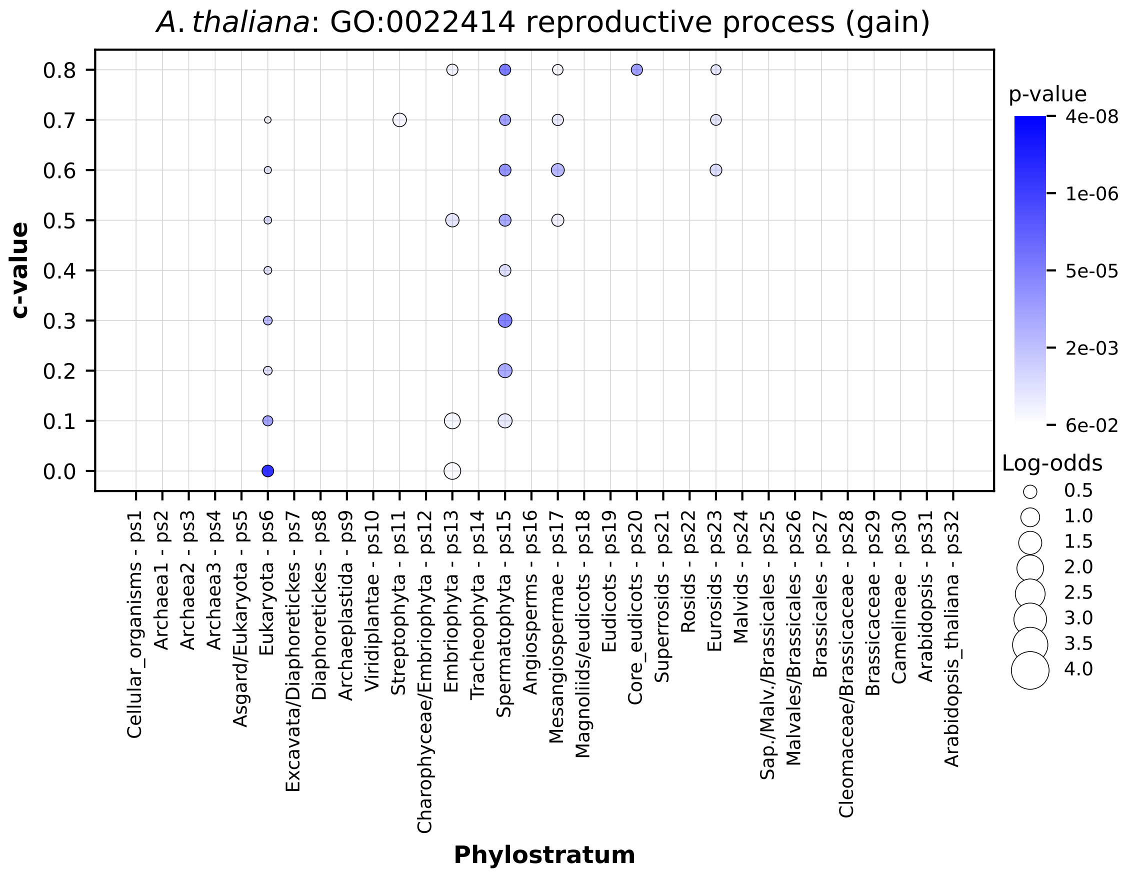

A. thaliana: GO:0022414 reproductive process (gain)
c-value
0.8
0.7
0.6
0.5
0.4
0.3
0.2
0.1
0.0
p-value
4e-08
1e-06
5e-05
2e-03
6e-02
Log-odds
0.5
1.0
1.5
2.0
2.5
3.0
3.5
4.0
Cellular_organisms - ps1
Archaea1 - ps2
Archaea2 - ps3
Archaea3 - ps4
Asgard/Eukaryota - ps5
Eukaryota - ps6
Excavata/Diaphoretickes - ps7
Diaphoretickes - ps8
Archaeplastida - ps9
Viridiplantae - ps10
Streptophyta - ps11
Charophyceae/Embriophyta - ps12
Embriophyta - ps13
Tracheophyta - ps14
Spermatophyta - ps15
Angiosperms - ps16
Mesangiospermae - ps17
Magnoliids/eudicots - ps18
Eudicots - ps19
Core_eudicots - ps20
Superrosids - ps21
Rosids - ps22
Eurosids - ps23
Malvids - ps24
Sap./Malv./Brassicales - ps25
Malvales/Brassicales - ps26
Brassicales - ps27
Cleomaceae/Brassicaceae - ps28
Brassicaceae - ps29
Camelineae - ps30
Arabidopsis - ps31
Arabidopsis_thaliana - ps32
Phylostratum

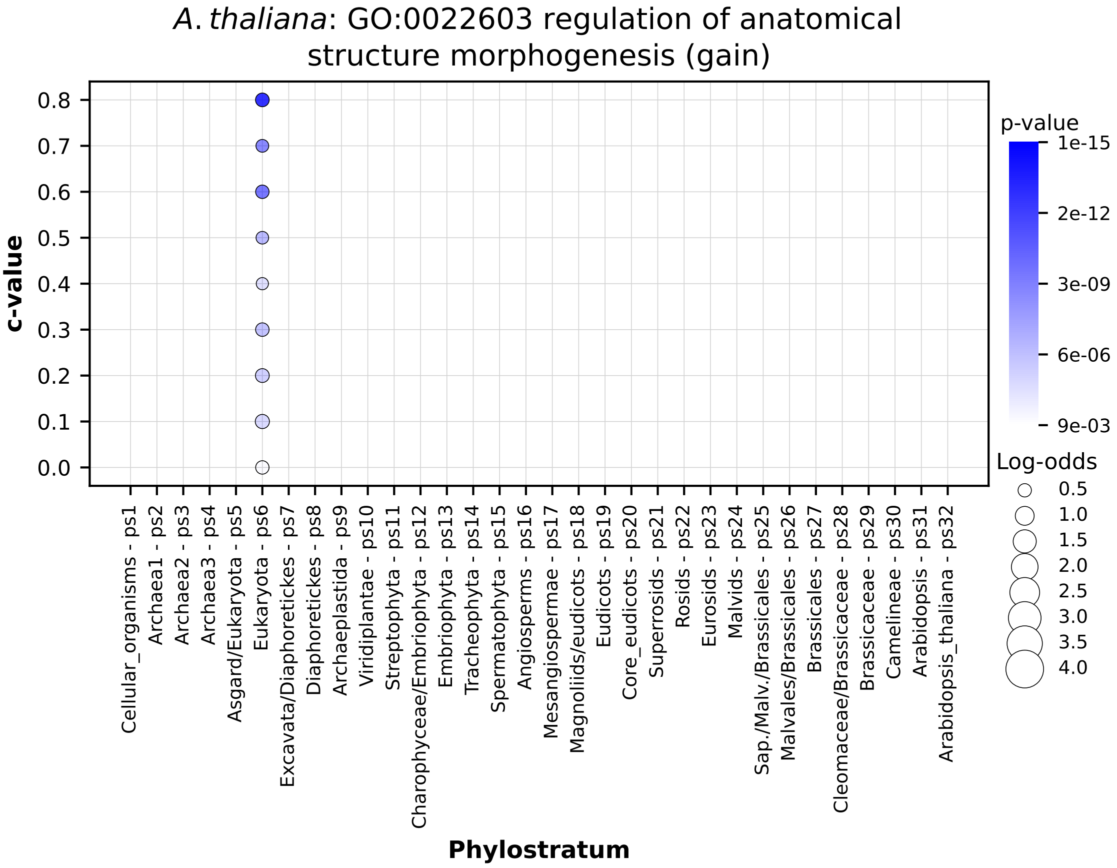

A. thaliana: GO:0022603 regulation of anatomical structure morphogenesis (gain)
c-value
0.8
0.7
0.6
0.5
0.4
0.3
0.2
0.1
0.0
p-value
1e-15
2e-12
3e-09
6e-06
9e-03
Log-odds
0.5
1.0
1.5
2.0
2.5
3.0
3.5
4.0
Cellular_organisms - ps1
Archaea1 - ps2
Archaea2 - ps3
Archaea3 - ps4
Asgard/Eukaryota - ps5
Eukaryota - ps6
Excavata/Diaphoretickes - ps7
Diaphoretickes - ps8
Archaeplastida - ps9
Viridiplantae - ps10
Streptophyta - ps11
Charophyceae/Embriophyta - ps12
Embriophyta - ps13
Tracheophyta - ps14
Spermatophyta - ps15
Angiosperms - ps16
Mesangiospermae - ps17
Magnoliids/eudicots - ps18
Eudicots - ps19
Core_eudicots - ps20
Superrosids - ps21
Rosids - ps22
Eurosids - ps23
Malvids - ps24
Sap./Malv./Brassicales - ps25
Malvales/Brassicales - ps26
Brassicales - ps27
Cleomaceae/Brassicaceae - ps28
Brassicaceae - ps29
Camelineae - ps30
Arabidopsis - ps31
Arabidopsis_thaliana - ps32
Phylostratum

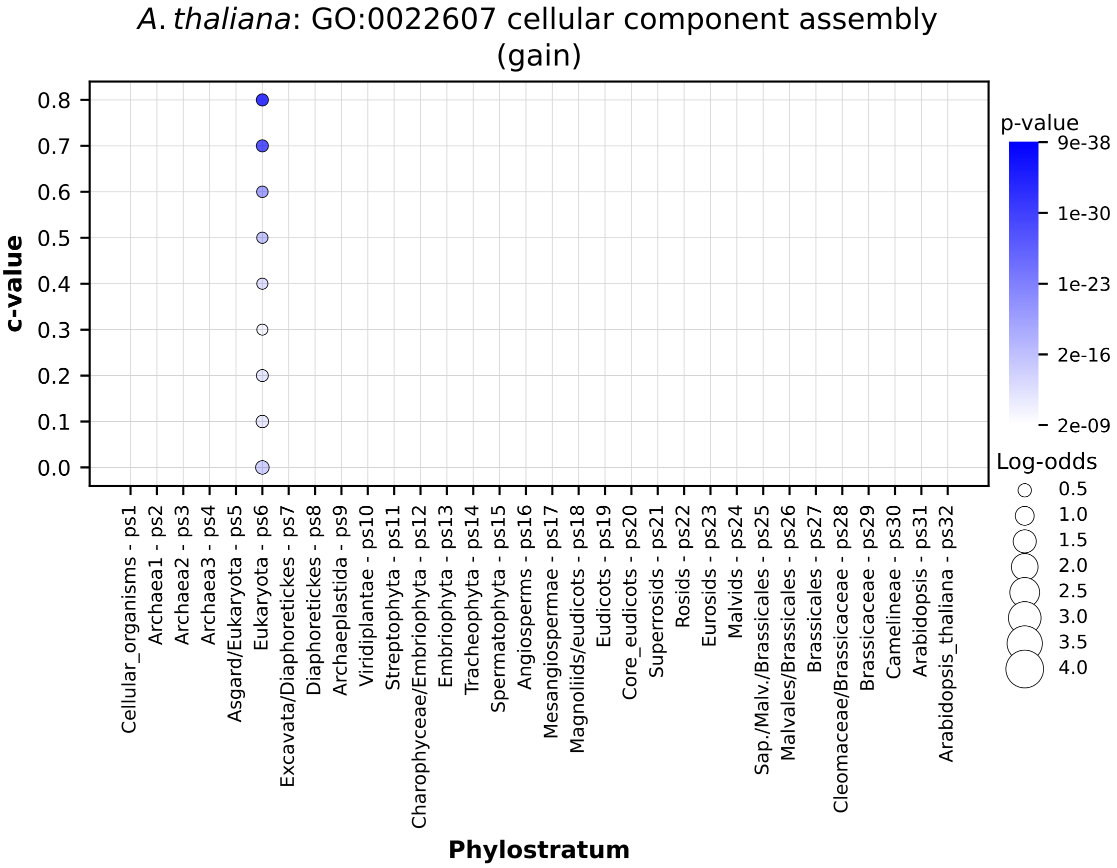

A. thaliana: GO:0022607 cellular component assembly
(gain)
c-value
0.8
0.7
0.6
0.5
0.4
0.3
0.2
0.1
0.0
Phylostratum
Cellular_organisms - ps1
Archaea1 - ps2
Archaea2 - ps3
Archaea3 - ps4
Asgard/Eukaryota - ps5
Eukaryota - ps6
Excavata/Diaphoretickes - ps7
Diaphoretickes - ps8
Archaeplastida - ps9
Viridiplantae - ps10
Streptophyta - ps11
Charophyceae/Embriophyta - ps12
Embriophyta - ps13
Tracheophyta - ps14
Spermatophyta - ps15
Angiosperms - ps16
Mesangiospermae - ps17
Magnoliids/eudicots - ps18
Eudicots - ps19
Core_eudicots - ps20
Superrosids - ps21
Rosids - ps22
Eurosids - ps23
Malvids - ps24
Sap./Malv./Brassicales - ps25
Malvales/Brassicales - ps26
Brassicales - ps27
Cleomaceae/Brassicaceae - ps28
Brassicaceae - ps29
Camelineae - ps30
Arabidopsis - ps31
Arabidopsis_thaliana - ps32
p-value
9e-38
1e-30
1e-23
2e-16
2e-09
Log-odds
0.5
1.0
1.5
2.0
2.5
3.0
3.5
4.0

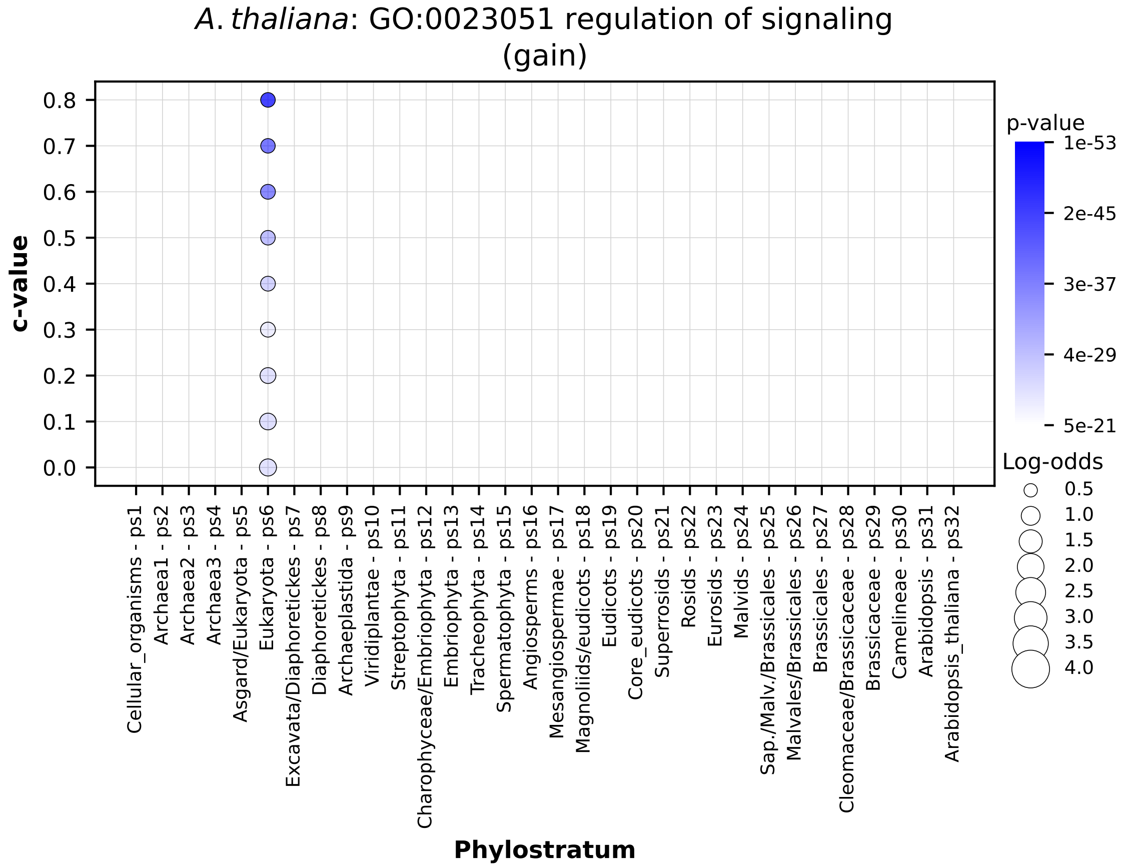

A. thaliana: GO:0023051 regulation of signaling (gain)
c-value
0.8
0.7
0.6
0.5
0.4
0.3
0.2
0.1
0.0
p-value
1e-53
2e-45
3e-37
4e-29
5e-21
Log-odds
0.5
1.0
1.5
2.0
2.5
3.0
3.5
4.0
Cellular_organisms - ps1
Archaea1 - ps2
Archaea2 - ps3
Archaea3 - ps4
Asgard/Eukaryota - ps5
Eukaryota - ps6
Excavata/Diaphoretickes - ps7
Diaphoretickes - ps8
Archaeplastida - ps9
Viridiplantae - ps10
Streptophyta - ps11
Charophyceae/Embriophyta - ps12
Embriophyta - ps13
Tracheophyta - ps14
Spermatophyta - ps15
Angiosperms - ps16
Mesangiospermae - ps17
Magnoliids/eudicots - ps18
Eudicots - ps19
Core_eudicots - ps20
Superrosids - ps21
Rosids - ps22
Eurosids - ps23
Malvids - ps24
Sap./Malv./Brassicales - ps25
Malvales/Brassicales - ps26
Brassicales - ps27
Cleomaceae/Brassicaceae - ps28
Brassicaceae - ps29
Camelineae - ps30
Arabidopsis - ps31
Arabidopsis_thaliana - ps32
Phylostratum

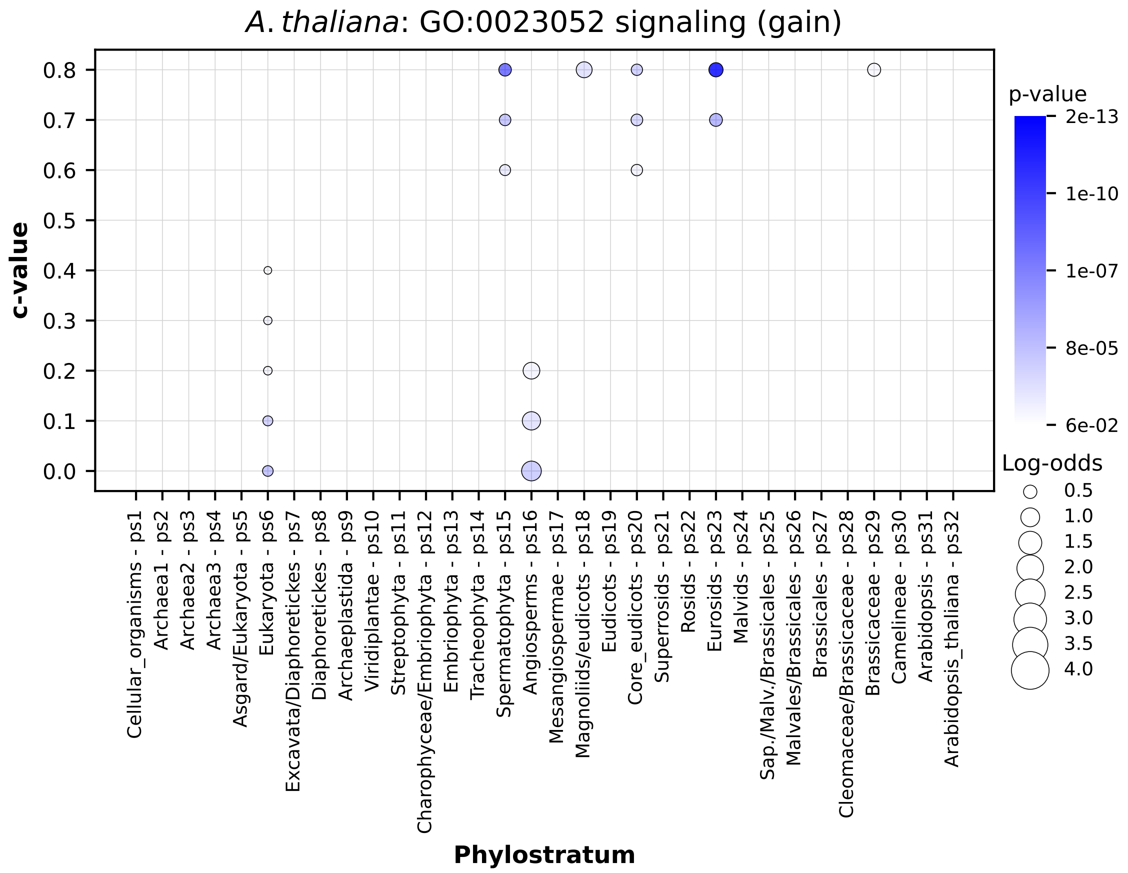
A. thaliana: GO:0023052 signaling (gain)
c-value
0.8
0.7
0.6
0.5
0.4
0.3
0.2
0.1
0.0
p-value
2e-13
1e-10
1e-07
8e-05
6e-02
Log-odds
0.5
1.0
1.5
2.0
2.5
3.0
3.5
4.0
Cellular_organisms - ps1
Archaea1 - ps2
Archaea2 - ps3
Archaea3 - ps4
Asgard/Eukaryota - ps5
Eukaryota - ps6
Excavata/Diaphoretickes - ps7
Diaphoretickes - ps8
Archaeplastida - ps9
Viridiplantae - ps10
Streptophyta - ps11
Charophyceae/Embriophyta - ps12
Embriophyta - ps13
Tracheophyta - ps14
Spermatophyta - ps15
Angiosperms - ps16
Mesangiospermae - ps17
Magnoliids/eudicots - ps18
Eudicots - ps19
Core_eudicots - ps20
Superrosids - ps21
Rosids - ps22
Eurosids - ps23
Malvids - ps24
Sap./Malv./Brassicales - ps25
Malvales/Brassicales - ps26
Brassicales - ps27
Cleomaceae/Brassicaceae - ps28
Brassicaceae - ps29
Camelineae - ps30
Arabidopsis - ps31
Arabidopsis_thaliana - ps32
Phylostratum

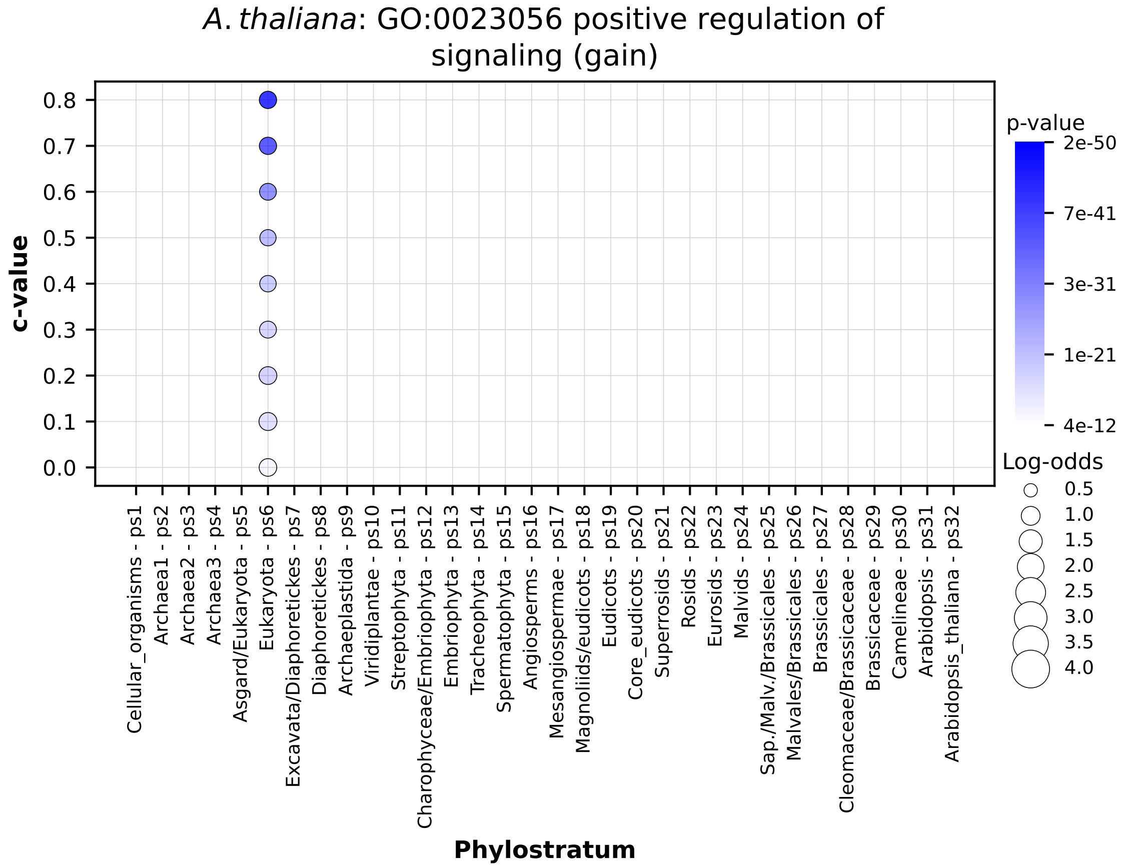

A. thaliana: GO:0023056 positive regulation of signaling (gain)
c-value
0.8
0.7
0.6
0.5
0.4
0.3
0.2
0.1
0.0
p-value
2e-50
7e-41
3e-31
1e-21
4e-12
Log-odds
0.5
1.0
1.5
2.0
2.5
3.0
3.5
4.0
Cellular_organisms - ps1
Archaea1 - ps2
Archaea2 - ps3
Archaea3 - ps4
Asgard/Eukaryota - ps5
Eukaryota - ps6
Excavata/Diaphoretickes - ps7
Diaphoretickes - ps8
Archaeplastida - ps9
Viridiplantae - ps10
Streptophyta - ps11
Charophyceae/Embriophyta - ps12
Embriophyta - ps13
Tracheophyta - ps14
Spermatophyta - ps15
Angiosperms - ps16
Mesangiospermae - ps17
Magnoliids/eudicots - ps18
Eudicots - ps19
Core_eudicots - ps20
Superrosids - ps21
Rosids - ps22
Eurosids - ps23
Malvids - ps24
Sap./Malv./Brassicales - ps25
Malvales/Brassicales - ps26
Brassicales - ps27
Cleomaceae/Brassicaceae - ps28
Brassicaceae - ps29
Camelineae - ps30
Arabidopsis - ps31
Arabidopsis_thaliana - ps32
Phylostratum

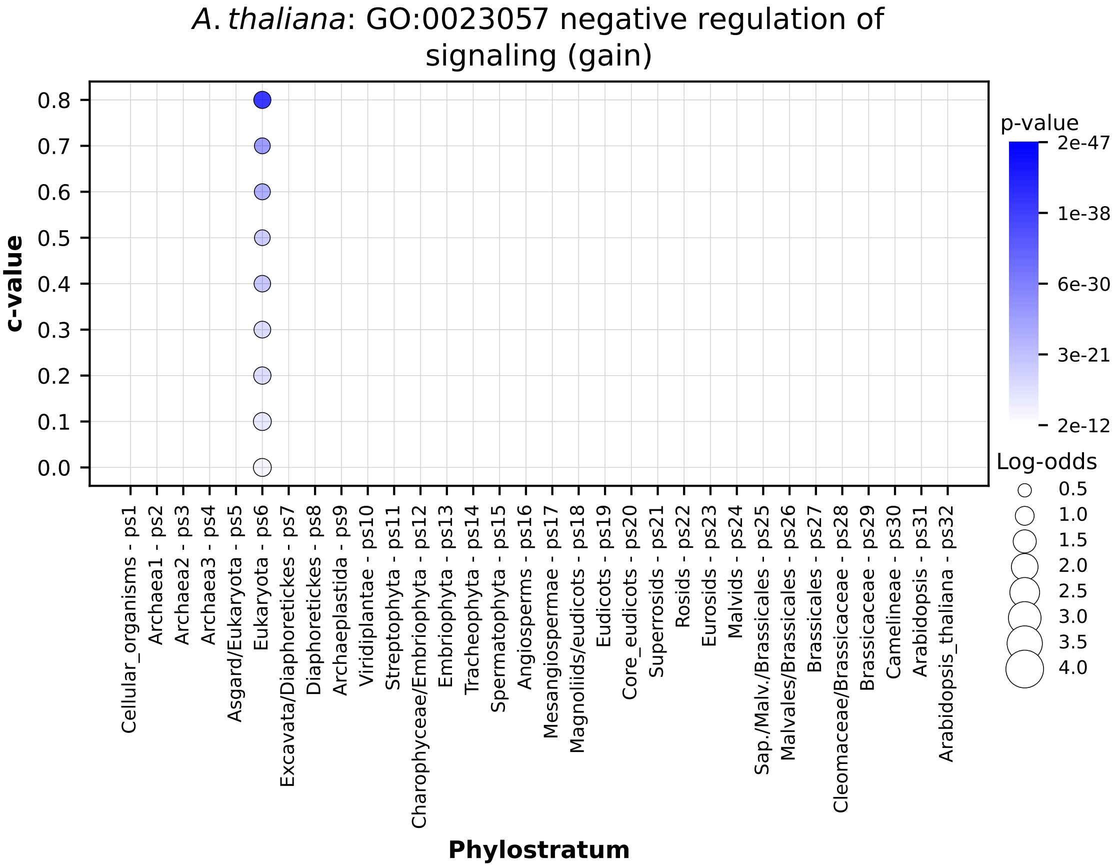
A. thaliana: GO:0023057 negative regulation of signaling (gain)
c-value
0.0
0.1
0.2
0.3
0.4
0.5
0.6
0.7
0.8
p-value
2e-47
1e-38
6e-30
3e-21
2e-12
Log-odds
0.5
1.0
1.5
2.0
2.5
3.0
3.5
4.0
Cellular_organisms - ps1
Archaea1 - ps2
Archaea2 - ps3
Archaea3 - ps4
Asgard/Eukaryota - ps5
Eukaryota - ps6
Excavata/Diaphoretickes - ps7
Diaphoretickes - ps8
Archaeplastida - ps9
Viridiplantae - ps10
Streptophyta - ps11
Charophyceae/Embriophyta - ps12
Embriophyta - ps13
Tracheophyta - ps14
Spermatophyta - ps15
Angiosperms - ps16
Mesangiospermae - ps17
Magnoliids/eudicots - ps18
Eudicots - ps19
Core_eudicots - ps20
Superrosids - ps21
Rosids - ps22
Eurosids - ps23
Malvids - ps24
Sap./Malv./Brassicales - ps25
Malvales/Brassicales - ps26
Brassicales - ps27
Cleomaceae/Brassicaceae - ps28
Brassicaceae - ps29
Camelineae - ps30
Arabidopsis - ps31
Arabidopsis_thaliana - ps32
Phylostratum

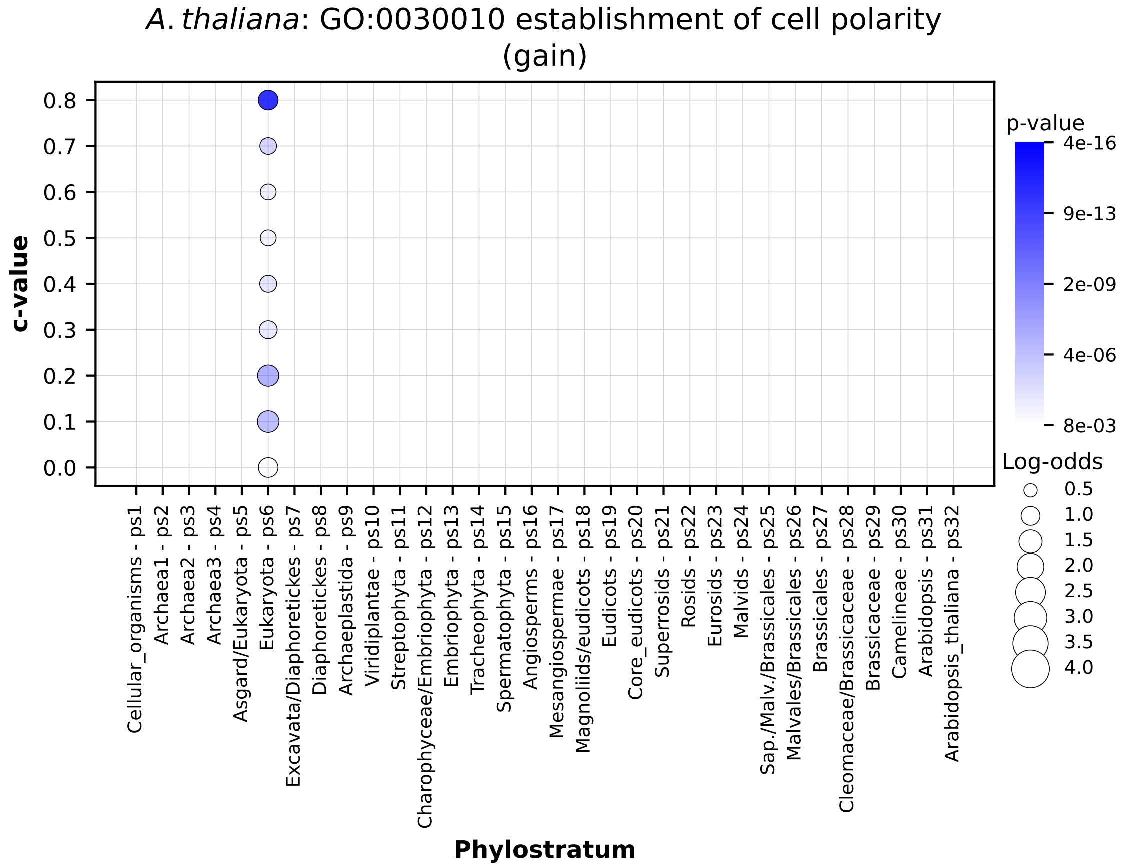

A. thaliana: GO:0030010 establishment of cell polarity
(gain)
c-value
0.8
0.7
0.6
0.5
0.4
0.3
0.2
0.1
0.0
p-value
4e-16
9e-13
2e-09
4e-06
8e-03
Log-odds
0.5
1.0
1.5
2.0
2.5
3.0
3.5
4.0
Cellular_organisms - ps1
Archaea1 - ps2
Archaea2 - ps3
Archaea3 - ps4
Asgard/Eukaryota - ps5
Eukaryota - ps6
Excavata/Diaphoretickes - ps7
Diaphoretickes - ps8
Archaeplastida - ps9
Viridiplantae - ps10
Streptophyta - ps11
Charophyceae/Embriophyta - ps12
Embriophyta - ps13
Tracheophyta - ps14
Spermatophyta - ps15
Angiosperms - ps16
Mesangiospermae - ps17
Magnoliids/eudicots - ps18
Eudicots - ps19
Core_eudicots - ps20
Superrosids - ps21
Rosids - ps22
Eurosids - ps23
Malvids - ps24
Sap./Malv./Brassicales - ps25
Malvales/Brassicales - ps26
Brassicales - ps27
Cleomaceae/Brassicaceae - ps28
Brassicaceae - ps29
Camelineae - ps30
Arabidopsis - ps31
Arabidopsis_thaliana - ps32
Phylostratum

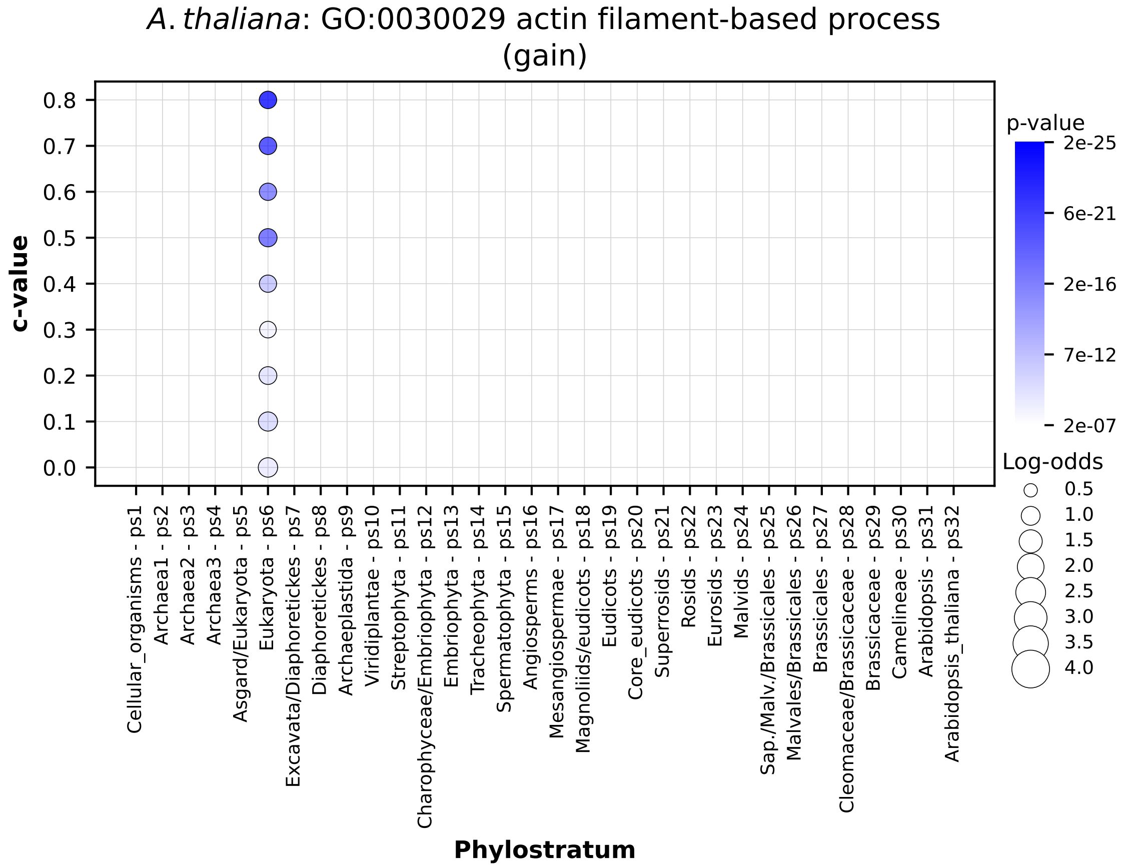
A. thaliana: GO:0030029 actin filament-based process
(gain)
c-value
0.8
0.7
0.6
0.5
0.4
0.3
0.2
0.1
0.0
p-value
2e-25
6e-21
2e-16
7e-12
2e-07
Log-odds
0.5
1.0
1.5
2.0
2.5
3.0
3.5
4.0
Cellular_organisms - ps1
Archaea1 - ps2
Archaea2 - ps3
Archaea3 - ps4
Asgard/Eukaryota - ps5
Eukaryota - ps6
Excavata/Diaphoretickes - ps7
Diaphoretickes - ps8
Archaeplastida - ps9
Viridiplantae - ps10
Streptophyta - ps11
Charophyceae/Embriophyta - ps12
Embriophyta - ps13
Tracheophyta - ps14
Spermatophyta - ps15
Angiosperms - ps16
Mesangiospermae - ps17
Magnoliids/eudicots - ps18
Eudicots - ps19
Core_eudicots - ps20
Superrosids - ps21
Rosids - ps22
Eurosids - ps23
Malvids - ps24
Sap./Malv./Brassicales - ps25
Malvales/Brassicales - ps26
Brassicales - ps27
Cleomaceae/Brassicaceae - ps28
Brassicaceae - ps29
Camelineae - ps30
Arabidopsis - ps31
Arabidopsis_thaliana - ps32
Phylostratum

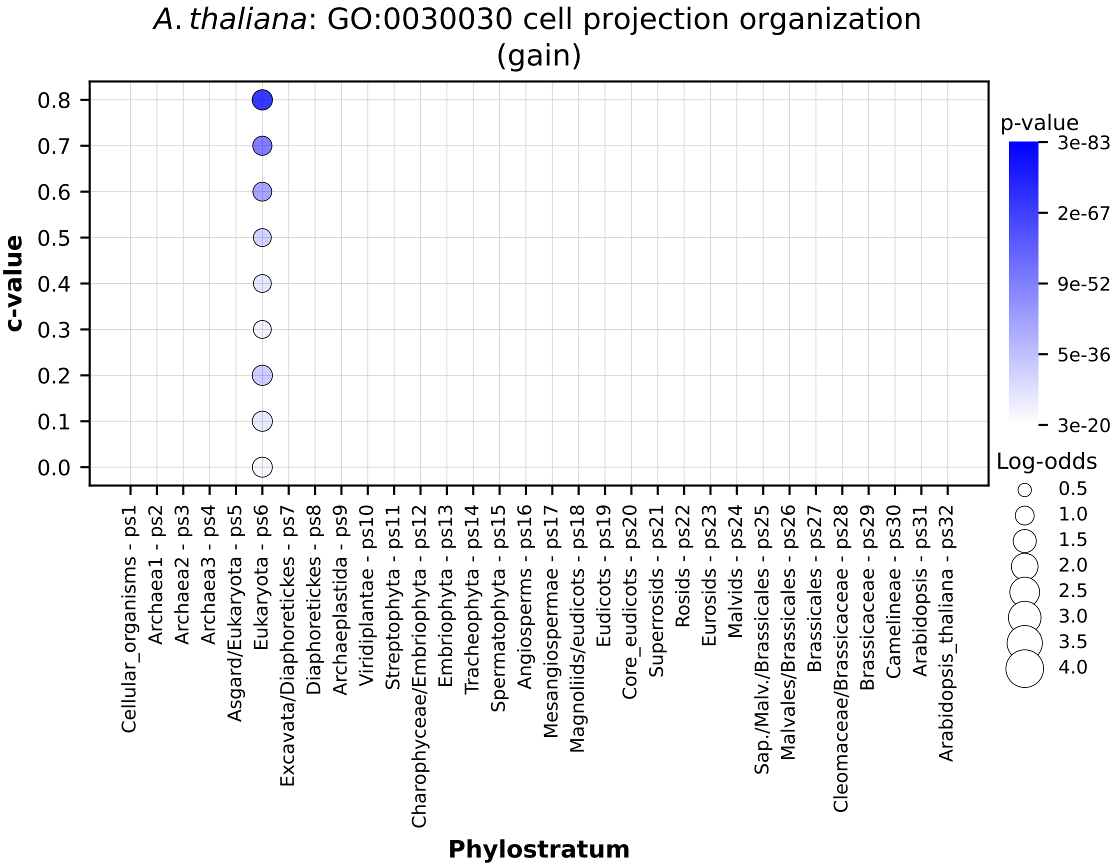

A. thaliana: GO:0030030 cell projection organization (gain)
c-value
0.8
0.7
0.6
0.5
0.4
0.3
0.2
0.1
0.0
p-value
3e-83
2e-67
9e-52
5e-36
3e-20
Log-odds
0.5
1.0
1.5
2.0
2.5
3.0
3.5
4.0
Cellular_organisms - ps1
Archaea1 - ps2
Archaea2 - ps3
Archaea3 - ps4
Asgard/Eukaryota - ps5
Eukaryota - ps6
Excavata/Diaphoretickes - ps7
Diaphoretickes - ps8
Archaeplastida - ps9
Viridiplantae - ps10
Streptophyta - ps11
Charophyceae/Embriophyta - ps12
Embriophyta - ps13
Tracheophyta - ps14
Spermatophyta - ps15
Angiosperms - ps16
Mesangiospermae - ps17
Magnoliids/eudicots - ps18
Eudicots - ps19
Core_eudicots - ps20
Superrosids - ps21
Rosids - ps22
Eurosids - ps23
Malvids - ps24
Sap./Malv./Brassicales - ps25
Malvales/Brassicales - ps26
Brassicales - ps27
Cleomaceae/Brassicaceae - ps28
Brassicaceae - ps29
Camelineae - ps30
Arabidopsis - ps31
Arabidopsis_thaliana - ps32
Phylostratum

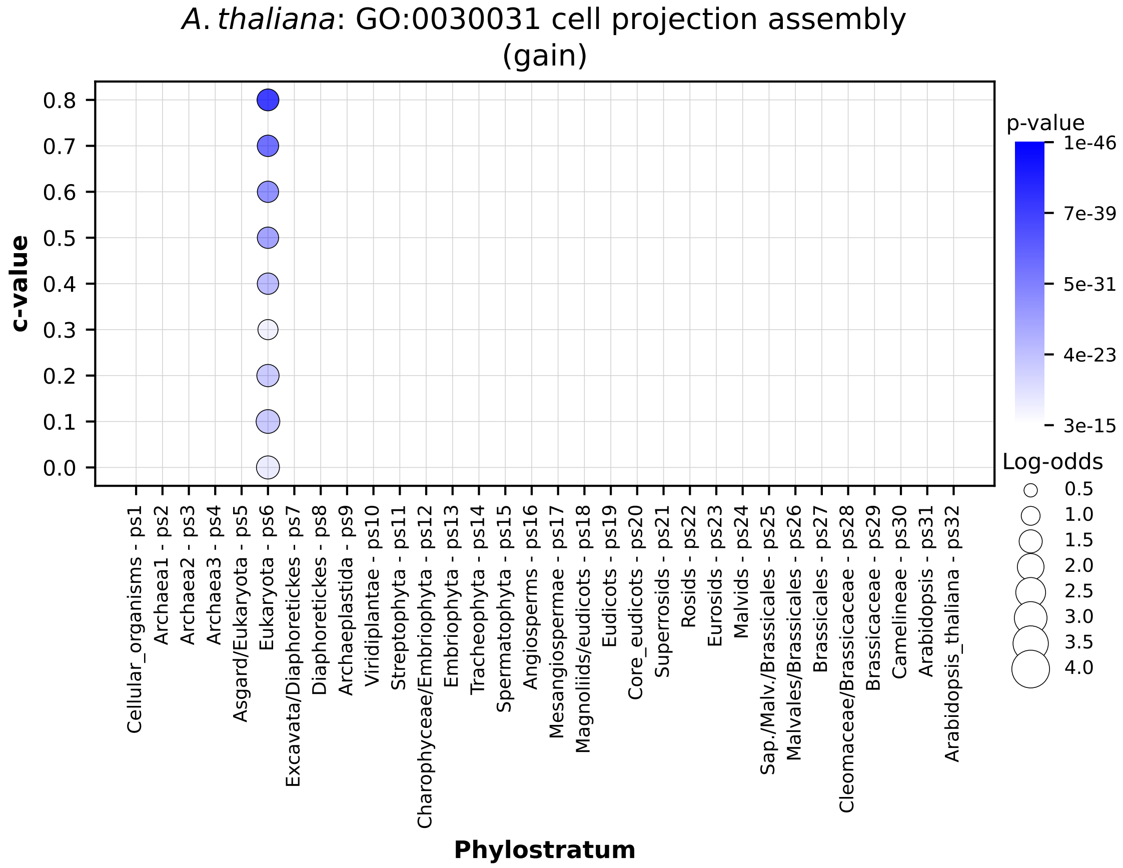
A. thaliana: GO:0030031 cell projection assembly
(gain)
c-value
0.0
0.1
0.2
0.3
0.4
0.5
0.6
0.7
0.8
p-value
1e-46
7e-39
5e-31
4e-23
3e-15
Log-odds
0.5
1.0
1.5
2.0
2.5
3.0
3.5
4.0
Cellular_organisms - ps1
Archaea1 - ps2
Archaea2 - ps3
Archaea3 - ps4
Asgard/Eukaryota - ps5
Eukaryota - ps6
Excavata/Diaphoretickes - ps7
Diaphoretickes - ps8
Archaeplastida - ps9
Viridiplantae - ps10
Streptophyta - ps11
Charophyceae/Embriophyta - ps12
Embriophyta - ps13
Tracheophyta - ps14
Spermatophyta - ps15
Angiosperms - ps16
Mesangiospermae - ps17
Magnoliids/eudicots - ps18
Eudicots - ps19
Core_eudicots - ps20
Superrosids - ps21
Rosids - ps22
Eurosids - ps23
Malvids - ps24
Sap./Malv./Brassicales - ps25
Malvales/Brassicales - ps26
Brassicales - ps27
Cleomaceae/Brassicaceae - ps28
Brassicaceae - ps29
Camelineae - ps30
Arabidopsis - ps31
Arabidopsis_thaliana - ps32
Phylostratum

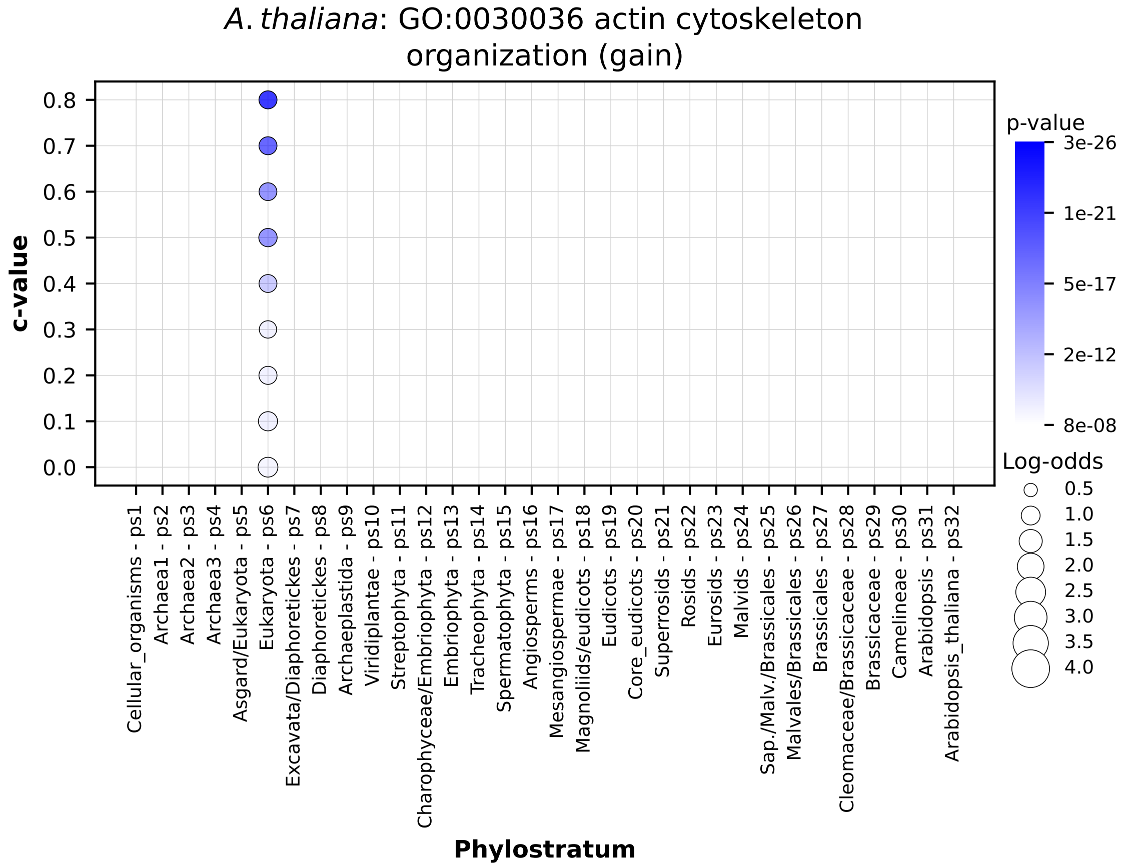
A. thaliana: GO:0030036 actin cytoskeleton organization (gain)
c-value
0.8
0.7
0.6
0.5
0.4
0.3
0.2
0.1
0.0
Phylostratum
Cellular_organisms - ps1
Archaea1 - ps2
Archaea2 - ps3
Archaea3 - ps4
Asgard/Eukaryota - ps5
Eukaryota - ps6
Excavata/Diaphoretickes - ps7
Diaphoretickes - ps8
Archaeplastida - ps9
Viridiplantae - ps10
Streptophyta - ps11
Charophyceae/Embriophyta - ps12
Embriophyta - ps13
Tracheophyta - ps14
Spermatophyta - ps15
Angiosperms - ps16
Mesangiospermae - ps17
Magnoliids/eudicots - ps18
Eudicots - ps19
Core_eudicots - ps20
Superrosids - ps21
Rosids - ps22
Eurosids - ps23
Malvids - ps24
Sap./Malv./Brassicales - ps25
Malvales/Brassicales - ps26
Brassicales - ps27
Cleomaceae/Brassicaceae - ps28
Brassicaceae - ps29
Camelineae - ps30
Arabidopsis - ps31
Arabidopsis_thaliana - ps32
p-value
3e-26
1e-21
5e-17
2e-12
8e-08
Log-odds
0.5
1.0
1.5
2.0
2.5
3.0
3.5
4.0

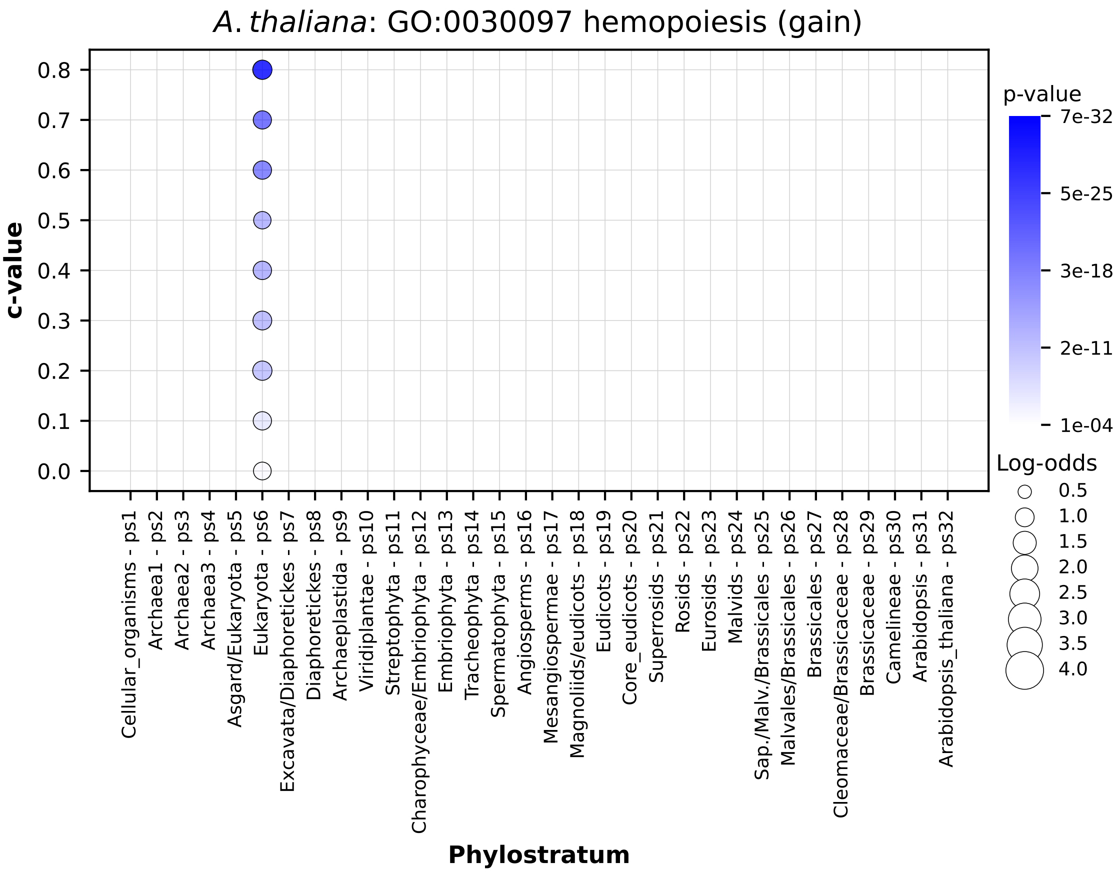

A. thaliana: GO:0030097 hemopoiesis (gain)
c-value
0.8
0.7
0.6
0.5
0.4
0.3
0.2
0.1
0.0
p-value
7e-32
5e-25
3e-18
2e-11
1e-04
Log-odds
0.5
1.0
1.5
2.0
2.5
3.0
3.5
4.0
Cellular_organisms - ps1
Archaea1 - ps2
Archaea2 - ps3
Archaea3 - ps4
Asgard/Eukaryota - ps5
Eukaryota - ps6
Excavata/Diaphoretickes - ps7
Diaphoretickes - ps8
Archaeplastida - ps9
Viridiplantae - ps10
Streptophyta - ps11
Charophyceae/Embriophyta - ps12
Embriophyta - ps13
Tracheophyta - ps14
Spermatophyta - ps15
Angiosperms - ps16
Mesangiospermae - ps17
Magnoliids/eudicots - ps18
Eudicots - ps19
Core_eudicots - ps20
Superrosids - ps21
Rosids - ps22
Eurosids - ps23
Malvids - ps24
Sap./Malv./Brassicales - ps25
Malvales/Brassicales - ps26
Brassicales - ps27
Cleomaceae/Brassicaceae - ps28
Brassicaceae - ps29
Camelineae - ps30
Arabidopsis - ps31
Arabidopsis_thaliana - ps32
Phylostratum

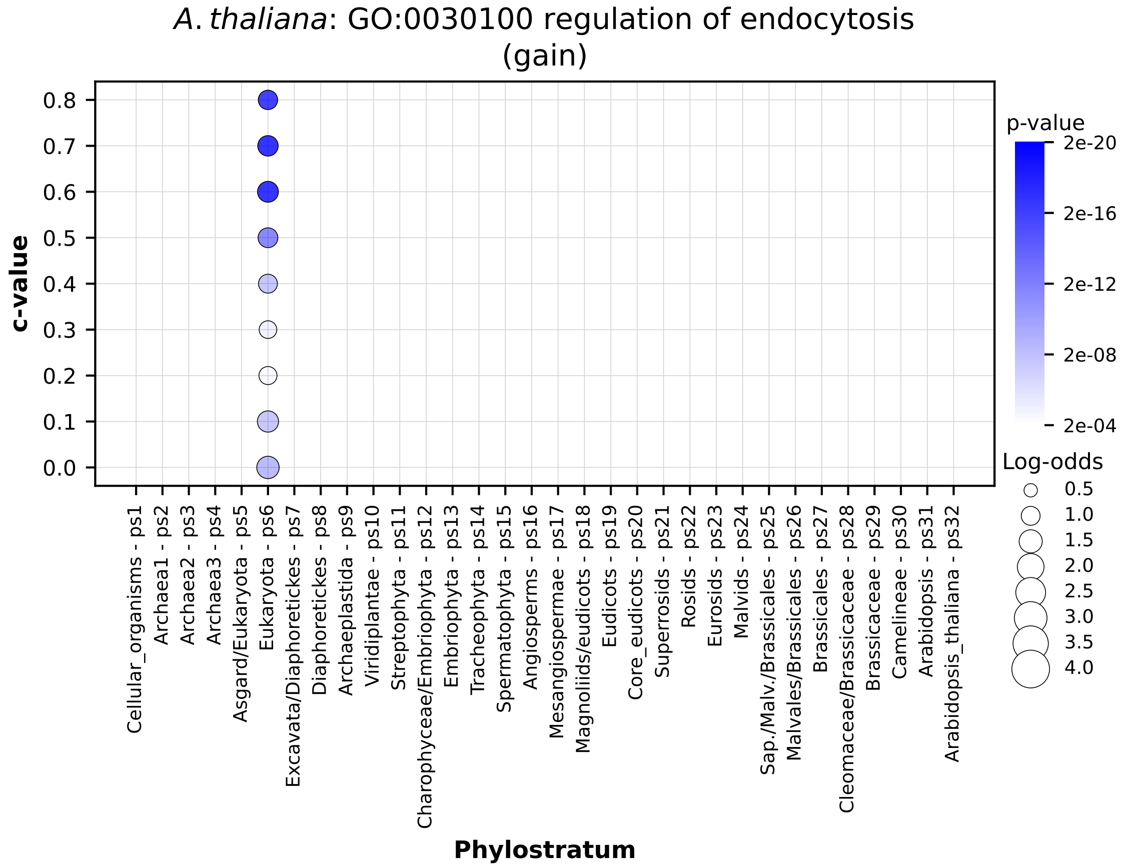
A. thaliana: GO:0030100 regulation of endocytosis
(gain)
c-value
0.8
0.7
0.6
0.5
0.4
0.3
0.2
0.1
0.0
p-value
2e-20
2e-16
2e-12
2e-08
2e-04
Log-odds
0.5
1.0
1.5
2.0
2.5
3.0
3.5
4.0
Cellular_organisms - ps1
Archaea1 - ps2
Archaea2 - ps3
Archaea3 - ps4
Asgard/Eukaryota - ps5
Eukaryota - ps6
Excavata/Diaphoretickes - ps7
Diaphoretickes - ps8
Archaeplastida - ps9
Viridiplantae - ps10
Streptophyta - ps11
Charophyceae/Embriophyta - ps12
Embriophyta - ps13
Tracheophyta - ps14
Spermatophyta - ps15
Angiosperms - ps16
Mesangiospermae - ps17
Magnoliids/eudicots - ps18
Eudicots - ps19
Core_eudicots - ps20
Superrosids - ps21
Rosids - ps22
Eurosids - ps23
Malvids - ps24
Sap./Malv./Brassicales - ps25
Malvales/Brassicales - ps26
Brassicales - ps27
Cleomaceae/Brassicaceae - ps28
Brassicaceae - ps29
Camelineae - ps30
Arabidopsis - ps31
Arabidopsis_thaliana - ps32
Phylostratum

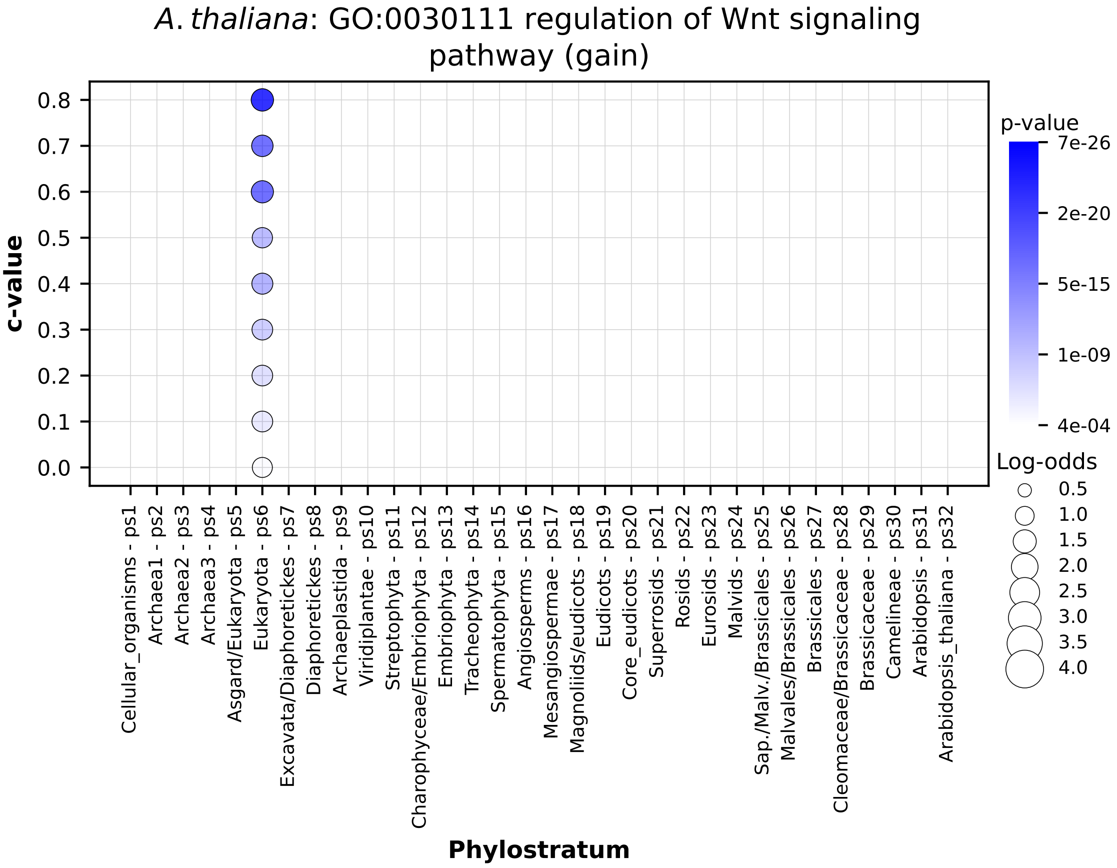

A. thaliana: GO:0030111 regulation of Wnt signaling pathway (gain)
c-value
0.8
0.7
0.6
0.5
0.4
0.3
0.2
0.1
0.0
p-value
7e-26
2e-20
5e-15
1e-09
4e-04
Log-odds
0.5
1.0
1.5
2.0
2.5
3.0
3.5
4.0
Cellular_organisms - ps1
Archaea1 - ps2
Archaea2 - ps3
Archaea3 - ps4
Asgard/Eukaryota - ps5
Eukaryota - ps6
Excavata/Diaphoretickes - ps7
Diaphoretickes - ps8
Archaeplastida - ps9
Viridiplantae - ps10
Streptophyta - ps11
Charophyceae/Embriophyta - ps12
Embriophyta - ps13
Tracheophyta - ps14
Spermatophyta - ps15
Angiosperms - ps16
Mesangiospermae - ps17
Magnoliids/eudicots - ps18
Eudicots - ps19
Core_eudicots - ps20
Superrosids - ps21
Rosids - ps22
Eurosids - ps23
Malvids - ps24
Sap./Malv./Brassicales - ps25
Malvales/Brassicales - ps26
Brassicales - ps27
Cleomaceae/Brassicaceae - ps28
Brassicaceae - ps29
Camelineae - ps30
Arabidopsis - ps31
Arabidopsis_thaliana - ps32
Phylostratum

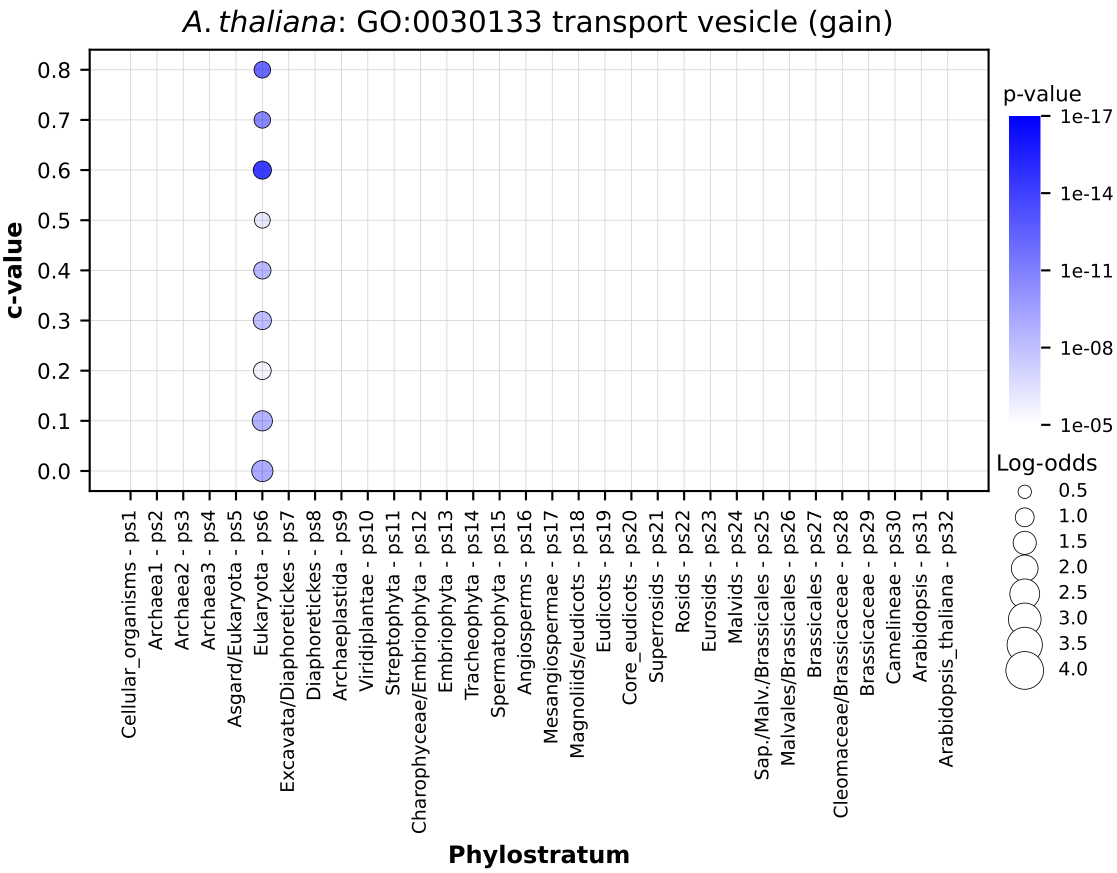

A. thaliana: GO:0030133 transport vesicle (gain)
c-value
0.8
0.7
0.6
0.5
0.4
0.3
0.2
0.1
0.0
p-value
1e-17
1e-14
1e-11
1e-08
1e-05
Log-odds
0.5
1.0
1.5
2.0
2.5
3.0
3.5
4.0
Cellular_organisms - ps1
Archaea1 - ps2
Archaea2 - ps3
Archaea3 - ps4
Asgard/Eukaryota - ps5
Eukaryota - ps6
Excavata/Diaphoretickes - ps7
Diaphoretickes - ps8
Archaeplastida - ps9
Viridiplantae - ps10
Streptophyta - ps11
Charophyceae/Embriophyta - ps12
Embriophyta - ps13
Tracheophyta - ps14
Spermatophyta - ps15
Angiosperms - ps16
Mesangiospermae - ps17
Magnoliids/eudicots - ps18
Eudicots - ps19
Core_eudicots - ps20
Superrosids - ps21
Rosids - ps22
Eurosids - ps23
Malvids - ps24
Sap./Malv./Brassicales - ps25
Malvales/Brassicales - ps26
Brassicales - ps27
Cleomaceae/Brassicaceae - ps28
Brassicaceae - ps29
Camelineae - ps30
Arabidopsis - ps31
Arabidopsis_thaliana - ps32
Phylostratum

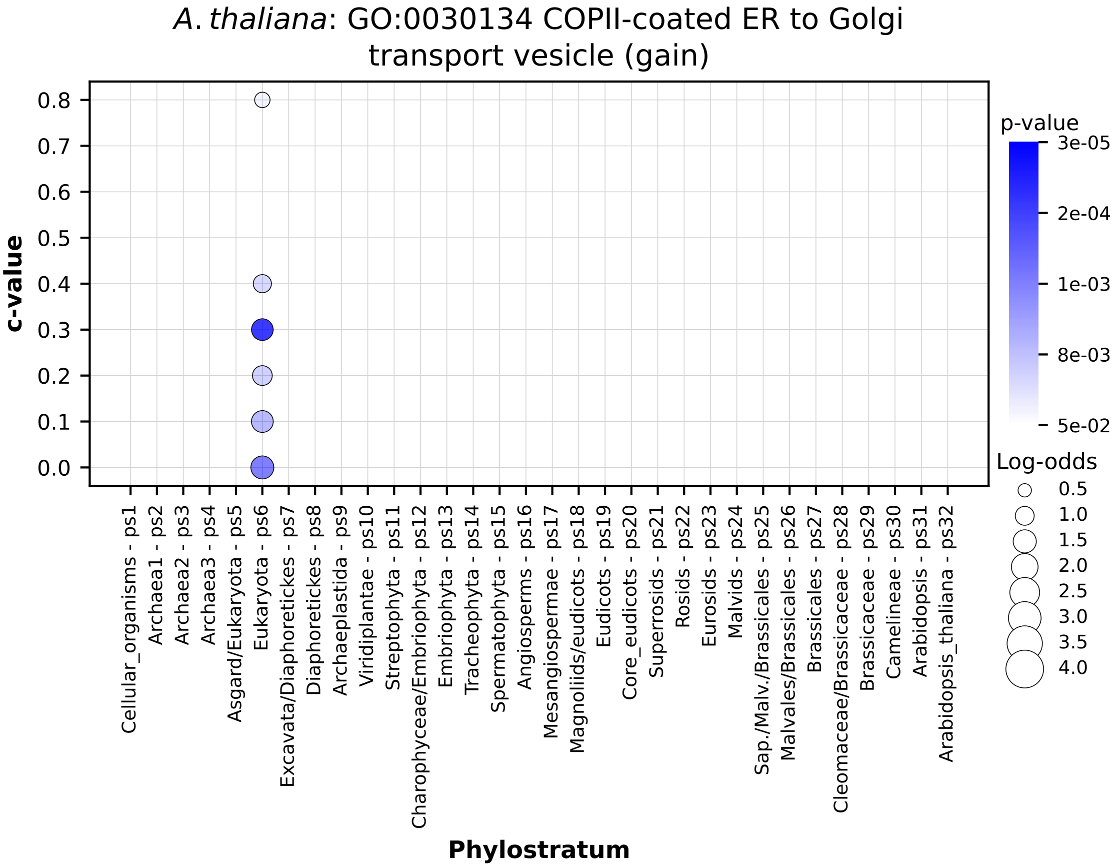

A. thaliana: GO:0030134 COPII-coated ER to Golgi transport vesicle (gain)
c-value
0.8
0.7
0.6
0.5
0.4
0.3
0.2
0.1
0.0
p-value
3e-05
2e-04
1e-03
8e-03
5e-02
Log-odds
0.5
1.0
1.5
2.0
2.5
3.0
3.5
4.0
Cellular_organisms - ps1
Archaea1 - ps2
Archaea2 - ps3
Archaea3 - ps4
Asgard/Eukaryota - ps5
Eukaryota - ps6
Excavata/Diaphoretickes - ps7
Diaphoretickes - ps8
Archaeplastida - ps9
Viridiplantae - ps10
Streptophyta - ps11
Charophyceae/Embriophyta - ps12
Embriophyta - ps13
Tracheophyta - ps14
Spermatophyta - ps15
Angiosperms - ps16
Mesangiospermae - ps17
Magnoliids/eudicots - ps18
Eudicots - ps19
Core_eudicots - ps20
Superrosids - ps21
Rosids - ps22
Eurosids - ps23
Malvids - ps24
Sap./Malv./Brassicales - ps25
Malvales/Brassicales - ps26
Brassicales - ps27
Cleomaceae/Brassicaceae - ps28
Brassicaceae - ps29
Camelineae - ps30
Arabidopsis - ps31
Arabidopsis_thaliana - ps32
Phylostratum

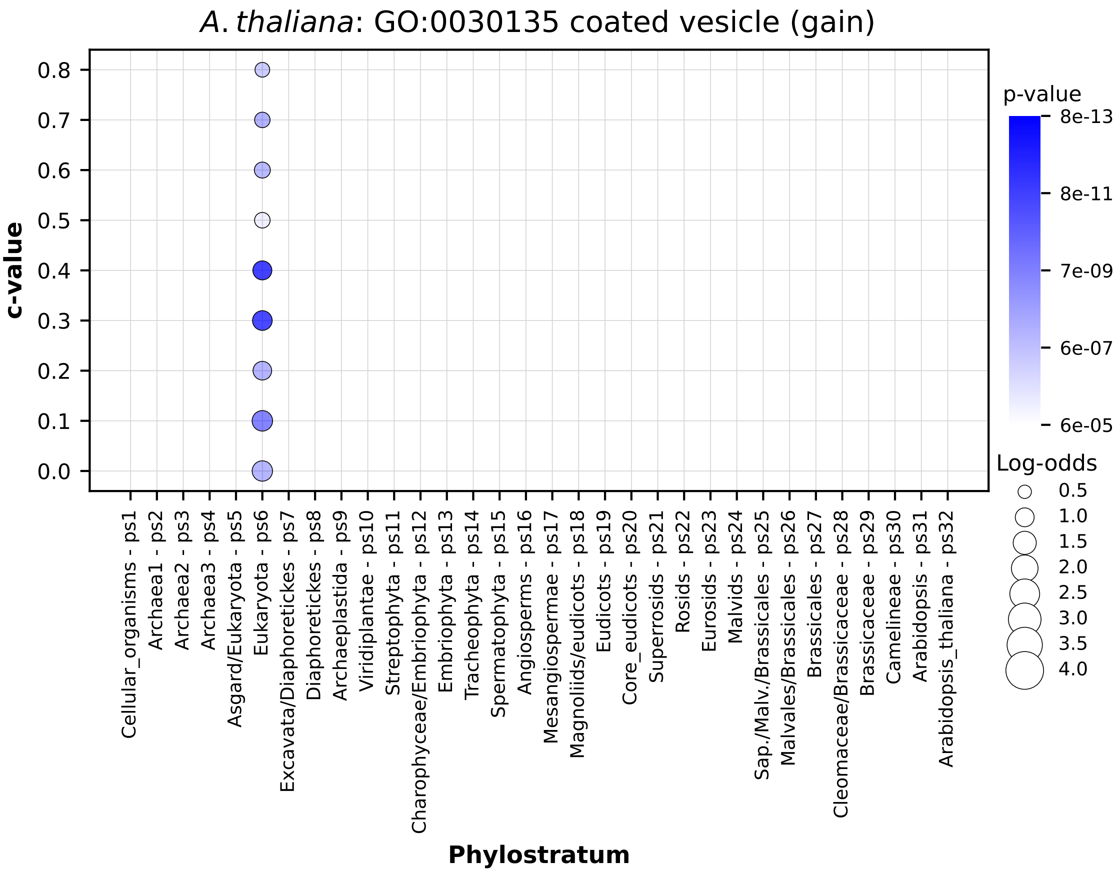
A. thaliana: GO:0030135 coated vesicle (gain)
c-value
0.8
0.7
0.6
0.5
0.4
0.3
0.2
0.1
0.0
Phylostratum
Cellular_organisms - ps1
Archaea1 - ps2
Archaea2 - ps3
Archaea3 - ps4
Asgard/Eukaryota - ps5
Eukaryota - ps6
Excavata/Diaphoretickes - ps7
Diaphoretickes - ps8
Archaeplastida - ps9
Viridiplantae - ps10
Streptophyta - ps11
Charophyceae/Embriophyta - ps12
Embriophyta - ps13
Tracheophyta - ps14
Spermatophyta - ps15
Angiosperms - ps16
Mesangiospermae - ps17
Magnoliids/eudicots - ps18
Eudicots - ps19
Core_eudicots - ps20
Superrosids - ps21
Rosids - ps22
Eurosids - ps23
Malvids - ps24
Sap./Malv./Brassicales - ps25
Malvales/Brassicales - ps26
Brassicales - ps27
Cleomaceae/Brassicaceae - ps28
Brassicaceae - ps29
Camelineae - ps30
Arabidopsis - ps31
Arabidopsis_thaliana - ps32
p-value
8e-13
8e-11
7e-09
6e-07
6e-05
Log-odds
0.5
1.0
1.5
2.0
2.5
3.0
3.5
4.0

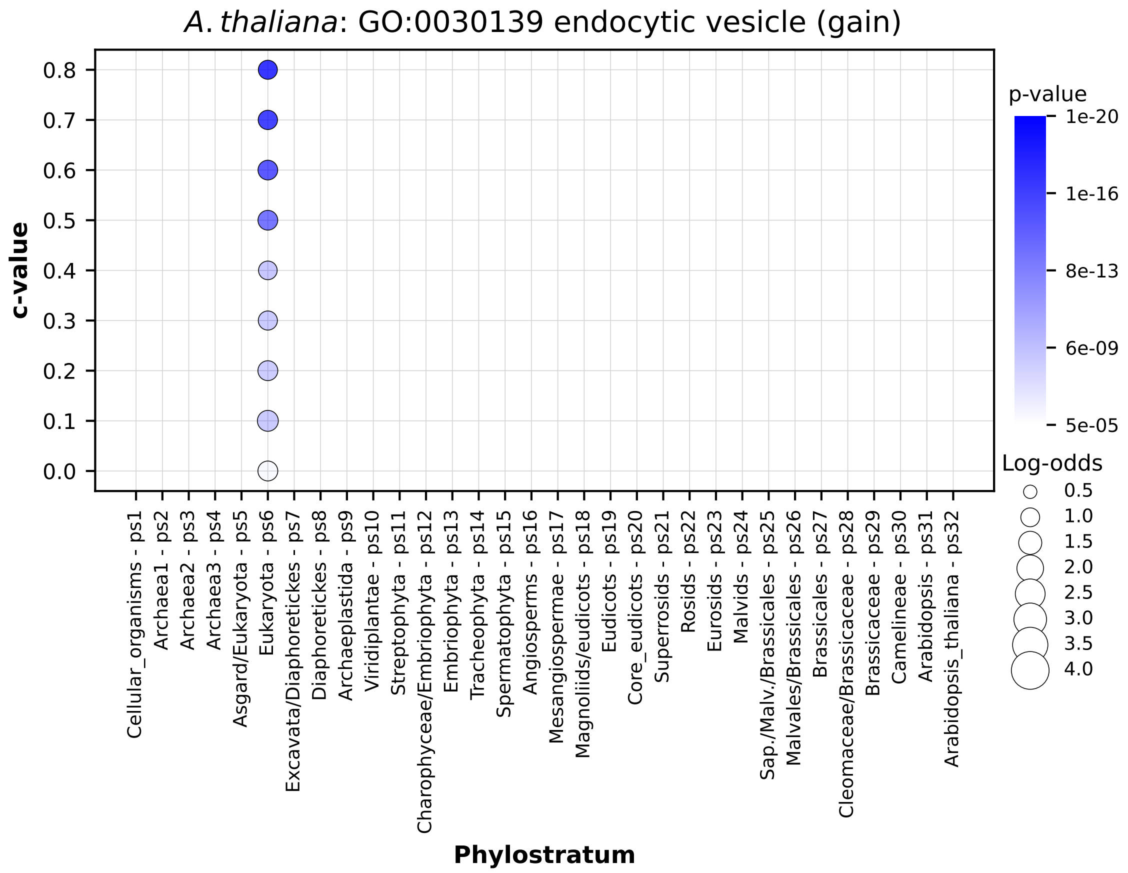

A. thaliana: GO:0030139 endocytic vesicle (gain)
c-value
0.8
0.7
0.6
0.5
0.4
0.3
0.2
0.1
0.0
Phylostratum
Cellular_organisms - ps1
Archaea1 - ps2
Archaea2 - ps3
Archaea3 - ps4
Asgard/Eukaryota - ps5
Eukaryota - ps6
Excavata/Diaphoretickes - ps7
Diaphoretickes - ps8
Archaeplastida - ps9
Viridiplantae - ps10
Streptophyta - ps11
Charophyceae/Embriophyta - ps12
Embriophyta - ps13
Tracheophyta - ps14
Spermatophyta - ps15
Angiosperms - ps16
Mesangiospermae - ps17
Magnoliids/eudicots - ps18
Eudicots - ps19
Core_eudicots - ps20
Superrosids - ps21
Rosids - ps22
Eurosids - ps23
Malvids - ps24
Sap./Malv./Brassicales - ps25
Malvales/Brassicales - ps26
Brassicales - ps27
Cleomaceae/Brassicaceae - ps28
Brassicaceae - ps29
Camelineae - ps30
Arabidopsis - ps31
Arabidopsis_thaliana - ps32
p-value
1e-20
1e-16
8e-13
6e-09
5e-05
Log-odds
0.5
1.0
1.5
2.0
2.5
3.0
3.5
4.0

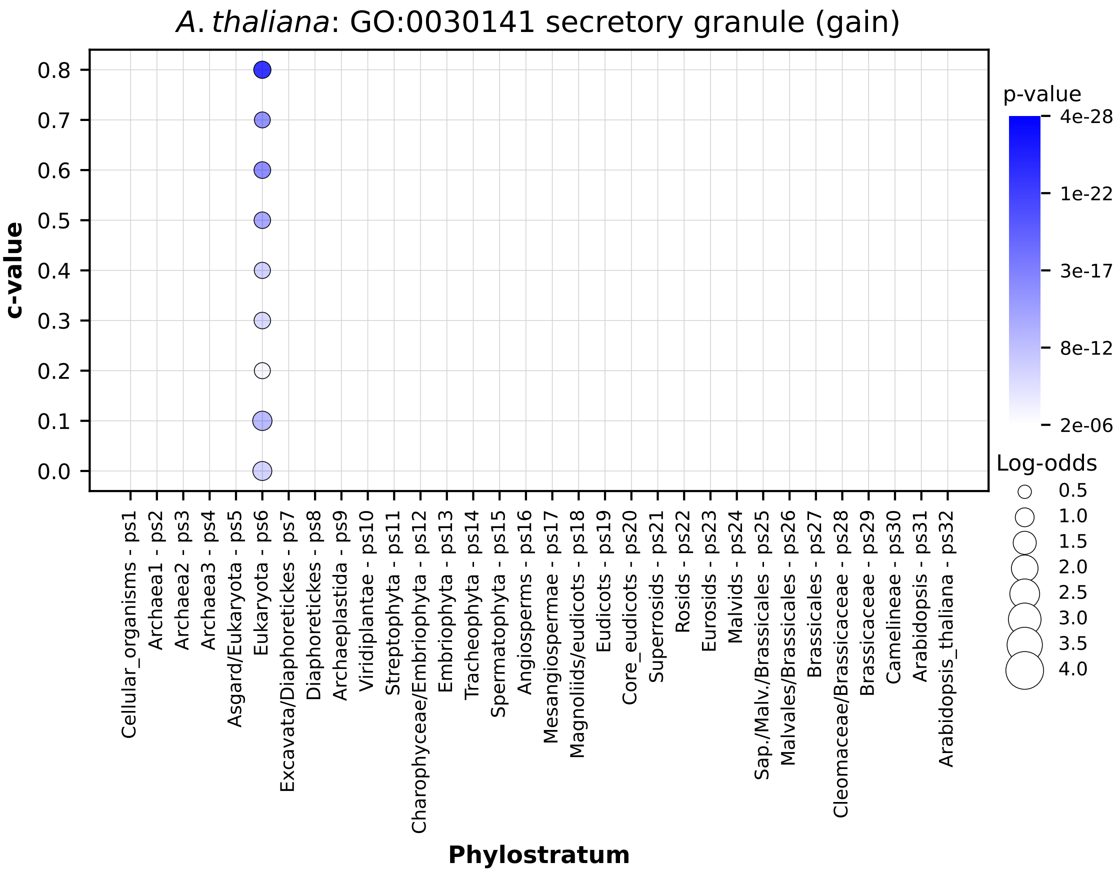
A. thaliana: GO:0030141 secretory granule (gain)
c-value
0.8
0.7
0.6
0.5
0.4
0.3
0.2
0.1
0.0
Phylostratum
Cellular_organisms - ps1
Archaea1 - ps2
Archaea2 - ps3
Archaea3 - ps4
Asgard/Eukaryota - ps5
Eukaryota - ps6
Excavata/Diaphoretickes - ps7
Diaphoretickes - ps8
Archaeplastida - ps9
Viridiplantae - ps10
Streptophyta - ps11
Charophyceae/Embriophyta - ps12
Embriophyta - ps13
Tracheophyta - ps14
Spermatophyta - ps15
Angiosperms - ps16
Mesangiospermae - ps17
Magnoliids/eudicots - ps18
Eudicots - ps19
Core_eudicots - ps20
Superrosids - ps21
Rosids - ps22
Eurosids - ps23
Malvids - ps24
Sap./Malv./Brassicales - ps25
Malvales/Brassicales - ps26
Brassicales - ps27
Cleomaceae/Brassicaceae - ps28
Brassicaceae - ps29
Camelineae - ps30
Arabidopsis - ps31
Arabidopsis_thaliana - ps32
p-value
4e-28
1e-22
3e-17
8e-12
2e-06
Log-odds
0.5
1.0
1.5
2.0
2.5
3.0
3.5
4.0

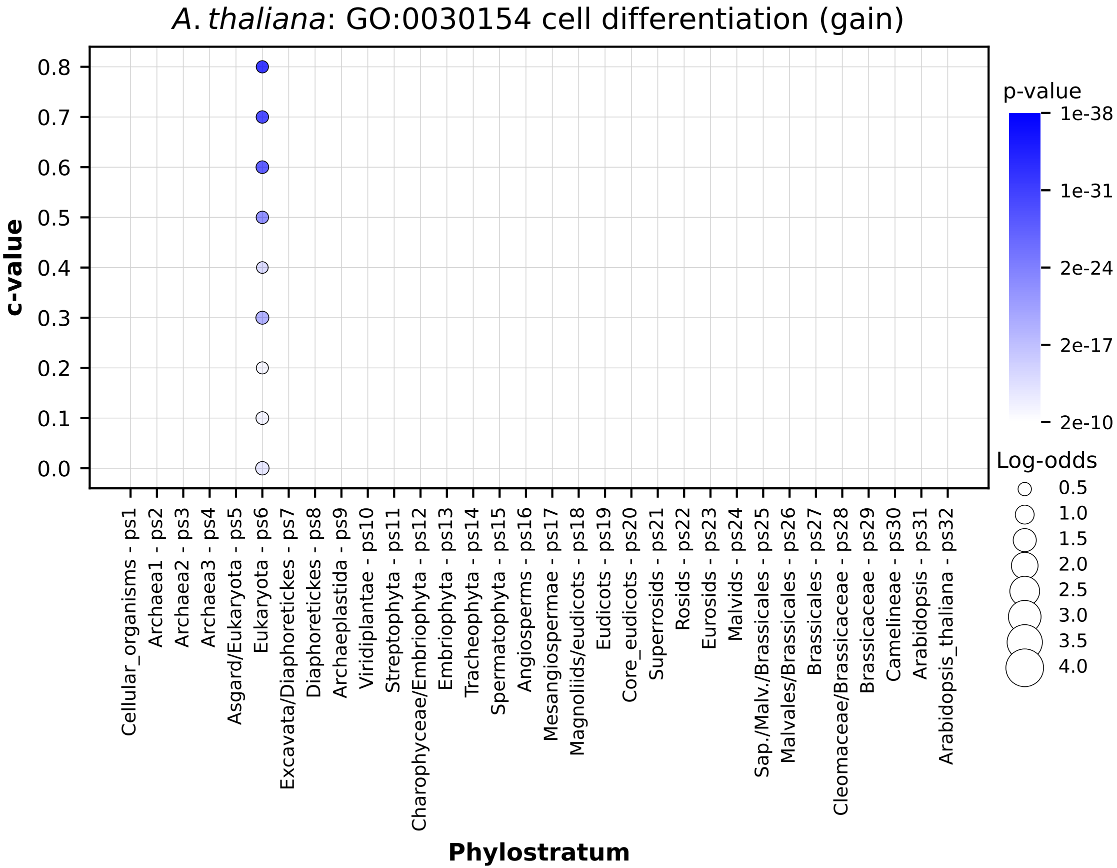

A. thaliana: GO:0030154 cell differentiation (gain)
c-value
0.8
0.7
0.6
0.5
0.4
0.3
0.2
0.1
0.0
p-value
1e-38
1e-31
2e-24
2e-17
2e-10
Log-odds
0.5
1.0
1.5
2.0
2.5
3.0
3.5
4.0
Cellular_organisms - ps1
Archaea1 - ps2
Archaea2 - ps3
Archaea3 - ps4
Asgard/Eukaryota - ps5
Eukaryota - ps6
Excavata/Diaphoretickes - ps7
Diaphoretickes - ps8
Archaeplastida - ps9
Viridiplantae - ps10
Streptophyta - ps11
Charophyceae/Embriophyta - ps12
Embriophyta - ps13
Tracheophyta - ps14
Spermatophyta - ps15
Angiosperms - ps16
Mesangiospermae - ps17
Magnoliids/eudicots - ps18
Eudicots - ps19
Core_eudicots - ps20
Superrosids - ps21
Rosids - ps22
Eurosids - ps23
Malvids - ps24
Sap./Malv./Brassicales - ps25
Malvales/Brassicales - ps26
Brassicales - ps27
Cleomaceae/Brassicaceae - ps28
Brassicaceae - ps29
Camelineae - ps30
Arabidopsis - ps31
Arabidopsis_thaliana - ps32
Phylostratum

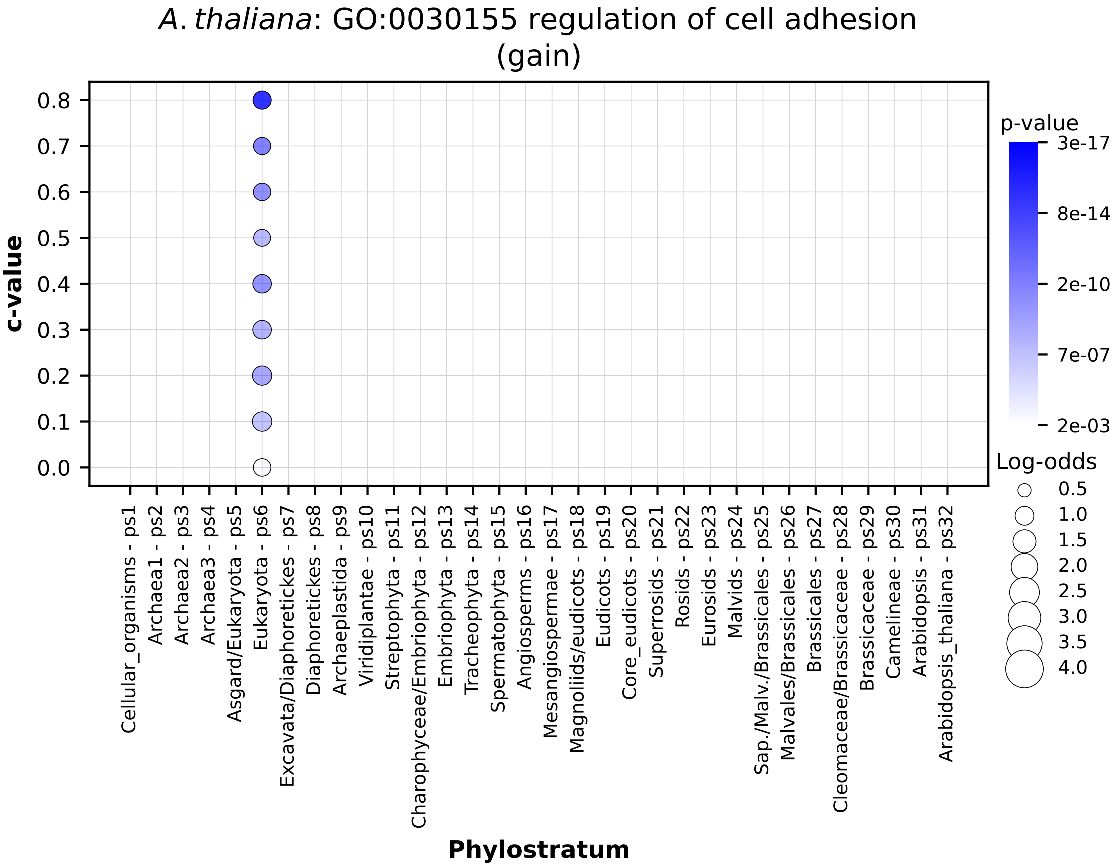

A. thaliana: GO:0030155 regulation of cell adhesion
(gain)
c-value
0.8
0.7
0.6
0.5
0.4
0.3
0.2
0.1
0.0
p-value
3e-17
8e-14
2e-10
7e-07
2e-03
Log-odds
0.5
1.0
1.5
2.0
2.5
3.0
3.5
4.0
Cellular_organisms - ps1
Archaea1 - ps2
Archaea2 - ps3
Archaea3 - ps4
Asgard/Eukaryota - ps5
Eukaryota - ps6
Excavata/Diaphoretickes - ps7
Diaphoretickes - ps8
Archaeplastida - ps9
Viridiplantae - ps10
Streptophyta - ps11
Charophyceae/Embriophyta - ps12
Embriophyta - ps13
Tracheophyta - ps14
Spermatophyta - ps15
Angiosperms - ps16
Mesangiospermae - ps17
Magnoliids/eudicots - ps18
Eudicots - ps19
Core_eudicots - ps20
Superrosids - ps21
Rosids - ps22
Eurosids - ps23
Malvids - ps24
Sap./Malv./Brassicales - ps25
Malvales/Brassicales - ps26
Brassicales - ps27
Cleomaceae/Brassicaceae - ps28
Brassicaceae - ps29
Camelineae - ps30
Arabidopsis - ps31
Arabidopsis_thaliana - ps32
Phylostratum

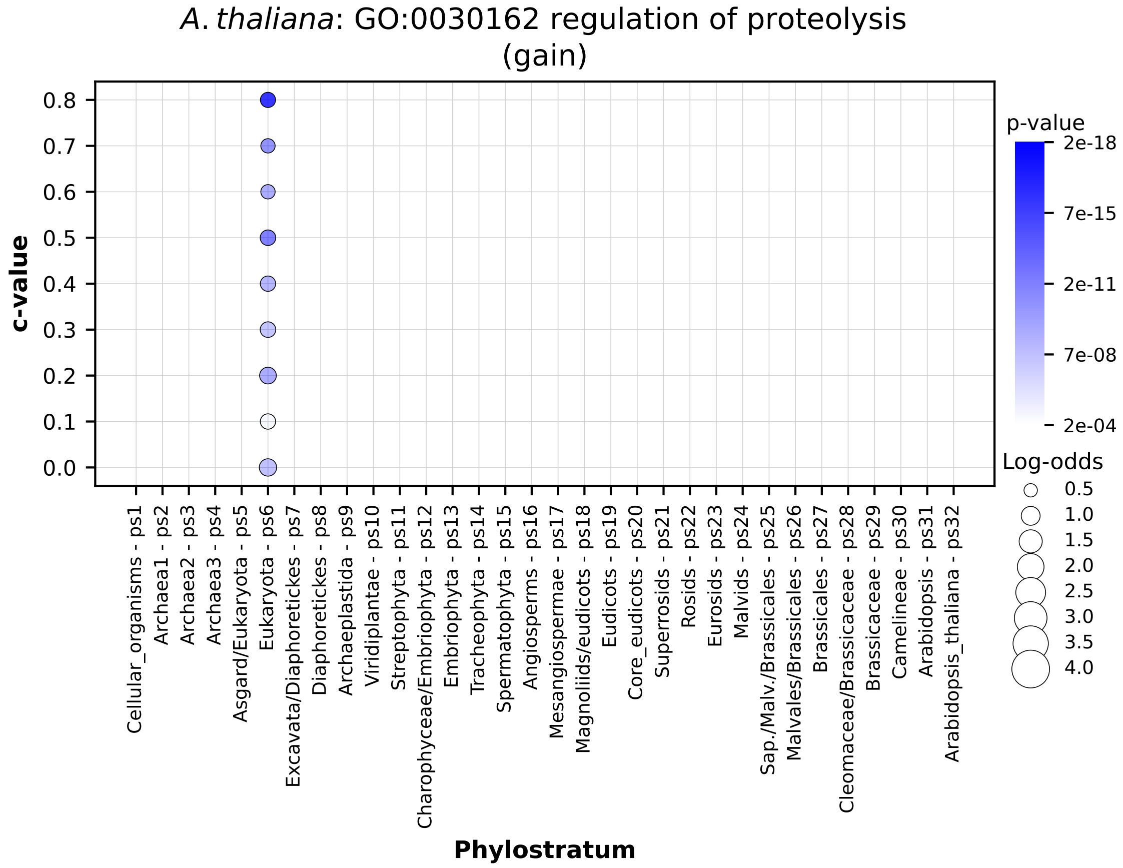

$A. thaliana$: GO:0030162 regulation of proteolysis
(gain)
c-value
0.8
0.7
0.6
0.5
0.4
0.3
0.2
0.1
0.0
p-value
2e-18
7e-15
2e-11
7e-08
2e-04
Log-odds
0.5
1.0
1.5
2.0
2.5
3.0
3.5
4.0
Cellular_organisms - ps1
Archaea1 - ps2
Archaea2 - ps3
Archaea3 - ps4
Asgard/Eukaryota - ps5
Eukaryota - ps6
Excavata/Diaphoretickes - ps7
Diaphoretickes - ps8
Archaeplastida - ps9
Viridiplantae - ps10
Streptophyta - ps11
Charophyceae/Embriophyta - ps12
Embriophyta - ps13
Tracheophyta - ps14
Spermatophyta - ps15
Angiosperms - ps16
Mesangiospermae - ps17
Magnoliids/eudicots - ps18
Eudicots - ps19
Core_eudicots - ps20
Superrosids - ps21
Rosids - ps22
Eurosids - ps23
Malvids - ps24
Sap./Malv./Brassicales - ps25
Malvales/Brassicales - ps26
Brassicales - ps27
Cleomaceae/Brassicaceae - ps28
Brassicaceae - ps29
Camelineae - ps30
Arabidopsis - ps31
Arabidopsis_thaliana - ps32
Phylostratum

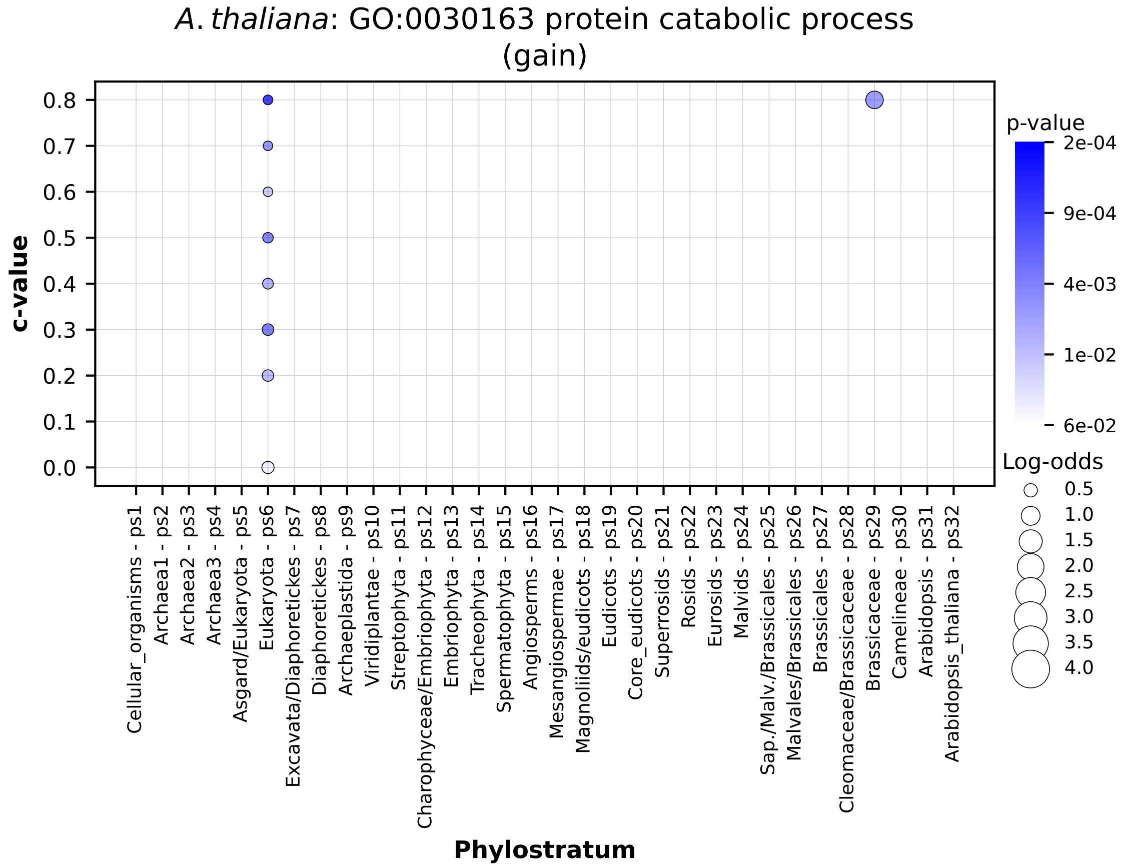

A. thaliana: GO:0030163 protein catabolic process
(gain)
c-value
0.8
0.7
0.6
0.5
0.4
0.3
0.2
0.1
0.0
p-value
2e-04
9e-04
4e-03
1e-02
6e-02
Log-odds
0.5
1.0
1.5
2.0
2.5
3.0
3.5
4.0
Cellular_organisms - ps1
Archaea1 - ps2
Archaea2 - ps3
Archaea3 - ps4
Asgard/Eukaryota - ps5
Eukaryota - ps6
Excavata/Diaphoretickes - ps7
Diaphoretickes - ps8
Archaeplastida - ps9
Viridiplantae - ps10
Streptophyta - ps11
Charophyceae/Embriophyta - ps12
Embriophyta - ps13
Tracheophyta - ps14
Spermatophyta - ps15
Angiosperms - ps16
Mesangiospermae - ps17
Magnoliids/eudicots - ps18
Eudicots - ps19
Core_eudicots - ps20
Superrosids - ps21
Rosids - ps22
Eurosids - ps23
Malvids - ps24
Sap./Malv./Brassicales - ps25
Malvales/Brassicales - ps26
Brassicales - ps27
Cleomaceae/Brassicaceae - ps28
Brassicaceae - ps29
Camelineae - ps30
Arabidopsis - ps31
Arabidopsis_thaliana - ps32
Phylostratum

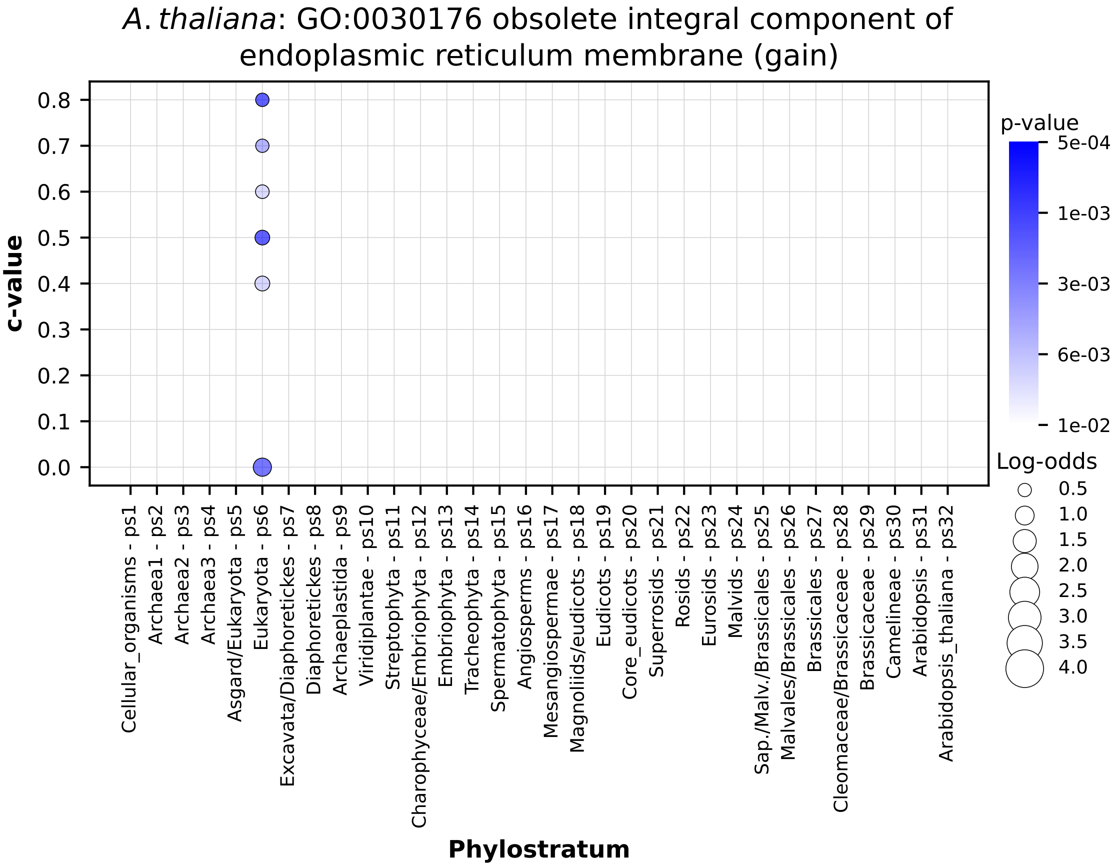

A. thaliana: GO:0030176 obsolete integral component of endoplasmic reticulum membrane (gain)
c-value
0.0
0.1
0.2
0.3
0.4
0.5
0.6
0.7
0.8
p-value
5e-04
1e-03
3e-03
6e-03
1e-02
Log-odds
0.5
1.0
1.5
2.0
2.5
3.0
3.5
4.0
Cellular_organisms - ps1
Archaea1 - ps2
Archaea2 - ps3
Archaea3 - ps4
Asgard/Eukaryota - ps5
Eukaryota - ps6
Excavata/Diaphoretickes - ps7
Diaphoretickes - ps8
Archaeplastida - ps9
Viridiplantae - ps10
Streptophyta - ps11
Charophyceae/Embriophyta - ps12
Embriophyta - ps13
Tracheophyta - ps14
Spermatophyta - ps15
Angiosperms - ps16
Mesangiospermae - ps17
Magnoliids/eudicots - ps18
Eudicots - ps19
Core_eudicots - ps20
Superrosids - ps21
Rosids - ps22
Eurosids - ps23
Malvids - ps24
Sap./Malv./Brassicales - ps25
Malvales/Brassicales - ps26
Brassicales - ps27
Cleomaceae/Brassicaceae - ps28
Brassicaceae - ps29
Camelineae - ps30
Arabidopsis - ps31
Arabidopsis_thaliana - ps32
Phylostratum

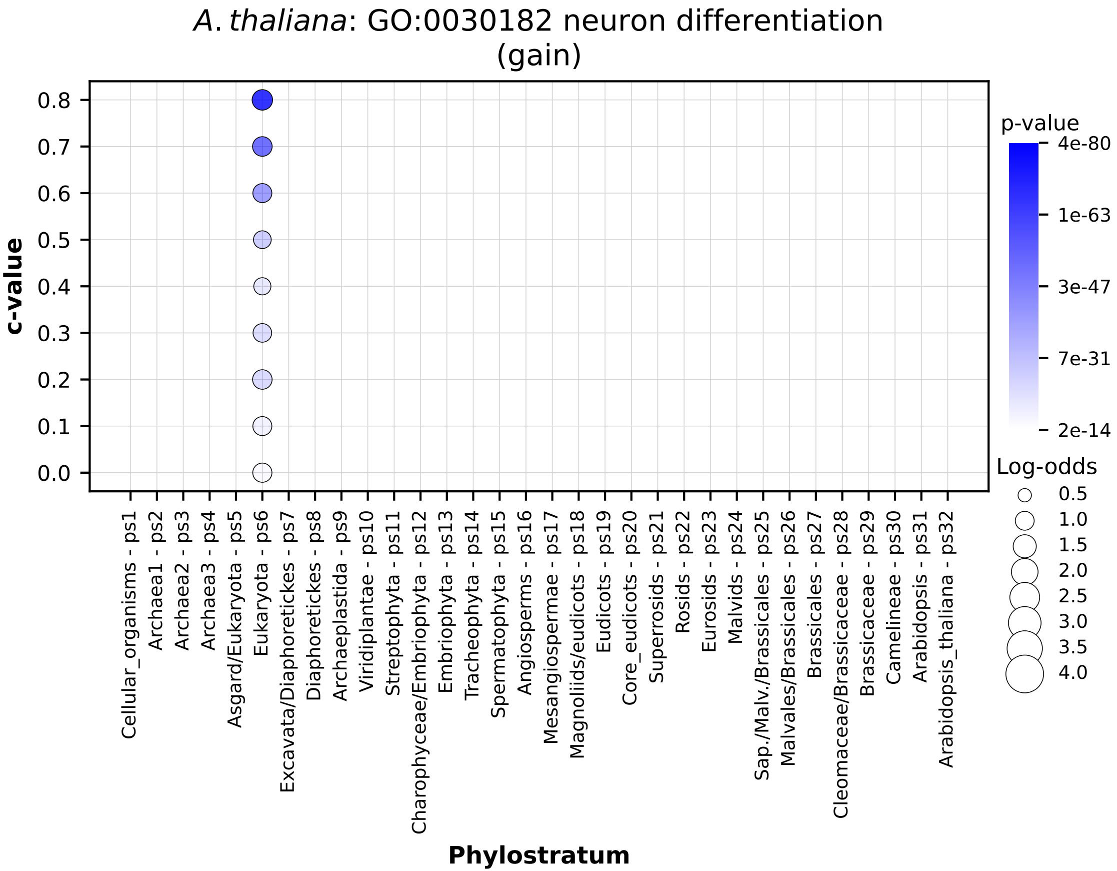

A. thaliana: GO:0030182 neuron differentiation
(gain)
c-value
0.8
0.7
0.6
0.5
0.4
0.3
0.2
0.1
0.0
p-value
4e-80
1e-63
3e-47
7e-31
2e-14
Log-odds
0.5
1.0
1.5
2.0
2.5
3.0
3.5
4.0
Cellular_organisms - ps1
Archaea1 - ps2
Archaea2 - ps3
Archaea3 - ps4
Asgard/Eukaryota - ps5
Eukaryota - ps6
Excavata/Diaphoretickes - ps7
Diaphoretickes - ps8
Archaeplastida - ps9
Viridiplantae - ps10
Streptophyta - ps11
Charophyceae/Embriophyta - ps12
Embriophyta - ps13
Tracheophyta - ps14
Spermatophyta - ps15
Angiosperms - ps16
Mesangiospermae - ps17
Magnoliids/eudicots - ps18
Eudicots - ps19
Core_eudicots - ps20
Superrosids - ps21
Rosids - ps22
Eurosids - ps23
Malvids - ps24
Sap./Malv./Brassicales - ps25
Malvales/Brassicales - ps26
Brassicales - ps27
Cleomaceae/Brassicaceae - ps28
Brassicaceae - ps29
Camelineae - ps30
Arabidopsis - ps31
Arabidopsis_thaliana - ps32
Phylostratum

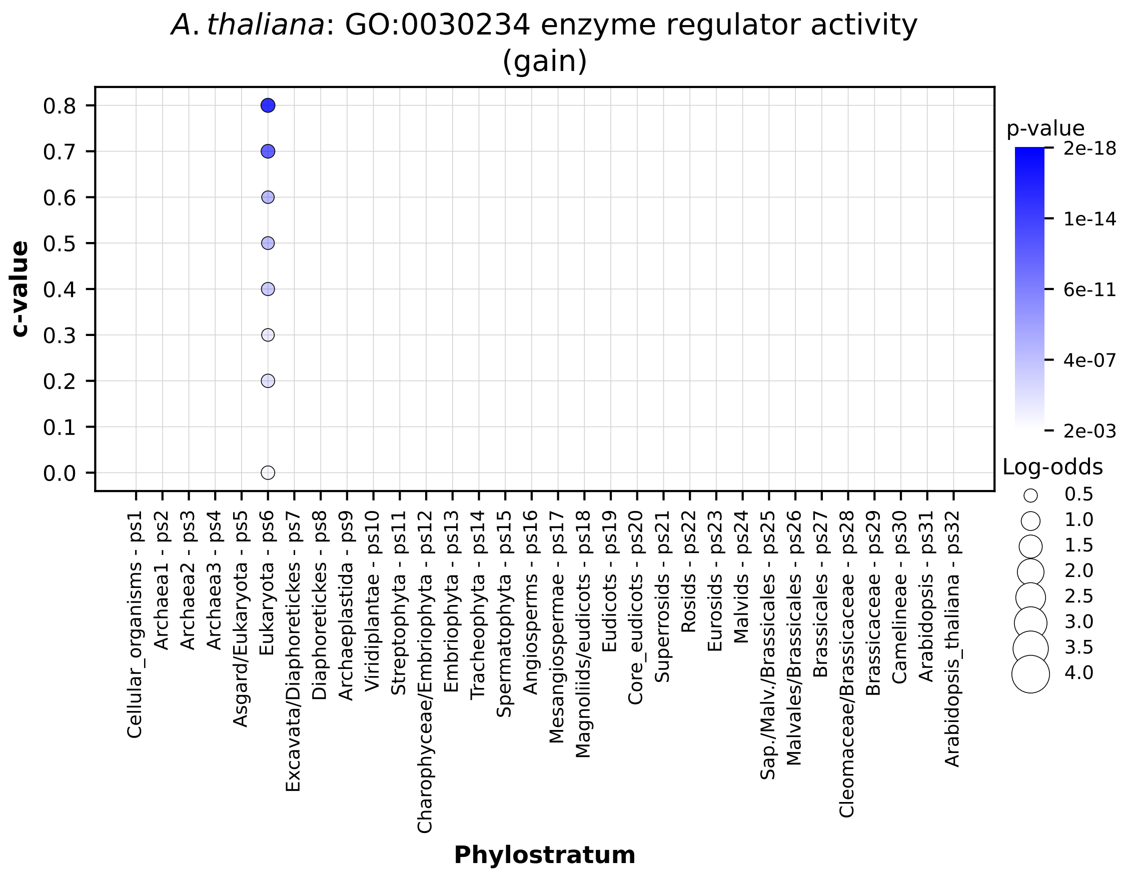

A. thaliana: GO:0030234 enzyme regulator activity
(gain)
c-value
0.8
0.7
0.6
0.5
0.4
0.3
0.2
0.1
0.0
p-value
2e-18
1e-14
6e-11
4e-07
2e-03
Log-odds
0.5
1.0
1.5
2.0
2.5
3.0
3.5
4.0
Cellular_organisms - ps1
Archaea1 - ps2
Archaea2 - ps3
Archaea3 - ps4
Asgard/Eukaryota - ps5
Eukaryota - ps6
Excavata/Diaphoretickes - ps7
Diaphoretickes - ps8
Archaeplastida - ps9
Viridiplantae - ps10
Streptophyta - ps11
Charophyceae/Embriophyta - ps12
Embriophyta - ps13
Tracheophyta - ps14
Spermatophyta - ps15
Angiosperms - ps16
Mesangiospermae - ps17
Magnoliids/eudicots - ps18
Eudicots - ps19
Core_eudicots - ps20
Superrosids - ps21
Rosids - ps22
Eurosids - ps23
Malvids - ps24
Sap./Malv./Brassicales - ps25
Malvales/Brassicales - ps26
Brassicales - ps27
Cleomaceae/Brassicaceae - ps28
Brassicaceae - ps29
Camelineae - ps30
Arabidopsis - ps31
Arabidopsis_thaliana - ps32
Phylostratum

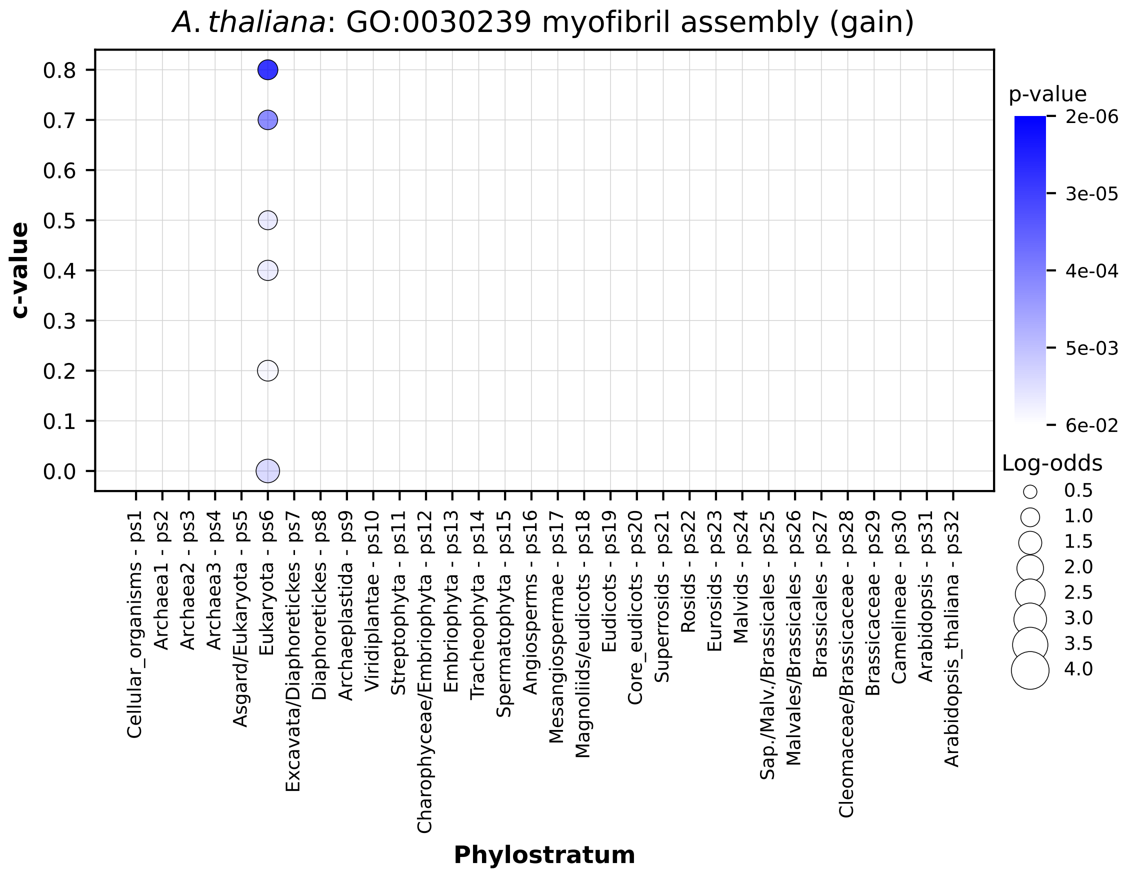

A. thaliana: GO:0030239 myofibril assembly (gain)
c-value
0.8
0.7
0.6
0.5
0.4
0.3
0.2
0.1
0.0
p-value
2e-06
3e-05
4e-04
5e-03
6e-02
Log-odds
0.5
1.0
1.5
2.0
2.5
3.0
3.5
4.0
Cellular_organisms - ps1
Archaea1 - ps2
Archaea2 - ps3
Archaea3 - ps4
Asgard/Eukaryota - ps5
Eukaryota - ps6
Excavata/Diaphoretickes - ps7
Diaphoretickes - ps8
Archaeplastida - ps9
Viridiplantae - ps10
Streptophyta - ps11
Charophyceae/Embriophyta - ps12
Embriophyta - ps13
Tracheophyta - ps14
Spermatophyta - ps15
Angiosperms - ps16
Mesangiospermae - ps17
Magnoliids/eudicots - ps18
Eudicots - ps19
Core_eudicots - ps20
Superrosids - ps21
Rosids - ps22
Eurosids - ps23
Malvids - ps24
Sap./Malv./Brassicales - ps25
Malvales/Brassicales - ps26
Brassicales - ps27
Cleomaceae/Brassicaceae - ps28
Brassicaceae - ps29
Camelineae - ps30
Arabidopsis - ps31
Arabidopsis_thaliana - ps32
Phylostratum

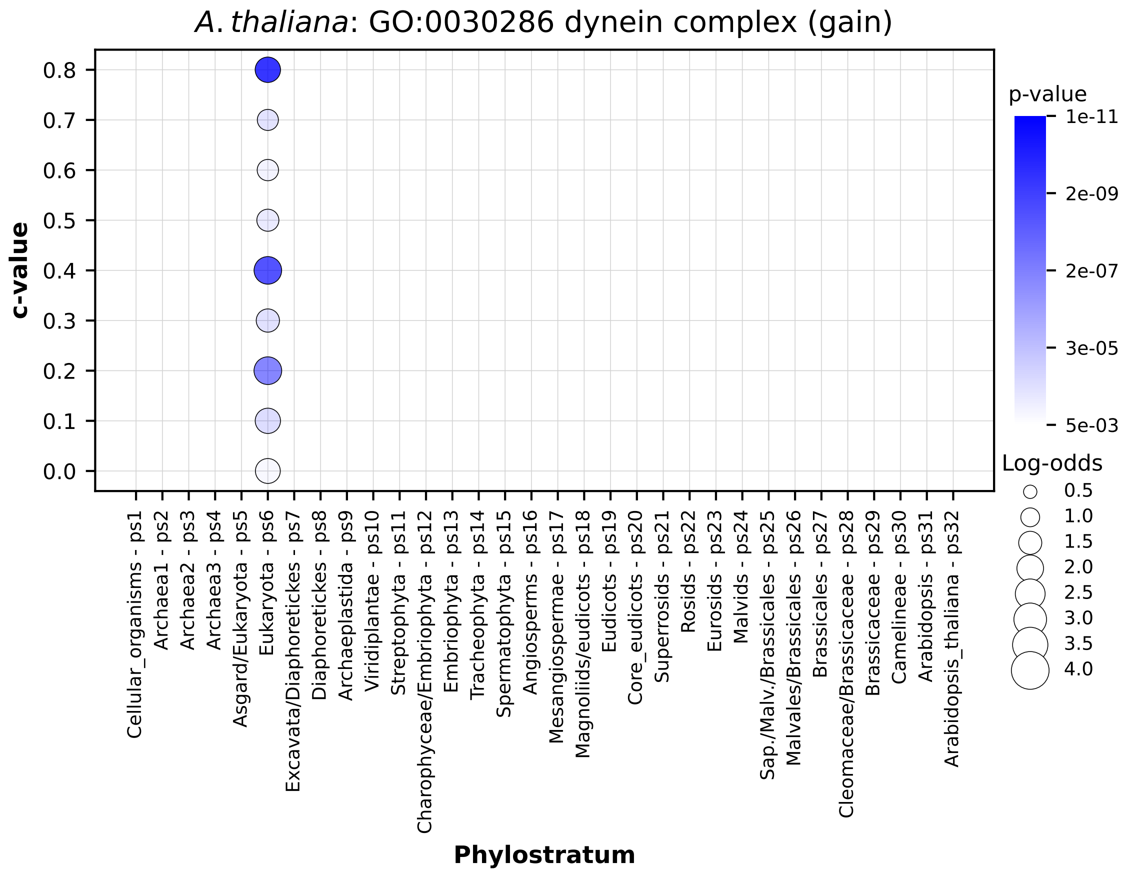

A. thaliana: GO:0030286 dynein complex (gain)
c-value
0.8
0.7
0.6
0.5
0.4
0.3
0.2
0.1
0.0
p-value
1e-11
2e-09
2e-07
3e-05
5e-03
Log-odds
0.5
1.0
1.5
2.0
2.5
3.0
3.5
4.0
Cellular_organisms - ps1
Archaea1 - ps2
Archaea2 - ps3
Archaea3 - ps4
Asgard/Eukaryota - ps5
Eukaryota - ps6
Excavata/Diaphoretickes - ps7
Diaphoretickes - ps8
Archaeplastida - ps9
Viridiplantae - ps10
Streptophyta - ps11
Charophyceae/Embriophyta - ps12
Embriophyta - ps13
Tracheophyta - ps14
Spermatophyta - ps15
Angiosperms - ps16
Mesangiospermae - ps17
Magnoliids/eudicots - ps18
Eudicots - ps19
Core_eudicots - ps20
Superrosids - ps21
Rosids - ps22
Eurosids - ps23
Malvids - ps24
Sap./Malv./Brassicales - ps25
Malvales/Brassicales - ps26
Brassicales - ps27
Cleomaceae/Brassicaceae - ps28
Brassicaceae - ps29
Camelineae - ps30
Arabidopsis - ps31
Arabidopsis_thaliana - ps32
Phylostratum

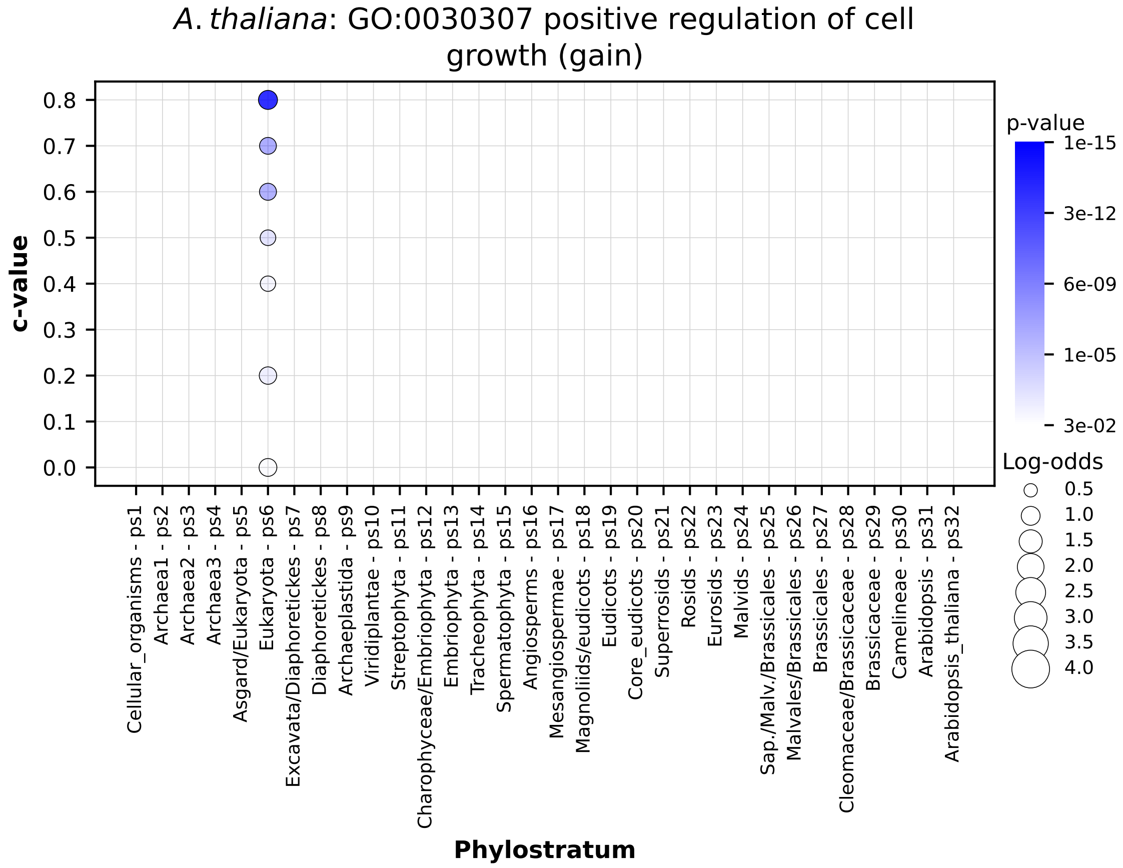
A. thaliana: GO:0030307 positive regulation of cell growth (gain)
c-value
0.8
0.7
0.6
0.5
0.4
0.3
0.2
0.1
0.0
p-value
1e-15
3e-12
6e-09
1e-05
3e-02
Log-odds
0.5
1.0
1.5
2.0
2.5
3.0
3.5
4.0
Cellular_organisms - ps1
Archaea1 - ps2
Archaea2 - ps3
Archaea3 - ps4
Asgard/Eukaryota - ps5
Eukaryota - ps6
Excavata/Diaphoretickes - ps7
Diaphoretickes - ps8
Archaeplastida - ps9
Viridiplantae - ps10
Streptophyta - ps11
Charophyceae/Embriophyta - ps12
Embriophyta - ps13
Tracheophyta - ps14
Spermatophyta - ps15
Angiosperms - ps16
Mesangiospermae - ps17
Magnoliids/eudicots - ps18
Eudicots - ps19
Core_eudicots - ps20
Superrosids - ps21
Rosids - ps22
Eurosids - ps23
Malvids - ps24
Sap./Malv./Brassicales - ps25
Malvales/Brassicales - ps26
Brassicales - ps27
Cleomaceae/Brassicaceae - ps28
Brassicaceae - ps29
Camelineae - ps30
Arabidopsis - ps31
Arabidopsis_thaliana - ps32
Phylostratum

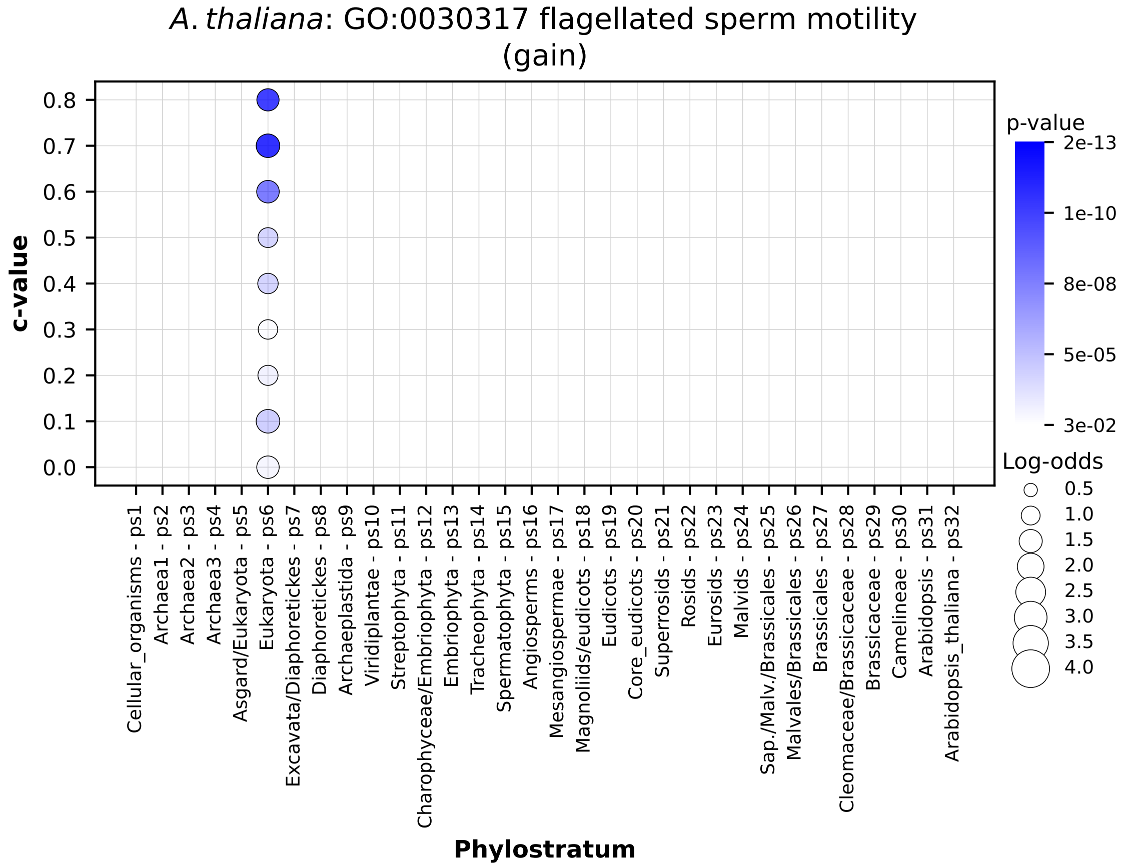
A. thaliana: GO:0030317 flagellated sperm motility
(gain)
c-value
0.8
0.7
0.6
0.5
0.4
0.3
0.2
0.1
0.0
p-value
2e-13
1e-10
8e-08
5e-05
3e-02
Log-odds
0.5
1.0
1.5
2.0
2.5
3.0
3.5
4.0
Cellular_organisms - ps1
Archaea1 - ps2
Archaea2 - ps3
Archaea3 - ps4
Asgard/Eukaryota - ps5
Eukaryota - ps6
Excavata/Diaphoretickes - ps7
Diaphoretickes - ps8
Archaeplastida - ps9
Viridiplantae - ps10
Streptophyta - ps11
Charophyceae/Embriophyta - ps12
Embriophyta - ps13
Tracheophyta - ps14
Spermatophyta - ps15
Angiosperms - ps16
Mesangiospermae - ps17
Magnoliids/eudicots - ps18
Eudicots - ps19
Core_eudicots - ps20
Superrosids - ps21
Rosids - ps22
Eurosids - ps23
Malvids - ps24
Sap./Malv./Brassicales - ps25
Malvales/Brassicales - ps26
Brassicales - ps27
Cleomaceae/Brassicaceae - ps28
Brassicaceae - ps29
Camelineae - ps30
Arabidopsis - ps31
Arabidopsis_thaliana - ps32
Phylostratum

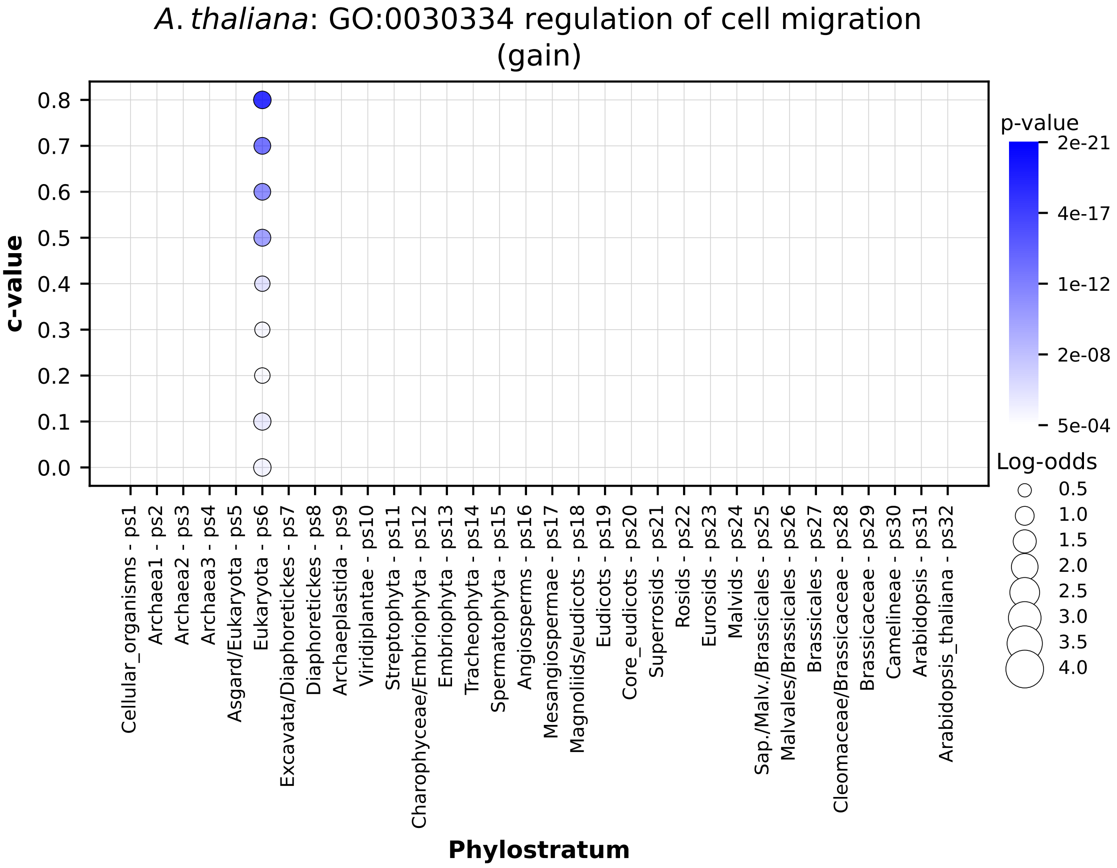
A. thaliana: GO:0030334 regulation of cell migration
(gain)
c-value
0.8
0.7
0.6
0.5
0.4
0.3
0.2
0.1
0.0
p-value
2e-21
4e-17
1e-12
2e-08
5e-04
Log-odds
0.5
1.0
1.5
2.0
2.5
3.0
3.5
4.0
Cellular_organisms - ps1
Archaea1 - ps2
Archaea2 - ps3
Archaea3 - ps4
Asgard/Eukaryota - ps5
Eukaryota - ps6
Excavata/Diaphoretickes - ps7
Diaphoretickes - ps8
Archaeplastida - ps9
Viridiplantae - ps10
Streptophyta - ps11
Charophyceae/Embriophyta - ps12
Embriophyta - ps13
Tracheophyta - ps14
Spermatophyta - ps15
Angiosperms - ps16
Mesangiospermae - ps17
Magnoliids/eudicots - ps18
Eudicots - ps19
Core_eudicots - ps20
Superrosids - ps21
Rosids - ps22
Eurosids - ps23
Malvids - ps24
Sap./Malv./Brassicales - ps25
Malvales/Brassicales - ps26
Brassicales - ps27
Cleomaceae/Brassicaceae - ps28
Brassicaceae - ps29
Camelineae - ps30
Arabidopsis - ps31
Arabidopsis_thaliana - ps32
Phylostratum

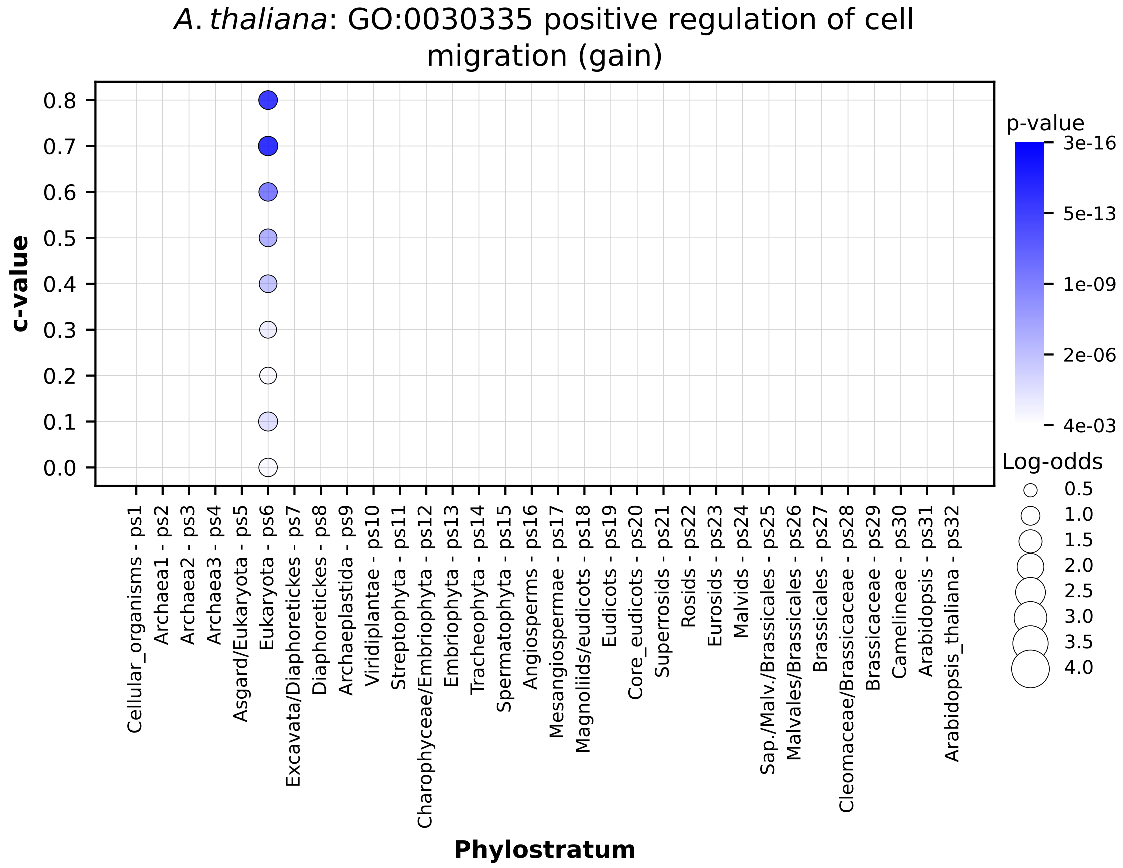

A. thaliana: GO:0030335 positive regulation of cell migration (gain)
c-value
0.8
0.7
0.6
0.5
0.4
0.3
0.2
0.1
0.0
Phylostratum
Cellular_organisms - ps1
Archaea1 - ps2
Archaea2 - ps3
Archaea3 - ps4
Asgard/Eukaryota - ps5
Eukaryota - ps6
Excavata/Diaphoretickes - ps7
Diaphoretickes - ps8
Archaeplastida - ps9
Viridiplantae - ps10
Streptophyta - ps11
Charophyceae/Embriophyta - ps12
Embriophyta - ps13
Tracheophyta - ps14
Spermatophyta - ps15
Angiosperms - ps16
Mesangiospermae - ps17
Magnoliids/eudicots - ps18
Eudicots - ps19
Core_eudicots - ps20
Superrosids - ps21
Rosids - ps22
Eurosids - ps23
Malvids - ps24
Sap./Malv./Brassicales - ps25
Malvales/Brassicales - ps26
Brassicales - ps27
Cleomaceae/Brassicaceae - ps28
Brassicaceae - ps29
Camelineae - ps30
Arabidopsis - ps31
Arabidopsis_thaliana - ps32
p-value
3e-16
5e-13
1e-09
2e-06
4e-03
Log-odds
0.5
1.0
1.5
2.0
2.5
3.0
3.5
4.0

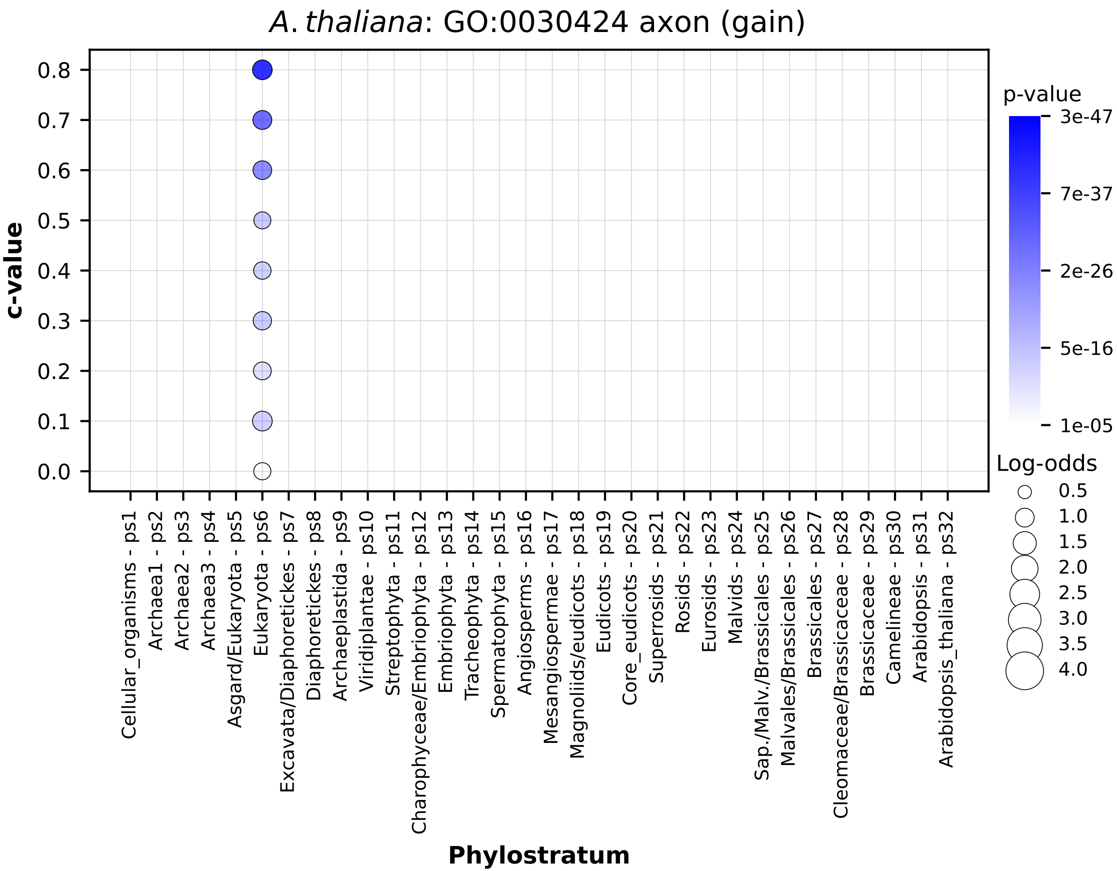

A. thaliana: GO:0030424 axon (gain)
c-value
0.0
0.1
0.2
0.3
0.4
0.5
0.6
0.7
0.8
p-value
3e-47
7e-37
2e-26
5e-16
1e-05
Log-odds
0.5
1.0
1.5
2.0
2.5
3.0
3.5
4.0
Cellular_organisms - ps1
Archaea1 - ps2
Archaea2 - ps3
Archaea3 - ps4
Asgard/Eukaryota - ps5
Eukaryota - ps6
Excavata/Diaphoretickes - ps7
Diaphoretickes - ps8
Archaeplastida - ps9
Viridiplantae - ps10
Streptophyta - ps11
Charophyceae/Embriophyta - ps12
Embriophyta - ps13
Tracheophyta - ps14
Spermatophyta - ps15
Angiosperms - ps16
Mesangiospermae - ps17
Magnoliids/eudicots - ps18
Eudicots - ps19
Core_eudicots - ps20
Superrosids - ps21
Rosids - ps22
Eurosids - ps23
Malvids - ps24
Sap./Malv./Brassicales - ps25
Malvales/Brassicales - ps26
Brassicales - ps27
Cleomaceae/Brassicaceae - ps28
Brassicaceae - ps29
Camelineae - ps30
Arabidopsis - ps31
Arabidopsis_thaliana - ps32
Phylostratum

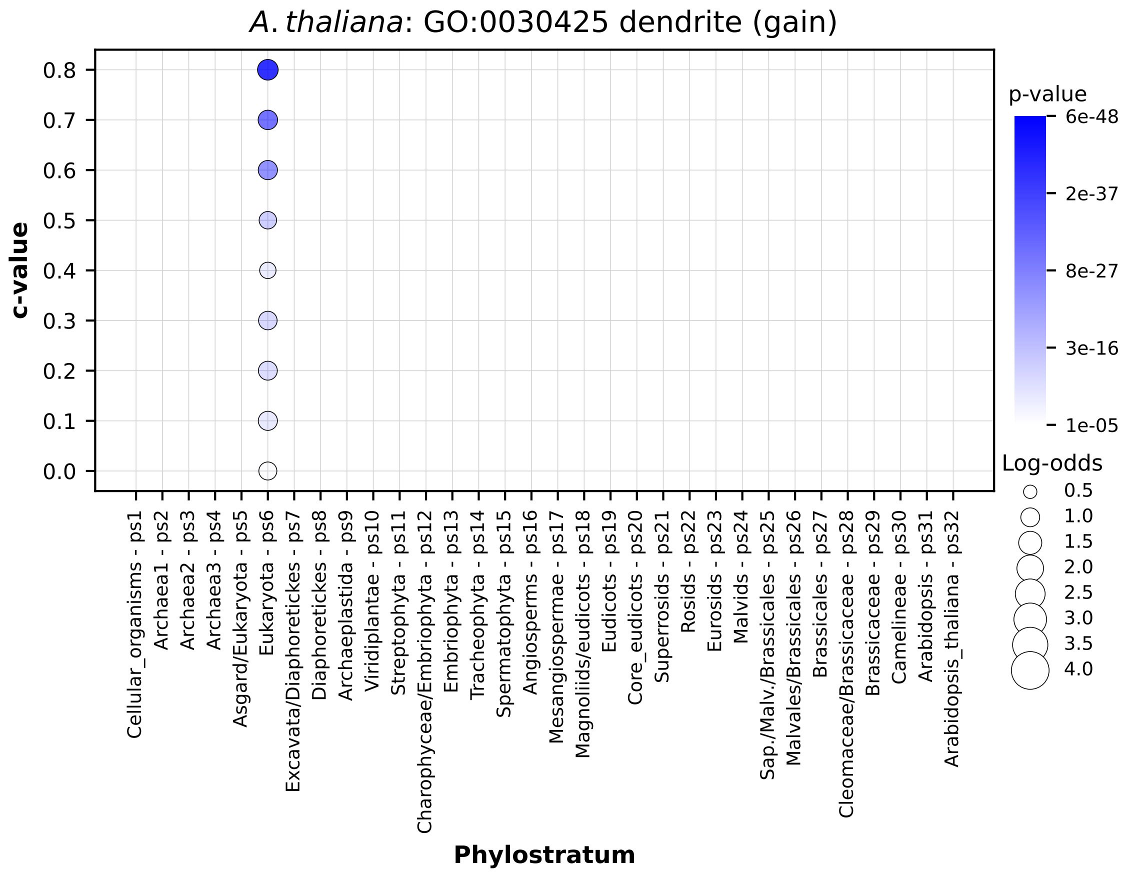

A. thaliana: GO:0030425 dendrite (gain)
c-value
0.8
0.7
0.6
0.5
0.4
0.3
0.2
0.1
0.0
p-value
6e-48
2e-37
8e-27
3e-16
1e-05
Log-odds
0.5
1.0
1.5
2.0
2.5
3.0
3.5
4.0
Cellular_organisms - ps1
Archaea1 - ps2
Archaea2 - ps3
Archaea3 - ps4
Asgard/Eukaryota - ps5
Eukaryota - ps6
Excavata/Diaphoretickes - ps7
Diaphoretickes - ps8
Archaeplastida - ps9
Viridiplantae - ps10
Streptophyta - ps11
Charophyceae/Embriophyta - ps12
Embriophyta - ps13
Tracheophyta - ps14
Spermatophyta - ps15
Angiosperms - ps16
Mesangiospermae - ps17
Magnoliids/eudicots - ps18
Eudicots - ps19
Core_eudicots - ps20
Superrosids - ps21
Rosids - ps22
Eurosids - ps23
Malvids - ps24
Sap./Malv./Brassicales - ps25
Malvales/Brassicales - ps26
Brassicales - ps27
Cleomaceae/Brassicaceae - ps28
Brassicaceae - ps29
Camelineae - ps30
Arabidopsis - ps31
Arabidopsis_thaliana - ps32
Phylostratum

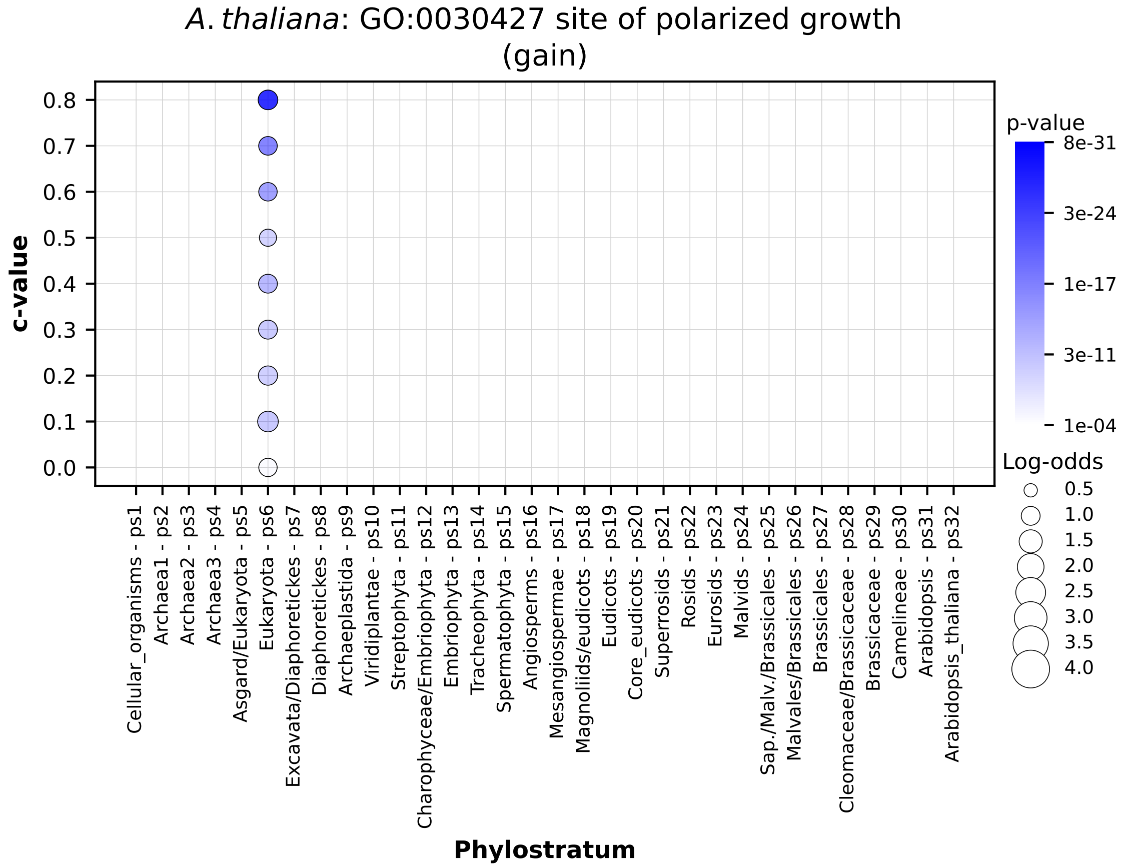

A. thaliana: GO:0030427 site of polarized growth (gain)
c-value
0.8
0.7
0.6
0.5
0.4
0.3
0.2
0.1
0.0
p-value
8e-31
3e-24
1e-17
3e-11
1e-04
Log-odds
0.5
1.0
1.5
2.0
2.5
3.0
3.5
4.0
Cellular_organisms - ps1
Archaea1 - ps2
Archaea2 - ps3
Archaea3 - ps4
Asgard/Eukaryota - ps5
Eukaryota - ps6
Excavata/Diaphoretickes - ps7
Diaphoretickes - ps8
Archaeplastida - ps9
Viridiplantae - ps10
Streptophyta - ps11
Charophyceae/Embriophyta - ps12
Embriophyta - ps13
Tracheophyta - ps14
Spermatophyta - ps15
Angiosperms - ps16
Mesangiospermae - ps17
Magnoliids/eudicots - ps18
Eudicots - ps19
Core_eudicots - ps20
Superrosids - ps21
Rosids - ps22
Eurosids - ps23
Malvids - ps24
Sap./Malv./Brassicales - ps25
Malvales/Brassicales - ps26
Brassicales - ps27
Cleomaceae/Brassicaceae - ps28
Brassicaceae - ps29
Camelineae - ps30
Arabidopsis - ps31
Arabidopsis_thaliana - ps32
Phylostratum

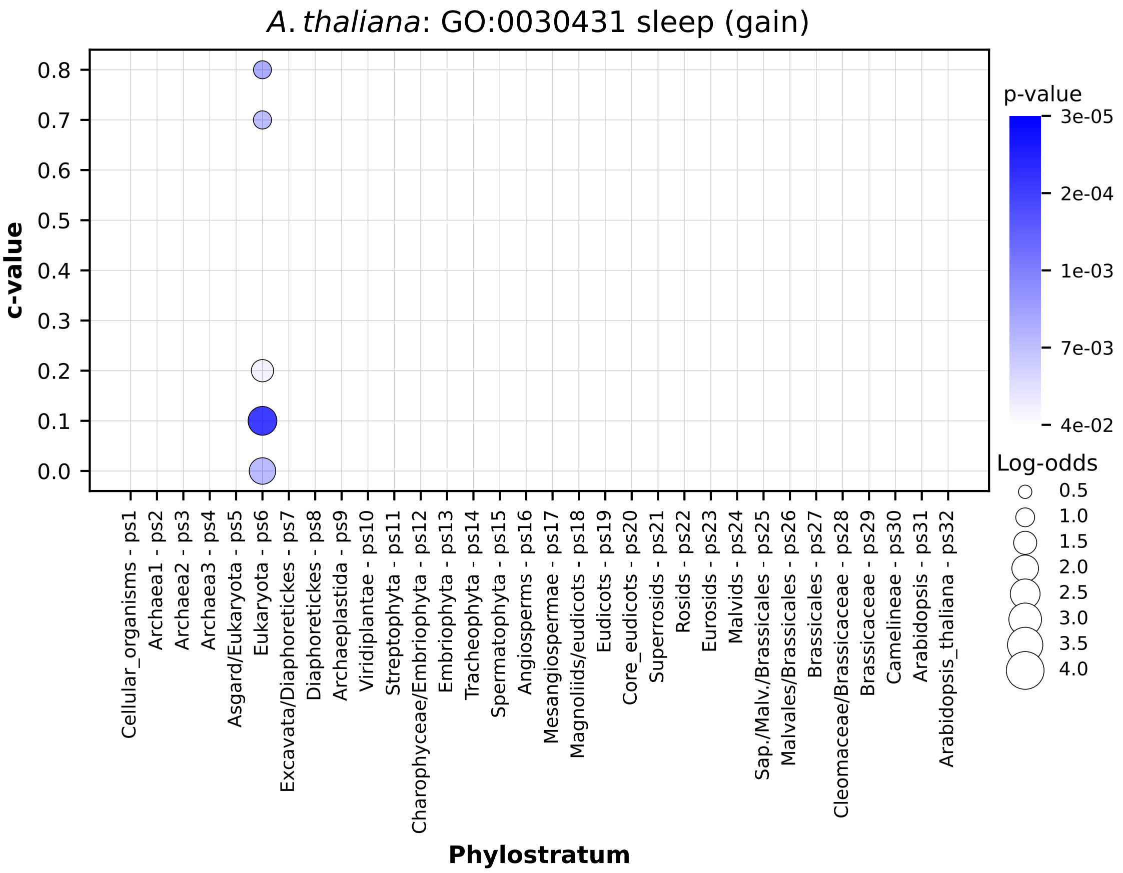

A. thaliana: GO:0030431 sleep (gain)
c-value
0.0
0.1
0.2
0.3
0.4
0.5
0.6
0.7
0.8
p-value
3e-05
2e-04
1e-03
7e-03
4e-02
Log-odds
0.5
1.0
1.5
2.0
2.5
3.0
3.5
4.0
Cellular_organisms - ps1
Archaea1 - ps2
Archaea2 - ps3
Archaea3 - ps4
Asgard/Eukaryota - ps5
Eukaryota - ps6
Excavata/Diaphoretickes - ps7
Diaphoretickes - ps8
Archaeplastida - ps9
Viridiplantae - ps10
Streptophyta - ps11
Charophyceae/Embriophyta - ps12
Embriophyta - ps13
Tracheophyta - ps14
Spermatophyta - ps15
Angiosperms - ps16
Mesangiospermae - ps17
Magnoliids/eudicots - ps18
Eudicots - ps19
Core_eudicots - ps20
Superrosids - ps21
Rosids - ps22
Eurosids - ps23
Malvids - ps24
Sap./Malv./Brassicales - ps25
Malvales/Brassicales - ps26
Brassicales - ps27
Cleomaceae/Brassicaceae - ps28
Brassicaceae - ps29
Camelineae - ps30
Arabidopsis - ps31
Arabidopsis_thaliana - ps32
Phylostratum

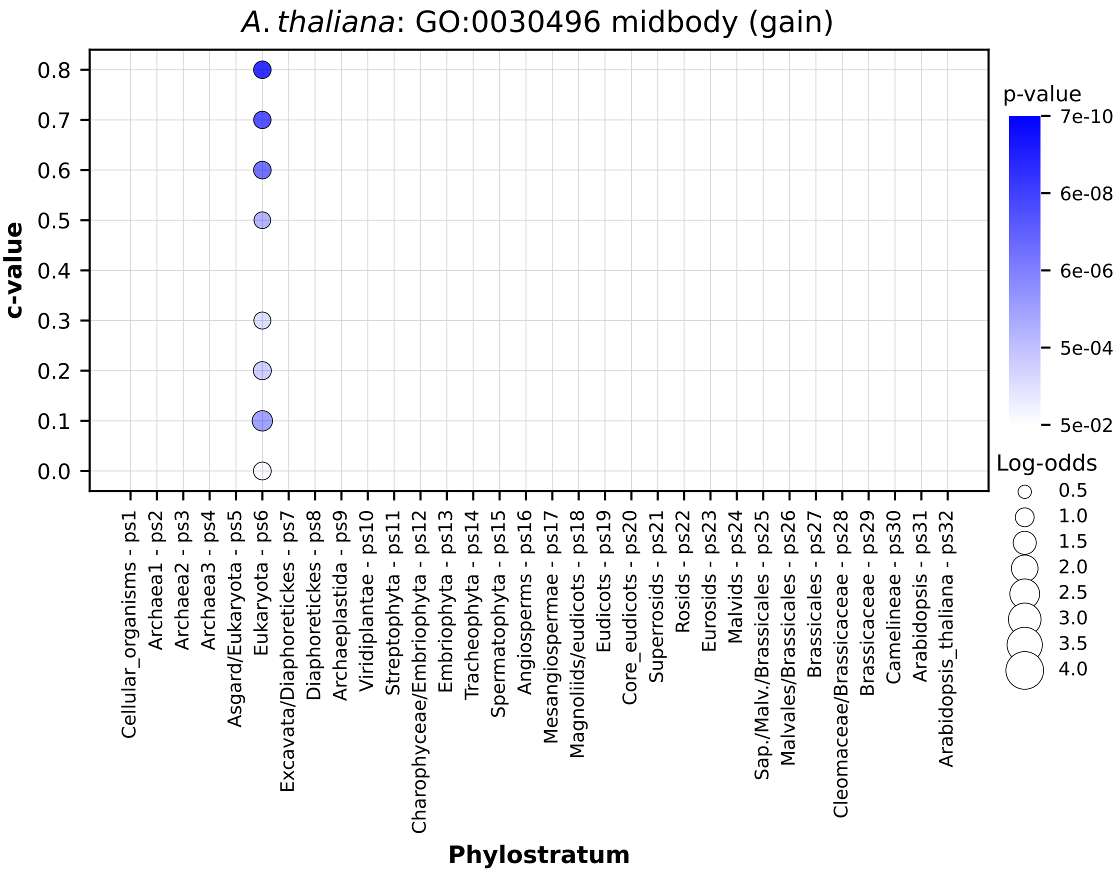

A. thaliana: GO:0030496 midbody (gain)
c-value
0.8
0.7
0.6
0.5
0.4
0.3
0.2
0.1
0.0
Phylostratum
Cellular_organisms - ps1
Archaea1 - ps2
Archaea2 - ps3
Archaea3 - ps4
Asgard/Eukaryota - ps5
Eukaryota - ps6
Excavata/Diaphoretickes - ps7
Diaphoretickes - ps8
Archaeplastida - ps9
Viridiplantae - ps10
Streptophyta - ps11
Charophyceae/Embriophyta - ps12
Embriophyta - ps13
Tracheophyta - ps14
Spermatophyta - ps15
Angiosperms - ps16
Mesangiospermae - ps17
Magnoliids/eudicots - ps18
Eudicots - ps19
Core_eudicots - ps20
Superrosids - ps21
Rosids - ps22
Eurosids - ps23
Malvids - ps24
Sap./Malv./Brassicales - ps25
Malvales/Brassicales - ps26
Brassicales - ps27
Cleomaceae/Brassicaceae - ps28
Brassicaceae - ps29
Camelineae - ps30
Arabidopsis - ps31
Arabidopsis_thaliana - ps32
p-value
7e-10
6e-08
6e-06
5e-04
5e-02
Log-odds
0.5
1.0
1.5
2.0
2.5
3.0
3.5
4.0

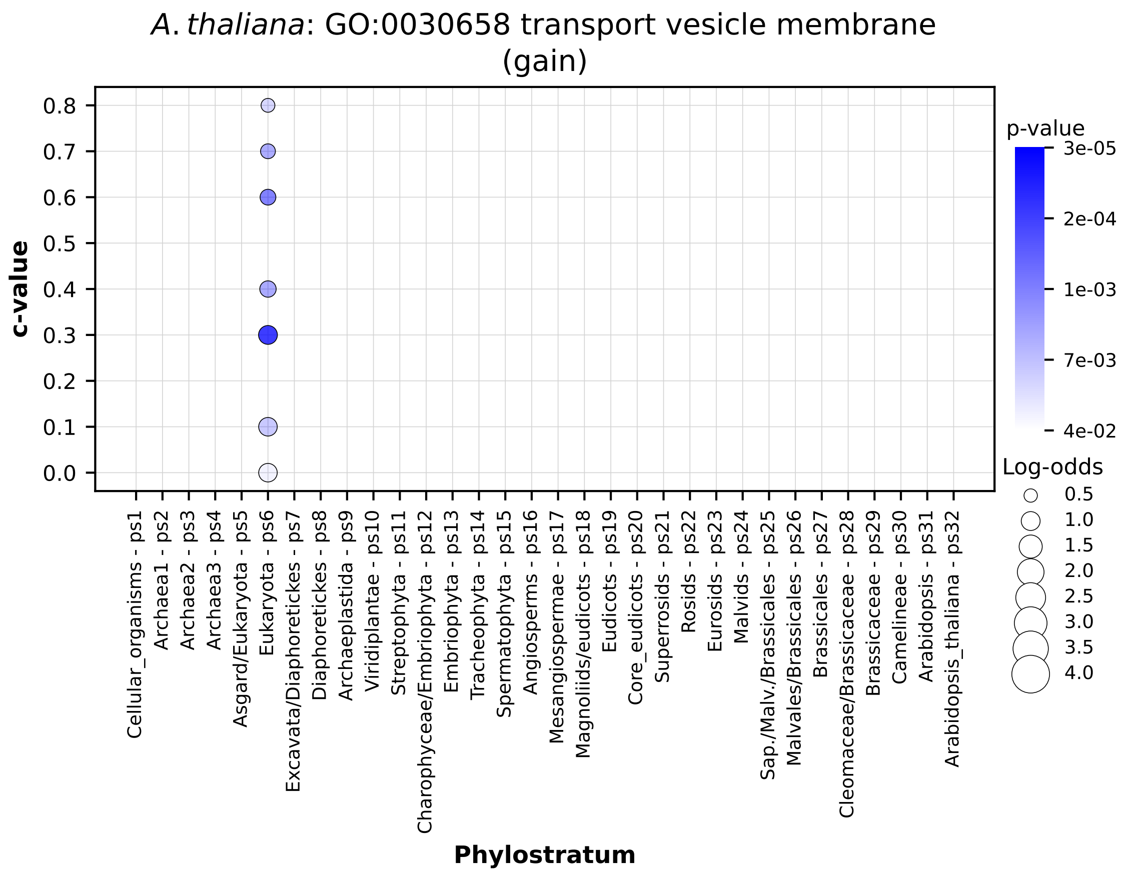

A. thaliana: GO:0030658 transport vesicle membrane (gain)
c-value
0.8
0.7
0.6
0.5
0.4
0.3
0.2
0.1
0.0
p-value
3e-05
2e-04
1e-03
7e-03
4e-02
Log-odds
0.5
1.0
1.5
2.0
2.5
3.0
3.5
4.0
Cellular_organisms - ps1
Archaea1 - ps2
Archaea2 - ps3
Archaea3 - ps4
Asgard/Eukaryota - ps5
Eukaryota - ps6
Excavata/Diaphoretickes - ps7
Diaphoretickes - ps8
Archaeplastida - ps9
Viridiplantae - ps10
Streptophyta - ps11
Charophyceae/Embriophyta - ps12
Embriophyta - ps13
Tracheophyta - ps14
Spermatophyta - ps15
Angiosperms - ps16
Mesangiospermae - ps17
Magnoliids/eudicots - ps18
Eudicots - ps19
Core_eudicots - ps20
Superrosids - ps21
Rosids - ps22
Eurosids - ps23
Malvids - ps24
Sap./Malv./Brassicales - ps25
Malvales/Brassicales - ps26
Brassicales - ps27
Cleomaceae/Brassicaceae - ps28
Brassicaceae - ps29
Camelineae - ps30
Arabidopsis - ps31
Arabidopsis_thaliana - ps32
Phylostratum

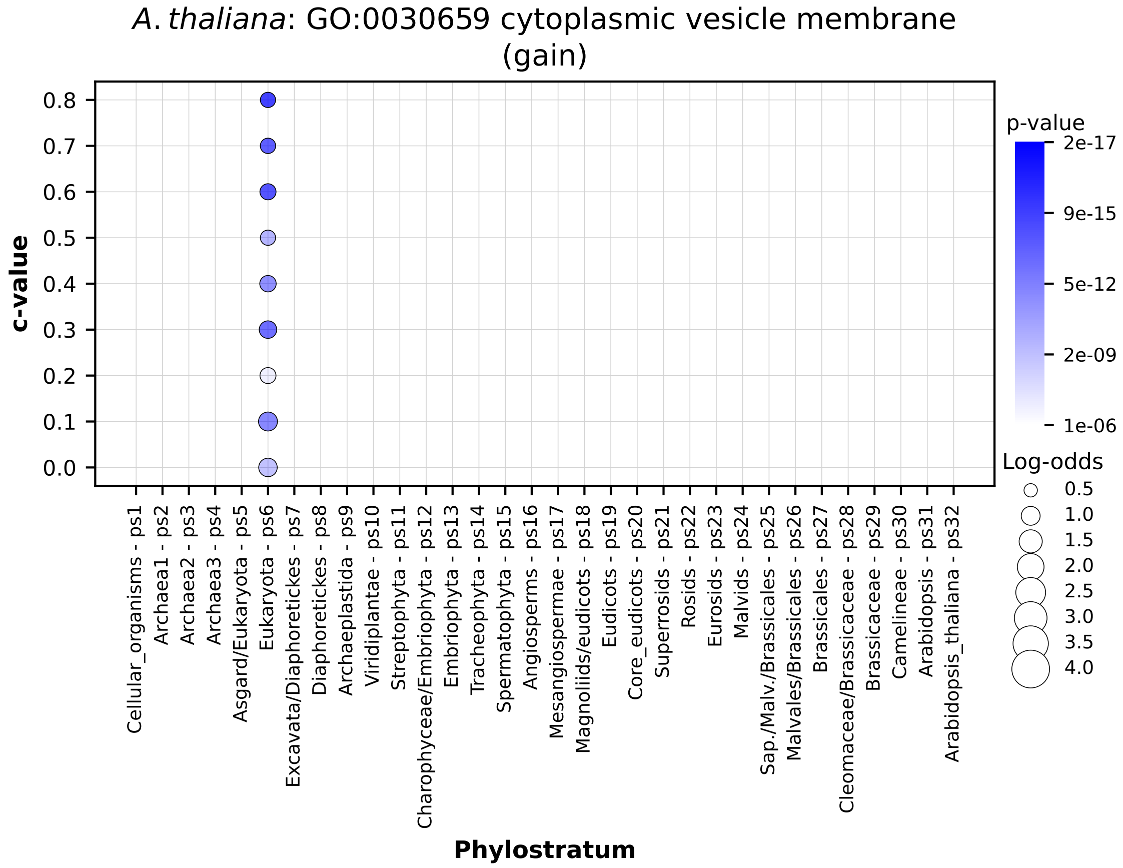
A. thaliana: GO:0030659 cytoplasmic vesicle membrane (gain)
c-value
0.8
0.7
0.6
0.5
0.4
0.3
0.2
0.1
0.0
Phylostratum
Cellular_organisms - ps1
Archaea1 - ps2
Archaea2 - ps3
Archaea3 - ps4
Asgard/Eukaryota - ps5
Eukaryota - ps6
Excavata/Diaphoretickes - ps7
Diaphoretickes - ps8
Archaeplastida - ps9
Viridiplantae - ps10
Streptophyta - ps11
Charophyceae/Embriophyta - ps12
Embriophyta - ps13
Tracheophyta - ps14
Spermatophyta - ps15
Angiosperms - ps16
Mesangiospermae - ps17
Magnoliids/eudicots - ps18
Eudicots - ps19
Core_eudicots - ps20
Superrosids - ps21
Rosids - ps22
Eurosids - ps23
Malvids - ps24
Sap./Malv./Brassicales - ps25
Malvales/Brassicales - ps26
Brassicales - ps27
Cleomaceae/Brassicaceae - ps28
Brassicaceae - ps29
Camelineae - ps30
Arabidopsis - ps31
Arabidopsis_thaliana - ps32
p-value
2e-17
9e-15
5e-12
2e-09
1e-06
Log-odds
0.5
1.0
1.5
2.0
2.5
3.0
3.5
4.0

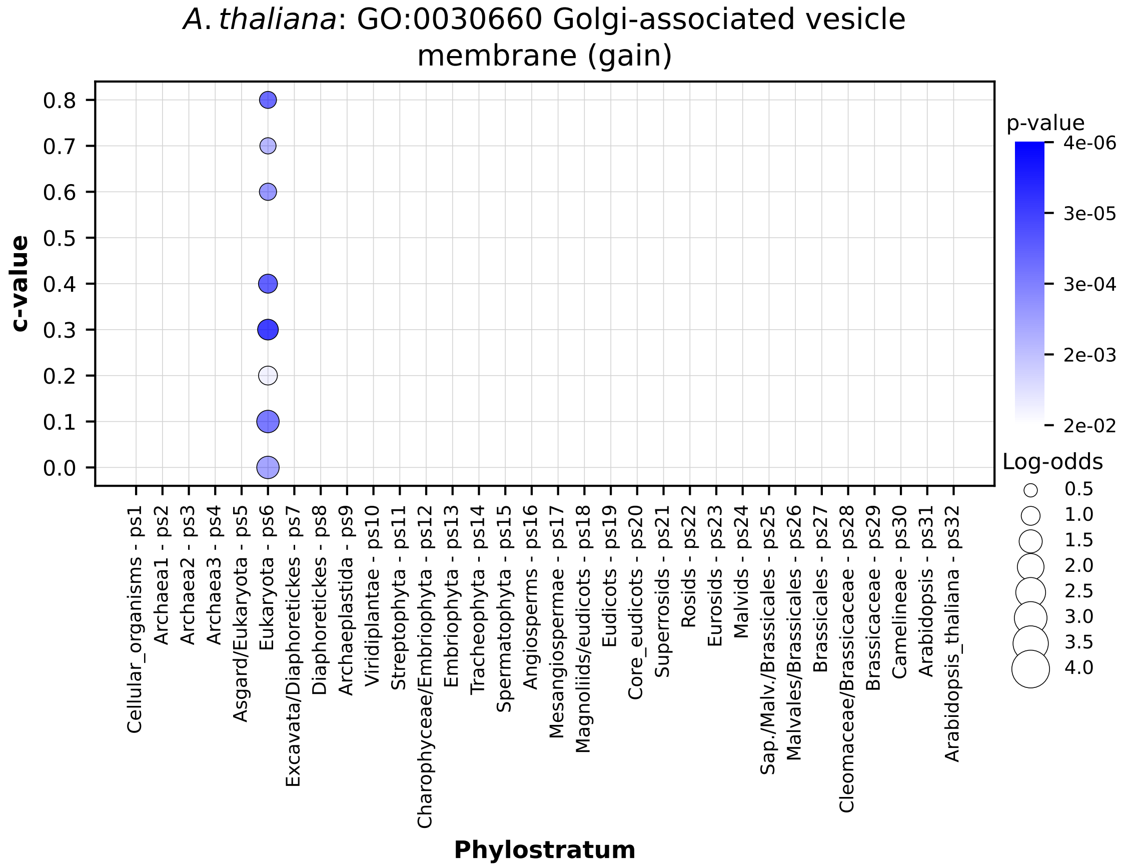

A. thaliana: GO:0030660 Golgi-associated vesicle membrane (gain)
c-value
0.8
0.7
0.6
0.5
0.4
0.3
0.2
0.1
0.0
p-value
4e-06
3e-05
3e-04
2e-03
2e-02
Log-odds
0.5
1.0
1.5
2.0
2.5
3.0
3.5
4.0
Cellular_organisms - ps1
Archaea1 - ps2
Archaea2 - ps3
Archaea3 - ps4
Asgard/Eukaryota - ps5
Eukaryota - ps6
Excavata/Diaphoretickes - ps7
Diaphoretickes - ps8
Archaeplastida - ps9
Viridiplantae - ps10
Streptophyta - ps11
Charophyceae/Embriophyta - ps12
Embriophyta - ps13
Tracheophyta - ps14
Spermatophyta - ps15
Angiosperms - ps16
Mesangiospermae - ps17
Magnoliids/eudicots - ps18
Eudicots - ps19
Core_eudicots - ps20
Superrosids - ps21
Rosids - ps22
Eurosids - ps23
Malvids - ps24
Sap./Malv./Brassicales - ps25
Malvales/Brassicales - ps26
Brassicales - ps27
Cleomaceae/Brassicaceae - ps28
Brassicaceae - ps29
Camelineae - ps30
Arabidopsis - ps31
Arabidopsis_thaliana - ps32
Phylostratum

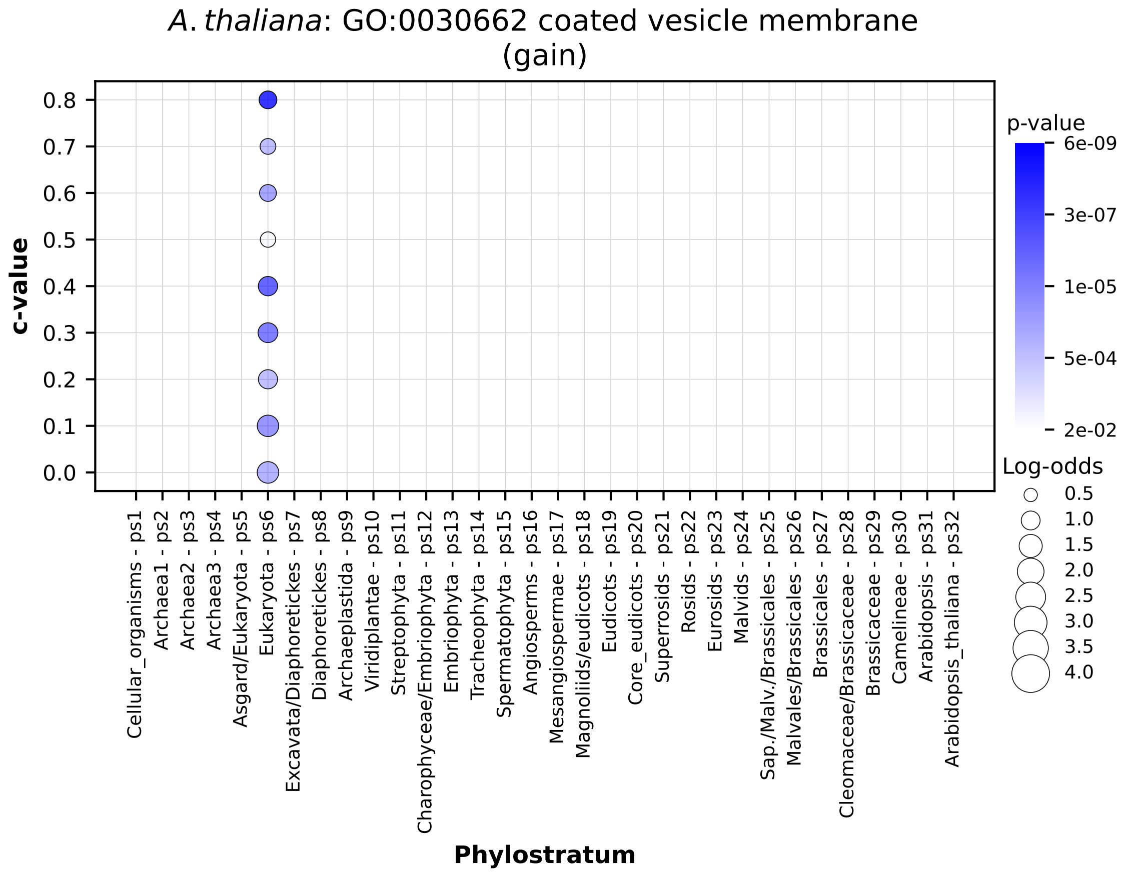

A. thaliana: GO:0030662 coated vesicle membrane (gain)
c-value
0.8
0.7
0.6
0.5
0.4
0.3
0.2
0.1
0.0
p-value
6e-09
3e-07
1e-05
5e-04
2e-02
Log-odds
0.5
1.0
1.5
2.0
2.5
3.0
3.5
4.0
Cellular_organisms - ps1
Archaea1 - ps2
Archaea2 - ps3
Archaea3 - ps4
Asgard/Eukaryota - ps5
Eukaryota - ps6
Excavata/Diaphoretickes - ps7
Diaphoretickes - ps8
Archaeplastida - ps9
Viridiplantae - ps10
Streptophyta - ps11
Charophyceae/Embriophyta - ps12
Embriophyta - ps13
Tracheophyta - ps14
Spermatophyta - ps15
Angiosperms - ps16
Mesangiospermae - ps17
Magnoliids/eudicots - ps18
Eudicots - ps19
Core_eudicots - ps20
Superrosids - ps21
Rosids - ps22
Eurosids - ps23
Malvids - ps24
Sap./Malv./Brassicales - ps25
Malvales/Brassicales - ps26
Brassicales - ps27
Cleomaceae/Brassicaceae - ps28
Brassicaceae - ps29
Camelineae - ps30
Arabidopsis - ps31
Arabidopsis_thaliana - ps32
Phylostratum

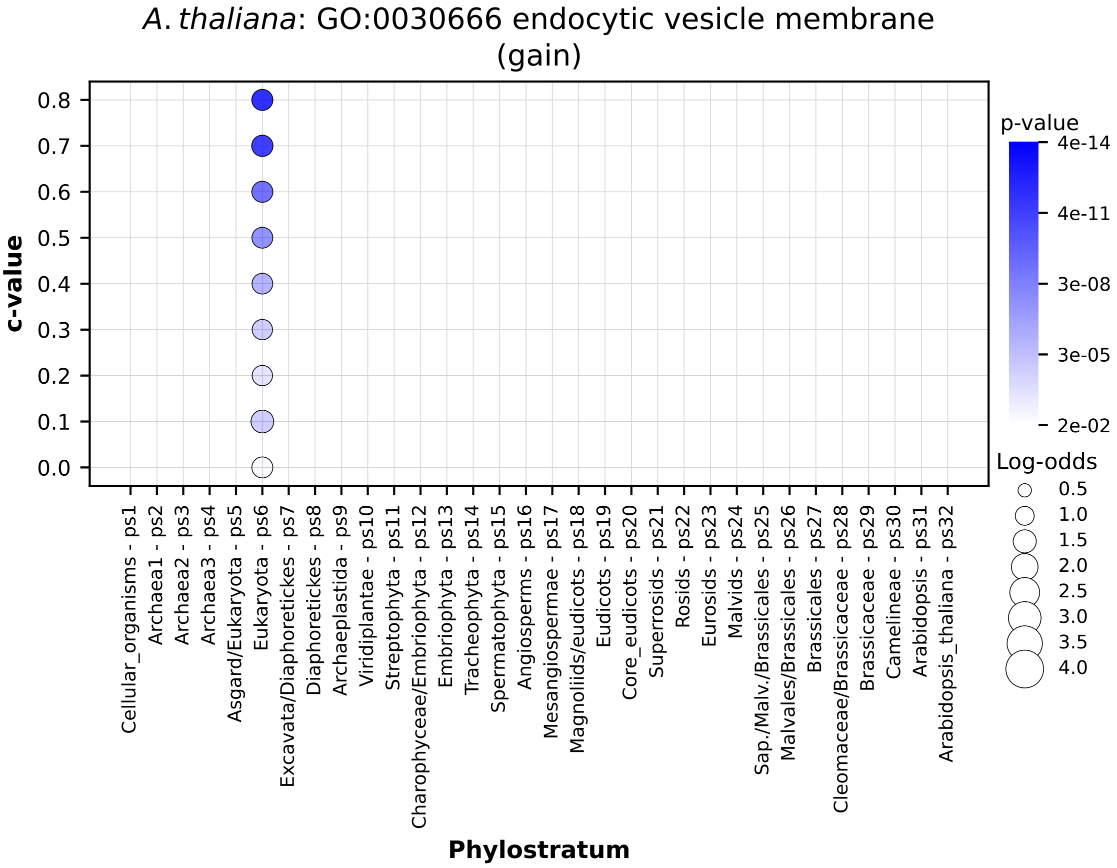

A. thaliana: GO:0030666 endocytic vesicle membrane
(gain)
c-value
0.8
0.7
0.6
0.5
0.4
0.3
0.2
0.1
0.0
p-value
4e-14
4e-11
3e-08
3e-05
2e-02
Log-odds
0.5
1.0
1.5
2.0
2.5
3.0
3.5
4.0
Cellular_organisms - ps1
Archaea1 - ps2
Archaea2 - ps3
Archaea3 - ps4
Asgard/Eukaryota - ps5
Eukaryota - ps6
Excavata/Diaphoretickes - ps7
Diaphoretickes - ps8
Archaeplastida - ps9
Viridiplantae - ps10
Streptophyta - ps11
Charophyceae/Embriophyta - ps12
Embriophyta - ps13
Tracheophyta - ps14
Spermatophyta - ps15
Angiosperms - ps16
Mesangiospermae - ps17
Magnoliids/eudicots - ps18
Eudicots - ps19
Core_eudicots - ps20
Superrosids - ps21
Rosids - ps22
Eurosids - ps23
Malvids - ps24
Sap./Malv./Brassicales - ps25
Malvales/Brassicales - ps26
Brassicales - ps27
Cleomaceae/Brassicaceae - ps28
Brassicaceae - ps29
Camelineae - ps30
Arabidopsis - ps31
Arabidopsis_thaliana - ps32
Phylostratum

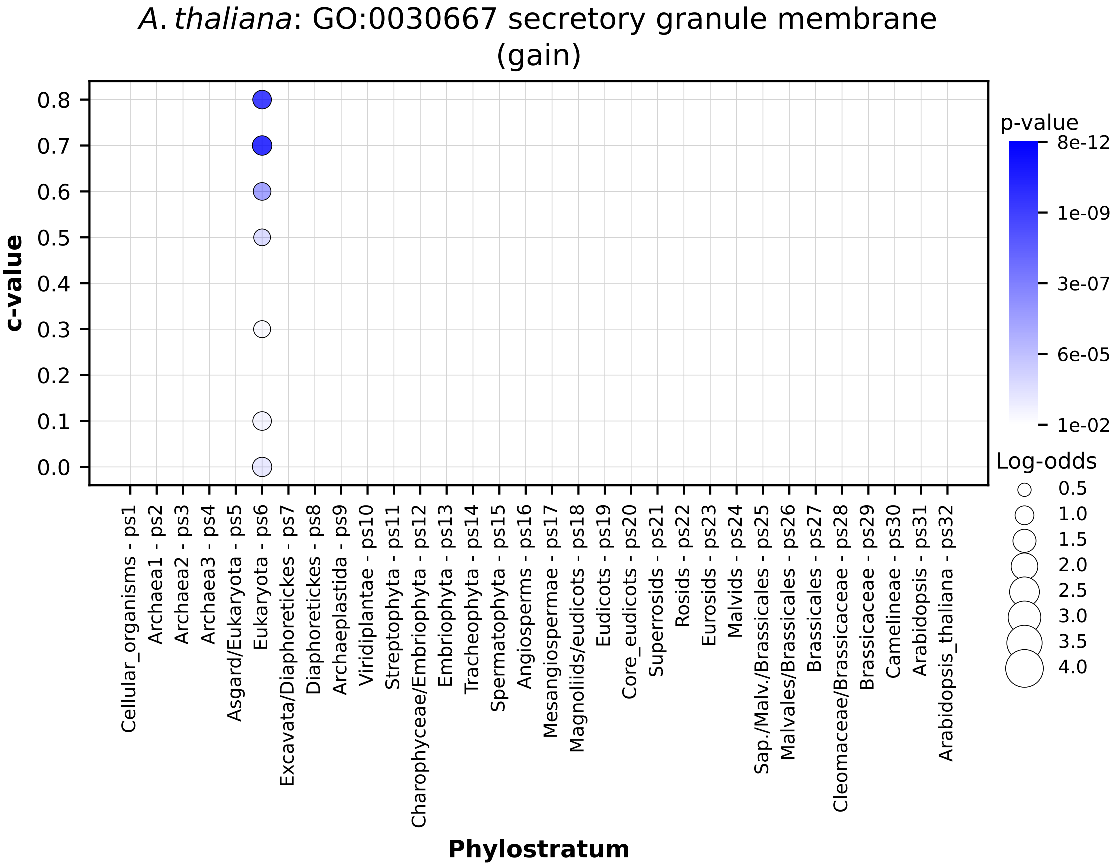

A. thaliana: GO:0030667 secretory granule membrane
(gain)
c-value
0.8
0.7
0.6
0.5
0.4
0.3
0.2
0.1
0.0
p-value
8e-12
1e-09
3e-07
6e-05
1e-02
Log-odds
0.5
1.0
1.5
2.0
2.5
3.0
3.5
4.0
Cellular_organisms - ps1
Archaea1 - ps2
Archaea2 - ps3
Archaea3 - ps4
Asgard/Eukaryota - ps5
Eukaryota - ps6
Excavata/Diaphoretickes - ps7
Diaphoretickes - ps8
Archaeplastida - ps9
Viridiplantae - ps10
Streptophyta - ps11
Charophyceae/Embriophyta - ps12
Embriophyta - ps13
Tracheophyta - ps14
Spermatophyta - ps15
Angiosperms - ps16
Mesangiospermae - ps17
Magnoliids/eudicots - ps18
Eudicots - ps19
Core_eudicots - ps20
Superrosids - ps21
Rosids - ps22
Eurosids - ps23
Malvids - ps24
Sap./Malv./Brassicales - ps25
Malvales/Brassicales - ps26
Brassicales - ps27
Cleomaceae/Brassicaceae - ps28
Brassicaceae - ps29
Camelineae - ps30
Arabidopsis - ps31
Arabidopsis_thaliana - ps32
Phylostratum

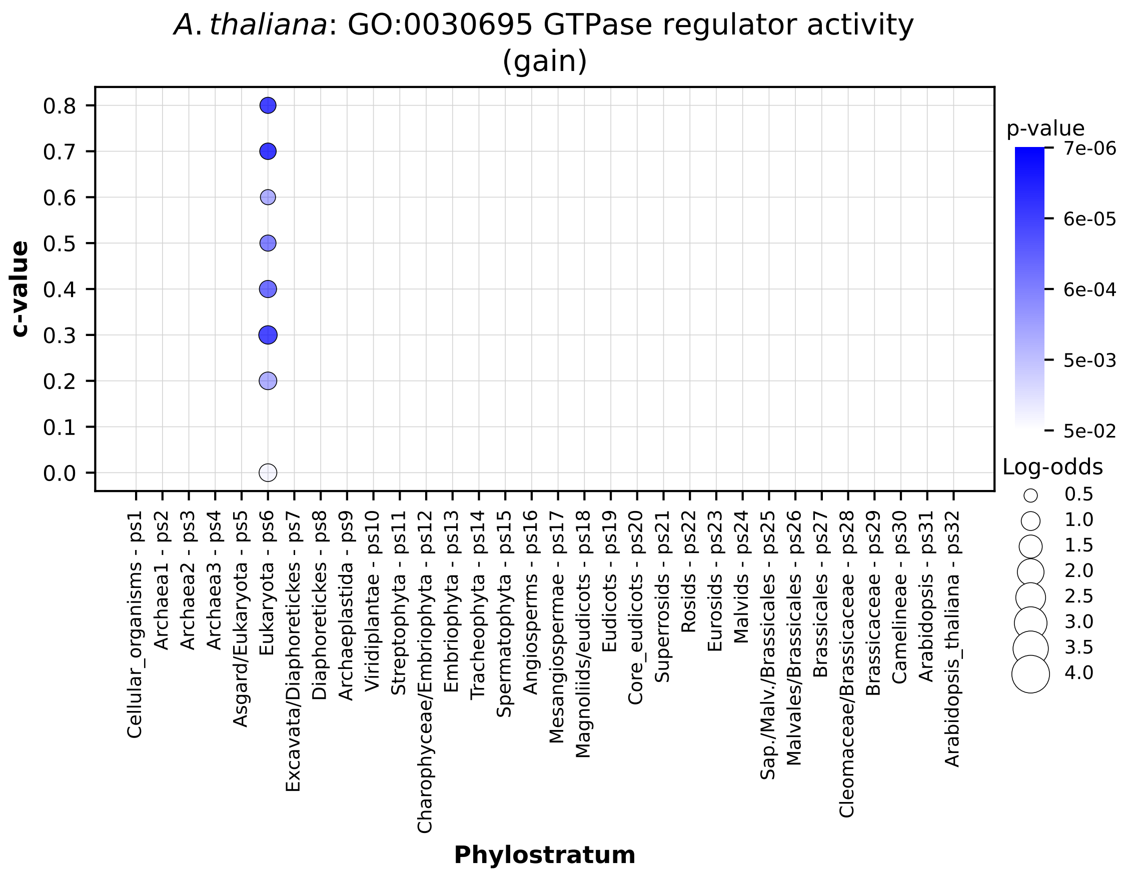
A. thaliana: GO:0030695 GTPase regulator activity
(gain)
c-value
0.8
0.7
0.6
0.5
0.4
0.3
0.2
0.1
0.0
Phylostratum
Cellular_organisms - ps1
Archaea1 - ps2
Archaea2 - ps3
Archaea3 - ps4
Asgard/Eukaryota - ps5
Eukaryota - ps6
Excavata/Diaphoretickes - ps7
Diaphoretickes - ps8
Archaeplastida - ps9
Viridiplantae - ps10
Streptophyta - ps11
Charophyceae/Embriophyta - ps12
Embriophyta - ps13
Tracheophyta - ps14
Spermatophyta - ps15
Angiosperms - ps16
Mesangiospermae - ps17
Magnoliids/eudicots - ps18
Eudicots - ps19
Core_eudicots - ps20
Superrosids - ps21
Rosids - ps22
Eurosids - ps23
Malvids - ps24
Sap./Malv./Brassicales - ps25
Malvales/Brassicales - ps26
Brassicales - ps27
Cleomaceae/Brassicaceae - ps28
Brassicaceae - ps29
Camelineae - ps30
Arabidopsis - ps31
Arabidopsis_thaliana - ps32
p-value
7e-06
6e-05
6e-04
5e-03
5e-02
Log-odds
0.5
1.0
1.5
2.0
2.5
3.0
3.5
4.0

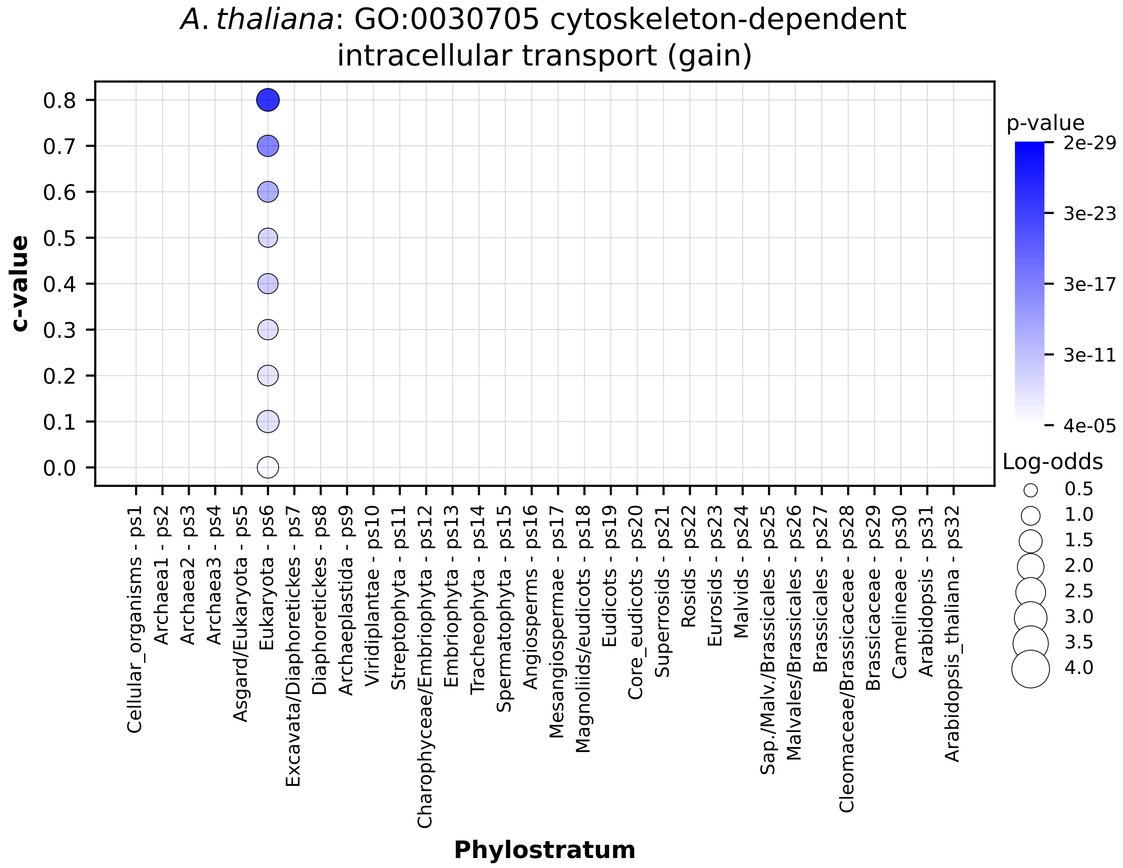
A. thaliana: GO:0030705 cytoskeleton-dependent intracellular transport (gain)
c-value
0.8
0.7
0.6
0.5
0.4
0.3
0.2
0.1
0.0
p-value
2e-29
3e-23
3e-17
3e-11
4e-05
Log-odds
0.5
1.0
1.5
2.0
2.5
3.0
3.5
4.0
Cellular_organisms - ps1
Archaea1 - ps2
Archaea2 - ps3
Archaea3 - ps4
Asgard/Eukaryota - ps5
Eukaryota - ps6
Excavata/Diaphoretickes - ps7
Diaphoretickes - ps8
Archaeplastida - ps9
Viridiplantae - ps10
Streptophyta - ps11
Charophyceae/Embriophyta - ps12
Embriophyta - ps13
Tracheophyta - ps14
Spermatophyta - ps15
Angiosperms - ps16
Mesangiospermae - ps17
Magnoliids/eudicots - ps18
Eudicots - ps19
Core_eudicots - ps20
Superrosids - ps21
Rosids - ps22
Eurosids - ps23
Malvids - ps24
Sap./Malv./Brassicales - ps25
Malvales/Brassicales - ps26
Brassicales - ps27
Cleomaceae/Brassicaceae - ps28
Brassicaceae - ps29
Camelineae - ps30
Arabidopsis - ps31
Arabidopsis_thaliana - ps32
Phylostratum

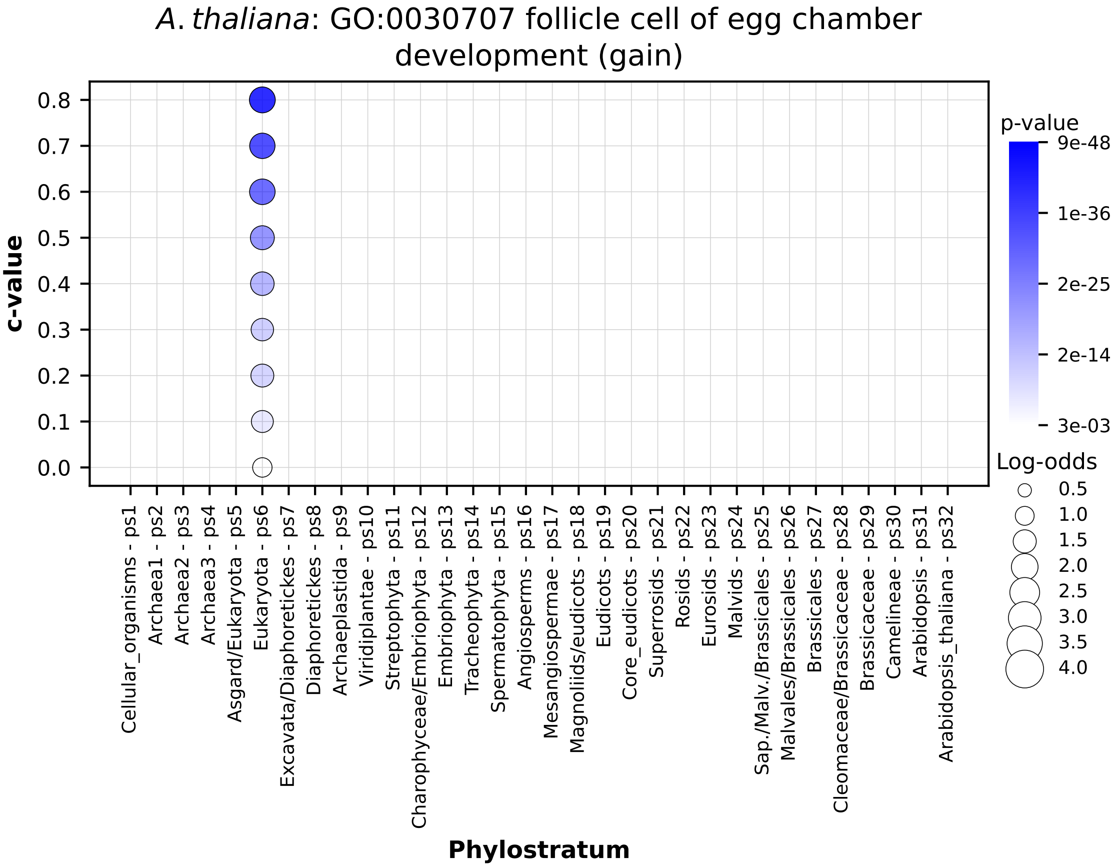

A. thaliana: GO:0030707 follicle cell of egg chamber development (gain)
c-value
0.0
0.1
0.2
0.3
0.4
0.5
0.6
0.7
0.8
p-value
9e-48
1e-36
2e-25
2e-14
3e-03
Log-odds
0.5
1.0
1.5
2.0
2.5
3.0
3.5
4.0
Cellular_organisms - ps1
Archaea1 - ps2
Archaea2 - ps3
Archaea3 - ps4
Asgard/Eukaryota - ps5
Eukaryota - ps6
Excavata/Diaphoretickes - ps7
Diaphoretickes - ps8
Archaeplastida - ps9
Viridiplantae - ps10
Streptophyta - ps11
Charophyceae/Embriophyta - ps12
Embriophyta - ps13
Tracheophyta - ps14
Spermatophyta - ps15
Angiosperms - ps16
Mesangiospermae - ps17
Magnoliids/eudicots - ps18
Eudicots - ps19
Core_eudicots - ps20
Superrosids - ps21
Rosids - ps22
Eurosids - ps23
Malvids - ps24
Sap./Malv./Brassicales - ps25
Malvales/Brassicales - ps26
Brassicales - ps27
Cleomaceae/Brassicaceae - ps28
Brassicaceae - ps29
Camelineae - ps30
Arabidopsis - ps31
Arabidopsis_thaliana - ps32
Phylostratum

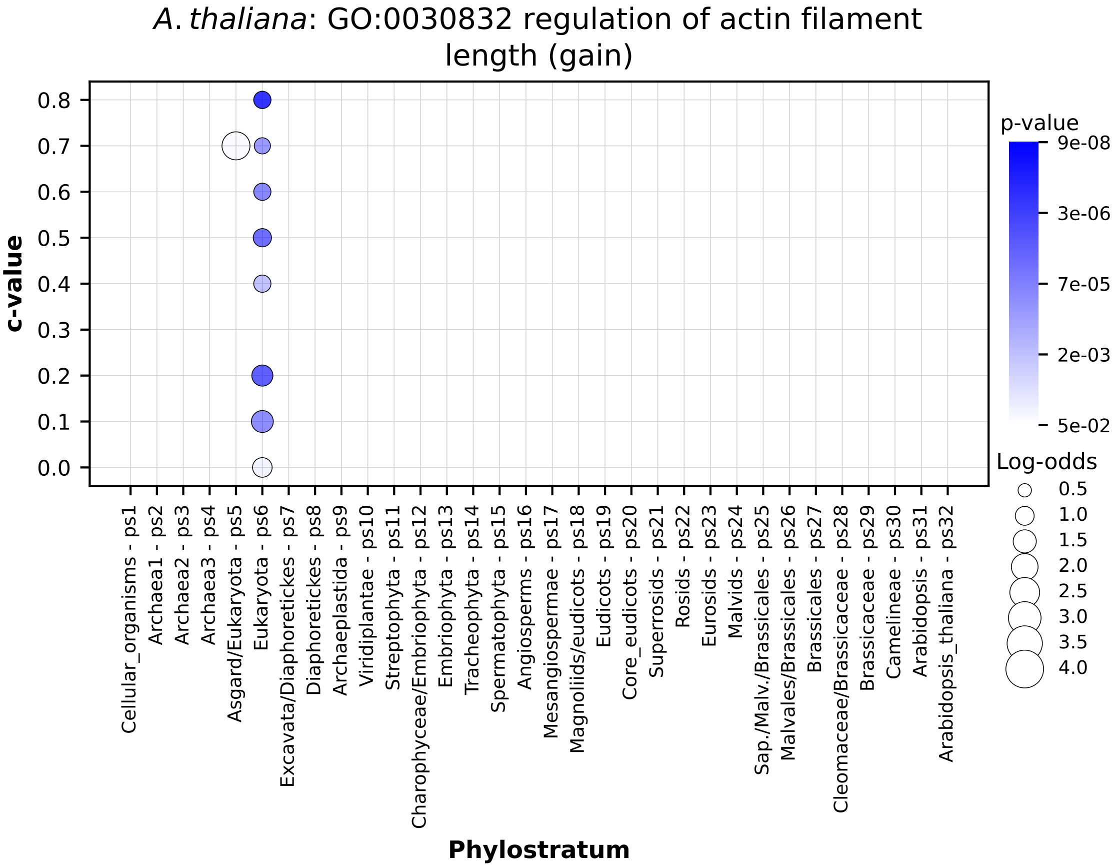
A. thaliana: GO:0030832 regulation of actin filament length (gain)
c-value
0.8
0.7
0.6
0.5
0.4
0.3
0.2
0.1
0.0
Phylostratum
Cellular_organisms - ps1
Archaea1 - ps2
Archaea2 - ps3
Archaea3 - ps4
Asgard/Eukaryota - ps5
Eukaryota - ps6
Excavata/Diaphoretickes - ps7
Diaphoretickes - ps8
Archaeplastida - ps9
Viridiplantae - ps10
Streptophyta - ps11
Charophyceae/Embriophyta - ps12
Embriophyta - ps13
Tracheophyta - ps14
Spermatophyta - ps15
Angiosperms - ps16
Mesangiospermae - ps17
Magnoliids/eudicots - ps18
Eudicots - ps19
Core_eudicots - ps20
Superrosids - ps21
Rosids - ps22
Eurosids - ps23
Malvids - ps24
Sap./Malv./Brassicales - ps25
Malvales/Brassicales - ps26
Brassicales - ps27
Cleomaceae/Brassicaceae - ps28
Brassicaceae - ps29
Camelineae - ps30
Arabidopsis - ps31
Arabidopsis_thaliana - ps32
p-value
9e-08
3e-06
7e-05
2e-03
5e-02
Log-odds
0.5
1.0
1.5
2.0
2.5
3.0
3.5
4.0

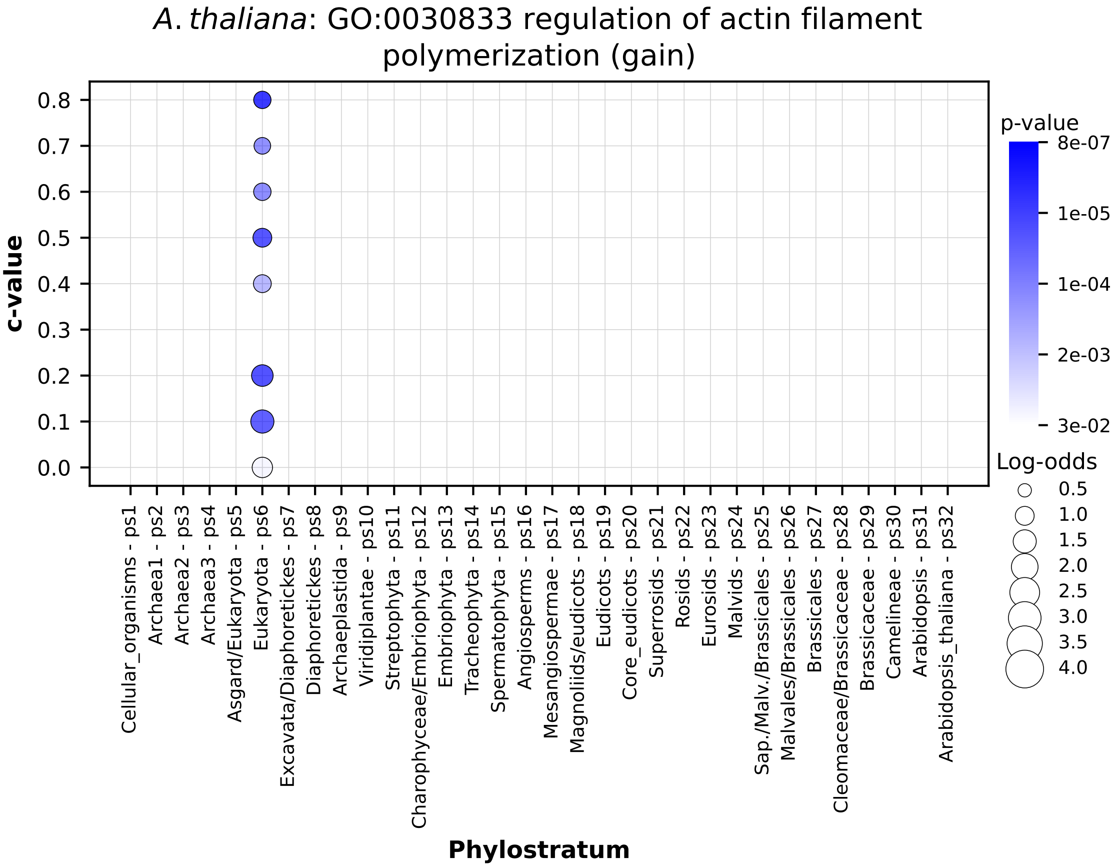

A. thaliana: GO:0030833 regulation of actin filament polymerization (gain)
c-value
0.8
0.7
0.6
0.5
0.4
0.3
0.2
0.1
0.0
Phylostratum
Cellular_organisms - ps1
Archaea1 - ps2
Archaea2 - ps3
Archaea3 - ps4
Asgard/Eukaryota - ps5
Eukaryota - ps6
Excavata/Diaphoretickes - ps7
Diaphoretickes - ps8
Archaeplastida - ps9
Viridiplantae - ps10
Streptophyta - ps11
Charophyceae/Embriophyta - ps12
Embriophyta - ps13
Tracheophyta - ps14
Spermatophyta - ps15
Angiosperms - ps16
Mesangiospermae - ps17
Magnoliids/eudicots - ps18
Eudicots - ps19
Core_eudicots - ps20
Superrosids - ps21
Rosids - ps22
Eurosids - ps23
Malvids - ps24
Sap./Malv./Brassicales - ps25
Malvales/Brassicales - ps26
Brassicales - ps27
Cleomaceae/Brassicaceae - ps28
Brassicaceae - ps29
Camelineae - ps30
Arabidopsis - ps31
Arabidopsis_thaliana - ps32
p-value
8e-07
1e-05
1e-04
2e-03
3e-02
Log-odds
0.5
1.0
1.5
2.0
2.5
3.0
3.5
4.0

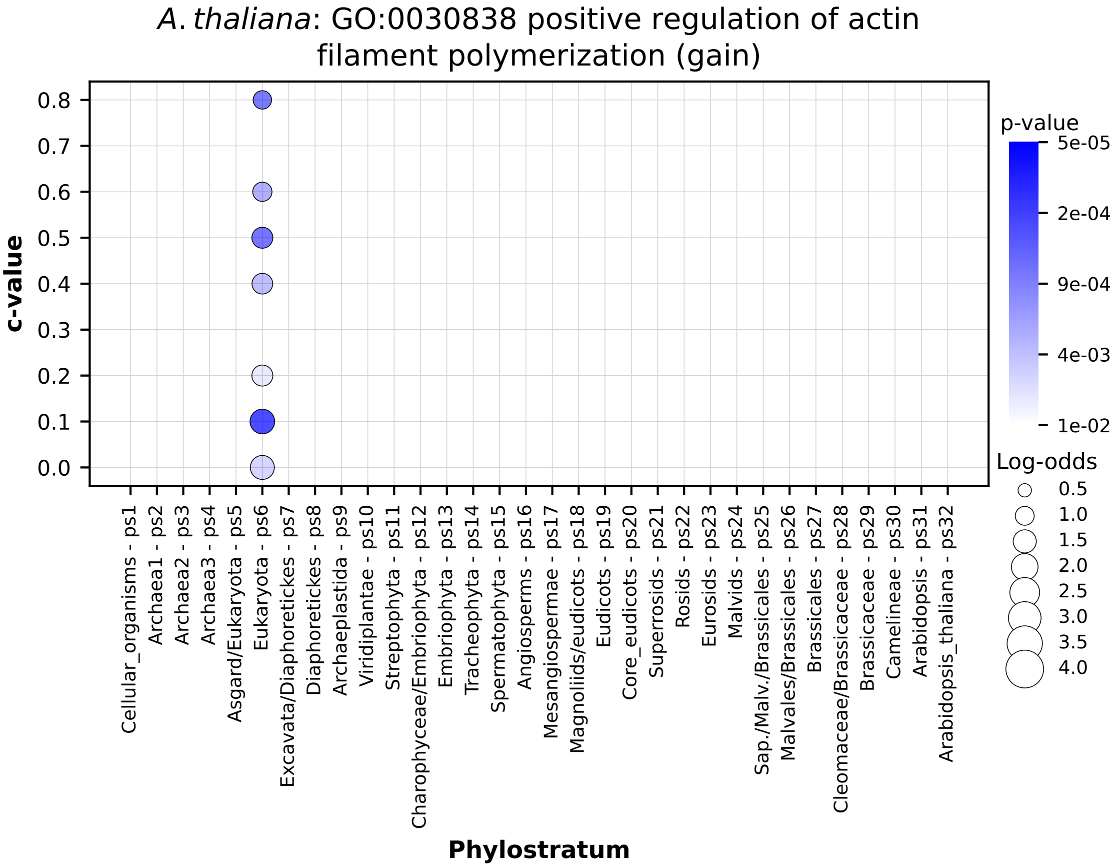

A. thaliana: GO:0030838 positive regulation of actin filament polymerization (gain)
c-value
0.8
0.7
0.6
0.5
0.4
0.3
0.2
0.1
0.0
p-value
5e-05
2e-04
9e-04
4e-03
1e-02
Log-odds
0.5
1.0
1.5
2.0
2.5
3.0
3.5
4.0
Cellular_organisms - ps1
Archaea1 - ps2
Archaea2 - ps3
Archaea3 - ps4
Asgard/Eukaryota - ps5
Eukaryota - ps6
Excavata/Diaphoretickes - ps7
Diaphoretickes - ps8
Archaeplastida - ps9
Viridiplantae - ps10
Streptophyta - ps11
Charophyceae/Embriophyta - ps12
Embriophyta - ps13
Tracheophyta - ps14
Spermatophyta - ps15
Angiosperms - ps16
Mesangiospermae - ps17
Magnoliids/eudicots - ps18
Eudicots - ps19
Core_eudicots - ps20
Superrosids - ps21
Rosids - ps22
Eurosids - ps23
Malvids - ps24
Sap./Malv./Brassicales - ps25
Malvales/Brassicales - ps26
Brassicales - ps27
Cleomaceae/Brassicaceae - ps28
Brassicaceae - ps29
Camelineae - ps30
Arabidopsis - ps31
Arabidopsis_thaliana - ps32
Phylostratum

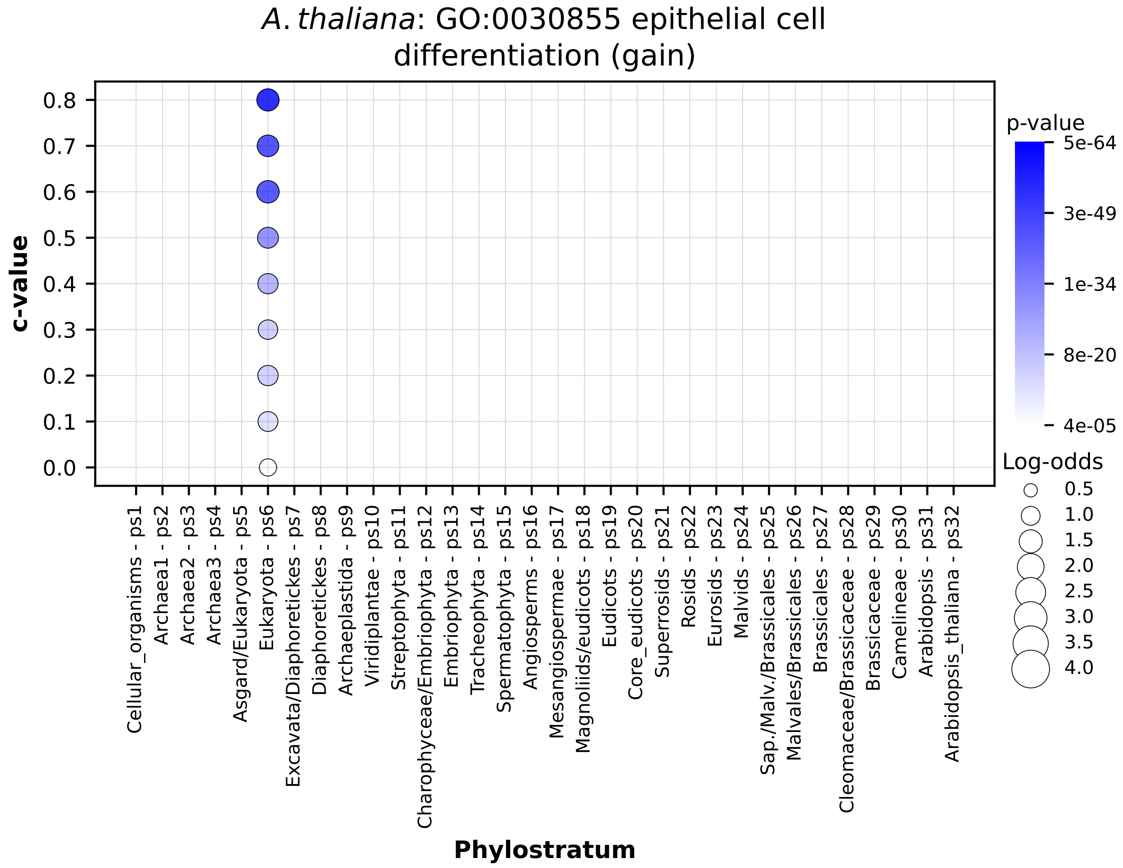

A. thaliana: GO:0030855 epithelial cell differentiation (gain)
c-value
0.8
0.7
0.6
0.5
0.4
0.3
0.2
0.1
0.0
p-value
5e-64
3e-49
1e-34
8e-20
4e-05
Log-odds
0.5
1.0
1.5
2.0
2.5
3.0
3.5
4.0
Cellular_organisms - ps1
Archaea1 - ps2
Archaea2 - ps3
Archaea3 - ps4
Asgard/Eukaryota - ps5
Eukaryota - ps6
Excavata/Diaphoretickes - ps7
Diaphoretickes - ps8
Archaeplastida - ps9
Viridiplantae - ps10
Streptophyta - ps11
Charophyceae/Embriophyta - ps12
Embriophyta - ps13
Tracheophyta - ps14
Spermatophyta - ps15
Angiosperms - ps16
Mesangiospermae - ps17
Magnoliids/eudicots - ps18
Eudicots - ps19
Core_eudicots - ps20
Superrosids - ps21
Rosids - ps22
Eurosids - ps23
Malvids - ps24
Sap./Malv./Brassicales - ps25
Malvales/Brassicales - ps26
Brassicales - ps27
Cleomaceae/Brassicaceae - ps28
Brassicaceae - ps29
Camelineae - ps30
Arabidopsis - ps31
Arabidopsis_thaliana - ps32
Phylostratum

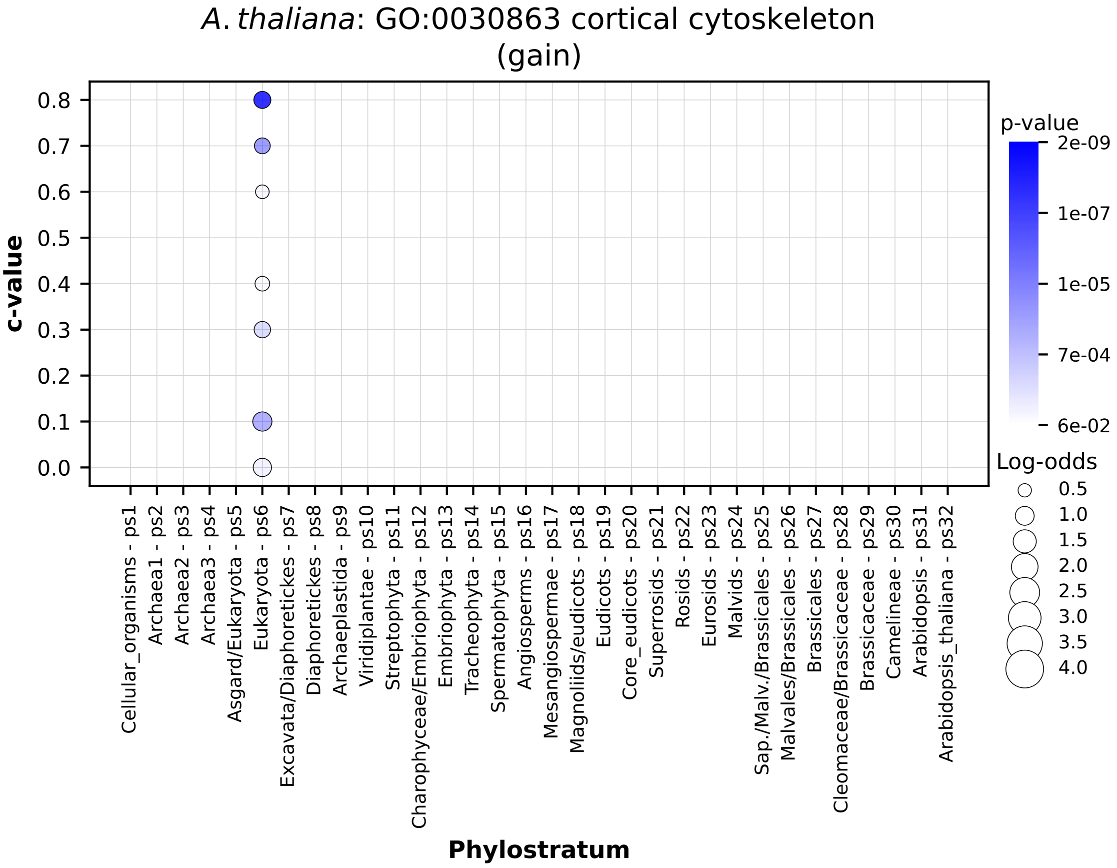

A. thaliana: GO:0030863 cortical cytoskeleton
(gain)
c-value
0.8
0.7
0.6
0.5
0.4
0.3
0.2
0.1
0.0
p-value
2e-09
1e-07
1e-05
7e-04
6e-02
Log-odds
0.5
1.0
1.5
2.0
2.5
3.0
3.5
4.0
Cellular_organisms - ps1
Archaea1 - ps2
Archaea2 - ps3
Archaea3 - ps4
Asgard/Eukaryota - ps5
Eukaryota - ps6
Excavata/Diaphoretickes - ps7
Diaphoretickes - ps8
Archaeplastida - ps9
Viridiplantae - ps10
Streptophyta - ps11
Charophyceae/Embriophyta - ps12
Embriophyta - ps13
Tracheophyta - ps14
Spermatophyta - ps15
Angiosperms - ps16
Mesangiospermae - ps17
Magnoliids/eudicots - ps18
Eudicots - ps19
Core_eudicots - ps20
Superrosids - ps21
Rosids - ps22
Eurosids - ps23
Malvids - ps24
Sap./Malv./Brassicales - ps25
Malvales/Brassicales - ps26
Brassicales - ps27
Cleomaceae/Brassicaceae - ps28
Brassicaceae - ps29
Camelineae - ps30
Arabidopsis - ps31
Arabidopsis_thaliana - ps32
Phylostratum

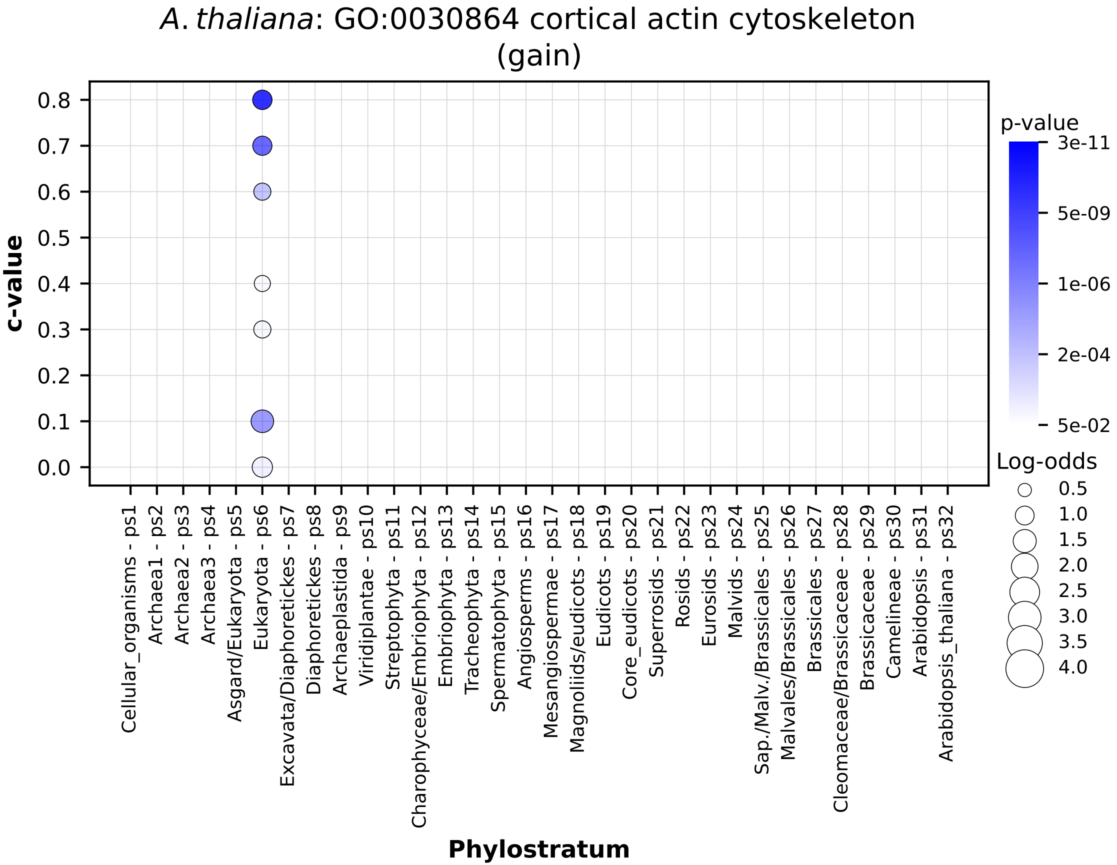

A. thaliana: GO:0030864 cortical actin cytoskeleton
(gain)
c-value
0.8
0.7
0.6
0.5
0.4
0.3
0.2
0.1
0.0
p-value
3e-11
5e-09
1e-06
2e-04
5e-02
Log-odds
0.5
1.0
1.5
2.0
2.5
3.0
3.5
4.0
Cellular_organisms - ps1
Archaea1 - ps2
Archaea2 - ps3
Archaea3 - ps4
Asgard/Eukaryota - ps5
Eukaryota - ps6
Excavata/Diaphoretickes - ps7
Diaphoretickes - ps8
Archaeplastida - ps9
Viridiplantae - ps10
Streptophyta - ps11
Charophyceae/Embriophyta - ps12
Embriophyta - ps13
Tracheophyta - ps14
Spermatophyta - ps15
Angiosperms - ps16
Mesangiospermae - ps17
Magnoliids/eudicots - ps18
Eudicots - ps19
Core_eudicots - ps20
Superrosids - ps21
Rosids - ps22
Eurosids - ps23
Malvids - ps24
Sap./Malv./Brassicales - ps25
Malvales/Brassicales - ps26
Brassicales - ps27
Cleomaceae/Brassicaceae - ps28
Brassicaceae - ps29
Camelineae - ps30
Arabidopsis - ps31
Arabidopsis_thaliana - ps32
Phylostratum

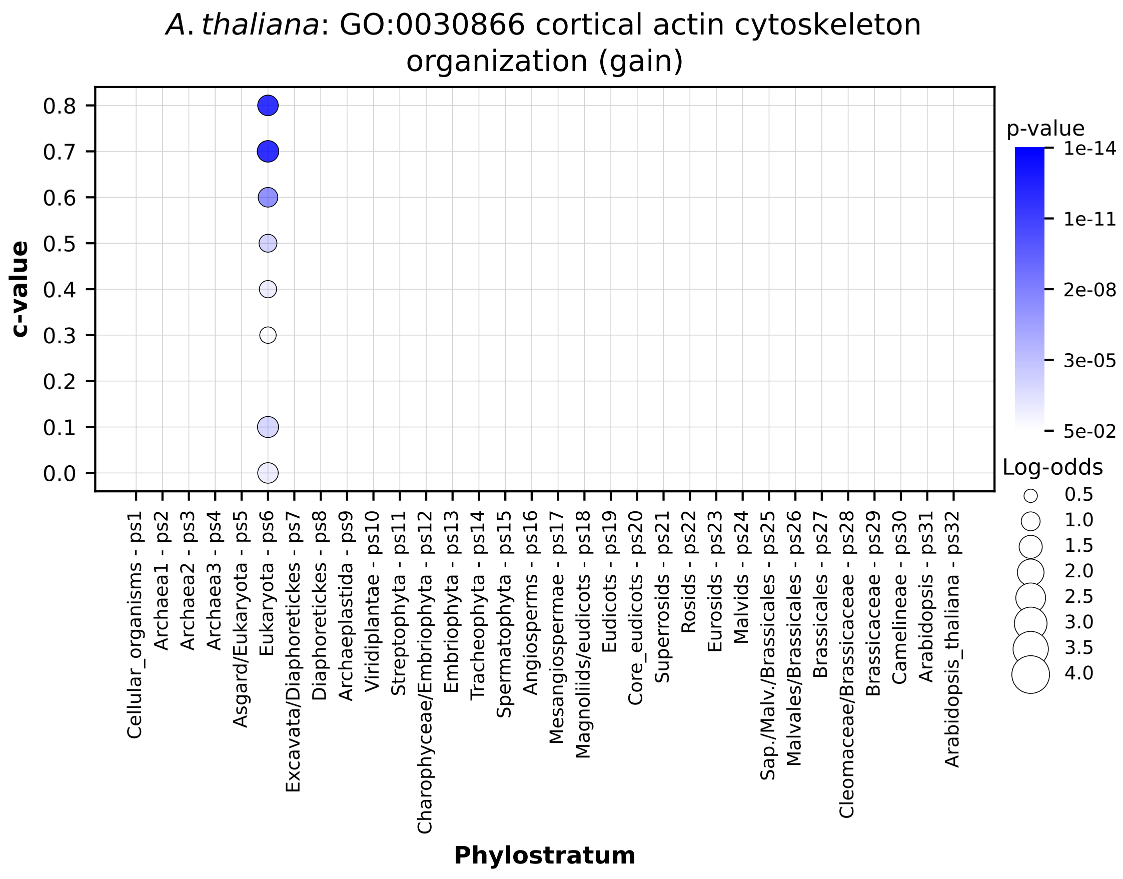

A. thaliana: GO:0030866 cortical actin cytoskeleton organization (gain)
c-value
0.0
0.1
0.2
0.3
0.4
0.5
0.6
0.7
0.8
p-value
1e-14
1e-11
2e-08
3e-05
5e-02
Log-odds
0.5
1.0
1.5
2.0
2.5
3.0
3.5
4.0
Cellular_organisms - ps1
Archaea1 - ps2
Archaea2 - ps3
Archaea3 - ps4
Asgard/Eukaryota - ps5
Eukaryota - ps6
Excavata/Diaphoretickes - ps7
Diaphoretickes - ps8
Archaeplastida - ps9
Viridiplantae - ps10
Streptophyta - ps11
Charophyceae/Embriophyta - ps12
Embriophyta - ps13
Tracheophyta - ps14
Spermatophyta - ps15
Angiosperms - ps16
Mesangiospermae - ps17
Magnoliids/eudicots - ps18
Eudicots - ps19
Core_eudicots - ps20
Superrosids - ps21
Rosids - ps22
Eurosids - ps23
Malvids - ps24
Sap./Malv./Brassicales - ps25
Malvales/Brassicales - ps26
Brassicales - ps27
Cleomaceae/Brassicaceae - ps28
Brassicaceae - ps29
Camelineae - ps30
Arabidopsis - ps31
Arabidopsis_thaliana - ps32
Phylostratum

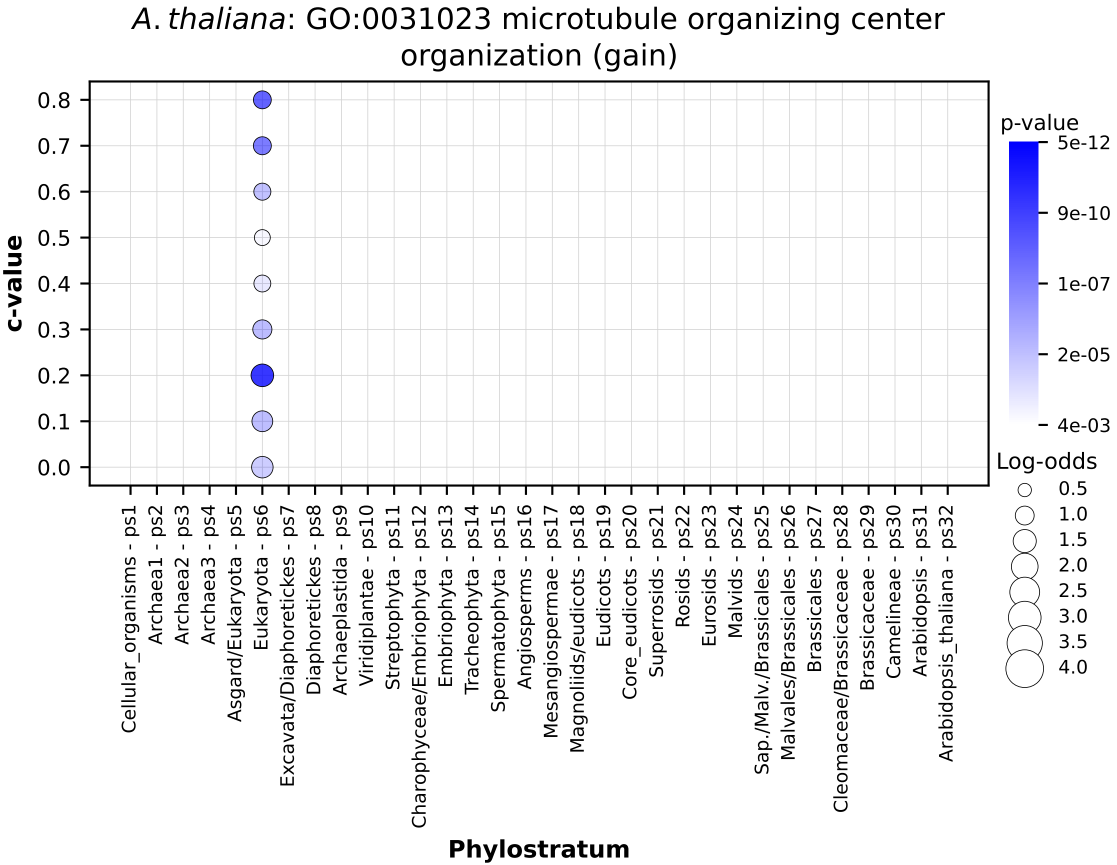

A. thaliana: GO:0031023 microtubule organizing center organization (gain)
c-value
0.8
0.7
0.6
0.5
0.4
0.3
0.2
0.1
0.0
p-value
5e-12
9e-10
1e-07
2e-05
4e-03
Log-odds
0.5
1.0
1.5
2.0
2.5
3.0
3.5
4.0
Cellular_organisms - ps1
Archaea1 - ps2
Archaea2 - ps3
Archaea3 - ps4
Asgard/Eukaryota - ps5
Eukaryota - ps6
Excavata/Diaphoretickes - ps7
Diaphoretickes - ps8
Archaeplastida - ps9
Viridiplantae - ps10
Streptophyta - ps11
Charophyceae/Embriophyta - ps12
Embriophyta - ps13
Tracheophyta - ps14
Spermatophyta - ps15
Angiosperms - ps16
Mesangiospermae - ps17
Magnoliids/eudicots - ps18
Eudicots - ps19
Core_eudicots - ps20
Superrosids - ps21
Rosids - ps22
Eurosids - ps23
Malvids - ps24
Sap./Malv./Brassicales - ps25
Malvales/Brassicales - ps26
Brassicales - ps27
Cleomaceae/Brassicaceae - ps28
Brassicaceae - ps29
Camelineae - ps30
Arabidopsis - ps31
Arabidopsis_thaliana - ps32
Phylostratum

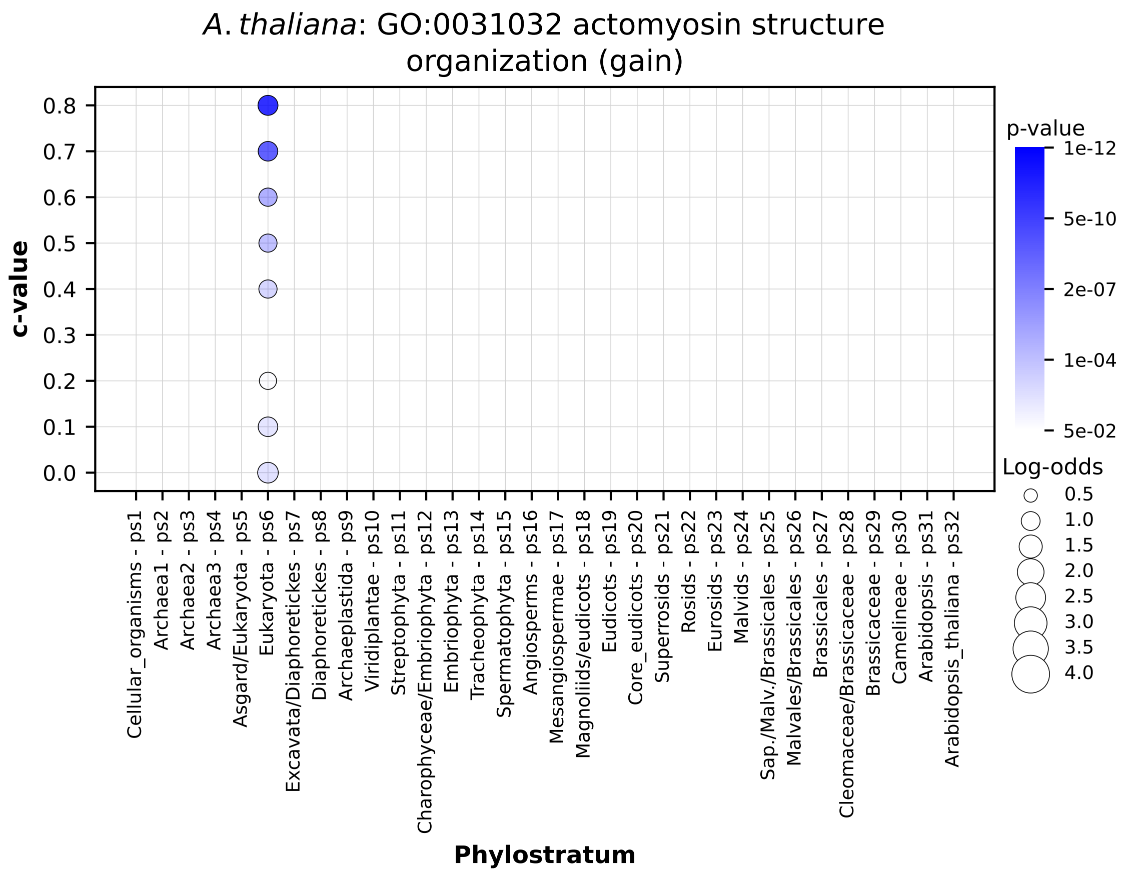
A. thaliana: GO:0031032 actomyosin structure organization (gain)
c-value
0.8
0.7
0.6
0.5
0.4
0.3
0.2
0.1
0.0
p-value
1e-12
5e-10
2e-07
1e-04
5e-02
Log-odds
0.5
1.0
1.5
2.0
2.5
3.0
3.5
4.0
Cellular_organisms - ps1
Archaea1 - ps2
Archaea2 - ps3
Archaea3 - ps4
Asgard/Eukaryota - ps5
Eukaryota - ps6
Excavata/Diaphoretickes - ps7
Diaphoretickes - ps8
Archaeplastida - ps9
Viridiplantae - ps10
Streptophyta - ps11
Charophyceae/Embriophyta - ps12
Embriophyta - ps13
Tracheophyta - ps14
Spermatophyta - ps15
Angiosperms - ps16
Mesangiospermae - ps17
Magnoliids/eudicots - ps18
Eudicots - ps19
Core_eudicots - ps20
Superrosids - ps21
Rosids - ps22
Eurosids - ps23
Malvids - ps24
Sap./Malv./Brassicales - ps25
Malvales/Brassicales - ps26
Brassicales - ps27
Cleomaceae/Brassicaceae - ps28
Brassicaceae - ps29
Camelineae - ps30
Arabidopsis - ps31
Arabidopsis_thaliana - ps32
Phylostratum

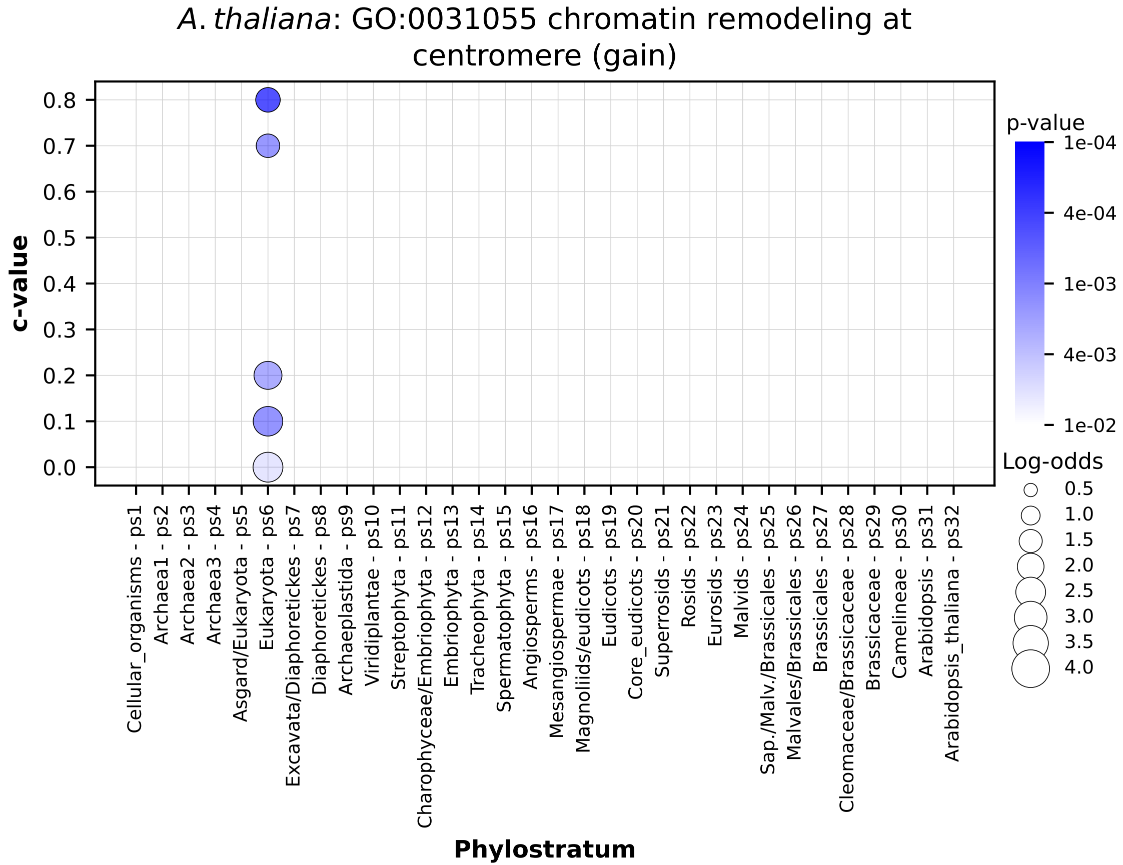

A. thaliana: GO:0031055 chromatin remodeling at centromere (gain)
c-value
0.8
0.7
0.6
0.5
0.4
0.3
0.2
0.1
0.0
p-value
1e-04
4e-04
1e-03
4e-03
1e-02
Log-odds
0.5
1.0
1.5
2.0
2.5
3.0
3.5
4.0
Cellular_organisms - ps1
Archaea1 - ps2
Archaea2 - ps3
Archaea3 - ps4
Asgard/Eukaryota - ps5
Eukaryota - ps6
Excavata/Diaphoretickes - ps7
Diaphoretickes - ps8
Archaeplastida - ps9
Viridiplantae - ps10
Streptophyta - ps11
Charophyceae/Embriophyta - ps12
Embriophyta - ps13
Tracheophyta - ps14
Spermatophyta - ps15
Angiosperms - ps16
Mesangiospermae - ps17
Magnoliids/eudicots - ps18
Eudicots - ps19
Core_eudicots - ps20
Superrosids - ps21
Rosids - ps22
Eurosids - ps23
Malvids - ps24
Sap./Malv./Brassicales - ps25
Malvales/Brassicales - ps26
Brassicales - ps27
Cleomaceae/Brassicaceae - ps28
Brassicaceae - ps29
Camelineae - ps30
Arabidopsis - ps31
Arabidopsis_thaliana - ps32
Phylostratum

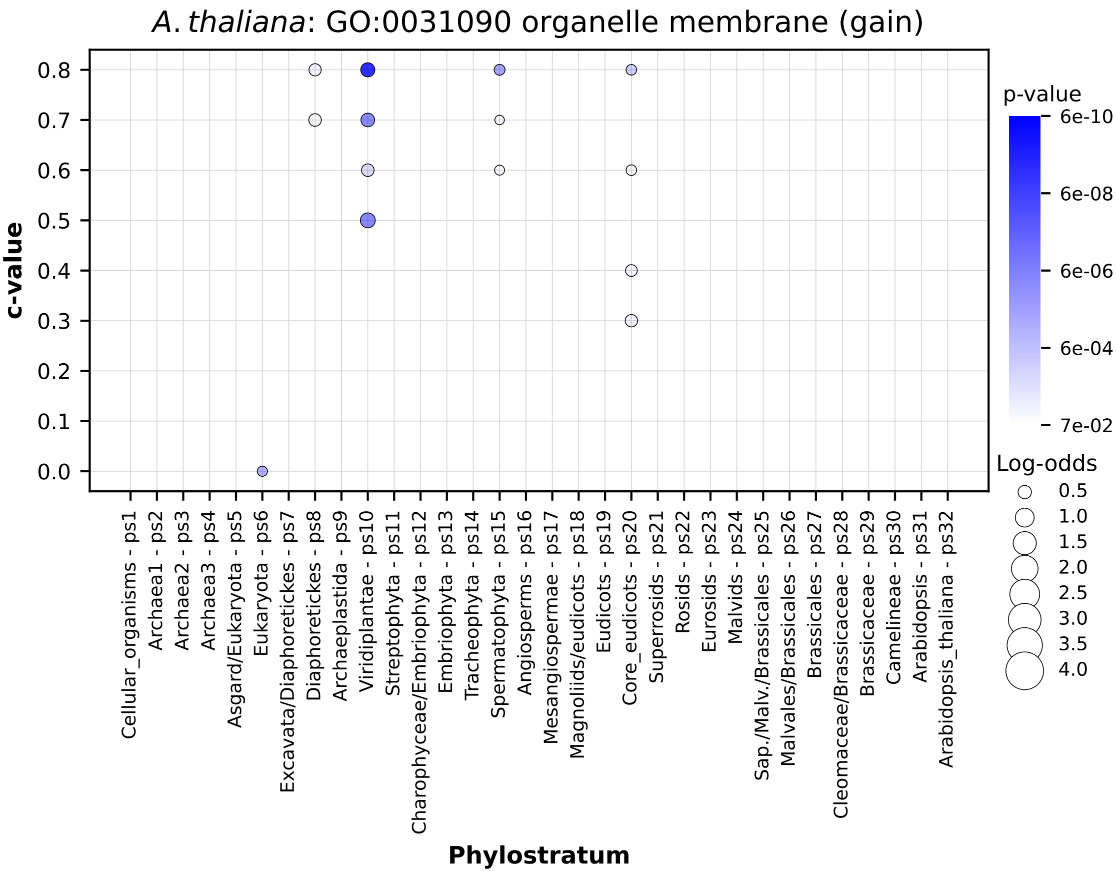

A. thaliana: GO:0031090 organelle membrane (gain)
c-value
0.0
0.1
0.2
0.3
0.4
0.5
0.6
0.7
0.8
p-value
6e-10
6e-08
6e-06
6e-04
7e-02
Log-odds
0.5
1.0
1.5
2.0
2.5
3.0
3.5
4.0
Cellular_organisms - ps1
Archaea1 - ps2
Archaea2 - ps3
Archaea3 - ps4
Asgard/Eukaryota - ps5
Eukaryota - ps6
Excavata/Diaphoretickes - ps7
Diaphoretickes - ps8
Archaeplastida - ps9
Viridiplantae - ps10
Streptophyta - ps11
Charophyceae/Embriophyta - ps12
Embriophyta - ps13
Tracheophyta - ps14
Spermatophyta - ps15
Angiosperms - ps16
Mesangiospermae - ps17
Magnoliids/eudicots - ps18
Eudicots - ps19
Core_eudicots - ps20
Superrosids - ps21
Rosids - ps22
Eurosids - ps23
Malvids - ps24
Sap./Malv./Brassicales - ps25
Malvales/Brassicales - ps26
Brassicales - ps27
Cleomaceae/Brassicaceae - ps28
Brassicaceae - ps29
Camelineae - ps30
Arabidopsis - ps31
Arabidopsis_thaliana - ps32
Phylostratum

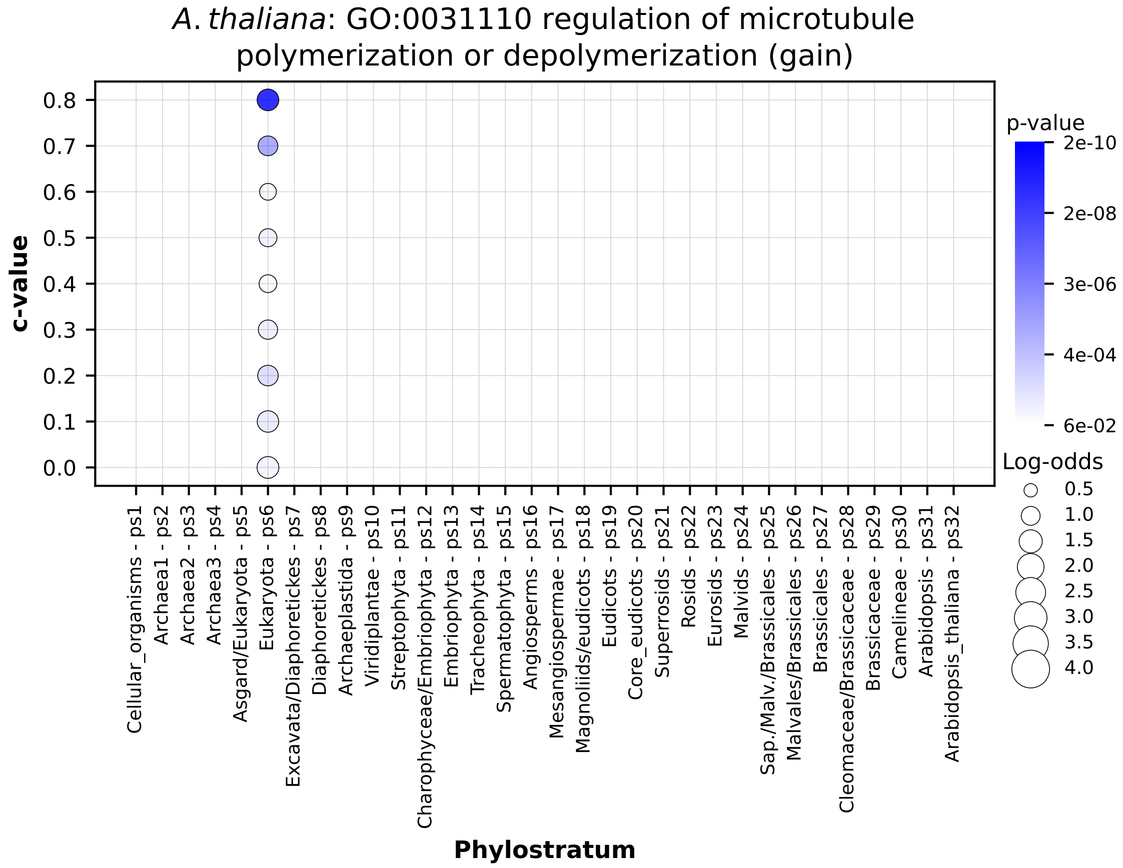
A. thaliana: GO:0031110 regulation of microtubule polymerization or depolymerization (gain)
c-value
0.8
0.7
0.6
0.5
0.4
0.3
0.2
0.1
0.0
p-value
2e-10
2e-08
3e-06
4e-04
6e-02
Log-odds
0.5
1.0
1.5
2.0
2.5
3.0
3.5
4.0
Cellular_organisms - ps1
Archaea1 - ps2
Archaea2 - ps3
Archaea3 - ps4
Asgard/Eukaryota - ps5
Eukaryota - ps6
Excavata/Diaphoretickes - ps7
Diaphoretickes - ps8
Archaeplastida - ps9
Viridiplantae - ps10
Streptophyta - ps11
Charophyceae/Embriophyta - ps12
Embriophyta - ps13
Tracheophyta - ps14
Spermatophyta - ps15
Angiosperms - ps16
Mesangiospermae - ps17
Magnoliids/eudicots - ps18
Eudicots - ps19
Core_eudicots - ps20
Superrosids - ps21
Rosids - ps22
Eurosids - ps23
Malvids - ps24
Sap./Malv./Brassicales - ps25
Malvales/Brassicales - ps26
Brassicales - ps27
Cleomaceae/Brassicaceae - ps28
Brassicaceae - ps29
Camelineae - ps30
Arabidopsis - ps31
Arabidopsis_thaliana - ps32
Phylostratum

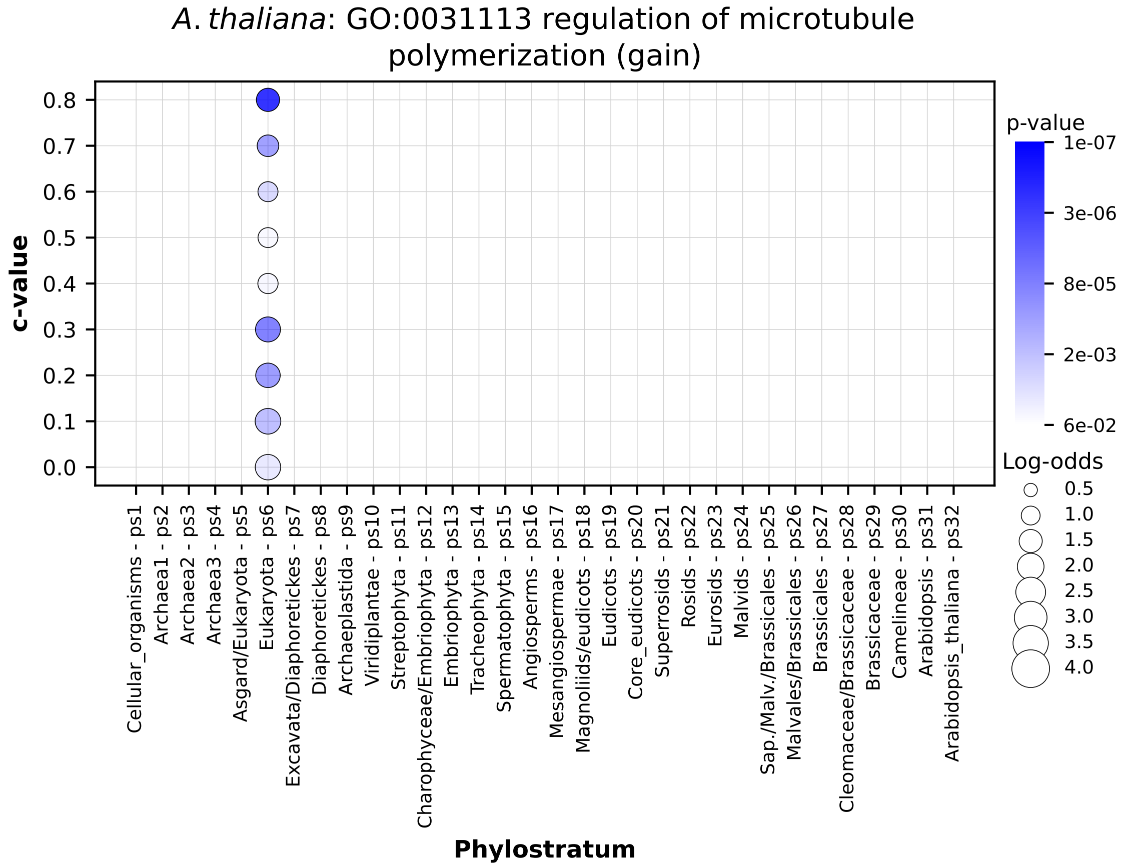

A. thaliana: GO:0031113 regulation of microtubule polymerization (gain)
c-value
0.8
0.7
0.6
0.5
0.4
0.3
0.2
0.1
0.0
p-value
1e-07
3e-06
8e-05
2e-03
6e-02
Log-odds
0.5
1.0
1.5
2.0
2.5
3.0
3.5
4.0
Cellular_organisms - ps1
Archaea1 - ps2
Archaea2 - ps3
Archaea3 - ps4
Asgard/Eukaryota - ps5
Eukaryota - ps6
Excavata/Diaphoretickes - ps7
Diaphoretickes - ps8
Archaeplastida - ps9
Viridiplantae - ps10
Streptophyta - ps11
Charophyceae/Embriophyta - ps12
Embriophyta - ps13
Tracheophyta - ps14
Spermatophyta - ps15
Angiosperms - ps16
Mesangiospermae - ps17
Magnoliids/eudicots - ps18
Eudicots - ps19
Core_eudicots - ps20
Superrosids - ps21
Rosids - ps22
Eurosids - ps23
Malvids - ps24
Sap./Malv./Brassicales - ps25
Malvales/Brassicales - ps26
Brassicales - ps27
Cleomaceae/Brassicaceae - ps28
Brassicaceae - ps29
Camelineae - ps30
Arabidopsis - ps31
Arabidopsis_thaliana - ps32
Phylostratum

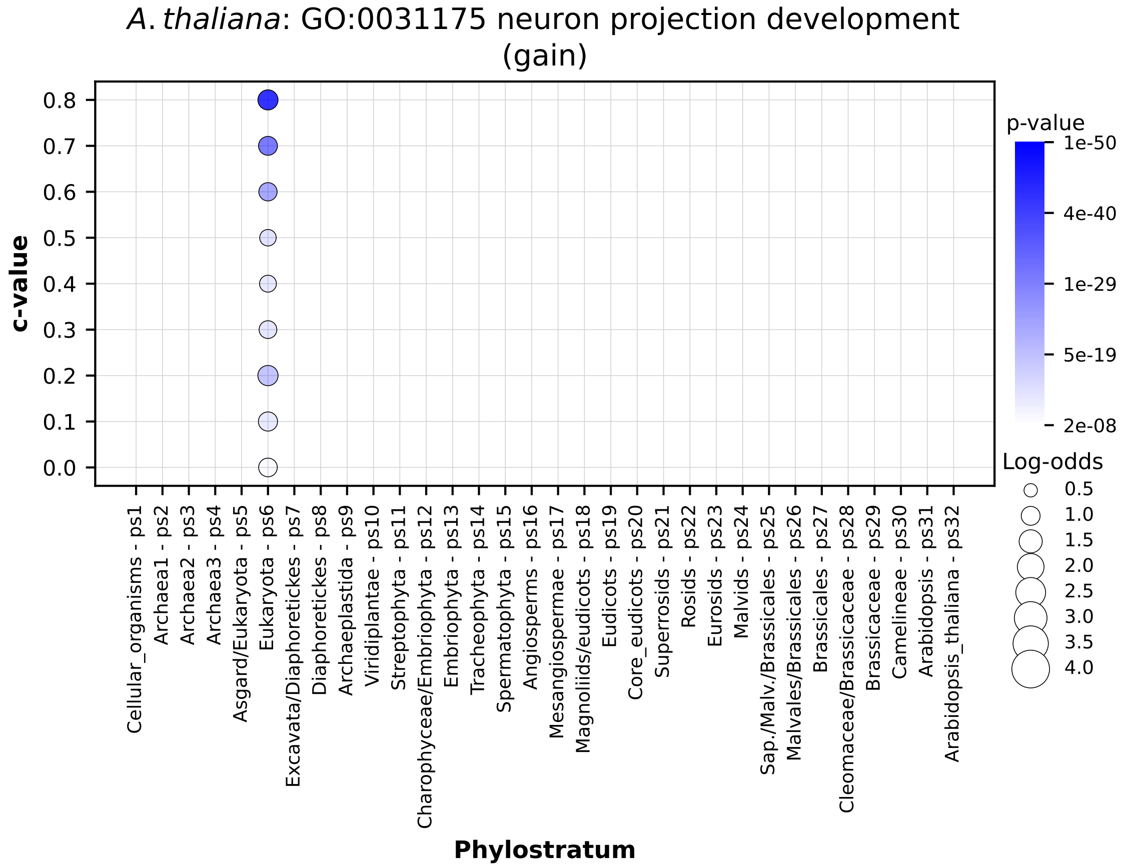

A. thaliana: GO:0031175 neuron projection development (gain)
c-value
0.8
0.7
0.6
0.5
0.4
0.3
0.2
0.1
0.0
Phylostratum
Cellular_organisms - ps1
Archaea1 - ps2
Archaea2 - ps3
Archaea3 - ps4
Asgard/Eukaryota - ps5
Eukaryota - ps6
Excavata/Diaphoretickes - ps7
Diaphoretickes - ps8
Archaeplastida - ps9
Viridiplantae - ps10
Streptophyta - ps11
Charophyceae/Embriophyta - ps12
Embriophyta - ps13
Tracheophyta - ps14
Spermatophyta - ps15
Angiosperms - ps16
Mesangiospermae - ps17
Magnoliids/eudicots - ps18
Eudicots - ps19
Core_eudicots - ps20
Superrosids - ps21
Rosids - ps22
Eurosids - ps23
Malvids - ps24
Sap./Malv./Brassicales - ps25
Malvales/Brassicales - ps26
Brassicales - ps27
Cleomaceae/Brassicaceae - ps28
Brassicaceae - ps29
Camelineae - ps30
Arabidopsis - ps31
Arabidopsis_thaliana - ps32
p-value
1e-50
4e-40
1e-29
5e-19
2e-08
Log-odds
0.5
1.0
1.5
2.0
2.5
3.0
3.5
4.0

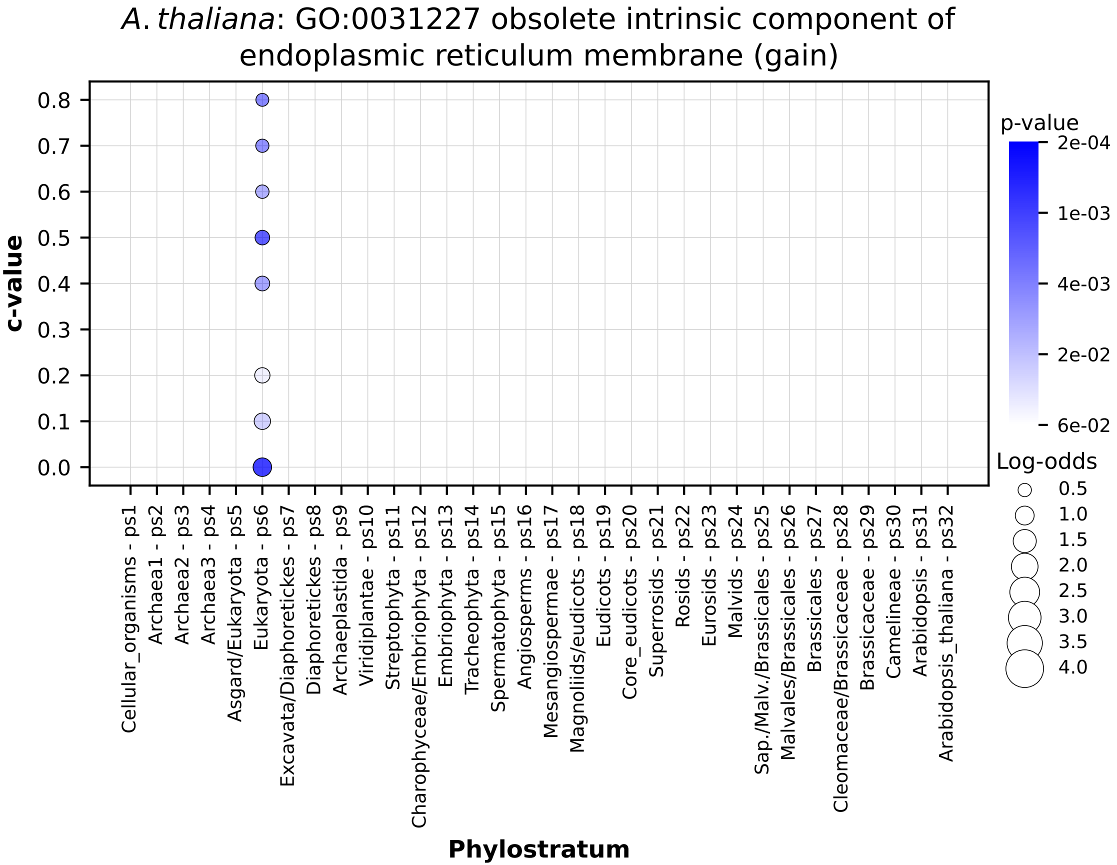

A. thaliana: GO:0031227 obsolete intrinsic component of endoplasmic reticulum membrane (gain)
c-value
0.8
0.7
0.6
0.5
0.4
0.3
0.2
0.1
0.0
p-value
2e-04
1e-03
4e-03
2e-02
6e-02
Log-odds
0.5
1.0
1.5
2.0
2.5
3.0
3.5
4.0
Cellular_organisms - ps1
Archaea1 - ps2
Archaea2 - ps3
Archaea3 - ps4
Asgard/Eukaryota - ps5
Eukaryota - ps6
Excavata/Diaphoretickes - ps7
Diaphoretickes - ps8
Archaeplastida - ps9
Viridiplantae - ps10
Streptophyta - ps11
Charophyceae/Embriophyta - ps12
Embriophyta - ps13
Tracheophyta - ps14
Spermatophyta - ps15
Angiosperms - ps16
Mesangiospermae - ps17
Magnoliids/eudicots - ps18
Eudicots - ps19
Core_eudicots - ps20
Superrosids - ps21
Rosids - ps22
Eurosids - ps23
Malvids - ps24
Sap./Malv./Brassicales - ps25
Malvales/Brassicales - ps26
Brassicales - ps27
Cleomaceae/Brassicaceae - ps28
Brassicaceae - ps29
Camelineae - ps30
Arabidopsis - ps31
Arabidopsis_thaliana - ps32
Phylostratum

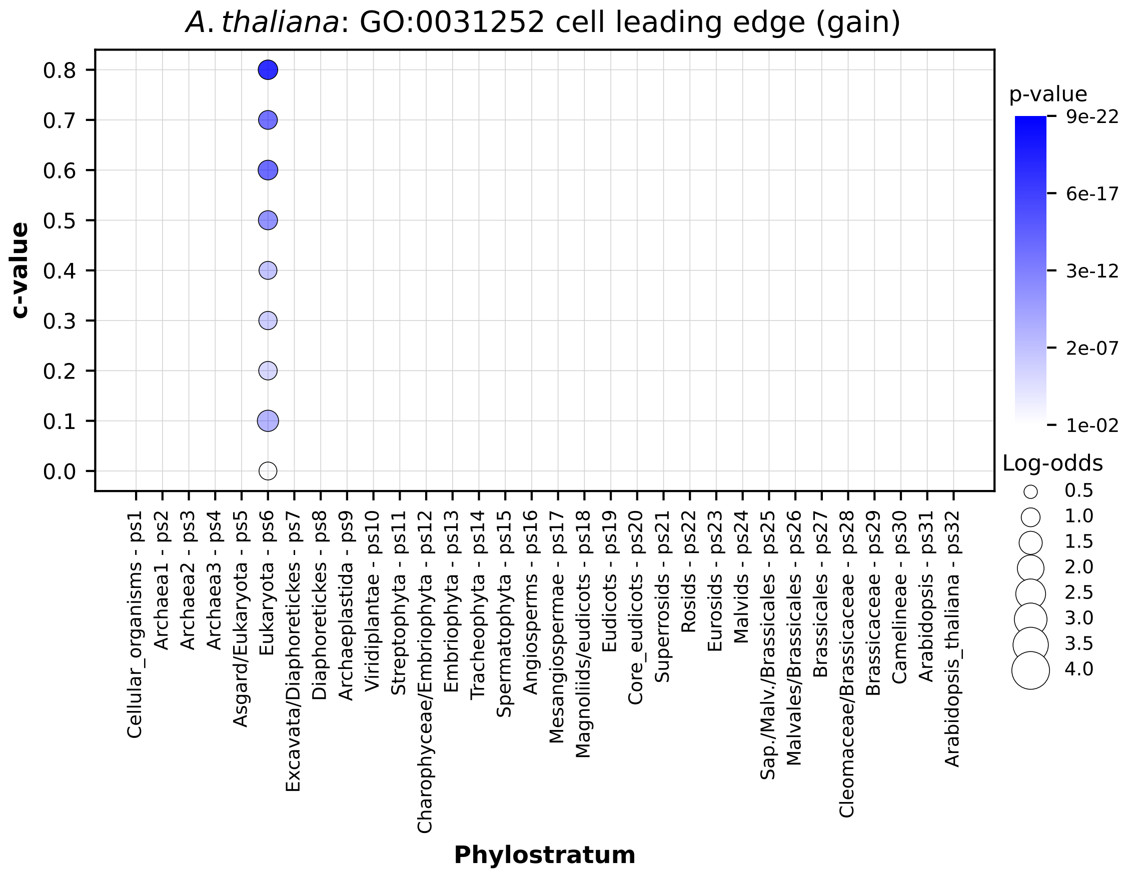

A. thaliana: GO:0031252 cell leading edge (gain)
c-value
0.8
0.7
0.6
0.5
0.4
0.3
0.2
0.1
0.0
p-value
9e-22
6e-17
3e-12
2e-07
1e-02
Log-odds
0.5
1.0
1.5
2.0
2.5
3.0
3.5
4.0
Cellular_organisms - ps1
Archaea1 - ps2
Archaea2 - ps3
Archaea3 - ps4
Asgard/Eukaryota - ps5
Eukaryota - ps6
Excavata/Diaphoretickes - ps7
Diaphoretickes - ps8
Archaeplastida - ps9
Viridiplantae - ps10
Streptophyta - ps11
Charophyceae/Embriophyta - ps12
Embriophyta - ps13
Tracheophyta - ps14
Spermatophyta - ps15
Angiosperms - ps16
Mesangiospermae - ps17
Magnoliids/eudicots - ps18
Eudicots - ps19
Core_eudicots - ps20
Superrosids - ps21
Rosids - ps22
Eurosids - ps23
Malvids - ps24
Sap./Malv./Brassicales - ps25
Malvales/Brassicales - ps26
Brassicales - ps27
Cleomaceae/Brassicaceae - ps28
Brassicaceae - ps29
Camelineae - ps30
Arabidopsis - ps31
Arabidopsis_thaliana - ps32
Phylostratum

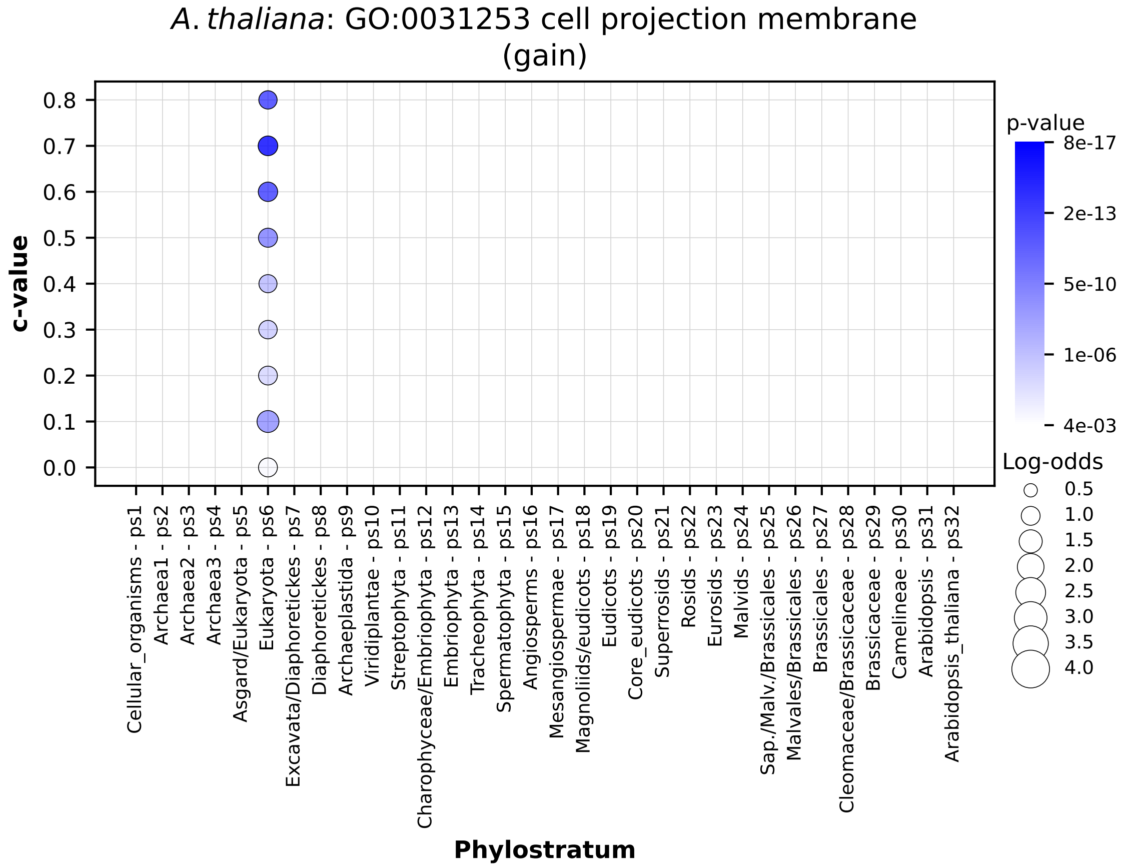
A. thaliana: GO:0031253 cell projection membrane
(gain)
c-value
0.8
0.7
0.6
0.5
0.4
0.3
0.2
0.1
0.0
p-value
8e-17
2e-13
5e-10
1e-06
4e-03
Log-odds
0.5
1.0
1.5
2.0
2.5
3.0
3.5
4.0
Cellular_organisms - ps1
Archaea1 - ps2
Archaea2 - ps3
Archaea3 - ps4
Asgard/Eukaryota - ps5
Eukaryota - ps6
Excavata/Diaphoretickes - ps7
Diaphoretickes - ps8
Archaeplastida - ps9
Viridiplantae - ps10
Streptophyta - ps11
Charophyceae/Embriophyta - ps12
Embriophyta - ps13
Tracheophyta - ps14
Spermatophyta - ps15
Angiosperms - ps16
Mesangiospermae - ps17
Magnoliids/eudicots - ps18
Eudicots - ps19
Core_eudicots - ps20
Superrosids - ps21
Rosids - ps22
Eurosids - ps23
Malvids - ps24
Sap./Malv./Brassicales - ps25
Malvales/Brassicales - ps26
Brassicales - ps27
Cleomaceae/Brassicaceae - ps28
Brassicaceae - ps29
Camelineae - ps30
Arabidopsis - ps31
Arabidopsis_thaliana - ps32
Phylostratum

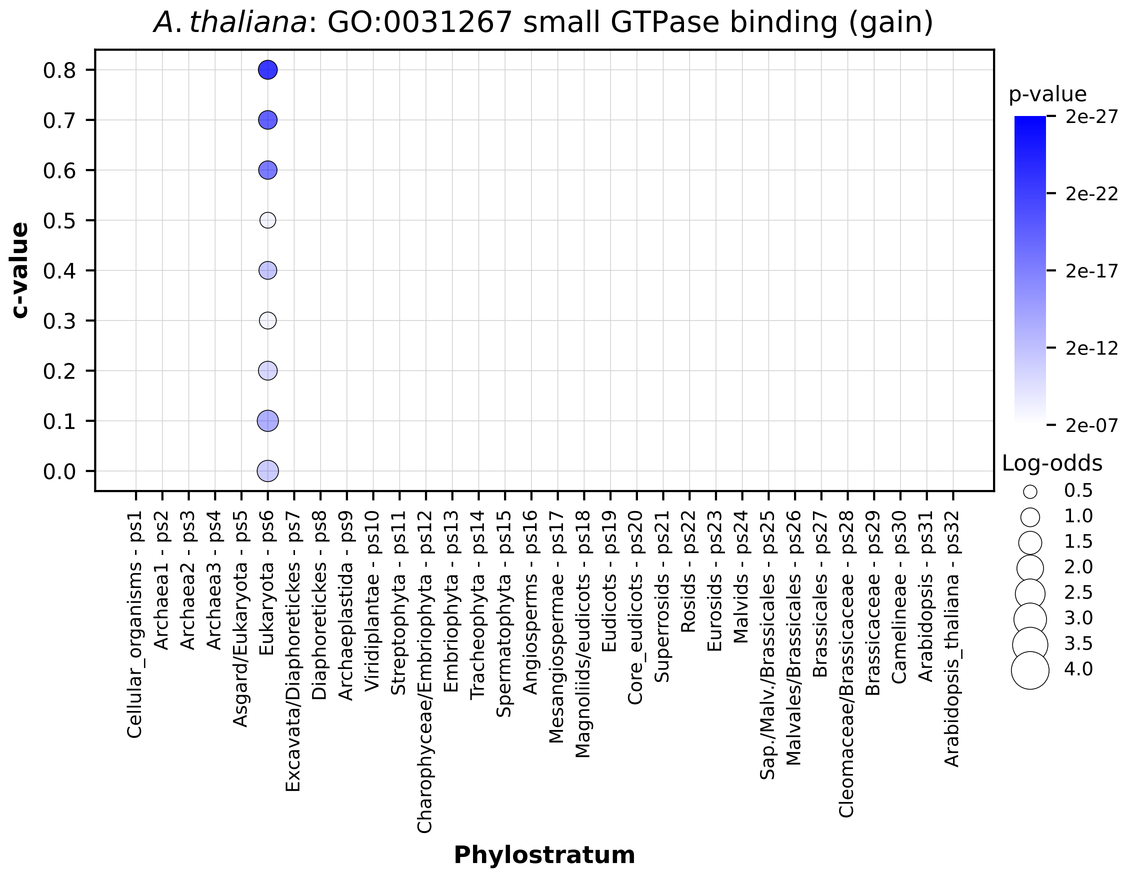

A. thaliana: GO:0031267 small GTPase binding (gain)
c-value
0.8
0.7
0.6
0.5
0.4
0.3
0.2
0.1
0.0
p-value
2e-27
2e-22
2e-17
2e-12
2e-07
Log-odds
0.5
1.0
1.5
2.0
2.5
3.0
3.5
4.0
Cellular_organisms - ps1
Archaea1 - ps2
Archaea2 - ps3
Archaea3 - ps4
Asgard/Eukaryota - ps5
Eukaryota - ps6
Excavata/Diaphoretickes - ps7
Diaphoretickes - ps8
Archaeplastida - ps9
Viridiplantae - ps10
Streptophyta - ps11
Charophyceae/Embriophyta - ps12
Embriophyta - ps13
Tracheophyta - ps14
Spermatophyta - ps15
Angiosperms - ps16
Mesangiospermae - ps17
Magnoliids/eudicots - ps18
Eudicots - ps19
Core_eudicots - ps20
Superrosids - ps21
Rosids - ps22
Eurosids - ps23
Malvids - ps24
Sap./Malv./Brassicales - ps25
Malvales/Brassicales - ps26
Brassicales - ps27
Cleomaceae/Brassicaceae - ps28
Brassicaceae - ps29
Camelineae - ps30
Arabidopsis - ps31
Arabidopsis_thaliana - ps32
Phylostratum

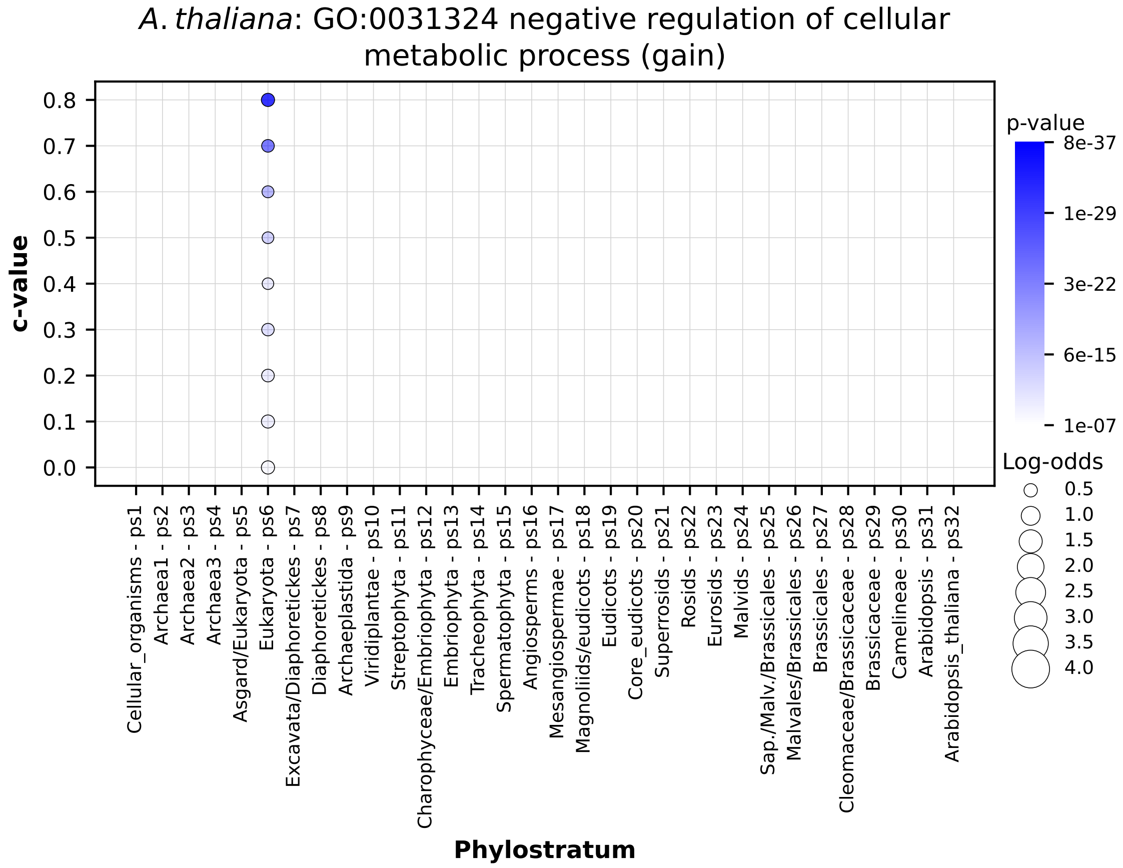

A. thaliana: GO:0031324 negative regulation of cellular metabolic process (gain)
c-value
0.0
0.1
0.2
0.3
0.4
0.5
0.6
0.7
0.8
Phylostratum
Cellular_organisms - ps1
Archaea1 - ps2
Archaea2 - ps3
Archaea3 - ps4
Asgard/Eukaryota - ps5
Eukaryota - ps6
Excavata/Diaphoretickes - ps7
Diaphoretickes - ps8
Archaeplastida - ps9
Viridiplantae - ps10
Streptophyta - ps11
Charophyceae/Embriophyta - ps12
Embriophyta - ps13
Tracheophyta - ps14
Spermatophyta - ps15
Angiosperms - ps16
Mesangiospermae - ps17
Magnoliids/eudicots - ps18
Eudicots - ps19
Core_eudicots - ps20
Superrosids - ps21
Rosids - ps22
Eurosids - ps23
Malvids - ps24
Sap./Malv./Brassicales - ps25
Malvales/Brassicales - ps26
Brassicales - ps27
Cleomaceae/Brassicaceae - ps28
Brassicaceae - ps29
Camelineae - ps30
Arabidopsis - ps31
Arabidopsis_thaliana - ps32
p-value
8e-37
1e-29
3e-22
6e-15
1e-07
Log-odds
0.5
1.0
1.5
2.0
2.5
3.0
3.5
4.0

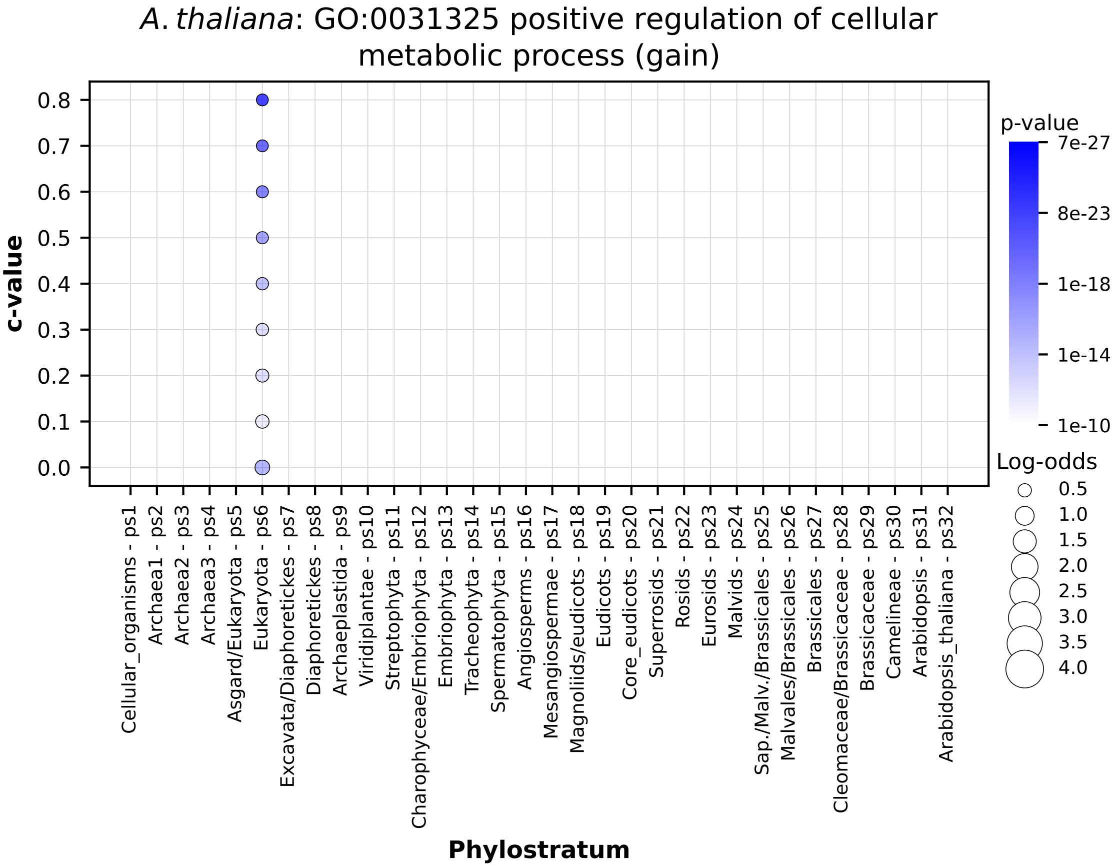

A. thaliana: GO:0031325 positive regulation of cellular metabolic process (gain)
c-value
0.0
0.1
0.2
0.3
0.4
0.5
0.6
0.7
0.8
p-value
7e-27
8e-23
1e-18
1e-14
1e-10
Log-odds
0.5
1.0
1.5
2.0
2.5
3.0
3.5
4.0
Cellular_organisms - ps1
Archaea1 - ps2
Archaea2 - ps3
Archaea3 - ps4
Asgard/Eukaryota - ps5
Eukaryota - ps6
Excavata/Diaphoretickes - ps7
Diaphoretickes - ps8
Archaeplastida - ps9
Viridiplantae - ps10
Streptophyta - ps11
Charophyceae/Embriophyta - ps12
Embriophyta - ps13
Tracheophyta - ps14
Spermatophyta - ps15
Angiosperms - ps16
Mesangiospermae - ps17
Magnoliids/eudicots - ps18
Eudicots - ps19
Core_eudicots - ps20
Superrosids - ps21
Rosids - ps22
Eurosids - ps23
Malvids - ps24
Sap./Malv./Brassicales - ps25
Malvales/Brassicales - ps26
Brassicales - ps27
Cleomaceae/Brassicaceae - ps28
Brassicaceae - ps29
Camelineae - ps30
Arabidopsis - ps31
Arabidopsis_thaliana - ps32
Phylostratum

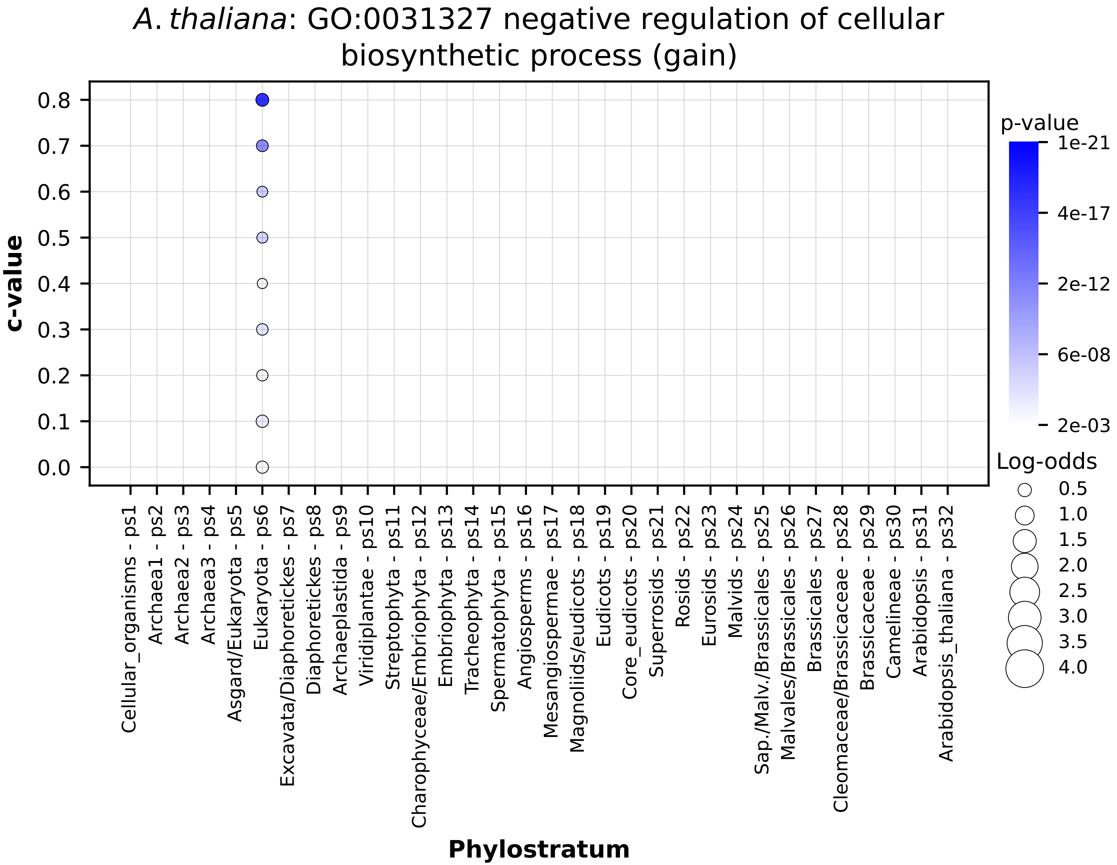

A. thaliana: GO:0031327 negative regulation of cellular biosynthetic process (gain)
c-value
0.8
0.7
0.6
0.5
0.4
0.3
0.2
0.1
0.0
p-value
1e-21
4e-17
2e-12
6e-08
2e-03
Log-odds
0.5
1.0
1.5
2.0
2.5
3.0
3.5
4.0
Cellular_organisms - ps1
Archaea1 - ps2
Archaea2 - ps3
Archaea3 - ps4
Asgard/Eukaryota - ps5
Eukaryota - ps6
Excavata/Diaphoretickes - ps7
Diaphoretickes - ps8
Archaeplastida - ps9
Viridiplantae - ps10
Streptophyta - ps11
Charophyceae/Embriophyta - ps12
Embriophyta - ps13
Tracheophyta - ps14
Spermatophyta - ps15
Angiosperms - ps16
Mesangiospermae - ps17
Magnoliids/eudicots - ps18
Eudicots - ps19
Core_eudicots - ps20
Superrosids - ps21
Rosids - ps22
Eurosids - ps23
Malvids - ps24
Sap./Malv./Brassicales - ps25
Malvales/Brassicales - ps26
Brassicales - ps27
Cleomaceae/Brassicaceae - ps28
Brassicaceae - ps29
Camelineae - ps30
Arabidopsis - ps31
Arabidopsis_thaliana - ps32
Phylostratum

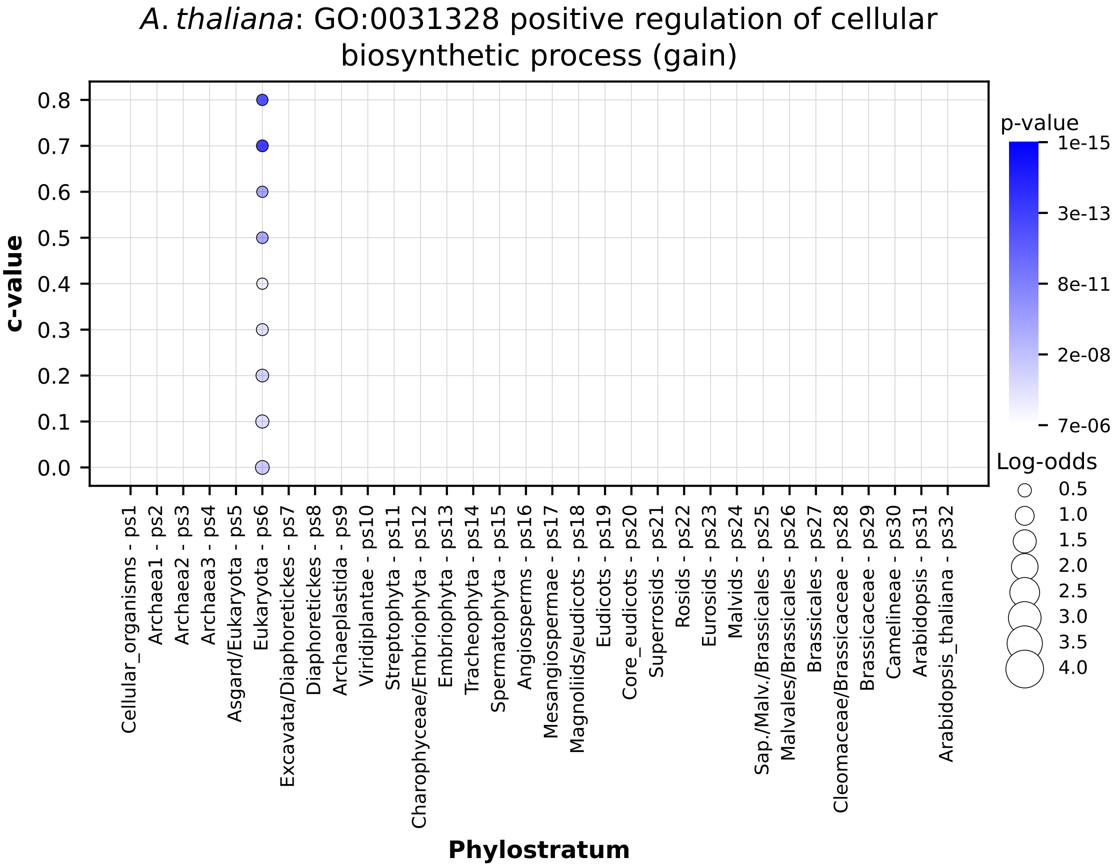

A. thaliana: GO:0031328 positive regulation of cellular biosynthetic process (gain)
c-value
0.8
0.7
0.6
0.5
0.4
0.3
0.2
0.1
0.0
Phylostratum
Cellular_organisms - ps1
Archaea1 - ps2
Archaea2 - ps3
Archaea3 - ps4
Asgard/Eukaryota - ps5
Eukaryota - ps6
Excavata/Diaphoretickes - ps7
Diaphoretickes - ps8
Archaeplastida - ps9
Viridiplantae - ps10
Streptophyta - ps11
Charophyceae/Embriophyta - ps12
Embriophyta - ps13
Tracheophyta - ps14
Spermatophyta - ps15
Angiosperms - ps16
Mesangiospermae - ps17
Magnoliids/eudicots - ps18
Eudicots - ps19
Core_eudicots - ps20
Superrosids - ps21
Rosids - ps22
Eurosids - ps23
Malvids - ps24
Sap./Malv./Brassicales - ps25
Malvales/Brassicales - ps26
Brassicales - ps27
Cleomaceae/Brassicaceae - ps28
Brassicaceae - ps29
Camelineae - ps30
Arabidopsis - ps31
Arabidopsis_thaliana - ps32
p-value
1e-15
3e-13
8e-11
2e-08
7e-06
Log-odds
0.5
1.0
1.5
2.0
2.5
3.0
3.5
4.0

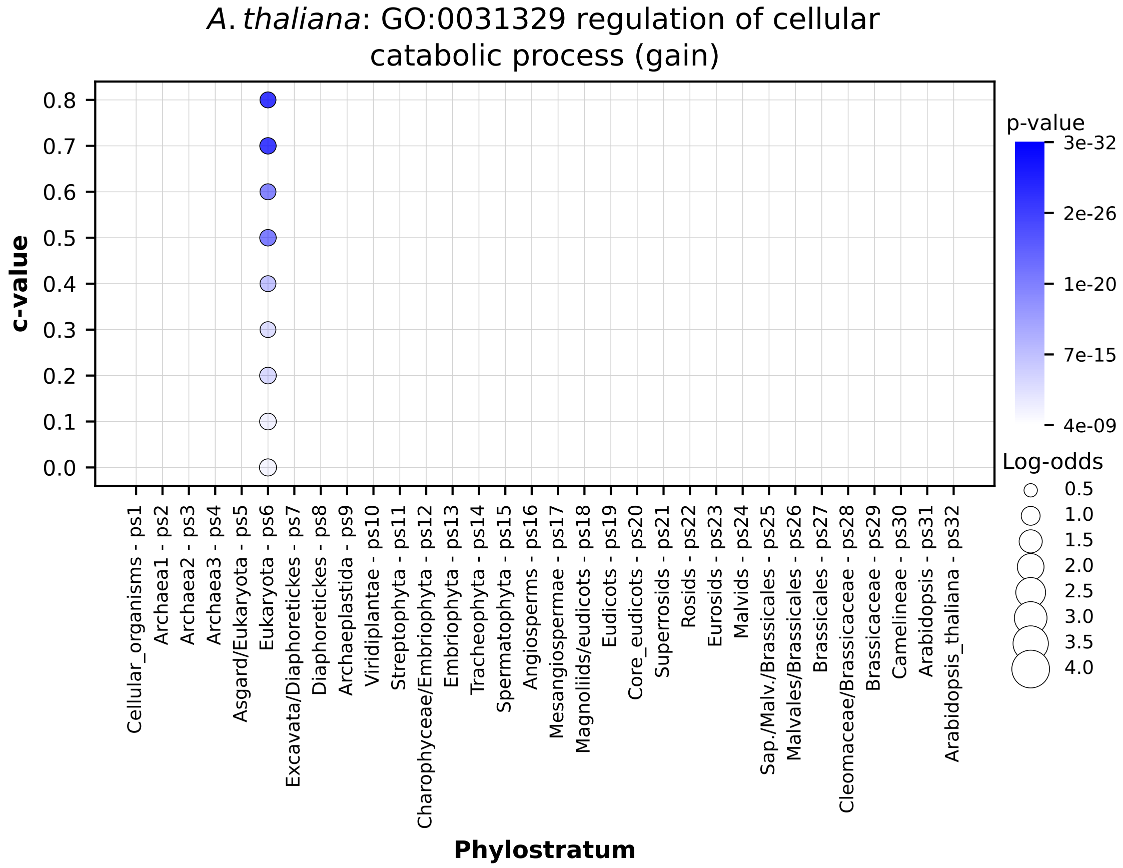

A. thaliana: GO:0031329 regulation of cellular catabolic process (gain)
c-value
0.8
0.7
0.6
0.5
0.4
0.3
0.2
0.1
0.0
p-value
3e-32
2e-26
1e-20
7e-15
4e-09
Log-odds
0.5
1.0
1.5
2.0
2.5
3.0
3.5
4.0
Cellular_organisms - ps1
Archaea1 - ps2
Archaea2 - ps3
Archaea3 - ps4
Asgard/Eukaryota - ps5
Eukaryota - ps6
Excavata/Diaphoretickes - ps7
Diaphoretickes - ps8
Archaeplastida - ps9
Viridiplantae - ps10
Streptophyta - ps11
Charophyceae/Embriophyta - ps12
Embriophyta - ps13
Tracheophyta - ps14
Spermatophyta - ps15
Angiosperms - ps16
Mesangiospermae - ps17
Magnoliids/eudicots - ps18
Eudicots - ps19
Core_eudicots - ps20
Superrosids - ps21
Rosids - ps22
Eurosids - ps23
Malvids - ps24
Sap./Malv./Brassicales - ps25
Malvales/Brassicales - ps26
Brassicales - ps27
Cleomaceae/Brassicaceae - ps28
Brassicaceae - ps29
Camelineae - ps30
Arabidopsis - ps31
Arabidopsis_thaliana - ps32
Phylostratum

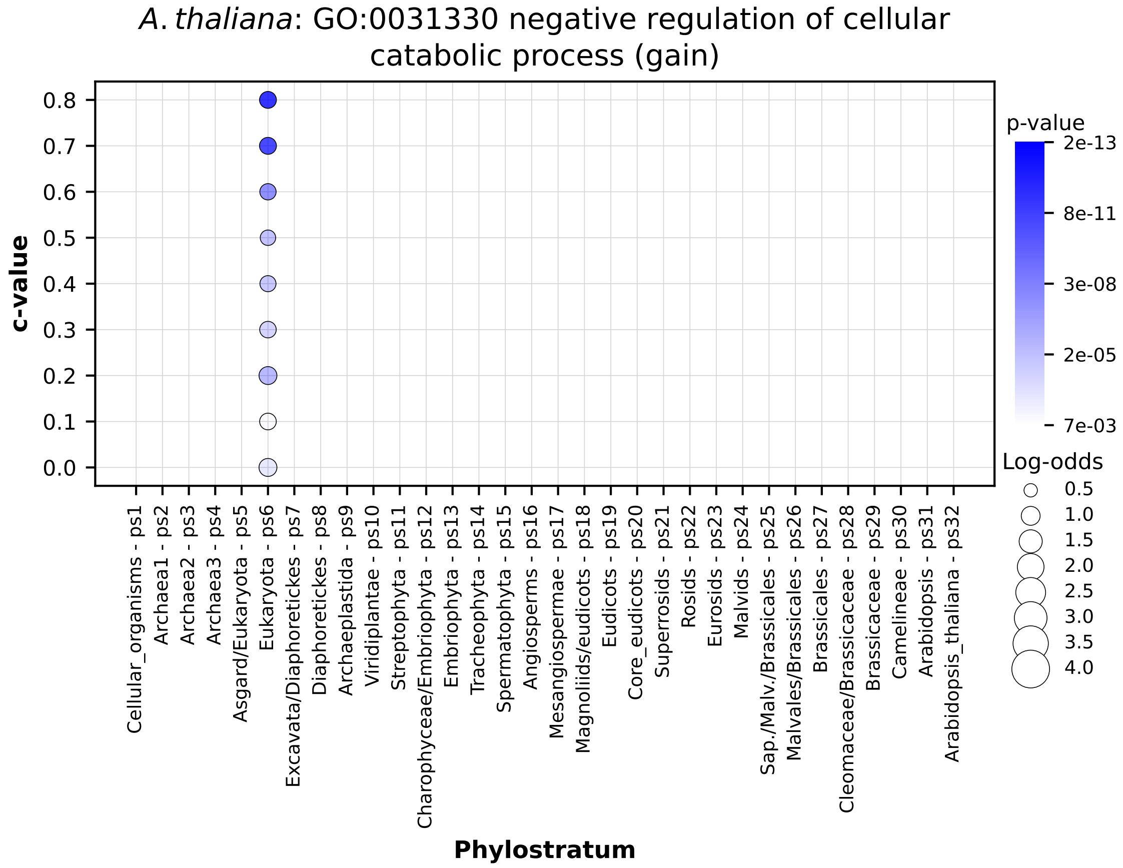

A. thaliana: GO:0031330 negative regulation of cellular catabolic process (gain)
c-value
0.8
0.7
0.6
0.5
0.4
0.3
0.2
0.1
0.0
p-value
2e-13
8e-11
3e-08
2e-05
7e-03
Log-odds
0.5
1.0
1.5
2.0
2.5
3.0
3.5
4.0
Cellular_organisms - ps1
Archaea1 - ps2
Archaea2 - ps3
Archaea3 - ps4
Asgard/Eukaryota - ps5
Eukaryota - ps6
Excavata/Diaphoretickes - ps7
Diaphoretickes - ps8
Archaeplastida - ps9
Viridiplantae - ps10
Streptophyta - ps11
Charophyceae/Embriophyta - ps12
Embriophyta - ps13
Tracheophyta - ps14
Spermatophyta - ps15
Angiosperms - ps16
Mesangiospermae - ps17
Magnoliids/eudicots - ps18
Eudicots - ps19
Core_eudicots - ps20
Superrosids - ps21
Rosids - ps22
Eurosids - ps23
Malvids - ps24
Sap./Malv./Brassicales - ps25
Malvales/Brassicales - ps26
Brassicales - ps27
Cleomaceae/Brassicaceae - ps28
Brassicaceae - ps29
Camelineae - ps30
Arabidopsis - ps31
Arabidopsis_thaliana - ps32
Phylostratum

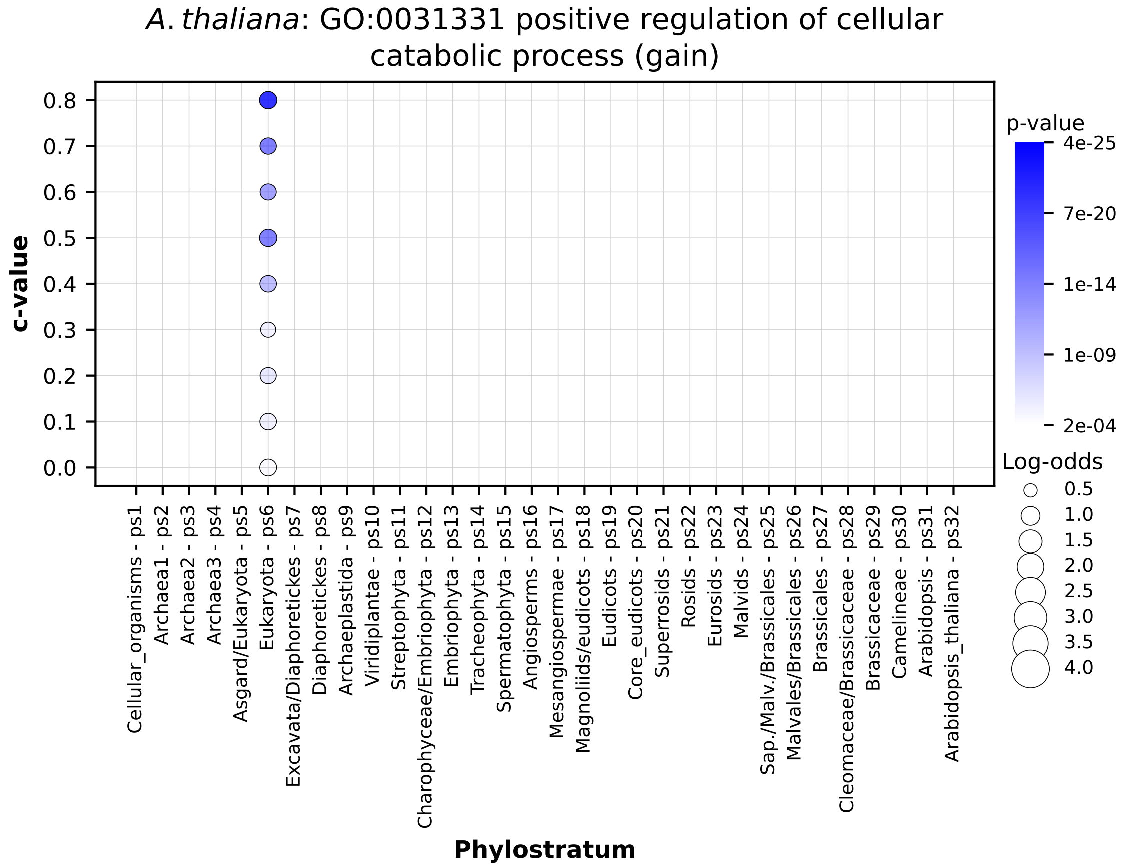

A. thaliana: GO:0031331 positive regulation of cellular catabolic process (gain)
c-value
0.8
0.7
0.6
0.5
0.4
0.3
0.2
0.1
0.0
Phylostratum
Cellular_organisms - ps1
Archaea1 - ps2
Archaea2 - ps3
Archaea3 - ps4
Asgard/Eukaryota - ps5
Eukaryota - ps6
Excavata/Diaphoretickes - ps7
Diaphoretickes - ps8
Archaeplastida - ps9
Viridiplantae - ps10
Streptophyta - ps11
Charophyceae/Embriophyta - ps12
Embriophyta - ps13
Tracheophyta - ps14
Spermatophyta - ps15
Angiosperms - ps16
Mesangiospermae - ps17
Magnoliids/eudicots - ps18
Eudicots - ps19
Core_eudicots - ps20
Superrosids - ps21
Rosids - ps22
Eurosids - ps23
Malvids - ps24
Sap./Malv./Brassicales - ps25
Malvales/Brassicales - ps26
Brassicales - ps27
Cleomaceae/Brassicaceae - ps28
Brassicaceae - ps29
Camelineae - ps30
Arabidopsis - ps31
Arabidopsis_thaliana - ps32
p-value
4e-25
7e-20
1e-14
1e-09
2e-04
Log-odds
0.5
1.0
1.5
2.0
2.5
3.0
3.5
4.0

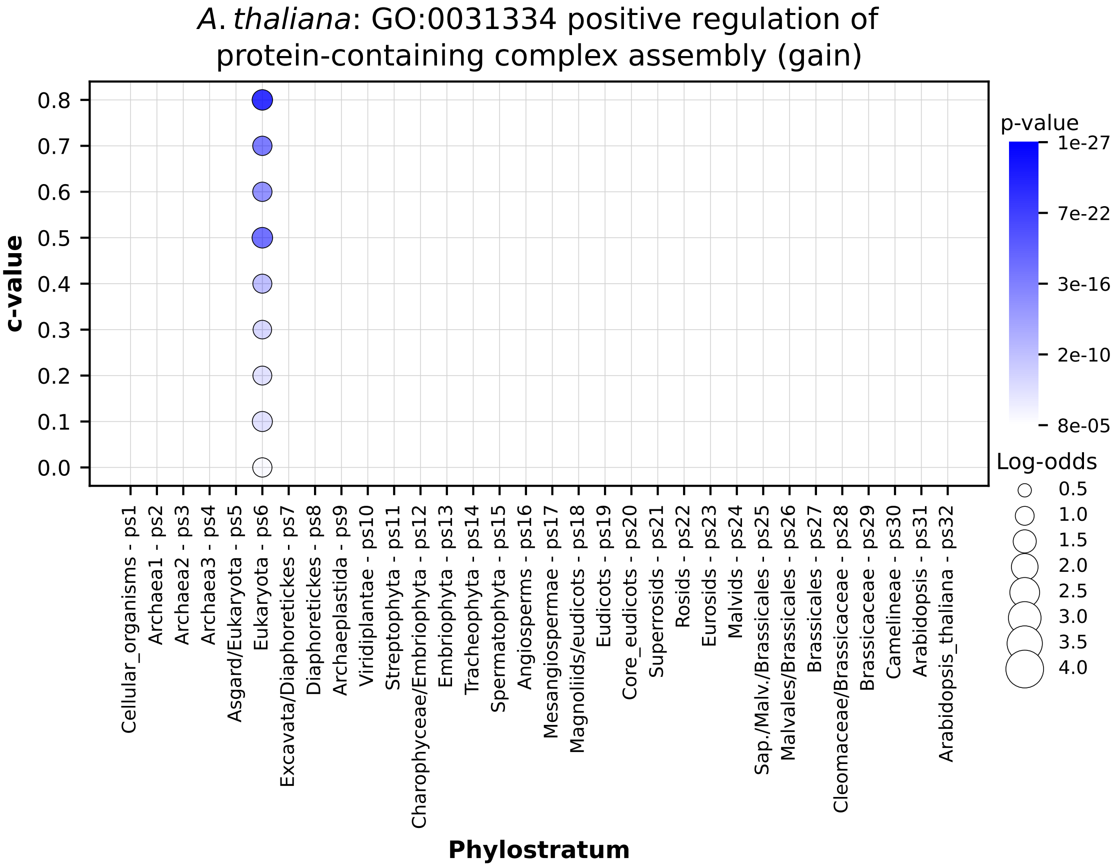

A. thaliana: GO:0031334 positive regulation of protein-containing complex assembly (gain)
c-value
0.8
0.7
0.6
0.5
0.4
0.3
0.2
0.1
0.0
p-value
1e-27
7e-22
3e-16
2e-10
8e-05
Log-odds
0.5
1.0
1.5
2.0
2.5
3.0
3.5
4.0
Cellular_organisms - ps1
Archaea1 - ps2
Archaea2 - ps3
Archaea3 - ps4
Asgard/Eukaryota - ps5
Eukaryota - ps6
Excavata/Diaphoretickes - ps7
Diaphoretickes - ps8
Archaeplastida - ps9
Viridiplantae - ps10
Streptophyta - ps11
Charophyceae/Embriophyta - ps12
Embriophyta - ps13
Tracheophyta - ps14
Spermatophyta - ps15
Angiosperms - ps16
Mesangiospermae - ps17
Magnoliids/eudicots - ps18
Eudicots - ps19
Core_eudicots - ps20
Superrosids - ps21
Rosids - ps22
Eurosids - ps23
Malvids - ps24
Sap./Malv./Brassicales - ps25
Malvales/Brassicales - ps26
Brassicales - ps27
Cleomaceae/Brassicaceae - ps28
Brassicaceae - ps29
Camelineae - ps30
Arabidopsis - ps31
Arabidopsis_thaliana - ps32
Phylostratum

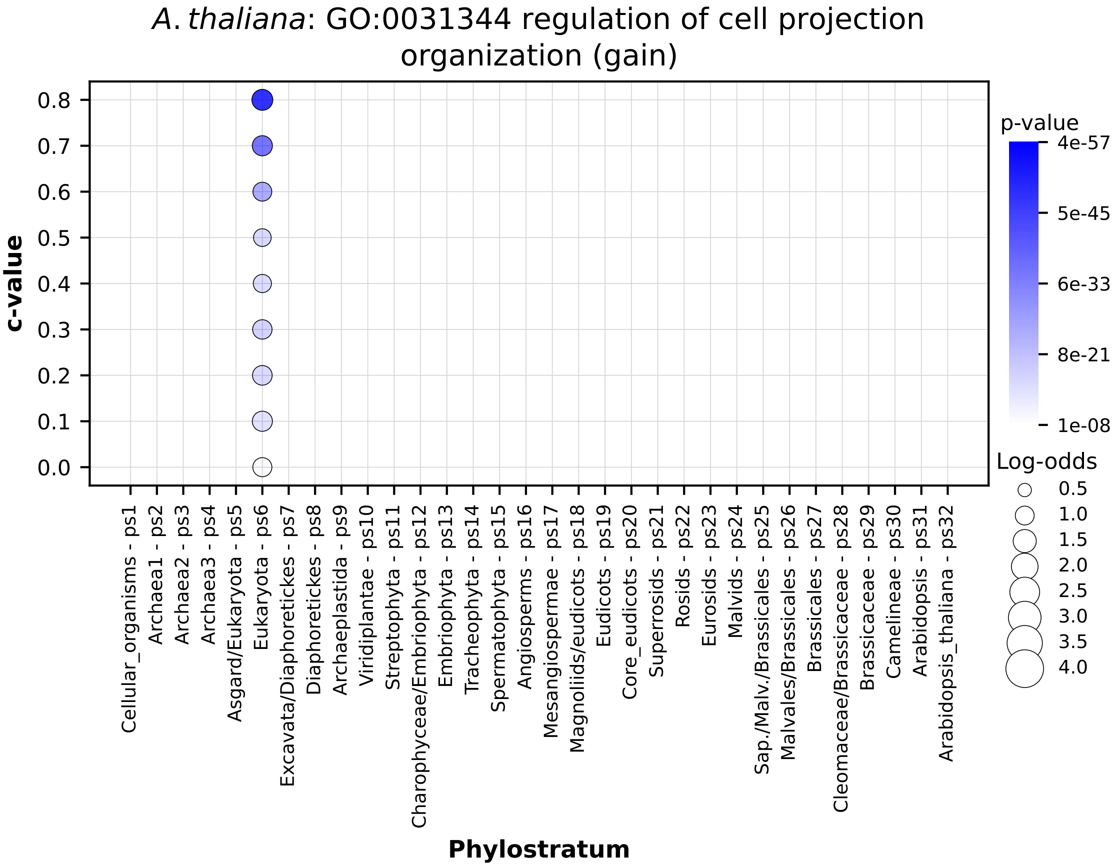

A. thaliana: GO:0031344 regulation of cell projection organization (gain)
c-value
0.8
0.7
0.6
0.5
0.4
0.3
0.2
0.1
0.0
p-value
4e-57
5e-45
6e-33
8e-21
1e-08
Log-odds
0.5
1.0
1.5
2.0
2.5
3.0
3.5
4.0
Cellular_organisms - ps1
Archaea1 - ps2
Archaea2 - ps3
Archaea3 - ps4
Asgard/Eukaryota - ps5
Eukaryota - ps6
Excavata/Diaphoretickes - ps7
Diaphoretickes - ps8
Archaeplastida - ps9
Viridiplantae - ps10
Streptophyta - ps11
Charophyceae/Embriophyta - ps12
Embriophyta - ps13
Tracheophyta - ps14
Spermatophyta - ps15
Angiosperms - ps16
Mesangiospermae - ps17
Magnoliids/eudicots - ps18
Eudicots - ps19
Core_eudicots - ps20
Superrosids - ps21
Rosids - ps22
Eurosids - ps23
Malvids - ps24
Sap./Malv./Brassicales - ps25
Malvales/Brassicales - ps26
Brassicales - ps27
Cleomaceae/Brassicaceae - ps28
Brassicaceae - ps29
Camelineae - ps30
Arabidopsis - ps31
Arabidopsis_thaliana - ps32
Phylostratum

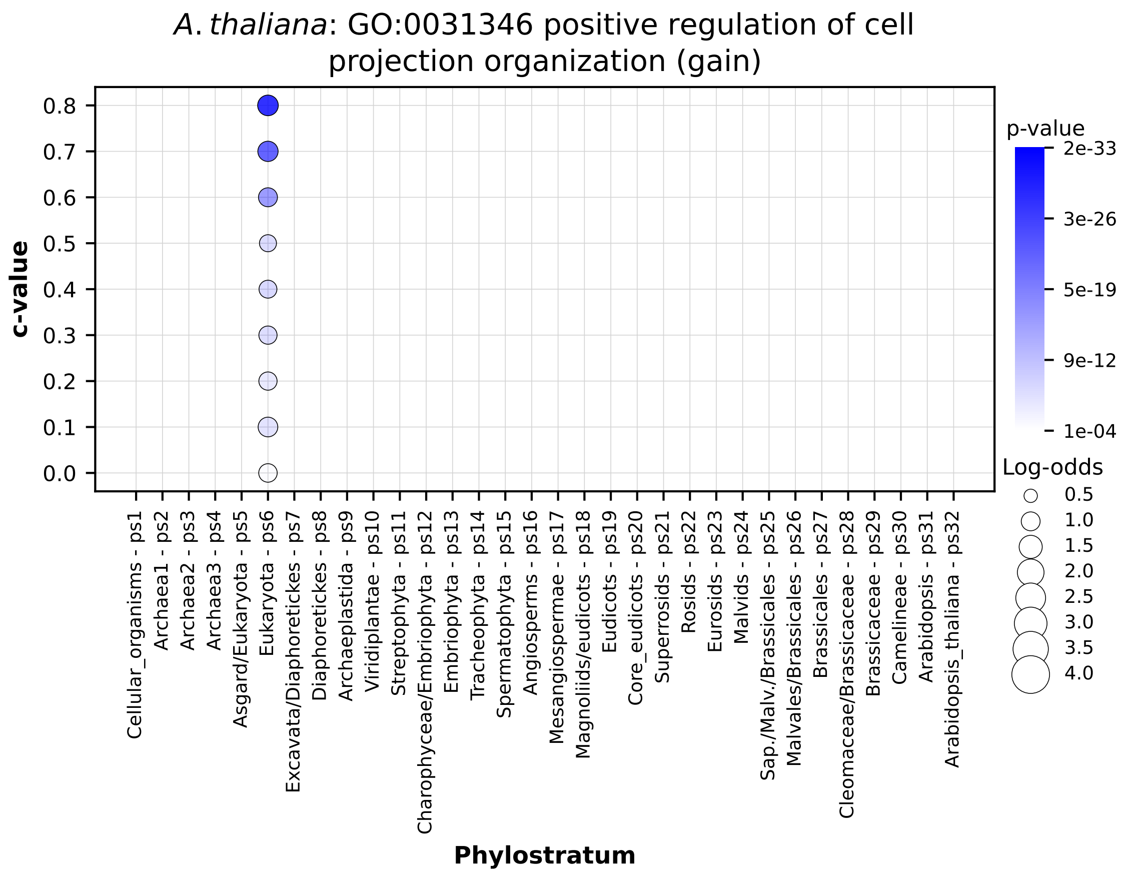

A. thaliana: GO:0031346 positive regulation of cell projection organization (gain)
c-value
0.8
0.7
0.6
0.5
0.4
0.3
0.2
0.1
0.0
p-value
2e-33
3e-26
5e-19
9e-12
1e-04
Log-odds
0.5
1.0
1.5
2.0
2.5
3.0
3.5
4.0
Cellular_organisms - ps1
Archaea1 - ps2
Archaea2 - ps3
Archaea3 - ps4
Asgard/Eukaryota - ps5
Eukaryota - ps6
Excavata/Diaphoretickes - ps7
Diaphoretickes - ps8
Archaeplastida - ps9
Viridiplantae - ps10
Streptophyta - ps11
Charophyceae/Embriophyta - ps12
Embriophyta - ps13
Tracheophyta - ps14
Spermatophyta - ps15
Angiosperms - ps16
Mesangiospermae - ps17
Magnoliids/eudicots - ps18
Eudicots - ps19
Core_eudicots - ps20
Superrosids - ps21
Rosids - ps22
Eurosids - ps23
Malvids - ps24
Sap./Malv./Brassicales - ps25
Malvales/Brassicales - ps26
Brassicales - ps27
Cleomaceae/Brassicaceae - ps28
Brassicaceae - ps29
Camelineae - ps30
Arabidopsis - ps31
Arabidopsis_thaliana - ps32
Phylostratum

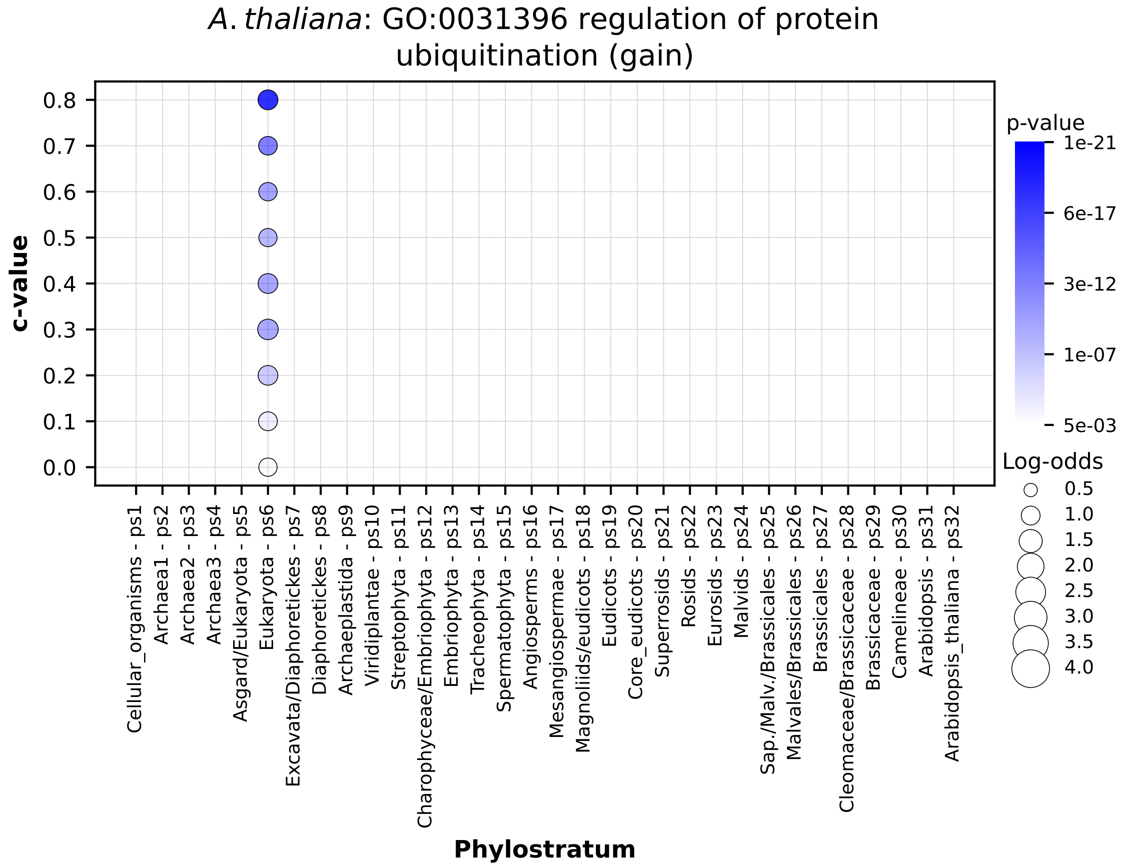

A. thaliana: GO:0031396 regulation of protein ubiquitination (gain)
c-value
0.8
0.7
0.6
0.5
0.4
0.3
0.2
0.1
0.0
p-value
1e-21
6e-17
3e-12
1e-07
5e-03
Log-odds
0.5
1.0
1.5
2.0
2.5
3.0
3.5
4.0
Cellular_organisms - ps1
Archaea1 - ps2
Archaea2 - ps3
Archaea3 - ps4
Asgard/Eukaryota - ps5
Eukaryota - ps6
Excavata/Diaphoretickes - ps7
Diaphoretickes - ps8
Archaeplastida - ps9
Viridiplantae - ps10
Streptophyta - ps11
Charophyceae/Embriophyta - ps12
Embriophyta - ps13
Tracheophyta - ps14
Spermatophyta - ps15
Angiosperms - ps16
Mesangiospermae - ps17
Magnoliids/eudicots - ps18
Eudicots - ps19
Core_eudicots - ps20
Superrosids - ps21
Rosids - ps22
Eurosids - ps23
Malvids - ps24
Sap./Malv./Brassicales - ps25
Malvales/Brassicales - ps26
Brassicales - ps27
Cleomaceae/Brassicaceae - ps28
Brassicaceae - ps29
Camelineae - ps30
Arabidopsis - ps31
Arabidopsis_thaliana - ps32
Phylostratum

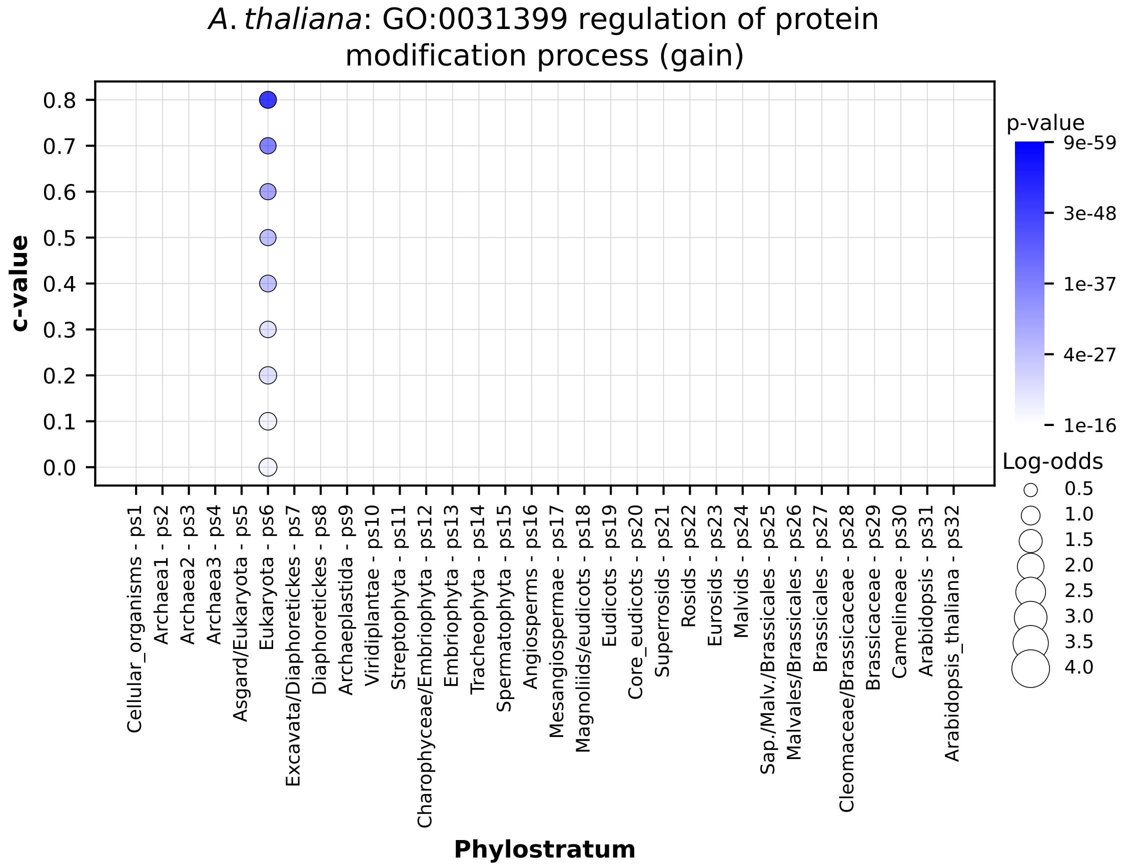

A. thaliana: GO:0031399 regulation of protein modification process (gain)
c-value
0.0
0.1
0.2
0.3
0.4
0.5
0.6
0.7
0.8
p-value
9e-59
3e-48
1e-37
4e-27
1e-16
Log-odds
0.5
1.0
1.5
2.0
2.5
3.0
3.5
4.0
Cellular_organisms - ps1
Archaea1 - ps2
Archaea2 - ps3
Archaea3 - ps4
Asgard/Eukaryota - ps5
Eukaryota - ps6
Excavata/Diaphoretickes - ps7
Diaphoretickes - ps8
Archaeplastida - ps9
Viridiplantae - ps10
Streptophyta - ps11
Charophyceae/Embriophyta - ps12
Embriophyta - ps13
Tracheophyta - ps14
Spermatophyta - ps15
Angiosperms - ps16
Mesangiospermae - ps17
Magnoliids/eudicots - ps18
Eudicots - ps19
Core_eudicots - ps20
Superrosids - ps21
Rosids - ps22
Eurosids - ps23
Malvids - ps24
Sap./Malv./Brassicales - ps25
Malvales/Brassicales - ps26
Brassicales - ps27
Cleomaceae/Brassicaceae - ps28
Brassicaceae - ps29
Camelineae - ps30
Arabidopsis - ps31
Arabidopsis_thaliana - ps32
Phylostratum

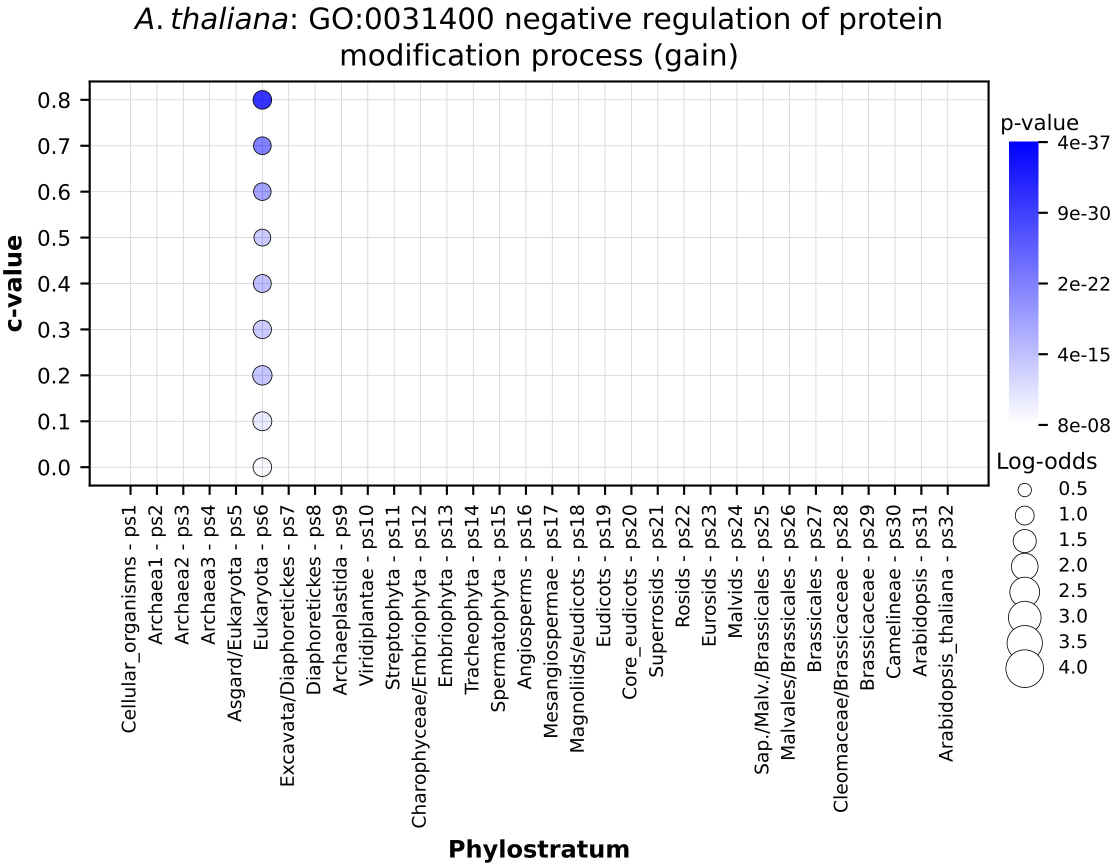

A. thaliana: GO:0031400 negative regulation of protein modification process (gain)
c-value
0.8
0.7
0.6
0.5
0.4
0.3
0.2
0.1
0.0
Phylostratum
Cellular_organisms - ps1
Archaea1 - ps2
Archaea2 - ps3
Archaea3 - ps4
Asgard/Eukaryota - ps5
Eukaryota - ps6
Excavata/Diaphoretickes - ps7
Diaphoretickes - ps8
Archaeplastida - ps9
Viridiplantae - ps10
Streptophyta - ps11
Charophyceae/Embriophyta - ps12
Embriophyta - ps13
Tracheophyta - ps14
Spermatophyta - ps15
Angiosperms - ps16
Mesangiospermae - ps17
Magnoliids/eudicots - ps18
Eudicots - ps19
Core_eudicots - ps20
Superrosids - ps21
Rosids - ps22
Eurosids - ps23
Malvids - ps24
Sap./Malv./Brassicales - ps25
Malvales/Brassicales - ps26
Brassicales - ps27
Cleomaceae/Brassicaceae - ps28
Brassicaceae - ps29
Camelineae - ps30
Arabidopsis - ps31
Arabidopsis_thaliana - ps32
p-value
4e-37
9e-30
2e-22
4e-15
8e-08
Log-odds
0.5
1.0
1.5
2.0
2.5
3.0
3.5
4.0

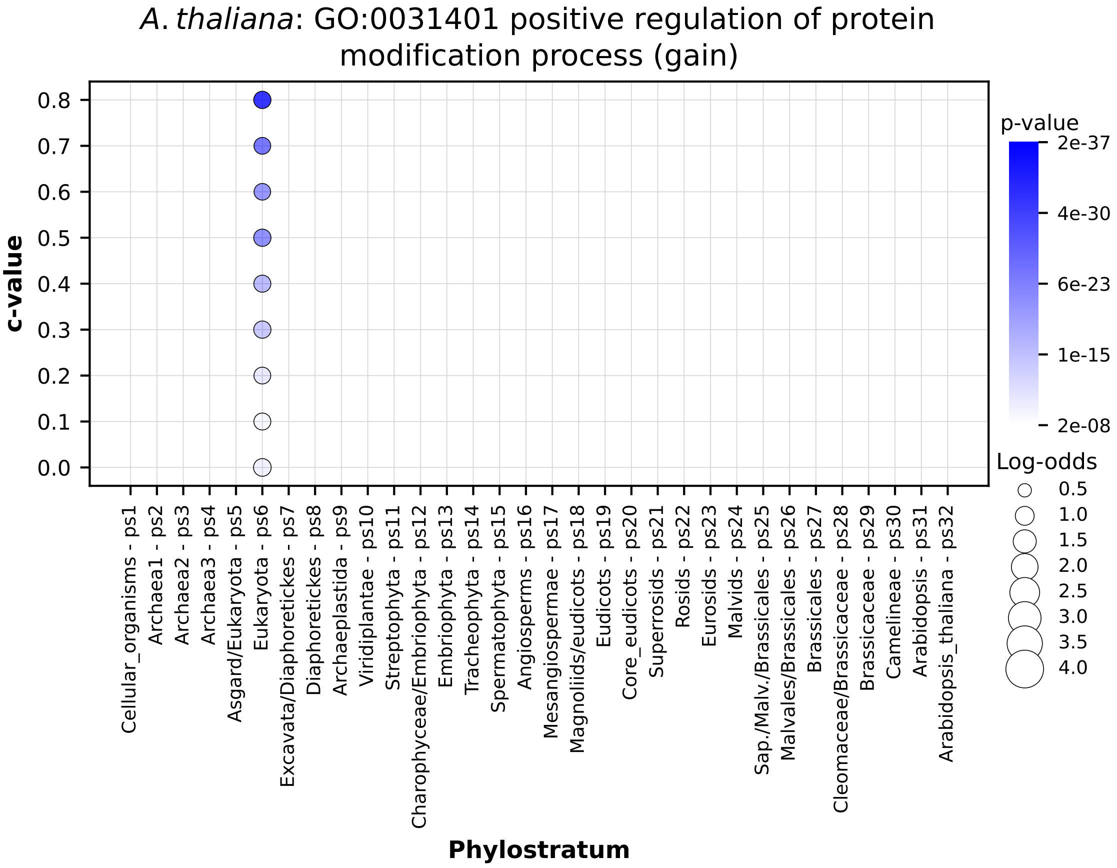

A. thaliana: GO:0031401 positive regulation of protein modification process (gain)
c-value
0.8
0.7
0.6
0.5
0.4
0.3
0.2
0.1
0.0
p-value
2e-37
4e-30
6e-23
1e-15
2e-08
Log-odds
0.5
1.0
1.5
2.0
2.5
3.0
3.5
4.0
Cellular_organisms - ps1
Archaea1 - ps2
Archaea2 - ps3
Archaea3 - ps4
Asgard/Eukaryota - ps5
Eukaryota - ps6
Excavata/Diaphoretickes - ps7
Diaphoretickes - ps8
Archaeplastida - ps9
Viridiplantae - ps10
Streptophyta - ps11
Charophyceae/Embriophyta - ps12
Embriophyta - ps13
Tracheophyta - ps14
Spermatophyta - ps15
Angiosperms - ps16
Mesangiospermae - ps17
Magnoliids/eudicots - ps18
Eudicots - ps19
Core_eudicots - ps20
Superrosids - ps21
Rosids - ps22
Eurosids - ps23
Malvids - ps24
Sap./Malv./Brassicales - ps25
Malvales/Brassicales - ps26
Brassicales - ps27
Cleomaceae/Brassicaceae - ps28
Brassicaceae - ps29
Camelineae - ps30
Arabidopsis - ps31
Arabidopsis_thaliana - ps32
Phylostratum

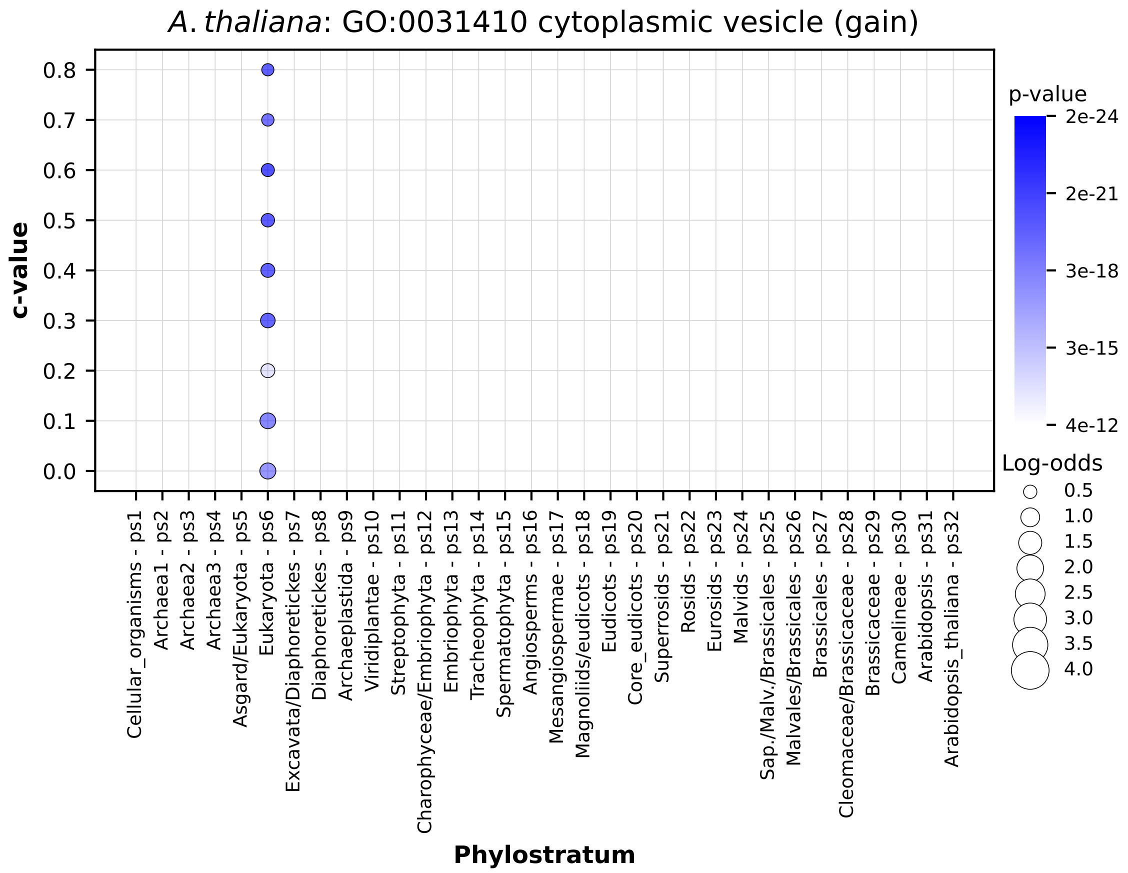

A. thaliana: GO:0031410 cytoplasmic vesicle (gain)
c-value
0.0
0.1
0.2
0.3
0.4
0.5
0.6
0.7
0.8
Phylostratum
Cellular_organisms - ps1
Archaea1 - ps2
Archaea2 - ps3
Archaea3 - ps4
Asgard/Eukaryota - ps5
Eukaryota - ps6
Excavata/Diaphoretickes - ps7
Diaphoretickes - ps8
Archaeplastida - ps9
Viridiplantae - ps10
Streptophyta - ps11
Charophyceae/Embriophyta - ps12
Embriophyta - ps13
Tracheophyta - ps14
Spermatophyta - ps15
Angiosperms - ps16
Mesangiospermae - ps17
Magnoliids/eudicots - ps18
Eudicots - ps19
Core_eudicots - ps20
Superrosids - ps21
Rosids - ps22
Eurosids - ps23
Malvids - ps24
Sap./Malv./Brassicales - ps25
Malvales/Brassicales - ps26
Brassicales - ps27
Cleomaceae/Brassicaceae - ps28
Brassicaceae - ps29
Camelineae - ps30
Arabidopsis - ps31
Arabidopsis_thaliana - ps32
p-value
2e-24
2e-21
3e-18
3e-15
4e-12
Log-odds
0.5
1.0
1.5
2.0
2.5
3.0
3.5
4.0

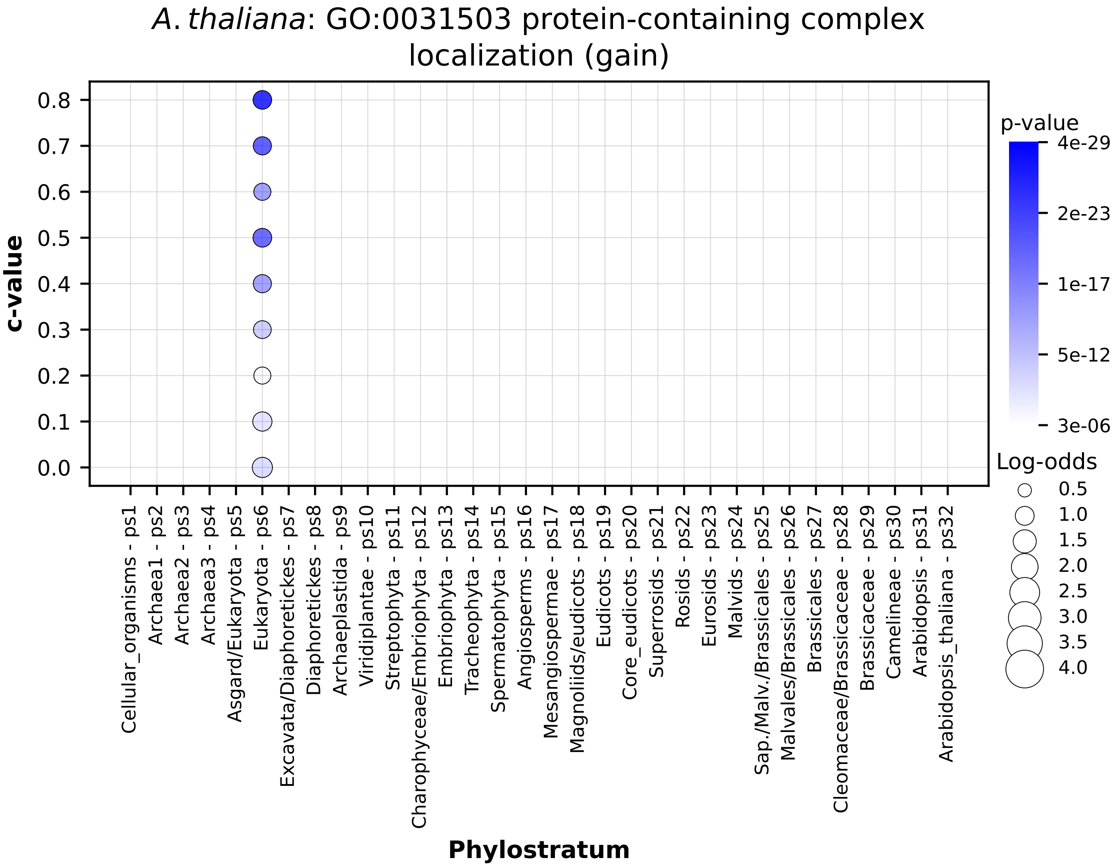
A. thaliana: GO:0031503 protein-containing complex localization (gain)
c-value
0.8
0.7
0.6
0.5
0.4
0.3
0.2
0.1
0.0
Phylostratum
Cellular_organisms - ps1
Archaea1 - ps2
Archaea2 - ps3
Archaea3 - ps4
Asgard/Eukaryota - ps5
Eukaryota - ps6
Excavata/Diaphoretickes - ps7
Diaphoretickes - ps8
Archaeplastida - ps9
Viridiplantae - ps10
Streptophyta - ps11
Charophyceae/Embriophyta - ps12
Embriophyta - ps13
Tracheophyta - ps14
Spermatophyta - ps15
Angiosperms - ps16
Mesangiospermae - ps17
Magnoliids/eudicots - ps18
Eudicots - ps19
Core_eudicots - ps20
Superrosids - ps21
Rosids - ps22
Eurosids - ps23
Malvids - ps24
Sap./Malv./Brassicales - ps25
Malvales/Brassicales - ps26
Brassicales - ps27
Cleomaceae/Brassicaceae - ps28
Brassicaceae - ps29
Camelineae - ps30
Arabidopsis - ps31
Arabidopsis_thaliana - ps32
p-value
4e-29
2e-23
1e-17
5e-12
3e-06
Log-odds
0.5
1.0
1.5
2.0
2.5
3.0
3.5
4.0

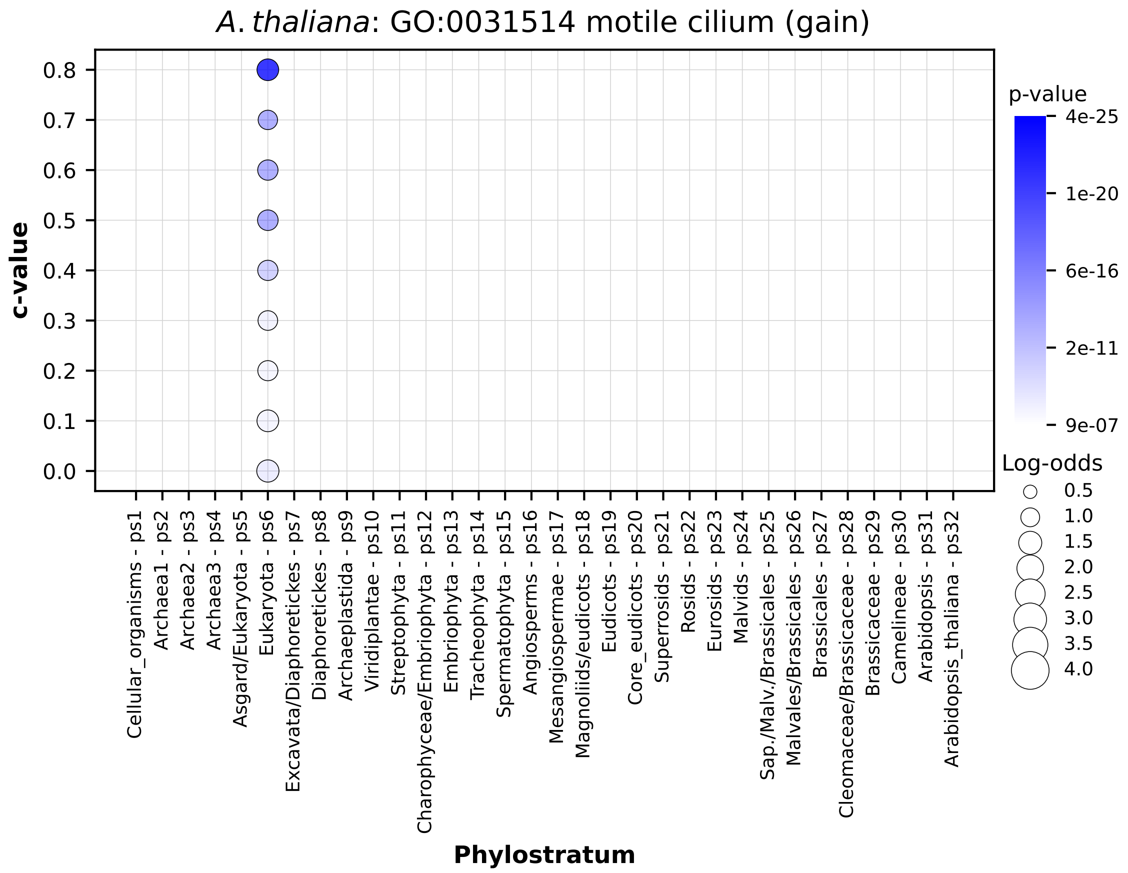

A. thaliana: GO:0031514 motile cilium (gain)
c-value
0.8
0.7
0.6
0.5
0.4
0.3
0.2
0.1
0.0
p-value
4e-25
1e-20
6e-16
2e-11
9e-07
Log-odds
0.5
1.0
1.5
2.0
2.5
3.0
3.5
4.0
Cellular_organisms - ps1
Archaea1 - ps2
Archaea2 - ps3
Archaea3 - ps4
Asgard/Eukaryota - ps5
Eukaryota - ps6
Excavata/Diaphoretickes - ps7
Diaphoretickes - ps8
Archaeplastida - ps9
Viridiplantae - ps10
Streptophyta - ps11
Charophyceae/Embriophyta - ps12
Embriophyta - ps13
Tracheophyta - ps14
Spermatophyta - ps15
Angiosperms - ps16
Mesangiospermae - ps17
Magnoliids/eudicots - ps18
Eudicots - ps19
Core_eudicots - ps20
Superrosids - ps21
Rosids - ps22
Eurosids - ps23
Malvids - ps24
Sap./Malv./Brassicales - ps25
Malvales/Brassicales - ps26
Brassicales - ps27
Cleomaceae/Brassicaceae - ps28
Brassicaceae - ps29
Camelineae - ps30
Arabidopsis - ps31
Arabidopsis_thaliana - ps32
Phylostratum

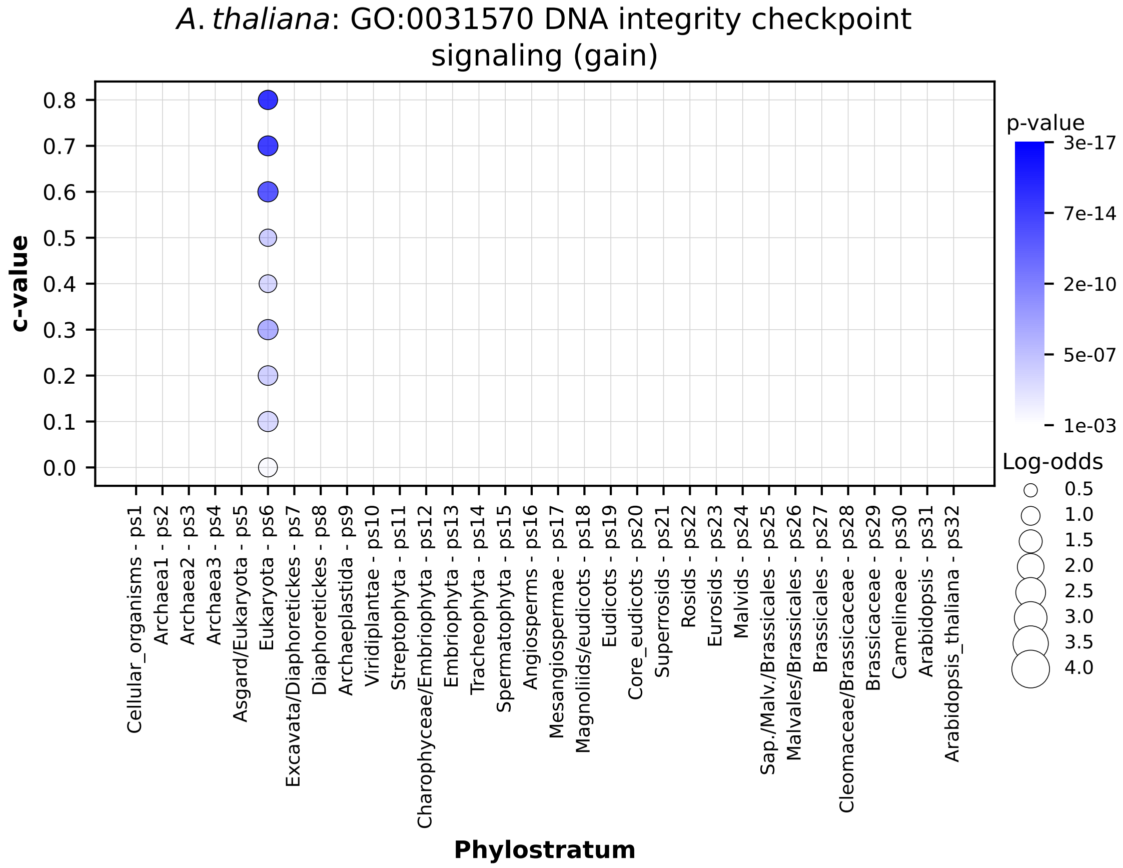

A. thaliana: GO:0031570 DNA integrity checkpoint signaling (gain)
c-value
0.8
0.7
0.6
0.5
0.4
0.3
0.2
0.1
0.0
p-value
3e-17
7e-14
2e-10
5e-07
1e-03
Log-odds
0.5
1.0
1.5
2.0
2.5
3.0
3.5
4.0
Cellular_organisms - ps1
Archaea1 - ps2
Archaea2 - ps3
Archaea3 - ps4
Asgard/Eukaryota - ps5
Eukaryota - ps6
Excavata/Diaphoretickes - ps7
Diaphoretickes - ps8
Archaeplastida - ps9
Viridiplantae - ps10
Streptophyta - ps11
Charophyceae/Embriophyta - ps12
Embriophyta - ps13
Tracheophyta - ps14
Spermatophyta - ps15
Angiosperms - ps16
Mesangiospermae - ps17
Magnoliids/eudicots - ps18
Eudicots - ps19
Core_eudicots - ps20
Superrosids - ps21
Rosids - ps22
Eurosids - ps23
Malvids - ps24
Sap./Malv./Brassicales - ps25
Malvales/Brassicales - ps26
Brassicales - ps27
Cleomaceae/Brassicaceae - ps28
Brassicaceae - ps29
Camelineae - ps30
Arabidopsis - ps31
Arabidopsis_thaliana - ps32
Phylostratum

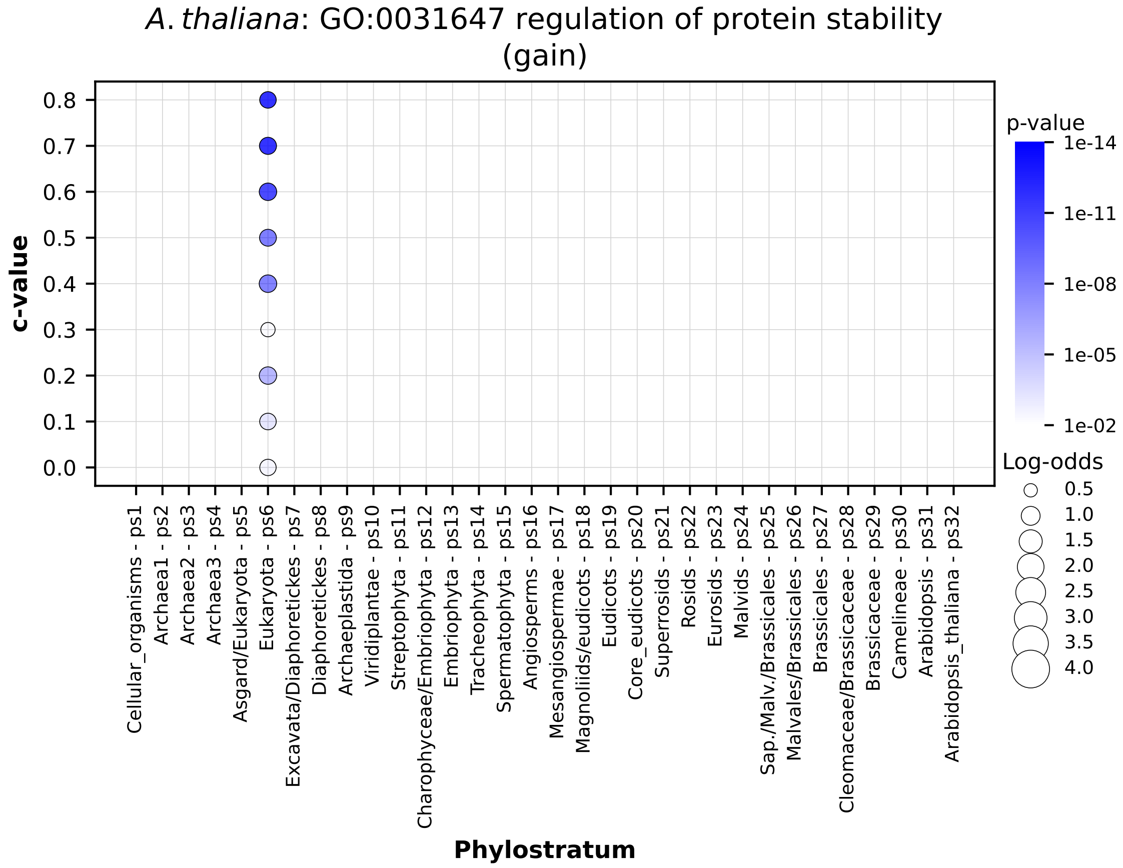

A. thaliana: GO:0031647 regulation of protein stability
(gain)
c-value
0.0
0.1
0.2
0.3
0.4
0.5
0.6
0.7
0.8
p-value
1e-14
1e-11
1e-08
1e-05
1e-02
Log-odds
0.5
1.0
1.5
2.0
2.5
3.0
3.5
4.0
Cellular_organisms - ps1
Archaea1 - ps2
Archaea2 - ps3
Archaea3 - ps4
Asgard/Eukaryota - ps5
Eukaryota - ps6
Excavata/Diaphoretickes - ps7
Diaphoretickes - ps8
Archaeplastida - ps9
Viridiplantae - ps10
Streptophyta - ps11
Charophyceae/Embriophyta - ps12
Embriophyta - ps13
Tracheophyta - ps14
Spermatophyta - ps15
Angiosperms - ps16
Mesangiospermae - ps17
Magnoliids/eudicots - ps18
Eudicots - ps19
Core_eudicots - ps20
Superrosids - ps21
Rosids - ps22
Eurosids - ps23
Malvids - ps24
Sap./Malv./Brassicales - ps25
Malvales/Brassicales - ps26
Brassicales - ps27
Cleomaceae/Brassicaceae - ps28
Brassicaceae - ps29
Camelineae - ps30
Arabidopsis - ps31
Arabidopsis_thaliana - ps32
Phylostratum

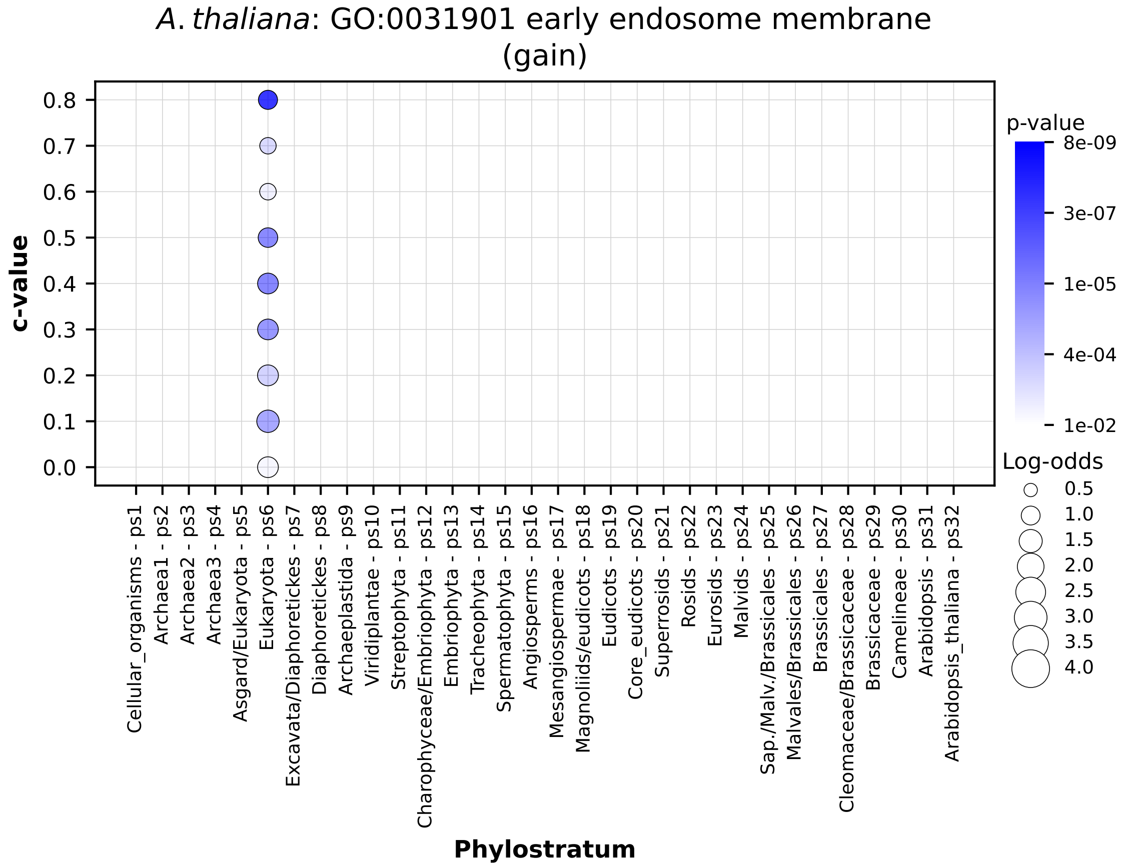

A. thaliana: GO:0031901 early endosome membrane (gain)
c-value
0.0
0.1
0.2
0.3
0.4
0.5
0.6
0.7
0.8
Phylostratum
Cellular_organisms - ps1
Archaea1 - ps2
Archaea2 - ps3
Archaea3 - ps4
Asgard/Eukaryota - ps5
Eukaryota - ps6
Excavata/Diaphoretickes - ps7
Diaphoretickes - ps8
Archaeplastida - ps9
Viridiplantae - ps10
Streptophyta - ps11
Charophyceae/Embriophyta - ps12
Embriophyta - ps13
Tracheophyta - ps14
Spermatophyta - ps15
Angiosperms - ps16
Mesangiospermae - ps17
Magnoliids/eudicots - ps18
Eudicots - ps19
Core_eudicots - ps20
Superrosids - ps21
Rosids - ps22
Eurosids - ps23
Malvids - ps24
Sap./Malv./Brassicales - ps25
Malvales/Brassicales - ps26
Brassicales - ps27
Cleomaceae/Brassicaceae - ps28
Brassicaceae - ps29
Camelineae - ps30
Arabidopsis - ps31
Arabidopsis_thaliana - ps32
p-value
8e-09
3e-07
1e-05
4e-04
1e-02
Log-odds
0.5
1.0
1.5
2.0
2.5
3.0
3.5
4.0

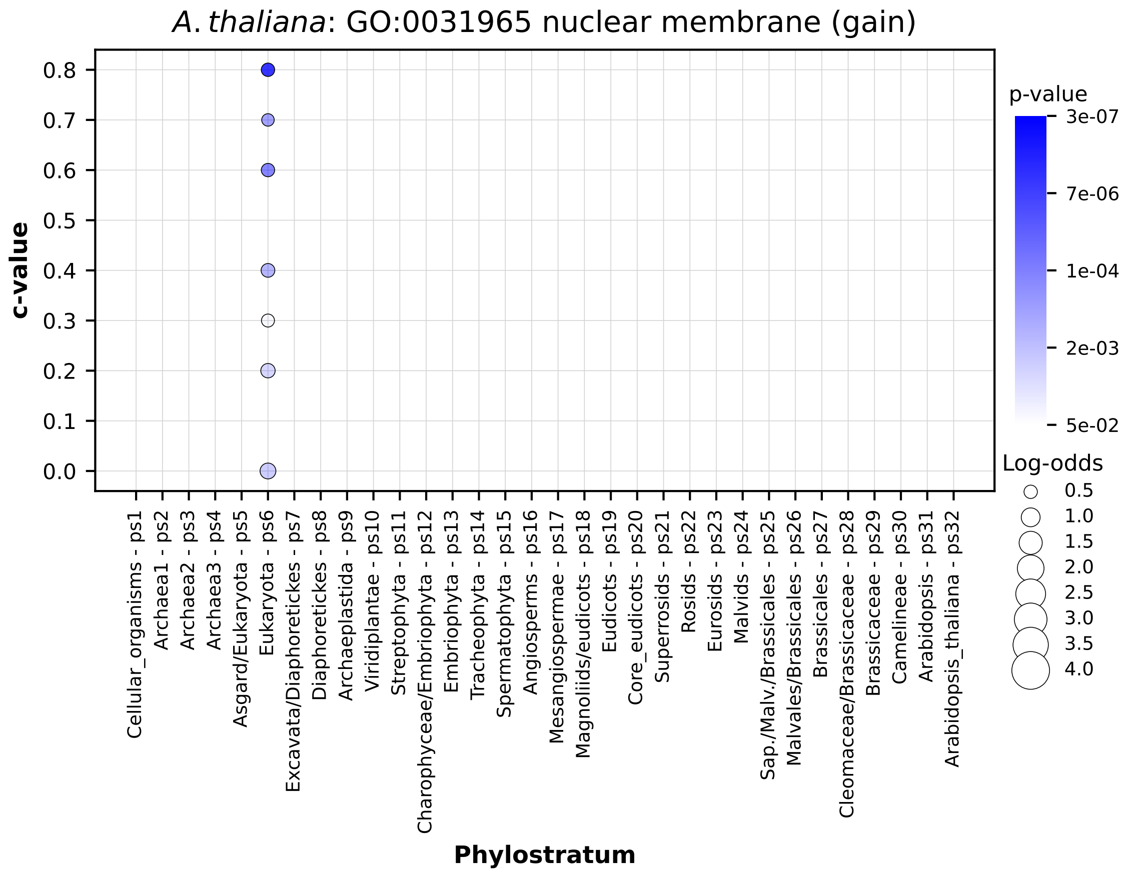

A. thaliana: GO:0031965 nuclear membrane (gain)
c-value
0.8
0.7
0.6
0.5
0.4
0.3
0.2
0.1
0.0
p-value
3e-07
7e-06
1e-04
2e-03
5e-02
Log-odds
0.5
1.0
1.5
2.0
2.5
3.0
3.5
4.0
Cellular_organisms - ps1
Archaea1 - ps2
Archaea2 - ps3
Archaea3 - ps4
Asgard/Eukaryota - ps5
Eukaryota - ps6
Excavata/Diaphoretickes - ps7
Diaphoretickes - ps8
Archaeplastida - ps9
Viridiplantae - ps10
Streptophyta - ps11
Charophyceae/Embriophyta - ps12
Embriophyta - ps13
Tracheophyta - ps14
Spermatophyta - ps15
Angiosperms - ps16
Mesangiospermae - ps17
Magnoliids/eudicots - ps18
Eudicots - ps19
Core_eudicots - ps20
Superrosids - ps21
Rosids - ps22
Eurosids - ps23
Malvids - ps24
Sap./Malv./Brassicales - ps25
Malvales/Brassicales - ps26
Brassicales - ps27
Cleomaceae/Brassicaceae - ps28
Brassicaceae - ps29
Camelineae - ps30
Arabidopsis - ps31
Arabidopsis_thaliana - ps32
Phylostratum

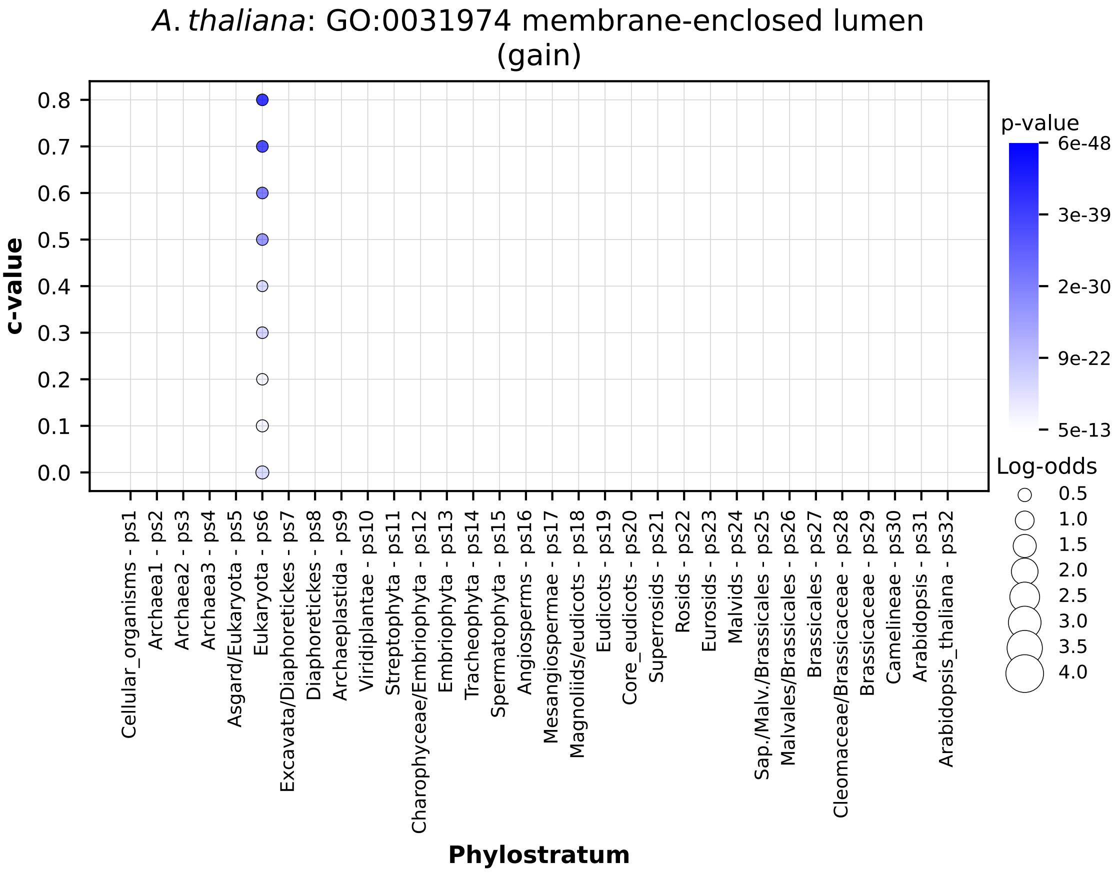
A. thaliana: GO:0031974 membrane-enclosed lumen (gain)
c-value
0.8
0.7
0.6
0.5
0.4
0.3
0.2
0.1
0.0
p-value
6e-48
3e-39
2e-30
9e-22
5e-13
Log-odds
0.5
1.0
1.5
2.0
2.5
3.0
3.5
4.0
Cellular_organisms - ps1
Archaea1 - ps2
Archaea2 - ps3
Archaea3 - ps4
Asgard/Eukaryota - ps5
Eukaryota - ps6
Excavata/Diaphoretickes - ps7
Diaphoretickes - ps8
Archaeplastida - ps9
Viridiplantae - ps10
Streptophyta - ps11
Charophyceae/Embriophyta - ps12
Embriophyta - ps13
Tracheophyta - ps14
Spermatophyta - ps15
Angiosperms - ps16
Mesangiospermae - ps17
Magnoliids/eudicots - ps18
Eudicots - ps19
Core_eudicots - ps20
Superrosids - ps21
Rosids - ps22
Eurosids - ps23
Malvids - ps24
Sap./Malv./Brassicales - ps25
Malvales/Brassicales - ps26
Brassicales - ps27
Cleomaceae/Brassicaceae - ps28
Brassicaceae - ps29
Camelineae - ps30
Arabidopsis - ps31
Arabidopsis_thaliana - ps32
Phylostratum

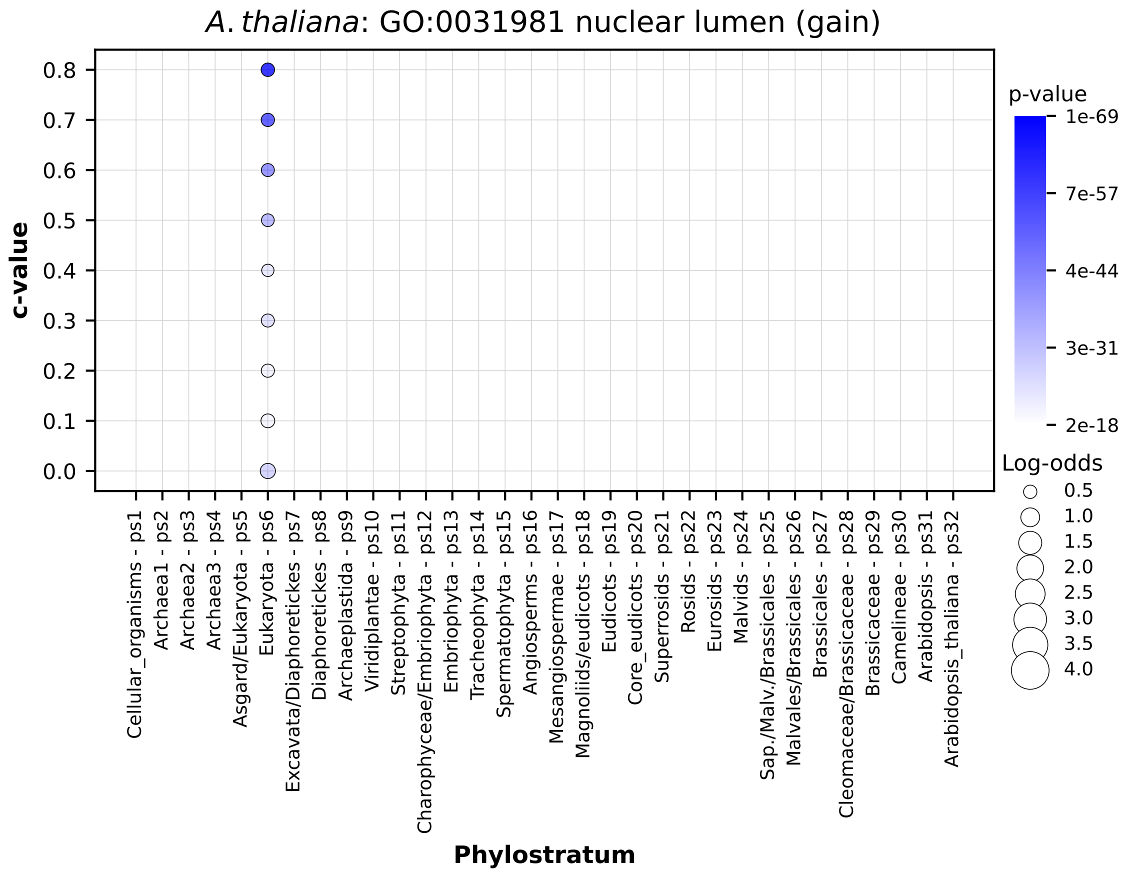

A. thaliana: GO:0031981 nuclear lumen (gain)
c-value
0.8
0.7
0.6
0.5
0.4
0.3
0.2
0.1
0.0
p-value
1e-69
7e-57
4e-44
3e-31
2e-18
Log-odds
0.5
1.0
1.5
2.0
2.5
3.0
3.5
4.0
Cellular_organisms - ps1
Archaea1 - ps2
Archaea2 - ps3
Archaea3 - ps4
Asgard/Eukaryota - ps5
Eukaryota - ps6
Excavata/Diaphoretickes - ps7
Diaphoretickes - ps8
Archaeplastida - ps9
Viridiplantae - ps10
Streptophyta - ps11
Charophyceae/Embriophyta - ps12
Embriophyta - ps13
Tracheophyta - ps14
Spermatophyta - ps15
Angiosperms - ps16
Mesangiospermae - ps17
Magnoliids/eudicots - ps18
Eudicots - ps19
Core_eudicots - ps20
Superrosids - ps21
Rosids - ps22
Eurosids - ps23
Malvids - ps24
Sap./Malv./Brassicales - ps25
Malvales/Brassicales - ps26
Brassicales - ps27
Cleomaceae/Brassicaceae - ps28
Brassicaceae - ps29
Camelineae - ps30
Arabidopsis - ps31
Arabidopsis_thaliana - ps32
Phylostratum

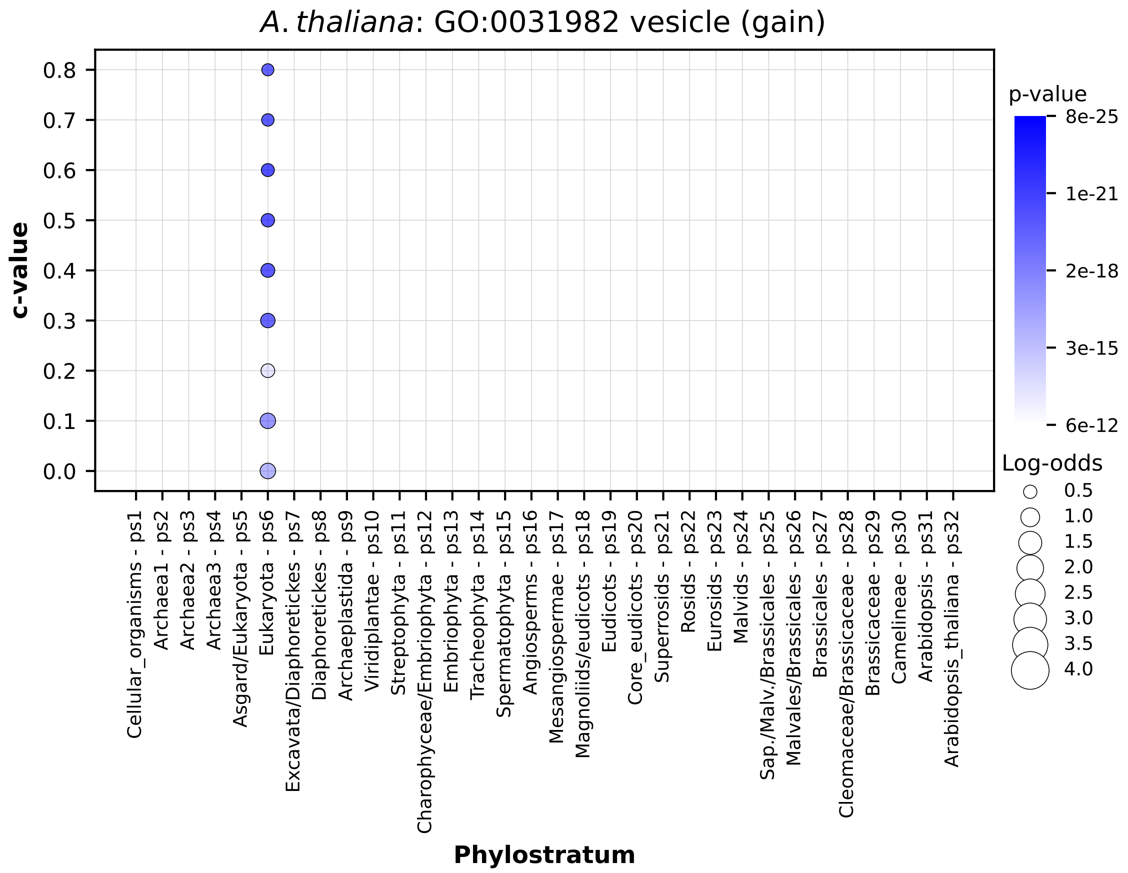
A. thaliana: GO:0031982 vesicle (gain)
c-value
0.0
0.1
0.2
0.3
0.4
0.5
0.6
0.7
0.8
Phylostratum
Cellular_organisms - ps1
Archaea1 - ps2
Archaea2 - ps3
Archaea3 - ps4
Asgard/Eukaryota - ps5
Eukaryota - ps6
Excavata/Diaphoretickes - ps7
Diaphoretickes - ps8
Archaeplastida - ps9
Viridiplantae - ps10
Streptophyta - ps11
Charophyceae/Embriophyta - ps12
Embriophyta - ps13
Tracheophyta - ps14
Spermatophyta - ps15
Angiosperms - ps16
Mesangiospermae - ps17
Magnoliids/eudicots - ps18
Eudicots - ps19
Core_eudicots - ps20
Superrosids - ps21
Rosids - ps22
Eurosids - ps23
Malvids - ps24
Sap./Malv./Brassicales - ps25
Malvales/Brassicales - ps26
Brassicales - ps27
Cleomaceae/Brassicaceae - ps28
Brassicaceae - ps29
Camelineae - ps30
Arabidopsis - ps31
Arabidopsis_thaliana - ps32
p-value
8e-25
1e-21
2e-18
3e-15
6e-12
Log-odds
0.5
1.0
1.5
2.0
2.5
3.0
3.5
4.0

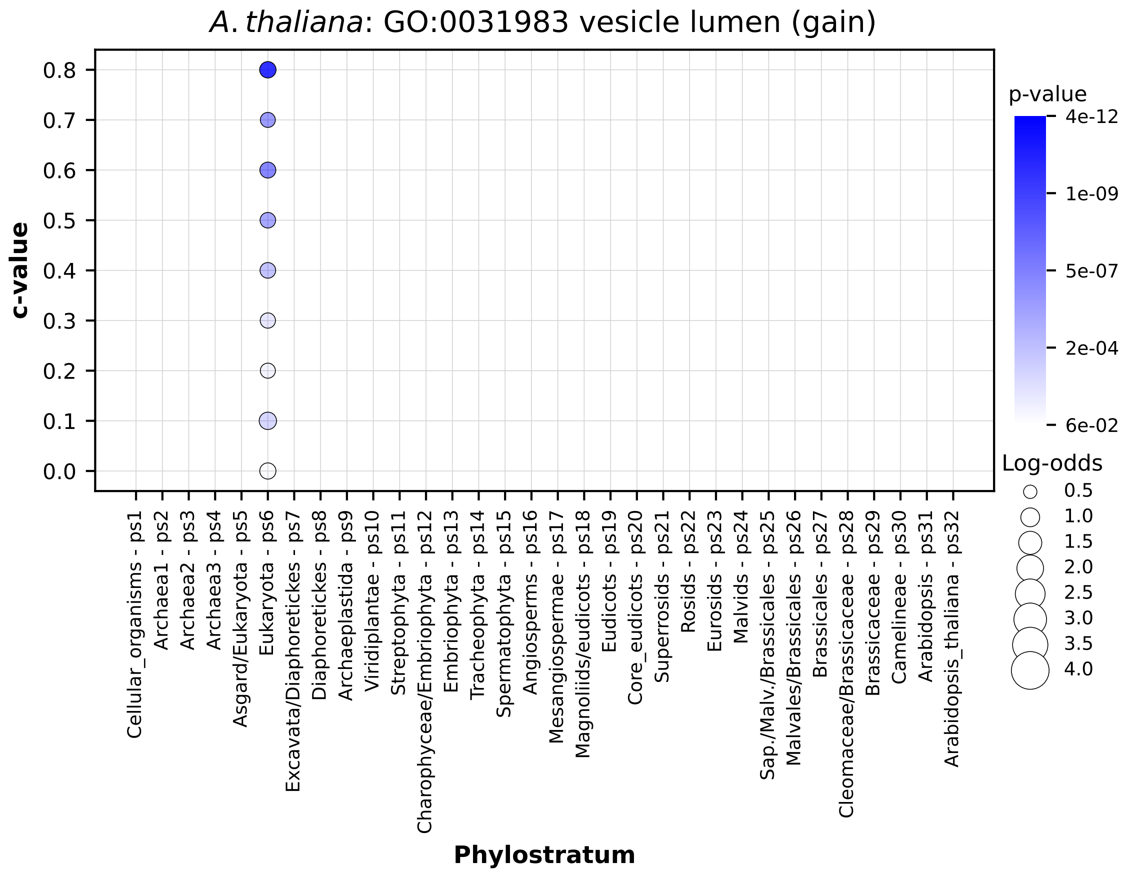
A. thaliana: GO:0031983 vesicle lumen (gain)
c-value
0.8
0.7
0.6
0.5
0.4
0.3
0.2
0.1
0.0
p-value
4e-12
1e-09
5e-07
2e-04
6e-02
Log-odds
0.5
1.0
1.5
2.0
2.5
3.0
3.5
4.0
Cellular_organisms - ps1
Archaea1 - ps2
Archaea2 - ps3
Archaea3 - ps4
Asgard/Eukaryota - ps5
Eukaryota - ps6
Excavata/Diaphoretickes - ps7
Diaphoretickes - ps8
Archaeplastida - ps9
Viridiplantae - ps10
Streptophyta - ps11
Charophyceae/Embriophyta - ps12
Embriophyta - ps13
Tracheophyta - ps14
Spermatophyta - ps15
Angiosperms - ps16
Mesangiospermae - ps17
Magnoliids/eudicots - ps18
Eudicots - ps19
Core_eudicots - ps20
Superrosids - ps21
Rosids - ps22
Eurosids - ps23
Malvids - ps24
Sap./Malv./Brassicales - ps25
Malvales/Brassicales - ps26
Brassicales - ps27
Cleomaceae/Brassicaceae - ps28
Brassicaceae - ps29
Camelineae - ps30
Arabidopsis - ps31
Arabidopsis_thaliana - ps32
Phylostratum

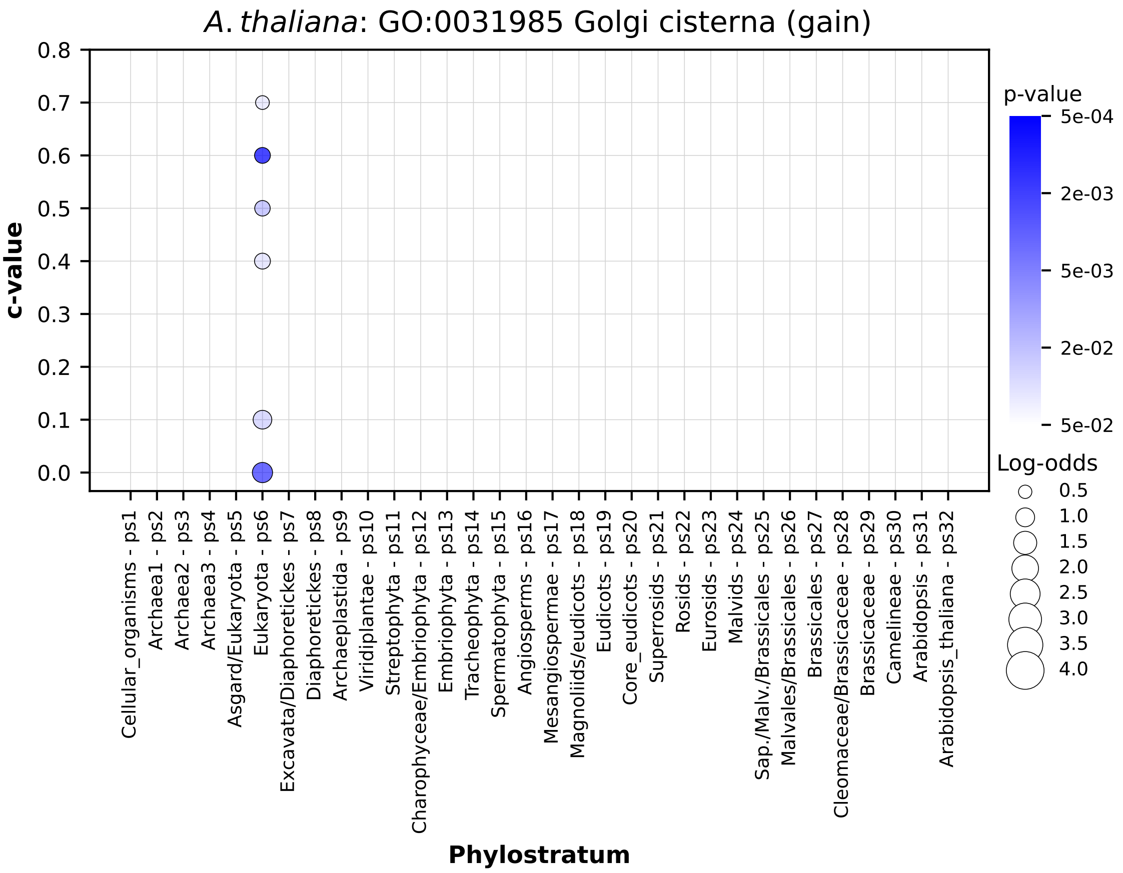

A. thaliana: GO:0031985 Golgi cisterna (gain)
c-value
0.8
0.7
0.6
0.5
0.4
0.3
0.2
0.1
0.0
p-value
5e-04
2e-03
5e-03
2e-02
5e-02
Log-odds
0.5
1.0
1.5
2.0
2.5
3.0
3.5
4.0
Cellular_organisms - ps1
Archaea1 - ps2
Archaea2 - ps3
Archaea3 - ps4
Asgard/Eukaryota - ps5
Eukaryota - ps6
Excavata/Diaphoretickes - ps7
Diaphoretickes - ps8
Archaeplastida - ps9
Viridiplantae - ps10
Streptophyta - ps11
Charophyceae/Embriophyta - ps12
Embriophyta - ps13
Tracheophyta - ps14
Spermatophyta - ps15
Angiosperms - ps16
Mesangiospermae - ps17
Magnoliids/eudicots - ps18
Eudicots - ps19
Core_eudicots - ps20
Superrosids - ps21
Rosids - ps22
Eurosids - ps23
Malvids - ps24
Sap./Malv./Brassicales - ps25
Malvales/Brassicales - ps26
Brassicales - ps27
Cleomaceae/Brassicaceae - ps28
Brassicaceae - ps29
Camelineae - ps30
Arabidopsis - ps31
Arabidopsis_thaliana - ps32
Phylostratum

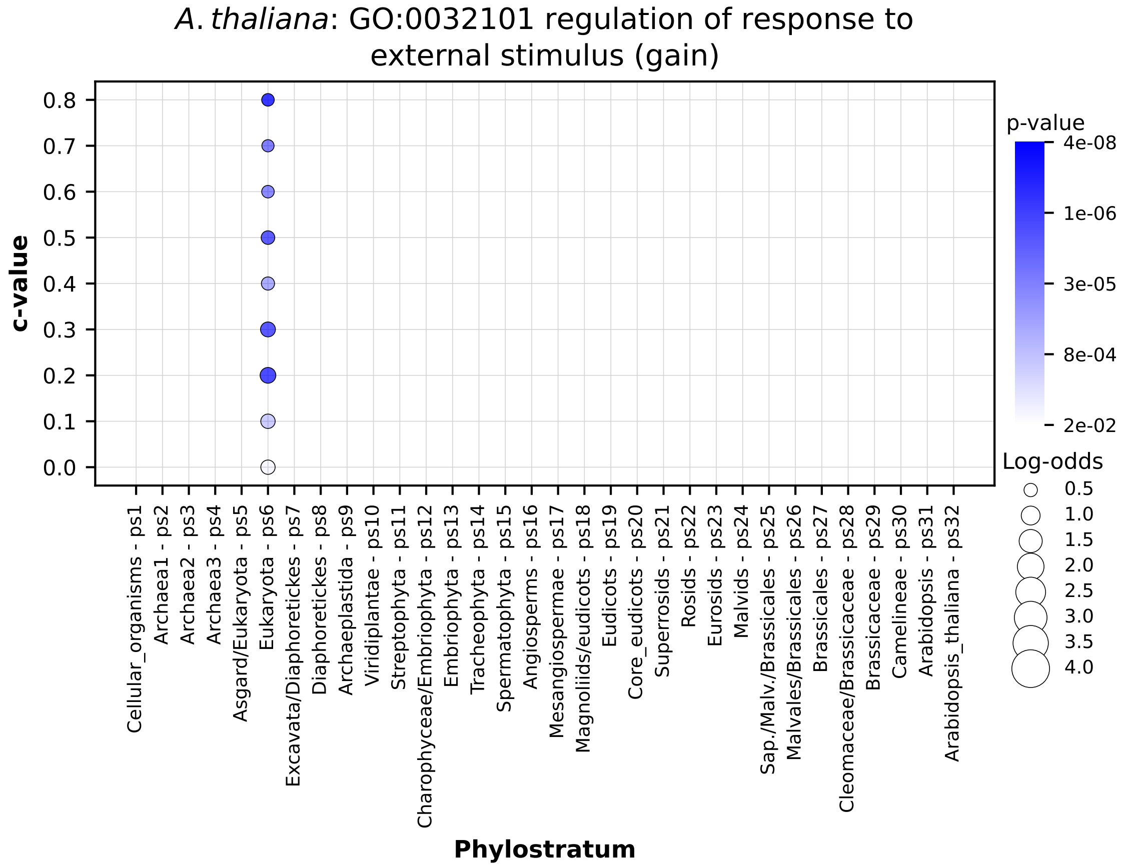
A. thaliana: GO:0032101 regulation of response to external stimulus (gain)
c-value
0.8
0.7
0.6
0.5
0.4
0.3
0.2
0.1
0.0
p-value
4e-08
1e-06
3e-05
8e-04
2e-02
Log-odds
0.5
1.0
1.5
2.0
2.5
3.0
3.5
4.0
Cellular_organisms - ps1
Archaea1 - ps2
Archaea2 - ps3
Archaea3 - ps4
Asgard/Eukaryota - ps5
Eukaryota - ps6
Excavata/Diaphoretickes - ps7
Diaphoretickes - ps8
Archaeplastida - ps9
Viridiplantae - ps10
Streptophyta - ps11
Charophyceae/Embriophyta - ps12
Embriophyta - ps13
Tracheophyta - ps14
Spermatophyta - ps15
Angiosperms - ps16
Mesangiospermae - ps17
Magnoliids/eudicots - ps18
Eudicots - ps19
Core_eudicots - ps20
Superrosids - ps21
Rosids - ps22
Eurosids - ps23
Malvids - ps24
Sap./Malv./Brassicales - ps25
Malvales/Brassicales - ps26
Brassicales - ps27
Cleomaceae/Brassicaceae - ps28
Brassicaceae - ps29
Camelineae - ps30
Arabidopsis - ps31
Arabidopsis_thaliana - ps32
Phylostratum

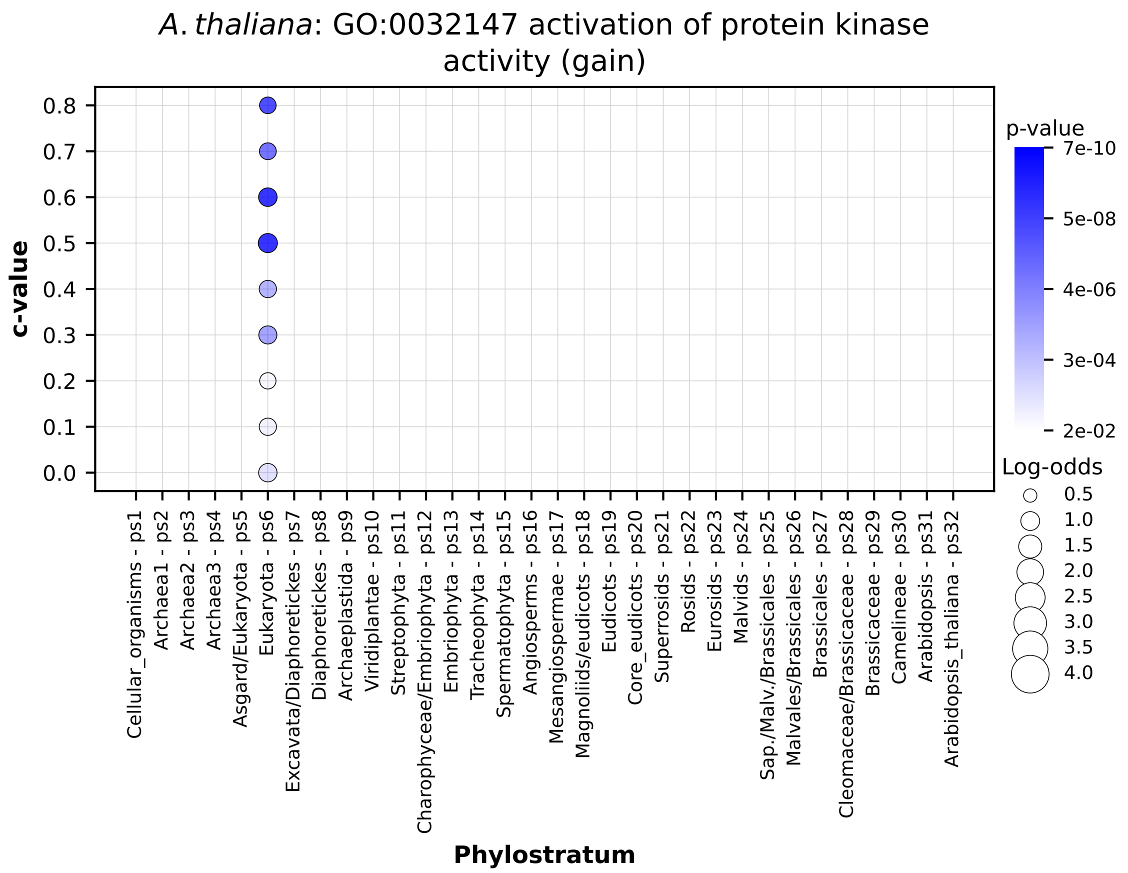

A. thaliana: GO:0032147 activation of protein kinase activity (gain)
c-value
0.8
0.7
0.6
0.5
0.4
0.3
0.2
0.1
0.0
Phylostratum
Cellular_organisms - ps1
Archaea1 - ps2
Archaea2 - ps3
Archaea3 - ps4
Asgard/Eukaryota - ps5
Eukaryota - ps6
Excavata/Diaphoretickes - ps7
Diaphoretickes - ps8
Archaeplastida - ps9
Viridiplantae - ps10
Streptophyta - ps11
Charophyceae/Embriophyta - ps12
Embriophyta - ps13
Tracheophyta - ps14
Spermatophyta - ps15
Angiosperms - ps16
Mesangiospermae - ps17
Magnoliids/eudicots - ps18
Eudicots - ps19
Core_eudicots - ps20
Superrosids - ps21
Rosids - ps22
Eurosids - ps23
Malvids - ps24
Sap./Malv./Brassicales - ps25
Malvales/Brassicales - ps26
Brassicales - ps27
Cleomaceae/Brassicaceae - ps28
Brassicaceae - ps29
Camelineae - ps30
Arabidopsis - ps31
Arabidopsis_thaliana - ps32
p-value
7e-10
5e-08
4e-06
3e-04
2e-02
Log-odds
0.5
1.0
1.5
2.0
2.5
3.0
3.5
4.0

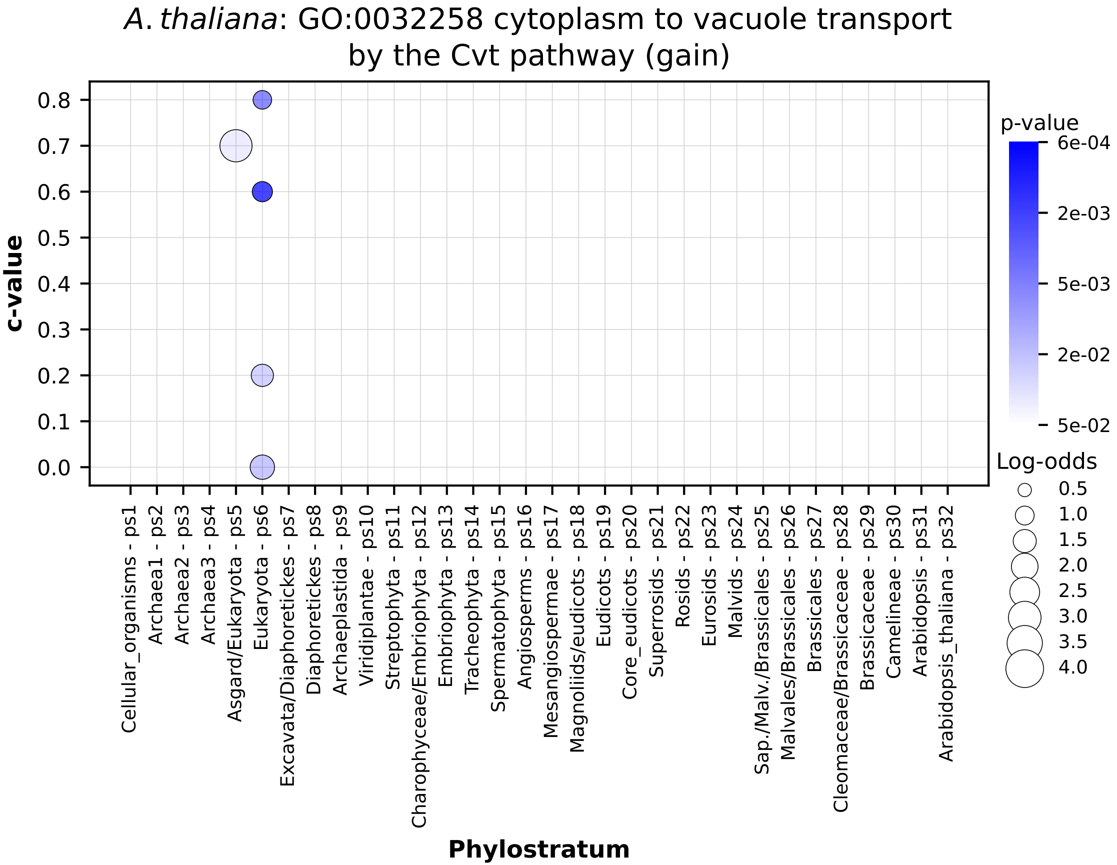

A. thaliana: GO:0032258 cytoplasm to vacuole transport by the Cvt pathway (gain)
c-value
0.8
0.7
0.6
0.5
0.4
0.3
0.2
0.1
0.0
p-value
6e-04
2e-03
5e-03
2e-02
5e-02
Log-odds
0.5
1.0
1.5
2.0
2.5
3.0
3.5
4.0
Cellular_organisms - ps1
Archaea1 - ps2
Archaea2 - ps3
Archaea3 - ps4
Asgard/Eukaryota - ps5
Eukaryota - ps6
Excavata/Diaphoretickes - ps7
Diaphoretickes - ps8
Archaeplastida - ps9
Viridiplantae - ps10
Streptophyta - ps11
Charophyceae/Embriophyta - ps12
Embriophyta - ps13
Tracheophyta - ps14
Spermatophyta - ps15
Angiosperms - ps16
Mesangiospermae - ps17
Magnoliids/eudicots - ps18
Eudicots - ps19
Core_eudicots - ps20
Superrosids - ps21
Rosids - ps22
Eurosids - ps23
Malvids - ps24
Sap./Malv./Brassicales - ps25
Malvales/Brassicales - ps26
Brassicales - ps27
Cleomaceae/Brassicaceae - ps28
Brassicaceae - ps29
Camelineae - ps30
Arabidopsis - ps31
Arabidopsis_thaliana - ps32
Phylostratum

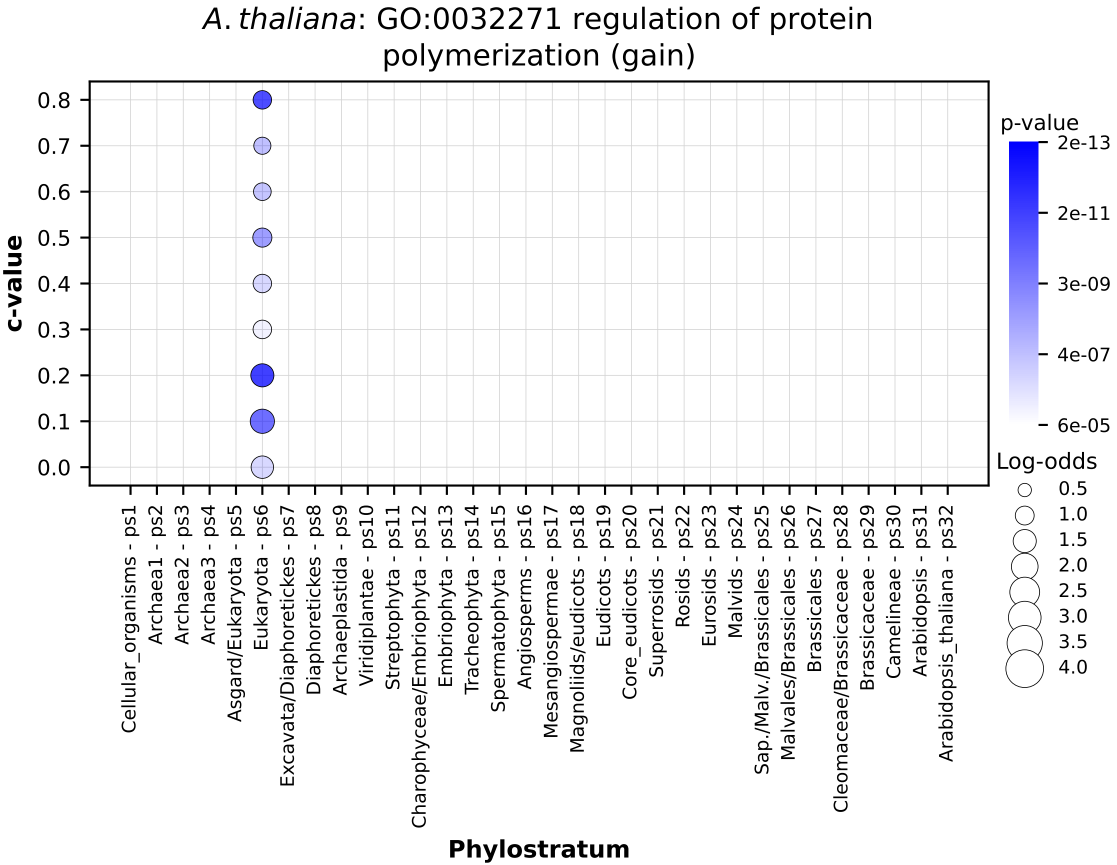
A. thaliana: GO:0032271 regulation of protein polymerization (gain)
c-value
0.0
0.1
0.2
0.3
0.4
0.5
0.6
0.7
0.8
Phylostratum
Cellular_organisms - ps1
Archaea1 - ps2
Archaea2 - ps3
Archaea3 - ps4
Asgard/Eukaryota - ps5
Eukaryota - ps6
Excavata/Diaphoretickes - ps7
Diaphoretickes - ps8
Archaeplastida - ps9
Viridiplantae - ps10
Streptophyta - ps11
Charophyceae/Embriophyta - ps12
Embriophyta - ps13
Tracheophyta - ps14
Spermatophyta - ps15
Angiosperms - ps16
Mesangiospermae - ps17
Magnoliids/eudicots - ps18
Eudicots - ps19
Core_eudicots - ps20
Superrosids - ps21
Rosids - ps22
Eurosids - ps23
Malvids - ps24
Sap./Malv./Brassicales - ps25
Malvales/Brassicales - ps26
Brassicales - ps27
Cleomaceae/Brassicaceae - ps28
Brassicaceae - ps29
Camelineae - ps30
Arabidopsis - ps31
Arabidopsis_thaliana - ps32
p-value
2e-13
2e-11
3e-09
4e-07
6e-05
Log-odds
0.5
1.0
1.5
2.0
2.5
3.0
3.5
4.0

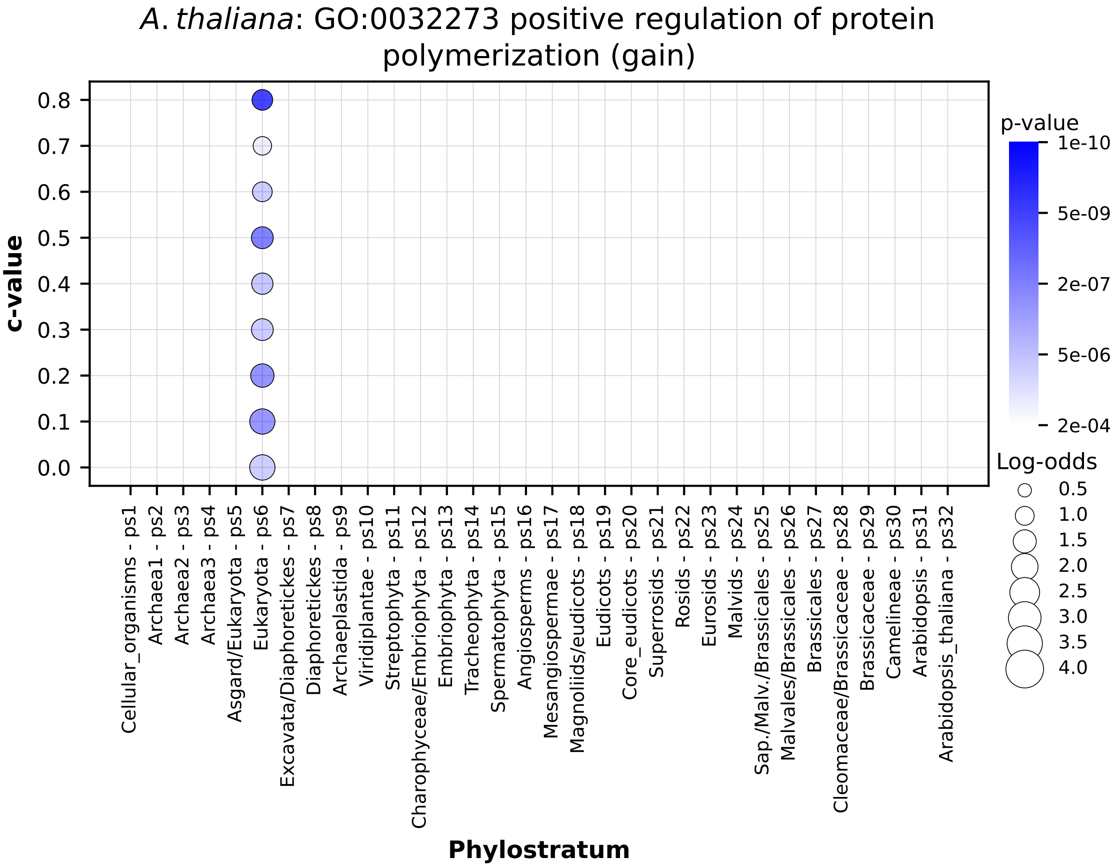

A. thaliana: GO:0032273 positive regulation of protein polymerization (gain)
c-value
0.8
0.7
0.6
0.5
0.4
0.3
0.2
0.1
0.0
p-value
1e-10
5e-09
2e-07
5e-06
2e-04
Log-odds
0.5
1.0
1.5
2.0
2.5
3.0
3.5
4.0
Cellular_organisms - ps1
Archaea1 - ps2
Archaea2 - ps3
Archaea3 - ps4
Asgard/Eukaryota - ps5
Eukaryota - ps6
Excavata/Diaphoretickes - ps7
Diaphoretickes - ps8
Archaeplastida - ps9
Viridiplantae - ps10
Streptophyta - ps11
Charophyceae/Embriophyta - ps12
Embriophyta - ps13
Tracheophyta - ps14
Spermatophyta - ps15
Angiosperms - ps16
Mesangiospermae - ps17
Magnoliids/eudicots - ps18
Eudicots - ps19
Core_eudicots - ps20
Superrosids - ps21
Rosids - ps22
Eurosids - ps23
Malvids - ps24
Sap./Malv./Brassicales - ps25
Malvales/Brassicales - ps26
Brassicales - ps27
Cleomaceae/Brassicaceae - ps28
Brassicaceae - ps29
Camelineae - ps30
Arabidopsis - ps31
Arabidopsis_thaliana - ps32
Phylostratum

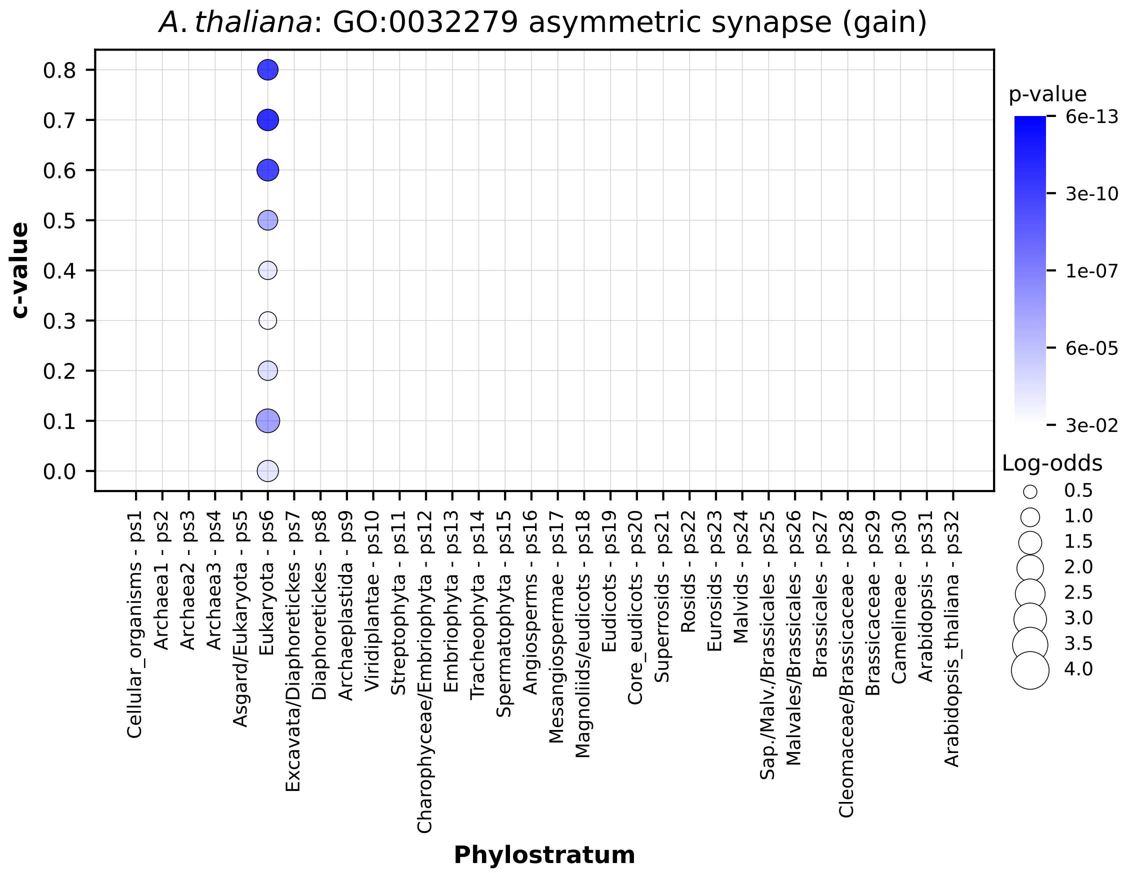
A. thaliana: GO:0032279 asymmetric synapse (gain)
c-value
0.8
0.7
0.6
0.5
0.4
0.3
0.2
0.1
0.0
p-value
6e-13
3e-10
1e-07
6e-05
3e-02
Log-odds
0.5
1.0
1.5
2.0
2.5
3.0
3.5
4.0
Cellular_organisms - ps1
Archaea1 - ps2
Archaea2 - ps3
Archaea3 - ps4
Asgard/Eukaryota - ps5
Eukaryota - ps6
Excavata/Diaphoretickes - ps7
Diaphoretickes - ps8
Archaeplastida - ps9
Viridiplantae - ps10
Streptophyta - ps11
Charophyceae/Embriophyta - ps12
Embriophyta - ps13
Tracheophyta - ps14
Spermatophyta - ps15
Angiosperms - ps16
Mesangiospermae - ps17
Magnoliids/eudicots - ps18
Eudicots - ps19
Core_eudicots - ps20
Superrosids - ps21
Rosids - ps22
Eurosids - ps23
Malvids - ps24
Sap./Malv./Brassicales - ps25
Malvales/Brassicales - ps26
Brassicales - ps27
Cleomaceae/Brassicaceae - ps28
Brassicaceae - ps29
Camelineae - ps30
Arabidopsis - ps31
Arabidopsis_thaliana - ps32
Phylostratum

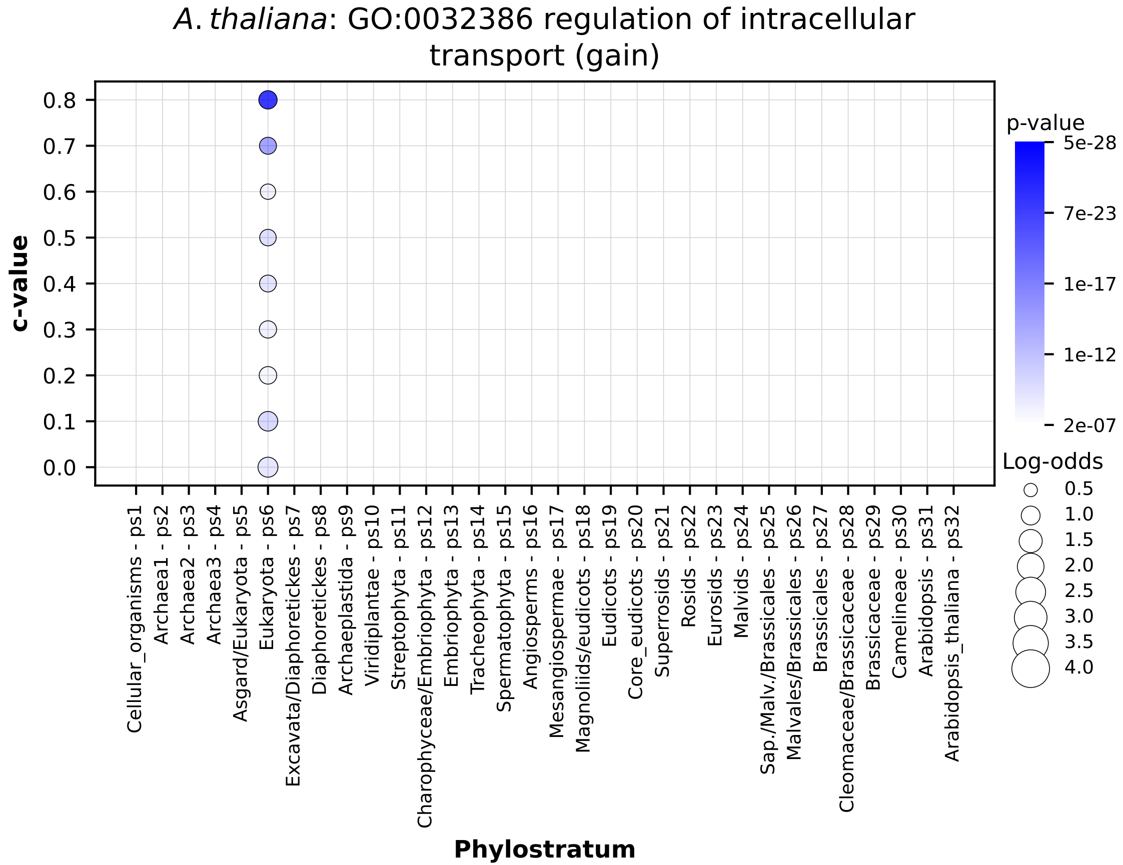

A. thaliana: GO:0032386 regulation of intracellular transport (gain)
c-value
0.8
0.7
0.6
0.5
0.4
0.3
0.2
0.1
0.0
p-value
5e-28
7e-23
1e-17
1e-12
2e-07
Log-odds
0.5
1.0
1.5
2.0
2.5
3.0
3.5
4.0
Cellular_organisms - ps1
Archaea1 - ps2
Archaea2 - ps3
Archaea3 - ps4
Asgard/Eukaryota - ps5
Eukaryota - ps6
Excavata/Diaphoretickes - ps7
Diaphoretickes - ps8
Archaeplastida - ps9
Viridiplantae - ps10
Streptophyta - ps11
Charophyceae/Embriophyta - ps12
Embriophyta - ps13
Tracheophyta - ps14
Spermatophyta - ps15
Angiosperms - ps16
Mesangiospermae - ps17
Magnoliids/eudicots - ps18
Eudicots - ps19
Core_eudicots - ps20
Superrosids - ps21
Rosids - ps22
Eurosids - ps23
Malvids - ps24
Sap./Malv./Brassicales - ps25
Malvales/Brassicales - ps26
Brassicales - ps27
Cleomaceae/Brassicaceae - ps28
Brassicaceae - ps29
Camelineae - ps30
Arabidopsis - ps31
Arabidopsis_thaliana - ps32
Phylostratum

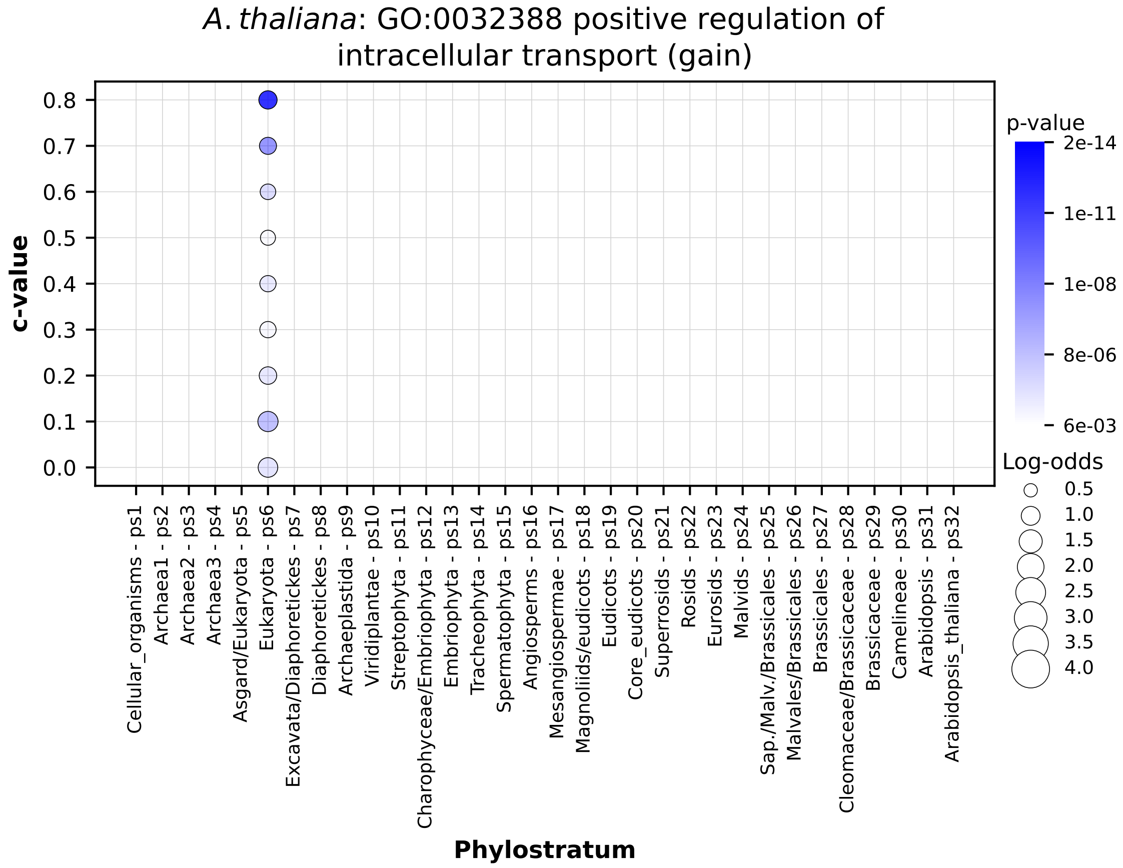

A. thaliana: GO:0032388 positive regulation of intracellular transport (gain)
c-value
0.8
0.7
0.6
0.5
0.4
0.3
0.2
0.1
0.0
p-value
2e-14
1e-11
1e-08
8e-06
6e-03
Log-odds
0.5
1.0
1.5
2.0
2.5
3.0
3.5
4.0
Cellular_organisms - ps1
Archaea1 - ps2
Archaea2 - ps3
Archaea3 - ps4
Asgard/Eukaryota - ps5
Eukaryota - ps6
Excavata/Diaphoretickes - ps7
Diaphoretickes - ps8
Archaeplastida - ps9
Viridiplantae - ps10
Streptophyta - ps11
Charophyceae/Embriophyta - ps12
Embriophyta - ps13
Tracheophyta - ps14
Spermatophyta - ps15
Angiosperms - ps16
Mesangiospermae - ps17
Magnoliids/eudicots - ps18
Eudicots - ps19
Core_eudicots - ps20
Superrosids - ps21
Rosids - ps22
Eurosids - ps23
Malvids - ps24
Sap./Malv./Brassicales - ps25
Malvales/Brassicales - ps26
Brassicales - ps27
Cleomaceae/Brassicaceae - ps28
Brassicaceae - ps29
Camelineae - ps30
Arabidopsis - ps31
Arabidopsis_thaliana - ps32
Phylostratum

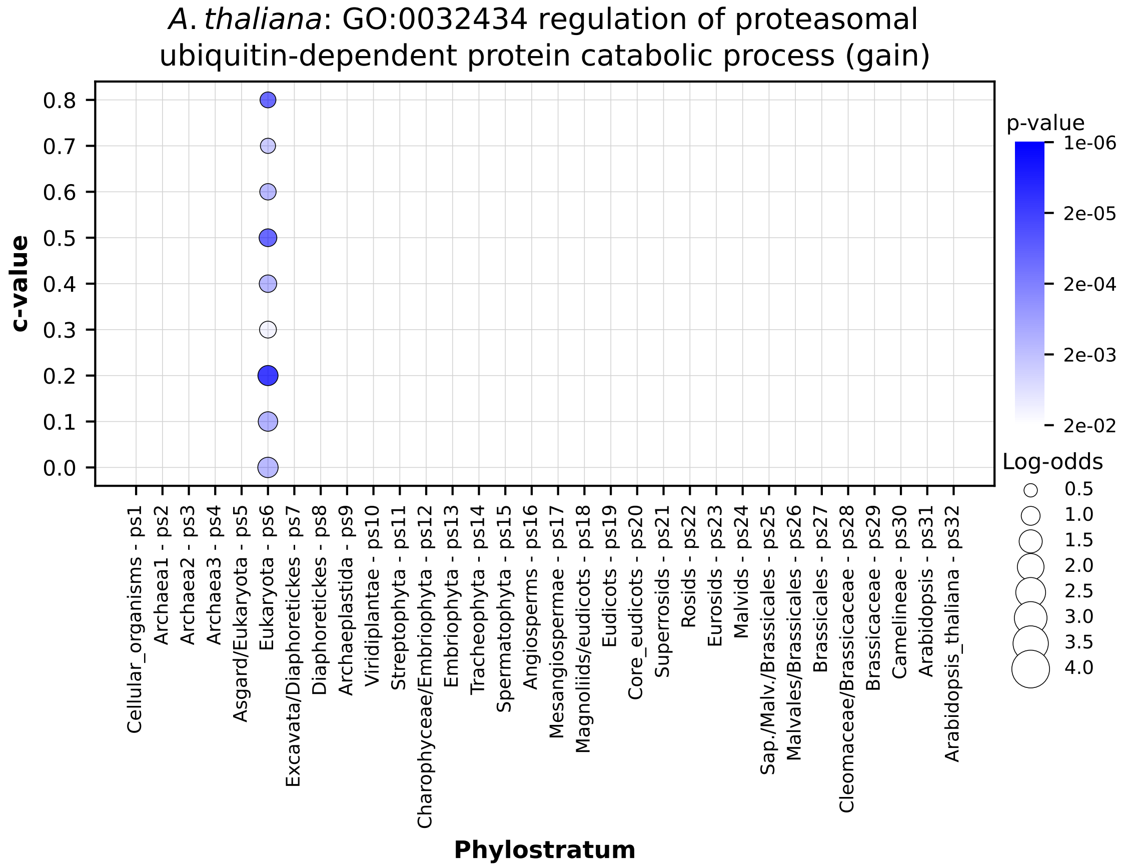

A. thaliana: GO:0032434 regulation of proteasomal ubiquitin-dependent protein catabolic process (gain)
c-value
0.8
0.7
0.6
0.5
0.4
0.3
0.2
0.1
0.0
p-value
1e-06
2e-05
2e-04
2e-03
2e-02
Log-odds
0.5
1.0
1.5
2.0
2.5
3.0
3.5
4.0
Cellular_organisms - ps1
Archaea1 - ps2
Archaea2 - ps3
Archaea3 - ps4
Asgard/Eukaryota - ps5
Eukaryota - ps6
Excavata/Diaphoretickes - ps7
Diaphoretickes - ps8
Archaeplastida - ps9
Viridiplantae - ps10
Streptophyta - ps11
Charophyceae/Embriophyta - ps12
Embriophyta - ps13
Tracheophyta - ps14
Spermatophyta - ps15
Angiosperms - ps16
Mesangiospermae - ps17
Magnoliids/eudicots - ps18
Eudicots - ps19
Core_eudicots - ps20
Superrosids - ps21
Rosids - ps22
Eurosids - ps23
Malvids - ps24
Sap./Malv./Brassicales - ps25
Malvales/Brassicales - ps26
Brassicales - ps27
Cleomaceae/Brassicaceae - ps28
Brassicaceae - ps29
Camelineae - ps30
Arabidopsis - ps31
Arabidopsis_thaliana - ps32
Phylostratum

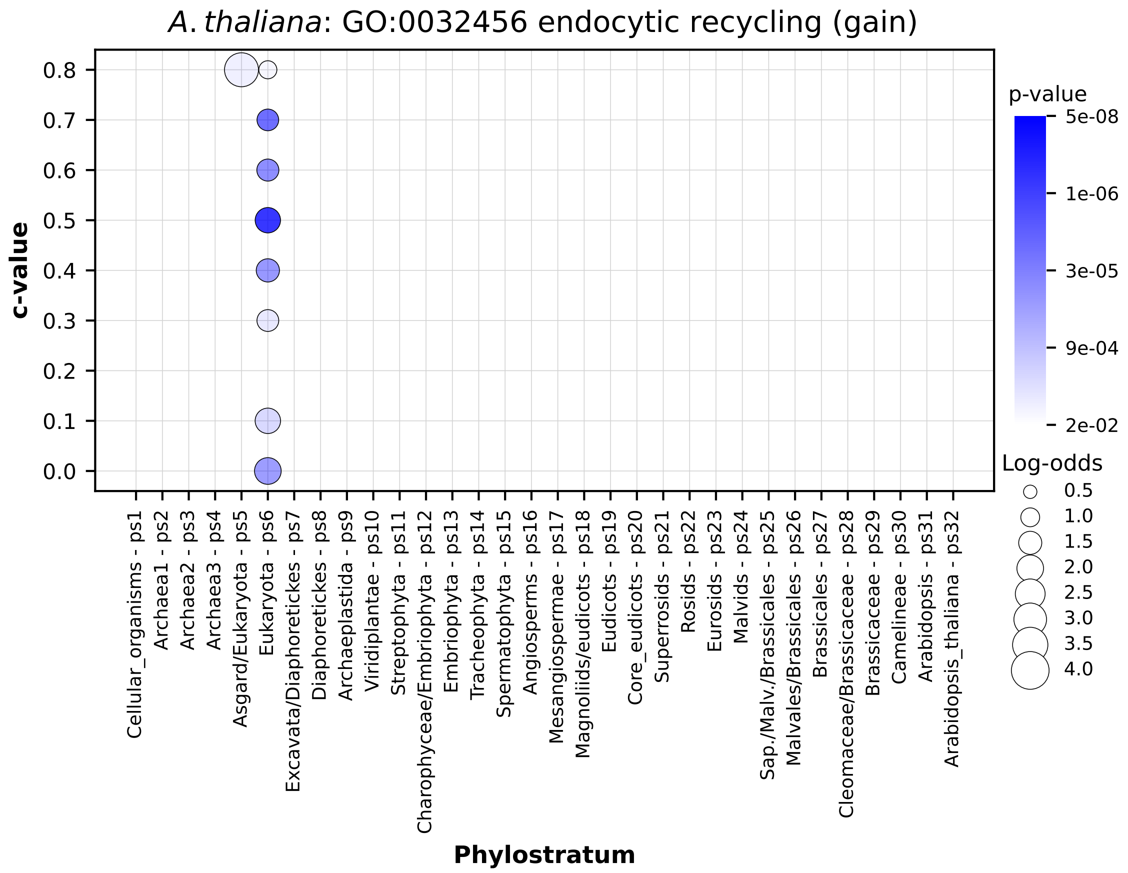

A. thaliana: GO:0032456 endocytic recycling (gain)
c-value
0.8
0.7
0.6
0.5
0.4
0.3
0.2
0.1
0.0
p-value
5e-08
1e-06
3e-05
9e-04
2e-02
Log-odds
0.5
1.0
1.5
2.0
2.5
3.0
3.5
4.0
Cellular_organisms - ps1
Archaea1 - ps2
Archaea2 - ps3
Archaea3 - ps4
Asgard/Eukaryota - ps5
Eukaryota - ps6
Excavata/Diaphoretickes - ps7
Diaphoretickes - ps8
Archaeplastida - ps9
Viridiplantae - ps10
Streptophyta - ps11
Charophyceae/Embriophyta - ps12
Embriophyta - ps13
Tracheophyta - ps14
Spermatophyta - ps15
Angiosperms - ps16
Mesangiospermae - ps17
Magnoliids/eudicots - ps18
Eudicots - ps19
Core_eudicots - ps20
Superrosids - ps21
Rosids - ps22
Eurosids - ps23
Malvids - ps24
Sap./Malv./Brassicales - ps25
Malvales/Brassicales - ps26
Brassicales - ps27
Cleomaceae/Brassicaceae - ps28
Brassicaceae - ps29
Camelineae - ps30
Arabidopsis - ps31
Arabidopsis_thaliana - ps32
Phylostratum

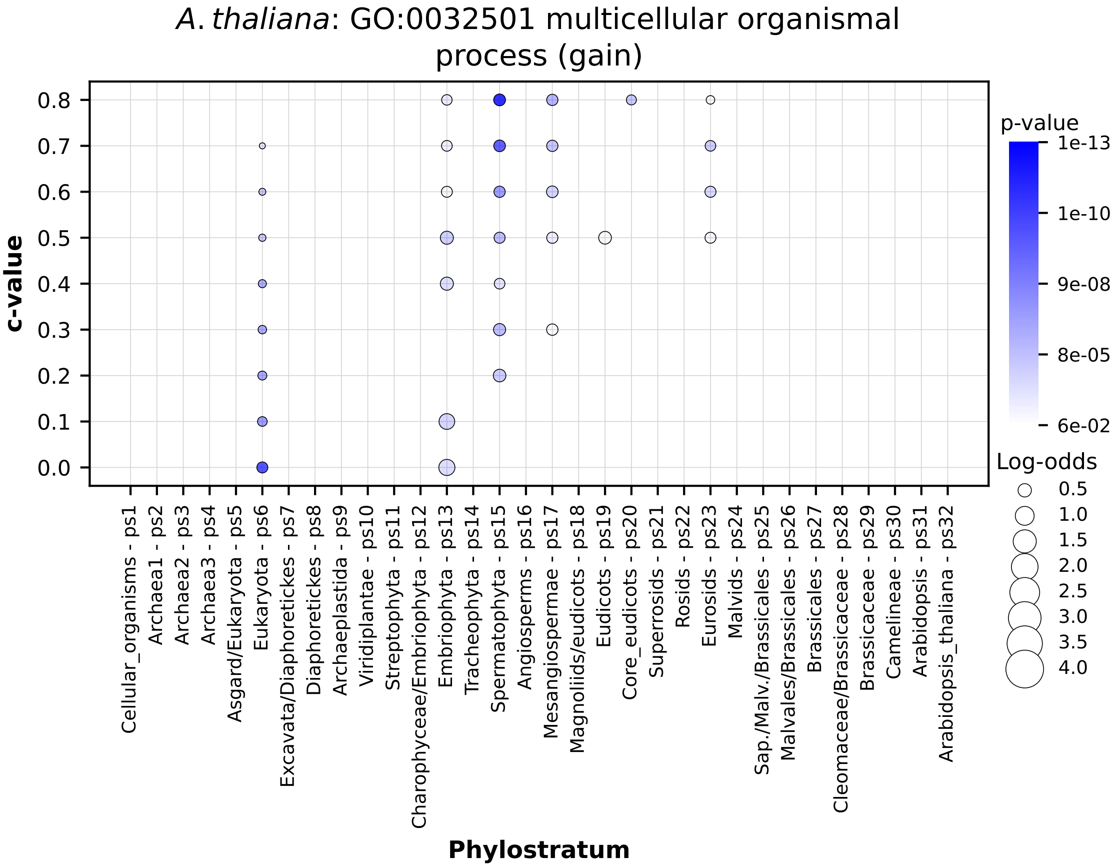
A. thaliana: GO:0032501 multicellular organismal process (gain)
c-value
0.8
0.7
0.6
0.5
0.4
0.3
0.2
0.1
0.0
Phylostratum
Cellular_organisms - ps1
Archaea1 - ps2
Archaea2 - ps3
Archaea3 - ps4
Asgard/Eukaryota - ps5
Eukaryota - ps6
Excavata/Diaphoretickes - ps7
Diaphoretickes - ps8
Archaeplastida - ps9
Viridiplantae - ps10
Streptophyta - ps11
Charophyceae/Embriophyta - ps12
Embriophyta - ps13
Tracheophyta - ps14
Spermatophyta - ps15
Angiosperms - ps16
Mesangiospermae - ps17
Magnoliids/eudicots - ps18
Eudicots - ps19
Core_eudicots - ps20
Superrosids - ps21
Rosids - ps22
Eurosids - ps23
Malvids - ps24
Sap./Malv./Brassicales - ps25
Malvales/Brassicales - ps26
Brassicales - ps27
Cleomaceae/Brassicaceae - ps28
Brassicaceae - ps29
Camelineae - ps30
Arabidopsis - ps31
Arabidopsis_thaliana - ps32
p-value
1e-13
1e-10
9e-08
8e-05
6e-02
Log-odds
0.5
1.0
1.5
2.0
2.5
3.0
3.5
4.0

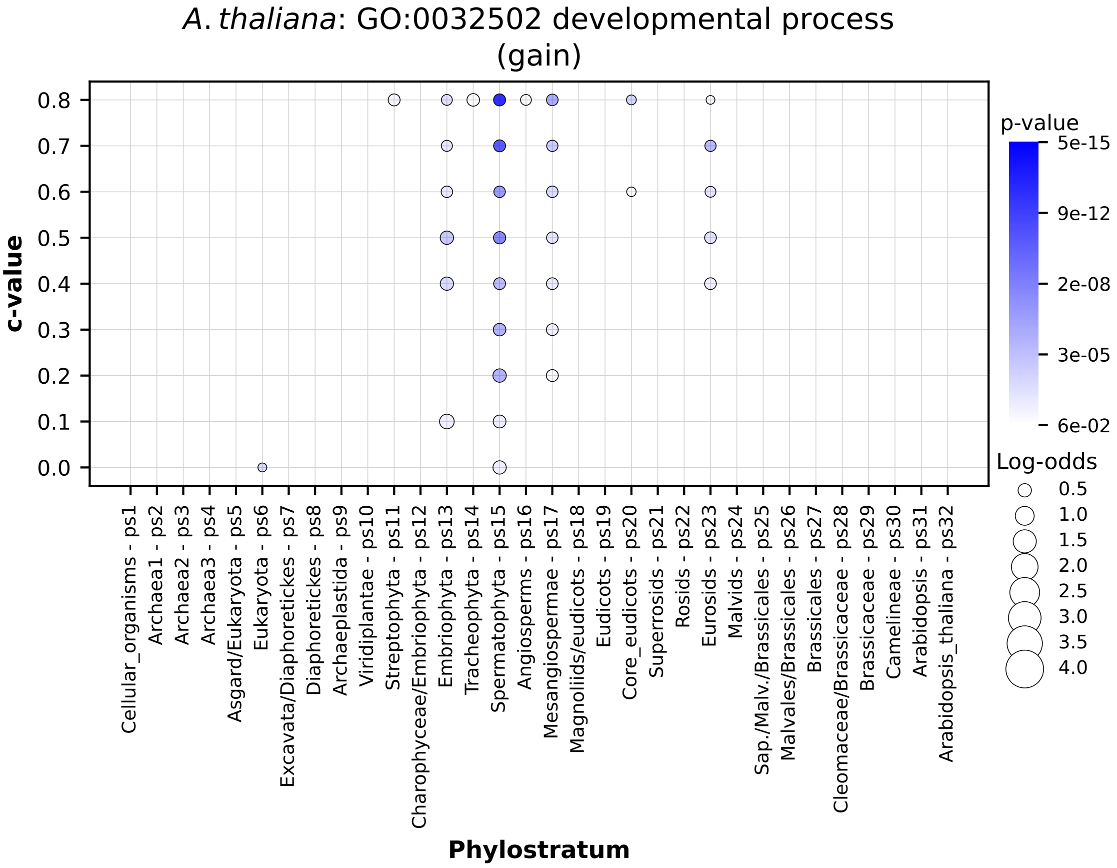

A. thaliana: GO:0032502 developmental process
(gain)
c-value
0.8
0.7
0.6
0.5
0.4
0.3
0.2
0.1
0.0
p-value
5e-15
9e-12
2e-08
3e-05
6e-02
Log-odds
0.5
1.0
1.5
2.0
2.5
3.0
3.5
4.0
Cellular_organisms - ps1
Archaea1 - ps2
Archaea2 - ps3
Archaea3 - ps4
Asgard/Eukaryota - ps5
Eukaryota - ps6
Excavata/Diaphoretickes - ps7
Diaphoretickes - ps8
Archaeplastida - ps9
Viridiplantae - ps10
Streptophyta - ps11
Charophyceae/Embriophyta - ps12
Embriophyta - ps13
Tracheophyta - ps14
Spermatophyta - ps15
Angiosperms - ps16
Mesangiospermae - ps17
Magnoliids/eudicots - ps18
Eudicots - ps19
Core_eudicots - ps20
Superrosids - ps21
Rosids - ps22
Eurosids - ps23
Malvids - ps24
Sap./Malv./Brassicales - ps25
Malvales/Brassicales - ps26
Brassicales - ps27
Cleomaceae/Brassicaceae - ps28
Brassicaceae - ps29
Camelineae - ps30
Arabidopsis - ps31
Arabidopsis_thaliana - ps32
Phylostratum

A. thaliana: GO:0032504 multicellular organism reproduction (gain)

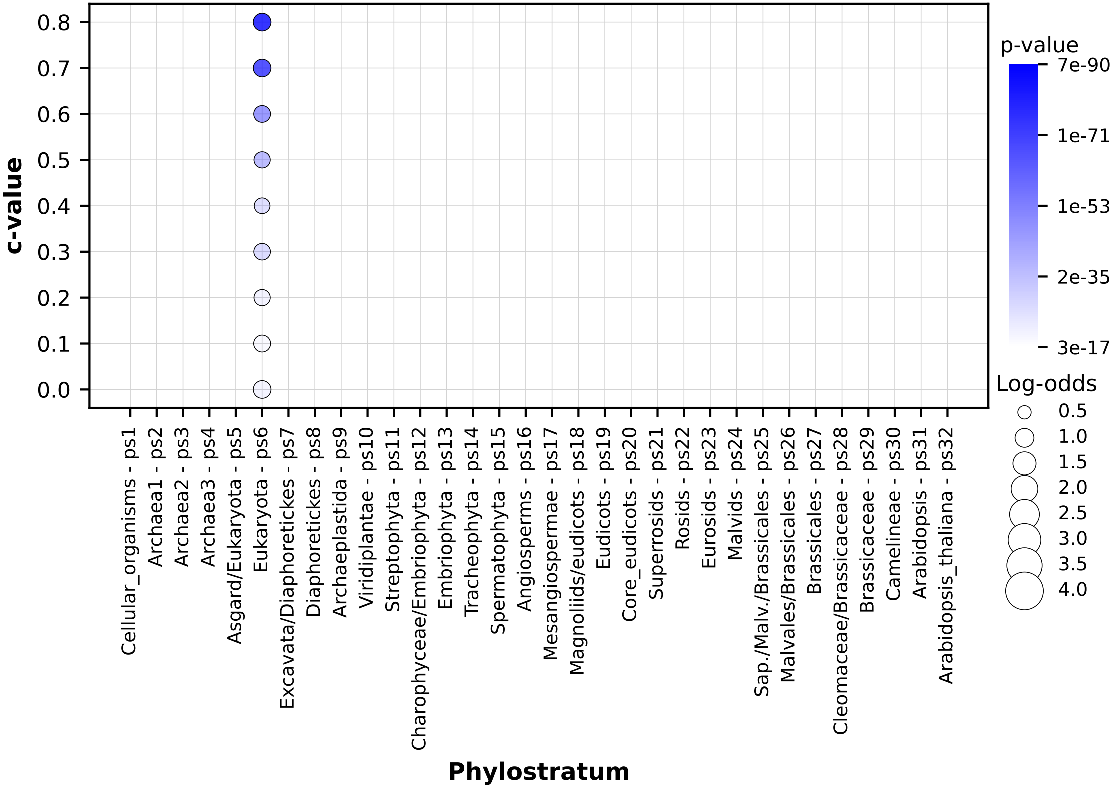

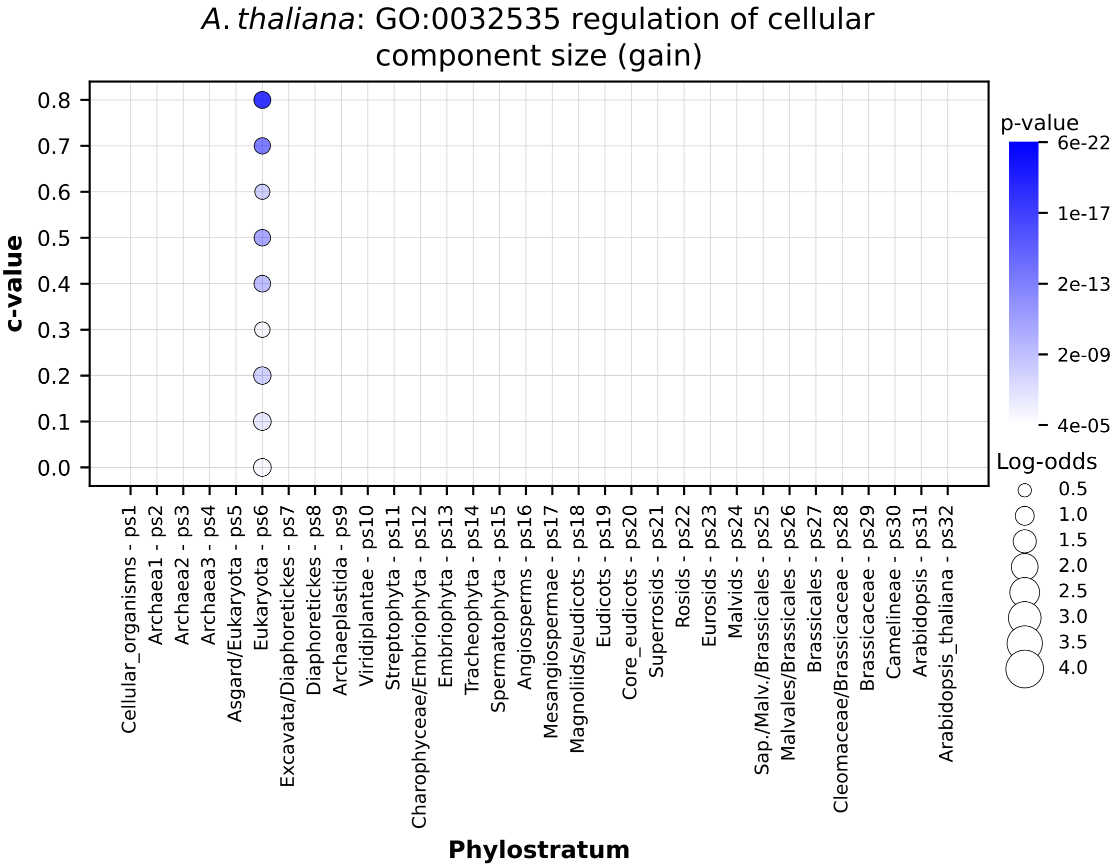

A. thaliana: GO:0032535 regulation of cellular component size (gain)
c-value
0.0
0.1
0.2
0.3
0.4
0.5
0.6
0.7
0.8
Phylostratum
Cellular_organisms - ps1
Archaea1 - ps2
Archaea2 - ps3
Archaea3 - ps4
Asgard/Eukaryota - ps5
Eukaryota - ps6
Excavata/Diaphoretickes - ps7
Diaphoretickes - ps8
Archaeplastida - ps9
Viridiplantae - ps10
Streptophyta - ps11
Charophyceae/Embriophyta - ps12
Embriophyta - ps13
Tracheophyta - ps14
Spermatophyta - ps15
Angiosperms - ps16
Mesangiospermae - ps17
Magnoliids/eudicots - ps18
Eudicots - ps19
Core_eudicots - ps20
Superrosids - ps21
Rosids - ps22
Eurosids - ps23
Malvids - ps24
Sap./Malv./Brassicales - ps25
Malvales/Brassicales - ps26
Brassicales - ps27
Cleomaceae/Brassicaceae - ps28
Brassicaceae - ps29
Camelineae - ps30
Arabidopsis - ps31
Arabidopsis_thaliana - ps32
p-value
6e-22
1e-17
2e-13
2e-09
4e-05
Log-odds
0.5
1.0
1.5
2.0
2.5
3.0
3.5
4.0

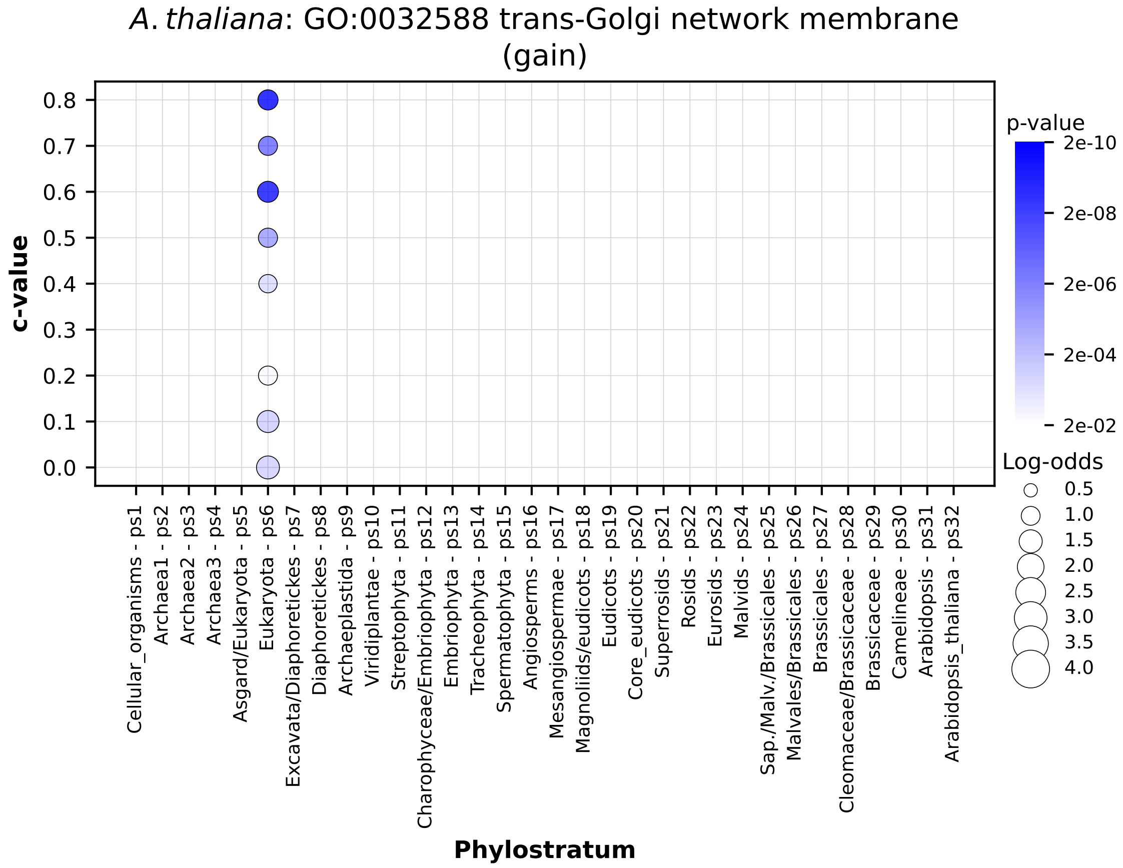

A. thaliana: GO:0032588 trans-Golgi network membrane
(gain)
c-value
0.8
0.7
0.6
0.5
0.4
0.3
0.2
0.1
0.0
p-value
2e-10
2e-08
2e-06
2e-04
2e-02
Log-odds
0.5
1.0
1.5
2.0
2.5
3.0
3.5
4.0
Cellular_organisms - ps1
Archaea1 - ps2
Archaea2 - ps3
Archaea3 - ps4
Asgard/Eukaryota - ps5
Eukaryota - ps6
Excavata/Diaphoretickes - ps7
Diaphoretickes - ps8
Archaeplastida - ps9
Viridiplantae - ps10
Streptophyta - ps11
Charophyceae/Embriophyta - ps12
Embriophyta - ps13
Tracheophyta - ps14
Spermatophyta - ps15
Angiosperms - ps16
Mesangiospermae - ps17
Magnoliids/eudicots - ps18
Eudicots - ps19
Core_eudicots - ps20
Superrosids - ps21
Rosids - ps22
Eurosids - ps23
Malvids - ps24
Sap./Malv./Brassicales - ps25
Malvales/Brassicales - ps26
Brassicales - ps27
Cleomaceae/Brassicaceae - ps28
Brassicaceae - ps29
Camelineae - ps30
Arabidopsis - ps31
Arabidopsis_thaliana - ps32
Phylostratum

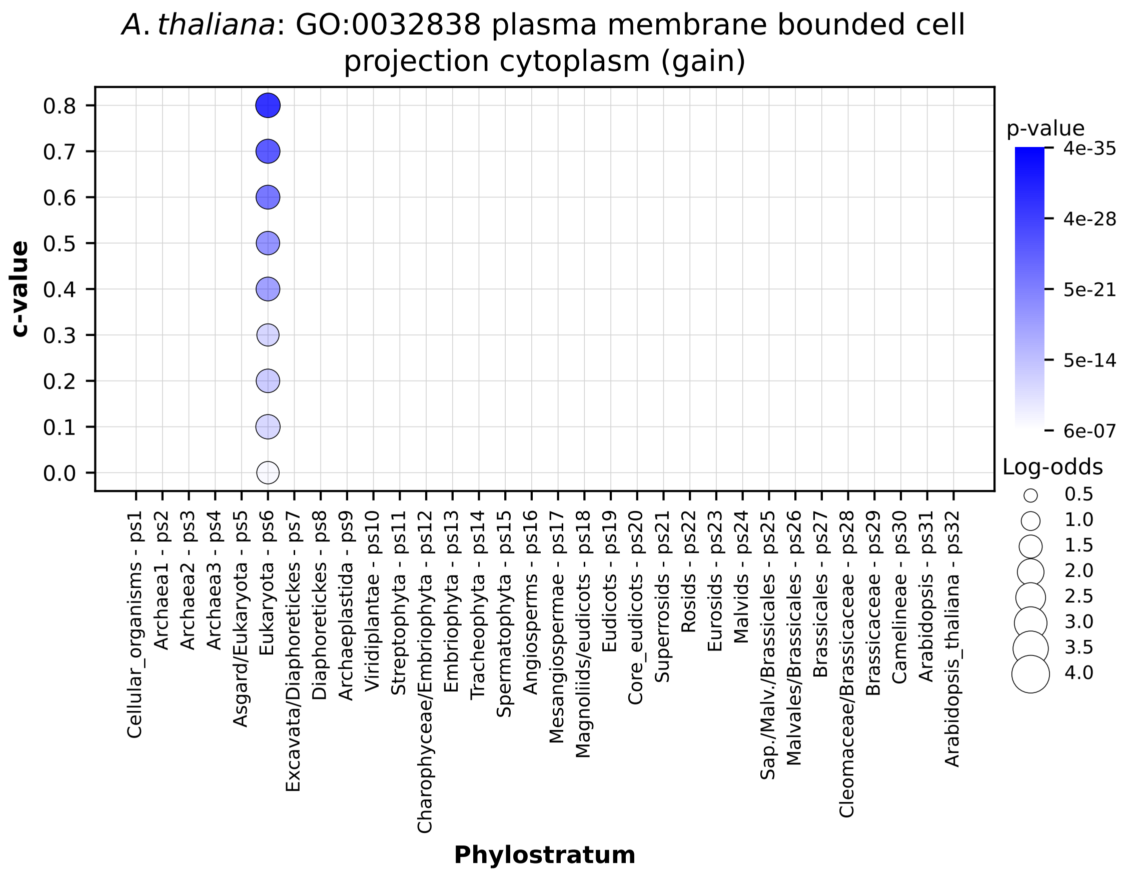

A. thaliana: GO:0032838 plasma membrane bounded cell projection cytoplasm (gain)
c-value
0.0
0.1
0.2
0.3
0.4
0.5
0.6
0.7
0.8
p-value
4e-35
4e-28
5e-21
5e-14
6e-07
Log-odds
0.5
1.0
1.5
2.0
2.5
3.0
3.5
4.0
Cellular_organisms - ps1
Archaea1 - ps2
Archaea2 - ps3
Archaea3 - ps4
Asgard/Eukaryota - ps5
Eukaryota - ps6
Excavata/Diaphoretickes - ps7
Diaphoretickes - ps8
Archaeplastida - ps9
Viridiplantae - ps10
Streptophyta - ps11
Charophyceae/Embriophyta - ps12
Embriophyta - ps13
Tracheophyta - ps14
Spermatophyta - ps15
Angiosperms - ps16
Mesangiospermae - ps17
Magnoliids/eudicots - ps18
Eudicots - ps19
Core_eudicots - ps20
Superrosids - ps21
Rosids - ps22
Eurosids - ps23
Malvids - ps24
Sap./Malv./Brassicales - ps25
Malvales/Brassicales - ps26
Brassicales - ps27
Cleomaceae/Brassicaceae - ps28
Brassicaceae - ps29
Camelineae - ps30
Arabidopsis - ps31
Arabidopsis_thaliana - ps32
Phylostratum

A. thaliana: GO:0032872 regulation of stress-activated MAPK cascade (gain)

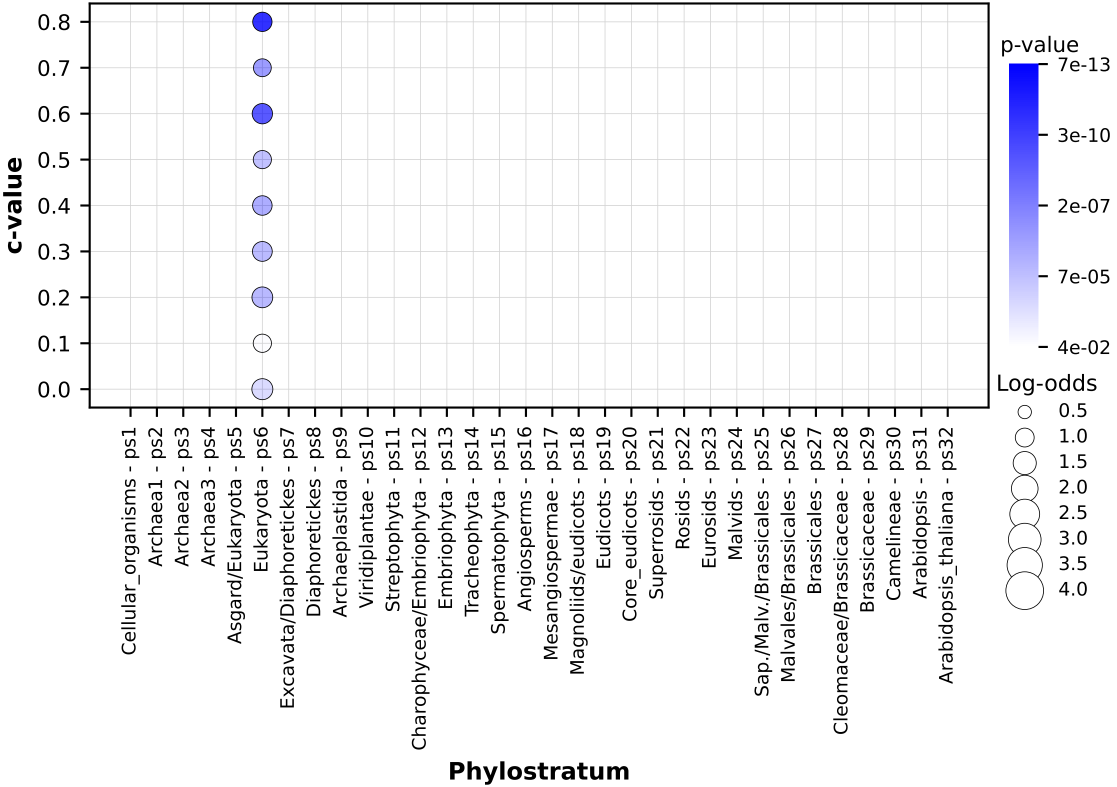

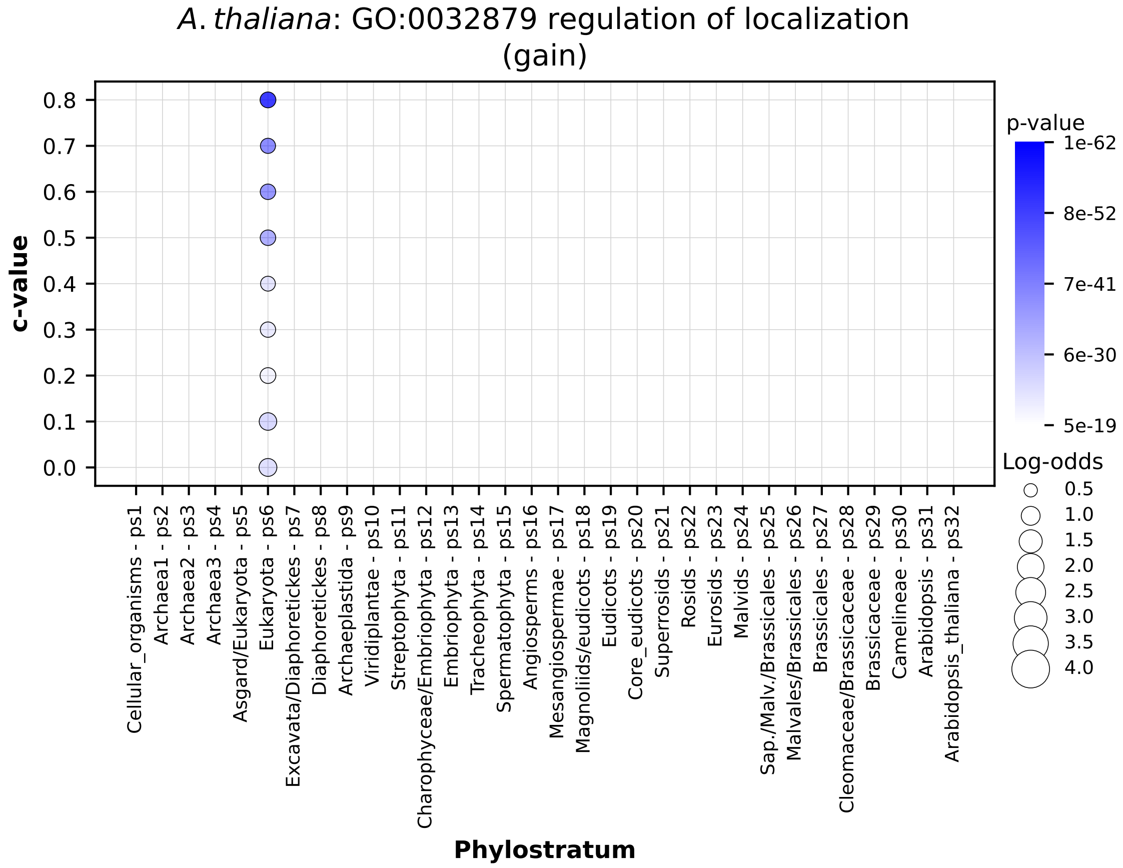

A. thaliana: GO:0032879 regulation of localization
(gain)
c-value
0.8
0.7
0.6
0.5
0.4
0.3
0.2
0.1
0.0
p-value
1e-62
8e-52
7e-41
6e-30
5e-19
Log-odds
0.5
1.0
1.5
2.0
2.5
3.0
3.5
4.0
Cellular_organisms - ps1
Archaea1 - ps2
Archaea2 - ps3
Archaea3 - ps4
Asgard/Eukaryota - ps5
Eukaryota - ps6
Excavata/Diaphoretickes - ps7
Diaphoretickes - ps8
Archaeplastida - ps9
Viridiplantae - ps10
Streptophyta - ps11
Charophyceae/Embriophyta - ps12
Embriophyta - ps13
Tracheophyta - ps14
Spermatophyta - ps15
Angiosperms - ps16
Mesangiospermae - ps17
Magnoliids/eudicots - ps18
Eudicots - ps19
Core_eudicots - ps20
Superrosids - ps21
Rosids - ps22
Eurosids - ps23
Malvids - ps24
Sap./Malv./Brassicales - ps25
Malvales/Brassicales - ps26
Brassicales - ps27
Cleomaceae/Brassicaceae - ps28
Brassicaceae - ps29
Camelineae - ps30
Arabidopsis - ps31
Arabidopsis_thaliana - ps32
Phylostratum

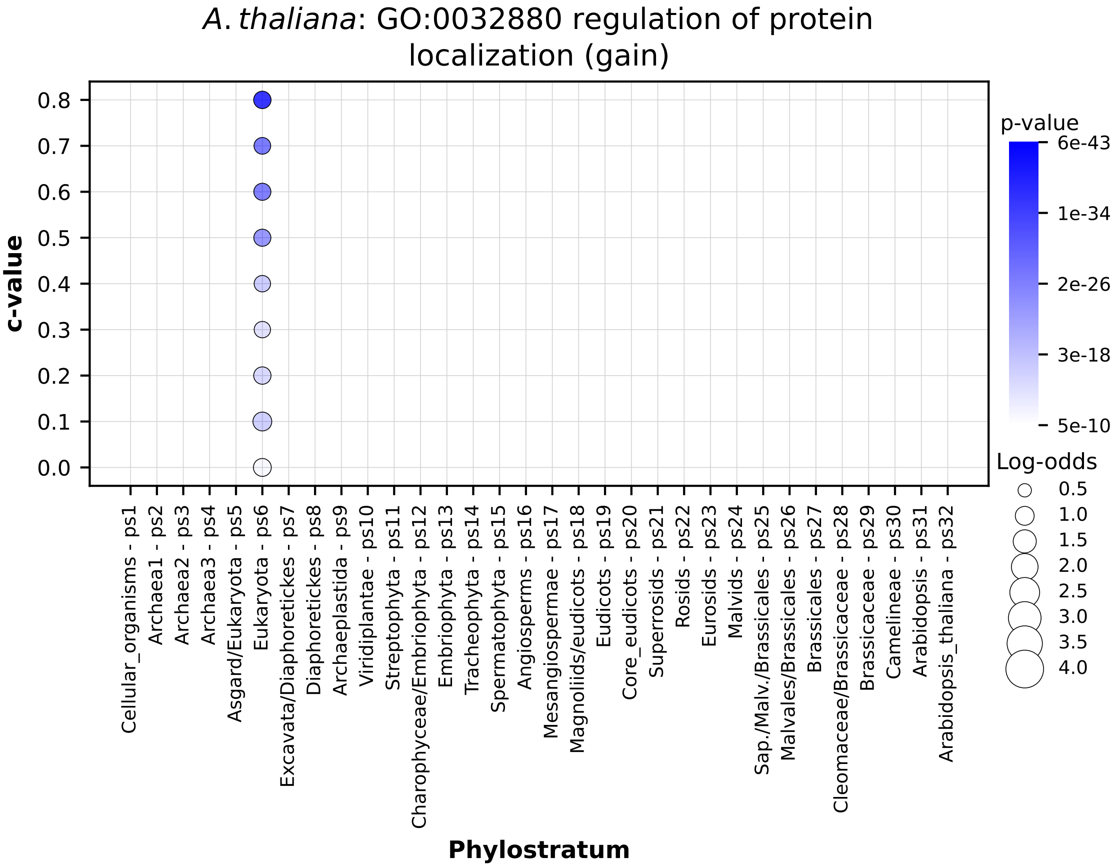
A. thaliana: GO:0032880 regulation of protein localization (gain)
c-value
0.8
0.7
0.6
0.5
0.4
0.3
0.2
0.1
0.0
p-value
6e-43
1e-34
2e-26
3e-18
5e-10
Log-odds
0.5
1.0
1.5
2.0
2.5
3.0
3.5
4.0
Cellular_organisms - ps1
Archaea1 - ps2
Archaea2 - ps3
Archaea3 - ps4
Asgard/Eukaryota - ps5
Eukaryota - ps6
Excavata/Diaphoretickes - ps7
Diaphoretickes - ps8
Archaeplastida - ps9
Viridiplantae - ps10
Streptophyta - ps11
Charophyceae/Embriophyta - ps12
Embriophyta - ps13
Tracheophyta - ps14
Spermatophyta - ps15
Angiosperms - ps16
Mesangiospermae - ps17
Magnoliids/eudicots - ps18
Eudicots - ps19
Core_eudicots - ps20
Superrosids - ps21
Rosids - ps22
Eurosids - ps23
Malvids - ps24
Sap./Malv./Brassicales - ps25
Malvales/Brassicales - ps26
Brassicales - ps27
Cleomaceae/Brassicaceae - ps28
Brassicaceae - ps29
Camelineae - ps30
Arabidopsis - ps31
Arabidopsis_thaliana - ps32
Phylostratum

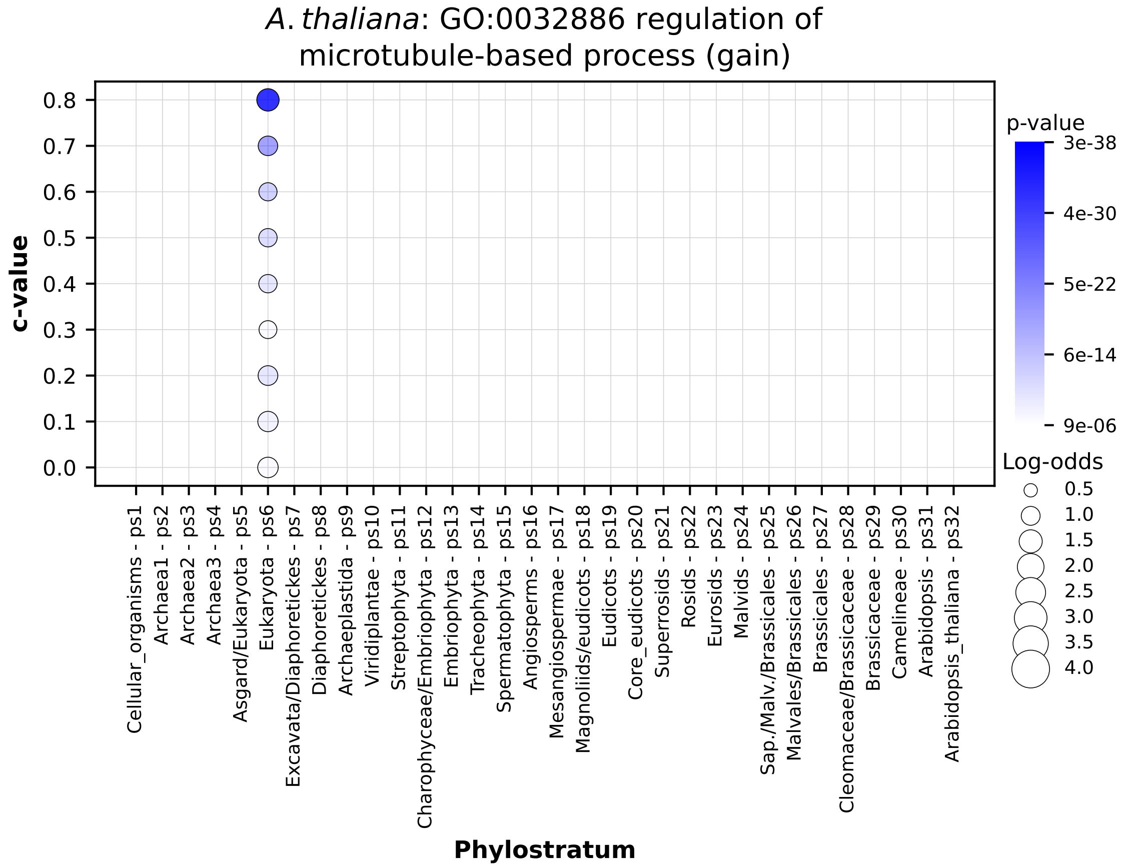

A. thaliana: GO:0032886 regulation of microtubule-based process (gain)
c-value
0.8
0.7
0.6
0.5
0.4
0.3
0.2
0.1
0.0
Phylostratum
Cellular_organisms - ps1
Archaea1 - ps2
Archaea2 - ps3
Archaea3 - ps4
Asgard/Eukaryota - ps5
Eukaryota - ps6
Excavata/Diaphoretickes - ps7
Diaphoretickes - ps8
Archaeplastida - ps9
Viridiplantae - ps10
Streptophyta - ps11
Charophyceae/Embriophyta - ps12
Embriophyta - ps13
Tracheophyta - ps14
Spermatophyta - ps15
Angiosperms - ps16
Mesangiospermae - ps17
Magnoliids/eudicots - ps18
Eudicots - ps19
Core_eudicots - ps20
Superrosids - ps21
Rosids - ps22
Eurosids - ps23
Malvids - ps24
Sap./Malv./Brassicales - ps25
Malvales/Brassicales - ps26
Brassicales - ps27
Cleomaceae/Brassicaceae - ps28
Brassicaceae - ps29
Camelineae - ps30
Arabidopsis - ps31
Arabidopsis_thaliana - ps32
p-value
3e-38
4e-30
5e-22
6e-14
9e-06
Log-odds
0.5
1.0
1.5
2.0
2.5
3.0
3.5
4.0

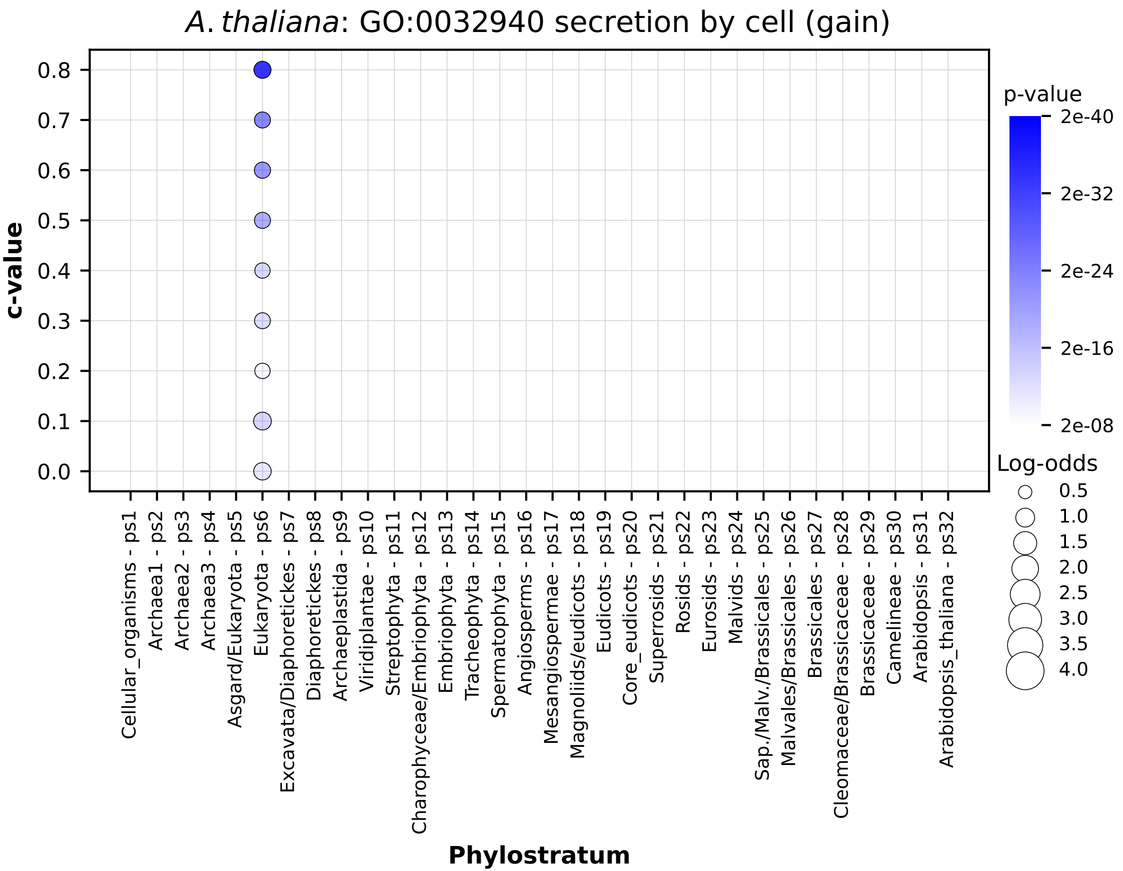

A. thaliana: GO:0032940 secretion by cell (gain)
c-value
0.0
0.1
0.2
0.3
0.4
0.5
0.6
0.7
0.8
p-value
2e-40
2e-32
2e-24
2e-16
2e-08
Log-odds
0.5
1.0
1.5
2.0
2.5
3.0
3.5
4.0
Cellular_organisms - ps1
Archaea1 - ps2
Archaea2 - ps3
Archaea3 - ps4
Asgard/Eukaryota - ps5
Eukaryota - ps6
Excavata/Diaphoretickes - ps7
Diaphoretickes - ps8
Archaeplastida - ps9
Viridiplantae - ps10
Streptophyta - ps11
Charophyceae/Embriophyta - ps12
Embriophyta - ps13
Tracheophyta - ps14
Spermatophyta - ps15
Angiosperms - ps16
Mesangiospermae - ps17
Magnoliids/eudicots - ps18
Eudicots - ps19
Core_eudicots - ps20
Superrosids - ps21
Rosids - ps22
Eurosids - ps23
Malvids - ps24
Sap./Malv./Brassicales - ps25
Malvales/Brassicales - ps26
Brassicales - ps27
Cleomaceae/Brassicaceae - ps28
Brassicaceae - ps29
Camelineae - ps30
Arabidopsis - ps31
Arabidopsis_thaliana - ps32
Phylostratum

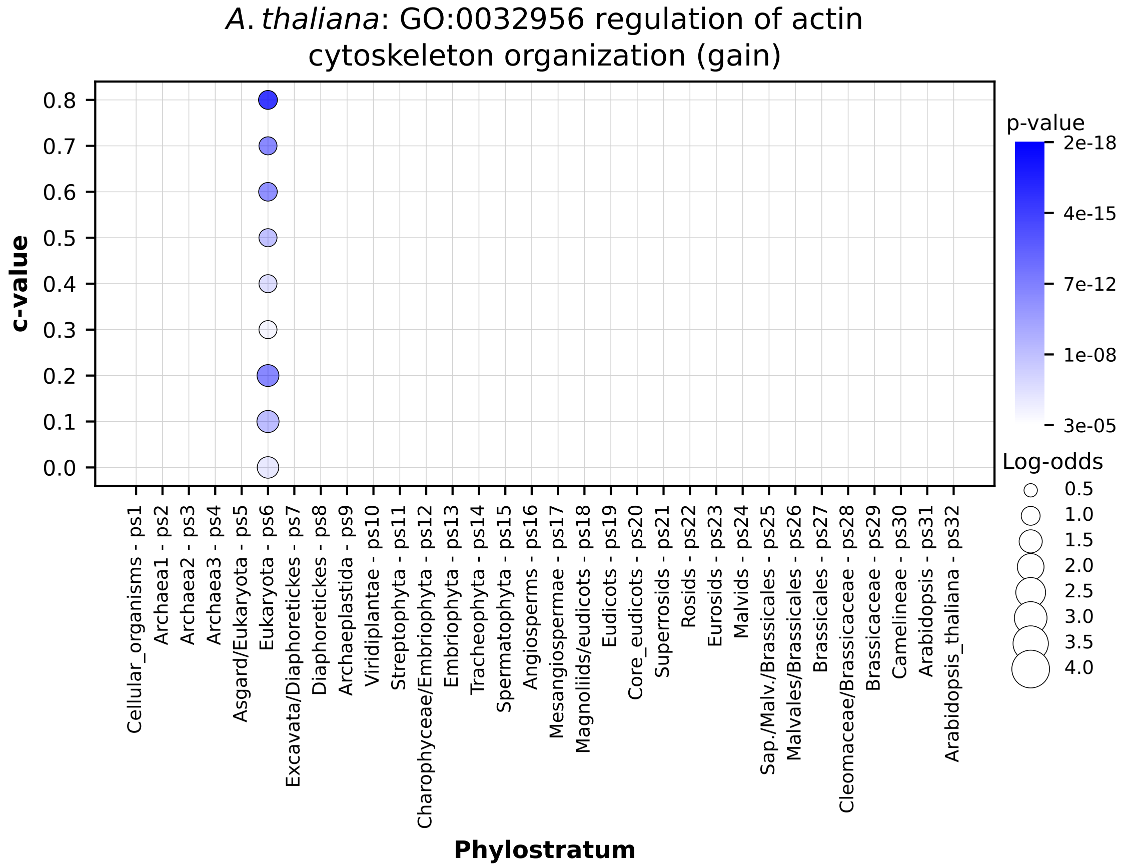

A. thaliana: GO:0032956 regulation of actin cytoskeleton organization (gain)
c-value
0.8
0.7
0.6
0.5
0.4
0.3
0.2
0.1
0.0
p-value
2e-18
4e-15
7e-12
1e-08
3e-05
Log-odds
0.5
1.0
1.5
2.0
2.5
3.0
3.5
4.0
Cellular_organisms - ps1
Archaea1 - ps2
Archaea2 - ps3
Archaea3 - ps4
Asgard/Eukaryota - ps5
Eukaryota - ps6
Excavata/Diaphoretickes - ps7
Diaphoretickes - ps8
Archaeplastida - ps9
Viridiplantae - ps10
Streptophyta - ps11
Charophyceae/Embriophyta - ps12
Embriophyta - ps13
Tracheophyta - ps14
Spermatophyta - ps15
Angiosperms - ps16
Mesangiospermae - ps17
Magnoliids/eudicots - ps18
Eudicots - ps19
Core_eudicots - ps20
Superrosids - ps21
Rosids - ps22
Eurosids - ps23
Malvids - ps24
Sap./Malv./Brassicales - ps25
Malvales/Brassicales - ps26
Brassicales - ps27
Cleomaceae/Brassicaceae - ps28
Brassicaceae - ps29
Camelineae - ps30
Arabidopsis - ps31
Arabidopsis_thaliana - ps32
Phylostratum

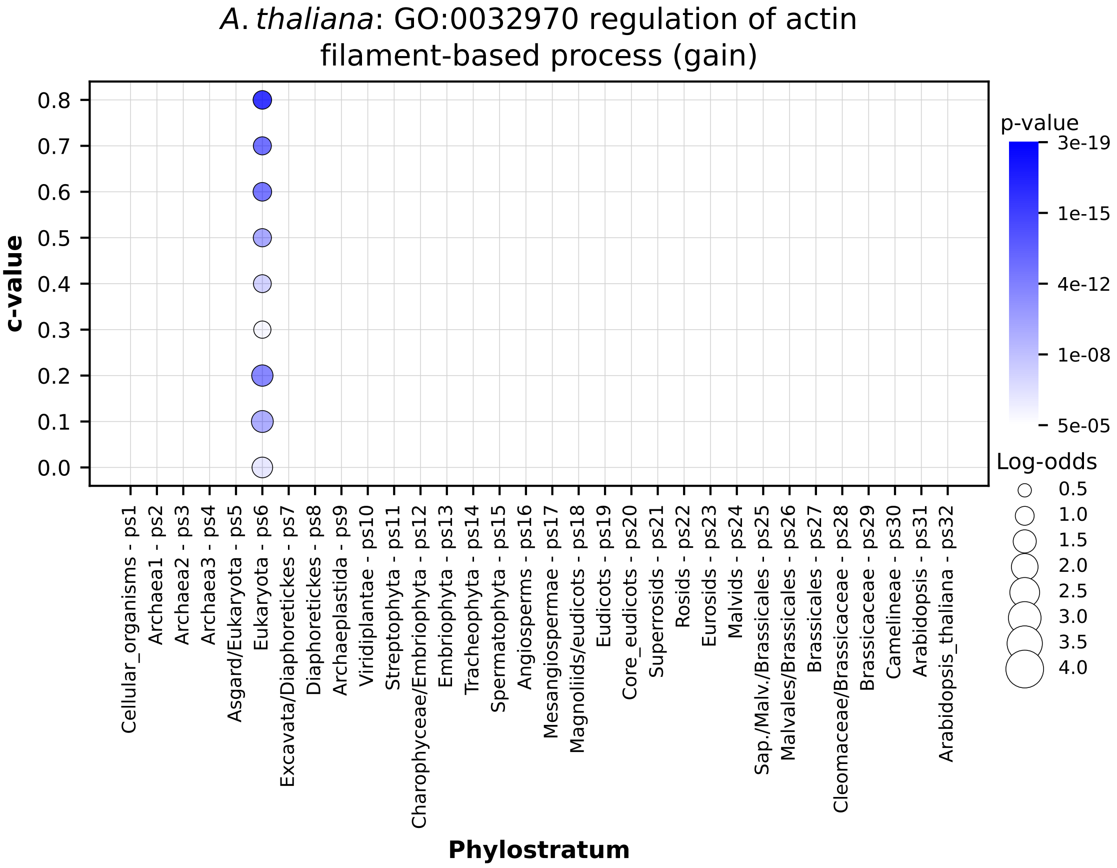

A. thaliana: GO:0032970 regulation of actin filament-based process (gain)
c-value
0.8
0.7
0.6
0.5
0.4
0.3
0.2
0.1
0.0
p-value
3e-19
1e-15
4e-12
1e-08
5e-05
Log-odds
0.5
1.0
1.5
2.0
2.5
3.0
3.5
4.0
Cellular_organisms - ps1
Archaea1 - ps2
Archaea2 - ps3
Archaea3 - ps4
Asgard/Eukaryota - ps5
Eukaryota - ps6
Excavata/Diaphoretickes - ps7
Diaphoretickes - ps8
Archaeplastida - ps9
Viridiplantae - ps10
Streptophyta - ps11
Charophyceae/Embriophyta - ps12
Embriophyta - ps13
Tracheophyta - ps14
Spermatophyta - ps15
Angiosperms - ps16
Mesangiospermae - ps17
Magnoliids/eudicots - ps18
Eudicots - ps19
Core_eudicots - ps20
Superrosids - ps21
Rosids - ps22
Eurosids - ps23
Malvids - ps24
Sap./Malv./Brassicales - ps25
Malvales/Brassicales - ps26
Brassicales - ps27
Cleomaceae/Brassicaceae - ps28
Brassicaceae - ps29
Camelineae - ps30
Arabidopsis - ps31
Arabidopsis_thaliana - ps32
Phylostratum

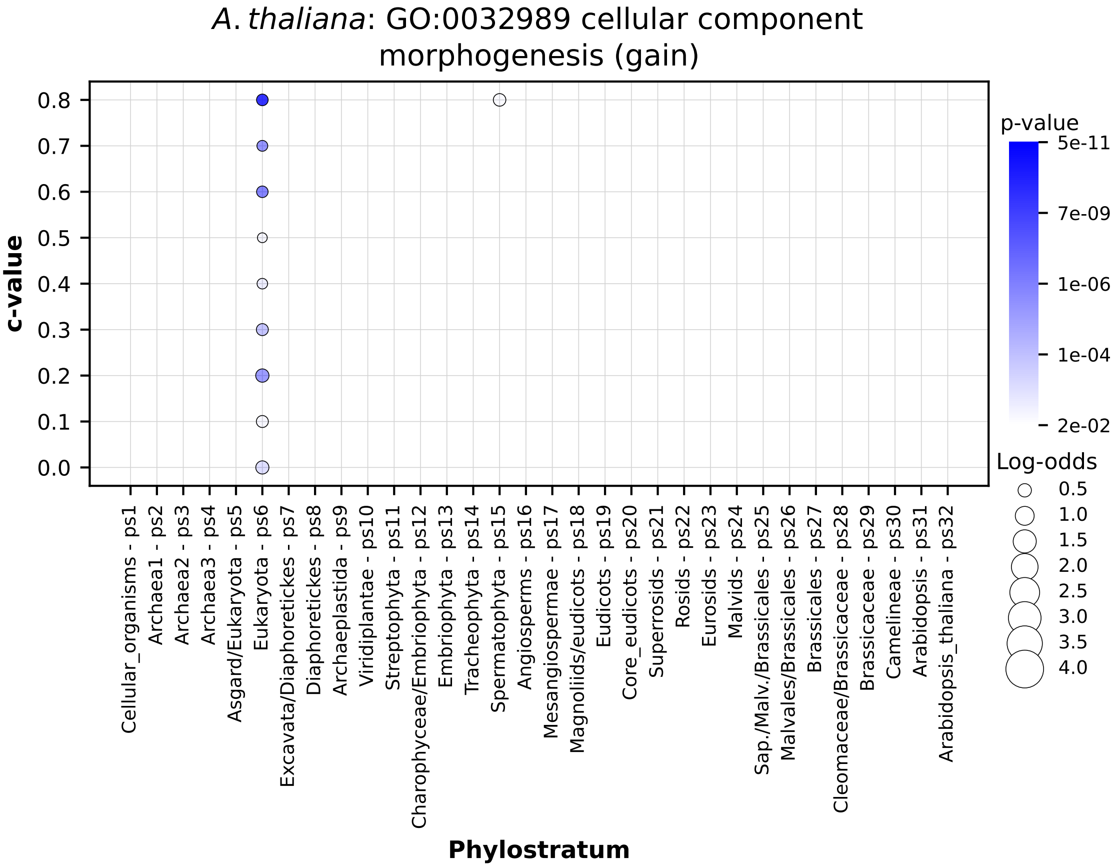
A. thaliana: GO:0032989 cellular component morphogenesis (gain)
c-value
0.8
0.7
0.6
0.5
0.4
0.3
0.2
0.1
0.0
p-value
5e-11
7e-09
1e-06
1e-04
2e-02
Log-odds
0.5
1.0
1.5
2.0
2.5
3.0
3.5
4.0
Cellular_organisms - ps1
Archaea1 - ps2
Archaea2 - ps3
Archaea3 - ps4
Asgard/Eukaryota - ps5
Eukaryota - ps6
Excavata/Diaphoretickes - ps7
Diaphoretickes - ps8
Archaeplastida - ps9
Viridiplantae - ps10
Streptophyta - ps11
Charophyceae/Embriophyta - ps12
Embriophyta - ps13
Tracheophyta - ps14
Spermatophyta - ps15
Angiosperms - ps16
Mesangiospermae - ps17
Magnoliids/eudicots - ps18
Eudicots - ps19
Core_eudicots - ps20
Superrosids - ps21
Rosids - ps22
Eurosids - ps23
Malvids - ps24
Sap./Malv./Brassicales - ps25
Malvales/Brassicales - ps26
Brassicales - ps27
Cleomaceae/Brassicaceae - ps28
Brassicaceae - ps29
Camelineae - ps30
Arabidopsis - ps31
Arabidopsis_thaliana - ps32
Phylostratum

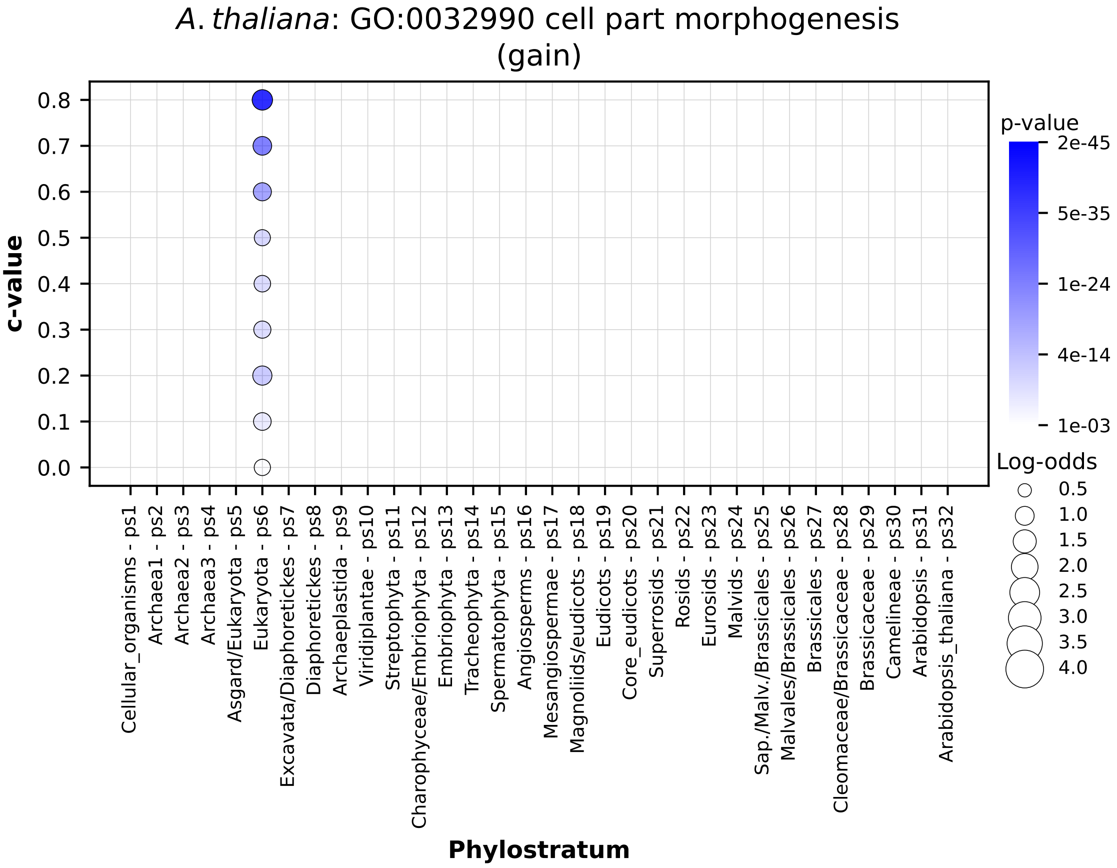

A. thaliana: GO:0032990 cell part morphogenesis
(gain)
c-value
0.8
0.7
0.6
0.5
0.4
0.3
0.2
0.1
0.0
Phylostratum
Cellular_organisms - ps1
Archaea1 - ps2
Archaea2 - ps3
Archaea3 - ps4
Asgard/Eukaryota - ps5
Eukaryota - ps6
Excavata/Diaphoretickes - ps7
Diaphoretickes - ps8
Archaeplastida - ps9
Viridiplantae - ps10
Streptophyta - ps11
Charophyceae/Embriophyta - ps12
Embriophyta - ps13
Tracheophyta - ps14
Spermatophyta - ps15
Angiosperms - ps16
Mesangiospermae - ps17
Magnoliids/eudicots - ps18
Eudicots - ps19
Core_eudicots - ps20
Superrosids - ps21
Rosids - ps22
Eurosids - ps23
Malvids - ps24
Sap./Malv./Brassicales - ps25
Malvales/Brassicales - ps26
Brassicales - ps27
Cleomaceae/Brassicaceae - ps28
Brassicaceae - ps29
Camelineae - ps30
Arabidopsis - ps31
Arabidopsis_thaliana - ps32
p-value
2e-45
5e-35
1e-24
4e-14
1e-03
Log-odds
0.5
1.0
1.5
2.0
2.5
3.0
3.5
4.0

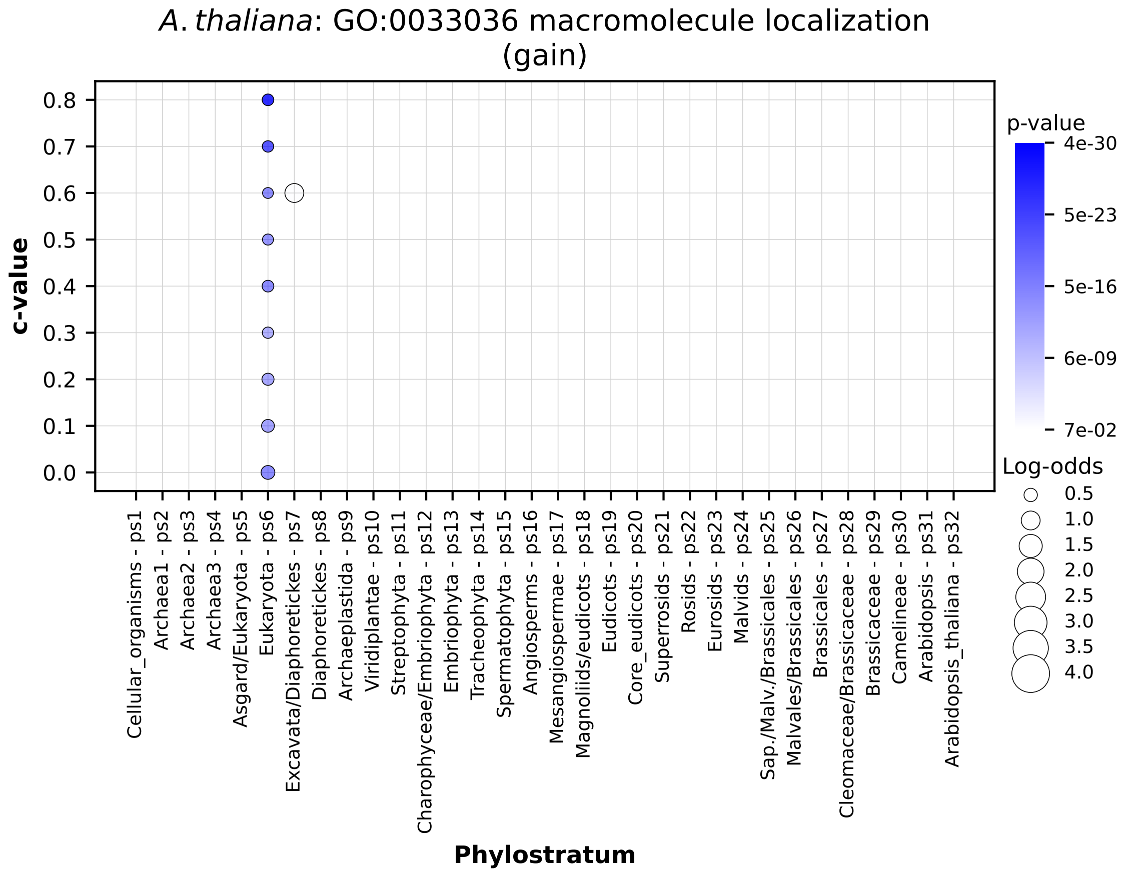

A. thaliana: GO:0033036 macromolecule localization
(gain)
c-value
0.8
0.7
0.6
0.5
0.4
0.3
0.2
0.1
0.0
p-value
4e-30
5e-23
5e-16
6e-09
7e-02
Log-odds
0.5
1.0
1.5
2.0
2.5
3.0
3.5
4.0
Cellular_organisms - ps1
Archaea1 - ps2
Archaea2 - ps3
Archaea3 - ps4
Asgard/Eukaryota - ps5
Eukaryota - ps6
Excavata/Diaphoretickes - ps7
Diaphoretickes - ps8
Archaeplastida - ps9
Viridiplantae - ps10
Streptophyta - ps11
Charophyceae/Embriophyta - ps12
Embriophyta - ps13
Tracheophyta - ps14
Spermatophyta - ps15
Angiosperms - ps16
Mesangiospermae - ps17
Magnoliids/eudicots - ps18
Eudicots - ps19
Core_eudicots - ps20
Superrosids - ps21
Rosids - ps22
Eurosids - ps23
Malvids - ps24
Sap./Malv./Brassicales - ps25
Malvales/Brassicales - ps26
Brassicales - ps27
Cleomaceae/Brassicaceae - ps28
Brassicaceae - ps29
Camelineae - ps30
Arabidopsis - ps31
Arabidopsis_thaliana - ps32
Phylostratum

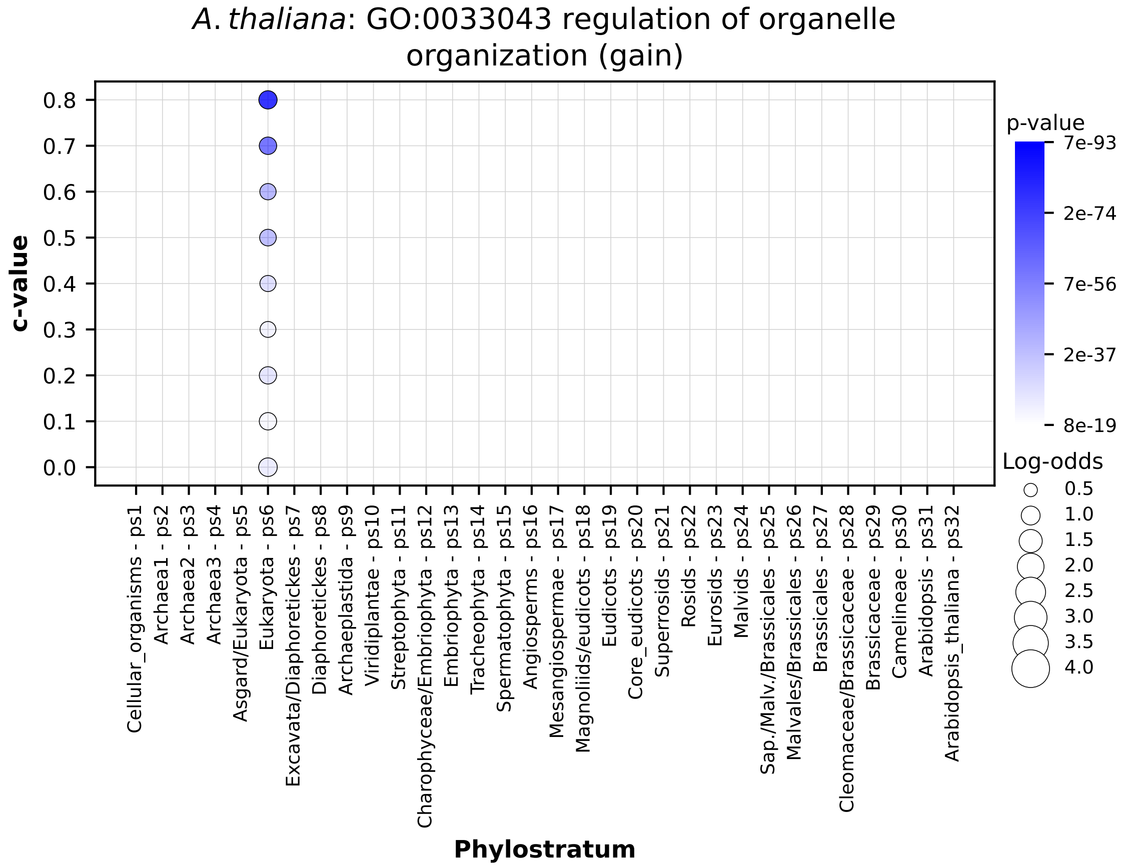

A. thaliana: GO:0033043 regulation of organelle organization (gain)
c-value
0.0
0.1
0.2
0.3
0.4
0.5
0.6
0.7
0.8
Phylostratum
Cellular_organisms - ps1
Archaea1 - ps2
Archaea2 - ps3
Archaea3 - ps4
Asgard/Eukaryota - ps5
Eukaryota - ps6
Excavata/Diaphoretickes - ps7
Diaphoretickes - ps8
Archaeplastida - ps9
Viridiplantae - ps10
Streptophyta - ps11
Charophyceae/Embriophyta - ps12
Embriophyta - ps13
Tracheophyta - ps14
Spermatophyta - ps15
Angiosperms - ps16
Mesangiospermae - ps17
Magnoliids/eudicots - ps18
Eudicots - ps19
Core_eudicots - ps20
Superrosids - ps21
Rosids - ps22
Eurosids - ps23
Malvids - ps24
Sap./Malv./Brassicales - ps25
Malvales/Brassicales - ps26
Brassicales - ps27
Cleomaceae/Brassicaceae - ps28
Brassicaceae - ps29
Camelineae - ps30
Arabidopsis - ps31
Arabidopsis_thaliana - ps32
p-value
7e-93
2e-74
7e-56
2e-37
8e-19
Log-odds
0.5
1.0
1.5
2.0
2.5
3.0
3.5
4.0

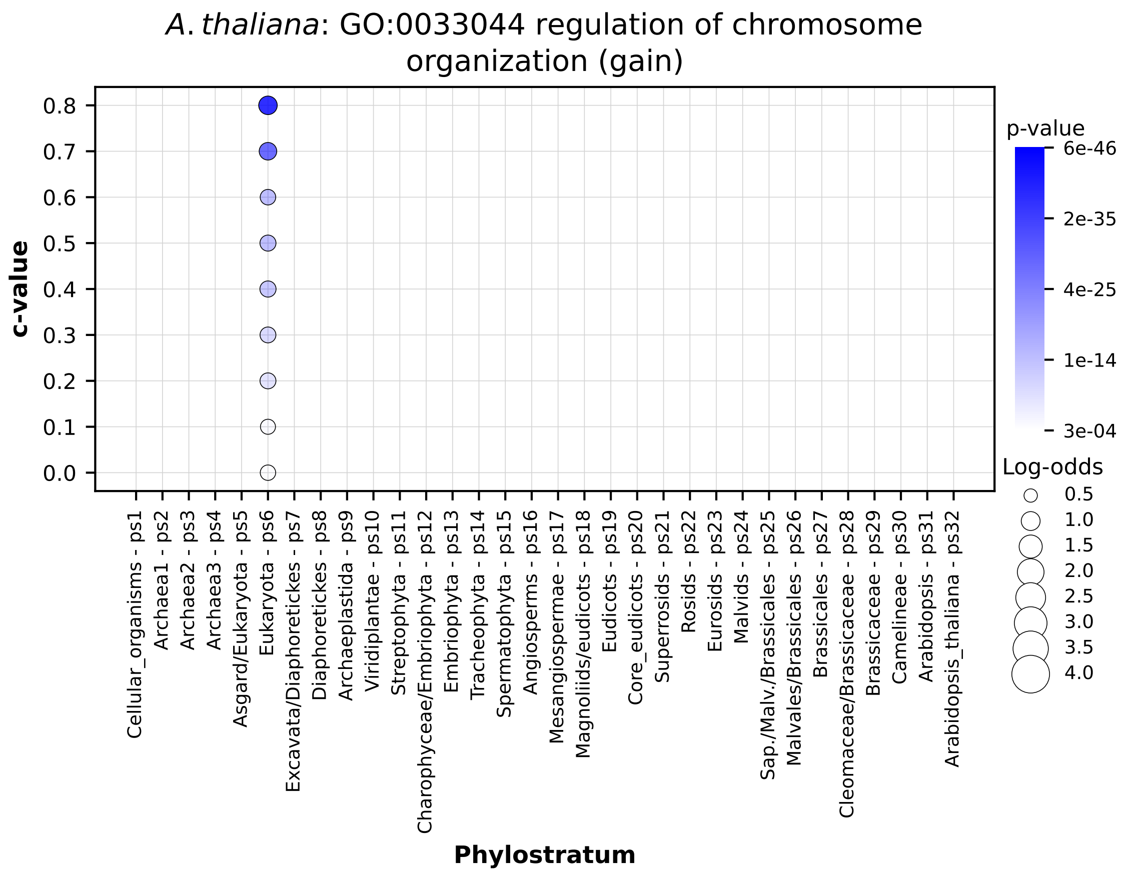
A. thaliana: GO:0033044 regulation of chromosome organization (gain)
c-value
0.8
0.7
0.6
0.5
0.4
0.3
0.2
0.1
0.0
Phylostratum
Cellular_organisms - ps1
Archaea1 - ps2
Archaea2 - ps3
Archaea3 - ps4
Asgard/Eukaryota - ps5
Eukaryota - ps6
Excavata/Diaphoretickes - ps7
Diaphoretickes - ps8
Archaeplastida - ps9
Viridiplantae - ps10
Streptophyta - ps11
Charophyceae/Embriophyta - ps12
Embriophyta - ps13
Tracheophyta - ps14
Spermatophyta - ps15
Angiosperms - ps16
Mesangiospermae - ps17
Magnoliids/eudicots - ps18
Eudicots - ps19
Core_eudicots - ps20
Superrosids - ps21
Rosids - ps22
Eurosids - ps23
Malvids - ps24
Sap./Malv./Brassicales - ps25
Malvales/Brassicales - ps26
Brassicales - ps27
Cleomaceae/Brassicaceae - ps28
Brassicaceae - ps29
Camelineae - ps30
Arabidopsis - ps31
Arabidopsis_thaliana - ps32
p-value
6e-46
2e-35
4e-25
1e-14
3e-04
Log-odds
0.5
1.0
1.5
2.0
2.5
3.0
3.5
4.0

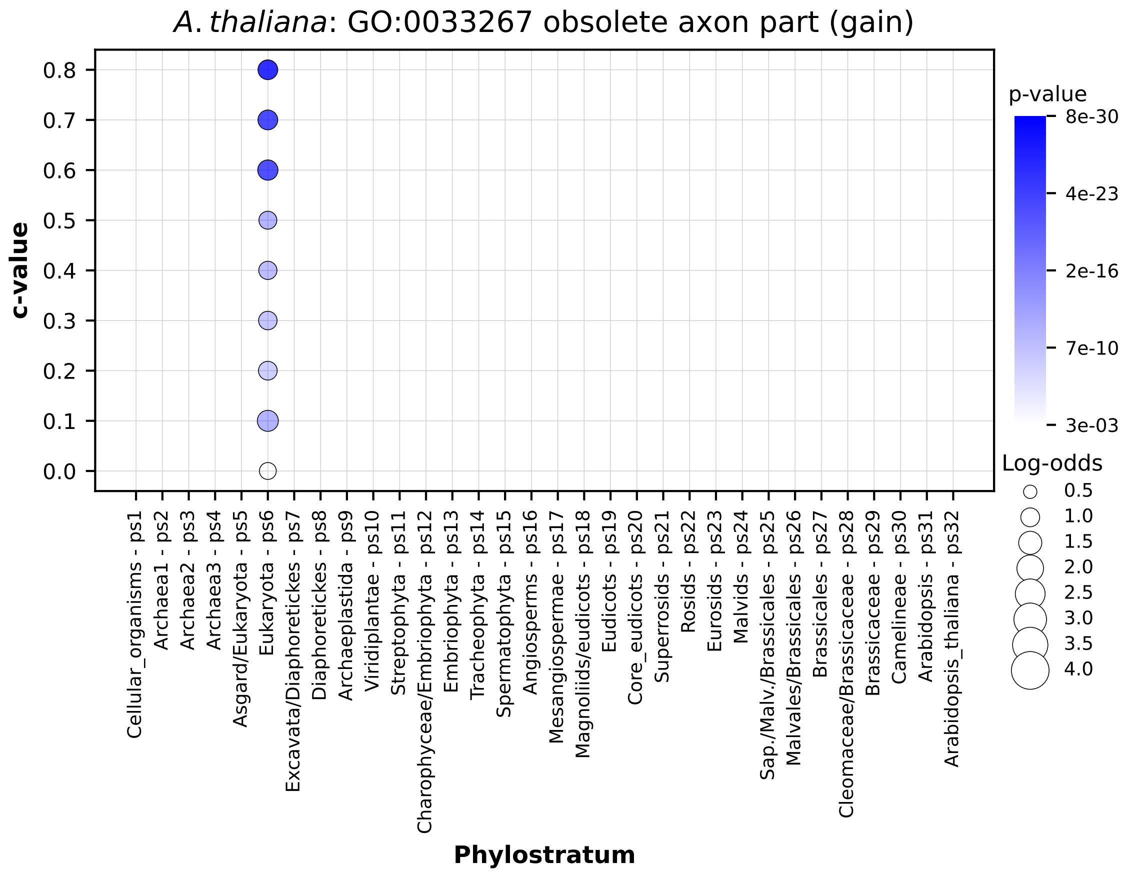
A. thaliana: GO:0033267 obsolete axon part (gain)
c-value
0.8
0.7
0.6
0.5
0.4
0.3
0.2
0.1
0.0
p-value
8e-30
4e-23
2e-16
7e-10
3e-03
Log-odds
0.5
1.0
1.5
2.0
2.5
3.0
3.5
4.0
Cellular_organisms - ps1
Archaea1 - ps2
Archaea2 - ps3
Archaea3 - ps4
Asgard/Eukaryota - ps5
Eukaryota - ps6
Excavata/Diaphoretickes - ps7
Diaphoretickes - ps8
Archaeplastida - ps9
Viridiplantae - ps10
Streptophyta - ps11
Charophyceae/Embriophyta - ps12
Embriophyta - ps13
Tracheophyta - ps14
Spermatophyta - ps15
Angiosperms - ps16
Mesangiospermae - ps17
Magnoliids/eudicots - ps18
Eudicots - ps19
Core_eudicots - ps20
Superrosids - ps21
Rosids - ps22
Eurosids - ps23
Malvids - ps24
Sap./Malv./Brassicales - ps25
Malvales/Brassicales - ps26
Brassicales - ps27
Cleomaceae/Brassicaceae - ps28
Brassicaceae - ps29
Camelineae - ps30
Arabidopsis - ps31
Arabidopsis_thaliana - ps32
Phylostratum

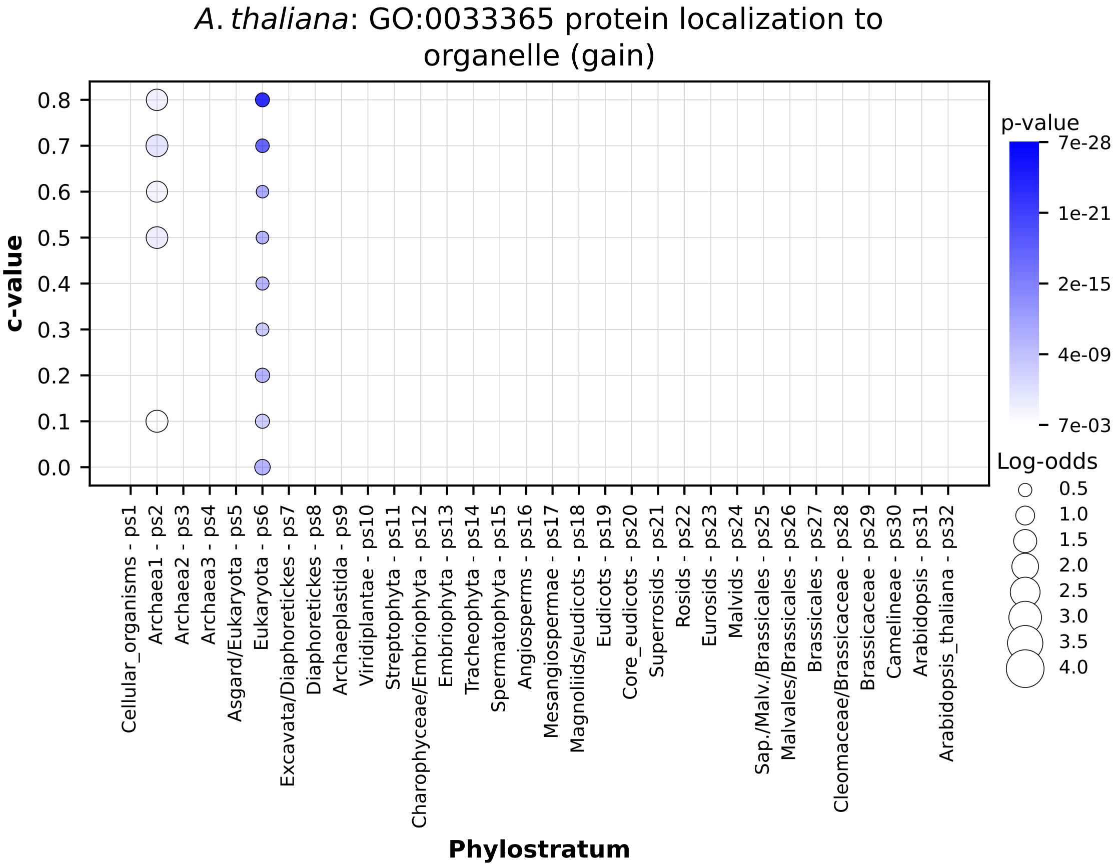

A. thaliana: GO:0033365 protein localization to organelle (gain)
c-value
0.8
0.7
0.6
0.5
0.4
0.3
0.2
0.1
0.0
Phylostratum
Cellular_organisms - ps1
Archaea1 - ps2
Archaea2 - ps3
Archaea3 - ps4
Asgard/Eukaryota - ps5
Eukaryota - ps6
Excavata/Diaphoretickes - ps7
Diaphoretickes - ps8
Archaeplastida - ps9
Viridiplantae - ps10
Streptophyta - ps11
Charophyceae/Embriophyta - ps12
Embriophyta - ps13
Tracheophyta - ps14
Spermatophyta - ps15
Angiosperms - ps16
Mesangiospermae - ps17
Magnoliids/eudicots - ps18
Eudicots - ps19
Core_eudicots - ps20
Superrosids - ps21
Rosids - ps22
Eurosids - ps23
Malvids - ps24
Sap./Malv./Brassicales - ps25
Malvales/Brassicales - ps26
Brassicales - ps27
Cleomaceae/Brassicaceae - ps28
Brassicaceae - ps29
Camelineae - ps30
Arabidopsis - ps31
Arabidopsis_thaliana - ps32
p-value
7e-28
1e-21
2e-15
4e-09
7e-03
Log-odds
0.5
1.0
1.5
2.0
2.5
3.0
3.5
4.0

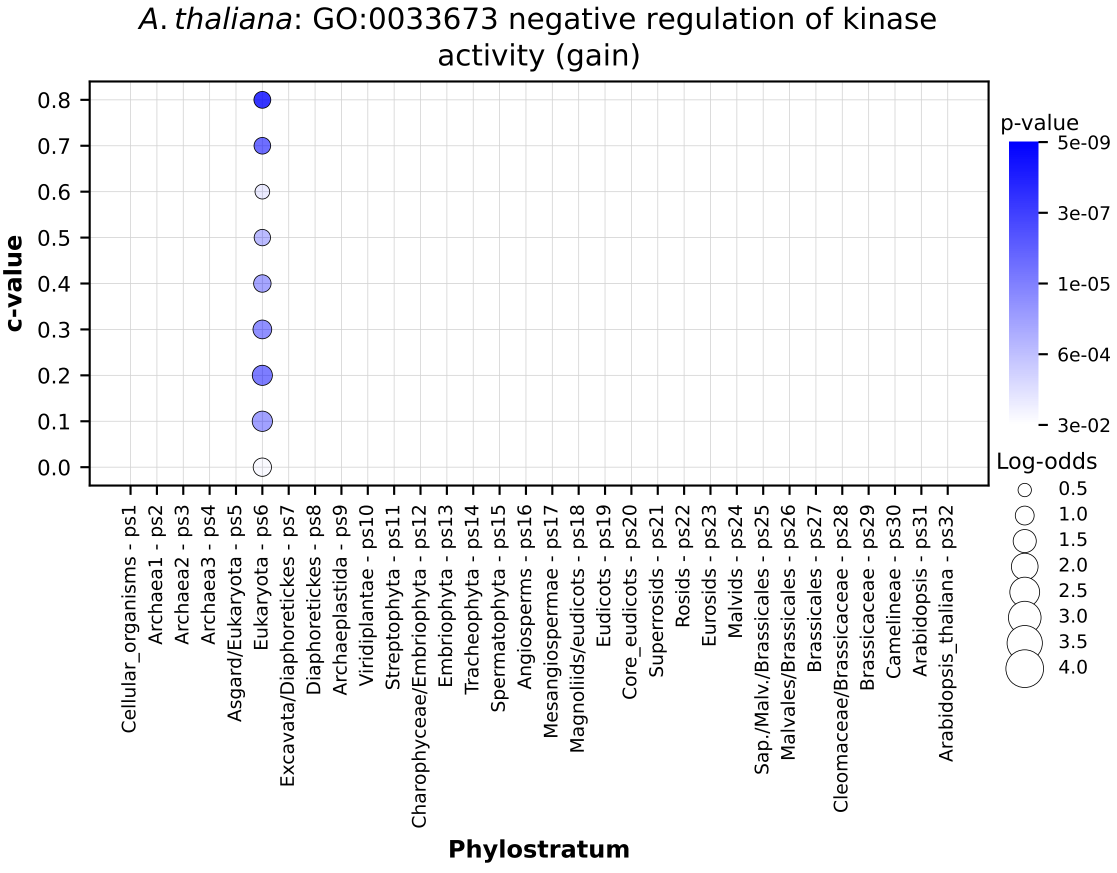
A. thaliana: GO:0033673 negative regulation of kinase activity (gain)
c-value
0.8
0.7
0.6
0.5
0.4
0.3
0.2
0.1
0.0
p-value
5e-09
3e-07
1e-05
6e-04
3e-02
Log-odds
0.5
1.0
1.5
2.0
2.5
3.0
3.5
4.0
Cellular_organisms - ps1
Archaea1 - ps2
Archaea2 - ps3
Archaea3 - ps4
Asgard/Eukaryota - ps5
Eukaryota - ps6
Excavata/Diaphoretickes - ps7
Diaphoretickes - ps8
Archaeplastida - ps9
Viridiplantae - ps10
Streptophyta - ps11
Charophyceae/Embriophyta - ps12
Embriophyta - ps13
Tracheophyta - ps14
Spermatophyta - ps15
Angiosperms - ps16
Mesangiospermae - ps17
Magnoliids/eudicots - ps18
Eudicots - ps19
Core_eudicots - ps20
Superrosids - ps21
Rosids - ps22
Eurosids - ps23
Malvids - ps24
Sap./Malv./Brassicales - ps25
Malvales/Brassicales - ps26
Brassicales - ps27
Cleomaceae/Brassicaceae - ps28
Brassicaceae - ps29
Camelineae - ps30
Arabidopsis - ps31
Arabidopsis_thaliana - ps32
Phylostratum

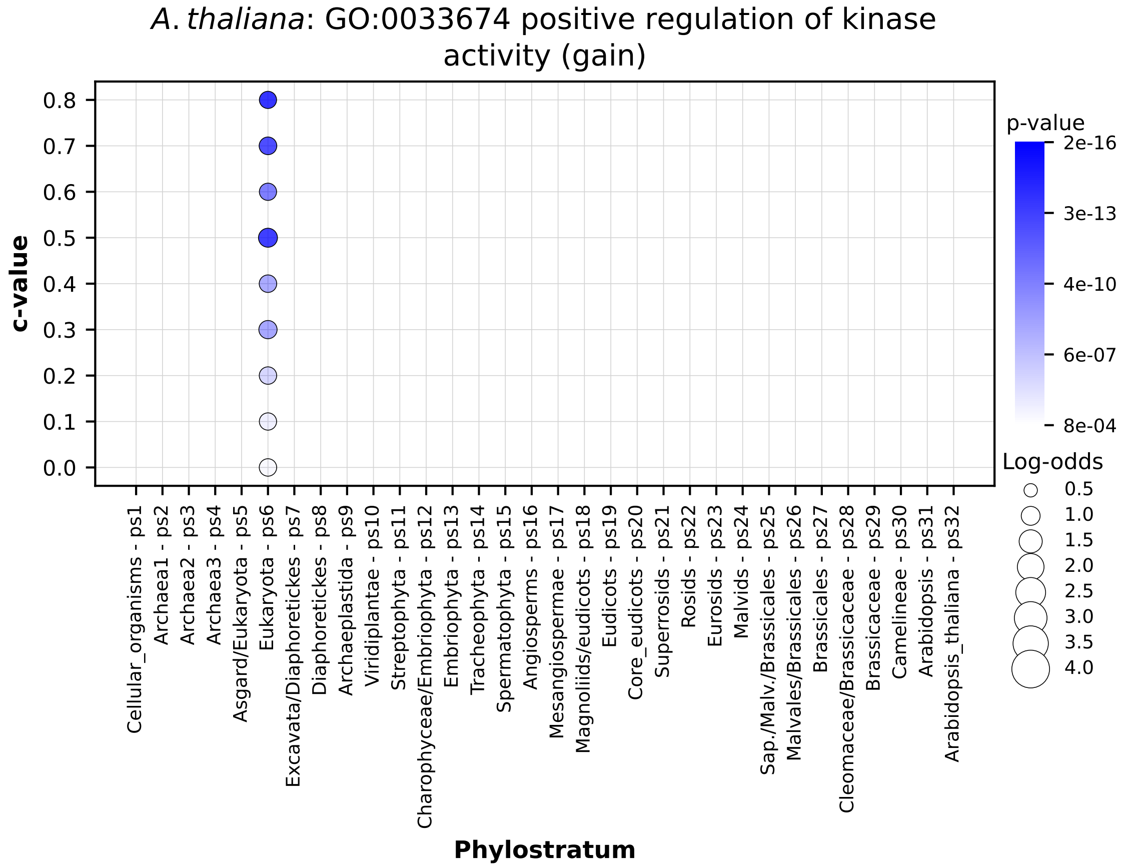

A. thaliana: GO:0033674 positive regulation of kinase activity (gain)
c-value
0.8
0.7
0.6
0.5
0.4
0.3
0.2
0.1
0.0
p-value
2e-16
3e-13
4e-10
6e-07
8e-04
Log-odds
0.5
1.0
1.5
2.0
2.5
3.0
3.5
4.0
Cellular_organisms - ps1
Archaea1 - ps2
Archaea2 - ps3
Archaea3 - ps4
Asgard/Eukaryota - ps5
Eukaryota - ps6
Excavata/Diaphoretickes - ps7
Diaphoretickes - ps8
Archaeplastida - ps9
Viridiplantae - ps10
Streptophyta - ps11
Charophyceae/Embriophyta - ps12
Embriophyta - ps13
Tracheophyta - ps14
Spermatophyta - ps15
Angiosperms - ps16
Mesangiospermae - ps17
Magnoliids/eudicots - ps18
Eudicots - ps19
Core_eudicots - ps20
Superrosids - ps21
Rosids - ps22
Eurosids - ps23
Malvids - ps24
Sap./Malv./Brassicales - ps25
Malvales/Brassicales - ps26
Brassicales - ps27
Cleomaceae/Brassicaceae - ps28
Brassicaceae - ps29
Camelineae - ps30
Arabidopsis - ps31
Arabidopsis_thaliana - ps32
Phylostratum

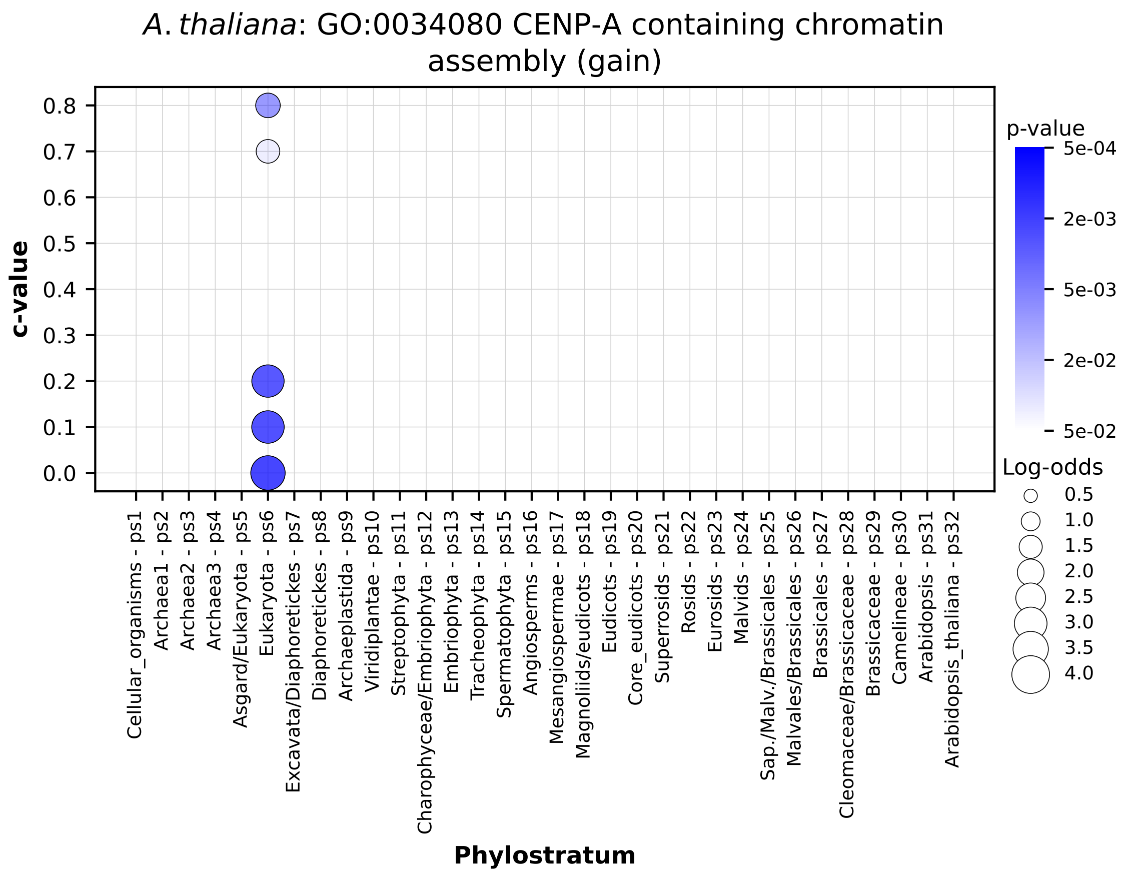

A. thaliana: GO:0034080 CENP-A containing chromatin assembly (gain)
c-value
0.0
0.1
0.2
0.3
0.4
0.5
0.6
0.7
0.8
p-value
5e-04
2e-03
5e-03
2e-02
5e-02
Log-odds
0.5
1.0
1.5
2.0
2.5
3.0
3.5
4.0
Cellular_organisms - ps1
Archaea1 - ps2
Archaea2 - ps3
Archaea3 - ps4
Asgard/Eukaryota - ps5
Eukaryota - ps6
Excavata/Diaphoretickes - ps7
Diaphoretickes - ps8
Archaeplastida - ps9
Viridiplantae - ps10
Streptophyta - ps11
Charophyceae/Embriophyta - ps12
Embriophyta - ps13
Tracheophyta - ps14
Spermatophyta - ps15
Angiosperms - ps16
Mesangiospermae - ps17
Magnoliids/eudicots - ps18
Eudicots - ps19
Core_eudicots - ps20
Superrosids - ps21
Rosids - ps22
Eurosids - ps23
Malvids - ps24
Sap./Malv./Brassicales - ps25
Malvales/Brassicales - ps26
Brassicales - ps27
Cleomaceae/Brassicaceae - ps28
Brassicaceae - ps29
Camelineae - ps30
Arabidopsis - ps31
Arabidopsis_thaliana - ps32
Phylostratum

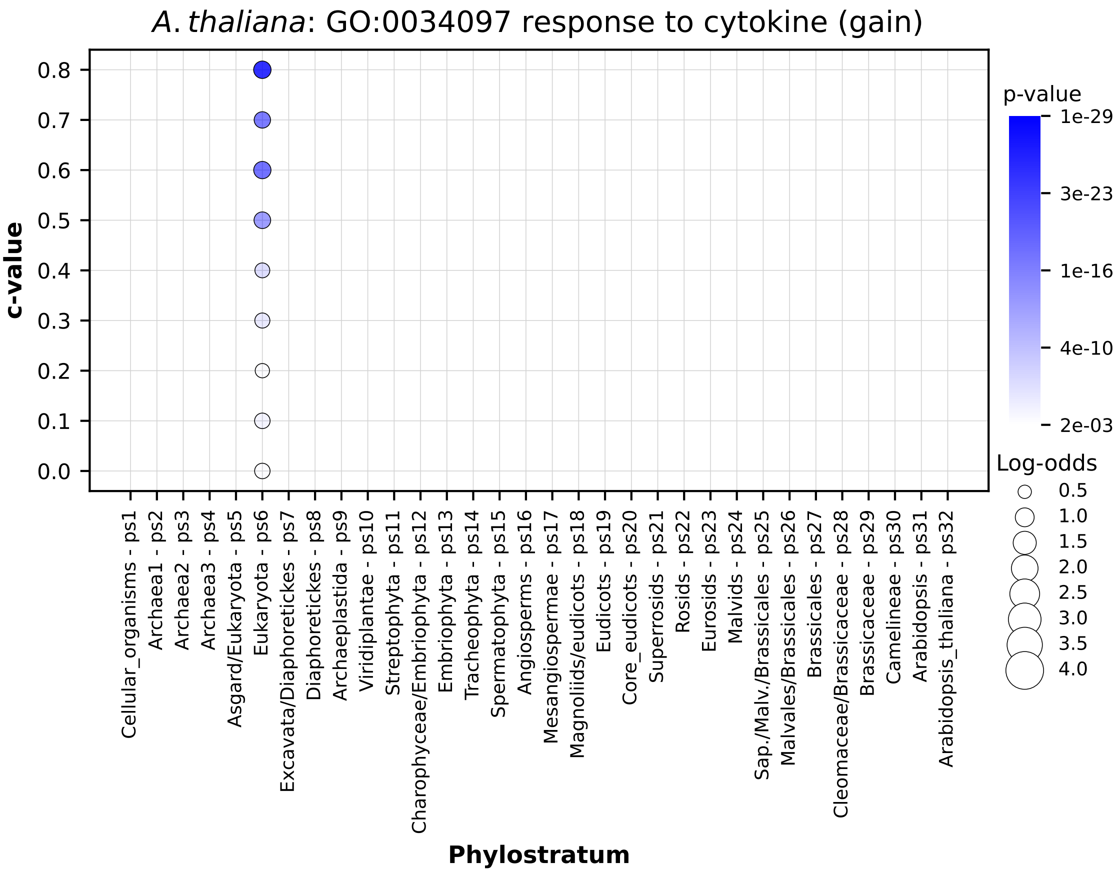
A. thaliana: GO:0034097 response to cytokine (gain)
c-value
0.0
0.1
0.2
0.3
0.4
0.5
0.6
0.7
0.8
Phylostratum
Cellular_organisms - ps1
Archaea1 - ps2
Archaea2 - ps3
Archaea3 - ps4
Asgard/Eukaryota - ps5
Eukaryota - ps6
Excavata/Diaphoretickes - ps7
Diaphoretickes - ps8
Archaeplastida - ps9
Viridiplantae - ps10
Streptophyta - ps11
Charophyceae/Embriophyta - ps12
Embriophyta - ps13
Tracheophyta - ps14
Spermatophyta - ps15
Angiosperms - ps16
Mesangiospermae - ps17
Magnoliids/eudicots - ps18
Eudicots - ps19
Core_eudicots - ps20
Superrosids - ps21
Rosids - ps22
Eurosids - ps23
Malvids - ps24
Sap./Malv./Brassicales - ps25
Malvales/Brassicales - ps26
Brassicales - ps27
Cleomaceae/Brassicaceae - ps28
Brassicaceae - ps29
Camelineae - ps30
Arabidopsis - ps31
Arabidopsis_thaliana - ps32
p-value
1e-29
3e-23
1e-16
4e-10
2e-03
Log-odds
0.5
1.0
1.5
2.0
2.5
3.0
3.5
4.0

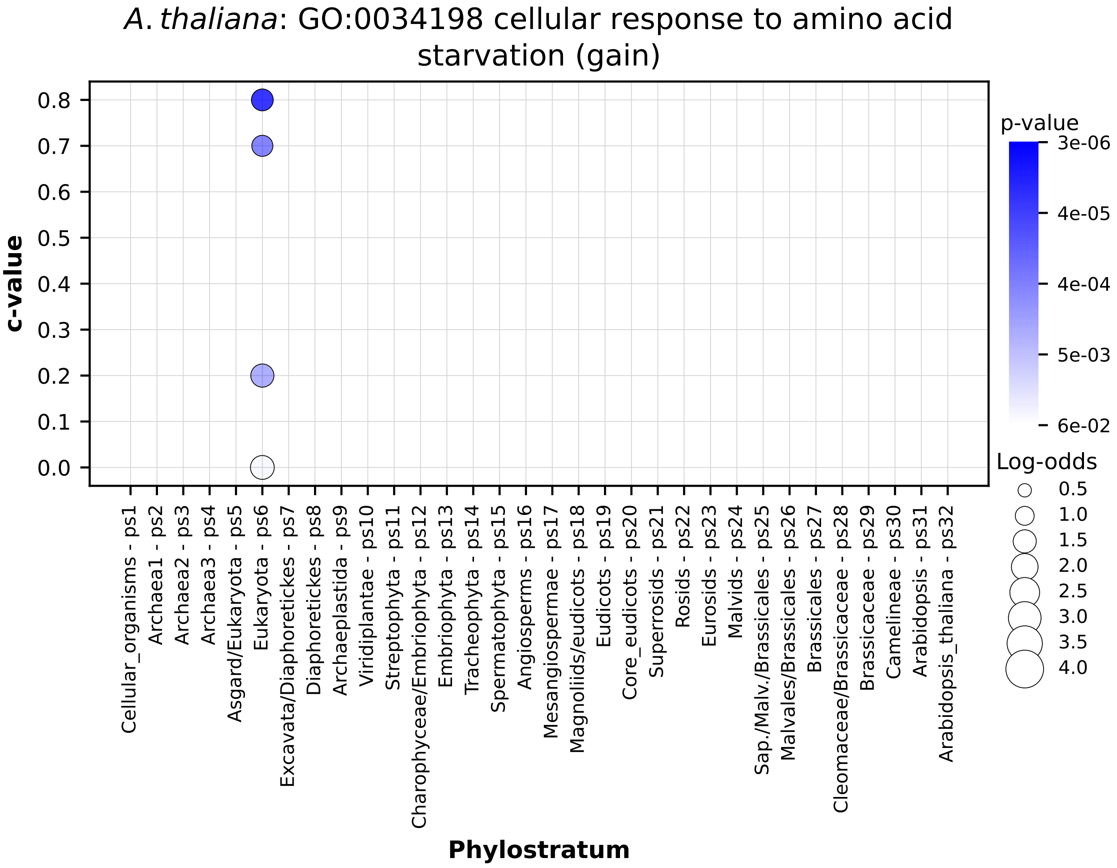

A. thaliana: GO:0034198 cellular response to amino acid starvation (gain)
c-value
0.8
0.7
0.6
0.5
0.4
0.3
0.2
0.1
0.0
p-value
3e-06
4e-05
4e-04
5e-03
6e-02
Log-odds
0.5
1.0
1.5
2.0
2.5
3.0
3.5
4.0
Cellular_organisms - ps1
Archaea1 - ps2
Archaea2 - ps3
Archaea3 - ps4
Asgard/Eukaryota - ps5
Eukaryota - ps6
Excavata/Diaphoretickes - ps7
Diaphoretickes - ps8
Archaeplastida - ps9
Viridiplantae - ps10
Streptophyta - ps11
Charophyceae/Embriophyta - ps12
Embriophyta - ps13
Tracheophyta - ps14
Spermatophyta - ps15
Angiosperms - ps16
Mesangiospermae - ps17
Magnoliids/eudicots - ps18
Eudicots - ps19
Core_eudicots - ps20
Superrosids - ps21
Rosids - ps22
Eurosids - ps23
Malvids - ps24
Sap./Malv./Brassicales - ps25
Malvales/Brassicales - ps26
Brassicales - ps27
Cleomaceae/Brassicaceae - ps28
Brassicaceae - ps29
Camelineae - ps30
Arabidopsis - ps31
Arabidopsis_thaliana - ps32
Phylostratum

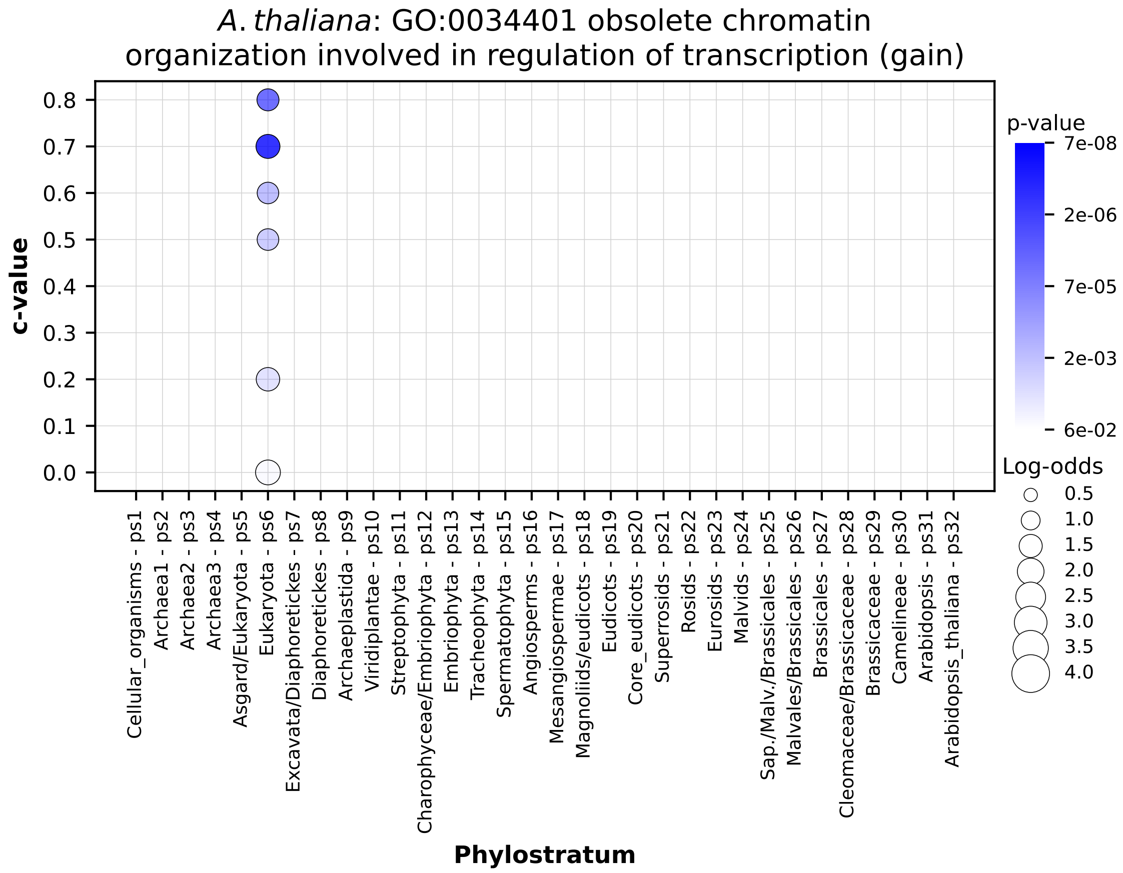

A. thaliana: GO:0034401 obsolete chromatin organization involved in regulation of transcription (gain)
c-value
0.8
0.7
0.6
0.5
0.4
0.3
0.2
0.1
0.0
p-value
7e-08
2e-06
7e-05
2e-03
6e-02
Log-odds
0.5
1.0
1.5
2.0
2.5
3.0
3.5
4.0
Cellular_organisms - ps1
Archaea1 - ps2
Archaea2 - ps3
Archaea3 - ps4
Asgard/Eukaryota - ps5
Eukaryota - ps6
Excavata/Diaphoretickes - ps7
Diaphoretickes - ps8
Archaeplastida - ps9
Viridiplantae - ps10
Streptophyta - ps11
Charophyceae/Embriophyta - ps12
Embriophyta - ps13
Tracheophyta - ps14
Spermatophyta - ps15
Angiosperms - ps16
Mesangiospermae - ps17
Magnoliids/eudicots - ps18
Eudicots - ps19
Core_eudicots - ps20
Superrosids - ps21
Rosids - ps22
Eurosids - ps23
Malvids - ps24
Sap./Malv./Brassicales - ps25
Malvales/Brassicales - ps26
Brassicales - ps27
Cleomaceae/Brassicaceae - ps28
Brassicaceae - ps29
Camelineae - ps30
Arabidopsis - ps31
Arabidopsis_thaliana - ps32
Phylostratum

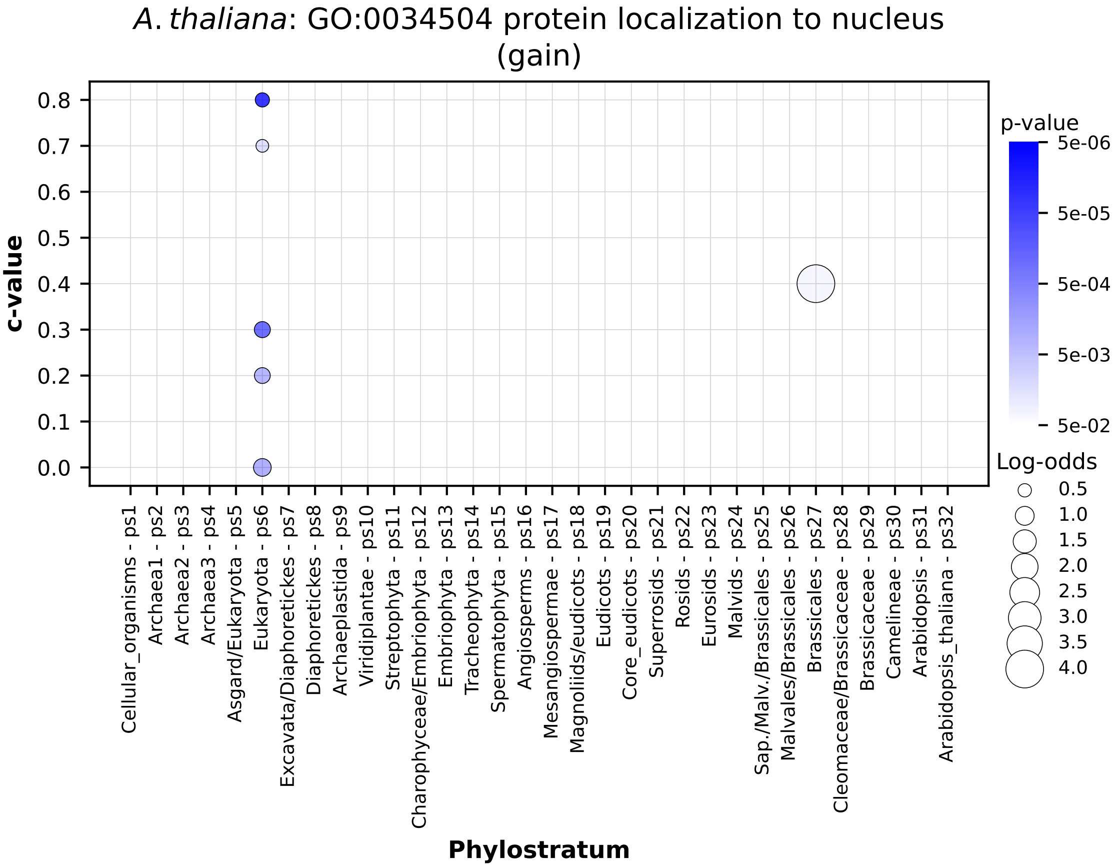
A. thaliana: GO:0034504 protein localization to nucleus
(gain)
c-value
0.8
0.7
0.6
0.5
0.4
0.3
0.2
0.1
0.0
p-value
5e-06
5e-05
5e-04
5e-03
5e-02
Log-odds
0.5
1.0
1.5
2.0
2.5
3.0
3.5
4.0
Cellular_organisms - ps1
Archaea1 - ps2
Archaea2 - ps3
Archaea3 - ps4
Asgard/Eukaryota - ps5
Eukaryota - ps6
Excavata/Diaphoretickes - ps7
Diaphoretickes - ps8
Archaeplastida - ps9
Viridiplantae - ps10
Streptophyta - ps11
Charophyceae/Embriophyta - ps12
Embriophyta - ps13
Tracheophyta - ps14
Spermatophyta - ps15
Angiosperms - ps16
Mesangiospermae - ps17
Magnoliids/eudicots - ps18
Eudicots - ps19
Core_eudicots - ps20
Superrosids - ps21
Rosids - ps22
Eurosids - ps23
Malvids - ps24
Sap./Malv./Brassicales - ps25
Malvales/Brassicales - ps26
Brassicales - ps27
Cleomaceae/Brassicaceae - ps28
Brassicaceae - ps29
Camelineae - ps30
Arabidopsis - ps31
Arabidopsis_thaliana - ps32
Phylostratum

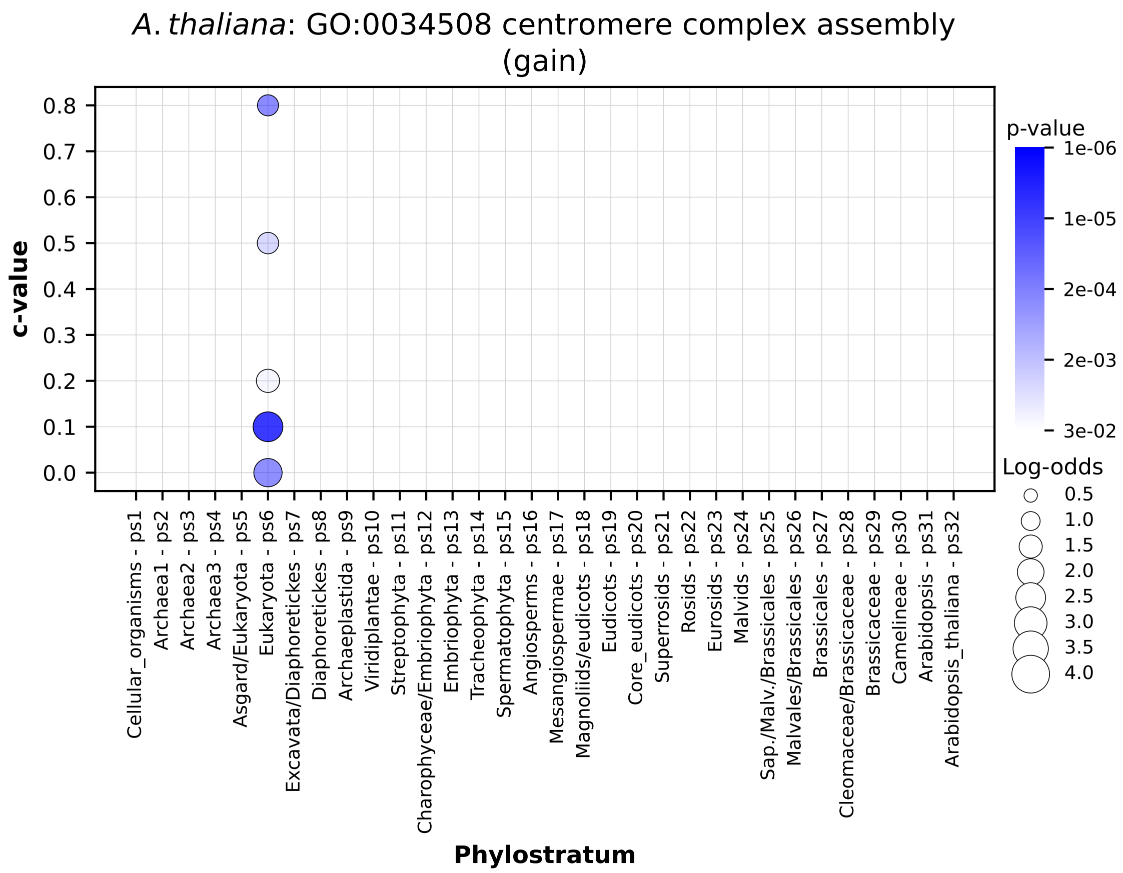

A. thaliana: GO:0034508 centromere complex assembly (gain)
c-value
0.0
0.1
0.2
0.3
0.4
0.5
0.6
0.7
0.8
p-value
1e-06
1e-05
2e-04
2e-03
3e-02
Log-odds
0.5
1.0
1.5
2.0
2.5
3.0
3.5
4.0
Cellular_organisms - ps1
Archaea1 - ps2
Archaea2 - ps3
Archaea3 - ps4
Asgard/Eukaryota - ps5
Eukaryota - ps6
Excavata/Diaphoretickes - ps7
Diaphoretickes - ps8
Archaeplastida - ps9
Viridiplantae - ps10
Streptophyta - ps11
Charophyceae/Embriophyta - ps12
Embriophyta - ps13
Tracheophyta - ps14
Spermatophyta - ps15
Angiosperms - ps16
Mesangiospermae - ps17
Magnoliids/eudicots - ps18
Eudicots - ps19
Core_eudicots - ps20
Superrosids - ps21
Rosids - ps22
Eurosids - ps23
Malvids - ps24
Sap./Malv./Brassicales - ps25
Malvales/Brassicales - ps26
Brassicales - ps27
Cleomaceae/Brassicaceae - ps28
Brassicaceae - ps29
Camelineae - ps30
Arabidopsis - ps31
Arabidopsis_thaliana - ps32
Phylostratum

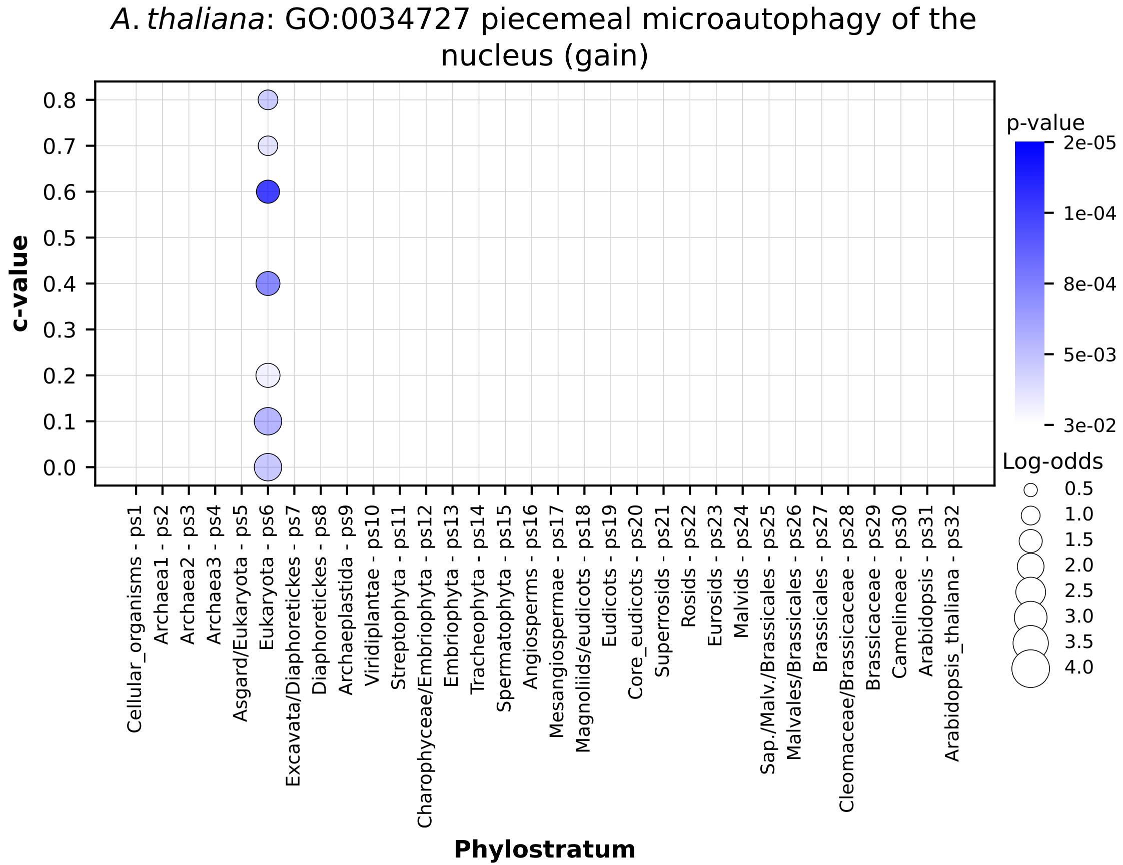

A. thaliana: GO:0034727 piecemeal microautophagy of the nucleus (gain)
c-value
0.8
0.7
0.6
0.5
0.4
0.3
0.2
0.1
0.0
Phylostratum
Cellular_organisms - ps1
Archaea1 - ps2
Archaea2 - ps3
Archaea3 - ps4
Asgard/Eukaryota - ps5
Eukaryota - ps6
Excavata/Diaphoretickes - ps7
Diaphoretickes - ps8
Archaeplastida - ps9
Viridiplantae - ps10
Streptophyta - ps11
Charophyceae/Embriophyta - ps12
Embriophyta - ps13
Tracheophyta - ps14
Spermatophyta - ps15
Angiosperms - ps16
Mesangiospermae - ps17
Magnoliids/eudicots - ps18
Eudicots - ps19
Core_eudicots - ps20
Superrosids - ps21
Rosids - ps22
Eurosids - ps23
Malvids - ps24
Sap./Malv./Brassicales - ps25
Malvales/Brassicales - ps26
Brassicales - ps27
Cleomaceae/Brassicaceae - ps28
Brassicaceae - ps29
Camelineae - ps30
Arabidopsis - ps31
Arabidopsis_thaliana - ps32
p-value
2e-05
1e-04
8e-04
5e-03
3e-02
Log-odds
0.5
1.0
1.5
2.0
2.5
3.0
3.5
4.0

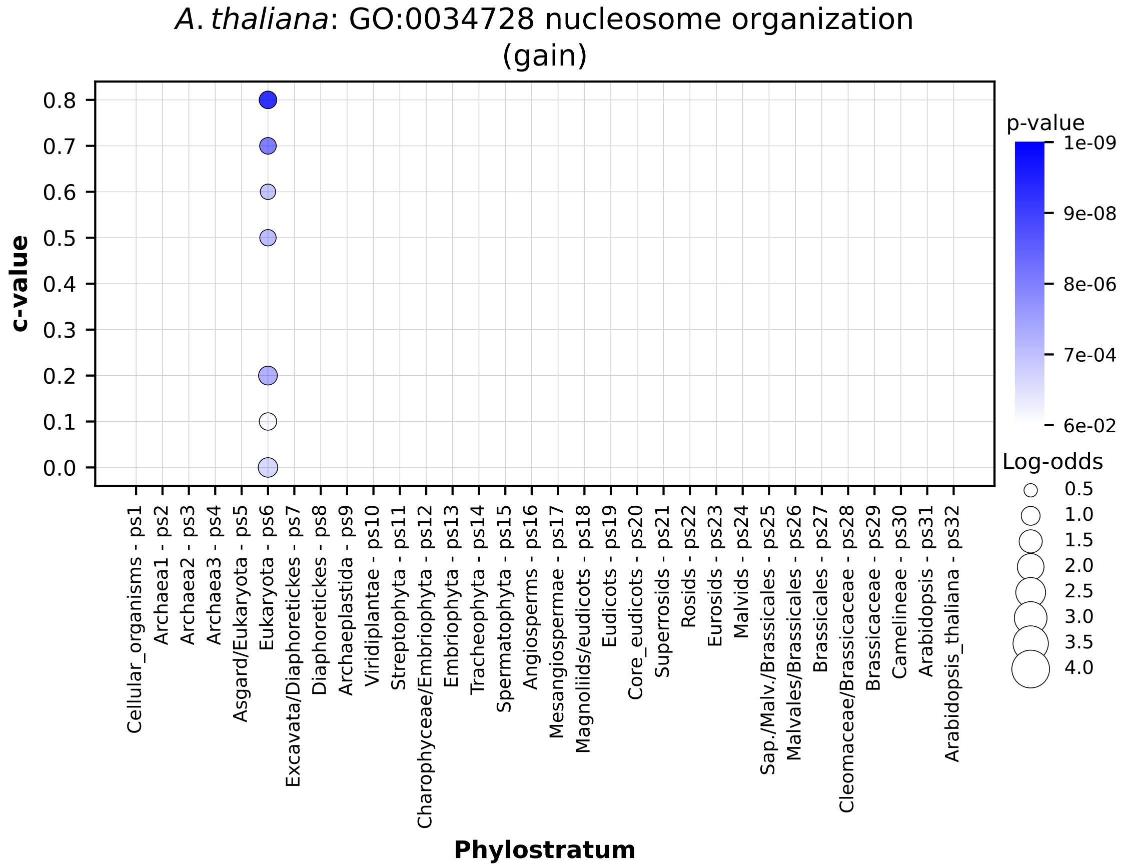
A. thaliana: GO:0034728 nucleosome organization
(gain)
c-value
0.0
0.1
0.2
0.3
0.4
0.5
0.6
0.7
0.8
p-value
1e-09
9e-08
8e-06
7e-04
6e-02
Log-odds
0.5
1.0
1.5
2.0
2.5
3.0
3.5
4.0
Cellular_organisms - ps1
Archaea1 - ps2
Archaea2 - ps3
Archaea3 - ps4
Asgard/Eukaryota - ps5
Eukaryota - ps6
Excavata/Diaphoretickes - ps7
Diaphoretickes - ps8
Archaeplastida - ps9
Viridiplantae - ps10
Streptophyta - ps11
Charophyceae/Embriophyta - ps12
Embriophyta - ps13
Tracheophyta - ps14
Spermatophyta - ps15
Angiosperms - ps16
Mesangiospermae - ps17
Magnoliids/eudicots - ps18
Eudicots - ps19
Core_eudicots - ps20
Superrosids - ps21
Rosids - ps22
Eurosids - ps23
Malvids - ps24
Sap./Malv./Brassicales - ps25
Malvales/Brassicales - ps26
Brassicales - ps27
Cleomaceae/Brassicaceae - ps28
Brassicaceae - ps29
Camelineae - ps30
Arabidopsis - ps31
Arabidopsis_thaliana - ps32
Phylostratum

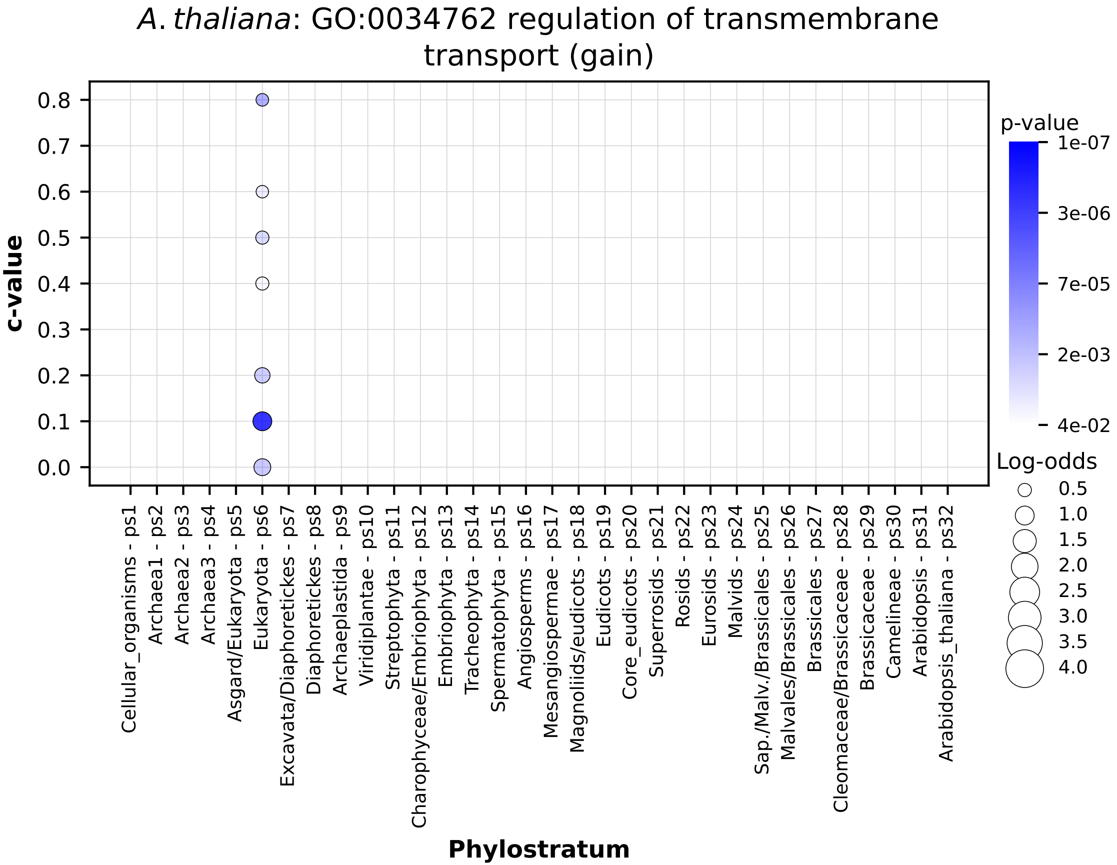
A. thaliana: GO:0034762 regulation of transmembrane transport (gain)
c-value
0.0
0.1
0.2
0.3
0.4
0.5
0.6
0.7
0.8
Phylostratum
Cellular_organisms - ps1
Archaea1 - ps2
Archaea2 - ps3
Archaea3 - ps4
Asgard/Eukaryota - ps5
Eukaryota - ps6
Excavata/Diaphoretickes - ps7
Diaphoretickes - ps8
Archaeplastida - ps9
Viridiplantae - ps10
Streptophyta - ps11
Charophyceae/Embriophyta - ps12
Embriophyta - ps13
Tracheophyta - ps14
Spermatophyta - ps15
Angiosperms - ps16
Mesangiospermae - ps17
Magnoliids/eudicots - ps18
Eudicots - ps19
Core_eudicots - ps20
Superrosids - ps21
Rosids - ps22
Eurosids - ps23
Malvids - ps24
Sap./Malv./Brassicales - ps25
Malvales/Brassicales - ps26
Brassicales - ps27
Cleomaceae/Brassicaceae - ps28
Brassicaceae - ps29
Camelineae - ps30
Arabidopsis - ps31
Arabidopsis_thaliana - ps32
p-value
1e-07
3e-06
7e-05
2e-03
4e-02
Log-odds
0.5
1.0
1.5
2.0
2.5
3.0
3.5
4.0

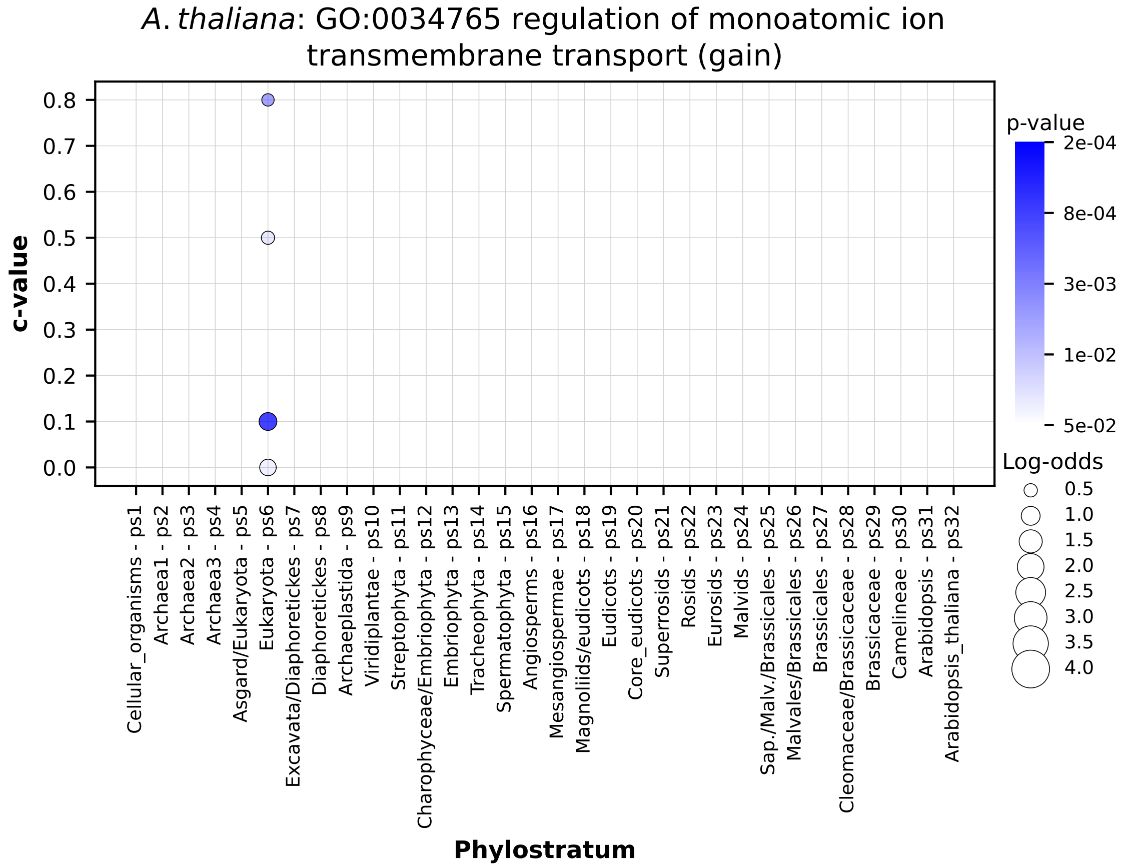

A. thaliana: GO:0034765 regulation of monoatomic ion transmembrane transport (gain)
c-value
0.8
0.7
0.6
0.5
0.4
0.3
0.2
0.1
0.0
Phylostratum
Cellular_organisms - ps1
Archaea1 - ps2
Archaea2 - ps3
Archaea3 - ps4
Asgard/Eukaryota - ps5
Eukaryota - ps6
Excavata/Diaphoretickes - ps7
Diaphoretickes - ps8
Archaeplastida - ps9
Viridiplantae - ps10
Streptophyta - ps11
Charophyceae/Embriophyta - ps12
Embriophyta - ps13
Tracheophyta - ps14
Spermatophyta - ps15
Angiosperms - ps16
Mesangiospermae - ps17
Magnoliids/eudicots - ps18
Eudicots - ps19
Core_eudicots - ps20
Superrosids - ps21
Rosids - ps22
Eurosids - ps23
Malvids - ps24
Sap./Malv./Brassicales - ps25
Malvales/Brassicales - ps26
Brassicales - ps27
Cleomaceae/Brassicaceae - ps28
Brassicaceae - ps29
Camelineae - ps30
Arabidopsis - ps31
Arabidopsis_thaliana - ps32
p-value
2e-04
8e-04
3e-03
1e-02
5e-02
Log-odds
0.5
1.0
1.5
2.0
2.5
3.0
3.5
4.0

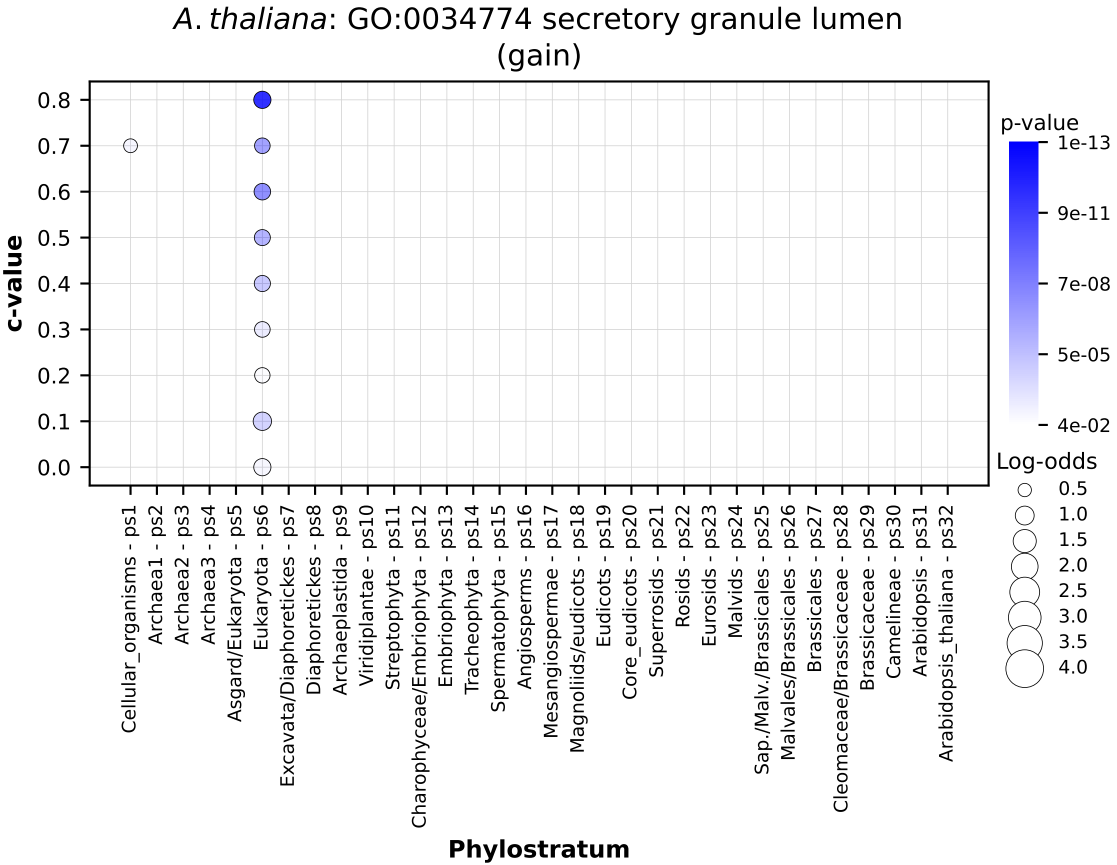

A. thaliana: GO:0034774 secretory granule lumen
(gain)
c-value
0.8
0.7
0.6
0.5
0.4
0.3
0.2
0.1
0.0
p-value
1e-13
9e-11
7e-08
5e-05
4e-02
Log-odds
0.5
1.0
1.5
2.0
2.5
3.0
3.5
4.0
Cellular_organisms - ps1
Archaea1 - ps2
Archaea2 - ps3
Archaea3 - ps4
Asgard/Eukaryota - ps5
Eukaryota - ps6
Excavata/Diaphoretickes - ps7
Diaphoretickes - ps8
Archaeplastida - ps9
Viridiplantae - ps10
Streptophyta - ps11
Charophyceae/Embriophyta - ps12
Embriophyta - ps13
Tracheophyta - ps14
Spermatophyta - ps15
Angiosperms - ps16
Mesangiospermae - ps17
Magnoliids/eudicots - ps18
Eudicots - ps19
Core_eudicots - ps20
Superrosids - ps21
Rosids - ps22
Eurosids - ps23
Malvids - ps24
Sap./Malv./Brassicales - ps25
Malvales/Brassicales - ps26
Brassicales - ps27
Cleomaceae/Brassicaceae - ps28
Brassicaceae - ps29
Camelineae - ps30
Arabidopsis - ps31
Arabidopsis_thaliana - ps32
Phylostratum

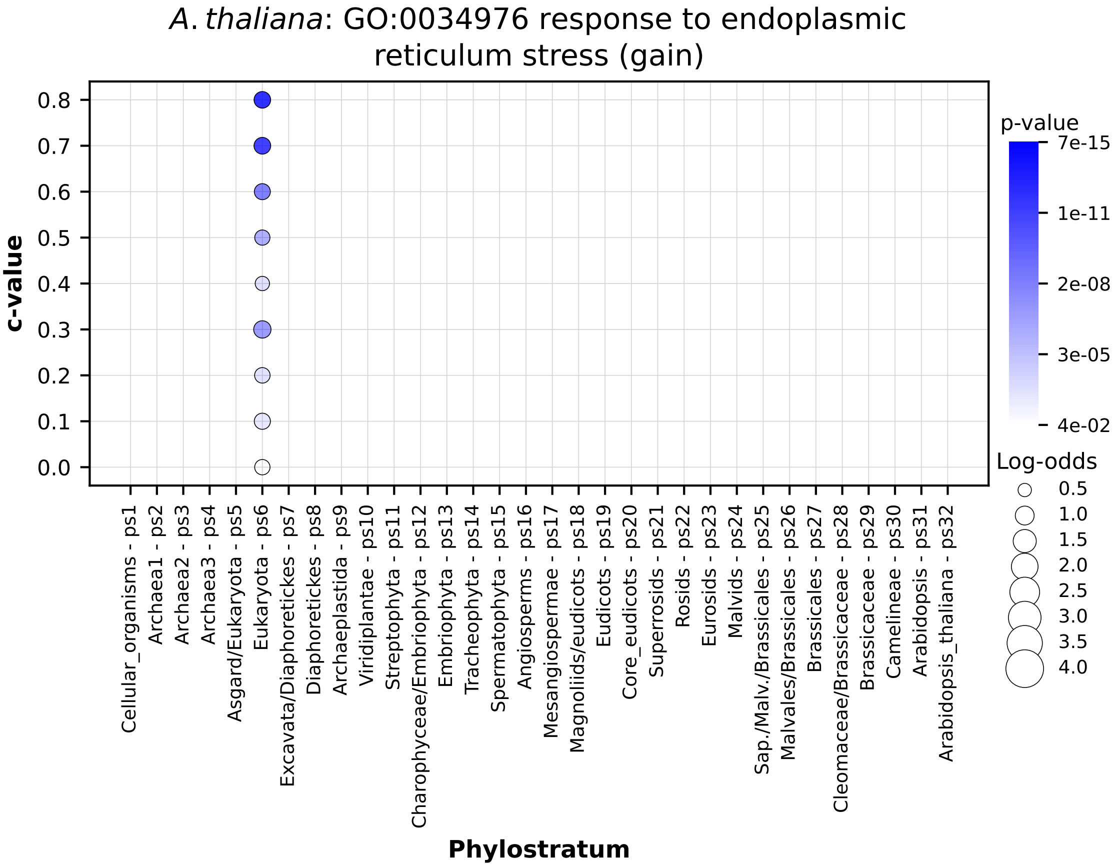

A. thaliana: GO:0034976 response to endoplasmic reticulum stress (gain)
c-value
0.8
0.7
0.6
0.5
0.4
0.3
0.2
0.1
0.0
p-value
7e-15
1e-11
2e-08
3e-05
4e-02
Log-odds
0.5
1.0
1.5
2.0
2.5
3.0
3.5
4.0
Cellular_organisms - ps1
Archaea1 - ps2
Archaea2 - ps3
Archaea3 - ps4
Asgard/Eukaryota - ps5
Eukaryota - ps6
Excavata/Diaphoretickes - ps7
Diaphoretickes - ps8
Archaeplastida - ps9
Viridiplantae - ps10
Streptophyta - ps11
Charophyceae/Embriophyta - ps12
Embriophyta - ps13
Tracheophyta - ps14
Spermatophyta - ps15
Angiosperms - ps16
Mesangiospermae - ps17
Magnoliids/eudicots - ps18
Eudicots - ps19
Core_eudicots - ps20
Superrosids - ps21
Rosids - ps22
Eurosids - ps23
Malvids - ps24
Sap./Malv./Brassicales - ps25
Malvales/Brassicales - ps26
Brassicales - ps27
Cleomaceae/Brassicaceae - ps28
Brassicaceae - ps29
Camelineae - ps30
Arabidopsis - ps31
Arabidopsis_thaliana - ps32
Phylostratum

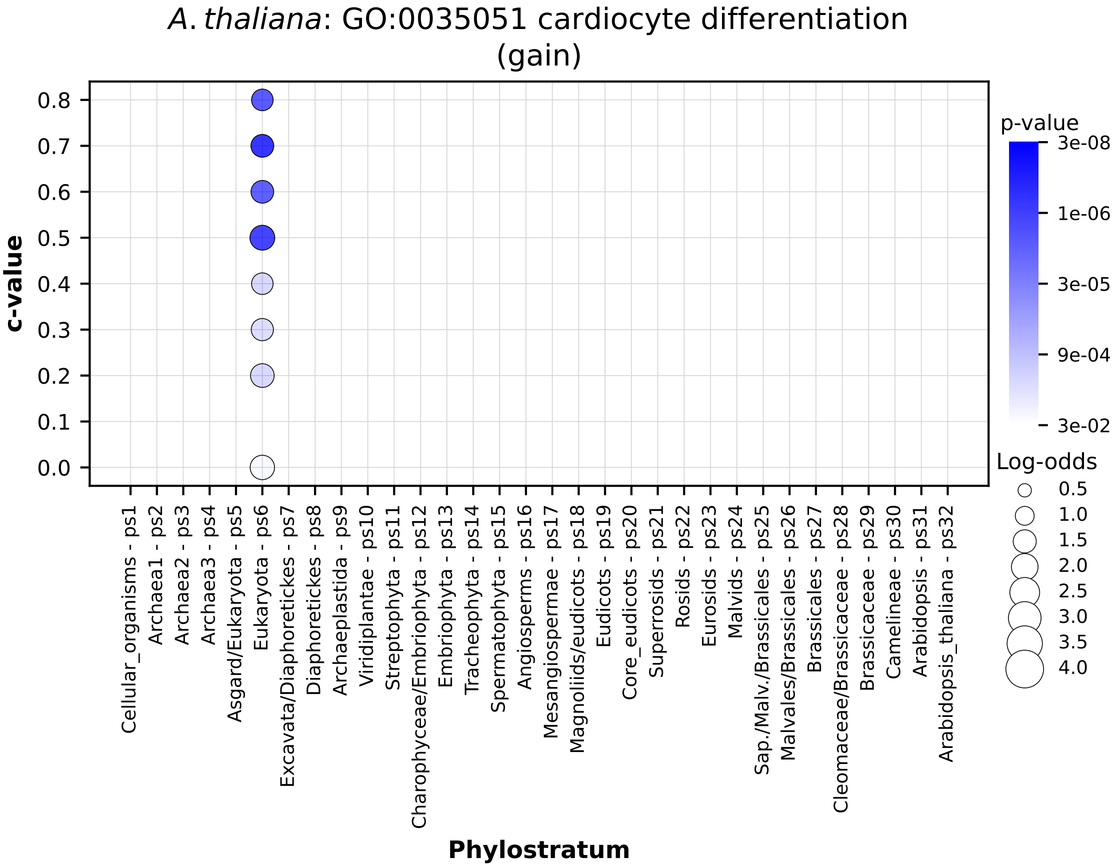

A. thaliana: GO:0035051 cardiocyte differentiation
(gain)
c-value
0.8
0.7
0.6
0.5
0.4
0.3
0.2
0.1
0.0
p-value
3e-08
1e-06
3e-05
9e-04
3e-02
Log-odds
0.5
1.0
1.5
2.0
2.5
3.0
3.5
4.0
Cellular_organisms - ps1
Archaea1 - ps2
Archaea2 - ps3
Archaea3 - ps4
Asgard/Eukaryota - ps5
Eukaryota - ps6
Excavata/Diaphoretickes - ps7
Diaphoretickes - ps8
Archaeplastida - ps9
Viridiplantae - ps10
Streptophyta - ps11
Charophyceae/Embriophyta - ps12
Embriophyta - ps13
Tracheophyta - ps14
Spermatophyta - ps15
Angiosperms - ps16
Mesangiospermae - ps17
Magnoliids/eudicots - ps18
Eudicots - ps19
Core_eudicots - ps20
Superrosids - ps21
Rosids - ps22
Eurosids - ps23
Malvids - ps24
Sap./Malv./Brassicales - ps25
Malvales/Brassicales - ps26
Brassicales - ps27
Cleomaceae/Brassicaceae - ps28
Brassicaceae - ps29
Camelineae - ps30
Arabidopsis - ps31
Arabidopsis_thaliana - ps32
Phylostratum

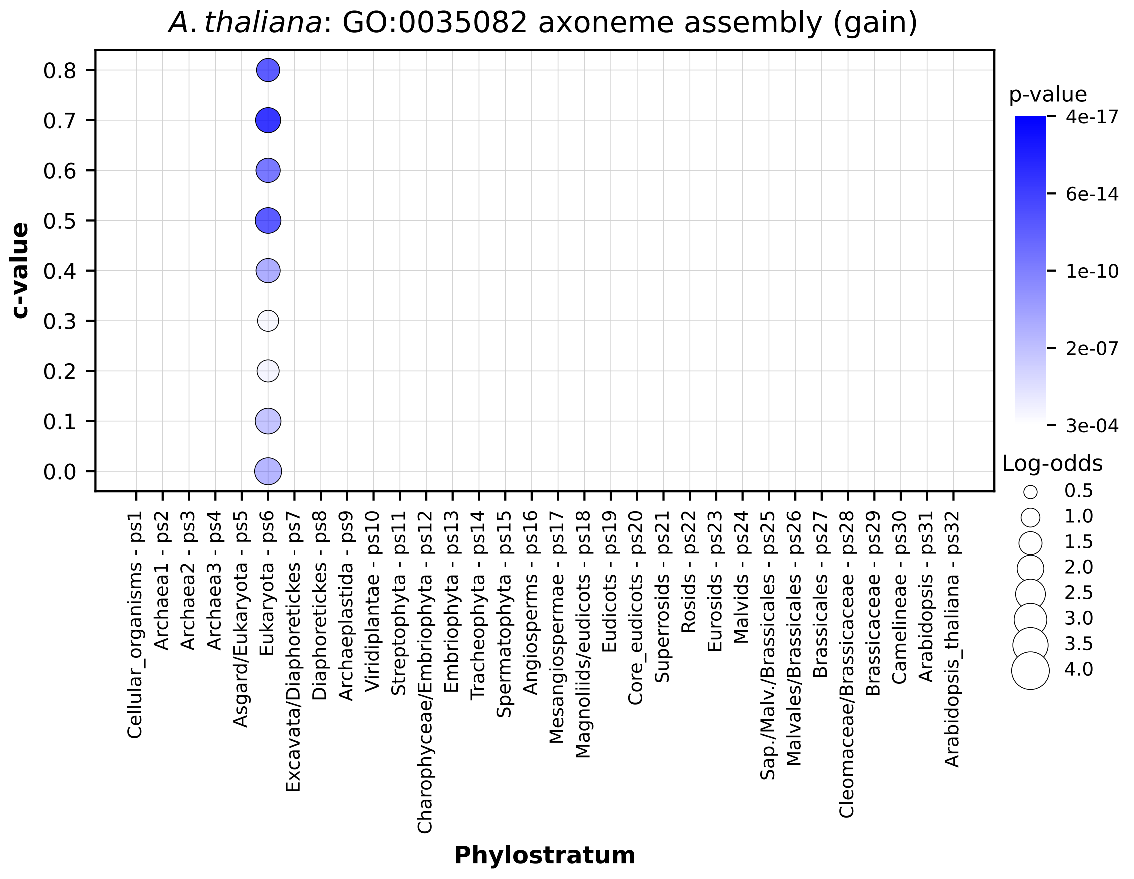

A. thaliana: GO:0035082 axoneme assembly (gain)
c-value
0.8
0.7
0.6
0.5
0.4
0.3
0.2
0.1
0.0
p-value
4e-17
6e-14
1e-10
2e-07
3e-04
Log-odds
0.5
1.0
1.5
2.0
2.5
3.0
3.5
4.0
Cellular_organisms - ps1
Archaea1 - ps2
Archaea2 - ps3
Archaea3 - ps4
Asgard/Eukaryota - ps5
Eukaryota - ps6
Excavata/Diaphoretickes - ps7
Diaphoretickes - ps8
Archaeplastida - ps9
Viridiplantae - ps10
Streptophyta - ps11
Charophyceae/Embriophyta - ps12
Embriophyta - ps13
Tracheophyta - ps14
Spermatophyta - ps15
Angiosperms - ps16
Mesangiospermae - ps17
Magnoliids/eudicots - ps18
Eudicots - ps19
Core_eudicots - ps20
Superrosids - ps21
Rosids - ps22
Eurosids - ps23
Malvids - ps24
Sap./Malv./Brassicales - ps25
Malvales/Brassicales - ps26
Brassicales - ps27
Cleomaceae/Brassicaceae - ps28
Brassicaceae - ps29
Camelineae - ps30
Arabidopsis - ps31
Arabidopsis_thaliana - ps32
Phylostratum

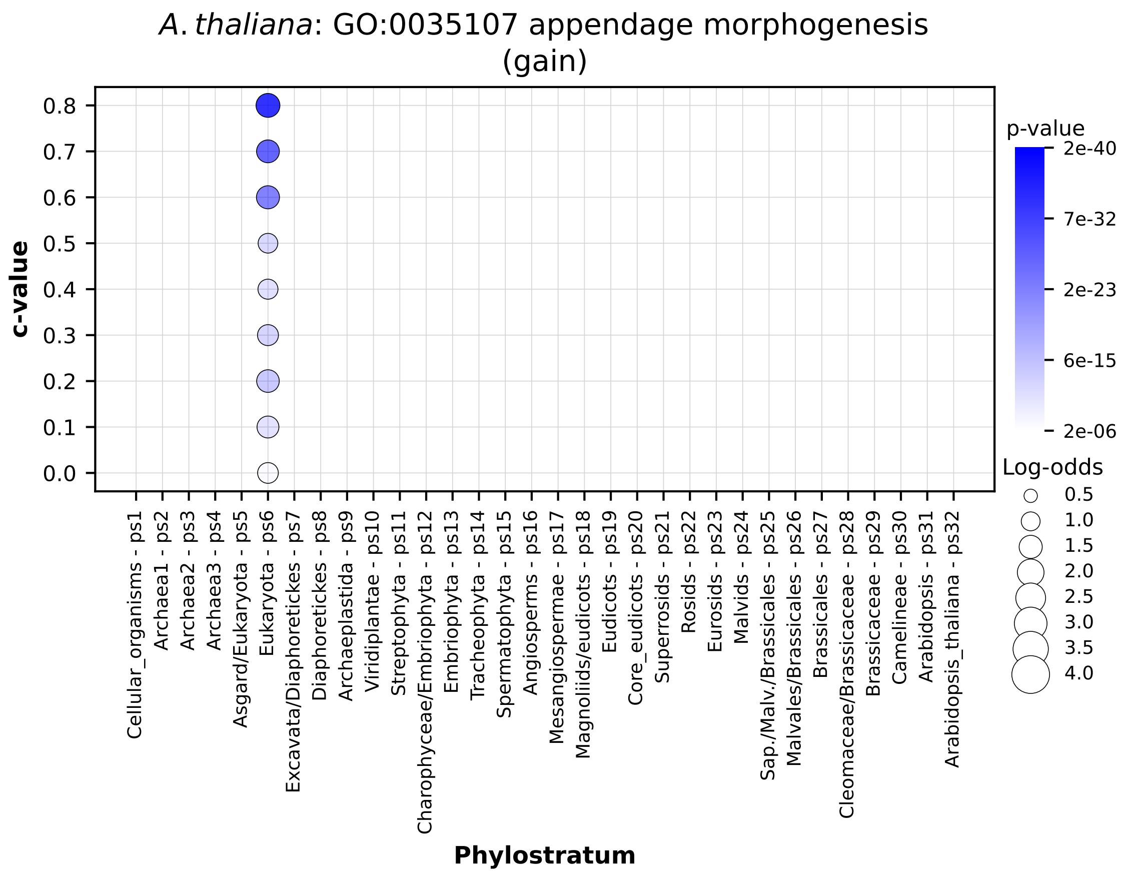

A. thaliana: GO:0035107 appendage morphogenesis
(gain)
c-value
0.0
0.1
0.2
0.3
0.4
0.5
0.6
0.7
0.8
Phylostratum
Cellular_organisms - ps1
Archaea1 - ps2
Archaea2 - ps3
Archaea3 - ps4
Asgard/Eukaryota - ps5
Eukaryota - ps6
Excavata/Diaphoretickes - ps7
Diaphoretickes - ps8
Archaeplastida - ps9
Viridiplantae - ps10
Streptophyta - ps11
Charophyceae/Embriophyta - ps12
Embriophyta - ps13
Tracheophyta - ps14
Spermatophyta - ps15
Angiosperms - ps16
Mesangiospermae - ps17
Magnoliids/eudicots - ps18
Eudicots - ps19
Core_eudicots - ps20
Superrosids - ps21
Rosids - ps22
Eurosids - ps23
Malvids - ps24
Sap./Malv./Brassicales - ps25
Malvales/Brassicales - ps26
Brassicales - ps27
Cleomaceae/Brassicaceae - ps28
Brassicaceae - ps29
Camelineae - ps30
Arabidopsis - ps31
Arabidopsis_thaliana - ps32
p-value
2e-40
7e-32
2e-23
6e-15
2e-06
Log-odds
0.5
1.0
1.5
2.0
2.5
3.0
3.5
4.0

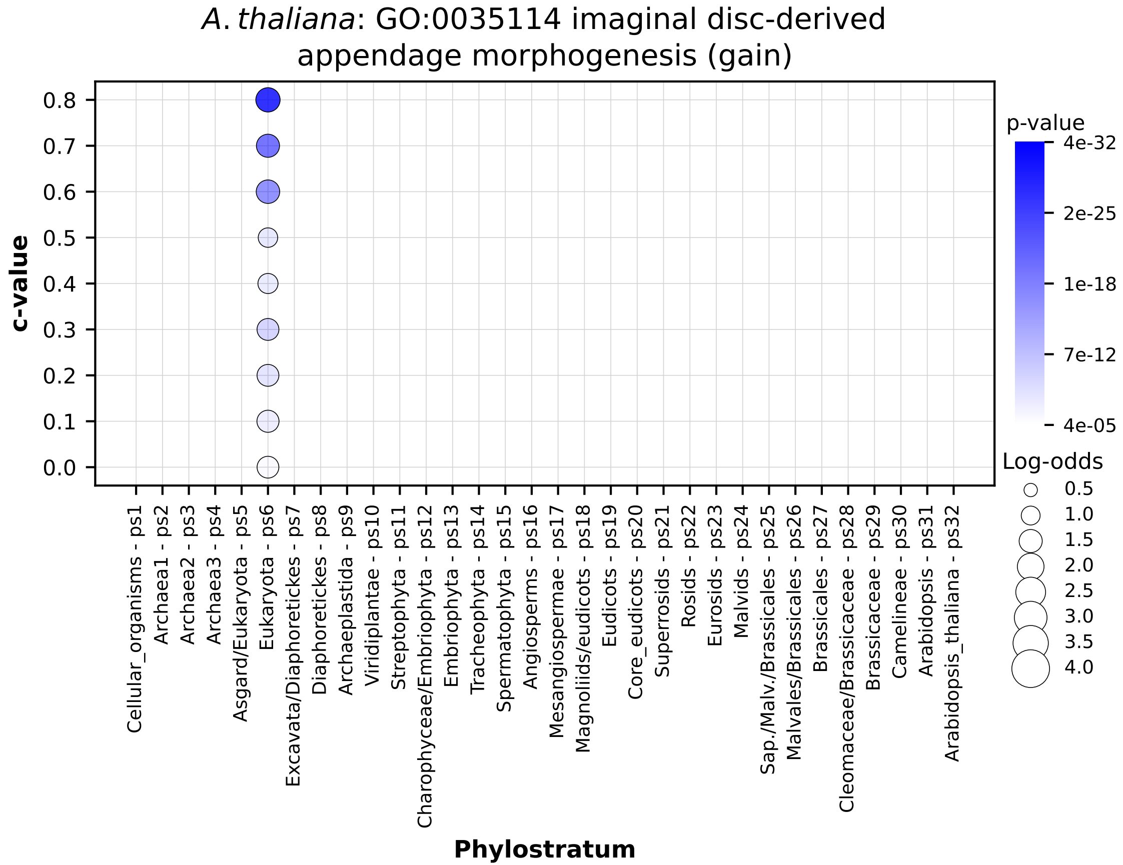
A. thaliana: GO:0035114 imaginal disc-derived appendage morphogenesis (gain)
c-value
0.8
0.7
0.6
0.5
0.4
0.3
0.2
0.1
0.0
p-value
4e-32
2e-25
1e-18
7e-12
4e-05
Log-odds
0.5
1.0
1.5
2.0
2.5
3.0
3.5
4.0
Cellular_organisms - ps1
Archaea1 - ps2
Archaea2 - ps3
Archaea3 - ps4
Asgard/Eukaryota - ps5
Eukaryota - ps6
Excavata/Diaphoretickes - ps7
Diaphoretickes - ps8
Archaeplastida - ps9
Viridiplantae - ps10
Streptophyta - ps11
Charophyceae/Embriophyta - ps12
Embriophyta - ps13
Tracheophyta - ps14
Spermatophyta - ps15
Angiosperms - ps16
Mesangiospermae - ps17
Magnoliids/eudicots - ps18
Eudicots - ps19
Core_eudicots - ps20
Superrosids - ps21
Rosids - ps22
Eurosids - ps23
Malvids - ps24
Sap./Malv./Brassicales - ps25
Malvales/Brassicales - ps26
Brassicales - ps27
Cleomaceae/Brassicaceae - ps28
Brassicaceae - ps29
Camelineae - ps30
Arabidopsis - ps31
Arabidopsis_thaliana - ps32
Phylostratum

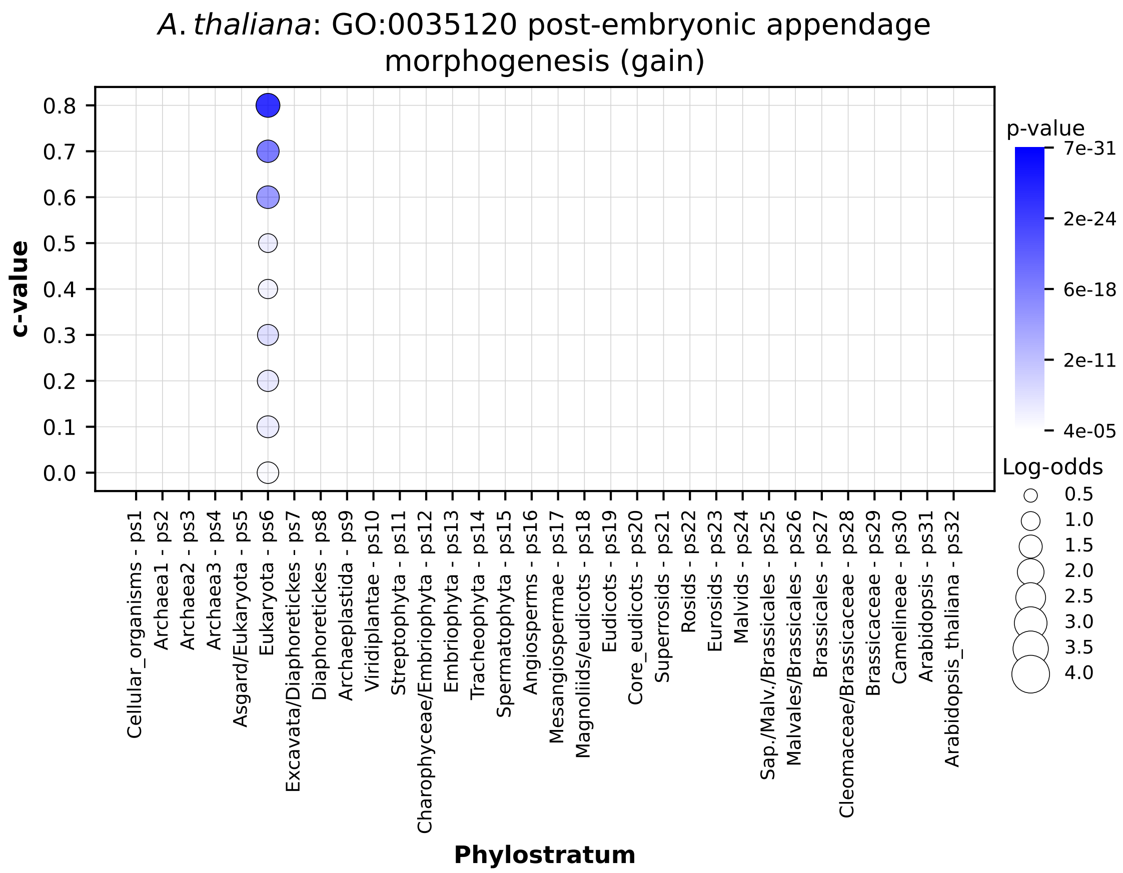

A. thaliana: GO:0035120 post-embryonic appendage morphogenesis (gain)
c-value
0.0
0.1
0.2
0.3
0.4
0.5
0.6
0.7
0.8
Phylostratum
Cellular_organisms - ps1
Archaea1 - ps2
Archaea2 - ps3
Archaea3 - ps4
Asgard/Eukaryota - ps5
Eukaryota - ps6
Excavata/Diaphoretickes - ps7
Diaphoretickes - ps8
Archaeplastida - ps9
Viridiplantae - ps10
Streptophyta - ps11
Charophyceae/Embriophyta - ps12
Embriophyta - ps13
Tracheophyta - ps14
Spermatophyta - ps15
Angiosperms - ps16
Mesangiospermae - ps17
Magnoliids/eudicots - ps18
Eudicots - ps19
Core_eudicots - ps20
Superrosids - ps21
Rosids - ps22
Eurosids - ps23
Malvids - ps24
Sap./Malv./Brassicales - ps25
Malvales/Brassicales - ps26
Brassicales - ps27
Cleomaceae/Brassicaceae - ps28
Brassicaceae - ps29
Camelineae - ps30
Arabidopsis - ps31
Arabidopsis_thaliana - ps32
p-value
7e-31
2e-24
6e-18
2e-11
4e-05
Log-odds
0.5
1.0
1.5
2.0
2.5
3.0
3.5
4.0

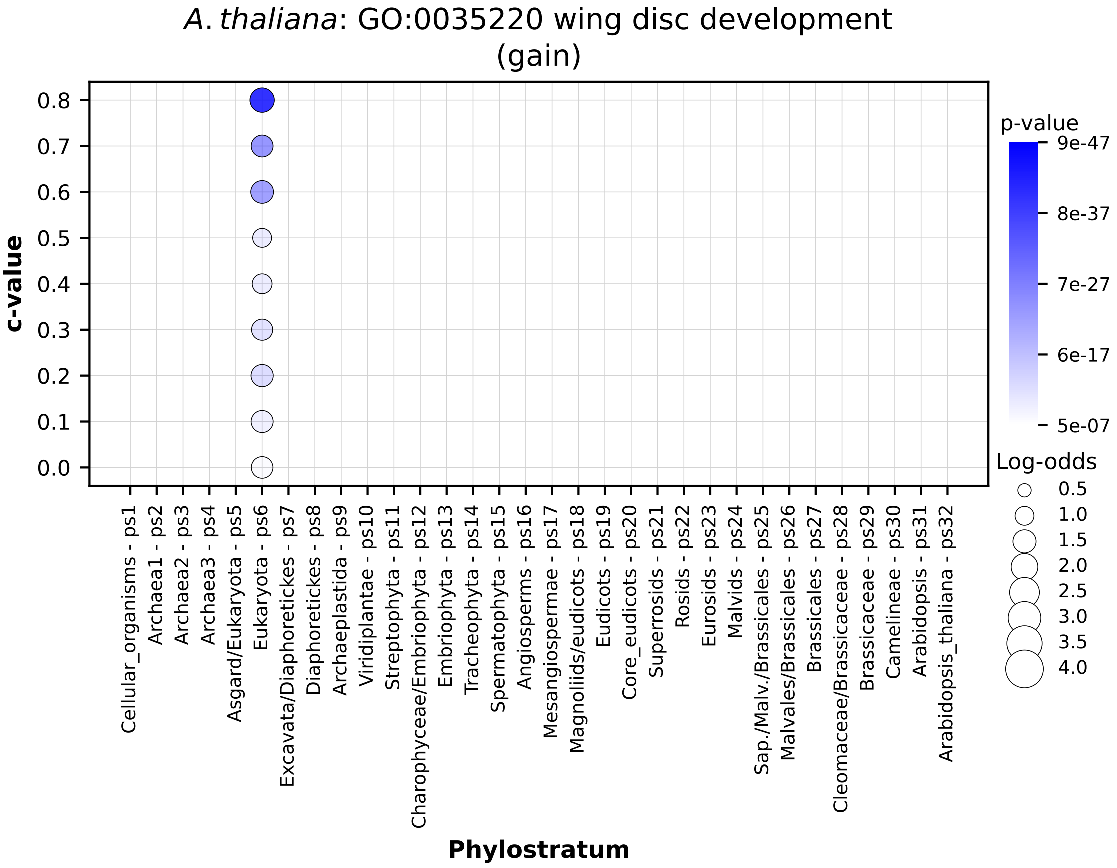

A. thaliana: GO:0035220 wing disc development
(gain)
c-value
0.8
0.7
0.6
0.5
0.4
0.3
0.2
0.1
0.0
p-value
9e-47
8e-37
7e-27
6e-17
5e-07
Log-odds
0.5
1.0
1.5
2.0
2.5
3.0
3.5
4.0
Cellular_organisms - ps1
Archaea1 - ps2
Archaea2 - ps3
Archaea3 - ps4
Asgard/Eukaryota - ps5
Eukaryota - ps6
Excavata/Diaphoretickes - ps7
Diaphoretickes - ps8
Archaeplastida - ps9
Viridiplantae - ps10
Streptophyta - ps11
Charophyceae/Embriophyta - ps12
Embriophyta - ps13
Tracheophyta - ps14
Spermatophyta - ps15
Angiosperms - ps16
Mesangiospermae - ps17
Magnoliids/eudicots - ps18
Eudicots - ps19
Core_eudicots - ps20
Superrosids - ps21
Rosids - ps22
Eurosids - ps23
Malvids - ps24
Sap./Malv./Brassicales - ps25
Malvales/Brassicales - ps26
Brassicales - ps27
Cleomaceae/Brassicaceae - ps28
Brassicaceae - ps29
Camelineae - ps30
Arabidopsis - ps31
Arabidopsis_thaliana - ps32
Phylostratum

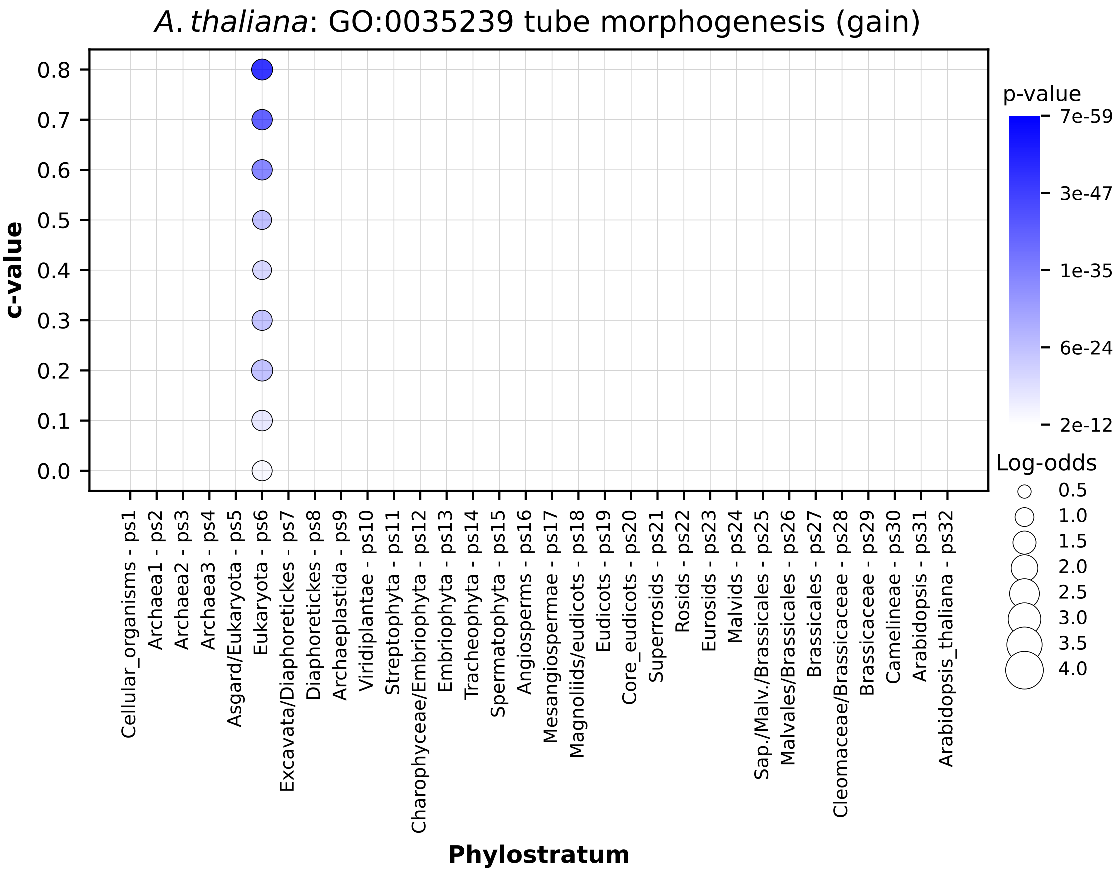

A. thaliana: GO:0035239 tube morphogenesis (gain)
c-value
0.8
0.7
0.6
0.5
0.4
0.3
0.2
0.1
0.0
p-value
7e-59
3e-47
1e-35
6e-24
2e-12
Log-odds
0.5
1.0
1.5
2.0
2.5
3.0
3.5
4.0
Cellular_organisms - ps1
Archaea1 - ps2
Archaea2 - ps3
Archaea3 - ps4
Asgard/Eukaryota - ps5
Eukaryota - ps6
Excavata/Diaphoretickes - ps7
Diaphoretickes - ps8
Archaeplastida - ps9
Viridiplantae - ps10
Streptophyta - ps11
Charophyceae/Embriophyta - ps12
Embriophyta - ps13
Tracheophyta - ps14
Spermatophyta - ps15
Angiosperms - ps16
Mesangiospermae - ps17
Magnoliids/eudicots - ps18
Eudicots - ps19
Core_eudicots - ps20
Superrosids - ps21
Rosids - ps22
Eurosids - ps23
Malvids - ps24
Sap./Malv./Brassicales - ps25
Malvales/Brassicales - ps26
Brassicales - ps27
Cleomaceae/Brassicaceae - ps28
Brassicaceae - ps29
Camelineae - ps30
Arabidopsis - ps31
Arabidopsis_thaliana - ps32
Phylostratum

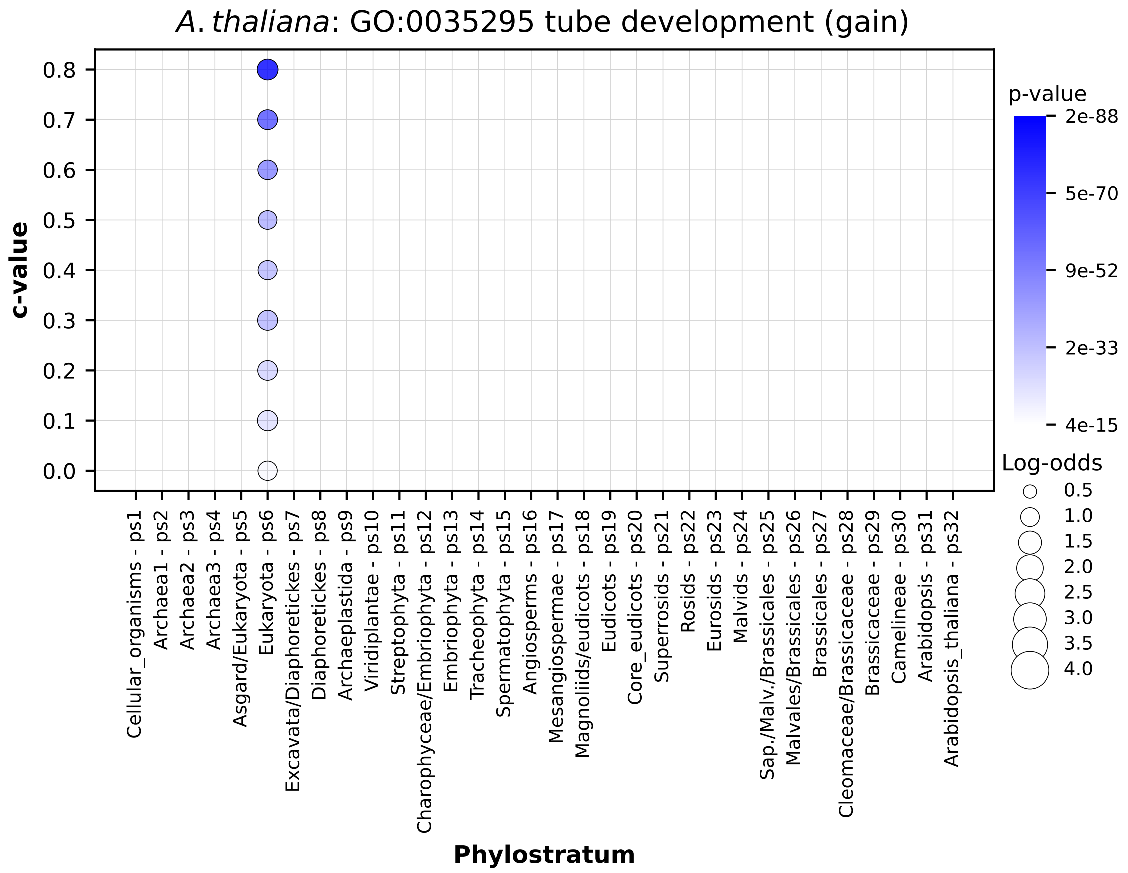

A. thaliana: GO:0035295 tube development (gain)
c-value
0.8
0.7
0.6
0.5
0.4
0.3
0.2
0.1
0.0
p-value
2e-88
5e-70
9e-52
2e-33
4e-15
Log-odds
0.5
1.0
1.5
2.0
2.5
3.0
3.5
4.0
Cellular_organisms - ps1
Archaea1 - ps2
Archaea2 - ps3
Archaea3 - ps4
Asgard/Eukaryota - ps5
Eukaryota - ps6
Excavata/Diaphoretickes - ps7
Diaphoretickes - ps8
Archaeplastida - ps9
Viridiplantae - ps10
Streptophyta - ps11
Charophyceae/Embriophyta - ps12
Embriophyta - ps13
Tracheophyta - ps14
Spermatophyta - ps15
Angiosperms - ps16
Mesangiospermae - ps17
Magnoliids/eudicots - ps18
Eudicots - ps19
Core_eudicots - ps20
Superrosids - ps21
Rosids - ps22
Eurosids - ps23
Malvids - ps24
Sap./Malv./Brassicales - ps25
Malvales/Brassicales - ps26
Brassicales - ps27
Cleomaceae/Brassicaceae - ps28
Brassicaceae - ps29
Camelineae - ps30
Arabidopsis - ps31
Arabidopsis_thaliana - ps32
Phylostratum

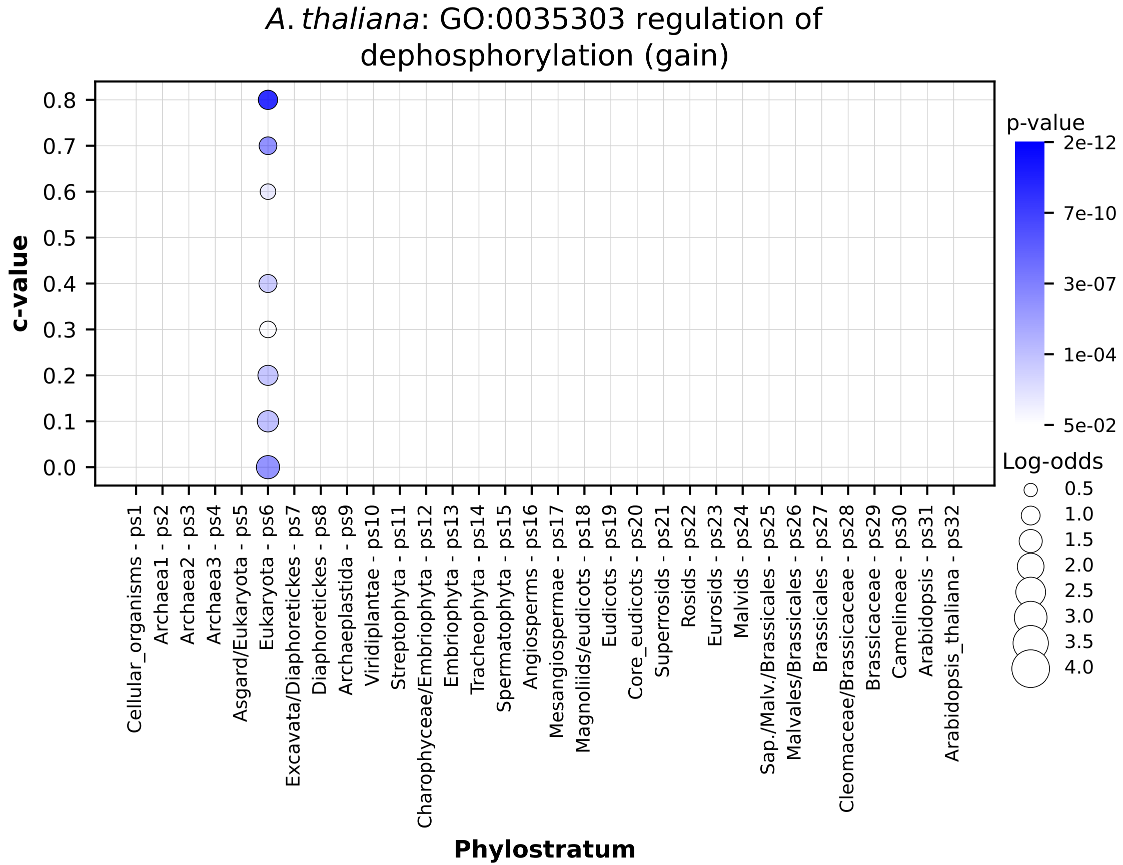

A. thaliana: GO:0035303 regulation of dephosphorylation (gain)
c-value
0.0
0.1
0.2
0.3
0.4
0.5
0.6
0.7
0.8
p-value
2e-12
7e-10
3e-07
1e-04
5e-02
Log-odds
0.5
1.0
1.5
2.0
2.5
3.0
3.5
4.0
Cellular_organisms - ps1
Archaea1 - ps2
Archaea2 - ps3
Archaea3 - ps4
Asgard/Eukaryota - ps5
Eukaryota - ps6
Excavata/Diaphoretickes - ps7
Diaphoretickes - ps8
Archaeplastida - ps9
Viridiplantae - ps10
Streptophyta - ps11
Charophyceae/Embriophyta - ps12
Embriophyta - ps13
Tracheophyta - ps14
Spermatophyta - ps15
Angiosperms - ps16
Mesangiospermae - ps17
Magnoliids/eudicots - ps18
Eudicots - ps19
Core_eudicots - ps20
Superrosids - ps21
Rosids - ps22
Eurosids - ps23
Malvids - ps24
Sap./Malv./Brassicales - ps25
Malvales/Brassicales - ps26
Brassicales - ps27
Cleomaceae/Brassicaceae - ps28
Brassicaceae - ps29
Camelineae - ps30
Arabidopsis - ps31
Arabidopsis_thaliana - ps32
Phylostratum

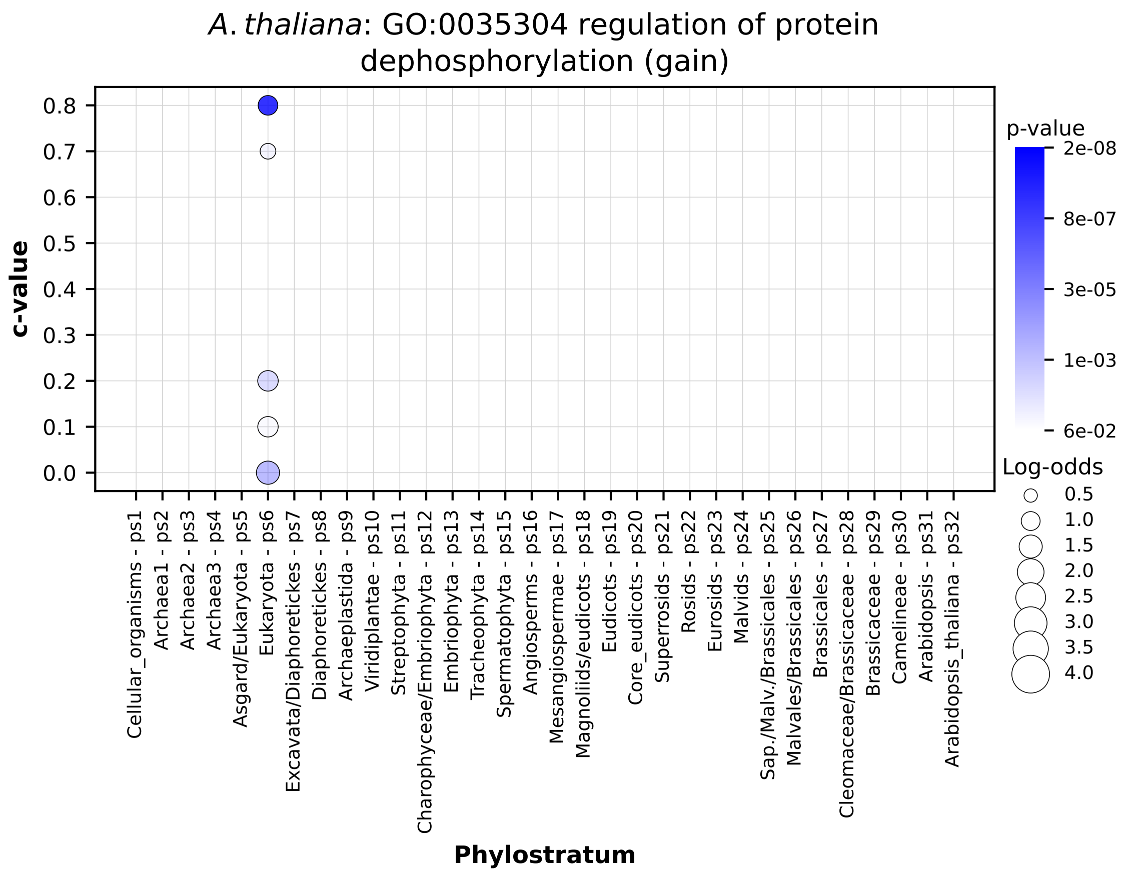

A. thaliana: GO:0035304 regulation of protein dephosphorylation (gain)
c-value
0.8
0.7
0.6
0.5
0.4
0.3
0.2
0.1
0.0
Phylostratum
Cellular_organisms - ps1
Archaea1 - ps2
Archaea2 - ps3
Archaea3 - ps4
Asgard/Eukaryota - ps5
Eukaryota - ps6
Excavata/Diaphoretickes - ps7
Diaphoretickes - ps8
Archaeplastida - ps9
Viridiplantae - ps10
Streptophyta - ps11
Charophyceae/Embriophyta - ps12
Embriophyta - ps13
Tracheophyta - ps14
Spermatophyta - ps15
Angiosperms - ps16
Mesangiospermae - ps17
Magnoliids/eudicots - ps18
Eudicots - ps19
Core_eudicots - ps20
Superrosids - ps21
Rosids - ps22
Eurosids - ps23
Malvids - ps24
Sap./Malv./Brassicales - ps25
Malvales/Brassicales - ps26
Brassicales - ps27
Cleomaceae/Brassicaceae - ps28
Brassicaceae - ps29
Camelineae - ps30
Arabidopsis - ps31
Arabidopsis_thaliana - ps32
p-value
2e-08
8e-07
3e-05
1e-03
6e-02
Log-odds
0.5
1.0
1.5
2.0
2.5
3.0
3.5
4.0

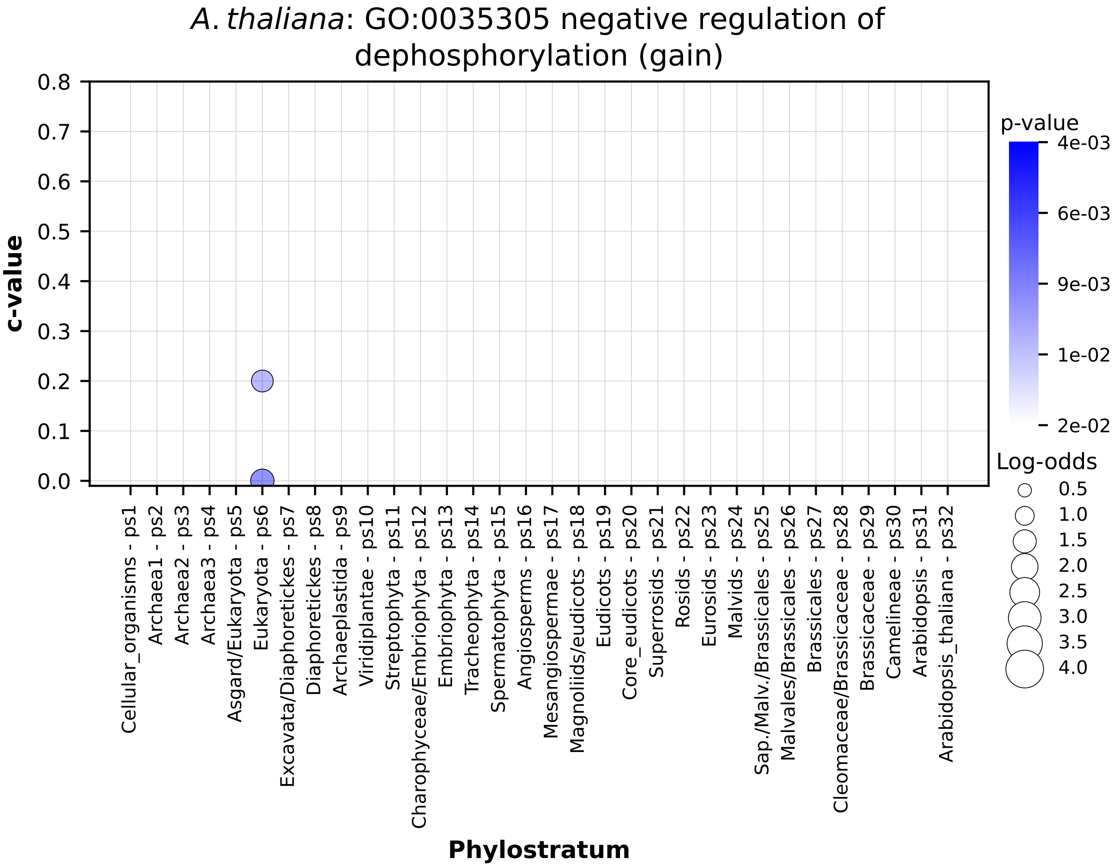

A. thaliana: GO:0035305 negative regulation of dephosphorylation (gain)
c-value
0.8
0.7
0.6
0.5
0.4
0.3
0.2
0.1
0.0
Phylostratum
Cellular_organisms - ps1
Archaea1 - ps2
Archaea2 - ps3
Archaea3 - ps4
Asgard/Eukaryota - ps5
Eukaryota - ps6
Excavata/Diaphoretickes - ps7
Diaphoretickes - ps8
Archaeplastida - ps9
Viridiplantae - ps10
Streptophyta - ps11
Charophyceae/Embriophyta - ps12
Embriophyta - ps13
Tracheophyta - ps14
Spermatophyta - ps15
Angiosperms - ps16
Mesangiospermae - ps17
Magnoliids/eudicots - ps18
Eudicots - ps19
Core_eudicots - ps20
Superrosids - ps21
Rosids - ps22
Eurosids - ps23
Malvids - ps24
Sap./Malv./Brassicales - ps25
Malvales/Brassicales - ps26
Brassicales - ps27
Cleomaceae/Brassicaceae - ps28
Brassicaceae - ps29
Camelineae - ps30
Arabidopsis - ps31
Arabidopsis_thaliana - ps32
p-value
4e-03
6e-03
9e-03
1e-02
2e-02
Log-odds
0.5
1.0
1.5
2.0
2.5
3.0
3.5
4.0

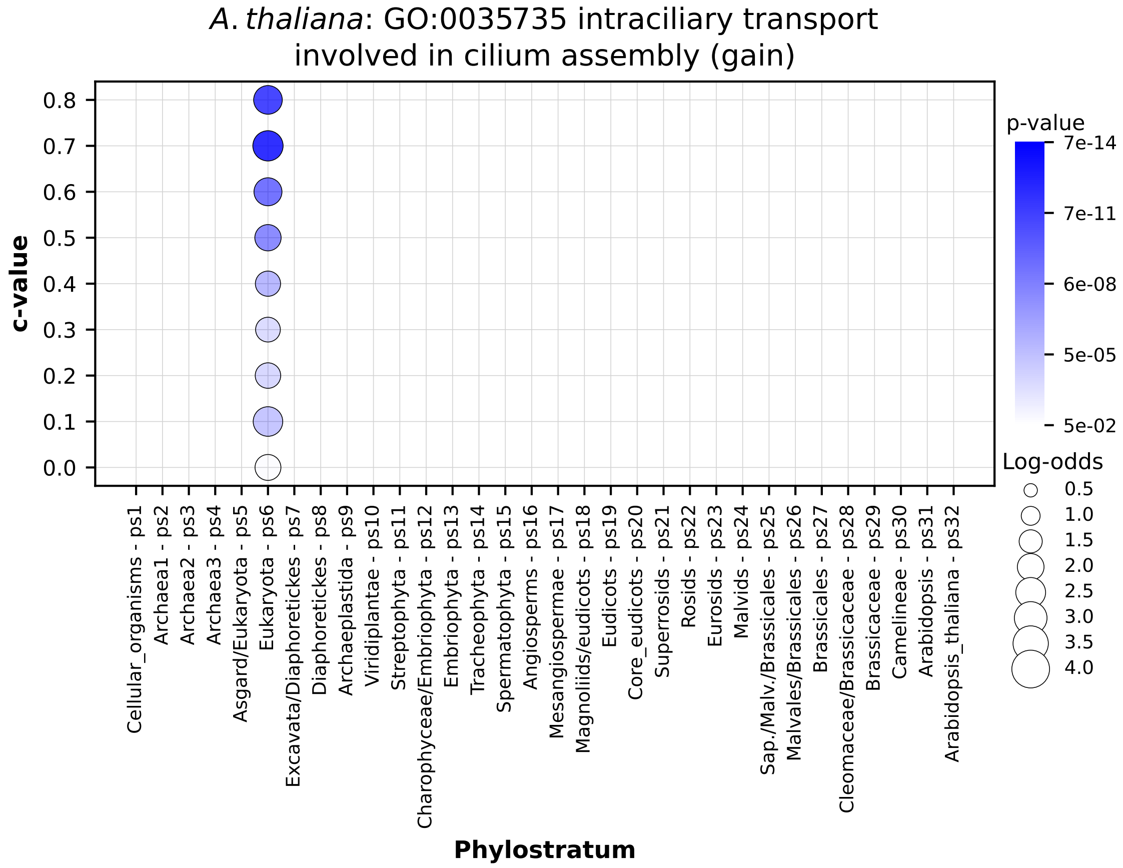

A. thaliana: GO:0035735 intraciliary transport involved in cilium assembly (gain)
c-value
0.8
0.7
0.6
0.5
0.4
0.3
0.2
0.1
0.0
Phylostratum
Cellular_organisms - ps1
Archaea1 - ps2
Archaea2 - ps3
Archaea3 - ps4
Asgard/Eukaryota - ps5
Eukaryota - ps6
Excavata/Diaphoretickes - ps7
Diaphoretickes - ps8
Archaeplastida - ps9
Viridiplantae - ps10
Streptophyta - ps11
Charophyceae/Embriophyta - ps12
Embriophyta - ps13
Tracheophyta - ps14
Spermatophyta - ps15
Angiosperms - ps16
Mesangiospermae - ps17
Magnoliids/eudicots - ps18
Eudicots - ps19
Core_eudicots - ps20
Superrosids - ps21
Rosids - ps22
Eurosids - ps23
Malvids - ps24
Sap./Malv./Brassicales - ps25
Malvales/Brassicales - ps26
Brassicales - ps27
Cleomaceae/Brassicaceae - ps28
Brassicaceae - ps29
Camelineae - ps30
Arabidopsis - ps31
Arabidopsis_thaliana - ps32
p-value
7e-14
7e-11
6e-08
5e-05
5e-02
Log-odds
0.5
1.0
1.5
2.0
2.5
3.0
3.5
4.0

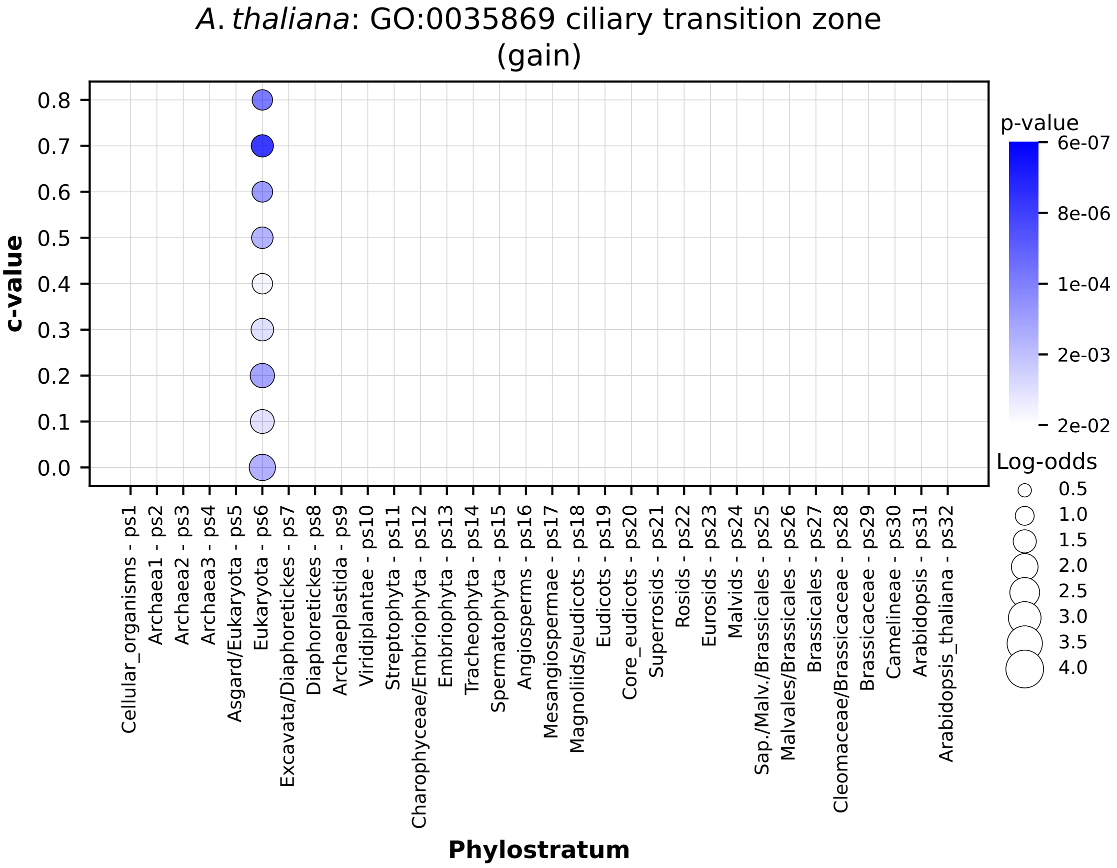
A. thaliana: GO:0035869 ciliary transition zone
(gain)
c-value
0.0
0.1
0.2
0.3
0.4
0.5
0.6
0.7
0.8
Phylostratum
Cellular_organisms - ps1
Archaea1 - ps2
Archaea2 - ps3
Archaea3 - ps4
Asgard/Eukaryota - ps5
Eukaryota - ps6
Excavata/Diaphoretickes - ps7
Diaphoretickes - ps8
Archaeplastida - ps9
Viridiplantae - ps10
Streptophyta - ps11
Charophyceae/Embriophyta - ps12
Embriophyta - ps13
Tracheophyta - ps14
Spermatophyta - ps15
Angiosperms - ps16
Mesangiospermae - ps17
Magnoliids/eudicots - ps18
Eudicots - ps19
Core_eudicots - ps20
Superrosids - ps21
Rosids - ps22
Eurosids - ps23
Malvids - ps24
Sap./Malv./Brassicales - ps25
Malvales/Brassicales - ps26
Brassicales - ps27
Cleomaceae/Brassicaceae - ps28
Brassicaceae - ps29
Camelineae - ps30
Arabidopsis - ps31
Arabidopsis_thaliana - ps32
p-value
6e-07
8e-06
1e-04
2e-03
2e-02
Log-odds
0.5
1.0
1.5
2.0
2.5
3.0
3.5
4.0

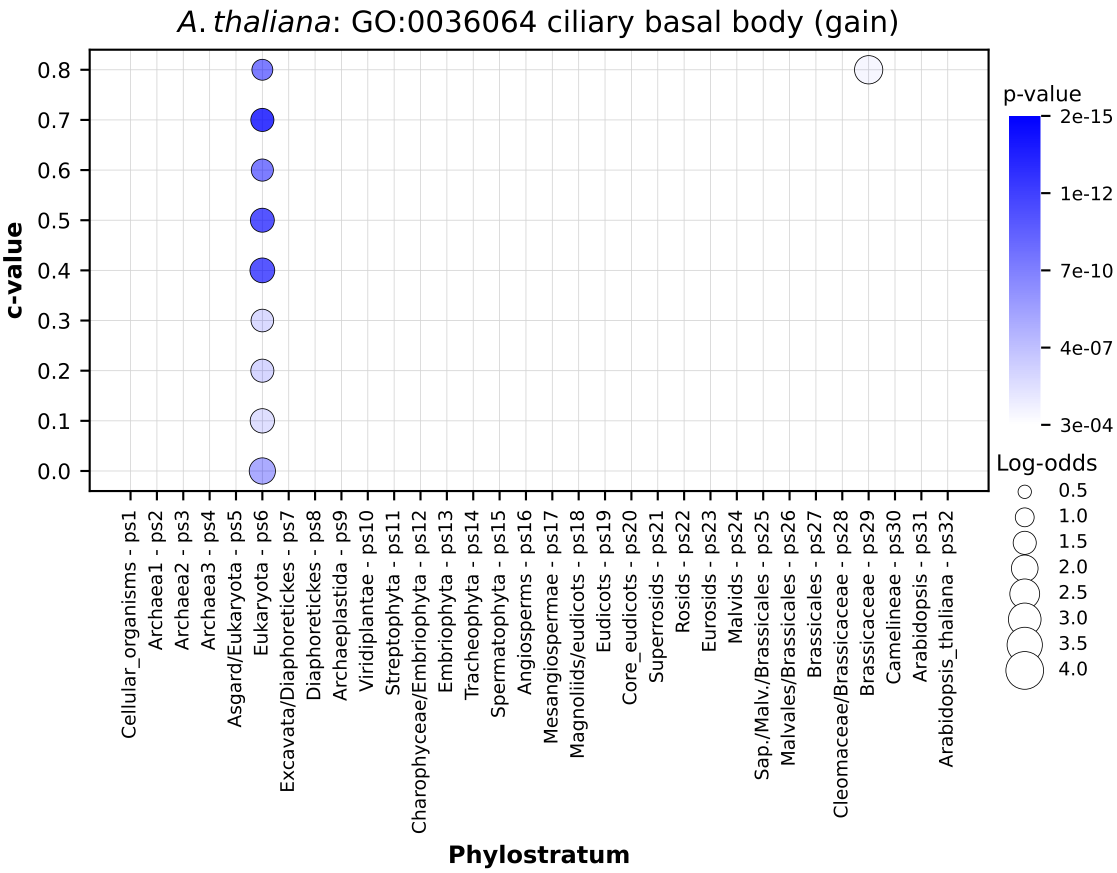
A. thaliana: GO:0036064 ciliary basal body (gain)
c-value
0.8
0.7
0.6
0.5
0.4
0.3
0.2
0.1
0.0
Phylostratum
Cellular_organisms - ps1
Archaea1 - ps2
Archaea2 - ps3
Archaea3 - ps4
Asgard/Eukaryota - ps5
Eukaryota - ps6
Excavata/Diaphoretickes - ps7
Diaphoretickes - ps8
Archaeplastida - ps9
Viridiplantae - ps10
Streptophyta - ps11
Charophyceae/Embriophyta - ps12
Embriophyta - ps13
Tracheophyta - ps14
Spermatophyta - ps15
Angiosperms - ps16
Mesangiospermae - ps17
Magnoliids/eudicots - ps18
Eudicots - ps19
Core_eudicots - ps20
Superrosids - ps21
Rosids - ps22
Eurosids - ps23
Malvids - ps24
Sap./Malv./Brassicales - ps25
Malvales/Brassicales - ps26
Brassicales - ps27
Cleomaceae/Brassicaceae - ps28
Brassicaceae - ps29
Camelineae - ps30
Arabidopsis - ps31
Arabidopsis_thaliana - ps32
p-value
2e-15
1e-12
7e-10
4e-07
3e-04
Log-odds
0.5
1.0
1.5
2.0
2.5
3.0
3.5
4.0

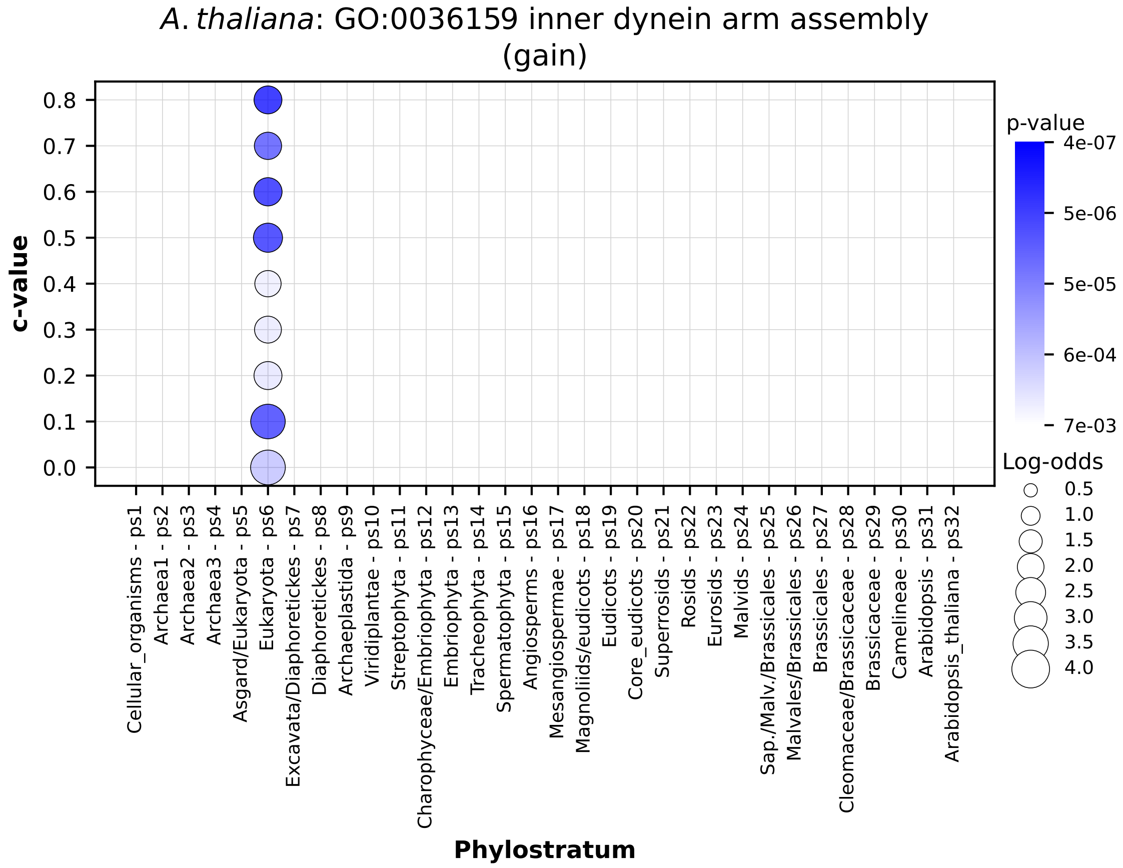
A. thaliana: GO:0036159 inner dynein arm assembly
(gain)
c-value
0.8
0.7
0.6
0.5
0.4
0.3
0.2
0.1
0.0
p-value
4e-07
5e-06
5e-05
6e-04
7e-03
Log-odds
0.5
1.0
1.5
2.0
2.5
3.0
3.5
4.0
Cellular_organisms - ps1
Archaea1 - ps2
Archaea2 - ps3
Archaea3 - ps4
Asgard/Eukaryota - ps5
Eukaryota - ps6
Excavata/Diaphoretickes - ps7
Diaphoretickes - ps8
Archaeplastida - ps9
Viridiplantae - ps10
Streptophyta - ps11
Charophyceae/Embriophyta - ps12
Embriophyta - ps13
Tracheophyta - ps14
Spermatophyta - ps15
Angiosperms - ps16
Mesangiospermae - ps17
Magnoliids/eudicots - ps18
Eudicots - ps19
Core_eudicots - ps20
Superrosids - ps21
Rosids - ps22
Eurosids - ps23
Malvids - ps24
Sap./Malv./Brassicales - ps25
Malvales/Brassicales - ps26
Brassicales - ps27
Cleomaceae/Brassicaceae - ps28
Brassicaceae - ps29
Camelineae - ps30
Arabidopsis - ps31
Arabidopsis_thaliana - ps32
Phylostratum

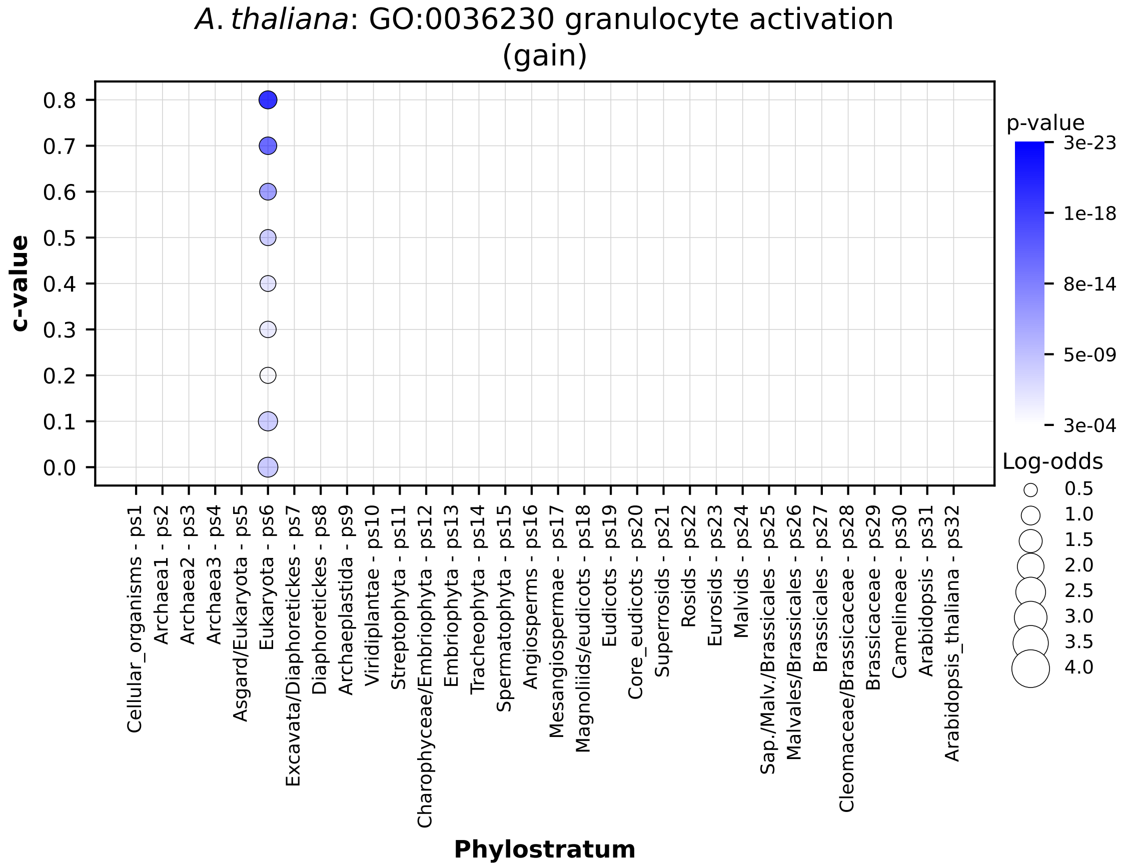

A. thaliana: GO:0036230 granulocyte activation
(gain)
c-value
0.8
0.7
0.6
0.5
0.4
0.3
0.2
0.1
0.0
Phylostratum
Cellular_organisms - ps1
Archaea1 - ps2
Archaea2 - ps3
Archaea3 - ps4
Asgard/Eukaryota - ps5
Eukaryota - ps6
Excavata/Diaphoretickes - ps7
Diaphoretickes - ps8
Archaeplastida - ps9
Viridiplantae - ps10
Streptophyta - ps11
Charophyceae/Embriophyta - ps12
Embriophyta - ps13
Tracheophyta - ps14
Spermatophyta - ps15
Angiosperms - ps16
Mesangiospermae - ps17
Magnoliids/eudicots - ps18
Eudicots - ps19
Core_eudicots - ps20
Superrosids - ps21
Rosids - ps22
Eurosids - ps23
Malvids - ps24
Sap./Malv./Brassicales - ps25
Malvales/Brassicales - ps26
Brassicales - ps27
Cleomaceae/Brassicaceae - ps28
Brassicaceae - ps29
Camelineae - ps30
Arabidopsis - ps31
Arabidopsis_thaliana - ps32
p-value
3e-23
1e-18
8e-14
5e-09
3e-04
Log-odds
0.5
1.0
1.5
2.0
2.5
3.0
3.5
4.0

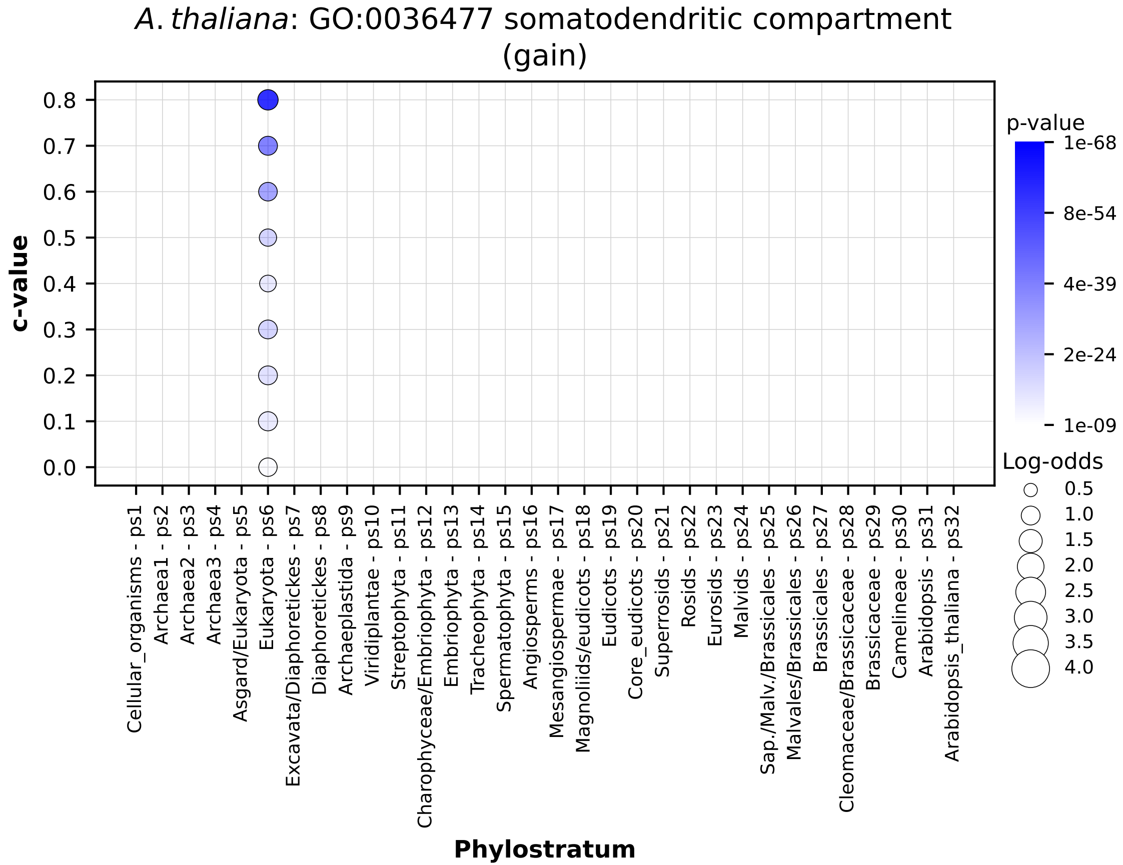

A. thaliana: GO:0036477 somatodendritic compartment (gain)
c-value
0.8
0.7
0.6
0.5
0.4
0.3
0.2
0.1
0.0
p-value
1e-68
8e-54
4e-39
2e-24
1e-09
Log-odds
0.5
1.0
1.5
2.0
2.5
3.0
3.5
4.0
Cellular_organisms - ps1
Archaea1 - ps2
Archaea2 - ps3
Archaea3 - ps4
Asgard/Eukaryota - ps5
Eukaryota - ps6
Excavata/Diaphoretickes - ps7
Diaphoretickes - ps8
Archaeplastida - ps9
Viridiplantae - ps10
Streptophyta - ps11
Charophyceae/Embriophyta - ps12
Embriophyta - ps13
Tracheophyta - ps14
Spermatophyta - ps15
Angiosperms - ps16
Mesangiospermae - ps17
Magnoliids/eudicots - ps18
Eudicots - ps19
Core_eudicots - ps20
Superrosids - ps21
Rosids - ps22
Eurosids - ps23
Malvids - ps24
Sap./Malv./Brassicales - ps25
Malvales/Brassicales - ps26
Brassicales - ps27
Cleomaceae/Brassicaceae - ps28
Brassicaceae - ps29
Camelineae - ps30
Arabidopsis - ps31
Arabidopsis_thaliana - ps32
Phylostratum

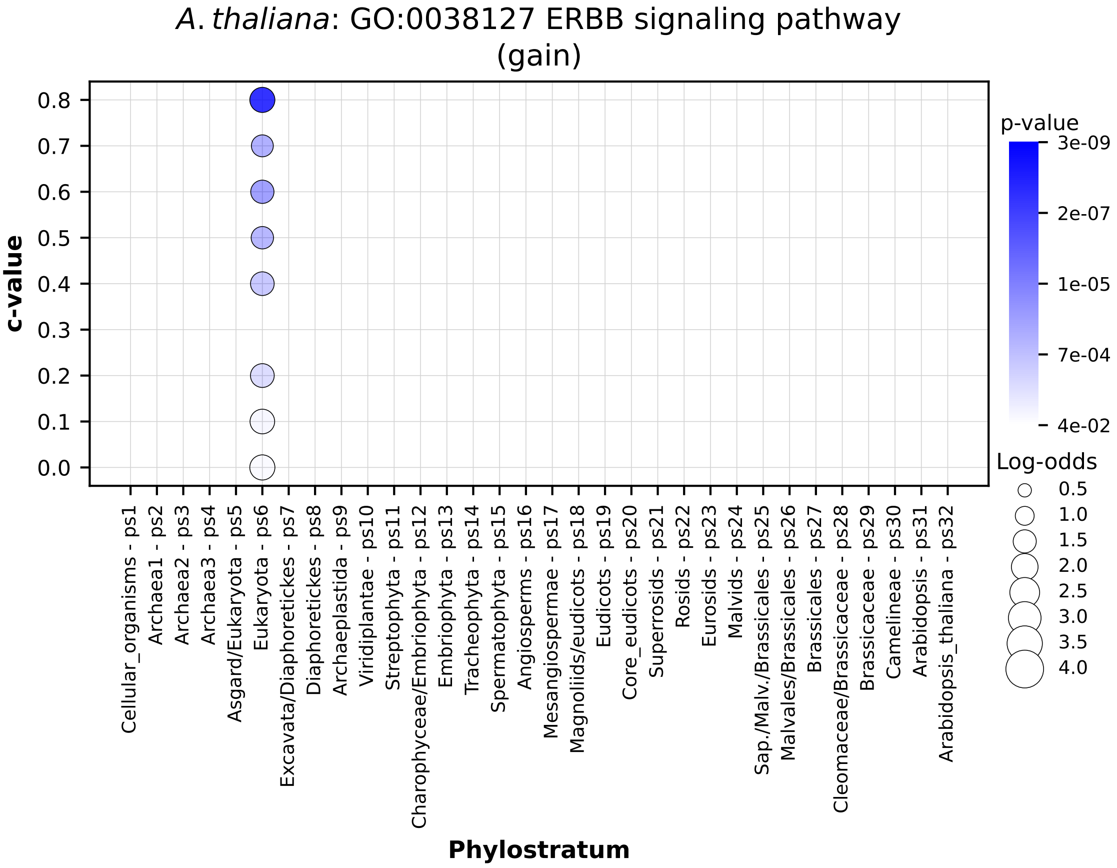
A. thaliana: GO:0038127 ERBB signaling pathway (gain)
c-value
0.8
0.7
0.6
0.5
0.4
0.3
0.2
0.1
0.0
p-value
3e-09
2e-07
1e-05
7e-04
4e-02
Log-odds
0.5
1.0
1.5
2.0
2.5
3.0
3.5
4.0
Cellular_organisms - ps1
Archaea1 - ps2
Archaea2 - ps3
Archaea3 - ps4
Asgard/Eukaryota - ps5
Eukaryota - ps6
Excavata/Diaphoretickes - ps7
Diaphoretickes - ps8
Archaeplastida - ps9
Viridiplantae - ps10
Streptophyta - ps11
Charophyceae/Embriophyta - ps12
Embriophyta - ps13
Tracheophyta - ps14
Spermatophyta - ps15
Angiosperms - ps16
Mesangiospermae - ps17
Magnoliids/eudicots - ps18
Eudicots - ps19
Core_eudicots - ps20
Superrosids - ps21
Rosids - ps22
Eurosids - ps23
Malvids - ps24
Sap./Malv./Brassicales - ps25
Malvales/Brassicales - ps26
Brassicales - ps27
Cleomaceae/Brassicaceae - ps28
Brassicaceae - ps29
Camelineae - ps30
Arabidopsis - ps31
Arabidopsis_thaliana - ps32
Phylostratum

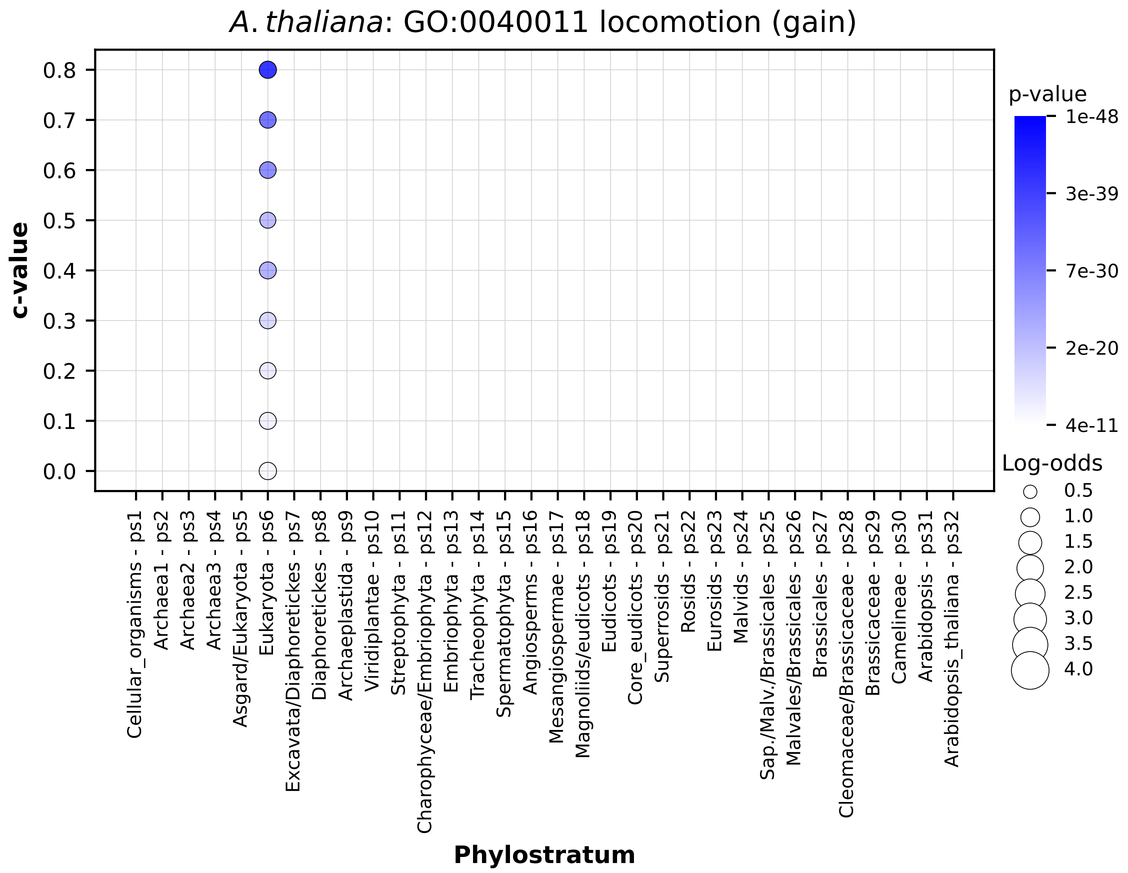
A. thaliana: GO:0040011 locomotion (gain)
c-value
0.8
0.7
0.6
0.5
0.4
0.3
0.2
0.1
0.0
p-value
1e-48
3e-39
7e-30
2e-20
4e-11
Log-odds
0.5
1.0
1.5
2.0
2.5
3.0
3.5
4.0
Cellular_organisms - ps1
Archaea1 - ps2
Archaea2 - ps3
Archaea3 - ps4
Asgard/Eukaryota - ps5
Eukaryota - ps6
Excavata/Diaphoretickes - ps7
Diaphoretickes - ps8
Archaeplastida - ps9
Viridiplantae - ps10
Streptophyta - ps11
Charophyceae/Embriophyta - ps12
Embriophyta - ps13
Tracheophyta - ps14
Spermatophyta - ps15
Angiosperms - ps16
Mesangiospermae - ps17
Magnoliids/eudicots - ps18
Eudicots - ps19
Core_eudicots - ps20
Superrosids - ps21
Rosids - ps22
Eurosids - ps23
Malvids - ps24
Sap./Malv./Brassicales - ps25
Malvales/Brassicales - ps26
Brassicales - ps27
Cleomaceae/Brassicaceae - ps28
Brassicaceae - ps29
Camelineae - ps30
Arabidopsis - ps31
Arabidopsis_thaliana - ps32
Phylostratum

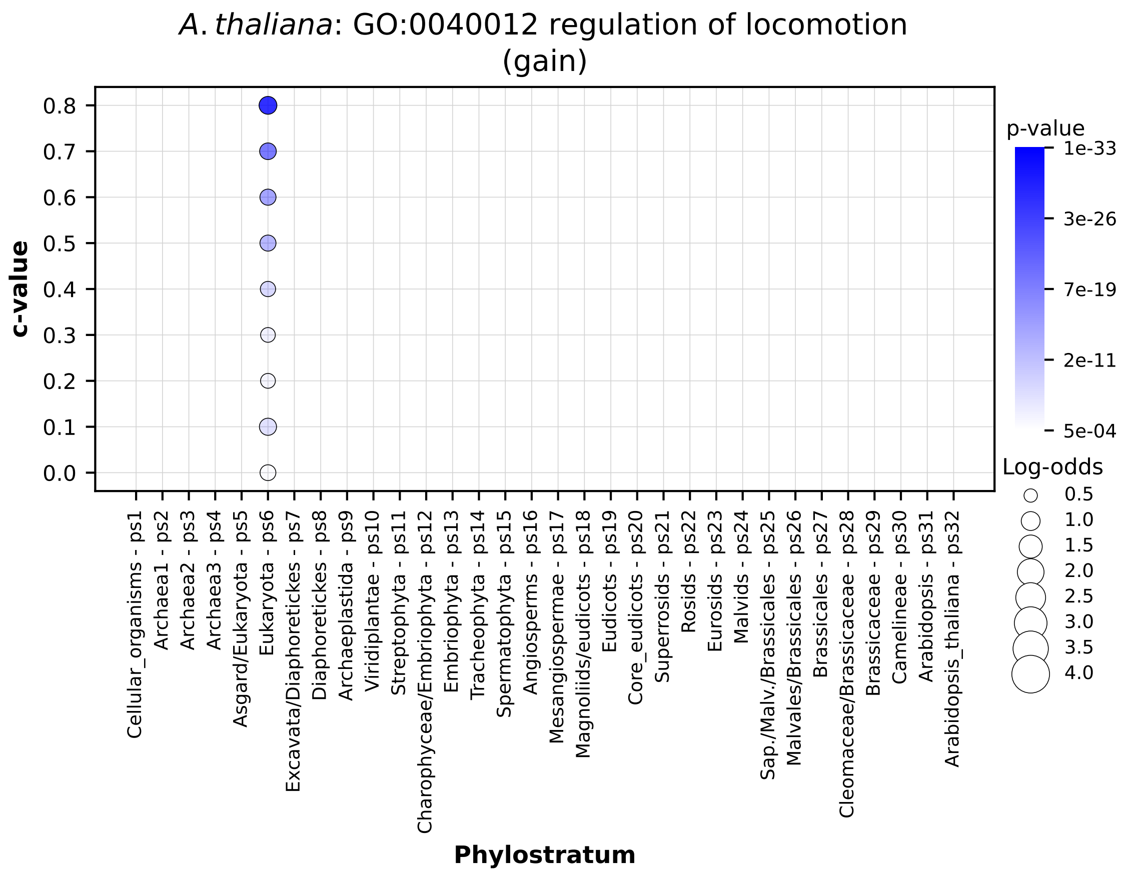

A. thaliana: GO:0040012 regulation of locomotion
(gain)
c-value
0.0
0.1
0.2
0.3
0.4
0.5
0.6
0.7
0.8
Phylostratum
Cellular_organisms - ps1
Archaea1 - ps2
Archaea2 - ps3
Archaea3 - ps4
Asgard/Eukaryota - ps5
Eukaryota - ps6
Excavata/Diaphoretickes - ps7
Diaphoretickes - ps8
Archaeplastida - ps9
Viridiplantae - ps10
Streptophyta - ps11
Charophyceae/Embriophyta - ps12
Embriophyta - ps13
Tracheophyta - ps14
Spermatophyta - ps15
Angiosperms - ps16
Mesangiospermae - ps17
Magnoliids/eudicots - ps18
Eudicots - ps19
Core_eudicots - ps20
Superrosids - ps21
Rosids - ps22
Eurosids - ps23
Malvids - ps24
Sap./Malv./Brassicales - ps25
Malvales/Brassicales - ps26
Brassicales - ps27
Cleomaceae/Brassicaceae - ps28
Brassicaceae - ps29
Camelineae - ps30
Arabidopsis - ps31
Arabidopsis_thaliana - ps32
p-value
1e-33
3e-26
7e-19
2e-11
5e-04
Log-odds
0.5
1.0
1.5
2.0
2.5
3.0
3.5
4.0

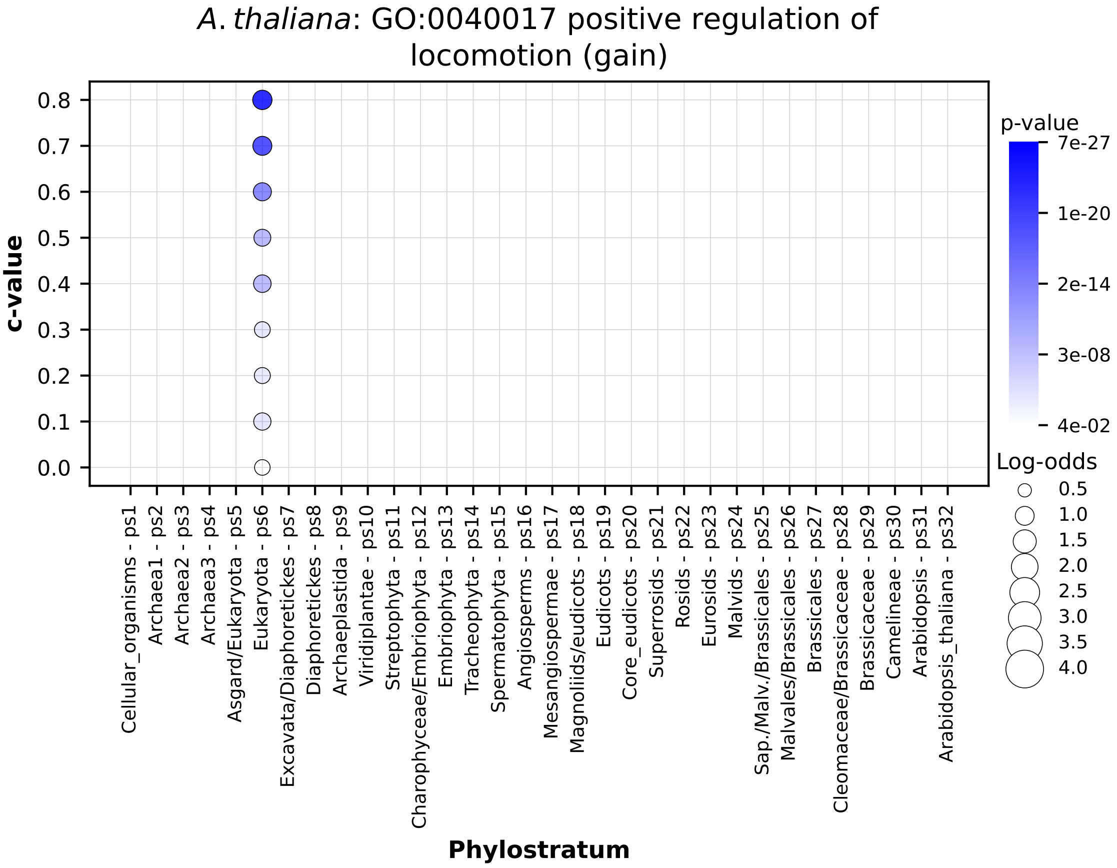

A. thaliana: GO:0040017 positive regulation of locomotion (gain)
c-value
0.0
0.1
0.2
0.3
0.4
0.5
0.6
0.7
0.8
p-value
7e-27
1e-20
2e-14
3e-08
4e-02
Log-odds
0.5
1.0
1.5
2.0
2.5
3.0
3.5
4.0
Cellular_organisms - ps1
Archaea1 - ps2
Archaea2 - ps3
Archaea3 - ps4
Asgard/Eukaryota - ps5
Eukaryota - ps6
Excavata/Diaphoretickes - ps7
Diaphoretickes - ps8
Archaeplastida - ps9
Viridiplantae - ps10
Streptophyta - ps11
Charophyceae/Embriophyta - ps12
Embriophyta - ps13
Tracheophyta - ps14
Spermatophyta - ps15
Angiosperms - ps16
Mesangiospermae - ps17
Magnoliids/eudicots - ps18
Eudicots - ps19
Core_eudicots - ps20
Superrosids - ps21
Rosids - ps22
Eurosids - ps23
Malvids - ps24
Sap./Malv./Brassicales - ps25
Malvales/Brassicales - ps26
Brassicales - ps27
Cleomaceae/Brassicaceae - ps28
Brassicaceae - ps29
Camelineae - ps30
Arabidopsis - ps31
Arabidopsis_thaliana - ps32
Phylostratum

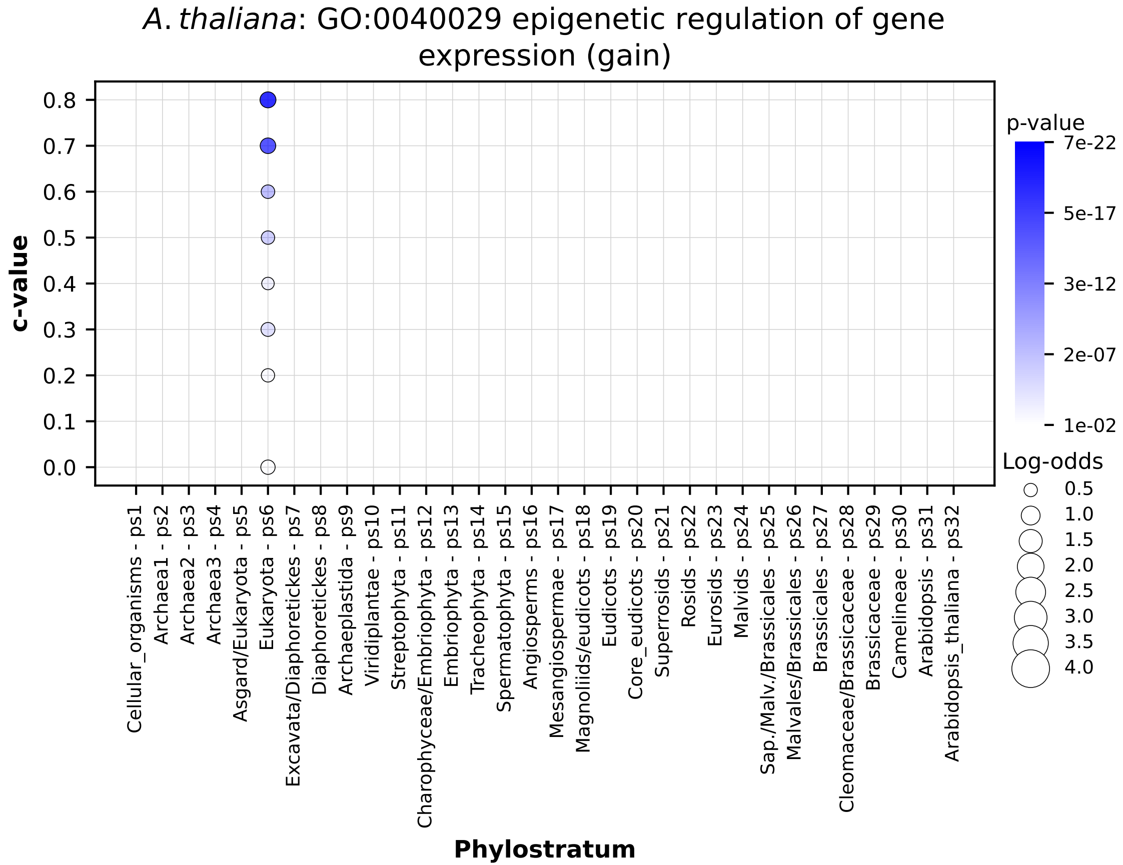

A. thaliana: GO:0040029 epigenetic regulation of gene expression (gain)
c-value
0.0
0.1
0.2
0.3
0.4
0.5
0.6
0.7
0.8
p-value
7e-22
5e-17
3e-12
2e-07
1e-02
Log-odds
0.5
1.0
1.5
2.0
2.5
3.0
3.5
4.0
Cellular_organisms - ps1
Archaea1 - ps2
Archaea2 - ps3
Archaea3 - ps4
Asgard/Eukaryota - ps5
Eukaryota - ps6
Excavata/Diaphoretickes - ps7
Diaphoretickes - ps8
Archaeplastida - ps9
Viridiplantae - ps10
Streptophyta - ps11
Charophyceae/Embriophyta - ps12
Embriophyta - ps13
Tracheophyta - ps14
Spermatophyta - ps15
Angiosperms - ps16
Mesangiospermae - ps17
Magnoliids/eudicots - ps18
Eudicots - ps19
Core_eudicots - ps20
Superrosids - ps21
Rosids - ps22
Eurosids - ps23
Malvids - ps24
Sap./Malv./Brassicales - ps25
Malvales/Brassicales - ps26
Brassicales - ps27
Cleomaceae/Brassicaceae - ps28
Brassicaceae - ps29
Camelineae - ps30
Arabidopsis - ps31
Arabidopsis_thaliana - ps32
Phylostratum

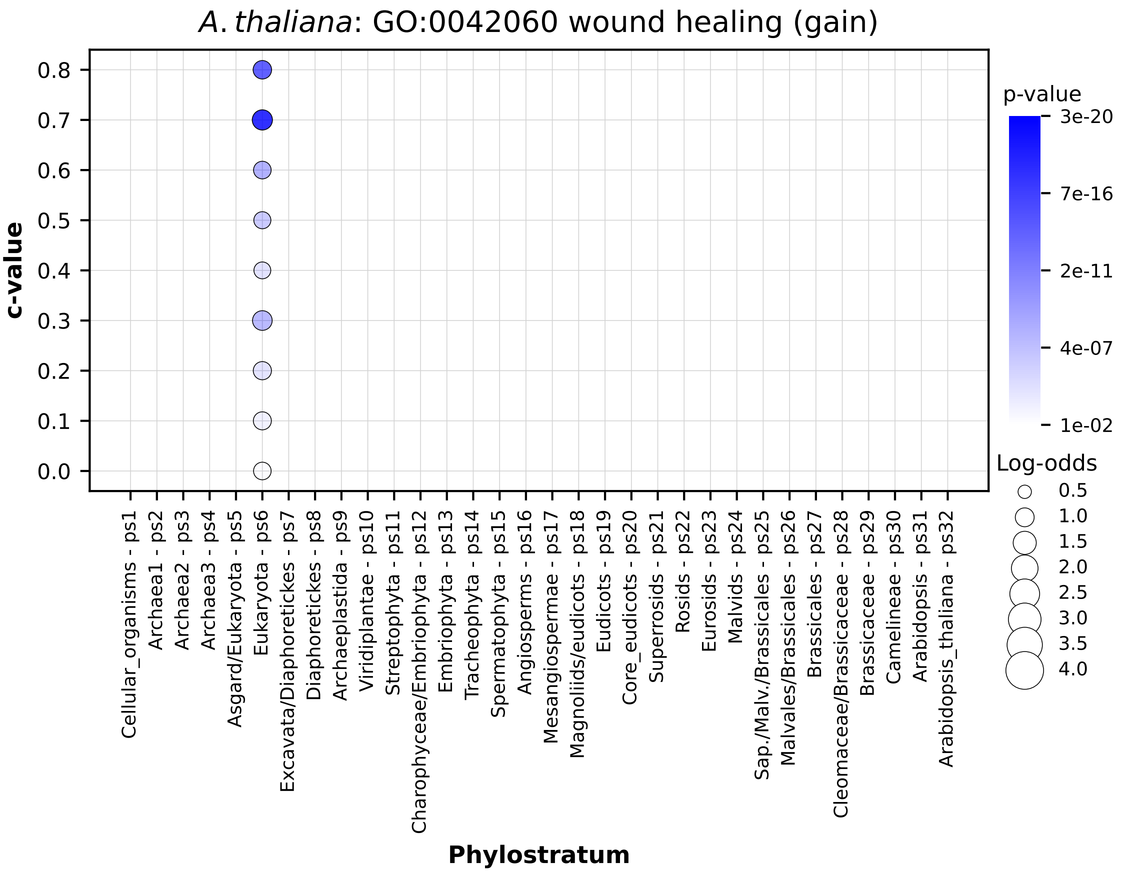
A. thaliana: GO:0042060 wound healing (gain)
c-value
0.0
0.1
0.2
0.3
0.4
0.5
0.6
0.7
0.8
Phylostratum
Cellular_organisms - ps1
Archaea1 - ps2
Archaea2 - ps3
Archaea3 - ps4
Asgard/Eukaryota - ps5
Eukaryota - ps6
Excavata/Diaphoretickes - ps7
Diaphoretickes - ps8
Archaeplastida - ps9
Viridiplantae - ps10
Streptophyta - ps11
Charophyceae/Embriophyta - ps12
Embriophyta - ps13
Tracheophyta - ps14
Spermatophyta - ps15
Angiosperms - ps16
Mesangiospermae - ps17
Magnoliids/eudicots - ps18
Eudicots - ps19
Core_eudicots - ps20
Superrosids - ps21
Rosids - ps22
Eurosids - ps23
Malvids - ps24
Sap./Malv./Brassicales - ps25
Malvales/Brassicales - ps26
Brassicales - ps27
Cleomaceae/Brassicaceae - ps28
Brassicaceae - ps29
Camelineae - ps30
Arabidopsis - ps31
Arabidopsis_thaliana - ps32
p-value
3e-20
7e-16
2e-11
4e-07
1e-02
Log-odds
0.5
1.0
1.5
2.0
2.5
3.0
3.5
4.0

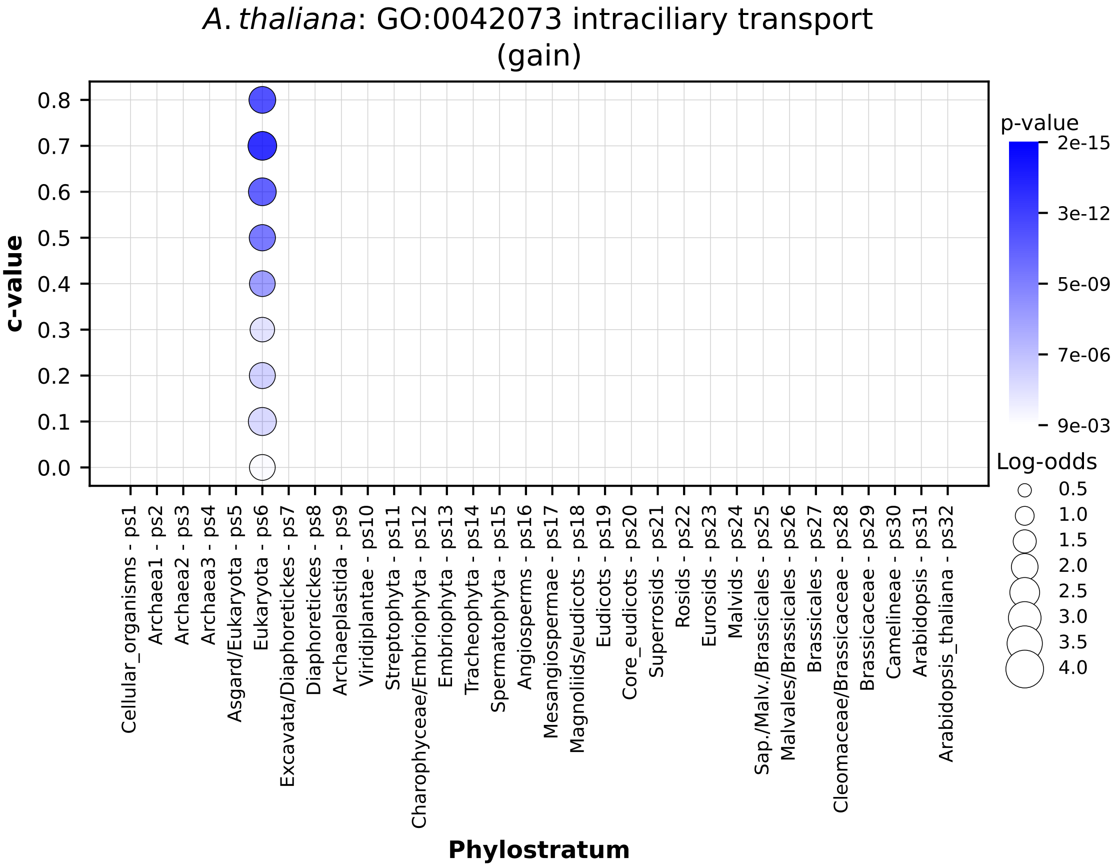

A. thaliana: GO:0042073 intraciliary transport
(gain)
c-value
0.8
0.7
0.6
0.5
0.4
0.3
0.2
0.1
0.0
p-value
2e-15
3e-12
5e-09
7e-06
9e-03
Log-odds
0.5
1.0
1.5
2.0
2.5
3.0
3.5
4.0
Cellular_organisms - ps1
Archaea1 - ps2
Archaea2 - ps3
Archaea3 - ps4
Asgard/Eukaryota - ps5
Eukaryota - ps6
Excavata/Diaphoretickes - ps7
Diaphoretickes - ps8
Archaeplastida - ps9
Viridiplantae - ps10
Streptophyta - ps11
Charophyceae/Embriophyta - ps12
Embriophyta - ps13
Tracheophyta - ps14
Spermatophyta - ps15
Angiosperms - ps16
Mesangiospermae - ps17
Magnoliids/eudicots - ps18
Eudicots - ps19
Core_eudicots - ps20
Superrosids - ps21
Rosids - ps22
Eurosids - ps23
Malvids - ps24
Sap./Malv./Brassicales - ps25
Malvales/Brassicales - ps26
Brassicales - ps27
Cleomaceae/Brassicaceae - ps28
Brassicaceae - ps29
Camelineae - ps30
Arabidopsis - ps31
Arabidopsis_thaliana - ps32
Phylostratum

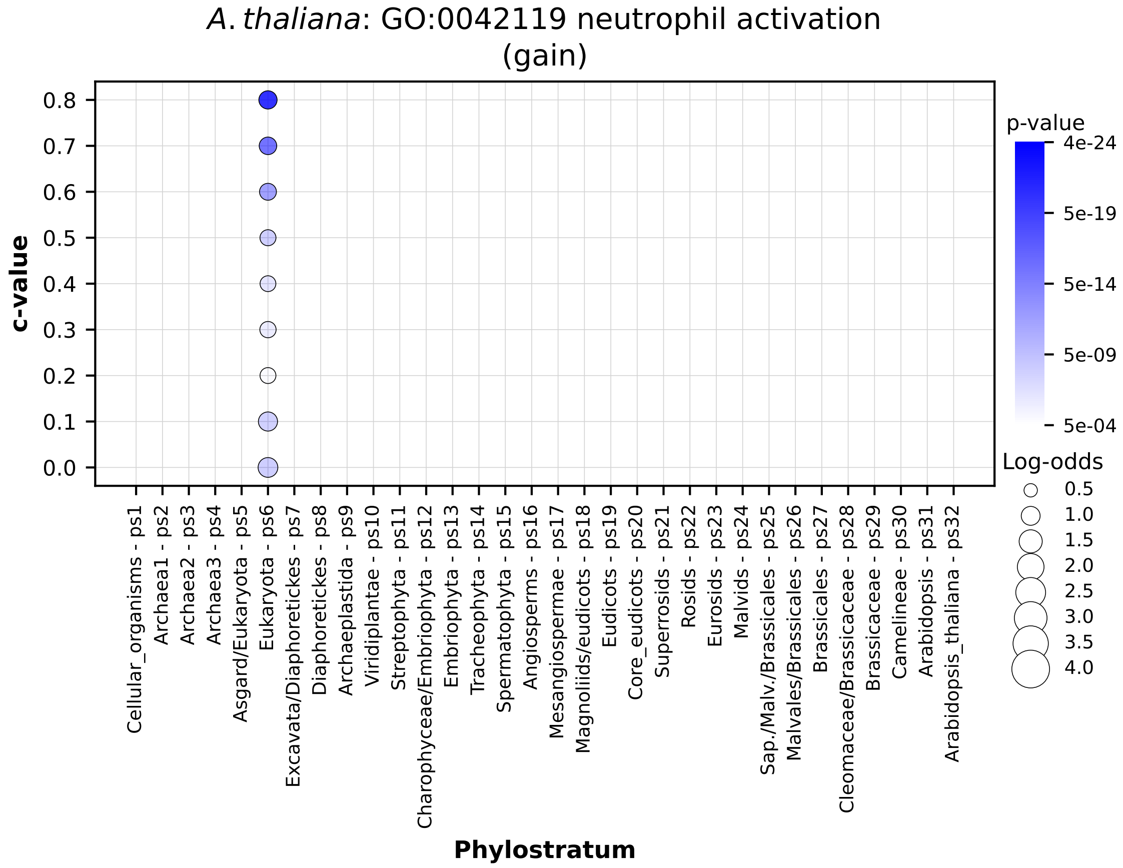

A. thaliana: GO:0042119 neutrophil activation
(gain)
c-value
0.8
0.7
0.6
0.5
0.4
0.3
0.2
0.1
0.0
p-value
4e-24
5e-19
5e-14
5e-09
5e-04
Log-odds
0.5
1.0
1.5
2.0
2.5
3.0
3.5
4.0
Cellular_organisms - ps1
Archaea1 - ps2
Archaea2 - ps3
Archaea3 - ps4
Asgard/Eukaryota - ps5
Eukaryota - ps6
Excavata/Diaphoretickes - ps7
Diaphoretickes - ps8
Archaeplastida - ps9
Viridiplantae - ps10
Streptophyta - ps11
Charophyceae/Embriophyta - ps12
Embriophyta - ps13
Tracheophyta - ps14
Spermatophyta - ps15
Angiosperms - ps16
Mesangiospermae - ps17
Magnoliids/eudicots - ps18
Eudicots - ps19
Core_eudicots - ps20
Superrosids - ps21
Rosids - ps22
Eurosids - ps23
Malvids - ps24
Sap./Malv./Brassicales - ps25
Malvales/Brassicales - ps26
Brassicales - ps27
Cleomaceae/Brassicaceae - ps28
Brassicaceae - ps29
Camelineae - ps30
Arabidopsis - ps31
Arabidopsis_thaliana - ps32
Phylostratum

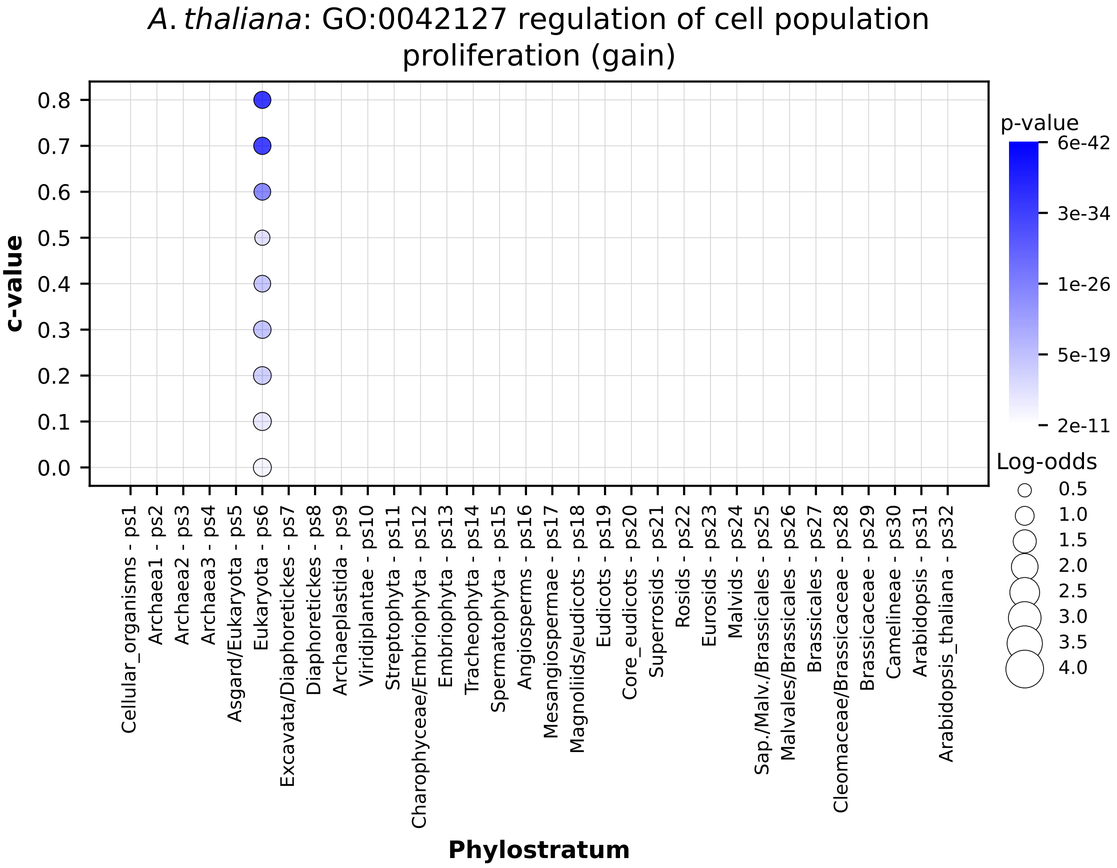

A. thaliana: GO:0042127 regulation of cell population proliferation (gain)
c-value
0.8
0.7
0.6
0.5
0.4
0.3
0.2
0.1
0.0
Phylostratum
Cellular_organisms - ps1
Archaea1 - ps2
Archaea2 - ps3
Archaea3 - ps4
Asgard/Eukaryota - ps5
Eukaryota - ps6
Excavata/Diaphoretickes - ps7
Diaphoretickes - ps8
Archaeplastida - ps9
Viridiplantae - ps10
Streptophyta - ps11
Charophyceae/Embriophyta - ps12
Embriophyta - ps13
Tracheophyta - ps14
Spermatophyta - ps15
Angiosperms - ps16
Mesangiospermae - ps17
Magnoliids/eudicots - ps18
Eudicots - ps19
Core_eudicots - ps20
Superrosids - ps21
Rosids - ps22
Eurosids - ps23
Malvids - ps24
Sap./Malv./Brassicales - ps25
Malvales/Brassicales - ps26
Brassicales - ps27
Cleomaceae/Brassicaceae - ps28
Brassicaceae - ps29
Camelineae - ps30
Arabidopsis - ps31
Arabidopsis_thaliana - ps32
p-value
6e-42
3e-34
1e-26
5e-19
2e-11
Log-odds
0.5
1.0
1.5
2.0
2.5
3.0
3.5
4.0

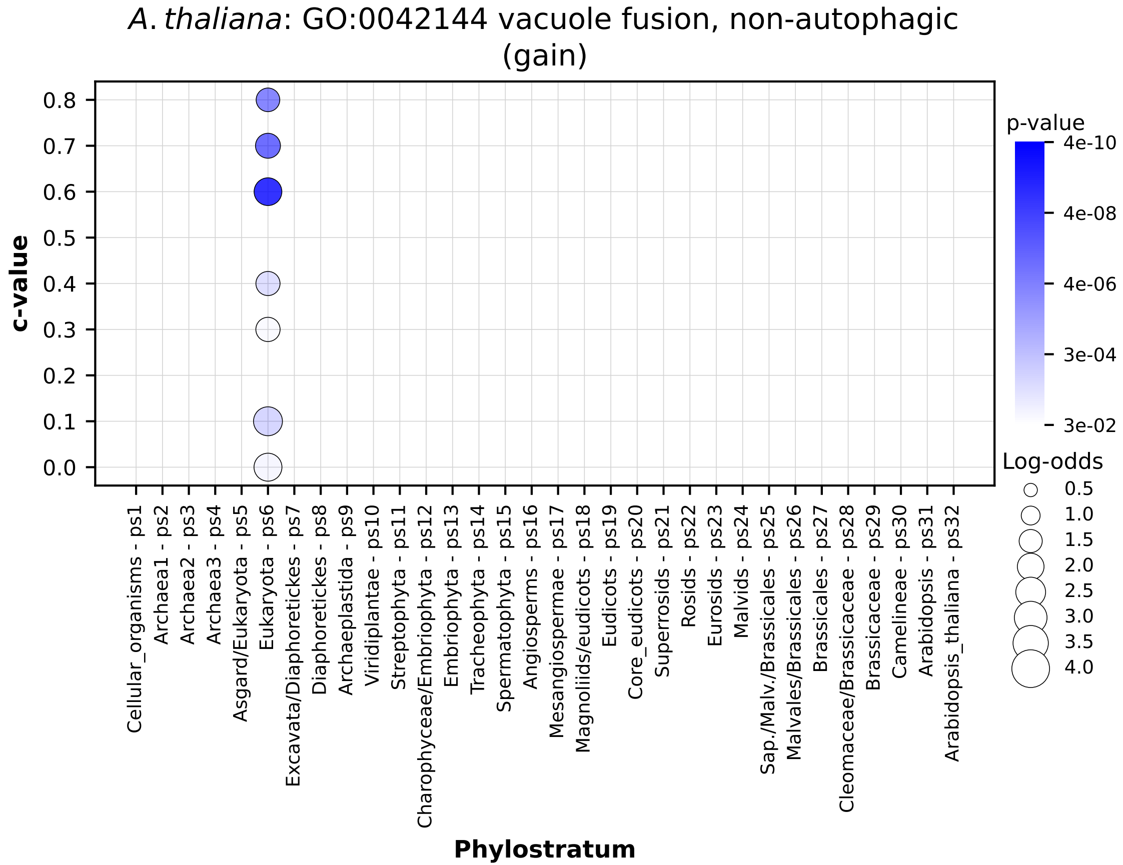

A. thaliana: GO:0042144 vacuole fusion, non-autophagic (gain)
c-value
0.8
0.7
0.6
0.5
0.4
0.3
0.2
0.1
0.0
p-value
4e-10
4e-08
4e-06
3e-04
3e-02
Log-odds
0.5
1.0
1.5
2.0
2.5
3.0
3.5
4.0
Cellular_organisms - ps1
Archaea1 - ps2
Archaea2 - ps3
Archaea3 - ps4
Asgard/Eukaryota - ps5
Eukaryota - ps6
Excavata/Diaphoretickes - ps7
Diaphoretickes - ps8
Archaeplastida - ps9
Viridiplantae - ps10
Streptophyta - ps11
Charophyceae/Embriophyta - ps12
Embriophyta - ps13
Tracheophyta - ps14
Spermatophyta - ps15
Angiosperms - ps16
Mesangiospermae - ps17
Magnoliids/eudicots - ps18
Eudicots - ps19
Core_eudicots - ps20
Superrosids - ps21
Rosids - ps22
Eurosids - ps23
Malvids - ps24
Sap./Malv./Brassicales - ps25
Malvales/Brassicales - ps26
Brassicales - ps27
Cleomaceae/Brassicaceae - ps28
Brassicaceae - ps29
Camelineae - ps30
Arabidopsis - ps31
Arabidopsis_thaliana - ps32
Phylostratum

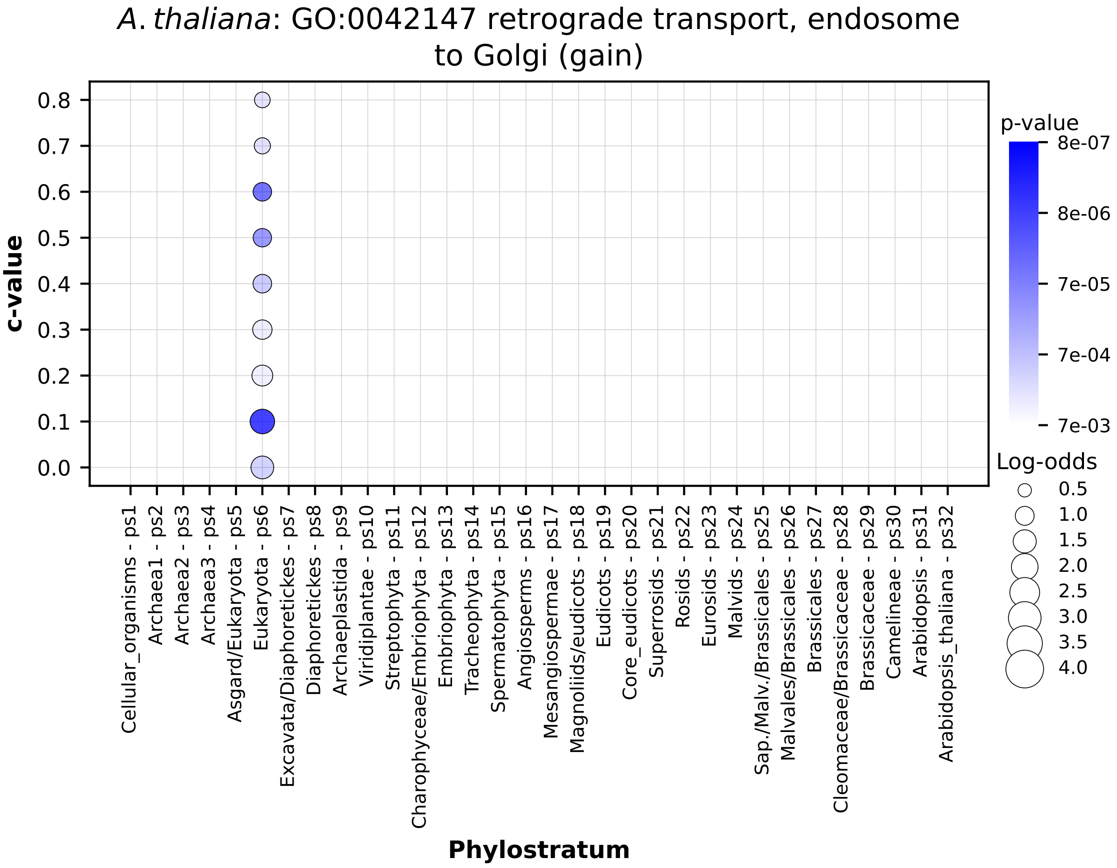

A. thaliana: GO:0042147 retrograde transport, endosome to Golgi (gain)
c-value
0.8
0.7
0.6
0.5
0.4
0.3
0.2
0.1
0.0
p-value
8e-07
8e-06
7e-05
7e-04
7e-03
Log-odds
0.5
1.0
1.5
2.0
2.5
3.0
3.5
4.0
Cellular_organisms - ps1
Archaea1 - ps2
Archaea2 - ps3
Archaea3 - ps4
Asgard/Eukaryota - ps5
Eukaryota - ps6
Excavata/Diaphoretickes - ps7
Diaphoretickes - ps8
Archaeplastida - ps9
Viridiplantae - ps10
Streptophyta - ps11
Charophyceae/Embriophyta - ps12
Embriophyta - ps13
Tracheophyta - ps14
Spermatophyta - ps15
Angiosperms - ps16
Mesangiospermae - ps17
Magnoliids/eudicots - ps18
Eudicots - ps19
Core_eudicots - ps20
Superrosids - ps21
Rosids - ps22
Eurosids - ps23
Malvids - ps24
Sap./Malv./Brassicales - ps25
Malvales/Brassicales - ps26
Brassicales - ps27
Cleomaceae/Brassicaceae - ps28
Brassicaceae - ps29
Camelineae - ps30
Arabidopsis - ps31
Arabidopsis_thaliana - ps32
Phylostratum

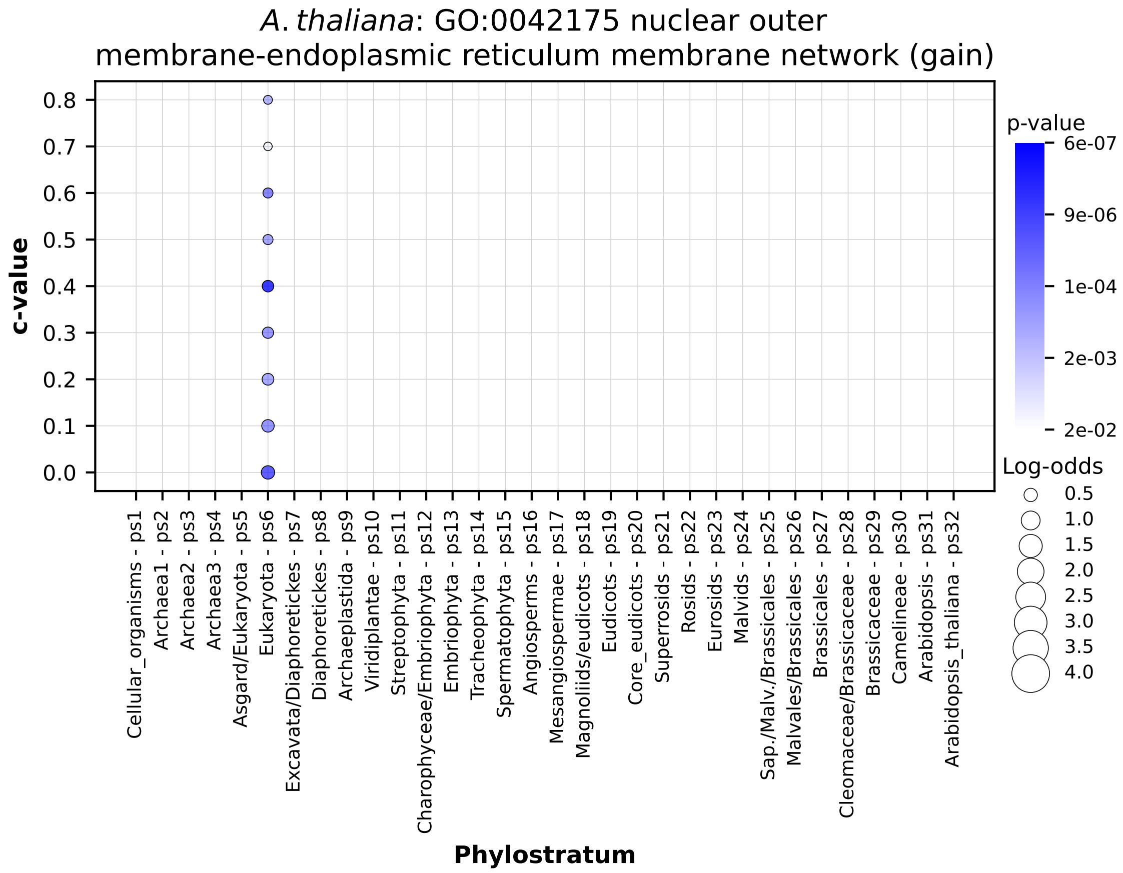
A. thaliana: GO:0042175 nuclear outer membrane-endoplasmic reticulum membrane network (gain)
c-value
0.8
0.7
0.6
0.5
0.4
0.3
0.2
0.1
0.0
p-value
6e-07
9e-06
1e-04
2e-03
2e-02
Log-odds
0.5
1.0
1.5
2.0
2.5
3.0
3.5
4.0
Cellular_organisms - ps1
Archaea1 - ps2
Archaea2 - ps3
Archaea3 - ps4
Asgard/Eukaryota - ps5
Eukaryota - ps6
Excavata/Diaphoretickes - ps7
Diaphoretickes - ps8
Archaeplastida - ps9
Viridiplantae - ps10
Streptophyta - ps11
Charophyceae/Embriophyta - ps12
Embriophyta - ps13
Tracheophyta - ps14
Spermatophyta - ps15
Angiosperms - ps16
Mesangiospermae - ps17
Magnoliids/eudicots - ps18
Eudicots - ps19
Core_eudicots - ps20
Superrosids - ps21
Rosids - ps22
Eurosids - ps23
Malvids - ps24
Sap./Malv./Brassicales - ps25
Malvales/Brassicales - ps26
Brassicales - ps27
Cleomaceae/Brassicaceae - ps28
Brassicaceae - ps29
Camelineae - ps30
Arabidopsis - ps31
Arabidopsis_thaliana - ps32
Phylostratum

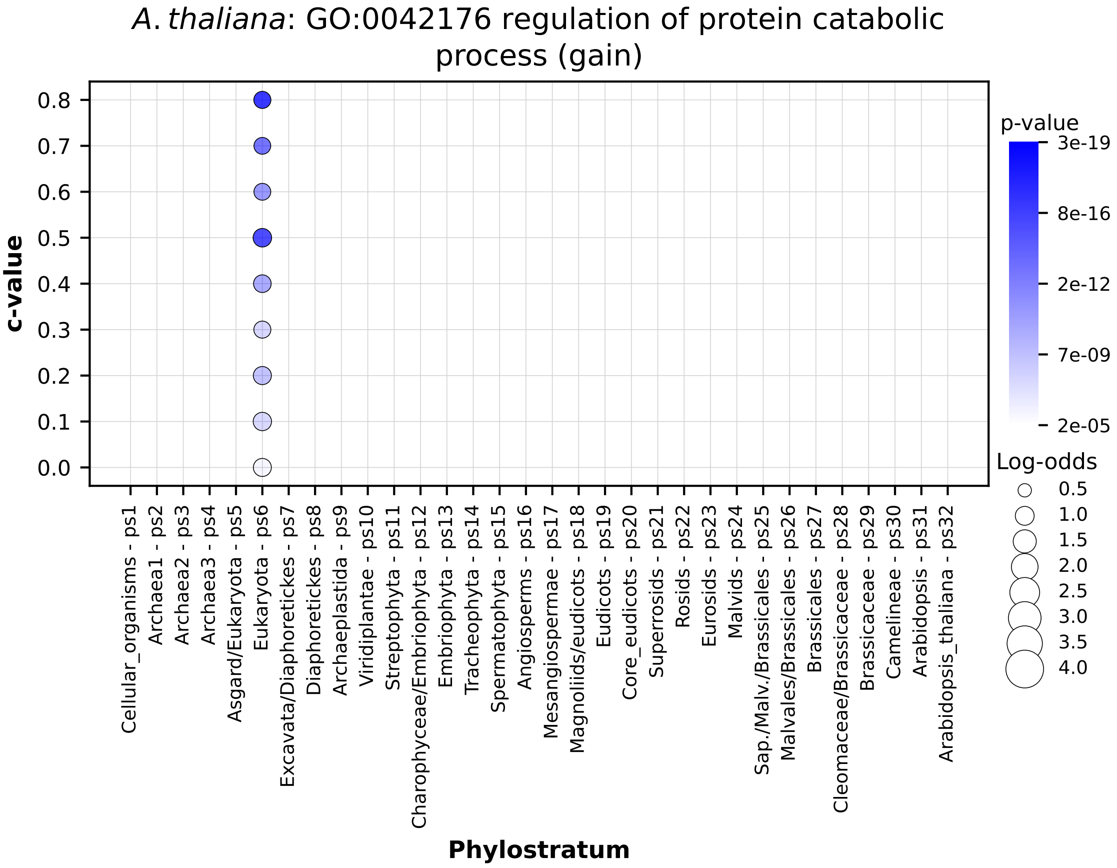

A. thaliana: GO:0042176 regulation of protein catabolic process (gain)
c-value
0.8
0.7
0.6
0.5
0.4
0.3
0.2
0.1
0.0
p-value
3e-19
8e-16
2e-12
7e-09
2e-05
Log-odds
0.5
1.0
1.5
2.0
2.5
3.0
3.5
4.0
Cellular_organisms - ps1
Archaea1 - ps2
Archaea2 - ps3
Archaea3 - ps4
Asgard/Eukaryota - ps5
Eukaryota - ps6
Excavata/Diaphoretickes - ps7
Diaphoretickes - ps8
Archaeplastida - ps9
Viridiplantae - ps10
Streptophyta - ps11
Charophyceae/Embriophyta - ps12
Embriophyta - ps13
Tracheophyta - ps14
Spermatophyta - ps15
Angiosperms - ps16
Mesangiospermae - ps17
Magnoliids/eudicots - ps18
Eudicots - ps19
Core_eudicots - ps20
Superrosids - ps21
Rosids - ps22
Eurosids - ps23
Malvids - ps24
Sap./Malv./Brassicales - ps25
Malvales/Brassicales - ps26
Brassicales - ps27
Cleomaceae/Brassicaceae - ps28
Brassicaceae - ps29
Camelineae - ps30
Arabidopsis - ps31
Arabidopsis_thaliana - ps32
Phylostratum

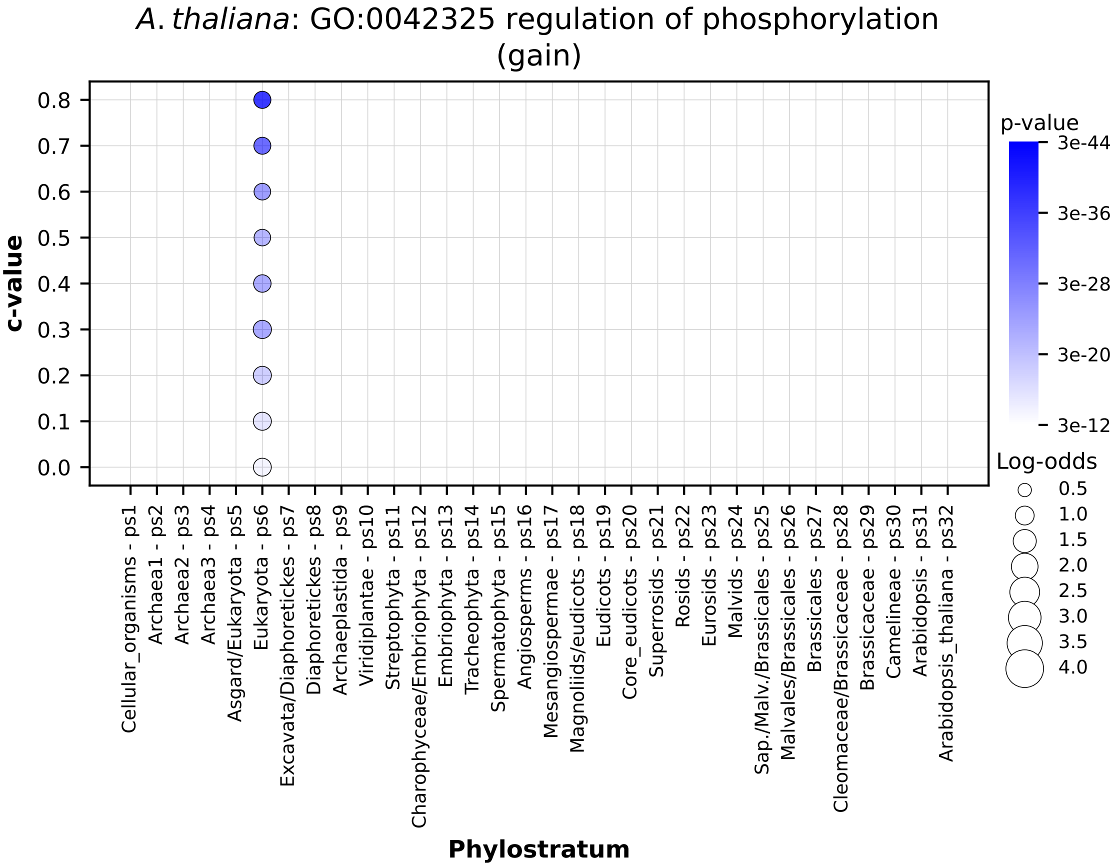
A. thaliana: GO:0042325 regulation of phosphorylation (gain)
c-value
0.8
0.7
0.6
0.5
0.4
0.3
0.2
0.1
0.0
p-value
3e-44
3e-36
3e-28
3e-20
3e-12
Log-odds
0.5
1.0
1.5
2.0
2.5
3.0
3.5
4.0
Cellular_organisms - ps1
Archaea1 - ps2
Archaea2 - ps3
Archaea3 - ps4
Asgard/Eukaryota - ps5
Eukaryota - ps6
Excavata/Diaphoretickes - ps7
Diaphoretickes - ps8
Archaeplastida - ps9
Viridiplantae - ps10
Streptophyta - ps11
Charophyceae/Embriophyta - ps12
Embriophyta - ps13
Tracheophyta - ps14
Spermatophyta - ps15
Angiosperms - ps16
Mesangiospermae - ps17
Magnoliids/eudicots - ps18
Eudicots - ps19
Core_eudicots - ps20
Superrosids - ps21
Rosids - ps22
Eurosids - ps23
Malvids - ps24
Sap./Malv./Brassicales - ps25
Malvales/Brassicales - ps26
Brassicales - ps27
Cleomaceae/Brassicaceae - ps28
Brassicaceae - ps29
Camelineae - ps30
Arabidopsis - ps31
Arabidopsis_thaliana - ps32
Phylostratum

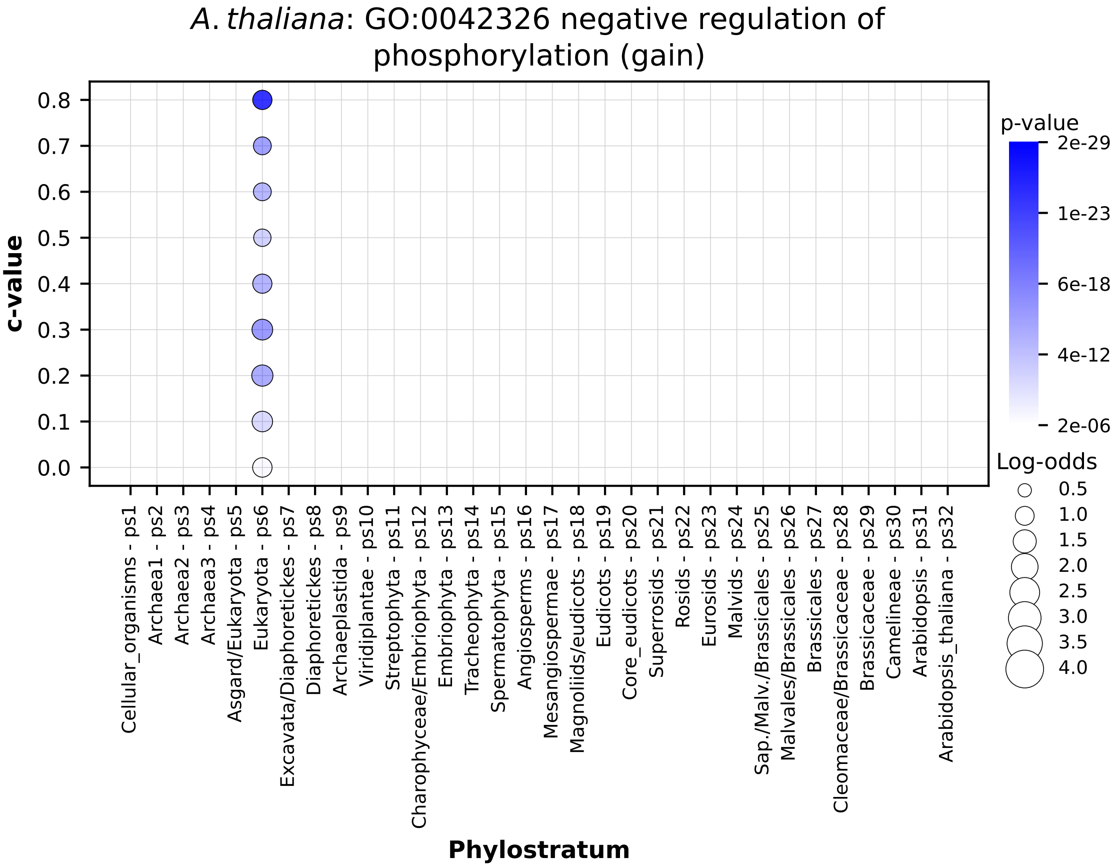

A. thaliana: GO:0042326 negative regulation of phosphorylation (gain)
c-value
0.8
0.7
0.6
0.5
0.4
0.3
0.2
0.1
0.0
p-value
2e-29
1e-23
6e-18
4e-12
2e-06
Log-odds
0.5
1.0
1.5
2.0
2.5
3.0
3.5
4.0
Cellular_organisms - ps1
Archaea1 - ps2
Archaea2 - ps3
Archaea3 - ps4
Asgard/Eukaryota - ps5
Eukaryota - ps6
Excavata/Diaphoretickes - ps7
Diaphoretickes - ps8
Archaeplastida - ps9
Viridiplantae - ps10
Streptophyta - ps11
Charophyceae/Embriophyta - ps12
Embriophyta - ps13
Tracheophyta - ps14
Spermatophyta - ps15
Angiosperms - ps16
Mesangiospermae - ps17
Magnoliids/eudicots - ps18
Eudicots - ps19
Core_eudicots - ps20
Superrosids - ps21
Rosids - ps22
Eurosids - ps23
Malvids - ps24
Sap./Malv./Brassicales - ps25
Malvales/Brassicales - ps26
Brassicales - ps27
Cleomaceae/Brassicaceae - ps28
Brassicaceae - ps29
Camelineae - ps30
Arabidopsis - ps31
Arabidopsis_thaliana - ps32
Phylostratum

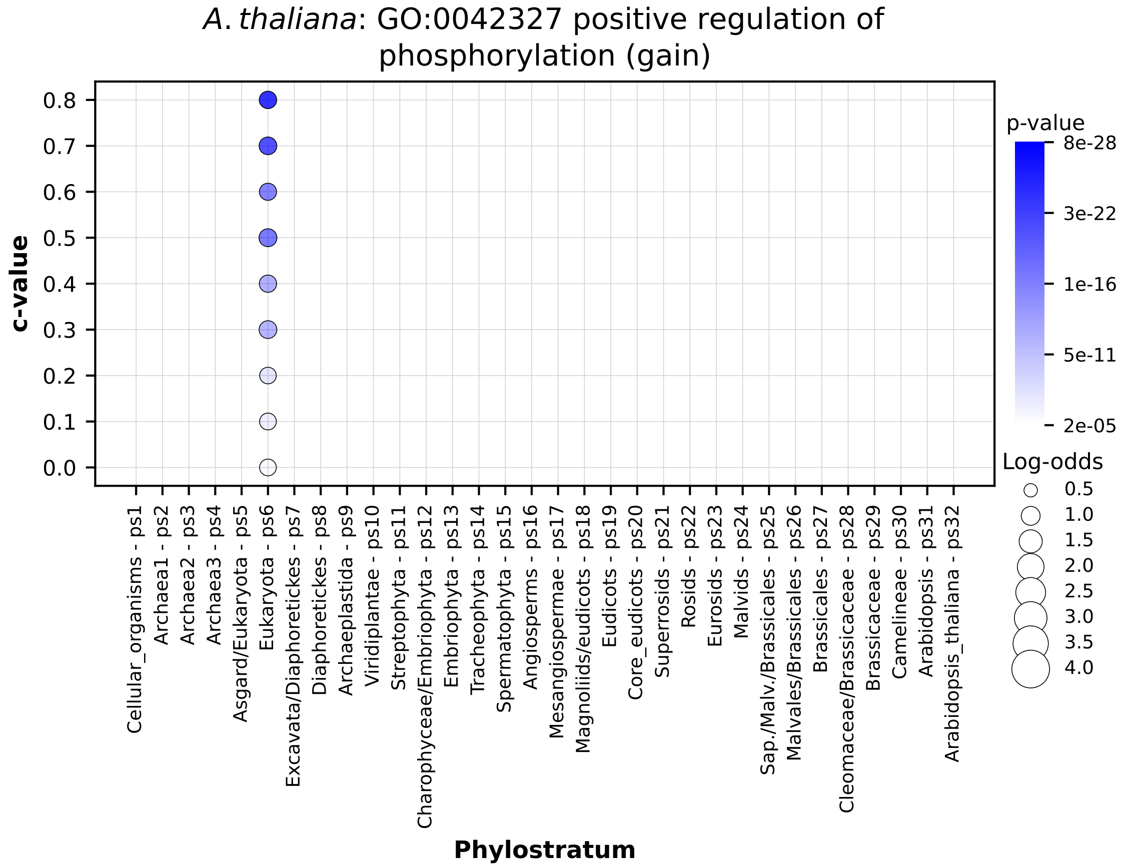

A. thaliana: GO:0042327 positive regulation of phosphorylation (gain)
c-value
0.8
0.7
0.6
0.5
0.4
0.3
0.2
0.1
0.0
p-value
8e-28
3e-22
1e-16
5e-11
2e-05
Log-odds
0.5
1.0
1.5
2.0
2.5
3.0
3.5
4.0
Cellular_organisms - ps1
Archaea1 - ps2
Archaea2 - ps3
Archaea3 - ps4
Asgard/Eukaryota - ps5
Eukaryota - ps6
Excavata/Diaphoretickes - ps7
Diaphoretickes - ps8
Archaeplastida - ps9
Viridiplantae - ps10
Streptophyta - ps11
Charophyceae/Embriophyta - ps12
Embriophyta - ps13
Tracheophyta - ps14
Spermatophyta - ps15
Angiosperms - ps16
Mesangiospermae - ps17
Magnoliids/eudicots - ps18
Eudicots - ps19
Core_eudicots - ps20
Superrosids - ps21
Rosids - ps22
Eurosids - ps23
Malvids - ps24
Sap./Malv./Brassicales - ps25
Malvales/Brassicales - ps26
Brassicales - ps27
Cleomaceae/Brassicaceae - ps28
Brassicaceae - ps29
Camelineae - ps30
Arabidopsis - ps31
Arabidopsis_thaliana - ps32
Phylostratum

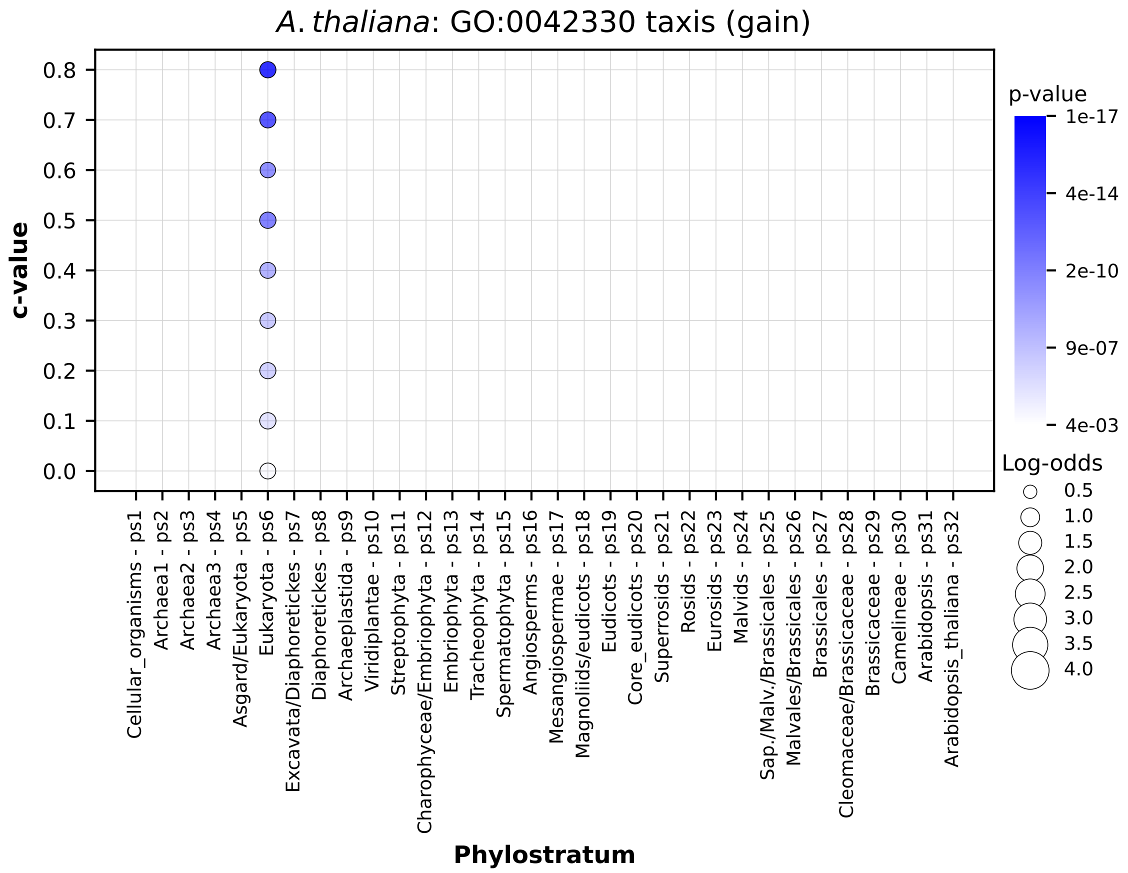
A. thaliana: GO:0042330 taxis (gain)
c-value
0.8
0.7
0.6
0.5
0.4
0.3
0.2
0.1
0.0
p-value
1e-17
4e-14
2e-10
9e-07
4e-03
Log-odds
0.5
1.0
1.5
2.0
2.5
3.0
3.5
4.0
Cellular_organisms - ps1
Archaea1 - ps2
Archaea2 - ps3
Archaea3 - ps4
Asgard/Eukaryota - ps5
Eukaryota - ps6
Excavata/Diaphoretickes - ps7
Diaphoretickes - ps8
Archaeplastida - ps9
Viridiplantae - ps10
Streptophyta - ps11
Charophyceae/Embriophyta - ps12
Embriophyta - ps13
Tracheophyta - ps14
Spermatophyta - ps15
Angiosperms - ps16
Mesangiospermae - ps17
Magnoliids/eudicots - ps18
Eudicots - ps19
Core_eudicots - ps20
Superrosids - ps21
Rosids - ps22
Eurosids - ps23
Malvids - ps24
Sap./Malv./Brassicales - ps25
Malvales/Brassicales - ps26
Brassicales - ps27
Cleomaceae/Brassicaceae - ps28
Brassicaceae - ps29
Camelineae - ps30
Arabidopsis - ps31
Arabidopsis_thaliana - ps32
Phylostratum

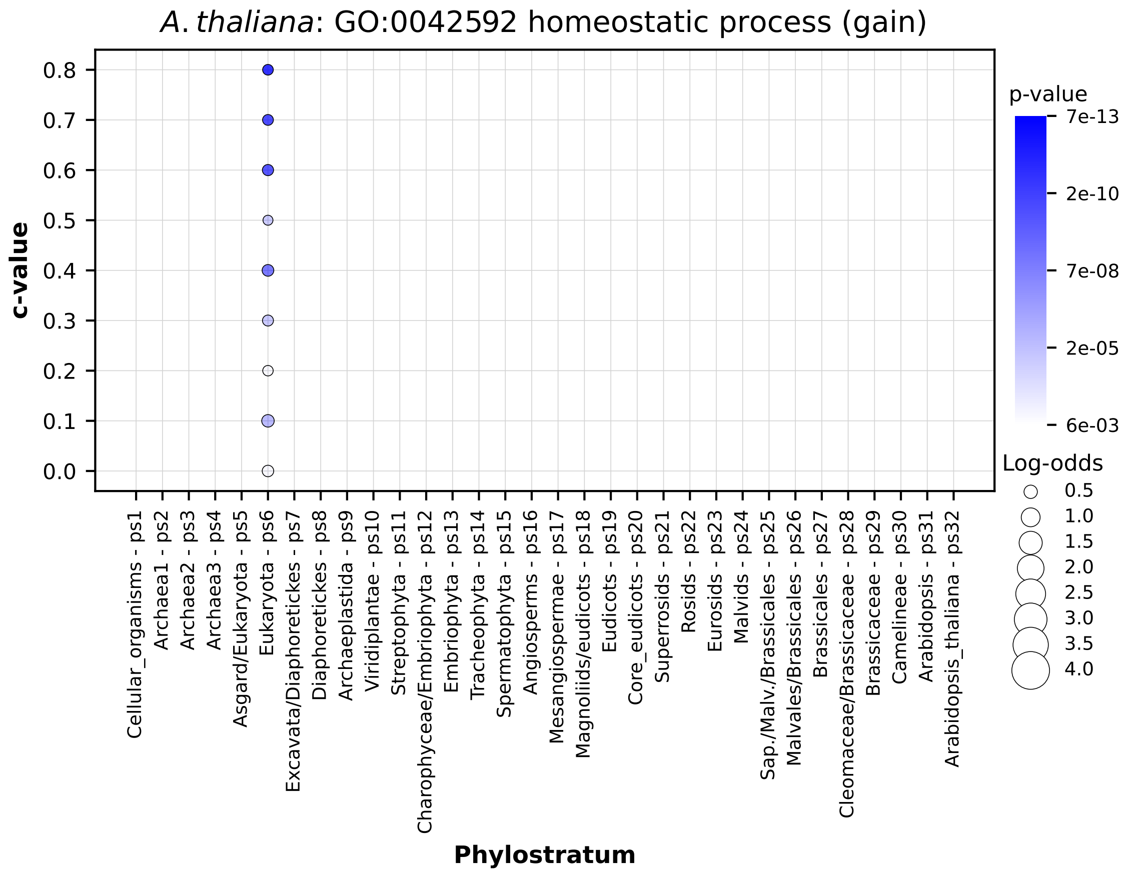
A. thaliana: GO:0042592 homeostatic process (gain)
c-value
0.8
0.7
0.6
0.5
0.4
0.3
0.2
0.1
0.0
p-value
7e-13
2e-10
7e-08
2e-05
6e-03
Log-odds
0.5
1.0
1.5
2.0
2.5
3.0
3.5
4.0
Cellular_organisms - ps1
Archaea1 - ps2
Archaea2 - ps3
Archaea3 - ps4
Asgard/Eukaryota - ps5
Eukaryota - ps6
Excavata/Diaphoretickes - ps7
Diaphoretickes - ps8
Archaeplastida - ps9
Viridiplantae - ps10
Streptophyta - ps11
Charophyceae/Embriophyta - ps12
Embriophyta - ps13
Tracheophyta - ps14
Spermatophyta - ps15
Angiosperms - ps16
Mesangiospermae - ps17
Magnoliids/eudicots - ps18
Eudicots - ps19
Core_eudicots - ps20
Superrosids - ps21
Rosids - ps22
Eurosids - ps23
Malvids - ps24
Sap./Malv./Brassicales - ps25
Malvales/Brassicales - ps26
Brassicales - ps27
Cleomaceae/Brassicaceae - ps28
Brassicaceae - ps29
Camelineae - ps30
Arabidopsis - ps31
Arabidopsis_thaliana - ps32
Phylostratum

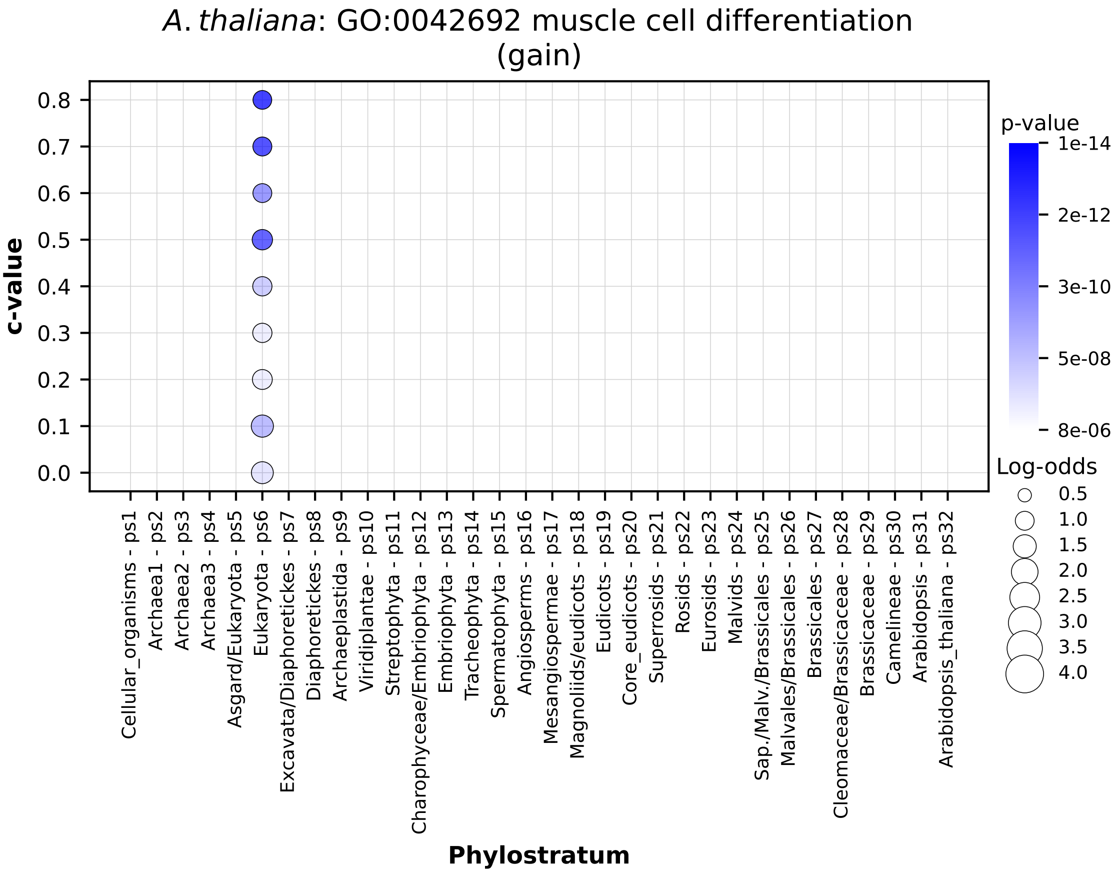

A. thaliana: GO:0042692 muscle cell differentiation
(gain)
c-value
0.8
0.7
0.6
0.5
0.4
0.3
0.2
0.1
0.0
p-value
1e-14
2e-12
3e-10
5e-08
8e-06
Log-odds
0.5
1.0
1.5
2.0
2.5
3.0
3.5
4.0
Cellular_organisms - ps1
Archaea1 - ps2
Archaea2 - ps3
Archaea3 - ps4
Asgard/Eukaryota - ps5
Eukaryota - ps6
Excavata/Diaphoretickes - ps7
Diaphoretickes - ps8
Archaeplastida - ps9
Viridiplantae - ps10
Streptophyta - ps11
Charophyceae/Embriophyta - ps12
Embriophyta - ps13
Tracheophyta - ps14
Spermatophyta - ps15
Angiosperms - ps16
Mesangiospermae - ps17
Magnoliids/eudicots - ps18
Eudicots - ps19
Core_eudicots - ps20
Superrosids - ps21
Rosids - ps22
Eurosids - ps23
Malvids - ps24
Sap./Malv./Brassicales - ps25
Malvales/Brassicales - ps26
Brassicales - ps27
Cleomaceae/Brassicaceae - ps28
Brassicaceae - ps29
Camelineae - ps30
Arabidopsis - ps31
Arabidopsis_thaliana - ps32
Phylostratum

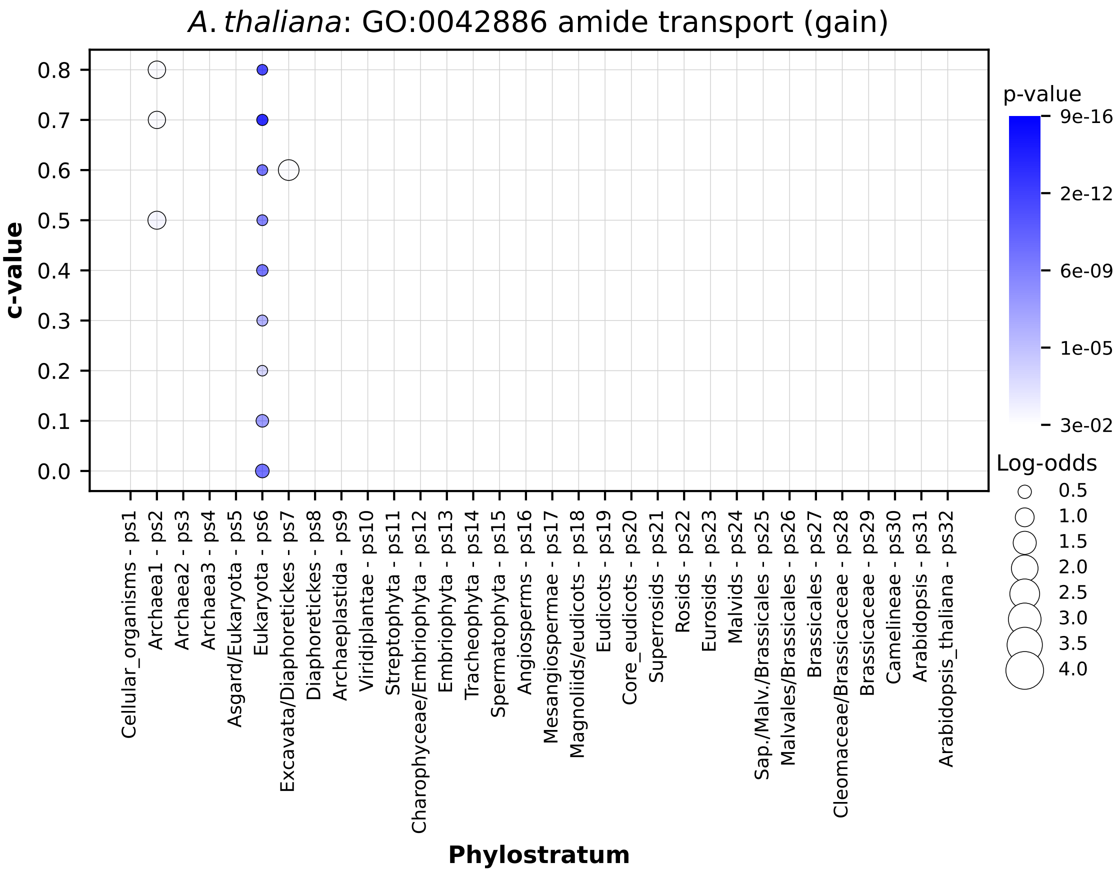
A. thaliana: GO:0042886 amide transport (gain)
c-value
0.0
0.1
0.2
0.3
0.4
0.5
0.6
0.7
0.8
Phylostratum
Cellular_organisms - ps1
Archaea1 - ps2
Archaea2 - ps3
Archaea3 - ps4
Asgard/Eukaryota - ps5
Eukaryota - ps6
Excavata/Diaphoretickes - ps7
Diaphoretickes - ps8
Archaeplastida - ps9
Viridiplantae - ps10
Streptophyta - ps11
Charophyceae/Embriophyta - ps12
Embriophyta - ps13
Tracheophyta - ps14
Spermatophyta - ps15
Angiosperms - ps16
Mesangiospermae - ps17
Magnoliids/eudicots - ps18
Eudicots - ps19
Core_eudicots - ps20
Superrosids - ps21
Rosids - ps22
Eurosids - ps23
Malvids - ps24
Sap./Malv./Brassicales - ps25
Malvales/Brassicales - ps26
Brassicales - ps27
Cleomaceae/Brassicaceae - ps28
Brassicaceae - ps29
Camelineae - ps30
Arabidopsis - ps31
Arabidopsis_thaliana - ps32
p-value
9e-16
2e-12
6e-09
1e-05
3e-02
Log-odds
0.5
1.0
1.5
2.0
2.5
3.0
3.5
4.0

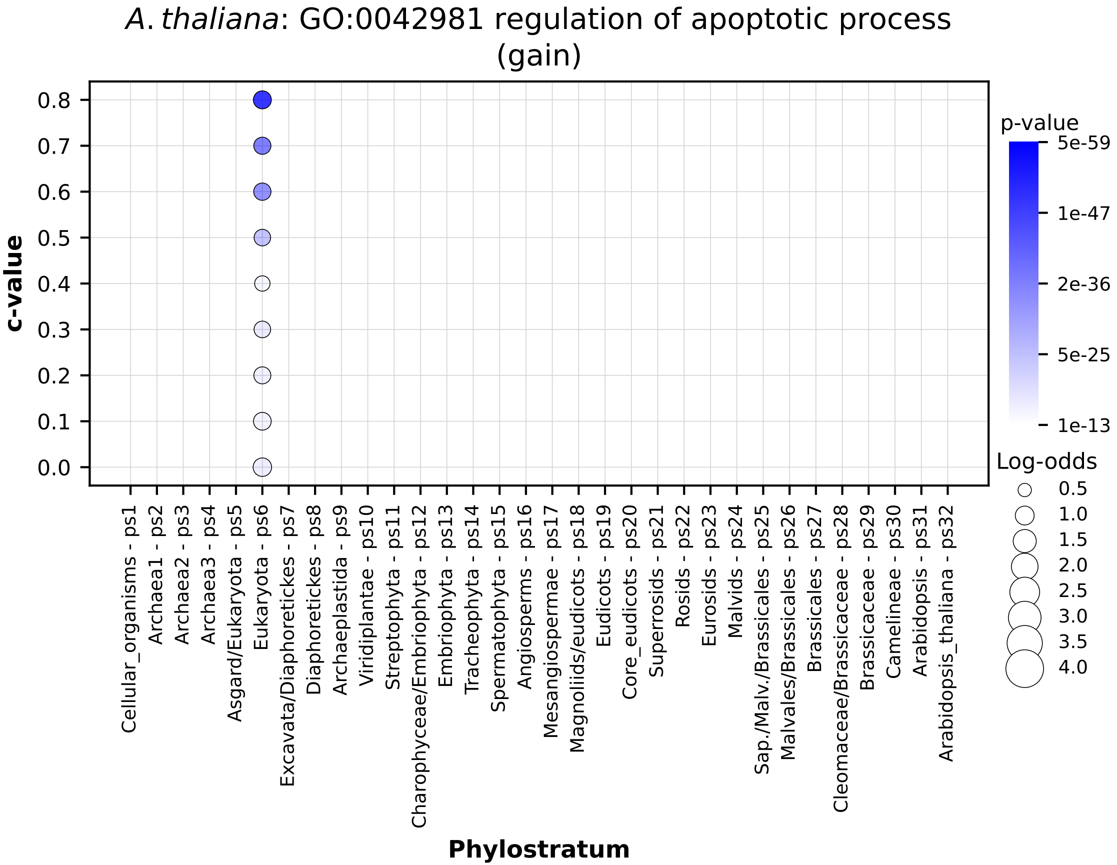

A. thaliana: GO:0042981 regulation of apoptotic process
(gain)
c-value
0.8
0.7
0.6
0.5
0.4
0.3
0.2
0.1
0.0
p-value
5e-59
1e-47
2e-36
5e-25
1e-13
Log-odds
0.5
1.0
1.5
2.0
2.5
3.0
3.5
4.0
Cellular_organisms - ps1
Archaea1 - ps2
Archaea2 - ps3
Archaea3 - ps4
Asgard/Eukaryota - ps5
Eukaryota - ps6
Excavata/Diaphoretickes - ps7
Diaphoretickes - ps8
Archaeplastida - ps9
Viridiplantae - ps10
Streptophyta - ps11
Charophyceae/Embriophyta - ps12
Embriophyta - ps13
Tracheophyta - ps14
Spermatophyta - ps15
Angiosperms - ps16
Mesangiospermae - ps17
Magnoliids/eudicots - ps18
Eudicots - ps19
Core_eudicots - ps20
Superrosids - ps21
Rosids - ps22
Eurosids - ps23
Malvids - ps24
Sap./Malv./Brassicales - ps25
Malvales/Brassicales - ps26
Brassicales - ps27
Cleomaceae/Brassicaceae - ps28
Brassicaceae - ps29
Camelineae - ps30
Arabidopsis - ps31
Arabidopsis_thaliana - ps32
Phylostratum

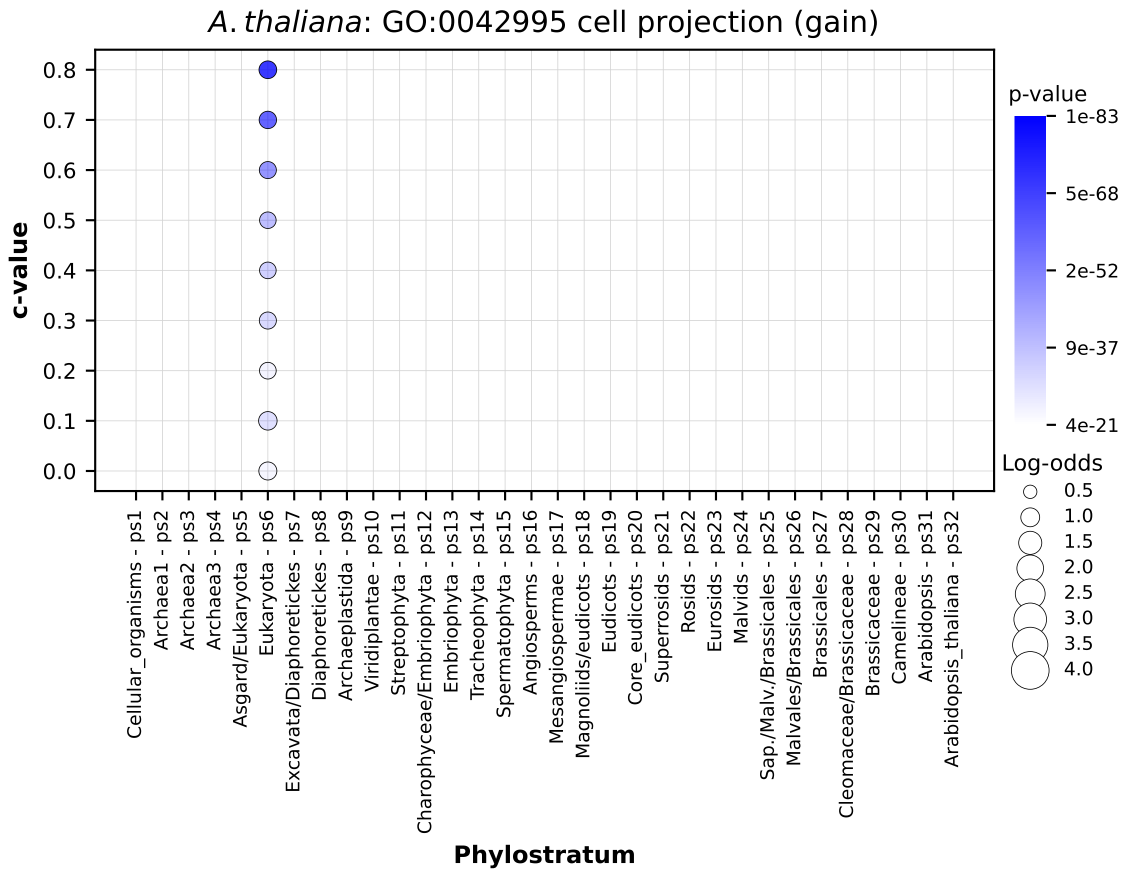

A. thaliana: GO:0042995 cell projection (gain)
c-value
0.8
0.7
0.6
0.5
0.4
0.3
0.2
0.1
0.0
p-value
1e-83
5e-68
2e-52
9e-37
4e-21
Log-odds
0.5
1.0
1.5
2.0
2.5
3.0
3.5
4.0
Cellular_organisms - ps1
Archaea1 - ps2
Archaea2 - ps3
Archaea3 - ps4
Asgard/Eukaryota - ps5
Eukaryota - ps6
Excavata/Diaphoretickes - ps7
Diaphoretickes - ps8
Archaeplastida - ps9
Viridiplantae - ps10
Streptophyta - ps11
Charophyceae/Embriophyta - ps12
Embriophyta - ps13
Tracheophyta - ps14
Spermatophyta - ps15
Angiosperms - ps16
Mesangiospermae - ps17
Magnoliids/eudicots - ps18
Eudicots - ps19
Core_eudicots - ps20
Superrosids - ps21
Rosids - ps22
Eurosids - ps23
Malvids - ps24
Sap./Malv./Brassicales - ps25
Malvales/Brassicales - ps26
Brassicales - ps27
Cleomaceae/Brassicaceae - ps28
Brassicaceae - ps29
Camelineae - ps30
Arabidopsis - ps31
Arabidopsis_thaliana - ps32
Phylostratum

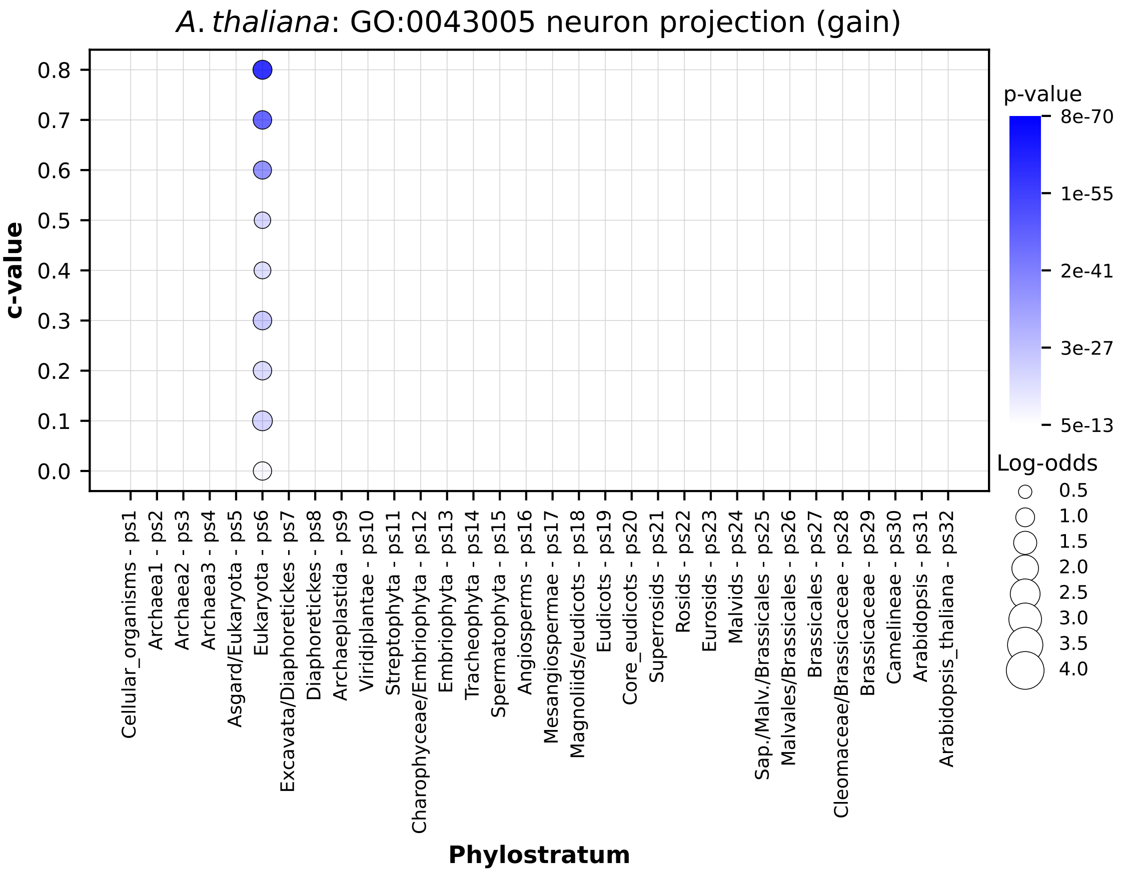

A. thaliana: GO:0043005 neuron projection (gain)
c-value
0.0
0.1
0.2
0.3
0.4
0.5
0.6
0.7
0.8
Phylostratum
Cellular_organisms - ps1
Archaea1 - ps2
Archaea2 - ps3
Archaea3 - ps4
Asgard/Eukaryota - ps5
Eukaryota - ps6
Excavata/Diaphoretickes - ps7
Diaphoretickes - ps8
Archaeplastida - ps9
Viridiplantae - ps10
Streptophyta - ps11
Charophyceae/Embriophyta - ps12
Embriophyta - ps13
Tracheophyta - ps14
Spermatophyta - ps15
Angiosperms - ps16
Mesangiospermae - ps17
Magnoliids/eudicots - ps18
Eudicots - ps19
Core_eudicots - ps20
Superrosids - ps21
Rosids - ps22
Eurosids - ps23
Malvids - ps24
Sap./Malv./Brassicales - ps25
Malvales/Brassicales - ps26
Brassicales - ps27
Cleomaceae/Brassicaceae - ps28
Brassicaceae - ps29
Camelineae - ps30
Arabidopsis - ps31
Arabidopsis_thaliana - ps32
p-value
8e-70
1e-55
2e-41
3e-27
5e-13
Log-odds
0.5
1.0
1.5
2.0
2.5
3.0
3.5
4.0

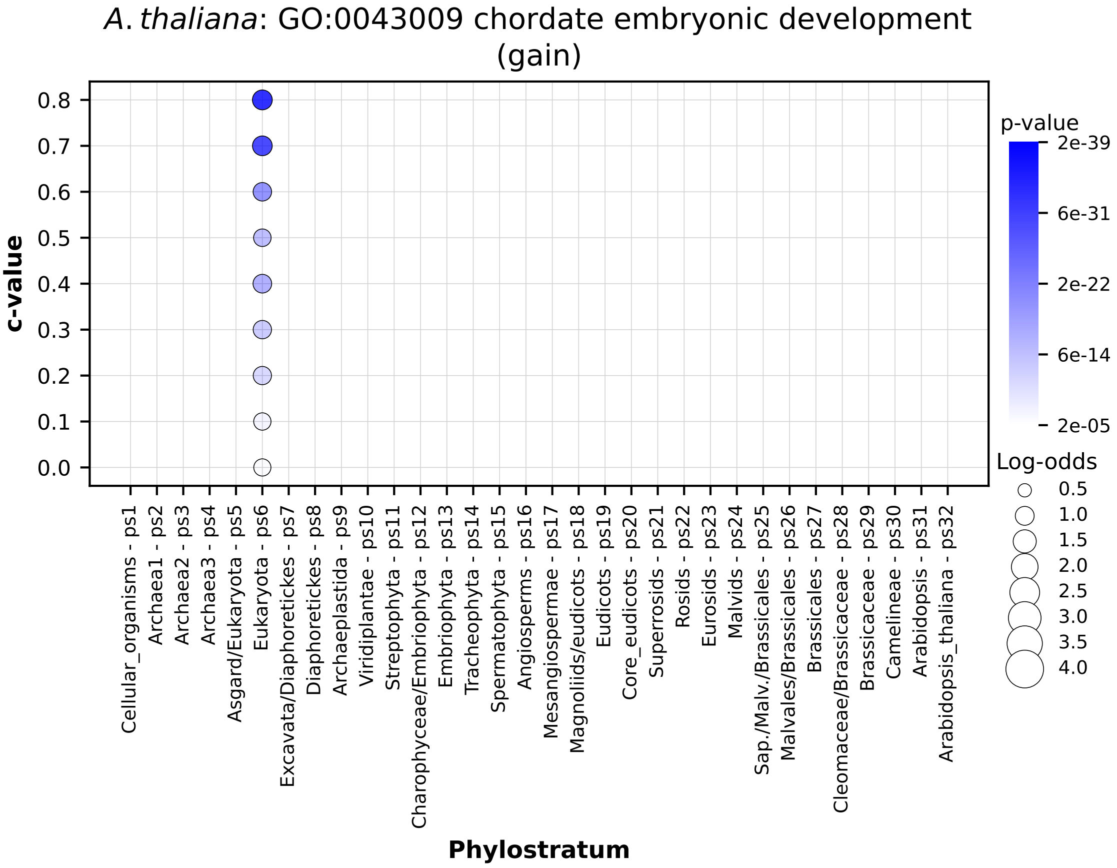
A. thaliana: GO:0043009 chordate embryonic development
(gain)
c-value
0.8
0.7
0.6
0.5
0.4
0.3
0.2
0.1
0.0
p-value
2e-39
6e-31
2e-22
6e-14
2e-05
Log-odds
0.5
1.0
1.5
2.0
2.5
3.0
3.5
4.0
Cellular_organisms - ps1
Archaea1 - ps2
Archaea2 - ps3
Archaea3 - ps4
Asgard/Eukaryota - ps5
Eukaryota - ps6
Excavata/Diaphoretickes - ps7
Diaphoretickes - ps8
Archaeplastida - ps9
Viridiplantae - ps10
Streptophyta - ps11
Charophyceae/Embriophyta - ps12
Embriophyta - ps13
Tracheophyta - ps14
Spermatophyta - ps15
Angiosperms - ps16
Mesangiospermae - ps17
Magnoliids/eudicots - ps18
Eudicots - ps19
Core_eudicots - ps20
Superrosids - ps21
Rosids - ps22
Eurosids - ps23
Malvids - ps24
Sap./Malv./Brassicales - ps25
Malvales/Brassicales - ps26
Brassicales - ps27
Cleomaceae/Brassicaceae - ps28
Brassicaceae - ps29
Camelineae - ps30
Arabidopsis - ps31
Arabidopsis_thaliana - ps32
Phylostratum

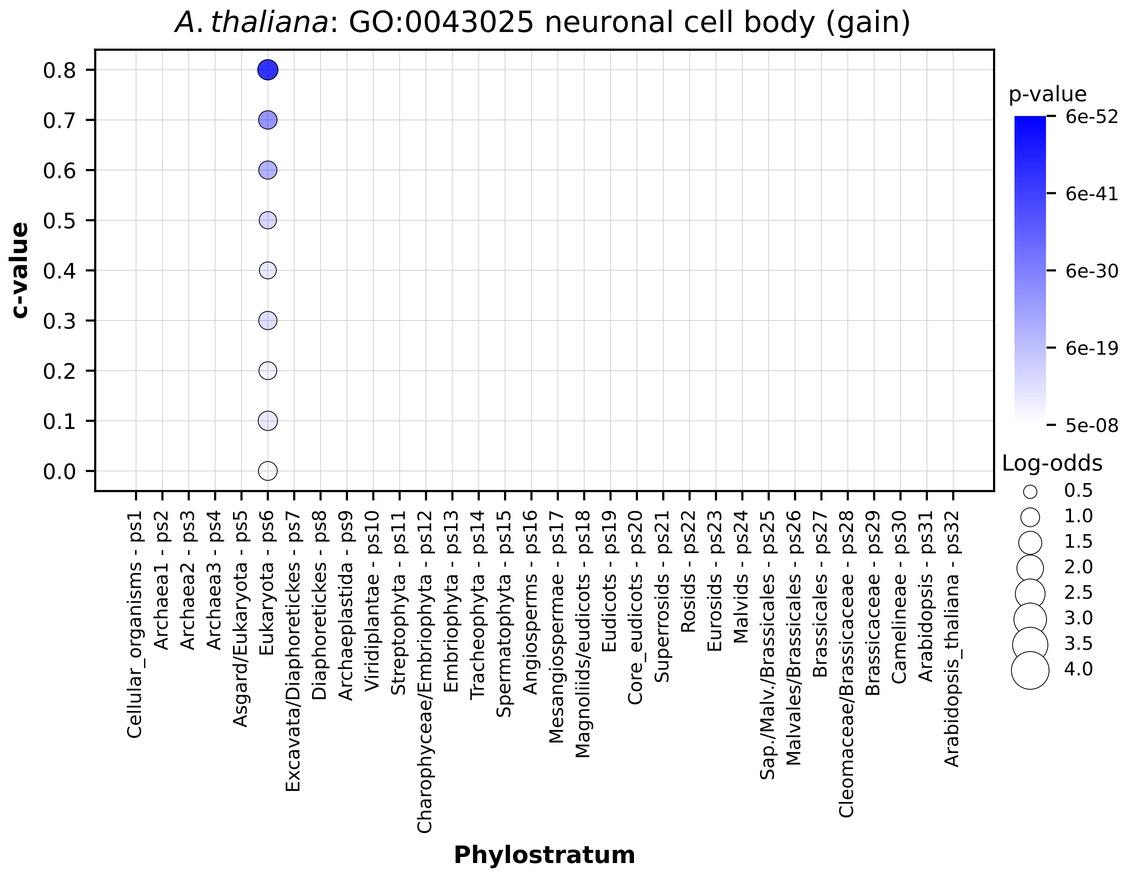

A. thaliana: GO:0043025 neuronal cell body (gain)
c-value
0.8
0.7
0.6
0.5
0.4
0.3
0.2
0.1
0.0
p-value
6e-52
6e-41
6e-30
6e-19
5e-08
Log-odds
0.5
1.0
1.5
2.0
2.5
3.0
3.5
4.0
Cellular_organisms - ps1
Archaea1 - ps2
Archaea2 - ps3
Archaea3 - ps4
Asgard/Eukaryota - ps5
Eukaryota - ps6
Excavata/Diaphoretickes - ps7
Diaphoretickes - ps8
Archaeplastida - ps9
Viridiplantae - ps10
Streptophyta - ps11
Charophyceae/Embriophyta - ps12
Embriophyta - ps13
Tracheophyta - ps14
Spermatophyta - ps15
Angiosperms - ps16
Mesangiospermae - ps17
Magnoliids/eudicots - ps18
Eudicots - ps19
Core_eudicots - ps20
Superrosids - ps21
Rosids - ps22
Eurosids - ps23
Malvids - ps24
Sap./Malv./Brassicales - ps25
Malvales/Brassicales - ps26
Brassicales - ps27
Cleomaceae/Brassicaceae - ps28
Brassicaceae - ps29
Camelineae - ps30
Arabidopsis - ps31
Arabidopsis_thaliana - ps32
Phylostratum

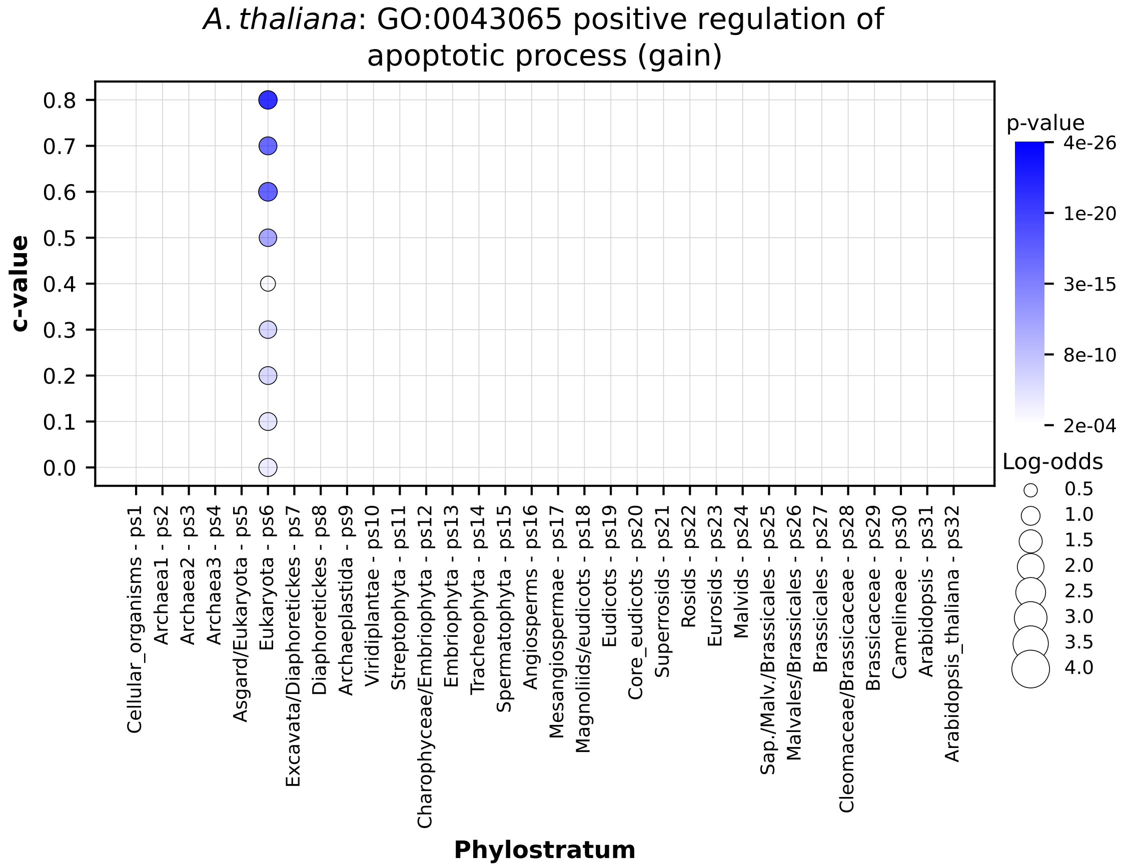
A. thaliana: GO:0043065 positive regulation of apoptotic process (gain)
c-value
0.8
0.7
0.6
0.5
0.4
0.3
0.2
0.1
0.0
p-value
4e-26
1e-20
3e-15
8e-10
2e-04
Log-odds
0.5
1.0
1.5
2.0
2.5
3.0
3.5
4.0
Cellular_organisms - ps1
Archaea1 - ps2
Archaea2 - ps3
Archaea3 - ps4
Asgard/Eukaryota - ps5
Eukaryota - ps6
Excavata/Diaphoretickes - ps7
Diaphoretickes - ps8
Archaeplastida - ps9
Viridiplantae - ps10
Streptophyta - ps11
Charophyceae/Embriophyta - ps12
Embriophyta - ps13
Tracheophyta - ps14
Spermatophyta - ps15
Angiosperms - ps16
Mesangiospermae - ps17
Magnoliids/eudicots - ps18
Eudicots - ps19
Core_eudicots - ps20
Superrosids - ps21
Rosids - ps22
Eurosids - ps23
Malvids - ps24
Sap./Malv./Brassicales - ps25
Malvales/Brassicales - ps26
Brassicales - ps27
Cleomaceae/Brassicaceae - ps28
Brassicaceae - ps29
Camelineae - ps30
Arabidopsis - ps31
Arabidopsis_thaliana - ps32
Phylostratum

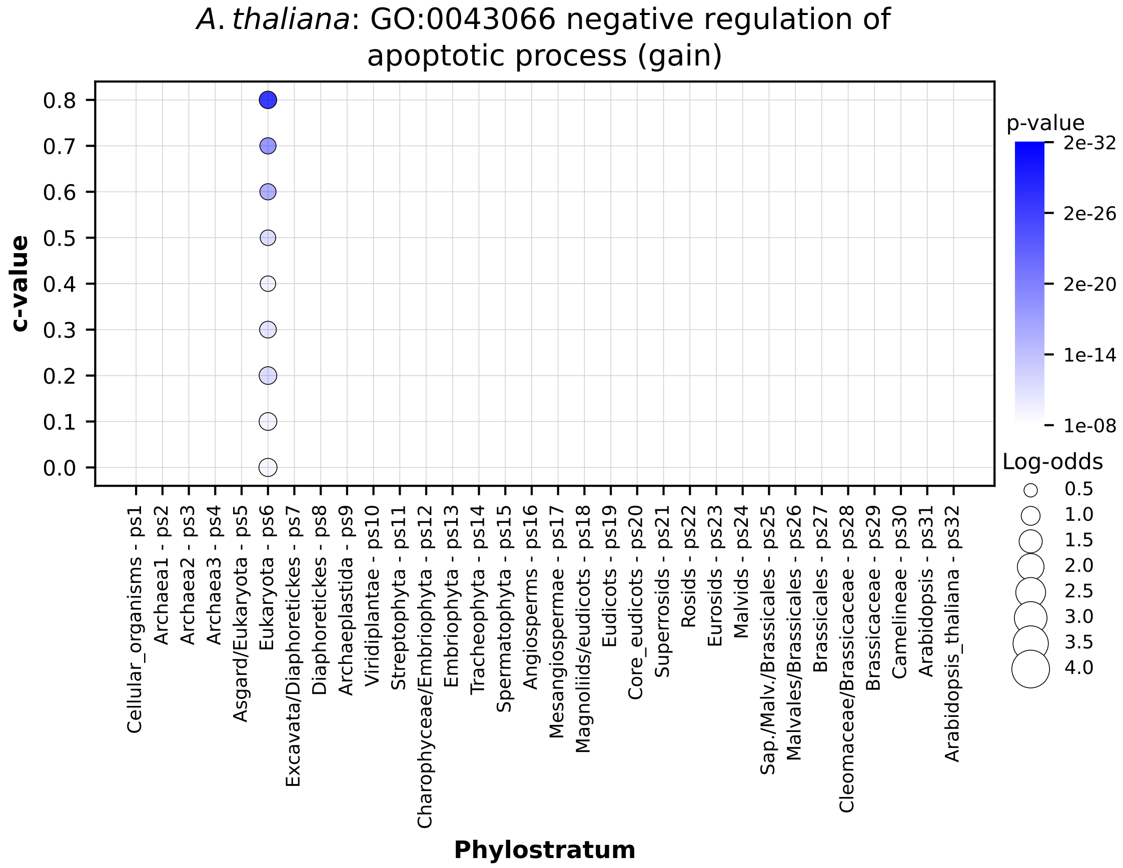

A. thaliana: GO:0043066 negative regulation of apoptotic process (gain)
c-value
0.8
0.7
0.6
0.5
0.4
0.3
0.2
0.1
0.0
p-value
2e-32
2e-26
2e-20
1e-14
1e-08
Log-odds
0.5
1.0
1.5
2.0
2.5
3.0
3.5
4.0
Cellular_organisms - ps1
Archaea1 - ps2
Archaea2 - ps3
Archaea3 - ps4
Asgard/Eukaryota - ps5
Eukaryota - ps6
Excavata/Diaphoretickes - ps7
Diaphoretickes - ps8
Archaeplastida - ps9
Viridiplantae - ps10
Streptophyta - ps11
Charophyceae/Embriophyta - ps12
Embriophyta - ps13
Tracheophyta - ps14
Spermatophyta - ps15
Angiosperms - ps16
Mesangiospermae - ps17
Magnoliids/eudicots - ps18
Eudicots - ps19
Core_eudicots - ps20
Superrosids - ps21
Rosids - ps22
Eurosids - ps23
Malvids - ps24
Sap./Malv./Brassicales - ps25
Malvales/Brassicales - ps26
Brassicales - ps27
Cleomaceae/Brassicaceae - ps28
Brassicaceae - ps29
Camelineae - ps30
Arabidopsis - ps31
Arabidopsis_thaliana - ps32
Phylostratum

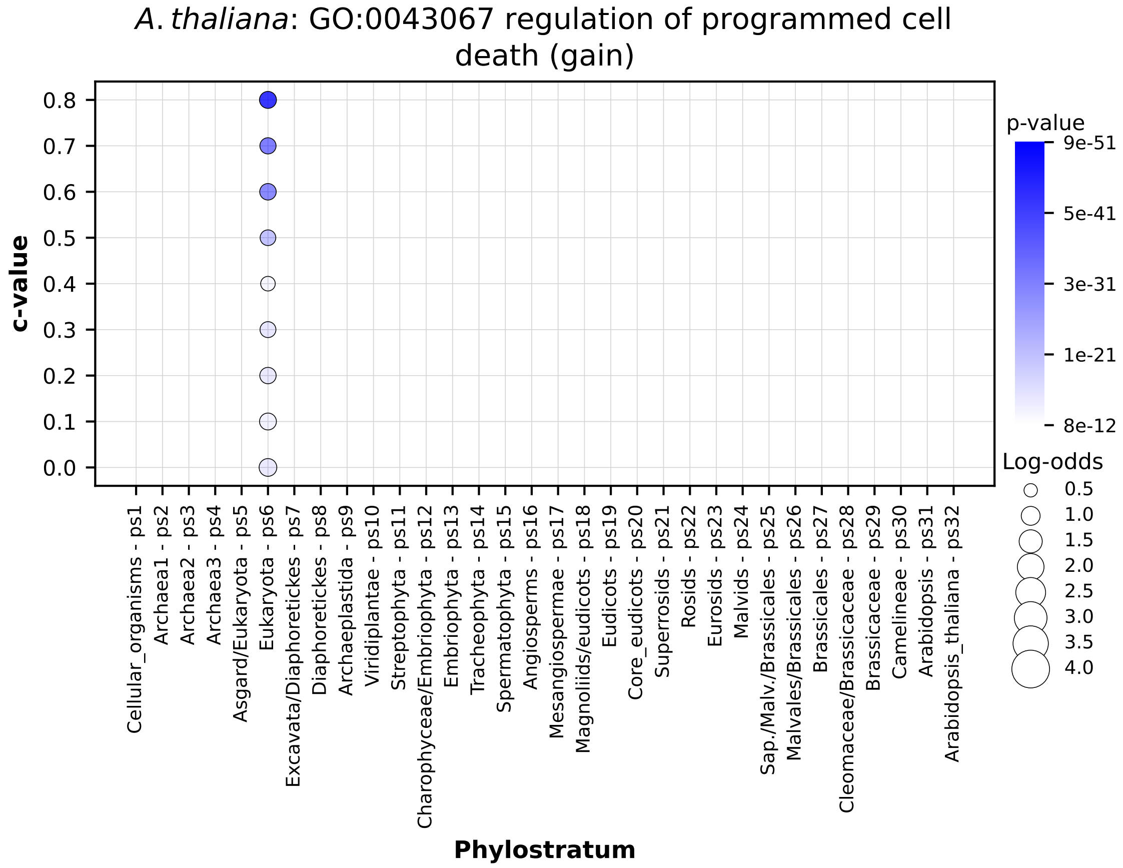
A. thaliana: GO:0043067 regulation of programmed cell death (gain)
c-value
0.8
0.7
0.6
0.5
0.4
0.3
0.2
0.1
0.0
Phylostratum
Cellular_organisms - ps1
Archaea1 - ps2
Archaea2 - ps3
Archaea3 - ps4
Asgard/Eukaryota - ps5
Eukaryota - ps6
Excavata/Diaphoretickes - ps7
Diaphoretickes - ps8
Archaeplastida - ps9
Viridiplantae - ps10
Streptophyta - ps11
Charophyceae/Embriophyta - ps12
Embriophyta - ps13
Tracheophyta - ps14
Spermatophyta - ps15
Angiosperms - ps16
Mesangiospermae - ps17
Magnoliids/eudicots - ps18
Eudicots - ps19
Core_eudicots - ps20
Superrosids - ps21
Rosids - ps22
Eurosids - ps23
Malvids - ps24
Sap./Malv./Brassicales - ps25
Malvales/Brassicales - ps26
Brassicales - ps27
Cleomaceae/Brassicaceae - ps28
Brassicaceae - ps29
Camelineae - ps30
Arabidopsis - ps31
Arabidopsis_thaliana - ps32
p-value
9e-51
5e-41
3e-31
1e-21
8e-12
Log-odds
0.5
1.0
1.5
2.0
2.5
3.0
3.5
4.0

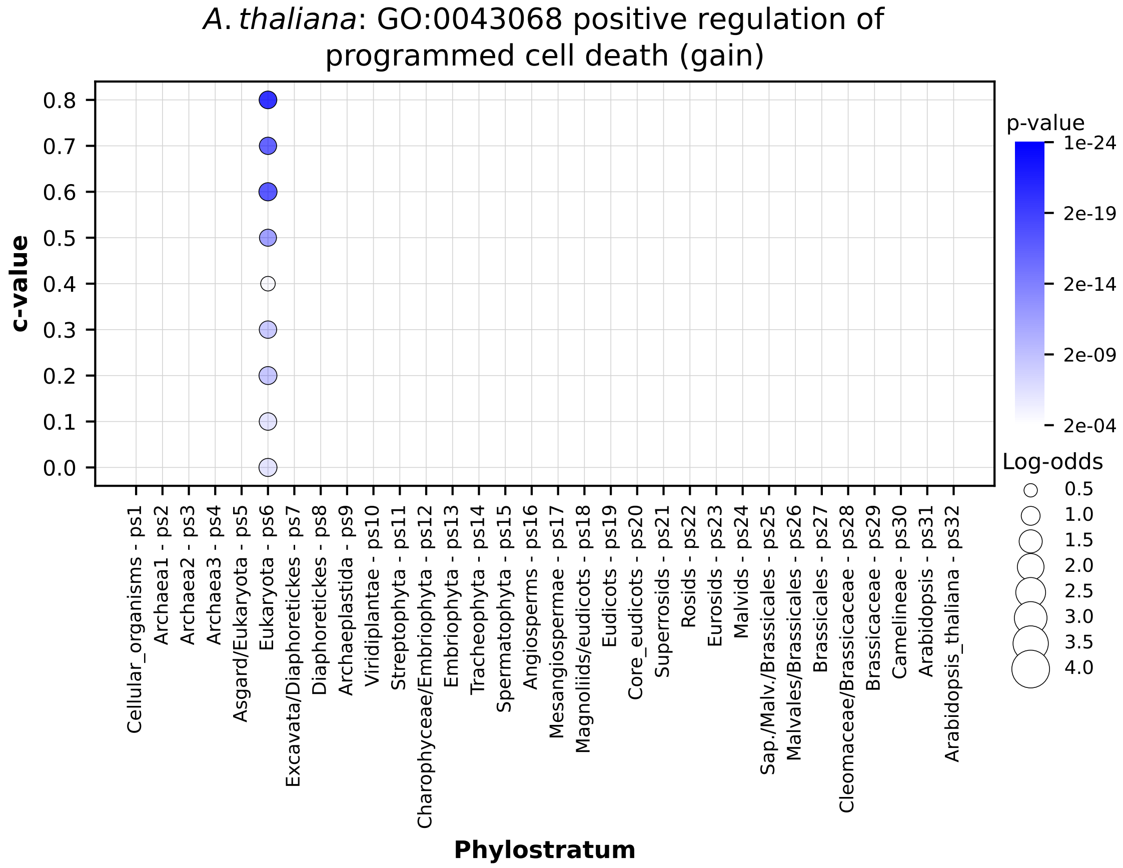

A. thaliana: GO:0043068 positive regulation of programmed cell death (gain)
c-value
0.8
0.7
0.6
0.5
0.4
0.3
0.2
0.1
0.0
Phylostratum
Cellular_organisms - ps1
Archaea1 - ps2
Archaea2 - ps3
Archaea3 - ps4
Asgard/Eukaryota - ps5
Eukaryota - ps6
Excavata/Diaphoretickes - ps7
Diaphoretickes - ps8
Archaeplastida - ps9
Viridiplantae - ps10
Streptophyta - ps11
Charophyceae/Embriophyta - ps12
Embriophyta - ps13
Tracheophyta - ps14
Spermatophyta - ps15
Angiosperms - ps16
Mesangiospermae - ps17
Magnoliids/eudicots - ps18
Eudicots - ps19
Core_eudicots - ps20
Superrosids - ps21
Rosids - ps22
Eurosids - ps23
Malvids - ps24
Sap./Malv./Brassicales - ps25
Malvales/Brassicales - ps26
Brassicales - ps27
Cleomaceae/Brassicaceae - ps28
Brassicaceae - ps29
Camelineae - ps30
Arabidopsis - ps31
Arabidopsis_thaliana - ps32
p-value
1e-24
2e-19
2e-14
2e-09
2e-04
Log-odds
0.5
1.0
1.5
2.0
2.5
3.0
3.5
4.0

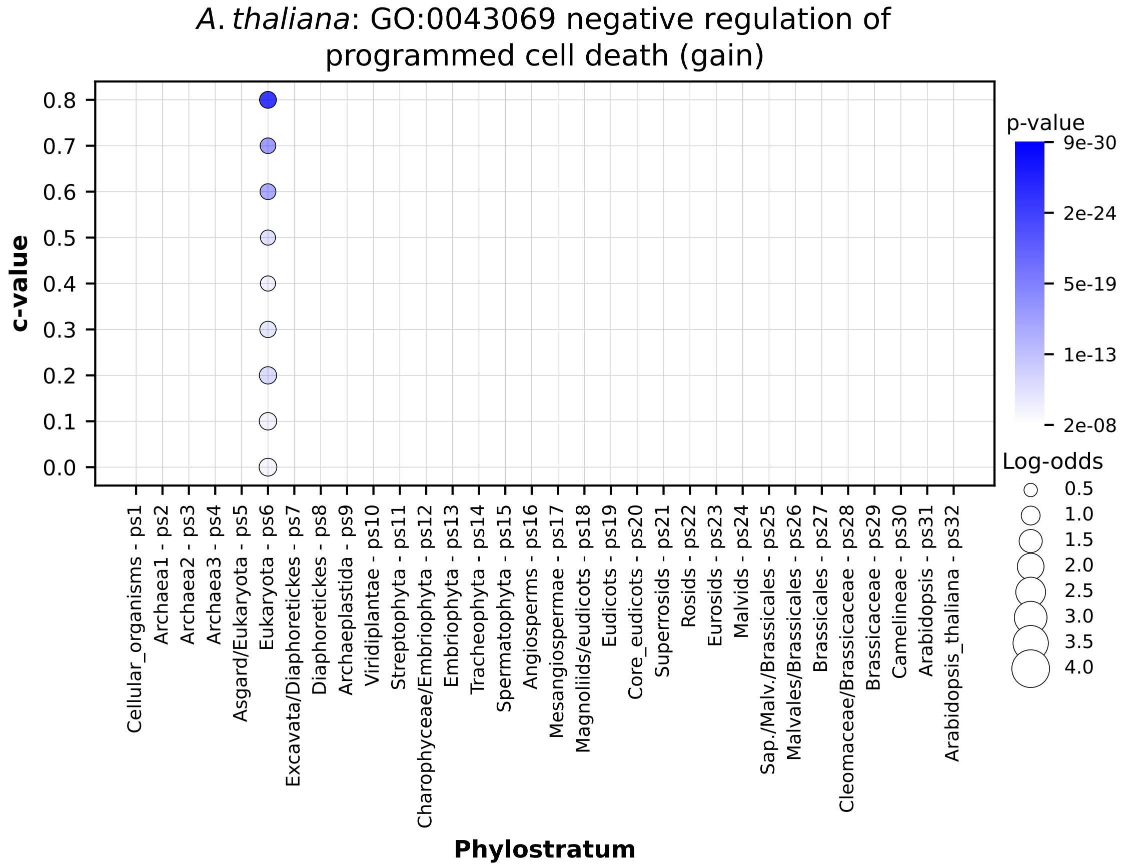
A. thaliana: GO:0043069 negative regulation of programmed cell death (gain)
c-value
0.8
0.7
0.6
0.5
0.4
0.3
0.2
0.1
0.0
p-value
9e-30
2e-24
5e-19
1e-13
2e-08
Log-odds
0.5
1.0
1.5
2.0
2.5
3.0
3.5
4.0
Cellular_organisms - ps1
Archaea1 - ps2
Archaea2 - ps3
Archaea3 - ps4
Asgard/Eukaryota - ps5
Eukaryota - ps6
Excavata/Diaphoretickes - ps7
Diaphoretickes - ps8
Archaeplastida - ps9
Viridiplantae - ps10
Streptophyta - ps11
Charophyceae/Embriophyta - ps12
Embriophyta - ps13
Tracheophyta - ps14
Spermatophyta - ps15
Angiosperms - ps16
Mesangiospermae - ps17
Magnoliids/eudicots - ps18
Eudicots - ps19
Core_eudicots - ps20
Superrosids - ps21
Rosids - ps22
Eurosids - ps23
Malvids - ps24
Sap./Malv./Brassicales - ps25
Malvales/Brassicales - ps26
Brassicales - ps27
Cleomaceae/Brassicaceae - ps28
Brassicaceae - ps29
Camelineae - ps30
Arabidopsis - ps31
Arabidopsis_thaliana - ps32
Phylostratum

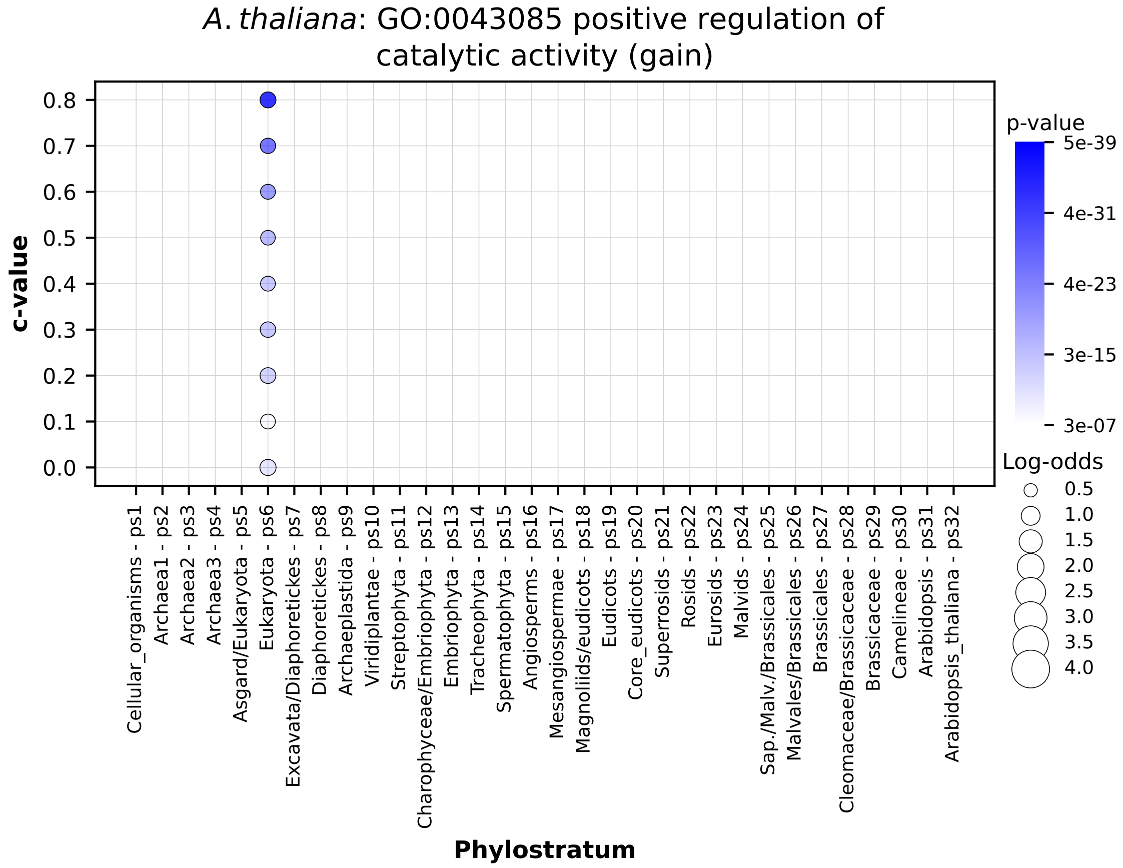

A. thaliana: GO:0043085 positive regulation of catalytic activity (gain)
c-value
0.8
0.7
0.6
0.5
0.4
0.3
0.2
0.1
0.0
p-value
5e-39
4e-31
4e-23
3e-15
3e-07
Log-odds
0.5
1.0
1.5
2.0
2.5
3.0
3.5
4.0
Cellular_organisms - ps1
Archaea1 - ps2
Archaea2 - ps3
Archaea3 - ps4
Asgard/Eukaryota - ps5
Eukaryota - ps6
Excavata/Diaphoretickes - ps7
Diaphoretickes - ps8
Archaeplastida - ps9
Viridiplantae - ps10
Streptophyta - ps11
Charophyceae/Embriophyta - ps12
Embriophyta - ps13
Tracheophyta - ps14
Spermatophyta - ps15
Angiosperms - ps16
Mesangiospermae - ps17
Magnoliids/eudicots - ps18
Eudicots - ps19
Core_eudicots - ps20
Superrosids - ps21
Rosids - ps22
Eurosids - ps23
Malvids - ps24
Sap./Malv./Brassicales - ps25
Malvales/Brassicales - ps26
Brassicales - ps27
Cleomaceae/Brassicaceae - ps28
Brassicaceae - ps29
Camelineae - ps30
Arabidopsis - ps31
Arabidopsis_thaliana - ps32
Phylostratum

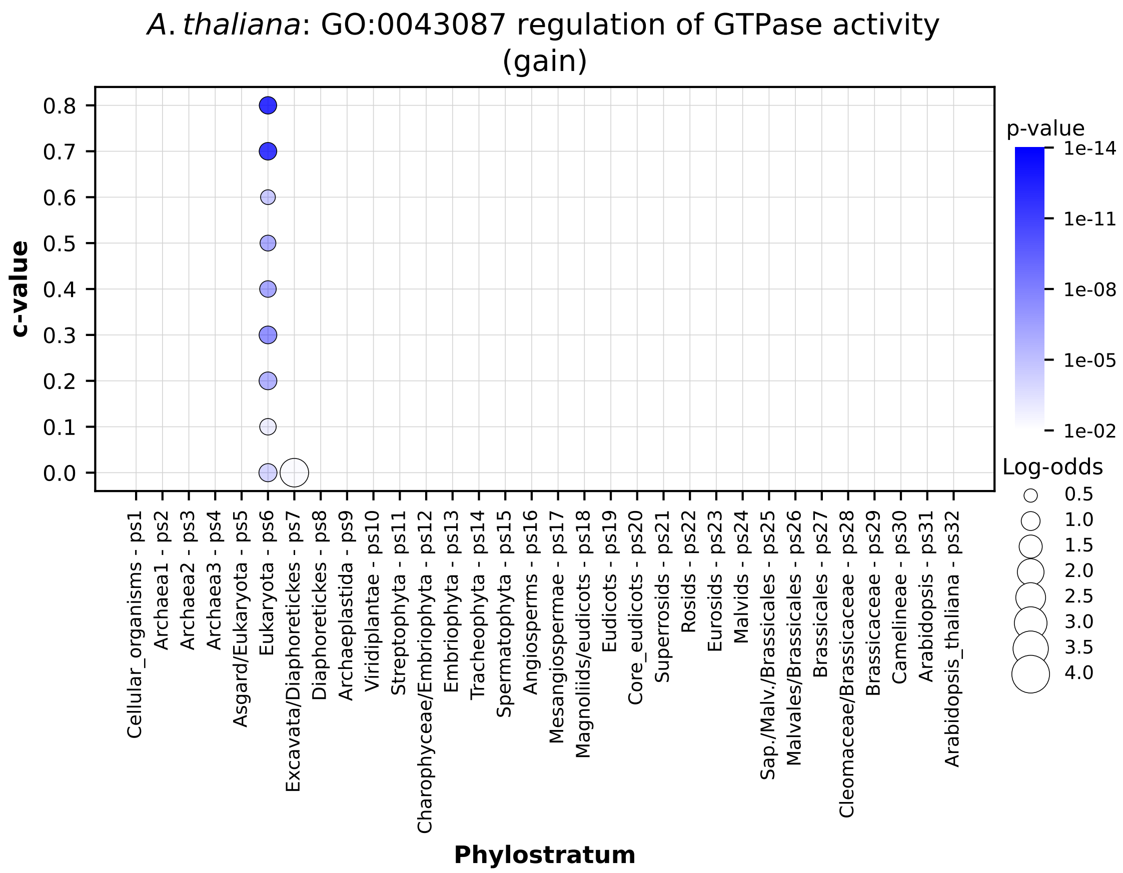

A. thaliana: GO:0043087 regulation of GTPase activity
(gain)
c-value
0.8
0.7
0.6
0.5
0.4
0.3
0.2
0.1
0.0
p-value
1e-14
1e-11
1e-08
1e-05
1e-02
Log-odds
0.5
1.0
1.5
2.0
2.5
3.0
3.5
4.0
Cellular_organisms - ps1
Archaea1 - ps2
Archaea2 - ps3
Archaea3 - ps4
Asgard/Eukaryota - ps5
Eukaryota - ps6
Excavata/Diaphoretickes - ps7
Diaphoretickes - ps8
Archaeplastida - ps9
Viridiplantae - ps10
Streptophyta - ps11
Charophyceae/Embriophyta - ps12
Embriophyta - ps13
Tracheophyta - ps14
Spermatophyta - ps15
Angiosperms - ps16
Mesangiospermae - ps17
Magnoliids/eudicots - ps18
Eudicots - ps19
Core_eudicots - ps20
Superrosids - ps21
Rosids - ps22
Eurosids - ps23
Malvids - ps24
Sap./Malv./Brassicales - ps25
Malvales/Brassicales - ps26
Brassicales - ps27
Cleomaceae/Brassicaceae - ps28
Brassicaceae - ps29
Camelineae - ps30
Arabidopsis - ps31
Arabidopsis_thaliana - ps32
Phylostratum

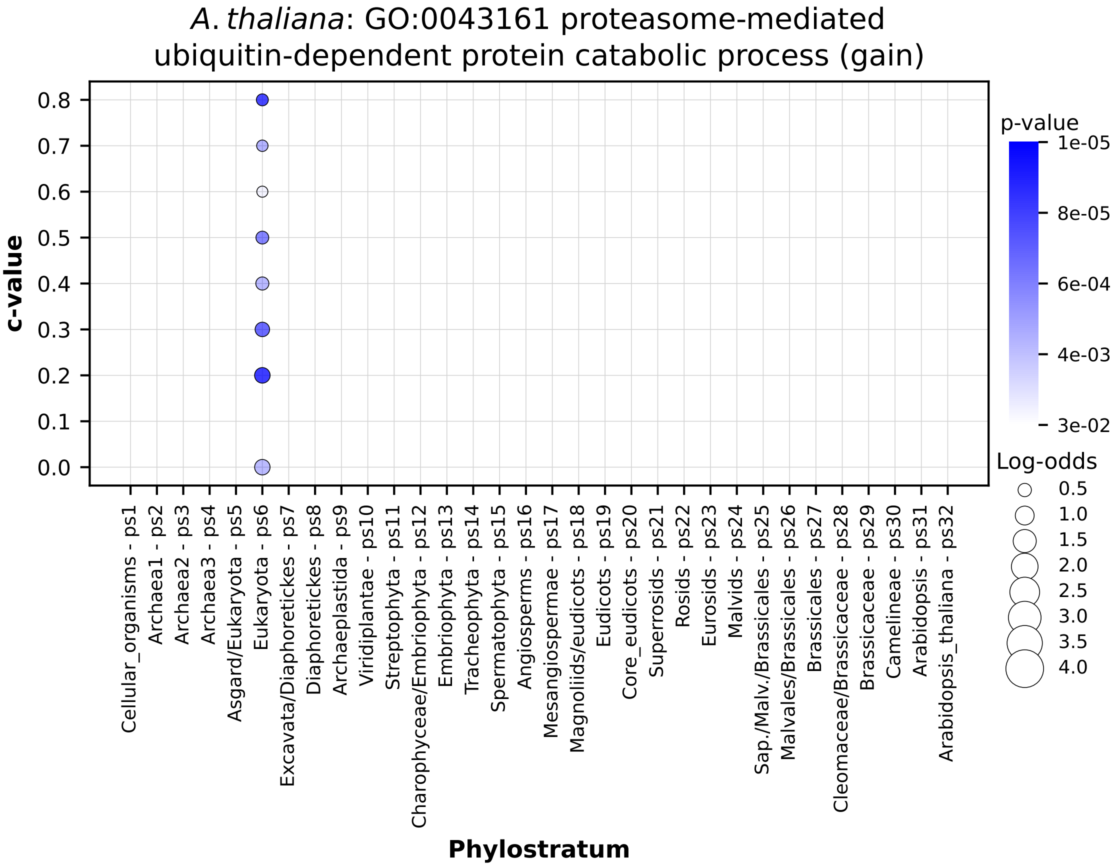

A. thaliana: GO:0043161 proteasome-mediated ubiquitin-dependent protein catabolic process (gain)
c-value
0.8
0.7
0.6
0.5
0.4
0.3
0.2
0.1
0.0
p-value
1e-05
8e-05
6e-04
4e-03
3e-02
Log-odds
0.5
1.0
1.5
2.0
2.5
3.0
3.5
4.0
Cellular_organisms - ps1
Archaea1 - ps2
Archaea2 - ps3
Archaea3 - ps4
Asgard/Eukaryota - ps5
Eukaryota - ps6
Excavata/Diaphoretickes - ps7
Diaphoretickes - ps8
Archaeplastida - ps9
Viridiplantae - ps10
Streptophyta - ps11
Charophyceae/Embriophyta - ps12
Embriophyta - ps13
Tracheophyta - ps14
Spermatophyta - ps15
Angiosperms - ps16
Mesangiospermae - ps17
Magnoliids/eudicots - ps18
Eudicots - ps19
Core_eudicots - ps20
Superrosids - ps21
Rosids - ps22
Eurosids - ps23
Malvids - ps24
Sap./Malv./Brassicales - ps25
Malvales/Brassicales - ps26
Brassicales - ps27
Cleomaceae/Brassicaceae - ps28
Brassicaceae - ps29
Camelineae - ps30
Arabidopsis - ps31
Arabidopsis_thaliana - ps32
Phylostratum

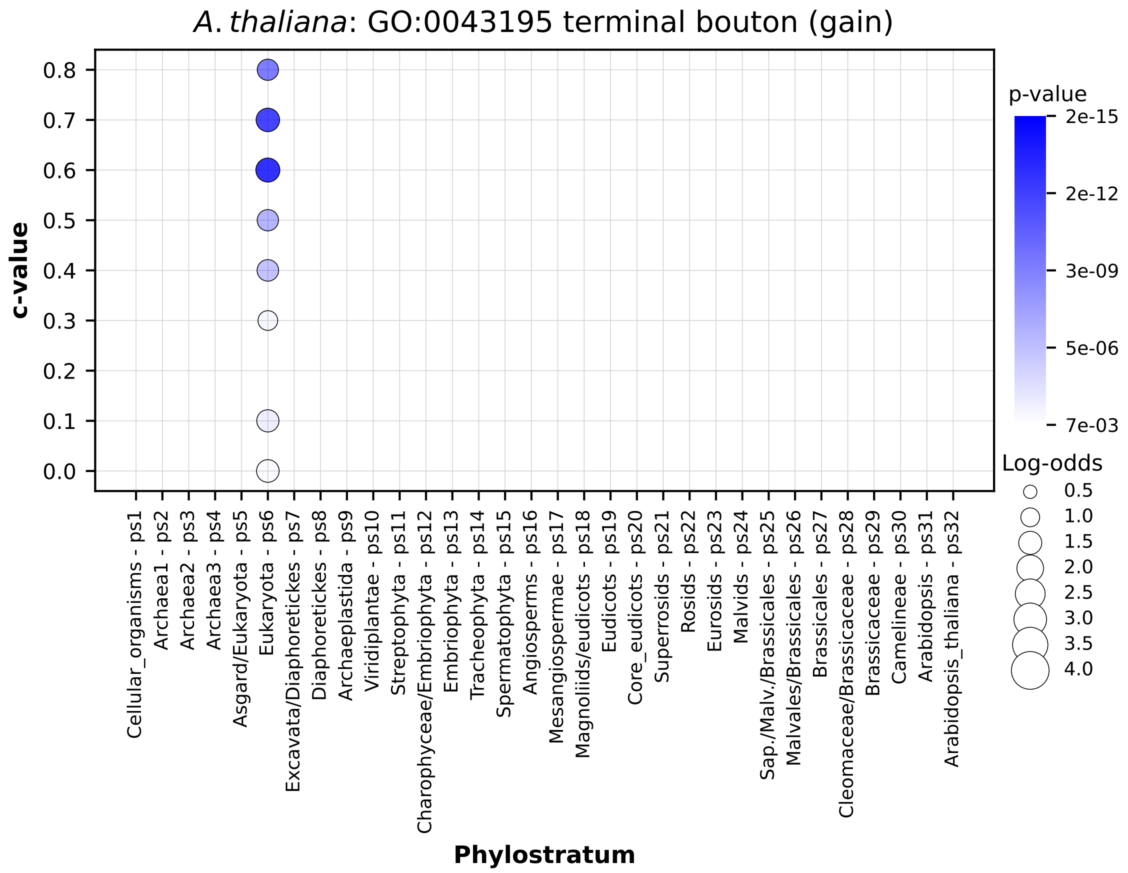
A. thaliana: GO:0043195 terminal bouton (gain)
c-value
0.8
0.7
0.6
0.5
0.4
0.3
0.2
0.1
0.0
p-value
2e-15
2e-12
3e-09
5e-06
7e-03
Log-odds
0.5
1.0
1.5
2.0
2.5
3.0
3.5
4.0
Cellular_organisms - ps1
Archaea1 - ps2
Archaea2 - ps3
Archaea3 - ps4
Asgard/Eukaryota - ps5
Eukaryota - ps6
Excavata/Diaphoretickes - ps7
Diaphoretickes - ps8
Archaeplastida - ps9
Viridiplantae - ps10
Streptophyta - ps11
Charophyceae/Embriophyta - ps12
Embriophyta - ps13
Tracheophyta - ps14
Spermatophyta - ps15
Angiosperms - ps16
Mesangiospermae - ps17
Magnoliids/eudicots - ps18
Eudicots - ps19
Core_eudicots - ps20
Superrosids - ps21
Rosids - ps22
Eurosids - ps23
Malvids - ps24
Sap./Malv./Brassicales - ps25
Malvales/Brassicales - ps26
Brassicales - ps27
Cleomaceae/Brassicaceae - ps28
Brassicaceae - ps29
Camelineae - ps30
Arabidopsis - ps31
Arabidopsis_thaliana - ps32
Phylostratum

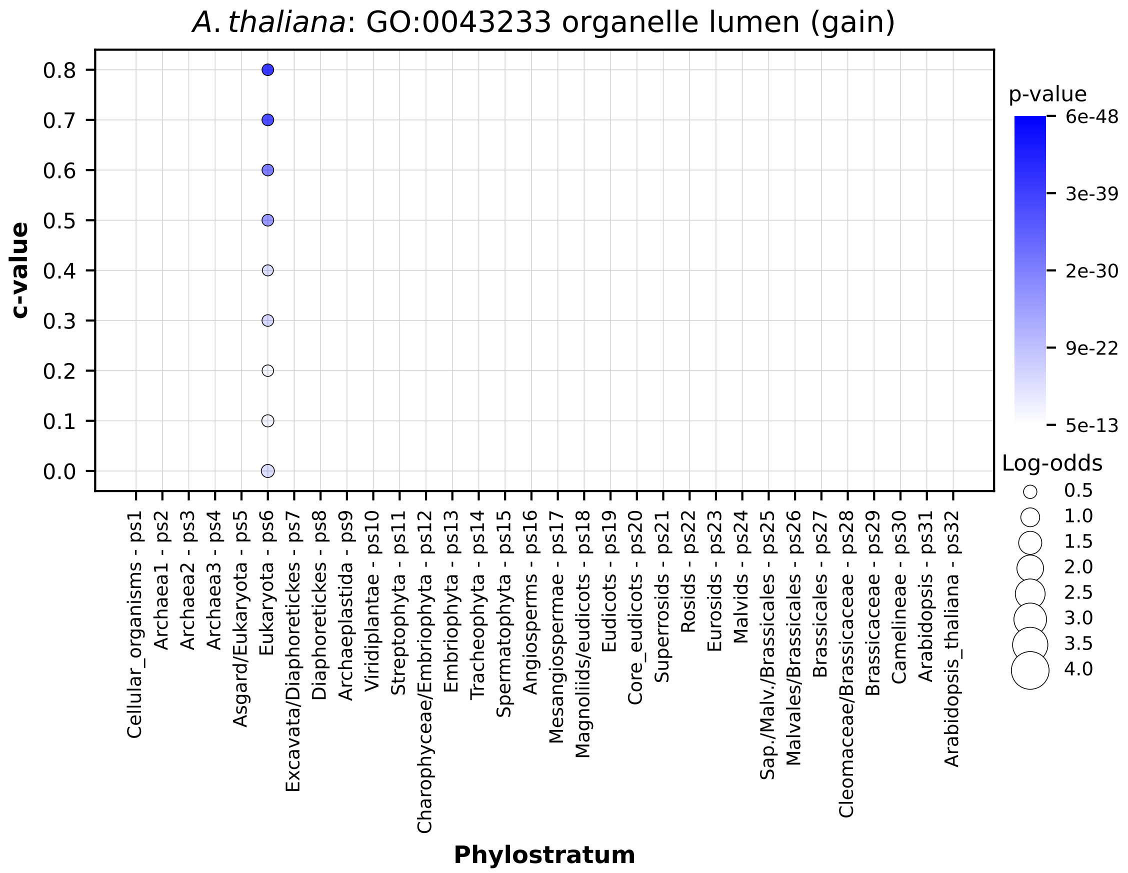

A. thaliana: GO:0043233 organelle lumen (gain)
c-value
0.0
0.1
0.2
0.3
0.4
0.5
0.6
0.7
0.8
p-value
6e-48
3e-39
2e-30
9e-22
5e-13
Log-odds
0.5
1.0
1.5
2.0
2.5
3.0
3.5
4.0
Cellular_organisms - ps1
Archaea1 - ps2
Archaea2 - ps3
Archaea3 - ps4
Asgard/Eukaryota - ps5
Eukaryota - ps6
Excavata/Diaphoretickes - ps7
Diaphoretickes - ps8
Archaeplastida - ps9
Viridiplantae - ps10
Streptophyta - ps11
Charophyceae/Embriophyta - ps12
Embriophyta - ps13
Tracheophyta - ps14
Spermatophyta - ps15
Angiosperms - ps16
Mesangiospermae - ps17
Magnoliids/eudicots - ps18
Eudicots - ps19
Core_eudicots - ps20
Superrosids - ps21
Rosids - ps22
Eurosids - ps23
Malvids - ps24
Sap./Malv./Brassicales - ps25
Malvales/Brassicales - ps26
Brassicales - ps27
Cleomaceae/Brassicaceae - ps28
Brassicaceae - ps29
Camelineae - ps30
Arabidopsis - ps31
Arabidopsis_thaliana - ps32
Phylostratum

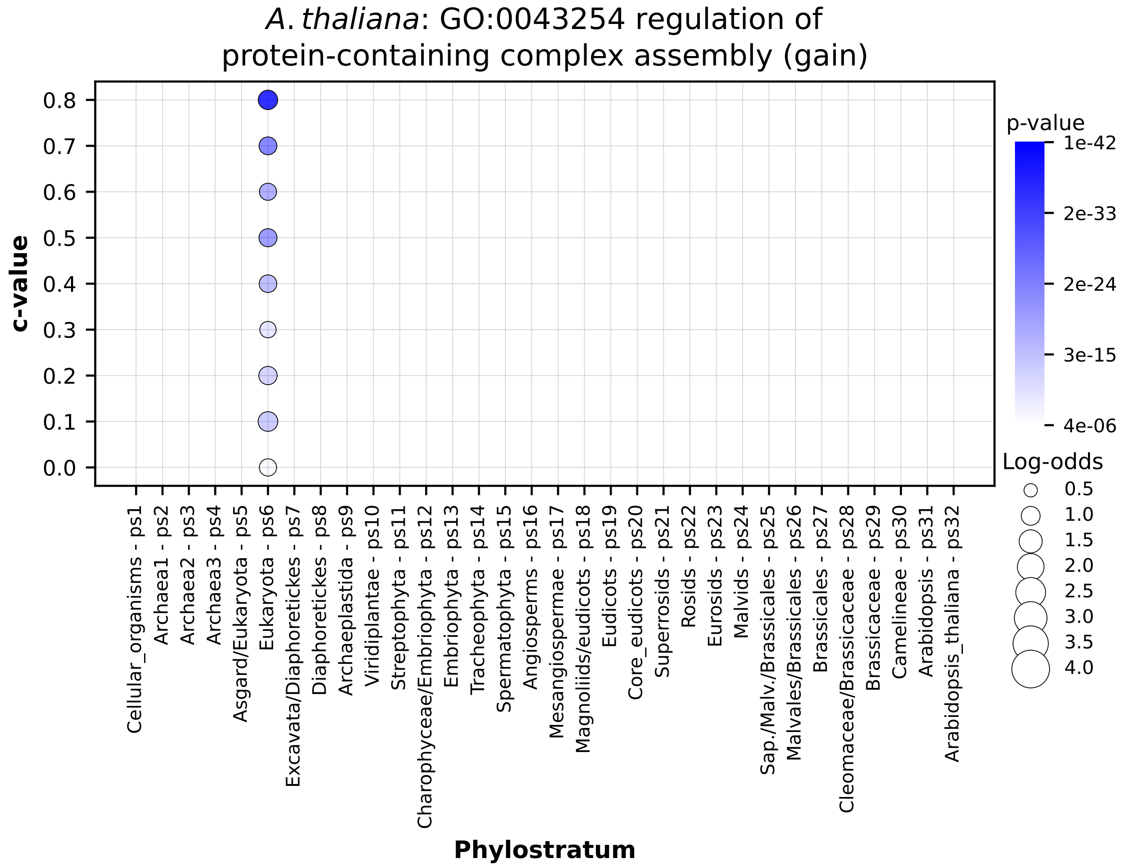
A. thaliana: GO:0043254 regulation of
protein-containing complex assembly (gain)
c-value
0.8
0.7
0.6
0.5
0.4
0.3
0.2
0.1
0.0
Phylostratum
Cellular_organisms - ps1
Archaea1 - ps2
Archaea2 - ps3
Archaea3 - ps4
Asgard/Eukaryota - ps5
Eukaryota - ps6
Excavata/Diaphoretickes - ps7
Diaphoretickes - ps8
Archaeplastida - ps9
Viridiplantae - ps10
Streptophyta - ps11
Charophyceae/Embriophyta - ps12
Embriophyta - ps13
Tracheophyta - ps14
Spermatophyta - ps15
Angiosperms - ps16
Mesangiospermae - ps17
Magnoliids/eudicots - ps18
Eudicots - ps19
Core_eudicots - ps20
Superrosids - ps21
Rosids - ps22
Eurosids - ps23
Malvids - ps24
Sap./Malv./Brassicales - ps25
Malvales/Brassicales - ps26
Brassicales - ps27
Cleomaceae/Brassicaceae - ps28
Brassicaceae - ps29
Camelineae - ps30
Arabidopsis - ps31
Arabidopsis_thaliana - ps32
p-value
1e-42
2e-33
2e-24
3e-15
4e-06
Log-odds
0.5
1.0
1.5
2.0
2.5
3.0
3.5
4.0

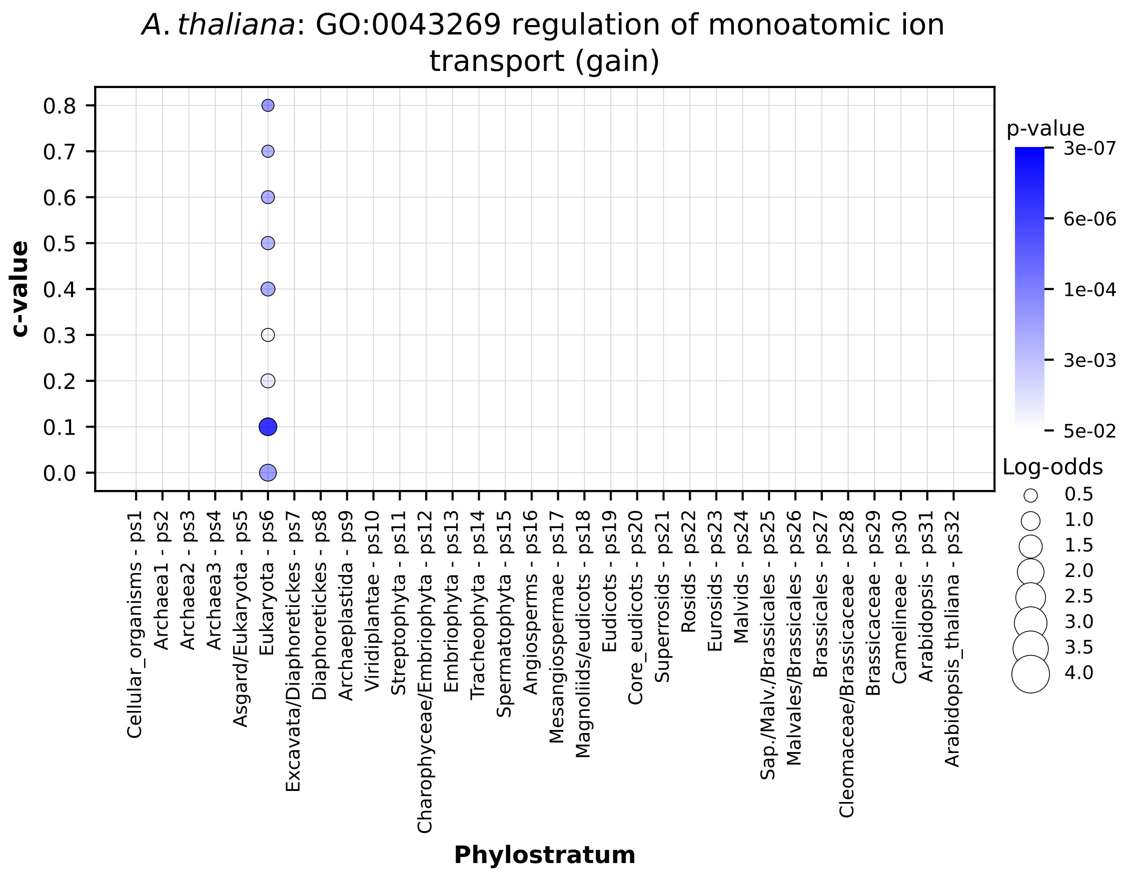
A. thaliana: GO:0043269 regulation of monoatomic ion transport (gain)
c-value
0.8
0.7
0.6
0.5
0.4
0.3
0.2
0.1
0.0
p-value
3e-07
6e-06
1e-04
3e-03
5e-02
Log-odds
0.5
1.0
1.5
2.0
2.5
3.0
3.5
4.0
Cellular_organisms - ps1
Archaea1 - ps2
Archaea2 - ps3
Archaea3 - ps4
Asgard/Eukaryota - ps5
Eukaryota - ps6
Excavata/Diaphoretickes - ps7
Diaphoretickes - ps8
Archaeplastida - ps9
Viridiplantae - ps10
Streptophyta - ps11
Charophyceae/Embriophyta - ps12
Embriophyta - ps13
Tracheophyta - ps14
Spermatophyta - ps15
Angiosperms - ps16
Mesangiospermae - ps17
Magnoliids/eudicots - ps18
Eudicots - ps19
Core_eudicots - ps20
Superrosids - ps21
Rosids - ps22
Eurosids - ps23
Malvids - ps24
Sap./Malv./Brassicales - ps25
Malvales/Brassicales - ps26
Brassicales - ps27
Cleomaceae/Brassicaceae - ps28
Brassicaceae - ps29
Camelineae - ps30
Arabidopsis - ps31
Arabidopsis_thaliana - ps32
Phylostratum

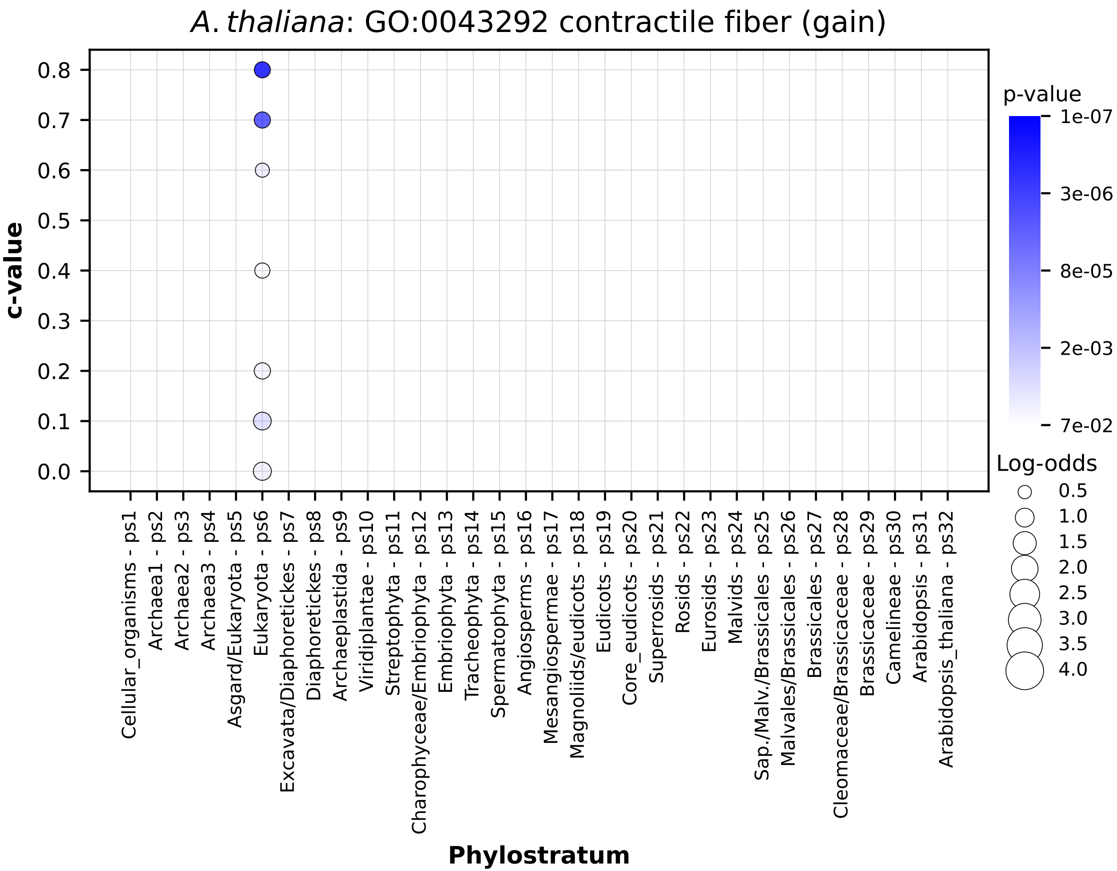

A. thaliana: GO:0043292 contractile fiber (gain)
c-value
0.8
0.7
0.6
0.5
0.4
0.3
0.2
0.1
0.0
Phylostratum
Cellular_organisms - ps1
Archaea1 - ps2
Archaea2 - ps3
Archaea3 - ps4
Asgard/Eukaryota - ps5
Eukaryota - ps6
Excavata/Diaphoretickes - ps7
Diaphoretickes - ps8
Archaeplastida - ps9
Viridiplantae - ps10
Streptophyta - ps11
Charophyceae/Embriophyta - ps12
Embriophyta - ps13
Tracheophyta - ps14
Spermatophyta - ps15
Angiosperms - ps16
Mesangiospermae - ps17
Magnoliids/eudicots - ps18
Eudicots - ps19
Core_eudicots - ps20
Superrosids - ps21
Rosids - ps22
Eurosids - ps23
Malvids - ps24
Sap./Malv./Brassicales - ps25
Malvales/Brassicales - ps26
Brassicales - ps27
Cleomaceae/Brassicaceae - ps28
Brassicaceae - ps29
Camelineae - ps30
Arabidopsis - ps31
Arabidopsis_thaliana - ps32
p-value
1e-07
3e-06
8e-05
2e-03
7e-02
Log-odds
0.5
1.0
1.5
2.0
2.5
3.0
3.5
4.0

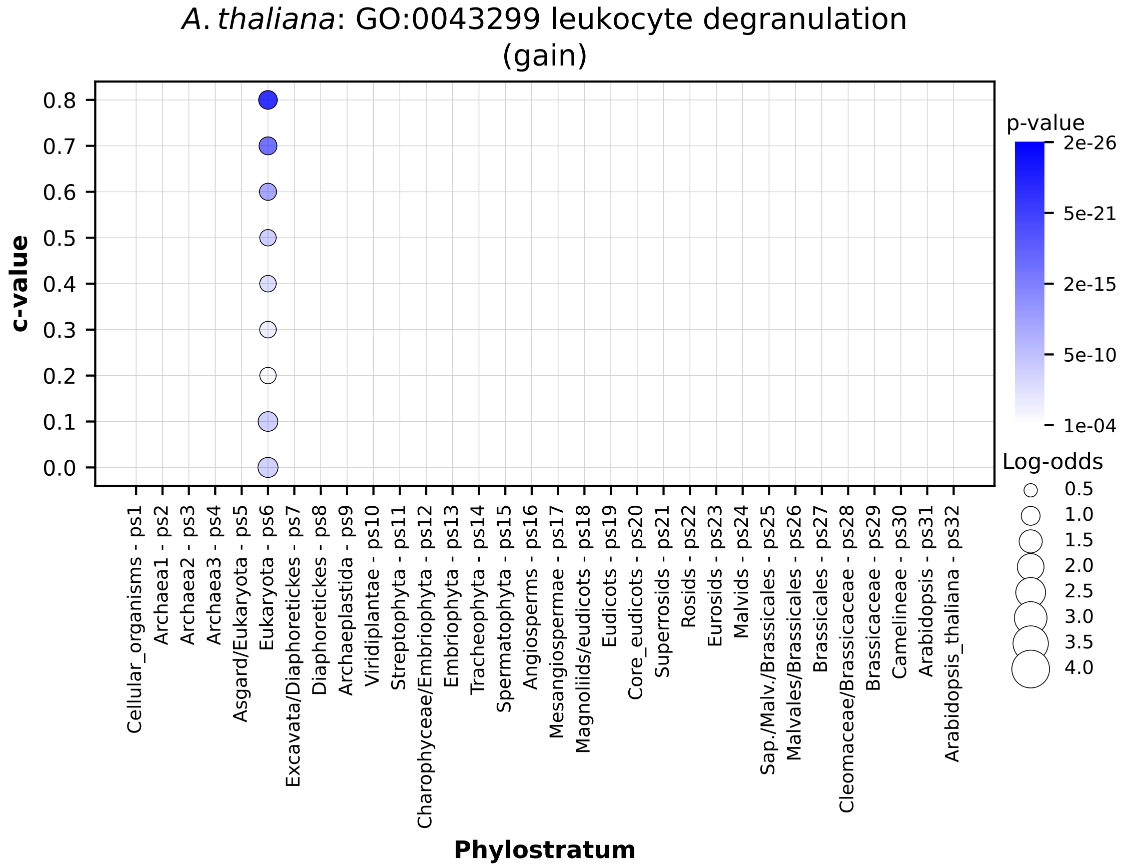

A. thaliana: GO:0043299 leukocyte degranulation
(gain)
c-value
0.8
0.7
0.6
0.5
0.4
0.3
0.2
0.1
0.0
p-value
2e-26
5e-21
2e-15
5e-10
1e-04
Log-odds
0.5
1.0
1.5
2.0
2.5
3.0
3.5
4.0
Cellular_organisms - ps1
Archaea1 - ps2
Archaea2 - ps3
Archaea3 - ps4
Asgard/Eukaryota - ps5
Eukaryota - ps6
Excavata/Diaphoretickes - ps7
Diaphoretickes - ps8
Archaeplastida - ps9
Viridiplantae - ps10
Streptophyta - ps11
Charophyceae/Embriophyta - ps12
Embriophyta - ps13
Tracheophyta - ps14
Spermatophyta - ps15
Angiosperms - ps16
Mesangiospermae - ps17
Magnoliids/eudicots - ps18
Eudicots - ps19
Core_eudicots - ps20
Superrosids - ps21
Rosids - ps22
Eurosids - ps23
Malvids - ps24
Sap./Malv./Brassicales - ps25
Malvales/Brassicales - ps26
Brassicales - ps27
Cleomaceae/Brassicaceae - ps28
Brassicaceae - ps29
Camelineae - ps30
Arabidopsis - ps31
Arabidopsis_thaliana - ps32
Phylostratum

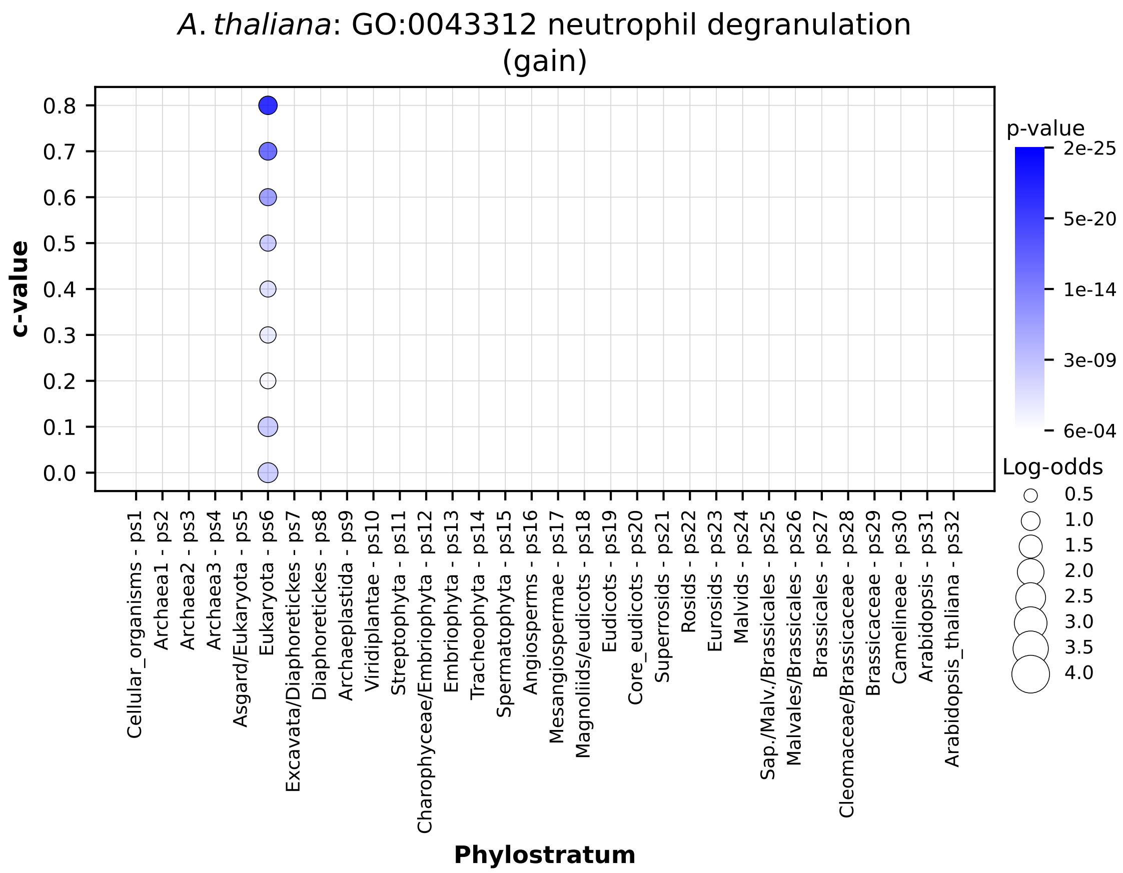

A. thaliana: GO:0043312 neutrophil degranulation
(gain)
c-value
0.8
0.7
0.6
0.5
0.4
0.3
0.2
0.1
0.0
p-value
2e-25
5e-20
1e-14
3e-09
6e-04
Log-odds
0.5
1.0
1.5
2.0
2.5
3.0
3.5
4.0
Cellular_organisms - ps1
Archaea1 - ps2
Archaea2 - ps3
Archaea3 - ps4
Asgard/Eukaryota - ps5
Eukaryota - ps6
Excavata/Diaphoretickes - ps7
Diaphoretickes - ps8
Archaeplastida - ps9
Viridiplantae - ps10
Streptophyta - ps11
Charophyceae/Embriophyta - ps12
Embriophyta - ps13
Tracheophyta - ps14
Spermatophyta - ps15
Angiosperms - ps16
Mesangiospermae - ps17
Magnoliids/eudicots - ps18
Eudicots - ps19
Core_eudicots - ps20
Superrosids - ps21
Rosids - ps22
Eurosids - ps23
Malvids - ps24
Sap./Malv./Brassicales - ps25
Malvales/Brassicales - ps26
Brassicales - ps27
Cleomaceae/Brassicaceae - ps28
Brassicaceae - ps29
Camelineae - ps30
Arabidopsis - ps31
Arabidopsis_thaliana - ps32
Phylostratum

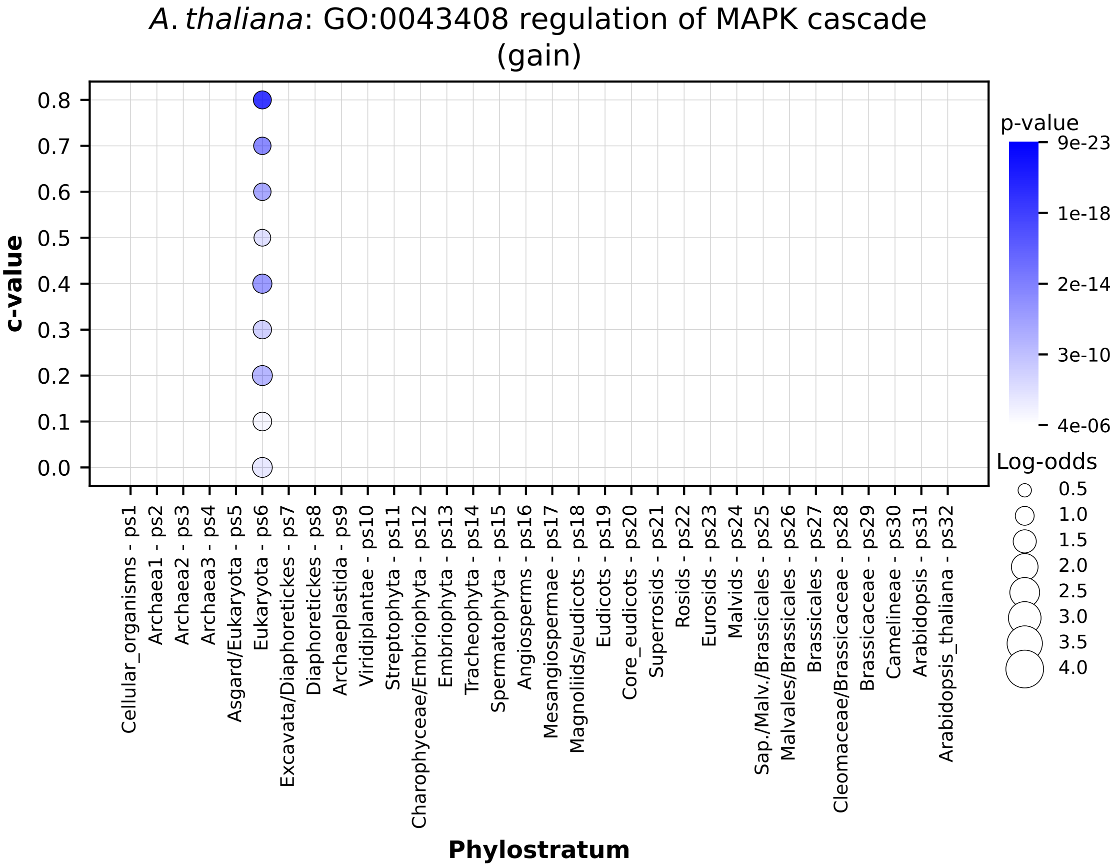

A. thaliana: GO:0043408 regulation of MAPK cascade (gain)
c-value
0.8
0.7
0.6
0.5
0.4
0.3
0.2
0.1
0.0
p-value
9e-23
1e-18
2e-14
3e-10
4e-06
Log-odds
0.5
1.0
1.5
2.0
2.5
3.0
3.5
4.0
Cellular_organisms - ps1
Archaea1 - ps2
Archaea2 - ps3
Archaea3 - ps4
Asgard/Eukaryota - ps5
Eukaryota - ps6
Excavata/Diaphoretickes - ps7
Diaphoretickes - ps8
Archaeplastida - ps9
Viridiplantae - ps10
Streptophyta - ps11
Charophyceae/Embriophyta - ps12
Embriophyta - ps13
Tracheophyta - ps14
Spermatophyta - ps15
Angiosperms - ps16
Mesangiospermae - ps17
Magnoliids/eudicots - ps18
Eudicots - ps19
Core_eudicots - ps20
Superrosids - ps21
Rosids - ps22
Eurosids - ps23
Malvids - ps24
Sap./Malv./Brassicales - ps25
Malvales/Brassicales - ps26
Brassicales - ps27
Cleomaceae/Brassicaceae - ps28
Brassicaceae - ps29
Camelineae - ps30
Arabidopsis - ps31
Arabidopsis_thaliana - ps32
Phylostratum

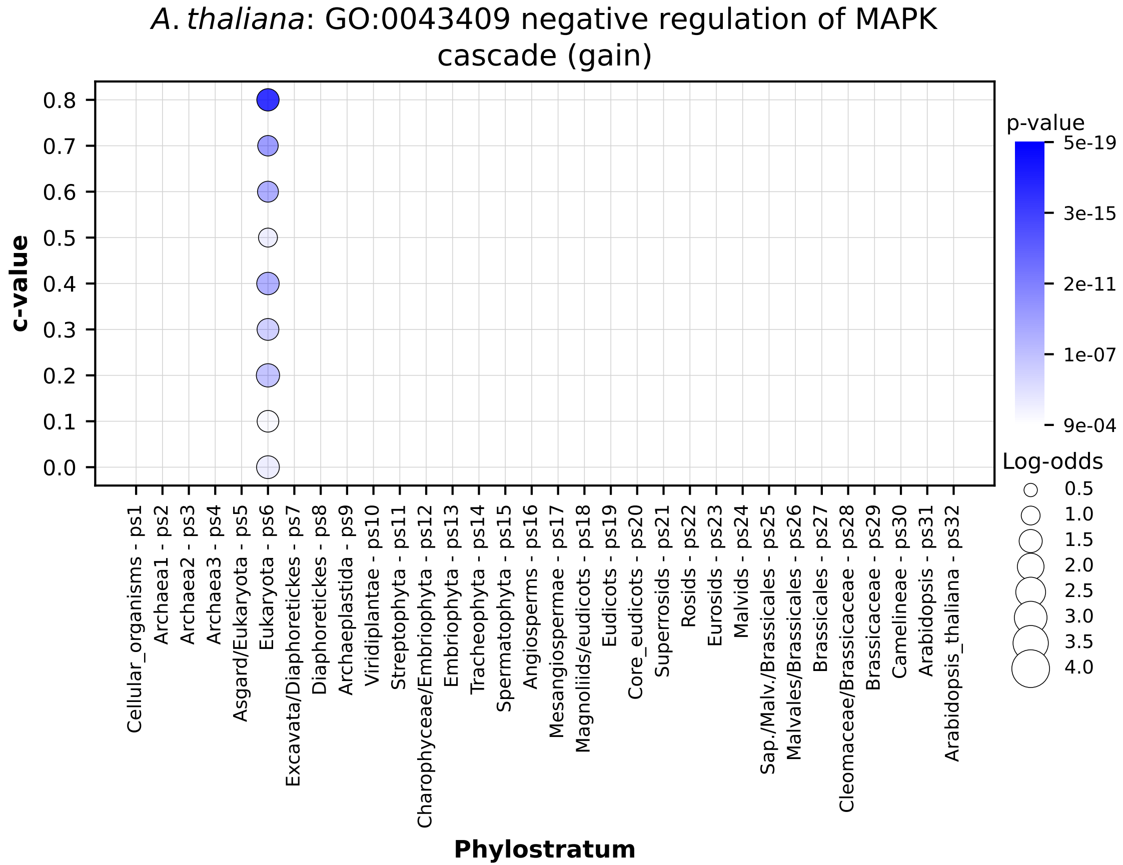
A. thaliana: GO:0043409 negative regulation of MAPK cascade (gain)
c-value
0.8
0.7
0.6
0.5
0.4
0.3
0.2
0.1
0.0
Phylostratum
Cellular_organisms - ps1
Archaea1 - ps2
Archaea2 - ps3
Archaea3 - ps4
Asgard/Eukaryota - ps5
Eukaryota - ps6
Excavata/Diaphoretickes - ps7
Diaphoretickes - ps8
Archaeplastida - ps9
Viridiplantae - ps10
Streptophyta - ps11
Charophyceae/Embriophyta - ps12
Embriophyta - ps13
Tracheophyta - ps14
Spermatophyta - ps15
Angiosperms - ps16
Mesangiospermae - ps17
Magnoliids/eudicots - ps18
Eudicots - ps19
Core_eudicots - ps20
Superrosids - ps21
Rosids - ps22
Eurosids - ps23
Malvids - ps24
Sap./Malv./Brassicales - ps25
Malvales/Brassicales - ps26
Brassicales - ps27
Cleomaceae/Brassicaceae - ps28
Brassicaceae - ps29
Camelineae - ps30
Arabidopsis - ps31
Arabidopsis_thaliana - ps32
p-value
5e-19
3e-15
2e-11
1e-07
9e-04
Log-odds
0.5
1.0
1.5
2.0
2.5
3.0
3.5
4.0

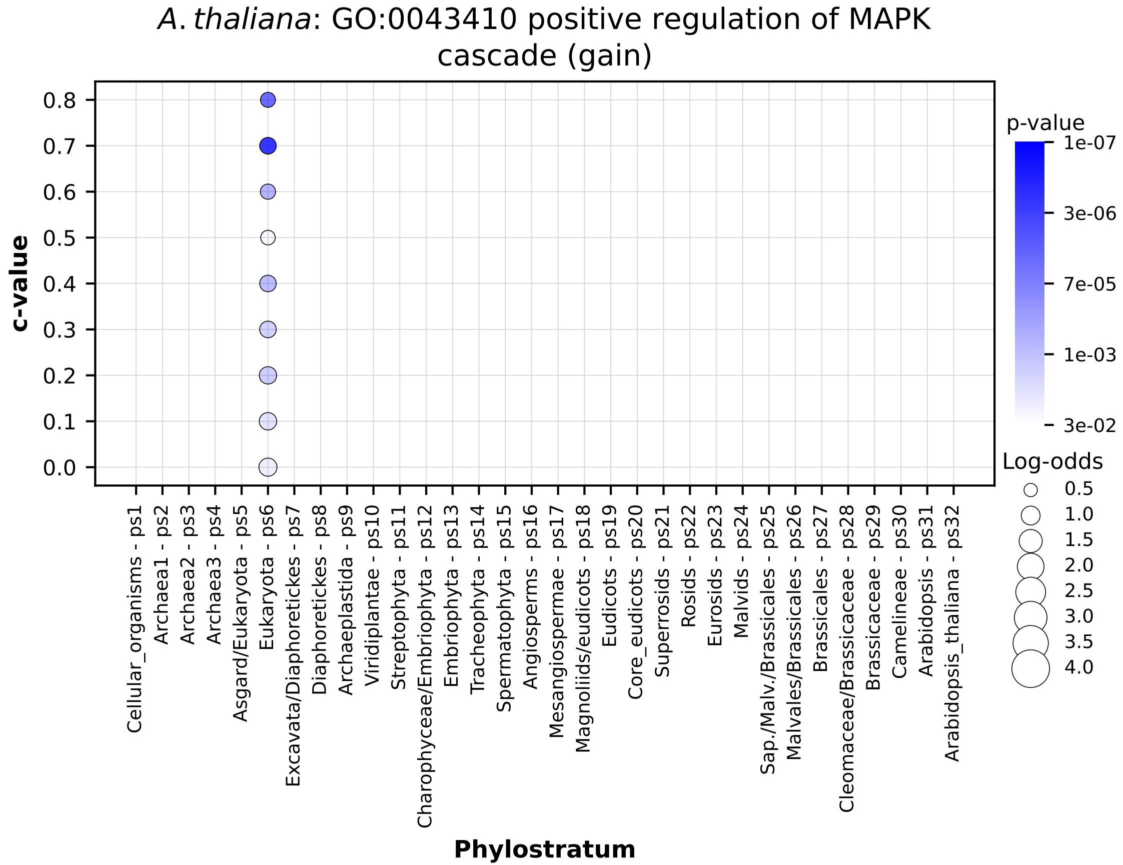
A. thaliana: GO:0043410 positive regulation of MAPK cascade (gain)
c-value
0.8
0.7
0.6
0.5
0.4
0.3
0.2
0.1
0.0
Phylostratum
Cellular_organisms - ps1
Archaea1 - ps2
Archaea2 - ps3
Archaea3 - ps4
Asgard/Eukaryota - ps5
Eukaryota - ps6
Excavata/Diaphoretickes - ps7
Diaphoretickes - ps8
Archaeplastida - ps9
Viridiplantae - ps10
Streptophyta - ps11
Charophyceae/Embriophyta - ps12
Embriophyta - ps13
Tracheophyta - ps14
Spermatophyta - ps15
Angiosperms - ps16
Mesangiospermae - ps17
Magnoliids/eudicots - ps18
Eudicots - ps19
Core_eudicots - ps20
Superrosids - ps21
Rosids - ps22
Eurosids - ps23
Malvids - ps24
Sap./Malv./Brassicales - ps25
Malvales/Brassicales - ps26
Brassicales - ps27
Cleomaceae/Brassicaceae - ps28
Brassicaceae - ps29
Camelineae - ps30
Arabidopsis - ps31
Arabidopsis_thaliana - ps32
p-value
1e-07
3e-06
7e-05
1e-03
3e-02
Log-odds
0.5
1.0
1.5
2.0
2.5
3.0
3.5
4.0

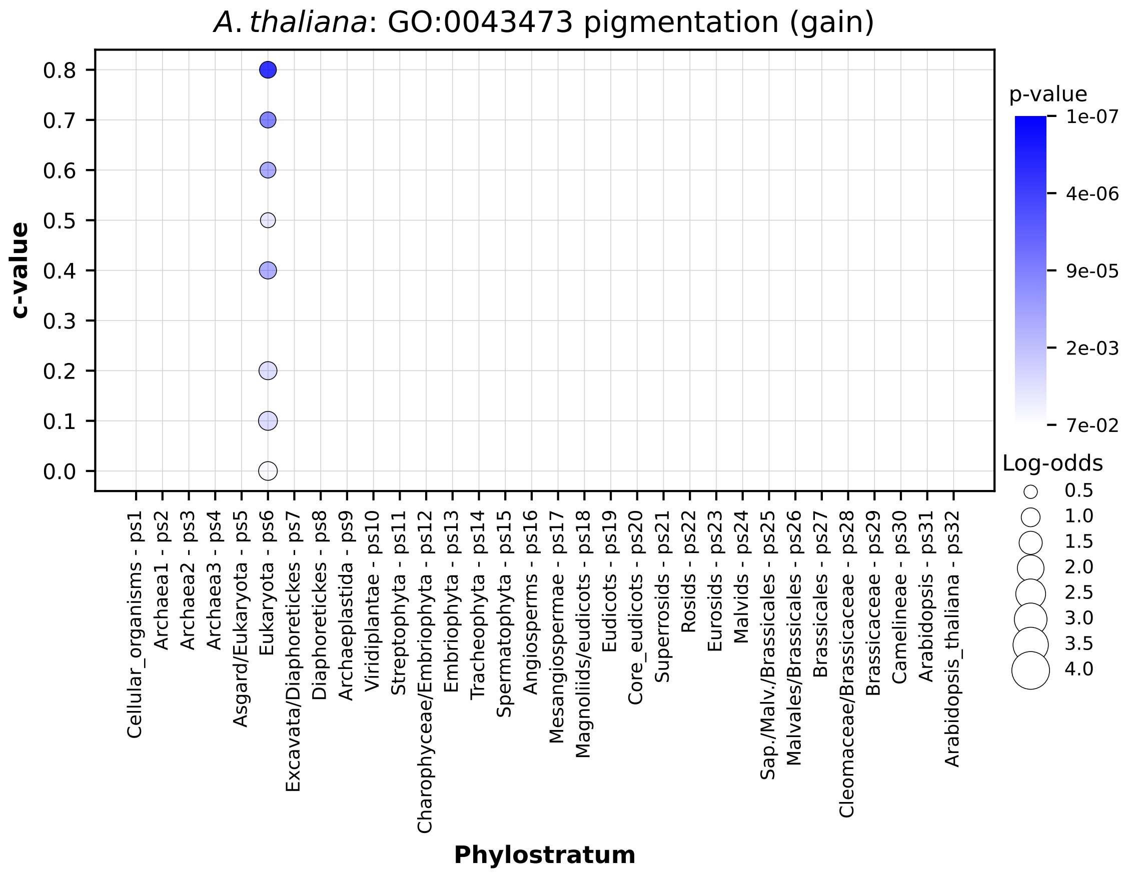

A. thaliana: GO:0043473 pigmentation (gain)
c-value
0.0
0.1
0.2
0.3
0.4
0.5
0.6
0.7
0.8
Phylostratum
Cellular_organisms - ps1
Archaea1 - ps2
Archaea2 - ps3
Archaea3 - ps4
Asgard/Eukaryota - ps5
Eukaryota - ps6
Excavata/Diaphoretickes - ps7
Diaphoretickes - ps8
Archaeplastida - ps9
Viridiplantae - ps10
Streptophyta - ps11
Charophyceae/Embriophyta - ps12
Embriophyta - ps13
Tracheophyta - ps14
Spermatophyta - ps15
Angiosperms - ps16
Mesangiospermae - ps17
Magnoliids/eudicots - ps18
Eudicots - ps19
Core_eudicots - ps20
Superrosids - ps21
Rosids - ps22
Eurosids - ps23
Malvids - ps24
Sap./Malv./Brassicales - ps25
Malvales/Brassicales - ps26
Brassicales - ps27
Cleomaceae/Brassicaceae - ps28
Brassicaceae - ps29
Camelineae - ps30
Arabidopsis - ps31
Arabidopsis_thaliana - ps32
p-value
1e-07
4e-06
9e-05
2e-03
7e-02
Log-odds
0.5
1.0
1.5
2.0
2.5
3.0
3.5
4.0

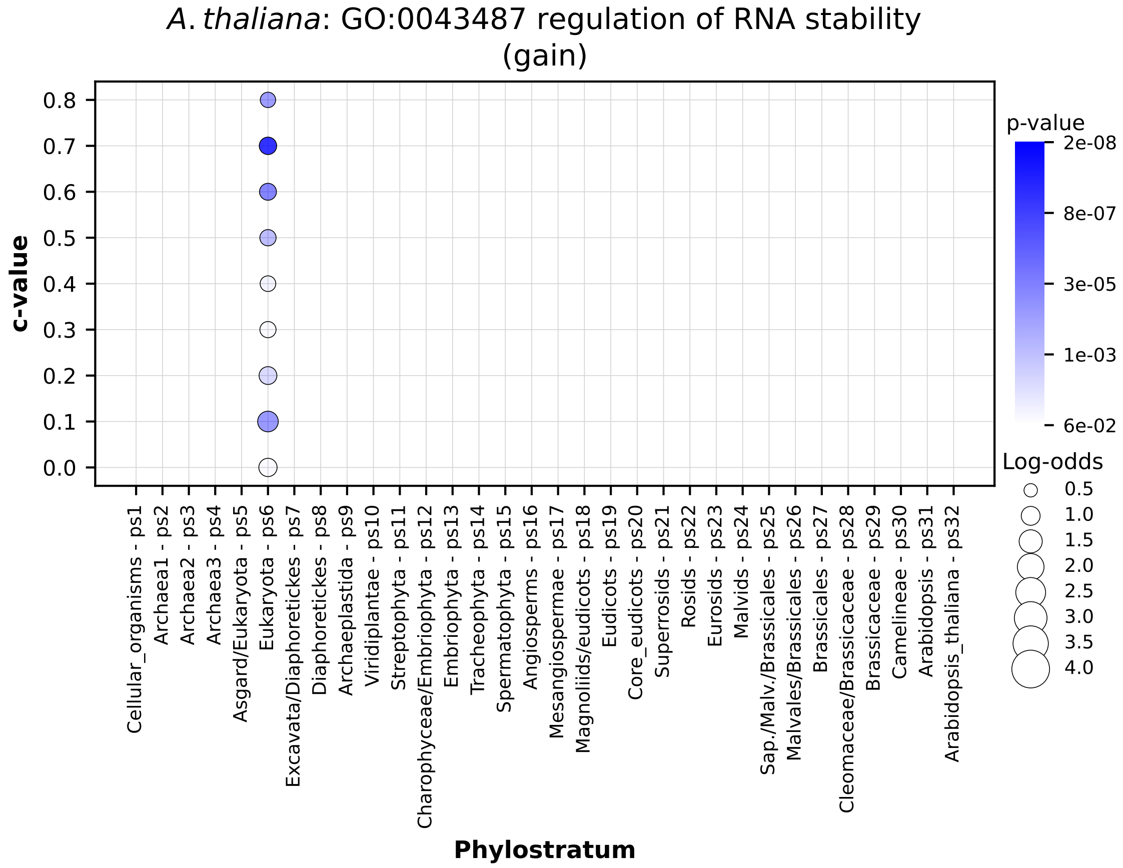

A. thaliana: GO:0043487 regulation of RNA stability
(gain)
c-value
0.8
0.7
0.6
0.5
0.4
0.3
0.2
0.1
0.0
p-value
2e-08
8e-07
3e-05
1e-03
6e-02
Log-odds
0.5
1.0
1.5
2.0
2.5
3.0
3.5
4.0
Cellular_organisms - ps1
Archaea1 - ps2
Archaea2 - ps3
Archaea3 - ps4
Asgard/Eukaryota - ps5
Eukaryota - ps6
Excavata/Diaphoretickes - ps7
Diaphoretickes - ps8
Archaeplastida - ps9
Viridiplantae - ps10
Streptophyta - ps11
Charophyceae/Embriophyta - ps12
Embriophyta - ps13
Tracheophyta - ps14
Spermatophyta - ps15
Angiosperms - ps16
Mesangiospermae - ps17
Magnoliids/eudicots - ps18
Eudicots - ps19
Core_eudicots - ps20
Superrosids - ps21
Rosids - ps22
Eurosids - ps23
Malvids - ps24
Sap./Malv./Brassicales - ps25
Malvales/Brassicales - ps26
Brassicales - ps27
Cleomaceae/Brassicaceae - ps28
Brassicaceae - ps29
Camelineae - ps30
Arabidopsis - ps31
Arabidopsis_thaliana - ps32
Phylostratum

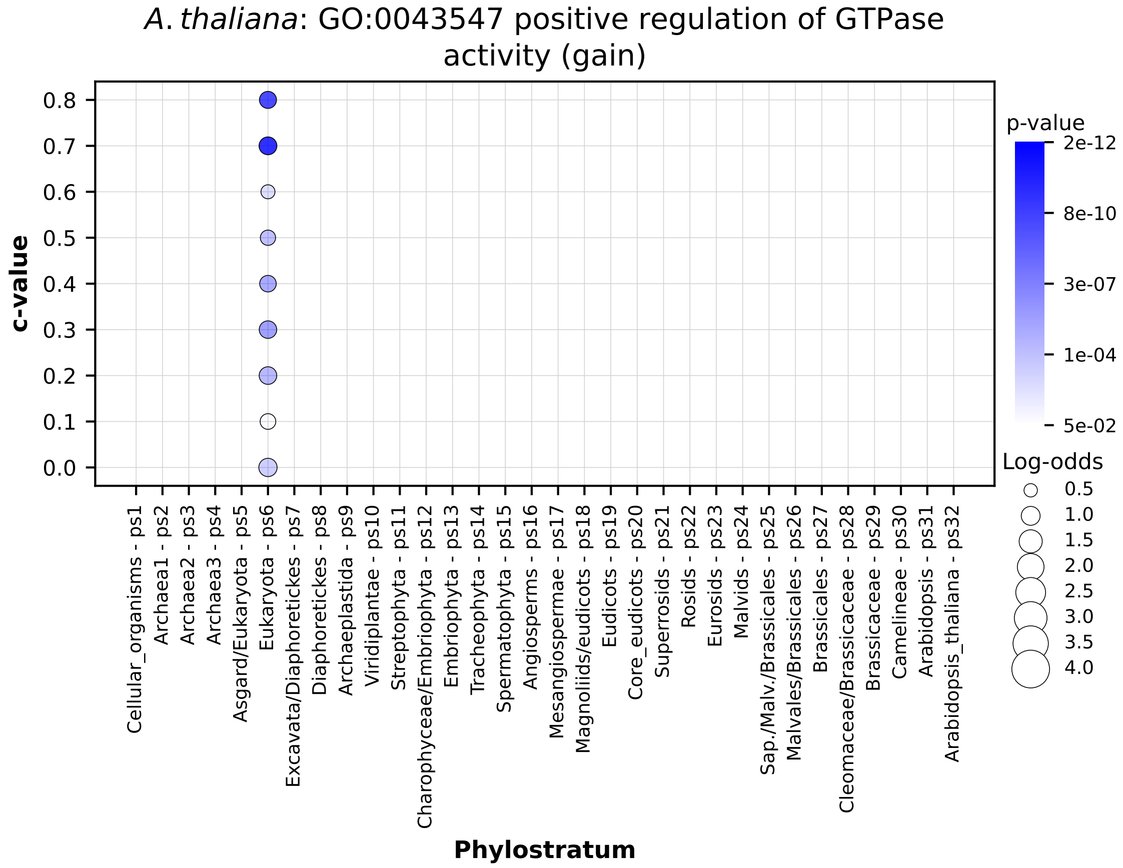
A. thaliana: GO:0043547 positive regulation of GTPase activity (gain)
c-value
0.8
0.7
0.6
0.5
0.4
0.3
0.2
0.1
0.0
Phylostratum
Cellular_organisms - ps1
Archaea1 - ps2
Archaea2 - ps3
Archaea3 - ps4
Asgard/Eukaryota - ps5
Eukaryota - ps6
Excavata/Diaphoretickes - ps7
Diaphoretickes - ps8
Archaeplastida - ps9
Viridiplantae - ps10
Streptophyta - ps11
Charophyceae/Embriophyta - ps12
Embriophyta - ps13
Tracheophyta - ps14
Spermatophyta - ps15
Angiosperms - ps16
Mesangiospermae - ps17
Magnoliids/eudicots - ps18
Eudicots - ps19
Core_eudicots - ps20
Superrosids - ps21
Rosids - ps22
Eurosids - ps23
Malvids - ps24
Sap./Malv./Brassicales - ps25
Malvales/Brassicales - ps26
Brassicales - ps27
Cleomaceae/Brassicaceae - ps28
Brassicaceae - ps29
Camelineae - ps30
Arabidopsis - ps31
Arabidopsis_thaliana - ps32
p-value
2e-12
8e-10
3e-07
1e-04
5e-02
Log-odds
0.5
1.0
1.5
2.0
2.5
3.0
3.5
4.0

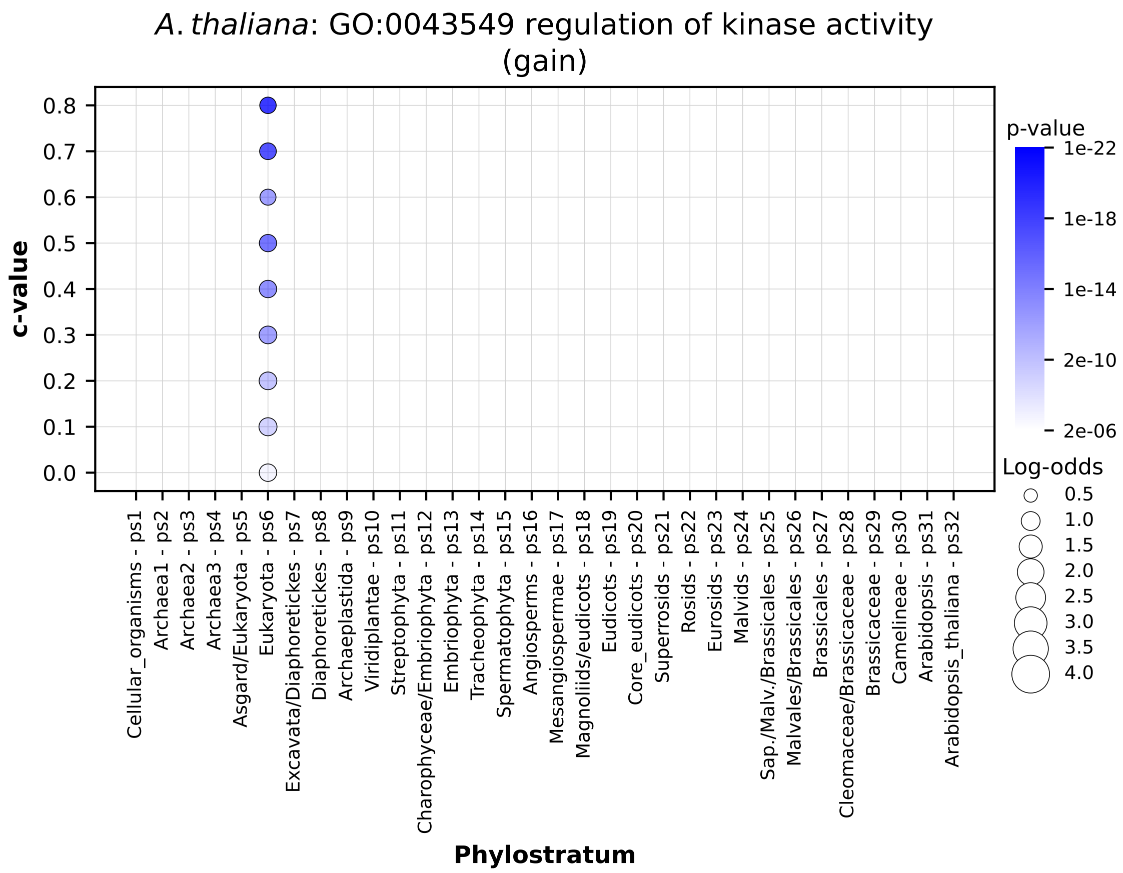

A. thaliana: GO:0043549 regulation of kinase activity (gain)
c-value
0.0
0.1
0.2
0.3
0.4
0.5
0.6
0.7
0.8
p-value
1e-22
1e-18
1e-14
2e-10
2e-06
Log-odds
0.5
1.0
1.5
2.0
2.5
3.0
3.5
4.0
Cellular_organisms - ps1
Archaea1 - ps2
Archaea2 - ps3
Archaea3 - ps4
Asgard/Eukaryota - ps5
Eukaryota - ps6
Excavata/Diaphoretickes - ps7
Diaphoretickes - ps8
Archaeplastida - ps9
Viridiplantae - ps10
Streptophyta - ps11
Charophyceae/Embriophyta - ps12
Embriophyta - ps13
Tracheophyta - ps14
Spermatophyta - ps15
Angiosperms - ps16
Mesangiospermae - ps17
Magnoliids/eudicots - ps18
Eudicots - ps19
Core_eudicots - ps20
Superrosids - ps21
Rosids - ps22
Eurosids - ps23
Malvids - ps24
Sap./Malv./Brassicales - ps25
Malvales/Brassicales - ps26
Brassicales - ps27
Cleomaceae/Brassicaceae - ps28
Brassicaceae - ps29
Camelineae - ps30
Arabidopsis - ps31
Arabidopsis_thaliana - ps32
Phylostratum

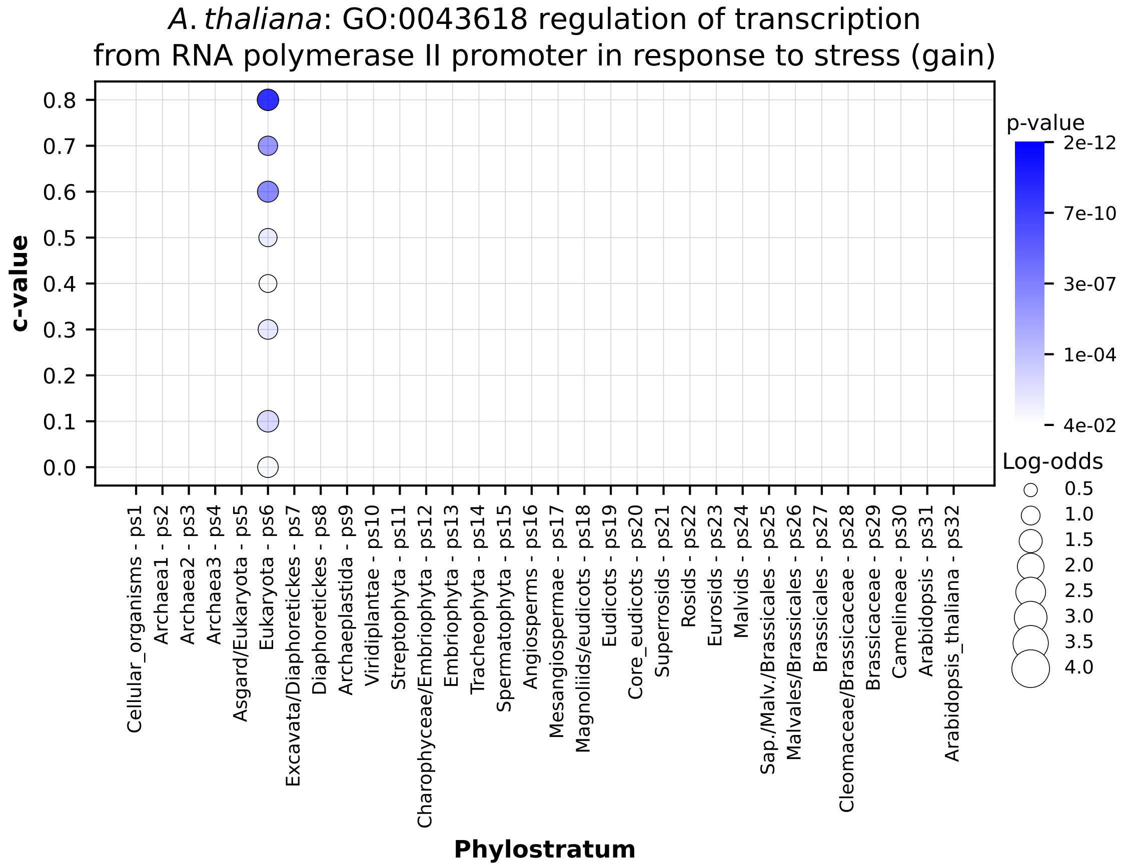

A. thaliana: GO:0043618 regulation of transcription from RNA polymerase II promoter in response to stress (gain)
c-value
0.8
0.7
0.6
0.5
0.4
0.3
0.2
0.1
0.0
Phylostratum
Cellular_organisms - ps1
Archaea1 - ps2
Archaea2 - ps3
Archaea3 - ps4
Asgard/Eukaryota - ps5
Eukaryota - ps6
Excavata/Diaphoretickes - ps7
Diaphoretickes - ps8
Archaeplastida - ps9
Viridiplantae - ps10
Streptophyta - ps11
Charophyceae/Embriophyta - ps12
Embriophyta - ps13
Tracheophyta - ps14
Spermatophyta - ps15
Angiosperms - ps16
Mesangiospermae - ps17
Magnoliids/eudicots - ps18
Eudicots - ps19
Core_eudicots - ps20
Superrosids - ps21
Rosids - ps22
Eurosids - ps23
Malvids - ps24
Sap./Malv./Brassicales - ps25
Malvales/Brassicales - ps26
Brassicales - ps27
Cleomaceae/Brassicaceae - ps28
Brassicaceae - ps29
Camelineae - ps30
Arabidopsis - ps31
Arabidopsis_thaliana - ps32
p-value
2e-12
7e-10
3e-07
1e-04
4e-02
Log-odds
0.5
1.0
1.5
2.0
2.5
3.0
3.5
4.0

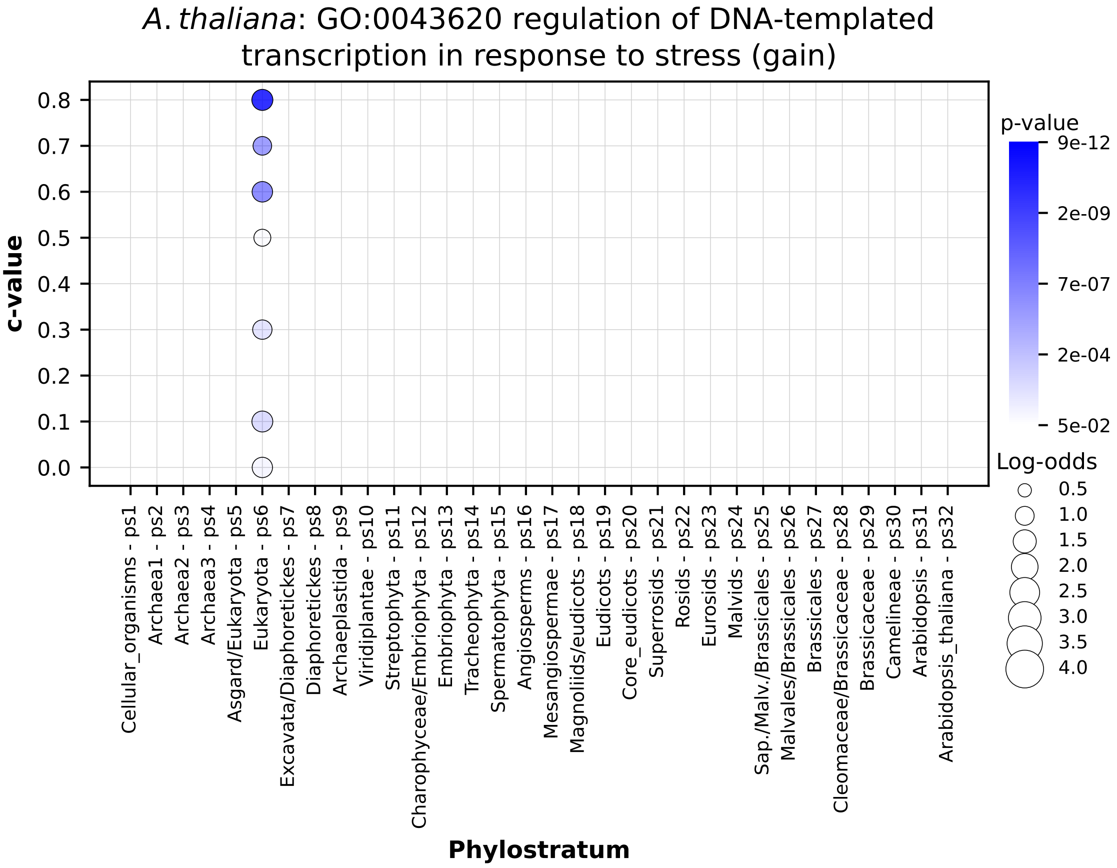

A. thaliana: GO:0043620 regulation of DNA-templated transcription in response to stress (gain)
c-value
0.0
0.1
0.2
0.3
0.4
0.5
0.6
0.7
0.8
Phylostratum
Cellular_organisms - ps1
Archaea1 - ps2
Archaea2 - ps3
Archaea3 - ps4
Asgard/Eukaryota - ps5
Eukaryota - ps6
Excavata/Diaphoretickes - ps7
Diaphoretickes - ps8
Archaeplastida - ps9
Viridiplantae - ps10
Streptophyta - ps11
Charophyceae/Embriophyta - ps12
Embriophyta - ps13
Tracheophyta - ps14
Spermatophyta - ps15
Angiosperms - ps16
Mesangiospermae - ps17
Magnoliids/eudicots - ps18
Eudicots - ps19
Core_eudicots - ps20
Superrosids - ps21
Rosids - ps22
Eurosids - ps23
Malvids - ps24
Sap./Malv./Brassicales - ps25
Malvales/Brassicales - ps26
Brassicales - ps27
Cleomaceae/Brassicaceae - ps28
Brassicaceae - ps29
Camelineae - ps30
Arabidopsis - ps31
Arabidopsis_thaliana - ps32
p-value
9e-12
2e-09
7e-07
2e-04
5e-02
Log-odds
0.5
1.0
1.5
2.0
2.5
3.0
3.5
4.0

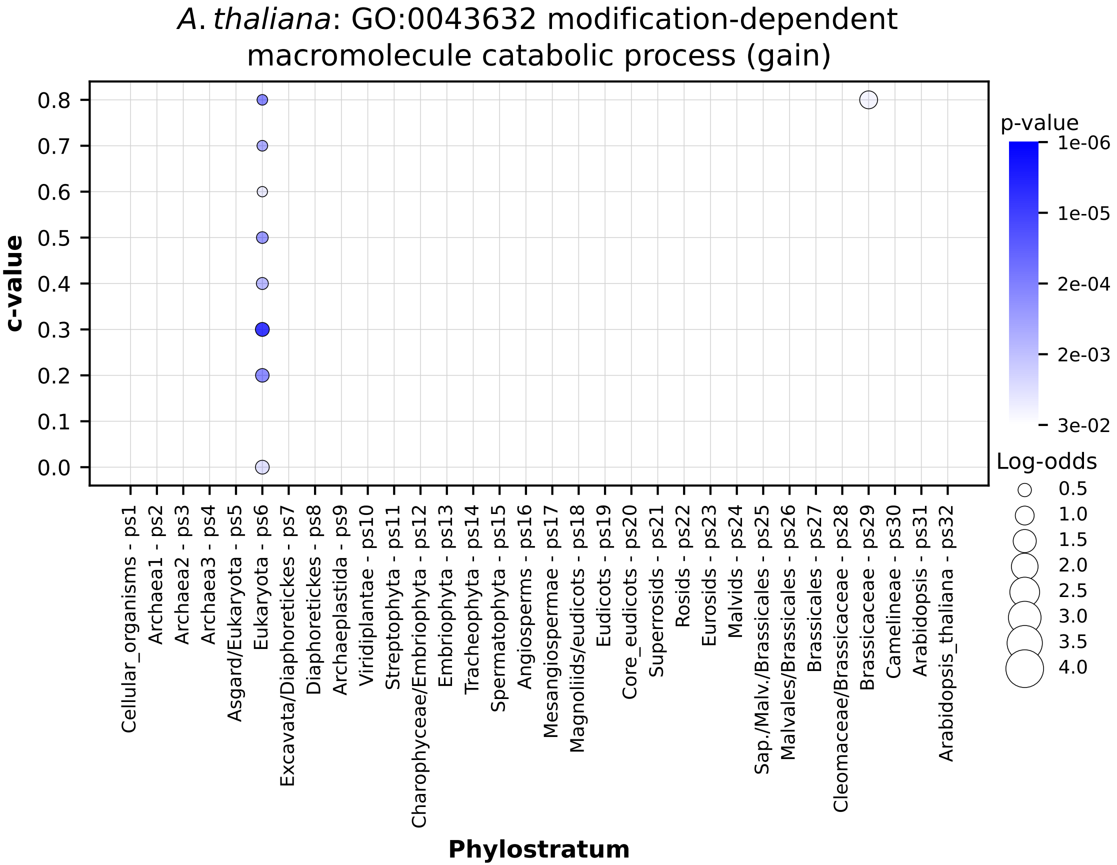

A. thaliana: GO:0043632 modification-dependent macromolecule catabolic process (gain)
c-value
0.8
0.7
0.6
0.5
0.4
0.3
0.2
0.1
0.0
p-value
1e-06
1e-05
2e-04
2e-03
3e-02
Log-odds
0.5
1.0
1.5
2.0
2.5
3.0
3.5
4.0
Cellular_organisms - ps1
Archaea1 - ps2
Archaea2 - ps3
Archaea3 - ps4
Asgard/Eukaryota - ps5
Eukaryota - ps6
Excavata/Diaphoretickes - ps7
Diaphoretickes - ps8
Archaeplastida - ps9
Viridiplantae - ps10
Streptophyta - ps11
Charophyceae/Embriophyta - ps12
Embriophyta - ps13
Tracheophyta - ps14
Spermatophyta - ps15
Angiosperms - ps16
Mesangiospermae - ps17
Magnoliids/eudicots - ps18
Eudicots - ps19
Core_eudicots - ps20
Superrosids - ps21
Rosids - ps22
Eurosids - ps23
Malvids - ps24
Sap./Malv./Brassicales - ps25
Malvales/Brassicales - ps26
Brassicales - ps27
Cleomaceae/Brassicaceae - ps28
Brassicaceae - ps29
Camelineae - ps30
Arabidopsis - ps31
Arabidopsis_thaliana - ps32
Phylostratum

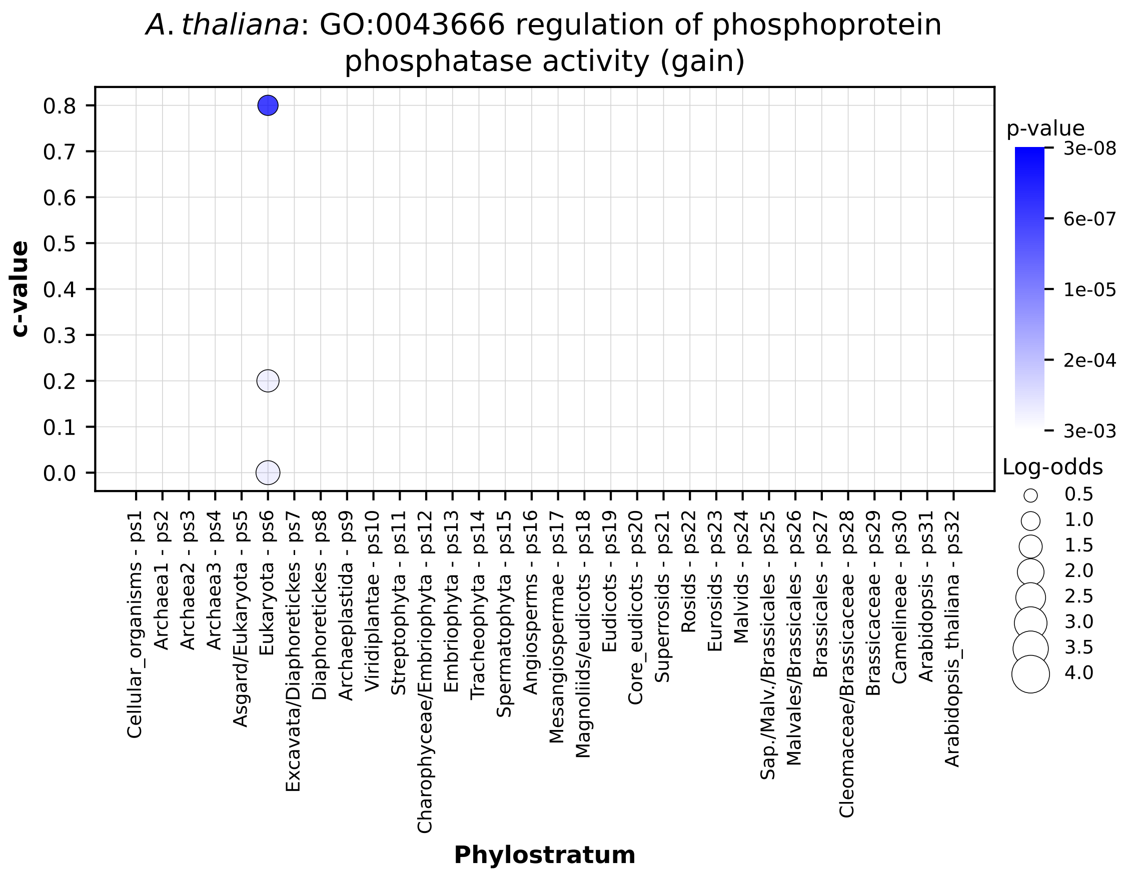

A. thaliana: GO:0043666 regulation of phosphoprotein phosphatase activity (gain)
c-value
0.0
0.1
0.2
0.3
0.4
0.5
0.6
0.7
0.8
p-value
3e-08
6e-07
1e-05
2e-04
3e-03
Log-odds
0.5
1.0
1.5
2.0
2.5
3.0
3.5
4.0
Cellular_organisms - ps1
Archaea1 - ps2
Archaea2 - ps3
Archaea3 - ps4
Asgard/Eukaryota - ps5
Eukaryota - ps6
Excavata/Diaphoretickes - ps7
Diaphoretickes - ps8
Archaeplastida - ps9
Viridiplantae - ps10
Streptophyta - ps11
Charophyceae/Embriophyta - ps12
Embriophyta - ps13
Tracheophyta - ps14
Spermatophyta - ps15
Angiosperms - ps16
Mesangiospermae - ps17
Magnoliids/eudicots - ps18
Eudicots - ps19
Core_eudicots - ps20
Superrosids - ps21
Rosids - ps22
Eurosids - ps23
Malvids - ps24
Sap./Malv./Brassicales - ps25
Malvales/Brassicales - ps26
Brassicales - ps27
Cleomaceae/Brassicaceae - ps28
Brassicaceae - ps29
Camelineae - ps30
Arabidopsis - ps31
Arabidopsis_thaliana - ps32
Phylostratum

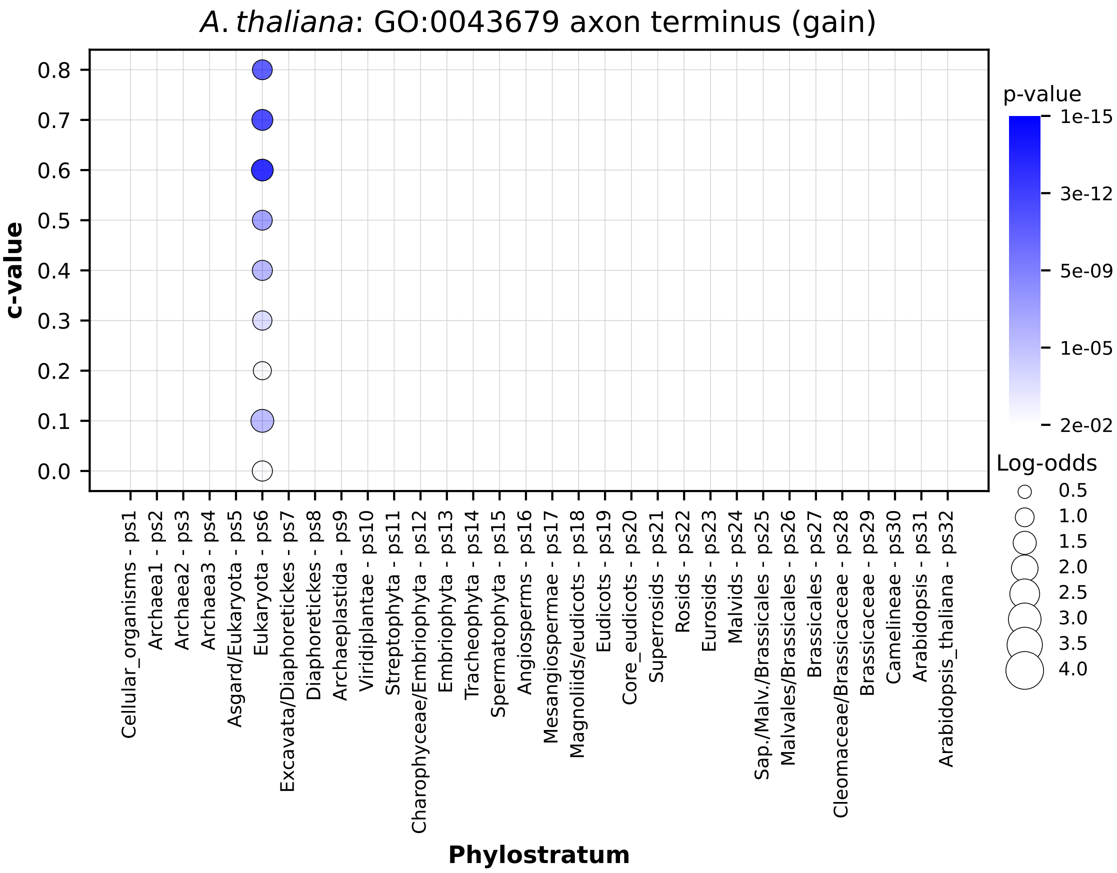

A. thaliana: GO:0043679 axon terminus (gain)
c-value
0.0
0.1
0.2
0.3
0.4
0.5
0.6
0.7
0.8
Phylostratum
Cellular_organisms - ps1
Archaea1 - ps2
Archaea2 - ps3
Archaea3 - ps4
Asgard/Eukaryota - ps5
Eukaryota - ps6
Excavata/Diaphoretickes - ps7
Diaphoretickes - ps8
Archaeplastida - ps9
Viridiplantae - ps10
Streptophyta - ps11
Charophyceae/Embriophyta - ps12
Embriophyta - ps13
Tracheophyta - ps14
Spermatophyta - ps15
Angiosperms - ps16
Mesangiospermae - ps17
Magnoliids/eudicots - ps18
Eudicots - ps19
Core_eudicots - ps20
Superrosids - ps21
Rosids - ps22
Eurosids - ps23
Malvids - ps24
Sap./Malv./Brassicales - ps25
Malvales/Brassicales - ps26
Brassicales - ps27
Cleomaceae/Brassicaceae - ps28
Brassicaceae - ps29
Camelineae - ps30
Arabidopsis - ps31
Arabidopsis_thaliana - ps32
p-value
1e-15
3e-12
5e-09
1e-05
2e-02
Log-odds
0.5
1.0
1.5
2.0
2.5
3.0
3.5
4.0

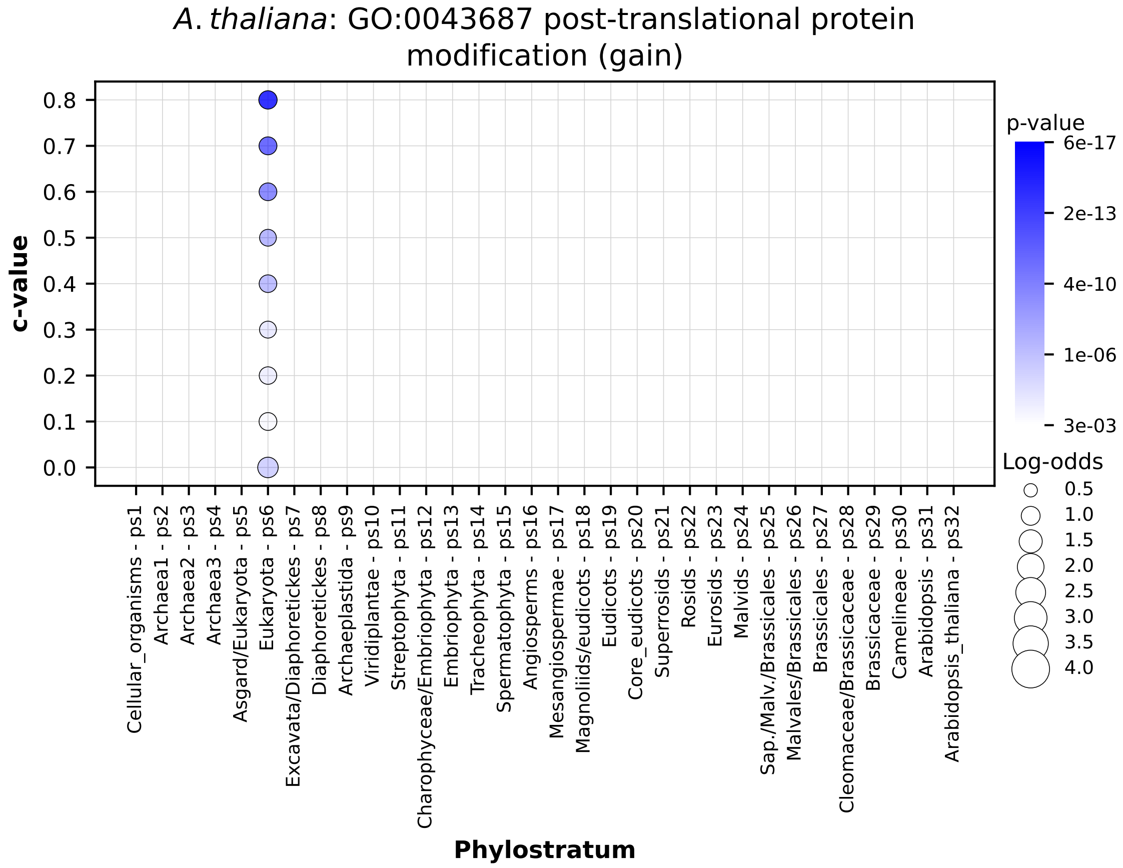
A. thaliana: GO:0043687 post-translational protein modification (gain)
c-value
0.8
0.7
0.6
0.5
0.4
0.3
0.2
0.1
0.0
p-value
6e-17
2e-13
4e-10
1e-06
3e-03
Log-odds
0.5
1.0
1.5
2.0
2.5
3.0
3.5
4.0
Cellular_organisms - ps1
Archaea1 - ps2
Archaea2 - ps3
Archaea3 - ps4
Asgard/Eukaryota - ps5
Eukaryota - ps6
Excavata/Diaphoretickes - ps7
Diaphoretickes - ps8
Archaeplastida - ps9
Viridiplantae - ps10
Streptophyta - ps11
Charophyceae/Embriophyta - ps12
Embriophyta - ps13
Tracheophyta - ps14
Spermatophyta - ps15
Angiosperms - ps16
Mesangiospermae - ps17
Magnoliids/eudicots - ps18
Eudicots - ps19
Core_eudicots - ps20
Superrosids - ps21
Rosids - ps22
Eurosids - ps23
Malvids - ps24
Sap./Malv./Brassicales - ps25
Malvales/Brassicales - ps26
Brassicales - ps27
Cleomaceae/Brassicaceae - ps28
Brassicaceae - ps29
Camelineae - ps30
Arabidopsis - ps31
Arabidopsis_thaliana - ps32
Phylostratum

A. thaliana: GO:0043933 protein-containing complex organization (gain)

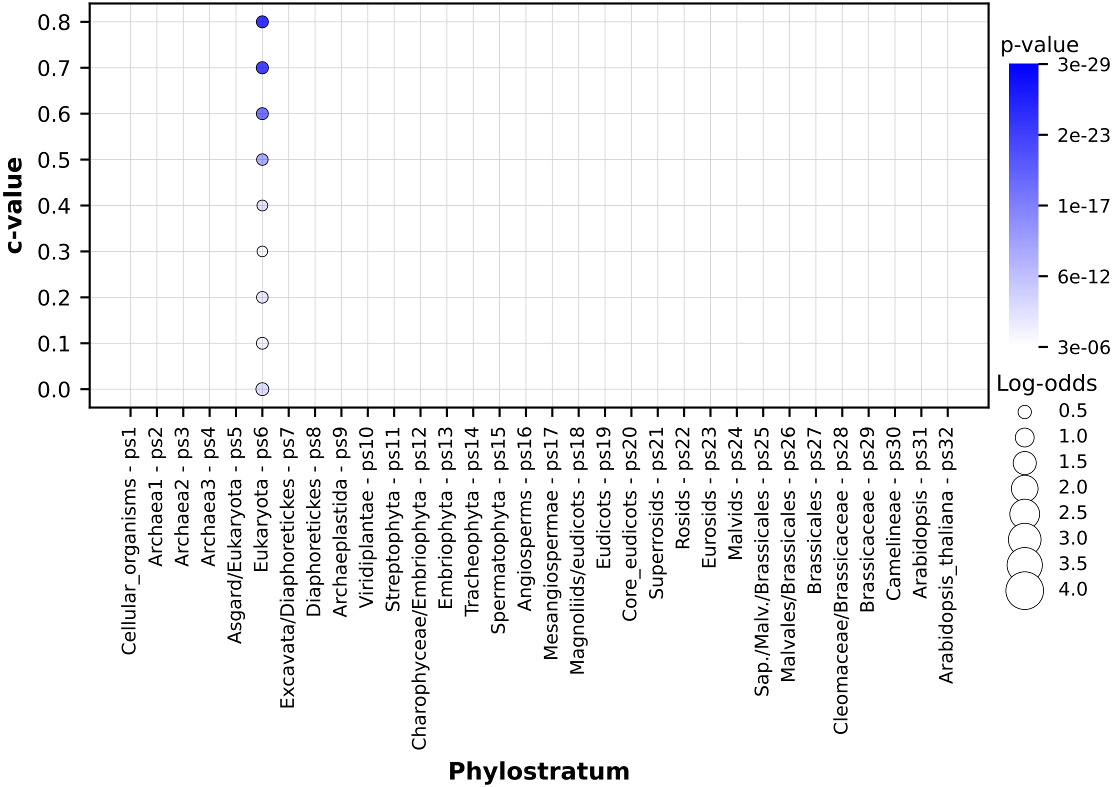

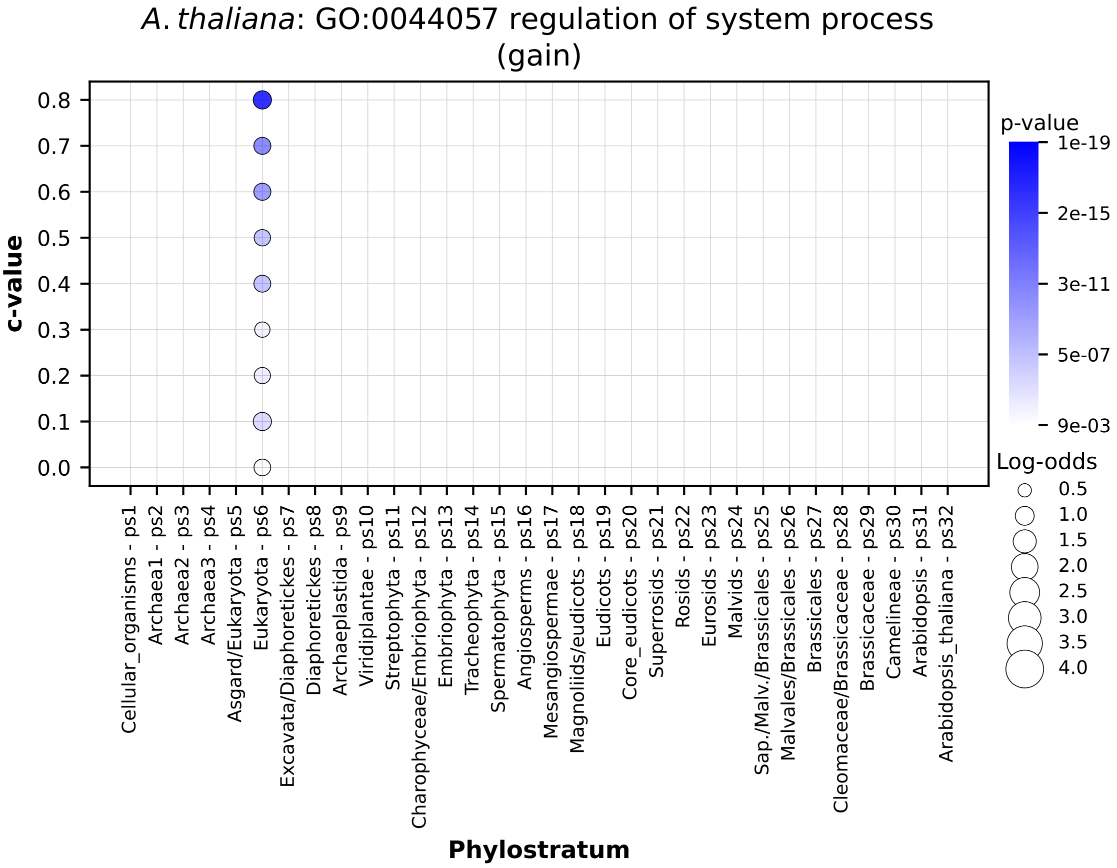

A. thaliana: GO:0044057 regulation of system process
(gain)
c-value
0.8
0.7
0.6
0.5
0.4
0.3
0.2
0.1
0.0
p-value
1e-19
2e-15
3e-11
5e-07
9e-03
Log-odds
0.5
1.0
1.5
2.0
2.5
3.0
3.5
4.0
Cellular_organisms - ps1
Archaea1 - ps2
Archaea2 - ps3
Archaea3 - ps4
Asgard/Eukaryota - ps5
Eukaryota - ps6
Excavata/Diaphoretickes - ps7
Diaphoretickes - ps8
Archaeplastida - ps9
Viridiplantae - ps10
Streptophyta - ps11
Charophyceae/Embriophyta - ps12
Embriophyta - ps13
Tracheophyta - ps14
Spermatophyta - ps15
Angiosperms - ps16
Mesangiospermae - ps17
Magnoliids/eudicots - ps18
Eudicots - ps19
Core_eudicots - ps20
Superrosids - ps21
Rosids - ps22
Eurosids - ps23
Malvids - ps24
Sap./Malv./Brassicales - ps25
Malvales/Brassicales - ps26
Brassicales - ps27
Cleomaceae/Brassicaceae - ps28
Brassicaceae - ps29
Camelineae - ps30
Arabidopsis - ps31
Arabidopsis_thaliana - ps32
Phylostratum

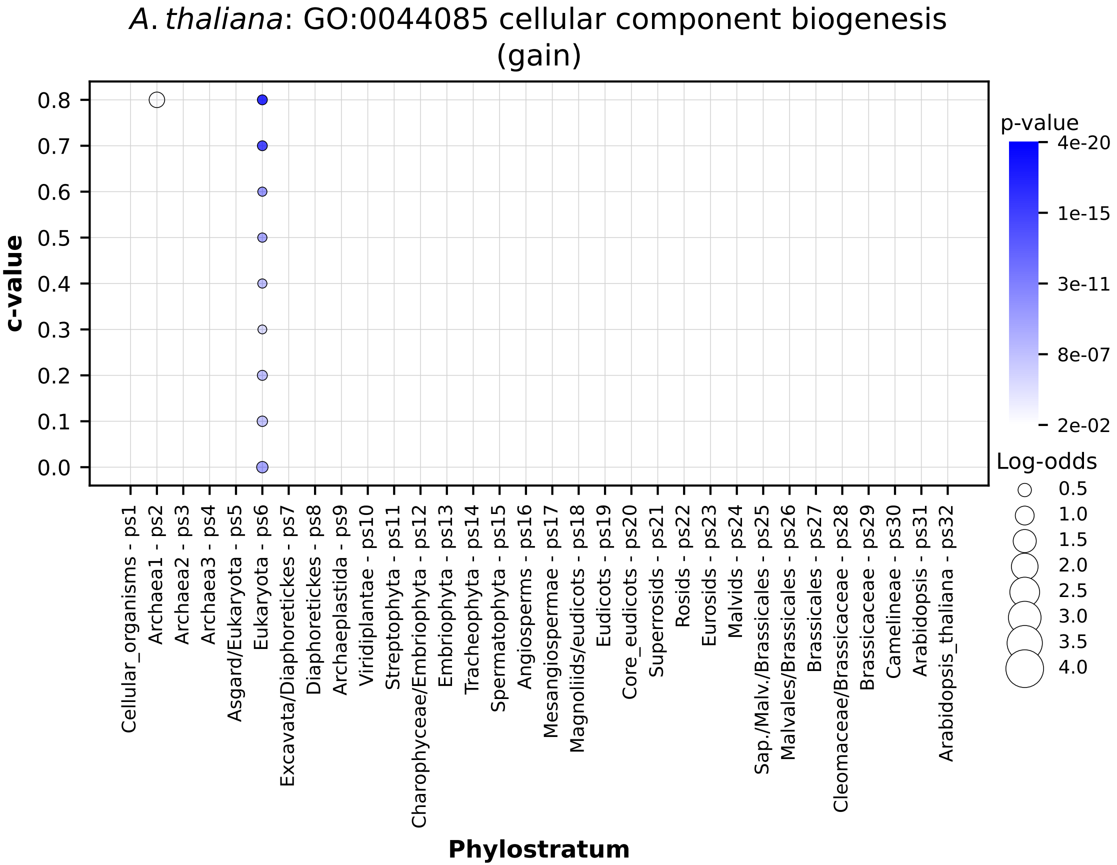
A. thaliana: GO:0044085 cellular component biogenesis (gain)
c-value
0.8
0.7
0.6
0.5
0.4
0.3
0.2
0.1
0.0
Phylostratum
Cellular_organisms - ps1
Archaea1 - ps2
Archaea2 - ps3
Archaea3 - ps4
Asgard/Eukaryota - ps5
Eukaryota - ps6
Excavata/Diaphoretickes - ps7
Diaphoretickes - ps8
Archaeplastida - ps9
Viridiplantae - ps10
Streptophyta - ps11
Charophyceae/Embriophyta - ps12
Embriophyta - ps13
Tracheophyta - ps14
Spermatophyta - ps15
Angiosperms - ps16
Mesangiospermae - ps17
Magnoliids/eudicots - ps18
Eudicots - ps19
Core_eudicots - ps20
Superrosids - ps21
Rosids - ps22
Eurosids - ps23
Malvids - ps24
Sap./Malv./Brassicales - ps25
Malvales/Brassicales - ps26
Brassicales - ps27
Cleomaceae/Brassicaceae - ps28
Brassicaceae - ps29
Camelineae - ps30
Arabidopsis - ps31
Arabidopsis_thaliana - ps32
p-value
4e-20
1e-15
3e-11
8e-07
2e-02
Log-odds
0.5
1.0
1.5
2.0
2.5
3.0
3.5
4.0

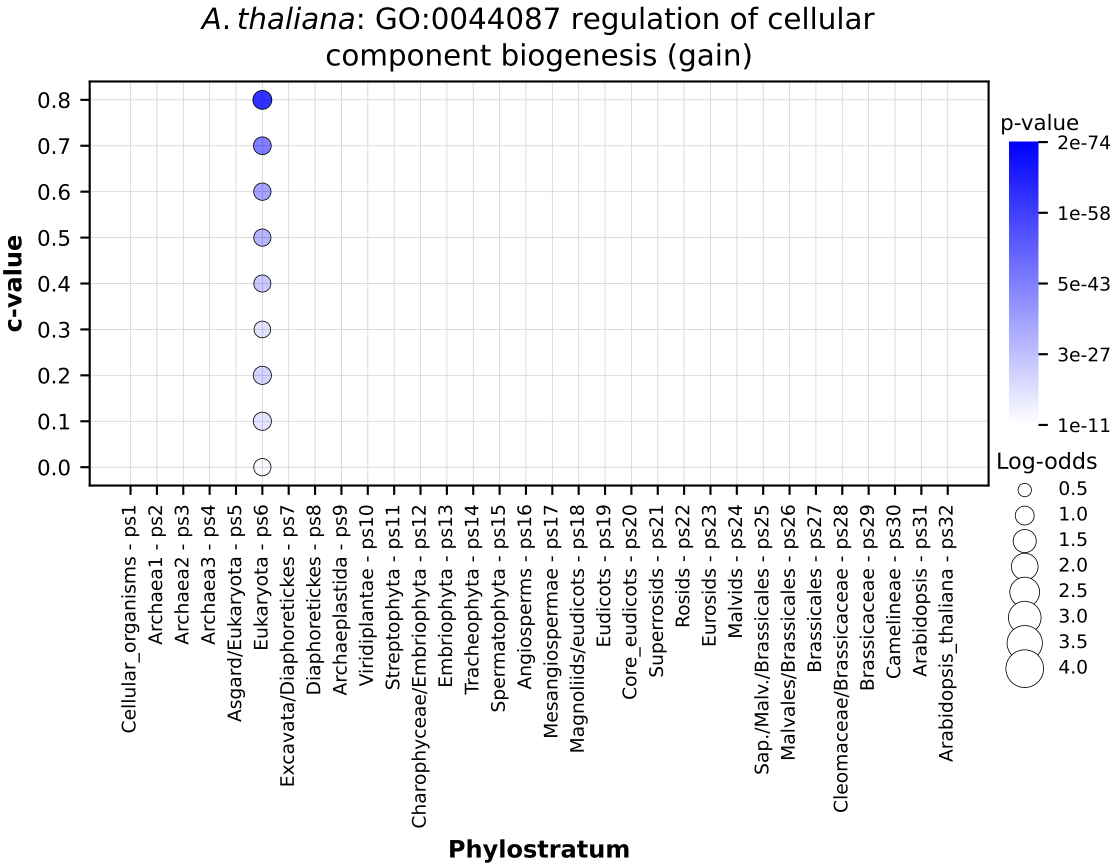

A. thaliana: GO:0044087 regulation of cellular component biogenesis (gain)
c-value
0.0
0.1
0.2
0.3
0.4
0.5
0.6
0.7
0.8
p-value
2e-74
1e-58
5e-43
3e-27
1e-11
Log-odds
0.5
1.0
1.5
2.0
2.5
3.0
3.5
4.0
Cellular_organisms - ps1
Archaea1 - ps2
Archaea2 - ps3
Archaea3 - ps4
Asgard/Eukaryota - ps5
Eukaryota - ps6
Excavata/Diaphoretickes - ps7
Diaphoretickes - ps8
Archaeplastida - ps9
Viridiplantae - ps10
Streptophyta - ps11
Charophyceae/Embriophyta - ps12
Embriophyta - ps13
Tracheophyta - ps14
Spermatophyta - ps15
Angiosperms - ps16
Mesangiospermae - ps17
Magnoliids/eudicots - ps18
Eudicots - ps19
Core_eudicots - ps20
Superrosids - ps21
Rosids - ps22
Eurosids - ps23
Malvids - ps24
Sap./Malv./Brassicales - ps25
Malvales/Brassicales - ps26
Brassicales - ps27
Cleomaceae/Brassicaceae - ps28
Brassicaceae - ps29
Camelineae - ps30
Arabidopsis - ps31
Arabidopsis_thaliana - ps32
Phylostratum

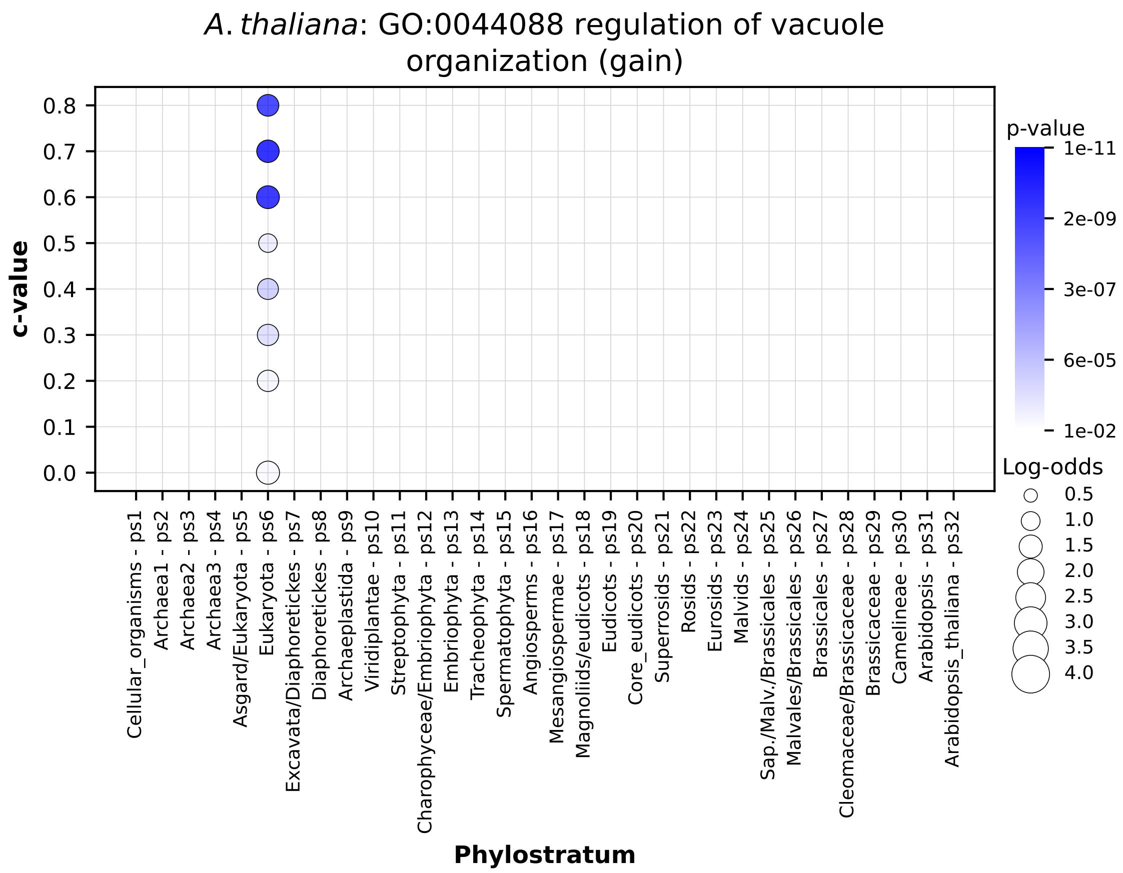

A. thaliana: GO:0044088 regulation of vacuole organization (gain)
c-value
0.8
0.7
0.6
0.5
0.4
0.3
0.2
0.1
0.0
p-value
1e-11
2e-09
3e-07
6e-05
1e-02
Log-odds
0.5
1.0
1.5
2.0
2.5
3.0
3.5
4.0
Cellular_organisms - ps1
Archaea1 - ps2
Archaea2 - ps3
Archaea3 - ps4
Asgard/Eukaryota - ps5
Eukaryota - ps6
Excavata/Diaphoretickes - ps7
Diaphoretickes - ps8
Archaeplastida - ps9
Viridiplantae - ps10
Streptophyta - ps11
Charophyceae/Embriophyta - ps12
Embriophyta - ps13
Tracheophyta - ps14
Spermatophyta - ps15
Angiosperms - ps16
Mesangiospermae - ps17
Magnoliids/eudicots - ps18
Eudicots - ps19
Core_eudicots - ps20
Superrosids - ps21
Rosids - ps22
Eurosids - ps23
Malvids - ps24
Sap./Malv./Brassicales - ps25
Malvales/Brassicales - ps26
Brassicales - ps27
Cleomaceae/Brassicaceae - ps28
Brassicaceae - ps29
Camelineae - ps30
Arabidopsis - ps31
Arabidopsis_thaliana - ps32
Phylostratum

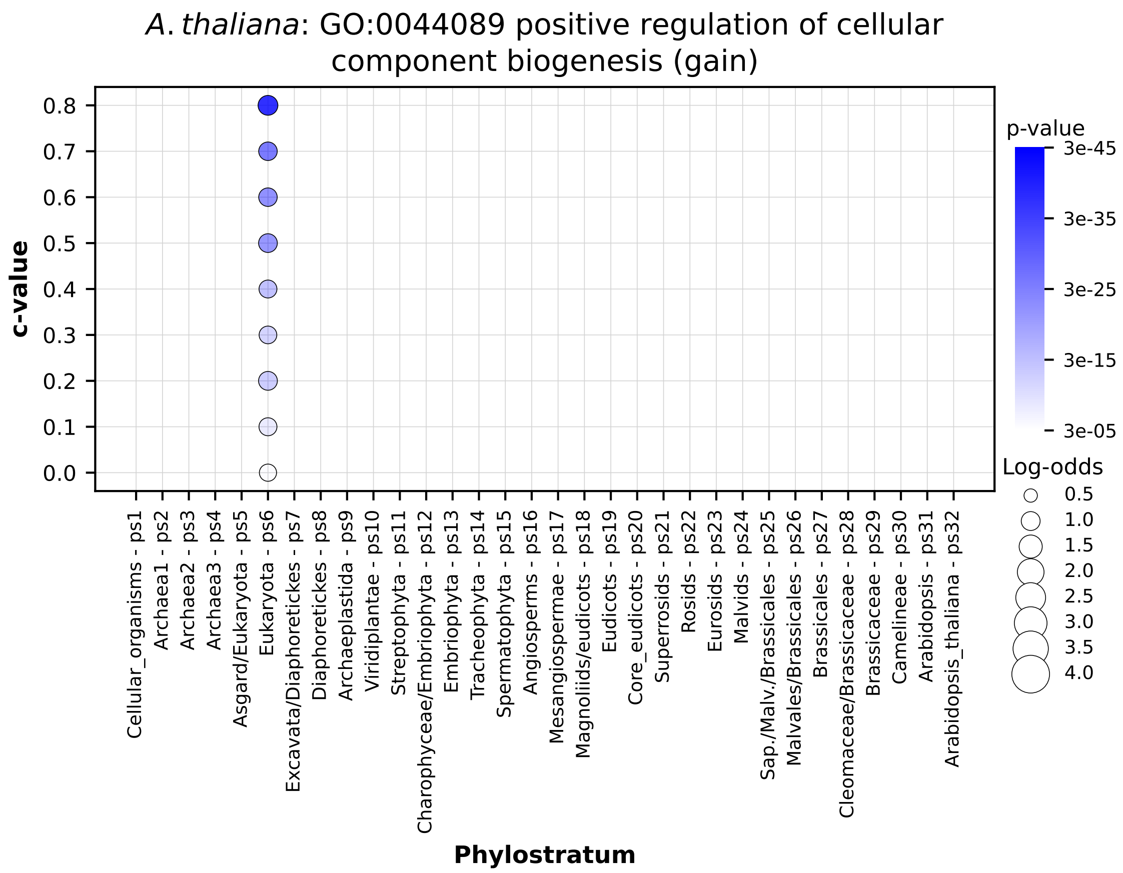
A. thaliana: GO:0044089 positive regulation of cellular component biogenesis (gain)
c-value
0.8
0.7
0.6
0.5
0.4
0.3
0.2
0.1
0.0
p-value
3e-45
3e-35
3e-25
3e-15
3e-05
Log-odds
0.5
1.0
1.5
2.0
2.5
3.0
3.5
4.0
Cellular_organisms - ps1
Archaea1 - ps2
Archaea2 - ps3
Archaea3 - ps4
Asgard/Eukaryota - ps5
Eukaryota - ps6
Excavata/Diaphoretickes - ps7
Diaphoretickes - ps8
Archaeplastida - ps9
Viridiplantae - ps10
Streptophyta - ps11
Charophyceae/Embriophyta - ps12
Embriophyta - ps13
Tracheophyta - ps14
Spermatophyta - ps15
Angiosperms - ps16
Mesangiospermae - ps17
Magnoliids/eudicots - ps18
Eudicots - ps19
Core_eudicots - ps20
Superrosids - ps21
Rosids - ps22
Eurosids - ps23
Malvids - ps24
Sap./Malv./Brassicales - ps25
Malvales/Brassicales - ps26
Brassicales - ps27
Cleomaceae/Brassicaceae - ps28
Brassicaceae - ps29
Camelineae - ps30
Arabidopsis - ps31
Arabidopsis_thaliana - ps32
Phylostratum

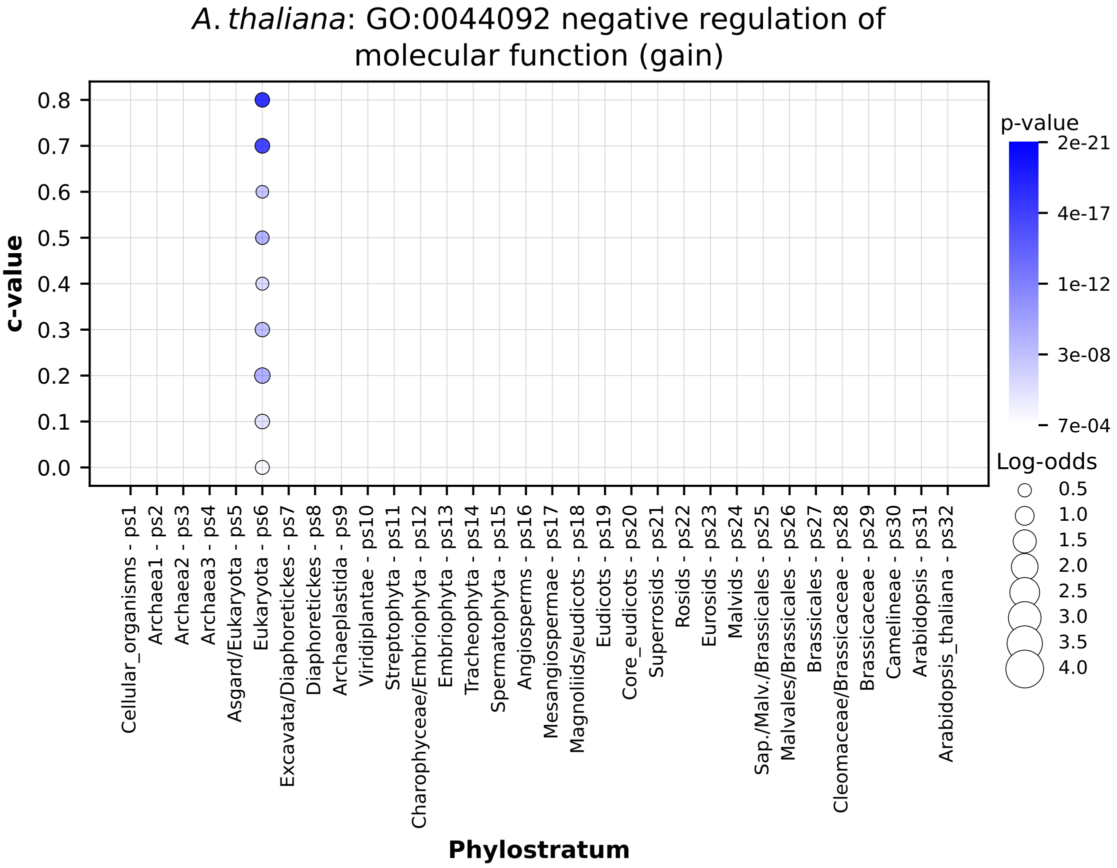

A. thaliana: GO:0044092 negative regulation of molecular function (gain)
c-value
0.8
0.7
0.6
0.5
0.4
0.3
0.2
0.1
0.0
p-value
2e-21
4e-17
1e-12
3e-08
7e-04
Log-odds
0.5
1.0
1.5
2.0
2.5
3.0
3.5
4.0
Cellular_organisms - ps1
Archaea1 - ps2
Archaea2 - ps3
Archaea3 - ps4
Asgard/Eukaryota - ps5
Eukaryota - ps6
Excavata/Diaphoretickes - ps7
Diaphoretickes - ps8
Archaeplastida - ps9
Viridiplantae - ps10
Streptophyta - ps11
Charophyceae/Embriophyta - ps12
Embriophyta - ps13
Tracheophyta - ps14
Spermatophyta - ps15
Angiosperms - ps16
Mesangiospermae - ps17
Magnoliids/eudicots - ps18
Eudicots - ps19
Core_eudicots - ps20
Superrosids - ps21
Rosids - ps22
Eurosids - ps23
Malvids - ps24
Sap./Malv./Brassicales - ps25
Malvales/Brassicales - ps26
Brassicales - ps27
Cleomaceae/Brassicaceae - ps28
Brassicaceae - ps29
Camelineae - ps30
Arabidopsis - ps31
Arabidopsis_thaliana - ps32
Phylostratum

A. thaliana: GO:0044093 positive regulation of molecular function (gain)

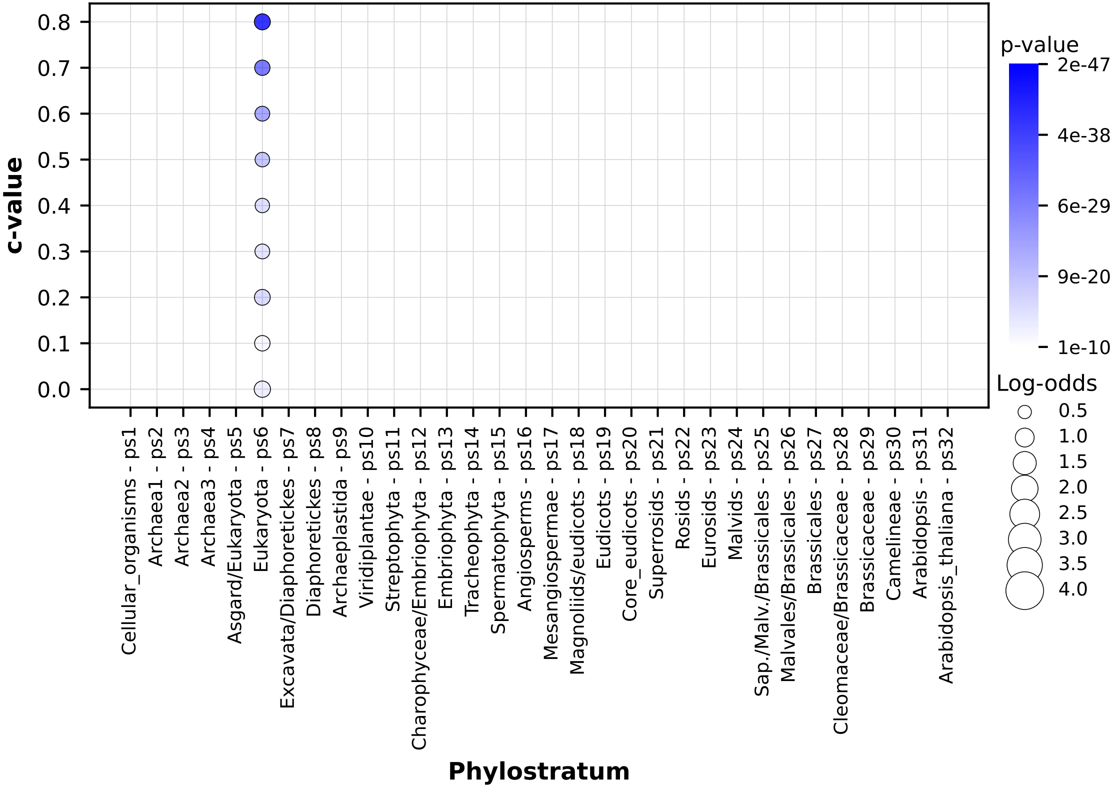

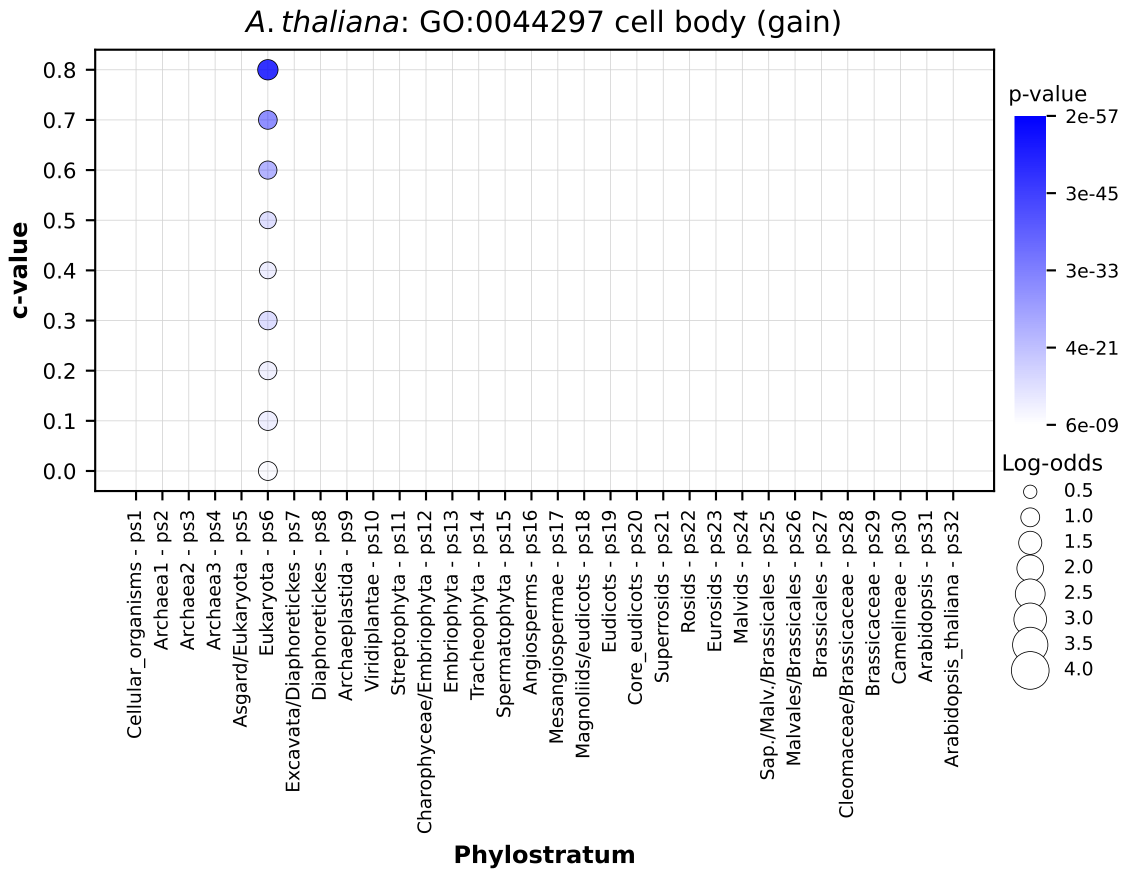

A. thaliana: GO:0044297 cell body (gain)
c-value
0.8
0.7
0.6
0.5
0.4
0.3
0.2
0.1
0.0
p-value
2e-57
3e-45
3e-33
4e-21
6e-09
Log-odds
0.5
1.0
1.5
2.0
2.5
3.0
3.5
4.0
Cellular_organisms - ps1
Archaea1 - ps2
Archaea2 - ps3
Archaea3 - ps4
Asgard/Eukaryota - ps5
Eukaryota - ps6
Excavata/Diaphoretickes - ps7
Diaphoretickes - ps8
Archaeplastida - ps9
Viridiplantae - ps10
Streptophyta - ps11
Charophyceae/Embriophyta - ps12
Embriophyta - ps13
Tracheophyta - ps14
Spermatophyta - ps15
Angiosperms - ps16
Mesangiospermae - ps17
Magnoliids/eudicots - ps18
Eudicots - ps19
Core_eudicots - ps20
Superrosids - ps21
Rosids - ps22
Eurosids - ps23
Malvids - ps24
Sap./Malv./Brassicales - ps25
Malvales/Brassicales - ps26
Brassicales - ps27
Cleomaceae/Brassicaceae - ps28
Brassicaceae - ps29
Camelineae - ps30
Arabidopsis - ps31
Arabidopsis_thaliana - ps32
Phylostratum

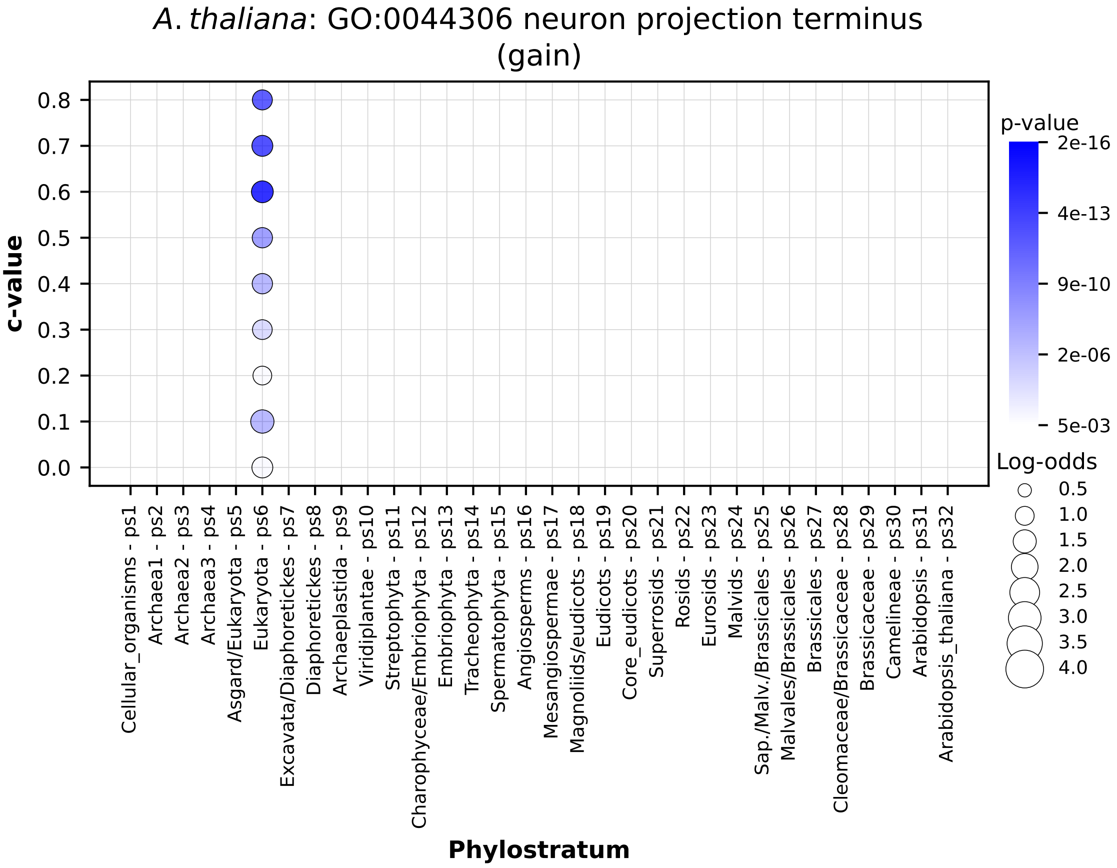

A. thaliana: GO:0044306 neuron projection terminus (gain)
c-value
0.8
0.7
0.6
0.5
0.4
0.3
0.2
0.1
0.0
p-value
2e-16
4e-13
9e-10
2e-06
5e-03
Log-odds
0.5
1.0
1.5
2.0
2.5
3.0
3.5
4.0
Cellular_organisms - ps1
Archaea1 - ps2
Archaea2 - ps3
Archaea3 - ps4
Asgard/Eukaryota - ps5
Eukaryota - ps6
Excavata/Diaphoretickes - ps7
Diaphoretickes - ps8
Archaeplastida - ps9
Viridiplantae - ps10
Streptophyta - ps11
Charophyceae/Embriophyta - ps12
Embriophyta - ps13
Tracheophyta - ps14
Spermatophyta - ps15
Angiosperms - ps16
Mesangiospermae - ps17
Magnoliids/eudicots - ps18
Eudicots - ps19
Core_eudicots - ps20
Superrosids - ps21
Rosids - ps22
Eurosids - ps23
Malvids - ps24
Sap./Malv./Brassicales - ps25
Malvales/Brassicales - ps26
Brassicales - ps27
Cleomaceae/Brassicaceae - ps28
Brassicaceae - ps29
Camelineae - ps30
Arabidopsis - ps31
Arabidopsis_thaliana - ps32
Phylostratum

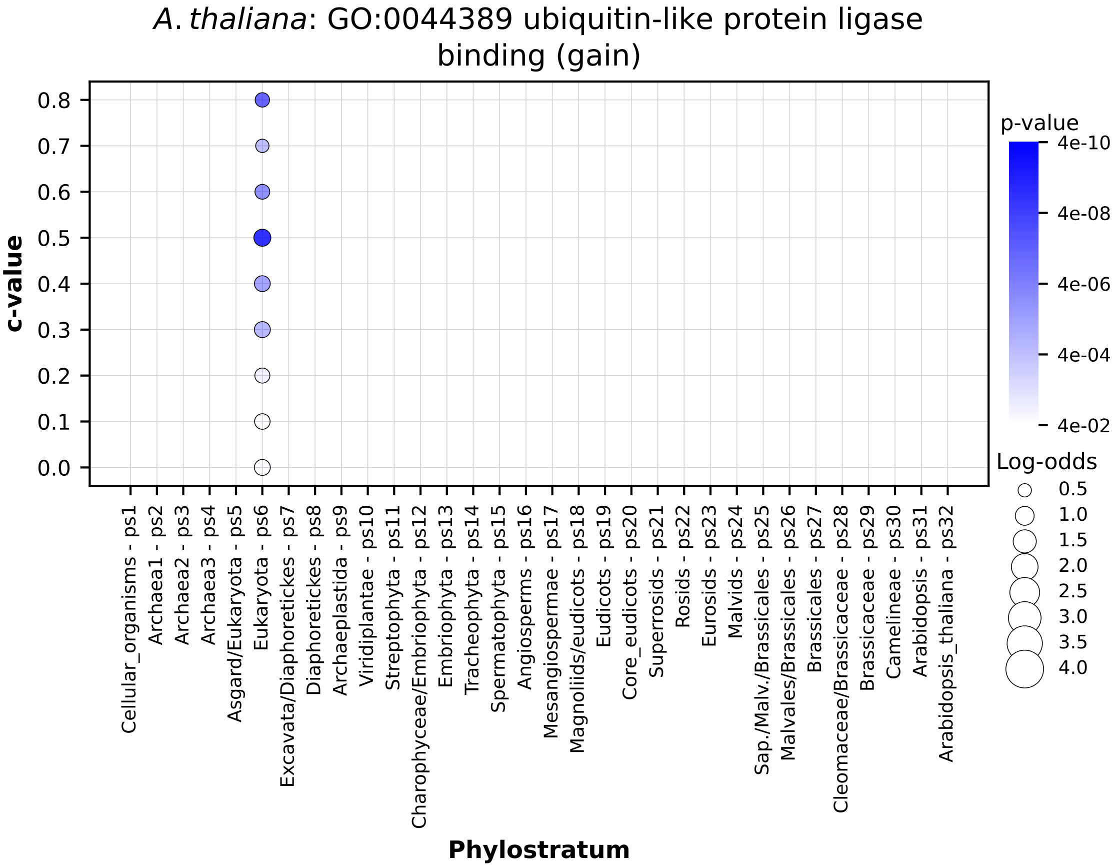

A. thaliana: GO:0044389 ubiquitin-like protein ligase binding (gain)
c-value
0.8
0.7
0.6
0.5
0.4
0.3
0.2
0.1
0.0
Phylostratum
Cellular_organisms - ps1
Archaea1 - ps2
Archaea2 - ps3
Archaea3 - ps4
Asgard/Eukaryota - ps5
Eukaryota - ps6
Excavata/Diaphoretickes - ps7
Diaphoretickes - ps8
Archaeplastida - ps9
Viridiplantae - ps10
Streptophyta - ps11
Charophyceae/Embriophyta - ps12
Embriophyta - ps13
Tracheophyta - ps14
Spermatophyta - ps15
Angiosperms - ps16
Mesangiospermae - ps17
Magnoliids/eudicots - ps18
Eudicots - ps19
Core_eudicots - ps20
Superrosids - ps21
Rosids - ps22
Eurosids - ps23
Malvids - ps24
Sap./Malv./Brassicales - ps25
Malvales/Brassicales - ps26
Brassicales - ps27
Cleomaceae/Brassicaceae - ps28
Brassicaceae - ps29
Camelineae - ps30
Arabidopsis - ps31
Arabidopsis_thaliana - ps32
p-value
4e-10
4e-08
4e-06
4e-04
4e-02
Log-odds
0.5
1.0
1.5
2.0
2.5
3.0
3.5
4.0

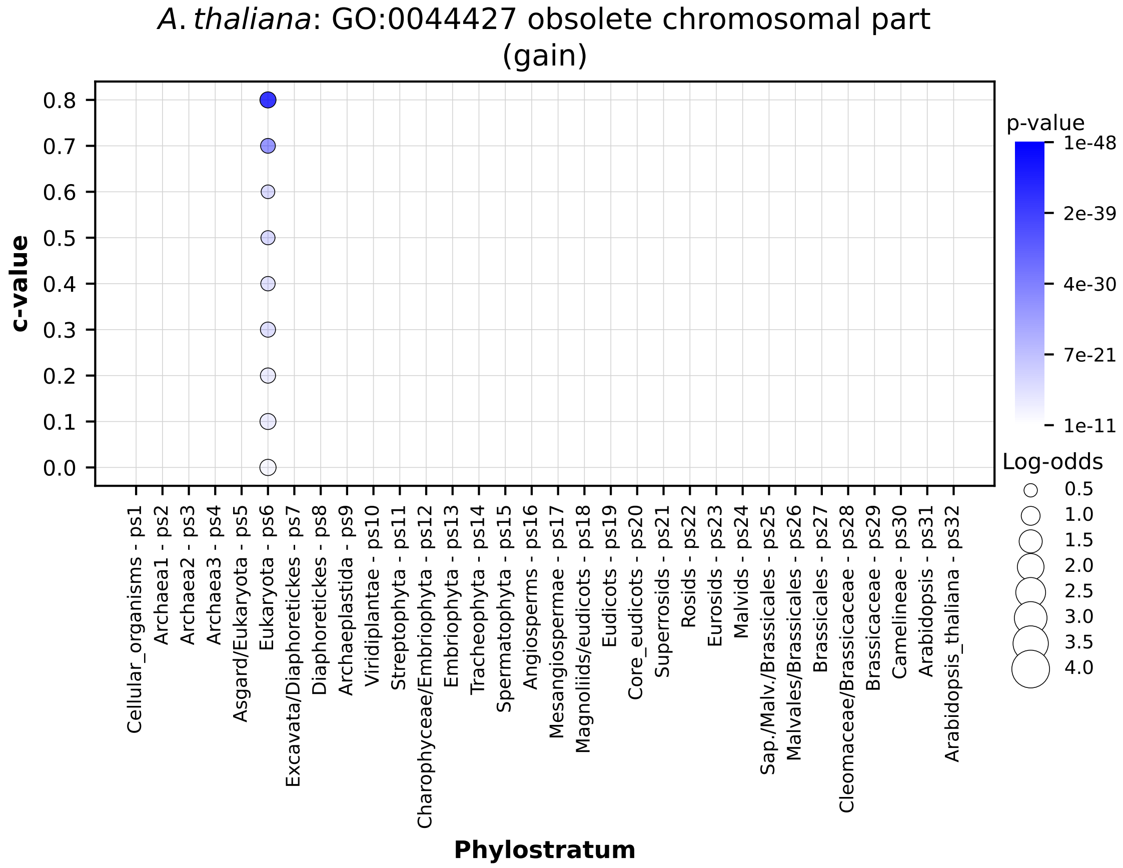
A. thaliana: GO:0044427 obsolete chromosomal part
(gain)
c-value
0.0
0.1
0.2
0.3
0.4
0.5
0.6
0.7
0.8
p-value
1e-48
2e-39
4e-30
7e-21
1e-11
Log-odds
0.5
1.0
1.5
2.0
2.5
3.0
3.5
4.0
Cellular_organisms - ps1
Archaea1 - ps2
Archaea2 - ps3
Archaea3 - ps4
Asgard/Eukaryota - ps5
Eukaryota - ps6
Excavata/Diaphoretickes - ps7
Diaphoretickes - ps8
Archaeplastida - ps9
Viridiplantae - ps10
Streptophyta - ps11
Charophyceae/Embriophyta - ps12
Embriophyta - ps13
Tracheophyta - ps14
Spermatophyta - ps15
Angiosperms - ps16
Mesangiospermae - ps17
Magnoliids/eudicots - ps18
Eudicots - ps19
Core_eudicots - ps20
Superrosids - ps21
Rosids - ps22
Eurosids - ps23
Malvids - ps24
Sap./Malv./Brassicales - ps25
Malvales/Brassicales - ps26
Brassicales - ps27
Cleomaceae/Brassicaceae - ps28
Brassicaceae - ps29
Camelineae - ps30
Arabidopsis - ps31
Arabidopsis_thaliana - ps32
Phylostratum

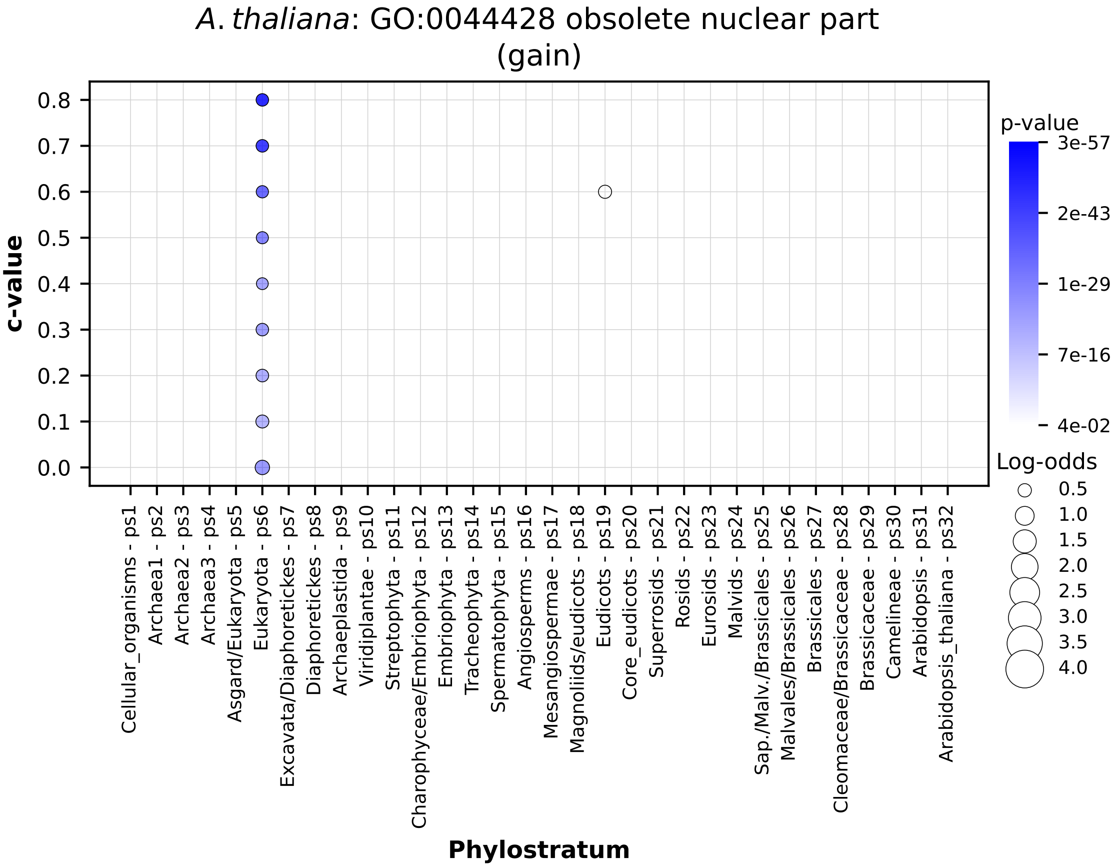

A. thaliana: GO:0044428 obsolete nuclear part
(gain)
c-value
0.8
0.7
0.6
0.5
0.4
0.3
0.2
0.1
0.0
p-value
3e-57
2e-43
1e-29
7e-16
4e-02
Log-odds
0.5
1.0
1.5
2.0
2.5
3.0
3.5
4.0
Cellular_organisms - ps1
Archaea1 - ps2
Archaea2 - ps3
Archaea3 - ps4
Asgard/Eukaryota - ps5
Eukaryota - ps6
Excavata/Diaphoretickes - ps7
Diaphoretickes - ps8
Archaeplastida - ps9
Viridiplantae - ps10
Streptophyta - ps11
Charophyceae/Embriophyta - ps12
Embriophyta - ps13
Tracheophyta - ps14
Spermatophyta - ps15
Angiosperms - ps16
Mesangiospermae - ps17
Magnoliids/eudicots - ps18
Eudicots - ps19
Core_eudicots - ps20
Superrosids - ps21
Rosids - ps22
Eurosids - ps23
Malvids - ps24
Sap./Malv./Brassicales - ps25
Malvales/Brassicales - ps26
Brassicales - ps27
Cleomaceae/Brassicaceae - ps28
Brassicaceae - ps29
Camelineae - ps30
Arabidopsis - ps31
Arabidopsis_thaliana - ps32
Phylostratum

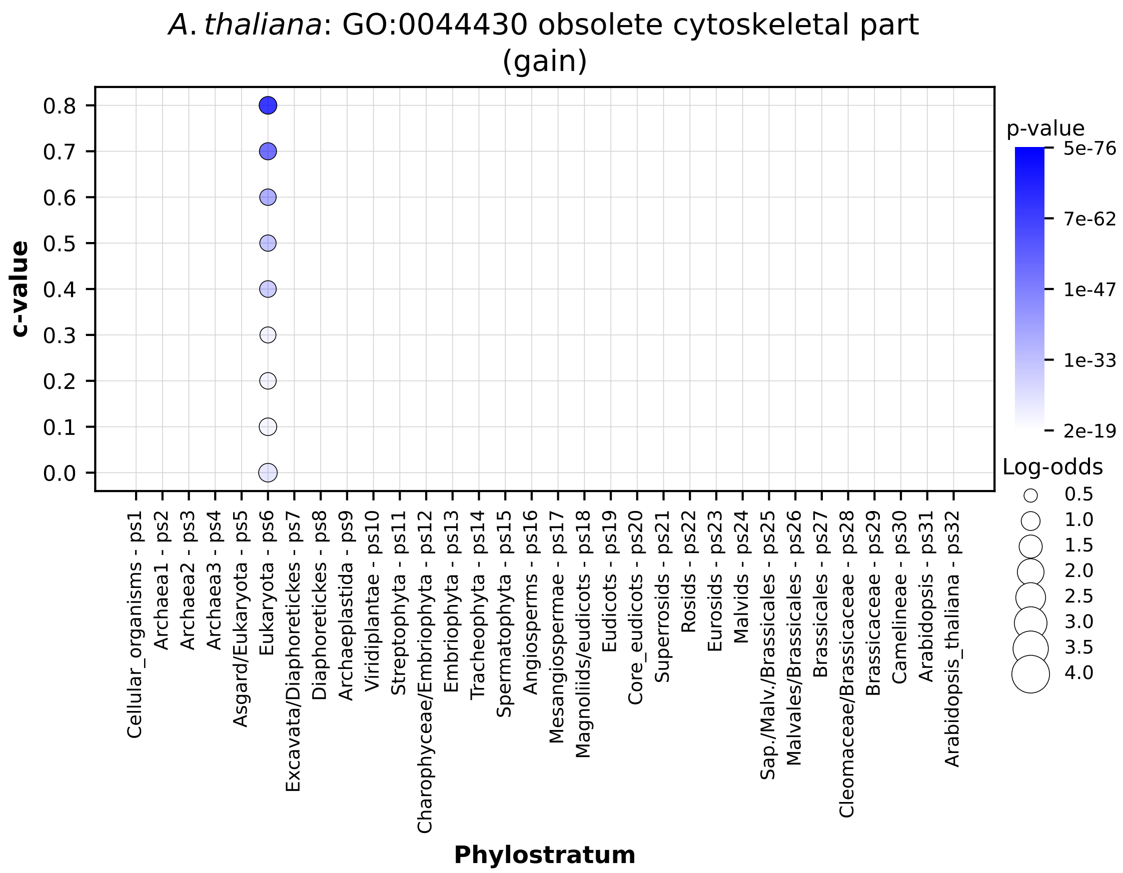

A. thaliana: GO:0044430 obsolete cytoskeletal part
(gain)
c-value
0.8
0.7
0.6
0.5
0.4
0.3
0.2
0.1
0.0
p-value
5e-76
7e-62
1e-47
1e-33
2e-19
Log-odds
0.5
1.0
1.5
2.0
2.5
3.0
3.5
4.0
Cellular_organisms - ps1
Archaea1 - ps2
Archaea2 - ps3
Archaea3 - ps4
Asgard/Eukaryota - ps5
Eukaryota - ps6
Excavata/Diaphoretickes - ps7
Diaphoretickes - ps8
Archaeplastida - ps9
Viridiplantae - ps10
Streptophyta - ps11
Charophyceae/Embriophyta - ps12
Embriophyta - ps13
Tracheophyta - ps14
Spermatophyta - ps15
Angiosperms - ps16
Mesangiospermae - ps17
Magnoliids/eudicots - ps18
Eudicots - ps19
Core_eudicots - ps20
Superrosids - ps21
Rosids - ps22
Eurosids - ps23
Malvids - ps24
Sap./Malv./Brassicales - ps25
Malvales/Brassicales - ps26
Brassicales - ps27
Cleomaceae/Brassicaceae - ps28
Brassicaceae - ps29
Camelineae - ps30
Arabidopsis - ps31
Arabidopsis_thaliana - ps32
Phylostratum

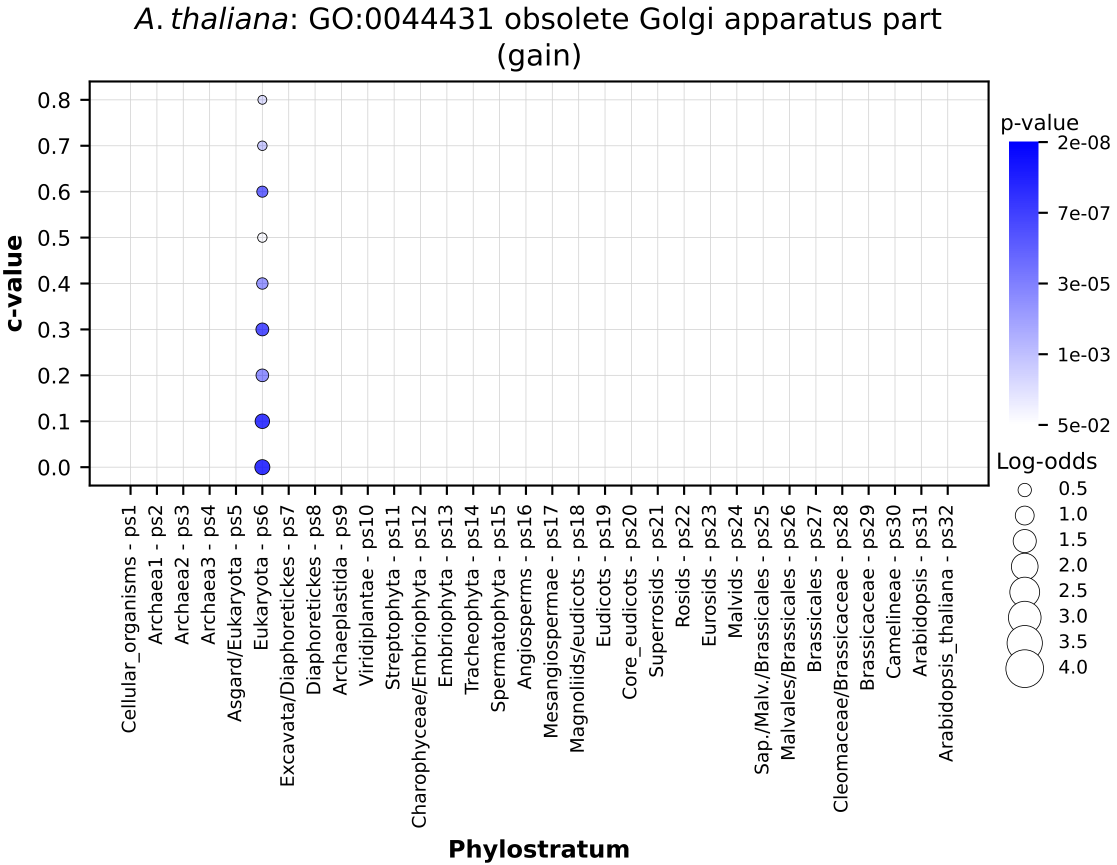

A. thaliana: GO:0044431 obsolete Golgi apparatus part
(gain)
c-value
0.8
0.7
0.6
0.5
0.4
0.3
0.2
0.1
0.0
p-value
2e-08
7e-07
3e-05
1e-03
5e-02
Log-odds
0.5
1.0
1.5
2.0
2.5
3.0
3.5
4.0
Cellular_organisms - ps1
Archaea1 - ps2
Archaea2 - ps3
Archaea3 - ps4
Asgard/Eukaryota - ps5
Eukaryota - ps6
Excavata/Diaphoretickes - ps7
Diaphoretickes - ps8
Archaeplastida - ps9
Viridiplantae - ps10
Streptophyta - ps11
Charophyceae/Embriophyta - ps12
Embriophyta - ps13
Tracheophyta - ps14
Spermatophyta - ps15
Angiosperms - ps16
Mesangiospermae - ps17
Magnoliids/eudicots - ps18
Eudicots - ps19
Core_eudicots - ps20
Superrosids - ps21
Rosids - ps22
Eurosids - ps23
Malvids - ps24
Sap./Malv./Brassicales - ps25
Malvales/Brassicales - ps26
Brassicales - ps27
Cleomaceae/Brassicaceae - ps28
Brassicaceae - ps29
Camelineae - ps30
Arabidopsis - ps31
Arabidopsis_thaliana - ps32
Phylostratum

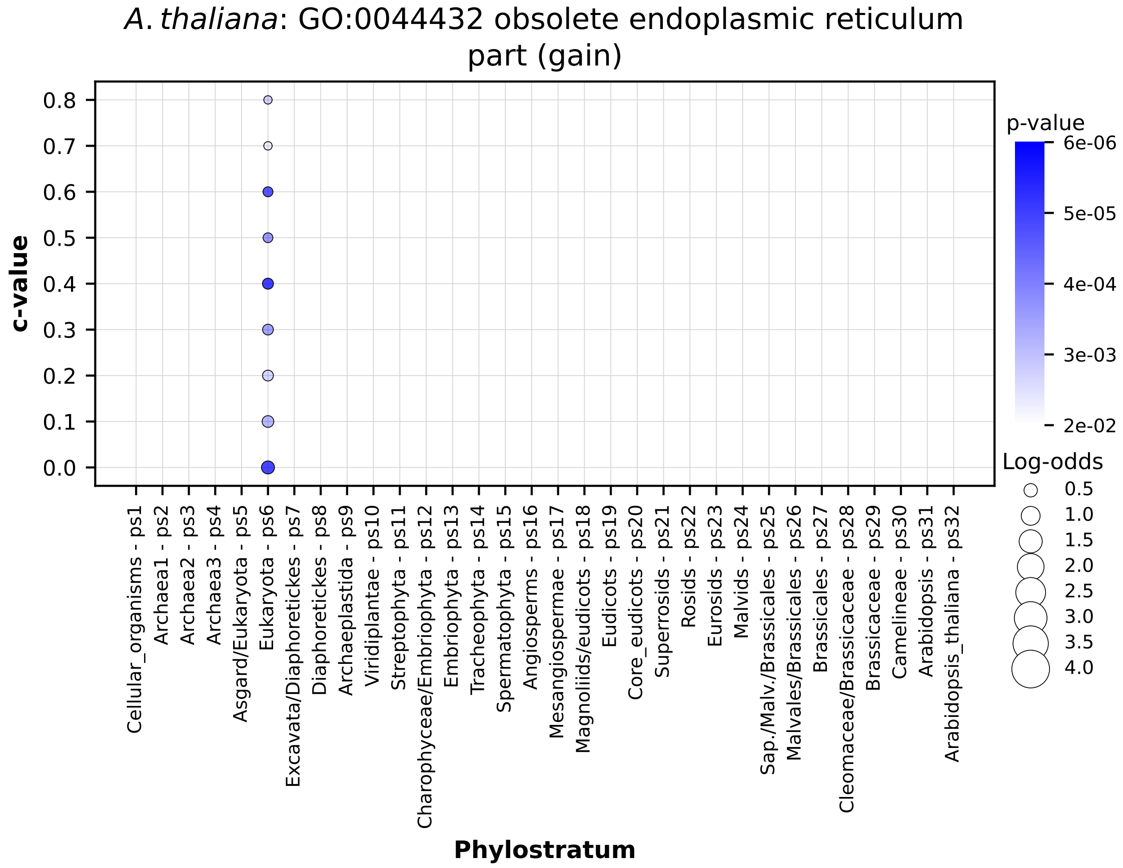

A. thaliana: GO:0044432 obsolete endoplasmic reticulum part (gain)
c-value
0.8
0.7
0.6
0.5
0.4
0.3
0.2
0.1
0.0
p-value
6e-06
5e-05
4e-04
3e-03
2e-02
Log-odds
0.5
1.0
1.5
2.0
2.5
3.0
3.5
4.0
Cellular_organisms - ps1
Archaea1 - ps2
Archaea2 - ps3
Archaea3 - ps4
Asgard/Eukaryota - ps5
Eukaryota - ps6
Excavata/Diaphoretickes - ps7
Diaphoretickes - ps8
Archaeplastida - ps9
Viridiplantae - ps10
Streptophyta - ps11
Charophyceae/Embriophyta - ps12
Embriophyta - ps13
Tracheophyta - ps14
Spermatophyta - ps15
Angiosperms - ps16
Mesangiospermae - ps17
Magnoliids/eudicots - ps18
Eudicots - ps19
Core_eudicots - ps20
Superrosids - ps21
Rosids - ps22
Eurosids - ps23
Malvids - ps24
Sap./Malv./Brassicales - ps25
Malvales/Brassicales - ps26
Brassicales - ps27
Cleomaceae/Brassicaceae - ps28
Brassicaceae - ps29
Camelineae - ps30
Arabidopsis - ps31
Arabidopsis_thaliana - ps32
Phylostratum

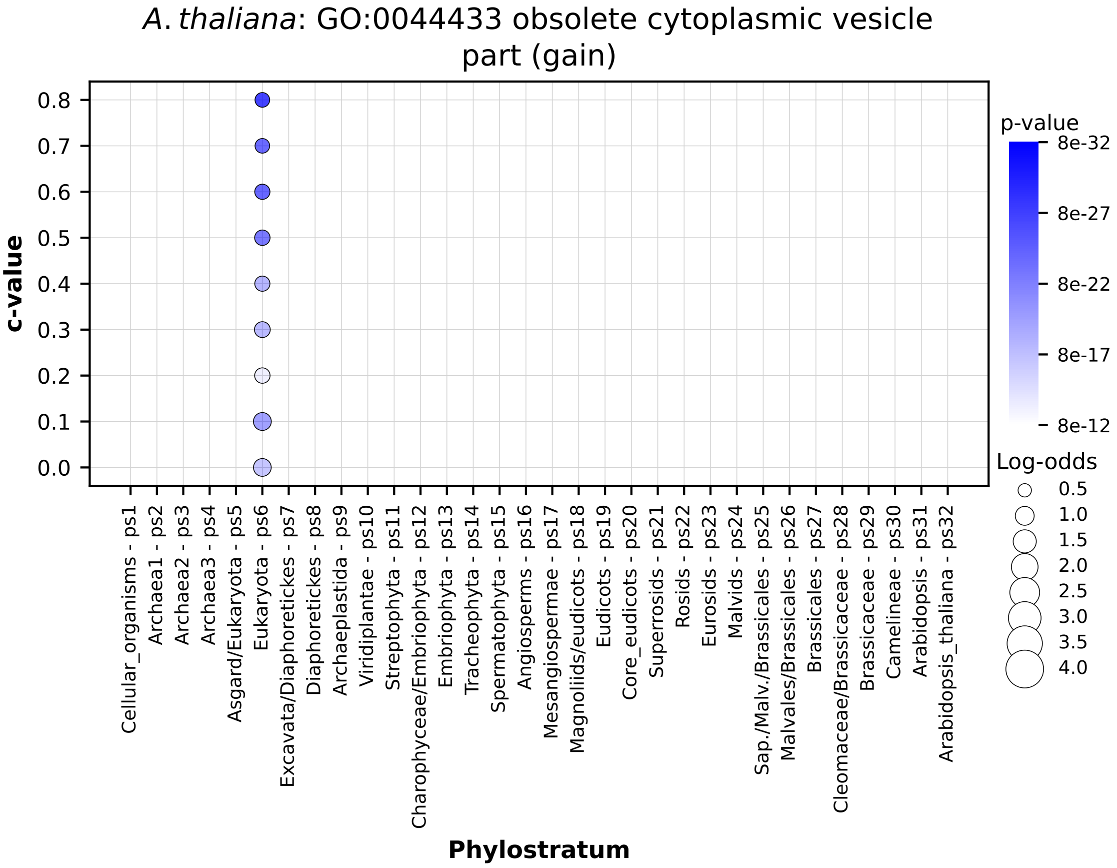

A. thaliana: GO:0044433 obsolete cytoplasmic vesicle part (gain)
c-value
0.8
0.7
0.6
0.5
0.4
0.3
0.2
0.1
0.0
p-value
8e-32
8e-27
8e-22
8e-17
8e-12
Log-odds
0.5
1.0
1.5
2.0
2.5
3.0
3.5
4.0
Cellular_organisms - ps1
Archaea1 - ps2
Archaea2 - ps3
Archaea3 - ps4
Asgard/Eukaryota - ps5
Eukaryota - ps6
Excavata/Diaphoretickes - ps7
Diaphoretickes - ps8
Archaeplastida - ps9
Viridiplantae - ps10
Streptophyta - ps11
Charophyceae/Embriophyta - ps12
Embriophyta - ps13
Tracheophyta - ps14
Spermatophyta - ps15
Angiosperms - ps16
Mesangiospermae - ps17
Magnoliids/eudicots - ps18
Eudicots - ps19
Core_eudicots - ps20
Superrosids - ps21
Rosids - ps22
Eurosids - ps23
Malvids - ps24
Sap./Malv./Brassicales - ps25
Malvales/Brassicales - ps26
Brassicales - ps27
Cleomaceae/Brassicaceae - ps28
Brassicaceae - ps29
Camelineae - ps30
Arabidopsis - ps31
Arabidopsis_thaliana - ps32
Phylostratum

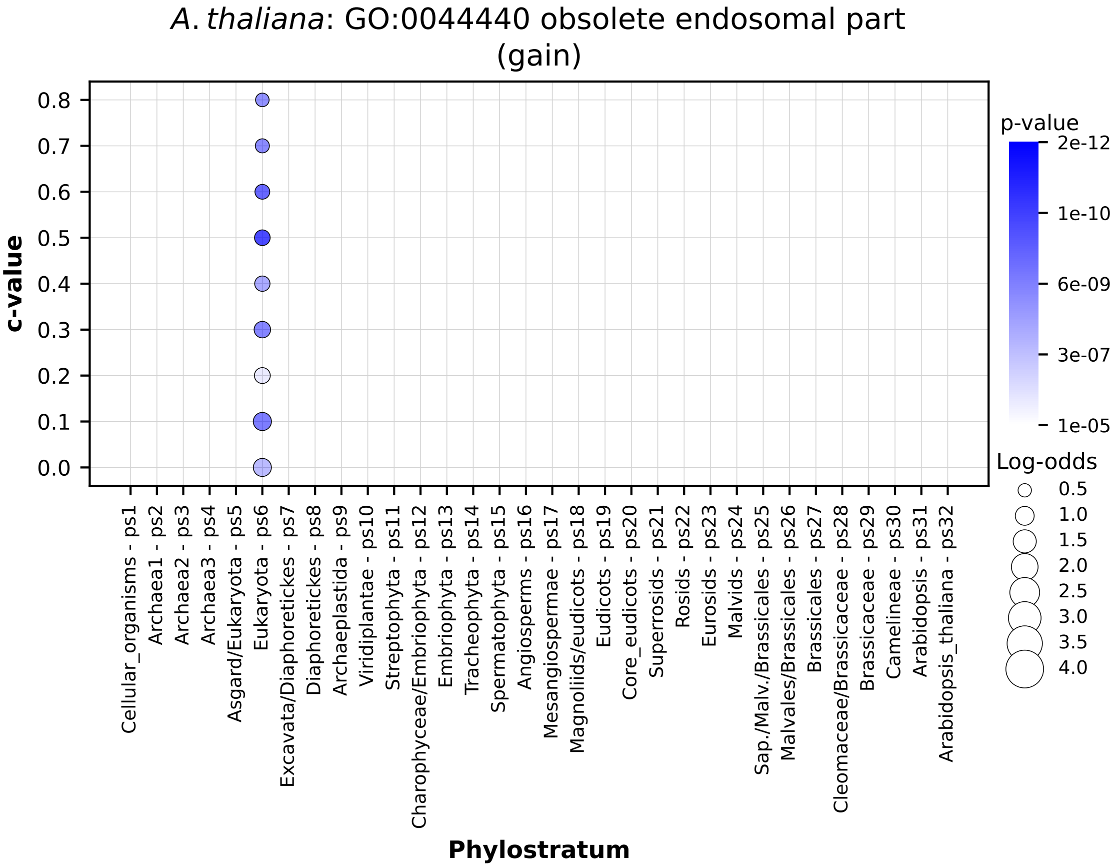

A. thaliana: GO:0044440 obsolete endosomal part
(gain)
c-value
0.8
0.7
0.6
0.5
0.4
0.3
0.2
0.1
0.0
p-value
2e-12
1e-10
6e-09
3e-07
1e-05
Log-odds
0.5
1.0
1.5
2.0
2.5
3.0
3.5
4.0
Cellular_organisms - ps1
Archaea1 - ps2
Archaea2 - ps3
Archaea3 - ps4
Asgard/Eukaryota - ps5
Eukaryota - ps6
Excavata/Diaphoretickes - ps7
Diaphoretickes - ps8
Archaeplastida - ps9
Viridiplantae - ps10
Streptophyta - ps11
Charophyceae/Embriophyta - ps12
Embriophyta - ps13
Tracheophyta - ps14
Spermatophyta - ps15
Angiosperms - ps16
Mesangiospermae - ps17
Magnoliids/eudicots - ps18
Eudicots - ps19
Core_eudicots - ps20
Superrosids - ps21
Rosids - ps22
Eurosids - ps23
Malvids - ps24
Sap./Malv./Brassicales - ps25
Malvales/Brassicales - ps26
Brassicales - ps27
Cleomaceae/Brassicaceae - ps28
Brassicaceae - ps29
Camelineae - ps30
Arabidopsis - ps31
Arabidopsis_thaliana - ps32
Phylostratum

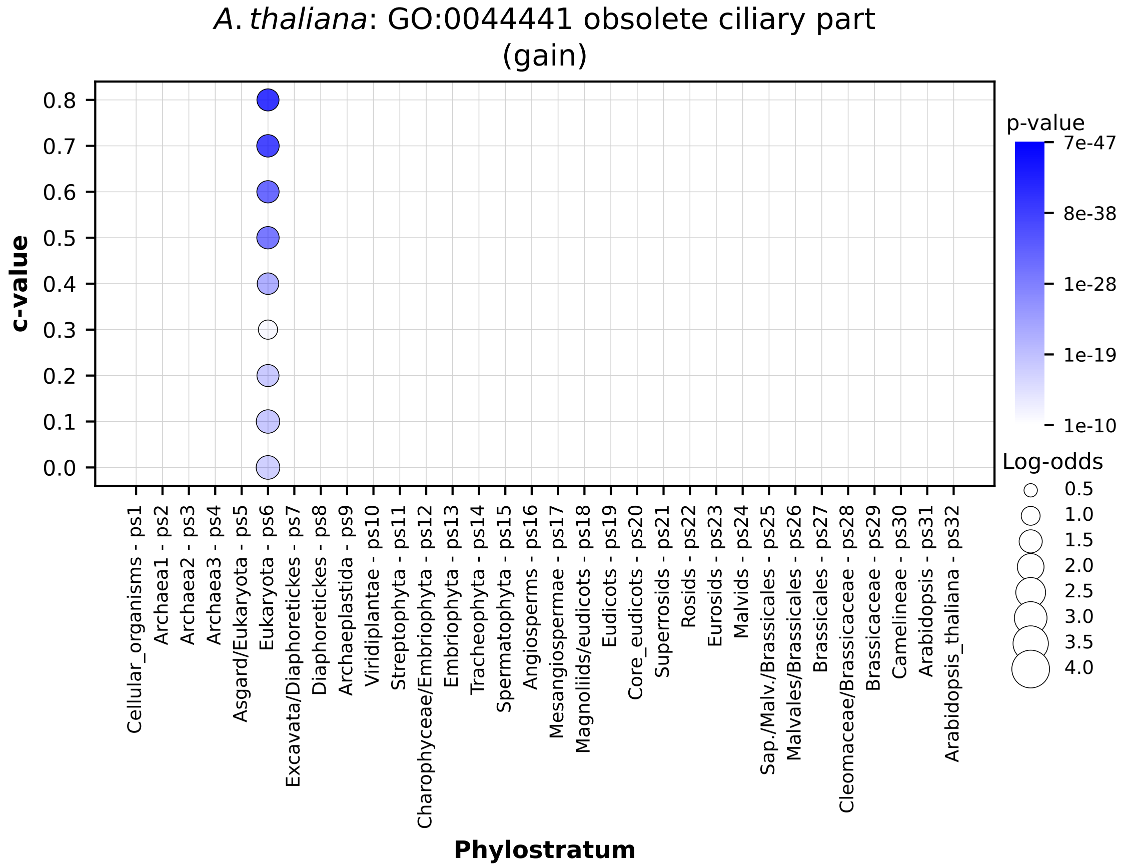

A. thaliana: GO:0044441 obsolete ciliary part
(gain)
c-value
0.0
0.1
0.2
0.3
0.4
0.5
0.6
0.7
0.8
Phylostratum
Cellular_organisms - ps1
Archaea1 - ps2
Archaea2 - ps3
Archaea3 - ps4
Asgard/Eukaryota - ps5
Eukaryota - ps6
Excavata/Diaphoretickes - ps7
Diaphoretickes - ps8
Archaeplastida - ps9
Viridiplantae - ps10
Streptophyta - ps11
Charophyceae/Embriophyta - ps12
Embriophyta - ps13
Tracheophyta - ps14
Spermatophyta - ps15
Angiosperms - ps16
Mesangiospermae - ps17
Magnoliids/eudicots - ps18
Eudicots - ps19
Core_eudicots - ps20
Superrosids - ps21
Rosids - ps22
Eurosids - ps23
Malvids - ps24
Sap./Malv./Brassicales - ps25
Malvales/Brassicales - ps26
Brassicales - ps27
Cleomaceae/Brassicaceae - ps28
Brassicaceae - ps29
Camelineae - ps30
Arabidopsis - ps31
Arabidopsis_thaliana - ps32
p-value
7e-47
8e-38
1e-28
1e-19
1e-10
Log-odds
0.5
1.0
1.5
2.0
2.5
3.0
3.5
4.0

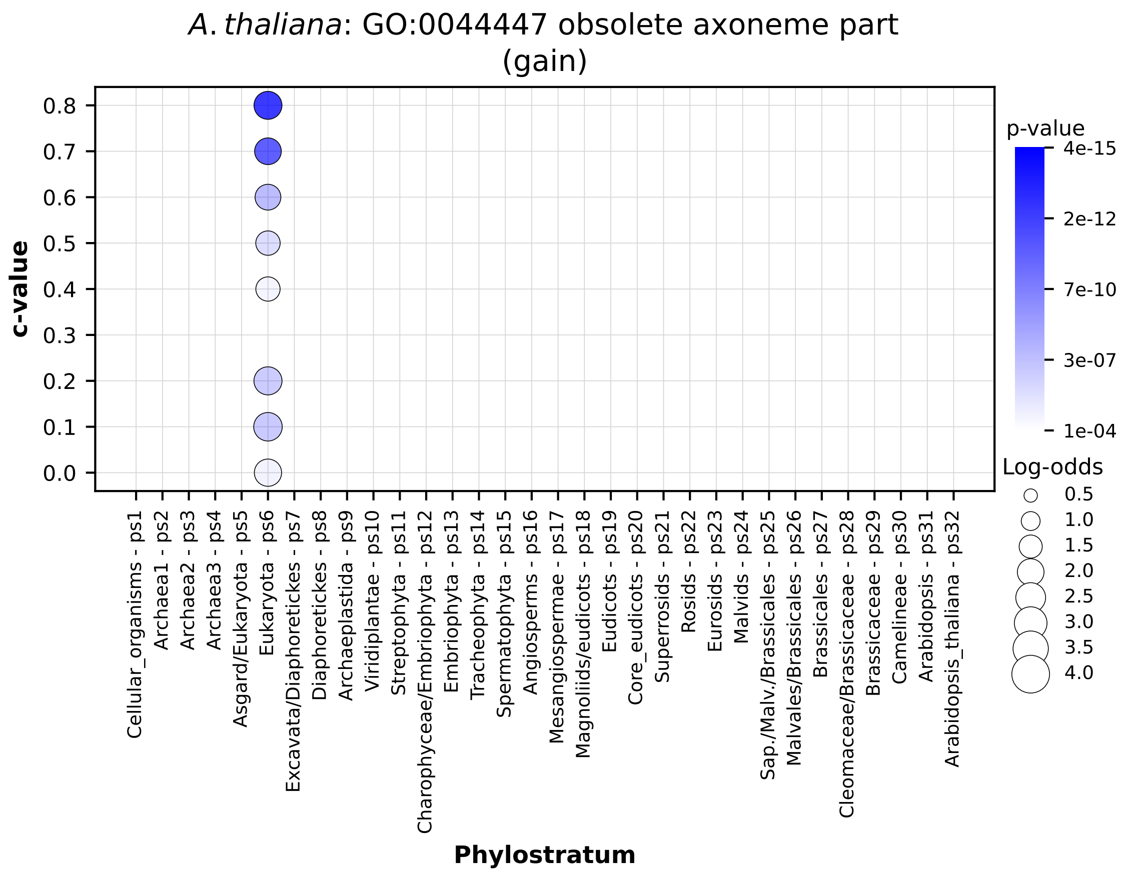

A. thaliana: GO:0044447 obsolete axoneme part
(gain)
c-value
0.8
0.7
0.6
0.5
0.4
0.3
0.2
0.1
0.0
p-value
4e-15
2e-12
7e-10
3e-07
1e-04
Log-odds
0.5
1.0
1.5
2.0
2.5
3.0
3.5
4.0
Cellular_organisms - ps1
Archaea1 - ps2
Archaea2 - ps3
Archaea3 - ps4
Asgard/Eukaryota - ps5
Eukaryota - ps6
Excavata/Diaphoretickes - ps7
Diaphoretickes - ps8
Archaeplastida - ps9
Viridiplantae - ps10
Streptophyta - ps11
Charophyceae/Embriophyta - ps12
Embriophyta - ps13
Tracheophyta - ps14
Spermatophyta - ps15
Angiosperms - ps16
Mesangiospermae - ps17
Magnoliids/eudicots - ps18
Eudicots - ps19
Core_eudicots - ps20
Superrosids - ps21
Rosids - ps22
Eurosids - ps23
Malvids - ps24
Sap./Malv./Brassicales - ps25
Malvales/Brassicales - ps26
Brassicales - ps27
Cleomaceae/Brassicaceae - ps28
Brassicaceae - ps29
Camelineae - ps30
Arabidopsis - ps31
Arabidopsis_thaliana - ps32
Phylostratum

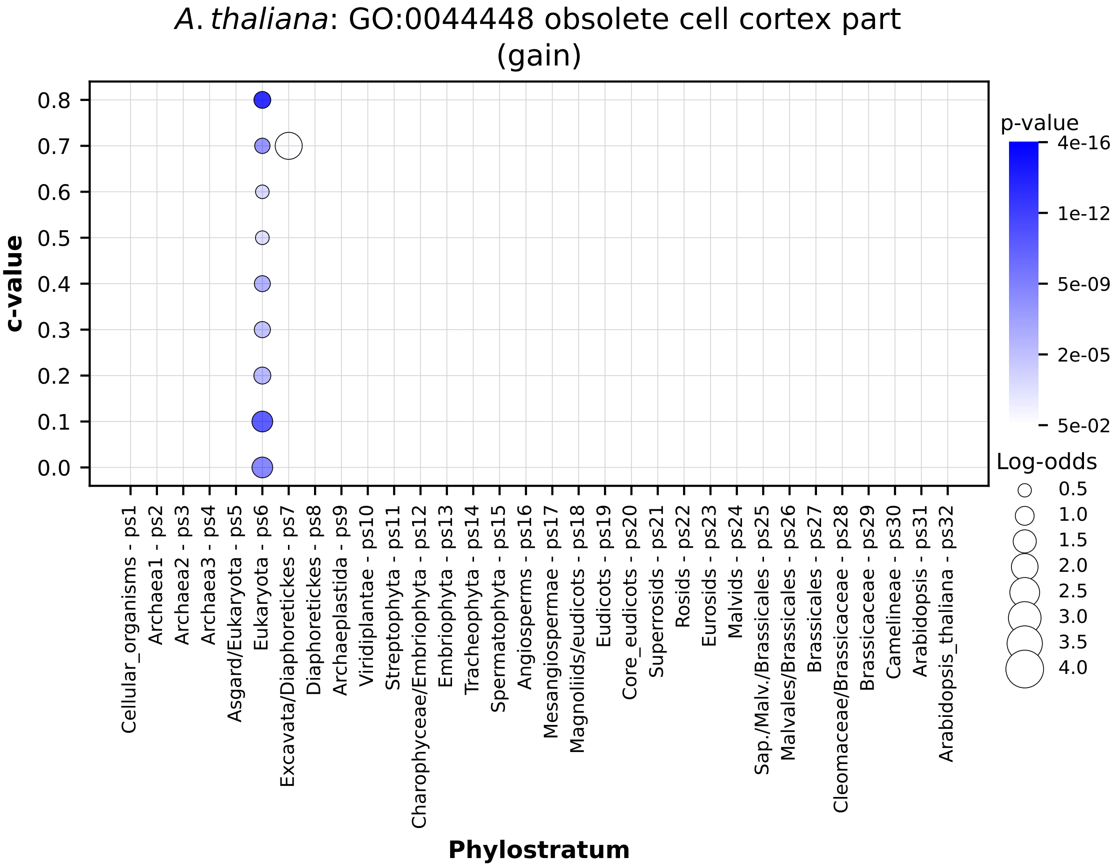

A. thaliana: GO:0044448 obsolete cell cortex part
(gain)
c-value
0.8
0.7
0.6
0.5
0.4
0.3
0.2
0.1
0.0
p-value
4e-16
1e-12
5e-09
2e-05
5e-02
Log-odds
0.5
1.0
1.5
2.0
2.5
3.0
3.5
4.0
Cellular_organisms - ps1
Archaea1 - ps2
Archaea2 - ps3
Archaea3 - ps4
Asgard/Eukaryota - ps5
Eukaryota - ps6
Excavata/Diaphoretickes - ps7
Diaphoretickes - ps8
Archaeplastida - ps9
Viridiplantae - ps10
Streptophyta - ps11
Charophyceae/Embriophyta - ps12
Embriophyta - ps13
Tracheophyta - ps14
Spermatophyta - ps15
Angiosperms - ps16
Mesangiospermae - ps17
Magnoliids/eudicots - ps18
Eudicots - ps19
Core_eudicots - ps20
Superrosids - ps21
Rosids - ps22
Eurosids - ps23
Malvids - ps24
Sap./Malv./Brassicales - ps25
Malvales/Brassicales - ps26
Brassicales - ps27
Cleomaceae/Brassicaceae - ps28
Brassicaceae - ps29
Camelineae - ps30
Arabidopsis - ps31
Arabidopsis_thaliana - ps32
Phylostratum

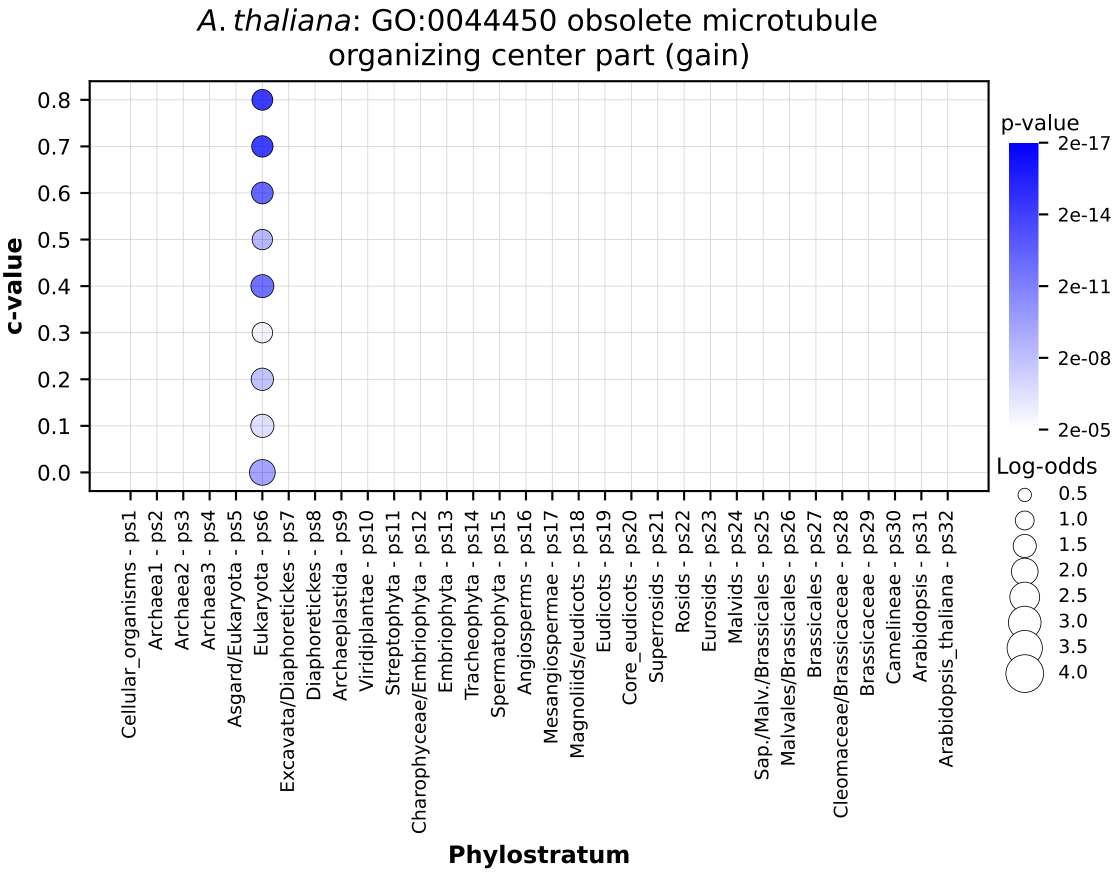
A. thaliana: GO:0044450 obsolete microtubule organizing center part (gain)
c-value
0.8
0.7
0.6
0.5
0.4
0.3
0.2
0.1
0.0
p-value
2e-17
2e-14
2e-11
2e-08
2e-05
Log-odds
0.5
1.0
1.5
2.0
2.5
3.0
3.5
4.0
Cellular_organisms - ps1
Archaea1 - ps2
Archaea2 - ps3
Archaea3 - ps4
Asgard/Eukaryota - ps5
Eukaryota - ps6
Excavata/Diaphoretickes - ps7
Diaphoretickes - ps8
Archaeplastida - ps9
Viridiplantae - ps10
Streptophyta - ps11
Charophyceae/Embriophyta - ps12
Embriophyta - ps13
Tracheophyta - ps14
Spermatophyta - ps15
Angiosperms - ps16
Mesangiospermae - ps17
Magnoliids/eudicots - ps18
Eudicots - ps19
Core_eudicots - ps20
Superrosids - ps21
Rosids - ps22
Eurosids - ps23
Malvids - ps24
Sap./Malv./Brassicales - ps25
Malvales/Brassicales - ps26
Brassicales - ps27
Cleomaceae/Brassicaceae - ps28
Brassicaceae - ps29
Camelineae - ps30
Arabidopsis - ps31
Arabidopsis_thaliana - ps32
Phylostratum

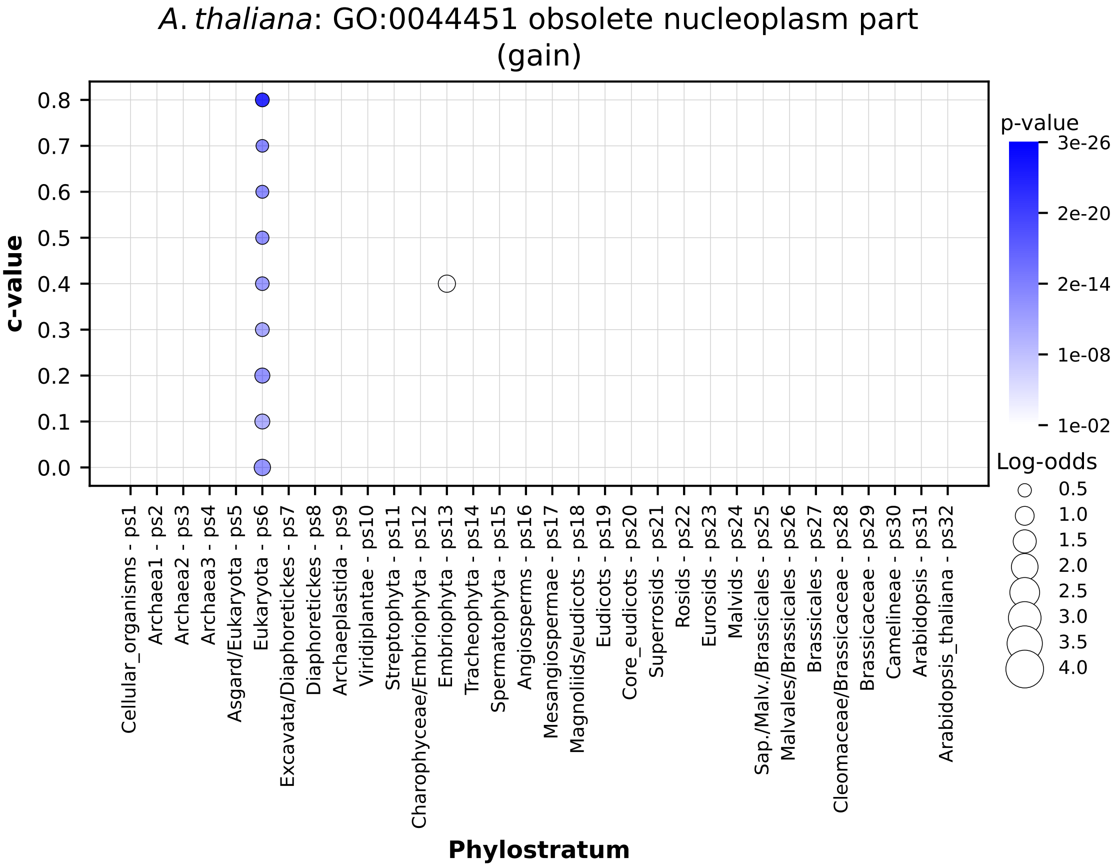

A. thaliana: GO:0044451 obsolete nucleoplasm part (gain)
c-value
0.8
0.7
0.6
0.5
0.4
0.3
0.2
0.1
0.0
p-value
3e-26
2e-20
2e-14
1e-08
1e-02
Log-odds
0.5
1.0
1.5
2.0
2.5
3.0
3.5
4.0
Cellular_organisms - ps1
Archaea1 - ps2
Archaea2 - ps3
Archaea3 - ps4
Asgard/Eukaryota - ps5
Eukaryota - ps6
Excavata/Diaphoretickes - ps7
Diaphoretickes - ps8
Archaeplastida - ps9
Viridiplantae - ps10
Streptophyta - ps11
Charophyceae/Embriophyta - ps12
Embriophyta - ps13
Tracheophyta - ps14
Spermatophyta - ps15
Angiosperms - ps16
Mesangiospermae - ps17
Magnoliids/eudicots - ps18
Eudicots - ps19
Core_eudicots - ps20
Superrosids - ps21
Rosids - ps22
Eurosids - ps23
Malvids - ps24
Sap./Malv./Brassicales - ps25
Malvales/Brassicales - ps26
Brassicales - ps27
Cleomaceae/Brassicaceae - ps28
Brassicaceae - ps29
Camelineae - ps30
Arabidopsis - ps31
Arabidopsis_thaliana - ps32
Phylostratum

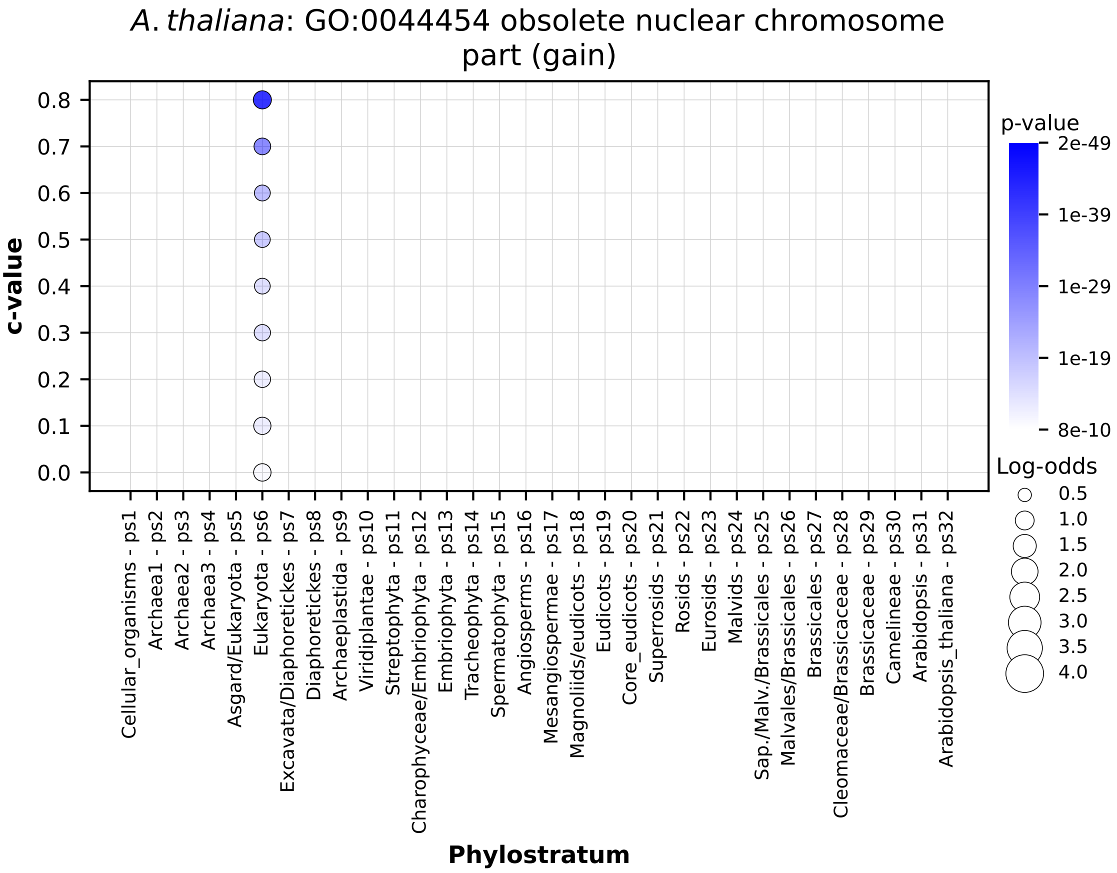

A. thaliana: GO:0044454 obsolete nuclear chromosome part (gain)
c-value
0.8
0.7
0.6
0.5
0.4
0.3
0.2
0.1
0.0
p-value
2e-49
1e-39
1e-29
1e-19
8e-10
Log-odds
0.5
1.0
1.5
2.0
2.5
3.0
3.5
4.0
Cellular_organisms - ps1
Archaea1 - ps2
Archaea2 - ps3
Archaea3 - ps4
Asgard/Eukaryota - ps5
Eukaryota - ps6
Excavata/Diaphoretickes - ps7
Diaphoretickes - ps8
Archaeplastida - ps9
Viridiplantae - ps10
Streptophyta - ps11
Charophyceae/Embriophyta - ps12
Embriophyta - ps13
Tracheophyta - ps14
Spermatophyta - ps15
Angiosperms - ps16
Mesangiospermae - ps17
Magnoliids/eudicots - ps18
Eudicots - ps19
Core_eudicots - ps20
Superrosids - ps21
Rosids - ps22
Eurosids - ps23
Malvids - ps24
Sap./Malv./Brassicales - ps25
Malvales/Brassicales - ps26
Brassicales - ps27
Cleomaceae/Brassicaceae - ps28
Brassicaceae - ps29
Camelineae - ps30
Arabidopsis - ps31
Arabidopsis_thaliana - ps32
Phylostratum

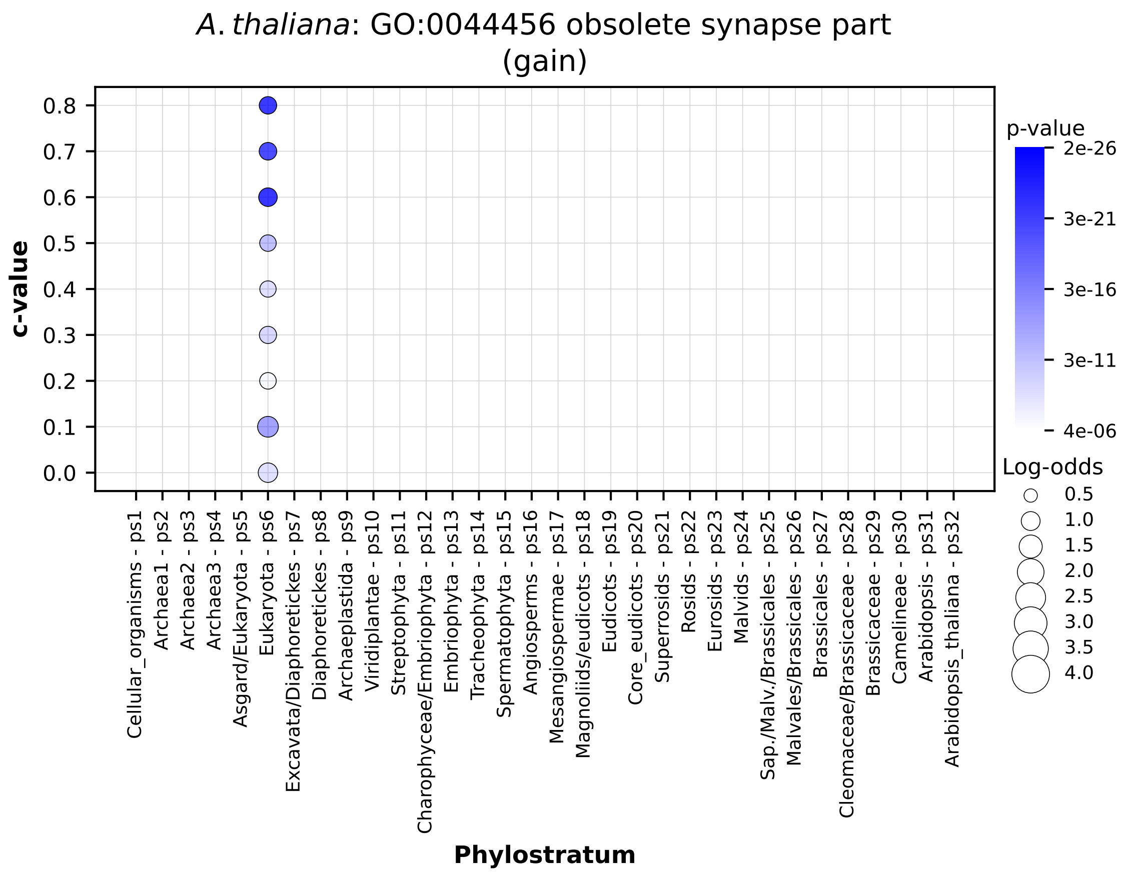

A. thaliana: GO:0044456 obsolete synapse part
(gain)
c-value
0.8
0.7
0.6
0.5
0.4
0.3
0.2
0.1
0.0
p-value
2e-26
3e-21
3e-16
3e-11
4e-06
Log-odds
0.5
1.0
1.5
2.0
2.5
3.0
3.5
4.0
Cellular_organisms - ps1
Archaea1 - ps2
Archaea2 - ps3
Archaea3 - ps4
Asgard/Eukaryota - ps5
Eukaryota - ps6
Excavata/Diaphoretickes - ps7
Diaphoretickes - ps8
Archaeplastida - ps9
Viridiplantae - ps10
Streptophyta - ps11
Charophyceae/Embriophyta - ps12
Embriophyta - ps13
Tracheophyta - ps14
Spermatophyta - ps15
Angiosperms - ps16
Mesangiospermae - ps17
Magnoliids/eudicots - ps18
Eudicots - ps19
Core_eudicots - ps20
Superrosids - ps21
Rosids - ps22
Eurosids - ps23
Malvids - ps24
Sap./Malv./Brassicales - ps25
Malvales/Brassicales - ps26
Brassicales - ps27
Cleomaceae/Brassicaceae - ps28
Brassicaceae - ps29
Camelineae - ps30
Arabidopsis - ps31
Arabidopsis_thaliana - ps32
Phylostratum

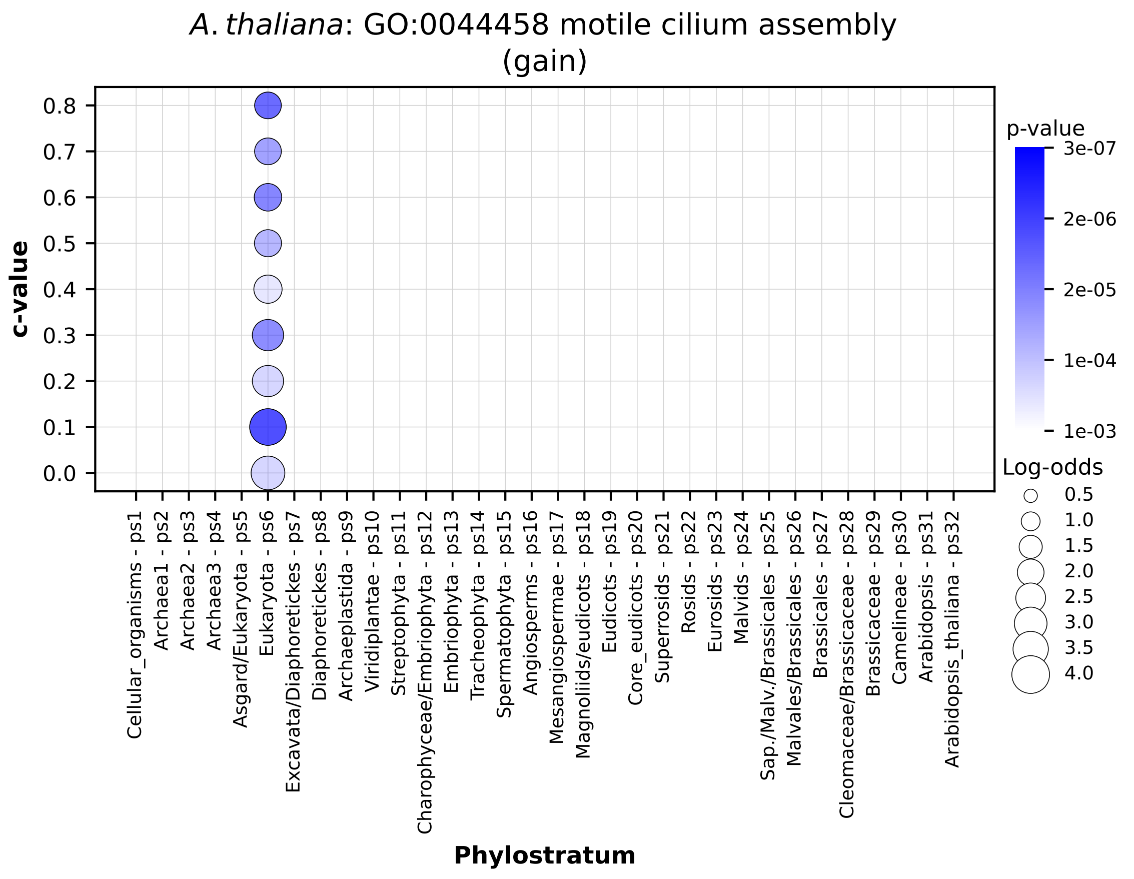

A. thaliana: GO:0044458 motile cilium assembly
(gain)
c-value
0.0
0.1
0.2
0.3
0.4
0.5
0.6
0.7
0.8
p-value
3e-07
2e-06
2e-05
1e-04
1e-03
Log-odds
0.5
1.0
1.5
2.0
2.5
3.0
3.5
4.0
Cellular_organisms - ps1
Archaea1 - ps2
Archaea2 - ps3
Archaea3 - ps4
Asgard/Eukaryota - ps5
Eukaryota - ps6
Excavata/Diaphoretickes - ps7
Diaphoretickes - ps8
Archaeplastida - ps9
Viridiplantae - ps10
Streptophyta - ps11
Charophyceae/Embriophyta - ps12
Embriophyta - ps13
Tracheophyta - ps14
Spermatophyta - ps15
Angiosperms - ps16
Mesangiospermae - ps17
Magnoliids/eudicots - ps18
Eudicots - ps19
Core_eudicots - ps20
Superrosids - ps21
Rosids - ps22
Eurosids - ps23
Malvids - ps24
Sap./Malv./Brassicales - ps25
Malvales/Brassicales - ps26
Brassicales - ps27
Cleomaceae/Brassicaceae - ps28
Brassicaceae - ps29
Camelineae - ps30
Arabidopsis - ps31
Arabidopsis_thaliana - ps32
Phylostratum

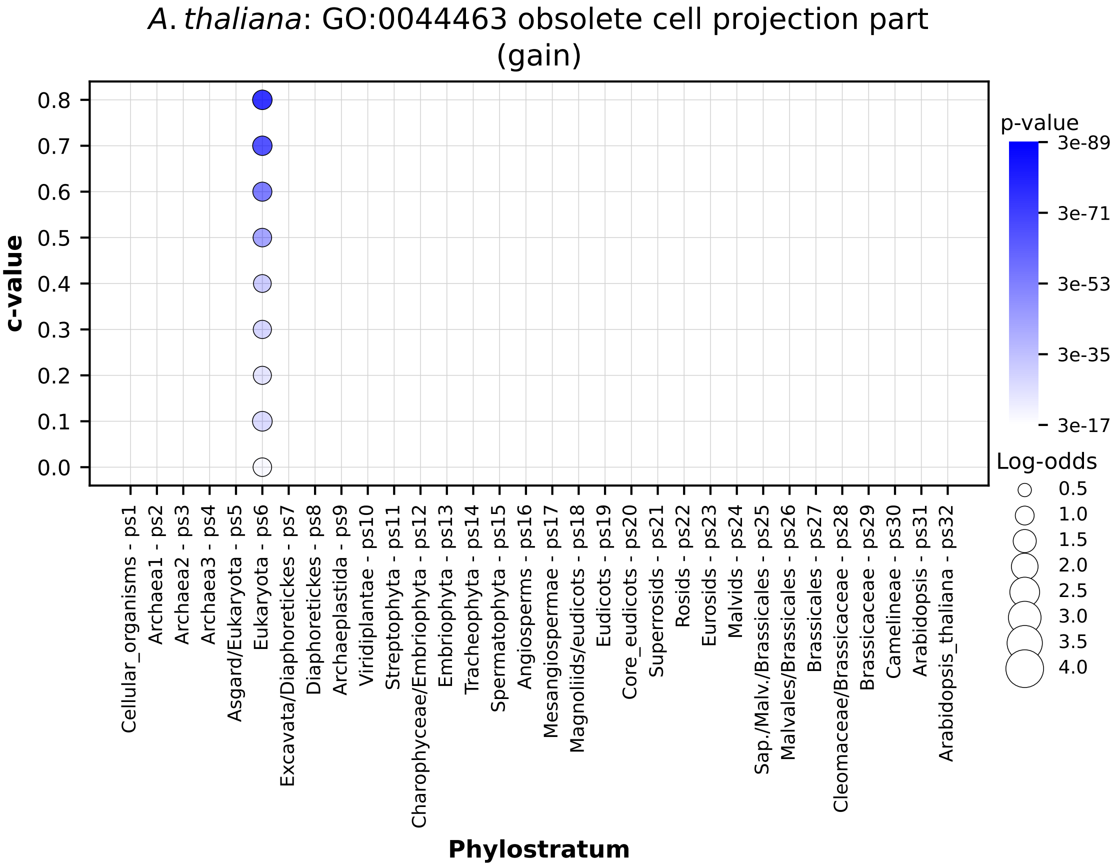

A. thaliana: GO:0044463 obsolete cell projection part
(gain)
c-value
0.8
0.7
0.6
0.5
0.4
0.3
0.2
0.1
0.0
p-value
3e-89
3e-71
3e-53
3e-35
3e-17
Log-odds
0.5
1.0
1.5
2.0
2.5
3.0
3.5
4.0
Cellular_organisms - ps1
Archaea1 - ps2
Archaea2 - ps3
Archaea3 - ps4
Asgard/Eukaryota - ps5
Eukaryota - ps6
Excavata/Diaphoretickes - ps7
Diaphoretickes - ps8
Archaeplastida - ps9
Viridiplantae - ps10
Streptophyta - ps11
Charophyceae/Embriophyta - ps12
Embriophyta - ps13
Tracheophyta - ps14
Spermatophyta - ps15
Angiosperms - ps16
Mesangiospermae - ps17
Magnoliids/eudicots - ps18
Eudicots - ps19
Core_eudicots - ps20
Superrosids - ps21
Rosids - ps22
Eurosids - ps23
Malvids - ps24
Sap./Malv./Brassicales - ps25
Malvales/Brassicales - ps26
Brassicales - ps27
Cleomaceae/Brassicaceae - ps28
Brassicaceae - ps29
Camelineae - ps30
Arabidopsis - ps31
Arabidopsis_thaliana - ps32
Phylostratum

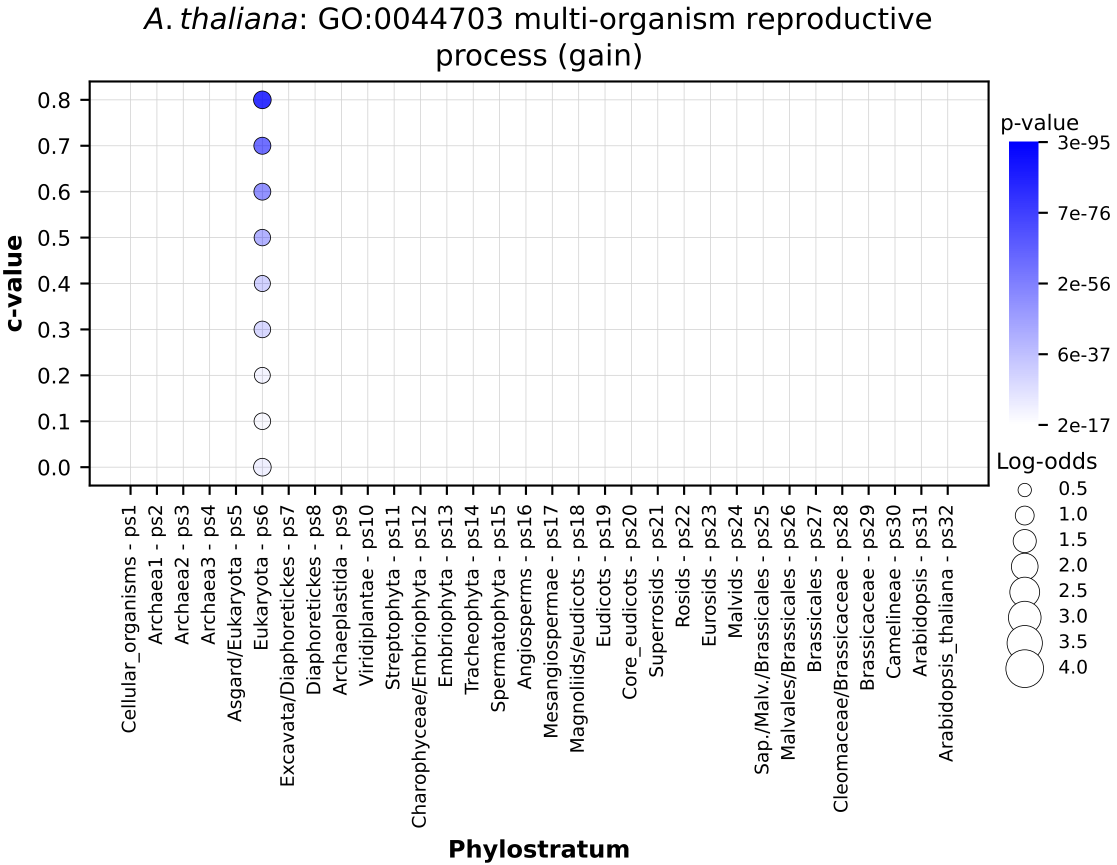
A. thaliana: GO:0044703 multi-organism reproductive process (gain)
c-value
0.8
0.7
0.6
0.5
0.4
0.3
0.2
0.1
0.0
p-value
3e-95
7e-76
2e-56
6e-37
2e-17
Log-odds
0.5
1.0
1.5
2.0
2.5
3.0
3.5
4.0
Cellular_organisms - ps1
Archaea1 - ps2
Archaea2 - ps3
Archaea3 - ps4
Asgard/Eukaryota - ps5
Eukaryota - ps6
Excavata/Diaphoretickes - ps7
Diaphoretickes - ps8
Archaeplastida - ps9
Viridiplantae - ps10
Streptophyta - ps11
Charophyceae/Embriophyta - ps12
Embriophyta - ps13
Tracheophyta - ps14
Spermatophyta - ps15
Angiosperms - ps16
Mesangiospermae - ps17
Magnoliids/eudicots - ps18
Eudicots - ps19
Core_eudicots - ps20
Superrosids - ps21
Rosids - ps22
Eurosids - ps23
Malvids - ps24
Sap./Malv./Brassicales - ps25
Malvales/Brassicales - ps26
Brassicales - ps27
Cleomaceae/Brassicaceae - ps28
Brassicaceae - ps29
Camelineae - ps30
Arabidopsis - ps31
Arabidopsis_thaliana - ps32
Phylostratum

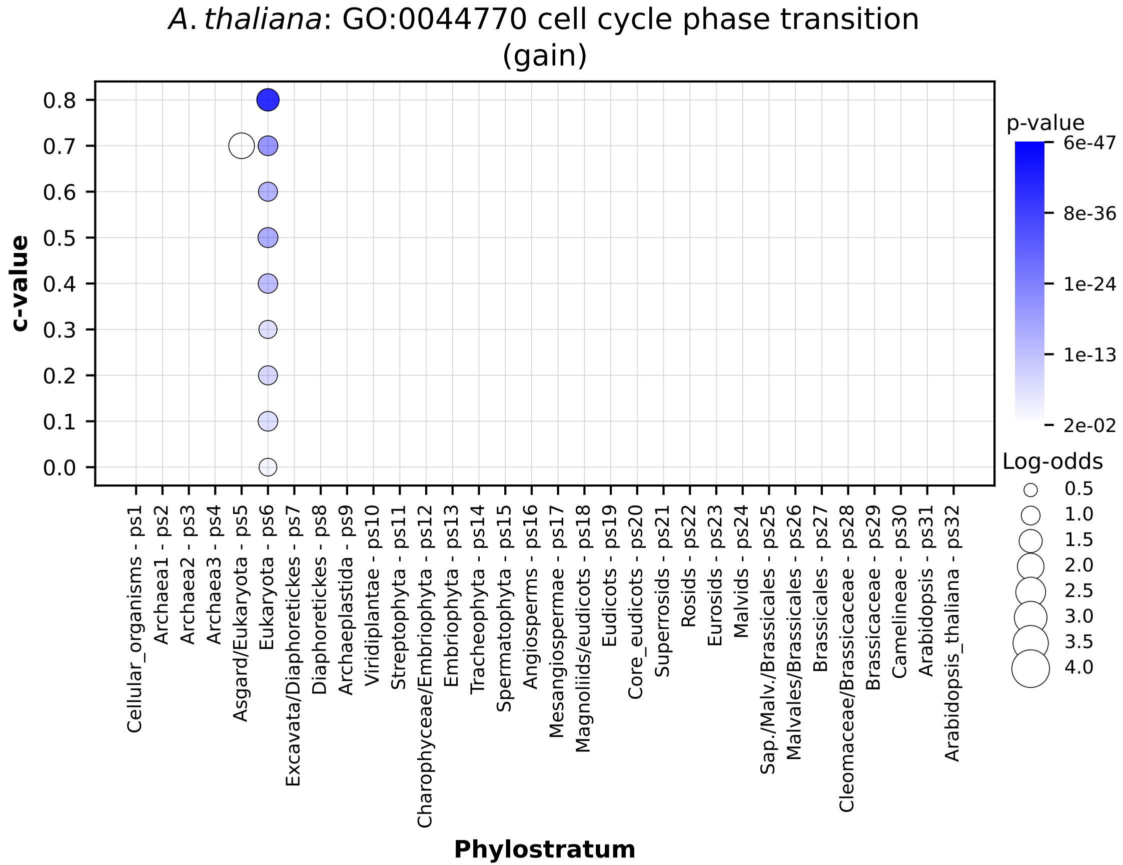

A. thaliana: GO:0044770 cell cycle phase transition (gain)
c-value
0.8
0.7
0.6
0.5
0.4
0.3
0.2
0.1
0.0
Phylostratum
Cellular_organisms - ps1
Archaea1 - ps2
Archaea2 - ps3
Archaea3 - ps4
Asgard/Eukaryota - ps5
Eukaryota - ps6
Excavata/Diaphoretickes - ps7
Diaphoretickes - ps8
Archaeplastida - ps9
Viridiplantae - ps10
Streptophyta - ps11
Charophyceae/Embriophyta - ps12
Embriophyta - ps13
Tracheophyta - ps14
Spermatophyta - ps15
Angiosperms - ps16
Mesangiospermae - ps17
Magnoliids/eudicots - ps18
Eudicots - ps19
Core_eudicots - ps20
Superrosids - ps21
Rosids - ps22
Eurosids - ps23
Malvids - ps24
Sap./Malv./Brassicales - ps25
Malvales/Brassicales - ps26
Brassicales - ps27
Cleomaceae/Brassicaceae - ps28
Brassicaceae - ps29
Camelineae - ps30
Arabidopsis - ps31
Arabidopsis_thaliana - ps32
p-value
6e-47
8e-36
1e-24
1e-13
2e-02
Log-odds
0.5
1.0
1.5
2.0
2.5
3.0
3.5
4.0

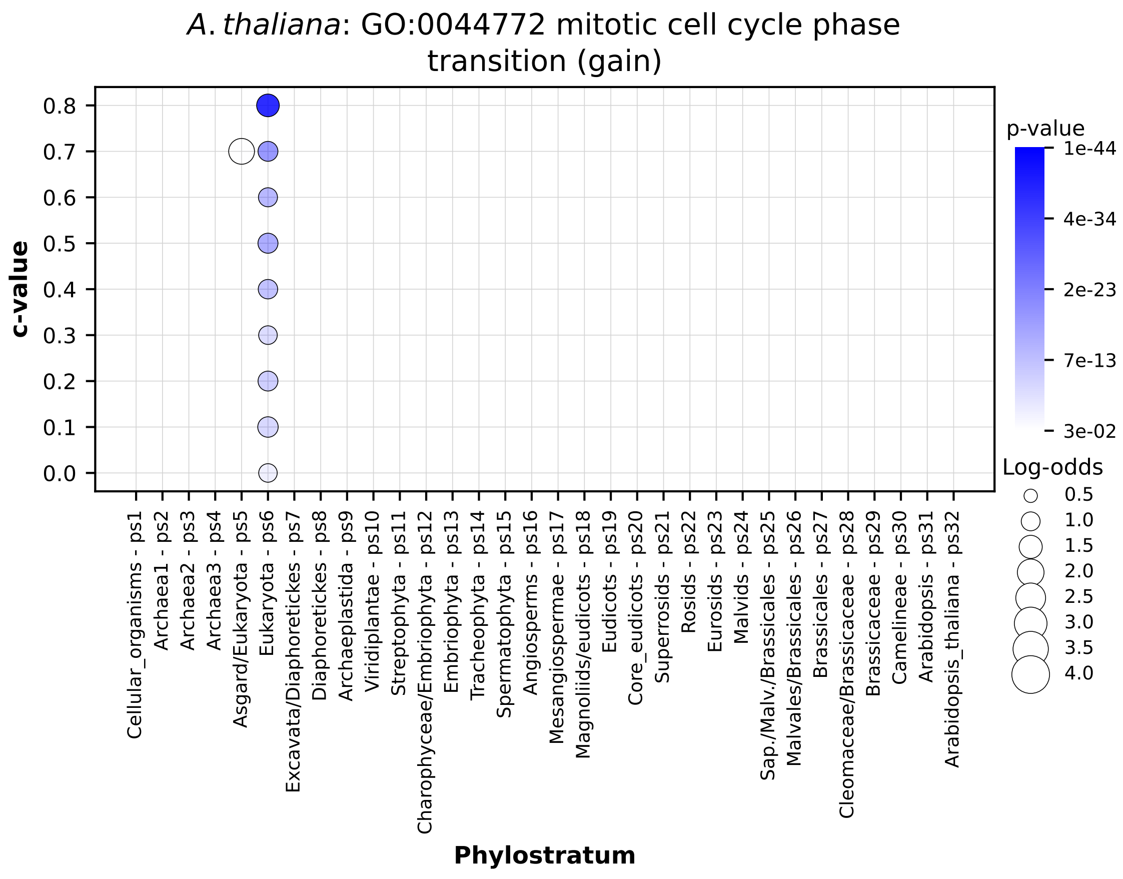
A. thaliana: GO:0044772 mitotic cell cycle phase transition (gain)
c-value
0.8
0.7
0.6
0.5
0.4
0.3
0.2
0.1
0.0
p-value
1e-44
4e-34
2e-23
7e-13
3e-02
Log-odds
0.5
1.0
1.5
2.0
2.5
3.0
3.5
4.0
Cellular_organisms - ps1
Archaea1 - ps2
Archaea2 - ps3
Archaea3 - ps4
Asgard/Eukaryota - ps5
Eukaryota - ps6
Excavata/Diaphoretickes - ps7
Diaphoretickes - ps8
Archaeplastida - ps9
Viridiplantae - ps10
Streptophyta - ps11
Charophyceae/Embriophyta - ps12
Embriophyta - ps13
Tracheophyta - ps14
Spermatophyta - ps15
Angiosperms - ps16
Mesangiospermae - ps17
Magnoliids/eudicots - ps18
Eudicots - ps19
Core_eudicots - ps20
Superrosids - ps21
Rosids - ps22
Eurosids - ps23
Malvids - ps24
Sap./Malv./Brassicales - ps25
Malvales/Brassicales - ps26
Brassicales - ps27
Cleomaceae/Brassicaceae - ps28
Brassicaceae - ps29
Camelineae - ps30
Arabidopsis - ps31
Arabidopsis_thaliana - ps32
Phylostratum

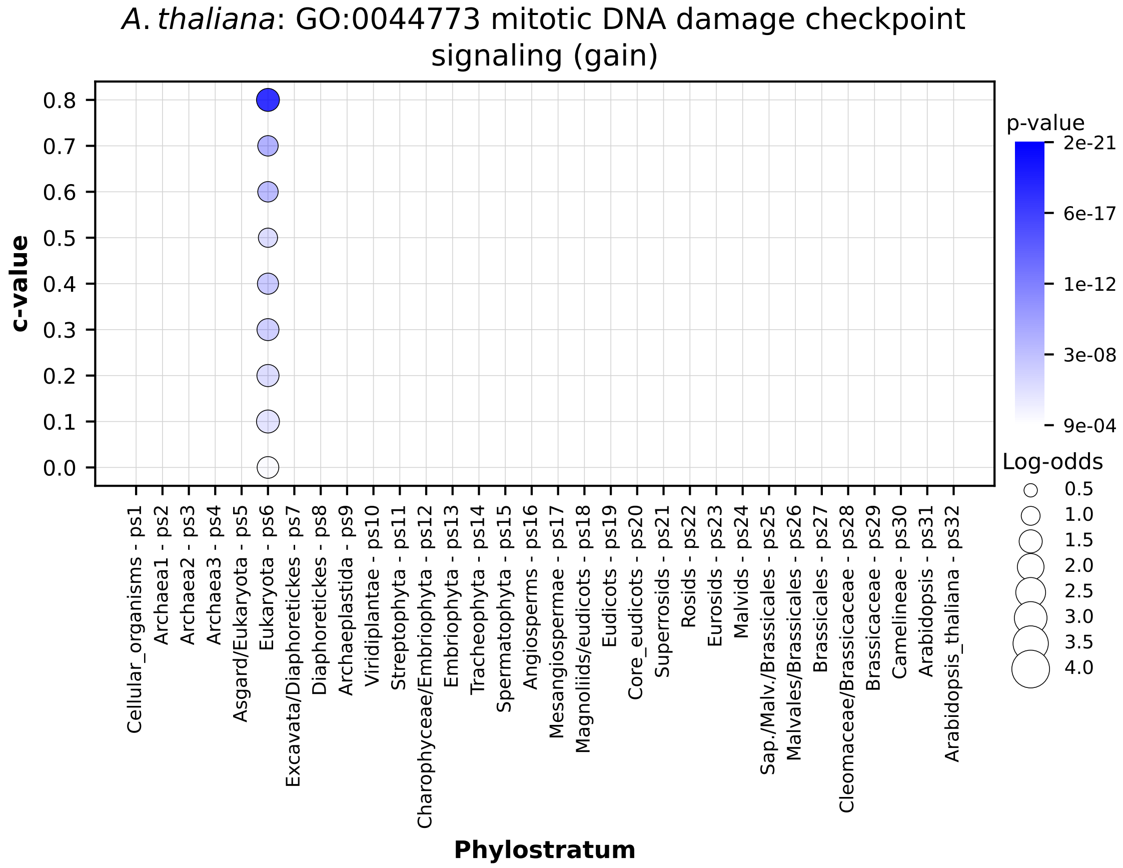

A. thaliana: GO:0044773 mitotic DNA damage checkpoint signaling (gain)
c-value
0.8
0.7
0.6
0.5
0.4
0.3
0.2
0.1
0.0
p-value
2e-21
6e-17
1e-12
3e-08
9e-04
Log-odds
0.5
1.0
1.5
2.0
2.5
3.0
3.5
4.0
Cellular_organisms - ps1
Archaea1 - ps2
Archaea2 - ps3
Archaea3 - ps4
Asgard/Eukaryota - ps5
Eukaryota - ps6
Excavata/Diaphoretickes - ps7
Diaphoretickes - ps8
Archaeplastida - ps9
Viridiplantae - ps10
Streptophyta - ps11
Charophyceae/Embriophyta - ps12
Embriophyta - ps13
Tracheophyta - ps14
Spermatophyta - ps15
Angiosperms - ps16
Mesangiospermae - ps17
Magnoliids/eudicots - ps18
Eudicots - ps19
Core_eudicots - ps20
Superrosids - ps21
Rosids - ps22
Eurosids - ps23
Malvids - ps24
Sap./Malv./Brassicales - ps25
Malvales/Brassicales - ps26
Brassicales - ps27
Cleomaceae/Brassicaceae - ps28
Brassicaceae - ps29
Camelineae - ps30
Arabidopsis - ps31
Arabidopsis_thaliana - ps32
Phylostratum

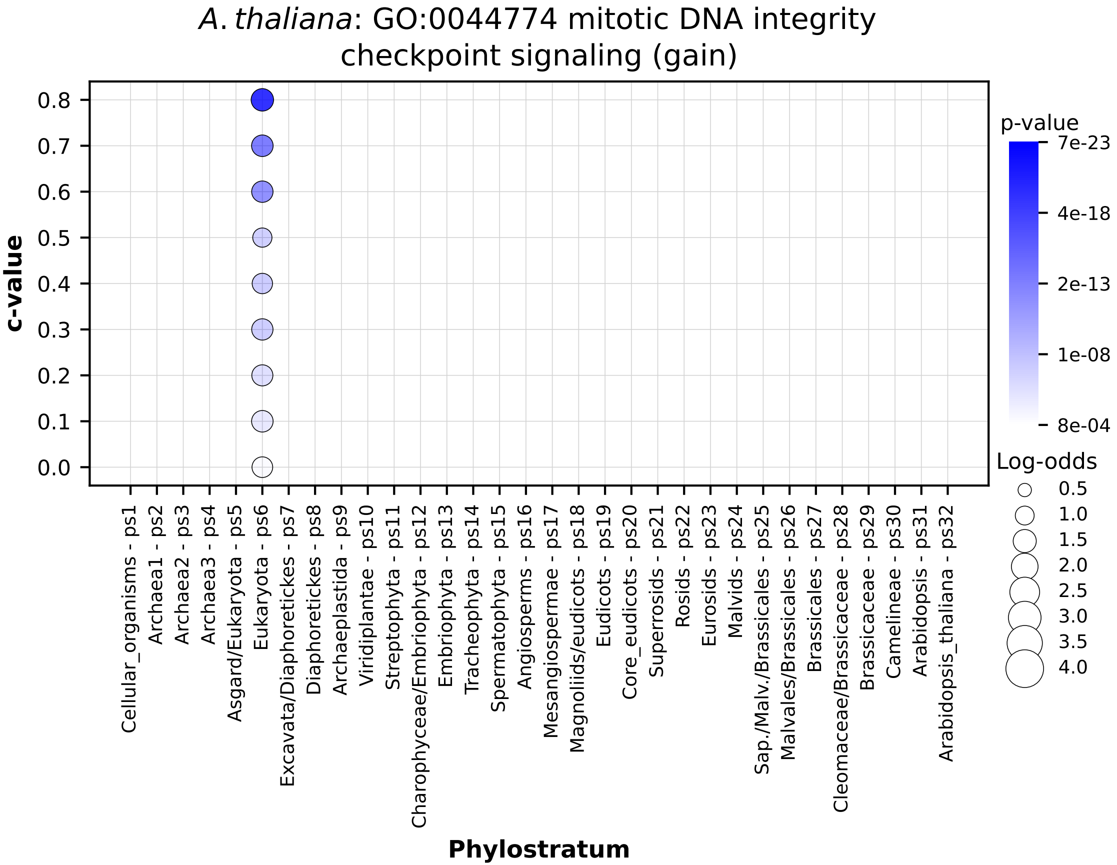
A. thaliana: GO:0044774 mitotic DNA integrity checkpoint signaling (gain)
c-value
0.8
0.7
0.6
0.5
0.4
0.3
0.2
0.1
0.0
p-value
7e-23
4e-18
2e-13
1e-08
8e-04
Log-odds
0.5
1.0
1.5
2.0
2.5
3.0
3.5
4.0
Cellular_organisms - ps1
Archaea1 - ps2
Archaea2 - ps3
Archaea3 - ps4
Asgard/Eukaryota - ps5
Eukaryota - ps6
Excavata/Diaphoretickes - ps7
Diaphoretickes - ps8
Archaeplastida - ps9
Viridiplantae - ps10
Streptophyta - ps11
Charophyceae/Embriophyta - ps12
Embriophyta - ps13
Tracheophyta - ps14
Spermatophyta - ps15
Angiosperms - ps16
Mesangiospermae - ps17
Magnoliids/eudicots - ps18
Eudicots - ps19
Core_eudicots - ps20
Superrosids - ps21
Rosids - ps22
Eurosids - ps23
Malvids - ps24
Sap./Malv./Brassicales - ps25
Malvales/Brassicales - ps26
Brassicales - ps27
Cleomaceae/Brassicaceae - ps28
Brassicaceae - ps29
Camelineae - ps30
Arabidopsis - ps31
Arabidopsis_thaliana - ps32
Phylostratum

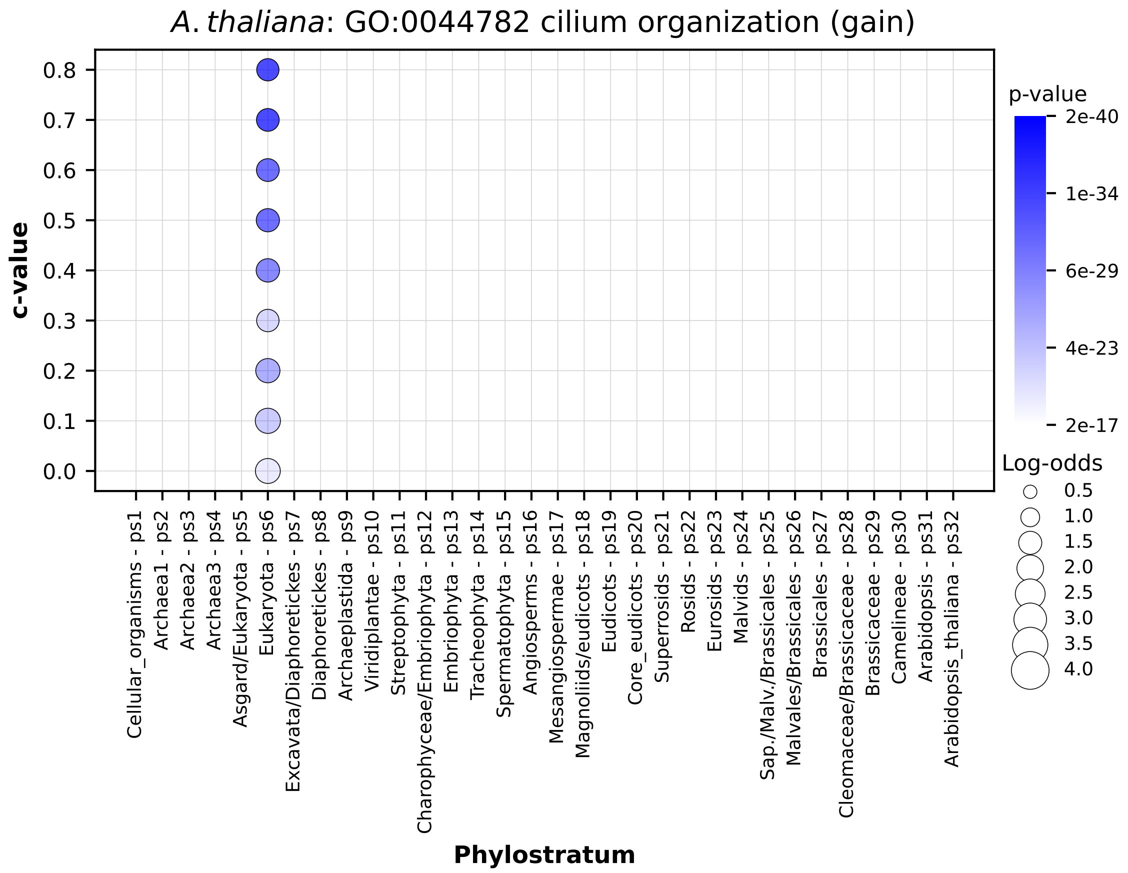
A. thaliana: GO:0044782 cilium organization (gain)
c-value
0.8
0.7
0.6
0.5
0.4
0.3
0.2
0.1
0.0
p-value
2e-40
1e-34
6e-29
4e-23
2e-17
Log-odds
0.5
1.0
1.5
2.0
2.5
3.0
3.5
4.0
Cellular_organisms - ps1
Archaea1 - ps2
Archaea2 - ps3
Archaea3 - ps4
Asgard/Eukaryota - ps5
Eukaryota - ps6
Excavata/Diaphoretickes - ps7
Diaphoretickes - ps8
Archaeplastida - ps9
Viridiplantae - ps10
Streptophyta - ps11
Charophyceae/Embriophyta - ps12
Embriophyta - ps13
Tracheophyta - ps14
Spermatophyta - ps15
Angiosperms - ps16
Mesangiospermae - ps17
Magnoliids/eudicots - ps18
Eudicots - ps19
Core_eudicots - ps20
Superrosids - ps21
Rosids - ps22
Eurosids - ps23
Malvids - ps24
Sap./Malv./Brassicales - ps25
Malvales/Brassicales - ps26
Brassicales - ps27
Cleomaceae/Brassicaceae - ps28
Brassicaceae - ps29
Camelineae - ps30
Arabidopsis - ps31
Arabidopsis_thaliana - ps32
Phylostratum

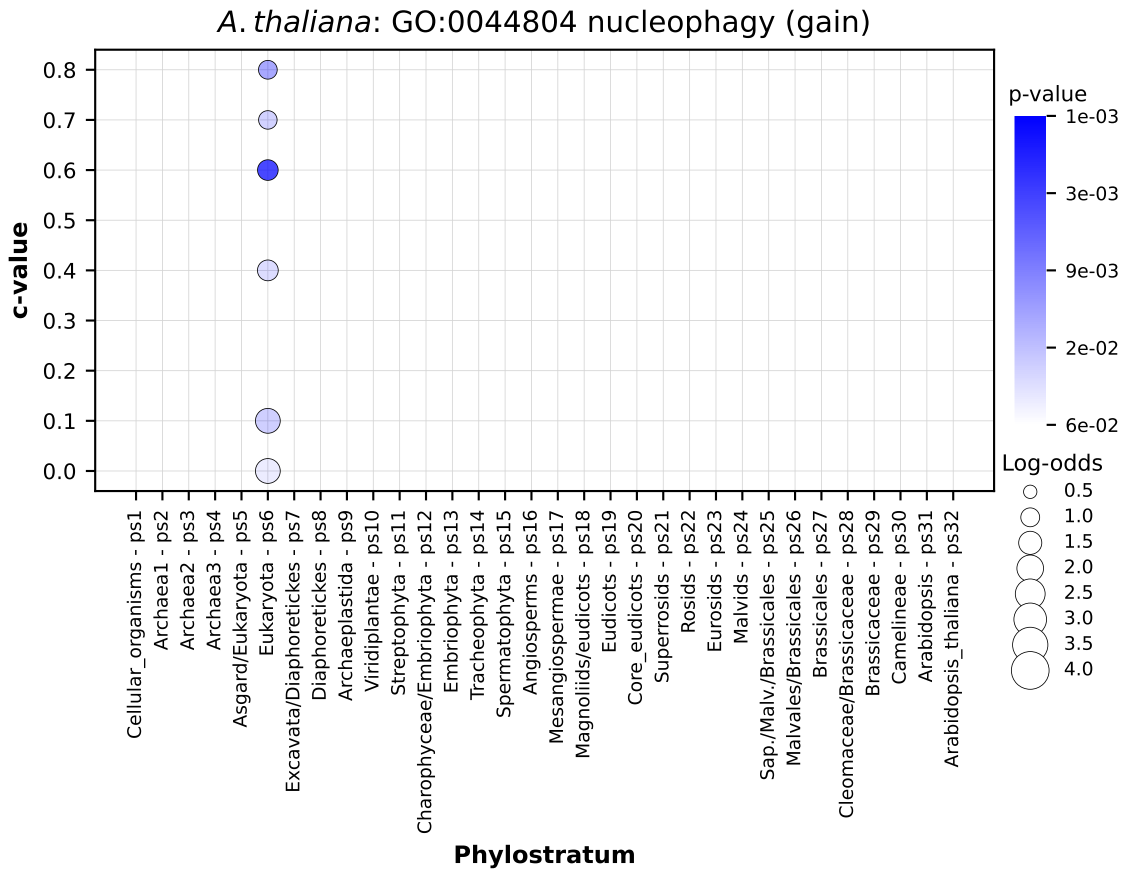

A. thaliana: GO:0044804 nucleophagy (gain)
c-value
0.0
0.1
0.2
0.3
0.4
0.5
0.6
0.7
0.8
p-value
1e-03
3e-03
9e-03
2e-02
6e-02
Log-odds
0.5
1.0
1.5
2.0
2.5
3.0
3.5
4.0
Cellular_organisms - ps1
Archaea1 - ps2
Archaea2 - ps3
Archaea3 - ps4
Asgard/Eukaryota - ps5
Eukaryota - ps6
Excavata/Diaphoretickes - ps7
Diaphoretickes - ps8
Archaeplastida - ps9
Viridiplantae - ps10
Streptophyta - ps11
Charophyceae/Embriophyta - ps12
Embriophyta - ps13
Tracheophyta - ps14
Spermatophyta - ps15
Angiosperms - ps16
Mesangiospermae - ps17
Magnoliids/eudicots - ps18
Eudicots - ps19
Core_eudicots - ps20
Superrosids - ps21
Rosids - ps22
Eurosids - ps23
Malvids - ps24
Sap./Malv./Brassicales - ps25
Malvales/Brassicales - ps26
Brassicales - ps27
Cleomaceae/Brassicaceae - ps28
Brassicaceae - ps29
Camelineae - ps30
Arabidopsis - ps31
Arabidopsis_thaliana - ps32
Phylostratum

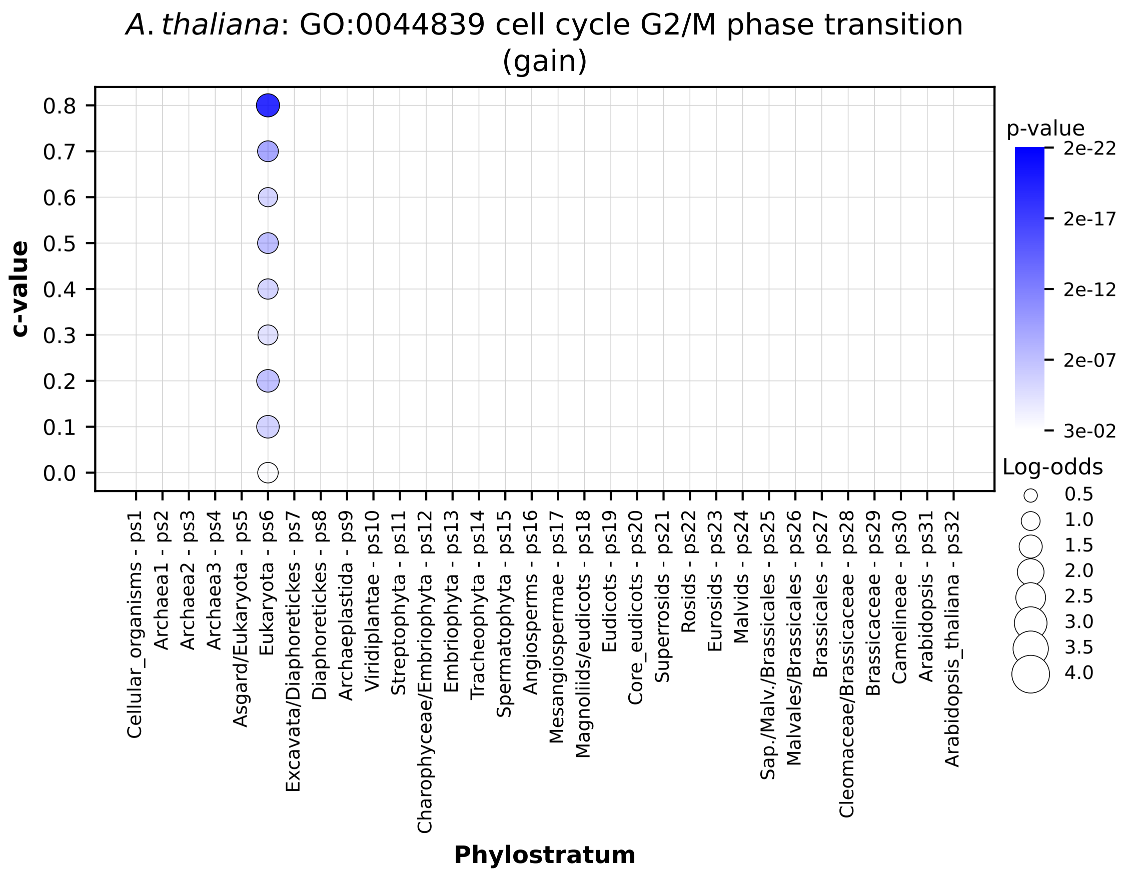

A. thaliana: GO:0044839 cell cycle G2/M phase transition (gain)
c-value
0.8
0.7
0.6
0.5
0.4
0.3
0.2
0.1
0.0
p-value
2e-22
2e-17
2e-12
2e-07
3e-02
Log-odds
0.5
1.0
1.5
2.0
2.5
3.0
3.5
4.0
Cellular_organisms - ps1
Archaea1 - ps2
Archaea2 - ps3
Archaea3 - ps4
Asgard/Eukaryota - ps5
Eukaryota - ps6
Excavata/Diaphoretickes - ps7
Diaphoretickes - ps8
Archaeplastida - ps9
Viridiplantae - ps10
Streptophyta - ps11
Charophyceae/Embriophyta - ps12
Embriophyta - ps13
Tracheophyta - ps14
Spermatophyta - ps15
Angiosperms - ps16
Mesangiospermae - ps17
Magnoliids/eudicots - ps18
Eudicots - ps19
Core_eudicots - ps20
Superrosids - ps21
Rosids - ps22
Eurosids - ps23
Malvids - ps24
Sap./Malv./Brassicales - ps25
Malvales/Brassicales - ps26
Brassicales - ps27
Cleomaceae/Brassicaceae - ps28
Brassicaceae - ps29
Camelineae - ps30
Arabidopsis - ps31
Arabidopsis_thaliana - ps32
Phylostratum

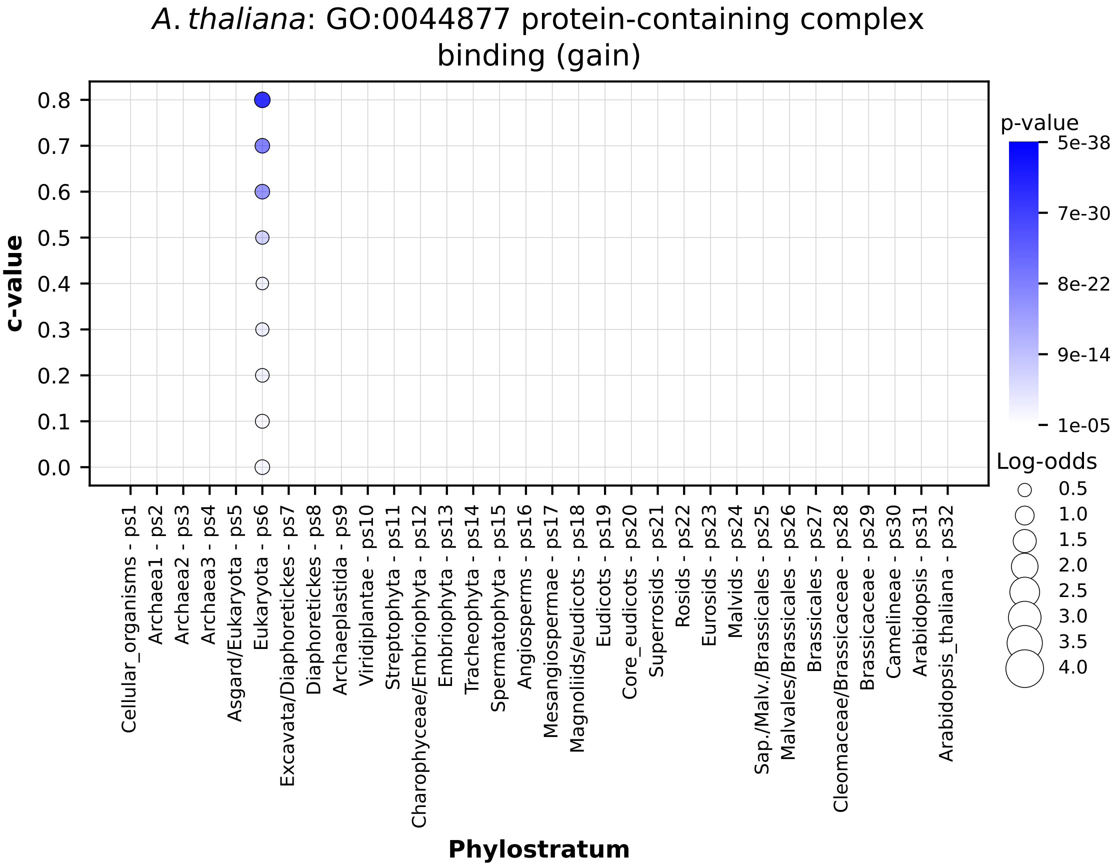

A. thaliana: GO:0044877 protein-containing complex binding (gain)
c-value
0.8
0.7
0.6
0.5
0.4
0.3
0.2
0.1
0.0
p-value
5e-38
7e-30
8e-22
9e-14
1e-05
Log-odds
0.5
1.0
1.5
2.0
2.5
3.0
3.5
4.0
Cellular_organisms - ps1
Archaea1 - ps2
Archaea2 - ps3
Archaea3 - ps4
Asgard/Eukaryota - ps5
Eukaryota - ps6
Excavata/Diaphoretickes - ps7
Diaphoretickes - ps8
Archaeplastida - ps9
Viridiplantae - ps10
Streptophyta - ps11
Charophyceae/Embriophyta - ps12
Embriophyta - ps13
Tracheophyta - ps14
Spermatophyta - ps15
Angiosperms - ps16
Mesangiospermae - ps17
Magnoliids/eudicots - ps18
Eudicots - ps19
Core_eudicots - ps20
Superrosids - ps21
Rosids - ps22
Eurosids - ps23
Malvids - ps24
Sap./Malv./Brassicales - ps25
Malvales/Brassicales - ps26
Brassicales - ps27
Cleomaceae/Brassicaceae - ps28
Brassicaceae - ps29
Camelineae - ps30
Arabidopsis - ps31
Arabidopsis_thaliana - ps32
Phylostratum

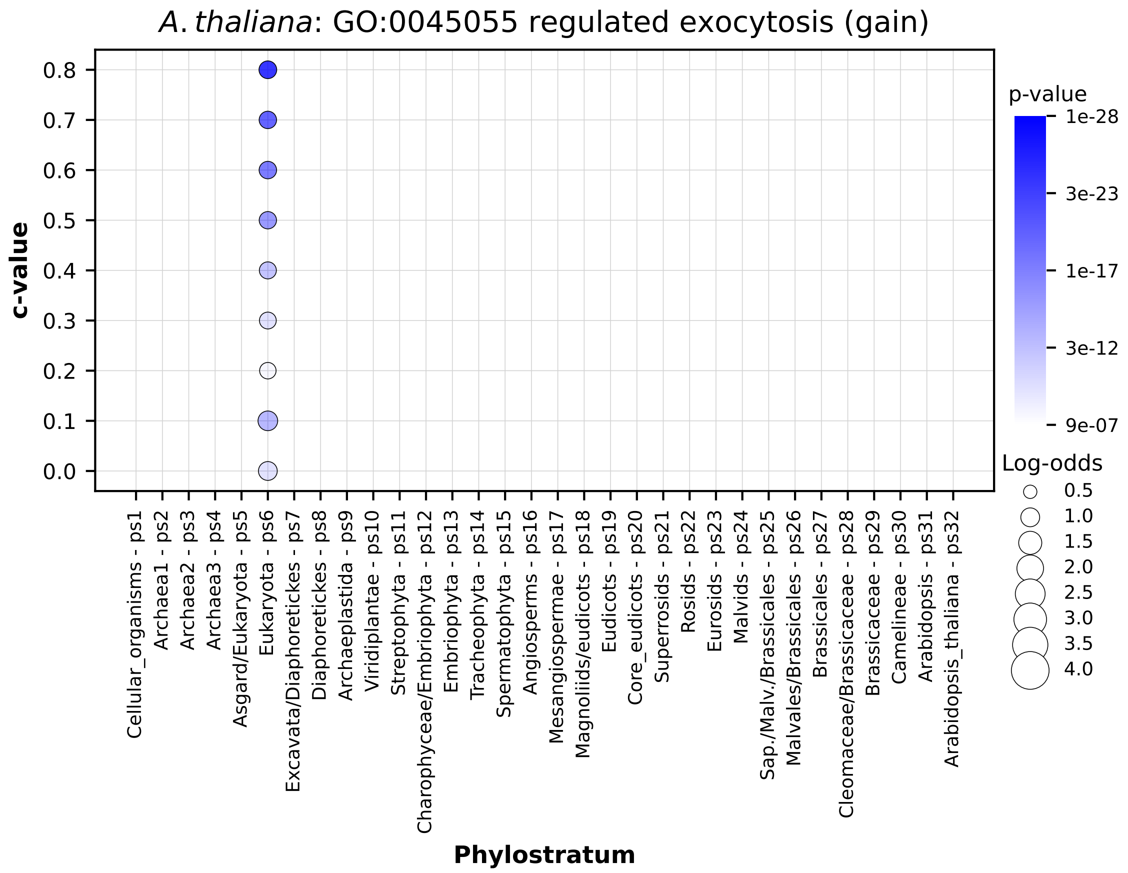
A. thaliana: GO:0045055 regulated exocytosis (gain)
c-value
Phylostratum
p-value
1e-28
3e-23
1e-17
3e-12
9e-07
Log-odds
0.5
1.0
1.5
2.0
2.5
3.0
3.5
4.0
Cellular_organisms - ps1
Archaea1 - ps2
Archaea2 - ps3
Archaea3 - ps4
Asgard/Eukaryota - ps5
Eukaryota - ps6
Excavata/Diaphoretickes - ps7
Diaphoretickes - ps8
Archaeplastida - ps9
Viridiplantae - ps10
Streptophyta - ps11
Charophyceae/Embriophyta - ps12
Embriophyta - ps13
Tracheophyta - ps14
Spermatophyta - ps15
Angiosperms - ps16
Mesangiospermae - ps17
Magnoliids/eudicots - ps18
Eudicots - ps19
Core_eudicots - ps20
Superrosids - ps21
Rosids - ps22
Eurosids - ps23
Malvids - ps24
Sap./Malv./Brassicales - ps25
Malvales/Brassicales - ps26
Brassicales - ps27
Cleomaceae/Brassicaceae - ps28
Brassicaceae - ps29
Camelineae - ps30
Arabidopsis - ps31
Arabidopsis_thaliana - ps32

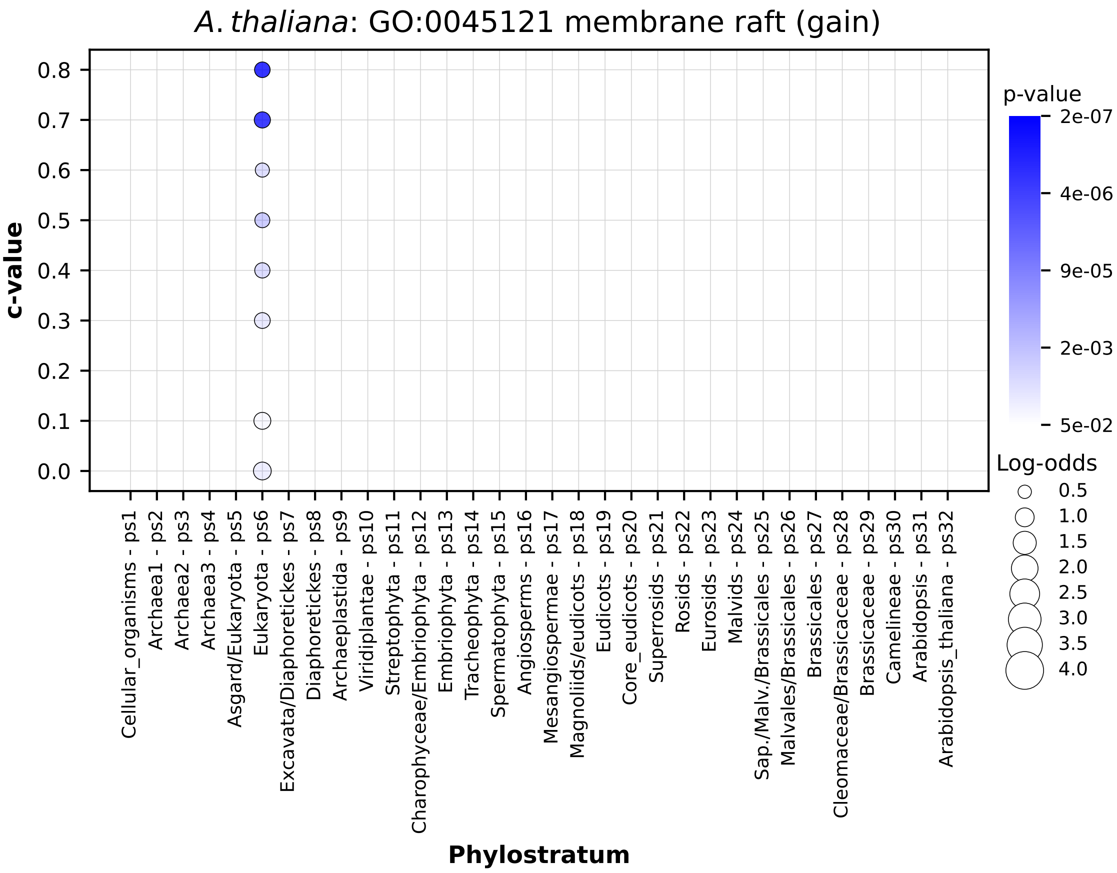

A. thaliana: GO:0045121 membrane raft (gain)
c-value
0.8
0.7
0.6
0.5
0.4
0.3
0.2
0.1
0.0
p-value
2e-07
4e-06
9e-05
2e-03
5e-02
Log-odds
0.5
1.0
1.5
2.0
2.5
3.0
3.5
4.0
Cellular_organisms - ps1
Archaea1 - ps2
Archaea2 - ps3
Archaea3 - ps4
Asgard/Eukaryota - ps5
Eukaryota - ps6
Excavata/Diaphoretickes - ps7
Diaphoretickes - ps8
Archaeplastida - ps9
Viridiplantae - ps10
Streptophyta - ps11
Charophyceae/Embriophyta - ps12
Embriophyta - ps13
Tracheophyta - ps14
Spermatophyta - ps15
Angiosperms - ps16
Mesangiospermae - ps17
Magnoliids/eudicots - ps18
Eudicots - ps19
Core_eudicots - ps20
Superrosids - ps21
Rosids - ps22
Eurosids - ps23
Malvids - ps24
Sap./Malv./Brassicales - ps25
Malvales/Brassicales - ps26
Brassicales - ps27
Cleomaceae/Brassicaceae - ps28
Brassicaceae - ps29
Camelineae - ps30
Arabidopsis - ps31
Arabidopsis_thaliana - ps32
Phylostratum

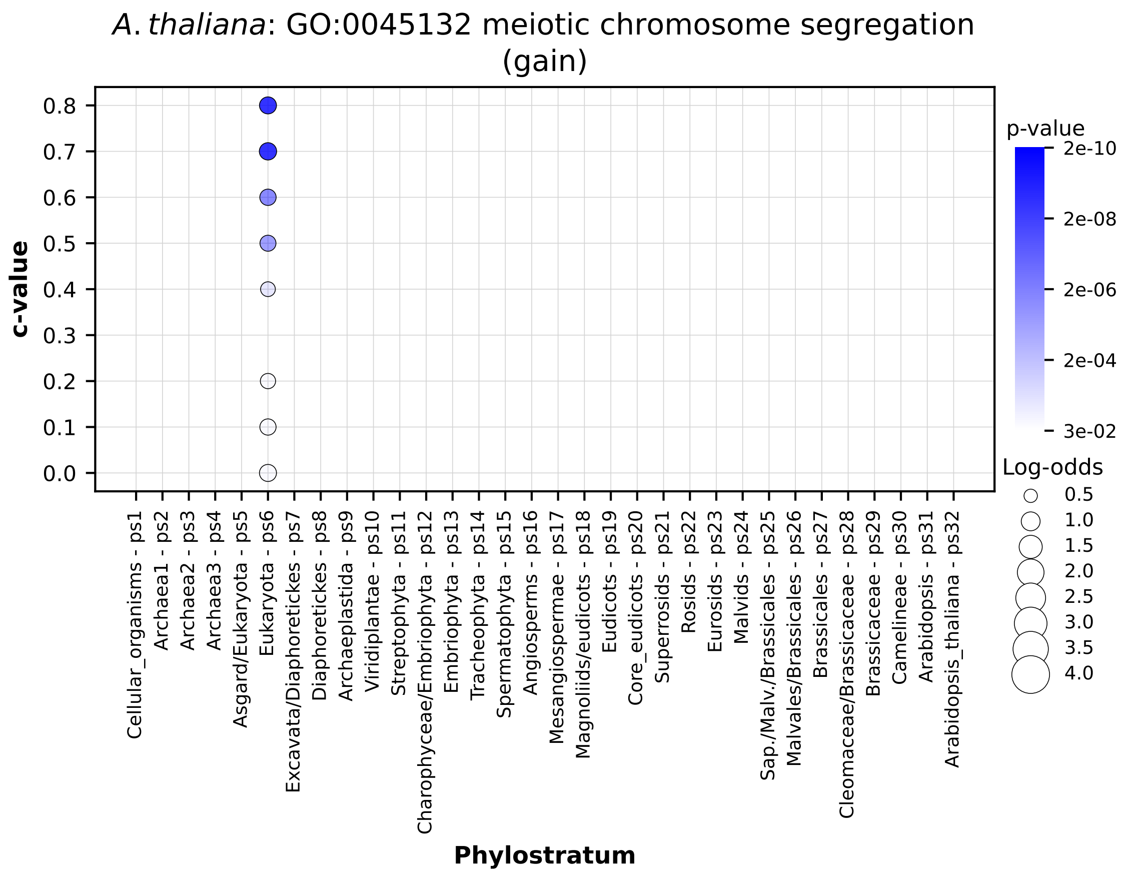

A. thaliana: GO:0045132 meiotic chromosome segregation (gain)
c-value
0.8
0.7
0.6
0.5
0.4
0.3
0.2
0.1
0.0
p-value
2e-10
2e-08
2e-06
2e-04
3e-02
Log-odds
0.5
1.0
1.5
2.0
2.5
3.0
3.5
4.0
Cellular_organisms - ps1
Archaea1 - ps2
Archaea2 - ps3
Archaea3 - ps4
Asgard/Eukaryota - ps5
Eukaryota - ps6
Excavata/Diaphoretickes - ps7
Diaphoretickes - ps8
Archaeplastida - ps9
Viridiplantae - ps10
Streptophyta - ps11
Charophyceae/Embriophyta - ps12
Embriophyta - ps13
Tracheophyta - ps14
Spermatophyta - ps15
Angiosperms - ps16
Mesangiospermae - ps17
Magnoliids/eudicots - ps18
Eudicots - ps19
Core_eudicots - ps20
Superrosids - ps21
Rosids - ps22
Eurosids - ps23
Malvids - ps24
Sap./Malv./Brassicales - ps25
Malvales/Brassicales - ps26
Brassicales - ps27
Cleomaceae/Brassicaceae - ps28
Brassicaceae - ps29
Camelineae - ps30
Arabidopsis - ps31
Arabidopsis_thaliana - ps32
Phylostratum

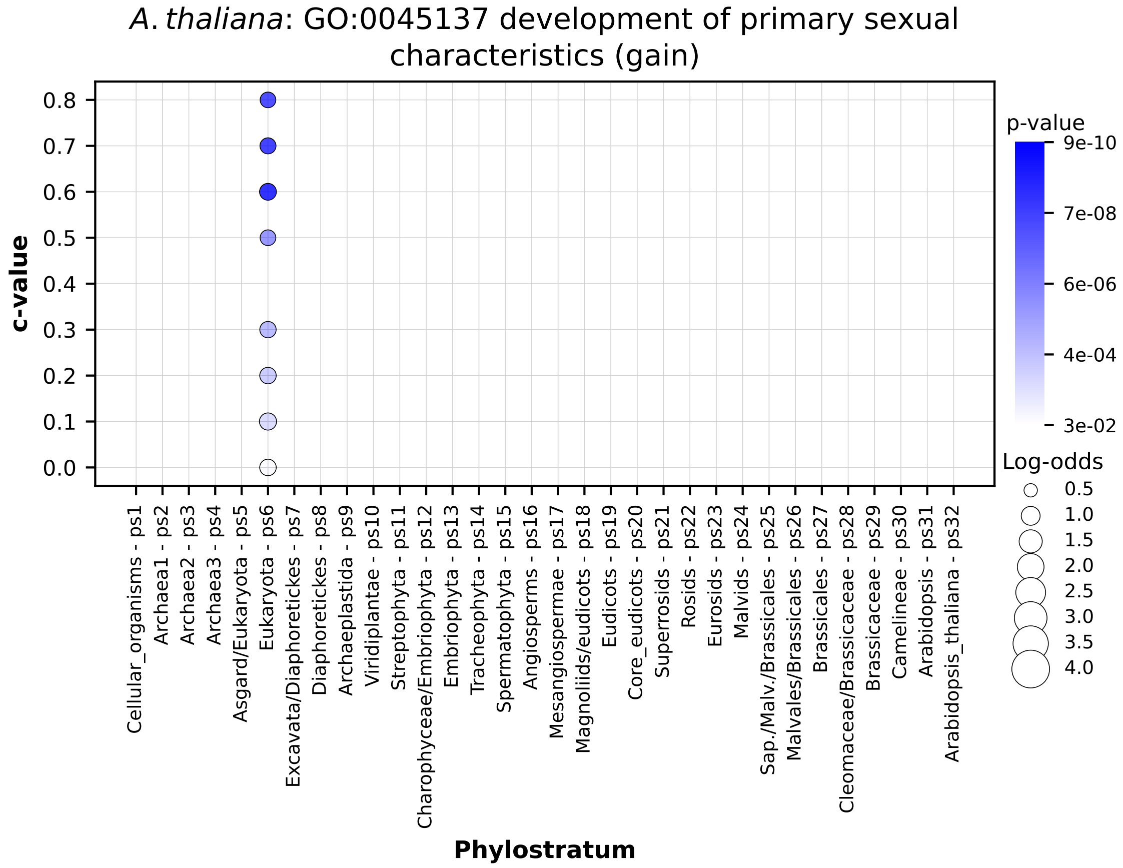

A. thaliana: GO:0045137 development of primary sexual characteristics (gain)
c-value
0.8
0.7
0.6
0.5
0.4
0.3
0.2
0.1
0.0
p-value
9e-10
7e-08
6e-06
4e-04
3e-02
Log-odds
0.5
1.0
1.5
2.0
2.5
3.0
3.5
4.0
Cellular_organisms - ps1
Archaea1 - ps2
Archaea2 - ps3
Archaea3 - ps4
Asgard/Eukaryota - ps5
Eukaryota - ps6
Excavata/Diaphoretickes - ps7
Diaphoretickes - ps8
Archaeplastida - ps9
Viridiplantae - ps10
Streptophyta - ps11
Charophyceae/Embriophyta - ps12
Embriophyta - ps13
Tracheophyta - ps14
Spermatophyta - ps15
Angiosperms - ps16
Mesangiospermae - ps17
Magnoliids/eudicots - ps18
Eudicots - ps19
Core_eudicots - ps20
Superrosids - ps21
Rosids - ps22
Eurosids - ps23
Malvids - ps24
Sap./Malv./Brassicales - ps25
Malvales/Brassicales - ps26
Brassicales - ps27
Cleomaceae/Brassicaceae - ps28
Brassicaceae - ps29
Camelineae - ps30
Arabidopsis - ps31
Arabidopsis_thaliana - ps32
Phylostratum

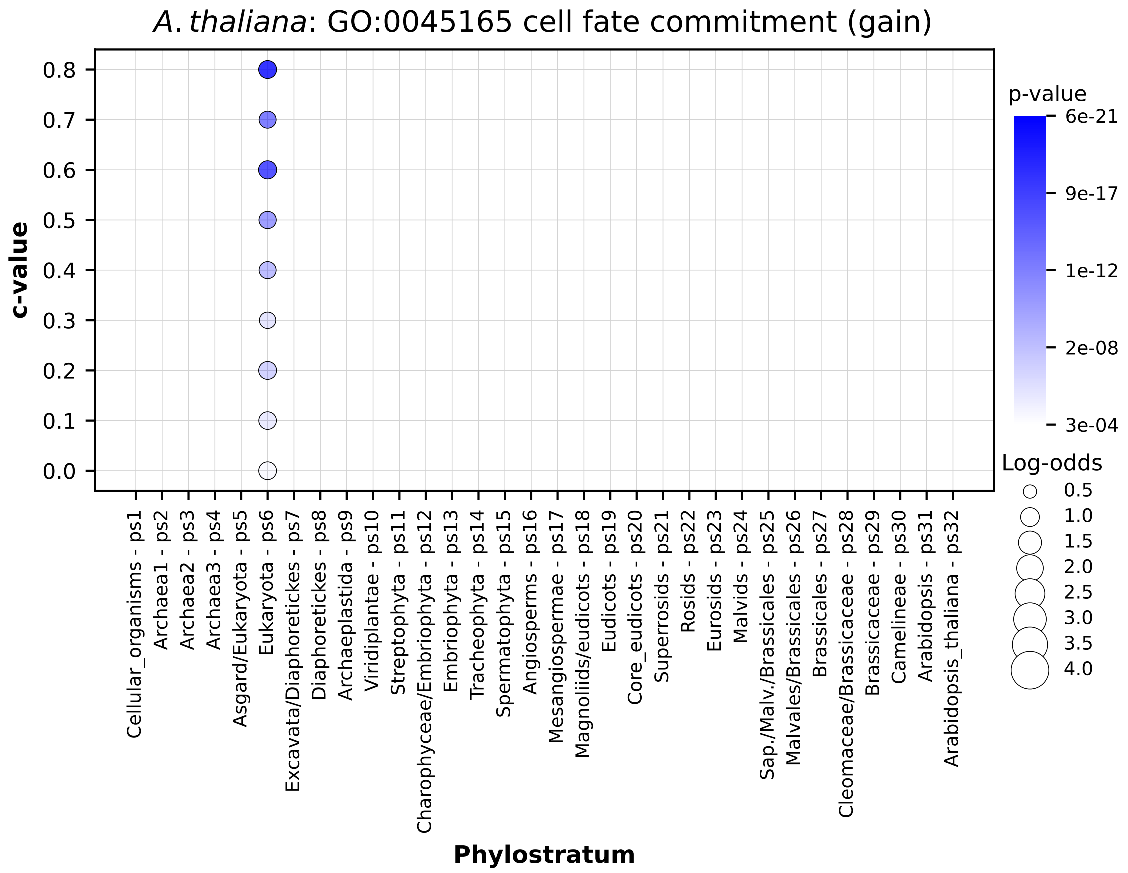
A. thaliana: GO:0045165 cell fate commitment (gain)
c-value
0.8
0.7
0.6
0.5
0.4
0.3
0.2
0.1
0.0
Phylostratum
Cellular_organisms - ps1
Archaea1 - ps2
Archaea2 - ps3
Archaea3 - ps4
Asgard/Eukaryota - ps5
Eukaryota - ps6
Excavata/Diaphoretickes - ps7
Diaphoretickes - ps8
Archaeplastida - ps9
Viridiplantae - ps10
Streptophyta - ps11
Charophyceae/Embriophyta - ps12
Embriophyta - ps13
Tracheophyta - ps14
Spermatophyta - ps15
Angiosperms - ps16
Mesangiospermae - ps17
Magnoliids/eudicots - ps18
Eudicots - ps19
Core_eudicots - ps20
Superrosids - ps21
Rosids - ps22
Eurosids - ps23
Malvids - ps24
Sap./Malv./Brassicales - ps25
Malvales/Brassicales - ps26
Brassicales - ps27
Cleomaceae/Brassicaceae - ps28
Brassicaceae - ps29
Camelineae - ps30
Arabidopsis - ps31
Arabidopsis_thaliana - ps32
p-value
6e-21
9e-17
1e-12
2e-08
3e-04
Log-odds
0.5
1.0
1.5
2.0
2.5
3.0
3.5
4.0

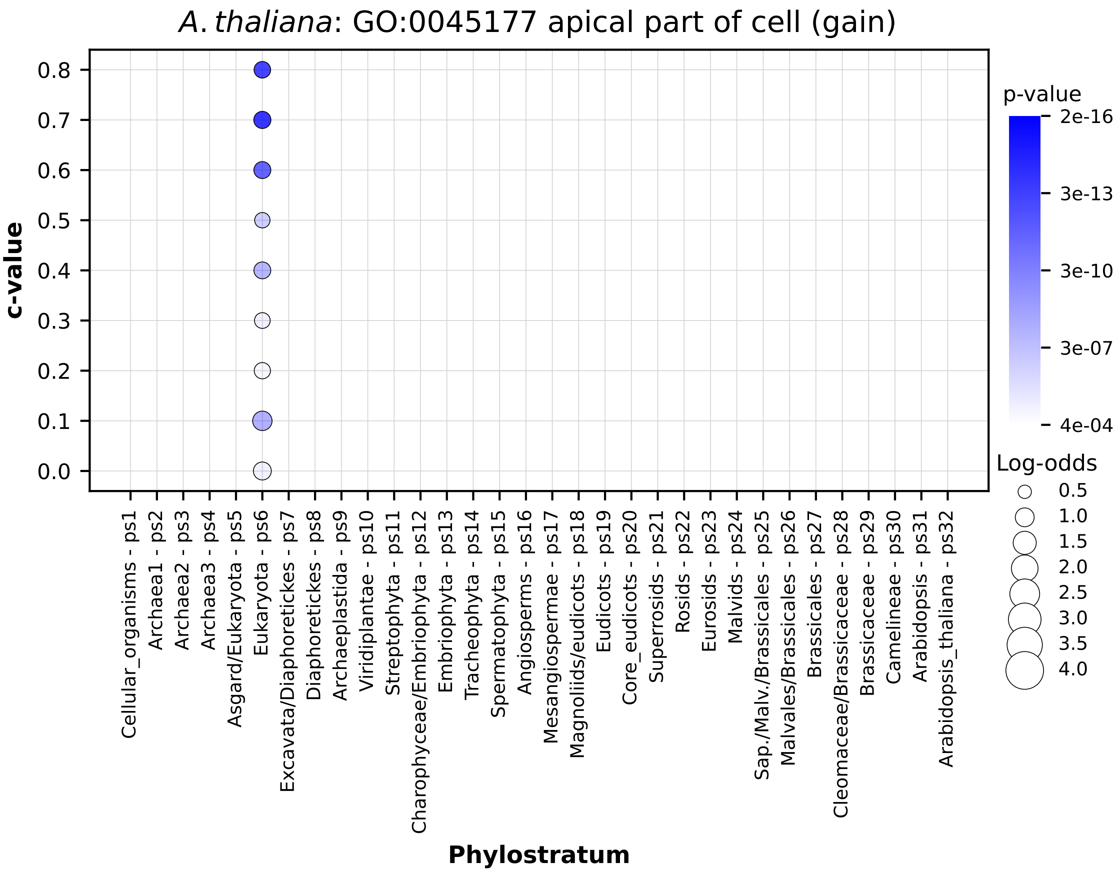

A. thaliana: GO:0045177 apical part of cell (gain)
c-value
0.8
0.7
0.6
0.5
0.4
0.3
0.2
0.1
0.0
p-value
2e-16
3e-13
3e-10
3e-07
4e-04
Log-odds
0.5
1.0
1.5
2.0
2.5
3.0
3.5
4.0
Cellular_organisms - ps1
Archaea1 - ps2
Archaea2 - ps3
Archaea3 - ps4
Asgard/Eukaryota - ps5
Eukaryota - ps6
Excavata/Diaphoretickes - ps7
Diaphoretickes - ps8
Archaeplastida - ps9
Viridiplantae - ps10
Streptophyta - ps11
Charophyceae/Embriophyta - ps12
Embriophyta - ps13
Tracheophyta - ps14
Spermatophyta - ps15
Angiosperms - ps16
Mesangiospermae - ps17
Magnoliids/eudicots - ps18
Eudicots - ps19
Core_eudicots - ps20
Superrosids - ps21
Rosids - ps22
Eurosids - ps23
Malvids - ps24
Sap./Malv./Brassicales - ps25
Malvales/Brassicales - ps26
Brassicales - ps27
Cleomaceae/Brassicaceae - ps28
Brassicaceae - ps29
Camelineae - ps30
Arabidopsis - ps31
Arabidopsis_thaliana - ps32
Phylostratum

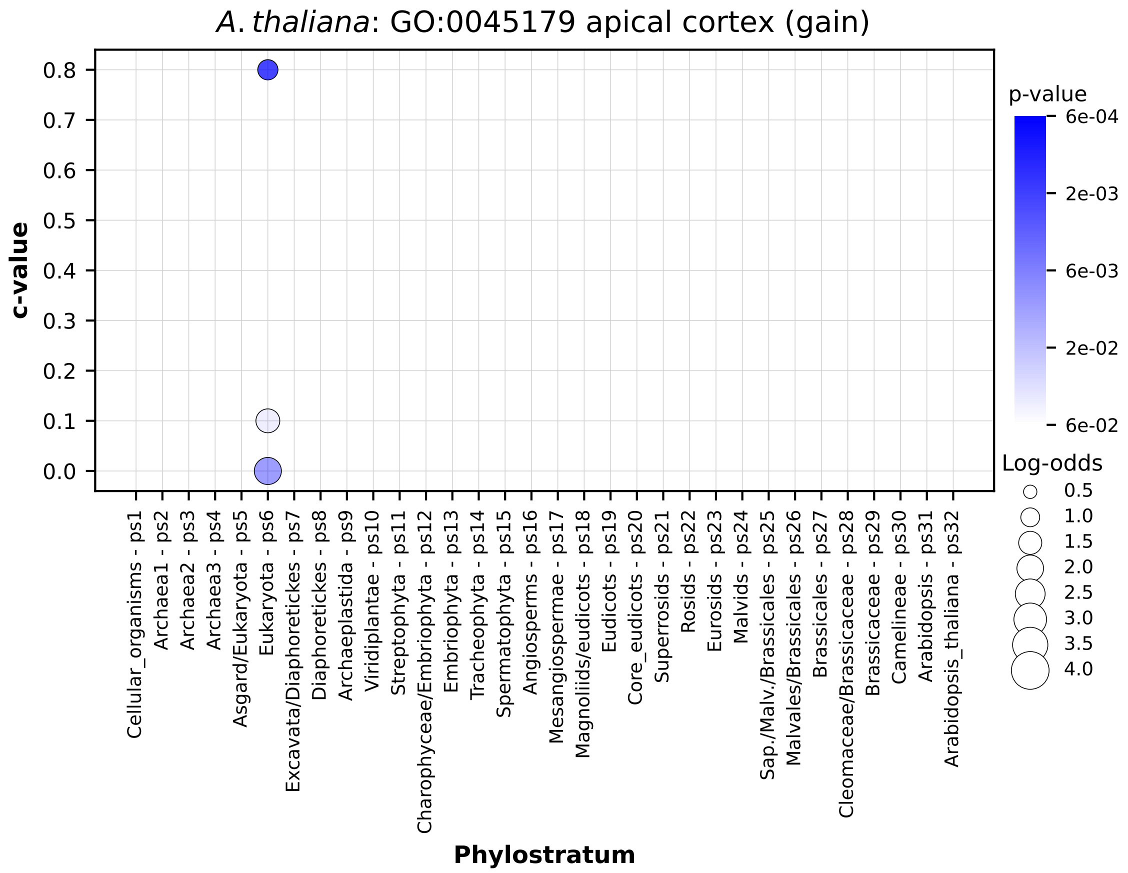

A. thaliana: GO:0045179 apical cortex (gain)
c-value
0.8
0.7
0.6
0.5
0.4
0.3
0.2
0.1
0.0
p-value
6e-04
2e-03
6e-03
2e-02
6e-02
Log-odds
0.5
1.0
1.5
2.0
2.5
3.0
3.5
4.0
Cellular_organisms - ps1
Archaea1 - ps2
Archaea2 - ps3
Archaea3 - ps4
Asgard/Eukaryota - ps5
Eukaryota - ps6
Excavata/Diaphoretickes - ps7
Diaphoretickes - ps8
Archaeplastida - ps9
Viridiplantae - ps10
Streptophyta - ps11
Charophyceae/Embriophyta - ps12
Embriophyta - ps13
Tracheophyta - ps14
Spermatophyta - ps15
Angiosperms - ps16
Mesangiospermae - ps17
Magnoliids/eudicots - ps18
Eudicots - ps19
Core_eudicots - ps20
Superrosids - ps21
Rosids - ps22
Eurosids - ps23
Malvids - ps24
Sap./Malv./Brassicales - ps25
Malvales/Brassicales - ps26
Brassicales - ps27
Cleomaceae/Brassicaceae - ps28
Brassicaceae - ps29
Camelineae - ps30
Arabidopsis - ps31
Arabidopsis_thaliana - ps32
Phylostratum

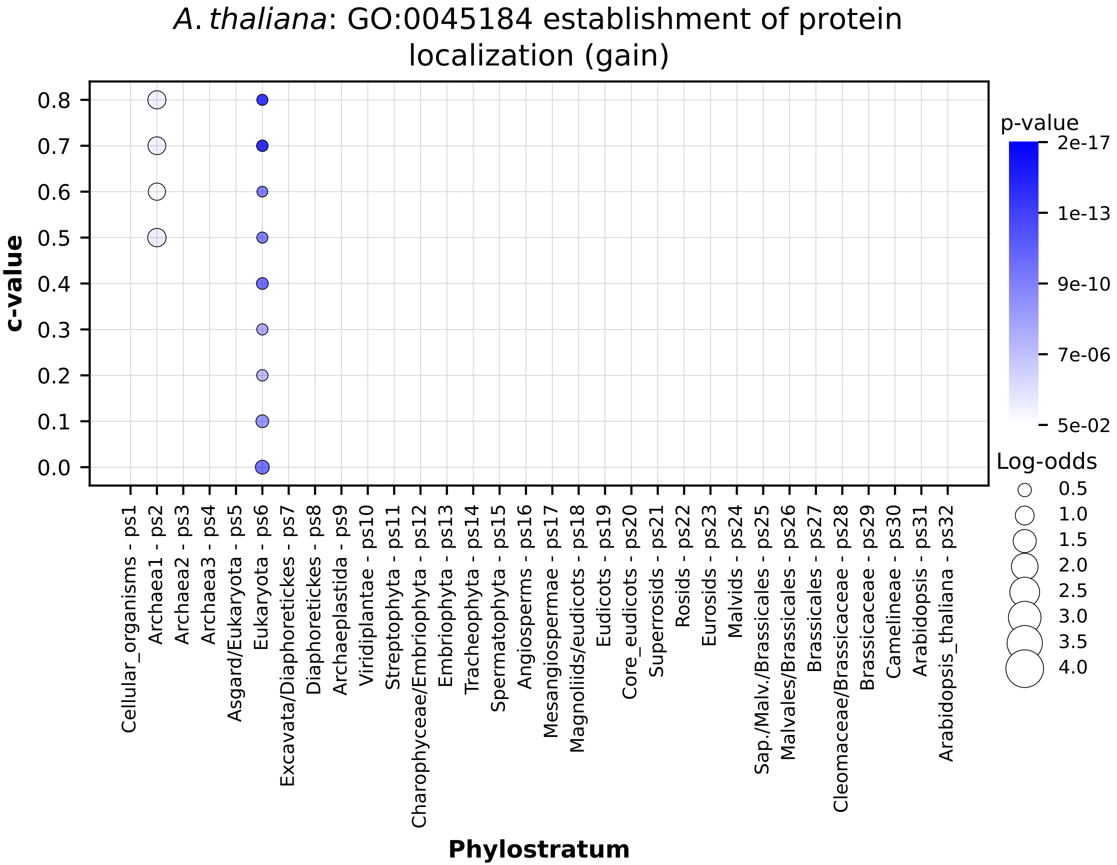
A. thaliana: GO:0045184 establishment of protein localization (gain)
c-value
0.0
0.1
0.2
0.3
0.4
0.5
0.6
0.7
0.8
Phylostratum
Cellular_organisms - ps1
Archaea1 - ps2
Archaea2 - ps3
Archaea3 - ps4
Asgard/Eukaryota - ps5
Eukaryota - ps6
Excavata/Diaphoretickes - ps7
Diaphoretickes - ps8
Archaeplastida - ps9
Viridiplantae - ps10
Streptophyta - ps11
Charophyceae/Embriophyta - ps12
Embriophyta - ps13
Tracheophyta - ps14
Spermatophyta - ps15
Angiosperms - ps16
Mesangiospermae - ps17
Magnoliids/eudicots - ps18
Eudicots - ps19
Core_eudicots - ps20
Superrosids - ps21
Rosids - ps22
Eurosids - ps23
Malvids - ps24
Sap./Malv./Brassicales - ps25
Malvales/Brassicales - ps26
Brassicales - ps27
Cleomaceae/Brassicaceae - ps28
Brassicaceae - ps29
Camelineae - ps30
Arabidopsis - ps31
Arabidopsis_thaliana - ps32
p-value
2e-17
1e-13
9e-10
7e-06
5e-02
Log-odds
0.5
1.0
1.5
2.0
2.5
3.0
3.5
4.0

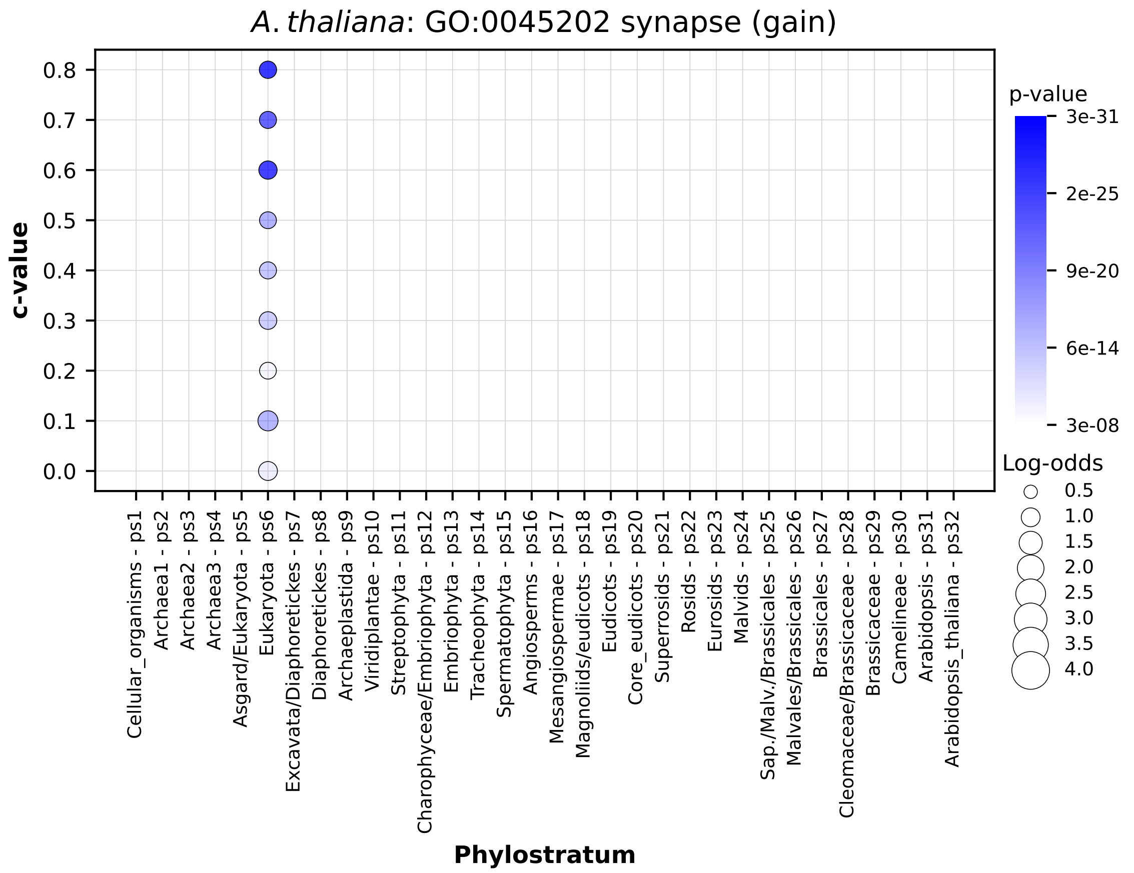
A. thaliana: GO:0045202 synapse (gain)
c-value
0.8
0.7
0.6
0.5
0.4
0.3
0.2
0.1
0.0
p-value
3e-31
2e-25
9e-20
6e-14
3e-08
Log-odds
0.5
1.0
1.5
2.0
2.5
3.0
3.5
4.0
Cellular_organisms - ps1
Archaea1 - ps2
Archaea2 - ps3
Archaea3 - ps4
Asgard/Eukaryota - ps5
Eukaryota - ps6
Excavata/Diaphoretickes - ps7
Diaphoretickes - ps8
Archaeplastida - ps9
Viridiplantae - ps10
Streptophyta - ps11
Charophyceae/Embriophyta - ps12
Embriophyta - ps13
Tracheophyta - ps14
Spermatophyta - ps15
Angiosperms - ps16
Mesangiospermae - ps17
Magnoliids/eudicots - ps18
Eudicots - ps19
Core_eudicots - ps20
Superrosids - ps21
Rosids - ps22
Eurosids - ps23
Malvids - ps24
Sap./Malv./Brassicales - ps25
Malvales/Brassicales - ps26
Brassicales - ps27
Cleomaceae/Brassicaceae - ps28
Brassicaceae - ps29
Camelineae - ps30
Arabidopsis - ps31
Arabidopsis_thaliana - ps32
Phylostratum

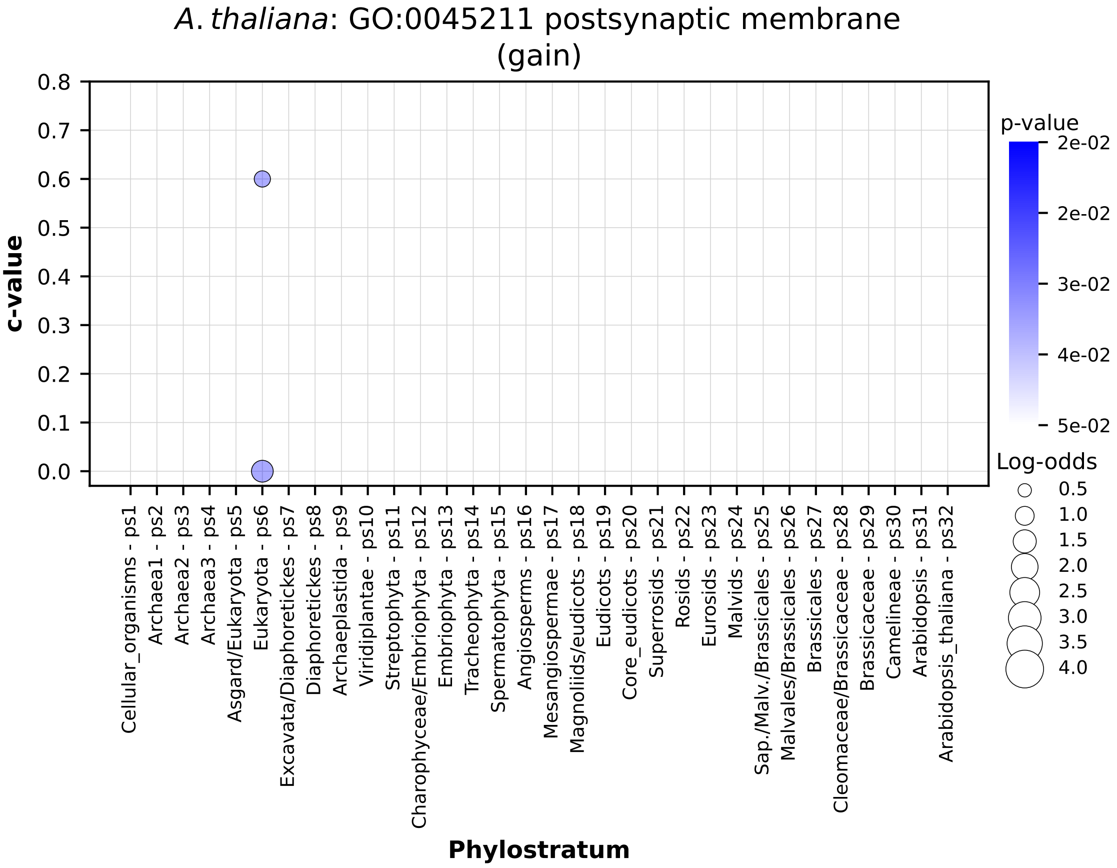
A. thaliana: GO:0045211 postsynaptic membrane
(gain)
c-value
0.8
0.7
0.6
0.5
0.4
0.3
0.2
0.1
0.0
Phylostratum
Cellular_organisms - ps1
Archaea1 - ps2
Archaea2 - ps3
Archaea3 - ps4
Asgard/Eukaryota - ps5
Eukaryota - ps6
Excavata/Diaphoretickes - ps7
Diaphoretickes - ps8
Archaeplastida - ps9
Viridiplantae - ps10
Streptophyta - ps11
Charophyceae/Embriophyta - ps12
Embriophyta - ps13
Tracheophyta - ps14
Spermatophyta - ps15
Angiosperms - ps16
Mesangiospermae - ps17
Magnoliids/eudicots - ps18
Eudicots - ps19
Core_eudicots - ps20
Superrosids - ps21
Rosids - ps22
Eurosids - ps23
Malvids - ps24
Sap./Malv./Brassicales - ps25
Malvales/Brassicales - ps26
Brassicales - ps27
Cleomaceae/Brassicaceae - ps28
Brassicaceae - ps29
Camelineae - ps30
Arabidopsis - ps31
Arabidopsis_thaliana - ps32
p-value
2e-02
2e-02
3e-02
4e-02
5e-02
Log-odds
0.5
1.0
1.5
2.0
2.5
3.0
3.5
4.0

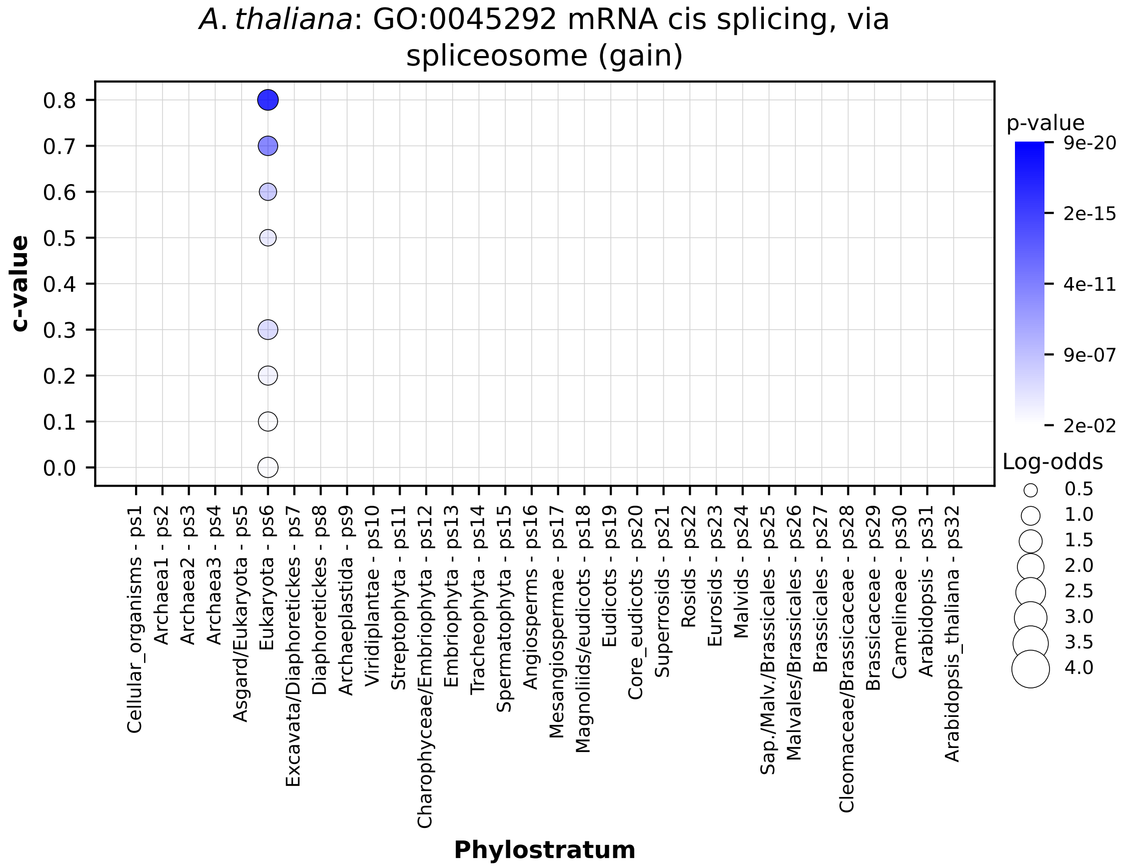

A. thaliana: GO:0045292 mRNA cis splicing, via spliceosome (gain)
c-value
0.8
0.7
0.6
0.5
0.4
0.3
0.2
0.1
0.0
p-value
9e-20
2e-15
4e-11
9e-07
2e-02
Log-odds
0.5
1.0
1.5
2.0
2.5
3.0
3.5
4.0
Cellular_organisms - ps1
Archaea1 - ps2
Archaea2 - ps3
Archaea3 - ps4
Asgard/Eukaryota - ps5
Eukaryota - ps6
Excavata/Diaphoretickes - ps7
Diaphoretickes - ps8
Archaeplastida - ps9
Viridiplantae - ps10
Streptophyta - ps11
Charophyceae/Embriophyta - ps12
Embriophyta - ps13
Tracheophyta - ps14
Spermatophyta - ps15
Angiosperms - ps16
Mesangiospermae - ps17
Magnoliids/eudicots - ps18
Eudicots - ps19
Core_eudicots - ps20
Superrosids - ps21
Rosids - ps22
Eurosids - ps23
Malvids - ps24
Sap./Malv./Brassicales - ps25
Malvales/Brassicales - ps26
Brassicales - ps27
Cleomaceae/Brassicaceae - ps28
Brassicaceae - ps29
Camelineae - ps30
Arabidopsis - ps31
Arabidopsis_thaliana - ps32
Phylostratum

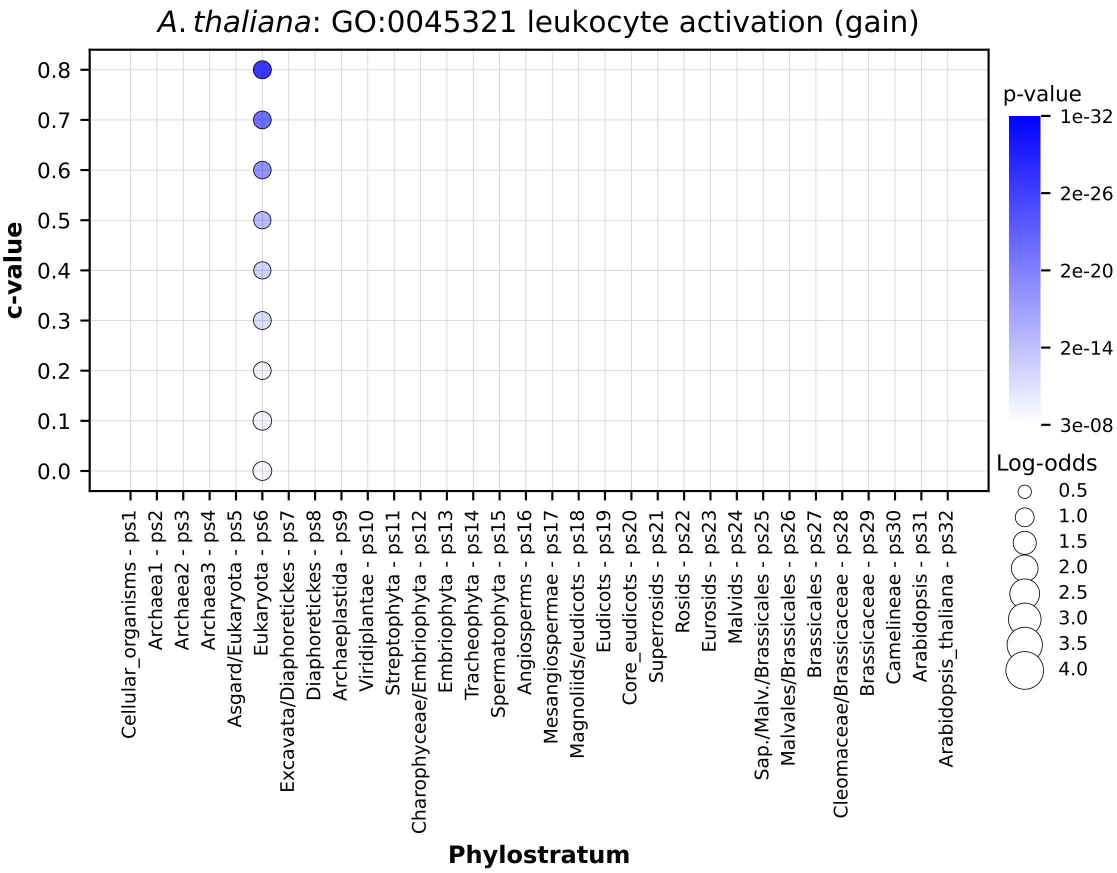

A. thaliana: GO:0045321 leukocyte activation (gain)
c-value
0.0
0.1
0.2
0.3
0.4
0.5
0.6
0.7
0.8
p-value
1e-32
2e-26
2e-20
2e-14
3e-08
Log-odds
0.5
1.0
1.5
2.0
2.5
3.0
3.5
4.0
Cellular_organisms - ps1
Archaea1 - ps2
Archaea2 - ps3
Archaea3 - ps4
Asgard/Eukaryota - ps5
Eukaryota - ps6
Excavata/Diaphoretickes - ps7
Diaphoretickes - ps8
Archaeplastida - ps9
Viridiplantae - ps10
Streptophyta - ps11
Charophyceae/Embriophyta - ps12
Embriophyta - ps13
Tracheophyta - ps14
Spermatophyta - ps15
Angiosperms - ps16
Mesangiospermae - ps17
Magnoliids/eudicots - ps18
Eudicots - ps19
Core_eudicots - ps20
Superrosids - ps21
Rosids - ps22
Eurosids - ps23
Malvids - ps24
Sap./Malv./Brassicales - ps25
Malvales/Brassicales - ps26
Brassicales - ps27
Cleomaceae/Brassicaceae - ps28
Brassicaceae - ps29
Camelineae - ps30
Arabidopsis - ps31
Arabidopsis_thaliana - ps32
Phylostratum

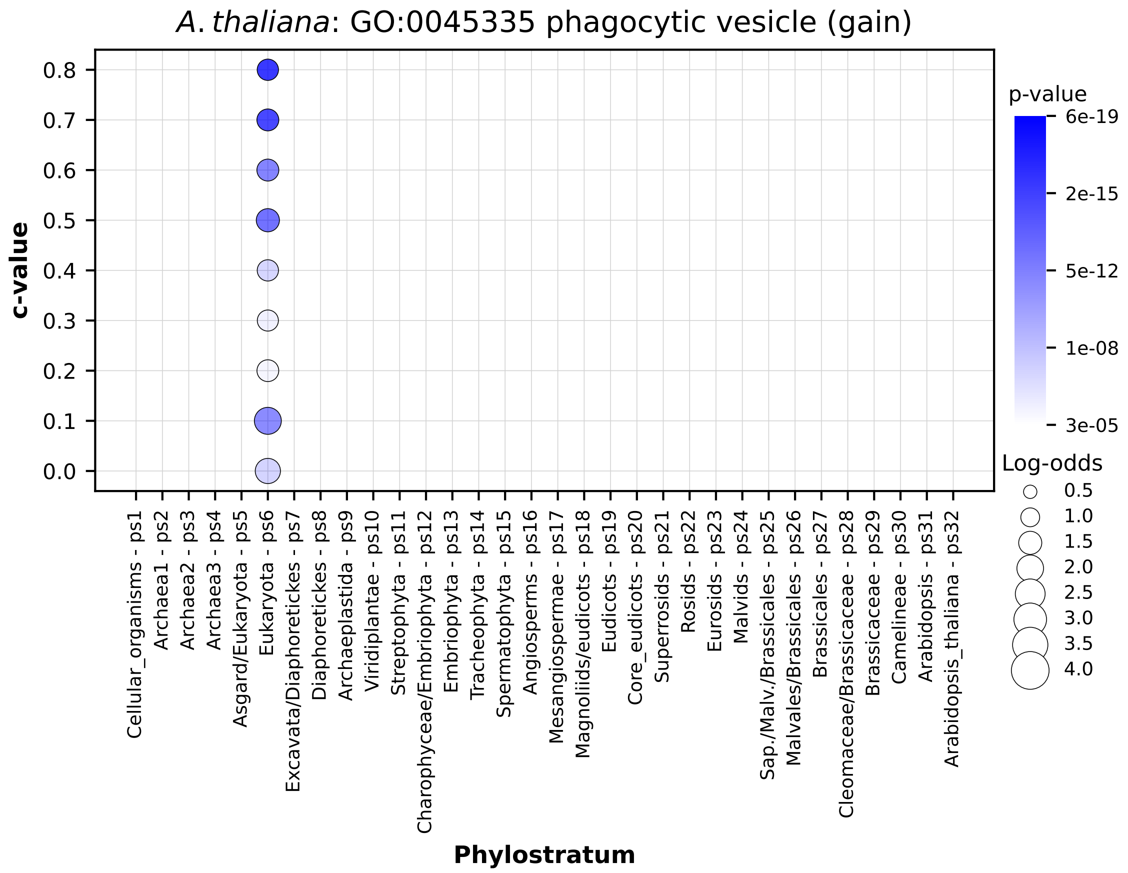

A. thaliana: GO:0045335 phagocytic vesicle (gain)
c-value
0.8
0.7
0.6
0.5
0.4
0.3
0.2
0.1
0.0
p-value
6e-19
2e-15
5e-12
1e-08
3e-05
Log-odds
0.5
1.0
1.5
2.0
2.5
3.0
3.5
4.0
Cellular_organisms - ps1
Archaea1 - ps2
Archaea2 - ps3
Archaea3 - ps4
Asgard/Eukaryota - ps5
Eukaryota - ps6
Excavata/Diaphoretickes - ps7
Diaphoretickes - ps8
Archaeplastida - ps9
Viridiplantae - ps10
Streptophyta - ps11
Charophyceae/Embriophyta - ps12
Embriophyta - ps13
Tracheophyta - ps14
Spermatophyta - ps15
Angiosperms - ps16
Mesangiospermae - ps17
Magnoliids/eudicots - ps18
Eudicots - ps19
Core_eudicots - ps20
Superrosids - ps21
Rosids - ps22
Eurosids - ps23
Malvids - ps24
Sap./Malv./Brassicales - ps25
Malvales/Brassicales - ps26
Brassicales - ps27
Cleomaceae/Brassicaceae - ps28
Brassicaceae - ps29
Camelineae - ps30
Arabidopsis - ps31
Arabidopsis_thaliana - ps32
Phylostratum

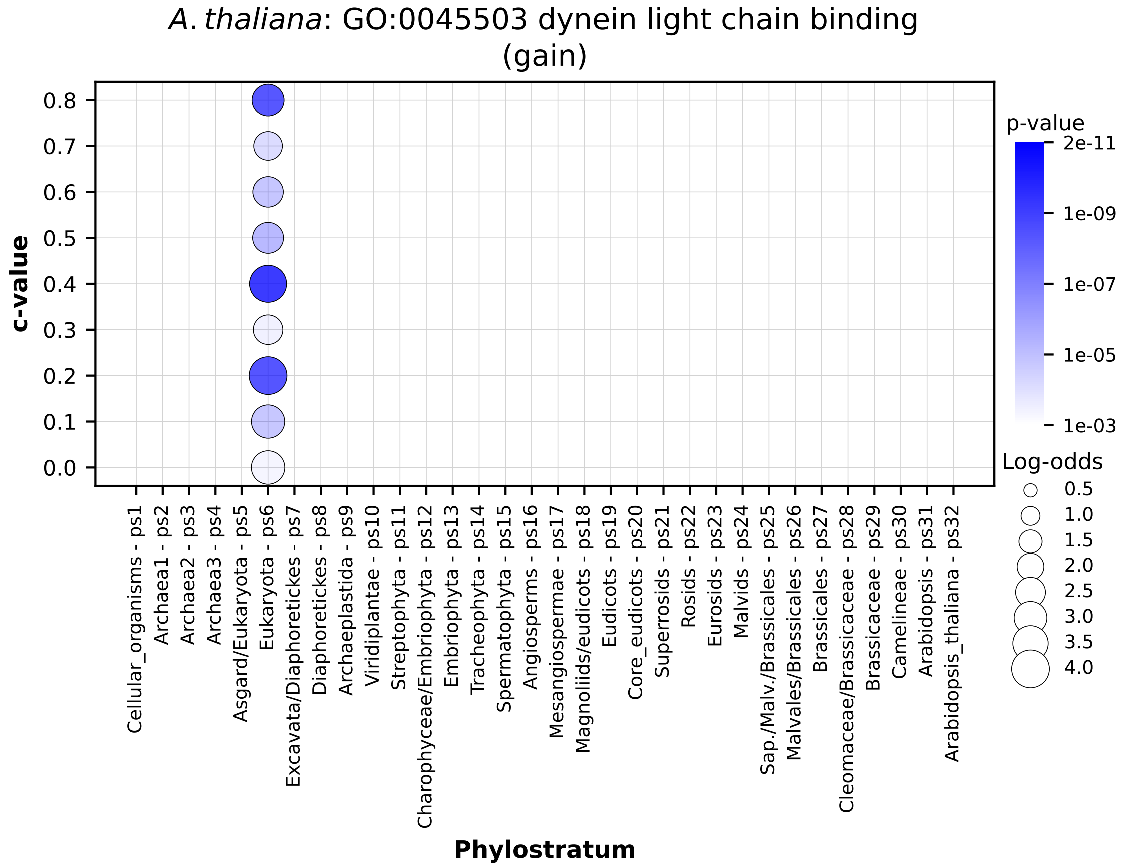

A. thaliana: GO:0045503 dynein light chain binding
(gain)
c-value
0.8
0.7
0.6
0.5
0.4
0.3
0.2
0.1
0.0
Phylostratum
Cellular_organisms - ps1
Archaea1 - ps2
Archaea2 - ps3
Archaea3 - ps4
Asgard/Eukaryota - ps5
Eukaryota - ps6
Excavata/Diaphoretickes - ps7
Diaphoretickes - ps8
Archaeplastida - ps9
Viridiplantae - ps10
Streptophyta - ps11
Charophyceae/Embriophyta - ps12
Embriophyta - ps13
Tracheophyta - ps14
Spermatophyta - ps15
Angiosperms - ps16
Mesangiospermae - ps17
Magnoliids/eudicots - ps18
Eudicots - ps19
Core_eudicots - ps20
Superrosids - ps21
Rosids - ps22
Eurosids - ps23
Malvids - ps24
Sap./Malv./Brassicales - ps25
Malvales/Brassicales - ps26
Brassicales - ps27
Cleomaceae/Brassicaceae - ps28
Brassicaceae - ps29
Camelineae - ps30
Arabidopsis - ps31
Arabidopsis_thaliana - ps32
p-value
2e-11
1e-09
1e-07
1e-05
1e-03
Log-odds
0.5
1.0
1.5
2.0
2.5
3.0
3.5
4.0

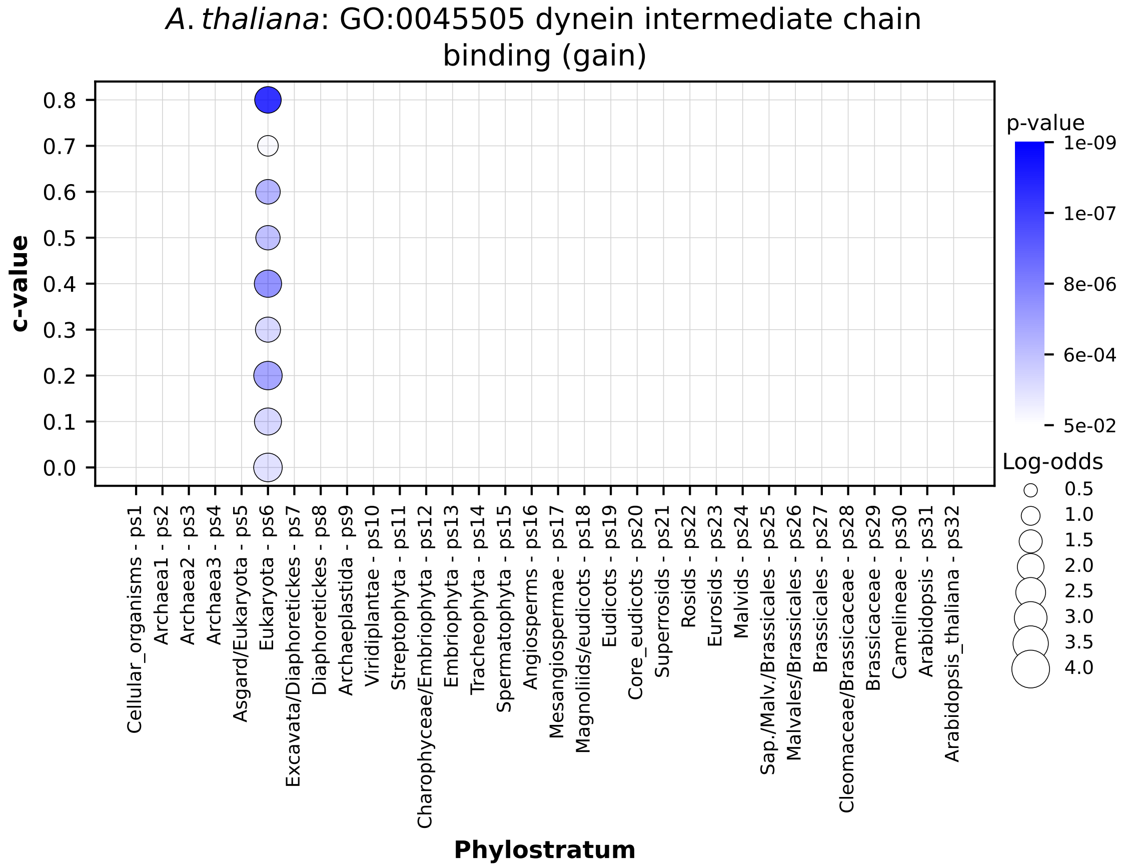
A. thaliana: GO:0045505 dynein intermediate chain binding (gain)
c-value
0.8
0.7
0.6
0.5
0.4
0.3
0.2
0.1
0.0
Phylostratum
Cellular_organisms - ps1
Archaea1 - ps2
Archaea2 - ps3
Archaea3 - ps4
Asgard/Eukaryota - ps5
Eukaryota - ps6
Excavata/Diaphoretickes - ps7
Diaphoretickes - ps8
Archaeplastida - ps9
Viridiplantae - ps10
Streptophyta - ps11
Charophyceae/Embriophyta - ps12
Embriophyta - ps13
Tracheophyta - ps14
Spermatophyta - ps15
Angiosperms - ps16
Mesangiospermae - ps17
Magnoliids/eudicots - ps18
Eudicots - ps19
Core_eudicots - ps20
Superrosids - ps21
Rosids - ps22
Eurosids - ps23
Malvids - ps24
Sap./Malv./Brassicales - ps25
Malvales/Brassicales - ps26
Brassicales - ps27
Cleomaceae/Brassicaceae - ps28
Brassicaceae - ps29
Camelineae - ps30
Arabidopsis - ps31
Arabidopsis_thaliana - ps32
p-value
1e-09
1e-07
8e-06
6e-04
5e-02
Log-odds
0.5
1.0
1.5
2.0
2.5
3.0
3.5
4.0

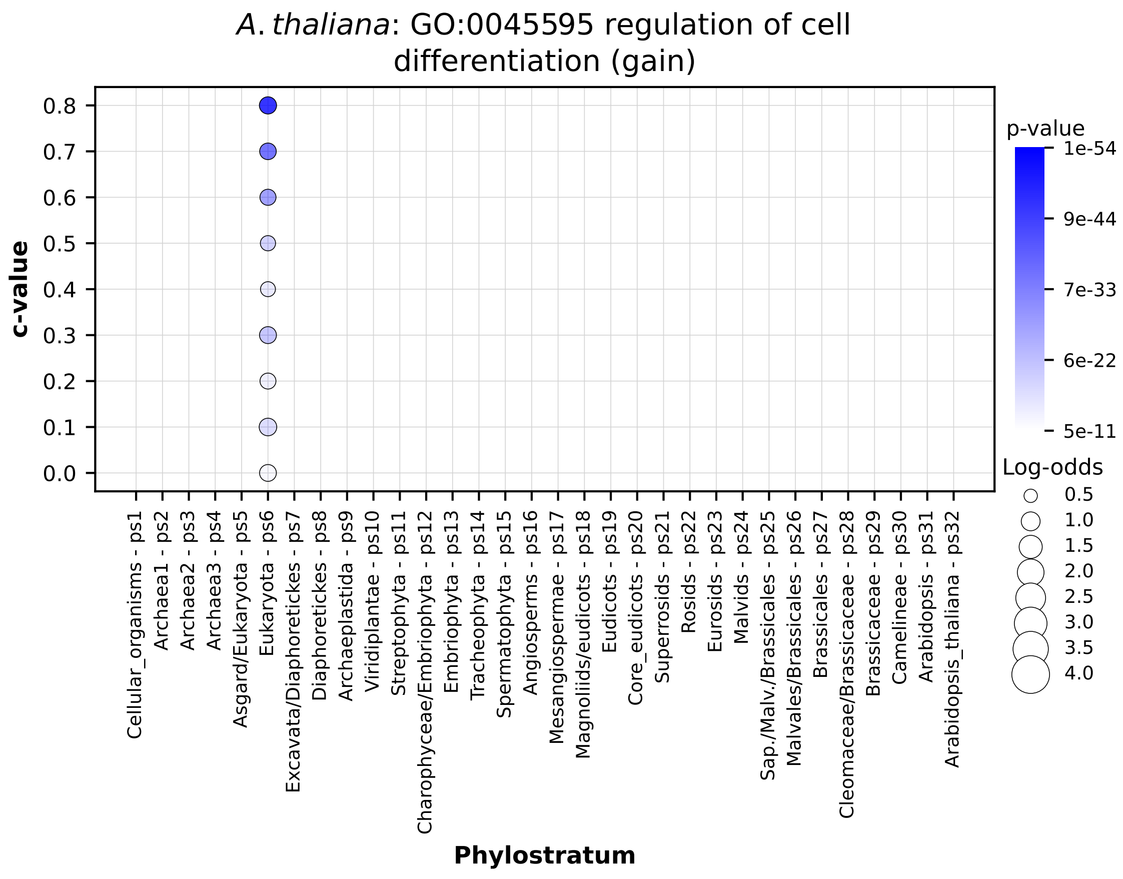

A. thaliana: GO:0045595 regulation of cell differentiation (gain)
c-value
0.8
0.7
0.6
0.5
0.4
0.3
0.2
0.1
0.0
p-value
1e-54
9e-44
7e-33
6e-22
5e-11
Log-odds
0.5
1.0
1.5
2.0
2.5
3.0
3.5
4.0
Cellular_organisms - ps1
Archaea1 - ps2
Archaea2 - ps3
Archaea3 - ps4
Asgard/Eukaryota - ps5
Eukaryota - ps6
Excavata/Diaphoretickes - ps7
Diaphoretickes - ps8
Archaeplastida - ps9
Viridiplantae - ps10
Streptophyta - ps11
Charophyceae/Embriophyta - ps12
Embriophyta - ps13
Tracheophyta - ps14
Spermatophyta - ps15
Angiosperms - ps16
Mesangiospermae - ps17
Magnoliids/eudicots - ps18
Eudicots - ps19
Core_eudicots - ps20
Superrosids - ps21
Rosids - ps22
Eurosids - ps23
Malvids - ps24
Sap./Malv./Brassicales - ps25
Malvales/Brassicales - ps26
Brassicales - ps27
Cleomaceae/Brassicaceae - ps28
Brassicaceae - ps29
Camelineae - ps30
Arabidopsis - ps31
Arabidopsis_thaliana - ps32
Phylostratum

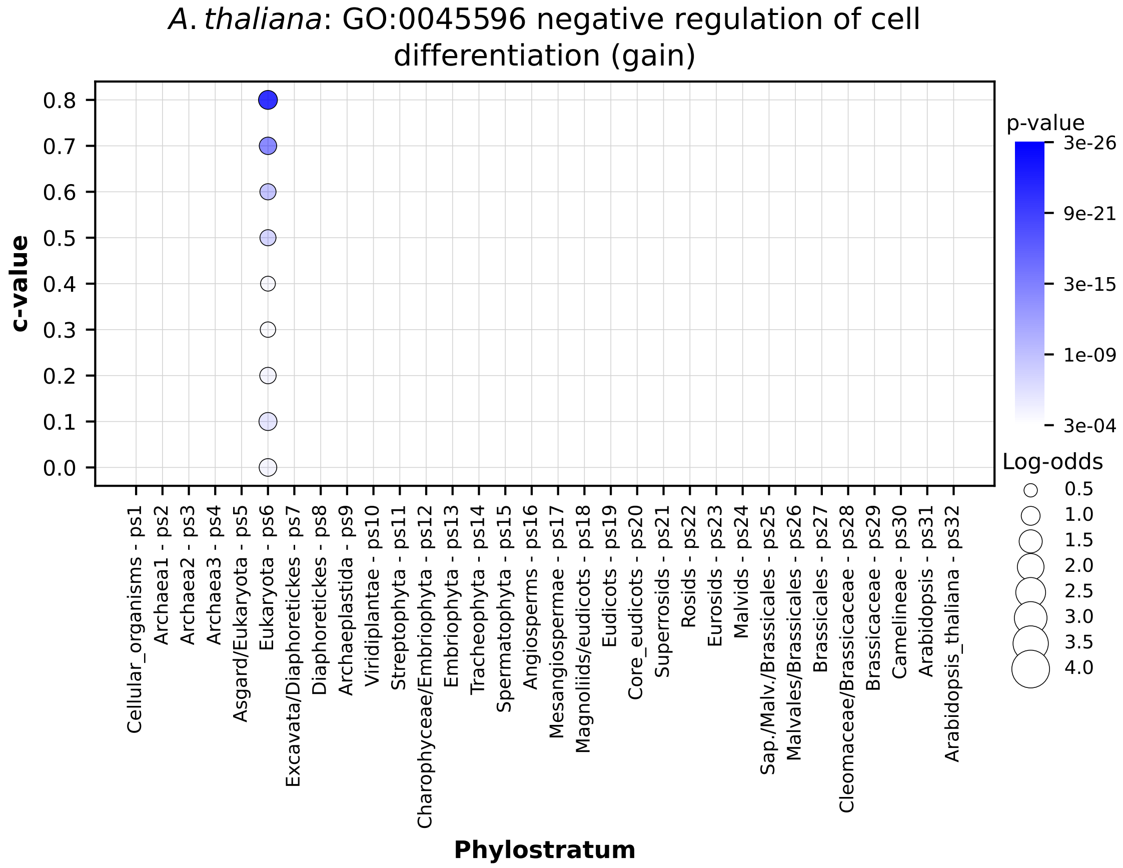

A. thaliana: GO:0045596 negative regulation of cell differentiation (gain)
c-value
0.8
0.7
0.6
0.5
0.4
0.3
0.2
0.1
0.0
p-value
3e-26
9e-21
3e-15
1e-09
3e-04
Log-odds
0.5
1.0
1.5
2.0
2.5
3.0
3.5
4.0
Cellular_organisms - ps1
Archaea1 - ps2
Archaea2 - ps3
Archaea3 - ps4
Asgard/Eukaryota - ps5
Eukaryota - ps6
Excavata/Diaphoretickes - ps7
Diaphoretickes - ps8
Archaeplastida - ps9
Viridiplantae - ps10
Streptophyta - ps11
Charophyceae/Embriophyta - ps12
Embriophyta - ps13
Tracheophyta - ps14
Spermatophyta - ps15
Angiosperms - ps16
Mesangiospermae - ps17
Magnoliids/eudicots - ps18
Eudicots - ps19
Core_eudicots - ps20
Superrosids - ps21
Rosids - ps22
Eurosids - ps23
Malvids - ps24
Sap./Malv./Brassicales - ps25
Malvales/Brassicales - ps26
Brassicales - ps27
Cleomaceae/Brassicaceae - ps28
Brassicaceae - ps29
Camelineae - ps30
Arabidopsis - ps31
Arabidopsis_thaliana - ps32
Phylostratum

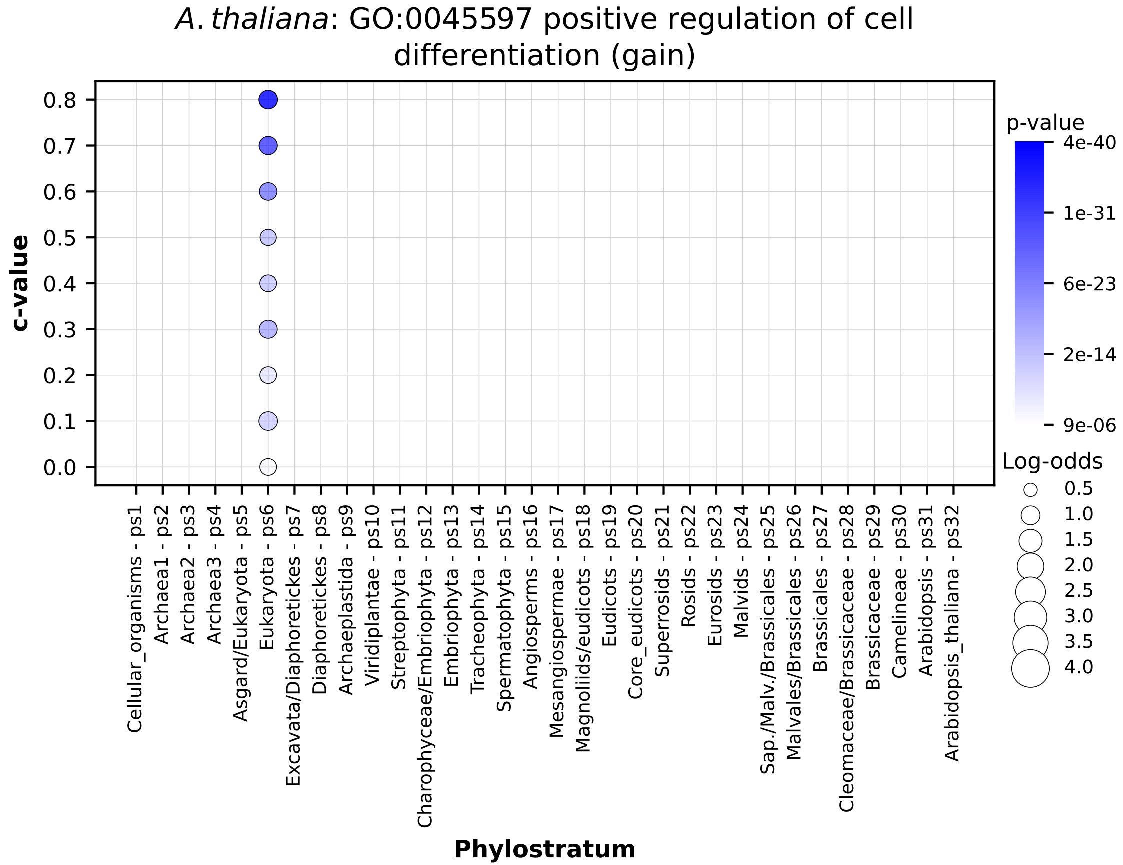

A. thaliana: GO:0045597 positive regulation of cell differentiation (gain)
c-value
0.8
0.7
0.6
0.5
0.4
0.3
0.2
0.1
0.0
p-value
4e-40
1e-31
6e-23
2e-14
9e-06
Log-odds
0.5
1.0
1.5
2.0
2.5
3.0
3.5
4.0
Cellular_organisms - ps1
Archaea1 - ps2
Archaea2 - ps3
Archaea3 - ps4
Asgard/Eukaryota - ps5
Eukaryota - ps6
Excavata/Diaphoretickes - ps7
Diaphoretickes - ps8
Archaeplastida - ps9
Viridiplantae - ps10
Streptophyta - ps11
Charophyceae/Embriophyta - ps12
Embriophyta - ps13
Tracheophyta - ps14
Spermatophyta - ps15
Angiosperms - ps16
Mesangiospermae - ps17
Magnoliids/eudicots - ps18
Eudicots - ps19
Core_eudicots - ps20
Superrosids - ps21
Rosids - ps22
Eurosids - ps23
Malvids - ps24
Sap./Malv./Brassicales - ps25
Malvales/Brassicales - ps26
Brassicales - ps27
Cleomaceae/Brassicaceae - ps28
Brassicaceae - ps29
Camelineae - ps30
Arabidopsis - ps31
Arabidopsis_thaliana - ps32
Phylostratum

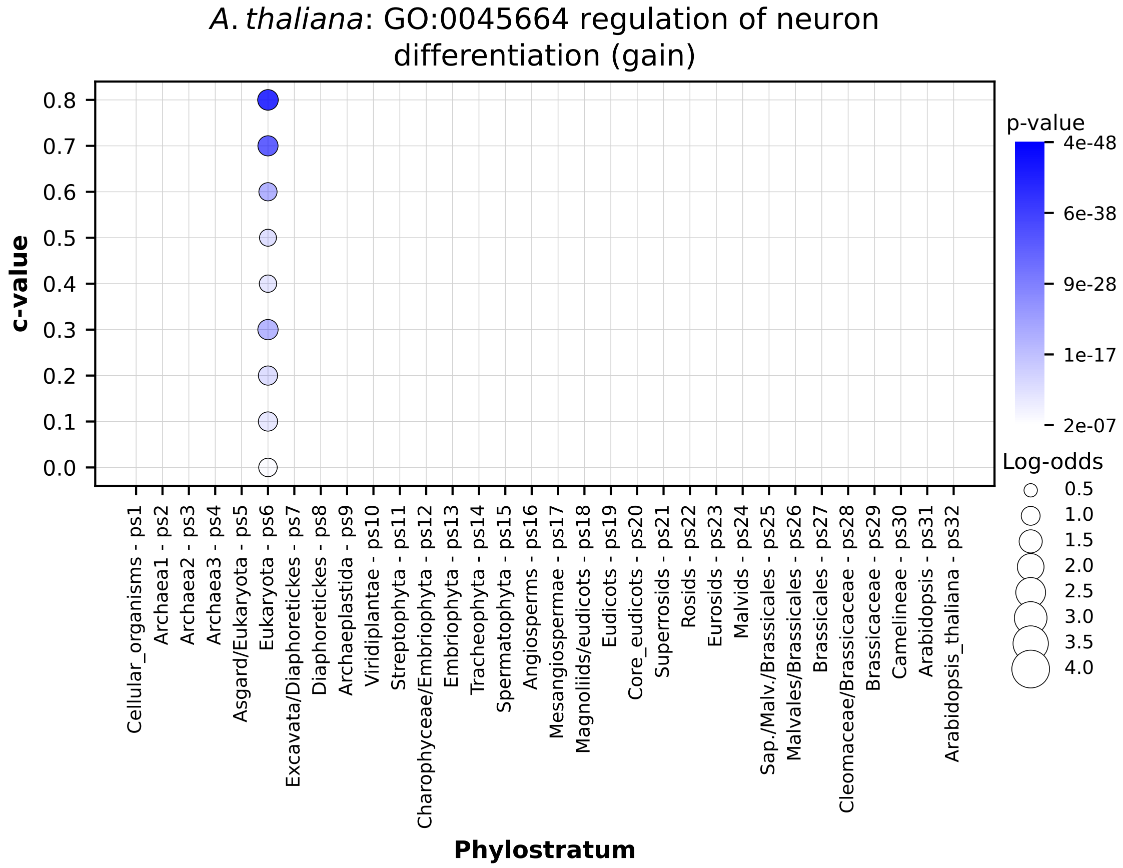

A. thaliana: GO:0045664 regulation of neuron differentiation (gain)
c-value
0.0
0.1
0.2
0.3
0.4
0.5
0.6
0.7
0.8
Phylostratum
Cellular_organisms - ps1
Archaea1 - ps2
Archaea2 - ps3
Archaea3 - ps4
Asgard/Eukaryota - ps5
Eukaryota - ps6
Excavata/Diaphoretickes - ps7
Diaphoretickes - ps8
Archaeplastida - ps9
Viridiplantae - ps10
Streptophyta - ps11
Charophyceae/Embriophyta - ps12
Embriophyta - ps13
Tracheophyta - ps14
Spermatophyta - ps15
Angiosperms - ps16
Mesangiospermae - ps17
Magnoliids/eudicots - ps18
Eudicots - ps19
Core_eudicots - ps20
Superrosids - ps21
Rosids - ps22
Eurosids - ps23
Malvids - ps24
Sap./Malv./Brassicales - ps25
Malvales/Brassicales - ps26
Brassicales - ps27
Cleomaceae/Brassicaceae - ps28
Brassicaceae - ps29
Camelineae - ps30
Arabidopsis - ps31
Arabidopsis_thaliana - ps32
p-value
4e-48
6e-38
9e-28
1e-17
2e-07
Log-odds
0.5
1.0
1.5
2.0
2.5
3.0
3.5
4.0

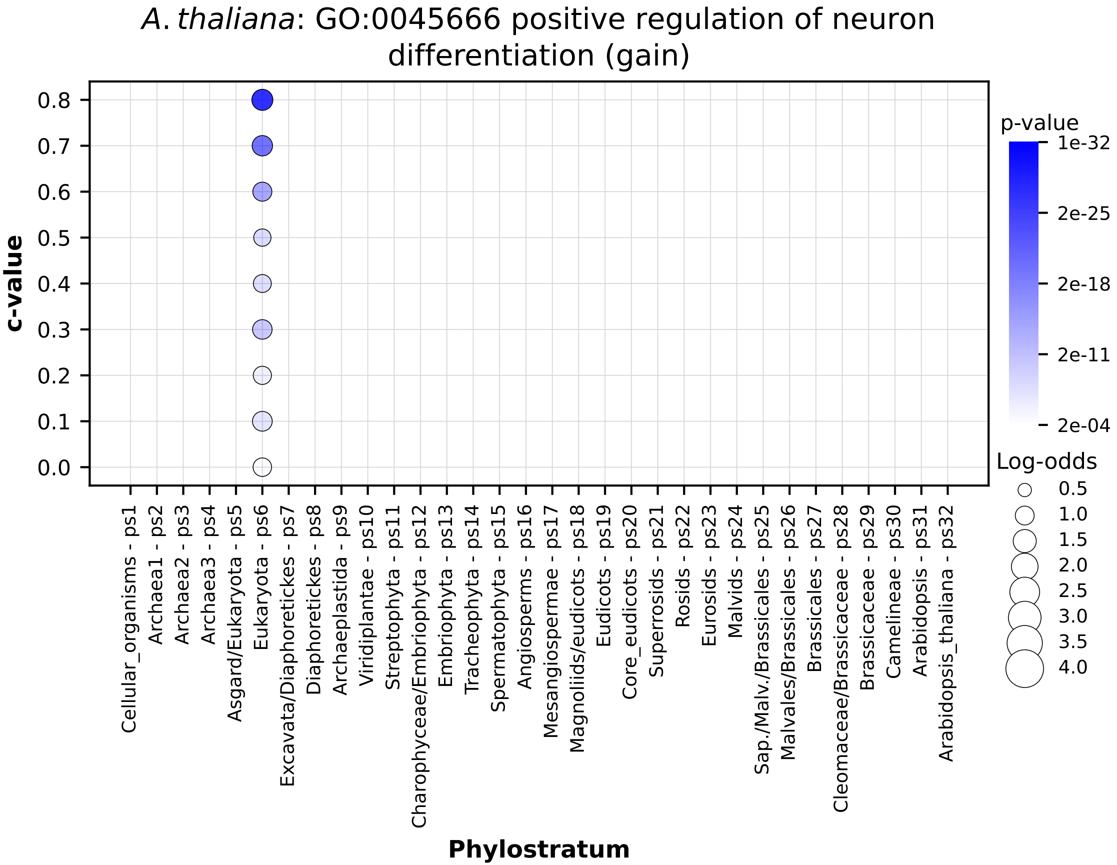

A. thaliana: GO:0045666 positive regulation of neuron differentiation (gain)
c-value
0.8
0.7
0.6
0.5
0.4
0.3
0.2
0.1
0.0
p-value
1e-32
2e-25
2e-18
2e-11
2e-04
Log-odds
0.5
1.0
1.5
2.0
2.5
3.0
3.5
4.0
Cellular_organisms - ps1
Archaea1 - ps2
Archaea2 - ps3
Archaea3 - ps4
Asgard/Eukaryota - ps5
Eukaryota - ps6
Excavata/Diaphoretickes - ps7
Diaphoretickes - ps8
Archaeplastida - ps9
Viridiplantae - ps10
Streptophyta - ps11
Charophyceae/Embriophyta - ps12
Embriophyta - ps13
Tracheophyta - ps14
Spermatophyta - ps15
Angiosperms - ps16
Mesangiospermae - ps17
Magnoliids/eudicots - ps18
Eudicots - ps19
Core_eudicots - ps20
Superrosids - ps21
Rosids - ps22
Eurosids - ps23
Malvids - ps24
Sap./Malv./Brassicales - ps25
Malvales/Brassicales - ps26
Brassicales - ps27
Cleomaceae/Brassicaceae - ps28
Brassicaceae - ps29
Camelineae - ps30
Arabidopsis - ps31
Arabidopsis_thaliana - ps32
Phylostratum

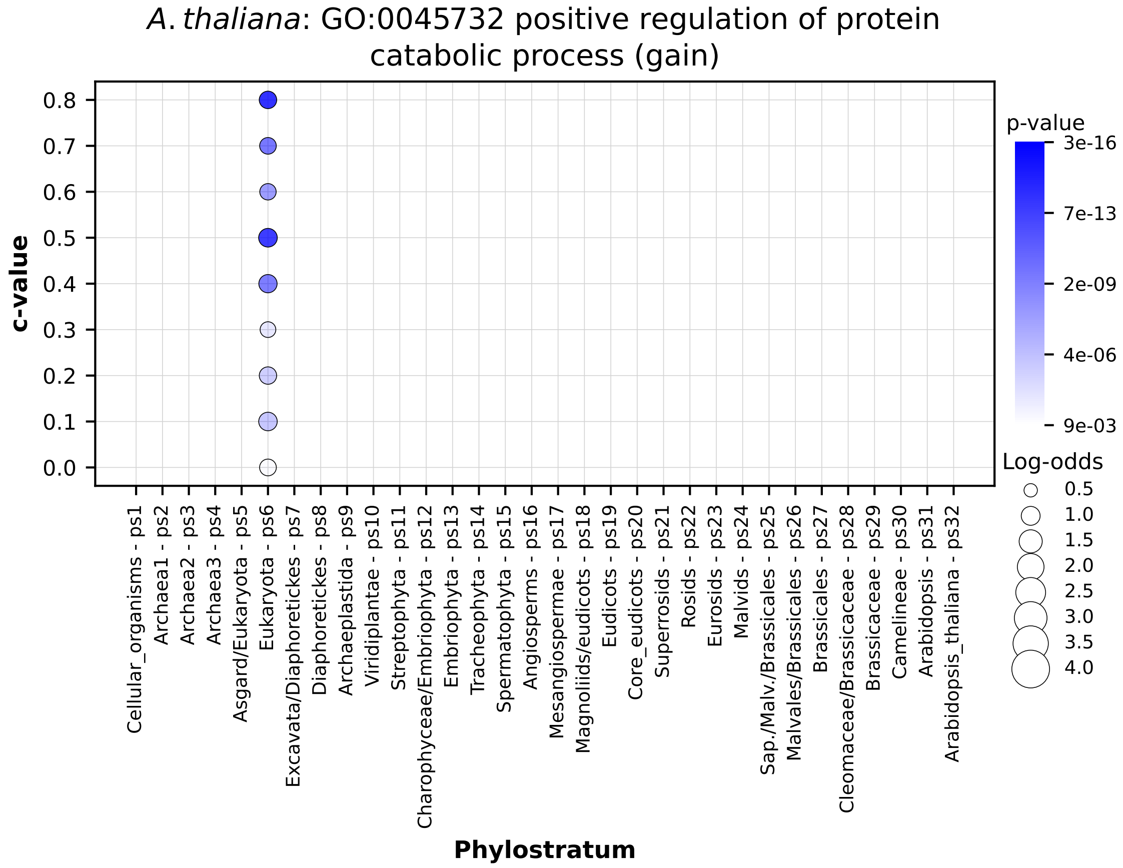

A. thaliana: GO:0045732 positive regulation of protein catabolic process (gain)
c-value
0.8
0.7
0.6
0.5
0.4
0.3
0.2
0.1
0.0
p-value
3e-16
7e-13
2e-09
4e-06
9e-03
Log-odds
0.5
1.0
1.5
2.0
2.5
3.0
3.5
4.0
Cellular_organisms - ps1
Archaea1 - ps2
Archaea2 - ps3
Archaea3 - ps4
Asgard/Eukaryota - ps5
Eukaryota - ps6
Excavata/Diaphoretickes - ps7
Diaphoretickes - ps8
Archaeplastida - ps9
Viridiplantae - ps10
Streptophyta - ps11
Charophyceae/Embriophyta - ps12
Embriophyta - ps13
Tracheophyta - ps14
Spermatophyta - ps15
Angiosperms - ps16
Mesangiospermae - ps17
Magnoliids/eudicots - ps18
Eudicots - ps19
Core_eudicots - ps20
Superrosids - ps21
Rosids - ps22
Eurosids - ps23
Malvids - ps24
Sap./Malv./Brassicales - ps25
Malvales/Brassicales - ps26
Brassicales - ps27
Cleomaceae/Brassicaceae - ps28
Brassicaceae - ps29
Camelineae - ps30
Arabidopsis - ps31
Arabidopsis_thaliana - ps32
Phylostratum

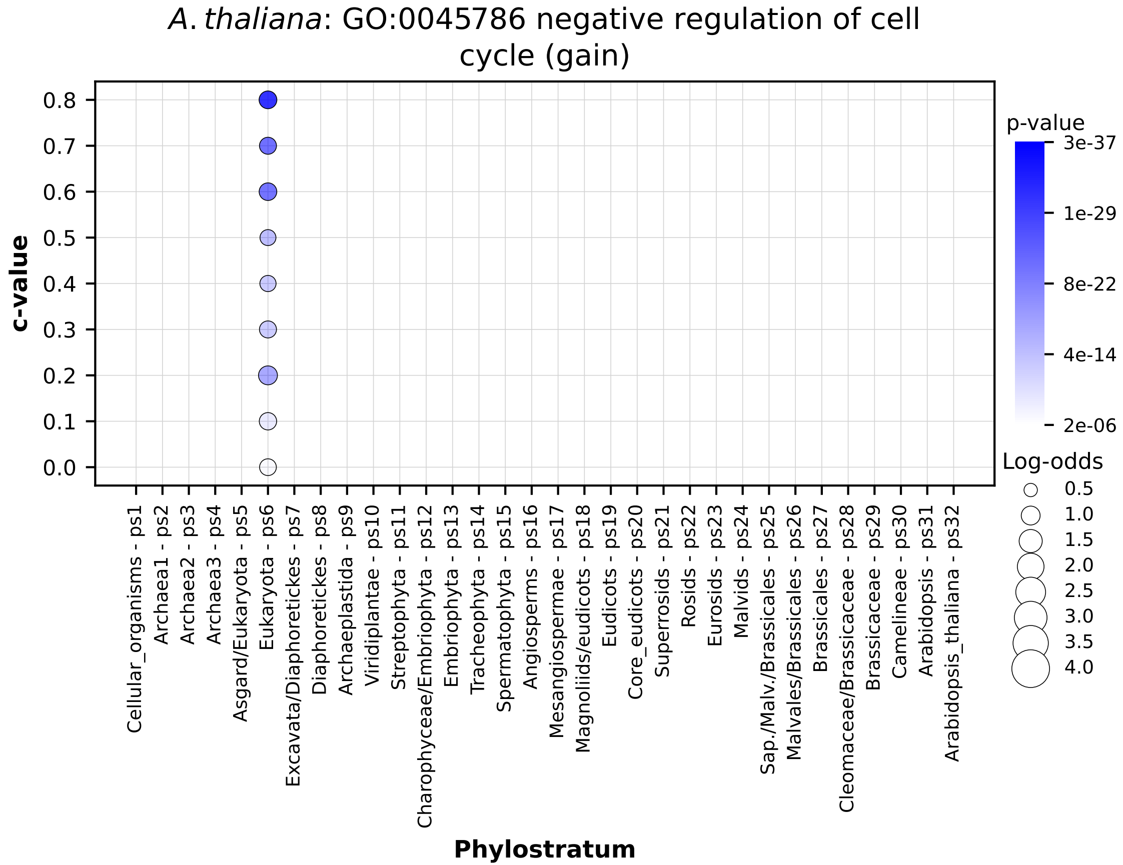
A. thaliana: GO:0045786 negative regulation of cell cycle (gain)
c-value
0.8
0.7
0.6
0.5
0.4
0.3
0.2
0.1
0.0
p-value
3e-37
1e-29
8e-22
4e-14
2e-06
Log-odds
0.5
1.0
1.5
2.0
2.5
3.0
3.5
4.0
Cellular_organisms - ps1
Archaea1 - ps2
Archaea2 - ps3
Archaea3 - ps4
Asgard/Eukaryota - ps5
Eukaryota - ps6
Excavata/Diaphoretickes - ps7
Diaphoretickes - ps8
Archaeplastida - ps9
Viridiplantae - ps10
Streptophyta - ps11
Charophyceae/Embriophyta - ps12
Embriophyta - ps13
Tracheophyta - ps14
Spermatophyta - ps15
Angiosperms - ps16
Mesangiospermae - ps17
Magnoliids/eudicots - ps18
Eudicots - ps19
Core_eudicots - ps20
Superrosids - ps21
Rosids - ps22
Eurosids - ps23
Malvids - ps24
Sap./Malv./Brassicales - ps25
Malvales/Brassicales - ps26
Brassicales - ps27
Cleomaceae/Brassicaceae - ps28
Brassicaceae - ps29
Camelineae - ps30
Arabidopsis - ps31
Arabidopsis_thaliana - ps32
Phylostratum

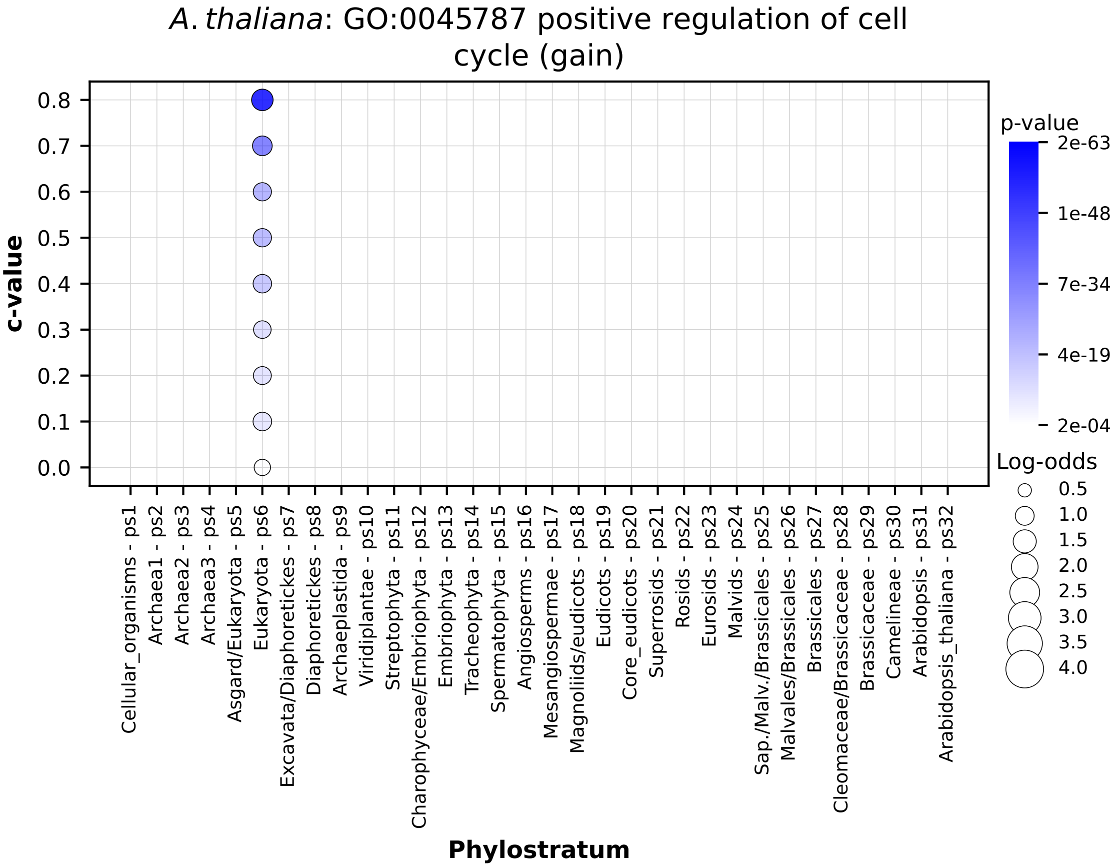

A. thaliana: GO:0045787 positive regulation of cell cycle (gain)
c-value
0.8
0.7
0.6
0.5
0.4
0.3
0.2
0.1
0.0
p-value
2e-63
1e-48
7e-34
4e-19
2e-04
Log-odds
0.5
1.0
1.5
2.0
2.5
3.0
3.5
4.0
Cellular_organisms - ps1
Archaea1 - ps2
Archaea2 - ps3
Archaea3 - ps4
Asgard/Eukaryota - ps5
Eukaryota - ps6
Excavata/Diaphoretickes - ps7
Diaphoretickes - ps8
Archaeplastida - ps9
Viridiplantae - ps10
Streptophyta - ps11
Charophyceae/Embriophyta - ps12
Embriophyta - ps13
Tracheophyta - ps14
Spermatophyta - ps15
Angiosperms - ps16
Mesangiospermae - ps17
Magnoliids/eudicots - ps18
Eudicots - ps19
Core_eudicots - ps20
Superrosids - ps21
Rosids - ps22
Eurosids - ps23
Malvids - ps24
Sap./Malv./Brassicales - ps25
Malvales/Brassicales - ps26
Brassicales - ps27
Cleomaceae/Brassicaceae - ps28
Brassicaceae - ps29
Camelineae - ps30
Arabidopsis - ps31
Arabidopsis_thaliana - ps32
Phylostratum

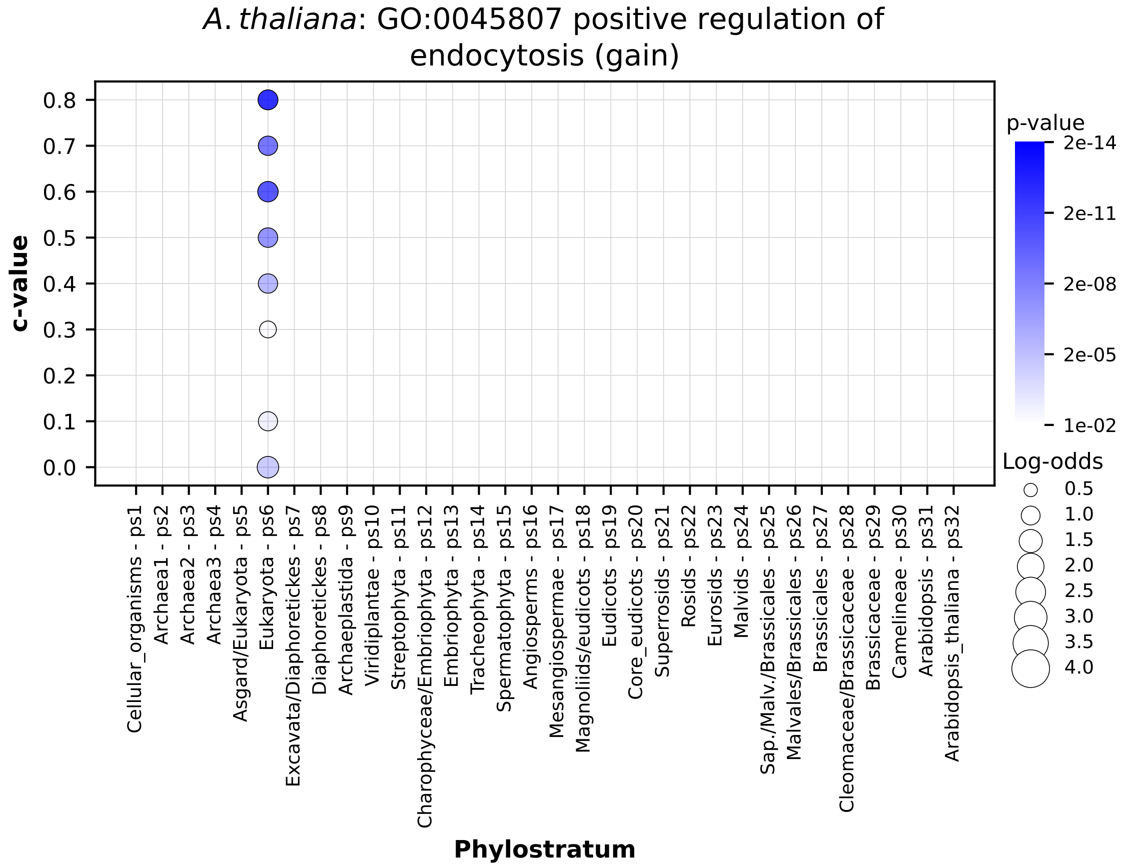

A. thaliana: GO:0045807 positive regulation of endocytosis (gain)
c-value
0.8
0.7
0.6
0.5
0.4
0.3
0.2
0.1
0.0
p-value
2e-14
2e-11
2e-08
2e-05
1e-02
Log-odds
0.5
1.0
1.5
2.0
2.5
3.0
3.5
4.0
Cellular_organisms - ps1
Archaea1 - ps2
Archaea2 - ps3
Archaea3 - ps4
Asgard/Eukaryota - ps5
Eukaryota - ps6
Excavata/Diaphoretickes - ps7
Diaphoretickes - ps8
Archaeplastida - ps9
Viridiplantae - ps10
Streptophyta - ps11
Charophyceae/Embriophyta - ps12
Embriophyta - ps13
Tracheophyta - ps14
Spermatophyta - ps15
Angiosperms - ps16
Mesangiospermae - ps17
Magnoliids/eudicots - ps18
Eudicots - ps19
Core_eudicots - ps20
Superrosids - ps21
Rosids - ps22
Eurosids - ps23
Malvids - ps24
Sap./Malv./Brassicales - ps25
Malvales/Brassicales - ps26
Brassicales - ps27
Cleomaceae/Brassicaceae - ps28
Brassicaceae - ps29
Camelineae - ps30
Arabidopsis - ps31
Arabidopsis_thaliana - ps32
Phylostratum

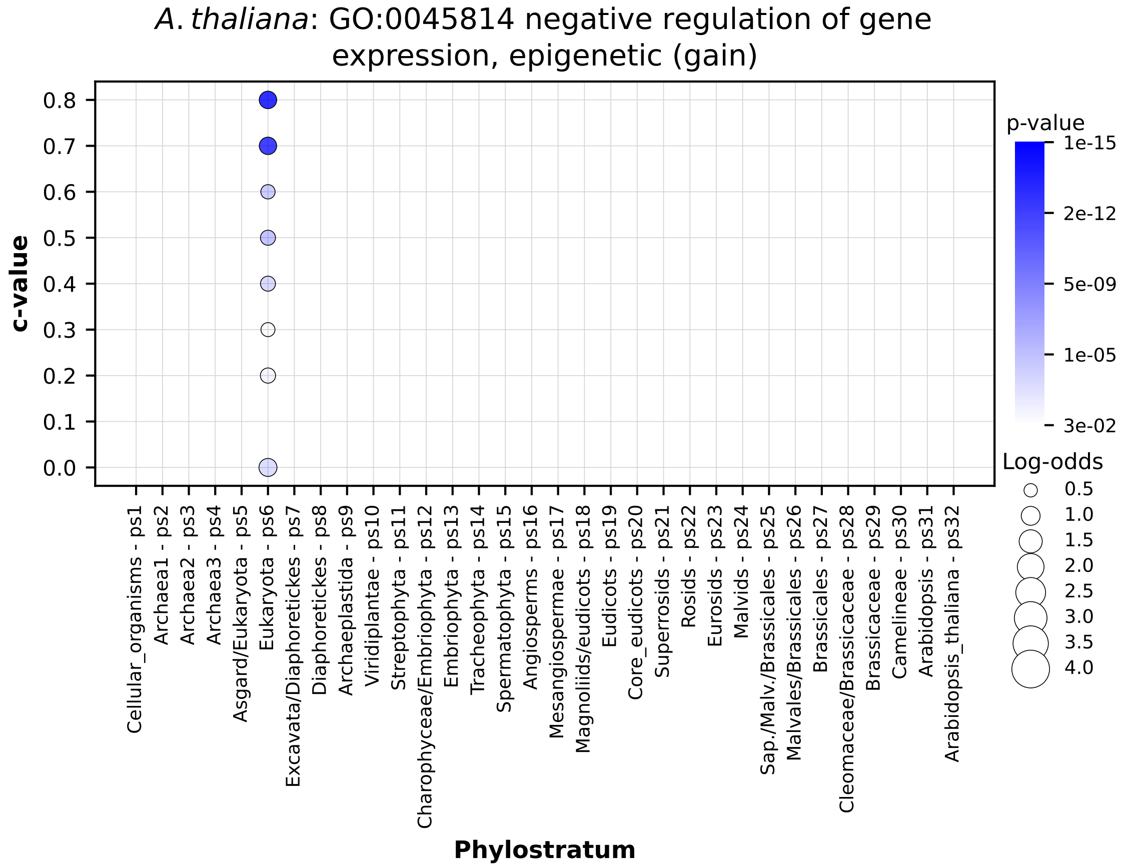
A. thaliana: GO:0045814 negative regulation of gene expression, epigenetic (gain)
c-value
0.8
0.7
0.6
0.5
0.4
0.3
0.2
0.1
0.0
p-value
1e-15
2e-12
5e-09
1e-05
3e-02
Log-odds
0.5
1.0
1.5
2.0
2.5
3.0
3.5
4.0
Cellular_organisms - ps1
Archaea1 - ps2
Archaea2 - ps3
Archaea3 - ps4
Asgard/Eukaryota - ps5
Eukaryota - ps6
Excavata/Diaphoretickes - ps7
Diaphoretickes - ps8
Archaeplastida - ps9
Viridiplantae - ps10
Streptophyta - ps11
Charophyceae/Embriophyta - ps12
Embriophyta - ps13
Tracheophyta - ps14
Spermatophyta - ps15
Angiosperms - ps16
Mesangiospermae - ps17
Magnoliids/eudicots - ps18
Eudicots - ps19
Core_eudicots - ps20
Superrosids - ps21
Rosids - ps22
Eurosids - ps23
Malvids - ps24
Sap./Malv./Brassicales - ps25
Malvales/Brassicales - ps26
Brassicales - ps27
Cleomaceae/Brassicaceae - ps28
Brassicaceae - ps29
Camelineae - ps30
Arabidopsis - ps31
Arabidopsis_thaliana - ps32
Phylostratum

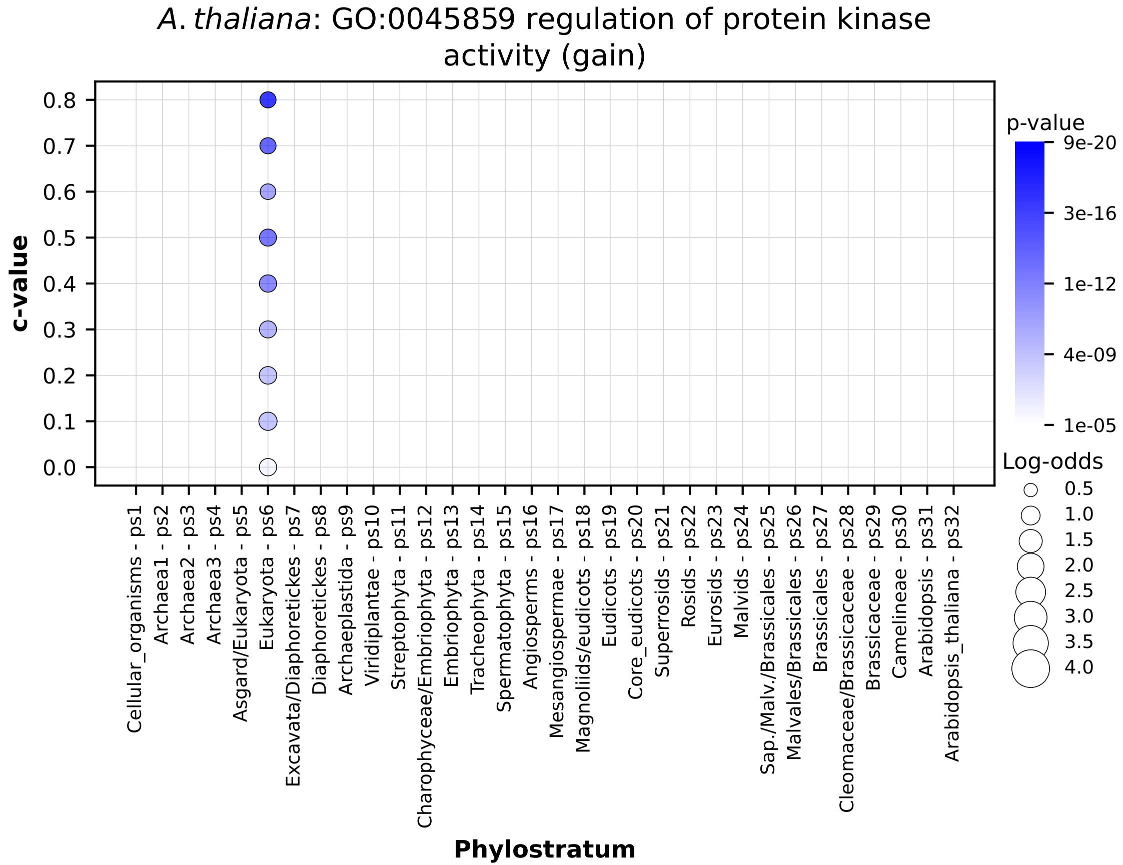

A. thaliana: GO:0045859 regulation of protein kinase activity (gain)
c-value
0.8
0.7
0.6
0.5
0.4
0.3
0.2
0.1
0.0
p-value
9e-20
3e-16
1e-12
4e-09
1e-05
Log-odds
0.5
1.0
1.5
2.0
2.5
3.0
3.5
4.0
Cellular_organisms - ps1
Archaea1 - ps2
Archaea2 - ps3
Archaea3 - ps4
Asgard/Eukaryota - ps5
Eukaryota - ps6
Excavata/Diaphoretickes - ps7
Diaphoretickes - ps8
Archaeplastida - ps9
Viridiplantae - ps10
Streptophyta - ps11
Charophyceae/Embriophyta - ps12
Embriophyta - ps13
Tracheophyta - ps14
Spermatophyta - ps15
Angiosperms - ps16
Mesangiospermae - ps17
Magnoliids/eudicots - ps18
Eudicots - ps19
Core_eudicots - ps20
Superrosids - ps21
Rosids - ps22
Eurosids - ps23
Malvids - ps24
Sap./Malv./Brassicales - ps25
Malvales/Brassicales - ps26
Brassicales - ps27
Cleomaceae/Brassicaceae - ps28
Brassicaceae - ps29
Camelineae - ps30
Arabidopsis - ps31
Arabidopsis_thaliana - ps32
Phylostratum

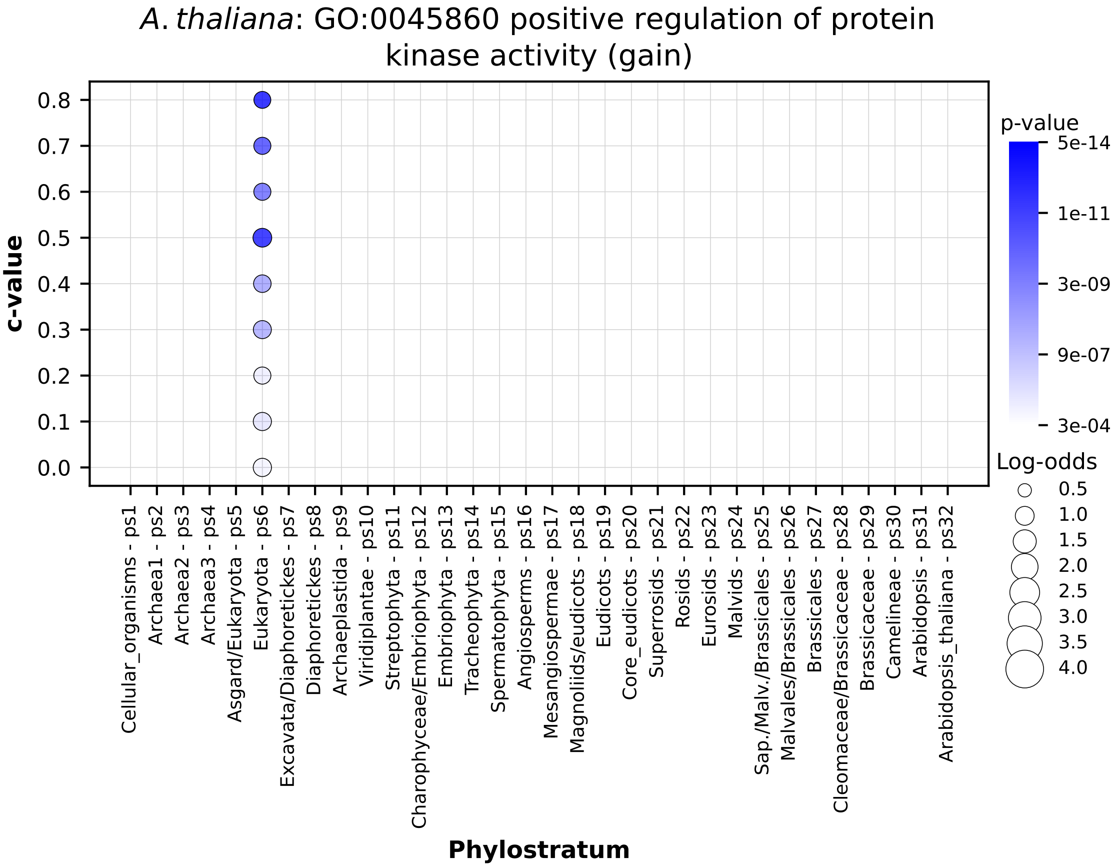

A. thaliana: GO:0045860 positive regulation of protein kinase activity (gain)
c-value
0.8
0.7
0.6
0.5
0.4
0.3
0.2
0.1
0.0
p-value
5e-14
1e-11
3e-09
9e-07
3e-04
Log-odds
0.5
1.0
1.5
2.0
2.5
3.0
3.5
4.0
Cellular_organisms - ps1
Archaea1 - ps2
Archaea2 - ps3
Archaea3 - ps4
Asgard/Eukaryota - ps5
Eukaryota - ps6
Excavata/Diaphoretickes - ps7
Diaphoretickes - ps8
Archaeplastida - ps9
Viridiplantae - ps10
Streptophyta - ps11
Charophyceae/Embriophyta - ps12
Embriophyta - ps13
Tracheophyta - ps14
Spermatophyta - ps15
Angiosperms - ps16
Mesangiospermae - ps17
Magnoliids/eudicots - ps18
Eudicots - ps19
Core_eudicots - ps20
Superrosids - ps21
Rosids - ps22
Eurosids - ps23
Malvids - ps24
Sap./Malv./Brassicales - ps25
Malvales/Brassicales - ps26
Brassicales - ps27
Cleomaceae/Brassicaceae - ps28
Brassicaceae - ps29
Camelineae - ps30
Arabidopsis - ps31
Arabidopsis_thaliana - ps32
Phylostratum

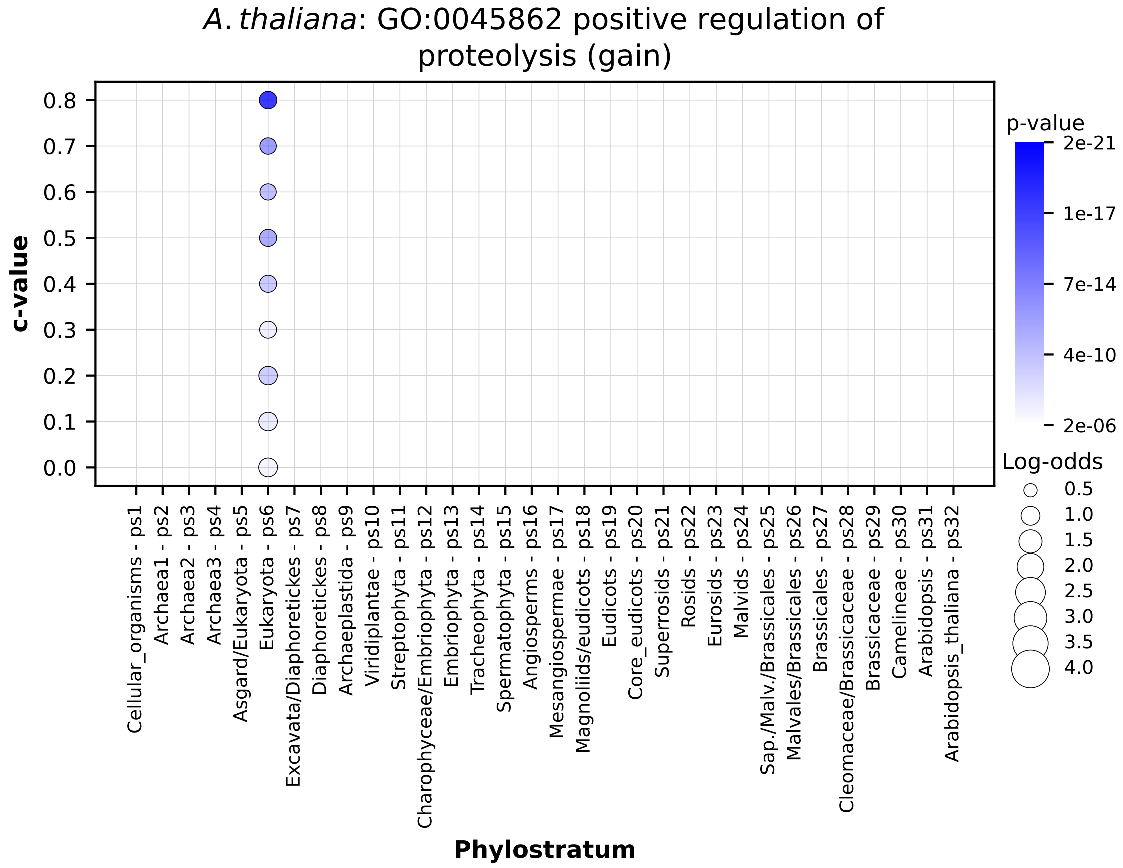

A. thaliana: GO:0045862 positive regulation of proteolysis (gain)
c-value
0.8
0.7
0.6
0.5
0.4
0.3
0.2
0.1
0.0
p-value
2e-21
1e-17
7e-14
4e-10
2e-06
Log-odds
0.5
1.0
1.5
2.0
2.5
3.0
3.5
4.0
Cellular_organisms - ps1
Archaea1 - ps2
Archaea2 - ps3
Archaea3 - ps4
Asgard/Eukaryota - ps5
Eukaryota - ps6
Excavata/Diaphoretickes - ps7
Diaphoretickes - ps8
Archaeplastida - ps9
Viridiplantae - ps10
Streptophyta - ps11
Charophyceae/Embriophyta - ps12
Embriophyta - ps13
Tracheophyta - ps14
Spermatophyta - ps15
Angiosperms - ps16
Mesangiospermae - ps17
Magnoliids/eudicots - ps18
Eudicots - ps19
Core_eudicots - ps20
Superrosids - ps21
Rosids - ps22
Eurosids - ps23
Malvids - ps24
Sap./Malv./Brassicales - ps25
Malvales/Brassicales - ps26
Brassicales - ps27
Cleomaceae/Brassicaceae - ps28
Brassicaceae - ps29
Camelineae - ps30
Arabidopsis - ps31
Arabidopsis_thaliana - ps32
Phylostratum

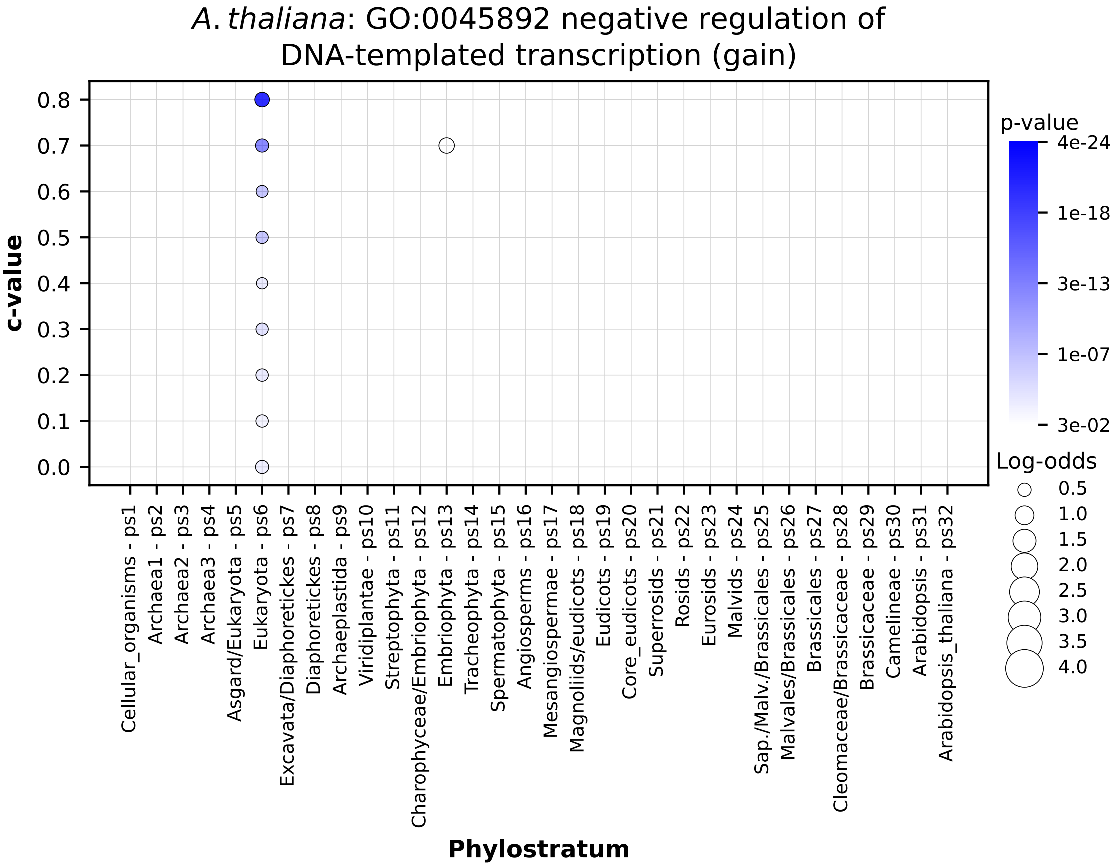

A. thaliana: GO:0045892 negative regulation of DNA-templated transcription (gain)
c-value
0.8
0.7
0.6
0.5
0.4
0.3
0.2
0.1
0.0
Phylostratum
Cellular_organisms - ps1
Archaea1 - ps2
Archaea2 - ps3
Archaea3 - ps4
Asgard/Eukaryota - ps5
Eukaryota - ps6
Excavata/Diaphoretickes - ps7
Diaphoretickes - ps8
Archaeplastida - ps9
Viridiplantae - ps10
Streptophyta - ps11
Charophyceae/Embriophyta - ps12
Embriophyta - ps13
Tracheophyta - ps14
Spermatophyta - ps15
Angiosperms - ps16
Mesangiospermae - ps17
Magnoliids/eudicots - ps18
Eudicots - ps19
Core_eudicots - ps20
Superrosids - ps21
Rosids - ps22
Eurosids - ps23
Malvids - ps24
Sap./Malv./Brassicales - ps25
Malvales/Brassicales - ps26
Brassicales - ps27
Cleomaceae/Brassicaceae - ps28
Brassicaceae - ps29
Camelineae - ps30
Arabidopsis - ps31
Arabidopsis_thaliana - ps32
p-value
4e-24
1e-18
3e-13
1e-07
3e-02
Log-odds
0.5
1.0
1.5
2.0
2.5
3.0
3.5
4.0

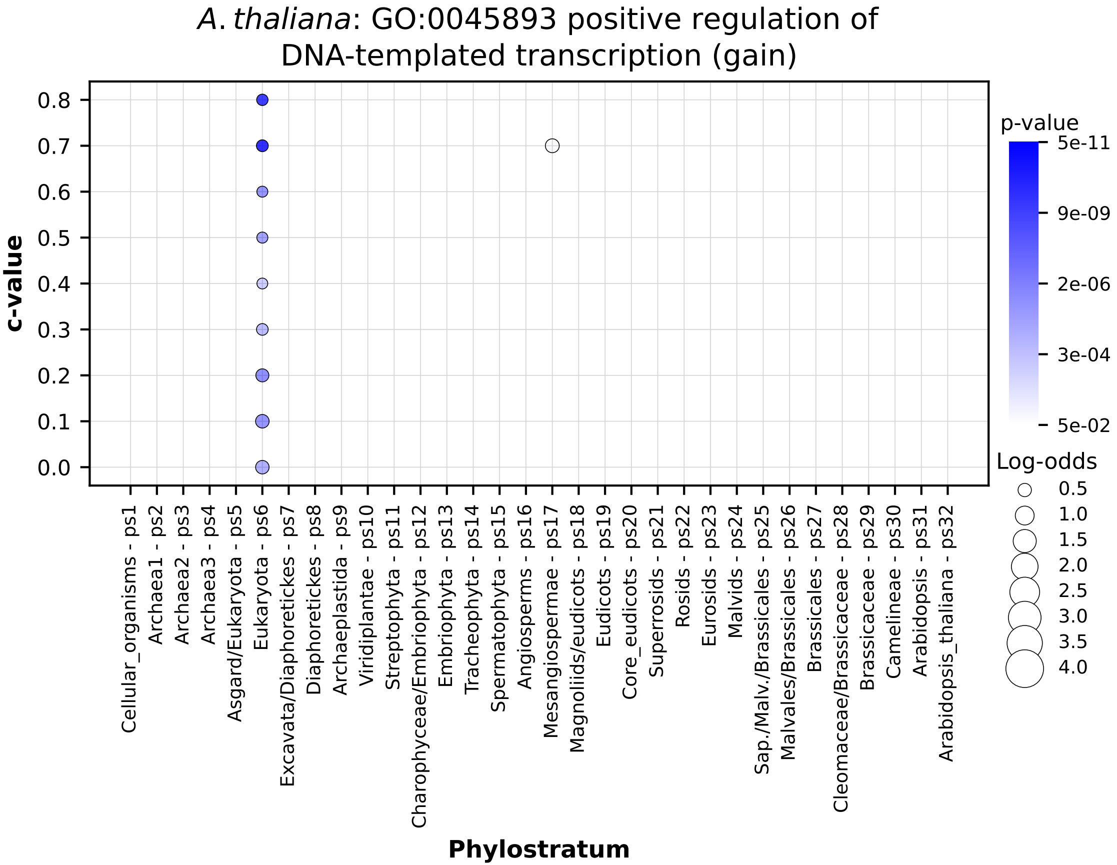

A. thaliana: GO:0045893 positive regulation of DNA-templated transcription (gain)
c-value
0.8
0.7
0.6
0.5
0.4
0.3
0.2
0.1
0.0
Phylostratum
Cellular_organisms - ps1
Archaea1 - ps2
Archaea2 - ps3
Archaea3 - ps4
Asgard/Eukaryota - ps5
Eukaryota - ps6
Excavata/Diaphoretickes - ps7
Diaphoretickes - ps8
Archaeplastida - ps9
Viridiplantae - ps10
Streptophyta - ps11
Charophyceae/Embriophyta - ps12
Embriophyta - ps13
Tracheophyta - ps14
Spermatophyta - ps15
Angiosperms - ps16
Mesangiospermae - ps17
Magnoliids/eudicots - ps18
Eudicots - ps19
Core_eudicots - ps20
Superrosids - ps21
Rosids - ps22
Eurosids - ps23
Malvids - ps24
Sap./Malv./Brassicales - ps25
Malvales/Brassicales - ps26
Brassicales - ps27
Cleomaceae/Brassicaceae - ps28
Brassicaceae - ps29
Camelineae - ps30
Arabidopsis - ps31
Arabidopsis_thaliana - ps32
p-value
5e-11
9e-09
2e-06
3e-04
5e-02
Log-odds
0.5
1.0
1.5
2.0
2.5
3.0
3.5
4.0

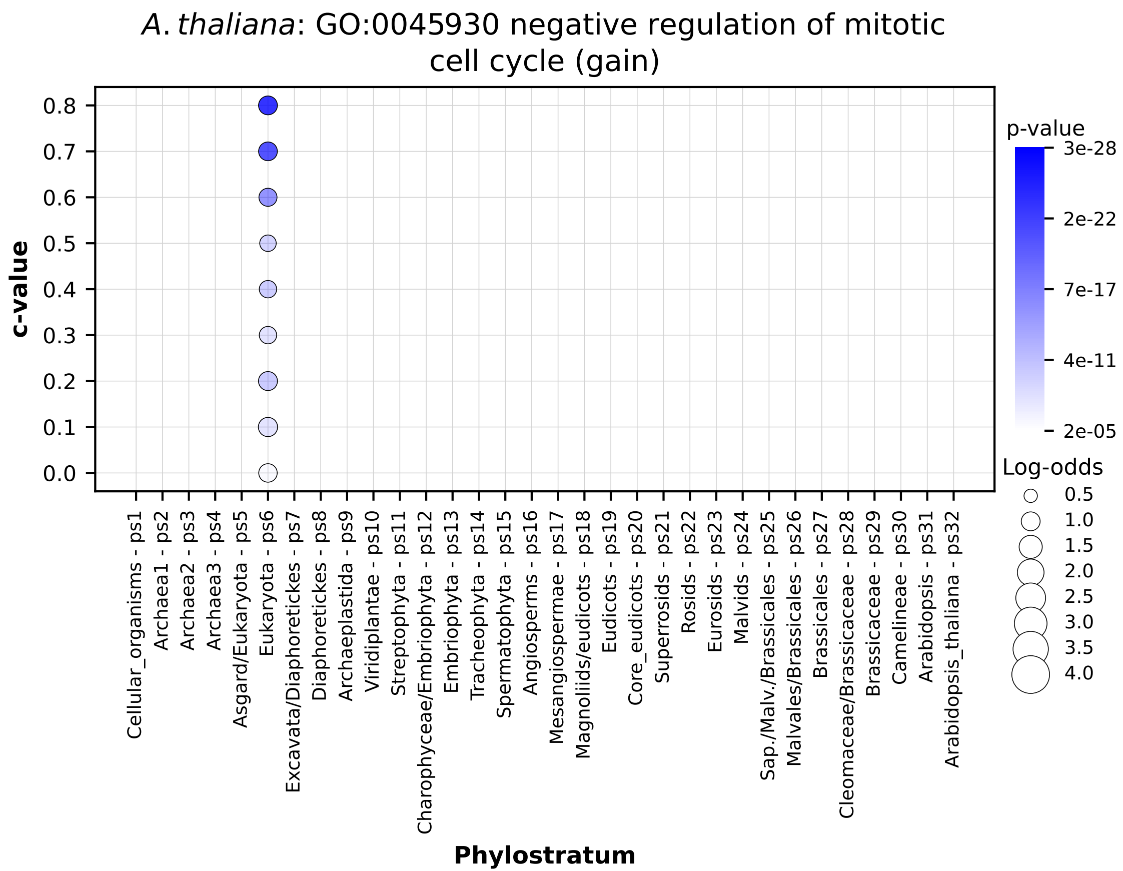

A. thaliana: GO:0045930 negative regulation of mitotic cell cycle (gain)
c-value
0.8
0.7
0.6
0.5
0.4
0.3
0.2
0.1
0.0
p-value
3e-28
2e-22
7e-17
4e-11
2e-05
Log-odds
0.5
1.0
1.5
2.0
2.5
3.0
3.5
4.0
Cellular_organisms - ps1
Archaea1 - ps2
Archaea2 - ps3
Archaea3 - ps4
Asgard/Eukaryota - ps5
Eukaryota - ps6
Excavata/Diaphoretickes - ps7
Diaphoretickes - ps8
Archaeplastida - ps9
Viridiplantae - ps10
Streptophyta - ps11
Charophyceae/Embriophyta - ps12
Embriophyta - ps13
Tracheophyta - ps14
Spermatophyta - ps15
Angiosperms - ps16
Mesangiospermae - ps17
Magnoliids/eudicots - ps18
Eudicots - ps19
Core_eudicots - ps20
Superrosids - ps21
Rosids - ps22
Eurosids - ps23
Malvids - ps24
Sap./Malv./Brassicales - ps25
Malvales/Brassicales - ps26
Brassicales - ps27
Cleomaceae/Brassicaceae - ps28
Brassicaceae - ps29
Camelineae - ps30
Arabidopsis - ps31
Arabidopsis_thaliana - ps32
Phylostratum

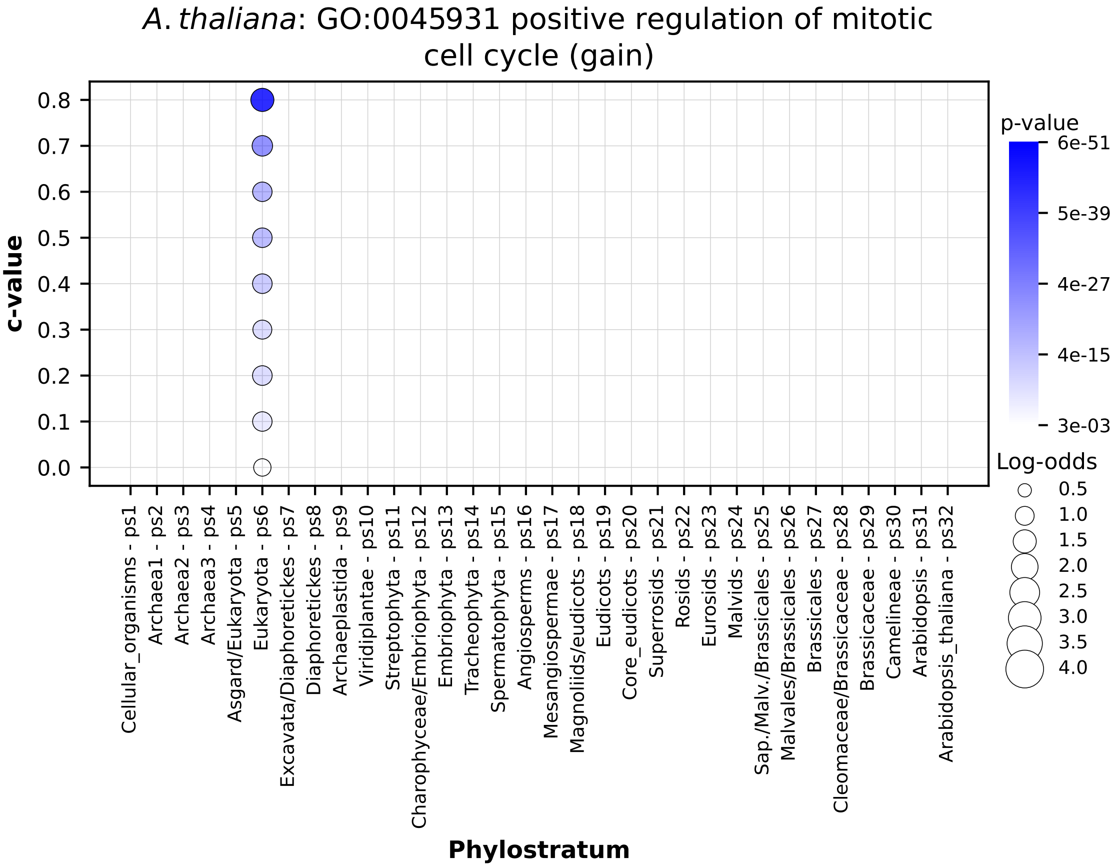
A. thaliana: GO:0045931 positive regulation of mitotic cell cycle (gain)
c-value
0.0
0.1
0.2
0.3
0.4
0.5
0.6
0.7
0.8
Phylostratum
Cellular_organisms - ps1
Archaea1 - ps2
Archaea2 - ps3
Archaea3 - ps4
Asgard/Eukaryota - ps5
Eukaryota - ps6
Excavata/Diaphoretickes - ps7
Diaphoretickes - ps8
Archaeplastida - ps9
Viridiplantae - ps10
Streptophyta - ps11
Charophyceae/Embriophyta - ps12
Embriophyta - ps13
Tracheophyta - ps14
Spermatophyta - ps15
Angiosperms - ps16
Mesangiospermae - ps17
Magnoliids/eudicots - ps18
Eudicots - ps19
Core_eudicots - ps20
Superrosids - ps21
Rosids - ps22
Eurosids - ps23
Malvids - ps24
Sap./Malv./Brassicales - ps25
Malvales/Brassicales - ps26
Brassicales - ps27
Cleomaceae/Brassicaceae - ps28
Brassicaceae - ps29
Camelineae - ps30
Arabidopsis - ps31
Arabidopsis_thaliana - ps32
p-value
6e-51
5e-39
4e-27
4e-15
3e-03
Log-odds
0.5
1.0
1.5
2.0
2.5
3.0
3.5
4.0

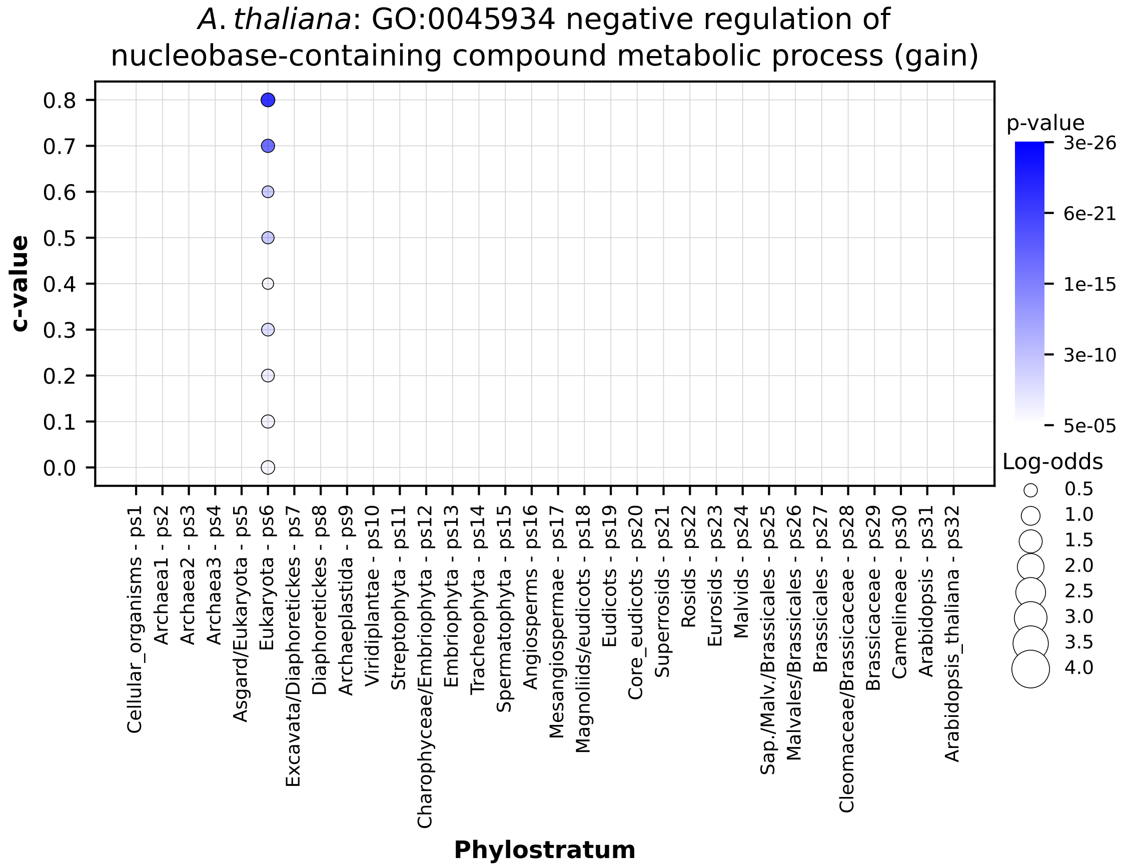

A. thaliana: GO:0045934 negative regulation of nucleobase-containing compound metabolic process (gain)
c-value
0.0
0.1
0.2
0.3
0.4
0.5
0.6
0.7
0.8
Phylostratum
Cellular_organisms - ps1
Archaea1 - ps2
Archaea2 - ps3
Archaea3 - ps4
Asgard/Eukaryota - ps5
Eukaryota - ps6
Excavata/Diaphoretickes - ps7
Diaphoretickes - ps8
Archaeplastida - ps9
Viridiplantae - ps10
Streptophyta - ps11
Charophyceae/Embriophyta - ps12
Embriophyta - ps13
Tracheophyta - ps14
Spermatophyta - ps15
Angiosperms - ps16
Mesangiospermae - ps17
Magnoliids/eudicots - ps18
Eudicots - ps19
Core_eudicots - ps20
Superrosids - ps21
Rosids - ps22
Eurosids - ps23
Malvids - ps24
Sap./Malv./Brassicales - ps25
Malvales/Brassicales - ps26
Brassicales - ps27
Cleomaceae/Brassicaceae - ps28
Brassicaceae - ps29
Camelineae - ps30
Arabidopsis - ps31
Arabidopsis_thaliana - ps32
p-value
3e-26
6e-21
1e-15
3e-10
5e-05
Log-odds
0.5
1.0
1.5
2.0
2.5
3.0
3.5
4.0

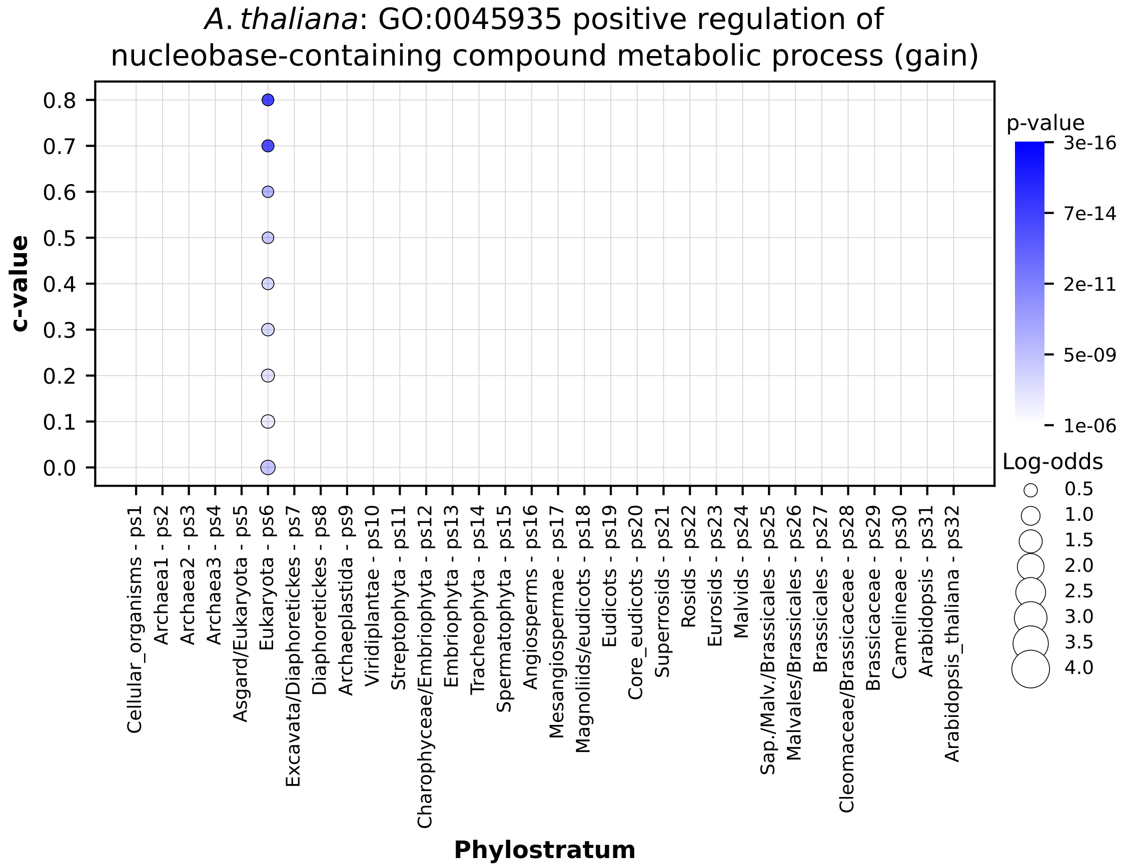

A. thaliana: GO:0045935 positive regulation of nucleobase-containing compound metabolic process (gain)
c-value
0.8
0.7
0.6
0.5
0.4
0.3
0.2
0.1
0.0
p-value
3e-16
7e-14
2e-11
5e-09
1e-06
Log-odds
0.5
1.0
1.5
2.0
2.5
3.0
3.5
4.0
Cellular_organisms - ps1
Archaea1 - ps2
Archaea2 - ps3
Archaea3 - ps4
Asgard/Eukaryota - ps5
Eukaryota - ps6
Excavata/Diaphoretickes - ps7
Diaphoretickes - ps8
Archaeplastida - ps9
Viridiplantae - ps10
Streptophyta - ps11
Charophyceae/Embriophyta - ps12
Embriophyta - ps13
Tracheophyta - ps14
Spermatophyta - ps15
Angiosperms - ps16
Mesangiospermae - ps17
Magnoliids/eudicots - ps18
Eudicots - ps19
Core_eudicots - ps20
Superrosids - ps21
Rosids - ps22
Eurosids - ps23
Malvids - ps24
Sap./Malv./Brassicales - ps25
Malvales/Brassicales - ps26
Brassicales - ps27
Cleomaceae/Brassicaceae - ps28
Brassicaceae - ps29
Camelineae - ps30
Arabidopsis - ps31
Arabidopsis_thaliana - ps32
Phylostratum

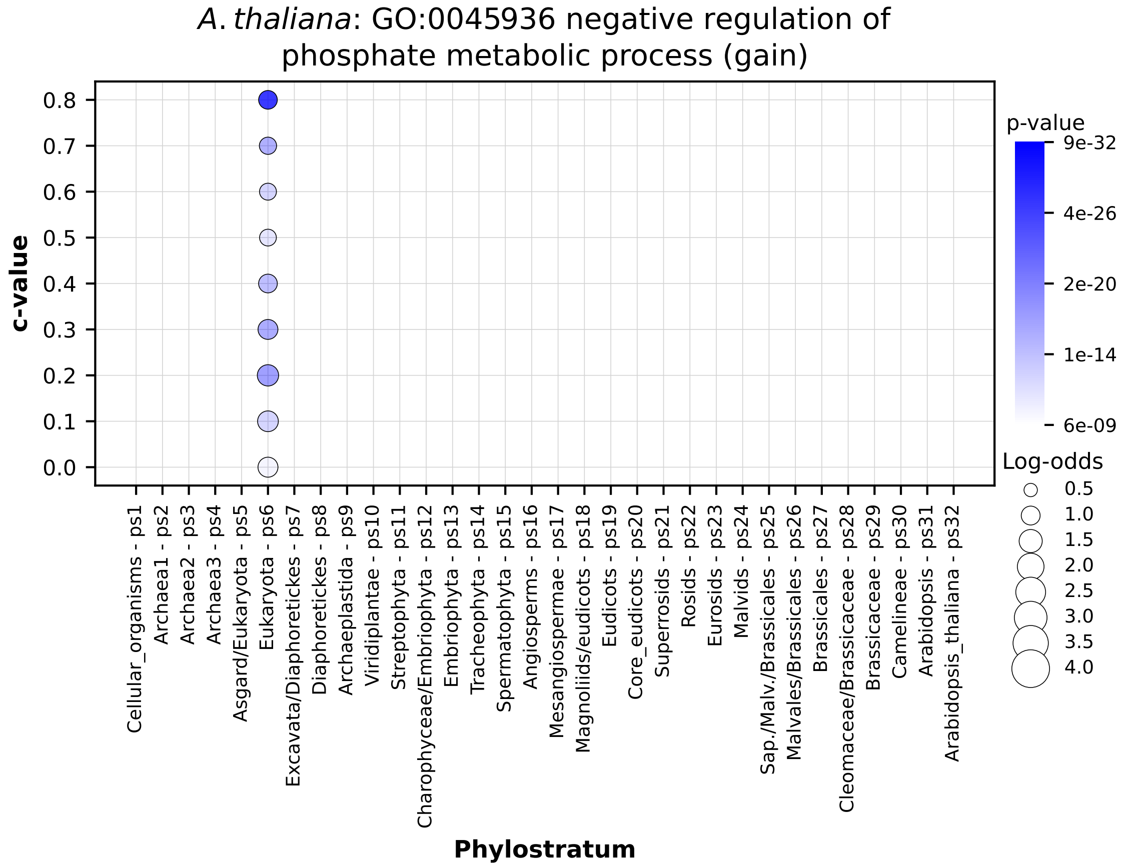

A. thaliana: GO:0045936 negative regulation of phosphate metabolic process (gain)
c-value
0.8
0.7
0.6
0.5
0.4
0.3
0.2
0.1
0.0
p-value
9e-32
4e-26
2e-20
1e-14
6e-09
Log-odds
0.5
1.0
1.5
2.0
2.5
3.0
3.5
4.0
Cellular_organisms - ps1
Archaea1 - ps2
Archaea2 - ps3
Archaea3 - ps4
Asgard/Eukaryota - ps5
Eukaryota - ps6
Excavata/Diaphoretickes - ps7
Diaphoretickes - ps8
Archaeplastida - ps9
Viridiplantae - ps10
Streptophyta - ps11
Charophyceae/Embriophyta - ps12
Embriophyta - ps13
Tracheophyta - ps14
Spermatophyta - ps15
Angiosperms - ps16
Mesangiospermae - ps17
Magnoliids/eudicots - ps18
Eudicots - ps19
Core_eudicots - ps20
Superrosids - ps21
Rosids - ps22
Eurosids - ps23
Malvids - ps24
Sap./Malv./Brassicales - ps25
Malvales/Brassicales - ps26
Brassicales - ps27
Cleomaceae/Brassicaceae - ps28
Brassicaceae - ps29
Camelineae - ps30
Arabidopsis - ps31
Arabidopsis_thaliana - ps32
Phylostratum

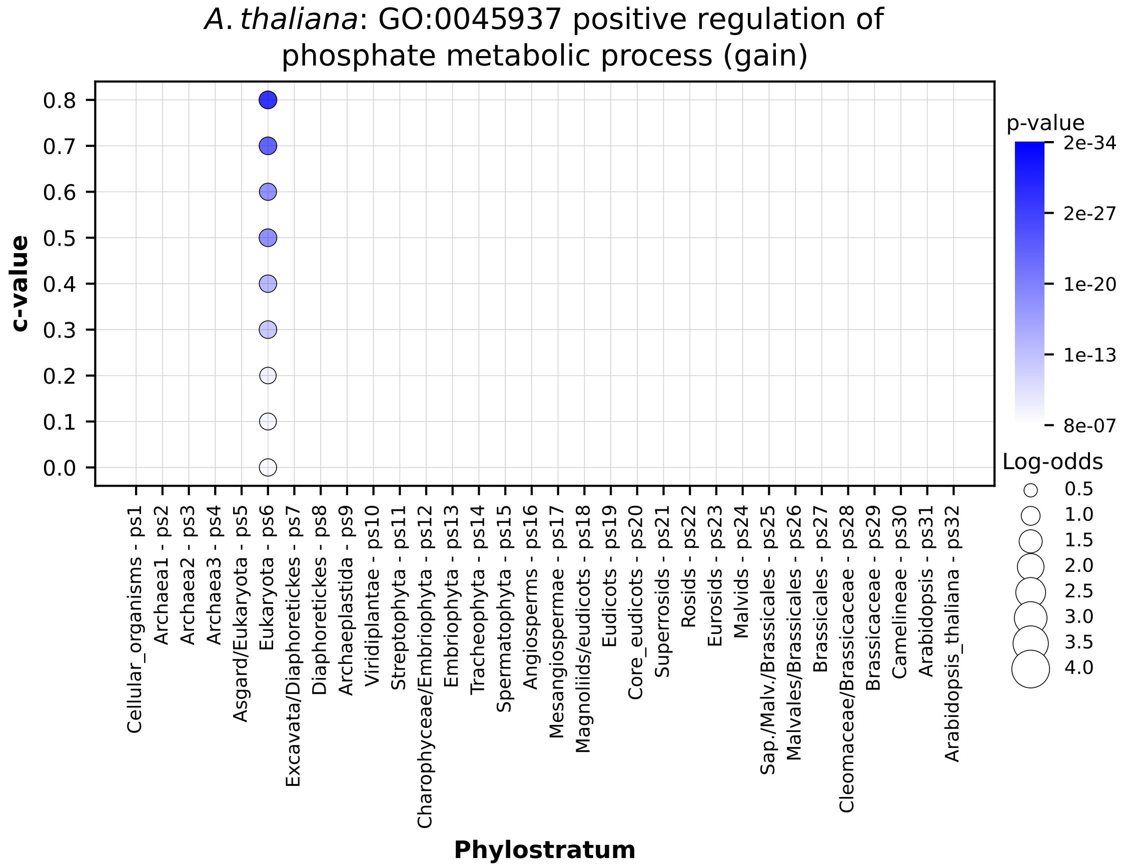

A. thaliana: GO:0045937 positive regulation of phosphate metabolic process (gain)
c-value
0.8
0.7
0.6
0.5
0.4
0.3
0.2
0.1
0.0
p-value
2e-34
2e-27
1e-20
1e-13
8e-07
Log-odds
0.5
1.0
1.5
2.0
2.5
3.0
3.5
4.0
Cellular_organisms - ps1
Archaea1 - ps2
Archaea2 - ps3
Archaea3 - ps4
Asgard/Eukaryota - ps5
Eukaryota - ps6
Excavata/Diaphoretickes - ps7
Diaphoretickes - ps8
Archaeplastida - ps9
Viridiplantae - ps10
Streptophyta - ps11
Charophyceae/Embriophyta - ps12
Embriophyta - ps13
Tracheophyta - ps14
Spermatophyta - ps15
Angiosperms - ps16
Mesangiospermae - ps17
Magnoliids/eudicots - ps18
Eudicots - ps19
Core_eudicots - ps20
Superrosids - ps21
Rosids - ps22
Eurosids - ps23
Malvids - ps24
Sap./Malv./Brassicales - ps25
Malvales/Brassicales - ps26
Brassicales - ps27
Cleomaceae/Brassicaceae - ps28
Brassicaceae - ps29
Camelineae - ps30
Arabidopsis - ps31
Arabidopsis_thaliana - ps32
Phylostratum

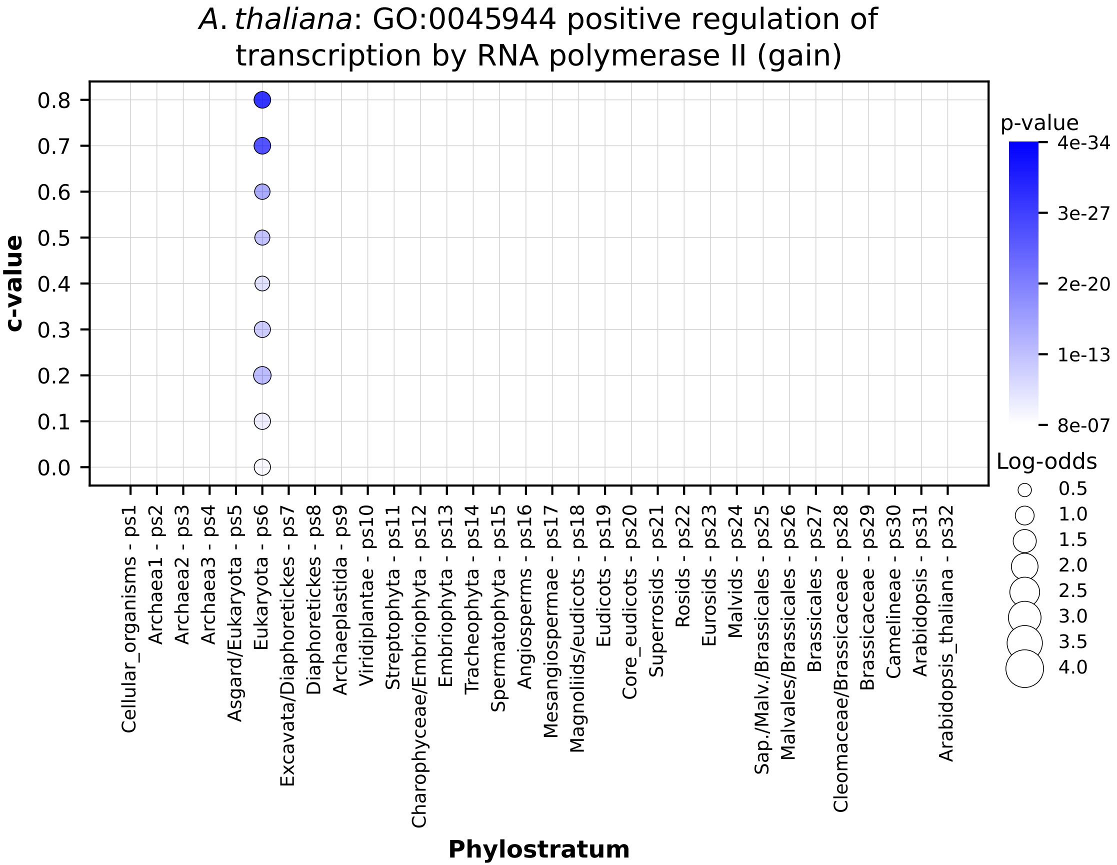

A. thaliana: GO:0045944 positive regulation of transcription by RNA polymerase II (gain)
c-value
0.8
0.7
0.6
0.5
0.4
0.3
0.2
0.1
0.0
Phylostratum
Cellular_organisms - ps1
Archaea1 - ps2
Archaea2 - ps3
Archaea3 - ps4
Asgard/Eukaryota - ps5
Eukaryota - ps6
Excavata/Diaphoretickes - ps7
Diaphoretickes - ps8
Archaeplastida - ps9
Viridiplantae - ps10
Streptophyta - ps11
Charophyceae/Embriophyta - ps12
Embriophyta - ps13
Tracheophyta - ps14
Spermatophyta - ps15
Angiosperms - ps16
Mesangiospermae - ps17
Magnoliids/eudicots - ps18
Eudicots - ps19
Core_eudicots - ps20
Superrosids - ps21
Rosids - ps22
Eurosids - ps23
Malvids - ps24
Sap./Malv./Brassicales - ps25
Malvales/Brassicales - ps26
Brassicales - ps27
Cleomaceae/Brassicaceae - ps28
Brassicaceae - ps29
Camelineae - ps30
Arabidopsis - ps31
Arabidopsis_thaliana - ps32
p-value
4e-34
3e-27
2e-20
1e-13
8e-07
Log-odds
0.5
1.0
1.5
2.0
2.5
3.0
3.5
4.0

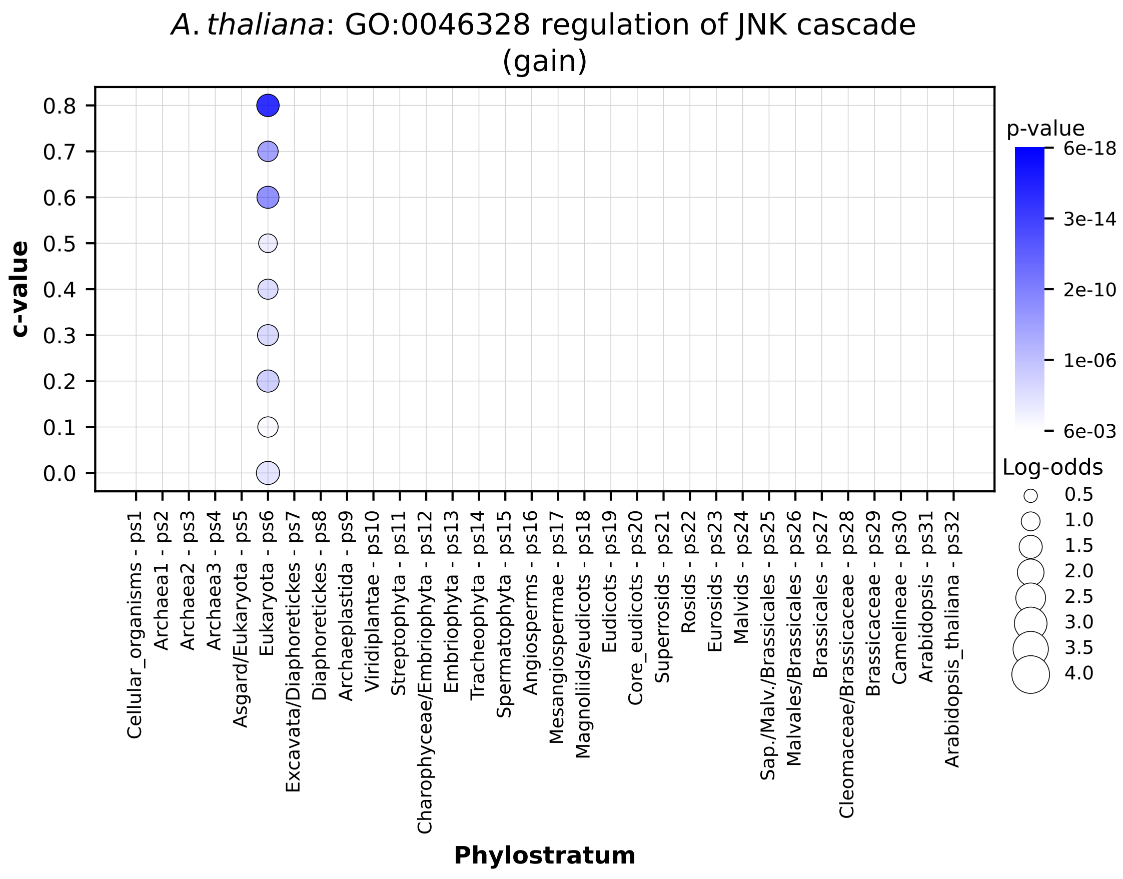

A. thaliana: GO:0046328 regulation of JNK cascade
(gain)
c-value
0.8
0.7
0.6
0.5
0.4
0.3
0.2
0.1
0.0
p-value
6e-18
3e-14
2e-10
1e-06
6e-03
Log-odds
0.5
1.0
1.5
2.0
2.5
3.0
3.5
4.0
Cellular_organisms - ps1
Archaea1 - ps2
Archaea2 - ps3
Archaea3 - ps4
Asgard/Eukaryota - ps5
Eukaryota - ps6
Excavata/Diaphoretickes - ps7
Diaphoretickes - ps8
Archaeplastida - ps9
Viridiplantae - ps10
Streptophyta - ps11
Charophyceae/Embriophyta - ps12
Embriophyta - ps13
Tracheophyta - ps14
Spermatophyta - ps15
Angiosperms - ps16
Mesangiospermae - ps17
Magnoliids/eudicots - ps18
Eudicots - ps19
Core_eudicots - ps20
Superrosids - ps21
Rosids - ps22
Eurosids - ps23
Malvids - ps24
Sap./Malv./Brassicales - ps25
Malvales/Brassicales - ps26
Brassicales - ps27
Cleomaceae/Brassicaceae - ps28
Brassicaceae - ps29
Camelineae - ps30
Arabidopsis - ps31
Arabidopsis_thaliana - ps32
Phylostratum

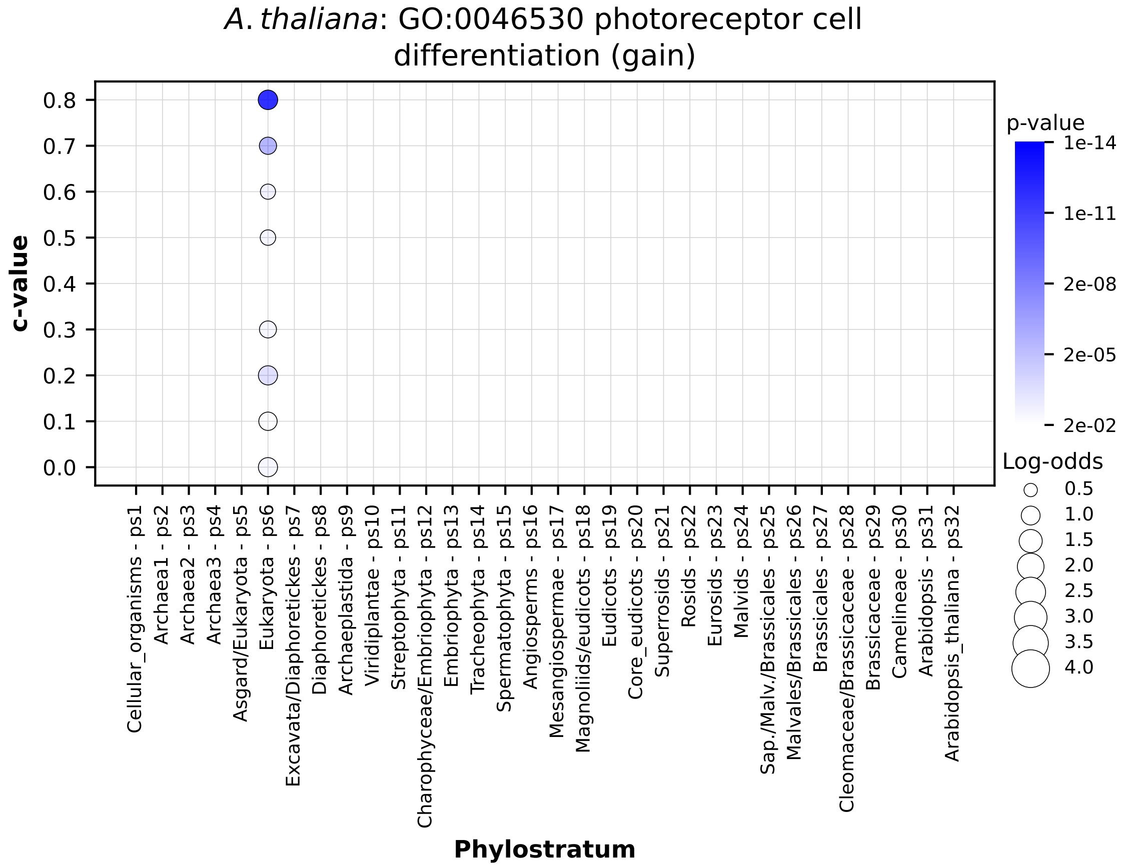

A. thaliana: GO:0046530 photoreceptor cell differentiation (gain)
c-value
0.8
0.7
0.6
0.5
0.4
0.3
0.2
0.1
0.0
p-value
1e-14
1e-11
2e-08
2e-05
2e-02
Log-odds
0.5
1.0
1.5
2.0
2.5
3.0
3.5
4.0
Cellular_organisms - ps1
Archaea1 - ps2
Archaea2 - ps3
Archaea3 - ps4
Asgard/Eukaryota - ps5
Eukaryota - ps6
Excavata/Diaphoretickes - ps7
Diaphoretickes - ps8
Archaeplastida - ps9
Viridiplantae - ps10
Streptophyta - ps11
Charophyceae/Embriophyta - ps12
Embriophyta - ps13
Tracheophyta - ps14
Spermatophyta - ps15
Angiosperms - ps16
Mesangiospermae - ps17
Magnoliids/eudicots - ps18
Eudicots - ps19
Core_eudicots - ps20
Superrosids - ps21
Rosids - ps22
Eurosids - ps23
Malvids - ps24
Sap./Malv./Brassicales - ps25
Malvales/Brassicales - ps26
Brassicales - ps27
Cleomaceae/Brassicaceae - ps28
Brassicaceae - ps29
Camelineae - ps30
Arabidopsis - ps31
Arabidopsis_thaliana - ps32
Phylostratum

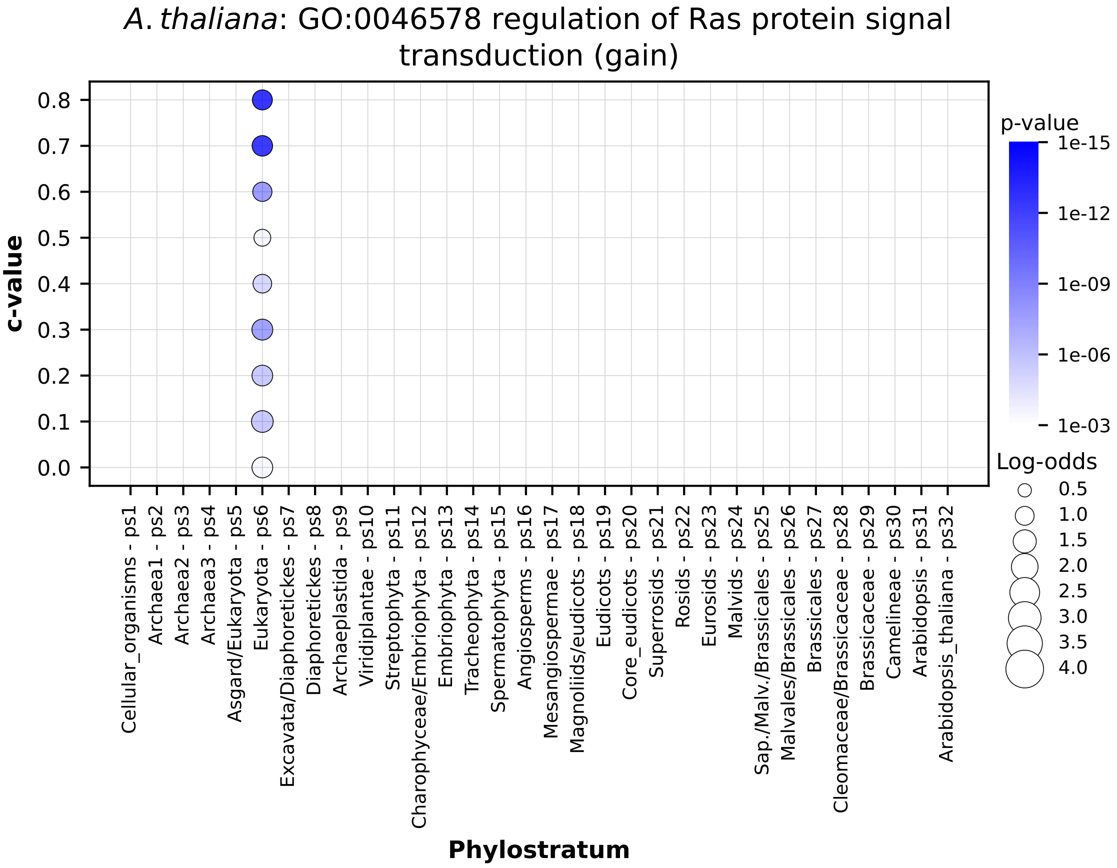
A. thaliana: GO:0046578 regulation of Ras protein signal transduction (gain)
c-value
0.8
0.7
0.6
0.5
0.4
0.3
0.2
0.1
0.0
p-value
1e-15
1e-12
1e-09
1e-06
1e-03
Log-odds
0.5
1.0
1.5
2.0
2.5
3.0
3.5
4.0
Cellular_organisms - ps1
Archaea1 - ps2
Archaea2 - ps3
Archaea3 - ps4
Asgard/Eukaryota - ps5
Eukaryota - ps6
Excavata/Diaphoretickes - ps7
Diaphoretickes - ps8
Archaeplastida - ps9
Viridiplantae - ps10
Streptophyta - ps11
Charophyceae/Embriophyta - ps12
Embriophyta - ps13
Tracheophyta - ps14
Spermatophyta - ps15
Angiosperms - ps16
Mesangiospermae - ps17
Magnoliids/eudicots - ps18
Eudicots - ps19
Core_eudicots - ps20
Superrosids - ps21
Rosids - ps22
Eurosids - ps23
Malvids - ps24
Sap./Malv./Brassicales - ps25
Malvales/Brassicales - ps26
Brassicales - ps27
Cleomaceae/Brassicaceae - ps28
Brassicaceae - ps29
Camelineae - ps30
Arabidopsis - ps31
Arabidopsis_thaliana - ps32
Phylostratum

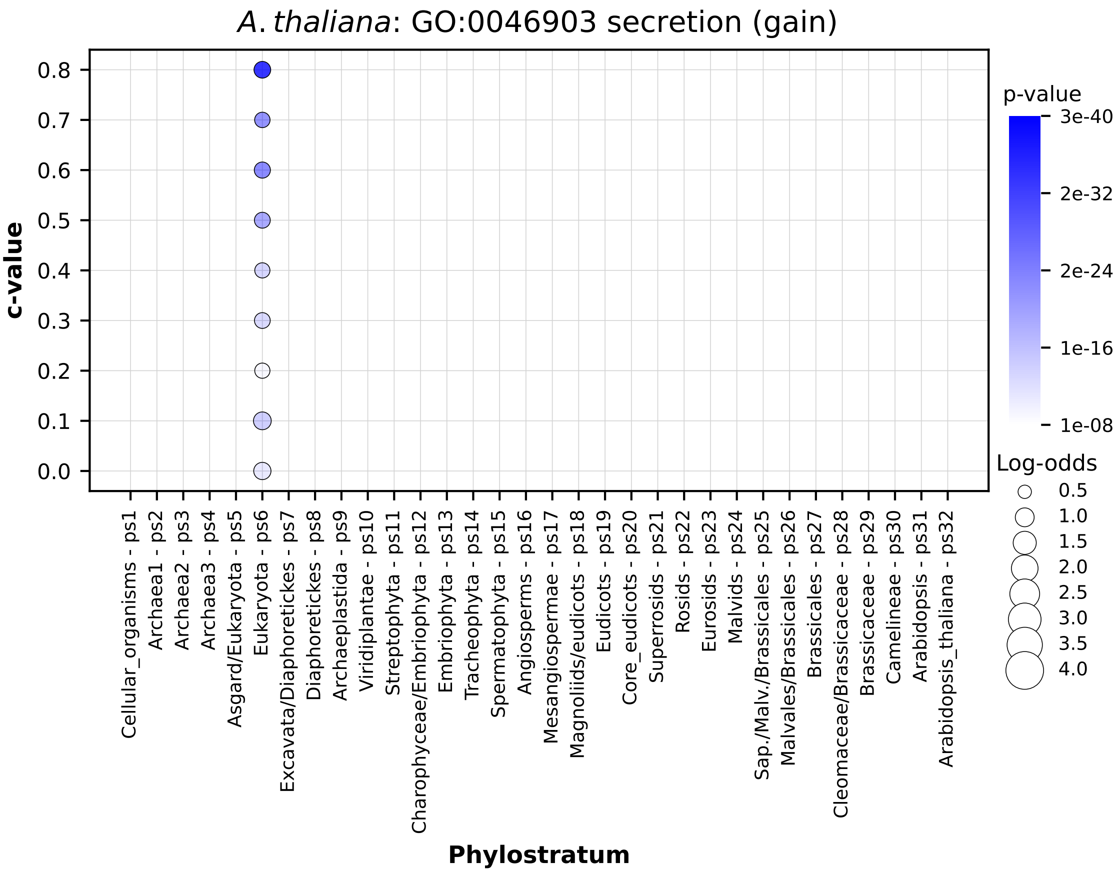

A. thaliana: GO:0046903 secretion (gain)
c-value
0.8
0.7
0.6
0.5
0.4
0.3
0.2
0.1
0.0
p-value
3e-40
2e-32
2e-24
1e-16
1e-08
Log-odds
0.5
1.0
1.5
2.0
2.5
3.0
3.5
4.0
Cellular_organisms - ps1
Archaea1 - ps2
Archaea2 - ps3
Archaea3 - ps4
Asgard/Eukaryota - ps5
Eukaryota - ps6
Excavata/Diaphoretickes - ps7
Diaphoretickes - ps8
Archaeplastida - ps9
Viridiplantae - ps10
Streptophyta - ps11
Charophyceae/Embriophyta - ps12
Embriophyta - ps13
Tracheophyta - ps14
Spermatophyta - ps15
Angiosperms - ps16
Mesangiospermae - ps17
Magnoliids/eudicots - ps18
Eudicots - ps19
Core_eudicots - ps20
Superrosids - ps21
Rosids - ps22
Eurosids - ps23
Malvids - ps24
Sap./Malv./Brassicales - ps25
Malvales/Brassicales - ps26
Brassicales - ps27
Cleomaceae/Brassicaceae - ps28
Brassicaceae - ps29
Camelineae - ps30
Arabidopsis - ps31
Arabidopsis_thaliana - ps32
Phylostratum

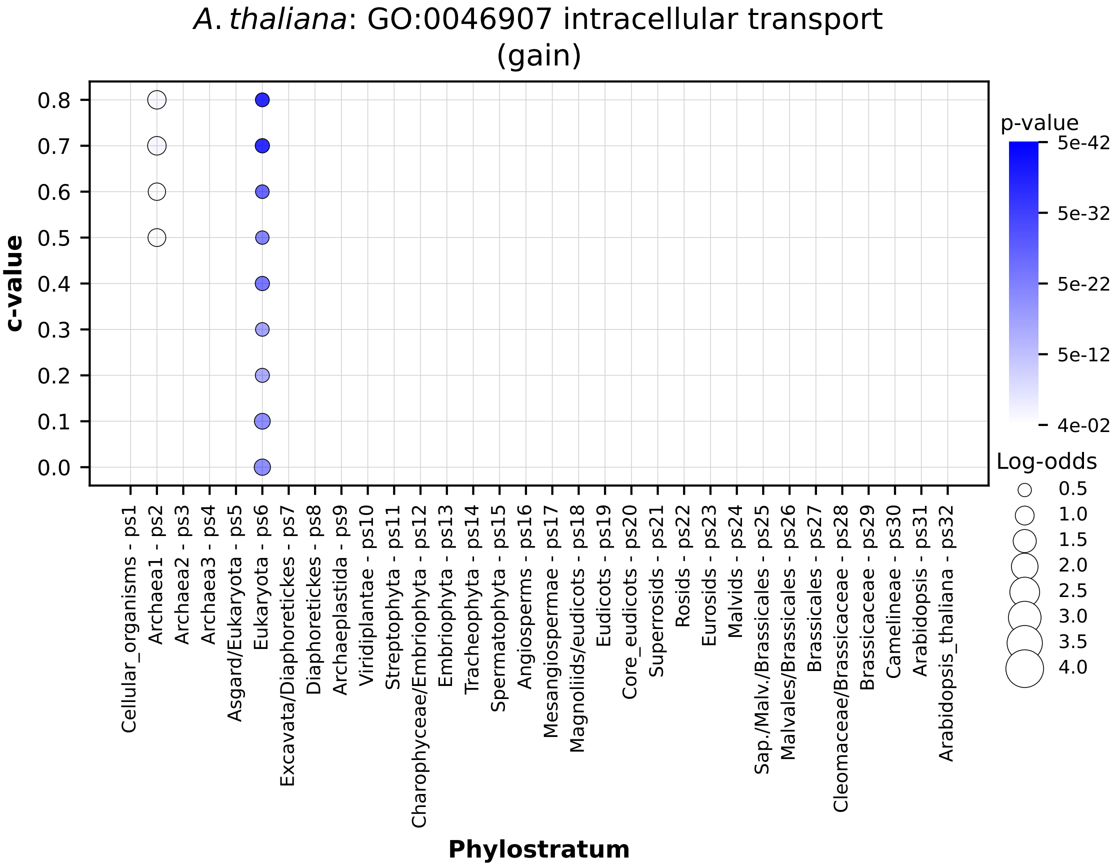
A. thaliana: GO:0046907 intracellular transport
(gain)
c-value
0.8
0.7
0.6
0.5
0.4
0.3
0.2
0.1
0.0
Phylostratum
Cellular_organisms - ps1
Archaea1 - ps2
Archaea2 - ps3
Archaea3 - ps4
Asgard/Eukaryota - ps5
Eukaryota - ps6
Excavata/Diaphoretickes - ps7
Diaphoretickes - ps8
Archaeplastida - ps9
Viridiplantae - ps10
Streptophyta - ps11
Charophyceae/Embriophyta - ps12
Embriophyta - ps13
Tracheophyta - ps14
Spermatophyta - ps15
Angiosperms - ps16
Mesangiospermae - ps17
Magnoliids/eudicots - ps18
Eudicots - ps19
Core_eudicots - ps20
Superrosids - ps21
Rosids - ps22
Eurosids - ps23
Malvids - ps24
Sap./Malv./Brassicales - ps25
Malvales/Brassicales - ps26
Brassicales - ps27
Cleomaceae/Brassicaceae - ps28
Brassicaceae - ps29
Camelineae - ps30
Arabidopsis - ps31
Arabidopsis_thaliana - ps32
p-value
5e-42
5e-32
5e-22
5e-12
4e-02
Log-odds
0.5
1.0
1.5
2.0
2.5
3.0
3.5
4.0

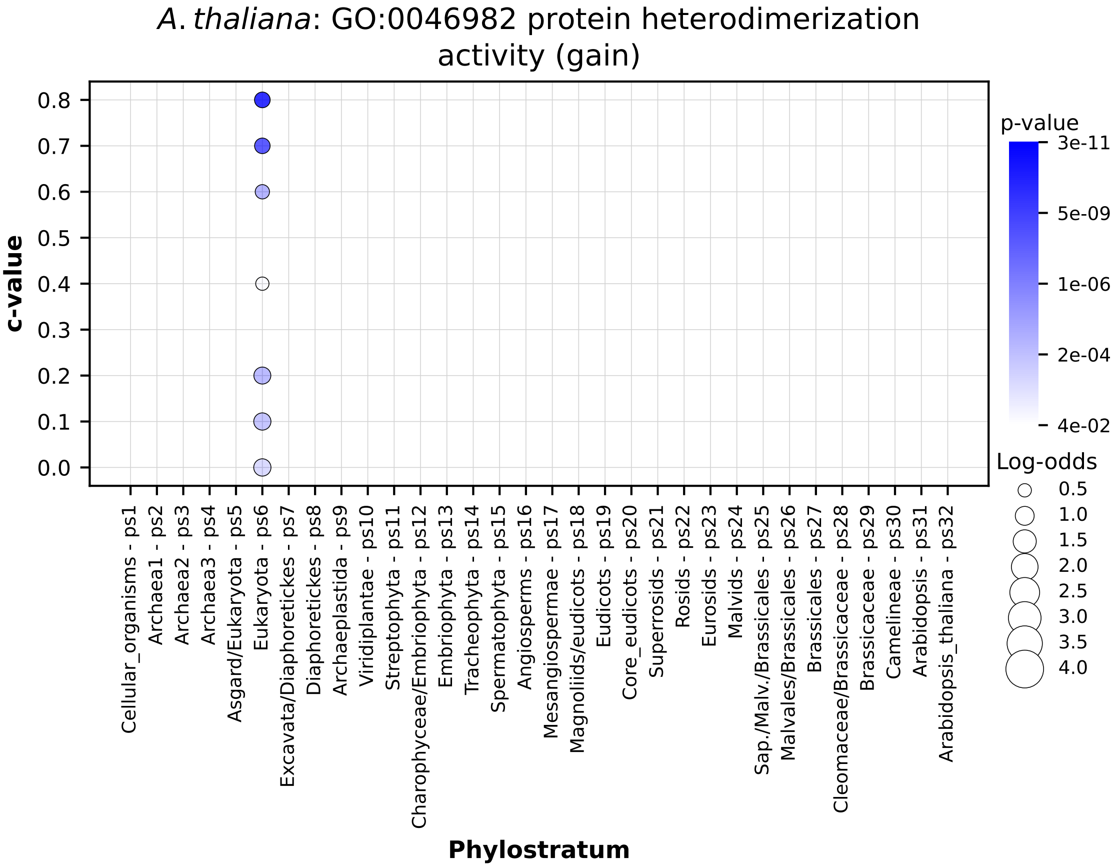

A. thaliana: GO:0046982 protein heterodimerization activity (gain)
c-value
0.0
0.1
0.2
0.3
0.4
0.5
0.6
0.7
0.8
p-value
3e-11
5e-09
1e-06
2e-04
4e-02
Log-odds
0.5
1.0
1.5
2.0
2.5
3.0
3.5
4.0
Cellular_organisms - ps1
Archaea1 - ps2
Archaea2 - ps3
Archaea3 - ps4
Asgard/Eukaryota - ps5
Eukaryota - ps6
Excavata/Diaphoretickes - ps7
Diaphoretickes - ps8
Archaeplastida - ps9
Viridiplantae - ps10
Streptophyta - ps11
Charophyceae/Embriophyta - ps12
Embriophyta - ps13
Tracheophyta - ps14
Spermatophyta - ps15
Angiosperms - ps16
Mesangiospermae - ps17
Magnoliids/eudicots - ps18
Eudicots - ps19
Core_eudicots - ps20
Superrosids - ps21
Rosids - ps22
Eurosids - ps23
Malvids - ps24
Sap./Malv./Brassicales - ps25
Malvales/Brassicales - ps26
Brassicales - ps27
Cleomaceae/Brassicaceae - ps28
Brassicaceae - ps29
Camelineae - ps30
Arabidopsis - ps31
Arabidopsis_thaliana - ps32
Phylostratum

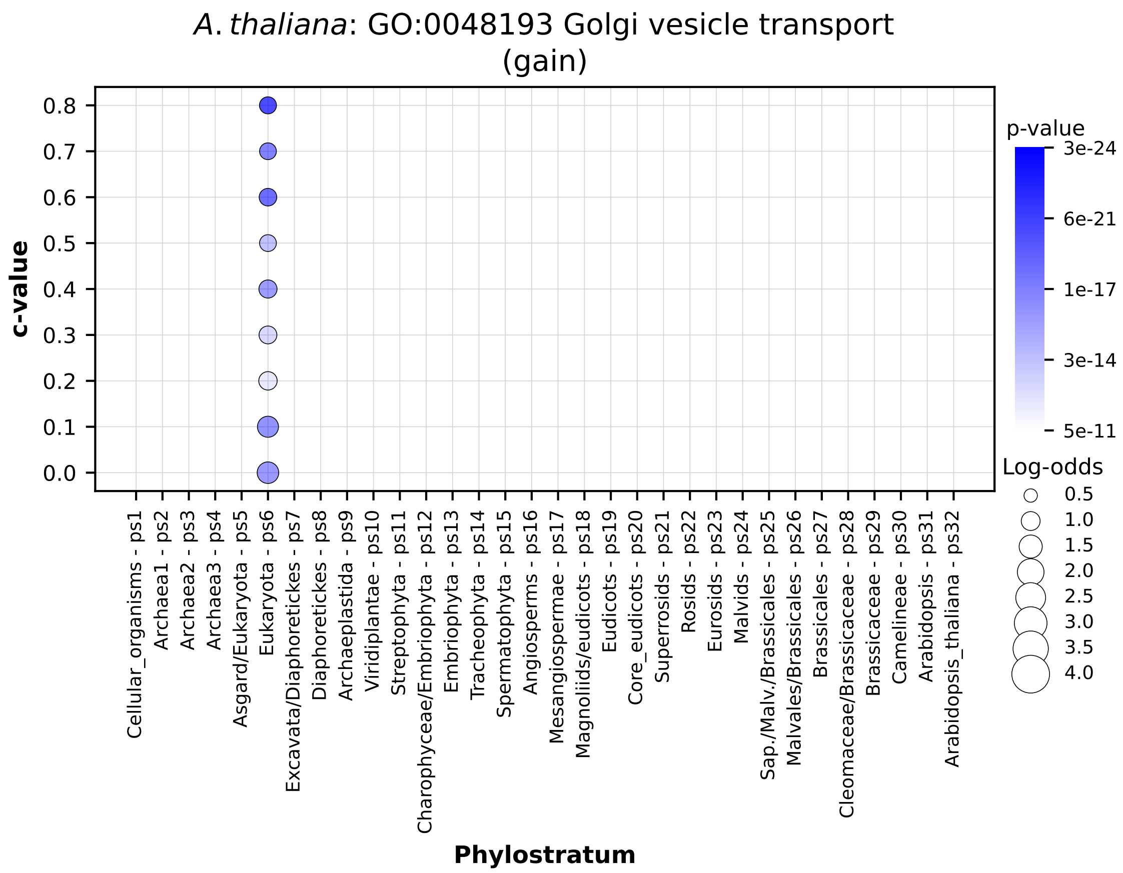
A. thaliana: GO:0048193 Golgi vesicle transport
(gain)
c-value
0.8
0.7
0.6
0.5
0.4
0.3
0.2
0.1
0.0
p-value
3e-24
6e-21
1e-17
3e-14
5e-11
Log-odds
0.5
1.0
1.5
2.0
2.5
3.0
3.5
4.0
Cellular_organisms - ps1
Archaea1 - ps2
Archaea2 - ps3
Archaea3 - ps4
Asgard/Eukaryota - ps5
Eukaryota - ps6
Excavata/Diaphoretickes - ps7
Diaphoretickes - ps8
Archaeplastida - ps9
Viridiplantae - ps10
Streptophyta - ps11
Charophyceae/Embriophyta - ps12
Embriophyta - ps13
Tracheophyta - ps14
Spermatophyta - ps15
Angiosperms - ps16
Mesangiospermae - ps17
Magnoliids/eudicots - ps18
Eudicots - ps19
Core_eudicots - ps20
Superrosids - ps21
Rosids - ps22
Eurosids - ps23
Malvids - ps24
Sap./Malv./Brassicales - ps25
Malvales/Brassicales - ps26
Brassicales - ps27
Cleomaceae/Brassicaceae - ps28
Brassicaceae - ps29
Camelineae - ps30
Arabidopsis - ps31
Arabidopsis_thaliana - ps32
Phylostratum

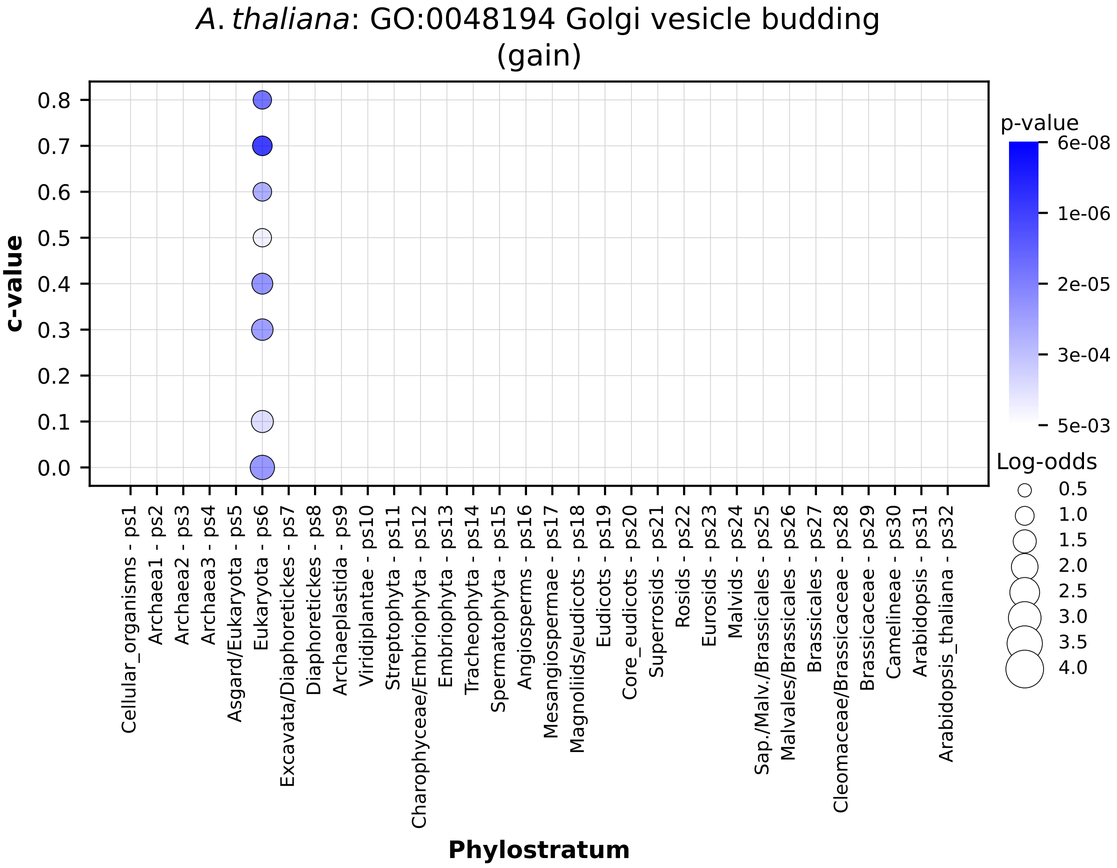

A. thaliana: GO:0048194 Golgi vesicle budding
(gain)
c-value
0.8
0.7
0.6
0.5
0.4
0.3
0.2
0.1
0.0
p-value
6e-08
1e-06
2e-05
3e-04
5e-03
Log-odds
0.5
1.0
1.5
2.0
2.5
3.0
3.5
4.0
Cellular_organisms - ps1
Archaea1 - ps2
Archaea2 - ps3
Archaea3 - ps4
Asgard/Eukaryota - ps5
Eukaryota - ps6
Excavata/Diaphoretickes - ps7
Diaphoretickes - ps8
Archaeplastida - ps9
Viridiplantae - ps10
Streptophyta - ps11
Charophyceae/Embriophyta - ps12
Embriophyta - ps13
Tracheophyta - ps14
Spermatophyta - ps15
Angiosperms - ps16
Mesangiospermae - ps17
Magnoliids/eudicots - ps18
Eudicots - ps19
Core_eudicots - ps20
Superrosids - ps21
Rosids - ps22
Eurosids - ps23
Malvids - ps24
Sap./Malv./Brassicales - ps25
Malvales/Brassicales - ps26
Brassicales - ps27
Cleomaceae/Brassicaceae - ps28
Brassicaceae - ps29
Camelineae - ps30
Arabidopsis - ps31
Arabidopsis_thaliana - ps32
Phylostratum

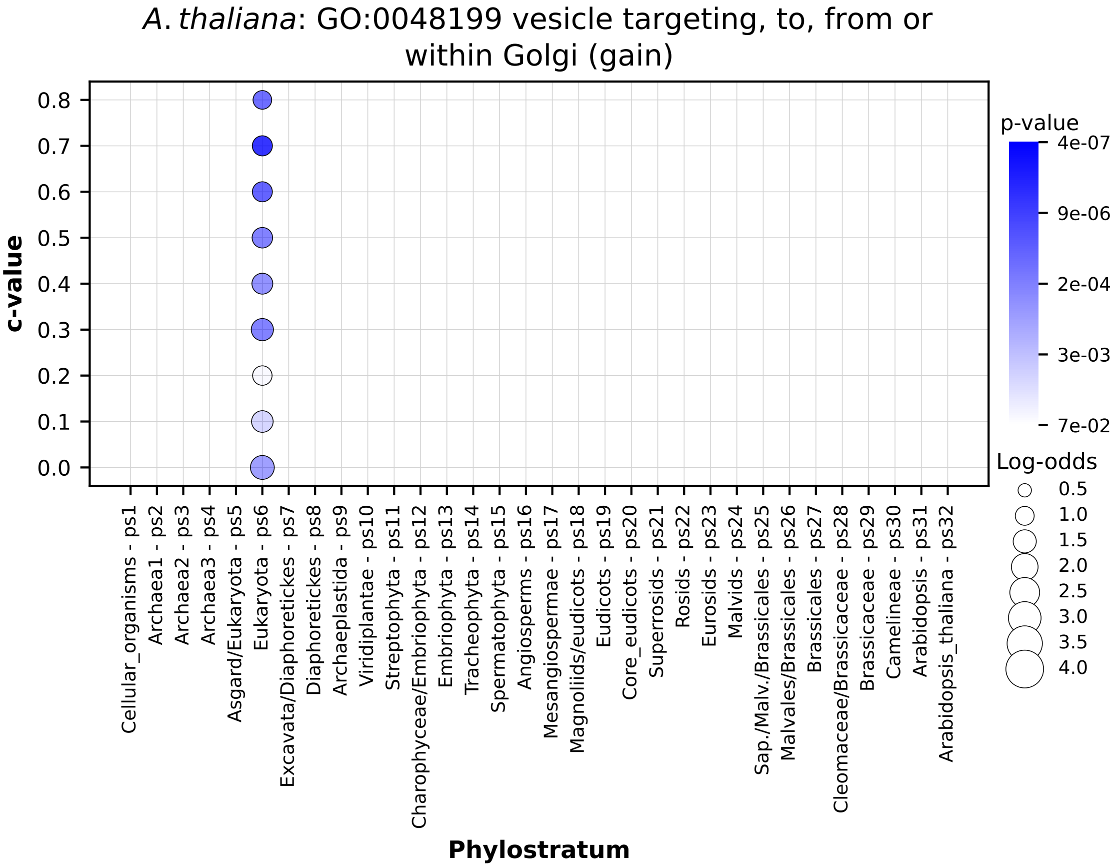
A. thaliana: GO:0048199 vesicle targeting, to, from or within Golgi (gain)
c-value
0.0
0.1
0.2
0.3
0.4
0.5
0.6
0.7
0.8
Phylostratum
Cellular_organisms - ps1
Archaea1 - ps2
Archaea2 - ps3
Archaea3 - ps4
Asgard/Eukaryota - ps5
Eukaryota - ps6
Excavata/Diaphoretickes - ps7
Diaphoretickes - ps8
Archaeplastida - ps9
Viridiplantae - ps10
Streptophyta - ps11
Charophyceae/Embriophyta - ps12
Embriophyta - ps13
Tracheophyta - ps14
Spermatophyta - ps15
Angiosperms - ps16
Mesangiospermae - ps17
Magnoliids/eudicots - ps18
Eudicots - ps19
Core_eudicots - ps20
Superrosids - ps21
Rosids - ps22
Eurosids - ps23
Malvids - ps24
Sap./Malv./Brassicales - ps25
Malvales/Brassicales - ps26
Brassicales - ps27
Cleomaceae/Brassicaceae - ps28
Brassicaceae - ps29
Camelineae - ps30
Arabidopsis - ps31
Arabidopsis_thaliana - ps32
p-value
4e-07
9e-06
2e-04
3e-03
7e-02
Log-odds
0.5
1.0
1.5
2.0
2.5
3.0
3.5
4.0

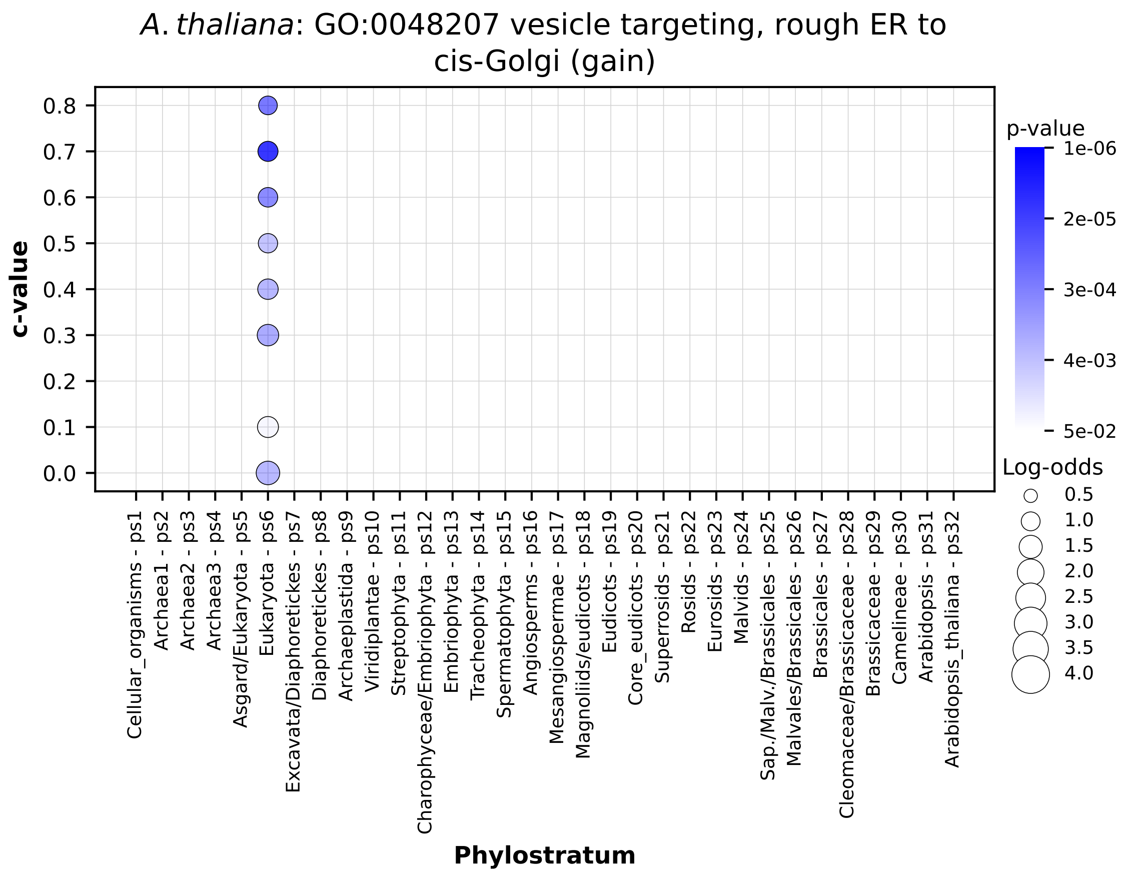

A. thaliana: GO:0048207 vesicle targeting, rough ER to cis-Golgi (gain)
c-value
0.8
0.7
0.6
0.5
0.4
0.3
0.2
0.1
0.0
p-value
1e-06
2e-05
3e-04
4e-03
5e-02
Log-odds
0.5
1.0
1.5
2.0
2.5
3.0
3.5
4.0
Cellular_organisms - ps1
Archaea1 - ps2
Archaea2 - ps3
Archaea3 - ps4
Asgard/Eukaryota - ps5
Eukaryota - ps6
Excavata/Diaphoretickes - ps7
Diaphoretickes - ps8
Archaeplastida - ps9
Viridiplantae - ps10
Streptophyta - ps11
Charophyceae/Embriophyta - ps12
Embriophyta - ps13
Tracheophyta - ps14
Spermatophyta - ps15
Angiosperms - ps16
Mesangiospermae - ps17
Magnoliids/eudicots - ps18
Eudicots - ps19
Core_eudicots - ps20
Superrosids - ps21
Rosids - ps22
Eurosids - ps23
Malvids - ps24
Sap./Malv./Brassicales - ps25
Malvales/Brassicales - ps26
Brassicales - ps27
Cleomaceae/Brassicaceae - ps28
Brassicaceae - ps29
Camelineae - ps30
Arabidopsis - ps31
Arabidopsis_thaliana - ps32
Phylostratum

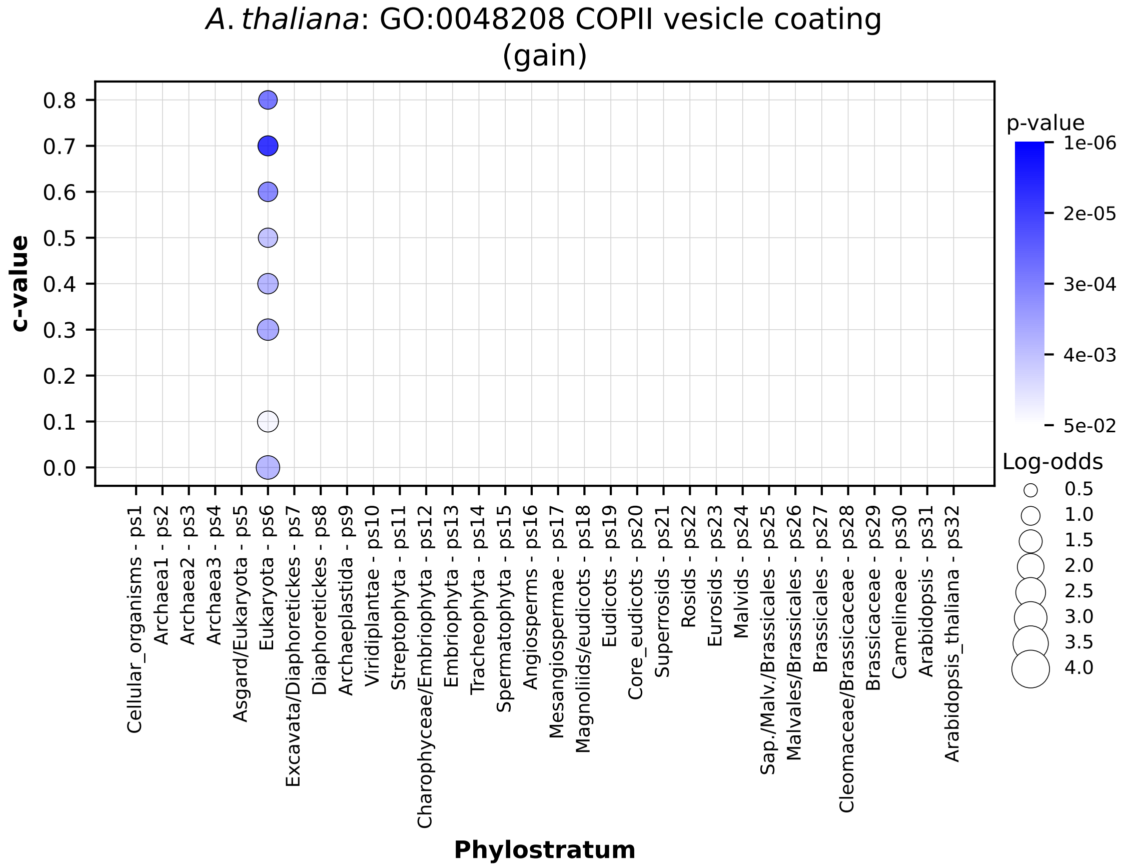

A. thaliana: GO:0048208 COPII vesicle coating
(gain)
c-value
0.8
0.7
0.6
0.5
0.4
0.3
0.2
0.1
0.0
p-value
1e-06
2e-05
3e-04
4e-03
5e-02
Log-odds
0.5
1.0
1.5
2.0
2.5
3.0
3.5
4.0
Cellular_organisms - ps1
Archaea1 - ps2
Archaea2 - ps3
Archaea3 - ps4
Asgard/Eukaryota - ps5
Eukaryota - ps6
Excavata/Diaphoretickes - ps7
Diaphoretickes - ps8
Archaeplastida - ps9
Viridiplantae - ps10
Streptophyta - ps11
Charophyceae/Embriophyta - ps12
Embriophyta - ps13
Tracheophyta - ps14
Spermatophyta - ps15
Angiosperms - ps16
Mesangiospermae - ps17
Magnoliids/eudicots - ps18
Eudicots - ps19
Core_eudicots - ps20
Superrosids - ps21
Rosids - ps22
Eurosids - ps23
Malvids - ps24
Sap./Malv./Brassicales - ps25
Malvales/Brassicales - ps26
Brassicales - ps27
Cleomaceae/Brassicaceae - ps28
Brassicaceae - ps29
Camelineae - ps30
Arabidopsis - ps31
Arabidopsis_thaliana - ps32
Phylostratum

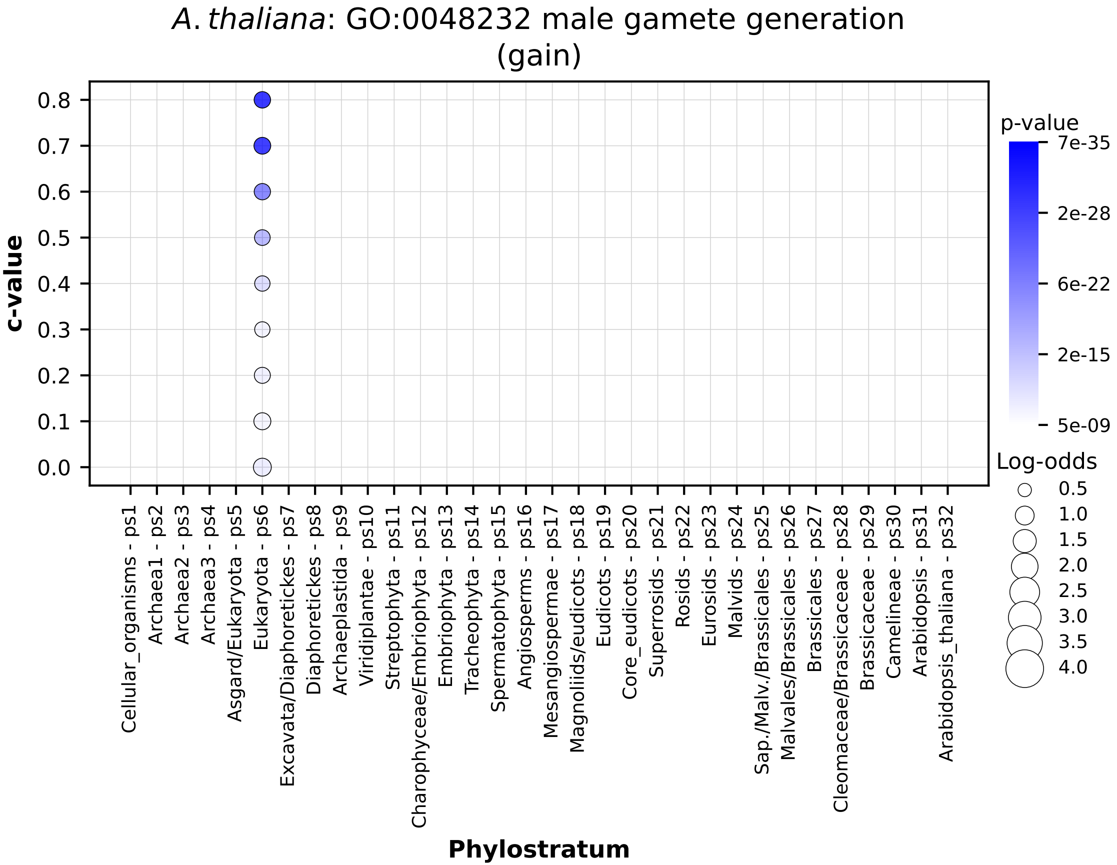

A. thaliana: GO:0048232 male gamete generation (gain)
c-value
0.8
0.7
0.6
0.5
0.4
0.3
0.2
0.1
0.0
p-value
7e-35
2e-28
6e-22
2e-15
5e-09
Log-odds
0.5
1.0
1.5
2.0
2.5
3.0
3.5
4.0
Cellular_organisms - ps1
Archaea1 - ps2
Archaea2 - ps3
Archaea3 - ps4
Asgard/Eukaryota - ps5
Eukaryota - ps6
Excavata/Diaphoretickes - ps7
Diaphoretickes - ps8
Archaeplastida - ps9
Viridiplantae - ps10
Streptophyta - ps11
Charophyceae/Embriophyta - ps12
Embriophyta - ps13
Tracheophyta - ps14
Spermatophyta - ps15
Angiosperms - ps16
Mesangiospermae - ps17
Magnoliids/eudicots - ps18
Eudicots - ps19
Core_eudicots - ps20
Superrosids - ps21
Rosids - ps22
Eurosids - ps23
Malvids - ps24
Sap./Malv./Brassicales - ps25
Malvales/Brassicales - ps26
Brassicales - ps27
Cleomaceae/Brassicaceae - ps28
Brassicaceae - ps29
Camelineae - ps30
Arabidopsis - ps31
Arabidopsis_thaliana - ps32
Phylostratum

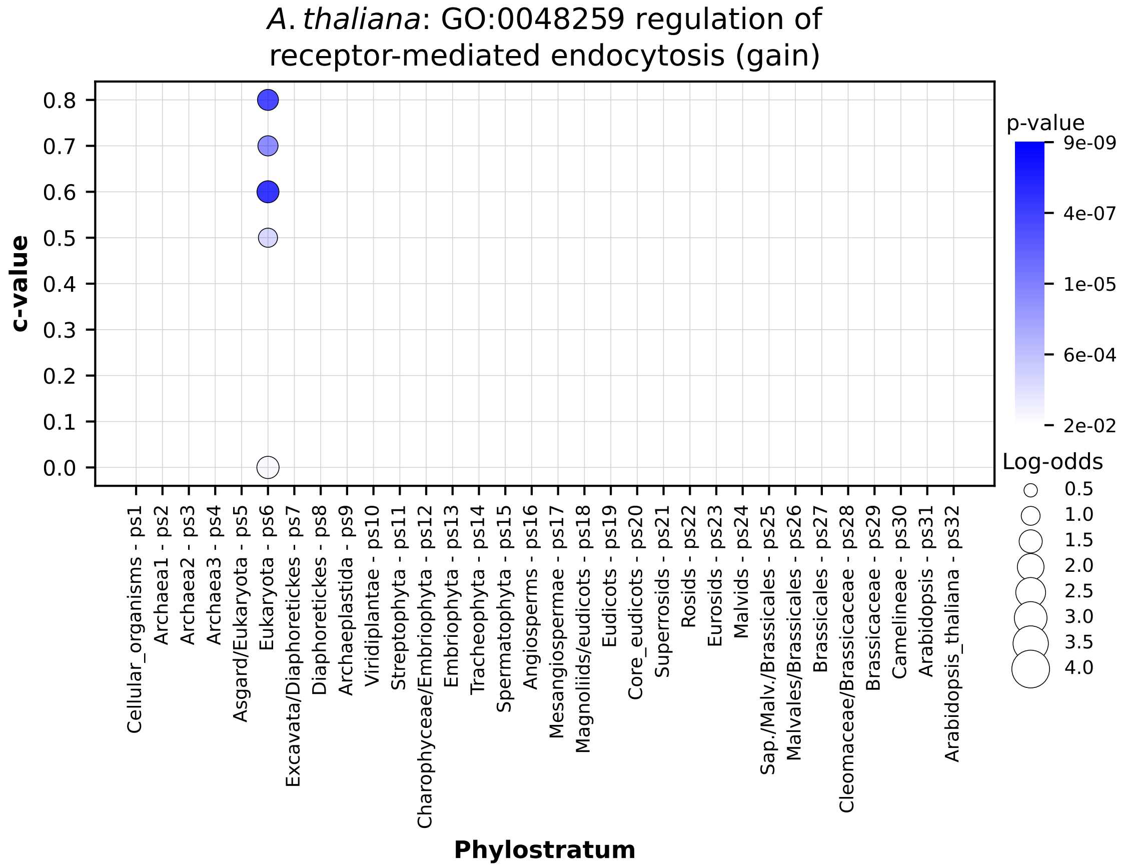

A. thaliana: GO:0048259 regulation of receptor-mediated endocytosis (gain)
c-value
0.8
0.7
0.6
0.5
0.4
0.3
0.2
0.1
0.0
p-value
9e-09
4e-07
1e-05
6e-04
2e-02
Log-odds
0.5
1.0
1.5
2.0
2.5
3.0
3.5
4.0
Cellular_organisms - ps1
Archaea1 - ps2
Archaea2 - ps3
Archaea3 - ps4
Asgard/Eukaryota - ps5
Eukaryota - ps6
Excavata/Diaphoretickes - ps7
Diaphoretickes - ps8
Archaeplastida - ps9
Viridiplantae - ps10
Streptophyta - ps11
Charophyceae/Embriophyta - ps12
Embriophyta - ps13
Tracheophyta - ps14
Spermatophyta - ps15
Angiosperms - ps16
Mesangiospermae - ps17
Magnoliids/eudicots - ps18
Eudicots - ps19
Core_eudicots - ps20
Superrosids - ps21
Rosids - ps22
Eurosids - ps23
Malvids - ps24
Sap./Malv./Brassicales - ps25
Malvales/Brassicales - ps26
Brassicales - ps27
Cleomaceae/Brassicaceae - ps28
Brassicaceae - ps29
Camelineae - ps30
Arabidopsis - ps31
Arabidopsis_thaliana - ps32
Phylostratum

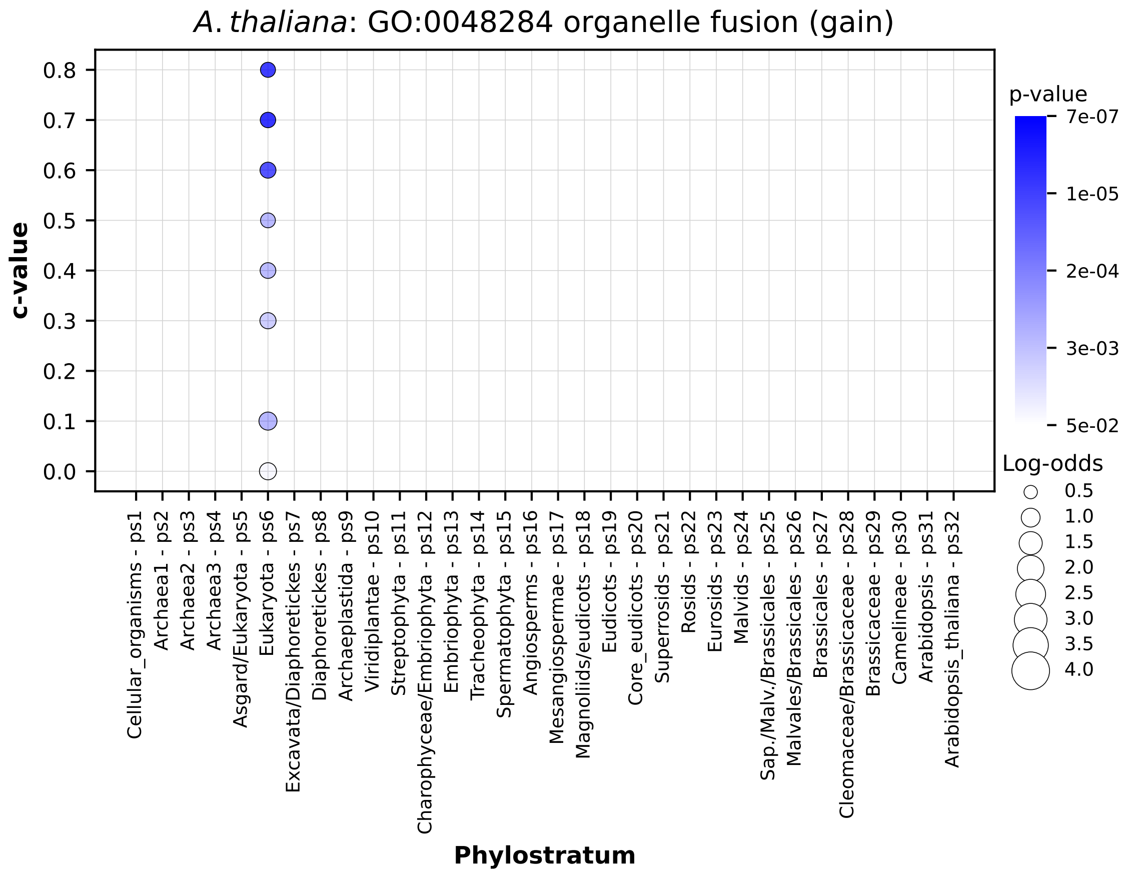
A. thaliana: GO:0048284 organelle fusion (gain)
c-value
0.8
0.7
0.6
0.5
0.4
0.3
0.2
0.1
0.0
p-value
7e-07
1e-05
2e-04
3e-03
5e-02
Log-odds
0.5
1.0
1.5
2.0
2.5
3.0
3.5
4.0
Cellular_organisms - ps1
Archaea1 - ps2
Archaea2 - ps3
Archaea3 - ps4
Asgard/Eukaryota - ps5
Eukaryota - ps6
Excavata/Diaphoretickes - ps7
Diaphoretickes - ps8
Archaeplastida - ps9
Viridiplantae - ps10
Streptophyta - ps11
Charophyceae/Embriophyta - ps12
Embriophyta - ps13
Tracheophyta - ps14
Spermatophyta - ps15
Angiosperms - ps16
Mesangiospermae - ps17
Magnoliids/eudicots - ps18
Eudicots - ps19
Core_eudicots - ps20
Superrosids - ps21
Rosids - ps22
Eurosids - ps23
Malvids - ps24
Sap./Malv./Brassicales - ps25
Malvales/Brassicales - ps26
Brassicales - ps27
Cleomaceae/Brassicaceae - ps28
Brassicaceae - ps29
Camelineae - ps30
Arabidopsis - ps31
Arabidopsis_thaliana - ps32
Phylostratum

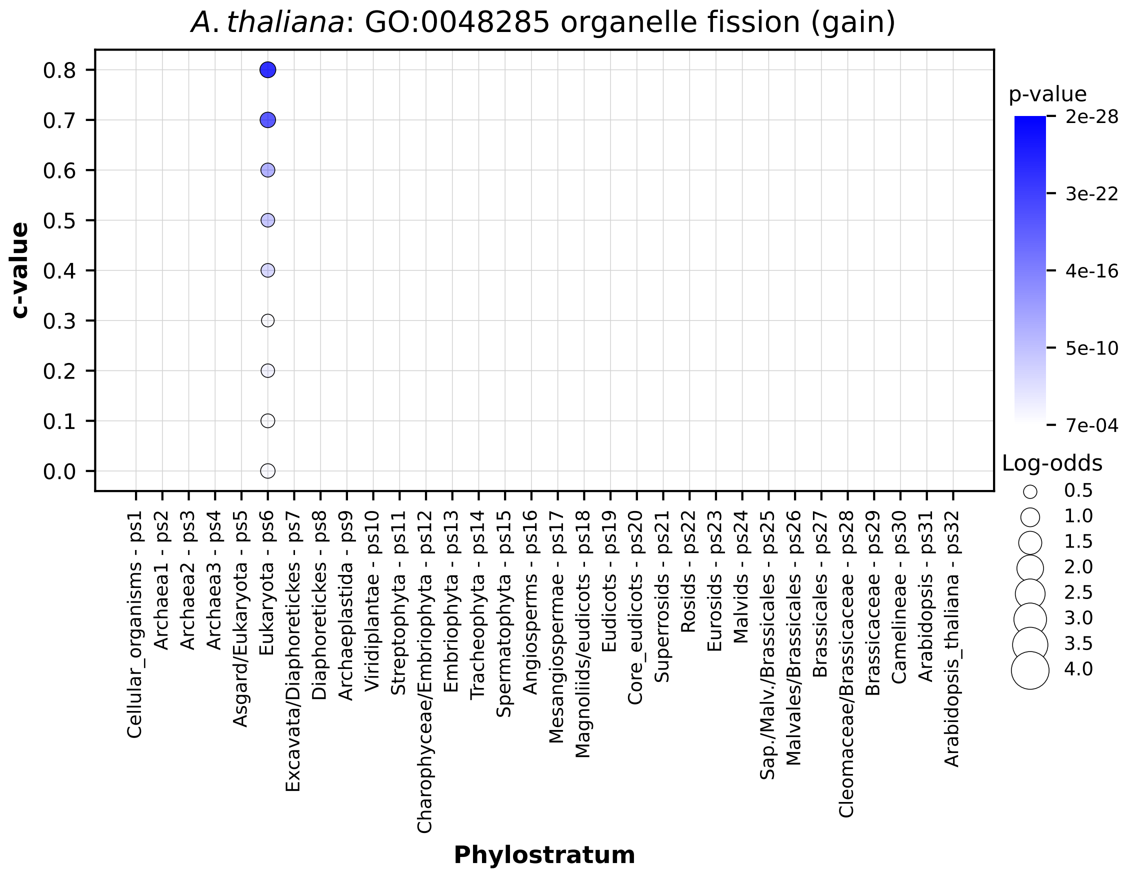
A. thaliana: GO:0048285 organelle fission (gain)
c-value
0.8
0.7
0.6
0.5
0.4
0.3
0.2
0.1
0.0
p-value
2e-28
3e-22
4e-16
5e-10
7e-04
Log-odds
0.5
1.0
1.5
2.0
2.5
3.0
3.5
4.0
Cellular_organisms - ps1
Archaea1 - ps2
Archaea2 - ps3
Archaea3 - ps4
Asgard/Eukaryota - ps5
Eukaryota - ps6
Excavata/Diaphoretickes - ps7
Diaphoretickes - ps8
Archaeplastida - ps9
Viridiplantae - ps10
Streptophyta - ps11
Charophyceae/Embriophyta - ps12
Embriophyta - ps13
Tracheophyta - ps14
Spermatophyta - ps15
Angiosperms - ps16
Mesangiospermae - ps17
Magnoliids/eudicots - ps18
Eudicots - ps19
Core_eudicots - ps20
Superrosids - ps21
Rosids - ps22
Eurosids - ps23
Malvids - ps24
Sap./Malv./Brassicales - ps25
Malvales/Brassicales - ps26
Brassicales - ps27
Cleomaceae/Brassicaceae - ps28
Brassicaceae - ps29
Camelineae - ps30
Arabidopsis - ps31
Arabidopsis_thaliana - ps32
Phylostratum

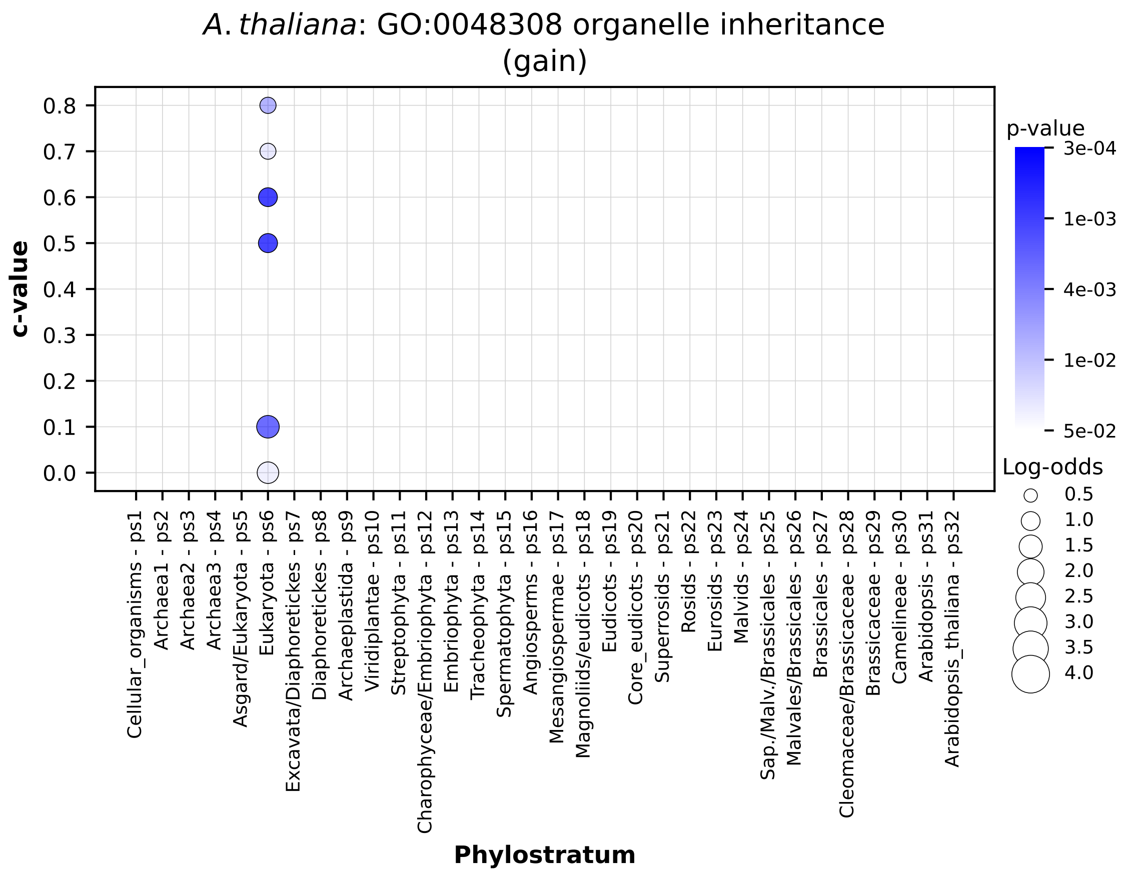

A. thaliana: GO:0048308 organelle inheritance
(gain)
c-value
0.0
0.1
0.2
0.3
0.4
0.5
0.6
0.7
0.8
p-value
3e-04
1e-03
4e-03
1e-02
5e-02
Log-odds
0.5
1.0
1.5
2.0
2.5
3.0
3.5
4.0
Cellular_organisms - ps1
Archaea1 - ps2
Archaea2 - ps3
Archaea3 - ps4
Asgard/Eukaryota - ps5
Eukaryota - ps6
Excavata/Diaphoretickes - ps7
Diaphoretickes - ps8
Archaeplastida - ps9
Viridiplantae - ps10
Streptophyta - ps11
Charophyceae/Embriophyta - ps12
Embriophyta - ps13
Tracheophyta - ps14
Spermatophyta - ps15
Angiosperms - ps16
Mesangiospermae - ps17
Magnoliids/eudicots - ps18
Eudicots - ps19
Core_eudicots - ps20
Superrosids - ps21
Rosids - ps22
Eurosids - ps23
Malvids - ps24
Sap./Malv./Brassicales - ps25
Malvales/Brassicales - ps26
Brassicales - ps27
Cleomaceae/Brassicaceae - ps28
Brassicaceae - ps29
Camelineae - ps30
Arabidopsis - ps31
Arabidopsis_thaliana - ps32
Phylostratum

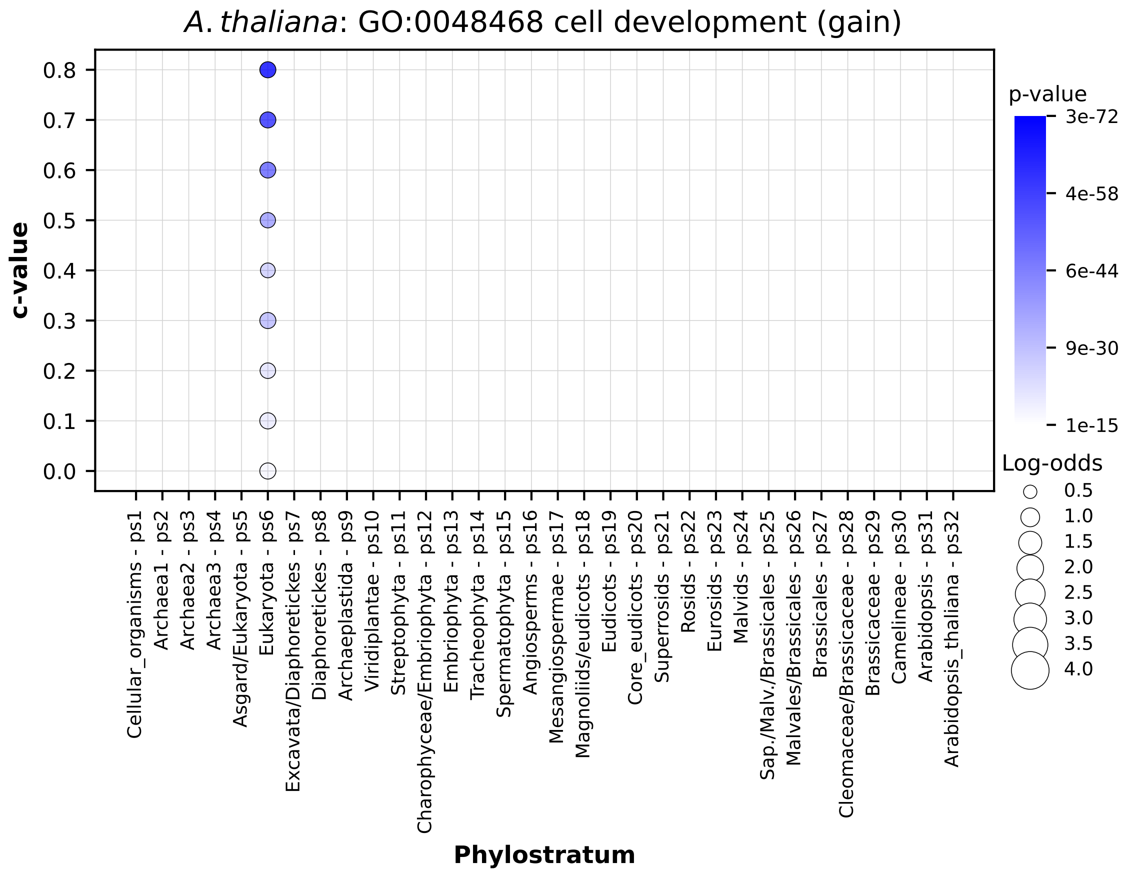
A. thaliana: GO:0048468 cell development (gain)
c-value
0.0
0.1
0.2
0.3
0.4
0.5
0.6
0.7
0.8
p-value
3e-72
4e-58
6e-44
9e-30
1e-15
Log-odds
0.5
1.0
1.5
2.0
2.5
3.0
3.5
4.0
Cellular_organisms - ps1
Archaea1 - ps2
Archaea2 - ps3
Archaea3 - ps4
Asgard/Eukaryota - ps5
Eukaryota - ps6
Excavata/Diaphoretickes - ps7
Diaphoretickes - ps8
Archaeplastida - ps9
Viridiplantae - ps10
Streptophyta - ps11
Charophyceae/Embriophyta - ps12
Embriophyta - ps13
Tracheophyta - ps14
Spermatophyta - ps15
Angiosperms - ps16
Mesangiospermae - ps17
Magnoliids/eudicots - ps18
Eudicots - ps19
Core_eudicots - ps20
Superrosids - ps21
Rosids - ps22
Eurosids - ps23
Malvids - ps24
Sap./Malv./Brassicales - ps25
Malvales/Brassicales - ps26
Brassicales - ps27
Cleomaceae/Brassicaceae - ps28
Brassicaceae - ps29
Camelineae - ps30
Arabidopsis - ps31
Arabidopsis_thaliana - ps32
Phylostratum

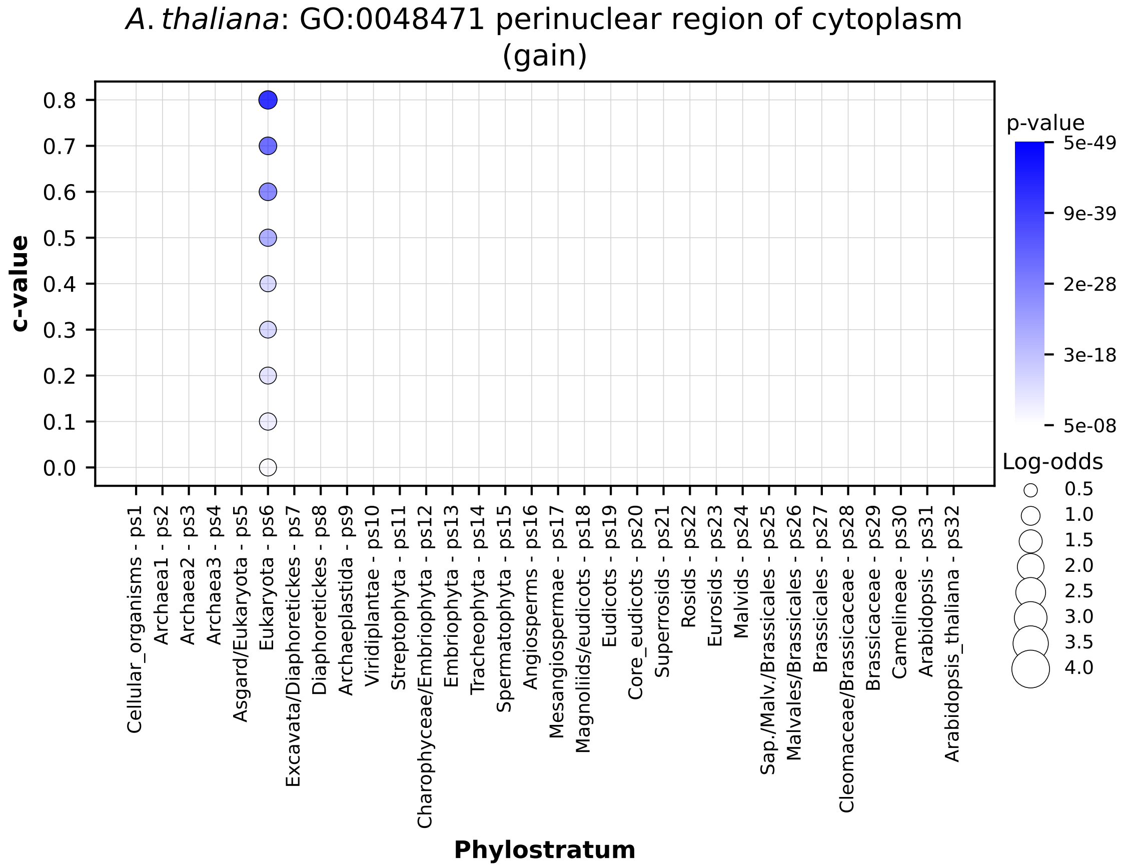

A. thaliana: GO:0048471 perinuclear region of cytoplasm (gain)
c-value
0.0
0.1
0.2
0.3
0.4
0.5
0.6
0.7
0.8
p-value
5e-49
9e-39
2e-28
3e-18
5e-08
Log-odds
0.5
1.0
1.5
2.0
2.5
3.0
3.5
4.0
Cellular_organisms - ps1
Archaea1 - ps2
Archaea2 - ps3
Archaea3 - ps4
Asgard/Eukaryota - ps5
Eukaryota - ps6
Excavata/Diaphoretickes - ps7
Diaphoretickes - ps8
Archaeplastida - ps9
Viridiplantae - ps10
Streptophyta - ps11
Charophyceae/Embriophyta - ps12
Embriophyta - ps13
Tracheophyta - ps14
Spermatophyta - ps15
Angiosperms - ps16
Mesangiospermae - ps17
Magnoliids/eudicots - ps18
Eudicots - ps19
Core_eudicots - ps20
Superrosids - ps21
Rosids - ps22
Eurosids - ps23
Malvids - ps24
Sap./Malv./Brassicales - ps25
Malvales/Brassicales - ps26
Brassicales - ps27
Cleomaceae/Brassicaceae - ps28
Brassicaceae - ps29
Camelineae - ps30
Arabidopsis - ps31
Arabidopsis_thaliana - ps32
Phylostratum

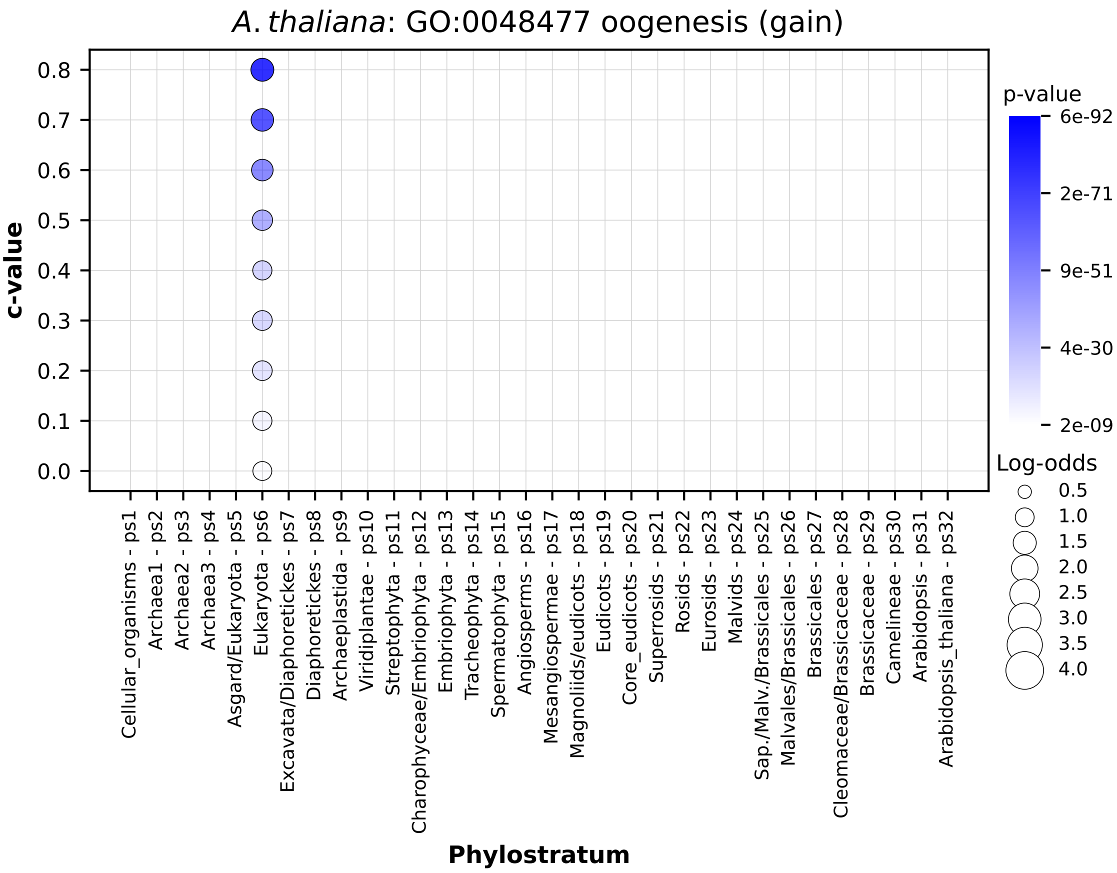

A. thaliana: GO:0048477 oogenesis (gain)
c-value
0.0
0.1
0.2
0.3
0.4
0.5
0.6
0.7
0.8
p-value
6e-92
2e-71
9e-51
4e-30
2e-09
Log-odds
0.5
1.0
1.5
2.0
2.5
3.0
3.5
4.0
Cellular_organisms - ps1
Archaea1 - ps2
Archaea2 - ps3
Archaea3 - ps4
Asgard/Eukaryota - ps5
Eukaryota - ps6
Excavata/Diaphoretickes - ps7
Diaphoretickes - ps8
Archaeplastida - ps9
Viridiplantae - ps10
Streptophyta - ps11
Charophyceae/Embriophyta - ps12
Embriophyta - ps13
Tracheophyta - ps14
Spermatophyta - ps15
Angiosperms - ps16
Mesangiospermae - ps17
Magnoliids/eudicots - ps18
Eudicots - ps19
Core_eudicots - ps20
Superrosids - ps21
Rosids - ps22
Eurosids - ps23
Malvids - ps24
Sap./Malv./Brassicales - ps25
Malvales/Brassicales - ps26
Brassicales - ps27
Cleomaceae/Brassicaceae - ps28
Brassicaceae - ps29
Camelineae - ps30
Arabidopsis - ps31
Arabidopsis_thaliana - ps32
Phylostratum

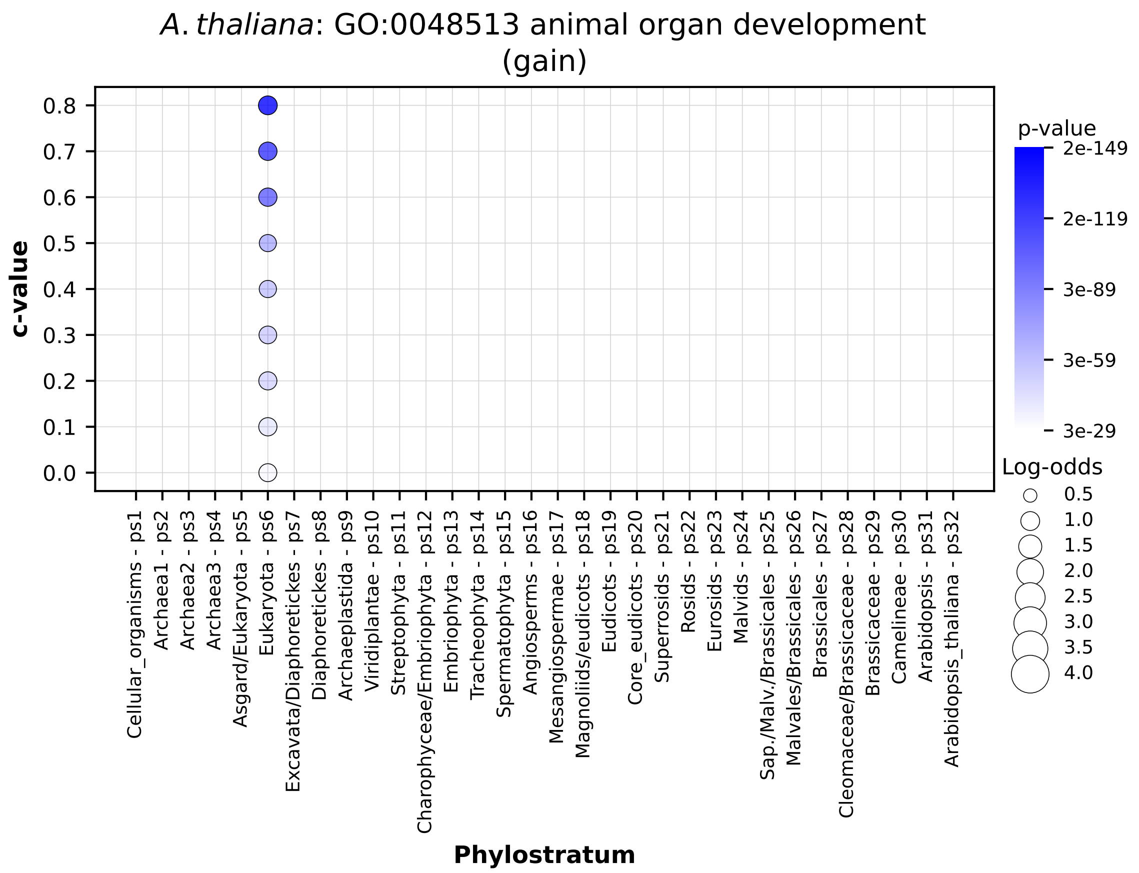

A. thaliana: GO:0048513 animal organ development
(gain)
c-value
0.8
0.7
0.6
0.5
0.4
0.3
0.2
0.1
0.0
p-value
2e-149
2e-119
3e-89
3e-59
3e-29
Log-odds
0.5
1.0
1.5
2.0
2.5
3.0
3.5
4.0
Cellular_organisms - ps1
Archaea1 - ps2
Archaea2 - ps3
Archaea3 - ps4
Asgard/Eukaryota - ps5
Eukaryota - ps6
Excavata/Diaphoretickes - ps7
Diaphoretickes - ps8
Archaeplastida - ps9
Viridiplantae - ps10
Streptophyta - ps11
Charophyceae/Embriophyta - ps12
Embriophyta - ps13
Tracheophyta - ps14
Spermatophyta - ps15
Angiosperms - ps16
Mesangiospermae - ps17
Magnoliids/eudicots - ps18
Eudicots - ps19
Core_eudicots - ps20
Superrosids - ps21
Rosids - ps22
Eurosids - ps23
Malvids - ps24
Sap./Malv./Brassicales - ps25
Malvales/Brassicales - ps26
Brassicales - ps27
Cleomaceae/Brassicaceae - ps28
Brassicaceae - ps29
Camelineae - ps30
Arabidopsis - ps31
Arabidopsis_thaliana - ps32
Phylostratum

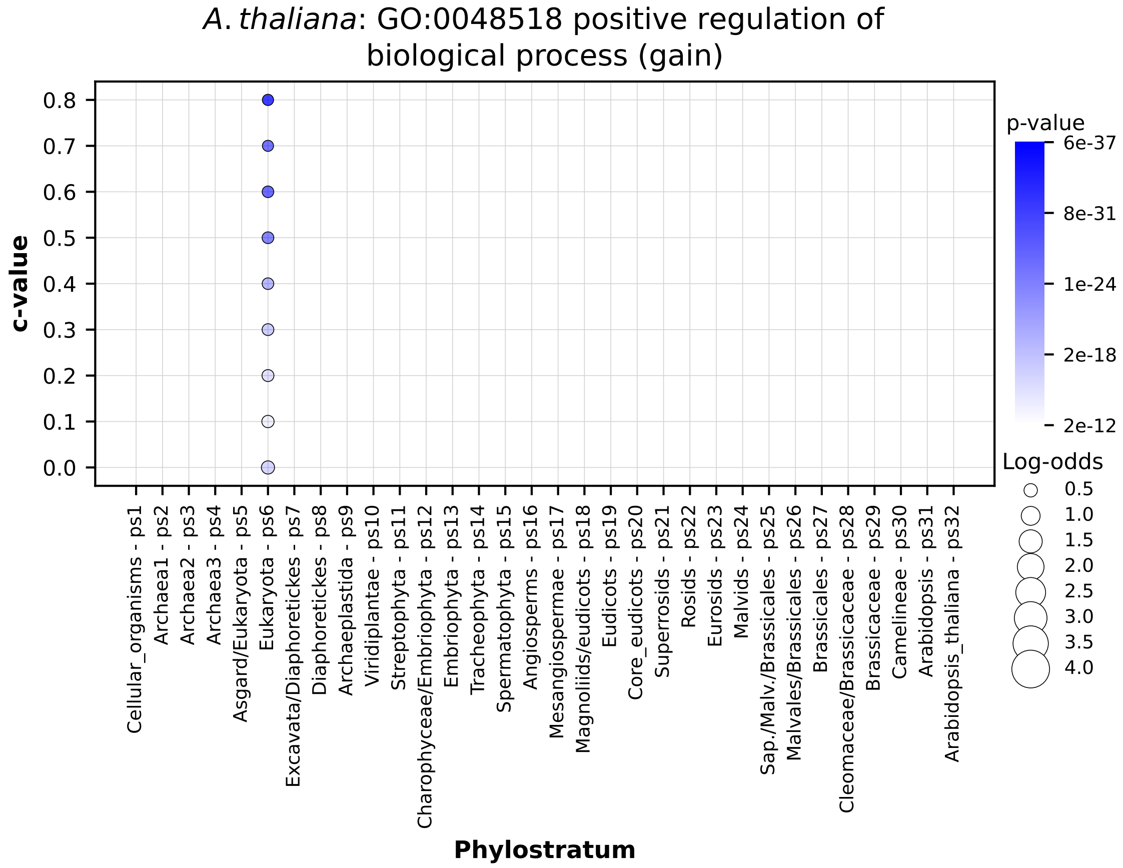
A. thaliana: GO:0048518 positive regulation of biological process (gain)
c-value
0.8
0.7
0.6
0.5
0.4
0.3
0.2
0.1
0.0
Phylostratum
Cellular_organisms - ps1
Archaea1 - ps2
Archaea2 - ps3
Archaea3 - ps4
Asgard/Eukaryota - ps5
Eukaryota - ps6
Excavata/Diaphoretickes - ps7
Diaphoretickes - ps8
Archaeplastida - ps9
Viridiplantae - ps10
Streptophyta - ps11
Charophyceae/Embriophyta - ps12
Embriophyta - ps13
Tracheophyta - ps14
Spermatophyta - ps15
Angiosperms - ps16
Mesangiospermae - ps17
Magnoliids/eudicots - ps18
Eudicots - ps19
Core_eudicots - ps20
Superrosids - ps21
Rosids - ps22
Eurosids - ps23
Malvids - ps24
Sap./Malv./Brassicales - ps25
Malvales/Brassicales - ps26
Brassicales - ps27
Cleomaceae/Brassicaceae - ps28
Brassicaceae - ps29
Camelineae - ps30
Arabidopsis - ps31
Arabidopsis_thaliana - ps32
p-value
6e-37
8e-31
1e-24
2e-18
2e-12
Log-odds
0.5
1.0
1.5
2.0
2.5
3.0
3.5
4.0

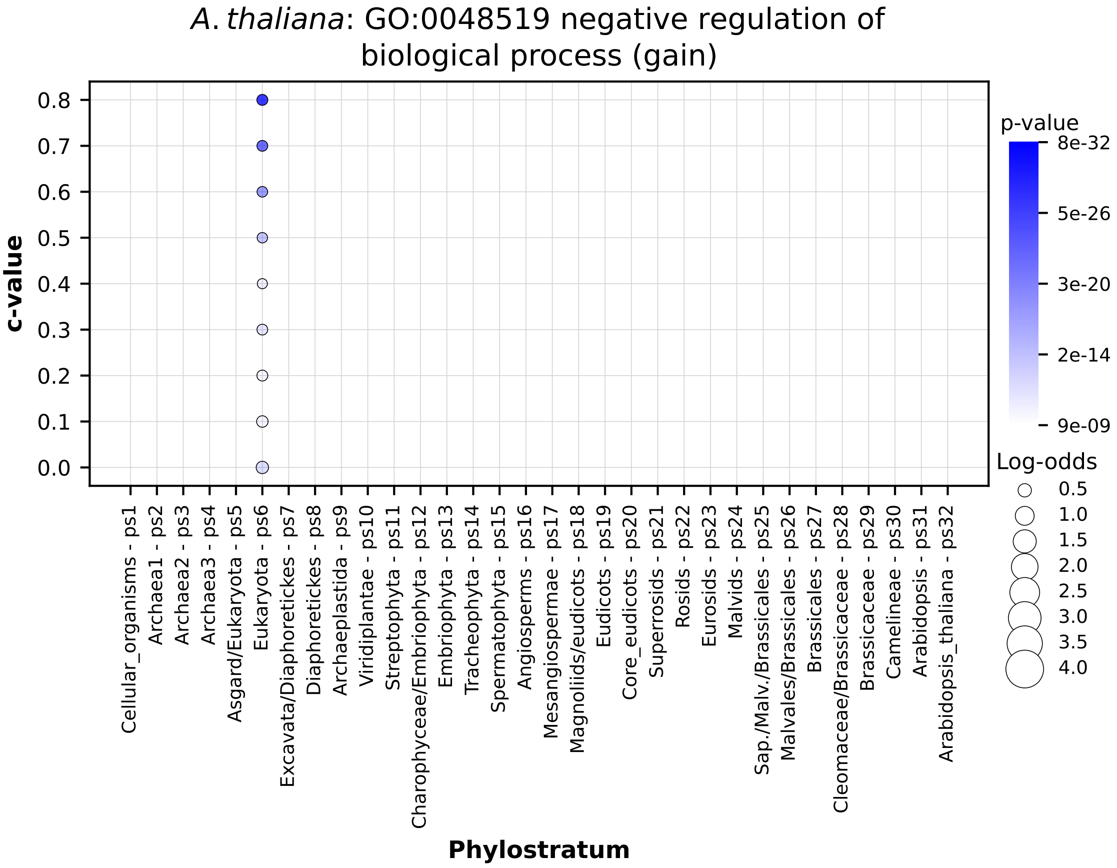

A. thaliana: GO:0048519 negative regulation of biological process (gain)
c-value
0.8
0.7
0.6
0.5
0.4
0.3
0.2
0.1
0.0
p-value
8e-32
5e-26
3e-20
2e-14
9e-09
Log-odds
0.5
1.0
1.5
2.0
2.5
3.0
3.5
4.0
Cellular_organisms - ps1
Archaea1 - ps2
Archaea2 - ps3
Archaea3 - ps4
Asgard/Eukaryota - ps5
Eukaryota - ps6
Excavata/Diaphoretickes - ps7
Diaphoretickes - ps8
Archaeplastida - ps9
Viridiplantae - ps10
Streptophyta - ps11
Charophyceae/Embriophyta - ps12
Embriophyta - ps13
Tracheophyta - ps14
Spermatophyta - ps15
Angiosperms - ps16
Mesangiospermae - ps17
Magnoliids/eudicots - ps18
Eudicots - ps19
Core_eudicots - ps20
Superrosids - ps21
Rosids - ps22
Eurosids - ps23
Malvids - ps24
Sap./Malv./Brassicales - ps25
Malvales/Brassicales - ps26
Brassicales - ps27
Cleomaceae/Brassicaceae - ps28
Brassicaceae - ps29
Camelineae - ps30
Arabidopsis - ps31
Arabidopsis_thaliana - ps32
Phylostratum

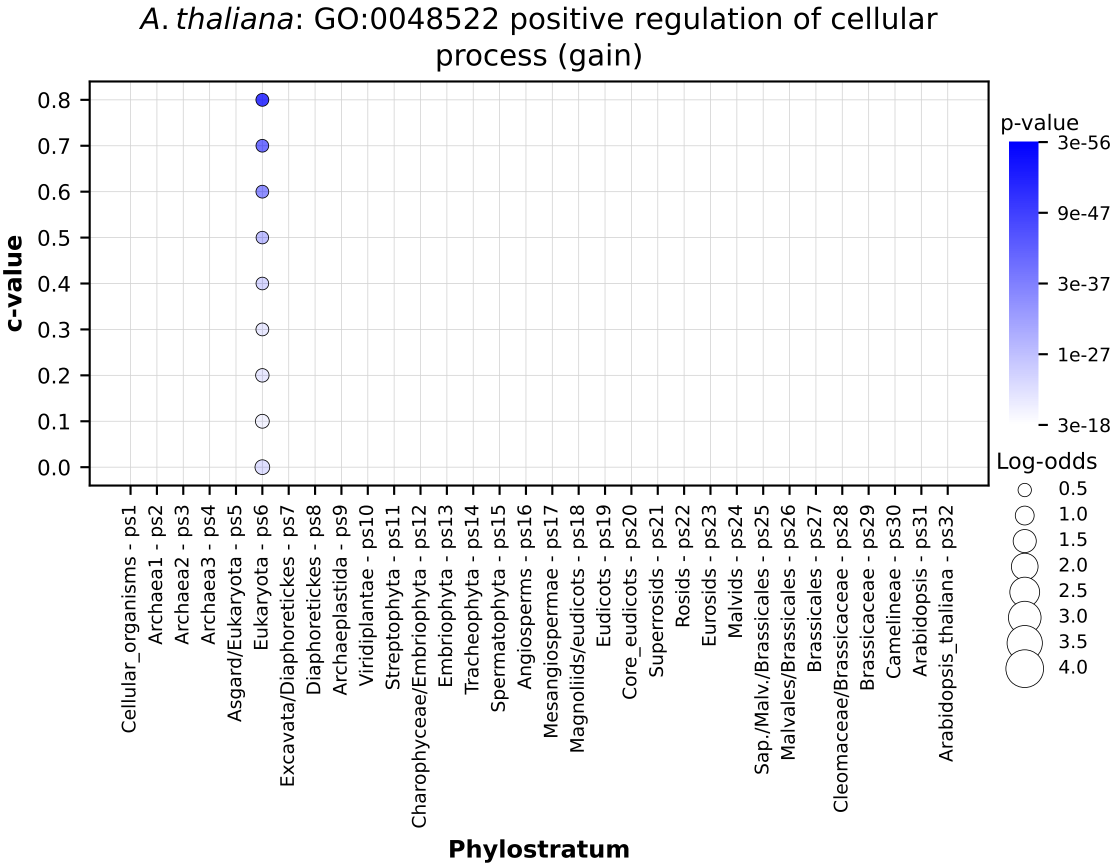

A. thaliana: GO:0048522 positive regulation of cellular process (gain)
c-value
0.8
0.7
0.6
0.5
0.4
0.3
0.2
0.1
0.0
p-value
3e-56
9e-47
3e-37
1e-27
3e-18
Log-odds
0.5
1.0
1.5
2.0
2.5
3.0
3.5
4.0
Cellular_organisms - ps1
Archaea1 - ps2
Archaea2 - ps3
Archaea3 - ps4
Asgard/Eukaryota - ps5
Eukaryota - ps6
Excavata/Diaphoretickes - ps7
Diaphoretickes - ps8
Archaeplastida - ps9
Viridiplantae - ps10
Streptophyta - ps11
Charophyceae/Embriophyta - ps12
Embriophyta - ps13
Tracheophyta - ps14
Spermatophyta - ps15
Angiosperms - ps16
Mesangiospermae - ps17
Magnoliids/eudicots - ps18
Eudicots - ps19
Core_eudicots - ps20
Superrosids - ps21
Rosids - ps22
Eurosids - ps23
Malvids - ps24
Sap./Malv./Brassicales - ps25
Malvales/Brassicales - ps26
Brassicales - ps27
Cleomaceae/Brassicaceae - ps28
Brassicaceae - ps29
Camelineae - ps30
Arabidopsis - ps31
Arabidopsis_thaliana - ps32
Phylostratum

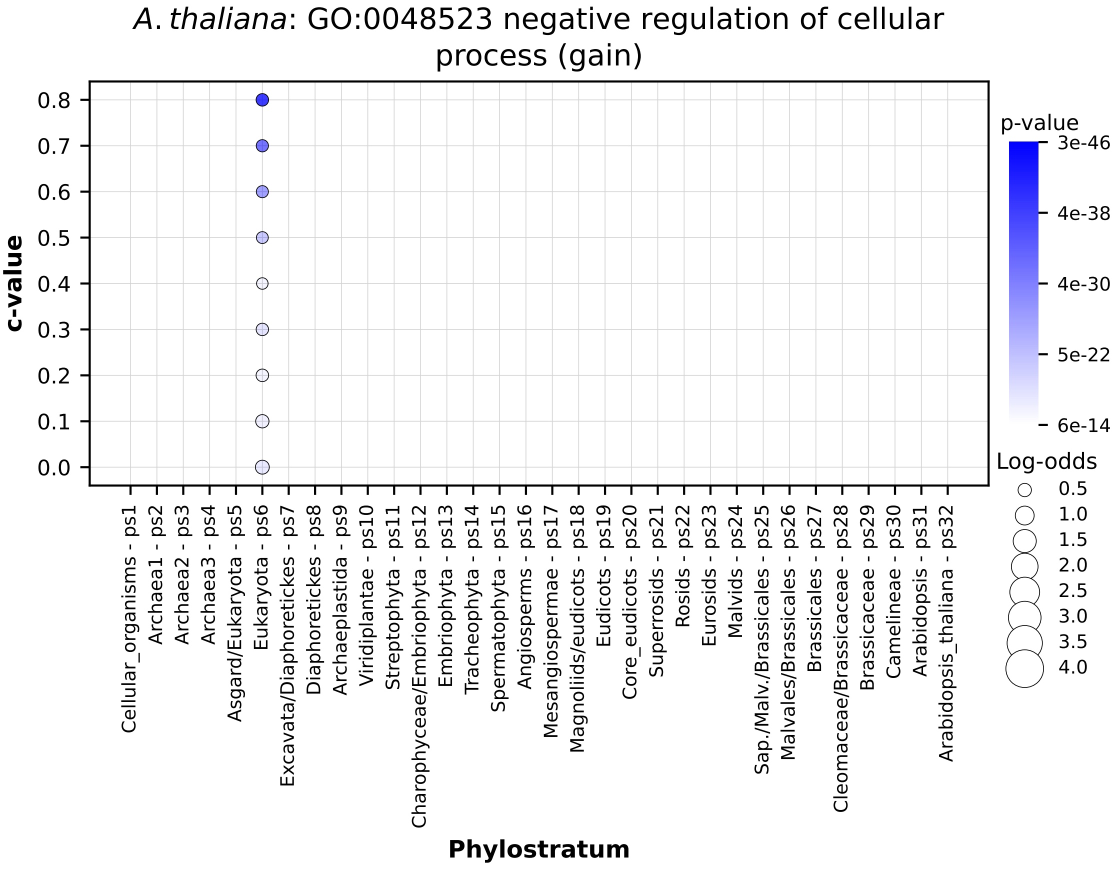
A. thaliana: GO:0048523 negative regulation of cellular process (gain)
c-value
0.8
0.7
0.6
0.5
0.4
0.3
0.2
0.1
0.0
p-value
3e-46
4e-38
4e-30
5e-22
6e-14
Log-odds
0.5
1.0
1.5
2.0
2.5
3.0
3.5
4.0
Cellular_organisms - ps1
Archaea1 - ps2
Archaea2 - ps3
Archaea3 - ps4
Asgard/Eukaryota - ps5
Eukaryota - ps6
Excavata/Diaphoretickes - ps7
Diaphoretickes - ps8
Archaeplastida - ps9
Viridiplantae - ps10
Streptophyta - ps11
Charophyceae/Embriophyta - ps12
Embriophyta - ps13
Tracheophyta - ps14
Spermatophyta - ps15
Angiosperms - ps16
Mesangiospermae - ps17
Magnoliids/eudicots - ps18
Eudicots - ps19
Core_eudicots - ps20
Superrosids - ps21
Rosids - ps22
Eurosids - ps23
Malvids - ps24
Sap./Malv./Brassicales - ps25
Malvales/Brassicales - ps26
Brassicales - ps27
Cleomaceae/Brassicaceae - ps28
Brassicaceae - ps29
Camelineae - ps30
Arabidopsis - ps31
Arabidopsis_thaliana - ps32
Phylostratum

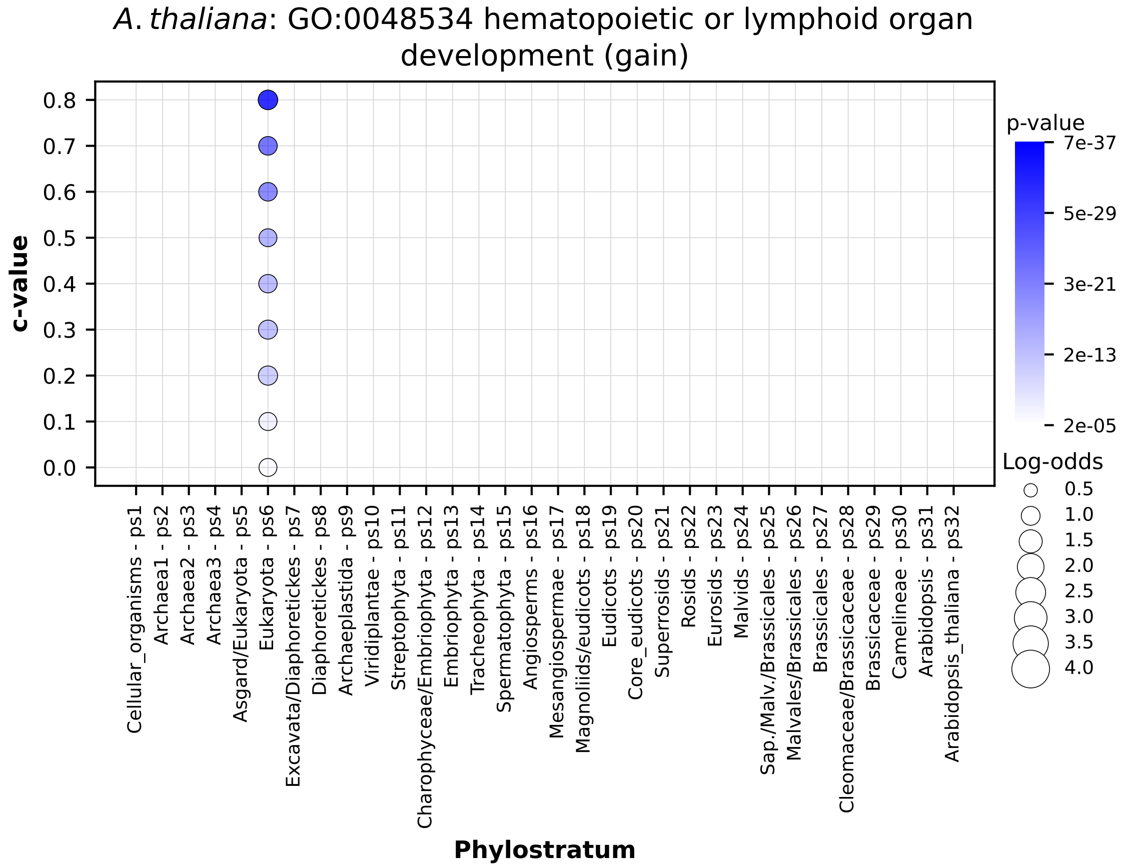
A. thaliana: GO:0048534 hematopoietic or lymphoid organ development (gain)
c-value
0.8
0.7
0.6
0.5
0.4
0.3
0.2
0.1
0.0
p-value
7e-37
5e-29
3e-21
2e-13
2e-05
Log-odds
0.5
1.0
1.5
2.0
2.5
3.0
3.5
4.0
Cellular_organisms - ps1
Archaea1 - ps2
Archaea2 - ps3
Archaea3 - ps4
Asgard/Eukaryota - ps5
Eukaryota - ps6
Excavata/Diaphoretickes - ps7
Diaphoretickes - ps8
Archaeplastida - ps9
Viridiplantae - ps10
Streptophyta - ps11
Charophyceae/Embriophyta - ps12
Embriophyta - ps13
Tracheophyta - ps14
Spermatophyta - ps15
Angiosperms - ps16
Mesangiospermae - ps17
Magnoliids/eudicots - ps18
Eudicots - ps19
Core_eudicots - ps20
Superrosids - ps21
Rosids - ps22
Eurosids - ps23
Malvids - ps24
Sap./Malv./Brassicales - ps25
Malvales/Brassicales - ps26
Brassicales - ps27
Cleomaceae/Brassicaceae - ps28
Brassicaceae - ps29
Camelineae - ps30
Arabidopsis - ps31
Arabidopsis_thaliana - ps32
Phylostratum

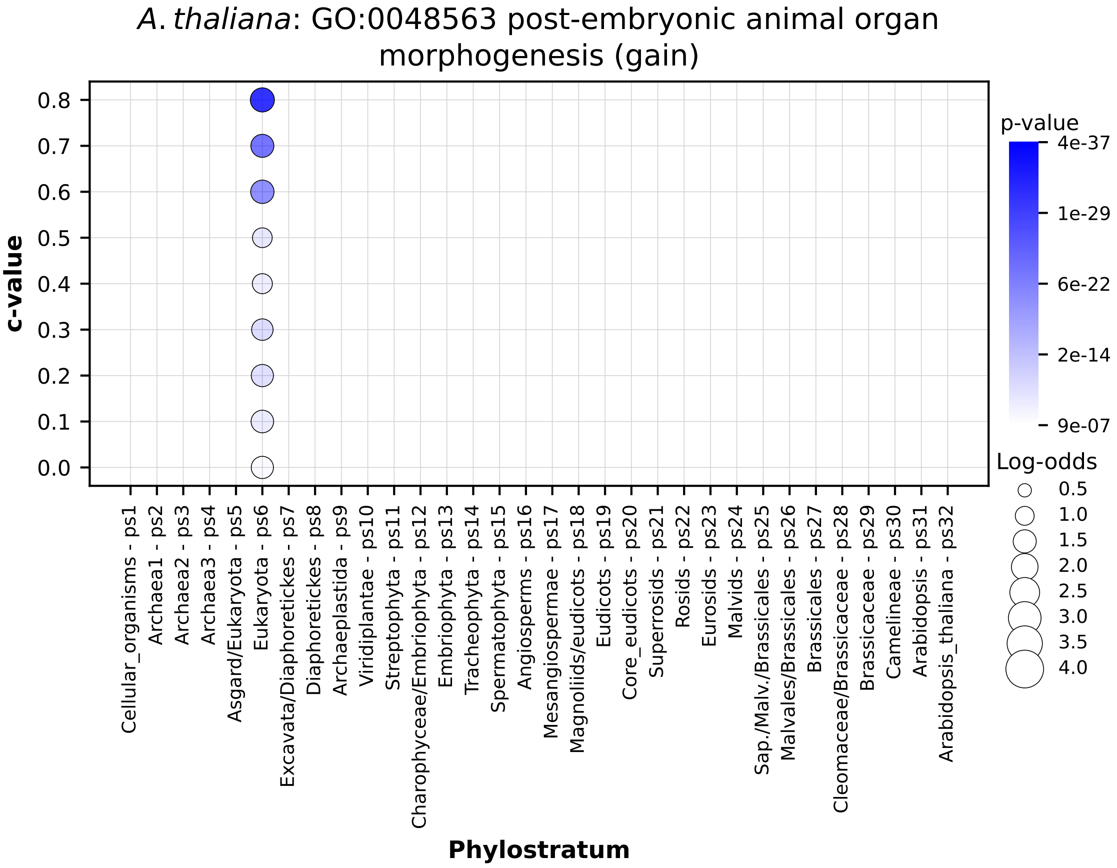

A. thaliana: GO:0048563 post-embryonic animal organ morphogenesis (gain)
c-value
0.8
0.7
0.6
0.5
0.4
0.3
0.2
0.1
0.0
p-value
4e-37
1e-29
6e-22
2e-14
9e-07
Log-odds
0.5
1.0
1.5
2.0
2.5
3.0
3.5
4.0
Cellular_organisms - ps1
Archaea1 - ps2
Archaea2 - ps3
Archaea3 - ps4
Asgard/Eukaryota - ps5
Eukaryota - ps6
Excavata/Diaphoretickes - ps7
Diaphoretickes - ps8
Archaeplastida - ps9
Viridiplantae - ps10
Streptophyta - ps11
Charophyceae/Embriophyta - ps12
Embriophyta - ps13
Tracheophyta - ps14
Spermatophyta - ps15
Angiosperms - ps16
Mesangiospermae - ps17
Magnoliids/eudicots - ps18
Eudicots - ps19
Core_eudicots - ps20
Superrosids - ps21
Rosids - ps22
Eurosids - ps23
Malvids - ps24
Sap./Malv./Brassicales - ps25
Malvales/Brassicales - ps26
Brassicales - ps27
Cleomaceae/Brassicaceae - ps28
Brassicaceae - ps29
Camelineae - ps30
Arabidopsis - ps31
Arabidopsis_thaliana - ps32
Phylostratum

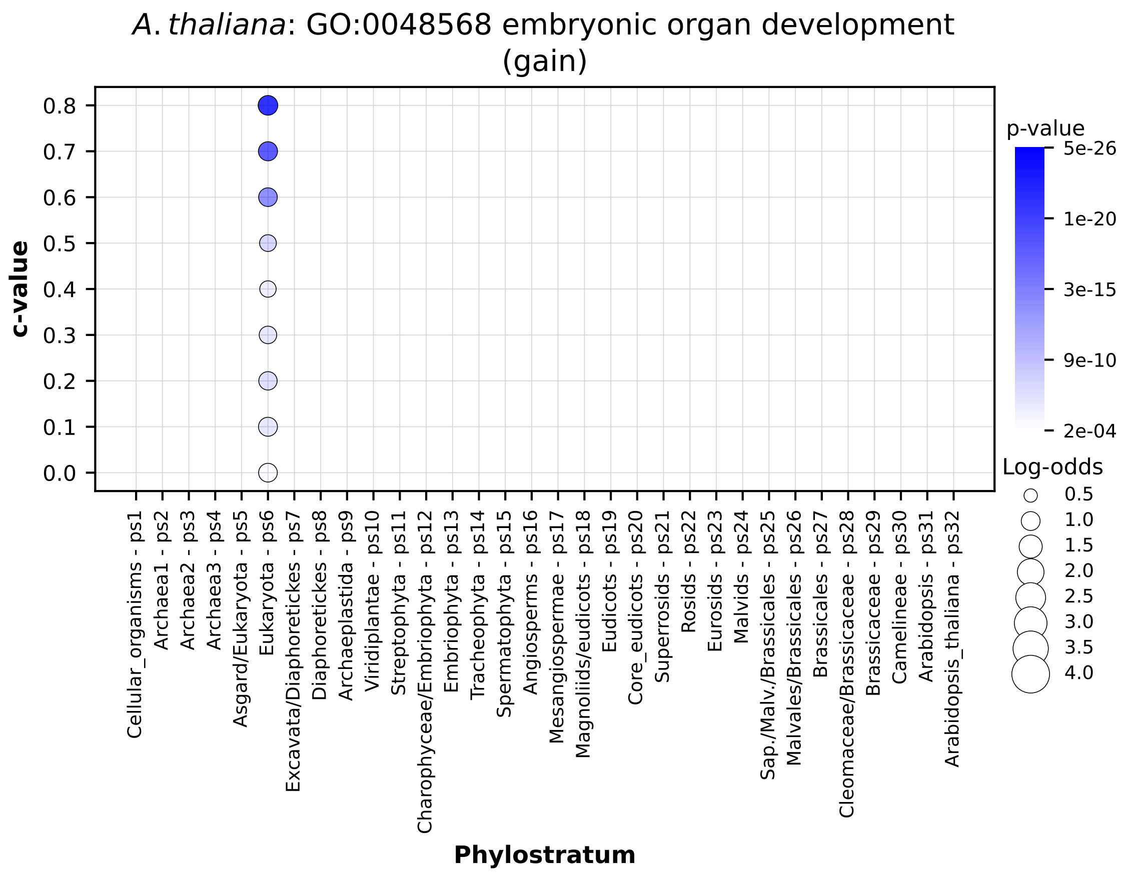

A. thaliana: GO:0048568 embryonic organ development
(gain)
c-value
0.8
0.7
0.6
0.5
0.4
0.3
0.2
0.1
0.0
p-value
5e-26
1e-20
3e-15
9e-10
2e-04
Log-odds
0.5
1.0
1.5
2.0
2.5
3.0
3.5
4.0
Cellular_organisms - ps1
Archaea1 - ps2
Archaea2 - ps3
Archaea3 - ps4
Asgard/Eukaryota - ps5
Eukaryota - ps6
Excavata/Diaphoretickes - ps7
Diaphoretickes - ps8
Archaeplastida - ps9
Viridiplantae - ps10
Streptophyta - ps11
Charophyceae/Embriophyta - ps12
Embriophyta - ps13
Tracheophyta - ps14
Spermatophyta - ps15
Angiosperms - ps16
Mesangiospermae - ps17
Magnoliids/eudicots - ps18
Eudicots - ps19
Core_eudicots - ps20
Superrosids - ps21
Rosids - ps22
Eurosids - ps23
Malvids - ps24
Sap./Malv./Brassicales - ps25
Malvales/Brassicales - ps26
Brassicales - ps27
Cleomaceae/Brassicaceae - ps28
Brassicaceae - ps29
Camelineae - ps30
Arabidopsis - ps31
Arabidopsis_thaliana - ps32
Phylostratum

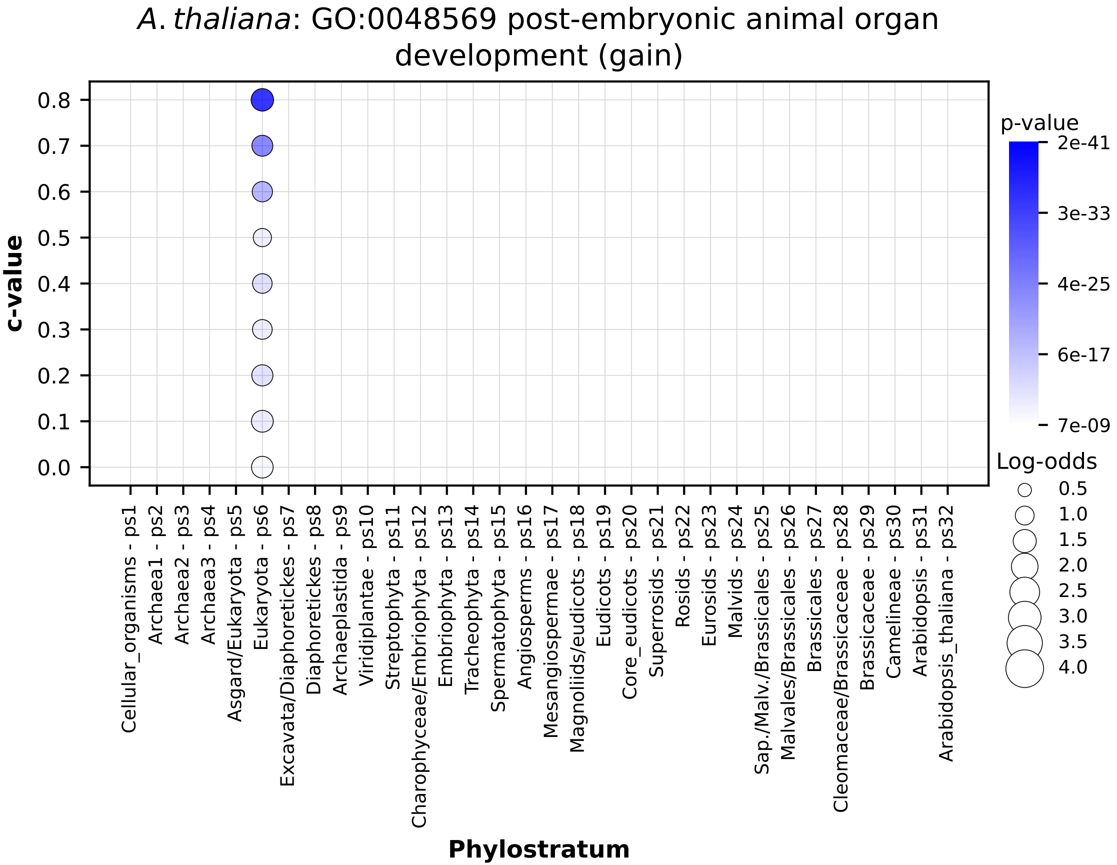
A. thaliana: GO:0048569 post-embryonic animal organ development (gain)
c-value
0.8
0.7
0.6
0.5
0.4
0.3
0.2
0.1
0.0
Phylostratum
Cellular_organisms - ps1
Archaea1 - ps2
Archaea2 - ps3
Archaea3 - ps4
Asgard/Eukaryota - ps5
Eukaryota - ps6
Excavata/Diaphoretickes - ps7
Diaphoretickes - ps8
Archaeplastida - ps9
Viridiplantae - ps10
Streptophyta - ps11
Charophyceae/Embriophyta - ps12
Embriophyta - ps13
Tracheophyta - ps14
Spermatophyta - ps15
Angiosperms - ps16
Mesangiospermae - ps17
Magnoliids/eudicots - ps18
Eudicots - ps19
Core_eudicots - ps20
Superrosids - ps21
Rosids - ps22
Eurosids - ps23
Malvids - ps24
Sap./Malv./Brassicales - ps25
Malvales/Brassicales - ps26
Brassicales - ps27
Cleomaceae/Brassicaceae - ps28
Brassicaceae - ps29
Camelineae - ps30
Arabidopsis - ps31
Arabidopsis_thaliana - ps32
p-value
2e-41
3e-33
4e-25
6e-17
7e-09
Log-odds
0.5
1.0
1.5
2.0
2.5
3.0
3.5
4.0

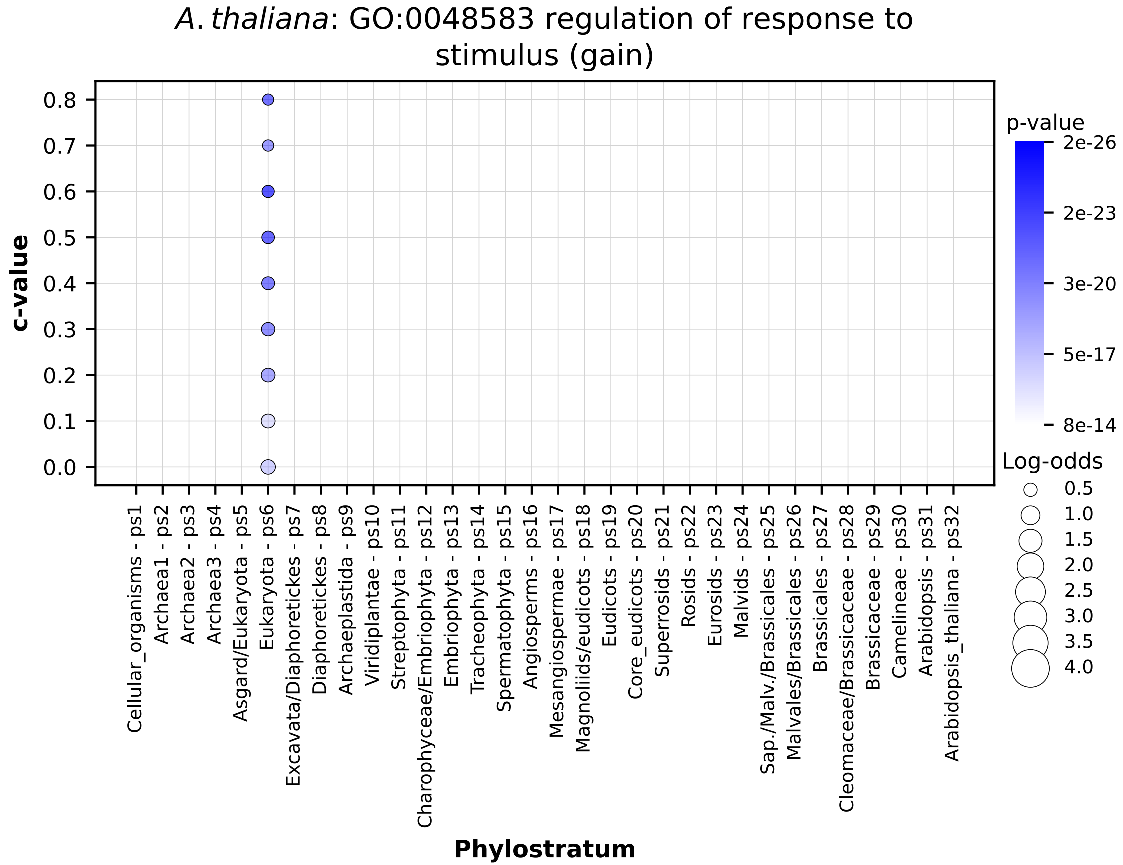

A. thaliana: GO:0048583 regulation of response to stimulus (gain)
c-value
0.8
0.7
0.6
0.5
0.4
0.3
0.2
0.1
0.0
p-value
2e-26
2e-23
3e-20
5e-17
8e-14
Log-odds
0.5
1.0
1.5
2.0
2.5
3.0
3.5
4.0
Cellular_organisms - ps1
Archaea1 - ps2
Archaea2 - ps3
Archaea3 - ps4
Asgard/Eukaryota - ps5
Eukaryota - ps6
Excavata/Diaphoretickes - ps7
Diaphoretickes - ps8
Archaeplastida - ps9
Viridiplantae - ps10
Streptophyta - ps11
Charophyceae/Embriophyta - ps12
Embriophyta - ps13
Tracheophyta - ps14
Spermatophyta - ps15
Angiosperms - ps16
Mesangiospermae - ps17
Magnoliids/eudicots - ps18
Eudicots - ps19
Core_eudicots - ps20
Superrosids - ps21
Rosids - ps22
Eurosids - ps23
Malvids - ps24
Sap./Malv./Brassicales - ps25
Malvales/Brassicales - ps26
Brassicales - ps27
Cleomaceae/Brassicaceae - ps28
Brassicaceae - ps29
Camelineae - ps30
Arabidopsis - ps31
Arabidopsis_thaliana - ps32
Phylostratum

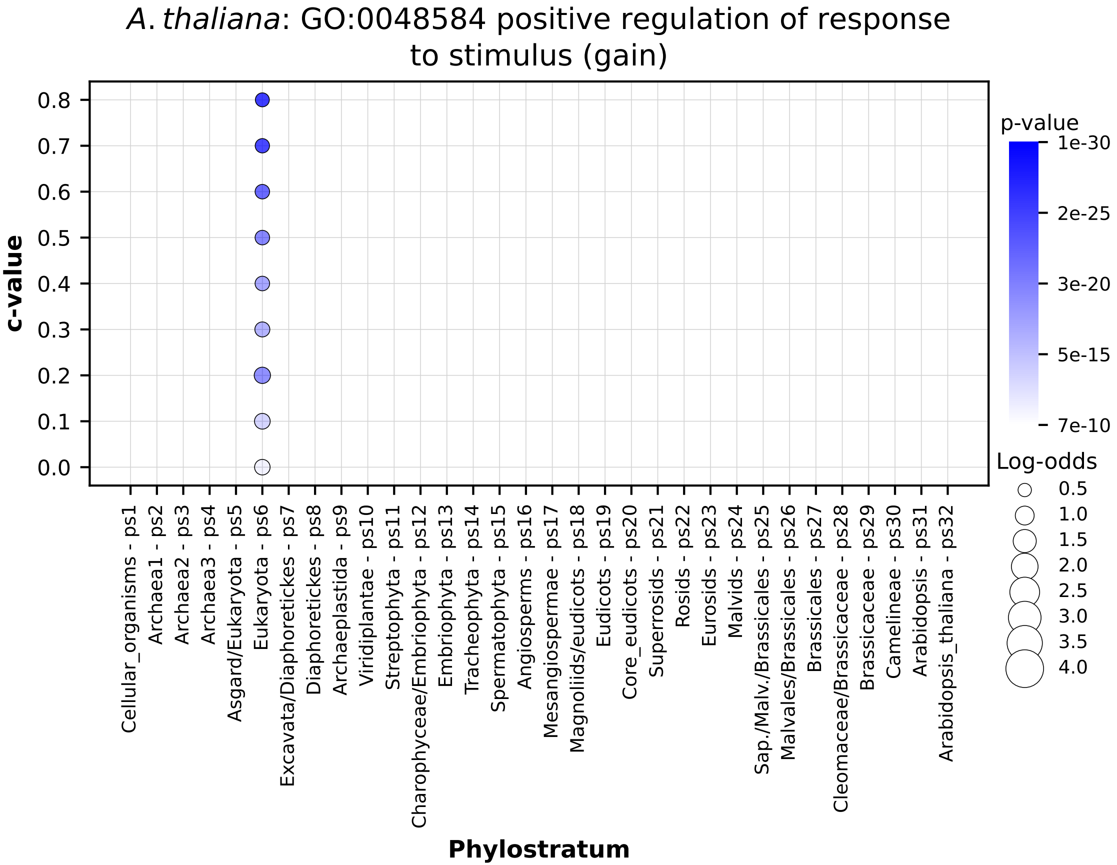

A. thaliana: GO:0048584 positive regulation of response to stimulus (gain)
c-value
0.8
0.7
0.6
0.5
0.4
0.3
0.2
0.1
0.0
Phylostratum
Cellular_organisms - ps1
Archaea1 - ps2
Archaea2 - ps3
Archaea3 - ps4
Asgard/Eukaryota - ps5
Eukaryota - ps6
Excavata/Diaphoretickes - ps7
Diaphoretickes - ps8
Archaeplastida - ps9
Viridiplantae - ps10
Streptophyta - ps11
Charophyceae/Embriophyta - ps12
Embriophyta - ps13
Tracheophyta - ps14
Spermatophyta - ps15
Angiosperms - ps16
Mesangiospermae - ps17
Magnoliids/eudicots - ps18
Eudicots - ps19
Core_eudicots - ps20
Superrosids - ps21
Rosids - ps22
Eurosids - ps23
Malvids - ps24
Sap./Malv./Brassicales - ps25
Malvales/Brassicales - ps26
Brassicales - ps27
Cleomaceae/Brassicaceae - ps28
Brassicaceae - ps29
Camelineae - ps30
Arabidopsis - ps31
Arabidopsis_thaliana - ps32
p-value
1e-30
2e-25
3e-20
5e-15
7e-10
Log-odds
0.5
1.0
1.5
2.0
2.5
3.0
3.5
4.0

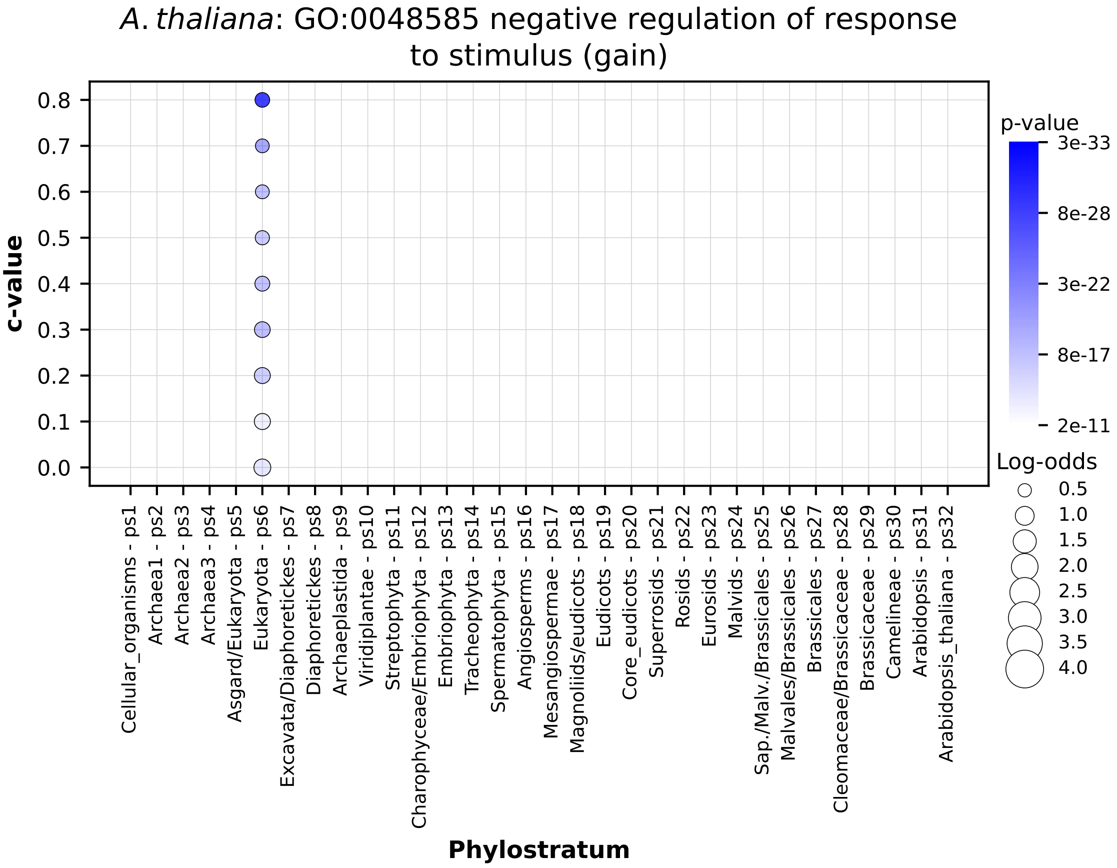

A. thaliana: GO:0048585 negative regulation of response to stimulus (gain)
c-value
0.8
0.7
0.6
0.5
0.4
0.3
0.2
0.1
0.0
Phylostratum
Cellular_organisms - ps1
Archaea1 - ps2
Archaea2 - ps3
Archaea3 - ps4
Asgard/Eukaryota - ps5
Eukaryota - ps6
Excavata/Diaphoretickes - ps7
Diaphoretickes - ps8
Archaeplastida - ps9
Viridiplantae - ps10
Streptophyta - ps11
Charophyceae/Embriophyta - ps12
Embriophyta - ps13
Tracheophyta - ps14
Spermatophyta - ps15
Angiosperms - ps16
Mesangiospermae - ps17
Magnoliids/eudicots - ps18
Eudicots - ps19
Core_eudicots - ps20
Superrosids - ps21
Rosids - ps22
Eurosids - ps23
Malvids - ps24
Sap./Malv./Brassicales - ps25
Malvales/Brassicales - ps26
Brassicales - ps27
Cleomaceae/Brassicaceae - ps28
Brassicaceae - ps29
Camelineae - ps30
Arabidopsis - ps31
Arabidopsis_thaliana - ps32
p-value
3e-33
8e-28
3e-22
8e-17
2e-11
Log-odds
0.5
1.0
1.5
2.0
2.5
3.0
3.5
4.0

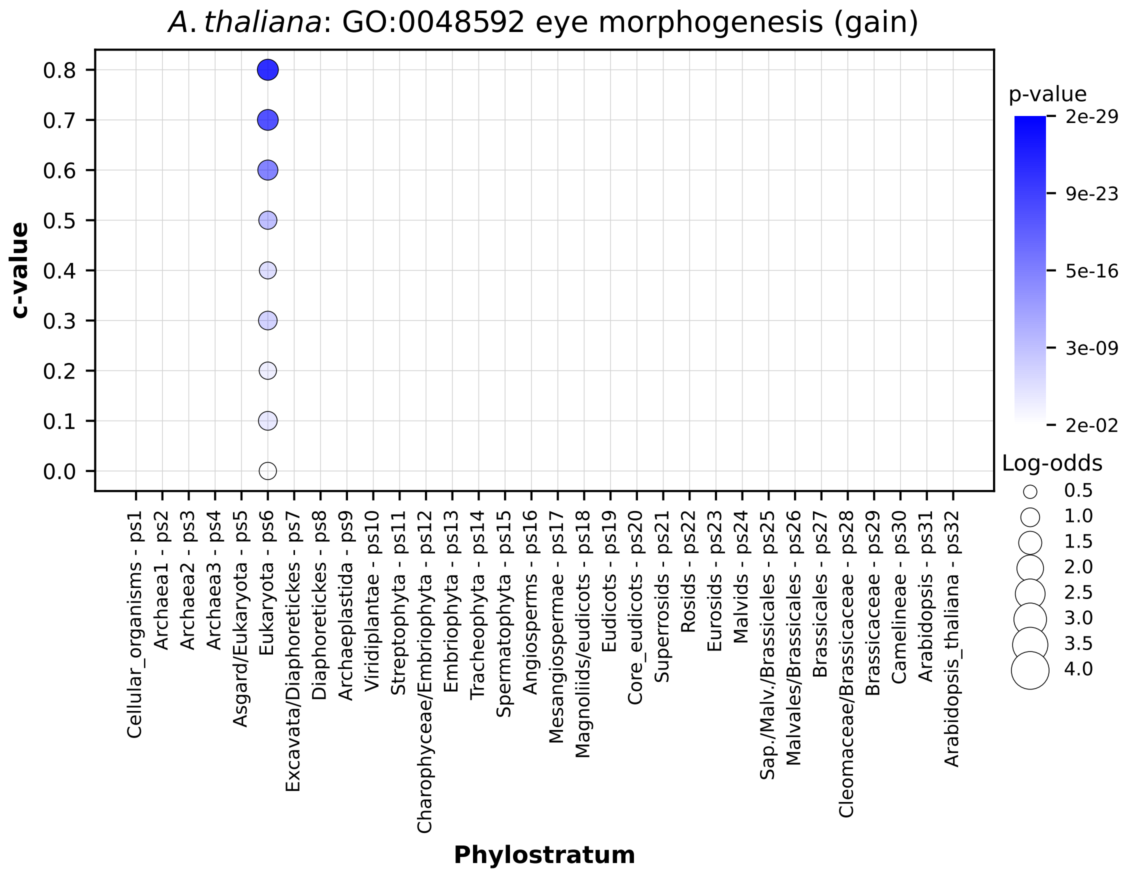
A. thaliana: GO:0048592 eye morphogenesis (gain)
c-value
0.8
0.7
0.6
0.5
0.4
0.3
0.2
0.1
0.0
p-value
2e-29
9e-23
5e-16
3e-09
2e-02
Log-odds
0.5
1.0
1.5
2.0
2.5
3.0
3.5
4.0
Cellular_organisms - ps1
Archaea1 - ps2
Archaea2 - ps3
Archaea3 - ps4
Asgard/Eukaryota - ps5
Eukaryota - ps6
Excavata/Diaphoretickes - ps7
Diaphoretickes - ps8
Archaeplastida - ps9
Viridiplantae - ps10
Streptophyta - ps11
Charophyceae/Embriophyta - ps12
Embriophyta - ps13
Tracheophyta - ps14
Spermatophyta - ps15
Angiosperms - ps16
Mesangiospermae - ps17
Magnoliids/eudicots - ps18
Eudicots - ps19
Core_eudicots - ps20
Superrosids - ps21
Rosids - ps22
Eurosids - ps23
Malvids - ps24
Sap./Malv./Brassicales - ps25
Malvales/Brassicales - ps26
Brassicales - ps27
Cleomaceae/Brassicaceae - ps28
Brassicaceae - ps29
Camelineae - ps30
Arabidopsis - ps31
Arabidopsis_thaliana - ps32
Phylostratum

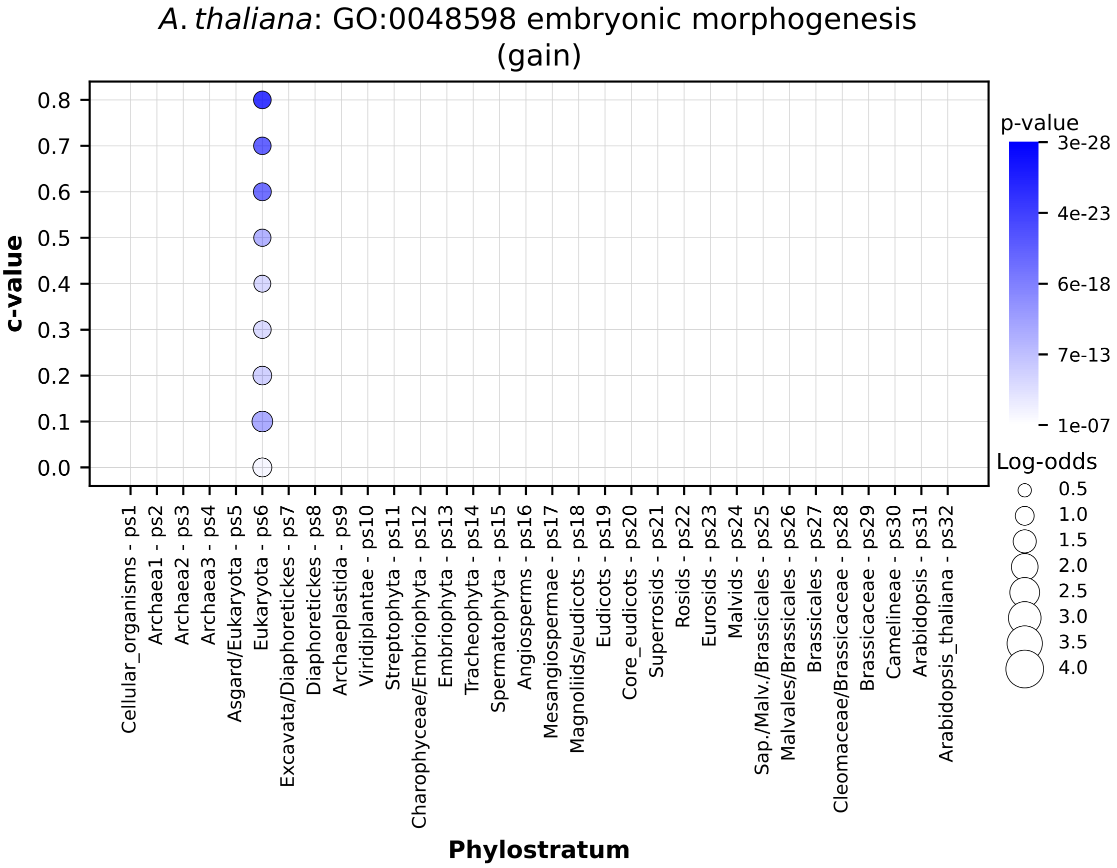
A. thaliana: GO:0048598 embryonic morphogenesis
(gain)
c-value
0.8
0.7
0.6
0.5
0.4
0.3
0.2
0.1
0.0
p-value
3e-28
4e-23
6e-18
7e-13
1e-07
Log-odds
0.5
1.0
1.5
2.0
2.5
3.0
3.5
4.0
Cellular_organisms - ps1
Archaea1 - ps2
Archaea2 - ps3
Archaea3 - ps4
Asgard/Eukaryota - ps5
Eukaryota - ps6
Excavata/Diaphoretickes - ps7
Diaphoretickes - ps8
Archaeplastida - ps9
Viridiplantae - ps10
Streptophyta - ps11
Charophyceae/Embriophyta - ps12
Embriophyta - ps13
Tracheophyta - ps14
Spermatophyta - ps15
Angiosperms - ps16
Mesangiospermae - ps17
Magnoliids/eudicots - ps18
Eudicots - ps19
Core_eudicots - ps20
Superrosids - ps21
Rosids - ps22
Eurosids - ps23
Malvids - ps24
Sap./Malv./Brassicales - ps25
Malvales/Brassicales - ps26
Brassicales - ps27
Cleomaceae/Brassicaceae - ps28
Brassicaceae - ps29
Camelineae - ps30
Arabidopsis - ps31
Arabidopsis_thaliana - ps32
Phylostratum

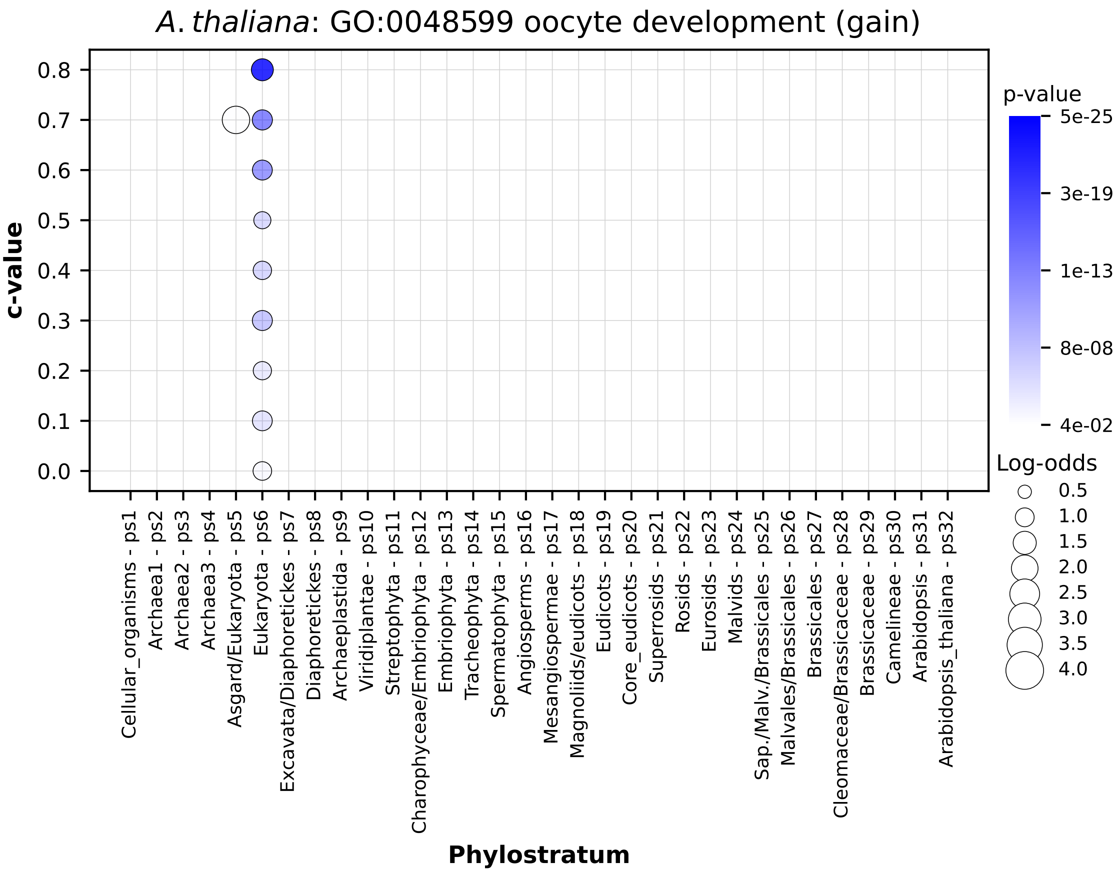
A. thaliana: GO:0048599 oocyte development (gain)
c-value
0.8
0.7
0.6
0.5
0.4
0.3
0.2
0.1
0.0
p-value
5e-25
3e-19
1e-13
8e-08
4e-02
Log-odds
0.5
1.0
1.5
2.0
2.5
3.0
3.5
4.0
Cellular_organisms - ps1
Archaea1 - ps2
Archaea2 - ps3
Archaea3 - ps4
Asgard/Eukaryota - ps5
Eukaryota - ps6
Excavata/Diaphoretickes - ps7
Diaphoretickes - ps8
Archaeplastida - ps9
Viridiplantae - ps10
Streptophyta - ps11
Charophyceae/Embriophyta - ps12
Embriophyta - ps13
Tracheophyta - ps14
Spermatophyta - ps15
Angiosperms - ps16
Mesangiospermae - ps17
Magnoliids/eudicots - ps18
Eudicots - ps19
Core_eudicots - ps20
Superrosids - ps21
Rosids - ps22
Eurosids - ps23
Malvids - ps24
Sap./Malv./Brassicales - ps25
Malvales/Brassicales - ps26
Brassicales - ps27
Cleomaceae/Brassicaceae - ps28
Brassicaceae - ps29
Camelineae - ps30
Arabidopsis - ps31
Arabidopsis_thaliana - ps32
Phylostratum

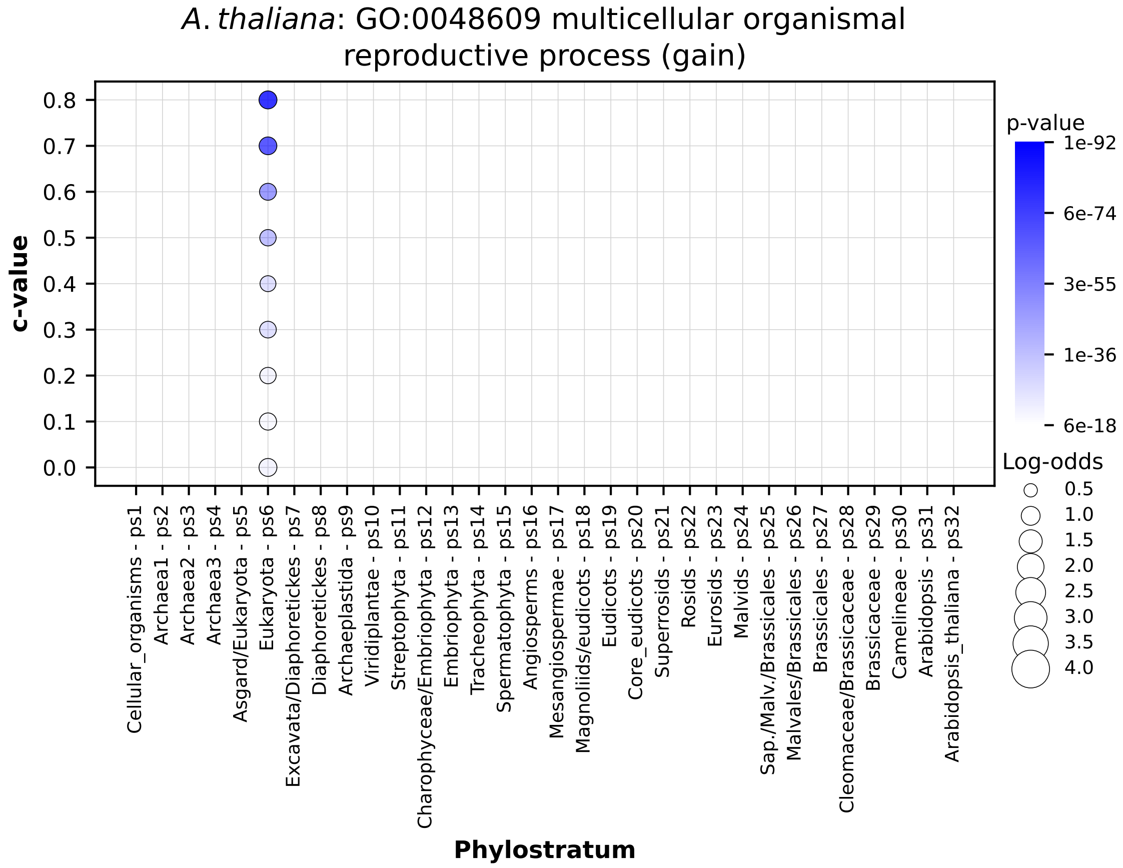
A. thaliana: GO:0048609 multicellular organismal reproductive process (gain)
c-value
0.8
0.7
0.6
0.5
0.4
0.3
0.2
0.1
0.0
p-value
1e-92
6e-74
3e-55
1e-36
6e-18
Log-odds
0.5
1.0
1.5
2.0
2.5
3.0
3.5
4.0
Cellular_organisms - ps1
Archaea1 - ps2
Archaea2 - ps3
Archaea3 - ps4
Asgard/Eukaryota - ps5
Eukaryota - ps6
Excavata/Diaphoretickes - ps7
Diaphoretickes - ps8
Archaeplastida - ps9
Viridiplantae - ps10
Streptophyta - ps11
Charophyceae/Embriophyta - ps12
Embriophyta - ps13
Tracheophyta - ps14
Spermatophyta - ps15
Angiosperms - ps16
Mesangiospermae - ps17
Magnoliids/eudicots - ps18
Eudicots - ps19
Core_eudicots - ps20
Superrosids - ps21
Rosids - ps22
Eurosids - ps23
Malvids - ps24
Sap./Malv./Brassicales - ps25
Malvales/Brassicales - ps26
Brassicales - ps27
Cleomaceae/Brassicaceae - ps28
Brassicaceae - ps29
Camelineae - ps30
Arabidopsis - ps31
Arabidopsis_thaliana - ps32
Phylostratum

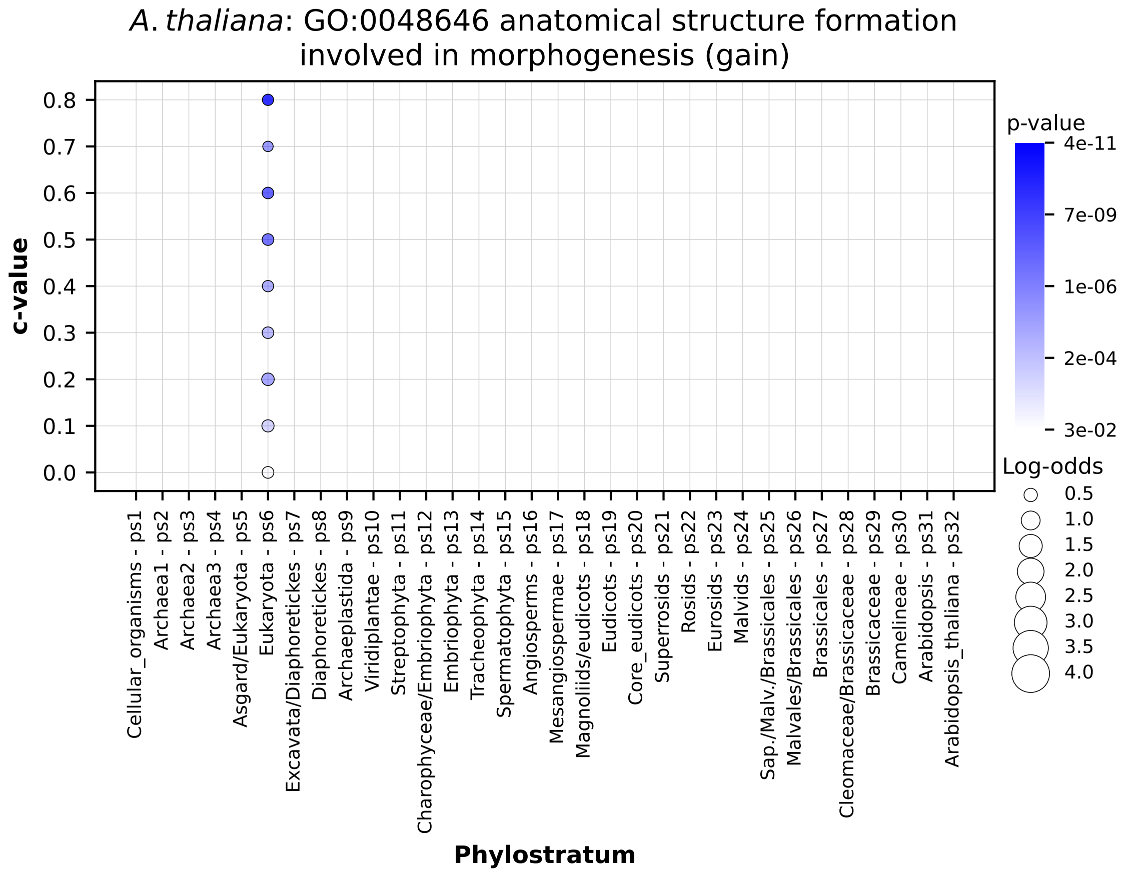
A. thaliana: GO:0048646 anatomical structure formation involved in morphogenesis (gain)
c-value
0.8
0.7
0.6
0.5
0.4
0.3
0.2
0.1
0.0
p-value
4e-11
7e-09
1e-06
2e-04
3e-02
Log-odds
0.5
1.0
1.5
2.0
2.5
3.0
3.5
4.0
Cellular_organisms - ps1
Archaea1 - ps2
Archaea2 - ps3
Archaea3 - ps4
Asgard/Eukaryota - ps5
Eukaryota - ps6
Excavata/Diaphoretickes - ps7
Diaphoretickes - ps8
Archaeplastida - ps9
Viridiplantae - ps10
Streptophyta - ps11
Charophyceae/Embriophyta - ps12
Embriophyta - ps13
Tracheophyta - ps14
Spermatophyta - ps15
Angiosperms - ps16
Mesangiospermae - ps17
Magnoliids/eudicots - ps18
Eudicots - ps19
Core_eudicots - ps20
Superrosids - ps21
Rosids - ps22
Eurosids - ps23
Malvids - ps24
Sap./Malv./Brassicales - ps25
Malvales/Brassicales - ps26
Brassicales - ps27
Cleomaceae/Brassicaceae - ps28
Brassicaceae - ps29
Camelineae - ps30
Arabidopsis - ps31
Arabidopsis_thaliana - ps32
Phylostratum

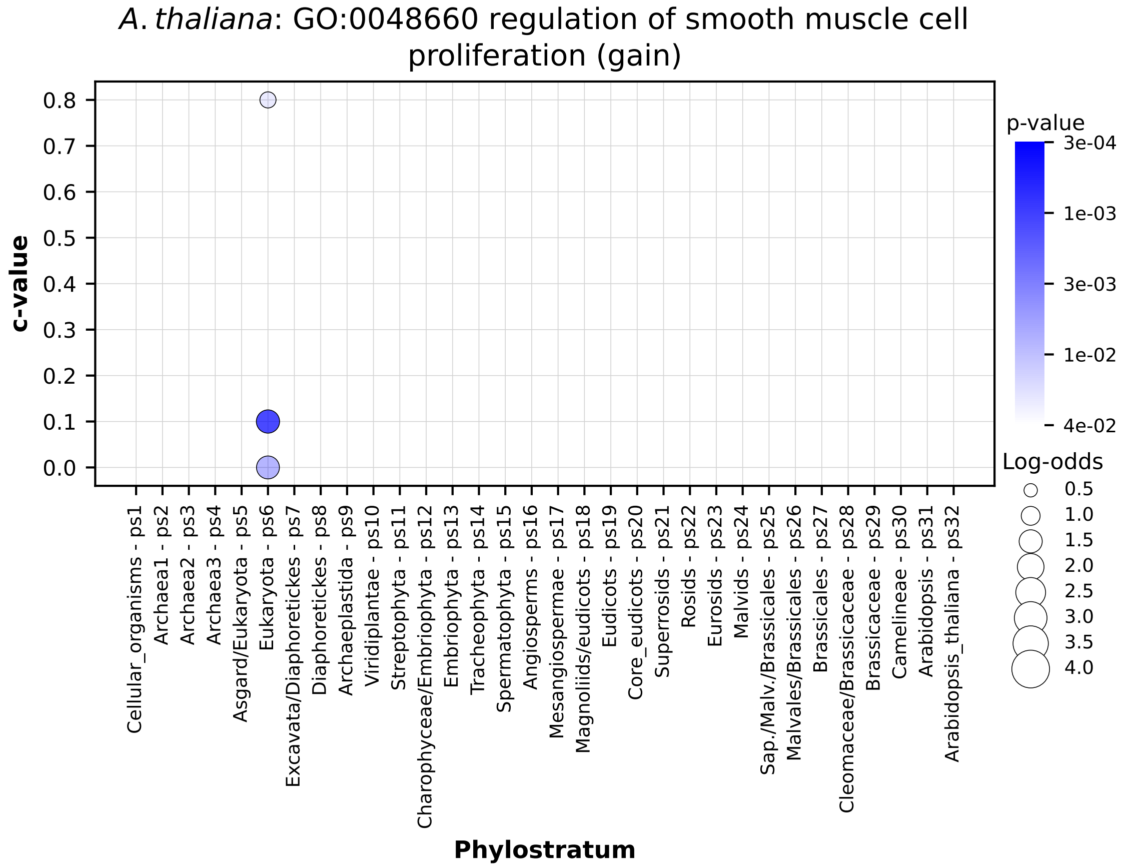
A. thaliana: GO:0048660 regulation of smooth muscle cell proliferation (gain)
c-value
0.8
0.7
0.6
0.5
0.4
0.3
0.2
0.1
0.0
Phylostratum
Cellular_organisms - ps1
Archaea1 - ps2
Archaea2 - ps3
Archaea3 - ps4
Asgard/Eukaryota - ps5
Eukaryota - ps6
Excavata/Diaphoretickes - ps7
Diaphoretickes - ps8
Archaeplastida - ps9
Viridiplantae - ps10
Streptophyta - ps11
Charophyceae/Embriophyta - ps12
Embriophyta - ps13
Tracheophyta - ps14
Spermatophyta - ps15
Angiosperms - ps16
Mesangiospermae - ps17
Magnoliids/eudicots - ps18
Eudicots - ps19
Core_eudicots - ps20
Superrosids - ps21
Rosids - ps22
Eurosids - ps23
Malvids - ps24
Sap./Malv./Brassicales - ps25
Malvales/Brassicales - ps26
Brassicales - ps27
Cleomaceae/Brassicaceae - ps28
Brassicaceae - ps29
Camelineae - ps30
Arabidopsis - ps31
Arabidopsis_thaliana - ps32
p-value
3e-04
1e-03
3e-03
1e-02
4e-02
Log-odds
0.5
1.0
1.5
2.0
2.5
3.0
3.5
4.0

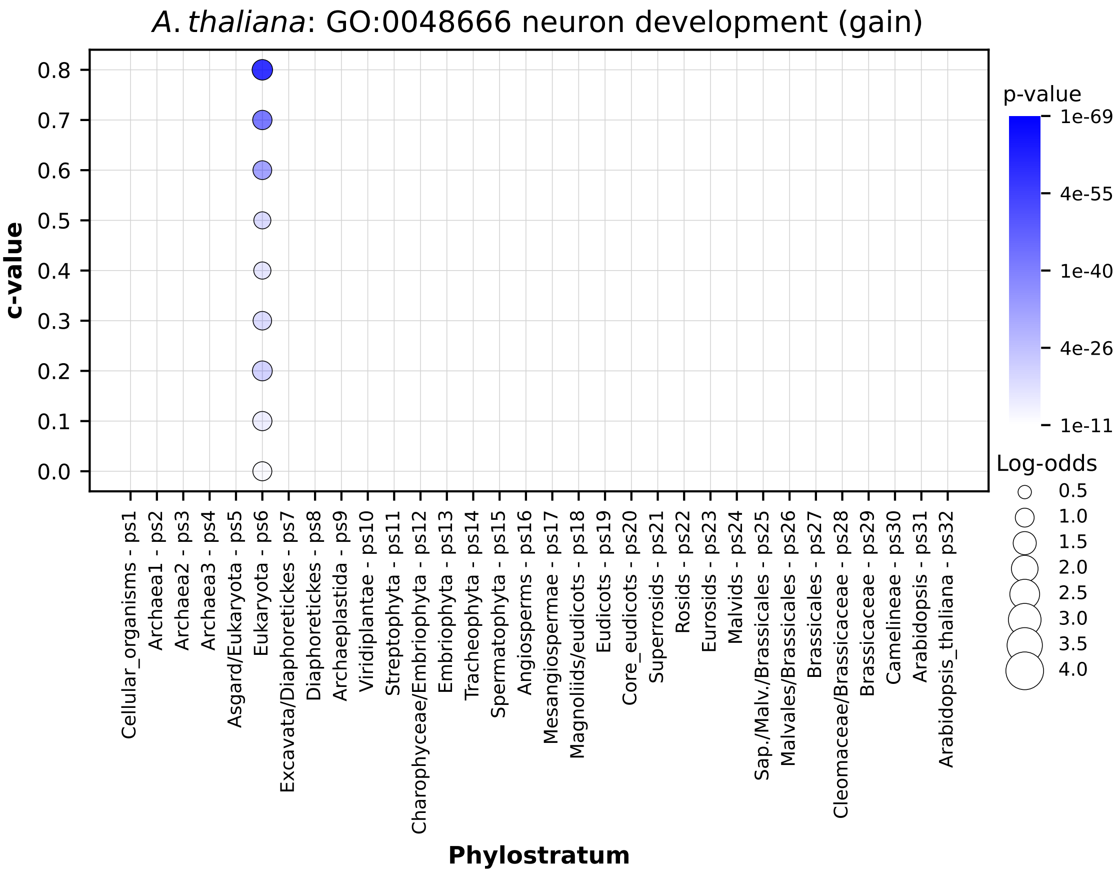
A. thaliana: GO:0048666 neuron development (gain)
c-value
0.0
0.1
0.2
0.3
0.4
0.5
0.6
0.7
0.8
Phylostratum
Cellular_organisms - ps1
Archaea1 - ps2
Archaea2 - ps3
Archaea3 - ps4
Asgard/Eukaryota - ps5
Eukaryota - ps6
Excavata/Diaphoretickes - ps7
Diaphoretickes - ps8
Archaeplastida - ps9
Viridiplantae - ps10
Streptophyta - ps11
Charophyceae/Embriophyta - ps12
Embriophyta - ps13
Tracheophyta - ps14
Spermatophyta - ps15
Angiosperms - ps16
Mesangiospermae - ps17
Magnoliids/eudicots - ps18
Eudicots - ps19
Core_eudicots - ps20
Superrosids - ps21
Rosids - ps22
Eurosids - ps23
Malvids - ps24
Sap./Malv./Brassicales - ps25
Malvales/Brassicales - ps26
Brassicales - ps27
Cleomaceae/Brassicaceae - ps28
Brassicaceae - ps29
Camelineae - ps30
Arabidopsis - ps31
Arabidopsis_thaliana - ps32
p-value
1e-69
4e-55
1e-40
4e-26
1e-11
Log-odds
0.5
1.0
1.5
2.0
2.5
3.0
3.5
4.0

A. thaliana: GO:0048667 cell morphogenesis involved in neuron differentiation (gain)

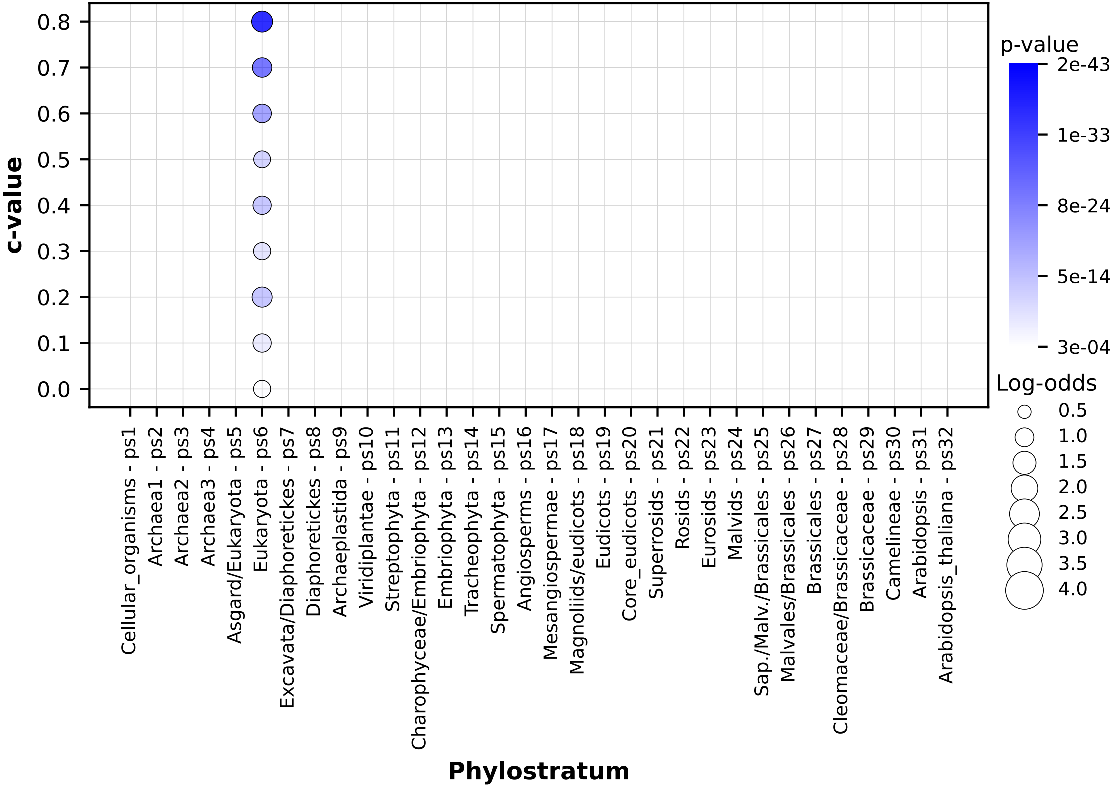

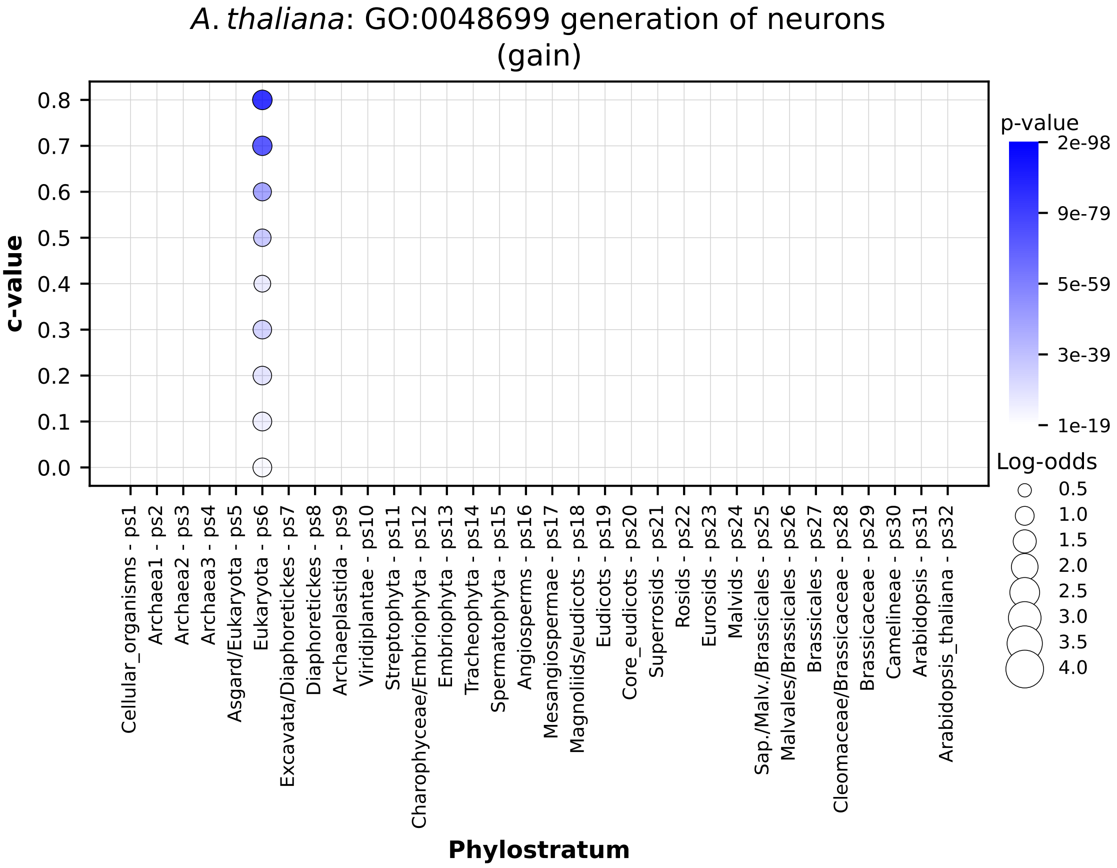

A. thaliana: GO:0048699 generation of neurons
(gain)
c-value
0.8
0.7
0.6
0.5
0.4
0.3
0.2
0.1
0.0
p-value
2e-98
9e-79
5e-59
3e-39
1e-19
Log-odds
0.5
1.0
1.5
2.0
2.5
3.0
3.5
4.0
Cellular_organisms - ps1
Archaea1 - ps2
Archaea2 - ps3
Archaea3 - ps4
Asgard/Eukaryota - ps5
Eukaryota - ps6
Excavata/Diaphoretickes - ps7
Diaphoretickes - ps8
Archaeplastida - ps9
Viridiplantae - ps10
Streptophyta - ps11
Charophyceae/Embriophyta - ps12
Embriophyta - ps13
Tracheophyta - ps14
Spermatophyta - ps15
Angiosperms - ps16
Mesangiospermae - ps17
Magnoliids/eudicots - ps18
Eudicots - ps19
Core_eudicots - ps20
Superrosids - ps21
Rosids - ps22
Eurosids - ps23
Malvids - ps24
Sap./Malv./Brassicales - ps25
Malvales/Brassicales - ps26
Brassicales - ps27
Cleomaceae/Brassicaceae - ps28
Brassicaceae - ps29
Camelineae - ps30
Arabidopsis - ps31
Arabidopsis_thaliana - ps32
Phylostratum

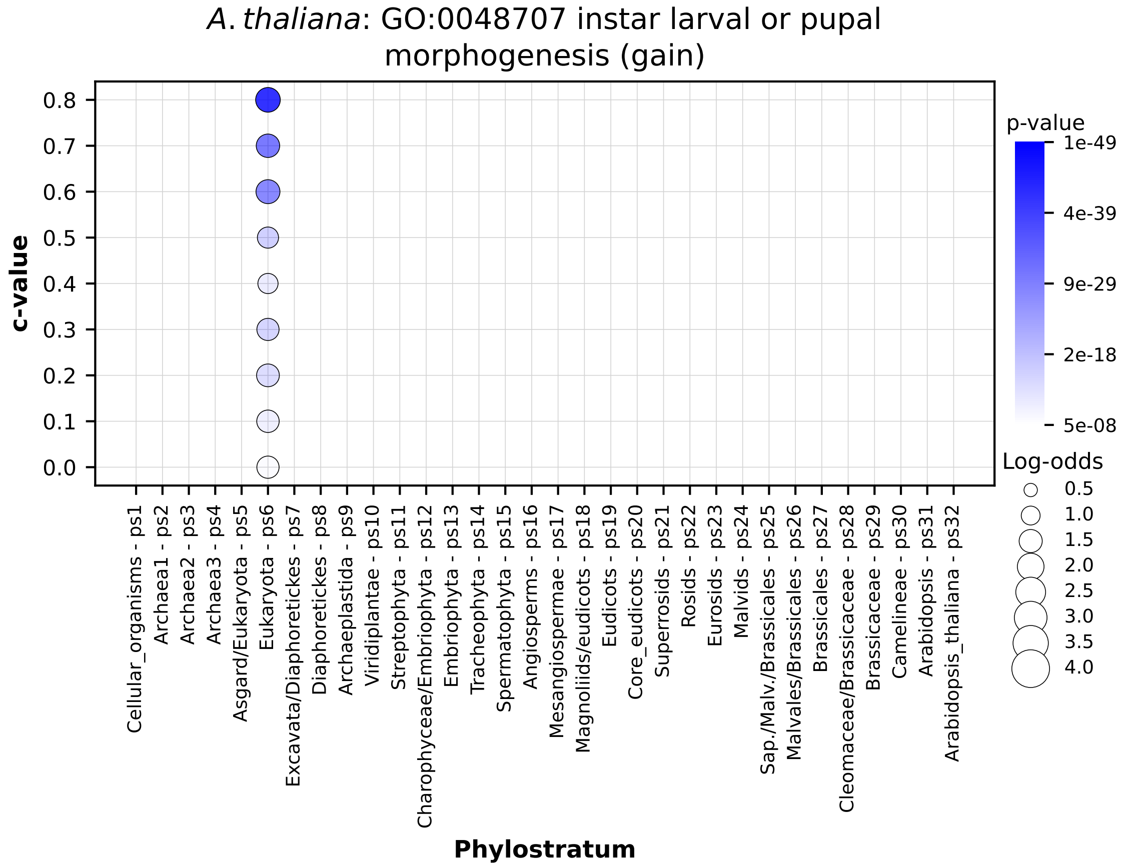
A. thaliana: GO:0048707 instar larval or pupal morphogenesis (gain)
c-value
0.0
0.1
0.2
0.3
0.4
0.5
0.6
0.7
0.8
p-value
1e-49
4e-39
9e-29
2e-18
5e-08
Log-odds
0.5
1.0
1.5
2.0
2.5
3.0
3.5
4.0
Cellular_organisms - ps1
Archaea1 - ps2
Archaea2 - ps3
Archaea3 - ps4
Asgard/Eukaryota - ps5
Eukaryota - ps6
Excavata/Diaphoretickes - ps7
Diaphoretickes - ps8
Archaeplastida - ps9
Viridiplantae - ps10
Streptophyta - ps11
Charophyceae/Embriophyta - ps12
Embriophyta - ps13
Tracheophyta - ps14
Spermatophyta - ps15
Angiosperms - ps16
Mesangiospermae - ps17
Magnoliids/eudicots - ps18
Eudicots - ps19
Core_eudicots - ps20
Superrosids - ps21
Rosids - ps22
Eurosids - ps23
Malvids - ps24
Sap./Malv./Brassicales - ps25
Malvales/Brassicales - ps26
Brassicales - ps27
Cleomaceae/Brassicaceae - ps28
Brassicaceae - ps29
Camelineae - ps30
Arabidopsis - ps31
Arabidopsis_thaliana - ps32
Phylostratum

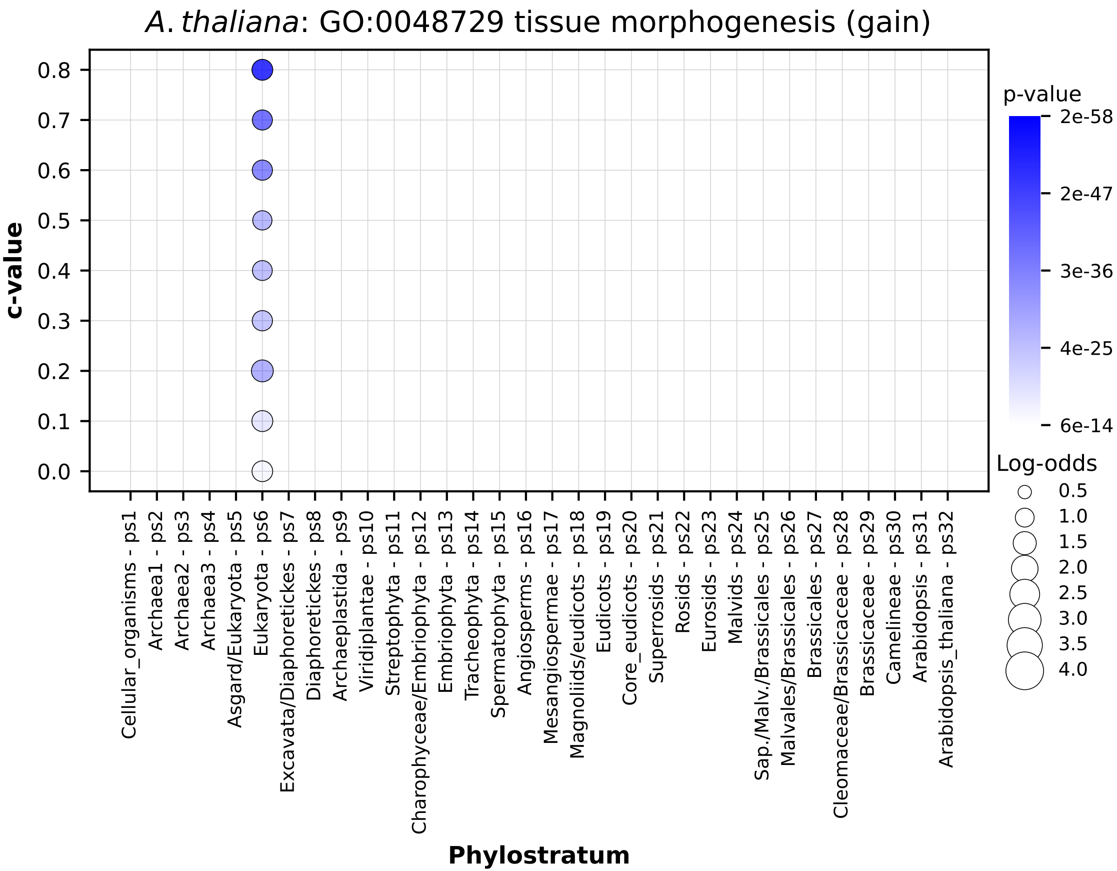

A. thaliana: GO:0048729 tissue morphogenesis (gain)
c-value
0.8
0.7
0.6
0.5
0.4
0.3
0.2
0.1
0.0
p-value
2e-58
2e-47
3e-36
4e-25
6e-14
Log-odds
0.5
1.0
1.5
2.0
2.5
3.0
3.5
4.0
Cellular_organisms - ps1
Archaea1 - ps2
Archaea2 - ps3
Archaea3 - ps4
Asgard/Eukaryota - ps5
Eukaryota - ps6
Excavata/Diaphoretickes - ps7
Diaphoretickes - ps8
Archaeplastida - ps9
Viridiplantae - ps10
Streptophyta - ps11
Charophyceae/Embriophyta - ps12
Embriophyta - ps13
Tracheophyta - ps14
Spermatophyta - ps15
Angiosperms - ps16
Mesangiospermae - ps17
Magnoliids/eudicots - ps18
Eudicots - ps19
Core_eudicots - ps20
Superrosids - ps21
Rosids - ps22
Eurosids - ps23
Malvids - ps24
Sap./Malv./Brassicales - ps25
Malvales/Brassicales - ps26
Brassicales - ps27
Cleomaceae/Brassicaceae - ps28
Brassicaceae - ps29
Camelineae - ps30
Arabidopsis - ps31
Arabidopsis_thaliana - ps32
Phylostratum

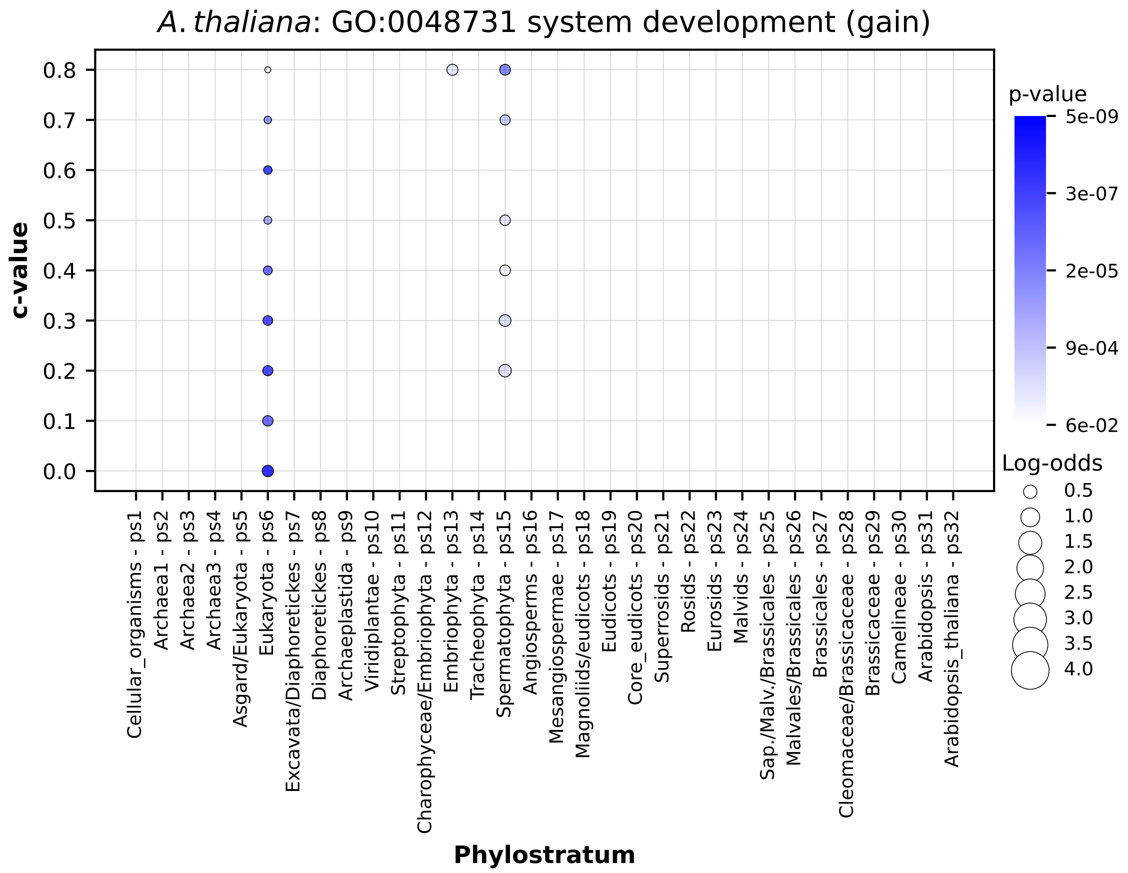
A. thaliana: GO:0048731 system development (gain)
c-value
0.0
0.1
0.2
0.3
0.4
0.5
0.6
0.7
0.8
Phylostratum
Cellular_organisms - ps1
Archaea1 - ps2
Archaea2 - ps3
Archaea3 - ps4
Asgard/Eukaryota - ps5
Eukaryota - ps6
Excavata/Diaphoretickes - ps7
Diaphoretickes - ps8
Archaeplastida - ps9
Viridiplantae - ps10
Streptophyta - ps11
Charophyceae/Embriophyta - ps12
Embriophyta - ps13
Tracheophyta - ps14
Spermatophyta - ps15
Angiosperms - ps16
Mesangiospermae - ps17
Magnoliids/eudicots - ps18
Eudicots - ps19
Core_eudicots - ps20
Superrosids - ps21
Rosids - ps22
Eurosids - ps23
Malvids - ps24
Sap./Malv./Brassicales - ps25
Malvales/Brassicales - ps26
Brassicales - ps27
Cleomaceae/Brassicaceae - ps28
Brassicaceae - ps29
Camelineae - ps30
Arabidopsis - ps31
Arabidopsis_thaliana - ps32
p-value
5e-09
3e-07
2e-05
9e-04
6e-02
Log-odds
0.5
1.0
1.5
2.0
2.5
3.0
3.5
4.0

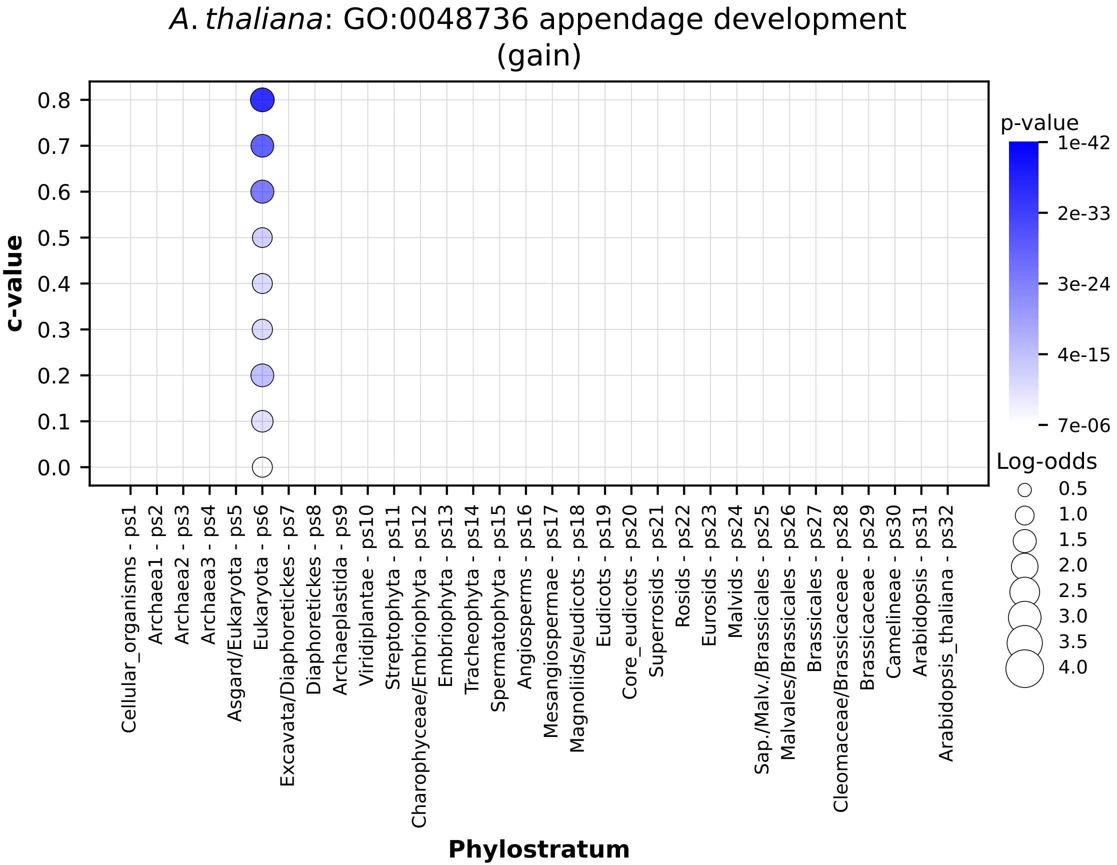
A. thaliana: GO:0048736 appendage development (gain)
c-value
0.0
0.1
0.2
0.3
0.4
0.5
0.6
0.7
0.8
Phylostratum
Cellular_organisms - ps1
Archaea1 - ps2
Archaea2 - ps3
Archaea3 - ps4
Asgard/Eukaryota - ps5
Eukaryota - ps6
Excavata/Diaphoretickes - ps7
Diaphoretickes - ps8
Archaeplastida - ps9
Viridiplantae - ps10
Streptophyta - ps11
Charophyceae/Embriophyta - ps12
Embriophyta - ps13
Tracheophyta - ps14
Spermatophyta - ps15
Angiosperms - ps16
Mesangiospermae - ps17
Magnoliids/eudicots - ps18
Eudicots - ps19
Core_eudicots - ps20
Superrosids - ps21
Rosids - ps22
Eurosids - ps23
Malvids - ps24
Sap./Malv./Brassicales - ps25
Malvales/Brassicales - ps26
Brassicales - ps27
Cleomaceae/Brassicaceae - ps28
Brassicaceae - ps29
Camelineae - ps30
Arabidopsis - ps31
Arabidopsis_thaliana - ps32
p-value
1e-42
2e-33
3e-24
4e-15
7e-06
Log-odds
0.5
1.0
1.5
2.0
2.5
3.0
3.5
4.0

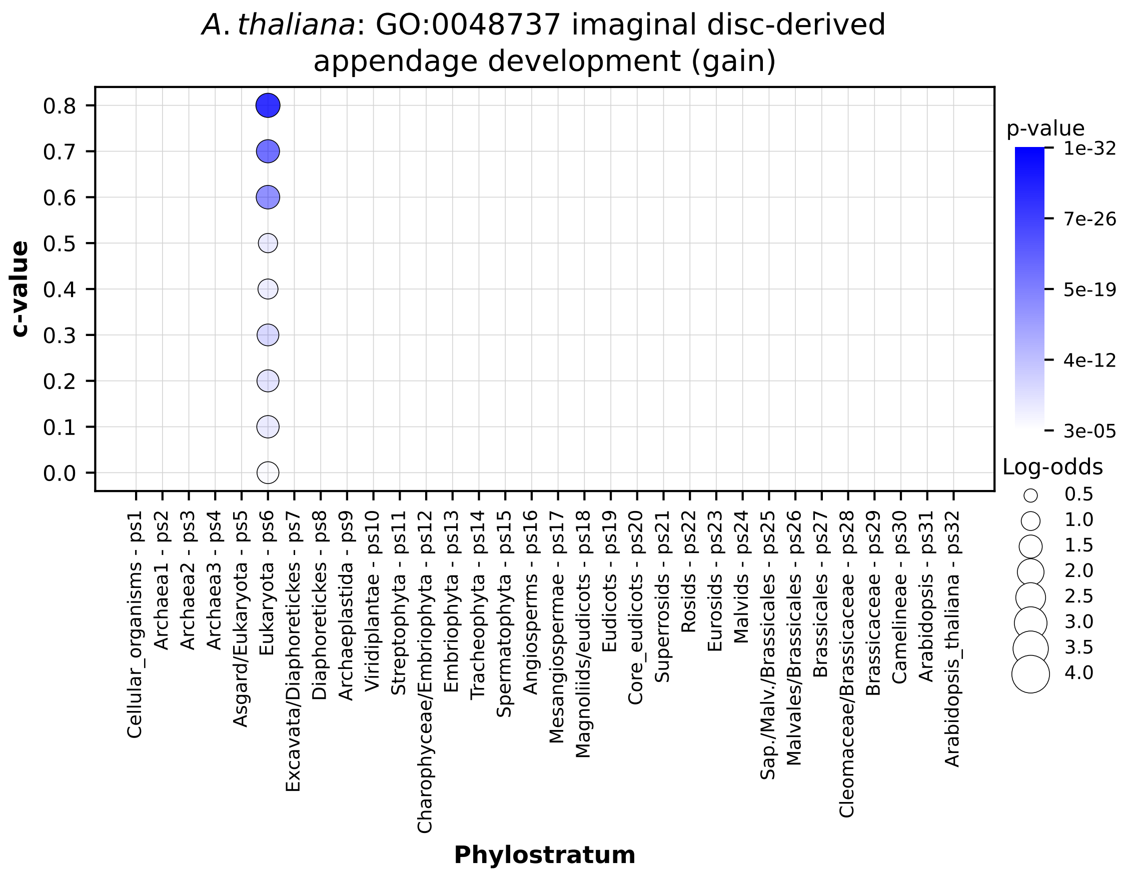

A. thaliana: GO:0048737 imaginal disc-derived appendage development (gain)
c-value
0.8
0.7
0.6
0.5
0.4
0.3
0.2
0.1
0.0
p-value
1e-32
7e-26
5e-19
4e-12
3e-05
Log-odds
0.5
1.0
1.5
2.0
2.5
3.0
3.5
4.0
Cellular_organisms - ps1
Archaea1 - ps2
Archaea2 - ps3
Archaea3 - ps4
Asgard/Eukaryota - ps5
Eukaryota - ps6
Excavata/Diaphoretickes - ps7
Diaphoretickes - ps8
Archaeplastida - ps9
Viridiplantae - ps10
Streptophyta - ps11
Charophyceae/Embriophyta - ps12
Embriophyta - ps13
Tracheophyta - ps14
Spermatophyta - ps15
Angiosperms - ps16
Mesangiospermae - ps17
Magnoliids/eudicots - ps18
Eudicots - ps19
Core_eudicots - ps20
Superrosids - ps21
Rosids - ps22
Eurosids - ps23
Malvids - ps24
Sap./Malv./Brassicales - ps25
Malvales/Brassicales - ps26
Brassicales - ps27
Cleomaceae/Brassicaceae - ps28
Brassicaceae - ps29
Camelineae - ps30
Arabidopsis - ps31
Arabidopsis_thaliana - ps32
Phylostratum

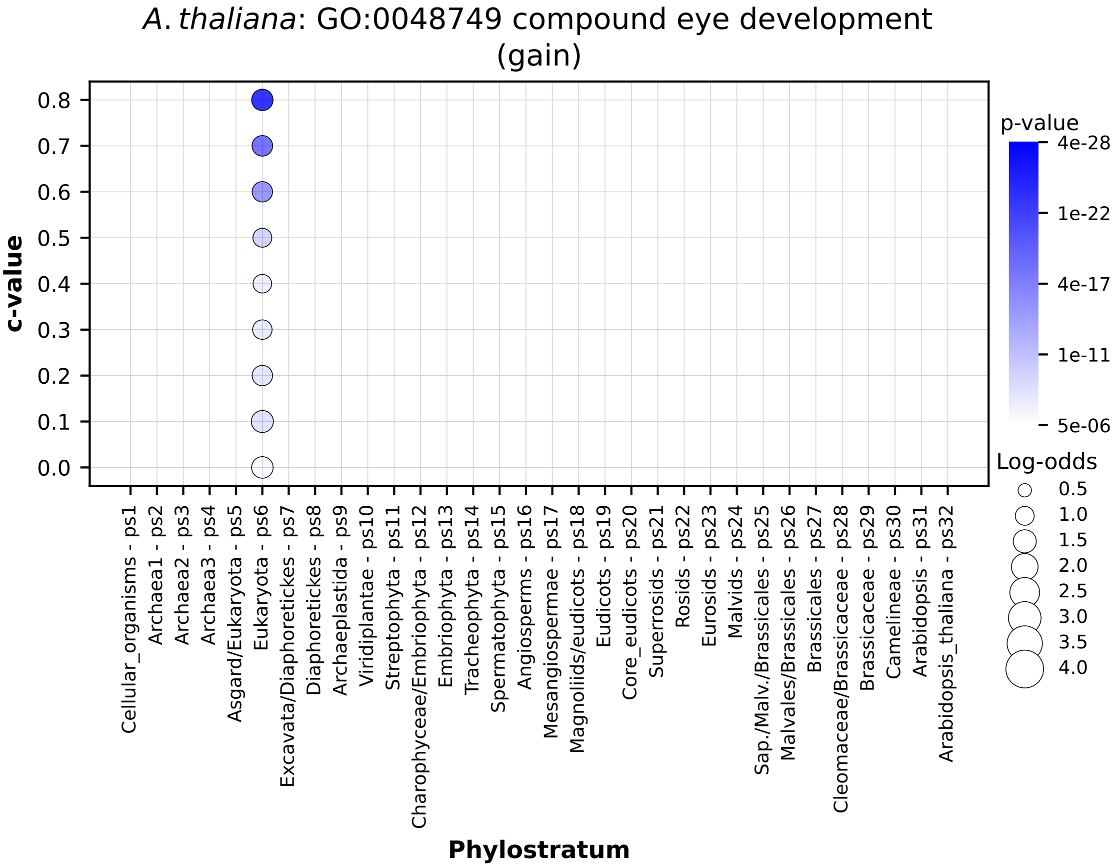

A. thaliana: GO:0048749 compound eye development
(gain)
c-value
0.8
0.7
0.6
0.5
0.4
0.3
0.2
0.1
0.0
p-value
4e-28
1e-22
4e-17
1e-11
5e-06
Log-odds
0.5
1.0
1.5
2.0
2.5
3.0
3.5
4.0
Cellular_organisms - ps1
Archaea1 - ps2
Archaea2 - ps3
Archaea3 - ps4
Asgard/Eukaryota - ps5
Eukaryota - ps6
Excavata/Diaphoretickes - ps7
Diaphoretickes - ps8
Archaeplastida - ps9
Viridiplantae - ps10
Streptophyta - ps11
Charophyceae/Embriophyta - ps12
Embriophyta - ps13
Tracheophyta - ps14
Spermatophyta - ps15
Angiosperms - ps16
Mesangiospermae - ps17
Magnoliids/eudicots - ps18
Eudicots - ps19
Core_eudicots - ps20
Superrosids - ps21
Rosids - ps22
Eurosids - ps23
Malvids - ps24
Sap./Malv./Brassicales - ps25
Malvales/Brassicales - ps26
Brassicales - ps27
Cleomaceae/Brassicaceae - ps28
Brassicaceae - ps29
Camelineae - ps30
Arabidopsis - ps31
Arabidopsis_thaliana - ps32
Phylostratum

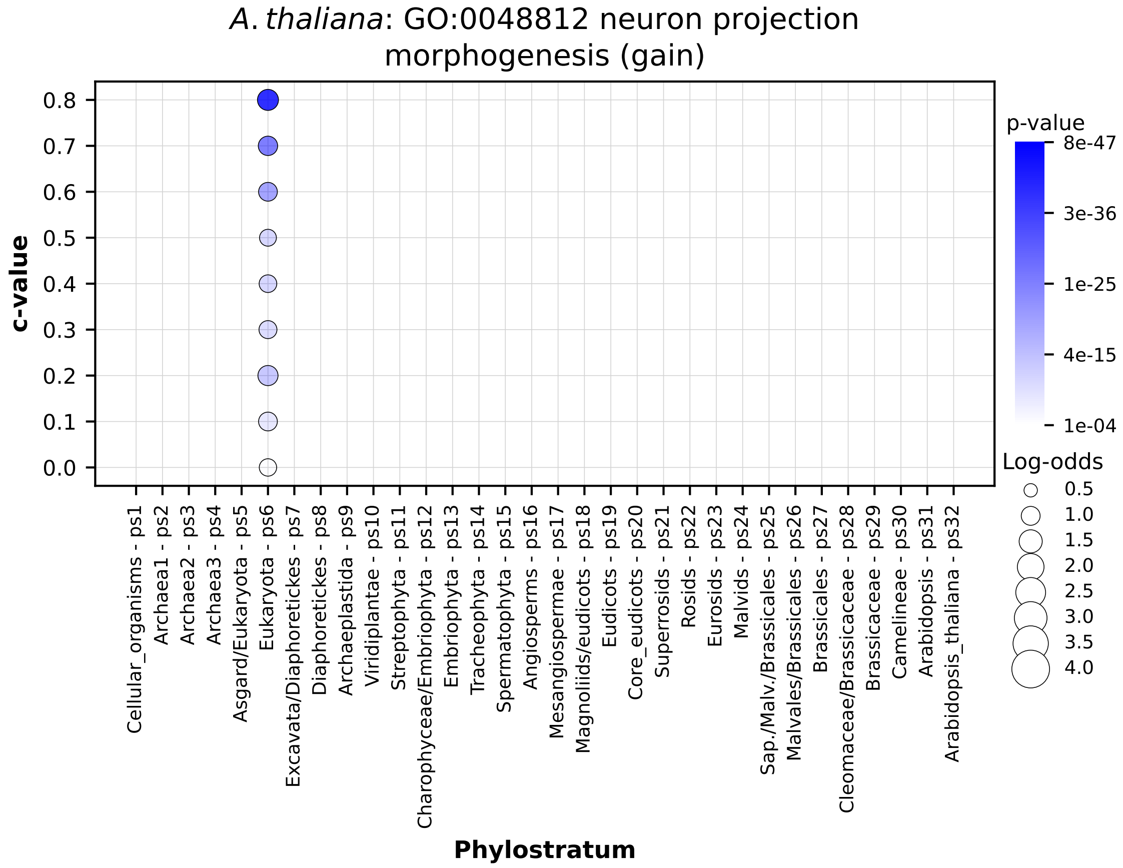
A. thaliana: GO:0048812 neuron projection morphogenesis (gain)
c-value
0.8
0.7
0.6
0.5
0.4
0.3
0.2
0.1
0.0
Phylostratum
Cellular_organisms - ps1
Archaea1 - ps2
Archaea2 - ps3
Archaea3 - ps4
Asgard/Eukaryota - ps5
Eukaryota - ps6
Excavata/Diaphoretickes - ps7
Diaphoretickes - ps8
Archaeplastida - ps9
Viridiplantae - ps10
Streptophyta - ps11
Charophyceae/Embriophyta - ps12
Embriophyta - ps13
Tracheophyta - ps14
Spermatophyta - ps15
Angiosperms - ps16
Mesangiospermae - ps17
Magnoliids/eudicots - ps18
Eudicots - ps19
Core_eudicots - ps20
Superrosids - ps21
Rosids - ps22
Eurosids - ps23
Malvids - ps24
Sap./Malv./Brassicales - ps25
Malvales/Brassicales - ps26
Brassicales - ps27
Cleomaceae/Brassicaceae - ps28
Brassicaceae - ps29
Camelineae - ps30
Arabidopsis - ps31
Arabidopsis_thaliana - ps32
p-value
8e-47
3e-36
1e-25
4e-15
1e-04
Log-odds
0.5
1.0
1.5
2.0
2.5
3.0
3.5
4.0

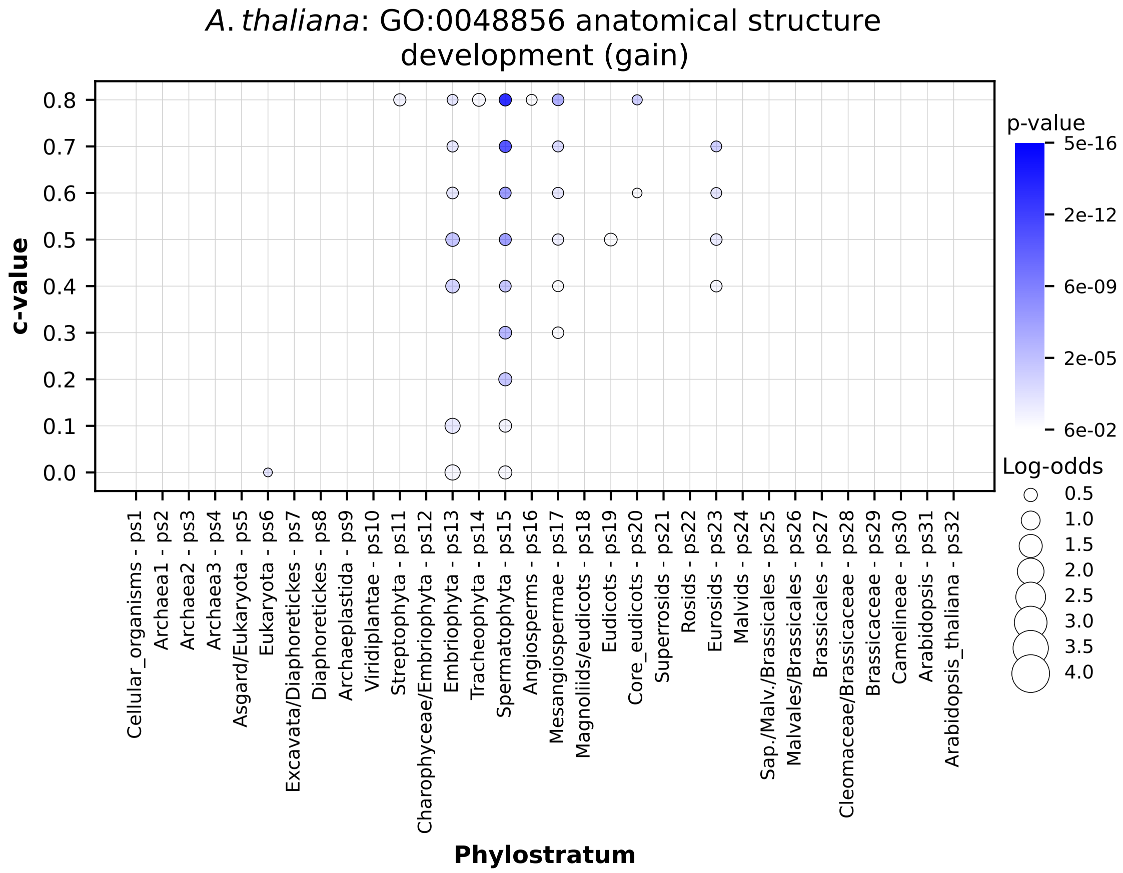

A. thaliana: GO:0048856 anatomical structure development (gain)
c-value
0.8
0.7
0.6
0.5
0.4
0.3
0.2
0.1
0.0
p-value
5e-16
2e-12
6e-09
2e-05
6e-02
Log-odds
0.5
1.0
1.5
2.0
2.5
3.0
3.5
4.0
Cellular_organisms - ps1
Archaea1 - ps2
Archaea2 - ps3
Archaea3 - ps4
Asgard/Eukaryota - ps5
Eukaryota - ps6
Excavata/Diaphoretickes - ps7
Diaphoretickes - ps8
Archaeplastida - ps9
Viridiplantae - ps10
Streptophyta - ps11
Charophyceae/Embriophyta - ps12
Embriophyta - ps13
Tracheophyta - ps14
Spermatophyta - ps15
Angiosperms - ps16
Mesangiospermae - ps17
Magnoliids/eudicots - ps18
Eudicots - ps19
Core_eudicots - ps20
Superrosids - ps21
Rosids - ps22
Eurosids - ps23
Malvids - ps24
Sap./Malv./Brassicales - ps25
Malvales/Brassicales - ps26
Brassicales - ps27
Cleomaceae/Brassicaceae - ps28
Brassicaceae - ps29
Camelineae - ps30
Arabidopsis - ps31
Arabidopsis_thaliana - ps32
Phylostratum

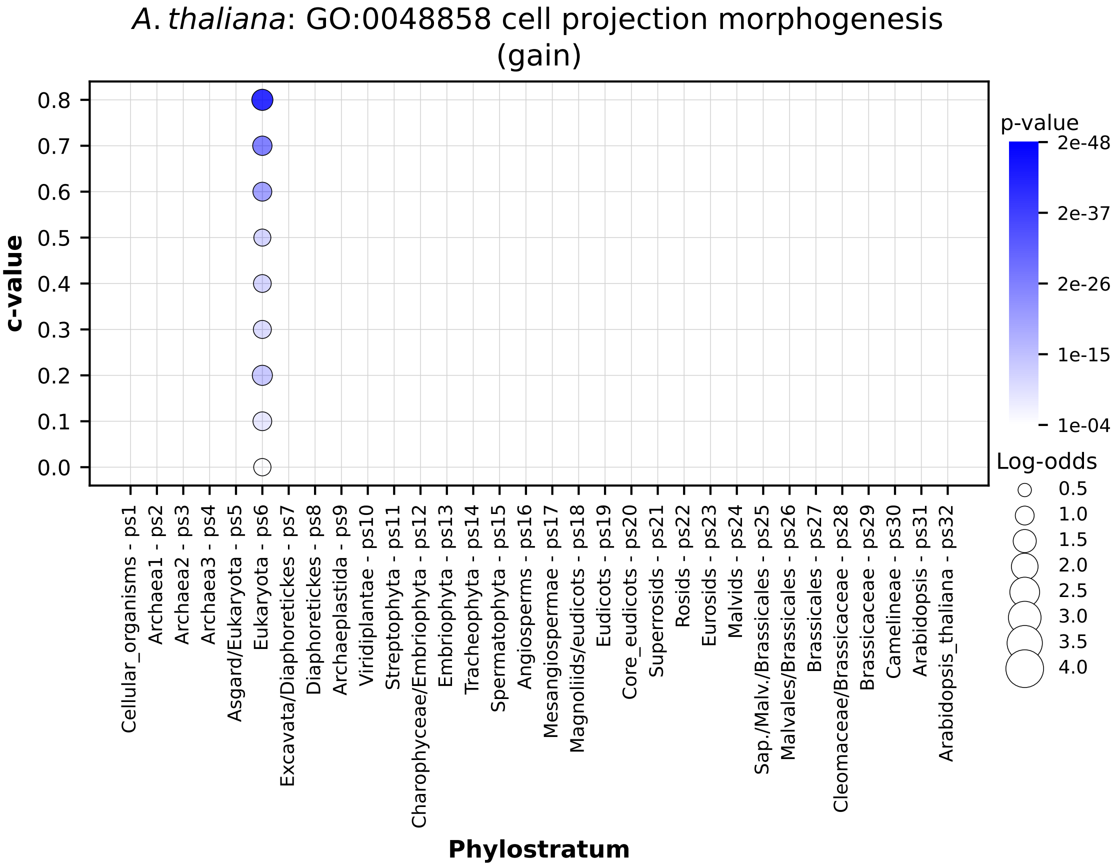

A. thaliana: GO:0048858 cell projection morphogenesis
(gain)
c-value
0.8
0.7
0.6
0.5
0.4
0.3
0.2
0.1
0.0
p-value
2e-48
2e-37
2e-26
1e-15
1e-04
Log-odds
0.5
1.0
1.5
2.0
2.5
3.0
3.5
4.0
Cellular_organisms - ps1
Archaea1 - ps2
Archaea2 - ps3
Archaea3 - ps4
Asgard/Eukaryota - ps5
Eukaryota - ps6
Excavata/Diaphoretickes - ps7
Diaphoretickes - ps8
Archaeplastida - ps9
Viridiplantae - ps10
Streptophyta - ps11
Charophyceae/Embriophyta - ps12
Embriophyta - ps13
Tracheophyta - ps14
Spermatophyta - ps15
Angiosperms - ps16
Mesangiospermae - ps17
Magnoliids/eudicots - ps18
Eudicots - ps19
Core_eudicots - ps20
Superrosids - ps21
Rosids - ps22
Eurosids - ps23
Malvids - ps24
Sap./Malv./Brassicales - ps25
Malvales/Brassicales - ps26
Brassicales - ps27
Cleomaceae/Brassicaceae - ps28
Brassicaceae - ps29
Camelineae - ps30
Arabidopsis - ps31
Arabidopsis_thaliana - ps32
Phylostratum

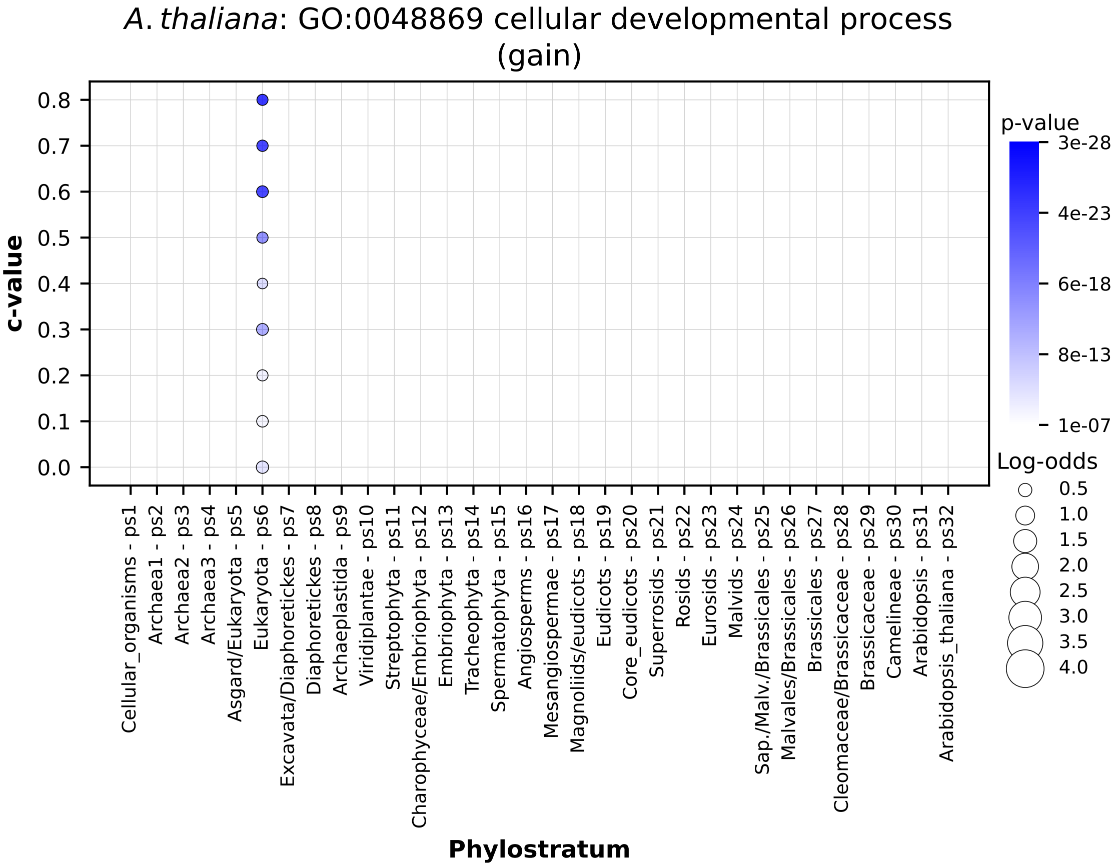

A. thaliana: GO:0048869 cellular developmental process (gain)
c-value
0.8
0.7
0.6
0.5
0.4
0.3
0.2
0.1
0.0
p-value
3e-28
4e-23
6e-18
8e-13
1e-07
Log-odds
0.5
1.0
1.5
2.0
2.5
3.0
3.5
4.0
Cellular_organisms - ps1
Archaea1 - ps2
Archaea2 - ps3
Archaea3 - ps4
Asgard/Eukaryota - ps5
Eukaryota - ps6
Excavata/Diaphoretickes - ps7
Diaphoretickes - ps8
Archaeplastida - ps9
Viridiplantae - ps10
Streptophyta - ps11
Charophyceae/Embriophyta - ps12
Embriophyta - ps13
Tracheophyta - ps14
Spermatophyta - ps15
Angiosperms - ps16
Mesangiospermae - ps17
Magnoliids/eudicots - ps18
Eudicots - ps19
Core_eudicots - ps20
Superrosids - ps21
Rosids - ps22
Eurosids - ps23
Malvids - ps24
Sap./Malv./Brassicales - ps25
Malvales/Brassicales - ps26
Brassicales - ps27
Cleomaceae/Brassicaceae - ps28
Brassicaceae - ps29
Camelineae - ps30
Arabidopsis - ps31
Arabidopsis_thaliana - ps32
Phylostratum

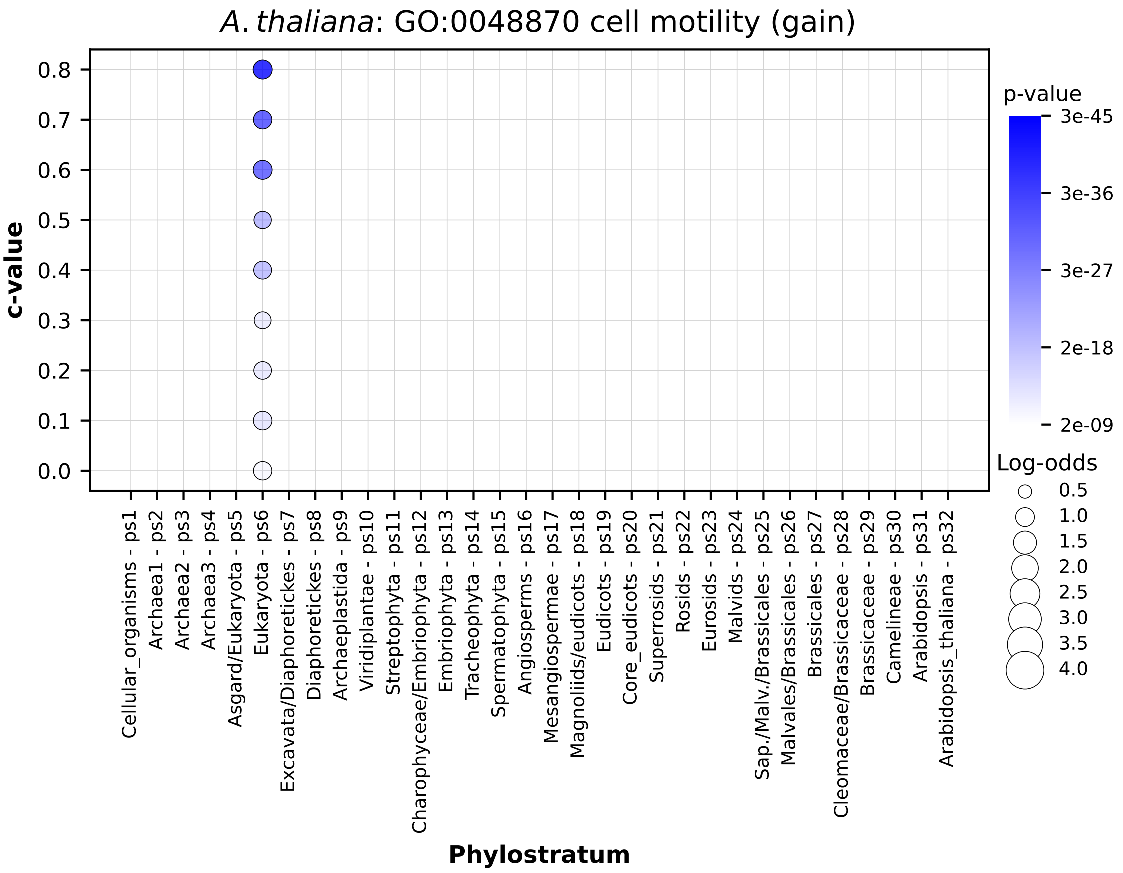

A. thaliana: GO:0048870 cell motility (gain)
c-value
0.8
0.7
0.6
0.5
0.4
0.3
0.2
0.1
0.0
p-value
3e-45
3e-36
3e-27
2e-18
2e-09
Log-odds
0.5
1.0
1.5
2.0
2.5
3.0
3.5
4.0
Cellular_organisms - ps1
Archaea1 - ps2
Archaea2 - ps3
Archaea3 - ps4
Asgard/Eukaryota - ps5
Eukaryota - ps6
Excavata/Diaphoretickes - ps7
Diaphoretickes - ps8
Archaeplastida - ps9
Viridiplantae - ps10
Streptophyta - ps11
Charophyceae/Embriophyta - ps12
Embriophyta - ps13
Tracheophyta - ps14
Spermatophyta - ps15
Angiosperms - ps16
Mesangiospermae - ps17
Magnoliids/eudicots - ps18
Eudicots - ps19
Core_eudicots - ps20
Superrosids - ps21
Rosids - ps22
Eurosids - ps23
Malvids - ps24
Sap./Malv./Brassicales - ps25
Malvales/Brassicales - ps26
Brassicales - ps27
Cleomaceae/Brassicaceae - ps28
Brassicaceae - ps29
Camelineae - ps30
Arabidopsis - ps31
Arabidopsis_thaliana - ps32
Phylostratum

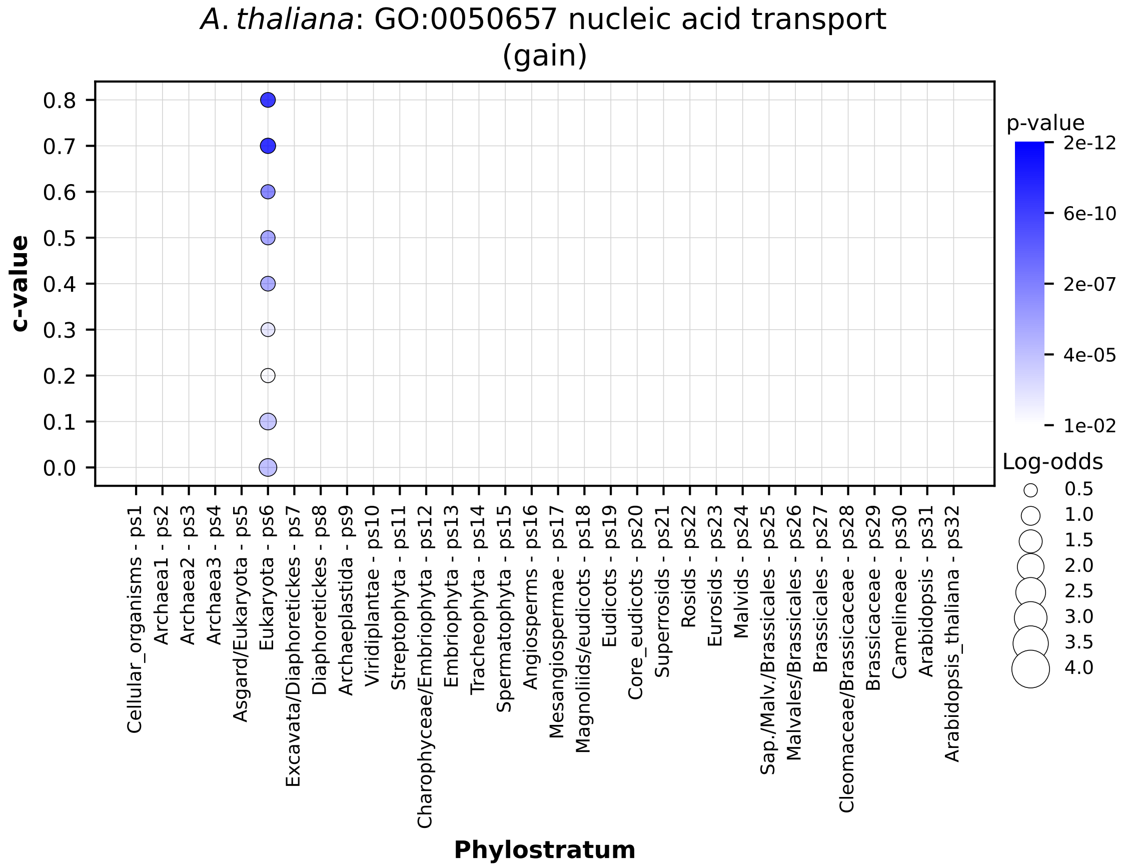

A. thaliana: GO:0050657 nucleic acid transport
(gain)
c-value
0.8
0.7
0.6
0.5
0.4
0.3
0.2
0.1
0.0
Phylostratum
Cellular_organisms - ps1
Archaea1 - ps2
Archaea2 - ps3
Archaea3 - ps4
Asgard/Eukaryota - ps5
Eukaryota - ps6
Excavata/Diaphoretickes - ps7
Diaphoretickes - ps8
Archaeplastida - ps9
Viridiplantae - ps10
Streptophyta - ps11
Charophyceae/Embriophyta - ps12
Embriophyta - ps13
Tracheophyta - ps14
Spermatophyta - ps15
Angiosperms - ps16
Mesangiospermae - ps17
Magnoliids/eudicots - ps18
Eudicots - ps19
Core_eudicots - ps20
Superrosids - ps21
Rosids - ps22
Eurosids - ps23
Malvids - ps24
Sap./Malv./Brassicales - ps25
Malvales/Brassicales - ps26
Brassicales - ps27
Cleomaceae/Brassicaceae - ps28
Brassicaceae - ps29
Camelineae - ps30
Arabidopsis - ps31
Arabidopsis_thaliana - ps32
p-value
2e-12
6e-10
2e-07
4e-05
1e-02
Log-odds
0.5
1.0
1.5
2.0
2.5
3.0
3.5
4.0

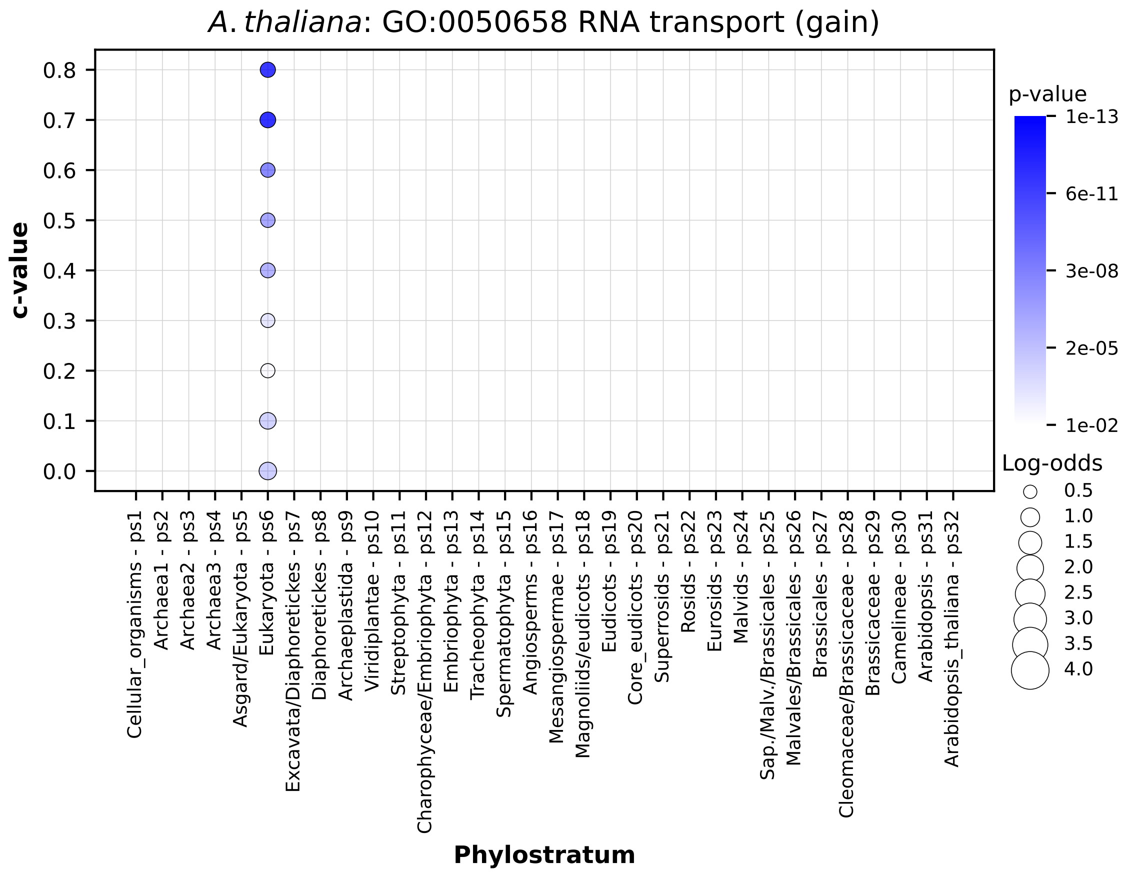

A. thaliana: GO:0050658 RNA transport (gain)
c-value
0.0
0.1
0.2
0.3
0.4
0.5
0.6
0.7
0.8
Phylostratum
Cellular_organisms - ps1
Archaea1 - ps2
Archaea2 - ps3
Archaea3 - ps4
Asgard/Eukaryota - ps5
Eukaryota - ps6
Excavata/Diaphoretickes - ps7
Diaphoretickes - ps8
Archaeplastida - ps9
Viridiplantae - ps10
Streptophyta - ps11
Charophyceae/Embriophyta - ps12
Embriophyta - ps13
Tracheophyta - ps14
Spermatophyta - ps15
Angiosperms - ps16
Mesangiospermae - ps17
Magnoliids/eudicots - ps18
Eudicots - ps19
Core_eudicots - ps20
Superrosids - ps21
Rosids - ps22
Eurosids - ps23
Malvids - ps24
Sap./Malv./Brassicales - ps25
Malvales/Brassicales - ps26
Brassicales - ps27
Cleomaceae/Brassicaceae - ps28
Brassicaceae - ps29
Camelineae - ps30
Arabidopsis - ps31
Arabidopsis_thaliana - ps32
p-value
1e-13
6e-11
3e-08
2e-05
1e-02
Log-odds
0.5
1.0
1.5
2.0
2.5
3.0
3.5
4.0

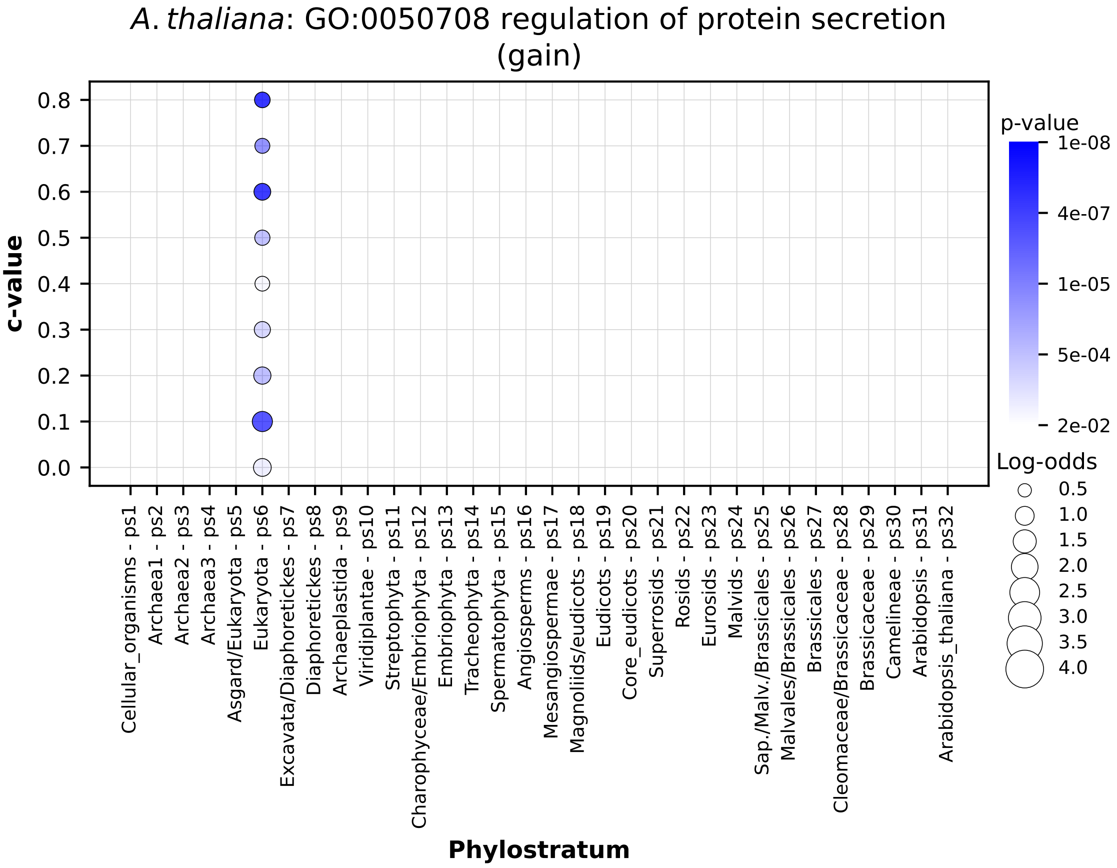

A. thaliana: GO:0050708 regulation of protein secretion
(gain)
c-value
0.8
0.7
0.6
0.5
0.4
0.3
0.2
0.1
0.0
p-value
1e-08
4e-07
1e-05
5e-04
2e-02
Log-odds
0.5
1.0
1.5
2.0
2.5
3.0
3.5
4.0
Cellular_organisms - ps1
Archaea1 - ps2
Archaea2 - ps3
Archaea3 - ps4
Asgard/Eukaryota - ps5
Eukaryota - ps6
Excavata/Diaphoretickes - ps7
Diaphoretickes - ps8
Archaeplastida - ps9
Viridiplantae - ps10
Streptophyta - ps11
Charophyceae/Embriophyta - ps12
Embriophyta - ps13
Tracheophyta - ps14
Spermatophyta - ps15
Angiosperms - ps16
Mesangiospermae - ps17
Magnoliids/eudicots - ps18
Eudicots - ps19
Core_eudicots - ps20
Superrosids - ps21
Rosids - ps22
Eurosids - ps23
Malvids - ps24
Sap./Malv./Brassicales - ps25
Malvales/Brassicales - ps26
Brassicales - ps27
Cleomaceae/Brassicaceae - ps28
Brassicaceae - ps29
Camelineae - ps30
Arabidopsis - ps31
Arabidopsis_thaliana - ps32
Phylostratum

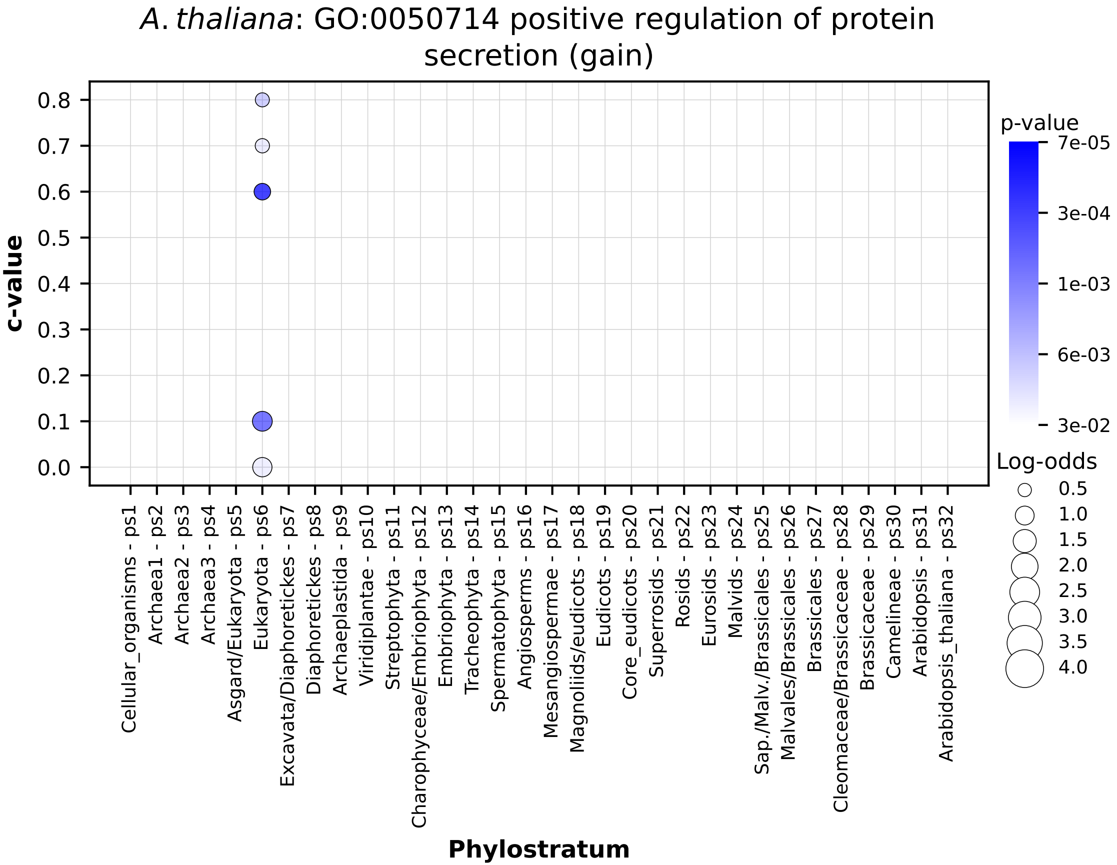

A. thaliana: GO:0050714 positive regulation of protein secretion (gain)
c-value
0.8
0.7
0.6
0.5
0.4
0.3
0.2
0.1
0.0
p-value
7e-05
3e-04
1e-03
6e-03
3e-02
Log-odds
0.5
1.0
1.5
2.0
2.5
3.0
3.5
4.0
Cellular_organisms - ps1
Archaea1 - ps2
Archaea2 - ps3
Archaea3 - ps4
Asgard/Eukaryota - ps5
Eukaryota - ps6
Excavata/Diaphoretickes - ps7
Diaphoretickes - ps8
Archaeplastida - ps9
Viridiplantae - ps10
Streptophyta - ps11
Charophyceae/Embriophyta - ps12
Embriophyta - ps13
Tracheophyta - ps14
Spermatophyta - ps15
Angiosperms - ps16
Mesangiospermae - ps17
Magnoliids/eudicots - ps18
Eudicots - ps19
Core_eudicots - ps20
Superrosids - ps21
Rosids - ps22
Eurosids - ps23
Malvids - ps24
Sap./Malv./Brassicales - ps25
Malvales/Brassicales - ps26
Brassicales - ps27
Cleomaceae/Brassicaceae - ps28
Brassicaceae - ps29
Camelineae - ps30
Arabidopsis - ps31
Arabidopsis_thaliana - ps32
Phylostratum

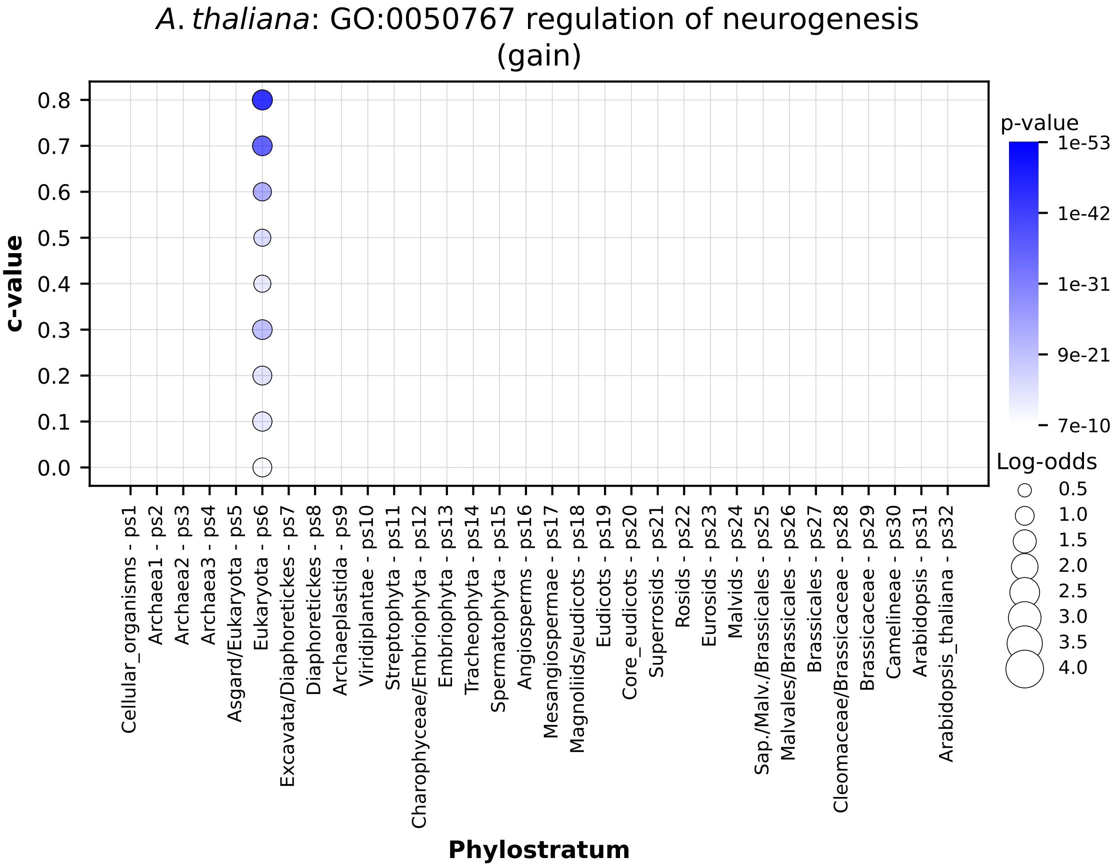
A. thaliana: GO:0050767 regulation of neurogenesis
(gain)
c-value
0.8
0.7
0.6
0.5
0.4
0.3
0.2
0.1
0.0
p-value
1e-53
1e-42
1e-31
9e-21
7e-10
Log-odds
0.5
1.0
1.5
2.0
2.5
3.0
3.5
4.0
Cellular_organisms - ps1
Archaea1 - ps2
Archaea2 - ps3
Archaea3 - ps4
Asgard/Eukaryota - ps5
Eukaryota - ps6
Excavata/Diaphoretickes - ps7
Diaphoretickes - ps8
Archaeplastida - ps9
Viridiplantae - ps10
Streptophyta - ps11
Charophyceae/Embriophyta - ps12
Embriophyta - ps13
Tracheophyta - ps14
Spermatophyta - ps15
Angiosperms - ps16
Mesangiospermae - ps17
Magnoliids/eudicots - ps18
Eudicots - ps19
Core_eudicots - ps20
Superrosids - ps21
Rosids - ps22
Eurosids - ps23
Malvids - ps24
Sap./Malv./Brassicales - ps25
Malvales/Brassicales - ps26
Brassicales - ps27
Cleomaceae/Brassicaceae - ps28
Brassicaceae - ps29
Camelineae - ps30
Arabidopsis - ps31
Arabidopsis_thaliana - ps32
Phylostratum

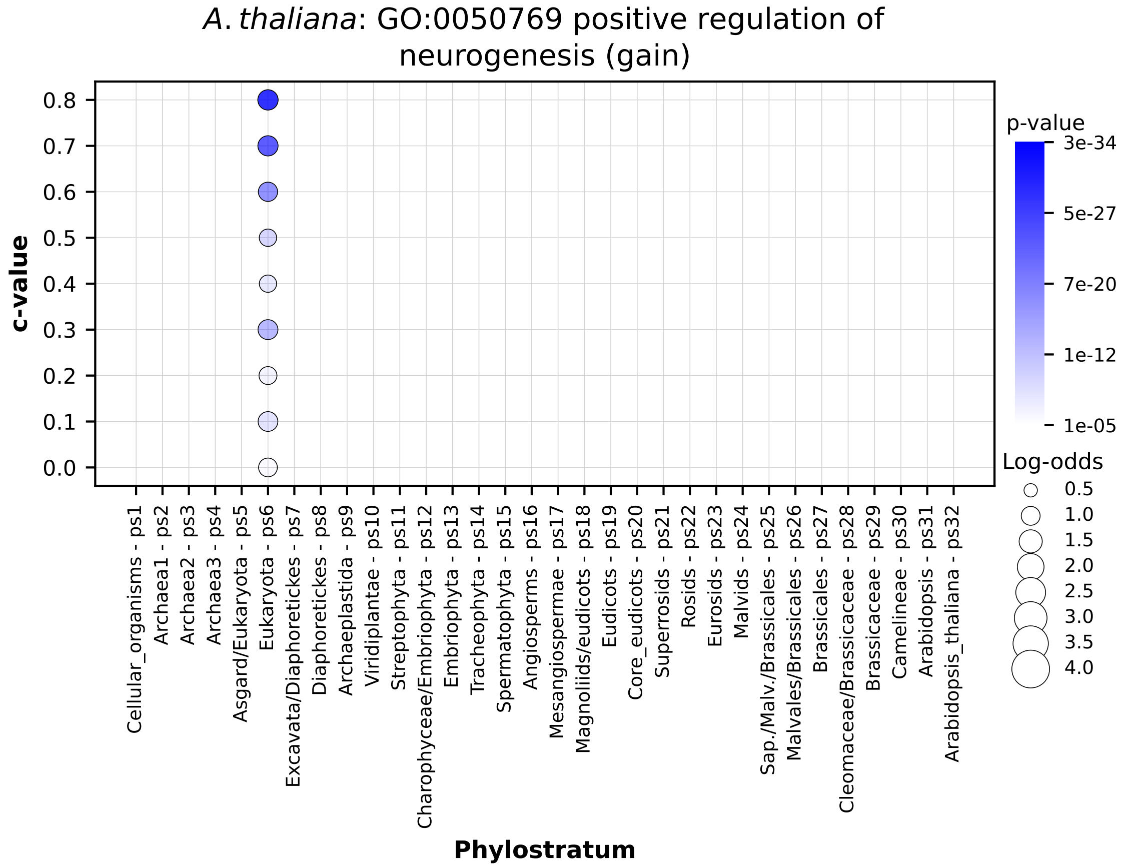
A. thaliana: GO:0050769 positive regulation of neurogenesis (gain)
c-value
0.8
0.7
0.6
0.5
0.4
0.3
0.2
0.1
0.0
p-value
3e-34
5e-27
7e-20
1e-12
1e-05
Log-odds
0.5
1.0
1.5
2.0
2.5
3.0
3.5
4.0
Cellular_organisms - ps1
Archaea1 - ps2
Archaea2 - ps3
Archaea3 - ps4
Asgard/Eukaryota - ps5
Eukaryota - ps6
Excavata/Diaphoretickes - ps7
Diaphoretickes - ps8
Archaeplastida - ps9
Viridiplantae - ps10
Streptophyta - ps11
Charophyceae/Embriophyta - ps12
Embriophyta - ps13
Tracheophyta - ps14
Spermatophyta - ps15
Angiosperms - ps16
Mesangiospermae - ps17
Magnoliids/eudicots - ps18
Eudicots - ps19
Core_eudicots - ps20
Superrosids - ps21
Rosids - ps22
Eurosids - ps23
Malvids - ps24
Sap./Malv./Brassicales - ps25
Malvales/Brassicales - ps26
Brassicales - ps27
Cleomaceae/Brassicaceae - ps28
Brassicaceae - ps29
Camelineae - ps30
Arabidopsis - ps31
Arabidopsis_thaliana - ps32
Phylostratum

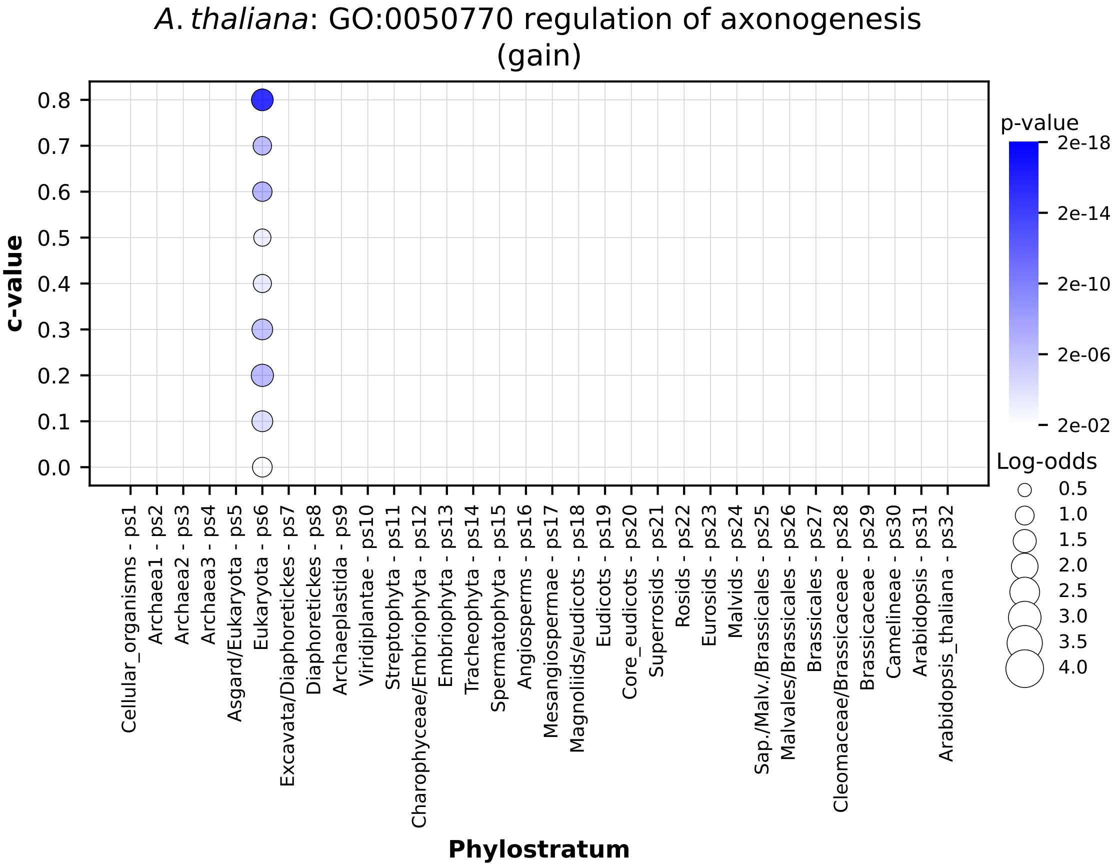

A. thaliana: GO:0050770 regulation of axonogenesis (gain)
c-value
0.8
0.7
0.6
0.5
0.4
0.3
0.2
0.1
0.0
p-value
2e-18
2e-14
2e-10
2e-06
2e-02
Log-odds
0.5
1.0
1.5
2.0
2.5
3.0
3.5
4.0
Cellular_organisms - ps1
Archaea1 - ps2
Archaea2 - ps3
Archaea3 - ps4
Asgard/Eukaryota - ps5
Eukaryota - ps6
Excavata/Diaphoretickes - ps7
Diaphoretickes - ps8
Archaeplastida - ps9
Viridiplantae - ps10
Streptophyta - ps11
Charophyceae/Embriophyta - ps12
Embriophyta - ps13
Tracheophyta - ps14
Spermatophyta - ps15
Angiosperms - ps16
Mesangiospermae - ps17
Magnoliids/eudicots - ps18
Eudicots - ps19
Core_eudicots - ps20
Superrosids - ps21
Rosids - ps22
Eurosids - ps23
Malvids - ps24
Sap./Malv./Brassicales - ps25
Malvales/Brassicales - ps26
Brassicales - ps27
Cleomaceae/Brassicaceae - ps28
Brassicaceae - ps29
Camelineae - ps30
Arabidopsis - ps31
Arabidopsis_thaliana - ps32
Phylostratum

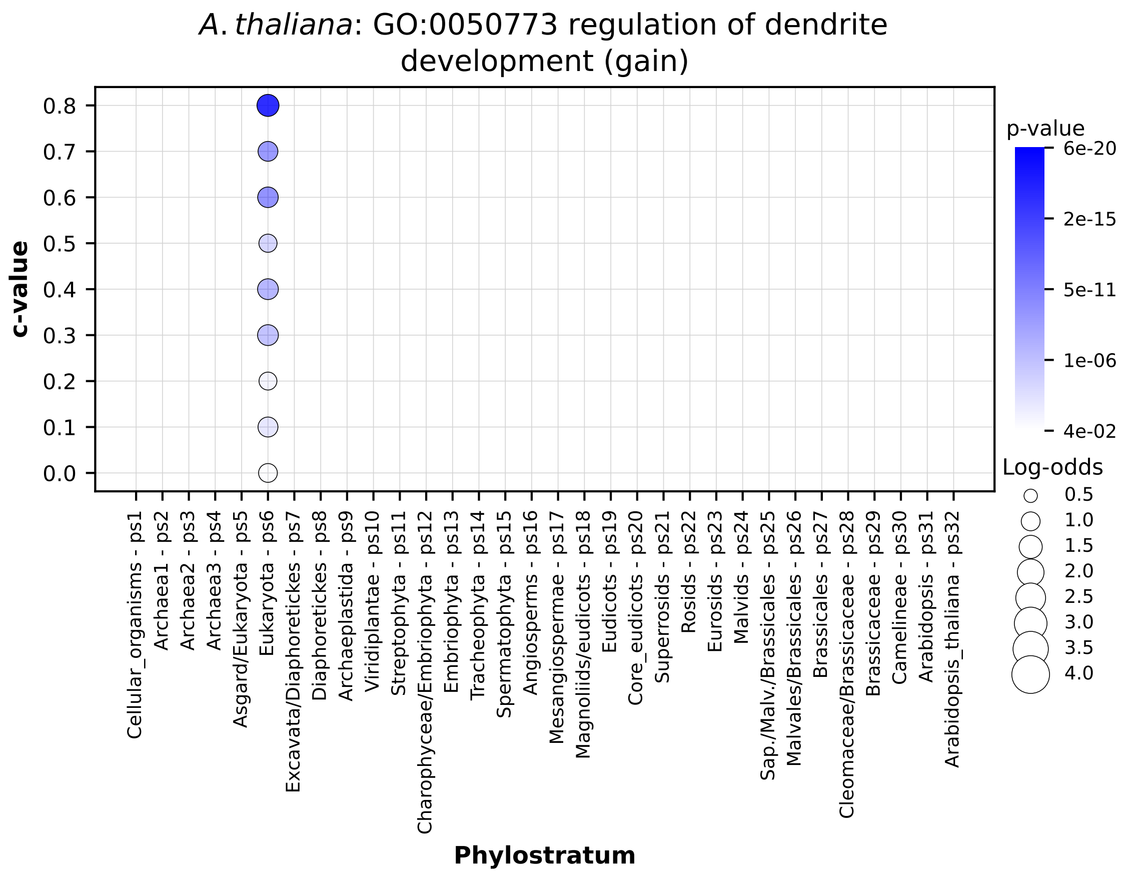
A. thaliana: GO:0050773 regulation of dendrite development (gain)
c-value
0.8
0.7
0.6
0.5
0.4
0.3
0.2
0.1
0.0
p-value
6e-20
2e-15
5e-11
1e-06
4e-02
Log-odds
0.5
1.0
1.5
2.0
2.5
3.0
3.5
4.0
Cellular_organisms - ps1
Archaea1 - ps2
Archaea2 - ps3
Archaea3 - ps4
Asgard/Eukaryota - ps5
Eukaryota - ps6
Excavata/Diaphoretickes - ps7
Diaphoretickes - ps8
Archaeplastida - ps9
Viridiplantae - ps10
Streptophyta - ps11
Charophyceae/Embriophyta - ps12
Embriophyta - ps13
Tracheophyta - ps14
Spermatophyta - ps15
Angiosperms - ps16
Mesangiospermae - ps17
Magnoliids/eudicots - ps18
Eudicots - ps19
Core_eudicots - ps20
Superrosids - ps21
Rosids - ps22
Eurosids - ps23
Malvids - ps24
Sap./Malv./Brassicales - ps25
Malvales/Brassicales - ps26
Brassicales - ps27
Cleomaceae/Brassicaceae - ps28
Brassicaceae - ps29
Camelineae - ps30
Arabidopsis - ps31
Arabidopsis_thaliana - ps32
Phylostratum

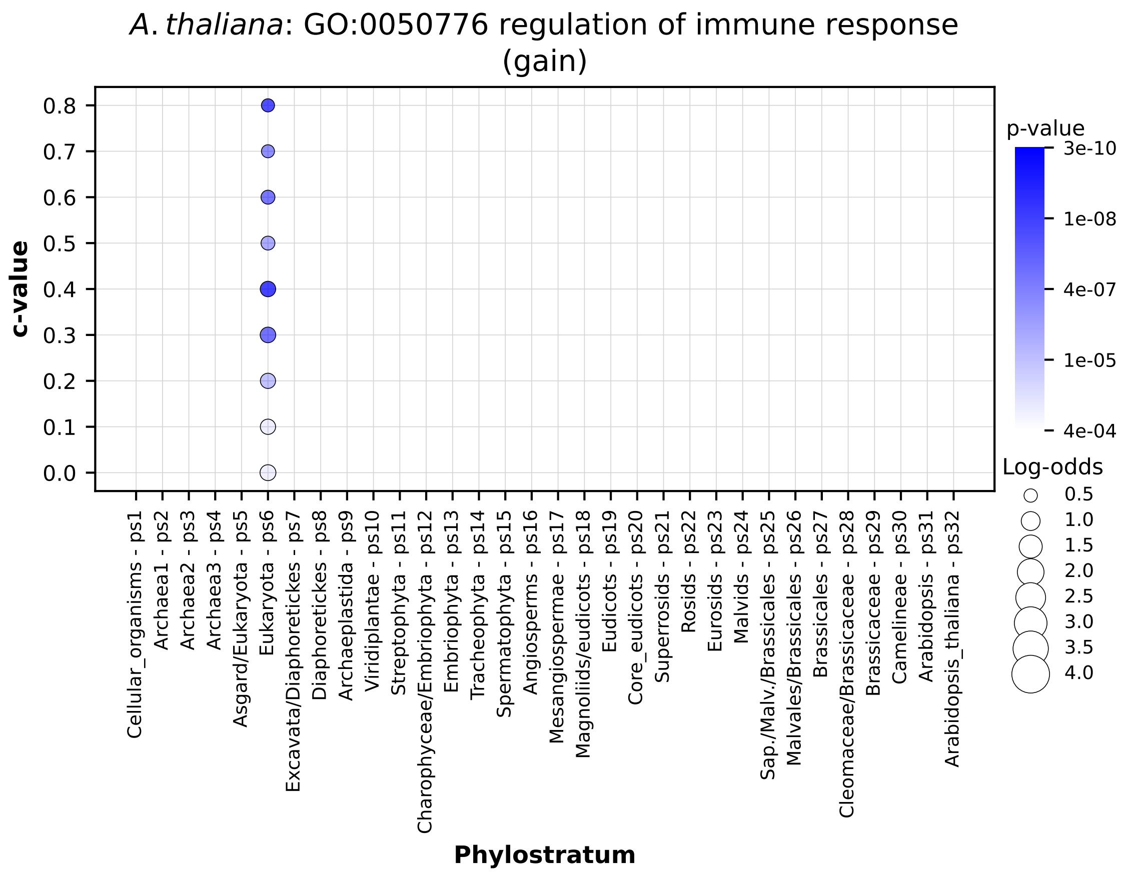

A. thaliana: GO:0050776 regulation of immune response (gain)
c-value
0.0
0.1
0.2
0.3
0.4
0.5
0.6
0.7
0.8
p-value
3e-10
1e-08
4e-07
1e-05
4e-04
Log-odds
0.5
1.0
1.5
2.0
2.5
3.0
3.5
4.0
Cellular_organisms - ps1
Archaea1 - ps2
Archaea2 - ps3
Archaea3 - ps4
Asgard/Eukaryota - ps5
Eukaryota - ps6
Excavata/Diaphoretickes - ps7
Diaphoretickes - ps8
Archaeplastida - ps9
Viridiplantae - ps10
Streptophyta - ps11
Charophyceae/Embriophyta - ps12
Embriophyta - ps13
Tracheophyta - ps14
Spermatophyta - ps15
Angiosperms - ps16
Mesangiospermae - ps17
Magnoliids/eudicots - ps18
Eudicots - ps19
Core_eudicots - ps20
Superrosids - ps21
Rosids - ps22
Eurosids - ps23
Malvids - ps24
Sap./Malv./Brassicales - ps25
Malvales/Brassicales - ps26
Brassicales - ps27
Cleomaceae/Brassicaceae - ps28
Brassicaceae - ps29
Camelineae - ps30
Arabidopsis - ps31
Arabidopsis_thaliana - ps32
Phylostratum

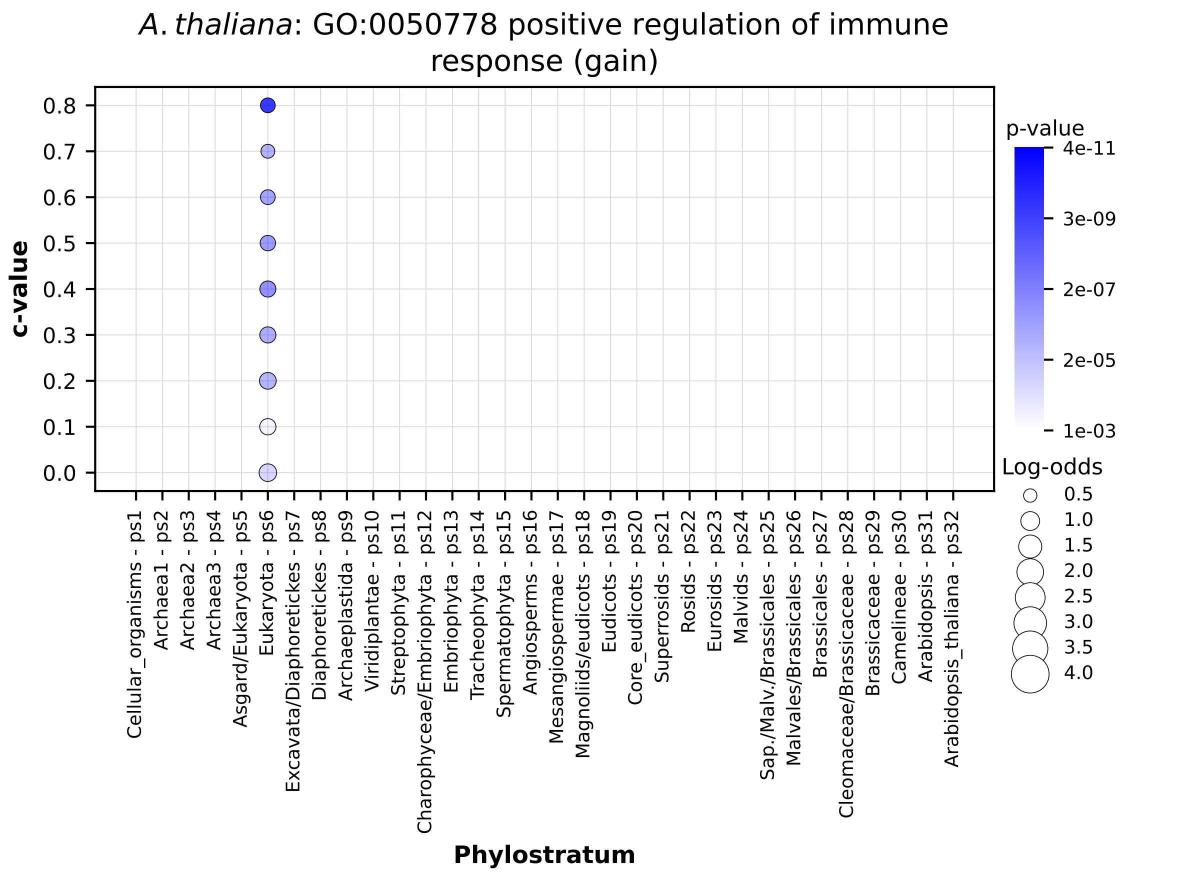

A. thaliana: GO:0050778 positive regulation of immune response (gain)
c-value
0.8
0.7
0.6
0.5
0.4
0.3
0.2
0.1
0.0
Phylostratum
Cellular_organisms - ps1
Archaea1 - ps2
Archaea2 - ps3
Archaea3 - ps4
Asgard/Eukaryota - ps5
Eukaryota - ps6
Excavata/Diaphoretickes - ps7
Diaphoretickes - ps8
Archaeplastida - ps9
Viridiplantae - ps10
Streptophyta - ps11
Charophyceae/Embriophyta - ps12
Embriophyta - ps13
Tracheophyta - ps14
Spermatophyta - ps15
Angiosperms - ps16
Mesangiospermae - ps17
Magnoliids/eudicots - ps18
Eudicots - ps19
Core_eudicots - ps20
Superrosids - ps21
Rosids - ps22
Eurosids - ps23
Malvids - ps24
Sap./Malv./Brassicales - ps25
Malvales/Brassicales - ps26
Brassicales - ps27
Cleomaceae/Brassicaceae - ps28
Brassicaceae - ps29
Camelineae - ps30
Arabidopsis - ps31
Arabidopsis_thaliana - ps32
p-value
4e-11
3e-09
2e-07
2e-05
1e-03
Log-odds
0.5
1.0
1.5
2.0
2.5
3.0
3.5
4.0

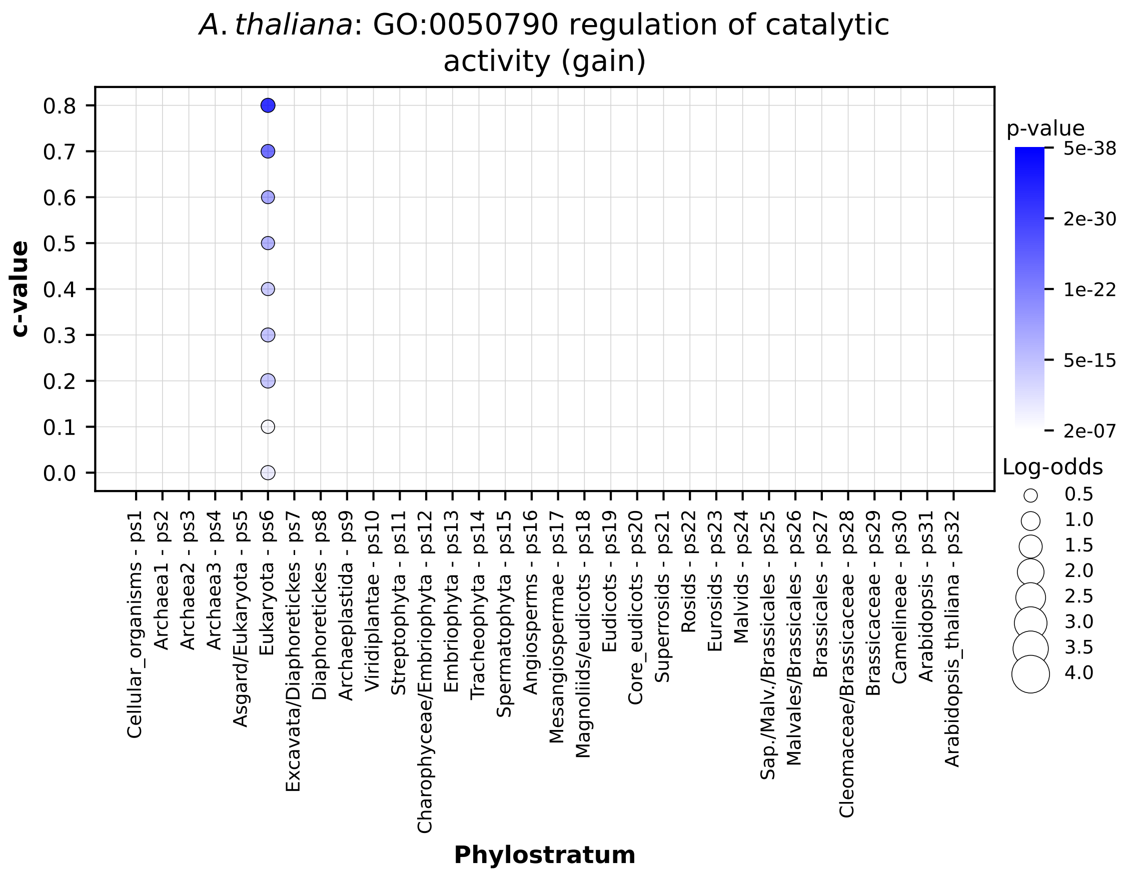
A. thaliana: GO:0050790 regulation of catalytic activity (gain)
c-value
0.8
0.7
0.6
0.5
0.4
0.3
0.2
0.1
0.0
p-value
5e-38
2e-30
1e-22
5e-15
2e-07
Log-odds
0.5
1.0
1.5
2.0
2.5
3.0
3.5
4.0
Cellular_organisms - ps1
Archaea1 - ps2
Archaea2 - ps3
Archaea3 - ps4
Asgard/Eukaryota - ps5
Eukaryota - ps6
Excavata/Diaphoretickes - ps7
Diaphoretickes - ps8
Archaeplastida - ps9
Viridiplantae - ps10
Streptophyta - ps11
Charophyceae/Embriophyta - ps12
Embriophyta - ps13
Tracheophyta - ps14
Spermatophyta - ps15
Angiosperms - ps16
Mesangiospermae - ps17
Magnoliids/eudicots - ps18
Eudicots - ps19
Core_eudicots - ps20
Superrosids - ps21
Rosids - ps22
Eurosids - ps23
Malvids - ps24
Sap./Malv./Brassicales - ps25
Malvales/Brassicales - ps26
Brassicales - ps27
Cleomaceae/Brassicaceae - ps28
Brassicaceae - ps29
Camelineae - ps30
Arabidopsis - ps31
Arabidopsis_thaliana - ps32
Phylostratum

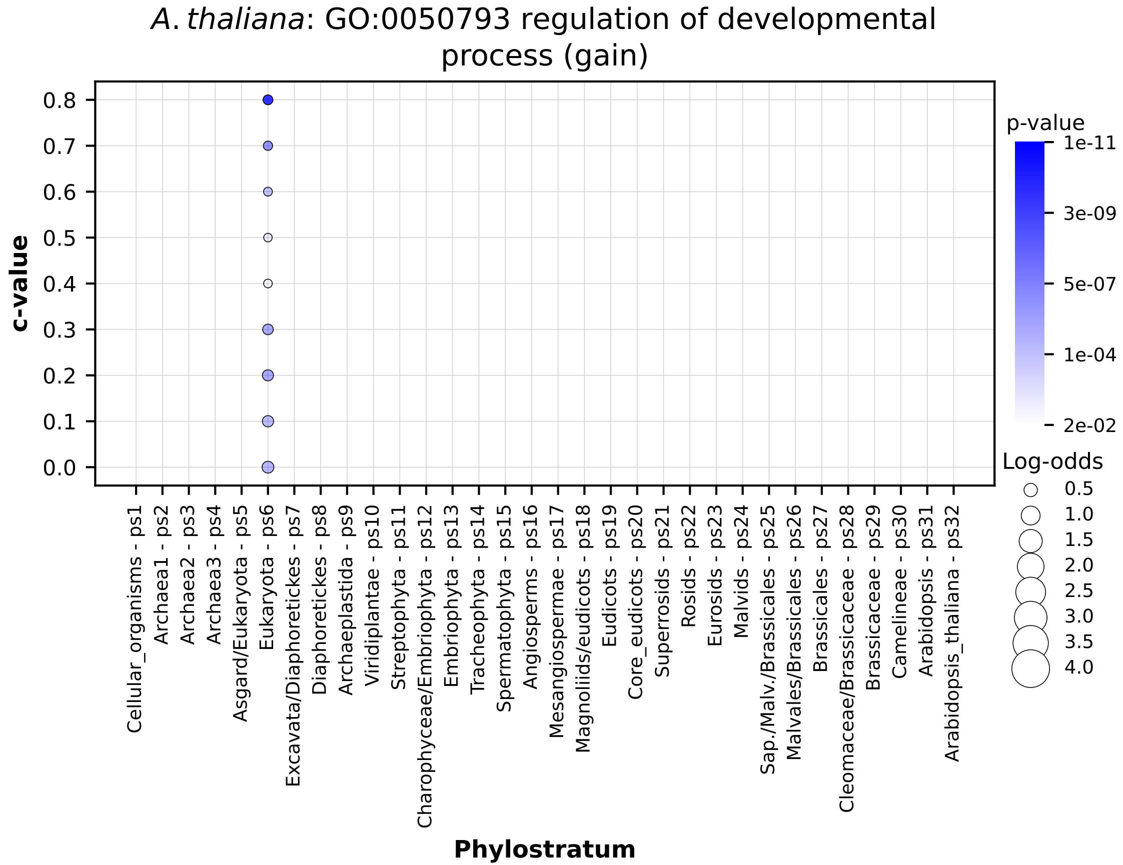
A. thaliana: GO:0050793 regulation of developmental process (gain)
c-value
0.8
0.7
0.6
0.5
0.4
0.3
0.2
0.1
0.0
Phylostratum
Cellular_organisms - ps1
Archaea1 - ps2
Archaea2 - ps3
Archaea3 - ps4
Asgard/Eukaryota - ps5
Eukaryota - ps6
Excavata/Diaphoretickes - ps7
Diaphoretickes - ps8
Archaeplastida - ps9
Viridiplantae - ps10
Streptophyta - ps11
Charophyceae/Embriophyta - ps12
Embriophyta - ps13
Tracheophyta - ps14
Spermatophyta - ps15
Angiosperms - ps16
Mesangiospermae - ps17
Magnoliids/eudicots - ps18
Eudicots - ps19
Core_eudicots - ps20
Superrosids - ps21
Rosids - ps22
Eurosids - ps23
Malvids - ps24
Sap./Malv./Brassicales - ps25
Malvales/Brassicales - ps26
Brassicales - ps27
Cleomaceae/Brassicaceae - ps28
Brassicaceae - ps29
Camelineae - ps30
Arabidopsis - ps31
Arabidopsis_thaliana - ps32
p-value
1e-11
3e-09
5e-07
1e-04
2e-02
Log-odds
0.5
1.0
1.5
2.0
2.5
3.0
3.5
4.0

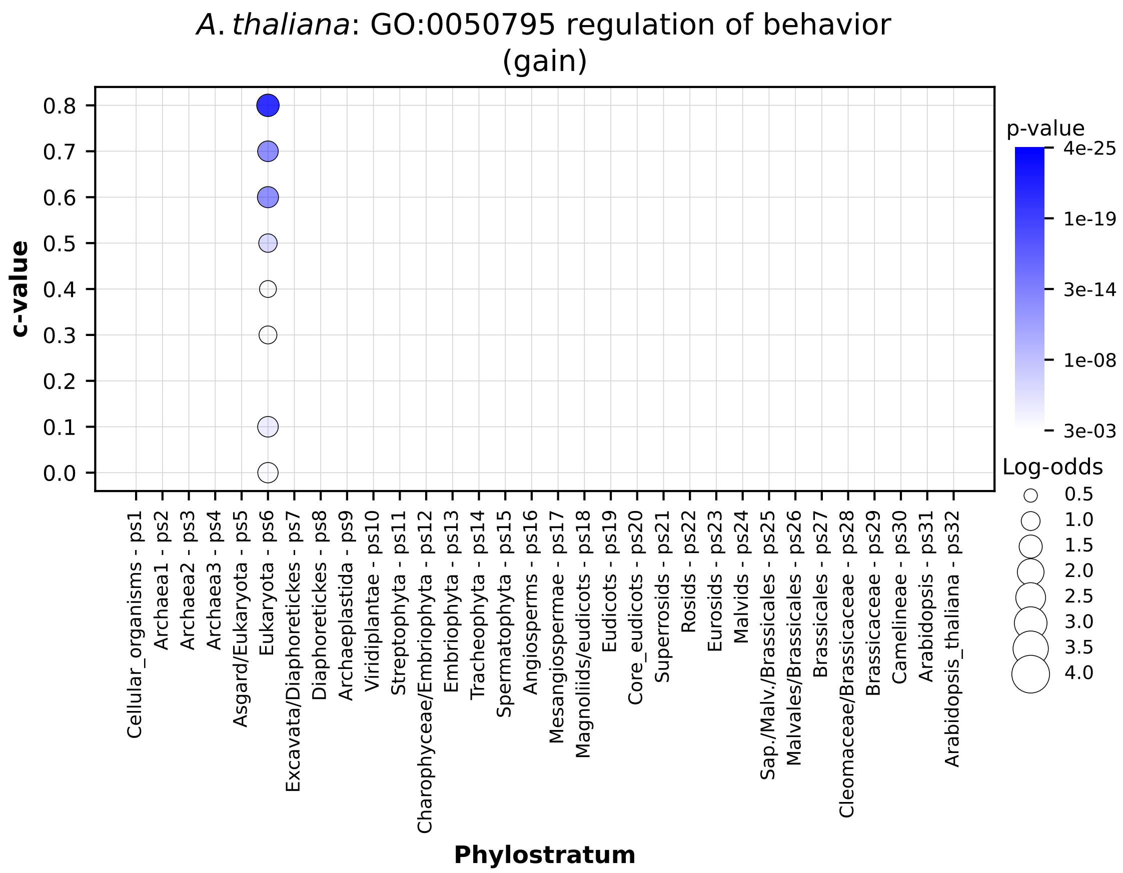
A. thaliana: GO:0050795 regulation of behavior
(gain)
c-value
0.8
0.7
0.6
0.5
0.4
0.3
0.2
0.1
0.0
p-value
4e-25
1e-19
3e-14
1e-08
3e-03
Log-odds
0.5
1.0
1.5
2.0
2.5
3.0
3.5
4.0
Cellular_organisms - ps1
Archaea1 - ps2
Archaea2 - ps3
Archaea3 - ps4
Asgard/Eukaryota - ps5
Eukaryota - ps6
Excavata/Diaphoretickes - ps7
Diaphoretickes - ps8
Archaeplastida - ps9
Viridiplantae - ps10
Streptophyta - ps11
Charophyceae/Embriophyta - ps12
Embriophyta - ps13
Tracheophyta - ps14
Spermatophyta - ps15
Angiosperms - ps16
Mesangiospermae - ps17
Magnoliids/eudicots - ps18
Eudicots - ps19
Core_eudicots - ps20
Superrosids - ps21
Rosids - ps22
Eurosids - ps23
Malvids - ps24
Sap./Malv./Brassicales - ps25
Malvales/Brassicales - ps26
Brassicales - ps27
Cleomaceae/Brassicaceae - ps28
Brassicaceae - ps29
Camelineae - ps30
Arabidopsis - ps31
Arabidopsis_thaliana - ps32
Phylostratum

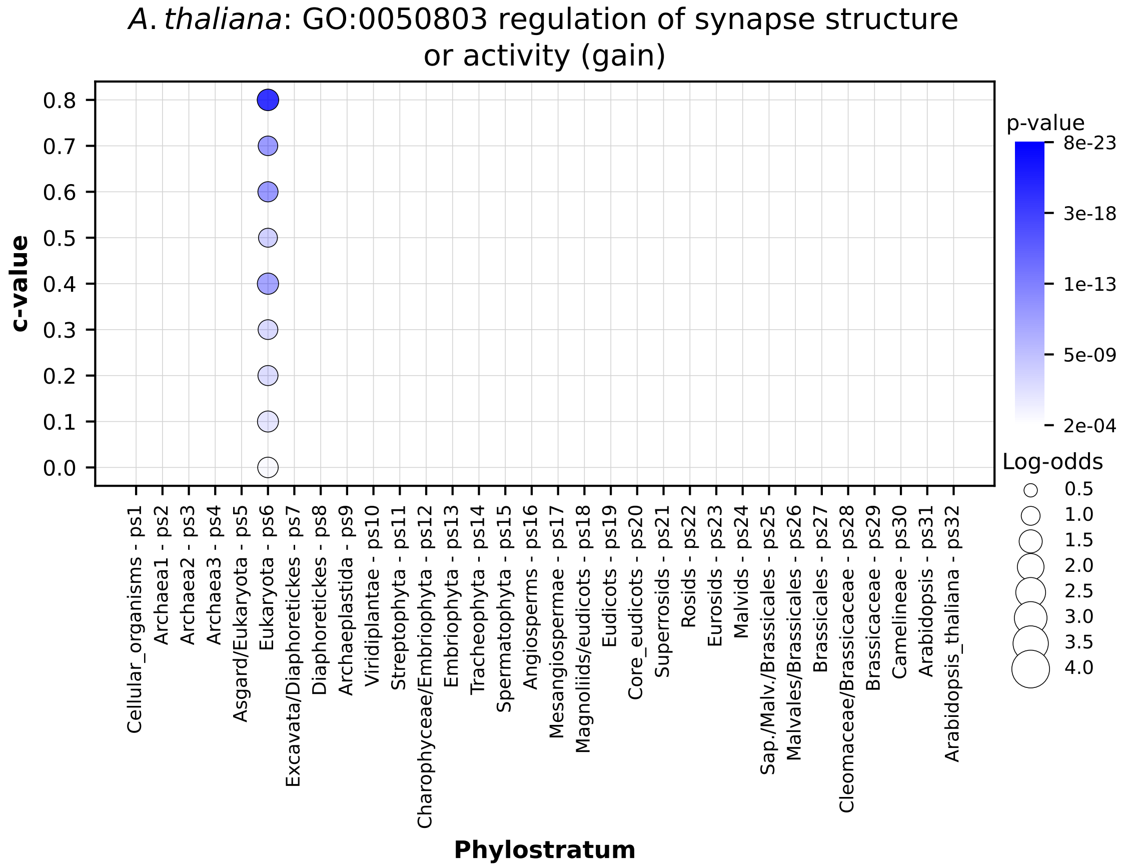
A. thaliana: GO:0050803 regulation of synapse structure or activity (gain)
c-value
0.8
0.7
0.6
0.5
0.4
0.3
0.2
0.1
0.0
Phylostratum
Cellular_organisms - ps1
Archaea1 - ps2
Archaea2 - ps3
Archaea3 - ps4
Asgard/Eukaryota - ps5
Eukaryota - ps6
Excavata/Diaphoretickes - ps7
Diaphoretickes - ps8
Archaeplastida - ps9
Viridiplantae - ps10
Streptophyta - ps11
Charophyceae/Embriophyta - ps12
Embriophyta - ps13
Tracheophyta - ps14
Spermatophyta - ps15
Angiosperms - ps16
Mesangiospermae - ps17
Magnoliids/eudicots - ps18
Eudicots - ps19
Core_eudicots - ps20
Superrosids - ps21
Rosids - ps22
Eurosids - ps23
Malvids - ps24
Sap./Malv./Brassicales - ps25
Malvales/Brassicales - ps26
Brassicales - ps27
Cleomaceae/Brassicaceae - ps28
Brassicaceae - ps29
Camelineae - ps30
Arabidopsis - ps31
Arabidopsis_thaliana - ps32
p-value
8e-23
3e-18
1e-13
5e-09
2e-04
Log-odds
0.5
1.0
1.5
2.0
2.5
3.0
3.5
4.0

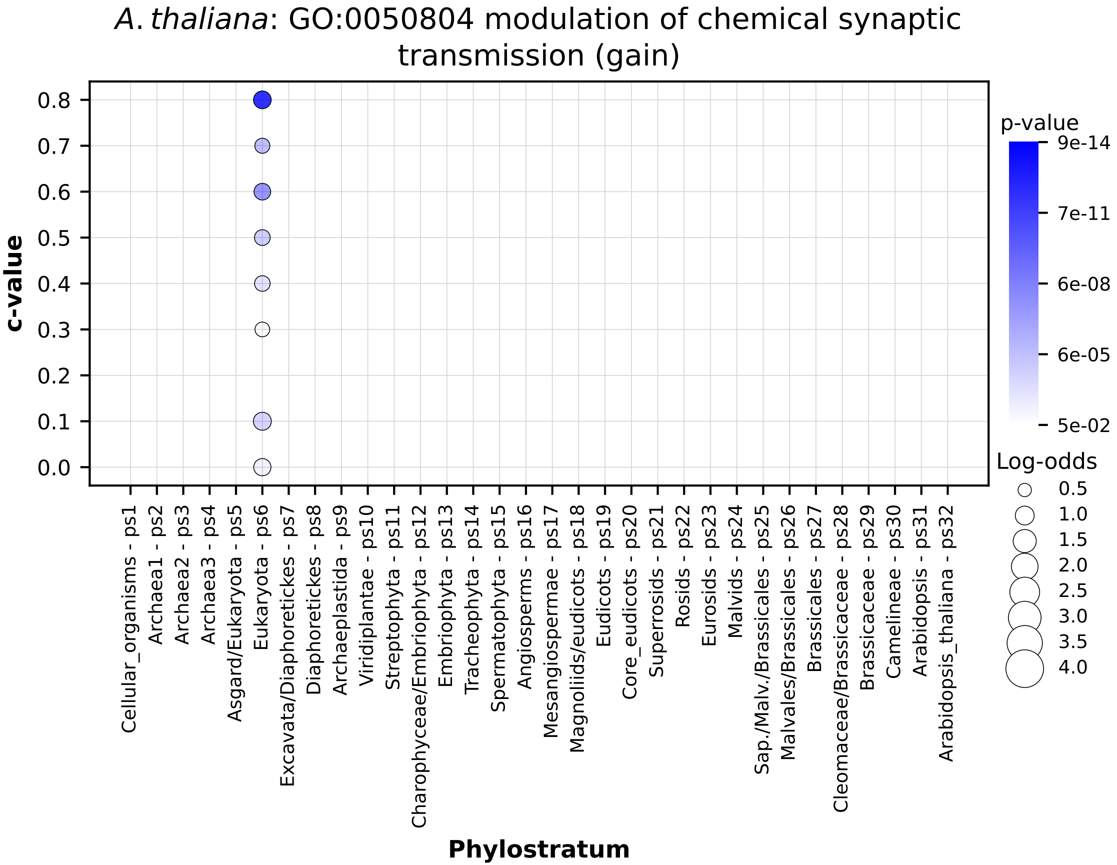
A. thaliana: GO:0050804 modulation of chemical synaptic transmission (gain)
c-value
0.8
0.7
0.6
0.5
0.4
0.3
0.2
0.1
0.0
p-value
9e-14
7e-11
6e-08
6e-05
5e-02
Log-odds
0.5
1.0
1.5
2.0
2.5
3.0
3.5
4.0
Cellular_organisms - ps1
Archaea1 - ps2
Archaea2 - ps3
Archaea3 - ps4
Asgard/Eukaryota - ps5
Eukaryota - ps6
Excavata/Diaphoretickes - ps7
Diaphoretickes - ps8
Archaeplastida - ps9
Viridiplantae - ps10
Streptophyta - ps11
Charophyceae/Embriophyta - ps12
Embriophyta - ps13
Tracheophyta - ps14
Spermatophyta - ps15
Angiosperms - ps16
Mesangiospermae - ps17
Magnoliids/eudicots - ps18
Eudicots - ps19
Core_eudicots - ps20
Superrosids - ps21
Rosids - ps22
Eurosids - ps23
Malvids - ps24
Sap./Malv./Brassicales - ps25
Malvales/Brassicales - ps26
Brassicales - ps27
Cleomaceae/Brassicaceae - ps28
Brassicaceae - ps29
Camelineae - ps30
Arabidopsis - ps31
Arabidopsis_thaliana - ps32
Phylostratum

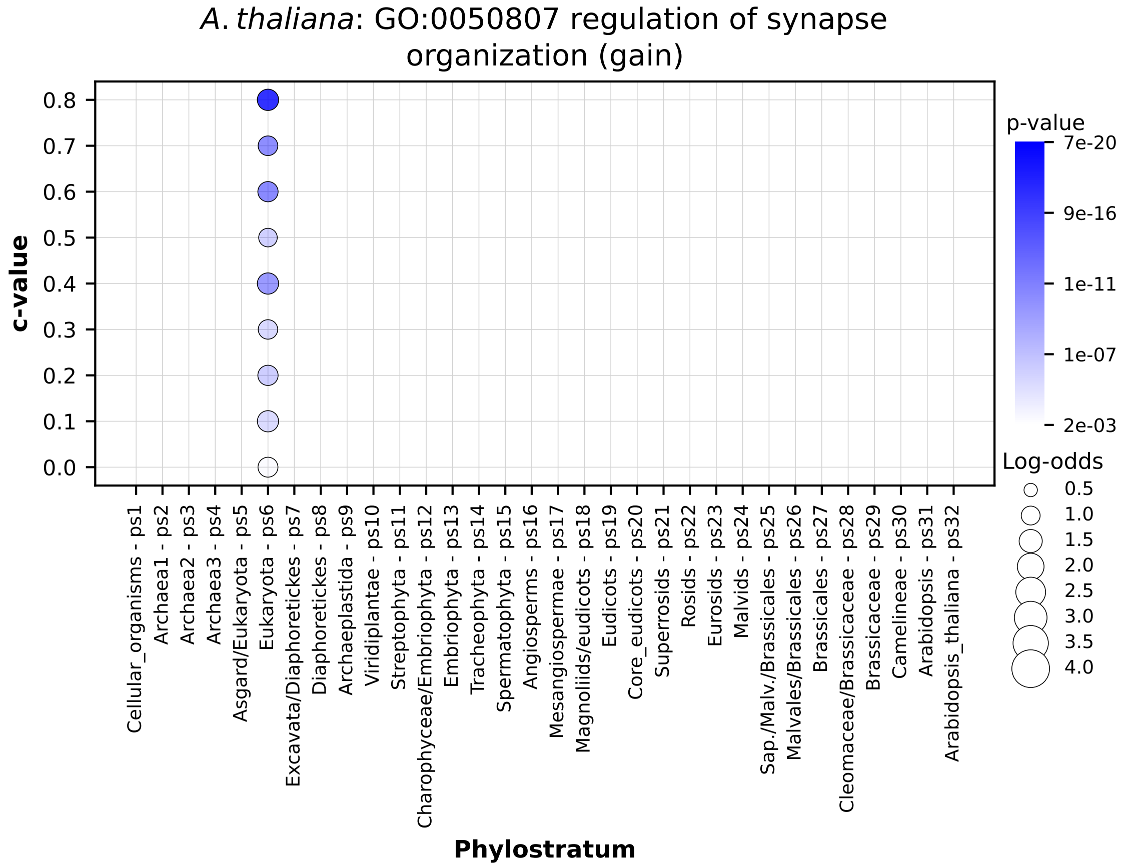

A. thaliana: GO:0050807 regulation of synapse organization (gain)
c-value
0.8
0.7
0.6
0.5
0.4
0.3
0.2
0.1
0.0
Phylostratum
Cellular_organisms - ps1
Archaea1 - ps2
Archaea2 - ps3
Archaea3 - ps4
Asgard/Eukaryota - ps5
Eukaryota - ps6
Excavata/Diaphoretickes - ps7
Diaphoretickes - ps8
Archaeplastida - ps9
Viridiplantae - ps10
Streptophyta - ps11
Charophyceae/Embriophyta - ps12
Embriophyta - ps13
Tracheophyta - ps14
Spermatophyta - ps15
Angiosperms - ps16
Mesangiospermae - ps17
Magnoliids/eudicots - ps18
Eudicots - ps19
Core_eudicots - ps20
Superrosids - ps21
Rosids - ps22
Eurosids - ps23
Malvids - ps24
Sap./Malv./Brassicales - ps25
Malvales/Brassicales - ps26
Brassicales - ps27
Cleomaceae/Brassicaceae - ps28
Brassicaceae - ps29
Camelineae - ps30
Arabidopsis - ps31
Arabidopsis_thaliana - ps32
p-value
7e-20
9e-16
1e-11
1e-07
2e-03
Log-odds
0.5
1.0
1.5
2.0
2.5
3.0
3.5
4.0

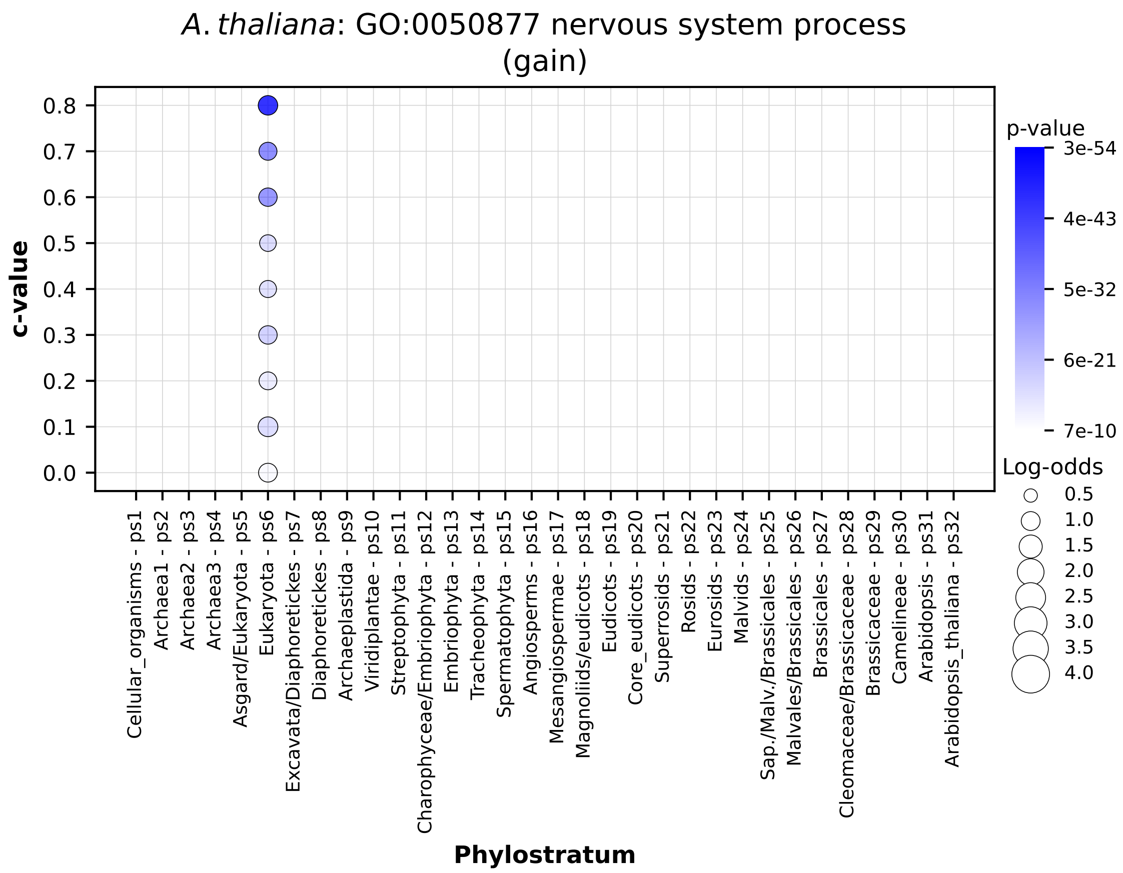

A. thaliana: GO:0050877 nervous system process
(gain)
c-value
0.8
0.7
0.6
0.5
0.4
0.3
0.2
0.1
0.0
p-value
3e-54
4e-43
5e-32
6e-21
7e-10
Log-odds
0.5
1.0
1.5
2.0
2.5
3.0
3.5
4.0
Cellular_organisms - ps1
Archaea1 - ps2
Archaea2 - ps3
Archaea3 - ps4
Asgard/Eukaryota - ps5
Eukaryota - ps6
Excavata/Diaphoretickes - ps7
Diaphoretickes - ps8
Archaeplastida - ps9
Viridiplantae - ps10
Streptophyta - ps11
Charophyceae/Embriophyta - ps12
Embriophyta - ps13
Tracheophyta - ps14
Spermatophyta - ps15
Angiosperms - ps16
Mesangiospermae - ps17
Magnoliids/eudicots - ps18
Eudicots - ps19
Core_eudicots - ps20
Superrosids - ps21
Rosids - ps22
Eurosids - ps23
Malvids - ps24
Sap./Malv./Brassicales - ps25
Malvales/Brassicales - ps26
Brassicales - ps27
Cleomaceae/Brassicaceae - ps28
Brassicaceae - ps29
Camelineae - ps30
Arabidopsis - ps31
Arabidopsis_thaliana - ps32
Phylostratum

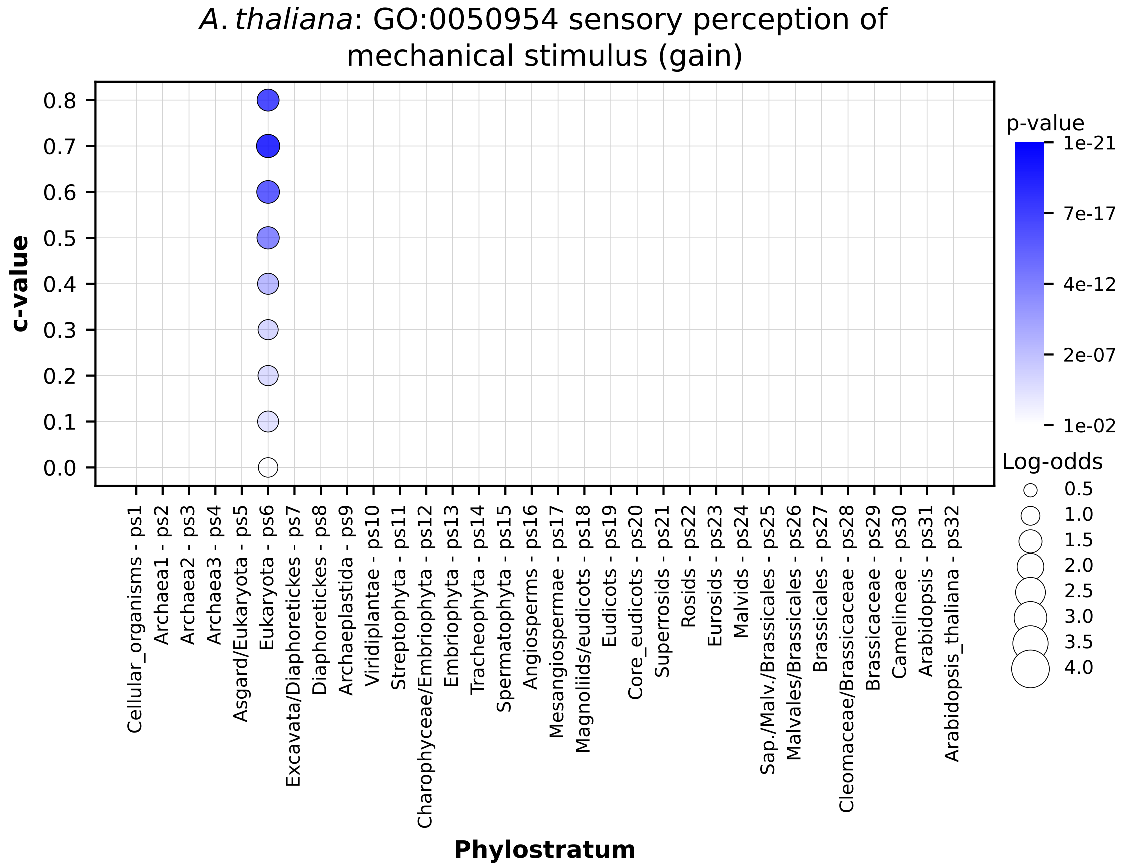
A. thaliana: GO:0050954 sensory perception of mechanical stimulus (gain)
c-value
0.8
0.7
0.6
0.5
0.4
0.3
0.2
0.1
0.0
p-value
1e-21
7e-17
4e-12
2e-07
1e-02
Log-odds
0.5
1.0
1.5
2.0
2.5
3.0
3.5
4.0
Cellular_organisms - ps1
Archaea1 - ps2
Archaea2 - ps3
Archaea3 - ps4
Asgard/Eukaryota - ps5
Eukaryota - ps6
Excavata/Diaphoretickes - ps7
Diaphoretickes - ps8
Archaeplastida - ps9
Viridiplantae - ps10
Streptophyta - ps11
Charophyceae/Embriophyta - ps12
Embriophyta - ps13
Tracheophyta - ps14
Spermatophyta - ps15
Angiosperms - ps16
Mesangiospermae - ps17
Magnoliids/eudicots - ps18
Eudicots - ps19
Core_eudicots - ps20
Superrosids - ps21
Rosids - ps22
Eurosids - ps23
Malvids - ps24
Sap./Malv./Brassicales - ps25
Malvales/Brassicales - ps26
Brassicales - ps27
Cleomaceae/Brassicaceae - ps28
Brassicaceae - ps29
Camelineae - ps30
Arabidopsis - ps31
Arabidopsis_thaliana - ps32
Phylostratum

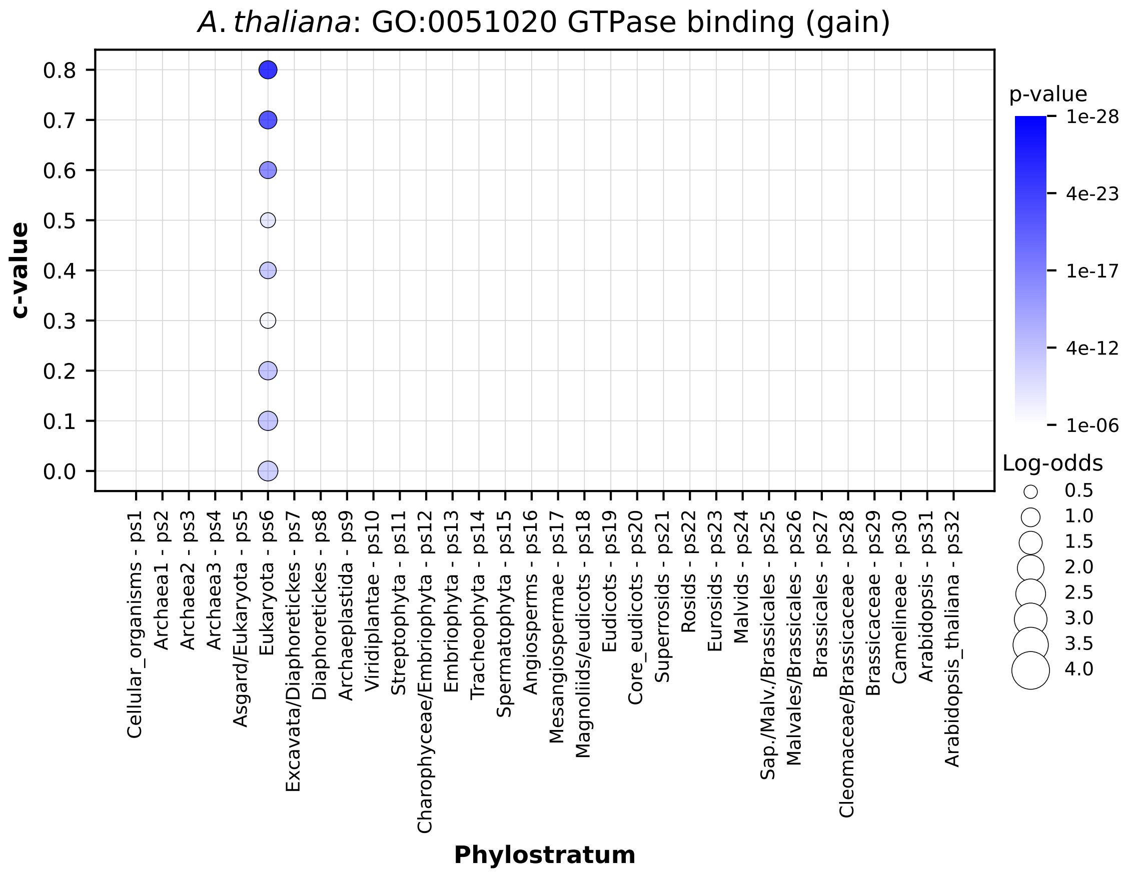

A. thaliana: GO:0051020 GTPase binding (gain)
c-value
0.8
0.7
0.6
0.5
0.4
0.3
0.2
0.1
0.0
p-value
1e-28
4e-23
1e-17
4e-12
1e-06
Log-odds
0.5
1.0
1.5
2.0
2.5
3.0
3.5
4.0
Cellular_organisms - ps1
Archaea1 - ps2
Archaea2 - ps3
Archaea3 - ps4
Asgard/Eukaryota - ps5
Eukaryota - ps6
Excavata/Diaphoretickes - ps7
Diaphoretickes - ps8
Archaeplastida - ps9
Viridiplantae - ps10
Streptophyta - ps11
Charophyceae/Embriophyta - ps12
Embriophyta - ps13
Tracheophyta - ps14
Spermatophyta - ps15
Angiosperms - ps16
Mesangiospermae - ps17
Magnoliids/eudicots - ps18
Eudicots - ps19
Core_eudicots - ps20
Superrosids - ps21
Rosids - ps22
Eurosids - ps23
Malvids - ps24
Sap./Malv./Brassicales - ps25
Malvales/Brassicales - ps26
Brassicales - ps27
Cleomaceae/Brassicaceae - ps28
Brassicaceae - ps29
Camelineae - ps30
Arabidopsis - ps31
Arabidopsis_thaliana - ps32
Phylostratum

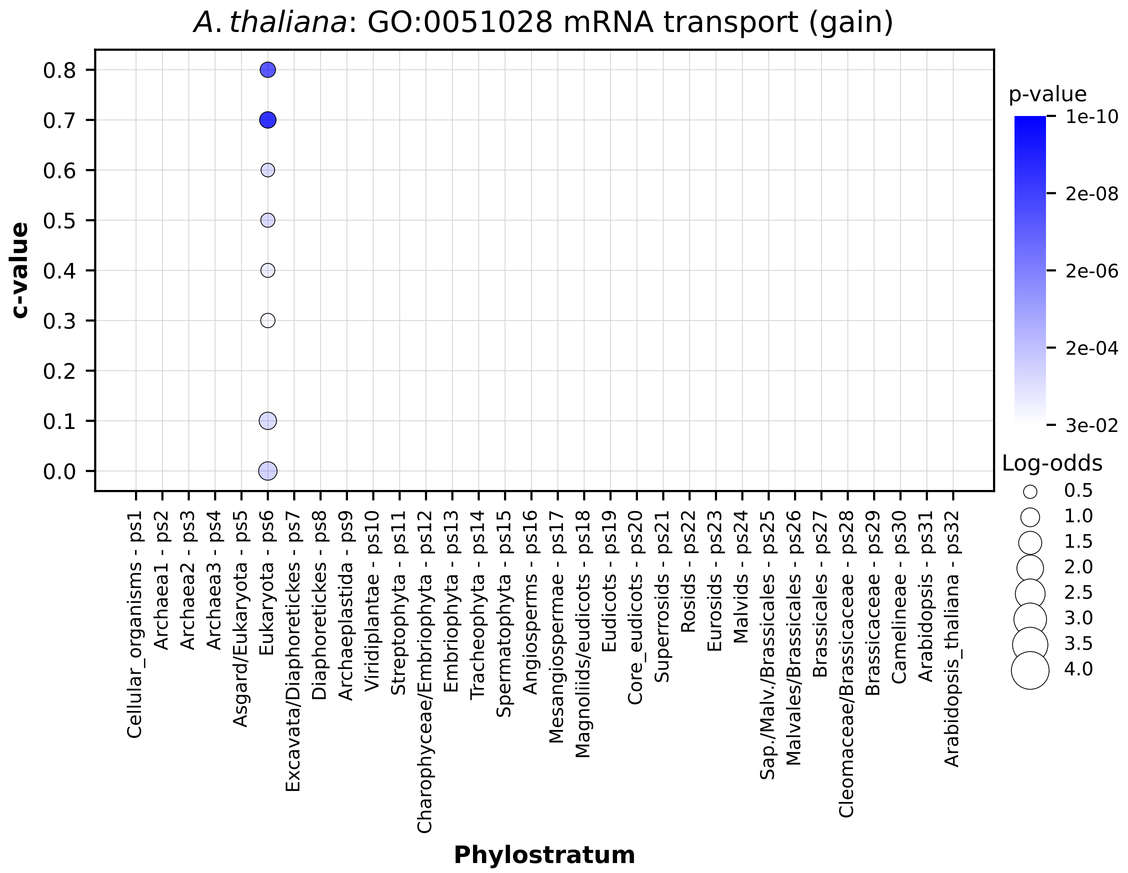

A. thaliana: GO:0051028 mRNA transport (gain)
c-value
0.8
0.7
0.6
0.5
0.4
0.3
0.2
0.1
0.0
Phylostratum
Cellular_organisms - ps1
Archaea1 - ps2
Archaea2 - ps3
Archaea3 - ps4
Asgard/Eukaryota - ps5
Eukaryota - ps6
Excavata/Diaphoretickes - ps7
Diaphoretickes - ps8
Archaeplastida - ps9
Viridiplantae - ps10
Streptophyta - ps11
Charophyceae/Embriophyta - ps12
Embriophyta - ps13
Tracheophyta - ps14
Spermatophyta - ps15
Angiosperms - ps16
Mesangiospermae - ps17
Magnoliids/eudicots - ps18
Eudicots - ps19
Core_eudicots - ps20
Superrosids - ps21
Rosids - ps22
Eurosids - ps23
Malvids - ps24
Sap./Malv./Brassicales - ps25
Malvales/Brassicales - ps26
Brassicales - ps27
Cleomaceae/Brassicaceae - ps28
Brassicaceae - ps29
Camelineae - ps30
Arabidopsis - ps31
Arabidopsis_thaliana - ps32
p-value
1e-10
2e-08
2e-06
2e-04
3e-02
Log-odds
0.5
1.0
1.5
2.0
2.5
3.0
3.5
4.0

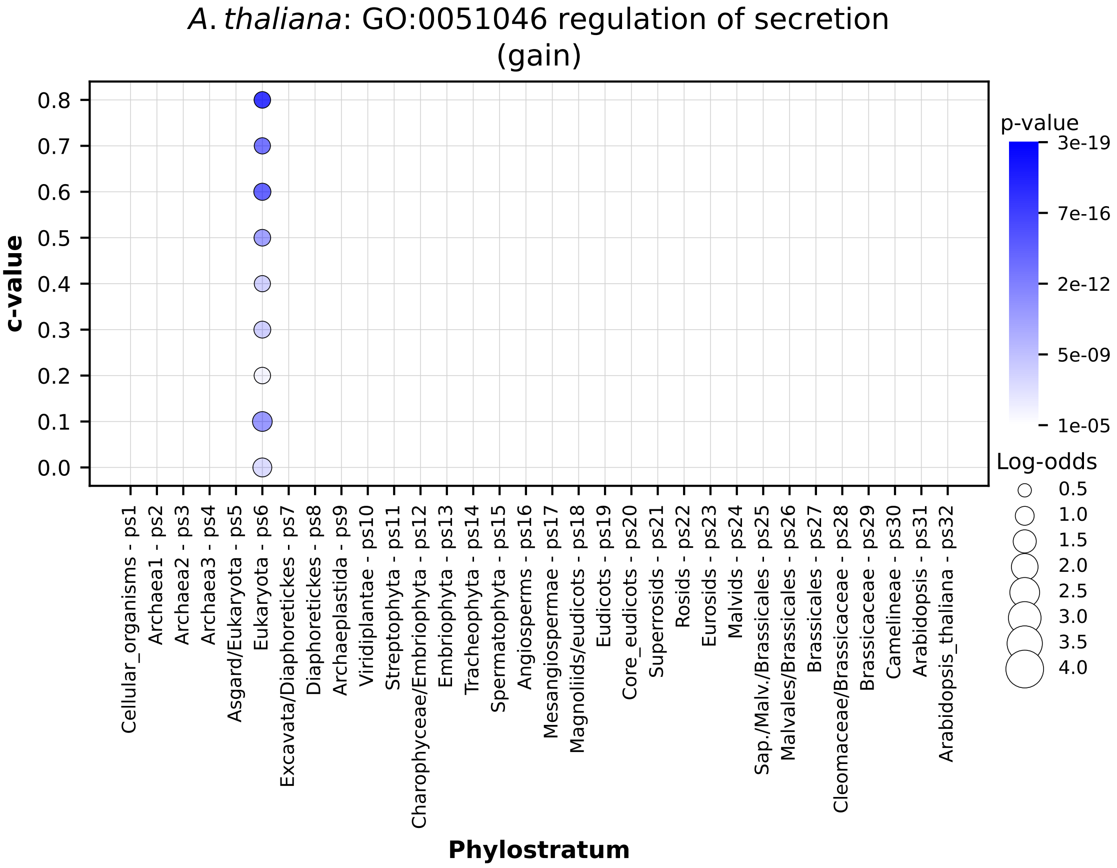
A. thaliana: GO:0051046 regulation of secretion (gain)
c-value
0.0
0.1
0.2
0.3
0.4
0.5
0.6
0.7
0.8
Phylostratum
Cellular_organisms - ps1
Archaea1 - ps2
Archaea2 - ps3
Archaea3 - ps4
Asgard/Eukaryota - ps5
Eukaryota - ps6
Excavata/Diaphoretickes - ps7
Diaphoretickes - ps8
Archaeplastida - ps9
Viridiplantae - ps10
Streptophyta - ps11
Charophyceae/Embriophyta - ps12
Embriophyta - ps13
Tracheophyta - ps14
Spermatophyta - ps15
Angiosperms - ps16
Mesangiospermae - ps17
Magnoliids/eudicots - ps18
Eudicots - ps19
Core_eudicots - ps20
Superrosids - ps21
Rosids - ps22
Eurosids - ps23
Malvids - ps24
Sap./Malv./Brassicales - ps25
Malvales/Brassicales - ps26
Brassicales - ps27
Cleomaceae/Brassicaceae - ps28
Brassicaceae - ps29
Camelineae - ps30
Arabidopsis - ps31
Arabidopsis_thaliana - ps32
p-value
3e-19
7e-16
2e-12
5e-09
1e-05
Log-odds
0.5
1.0
1.5
2.0
2.5
3.0
3.5
4.0

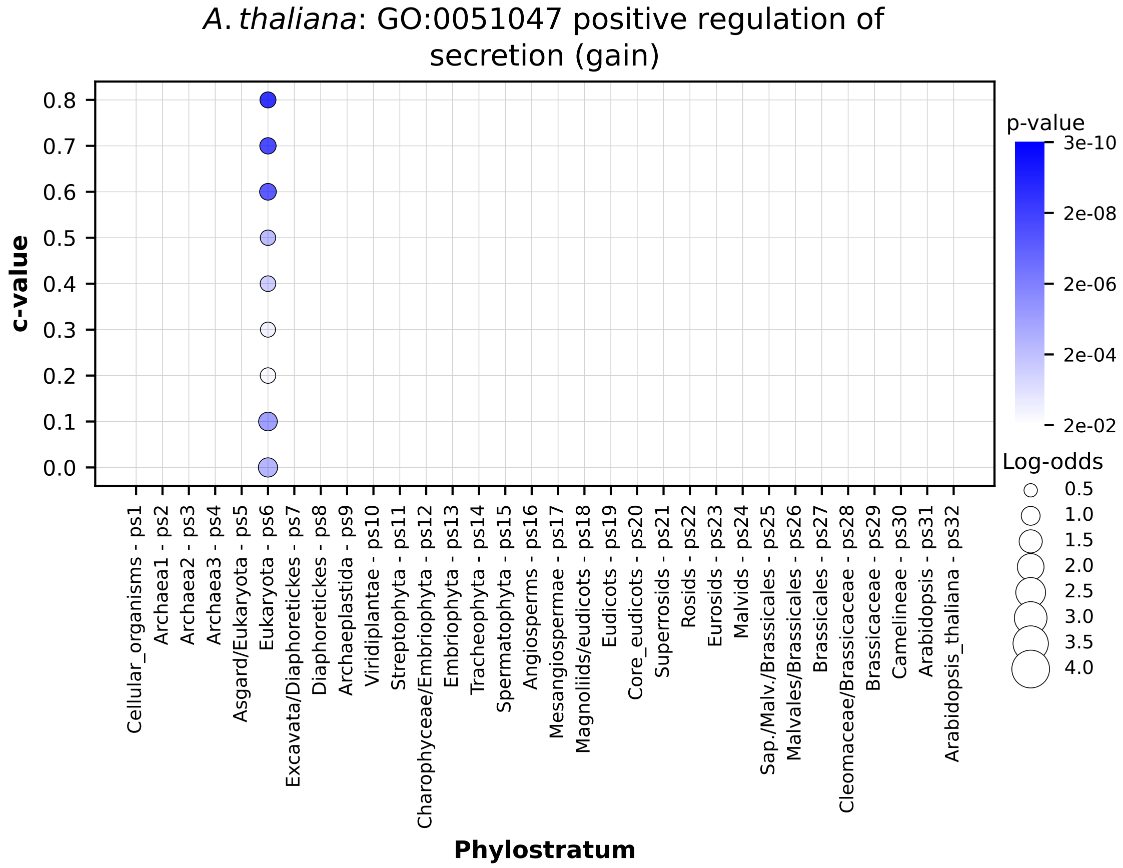

A. thaliana: GO:0051047 positive regulation of secretion (gain)
c-value
0.8
0.7
0.6
0.5
0.4
0.3
0.2
0.1
0.0
Phylostratum
Cellular_organisms - ps1
Archaea1 - ps2
Archaea2 - ps3
Archaea3 - ps4
Asgard/Eukaryota - ps5
Eukaryota - ps6
Excavata/Diaphoretickes - ps7
Diaphoretickes - ps8
Archaeplastida - ps9
Viridiplantae - ps10
Streptophyta - ps11
Charophyceae/Embriophyta - ps12
Embriophyta - ps13
Tracheophyta - ps14
Spermatophyta - ps15
Angiosperms - ps16
Mesangiospermae - ps17
Magnoliids/eudicots - ps18
Eudicots - ps19
Core_eudicots - ps20
Superrosids - ps21
Rosids - ps22
Eurosids - ps23
Malvids - ps24
Sap./Malv./Brassicales - ps25
Malvales/Brassicales - ps26
Brassicales - ps27
Cleomaceae/Brassicaceae - ps28
Brassicaceae - ps29
Camelineae - ps30
Arabidopsis - ps31
Arabidopsis_thaliana - ps32
p-value
3e-10
2e-08
2e-06
2e-04
2e-02
Log-odds
0.5
1.0
1.5
2.0
2.5
3.0
3.5
4.0

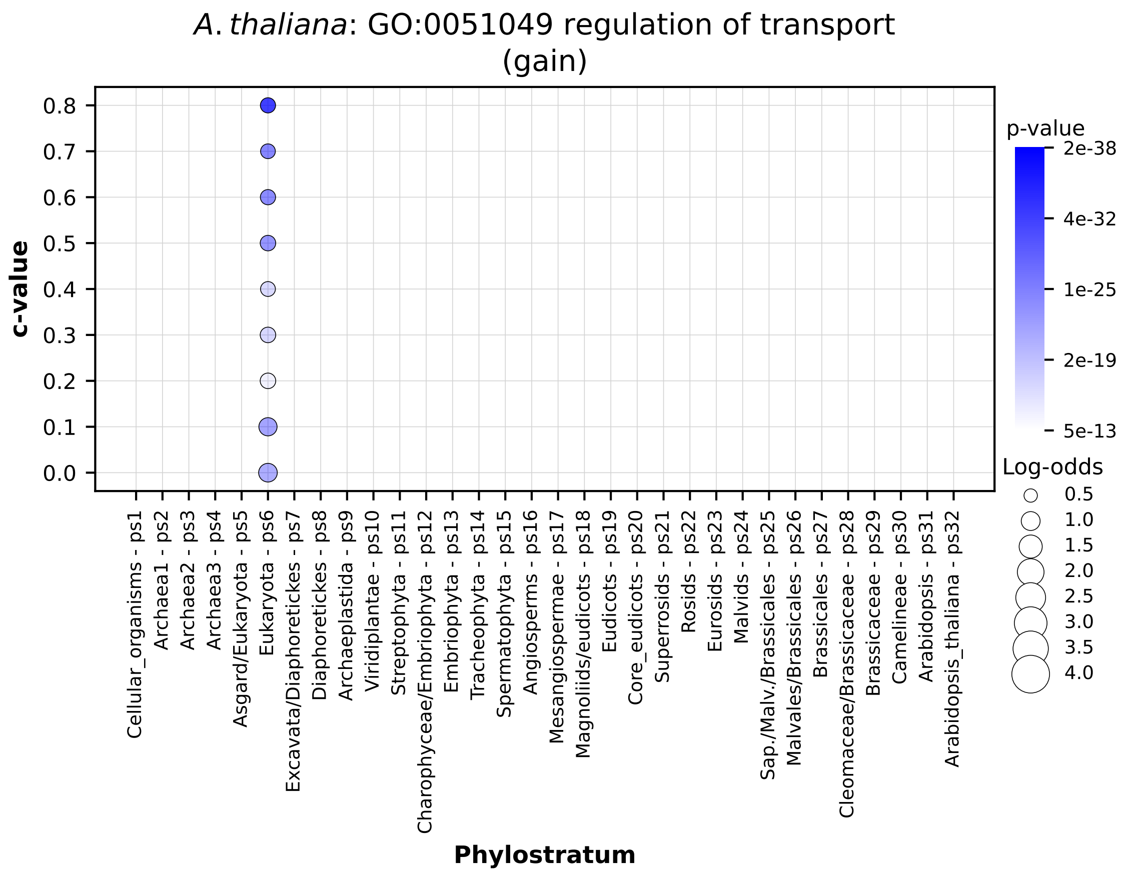

A. thaliana: GO:0051049 regulation of transport
(gain)
c-value
0.8
0.7
0.6
0.5
0.4
0.3
0.2
0.1
0.0
p-value
2e-38
4e-32
1e-25
2e-19
5e-13
Log-odds
0.5
1.0
1.5
2.0
2.5
3.0
3.5
4.0
Cellular_organisms - ps1
Archaea1 - ps2
Archaea2 - ps3
Archaea3 - ps4
Asgard/Eukaryota - ps5
Eukaryota - ps6
Excavata/Diaphoretickes - ps7
Diaphoretickes - ps8
Archaeplastida - ps9
Viridiplantae - ps10
Streptophyta - ps11
Charophyceae/Embriophyta - ps12
Embriophyta - ps13
Tracheophyta - ps14
Spermatophyta - ps15
Angiosperms - ps16
Mesangiospermae - ps17
Magnoliids/eudicots - ps18
Eudicots - ps19
Core_eudicots - ps20
Superrosids - ps21
Rosids - ps22
Eurosids - ps23
Malvids - ps24
Sap./Malv./Brassicales - ps25
Malvales/Brassicales - ps26
Brassicales - ps27
Cleomaceae/Brassicaceae - ps28
Brassicaceae - ps29
Camelineae - ps30
Arabidopsis - ps31
Arabidopsis_thaliana - ps32
Phylostratum

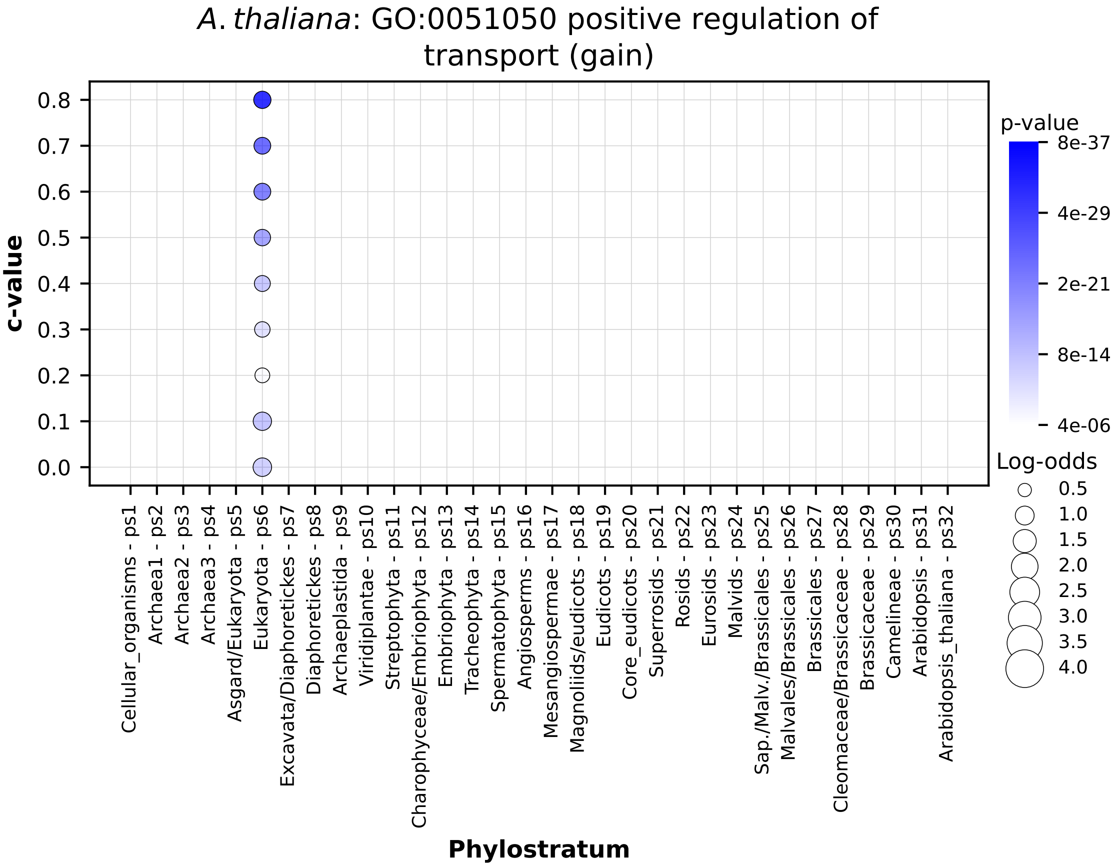

A. thaliana: GO:0051050 positive regulation of transport (gain)
c-value
0.8
0.7
0.6
0.5
0.4
0.3
0.2
0.1
0.0
p-value
8e-37
4e-29
2e-21
8e-14
4e-06
Log-odds
0.5
1.0
1.5
2.0
2.5
3.0
3.5
4.0
Cellular_organisms - ps1
Archaea1 - ps2
Archaea2 - ps3
Archaea3 - ps4
Asgard/Eukaryota - ps5
Eukaryota - ps6
Excavata/Diaphoretickes - ps7
Diaphoretickes - ps8
Archaeplastida - ps9
Viridiplantae - ps10
Streptophyta - ps11
Charophyceae/Embriophyta - ps12
Embriophyta - ps13
Tracheophyta - ps14
Spermatophyta - ps15
Angiosperms - ps16
Mesangiospermae - ps17
Magnoliids/eudicots - ps18
Eudicots - ps19
Core_eudicots - ps20
Superrosids - ps21
Rosids - ps22
Eurosids - ps23
Malvids - ps24
Sap./Malv./Brassicales - ps25
Malvales/Brassicales - ps26
Brassicales - ps27
Cleomaceae/Brassicaceae - ps28
Brassicaceae - ps29
Camelineae - ps30
Arabidopsis - ps31
Arabidopsis_thaliana - ps32
Phylostratum

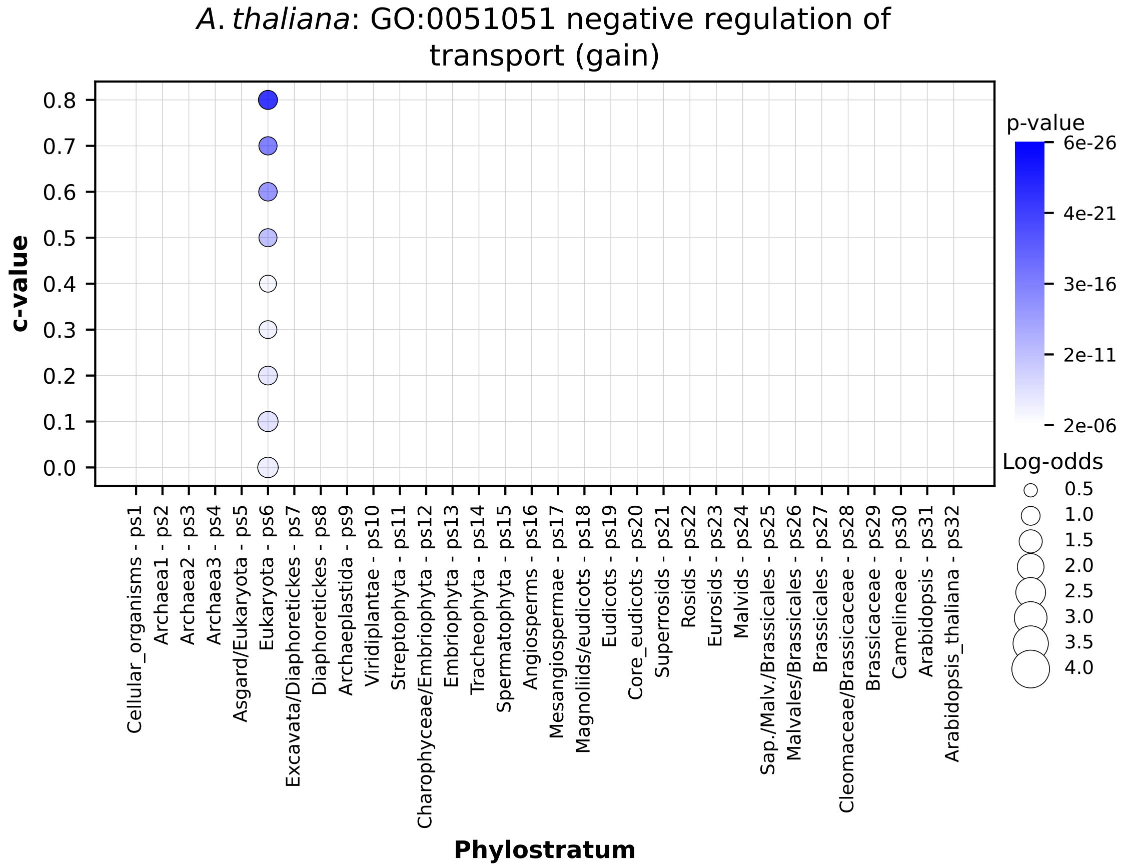

A. thaliana: GO:0051051 negative regulation of transport (gain)
c-value
0.0
0.1
0.2
0.3
0.4
0.5
0.6
0.7
0.8
p-value
6e-26
4e-21
3e-16
2e-11
2e-06
Log-odds
0.5
1.0
1.5
2.0
2.5
3.0
3.5
4.0
Cellular_organisms - ps1
Archaea1 - ps2
Archaea2 - ps3
Archaea3 - ps4
Asgard/Eukaryota - ps5
Eukaryota - ps6
Excavata/Diaphoretickes - ps7
Diaphoretickes - ps8
Archaeplastida - ps9
Viridiplantae - ps10
Streptophyta - ps11
Charophyceae/Embriophyta - ps12
Embriophyta - ps13
Tracheophyta - ps14
Spermatophyta - ps15
Angiosperms - ps16
Mesangiospermae - ps17
Magnoliids/eudicots - ps18
Eudicots - ps19
Core_eudicots - ps20
Superrosids - ps21
Rosids - ps22
Eurosids - ps23
Malvids - ps24
Sap./Malv./Brassicales - ps25
Malvales/Brassicales - ps26
Brassicales - ps27
Cleomaceae/Brassicaceae - ps28
Brassicaceae - ps29
Camelineae - ps30
Arabidopsis - ps31
Arabidopsis_thaliana - ps32
Phylostratum

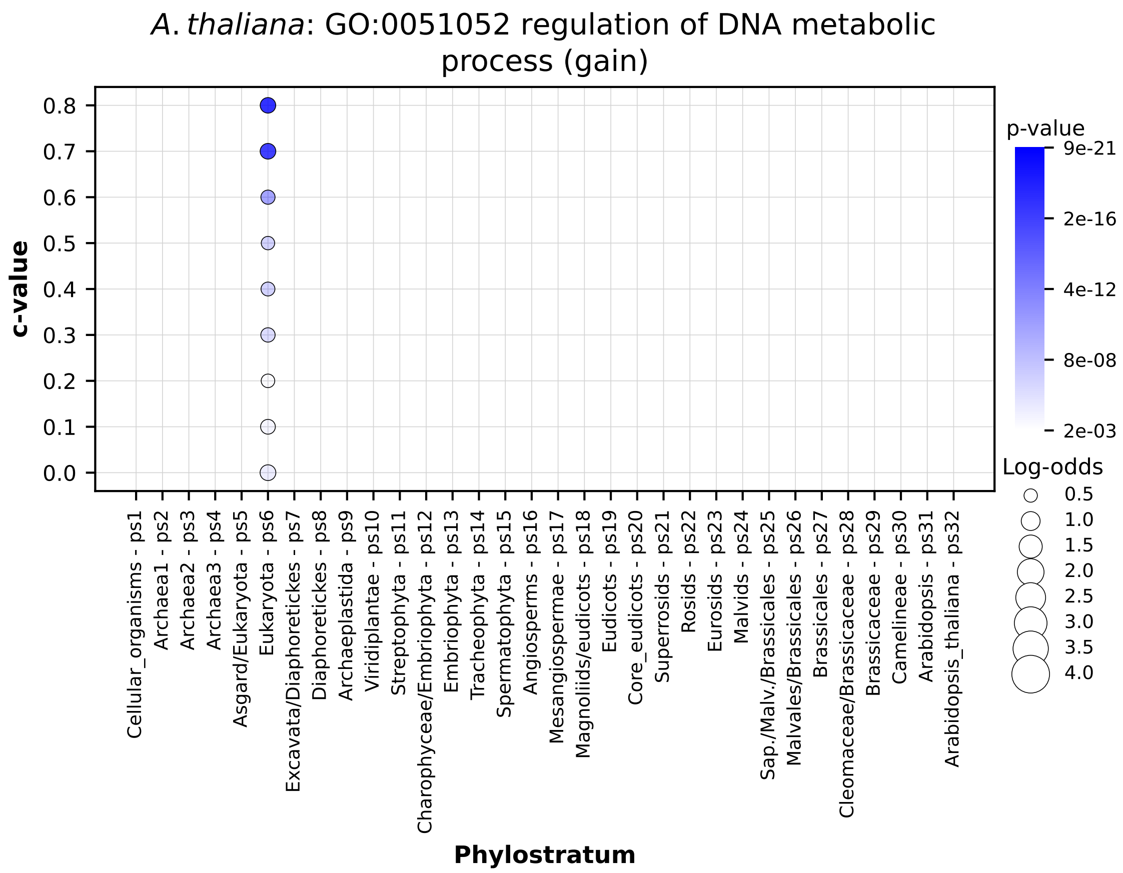
A. thaliana: GO:0051052 regulation of DNA metabolic process (gain)
c-value
0.8
0.7
0.6
0.5
0.4
0.3
0.2
0.1
0.0
p-value
9e-21
2e-16
4e-12
8e-08
2e-03
Log-odds
0.5
1.0
1.5
2.0
2.5
3.0
3.5
4.0
Cellular_organisms - ps1
Archaea1 - ps2
Archaea2 - ps3
Archaea3 - ps4
Asgard/Eukaryota - ps5
Eukaryota - ps6
Excavata/Diaphoretickes - ps7
Diaphoretickes - ps8
Archaeplastida - ps9
Viridiplantae - ps10
Streptophyta - ps11
Charophyceae/Embriophyta - ps12
Embriophyta - ps13
Tracheophyta - ps14
Spermatophyta - ps15
Angiosperms - ps16
Mesangiospermae - ps17
Magnoliids/eudicots - ps18
Eudicots - ps19
Core_eudicots - ps20
Superrosids - ps21
Rosids - ps22
Eurosids - ps23
Malvids - ps24
Sap./Malv./Brassicales - ps25
Malvales/Brassicales - ps26
Brassicales - ps27
Cleomaceae/Brassicaceae - ps28
Brassicaceae - ps29
Camelineae - ps30
Arabidopsis - ps31
Arabidopsis_thaliana - ps32
Phylostratum

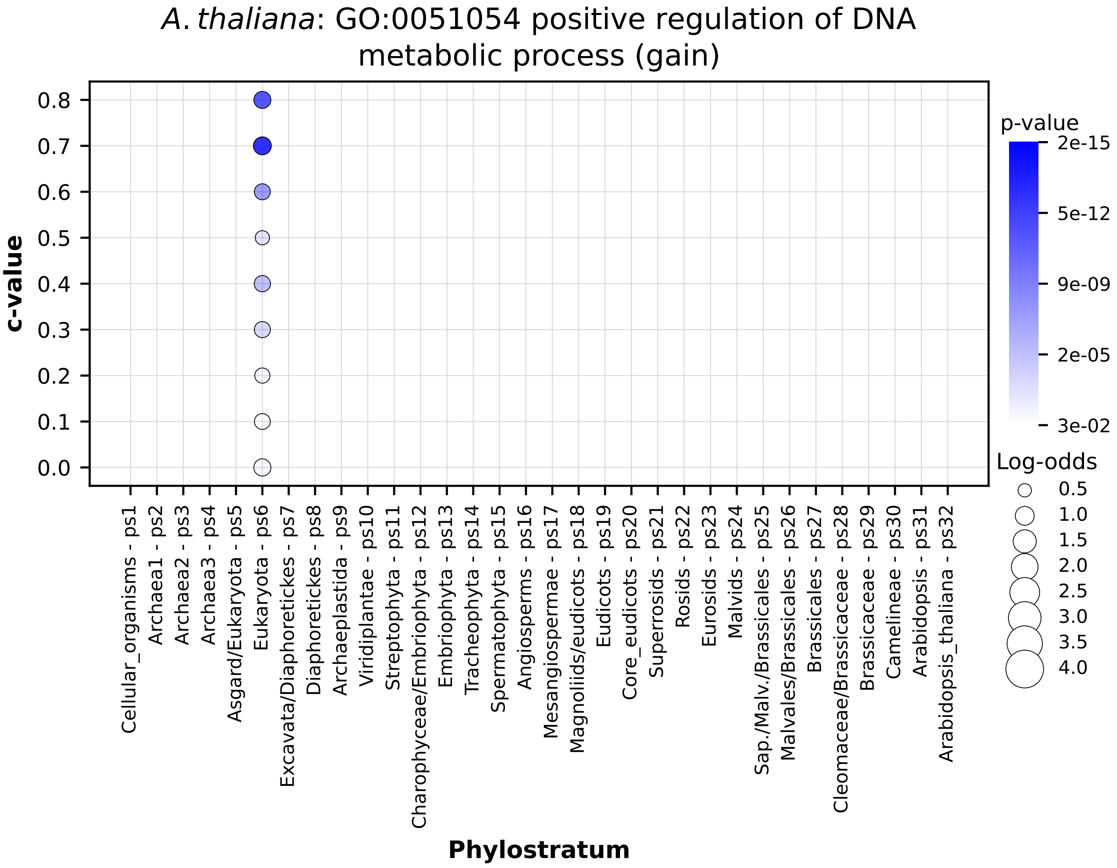

A. thaliana: GO:0051054 positive regulation of DNA metabolic process (gain)
c-value
0.8
0.7
0.6
0.5
0.4
0.3
0.2
0.1
0.0
p-value
2e-15
5e-12
9e-09
2e-05
3e-02
Log-odds
0.5
1.0
1.5
2.0
2.5
3.0
3.5
4.0
Cellular_organisms - ps1
Archaea1 - ps2
Archaea2 - ps3
Archaea3 - ps4
Asgard/Eukaryota - ps5
Eukaryota - ps6
Excavata/Diaphoretickes - ps7
Diaphoretickes - ps8
Archaeplastida - ps9
Viridiplantae - ps10
Streptophyta - ps11
Charophyceae/Embriophyta - ps12
Embriophyta - ps13
Tracheophyta - ps14
Spermatophyta - ps15
Angiosperms - ps16
Mesangiospermae - ps17
Magnoliids/eudicots - ps18
Eudicots - ps19
Core_eudicots - ps20
Superrosids - ps21
Rosids - ps22
Eurosids - ps23
Malvids - ps24
Sap./Malv./Brassicales - ps25
Malvales/Brassicales - ps26
Brassicales - ps27
Cleomaceae/Brassicaceae - ps28
Brassicaceae - ps29
Camelineae - ps30
Arabidopsis - ps31
Arabidopsis_thaliana - ps32
Phylostratum

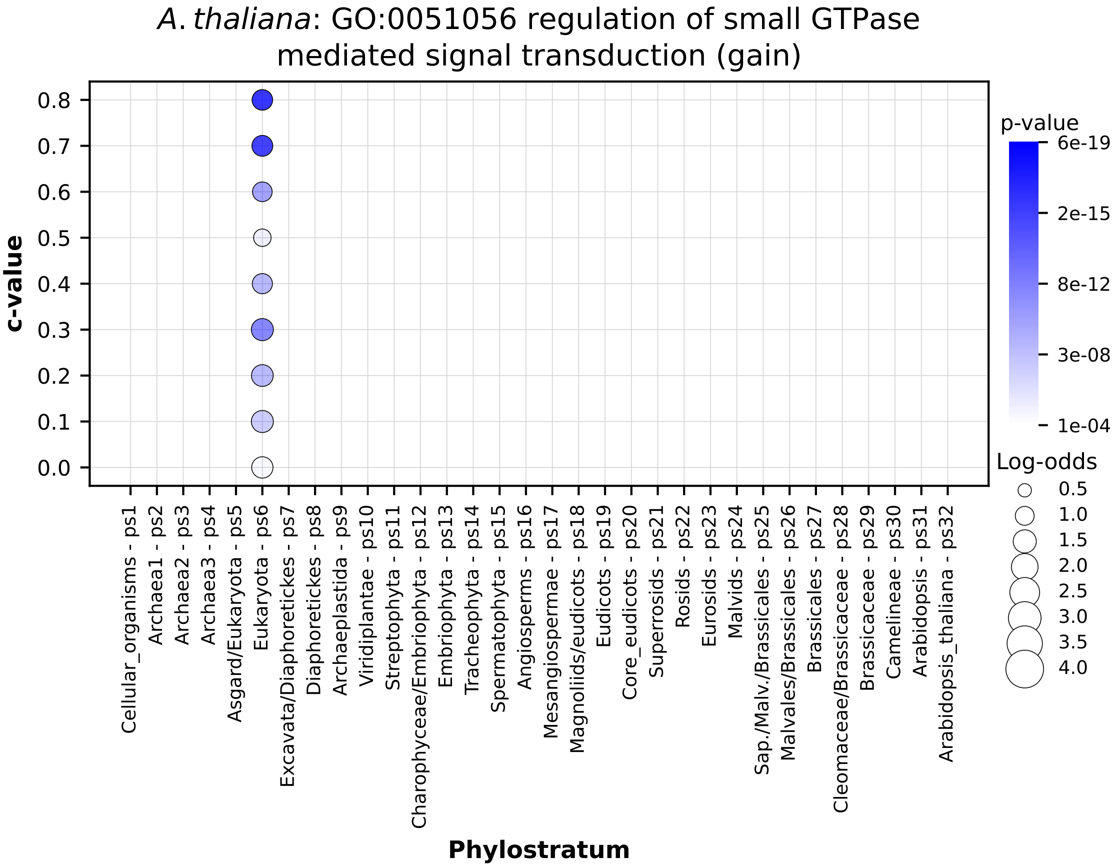

A. thaliana: GO:0051056 regulation of small GTPase mediated signal transduction (gain)
c-value
0.8
0.7
0.6
0.5
0.4
0.3
0.2
0.1
0.0
p-value
6e-19
2e-15
8e-12
3e-08
1e-04
Log-odds
0.5
1.0
1.5
2.0
2.5
3.0
3.5
4.0
Cellular_organisms - ps1
Archaea1 - ps2
Archaea2 - ps3
Archaea3 - ps4
Asgard/Eukaryota - ps5
Eukaryota - ps6
Excavata/Diaphoretickes - ps7
Diaphoretickes - ps8
Archaeplastida - ps9
Viridiplantae - ps10
Streptophyta - ps11
Charophyceae/Embriophyta - ps12
Embriophyta - ps13
Tracheophyta - ps14
Spermatophyta - ps15
Angiosperms - ps16
Mesangiospermae - ps17
Magnoliids/eudicots - ps18
Eudicots - ps19
Core_eudicots - ps20
Superrosids - ps21
Rosids - ps22
Eurosids - ps23
Malvids - ps24
Sap./Malv./Brassicales - ps25
Malvales/Brassicales - ps26
Brassicales - ps27
Cleomaceae/Brassicaceae - ps28
Brassicaceae - ps29
Camelineae - ps30
Arabidopsis - ps31
Arabidopsis_thaliana - ps32
Phylostratum

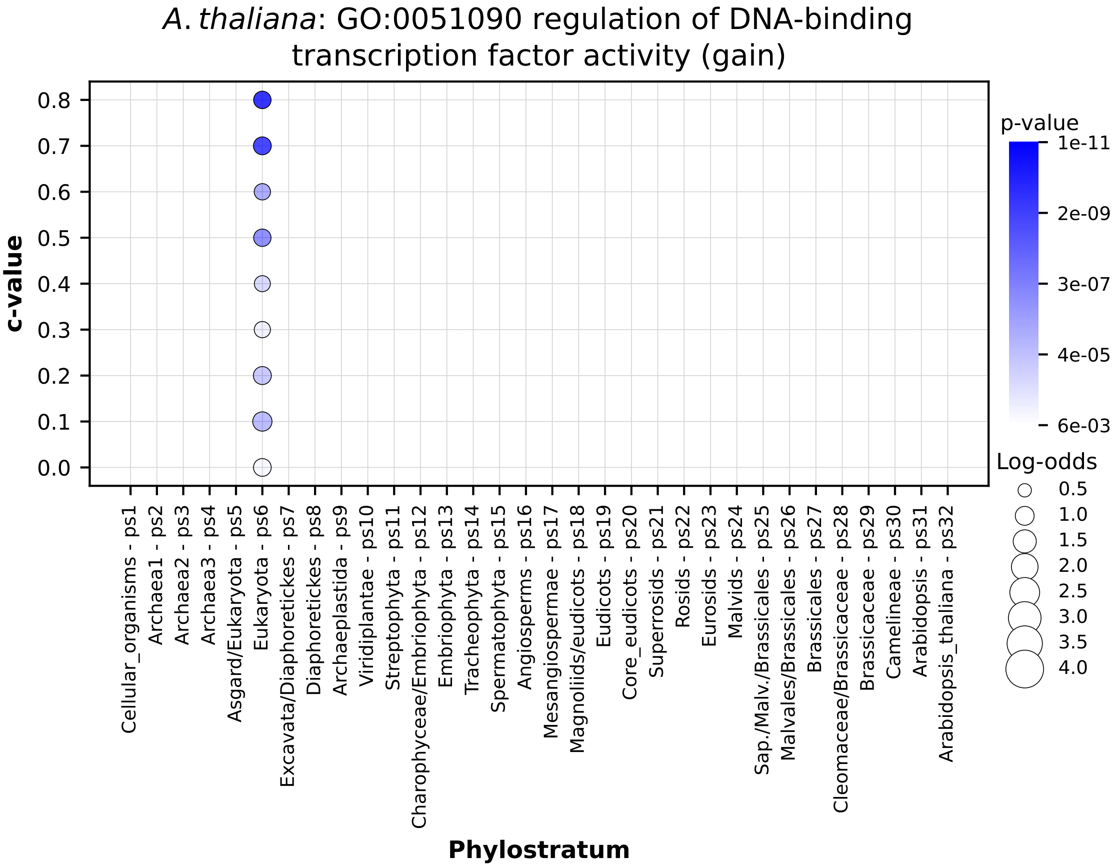

A. thaliana: GO:0051090 regulation of DNA-binding transcription factor activity (gain)
c-value
0.8
0.7
0.6
0.5
0.4
0.3
0.2
0.1
0.0
Phylostratum
Cellular_organisms - ps1
Archaea1 - ps2
Archaea2 - ps3
Archaea3 - ps4
Asgard/Eukaryota - ps5
Eukaryota - ps6
Excavata/Diaphoretickes - ps7
Diaphoretickes - ps8
Archaeplastida - ps9
Viridiplantae - ps10
Streptophyta - ps11
Charophyceae/Embriophyta - ps12
Embriophyta - ps13
Tracheophyta - ps14
Spermatophyta - ps15
Angiosperms - ps16
Mesangiospermae - ps17
Magnoliids/eudicots - ps18
Eudicots - ps19
Core_eudicots - ps20
Superrosids - ps21
Rosids - ps22
Eurosids - ps23
Malvids - ps24
Sap./Malv./Brassicales - ps25
Malvales/Brassicales - ps26
Brassicales - ps27
Cleomaceae/Brassicaceae - ps28
Brassicaceae - ps29
Camelineae - ps30
Arabidopsis - ps31
Arabidopsis_thaliana - ps32
p-value
1e-11
2e-09
3e-07
4e-05
6e-03
Log-odds
0.5
1.0
1.5
2.0
2.5
3.0
3.5
4.0

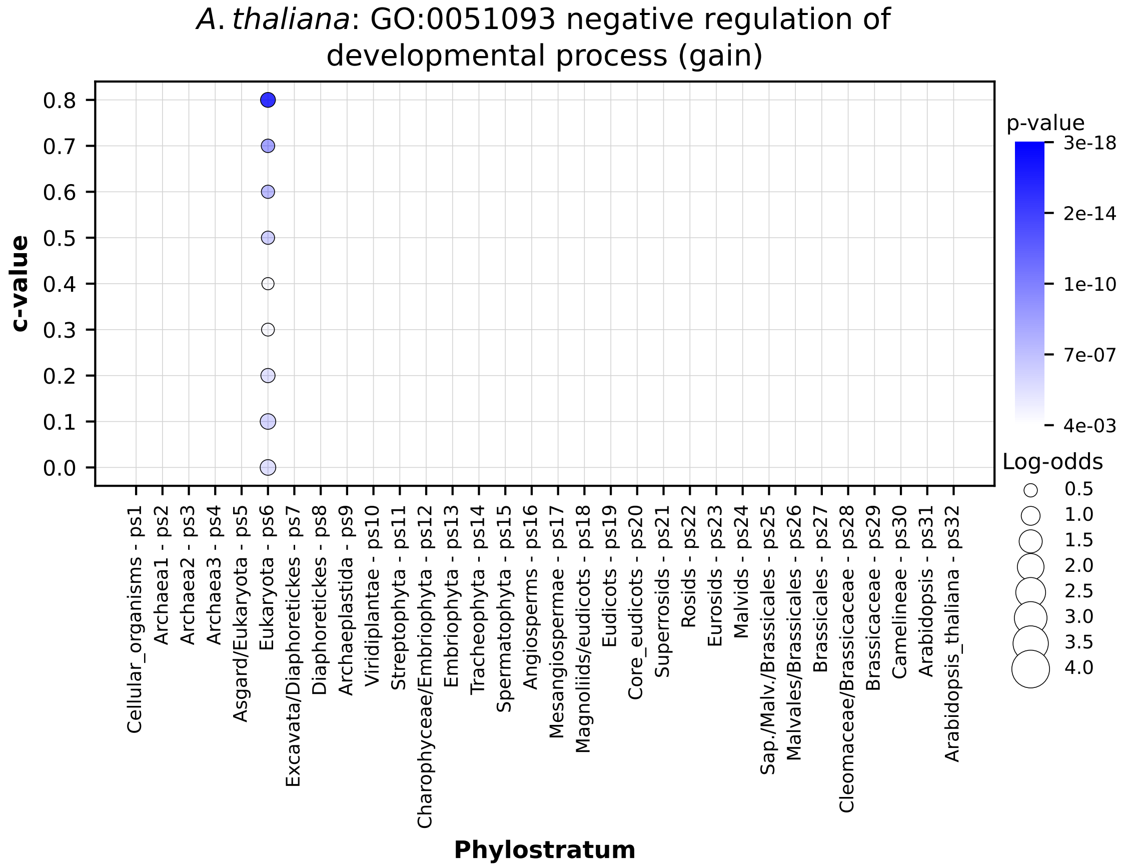

A. thaliana: GO:0051093 negative regulation of developmental process (gain)
c-value
0.8
0.7
0.6
0.5
0.4
0.3
0.2
0.1
0.0
p-value
3e-18
2e-14
1e-10
7e-07
4e-03
Log-odds
0.5
1.0
1.5
2.0
2.5
3.0
3.5
4.0
Cellular_organisms - ps1
Archaea1 - ps2
Archaea2 - ps3
Archaea3 - ps4
Asgard/Eukaryota - ps5
Eukaryota - ps6
Excavata/Diaphoretickes - ps7
Diaphoretickes - ps8
Archaeplastida - ps9
Viridiplantae - ps10
Streptophyta - ps11
Charophyceae/Embriophyta - ps12
Embriophyta - ps13
Tracheophyta - ps14
Spermatophyta - ps15
Angiosperms - ps16
Mesangiospermae - ps17
Magnoliids/eudicots - ps18
Eudicots - ps19
Core_eudicots - ps20
Superrosids - ps21
Rosids - ps22
Eurosids - ps23
Malvids - ps24
Sap./Malv./Brassicales - ps25
Malvales/Brassicales - ps26
Brassicales - ps27
Cleomaceae/Brassicaceae - ps28
Brassicaceae - ps29
Camelineae - ps30
Arabidopsis - ps31
Arabidopsis_thaliana - ps32
Phylostratum

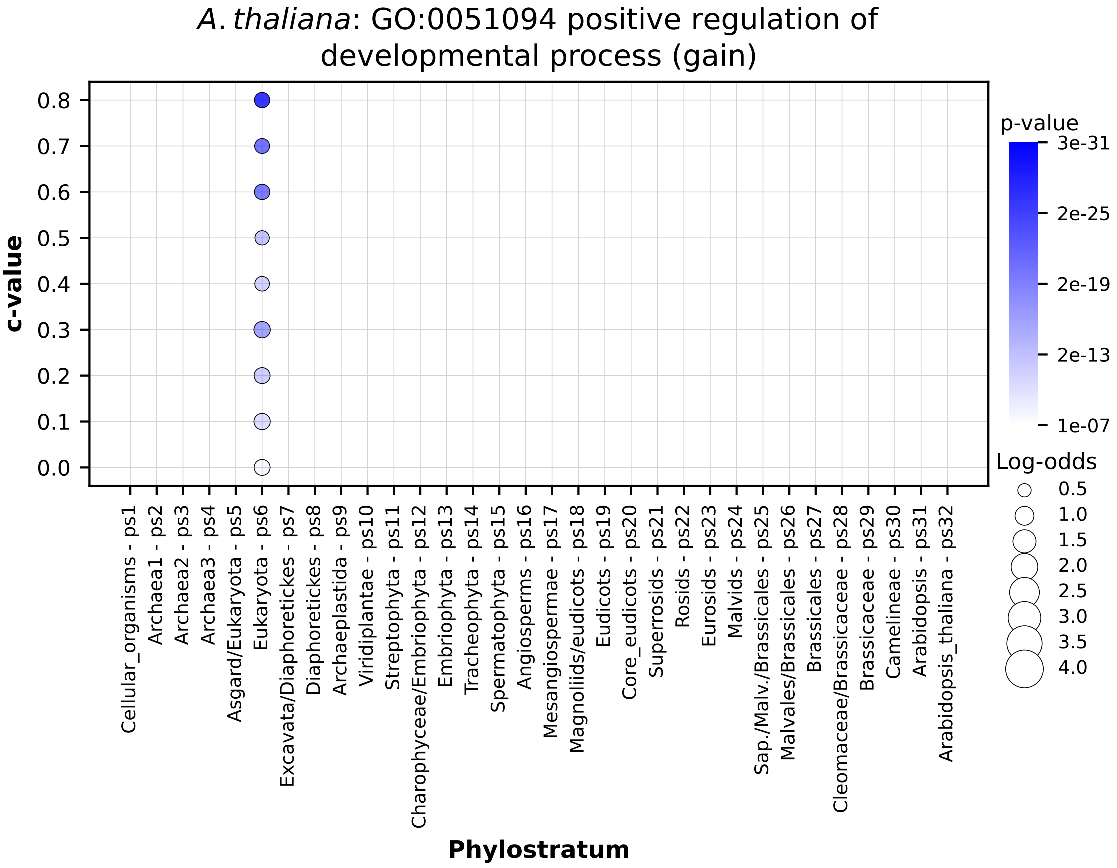

A. thaliana: GO:0051094 positive regulation of developmental process (gain)
c-value
0.8
0.7
0.6
0.5
0.4
0.3
0.2
0.1
0.0
p-value
3e-31
2e-25
2e-19
2e-13
1e-07
Log-odds
0.5
1.0
1.5
2.0
2.5
3.0
3.5
4.0
Cellular_organisms - ps1
Archaea1 - ps2
Archaea2 - ps3
Archaea3 - ps4
Asgard/Eukaryota - ps5
Eukaryota - ps6
Excavata/Diaphoretickes - ps7
Diaphoretickes - ps8
Archaeplastida - ps9
Viridiplantae - ps10
Streptophyta - ps11
Charophyceae/Embriophyta - ps12
Embriophyta - ps13
Tracheophyta - ps14
Spermatophyta - ps15
Angiosperms - ps16
Mesangiospermae - ps17
Magnoliids/eudicots - ps18
Eudicots - ps19
Core_eudicots - ps20
Superrosids - ps21
Rosids - ps22
Eurosids - ps23
Malvids - ps24
Sap./Malv./Brassicales - ps25
Malvales/Brassicales - ps26
Brassicales - ps27
Cleomaceae/Brassicaceae - ps28
Brassicaceae - ps29
Camelineae - ps30
Arabidopsis - ps31
Arabidopsis_thaliana - ps32
Phylostratum

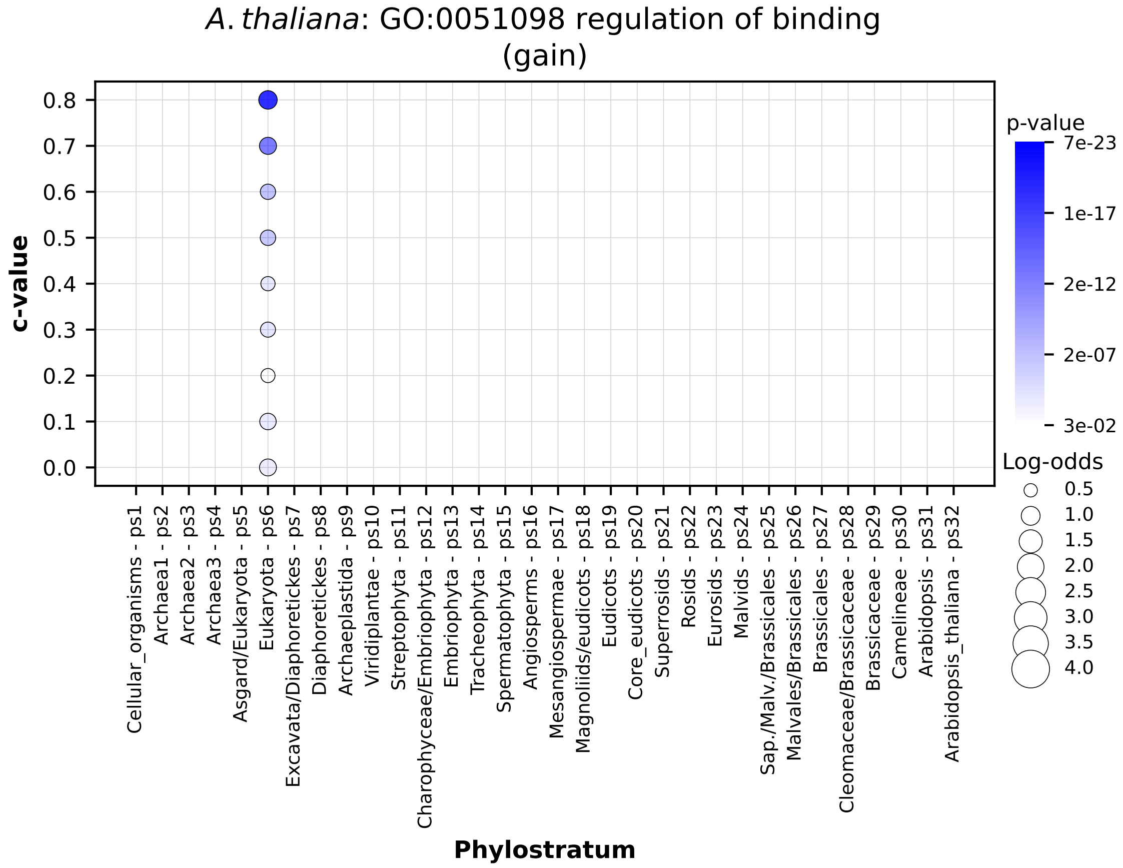

A. thaliana: GO:0051098 regulation of binding
(gain)
c-value
0.8
0.7
0.6
0.5
0.4
0.3
0.2
0.1
0.0
p-value
7e-23
1e-17
2e-12
2e-07
3e-02
Log-odds
0.5
1.0
1.5
2.0
2.5
3.0
3.5
4.0
Cellular_organisms - ps1
Archaea1 - ps2
Archaea2 - ps3
Archaea3 - ps4
Asgard/Eukaryota - ps5
Eukaryota - ps6
Excavata/Diaphoretickes - ps7
Diaphoretickes - ps8
Archaeplastida - ps9
Viridiplantae - ps10
Streptophyta - ps11
Charophyceae/Embriophyta - ps12
Embriophyta - ps13
Tracheophyta - ps14
Spermatophyta - ps15
Angiosperms - ps16
Mesangiospermae - ps17
Magnoliids/eudicots - ps18
Eudicots - ps19
Core_eudicots - ps20
Superrosids - ps21
Rosids - ps22
Eurosids - ps23
Malvids - ps24
Sap./Malv./Brassicales - ps25
Malvales/Brassicales - ps26
Brassicales - ps27
Cleomaceae/Brassicaceae - ps28
Brassicaceae - ps29
Camelineae - ps30
Arabidopsis - ps31
Arabidopsis_thaliana - ps32
Phylostratum

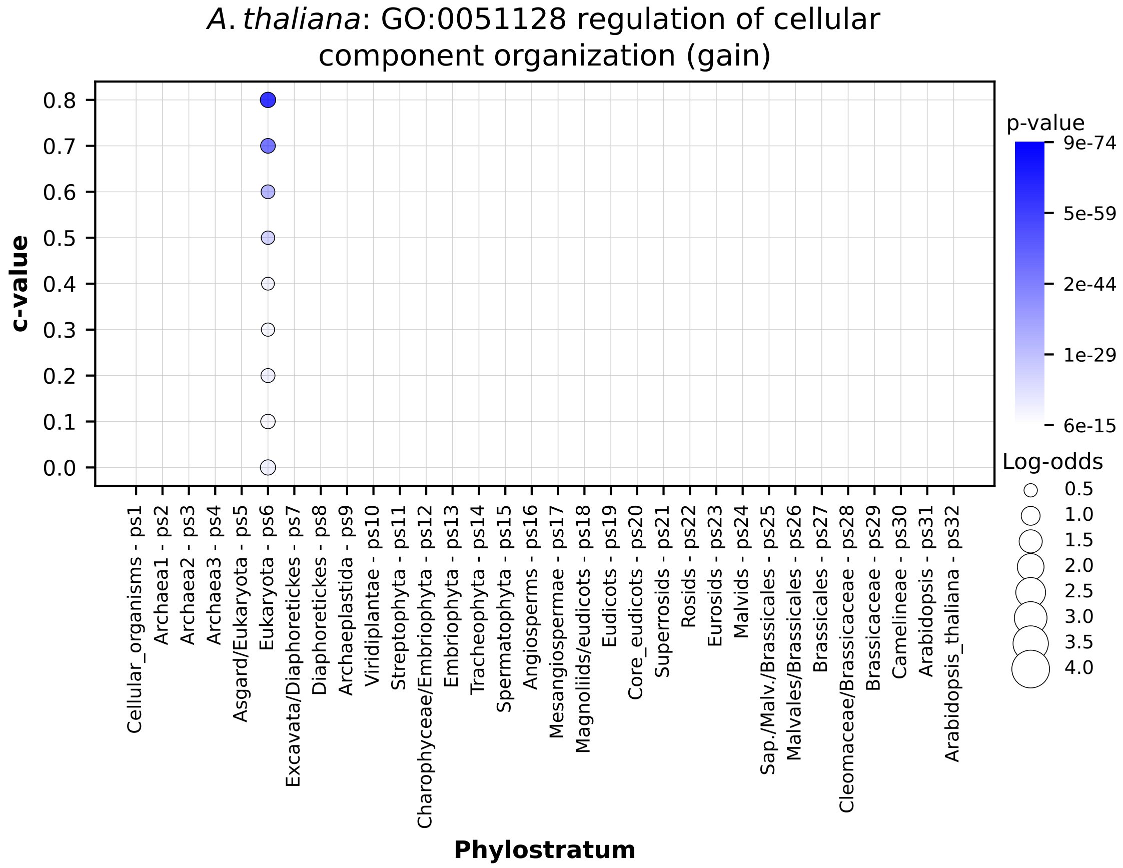

A. thaliana: GO:0051128 regulation of cellular component organization (gain)
c-value
0.0
0.1
0.2
0.3
0.4
0.5
0.6
0.7
0.8
p-value
9e-74
5e-59
2e-44
1e-29
6e-15
Log-odds
0.5
1.0
1.5
2.0
2.5
3.0
3.5
4.0
Cellular_organisms - ps1
Archaea1 - ps2
Archaea2 - ps3
Archaea3 - ps4
Asgard/Eukaryota - ps5
Eukaryota - ps6
Excavata/Diaphoretickes - ps7
Diaphoretickes - ps8
Archaeplastida - ps9
Viridiplantae - ps10
Streptophyta - ps11
Charophyceae/Embriophyta - ps12
Embriophyta - ps13
Tracheophyta - ps14
Spermatophyta - ps15
Angiosperms - ps16
Mesangiospermae - ps17
Magnoliids/eudicots - ps18
Eudicots - ps19
Core_eudicots - ps20
Superrosids - ps21
Rosids - ps22
Eurosids - ps23
Malvids - ps24
Sap./Malv./Brassicales - ps25
Malvales/Brassicales - ps26
Brassicales - ps27
Cleomaceae/Brassicaceae - ps28
Brassicaceae - ps29
Camelineae - ps30
Arabidopsis - ps31
Arabidopsis_thaliana - ps32
Phylostratum

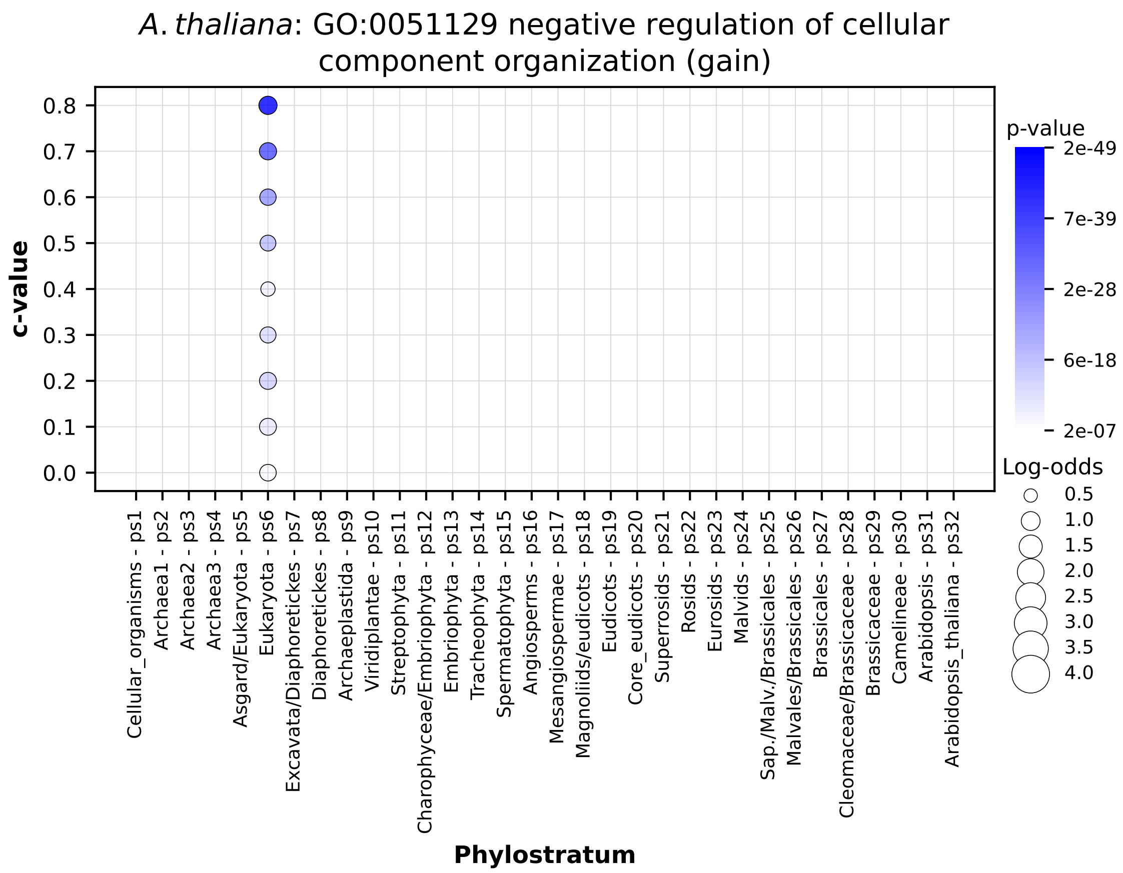

A. thaliana: GO:0051129 negative regulation of cellular component organization (gain)
c-value
0.8
0.7
0.6
0.5
0.4
0.3
0.2
0.1
0.0
Phylostratum
Cellular_organisms - ps1
Archaea1 - ps2
Archaea2 - ps3
Archaea3 - ps4
Asgard/Eukaryota - ps5
Eukaryota - ps6
Excavata/Diaphoretickes - ps7
Diaphoretickes - ps8
Archaeplastida - ps9
Viridiplantae - ps10
Streptophyta - ps11
Charophyceae/Embriophyta - ps12
Embriophyta - ps13
Tracheophyta - ps14
Spermatophyta - ps15
Angiosperms - ps16
Mesangiospermae - ps17
Magnoliids/eudicots - ps18
Eudicots - ps19
Core_eudicots - ps20
Superrosids - ps21
Rosids - ps22
Eurosids - ps23
Malvids - ps24
Sap./Malv./Brassicales - ps25
Malvales/Brassicales - ps26
Brassicales - ps27
Cleomaceae/Brassicaceae - ps28
Brassicaceae - ps29
Camelineae - ps30
Arabidopsis - ps31
Arabidopsis_thaliana - ps32
p-value
2e-49
7e-39
2e-28
6e-18
2e-07
Log-odds
0.5
1.0
1.5
2.0
2.5
3.0
3.5
4.0

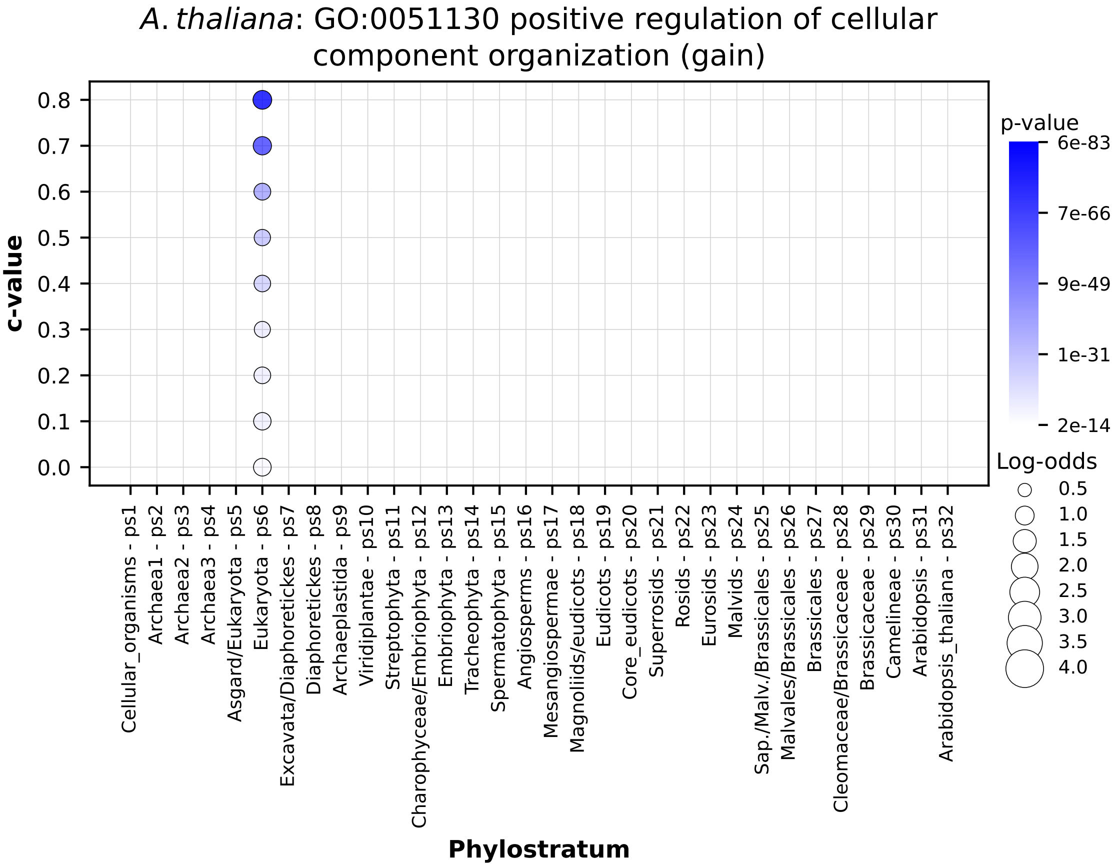

A. thaliana: GO:0051130 positive regulation of cellular component organization (gain)
c-value
0.8
0.7
0.6
0.5
0.4
0.3
0.2
0.1
0.0
p-value
6e-83
7e-66
9e-49
1e-31
2e-14
Log-odds
0.5
1.0
1.5
2.0
2.5
3.0
3.5
4.0
Cellular_organisms - ps1
Archaea1 - ps2
Archaea2 - ps3
Archaea3 - ps4
Asgard/Eukaryota - ps5
Eukaryota - ps6
Excavata/Diaphoretickes - ps7
Diaphoretickes - ps8
Archaeplastida - ps9
Viridiplantae - ps10
Streptophyta - ps11
Charophyceae/Embriophyta - ps12
Embriophyta - ps13
Tracheophyta - ps14
Spermatophyta - ps15
Angiosperms - ps16
Mesangiospermae - ps17
Magnoliids/eudicots - ps18
Eudicots - ps19
Core_eudicots - ps20
Superrosids - ps21
Rosids - ps22
Eurosids - ps23
Malvids - ps24
Sap./Malv./Brassicales - ps25
Malvales/Brassicales - ps26
Brassicales - ps27
Cleomaceae/Brassicaceae - ps28
Brassicaceae - ps29
Camelineae - ps30
Arabidopsis - ps31
Arabidopsis_thaliana - ps32
Phylostratum

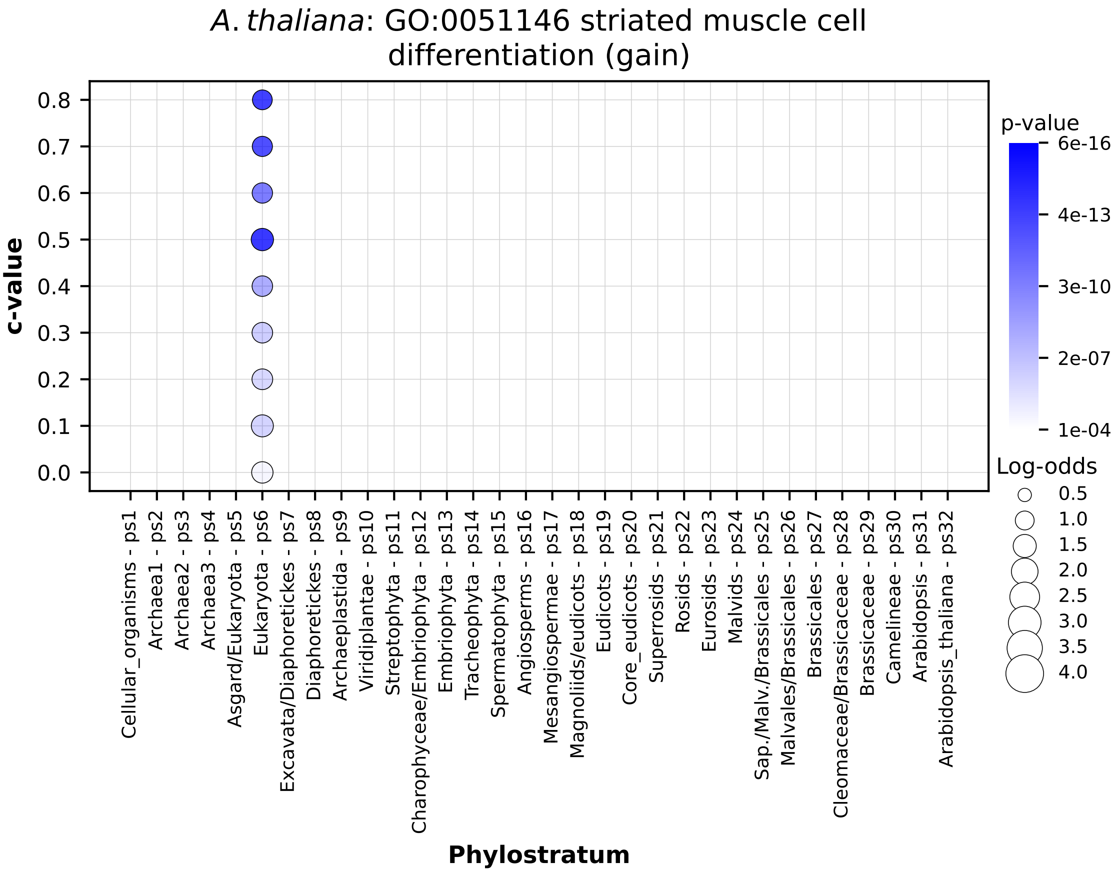

A. thaliana: GO:0051146 striated muscle cell differentiation (gain)
c-value
0.8
0.7
0.6
0.5
0.4
0.3
0.2
0.1
0.0
p-value
6e-16
4e-13
3e-10
2e-07
1e-04
Log-odds
0.5
1.0
1.5
2.0
2.5
3.0
3.5
4.0
Cellular_organisms - ps1
Archaea1 - ps2
Archaea2 - ps3
Archaea3 - ps4
Asgard/Eukaryota - ps5
Eukaryota - ps6
Excavata/Diaphoretickes - ps7
Diaphoretickes - ps8
Archaeplastida - ps9
Viridiplantae - ps10
Streptophyta - ps11
Charophyceae/Embriophyta - ps12
Embriophyta - ps13
Tracheophyta - ps14
Spermatophyta - ps15
Angiosperms - ps16
Mesangiospermae - ps17
Magnoliids/eudicots - ps18
Eudicots - ps19
Core_eudicots - ps20
Superrosids - ps21
Rosids - ps22
Eurosids - ps23
Malvids - ps24
Sap./Malv./Brassicales - ps25
Malvales/Brassicales - ps26
Brassicales - ps27
Cleomaceae/Brassicaceae - ps28
Brassicaceae - ps29
Camelineae - ps30
Arabidopsis - ps31
Arabidopsis_thaliana - ps32
Phylostratum

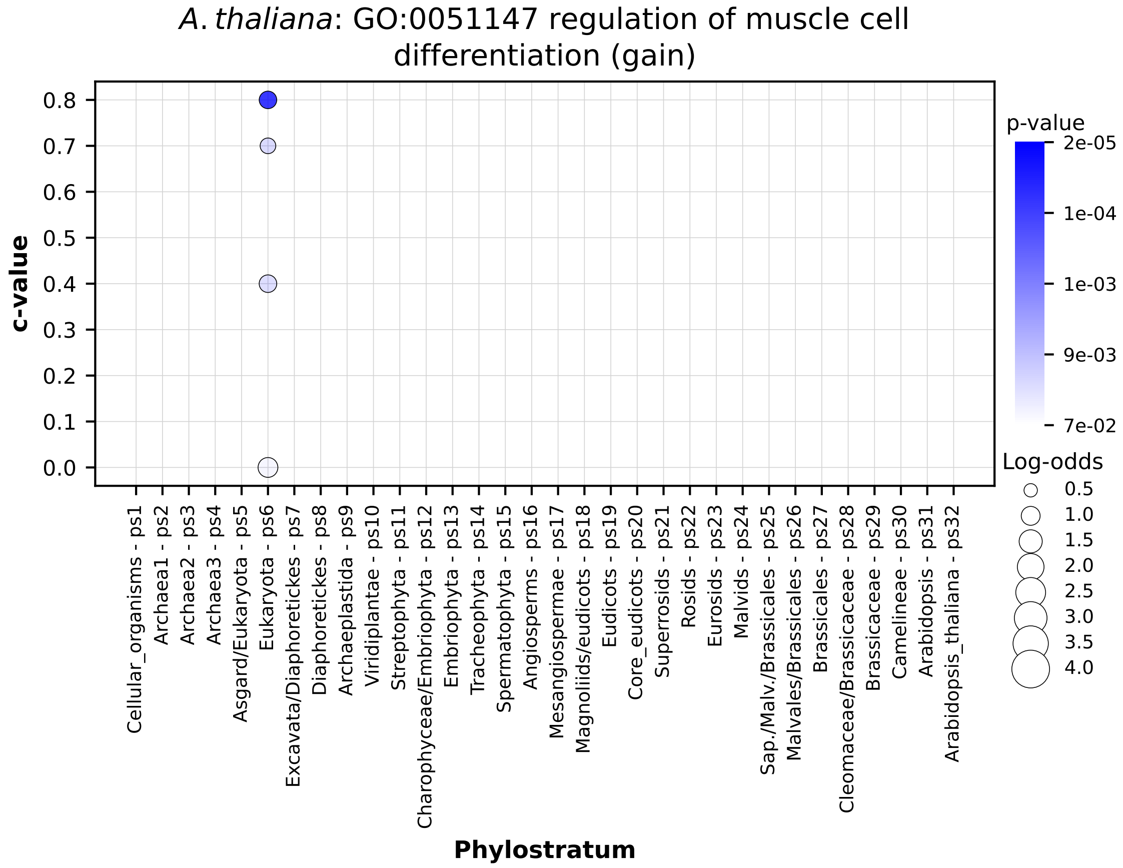

A. thaliana: GO:0051147 regulation of muscle cell differentiation (gain)
c-value
0.8
0.7
0.6
0.5
0.4
0.3
0.2
0.1
0.0
p-value
2e-05
1e-04
1e-03
9e-03
7e-02
Log-odds
0.5
1.0
1.5
2.0
2.5
3.0
3.5
4.0
Cellular_organisms - ps1
Archaea1 - ps2
Archaea2 - ps3
Archaea3 - ps4
Asgard/Eukaryota - ps5
Eukaryota - ps6
Excavata/Diaphoretickes - ps7
Diaphoretickes - ps8
Archaeplastida - ps9
Viridiplantae - ps10
Streptophyta - ps11
Charophyceae/Embriophyta - ps12
Embriophyta - ps13
Tracheophyta - ps14
Spermatophyta - ps15
Angiosperms - ps16
Mesangiospermae - ps17
Magnoliids/eudicots - ps18
Eudicots - ps19
Core_eudicots - ps20
Superrosids - ps21
Rosids - ps22
Eurosids - ps23
Malvids - ps24
Sap./Malv./Brassicales - ps25
Malvales/Brassicales - ps26
Brassicales - ps27
Cleomaceae/Brassicaceae - ps28
Brassicaceae - ps29
Camelineae - ps30
Arabidopsis - ps31
Arabidopsis_thaliana - ps32
Phylostratum

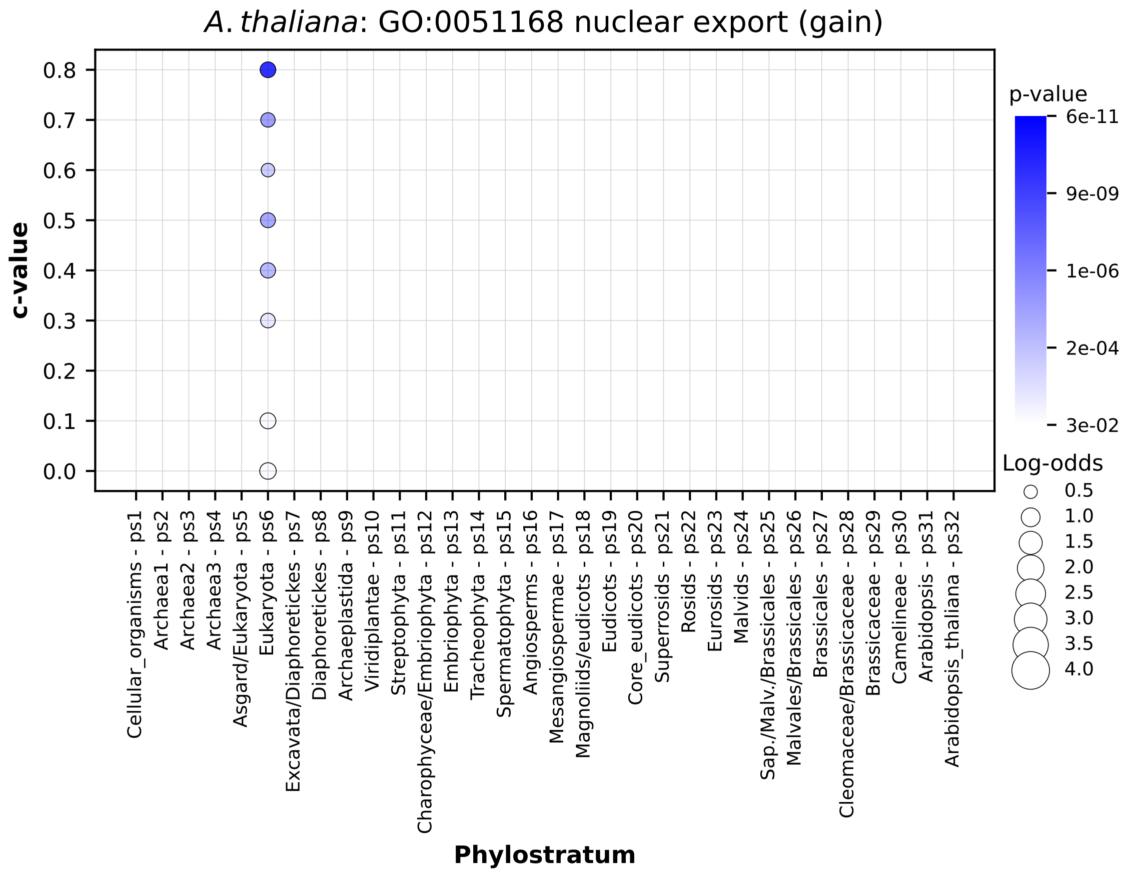
A. thaliana: GO:0051168 nuclear export (gain)
c-value
0.0
0.1
0.2
0.3
0.4
0.5
0.6
0.7
0.8
p-value
6e-11
9e-09
1e-06
2e-04
3e-02
Log-odds
0.5
1.0
1.5
2.0
2.5
3.0
3.5
4.0
Cellular_organisms - ps1
Archaea1 - ps2
Archaea2 - ps3
Archaea3 - ps4
Asgard/Eukaryota - ps5
Eukaryota - ps6
Excavata/Diaphoretickes - ps7
Diaphoretickes - ps8
Archaeplastida - ps9
Viridiplantae - ps10
Streptophyta - ps11
Charophyceae/Embriophyta - ps12
Embriophyta - ps13
Tracheophyta - ps14
Spermatophyta - ps15
Angiosperms - ps16
Mesangiospermae - ps17
Magnoliids/eudicots - ps18
Eudicots - ps19
Core_eudicots - ps20
Superrosids - ps21
Rosids - ps22
Eurosids - ps23
Malvids - ps24
Sap./Malv./Brassicales - ps25
Malvales/Brassicales - ps26
Brassicales - ps27
Cleomaceae/Brassicaceae - ps28
Brassicaceae - ps29
Camelineae - ps30
Arabidopsis - ps31
Arabidopsis_thaliana - ps32
Phylostratum

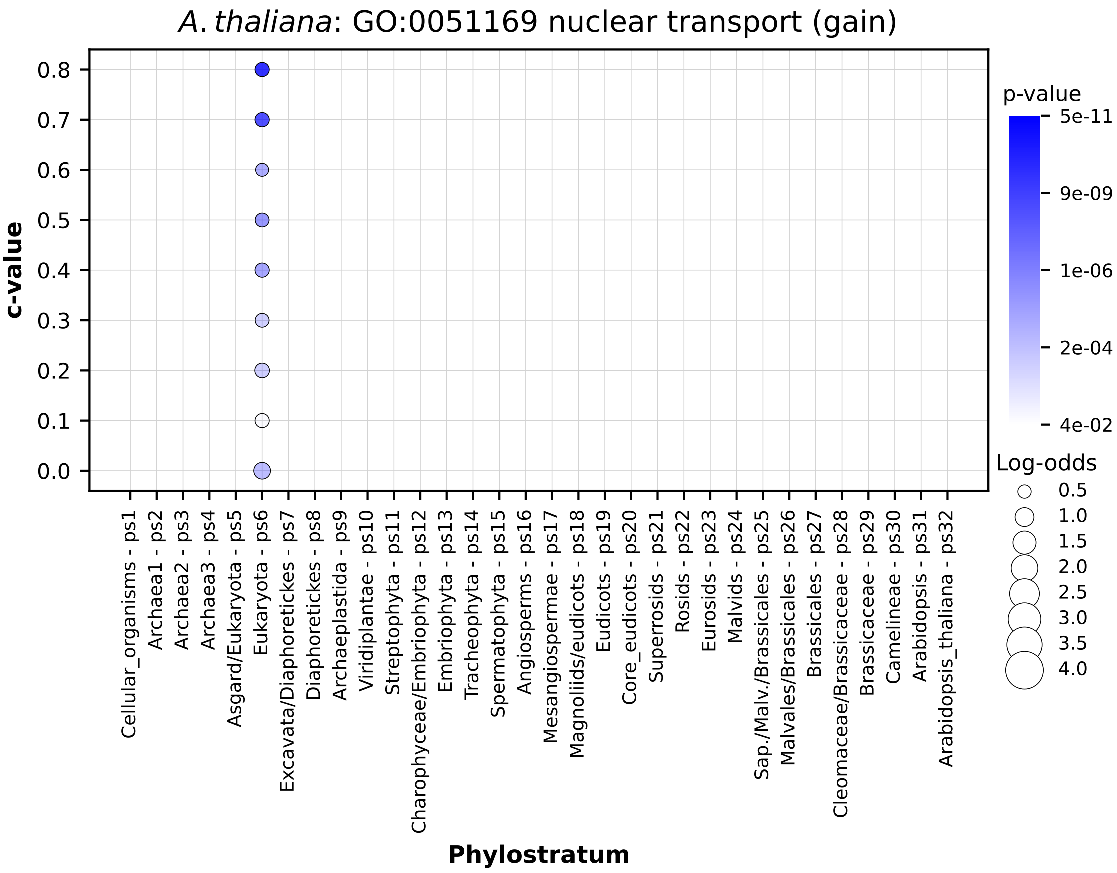

A. thaliana: GO:0051169 nuclear transport (gain)
c-value
0.0
0.1
0.2
0.3
0.4
0.5
0.6
0.7
0.8
p-value
5e-11
9e-09
1e-06
2e-04
4e-02
Log-odds
0.5
1.0
1.5
2.0
2.5
3.0
3.5
4.0
Cellular_organisms - ps1
Archaea1 - ps2
Archaea2 - ps3
Archaea3 - ps4
Asgard/Eukaryota - ps5
Eukaryota - ps6
Excavata/Diaphoretickes - ps7
Diaphoretickes - ps8
Archaeplastida - ps9
Viridiplantae - ps10
Streptophyta - ps11
Charophyceae/Embriophyta - ps12
Embriophyta - ps13
Tracheophyta - ps14
Spermatophyta - ps15
Angiosperms - ps16
Mesangiospermae - ps17
Magnoliids/eudicots - ps18
Eudicots - ps19
Core_eudicots - ps20
Superrosids - ps21
Rosids - ps22
Eurosids - ps23
Malvids - ps24
Sap./Malv./Brassicales - ps25
Malvales/Brassicales - ps26
Brassicales - ps27
Cleomaceae/Brassicaceae - ps28
Brassicaceae - ps29
Camelineae - ps30
Arabidopsis - ps31
Arabidopsis_thaliana - ps32
Phylostratum

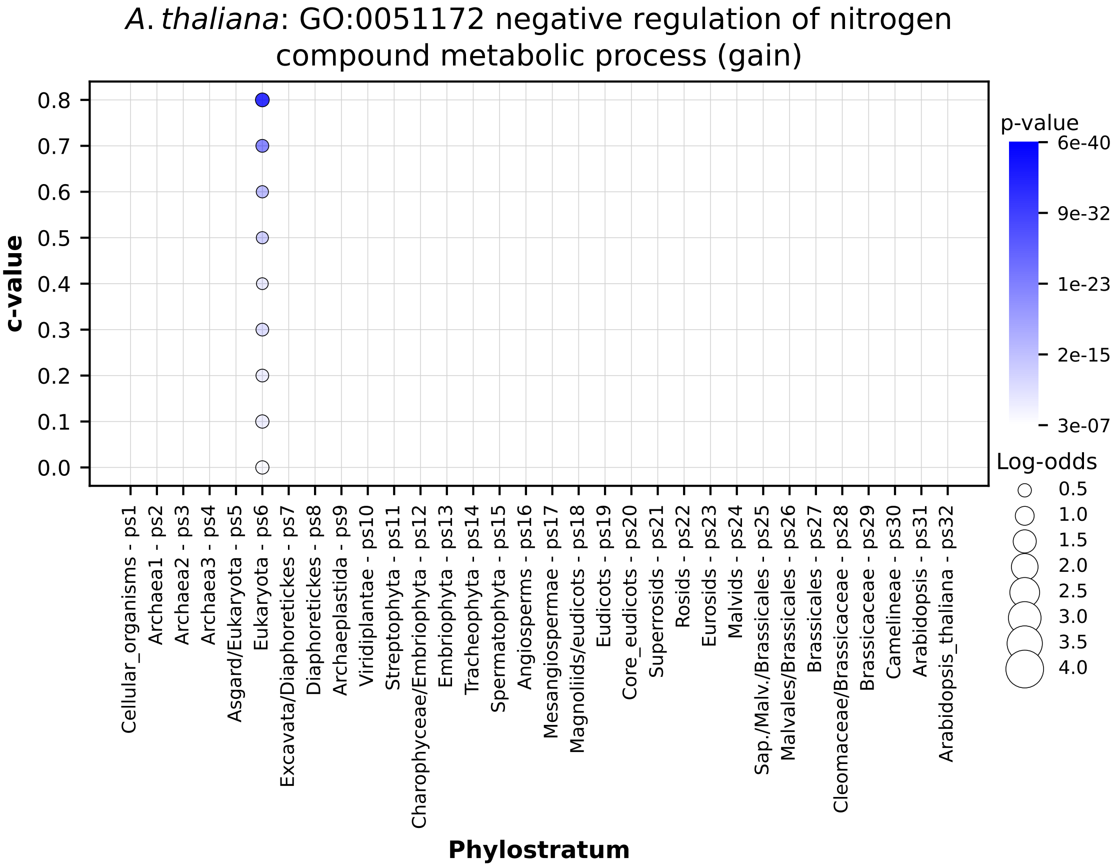

A. thaliana: GO:0051172 negative regulation of nitrogen compound metabolic process (gain)
c-value
0.8
0.7
0.6
0.5
0.4
0.3
0.2
0.1
0.0
p-value
6e-40
9e-32
1e-23
2e-15
3e-07
Log-odds
0.5
1.0
1.5
2.0
2.5
3.0
3.5
4.0
Cellular_organisms - ps1
Archaea1 - ps2
Archaea2 - ps3
Archaea3 - ps4
Asgard/Eukaryota - ps5
Eukaryota - ps6
Excavata/Diaphoretickes - ps7
Diaphoretickes - ps8
Archaeplastida - ps9
Viridiplantae - ps10
Streptophyta - ps11
Charophyceae/Embriophyta - ps12
Embriophyta - ps13
Tracheophyta - ps14
Spermatophyta - ps15
Angiosperms - ps16
Mesangiospermae - ps17
Magnoliids/eudicots - ps18
Eudicots - ps19
Core_eudicots - ps20
Superrosids - ps21
Rosids - ps22
Eurosids - ps23
Malvids - ps24
Sap./Malv./Brassicales - ps25
Malvales/Brassicales - ps26
Brassicales - ps27
Cleomaceae/Brassicaceae - ps28
Brassicaceae - ps29
Camelineae - ps30
Arabidopsis - ps31
Arabidopsis_thaliana - ps32
Phylostratum

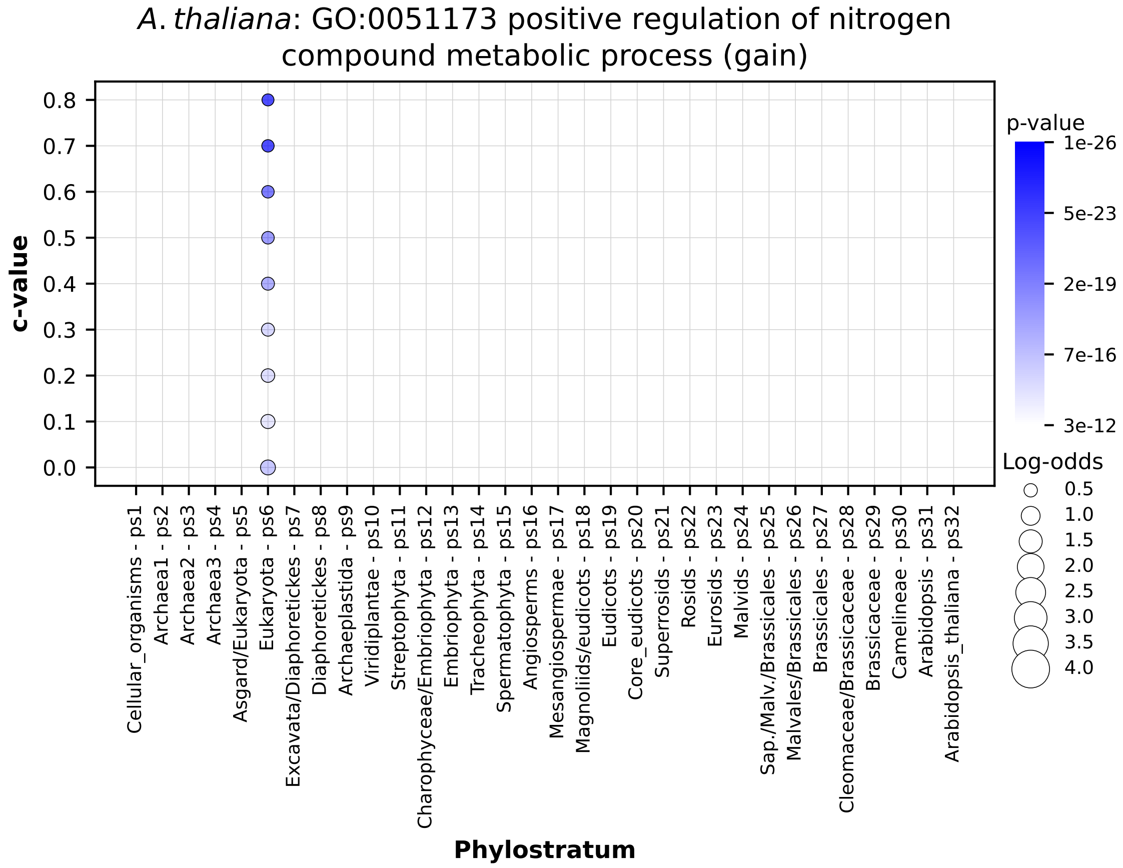
A. thaliana: GO:0051173 positive regulation of nitrogen compound metabolic process (gain)
c-value
0.8
0.7
0.6
0.5
0.4
0.3
0.2
0.1
0.0
Phylostratum
Cellular_organisms - ps1
Archaea1 - ps2
Archaea2 - ps3
Archaea3 - ps4
Asgard/Eukaryota - ps5
Eukaryota - ps6
Excavata/Diaphoretickes - ps7
Diaphoretickes - ps8
Archaeplastida - ps9
Viridiplantae - ps10
Streptophyta - ps11
Charophyceae/Embriophyta - ps12
Embriophyta - ps13
Tracheophyta - ps14
Spermatophyta - ps15
Angiosperms - ps16
Mesangiospermae - ps17
Magnoliids/eudicots - ps18
Eudicots - ps19
Core_eudicots - ps20
Superrosids - ps21
Rosids - ps22
Eurosids - ps23
Malvids - ps24
Sap./Malv./Brassicales - ps25
Malvales/Brassicales - ps26
Brassicales - ps27
Cleomaceae/Brassicaceae - ps28
Brassicaceae - ps29
Camelineae - ps30
Arabidopsis - ps31
Arabidopsis_thaliana - ps32
p-value
1e-26
5e-23
2e-19
7e-16
3e-12
Log-odds
0.5
1.0
1.5
2.0
2.5
3.0
3.5
4.0

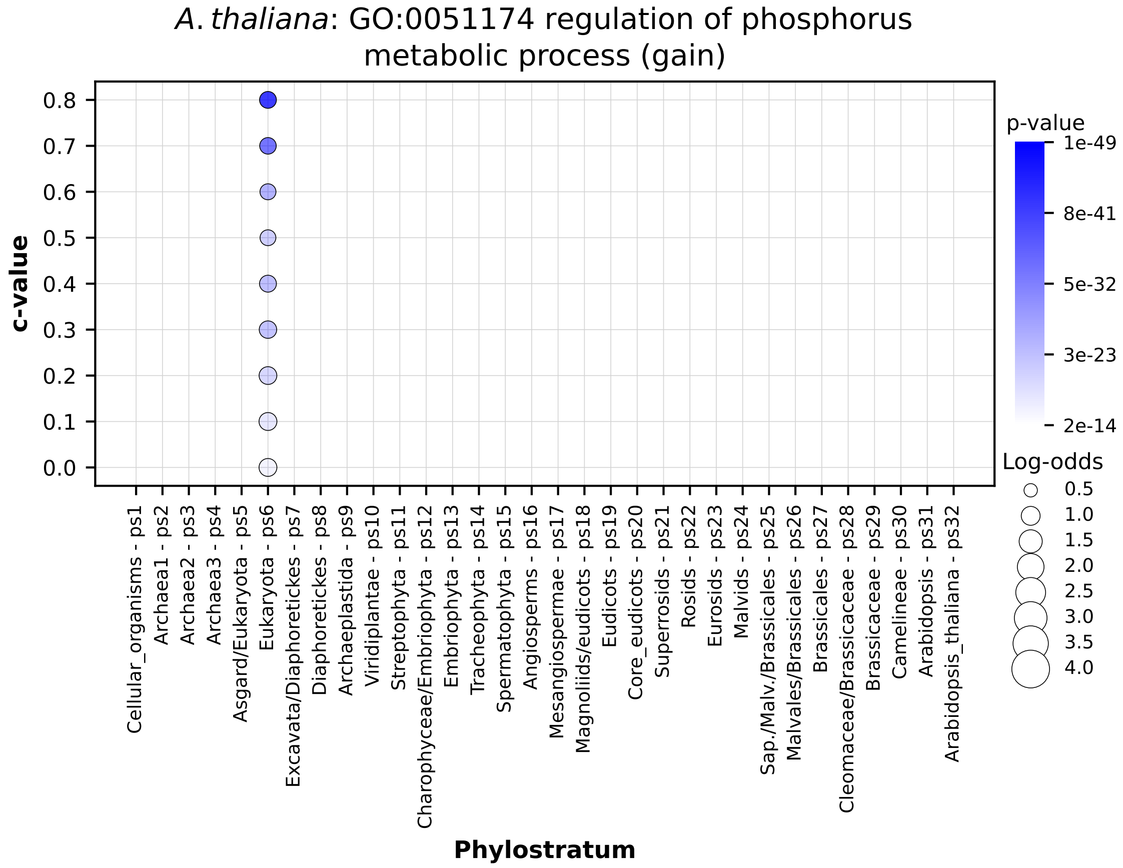

A. thaliana: GO:0051174 regulation of phosphorus metabolic process (gain)
c-value
0.8
0.7
0.6
0.5
0.4
0.3
0.2
0.1
0.0
Phylostratum
Cellular_organisms - ps1
Archaea1 - ps2
Archaea2 - ps3
Archaea3 - ps4
Asgard/Eukaryota - ps5
Eukaryota - ps6
Excavata/Diaphoretickes - ps7
Diaphoretickes - ps8
Archaeplastida - ps9
Viridiplantae - ps10
Streptophyta - ps11
Charophyceae/Embriophyta - ps12
Embriophyta - ps13
Tracheophyta - ps14
Spermatophyta - ps15
Angiosperms - ps16
Mesangiospermae - ps17
Magnoliids/eudicots - ps18
Eudicots - ps19
Core_eudicots - ps20
Superrosids - ps21
Rosids - ps22
Eurosids - ps23
Malvids - ps24
Sap./Malv./Brassicales - ps25
Malvales/Brassicales - ps26
Brassicales - ps27
Cleomaceae/Brassicaceae - ps28
Brassicaceae - ps29
Camelineae - ps30
Arabidopsis - ps31
Arabidopsis_thaliana - ps32
p-value
1e-49
8e-41
5e-32
3e-23
2e-14
Log-odds
0.5
1.0
1.5
2.0
2.5
3.0
3.5
4.0

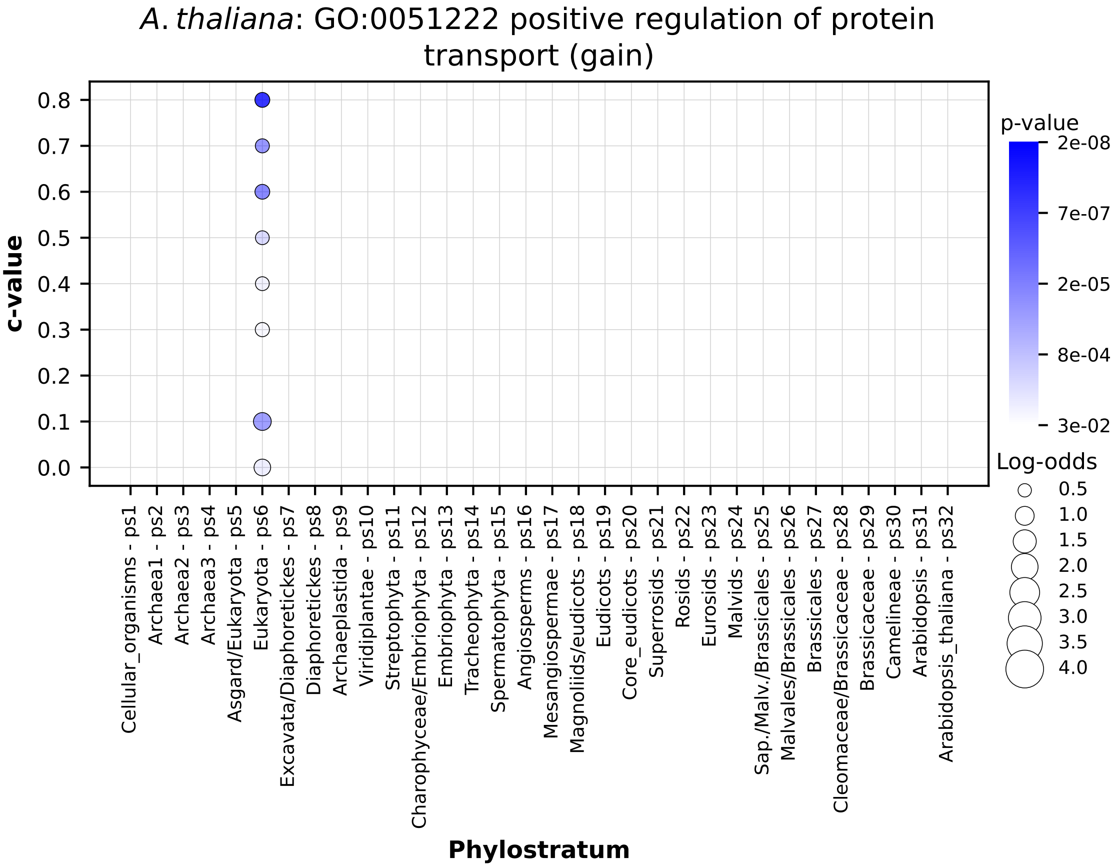

A. thaliana: GO:0051222 positive regulation of protein transport (gain)
c-value
0.8
0.7
0.6
0.5
0.4
0.3
0.2
0.1
0.0
p-value
2e-08
7e-07
2e-05
8e-04
3e-02
Log-odds
0.5
1.0
1.5
2.0
2.5
3.0
3.5
4.0
Cellular_organisms - ps1
Archaea1 - ps2
Archaea2 - ps3
Archaea3 - ps4
Asgard/Eukaryota - ps5
Eukaryota - ps6
Excavata/Diaphoretickes - ps7
Diaphoretickes - ps8
Archaeplastida - ps9
Viridiplantae - ps10
Streptophyta - ps11
Charophyceae/Embriophyta - ps12
Embriophyta - ps13
Tracheophyta - ps14
Spermatophyta - ps15
Angiosperms - ps16
Mesangiospermae - ps17
Magnoliids/eudicots - ps18
Eudicots - ps19
Core_eudicots - ps20
Superrosids - ps21
Rosids - ps22
Eurosids - ps23
Malvids - ps24
Sap./Malv./Brassicales - ps25
Malvales/Brassicales - ps26
Brassicales - ps27
Cleomaceae/Brassicaceae - ps28
Brassicaceae - ps29
Camelineae - ps30
Arabidopsis - ps31
Arabidopsis_thaliana - ps32
Phylostratum

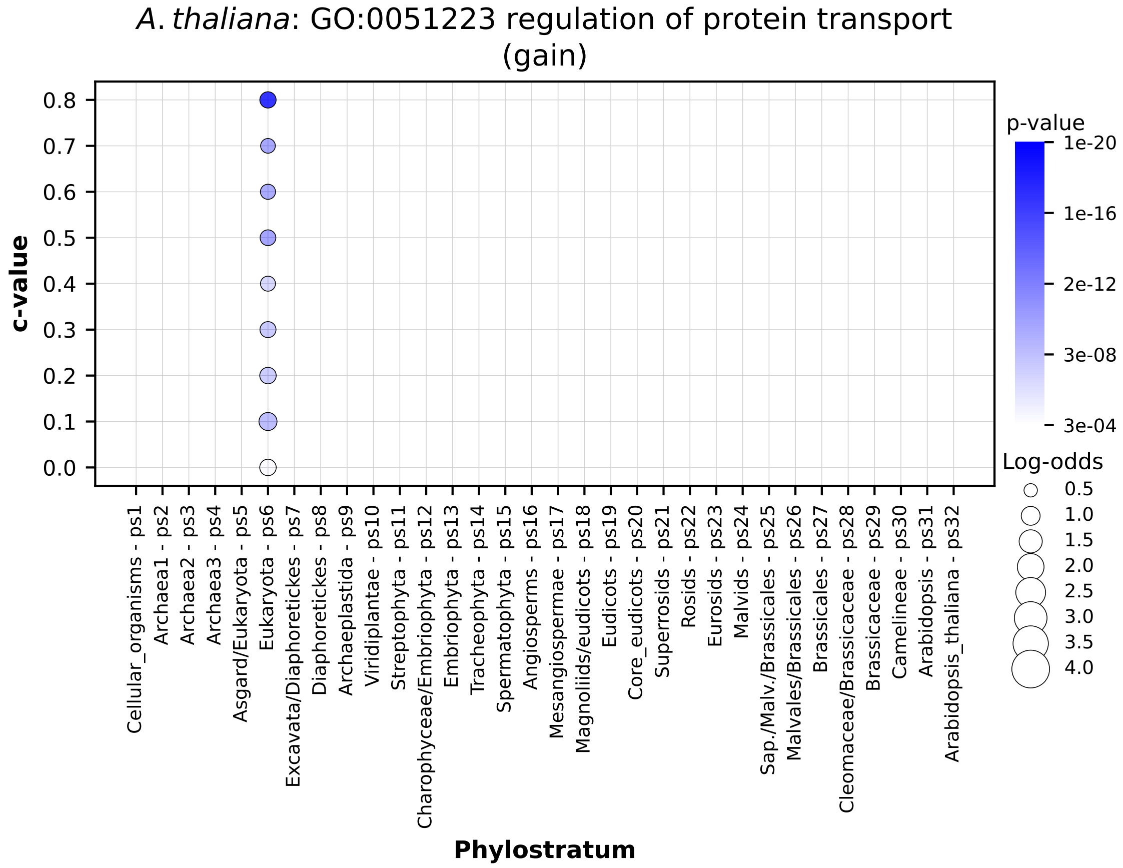

A. thaliana: GO:0051223 regulation of protein transport (gain)
c-value
0.8
0.7
0.6
0.5
0.4
0.3
0.2
0.1
0.0
Phylostratum
Cellular_organisms - ps1
Archaea1 - ps2
Archaea2 - ps3
Archaea3 - ps4
Asgard/Eukaryota - ps5
Eukaryota - ps6
Excavata/Diaphoretickes - ps7
Diaphoretickes - ps8
Archaeplastida - ps9
Viridiplantae - ps10
Streptophyta - ps11
Charophyceae/Embriophyta - ps12
Embriophyta - ps13
Tracheophyta - ps14
Spermatophyta - ps15
Angiosperms - ps16
Mesangiospermae - ps17
Magnoliids/eudicots - ps18
Eudicots - ps19
Core_eudicots - ps20
Superrosids - ps21
Rosids - ps22
Eurosids - ps23
Malvids - ps24
Sap./Malv./Brassicales - ps25
Malvales/Brassicales - ps26
Brassicales - ps27
Cleomaceae/Brassicaceae - ps28
Brassicaceae - ps29
Camelineae - ps30
Arabidopsis - ps31
Arabidopsis_thaliana - ps32
p-value
1e-20
1e-16
2e-12
3e-08
3e-04
Log-odds
0.5
1.0
1.5
2.0
2.5
3.0
3.5
4.0

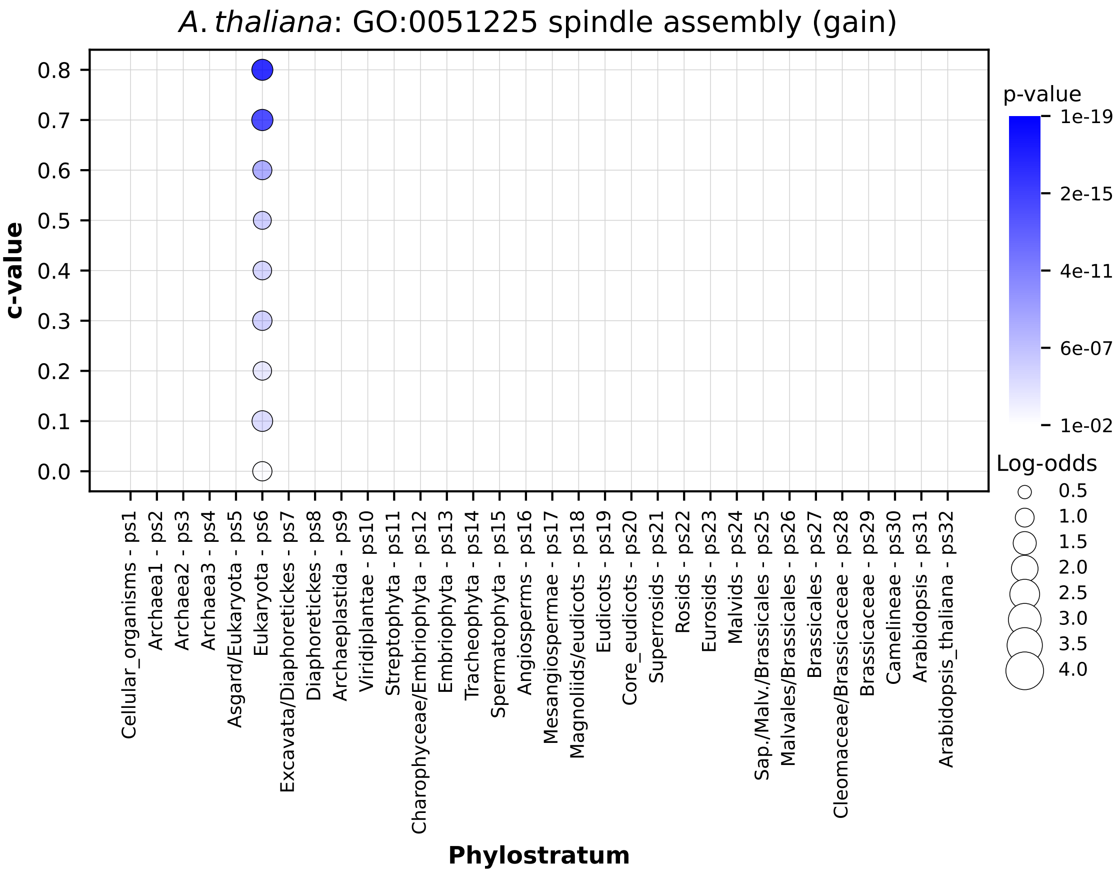

A. thaliana: GO:0051225 spindle assembly (gain)
c-value
0.8
0.7
0.6
0.5
0.4
0.3
0.2
0.1
0.0
Phylostratum
Cellular_organisms - ps1
Archaea1 - ps2
Archaea2 - ps3
Archaea3 - ps4
Asgard/Eukaryota - ps5
Eukaryota - ps6
Excavata/Diaphoretickes - ps7
Diaphoretickes - ps8
Archaeplastida - ps9
Viridiplantae - ps10
Streptophyta - ps11
Charophyceae/Embriophyta - ps12
Embriophyta - ps13
Tracheophyta - ps14
Spermatophyta - ps15
Angiosperms - ps16
Mesangiospermae - ps17
Magnoliids/eudicots - ps18
Eudicots - ps19
Core_eudicots - ps20
Superrosids - ps21
Rosids - ps22
Eurosids - ps23
Malvids - ps24
Sap./Malv./Brassicales - ps25
Malvales/Brassicales - ps26
Brassicales - ps27
Cleomaceae/Brassicaceae - ps28
Brassicaceae - ps29
Camelineae - ps30
Arabidopsis - ps31
Arabidopsis_thaliana - ps32
p-value
1e-19
2e-15
4e-11
6e-07
1e-02
Log-odds
0.5
1.0
1.5
2.0
2.5
3.0
3.5
4.0

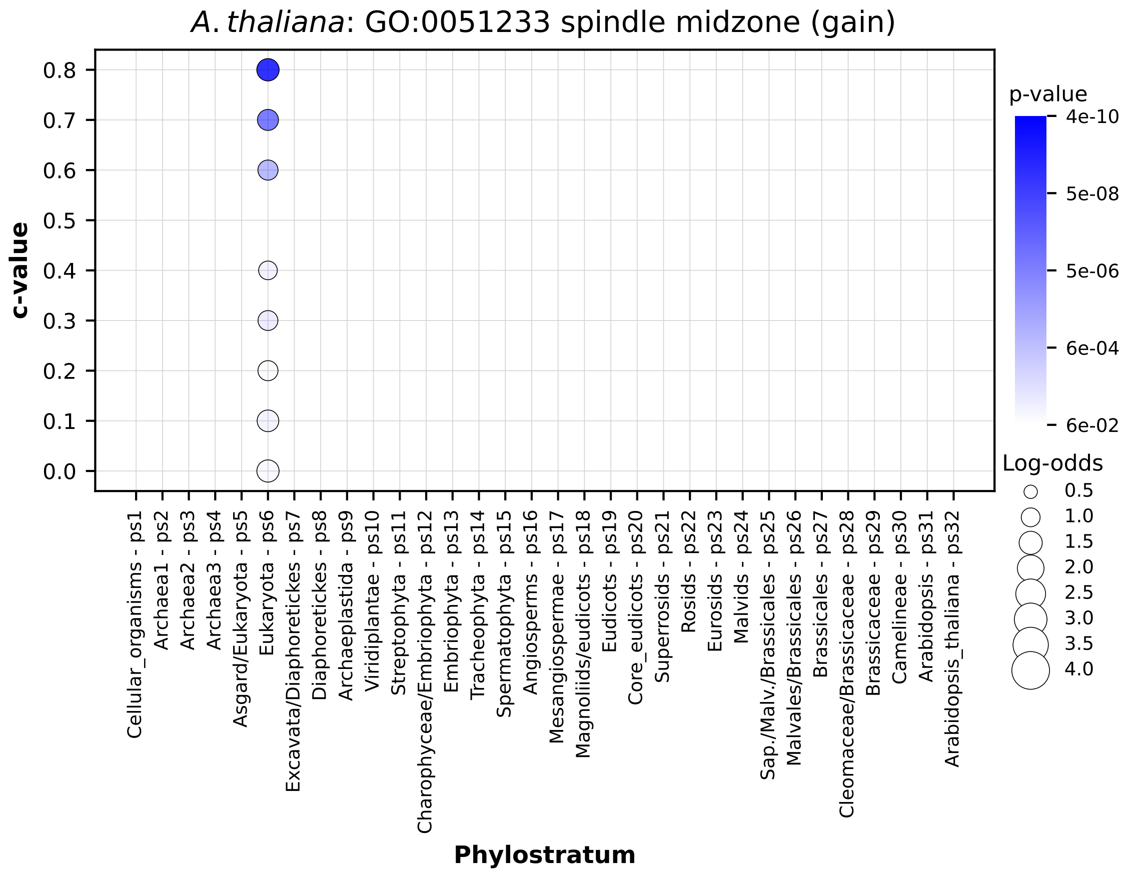

A. thaliana: GO:0051233 spindle midzone (gain)
c-value
0.0
0.1
0.2
0.3
0.4
0.5
0.6
0.7
0.8
p-value
4e-10
5e-08
5e-06
6e-04
6e-02
Log-odds
0.5
1.0
1.5
2.0
2.5
3.0
3.5
4.0
Cellular_organisms - ps1
Archaea1 - ps2
Archaea2 - ps3
Archaea3 - ps4
Asgard/Eukaryota - ps5
Eukaryota - ps6
Excavata/Diaphoretickes - ps7
Diaphoretickes - ps8
Archaeplastida - ps9
Viridiplantae - ps10
Streptophyta - ps11
Charophyceae/Embriophyta - ps12
Embriophyta - ps13
Tracheophyta - ps14
Spermatophyta - ps15
Angiosperms - ps16
Mesangiospermae - ps17
Magnoliids/eudicots - ps18
Eudicots - ps19
Core_eudicots - ps20
Superrosids - ps21
Rosids - ps22
Eurosids - ps23
Malvids - ps24
Sap./Malv./Brassicales - ps25
Malvales/Brassicales - ps26
Brassicales - ps27
Cleomaceae/Brassicaceae - ps28
Brassicaceae - ps29
Camelineae - ps30
Arabidopsis - ps31
Arabidopsis_thaliana - ps32
Phylostratum

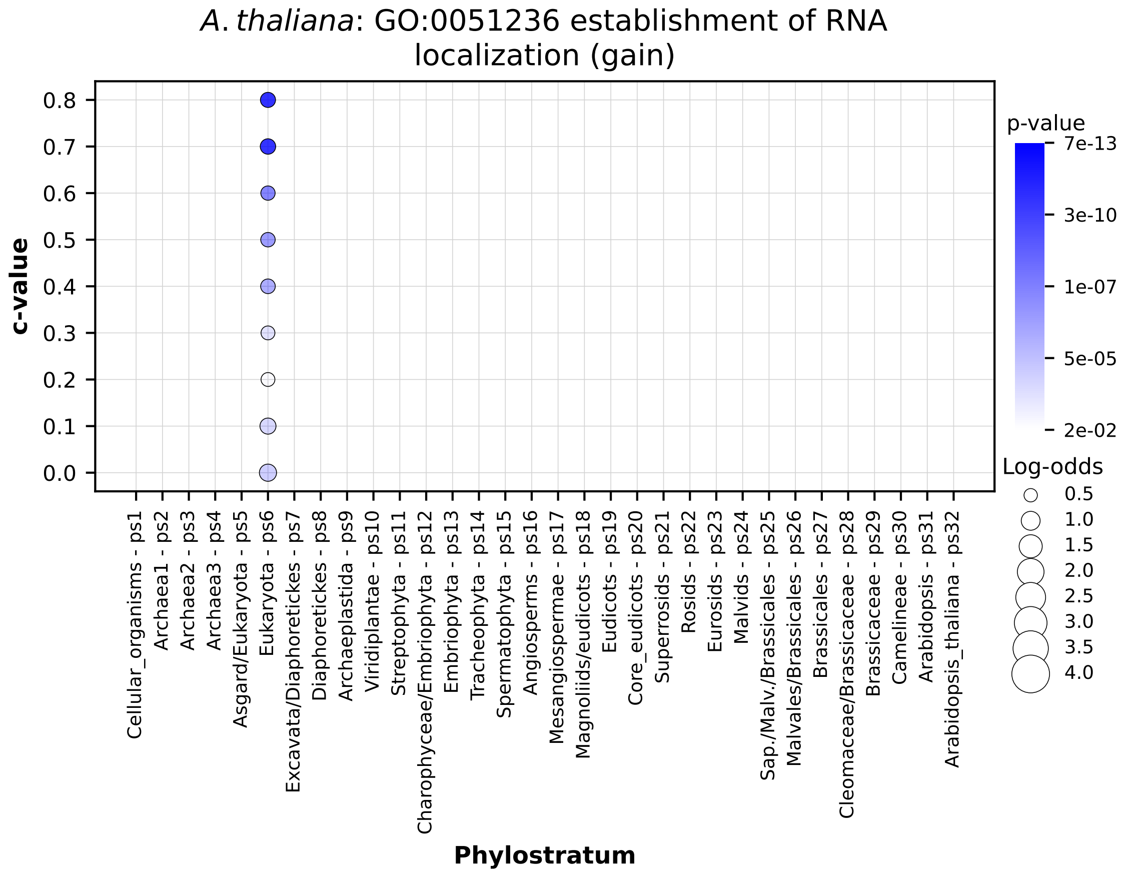

A. thaliana: GO:0051236 establishment of RNA localization (gain)
c-value
0.8
0.7
0.6
0.5
0.4
0.3
0.2
0.1
0.0
p-value
7e-13
3e-10
1e-07
5e-05
2e-02
Log-odds
0.5
1.0
1.5
2.0
2.5
3.0
3.5
4.0
Cellular_organisms - ps1
Archaea1 - ps2
Archaea2 - ps3
Archaea3 - ps4
Asgard/Eukaryota - ps5
Eukaryota - ps6
Excavata/Diaphoretickes - ps7
Diaphoretickes - ps8
Archaeplastida - ps9
Viridiplantae - ps10
Streptophyta - ps11
Charophyceae/Embriophyta - ps12
Embriophyta - ps13
Tracheophyta - ps14
Spermatophyta - ps15
Angiosperms - ps16
Mesangiospermae - ps17
Magnoliids/eudicots - ps18
Eudicots - ps19
Core_eudicots - ps20
Superrosids - ps21
Rosids - ps22
Eurosids - ps23
Malvids - ps24
Sap./Malv./Brassicales - ps25
Malvales/Brassicales - ps26
Brassicales - ps27
Cleomaceae/Brassicaceae - ps28
Brassicaceae - ps29
Camelineae - ps30
Arabidopsis - ps31
Arabidopsis_thaliana - ps32
Phylostratum

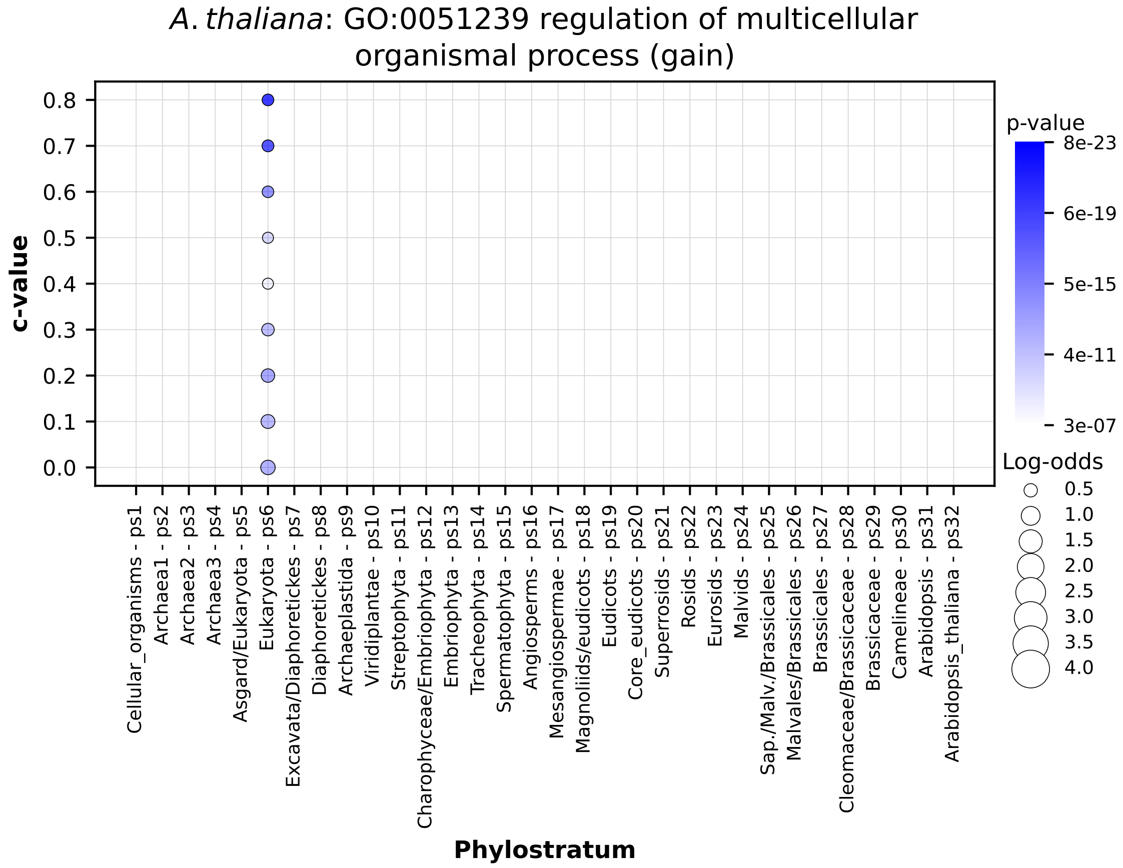

A. thaliana: GO:0051239 regulation of multicellular organismal process (gain)
c-value
0.8
0.7
0.6
0.5
0.4
0.3
0.2
0.1
0.0
p-value
8e-23
6e-19
5e-15
4e-11
3e-07
Log-odds
0.5
1.0
1.5
2.0
2.5
3.0
3.5
4.0
Cellular_organisms - ps1
Archaea1 - ps2
Archaea2 - ps3
Archaea3 - ps4
Asgard/Eukaryota - ps5
Eukaryota - ps6
Excavata/Diaphoretickes - ps7
Diaphoretickes - ps8
Archaeplastida - ps9
Viridiplantae - ps10
Streptophyta - ps11
Charophyceae/Embriophyta - ps12
Embriophyta - ps13
Tracheophyta - ps14
Spermatophyta - ps15
Angiosperms - ps16
Mesangiospermae - ps17
Magnoliids/eudicots - ps18
Eudicots - ps19
Core_eudicots - ps20
Superrosids - ps21
Rosids - ps22
Eurosids - ps23
Malvids - ps24
Sap./Malv./Brassicales - ps25
Malvales/Brassicales - ps26
Brassicales - ps27
Cleomaceae/Brassicaceae - ps28
Brassicaceae - ps29
Camelineae - ps30
Arabidopsis - ps31
Arabidopsis_thaliana - ps32
Phylostratum

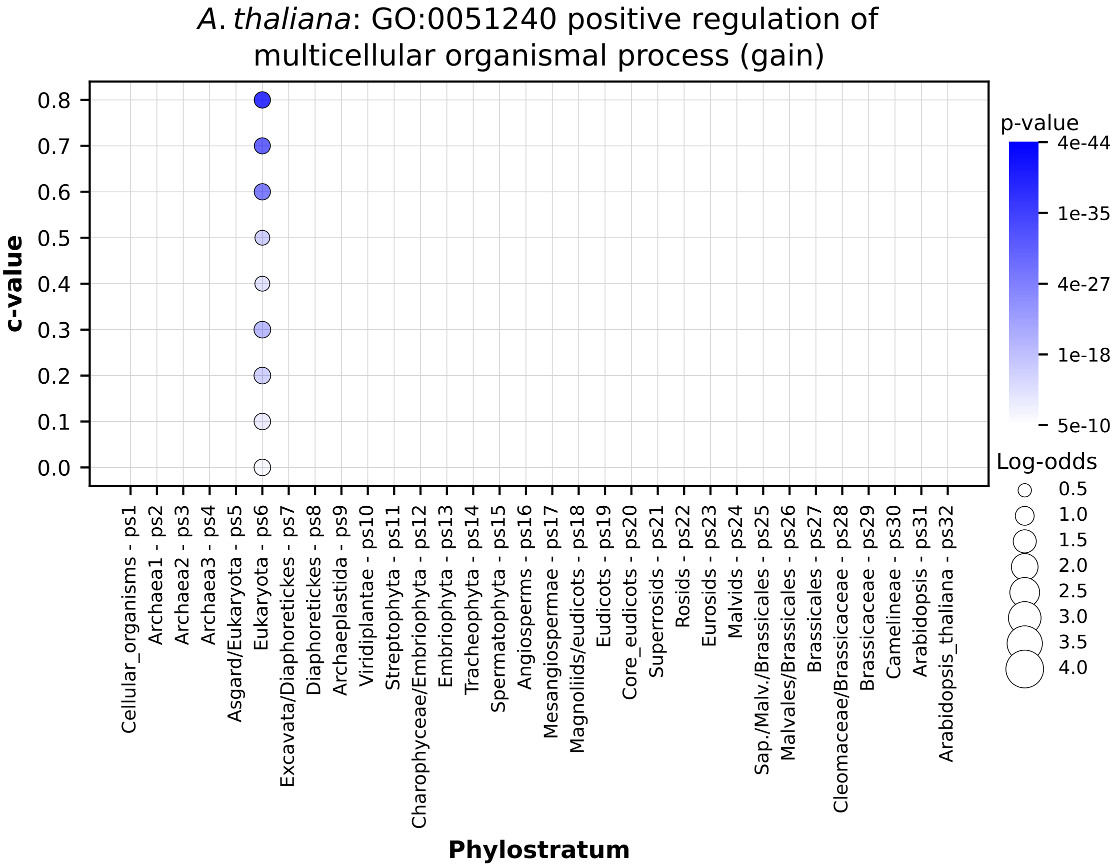
A. thaliana: GO:0051240 positive regulation of multicellular organismal process (gain)
c-value
0.8
0.7
0.6
0.5
0.4
0.3
0.2
0.1
0.0
p-value
4e-44
1e-35
4e-27
1e-18
5e-10
Log-odds
0.5
1.0
1.5
2.0
2.5
3.0
3.5
4.0
Cellular_organisms - ps1
Archaea1 - ps2
Archaea2 - ps3
Archaea3 - ps4
Asgard/Eukaryota - ps5
Eukaryota - ps6
Excavata/Diaphoretickes - ps7
Diaphoretickes - ps8
Archaeplastida - ps9
Viridiplantae - ps10
Streptophyta - ps11
Charophyceae/Embriophyta - ps12
Embriophyta - ps13
Tracheophyta - ps14
Spermatophyta - ps15
Angiosperms - ps16
Mesangiospermae - ps17
Magnoliids/eudicots - ps18
Eudicots - ps19
Core_eudicots - ps20
Superrosids - ps21
Rosids - ps22
Eurosids - ps23
Malvids - ps24
Sap./Malv./Brassicales - ps25
Malvales/Brassicales - ps26
Brassicales - ps27
Cleomaceae/Brassicaceae - ps28
Brassicaceae - ps29
Camelineae - ps30
Arabidopsis - ps31
Arabidopsis_thaliana - ps32
Phylostratum

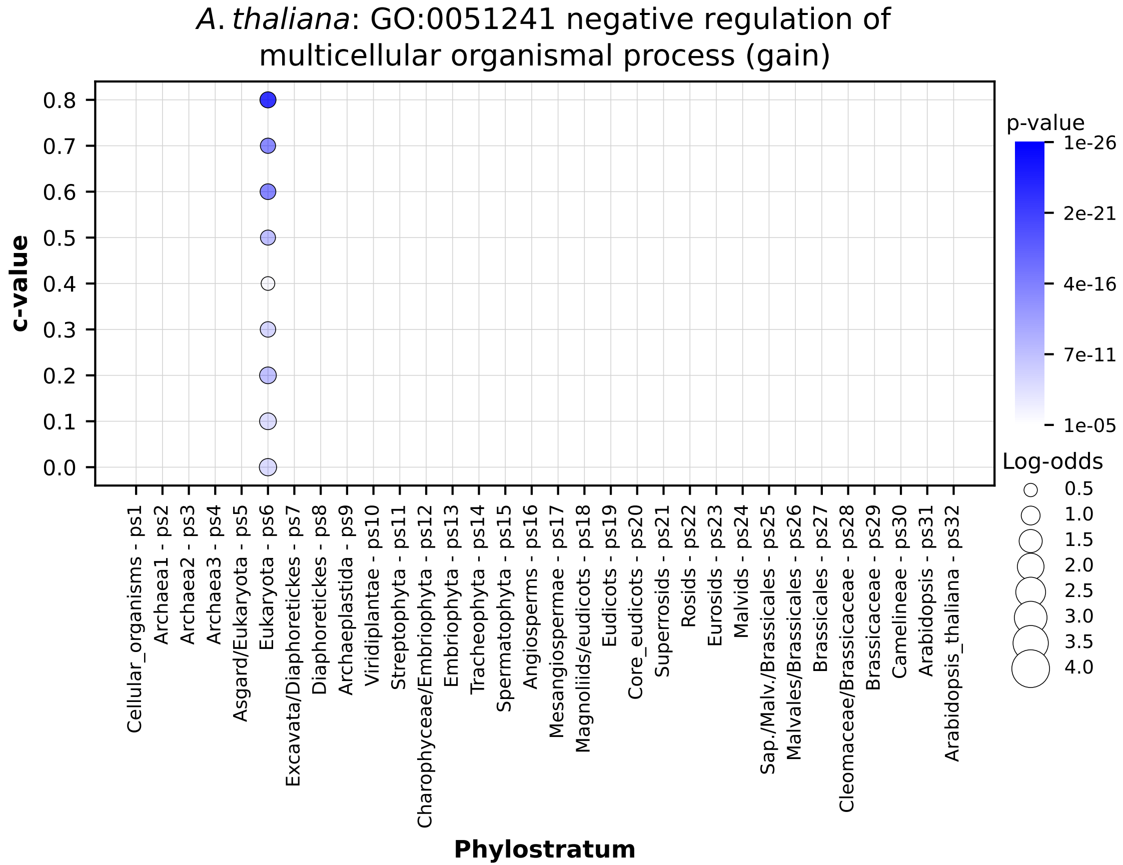

A. thaliana: GO:0051241 negative regulation of multicellular organismal process (gain)
c-value
0.8
0.7
0.6
0.5
0.4
0.3
0.2
0.1
0.0
p-value
1e-26
2e-21
4e-16
7e-11
1e-05
Log-odds
0.5
1.0
1.5
2.0
2.5
3.0
3.5
4.0
Cellular_organisms - ps1
Archaea1 - ps2
Archaea2 - ps3
Archaea3 - ps4
Asgard/Eukaryota - ps5
Eukaryota - ps6
Excavata/Diaphoretickes - ps7
Diaphoretickes - ps8
Archaeplastida - ps9
Viridiplantae - ps10
Streptophyta - ps11
Charophyceae/Embriophyta - ps12
Embriophyta - ps13
Tracheophyta - ps14
Spermatophyta - ps15
Angiosperms - ps16
Mesangiospermae - ps17
Magnoliids/eudicots - ps18
Eudicots - ps19
Core_eudicots - ps20
Superrosids - ps21
Rosids - ps22
Eurosids - ps23
Malvids - ps24
Sap./Malv./Brassicales - ps25
Malvales/Brassicales - ps26
Brassicales - ps27
Cleomaceae/Brassicaceae - ps28
Brassicaceae - ps29
Camelineae - ps30
Arabidopsis - ps31
Arabidopsis_thaliana - ps32
Phylostratum

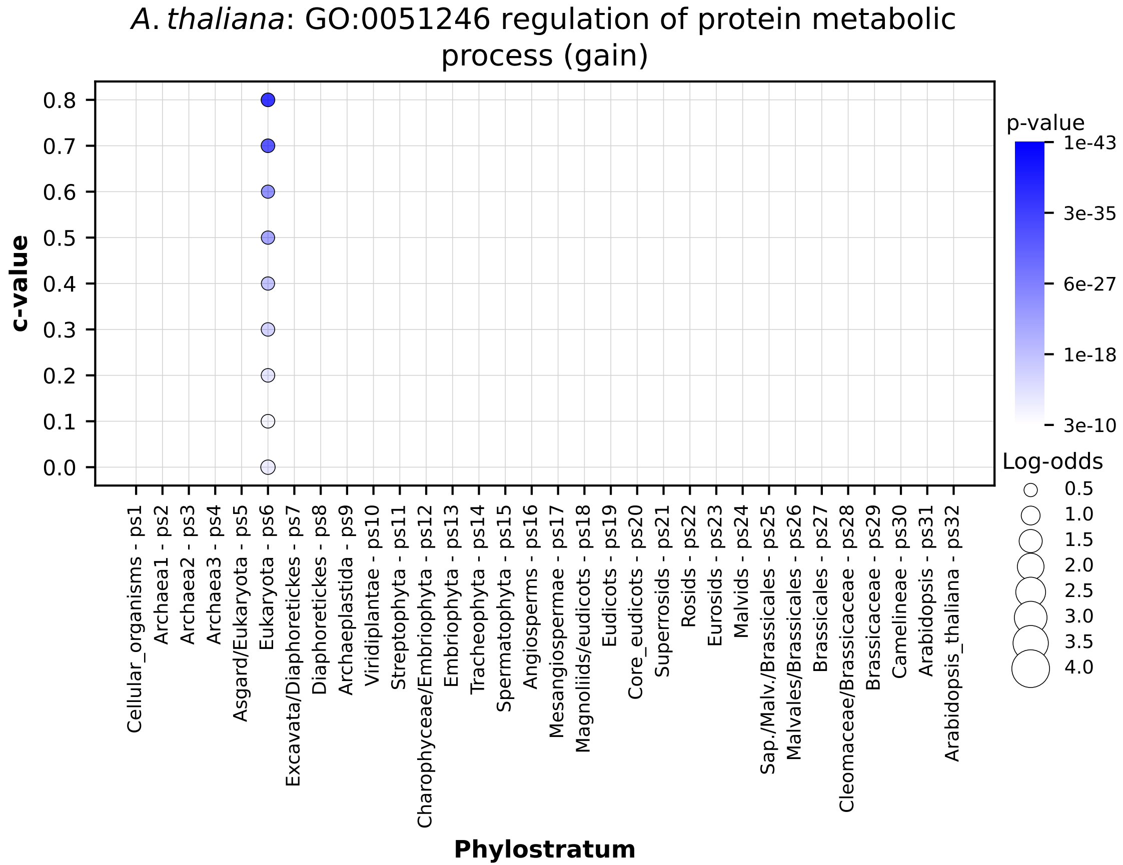

A. thaliana: GO:0051246 regulation of protein metabolic process (gain)
c-value
0.8
0.7
0.6
0.5
0.4
0.3
0.2
0.1
0.0
p-value
1e-43
3e-35
6e-27
1e-18
3e-10
Log-odds
0.5
1.0
1.5
2.0
2.5
3.0
3.5
4.0
Cellular_organisms - ps1
Archaea1 - ps2
Archaea2 - ps3
Archaea3 - ps4
Asgard/Eukaryota - ps5
Eukaryota - ps6
Excavata/Diaphoretickes - ps7
Diaphoretickes - ps8
Archaeplastida - ps9
Viridiplantae - ps10
Streptophyta - ps11
Charophyceae/Embriophyta - ps12
Embriophyta - ps13
Tracheophyta - ps14
Spermatophyta - ps15
Angiosperms - ps16
Mesangiospermae - ps17
Magnoliids/eudicots - ps18
Eudicots - ps19
Core_eudicots - ps20
Superrosids - ps21
Rosids - ps22
Eurosids - ps23
Malvids - ps24
Sap./Malv./Brassicales - ps25
Malvales/Brassicales - ps26
Brassicales - ps27
Cleomaceae/Brassicaceae - ps28
Brassicaceae - ps29
Camelineae - ps30
Arabidopsis - ps31
Arabidopsis_thaliana - ps32
Phylostratum

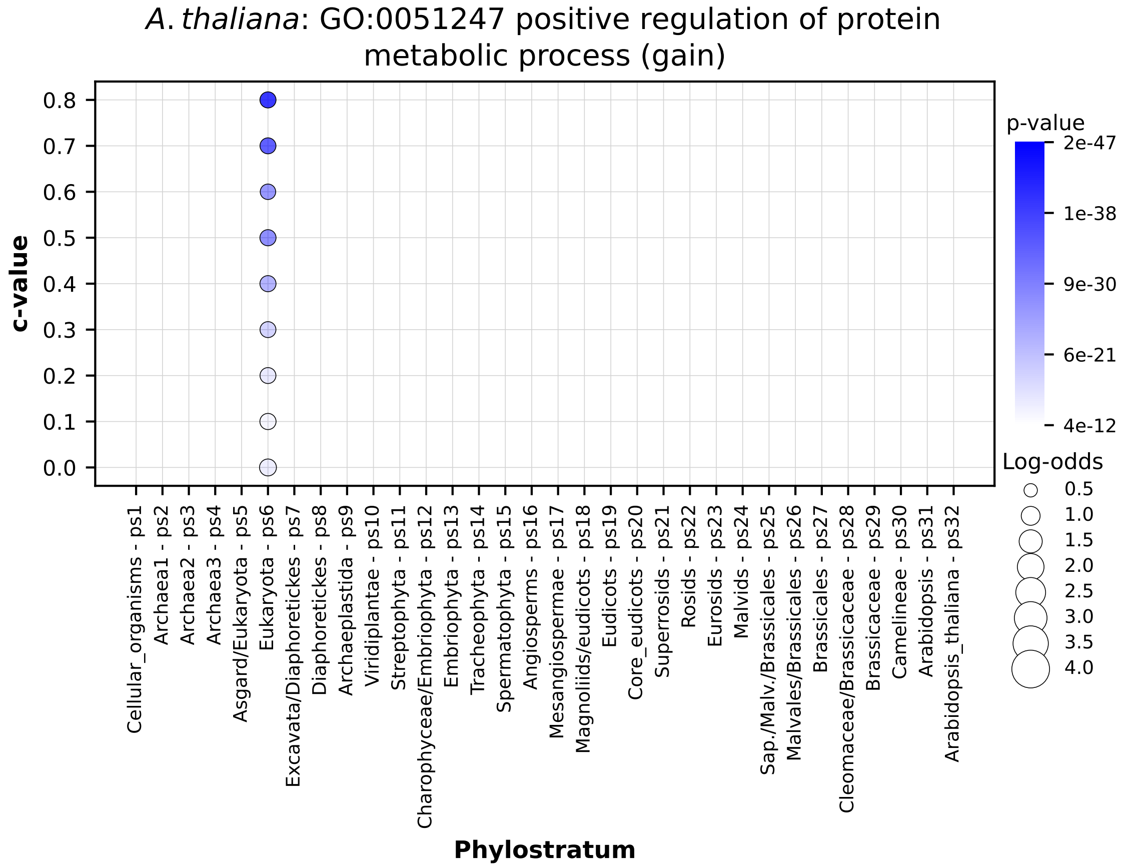
A. thaliana: GO:0051247 positive regulation of protein metabolic process (gain)
c-value
0.8
0.7
0.6
0.5
0.4
0.3
0.2
0.1
0.0
p-value
2e-47
1e-38
9e-30
6e-21
4e-12
Log-odds
0.5
1.0
1.5
2.0
2.5
3.0
3.5
4.0
Cellular_organisms - ps1
Archaea1 - ps2
Archaea2 - ps3
Archaea3 - ps4
Asgard/Eukaryota - ps5
Eukaryota - ps6
Excavata/Diaphoretickes - ps7
Diaphoretickes - ps8
Archaeplastida - ps9
Viridiplantae - ps10
Streptophyta - ps11
Charophyceae/Embriophyta - ps12
Embriophyta - ps13
Tracheophyta - ps14
Spermatophyta - ps15
Angiosperms - ps16
Mesangiospermae - ps17
Magnoliids/eudicots - ps18
Eudicots - ps19
Core_eudicots - ps20
Superrosids - ps21
Rosids - ps22
Eurosids - ps23
Malvids - ps24
Sap./Malv./Brassicales - ps25
Malvales/Brassicales - ps26
Brassicales - ps27
Cleomaceae/Brassicaceae - ps28
Brassicaceae - ps29
Camelineae - ps30
Arabidopsis - ps31
Arabidopsis_thaliana - ps32
Phylostratum

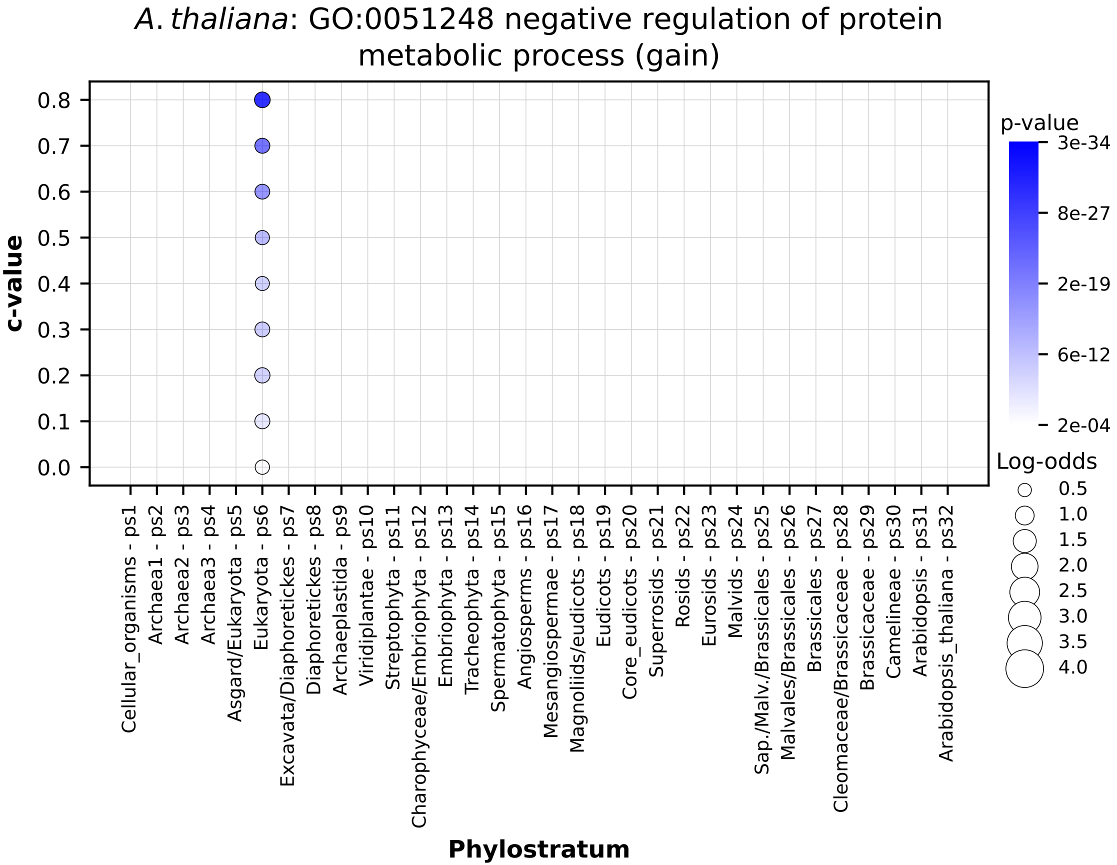
A. thaliana: GO:0051248 negative regulation of protein metabolic process (gain)
c-value
0.8
0.7
0.6
0.5
0.4
0.3
0.2
0.1
0.0
Phylostratum
Cellular_organisms - ps1
Archaea1 - ps2
Archaea2 - ps3
Archaea3 - ps4
Asgard/Eukaryota - ps5
Eukaryota - ps6
Excavata/Diaphoretickes - ps7
Diaphoretickes - ps8
Archaeplastida - ps9
Viridiplantae - ps10
Streptophyta - ps11
Charophyceae/Embriophyta - ps12
Embriophyta - ps13
Tracheophyta - ps14
Spermatophyta - ps15
Angiosperms - ps16
Mesangiospermae - ps17
Magnoliids/eudicots - ps18
Eudicots - ps19
Core_eudicots - ps20
Superrosids - ps21
Rosids - ps22
Eurosids - ps23
Malvids - ps24
Sap./Malv./Brassicales - ps25
Malvales/Brassicales - ps26
Brassicales - ps27
Cleomaceae/Brassicaceae - ps28
Brassicaceae - ps29
Camelineae - ps30
Arabidopsis - ps31
Arabidopsis_thaliana - ps32
p-value
3e-34
8e-27
2e-19
6e-12
2e-04
Log-odds
0.5
1.0
1.5
2.0
2.5
3.0
3.5
4.0

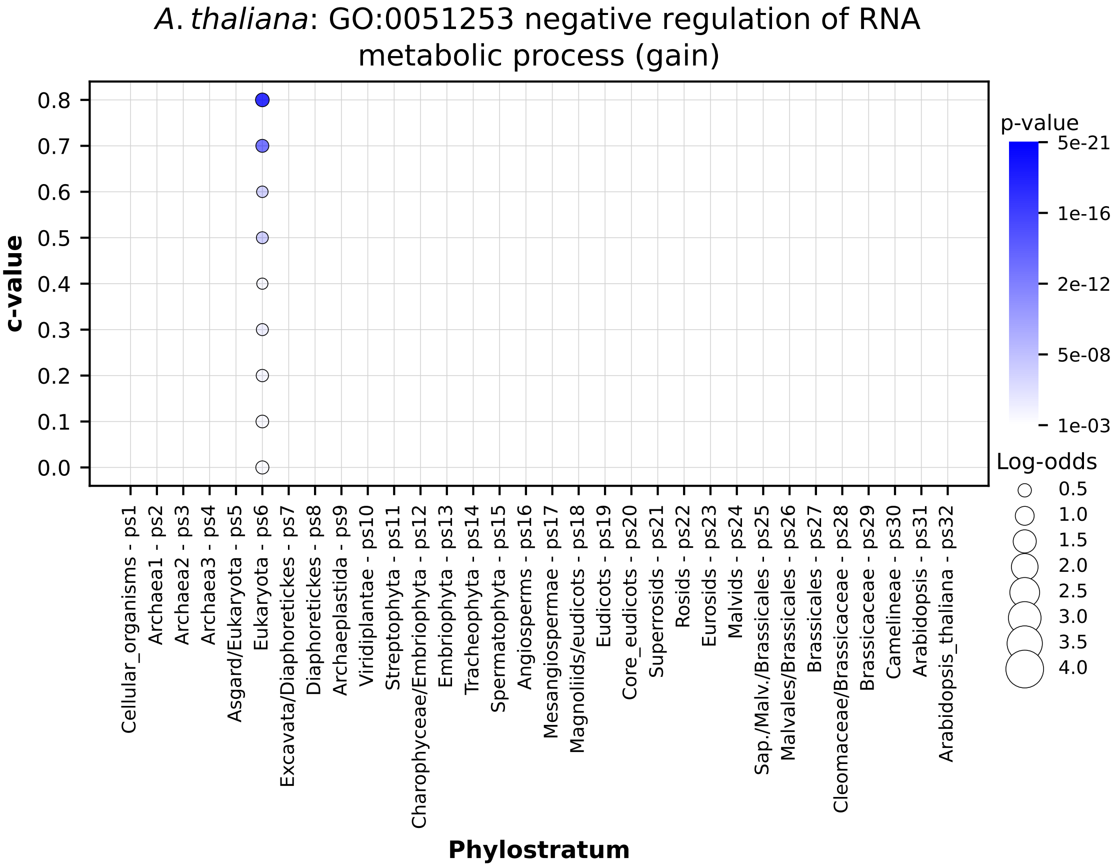

A. thaliana: GO:0051253 negative regulation of RNA metabolic process (gain)
c-value
0.8
0.7
0.6
0.5
0.4
0.3
0.2
0.1
0.0
Phylostratum
Cellular_organisms - ps1
Archaea1 - ps2
Archaea2 - ps3
Archaea3 - ps4
Asgard/Eukaryota - ps5
Eukaryota - ps6
Excavata/Diaphoretickes - ps7
Diaphoretickes - ps8
Archaeplastida - ps9
Viridiplantae - ps10
Streptophyta - ps11
Charophyceae/Embriophyta - ps12
Embriophyta - ps13
Tracheophyta - ps14
Spermatophyta - ps15
Angiosperms - ps16
Mesangiospermae - ps17
Magnoliids/eudicots - ps18
Eudicots - ps19
Core_eudicots - ps20
Superrosids - ps21
Rosids - ps22
Eurosids - ps23
Malvids - ps24
Sap./Malv./Brassicales - ps25
Malvales/Brassicales - ps26
Brassicales - ps27
Cleomaceae/Brassicaceae - ps28
Brassicaceae - ps29
Camelineae - ps30
Arabidopsis - ps31
Arabidopsis_thaliana - ps32
p-value
5e-21
1e-16
2e-12
5e-08
1e-03
Log-odds
0.5
1.0
1.5
2.0
2.5
3.0
3.5
4.0

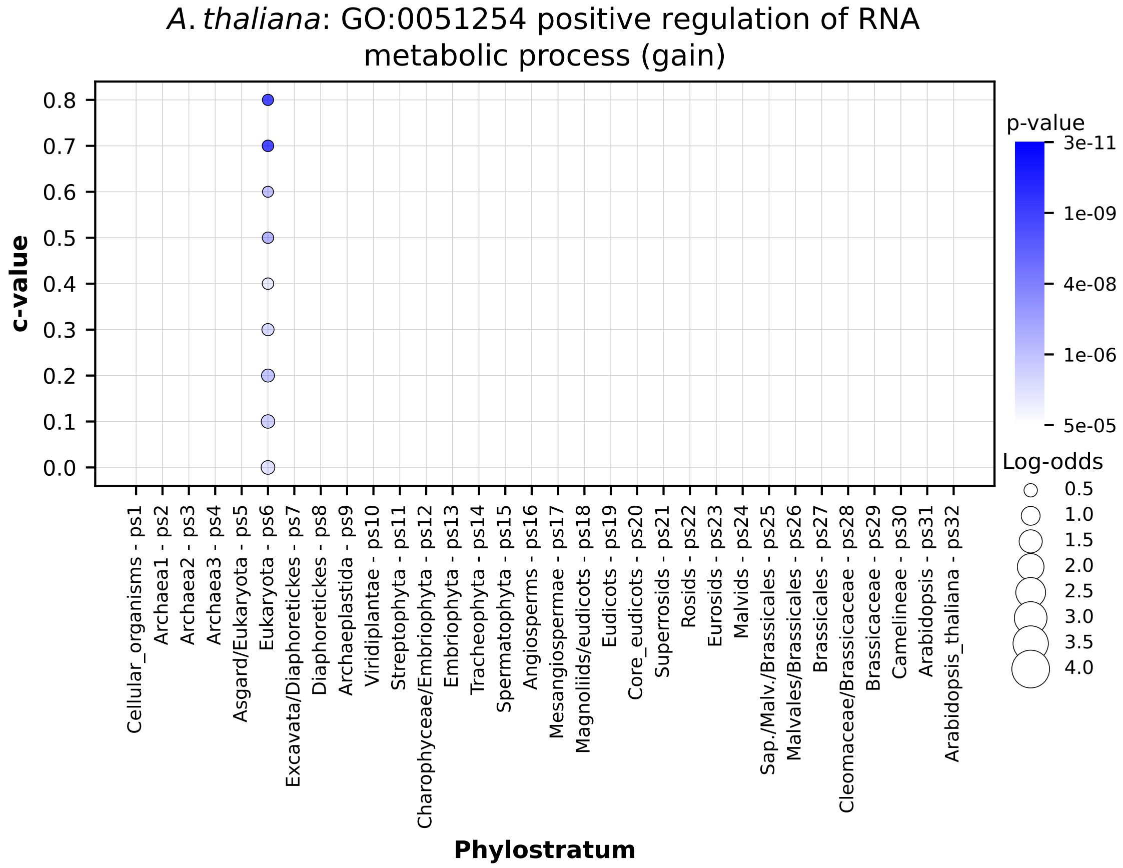
A. thaliana: GO:0051254 positive regulation of RNA metabolic process (gain)
c-value
0.8
0.7
0.6
0.5
0.4
0.3
0.2
0.1
0.0
Phylostratum
Cellular_organisms - ps1
Archaea1 - ps2
Archaea2 - ps3
Archaea3 - ps4
Asgard/Eukaryota - ps5
Eukaryota - ps6
Excavata/Diaphoretickes - ps7
Diaphoretickes - ps8
Archaeplastida - ps9
Viridiplantae - ps10
Streptophyta - ps11
Charophyceae/Embriophyta - ps12
Embriophyta - ps13
Tracheophyta - ps14
Spermatophyta - ps15
Angiosperms - ps16
Mesangiospermae - ps17
Magnoliids/eudicots - ps18
Eudicots - ps19
Core_eudicots - ps20
Superrosids - ps21
Rosids - ps22
Eurosids - ps23
Malvids - ps24
Sap./Malv./Brassicales - ps25
Malvales/Brassicales - ps26
Brassicales - ps27
Cleomaceae/Brassicaceae - ps28
Brassicaceae - ps29
Camelineae - ps30
Arabidopsis - ps31
Arabidopsis_thaliana - ps32
p-value
3e-11
1e-09
4e-08
1e-06
5e-05
Log-odds
0.5
1.0
1.5
2.0
2.5
3.0
3.5
4.0

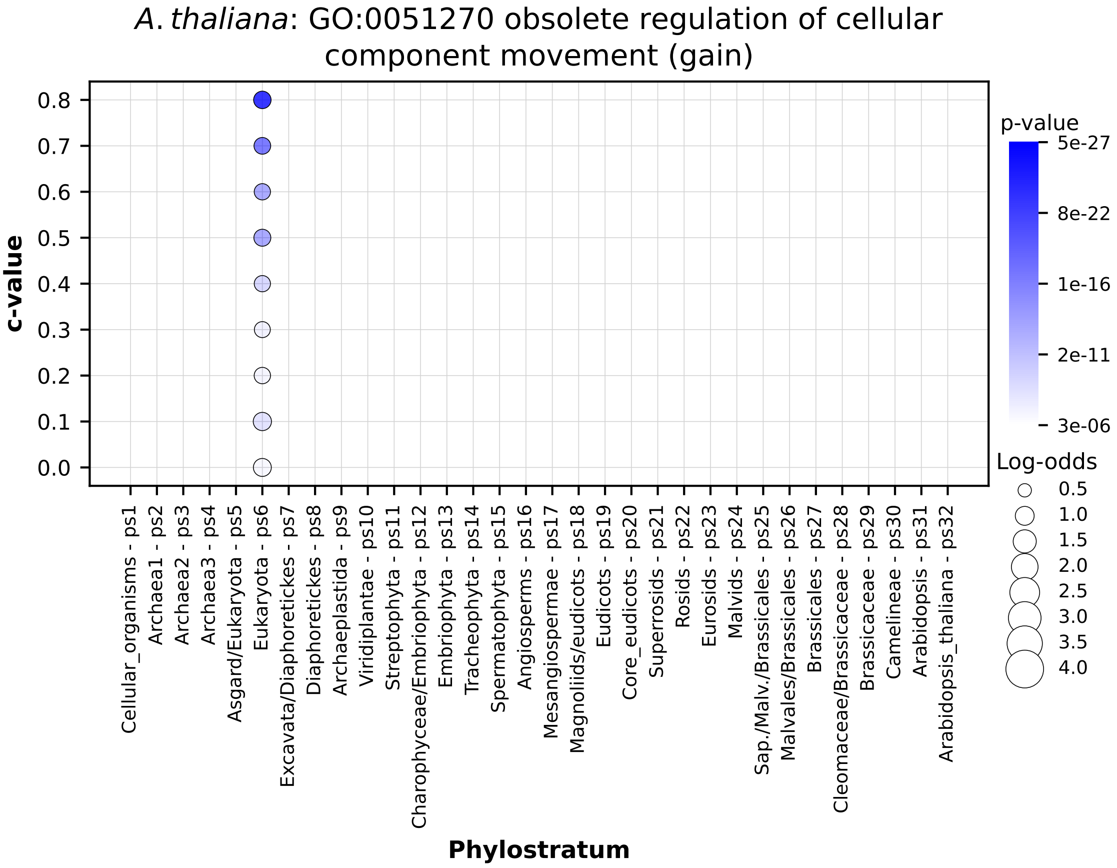

A. thaliana: GO:0051270 obsolete regulation of cellular component movement (gain)
c-value
0.8
0.7
0.6
0.5
0.4
0.3
0.2
0.1
0.0
p-value
5e-27
8e-22
1e-16
2e-11
3e-06
Log-odds
0.5
1.0
1.5
2.0
2.5
3.0
3.5
4.0
Cellular_organisms - ps1
Archaea1 - ps2
Archaea2 - ps3
Archaea3 - ps4
Asgard/Eukaryota - ps5
Eukaryota - ps6
Excavata/Diaphoretickes - ps7
Diaphoretickes - ps8
Archaeplastida - ps9
Viridiplantae - ps10
Streptophyta - ps11
Charophyceae/Embriophyta - ps12
Embriophyta - ps13
Tracheophyta - ps14
Spermatophyta - ps15
Angiosperms - ps16
Mesangiospermae - ps17
Magnoliids/eudicots - ps18
Eudicots - ps19
Core_eudicots - ps20
Superrosids - ps21
Rosids - ps22
Eurosids - ps23
Malvids - ps24
Sap./Malv./Brassicales - ps25
Malvales/Brassicales - ps26
Brassicales - ps27
Cleomaceae/Brassicaceae - ps28
Brassicaceae - ps29
Camelineae - ps30
Arabidopsis - ps31
Arabidopsis_thaliana - ps32
Phylostratum

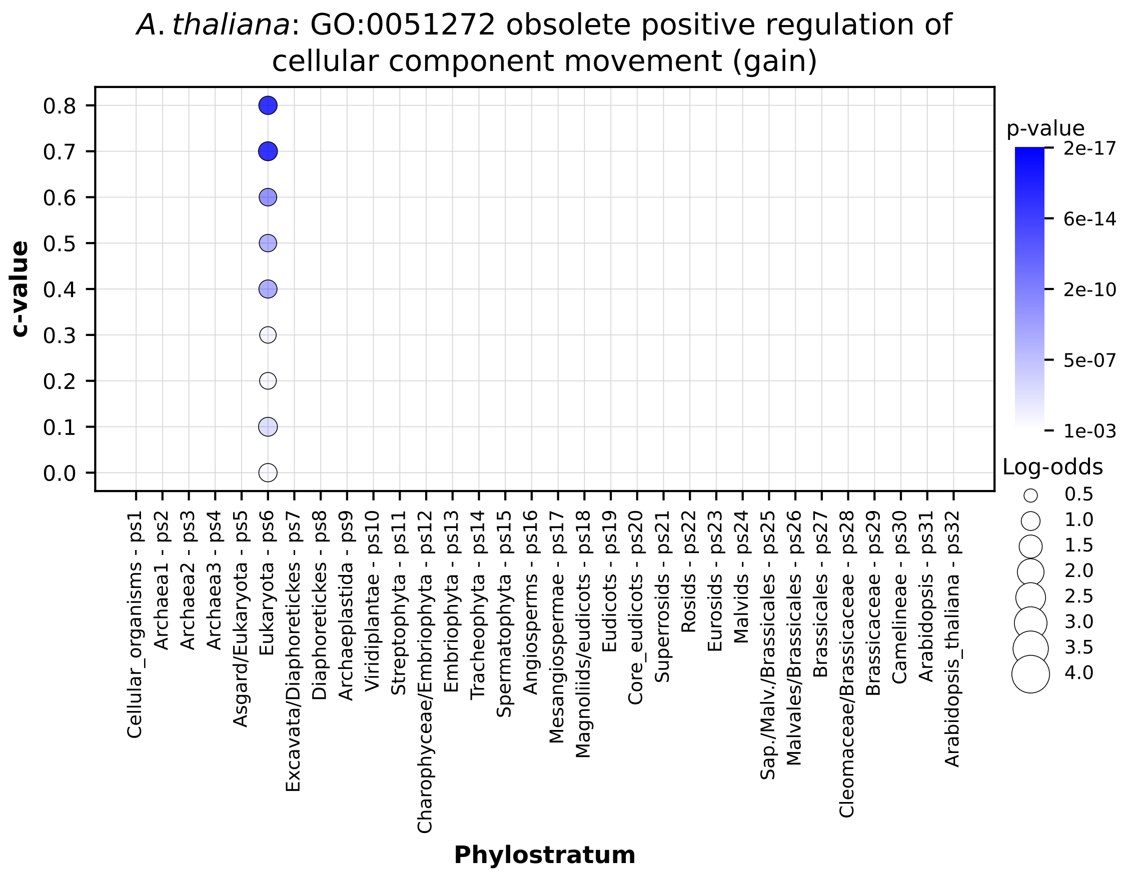

A. thaliana: GO:0051272 obsolete positive regulation of cellular component movement (gain)
c-value
0.8
0.7
0.6
0.5
0.4
0.3
0.2
0.1
0.0
p-value
2e-17
6e-14
2e-10
5e-07
1e-03
Log-odds
0.5
1.0
1.5
2.0
2.5
3.0
3.5
4.0
Cellular_organisms - ps1
Archaea1 - ps2
Archaea2 - ps3
Archaea3 - ps4
Asgard/Eukaryota - ps5
Eukaryota - ps6
Excavata/Diaphoretickes - ps7
Diaphoretickes - ps8
Archaeplastida - ps9
Viridiplantae - ps10
Streptophyta - ps11
Charophyceae/Embriophyta - ps12
Embriophyta - ps13
Tracheophyta - ps14
Spermatophyta - ps15
Angiosperms - ps16
Mesangiospermae - ps17
Magnoliids/eudicots - ps18
Eudicots - ps19
Core_eudicots - ps20
Superrosids - ps21
Rosids - ps22
Eurosids - ps23
Malvids - ps24
Sap./Malv./Brassicales - ps25
Malvales/Brassicales - ps26
Brassicales - ps27
Cleomaceae/Brassicaceae - ps28
Brassicaceae - ps29
Camelineae - ps30
Arabidopsis - ps31
Arabidopsis_thaliana - ps32
Phylostratum

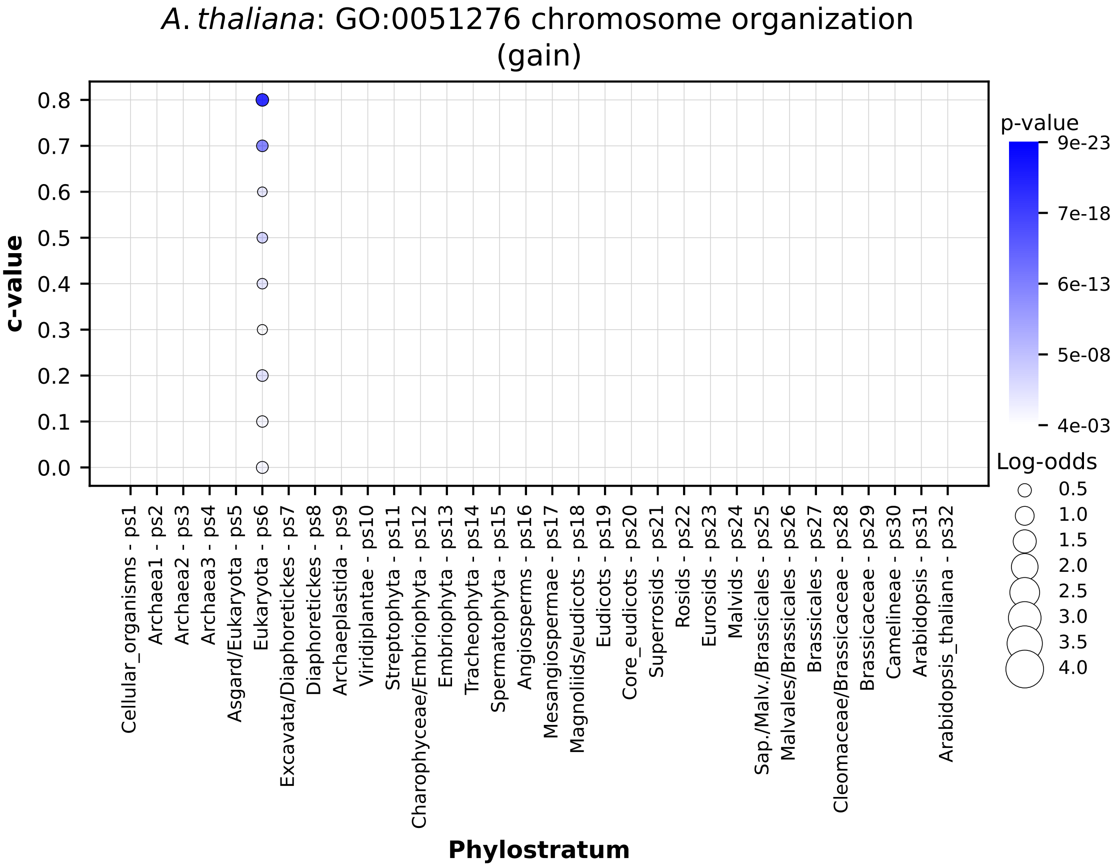
A. thaliana: GO:0051276 chromosome organization
(gain)
c-value
0.0
0.1
0.2
0.3
0.4
0.5
0.6
0.7
0.8
p-value
9e-23
7e-18
6e-13
5e-08
4e-03
Log-odds
0.5
1.0
1.5
2.0
2.5
3.0
3.5
4.0
Cellular_organisms - ps1
Archaea1 - ps2
Archaea2 - ps3
Archaea3 - ps4
Asgard/Eukaryota - ps5
Eukaryota - ps6
Excavata/Diaphoretickes - ps7
Diaphoretickes - ps8
Archaeplastida - ps9
Viridiplantae - ps10
Streptophyta - ps11
Charophyceae/Embriophyta - ps12
Embriophyta - ps13
Tracheophyta - ps14
Spermatophyta - ps15
Angiosperms - ps16
Mesangiospermae - ps17
Magnoliids/eudicots - ps18
Eudicots - ps19
Core_eudicots - ps20
Superrosids - ps21
Rosids - ps22
Eurosids - ps23
Malvids - ps24
Sap./Malv./Brassicales - ps25
Malvales/Brassicales - ps26
Brassicales - ps27
Cleomaceae/Brassicaceae - ps28
Brassicaceae - ps29
Camelineae - ps30
Arabidopsis - ps31
Arabidopsis_thaliana - ps32
Phylostratum

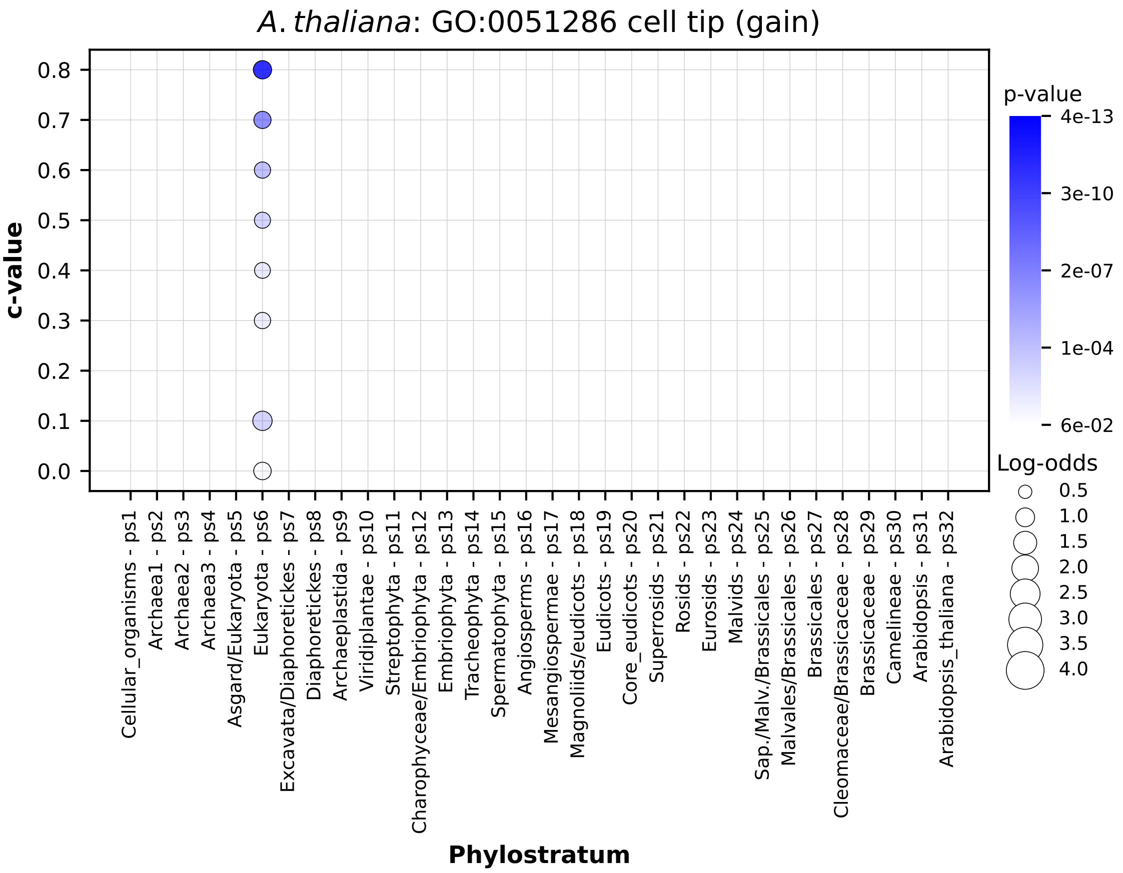

A. thaliana: GO:0051286 cell tip (gain)
c-value
0.0
0.1
0.2
0.3
0.4
0.5
0.6
0.7
0.8
p-value
4e-13
3e-10
2e-07
1e-04
6e-02
Log-odds
0.5
1.0
1.5
2.0
2.5
3.0
3.5
4.0
Cellular_organisms - ps1
Archaea1 - ps2
Archaea2 - ps3
Archaea3 - ps4
Asgard/Eukaryota - ps5
Eukaryota - ps6
Excavata/Diaphoretickes - ps7
Diaphoretickes - ps8
Archaeplastida - ps9
Viridiplantae - ps10
Streptophyta - ps11
Charophyceae/Embriophyta - ps12
Embriophyta - ps13
Tracheophyta - ps14
Spermatophyta - ps15
Angiosperms - ps16
Mesangiospermae - ps17
Magnoliids/eudicots - ps18
Eudicots - ps19
Core_eudicots - ps20
Superrosids - ps21
Rosids - ps22
Eurosids - ps23
Malvids - ps24
Sap./Malv./Brassicales - ps25
Malvales/Brassicales - ps26
Brassicales - ps27
Cleomaceae/Brassicaceae - ps28
Brassicaceae - ps29
Camelineae - ps30
Arabidopsis - ps31
Arabidopsis_thaliana - ps32
Phylostratum

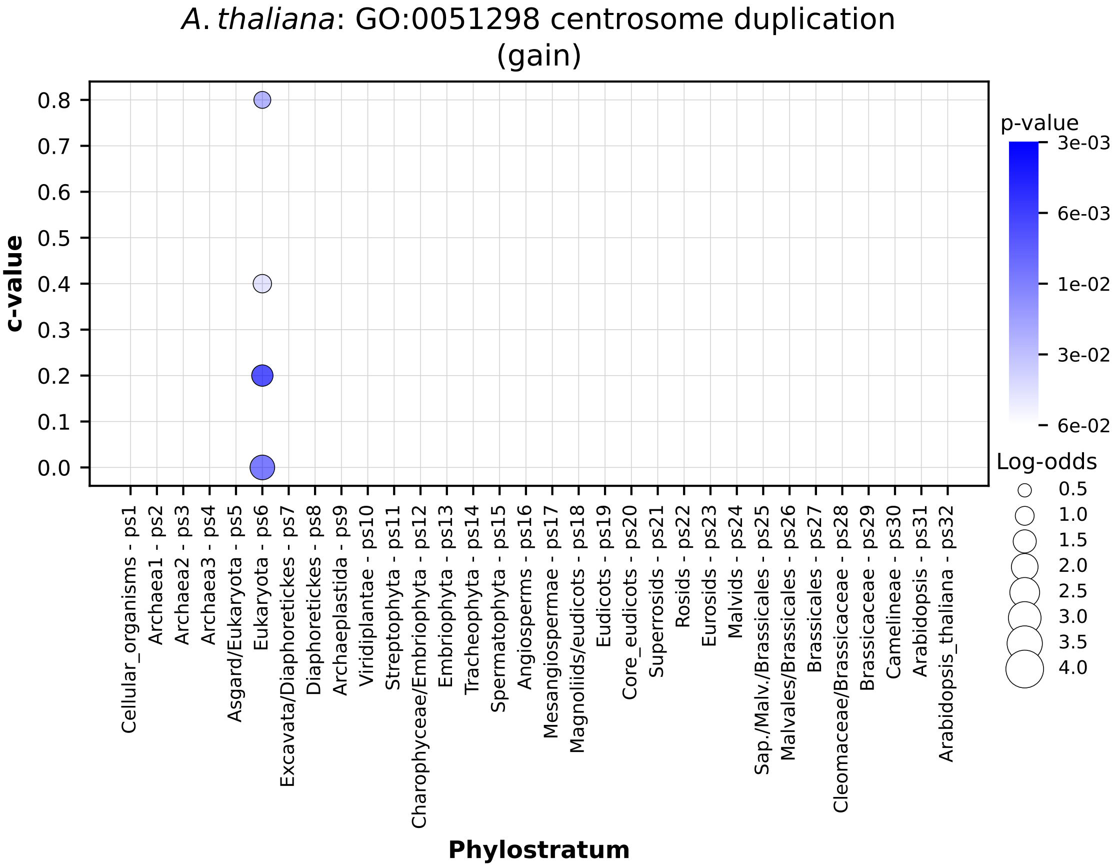

A. thaliana: GO:0051298 centrosome duplication
(gain)
c-value
0.0
0.1
0.2
0.3
0.4
0.5
0.6
0.7
0.8
Phylostratum
Cellular_organisms - ps1
Archaea1 - ps2
Archaea2 - ps3
Archaea3 - ps4
Asgard/Eukaryota - ps5
Eukaryota - ps6
Excavata/Diaphoretickes - ps7
Diaphoretickes - ps8
Archaeplastida - ps9
Viridiplantae - ps10
Streptophyta - ps11
Charophyceae/Embriophyta - ps12
Embriophyta - ps13
Tracheophyta - ps14
Spermatophyta - ps15
Angiosperms - ps16
Mesangiospermae - ps17
Magnoliids/eudicots - ps18
Eudicots - ps19
Core_eudicots - ps20
Superrosids - ps21
Rosids - ps22
Eurosids - ps23
Malvids - ps24
Sap./Malv./Brassicales - ps25
Malvales/Brassicales - ps26
Brassicales - ps27
Cleomaceae/Brassicaceae - ps28
Brassicaceae - ps29
Camelineae - ps30
Arabidopsis - ps31
Arabidopsis_thaliana - ps32
p-value
3e-03
6e-03
1e-02
3e-02
6e-02
Log-odds
0.5
1.0
1.5
2.0
2.5
3.0
3.5
4.0

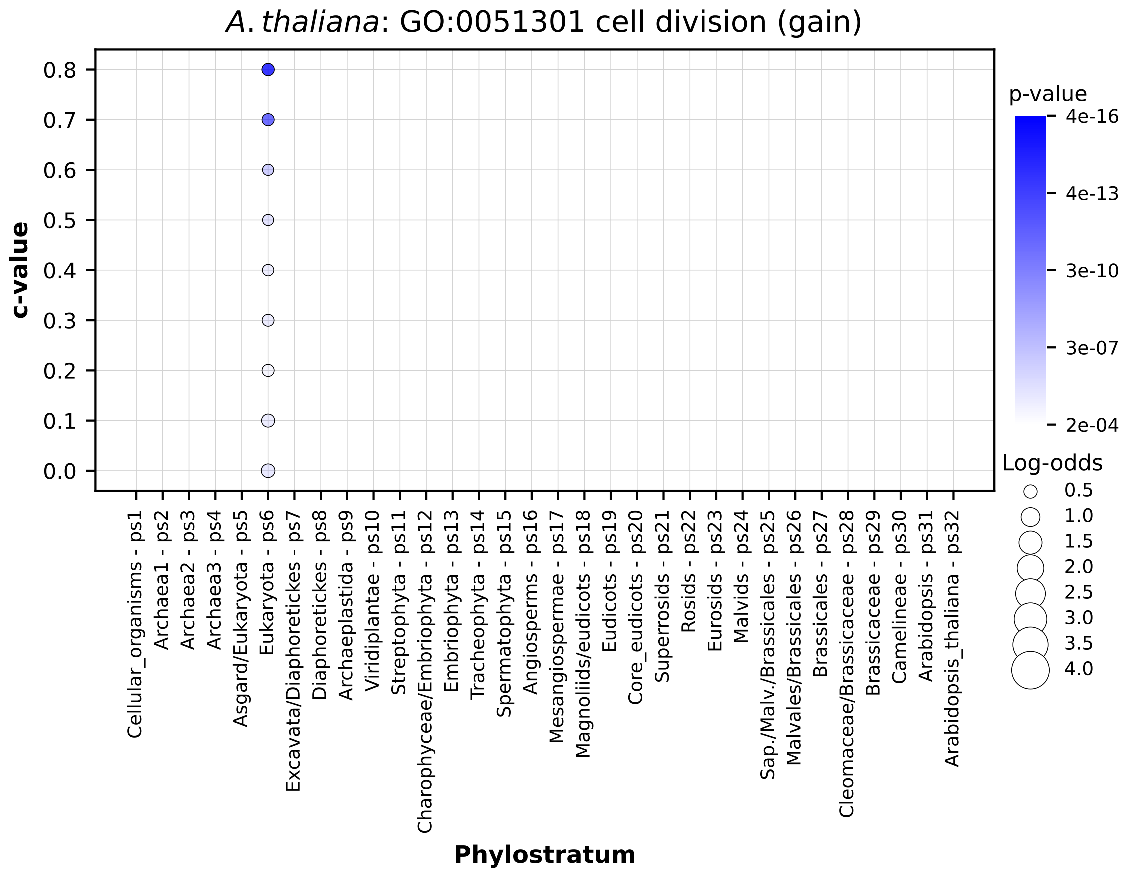
A. thaliana: GO:0051301 cell division (gain)
c-value
0.0
0.1
0.2
0.3
0.4
0.5
0.6
0.7
0.8
Phylostratum
Cellular_organisms - ps1
Archaea1 - ps2
Archaea2 - ps3
Archaea3 - ps4
Asgard/Eukaryota - ps5
Eukaryota - ps6
Excavata/Diaphoretickes - ps7
Diaphoretickes - ps8
Archaeplastida - ps9
Viridiplantae - ps10
Streptophyta - ps11
Charophyceae/Embriophyta - ps12
Embriophyta - ps13
Tracheophyta - ps14
Spermatophyta - ps15
Angiosperms - ps16
Mesangiospermae - ps17
Magnoliids/eudicots - ps18
Eudicots - ps19
Core_eudicots - ps20
Superrosids - ps21
Rosids - ps22
Eurosids - ps23
Malvids - ps24
Sap./Malv./Brassicales - ps25
Malvales/Brassicales - ps26
Brassicales - ps27
Cleomaceae/Brassicaceae - ps28
Brassicaceae - ps29
Camelineae - ps30
Arabidopsis - ps31
Arabidopsis_thaliana - ps32
p-value
4e-16
4e-13
3e-10
3e-07
2e-04
Log-odds
0.5
1.0
1.5
2.0
2.5
3.0
3.5
4.0

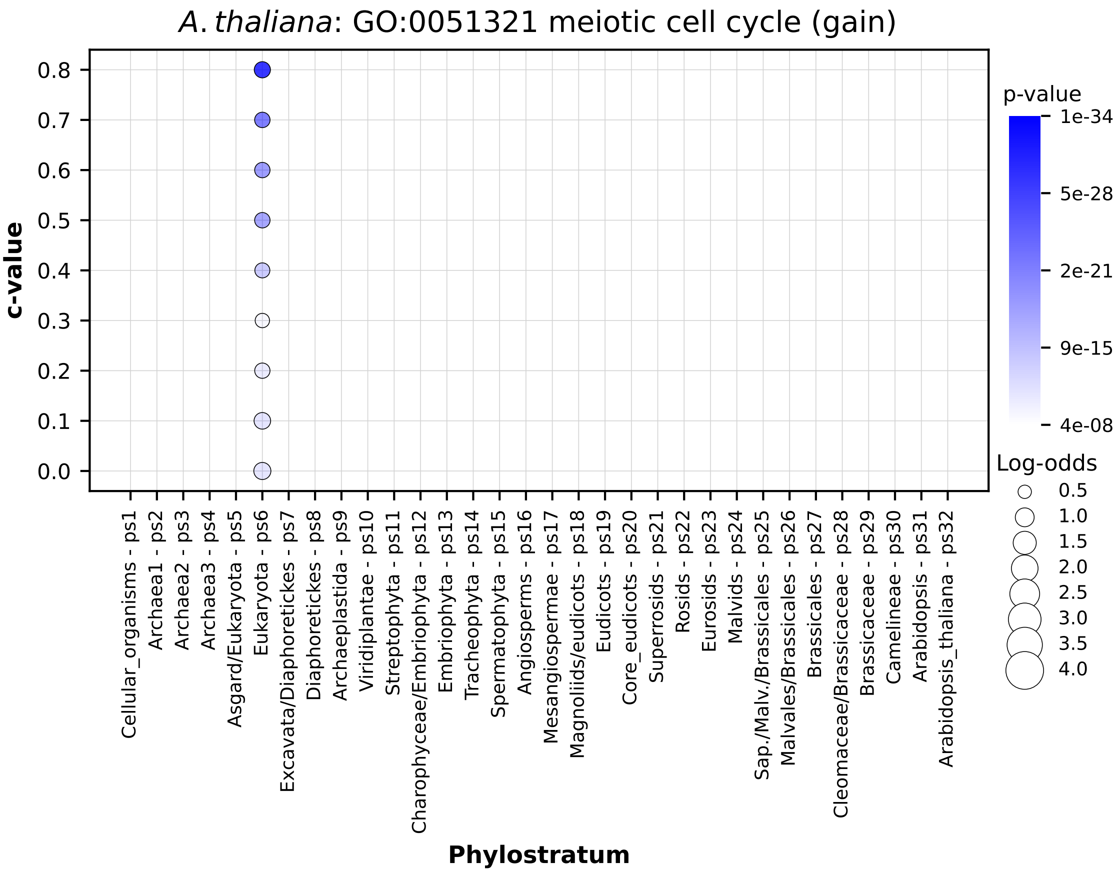
A. thaliana: GO:0051321 meiotic cell cycle (gain)
c-value
0.0
0.1
0.2
0.3
0.4
0.5
0.6
0.7
0.8
p-value
1e-34
5e-28
2e-21
9e-15
4e-08
Log-odds
0.5
1.0
1.5
2.0
2.5
3.0
3.5
4.0
Cellular_organisms - ps1
Archaea1 - ps2
Archaea2 - ps3
Archaea3 - ps4
Asgard/Eukaryota - ps5
Eukaryota - ps6
Excavata/Diaphoretickes - ps7
Diaphoretickes - ps8
Archaeplastida - ps9
Viridiplantae - ps10
Streptophyta - ps11
Charophyceae/Embriophyta - ps12
Embriophyta - ps13
Tracheophyta - ps14
Spermatophyta - ps15
Angiosperms - ps16
Mesangiospermae - ps17
Magnoliids/eudicots - ps18
Eudicots - ps19
Core_eudicots - ps20
Superrosids - ps21
Rosids - ps22
Eurosids - ps23
Malvids - ps24
Sap./Malv./Brassicales - ps25
Malvales/Brassicales - ps26
Brassicales - ps27
Cleomaceae/Brassicaceae - ps28
Brassicaceae - ps29
Camelineae - ps30
Arabidopsis - ps31
Arabidopsis_thaliana - ps32
Phylostratum

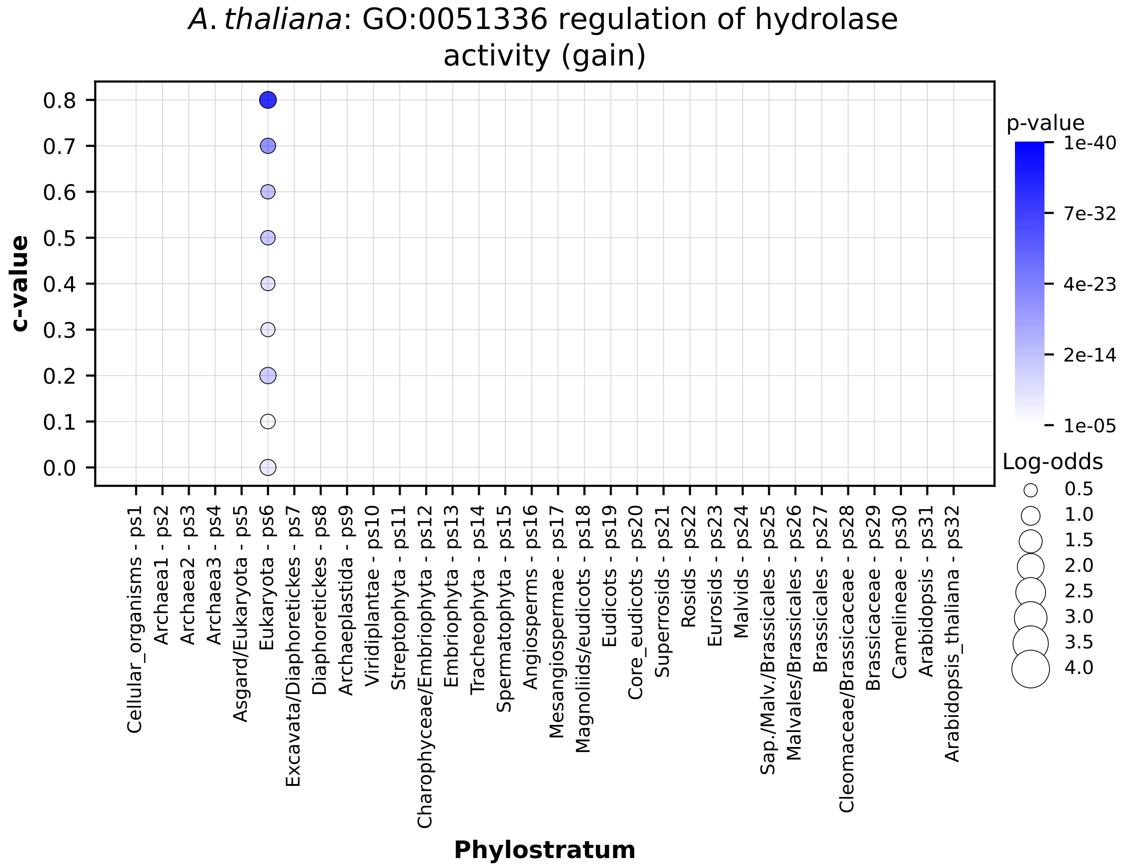

A. thaliana: GO:0051336 regulation of hydrolase activity (gain)
c-value
0.0
0.1
0.2
0.3
0.4
0.5
0.6
0.7
0.8
p-value
1e-40
7e-32
4e-23
2e-14
1e-05
Log-odds
0.5
1.0
1.5
2.0
2.5
3.0
3.5
4.0
Cellular_organisms - ps1
Archaea1 - ps2
Archaea2 - ps3
Archaea3 - ps4
Asgard/Eukaryota - ps5
Eukaryota - ps6
Excavata/Diaphoretickes - ps7
Diaphoretickes - ps8
Archaeplastida - ps9
Viridiplantae - ps10
Streptophyta - ps11
Charophyceae/Embriophyta - ps12
Embriophyta - ps13
Tracheophyta - ps14
Spermatophyta - ps15
Angiosperms - ps16
Mesangiospermae - ps17
Magnoliids/eudicots - ps18
Eudicots - ps19
Core_eudicots - ps20
Superrosids - ps21
Rosids - ps22
Eurosids - ps23
Malvids - ps24
Sap./Malv./Brassicales - ps25
Malvales/Brassicales - ps26
Brassicales - ps27
Cleomaceae/Brassicaceae - ps28
Brassicaceae - ps29
Camelineae - ps30
Arabidopsis - ps31
Arabidopsis_thaliana - ps32
Phylostratum

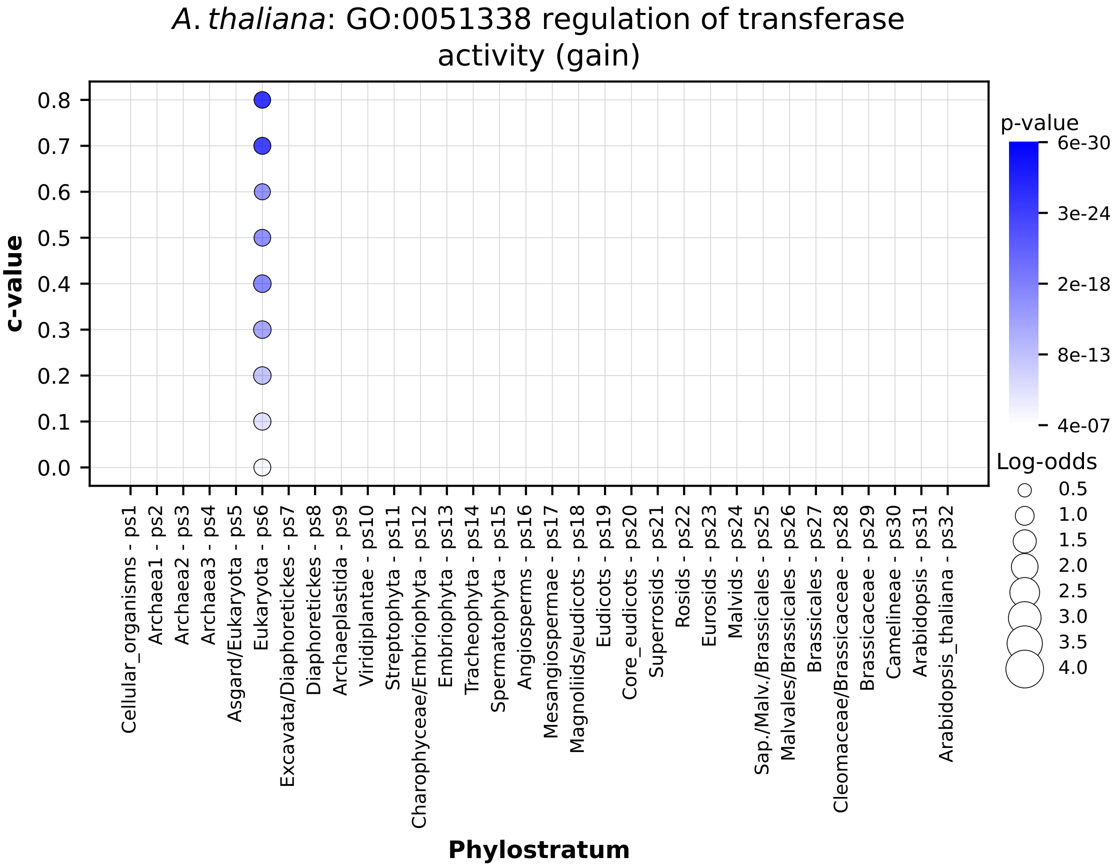
A. thaliana: GO:0051338 regulation of transferase activity (gain)
c-value
0.8
0.7
0.6
0.5
0.4
0.3
0.2
0.1
0.0
p-value
6e-30
3e-24
2e-18
8e-13
4e-07
Log-odds
0.5
1.0
1.5
2.0
2.5
3.0
3.5
4.0
Cellular_organisms - ps1
Archaea1 - ps2
Archaea2 - ps3
Archaea3 - ps4
Asgard/Eukaryota - ps5
Eukaryota - ps6
Excavata/Diaphoretickes - ps7
Diaphoretickes - ps8
Archaeplastida - ps9
Viridiplantae - ps10
Streptophyta - ps11
Charophyceae/Embriophyta - ps12
Embriophyta - ps13
Tracheophyta - ps14
Spermatophyta - ps15
Angiosperms - ps16
Mesangiospermae - ps17
Magnoliids/eudicots - ps18
Eudicots - ps19
Core_eudicots - ps20
Superrosids - ps21
Rosids - ps22
Eurosids - ps23
Malvids - ps24
Sap./Malv./Brassicales - ps25
Malvales/Brassicales - ps26
Brassicales - ps27
Cleomaceae/Brassicaceae - ps28
Brassicaceae - ps29
Camelineae - ps30
Arabidopsis - ps31
Arabidopsis_thaliana - ps32
Phylostratum

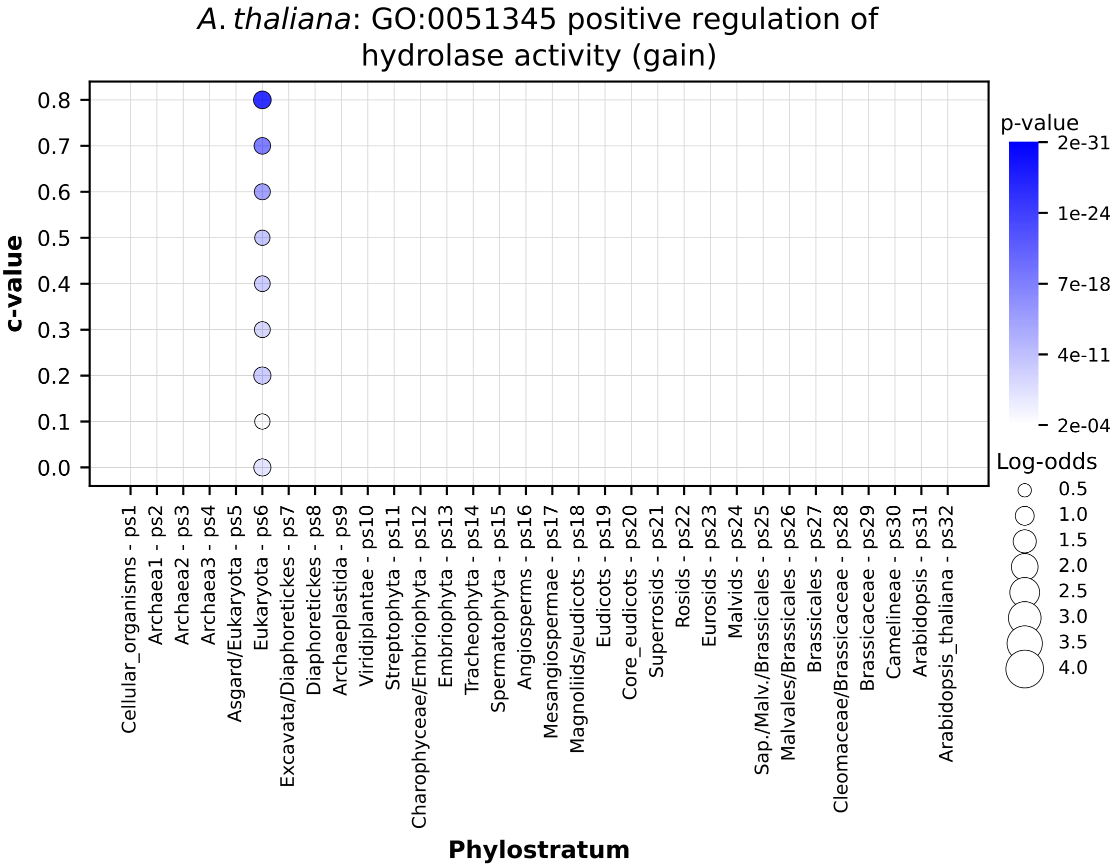

A. thaliana: GO:0051345 positive regulation of hydrolase activity (gain)
c-value
0.8
0.7
0.6
0.5
0.4
0.3
0.2
0.1
0.0
p-value
2e-31
1e-24
7e-18
4e-11
2e-04
Log-odds
0.5
1.0
1.5
2.0
2.5
3.0
3.5
4.0
Cellular_organisms - ps1
Archaea1 - ps2
Archaea2 - ps3
Archaea3 - ps4
Asgard/Eukaryota - ps5
Eukaryota - ps6
Excavata/Diaphoretickes - ps7
Diaphoretickes - ps8
Archaeplastida - ps9
Viridiplantae - ps10
Streptophyta - ps11
Charophyceae/Embriophyta - ps12
Embriophyta - ps13
Tracheophyta - ps14
Spermatophyta - ps15
Angiosperms - ps16
Mesangiospermae - ps17
Magnoliids/eudicots - ps18
Eudicots - ps19
Core_eudicots - ps20
Superrosids - ps21
Rosids - ps22
Eurosids - ps23
Malvids - ps24
Sap./Malv./Brassicales - ps25
Malvales/Brassicales - ps26
Brassicales - ps27
Cleomaceae/Brassicaceae - ps28
Brassicaceae - ps29
Camelineae - ps30
Arabidopsis - ps31
Arabidopsis_thaliana - ps32
Phylostratum

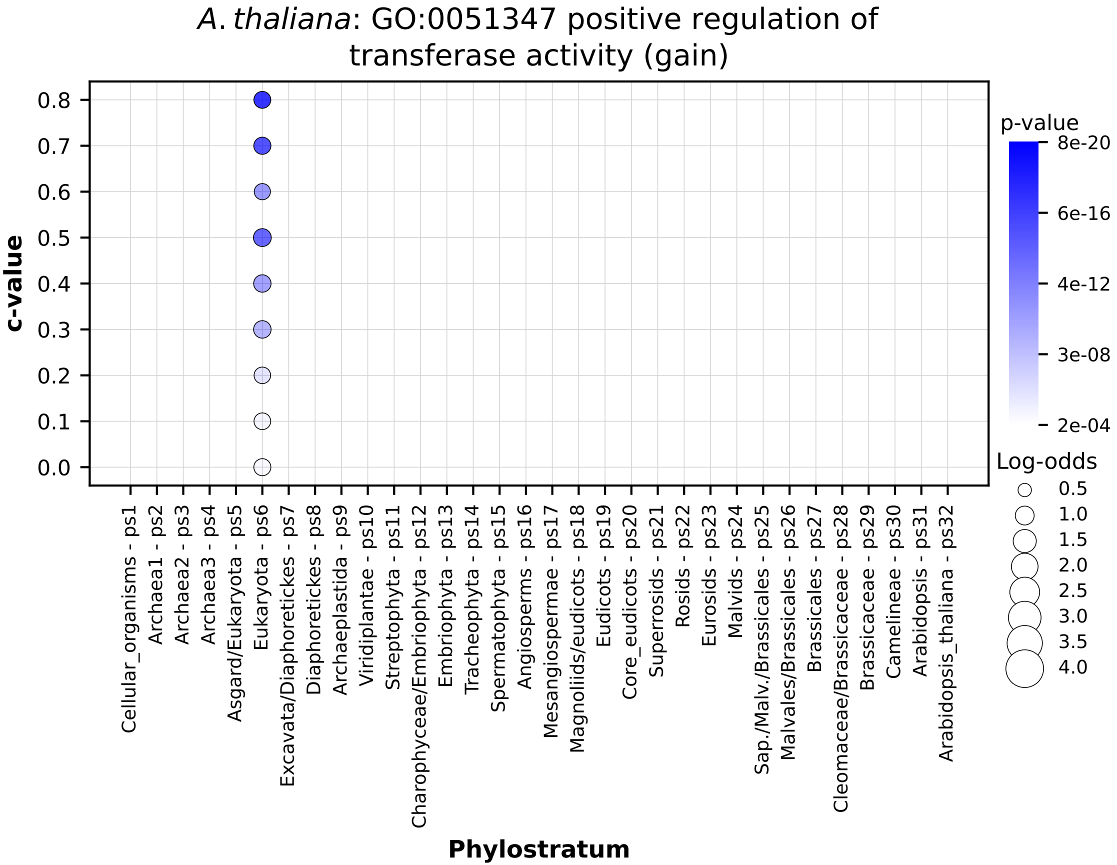

A. thaliana: GO:0051347 positive regulation of transferase activity (gain)
c-value
0.8
0.7
0.6
0.5
0.4
0.3
0.2
0.1
0.0
p-value
8e-20
6e-16
4e-12
3e-08
2e-04
Log-odds
0.5
1.0
1.5
2.0
2.5
3.0
3.5
4.0
Cellular_organisms - ps1
Archaea1 - ps2
Archaea2 - ps3
Archaea3 - ps4
Asgard/Eukaryota - ps5
Eukaryota - ps6
Excavata/Diaphoretickes - ps7
Diaphoretickes - ps8
Archaeplastida - ps9
Viridiplantae - ps10
Streptophyta - ps11
Charophyceae/Embriophyta - ps12
Embriophyta - ps13
Tracheophyta - ps14
Spermatophyta - ps15
Angiosperms - ps16
Mesangiospermae - ps17
Magnoliids/eudicots - ps18
Eudicots - ps19
Core_eudicots - ps20
Superrosids - ps21
Rosids - ps22
Eurosids - ps23
Malvids - ps24
Sap./Malv./Brassicales - ps25
Malvales/Brassicales - ps26
Brassicales - ps27
Cleomaceae/Brassicaceae - ps28
Brassicaceae - ps29
Camelineae - ps30
Arabidopsis - ps31
Arabidopsis_thaliana - ps32
Phylostratum

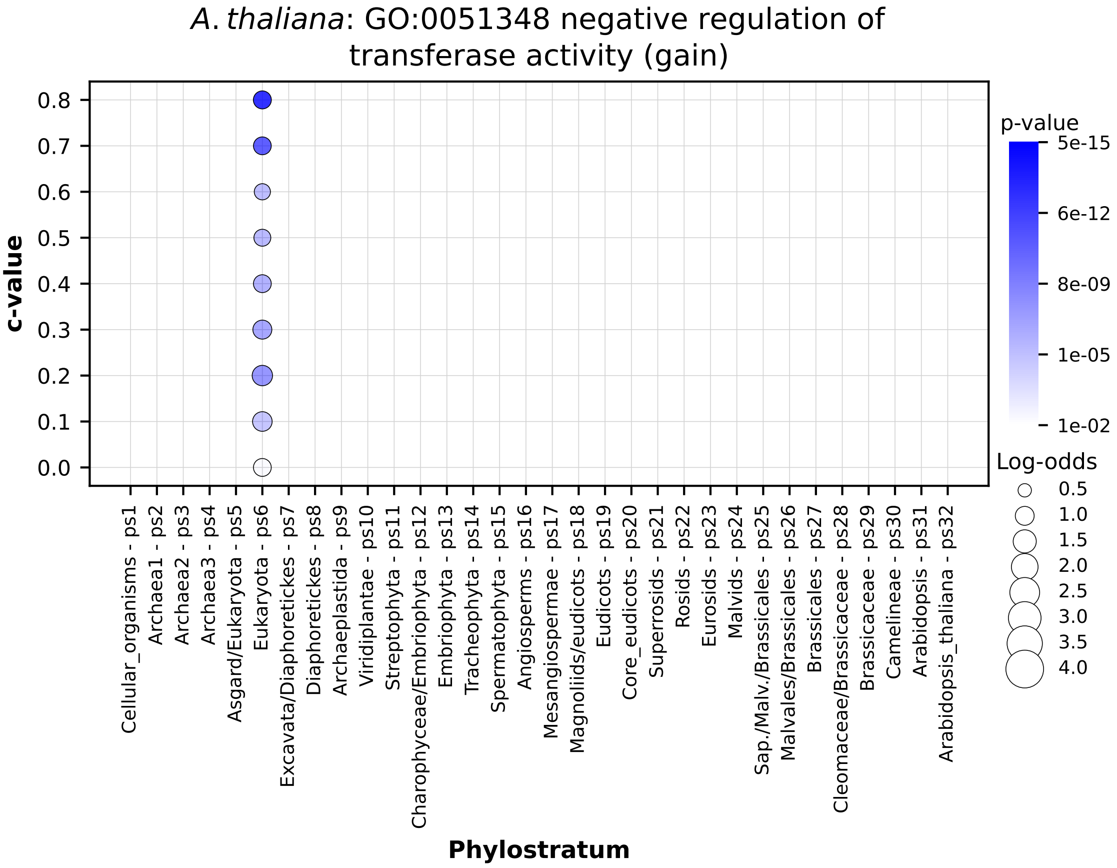

A. thaliana: GO:0051348 negative regulation of transferase activity (gain)
c-value
0.8
0.7
0.6
0.5
0.4
0.3
0.2
0.1
0.0
p-value
5e-15
6e-12
8e-09
1e-05
1e-02
Log-odds
0.5
1.0
1.5
2.0
2.5
3.0
3.5
4.0
Cellular_organisms - ps1
Archaea1 - ps2
Archaea2 - ps3
Archaea3 - ps4
Asgard/Eukaryota - ps5
Eukaryota - ps6
Excavata/Diaphoretickes - ps7
Diaphoretickes - ps8
Archaeplastida - ps9
Viridiplantae - ps10
Streptophyta - ps11
Charophyceae/Embriophyta - ps12
Embriophyta - ps13
Tracheophyta - ps14
Spermatophyta - ps15
Angiosperms - ps16
Mesangiospermae - ps17
Magnoliids/eudicots - ps18
Eudicots - ps19
Core_eudicots - ps20
Superrosids - ps21
Rosids - ps22
Eurosids - ps23
Malvids - ps24
Sap./Malv./Brassicales - ps25
Malvales/Brassicales - ps26
Brassicales - ps27
Cleomaceae/Brassicaceae - ps28
Brassicaceae - ps29
Camelineae - ps30
Arabidopsis - ps31
Arabidopsis_thaliana - ps32
Phylostratum

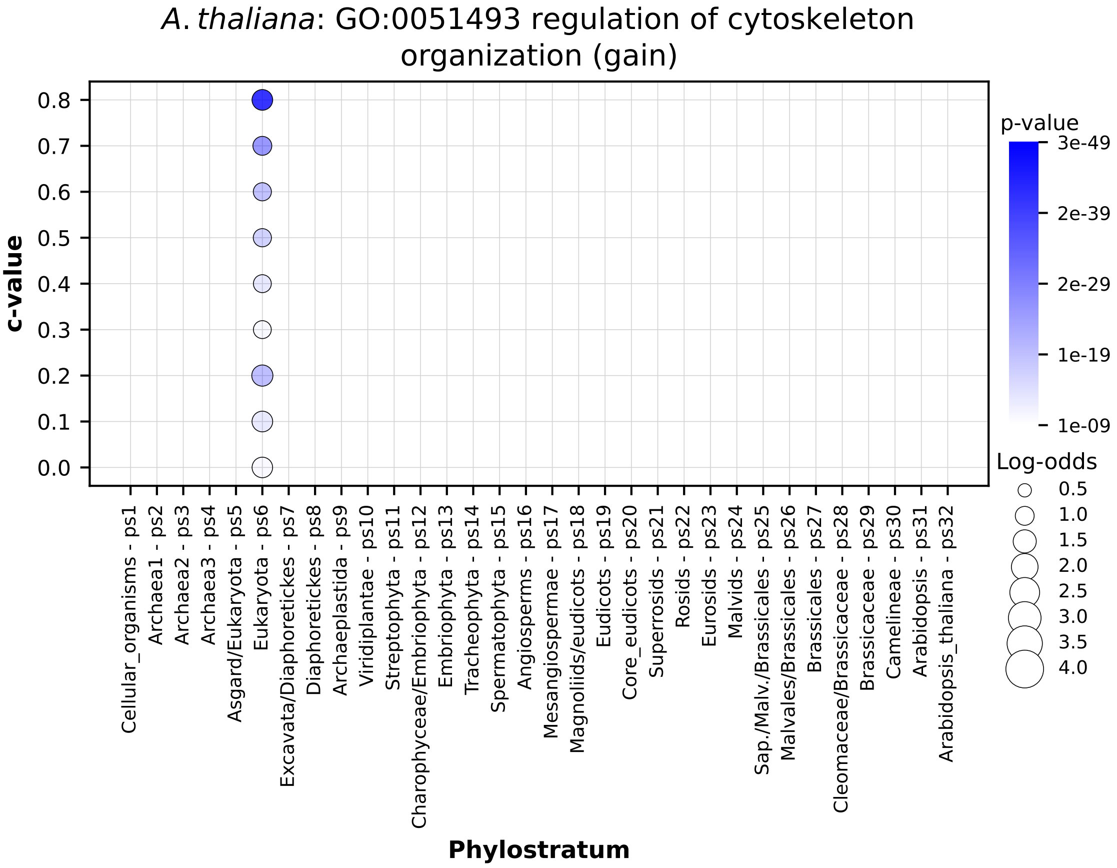

A. thaliana: GO:0051493 regulation of cytoskeleton organization (gain)
c-value
0.8
0.7
0.6
0.5
0.4
0.3
0.2
0.1
0.0
Phylostratum
Cellular_organisms - ps1
Archaea1 - ps2
Archaea2 - ps3
Archaea3 - ps4
Asgard/Eukaryota - ps5
Eukaryota - ps6
Excavata/Diaphoretickes - ps7
Diaphoretickes - ps8
Archaeplastida - ps9
Viridiplantae - ps10
Streptophyta - ps11
Charophyceae/Embriophyta - ps12
Embriophyta - ps13
Tracheophyta - ps14
Spermatophyta - ps15
Angiosperms - ps16
Mesangiospermae - ps17
Magnoliids/eudicots - ps18
Eudicots - ps19
Core_eudicots - ps20
Superrosids - ps21
Rosids - ps22
Eurosids - ps23
Malvids - ps24
Sap./Malv./Brassicales - ps25
Malvales/Brassicales - ps26
Brassicales - ps27
Cleomaceae/Brassicaceae - ps28
Brassicaceae - ps29
Camelineae - ps30
Arabidopsis - ps31
Arabidopsis_thaliana - ps32
p-value
3e-49
2e-39
2e-29
1e-19
1e-09
Log-odds
0.5
1.0
1.5
2.0
2.5
3.0
3.5
4.0

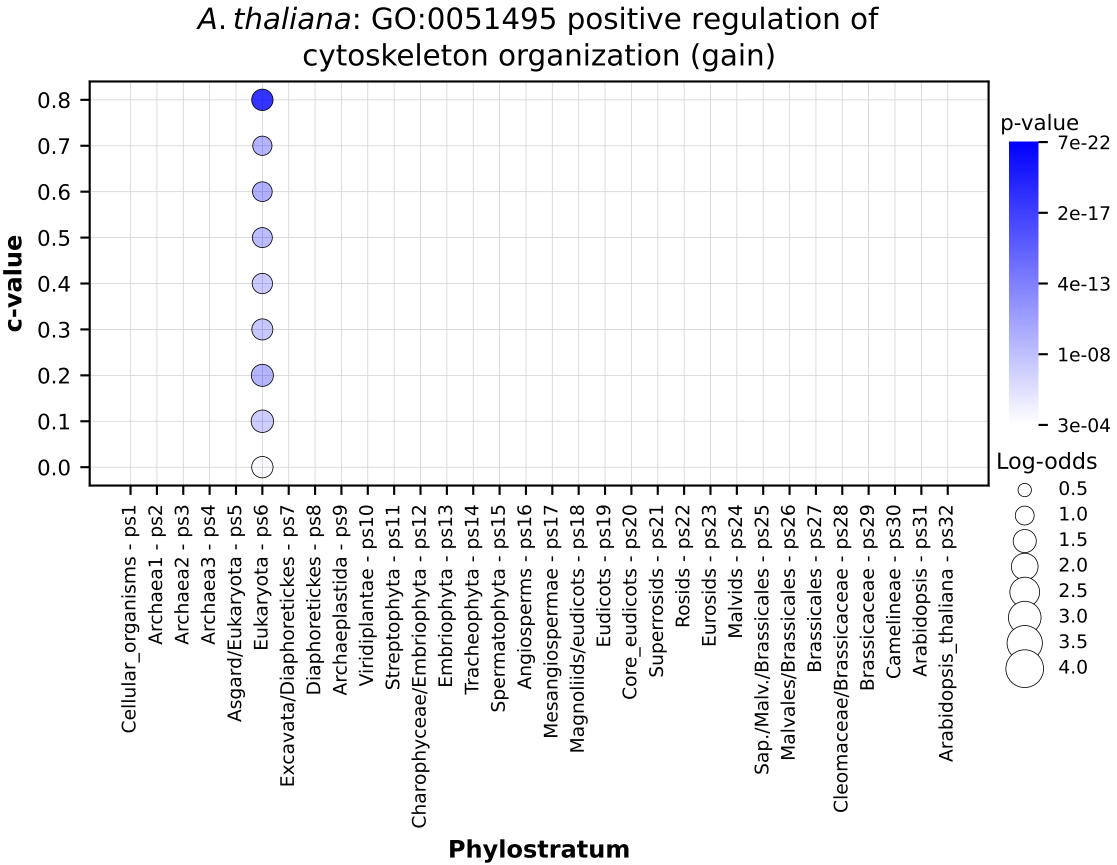

A. thaliana: GO:0051495 positive regulation of cytoskeleton organization (gain)
c-value
0.8
0.7
0.6
0.5
0.4
0.3
0.2
0.1
0.0
p-value
7e-22
2e-17
4e-13
1e-08
3e-04
Log-odds
0.5
1.0
1.5
2.0
2.5
3.0
3.5
4.0
Cellular_organisms - ps1
Archaea1 - ps2
Archaea2 - ps3
Archaea3 - ps4
Asgard/Eukaryota - ps5
Eukaryota - ps6
Excavata/Diaphoretickes - ps7
Diaphoretickes - ps8
Archaeplastida - ps9
Viridiplantae - ps10
Streptophyta - ps11
Charophyceae/Embriophyta - ps12
Embriophyta - ps13
Tracheophyta - ps14
Spermatophyta - ps15
Angiosperms - ps16
Mesangiospermae - ps17
Magnoliids/eudicots - ps18
Eudicots - ps19
Core_eudicots - ps20
Superrosids - ps21
Rosids - ps22
Eurosids - ps23
Malvids - ps24
Sap./Malv./Brassicales - ps25
Malvales/Brassicales - ps26
Brassicales - ps27
Cleomaceae/Brassicaceae - ps28
Brassicaceae - ps29
Camelineae - ps30
Arabidopsis - ps31
Arabidopsis_thaliana - ps32
Phylostratum

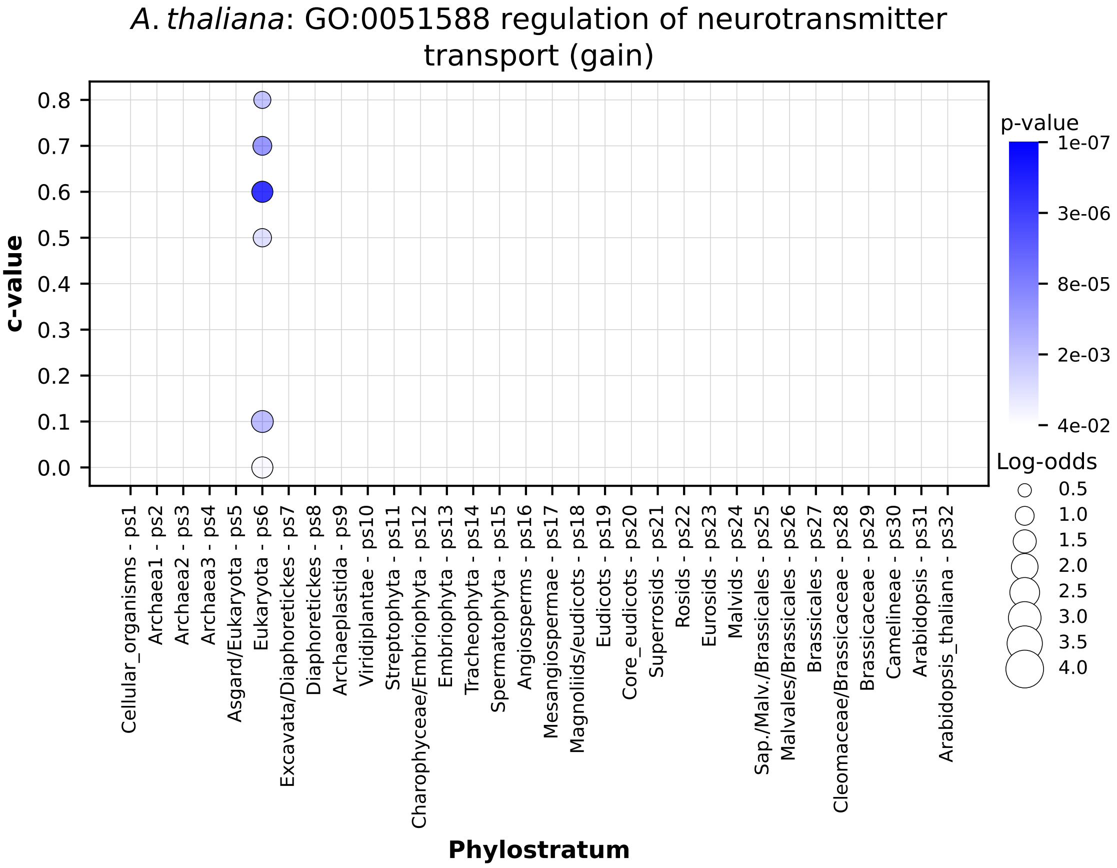

A. thaliana: GO:0051588 regulation of neurotransmitter transport (gain)
c-value
0.0
0.1
0.2
0.3
0.4
0.5
0.6
0.7
0.8
p-value
1e-07
3e-06
8e-05
2e-03
4e-02
Log-odds
0.5
1.0
1.5
2.0
2.5
3.0
3.5
4.0
Cellular_organisms - ps1
Archaea1 - ps2
Archaea2 - ps3
Archaea3 - ps4
Asgard/Eukaryota - ps5
Eukaryota - ps6
Excavata/Diaphoretickes - ps7
Diaphoretickes - ps8
Archaeplastida - ps9
Viridiplantae - ps10
Streptophyta - ps11
Charophyceae/Embriophyta - ps12
Embriophyta - ps13
Tracheophyta - ps14
Spermatophyta - ps15
Angiosperms - ps16
Mesangiospermae - ps17
Magnoliids/eudicots - ps18
Eudicots - ps19
Core_eudicots - ps20
Superrosids - ps21
Rosids - ps22
Eurosids - ps23
Malvids - ps24
Sap./Malv./Brassicales - ps25
Malvales/Brassicales - ps26
Brassicales - ps27
Cleomaceae/Brassicaceae - ps28
Brassicaceae - ps29
Camelineae - ps30
Arabidopsis - ps31
Arabidopsis_thaliana - ps32
Phylostratum

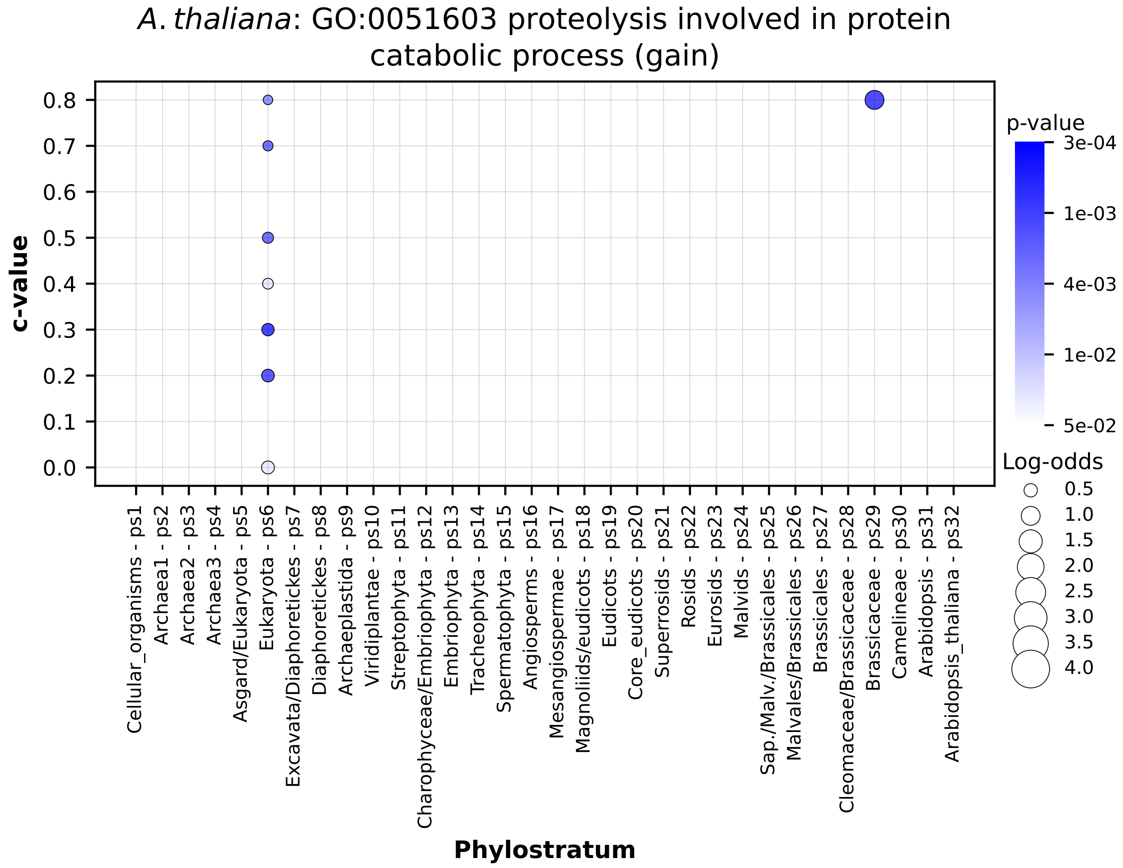
A. thaliana: GO:0051603 proteolysis involved in protein catabolic process (gain)
c-value
0.8
0.7
0.6
0.5
0.4
0.3
0.2
0.1
0.0
p-value
3e-04
1e-03
4e-03
1e-02
5e-02
Log-odds
0.5
1.0
1.5
2.0
2.5
3.0
3.5
4.0
Cellular_organisms - ps1
Archaea1 - ps2
Archaea2 - ps3
Archaea3 - ps4
Asgard/Eukaryota - ps5
Eukaryota - ps6
Excavata/Diaphoretickes - ps7
Diaphoretickes - ps8
Archaeplastida - ps9
Viridiplantae - ps10
Streptophyta - ps11
Charophyceae/Embriophyta - ps12
Embriophyta - ps13
Tracheophyta - ps14
Spermatophyta - ps15
Angiosperms - ps16
Mesangiospermae - ps17
Magnoliids/eudicots - ps18
Eudicots - ps19
Core_eudicots - ps20
Superrosids - ps21
Rosids - ps22
Eurosids - ps23
Malvids - ps24
Sap./Malv./Brassicales - ps25
Malvales/Brassicales - ps26
Brassicales - ps27
Cleomaceae/Brassicaceae - ps28
Brassicaceae - ps29
Camelineae - ps30
Arabidopsis - ps31
Arabidopsis_thaliana - ps32
Phylostratum

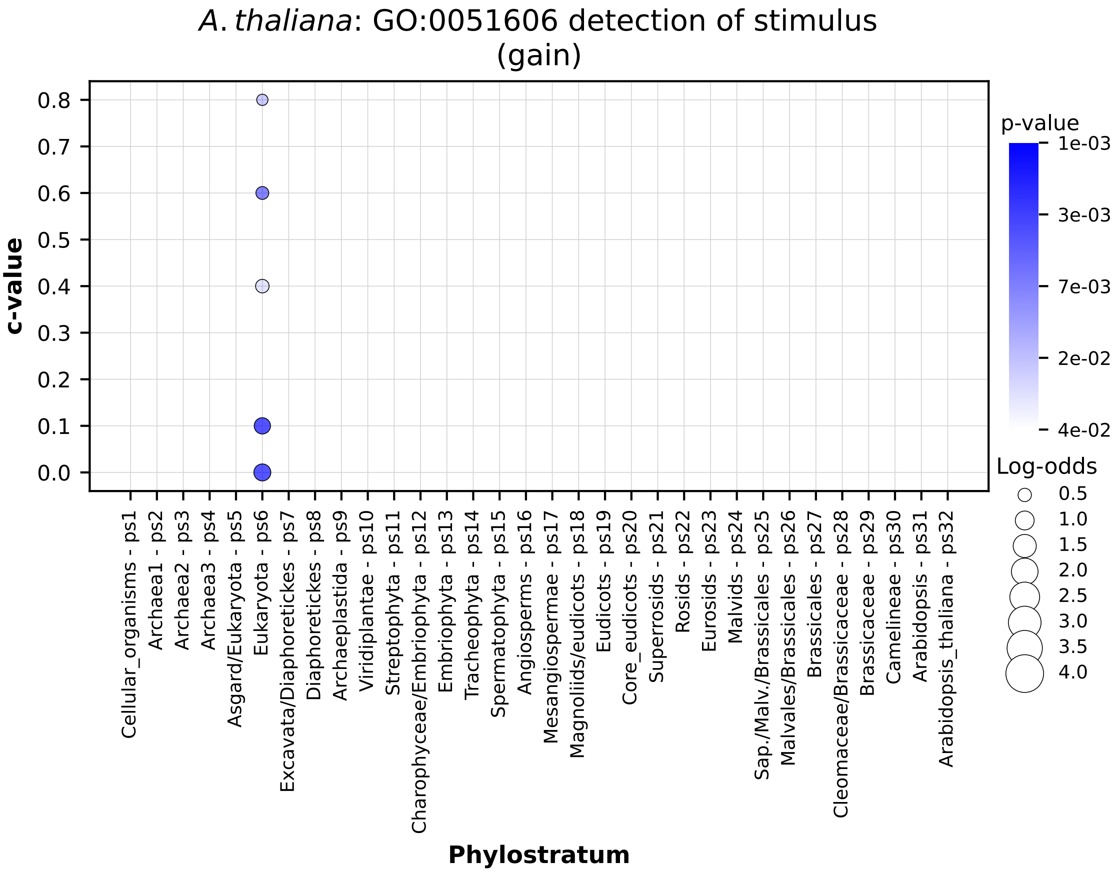

A. thaliana: GO:0051606 detection of stimulus
(gain)
c-value
0.8
0.7
0.6
0.5
0.4
0.3
0.2
0.1
0.0
p-value
1e-03
3e-03
7e-03
2e-02
4e-02
Log-odds
0.5
1.0
1.5
2.0
2.5
3.0
3.5
4.0
Cellular_organisms - ps1
Archaea1 - ps2
Archaea2 - ps3
Archaea3 - ps4
Asgard/Eukaryota - ps5
Eukaryota - ps6
Excavata/Diaphoretickes - ps7
Diaphoretickes - ps8
Archaeplastida - ps9
Viridiplantae - ps10
Streptophyta - ps11
Charophyceae/Embriophyta - ps12
Embriophyta - ps13
Tracheophyta - ps14
Spermatophyta - ps15
Angiosperms - ps16
Mesangiospermae - ps17
Magnoliids/eudicots - ps18
Eudicots - ps19
Core_eudicots - ps20
Superrosids - ps21
Rosids - ps22
Eurosids - ps23
Malvids - ps24
Sap./Malv./Brassicales - ps25
Malvales/Brassicales - ps26
Brassicales - ps27
Cleomaceae/Brassicaceae - ps28
Brassicaceae - ps29
Camelineae - ps30
Arabidopsis - ps31
Arabidopsis_thaliana - ps32
Phylostratum

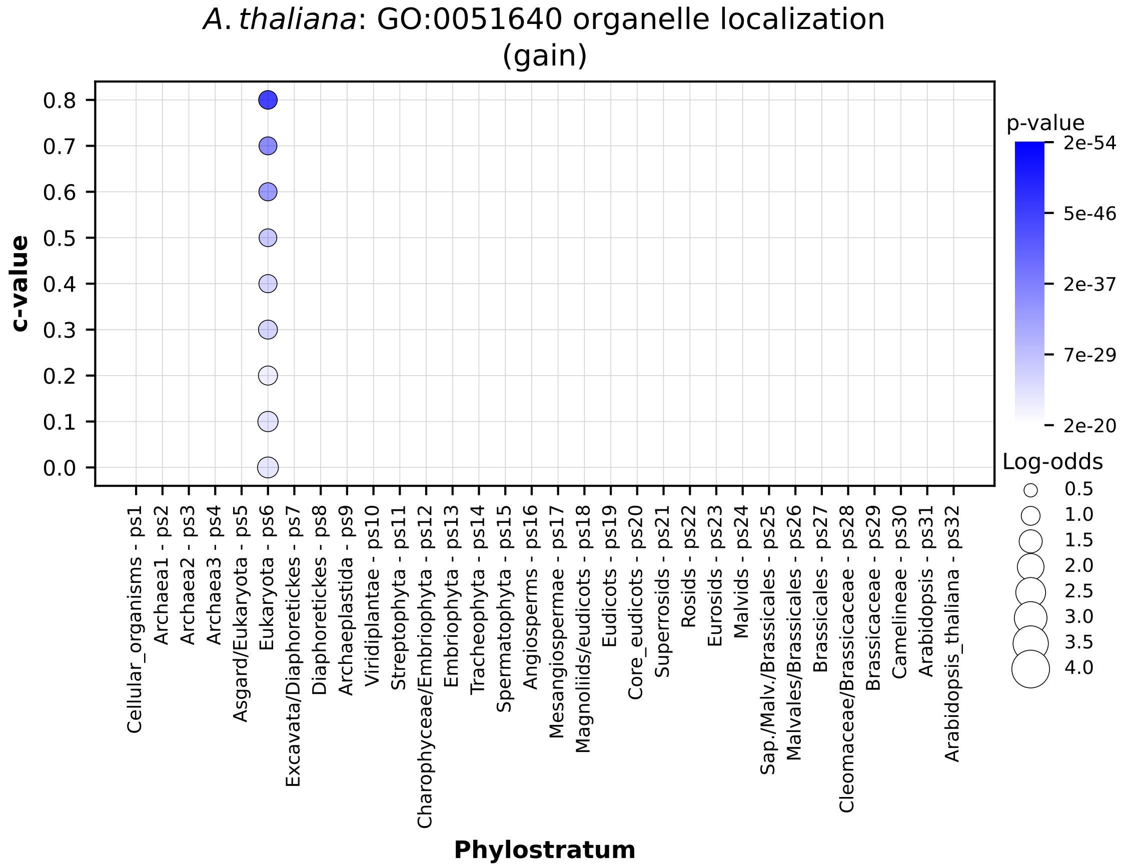

A. thaliana: GO:0051640 organelle localization
(gain)
c-value
0.8
0.7
0.6
0.5
0.4
0.3
0.2
0.1
0.0
p-value
2e-54
5e-46
2e-37
7e-29
2e-20
Log-odds
0.5
1.0
1.5
2.0
2.5
3.0
3.5
4.0
Cellular_organisms - ps1
Archaea1 - ps2
Archaea2 - ps3
Archaea3 - ps4
Asgard/Eukaryota - ps5
Eukaryota - ps6
Excavata/Diaphoretickes - ps7
Diaphoretickes - ps8
Archaeplastida - ps9
Viridiplantae - ps10
Streptophyta - ps11
Charophyceae/Embriophyta - ps12
Embriophyta - ps13
Tracheophyta - ps14
Spermatophyta - ps15
Angiosperms - ps16
Mesangiospermae - ps17
Magnoliids/eudicots - ps18
Eudicots - ps19
Core_eudicots - ps20
Superrosids - ps21
Rosids - ps22
Eurosids - ps23
Malvids - ps24
Sap./Malv./Brassicales - ps25
Malvales/Brassicales - ps26
Brassicales - ps27
Cleomaceae/Brassicaceae - ps28
Brassicaceae - ps29
Camelineae - ps30
Arabidopsis - ps31
Arabidopsis_thaliana - ps32
Phylostratum

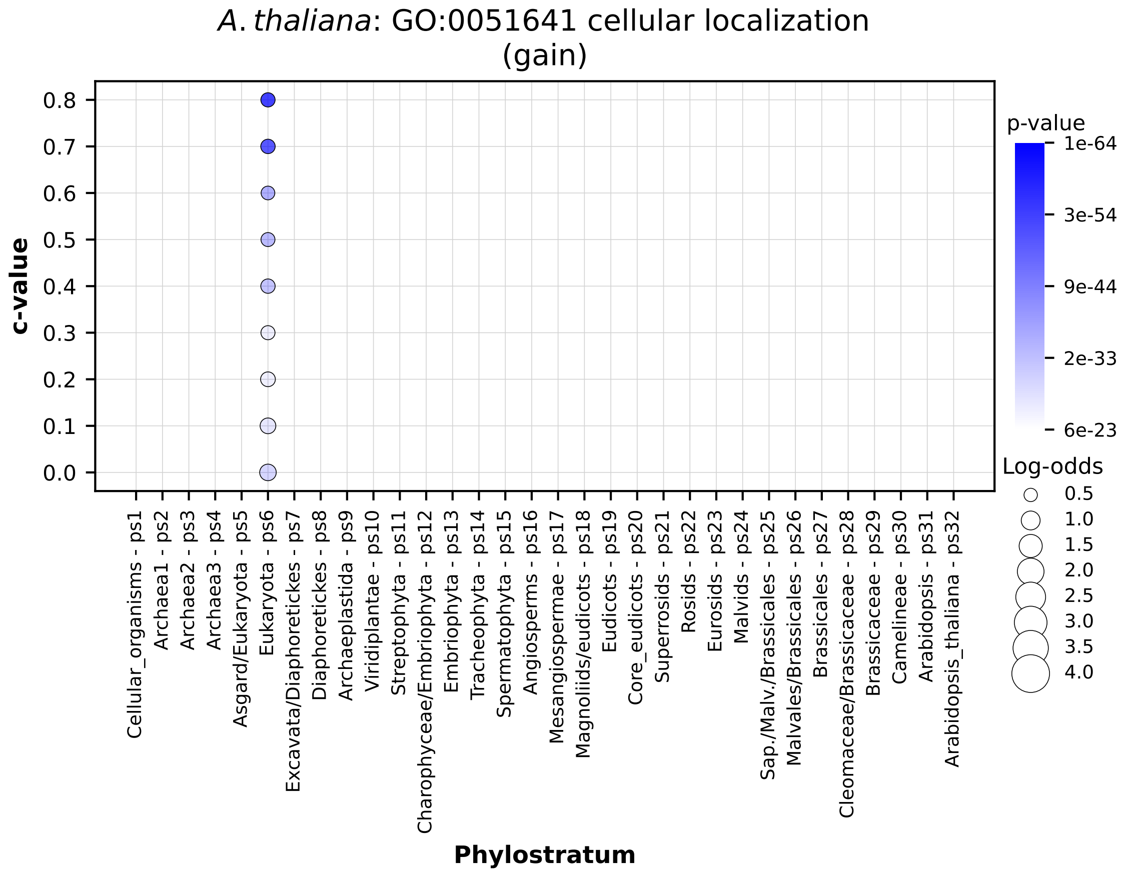

A. thaliana: GO:0051641 cellular localization
(gain)
c-value
0.8
0.7
0.6
0.5
0.4
0.3
0.2
0.1
0.0
p-value
1e-64
3e-54
9e-44
2e-33
6e-23
Log-odds
0.5
1.0
1.5
2.0
2.5
3.0
3.5
4.0
Cellular_organisms - ps1
Archaea1 - ps2
Archaea2 - ps3
Archaea3 - ps4
Asgard/Eukaryota - ps5
Eukaryota - ps6
Excavata/Diaphoretickes - ps7
Diaphoretickes - ps8
Archaeplastida - ps9
Viridiplantae - ps10
Streptophyta - ps11
Charophyceae/Embriophyta - ps12
Embriophyta - ps13
Tracheophyta - ps14
Spermatophyta - ps15
Angiosperms - ps16
Mesangiospermae - ps17
Magnoliids/eudicots - ps18
Eudicots - ps19
Core_eudicots - ps20
Superrosids - ps21
Rosids - ps22
Eurosids - ps23
Malvids - ps24
Sap./Malv./Brassicales - ps25
Malvales/Brassicales - ps26
Brassicales - ps27
Cleomaceae/Brassicaceae - ps28
Brassicaceae - ps29
Camelineae - ps30
Arabidopsis - ps31
Arabidopsis_thaliana - ps32
Phylostratum

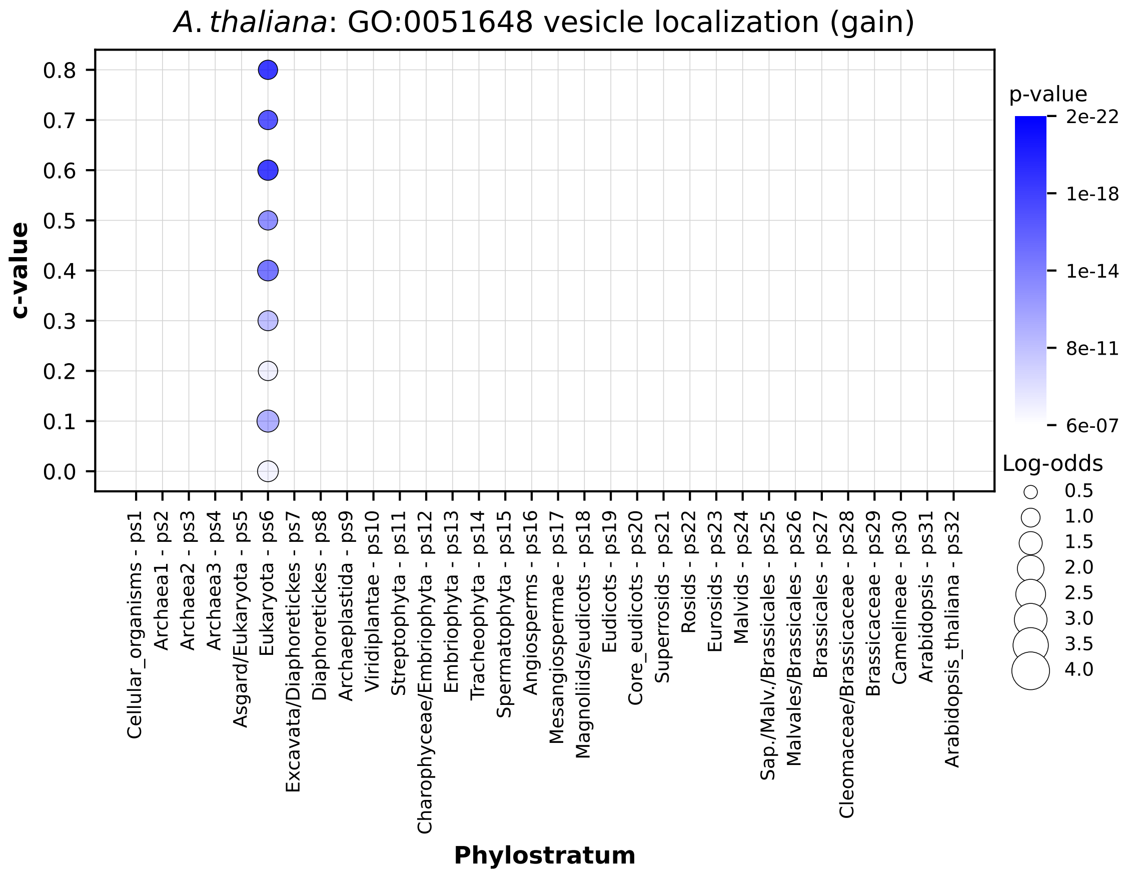

A. thaliana: GO:0051648 vesicle localization (gain)
c-value
0.8
0.7
0.6
0.5
0.4
0.3
0.2
0.1
0.0
p-value
2e-22
1e-18
1e-14
8e-11
6e-07
Log-odds
0.5
1.0
1.5
2.0
2.5
3.0
3.5
4.0
Cellular_organisms - ps1
Archaea1 - ps2
Archaea2 - ps3
Archaea3 - ps4
Asgard/Eukaryota - ps5
Eukaryota - ps6
Excavata/Diaphoretickes - ps7
Diaphoretickes - ps8
Archaeplastida - ps9
Viridiplantae - ps10
Streptophyta - ps11
Charophyceae/Embriophyta - ps12
Embriophyta - ps13
Tracheophyta - ps14
Spermatophyta - ps15
Angiosperms - ps16
Mesangiospermae - ps17
Magnoliids/eudicots - ps18
Eudicots - ps19
Core_eudicots - ps20
Superrosids - ps21
Rosids - ps22
Eurosids - ps23
Malvids - ps24
Sap./Malv./Brassicales - ps25
Malvales/Brassicales - ps26
Brassicales - ps27
Cleomaceae/Brassicaceae - ps28
Brassicaceae - ps29
Camelineae - ps30
Arabidopsis - ps31
Arabidopsis_thaliana - ps32
Phylostratum

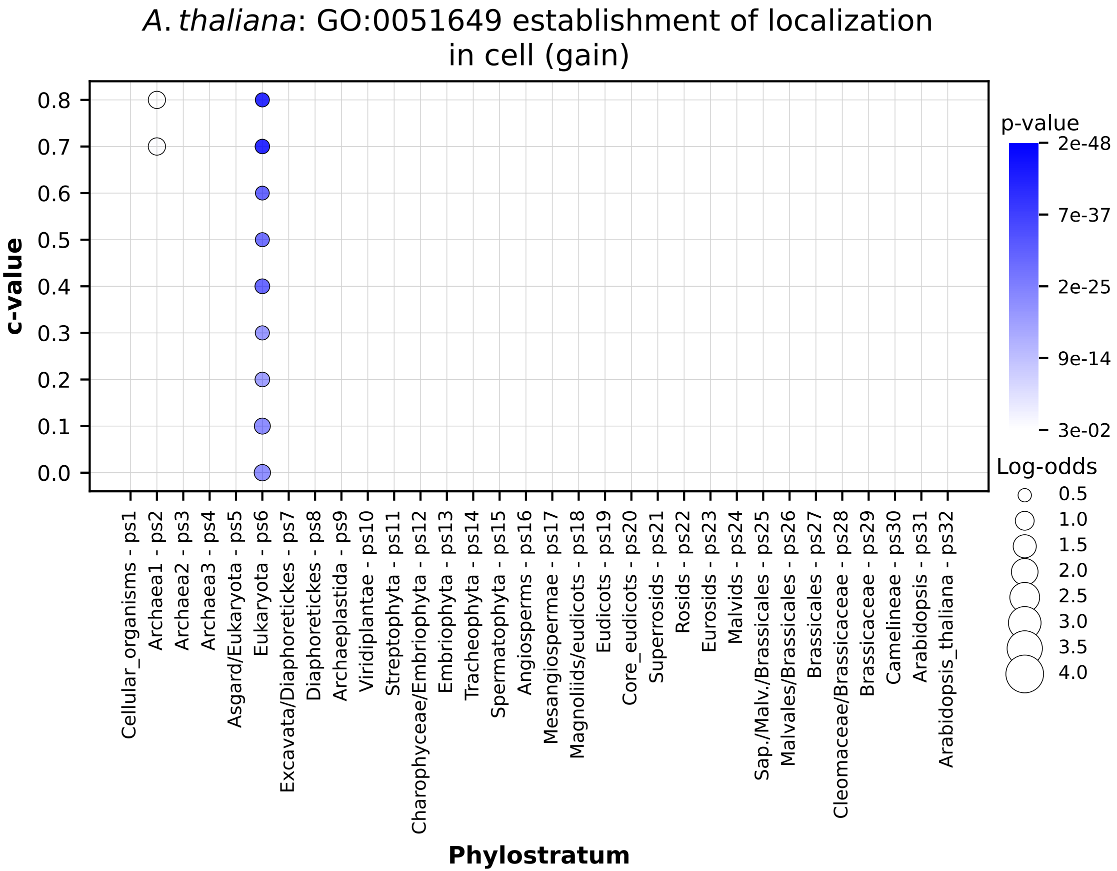

A. thaliana: GO:0051649 establishment of localization in cell (gain)
c-value
0.8
0.7
0.6
0.5
0.4
0.3
0.2
0.1
0.0
Phylostratum
Cellular_organisms - ps1
Archaea1 - ps2
Archaea2 - ps3
Archaea3 - ps4
Asgard/Eukaryota - ps5
Eukaryota - ps6
Excavata/Diaphoretickes - ps7
Diaphoretickes - ps8
Archaeplastida - ps9
Viridiplantae - ps10
Streptophyta - ps11
Charophyceae/Embriophyta - ps12
Embriophyta - ps13
Tracheophyta - ps14
Spermatophyta - ps15
Angiosperms - ps16
Mesangiospermae - ps17
Magnoliids/eudicots - ps18
Eudicots - ps19
Core_eudicots - ps20
Superrosids - ps21
Rosids - ps22
Eurosids - ps23
Malvids - ps24
Sap./Malv./Brassicales - ps25
Malvales/Brassicales - ps26
Brassicales - ps27
Cleomaceae/Brassicaceae - ps28
Brassicaceae - ps29
Camelineae - ps30
Arabidopsis - ps31
Arabidopsis_thaliana - ps32
p-value
2e-48
7e-37
2e-25
9e-14
3e-02
Log-odds
0.5
1.0
1.5
2.0
2.5
3.0
3.5
4.0

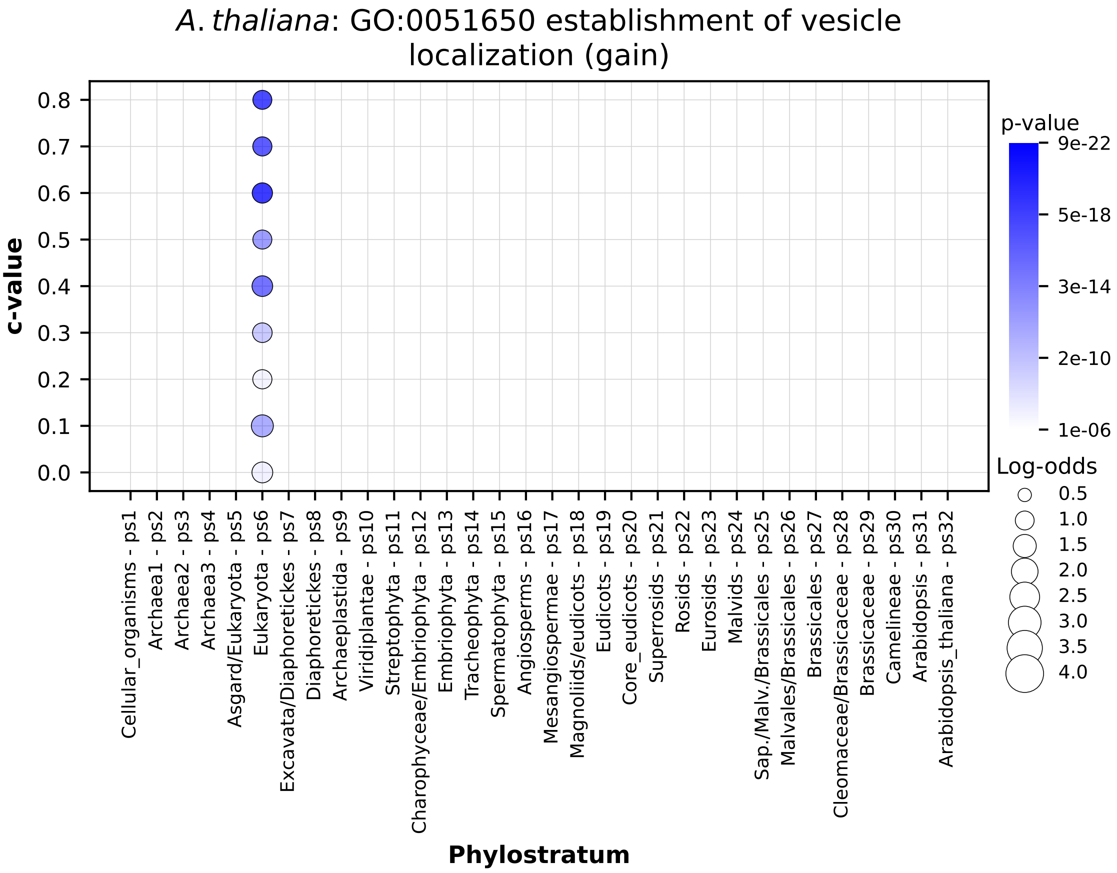

A. thaliana: GO:0051650 establishment of vesicle localization (gain)
c-value
0.8
0.7
0.6
0.5
0.4
0.3
0.2
0.1
0.0
p-value
9e-22
5e-18
3e-14
2e-10
1e-06
Log-odds
0.5
1.0
1.5
2.0
2.5
3.0
3.5
4.0
Cellular_organisms - ps1
Archaea1 - ps2
Archaea2 - ps3
Archaea3 - ps4
Asgard/Eukaryota - ps5
Eukaryota - ps6
Excavata/Diaphoretickes - ps7
Diaphoretickes - ps8
Archaeplastida - ps9
Viridiplantae - ps10
Streptophyta - ps11
Charophyceae/Embriophyta - ps12
Embriophyta - ps13
Tracheophyta - ps14
Spermatophyta - ps15
Angiosperms - ps16
Mesangiospermae - ps17
Magnoliids/eudicots - ps18
Eudicots - ps19
Core_eudicots - ps20
Superrosids - ps21
Rosids - ps22
Eurosids - ps23
Malvids - ps24
Sap./Malv./Brassicales - ps25
Malvales/Brassicales - ps26
Brassicales - ps27
Cleomaceae/Brassicaceae - ps28
Brassicaceae - ps29
Camelineae - ps30
Arabidopsis - ps31
Arabidopsis_thaliana - ps32
Phylostratum

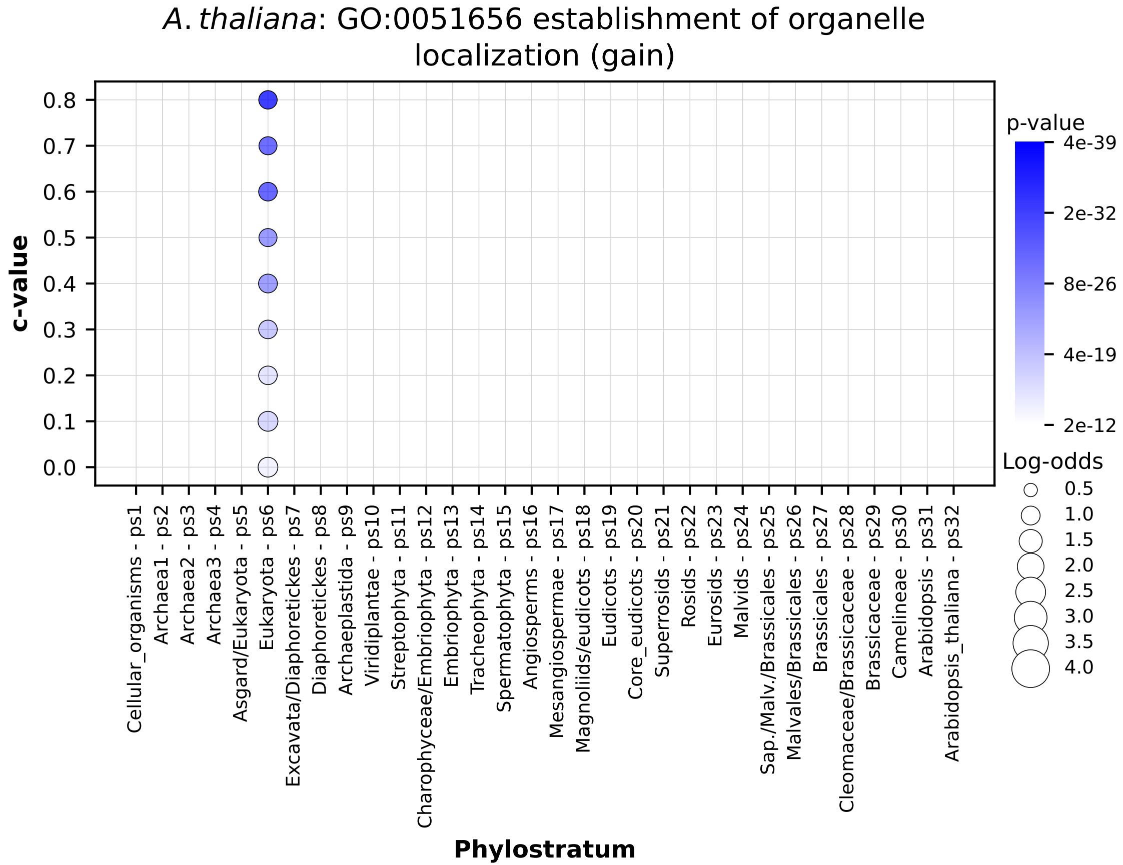

A. thaliana: GO:0051656 establishment of organelle localization (gain)
c-value
0.8
0.7
0.6
0.5
0.4
0.3
0.2
0.1
0.0
p-value
4e-39
2e-32
8e-26
4e-19
2e-12
Log-odds
0.5
1.0
1.5
2.0
2.5
3.0
3.5
4.0
Cellular_organisms - ps1
Archaea1 - ps2
Archaea2 - ps3
Archaea3 - ps4
Asgard/Eukaryota - ps5
Eukaryota - ps6
Excavata/Diaphoretickes - ps7
Diaphoretickes - ps8
Archaeplastida - ps9
Viridiplantae - ps10
Streptophyta - ps11
Charophyceae/Embriophyta - ps12
Embriophyta - ps13
Tracheophyta - ps14
Spermatophyta - ps15
Angiosperms - ps16
Mesangiospermae - ps17
Magnoliids/eudicots - ps18
Eudicots - ps19
Core_eudicots - ps20
Superrosids - ps21
Rosids - ps22
Eurosids - ps23
Malvids - ps24
Sap./Malv./Brassicales - ps25
Malvales/Brassicales - ps26
Brassicales - ps27
Cleomaceae/Brassicaceae - ps28
Brassicaceae - ps29
Camelineae - ps30
Arabidopsis - ps31
Arabidopsis_thaliana - ps32
Phylostratum

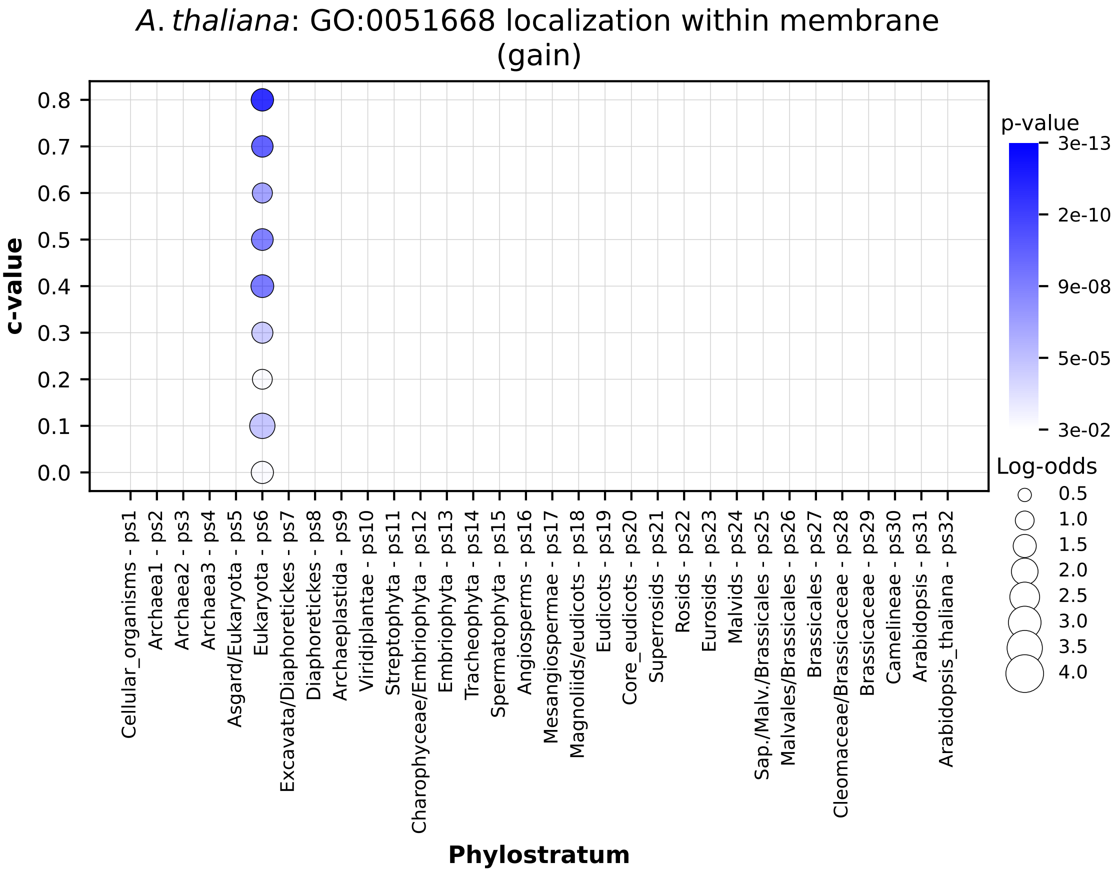

A. thaliana: GO:0051668 localization within membrane
(gain)
c-value
0.8
0.7
0.6
0.5
0.4
0.3
0.2
0.1
0.0
p-value
3e-13
2e-10
9e-08
5e-05
3e-02
Log-odds
0.5
1.0
1.5
2.0
2.5
3.0
3.5
4.0
Cellular_organisms - ps1
Archaea1 - ps2
Archaea2 - ps3
Archaea3 - ps4
Asgard/Eukaryota - ps5
Eukaryota - ps6
Excavata/Diaphoretickes - ps7
Diaphoretickes - ps8
Archaeplastida - ps9
Viridiplantae - ps10
Streptophyta - ps11
Charophyceae/Embriophyta - ps12
Embriophyta - ps13
Tracheophyta - ps14
Spermatophyta - ps15
Angiosperms - ps16
Mesangiospermae - ps17
Magnoliids/eudicots - ps18
Eudicots - ps19
Core_eudicots - ps20
Superrosids - ps21
Rosids - ps22
Eurosids - ps23
Malvids - ps24
Sap./Malv./Brassicales - ps25
Malvales/Brassicales - ps26
Brassicales - ps27
Cleomaceae/Brassicaceae - ps28
Brassicaceae - ps29
Camelineae - ps30
Arabidopsis - ps31
Arabidopsis_thaliana - ps32
Phylostratum

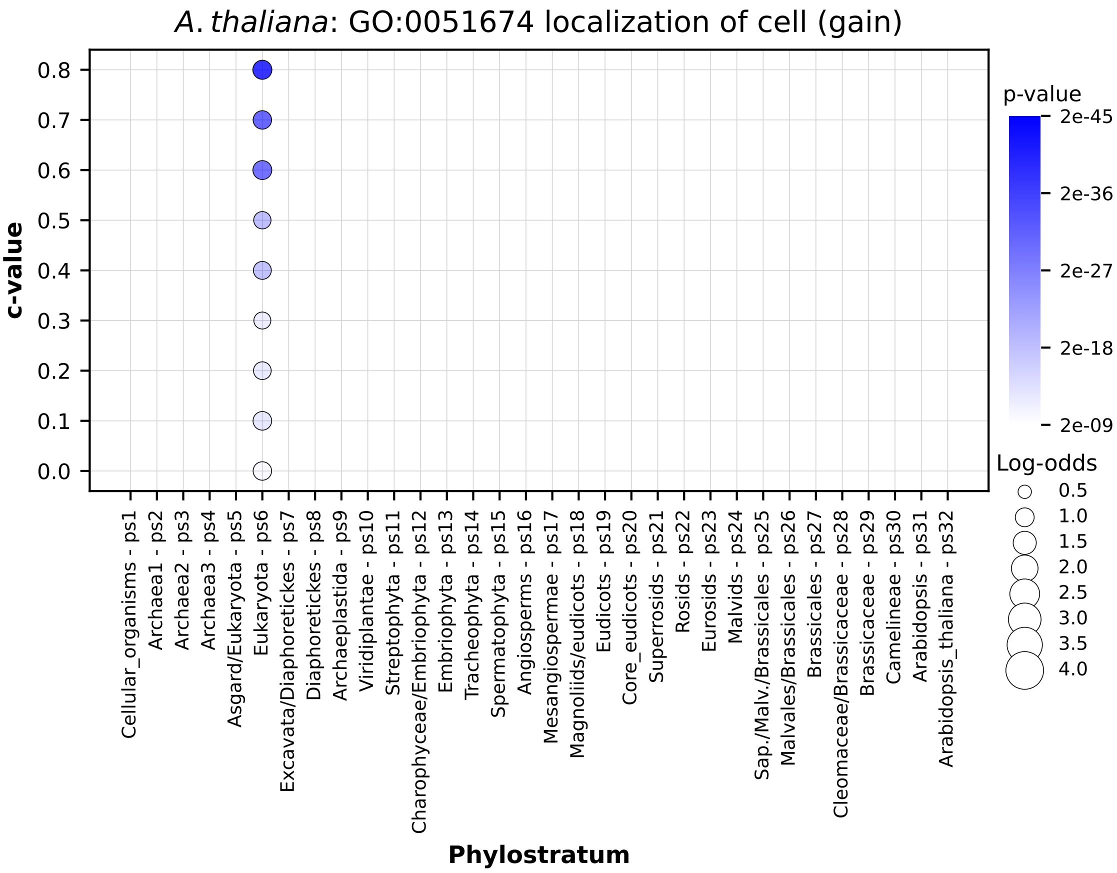
A. thaliana: GO:0051674 localization of cell (gain)
c-value
0.0
0.1
0.2
0.3
0.4
0.5
0.6
0.7
0.8
Phylostratum
Cellular_organisms - ps1
Archaea1 - ps2
Archaea2 - ps3
Archaea3 - ps4
Asgard/Eukaryota - ps5
Eukaryota - ps6
Excavata/Diaphoretickes - ps7
Diaphoretickes - ps8
Archaeplastida - ps9
Viridiplantae - ps10
Streptophyta - ps11
Charophyceae/Embriophyta - ps12
Embriophyta - ps13
Tracheophyta - ps14
Spermatophyta - ps15
Angiosperms - ps16
Mesangiospermae - ps17
Magnoliids/eudicots - ps18
Eudicots - ps19
Core_eudicots - ps20
Superrosids - ps21
Rosids - ps22
Eurosids - ps23
Malvids - ps24
Sap./Malv./Brassicales - ps25
Malvales/Brassicales - ps26
Brassicales - ps27
Cleomaceae/Brassicaceae - ps28
Brassicaceae - ps29
Camelineae - ps30
Arabidopsis - ps31
Arabidopsis_thaliana - ps32
p-value
2e-45
2e-36
2e-27
2e-18
2e-09
Log-odds
0.5
1.0
1.5
2.0
2.5
3.0
3.5
4.0

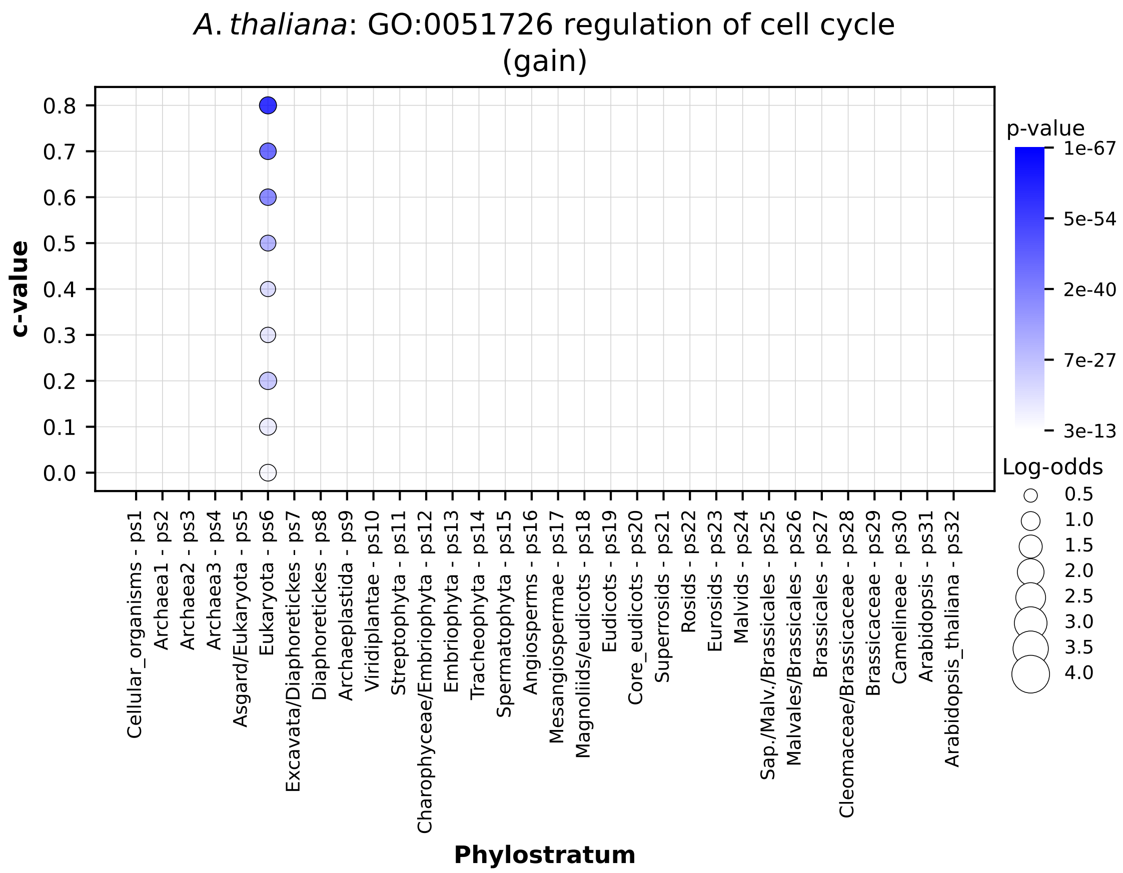

A. thaliana: GO:0051726 regulation of cell cycle
(gain)
c-value
0.8
0.7
0.6
0.5
0.4
0.3
0.2
0.1
0.0
p-value
1e-67
5e-54
2e-40
7e-27
3e-13
Log-odds
0.5
1.0
1.5
2.0
2.5
3.0
3.5
4.0
Cellular_organisms - ps1
Archaea1 - ps2
Archaea2 - ps3
Archaea3 - ps4
Asgard/Eukaryota - ps5
Eukaryota - ps6
Excavata/Diaphoretickes - ps7
Diaphoretickes - ps8
Archaeplastida - ps9
Viridiplantae - ps10
Streptophyta - ps11
Charophyceae/Embriophyta - ps12
Embriophyta - ps13
Tracheophyta - ps14
Spermatophyta - ps15
Angiosperms - ps16
Mesangiospermae - ps17
Magnoliids/eudicots - ps18
Eudicots - ps19
Core_eudicots - ps20
Superrosids - ps21
Rosids - ps22
Eurosids - ps23
Malvids - ps24
Sap./Malv./Brassicales - ps25
Malvales/Brassicales - ps26
Brassicales - ps27
Cleomaceae/Brassicaceae - ps28
Brassicaceae - ps29
Camelineae - ps30
Arabidopsis - ps31
Arabidopsis_thaliana - ps32
Phylostratum

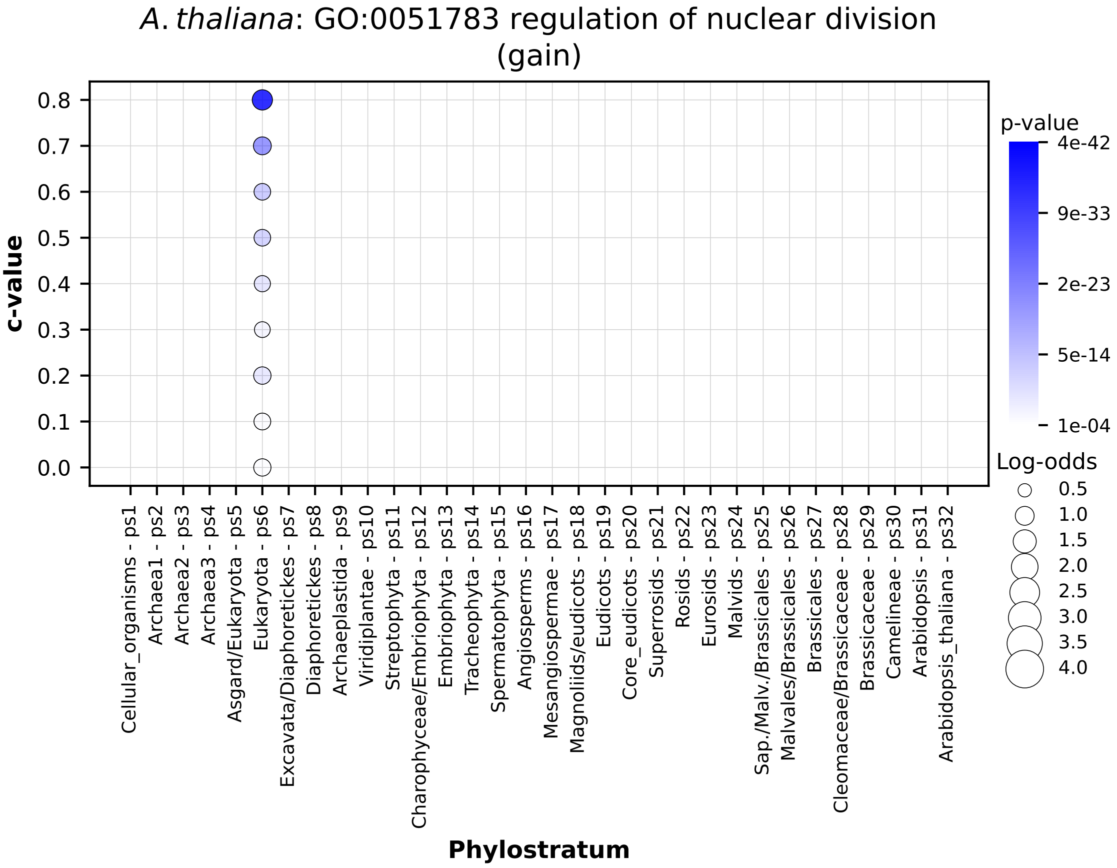

A. thaliana: GO:0051783 regulation of nuclear division
(gain)
c-value
0.8
0.7
0.6
0.5
0.4
0.3
0.2
0.1
0.0
p-value
4e-42
9e-33
2e-23
5e-14
1e-04
Log-odds
0.5
1.0
1.5
2.0
2.5
3.0
3.5
4.0
Cellular_organisms - ps1
Archaea1 - ps2
Archaea2 - ps3
Archaea3 - ps4
Asgard/Eukaryota - ps5
Eukaryota - ps6
Excavata/Diaphoretickes - ps7
Diaphoretickes - ps8
Archaeplastida - ps9
Viridiplantae - ps10
Streptophyta - ps11
Charophyceae/Embriophyta - ps12
Embriophyta - ps13
Tracheophyta - ps14
Spermatophyta - ps15
Angiosperms - ps16
Mesangiospermae - ps17
Magnoliids/eudicots - ps18
Eudicots - ps19
Core_eudicots - ps20
Superrosids - ps21
Rosids - ps22
Eurosids - ps23
Malvids - ps24
Sap./Malv./Brassicales - ps25
Malvales/Brassicales - ps26
Brassicales - ps27
Cleomaceae/Brassicaceae - ps28
Brassicaceae - ps29
Camelineae - ps30
Arabidopsis - ps31
Arabidopsis_thaliana - ps32
Phylostratum

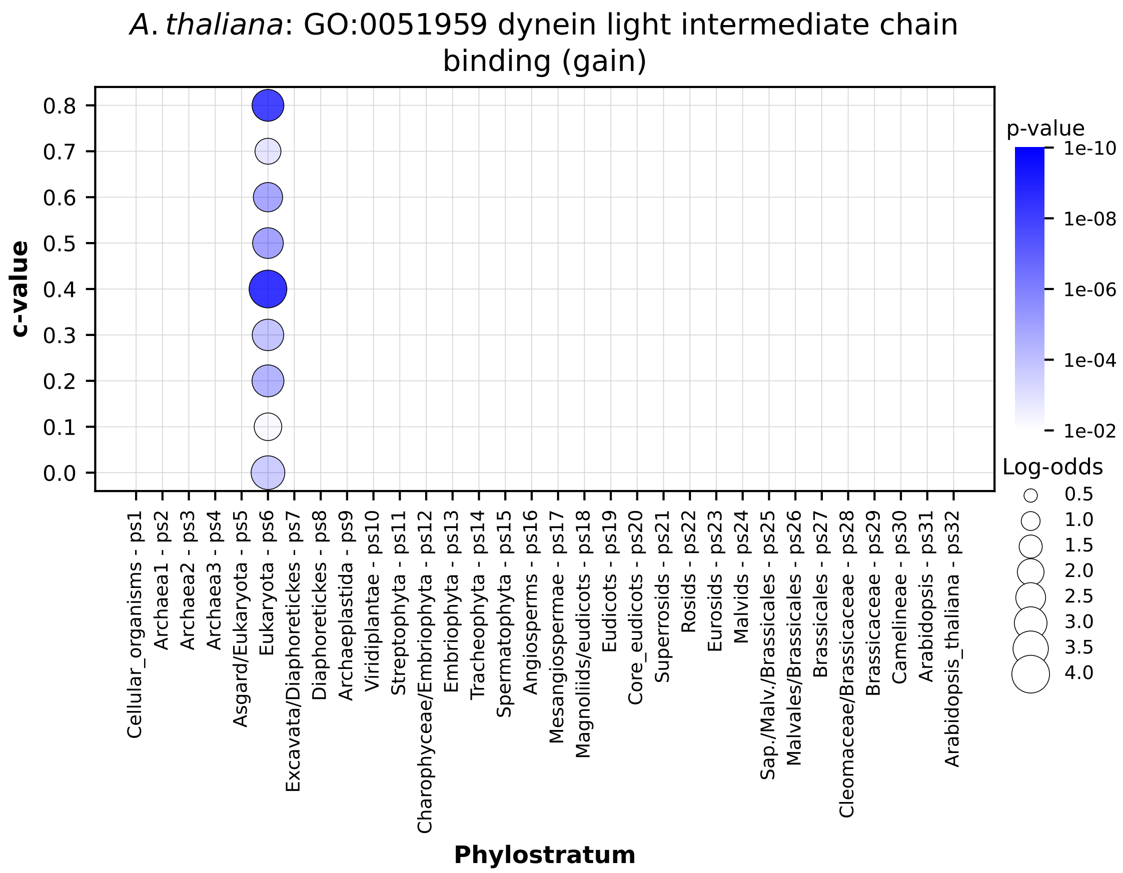

A. thaliana: GO:0051959 dynein light intermediate chain binding (gain)
c-value
0.8
0.7
0.6
0.5
0.4
0.3
0.2
0.1
0.0
p-value
1e-10
1e-08
1e-06
1e-04
1e-02
Log-odds
0.5
1.0
1.5
2.0
2.5
3.0
3.5
4.0
Cellular_organisms - ps1
Archaea1 - ps2
Archaea2 - ps3
Archaea3 - ps4
Asgard/Eukaryota - ps5
Eukaryota - ps6
Excavata/Diaphoretickes - ps7
Diaphoretickes - ps8
Archaeplastida - ps9
Viridiplantae - ps10
Streptophyta - ps11
Charophyceae/Embriophyta - ps12
Embriophyta - ps13
Tracheophyta - ps14
Spermatophyta - ps15
Angiosperms - ps16
Mesangiospermae - ps17
Magnoliids/eudicots - ps18
Eudicots - ps19
Core_eudicots - ps20
Superrosids - ps21
Rosids - ps22
Eurosids - ps23
Malvids - ps24
Sap./Malv./Brassicales - ps25
Malvales/Brassicales - ps26
Brassicales - ps27
Cleomaceae/Brassicaceae - ps28
Brassicaceae - ps29
Camelineae - ps30
Arabidopsis - ps31
Arabidopsis_thaliana - ps32
Phylostratum

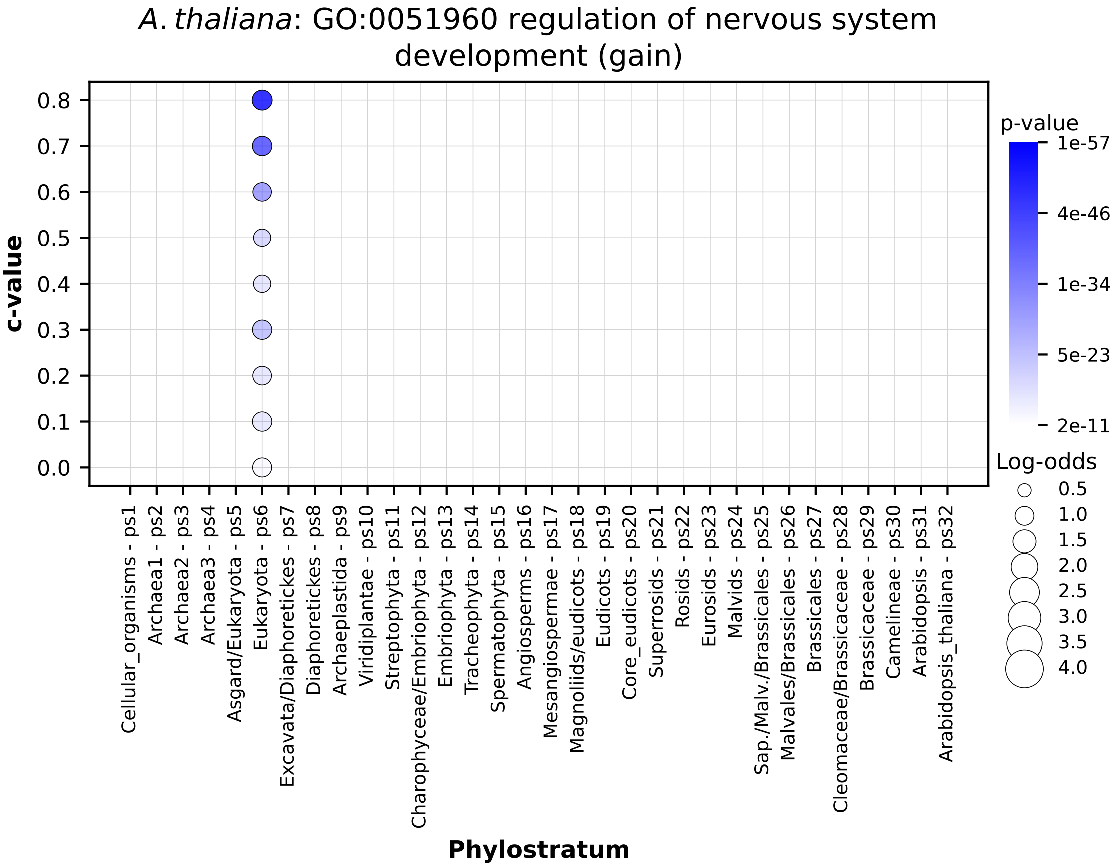

A. thaliana: GO:0051960 regulation of nervous system development (gain)
c-value
0.8
0.7
0.6
0.5
0.4
0.3
0.2
0.1
0.0
p-value
1e-57
4e-46
1e-34
5e-23
2e-11
Log-odds
0.5
1.0
1.5
2.0
2.5
3.0
3.5
4.0
Cellular_organisms - ps1
Archaea1 - ps2
Archaea2 - ps3
Archaea3 - ps4
Asgard/Eukaryota - ps5
Eukaryota - ps6
Excavata/Diaphoretickes - ps7
Diaphoretickes - ps8
Archaeplastida - ps9
Viridiplantae - ps10
Streptophyta - ps11
Charophyceae/Embriophyta - ps12
Embriophyta - ps13
Tracheophyta - ps14
Spermatophyta - ps15
Angiosperms - ps16
Mesangiospermae - ps17
Magnoliids/eudicots - ps18
Eudicots - ps19
Core_eudicots - ps20
Superrosids - ps21
Rosids - ps22
Eurosids - ps23
Malvids - ps24
Sap./Malv./Brassicales - ps25
Malvales/Brassicales - ps26
Brassicales - ps27
Cleomaceae/Brassicaceae - ps28
Brassicaceae - ps29
Camelineae - ps30
Arabidopsis - ps31
Arabidopsis_thaliana - ps32
Phylostratum

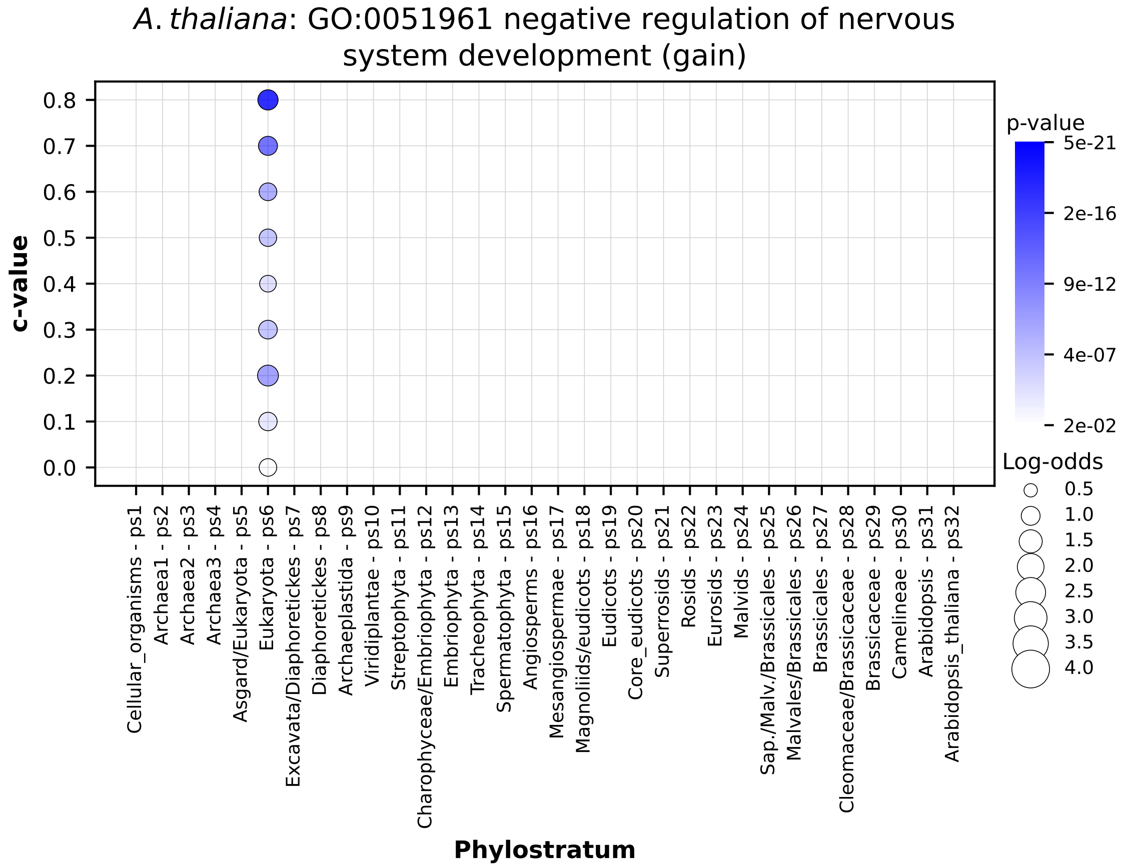
A. thaliana: GO:0051961 negative regulation of nervous system development (gain)
c-value
0.8
0.7
0.6
0.5
0.4
0.3
0.2
0.1
0.0
p-value
5e-21
2e-16
9e-12
4e-07
2e-02
Log-odds
0.5
1.0
1.5
2.0
2.5
3.0
3.5
4.0
Cellular_organisms - ps1
Archaea1 - ps2
Archaea2 - ps3
Archaea3 - ps4
Asgard/Eukaryota - ps5
Eukaryota - ps6
Excavata/Diaphoretickes - ps7
Diaphoretickes - ps8
Archaeplastida - ps9
Viridiplantae - ps10
Streptophyta - ps11
Charophyceae/Embriophyta - ps12
Embriophyta - ps13
Tracheophyta - ps14
Spermatophyta - ps15
Angiosperms - ps16
Mesangiospermae - ps17
Magnoliids/eudicots - ps18
Eudicots - ps19
Core_eudicots - ps20
Superrosids - ps21
Rosids - ps22
Eurosids - ps23
Malvids - ps24
Sap./Malv./Brassicales - ps25
Malvales/Brassicales - ps26
Brassicales - ps27
Cleomaceae/Brassicaceae - ps28
Brassicaceae - ps29
Camelineae - ps30
Arabidopsis - ps31
Arabidopsis_thaliana - ps32
Phylostratum

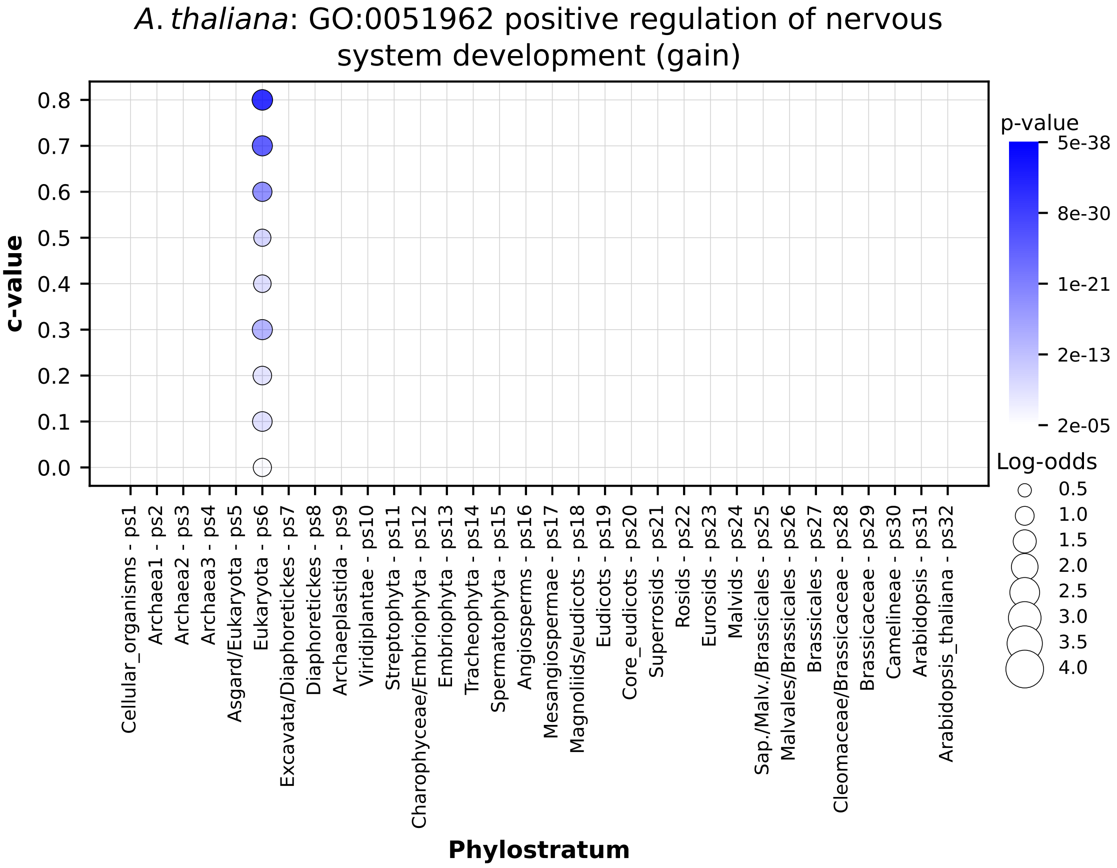
A. thaliana: GO:0051962 positive regulation of nervous system development (gain)
c-value
0.8
0.7
0.6
0.5
0.4
0.3
0.2
0.1
0.0
p-value
5e-38
8e-30
1e-21
2e-13
2e-05
Log-odds
0.5
1.0
1.5
2.0
2.5
3.0
3.5
4.0
Cellular_organisms - ps1
Archaea1 - ps2
Archaea2 - ps3
Archaea3 - ps4
Asgard/Eukaryota - ps5
Eukaryota - ps6
Excavata/Diaphoretickes - ps7
Diaphoretickes - ps8
Archaeplastida - ps9
Viridiplantae - ps10
Streptophyta - ps11
Charophyceae/Embriophyta - ps12
Embriophyta - ps13
Tracheophyta - ps14
Spermatophyta - ps15
Angiosperms - ps16
Mesangiospermae - ps17
Magnoliids/eudicots - ps18
Eudicots - ps19
Core_eudicots - ps20
Superrosids - ps21
Rosids - ps22
Eurosids - ps23
Malvids - ps24
Sap./Malv./Brassicales - ps25
Malvales/Brassicales - ps26
Brassicales - ps27
Cleomaceae/Brassicaceae - ps28
Brassicaceae - ps29
Camelineae - ps30
Arabidopsis - ps31
Arabidopsis_thaliana - ps32
Phylostratum

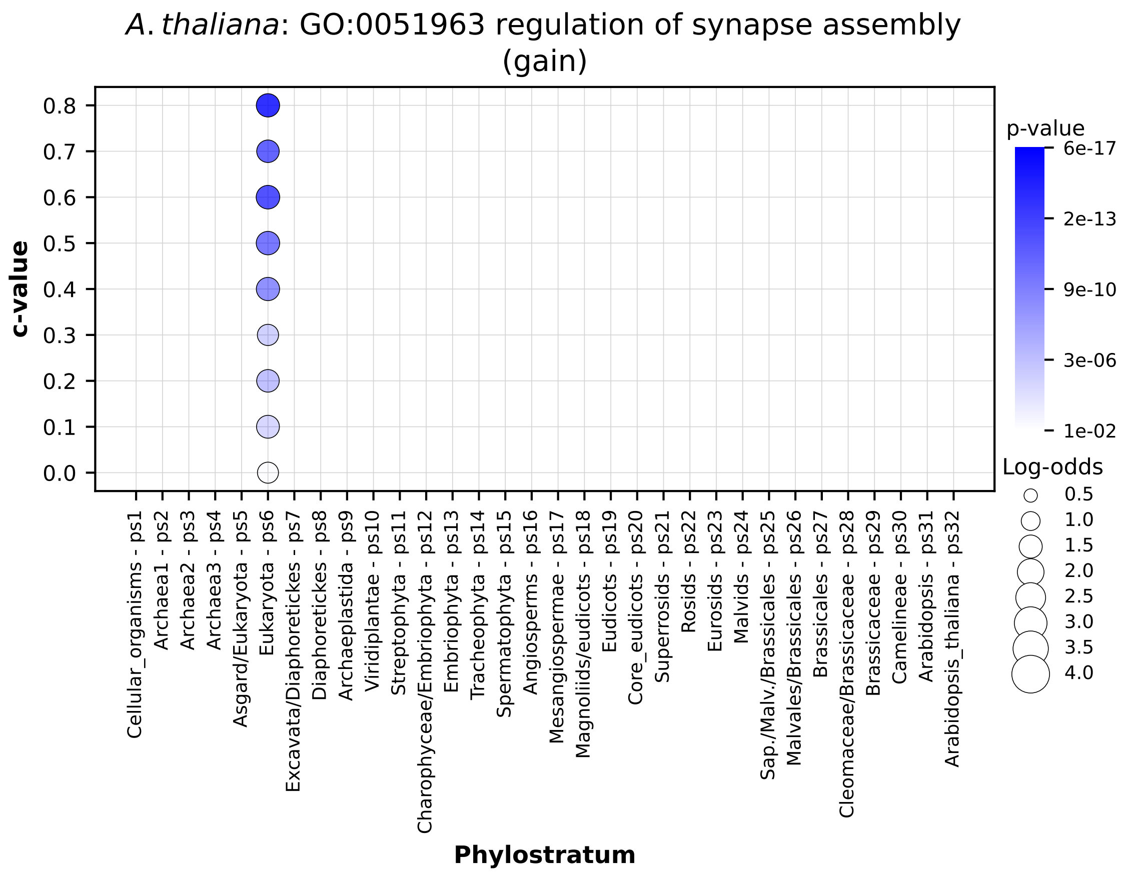

A. thaliana: GO:0051963 regulation of synapse assembly
(gain)
c-value
0.8
0.7
0.6
0.5
0.4
0.3
0.2
0.1
0.0
p-value
6e-17
2e-13
9e-10
3e-06
1e-02
Log-odds
0.5
1.0
1.5
2.0
2.5
3.0
3.5
4.0
Cellular_organisms - ps1
Archaea1 - ps2
Archaea2 - ps3
Archaea3 - ps4
Asgard/Eukaryota - ps5
Eukaryota - ps6
Excavata/Diaphoretickes - ps7
Diaphoretickes - ps8
Archaeplastida - ps9
Viridiplantae - ps10
Streptophyta - ps11
Charophyceae/Embriophyta - ps12
Embriophyta - ps13
Tracheophyta - ps14
Spermatophyta - ps15
Angiosperms - ps16
Mesangiospermae - ps17
Magnoliids/eudicots - ps18
Eudicots - ps19
Core_eudicots - ps20
Superrosids - ps21
Rosids - ps22
Eurosids - ps23
Malvids - ps24
Sap./Malv./Brassicales - ps25
Malvales/Brassicales - ps26
Brassicales - ps27
Cleomaceae/Brassicaceae - ps28
Brassicaceae - ps29
Camelineae - ps30
Arabidopsis - ps31
Arabidopsis_thaliana - ps32
Phylostratum

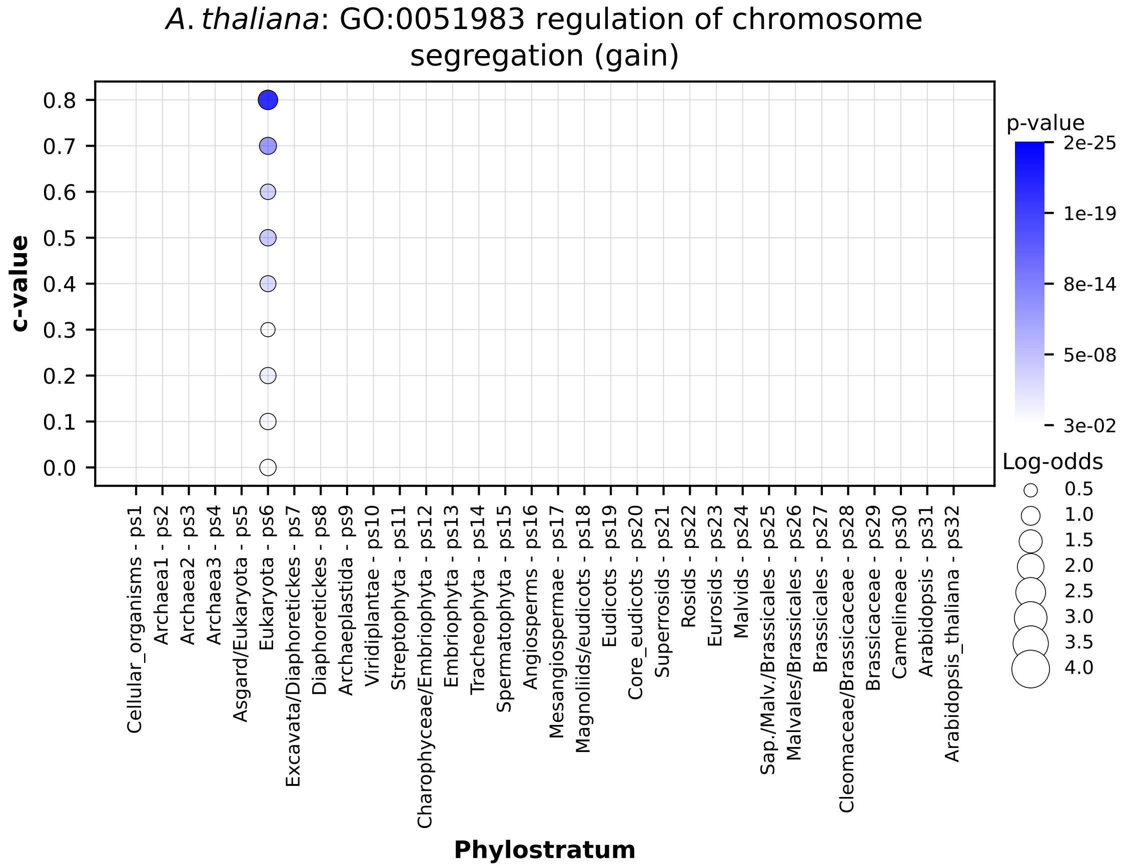
A. thaliana: GO:0051983 regulation of chromosome segregation (gain)
c-value
0.8
0.7
0.6
0.5
0.4
0.3
0.2
0.1
0.0
Phylostratum
Cellular_organisms - ps1
Archaea1 - ps2
Archaea2 - ps3
Archaea3 - ps4
Asgard/Eukaryota - ps5
Eukaryota - ps6
Excavata/Diaphoretickes - ps7
Diaphoretickes - ps8
Archaeplastida - ps9
Viridiplantae - ps10
Streptophyta - ps11
Charophyceae/Embriophyta - ps12
Embriophyta - ps13
Tracheophyta - ps14
Spermatophyta - ps15
Angiosperms - ps16
Mesangiospermae - ps17
Magnoliids/eudicots - ps18
Eudicots - ps19
Core_eudicots - ps20
Superrosids - ps21
Rosids - ps22
Eurosids - ps23
Malvids - ps24
Sap./Malv./Brassicales - ps25
Malvales/Brassicales - ps26
Brassicales - ps27
Cleomaceae/Brassicaceae - ps28
Brassicaceae - ps29
Camelineae - ps30
Arabidopsis - ps31
Arabidopsis_thaliana - ps32
p-value
2e-25
1e-19
8e-14
5e-08
3e-02
Log-odds
0.5
1.0
1.5
2.0
2.5
3.0
3.5
4.0

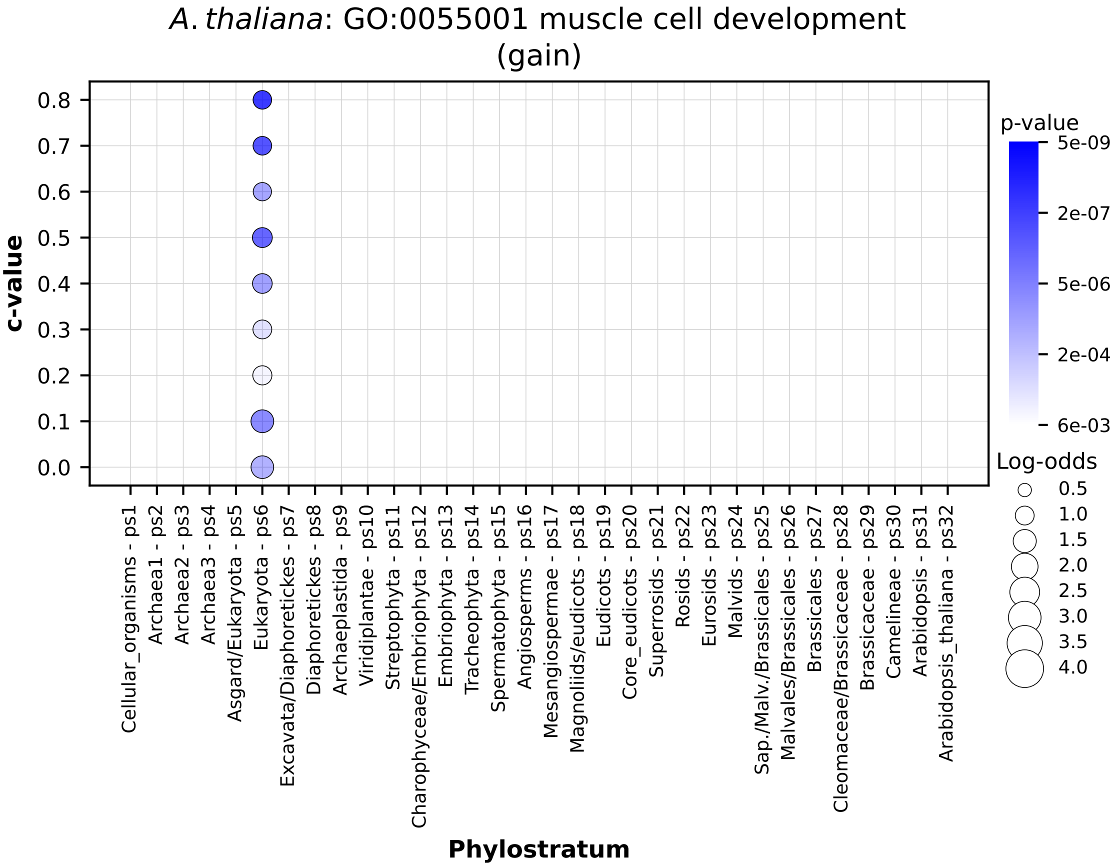

A. thaliana: GO:0055001 muscle cell development
(gain)
c-value
0.8
0.7
0.6
0.5
0.4
0.3
0.2
0.1
0.0
p-value
5e-09
2e-07
5e-06
2e-04
6e-03
Log-odds
0.5
1.0
1.5
2.0
2.5
3.0
3.5
4.0
Cellular_organisms - ps1
Archaea1 - ps2
Archaea2 - ps3
Archaea3 - ps4
Asgard/Eukaryota - ps5
Eukaryota - ps6
Excavata/Diaphoretickes - ps7
Diaphoretickes - ps8
Archaeplastida - ps9
Viridiplantae - ps10
Streptophyta - ps11
Charophyceae/Embriophyta - ps12
Embriophyta - ps13
Tracheophyta - ps14
Spermatophyta - ps15
Angiosperms - ps16
Mesangiospermae - ps17
Magnoliids/eudicots - ps18
Eudicots - ps19
Core_eudicots - ps20
Superrosids - ps21
Rosids - ps22
Eurosids - ps23
Malvids - ps24
Sap./Malv./Brassicales - ps25
Malvales/Brassicales - ps26
Brassicales - ps27
Cleomaceae/Brassicaceae - ps28
Brassicaceae - ps29
Camelineae - ps30
Arabidopsis - ps31
Arabidopsis_thaliana - ps32
Phylostratum

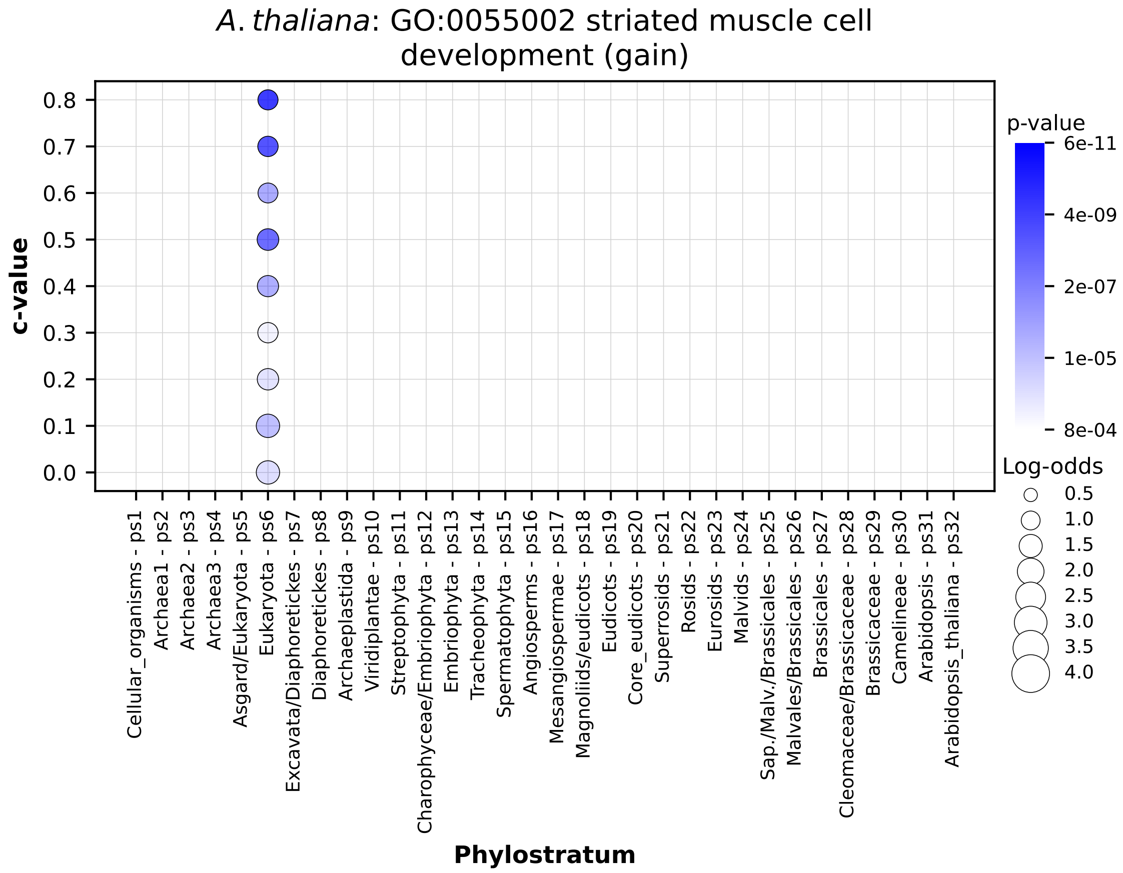

A. thaliana: GO:0055002 striated muscle cell development (gain)
c-value
0.8
0.7
0.6
0.5
0.4
0.3
0.2
0.1
0.0
p-value
6e-11
4e-09
2e-07
1e-05
8e-04
Log-odds
0.5
1.0
1.5
2.0
2.5
3.0
3.5
4.0
Cellular_organisms - ps1
Archaea1 - ps2
Archaea2 - ps3
Archaea3 - ps4
Asgard/Eukaryota - ps5
Eukaryota - ps6
Excavata/Diaphoretickes - ps7
Diaphoretickes - ps8
Archaeplastida - ps9
Viridiplantae - ps10
Streptophyta - ps11
Charophyceae/Embriophyta - ps12
Embriophyta - ps13
Tracheophyta - ps14
Spermatophyta - ps15
Angiosperms - ps16
Mesangiospermae - ps17
Magnoliids/eudicots - ps18
Eudicots - ps19
Core_eudicots - ps20
Superrosids - ps21
Rosids - ps22
Eurosids - ps23
Malvids - ps24
Sap./Malv./Brassicales - ps25
Malvales/Brassicales - ps26
Brassicales - ps27
Cleomaceae/Brassicaceae - ps28
Brassicaceae - ps29
Camelineae - ps30
Arabidopsis - ps31
Arabidopsis_thaliana - ps32
Phylostratum

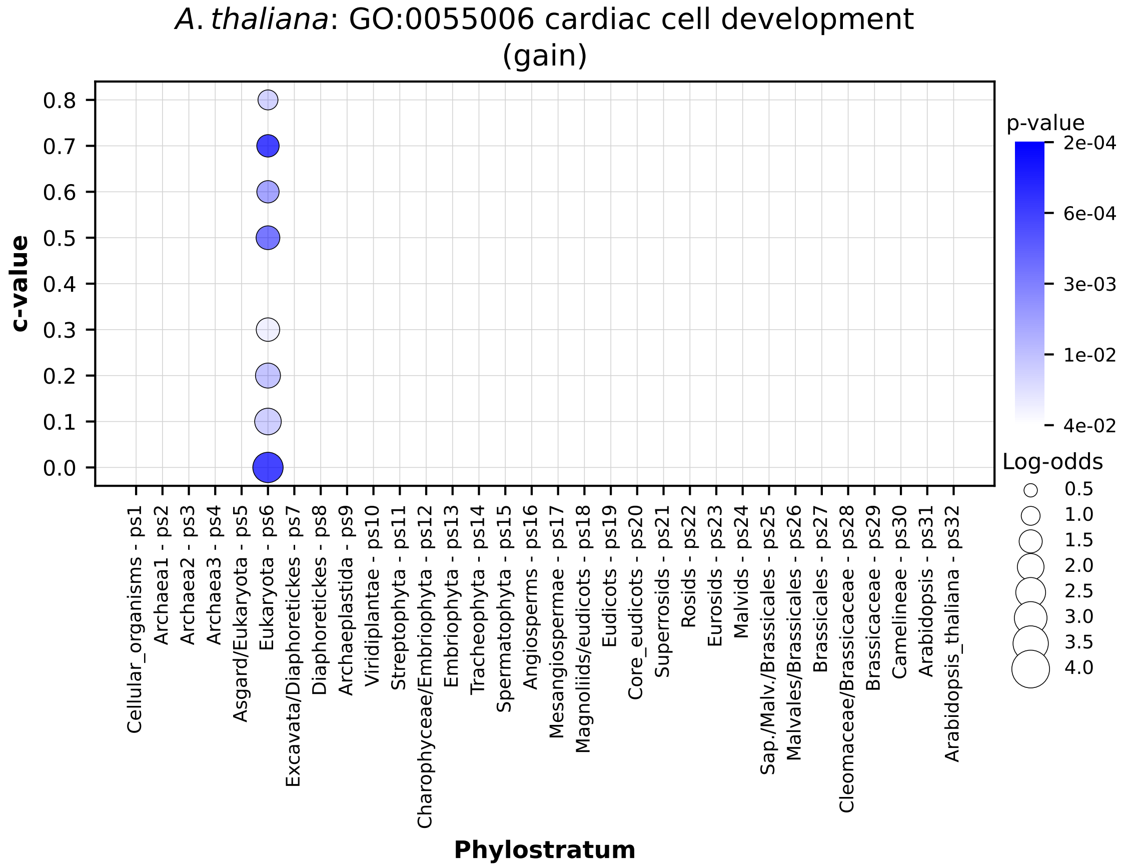
A. thaliana: GO:0055006 cardiac cell development (gain)
c-value
0.8
0.7
0.6
0.5
0.4
0.3
0.2
0.1
0.0
p-value
2e-04
6e-04
3e-03
1e-02
4e-02
Log-odds
0.5
1.0
1.5
2.0
2.5
3.0
3.5
4.0
Cellular_organisms - ps1
Archaea1 - ps2
Archaea2 - ps3
Archaea3 - ps4
Asgard/Eukaryota - ps5
Eukaryota - ps6
Excavata/Diaphoretickes - ps7
Diaphoretickes - ps8
Archaeplastida - ps9
Viridiplantae - ps10
Streptophyta - ps11
Charophyceae/Embriophyta - ps12
Embriophyta - ps13
Tracheophyta - ps14
Spermatophyta - ps15
Angiosperms - ps16
Mesangiospermae - ps17
Magnoliids/eudicots - ps18
Eudicots - ps19
Core_eudicots - ps20
Superrosids - ps21
Rosids - ps22
Eurosids - ps23
Malvids - ps24
Sap./Malv./Brassicales - ps25
Malvales/Brassicales - ps26
Brassicales - ps27
Cleomaceae/Brassicaceae - ps28
Brassicaceae - ps29
Camelineae - ps30
Arabidopsis - ps31
Arabidopsis_thaliana - ps32
Phylostratum

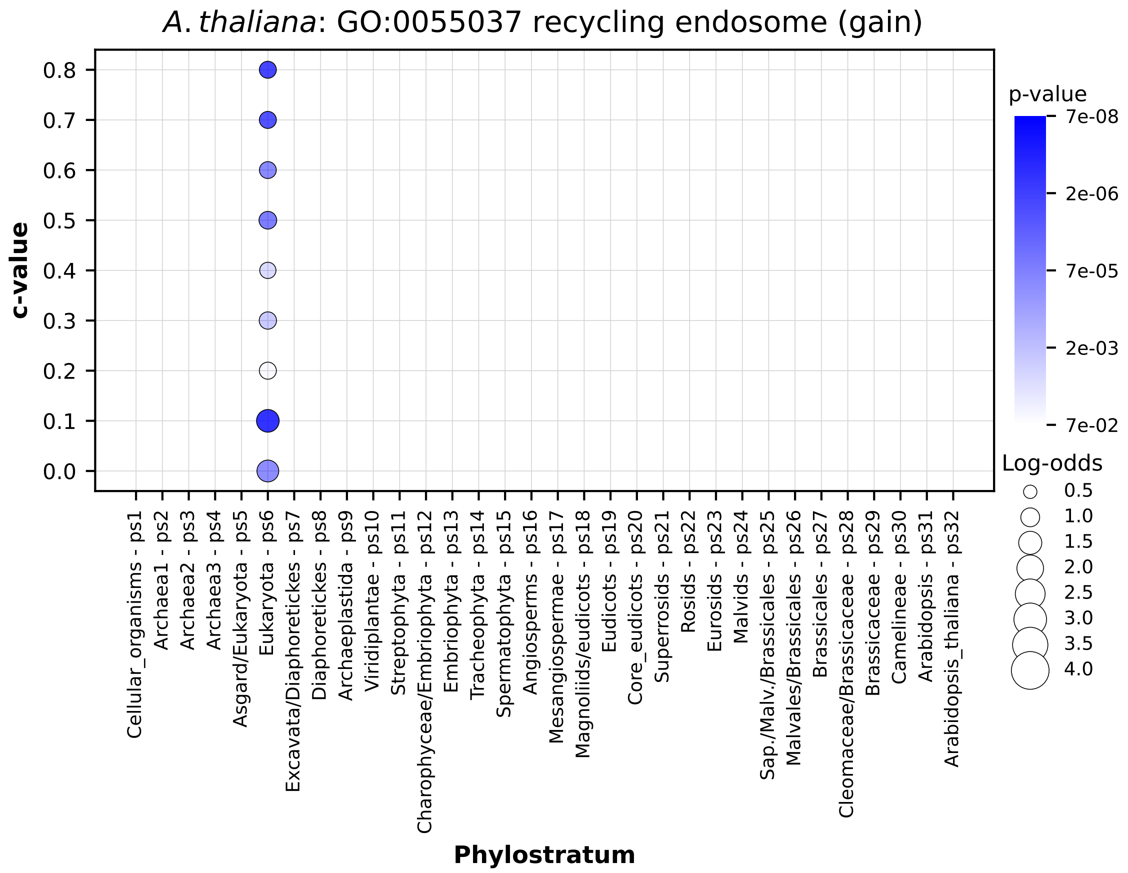
A. thaliana: GO:0055037 recycling endosome (gain)
c-value
0.8
0.7
0.6
0.5
0.4
0.3
0.2
0.1
0.0
Phylostratum
Cellular_organisms - ps1
Archaea1 - ps2
Archaea2 - ps3
Archaea3 - ps4
Asgard/Eukaryota - ps5
Eukaryota - ps6
Excavata/Diaphoretickes - ps7
Diaphoretickes - ps8
Archaeplastida - ps9
Viridiplantae - ps10
Streptophyta - ps11
Charophyceae/Embriophyta - ps12
Embriophyta - ps13
Tracheophyta - ps14
Spermatophyta - ps15
Angiosperms - ps16
Mesangiospermae - ps17
Magnoliids/eudicots - ps18
Eudicots - ps19
Core_eudicots - ps20
Superrosids - ps21
Rosids - ps22
Eurosids - ps23
Malvids - ps24
Sap./Malv./Brassicales - ps25
Malvales/Brassicales - ps26
Brassicales - ps27
Cleomaceae/Brassicaceae - ps28
Brassicaceae - ps29
Camelineae - ps30
Arabidopsis - ps31
Arabidopsis_thaliana - ps32
p-value
7e-08
2e-06
7e-05
2e-03
7e-02
Log-odds
0.5
1.0
1.5
2.0
2.5
3.0
3.5
4.0

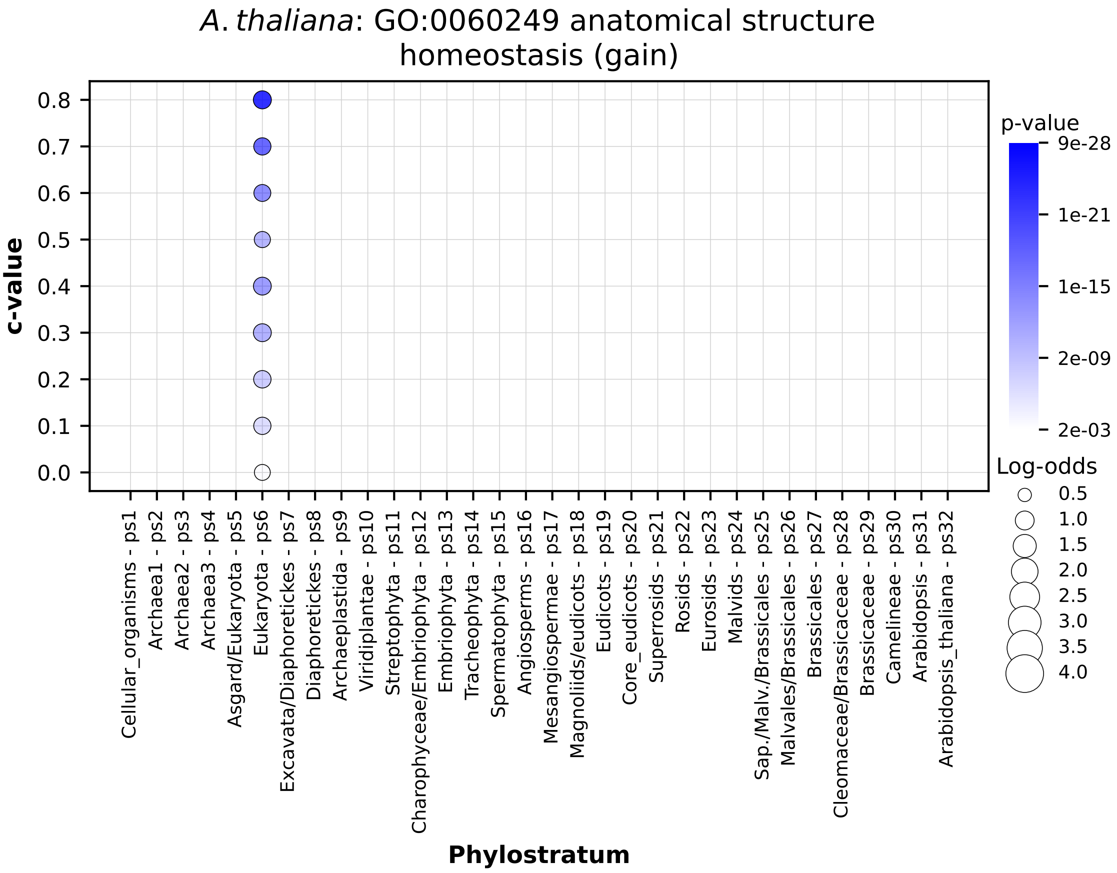

A. thaliana: GO:0060249 anatomical structure homeostasis (gain)
c-value
0.8
0.7
0.6
0.5
0.4
0.3
0.2
0.1
0.0
Phylostratum
Cellular_organisms - ps1
Archaea1 - ps2
Archaea2 - ps3
Archaea3 - ps4
Asgard/Eukaryota - ps5
Eukaryota - ps6
Excavata/Diaphoretickes - ps7
Diaphoretickes - ps8
Archaeplastida - ps9
Viridiplantae - ps10
Streptophyta - ps11
Charophyceae/Embriophyta - ps12
Embriophyta - ps13
Tracheophyta - ps14
Spermatophyta - ps15
Angiosperms - ps16
Mesangiospermae - ps17
Magnoliids/eudicots - ps18
Eudicots - ps19
Core_eudicots - ps20
Superrosids - ps21
Rosids - ps22
Eurosids - ps23
Malvids - ps24
Sap./Malv./Brassicales - ps25
Malvales/Brassicales - ps26
Brassicales - ps27
Cleomaceae/Brassicaceae - ps28
Brassicaceae - ps29
Camelineae - ps30
Arabidopsis - ps31
Arabidopsis_thaliana - ps32
p-value
9e-28
1e-21
1e-15
2e-09
2e-03
Log-odds
0.5
1.0
1.5
2.0
2.5
3.0
3.5
4.0

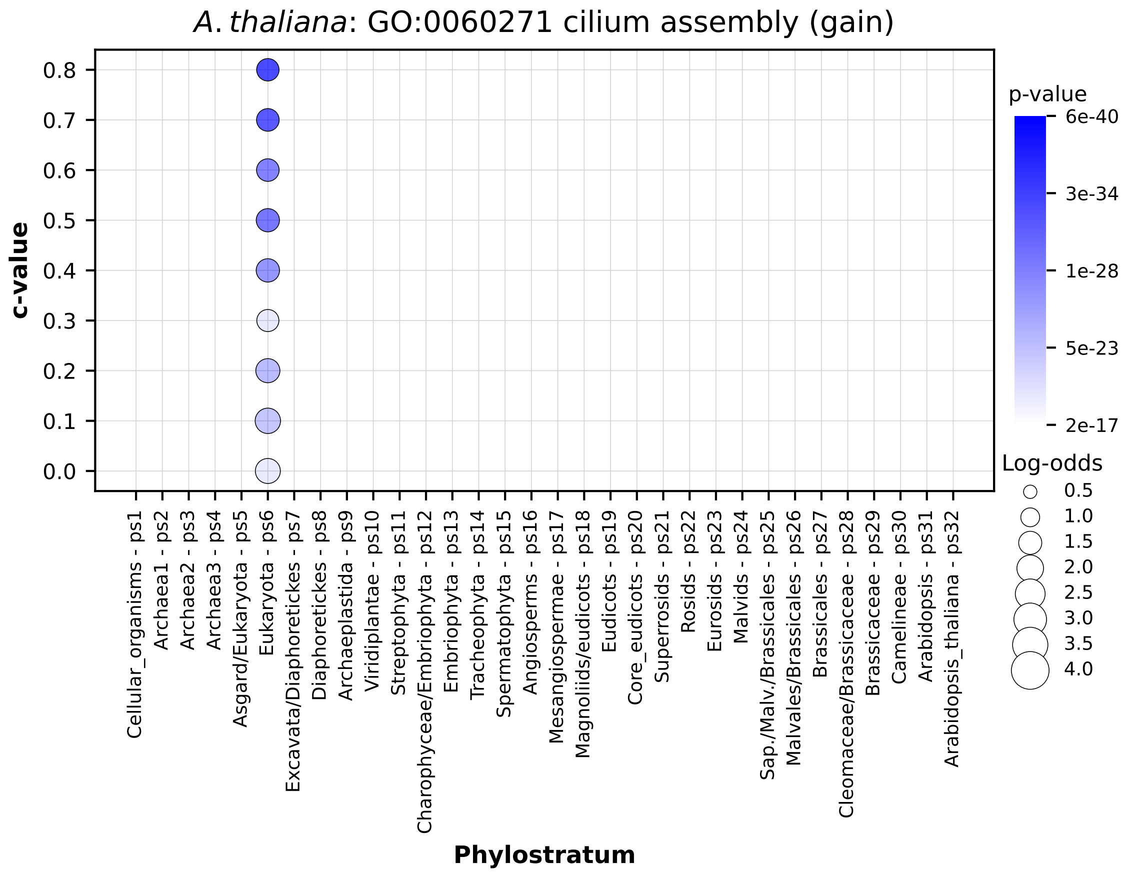
A. thaliana: GO:0060271 cilium assembly (gain)
c-value
0.0
0.1
0.2
0.3
0.4
0.5
0.6
0.7
0.8
Phylostratum
Cellular_organisms - ps1
Archaea1 - ps2
Archaea2 - ps3
Archaea3 - ps4
Asgard/Eukaryota - ps5
Eukaryota - ps6
Excavata/Diaphoretickes - ps7
Diaphoretickes - ps8
Archaeplastida - ps9
Viridiplantae - ps10
Streptophyta - ps11
Charophyceae/Embriophyta - ps12
Embriophyta - ps13
Tracheophyta - ps14
Spermatophyta - ps15
Angiosperms - ps16
Mesangiospermae - ps17
Magnoliids/eudicots - ps18
Eudicots - ps19
Core_eudicots - ps20
Superrosids - ps21
Rosids - ps22
Eurosids - ps23
Malvids - ps24
Sap./Malv./Brassicales - ps25
Malvales/Brassicales - ps26
Brassicales - ps27
Cleomaceae/Brassicaceae - ps28
Brassicaceae - ps29
Camelineae - ps30
Arabidopsis - ps31
Arabidopsis_thaliana - ps32
p-value
6e-40
3e-34
1e-28
5e-23
2e-17
Log-odds
0.5
1.0
1.5
2.0
2.5
3.0
3.5
4.0

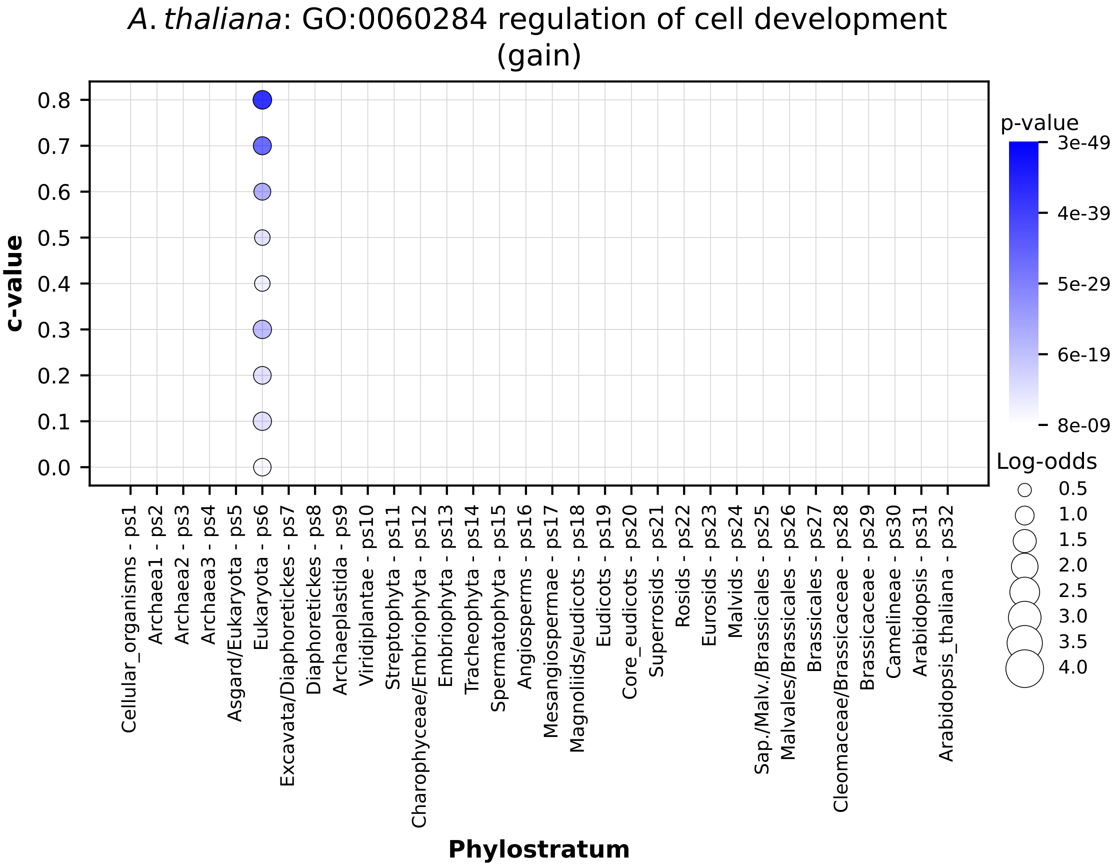

A. thaliana: GO:0060284 regulation of cell development
(gain)
c-value
0.0
0.1
0.2
0.3
0.4
0.5
0.6
0.7
0.8
p-value
3e-49
4e-39
5e-29
6e-19
8e-09
Log-odds
0.5
1.0
1.5
2.0
2.5
3.0
3.5
4.0
Cellular_organisms - ps1
Archaea1 - ps2
Archaea2 - ps3
Archaea3 - ps4
Asgard/Eukaryota - ps5
Eukaryota - ps6
Excavata/Diaphoretickes - ps7
Diaphoretickes - ps8
Archaeplastida - ps9
Viridiplantae - ps10
Streptophyta - ps11
Charophyceae/Embriophyta - ps12
Embriophyta - ps13
Tracheophyta - ps14
Spermatophyta - ps15
Angiosperms - ps16
Mesangiospermae - ps17
Magnoliids/eudicots - ps18
Eudicots - ps19
Core_eudicots - ps20
Superrosids - ps21
Rosids - ps22
Eurosids - ps23
Malvids - ps24
Sap./Malv./Brassicales - ps25
Malvales/Brassicales - ps26
Brassicales - ps27
Cleomaceae/Brassicaceae - ps28
Brassicaceae - ps29
Camelineae - ps30
Arabidopsis - ps31
Arabidopsis_thaliana - ps32
Phylostratum

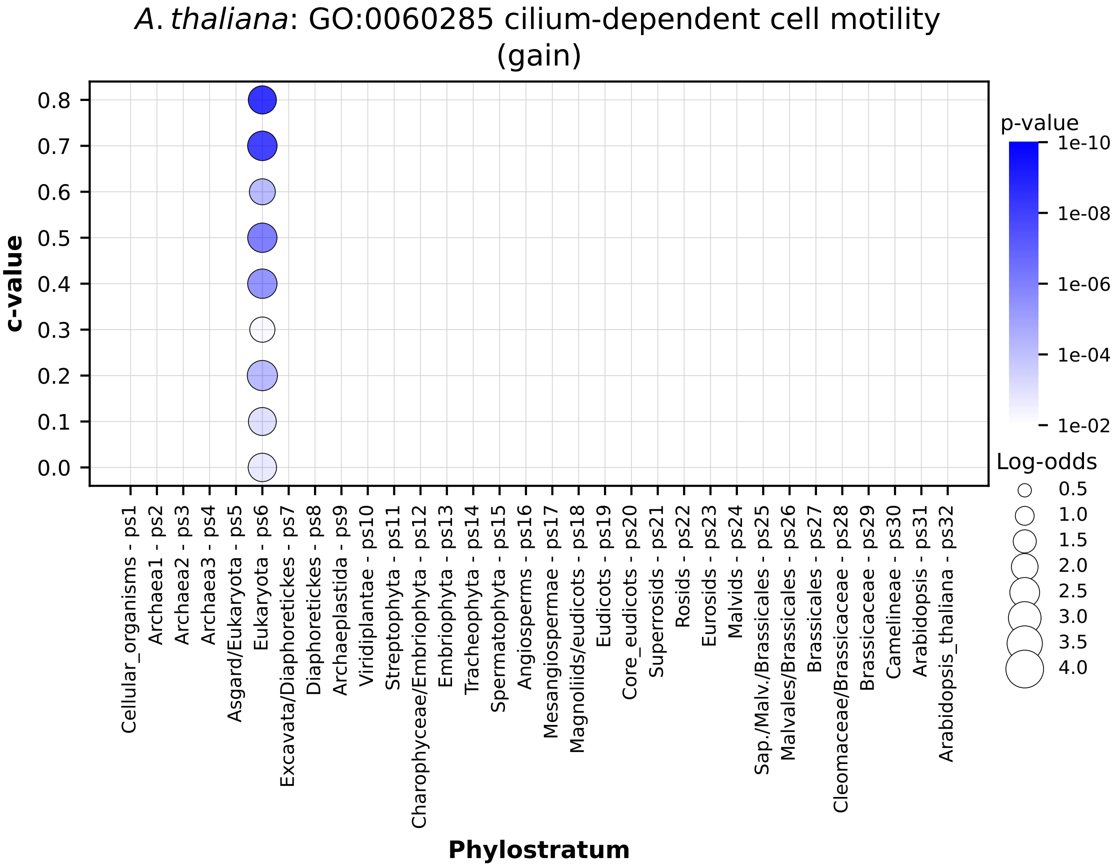
A. thaliana: GO:0060285 cilium-dependent cell motility
(gain)
c-value
0.8
0.7
0.6
0.5
0.4
0.3
0.2
0.1
0.0
Phylostratum
Cellular_organisms - ps1
Archaea1 - ps2
Archaea2 - ps3
Archaea3 - ps4
Asgard/Eukaryota - ps5
Eukaryota - ps6
Excavata/Diaphoretickes - ps7
Diaphoretickes - ps8
Archaeplastida - ps9
Viridiplantae - ps10
Streptophyta - ps11
Charophyceae/Embriophyta - ps12
Embriophyta - ps13
Tracheophyta - ps14
Spermatophyta - ps15
Angiosperms - ps16
Mesangiospermae - ps17
Magnoliids/eudicots - ps18
Eudicots - ps19
Core_eudicots - ps20
Superrosids - ps21
Rosids - ps22
Eurosids - ps23
Malvids - ps24
Sap./Malv./Brassicales - ps25
Malvales/Brassicales - ps26
Brassicales - ps27
Cleomaceae/Brassicaceae - ps28
Brassicaceae - ps29
Camelineae - ps30
Arabidopsis - ps31
Arabidopsis_thaliana - ps32
p-value
1e-10
1e-08
1e-06
1e-04
1e-02
Log-odds
0.5
1.0
1.5
2.0
2.5
3.0
3.5
4.0

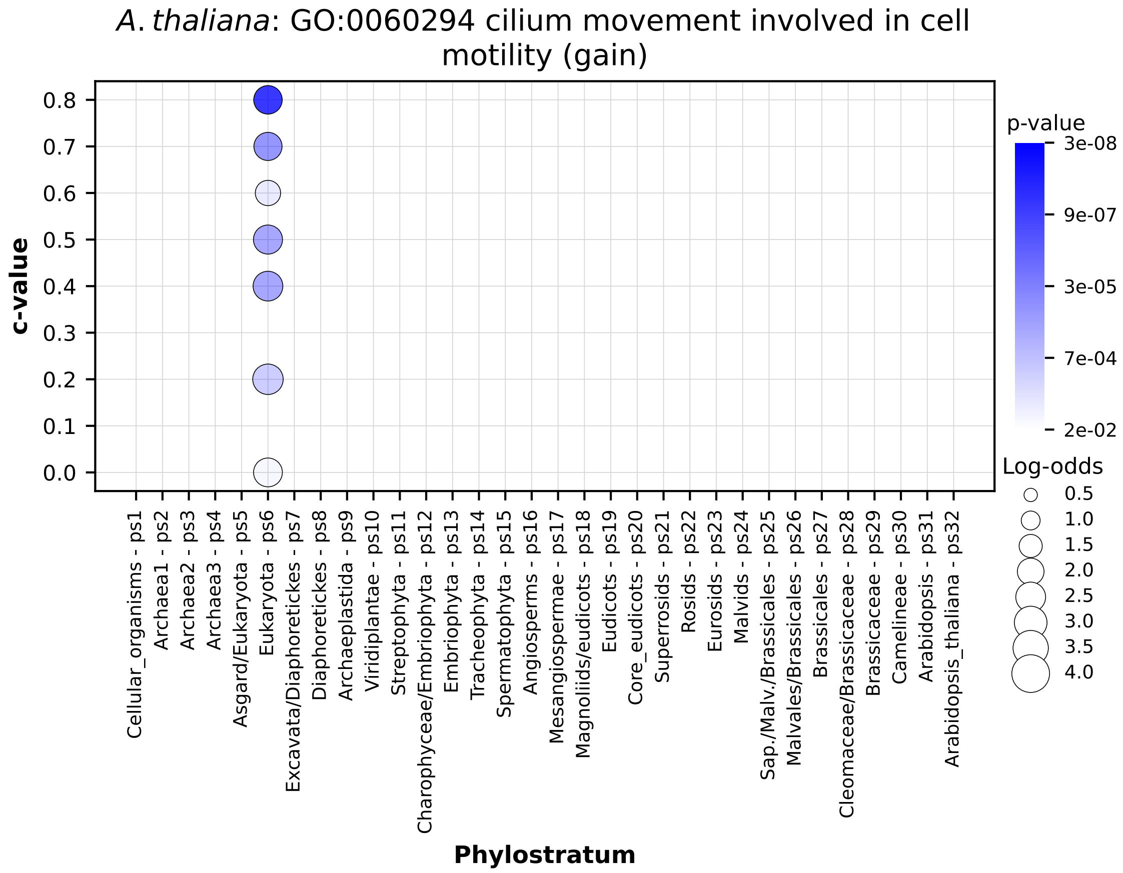
A. thaliana: GO:0060294 cilium movement involved in cell motility (gain)
c-value
0.8
0.7
0.6
0.5
0.4
0.3
0.2
0.1
0.0
Phylostratum
Cellular_organisms - ps1
Archaea1 - ps2
Archaea2 - ps3
Archaea3 - ps4
Asgard/Eukaryota - ps5
Eukaryota - ps6
Excavata/Diaphoretickes - ps7
Diaphoretickes - ps8
Archaeplastida - ps9
Viridiplantae - ps10
Streptophyta - ps11
Charophyceae/Embriophyta - ps12
Embriophyta - ps13
Tracheophyta - ps14
Spermatophyta - ps15
Angiosperms - ps16
Mesangiospermae - ps17
Magnoliids/eudicots - ps18
Eudicots - ps19
Core_eudicots - ps20
Superrosids - ps21
Rosids - ps22
Eurosids - ps23
Malvids - ps24
Sap./Malv./Brassicales - ps25
Malvales/Brassicales - ps26
Brassicales - ps27
Cleomaceae/Brassicaceae - ps28
Brassicaceae - ps29
Camelineae - ps30
Arabidopsis - ps31
Arabidopsis_thaliana - ps32
p-value
3e-08
9e-07
3e-05
7e-04
2e-02
Log-odds
0.5
1.0
1.5
2.0
2.5
3.0
3.5
4.0

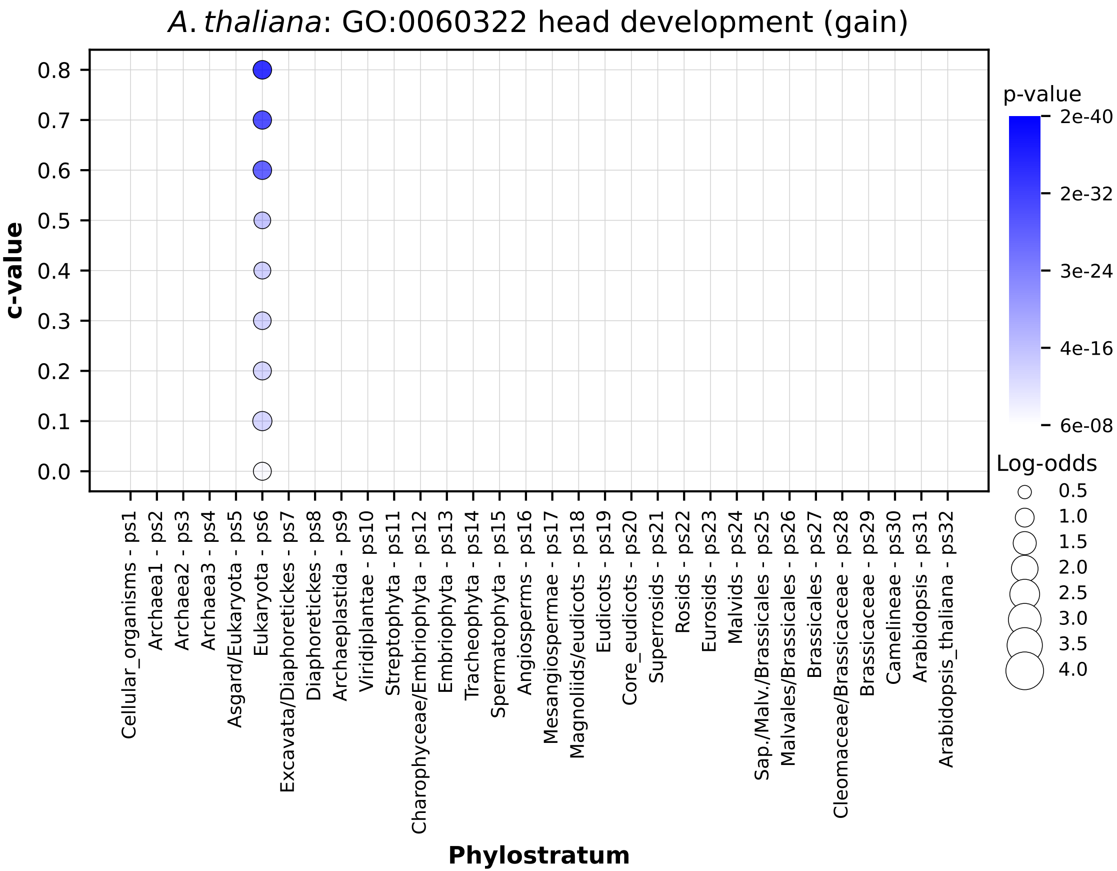

A. thaliana: GO:0060322 head development (gain)
c-value
0.0
0.1
0.2
0.3
0.4
0.5
0.6
0.7
0.8
p-value
2e-40
2e-32
3e-24
4e-16
6e-08
Log-odds
0.5
1.0
1.5
2.0
2.5
3.0
3.5
4.0
Cellular_organisms - ps1
Archaea1 - ps2
Archaea2 - ps3
Archaea3 - ps4
Asgard/Eukaryota - ps5
Eukaryota - ps6
Excavata/Diaphoretickes - ps7
Diaphoretickes - ps8
Archaeplastida - ps9
Viridiplantae - ps10
Streptophyta - ps11
Charophyceae/Embriophyta - ps12
Embriophyta - ps13
Tracheophyta - ps14
Spermatophyta - ps15
Angiosperms - ps16
Mesangiospermae - ps17
Magnoliids/eudicots - ps18
Eudicots - ps19
Core_eudicots - ps20
Superrosids - ps21
Rosids - ps22
Eurosids - ps23
Malvids - ps24
Sap./Malv./Brassicales - ps25
Malvales/Brassicales - ps26
Brassicales - ps27
Cleomaceae/Brassicaceae - ps28
Brassicaceae - ps29
Camelineae - ps30
Arabidopsis - ps31
Arabidopsis_thaliana - ps32
Phylostratum

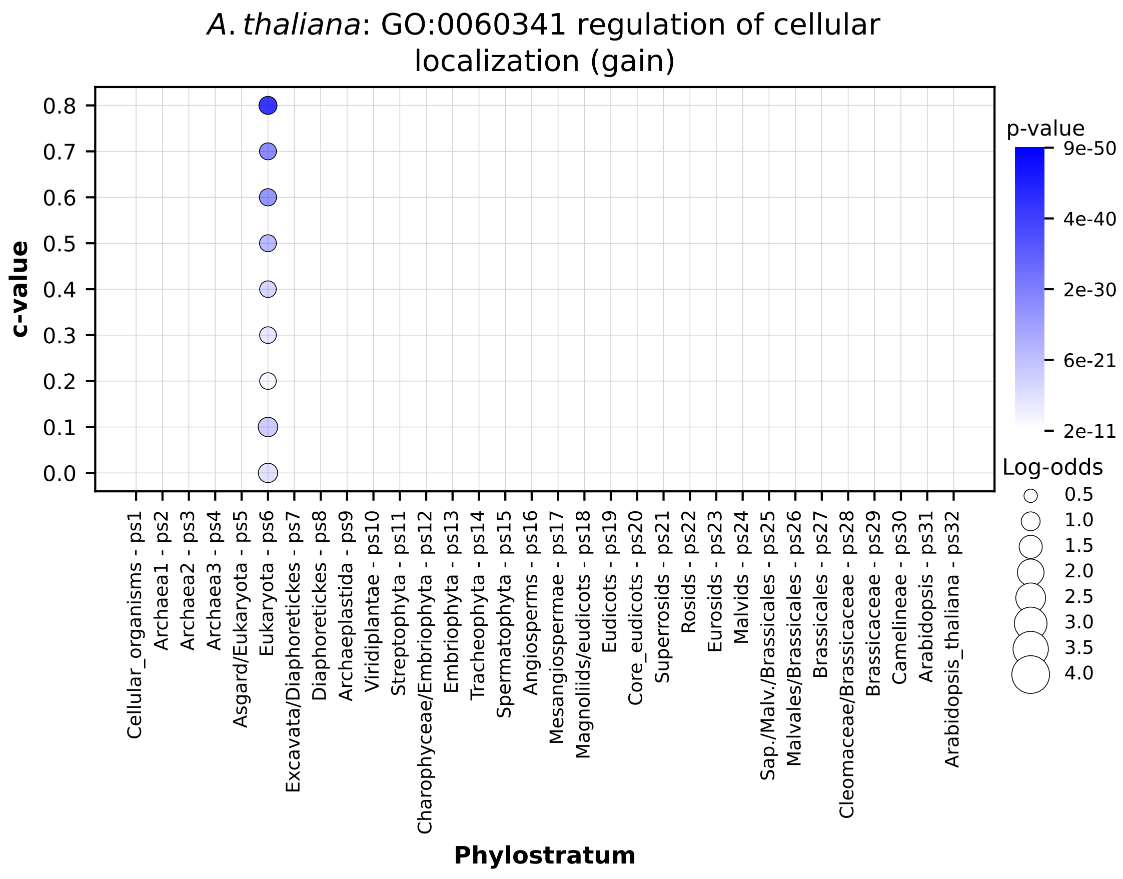

A. thaliana: GO:0060341 regulation of cellular localization (gain)
c-value
0.8
0.7
0.6
0.5
0.4
0.3
0.2
0.1
0.0
p-value
9e-50
4e-40
2e-30
6e-21
2e-11
Log-odds
0.5
1.0
1.5
2.0
2.5
3.0
3.5
4.0
Cellular_organisms - ps1
Archaea1 - ps2
Archaea2 - ps3
Archaea3 - ps4
Asgard/Eukaryota - ps5
Eukaryota - ps6
Excavata/Diaphoretickes - ps7
Diaphoretickes - ps8
Archaeplastida - ps9
Viridiplantae - ps10
Streptophyta - ps11
Charophyceae/Embriophyta - ps12
Embriophyta - ps13
Tracheophyta - ps14
Spermatophyta - ps15
Angiosperms - ps16
Mesangiospermae - ps17
Magnoliids/eudicots - ps18
Eudicots - ps19
Core_eudicots - ps20
Superrosids - ps21
Rosids - ps22
Eurosids - ps23
Malvids - ps24
Sap./Malv./Brassicales - ps25
Malvales/Brassicales - ps26
Brassicales - ps27
Cleomaceae/Brassicaceae - ps28
Brassicaceae - ps29
Camelineae - ps30
Arabidopsis - ps31
Arabidopsis_thaliana - ps32
Phylostratum

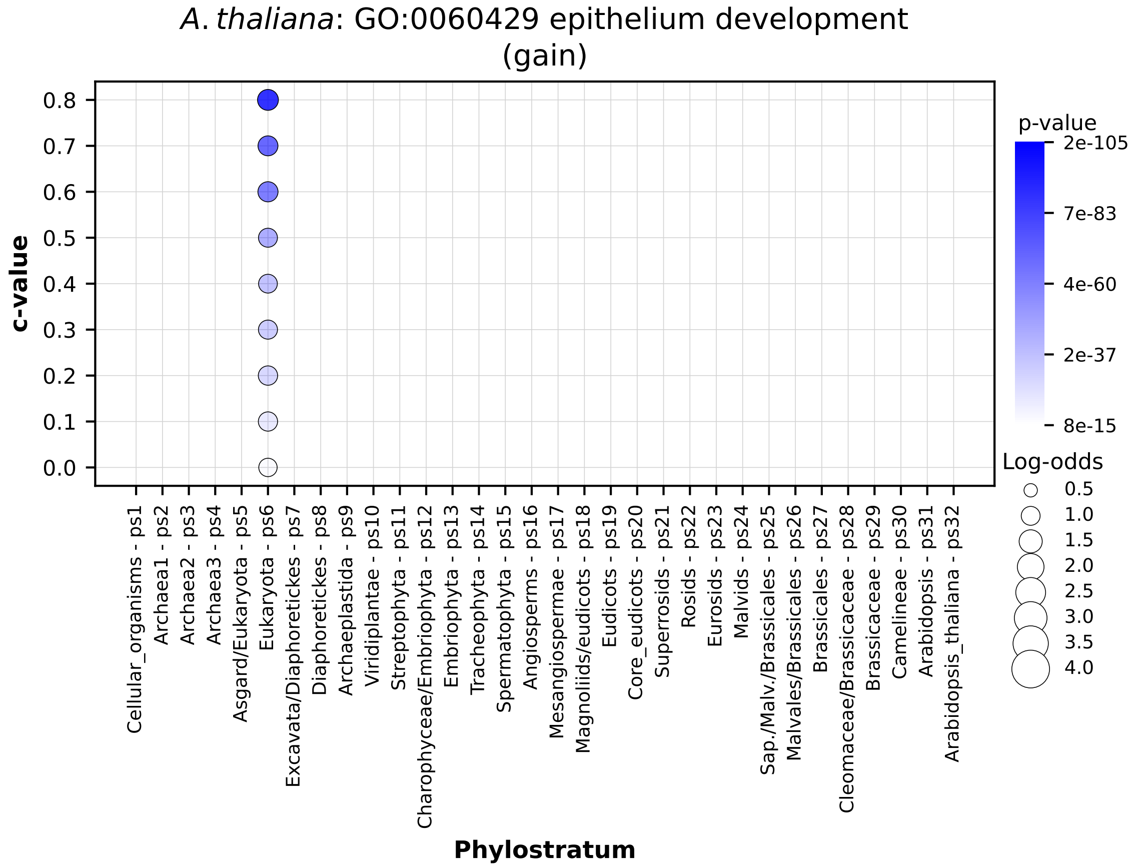
A. thaliana: GO:0060429 epithelium development
(gain)
c-value
0.8
0.7
0.6
0.5
0.4
0.3
0.2
0.1
0.0
p-value
2e-105
7e-83
4e-60
2e-37
8e-15
Log-odds
0.5
1.0
1.5
2.0
2.5
3.0
3.5
4.0
Cellular_organisms - ps1
Archaea1 - ps2
Archaea2 - ps3
Archaea3 - ps4
Asgard/Eukaryota - ps5
Eukaryota - ps6
Excavata/Diaphoretickes - ps7
Diaphoretickes - ps8
Archaeplastida - ps9
Viridiplantae - ps10
Streptophyta - ps11
Charophyceae/Embriophyta - ps12
Embriophyta - ps13
Tracheophyta - ps14
Spermatophyta - ps15
Angiosperms - ps16
Mesangiospermae - ps17
Magnoliids/eudicots - ps18
Eudicots - ps19
Core_eudicots - ps20
Superrosids - ps21
Rosids - ps22
Eurosids - ps23
Malvids - ps24
Sap./Malv./Brassicales - ps25
Malvales/Brassicales - ps26
Brassicales - ps27
Cleomaceae/Brassicaceae - ps28
Brassicaceae - ps29
Camelineae - ps30
Arabidopsis - ps31
Arabidopsis_thaliana - ps32
Phylostratum

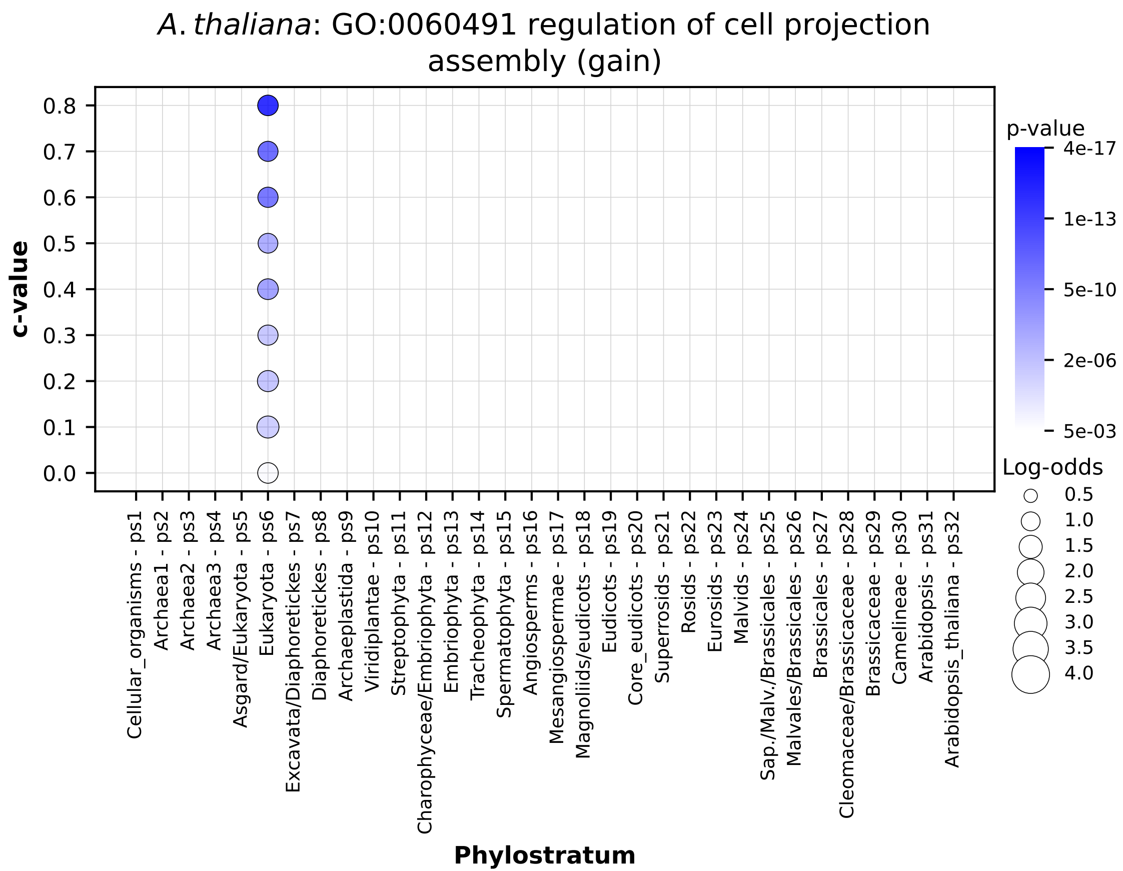
A. thaliana: GO:0060491 regulation of cell projection assembly (gain)
c-value
0.8
0.7
0.6
0.5
0.4
0.3
0.2
0.1
0.0
p-value
4e-17
1e-13
5e-10
2e-06
5e-03
Log-odds
0.5
1.0
1.5
2.0
2.5
3.0
3.5
4.0
Cellular_organisms - ps1
Archaea1 - ps2
Archaea2 - ps3
Archaea3 - ps4
Asgard/Eukaryota - ps5
Eukaryota - ps6
Excavata/Diaphoretickes - ps7
Diaphoretickes - ps8
Archaeplastida - ps9
Viridiplantae - ps10
Streptophyta - ps11
Charophyceae/Embriophyta - ps12
Embriophyta - ps13
Tracheophyta - ps14
Spermatophyta - ps15
Angiosperms - ps16
Mesangiospermae - ps17
Magnoliids/eudicots - ps18
Eudicots - ps19
Core_eudicots - ps20
Superrosids - ps21
Rosids - ps22
Eurosids - ps23
Malvids - ps24
Sap./Malv./Brassicales - ps25
Malvales/Brassicales - ps26
Brassicales - ps27
Cleomaceae/Brassicaceae - ps28
Brassicaceae - ps29
Camelineae - ps30
Arabidopsis - ps31
Arabidopsis_thaliana - ps32
Phylostratum

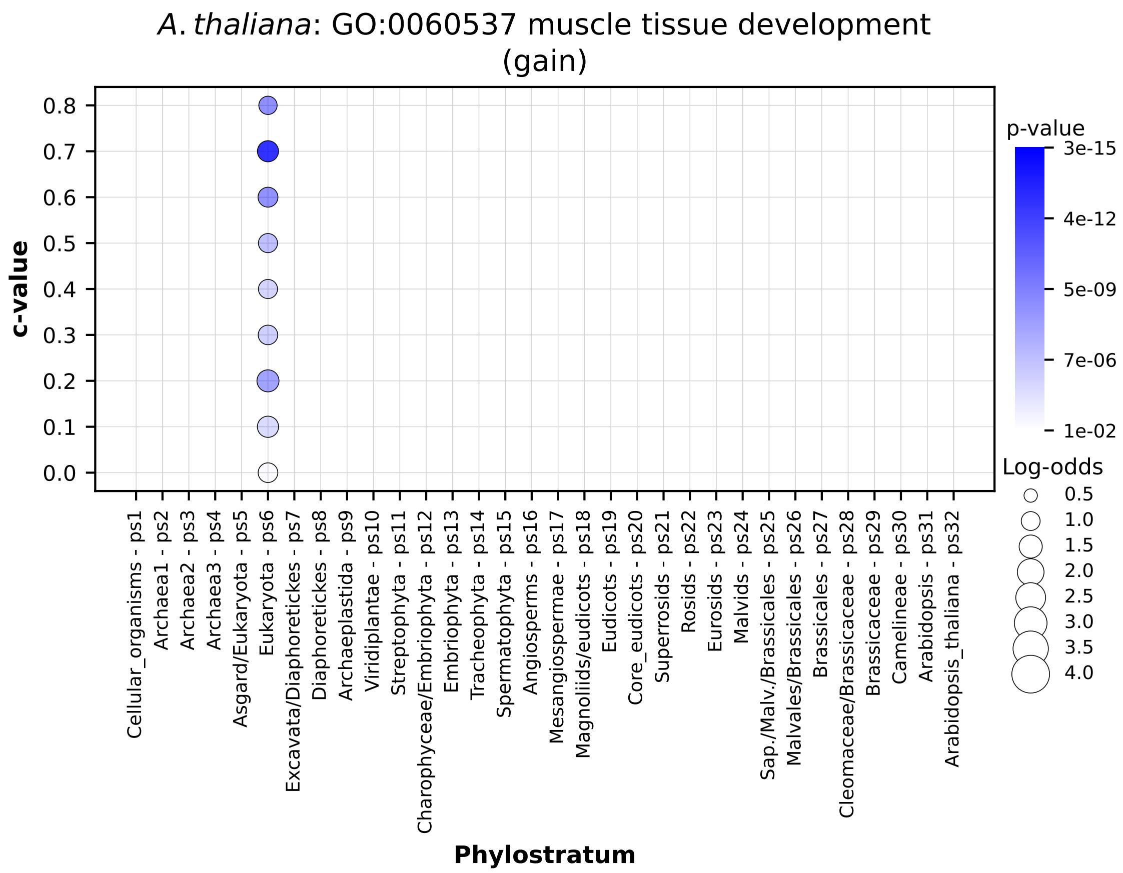

A. thaliana: GO:0060537 muscle tissue development
(gain)
c-value
0.8
0.7
0.6
0.5
0.4
0.3
0.2
0.1
0.0
p-value
3e-15
4e-12
5e-09
7e-06
1e-02
Log-odds
0.5
1.0
1.5
2.0
2.5
3.0
3.5
4.0
Cellular_organisms - ps1
Archaea1 - ps2
Archaea2 - ps3
Archaea3 - ps4
Asgard/Eukaryota - ps5
Eukaryota - ps6
Excavata/Diaphoretickes - ps7
Diaphoretickes - ps8
Archaeplastida - ps9
Viridiplantae - ps10
Streptophyta - ps11
Charophyceae/Embriophyta - ps12
Embriophyta - ps13
Tracheophyta - ps14
Spermatophyta - ps15
Angiosperms - ps16
Mesangiospermae - ps17
Magnoliids/eudicots - ps18
Eudicots - ps19
Core_eudicots - ps20
Superrosids - ps21
Rosids - ps22
Eurosids - ps23
Malvids - ps24
Sap./Malv./Brassicales - ps25
Malvales/Brassicales - ps26
Brassicales - ps27
Cleomaceae/Brassicaceae - ps28
Brassicaceae - ps29
Camelineae - ps30
Arabidopsis - ps31
Arabidopsis_thaliana - ps32
Phylostratum

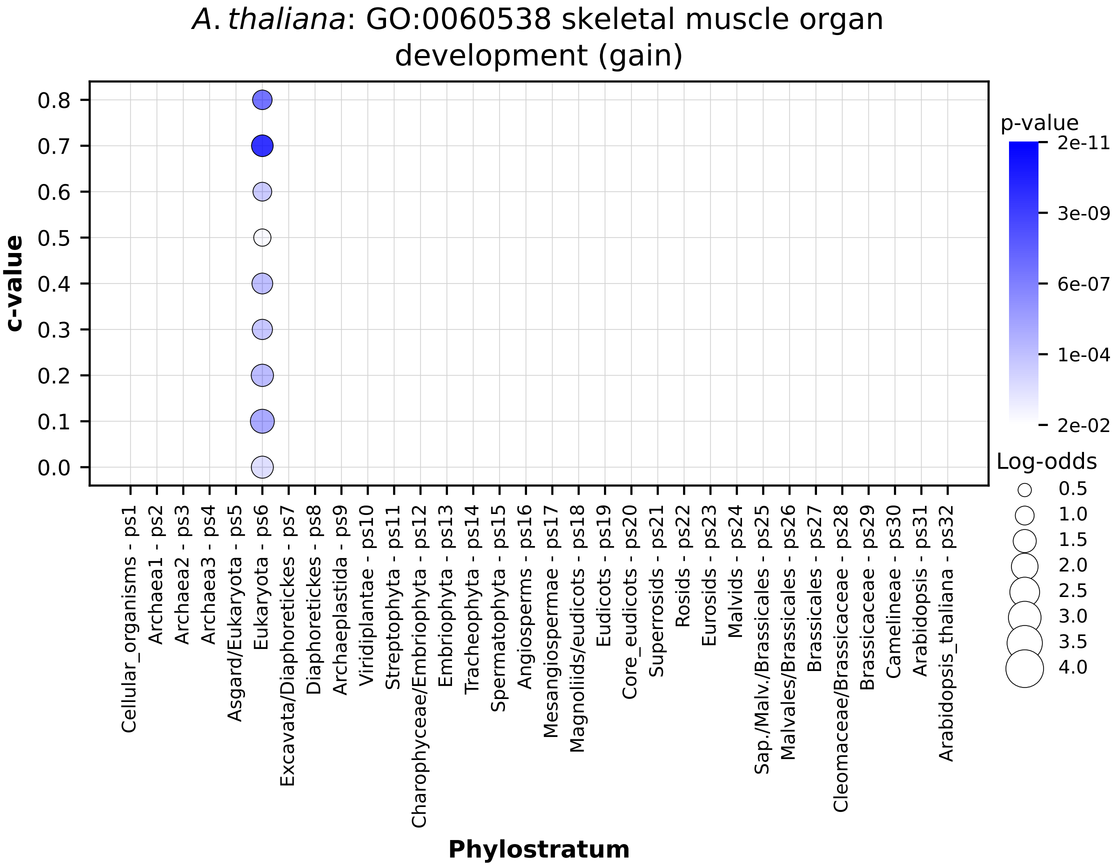

A. thaliana: GO:0060538 skeletal muscle organ development (gain)
c-value
0.8
0.7
0.6
0.5
0.4
0.3
0.2
0.1
0.0
p-value
2e-11
3e-09
6e-07
1e-04
2e-02
Log-odds
0.5
1.0
1.5
2.0
2.5
3.0
3.5
4.0
Cellular_organisms - ps1
Archaea1 - ps2
Archaea2 - ps3
Archaea3 - ps4
Asgard/Eukaryota - ps5
Eukaryota - ps6
Excavata/Diaphoretickes - ps7
Diaphoretickes - ps8
Archaeplastida - ps9
Viridiplantae - ps10
Streptophyta - ps11
Charophyceae/Embriophyta - ps12
Embriophyta - ps13
Tracheophyta - ps14
Spermatophyta - ps15
Angiosperms - ps16
Mesangiospermae - ps17
Magnoliids/eudicots - ps18
Eudicots - ps19
Core_eudicots - ps20
Superrosids - ps21
Rosids - ps22
Eurosids - ps23
Malvids - ps24
Sap./Malv./Brassicales - ps25
Malvales/Brassicales - ps26
Brassicales - ps27
Cleomaceae/Brassicaceae - ps28
Brassicaceae - ps29
Camelineae - ps30
Arabidopsis - ps31
Arabidopsis_thaliana - ps32
Phylostratum

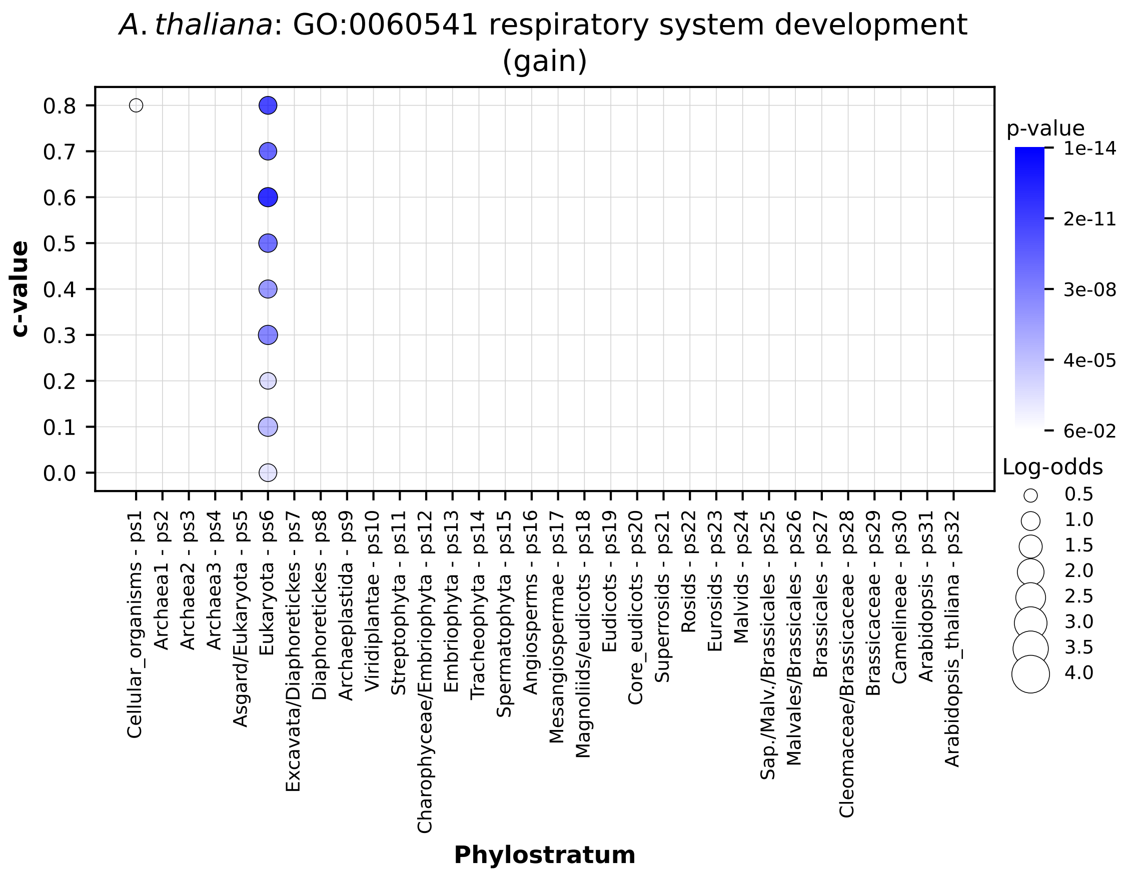
A. thaliana: GO:0060541 respiratory system development
(gain)
c-value
0.8
0.7
0.6
0.5
0.4
0.3
0.2
0.1
0.0
p-value
1e-14
2e-11
3e-08
4e-05
6e-02
Log-odds
0.5
1.0
1.5
2.0
2.5
3.0
3.5
4.0
Cellular_organisms - ps1
Archaea1 - ps2
Archaea2 - ps3
Archaea3 - ps4
Asgard/Eukaryota - ps5
Eukaryota - ps6
Excavata/Diaphoretickes - ps7
Diaphoretickes - ps8
Archaeplastida - ps9
Viridiplantae - ps10
Streptophyta - ps11
Charophyceae/Embriophyta - ps12
Embriophyta - ps13
Tracheophyta - ps14
Spermatophyta - ps15
Angiosperms - ps16
Mesangiospermae - ps17
Magnoliids/eudicots - ps18
Eudicots - ps19
Core_eudicots - ps20
Superrosids - ps21
Rosids - ps22
Eurosids - ps23
Malvids - ps24
Sap./Malv./Brassicales - ps25
Malvales/Brassicales - ps26
Brassicales - ps27
Cleomaceae/Brassicaceae - ps28
Brassicaceae - ps29
Camelineae - ps30
Arabidopsis - ps31
Arabidopsis_thaliana - ps32
Phylostratum

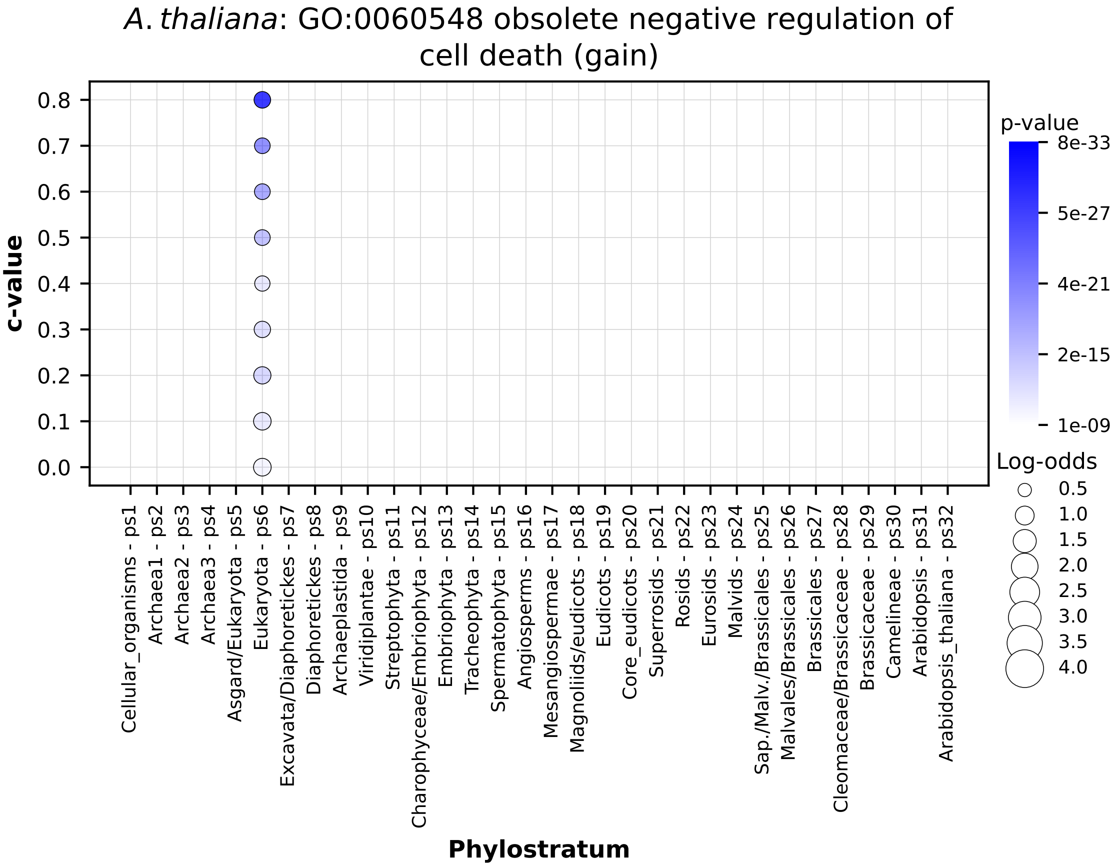

A. thaliana: GO:0060548 obsolete negative regulation of cell death (gain)
c-value
0.8
0.7
0.6
0.5
0.4
0.3
0.2
0.1
0.0
p-value
8e-33
5e-27
4e-21
2e-15
1e-09
Log-odds
0.5
1.0
1.5
2.0
2.5
3.0
3.5
4.0
Cellular_organisms - ps1
Archaea1 - ps2
Archaea2 - ps3
Archaea3 - ps4
Asgard/Eukaryota - ps5
Eukaryota - ps6
Excavata/Diaphoretickes - ps7
Diaphoretickes - ps8
Archaeplastida - ps9
Viridiplantae - ps10
Streptophyta - ps11
Charophyceae/Embriophyta - ps12
Embriophyta - ps13
Tracheophyta - ps14
Spermatophyta - ps15
Angiosperms - ps16
Mesangiospermae - ps17
Magnoliids/eudicots - ps18
Eudicots - ps19
Core_eudicots - ps20
Superrosids - ps21
Rosids - ps22
Eurosids - ps23
Malvids - ps24
Sap./Malv./Brassicales - ps25
Malvales/Brassicales - ps26
Brassicales - ps27
Cleomaceae/Brassicaceae - ps28
Brassicaceae - ps29
Camelineae - ps30
Arabidopsis - ps31
Arabidopsis_thaliana - ps32
Phylostratum

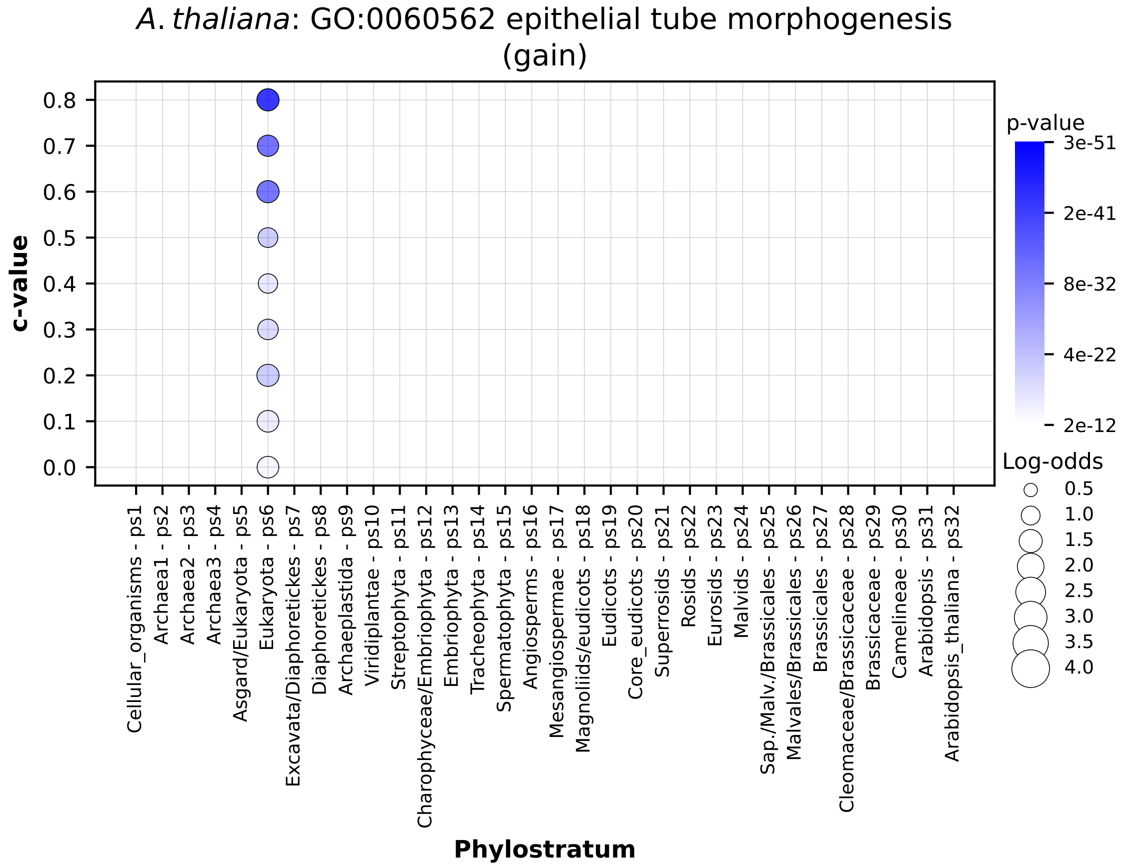

A. thaliana: GO:0060562 epithelial tube morphogenesis
(gain)
c-value
0.8
0.7
0.6
0.5
0.4
0.3
0.2
0.1
0.0
p-value
3e-51
2e-41
8e-32
4e-22
2e-12
Log-odds
0.5
1.0
1.5
2.0
2.5
3.0
3.5
4.0
Cellular_organisms - ps1
Archaea1 - ps2
Archaea2 - ps3
Archaea3 - ps4
Asgard/Eukaryota - ps5
Eukaryota - ps6
Excavata/Diaphoretickes - ps7
Diaphoretickes - ps8
Archaeplastida - ps9
Viridiplantae - ps10
Streptophyta - ps11
Charophyceae/Embriophyta - ps12
Embriophyta - ps13
Tracheophyta - ps14
Spermatophyta - ps15
Angiosperms - ps16
Mesangiospermae - ps17
Magnoliids/eudicots - ps18
Eudicots - ps19
Core_eudicots - ps20
Superrosids - ps21
Rosids - ps22
Eurosids - ps23
Malvids - ps24
Sap./Malv./Brassicales - ps25
Malvales/Brassicales - ps26
Brassicales - ps27
Cleomaceae/Brassicaceae - ps28
Brassicaceae - ps29
Camelineae - ps30
Arabidopsis - ps31
Arabidopsis_thaliana - ps32
Phylostratum

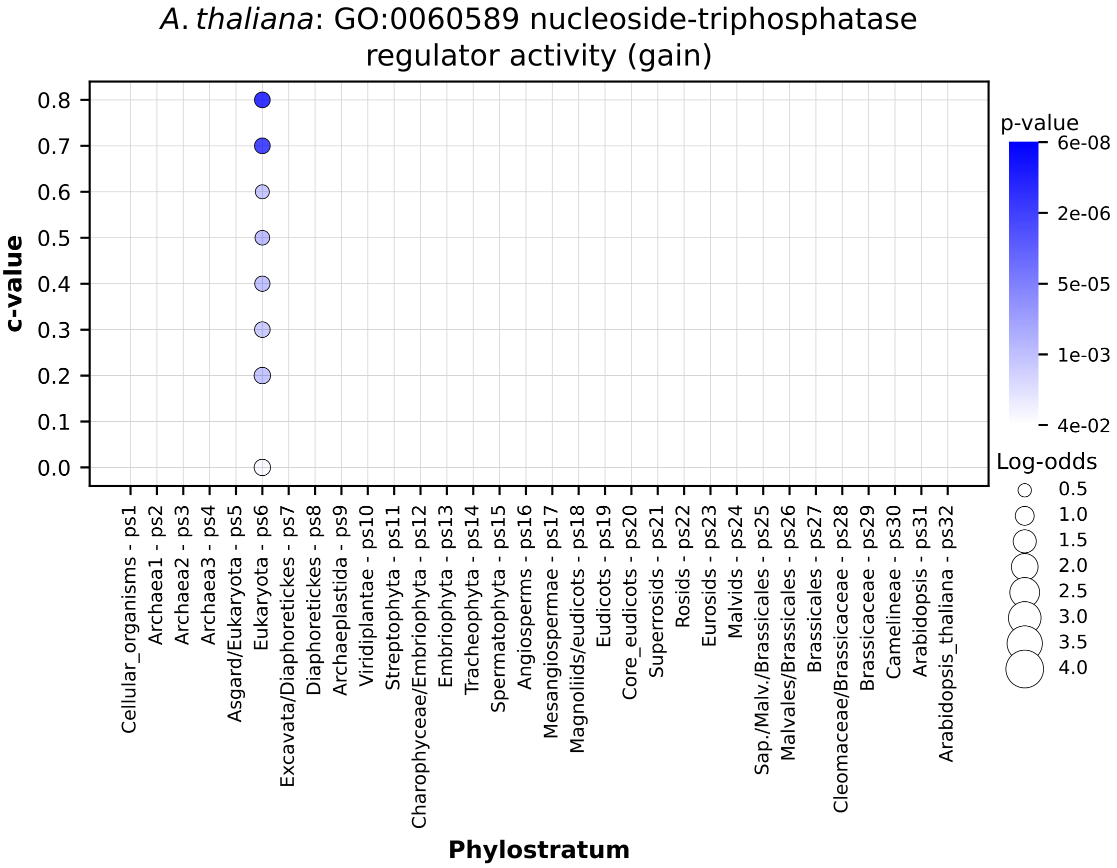

A. thaliana: GO:0060589 nucleoside-triphosphatase regulator activity (gain)
c-value
0.8
0.7
0.6
0.5
0.4
0.3
0.2
0.1
0.0
p-value
6e-08
2e-06
5e-05
1e-03
4e-02
Log-odds
0.5
1.0
1.5
2.0
2.5
3.0
3.5
4.0
Cellular_organisms - ps1
Archaea1 - ps2
Archaea2 - ps3
Archaea3 - ps4
Asgard/Eukaryota - ps5
Eukaryota - ps6
Excavata/Diaphoretickes - ps7
Diaphoretickes - ps8
Archaeplastida - ps9
Viridiplantae - ps10
Streptophyta - ps11
Charophyceae/Embriophyta - ps12
Embriophyta - ps13
Tracheophyta - ps14
Spermatophyta - ps15
Angiosperms - ps16
Mesangiospermae - ps17
Magnoliids/eudicots - ps18
Eudicots - ps19
Core_eudicots - ps20
Superrosids - ps21
Rosids - ps22
Eurosids - ps23
Malvids - ps24
Sap./Malv./Brassicales - ps25
Malvales/Brassicales - ps26
Brassicales - ps27
Cleomaceae/Brassicaceae - ps28
Brassicaceae - ps29
Camelineae - ps30
Arabidopsis - ps31
Arabidopsis_thaliana - ps32
Phylostratum

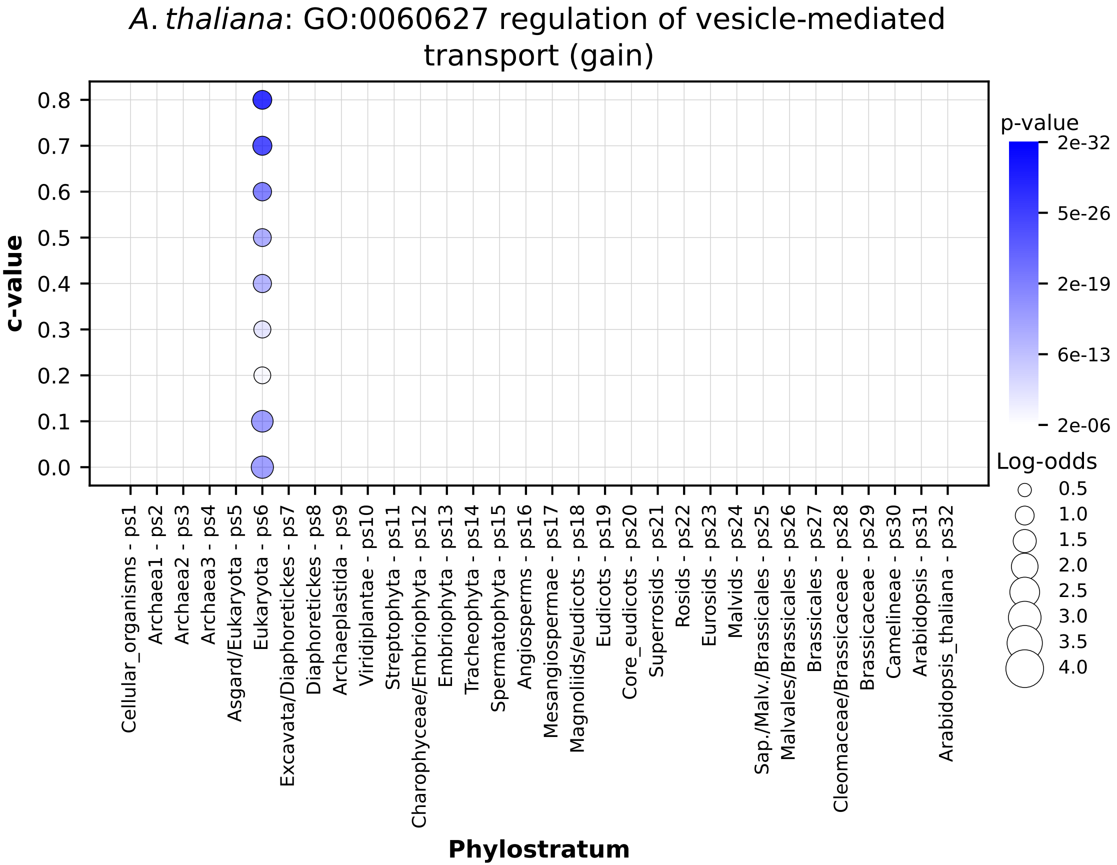

A. thaliana: GO:0060627 regulation of vesicle-mediated transport (gain)
c-value
0.8
0.7
0.6
0.5
0.4
0.3
0.2
0.1
0.0
p-value
2e-32
5e-26
2e-19
6e-13
2e-06
Log-odds
0.5
1.0
1.5
2.0
2.5
3.0
3.5
4.0
Cellular_organisms - ps1
Archaea1 - ps2
Archaea2 - ps3
Archaea3 - ps4
Asgard/Eukaryota - ps5
Eukaryota - ps6
Excavata/Diaphoretickes - ps7
Diaphoretickes - ps8
Archaeplastida - ps9
Viridiplantae - ps10
Streptophyta - ps11
Charophyceae/Embriophyta - ps12
Embriophyta - ps13
Tracheophyta - ps14
Spermatophyta - ps15
Angiosperms - ps16
Mesangiospermae - ps17
Magnoliids/eudicots - ps18
Eudicots - ps19
Core_eudicots - ps20
Superrosids - ps21
Rosids - ps22
Eurosids - ps23
Malvids - ps24
Sap./Malv./Brassicales - ps25
Malvales/Brassicales - ps26
Brassicales - ps27
Cleomaceae/Brassicaceae - ps28
Brassicaceae - ps29
Camelineae - ps30
Arabidopsis - ps31
Arabidopsis_thaliana - ps32
Phylostratum

A. thaliana: GO:0060828 regulation of canonical Wnt signaling pathway (gain)

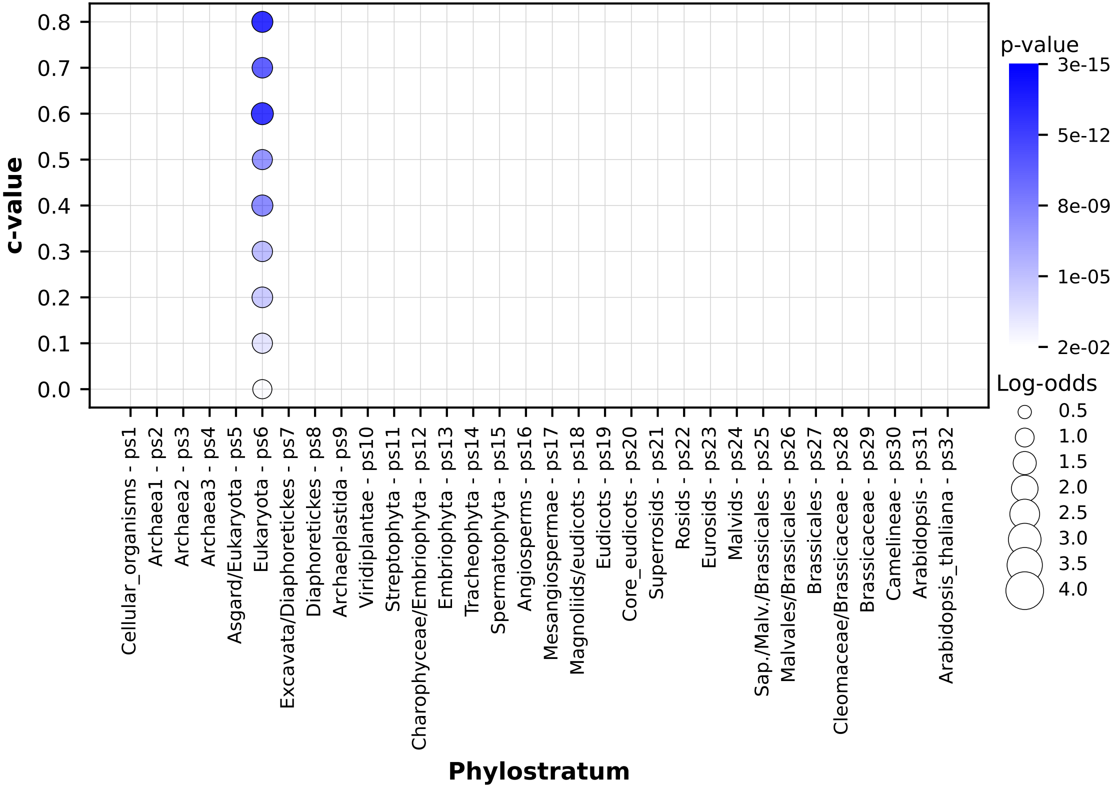

A. thaliana: GO:0061013 regulation of mRNA catabolic process (gain)

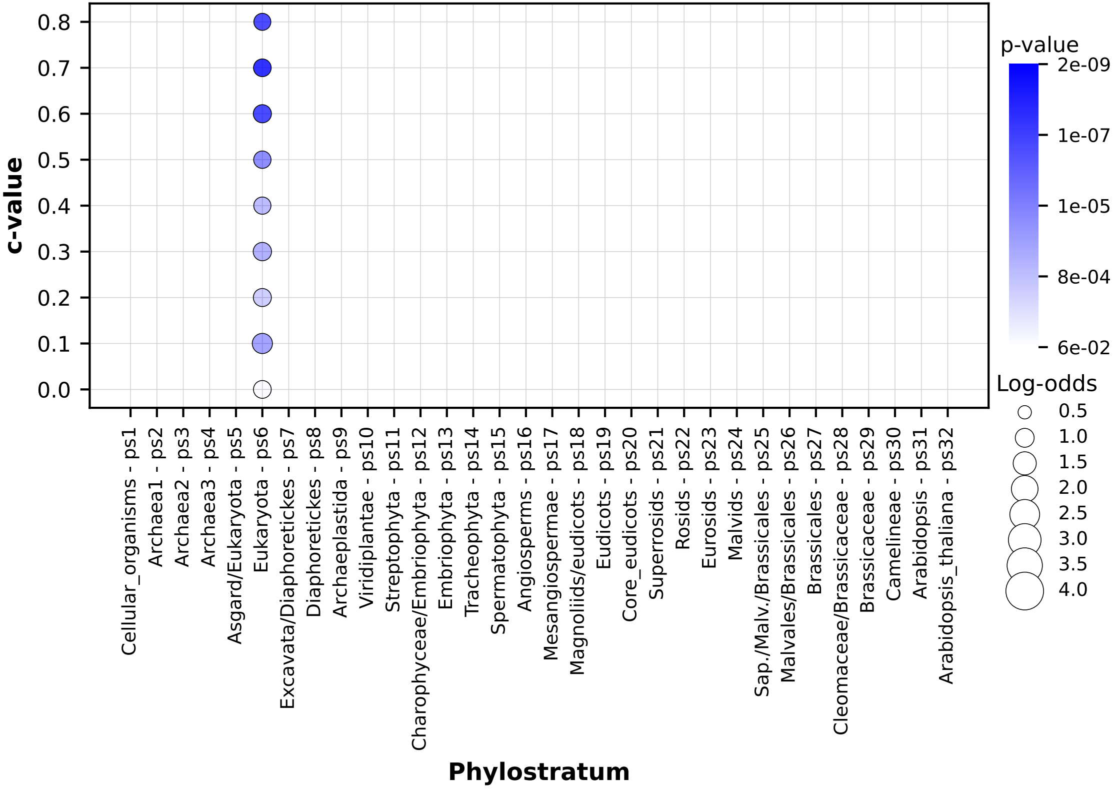

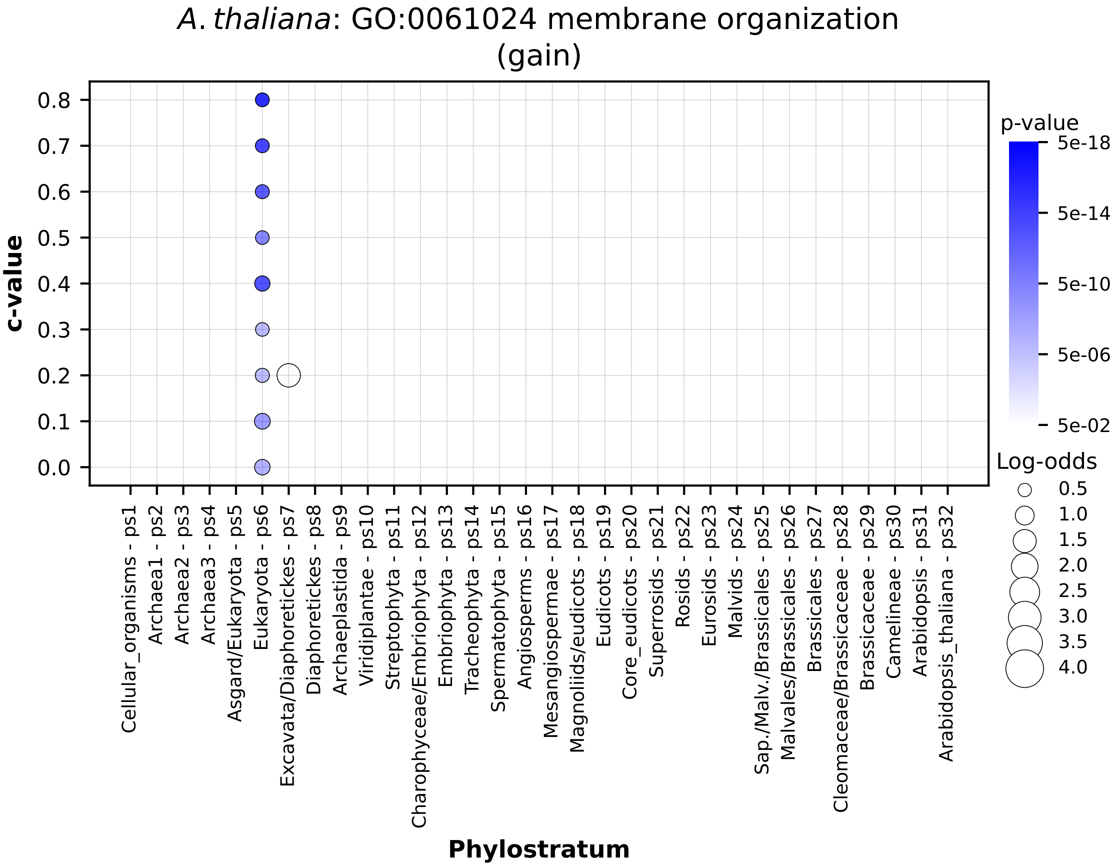

A. thaliana: GO:0061024 membrane organization
(gain)
c-value
0.0
0.1
0.2
0.3
0.4
0.5
0.6
0.7
0.8
Phylostratum
Cellular_organisms - ps1
Archaea1 - ps2
Archaea2 - ps3
Archaea3 - ps4
Asgard/Eukaryota - ps5
Eukaryota - ps6
Excavata/Diaphoretickes - ps7
Diaphoretickes - ps8
Archaeplastida - ps9
Viridiplantae - ps10
Streptophyta - ps11
Charophyceae/Embriophyta - ps12
Embriophyta - ps13
Tracheophyta - ps14
Spermatophyta - ps15
Angiosperms - ps16
Mesangiospermae - ps17
Magnoliids/eudicots - ps18
Eudicots - ps19
Core_eudicots - ps20
Superrosids - ps21
Rosids - ps22
Eurosids - ps23
Malvids - ps24
Sap./Malv./Brassicales - ps25
Malvales/Brassicales - ps26
Brassicales - ps27
Cleomaceae/Brassicaceae - ps28
Brassicaceae - ps29
Camelineae - ps30
Arabidopsis - ps31
Arabidopsis_thaliana - ps32
p-value
5e-18
5e-14
5e-10
5e-06
5e-02
Log-odds
0.5
1.0
1.5
2.0
2.5
3.0
3.5
4.0

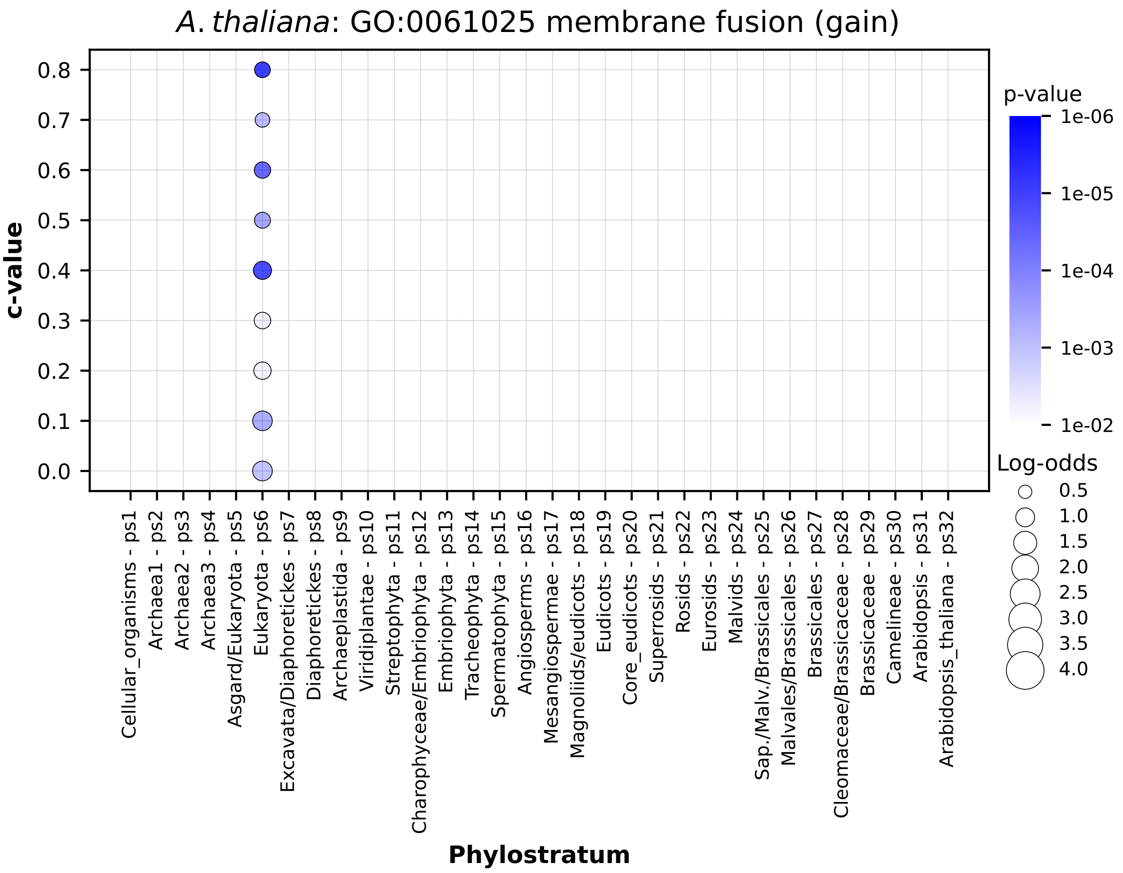

A. thaliana: GO:0061025 membrane fusion (gain)
c-value
0.8
0.7
0.6
0.5
0.4
0.3
0.2
0.1
0.0
p-value
1e-06
1e-05
1e-04
1e-03
1e-02
Log-odds
0.5
1.0
1.5
2.0
2.5
3.0
3.5
4.0
Cellular_organisms - ps1
Archaea1 - ps2
Archaea2 - ps3
Archaea3 - ps4
Asgard/Eukaryota - ps5
Eukaryota - ps6
Excavata/Diaphoretickes - ps7
Diaphoretickes - ps8
Archaeplastida - ps9
Viridiplantae - ps10
Streptophyta - ps11
Charophyceae/Embriophyta - ps12
Embriophyta - ps13
Tracheophyta - ps14
Spermatophyta - ps15
Angiosperms - ps16
Mesangiospermae - ps17
Magnoliids/eudicots - ps18
Eudicots - ps19
Core_eudicots - ps20
Superrosids - ps21
Rosids - ps22
Eurosids - ps23
Malvids - ps24
Sap./Malv./Brassicales - ps25
Malvales/Brassicales - ps26
Brassicales - ps27
Cleomaceae/Brassicaceae - ps28
Brassicaceae - ps29
Camelineae - ps30
Arabidopsis - ps31
Arabidopsis_thaliana - ps32
Phylostratum

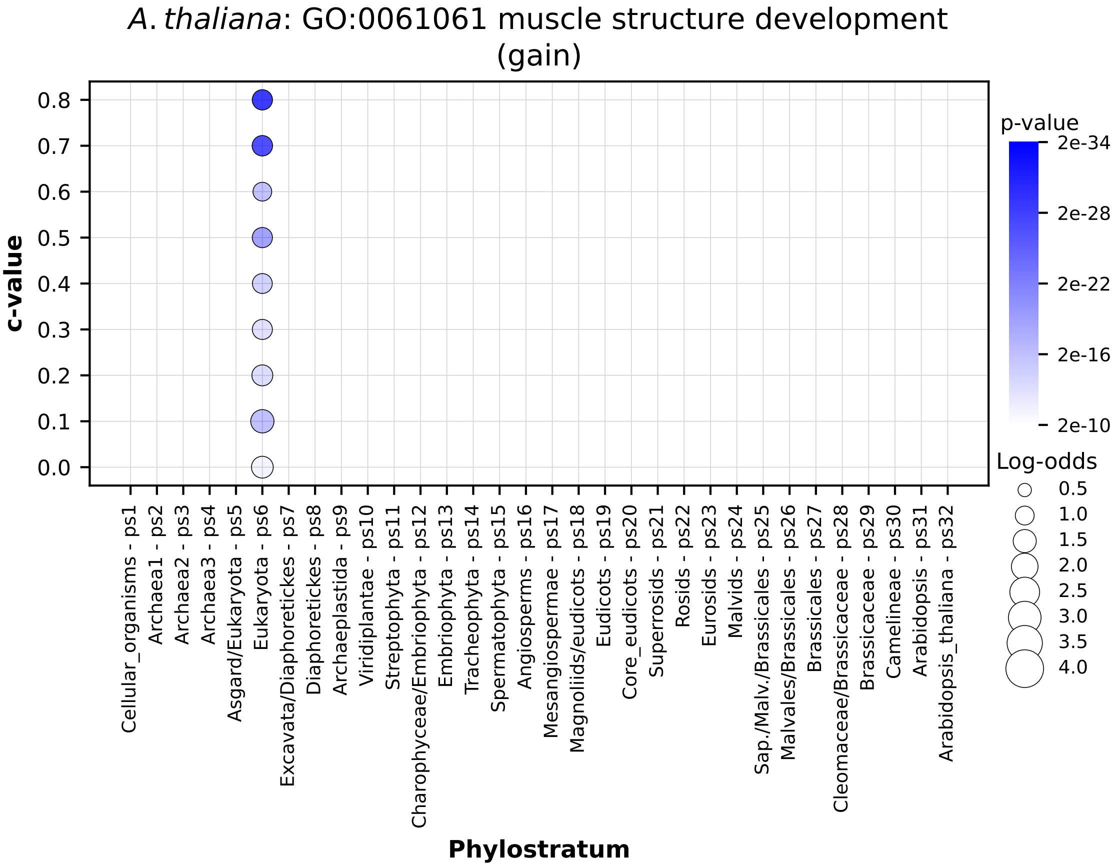

A. thaliana: GO:0061061 muscle structure development
(gain)
c-value
0.8
0.7
0.6
0.5
0.4
0.3
0.2
0.1
0.0
p-value
2e-34
2e-28
2e-22
2e-16
2e-10
Log-odds
0.5
1.0
1.5
2.0
2.5
3.0
3.5
4.0
Cellular_organisms - ps1
Archaea1 - ps2
Archaea2 - ps3
Archaea3 - ps4
Asgard/Eukaryota - ps5
Eukaryota - ps6
Excavata/Diaphoretickes - ps7
Diaphoretickes - ps8
Archaeplastida - ps9
Viridiplantae - ps10
Streptophyta - ps11
Charophyceae/Embriophyta - ps12
Embriophyta - ps13
Tracheophyta - ps14
Spermatophyta - ps15
Angiosperms - ps16
Mesangiospermae - ps17
Magnoliids/eudicots - ps18
Eudicots - ps19
Core_eudicots - ps20
Superrosids - ps21
Rosids - ps22
Eurosids - ps23
Malvids - ps24
Sap./Malv./Brassicales - ps25
Malvales/Brassicales - ps26
Brassicales - ps27
Cleomaceae/Brassicaceae - ps28
Brassicaceae - ps29
Camelineae - ps30
Arabidopsis - ps31
Arabidopsis_thaliana - ps32
Phylostratum

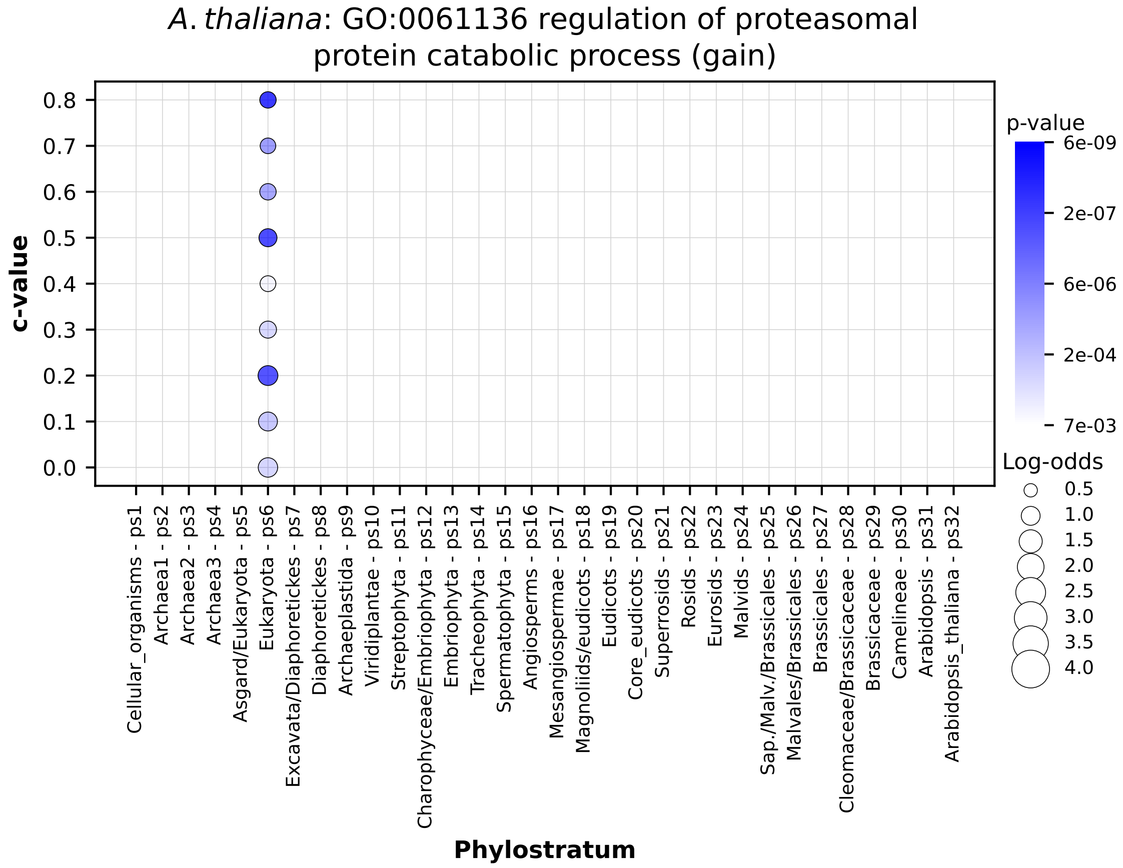

$A. thaliana$: GO:0061136 regulation of proteasomal protein catabolic process (gain)
c-value
0.0
0.1
0.2
0.3
0.4
0.5
0.6
0.7
0.8
p-value
6e-09
2e-07
6e-06
2e-04
7e-03
Log-odds
0.5
1.0
1.5
2.0
2.5
3.0
3.5
4.0
Cellular_organisms - ps1
Archaea1 - ps2
Archaea2 - ps3
Archaea3 - ps4
Asgard/Eukaryota - ps5
Eukaryota - ps6
Excavata/Diaphoretickes - ps7
Diaphoretickes - ps8
Archaeplastida - ps9
Viridiplantae - ps10
Streptophyta - ps11
Charophyceae/Embriophyta - ps12
Embriophyta - ps13
Tracheophyta - ps14
Spermatophyta - ps15
Angiosperms - ps16
Mesangiospermae - ps17
Magnoliids/eudicots - ps18
Eudicots - ps19
Core_eudicots - ps20
Superrosids - ps21
Rosids - ps22
Eurosids - ps23
Malvids - ps24
Sap./Malv./Brassicales - ps25
Malvales/Brassicales - ps26
Brassicales - ps27
Cleomaceae/Brassicaceae - ps28
Brassicaceae - ps29
Camelineae - ps30
Arabidopsis - ps31
Arabidopsis_thaliana - ps32
Phylostratum

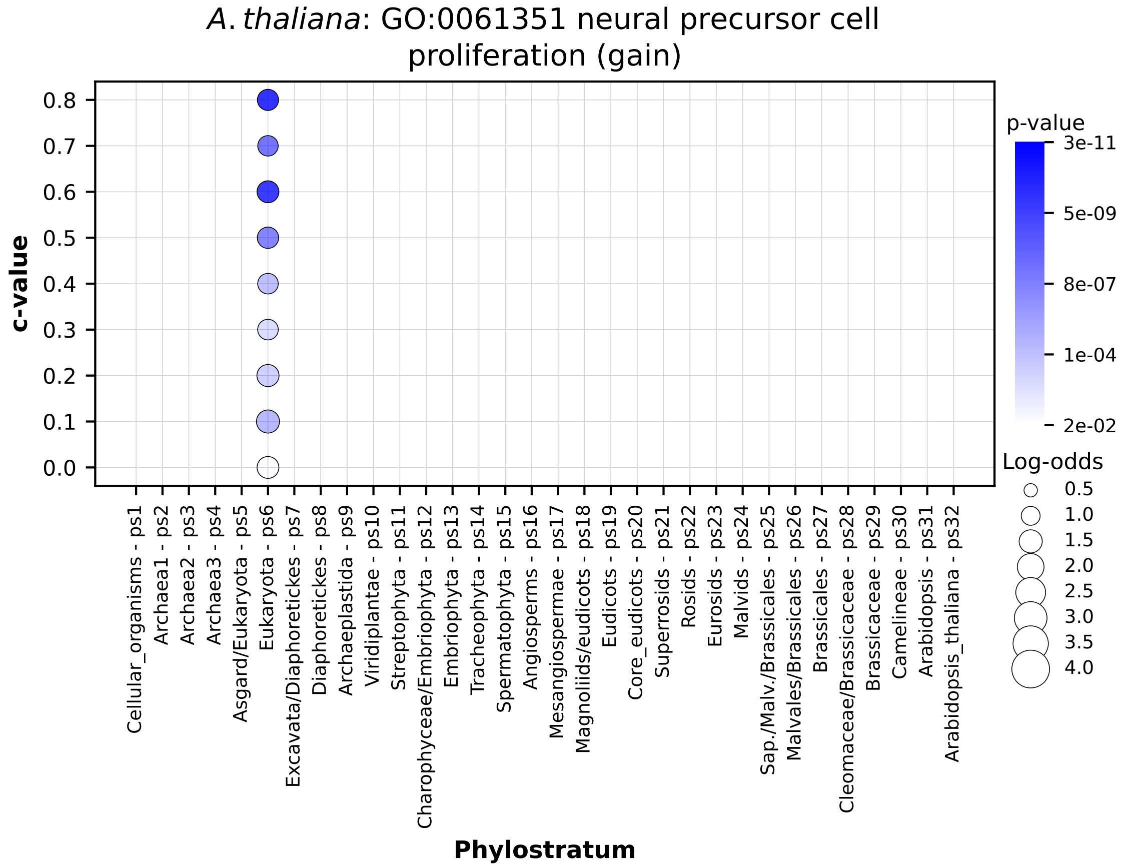

A. thaliana: GO:0061351 neural precursor cell proliferation (gain)
c-value
0.8
0.7
0.6
0.5
0.4
0.3
0.2
0.1
0.0
Phylostratum
Cellular_organisms - ps1
Archaea1 - ps2
Archaea2 - ps3
Archaea3 - ps4
Asgard/Eukaryota - ps5
Eukaryota - ps6
Excavata/Diaphoretickes - ps7
Diaphoretickes - ps8
Archaeplastida - ps9
Viridiplantae - ps10
Streptophyta - ps11
Charophyceae/Embriophyta - ps12
Embriophyta - ps13
Tracheophyta - ps14
Spermatophyta - ps15
Angiosperms - ps16
Mesangiospermae - ps17
Magnoliids/eudicots - ps18
Eudicots - ps19
Core_eudicots - ps20
Superrosids - ps21
Rosids - ps22
Eurosids - ps23
Malvids - ps24
Sap./Malv./Brassicales - ps25
Malvales/Brassicales - ps26
Brassicales - ps27
Cleomaceae/Brassicaceae - ps28
Brassicaceae - ps29
Camelineae - ps30
Arabidopsis - ps31
Arabidopsis_thaliana - ps32
p-value
3e-11
5e-09
8e-07
1e-04
2e-02
Log-odds
0.5
1.0
1.5
2.0
2.5
3.0
3.5
4.0

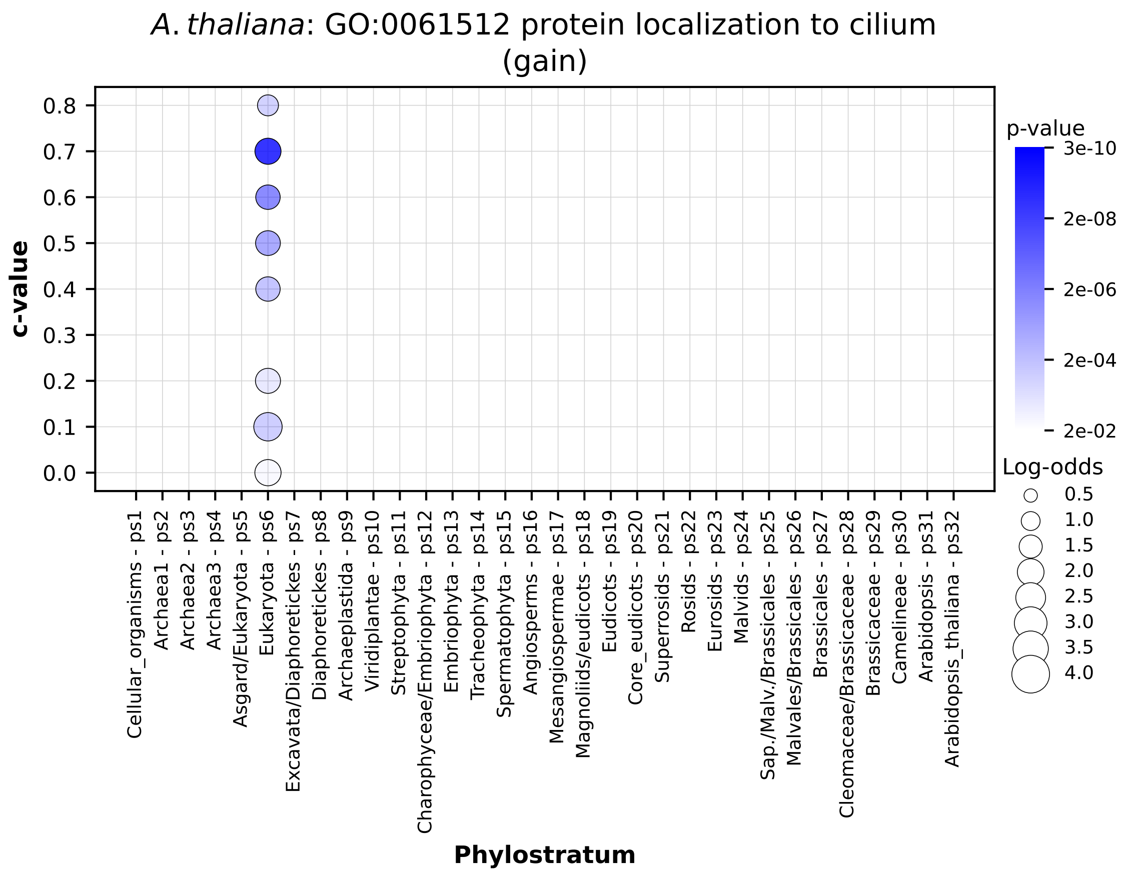

A. thaliana: GO:0061512 protein localization to cilium
(gain)
c-value
0.8
0.7
0.6
0.5
0.4
0.3
0.2
0.1
0.0
p-value
3e-10
2e-08
2e-06
2e-04
2e-02
Log-odds
0.5
1.0
1.5
2.0
2.5
3.0
3.5
4.0
Cellular_organisms - ps1
Archaea1 - ps2
Archaea2 - ps3
Archaea3 - ps4
Asgard/Eukaryota - ps5
Eukaryota - ps6
Excavata/Diaphoretickes - ps7
Diaphoretickes - ps8
Archaeplastida - ps9
Viridiplantae - ps10
Streptophyta - ps11
Charophyceae/Embriophyta - ps12
Embriophyta - ps13
Tracheophyta - ps14
Spermatophyta - ps15
Angiosperms - ps16
Mesangiospermae - ps17
Magnoliids/eudicots - ps18
Eudicots - ps19
Core_eudicots - ps20
Superrosids - ps21
Rosids - ps22
Eurosids - ps23
Malvids - ps24
Sap./Malv./Brassicales - ps25
Malvales/Brassicales - ps26
Brassicales - ps27
Cleomaceae/Brassicaceae - ps28
Brassicaceae - ps29
Camelineae - ps30
Arabidopsis - ps31
Arabidopsis_thaliana - ps32
Phylostratum

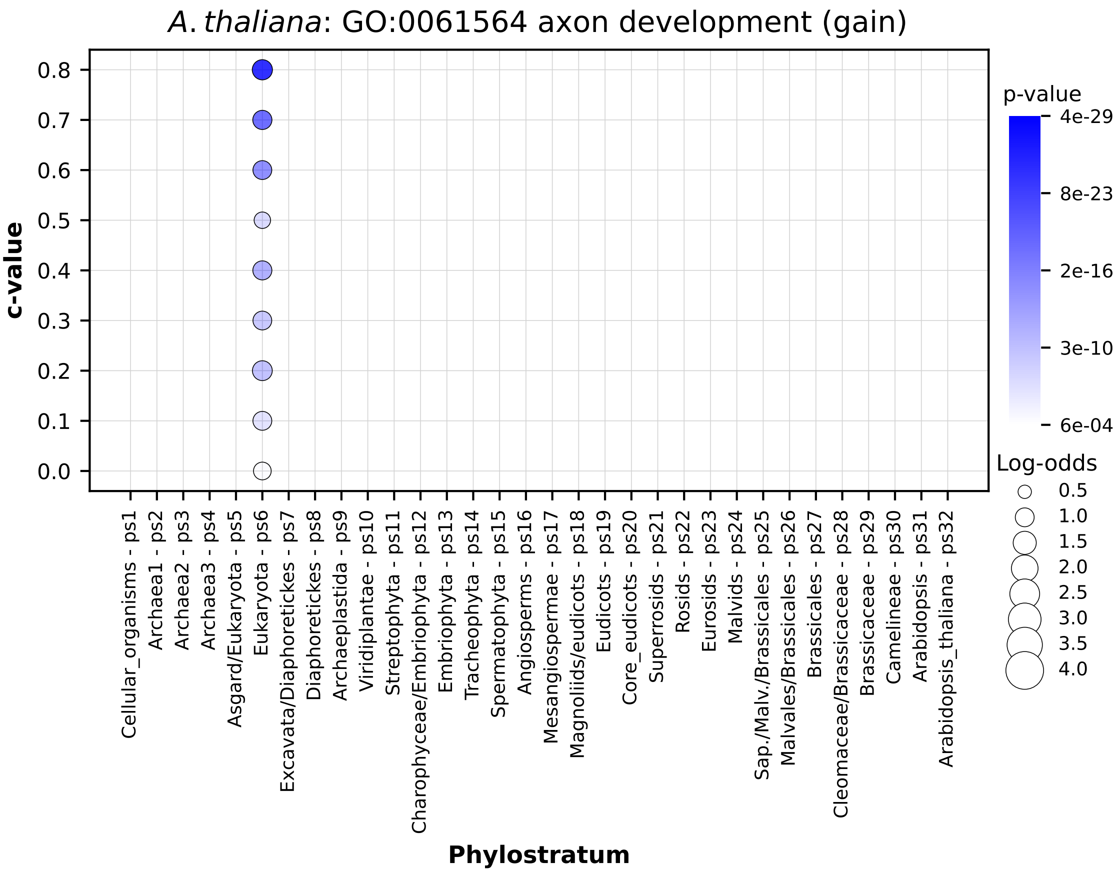

A. thaliana: GO:0061564 axon development (gain)
c-value
0.8
0.7
0.6
0.5
0.4
0.3
0.2
0.1
0.0
p-value
4e-29
8e-23
2e-16
3e-10
6e-04
Log-odds
0.5
1.0
1.5
2.0
2.5
3.0
3.5
4.0
Cellular_organisms - ps1
Archaea1 - ps2
Archaea2 - ps3
Archaea3 - ps4
Asgard/Eukaryota - ps5
Eukaryota - ps6
Excavata/Diaphoretickes - ps7
Diaphoretickes - ps8
Archaeplastida - ps9
Viridiplantae - ps10
Streptophyta - ps11
Charophyceae/Embriophyta - ps12
Embriophyta - ps13
Tracheophyta - ps14
Spermatophyta - ps15
Angiosperms - ps16
Mesangiospermae - ps17
Magnoliids/eudicots - ps18
Eudicots - ps19
Core_eudicots - ps20
Superrosids - ps21
Rosids - ps22
Eurosids - ps23
Malvids - ps24
Sap./Malv./Brassicales - ps25
Malvales/Brassicales - ps26
Brassicales - ps27
Cleomaceae/Brassicaceae - ps28
Brassicaceae - ps29
Camelineae - ps30
Arabidopsis - ps31
Arabidopsis_thaliana - ps32
Phylostratum

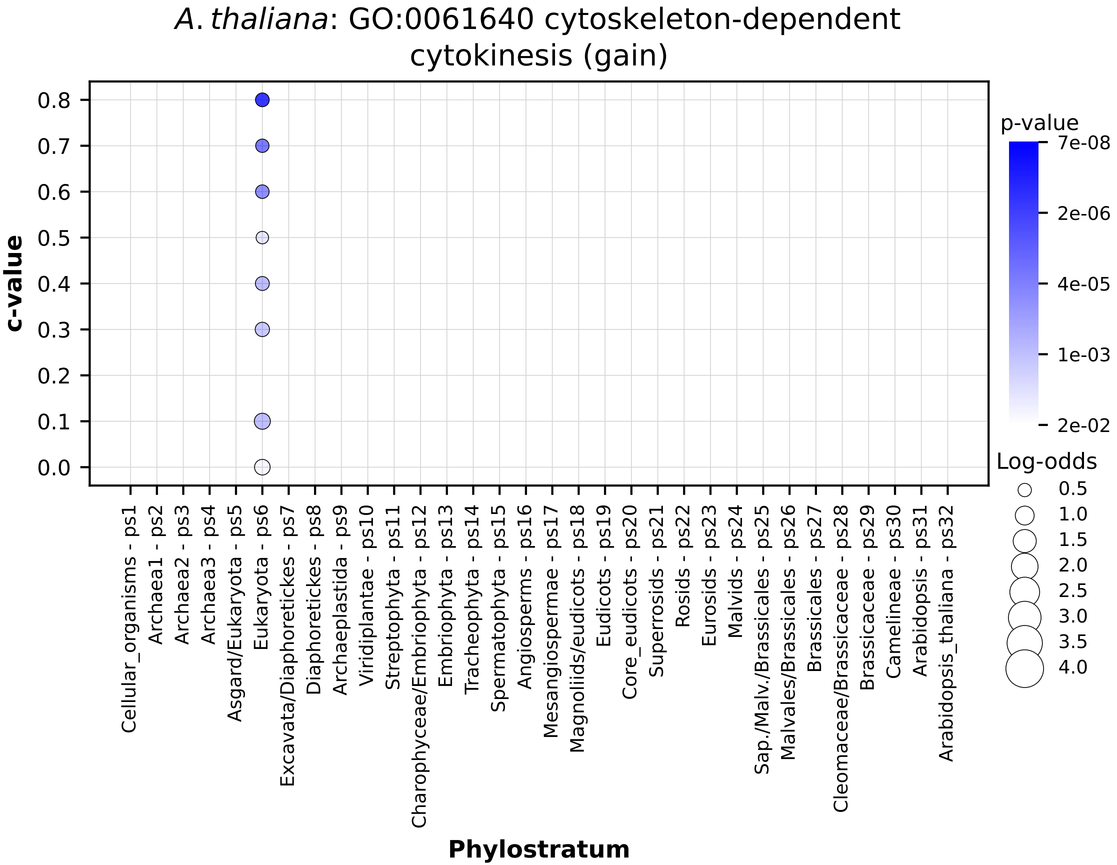

A. thaliana: GO:0061640 cytoskeleton-dependent cytokinesis (gain)
c-value
0.0
0.1
0.2
0.3
0.4
0.5
0.6
0.7
0.8
Phylostratum
Cellular_organisms - ps1
Archaea1 - ps2
Archaea2 - ps3
Archaea3 - ps4
Asgard/Eukaryota - ps5
Eukaryota - ps6
Excavata/Diaphoretickes - ps7
Diaphoretickes - ps8
Archaeplastida - ps9
Viridiplantae - ps10
Streptophyta - ps11
Charophyceae/Embriophyta - ps12
Embriophyta - ps13
Tracheophyta - ps14
Spermatophyta - ps15
Angiosperms - ps16
Mesangiospermae - ps17
Magnoliids/eudicots - ps18
Eudicots - ps19
Core_eudicots - ps20
Superrosids - ps21
Rosids - ps22
Eurosids - ps23
Malvids - ps24
Sap./Malv./Brassicales - ps25
Malvales/Brassicales - ps26
Brassicales - ps27
Cleomaceae/Brassicaceae - ps28
Brassicaceae - ps29
Camelineae - ps30
Arabidopsis - ps31
Arabidopsis_thaliana - ps32
p-value
7e-08
2e-06
4e-05
1e-03
2e-02
Log-odds
0.5
1.0
1.5
2.0
2.5
3.0
3.5
4.0

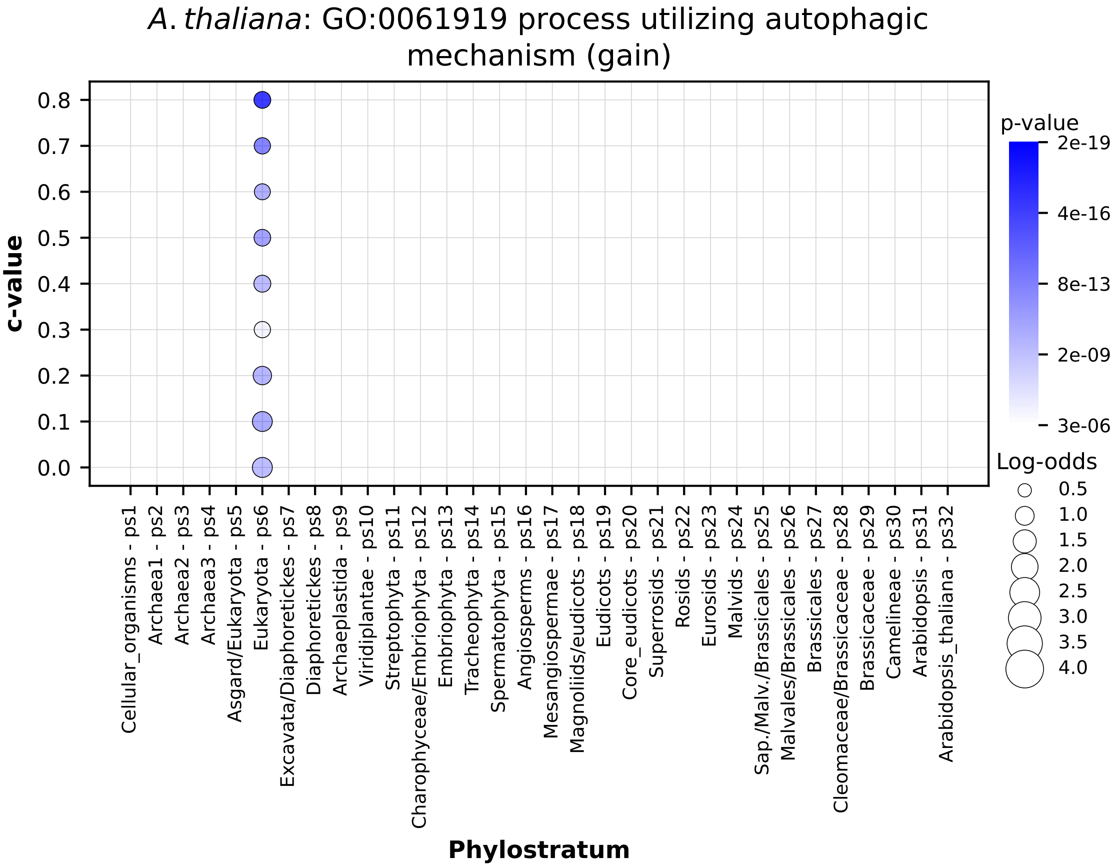
A. thaliana: GO:0061919 process utilizing autophagic mechanism (gain)
c-value
0.8
0.7
0.6
0.5
0.4
0.3
0.2
0.1
0.0
p-value
2e-19
4e-16
8e-13
2e-09
3e-06
Log-odds
0.5
1.0
1.5
2.0
2.5
3.0
3.5
4.0
Cellular_organisms - ps1
Archaea1 - ps2
Archaea2 - ps3
Archaea3 - ps4
Asgard/Eukaryota - ps5
Eukaryota - ps6
Excavata/Diaphoretickes - ps7
Diaphoretickes - ps8
Archaeplastida - ps9
Viridiplantae - ps10
Streptophyta - ps11
Charophyceae/Embriophyta - ps12
Embriophyta - ps13
Tracheophyta - ps14
Spermatophyta - ps15
Angiosperms - ps16
Mesangiospermae - ps17
Magnoliids/eudicots - ps18
Eudicots - ps19
Core_eudicots - ps20
Superrosids - ps21
Rosids - ps22
Eurosids - ps23
Malvids - ps24
Sap./Malv./Brassicales - ps25
Malvales/Brassicales - ps26
Brassicales - ps27
Cleomaceae/Brassicaceae - ps28
Brassicaceae - ps29
Camelineae - ps30
Arabidopsis - ps31
Arabidopsis_thaliana - ps32
Phylostratum

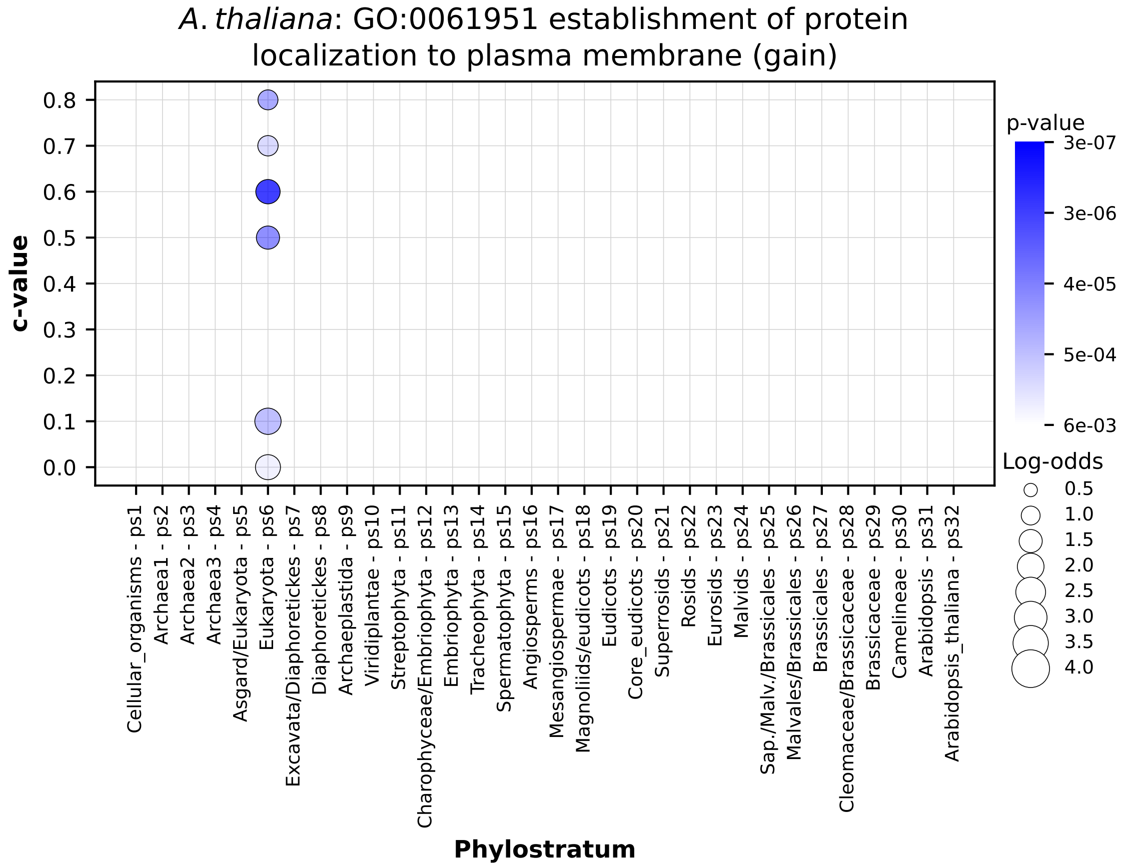

A. thaliana: GO:0061951 establishment of protein localization to plasma membrane (gain)
c-value
0.0
0.1
0.2
0.3
0.4
0.5
0.6
0.7
0.8
Phylostratum
Cellular_organisms - ps1
Archaea1 - ps2
Archaea2 - ps3
Archaea3 - ps4
Asgard/Eukaryota - ps5
Eukaryota - ps6
Excavata/Diaphoretickes - ps7
Diaphoretickes - ps8
Archaeplastida - ps9
Viridiplantae - ps10
Streptophyta - ps11
Charophyceae/Embriophyta - ps12
Embriophyta - ps13
Tracheophyta - ps14
Spermatophyta - ps15
Angiosperms - ps16
Mesangiospermae - ps17
Magnoliids/eudicots - ps18
Eudicots - ps19
Core_eudicots - ps20
Superrosids - ps21
Rosids - ps22
Eurosids - ps23
Malvids - ps24
Sap./Malv./Brassicales - ps25
Malvales/Brassicales - ps26
Brassicales - ps27
Cleomaceae/Brassicaceae - ps28
Brassicaceae - ps29
Camelineae - ps30
Arabidopsis - ps31
Arabidopsis_thaliana - ps32
p-value
3e-07
3e-06
4e-05
5e-04
6e-03
Log-odds
0.5
1.0
1.5
2.0
2.5
3.0
3.5
4.0

A. thaliana: GO:0065003 protein-containing complex assembly (gain)

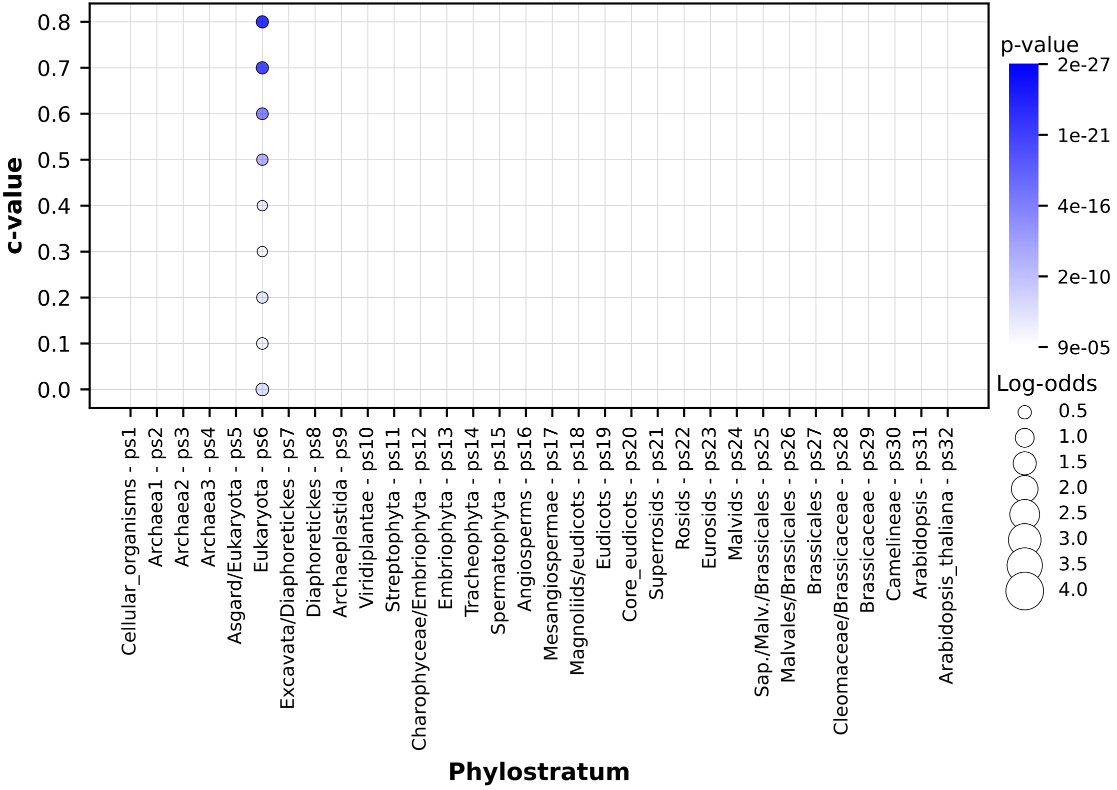

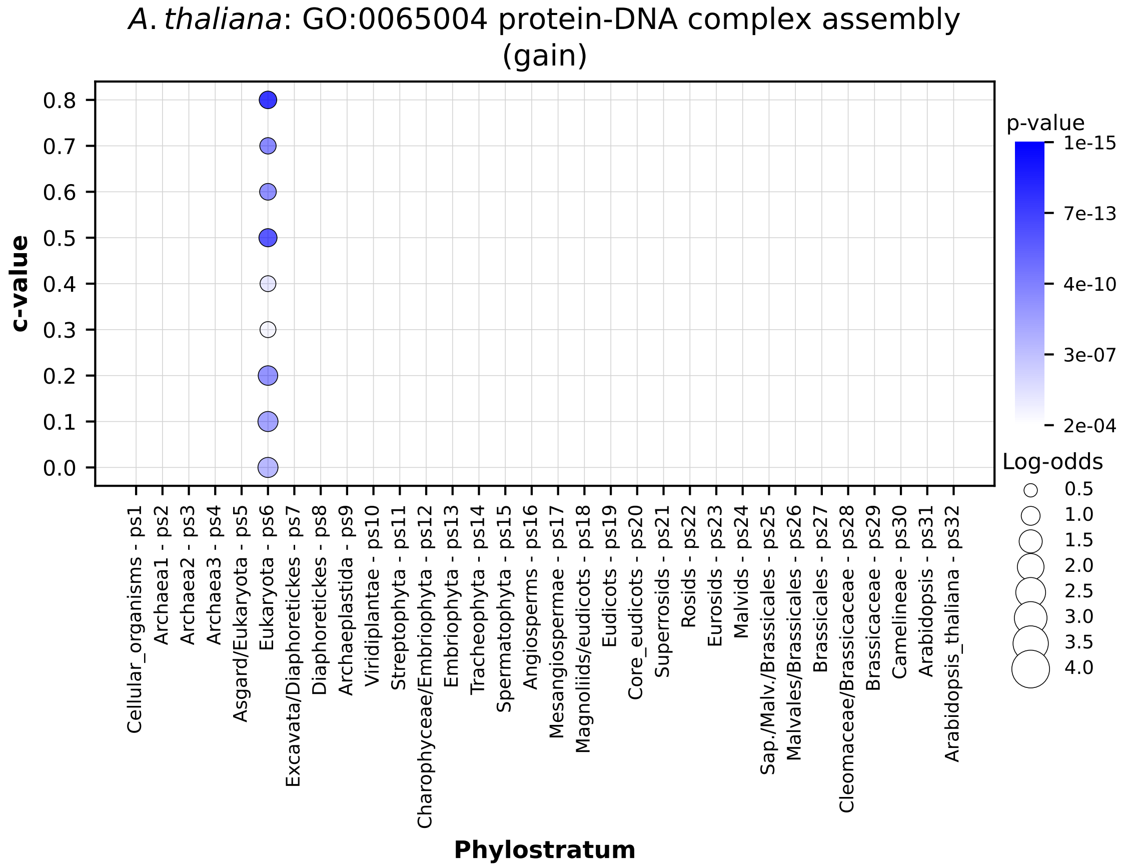

A. thaliana: GO:0065004 protein-DNA complex assembly (gain)
c-value
0.0
0.1
0.2
0.3
0.4
0.5
0.6
0.7
0.8
Phylostratum
Cellular_organisms - ps1
Archaea1 - ps2
Archaea2 - ps3
Archaea3 - ps4
Asgard/Eukaryota - ps5
Eukaryota - ps6
Excavata/Diaphoretickes - ps7
Diaphoretickes - ps8
Archaeplastida - ps9
Viridiplantae - ps10
Streptophyta - ps11
Charophyceae/Embriophyta - ps12
Embriophyta - ps13
Tracheophyta - ps14
Spermatophyta - ps15
Angiosperms - ps16
Mesangiospermae - ps17
Magnoliids/eudicots - ps18
Eudicots - ps19
Core_eudicots - ps20
Superrosids - ps21
Rosids - ps22
Eurosids - ps23
Malvids - ps24
Sap./Malv./Brassicales - ps25
Malvales/Brassicales - ps26
Brassicales - ps27
Cleomaceae/Brassicaceae - ps28
Brassicaceae - ps29
Camelineae - ps30
Arabidopsis - ps31
Arabidopsis_thaliana - ps32
p-value
1e-15
7e-13
4e-10
3e-07
2e-04
Log-odds
0.5
1.0
1.5
2.0
2.5
3.0
3.5
4.0

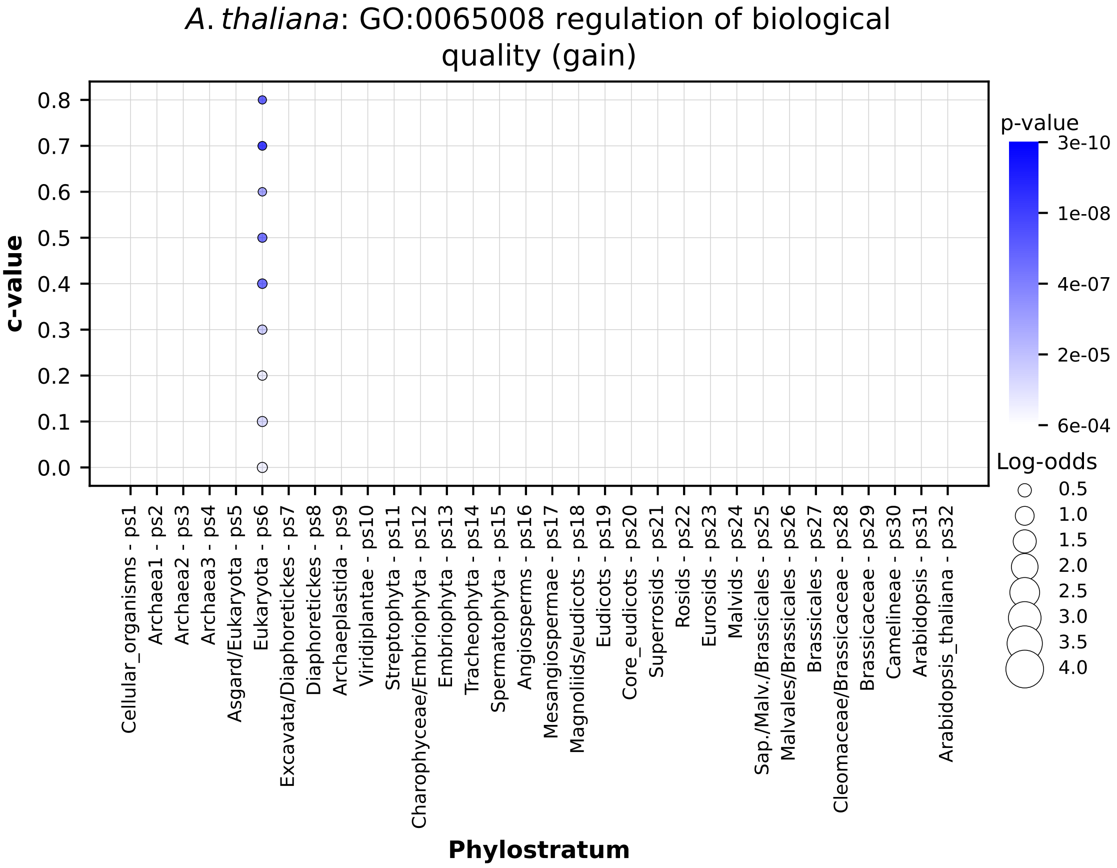

A. thaliana: GO:0065008 regulation of biological quality (gain)
c-value
0.8
0.7
0.6
0.5
0.4
0.3
0.2
0.1
0.0
p-value
3e-10
1e-08
4e-07
2e-05
6e-04
Log-odds
0.5
1.0
1.5
2.0
2.5
3.0
3.5
4.0
Cellular_organisms - ps1
Archaea1 - ps2
Archaea2 - ps3
Archaea3 - ps4
Asgard/Eukaryota - ps5
Eukaryota - ps6
Excavata/Diaphoretickes - ps7
Diaphoretickes - ps8
Archaeplastida - ps9
Viridiplantae - ps10
Streptophyta - ps11
Charophyceae/Embriophyta - ps12
Embriophyta - ps13
Tracheophyta - ps14
Spermatophyta - ps15
Angiosperms - ps16
Mesangiospermae - ps17
Magnoliids/eudicots - ps18
Eudicots - ps19
Core_eudicots - ps20
Superrosids - ps21
Rosids - ps22
Eurosids - ps23
Malvids - ps24
Sap./Malv./Brassicales - ps25
Malvales/Brassicales - ps26
Brassicales - ps27
Cleomaceae/Brassicaceae - ps28
Brassicaceae - ps29
Camelineae - ps30
Arabidopsis - ps31
Arabidopsis_thaliana - ps32
Phylostratum

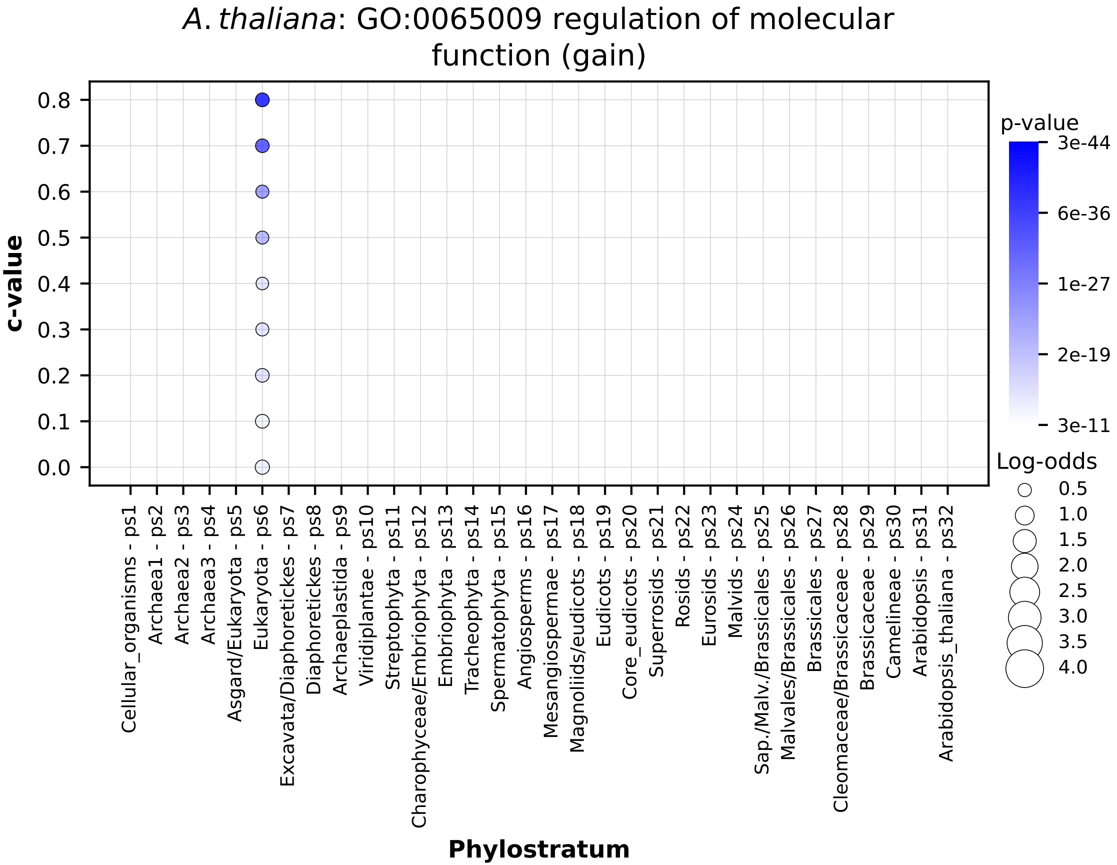

A. thaliana: GO:0065009 regulation of molecular function (gain)
c-value
0.8
0.7
0.6
0.5
0.4
0.3
0.2
0.1
0.0
p-value
3e-44
6e-36
1e-27
2e-19
3e-11
Log-odds
0.5
1.0
1.5
2.0
2.5
3.0
3.5
4.0
Cellular_organisms - ps1
Archaea1 - ps2
Archaea2 - ps3
Archaea3 - ps4
Asgard/Eukaryota - ps5
Eukaryota - ps6
Excavata/Diaphoretickes - ps7
Diaphoretickes - ps8
Archaeplastida - ps9
Viridiplantae - ps10
Streptophyta - ps11
Charophyceae/Embriophyta - ps12
Embriophyta - ps13
Tracheophyta - ps14
Spermatophyta - ps15
Angiosperms - ps16
Mesangiospermae - ps17
Magnoliids/eudicots - ps18
Eudicots - ps19
Core_eudicots - ps20
Superrosids - ps21
Rosids - ps22
Eurosids - ps23
Malvids - ps24
Sap./Malv./Brassicales - ps25
Malvales/Brassicales - ps26
Brassicales - ps27
Cleomaceae/Brassicaceae - ps28
Brassicaceae - ps29
Camelineae - ps30
Arabidopsis - ps31
Arabidopsis_thaliana - ps32
Phylostratum

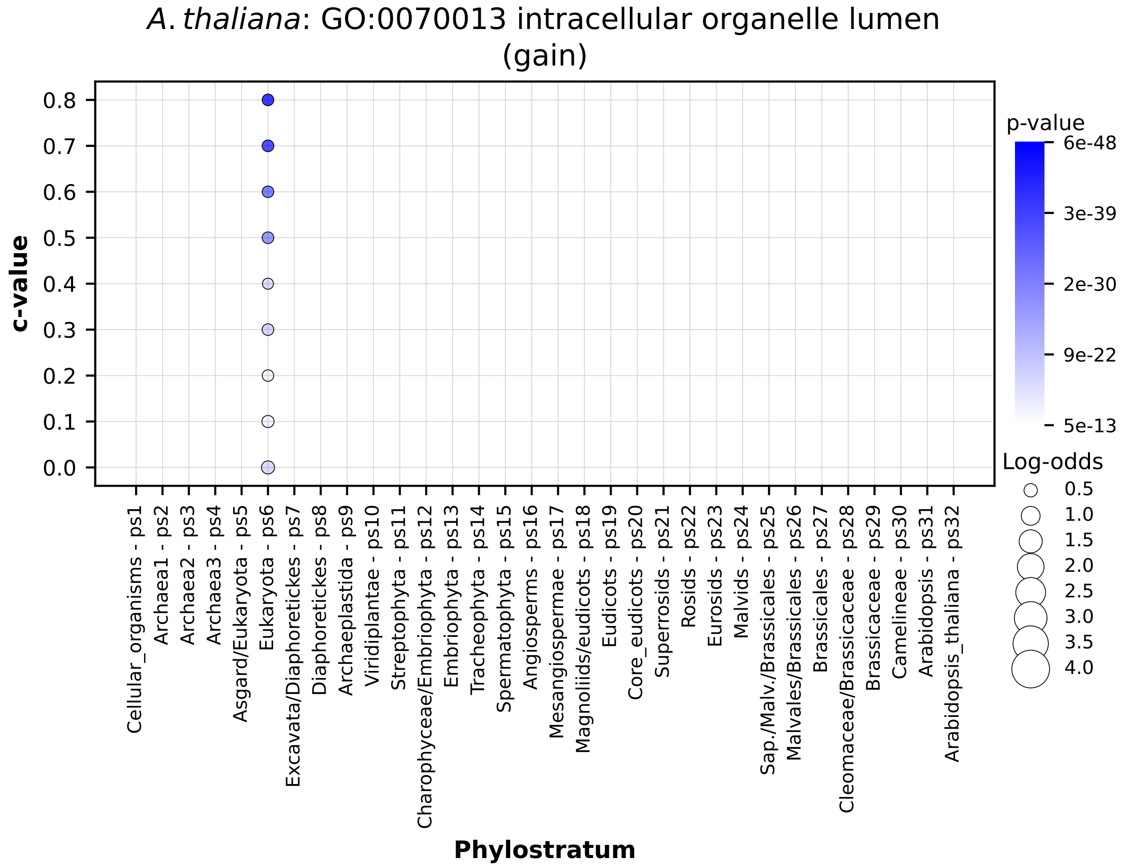

A. thaliana: GO:0070013 intracellular organelle lumen
(gain)
c-value
0.8
0.7
0.6
0.5
0.4
0.3
0.2
0.1
0.0
p-value
6e-48
3e-39
2e-30
9e-22
5e-13
Log-odds
0.5
1.0
1.5
2.0
2.5
3.0
3.5
4.0
Cellular_organisms - ps1
Archaea1 - ps2
Archaea2 - ps3
Archaea3 - ps4
Asgard/Eukaryota - ps5
Eukaryota - ps6
Excavata/Diaphoretickes - ps7
Diaphoretickes - ps8
Archaeplastida - ps9
Viridiplantae - ps10
Streptophyta - ps11
Charophyceae/Embriophyta - ps12
Embriophyta - ps13
Tracheophyta - ps14
Spermatophyta - ps15
Angiosperms - ps16
Mesangiospermae - ps17
Magnoliids/eudicots - ps18
Eudicots - ps19
Core_eudicots - ps20
Superrosids - ps21
Rosids - ps22
Eurosids - ps23
Malvids - ps24
Sap./Malv./Brassicales - ps25
Malvales/Brassicales - ps26
Brassicales - ps27
Cleomaceae/Brassicaceae - ps28
Brassicaceae - ps29
Camelineae - ps30
Arabidopsis - ps31
Arabidopsis_thaliana - ps32
Phylostratum

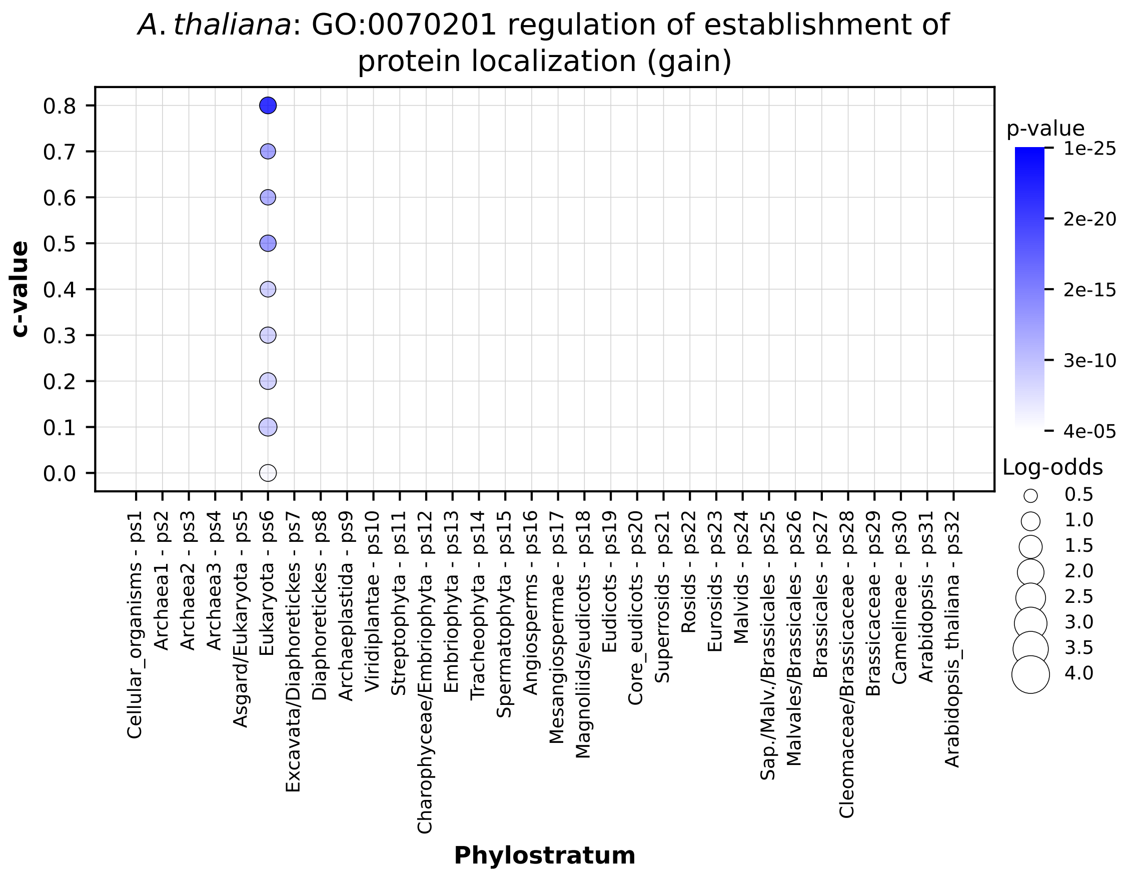

A. thaliana: GO:0070201 regulation of establishment of protein localization (gain)
c-value
0.8
0.7
0.6
0.5
0.4
0.3
0.2
0.1
0.0
p-value
1e-25
2e-20
2e-15
3e-10
4e-05
Log-odds
0.5
1.0
1.5
2.0
2.5
3.0
3.5
4.0
Cellular_organisms - ps1
Archaea1 - ps2
Archaea2 - ps3
Archaea3 - ps4
Asgard/Eukaryota - ps5
Eukaryota - ps6
Excavata/Diaphoretickes - ps7
Diaphoretickes - ps8
Archaeplastida - ps9
Viridiplantae - ps10
Streptophyta - ps11
Charophyceae/Embriophyta - ps12
Embriophyta - ps13
Tracheophyta - ps14
Spermatophyta - ps15
Angiosperms - ps16
Mesangiospermae - ps17
Magnoliids/eudicots - ps18
Eudicots - ps19
Core_eudicots - ps20
Superrosids - ps21
Rosids - ps22
Eurosids - ps23
Malvids - ps24
Sap./Malv./Brassicales - ps25
Malvales/Brassicales - ps26
Brassicales - ps27
Cleomaceae/Brassicaceae - ps28
Brassicaceae - ps29
Camelineae - ps30
Arabidopsis - ps31
Arabidopsis_thaliana - ps32
Phylostratum

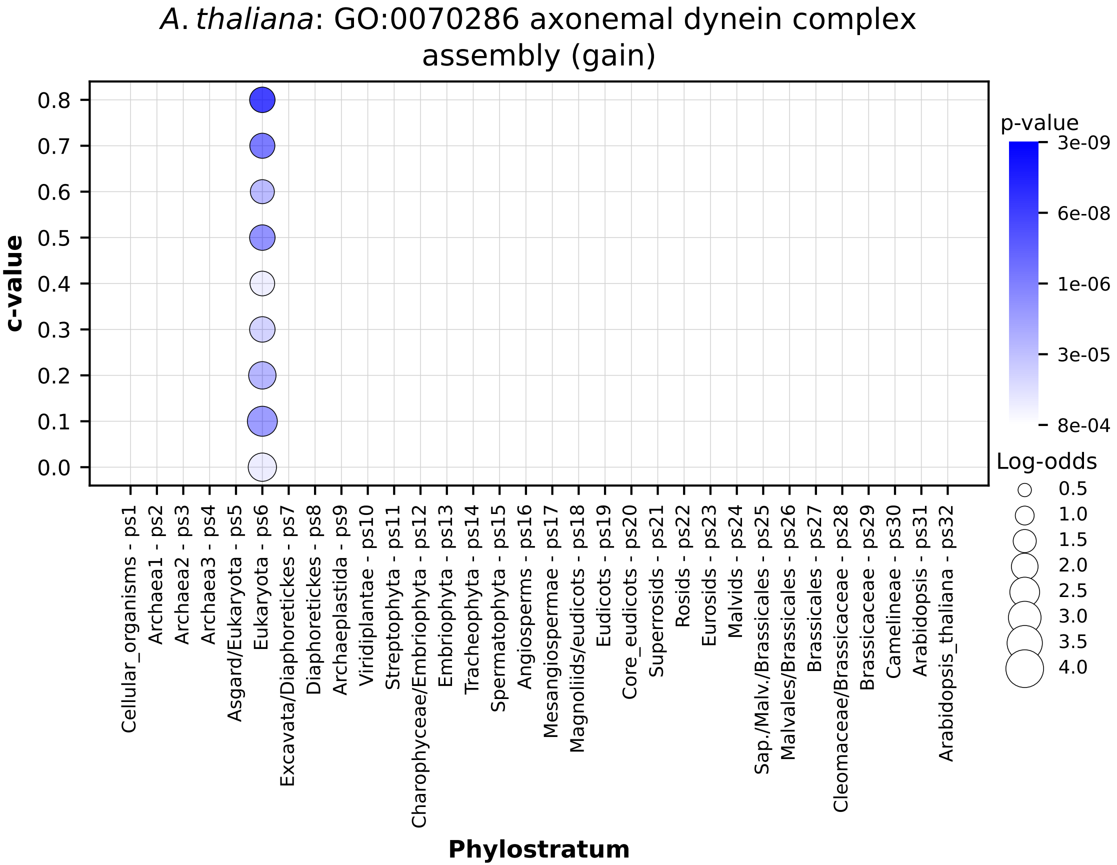
A. thaliana: GO:0070286 axonemal dynein complex assembly (gain)
c-value
0.8
0.7
0.6
0.5
0.4
0.3
0.2
0.1
0.0
p-value
3e-09
6e-08
1e-06
3e-05
8e-04
Log-odds
0.5
1.0
1.5
2.0
2.5
3.0
3.5
4.0
Cellular_organisms - ps1
Archaea1 - ps2
Archaea2 - ps3
Archaea3 - ps4
Asgard/Eukaryota - ps5
Eukaryota - ps6
Excavata/Diaphoretickes - ps7
Diaphoretickes - ps8
Archaeplastida - ps9
Viridiplantae - ps10
Streptophyta - ps11
Charophyceae/Embriophyta - ps12
Embriophyta - ps13
Tracheophyta - ps14
Spermatophyta - ps15
Angiosperms - ps16
Mesangiospermae - ps17
Magnoliids/eudicots - ps18
Eudicots - ps19
Core_eudicots - ps20
Superrosids - ps21
Rosids - ps22
Eurosids - ps23
Malvids - ps24
Sap./Malv./Brassicales - ps25
Malvales/Brassicales - ps26
Brassicales - ps27
Cleomaceae/Brassicaceae - ps28
Brassicaceae - ps29
Camelineae - ps30
Arabidopsis - ps31
Arabidopsis_thaliana - ps32
Phylostratum

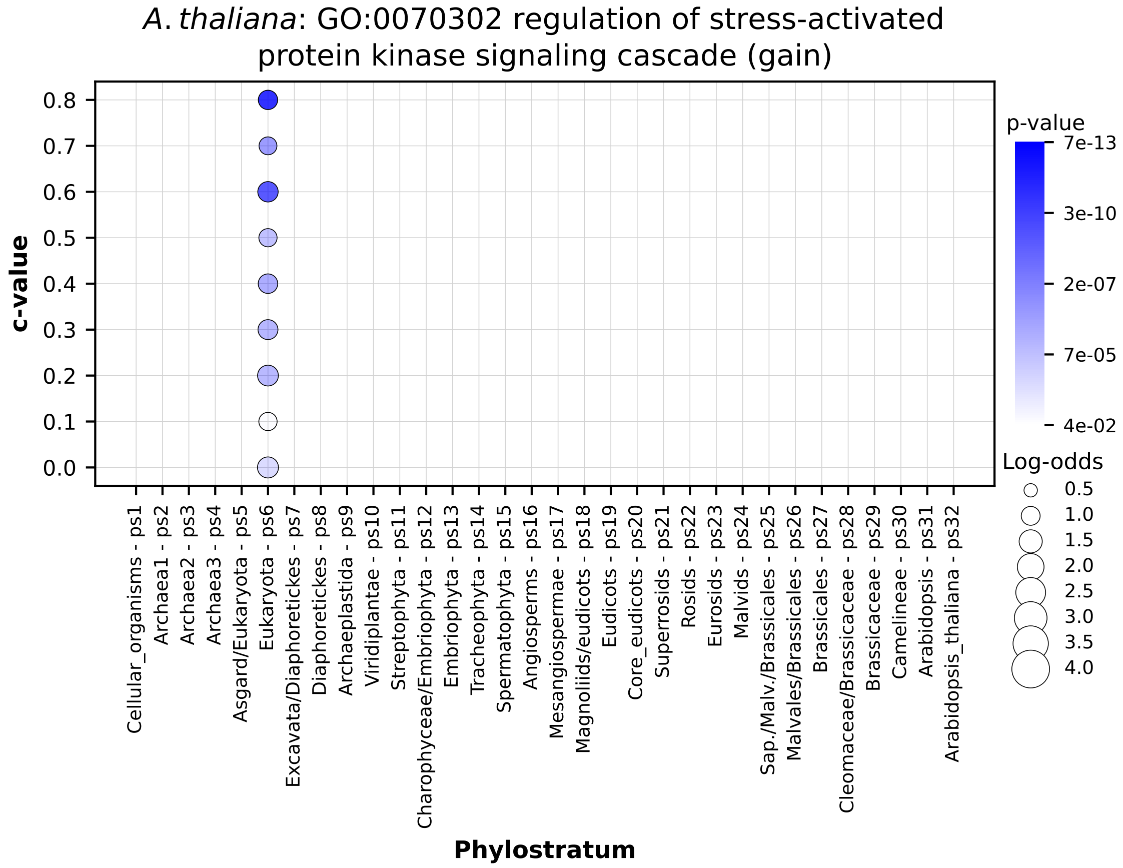
A. thaliana: GO:0070302 regulation of stress-activated protein kinase signaling cascade (gain)
c-value
0.8
0.7
0.6
0.5
0.4
0.3
0.2
0.1
0.0
p-value
7e-13
3e-10
2e-07
7e-05
4e-02
Log-odds
0.5
1.0
1.5
2.0
2.5
3.0
3.5
4.0
Cellular_organisms - ps1
Archaea1 - ps2
Archaea2 - ps3
Archaea3 - ps4
Asgard/Eukaryota - ps5
Eukaryota - ps6
Excavata/Diaphoretickes - ps7
Diaphoretickes - ps8
Archaeplastida - ps9
Viridiplantae - ps10
Streptophyta - ps11
Charophyceae/Embriophyta - ps12
Embriophyta - ps13
Tracheophyta - ps14
Spermatophyta - ps15
Angiosperms - ps16
Mesangiospermae - ps17
Magnoliids/eudicots - ps18
Eudicots - ps19
Core_eudicots - ps20
Superrosids - ps21
Rosids - ps22
Eurosids - ps23
Malvids - ps24
Sap./Malv./Brassicales - ps25
Malvales/Brassicales - ps26
Brassicales - ps27
Cleomaceae/Brassicaceae - ps28
Brassicaceae - ps29
Camelineae - ps30
Arabidopsis - ps31
Arabidopsis_thaliana - ps32
Phylostratum

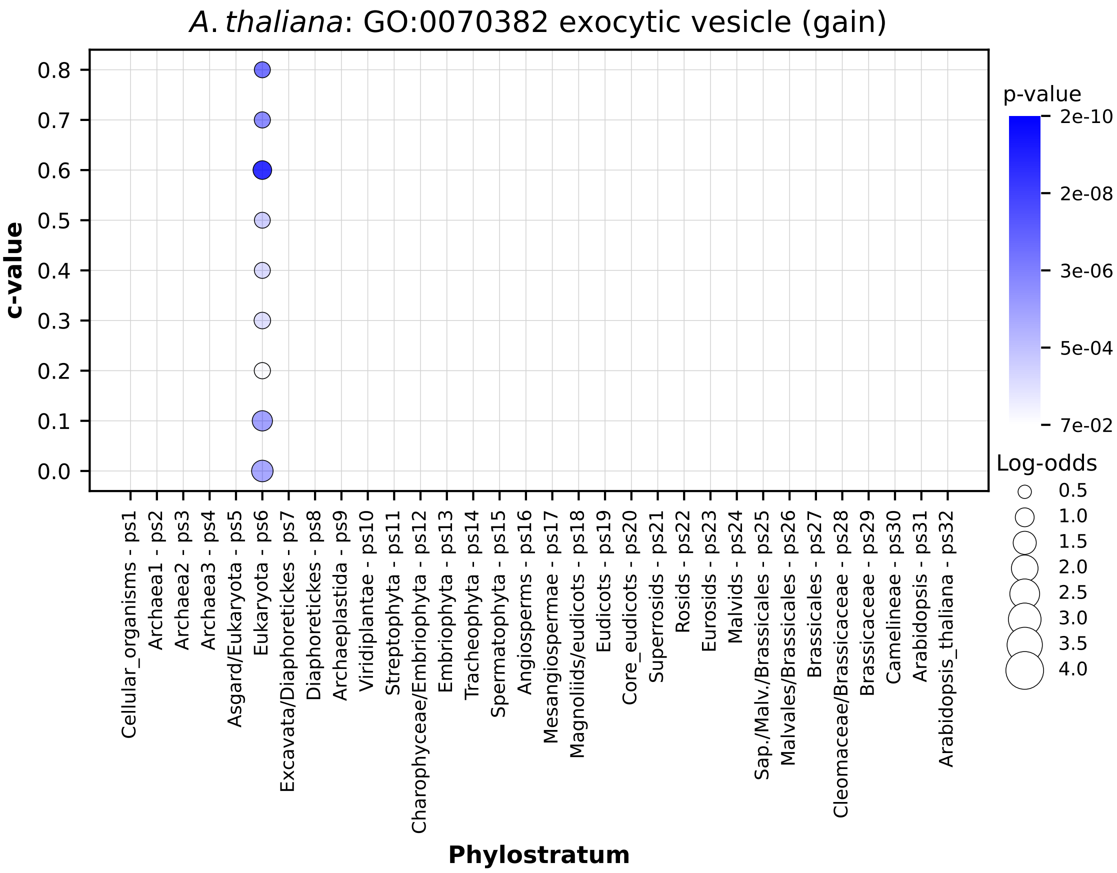
A. thaliana: GO:0070382 exocytic vesicle (gain)
c-value
0.8
0.7
0.6
0.5
0.4
0.3
0.2
0.1
0.0
Phylostratum
Cellular_organisms - ps1
Archaea1 - ps2
Archaea2 - ps3
Archaea3 - ps4
Asgard/Eukaryota - ps5
Eukaryota - ps6
Excavata/Diaphoretickes - ps7
Diaphoretickes - ps8
Archaeplastida - ps9
Viridiplantae - ps10
Streptophyta - ps11
Charophyceae/Embriophyta - ps12
Embriophyta - ps13
Tracheophyta - ps14
Spermatophyta - ps15
Angiosperms - ps16
Mesangiospermae - ps17
Magnoliids/eudicots - ps18
Eudicots - ps19
Core_eudicots - ps20
Superrosids - ps21
Rosids - ps22
Eurosids - ps23
Malvids - ps24
Sap./Malv./Brassicales - ps25
Malvales/Brassicales - ps26
Brassicales - ps27
Cleomaceae/Brassicaceae - ps28
Brassicaceae - ps29
Camelineae - ps30
Arabidopsis - ps31
Arabidopsis_thaliana - ps32
p-value
2e-10
2e-08
3e-06
5e-04
7e-02
Log-odds
0.5
1.0
1.5
2.0
2.5
3.0
3.5
4.0

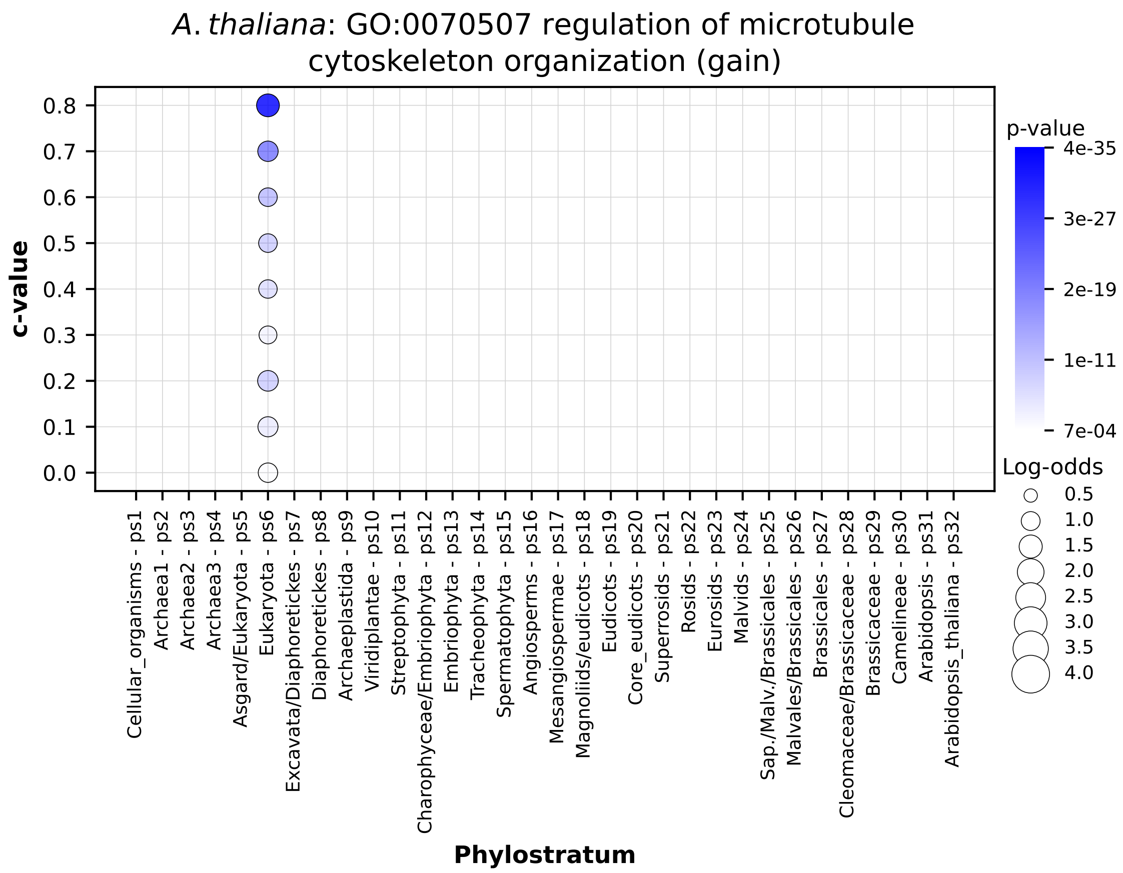
A. thaliana: GO:0070507 regulation of microtubule cytoskeleton organization (gain)
c-value
0.8
0.7
0.6
0.5
0.4
0.3
0.2
0.1
0.0
p-value
4e-35
3e-27
2e-19
1e-11
7e-04
Log-odds
0.5
1.0
1.5
2.0
2.5
3.0
3.5
4.0
Cellular_organisms - ps1
Archaea1 - ps2
Archaea2 - ps3
Archaea3 - ps4
Asgard/Eukaryota - ps5
Eukaryota - ps6
Excavata/Diaphoretickes - ps7
Diaphoretickes - ps8
Archaeplastida - ps9
Viridiplantae - ps10
Streptophyta - ps11
Charophyceae/Embriophyta - ps12
Embriophyta - ps13
Tracheophyta - ps14
Spermatophyta - ps15
Angiosperms - ps16
Mesangiospermae - ps17
Magnoliids/eudicots - ps18
Eudicots - ps19
Core_eudicots - ps20
Superrosids - ps21
Rosids - ps22
Eurosids - ps23
Malvids - ps24
Sap./Malv./Brassicales - ps25
Malvales/Brassicales - ps26
Brassicales - ps27
Cleomaceae/Brassicaceae - ps28
Brassicaceae - ps29
Camelineae - ps30
Arabidopsis - ps31
Arabidopsis_thaliana - ps32
Phylostratum

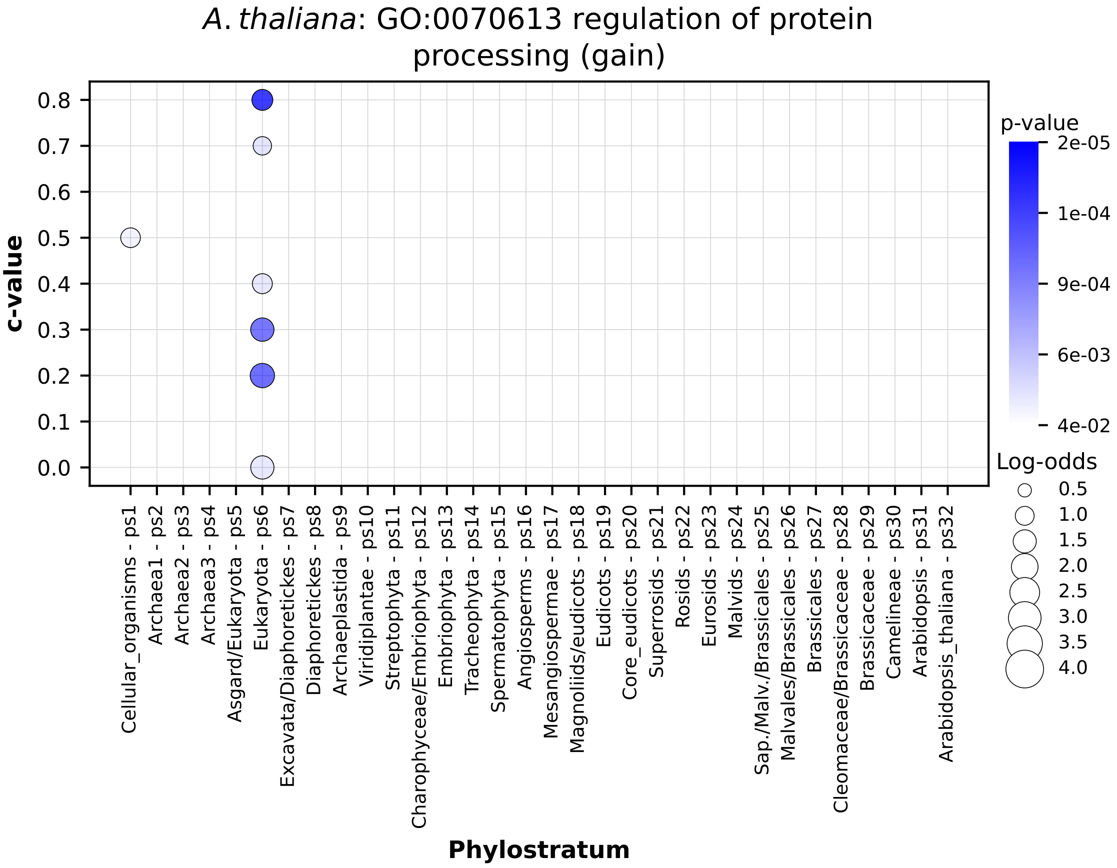

A. thaliana: GO:0070613 regulation of protein processing (gain)
c-value
0.8
0.7
0.6
0.5
0.4
0.3
0.2
0.1
0.0
p-value
2e-05
1e-04
9e-04
6e-03
4e-02
Log-odds
0.5
1.0
1.5
2.0
2.5
3.0
3.5
4.0
Cellular_organisms - ps1
Archaea1 - ps2
Archaea2 - ps3
Archaea3 - ps4
Asgard/Eukaryota - ps5
Eukaryota - ps6
Excavata/Diaphoretickes - ps7
Diaphoretickes - ps8
Archaeplastida - ps9
Viridiplantae - ps10
Streptophyta - ps11
Charophyceae/Embriophyta - ps12
Embriophyta - ps13
Tracheophyta - ps14
Spermatophyta - ps15
Angiosperms - ps16
Mesangiospermae - ps17
Magnoliids/eudicots - ps18
Eudicots - ps19
Core_eudicots - ps20
Superrosids - ps21
Rosids - ps22
Eurosids - ps23
Malvids - ps24
Sap./Malv./Brassicales - ps25
Malvales/Brassicales - ps26
Brassicales - ps27
Cleomaceae/Brassicaceae - ps28
Brassicaceae - ps29
Camelineae - ps30
Arabidopsis - ps31
Arabidopsis_thaliana - ps32
Phylostratum

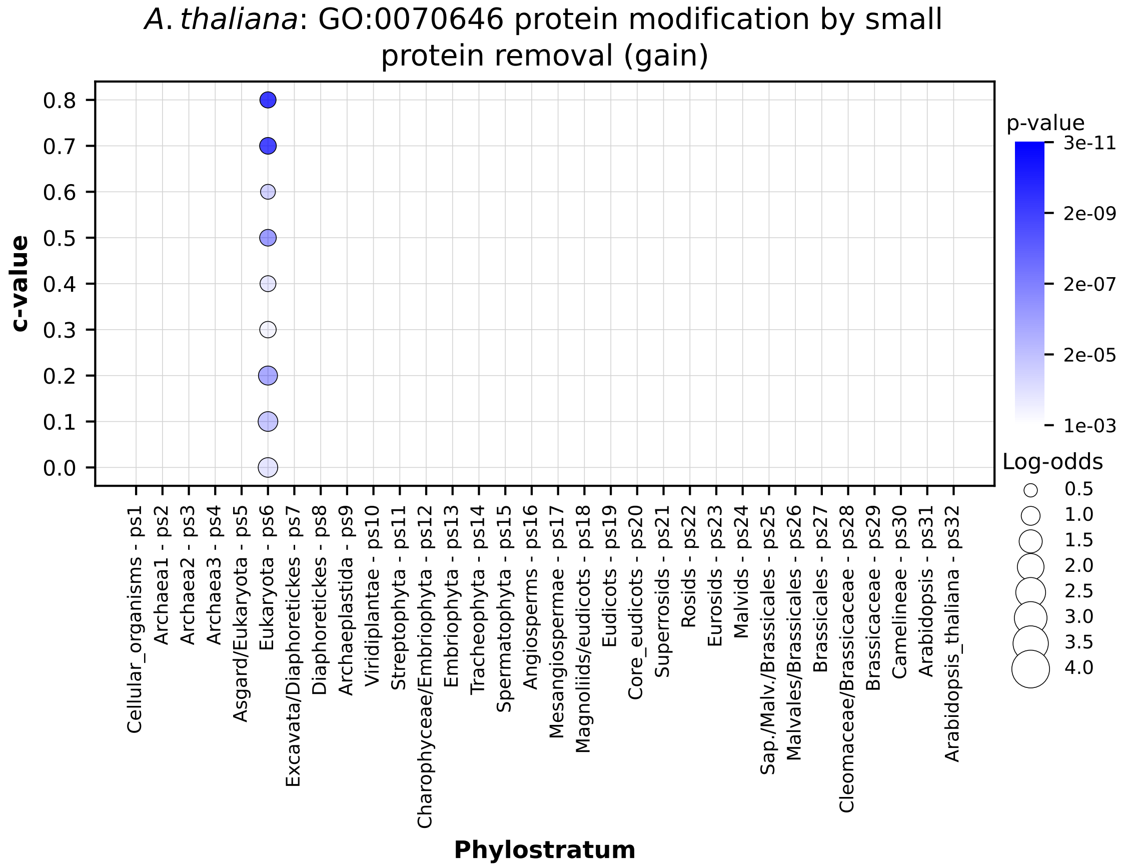

A. thaliana: GO:0070646 protein modification by small protein removal (gain)
c-value
0.8
0.7
0.6
0.5
0.4
0.3
0.2
0.1
0.0
p-value
3e-11
2e-09
2e-07
2e-05
1e-03
Log-odds
0.5
1.0
1.5
2.0
2.5
3.0
3.5
4.0
Cellular_organisms - ps1
Archaea1 - ps2
Archaea2 - ps3
Archaea3 - ps4
Asgard/Eukaryota - ps5
Eukaryota - ps6
Excavata/Diaphoretickes - ps7
Diaphoretickes - ps8
Archaeplastida - ps9
Viridiplantae - ps10
Streptophyta - ps11
Charophyceae/Embriophyta - ps12
Embriophyta - ps13
Tracheophyta - ps14
Spermatophyta - ps15
Angiosperms - ps16
Mesangiospermae - ps17
Magnoliids/eudicots - ps18
Eudicots - ps19
Core_eudicots - ps20
Superrosids - ps21
Rosids - ps22
Eurosids - ps23
Malvids - ps24
Sap./Malv./Brassicales - ps25
Malvales/Brassicales - ps26
Brassicales - ps27
Cleomaceae/Brassicaceae - ps28
Brassicaceae - ps29
Camelineae - ps30
Arabidopsis - ps31
Arabidopsis_thaliana - ps32
Phylostratum

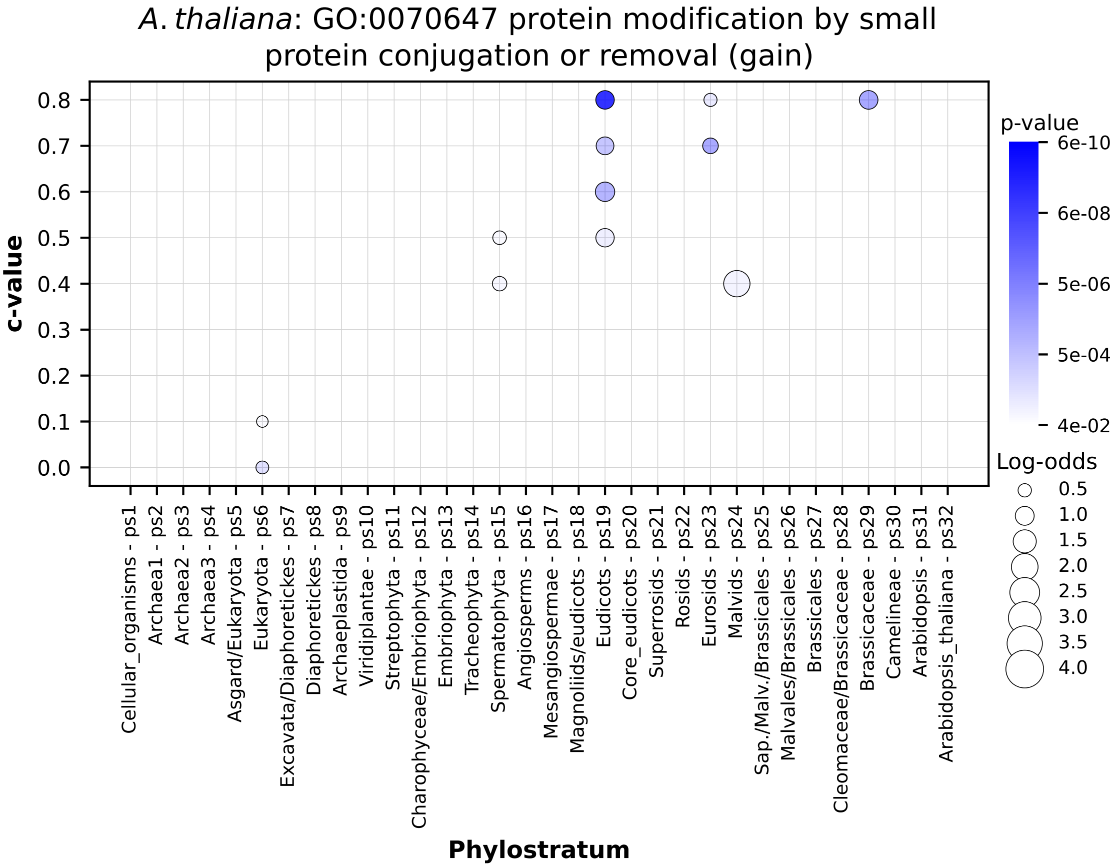

A. thaliana: GO:0070647 protein modification by small protein conjugation or removal (gain)
c-value
0.8
0.7
0.6
0.5
0.4
0.3
0.2
0.1
0.0
p-value
6e-10
6e-08
5e-06
5e-04
4e-02
Log-odds
0.5
1.0
1.5
2.0
2.5
3.0
3.5
4.0
Cellular_organisms - ps1
Archaea1 - ps2
Archaea2 - ps3
Archaea3 - ps4
Asgard/Eukaryota - ps5
Eukaryota - ps6
Excavata/Diaphoretickes - ps7
Diaphoretickes - ps8
Archaeplastida - ps9
Viridiplantae - ps10
Streptophyta - ps11
Charophyceae/Embriophyta - ps12
Embriophyta - ps13
Tracheophyta - ps14
Spermatophyta - ps15
Angiosperms - ps16
Mesangiospermae - ps17
Magnoliids/eudicots - ps18
Eudicots - ps19
Core_eudicots - ps20
Superrosids - ps21
Rosids - ps22
Eurosids - ps23
Malvids - ps24
Sap./Malv./Brassicales - ps25
Malvales/Brassicales - ps26
Brassicales - ps27
Cleomaceae/Brassicaceae - ps28
Brassicaceae - ps29
Camelineae - ps30
Arabidopsis - ps31
Arabidopsis_thaliana - ps32
Phylostratum

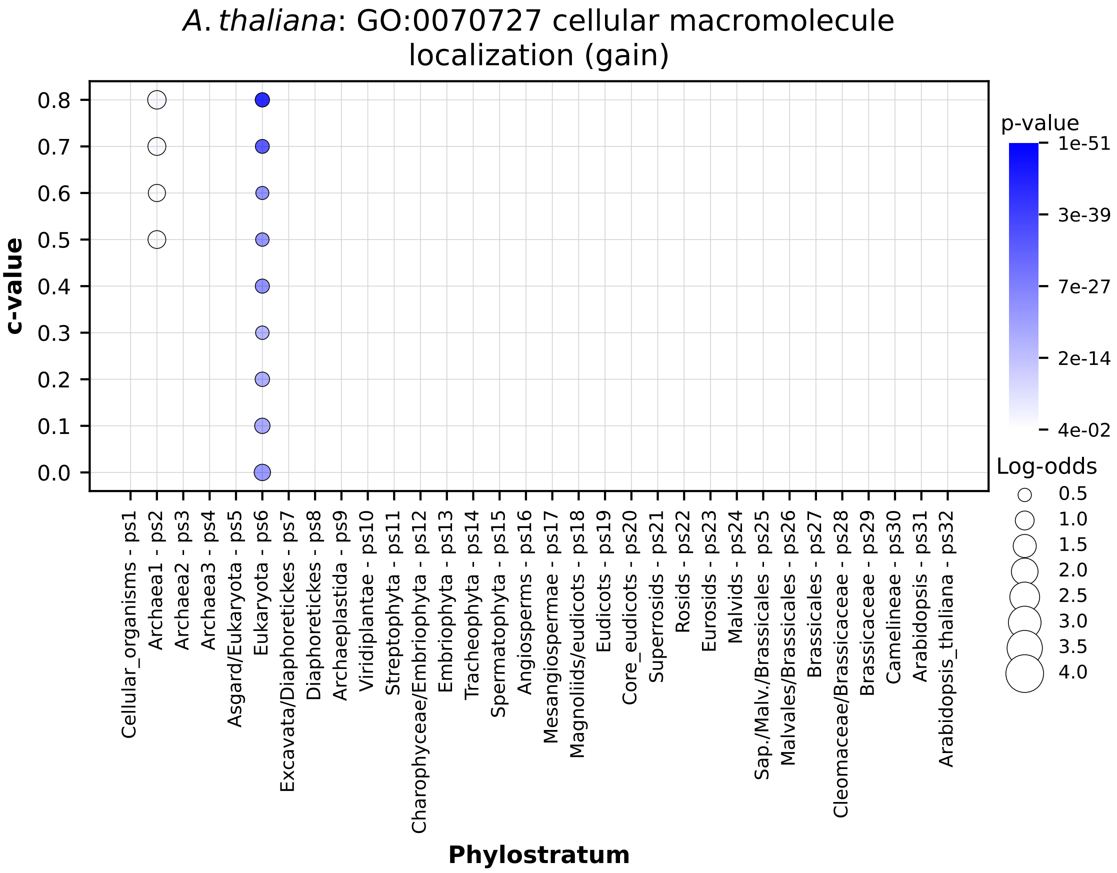

A. thaliana: GO:0070727 cellular macromolecule localization (gain)
c-value
0.8
0.7
0.6
0.5
0.4
0.3
0.2
0.1
0.0
p-value
1e-51
3e-39
7e-27
2e-14
4e-02
Log-odds
0.5
1.0
1.5
2.0
2.5
3.0
3.5
4.0
Cellular_organisms - ps1
Archaea1 - ps2
Archaea2 - ps3
Archaea3 - ps4
Asgard/Eukaryota - ps5
Eukaryota - ps6
Excavata/Diaphoretickes - ps7
Diaphoretickes - ps8
Archaeplastida - ps9
Viridiplantae - ps10
Streptophyta - ps11
Charophyceae/Embriophyta - ps12
Embriophyta - ps13
Tracheophyta - ps14
Spermatophyta - ps15
Angiosperms - ps16
Mesangiospermae - ps17
Magnoliids/eudicots - ps18
Eudicots - ps19
Core_eudicots - ps20
Superrosids - ps21
Rosids - ps22
Eurosids - ps23
Malvids - ps24
Sap./Malv./Brassicales - ps25
Malvales/Brassicales - ps26
Brassicales - ps27
Cleomaceae/Brassicaceae - ps28
Brassicaceae - ps29
Camelineae - ps30
Arabidopsis - ps31
Arabidopsis_thaliana - ps32
Phylostratum

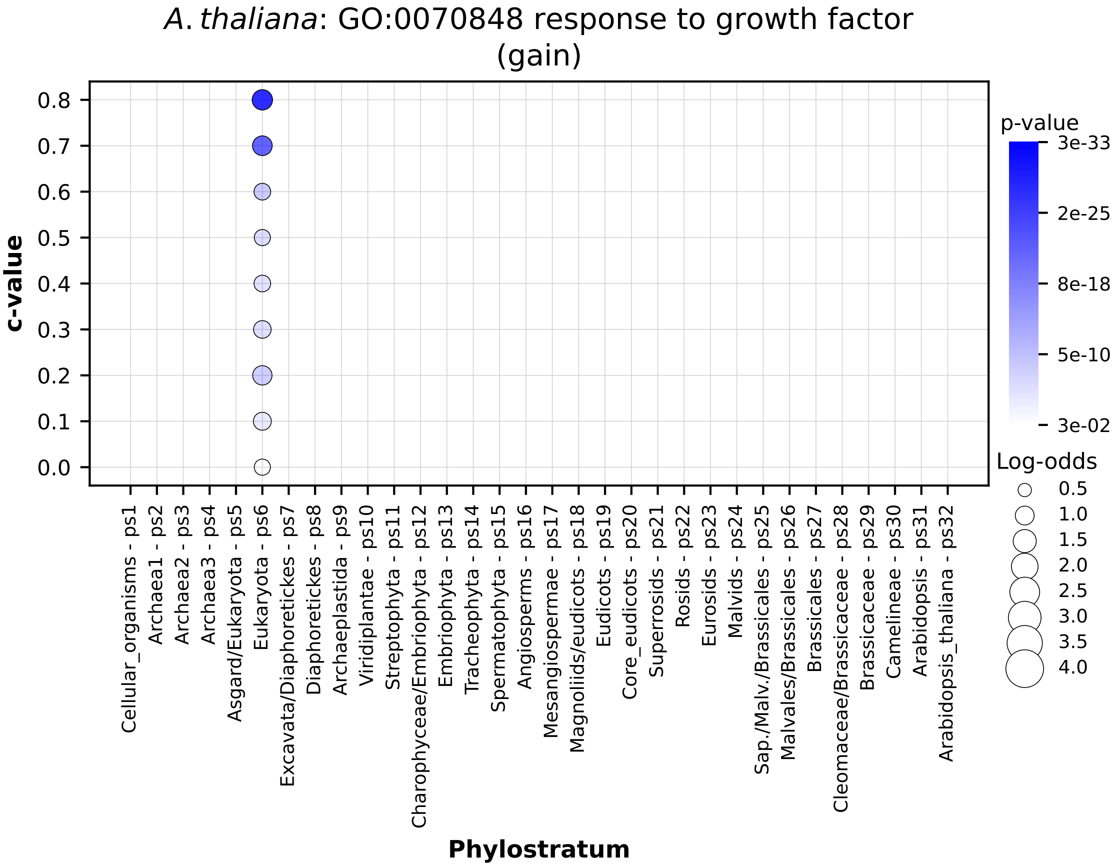
A. thaliana: GO:0070848 response to growth factor
(gain)
c-value
0.8
0.7
0.6
0.5
0.4
0.3
0.2
0.1
0.0
p-value
3e-33
2e-25
8e-18
5e-10
3e-02
Log-odds
0.5
1.0
1.5
2.0
2.5
3.0
3.5
4.0
Cellular_organisms - ps1
Archaea1 - ps2
Archaea2 - ps3
Archaea3 - ps4
Asgard/Eukaryota - ps5
Eukaryota - ps6
Excavata/Diaphoretickes - ps7
Diaphoretickes - ps8
Archaeplastida - ps9
Viridiplantae - ps10
Streptophyta - ps11
Charophyceae/Embriophyta - ps12
Embriophyta - ps13
Tracheophyta - ps14
Spermatophyta - ps15
Angiosperms - ps16
Mesangiospermae - ps17
Magnoliids/eudicots - ps18
Eudicots - ps19
Core_eudicots - ps20
Superrosids - ps21
Rosids - ps22
Eurosids - ps23
Malvids - ps24
Sap./Malv./Brassicales - ps25
Malvales/Brassicales - ps26
Brassicales - ps27
Cleomaceae/Brassicaceae - ps28
Brassicaceae - ps29
Camelineae - ps30
Arabidopsis - ps31
Arabidopsis_thaliana - ps32
Phylostratum

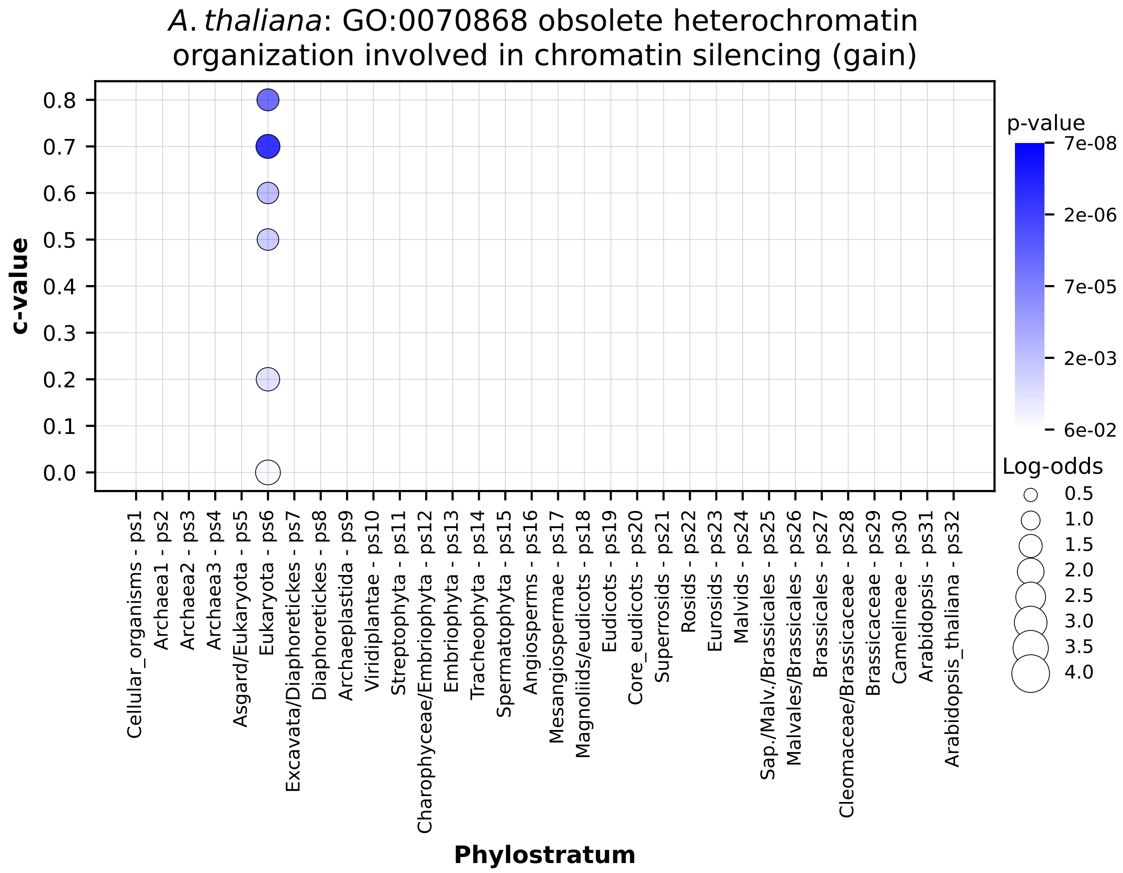
A. thaliana: GO:0070868 obsolete heterochromatin organization involved in chromatin silencing (gain)
c-value
0.0
0.1
0.2
0.3
0.4
0.5
0.6
0.7
0.8
p-value
7e-08
2e-06
7e-05
2e-03
6e-02
Log-odds
0.5
1.0
1.5
2.0
2.5
3.0
3.5
4.0
Cellular_organisms - ps1
Archaea1 - ps2
Archaea2 - ps3
Archaea3 - ps4
Asgard/Eukaryota - ps5
Eukaryota - ps6
Excavata/Diaphoretickes - ps7
Diaphoretickes - ps8
Archaeplastida - ps9
Viridiplantae - ps10
Streptophyta - ps11
Charophyceae/Embriophyta - ps12
Embriophyta - ps13
Tracheophyta - ps14
Spermatophyta - ps15
Angiosperms - ps16
Mesangiospermae - ps17
Magnoliids/eudicots - ps18
Eudicots - ps19
Core_eudicots - ps20
Superrosids - ps21
Rosids - ps22
Eurosids - ps23
Malvids - ps24
Sap./Malv./Brassicales - ps25
Malvales/Brassicales - ps26
Brassicales - ps27
Cleomaceae/Brassicaceae - ps28
Brassicaceae - ps29
Camelineae - ps30
Arabidopsis - ps31
Arabidopsis_thaliana - ps32
Phylostratum

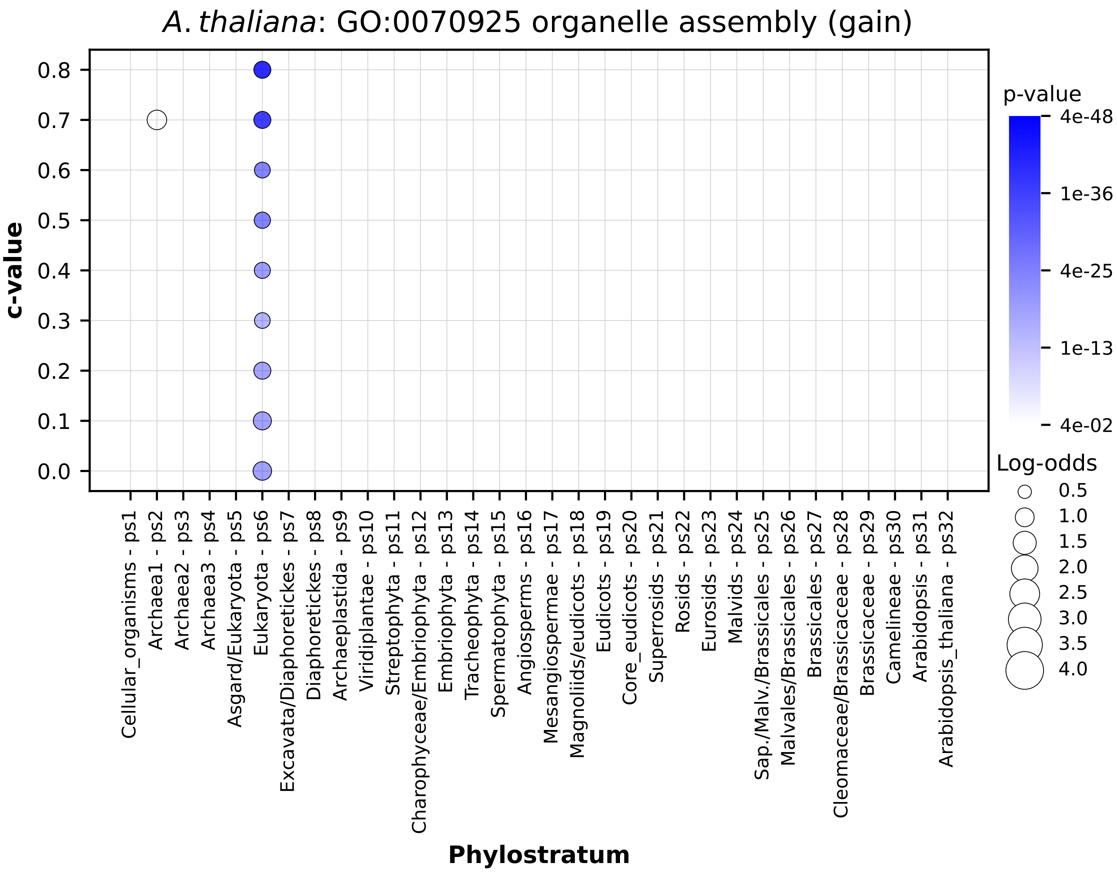

A. thaliana: GO:0070925 organelle assembly (gain)
c-value
0.8
0.7
0.6
0.5
0.4
0.3
0.2
0.1
0.0
Phylostratum
Cellular_organisms - ps1
Archaea1 - ps2
Archaea2 - ps3
Archaea3 - ps4
Asgard/Eukaryota - ps5
Eukaryota - ps6
Excavata/Diaphoretickes - ps7
Diaphoretickes - ps8
Archaeplastida - ps9
Viridiplantae - ps10
Streptophyta - ps11
Charophyceae/Embriophyta - ps12
Embriophyta - ps13
Tracheophyta - ps14
Spermatophyta - ps15
Angiosperms - ps16
Mesangiospermae - ps17
Magnoliids/eudicots - ps18
Eudicots - ps19
Core_eudicots - ps20
Superrosids - ps21
Rosids - ps22
Eurosids - ps23
Malvids - ps24
Sap./Malv./Brassicales - ps25
Malvales/Brassicales - ps26
Brassicales - ps27
Cleomaceae/Brassicaceae - ps28
Brassicaceae - ps29
Camelineae - ps30
Arabidopsis - ps31
Arabidopsis_thaliana - ps32
p-value
4e-48
1e-36
4e-25
1e-13
4e-02
Log-odds
0.5
1.0
1.5
2.0
2.5
3.0
3.5
4.0

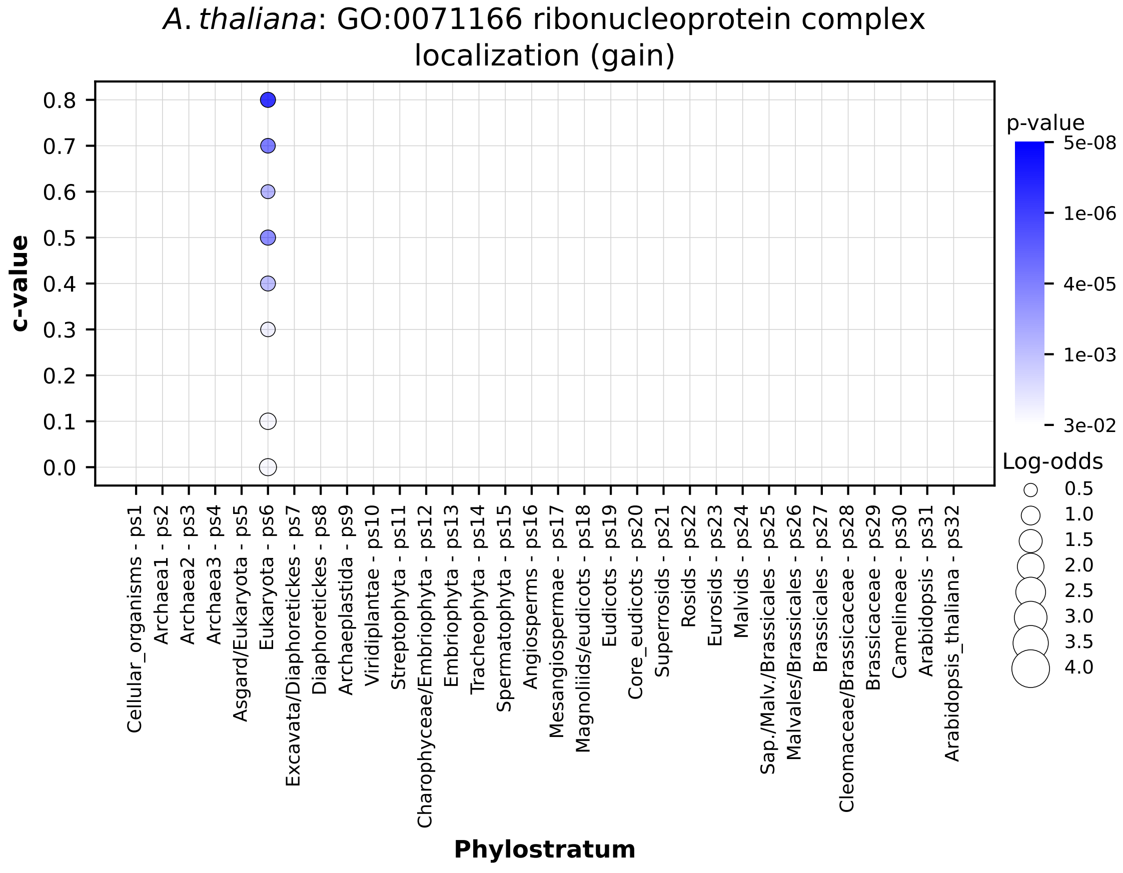

A. thaliana: GO:0071166 ribonucleoprotein complex localization (gain)
c-value
0.8
0.7
0.6
0.5
0.4
0.3
0.2
0.1
0.0
p-value
5e-08
1e-06
4e-05
1e-03
3e-02
Log-odds
0.5
1.0
1.5
2.0
2.5
3.0
3.5
4.0
Cellular_organisms - ps1
Archaea1 - ps2
Archaea2 - ps3
Archaea3 - ps4
Asgard/Eukaryota - ps5
Eukaryota - ps6
Excavata/Diaphoretickes - ps7
Diaphoretickes - ps8
Archaeplastida - ps9
Viridiplantae - ps10
Streptophyta - ps11
Charophyceae/Embriophyta - ps12
Embriophyta - ps13
Tracheophyta - ps14
Spermatophyta - ps15
Angiosperms - ps16
Mesangiospermae - ps17
Magnoliids/eudicots - ps18
Eudicots - ps19
Core_eudicots - ps20
Superrosids - ps21
Rosids - ps22
Eurosids - ps23
Malvids - ps24
Sap./Malv./Brassicales - ps25
Malvales/Brassicales - ps26
Brassicales - ps27
Cleomaceae/Brassicaceae - ps28
Brassicaceae - ps29
Camelineae - ps30
Arabidopsis - ps31
Arabidopsis_thaliana - ps32
Phylostratum

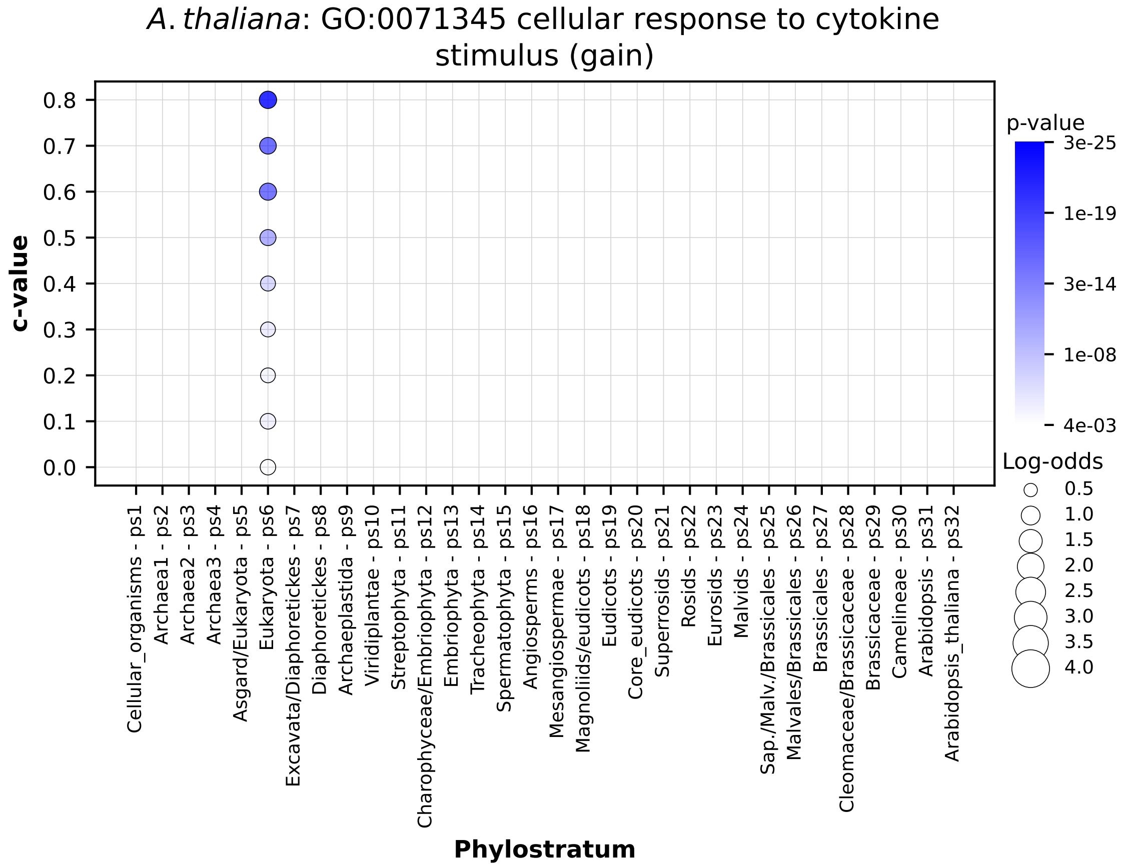

A. thaliana: GO:0071345 cellular response to cytokine stimulus (gain)
c-value
0.8
0.7
0.6
0.5
0.4
0.3
0.2
0.1
0.0
p-value
3e-25
1e-19
3e-14
1e-08
4e-03
Log-odds
0.5
1.0
1.5
2.0
2.5
3.0
3.5
4.0
Cellular_organisms - ps1
Archaea1 - ps2
Archaea2 - ps3
Archaea3 - ps4
Asgard/Eukaryota - ps5
Eukaryota - ps6
Excavata/Diaphoretickes - ps7
Diaphoretickes - ps8
Archaeplastida - ps9
Viridiplantae - ps10
Streptophyta - ps11
Charophyceae/Embriophyta - ps12
Embriophyta - ps13
Tracheophyta - ps14
Spermatophyta - ps15
Angiosperms - ps16
Mesangiospermae - ps17
Magnoliids/eudicots - ps18
Eudicots - ps19
Core_eudicots - ps20
Superrosids - ps21
Rosids - ps22
Eurosids - ps23
Malvids - ps24
Sap./Malv./Brassicales - ps25
Malvales/Brassicales - ps26
Brassicales - ps27
Cleomaceae/Brassicaceae - ps28
Brassicaceae - ps29
Camelineae - ps30
Arabidopsis - ps31
Arabidopsis_thaliana - ps32
Phylostratum

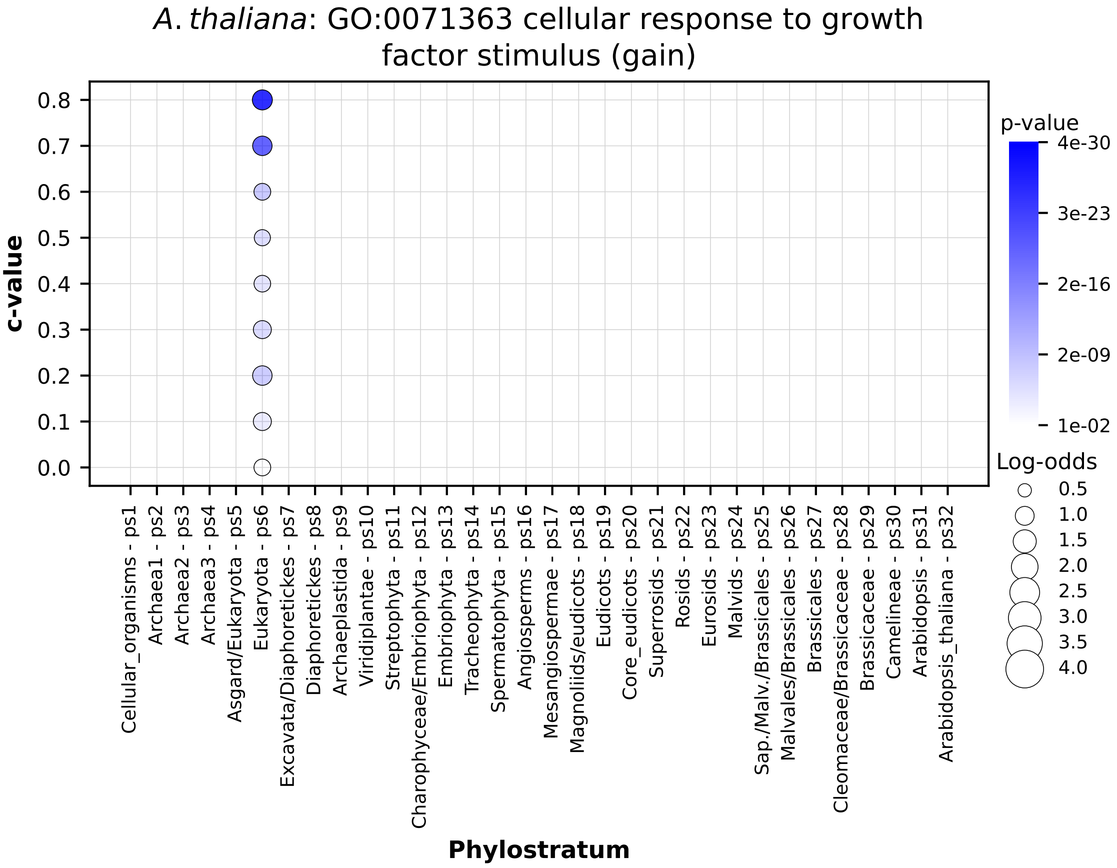
A. thaliana: GO:0071363 cellular response to growth factor stimulus (gain)
c-value
0.8
0.7
0.6
0.5
0.4
0.3
0.2
0.1
0.0
Phylostratum
Cellular_organisms - ps1
Archaea1 - ps2
Archaea2 - ps3
Archaea3 - ps4
Asgard/Eukaryota - ps5
Eukaryota - ps6
Excavata/Diaphoretickes - ps7
Diaphoretickes - ps8
Archaeplastida - ps9
Viridiplantae - ps10
Streptophyta - ps11
Charophyceae/Embriophyta - ps12
Embriophyta - ps13
Tracheophyta - ps14
Spermatophyta - ps15
Angiosperms - ps16
Mesangiospermae - ps17
Magnoliids/eudicots - ps18
Eudicots - ps19
Core_eudicots - ps20
Superrosids - ps21
Rosids - ps22
Eurosids - ps23
Malvids - ps24
Sap./Malv./Brassicales - ps25
Malvales/Brassicales - ps26
Brassicales - ps27
Cleomaceae/Brassicaceae - ps28
Brassicaceae - ps29
Camelineae - ps30
Arabidopsis - ps31
Arabidopsis_thaliana - ps32
p-value
4e-30
3e-23
2e-16
2e-09
1e-02
Log-odds
0.5
1.0
1.5
2.0
2.5
3.0
3.5
4.0

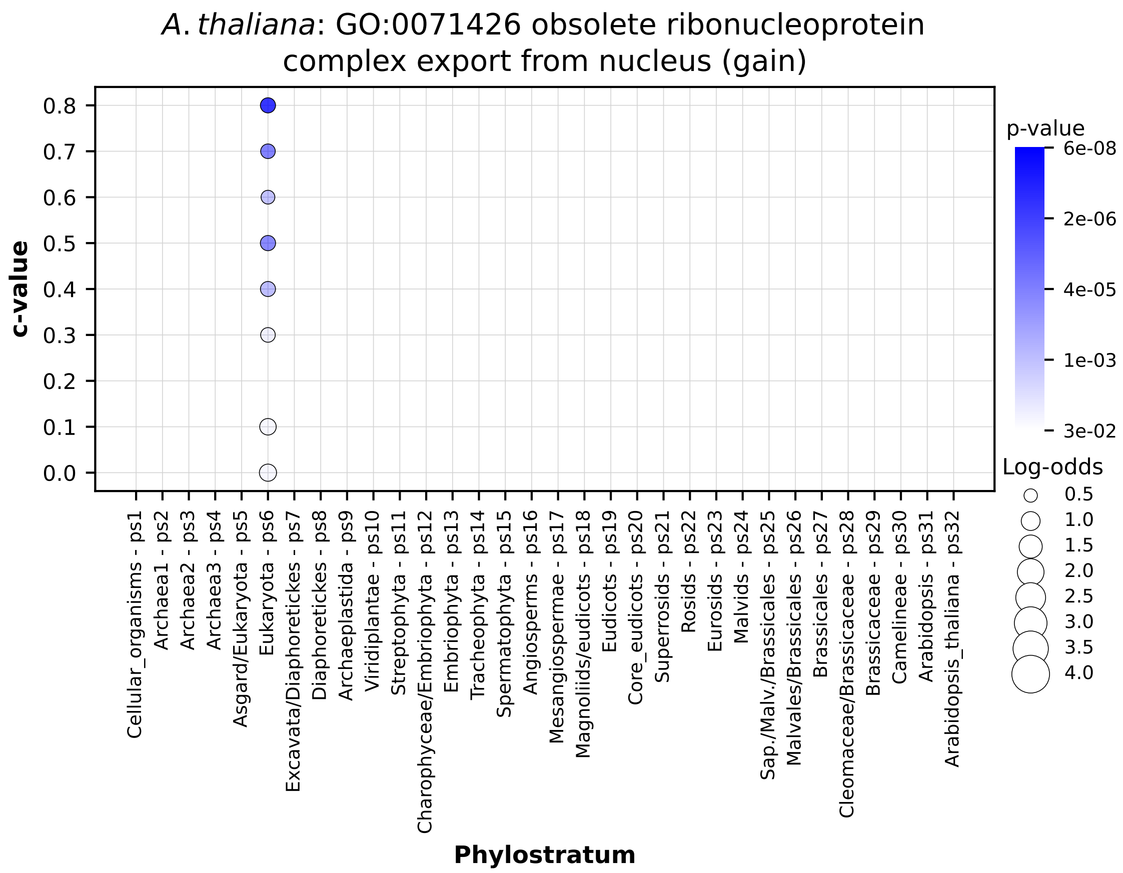
A. thaliana: GO:0071426 obsolete ribonucleoprotein complex export from nucleus (gain)
c-value
0.8
0.7
0.6
0.5
0.4
0.3
0.2
0.1
0.0
p-value
6e-08
2e-06
4e-05
1e-03
3e-02
Log-odds
0.5
1.0
1.5
2.0
2.5
3.0
3.5
4.0
Cellular_organisms - ps1
Archaea1 - ps2
Archaea2 - ps3
Archaea3 - ps4
Asgard/Eukaryota - ps5
Eukaryota - ps6
Excavata/Diaphoretickes - ps7
Diaphoretickes - ps8
Archaeplastida - ps9
Viridiplantae - ps10
Streptophyta - ps11
Charophyceae/Embriophyta - ps12
Embriophyta - ps13
Tracheophyta - ps14
Spermatophyta - ps15
Angiosperms - ps16
Mesangiospermae - ps17
Magnoliids/eudicots - ps18
Eudicots - ps19
Core_eudicots - ps20
Superrosids - ps21
Rosids - ps22
Eurosids - ps23
Malvids - ps24
Sap./Malv./Brassicales - ps25
Malvales/Brassicales - ps26
Brassicales - ps27
Cleomaceae/Brassicaceae - ps28
Brassicaceae - ps29
Camelineae - ps30
Arabidopsis - ps31
Arabidopsis_thaliana - ps32
Phylostratum

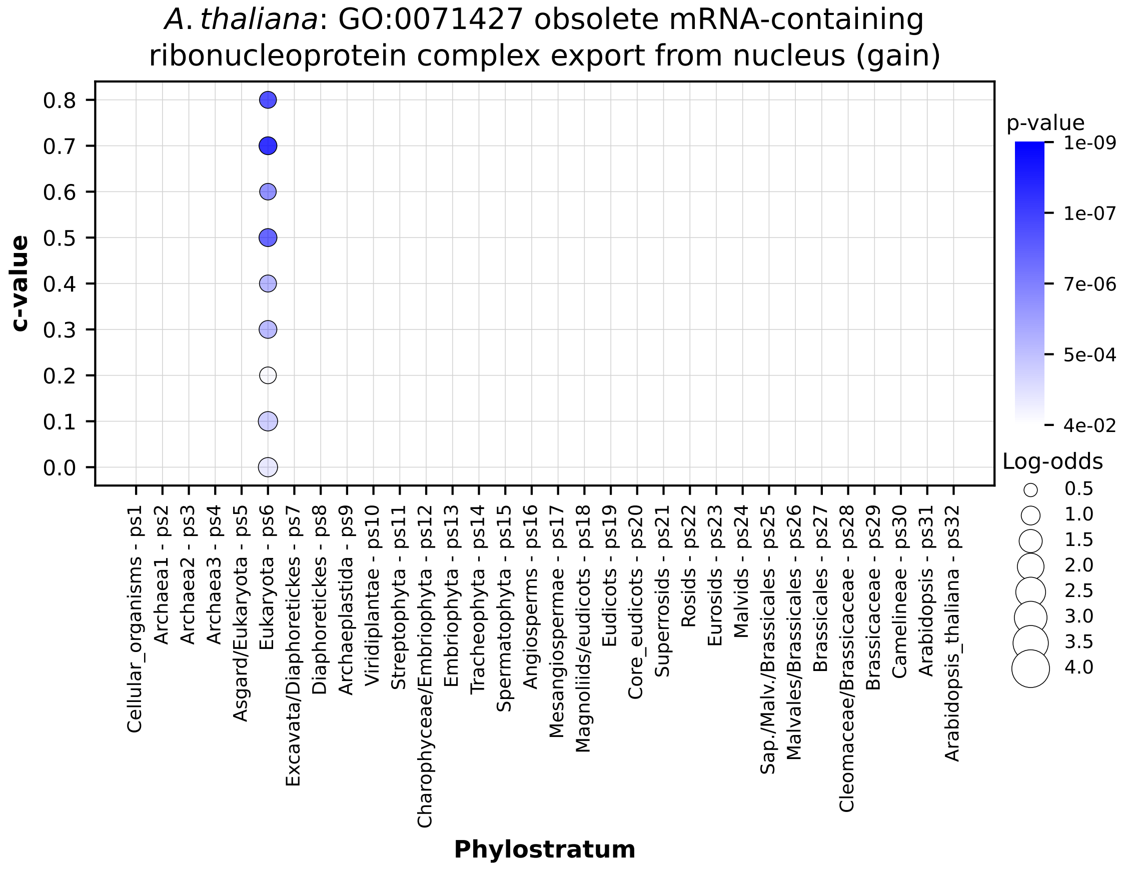

A. thaliana: GO:0071427 obsolete mRNA-containing ribonucleoprotein complex export from nucleus (gain)
c-value
0.8
0.7
0.6
0.5
0.4
0.3
0.2
0.1
0.0
p-value
1e-09
1e-07
7e-06
5e-04
4e-02
Log-odds
0.5
1.0
1.5
2.0
2.5
3.0
3.5
4.0
Cellular_organisms - ps1
Archaea1 - ps2
Archaea2 - ps3
Archaea3 - ps4
Asgard/Eukaryota - ps5
Eukaryota - ps6
Excavata/Diaphoretickes - ps7
Diaphoretickes - ps8
Archaeplastida - ps9
Viridiplantae - ps10
Streptophyta - ps11
Charophyceae/Embriophyta - ps12
Embriophyta - ps13
Tracheophyta - ps14
Spermatophyta - ps15
Angiosperms - ps16
Mesangiospermae - ps17
Magnoliids/eudicots - ps18
Eudicots - ps19
Core_eudicots - ps20
Superrosids - ps21
Rosids - ps22
Eurosids - ps23
Malvids - ps24
Sap./Malv./Brassicales - ps25
Malvales/Brassicales - ps26
Brassicales - ps27
Cleomaceae/Brassicaceae - ps28
Brassicaceae - ps29
Camelineae - ps30
Arabidopsis - ps31
Arabidopsis_thaliana - ps32
Phylostratum

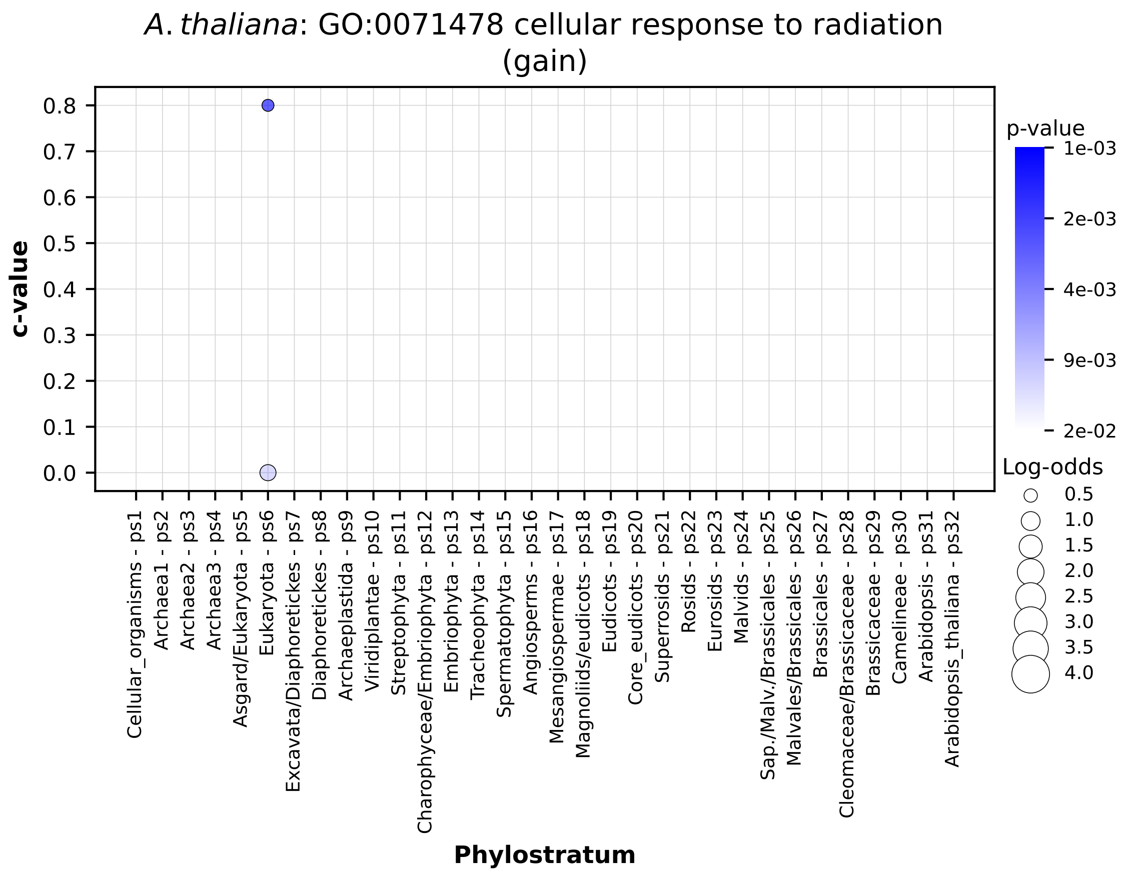
A. thaliana: GO:0071478 cellular response to radiation
(gain)
c-value
0.8
0.7
0.6
0.5
0.4
0.3
0.2
0.1
0.0
p-value
1e-03
2e-03
4e-03
9e-03
2e-02
Log-odds
0.5
1.0
1.5
2.0
2.5
3.0
3.5
4.0
Cellular_organisms - ps1
Archaea1 - ps2
Archaea2 - ps3
Archaea3 - ps4
Asgard/Eukaryota - ps5
Eukaryota - ps6
Excavata/Diaphoretickes - ps7
Diaphoretickes - ps8
Archaeplastida - ps9
Viridiplantae - ps10
Streptophyta - ps11
Charophyceae/Embriophyta - ps12
Embriophyta - ps13
Tracheophyta - ps14
Spermatophyta - ps15
Angiosperms - ps16
Mesangiospermae - ps17
Magnoliids/eudicots - ps18
Eudicots - ps19
Core_eudicots - ps20
Superrosids - ps21
Rosids - ps22
Eurosids - ps23
Malvids - ps24
Sap./Malv./Brassicales - ps25
Malvales/Brassicales - ps26
Brassicales - ps27
Cleomaceae/Brassicaceae - ps28
Brassicaceae - ps29
Camelineae - ps30
Arabidopsis - ps31
Arabidopsis_thaliana - ps32
Phylostratum

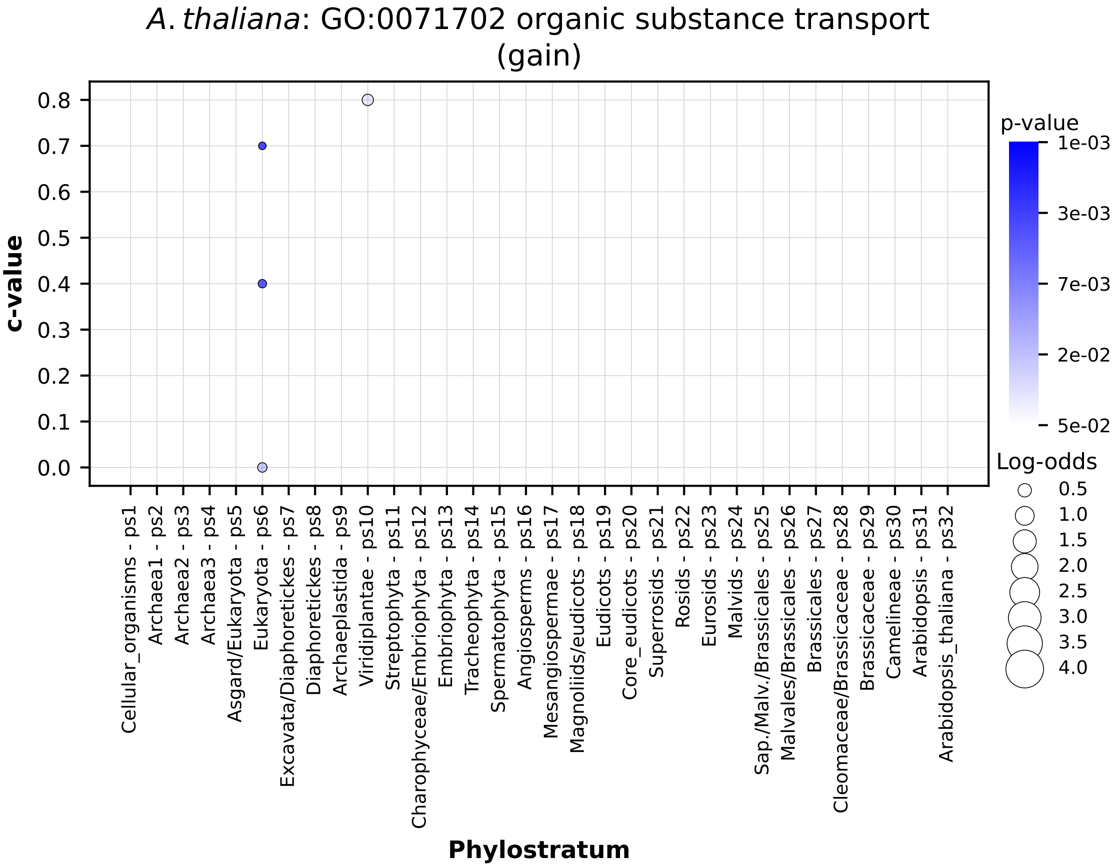
A. thaliana: GO:0071702 organic substance transport
(gain)
c-value
0.8
0.7
0.6
0.5
0.4
0.3
0.2
0.1
0.0
p-value
1e-03
3e-03
7e-03
2e-02
5e-02
Log-odds
0.5
1.0
1.5
2.0
2.5
3.0
3.5
4.0
Cellular_organisms - ps1
Archaea1 - ps2
Archaea2 - ps3
Archaea3 - ps4
Asgard/Eukaryota - ps5
Eukaryota - ps6
Excavata/Diaphoretickes - ps7
Diaphoretickes - ps8
Archaeplastida - ps9
Viridiplantae - ps10
Streptophyta - ps11
Charophyceae/Embriophyta - ps12
Embriophyta - ps13
Tracheophyta - ps14
Spermatophyta - ps15
Angiosperms - ps16
Mesangiospermae - ps17
Magnoliids/eudicots - ps18
Eudicots - ps19
Core_eudicots - ps20
Superrosids - ps21
Rosids - ps22
Eurosids - ps23
Malvids - ps24
Sap./Malv./Brassicales - ps25
Malvales/Brassicales - ps26
Brassicales - ps27
Cleomaceae/Brassicaceae - ps28
Brassicaceae - ps29
Camelineae - ps30
Arabidopsis - ps31
Arabidopsis_thaliana - ps32
Phylostratum

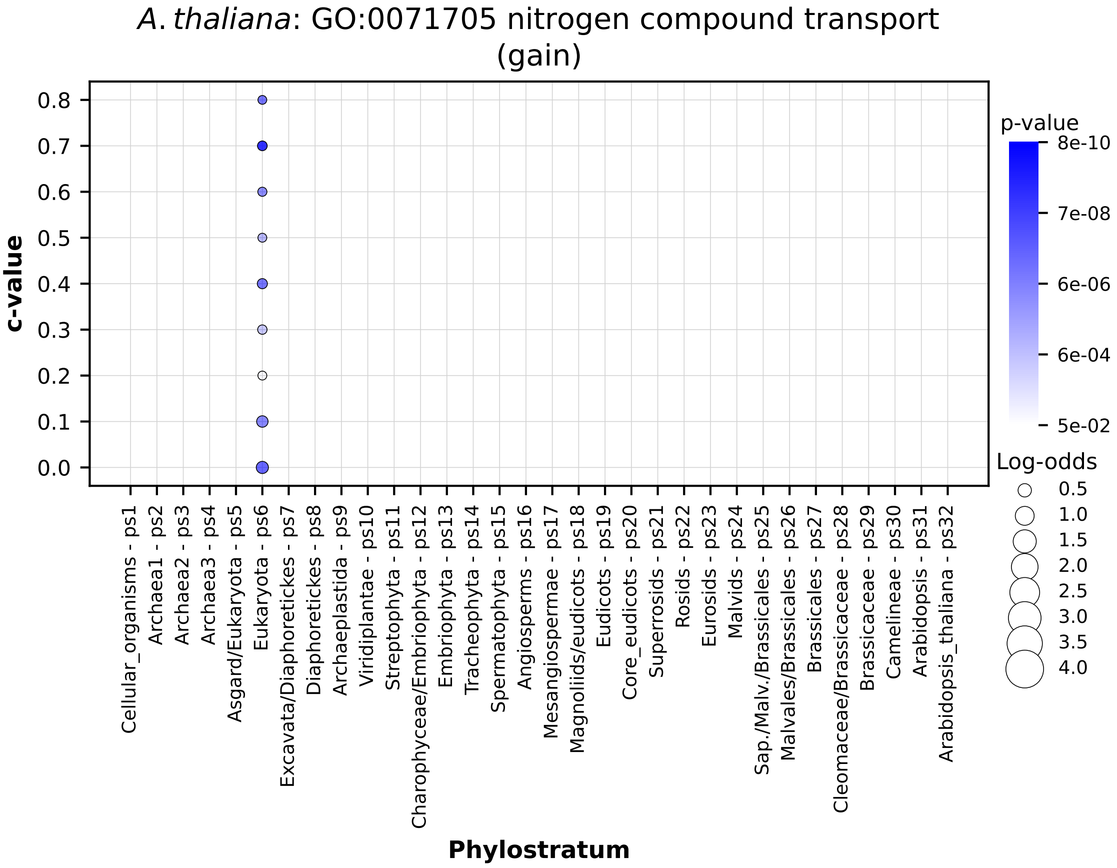

A. thaliana: GO:0071705 nitrogen compound transport
(gain)
c-value
0.8
0.7
0.6
0.5
0.4
0.3
0.2
0.1
0.0
p-value
8e-10
7e-08
6e-06
6e-04
5e-02
Log-odds
0.5
1.0
1.5
2.0
2.5
3.0
3.5
4.0
Cellular_organisms - ps1
Archaea1 - ps2
Archaea2 - ps3
Archaea3 - ps4
Asgard/Eukaryota - ps5
Eukaryota - ps6
Excavata/Diaphoretickes - ps7
Diaphoretickes - ps8
Archaeplastida - ps9
Viridiplantae - ps10
Streptophyta - ps11
Charophyceae/Embriophyta - ps12
Embriophyta - ps13
Tracheophyta - ps14
Spermatophyta - ps15
Angiosperms - ps16
Mesangiospermae - ps17
Magnoliids/eudicots - ps18
Eudicots - ps19
Core_eudicots - ps20
Superrosids - ps21
Rosids - ps22
Eurosids - ps23
Malvids - ps24
Sap./Malv./Brassicales - ps25
Malvales/Brassicales - ps26
Brassicales - ps27
Cleomaceae/Brassicaceae - ps28
Brassicaceae - ps29
Camelineae - ps30
Arabidopsis - ps31
Arabidopsis_thaliana - ps32
Phylostratum

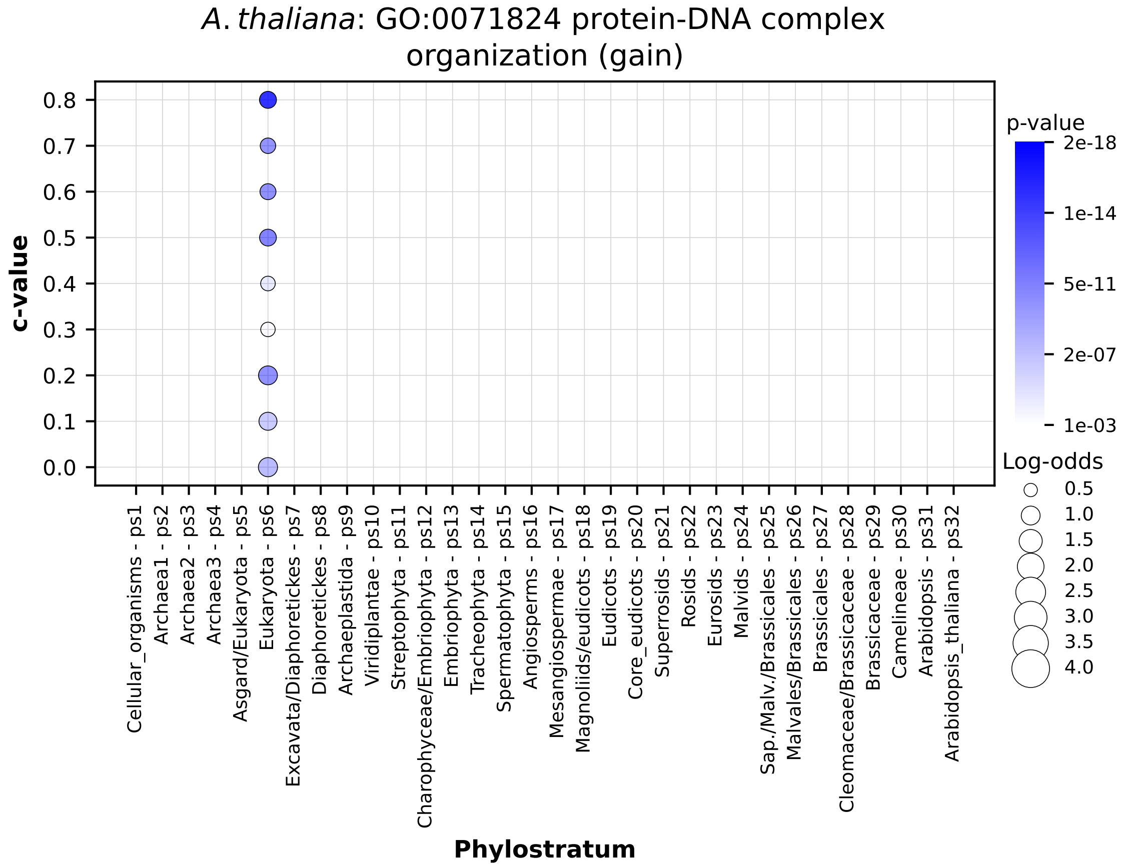

A. thaliana: GO:0071824 protein-DNA complex organization (gain)
c-value
0.8
0.7
0.6
0.5
0.4
0.3
0.2
0.1
0.0
Phylostratum
Cellular_organisms - ps1
Archaea1 - ps2
Archaea2 - ps3
Archaea3 - ps4
Asgard/Eukaryota - ps5
Eukaryota - ps6
Excavata/Diaphoretickes - ps7
Diaphoretickes - ps8
Archaeplastida - ps9
Viridiplantae - ps10
Streptophyta - ps11
Charophyceae/Embriophyta - ps12
Embriophyta - ps13
Tracheophyta - ps14
Spermatophyta - ps15
Angiosperms - ps16
Mesangiospermae - ps17
Magnoliids/eudicots - ps18
Eudicots - ps19
Core_eudicots - ps20
Superrosids - ps21
Rosids - ps22
Eurosids - ps23
Malvids - ps24
Sap./Malv./Brassicales - ps25
Malvales/Brassicales - ps26
Brassicales - ps27
Cleomaceae/Brassicaceae - ps28
Brassicaceae - ps29
Camelineae - ps30
Arabidopsis - ps31
Arabidopsis_thaliana - ps32
p-value
2e-18
1e-14
5e-11
2e-07
1e-03
Log-odds
0.5
1.0
1.5
2.0
2.5
3.0
3.5
4.0

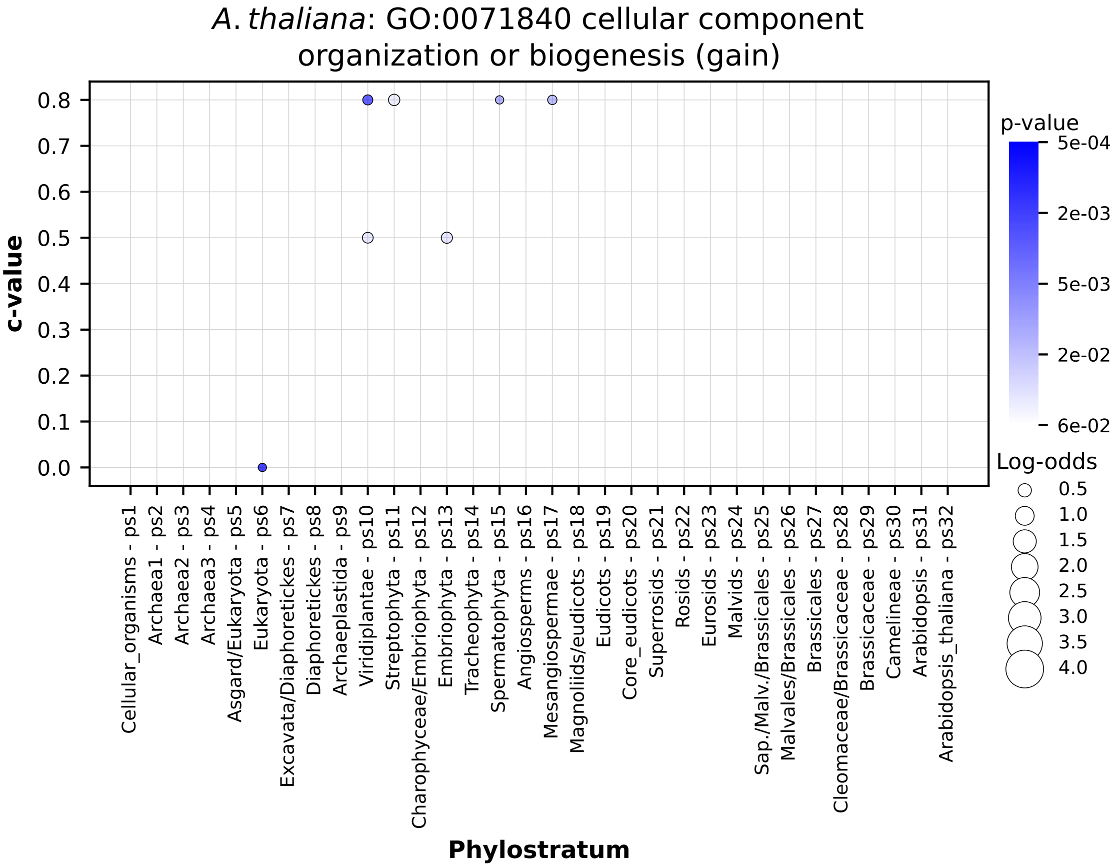
A. thaliana: GO:0071840 cellular component organization or biogenesis (gain)
c-value
0.8
0.7
0.6
0.5
0.4
0.3
0.2
0.1
0.0
Phylostratum
Cellular_organisms - ps1
Archaea1 - ps2
Archaea2 - ps3
Archaea3 - ps4
Asgard/Eukaryota - ps5
Eukaryota - ps6
Excavata/Diaphoretickes - ps7
Diaphoretickes - ps8
Archaeplastida - ps9
Viridiplantae - ps10
Streptophyta - ps11
Charophyceae/Embriophyta - ps12
Embriophyta - ps13
Tracheophyta - ps14
Spermatophyta - ps15
Angiosperms - ps16
Mesangiospermae - ps17
Magnoliids/eudicots - ps18
Eudicots - ps19
Core_eudicots - ps20
Superrosids - ps21
Rosids - ps22
Eurosids - ps23
Malvids - ps24
Sap./Malv./Brassicales - ps25
Malvales/Brassicales - ps26
Brassicales - ps27
Cleomaceae/Brassicaceae - ps28
Brassicaceae - ps29
Camelineae - ps30
Arabidopsis - ps31
Arabidopsis_thaliana - ps32
p-value
5e-04
2e-03
5e-03
2e-02
6e-02
Log-odds
0.5
1.0
1.5
2.0
2.5
3.0
3.5
4.0

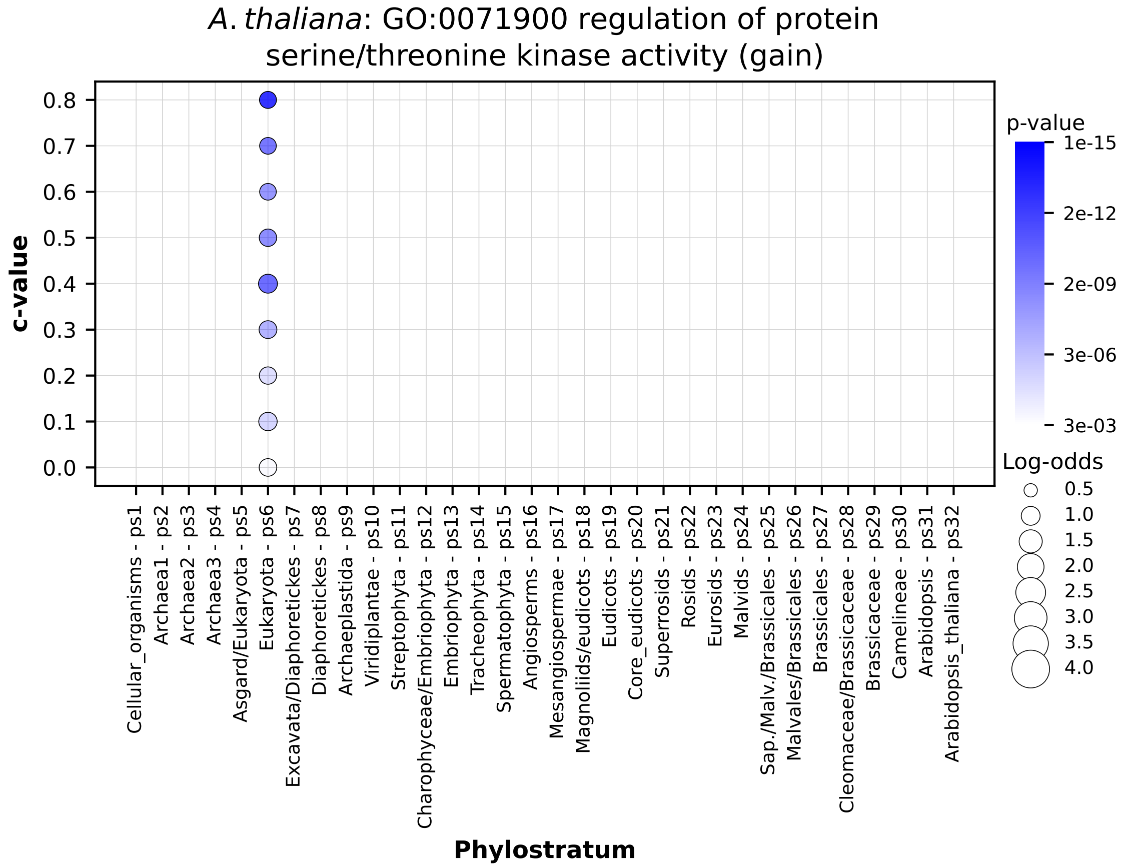

A. thaliana: GO:0071900 regulation of protein serine/threonine kinase activity (gain)
c-value
0.8
0.7
0.6
0.5
0.4
0.3
0.2
0.1
0.0
Phylostratum
Cellular_organisms - ps1
Archaea1 - ps2
Archaea2 - ps3
Archaea3 - ps4
Asgard/Eukaryota - ps5
Eukaryota - ps6
Excavata/Diaphoretickes - ps7
Diaphoretickes - ps8
Archaeplastida - ps9
Viridiplantae - ps10
Streptophyta - ps11
Charophyceae/Embriophyta - ps12
Embriophyta - ps13
Tracheophyta - ps14
Spermatophyta - ps15
Angiosperms - ps16
Mesangiospermae - ps17
Magnoliids/eudicots - ps18
Eudicots - ps19
Core_eudicots - ps20
Superrosids - ps21
Rosids - ps22
Eurosids - ps23
Malvids - ps24
Sap./Malv./Brassicales - ps25
Malvales/Brassicales - ps26
Brassicales - ps27
Cleomaceae/Brassicaceae - ps28
Brassicaceae - ps29
Camelineae - ps30
Arabidopsis - ps31
Arabidopsis_thaliana - ps32
p-value
1e-15
2e-12
2e-09
3e-06
3e-03
Log-odds
0.5
1.0
1.5
2.0
2.5
3.0
3.5
4.0

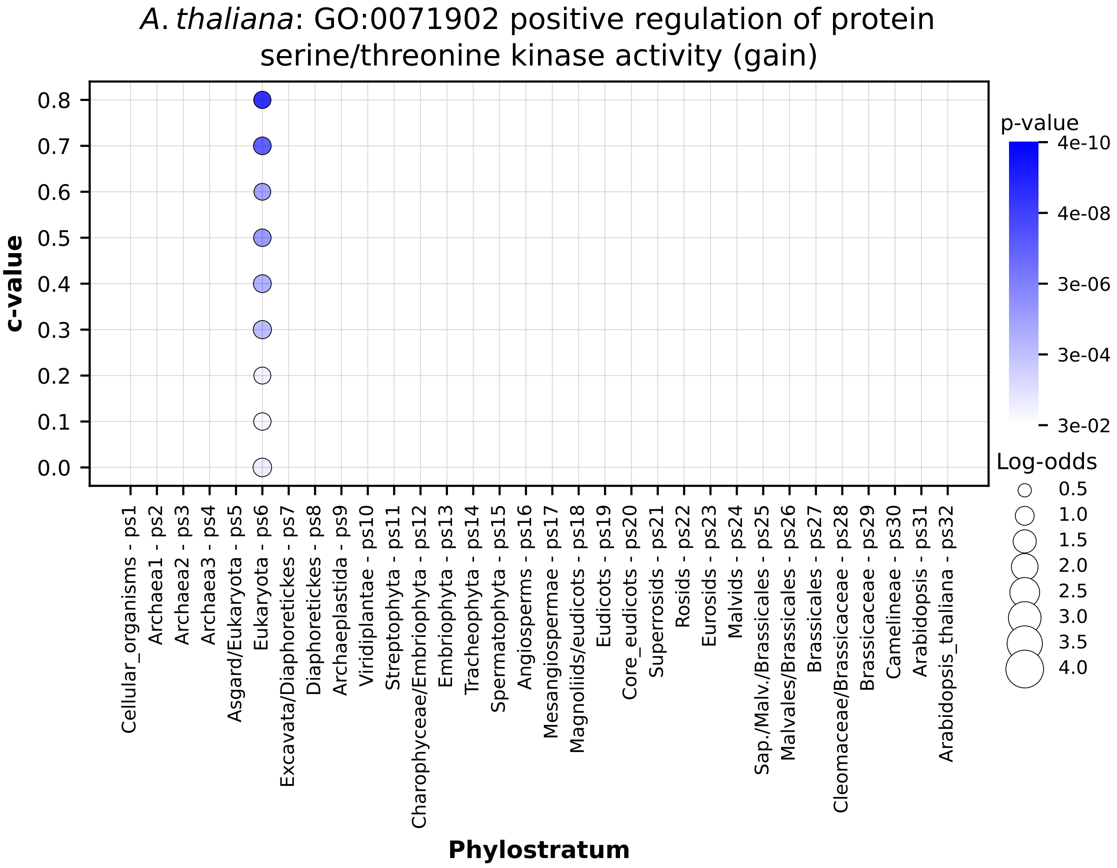
A. thaliana: GO:0071902 positive regulation of protein serine/threonine kinase activity (gain)
c-value
0.8
0.7
0.6
0.5
0.4
0.3
0.2
0.1
0.0
p-value
4e-10
4e-08
3e-06
3e-04
3e-02
Log-odds
0.5
1.0
1.5
2.0
2.5
3.0
3.5
4.0
Cellular_organisms - ps1
Archaea1 - ps2
Archaea2 - ps3
Archaea3 - ps4
Asgard/Eukaryota - ps5
Eukaryota - ps6
Excavata/Diaphoretickes - ps7
Diaphoretickes - ps8
Archaeplastida - ps9
Viridiplantae - ps10
Streptophyta - ps11
Charophyceae/Embriophyta - ps12
Embriophyta - ps13
Tracheophyta - ps14
Spermatophyta - ps15
Angiosperms - ps16
Mesangiospermae - ps17
Magnoliids/eudicots - ps18
Eudicots - ps19
Core_eudicots - ps20
Superrosids - ps21
Rosids - ps22
Eurosids - ps23
Malvids - ps24
Sap./Malv./Brassicales - ps25
Malvales/Brassicales - ps26
Brassicales - ps27
Cleomaceae/Brassicaceae - ps28
Brassicaceae - ps29
Camelineae - ps30
Arabidopsis - ps31
Arabidopsis_thaliana - ps32
Phylostratum

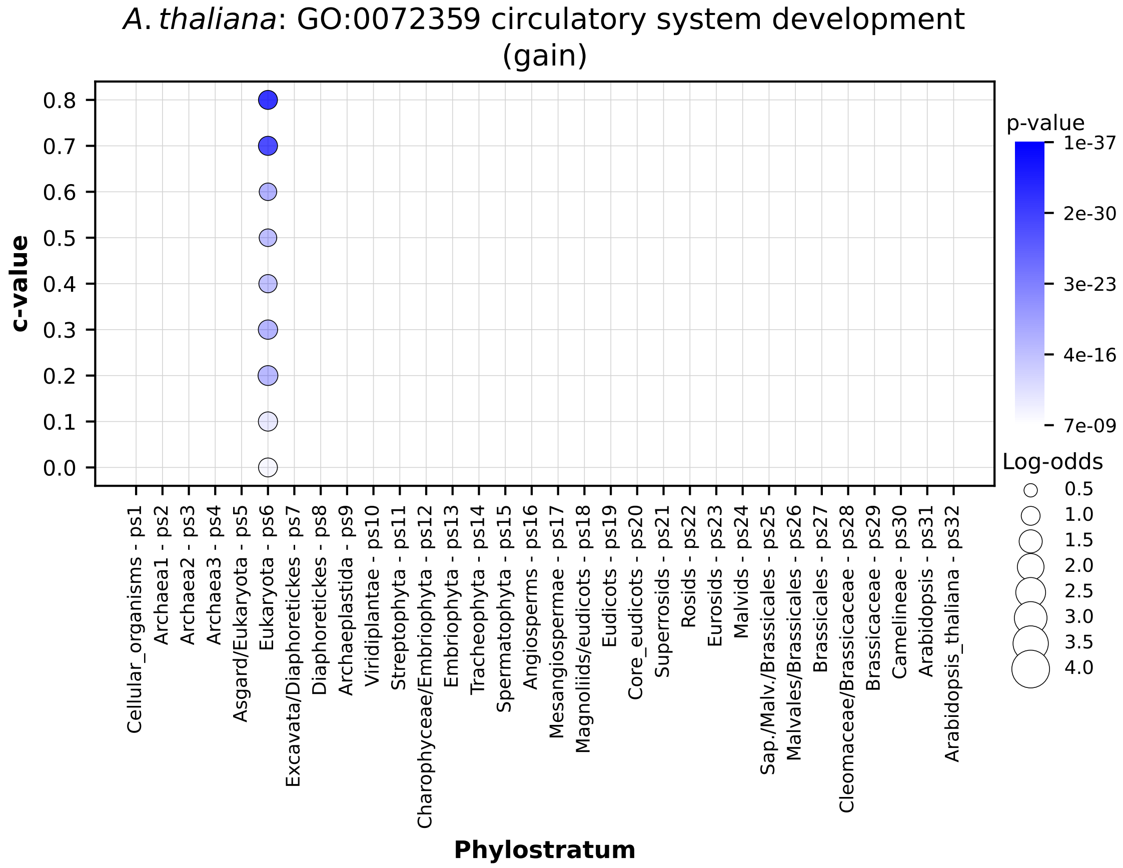

A. thaliana: GO:0072359 circulatory system development (gain)
c-value
0.0
0.1
0.2
0.3
0.4
0.5
0.6
0.7
0.8
p-value
1e-37
2e-30
3e-23
4e-16
7e-09
Log-odds
0.5
1.0
1.5
2.0
2.5
3.0
3.5
4.0
Cellular_organisms - ps1
Archaea1 - ps2
Archaea2 - ps3
Archaea3 - ps4
Asgard/Eukaryota - ps5
Eukaryota - ps6
Excavata/Diaphoretickes - ps7
Diaphoretickes - ps8
Archaeplastida - ps9
Viridiplantae - ps10
Streptophyta - ps11
Charophyceae/Embriophyta - ps12
Embriophyta - ps13
Tracheophyta - ps14
Spermatophyta - ps15
Angiosperms - ps16
Mesangiospermae - ps17
Magnoliids/eudicots - ps18
Eudicots - ps19
Core_eudicots - ps20
Superrosids - ps21
Rosids - ps22
Eurosids - ps23
Malvids - ps24
Sap./Malv./Brassicales - ps25
Malvales/Brassicales - ps26
Brassicales - ps27
Cleomaceae/Brassicaceae - ps28
Brassicaceae - ps29
Camelineae - ps30
Arabidopsis - ps31
Arabidopsis_thaliana - ps32
Phylostratum

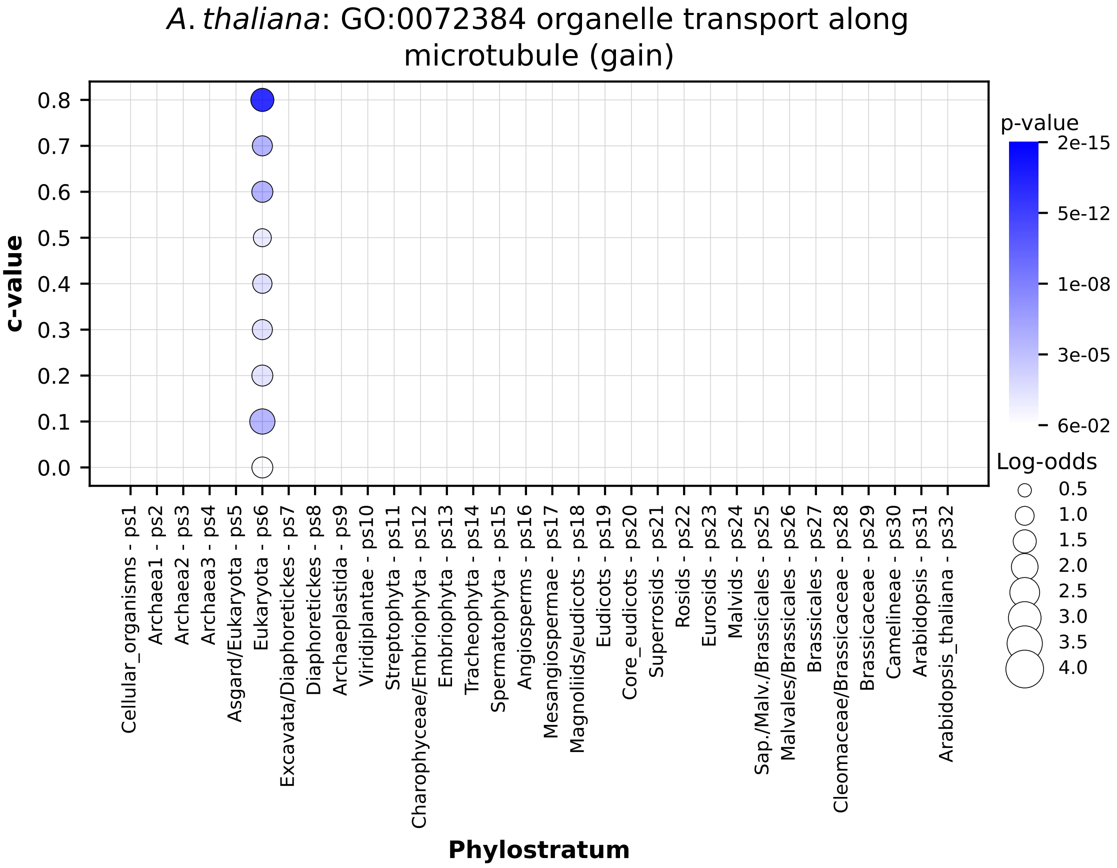

A. thaliana: GO:0072384 organelle transport along microtubule (gain)
c-value
0.8
0.7
0.6
0.5
0.4
0.3
0.2
0.1
0.0
Phylostratum
Cellular_organisms - ps1
Archaea1 - ps2
Archaea2 - ps3
Archaea3 - ps4
Asgard/Eukaryota - ps5
Eukaryota - ps6
Excavata/Diaphoretickes - ps7
Diaphoretickes - ps8
Archaeplastida - ps9
Viridiplantae - ps10
Streptophyta - ps11
Charophyceae/Embriophyta - ps12
Embriophyta - ps13
Tracheophyta - ps14
Spermatophyta - ps15
Angiosperms - ps16
Mesangiospermae - ps17
Magnoliids/eudicots - ps18
Eudicots - ps19
Core_eudicots - ps20
Superrosids - ps21
Rosids - ps22
Eurosids - ps23
Malvids - ps24
Sap./Malv./Brassicales - ps25
Malvales/Brassicales - ps26
Brassicales - ps27
Cleomaceae/Brassicaceae - ps28
Brassicaceae - ps29
Camelineae - ps30
Arabidopsis - ps31
Arabidopsis_thaliana - ps32
p-value
2e-15
5e-12
1e-08
3e-05
6e-02
Log-odds
0.5
1.0
1.5
2.0
2.5
3.0
3.5
4.0

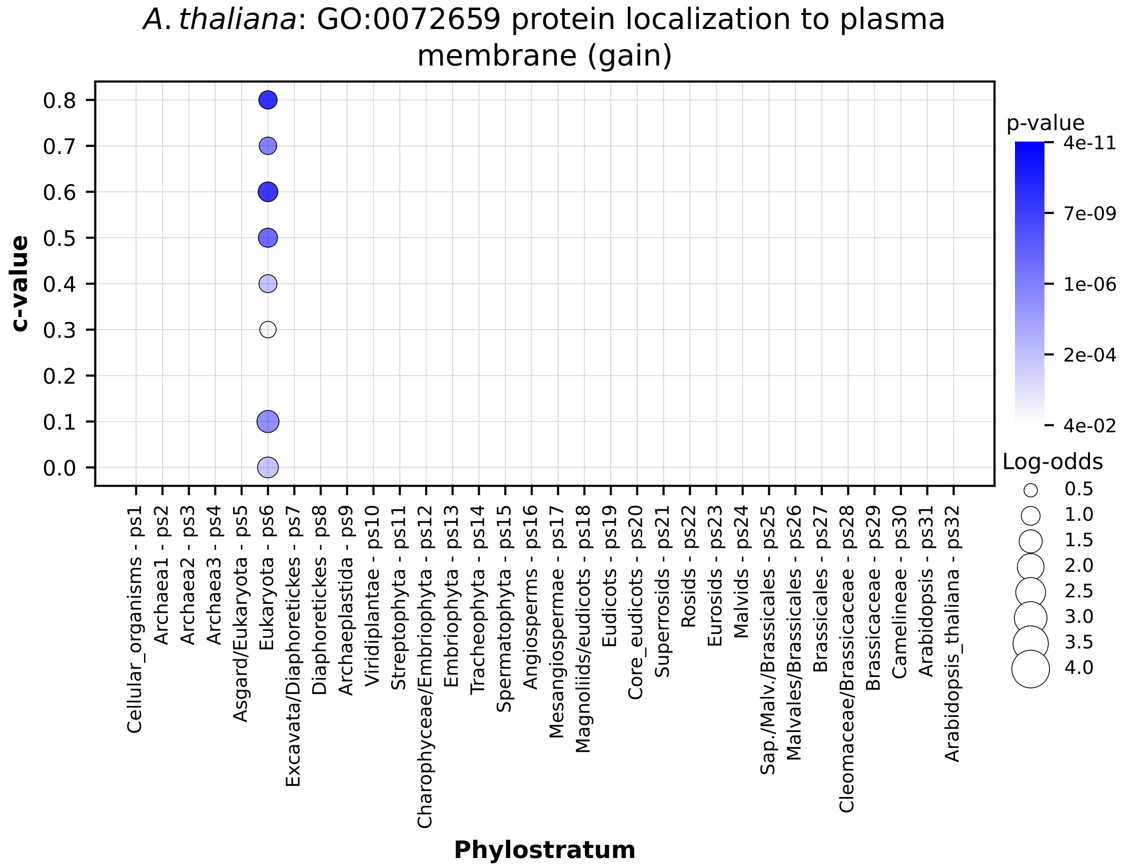

A. thaliana: GO:0072659 protein localization to plasma membrane (gain)
c-value
0.8
0.7
0.6
0.5
0.4
0.3
0.2
0.1
0.0
p-value
4e-11
7e-09
1e-06
2e-04
4e-02
Log-odds
0.5
1.0
1.5
2.0
2.5
3.0
3.5
4.0
Cellular_organisms - ps1
Archaea1 - ps2
Archaea2 - ps3
Archaea3 - ps4
Asgard/Eukaryota - ps5
Eukaryota - ps6
Excavata/Diaphoretickes - ps7
Diaphoretickes - ps8
Archaeplastida - ps9
Viridiplantae - ps10
Streptophyta - ps11
Charophyceae/Embriophyta - ps12
Embriophyta - ps13
Tracheophyta - ps14
Spermatophyta - ps15
Angiosperms - ps16
Mesangiospermae - ps17
Magnoliids/eudicots - ps18
Eudicots - ps19
Core_eudicots - ps20
Superrosids - ps21
Rosids - ps22
Eurosids - ps23
Malvids - ps24
Sap./Malv./Brassicales - ps25
Malvales/Brassicales - ps26
Brassicales - ps27
Cleomaceae/Brassicaceae - ps28
Brassicaceae - ps29
Camelineae - ps30
Arabidopsis - ps31
Arabidopsis_thaliana - ps32
Phylostratum

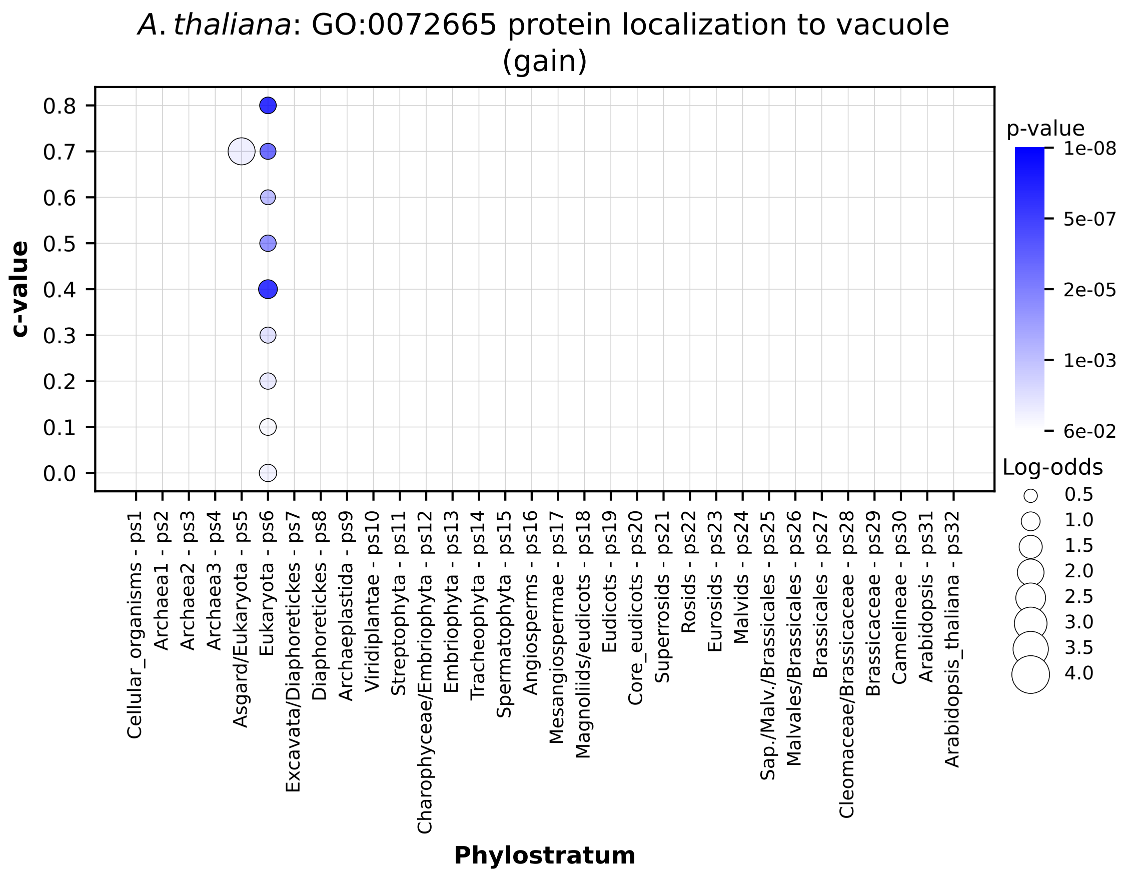

A. thaliana: GO:0072665 protein localization to vacuole
(gain)
c-value
0.8
0.7
0.6
0.5
0.4
0.3
0.2
0.1
0.0
p-value
1e-08
5e-07
2e-05
1e-03
6e-02
Log-odds
0.5
1.0
1.5
2.0
2.5
3.0
3.5
4.0
Cellular_organisms - ps1
Archaea1 - ps2
Archaea2 - ps3
Archaea3 - ps4
Asgard/Eukaryota - ps5
Eukaryota - ps6
Excavata/Diaphoretickes - ps7
Diaphoretickes - ps8
Archaeplastida - ps9
Viridiplantae - ps10
Streptophyta - ps11
Charophyceae/Embriophyta - ps12
Embriophyta - ps13
Tracheophyta - ps14
Spermatophyta - ps15
Angiosperms - ps16
Mesangiospermae - ps17
Magnoliids/eudicots - ps18
Eudicots - ps19
Core_eudicots - ps20
Superrosids - ps21
Rosids - ps22
Eurosids - ps23
Malvids - ps24
Sap./Malv./Brassicales - ps25
Malvales/Brassicales - ps26
Brassicales - ps27
Cleomaceae/Brassicaceae - ps28
Brassicaceae - ps29
Camelineae - ps30
Arabidopsis - ps31
Arabidopsis_thaliana - ps32
Phylostratum

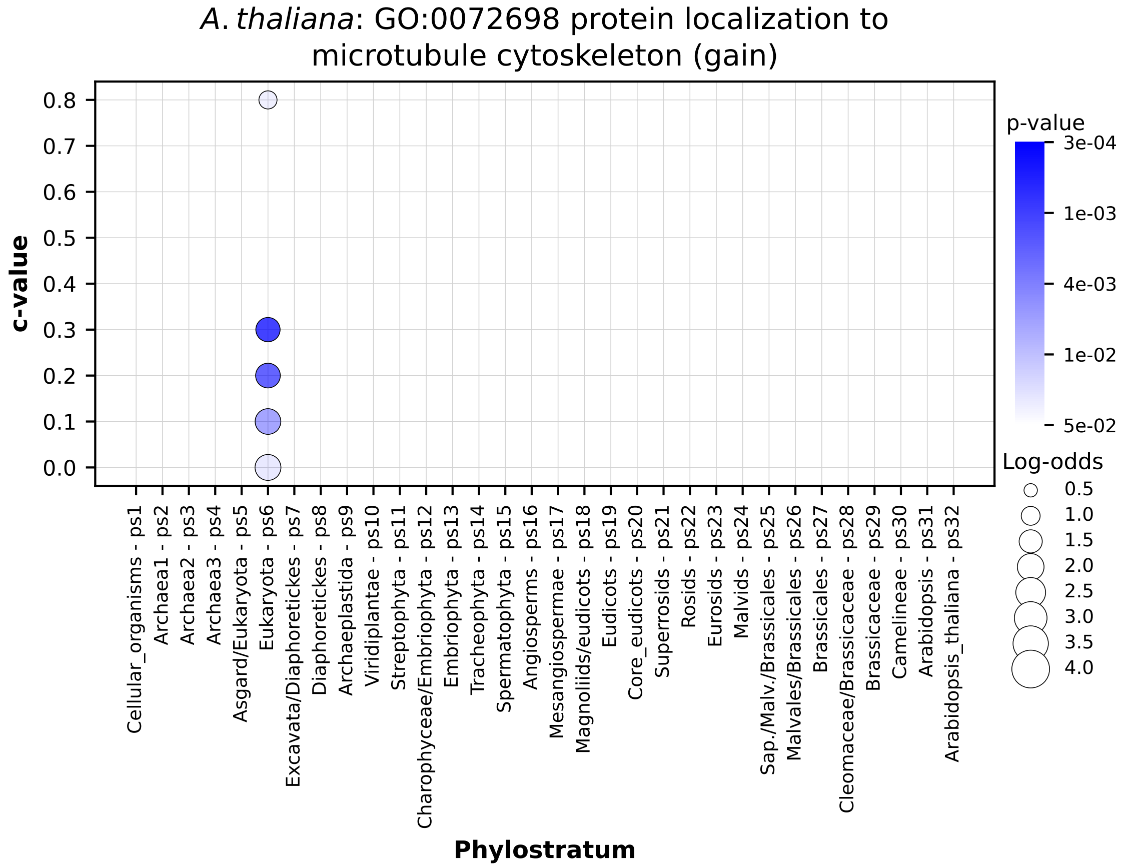

A. thaliana: GO:0072698 protein localization to microtubule cytoskeleton (gain)
c-value
0.8
0.7
0.6
0.5
0.4
0.3
0.2
0.1
0.0
Phylostratum
Cellular_organisms - ps1
Archaea1 - ps2
Archaea2 - ps3
Archaea3 - ps4
Asgard/Eukaryota - ps5
Eukaryota - ps6
Excavata/Diaphoretickes - ps7
Diaphoretickes - ps8
Archaeplastida - ps9
Viridiplantae - ps10
Streptophyta - ps11
Charophyceae/Embriophyta - ps12
Embriophyta - ps13
Tracheophyta - ps14
Spermatophyta - ps15
Angiosperms - ps16
Mesangiospermae - ps17
Magnoliids/eudicots - ps18
Eudicots - ps19
Core_eudicots - ps20
Superrosids - ps21
Rosids - ps22
Eurosids - ps23
Malvids - ps24
Sap./Malv./Brassicales - ps25
Malvales/Brassicales - ps26
Brassicales - ps27
Cleomaceae/Brassicaceae - ps28
Brassicaceae - ps29
Camelineae - ps30
Arabidopsis - ps31
Arabidopsis_thaliana - ps32
p-value
3e-04
1e-03
4e-03
1e-02
5e-02
Log-odds
0.5
1.0
1.5
2.0
2.5
3.0
3.5
4.0

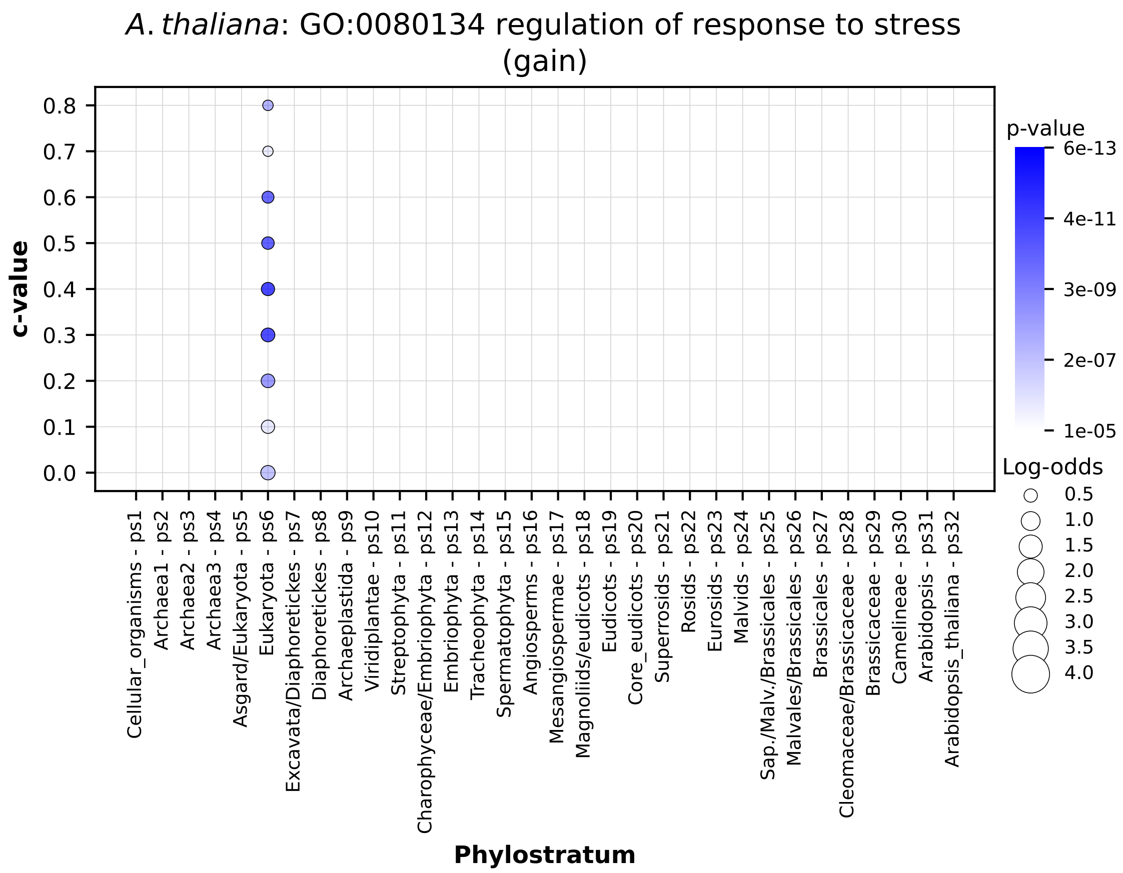

A. thaliana: GO:0080134 regulation of response to stress (gain)
c-value
0.8
0.7
0.6
0.5
0.4
0.3
0.2
0.1
0.0
p-value
6e-13
4e-11
3e-09
2e-07
1e-05
Log-odds
0.5
1.0
1.5
2.0
2.5
3.0
3.5
4.0
Cellular_organisms - ps1
Archaea1 - ps2
Archaea2 - ps3
Archaea3 - ps4
Asgard/Eukaryota - ps5
Eukaryota - ps6
Excavata/Diaphoretickes - ps7
Diaphoretickes - ps8
Archaeplastida - ps9
Viridiplantae - ps10
Streptophyta - ps11
Charophyceae/Embriophyta - ps12
Embriophyta - ps13
Tracheophyta - ps14
Spermatophyta - ps15
Angiosperms - ps16
Mesangiospermae - ps17
Magnoliids/eudicots - ps18
Eudicots - ps19
Core_eudicots - ps20
Superrosids - ps21
Rosids - ps22
Eurosids - ps23
Malvids - ps24
Sap./Malv./Brassicales - ps25
Malvales/Brassicales - ps26
Brassicales - ps27
Cleomaceae/Brassicaceae - ps28
Brassicaceae - ps29
Camelineae - ps30
Arabidopsis - ps31
Arabidopsis_thaliana - ps32
Phylostratum

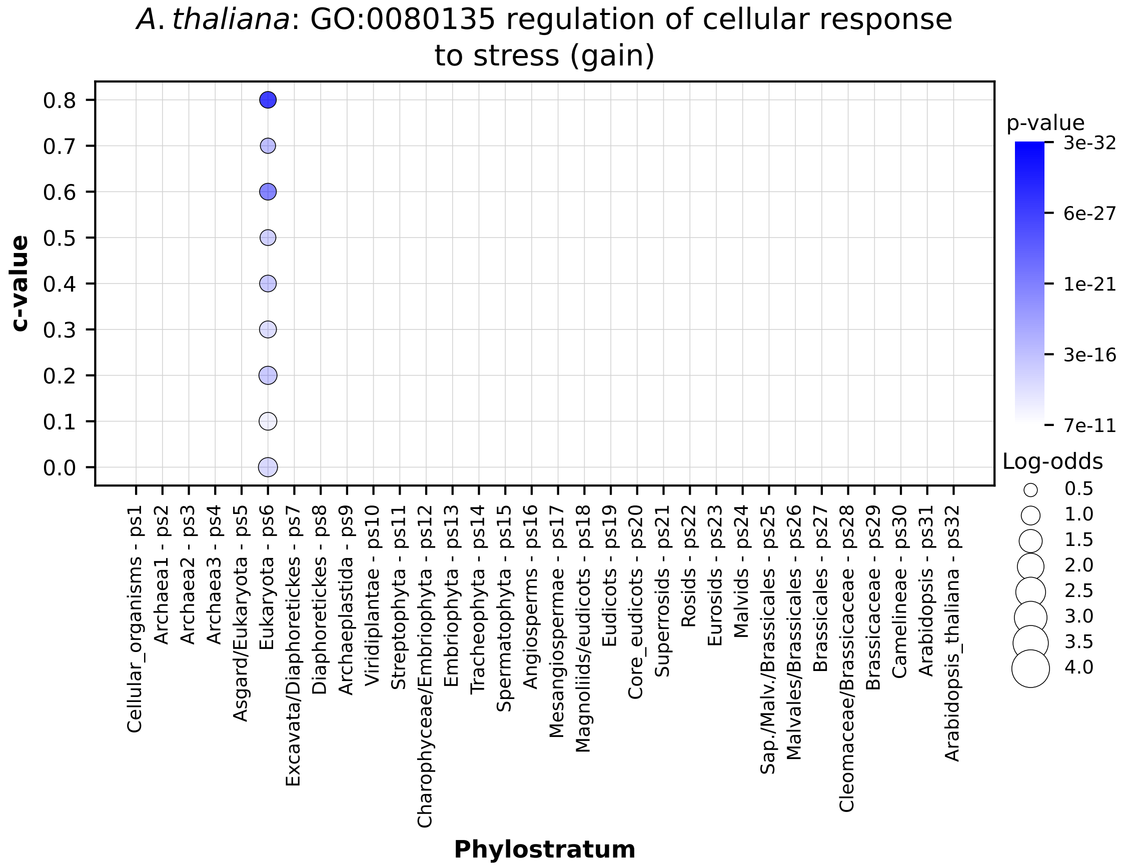

A. thaliana: GO:0080135 regulation of cellular response to stress (gain)
c-value
0.8
0.7
0.6
0.5
0.4
0.3
0.2
0.1
0.0
p-value
3e-32
6e-27
1e-21
3e-16
7e-11
Log-odds
0.5
1.0
1.5
2.0
2.5
3.0
3.5
4.0
Cellular_organisms - ps1
Archaea1 - ps2
Archaea2 - ps3
Archaea3 - ps4
Asgard/Eukaryota - ps5
Eukaryota - ps6
Excavata/Diaphoretickes - ps7
Diaphoretickes - ps8
Archaeplastida - ps9
Viridiplantae - ps10
Streptophyta - ps11
Charophyceae/Embriophyta - ps12
Embriophyta - ps13
Tracheophyta - ps14
Spermatophyta - ps15
Angiosperms - ps16
Mesangiospermae - ps17
Magnoliids/eudicots - ps18
Eudicots - ps19
Core_eudicots - ps20
Superrosids - ps21
Rosids - ps22
Eurosids - ps23
Malvids - ps24
Sap./Malv./Brassicales - ps25
Malvales/Brassicales - ps26
Brassicales - ps27
Cleomaceae/Brassicaceae - ps28
Brassicaceae - ps29
Camelineae - ps30
Arabidopsis - ps31
Arabidopsis_thaliana - ps32
Phylostratum

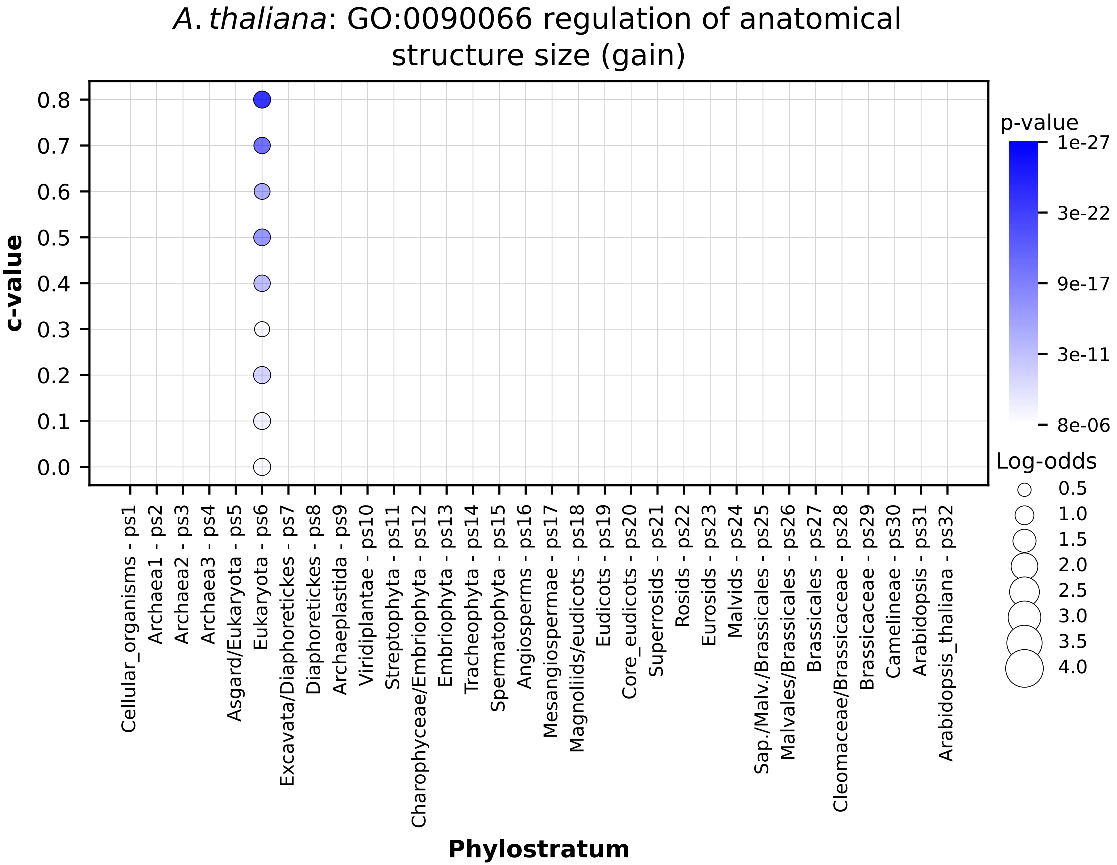

A. thaliana: GO:0090066 regulation of anatomical structure size (gain)
c-value
0.8
0.7
0.6
0.5
0.4
0.3
0.2
0.1
0.0
p-value
1e-27
3e-22
9e-17
3e-11
8e-06
Log-odds
0.5
1.0
1.5
2.0
2.5
3.0
3.5
4.0
Cellular_organisms - ps1
Archaea1 - ps2
Archaea2 - ps3
Archaea3 - ps4
Asgard/Eukaryota - ps5
Eukaryota - ps6
Excavata/Diaphoretickes - ps7
Diaphoretickes - ps8
Archaeplastida - ps9
Viridiplantae - ps10
Streptophyta - ps11
Charophyceae/Embriophyta - ps12
Embriophyta - ps13
Tracheophyta - ps14
Spermatophyta - ps15
Angiosperms - ps16
Mesangiospermae - ps17
Magnoliids/eudicots - ps18
Eudicots - ps19
Core_eudicots - ps20
Superrosids - ps21
Rosids - ps22
Eurosids - ps23
Malvids - ps24
Sap./Malv./Brassicales - ps25
Malvales/Brassicales - ps26
Brassicales - ps27
Cleomaceae/Brassicaceae - ps28
Brassicaceae - ps29
Camelineae - ps30
Arabidopsis - ps31
Arabidopsis_thaliana - ps32
Phylostratum

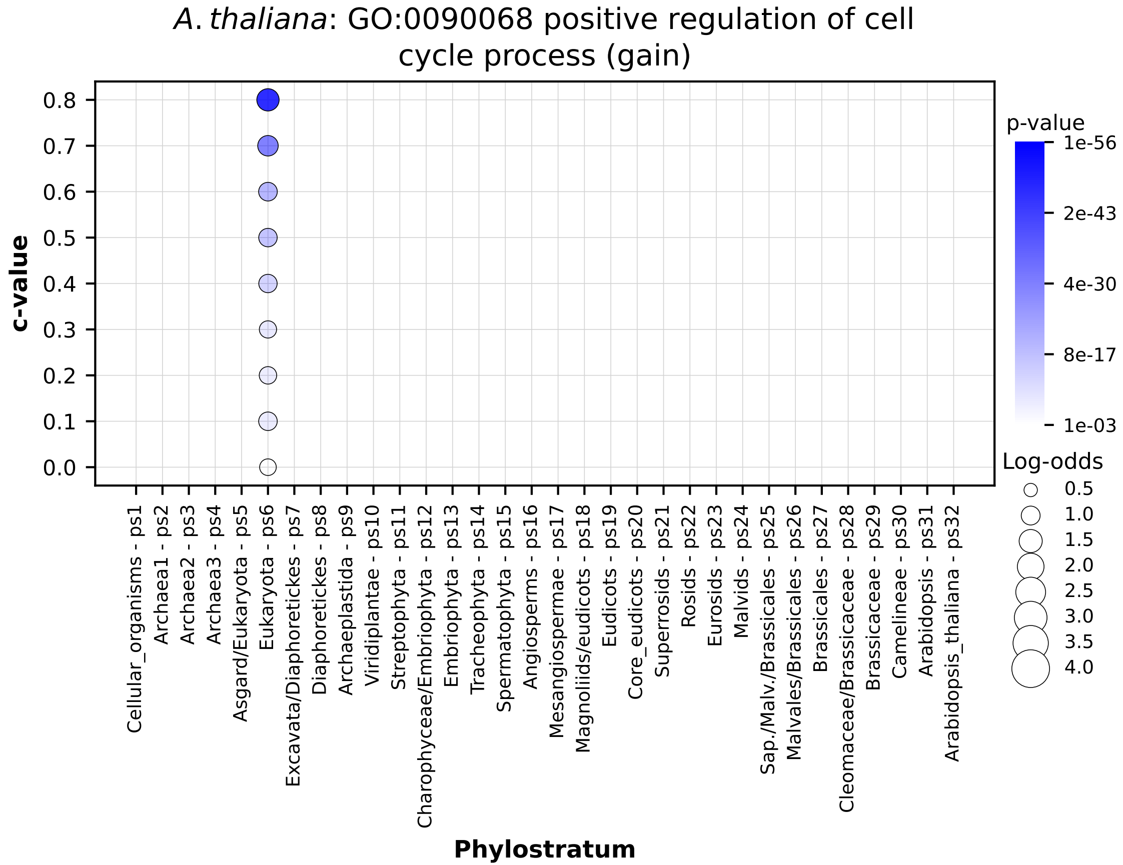
A. thaliana: GO:0090068 positive regulation of cell cycle process (gain)
c-value
0.8
0.7
0.6
0.5
0.4
0.3
0.2
0.1
0.0
p-value
1e-56
2e-43
4e-30
8e-17
1e-03
Log-odds
0.5
1.0
1.5
2.0
2.5
3.0
3.5
4.0
Cellular_organisms - ps1
Archaea1 - ps2
Archaea2 - ps3
Archaea3 - ps4
Asgard/Eukaryota - ps5
Eukaryota - ps6
Excavata/Diaphoretickes - ps7
Diaphoretickes - ps8
Archaeplastida - ps9
Viridiplantae - ps10
Streptophyta - ps11
Charophyceae/Embriophyta - ps12
Embriophyta - ps13
Tracheophyta - ps14
Spermatophyta - ps15
Angiosperms - ps16
Mesangiospermae - ps17
Magnoliids/eudicots - ps18
Eudicots - ps19
Core_eudicots - ps20
Superrosids - ps21
Rosids - ps22
Eurosids - ps23
Malvids - ps24
Sap./Malv./Brassicales - ps25
Malvales/Brassicales - ps26
Brassicales - ps27
Cleomaceae/Brassicaceae - ps28
Brassicaceae - ps29
Camelineae - ps30
Arabidopsis - ps31
Arabidopsis_thaliana - ps32
Phylostratum

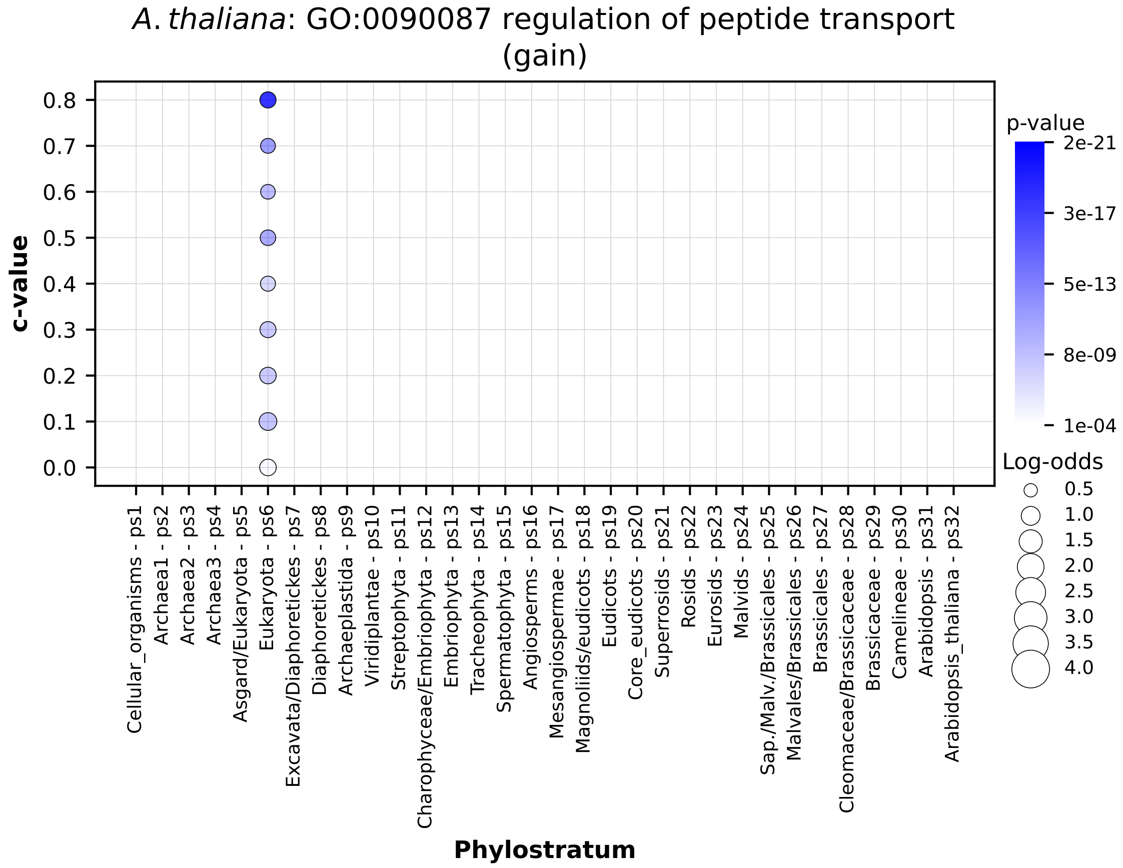
A. thaliana: GO:0090087 regulation of peptide transport
(gain)
c-value
0.8
0.7
0.6
0.5
0.4
0.3
0.2
0.1
0.0
p-value
2e-21
3e-17
5e-13
8e-09
1e-04
Log-odds
0.5
1.0
1.5
2.0
2.5
3.0
3.5
4.0
Cellular_organisms - ps1
Archaea1 - ps2
Archaea2 - ps3
Archaea3 - ps4
Asgard/Eukaryota - ps5
Eukaryota - ps6
Excavata/Diaphoretickes - ps7
Diaphoretickes - ps8
Archaeplastida - ps9
Viridiplantae - ps10
Streptophyta - ps11
Charophyceae/Embriophyta - ps12
Embriophyta - ps13
Tracheophyta - ps14
Spermatophyta - ps15
Angiosperms - ps16
Mesangiospermae - ps17
Magnoliids/eudicots - ps18
Eudicots - ps19
Core_eudicots - ps20
Superrosids - ps21
Rosids - ps22
Eurosids - ps23
Malvids - ps24
Sap./Malv./Brassicales - ps25
Malvales/Brassicales - ps26
Brassicales - ps27
Cleomaceae/Brassicaceae - ps28
Brassicaceae - ps29
Camelineae - ps30
Arabidopsis - ps31
Arabidopsis_thaliana - ps32
Phylostratum

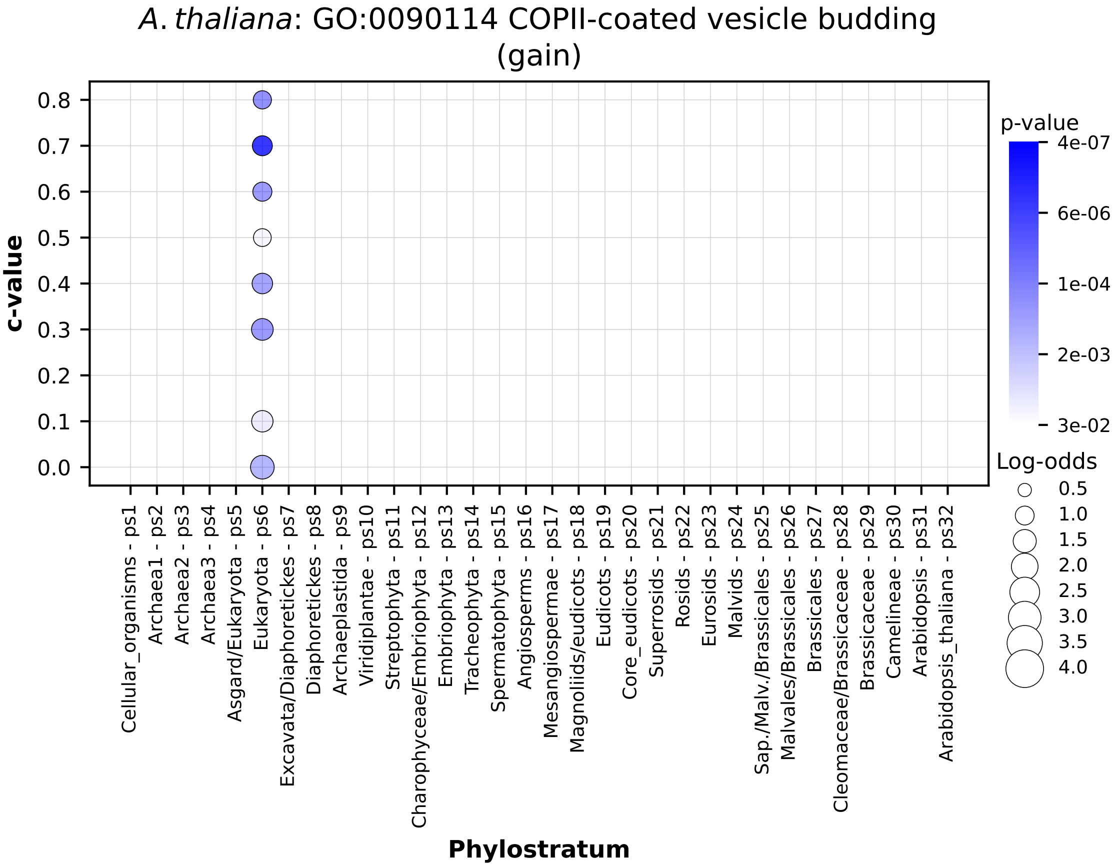

A. thaliana: GO:0090114 COPII-coated vesicle budding
(gain)
c-value
0.8
0.7
0.6
0.5
0.4
0.3
0.2
0.1
0.0
p-value
4e-07
6e-06
1e-04
2e-03
3e-02
Log-odds
0.5
1.0
1.5
2.0
2.5
3.0
3.5
4.0
Cellular_organisms - ps1
Archaea1 - ps2
Archaea2 - ps3
Archaea3 - ps4
Asgard/Eukaryota - ps5
Eukaryota - ps6
Excavata/Diaphoretickes - ps7
Diaphoretickes - ps8
Archaeplastida - ps9
Viridiplantae - ps10
Streptophyta - ps11
Charophyceae/Embriophyta - ps12
Embriophyta - ps13
Tracheophyta - ps14
Spermatophyta - ps15
Angiosperms - ps16
Mesangiospermae - ps17
Magnoliids/eudicots - ps18
Eudicots - ps19
Core_eudicots - ps20
Superrosids - ps21
Rosids - ps22
Eurosids - ps23
Malvids - ps24
Sap./Malv./Brassicales - ps25
Malvales/Brassicales - ps26
Brassicales - ps27
Cleomaceae/Brassicaceae - ps28
Brassicaceae - ps29
Camelineae - ps30
Arabidopsis - ps31
Arabidopsis_thaliana - ps32
Phylostratum

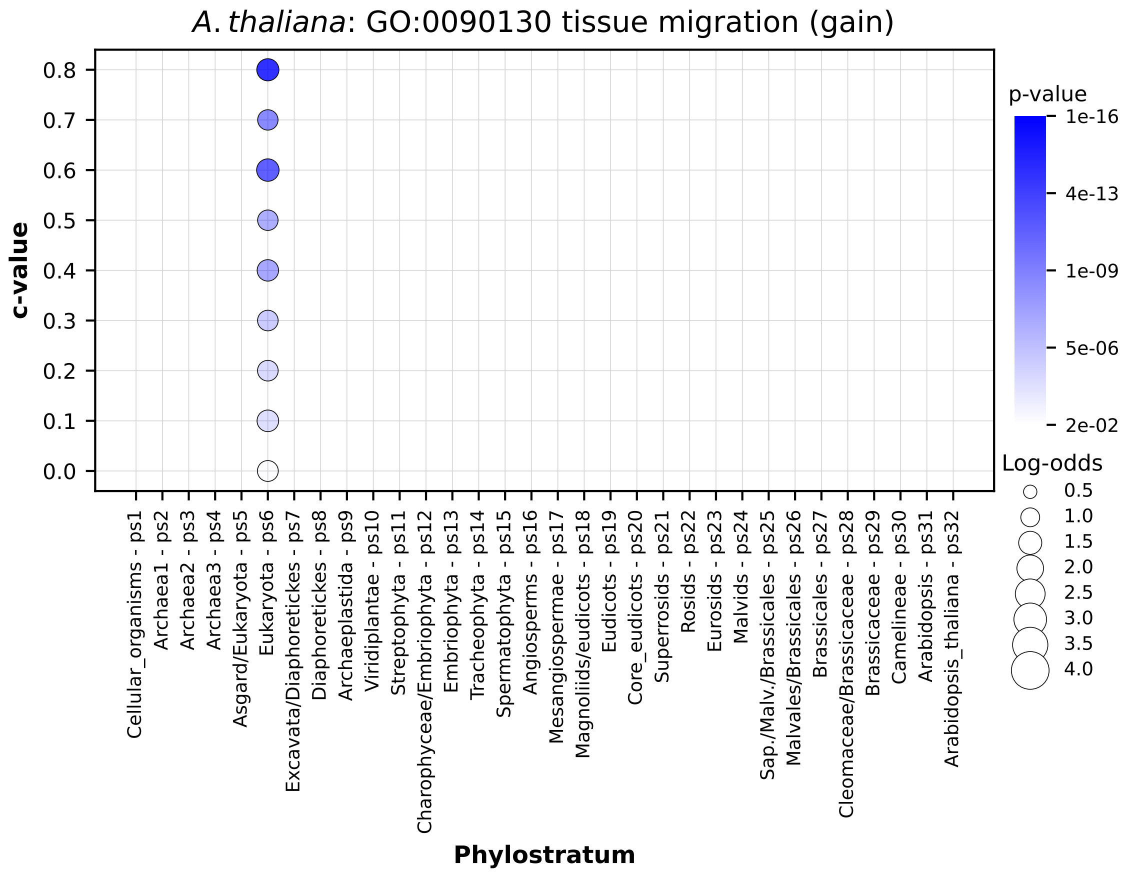

A. thaliana: GO:0090130 tissue migration (gain)
c-value
0.0
0.1
0.2
0.3
0.4
0.5
0.6
0.7
0.8
Phylostratum
Cellular_organisms - ps1
Archaea1 - ps2
Archaea2 - ps3
Archaea3 - ps4
Asgard/Eukaryota - ps5
Eukaryota - ps6
Excavata/Diaphoretickes - ps7
Diaphoretickes - ps8
Archaeplastida - ps9
Viridiplantae - ps10
Streptophyta - ps11
Charophyceae/Embriophyta - ps12
Embriophyta - ps13
Tracheophyta - ps14
Spermatophyta - ps15
Angiosperms - ps16
Mesangiospermae - ps17
Magnoliids/eudicots - ps18
Eudicots - ps19
Core_eudicots - ps20
Superrosids - ps21
Rosids - ps22
Eurosids - ps23
Malvids - ps24
Sap./Malv./Brassicales - ps25
Malvales/Brassicales - ps26
Brassicales - ps27
Cleomaceae/Brassicaceae - ps28
Brassicaceae - ps29
Camelineae - ps30
Arabidopsis - ps31
Arabidopsis_thaliana - ps32
p-value
1e-16
4e-13
1e-09
5e-06
2e-02
Log-odds
0.5
1.0
1.5
2.0
2.5
3.0
3.5
4.0

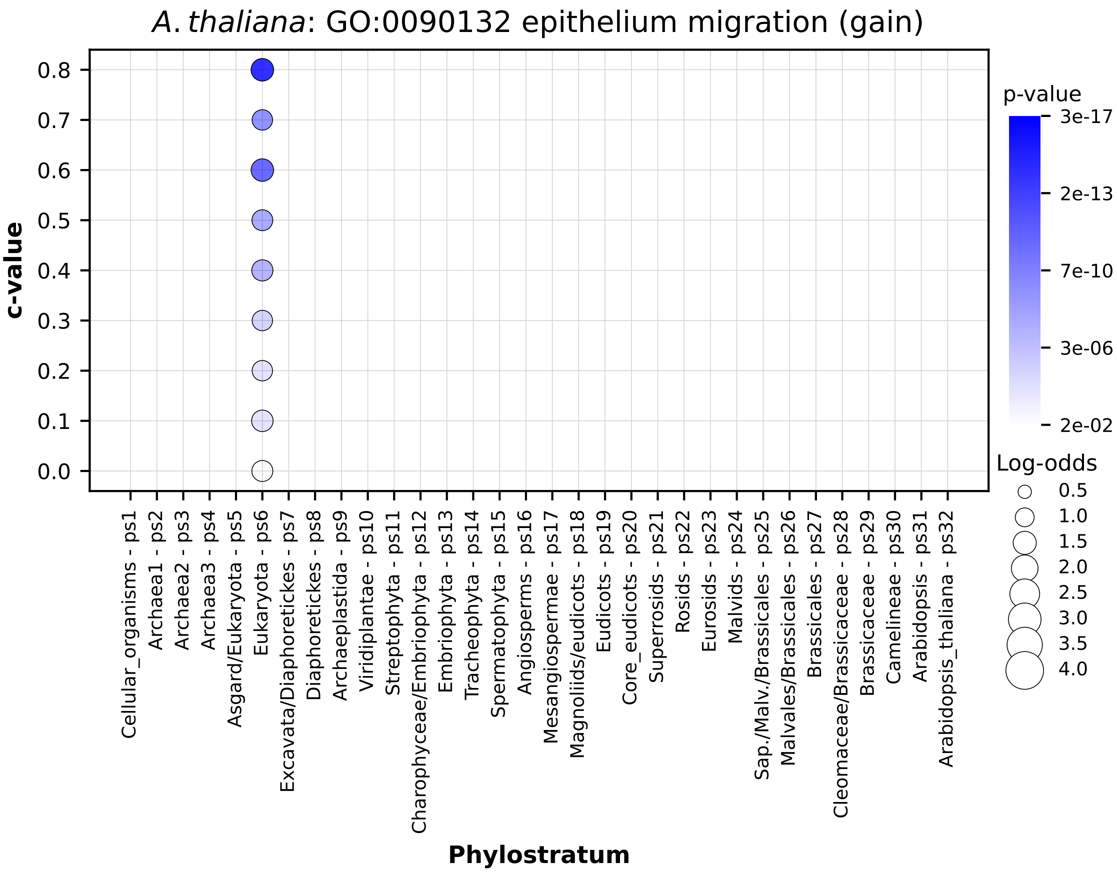

A. thaliana: GO:0090132 epithelium migration (gain)
c-value
0.8
0.7
0.6
0.5
0.4
0.3
0.2
0.1
0.0
Phylostratum
Cellular_organisms - ps1
Archaea1 - ps2
Archaea2 - ps3
Archaea3 - ps4
Asgard/Eukaryota - ps5
Eukaryota - ps6
Excavata/Diaphoretickes - ps7
Diaphoretickes - ps8
Archaeplastida - ps9
Viridiplantae - ps10
Streptophyta - ps11
Charophyceae/Embriophyta - ps12
Embriophyta - ps13
Tracheophyta - ps14
Spermatophyta - ps15
Angiosperms - ps16
Mesangiospermae - ps17
Magnoliids/eudicots - ps18
Eudicots - ps19
Core_eudicots - ps20
Superrosids - ps21
Rosids - ps22
Eurosids - ps23
Malvids - ps24
Sap./Malv./Brassicales - ps25
Malvales/Brassicales - ps26
Brassicales - ps27
Cleomaceae/Brassicaceae - ps28
Brassicaceae - ps29
Camelineae - ps30
Arabidopsis - ps31
Arabidopsis_thaliana - ps32
p-value
3e-17
2e-13
7e-10
3e-06
2e-02
Log-odds
0.5
1.0
1.5
2.0
2.5
3.0
3.5
4.0

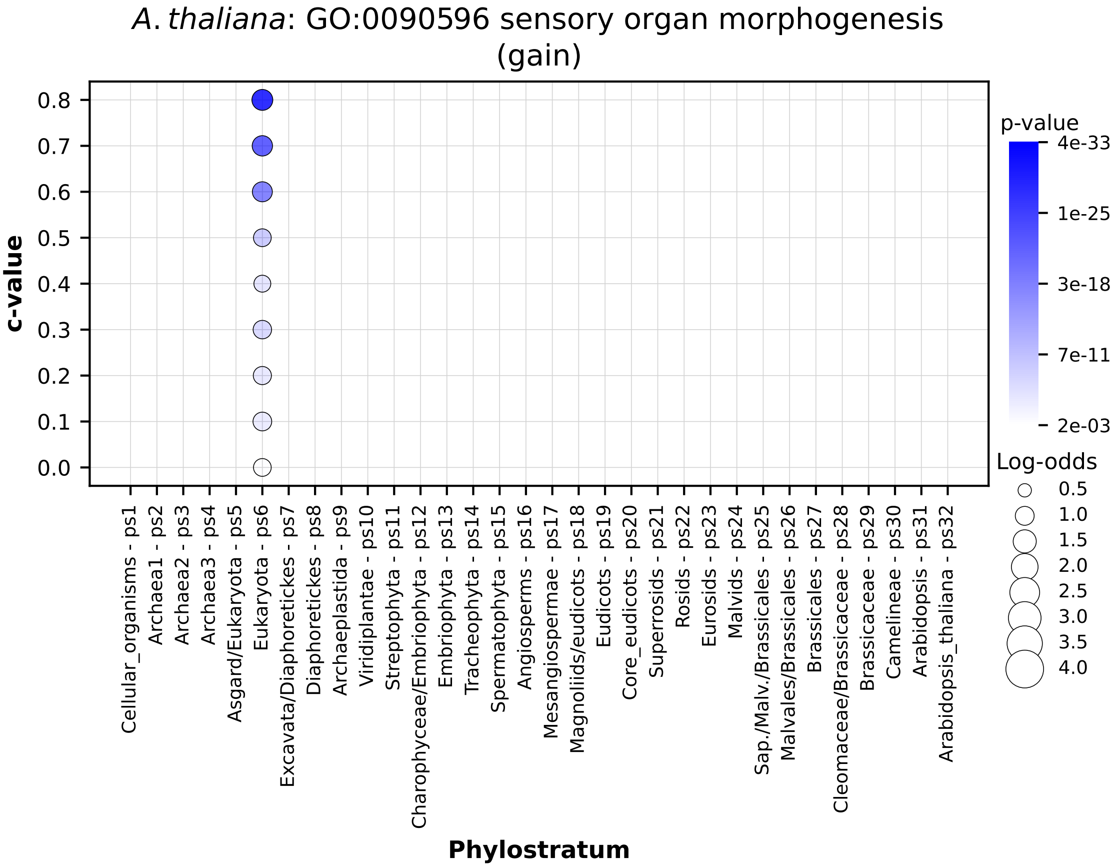

A. thaliana: GO:0090596 sensory organ morphogenesis (gain)
c-value
0.8
0.7
0.6
0.5
0.4
0.3
0.2
0.1
0.0
p-value
4e-33
1e-25
3e-18
7e-11
2e-03
Log-odds
0.5
1.0
1.5
2.0
2.5
3.0
3.5
4.0
Cellular_organisms - ps1
Archaea1 - ps2
Archaea2 - ps3
Archaea3 - ps4
Asgard/Eukaryota - ps5
Eukaryota - ps6
Excavata/Diaphoretickes - ps7
Diaphoretickes - ps8
Archaeplastida - ps9
Viridiplantae - ps10
Streptophyta - ps11
Charophyceae/Embriophyta - ps12
Embriophyta - ps13
Tracheophyta - ps14
Spermatophyta - ps15
Angiosperms - ps16
Mesangiospermae - ps17
Magnoliids/eudicots - ps18
Eudicots - ps19
Core_eudicots - ps20
Superrosids - ps21
Rosids - ps22
Eurosids - ps23
Malvids - ps24
Sap./Malv./Brassicales - ps25
Malvales/Brassicales - ps26
Brassicales - ps27
Cleomaceae/Brassicaceae - ps28
Brassicaceae - ps29
Camelineae - ps30
Arabidopsis - ps31
Arabidopsis_thaliana - ps32
Phylostratum

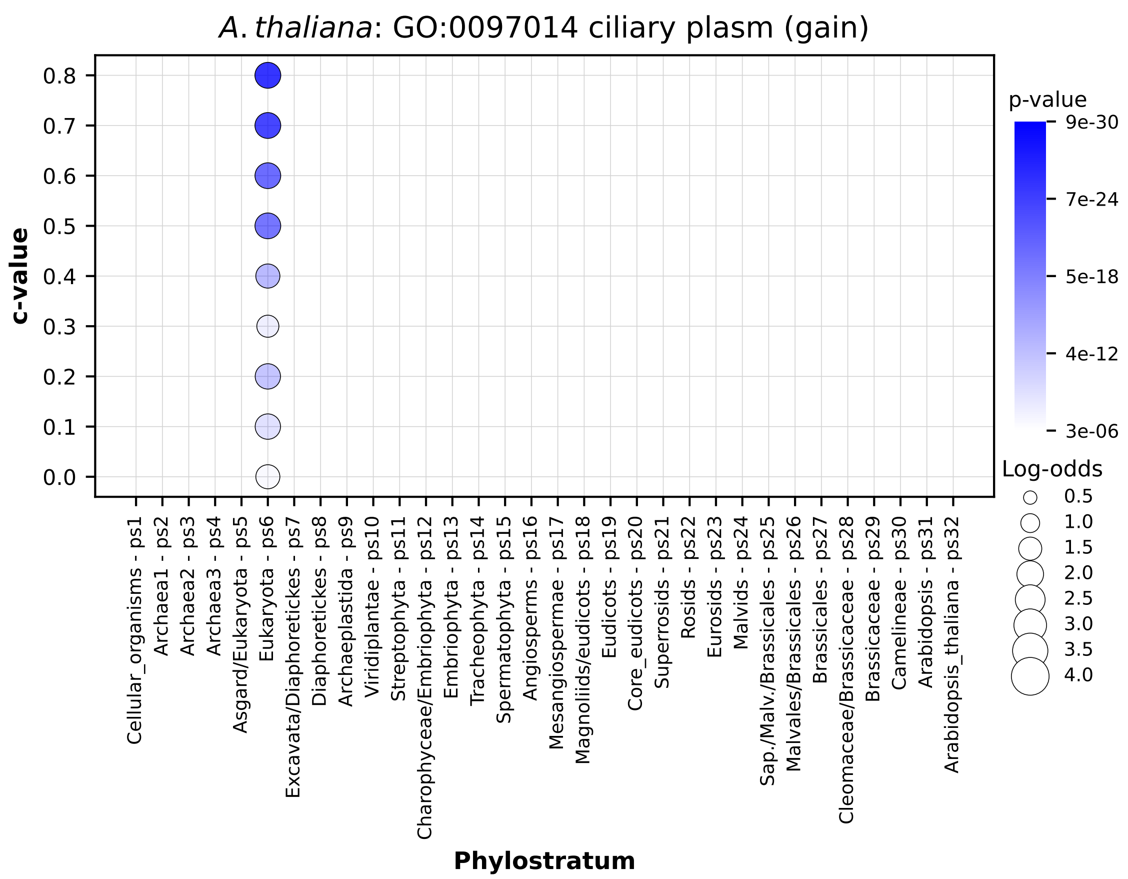

A. thaliana: GO:0097014 ciliary plasm (gain)
c-value
0.8
0.7
0.6
0.5
0.4
0.3
0.2
0.1
0.0
p-value
9e-30
7e-24
5e-18
4e-12
3e-06
Log-odds
0.5
1.0
1.5
2.0
2.5
3.0
3.5
4.0
Cellular_organisms - ps1
Archaea1 - ps2
Archaea2 - ps3
Archaea3 - ps4
Asgard/Eukaryota - ps5
Eukaryota - ps6
Excavata/Diaphoretickes - ps7
Diaphoretickes - ps8
Archaeplastida - ps9
Viridiplantae - ps10
Streptophyta - ps11
Charophyceae/Embriophyta - ps12
Embriophyta - ps13
Tracheophyta - ps14
Spermatophyta - ps15
Angiosperms - ps16
Mesangiospermae - ps17
Magnoliids/eudicots - ps18
Eudicots - ps19
Core_eudicots - ps20
Superrosids - ps21
Rosids - ps22
Eurosids - ps23
Malvids - ps24
Sap./Malv./Brassicales - ps25
Malvales/Brassicales - ps26
Brassicales - ps27
Cleomaceae/Brassicaceae - ps28
Brassicaceae - ps29
Camelineae - ps30
Arabidopsis - ps31
Arabidopsis_thaliana - ps32
Phylostratum

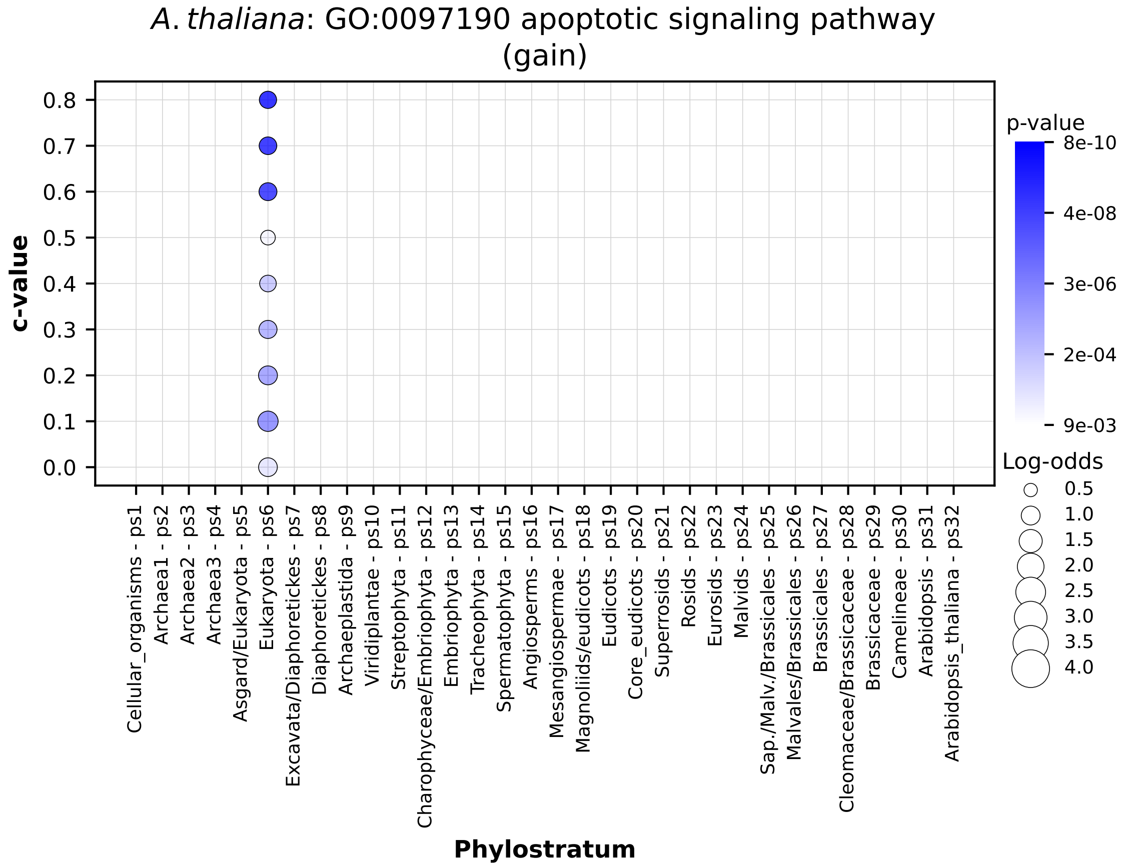

A. thaliana: GO:0097190 apoptotic signaling pathway
(gain)
c-value
0.8
0.7
0.6
0.5
0.4
0.3
0.2
0.1
0.0
p-value
8e-10
4e-08
3e-06
2e-04
9e-03
Log-odds
0.5
1.0
1.5
2.0
2.5
3.0
3.5
4.0
Cellular_organisms - ps1
Archaea1 - ps2
Archaea2 - ps3
Archaea3 - ps4
Asgard/Eukaryota - ps5
Eukaryota - ps6
Excavata/Diaphoretickes - ps7
Diaphoretickes - ps8
Archaeplastida - ps9
Viridiplantae - ps10
Streptophyta - ps11
Charophyceae/Embriophyta - ps12
Embriophyta - ps13
Tracheophyta - ps14
Spermatophyta - ps15
Angiosperms - ps16
Mesangiospermae - ps17
Magnoliids/eudicots - ps18
Eudicots - ps19
Core_eudicots - ps20
Superrosids - ps21
Rosids - ps22
Eurosids - ps23
Malvids - ps24
Sap./Malv./Brassicales - ps25
Malvales/Brassicales - ps26
Brassicales - ps27
Cleomaceae/Brassicaceae - ps28
Brassicaceae - ps29
Camelineae - ps30
Arabidopsis - ps31
Arabidopsis_thaliana - ps32
Phylostratum

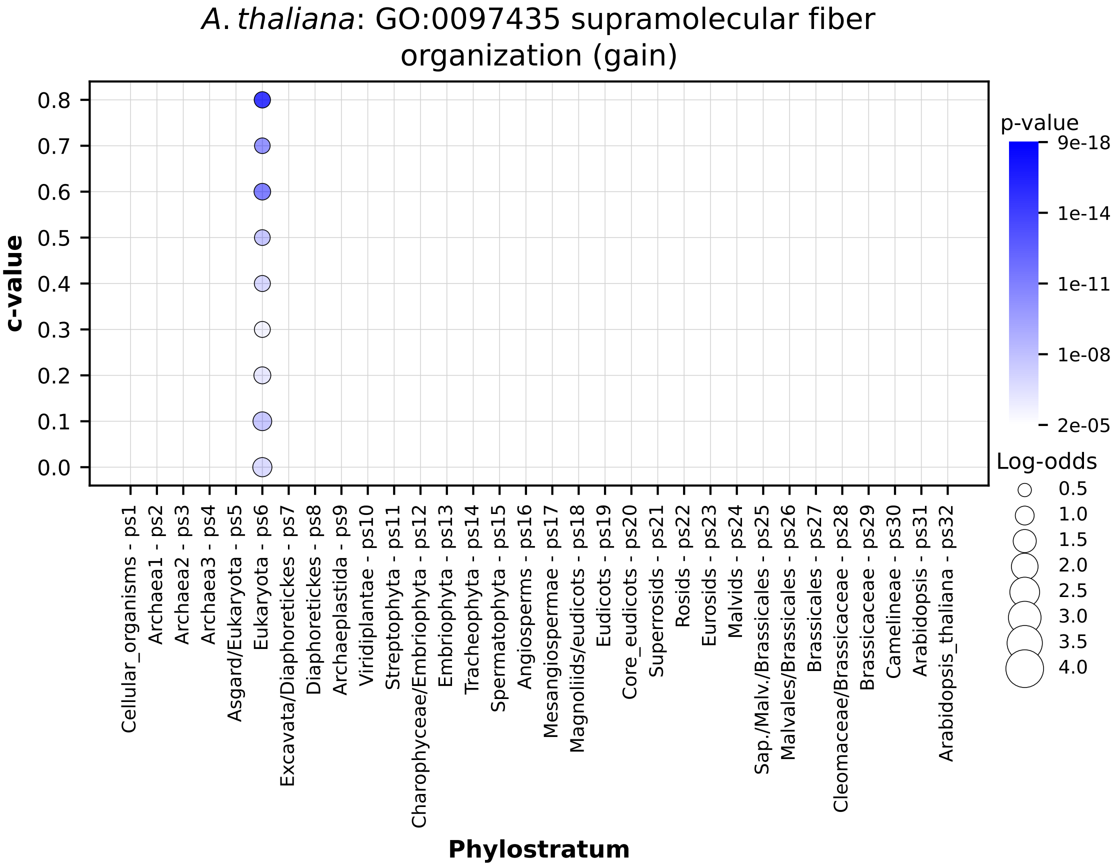
A. thaliana: GO:0097435 supramolecular fiber organization (gain)
c-value
0.0
0.1
0.2
0.3
0.4
0.5
0.6
0.7
0.8
Phylostratum
Cellular_organisms - ps1
Archaea1 - ps2
Archaea2 - ps3
Archaea3 - ps4
Asgard/Eukaryota - ps5
Eukaryota - ps6
Excavata/Diaphoretickes - ps7
Diaphoretickes - ps8
Archaeplastida - ps9
Viridiplantae - ps10
Streptophyta - ps11
Charophyceae/Embriophyta - ps12
Embriophyta - ps13
Tracheophyta - ps14
Spermatophyta - ps15
Angiosperms - ps16
Mesangiospermae - ps17
Magnoliids/eudicots - ps18
Eudicots - ps19
Core_eudicots - ps20
Superrosids - ps21
Rosids - ps22
Eurosids - ps23
Malvids - ps24
Sap./Malv./Brassicales - ps25
Malvales/Brassicales - ps26
Brassicales - ps27
Cleomaceae/Brassicaceae - ps28
Brassicaceae - ps29
Camelineae - ps30
Arabidopsis - ps31
Arabidopsis_thaliana - ps32
p-value
9e-18
1e-14
1e-11
1e-08
2e-05
Log-odds
0.5
1.0
1.5
2.0
2.5
3.0
3.5
4.0

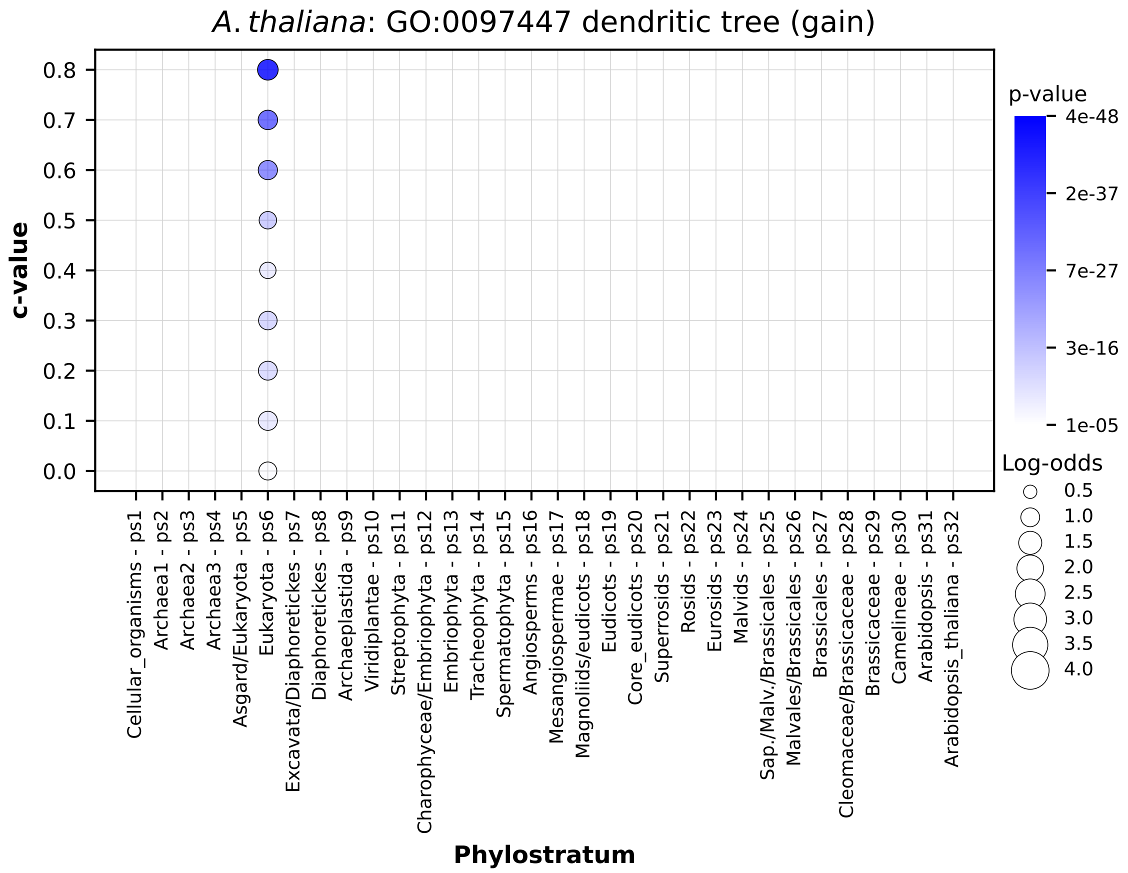

A. thaliana: GO:0097447 dendritic tree (gain)
c-value
0.8
0.7
0.6
0.5
0.4
0.3
0.2
0.1
0.0
p-value
4e-48
2e-37
7e-27
3e-16
1e-05
Log-odds
0.5
1.0
1.5
2.0
2.5
3.0
3.5
4.0
Cellular_organisms - ps1
Archaea1 - ps2
Archaea2 - ps3
Archaea3 - ps4
Asgard/Eukaryota - ps5
Eukaryota - ps6
Excavata/Diaphoretickes - ps7
Diaphoretickes - ps8
Archaeplastida - ps9
Viridiplantae - ps10
Streptophyta - ps11
Charophyceae/Embriophyta - ps12
Embriophyta - ps13
Tracheophyta - ps14
Spermatophyta - ps15
Angiosperms - ps16
Mesangiospermae - ps17
Magnoliids/eudicots - ps18
Eudicots - ps19
Core_eudicots - ps20
Superrosids - ps21
Rosids - ps22
Eurosids - ps23
Malvids - ps24
Sap./Malv./Brassicales - ps25
Malvales/Brassicales - ps26
Brassicales - ps27
Cleomaceae/Brassicaceae - ps28
Brassicaceae - ps29
Camelineae - ps30
Arabidopsis - ps31
Arabidopsis_thaliana - ps32
Phylostratum

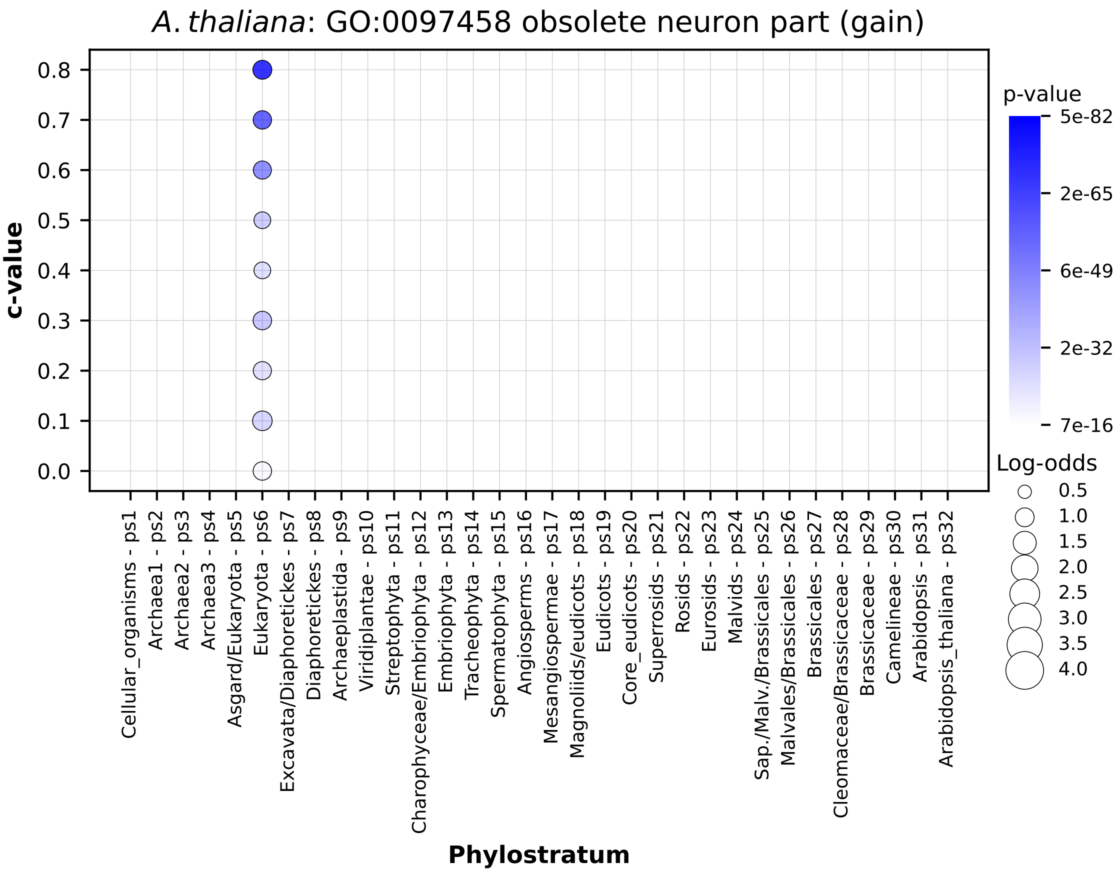

A. thaliana: GO:0097458 obsolete neuron part (gain)
c-value
0.8
0.7
0.6
0.5
0.4
0.3
0.2
0.1
0.0
p-value
5e-82
2e-65
6e-49
2e-32
7e-16
Log-odds
0.5
1.0
1.5
2.0
2.5
3.0
3.5
4.0
Cellular_organisms - ps1
Archaea1 - ps2
Archaea2 - ps3
Archaea3 - ps4
Asgard/Eukaryota - ps5
Eukaryota - ps6
Excavata/Diaphoretickes - ps7
Diaphoretickes - ps8
Archaeplastida - ps9
Viridiplantae - ps10
Streptophyta - ps11
Charophyceae/Embriophyta - ps12
Embriophyta - ps13
Tracheophyta - ps14
Spermatophyta - ps15
Angiosperms - ps16
Mesangiospermae - ps17
Magnoliids/eudicots - ps18
Eudicots - ps19
Core_eudicots - ps20
Superrosids - ps21
Rosids - ps22
Eurosids - ps23
Malvids - ps24
Sap./Malv./Brassicales - ps25
Malvales/Brassicales - ps26
Brassicales - ps27
Cleomaceae/Brassicaceae - ps28
Brassicaceae - ps29
Camelineae - ps30
Arabidopsis - ps31
Arabidopsis_thaliana - ps32
Phylostratum

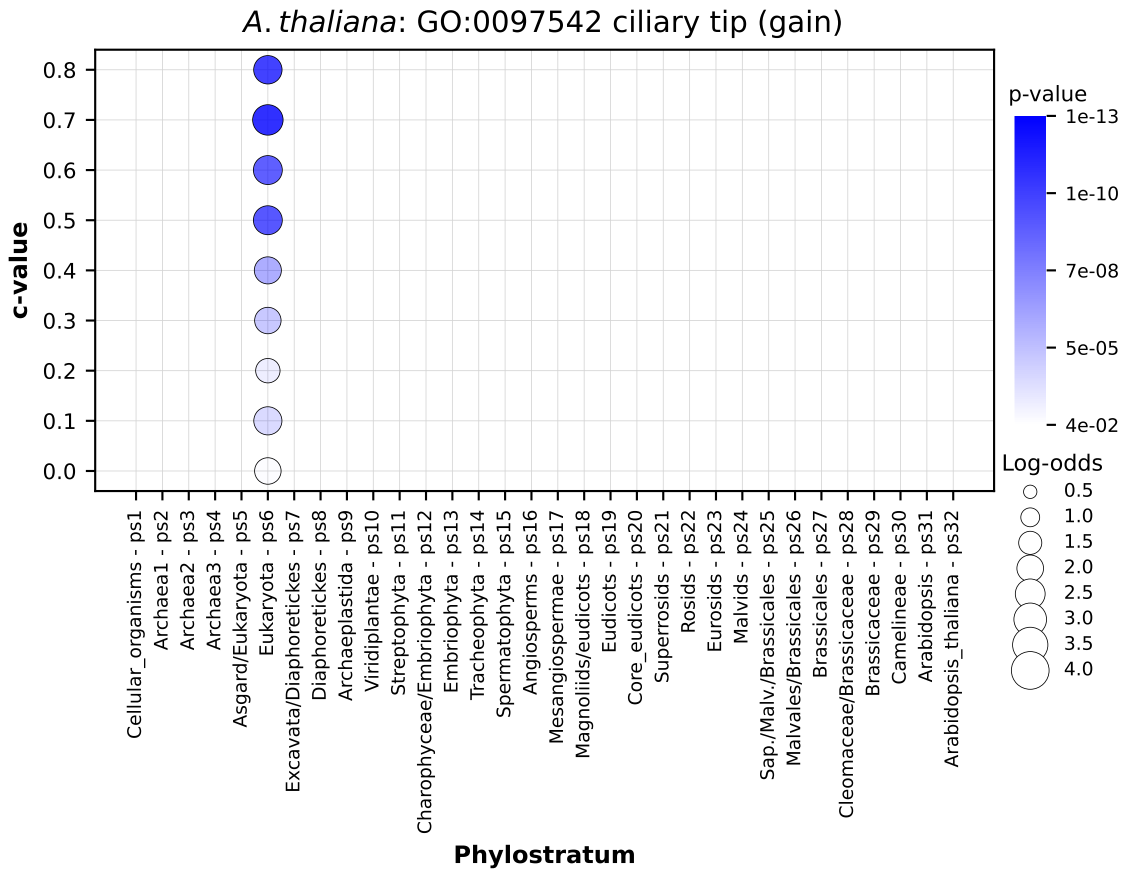

A. thaliana: GO:0097542 ciliary tip (gain)
c-value
0.0
0.1
0.2
0.3
0.4
0.5
0.6
0.7
0.8
Phylostratum
Cellular_organisms - ps1
Archaea1 - ps2
Archaea2 - ps3
Archaea3 - ps4
Asgard/Eukaryota - ps5
Eukaryota - ps6
Excavata/Diaphoretickes - ps7
Diaphoretickes - ps8
Archaeplastida - ps9
Viridiplantae - ps10
Streptophyta - ps11
Charophyceae/Embriophyta - ps12
Embriophyta - ps13
Tracheophyta - ps14
Spermatophyta - ps15
Angiosperms - ps16
Mesangiospermae - ps17
Magnoliids/eudicots - ps18
Eudicots - ps19
Core_eudicots - ps20
Superrosids - ps21
Rosids - ps22
Eurosids - ps23
Malvids - ps24
Sap./Malv./Brassicales - ps25
Malvales/Brassicales - ps26
Brassicales - ps27
Cleomaceae/Brassicaceae - ps28
Brassicaceae - ps29
Camelineae - ps30
Arabidopsis - ps31
Arabidopsis_thaliana - ps32
p-value
1e-13
1e-10
7e-08
5e-05
4e-02
Log-odds
0.5
1.0
1.5
2.0
2.5
3.0
3.5
4.0

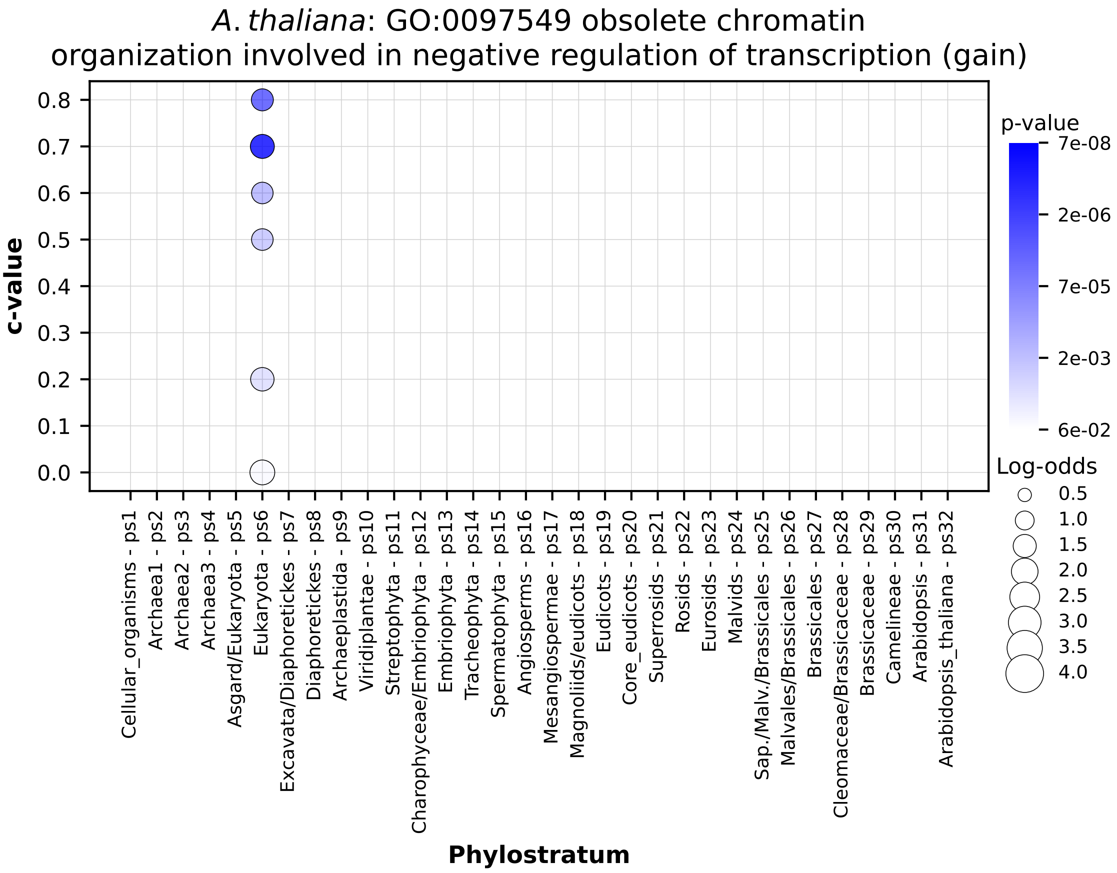
A. thaliana: GO:0097549 obsolete chromatin organization involved in negative regulation of transcription (gain)
c-value
0.8
0.7
0.6
0.5
0.4
0.3
0.2
0.1
0.0
p-value
7e-08
2e-06
7e-05
2e-03
6e-02
Log-odds
0.5
1.0
1.5
2.0
2.5
3.0
3.5
4.0
Cellular_organisms - ps1
Archaea1 - ps2
Archaea2 - ps3
Archaea3 - ps4
Asgard/Eukaryota - ps5
Eukaryota - ps6
Excavata/Diaphoretickes - ps7
Diaphoretickes - ps8
Archaeplastida - ps9
Viridiplantae - ps10
Streptophyta - ps11
Charophyceae/Embriophyta - ps12
Embriophyta - ps13
Tracheophyta - ps14
Spermatophyta - ps15
Angiosperms - ps16
Mesangiospermae - ps17
Magnoliids/eudicots - ps18
Eudicots - ps19
Core_eudicots - ps20
Superrosids - ps21
Rosids - ps22
Eurosids - ps23
Malvids - ps24
Sap./Malv./Brassicales - ps25
Malvales/Brassicales - ps26
Brassicales - ps27
Cleomaceae/Brassicaceae - ps28
Brassicaceae - ps29
Camelineae - ps30
Arabidopsis - ps31
Arabidopsis_thaliana - ps32
Phylostratum

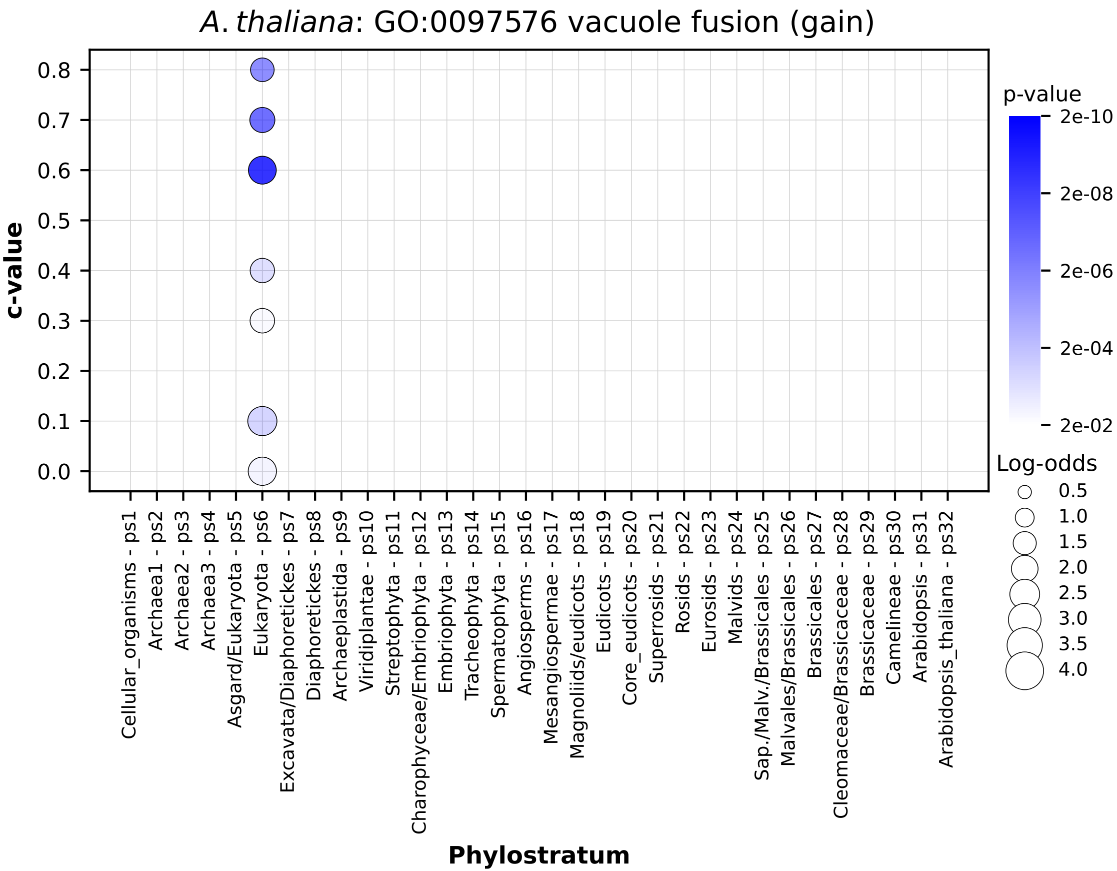
A. thaliana: GO:0097576 vacuole fusion (gain)
c-value
0.8
0.7
0.6
0.5
0.4
0.3
0.2
0.1
0.0
p-value
2e-10
2e-08
2e-06
2e-04
2e-02
Log-odds
0.5
1.0
1.5
2.0
2.5
3.0
3.5
4.0
Cellular_organisms - ps1
Archaea1 - ps2
Archaea2 - ps3
Archaea3 - ps4
Asgard/Eukaryota - ps5
Eukaryota - ps6
Excavata/Diaphoretickes - ps7
Diaphoretickes - ps8
Archaeplastida - ps9
Viridiplantae - ps10
Streptophyta - ps11
Charophyceae/Embriophyta - ps12
Embriophyta - ps13
Tracheophyta - ps14
Spermatophyta - ps15
Angiosperms - ps16
Mesangiospermae - ps17
Magnoliids/eudicots - ps18
Eudicots - ps19
Core_eudicots - ps20
Superrosids - ps21
Rosids - ps22
Eurosids - ps23
Malvids - ps24
Sap./Malv./Brassicales - ps25
Malvales/Brassicales - ps26
Brassicales - ps27
Cleomaceae/Brassicaceae - ps28
Brassicaceae - ps29
Camelineae - ps30
Arabidopsis - ps31
Arabidopsis_thaliana - ps32
Phylostratum

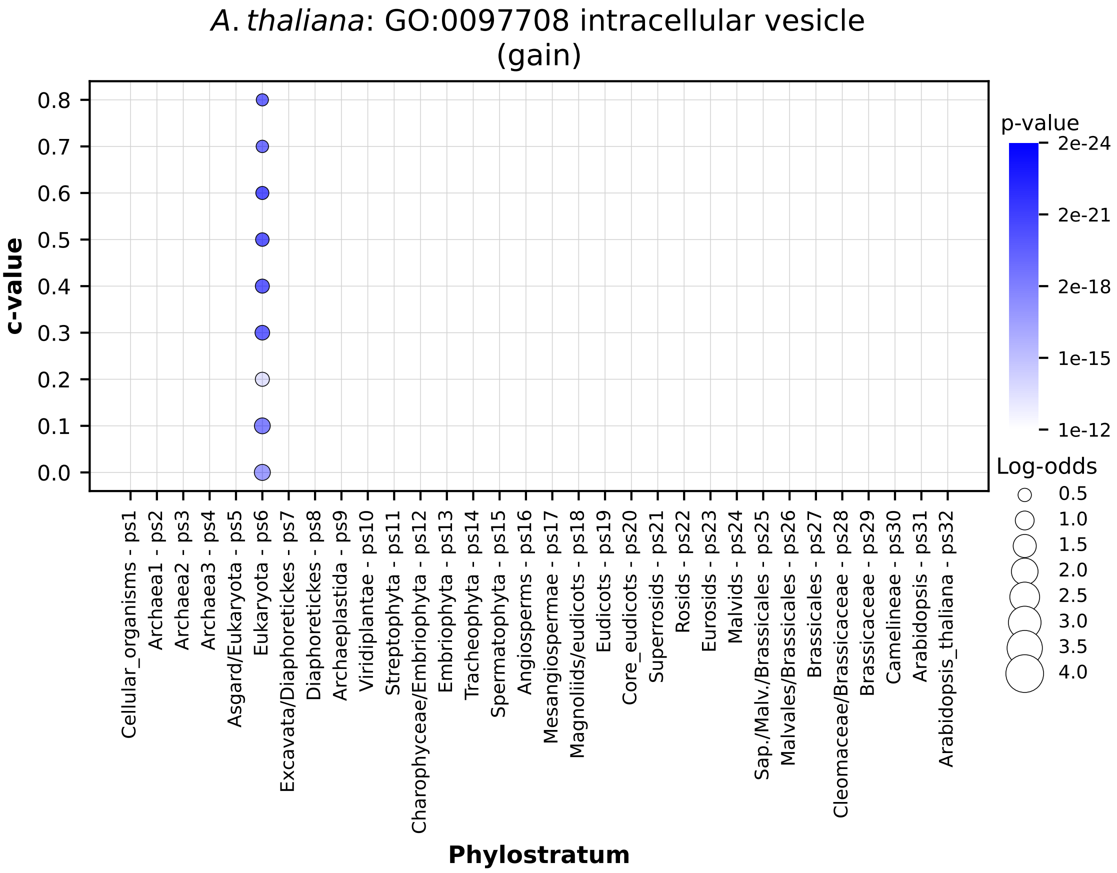
A. thaliana: GO:0097708 intracellular vesicle
(gain)
c-value
0.8
0.7
0.6
0.5
0.4
0.3
0.2
0.1
0.0
Phylostratum
Cellular_organisms - ps1
Archaea1 - ps2
Archaea2 - ps3
Archaea3 - ps4
Asgard/Eukaryota - ps5
Eukaryota - ps6
Excavata/Diaphoretickes - ps7
Diaphoretickes - ps8
Archaeplastida - ps9
Viridiplantae - ps10
Streptophyta - ps11
Charophyceae/Embriophyta - ps12
Embriophyta - ps13
Tracheophyta - ps14
Spermatophyta - ps15
Angiosperms - ps16
Mesangiospermae - ps17
Magnoliids/eudicots - ps18
Eudicots - ps19
Core_eudicots - ps20
Superrosids - ps21
Rosids - ps22
Eurosids - ps23
Malvids - ps24
Sap./Malv./Brassicales - ps25
Malvales/Brassicales - ps26
Brassicales - ps27
Cleomaceae/Brassicaceae - ps28
Brassicaceae - ps29
Camelineae - ps30
Arabidopsis - ps31
Arabidopsis_thaliana - ps32
p-value
2e-24
2e-21
2e-18
1e-15
1e-12
Log-odds
0.5
1.0
1.5
2.0
2.5
3.0
3.5
4.0

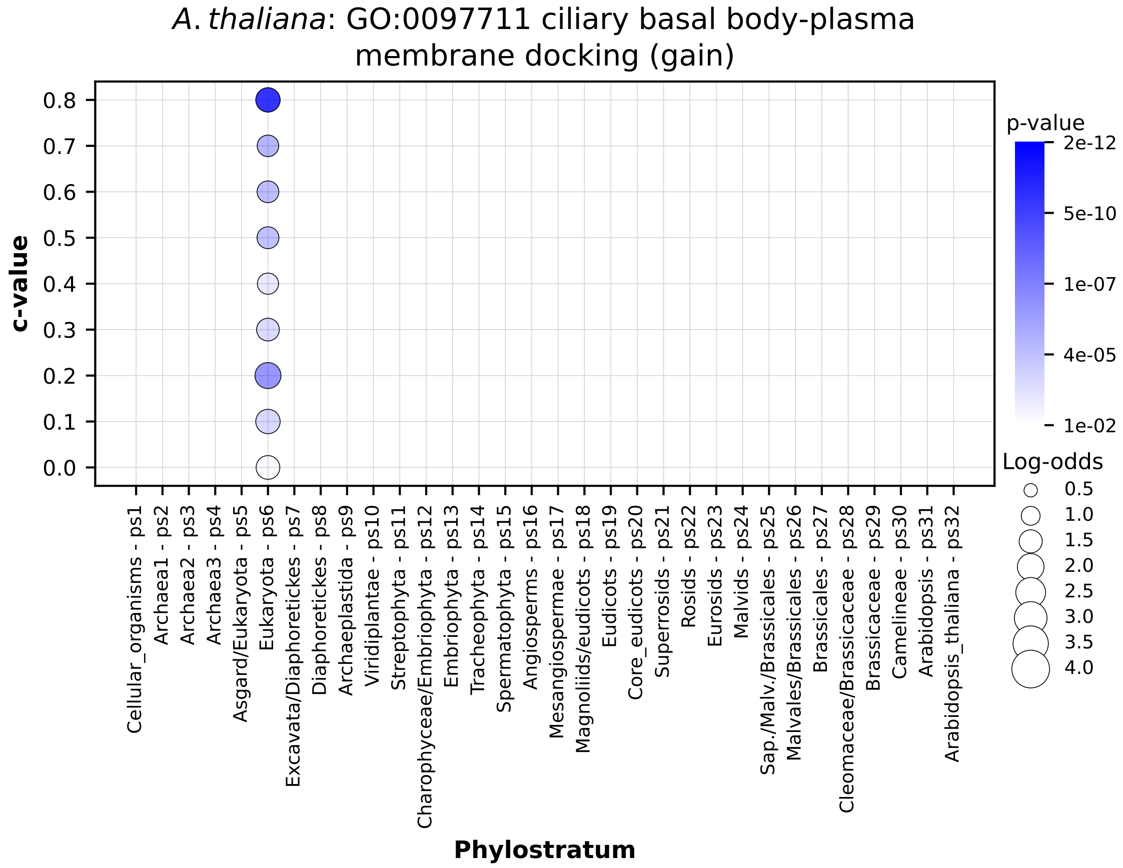
A. thaliana: GO:0097711 ciliary basal body-plasma membrane docking (gain)
c-value
Phylostratum
p-value
2e-12
5e-10
1e-07
4e-05
1e-02
Log-odds
0.5
1.0
1.5
2.0
2.5
3.0
3.5
4.0
Cellular_organisms - ps1
Archaea1 - ps2
Archaea2 - ps3
Archaea3 - ps4
Asgard/Eukaryota - ps5
Eukaryota - ps6
Excavata/Diaphoretickes - ps7
Diaphoretickes - ps8
Archaeplastida - ps9
Viridiplantae - ps10
Streptophyta - ps11
Charophyceae/Embriophyta - ps12
Embriophyta - ps13
Tracheophyta - ps14
Spermatophyta - ps15
Angiosperms - ps16
Mesangiospermae - ps17
Magnoliids/eudicots - ps18
Eudicots - ps19
Core_eudicots - ps20
Superrosids - ps21
Rosids - ps22
Eurosids - ps23
Malvids - ps24
Sap./Malv./Brassicales - ps25
Malvales/Brassicales - ps26
Brassicales - ps27
Cleomaceae/Brassicaceae - ps28
Brassicaceae - ps29
Camelineae - ps30
Arabidopsis - ps31
Arabidopsis_thaliana - ps32

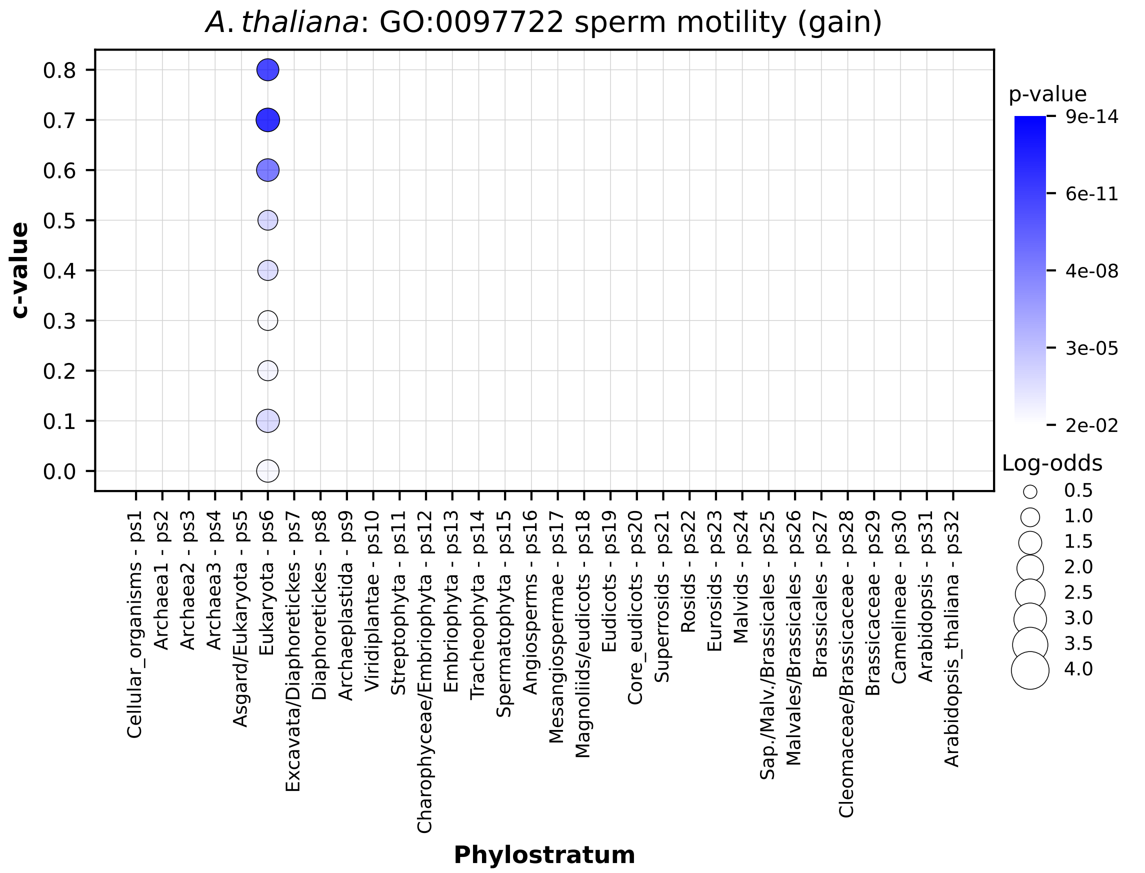
A. thaliana: GO:0097722 sperm motility (gain)
c-value
0.8
0.7
0.6
0.5
0.4
0.3
0.2
0.1
0.0
p-value
9e-14
6e-11
4e-08
3e-05
2e-02
Log-odds
0.5
1.0
1.5
2.0
2.5
3.0
3.5
4.0
Cellular_organisms - ps1
Archaea1 - ps2
Archaea2 - ps3
Archaea3 - ps4
Asgard/Eukaryota - ps5
Eukaryota - ps6
Excavata/Diaphoretickes - ps7
Diaphoretickes - ps8
Archaeplastida - ps9
Viridiplantae - ps10
Streptophyta - ps11
Charophyceae/Embriophyta - ps12
Embriophyta - ps13
Tracheophyta - ps14
Spermatophyta - ps15
Angiosperms - ps16
Mesangiospermae - ps17
Magnoliids/eudicots - ps18
Eudicots - ps19
Core_eudicots - ps20
Superrosids - ps21
Rosids - ps22
Eurosids - ps23
Malvids - ps24
Sap./Malv./Brassicales - ps25
Malvales/Brassicales - ps26
Brassicales - ps27
Cleomaceae/Brassicaceae - ps28
Brassicaceae - ps29
Camelineae - ps30
Arabidopsis - ps31
Arabidopsis_thaliana - ps32
Phylostratum

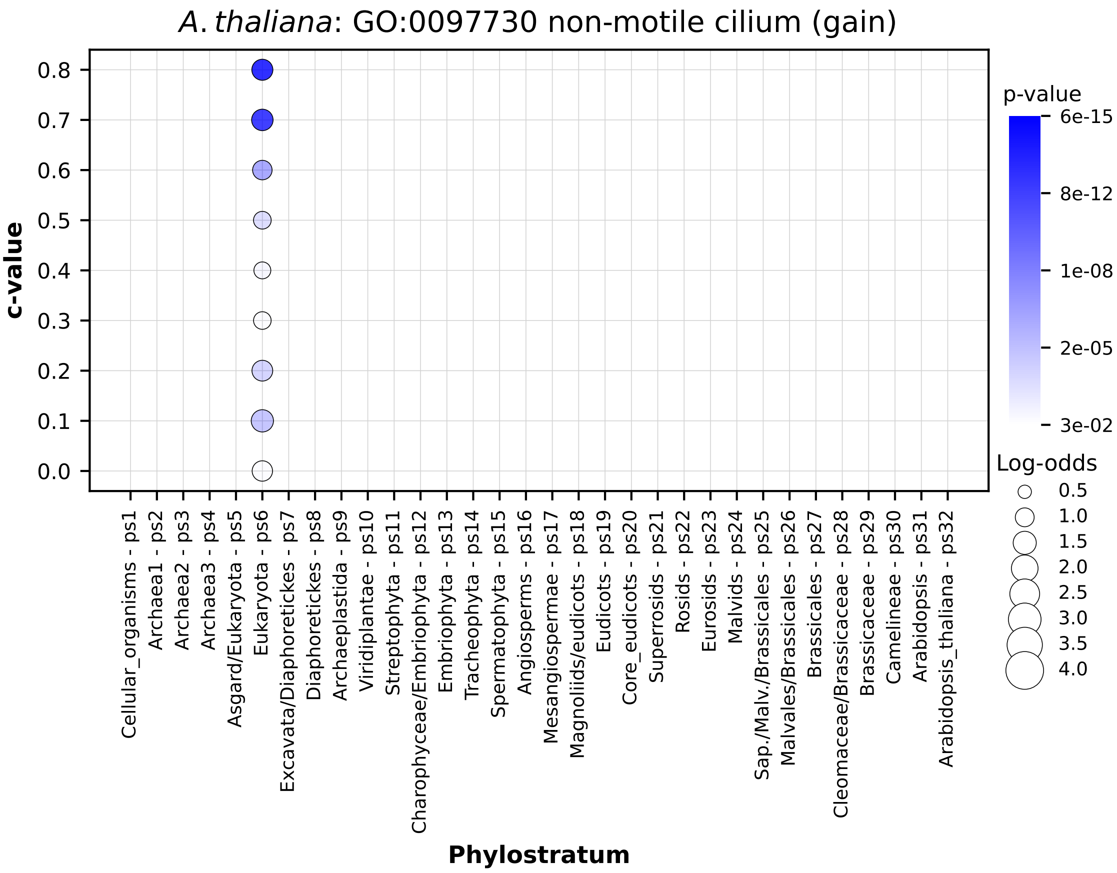

A. thaliana: GO:0097730 non-motile cilium (gain)
c-value
0.8
0.7
0.6
0.5
0.4
0.3
0.2
0.1
0.0
p-value
6e-15
8e-12
1e-08
2e-05
3e-02
Log-odds
0.5
1.0
1.5
2.0
2.5
3.0
3.5
4.0
Cellular_organisms - ps1
Archaea1 - ps2
Archaea2 - ps3
Archaea3 - ps4
Asgard/Eukaryota - ps5
Eukaryota - ps6
Excavata/Diaphoretickes - ps7
Diaphoretickes - ps8
Archaeplastida - ps9
Viridiplantae - ps10
Streptophyta - ps11
Charophyceae/Embriophyta - ps12
Embriophyta - ps13
Tracheophyta - ps14
Spermatophyta - ps15
Angiosperms - ps16
Mesangiospermae - ps17
Magnoliids/eudicots - ps18
Eudicots - ps19
Core_eudicots - ps20
Superrosids - ps21
Rosids - ps22
Eurosids - ps23
Malvids - ps24
Sap./Malv./Brassicales - ps25
Malvales/Brassicales - ps26
Brassicales - ps27
Cleomaceae/Brassicaceae - ps28
Brassicaceae - ps29
Camelineae - ps30
Arabidopsis - ps31
Arabidopsis_thaliana - ps32
Phylostratum

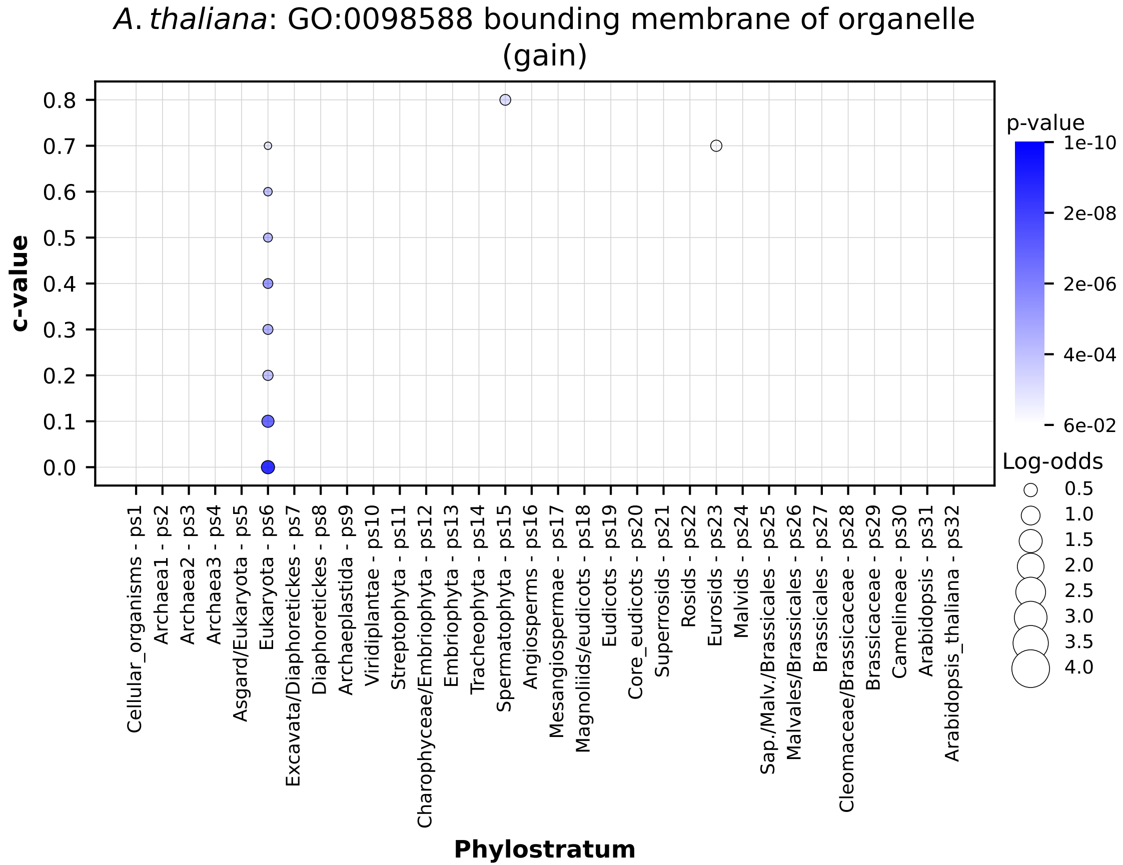

A. thaliana: GO:0098588 bounding membrane of organelle (gain)
c-value
0.0
0.1
0.2
0.3
0.4
0.5
0.6
0.7
0.8
Phylostratum
Cellular_organisms - ps1
Archaea1 - ps2
Archaea2 - ps3
Archaea3 - ps4
Asgard/Eukaryota - ps5
Eukaryota - ps6
Excavata/Diaphoretickes - ps7
Diaphoretickes - ps8
Archaeplastida - ps9
Viridiplantae - ps10
Streptophyta - ps11
Charophyceae/Embriophyta - ps12
Embriophyta - ps13
Tracheophyta - ps14
Spermatophyta - ps15
Angiosperms - ps16
Mesangiospermae - ps17
Magnoliids/eudicots - ps18
Eudicots - ps19
Core_eudicots - ps20
Superrosids - ps21
Rosids - ps22
Eurosids - ps23
Malvids - ps24
Sap./Malv./Brassicales - ps25
Malvales/Brassicales - ps26
Brassicales - ps27
Cleomaceae/Brassicaceae - ps28
Brassicaceae - ps29
Camelineae - ps30
Arabidopsis - ps31
Arabidopsis_thaliana - ps32
p-value
1e-10
2e-08
2e-06
4e-04
6e-02
Log-odds
0.5
1.0
1.5
2.0
2.5
3.0
3.5
4.0

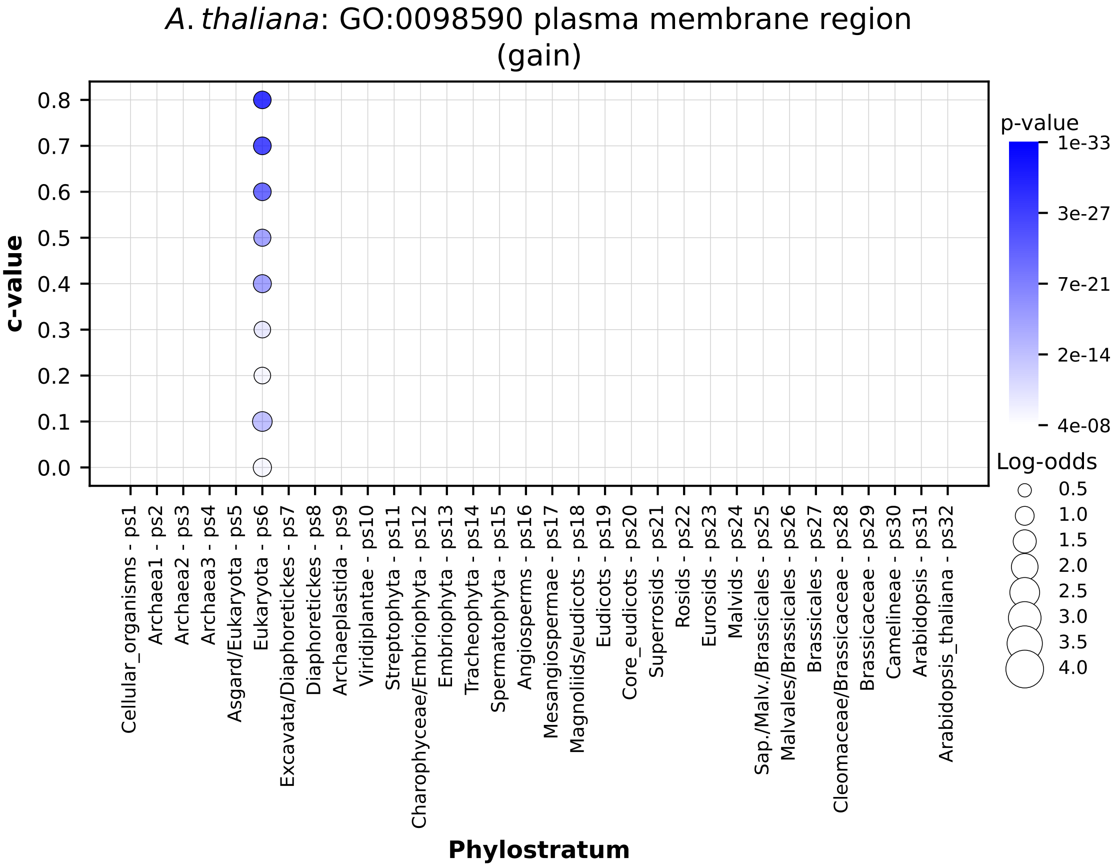

A. thaliana: GO:0098590 plasma membrane region (gain)
c-value
0.8
0.7
0.6
0.5
0.4
0.3
0.2
0.1
0.0
Phylostratum
Cellular_organisms - ps1
Archaea1 - ps2
Archaea2 - ps3
Archaea3 - ps4
Asgard/Eukaryota - ps5
Eukaryota - ps6
Excavata/Diaphoretickes - ps7
Diaphoretickes - ps8
Archaeplastida - ps9
Viridiplantae - ps10
Streptophyta - ps11
Charophyceae/Embriophyta - ps12
Embriophyta - ps13
Tracheophyta - ps14
Spermatophyta - ps15
Angiosperms - ps16
Mesangiospermae - ps17
Magnoliids/eudicots - ps18
Eudicots - ps19
Core_eudicots - ps20
Superrosids - ps21
Rosids - ps22
Eurosids - ps23
Malvids - ps24
Sap./Malv./Brassicales - ps25
Malvales/Brassicales - ps26
Brassicales - ps27
Cleomaceae/Brassicaceae - ps28
Brassicaceae - ps29
Camelineae - ps30
Arabidopsis - ps31
Arabidopsis_thaliana - ps32
p-value
1e-33
3e-27
7e-21
2e-14
4e-08
Log-odds
0.5
1.0
1.5
2.0
2.5
3.0
3.5
4.0

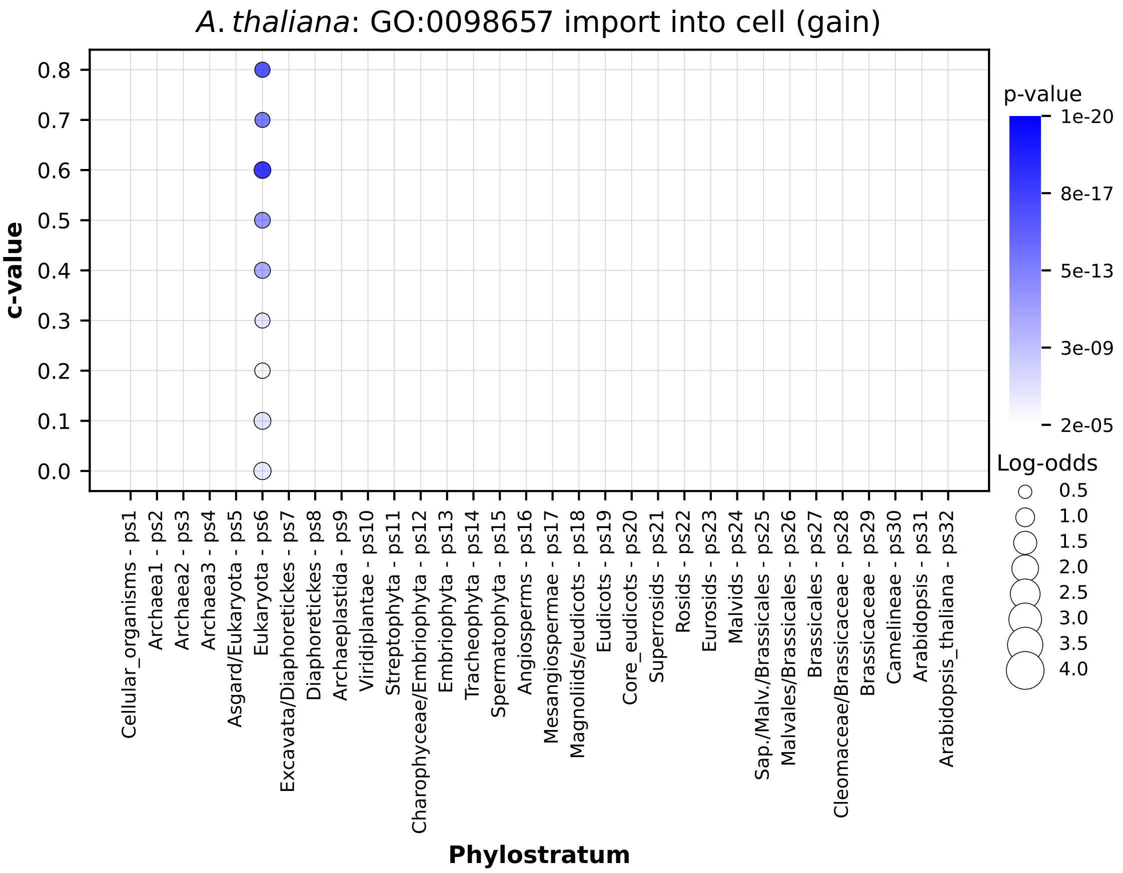

A. thaliana: GO:0098657 import into cell (gain)
c-value
0.8
0.7
0.6
0.5
0.4
0.3
0.2
0.1
0.0
p-value
1e-20
8e-17
5e-13
3e-09
2e-05
Log-odds
0.5
1.0
1.5
2.0
2.5
3.0
3.5
4.0
Cellular_organisms - ps1
Archaea1 - ps2
Archaea2 - ps3
Archaea3 - ps4
Asgard/Eukaryota - ps5
Eukaryota - ps6
Excavata/Diaphoretickes - ps7
Diaphoretickes - ps8
Archaeplastida - ps9
Viridiplantae - ps10
Streptophyta - ps11
Charophyceae/Embriophyta - ps12
Embriophyta - ps13
Tracheophyta - ps14
Spermatophyta - ps15
Angiosperms - ps16
Mesangiospermae - ps17
Magnoliids/eudicots - ps18
Eudicots - ps19
Core_eudicots - ps20
Superrosids - ps21
Rosids - ps22
Eurosids - ps23
Malvids - ps24
Sap./Malv./Brassicales - ps25
Malvales/Brassicales - ps26
Brassicales - ps27
Cleomaceae/Brassicaceae - ps28
Brassicaceae - ps29
Camelineae - ps30
Arabidopsis - ps31
Arabidopsis_thaliana - ps32
Phylostratum

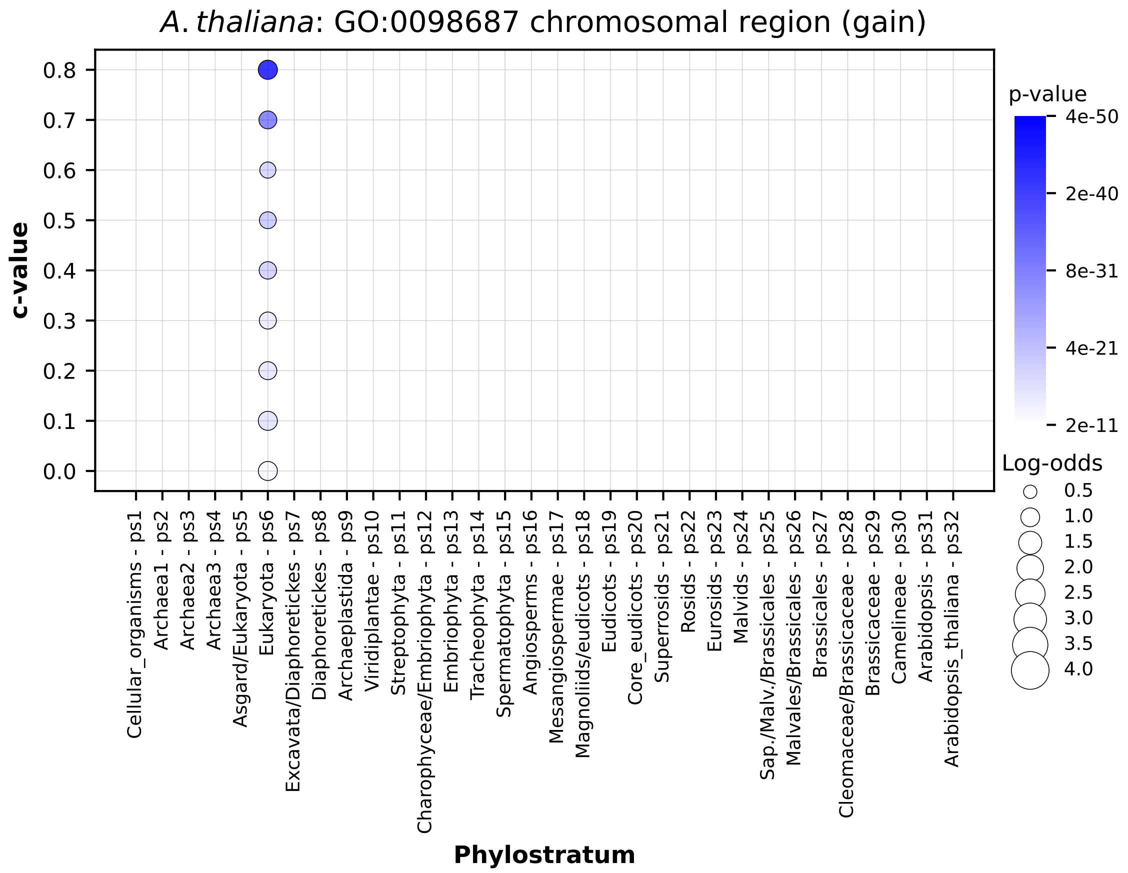
A. thaliana: GO:0098687 chromosomal region (gain)
c-value
0.0
0.1
0.2
0.3
0.4
0.5
0.6
0.7
0.8
Phylostratum
Cellular_organisms - ps1
Archaea1 - ps2
Archaea2 - ps3
Archaea3 - ps4
Asgard/Eukaryota - ps5
Eukaryota - ps6
Excavata/Diaphoretickes - ps7
Diaphoretickes - ps8
Archaeplastida - ps9
Viridiplantae - ps10
Streptophyta - ps11
Charophyceae/Embriophyta - ps12
Embriophyta - ps13
Tracheophyta - ps14
Spermatophyta - ps15
Angiosperms - ps16
Mesangiospermae - ps17
Magnoliids/eudicots - ps18
Eudicots - ps19
Core_eudicots - ps20
Superrosids - ps21
Rosids - ps22
Eurosids - ps23
Malvids - ps24
Sap./Malv./Brassicales - ps25
Malvales/Brassicales - ps26
Brassicales - ps27
Cleomaceae/Brassicaceae - ps28
Brassicaceae - ps29
Camelineae - ps30
Arabidopsis - ps31
Arabidopsis_thaliana - ps32
p-value
4e-50
2e-40
8e-31
4e-21
2e-11
Log-odds
0.5
1.0
1.5
2.0
2.5
3.0
3.5
4.0

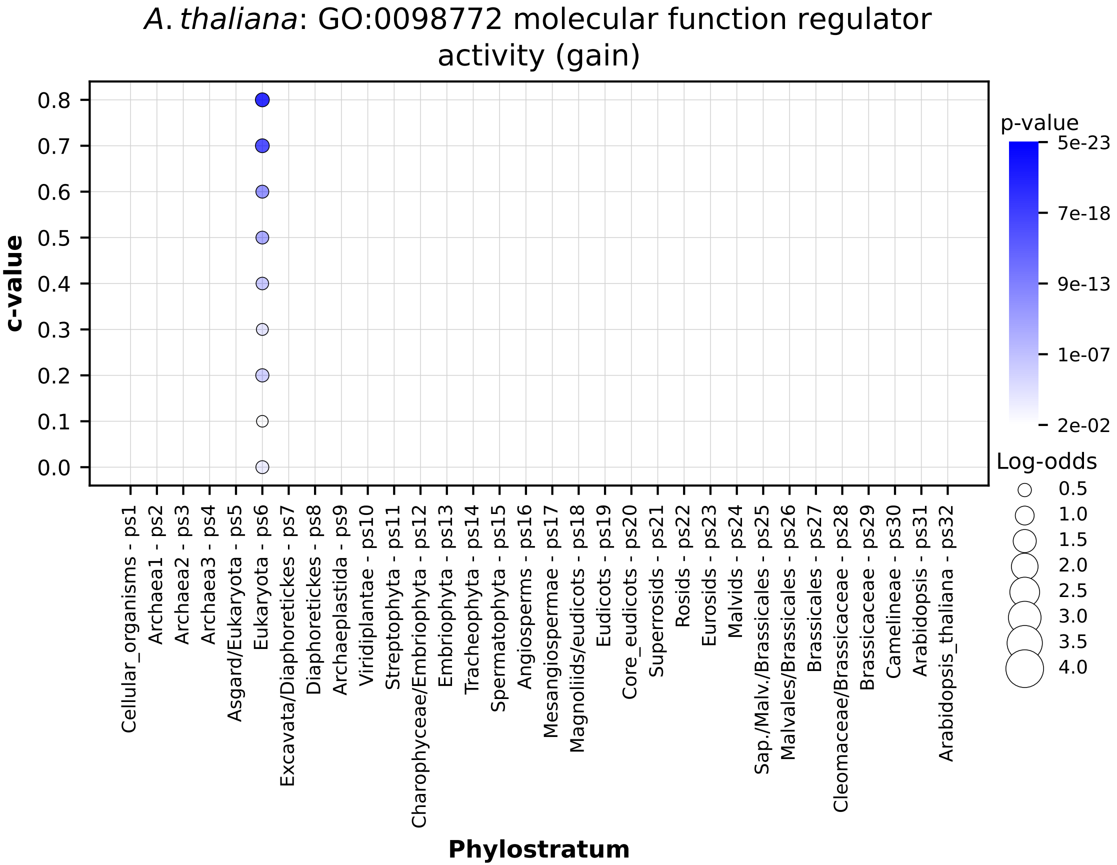

A. thaliana: GO:0098772 molecular function regulator activity (gain)
c-value
0.8
0.7
0.6
0.5
0.4
0.3
0.2
0.1
0.0
p-value
5e-23
7e-18
9e-13
1e-07
2e-02
Log-odds
0.5
1.0
1.5
2.0
2.5
3.0
3.5
4.0
Cellular_organisms - ps1
Archaea1 - ps2
Archaea2 - ps3
Archaea3 - ps4
Asgard/Eukaryota - ps5
Eukaryota - ps6
Excavata/Diaphoretickes - ps7
Diaphoretickes - ps8
Archaeplastida - ps9
Viridiplantae - ps10
Streptophyta - ps11
Charophyceae/Embriophyta - ps12
Embriophyta - ps13
Tracheophyta - ps14
Spermatophyta - ps15
Angiosperms - ps16
Mesangiospermae - ps17
Magnoliids/eudicots - ps18
Eudicots - ps19
Core_eudicots - ps20
Superrosids - ps21
Rosids - ps22
Eurosids - ps23
Malvids - ps24
Sap./Malv./Brassicales - ps25
Malvales/Brassicales - ps26
Brassicales - ps27
Cleomaceae/Brassicaceae - ps28
Brassicaceae - ps29
Camelineae - ps30
Arabidopsis - ps31
Arabidopsis_thaliana - ps32
Phylostratum

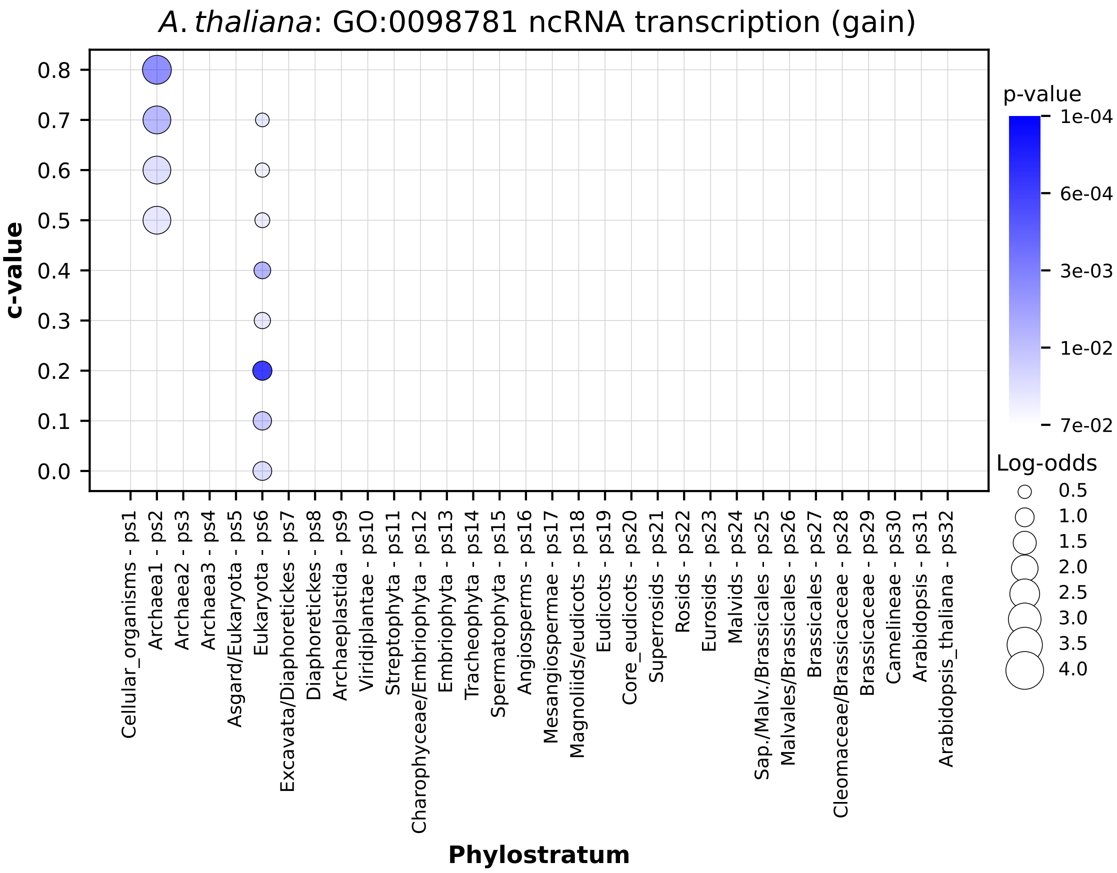

A. thaliana: GO:0098781 ncRNA transcription (gain)
c-value
0.8
0.7
0.6
0.5
0.4
0.3
0.2
0.1
0.0
p-value
1e-04
6e-04
3e-03
1e-02
7e-02
Log-odds
0.5
1.0
1.5
2.0
2.5
3.0
3.5
4.0
Cellular_organisms - ps1
Archaea1 - ps2
Archaea2 - ps3
Archaea3 - ps4
Asgard/Eukaryota - ps5
Eukaryota - ps6
Excavata/Diaphoretickes - ps7
Diaphoretickes - ps8
Archaeplastida - ps9
Viridiplantae - ps10
Streptophyta - ps11
Charophyceae/Embriophyta - ps12
Embriophyta - ps13
Tracheophyta - ps14
Spermatophyta - ps15
Angiosperms - ps16
Mesangiospermae - ps17
Magnoliids/eudicots - ps18
Eudicots - ps19
Core_eudicots - ps20
Superrosids - ps21
Rosids - ps22
Eurosids - ps23
Malvids - ps24
Sap./Malv./Brassicales - ps25
Malvales/Brassicales - ps26
Brassicales - ps27
Cleomaceae/Brassicaceae - ps28
Brassicaceae - ps29
Camelineae - ps30
Arabidopsis - ps31
Arabidopsis_thaliana - ps32
Phylostratum

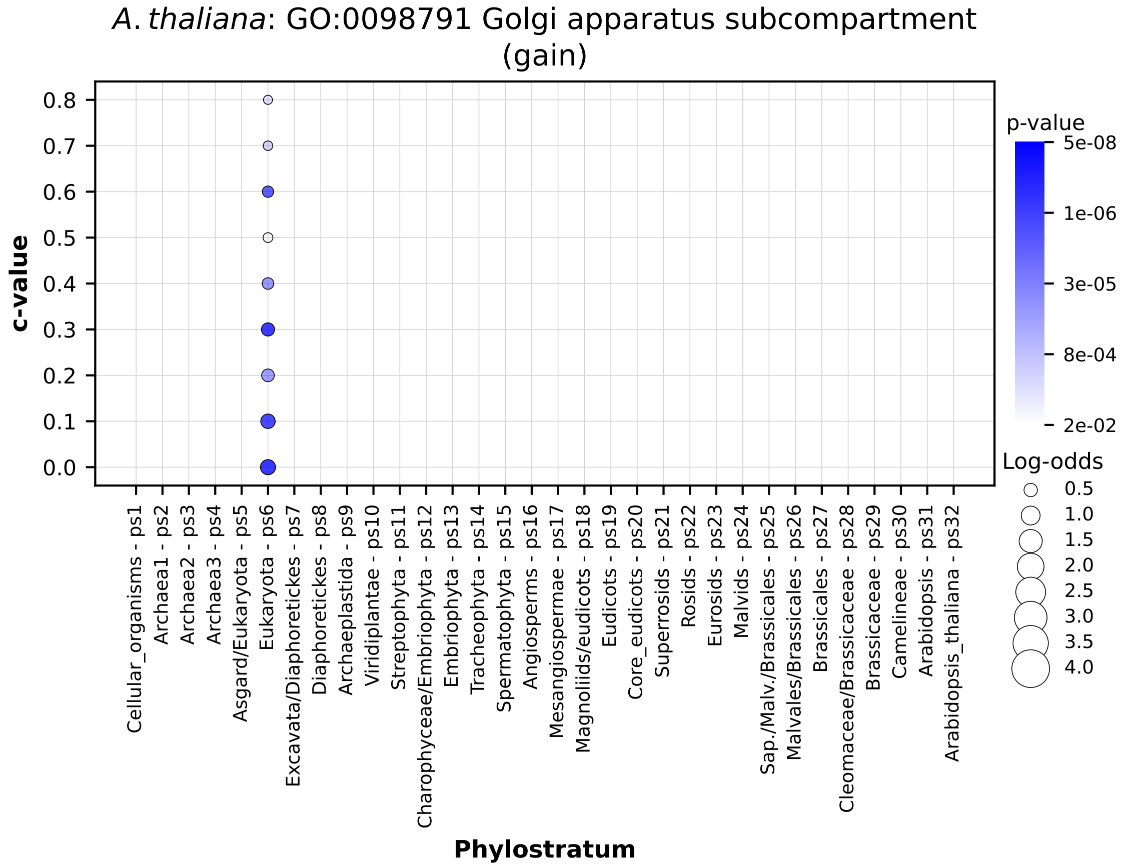

A. thaliana: GO:0098791 Golgi apparatus subcompartment (gain)
c-value
0.8
0.7
0.6
0.5
0.4
0.3
0.2
0.1
0.0
p-value
5e-08
1e-06
3e-05
8e-04
2e-02
Log-odds
0.5
1.0
1.5
2.0
2.5
3.0
3.5
4.0
Cellular_organisms - ps1
Archaea1 - ps2
Archaea2 - ps3
Archaea3 - ps4
Asgard/Eukaryota - ps5
Eukaryota - ps6
Excavata/Diaphoretickes - ps7
Diaphoretickes - ps8
Archaeplastida - ps9
Viridiplantae - ps10
Streptophyta - ps11
Charophyceae/Embriophyta - ps12
Embriophyta - ps13
Tracheophyta - ps14
Spermatophyta - ps15
Angiosperms - ps16
Mesangiospermae - ps17
Magnoliids/eudicots - ps18
Eudicots - ps19
Core_eudicots - ps20
Superrosids - ps21
Rosids - ps22
Eurosids - ps23
Malvids - ps24
Sap./Malv./Brassicales - ps25
Malvales/Brassicales - ps26
Brassicales - ps27
Cleomaceae/Brassicaceae - ps28
Brassicaceae - ps29
Camelineae - ps30
Arabidopsis - ps31
Arabidopsis_thaliana - ps32
Phylostratum

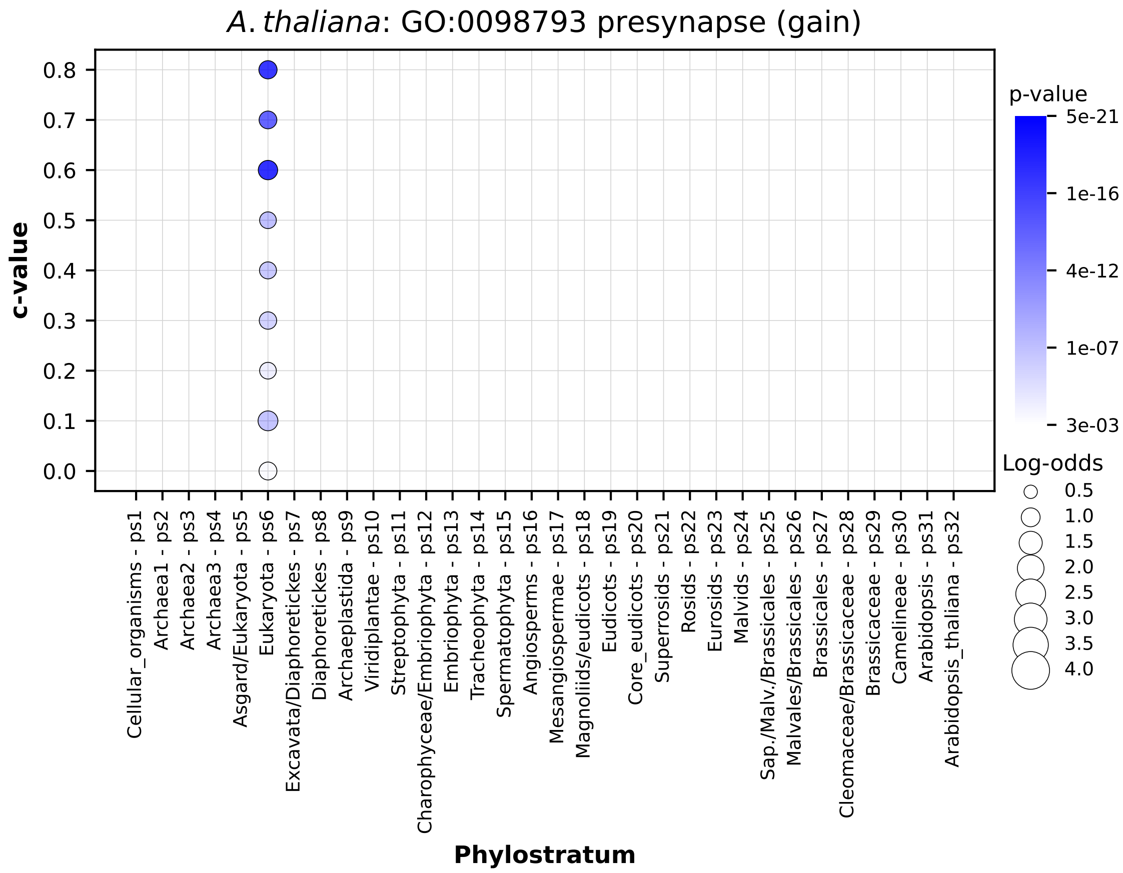

A. thaliana: GO:0098793 presynapse (gain)
c-value
0.0
0.1
0.2
0.3
0.4
0.5
0.6
0.7
0.8
p-value
5e-21
1e-16
4e-12
1e-07
3e-03
Log-odds
0.5
1.0
1.5
2.0
2.5
3.0
3.5
4.0
Cellular_organisms - ps1
Archaea1 - ps2
Archaea2 - ps3
Archaea3 - ps4
Asgard/Eukaryota - ps5
Eukaryota - ps6
Excavata/Diaphoretickes - ps7
Diaphoretickes - ps8
Archaeplastida - ps9
Viridiplantae - ps10
Streptophyta - ps11
Charophyceae/Embriophyta - ps12
Embriophyta - ps13
Tracheophyta - ps14
Spermatophyta - ps15
Angiosperms - ps16
Mesangiospermae - ps17
Magnoliids/eudicots - ps18
Eudicots - ps19
Core_eudicots - ps20
Superrosids - ps21
Rosids - ps22
Eurosids - ps23
Malvids - ps24
Sap./Malv./Brassicales - ps25
Malvales/Brassicales - ps26
Brassicales - ps27
Cleomaceae/Brassicaceae - ps28
Brassicaceae - ps29
Camelineae - ps30
Arabidopsis - ps31
Arabidopsis_thaliana - ps32
Phylostratum

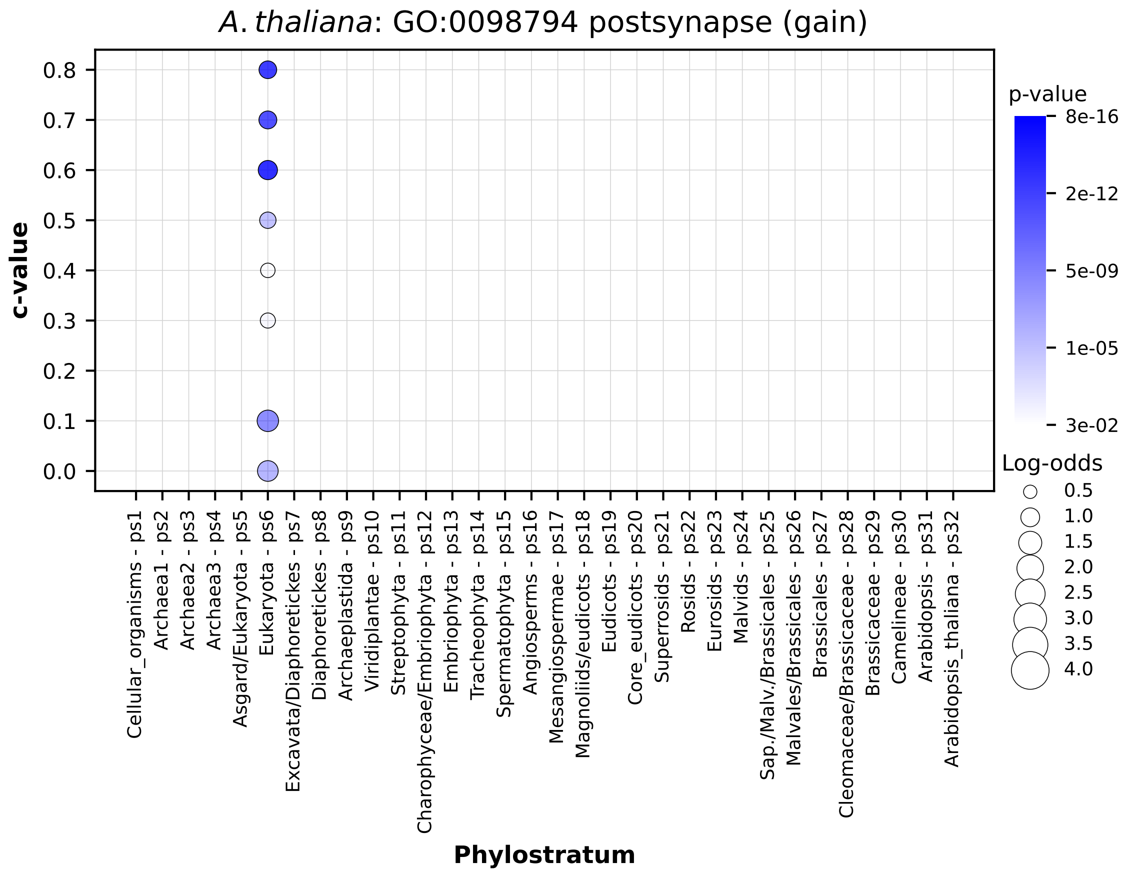

A. thaliana: GO:0098794 postsynapse (gain)
c-value
0.0
0.1
0.2
0.3
0.4
0.5
0.6
0.7
0.8
Phylostratum
Cellular_organisms - ps1
Archaea1 - ps2
Archaea2 - ps3
Archaea3 - ps4
Asgard/Eukaryota - ps5
Eukaryota - ps6
Excavata/Diaphoretickes - ps7
Diaphoretickes - ps8
Archaeplastida - ps9
Viridiplantae - ps10
Streptophyta - ps11
Charophyceae/Embriophyta - ps12
Embriophyta - ps13
Tracheophyta - ps14
Spermatophyta - ps15
Angiosperms - ps16
Mesangiospermae - ps17
Magnoliids/eudicots - ps18
Eudicots - ps19
Core_eudicots - ps20
Superrosids - ps21
Rosids - ps22
Eurosids - ps23
Malvids - ps24
Sap./Malv./Brassicales - ps25
Malvales/Brassicales - ps26
Brassicales - ps27
Cleomaceae/Brassicaceae - ps28
Brassicaceae - ps29
Camelineae - ps30
Arabidopsis - ps31
Arabidopsis_thaliana - ps32
p-value
8e-16
2e-12
5e-09
1e-05
3e-02
Log-odds
0.5
1.0
1.5
2.0
2.5
3.0
3.5
4.0

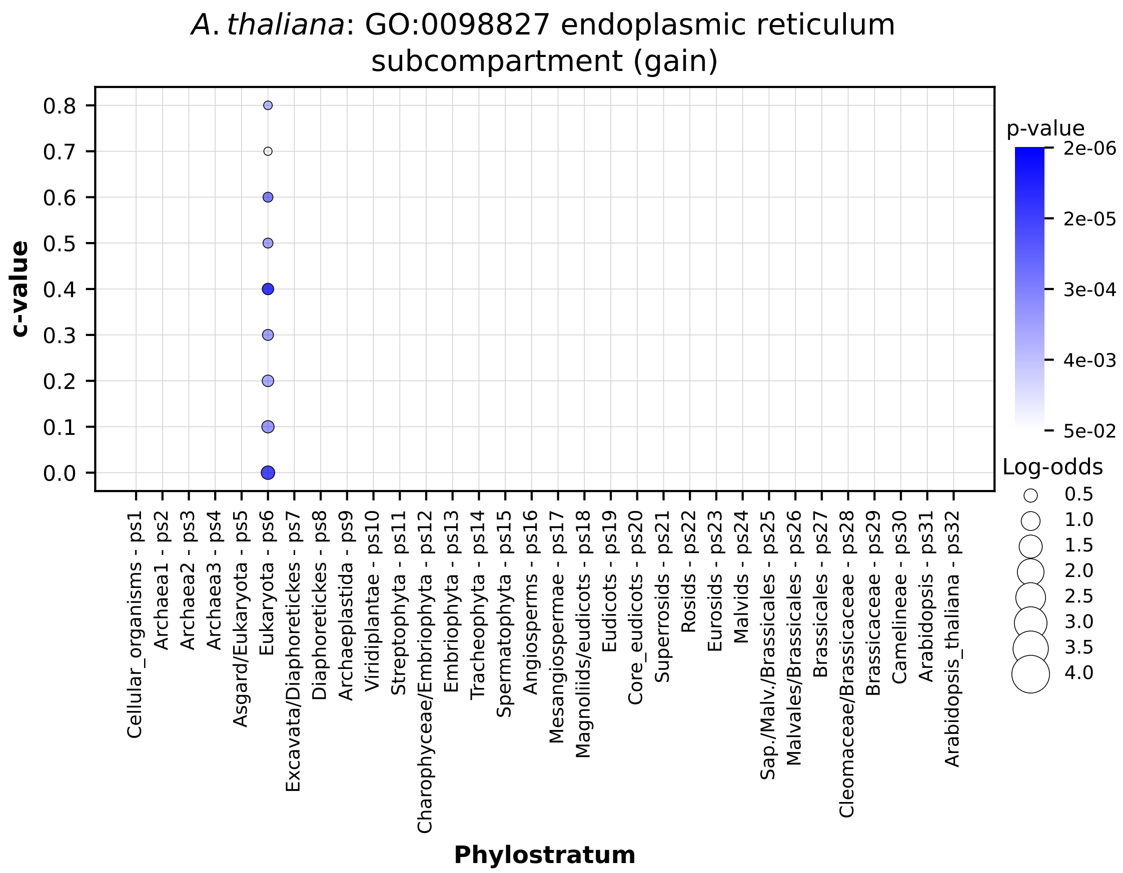

A. thaliana: GO:0098827 endoplasmic reticulum subcompartment (gain)
c-value
0.8
0.7
0.6
0.5
0.4
0.3
0.2
0.1
0.0
p-value
2e-06
2e-05
3e-04
4e-03
5e-02
Log-odds
0.5
1.0
1.5
2.0
2.5
3.0
3.5
4.0
Cellular_organisms - ps1
Archaea1 - ps2
Archaea2 - ps3
Archaea3 - ps4
Asgard/Eukaryota - ps5
Eukaryota - ps6
Excavata/Diaphoretickes - ps7
Diaphoretickes - ps8
Archaeplastida - ps9
Viridiplantae - ps10
Streptophyta - ps11
Charophyceae/Embriophyta - ps12
Embriophyta - ps13
Tracheophyta - ps14
Spermatophyta - ps15
Angiosperms - ps16
Mesangiospermae - ps17
Magnoliids/eudicots - ps18
Eudicots - ps19
Core_eudicots - ps20
Superrosids - ps21
Rosids - ps22
Eurosids - ps23
Malvids - ps24
Sap./Malv./Brassicales - ps25
Malvales/Brassicales - ps26
Brassicales - ps27
Cleomaceae/Brassicaceae - ps28
Brassicaceae - ps29
Camelineae - ps30
Arabidopsis - ps31
Arabidopsis_thaliana - ps32
Phylostratum

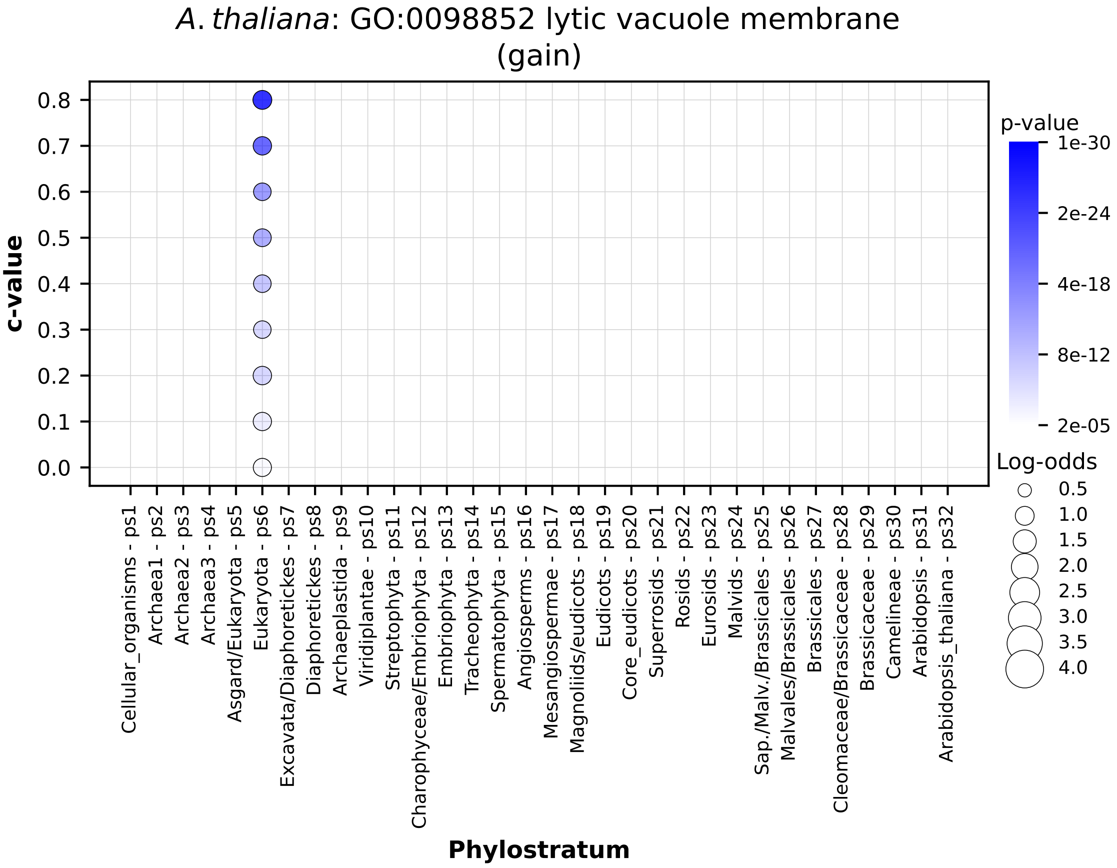

A. thaliana: GO:0098852 lytic vacuole membrane (gain)
c-value
0.8
0.7
0.6
0.5
0.4
0.3
0.2
0.1
0.0
p-value
1e-30
2e-24
4e-18
8e-12
2e-05
Log-odds
0.5
1.0
1.5
2.0
2.5
3.0
3.5
4.0
Cellular_organisms - ps1
Archaea1 - ps2
Archaea2 - ps3
Archaea3 - ps4
Asgard/Eukaryota - ps5
Eukaryota - ps6
Excavata/Diaphoretickes - ps7
Diaphoretickes - ps8
Archaeplastida - ps9
Viridiplantae - ps10
Streptophyta - ps11
Charophyceae/Embriophyta - ps12
Embriophyta - ps13
Tracheophyta - ps14
Spermatophyta - ps15
Angiosperms - ps16
Mesangiospermae - ps17
Magnoliids/eudicots - ps18
Eudicots - ps19
Core_eudicots - ps20
Superrosids - ps21
Rosids - ps22
Eurosids - ps23
Malvids - ps24
Sap./Malv./Brassicales - ps25
Malvales/Brassicales - ps26
Brassicales - ps27
Cleomaceae/Brassicaceae - ps28
Brassicaceae - ps29
Camelineae - ps30
Arabidopsis - ps31
Arabidopsis_thaliana - ps32
Phylostratum

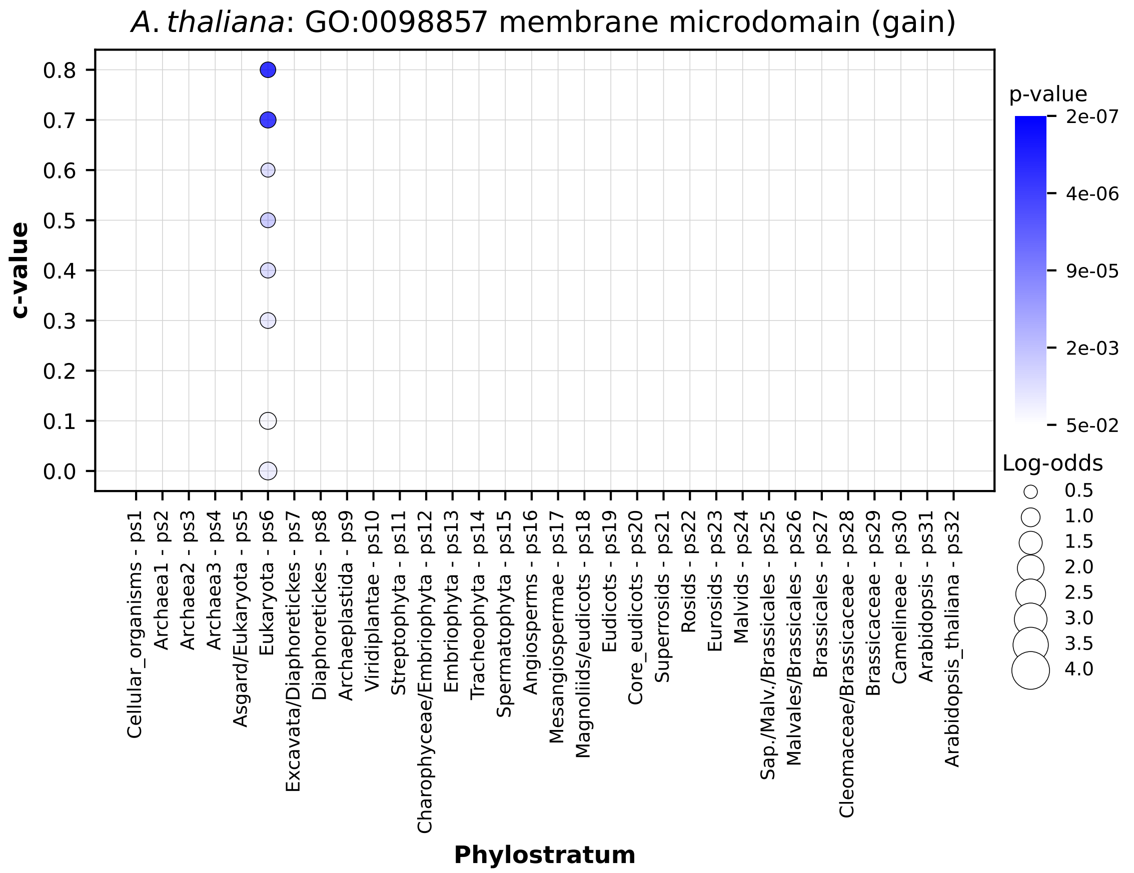
A. thaliana: GO:0098857 membrane microdomain (gain)
c-value
0.8
0.7
0.6
0.5
0.4
0.3
0.2
0.1
0.0
p-value
2e-07
4e-06
9e-05
2e-03
5e-02
Log-odds
0.5
1.0
1.5
2.0
2.5
3.0
3.5
4.0
Cellular_organisms - ps1
Archaea1 - ps2
Archaea2 - ps3
Archaea3 - ps4
Asgard/Eukaryota - ps5
Eukaryota - ps6
Excavata/Diaphoretickes - ps7
Diaphoretickes - ps8
Archaeplastida - ps9
Viridiplantae - ps10
Streptophyta - ps11
Charophyceae/Embriophyta - ps12
Embriophyta - ps13
Tracheophyta - ps14
Spermatophyta - ps15
Angiosperms - ps16
Mesangiospermae - ps17
Magnoliids/eudicots - ps18
Eudicots - ps19
Core_eudicots - ps20
Superrosids - ps21
Rosids - ps22
Eurosids - ps23
Malvids - ps24
Sap./Malv./Brassicales - ps25
Malvales/Brassicales - ps26
Brassicales - ps27
Cleomaceae/Brassicaceae - ps28
Brassicaceae - ps29
Camelineae - ps30
Arabidopsis - ps31
Arabidopsis_thaliana - ps32
Phylostratum

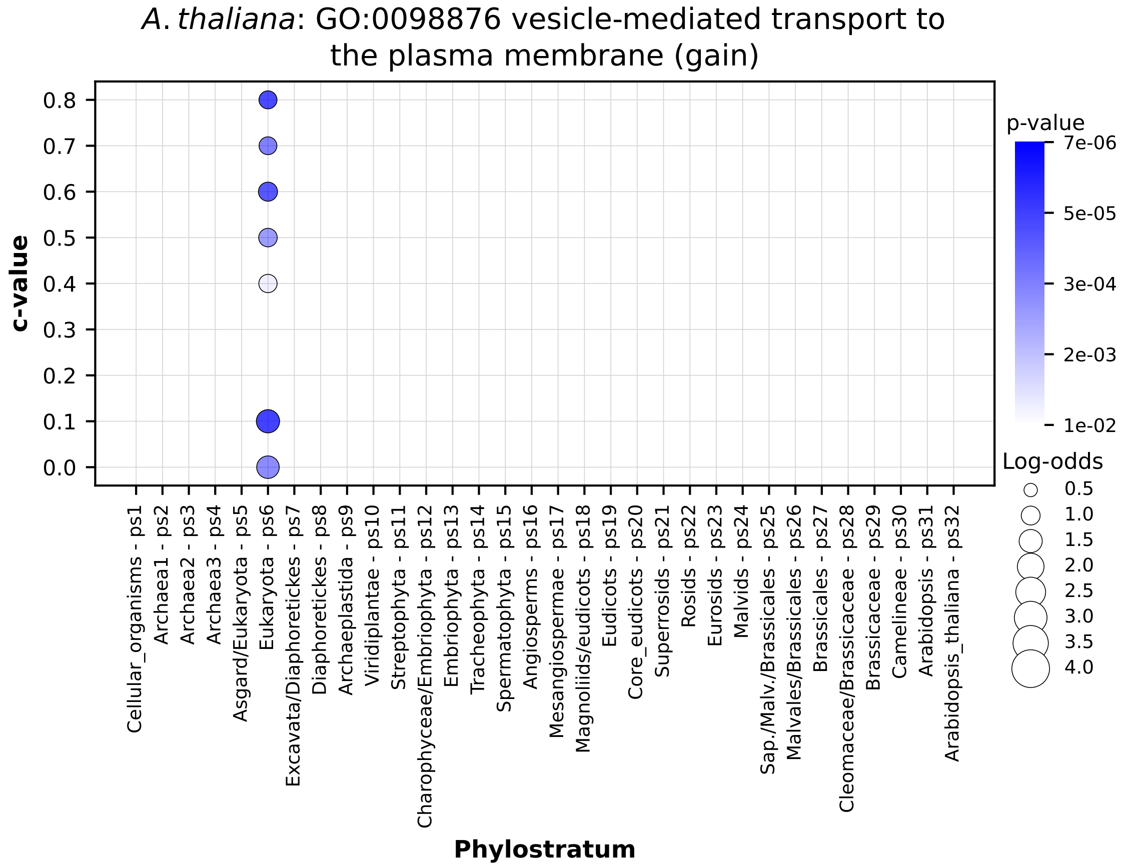
A. thaliana: GO:0098876 vesicle-mediated transport to the plasma membrane (gain)
c-value
0.8
0.7
0.6
0.5
0.4
0.3
0.2
0.1
0.0
p-value
7e-06
5e-05
3e-04
2e-03
1e-02
Log-odds
0.5
1.0
1.5
2.0
2.5
3.0
3.5
4.0
Cellular_organisms - ps1
Archaea1 - ps2
Archaea2 - ps3
Archaea3 - ps4
Asgard/Eukaryota - ps5
Eukaryota - ps6
Excavata/Diaphoretickes - ps7
Diaphoretickes - ps8
Archaeplastida - ps9
Viridiplantae - ps10
Streptophyta - ps11
Charophyceae/Embriophyta - ps12
Embriophyta - ps13
Tracheophyta - ps14
Spermatophyta - ps15
Angiosperms - ps16
Mesangiospermae - ps17
Magnoliids/eudicots - ps18
Eudicots - ps19
Core_eudicots - ps20
Superrosids - ps21
Rosids - ps22
Eurosids - ps23
Malvids - ps24
Sap./Malv./Brassicales - ps25
Malvales/Brassicales - ps26
Brassicales - ps27
Cleomaceae/Brassicaceae - ps28
Brassicaceae - ps29
Camelineae - ps30
Arabidopsis - ps31
Arabidopsis_thaliana - ps32
Phylostratum

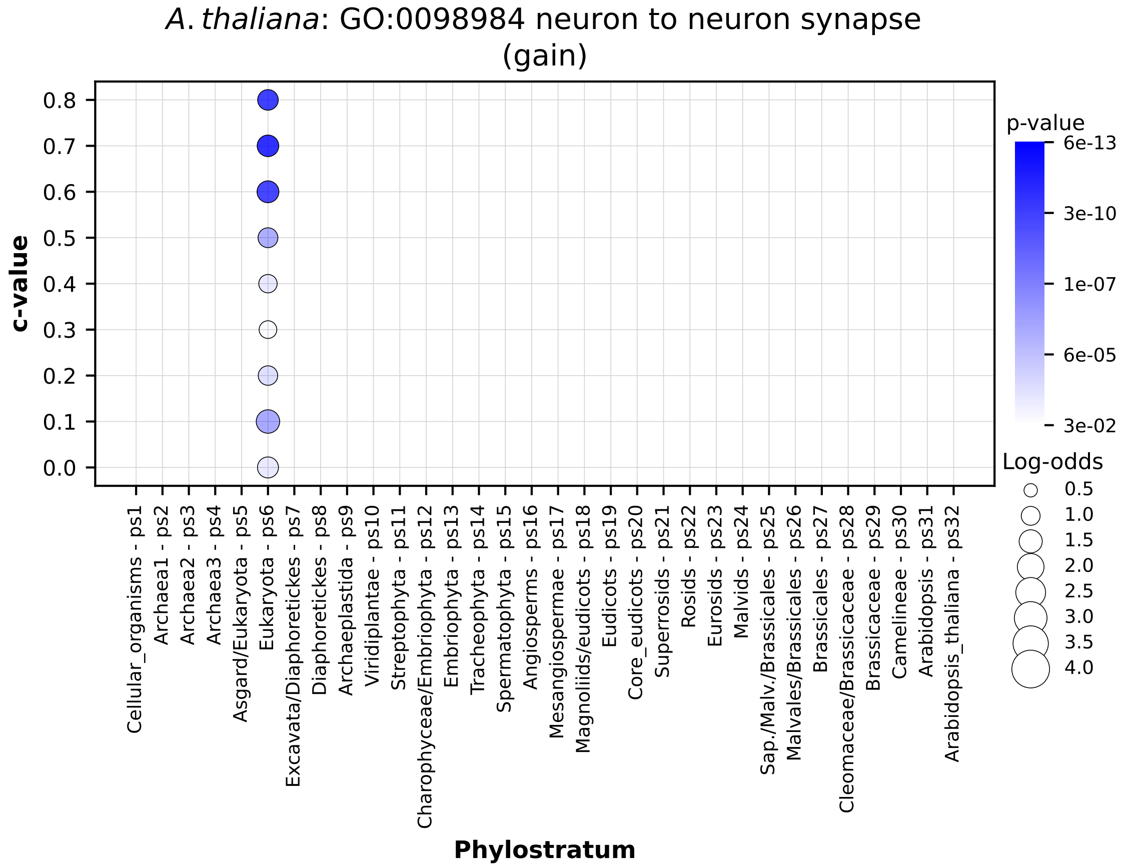

A. thaliana: GO:0098984 neuron to neuron synapse
(gain)
c-value
0.8
0.7
0.6
0.5
0.4
0.3
0.2
0.1
0.0
p-value
6e-13
3e-10
1e-07
6e-05
3e-02
Log-odds
0.5
1.0
1.5
2.0
2.5
3.0
3.5
4.0
Cellular_organisms - ps1
Archaea1 - ps2
Archaea2 - ps3
Archaea3 - ps4
Asgard/Eukaryota - ps5
Eukaryota - ps6
Excavata/Diaphoretickes - ps7
Diaphoretickes - ps8
Archaeplastida - ps9
Viridiplantae - ps10
Streptophyta - ps11
Charophyceae/Embriophyta - ps12
Embriophyta - ps13
Tracheophyta - ps14
Spermatophyta - ps15
Angiosperms - ps16
Mesangiospermae - ps17
Magnoliids/eudicots - ps18
Eudicots - ps19
Core_eudicots - ps20
Superrosids - ps21
Rosids - ps22
Eurosids - ps23
Malvids - ps24
Sap./Malv./Brassicales - ps25
Malvales/Brassicales - ps26
Brassicales - ps27
Cleomaceae/Brassicaceae - ps28
Brassicaceae - ps29
Camelineae - ps30
Arabidopsis - ps31
Arabidopsis_thaliana - ps32
Phylostratum

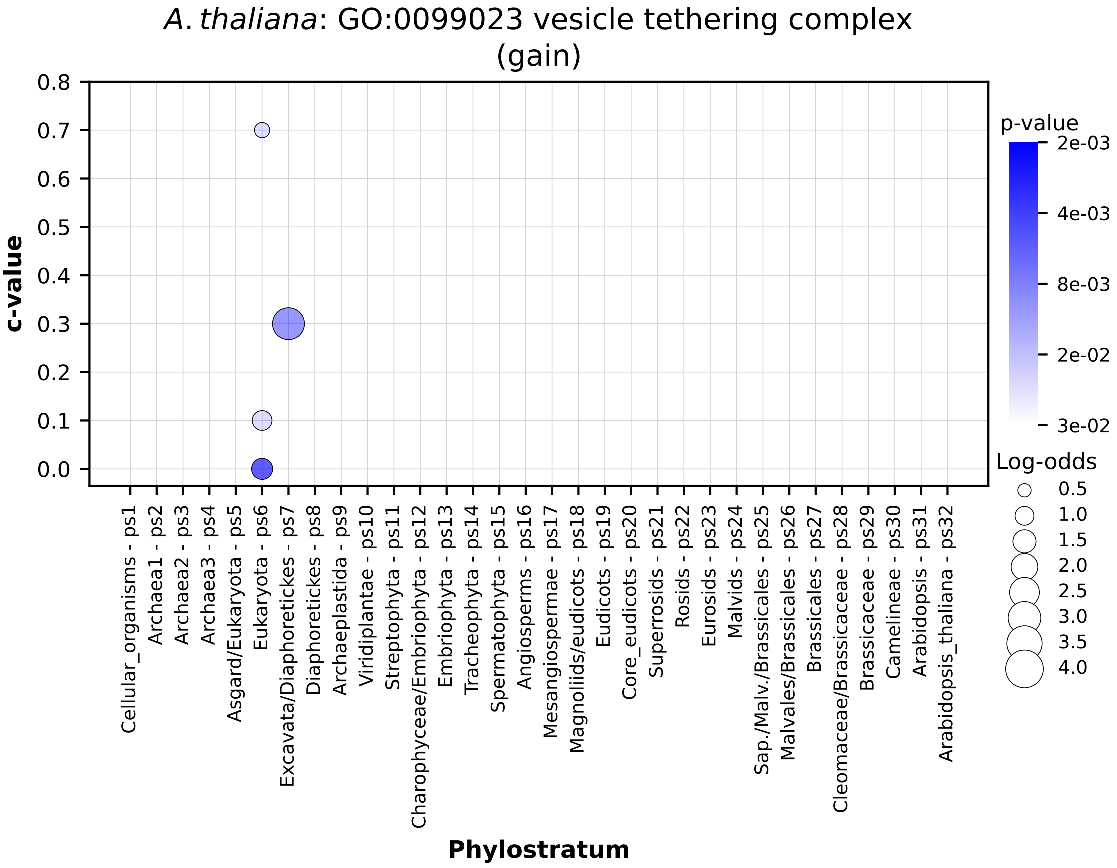

A. thaliana: GO:0099023 vesicle tethering complex
(gain)
c-value
0.8
0.7
0.6
0.5
0.4
0.3
0.2
0.1
0.0
p-value
2e-03
4e-03
8e-03
2e-02
3e-02
Log-odds
0.5
1.0
1.5
2.0
2.5
3.0
3.5
4.0
Cellular_organisms - ps1
Archaea1 - ps2
Archaea2 - ps3
Archaea3 - ps4
Asgard/Eukaryota - ps5
Eukaryota - ps6
Excavata/Diaphoretickes - ps7
Diaphoretickes - ps8
Archaeplastida - ps9
Viridiplantae - ps10
Streptophyta - ps11
Charophyceae/Embriophyta - ps12
Embriophyta - ps13
Tracheophyta - ps14
Spermatophyta - ps15
Angiosperms - ps16
Mesangiospermae - ps17
Magnoliids/eudicots - ps18
Eudicots - ps19
Core_eudicots - ps20
Superrosids - ps21
Rosids - ps22
Eurosids - ps23
Malvids - ps24
Sap./Malv./Brassicales - ps25
Malvales/Brassicales - ps26
Brassicales - ps27
Cleomaceae/Brassicaceae - ps28
Brassicaceae - ps29
Camelineae - ps30
Arabidopsis - ps31
Arabidopsis_thaliana - ps32
Phylostratum

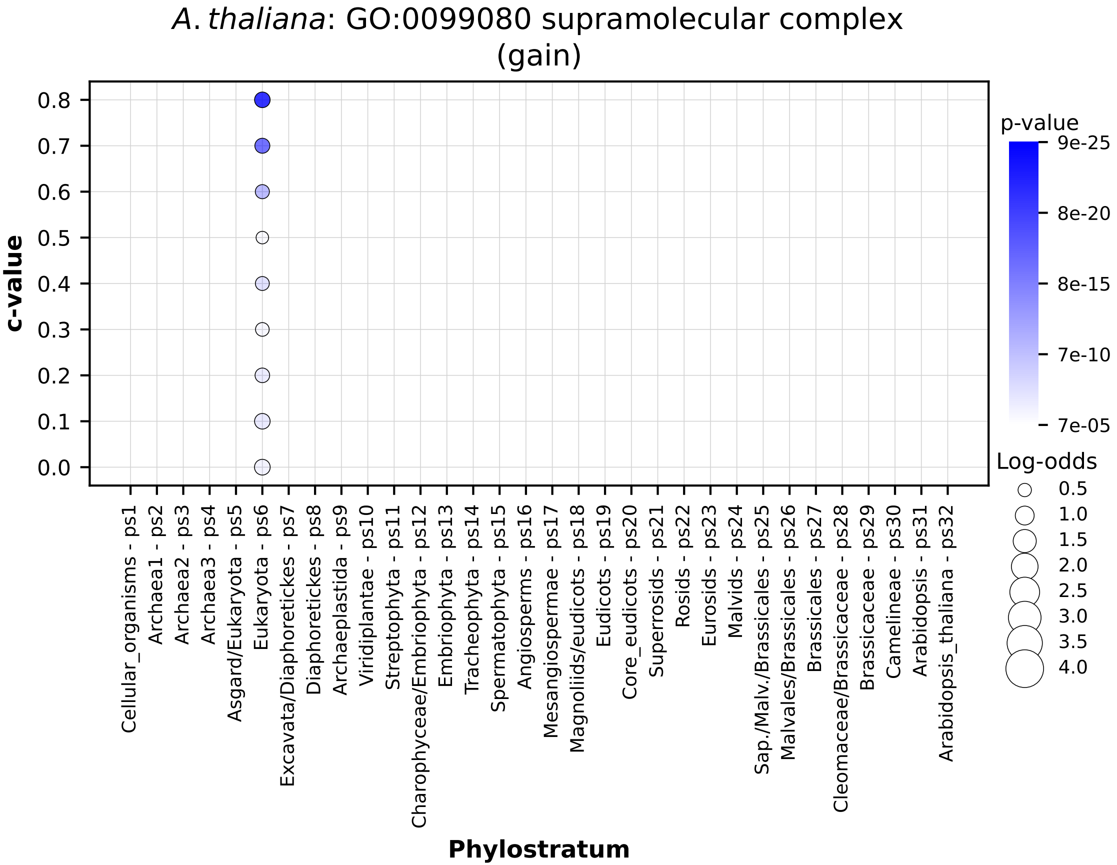

A. thaliana: GO:0099080 supramolecular complex
(gain)
c-value
0.8
0.7
0.6
0.5
0.4
0.3
0.2
0.1
0.0
p-value
9e-25
8e-20
8e-15
7e-10
7e-05
Log-odds
0.5
1.0
1.5
2.0
2.5
3.0
3.5
4.0
Cellular_organisms - ps1
Archaea1 - ps2
Archaea2 - ps3
Archaea3 - ps4
Asgard/Eukaryota - ps5
Eukaryota - ps6
Excavata/Diaphoretickes - ps7
Diaphoretickes - ps8
Archaeplastida - ps9
Viridiplantae - ps10
Streptophyta - ps11
Charophyceae/Embriophyta - ps12
Embriophyta - ps13
Tracheophyta - ps14
Spermatophyta - ps15
Angiosperms - ps16
Mesangiospermae - ps17
Magnoliids/eudicots - ps18
Eudicots - ps19
Core_eudicots - ps20
Superrosids - ps21
Rosids - ps22
Eurosids - ps23
Malvids - ps24
Sap./Malv./Brassicales - ps25
Malvales/Brassicales - ps26
Brassicales - ps27
Cleomaceae/Brassicaceae - ps28
Brassicaceae - ps29
Camelineae - ps30
Arabidopsis - ps31
Arabidopsis_thaliana - ps32
Phylostratum

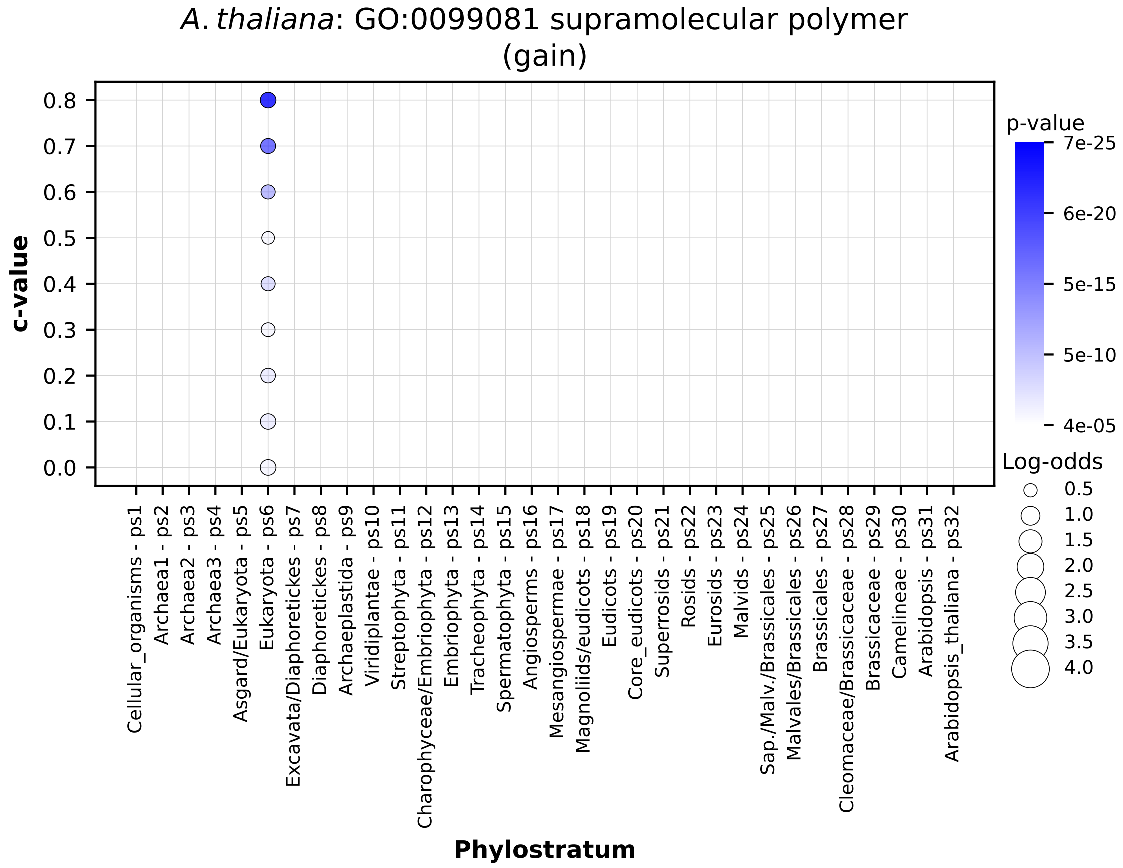
A. thaliana: GO:0099081 supramolecular polymer
(gain)
c-value
0.8
0.7
0.6
0.5
0.4
0.3
0.2
0.1
0.0
p-value
7e-25
6e-20
5e-15
5e-10
4e-05
Log-odds
0.5
1.0
1.5
2.0
2.5
3.0
3.5
4.0
Cellular_organisms - ps1
Archaea1 - ps2
Archaea2 - ps3
Archaea3 - ps4
Asgard/Eukaryota - ps5
Eukaryota - ps6
Excavata/Diaphoretickes - ps7
Diaphoretickes - ps8
Archaeplastida - ps9
Viridiplantae - ps10
Streptophyta - ps11
Charophyceae/Embriophyta - ps12
Embriophyta - ps13
Tracheophyta - ps14
Spermatophyta - ps15
Angiosperms - ps16
Mesangiospermae - ps17
Magnoliids/eudicots - ps18
Eudicots - ps19
Core_eudicots - ps20
Superrosids - ps21
Rosids - ps22
Eurosids - ps23
Malvids - ps24
Sap./Malv./Brassicales - ps25
Malvales/Brassicales - ps26
Brassicales - ps27
Cleomaceae/Brassicaceae - ps28
Brassicaceae - ps29
Camelineae - ps30
Arabidopsis - ps31
Arabidopsis_thaliana - ps32
Phylostratum

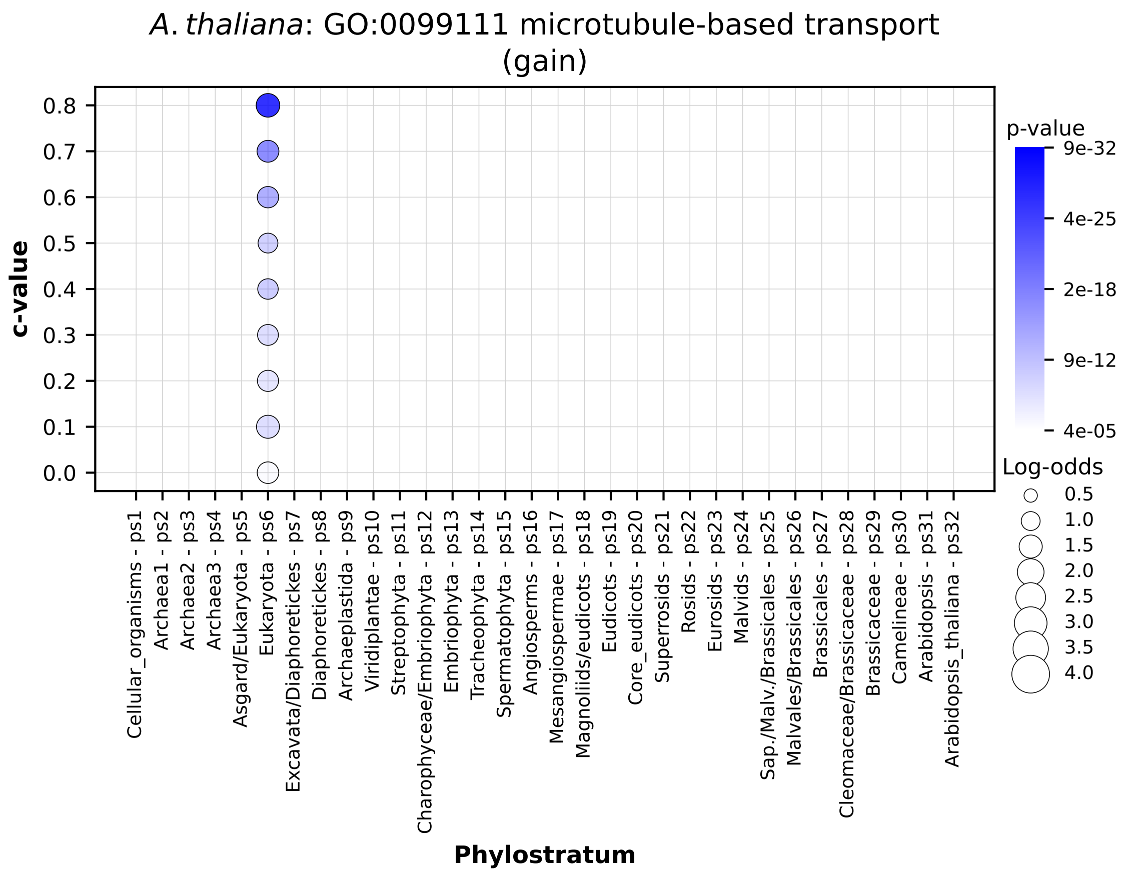

A. thaliana: GO:0099111 microtubule-based transport
(gain)
c-value
0.8
0.7
0.6
0.5
0.4
0.3
0.2
0.1
0.0
p-value
9e-32
4e-25
2e-18
9e-12
4e-05
Log-odds
0.5
1.0
1.5
2.0
2.5
3.0
3.5
4.0
Cellular_organisms - ps1
Archaea1 - ps2
Archaea2 - ps3
Archaea3 - ps4
Asgard/Eukaryota - ps5
Eukaryota - ps6
Excavata/Diaphoretickes - ps7
Diaphoretickes - ps8
Archaeplastida - ps9
Viridiplantae - ps10
Streptophyta - ps11
Charophyceae/Embriophyta - ps12
Embriophyta - ps13
Tracheophyta - ps14
Spermatophyta - ps15
Angiosperms - ps16
Mesangiospermae - ps17
Magnoliids/eudicots - ps18
Eudicots - ps19
Core_eudicots - ps20
Superrosids - ps21
Rosids - ps22
Eurosids - ps23
Malvids - ps24
Sap./Malv./Brassicales - ps25
Malvales/Brassicales - ps26
Brassicales - ps27
Cleomaceae/Brassicaceae - ps28
Brassicaceae - ps29
Camelineae - ps30
Arabidopsis - ps31
Arabidopsis_thaliana - ps32
Phylostratum

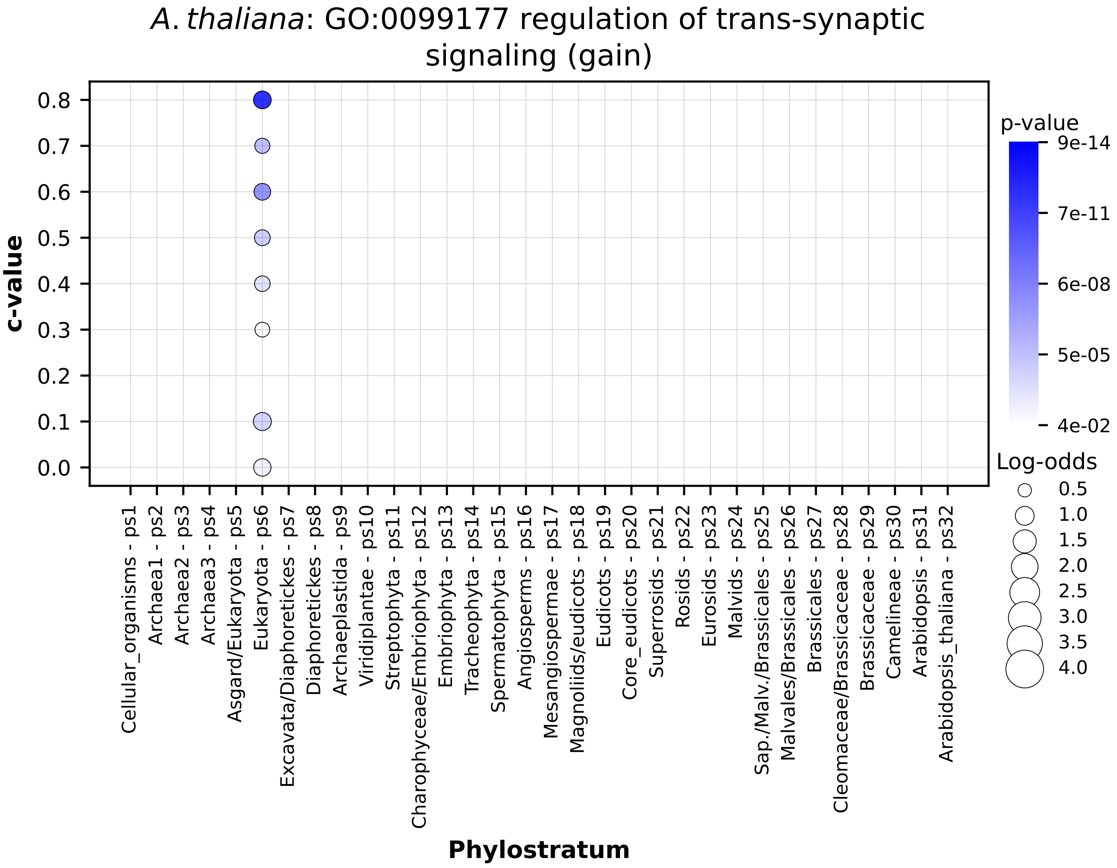

A. thaliana: GO:0099177 regulation of trans-synaptic signaling (gain)
c-value
0.8
0.7
0.6
0.5
0.4
0.3
0.2
0.1
0.0
p-value
9e-14
7e-11
6e-08
5e-05
4e-02
Log-odds
0.5
1.0
1.5
2.0
2.5
3.0
3.5
4.0
Cellular_organisms - ps1
Archaea1 - ps2
Archaea2 - ps3
Archaea3 - ps4
Asgard/Eukaryota - ps5
Eukaryota - ps6
Excavata/Diaphoretickes - ps7
Diaphoretickes - ps8
Archaeplastida - ps9
Viridiplantae - ps10
Streptophyta - ps11
Charophyceae/Embriophyta - ps12
Embriophyta - ps13
Tracheophyta - ps14
Spermatophyta - ps15
Angiosperms - ps16
Mesangiospermae - ps17
Magnoliids/eudicots - ps18
Eudicots - ps19
Core_eudicots - ps20
Superrosids - ps21
Rosids - ps22
Eurosids - ps23
Malvids - ps24
Sap./Malv./Brassicales - ps25
Malvales/Brassicales - ps26
Brassicales - ps27
Cleomaceae/Brassicaceae - ps28
Brassicaceae - ps29
Camelineae - ps30
Arabidopsis - ps31
Arabidopsis_thaliana - ps32
Phylostratum

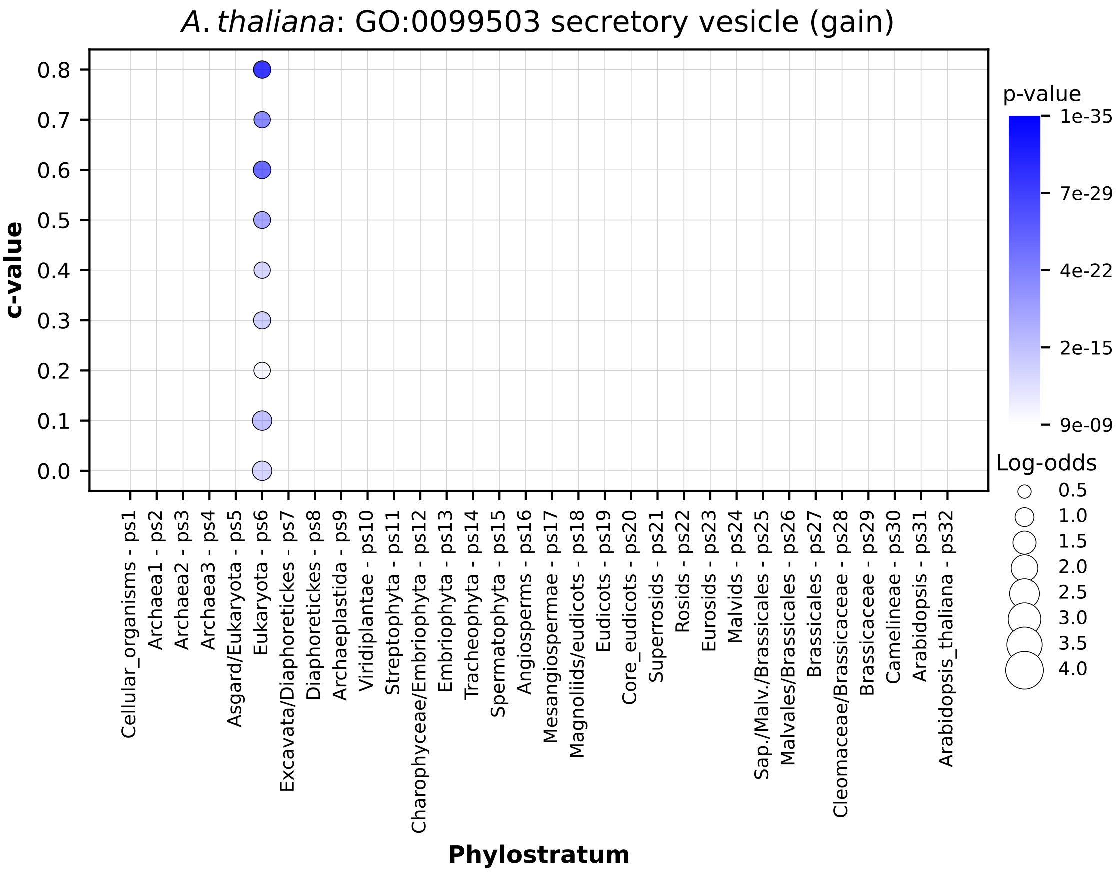
A. thaliana: GO:0099503 secretory vesicle (gain)
c-value
0.8
0.7
0.6
0.5
0.4
0.3
0.2
0.1
0.0
p-value
1e-35
7e-29
4e-22
2e-15
9e-09
Log-odds
0.5
1.0
1.5
2.0
2.5
3.0
3.5
4.0
Cellular_organisms - ps1
Archaea1 - ps2
Archaea2 - ps3
Archaea3 - ps4
Asgard/Eukaryota - ps5
Eukaryota - ps6
Excavata/Diaphoretickes - ps7
Diaphoretickes - ps8
Archaeplastida - ps9
Viridiplantae - ps10
Streptophyta - ps11
Charophyceae/Embriophyta - ps12
Embriophyta - ps13
Tracheophyta - ps14
Spermatophyta - ps15
Angiosperms - ps16
Mesangiospermae - ps17
Magnoliids/eudicots - ps18
Eudicots - ps19
Core_eudicots - ps20
Superrosids - ps21
Rosids - ps22
Eurosids - ps23
Malvids - ps24
Sap./Malv./Brassicales - ps25
Malvales/Brassicales - ps26
Brassicales - ps27
Cleomaceae/Brassicaceae - ps28
Brassicaceae - ps29
Camelineae - ps30
Arabidopsis - ps31
Arabidopsis_thaliana - ps32
Phylostratum

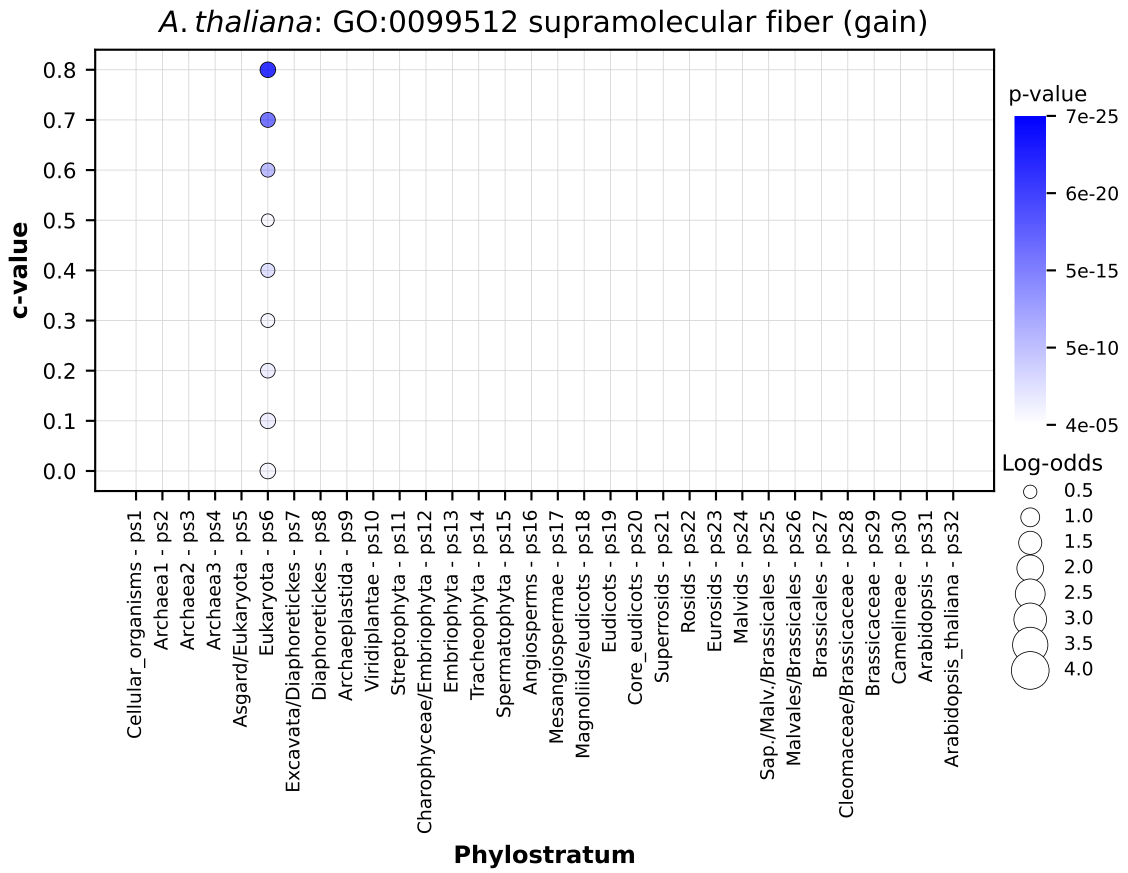

A. thaliana: GO:0099512 supramolecular fiber (gain)
c-value
0.0
0.1
0.2
0.3
0.4
0.5
0.6
0.7
0.8
p-value
7e-25
6e-20
5e-15
5e-10
4e-05
Log-odds
0.5
1.0
1.5
2.0
2.5
3.0
3.5
4.0
Cellular_organisms - ps1
Archaea1 - ps2
Archaea2 - ps3
Archaea3 - ps4
Asgard/Eukaryota - ps5
Eukaryota - ps6
Excavata/Diaphoretickes - ps7
Diaphoretickes - ps8
Archaeplastida - ps9
Viridiplantae - ps10
Streptophyta - ps11
Charophyceae/Embriophyta - ps12
Embriophyta - ps13
Tracheophyta - ps14
Spermatophyta - ps15
Angiosperms - ps16
Mesangiospermae - ps17
Magnoliids/eudicots - ps18
Eudicots - ps19
Core_eudicots - ps20
Superrosids - ps21
Rosids - ps22
Eurosids - ps23
Malvids - ps24
Sap./Malv./Brassicales - ps25
Malvales/Brassicales - ps26
Brassicales - ps27
Cleomaceae/Brassicaceae - ps28
Brassicaceae - ps29
Camelineae - ps30
Arabidopsis - ps31
Arabidopsis_thaliana - ps32
Phylostratum

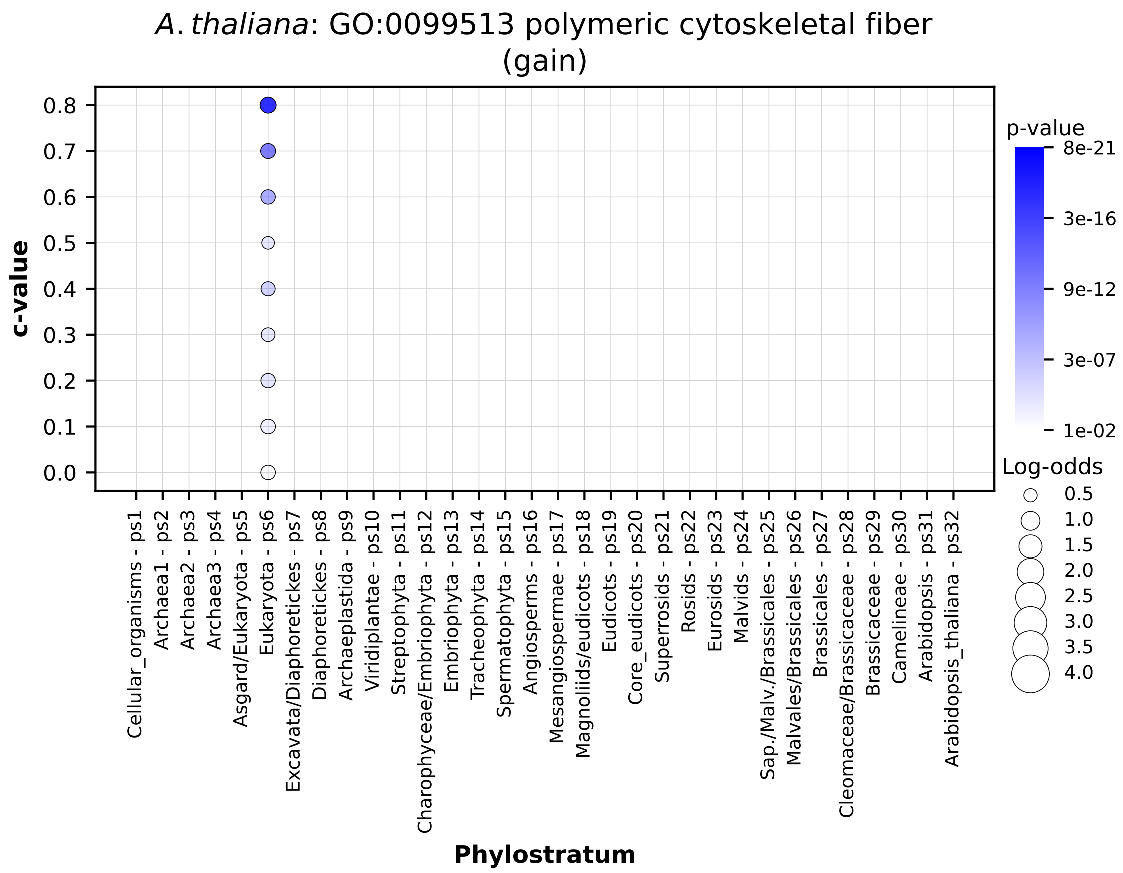

A. thaliana: GO:0099513 polymeric cytoskeletal fiber
(gain)
c-value
0.8
0.7
0.6
0.5
0.4
0.3
0.2
0.1
0.0
p-value
8e-21
3e-16
9e-12
3e-07
1e-02
Log-odds
0.5
1.0
1.5
2.0
2.5
3.0
3.5
4.0
Cellular_organisms - ps1
Archaea1 - ps2
Archaea2 - ps3
Archaea3 - ps4
Asgard/Eukaryota - ps5
Eukaryota - ps6
Excavata/Diaphoretickes - ps7
Diaphoretickes - ps8
Archaeplastida - ps9
Viridiplantae - ps10
Streptophyta - ps11
Charophyceae/Embriophyta - ps12
Embriophyta - ps13
Tracheophyta - ps14
Spermatophyta - ps15
Angiosperms - ps16
Mesangiospermae - ps17
Magnoliids/eudicots - ps18
Eudicots - ps19
Core_eudicots - ps20
Superrosids - ps21
Rosids - ps22
Eurosids - ps23
Malvids - ps24
Sap./Malv./Brassicales - ps25
Malvales/Brassicales - ps26
Brassicales - ps27
Cleomaceae/Brassicaceae - ps28
Brassicaceae - ps29
Camelineae - ps30
Arabidopsis - ps31
Arabidopsis_thaliana - ps32
Phylostratum

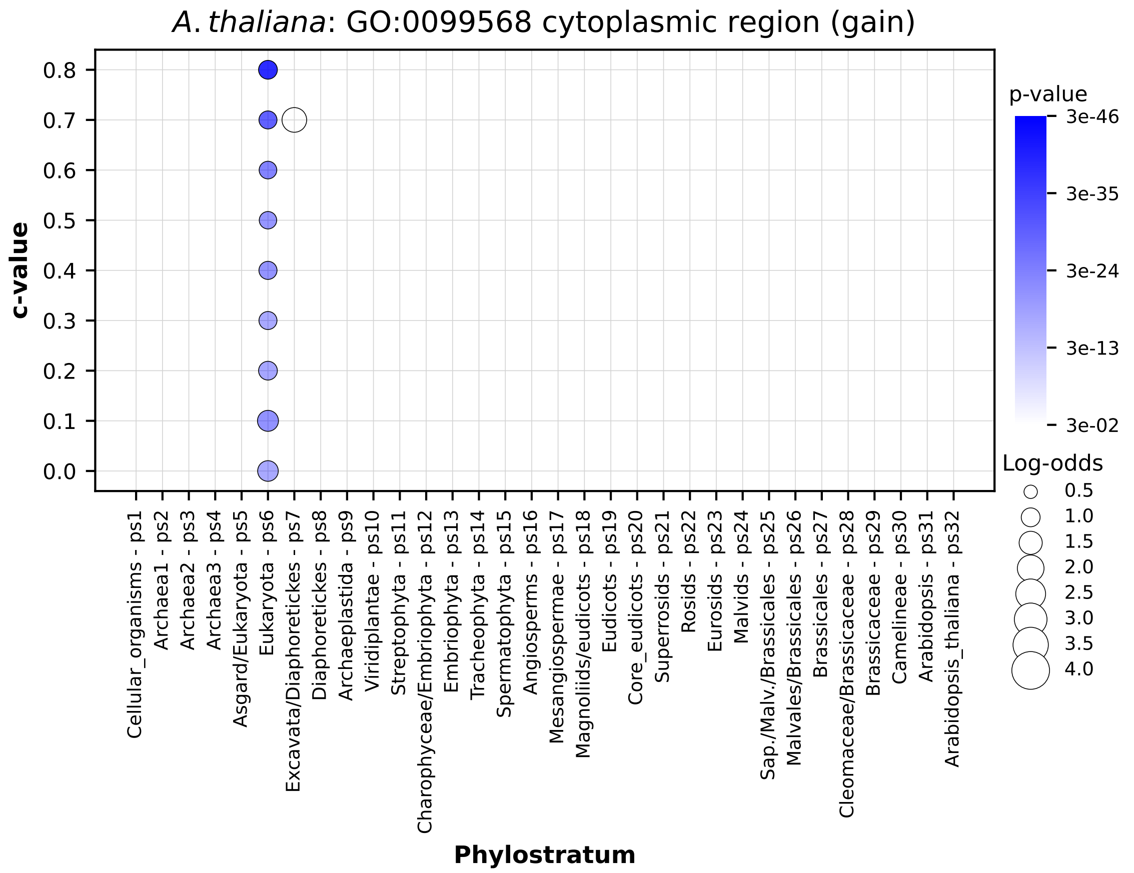
A. thaliana: GO:0099568 cytoplasmic region (gain)
c-value
0.8
0.7
0.6
0.5
0.4
0.3
0.2
0.1
0.0
p-value
3e-46
3e-35
3e-24
3e-13
3e-02
Log-odds
0.5
1.0
1.5
2.0
2.5
3.0
3.5
4.0
Cellular_organisms - ps1
Archaea1 - ps2
Archaea2 - ps3
Archaea3 - ps4
Asgard/Eukaryota - ps5
Eukaryota - ps6
Excavata/Diaphoretickes - ps7
Diaphoretickes - ps8
Archaeplastida - ps9
Viridiplantae - ps10
Streptophyta - ps11
Charophyceae/Embriophyta - ps12
Embriophyta - ps13
Tracheophyta - ps14
Spermatophyta - ps15
Angiosperms - ps16
Mesangiospermae - ps17
Magnoliids/eudicots - ps18
Eudicots - ps19
Core_eudicots - ps20
Superrosids - ps21
Rosids - ps22
Eurosids - ps23
Malvids - ps24
Sap./Malv./Brassicales - ps25
Malvales/Brassicales - ps26
Brassicales - ps27
Cleomaceae/Brassicaceae - ps28
Brassicaceae - ps29
Camelineae - ps30
Arabidopsis - ps31
Arabidopsis_thaliana - ps32
Phylostratum

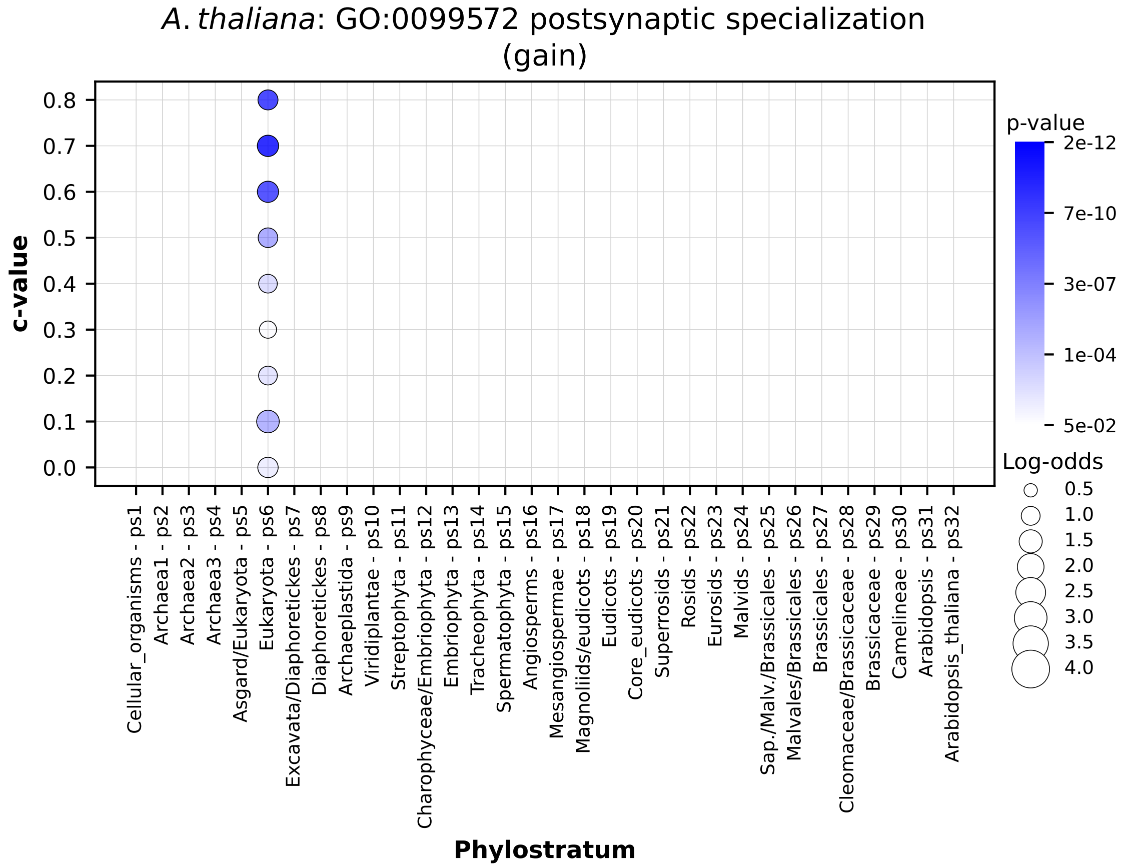

A. thaliana: GO:0099572 postsynaptic specialization
(gain)
c-value
0.8
0.7
0.6
0.5
0.4
0.3
0.2
0.1
0.0
Phylostratum
Cellular_organisms - ps1
Archaea1 - ps2
Archaea2 - ps3
Archaea3 - ps4
Asgard/Eukaryota - ps5
Eukaryota - ps6
Excavata/Diaphoretickes - ps7
Diaphoretickes - ps8
Archaeplastida - ps9
Viridiplantae - ps10
Streptophyta - ps11
Charophyceae/Embriophyta - ps12
Embriophyta - ps13
Tracheophyta - ps14
Spermatophyta - ps15
Angiosperms - ps16
Mesangiospermae - ps17
Magnoliids/eudicots - ps18
Eudicots - ps19
Core_eudicots - ps20
Superrosids - ps21
Rosids - ps22
Eurosids - ps23
Malvids - ps24
Sap./Malv./Brassicales - ps25
Malvales/Brassicales - ps26
Brassicales - ps27
Cleomaceae/Brassicaceae - ps28
Brassicaceae - ps29
Camelineae - ps30
Arabidopsis - ps31
Arabidopsis_thaliana - ps32
p-value
2e-12
7e-10
3e-07
1e-04
5e-02
Log-odds
0.5
1.0
1.5
2.0
2.5
3.0
3.5
4.0

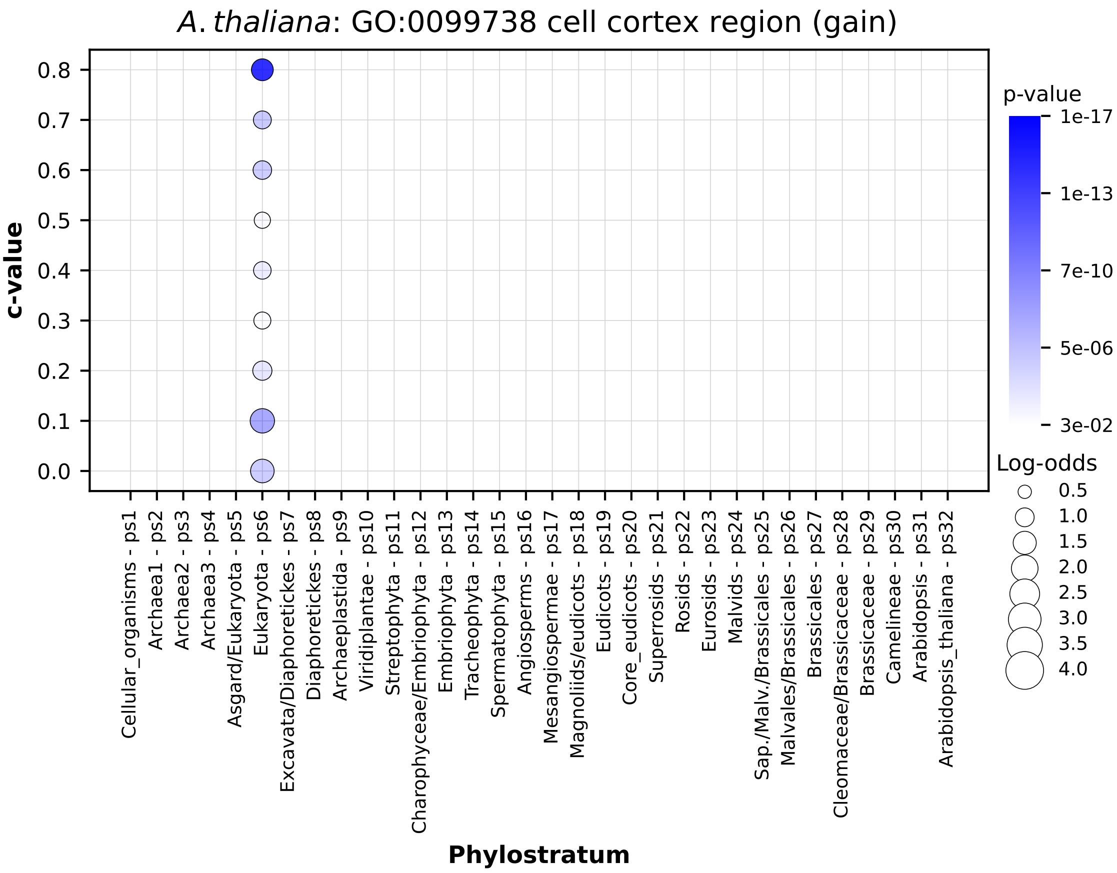
A. thaliana: GO:0099738 cell cortex region (gain)
c-value
0.0
0.1
0.2
0.3
0.4
0.5
0.6
0.7
0.8
p-value
1e-17
1e-13
7e-10
5e-06
3e-02
Log-odds
0.5
1.0
1.5
2.0
2.5
3.0
3.5
4.0
Cellular_organisms - ps1
Archaea1 - ps2
Archaea2 - ps3
Archaea3 - ps4
Asgard/Eukaryota - ps5
Eukaryota - ps6
Excavata/Diaphoretickes - ps7
Diaphoretickes - ps8
Archaeplastida - ps9
Viridiplantae - ps10
Streptophyta - ps11
Charophyceae/Embriophyta - ps12
Embriophyta - ps13
Tracheophyta - ps14
Spermatophyta - ps15
Angiosperms - ps16
Mesangiospermae - ps17
Magnoliids/eudicots - ps18
Eudicots - ps19
Core_eudicots - ps20
Superrosids - ps21
Rosids - ps22
Eurosids - ps23
Malvids - ps24
Sap./Malv./Brassicales - ps25
Malvales/Brassicales - ps26
Brassicales - ps27
Cleomaceae/Brassicaceae - ps28
Brassicaceae - ps29
Camelineae - ps30
Arabidopsis - ps31
Arabidopsis_thaliana - ps32
Phylostratum

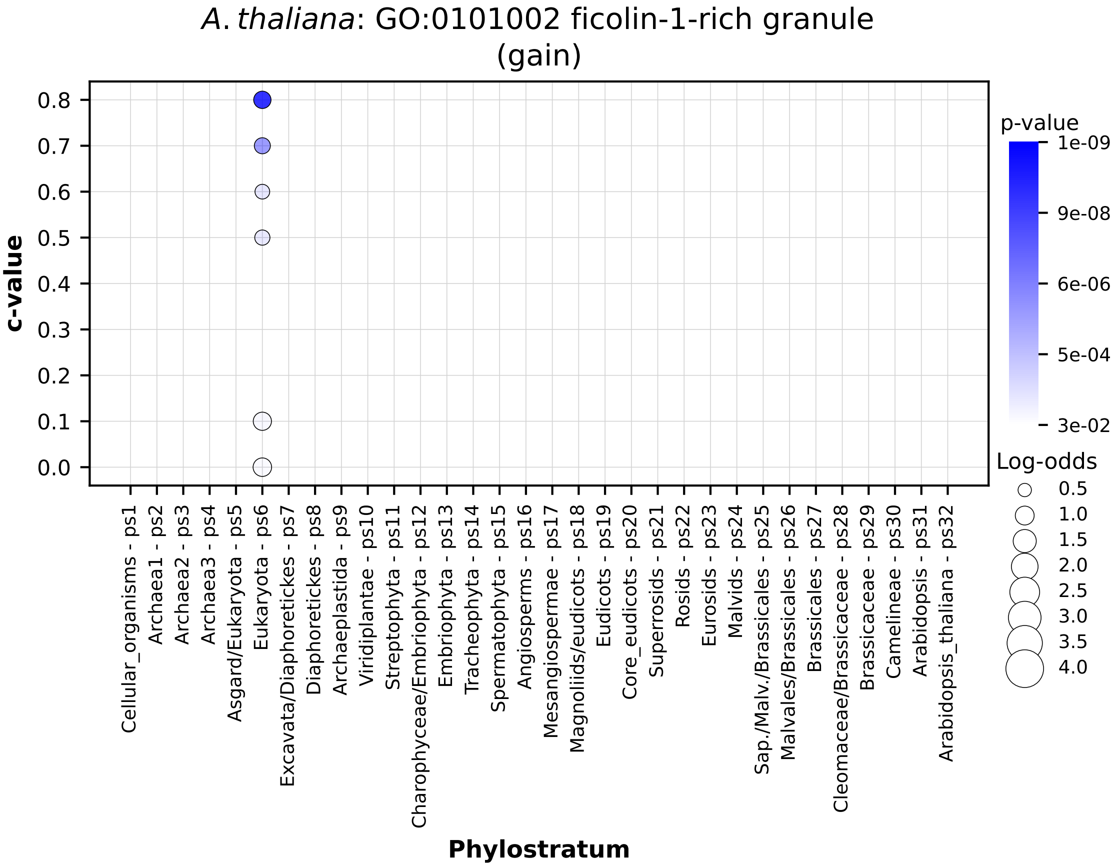

A. thaliana: GO:0101002 ficolin-1-rich granule
(gain)
c-value
0.8
0.7
0.6
0.5
0.4
0.3
0.2
0.1
0.0
p-value
1e-09
9e-08
6e-06
5e-04
3e-02
Log-odds
0.5
1.0
1.5
2.0
2.5
3.0
3.5
4.0
Cellular_organisms - ps1
Archaea1 - ps2
Archaea2 - ps3
Archaea3 - ps4
Asgard/Eukaryota - ps5
Eukaryota - ps6
Excavata/Diaphoretickes - ps7
Diaphoretickes - ps8
Archaeplastida - ps9
Viridiplantae - ps10
Streptophyta - ps11
Charophyceae/Embriophyta - ps12
Embriophyta - ps13
Tracheophyta - ps14
Spermatophyta - ps15
Angiosperms - ps16
Mesangiospermae - ps17
Magnoliids/eudicots - ps18
Eudicots - ps19
Core_eudicots - ps20
Superrosids - ps21
Rosids - ps22
Eurosids - ps23
Malvids - ps24
Sap./Malv./Brassicales - ps25
Malvales/Brassicales - ps26
Brassicales - ps27
Cleomaceae/Brassicaceae - ps28
Brassicaceae - ps29
Camelineae - ps30
Arabidopsis - ps31
Arabidopsis_thaliana - ps32
Phylostratum

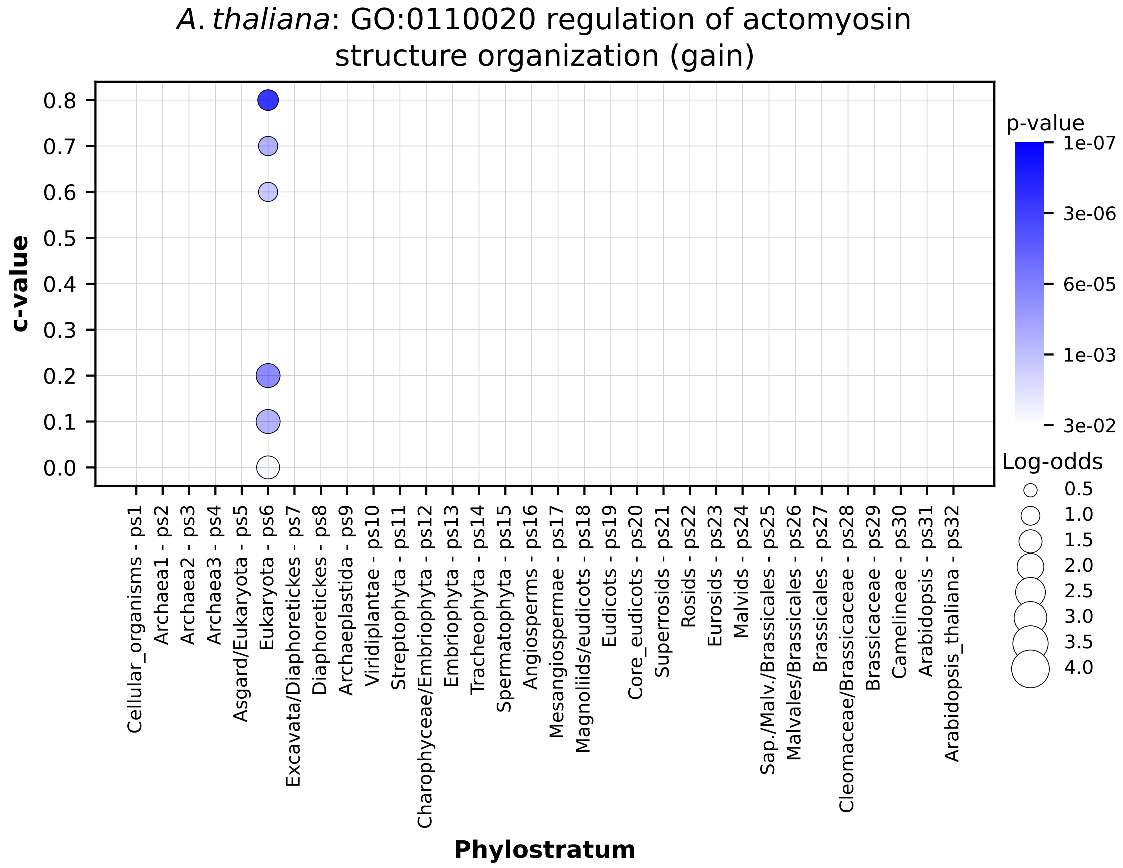

A. thaliana: GO:0110020 regulation of actomyosin structure organization (gain)
c-value
0.8
0.7
0.6
0.5
0.4
0.3
0.2
0.1
0.0
p-value
1e-07
3e-06
6e-05
1e-03
3e-02
Log-odds
0.5
1.0
1.5
2.0
2.5
3.0
3.5
4.0
Cellular_organisms - ps1
Archaea1 - ps2
Archaea2 - ps3
Archaea3 - ps4
Asgard/Eukaryota - ps5
Eukaryota - ps6
Excavata/Diaphoretickes - ps7
Diaphoretickes - ps8
Archaeplastida - ps9
Viridiplantae - ps10
Streptophyta - ps11
Charophyceae/Embriophyta - ps12
Embriophyta - ps13
Tracheophyta - ps14
Spermatophyta - ps15
Angiosperms - ps16
Mesangiospermae - ps17
Magnoliids/eudicots - ps18
Eudicots - ps19
Core_eudicots - ps20
Superrosids - ps21
Rosids - ps22
Eurosids - ps23
Malvids - ps24
Sap./Malv./Brassicales - ps25
Malvales/Brassicales - ps26
Brassicales - ps27
Cleomaceae/Brassicaceae - ps28
Brassicaceae - ps29
Camelineae - ps30
Arabidopsis - ps31
Arabidopsis_thaliana - ps32
Phylostratum

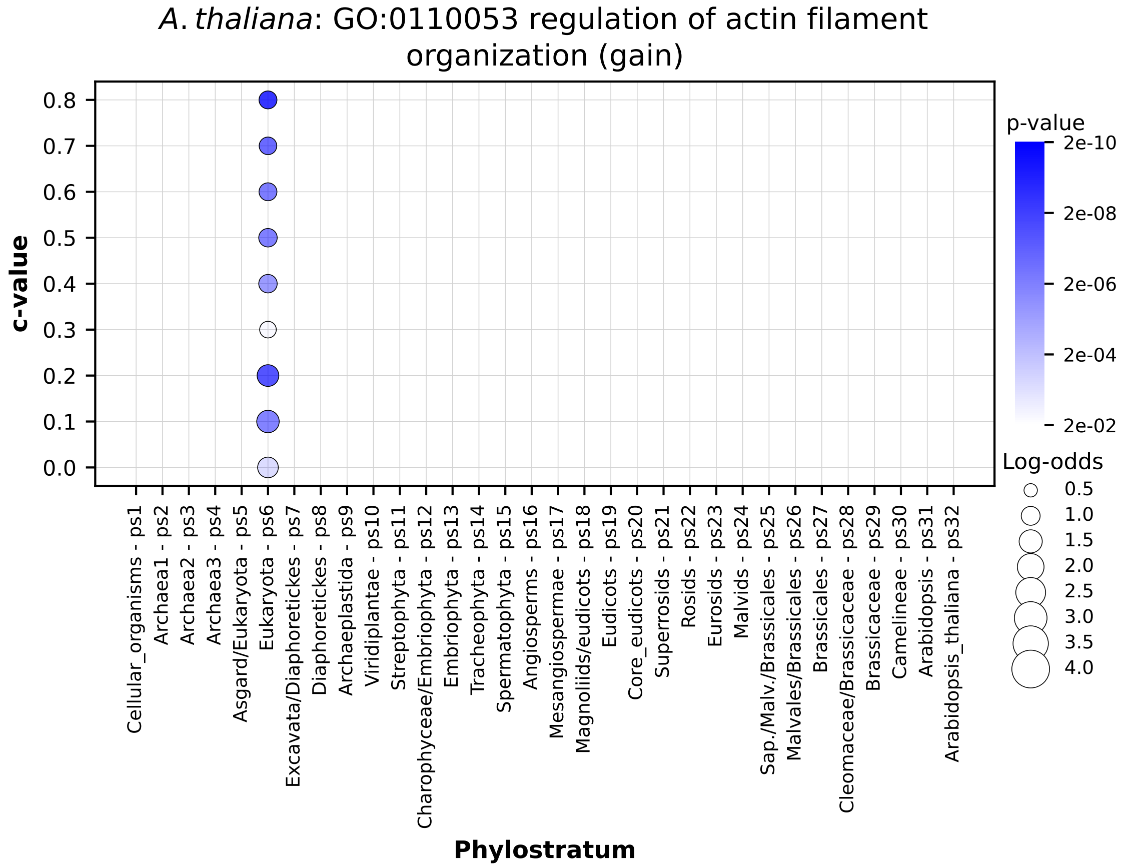
A. thaliana: GO:0110053 regulation of actin filament organization (gain)
c-value
0.8
0.7
0.6
0.5
0.4
0.3
0.2
0.1
0.0
Phylostratum
Cellular_organisms - ps1
Archaea1 - ps2
Archaea2 - ps3
Archaea3 - ps4
Asgard/Eukaryota - ps5
Eukaryota - ps6
Excavata/Diaphoretickes - ps7
Diaphoretickes - ps8
Archaeplastida - ps9
Viridiplantae - ps10
Streptophyta - ps11
Charophyceae/Embriophyta - ps12
Embriophyta - ps13
Tracheophyta - ps14
Spermatophyta - ps15
Angiosperms - ps16
Mesangiospermae - ps17
Magnoliids/eudicots - ps18
Eudicots - ps19
Core_eudicots - ps20
Superrosids - ps21
Rosids - ps22
Eurosids - ps23
Malvids - ps24
Sap./Malv./Brassicales - ps25
Malvales/Brassicales - ps26
Brassicales - ps27
Cleomaceae/Brassicaceae - ps28
Brassicaceae - ps29
Camelineae - ps30
Arabidopsis - ps31
Arabidopsis_thaliana - ps32
p-value
2e-10
2e-08
2e-06
2e-04
2e-02
Log-odds
0.5
1.0
1.5
2.0
2.5
3.0
3.5
4.0

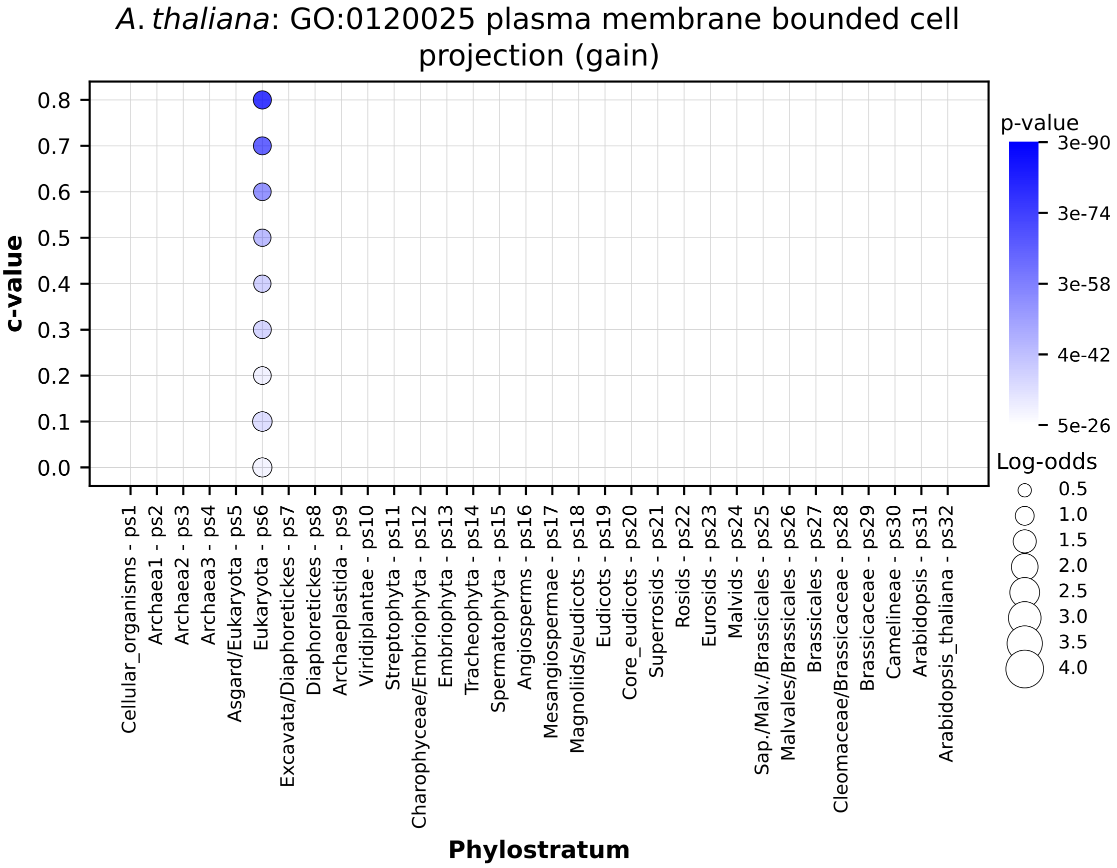
A. thaliana: GO:0120025 plasma membrane bounded cell projection (gain)
c-value
0.8
0.7
0.6
0.5
0.4
0.3
0.2
0.1
0.0
Phylostratum
Cellular_organisms - ps1
Archaea1 - ps2
Archaea2 - ps3
Archaea3 - ps4
Asgard/Eukaryota - ps5
Eukaryota - ps6
Excavata/Diaphoretickes - ps7
Diaphoretickes - ps8
Archaeplastida - ps9
Viridiplantae - ps10
Streptophyta - ps11
Charophyceae/Embriophyta - ps12
Embriophyta - ps13
Tracheophyta - ps14
Spermatophyta - ps15
Angiosperms - ps16
Mesangiospermae - ps17
Magnoliids/eudicots - ps18
Eudicots - ps19
Core_eudicots - ps20
Superrosids - ps21
Rosids - ps22
Eurosids - ps23
Malvids - ps24
Sap./Malv./Brassicales - ps25
Malvales/Brassicales - ps26
Brassicales - ps27
Cleomaceae/Brassicaceae - ps28
Brassicaceae - ps29
Camelineae - ps30
Arabidopsis - ps31
Arabidopsis_thaliana - ps32
p-value
3e-90
3e-74
3e-58
4e-42
5e-26
Log-odds
0.5
1.0
1.5
2.0
2.5
3.0
3.5
4.0

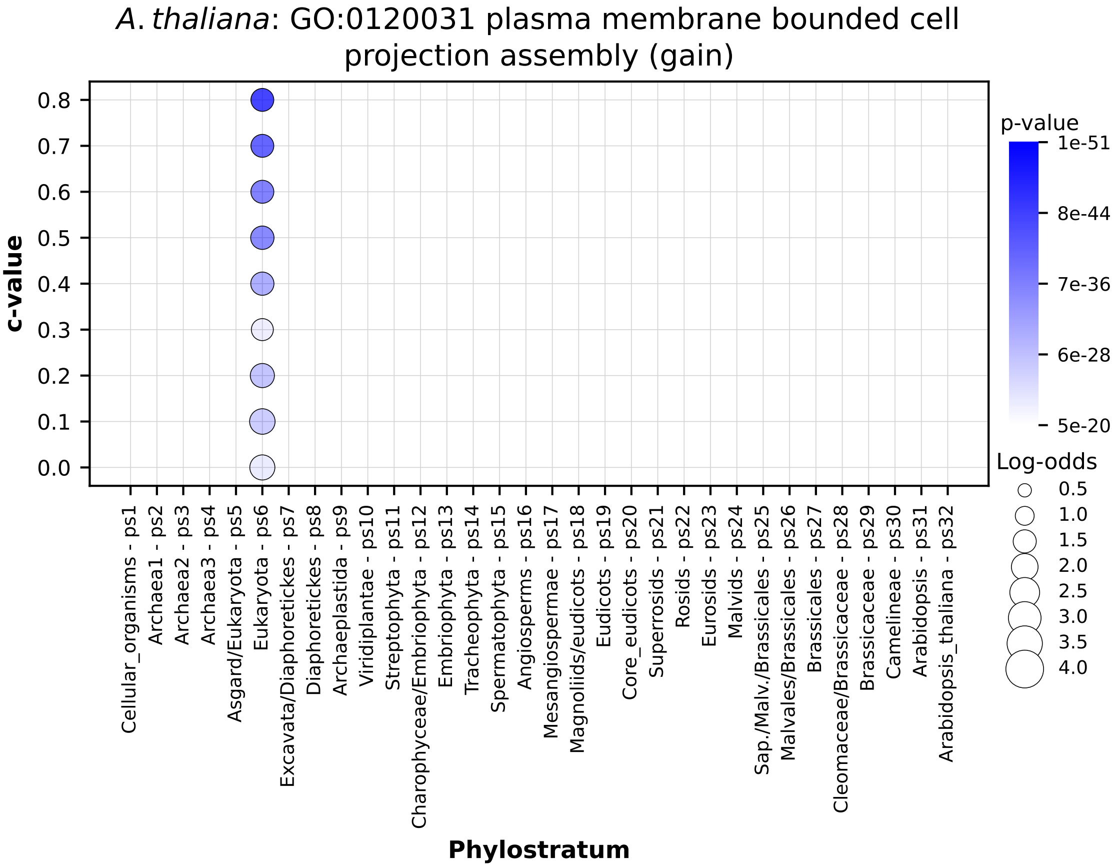
A. thaliana: GO:0120031 plasma membrane bounded cell projection assembly (gain)
c-value
0.8
0.7
0.6
0.5
0.4
0.3
0.2
0.1
0.0
p-value
1e-51
8e-44
7e-36
6e-28
5e-20
Log-odds
0.5
1.0
1.5
2.0
2.5
3.0
3.5
4.0
Cellular_organisms - ps1
Archaea1 - ps2
Archaea2 - ps3
Archaea3 - ps4
Asgard/Eukaryota - ps5
Eukaryota - ps6
Excavata/Diaphoretickes - ps7
Diaphoretickes - ps8
Archaeplastida - ps9
Viridiplantae - ps10
Streptophyta - ps11
Charophyceae/Embriophyta - ps12
Embriophyta - ps13
Tracheophyta - ps14
Spermatophyta - ps15
Angiosperms - ps16
Mesangiospermae - ps17
Magnoliids/eudicots - ps18
Eudicots - ps19
Core_eudicots - ps20
Superrosids - ps21
Rosids - ps22
Eurosids - ps23
Malvids - ps24
Sap./Malv./Brassicales - ps25
Malvales/Brassicales - ps26
Brassicales - ps27
Cleomaceae/Brassicaceae - ps28
Brassicaceae - ps29
Camelineae - ps30
Arabidopsis - ps31
Arabidopsis_thaliana - ps32
Phylostratum

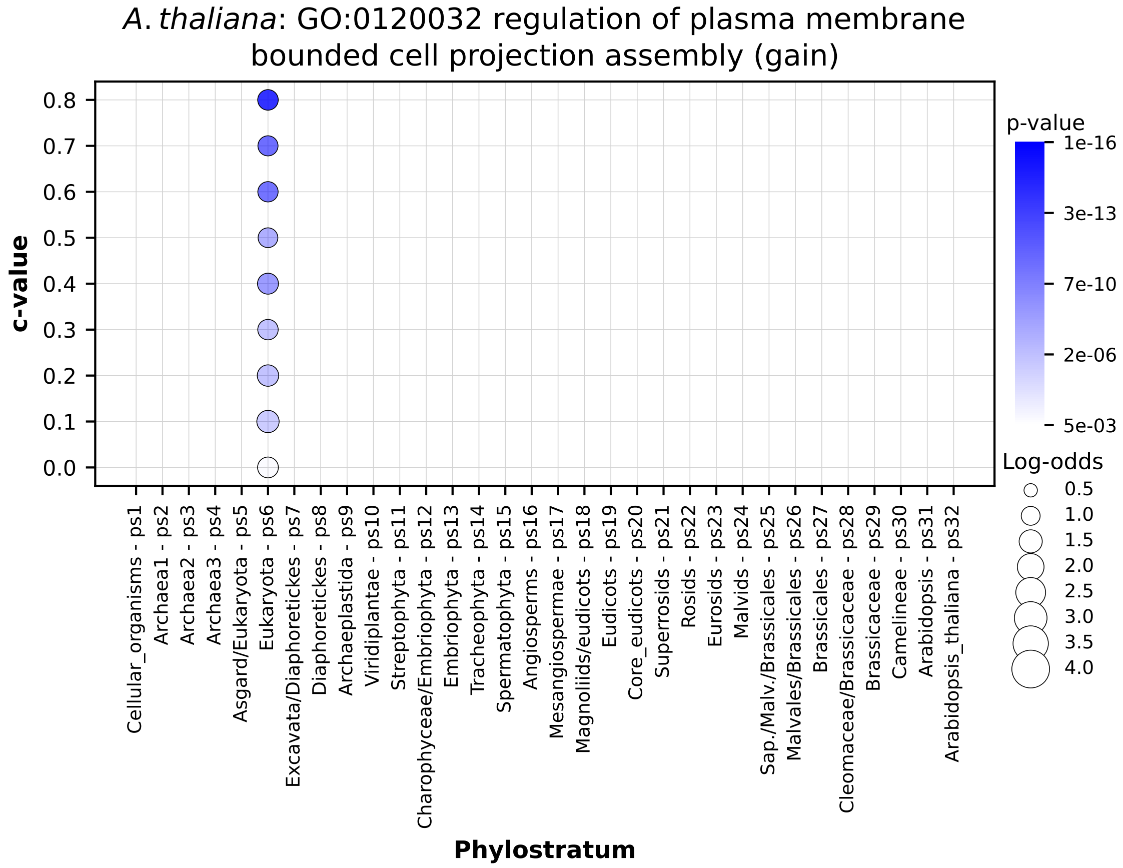
A. thaliana: GO:0120032 regulation of plasma membrane bounded cell projection assembly (gain)
c-value
0.8
0.7
0.6
0.5
0.4
0.3
0.2
0.1
0.0
p-value
1e-16
3e-13
7e-10
2e-06
5e-03
Log-odds
0.5
1.0
1.5
2.0
2.5
3.0
3.5
4.0
Cellular_organisms - ps1
Archaea1 - ps2
Archaea2 - ps3
Archaea3 - ps4
Asgard/Eukaryota - ps5
Eukaryota - ps6
Excavata/Diaphoretickes - ps7
Diaphoretickes - ps8
Archaeplastida - ps9
Viridiplantae - ps10
Streptophyta - ps11
Charophyceae/Embriophyta - ps12
Embriophyta - ps13
Tracheophyta - ps14
Spermatophyta - ps15
Angiosperms - ps16
Mesangiospermae - ps17
Magnoliids/eudicots - ps18
Eudicots - ps19
Core_eudicots - ps20
Superrosids - ps21
Rosids - ps22
Eurosids - ps23
Malvids - ps24
Sap./Malv./Brassicales - ps25
Malvales/Brassicales - ps26
Brassicales - ps27
Cleomaceae/Brassicaceae - ps28
Brassicaceae - ps29
Camelineae - ps30
Arabidopsis - ps31
Arabidopsis_thaliana - ps32
Phylostratum

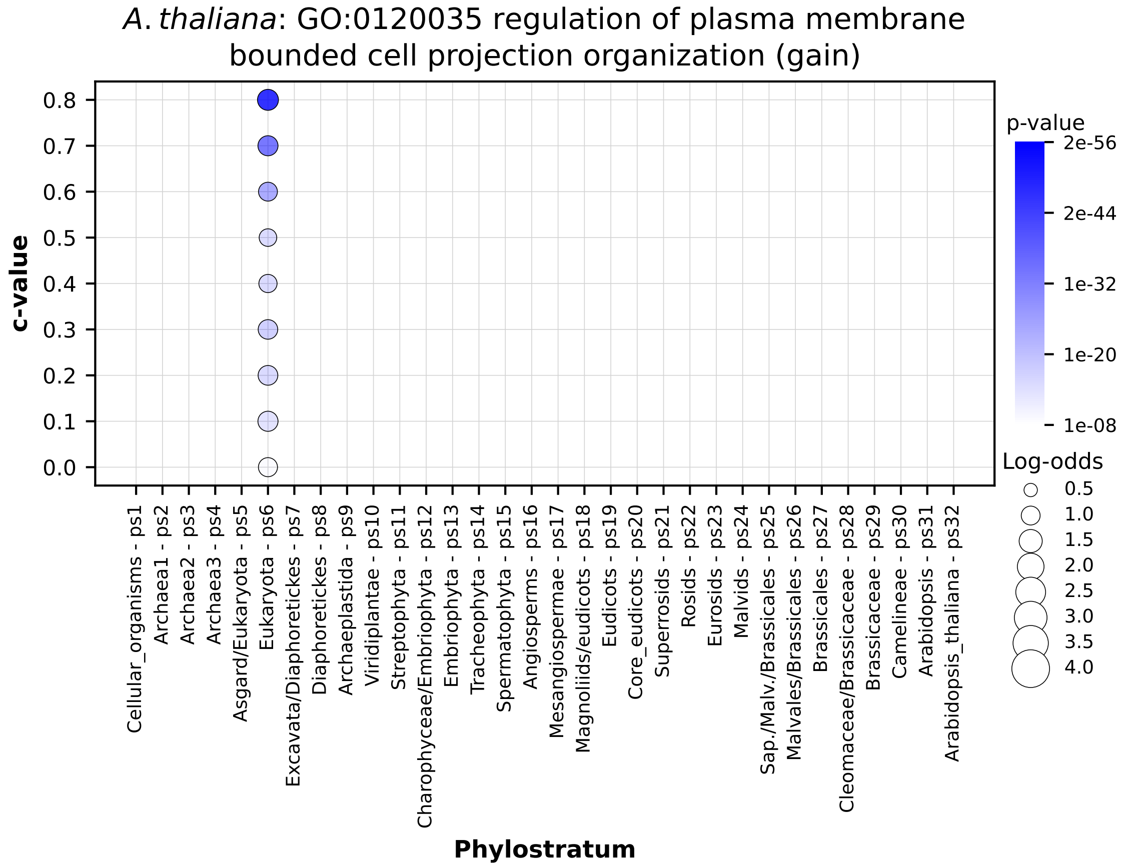
A. thaliana: GO:0120035 regulation of plasma membrane bounded cell projection organization (gain)
c-value
0.8
0.7
0.6
0.5
0.4
0.3
0.2
0.1
0.0
p-value
2e-56
2e-44
1e-32
1e-20
1e-08
Log-odds
0.5
1.0
1.5
2.0
2.5
3.0
3.5
4.0
Cellular_organisms - ps1
Archaea1 - ps2
Archaea2 - ps3
Archaea3 - ps4
Asgard/Eukaryota - ps5
Eukaryota - ps6
Excavata/Diaphoretickes - ps7
Diaphoretickes - ps8
Archaeplastida - ps9
Viridiplantae - ps10
Streptophyta - ps11
Charophyceae/Embriophyta - ps12
Embriophyta - ps13
Tracheophyta - ps14
Spermatophyta - ps15
Angiosperms - ps16
Mesangiospermae - ps17
Magnoliids/eudicots - ps18
Eudicots - ps19
Core_eudicots - ps20
Superrosids - ps21
Rosids - ps22
Eurosids - ps23
Malvids - ps24
Sap./Malv./Brassicales - ps25
Malvales/Brassicales - ps26
Brassicales - ps27
Cleomaceae/Brassicaceae - ps28
Brassicaceae - ps29
Camelineae - ps30
Arabidopsis - ps31
Arabidopsis_thaliana - ps32
Phylostratum

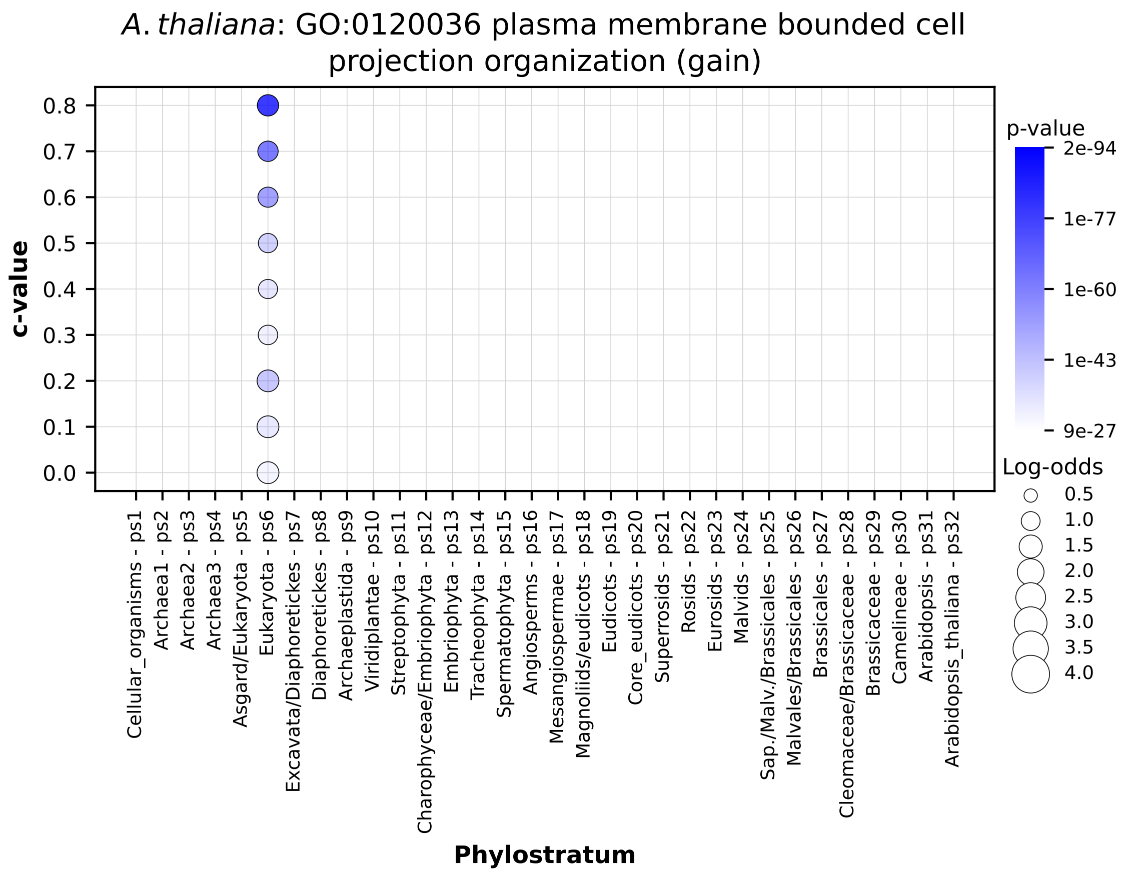

A. thaliana: GO:0120036 plasma membrane bounded cell projection organization (gain)
c-value
0.0
0.1
0.2
0.3
0.4
0.5
0.6
0.7
0.8
Phylostratum
Cellular_organisms - ps1
Archaea1 - ps2
Archaea2 - ps3
Archaea3 - ps4
Asgard/Eukaryota - ps5
Eukaryota - ps6
Excavata/Diaphoretickes - ps7
Diaphoretickes - ps8
Archaeplastida - ps9
Viridiplantae - ps10
Streptophyta - ps11
Charophyceae/Embriophyta - ps12
Embriophyta - ps13
Tracheophyta - ps14
Spermatophyta - ps15
Angiosperms - ps16
Mesangiospermae - ps17
Magnoliids/eudicots - ps18
Eudicots - ps19
Core_eudicots - ps20
Superrosids - ps21
Rosids - ps22
Eurosids - ps23
Malvids - ps24
Sap./Malv./Brassicales - ps25
Malvales/Brassicales - ps26
Brassicales - ps27
Cleomaceae/Brassicaceae - ps28
Brassicaceae - ps29
Camelineae - ps30
Arabidopsis - ps31
Arabidopsis_thaliana - ps32
p-value
2e-94
1e-77
1e-60
1e-43
9e-27
Log-odds
0.5
1.0
1.5
2.0
2.5
3.0
3.5
4.0

A. thaliana: GO:0120038 obsolete plasma membrane bounded cell projection part (gain)

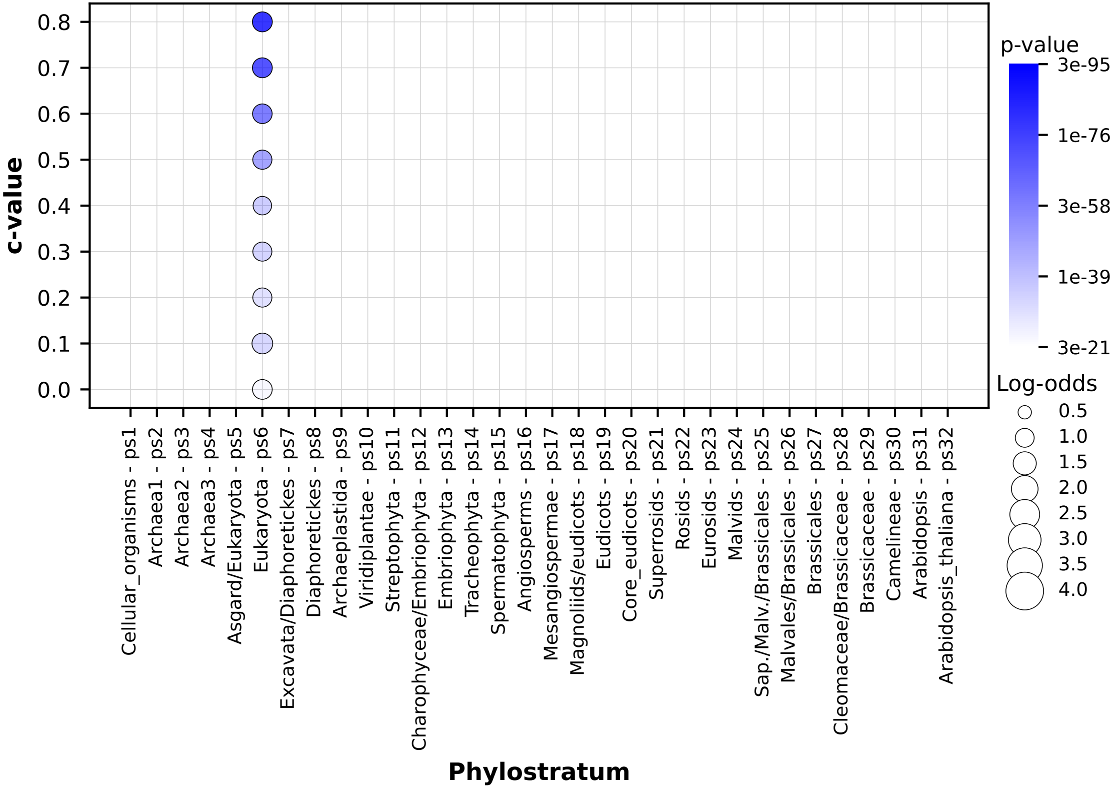

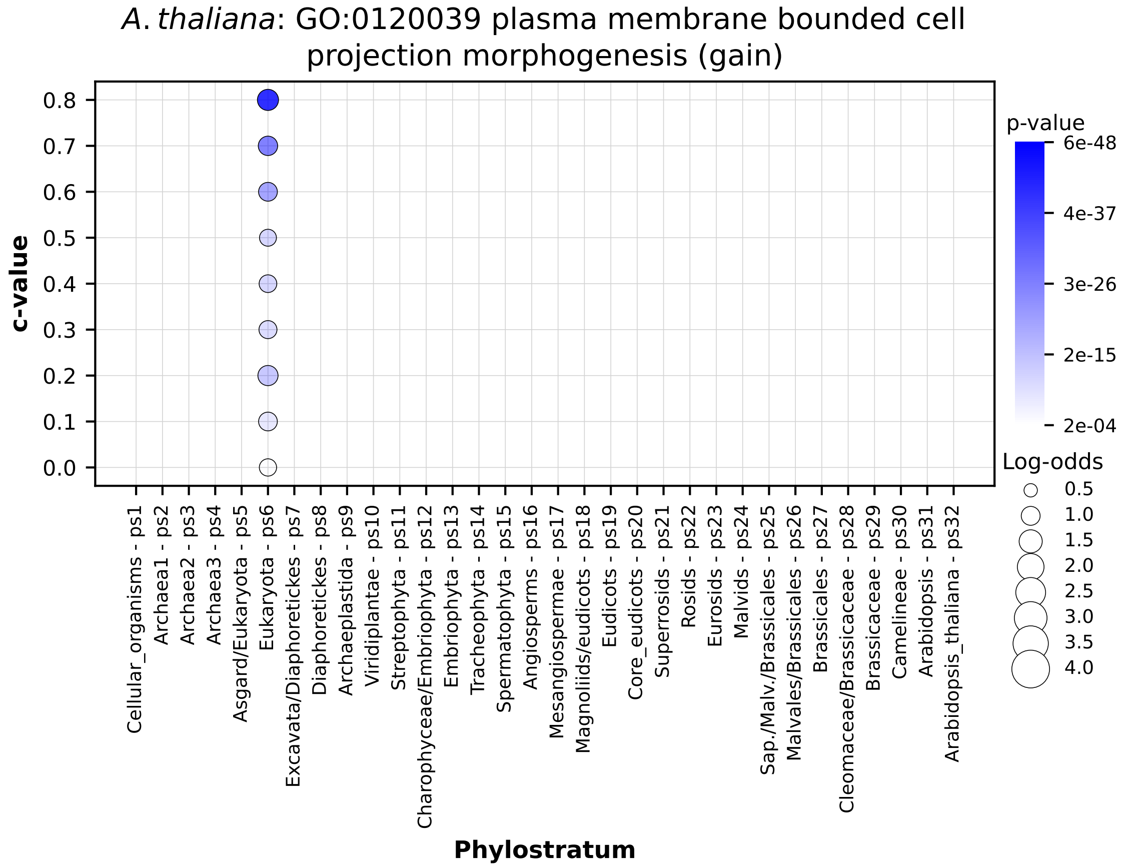

A. thaliana: GO:0120039 plasma membrane bounded cell projection morphogenesis (gain)
c-value
0.8
0.7
0.6
0.5
0.4
0.3
0.2
0.1
0.0
p-value
6e-48
4e-37
3e-26
2e-15
2e-04
Log-odds
0.5
1.0
1.5
2.0
2.5
3.0
3.5
4.0
Cellular_organisms - ps1
Archaea1 - ps2
Archaea2 - ps3
Archaea3 - ps4
Asgard/Eukaryota - ps5
Eukaryota - ps6
Excavata/Diaphoretickes - ps7
Diaphoretickes - ps8
Archaeplastida - ps9
Viridiplantae - ps10
Streptophyta - ps11
Charophyceae/Embriophyta - ps12
Embriophyta - ps13
Tracheophyta - ps14
Spermatophyta - ps15
Angiosperms - ps16
Mesangiospermae - ps17
Magnoliids/eudicots - ps18
Eudicots - ps19
Core_eudicots - ps20
Superrosids - ps21
Rosids - ps22
Eurosids - ps23
Malvids - ps24
Sap./Malv./Brassicales - ps25
Malvales/Brassicales - ps26
Brassicales - ps27
Cleomaceae/Brassicaceae - ps28
Brassicaceae - ps29
Camelineae - ps30
Arabidopsis - ps31
Arabidopsis_thaliana - ps32
Phylostratum

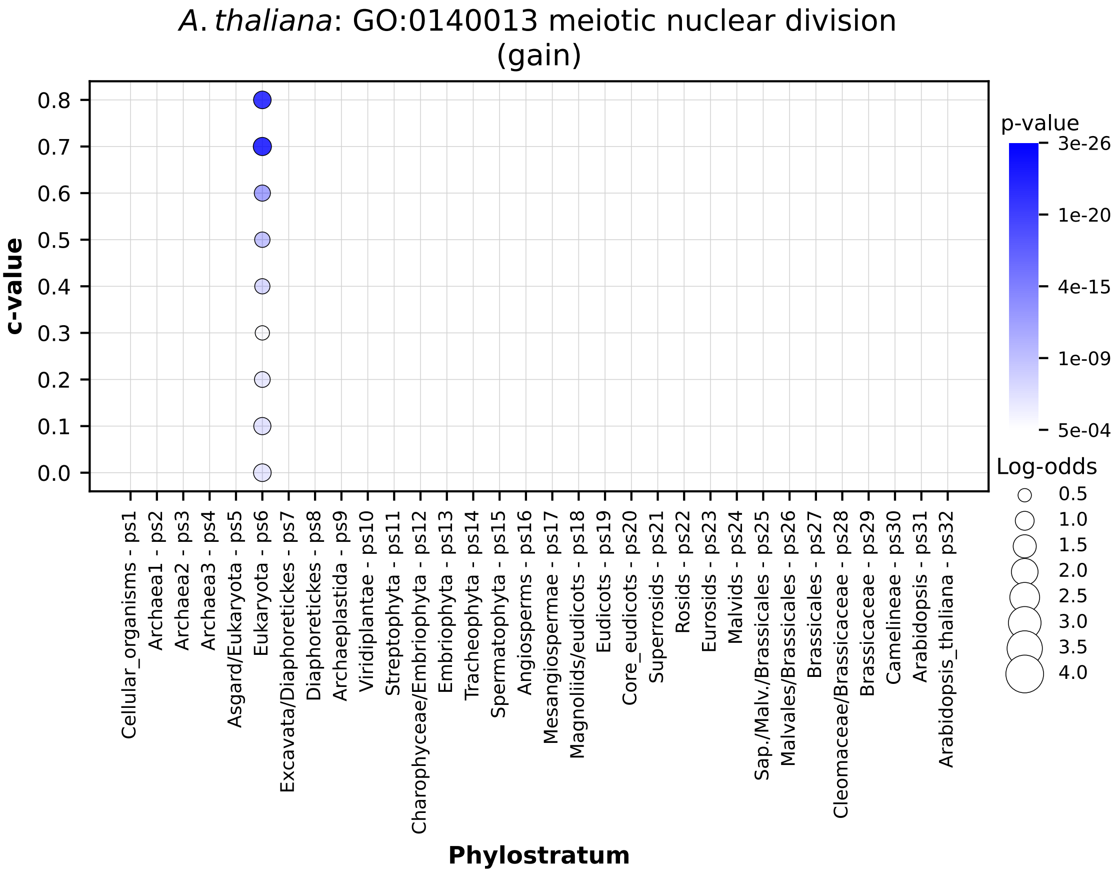

A. thaliana: GO:0140013 meiotic nuclear division
(gain)
c-value
0.8
0.7
0.6
0.5
0.4
0.3
0.2
0.1
0.0
p-value
3e-26
1e-20
4e-15
1e-09
5e-04
Log-odds
0.5
1.0
1.5
2.0
2.5
3.0
3.5
4.0
Cellular_organisms - ps1
Archaea1 - ps2
Archaea2 - ps3
Archaea3 - ps4
Asgard/Eukaryota - ps5
Eukaryota - ps6
Excavata/Diaphoretickes - ps7
Diaphoretickes - ps8
Archaeplastida - ps9
Viridiplantae - ps10
Streptophyta - ps11
Charophyceae/Embriophyta - ps12
Embriophyta - ps13
Tracheophyta - ps14
Spermatophyta - ps15
Angiosperms - ps16
Mesangiospermae - ps17
Magnoliids/eudicots - ps18
Eudicots - ps19
Core_eudicots - ps20
Superrosids - ps21
Rosids - ps22
Eurosids - ps23
Malvids - ps24
Sap./Malv./Brassicales - ps25
Malvales/Brassicales - ps26
Brassicales - ps27
Cleomaceae/Brassicaceae - ps28
Brassicaceae - ps29
Camelineae - ps30
Arabidopsis - ps31
Arabidopsis_thaliana - ps32
Phylostratum

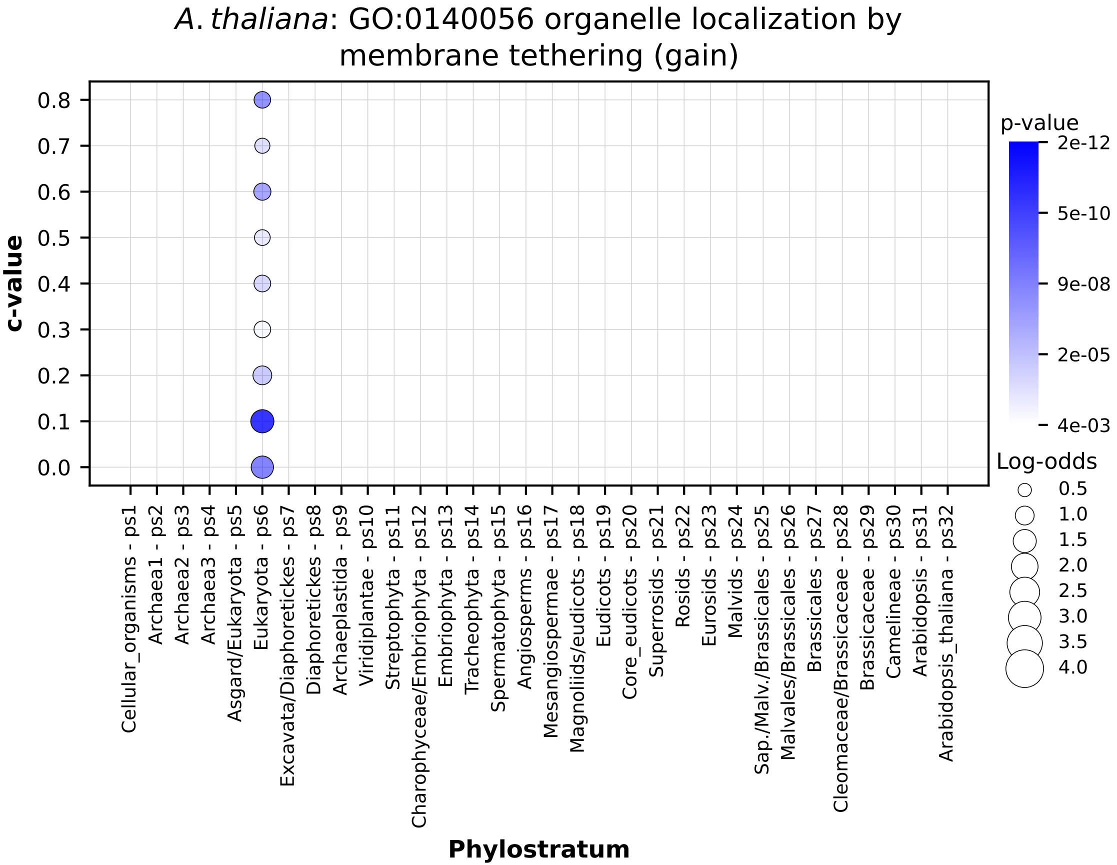
A. thaliana: GO:0140056 organelle localization by membrane tethering (gain)
c-value
0.0
0.1
0.2
0.3
0.4
0.5
0.6
0.7
0.8
Phylostratum
Cellular_organisms - ps1
Archaea1 - ps2
Archaea2 - ps3
Archaea3 - ps4
Asgard/Eukaryota - ps5
Eukaryota - ps6
Excavata/Diaphoretickes - ps7
Diaphoretickes - ps8
Archaeplastida - ps9
Viridiplantae - ps10
Streptophyta - ps11
Charophyceae/Embriophyta - ps12
Embriophyta - ps13
Tracheophyta - ps14
Spermatophyta - ps15
Angiosperms - ps16
Mesangiospermae - ps17
Magnoliids/eudicots - ps18
Eudicots - ps19
Core_eudicots - ps20
Superrosids - ps21
Rosids - ps22
Eurosids - ps23
Malvids - ps24
Sap./Malv./Brassicales - ps25
Malvales/Brassicales - ps26
Brassicales - ps27
Cleomaceae/Brassicaceae - ps28
Brassicaceae - ps29
Camelineae - ps30
Arabidopsis - ps31
Arabidopsis_thaliana - ps32
p-value
2e-12
5e-10
9e-08
2e-05
4e-03
Log-odds
0.5
1.0
1.5
2.0
2.5
3.0
3.5
4.0

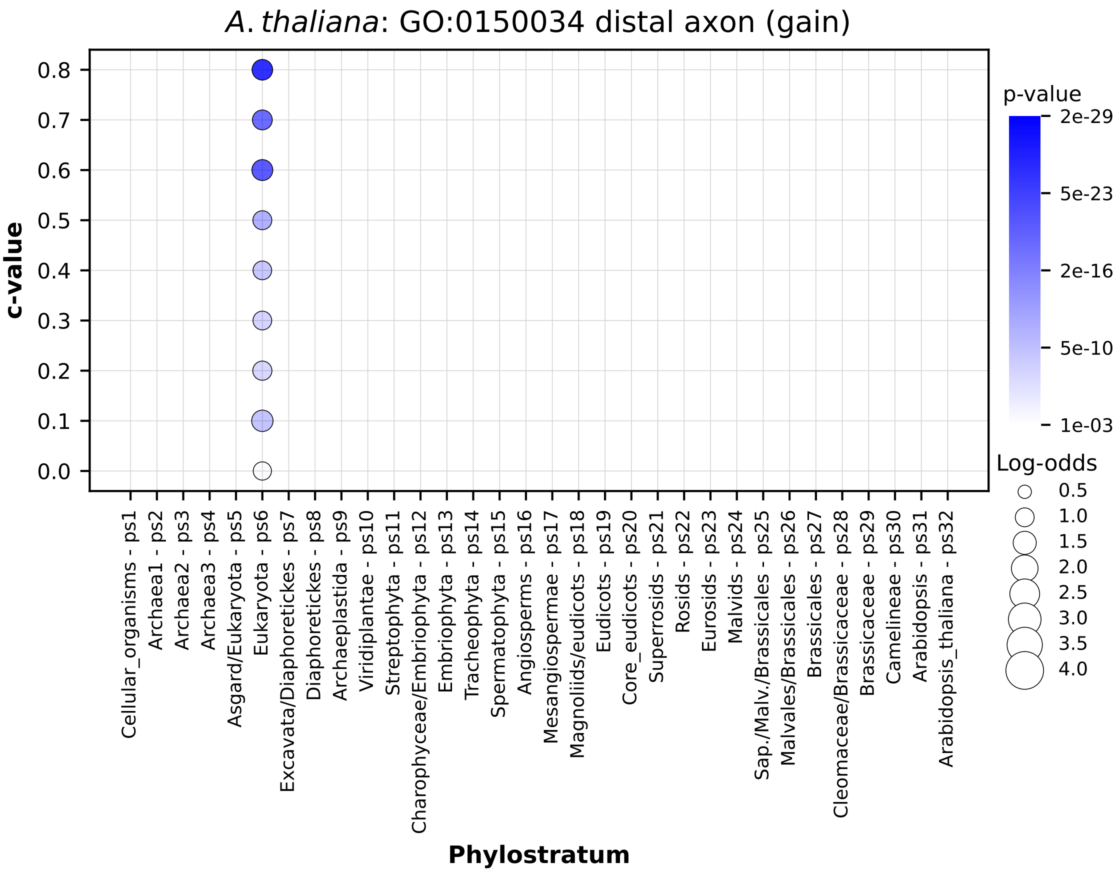
A. thaliana: GO:0150034 distal axon (gain)
c-value
0.0
0.1
0.2
0.3
0.4
0.5
0.6
0.7
0.8
Phylostratum
Cellular_organisms - ps1
Archaea1 - ps2
Archaea2 - ps3
Archaea3 - ps4
Asgard/Eukaryota - ps5
Eukaryota - ps6
Excavata/Diaphoretickes - ps7
Diaphoretickes - ps8
Archaeplastida - ps9
Viridiplantae - ps10
Streptophyta - ps11
Charophyceae/Embriophyta - ps12
Embriophyta - ps13
Tracheophyta - ps14
Spermatophyta - ps15
Angiosperms - ps16
Mesangiospermae - ps17
Magnoliids/eudicots - ps18
Eudicots - ps19
Core_eudicots - ps20
Superrosids - ps21
Rosids - ps22
Eurosids - ps23
Malvids - ps24
Sap./Malv./Brassicales - ps25
Malvales/Brassicales - ps26
Brassicales - ps27
Cleomaceae/Brassicaceae - ps28
Brassicaceae - ps29
Camelineae - ps30
Arabidopsis - ps31
Arabidopsis_thaliana - ps32
p-value
2e-29
5e-23
2e-16
5e-10
1e-03
Log-odds
0.5
1.0
1.5
2.0
2.5
3.0
3.5
4.0

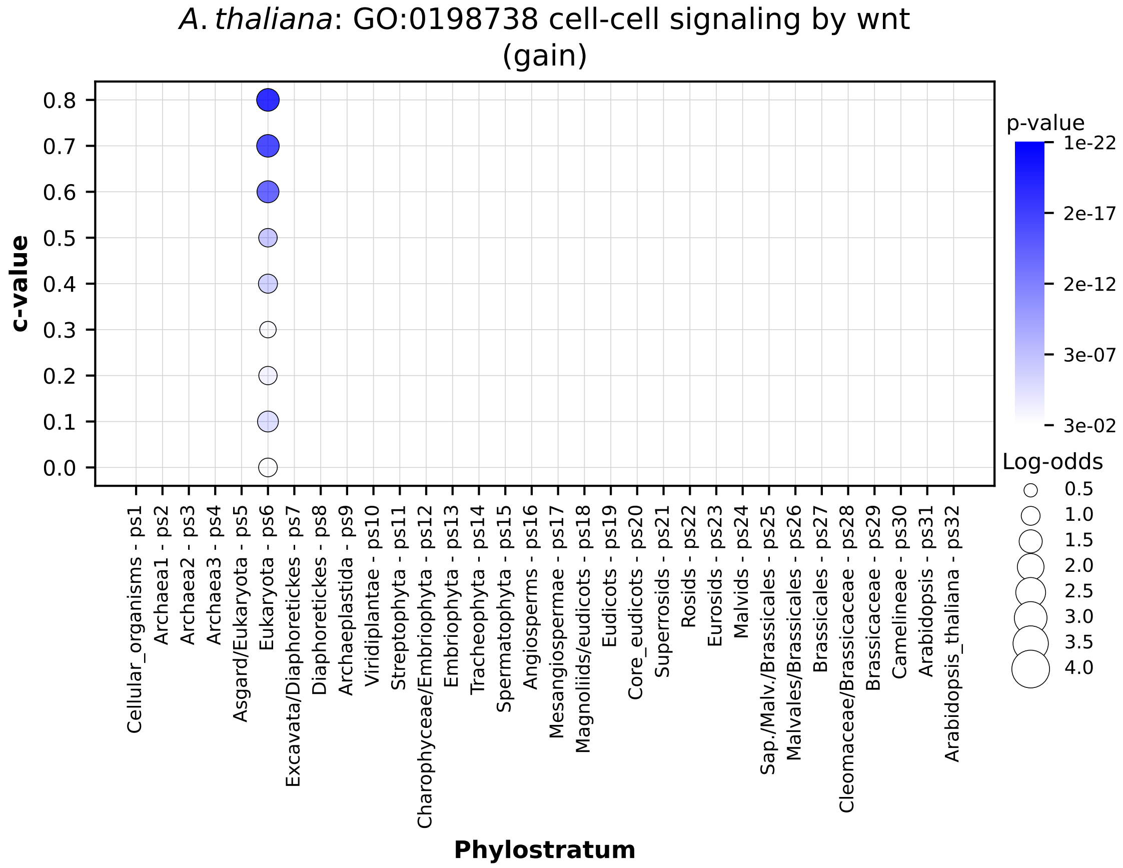
A. thaliana: GO:0198738 cell-cell signaling by wnt
(gain)
c-value
0.8
0.7
0.6
0.5
0.4
0.3
0.2
0.1
0.0
p-value
1e-22
2e-17
2e-12
3e-07
3e-02
Log-odds
0.5
1.0
1.5
2.0
2.5
3.0
3.5
4.0
Cellular_organisms - ps1
Archaea1 - ps2
Archaea2 - ps3
Archaea3 - ps4
Asgard/Eukaryota - ps5
Eukaryota - ps6
Excavata/Diaphoretickes - ps7
Diaphoretickes - ps8
Archaeplastida - ps9
Viridiplantae - ps10
Streptophyta - ps11
Charophyceae/Embriophyta - ps12
Embriophyta - ps13
Tracheophyta - ps14
Spermatophyta - ps15
Angiosperms - ps16
Mesangiospermae - ps17
Magnoliids/eudicots - ps18
Eudicots - ps19
Core_eudicots - ps20
Superrosids - ps21
Rosids - ps22
Eurosids - ps23
Malvids - ps24
Sap./Malv./Brassicales - ps25
Malvales/Brassicales - ps26
Brassicales - ps27
Cleomaceae/Brassicaceae - ps28
Brassicaceae - ps29
Camelineae - ps30
Arabidopsis - ps31
Arabidopsis_thaliana - ps32
Phylostratum

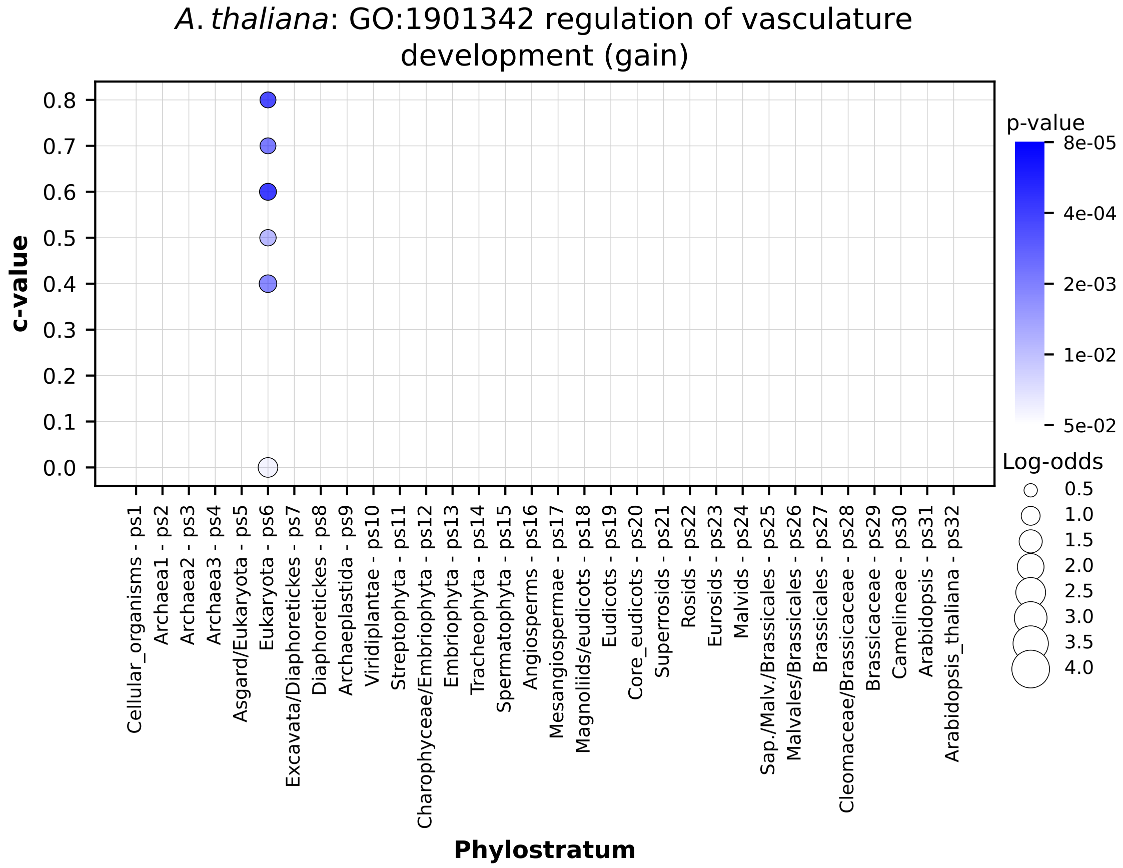
A. thaliana: GO:1901342 regulation of vasculature development (gain)
c-value
0.8
0.7
0.6
0.5
0.4
0.3
0.2
0.1
0.0
Phylostratum
Cellular_organisms - ps1
Archaea1 - ps2
Archaea2 - ps3
Archaea3 - ps4
Asgard/Eukaryota - ps5
Eukaryota - ps6
Excavata/Diaphoretickes - ps7
Diaphoretickes - ps8
Archaeplastida - ps9
Viridiplantae - ps10
Streptophyta - ps11
Charophyceae/Embriophyta - ps12
Embriophyta - ps13
Tracheophyta - ps14
Spermatophyta - ps15
Angiosperms - ps16
Mesangiospermae - ps17
Magnoliids/eudicots - ps18
Eudicots - ps19
Core_eudicots - ps20
Superrosids - ps21
Rosids - ps22
Eurosids - ps23
Malvids - ps24
Sap./Malv./Brassicales - ps25
Malvales/Brassicales - ps26
Brassicales - ps27
Cleomaceae/Brassicaceae - ps28
Brassicaceae - ps29
Camelineae - ps30
Arabidopsis - ps31
Arabidopsis_thaliana - ps32
p-value
8e-05
4e-04
2e-03
1e-02
5e-02
Log-odds
0.5
1.0
1.5
2.0
2.5
3.0
3.5
4.0

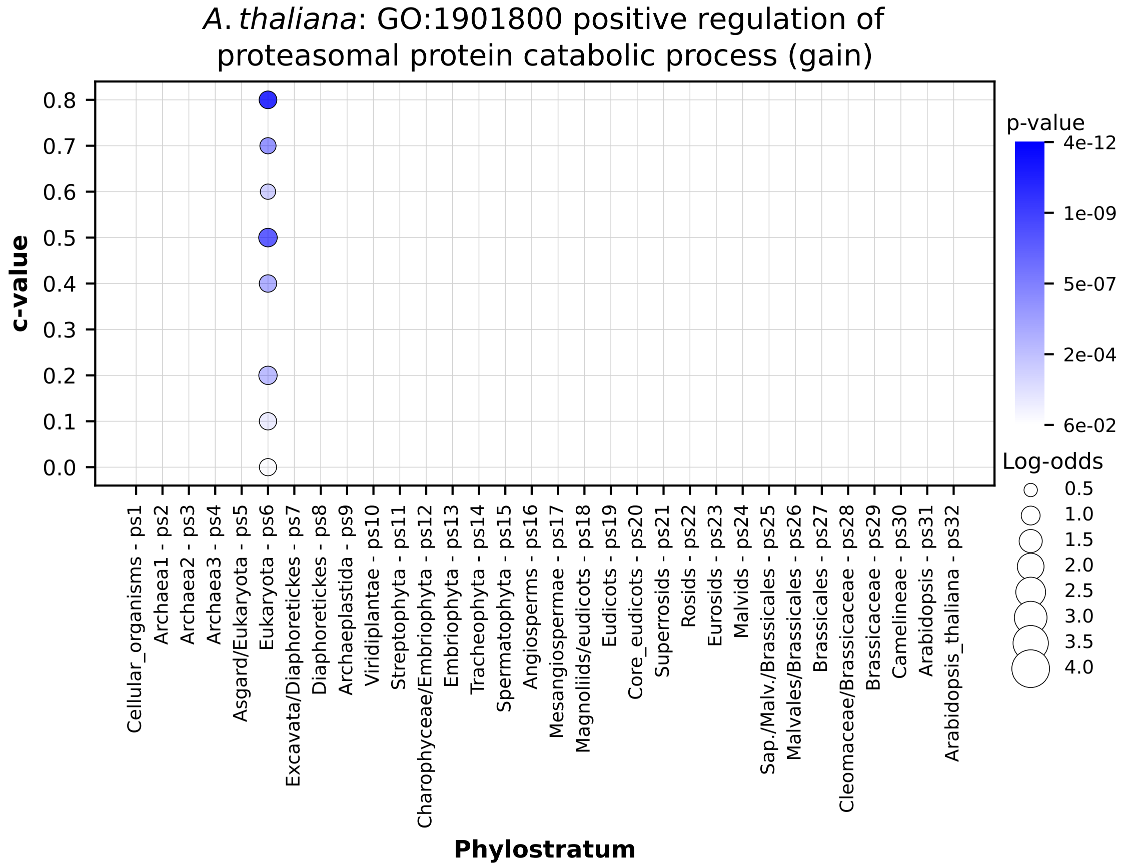

A. thaliana: GO:1901800 positive regulation of proteasomal protein catabolic process (gain)
c-value
0.8
0.7
0.6
0.5
0.4
0.3
0.2
0.1
0.0
p-value
4e-12
1e-09
5e-07
2e-04
6e-02
Log-odds
0.5
1.0
1.5
2.0
2.5
3.0
3.5
4.0
Cellular_organisms - ps1
Archaea1 - ps2
Archaea2 - ps3
Archaea3 - ps4
Asgard/Eukaryota - ps5
Eukaryota - ps6
Excavata/Diaphoretickes - ps7
Diaphoretickes - ps8
Archaeplastida - ps9
Viridiplantae - ps10
Streptophyta - ps11
Charophyceae/Embriophyta - ps12
Embriophyta - ps13
Tracheophyta - ps14
Spermatophyta - ps15
Angiosperms - ps16
Mesangiospermae - ps17
Magnoliids/eudicots - ps18
Eudicots - ps19
Core_eudicots - ps20
Superrosids - ps21
Rosids - ps22
Eurosids - ps23
Malvids - ps24
Sap./Malv./Brassicales - ps25
Malvales/Brassicales - ps26
Brassicales - ps27
Cleomaceae/Brassicaceae - ps28
Brassicaceae - ps29
Camelineae - ps30
Arabidopsis - ps31
Arabidopsis_thaliana - ps32
Phylostratum

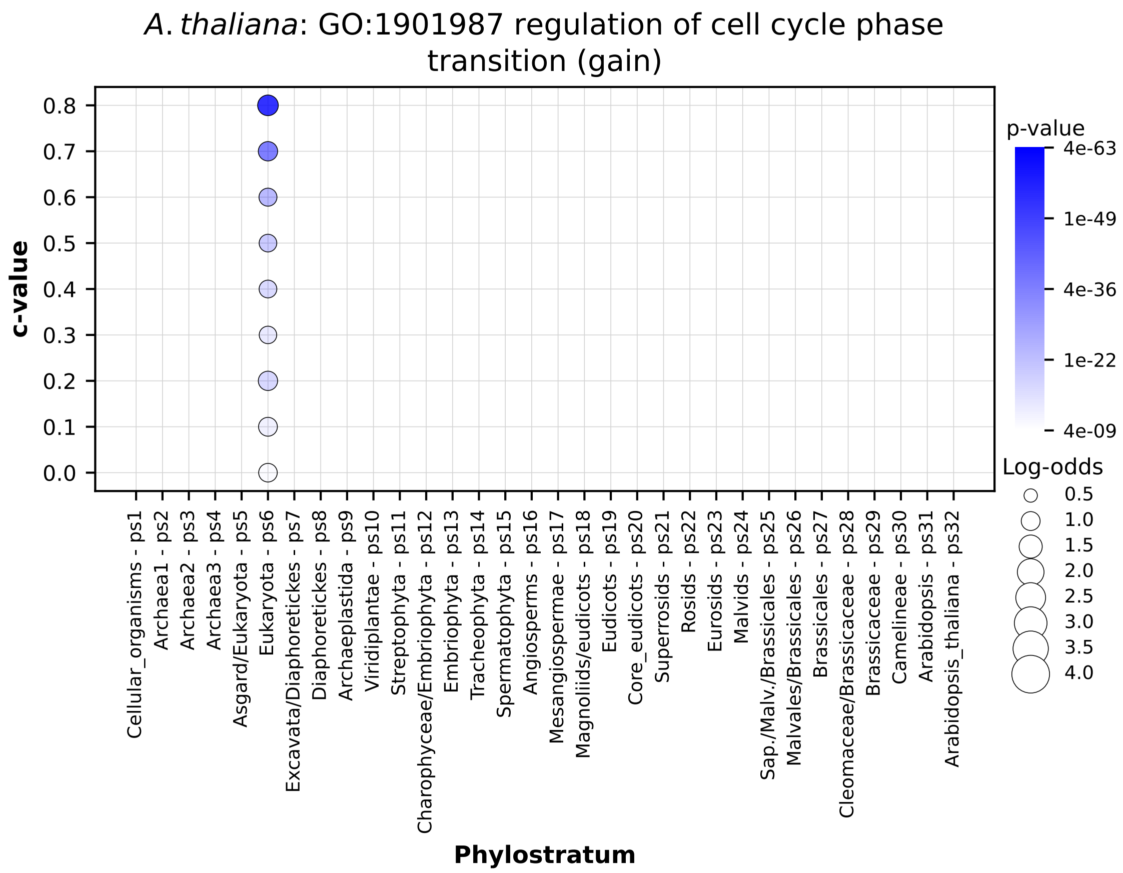
A. thaliana: GO:1901987 regulation of cell cycle phase transition (gain)
c-value
0.0
0.1
0.2
0.3
0.4
0.5
0.6
0.7
0.8
Phylostratum
Cellular_organisms - ps1
Archaea1 - ps2
Archaea2 - ps3
Archaea3 - ps4
Asgard/Eukaryota - ps5
Eukaryota - ps6
Excavata/Diaphoretickes - ps7
Diaphoretickes - ps8
Archaeplastida - ps9
Viridiplantae - ps10
Streptophyta - ps11
Charophyceae/Embriophyta - ps12
Embriophyta - ps13
Tracheophyta - ps14
Spermatophyta - ps15
Angiosperms - ps16
Mesangiospermae - ps17
Magnoliids/eudicots - ps18
Eudicots - ps19
Core_eudicots - ps20
Superrosids - ps21
Rosids - ps22
Eurosids - ps23
Malvids - ps24
Sap./Malv./Brassicales - ps25
Malvales/Brassicales - ps26
Brassicales - ps27
Cleomaceae/Brassicaceae - ps28
Brassicaceae - ps29
Camelineae - ps30
Arabidopsis - ps31
Arabidopsis_thaliana - ps32
p-value
4e-63
1e-49
4e-36
1e-22
4e-09
Log-odds
0.5
1.0
1.5
2.0
2.5
3.0
3.5
4.0

A. thaliana: GO:1901988 negative regulation of cell cycle phase transition (gain)

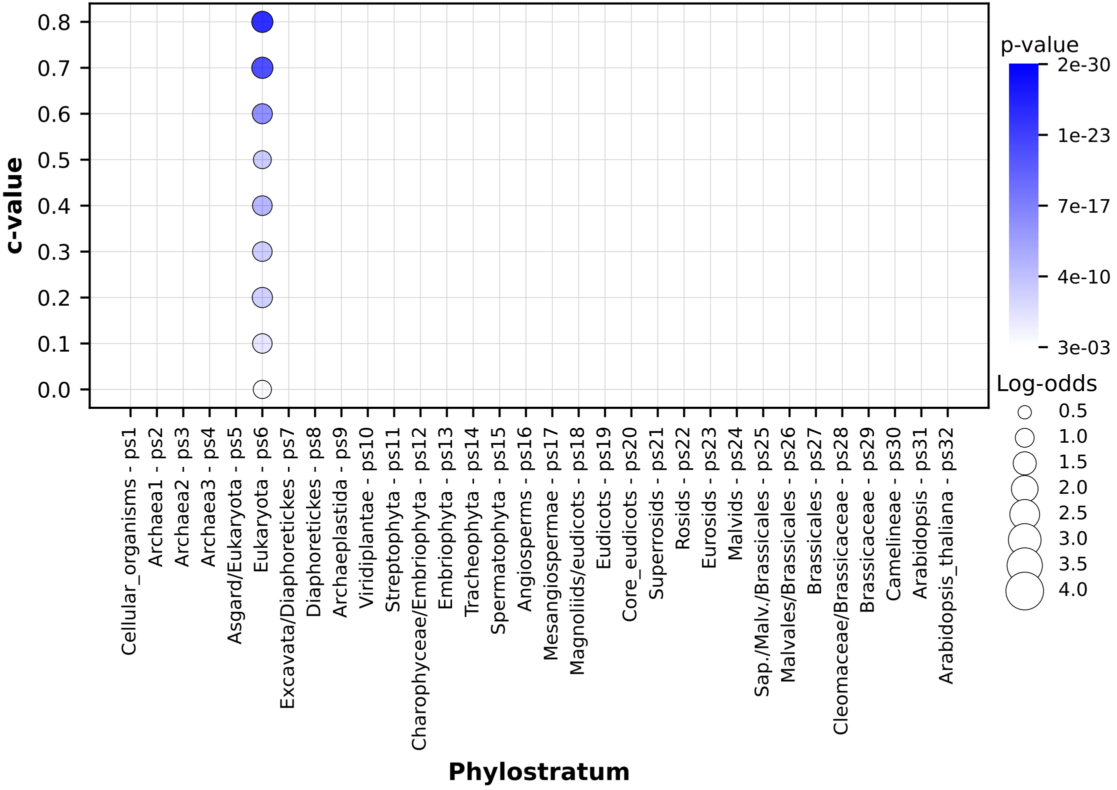

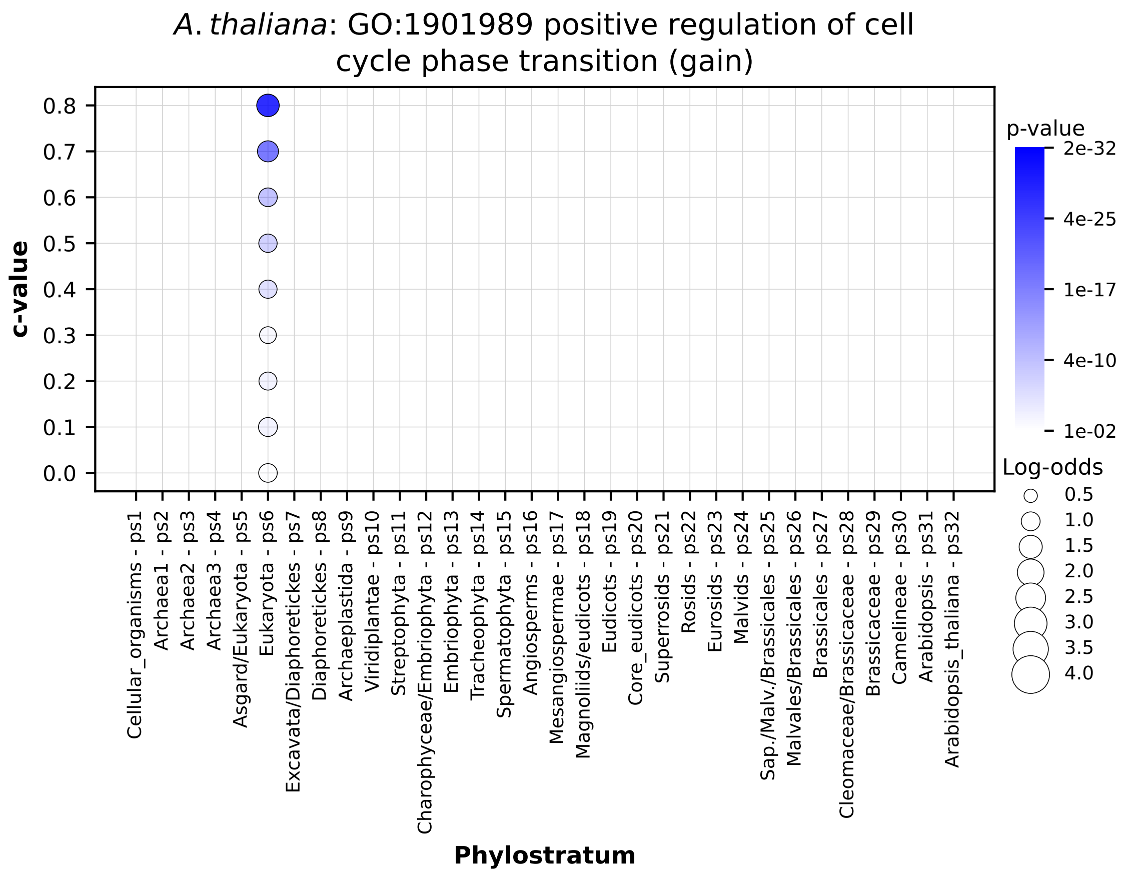

A. thaliana: GO:1901989 positive regulation of cell cycle phase transition (gain)
c-value
0.0
0.1
0.2
0.3
0.4
0.5
0.6
0.7
0.8
p-value
2e-32
4e-25
1e-17
4e-10
1e-02
Log-odds
0.5
1.0
1.5
2.0
2.5
3.0
3.5
4.0
Cellular_organisms - ps1
Archaea1 - ps2
Archaea2 - ps3
Archaea3 - ps4
Asgard/Eukaryota - ps5
Eukaryota - ps6
Excavata/Diaphoretickes - ps7
Diaphoretickes - ps8
Archaeplastida - ps9
Viridiplantae - ps10
Streptophyta - ps11
Charophyceae/Embriophyta - ps12
Embriophyta - ps13
Tracheophyta - ps14
Spermatophyta - ps15
Angiosperms - ps16
Mesangiospermae - ps17
Magnoliids/eudicots - ps18
Eudicots - ps19
Core_eudicots - ps20
Superrosids - ps21
Rosids - ps22
Eurosids - ps23
Malvids - ps24
Sap./Malv./Brassicales - ps25
Malvales/Brassicales - ps26
Brassicales - ps27
Cleomaceae/Brassicaceae - ps28
Brassicaceae - ps29
Camelineae - ps30
Arabidopsis - ps31
Arabidopsis_thaliana - ps32
Phylostratum

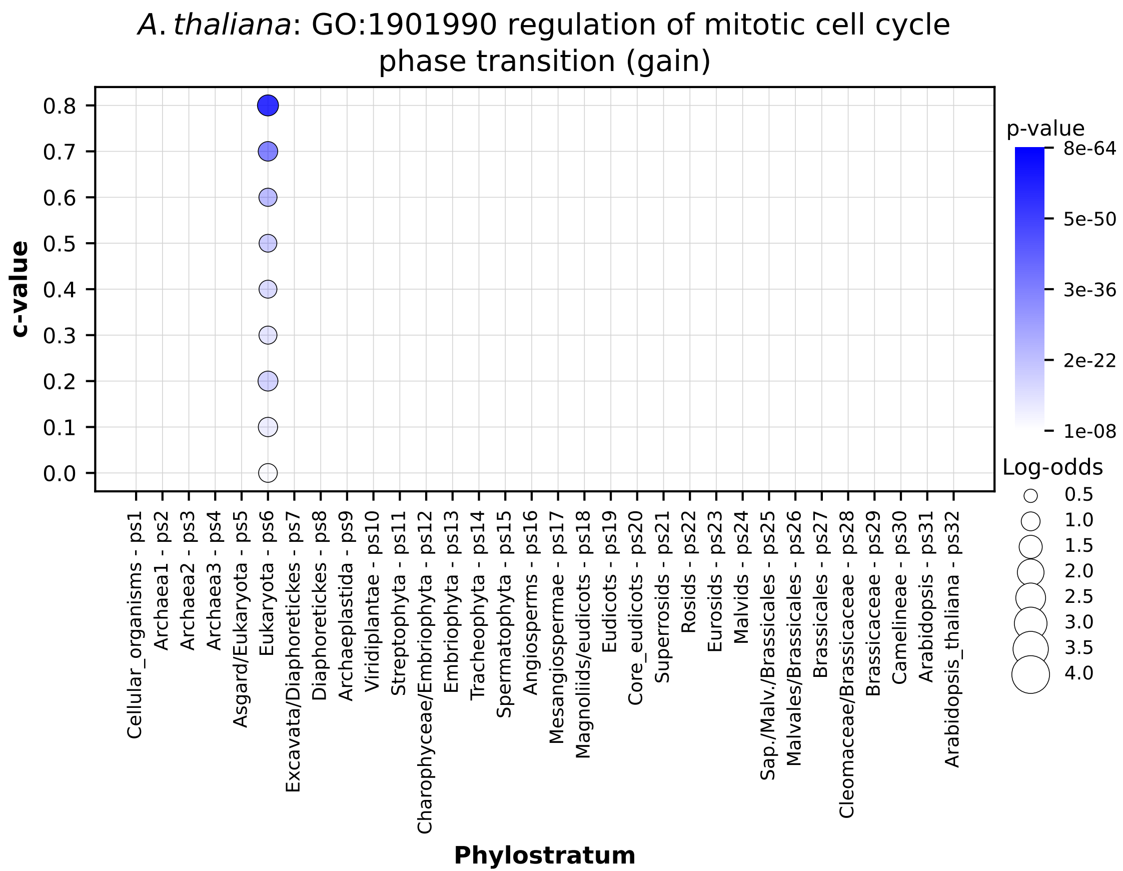
A. thaliana: GO:1901990 regulation of mitotic cell cycle phase transition (gain)
c-value
0.8
0.7
0.6
0.5
0.4
0.3
0.2
0.1
0.0
Phylostratum
Cellular_organisms - ps1
Archaea1 - ps2
Archaea2 - ps3
Archaea3 - ps4
Asgard/Eukaryota - ps5
Eukaryota - ps6
Excavata/Diaphoretickes - ps7
Diaphoretickes - ps8
Archaeplastida - ps9
Viridiplantae - ps10
Streptophyta - ps11
Charophyceae/Embriophyta - ps12
Embriophyta - ps13
Tracheophyta - ps14
Spermatophyta - ps15
Angiosperms - ps16
Mesangiospermae - ps17
Magnoliids/eudicots - ps18
Eudicots - ps19
Core_eudicots - ps20
Superrosids - ps21
Rosids - ps22
Eurosids - ps23
Malvids - ps24
Sap./Malv./Brassicales - ps25
Malvales/Brassicales - ps26
Brassicales - ps27
Cleomaceae/Brassicaceae - ps28
Brassicaceae - ps29
Camelineae - ps30
Arabidopsis - ps31
Arabidopsis_thaliana - ps32
p-value
8e-64
5e-50
3e-36
2e-22
1e-08
Log-odds
0.5
1.0
1.5
2.0
2.5
3.0
3.5
4.0

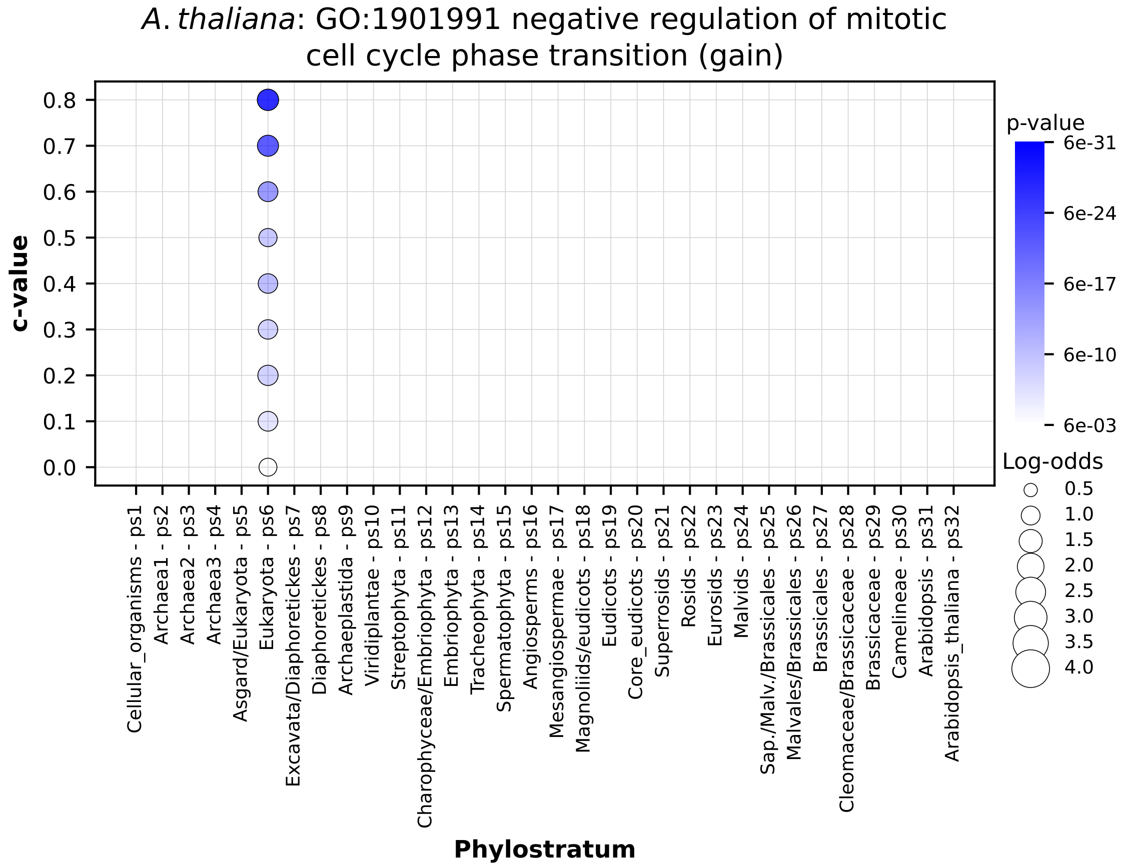

A. thaliana: GO:1901991 negative regulation of mitotic cell cycle phase transition (gain)
c-value
0.8
0.7
0.6
0.5
0.4
0.3
0.2
0.1
0.0
p-value
6e-31
6e-24
6e-17
6e-10
6e-03
Log-odds
0.5
1.0
1.5
2.0
2.5
3.0
3.5
4.0
Cellular_organisms - ps1
Archaea1 - ps2
Archaea2 - ps3
Archaea3 - ps4
Asgard/Eukaryota - ps5
Eukaryota - ps6
Excavata/Diaphoretickes - ps7
Diaphoretickes - ps8
Archaeplastida - ps9
Viridiplantae - ps10
Streptophyta - ps11
Charophyceae/Embriophyta - ps12
Embriophyta - ps13
Tracheophyta - ps14
Spermatophyta - ps15
Angiosperms - ps16
Mesangiospermae - ps17
Magnoliids/eudicots - ps18
Eudicots - ps19
Core_eudicots - ps20
Superrosids - ps21
Rosids - ps22
Eurosids - ps23
Malvids - ps24
Sap./Malv./Brassicales - ps25
Malvales/Brassicales - ps26
Brassicales - ps27
Cleomaceae/Brassicaceae - ps28
Brassicaceae - ps29
Camelineae - ps30
Arabidopsis - ps31
Arabidopsis_thaliana - ps32
Phylostratum

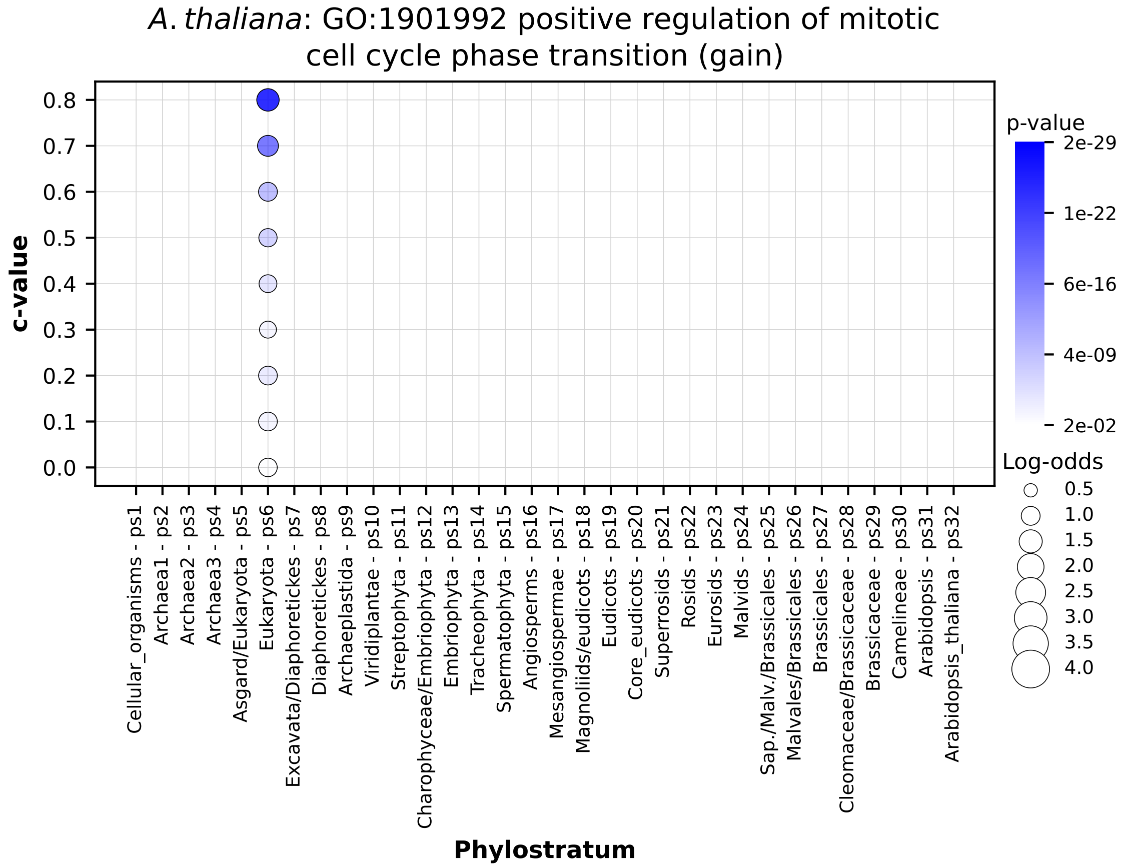

A. thaliana: GO:1901992 positive regulation of mitotic cell cycle phase transition (gain)
c-value
0.0
0.1
0.2
0.3
0.4
0.5
0.6
0.7
0.8
Phylostratum
Cellular_organisms - ps1
Archaea1 - ps2
Archaea2 - ps3
Archaea3 - ps4
Asgard/Eukaryota - ps5
Eukaryota - ps6
Excavata/Diaphoretickes - ps7
Diaphoretickes - ps8
Archaeplastida - ps9
Viridiplantae - ps10
Streptophyta - ps11
Charophyceae/Embriophyta - ps12
Embriophyta - ps13
Tracheophyta - ps14
Spermatophyta - ps15
Angiosperms - ps16
Mesangiospermae - ps17
Magnoliids/eudicots - ps18
Eudicots - ps19
Core_eudicots - ps20
Superrosids - ps21
Rosids - ps22
Eurosids - ps23
Malvids - ps24
Sap./Malv./Brassicales - ps25
Malvales/Brassicales - ps26
Brassicales - ps27
Cleomaceae/Brassicaceae - ps28
Brassicaceae - ps29
Camelineae - ps30
Arabidopsis - ps31
Arabidopsis_thaliana - ps32
p-value
2e-29
1e-22
6e-16
4e-09
2e-02
Log-odds
0.5
1.0
1.5
2.0
2.5
3.0
3.5
4.0

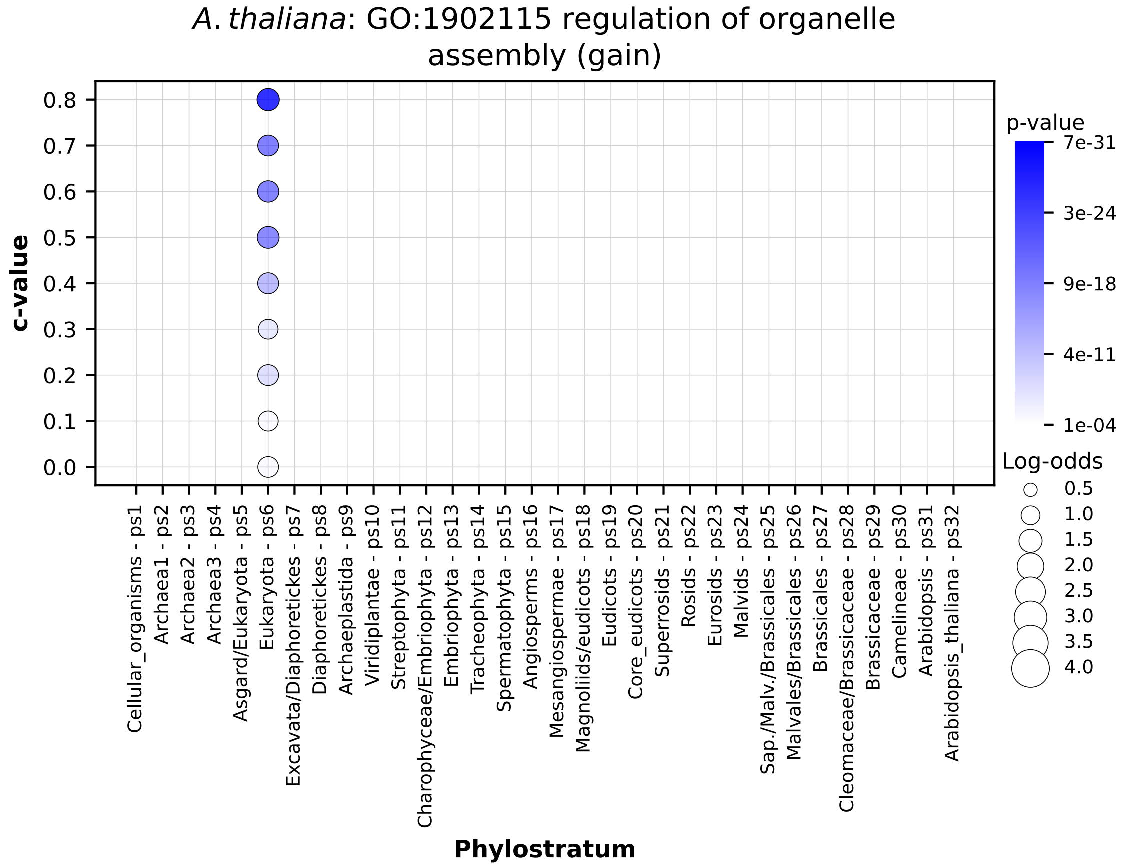

A. thaliana: GO:1902115 regulation of organelle assembly (gain)
c-value
0.8
0.7
0.6
0.5
0.4
0.3
0.2
0.1
0.0
p-value
7e-31
3e-24
9e-18
4e-11
1e-04
Log-odds
0.5
1.0
1.5
2.0
2.5
3.0
3.5
4.0
Cellular_organisms - ps1
Archaea1 - ps2
Archaea2 - ps3
Archaea3 - ps4
Asgard/Eukaryota - ps5
Eukaryota - ps6
Excavata/Diaphoretickes - ps7
Diaphoretickes - ps8
Archaeplastida - ps9
Viridiplantae - ps10
Streptophyta - ps11
Charophyceae/Embriophyta - ps12
Embriophyta - ps13
Tracheophyta - ps14
Spermatophyta - ps15
Angiosperms - ps16
Mesangiospermae - ps17
Magnoliids/eudicots - ps18
Eudicots - ps19
Core_eudicots - ps20
Superrosids - ps21
Rosids - ps22
Eurosids - ps23
Malvids - ps24
Sap./Malv./Brassicales - ps25
Malvales/Brassicales - ps26
Brassicales - ps27
Cleomaceae/Brassicaceae - ps28
Brassicaceae - ps29
Camelineae - ps30
Arabidopsis - ps31
Arabidopsis_thaliana - ps32
Phylostratum

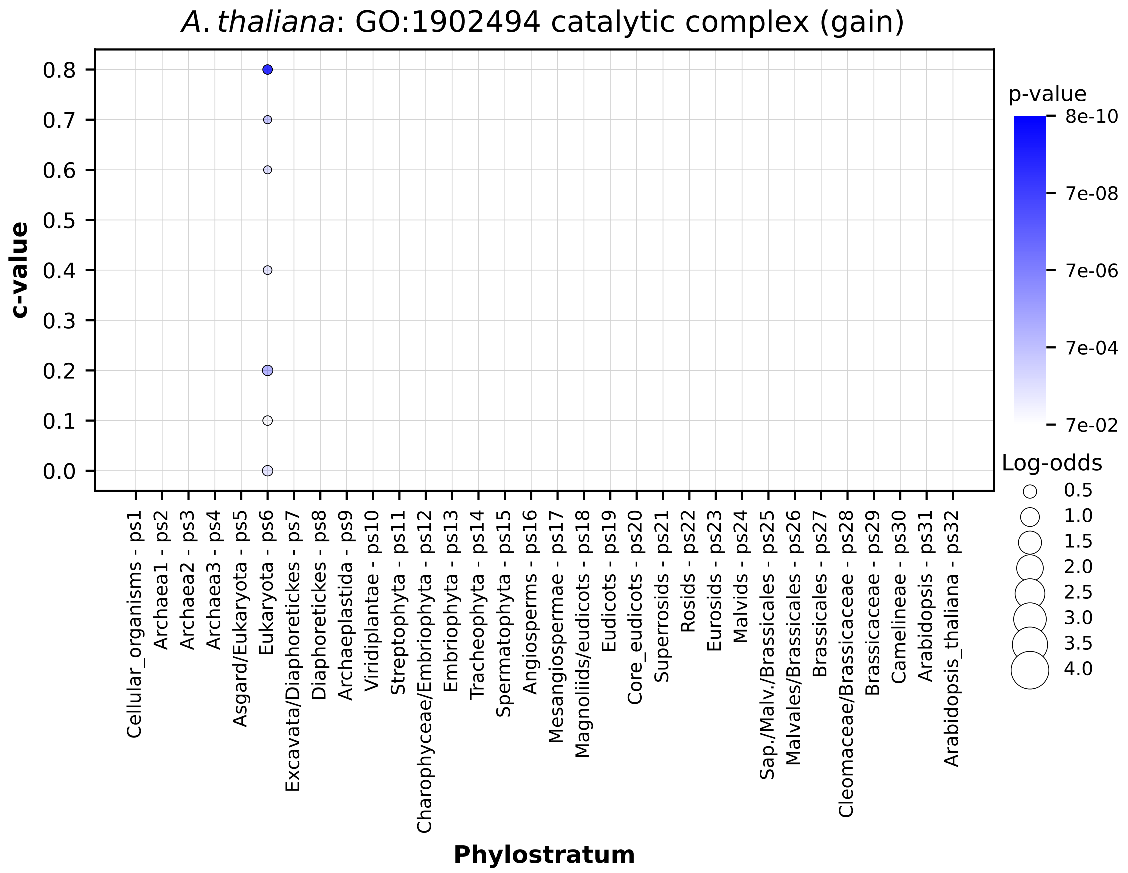
A. thaliana: GO:1902494 catalytic complex (gain)
c-value
0.8
0.7
0.6
0.5
0.4
0.3
0.2
0.1
0.0
p-value
8e-10
7e-08
7e-06
7e-04
7e-02
Log-odds
0.5
1.0
1.5
2.0
2.5
3.0
3.5
4.0
Cellular_organisms - ps1
Archaea1 - ps2
Archaea2 - ps3
Archaea3 - ps4
Asgard/Eukaryota - ps5
Eukaryota - ps6
Excavata/Diaphoretickes - ps7
Diaphoretickes - ps8
Archaeplastida - ps9
Viridiplantae - ps10
Streptophyta - ps11
Charophyceae/Embriophyta - ps12
Embriophyta - ps13
Tracheophyta - ps14
Spermatophyta - ps15
Angiosperms - ps16
Mesangiospermae - ps17
Magnoliids/eudicots - ps18
Eudicots - ps19
Core_eudicots - ps20
Superrosids - ps21
Rosids - ps22
Eurosids - ps23
Malvids - ps24
Sap./Malv./Brassicales - ps25
Malvales/Brassicales - ps26
Brassicales - ps27
Cleomaceae/Brassicaceae - ps28
Brassicaceae - ps29
Camelineae - ps30
Arabidopsis - ps31
Arabidopsis_thaliana - ps32
Phylostratum

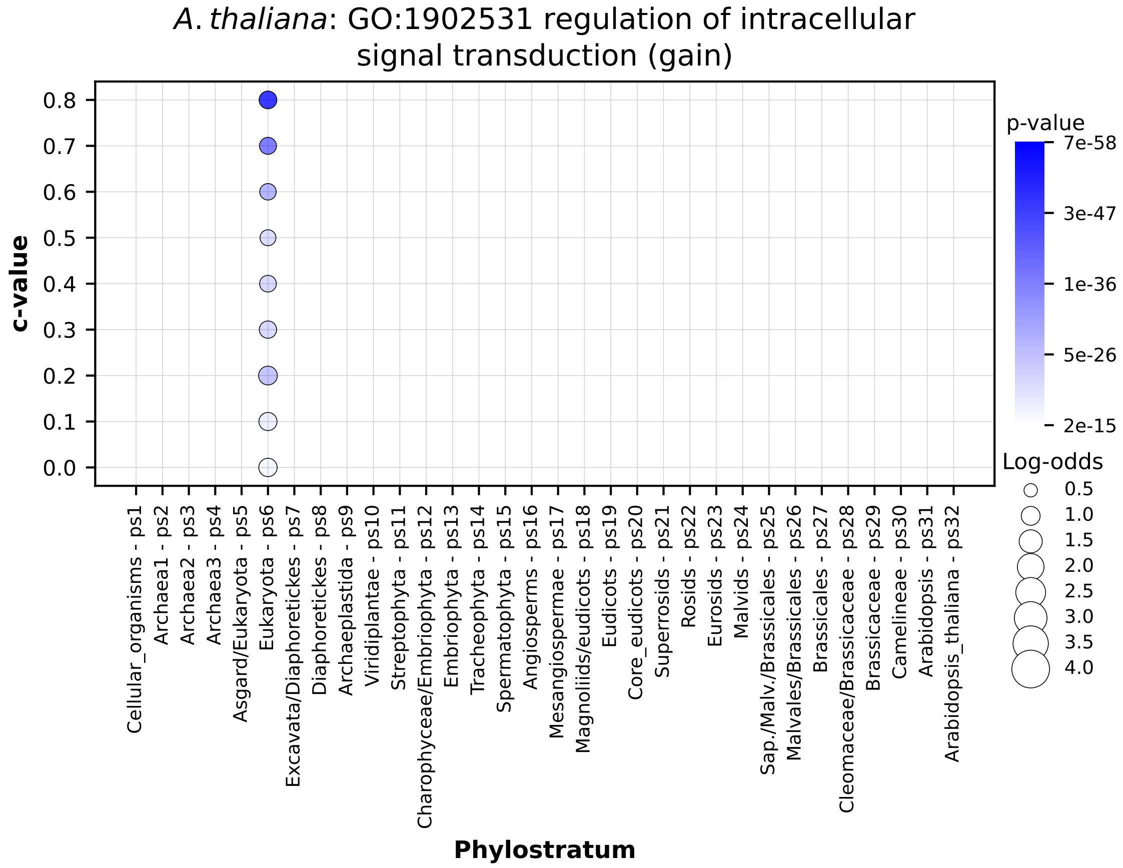
A. thaliana: GO:1902531 regulation of intracellular signal transduction (gain)
c-value
0.8
0.7
0.6
0.5
0.4
0.3
0.2
0.1
0.0
p-value
7e-58
3e-47
1e-36
5e-26
2e-15
Log-odds
0.5
1.0
1.5
2.0
2.5
3.0
3.5
4.0
Cellular_organisms - ps1
Archaea1 - ps2
Archaea2 - ps3
Archaea3 - ps4
Asgard/Eukaryota - ps5
Eukaryota - ps6
Excavata/Diaphoretickes - ps7
Diaphoretickes - ps8
Archaeplastida - ps9
Viridiplantae - ps10
Streptophyta - ps11
Charophyceae/Embriophyta - ps12
Embriophyta - ps13
Tracheophyta - ps14
Spermatophyta - ps15
Angiosperms - ps16
Mesangiospermae - ps17
Magnoliids/eudicots - ps18
Eudicots - ps19
Core_eudicots - ps20
Superrosids - ps21
Rosids - ps22
Eurosids - ps23
Malvids - ps24
Sap./Malv./Brassicales - ps25
Malvales/Brassicales - ps26
Brassicales - ps27
Cleomaceae/Brassicaceae - ps28
Brassicaceae - ps29
Camelineae - ps30
Arabidopsis - ps31
Arabidopsis_thaliana - ps32
Phylostratum

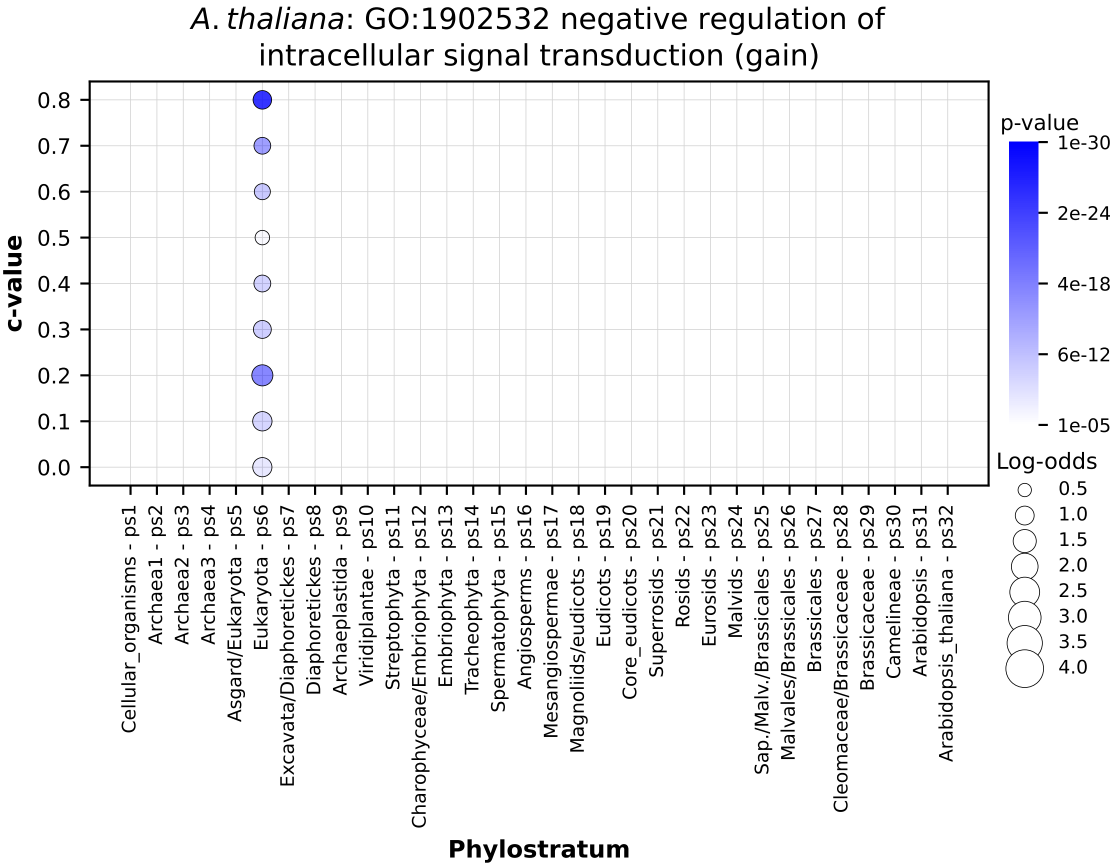
A. thaliana: GO:1902532 negative regulation of intracellular signal transduction (gain)
c-value
0.8
0.7
0.6
0.5
0.4
0.3
0.2
0.1
0.0
p-value
1e-30
2e-24
4e-18
6e-12
1e-05
Log-odds
0.5
1.0
1.5
2.0
2.5
3.0
3.5
4.0
Cellular_organisms - ps1
Archaea1 - ps2
Archaea2 - ps3
Archaea3 - ps4
Asgard/Eukaryota - ps5
Eukaryota - ps6
Excavata/Diaphoretickes - ps7
Diaphoretickes - ps8
Archaeplastida - ps9
Viridiplantae - ps10
Streptophyta - ps11
Charophyceae/Embriophyta - ps12
Embriophyta - ps13
Tracheophyta - ps14
Spermatophyta - ps15
Angiosperms - ps16
Mesangiospermae - ps17
Magnoliids/eudicots - ps18
Eudicots - ps19
Core_eudicots - ps20
Superrosids - ps21
Rosids - ps22
Eurosids - ps23
Malvids - ps24
Sap./Malv./Brassicales - ps25
Malvales/Brassicales - ps26
Brassicales - ps27
Cleomaceae/Brassicaceae - ps28
Brassicaceae - ps29
Camelineae - ps30
Arabidopsis - ps31
Arabidopsis_thaliana - ps32
Phylostratum

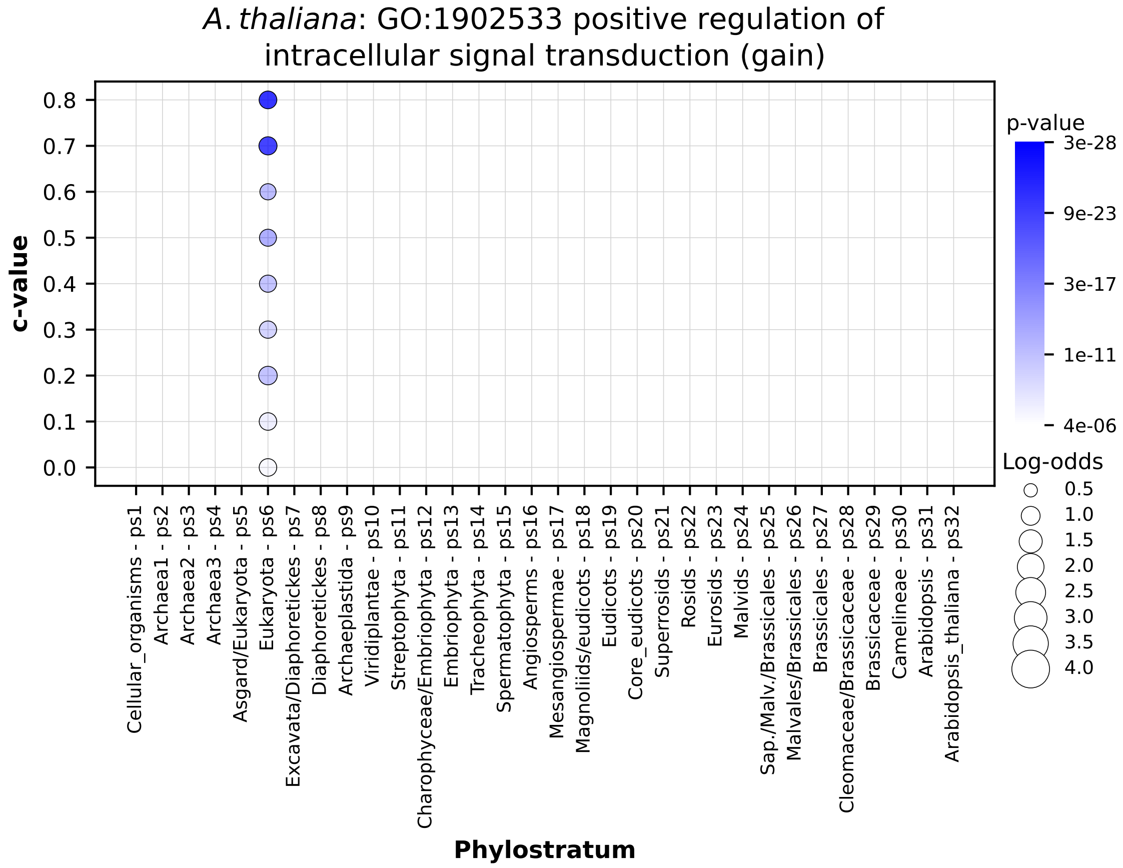

A. thaliana: GO:1902533 positive regulation of
intracellular signal transduction (gain)
c-value
0.8
0.7
0.6
0.5
0.4
0.3
0.2
0.1
0.0
p-value
3e-28
9e-23
3e-17
1e-11
4e-06
Log-odds
0.5
1.0
1.5
2.0
2.5
3.0
3.5
4.0
Cellular_organisms - ps1
Archaea1 - ps2
Archaea2 - ps3
Archaea3 - ps4
Asgard/Eukaryota - ps5
Eukaryota - ps6
Excavata/Diaphoretickes - ps7
Diaphoretickes - ps8
Archaeplastida - ps9
Viridiplantae - ps10
Streptophyta - ps11
Charophyceae/Embriophyta - ps12
Embriophyta - ps13
Tracheophyta - ps14
Spermatophyta - ps15
Angiosperms - ps16
Mesangiospermae - ps17
Magnoliids/eudicots - ps18
Eudicots - ps19
Core_eudicots - ps20
Superrosids - ps21
Rosids - ps22
Eurosids - ps23
Malvids - ps24
Sap./Malv./Brassicales - ps25
Malvales/Brassicales - ps26
Brassicales - ps27
Cleomaceae/Brassicaceae - ps28
Brassicaceae - ps29
Camelineae - ps30
Arabidopsis - ps31
Arabidopsis_thaliana - ps32
Phylostratum

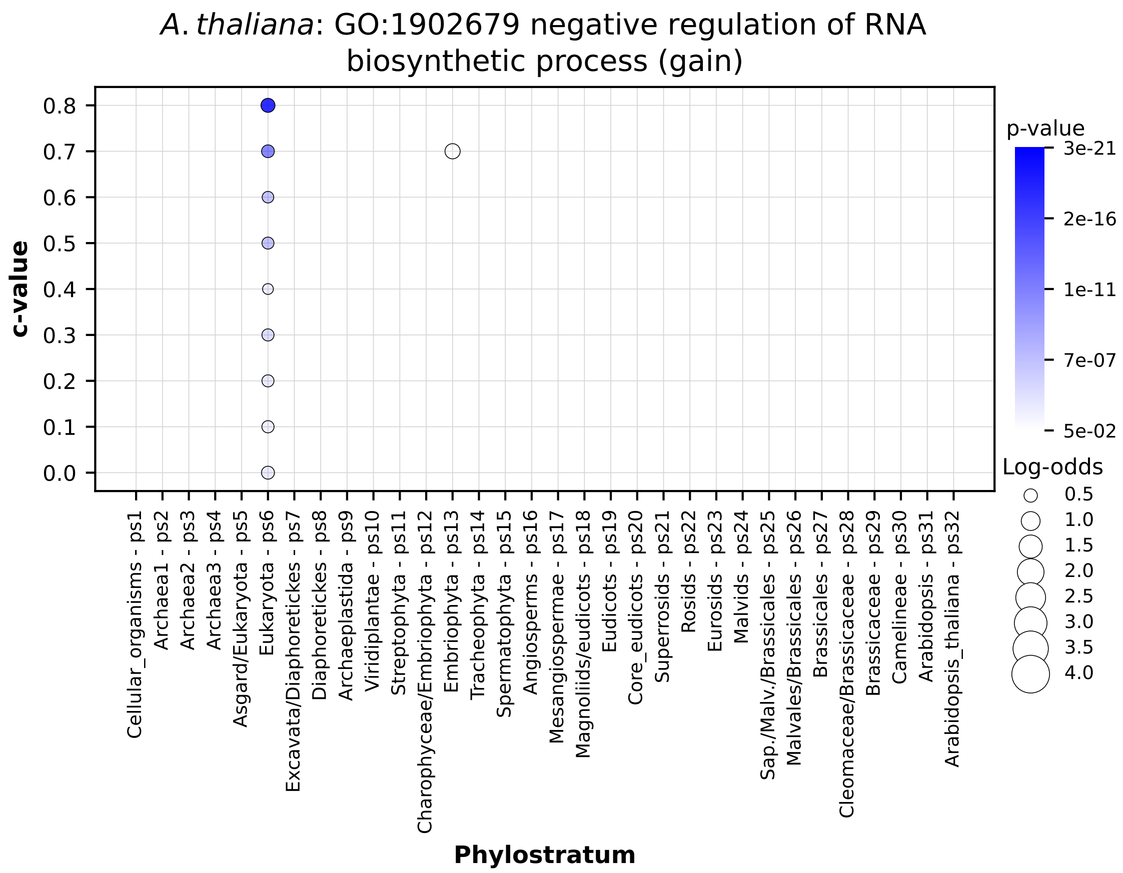

A. thaliana: GO:1902679 negative regulation of RNA biosynthetic process (gain)
c-value
0.8
0.7
0.6
0.5
0.4
0.3
0.2
0.1
0.0
p-value
3e-21
2e-16
1e-11
7e-07
5e-02
Log-odds
0.5
1.0
1.5
2.0
2.5
3.0
3.5
4.0
Cellular_organisms - ps1
Archaea1 - ps2
Archaea2 - ps3
Archaea3 - ps4
Asgard/Eukaryota - ps5
Eukaryota - ps6
Excavata/Diaphoretickes - ps7
Diaphoretickes - ps8
Archaeplastida - ps9
Viridiplantae - ps10
Streptophyta - ps11
Charophyceae/Embriophyta - ps12
Embriophyta - ps13
Tracheophyta - ps14
Spermatophyta - ps15
Angiosperms - ps16
Mesangiospermae - ps17
Magnoliids/eudicots - ps18
Eudicots - ps19
Core_eudicots - ps20
Superrosids - ps21
Rosids - ps22
Eurosids - ps23
Malvids - ps24
Sap./Malv./Brassicales - ps25
Malvales/Brassicales - ps26
Brassicales - ps27
Cleomaceae/Brassicaceae - ps28
Brassicaceae - ps29
Camelineae - ps30
Arabidopsis - ps31
Arabidopsis_thaliana - ps32
Phylostratum

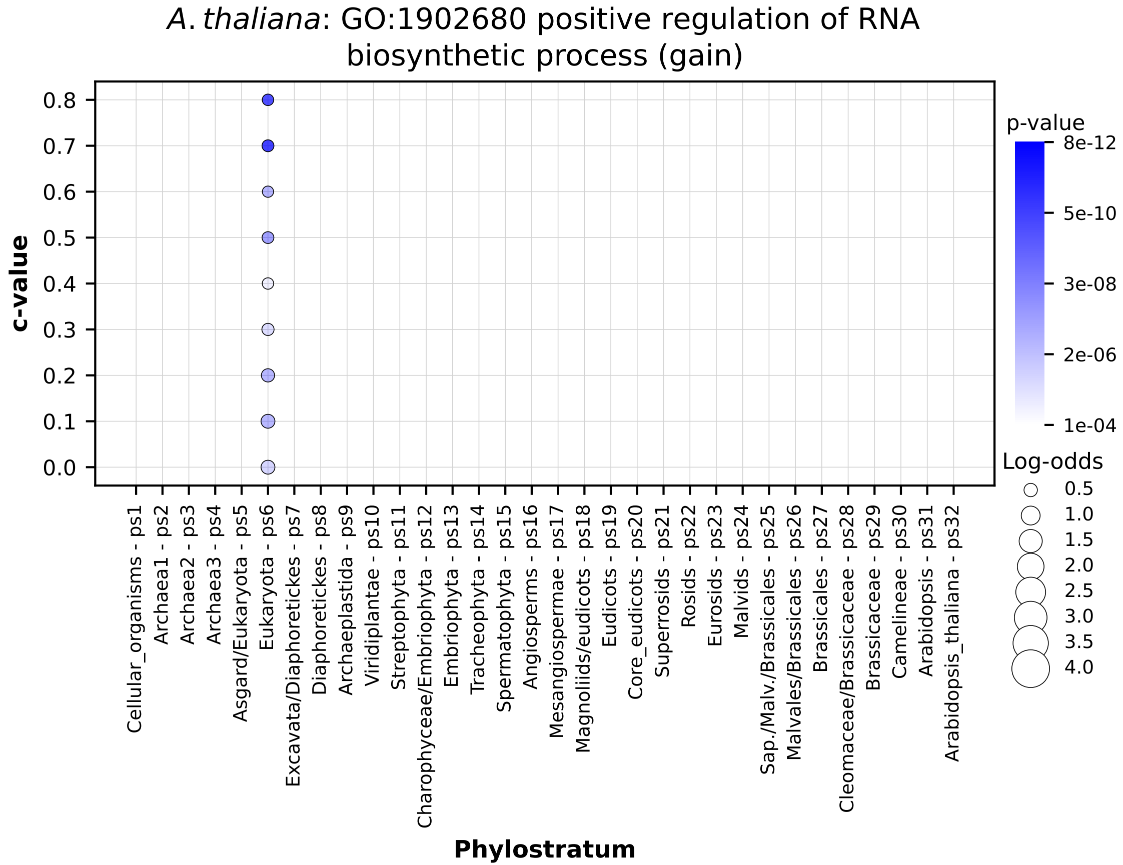

A. thaliana: GO:1902680 positive regulation of RNA biosynthetic process (gain)
c-value
0.8
0.7
0.6
0.5
0.4
0.3
0.2
0.1
0.0
p-value
8e-12
5e-10
3e-08
2e-06
1e-04
Log-odds
0.5
1.0
1.5
2.0
2.5
3.0
3.5
4.0
Cellular_organisms - ps1
Archaea1 - ps2
Archaea2 - ps3
Archaea3 - ps4
Asgard/Eukaryota - ps5
Eukaryota - ps6
Excavata/Diaphoretickes - ps7
Diaphoretickes - ps8
Archaeplastida - ps9
Viridiplantae - ps10
Streptophyta - ps11
Charophyceae/Embriophyta - ps12
Embriophyta - ps13
Tracheophyta - ps14
Spermatophyta - ps15
Angiosperms - ps16
Mesangiospermae - ps17
Magnoliids/eudicots - ps18
Eudicots - ps19
Core_eudicots - ps20
Superrosids - ps21
Rosids - ps22
Eurosids - ps23
Malvids - ps24
Sap./Malv./Brassicales - ps25
Malvales/Brassicales - ps26
Brassicales - ps27
Cleomaceae/Brassicaceae - ps28
Brassicaceae - ps29
Camelineae - ps30
Arabidopsis - ps31
Arabidopsis_thaliana - ps32
Phylostratum

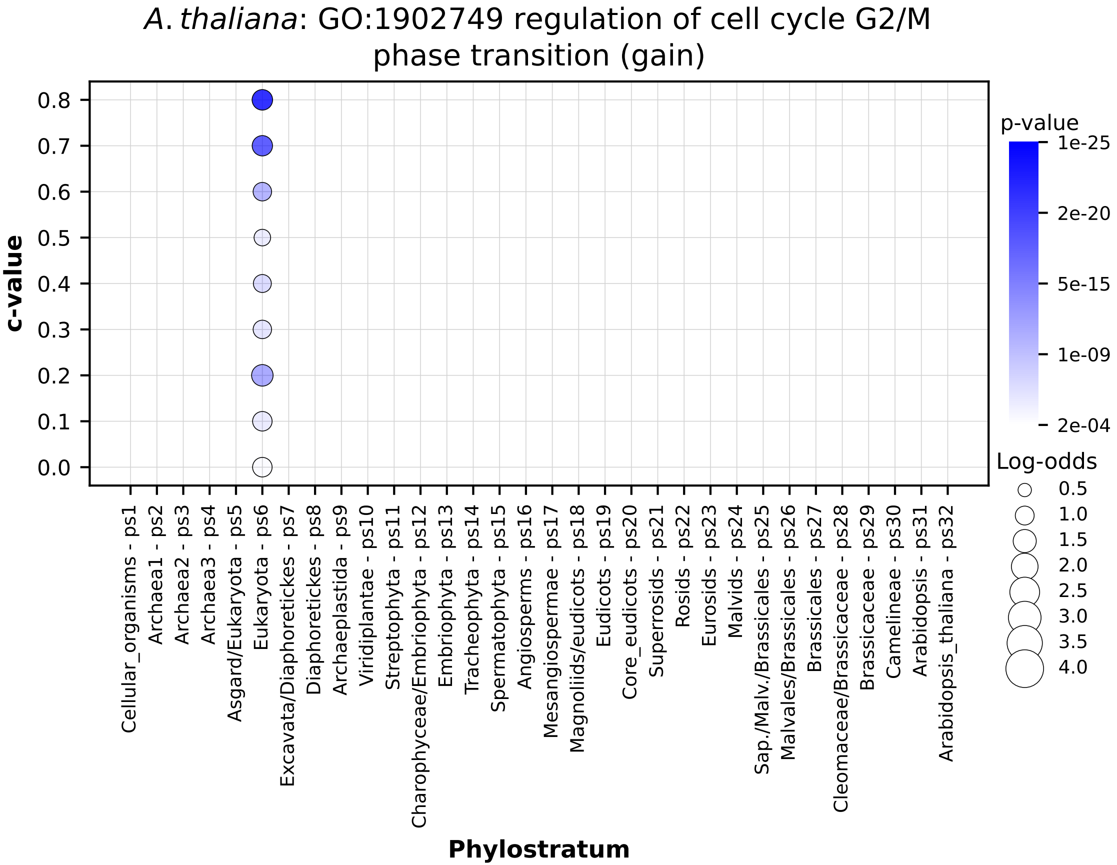
A. thaliana: GO:1902749 regulation of cell cycle G2/M phase transition (gain)
c-value
0.8
0.7
0.6
0.5
0.4
0.3
0.2
0.1
0.0
p-value
1e-25
2e-20
5e-15
1e-09
2e-04
Log-odds
0.5
1.0
1.5
2.0
2.5
3.0
3.5
4.0
Cellular_organisms - ps1
Archaea1 - ps2
Archaea2 - ps3
Archaea3 - ps4
Asgard/Eukaryota - ps5
Eukaryota - ps6
Excavata/Diaphoretickes - ps7
Diaphoretickes - ps8
Archaeplastida - ps9
Viridiplantae - ps10
Streptophyta - ps11
Charophyceae/Embriophyta - ps12
Embriophyta - ps13
Tracheophyta - ps14
Spermatophyta - ps15
Angiosperms - ps16
Mesangiospermae - ps17
Magnoliids/eudicots - ps18
Eudicots - ps19
Core_eudicots - ps20
Superrosids - ps21
Rosids - ps22
Eurosids - ps23
Malvids - ps24
Sap./Malv./Brassicales - ps25
Malvales/Brassicales - ps26
Brassicales - ps27
Cleomaceae/Brassicaceae - ps28
Brassicaceae - ps29
Camelineae - ps30
Arabidopsis - ps31
Arabidopsis_thaliana - ps32
Phylostratum

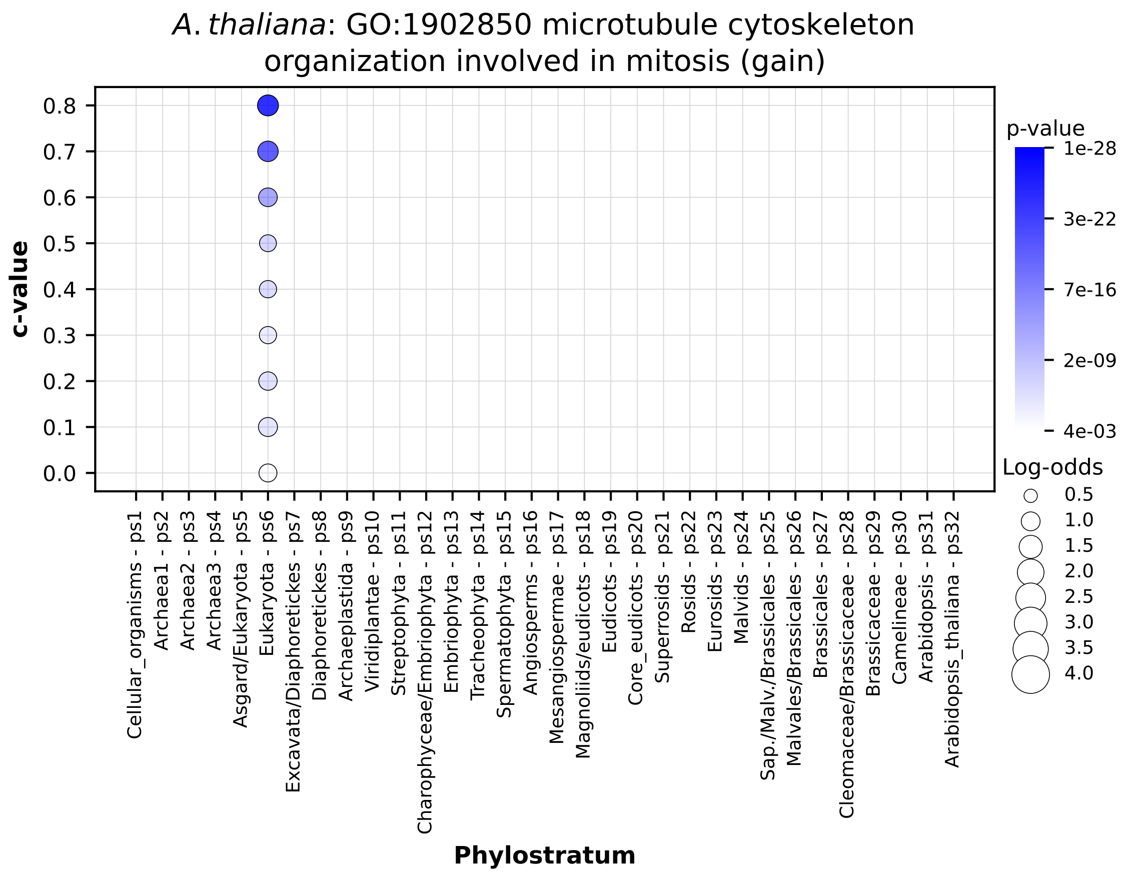

A. thaliana: GO:1902850 microtubule cytoskeleton organization involved in mitosis (gain)
c-value
0.8
0.7
0.6
0.5
0.4
0.3
0.2
0.1
0.0
p-value
1e-28
3e-22
7e-16
2e-09
4e-03
Log-odds
0.5
1.0
1.5
2.0
2.5
3.0
3.5
4.0
Cellular_organisms - ps1
Archaea1 - ps2
Archaea2 - ps3
Archaea3 - ps4
Asgard/Eukaryota - ps5
Eukaryota - ps6
Excavata/Diaphoretickes - ps7
Diaphoretickes - ps8
Archaeplastida - ps9
Viridiplantae - ps10
Streptophyta - ps11
Charophyceae/Embriophyta - ps12
Embriophyta - ps13
Tracheophyta - ps14
Spermatophyta - ps15
Angiosperms - ps16
Mesangiospermae - ps17
Magnoliids/eudicots - ps18
Eudicots - ps19
Core_eudicots - ps20
Superrosids - ps21
Rosids - ps22
Eurosids - ps23
Malvids - ps24
Sap./Malv./Brassicales - ps25
Malvales/Brassicales - ps26
Brassicales - ps27
Cleomaceae/Brassicaceae - ps28
Brassicaceae - ps29
Camelineae - ps30
Arabidopsis - ps31
Arabidopsis_thaliana - ps32
Phylostratum

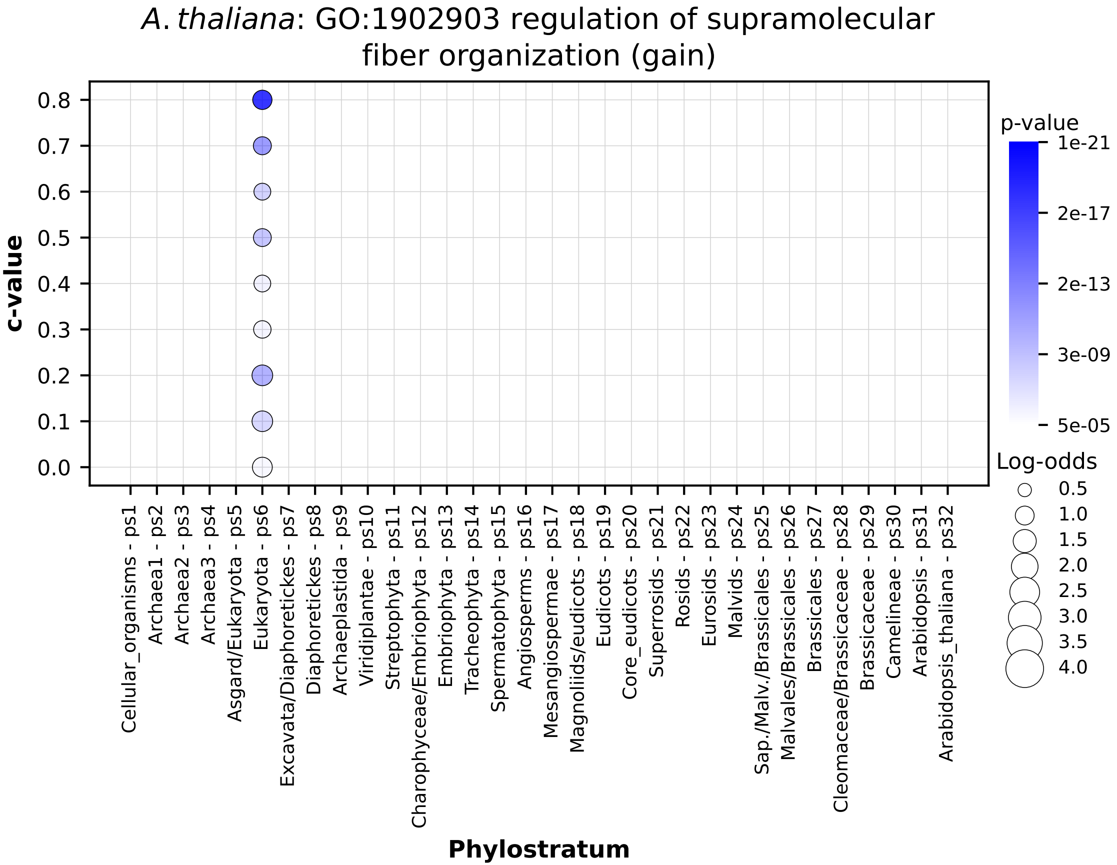
A. thaliana: GO:1902903 regulation of supramolecular fiber organization (gain)
c-value
0.8
0.7
0.6
0.5
0.4
0.3
0.2
0.1
0.0
p-value
1e-21
2e-17
2e-13
3e-09
5e-05
Log-odds
0.5
1.0
1.5
2.0
2.5
3.0
3.5
4.0
Cellular_organisms - ps1
Archaea1 - ps2
Archaea2 - ps3
Archaea3 - ps4
Asgard/Eukaryota - ps5
Eukaryota - ps6
Excavata/Diaphoretickes - ps7
Diaphoretickes - ps8
Archaeplastida - ps9
Viridiplantae - ps10
Streptophyta - ps11
Charophyceae/Embriophyta - ps12
Embriophyta - ps13
Tracheophyta - ps14
Spermatophyta - ps15
Angiosperms - ps16
Mesangiospermae - ps17
Magnoliids/eudicots - ps18
Eudicots - ps19
Core_eudicots - ps20
Superrosids - ps21
Rosids - ps22
Eurosids - ps23
Malvids - ps24
Sap./Malv./Brassicales - ps25
Malvales/Brassicales - ps26
Brassicales - ps27
Cleomaceae/Brassicaceae - ps28
Brassicaceae - ps29
Camelineae - ps30
Arabidopsis - ps31
Arabidopsis_thaliana - ps32
Phylostratum

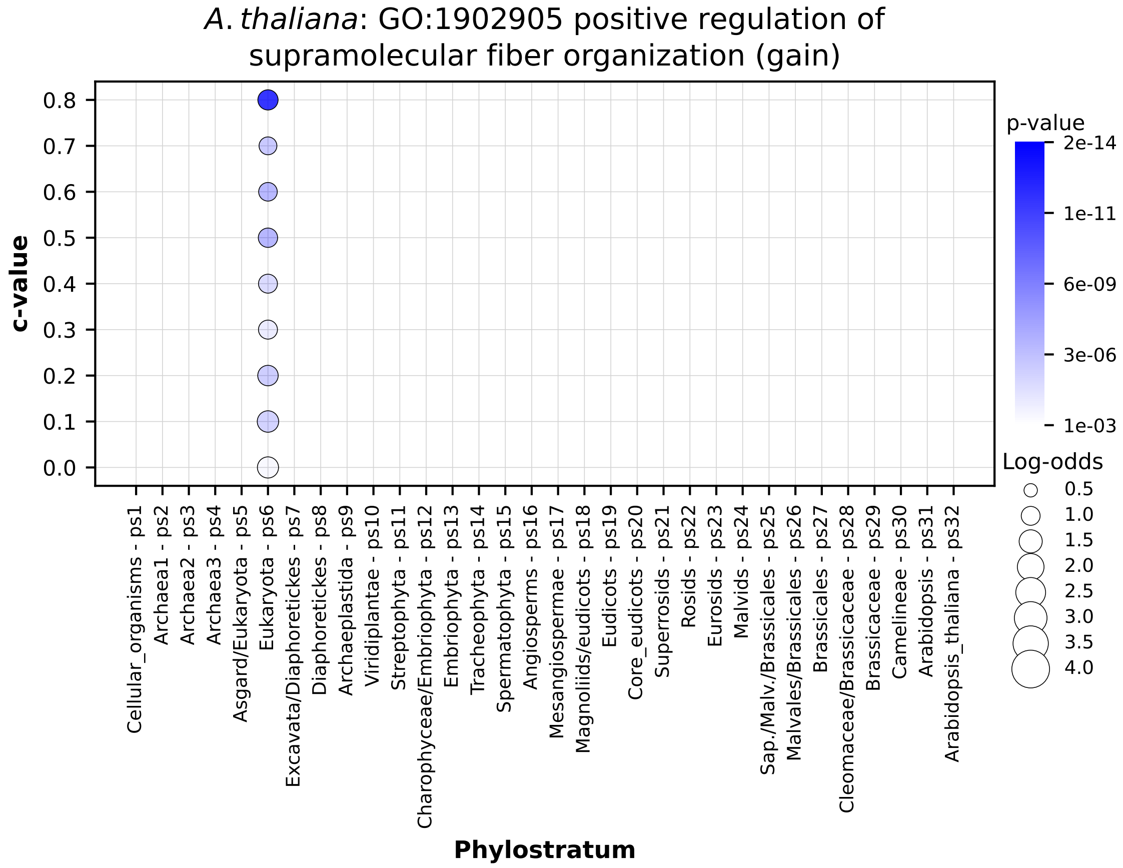

A. thaliana: GO:1902905 positive regulation of supramolecular fiber organization (gain)
c-value
0.8
0.7
0.6
0.5
0.4
0.3
0.2
0.1
0.0
p-value
2e-14
1e-11
6e-09
3e-06
1e-03
Log-odds
0.5
1.0
1.5
2.0
2.5
3.0
3.5
4.0
Cellular_organisms - ps1
Archaea1 - ps2
Archaea2 - ps3
Archaea3 - ps4
Asgard/Eukaryota - ps5
Eukaryota - ps6
Excavata/Diaphoretickes - ps7
Diaphoretickes - ps8
Archaeplastida - ps9
Viridiplantae - ps10
Streptophyta - ps11
Charophyceae/Embriophyta - ps12
Embriophyta - ps13
Tracheophyta - ps14
Spermatophyta - ps15
Angiosperms - ps16
Mesangiospermae - ps17
Magnoliids/eudicots - ps18
Eudicots - ps19
Core_eudicots - ps20
Superrosids - ps21
Rosids - ps22
Eurosids - ps23
Malvids - ps24
Sap./Malv./Brassicales - ps25
Malvales/Brassicales - ps26
Brassicales - ps27
Cleomaceae/Brassicaceae - ps28
Brassicaceae - ps29
Camelineae - ps30
Arabidopsis - ps31
Arabidopsis_thaliana - ps32
Phylostratum

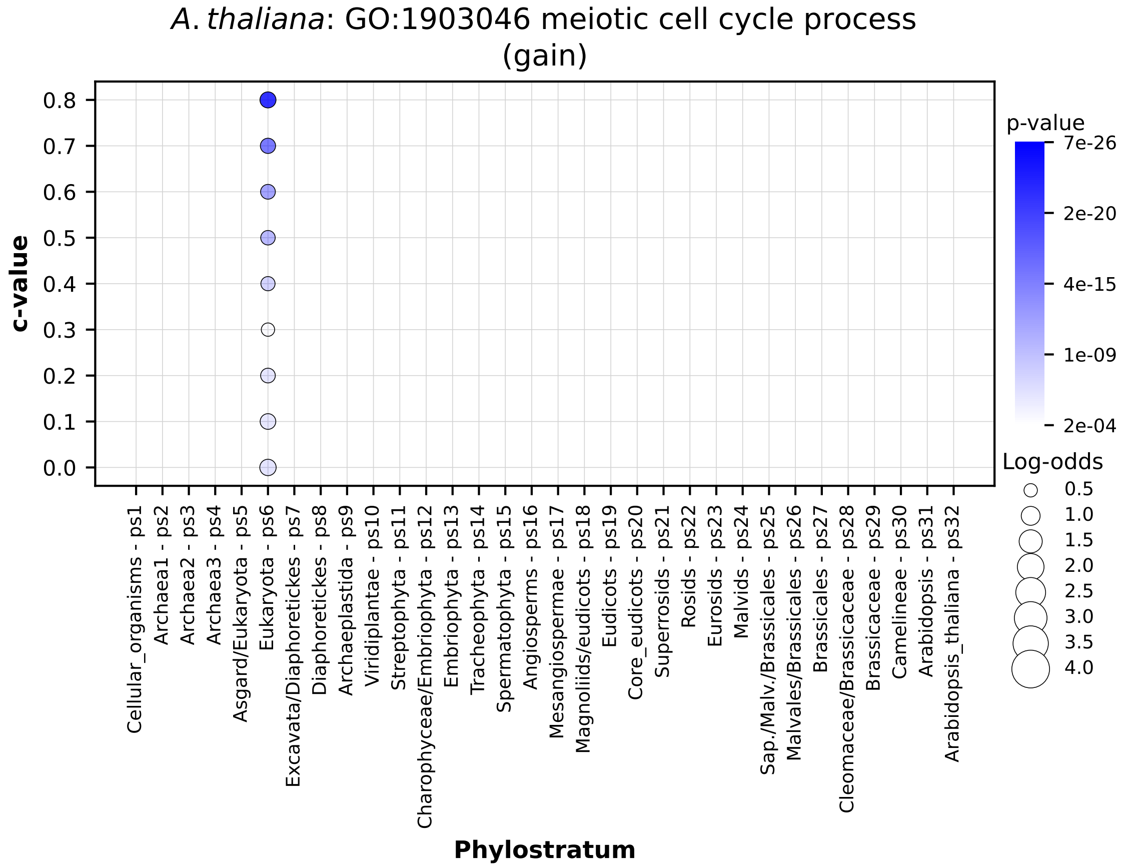
A. thaliana: GO:1903046 meiotic cell cycle process
(gain)
c-value
0.8
0.7
0.6
0.5
0.4
0.3
0.2
0.1
0.0
p-value
7e-26
2e-20
4e-15
1e-09
2e-04
Log-odds
0.5
1.0
1.5
2.0
2.5
3.0
3.5
4.0
Cellular_organisms - ps1
Archaea1 - ps2
Archaea2 - ps3
Archaea3 - ps4
Asgard/Eukaryota - ps5
Eukaryota - ps6
Excavata/Diaphoretickes - ps7
Diaphoretickes - ps8
Archaeplastida - ps9
Viridiplantae - ps10
Streptophyta - ps11
Charophyceae/Embriophyta - ps12
Embriophyta - ps13
Tracheophyta - ps14
Spermatophyta - ps15
Angiosperms - ps16
Mesangiospermae - ps17
Magnoliids/eudicots - ps18
Eudicots - ps19
Core_eudicots - ps20
Superrosids - ps21
Rosids - ps22
Eurosids - ps23
Malvids - ps24
Sap./Malv./Brassicales - ps25
Malvales/Brassicales - ps26
Brassicales - ps27
Cleomaceae/Brassicaceae - ps28
Brassicaceae - ps29
Camelineae - ps30
Arabidopsis - ps31
Arabidopsis_thaliana - ps32
Phylostratum

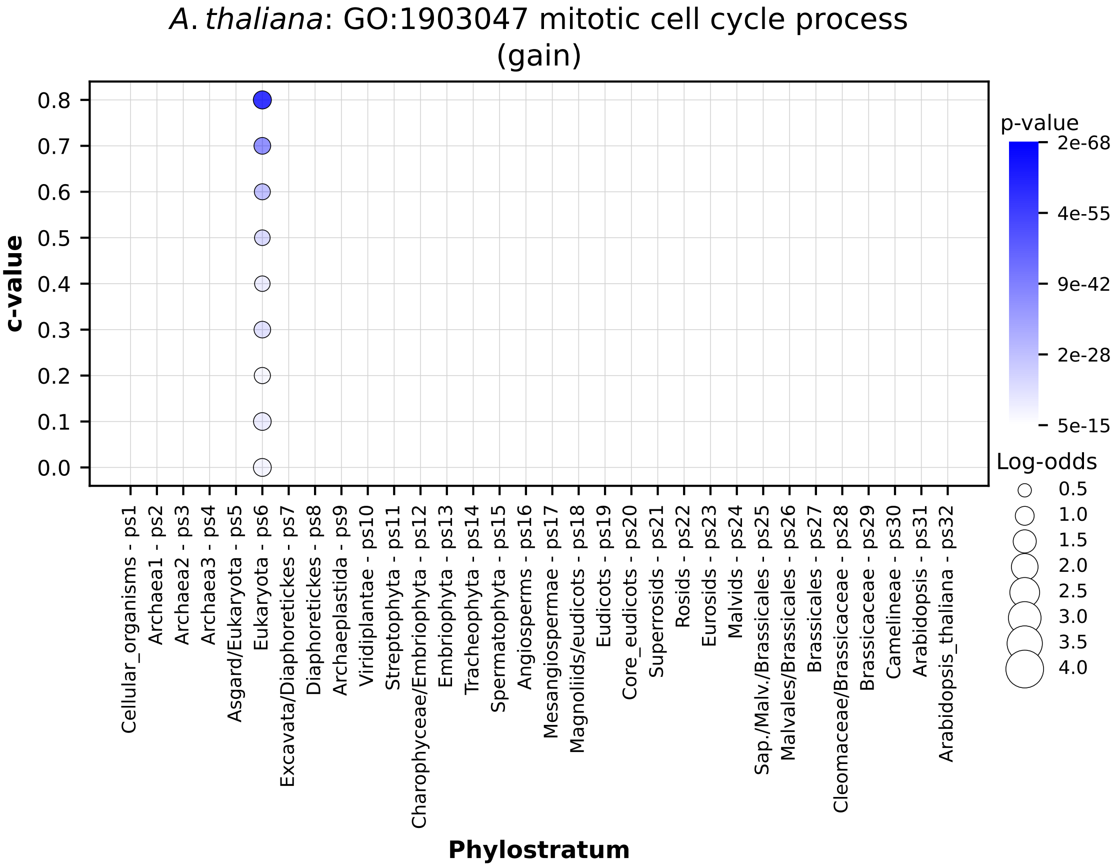

A. thaliana: GO:1903047 mitotic cell cycle process
(gain)
c-value
0.8
0.7
0.6
0.5
0.4
0.3
0.2
0.1
0.0
p-value
2e-68
4e-55
9e-42
2e-28
5e-15
Log-odds
0.5
1.0
1.5
2.0
2.5
3.0
3.5
4.0
Cellular_organisms - ps1
Archaea1 - ps2
Archaea2 - ps3
Archaea3 - ps4
Asgard/Eukaryota - ps5
Eukaryota - ps6
Excavata/Diaphoretickes - ps7
Diaphoretickes - ps8
Archaeplastida - ps9
Viridiplantae - ps10
Streptophyta - ps11
Charophyceae/Embriophyta - ps12
Embriophyta - ps13
Tracheophyta - ps14
Spermatophyta - ps15
Angiosperms - ps16
Mesangiospermae - ps17
Magnoliids/eudicots - ps18
Eudicots - ps19
Core_eudicots - ps20
Superrosids - ps21
Rosids - ps22
Eurosids - ps23
Malvids - ps24
Sap./Malv./Brassicales - ps25
Malvales/Brassicales - ps26
Brassicales - ps27
Cleomaceae/Brassicaceae - ps28
Brassicaceae - ps29
Camelineae - ps30
Arabidopsis - ps31
Arabidopsis_thaliana - ps32
Phylostratum

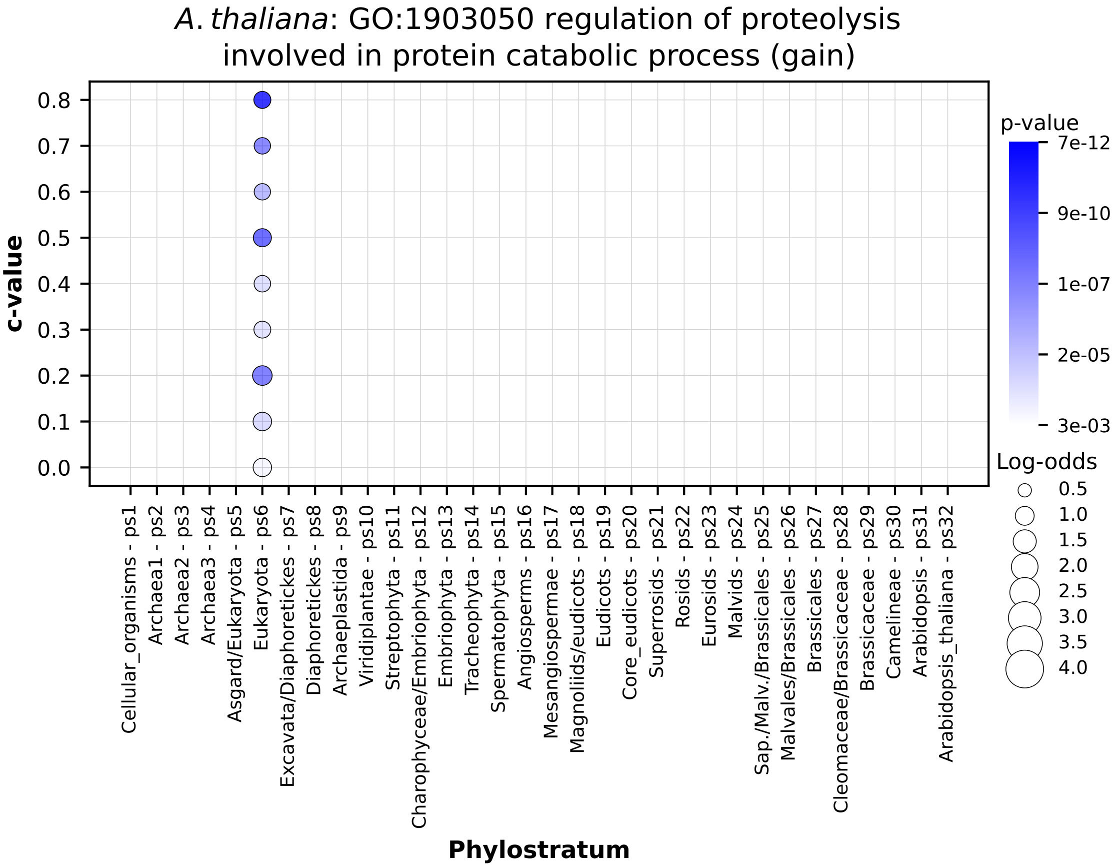

A. thaliana: GO:1903050 regulation of proteolysis involved in protein catabolic process (gain)
c-value
0.8
0.7
0.6
0.5
0.4
0.3
0.2
0.1
0.0
p-value
7e-12
9e-10
1e-07
2e-05
3e-03
Log-odds
0.5
1.0
1.5
2.0
2.5
3.0
3.5
4.0
Cellular_organisms - ps1
Archaea1 - ps2
Archaea2 - ps3
Archaea3 - ps4
Asgard/Eukaryota - ps5
Eukaryota - ps6
Excavata/Diaphoretickes - ps7
Diaphoretickes - ps8
Archaeplastida - ps9
Viridiplantae - ps10
Streptophyta - ps11
Charophyceae/Embriophyta - ps12
Embriophyta - ps13
Tracheophyta - ps14
Spermatophyta - ps15
Angiosperms - ps16
Mesangiospermae - ps17
Magnoliids/eudicots - ps18
Eudicots - ps19
Core_eudicots - ps20
Superrosids - ps21
Rosids - ps22
Eurosids - ps23
Malvids - ps24
Sap./Malv./Brassicales - ps25
Malvales/Brassicales - ps26
Brassicales - ps27
Cleomaceae/Brassicaceae - ps28
Brassicaceae - ps29
Camelineae - ps30
Arabidopsis - ps31
Arabidopsis_thaliana - ps32
Phylostratum

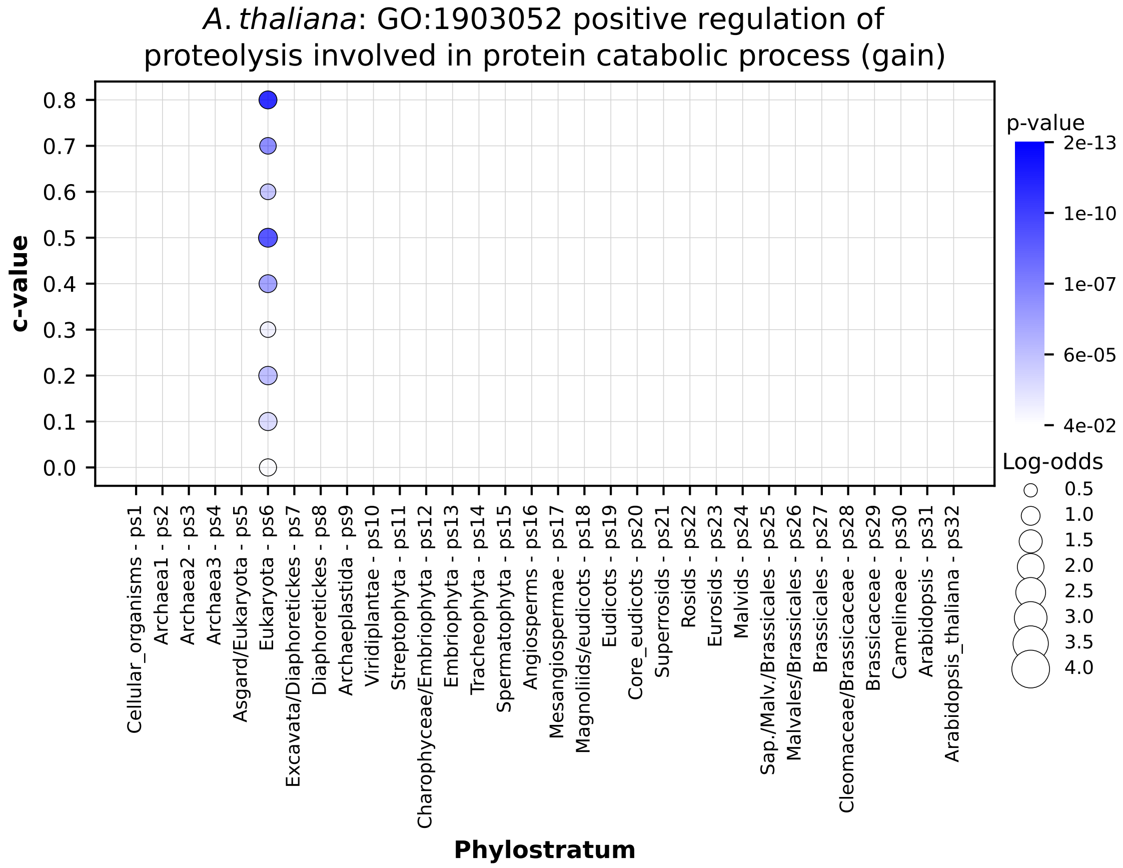

A. thaliana: GO:1903052 positive regulation of proteolysis involved in protein catabolic process (gain)
c-value
0.0
0.1
0.2
0.3
0.4
0.5
0.6
0.7
0.8
Phylostratum
Cellular_organisms - ps1
Archaea1 - ps2
Archaea2 - ps3
Archaea3 - ps4
Asgard/Eukaryota - ps5
Eukaryota - ps6
Excavata/Diaphoretickes - ps7
Diaphoretickes - ps8
Archaeplastida - ps9
Viridiplantae - ps10
Streptophyta - ps11
Charophyceae/Embriophyta - ps12
Embriophyta - ps13
Tracheophyta - ps14
Spermatophyta - ps15
Angiosperms - ps16
Mesangiospermae - ps17
Magnoliids/eudicots - ps18
Eudicots - ps19
Core_eudicots - ps20
Superrosids - ps21
Rosids - ps22
Eurosids - ps23
Malvids - ps24
Sap./Malv./Brassicales - ps25
Malvales/Brassicales - ps26
Brassicales - ps27
Cleomaceae/Brassicaceae - ps28
Brassicaceae - ps29
Camelineae - ps30
Arabidopsis - ps31
Arabidopsis_thaliana - ps32
p-value
2e-13
1e-10
1e-07
6e-05
4e-02
Log-odds
0.5
1.0
1.5
2.0
2.5
3.0
3.5
4.0

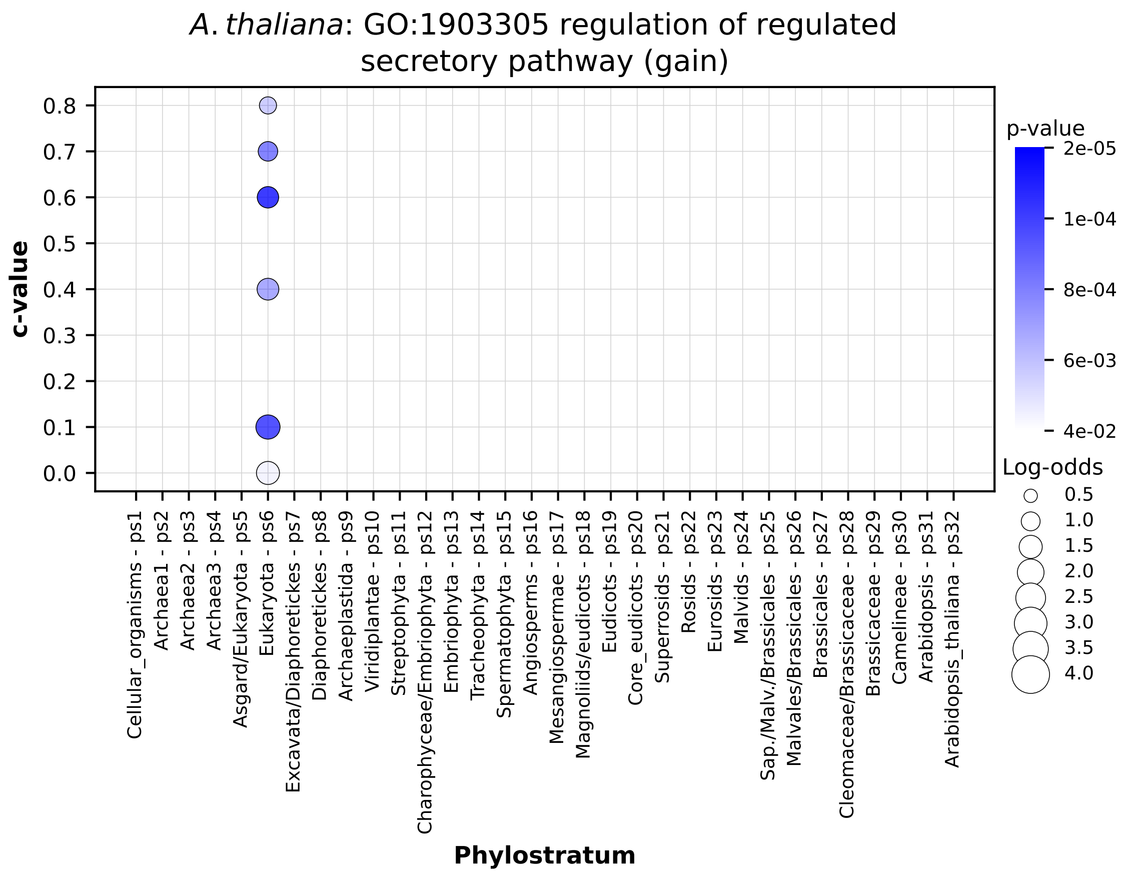

A. thaliana: GO:1903305 regulation of regulated secretory pathway (gain)
c-value
0.8
0.7
0.6
0.5
0.4
0.3
0.2
0.1
0.0
p-value
2e-05
1e-04
8e-04
6e-03
4e-02
Log-odds
0.5
1.0
1.5
2.0
2.5
3.0
3.5
4.0
Cellular_organisms - ps1
Archaea1 - ps2
Archaea2 - ps3
Archaea3 - ps4
Asgard/Eukaryota - ps5
Eukaryota - ps6
Excavata/Diaphoretickes - ps7
Diaphoretickes - ps8
Archaeplastida - ps9
Viridiplantae - ps10
Streptophyta - ps11
Charophyceae/Embriophyta - ps12
Embriophyta - ps13
Tracheophyta - ps14
Spermatophyta - ps15
Angiosperms - ps16
Mesangiospermae - ps17
Magnoliids/eudicots - ps18
Eudicots - ps19
Core_eudicots - ps20
Superrosids - ps21
Rosids - ps22
Eurosids - ps23
Malvids - ps24
Sap./Malv./Brassicales - ps25
Malvales/Brassicales - ps26
Brassicales - ps27
Cleomaceae/Brassicaceae - ps28
Brassicaceae - ps29
Camelineae - ps30
Arabidopsis - ps31
Arabidopsis_thaliana - ps32
Phylostratum

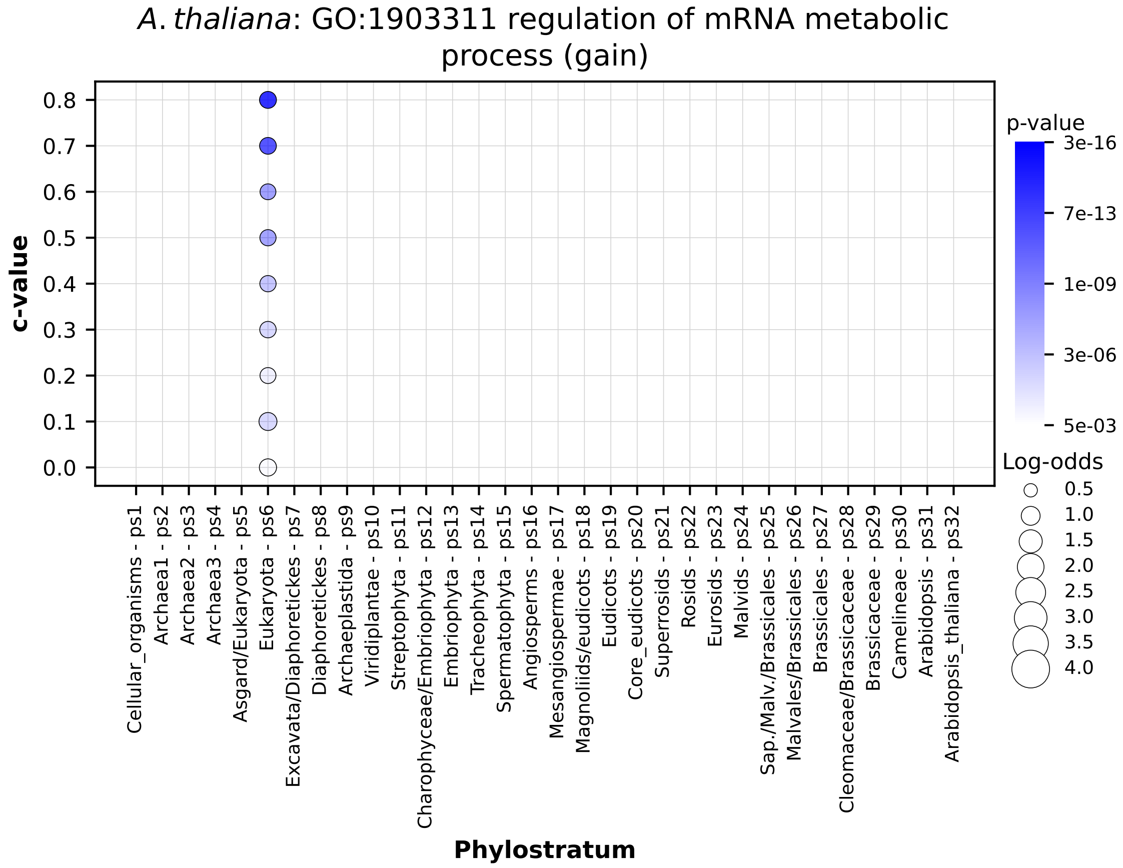
A. thaliana: GO:1903311 regulation of mRNA metabolic process (gain)
c-value
0.8
0.7
0.6
0.5
0.4
0.3
0.2
0.1
0.0
p-value
3e-16
7e-13
1e-09
3e-06
5e-03
Log-odds
0.5
1.0
1.5
2.0
2.5
3.0
3.5
4.0
Cellular_organisms - ps1
Archaea1 - ps2
Archaea2 - ps3
Archaea3 - ps4
Asgard/Eukaryota - ps5
Eukaryota - ps6
Excavata/Diaphoretickes - ps7
Diaphoretickes - ps8
Archaeplastida - ps9
Viridiplantae - ps10
Streptophyta - ps11
Charophyceae/Embriophyta - ps12
Embriophyta - ps13
Tracheophyta - ps14
Spermatophyta - ps15
Angiosperms - ps16
Mesangiospermae - ps17
Magnoliids/eudicots - ps18
Eudicots - ps19
Core_eudicots - ps20
Superrosids - ps21
Rosids - ps22
Eurosids - ps23
Malvids - ps24
Sap./Malv./Brassicales - ps25
Malvales/Brassicales - ps26
Brassicales - ps27
Cleomaceae/Brassicaceae - ps28
Brassicaceae - ps29
Camelineae - ps30
Arabidopsis - ps31
Arabidopsis_thaliana - ps32
Phylostratum

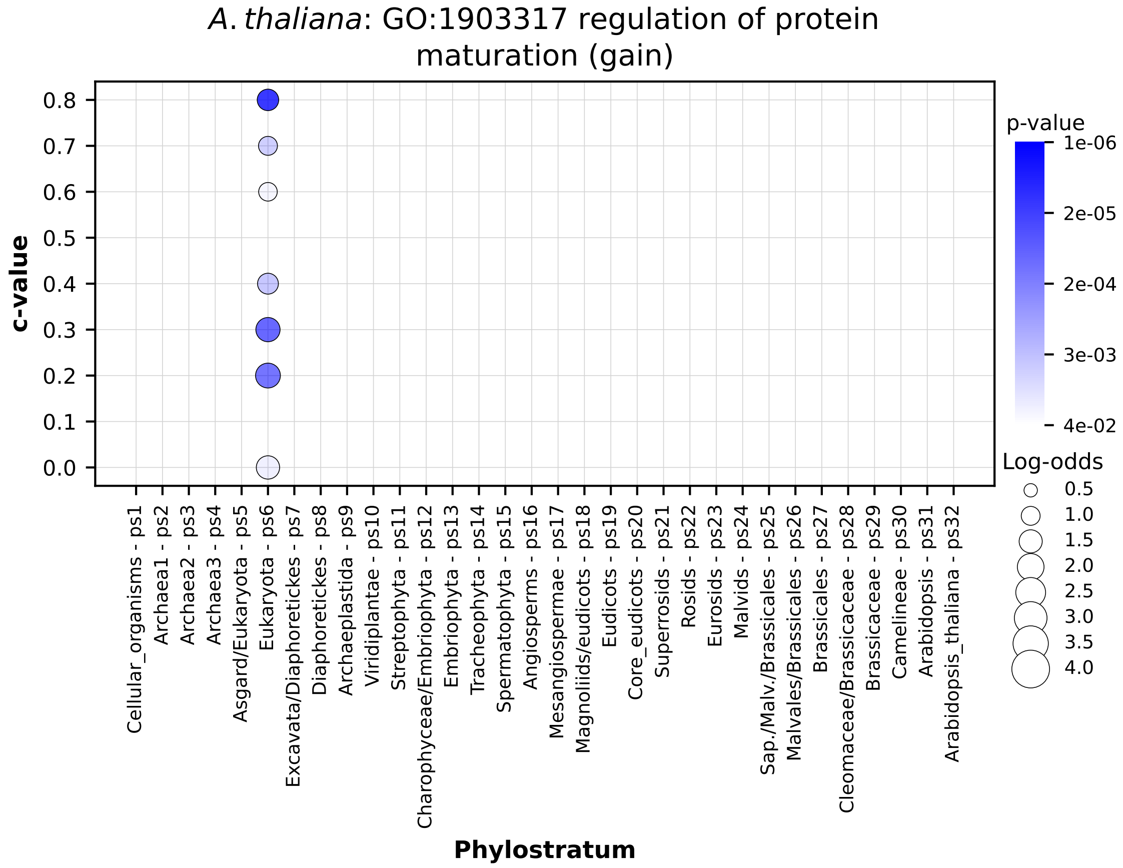

A. thaliana: GO:1903317 regulation of protein maturation (gain)
c-value
0.8
0.7
0.6
0.5
0.4
0.3
0.2
0.1
0.0
p-value
1e-06
2e-05
2e-04
3e-03
4e-02
Log-odds
0.5
1.0
1.5
2.0
2.5
3.0
3.5
4.0
Cellular_organisms - ps1
Archaea1 - ps2
Archaea2 - ps3
Archaea3 - ps4
Asgard/Eukaryota - ps5
Eukaryota - ps6
Excavata/Diaphoretickes - ps7
Diaphoretickes - ps8
Archaeplastida - ps9
Viridiplantae - ps10
Streptophyta - ps11
Charophyceae/Embriophyta - ps12
Embriophyta - ps13
Tracheophyta - ps14
Spermatophyta - ps15
Angiosperms - ps16
Mesangiospermae - ps17
Magnoliids/eudicots - ps18
Eudicots - ps19
Core_eudicots - ps20
Superrosids - ps21
Rosids - ps22
Eurosids - ps23
Malvids - ps24
Sap./Malv./Brassicales - ps25
Malvales/Brassicales - ps26
Brassicales - ps27
Cleomaceae/Brassicaceae - ps28
Brassicaceae - ps29
Camelineae - ps30
Arabidopsis - ps31
Arabidopsis_thaliana - ps32
Phylostratum

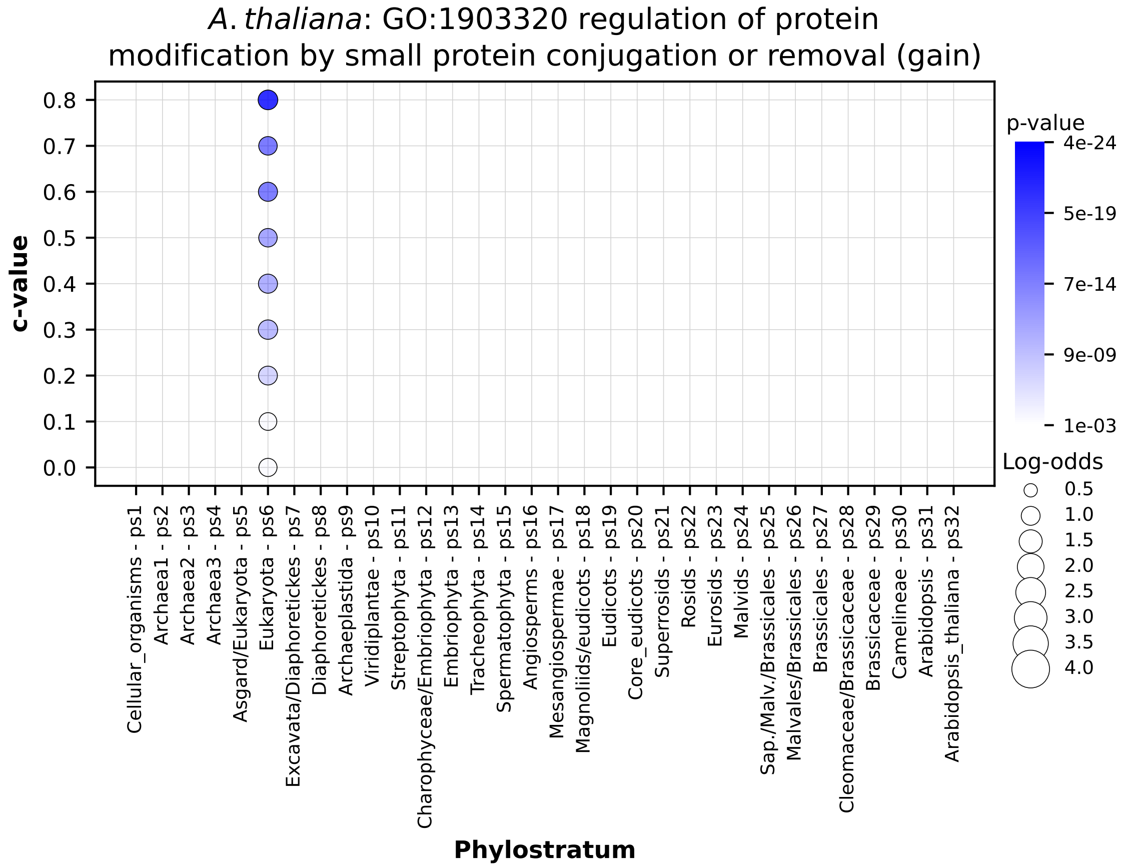

A. thaliana: GO:1903320 regulation of protein modification by small protein conjugation or removal (gain)
c-value
0.0
0.1
0.2
0.3
0.4
0.5
0.6
0.7
0.8
Phylostratum
Cellular_organisms - ps1
Archaea1 - ps2
Archaea2 - ps3
Archaea3 - ps4
Asgard/Eukaryota - ps5
Eukaryota - ps6
Excavata/Diaphoretickes - ps7
Diaphoretickes - ps8
Archaeplastida - ps9
Viridiplantae - ps10
Streptophyta - ps11
Charophyceae/Embriophyta - ps12
Embriophyta - ps13
Tracheophyta - ps14
Spermatophyta - ps15
Angiosperms - ps16
Mesangiospermae - ps17
Magnoliids/eudicots - ps18
Eudicots - ps19
Core_eudicots - ps20
Superrosids - ps21
Rosids - ps22
Eurosids - ps23
Malvids - ps24
Sap./Malv./Brassicales - ps25
Malvales/Brassicales - ps26
Brassicales - ps27
Cleomaceae/Brassicaceae - ps28
Brassicaceae - ps29
Camelineae - ps30
Arabidopsis - ps31
Arabidopsis_thaliana - ps32
p-value
4e-24
5e-19
7e-14
9e-09
1e-03
Log-odds
0.5
1.0
1.5
2.0
2.5
3.0
3.5
4.0

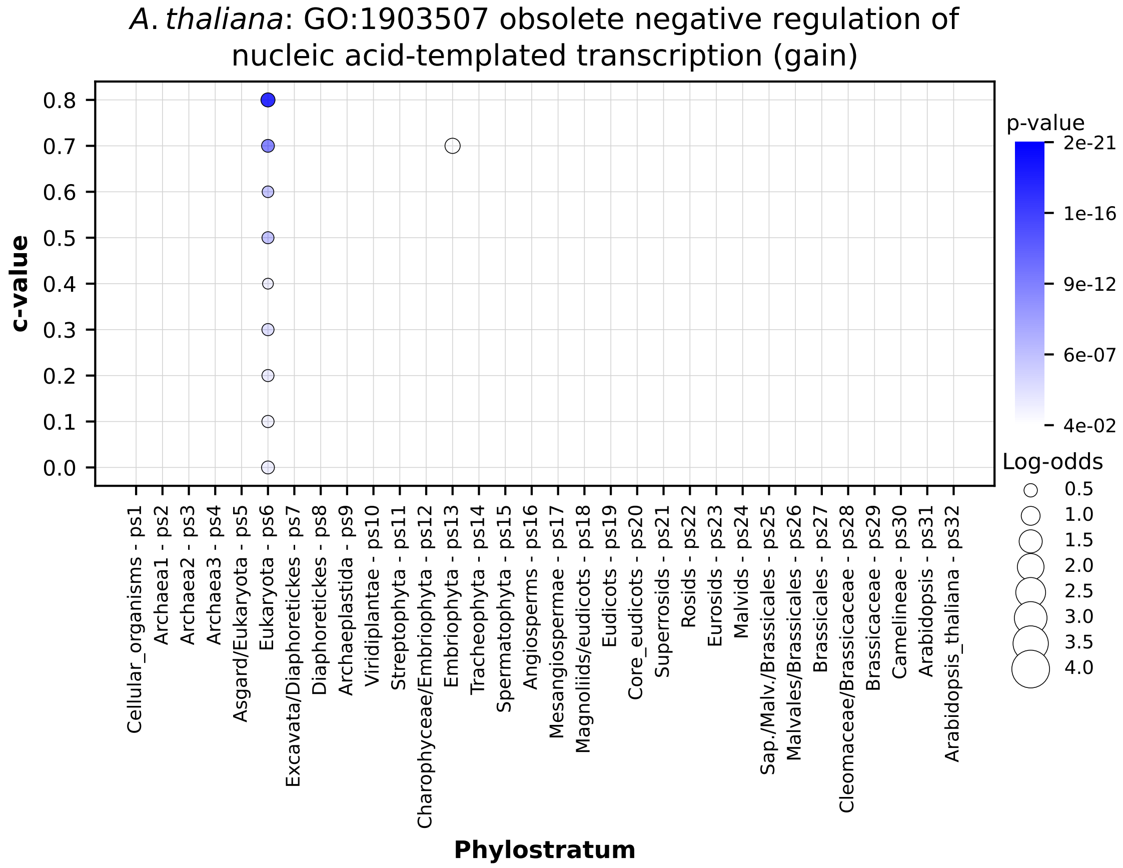
A. thaliana: GO:1903507 obsolete negative regulation of nucleic acid-templated transcription (gain)
c-value
0.0
0.1
0.2
0.3
0.4
0.5
0.6
0.7
0.8
Phylostratum
Cellular_organisms - ps1
Archaea1 - ps2
Archaea2 - ps3
Archaea3 - ps4
Asgard/Eukaryota - ps5
Eukaryota - ps6
Excavata/Diaphoretickes - ps7
Diaphoretickes - ps8
Archaeplastida - ps9
Viridiplantae - ps10
Streptophyta - ps11
Charophyceae/Embriophyta - ps12
Embriophyta - ps13
Tracheophyta - ps14
Spermatophyta - ps15
Angiosperms - ps16
Mesangiospermae - ps17
Magnoliids/eudicots - ps18
Eudicots - ps19
Core_eudicots - ps20
Superrosids - ps21
Rosids - ps22
Eurosids - ps23
Malvids - ps24
Sap./Malv./Brassicales - ps25
Malvales/Brassicales - ps26
Brassicales - ps27
Cleomaceae/Brassicaceae - ps28
Brassicaceae - ps29
Camelineae - ps30
Arabidopsis - ps31
Arabidopsis_thaliana - ps32
p-value
2e-21
1e-16
9e-12
6e-07
4e-02
Log-odds
0.5
1.0
1.5
2.0
2.5
3.0
3.5
4.0

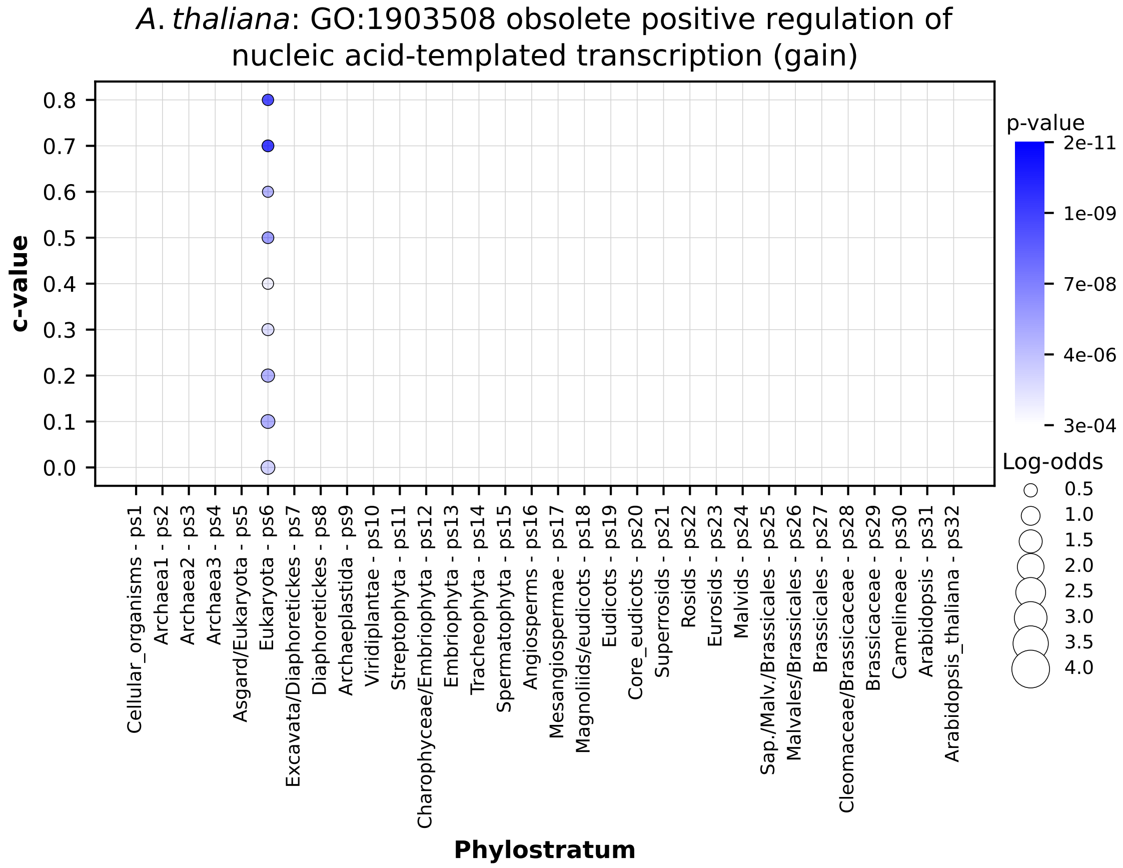
A. thaliana: GO:1903508 obsolete positive regulation of nucleic acid-templated transcription (gain)
c-value
0.8
0.7
0.6
0.5
0.4
0.3
0.2
0.1
0.0
Phylostratum
Cellular_organisms - ps1
Archaea1 - ps2
Archaea2 - ps3
Archaea3 - ps4
Asgard/Eukaryota - ps5
Eukaryota - ps6
Excavata/Diaphoretickes - ps7
Diaphoretickes - ps8
Archaeplastida - ps9
Viridiplantae - ps10
Streptophyta - ps11
Charophyceae/Embriophyta - ps12
Embriophyta - ps13
Tracheophyta - ps14
Spermatophyta - ps15
Angiosperms - ps16
Mesangiospermae - ps17
Magnoliids/eudicots - ps18
Eudicots - ps19
Core_eudicots - ps20
Superrosids - ps21
Rosids - ps22
Eurosids - ps23
Malvids - ps24
Sap./Malv./Brassicales - ps25
Malvales/Brassicales - ps26
Brassicales - ps27
Cleomaceae/Brassicaceae - ps28
Brassicaceae - ps29
Camelineae - ps30
Arabidopsis - ps31
Arabidopsis_thaliana - ps32
p-value
2e-11
1e-09
7e-08
4e-06
3e-04
Log-odds
0.5
1.0
1.5
2.0
2.5
3.0
3.5
4.0

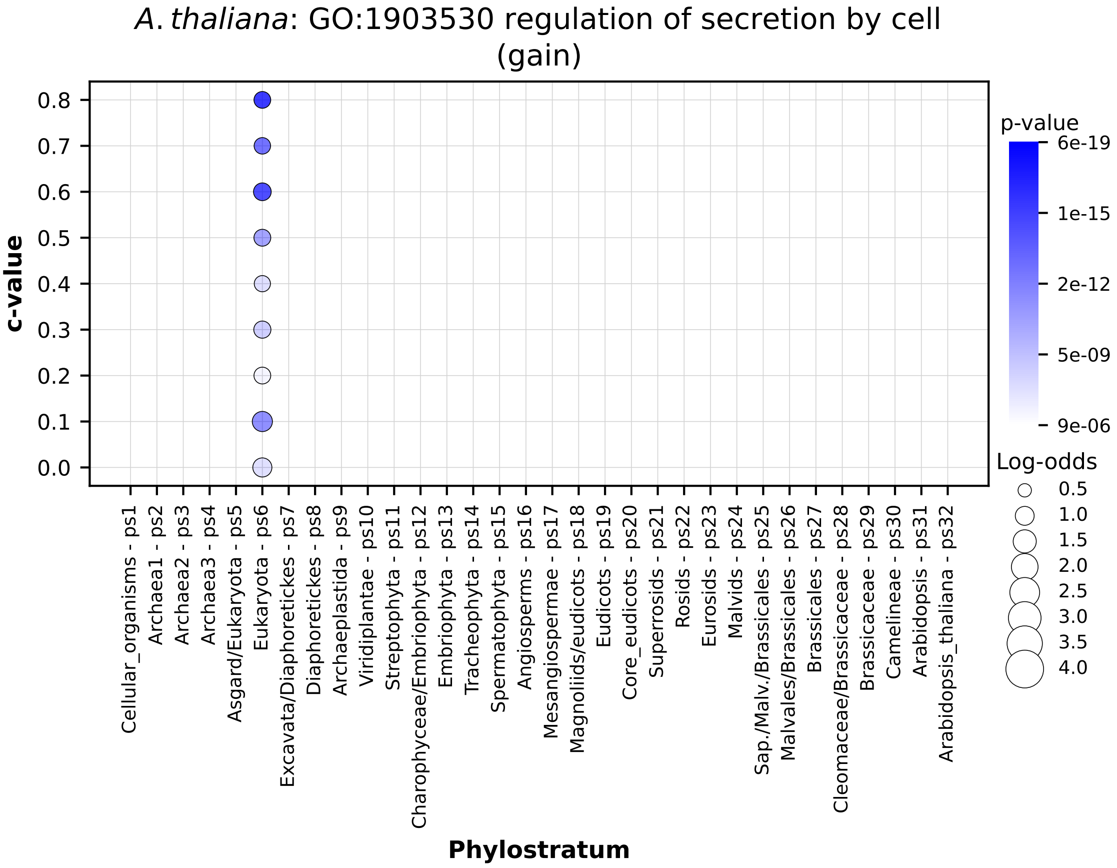

$A. thaliana$: GO:1903530 regulation of secretion by cell
(gain)
c-value
0.8
0.7
0.6
0.5
0.4
0.3
0.2
0.1
0.0
p-value
6e-19
1e-15
2e-12
5e-09
9e-06
Log-odds
0.5
1.0
1.5
2.0
2.5
3.0
3.5
4.0
Cellular_organisms - ps1
Archaea1 - ps2
Archaea2 - ps3
Archaea3 - ps4
Asgard/Eukaryota - ps5
Eukaryota - ps6
Excavata/Diaphoretickes - ps7
Diaphoretickes - ps8
Archaeplastida - ps9
Viridiplantae - ps10
Streptophyta - ps11
Charophyceae/Embriophyta - ps12
Embriophyta - ps13
Tracheophyta - ps14
Spermatophyta - ps15
Angiosperms - ps16
Mesangiospermae - ps17
Magnoliids/eudicots - ps18
Eudicots - ps19
Core_eudicots - ps20
Superrosids - ps21
Rosids - ps22
Eurosids - ps23
Malvids - ps24
Sap./Malv./Brassicales - ps25
Malvales/Brassicales - ps26
Brassicales - ps27
Cleomaceae/Brassicaceae - ps28
Brassicaceae - ps29
Camelineae - ps30
Arabidopsis - ps31
Arabidopsis_thaliana - ps32
Phylostratum

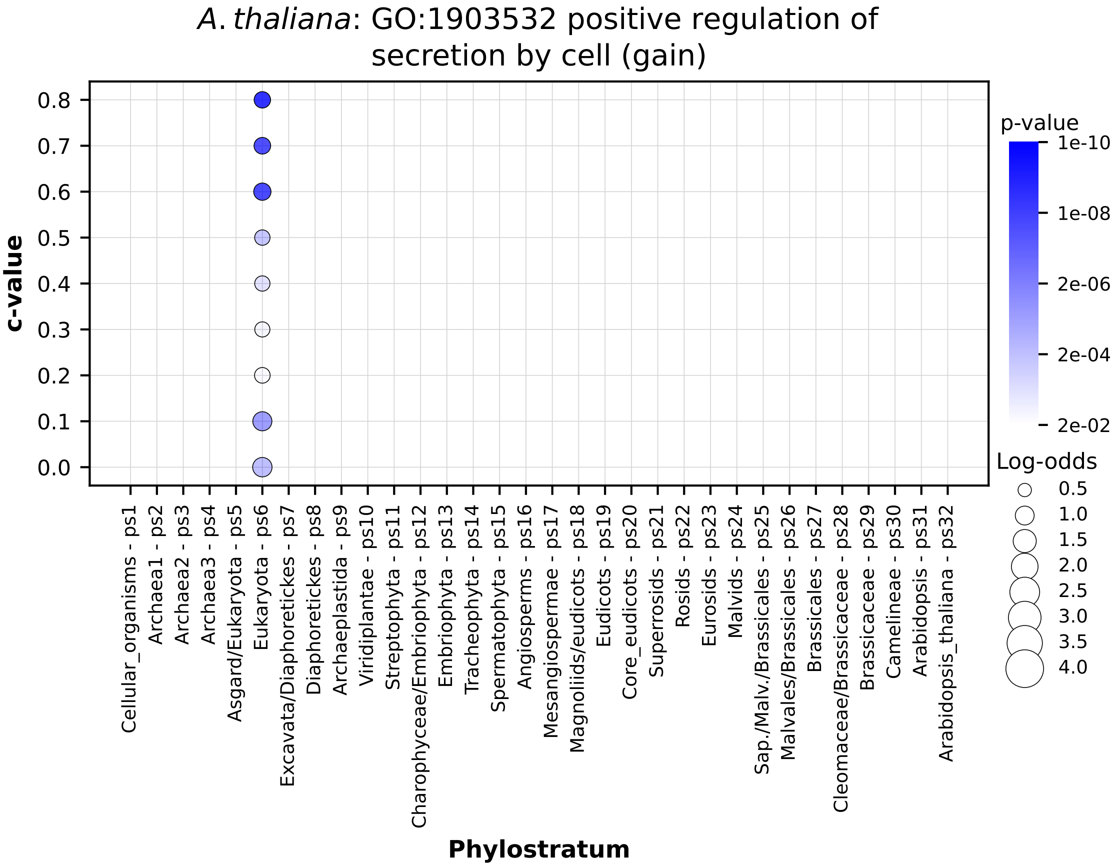

A. thaliana: GO:1903532 positive regulation of secretion by cell (gain)
c-value
0.8
0.7
0.6
0.5
0.4
0.3
0.2
0.1
0.0
Phylostratum
Cellular_organisms - ps1
Archaea1 - ps2
Archaea2 - ps3
Archaea3 - ps4
Asgard/Eukaryota - ps5
Eukaryota - ps6
Excavata/Diaphoretickes - ps7
Diaphoretickes - ps8
Archaeplastida - ps9
Viridiplantae - ps10
Streptophyta - ps11
Charophyceae/Embriophyta - ps12
Embriophyta - ps13
Tracheophyta - ps14
Spermatophyta - ps15
Angiosperms - ps16
Mesangiospermae - ps17
Magnoliids/eudicots - ps18
Eudicots - ps19
Core_eudicots - ps20
Superrosids - ps21
Rosids - ps22
Eurosids - ps23
Malvids - ps24
Sap./Malv./Brassicales - ps25
Malvales/Brassicales - ps26
Brassicales - ps27
Cleomaceae/Brassicaceae - ps28
Brassicaceae - ps29
Camelineae - ps30
Arabidopsis - ps31
Arabidopsis_thaliana - ps32
p-value
1e-10
1e-08
2e-06
2e-04
2e-02
Log-odds
0.5
1.0
1.5
2.0
2.5
3.0
3.5
4.0

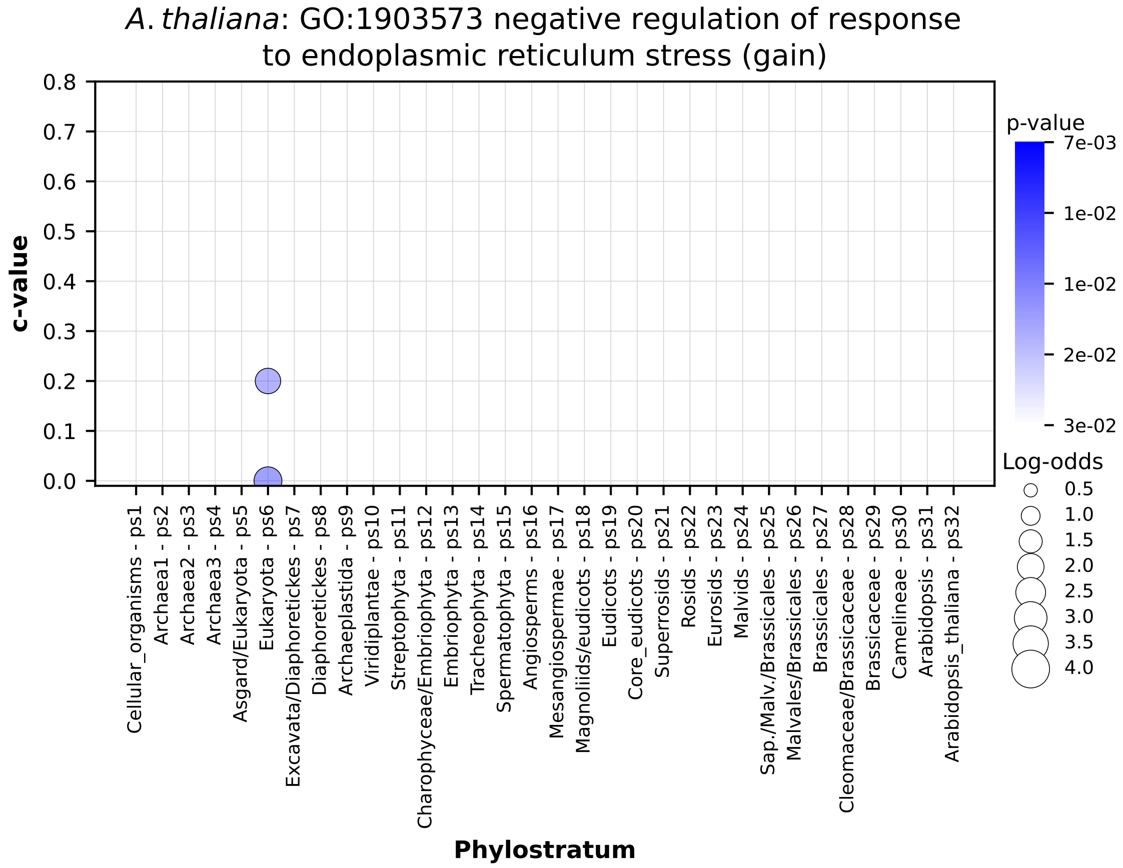

A. thaliana: GO:1903573 negative regulation of response to endoplasmic reticulum stress (gain)
c-value
0.0
0.1
0.2
0.3
0.4
0.5
0.6
0.7
0.8
Phylostratum
Cellular_organisms - ps1
Archaea1 - ps2
Archaea2 - ps3
Archaea3 - ps4
Asgard/Eukaryota - ps5
Eukaryota - ps6
Excavata/Diaphoretickes - ps7
Diaphoretickes - ps8
Archaeplastida - ps9
Viridiplantae - ps10
Streptophyta - ps11
Charophyceae/Embriophyta - ps12
Embriophyta - ps13
Tracheophyta - ps14
Spermatophyta - ps15
Angiosperms - ps16
Mesangiospermae - ps17
Magnoliids/eudicots - ps18
Eudicots - ps19
Core_eudicots - ps20
Superrosids - ps21
Rosids - ps22
Eurosids - ps23
Malvids - ps24
Sap./Malv./Brassicales - ps25
Malvales/Brassicales - ps26
Brassicales - ps27
Cleomaceae/Brassicaceae - ps28
Brassicaceae - ps29
Camelineae - ps30
Arabidopsis - ps31
Arabidopsis_thaliana - ps32
p-value
7e-03
1e-02
1e-02
2e-02
3e-02
Log-odds
0.5
1.0
1.5
2.0
2.5
3.0
3.5
4.0

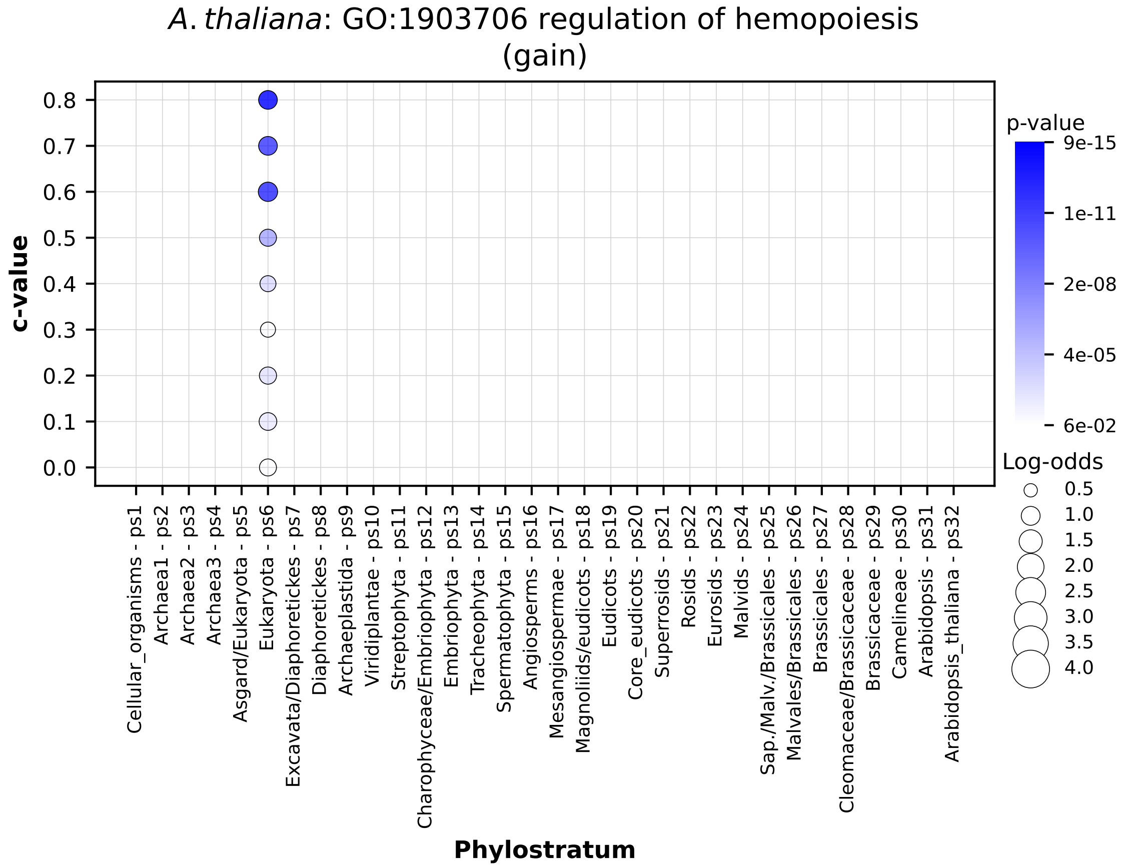

A. thaliana: GO:1903706 regulation of hemopoiesis
(gain)
c-value
0.8
0.7
0.6
0.5
0.4
0.3
0.2
0.1
0.0
p-value
9e-15
1e-11
2e-08
4e-05
6e-02
Log-odds
0.5
1.0
1.5
2.0
2.5
3.0
3.5
4.0
Cellular_organisms - ps1
Archaea1 - ps2
Archaea2 - ps3
Archaea3 - ps4
Asgard/Eukaryota - ps5
Eukaryota - ps6
Excavata/Diaphoretickes - ps7
Diaphoretickes - ps8
Archaeplastida - ps9
Viridiplantae - ps10
Streptophyta - ps11
Charophyceae/Embriophyta - ps12
Embriophyta - ps13
Tracheophyta - ps14
Spermatophyta - ps15
Angiosperms - ps16
Mesangiospermae - ps17
Magnoliids/eudicots - ps18
Eudicots - ps19
Core_eudicots - ps20
Superrosids - ps21
Rosids - ps22
Eurosids - ps23
Malvids - ps24
Sap./Malv./Brassicales - ps25
Malvales/Brassicales - ps26
Brassicales - ps27
Cleomaceae/Brassicaceae - ps28
Brassicaceae - ps29
Camelineae - ps30
Arabidopsis - ps31
Arabidopsis_thaliana - ps32
Phylostratum

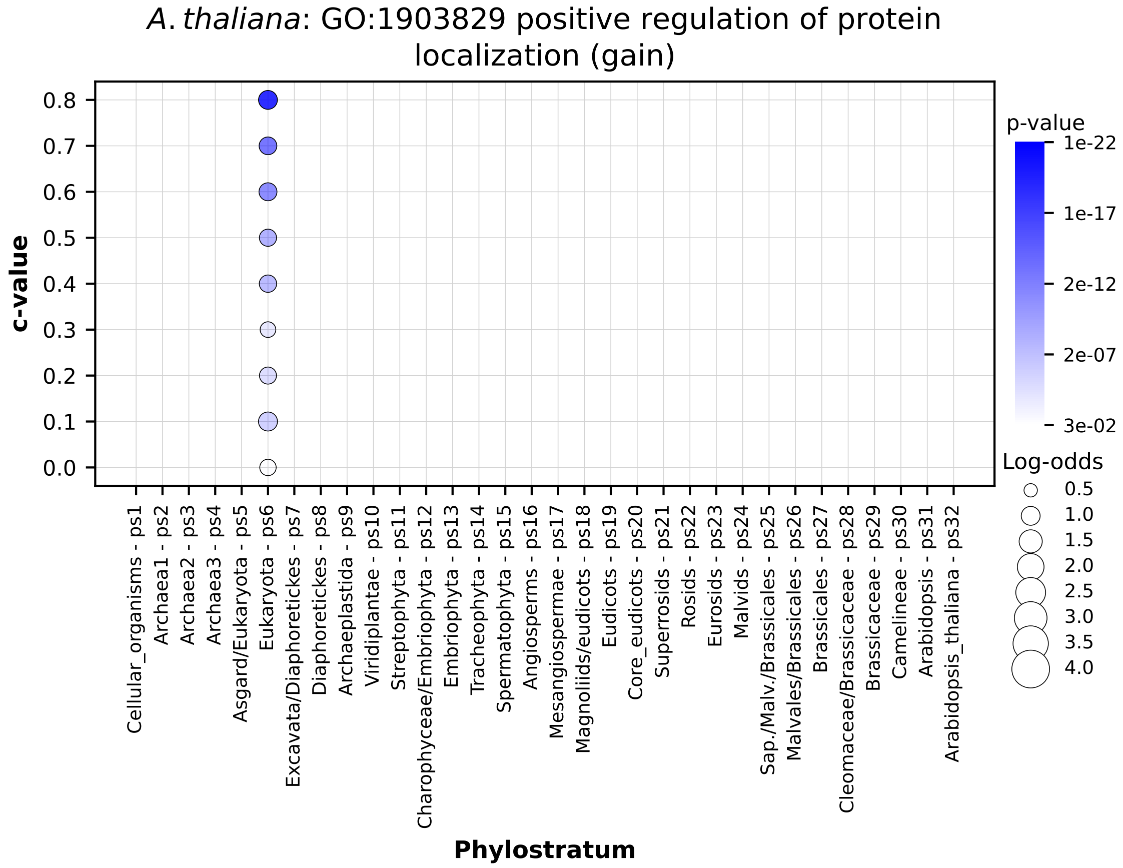

A. thaliana: GO:1903829 positive regulation of protein localization (gain)
c-value
0.8
0.7
0.6
0.5
0.4
0.3
0.2
0.1
0.0
p-value
1e-22
1e-17
2e-12
2e-07
3e-02
Log-odds
0.5
1.0
1.5
2.0
2.5
3.0
3.5
4.0
Cellular_organisms - ps1
Archaea1 - ps2
Archaea2 - ps3
Archaea3 - ps4
Asgard/Eukaryota - ps5
Eukaryota - ps6
Excavata/Diaphoretickes - ps7
Diaphoretickes - ps8
Archaeplastida - ps9
Viridiplantae - ps10
Streptophyta - ps11
Charophyceae/Embriophyta - ps12
Embriophyta - ps13
Tracheophyta - ps14
Spermatophyta - ps15
Angiosperms - ps16
Mesangiospermae - ps17
Magnoliids/eudicots - ps18
Eudicots - ps19
Core_eudicots - ps20
Superrosids - ps21
Rosids - ps22
Eurosids - ps23
Malvids - ps24
Sap./Malv./Brassicales - ps25
Malvales/Brassicales - ps26
Brassicales - ps27
Cleomaceae/Brassicaceae - ps28
Brassicaceae - ps29
Camelineae - ps30
Arabidopsis - ps31
Arabidopsis_thaliana - ps32
Phylostratum

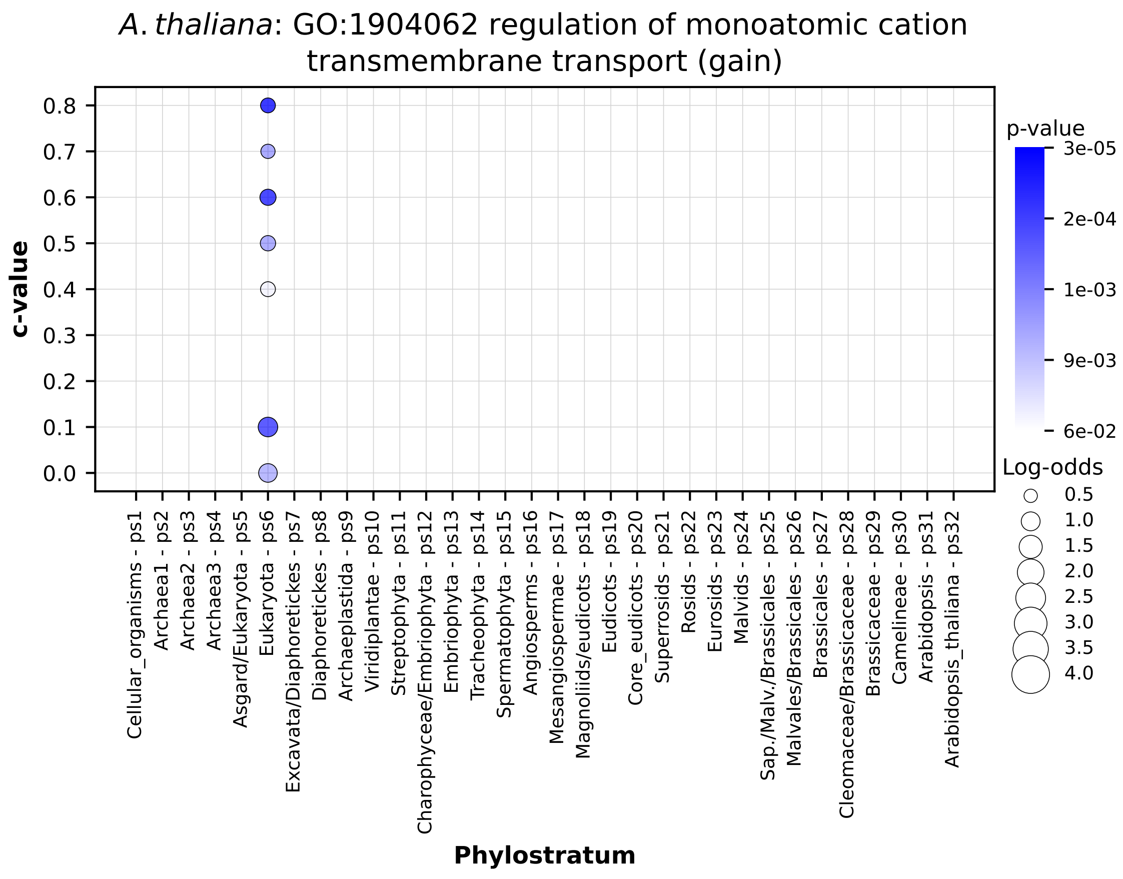

A. thaliana: GO:1904062 regulation of monoatomic cation transmembrane transport (gain)
c-value
0.8
0.7
0.6
0.5
0.4
0.3
0.2
0.1
0.0
Phylostratum
Cellular_organisms - ps1
Archaea1 - ps2
Archaea2 - ps3
Archaea3 - ps4
Asgard/Eukaryota - ps5
Eukaryota - ps6
Excavata/Diaphoretickes - ps7
Diaphoretickes - ps8
Archaeplastida - ps9
Viridiplantae - ps10
Streptophyta - ps11
Charophyceae/Embriophyta - ps12
Embriophyta - ps13
Tracheophyta - ps14
Spermatophyta - ps15
Angiosperms - ps16
Mesangiospermae - ps17
Magnoliids/eudicots - ps18
Eudicots - ps19
Core_eudicots - ps20
Superrosids - ps21
Rosids - ps22
Eurosids - ps23
Malvids - ps24
Sap./Malv./Brassicales - ps25
Malvales/Brassicales - ps26
Brassicales - ps27
Cleomaceae/Brassicaceae - ps28
Brassicaceae - ps29
Camelineae - ps30
Arabidopsis - ps31
Arabidopsis_thaliana - ps32
p-value
3e-05
2e-04
1e-03
9e-03
6e-02
Log-odds
0.5
1.0
1.5
2.0
2.5
3.0
3.5
4.0

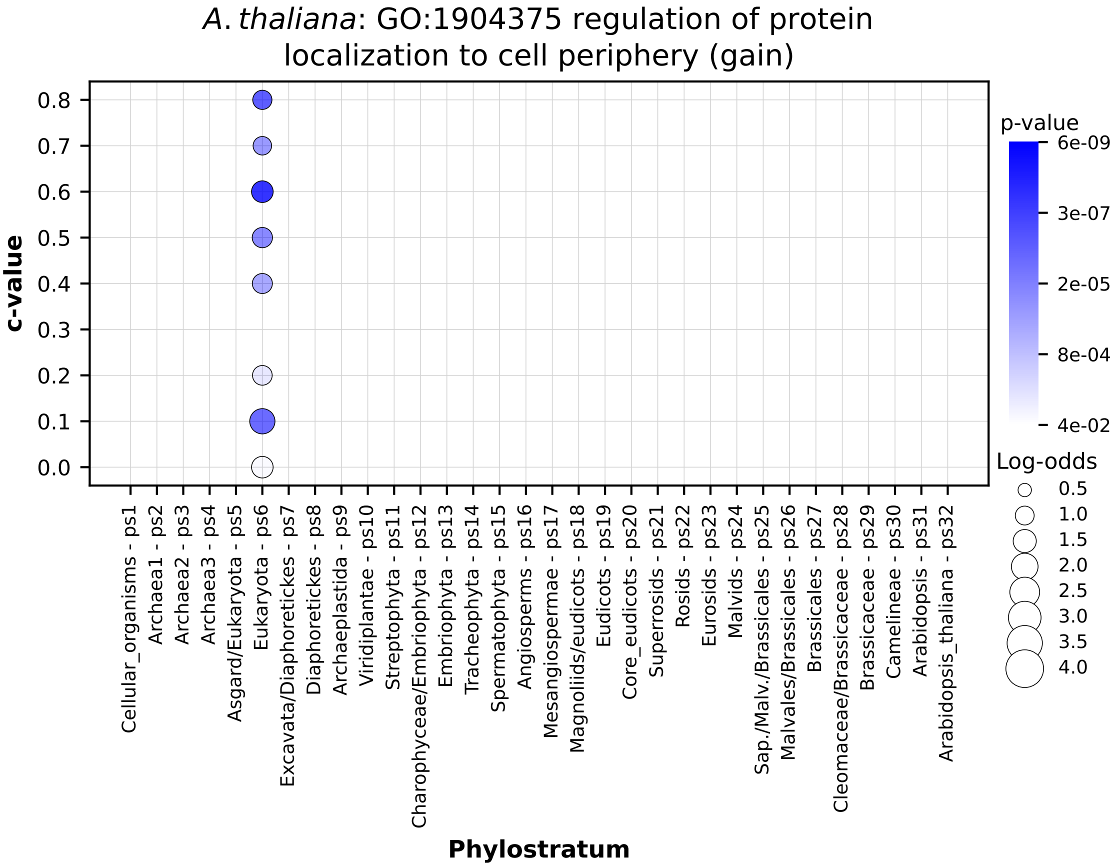

A. thaliana: GO:1904375 regulation of protein localization to cell periphery (gain)
c-value
0.8
0.7
0.6
0.5
0.4
0.3
0.2
0.1
0.0
p-value
6e-09
3e-07
2e-05
8e-04
4e-02
Log-odds
0.5
1.0
1.5
2.0
2.5
3.0
3.5
4.0
Cellular_organisms - ps1
Archaea1 - ps2
Archaea2 - ps3
Archaea3 - ps4
Asgard/Eukaryota - ps5
Eukaryota - ps6
Excavata/Diaphoretickes - ps7
Diaphoretickes - ps8
Archaeplastida - ps9
Viridiplantae - ps10
Streptophyta - ps11
Charophyceae/Embriophyta - ps12
Embriophyta - ps13
Tracheophyta - ps14
Spermatophyta - ps15
Angiosperms - ps16
Mesangiospermae - ps17
Magnoliids/eudicots - ps18
Eudicots - ps19
Core_eudicots - ps20
Superrosids - ps21
Rosids - ps22
Eurosids - ps23
Malvids - ps24
Sap./Malv./Brassicales - ps25
Malvales/Brassicales - ps26
Brassicales - ps27
Cleomaceae/Brassicaceae - ps28
Brassicaceae - ps29
Camelineae - ps30
Arabidopsis - ps31
Arabidopsis_thaliana - ps32
Phylostratum

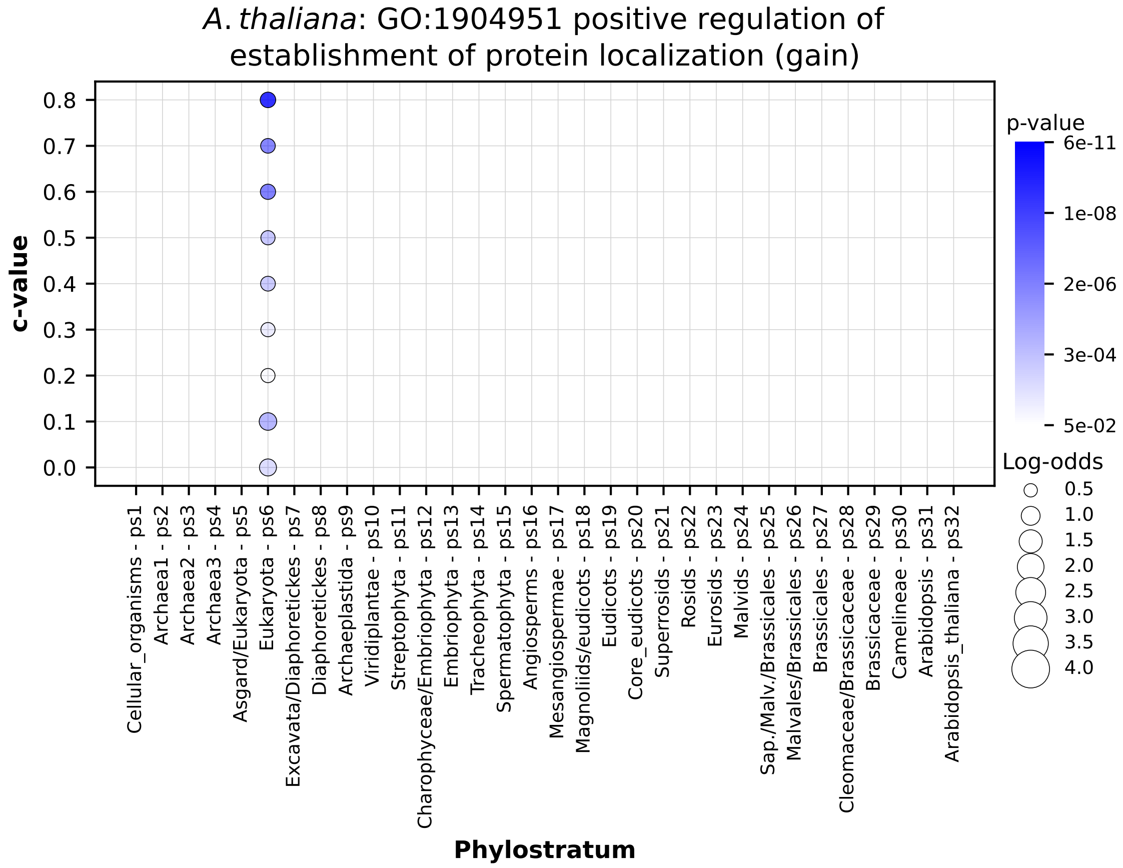

A. thaliana: GO:1904951 positive regulation of establishment of protein localization (gain)
c-value
0.8
0.7
0.6
0.5
0.4
0.3
0.2
0.1
0.0
Phylostratum
Cellular_organisms - ps1
Archaea1 - ps2
Archaea2 - ps3
Archaea3 - ps4
Asgard/Eukaryota - ps5
Eukaryota - ps6
Excavata/Diaphoretickes - ps7
Diaphoretickes - ps8
Archaeplastida - ps9
Viridiplantae - ps10
Streptophyta - ps11
Charophyceae/Embriophyta - ps12
Embriophyta - ps13
Tracheophyta - ps14
Spermatophyta - ps15
Angiosperms - ps16
Mesangiospermae - ps17
Magnoliids/eudicots - ps18
Eudicots - ps19
Core_eudicots - ps20
Superrosids - ps21
Rosids - ps22
Eurosids - ps23
Malvids - ps24
Sap./Malv./Brassicales - ps25
Malvales/Brassicales - ps26
Brassicales - ps27
Cleomaceae/Brassicaceae - ps28
Brassicaceae - ps29
Camelineae - ps30
Arabidopsis - ps31
Arabidopsis_thaliana - ps32
p-value
6e-11
1e-08
2e-06
3e-04
5e-02
Log-odds
0.5
1.0
1.5
2.0
2.5
3.0
3.5
4.0

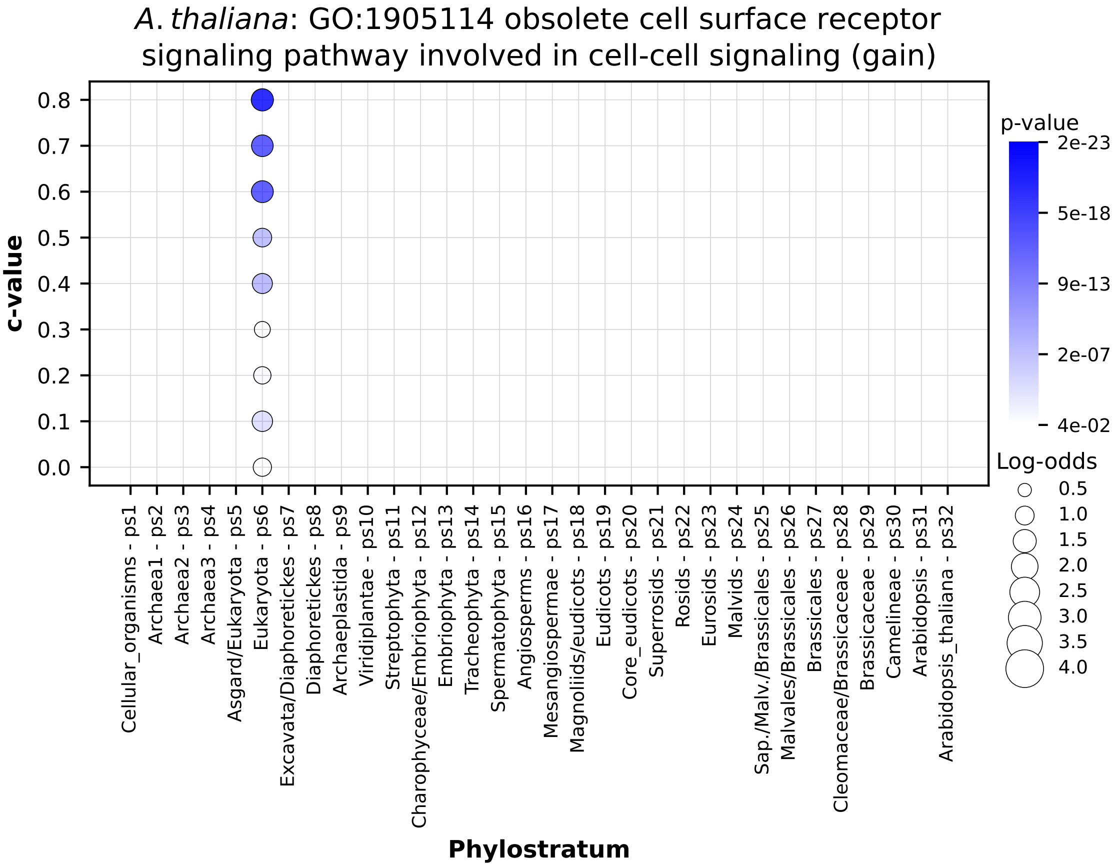

A. thaliana: GO:1905114 obsolete cell surface receptor signaling pathway involved in cell-cell signaling (gain)
c-value
0.8
0.7
0.6
0.5
0.4
0.3
0.2
0.1
0.0
p-value
2e-23
5e-18
9e-13
2e-07
4e-02
Log-odds
0.5
1.0
1.5
2.0
2.5
3.0
3.5
4.0
Cellular_organisms - ps1
Archaea1 - ps2
Archaea2 - ps3
Archaea3 - ps4
Asgard/Eukaryota - ps5
Eukaryota - ps6
Excavata/Diaphoretickes - ps7
Diaphoretickes - ps8
Archaeplastida - ps9
Viridiplantae - ps10
Streptophyta - ps11
Charophyceae/Embriophyta - ps12
Embriophyta - ps13
Tracheophyta - ps14
Spermatophyta - ps15
Angiosperms - ps16
Mesangiospermae - ps17
Magnoliids/eudicots - ps18
Eudicots - ps19
Core_eudicots - ps20
Superrosids - ps21
Rosids - ps22
Eurosids - ps23
Malvids - ps24
Sap./Malv./Brassicales - ps25
Malvales/Brassicales - ps26
Brassicales - ps27
Cleomaceae/Brassicaceae - ps28
Brassicaceae - ps29
Camelineae - ps30
Arabidopsis - ps31
Arabidopsis_thaliana - ps32
Phylostratum

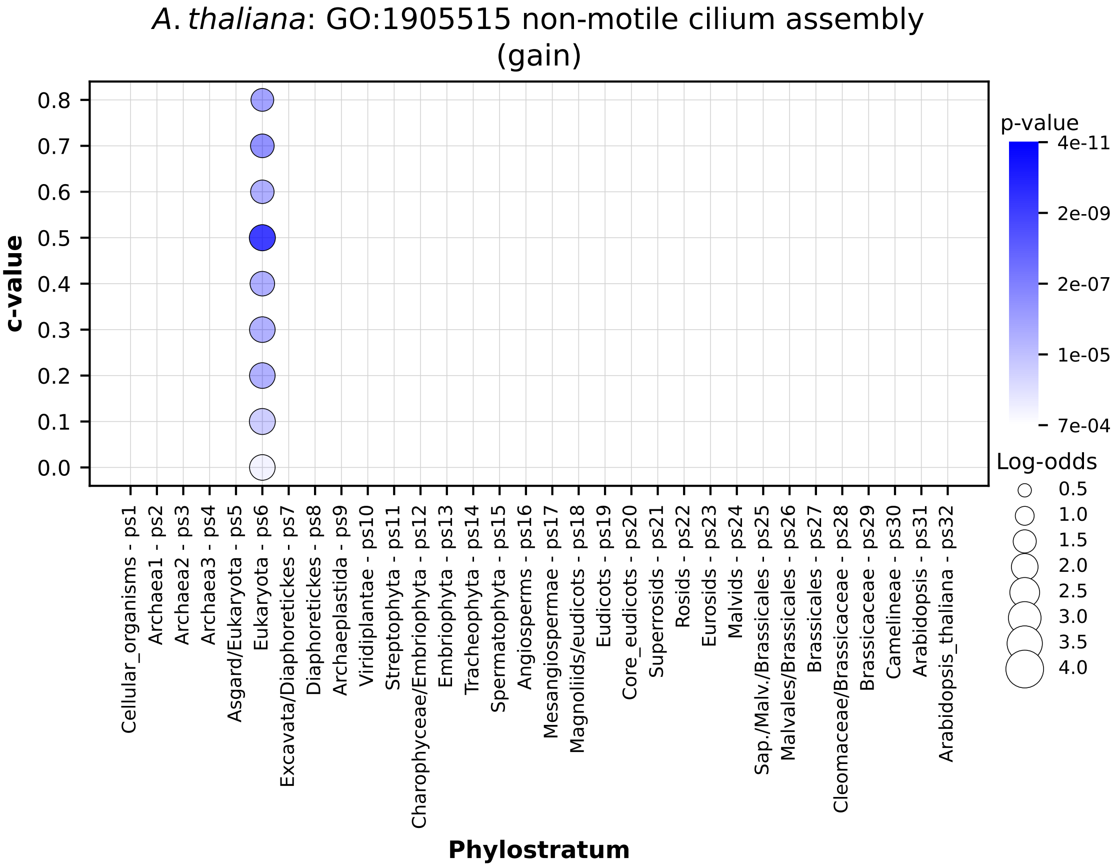
A. thaliana: GO:1905515 non-motile cilium assembly
(gain)
c-value
0.8
0.7
0.6
0.5
0.4
0.3
0.2
0.1
0.0
p-value
4e-11
2e-09
2e-07
1e-05
7e-04
Log-odds
0.5
1.0
1.5
2.0
2.5
3.0
3.5
4.0
Cellular_organisms - ps1
Archaea1 - ps2
Archaea2 - ps3
Archaea3 - ps4
Asgard/Eukaryota - ps5
Eukaryota - ps6
Excavata/Diaphoretickes - ps7
Diaphoretickes - ps8
Archaeplastida - ps9
Viridiplantae - ps10
Streptophyta - ps11
Charophyceae/Embriophyta - ps12
Embriophyta - ps13
Tracheophyta - ps14
Spermatophyta - ps15
Angiosperms - ps16
Mesangiospermae - ps17
Magnoliids/eudicots - ps18
Eudicots - ps19
Core_eudicots - ps20
Superrosids - ps21
Rosids - ps22
Eurosids - ps23
Malvids - ps24
Sap./Malv./Brassicales - ps25
Malvales/Brassicales - ps26
Brassicales - ps27
Cleomaceae/Brassicaceae - ps28
Brassicaceae - ps29
Camelineae - ps30
Arabidopsis - ps31
Arabidopsis_thaliana - ps32
Phylostratum

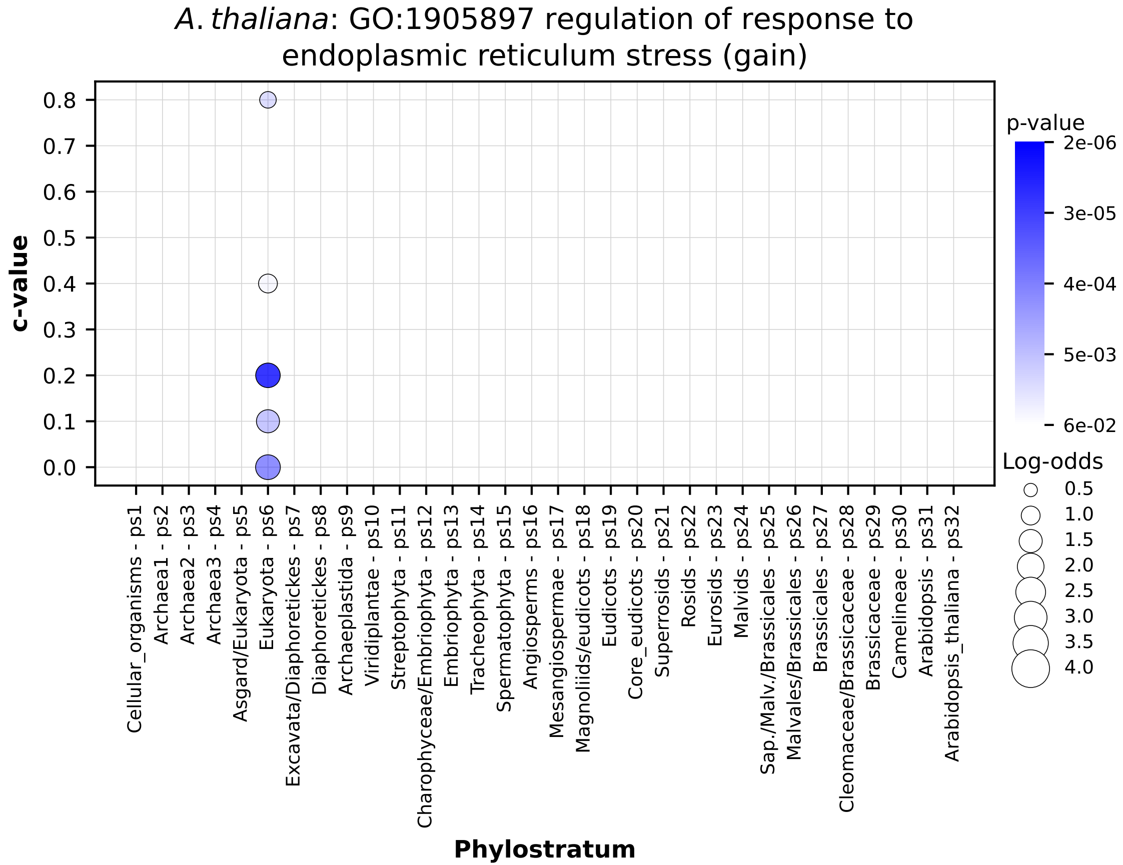

A. thaliana: GO:1905897 regulation of response to endoplasmic reticulum stress (gain)
c-value
0.8
0.7
0.6
0.5
0.4
0.3
0.2
0.1
0.0
p-value
2e-06
3e-05
4e-04
5e-03
6e-02
Log-odds
0.5
1.0
1.5
2.0
2.5
3.0
3.5
4.0
Cellular_organisms - ps1
Archaea1 - ps2
Archaea2 - ps3
Archaea3 - ps4
Asgard/Eukaryota - ps5
Eukaryota - ps6
Excavata/Diaphoretickes - ps7
Diaphoretickes - ps8
Archaeplastida - ps9
Viridiplantae - ps10
Streptophyta - ps11
Charophyceae/Embriophyta - ps12
Embriophyta - ps13
Tracheophyta - ps14
Spermatophyta - ps15
Angiosperms - ps16
Mesangiospermae - ps17
Magnoliids/eudicots - ps18
Eudicots - ps19
Core_eudicots - ps20
Superrosids - ps21
Rosids - ps22
Eurosids - ps23
Malvids - ps24
Sap./Malv./Brassicales - ps25
Malvales/Brassicales - ps26
Brassicales - ps27
Cleomaceae/Brassicaceae - ps28
Brassicaceae - ps29
Camelineae - ps30
Arabidopsis - ps31
Arabidopsis_thaliana - ps32
Phylostratum

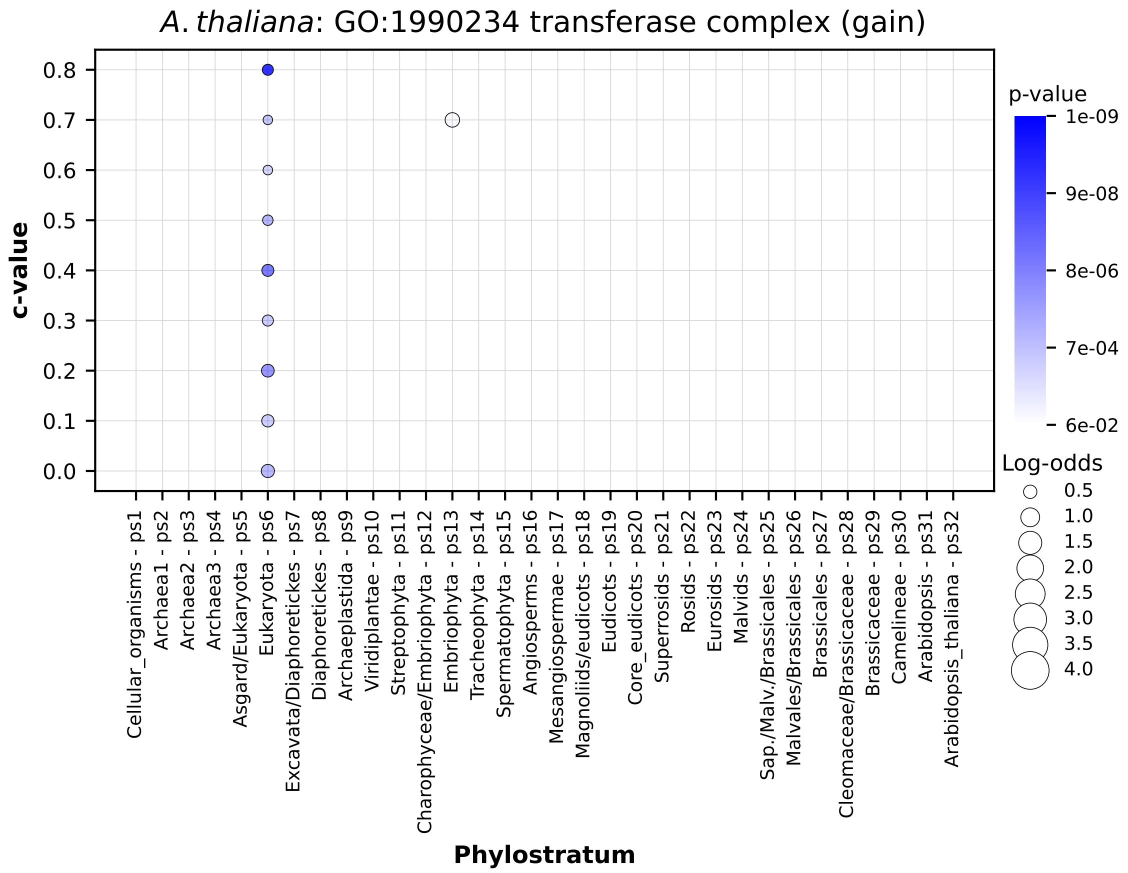

A. thaliana: GO:1990234 transferase complex (gain)
c-value
0.8
0.7
0.6
0.5
0.4
0.3
0.2
0.1
0.0
p-value
1e-09
9e-08
8e-06
7e-04
6e-02
Log-odds
0.5
1.0
1.5
2.0
2.5
3.0
3.5
4.0
Cellular_organisms - ps1
Archaea1 - ps2
Archaea2 - ps3
Archaea3 - ps4
Asgard/Eukaryota - ps5
Eukaryota - ps6
Excavata/Diaphoretickes - ps7
Diaphoretickes - ps8
Archaeplastida - ps9
Viridiplantae - ps10
Streptophyta - ps11
Charophyceae/Embriophyta - ps12
Embriophyta - ps13
Tracheophyta - ps14
Spermatophyta - ps15
Angiosperms - ps16
Mesangiospermae - ps17
Magnoliids/eudicots - ps18
Eudicots - ps19
Core_eudicots - ps20
Superrosids - ps21
Rosids - ps22
Eurosids - ps23
Malvids - ps24
Sap./Malv./Brassicales - ps25
Malvales/Brassicales - ps26
Brassicales - ps27
Cleomaceae/Brassicaceae - ps28
Brassicaceae - ps29
Camelineae - ps30
Arabidopsis - ps31
Arabidopsis_thaliana - ps32
Phylostratum

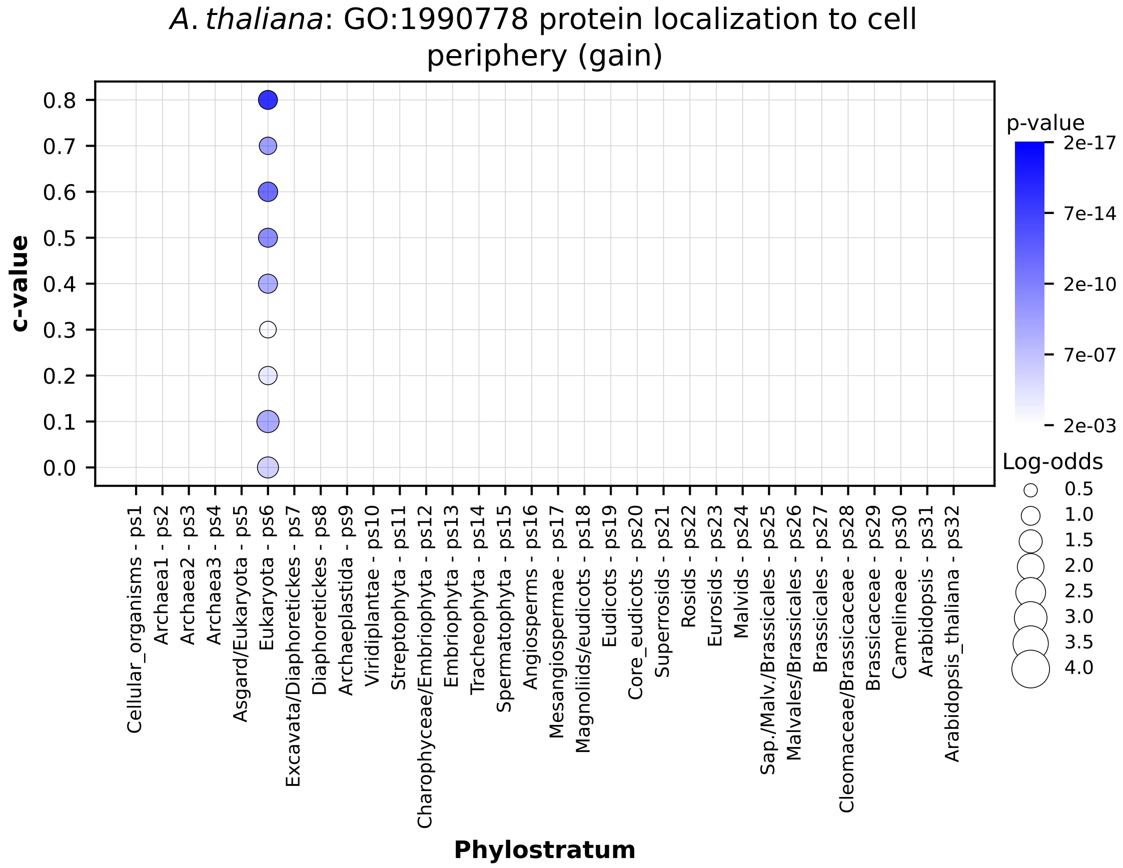

A. thaliana: GO:1990778 protein localization to cell periphery (gain)
c-value
0.8
0.7
0.6
0.5
0.4
0.3
0.2
0.1
0.0
p-value
2e-17
7e-14
2e-10
7e-07
2e-03
Log-odds
0.5
1.0
1.5
2.0
2.5
3.0
3.5
4.0
Cellular_organisms - ps1
Archaea1 - ps2
Archaea2 - ps3
Archaea3 - ps4
Asgard/Eukaryota - ps5
Eukaryota - ps6
Excavata/Diaphoretickes - ps7
Diaphoretickes - ps8
Archaeplastida - ps9
Viridiplantae - ps10
Streptophyta - ps11
Charophyceae/Embriophyta - ps12
Embriophyta - ps13
Tracheophyta - ps14
Spermatophyta - ps15
Angiosperms - ps16
Mesangiospermae - ps17
Magnoliids/eudicots - ps18
Eudicots - ps19
Core_eudicots - ps20
Superrosids - ps21
Rosids - ps22
Eurosids - ps23
Malvids - ps24
Sap./Malv./Brassicales - ps25
Malvales/Brassicales - ps26
Brassicales - ps27
Cleomaceae/Brassicaceae - ps28
Brassicaceae - ps29
Camelineae - ps30
Arabidopsis - ps31
Arabidopsis_thaliana - ps32
Phylostratum

A. thaliana: GO:2000026 regulation of multicellular organismal development (gain)

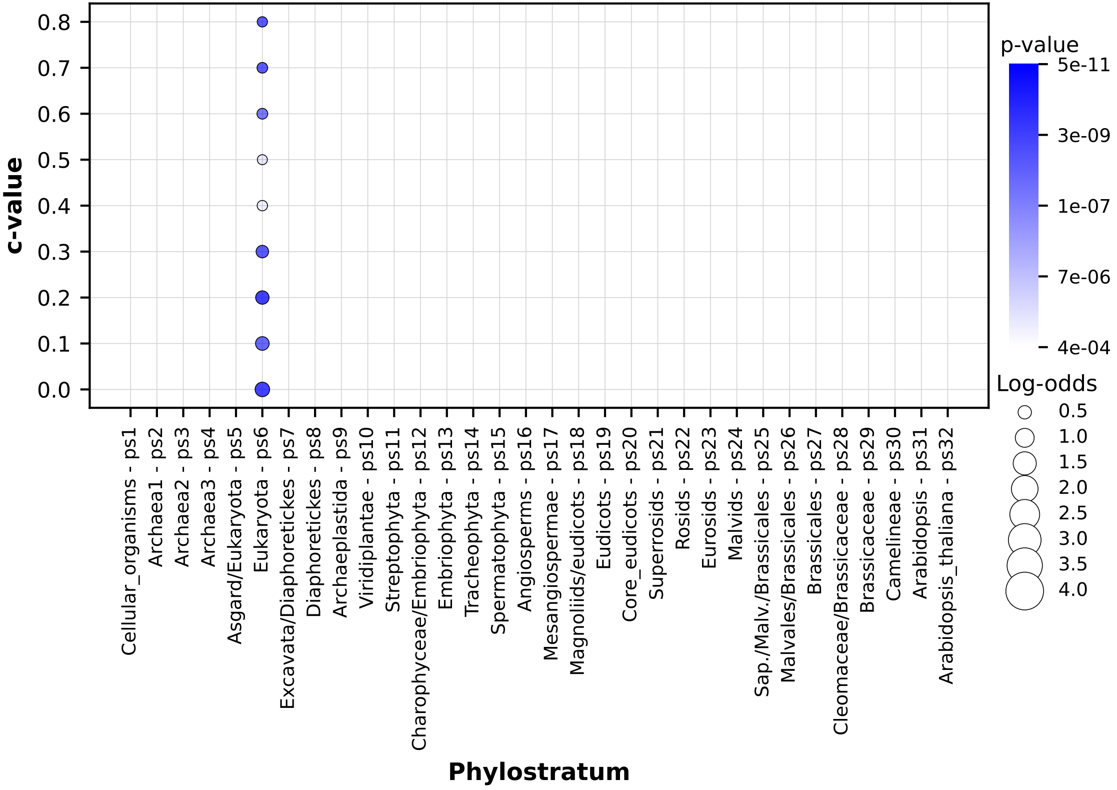

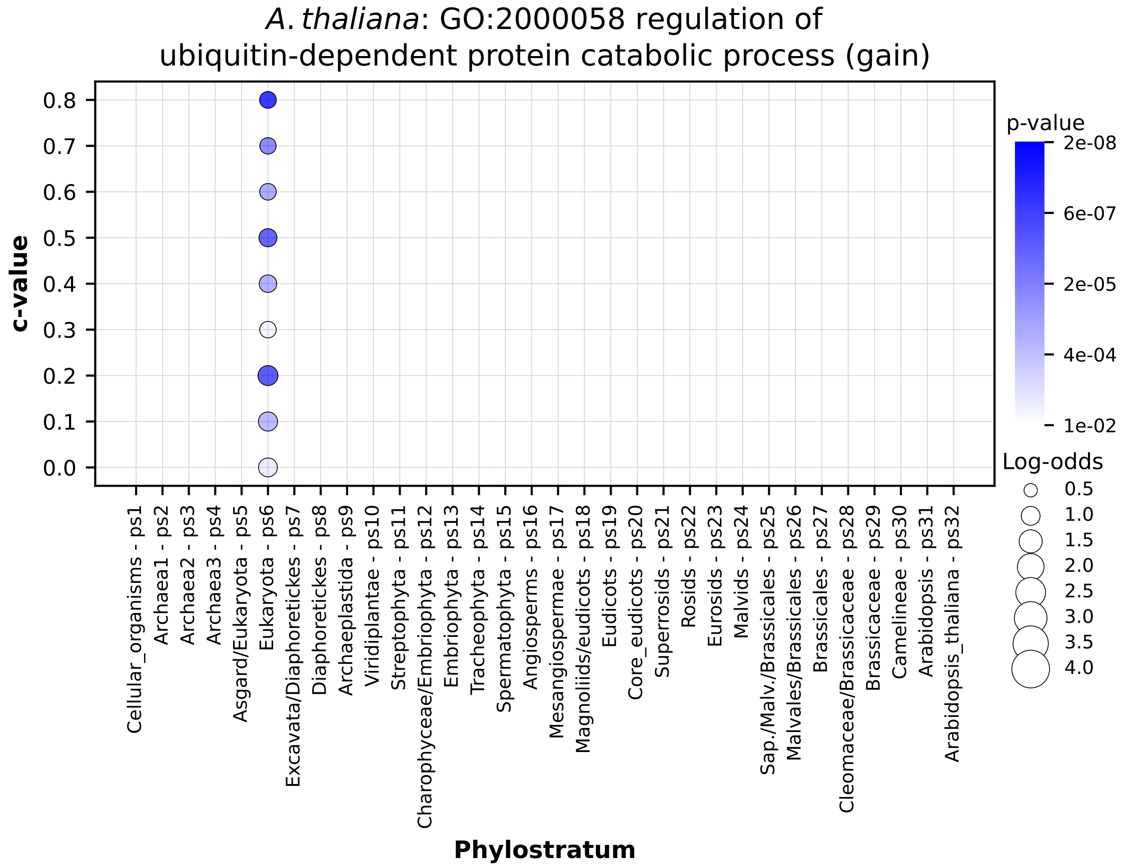
A. thaliana: GO:2000058 regulation of ubiquitin-dependent protein catabolic process (gain)
c-value
0.8
0.7
0.6
0.5
0.4
0.3
0.2
0.1
0.0
p-value
2e-08
6e-07
2e-05
4e-04
1e-02
Log-odds
0.5
1.0
1.5
2.0
2.5
3.0
3.5
4.0
Cellular_organisms - ps1
Archaea1 - ps2
Archaea2 - ps3
Archaea3 - ps4
Asgard/Eukaryota - ps5
Eukaryota - ps6
Excavata/Diaphoretickes - ps7
Diaphoretickes - ps8
Archaeplastida - ps9
Viridiplantae - ps10
Streptophyta - ps11
Charophyceae/Embriophyta - ps12
Embriophyta - ps13
Tracheophyta - ps14
Spermatophyta - ps15
Angiosperms - ps16
Mesangiospermae - ps17
Magnoliids/eudicots - ps18
Eudicots - ps19
Core_eudicots - ps20
Superrosids - ps21
Rosids - ps22
Eurosids - ps23
Malvids - ps24
Sap./Malv./Brassicales - ps25
Malvales/Brassicales - ps26
Brassicales - ps27
Cleomaceae/Brassicaceae - ps28
Brassicaceae - ps29
Camelineae - ps30
Arabidopsis - ps31
Arabidopsis_thaliana - ps32
Phylostratum

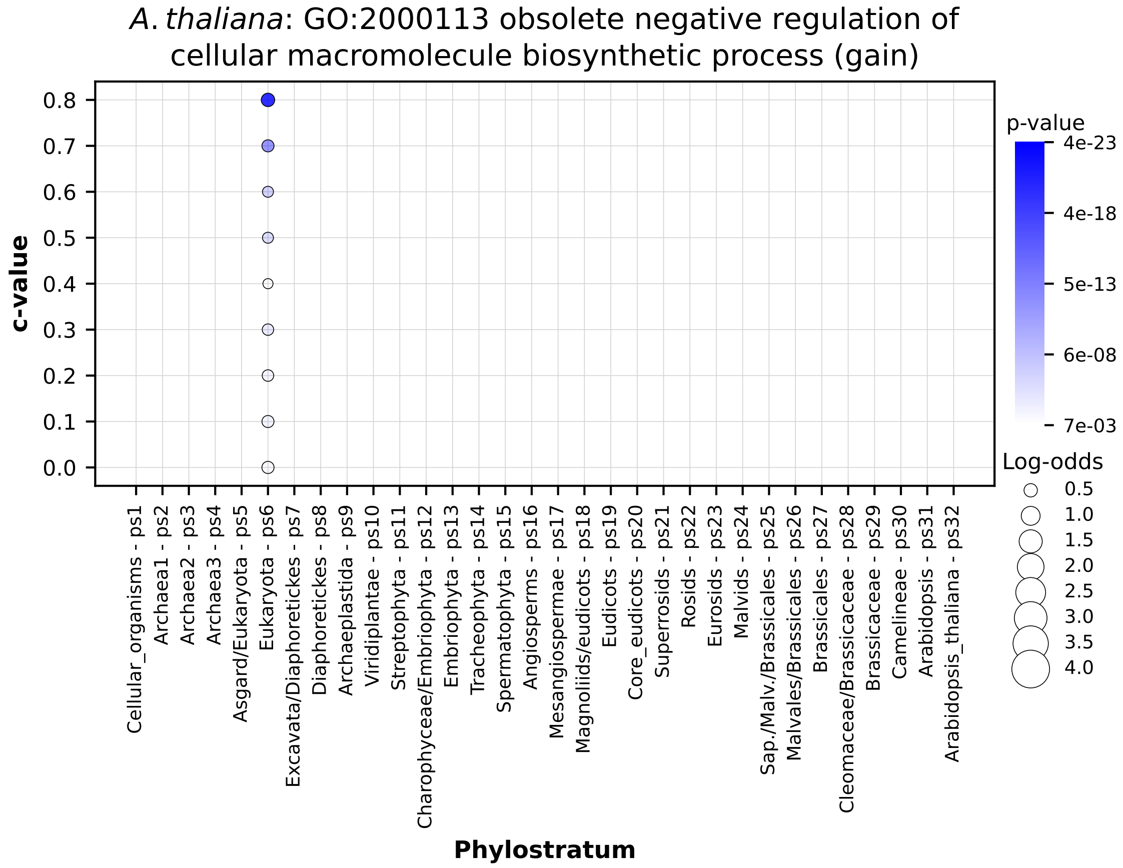

A. thaliana: GO:2000113 obsolete negative regulation of cellular macromolecule biosynthetic process (gain)
c-value
0.8
0.7
0.6
0.5
0.4
0.3
0.2
0.1
0.0
p-value
4e-23
4e-18
5e-13
6e-08
7e-03
Log-odds
0.5
1.0
1.5
2.0
2.5
3.0
3.5
4.0
Cellular_organisms - ps1
Archaea1 - ps2
Archaea2 - ps3
Archaea3 - ps4
Asgard/Eukaryota - ps5
Eukaryota - ps6
Excavata/Diaphoretickes - ps7
Diaphoretickes - ps8
Archaeplastida - ps9
Viridiplantae - ps10
Streptophyta - ps11
Charophyceae/Embriophyta - ps12
Embriophyta - ps13
Tracheophyta - ps14
Spermatophyta - ps15
Angiosperms - ps16
Mesangiospermae - ps17
Magnoliids/eudicots - ps18
Eudicots - ps19
Core_eudicots - ps20
Superrosids - ps21
Rosids - ps22
Eurosids - ps23
Malvids - ps24
Sap./Malv./Brassicales - ps25
Malvales/Brassicales - ps26
Brassicales - ps27
Cleomaceae/Brassicaceae - ps28
Brassicaceae - ps29
Camelineae - ps30
Arabidopsis - ps31
Arabidopsis_thaliana - ps32
Phylostratum

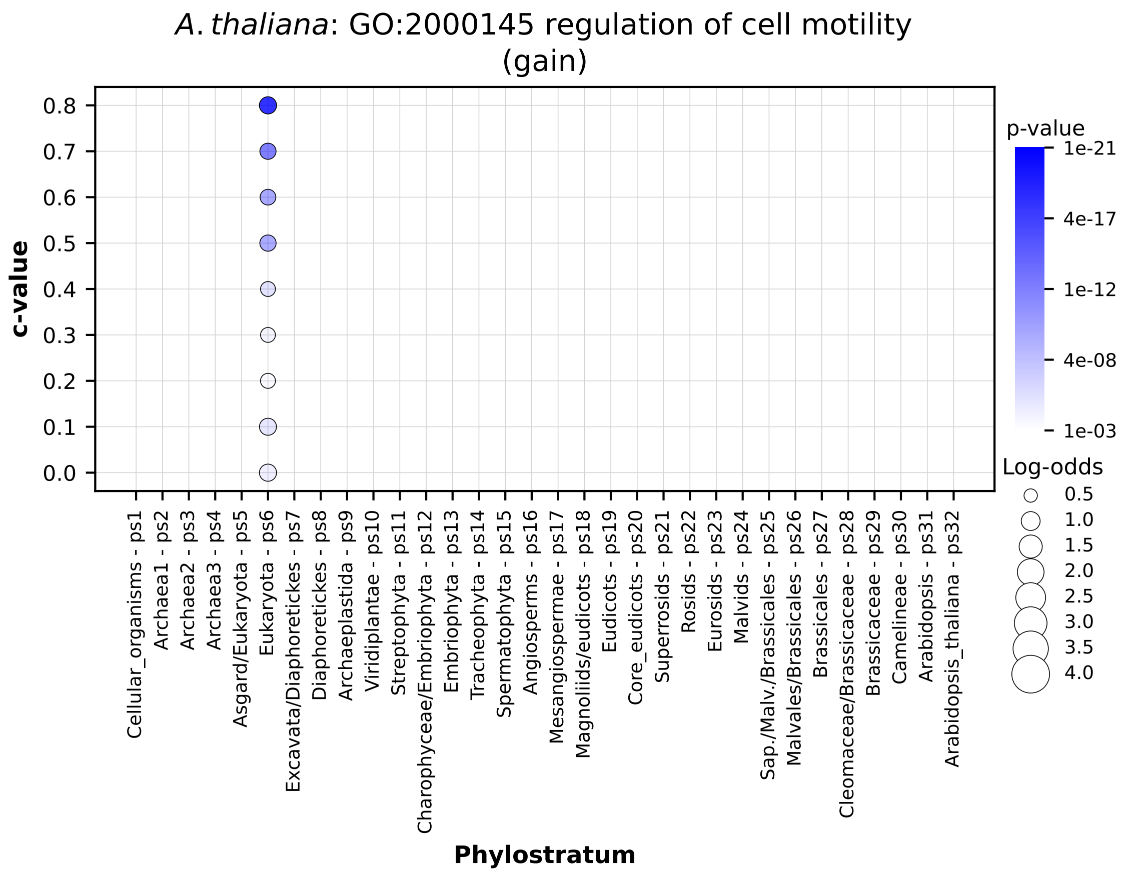

A. thaliana: GO:2000145 regulation of cell motility
(gain)
c-value
0.8
0.7
0.6
0.5
0.4
0.3
0.2
0.1
0.0
p-value
1e-21
4e-17
1e-12
4e-08
1e-03
Log-odds
0.5
1.0
1.5
2.0
2.5
3.0
3.5
4.0
Cellular_organisms - ps1
Archaea1 - ps2
Archaea2 - ps3
Archaea3 - ps4
Asgard/Eukaryota - ps5
Eukaryota - ps6
Excavata/Diaphoretickes - ps7
Diaphoretickes - ps8
Archaeplastida - ps9
Viridiplantae - ps10
Streptophyta - ps11
Charophyceae/Embriophyta - ps12
Embriophyta - ps13
Tracheophyta - ps14
Spermatophyta - ps15
Angiosperms - ps16
Mesangiospermae - ps17
Magnoliids/eudicots - ps18
Eudicots - ps19
Core_eudicots - ps20
Superrosids - ps21
Rosids - ps22
Eurosids - ps23
Malvids - ps24
Sap./Malv./Brassicales - ps25
Malvales/Brassicales - ps26
Brassicales - ps27
Cleomaceae/Brassicaceae - ps28
Brassicaceae - ps29
Camelineae - ps30
Arabidopsis - ps31
Arabidopsis_thaliana - ps32
Phylostratum

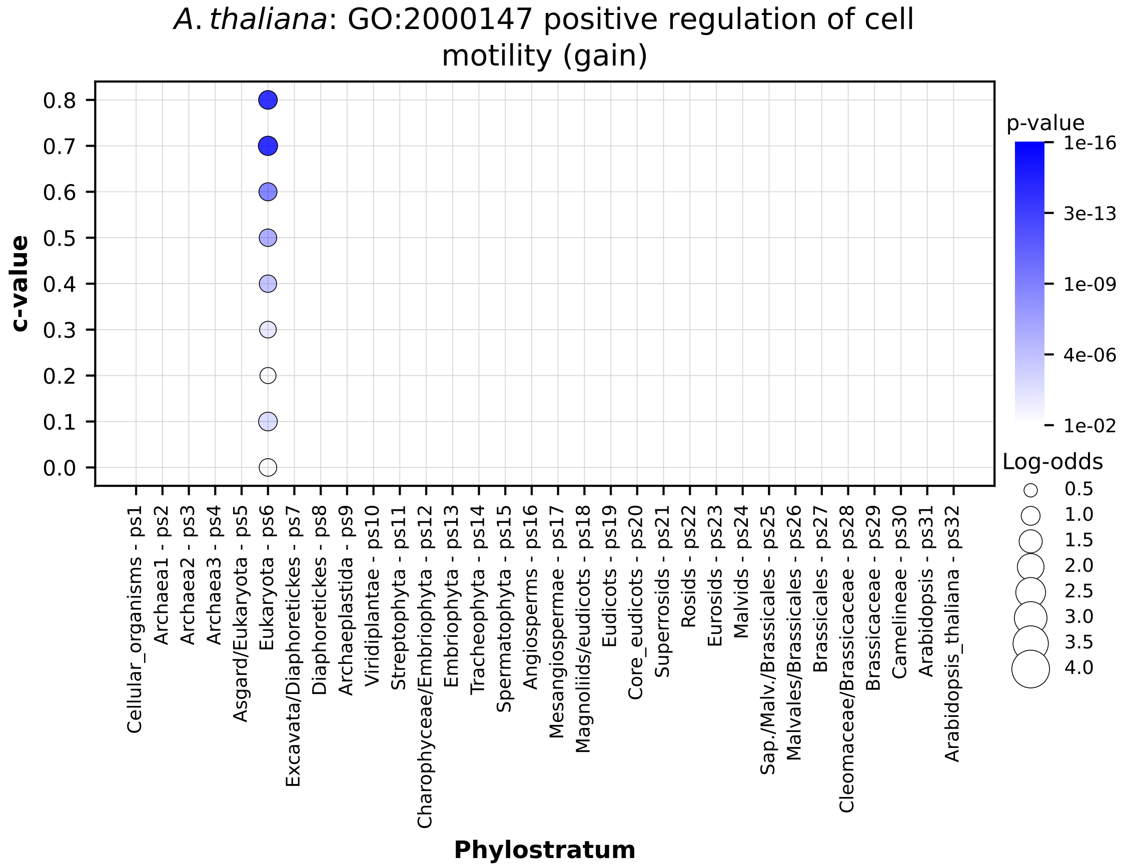

A. thaliana: GO:2000147 positive regulation of cell motility (gain)
c-value
0.8
0.7
0.6
0.5
0.4
0.3
0.2
0.1
0.0
p-value
1e-16
3e-13
1e-09
4e-06
1e-02
Log-odds
0.5
1.0
1.5
2.0
2.5
3.0
3.5
4.0
Cellular_organisms - ps1
Archaea1 - ps2
Archaea2 - ps3
Archaea3 - ps4
Asgard/Eukaryota - ps5
Eukaryota - ps6
Excavata/Diaphoretickes - ps7
Diaphoretickes - ps8
Archaeplastida - ps9
Viridiplantae - ps10
Streptophyta - ps11
Charophyceae/Embriophyta - ps12
Embriophyta - ps13
Tracheophyta - ps14
Spermatophyta - ps15
Angiosperms - ps16
Mesangiospermae - ps17
Magnoliids/eudicots - ps18
Eudicots - ps19
Core_eudicots - ps20
Superrosids - ps21
Rosids - ps22
Eurosids - ps23
Malvids - ps24
Sap./Malv./Brassicales - ps25
Malvales/Brassicales - ps26
Brassicales - ps27
Cleomaceae/Brassicaceae - ps28
Brassicaceae - ps29
Camelineae - ps30
Arabidopsis - ps31
Arabidopsis_thaliana - ps32
Phylostratum

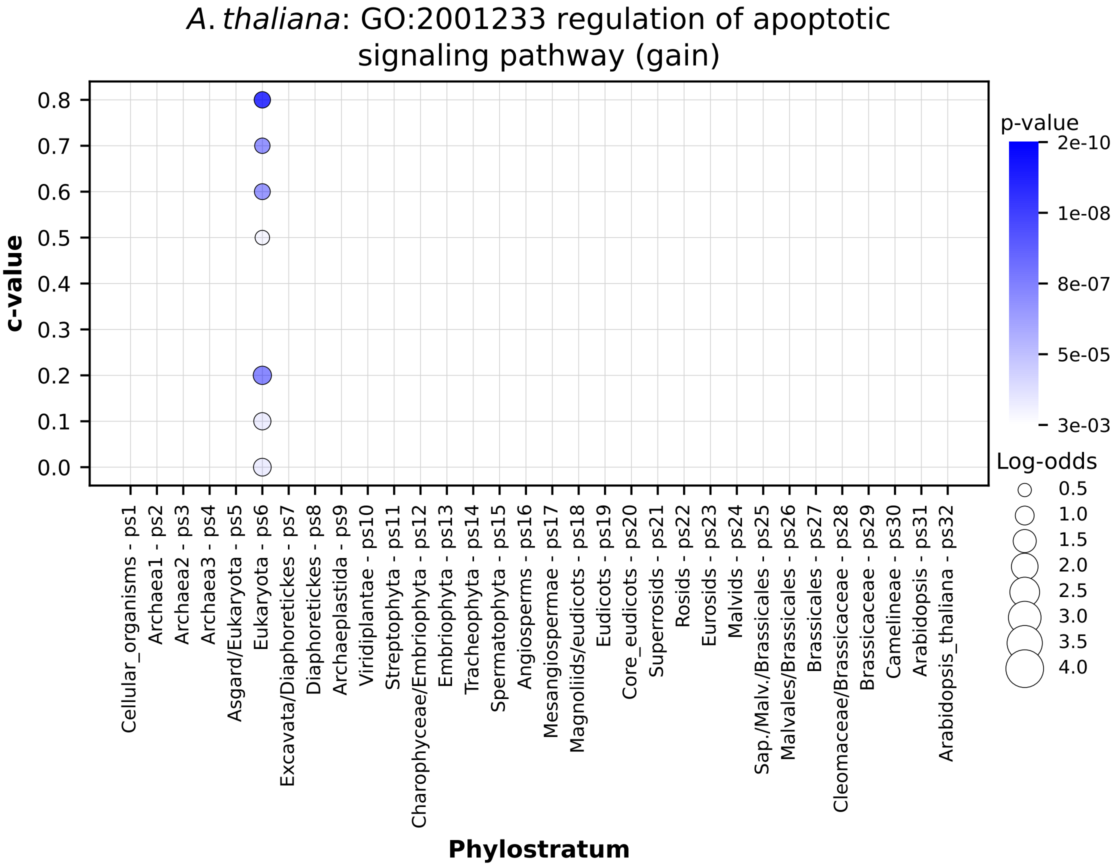

A. thaliana: GO:2001233 regulation of apoptotic signaling pathway (gain)
c-value
0.8
0.7
0.6
0.5
0.4
0.3
0.2
0.1
0.0
p-value
2e-10
1e-08
8e-07
5e-05
3e-03
Log-odds
0.5
1.0
1.5
2.0
2.5
3.0
3.5
4.0
Cellular_organisms - ps1
Archaea1 - ps2
Archaea2 - ps3
Archaea3 - ps4
Asgard/Eukaryota - ps5
Eukaryota - ps6
Excavata/Diaphoretickes - ps7
Diaphoretickes - ps8
Archaeplastida - ps9
Viridiplantae - ps10
Streptophyta - ps11
Charophyceae/Embriophyta - ps12
Embriophyta - ps13
Tracheophyta - ps14
Spermatophyta - ps15
Angiosperms - ps16
Mesangiospermae - ps17
Magnoliids/eudicots - ps18
Eudicots - ps19
Core_eudicots - ps20
Superrosids - ps21
Rosids - ps22
Eurosids - ps23
Malvids - ps24
Sap./Malv./Brassicales - ps25
Malvales/Brassicales - ps26
Brassicales - ps27
Cleomaceae/Brassicaceae - ps28
Brassicaceae - ps29
Camelineae - ps30
Arabidopsis - ps31
Arabidopsis_thaliana - ps32
Phylostratum

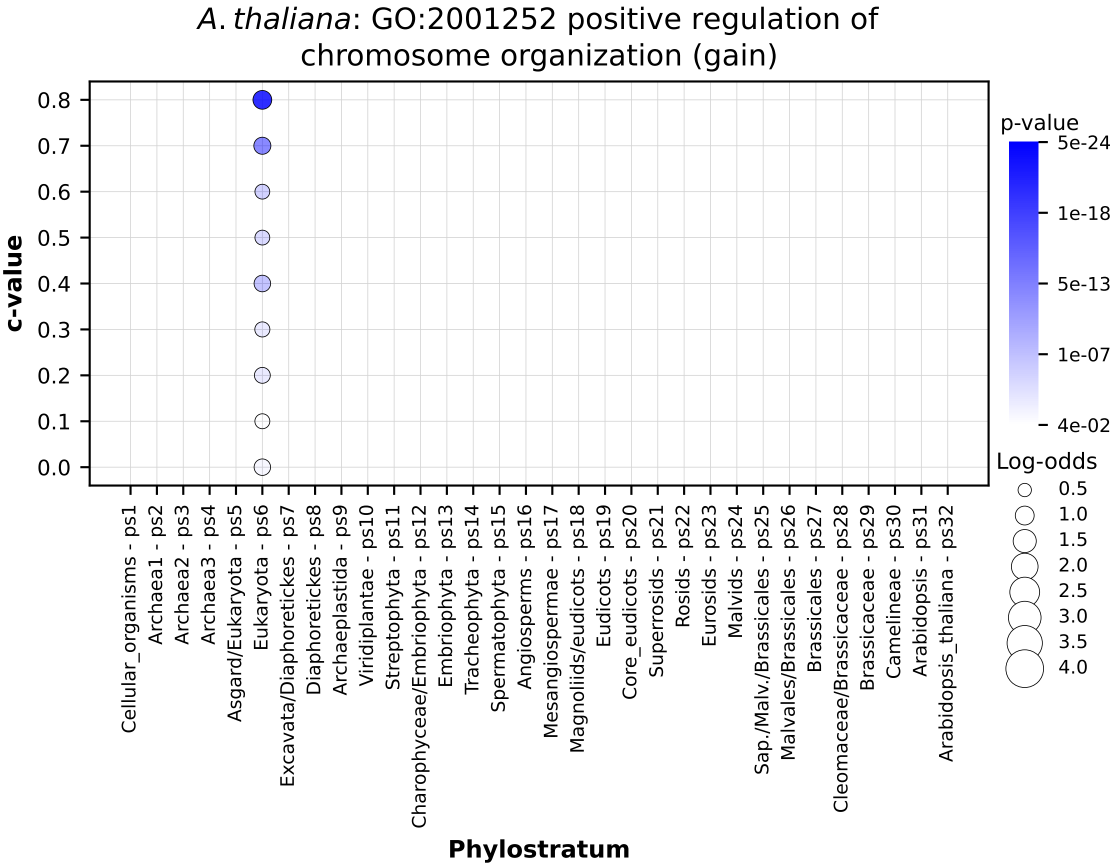

A. thaliana: GO:2001252 positive regulation of chromosome organization (gain)
c-value
0.8
0.7
0.6
0.5
0.4
0.3
0.2
0.1
0.0
p-value
5e-24
1e-18
5e-13
1e-07
4e-02
Log-odds
0.5
1.0
1.5
2.0
2.5
3.0
3.5
4.0
Cellular_organisms - ps1
Archaea1 - ps2
Archaea2 - ps3
Archaea3 - ps4
Asgard/Eukaryota - ps5
Eukaryota - ps6
Excavata/Diaphoretickes - ps7
Diaphoretickes - ps8
Archaeplastida - ps9
Viridiplantae - ps10
Streptophyta - ps11
Charophyceae/Embriophyta - ps12
Embriophyta - ps13
Tracheophyta - ps14
Spermatophyta - ps15
Angiosperms - ps16
Mesangiospermae - ps17
Magnoliids/eudicots - ps18
Eudicots - ps19
Core_eudicots - ps20
Superrosids - ps21
Rosids - ps22
Eurosids - ps23
Malvids - ps24
Sap./Malv./Brassicales - ps25
Malvales/Brassicales - ps26
Brassicales - ps27
Cleomaceae/Brassicaceae - ps28
Brassicaceae - ps29
Camelineae - ps30
Arabidopsis - ps31
Arabidopsis_thaliana - ps32
Phylostratum

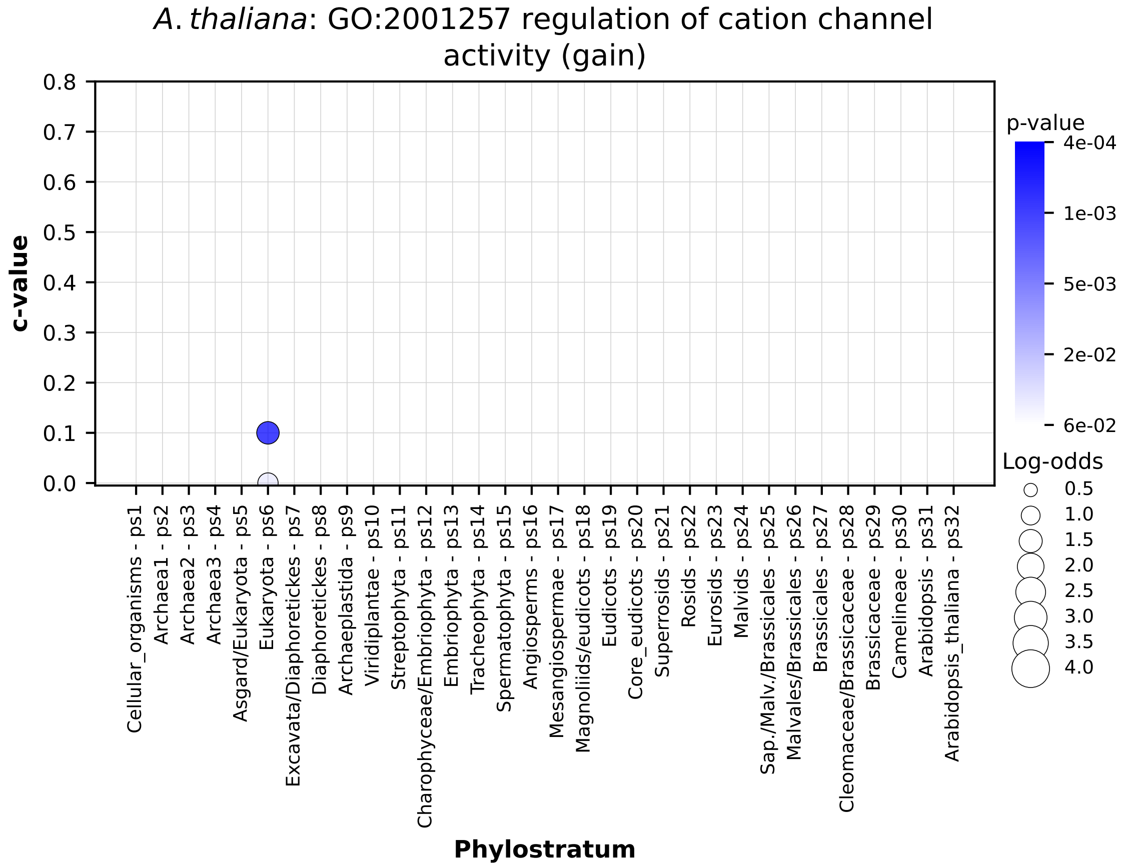
A. thaliana: GO:2001257 regulation of cation channel activity (gain)
c-value
0.8
0.7
0.6
0.5
0.4
0.3
0.2
0.1
0.0
Cellular_organisms - ps1
Archaea1 - ps2
Archaea2 - ps3
Archaea3 - ps4
Asgard/Eukaryota - ps5
Eukaryota - ps6
Excavata/Diaphoretickes - ps7
Diaphoretickes - ps8
Archaeplastida - ps9
Viridiplantae - ps10
Streptophyta - ps11
Charophyceae/Embriophyta - ps12
Embriophyta - ps13
Tracheophyta - ps14
Spermatophyta - ps15
Angiosperms - ps16
Mesangiospermae - ps17
Magnoliids/eudicots - ps18
Eudicots - ps19
Core_eudicots - ps20
Superrosids - ps21
Rosids - ps22
Eurosids - ps23
Malvids - ps24
Sap./Malv./Brassicales - ps25
Malvales/Brassicales - ps26
Brassicales - ps27
Cleomaceae/Brassicaceae - ps28
Brassicaceae - ps29
Camelineae - ps30
Arabidopsis - ps31
Arabidopsis_thaliana - ps32
Phylostratum
p-value
4e-04
1e-03
5e-03
2e-02
6e-02
Log-odds
0.5
1.0
1.5
2.0
2.5
3.0
3.5
4.0

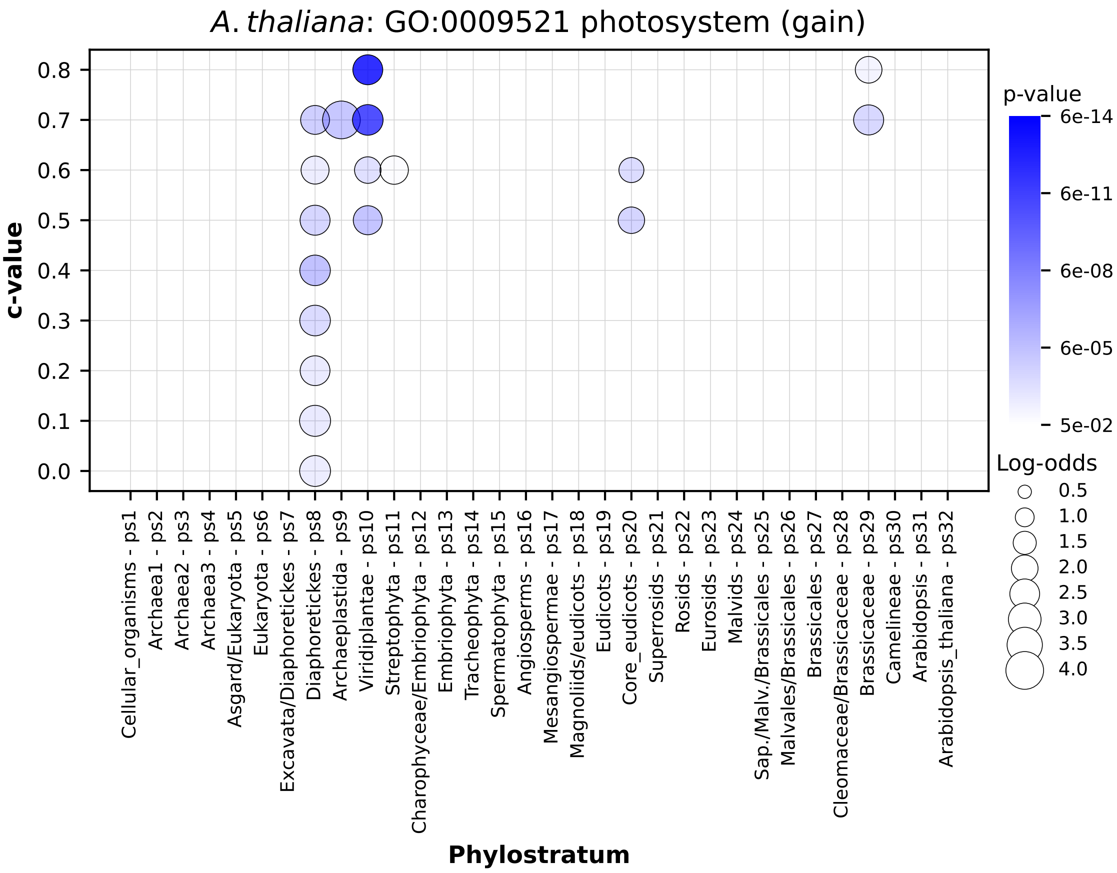
A. thaliana: GO:0009521 photosystem (gain)
c-value
0.8
0.7
0.6
0.5
0.4
0.3
0.2
0.1
0.0
p-value
6e-14
6e-11
6e-08
6e-05
5e-02
Log-odds
0.5
1.0
1.5
2.0
2.5
3.0
3.5
4.0
Cellular_organisms - ps1
Archaea1 - ps2
Archaea2 - ps3
Archaea3 - ps4
Asgard/Eukaryota - ps5
Eukaryota - ps6
Excavata/Diaphoretickes - ps7
Diaphoretickes - ps8
Archaeplastida - ps9
Viridiplantae - ps10
Streptophyta - ps11
Charophyceae/Embriophyta - ps12
Embriophyta - ps13
Tracheophyta - ps14
Spermatophyta - ps15
Angiosperms - ps16
Mesangiospermae - ps17
Magnoliids/eudicots - ps18
Eudicots - ps19
Core_eudicots - ps20
Superrosids - ps21
Rosids - ps22
Eurosids - ps23
Malvids - ps24
Sap./Malv./Brassicales - ps25
Malvales/Brassicales - ps26
Brassicales - ps27
Cleomaceae/Brassicaceae - ps28
Brassicaceae - ps29
Camelineae - ps30
Arabidopsis - ps31
Arabidopsis_thaliana - ps32
Phylostratum

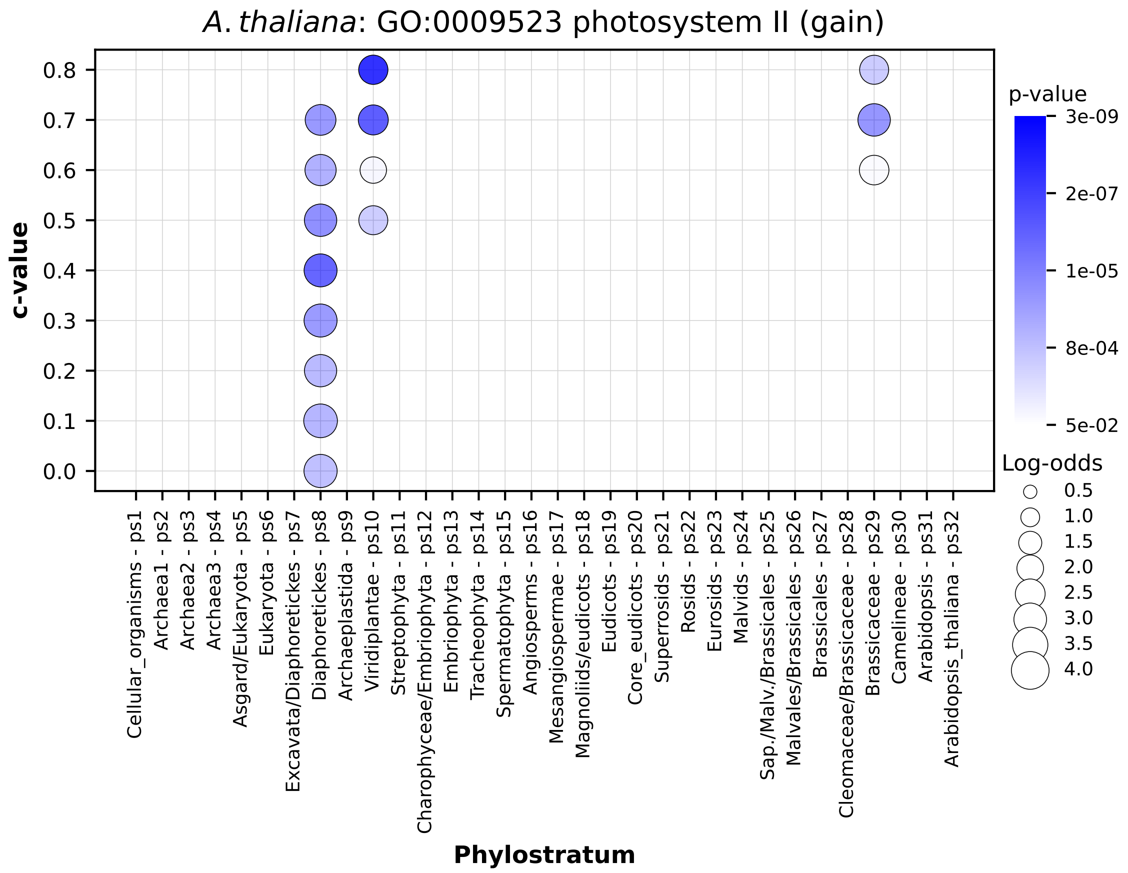

A. thaliana: GO:0009523 photosystem II (gain)
c-value
0.8
0.7
0.6
0.5
0.4
0.3
0.2
0.1
0.0
p-value
3e-09
2e-07
1e-05
8e-04
5e-02
Log-odds
0.5
1.0
1.5
2.0
2.5
3.0
3.5
4.0
Cellular_organisms - ps1
Archaea1 - ps2
Archaea2 - ps3
Archaea3 - ps4
Asgard/Eukaryota - ps5
Eukaryota - ps6
Excavata/Diaphoretickes - ps7
Diaphoretickes - ps8
Archaeplastida - ps9
Viridiplantae - ps10
Streptophyta - ps11
Charophyceae/Embriophyta - ps12
Embriophyta - ps13
Tracheophyta - ps14
Spermatophyta - ps15
Angiosperms - ps16
Mesangiospermae - ps17
Magnoliids/eudicots - ps18
Eudicots - ps19
Core_eudicots - ps20
Superrosids - ps21
Rosids - ps22
Eurosids - ps23
Malvids - ps24
Sap./Malv./Brassicales - ps25
Malvales/Brassicales - ps26
Brassicales - ps27
Cleomaceae/Brassicaceae - ps28
Brassicaceae - ps29
Camelineae - ps30
Arabidopsis - ps31
Arabidopsis_thaliana - ps32
Phylostratum

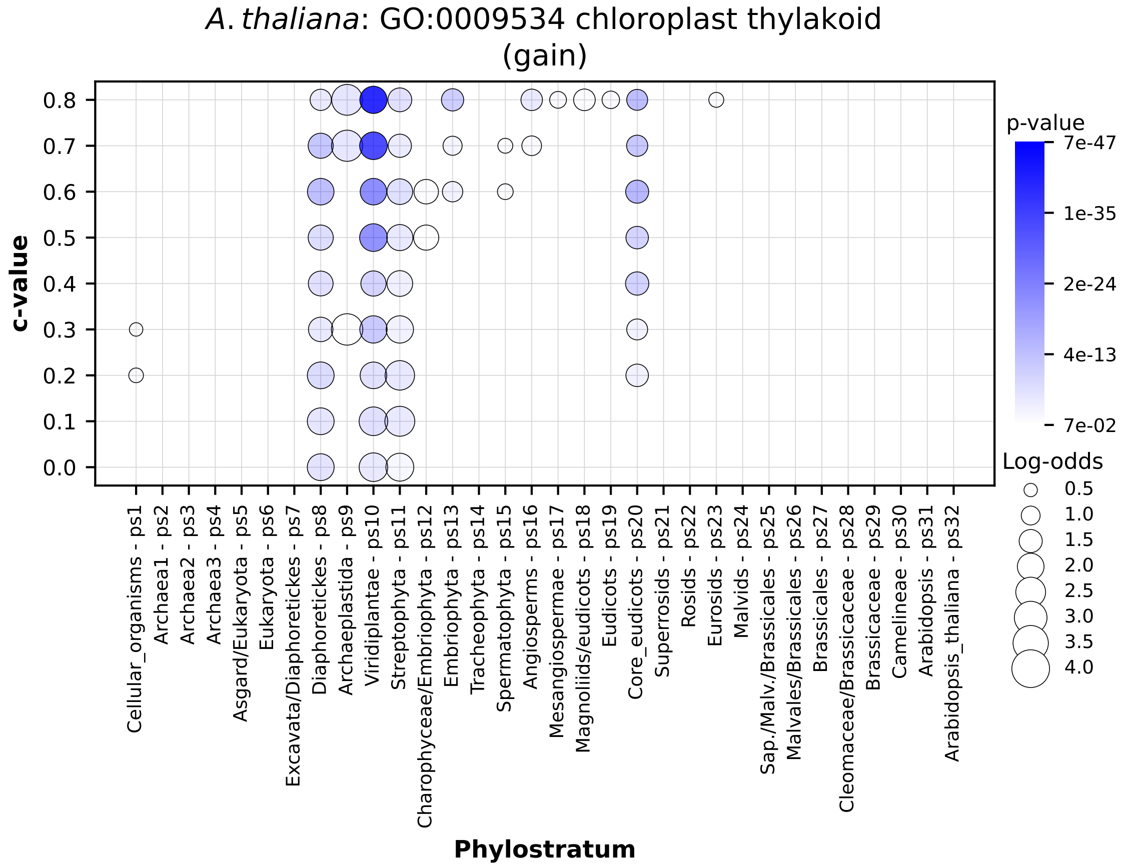

A. thaliana: GO:0009534 chloroplast thylakoid
(gain)
c-value
0.8
0.7
0.6
0.5
0.4
0.3
0.2
0.1
0.0
Phylostratum
Cellular_organisms - ps1
Archaea1 - ps2
Archaea2 - ps3
Archaea3 - ps4
Asgard/Eukaryota - ps5
Eukaryota - ps6
Excavata/Diaphoretickes - ps7
Diaphoretickes - ps8
Archaeplastida - ps9
Viridiplantae - ps10
Streptophyta - ps11
Charophyceae/Embriophyta - ps12
Embriophyta - ps13
Tracheophyta - ps14
Spermatophyta - ps15
Angiosperms - ps16
Mesangiospermae - ps17
Magnoliids/eudicots - ps18
Eudicots - ps19
Core_eudicots - ps20
Superrosids - ps21
Rosids - ps22
Eurosids - ps23
Malvids - ps24
Sap./Malv./Brassicales - ps25
Malvales/Brassicales - ps26
Brassicales - ps27
Cleomaceae/Brassicaceae - ps28
Brassicaceae - ps29
Camelineae - ps30
Arabidopsis - ps31
Arabidopsis_thaliana - ps32
p-value
7e-47
1e-35
2e-24
4e-13
7e-02
Log-odds
0.5
1.0
1.5
2.0
2.5
3.0
3.5
4.0

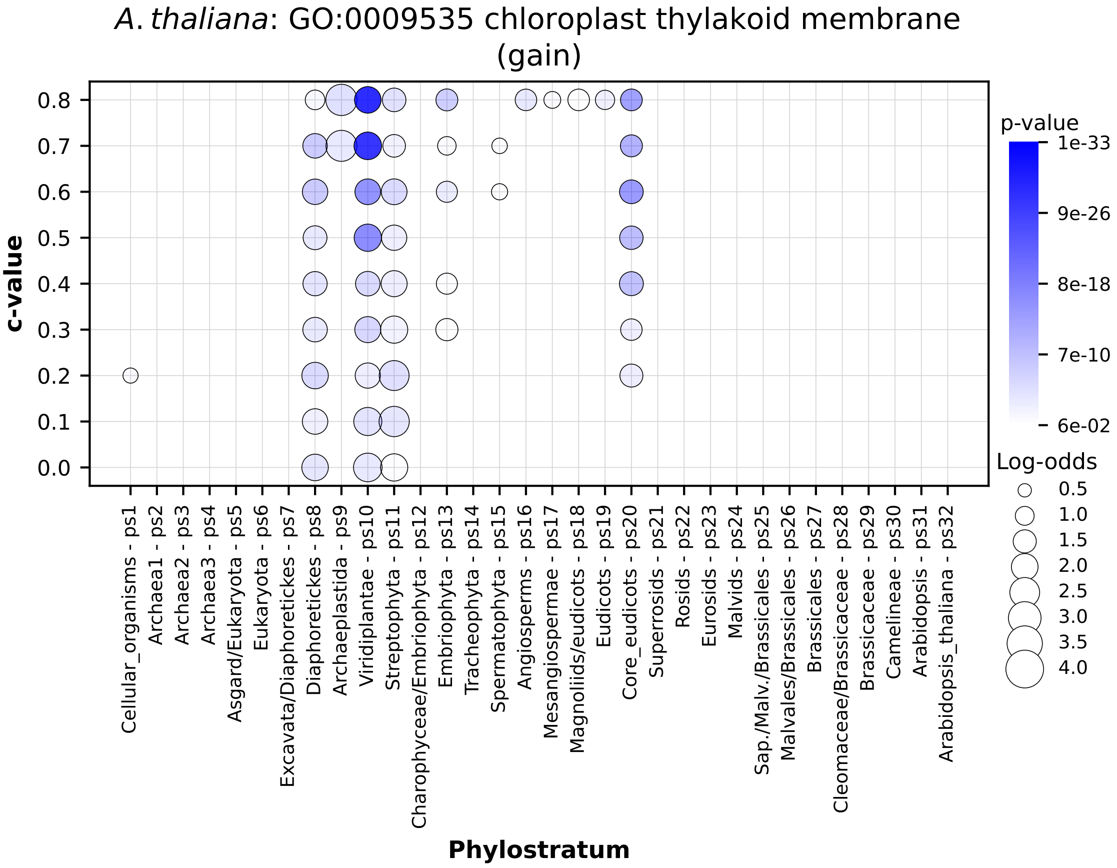

A. thaliana: GO:0009535 chloroplast thylakoid membrane (gain)
c-value
0.8
0.7
0.6
0.5
0.4
0.3
0.2
0.1
0.0
Phylostratum
Cellular_organisms - ps1
Archaea1 - ps2
Archaea2 - ps3
Archaea3 - ps4
Asgard/Eukaryota - ps5
Eukaryota - ps6
Excavata/Diaphoretickes - ps7
Diaphoretickes - ps8
Archaeplastida - ps9
Viridiplantae - ps10
Streptophyta - ps11
Charophyceae/Embriophyta - ps12
Embriophyta - ps13
Tracheophyta - ps14
Spermatophyta - ps15
Angiosperms - ps16
Mesangiospermae - ps17
Magnoliids/eudicots - ps18
Eudicots - ps19
Core_eudicots - ps20
Superrosids - ps21
Rosids - ps22
Eurosids - ps23
Malvids - ps24
Sap./Malv./Brassicales - ps25
Malvales/Brassicales - ps26
Brassicales - ps27
Cleomaceae/Brassicaceae - ps28
Brassicaceae - ps29
Camelineae - ps30
Arabidopsis - ps31
Arabidopsis_thaliana - ps32
p-value
1e-33
9e-26
8e-18
7e-10
6e-02
Log-odds
0.5
1.0
1.5
2.0
2.5
3.0
3.5
4.0

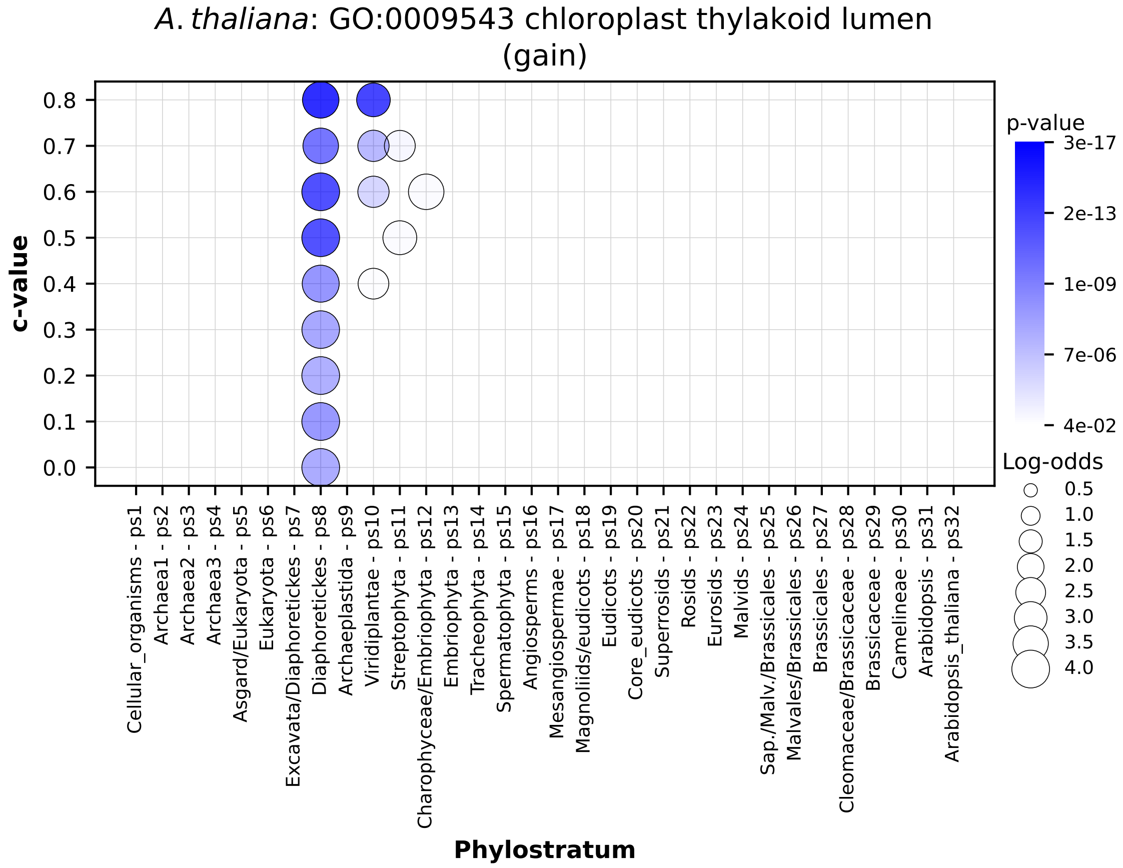

A. thaliana: GO:0009543 chloroplast thylakoid lumen (gain)
c-value
0.8
0.7
0.6
0.5
0.4
0.3
0.2
0.1
0.0
Phylostratum
Cellular_organisms - ps1
Archaea1 - ps2
Archaea2 - ps3
Archaea3 - ps4
Asgard/Eukaryota - ps5
Eukaryota - ps6
Excavata/Diaphoretickes - ps7
Diaphoretickes - ps8
Archaeplastida - ps9
Viridiplantae - ps10
Streptophyta - ps11
Charophyceae/Embriophyta - ps12
Embriophyta - ps13
Tracheophyta - ps14
Spermatophyta - ps15
Angiosperms - ps16
Mesangiospermae - ps17
Magnoliids/eudicots - ps18
Eudicots - ps19
Core_eudicots - ps20
Superrosids - ps21
Rosids - ps22
Eurosids - ps23
Malvids - ps24
Sap./Malv./Brassicales - ps25
Malvales/Brassicales - ps26
Brassicales - ps27
Cleomaceae/Brassicaceae - ps28
Brassicaceae - ps29
Camelineae - ps30
Arabidopsis - ps31
Arabidopsis_thaliana - ps32
p-value
3e-17
2e-13
1e-09
7e-06
4e-02
Log-odds
0.5
1.0
1.5
2.0
2.5
3.0
3.5
4.0

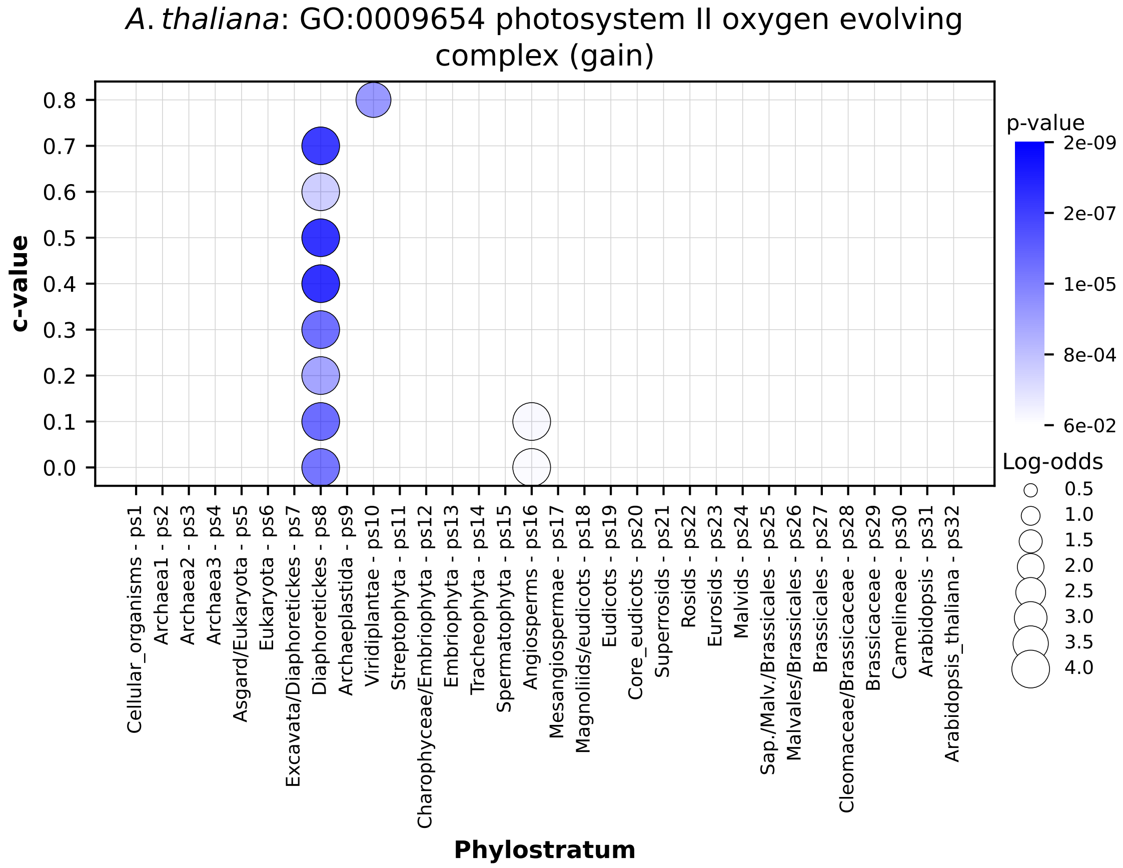
A. thaliana: GO:0009654 photosystem II oxygen evolving complex (gain)
c-value
0.8
0.7
0.6
0.5
0.4
0.3
0.2
0.1
0.0
p-value
2e-09
2e-07
1e-05
8e-04
6e-02
Log-odds
0.5
1.0
1.5
2.0
2.5
3.0
3.5
4.0
Cellular_organisms - ps1
Archaea1 - ps2
Archaea2 - ps3
Archaea3 - ps4
Asgard/Eukaryota - ps5
Eukaryota - ps6
Excavata/Diaphoretickes - ps7
Diaphoretickes - ps8
Archaeplastida - ps9
Viridiplantae - ps10
Streptophyta - ps11
Charophyceae/Embriophyta - ps12
Embriophyta - ps13
Tracheophyta - ps14
Spermatophyta - ps15
Angiosperms - ps16
Mesangiospermae - ps17
Magnoliids/eudicots - ps18
Eudicots - ps19
Core_eudicots - ps20
Superrosids - ps21
Rosids - ps22
Eurosids - ps23
Malvids - ps24
Sap./Malv./Brassicales - ps25
Malvales/Brassicales - ps26
Brassicales - ps27
Cleomaceae/Brassicaceae - ps28
Brassicaceae - ps29
Camelineae - ps30
Arabidopsis - ps31
Arabidopsis_thaliana - ps32
Phylostratum

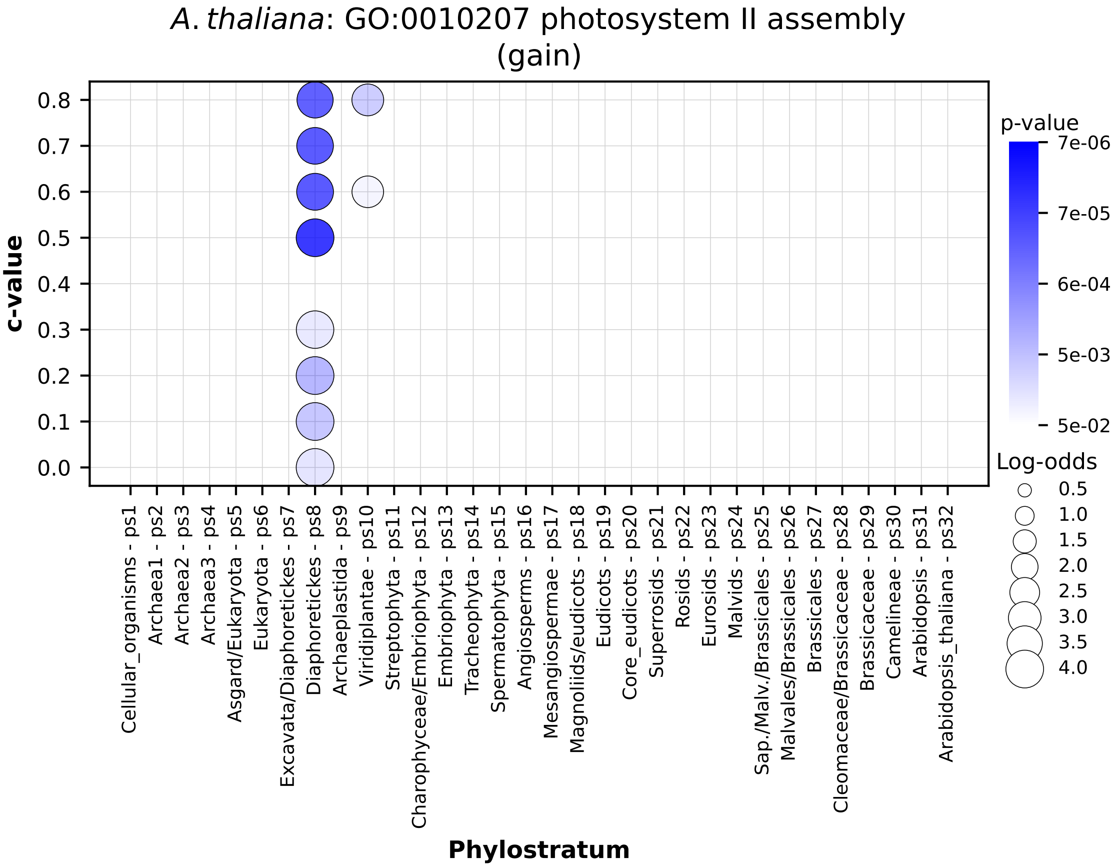

A. thaliana: GO:0010207 photosystem II assembly
(gain)
c-value
0.8
0.7
0.6
0.5
0.4
0.3
0.2
0.1
0.0
p-value
7e-06
7e-05
6e-04
5e-03
5e-02
Log-odds
0.5
1.0
1.5
2.0
2.5
3.0
3.5
4.0
Cellular_organisms - ps1
Archaea1 - ps2
Archaea2 - ps3
Archaea3 - ps4
Asgard/Eukaryota - ps5
Eukaryota - ps6
Excavata/Diaphoretickes - ps7
Diaphoretickes - ps8
Archaeplastida - ps9
Viridiplantae - ps10
Streptophyta - ps11
Charophyceae/Embriophyta - ps12
Embriophyta - ps13
Tracheophyta - ps14
Spermatophyta - ps15
Angiosperms - ps16
Mesangiospermae - ps17
Magnoliids/eudicots - ps18
Eudicots - ps19
Core_eudicots - ps20
Superrosids - ps21
Rosids - ps22
Eurosids - ps23
Malvids - ps24
Sap./Malv./Brassicales - ps25
Malvales/Brassicales - ps26
Brassicales - ps27
Cleomaceae/Brassicaceae - ps28
Brassicaceae - ps29
Camelineae - ps30
Arabidopsis - ps31
Arabidopsis_thaliana - ps32
Phylostratum

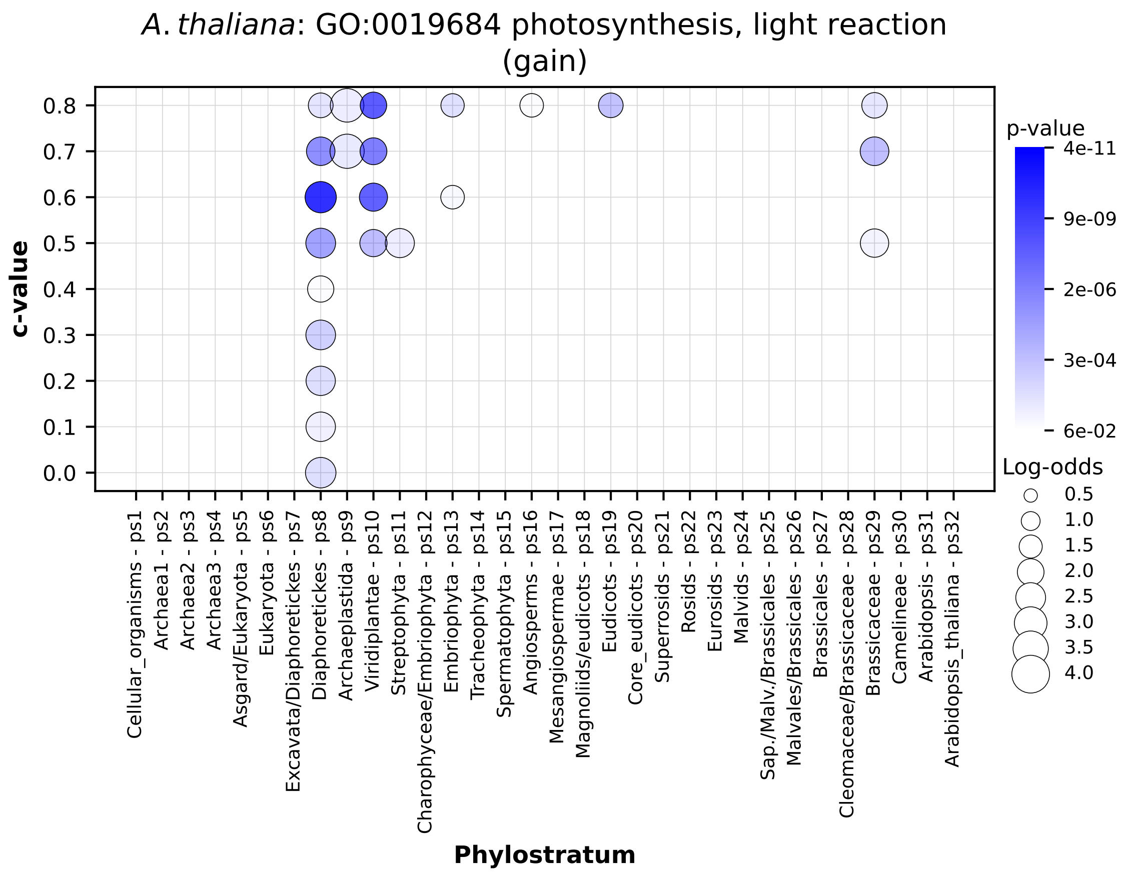

A. thaliana: GO:0019684 photosynthesis, light reaction
(gain)
c-value
0.8
0.7
0.6
0.5
0.4
0.3
0.2
0.1
0.0
p-value
4e-11
9e-09
2e-06
3e-04
6e-02
Log-odds
0.5
1.0
1.5
2.0
2.5
3.0
3.5
4.0
Cellular_organisms - ps1
Archaea1 - ps2
Archaea2 - ps3
Archaea3 - ps4
Asgard/Eukaryota - ps5
Eukaryota - ps6
Excavata/Diaphoretickes - ps7
Diaphoretickes - ps8
Archaeplastida - ps9
Viridiplantae - ps10
Streptophyta - ps11
Charophyceae/Embriophyta - ps12
Embriophyta - ps13
Tracheophyta - ps14
Spermatophyta - ps15
Angiosperms - ps16
Mesangiospermae - ps17
Magnoliids/eudicots - ps18
Eudicots - ps19
Core_eudicots - ps20
Superrosids - ps21
Rosids - ps22
Eurosids - ps23
Malvids - ps24
Sap./Malv./Brassicales - ps25
Malvales/Brassicales - ps26
Brassicales - ps27
Cleomaceae/Brassicaceae - ps28
Brassicaceae - ps29
Camelineae - ps30
Arabidopsis - ps31
Arabidopsis_thaliana - ps32
Phylostratum

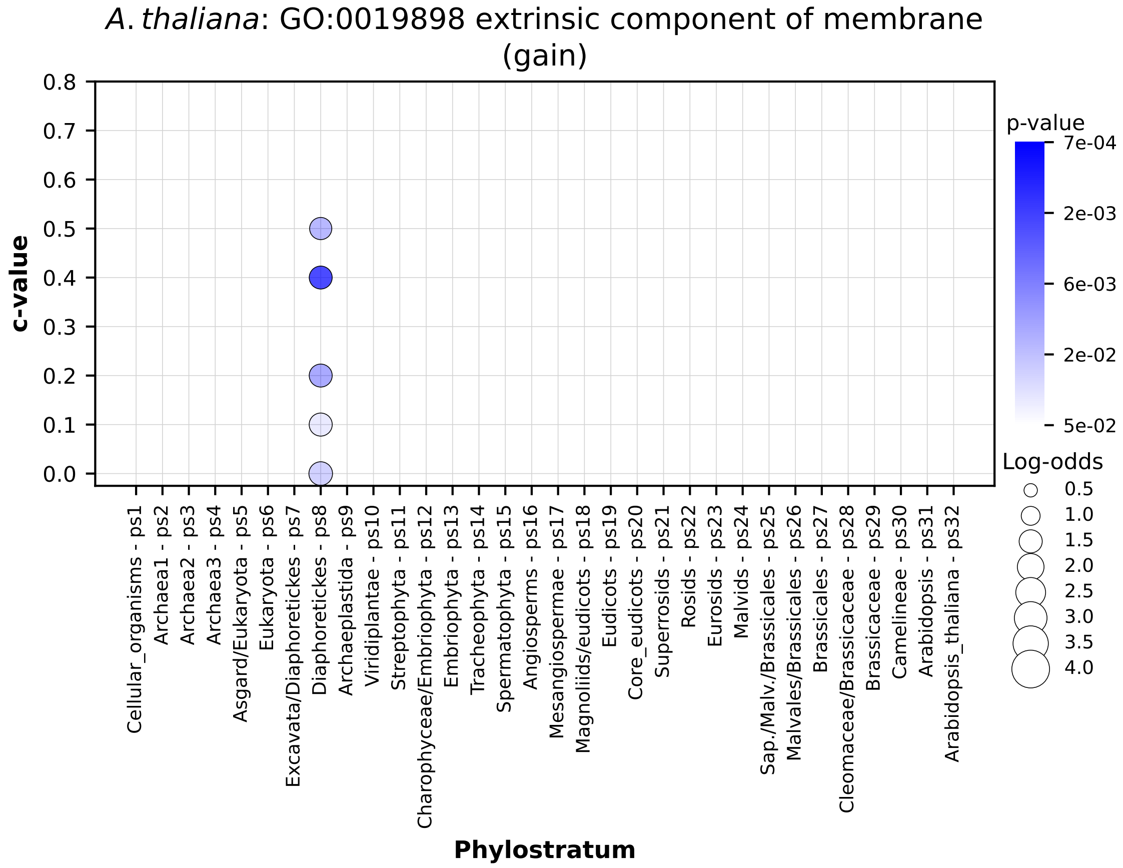
A. thaliana: GO:0019898 extrinsic component of membrane
(gain)
c-value
0.8
0.7
0.6
0.5
0.4
0.3
0.2
0.1
0.0
p-value
7e-04
2e-03
6e-03
2e-02
5e-02
Log-odds
0.5
1.0
1.5
2.0
2.5
3.0
3.5
4.0
Cellular_organisms - ps1
Archaea1 - ps2
Archaea2 - ps3
Archaea3 - ps4
Asgard/Eukaryota - ps5
Eukaryota - ps6
Excavata/Diaphoretickes - ps7
Diaphoretickes - ps8
Archaeplastida - ps9
Viridiplantae - ps10
Streptophyta - ps11
Charophyceae/Embriophyta - ps12
Embriophyta - ps13
Tracheophyta - ps14
Spermatophyta - ps15
Angiosperms - ps16
Mesangiospermae - ps17
Magnoliids/eudicots - ps18
Eudicots - ps19
Core_eudicots - ps20
Superrosids - ps21
Rosids - ps22
Eurosids - ps23
Malvids - ps24
Sap./Malv./Brassicales - ps25
Malvales/Brassicales - ps26
Brassicales - ps27
Cleomaceae/Brassicaceae - ps28
Brassicaceae - ps29
Camelineae - ps30
Arabidopsis - ps31
Arabidopsis_thaliana - ps32
Phylostratum

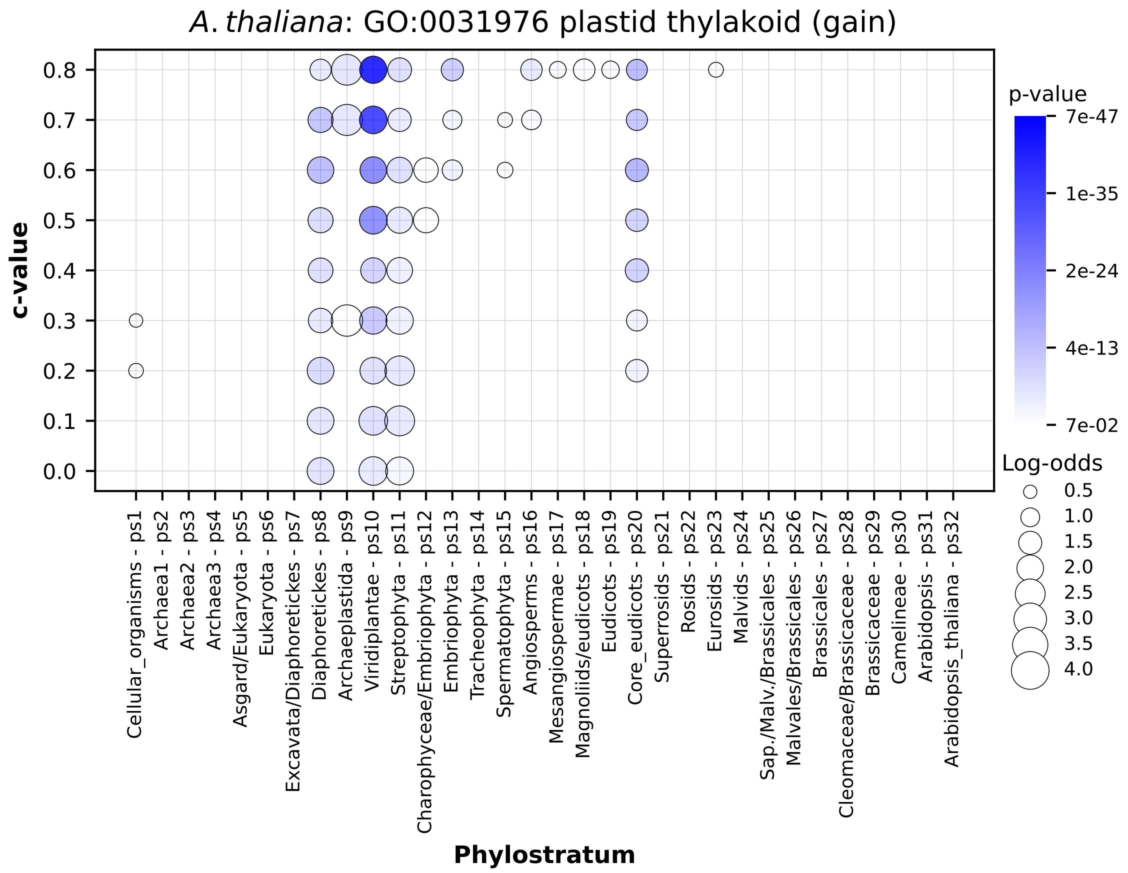
A. thaliana: GO:0031976 plastid thylakoid (gain)
c-value
0.0
0.1
0.2
0.3
0.4
0.5
0.6
0.7
0.8
Phylostratum
Cellular_organisms - ps1
Archaea1 - ps2
Archaea2 - ps3
Archaea3 - ps4
Asgard/Eukaryota - ps5
Eukaryota - ps6
Excavata/Diaphoretickes - ps7
Diaphoretickes - ps8
Archaeplastida - ps9
Viridiplantae - ps10
Streptophyta - ps11
Charophyceae/Embriophyta - ps12
Embriophyta - ps13
Tracheophyta - ps14
Spermatophyta - ps15
Angiosperms - ps16
Mesangiospermae - ps17
Magnoliids/eudicots - ps18
Eudicots - ps19
Core_eudicots - ps20
Superrosids - ps21
Rosids - ps22
Eurosids - ps23
Malvids - ps24
Sap./Malv./Brassicales - ps25
Malvales/Brassicales - ps26
Brassicales - ps27
Cleomaceae/Brassicaceae - ps28
Brassicaceae - ps29
Camelineae - ps30
Arabidopsis - ps31
Arabidopsis_thaliana - ps32
p-value
7e-47
1e-35
2e-24
4e-13
7e-02
Log-odds
0.5
1.0
1.5
2.0
2.5
3.0
3.5
4.0

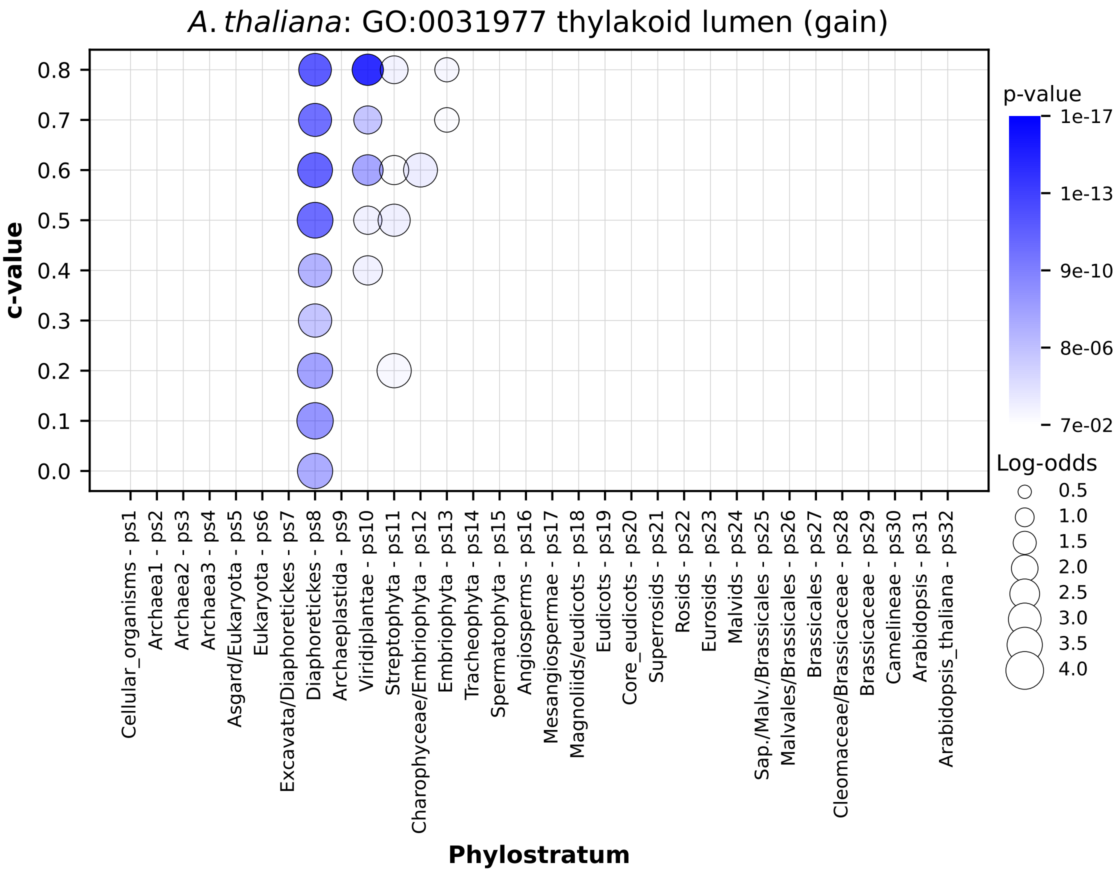
A. thaliana: GO:0031977 thylakoid lumen (gain)
c-value
0.8
0.7
0.6
0.5
0.4
0.3
0.2
0.1
0.0
Phylostratum
Cellular_organisms - ps1
Archaea1 - ps2
Archaea2 - ps3
Archaea3 - ps4
Asgard/Eukaryota - ps5
Eukaryota - ps6
Excavata/Diaphoretickes - ps7
Diaphoretickes - ps8
Archaeplastida - ps9
Viridiplantae - ps10
Streptophyta - ps11
Charophyceae/Embriophyta - ps12
Embriophyta - ps13
Tracheophyta - ps14
Spermatophyta - ps15
Angiosperms - ps16
Mesangiospermae - ps17
Magnoliids/eudicots - ps18
Eudicots - ps19
Core_eudicots - ps20
Superrosids - ps21
Rosids - ps22
Eurosids - ps23
Malvids - ps24
Sap./Malv./Brassicales - ps25
Malvales/Brassicales - ps26
Brassicales - ps27
Cleomaceae/Brassicaceae - ps28
Brassicaceae - ps29
Camelineae - ps30
Arabidopsis - ps31
Arabidopsis_thaliana - ps32
p-value
1e-17
1e-13
9e-10
8e-06
7e-02
Log-odds
0.5
1.0
1.5
2.0
2.5
3.0
3.5
4.0

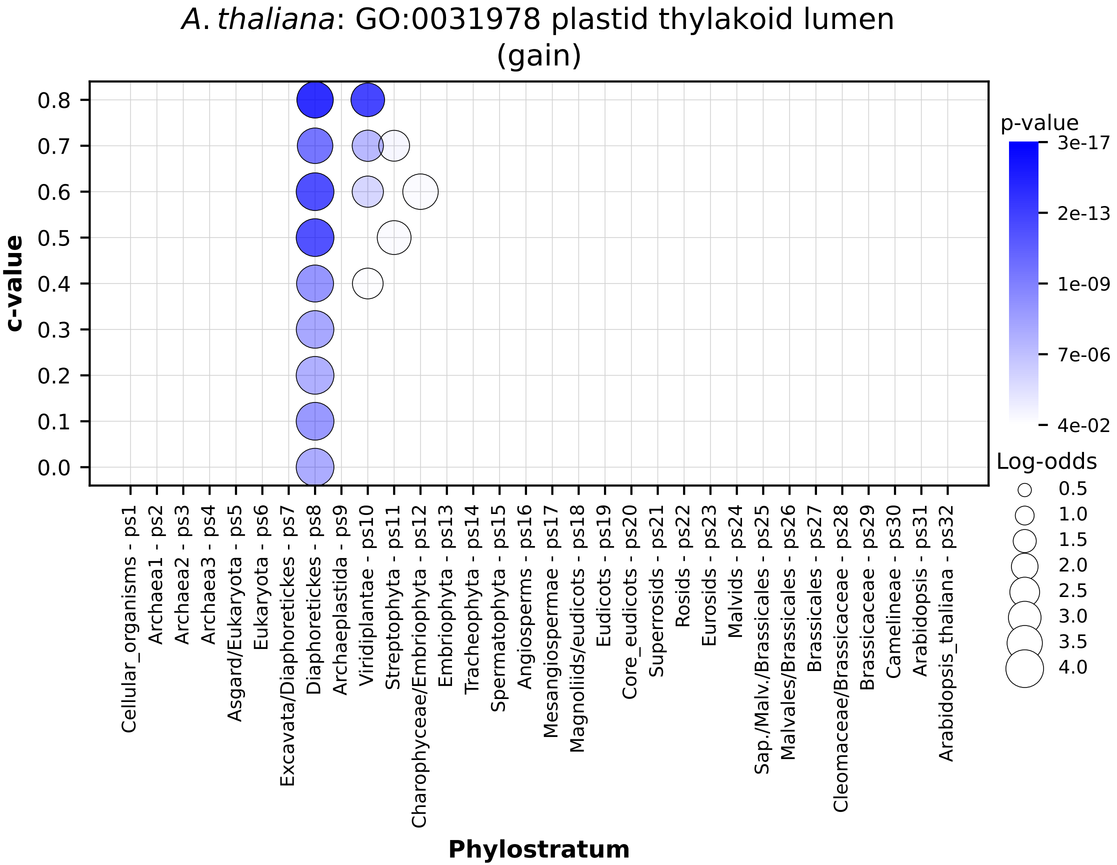

A. thaliana: GO:0031978 plastid thylakoid lumen
(gain)
c-value
0.8
0.7
0.6
0.5
0.4
0.3
0.2
0.1
0.0
p-value
3e-17
2e-13
1e-09
7e-06
4e-02
Log-odds
0.5
1.0
1.5
2.0
2.5
3.0
3.5
4.0
Cellular_organisms - ps1
Archaea1 - ps2
Archaea2 - ps3
Archaea3 - ps4
Asgard/Eukaryota - ps5
Eukaryota - ps6
Excavata/Diaphoretickes - ps7
Diaphoretickes - ps8
Archaeplastida - ps9
Viridiplantae - ps10
Streptophyta - ps11
Charophyceae/Embriophyta - ps12
Embriophyta - ps13
Tracheophyta - ps14
Spermatophyta - ps15
Angiosperms - ps16
Mesangiospermae - ps17
Magnoliids/eudicots - ps18
Eudicots - ps19
Core_eudicots - ps20
Superrosids - ps21
Rosids - ps22
Eurosids - ps23
Malvids - ps24
Sap./Malv./Brassicales - ps25
Malvales/Brassicales - ps26
Brassicales - ps27
Cleomaceae/Brassicaceae - ps28
Brassicaceae - ps29
Camelineae - ps30
Arabidopsis - ps31
Arabidopsis_thaliana - ps32
Phylostratum

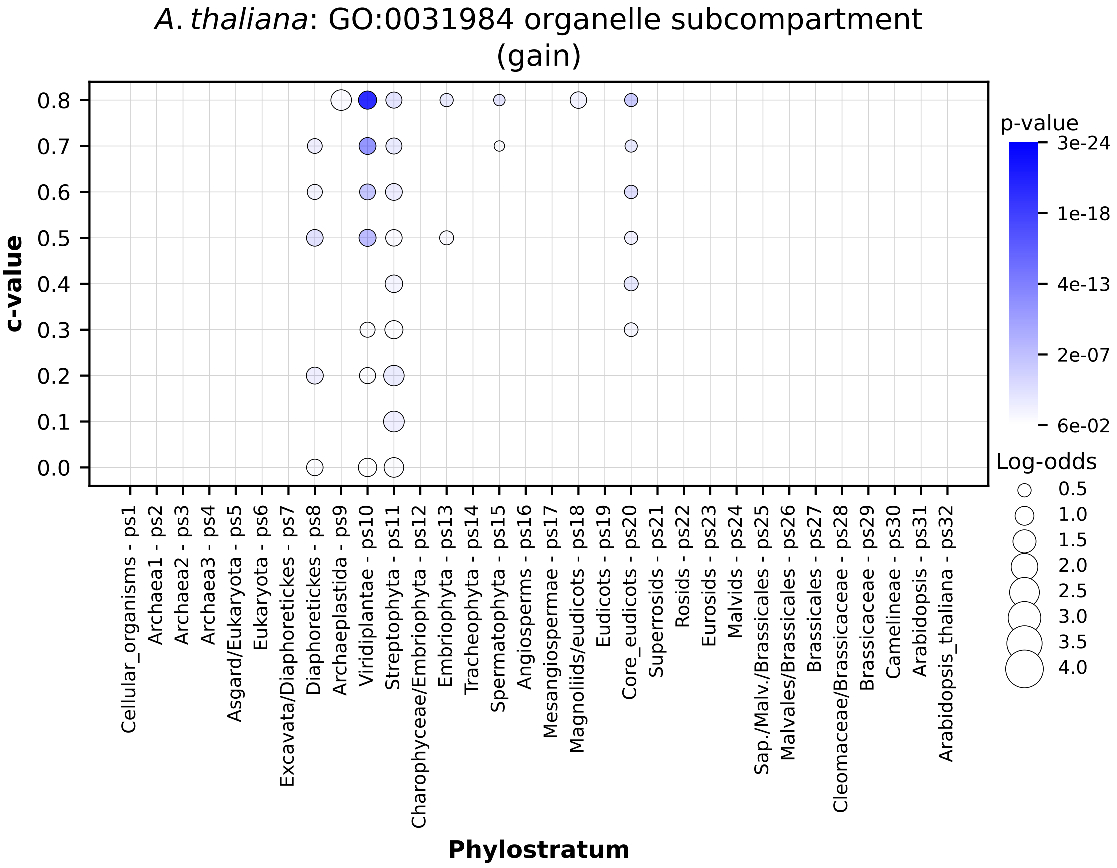

A. thaliana: GO:0031984 organelle subcompartment (gain)
c-value
0.8
0.7
0.6
0.5
0.4
0.3
0.2
0.1
0.0
p-value
3e-24
1e-18
4e-13
2e-07
6e-02
Log-odds
0.5
1.0
1.5
2.0
2.5
3.0
3.5
4.0
Cellular_organisms - ps1
Archaea1 - ps2
Archaea2 - ps3
Archaea3 - ps4
Asgard/Eukaryota - ps5
Eukaryota - ps6
Excavata/Diaphoretickes - ps7
Diaphoretickes - ps8
Archaeplastida - ps9
Viridiplantae - ps10
Streptophyta - ps11
Charophyceae/Embriophyta - ps12
Embriophyta - ps13
Tracheophyta - ps14
Spermatophyta - ps15
Angiosperms - ps16
Mesangiospermae - ps17
Magnoliids/eudicots - ps18
Eudicots - ps19
Core_eudicots - ps20
Superrosids - ps21
Rosids - ps22
Eurosids - ps23
Malvids - ps24
Sap./Malv./Brassicales - ps25
Malvales/Brassicales - ps26
Brassicales - ps27
Cleomaceae/Brassicaceae - ps28
Brassicaceae - ps29
Camelineae - ps30
Arabidopsis - ps31
Arabidopsis_thaliana - ps32
Phylostratum

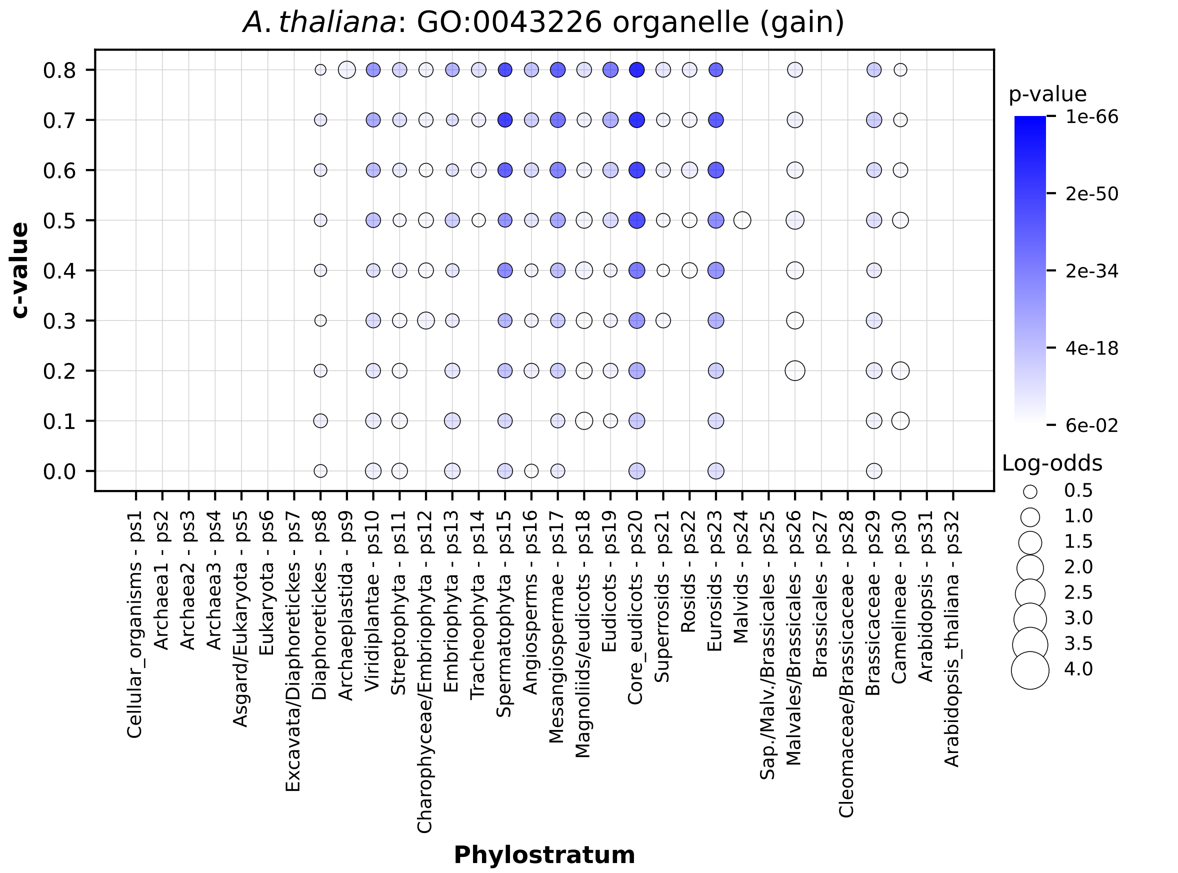

A. thaliana: GO:0043226 organelle (gain)
c-value
0.8
0.7
0.6
0.5
0.4
0.3
0.2
0.1
0.0
p-value
1e-66
2e-50
2e-34
4e-18
6e-02
Log-odds
0.5
1.0
1.5
2.0
2.5
3.0
3.5
4.0
Cellular_organisms - ps1
Archaea1 - ps2
Archaea2 - ps3
Archaea3 - ps4
Asgard/Eukaryota - ps5
Eukaryota - ps6
Excavata/Diaphoretickes - ps7
Diaphoretickes - ps8
Archaeplastida - ps9
Viridiplantae - ps10
Streptophyta - ps11
Charophyceae/Embriophyta - ps12
Embriophyta - ps13
Tracheophyta - ps14
Spermatophyta - ps15
Angiosperms - ps16
Mesangiospermae - ps17
Magnoliids/eudicots - ps18
Eudicots - ps19
Core_eudicots - ps20
Superrosids - ps21
Rosids - ps22
Eurosids - ps23
Malvids - ps24
Sap./Malv./Brassicales - ps25
Malvales/Brassicales - ps26
Brassicales - ps27
Cleomaceae/Brassicaceae - ps28
Brassicaceae - ps29
Camelineae - ps30
Arabidopsis - ps31
Arabidopsis_thaliana - ps32
Phylostratum

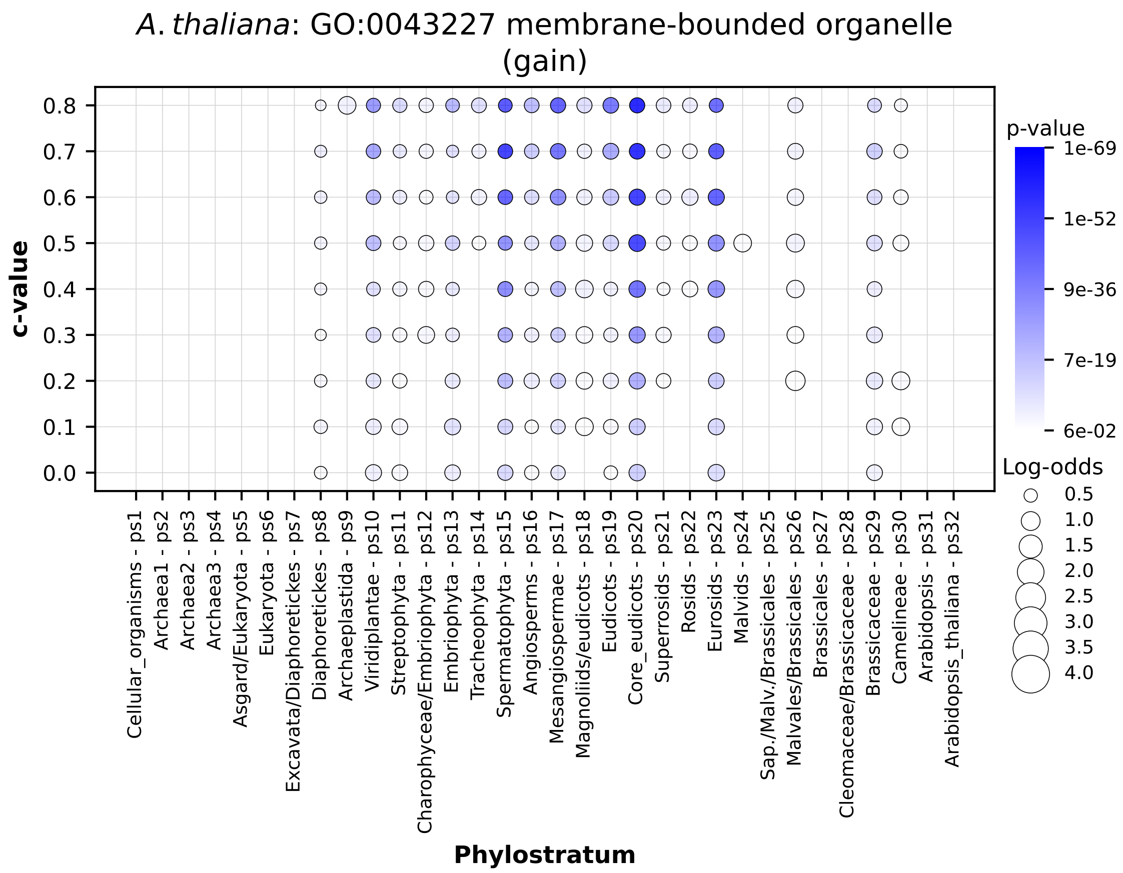

A. thaliana: GO:0043227 membrane-bounded organelle (gain)
c-value
0.8
0.7
0.6
0.5
0.4
0.3
0.2
0.1
0.0
Phylostratum
Cellular_organisms - ps1
Archaea1 - ps2
Archaea2 - ps3
Archaea3 - ps4
Asgard/Eukaryota - ps5
Eukaryota - ps6
Excavata/Diaphoretickes - ps7
Diaphoretickes - ps8
Archaeplastida - ps9
Viridiplantae - ps10
Streptophyta - ps11
Charophyceae/Embriophyta - ps12
Embriophyta - ps13
Tracheophyta - ps14
Spermatophyta - ps15
Angiosperms - ps16
Mesangiospermae - ps17
Magnoliids/eudicots - ps18
Eudicots - ps19
Core_eudicots - ps20
Superrosids - ps21
Rosids - ps22
Eurosids - ps23
Malvids - ps24
Sap./Malv./Brassicales - ps25
Malvales/Brassicales - ps26
Brassicales - ps27
Cleomaceae/Brassicaceae - ps28
Brassicaceae - ps29
Camelineae - ps30
Arabidopsis - ps31
Arabidopsis_thaliana - ps32
p-value
1e-69
1e-52
9e-36
7e-19
6e-02
Log-odds
0.5
1.0
1.5
2.0
2.5
3.0
3.5
4.0

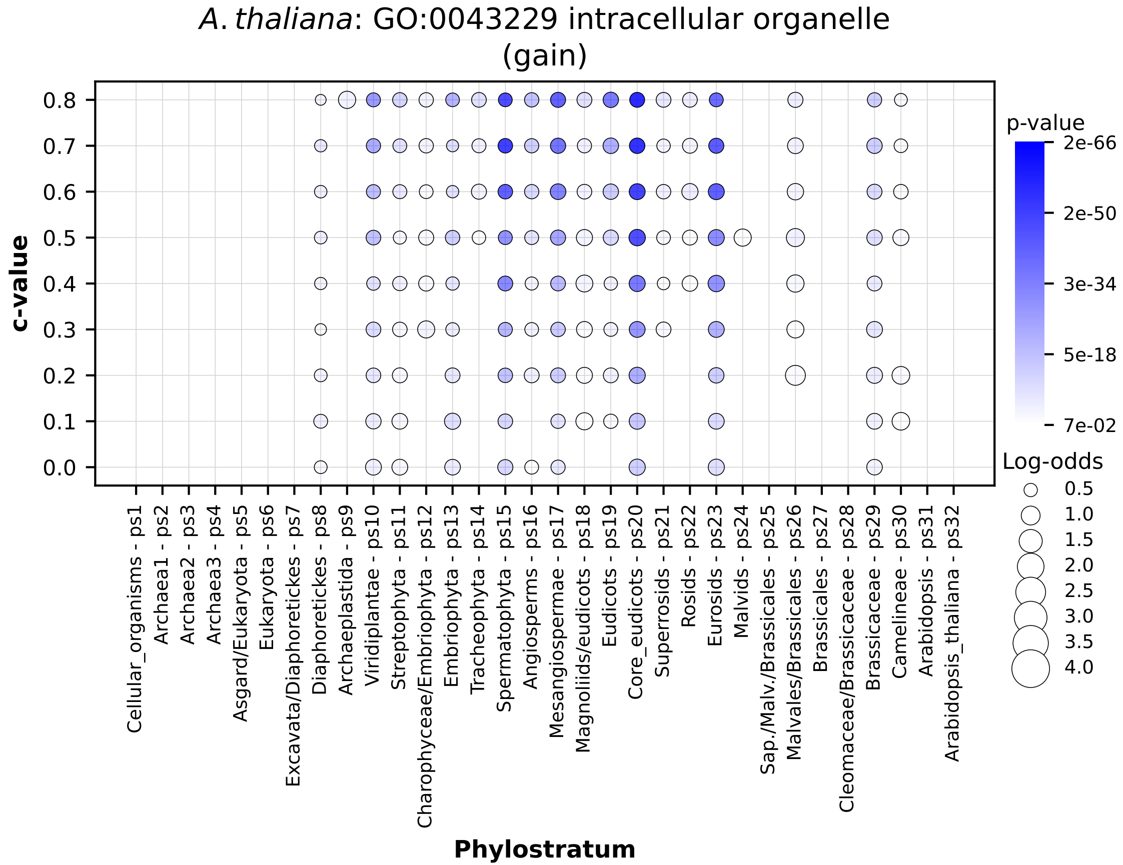

A. thaliana: GO:0043229 intracellular organelle
(gain)
c-value
0.8
0.7
0.6
0.5
0.4
0.3
0.2
0.1
0.0
Phylostratum
Cellular_organisms - ps1
Archaea1 - ps2
Archaea2 - ps3
Archaea3 - ps4
Asgard/Eukaryota - ps5
Eukaryota - ps6
Excavata/Diaphoretickes - ps7
Diaphoretickes - ps8
Archaeplastida - ps9
Viridiplantae - ps10
Streptophyta - ps11
Charophyceae/Embriophyta - ps12
Embriophyta - ps13
Tracheophyta - ps14
Spermatophyta - ps15
Angiosperms - ps16
Mesangiospermae - ps17
Magnoliids/eudicots - ps18
Eudicots - ps19
Core_eudicots - ps20
Superrosids - ps21
Rosids - ps22
Eurosids - ps23
Malvids - ps24
Sap./Malv./Brassicales - ps25
Malvales/Brassicales - ps26
Brassicales - ps27
Cleomaceae/Brassicaceae - ps28
Brassicaceae - ps29
Camelineae - ps30
Arabidopsis - ps31
Arabidopsis_thaliana - ps32
p-value
2e-66
2e-50
3e-34
5e-18
7e-02
Log-odds
0.5
1.0
1.5
2.0
2.5
3.0
3.5
4.0

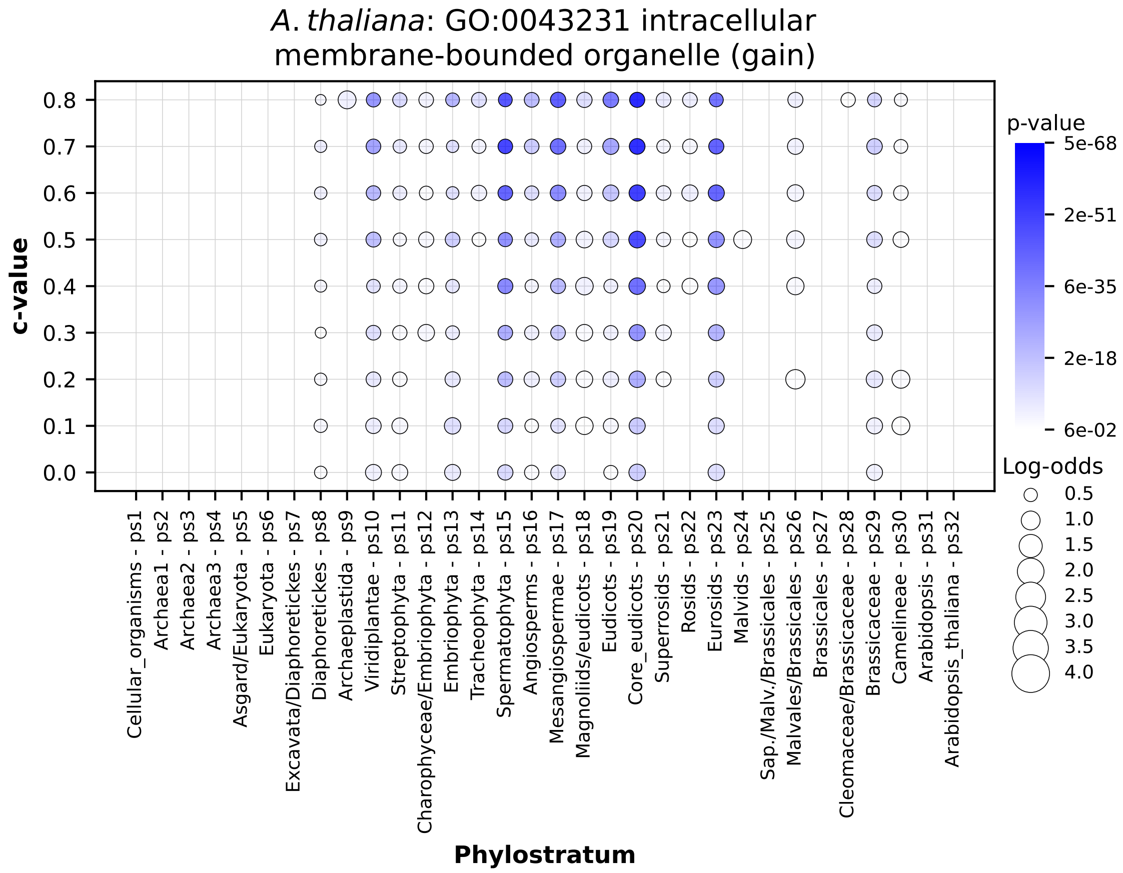
A. thaliana: GO:0043231 intracellular membrane-bounded organelle (gain)
c-value
0.8
0.7
0.6
0.5
0.4
0.3
0.2
0.1
0.0
p-value
5e-68
2e-51
6e-35
2e-18
6e-02
Log-odds
0.5
1.0
1.5
2.0
2.5
3.0
3.5
4.0
Cellular_organisms - ps1
Archaea1 - ps2
Archaea2 - ps3
Archaea3 - ps4
Asgard/Eukaryota - ps5
Eukaryota - ps6
Excavata/Diaphoretickes - ps7
Diaphoretickes - ps8
Archaeplastida - ps9
Viridiplantae - ps10
Streptophyta - ps11
Charophyceae/Embriophyta - ps12
Embriophyta - ps13
Tracheophyta - ps14
Spermatophyta - ps15
Angiosperms - ps16
Mesangiospermae - ps17
Magnoliids/eudicots - ps18
Eudicots - ps19
Core_eudicots - ps20
Superrosids - ps21
Rosids - ps22
Eurosids - ps23
Malvids - ps24
Sap./Malv./Brassicales - ps25
Malvales/Brassicales - ps26
Brassicales - ps27
Cleomaceae/Brassicaceae - ps28
Brassicaceae - ps29
Camelineae - ps30
Arabidopsis - ps31
Arabidopsis_thaliana - ps32
Phylostratum

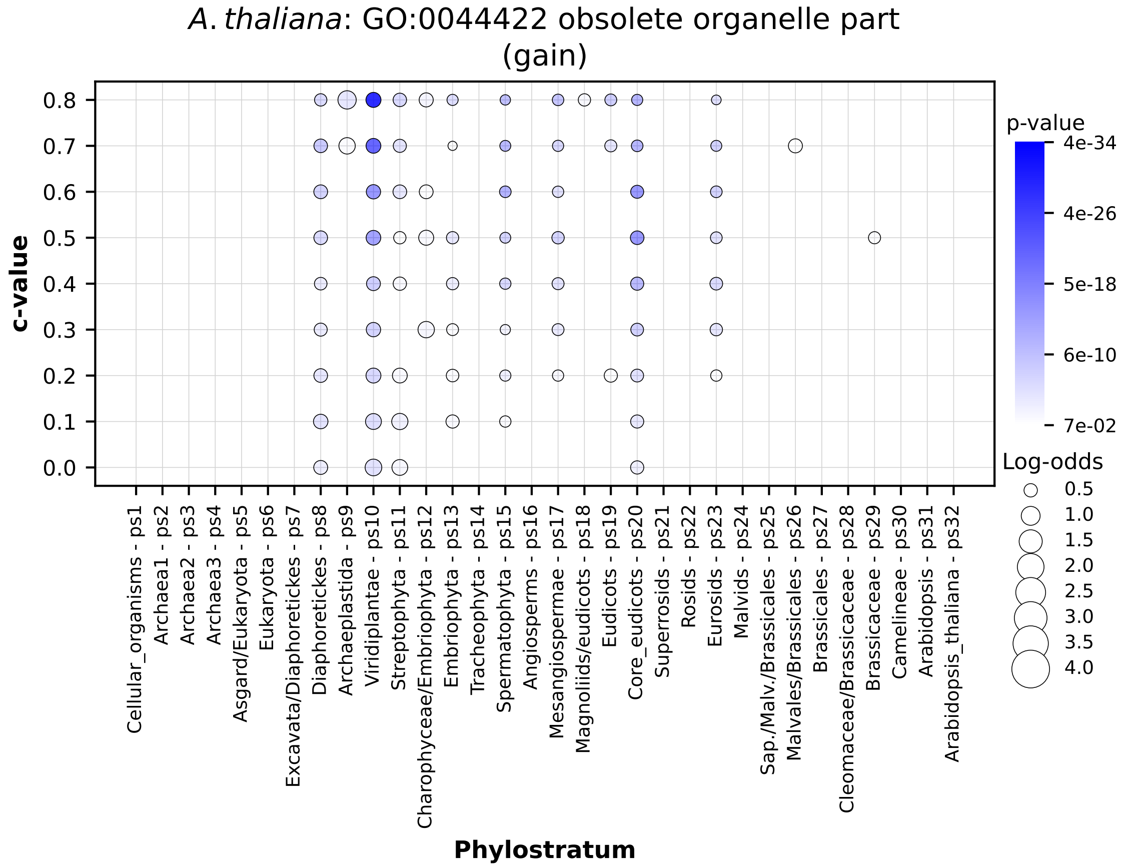
A. thaliana: GO:0044422 obsolete organelle part
(gain)
c-value
0.8
0.7
0.6
0.5
0.4
0.3
0.2
0.1
0.0
p-value
4e-34
4e-26
5e-18
6e-10
7e-02
Log-odds
0.5
1.0
1.5
2.0
2.5
3.0
3.5
4.0
Cellular_organisms - ps1
Archaea1 - ps2
Archaea2 - ps3
Archaea3 - ps4
Asgard/Eukaryota - ps5
Eukaryota - ps6
Excavata/Diaphoretickes - ps7
Diaphoretickes - ps8
Archaeplastida - ps9
Viridiplantae - ps10
Streptophyta - ps11
Charophyceae/Embriophyta - ps12
Embriophyta - ps13
Tracheophyta - ps14
Spermatophyta - ps15
Angiosperms - ps16
Mesangiospermae - ps17
Magnoliids/eudicots - ps18
Eudicots - ps19
Core_eudicots - ps20
Superrosids - ps21
Rosids - ps22
Eurosids - ps23
Malvids - ps24
Sap./Malv./Brassicales - ps25
Malvales/Brassicales - ps26
Brassicales - ps27
Cleomaceae/Brassicaceae - ps28
Brassicaceae - ps29
Camelineae - ps30
Arabidopsis - ps31
Arabidopsis_thaliana - ps32
Phylostratum

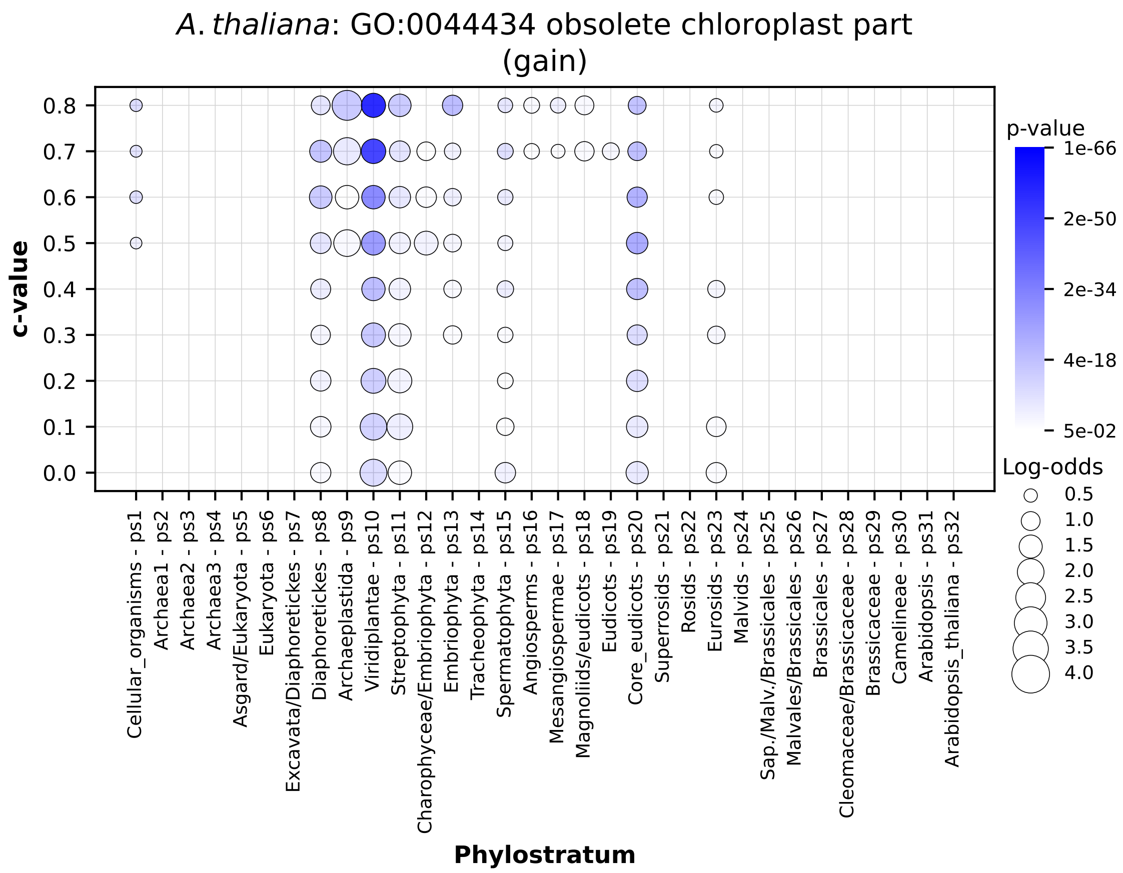
A. thaliana: GO:0044434 obsolete chloroplast part
(gain)
c-value
0.8
0.7
0.6
0.5
0.4
0.3
0.2
0.1
0.0
p-value
1e-66
2e-50
2e-34
4e-18
5e-02
Log-odds
0.5
1.0
1.5
2.0
2.5
3.0
3.5
4.0
Cellular_organisms - ps1
Archaea1 - ps2
Archaea2 - ps3
Archaea3 - ps4
Asgard/Eukaryota - ps5
Eukaryota - ps6
Excavata/Diaphoretickes - ps7
Diaphoretickes - ps8
Archaeplastida - ps9
Viridiplantae - ps10
Streptophyta - ps11
Charophyceae/Embriophyta - ps12
Embriophyta - ps13
Tracheophyta - ps14
Spermatophyta - ps15
Angiosperms - ps16
Mesangiospermae - ps17
Magnoliids/eudicots - ps18
Eudicots - ps19
Core_eudicots - ps20
Superrosids - ps21
Rosids - ps22
Eurosids - ps23
Malvids - ps24
Sap./Malv./Brassicales - ps25
Malvales/Brassicales - ps26
Brassicales - ps27
Cleomaceae/Brassicaceae - ps28
Brassicaceae - ps29
Camelineae - ps30
Arabidopsis - ps31
Arabidopsis_thaliana - ps32
Phylostratum

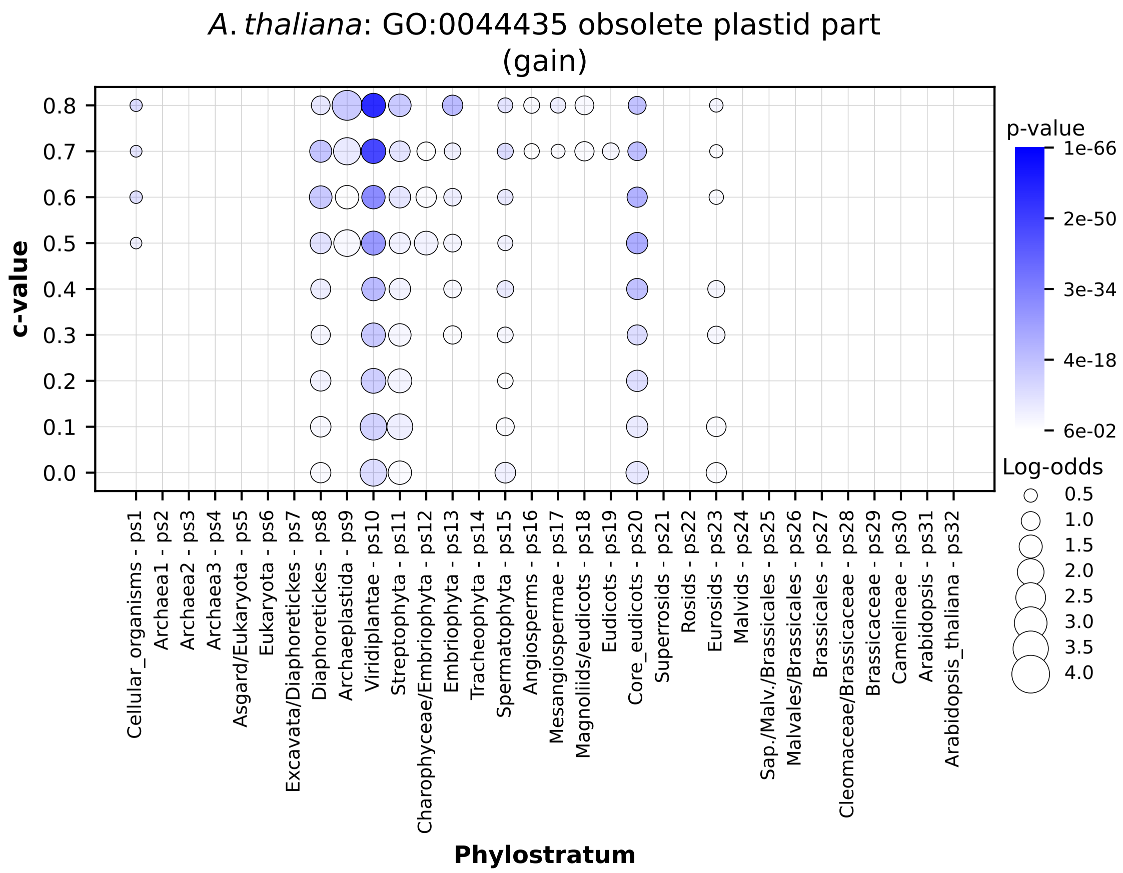

A. thaliana: GO:0044435 obsolete plastid part
(gain)
c-value
0.8
0.7
0.6
0.5
0.4
0.3
0.2
0.1
0.0
p-value
1e-66
2e-50
3e-34
4e-18
6e-02
Log-odds
0.5
1.0
1.5
2.0
2.5
3.0
3.5
4.0
Cellular_organisms - ps1
Archaea1 - ps2
Archaea2 - ps3
Archaea3 - ps4
Asgard/Eukaryota - ps5
Eukaryota - ps6
Excavata/Diaphoretickes - ps7
Diaphoretickes - ps8
Archaeplastida - ps9
Viridiplantae - ps10
Streptophyta - ps11
Charophyceae/Embriophyta - ps12
Embriophyta - ps13
Tracheophyta - ps14
Spermatophyta - ps15
Angiosperms - ps16
Mesangiospermae - ps17
Magnoliids/eudicots - ps18
Eudicots - ps19
Core_eudicots - ps20
Superrosids - ps21
Rosids - ps22
Eurosids - ps23
Malvids - ps24
Sap./Malv./Brassicales - ps25
Malvales/Brassicales - ps26
Brassicales - ps27
Cleomaceae/Brassicaceae - ps28
Brassicaceae - ps29
Camelineae - ps30
Arabidopsis - ps31
Arabidopsis_thaliana - ps32
Phylostratum

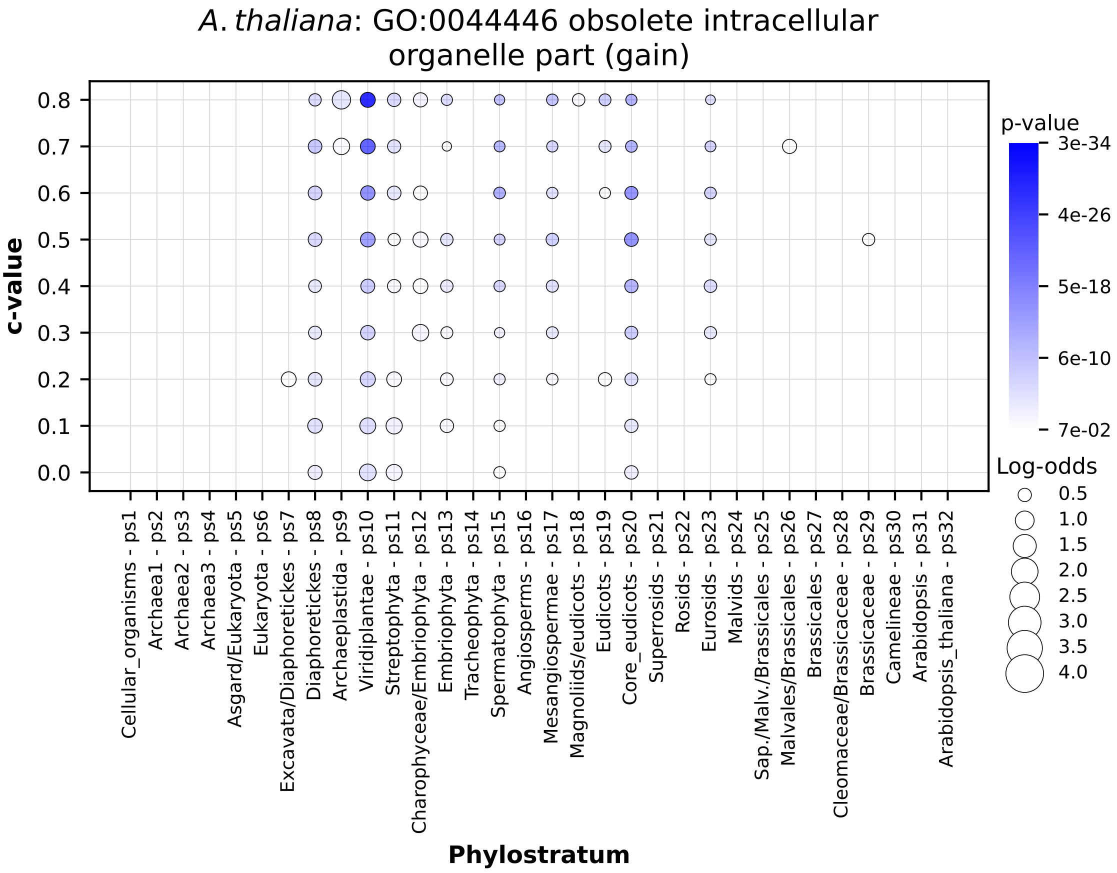

A. thaliana: GO:0044446 obsolete intracellular organelle part (gain)
c-value
0.8
0.7
0.6
0.5
0.4
0.3
0.2
0.1
0.0
Phylostratum
Cellular_organisms - ps1
Archaea1 - ps2
Archaea2 - ps3
Archaea3 - ps4
Asgard/Eukaryota - ps5
Eukaryota - ps6
Excavata/Diaphoretickes - ps7
Diaphoretickes - ps8
Archaeplastida - ps9
Viridiplantae - ps10
Streptophyta - ps11
Charophyceae/Embriophyta - ps12
Embriophyta - ps13
Tracheophyta - ps14
Spermatophyta - ps15
Angiosperms - ps16
Mesangiospermae - ps17
Magnoliids/eudicots - ps18
Eudicots - ps19
Core_eudicots - ps20
Superrosids - ps21
Rosids - ps22
Eurosids - ps23
Malvids - ps24
Sap./Malv./Brassicales - ps25
Malvales/Brassicales - ps26
Brassicales - ps27
Cleomaceae/Brassicaceae - ps28
Brassicaceae - ps29
Camelineae - ps30
Arabidopsis - ps31
Arabidopsis_thaliana - ps32
p-value
3e-34
4e-26
5e-18
6e-10
7e-02
Log-odds
0.5
1.0
1.5
2.0
2.5
3.0
3.5
4.0

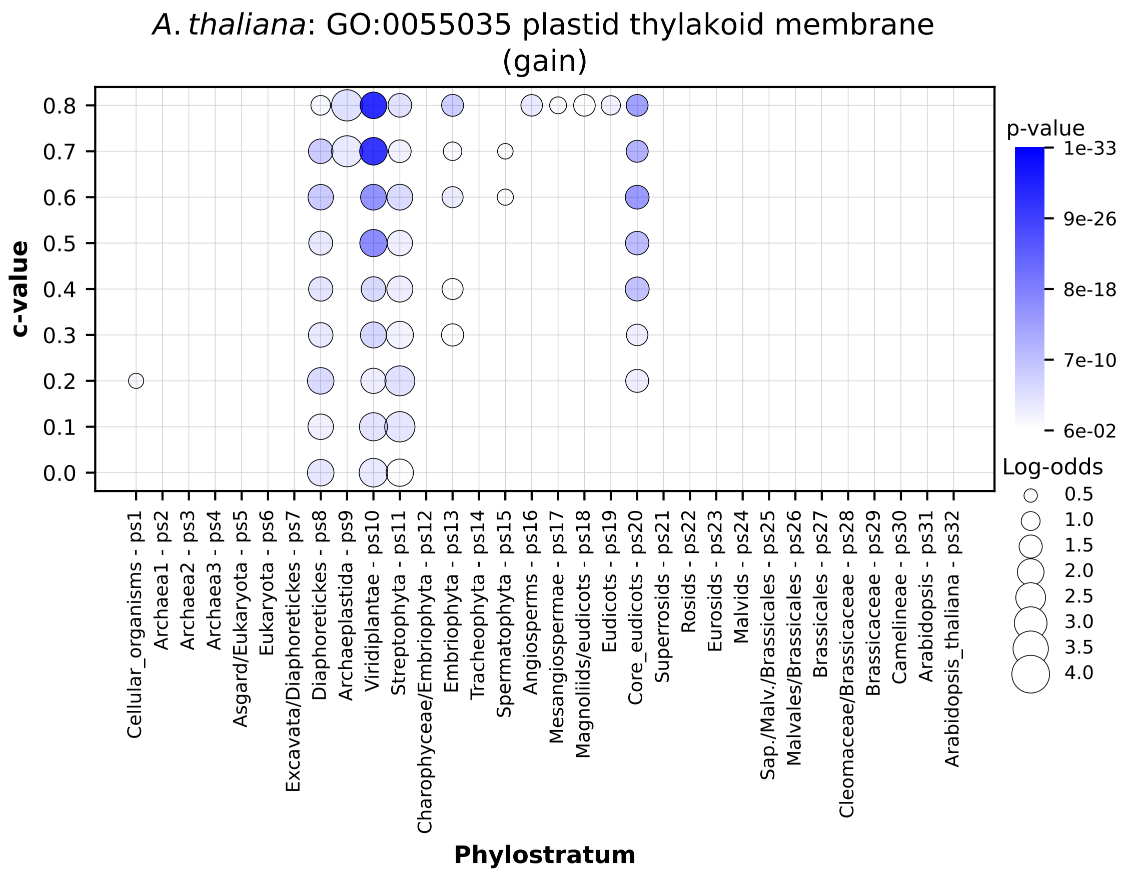

A. thaliana: GO:0055035 plastid thylakoid membrane (gain)
c-value
0.8
0.7
0.6
0.5
0.4
0.3
0.2
0.1
0.0
p-value
1e-33
9e-26
8e-18
7e-10
6e-02
Log-odds
0.5
1.0
1.5
2.0
2.5
3.0
3.5
4.0
Cellular_organisms - ps1
Archaea1 - ps2
Archaea2 - ps3
Archaea3 - ps4
Asgard/Eukaryota - ps5
Eukaryota - ps6
Excavata/Diaphoretickes - ps7
Diaphoretickes - ps8
Archaeplastida - ps9
Viridiplantae - ps10
Streptophyta - ps11
Charophyceae/Embriophyta - ps12
Embriophyta - ps13
Tracheophyta - ps14
Spermatophyta - ps15
Angiosperms - ps16
Mesangiospermae - ps17
Magnoliids/eudicots - ps18
Eudicots - ps19
Core_eudicots - ps20
Superrosids - ps21
Rosids - ps22
Eurosids - ps23
Malvids - ps24
Sap./Malv./Brassicales - ps25
Malvales/Brassicales - ps26
Brassicales - ps27
Cleomaceae/Brassicaceae - ps28
Brassicaceae - ps29
Camelineae - ps30
Arabidopsis - ps31
Arabidopsis_thaliana - ps32
Phylostratum

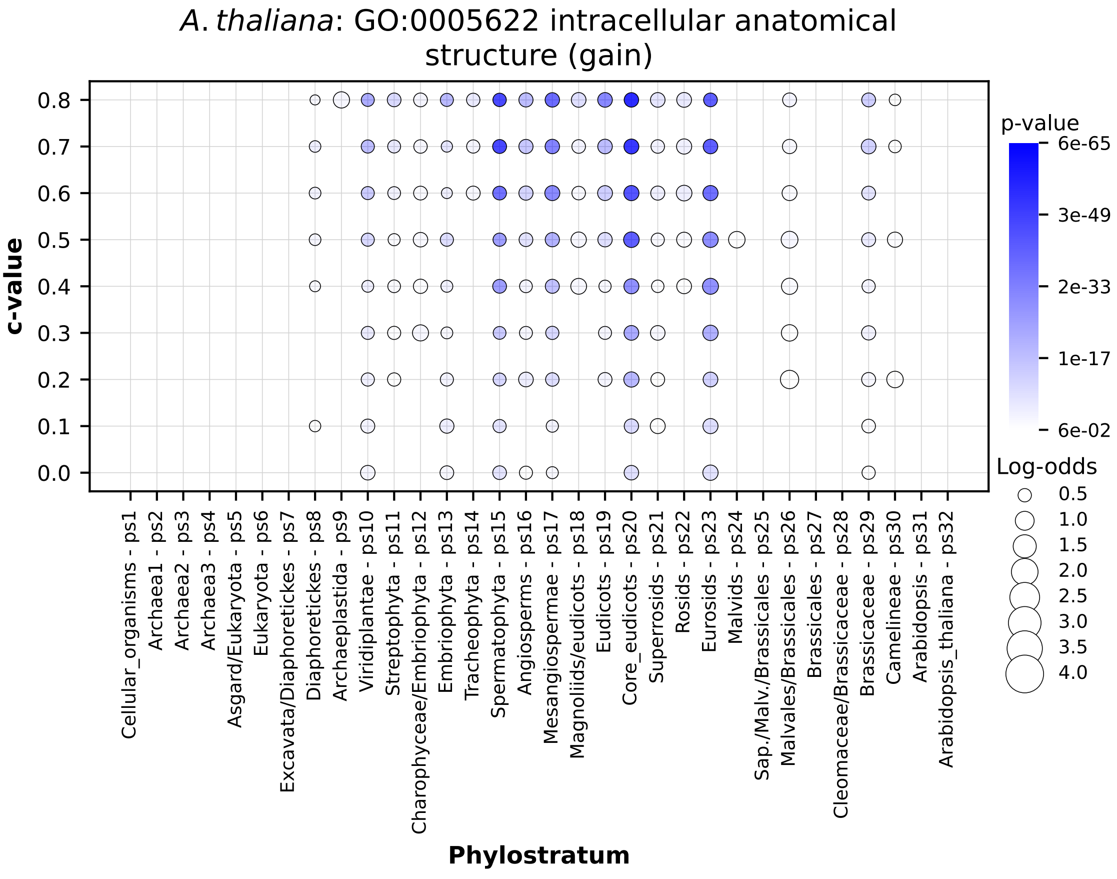

A. thaliana: GO:0005622 intracellular anatomical structure (gain)
c-value
0.8
0.7
0.6
0.5
0.4
0.3
0.2
0.1
0.0
p-value
6e-65
3e-49
2e-33
1e-17
6e-02
Log-odds
0.5
1.0
1.5
2.0
2.5
3.0
3.5
4.0
Cellular_organisms - ps1
Archaea1 - ps2
Archaea2 - ps3
Archaea3 - ps4
Asgard/Eukaryota - ps5
Eukaryota - ps6
Excavata/Diaphoretickes - ps7
Diaphoretickes - ps8
Archaeplastida - ps9
Viridiplantae - ps10
Streptophyta - ps11
Charophyceae/Embriophyta - ps12
Embriophyta - ps13
Tracheophyta - ps14
Spermatophyta - ps15
Angiosperms - ps16
Mesangiospermae - ps17
Magnoliids/eudicots - ps18
Eudicots - ps19
Core_eudicots - ps20
Superrosids - ps21
Rosids - ps22
Eurosids - ps23
Malvids - ps24
Sap./Malv./Brassicales - ps25
Malvales/Brassicales - ps26
Brassicales - ps27
Cleomaceae/Brassicaceae - ps28
Brassicaceae - ps29
Camelineae - ps30
Arabidopsis - ps31
Arabidopsis_thaliana - ps32
Phylostratum

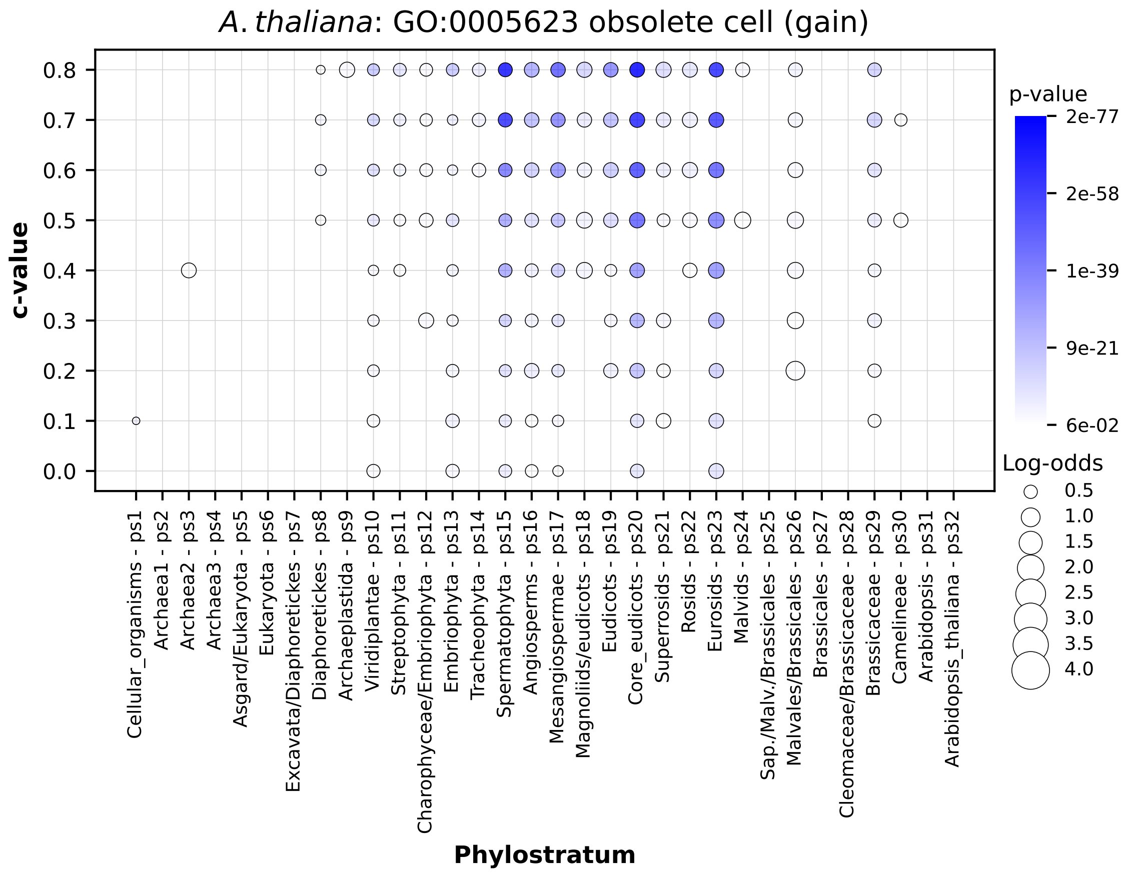

A. thaliana: GO:0005623 obsolete cell (gain)
c-value
0.8
0.7
0.6
0.5
0.4
0.3
0.2
0.1
0.0
Phylostratum
Cellular_organisms - ps1
Archaea1 - ps2
Archaea2 - ps3
Archaea3 - ps4
Asgard/Eukaryota - ps5
Eukaryota - ps6
Excavata/Diaphoretickes - ps7
Diaphoretickes - ps8
Archaeplastida - ps9
Viridiplantae - ps10
Streptophyta - ps11
Charophyceae/Embriophyta - ps12
Embriophyta - ps13
Tracheophyta - ps14
Spermatophyta - ps15
Angiosperms - ps16
Mesangiospermae - ps17
Magnoliids/eudicots - ps18
Eudicots - ps19
Core_eudicots - ps20
Superrosids - ps21
Rosids - ps22
Eurosids - ps23
Malvids - ps24
Sap./Malv./Brassicales - ps25
Malvales/Brassicales - ps26
Brassicales - ps27
Cleomaceae/Brassicaceae - ps28
Brassicaceae - ps29
Camelineae - ps30
Arabidopsis - ps31
Arabidopsis_thaliana - ps32
p-value
2e-77
2e-58
1e-39
9e-21
6e-02
Log-odds
0.5
1.0
1.5
2.0
2.5
3.0
3.5
4.0

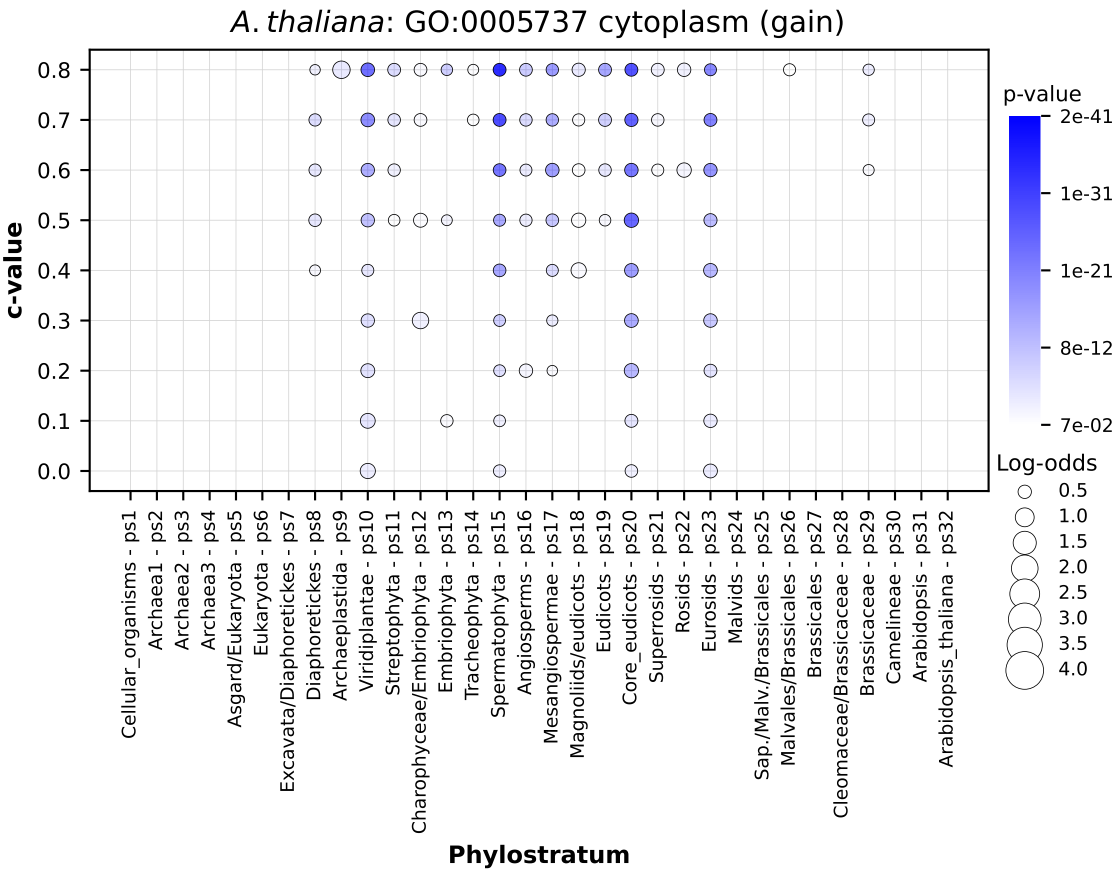

A. thaliana: GO:0005737 cytoplasm (gain)
c-value
0.8
0.7
0.6
0.5
0.4
0.3
0.2
0.1
0.0
p-value
2e-41
1e-31
1e-21
8e-12
7e-02
Log-odds
0.5
1.0
1.5
2.0
2.5
3.0
3.5
4.0
Cellular_organisms - ps1
Archaea1 - ps2
Archaea2 - ps3
Archaea3 - ps4
Asgard/Eukaryota - ps5
Eukaryota - ps6
Excavata/Diaphoretickes - ps7
Diaphoretickes - ps8
Archaeplastida - ps9
Viridiplantae - ps10
Streptophyta - ps11
Charophyceae/Embriophyta - ps12
Embriophyta - ps13
Tracheophyta - ps14
Spermatophyta - ps15
Angiosperms - ps16
Mesangiospermae - ps17
Magnoliids/eudicots - ps18
Eudicots - ps19
Core_eudicots - ps20
Superrosids - ps21
Rosids - ps22
Eurosids - ps23
Malvids - ps24
Sap./Malv./Brassicales - ps25
Malvales/Brassicales - ps26
Brassicales - ps27
Cleomaceae/Brassicaceae - ps28
Brassicaceae - ps29
Camelineae - ps30
Arabidopsis - ps31
Arabidopsis_thaliana - ps32
Phylostratum

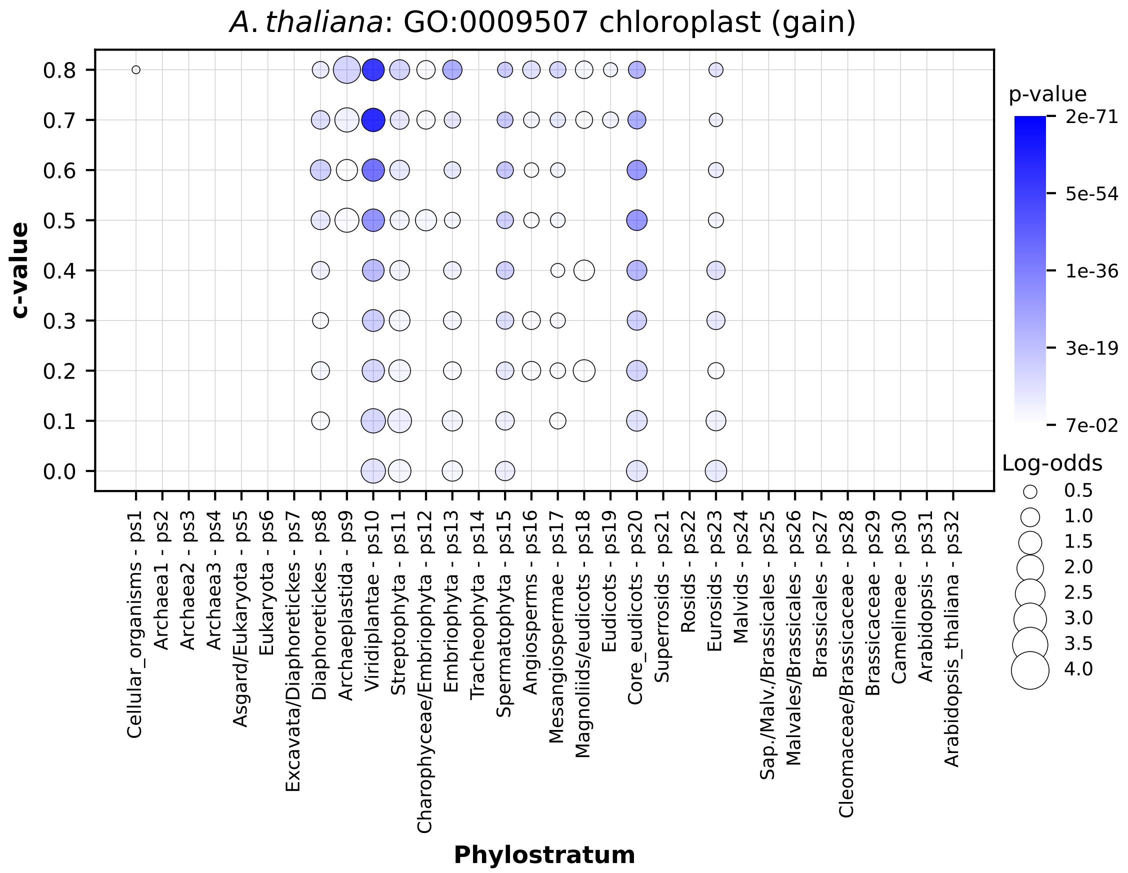

A. thaliana: GO:0009507 chloroplast (gain)
c-value
0.8
0.7
0.6
0.5
0.4
0.3
0.2
0.1
0.0
Phylostratum
Cellular_organisms - ps1
Archaea1 - ps2
Archaea2 - ps3
Archaea3 - ps4
Asgard/Eukaryota - ps5
Eukaryota - ps6
Excavata/Diaphoretickes - ps7
Diaphoretickes - ps8
Archaeplastida - ps9
Viridiplantae - ps10
Streptophyta - ps11
Charophyceae/Embriophyta - ps12
Embriophyta - ps13
Tracheophyta - ps14
Spermatophyta - ps15
Angiosperms - ps16
Mesangiospermae - ps17
Magnoliids/eudicots - ps18
Eudicots - ps19
Core_eudicots - ps20
Superrosids - ps21
Rosids - ps22
Eurosids - ps23
Malvids - ps24
Sap./Malv./Brassicales - ps25
Malvales/Brassicales - ps26
Brassicales - ps27
Cleomaceae/Brassicaceae - ps28
Brassicaceae - ps29
Camelineae - ps30
Arabidopsis - ps31
Arabidopsis_thaliana - ps32
p-value
2e-71
5e-54
1e-36
3e-19
7e-02
Log-odds
0.5
1.0
1.5
2.0
2.5
3.0
3.5
4.0

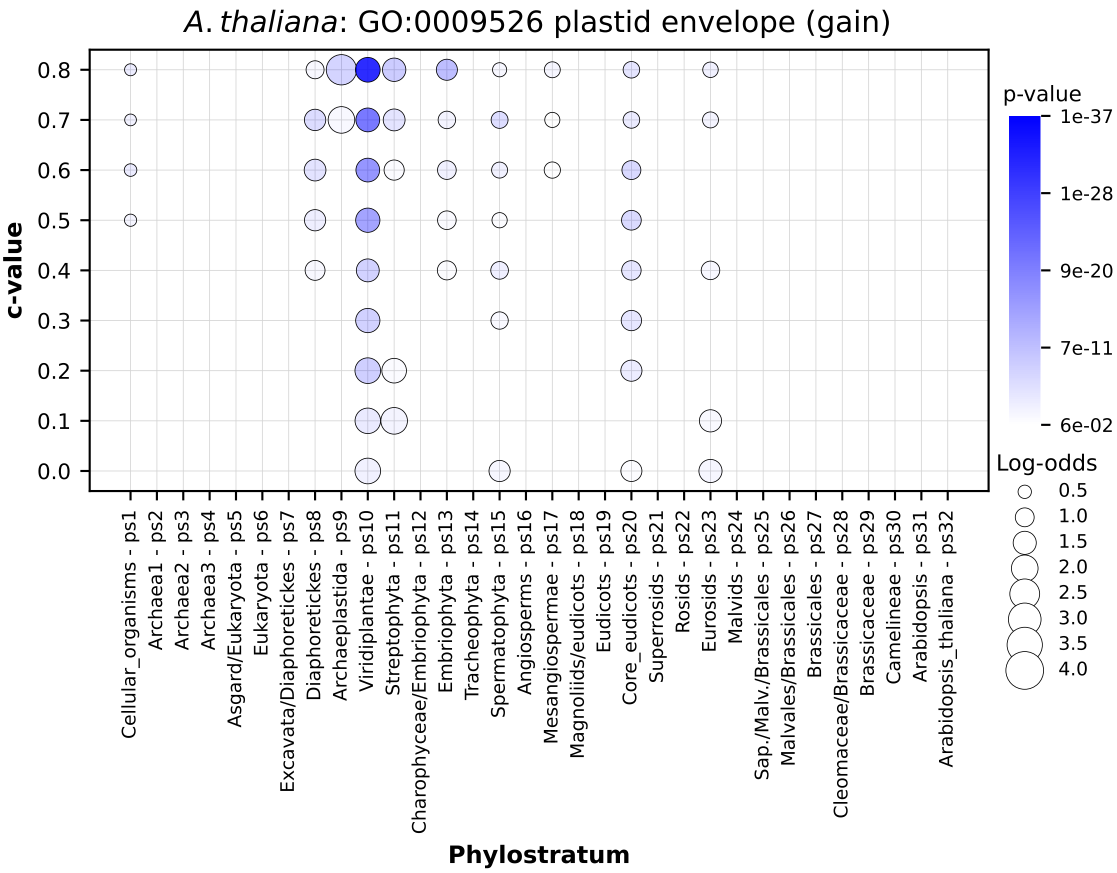

A. thaliana: GO:0009526 plastid envelope (gain)
c-value
0.8
0.7
0.6
0.5
0.4
0.3
0.2
0.1
0.0
Phylostratum
Cellular_organisms - ps1
Archaea1 - ps2
Archaea2 - ps3
Archaea3 - ps4
Asgard/Eukaryota - ps5
Eukaryota - ps6
Excavata/Diaphoretickes - ps7
Diaphoretickes - ps8
Archaeplastida - ps9
Viridiplantae - ps10
Streptophyta - ps11
Charophyceae/Embriophyta - ps12
Embriophyta - ps13
Tracheophyta - ps14
Spermatophyta - ps15
Angiosperms - ps16
Mesangiospermae - ps17
Magnoliids/eudicots - ps18
Eudicots - ps19
Core_eudicots - ps20
Superrosids - ps21
Rosids - ps22
Eurosids - ps23
Malvids - ps24
Sap./Malv./Brassicales - ps25
Malvales/Brassicales - ps26
Brassicales - ps27
Cleomaceae/Brassicaceae - ps28
Brassicaceae - ps29
Camelineae - ps30
Arabidopsis - ps31
Arabidopsis_thaliana - ps32
p-value
1e-37
1e-28
9e-20
7e-11
6e-02
Log-odds
0.5
1.0
1.5
2.0
2.5
3.0
3.5
4.0

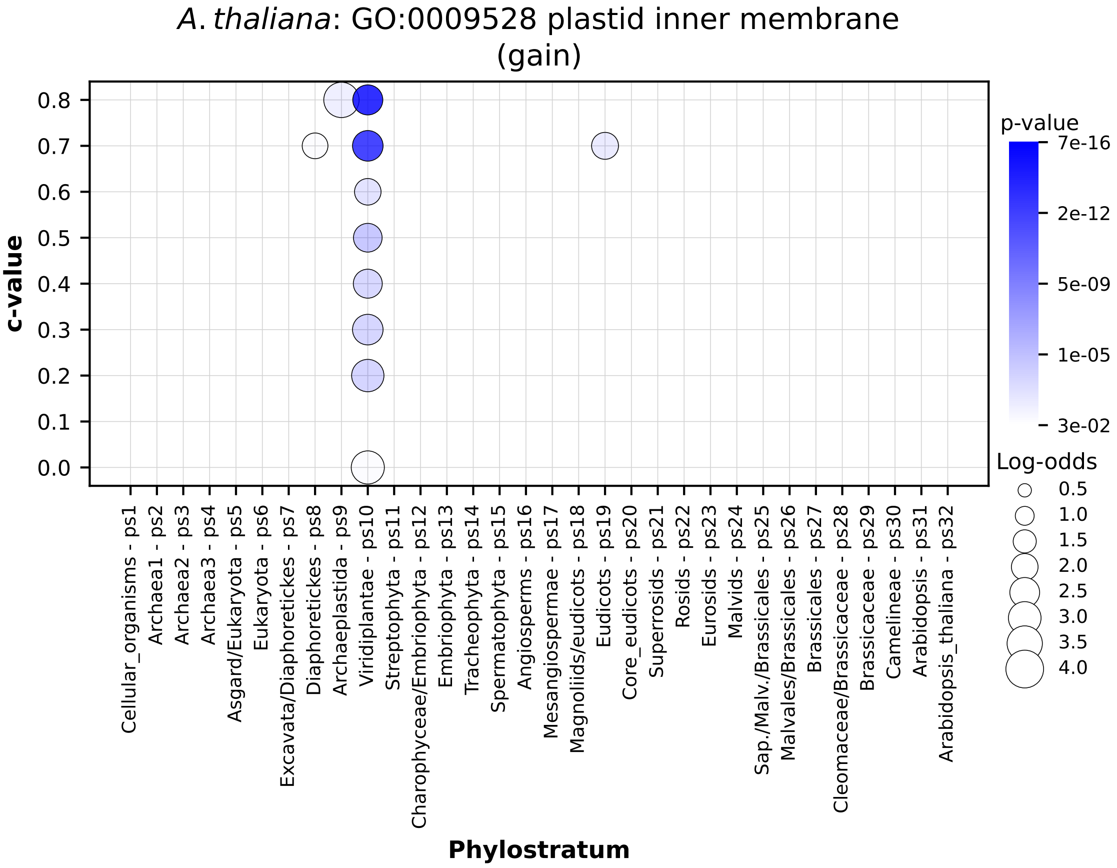

A. thaliana: GO:0009528 plastid inner membrane
(gain)
c-value
0.8
0.7
0.6
0.5
0.4
0.3
0.2
0.1
0.0
Phylostratum
Cellular_organisms - ps1
Archaea1 - ps2
Archaea2 - ps3
Archaea3 - ps4
Asgard/Eukaryota - ps5
Eukaryota - ps6
Excavata/Diaphoretickes - ps7
Diaphoretickes - ps8
Archaeplastida - ps9
Viridiplantae - ps10
Streptophyta - ps11
Charophyceae/Embriophyta - ps12
Embriophyta - ps13
Tracheophyta - ps14
Spermatophyta - ps15
Angiosperms - ps16
Mesangiospermae - ps17
Magnoliids/eudicots - ps18
Eudicots - ps19
Core_eudicots - ps20
Superrosids - ps21
Rosids - ps22
Eurosids - ps23
Malvids - ps24
Sap./Malv./Brassicales - ps25
Malvales/Brassicales - ps26
Brassicales - ps27
Cleomaceae/Brassicaceae - ps28
Brassicaceae - ps29
Camelineae - ps30
Arabidopsis - ps31
Arabidopsis_thaliana - ps32
p-value
7e-16
2e-12
5e-09
1e-05
3e-02
Log-odds
0.5
1.0
1.5
2.0
2.5
3.0
3.5
4.0

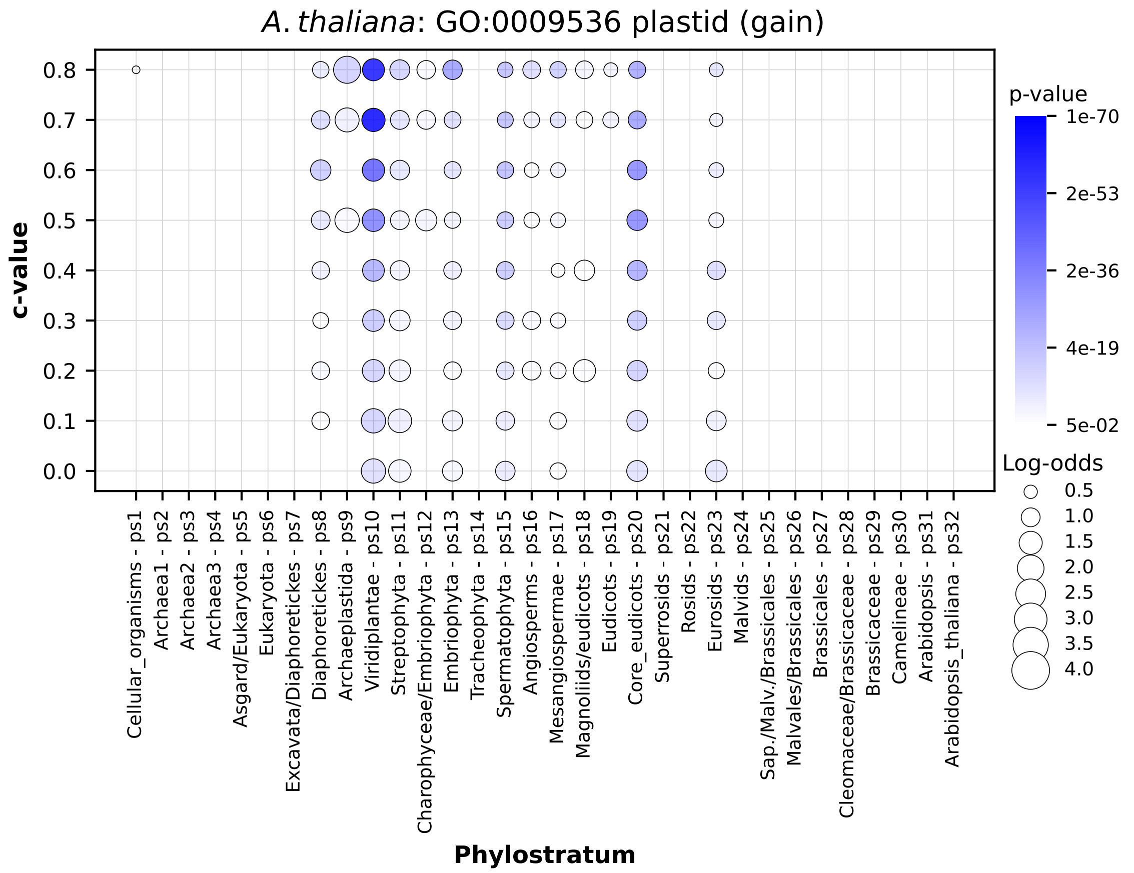

A. thaliana: GO:0009536 plastid (gain)
c-value
0.8
0.7
0.6
0.5
0.4
0.3
0.2
0.1
0.0
p-value
1e-70
2e-53
2e-36
4e-19
5e-02
Log-odds
0.5
1.0
1.5
2.0
2.5
3.0
3.5
4.0
Cellular_organisms - ps1
Archaea1 - ps2
Archaea2 - ps3
Archaea3 - ps4
Asgard/Eukaryota - ps5
Eukaryota - ps6
Excavata/Diaphoretickes - ps7
Diaphoretickes - ps8
Archaeplastida - ps9
Viridiplantae - ps10
Streptophyta - ps11
Charophyceae/Embriophyta - ps12
Embriophyta - ps13
Tracheophyta - ps14
Spermatophyta - ps15
Angiosperms - ps16
Mesangiospermae - ps17
Magnoliids/eudicots - ps18
Eudicots - ps19
Core_eudicots - ps20
Superrosids - ps21
Rosids - ps22
Eurosids - ps23
Malvids - ps24
Sap./Malv./Brassicales - ps25
Malvales/Brassicales - ps26
Brassicales - ps27
Cleomaceae/Brassicaceae - ps28
Brassicaceae - ps29
Camelineae - ps30
Arabidopsis - ps31
Arabidopsis_thaliana - ps32
Phylostratum

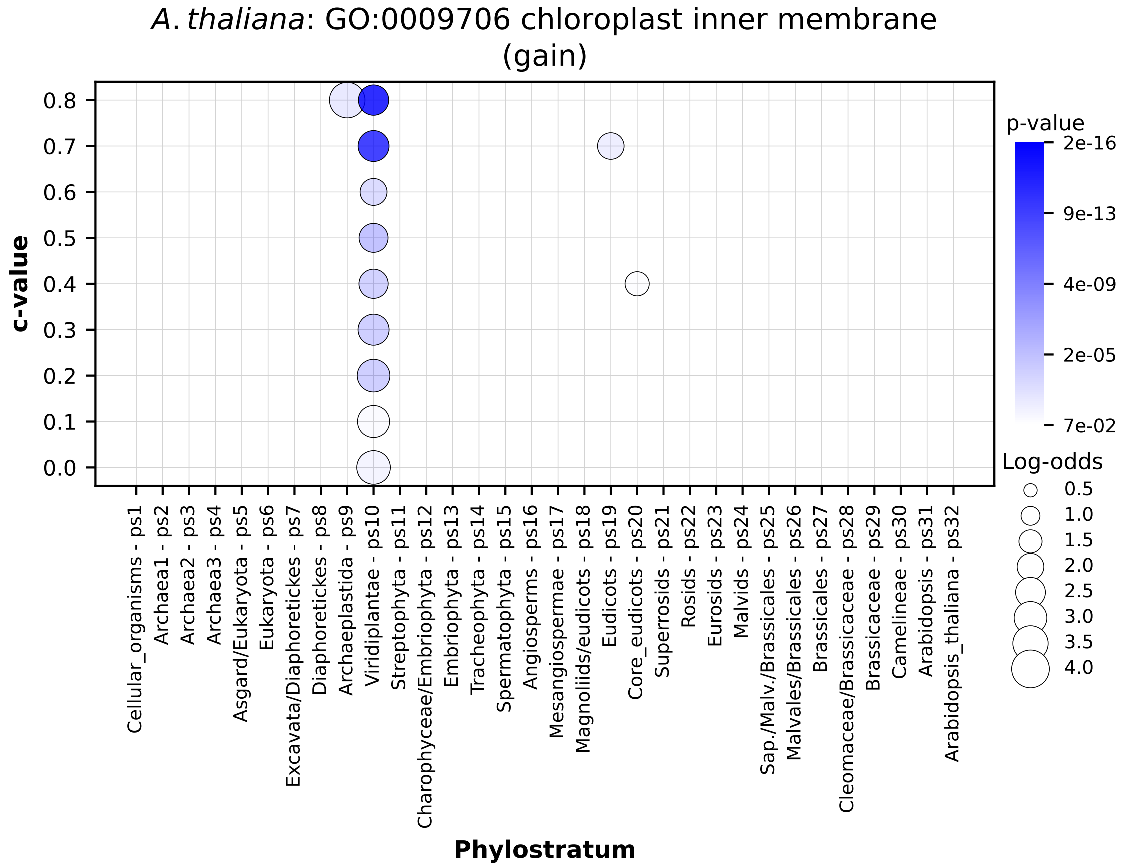
A. thaliana: GO:0009706 chloroplast inner membrane (gain)
c-value
0.8
0.7
0.6
0.5
0.4
0.3
0.2
0.1
0.0
Phylostratum
Cellular_organisms - ps1
Archaea1 - ps2
Archaea2 - ps3
Archaea3 - ps4
Asgard/Eukaryota - ps5
Eukaryota - ps6
Excavata/Diaphoretickes - ps7
Diaphoretickes - ps8
Archaeplastida - ps9
Viridiplantae - ps10
Streptophyta - ps11
Charophyceae/Embriophyta - ps12
Embriophyta - ps13
Tracheophyta - ps14
Spermatophyta - ps15
Angiosperms - ps16
Mesangiospermae - ps17
Magnoliids/eudicots - ps18
Eudicots - ps19
Core_eudicots - ps20
Superrosids - ps21
Rosids - ps22
Eurosids - ps23
Malvids - ps24
Sap./Malv./Brassicales - ps25
Malvales/Brassicales - ps26
Brassicales - ps27
Cleomaceae/Brassicaceae - ps28
Brassicaceae - ps29
Camelineae - ps30
Arabidopsis - ps31
Arabidopsis_thaliana - ps32
p-value
2e-16
9e-13
4e-09
2e-05
7e-02
Log-odds
0.5
1.0
1.5
2.0
2.5
3.0
3.5
4.0

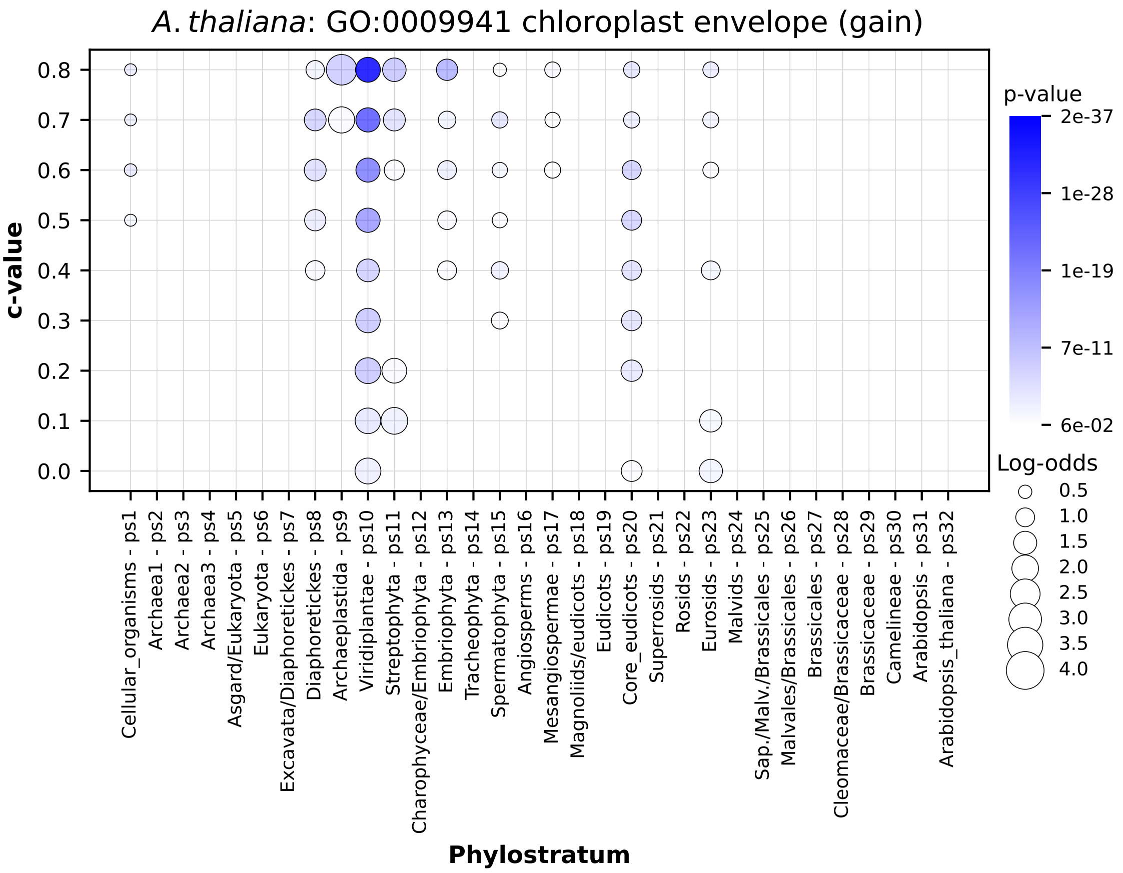

A. thaliana: GO:0009941 chloroplast envelope (gain)
c-value
0.8
0.7
0.6
0.5
0.4
0.3
0.2
0.1
0.0
Phylostratum
Cellular_organisms - ps1
Archaea1 - ps2
Archaea2 - ps3
Archaea3 - ps4
Asgard/Eukaryota - ps5
Eukaryota - ps6
Excavata/Diaphoretickes - ps7
Diaphoretickes - ps8
Archaeplastida - ps9
Viridiplantae - ps10
Streptophyta - ps11
Charophyceae/Embriophyta - ps12
Embriophyta - ps13
Tracheophyta - ps14
Spermatophyta - ps15
Angiosperms - ps16
Mesangiospermae - ps17
Magnoliids/eudicots - ps18
Eudicots - ps19
Core_eudicots - ps20
Superrosids - ps21
Rosids - ps22
Eurosids - ps23
Malvids - ps24
Sap./Malv./Brassicales - ps25
Malvales/Brassicales - ps26
Brassicales - ps27
Cleomaceae/Brassicaceae - ps28
Brassicaceae - ps29
Camelineae - ps30
Arabidopsis - ps31
Arabidopsis_thaliana - ps32
p-value
2e-37
1e-28
1e-19
7e-11
6e-02
Log-odds
0.5
1.0
1.5
2.0
2.5
3.0
3.5
4.0

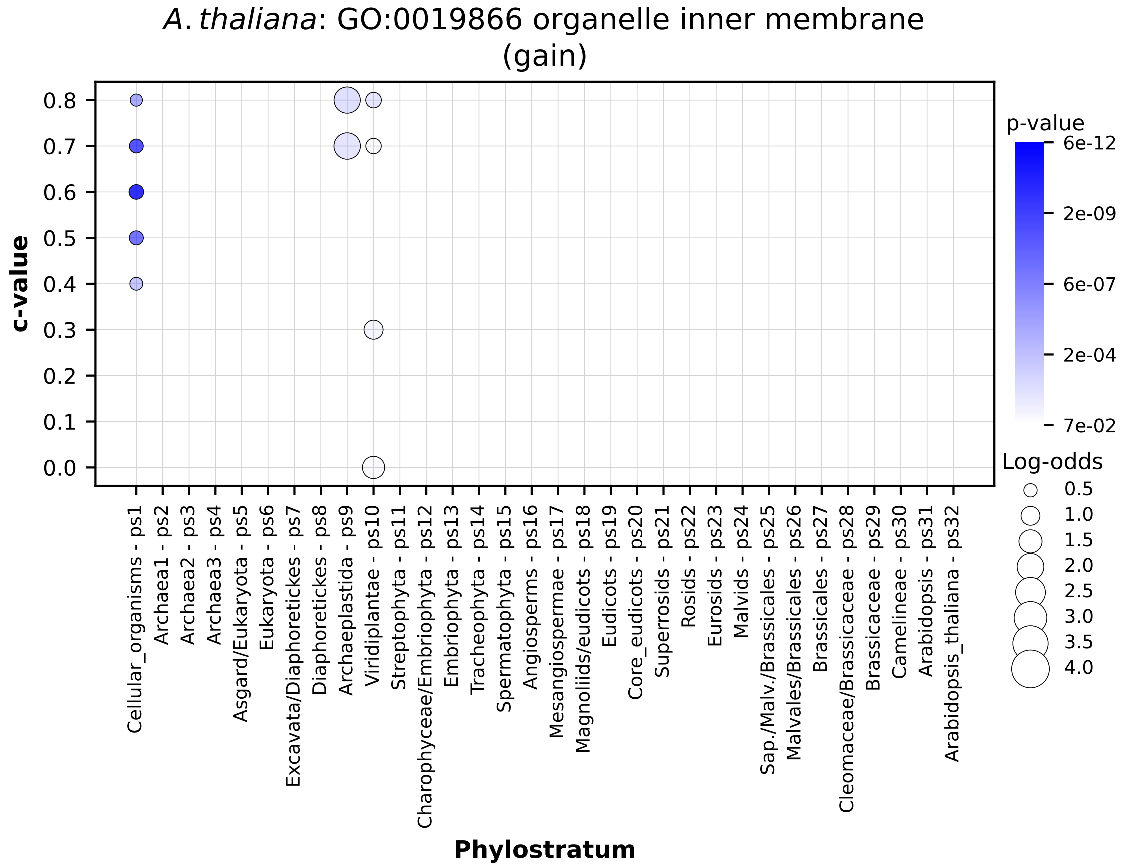

A. thaliana: GO:0019866 organelle inner membrane
(gain)
c-value
0.8
0.7
0.6
0.5
0.4
0.3
0.2
0.1
0.0
p-value
6e-12
2e-09
6e-07
2e-04
7e-02
Log-odds
0.5
1.0
1.5
2.0
2.5
3.0
3.5
4.0
Cellular_organisms - ps1
Archaea1 - ps2
Archaea2 - ps3
Archaea3 - ps4
Asgard/Eukaryota - ps5
Eukaryota - ps6
Excavata/Diaphoretickes - ps7
Diaphoretickes - ps8
Archaeplastida - ps9
Viridiplantae - ps10
Streptophyta - ps11
Charophyceae/Embriophyta - ps12
Embriophyta - ps13
Tracheophyta - ps14
Spermatophyta - ps15
Angiosperms - ps16
Mesangiospermae - ps17
Magnoliids/eudicots - ps18
Eudicots - ps19
Core_eudicots - ps20
Superrosids - ps21
Rosids - ps22
Eurosids - ps23
Malvids - ps24
Sap./Malv./Brassicales - ps25
Malvales/Brassicales - ps26
Brassicales - ps27
Cleomaceae/Brassicaceae - ps28
Brassicaceae - ps29
Camelineae - ps30
Arabidopsis - ps31
Arabidopsis_thaliana - ps32
Phylostratum

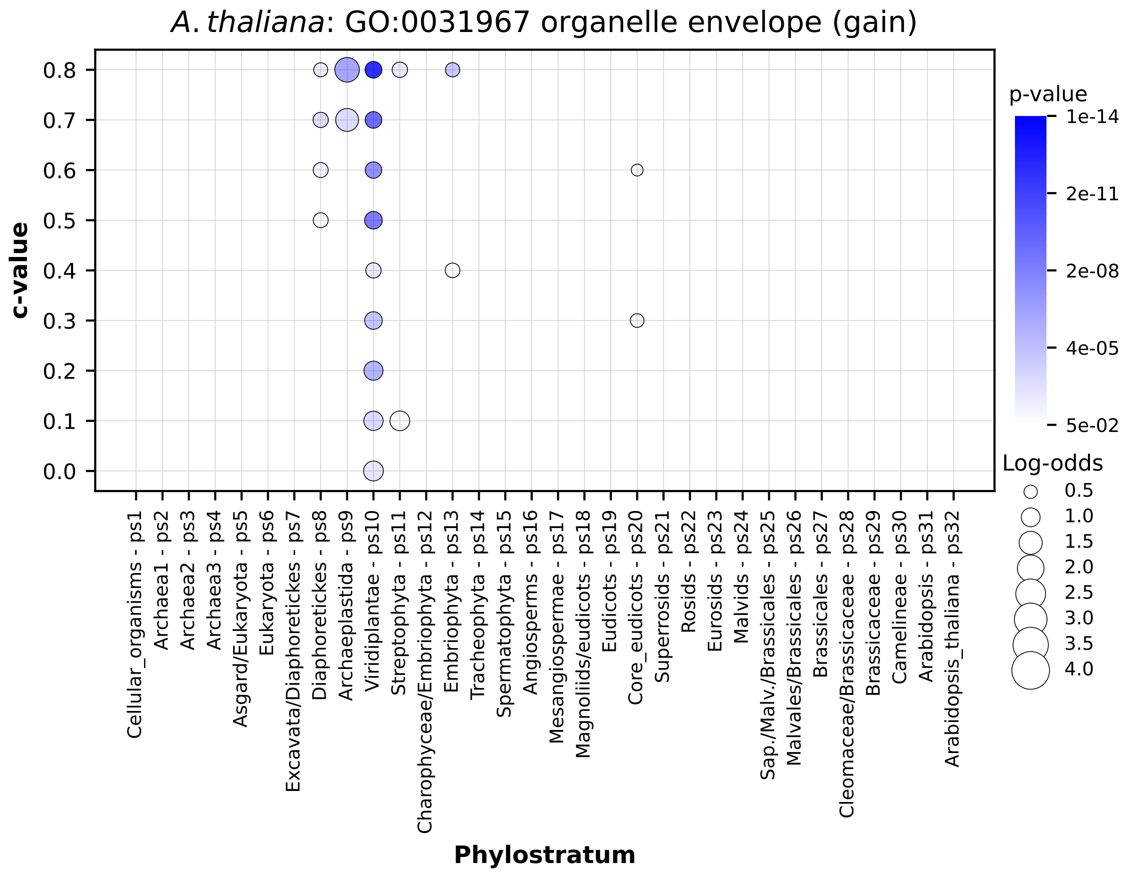
A. thaliana: GO:0031967 organelle envelope (gain)
c-value
0.8
0.7
0.6
0.5
0.4
0.3
0.2
0.1
0.0
p-value
1e-14
2e-11
2e-08
4e-05
5e-02
Log-odds
0.5
1.0
1.5
2.0
2.5
3.0
3.5
4.0
Cellular_organisms - ps1
Archaea1 - ps2
Archaea2 - ps3
Archaea3 - ps4
Asgard/Eukaryota - ps5
Eukaryota - ps6
Excavata/Diaphoretickes - ps7
Diaphoretickes - ps8
Archaeplastida - ps9
Viridiplantae - ps10
Streptophyta - ps11
Charophyceae/Embriophyta - ps12
Embriophyta - ps13
Tracheophyta - ps14
Spermatophyta - ps15
Angiosperms - ps16
Mesangiospermae - ps17
Magnoliids/eudicots - ps18
Eudicots - ps19
Core_eudicots - ps20
Superrosids - ps21
Rosids - ps22
Eurosids - ps23
Malvids - ps24
Sap./Malv./Brassicales - ps25
Malvales/Brassicales - ps26
Brassicales - ps27
Cleomaceae/Brassicaceae - ps28
Brassicaceae - ps29
Camelineae - ps30
Arabidopsis - ps31
Arabidopsis_thaliana - ps32
Phylostratum

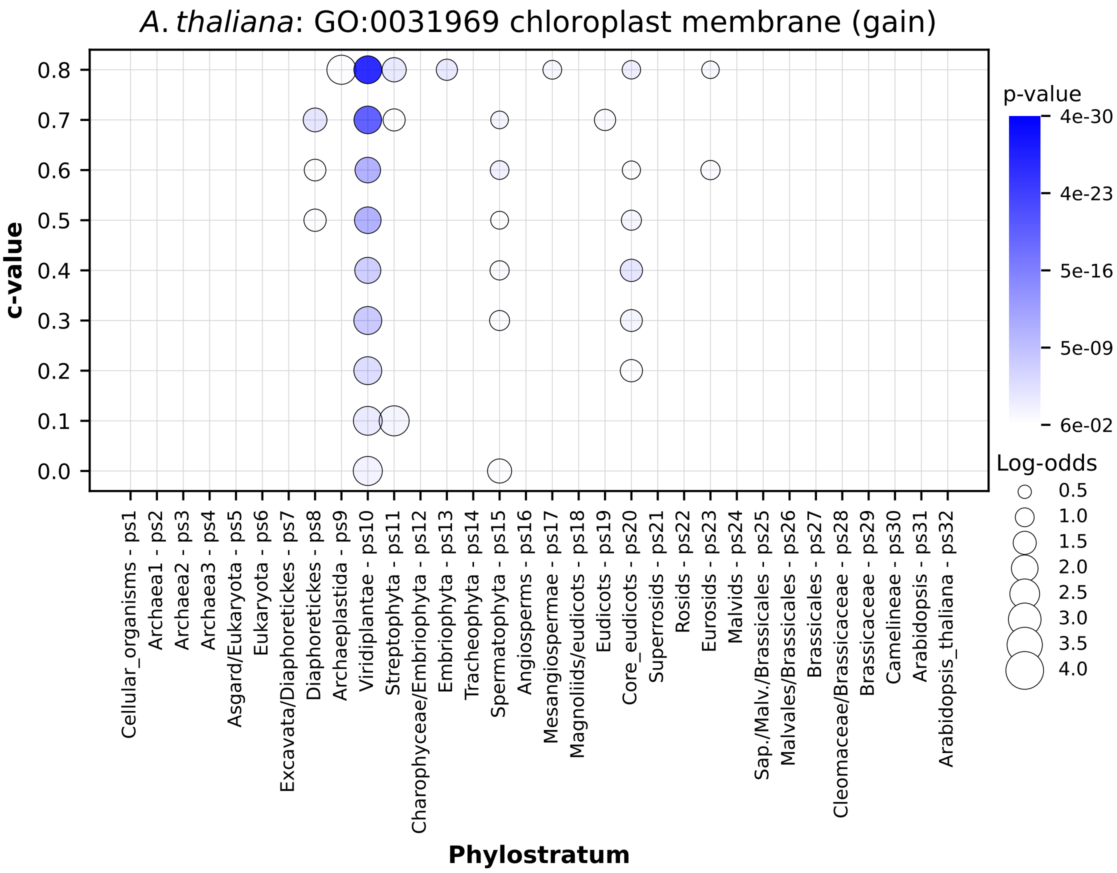

A. thaliana: GO:0031969 chloroplast membrane (gain)
c-value
0.8
0.7
0.6
0.5
0.4
0.3
0.2
0.1
0.0
p-value
4e-30
4e-23
5e-16
5e-09
6e-02
Log-odds
0.5
1.0
1.5
2.0
2.5
3.0
3.5
4.0
Cellular_organisms - ps1
Archaea1 - ps2
Archaea2 - ps3
Archaea3 - ps4
Asgard/Eukaryota - ps5
Eukaryota - ps6
Excavata/Diaphoretickes - ps7
Diaphoretickes - ps8
Archaeplastida - ps9
Viridiplantae - ps10
Streptophyta - ps11
Charophyceae/Embriophyta - ps12
Embriophyta - ps13
Tracheophyta - ps14
Spermatophyta - ps15
Angiosperms - ps16
Mesangiospermae - ps17
Magnoliids/eudicots - ps18
Eudicots - ps19
Core_eudicots - ps20
Superrosids - ps21
Rosids - ps22
Eurosids - ps23
Malvids - ps24
Sap./Malv./Brassicales - ps25
Malvales/Brassicales - ps26
Brassicales - ps27
Cleomaceae/Brassicaceae - ps28
Brassicaceae - ps29
Camelineae - ps30
Arabidopsis - ps31
Arabidopsis_thaliana - ps32
Phylostratum

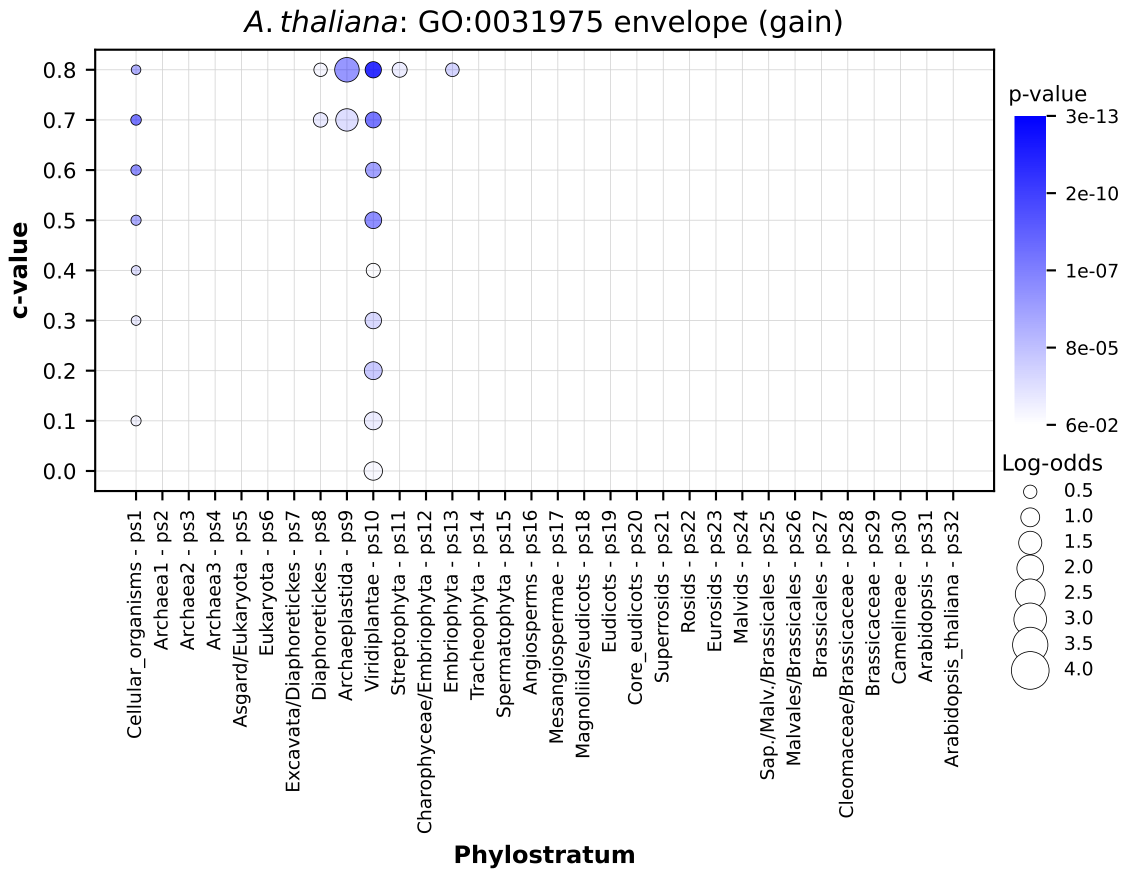
A. thaliana: GO:0031975 envelope (gain)
c-value
0.8
0.7
0.6
0.5
0.4
0.3
0.2
0.1
0.0
Phylostratum
Cellular_organisms - ps1
Archaea1 - ps2
Archaea2 - ps3
Archaea3 - ps4
Asgard/Eukaryota - ps5
Eukaryota - ps6
Excavata/Diaphoretickes - ps7
Diaphoretickes - ps8
Archaeplastida - ps9
Viridiplantae - ps10
Streptophyta - ps11
Charophyceae/Embriophyta - ps12
Embriophyta - ps13
Tracheophyta - ps14
Spermatophyta - ps15
Angiosperms - ps16
Mesangiospermae - ps17
Magnoliids/eudicots - ps18
Eudicots - ps19
Core_eudicots - ps20
Superrosids - ps21
Rosids - ps22
Eurosids - ps23
Malvids - ps24
Sap./Malv./Brassicales - ps25
Malvales/Brassicales - ps26
Brassicales - ps27
Cleomaceae/Brassicaceae - ps28
Brassicaceae - ps29
Camelineae - ps30
Arabidopsis - ps31
Arabidopsis_thaliana - ps32
p-value
3e-13
2e-10
1e-07
8e-05
6e-02
Log-odds
0.5
1.0
1.5
2.0
2.5
3.0
3.5
4.0

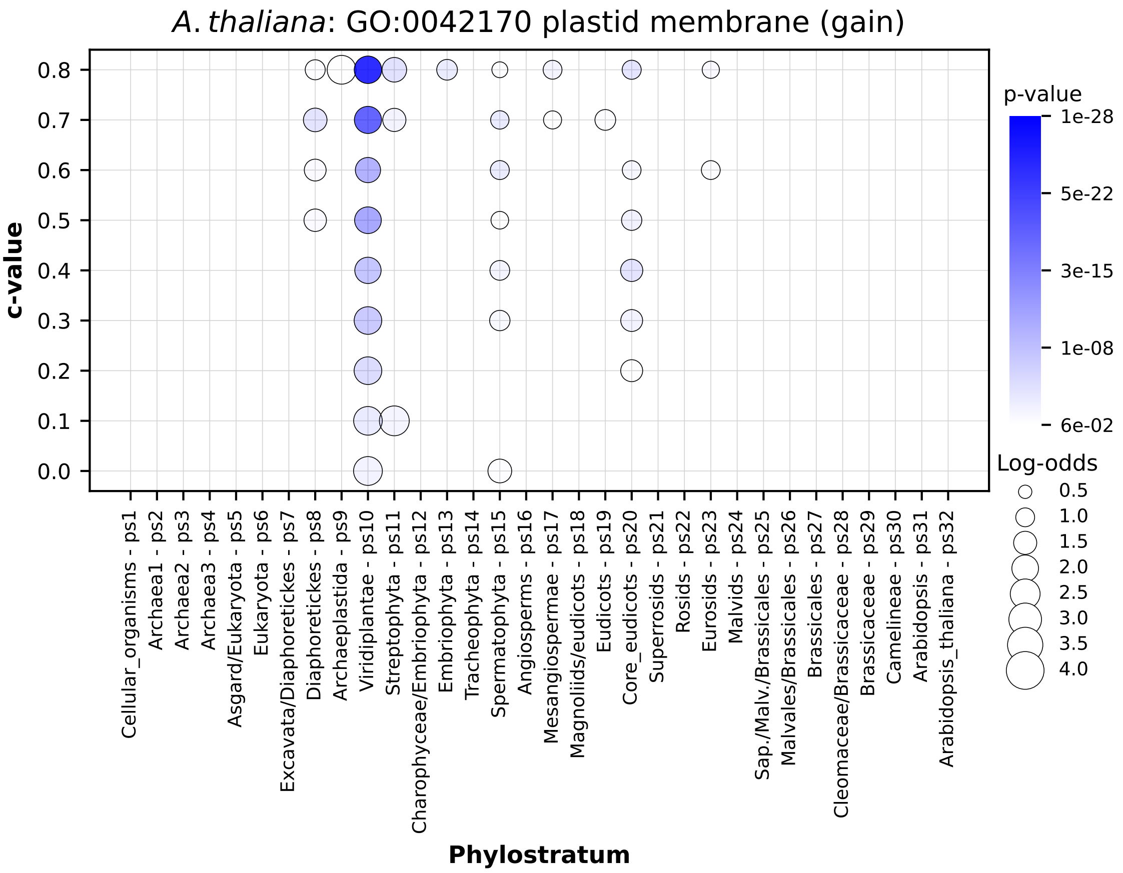
A. thaliana: GO:0042170 plastid membrane (gain)
c-value
0.8
0.7
0.6
0.5
0.4
0.3
0.2
0.1
0.0
p-value
1e-28
5e-22
3e-15
1e-08
6e-02
Log-odds
0.5
1.0
1.5
2.0
2.5
3.0
3.5
4.0
Cellular_organisms - ps1
Archaea1 - ps2
Archaea2 - ps3
Archaea3 - ps4
Asgard/Eukaryota - ps5
Eukaryota - ps6
Excavata/Diaphoretickes - ps7
Diaphoretickes - ps8
Archaeplastida - ps9
Viridiplantae - ps10
Streptophyta - ps11
Charophyceae/Embriophyta - ps12
Embriophyta - ps13
Tracheophyta - ps14
Spermatophyta - ps15
Angiosperms - ps16
Mesangiospermae - ps17
Magnoliids/eudicots - ps18
Eudicots - ps19
Core_eudicots - ps20
Superrosids - ps21
Rosids - ps22
Eurosids - ps23
Malvids - ps24
Sap./Malv./Brassicales - ps25
Malvales/Brassicales - ps26
Brassicales - ps27
Cleomaceae/Brassicaceae - ps28
Brassicaceae - ps29
Camelineae - ps30
Arabidopsis - ps31
Arabidopsis_thaliana - ps32
Phylostratum

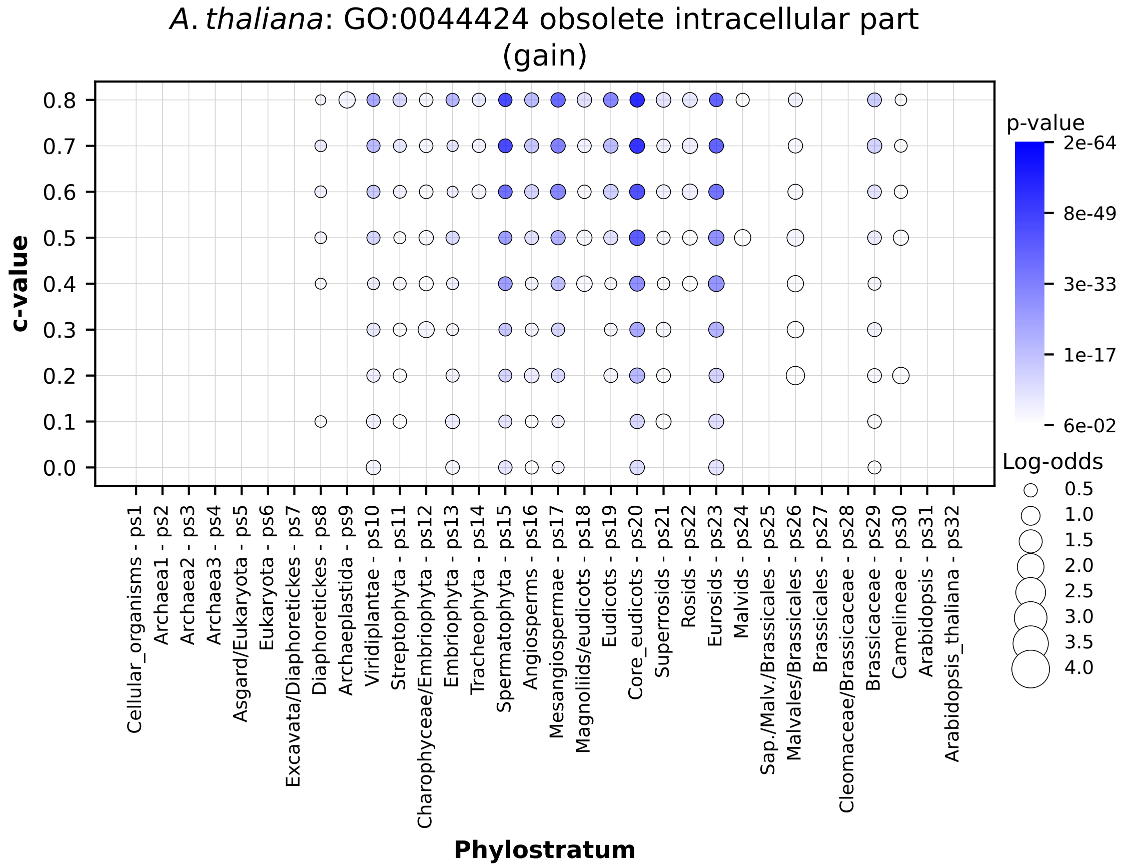

A. thaliana: GO:0044424 obsolete intracellular part
(gain)
c-value
0.8
0.7
0.6
0.5
0.4
0.3
0.2
0.1
0.0
p-value
2e-64
8e-49
3e-33
1e-17
6e-02
Log-odds
0.5
1.0
1.5
2.0
2.5
3.0
3.5
4.0
Cellular_organisms - ps1
Archaea1 - ps2
Archaea2 - ps3
Archaea3 - ps4
Asgard/Eukaryota - ps5
Eukaryota - ps6
Excavata/Diaphoretickes - ps7
Diaphoretickes - ps8
Archaeplastida - ps9
Viridiplantae - ps10
Streptophyta - ps11
Charophyceae/Embriophyta - ps12
Embriophyta - ps13
Tracheophyta - ps14
Spermatophyta - ps15
Angiosperms - ps16
Mesangiospermae - ps17
Magnoliids/eudicots - ps18
Eudicots - ps19
Core_eudicots - ps20
Superrosids - ps21
Rosids - ps22
Eurosids - ps23
Malvids - ps24
Sap./Malv./Brassicales - ps25
Malvales/Brassicales - ps26
Brassicales - ps27
Cleomaceae/Brassicaceae - ps28
Brassicaceae - ps29
Camelineae - ps30
Arabidopsis - ps31
Arabidopsis_thaliana - ps32
Phylostratum

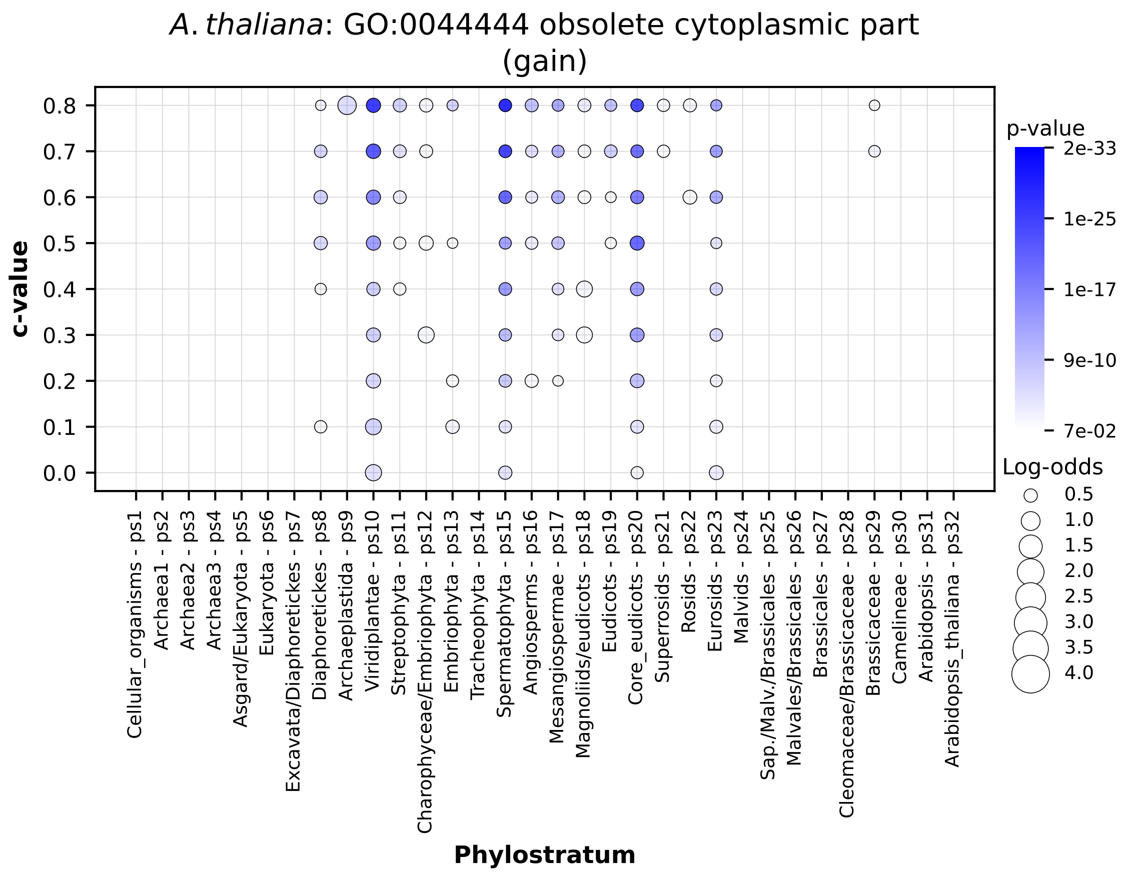
A. thaliana: GO:0044444 obsolete cytoplasmic part
(gain)
c-value
0.8
0.7
0.6
0.5
0.4
0.3
0.2
0.1
0.0
Phylostratum
Cellular_organisms - ps1
Archaea1 - ps2
Archaea2 - ps3
Archaea3 - ps4
Asgard/Eukaryota - ps5
Eukaryota - ps6
Excavata/Diaphoretickes - ps7
Diaphoretickes - ps8
Archaeplastida - ps9
Viridiplantae - ps10
Streptophyta - ps11
Charophyceae/Embriophyta - ps12
Embriophyta - ps13
Tracheophyta - ps14
Spermatophyta - ps15
Angiosperms - ps16
Mesangiospermae - ps17
Magnoliids/eudicots - ps18
Eudicots - ps19
Core_eudicots - ps20
Superrosids - ps21
Rosids - ps22
Eurosids - ps23
Malvids - ps24
Sap./Malv./Brassicales - ps25
Malvales/Brassicales - ps26
Brassicales - ps27
Cleomaceae/Brassicaceae - ps28
Brassicaceae - ps29
Camelineae - ps30
Arabidopsis - ps31
Arabidopsis_thaliana - ps32
p-value
2e-33
1e-25
1e-17
9e-10
7e-02
Log-odds
0.5
1.0
1.5
2.0
2.5
3.0
3.5
4.0

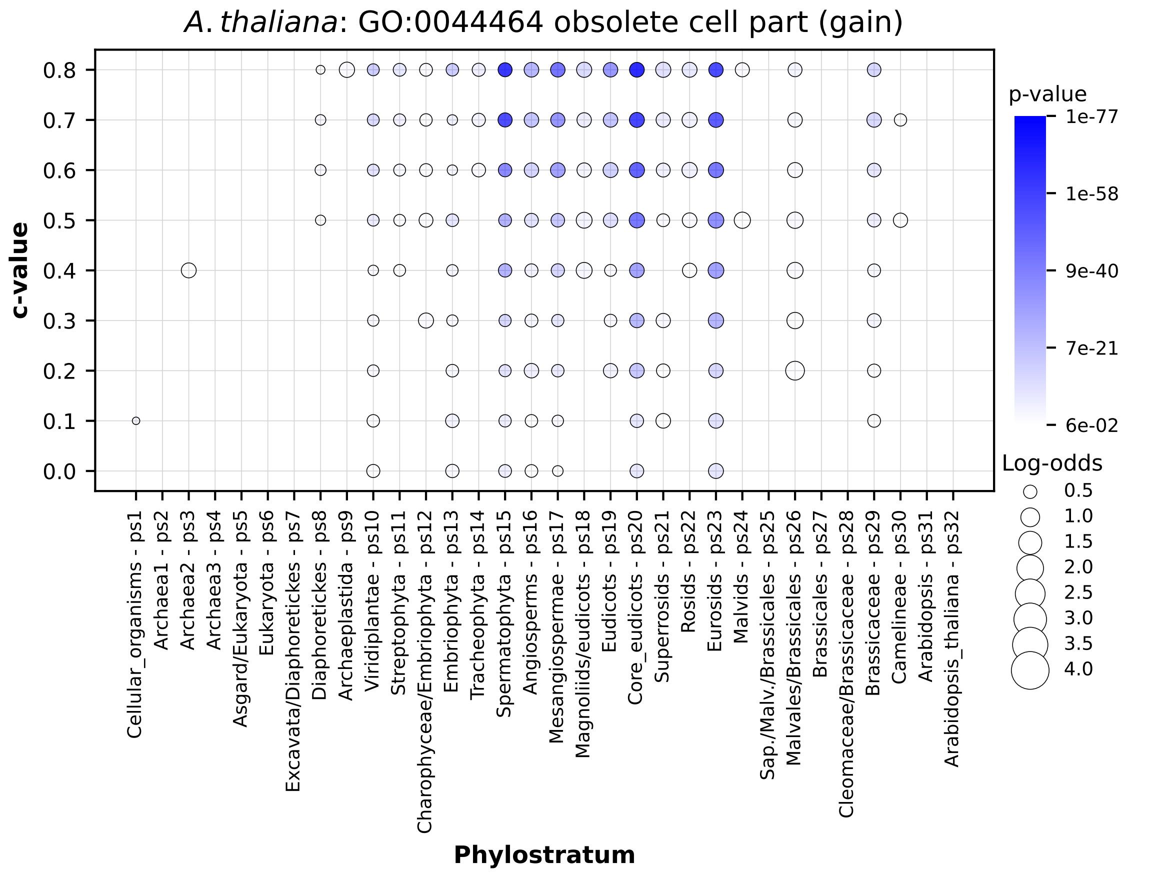

A. thaliana: GO:0044464 obsolete cell part (gain)
c-value
0.8
0.7
0.6
0.5
0.4
0.3
0.2
0.1
0.0
p-value
1e-77
1e-58
9e-40
7e-21
6e-02
Log-odds
0.5
1.0
1.5
2.0
2.5
3.0
3.5
4.0
Cellular_organisms - ps1
Archaea1 - ps2
Archaea2 - ps3
Archaea3 - ps4
Asgard/Eukaryota - ps5
Eukaryota - ps6
Excavata/Diaphoretickes - ps7
Diaphoretickes - ps8
Archaeplastida - ps9
Viridiplantae - ps10
Streptophyta - ps11
Charophyceae/Embriophyta - ps12
Embriophyta - ps13
Tracheophyta - ps14
Spermatophyta - ps15
Angiosperms - ps16
Mesangiospermae - ps17
Magnoliids/eudicots - ps18
Eudicots - ps19
Core_eudicots - ps20
Superrosids - ps21
Rosids - ps22
Eurosids - ps23
Malvids - ps24
Sap./Malv./Brassicales - ps25
Malvales/Brassicales - ps26
Brassicales - ps27
Cleomaceae/Brassicaceae - ps28
Brassicaceae - ps29
Camelineae - ps30
Arabidopsis - ps31
Arabidopsis_thaliana - ps32
Phylostratum

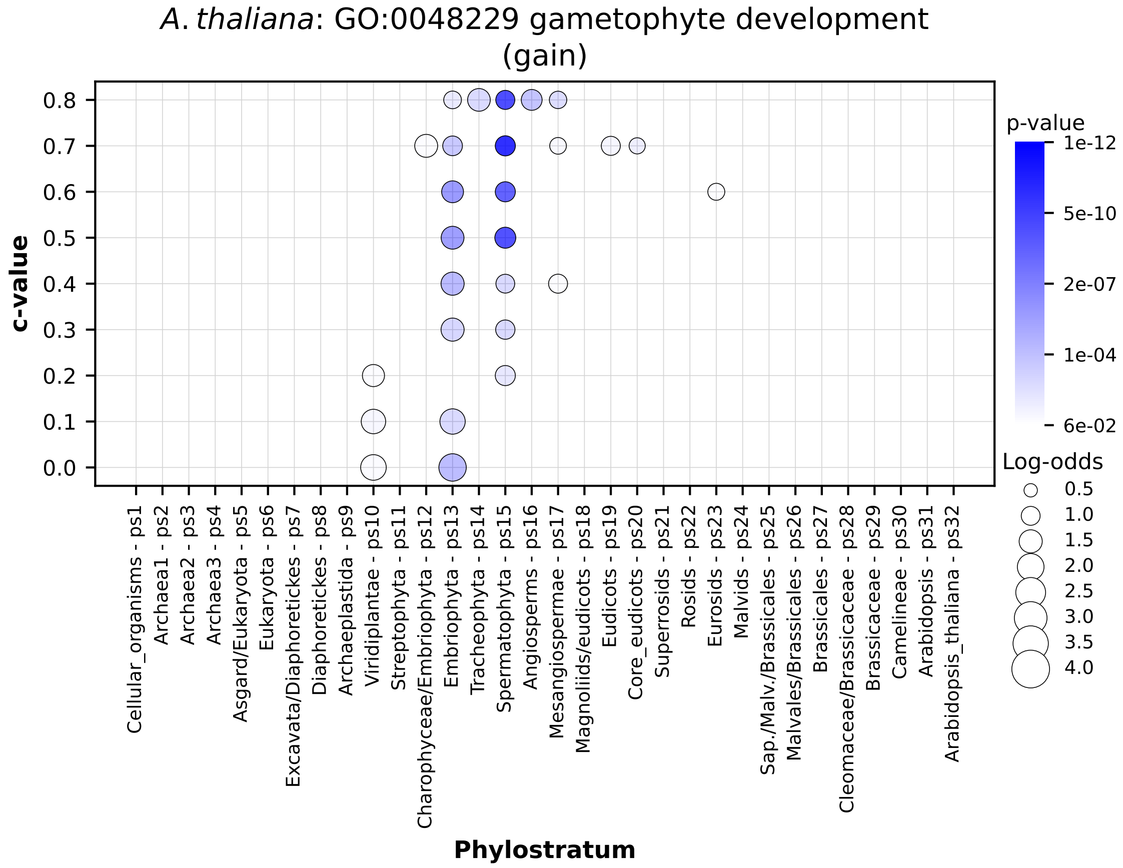

A. thaliana: GO:0048229 gametophyte development
(gain)
c-value
0.8
0.7
0.6
0.5
0.4
0.3
0.2
0.1
0.0
p-value
1e-12
5e-10
2e-07
1e-04
6e-02
Log-odds
0.5
1.0
1.5
2.0
2.5
3.0
3.5
4.0
Cellular_organisms - ps1
Archaea1 - ps2
Archaea2 - ps3
Archaea3 - ps4
Asgard/Eukaryota - ps5
Eukaryota - ps6
Excavata/Diaphoretickes - ps7
Diaphoretickes - ps8
Archaeplastida - ps9
Viridiplantae - ps10
Streptophyta - ps11
Charophyceae/Embriophyta - ps12
Embriophyta - ps13
Tracheophyta - ps14
Spermatophyta - ps15
Angiosperms - ps16
Mesangiospermae - ps17
Magnoliids/eudicots - ps18
Eudicots - ps19
Core_eudicots - ps20
Superrosids - ps21
Rosids - ps22
Eurosids - ps23
Malvids - ps24
Sap./Malv./Brassicales - ps25
Malvales/Brassicales - ps26
Brassicales - ps27
Cleomaceae/Brassicaceae - ps28
Brassicaceae - ps29
Camelineae - ps30
Arabidopsis - ps31
Arabidopsis_thaliana - ps32
Phylostratum

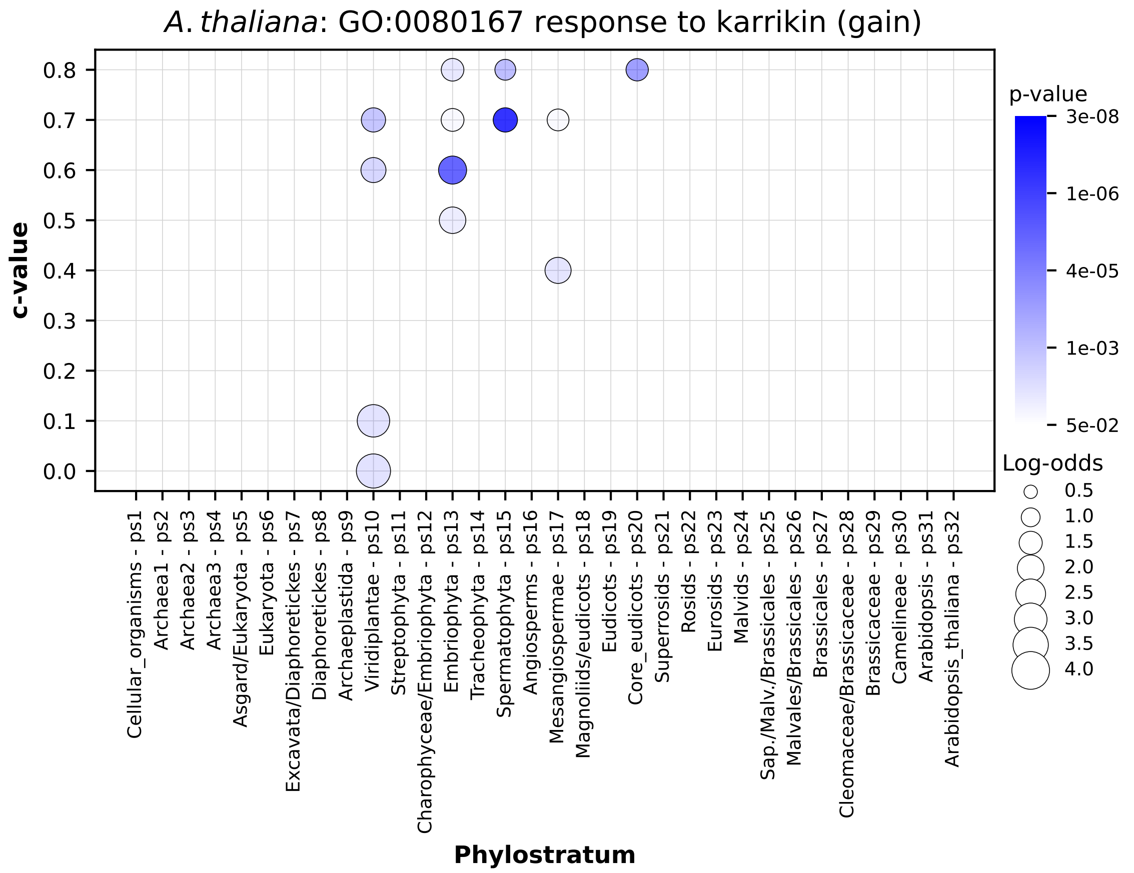

A. thaliana: GO:0080167 response to karrikin (gain)
c-value
0.8
0.7
0.6
0.5
0.4
0.3
0.2
0.1
0.0
Phylostratum
Cellular_organisms - ps1
Archaea1 - ps2
Archaea2 - ps3
Archaea3 - ps4
Asgard/Eukaryota - ps5
Eukaryota - ps6
Excavata/Diaphoretickes - ps7
Diaphoretickes - ps8
Archaeplastida - ps9
Viridiplantae - ps10
Streptophyta - ps11
Charophyceae/Embriophyta - ps12
Embriophyta - ps13
Tracheophyta - ps14
Spermatophyta - ps15
Angiosperms - ps16
Mesangiospermae - ps17
Magnoliids/eudicots - ps18
Eudicots - ps19
Core_eudicots - ps20
Superrosids - ps21
Rosids - ps22
Eurosids - ps23
Malvids - ps24
Sap./Malv./Brassicales - ps25
Malvales/Brassicales - ps26
Brassicales - ps27
Cleomaceae/Brassicaceae - ps28
Brassicaceae - ps29
Camelineae - ps30
Arabidopsis - ps31
Arabidopsis_thaliana - ps32
p-value
3e-08
1e-06
4e-05
1e-03
5e-02
Log-odds
0.5
1.0
1.5
2.0
2.5
3.0
3.5
4.0

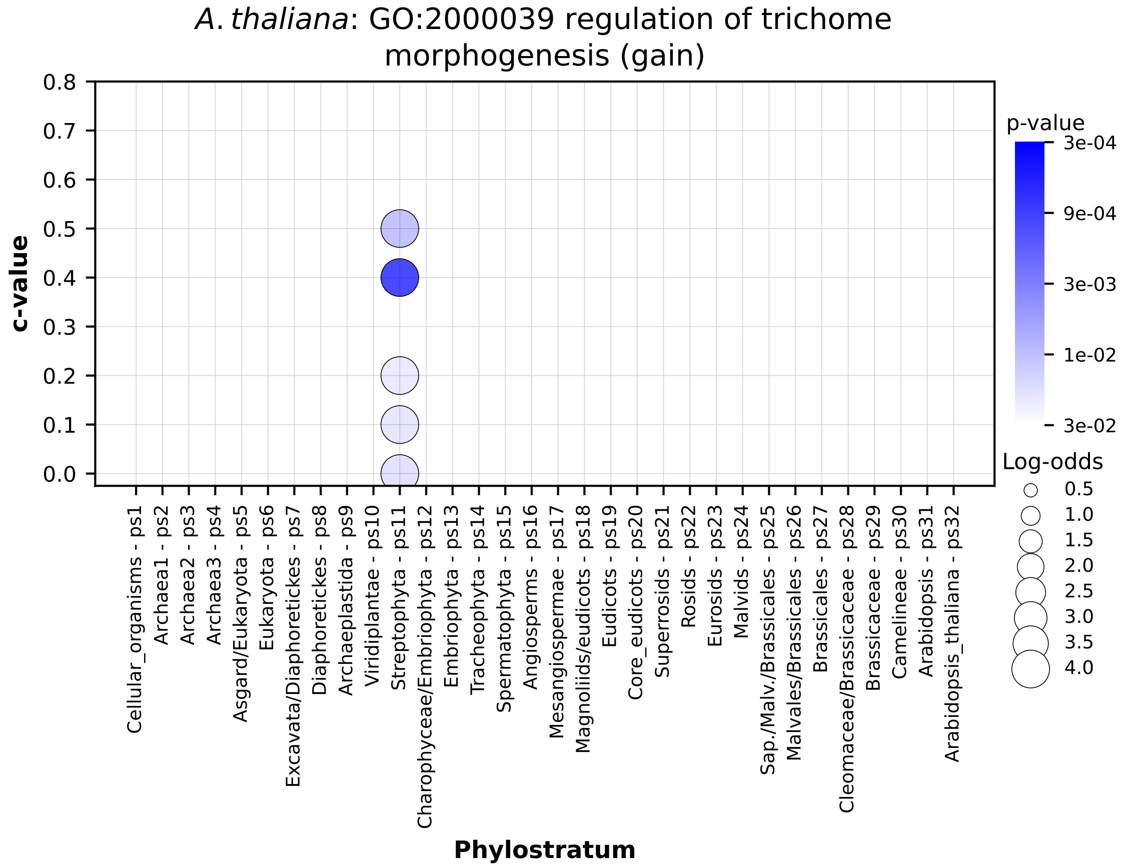

A. thaliana: GO:2000039 regulation of trichome morphogenesis (gain)
c-value
0.8
0.7
0.6
0.5
0.4
0.3
0.2
0.1
0.0
Phylostratum
Cellular_organisms - ps1
Archaea1 - ps2
Archaea2 - ps3
Archaea3 - ps4
Asgard/Eukaryota - ps5
Eukaryota - ps6
Excavata/Diaphoretickes - ps7
Diaphoretickes - ps8
Archaeplastida - ps9
Viridiplantae - ps10
Streptophyta - ps11
Charophyceae/Embriophyta - ps12
Embriophyta - ps13
Tracheophyta - ps14
Spermatophyta - ps15
Angiosperms - ps16
Mesangiospermae - ps17
Magnoliids/eudicots - ps18
Eudicots - ps19
Core_eudicots - ps20
Superrosids - ps21
Rosids - ps22
Eurosids - ps23
Malvids - ps24
Sap./Malv./Brassicales - ps25
Malvales/Brassicales - ps26
Brassicales - ps27
Cleomaceae/Brassicaceae - ps28
Brassicaceae - ps29
Camelineae - ps30
Arabidopsis - ps31
Arabidopsis_thaliana - ps32
p-value
3e-04
9e-04
3e-03
1e-02
3e-02
Log-odds
0.5
1.0
1.5
2.0
2.5
3.0
3.5
4.0

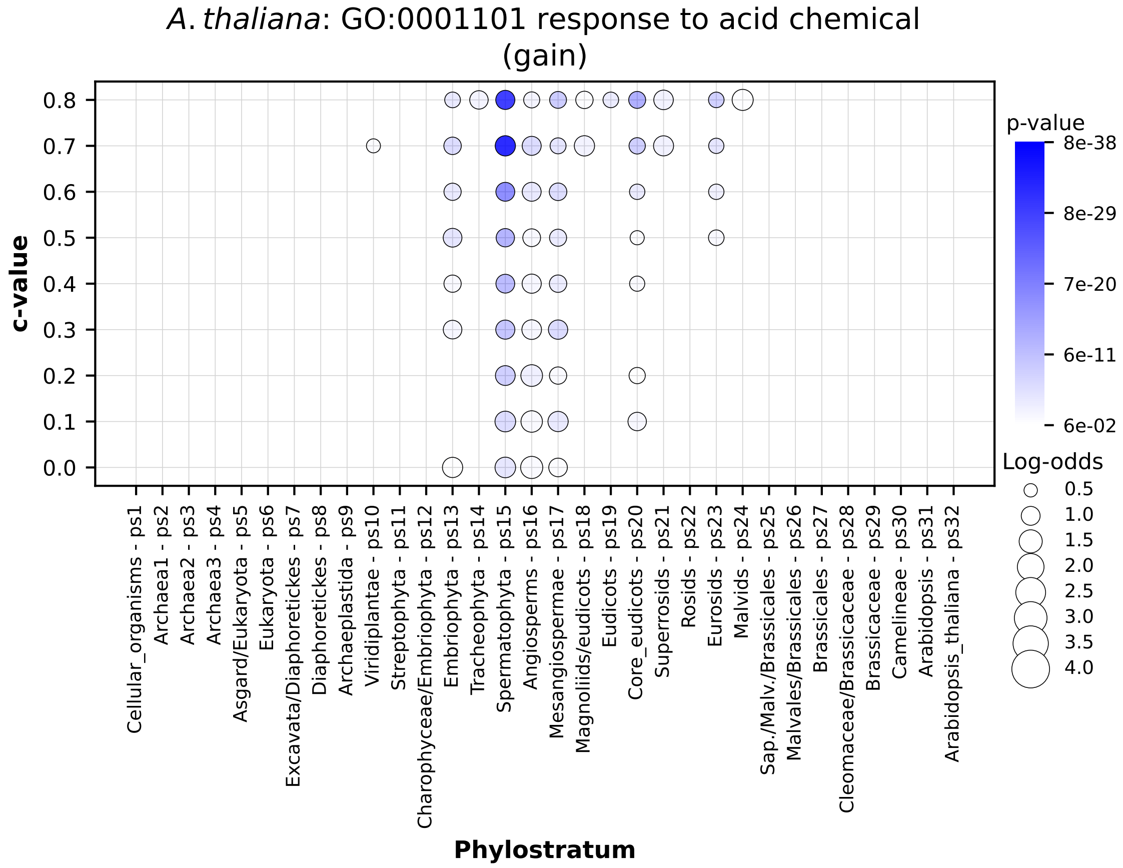
A. thaliana: GO:0001101 response to acid chemical (gain)
c-value
0.8
0.7
0.6
0.5
0.4
0.3
0.2
0.1
0.0
Phylostratum
Cellular_organisms - ps1
Archaea1 - ps2
Archaea2 - ps3
Archaea3 - ps4
Asgard/Eukaryota - ps5
Eukaryota - ps6
Excavata/Diaphoretickes - ps7
Diaphoretickes - ps8
Archaeplastida - ps9
Viridiplantae - ps10
Streptophyta - ps11
Charophyceae/Embriophyta - ps12
Embriophyta - ps13
Tracheophyta - ps14
Spermatophyta - ps15
Angiosperms - ps16
Mesangiospermae - ps17
Magnoliids/eudicots - ps18
Eudicots - ps19
Core_eudicots - ps20
Superrosids - ps21
Rosids - ps22
Eurosids - ps23
Malvids - ps24
Sap./Malv./Brassicales - ps25
Malvales/Brassicales - ps26
Brassicales - ps27
Cleomaceae/Brassicaceae - ps28
Brassicaceae - ps29
Camelineae - ps30
Arabidopsis - ps31
Arabidopsis_thaliana - ps32
p-value
8e-38
8e-29
7e-20
6e-11
6e-02
Log-odds
0.5
1.0
1.5
2.0
2.5
3.0
3.5
4.0

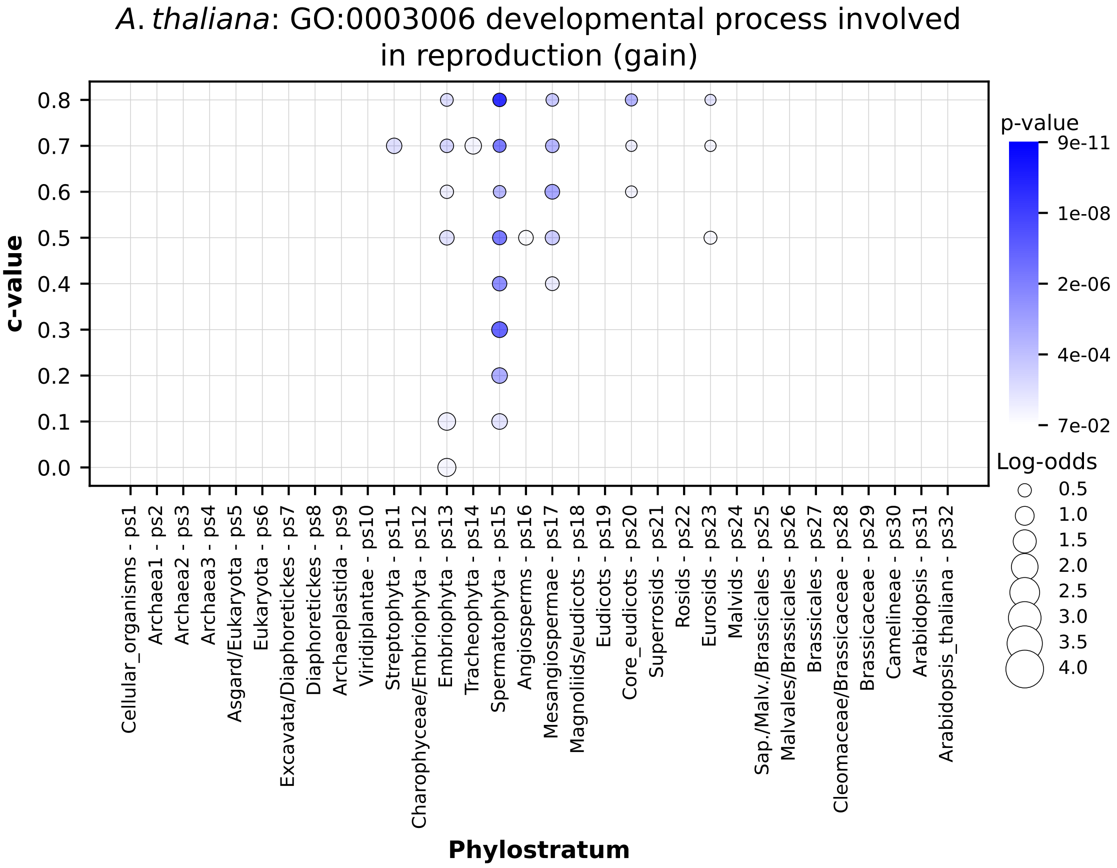
A. thaliana: GO:0003006 developmental process involved in reproduction (gain)
c-value
0.8
0.7
0.6
0.5
0.4
0.3
0.2
0.1
0.0
p-value
9e-11
1e-08
2e-06
4e-04
7e-02
Log-odds
0.5
1.0
1.5
2.0
2.5
3.0
3.5
4.0
Cellular_organisms - ps1
Archaea1 - ps2
Archaea2 - ps3
Archaea3 - ps4
Asgard/Eukaryota - ps5
Eukaryota - ps6
Excavata/Diaphoretickes - ps7
Diaphoretickes - ps8
Archaeplastida - ps9
Viridiplantae - ps10
Streptophyta - ps11
Charophyceae/Embriophyta - ps12
Embriophyta - ps13
Tracheophyta - ps14
Spermatophyta - ps15
Angiosperms - ps16
Mesangiospermae - ps17
Magnoliids/eudicots - ps18
Eudicots - ps19
Core_eudicots - ps20
Superrosids - ps21
Rosids - ps22
Eurosids - ps23
Malvids - ps24
Sap./Malv./Brassicales - ps25
Malvales/Brassicales - ps26
Brassicales - ps27
Cleomaceae/Brassicaceae - ps28
Brassicaceae - ps29
Camelineae - ps30
Arabidopsis - ps31
Arabidopsis_thaliana - ps32
Phylostratum

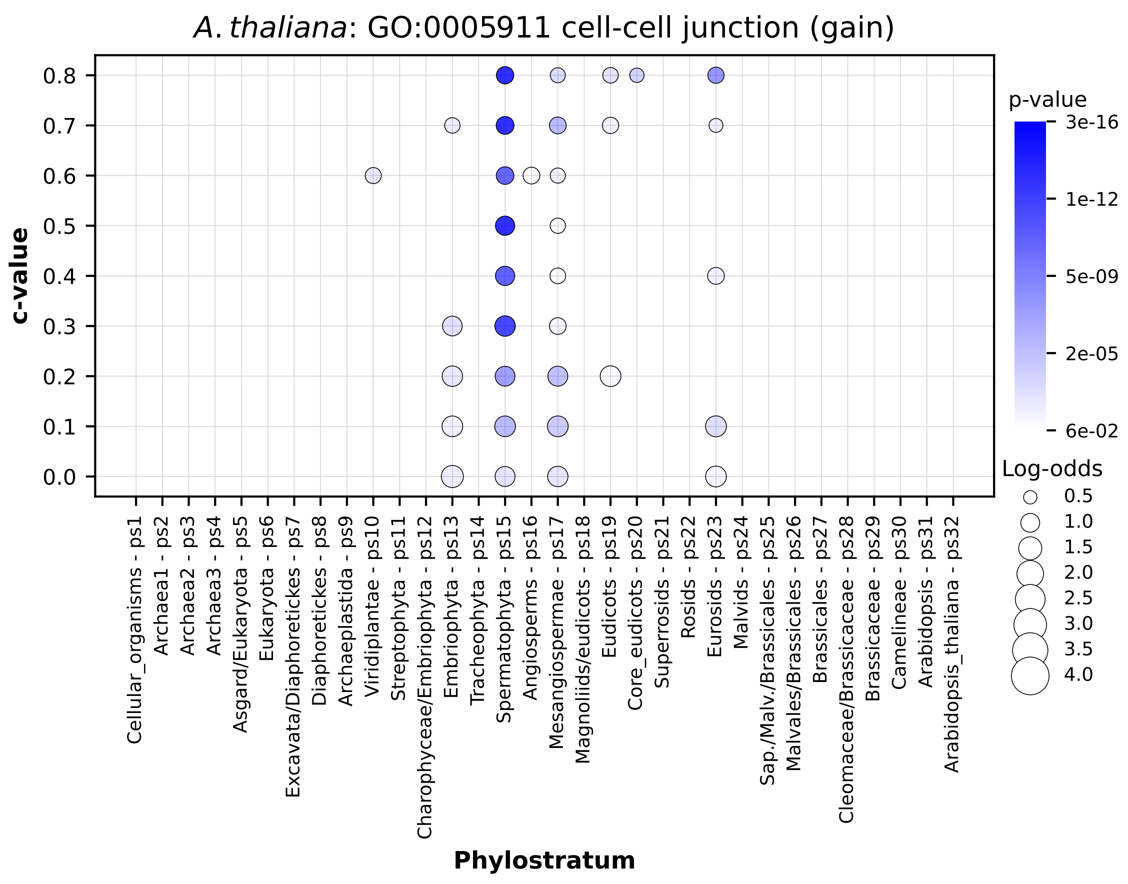
A. thaliana: GO:0005911 cell-cell junction (gain)
c-value
0.8
0.7
0.6
0.5
0.4
0.3
0.2
0.1
0.0
p-value
3e-16
1e-12
5e-09
2e-05
6e-02
Log-odds
0.5
1.0
1.5
2.0
2.5
3.0
3.5
4.0
Cellular_organisms - ps1
Archaea1 - ps2
Archaea2 - ps3
Archaea3 - ps4
Asgard/Eukaryota - ps5
Eukaryota - ps6
Excavata/Diaphoretickes - ps7
Diaphoretickes - ps8
Archaeplastida - ps9
Viridiplantae - ps10
Streptophyta - ps11
Charophyceae/Embriophyta - ps12
Embriophyta - ps13
Tracheophyta - ps14
Spermatophyta - ps15
Angiosperms - ps16
Mesangiospermae - ps17
Magnoliids/eudicots - ps18
Eudicots - ps19
Core_eudicots - ps20
Superrosids - ps21
Rosids - ps22
Eurosids - ps23
Malvids - ps24
Sap./Malv./Brassicales - ps25
Malvales/Brassicales - ps26
Brassicales - ps27
Cleomaceae/Brassicaceae - ps28
Brassicaceae - ps29
Camelineae - ps30
Arabidopsis - ps31
Arabidopsis_thaliana - ps32
Phylostratum

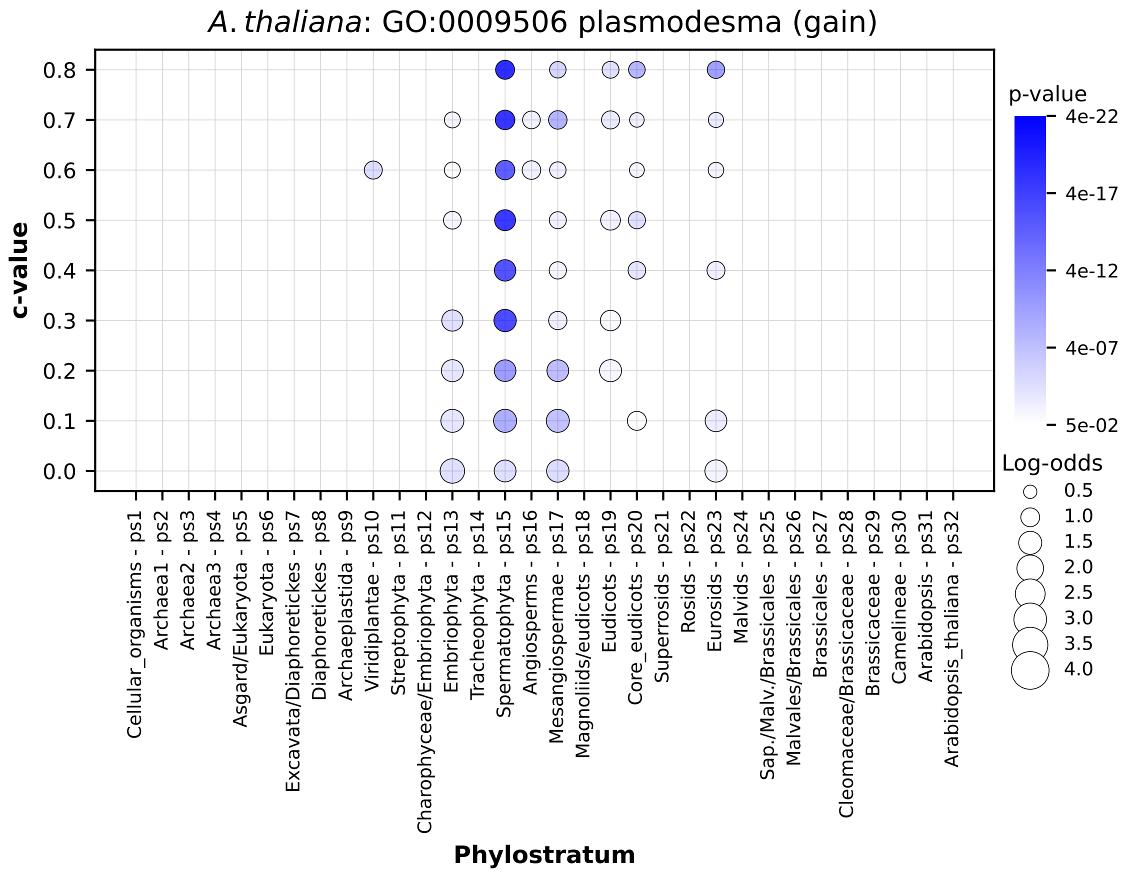

A. thaliana: GO:0009506 plasmodesma (gain)
c-value
0.8
0.7
0.6
0.5
0.4
0.3
0.2
0.1
0.0
p-value
4e-22
4e-17
4e-12
4e-07
5e-02
Log-odds
0.5
1.0
1.5
2.0
2.5
3.0
3.5
4.0
Cellular_organisms - ps1
Archaea1 - ps2
Archaea2 - ps3
Archaea3 - ps4
Asgard/Eukaryota - ps5
Eukaryota - ps6
Excavata/Diaphoretickes - ps7
Diaphoretickes - ps8
Archaeplastida - ps9
Viridiplantae - ps10
Streptophyta - ps11
Charophyceae/Embriophyta - ps12
Embriophyta - ps13
Tracheophyta - ps14
Spermatophyta - ps15
Angiosperms - ps16
Mesangiospermae - ps17
Magnoliids/eudicots - ps18
Eudicots - ps19
Core_eudicots - ps20
Superrosids - ps21
Rosids - ps22
Eurosids - ps23
Malvids - ps24
Sap./Malv./Brassicales - ps25
Malvales/Brassicales - ps26
Brassicales - ps27
Cleomaceae/Brassicaceae - ps28
Brassicaceae - ps29
Camelineae - ps30
Arabidopsis - ps31
Arabidopsis_thaliana - ps32
Phylostratum

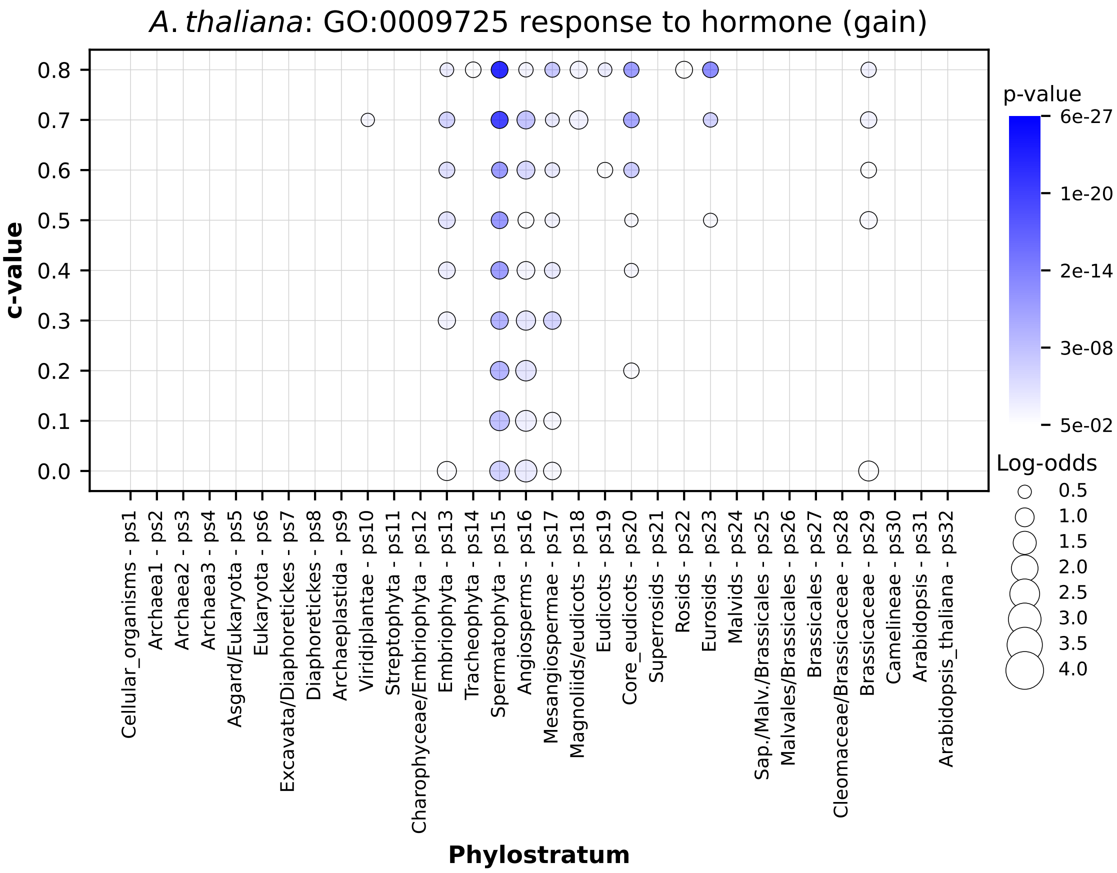

A. thaliana: GO:0009725 response to hormone (gain)
c-value
0.8
0.7
0.6
0.5
0.4
0.3
0.2
0.1
0.0
Phylostratum
Cellular_organisms - ps1
Archaea1 - ps2
Archaea2 - ps3
Archaea3 - ps4
Asgard/Eukaryota - ps5
Eukaryota - ps6
Excavata/Diaphoretickes - ps7
Diaphoretickes - ps8
Archaeplastida - ps9
Viridiplantae - ps10
Streptophyta - ps11
Charophyceae/Embriophyta - ps12
Embriophyta - ps13
Tracheophyta - ps14
Spermatophyta - ps15
Angiosperms - ps16
Mesangiospermae - ps17
Magnoliids/eudicots - ps18
Eudicots - ps19
Core_eudicots - ps20
Superrosids - ps21
Rosids - ps22
Eurosids - ps23
Malvids - ps24
Sap./Malv./Brassicales - ps25
Malvales/Brassicales - ps26
Brassicales - ps27
Cleomaceae/Brassicaceae - ps28
Brassicaceae - ps29
Camelineae - ps30
Arabidopsis - ps31
Arabidopsis_thaliana - ps32
p-value
6e-27
1e-20
2e-14
3e-08
5e-02
Log-odds
0.5
1.0
1.5
2.0
2.5
3.0
3.5
4.0

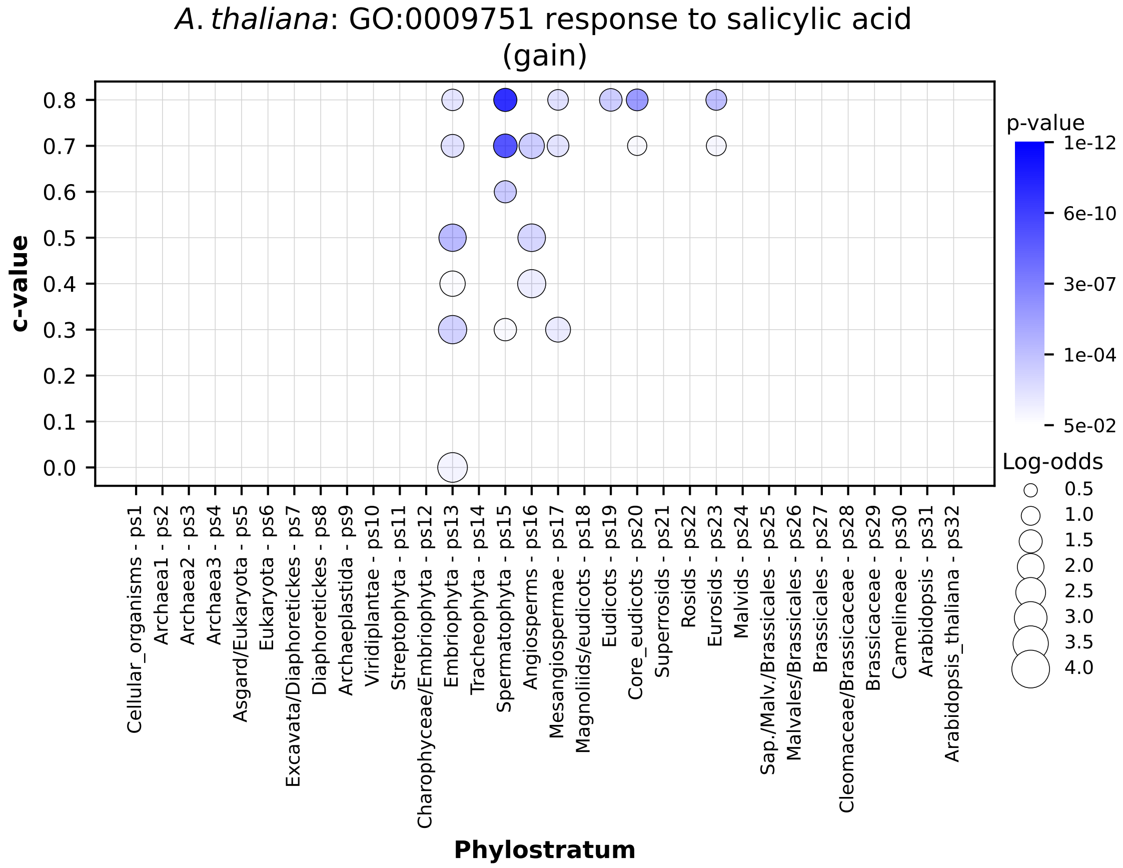

A. thaliana: GO:0009751 response to salicylic acid
(gain)
c-value
0.8
0.7
0.6
0.5
0.4
0.3
0.2
0.1
0.0
Phylostratum
Cellular_organisms - ps1
Archaea1 - ps2
Archaea2 - ps3
Archaea3 - ps4
Asgard/Eukaryota - ps5
Eukaryota - ps6
Excavata/Diaphoretickes - ps7
Diaphoretickes - ps8
Archaeplastida - ps9
Viridiplantae - ps10
Streptophyta - ps11
Charophyceae/Embriophyta - ps12
Embriophyta - ps13
Tracheophyta - ps14
Spermatophyta - ps15
Angiosperms - ps16
Mesangiospermae - ps17
Magnoliids/eudicots - ps18
Eudicots - ps19
Core_eudicots - ps20
Superrosids - ps21
Rosids - ps22
Eurosids - ps23
Malvids - ps24
Sap./Malv./Brassicales - ps25
Malvales/Brassicales - ps26
Brassicales - ps27
Cleomaceae/Brassicaceae - ps28
Brassicaceae - ps29
Camelineae - ps30
Arabidopsis - ps31
Arabidopsis_thaliana - ps32
p-value
1e-12
6e-10
3e-07
1e-04
5e-02
Log-odds
0.5
1.0
1.5
2.0
2.5
3.0
3.5
4.0

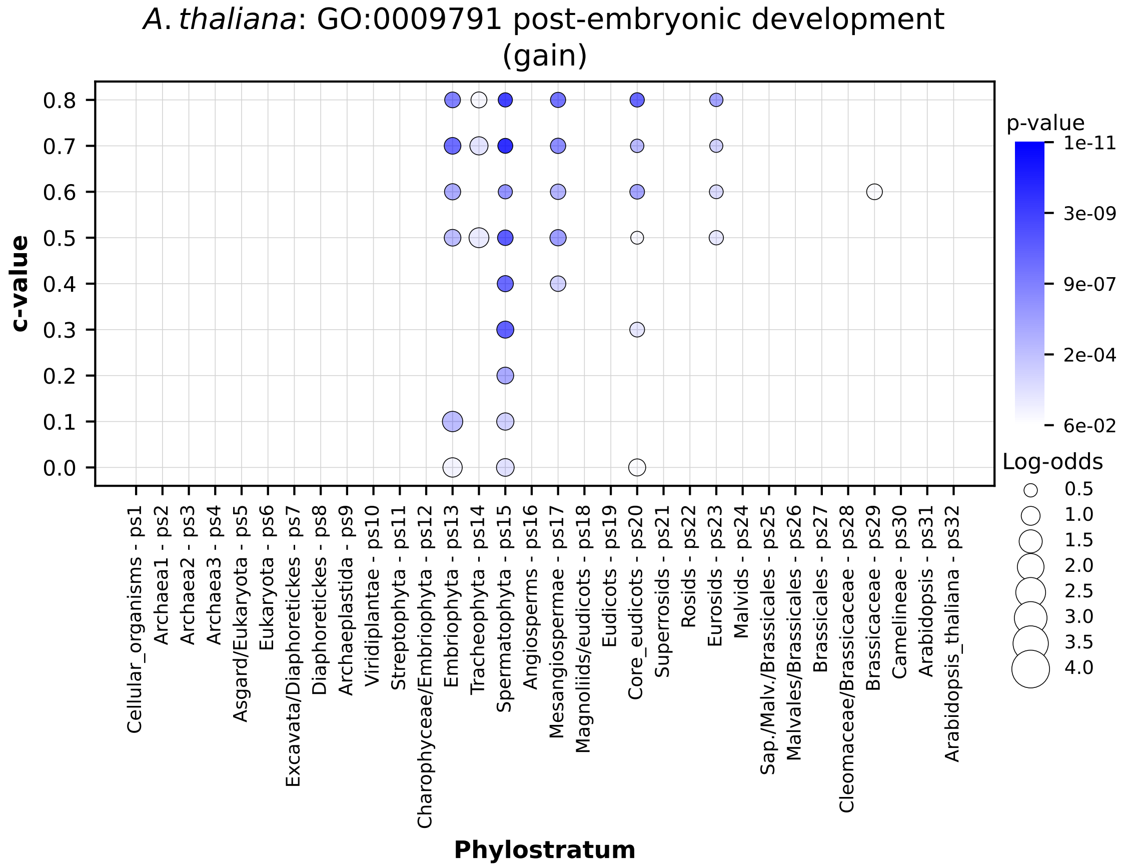
A. thaliana: GO:0009791 post-embryonic development (gain)
c-value
0.8
0.7
0.6
0.5
0.4
0.3
0.2
0.1
0.0
Phylostratum
Cellular_organisms - ps1
Archaea1 - ps2
Archaea2 - ps3
Archaea3 - ps4
Asgard/Eukaryota - ps5
Eukaryota - ps6
Excavata/Diaphoretickes - ps7
Diaphoretickes - ps8
Archaeplastida - ps9
Viridiplantae - ps10
Streptophyta - ps11
Charophyceae/Embriophyta - ps12
Embriophyta - ps13
Tracheophyta - ps14
Spermatophyta - ps15
Angiosperms - ps16
Mesangiospermae - ps17
Magnoliids/eudicots - ps18
Eudicots - ps19
Core_eudicots - ps20
Superrosids - ps21
Rosids - ps22
Eurosids - ps23
Malvids - ps24
Sap./Malv./Brassicales - ps25
Malvales/Brassicales - ps26
Brassicales - ps27
Cleomaceae/Brassicaceae - ps28
Brassicaceae - ps29
Camelineae - ps30
Arabidopsis - ps31
Arabidopsis_thaliana - ps32
p-value
1e-11
3e-09
9e-07
2e-04
6e-02
Log-odds
0.5
1.0
1.5
2.0
2.5
3.0
3.5
4.0

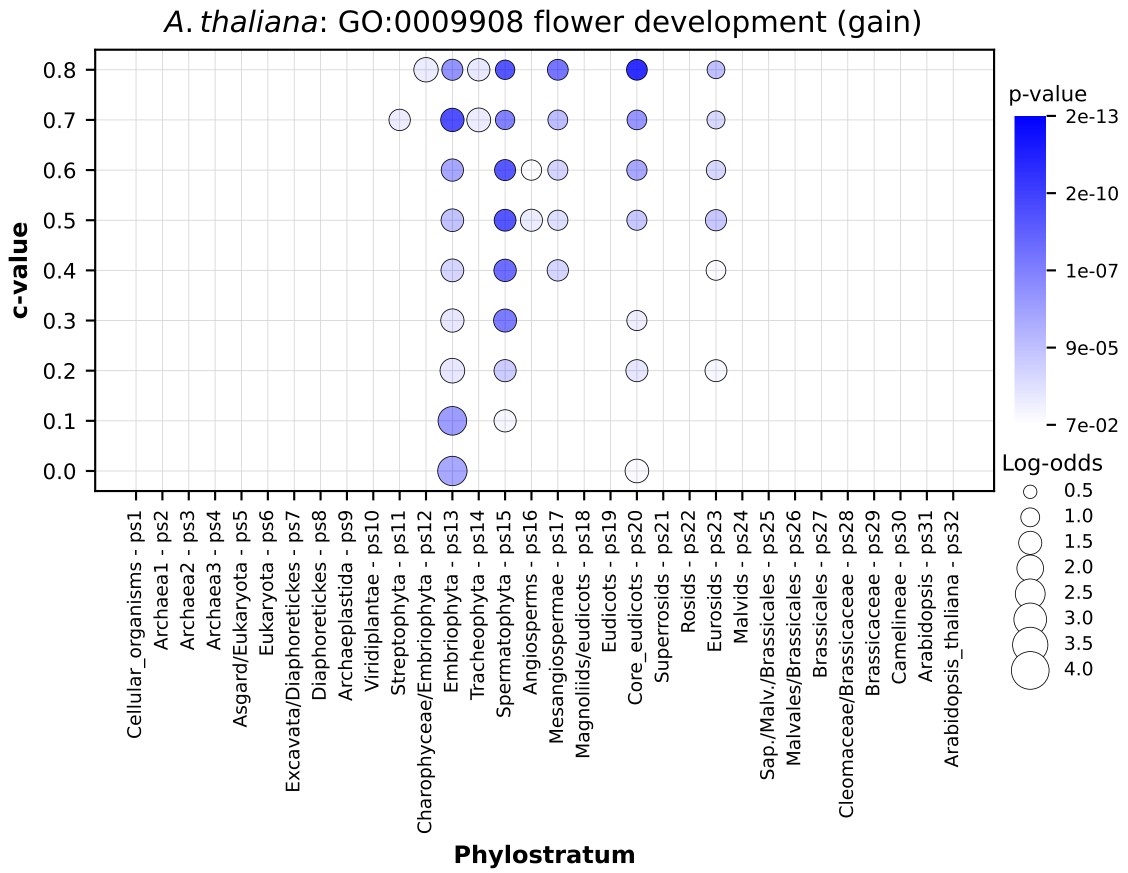

A. thaliana: GO:0009908 flower development (gain)
c-value
0.8
0.7
0.6
0.5
0.4
0.3
0.2
0.1
0.0
Phylostratum
Cellular_organisms - ps1
Archaea1 - ps2
Archaea2 - ps3
Archaea3 - ps4
Asgard/Eukaryota - ps5
Eukaryota - ps6
Excavata/Diaphoretickes - ps7
Diaphoretickes - ps8
Archaeplastida - ps9
Viridiplantae - ps10
Streptophyta - ps11
Charophyceae/Embriophyta - ps12
Embriophyta - ps13
Tracheophyta - ps14
Spermatophyta - ps15
Angiosperms - ps16
Mesangiospermae - ps17
Magnoliids/eudicots - ps18
Eudicots - ps19
Core_eudicots - ps20
Superrosids - ps21
Rosids - ps22
Eurosids - ps23
Malvids - ps24
Sap./Malv./Brassicales - ps25
Malvales/Brassicales - ps26
Brassicales - ps27
Cleomaceae/Brassicaceae - ps28
Brassicaceae - ps29
Camelineae - ps30
Arabidopsis - ps31
Arabidopsis_thaliana - ps32
p-value
2e-13
2e-10
1e-07
9e-05
7e-02
Log-odds
0.5
1.0
1.5
2.0
2.5
3.0
3.5
4.0

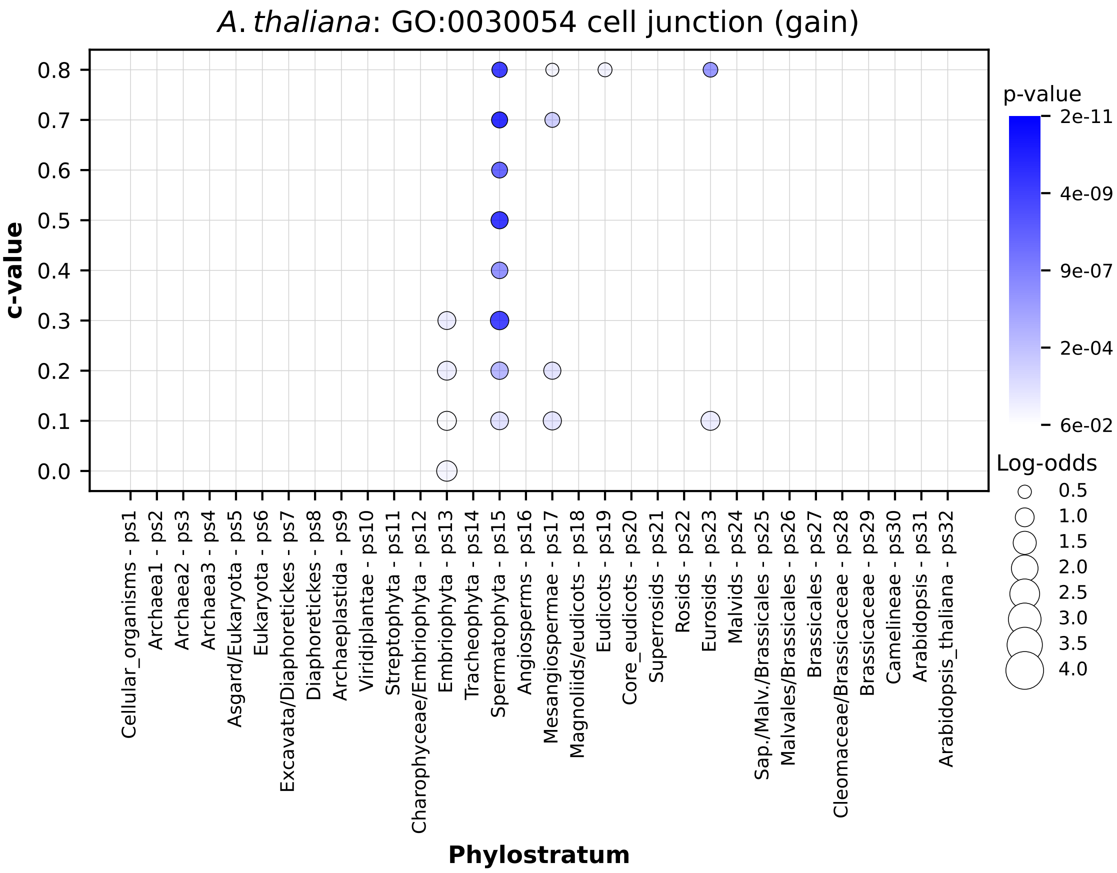
A. thaliana: GO:0030054 cell junction (gain)
c-value
0.8
0.7
0.6
0.5
0.4
0.3
0.2
0.1
0.0
p-value
2e-11
4e-09
9e-07
2e-04
6e-02
Log-odds
0.5
1.0
1.5
2.0
2.5
3.0
3.5
4.0
Cellular_organisms - ps1
Archaea1 - ps2
Archaea2 - ps3
Archaea3 - ps4
Asgard/Eukaryota - ps5
Eukaryota - ps6
Excavata/Diaphoretickes - ps7
Diaphoretickes - ps8
Archaeplastida - ps9
Viridiplantae - ps10
Streptophyta - ps11
Charophyceae/Embriophyta - ps12
Embriophyta - ps13
Tracheophyta - ps14
Spermatophyta - ps15
Angiosperms - ps16
Mesangiospermae - ps17
Magnoliids/eudicots - ps18
Eudicots - ps19
Core_eudicots - ps20
Superrosids - ps21
Rosids - ps22
Eurosids - ps23
Malvids - ps24
Sap./Malv./Brassicales - ps25
Malvales/Brassicales - ps26
Brassicales - ps27
Cleomaceae/Brassicaceae - ps28
Brassicaceae - ps29
Camelineae - ps30
Arabidopsis - ps31
Arabidopsis_thaliana - ps32
Phylostratum

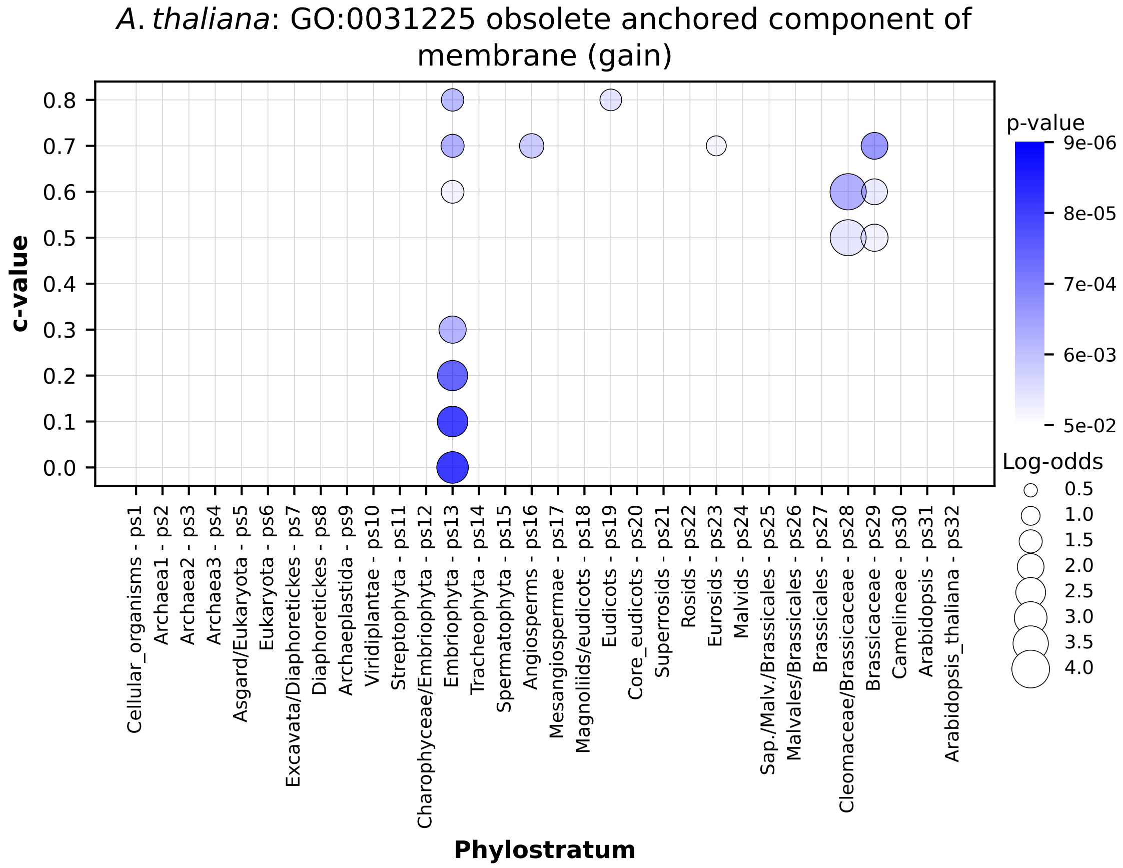
A. thaliana: GO:0031225 obsolete anchored component of membrane (gain)
c-value
0.8
0.7
0.6
0.5
0.4
0.3
0.2
0.1
0.0
Phylostratum
Cellular_organisms - ps1
Archaea1 - ps2
Archaea2 - ps3
Archaea3 - ps4
Asgard/Eukaryota - ps5
Eukaryota - ps6
Excavata/Diaphoretickes - ps7
Diaphoretickes - ps8
Archaeplastida - ps9
Viridiplantae - ps10
Streptophyta - ps11
Charophyceae/Embriophyta - ps12
Embriophyta - ps13
Tracheophyta - ps14
Spermatophyta - ps15
Angiosperms - ps16
Mesangiospermae - ps17
Magnoliids/eudicots - ps18
Eudicots - ps19
Core_eudicots - ps20
Superrosids - ps21
Rosids - ps22
Eurosids - ps23
Malvids - ps24
Sap./Malv./Brassicales - ps25
Malvales/Brassicales - ps26
Brassicales - ps27
Cleomaceae/Brassicaceae - ps28
Brassicaceae - ps29
Camelineae - ps30
Arabidopsis - ps31
Arabidopsis_thaliana - ps32
p-value
9e-06
8e-05
7e-04
6e-03
5e-02
Log-odds
0.5
1.0
1.5
2.0
2.5
3.0
3.5
4.0

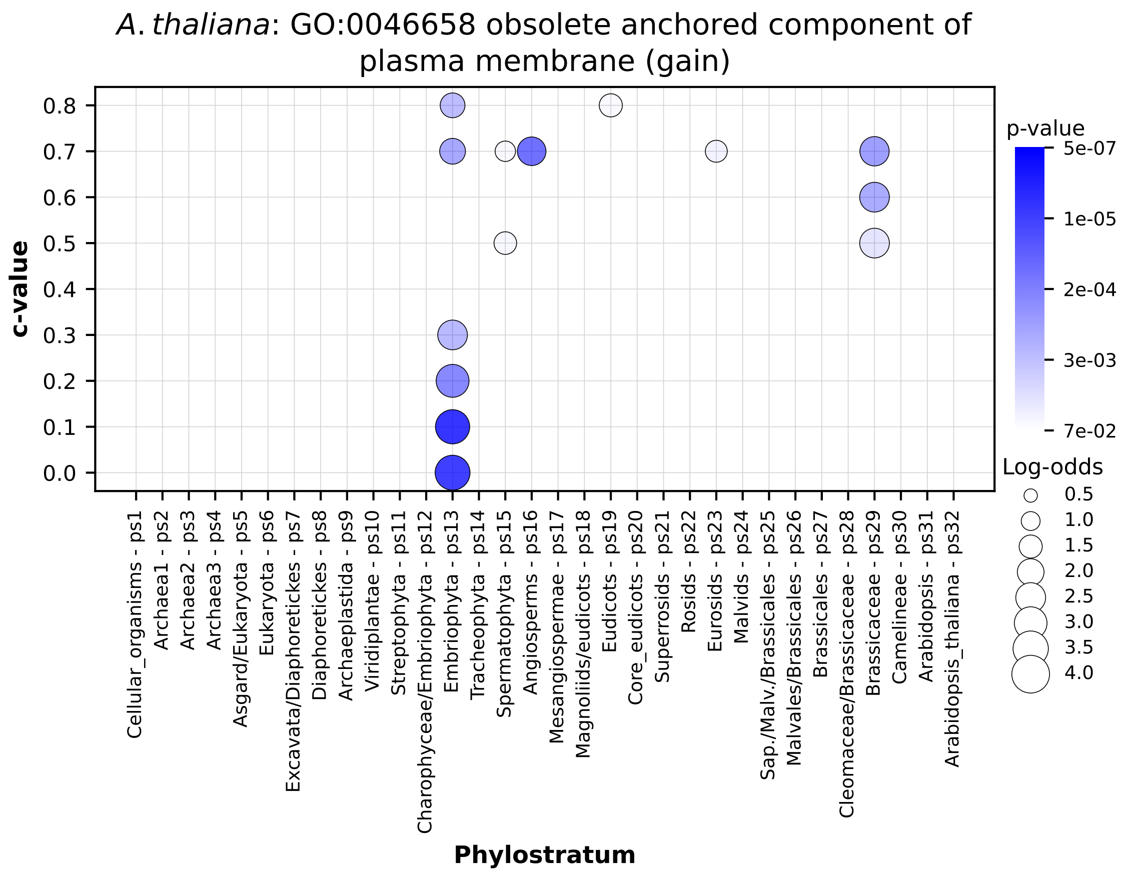
A. thaliana: GO:0046658 obsolete anchored component of plasma membrane (gain)
c-value
0.8
0.7
0.6
0.5
0.4
0.3
0.2
0.1
0.0
Phylostratum
Cellular_organisms - ps1
Archaea1 - ps2
Archaea2 - ps3
Archaea3 - ps4
Asgard/Eukaryota - ps5
Eukaryota - ps6
Excavata/Diaphoretickes - ps7
Diaphoretickes - ps8
Archaeplastida - ps9
Viridiplantae - ps10
Streptophyta - ps11
Charophyceae/Embriophyta - ps12
Embriophyta - ps13
Tracheophyta - ps14
Spermatophyta - ps15
Angiosperms - ps16
Mesangiospermae - ps17
Magnoliids/eudicots - ps18
Eudicots - ps19
Core_eudicots - ps20
Superrosids - ps21
Rosids - ps22
Eurosids - ps23
Malvids - ps24
Sap./Malv./Brassicales - ps25
Malvales/Brassicales - ps26
Brassicales - ps27
Cleomaceae/Brassicaceae - ps28
Brassicaceae - ps29
Camelineae - ps30
Arabidopsis - ps31
Arabidopsis_thaliana - ps32
p-value
5e-07
1e-05
2e-04
3e-03
7e-02
Log-odds
0.5
1.0
1.5
2.0
2.5
3.0
3.5
4.0

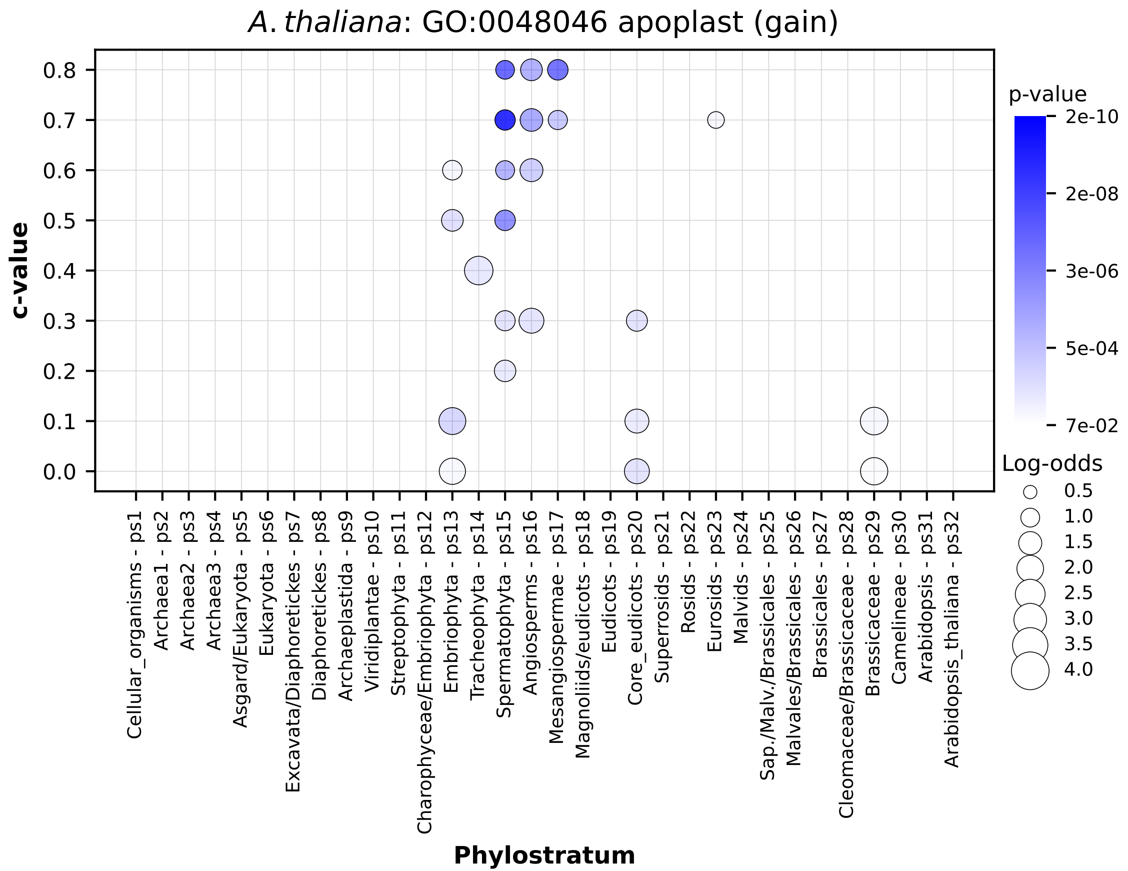
A. thaliana: GO:0048046 apoplast (gain)
c-value
0.8
0.7
0.6
0.5
0.4
0.3
0.2
0.1
0.0
p-value
2e-10
2e-08
3e-06
5e-04
7e-02
Log-odds
0.5
1.0
1.5
2.0
2.5
3.0
3.5
4.0
Cellular_organisms - ps1
Archaea1 - ps2
Archaea2 - ps3
Archaea3 - ps4
Asgard/Eukaryota - ps5
Eukaryota - ps6
Excavata/Diaphoretickes - ps7
Diaphoretickes - ps8
Archaeplastida - ps9
Viridiplantae - ps10
Streptophyta - ps11
Charophyceae/Embriophyta - ps12
Embriophyta - ps13
Tracheophyta - ps14
Spermatophyta - ps15
Angiosperms - ps16
Mesangiospermae - ps17
Magnoliids/eudicots - ps18
Eudicots - ps19
Core_eudicots - ps20
Superrosids - ps21
Rosids - ps22
Eurosids - ps23
Malvids - ps24
Sap./Malv./Brassicales - ps25
Malvales/Brassicales - ps26
Brassicales - ps27
Cleomaceae/Brassicaceae - ps28
Brassicaceae - ps29
Camelineae - ps30
Arabidopsis - ps31
Arabidopsis_thaliana - ps32
Phylostratum

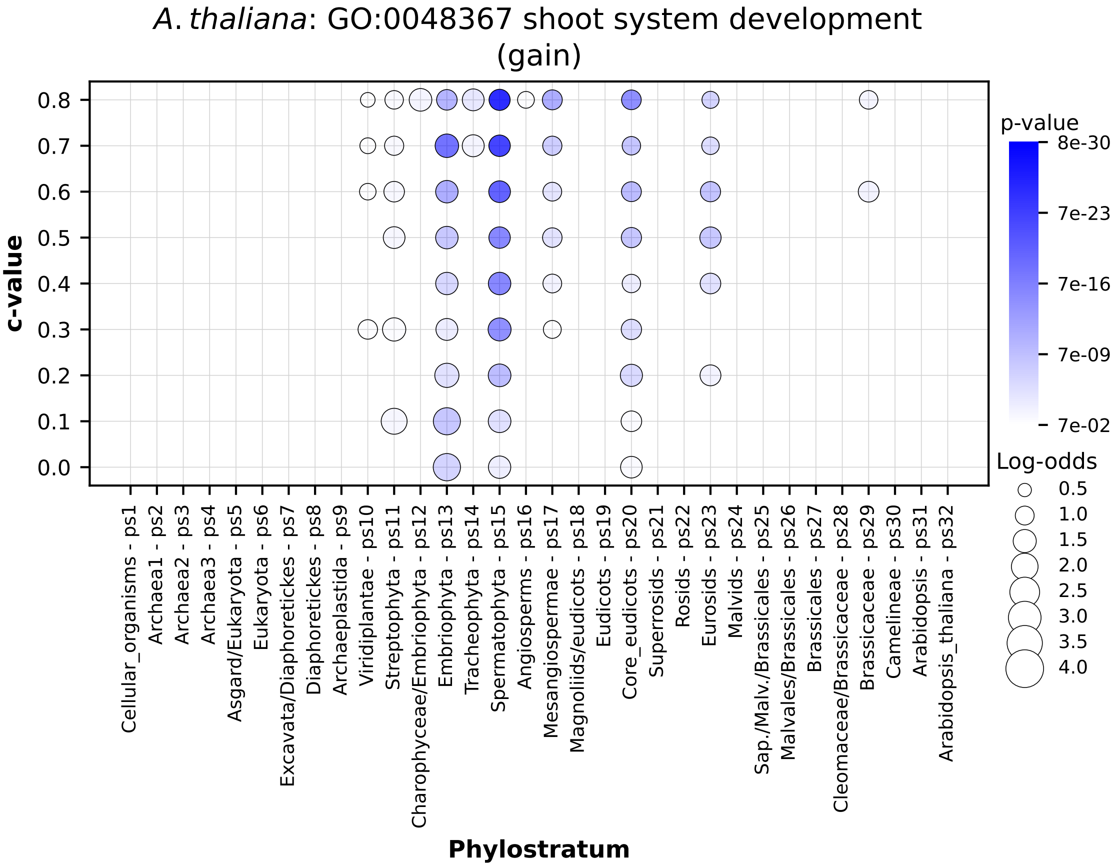
A. thaliana: GO:0048367 shoot system development
(gain)
c-value
0.8
0.7
0.6
0.5
0.4
0.3
0.2
0.1
0.0
p-value
8e-30
7e-23
7e-16
7e-09
7e-02
Log-odds
0.5
1.0
1.5
2.0
2.5
3.0
3.5
4.0
Cellular_organisms - ps1
Archaea1 - ps2
Archaea2 - ps3
Archaea3 - ps4
Asgard/Eukaryota - ps5
Eukaryota - ps6
Excavata/Diaphoretickes - ps7
Diaphoretickes - ps8
Archaeplastida - ps9
Viridiplantae - ps10
Streptophyta - ps11
Charophyceae/Embriophyta - ps12
Embriophyta - ps13
Tracheophyta - ps14
Spermatophyta - ps15
Angiosperms - ps16
Mesangiospermae - ps17
Magnoliids/eudicots - ps18
Eudicots - ps19
Core_eudicots - ps20
Superrosids - ps21
Rosids - ps22
Eurosids - ps23
Malvids - ps24
Sap./Malv./Brassicales - ps25
Malvales/Brassicales - ps26
Brassicales - ps27
Cleomaceae/Brassicaceae - ps28
Brassicaceae - ps29
Camelineae - ps30
Arabidopsis - ps31
Arabidopsis_thaliana - ps32
Phylostratum

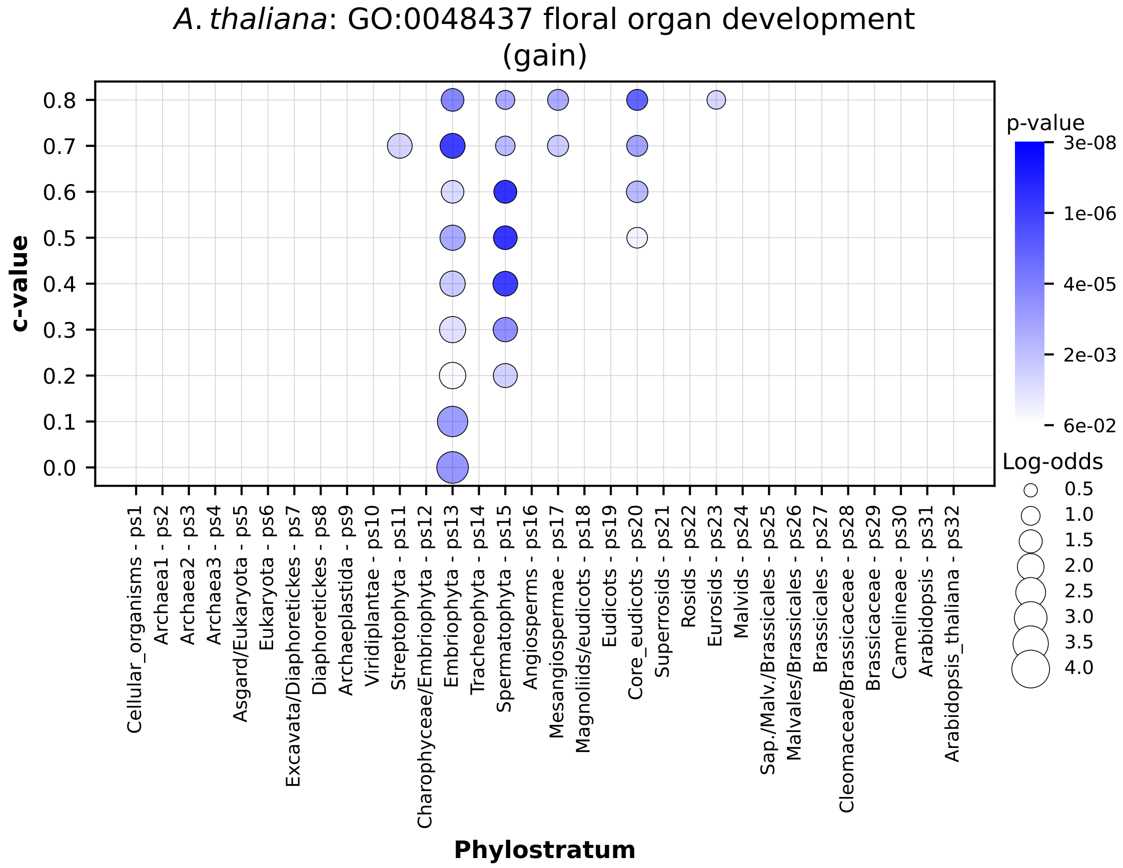
A. thaliana: GO:0048437 floral organ development
(gain)
c-value
0.8
0.7
0.6
0.5
0.4
0.3
0.2
0.1
0.0
Phylostratum
Cellular_organisms - ps1
Archaea1 - ps2
Archaea2 - ps3
Archaea3 - ps4
Asgard/Eukaryota - ps5
Eukaryota - ps6
Excavata/Diaphoretickes - ps7
Diaphoretickes - ps8
Archaeplastida - ps9
Viridiplantae - ps10
Streptophyta - ps11
Charophyceae/Embriophyta - ps12
Embriophyta - ps13
Tracheophyta - ps14
Spermatophyta - ps15
Angiosperms - ps16
Mesangiospermae - ps17
Magnoliids/eudicots - ps18
Eudicots - ps19
Core_eudicots - ps20
Superrosids - ps21
Rosids - ps22
Eurosids - ps23
Malvids - ps24
Sap./Malv./Brassicales - ps25
Malvales/Brassicales - ps26
Brassicales - ps27
Cleomaceae/Brassicaceae - ps28
Brassicaceae - ps29
Camelineae - ps30
Arabidopsis - ps31
Arabidopsis_thaliana - ps32
p-value
3e-08
1e-06
4e-05
2e-03
6e-02
Log-odds
0.5
1.0
1.5
2.0
2.5
3.0
3.5
4.0

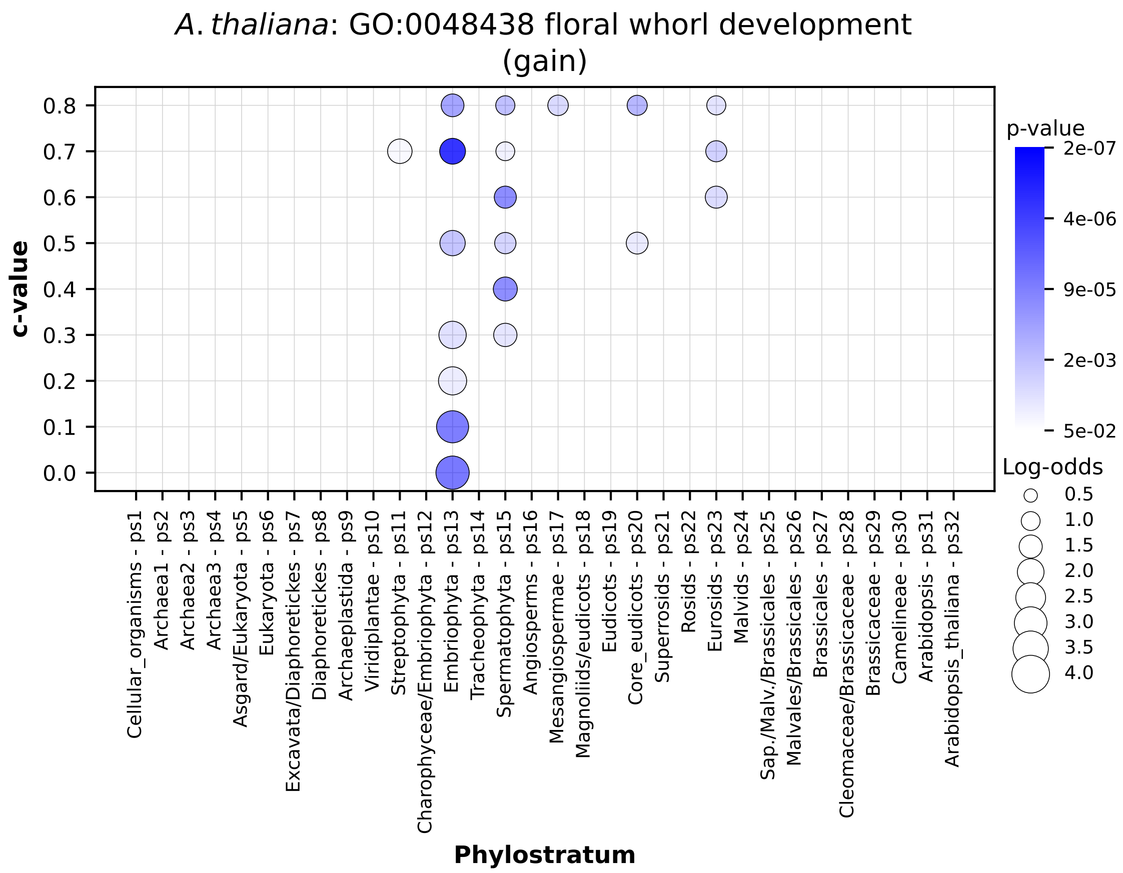

A. thaliana: GO:0048438 floral whorl development
(gain)
c-value
0.8
0.7
0.6
0.5
0.4
0.3
0.2
0.1
0.0
Phylostratum
Cellular_organisms - ps1
Archaea1 - ps2
Archaea2 - ps3
Archaea3 - ps4
Asgard/Eukaryota - ps5
Eukaryota - ps6
Excavata/Diaphoretickes - ps7
Diaphoretickes - ps8
Archaeplastida - ps9
Viridiplantae - ps10
Streptophyta - ps11
Charophyceae/Embriophyta - ps12
Embriophyta - ps13
Tracheophyta - ps14
Spermatophyta - ps15
Angiosperms - ps16
Mesangiospermae - ps17
Magnoliids/eudicots - ps18
Eudicots - ps19
Core_eudicots - ps20
Superrosids - ps21
Rosids - ps22
Eurosids - ps23
Malvids - ps24
Sap./Malv./Brassicales - ps25
Malvales/Brassicales - ps26
Brassicales - ps27
Cleomaceae/Brassicaceae - ps28
Brassicaceae - ps29
Camelineae - ps30
Arabidopsis - ps31
Arabidopsis_thaliana - ps32
p-value
2e-07
4e-06
9e-05
2e-03
5e-02
Log-odds
0.5
1.0
1.5
2.0
2.5
3.0
3.5
4.0

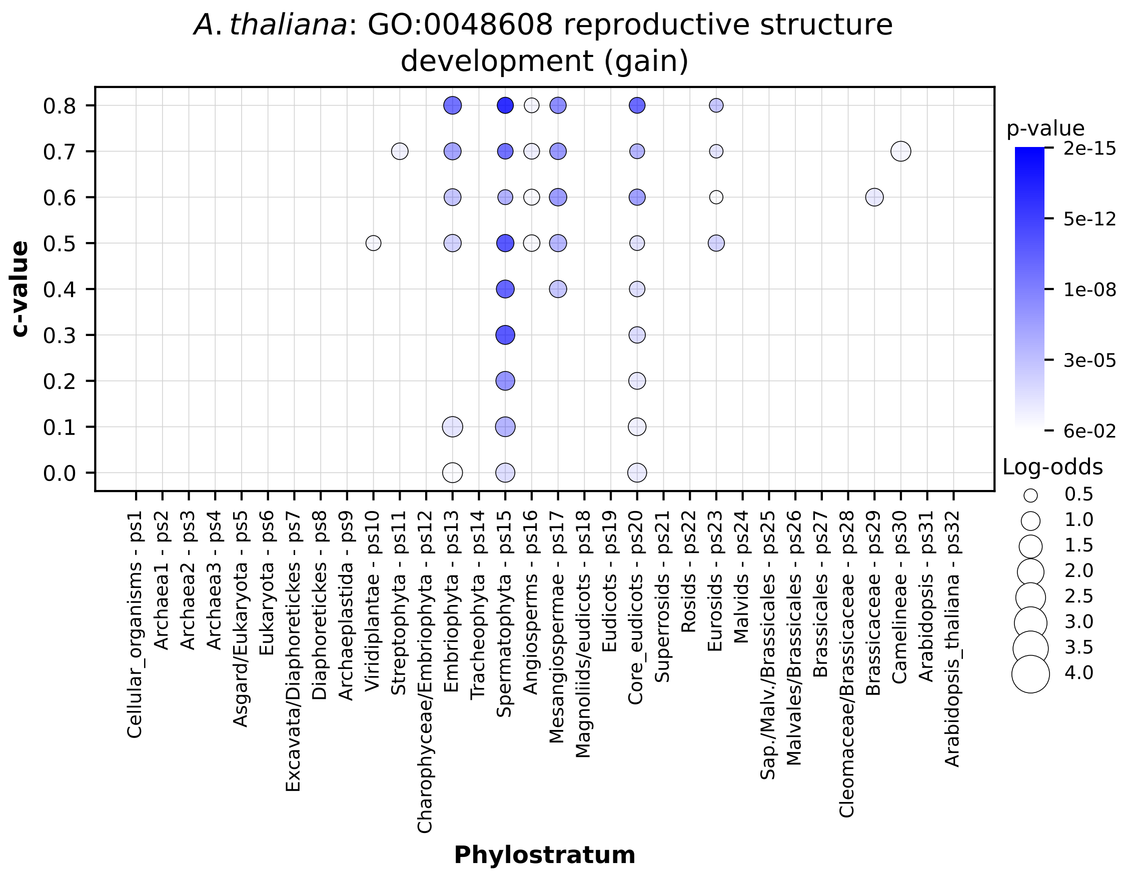
A. thaliana: GO:0048608 reproductive structure development (gain)
c-value
0.8
0.7
0.6
0.5
0.4
0.3
0.2
0.1
0.0
p-value
2e-15
5e-12
1e-08
3e-05
6e-02
Log-odds
0.5
1.0
1.5
2.0
2.5
3.0
3.5
4.0
Cellular_organisms - ps1
Archaea1 - ps2
Archaea2 - ps3
Archaea3 - ps4
Asgard/Eukaryota - ps5
Eukaryota - ps6
Excavata/Diaphoretickes - ps7
Diaphoretickes - ps8
Archaeplastida - ps9
Viridiplantae - ps10
Streptophyta - ps11
Charophyceae/Embriophyta - ps12
Embriophyta - ps13
Tracheophyta - ps14
Spermatophyta - ps15
Angiosperms - ps16
Mesangiospermae - ps17
Magnoliids/eudicots - ps18
Eudicots - ps19
Core_eudicots - ps20
Superrosids - ps21
Rosids - ps22
Eurosids - ps23
Malvids - ps24
Sap./Malv./Brassicales - ps25
Malvales/Brassicales - ps26
Brassicales - ps27
Cleomaceae/Brassicaceae - ps28
Brassicaceae - ps29
Camelineae - ps30
Arabidopsis - ps31
Arabidopsis_thaliana - ps32
Phylostratum

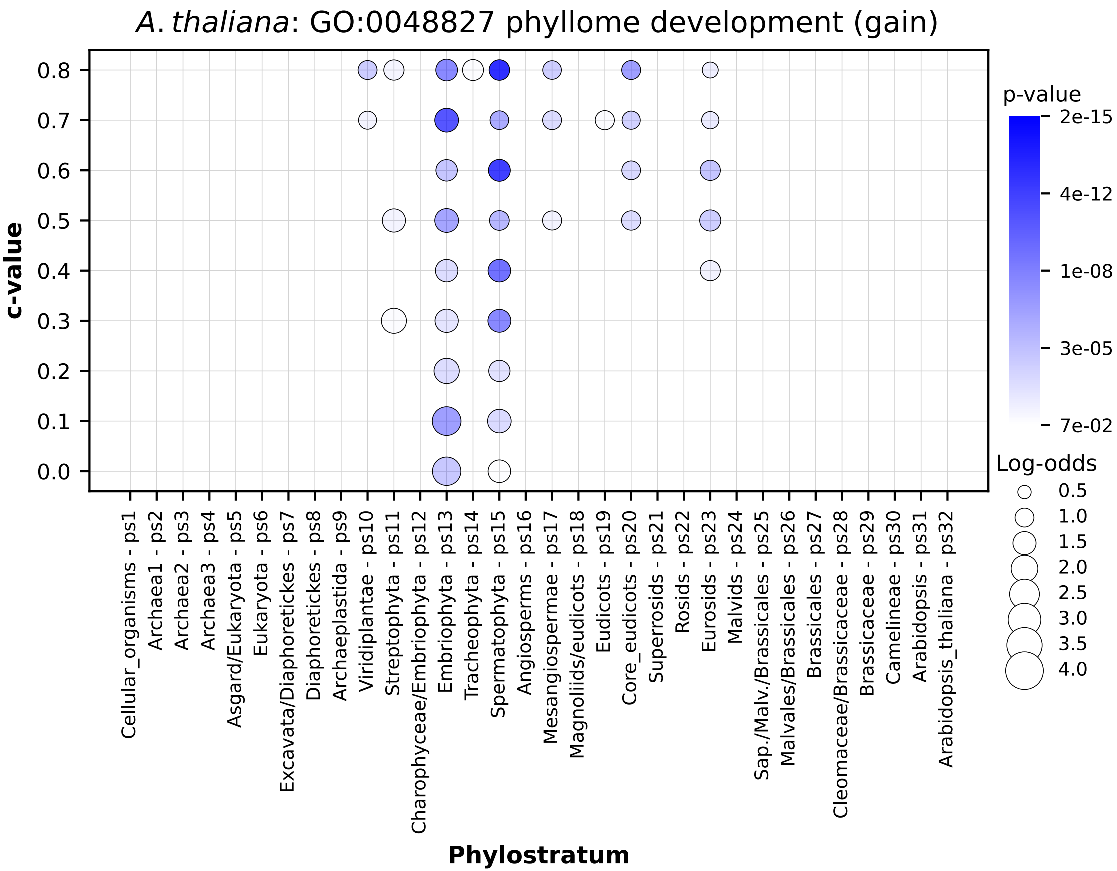

A. thaliana: GO:0048827 phyllome development (gain)
c-value
0.8
0.7
0.6
0.5
0.4
0.3
0.2
0.1
0.0
p-value
2e-15
4e-12
1e-08
3e-05
7e-02
Log-odds
0.5
1.0
1.5
2.0
2.5
3.0
3.5
4.0
Cellular_organisms - ps1
Archaea1 - ps2
Archaea2 - ps3
Archaea3 - ps4
Asgard/Eukaryota - ps5
Eukaryota - ps6
Excavata/Diaphoretickes - ps7
Diaphoretickes - ps8
Archaeplastida - ps9
Viridiplantae - ps10
Streptophyta - ps11
Charophyceae/Embriophyta - ps12
Embriophyta - ps13
Tracheophyta - ps14
Spermatophyta - ps15
Angiosperms - ps16
Mesangiospermae - ps17
Magnoliids/eudicots - ps18
Eudicots - ps19
Core_eudicots - ps20
Superrosids - ps21
Rosids - ps22
Eurosids - ps23
Malvids - ps24
Sap./Malv./Brassicales - ps25
Malvales/Brassicales - ps26
Brassicales - ps27
Cleomaceae/Brassicaceae - ps28
Brassicaceae - ps29
Camelineae - ps30
Arabidopsis - ps31
Arabidopsis_thaliana - ps32
Phylostratum

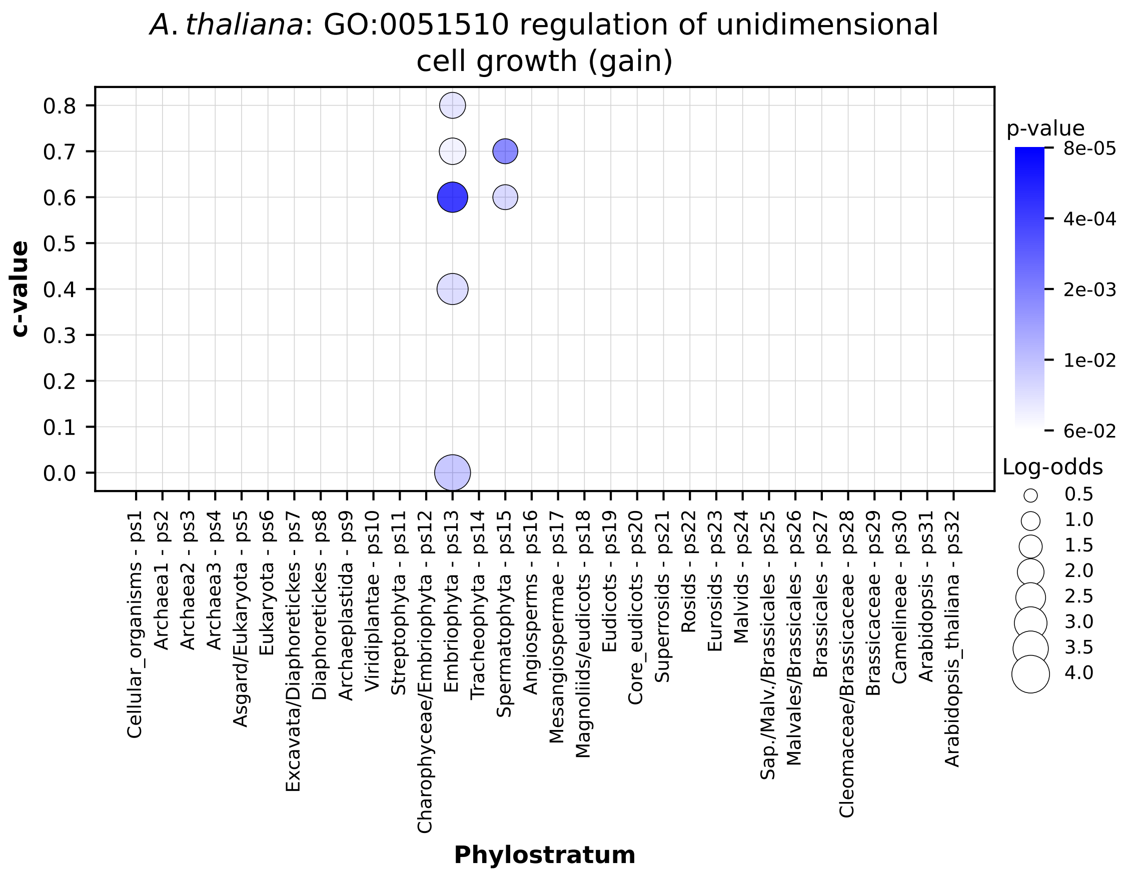

A. thaliana: GO:0051510 regulation of unidimensional cell growth (gain)
c-value
0.8
0.7
0.6
0.5
0.4
0.3
0.2
0.1
0.0
p-value
8e-05
4e-04
2e-03
1e-02
6e-02
Log-odds
0.5
1.0
1.5
2.0
2.5
3.0
3.5
4.0
Cellular_organisms - ps1
Archaea1 - ps2
Archaea2 - ps3
Archaea3 - ps4
Asgard/Eukaryota - ps5
Eukaryota - ps6
Excavata/Diaphoretickes - ps7
Diaphoretickes - ps8
Archaeplastida - ps9
Viridiplantae - ps10
Streptophyta - ps11
Charophyceae/Embriophyta - ps12
Embriophyta - ps13
Tracheophyta - ps14
Spermatophyta - ps15
Angiosperms - ps16
Mesangiospermae - ps17
Magnoliids/eudicots - ps18
Eudicots - ps19
Core_eudicots - ps20
Superrosids - ps21
Rosids - ps22
Eurosids - ps23
Malvids - ps24
Sap./Malv./Brassicales - ps25
Malvales/Brassicales - ps26
Brassicales - ps27
Cleomaceae/Brassicaceae - ps28
Brassicaceae - ps29
Camelineae - ps30
Arabidopsis - ps31
Arabidopsis_thaliana - ps32
Phylostratum

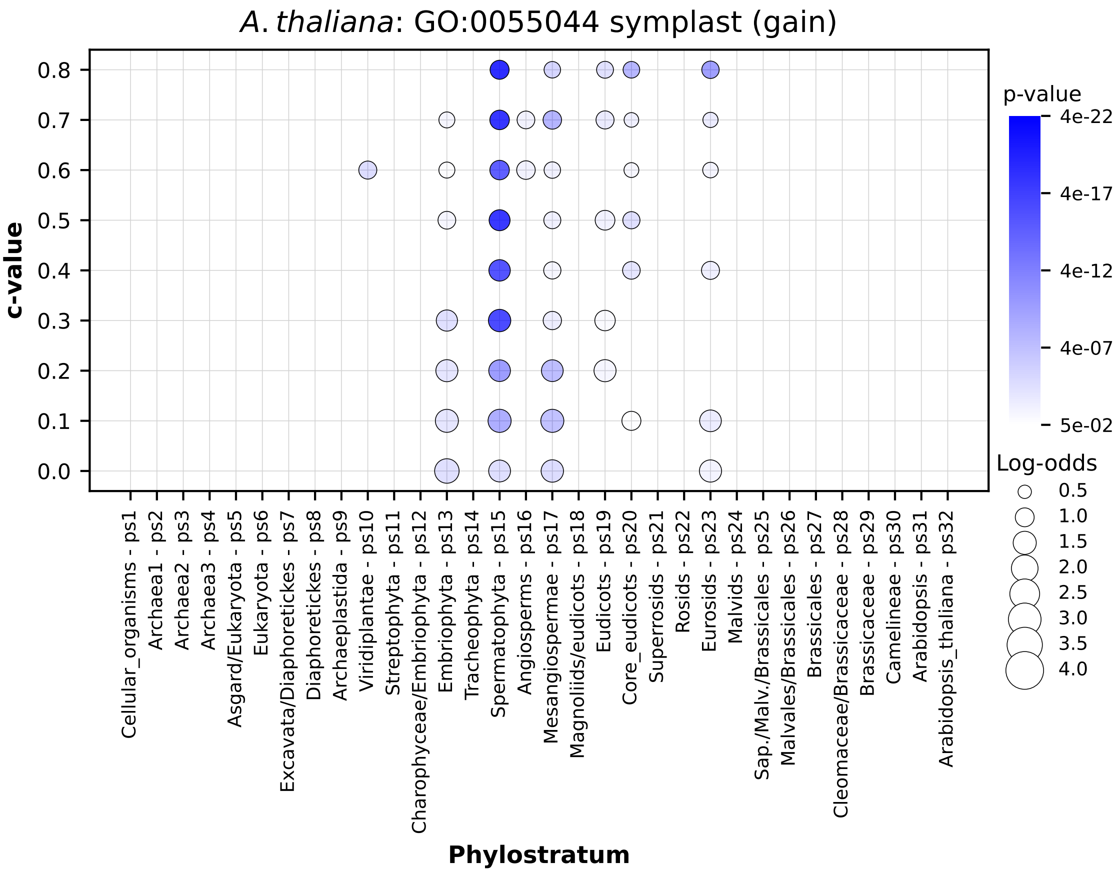

A. thaliana: GO:0055044 symplast (gain)
c-value
0.8
0.7
0.6
0.5
0.4
0.3
0.2
0.1
0.0
p-value
4e-22
4e-17
4e-12
4e-07
5e-02
Log-odds
0.5
1.0
1.5
2.0
2.5
3.0
3.5
4.0
Cellular_organisms - ps1
Archaea1 - ps2
Archaea2 - ps3
Archaea3 - ps4
Asgard/Eukaryota - ps5
Eukaryota - ps6
Excavata/Diaphoretickes - ps7
Diaphoretickes - ps8
Archaeplastida - ps9
Viridiplantae - ps10
Streptophyta - ps11
Charophyceae/Embriophyta - ps12
Embriophyta - ps13
Tracheophyta - ps14
Spermatophyta - ps15
Angiosperms - ps16
Mesangiospermae - ps17
Magnoliids/eudicots - ps18
Eudicots - ps19
Core_eudicots - ps20
Superrosids - ps21
Rosids - ps22
Eurosids - ps23
Malvids - ps24
Sap./Malv./Brassicales - ps25
Malvales/Brassicales - ps26
Brassicales - ps27
Cleomaceae/Brassicaceae - ps28
Brassicaceae - ps29
Camelineae - ps30
Arabidopsis - ps31
Arabidopsis_thaliana - ps32
Phylostratum

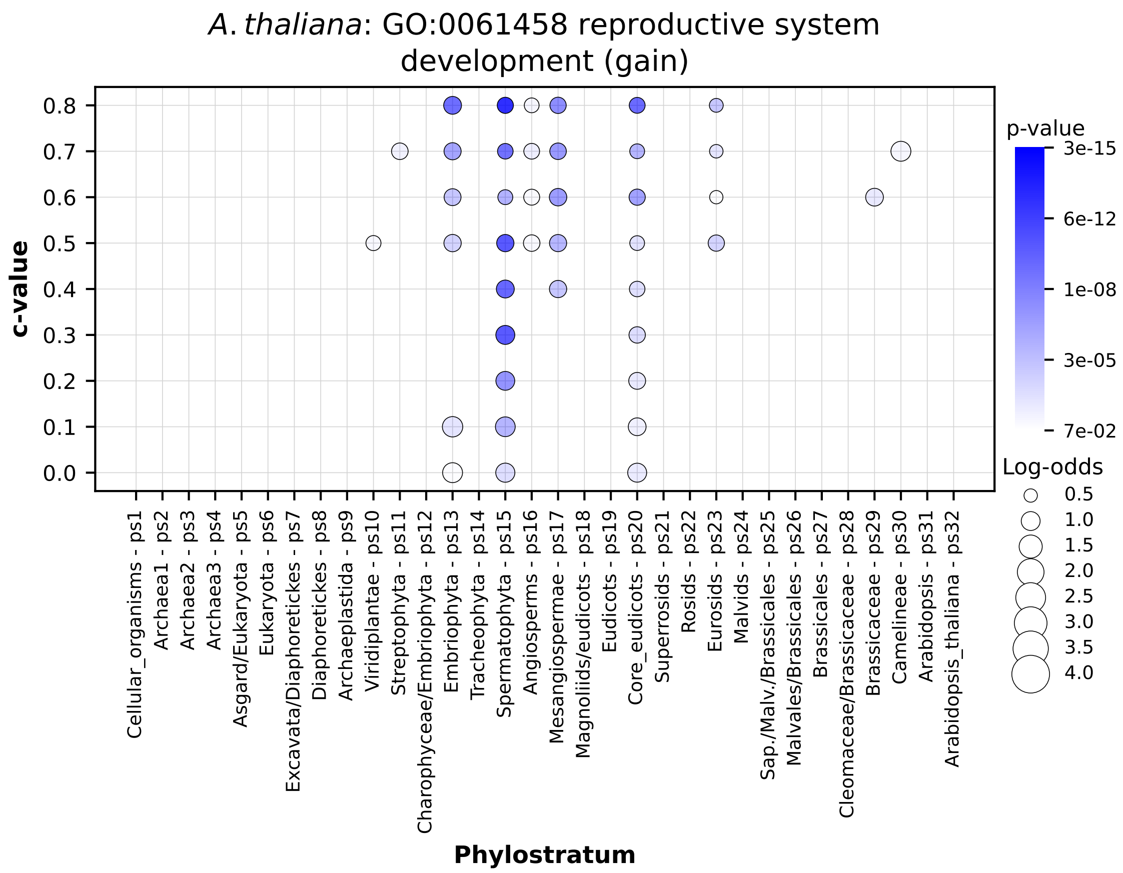
A. thaliana: GO:0061458 reproductive system development (gain)
c-value
0.8
0.7
0.6
0.5
0.4
0.3
0.2
0.1
0.0
p-value
3e-15
6e-12
1e-08
3e-05
7e-02
Log-odds
0.5
1.0
1.5
2.0
2.5
3.0
3.5
4.0
Cellular_organisms - ps1
Archaea1 - ps2
Archaea2 - ps3
Archaea3 - ps4
Asgard/Eukaryota - ps5
Eukaryota - ps6
Excavata/Diaphoretickes - ps7
Diaphoretickes - ps8
Archaeplastida - ps9
Viridiplantae - ps10
Streptophyta - ps11
Charophyceae/Embriophyta - ps12
Embriophyta - ps13
Tracheophyta - ps14
Spermatophyta - ps15
Angiosperms - ps16
Mesangiospermae - ps17
Magnoliids/eudicots - ps18
Eudicots - ps19
Core_eudicots - ps20
Superrosids - ps21
Rosids - ps22
Eurosids - ps23
Malvids - ps24
Sap./Malv./Brassicales - ps25
Malvales/Brassicales - ps26
Brassicales - ps27
Cleomaceae/Brassicaceae - ps28
Brassicaceae - ps29
Camelineae - ps30
Arabidopsis - ps31
Arabidopsis_thaliana - ps32
Phylostratum

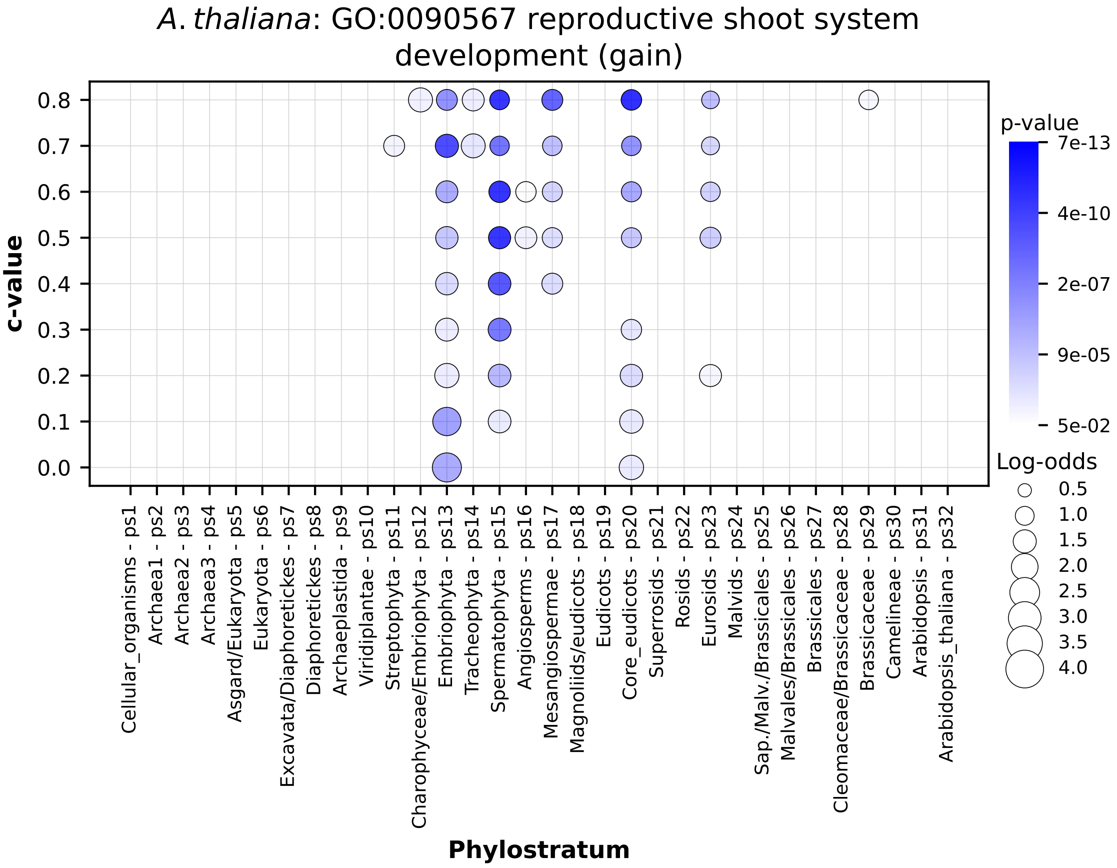

A. thaliana: GO:0090567 reproductive shoot system development (gain)
c-value
0.8
0.7
0.6
0.5
0.4
0.3
0.2
0.1
0.0
p-value
7e-13
4e-10
2e-07
9e-05
5e-02
Log-odds
0.5
1.0
1.5
2.0
2.5
3.0
3.5
4.0
Cellular_organisms - ps1
Archaea1 - ps2
Archaea2 - ps3
Archaea3 - ps4
Asgard/Eukaryota - ps5
Eukaryota - ps6
Excavata/Diaphoretickes - ps7
Diaphoretickes - ps8
Archaeplastida - ps9
Viridiplantae - ps10
Streptophyta - ps11
Charophyceae/Embriophyta - ps12
Embriophyta - ps13
Tracheophyta - ps14
Spermatophyta - ps15
Angiosperms - ps16
Mesangiospermae - ps17
Magnoliids/eudicots - ps18
Eudicots - ps19
Core_eudicots - ps20
Superrosids - ps21
Rosids - ps22
Eurosids - ps23
Malvids - ps24
Sap./Malv./Brassicales - ps25
Malvales/Brassicales - ps26
Brassicales - ps27
Cleomaceae/Brassicaceae - ps28
Brassicaceae - ps29
Camelineae - ps30
Arabidopsis - ps31
Arabidopsis_thaliana - ps32
Phylostratum

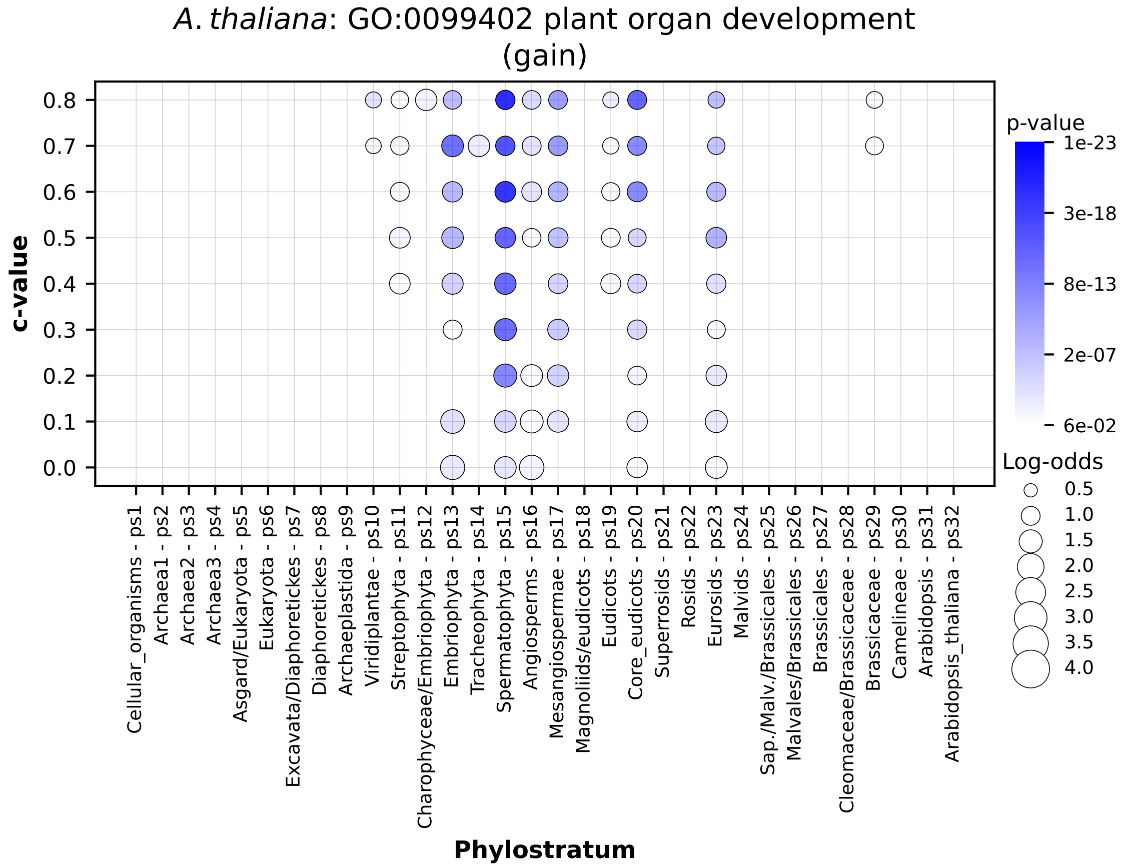

A. thaliana: GO:0099402 plant organ development
(gain)
c-value
0.8
0.7
0.6
0.5
0.4
0.3
0.2
0.1
0.0
p-value
1e-23
3e-18
8e-13
2e-07
6e-02
Log-odds
0.5
1.0
1.5
2.0
2.5
3.0
3.5
4.0
Cellular_organisms - ps1
Archaea1 - ps2
Archaea2 - ps3
Archaea3 - ps4
Asgard/Eukaryota - ps5
Eukaryota - ps6
Excavata/Diaphoretickes - ps7
Diaphoretickes - ps8
Archaeplastida - ps9
Viridiplantae - ps10
Streptophyta - ps11
Charophyceae/Embriophyta - ps12
Embriophyta - ps13
Tracheophyta - ps14
Spermatophyta - ps15
Angiosperms - ps16
Mesangiospermae - ps17
Magnoliids/eudicots - ps18
Eudicots - ps19
Core_eudicots - ps20
Superrosids - ps21
Rosids - ps22
Eurosids - ps23
Malvids - ps24
Sap./Malv./Brassicales - ps25
Malvales/Brassicales - ps26
Brassicales - ps27
Cleomaceae/Brassicaceae - ps28
Brassicaceae - ps29
Camelineae - ps30
Arabidopsis - ps31
Arabidopsis_thaliana - ps32
Phylostratum

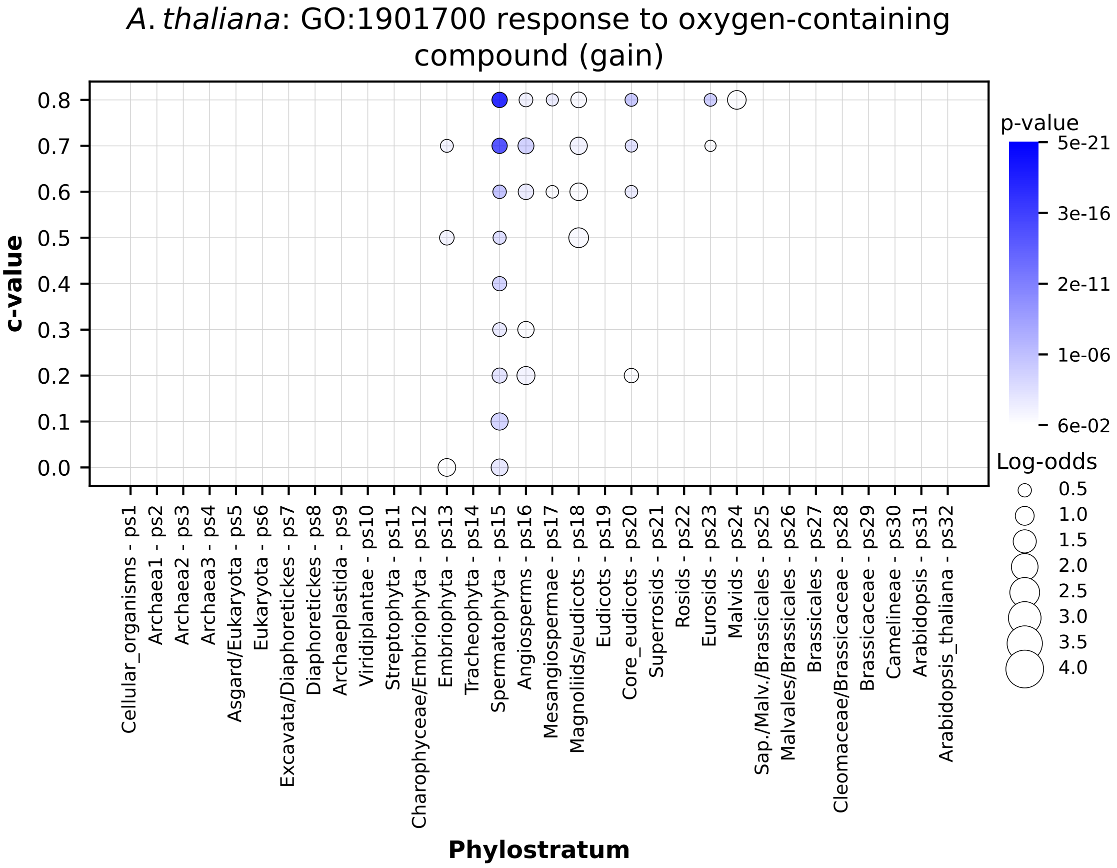
A. thaliana: GO:1901700 response to oxygen-containing compound (gain)
c-value
0.8
0.7
0.6
0.5
0.4
0.3
0.2
0.1
0.0
Phylostratum
Cellular_organisms - ps1
Archaea1 - ps2
Archaea2 - ps3
Archaea3 - ps4
Asgard/Eukaryota - ps5
Eukaryota - ps6
Excavata/Diaphoretickes - ps7
Diaphoretickes - ps8
Archaeplastida - ps9
Viridiplantae - ps10
Streptophyta - ps11
Charophyceae/Embriophyta - ps12
Embriophyta - ps13
Tracheophyta - ps14
Spermatophyta - ps15
Angiosperms - ps16
Mesangiospermae - ps17
Magnoliids/eudicots - ps18
Eudicots - ps19
Core_eudicots - ps20
Superrosids - ps21
Rosids - ps22
Eurosids - ps23
Malvids - ps24
Sap./Malv./Brassicales - ps25
Malvales/Brassicales - ps26
Brassicales - ps27
Cleomaceae/Brassicaceae - ps28
Brassicaceae - ps29
Camelineae - ps30
Arabidopsis - ps31
Arabidopsis_thaliana - ps32
p-value
5e-21
3e-16
2e-11
1e-06
6e-02
Log-odds
0.5
1.0
1.5
2.0
2.5
3.0
3.5
4.0

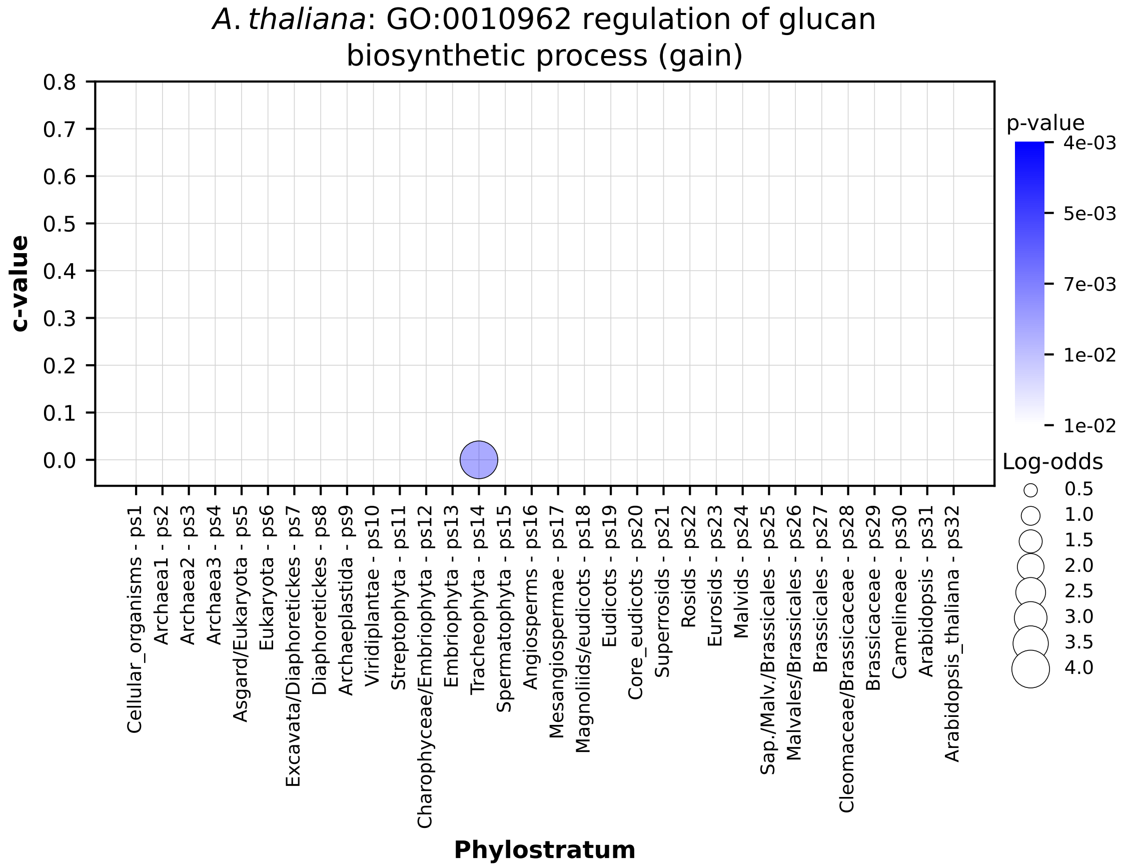

A. thaliana: GO:0010962 regulation of glucan biosynthetic process (gain)
c-value
0.8
0.7
0.6
0.5
0.4
0.3
0.2
0.1
0.0
p-value
4e-03
5e-03
7e-03
1e-02
1e-02
Log-odds
0.5
1.0
1.5
2.0
2.5
3.0
3.5
4.0
Cellular_organisms - ps1
Archaea1 - ps2
Archaea2 - ps3
Archaea3 - ps4
Asgard/Eukaryota - ps5
Eukaryota - ps6
Excavata/Diaphoretickes - ps7
Diaphoretickes - ps8
Archaeplastida - ps9
Viridiplantae - ps10
Streptophyta - ps11
Charophyceae/Embriophyta - ps12
Embriophyta - ps13
Tracheophyta - ps14
Spermatophyta - ps15
Angiosperms - ps16
Mesangiospermae - ps17
Magnoliids/eudicots - ps18
Eudicots - ps19
Core_eudicots - ps20
Superrosids - ps21
Rosids - ps22
Eurosids - ps23
Malvids - ps24
Sap./Malv./Brassicales - ps25
Malvales/Brassicales - ps26
Brassicales - ps27
Cleomaceae/Brassicaceae - ps28
Brassicaceae - ps29
Camelineae - ps30
Arabidopsis - ps31
Arabidopsis_thaliana - ps32
Phylostratum

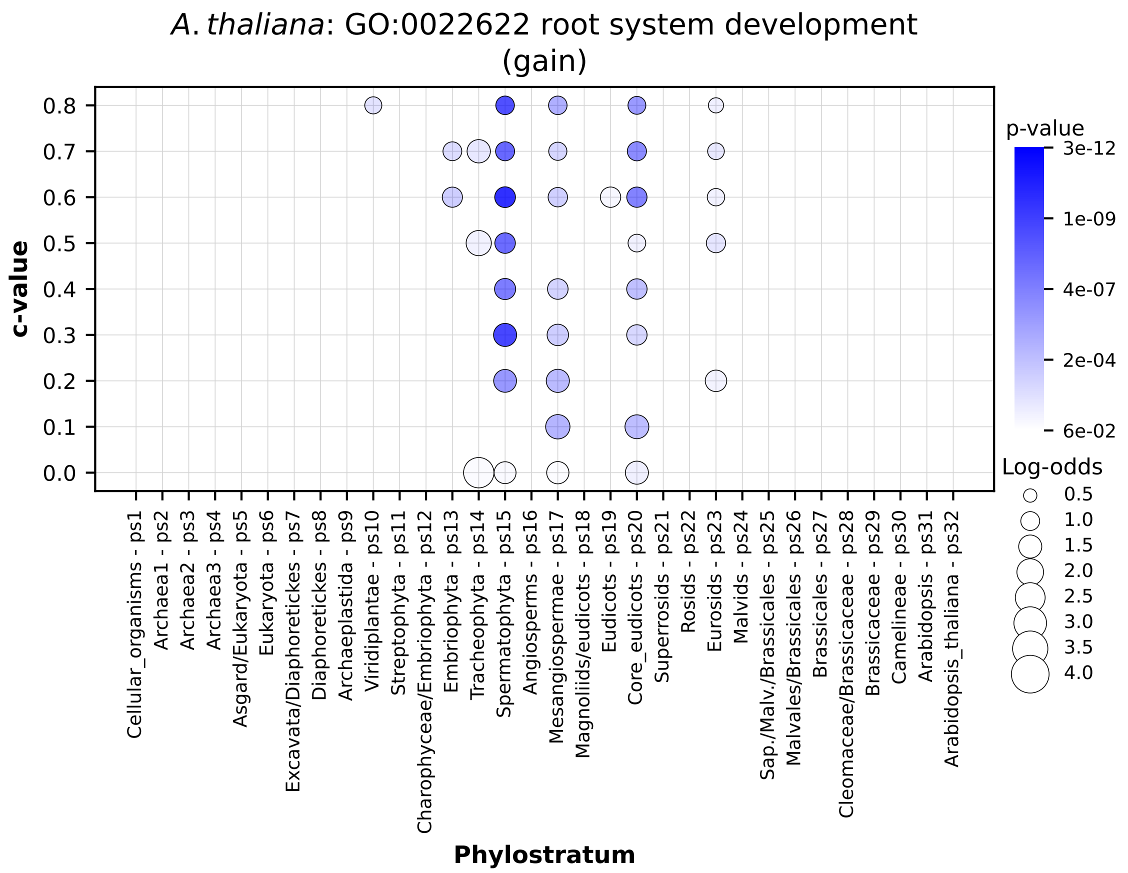

A. thaliana: GO:0022622 root system development
(gain)
c-value
0.8
0.7
0.6
0.5
0.4
0.3
0.2
0.1
0.0
p-value
3e-12
1e-09
4e-07
2e-04
6e-02
Log-odds
0.5
1.0
1.5
2.0
2.5
3.0
3.5
4.0
Cellular_organisms - ps1
Archaea1 - ps2
Archaea2 - ps3
Archaea3 - ps4
Asgard/Eukaryota - ps5
Eukaryota - ps6
Excavata/Diaphoretickes - ps7
Diaphoretickes - ps8
Archaeplastida - ps9
Viridiplantae - ps10
Streptophyta - ps11
Charophyceae/Embriophyta - ps12
Embriophyta - ps13
Tracheophyta - ps14
Spermatophyta - ps15
Angiosperms - ps16
Mesangiospermae - ps17
Magnoliids/eudicots - ps18
Eudicots - ps19
Core_eudicots - ps20
Superrosids - ps21
Rosids - ps22
Eurosids - ps23
Malvids - ps24
Sap./Malv./Brassicales - ps25
Malvales/Brassicales - ps26
Brassicales - ps27
Cleomaceae/Brassicaceae - ps28
Brassicaceae - ps29
Camelineae - ps30
Arabidopsis - ps31
Arabidopsis_thaliana - ps32
Phylostratum

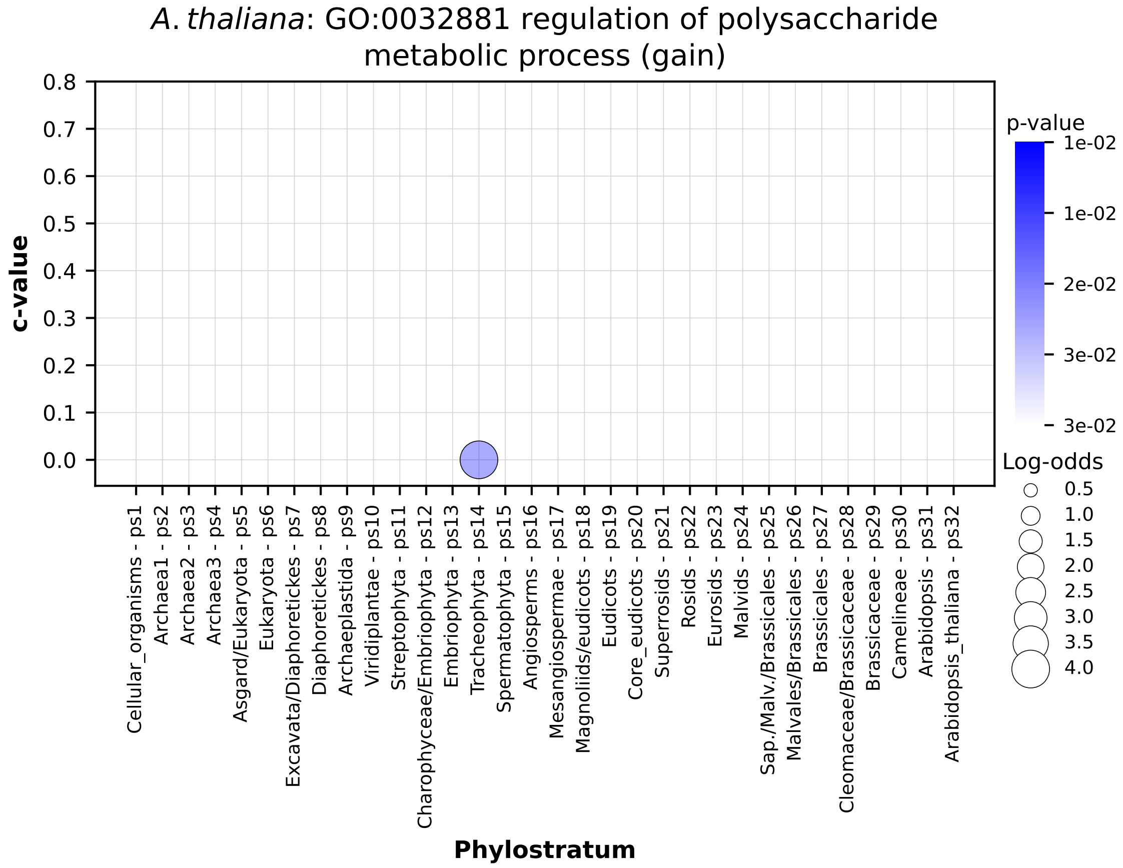

A. thaliana: GO:0032881 regulation of polysaccharide metabolic process (gain)
c-value
0.8
0.7
0.6
0.5
0.4
0.3
0.2
0.1
0.0
p-value
1e-02
1e-02
2e-02
3e-02
3e-02
Log-odds
0.5
1.0
1.5
2.0
2.5
3.0
3.5
4.0
Cellular_organisms - ps1
Archaea1 - ps2
Archaea2 - ps3
Archaea3 - ps4
Asgard/Eukaryota - ps5
Eukaryota - ps6
Excavata/Diaphoretickes - ps7
Diaphoretickes - ps8
Archaeplastida - ps9
Viridiplantae - ps10
Streptophyta - ps11
Charophyceae/Embriophyta - ps12
Embriophyta - ps13
Tracheophyta - ps14
Spermatophyta - ps15
Angiosperms - ps16
Mesangiospermae - ps17
Magnoliids/eudicots - ps18
Eudicots - ps19
Core_eudicots - ps20
Superrosids - ps21
Rosids - ps22
Eurosids - ps23
Malvids - ps24
Sap./Malv./Brassicales - ps25
Malvales/Brassicales - ps26
Brassicales - ps27
Cleomaceae/Brassicaceae - ps28
Brassicaceae - ps29
Camelineae - ps30
Arabidopsis - ps31
Arabidopsis_thaliana - ps32
Phylostratum

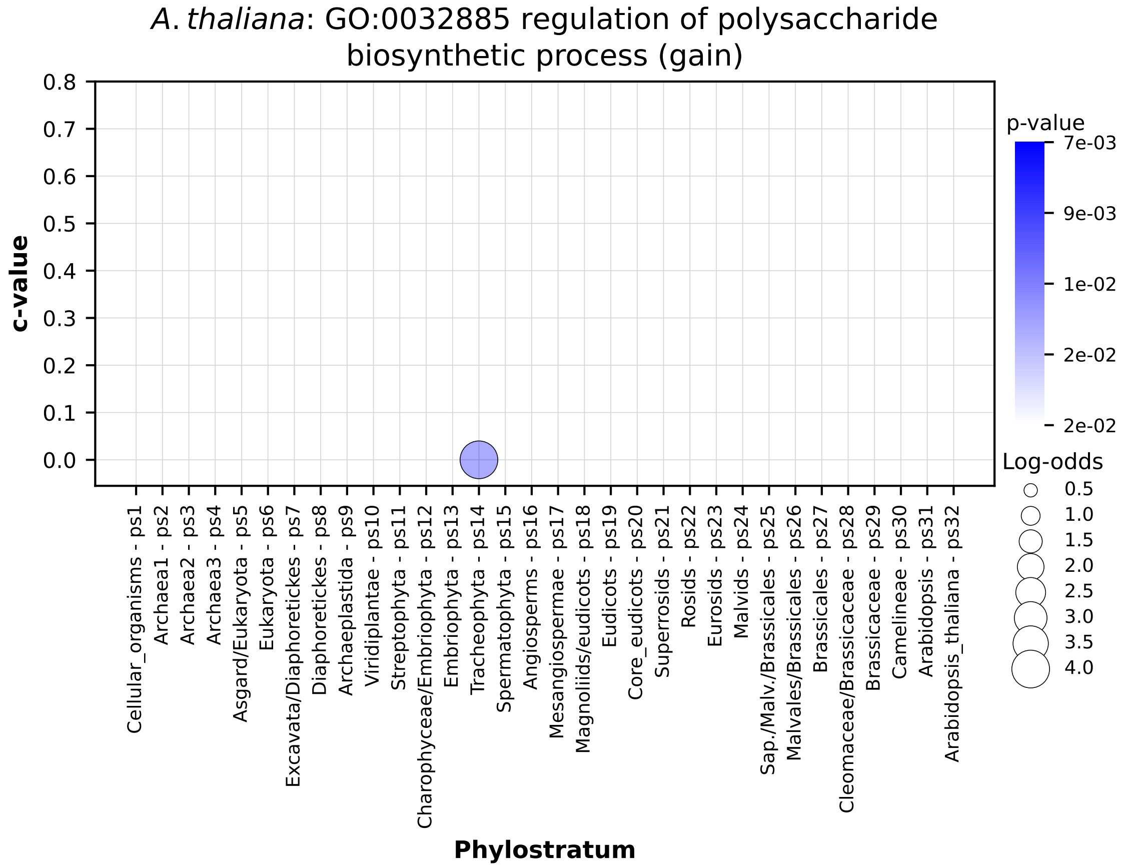

A. thaliana: GO:0032885 regulation of polysaccharide biosynthetic process (gain)
c-value
0.8
0.7
0.6
0.5
0.4
0.3
0.2
0.1
0.0
Phylostratum
Cellular_organisms - ps1
Archaea1 - ps2
Archaea2 - ps3
Archaea3 - ps4
Asgard/Eukaryota - ps5
Eukaryota - ps6
Excavata/Diaphoretickes - ps7
Diaphoretickes - ps8
Archaeplastida - ps9
Viridiplantae - ps10
Streptophyta - ps11
Charophyceae/Embriophyta - ps12
Embriophyta - ps13
Tracheophyta - ps14
Spermatophyta - ps15
Angiosperms - ps16
Mesangiospermae - ps17
Magnoliids/eudicots - ps18
Eudicots - ps19
Core_eudicots - ps20
Superrosids - ps21
Rosids - ps22
Eurosids - ps23
Malvids - ps24
Sap./Malv./Brassicales - ps25
Malvales/Brassicales - ps26
Brassicales - ps27
Cleomaceae/Brassicaceae - ps28
Brassicaceae - ps29
Camelineae - ps30
Arabidopsis - ps31
Arabidopsis_thaliana - ps32
p-value
7e-03
9e-03
1e-02
2e-02
2e-02
Log-odds
0.5
1.0
1.5
2.0
2.5
3.0
3.5
4.0

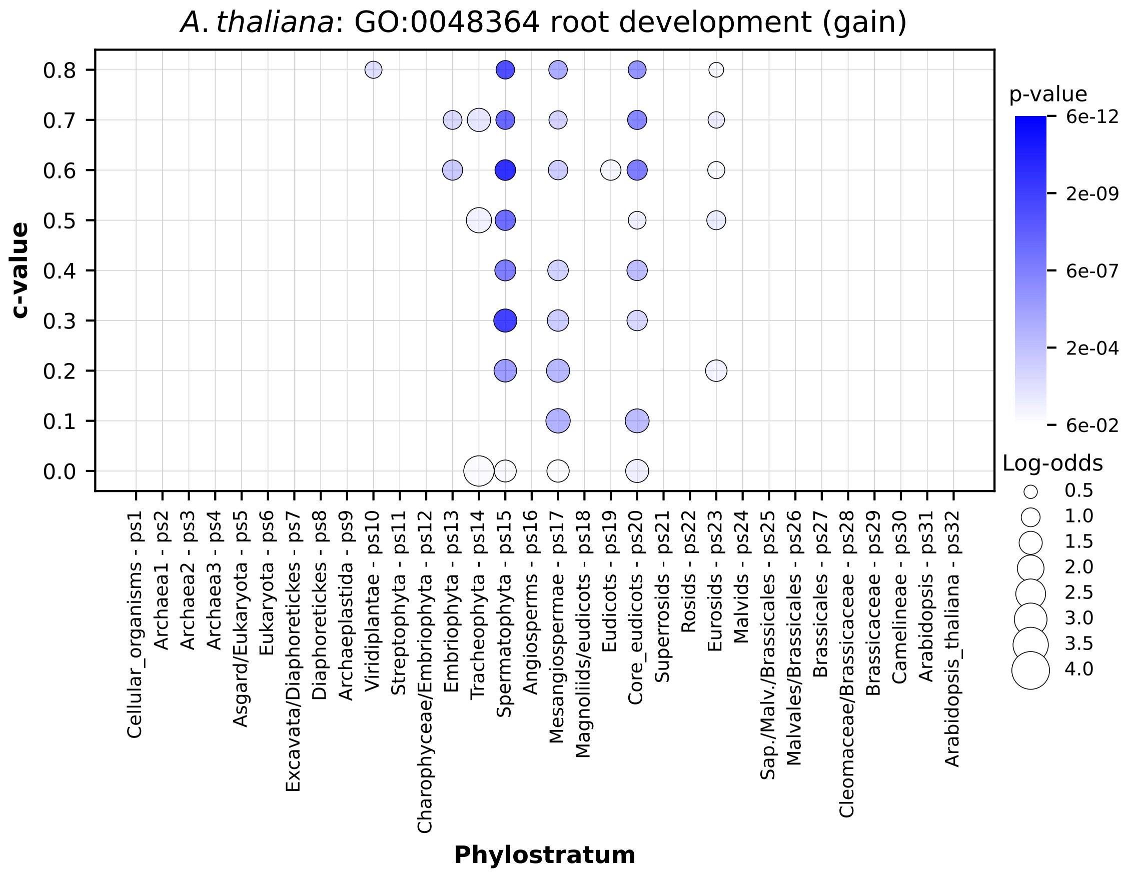
A. thaliana: GO:0048364 root development (gain)
c-value
0.8
0.7
0.6
0.5
0.4
0.3
0.2
0.1
0.0
Phylostratum
Cellular_organisms - ps1
Archaea1 - ps2
Archaea2 - ps3
Archaea3 - ps4
Asgard/Eukaryota - ps5
Eukaryota - ps6
Excavata/Diaphoretickes - ps7
Diaphoretickes - ps8
Archaeplastida - ps9
Viridiplantae - ps10
Streptophyta - ps11
Charophyceae/Embriophyta - ps12
Embriophyta - ps13
Tracheophyta - ps14
Spermatophyta - ps15
Angiosperms - ps16
Mesangiospermae - ps17
Magnoliids/eudicots - ps18
Eudicots - ps19
Core_eudicots - ps20
Superrosids - ps21
Rosids - ps22
Eurosids - ps23
Malvids - ps24
Sap./Malv./Brassicales - ps25
Malvales/Brassicales - ps26
Brassicales - ps27
Cleomaceae/Brassicaceae - ps28
Brassicaceae - ps29
Camelineae - ps30
Arabidopsis - ps31
Arabidopsis_thaliana - ps32
p-value
6e-12
2e-09
6e-07
2e-04
6e-02
Log-odds
0.5
1.0
1.5
2.0
2.5
3.0
3.5
4.0

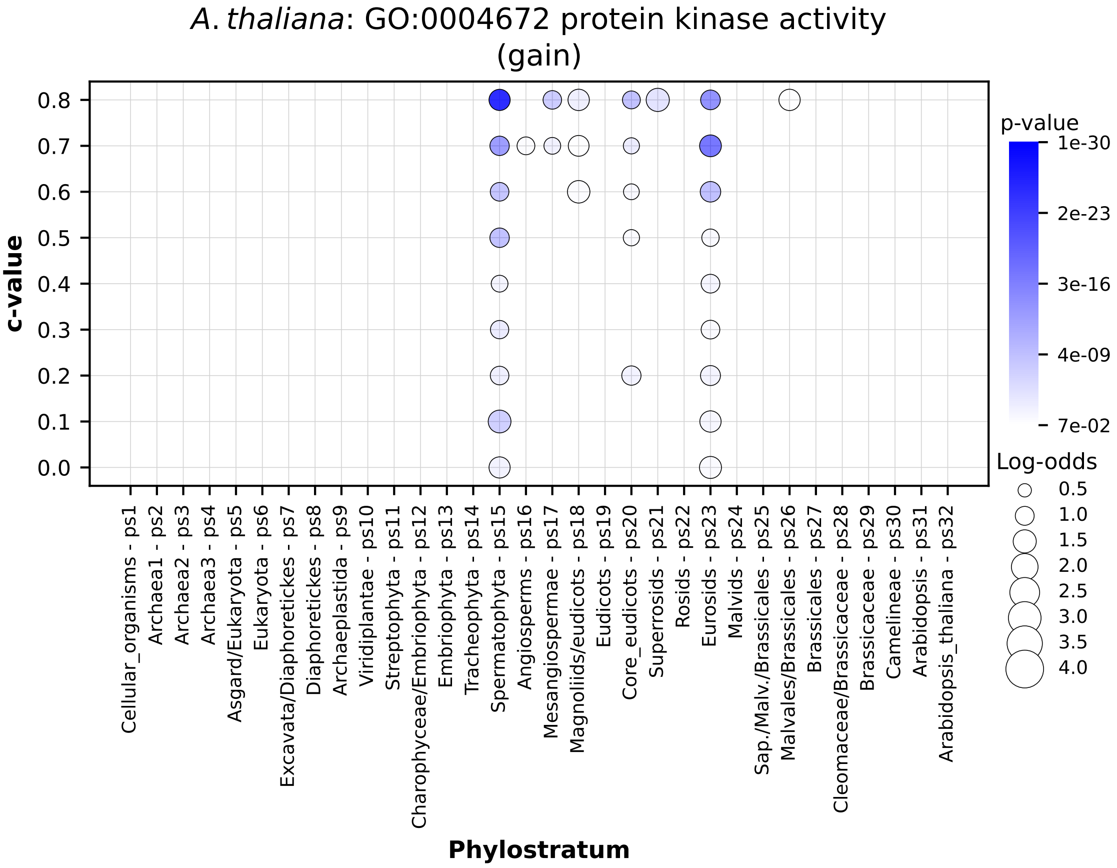
A. thaliana: GO:0004672 protein kinase activity
(gain)
c-value
0.8
0.7
0.6
0.5
0.4
0.3
0.2
0.1
0.0
p-value
1e-30
2e-23
3e-16
4e-09
7e-02
Log-odds
0.5
1.0
1.5
2.0
2.5
3.0
3.5
4.0
Cellular_organisms - ps1
Archaea1 - ps2
Archaea2 - ps3
Archaea3 - ps4
Asgard/Eukaryota - ps5
Eukaryota - ps6
Excavata/Diaphoretickes - ps7
Diaphoretickes - ps8
Archaeplastida - ps9
Viridiplantae - ps10
Streptophyta - ps11
Charophyceae/Embriophyta - ps12
Embriophyta - ps13
Tracheophyta - ps14
Spermatophyta - ps15
Angiosperms - ps16
Mesangiospermae - ps17
Magnoliids/eudicots - ps18
Eudicots - ps19
Core_eudicots - ps20
Superrosids - ps21
Rosids - ps22
Eurosids - ps23
Malvids - ps24
Sap./Malv./Brassicales - ps25
Malvales/Brassicales - ps26
Brassicales - ps27
Cleomaceae/Brassicaceae - ps28
Brassicaceae - ps29
Camelineae - ps30
Arabidopsis - ps31
Arabidopsis_thaliana - ps32
Phylostratum

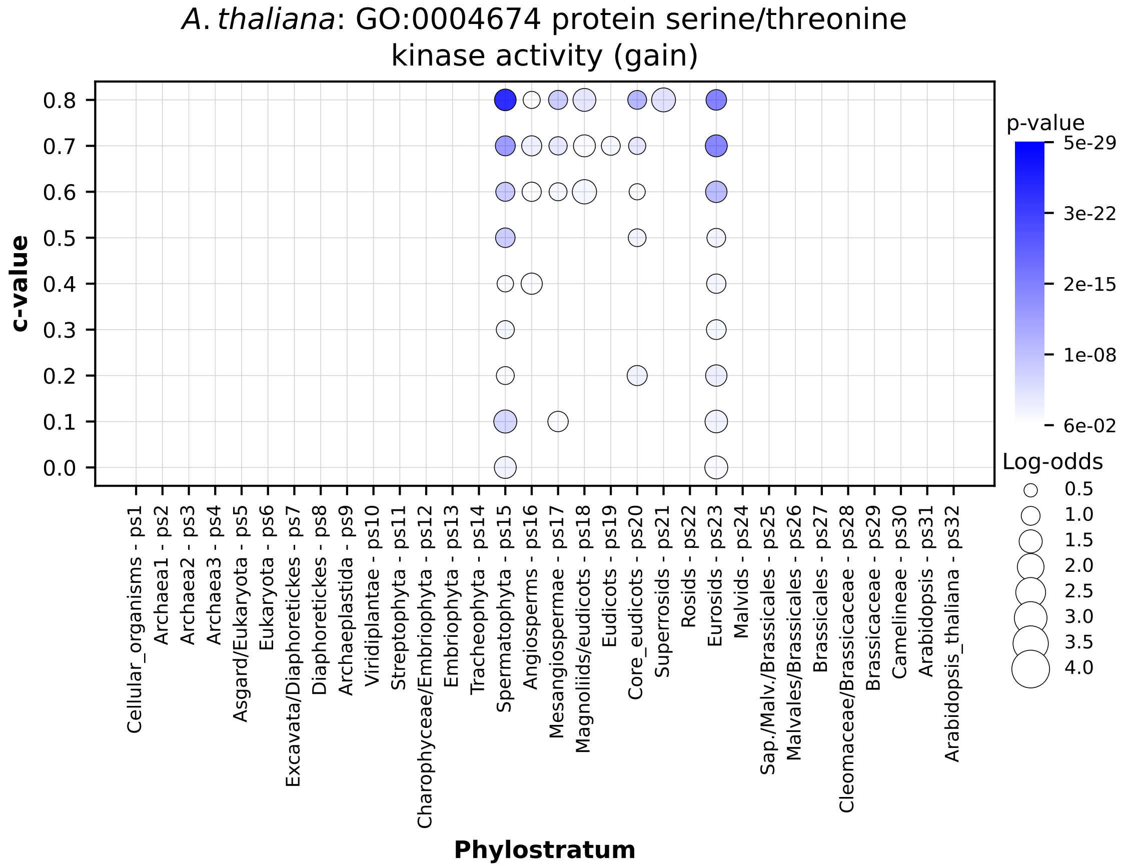
A. thaliana: GO:0004674 protein serine/threonine kinase activity (gain)
c-value
0.8
0.7
0.6
0.5
0.4
0.3
0.2
0.1
0.0
p-value
5e-29
3e-22
2e-15
1e-08
6e-02
Log-odds
0.5
1.0
1.5
2.0
2.5
3.0
3.5
4.0
Cellular_organisms - ps1
Archaea1 - ps2
Archaea2 - ps3
Archaea3 - ps4
Asgard/Eukaryota - ps5
Eukaryota - ps6
Excavata/Diaphoretickes - ps7
Diaphoretickes - ps8
Archaeplastida - ps9
Viridiplantae - ps10
Streptophyta - ps11
Charophyceae/Embriophyta - ps12
Embriophyta - ps13
Tracheophyta - ps14
Spermatophyta - ps15
Angiosperms - ps16
Mesangiospermae - ps17
Magnoliids/eudicots - ps18
Eudicots - ps19
Core_eudicots - ps20
Superrosids - ps21
Rosids - ps22
Eurosids - ps23
Malvids - ps24
Sap./Malv./Brassicales - ps25
Malvales/Brassicales - ps26
Brassicales - ps27
Cleomaceae/Brassicaceae - ps28
Brassicaceae - ps29
Camelineae - ps30
Arabidopsis - ps31
Arabidopsis_thaliana - ps32
Phylostratum

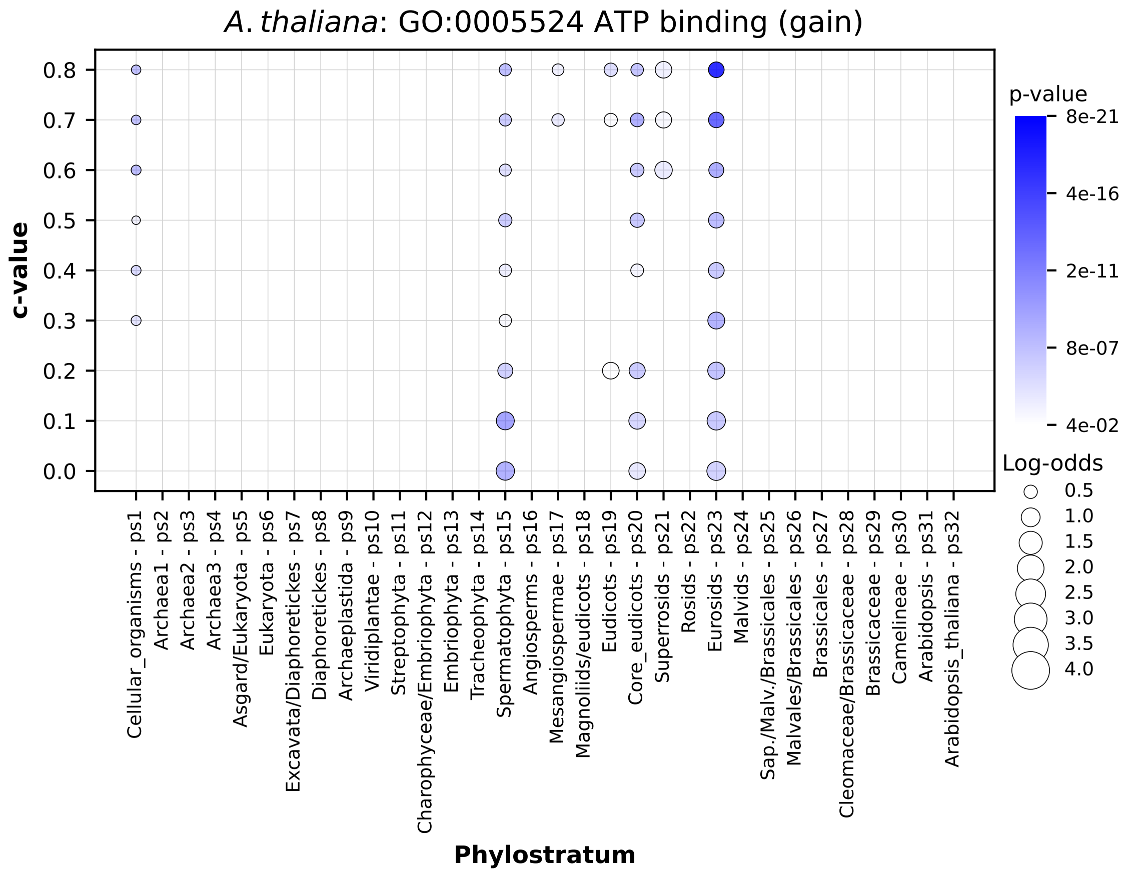

A. thaliana: GO:0005524 ATP binding (gain)
c-value
0.8
0.7
0.6
0.5
0.4
0.3
0.2
0.1
0.0
Phylostratum
Cellular_organisms - ps1
Archaea1 - ps2
Archaea2 - ps3
Archaea3 - ps4
Asgard/Eukaryota - ps5
Eukaryota - ps6
Excavata/Diaphoretickes - ps7
Diaphoretickes - ps8
Archaeplastida - ps9
Viridiplantae - ps10
Streptophyta - ps11
Charophyceae/Embriophyta - ps12
Embriophyta - ps13
Tracheophyta - ps14
Spermatophyta - ps15
Angiosperms - ps16
Mesangiospermae - ps17
Magnoliids/eudicots - ps18
Eudicots - ps19
Core_eudicots - ps20
Superrosids - ps21
Rosids - ps22
Eurosids - ps23
Malvids - ps24
Sap./Malv./Brassicales - ps25
Malvales/Brassicales - ps26
Brassicales - ps27
Cleomaceae/Brassicaceae - ps28
Brassicaceae - ps29
Camelineae - ps30
Arabidopsis - ps31
Arabidopsis_thaliana - ps32
p-value
8e-21
4e-16
2e-11
8e-07
4e-02
Log-odds
0.5
1.0
1.5
2.0
2.5
3.0
3.5
4.0

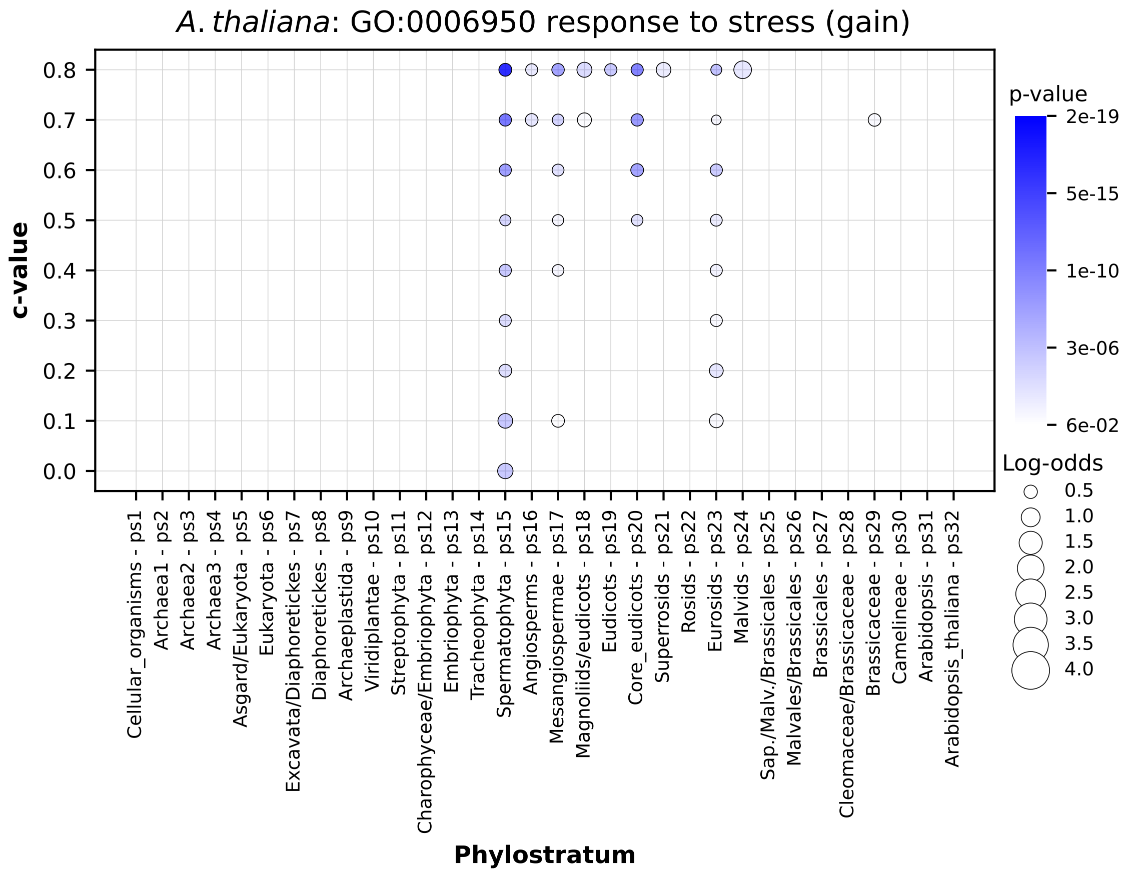
A. thaliana: GO:0006950 response to stress (gain)
c-value
0.8
0.7
0.6
0.5
0.4
0.3
0.2
0.1
0.0
Phylostratum
Cellular_organisms - ps1
Archaea1 - ps2
Archaea2 - ps3
Archaea3 - ps4
Asgard/Eukaryota - ps5
Eukaryota - ps6
Excavata/Diaphoretickes - ps7
Diaphoretickes - ps8
Archaeplastida - ps9
Viridiplantae - ps10
Streptophyta - ps11
Charophyceae/Embriophyta - ps12
Embriophyta - ps13
Tracheophyta - ps14
Spermatophyta - ps15
Angiosperms - ps16
Mesangiospermae - ps17
Magnoliids/eudicots - ps18
Eudicots - ps19
Core_eudicots - ps20
Superrosids - ps21
Rosids - ps22
Eurosids - ps23
Malvids - ps24
Sap./Malv./Brassicales - ps25
Malvales/Brassicales - ps26
Brassicales - ps27
Cleomaceae/Brassicaceae - ps28
Brassicaceae - ps29
Camelineae - ps30
Arabidopsis - ps31
Arabidopsis_thaliana - ps32
p-value
2e-19
5e-15
1e-10
3e-06
6e-02
Log-odds
0.5
1.0
1.5
2.0
2.5
3.0
3.5
4.0

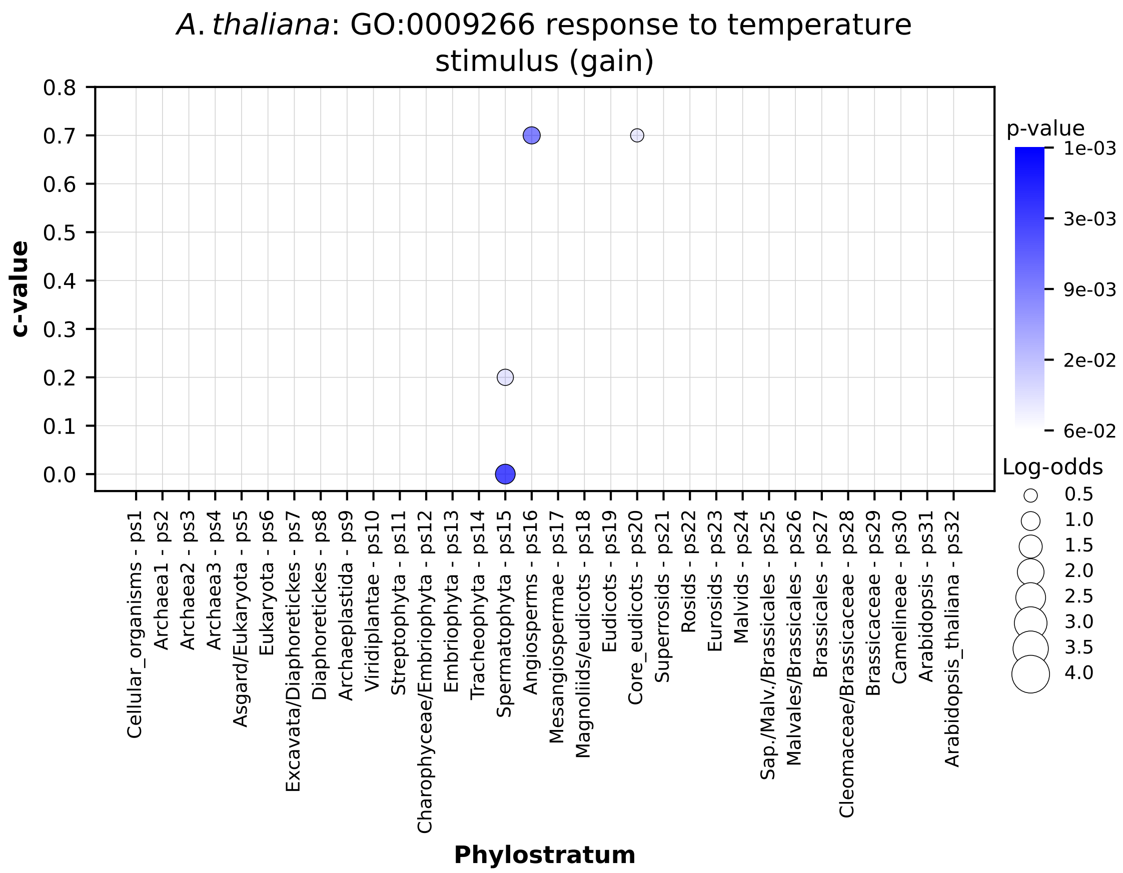

A. thaliana: GO:0009266 response to temperature stimulus (gain)
c-value
0.8
0.7
0.6
0.5
0.4
0.3
0.2
0.1
0.0
p-value
1e-03
3e-03
9e-03
2e-02
6e-02
Log-odds
0.5
1.0
1.5
2.0
2.5
3.0
3.5
4.0
Cellular_organisms - ps1
Archaea1 - ps2
Archaea2 - ps3
Archaea3 - ps4
Asgard/Eukaryota - ps5
Eukaryota - ps6
Excavata/Diaphoretickes - ps7
Diaphoretickes - ps8
Archaeplastida - ps9
Viridiplantae - ps10
Streptophyta - ps11
Charophyceae/Embriophyta - ps12
Embriophyta - ps13
Tracheophyta - ps14
Spermatophyta - ps15
Angiosperms - ps16
Mesangiospermae - ps17
Magnoliids/eudicots - ps18
Eudicots - ps19
Core_eudicots - ps20
Superrosids - ps21
Rosids - ps22
Eurosids - ps23
Malvids - ps24
Sap./Malv./Brassicales - ps25
Malvales/Brassicales - ps26
Brassicales - ps27
Cleomaceae/Brassicaceae - ps28
Brassicaceae - ps29
Camelineae - ps30
Arabidopsis - ps31
Arabidopsis_thaliana - ps32
Phylostratum

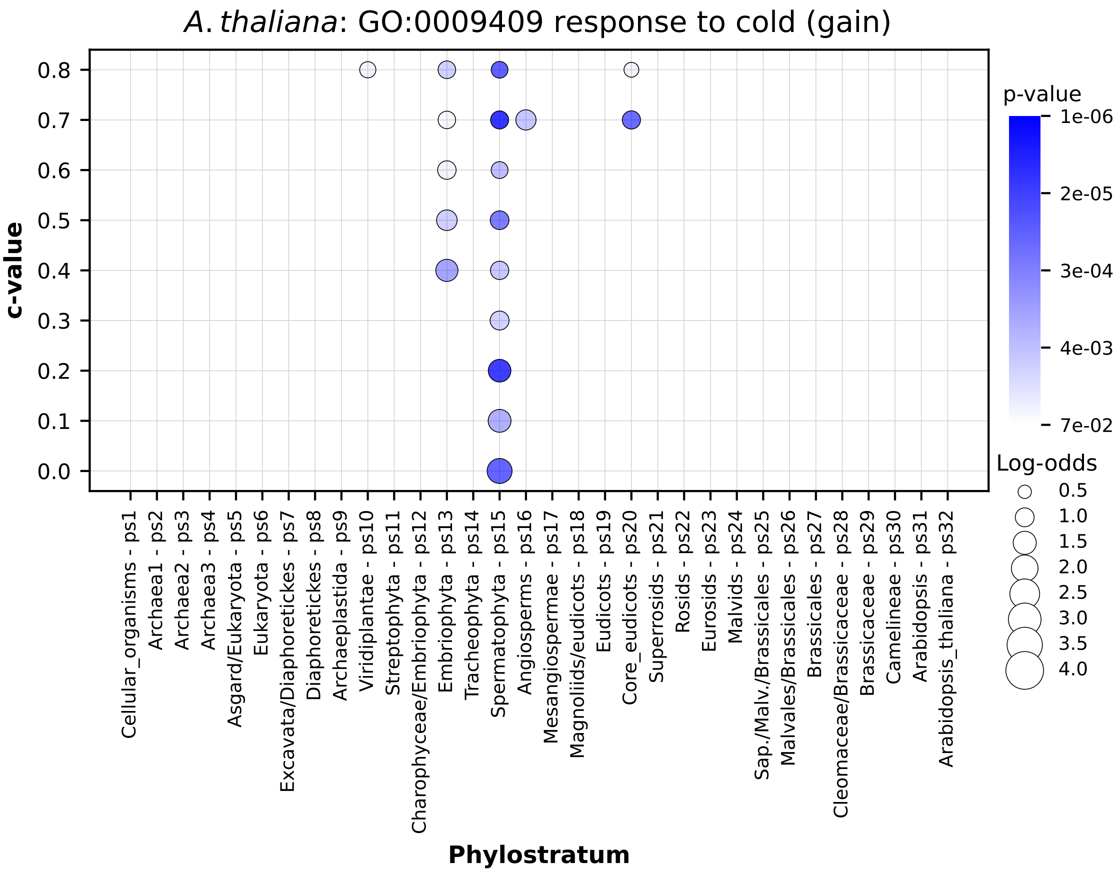

A. thaliana: GO:0009409 response to cold (gain)
c-value
0.8
0.7
0.6
0.5
0.4
0.3
0.2
0.1
0.0
p-value
1e-06
2e-05
3e-04
4e-03
7e-02
Log-odds
0.5
1.0
1.5
2.0
2.5
3.0
3.5
4.0
Cellular_organisms - ps1
Archaea1 - ps2
Archaea2 - ps3
Archaea3 - ps4
Asgard/Eukaryota - ps5
Eukaryota - ps6
Excavata/Diaphoretickes - ps7
Diaphoretickes - ps8
Archaeplastida - ps9
Viridiplantae - ps10
Streptophyta - ps11
Charophyceae/Embriophyta - ps12
Embriophyta - ps13
Tracheophyta - ps14
Spermatophyta - ps15
Angiosperms - ps16
Mesangiospermae - ps17
Magnoliids/eudicots - ps18
Eudicots - ps19
Core_eudicots - ps20
Superrosids - ps21
Rosids - ps22
Eurosids - ps23
Malvids - ps24
Sap./Malv./Brassicales - ps25
Malvales/Brassicales - ps26
Brassicales - ps27
Cleomaceae/Brassicaceae - ps28
Brassicaceae - ps29
Camelineae - ps30
Arabidopsis - ps31
Arabidopsis_thaliana - ps32
Phylostratum

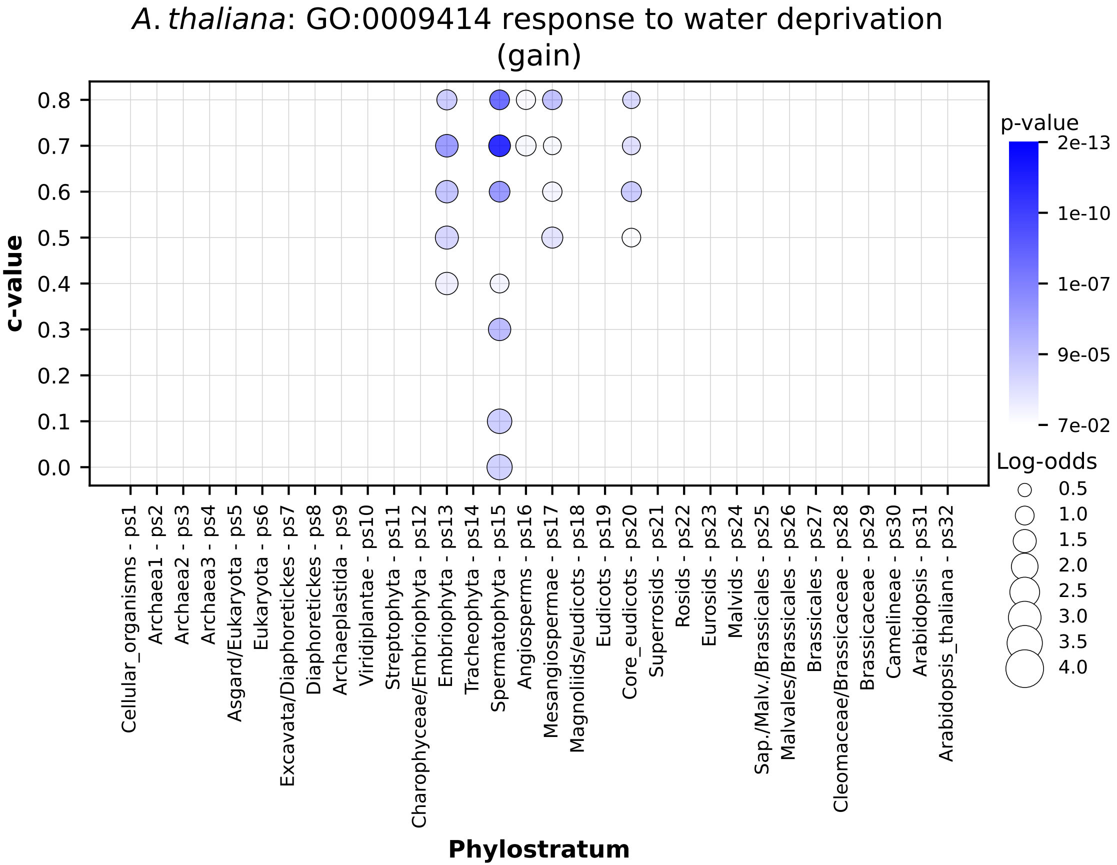
A. thaliana: GO:0009414 response to water deprivation
(gain)
c-value
0.8
0.7
0.6
0.5
0.4
0.3
0.2
0.1
0.0
p-value
2e-13
1e-10
1e-07
9e-05
7e-02
Log-odds
0.5
1.0
1.5
2.0
2.5
3.0
3.5
4.0
Cellular_organisms - ps1
Archaea1 - ps2
Archaea2 - ps3
Archaea3 - ps4
Asgard/Eukaryota - ps5
Eukaryota - ps6
Excavata/Diaphoretickes - ps7
Diaphoretickes - ps8
Archaeplastida - ps9
Viridiplantae - ps10
Streptophyta - ps11
Charophyceae/Embriophyta - ps12
Embriophyta - ps13
Tracheophyta - ps14
Spermatophyta - ps15
Angiosperms - ps16
Mesangiospermae - ps17
Magnoliids/eudicots - ps18
Eudicots - ps19
Core_eudicots - ps20
Superrosids - ps21
Rosids - ps22
Eurosids - ps23
Malvids - ps24
Sap./Malv./Brassicales - ps25
Malvales/Brassicales - ps26
Brassicales - ps27
Cleomaceae/Brassicaceae - ps28
Brassicaceae - ps29
Camelineae - ps30
Arabidopsis - ps31
Arabidopsis_thaliana - ps32
Phylostratum

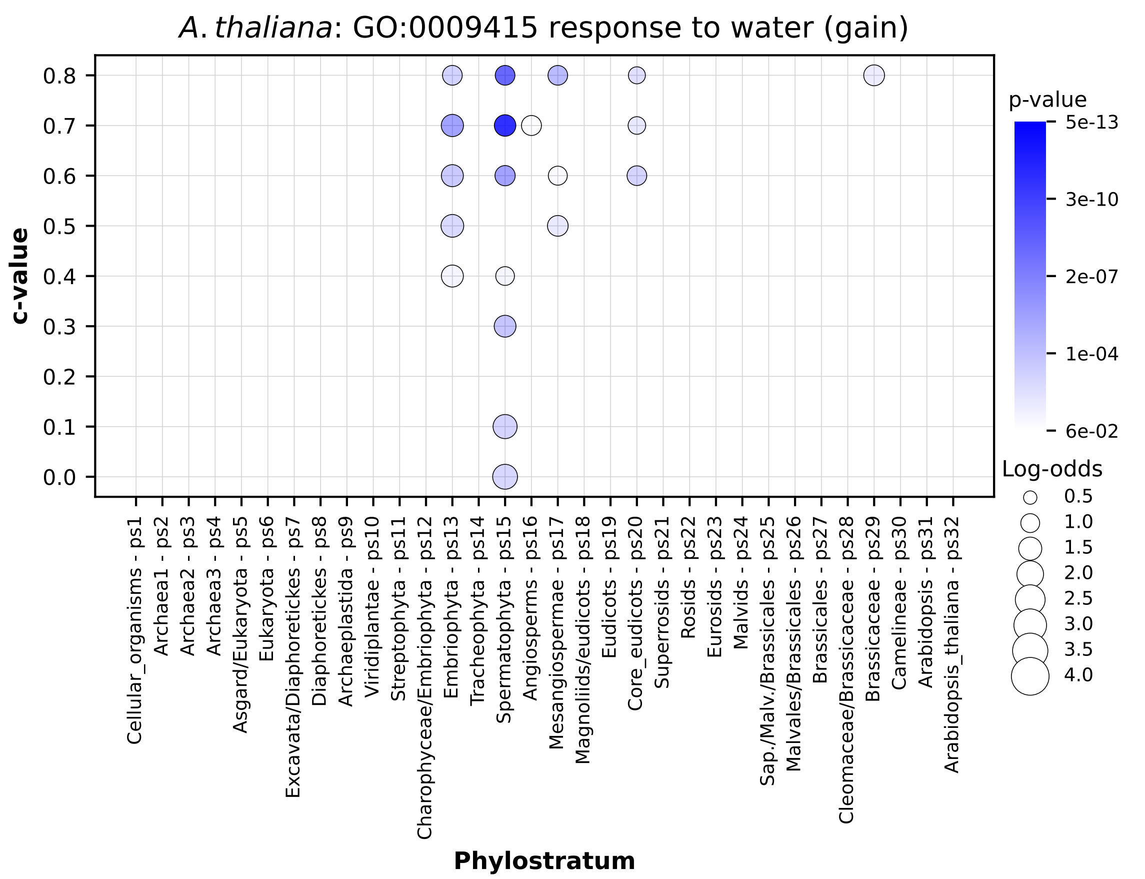

A. thaliana: GO:0009415 response to water (gain)
c-value
0.8
0.7
0.6
0.5
0.4
0.3
0.2
0.1
0.0
p-value
5e-13
3e-10
2e-07
1e-04
6e-02
Log-odds
0.5
1.0
1.5
2.0
2.5
3.0
3.5
4.0
Cellular_organisms - ps1
Archaea1 - ps2
Archaea2 - ps3
Archaea3 - ps4
Asgard/Eukaryota - ps5
Eukaryota - ps6
Excavata/Diaphoretickes - ps7
Diaphoretickes - ps8
Archaeplastida - ps9
Viridiplantae - ps10
Streptophyta - ps11
Charophyceae/Embriophyta - ps12
Embriophyta - ps13
Tracheophyta - ps14
Spermatophyta - ps15
Angiosperms - ps16
Mesangiospermae - ps17
Magnoliids/eudicots - ps18
Eudicots - ps19
Core_eudicots - ps20
Superrosids - ps21
Rosids - ps22
Eurosids - ps23
Malvids - ps24
Sap./Malv./Brassicales - ps25
Malvales/Brassicales - ps26
Brassicales - ps27
Cleomaceae/Brassicaceae - ps28
Brassicaceae - ps29
Camelineae - ps30
Arabidopsis - ps31
Arabidopsis_thaliana - ps32
Phylostratum

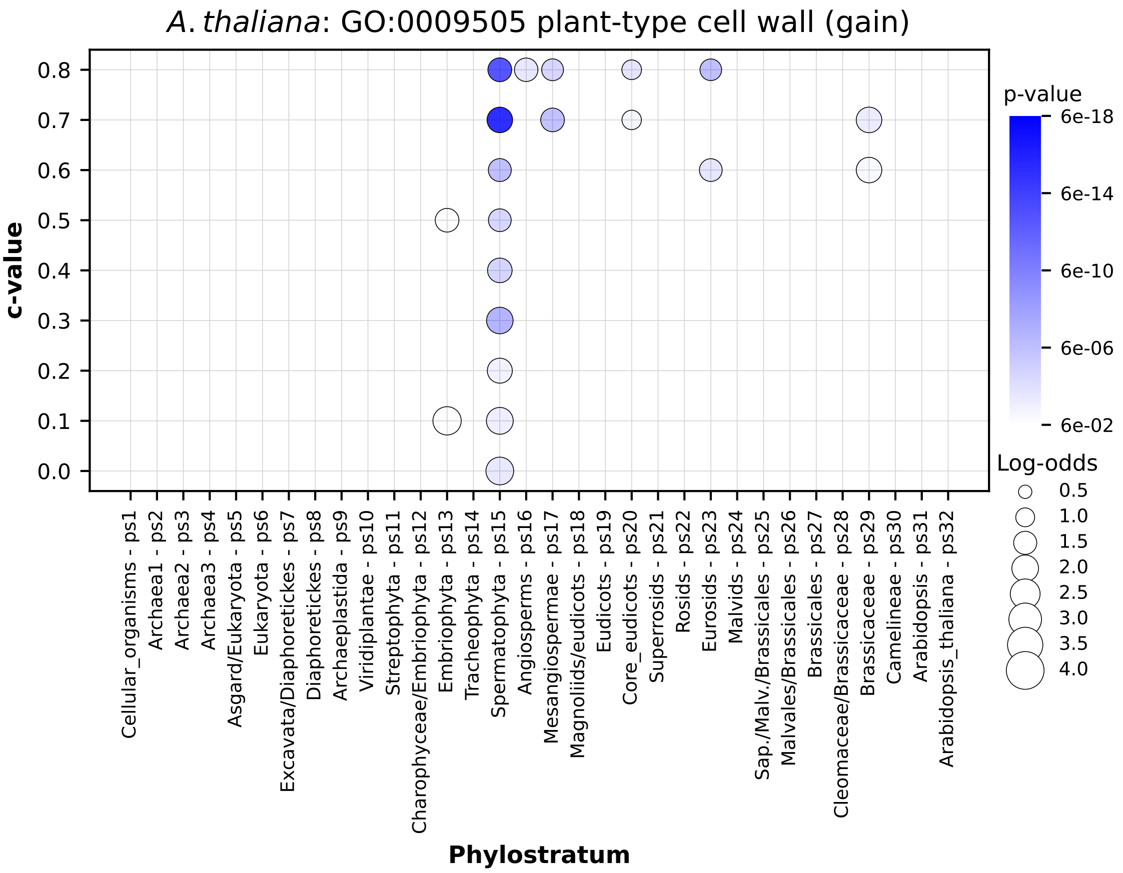

A. thaliana: GO:0009505 plant-type cell wall (gain)
c-value
0.8
0.7
0.6
0.5
0.4
0.3
0.2
0.1
0.0
p-value
6e-18
6e-14
6e-10
6e-06
6e-02
Log-odds
0.5
1.0
1.5
2.0
2.5
3.0
3.5
4.0
Cellular_organisms - ps1
Archaea1 - ps2
Archaea2 - ps3
Archaea3 - ps4
Asgard/Eukaryota - ps5
Eukaryota - ps6
Excavata/Diaphoretickes - ps7
Diaphoretickes - ps8
Archaeplastida - ps9
Viridiplantae - ps10
Streptophyta - ps11
Charophyceae/Embriophyta - ps12
Embriophyta - ps13
Tracheophyta - ps14
Spermatophyta - ps15
Angiosperms - ps16
Mesangiospermae - ps17
Magnoliids/eudicots - ps18
Eudicots - ps19
Core_eudicots - ps20
Superrosids - ps21
Rosids - ps22
Eurosids - ps23
Malvids - ps24
Sap./Malv./Brassicales - ps25
Malvales/Brassicales - ps26
Brassicales - ps27
Cleomaceae/Brassicaceae - ps28
Brassicaceae - ps29
Camelineae - ps30
Arabidopsis - ps31
Arabidopsis_thaliana - ps32
Phylostratum

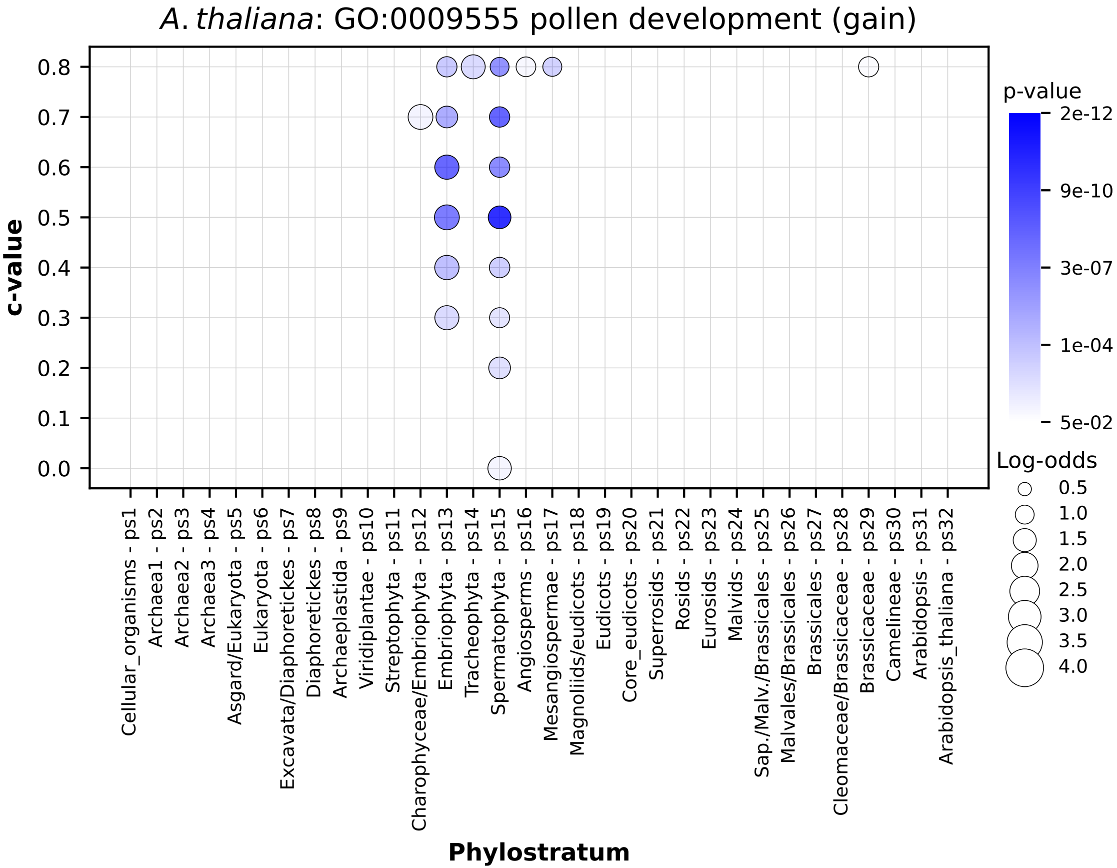

A. thaliana: GO:0009555 pollen development (gain)
c-value
0.8
0.7
0.6
0.5
0.4
0.3
0.2
0.1
0.0
Phylostratum
Cellular_organisms - ps1
Archaea1 - ps2
Archaea2 - ps3
Archaea3 - ps4
Asgard/Eukaryota - ps5
Eukaryota - ps6
Excavata/Diaphoretickes - ps7
Diaphoretickes - ps8
Archaeplastida - ps9
Viridiplantae - ps10
Streptophyta - ps11
Charophyceae/Embriophyta - ps12
Embriophyta - ps13
Tracheophyta - ps14
Spermatophyta - ps15
Angiosperms - ps16
Mesangiospermae - ps17
Magnoliids/eudicots - ps18
Eudicots - ps19
Core_eudicots - ps20
Superrosids - ps21
Rosids - ps22
Eurosids - ps23
Malvids - ps24
Sap./Malv./Brassicales - ps25
Malvales/Brassicales - ps26
Brassicales - ps27
Cleomaceae/Brassicaceae - ps28
Brassicaceae - ps29
Camelineae - ps30
Arabidopsis - ps31
Arabidopsis_thaliana - ps32
p-value
2e-12
9e-10
3e-07
1e-04
5e-02
Log-odds
0.5
1.0
1.5
2.0
2.5
3.0
3.5
4.0

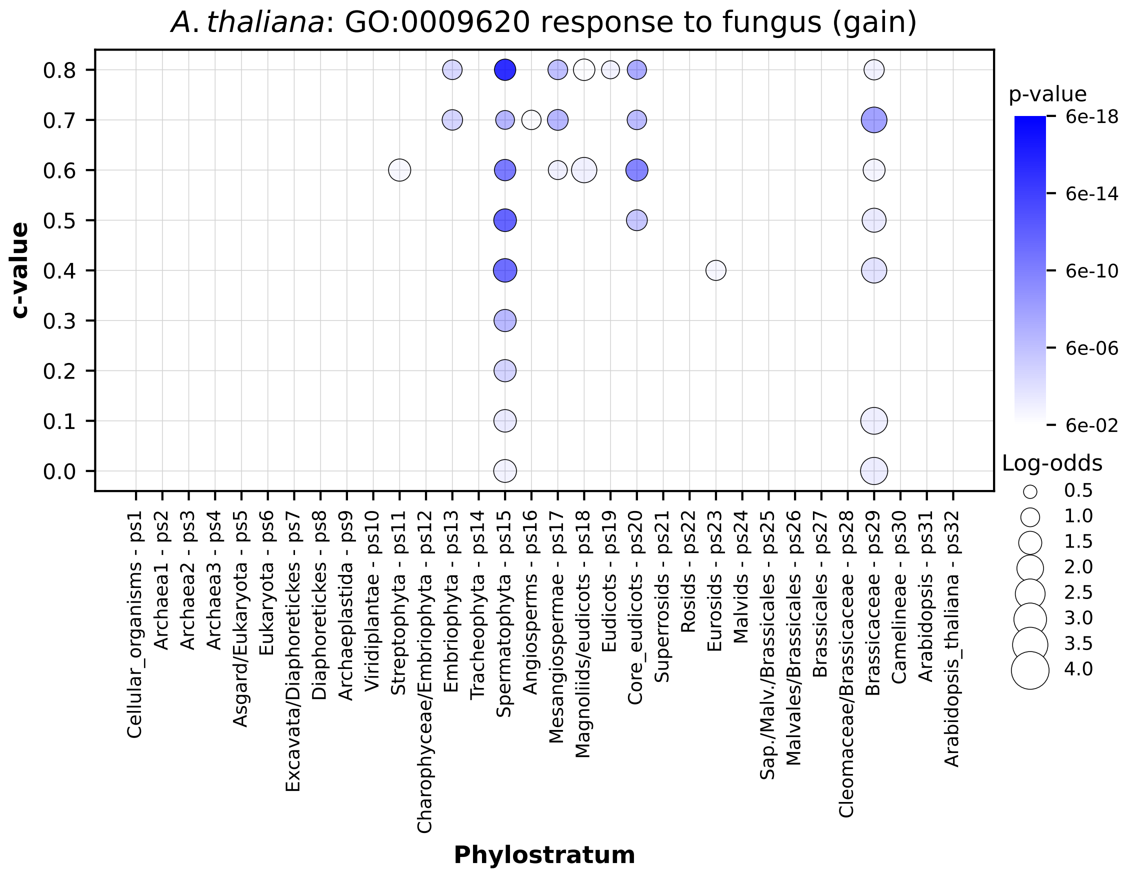

A. thaliana: GO:0009620 response to fungus (gain)
c-value
0.8
0.7
0.6
0.5
0.4
0.3
0.2
0.1
0.0
Phylostratum
Cellular_organisms - ps1
Archaea1 - ps2
Archaea2 - ps3
Archaea3 - ps4
Asgard/Eukaryota - ps5
Eukaryota - ps6
Excavata/Diaphoretickes - ps7
Diaphoretickes - ps8
Archaeplastida - ps9
Viridiplantae - ps10
Streptophyta - ps11
Charophyceae/Embriophyta - ps12
Embriophyta - ps13
Tracheophyta - ps14
Spermatophyta - ps15
Angiosperms - ps16
Mesangiospermae - ps17
Magnoliids/eudicots - ps18
Eudicots - ps19
Core_eudicots - ps20
Superrosids - ps21
Rosids - ps22
Eurosids - ps23
Malvids - ps24
Sap./Malv./Brassicales - ps25
Malvales/Brassicales - ps26
Brassicales - ps27
Cleomaceae/Brassicaceae - ps28
Brassicaceae - ps29
Camelineae - ps30
Arabidopsis - ps31
Arabidopsis_thaliana - ps32
p-value
6e-18
6e-14
6e-10
6e-06
6e-02
Log-odds
0.5
1.0
1.5
2.0
2.5
3.0
3.5
4.0

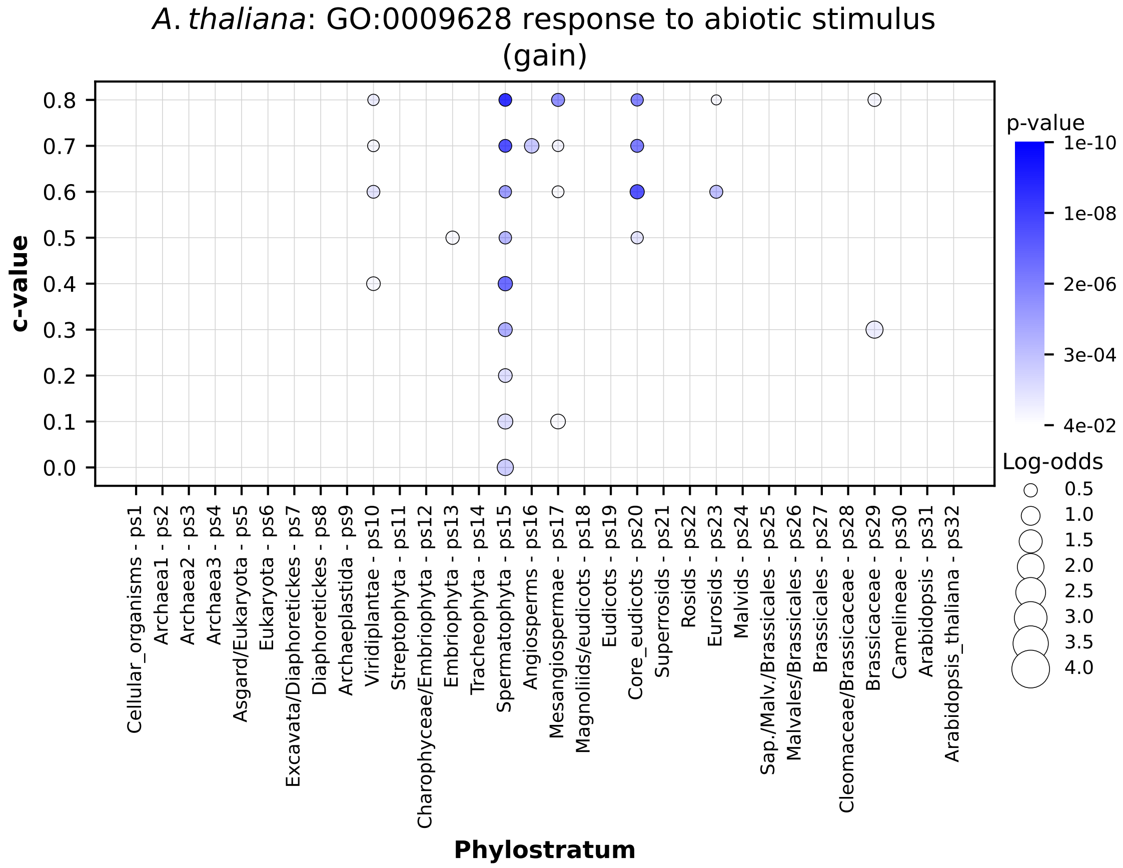

A. thaliana: GO:0009628 response to abiotic stimulus (gain)
c-value
0.8
0.7
0.6
0.5
0.4
0.3
0.2
0.1
0.0
Phylostratum
Cellular_organisms - ps1
Archaea1 - ps2
Archaea2 - ps3
Archaea3 - ps4
Asgard/Eukaryota - ps5
Eukaryota - ps6
Excavata/Diaphoretickes - ps7
Diaphoretickes - ps8
Archaeplastida - ps9
Viridiplantae - ps10
Streptophyta - ps11
Charophyceae/Embriophyta - ps12
Embriophyta - ps13
Tracheophyta - ps14
Spermatophyta - ps15
Angiosperms - ps16
Mesangiospermae - ps17
Magnoliids/eudicots - ps18
Eudicots - ps19
Core_eudicots - ps20
Superrosids - ps21
Rosids - ps22
Eurosids - ps23
Malvids - ps24
Sap./Malv./Brassicales - ps25
Malvales/Brassicales - ps26
Brassicales - ps27
Cleomaceae/Brassicaceae - ps28
Brassicaceae - ps29
Camelineae - ps30
Arabidopsis - ps31
Arabidopsis_thaliana - ps32
p-value
1e-10
1e-08
2e-06
3e-04
4e-02
Log-odds
0.5
1.0
1.5
2.0
2.5
3.0
3.5
4.0

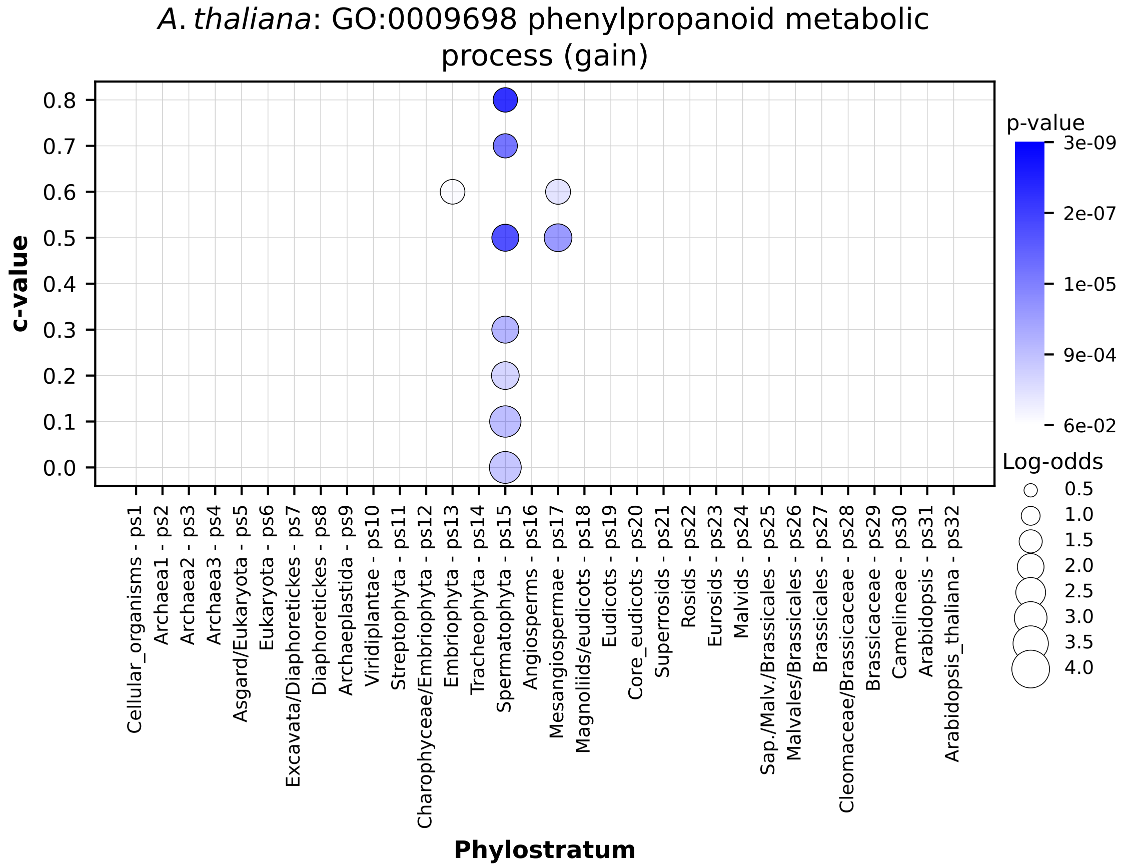

A. thaliana: GO:0009698 phenylpropanoid metabolic process (gain)
c-value
0.8
0.7
0.6
0.5
0.4
0.3
0.2
0.1
0.0
p-value
3e-09
2e-07
1e-05
9e-04
6e-02
Log-odds
0.5
1.0
1.5
2.0
2.5
3.0
3.5
4.0
Cellular_organisms - ps1
Archaea1 - ps2
Archaea2 - ps3
Archaea3 - ps4
Asgard/Eukaryota - ps5
Eukaryota - ps6
Excavata/Diaphoretickes - ps7
Diaphoretickes - ps8
Archaeplastida - ps9
Viridiplantae - ps10
Streptophyta - ps11
Charophyceae/Embriophyta - ps12
Embriophyta - ps13
Tracheophyta - ps14
Spermatophyta - ps15
Angiosperms - ps16
Mesangiospermae - ps17
Magnoliids/eudicots - ps18
Eudicots - ps19
Core_eudicots - ps20
Superrosids - ps21
Rosids - ps22
Eurosids - ps23
Malvids - ps24
Sap./Malv./Brassicales - ps25
Malvales/Brassicales - ps26
Brassicales - ps27
Cleomaceae/Brassicaceae - ps28
Brassicaceae - ps29
Camelineae - ps30
Arabidopsis - ps31
Arabidopsis_thaliana - ps32
Phylostratum

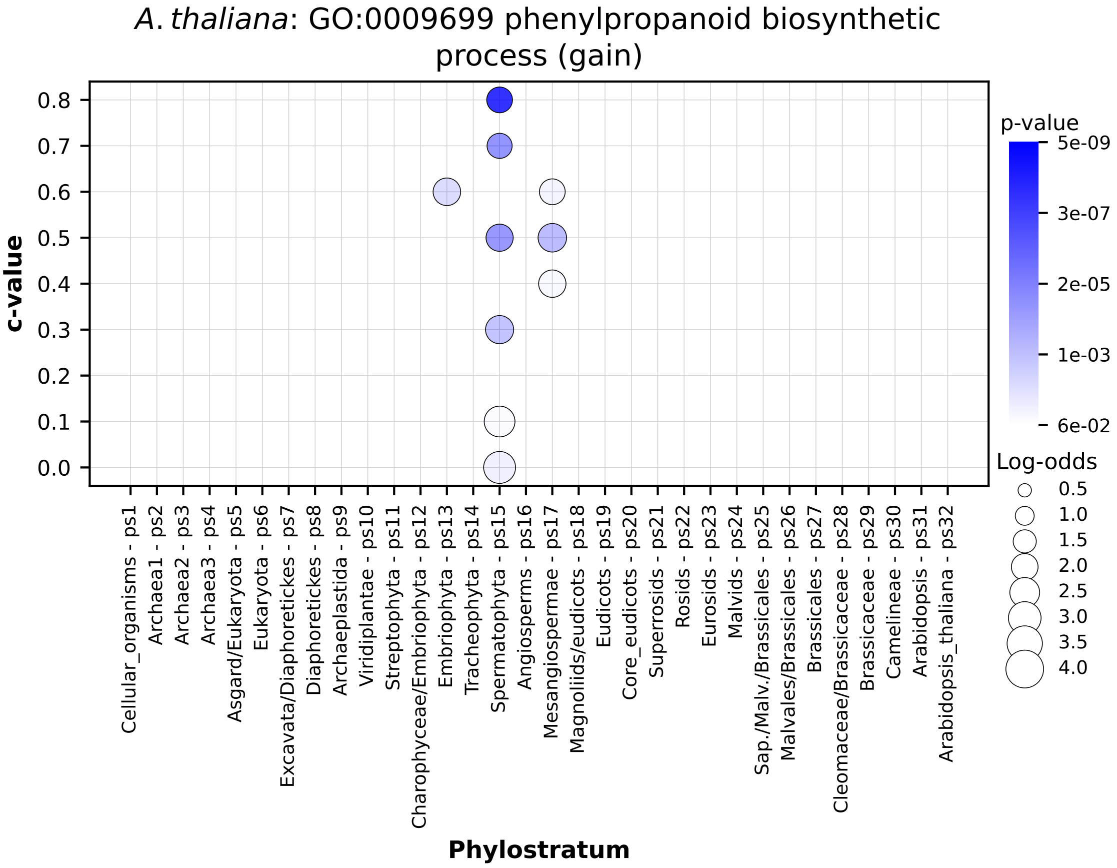
A. thaliana: GO:0009699 phenylpropanoid biosynthetic process (gain)
c-value
0.8
0.7
0.6
0.5
0.4
0.3
0.2
0.1
0.0
p-value
5e-09
3e-07
2e-05
1e-03
6e-02
Log-odds
0.5
1.0
1.5
2.0
2.5
3.0
3.5
4.0
Cellular_organisms - ps1
Archaea1 - ps2
Archaea2 - ps3
Archaea3 - ps4
Asgard/Eukaryota - ps5
Eukaryota - ps6
Excavata/Diaphoretickes - ps7
Diaphoretickes - ps8
Archaeplastida - ps9
Viridiplantae - ps10
Streptophyta - ps11
Charophyceae/Embriophyta - ps12
Embriophyta - ps13
Tracheophyta - ps14
Spermatophyta - ps15
Angiosperms - ps16
Mesangiospermae - ps17
Magnoliids/eudicots - ps18
Eudicots - ps19
Core_eudicots - ps20
Superrosids - ps21
Rosids - ps22
Eurosids - ps23
Malvids - ps24
Sap./Malv./Brassicales - ps25
Malvales/Brassicales - ps26
Brassicales - ps27
Cleomaceae/Brassicaceae - ps28
Brassicaceae - ps29
Camelineae - ps30
Arabidopsis - ps31
Arabidopsis_thaliana - ps32
Phylostratum

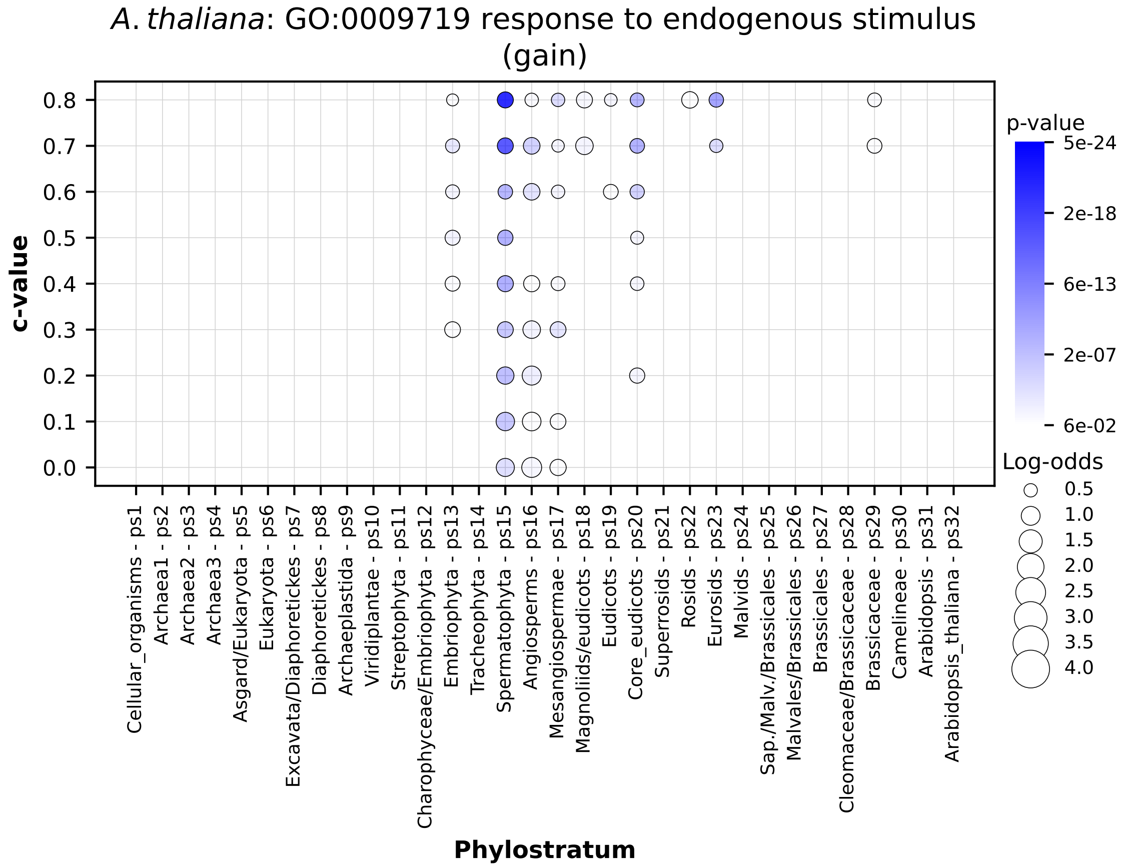

A. thaliana: GO:0009719 response to endogenous stimulus
(gain)
c-value
0.8
0.7
0.6
0.5
0.4
0.3
0.2
0.1
0.0
p-value
5e-24
2e-18
6e-13
2e-07
6e-02
Log-odds
0.5
1.0
1.5
2.0
2.5
3.0
3.5
4.0
Cellular_organisms - ps1
Archaea1 - ps2
Archaea2 - ps3
Archaea3 - ps4
Asgard/Eukaryota - ps5
Eukaryota - ps6
Excavata/Diaphoretickes - ps7
Diaphoretickes - ps8
Archaeplastida - ps9
Viridiplantae - ps10
Streptophyta - ps11
Charophyceae/Embriophyta - ps12
Embriophyta - ps13
Tracheophyta - ps14
Spermatophyta - ps15
Angiosperms - ps16
Mesangiospermae - ps17
Magnoliids/eudicots - ps18
Eudicots - ps19
Core_eudicots - ps20
Superrosids - ps21
Rosids - ps22
Eurosids - ps23
Malvids - ps24
Sap./Malv./Brassicales - ps25
Malvales/Brassicales - ps26
Brassicales - ps27
Cleomaceae/Brassicaceae - ps28
Brassicaceae - ps29
Camelineae - ps30
Arabidopsis - ps31
Arabidopsis_thaliana - ps32
Phylostratum

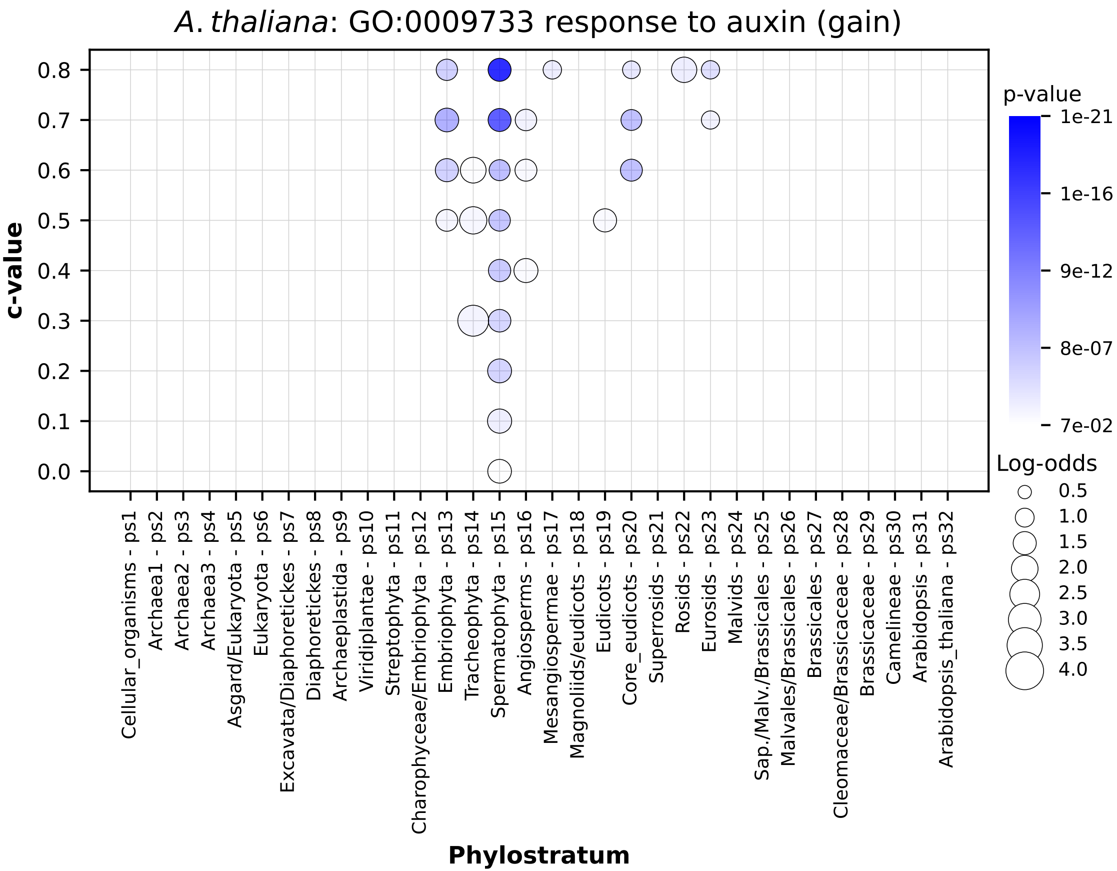
A. thaliana: GO:0009733 response to auxin (gain)
c-value
0.0
0.1
0.2
0.3
0.4
0.5
0.6
0.7
0.8
Phylostratum
Cellular_organisms - ps1
Archaea1 - ps2
Archaea2 - ps3
Archaea3 - ps4
Asgard/Eukaryota - ps5
Eukaryota - ps6
Excavata/Diaphoretickes - ps7
Diaphoretickes - ps8
Archaeplastida - ps9
Viridiplantae - ps10
Streptophyta - ps11
Charophyceae/Embriophyta - ps12
Embriophyta - ps13
Tracheophyta - ps14
Spermatophyta - ps15
Angiosperms - ps16
Mesangiospermae - ps17
Magnoliids/eudicots - ps18
Eudicots - ps19
Core_eudicots - ps20
Superrosids - ps21
Rosids - ps22
Eurosids - ps23
Malvids - ps24
Sap./Malv./Brassicales - ps25
Malvales/Brassicales - ps26
Brassicales - ps27
Cleomaceae/Brassicaceae - ps28
Brassicaceae - ps29
Camelineae - ps30
Arabidopsis - ps31
Arabidopsis_thaliana - ps32
p-value
1e-21
1e-16
9e-12
8e-07
7e-02
Log-odds
0.5
1.0
1.5
2.0
2.5
3.0
3.5
4.0

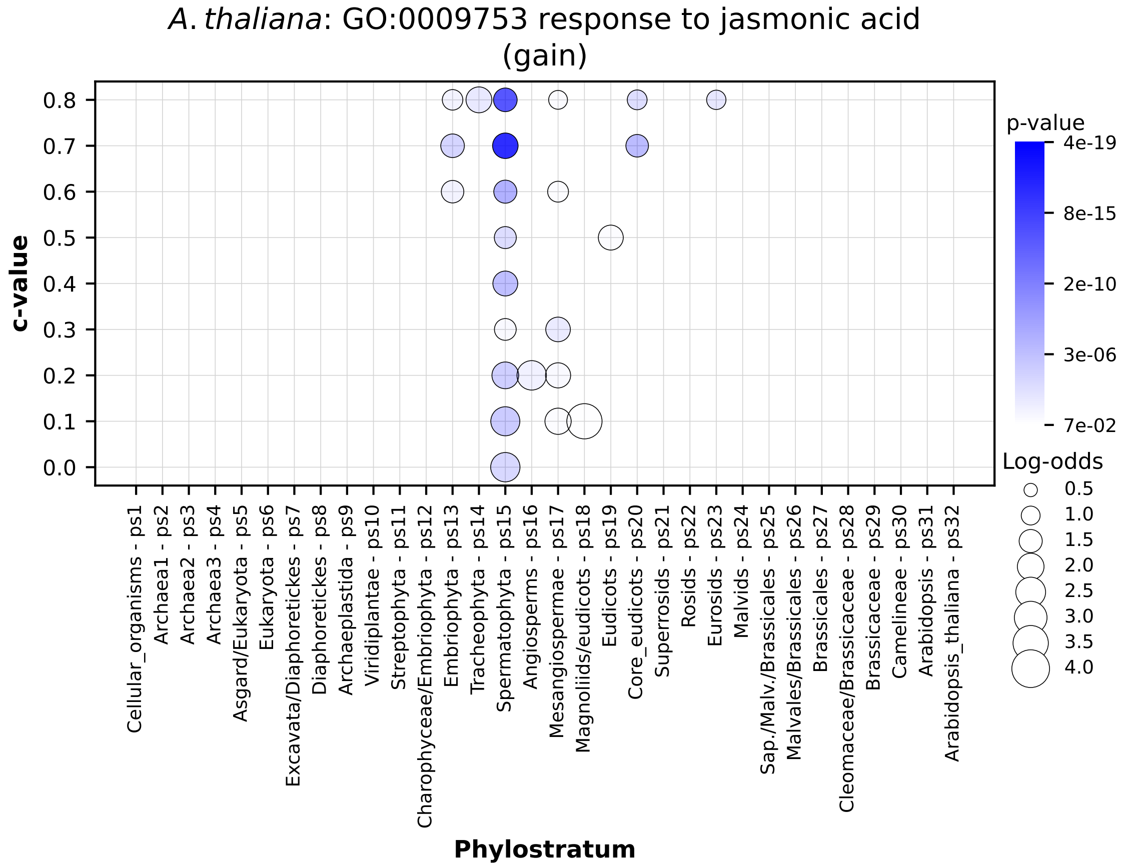

A. thaliana: GO:0009753 response to jasmonic acid (gain)
c-value
0.8
0.7
0.6
0.5
0.4
0.3
0.2
0.1
0.0
p-value
4e-19
8e-15
2e-10
3e-06
7e-02
Log-odds
0.5
1.0
1.5
2.0
2.5
3.0
3.5
4.0
Cellular_organisms - ps1
Archaea1 - ps2
Archaea2 - ps3
Archaea3 - ps4
Asgard/Eukaryota - ps5
Eukaryota - ps6
Excavata/Diaphoretickes - ps7
Diaphoretickes - ps8
Archaeplastida - ps9
Viridiplantae - ps10
Streptophyta - ps11
Charophyceae/Embriophyta - ps12
Embriophyta - ps13
Tracheophyta - ps14
Spermatophyta - ps15
Angiosperms - ps16
Mesangiospermae - ps17
Magnoliids/eudicots - ps18
Eudicots - ps19
Core_eudicots - ps20
Superrosids - ps21
Rosids - ps22
Eurosids - ps23
Malvids - ps24
Sap./Malv./Brassicales - ps25
Malvales/Brassicales - ps26
Brassicales - ps27
Cleomaceae/Brassicaceae - ps28
Brassicaceae - ps29
Camelineae - ps30
Arabidopsis - ps31
Arabidopsis_thaliana - ps32
Phylostratum

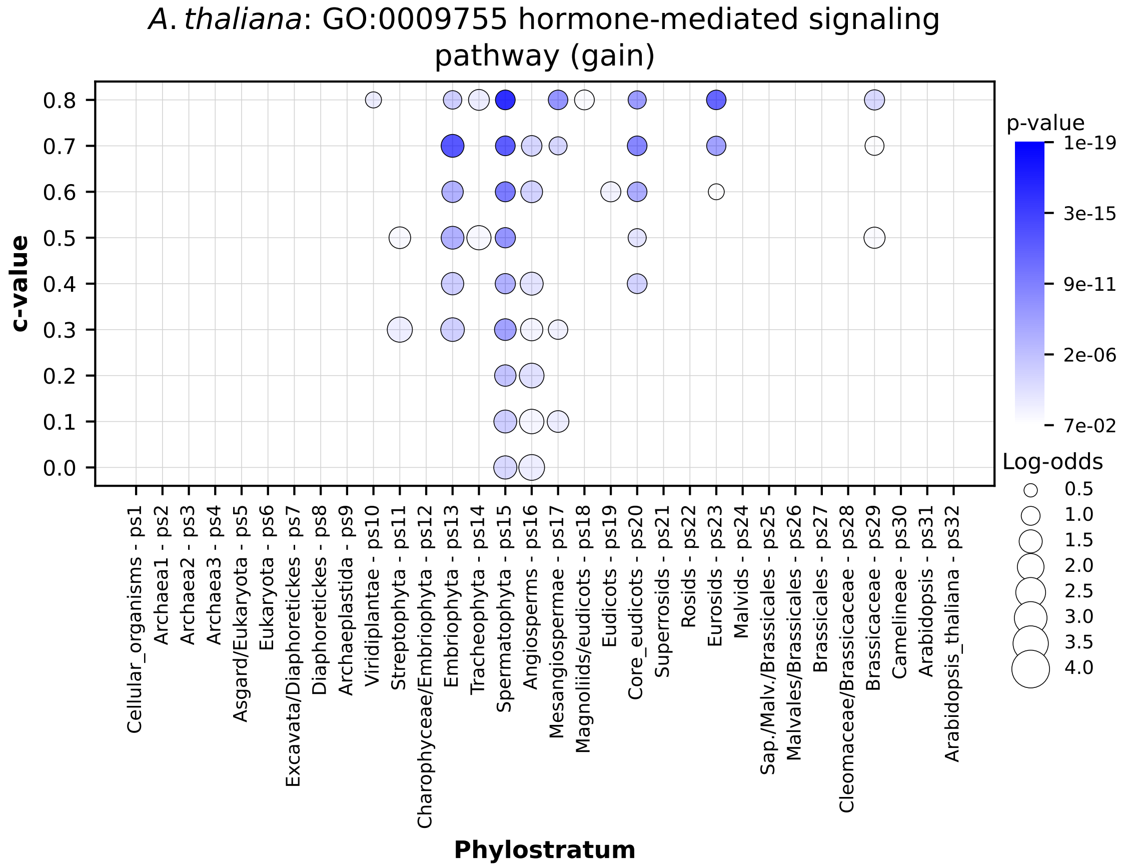

$A. thaliana$: GO:0009755 hormone-mediated signaling pathway (gain)
c-value
0.8
0.7
0.6
0.5
0.4
0.3
0.2
0.1
0.0
p-value
1e-19
3e-15
9e-11
2e-06
7e-02
Log-odds
0.5
1.0
1.5
2.0
2.5
3.0
3.5
4.0
Cellular_organisms - ps1
Archaea1 - ps2
Archaea2 - ps3
Archaea3 - ps4
Asgard/Eukaryota - ps5
Eukaryota - ps6
Excavata/Diaphoretickes - ps7
Diaphoretickes - ps8
Archaeplastida - ps9
Viridiplantae - ps10
Streptophyta - ps11
Charophyceae/Embriophyta - ps12
Embriophyta - ps13
Tracheophyta - ps14
Spermatophyta - ps15
Angiosperms - ps16
Mesangiospermae - ps17
Magnoliids/eudicots - ps18
Eudicots - ps19
Core_eudicots - ps20
Superrosids - ps21
Rosids - ps22
Eurosids - ps23
Malvids - ps24
Sap./Malv./Brassicales - ps25
Malvales/Brassicales - ps26
Brassicales - ps27
Cleomaceae/Brassicaceae - ps28
Brassicaceae - ps29
Camelineae - ps30
Arabidopsis - ps31
Arabidopsis_thaliana - ps32
Phylostratum

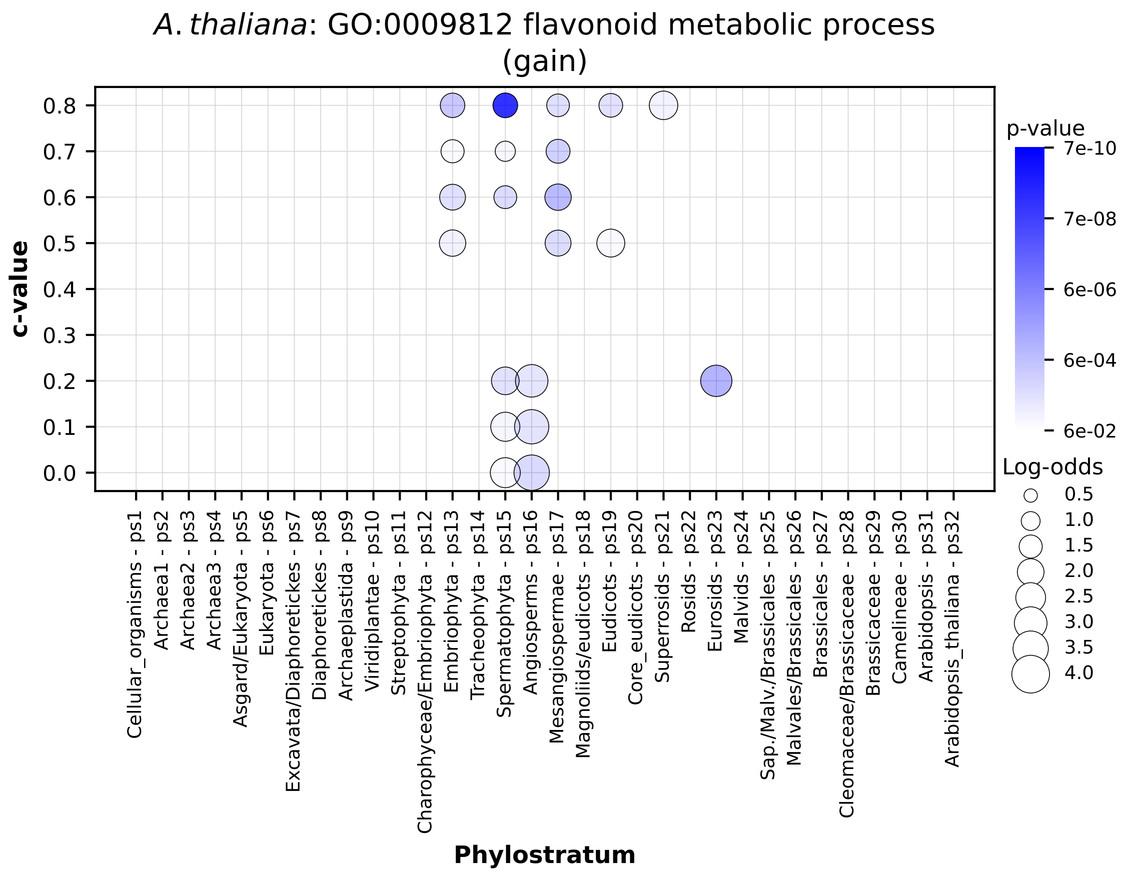

A. thaliana: GO:0009812 flavonoid metabolic process (gain)
c-value
0.8
0.7
0.6
0.5
0.4
0.3
0.2
0.1
0.0
p-value
7e-10
7e-08
6e-06
6e-04
6e-02
Log-odds
0.5
1.0
1.5
2.0
2.5
3.0
3.5
4.0
Cellular_organisms - ps1
Archaea1 - ps2
Archaea2 - ps3
Archaea3 - ps4
Asgard/Eukaryota - ps5
Eukaryota - ps6
Excavata/Diaphoretickes - ps7
Diaphoretickes - ps8
Archaeplastida - ps9
Viridiplantae - ps10
Streptophyta - ps11
Charophyceae/Embriophyta - ps12
Embriophyta - ps13
Tracheophyta - ps14
Spermatophyta - ps15
Angiosperms - ps16
Mesangiospermae - ps17
Magnoliids/eudicots - ps18
Eudicots - ps19
Core_eudicots - ps20
Superrosids - ps21
Rosids - ps22
Eurosids - ps23
Malvids - ps24
Sap./Malv./Brassicales - ps25
Malvales/Brassicales - ps26
Brassicales - ps27
Cleomaceae/Brassicaceae - ps28
Brassicaceae - ps29
Camelineae - ps30
Arabidopsis - ps31
Arabidopsis_thaliana - ps32
Phylostratum

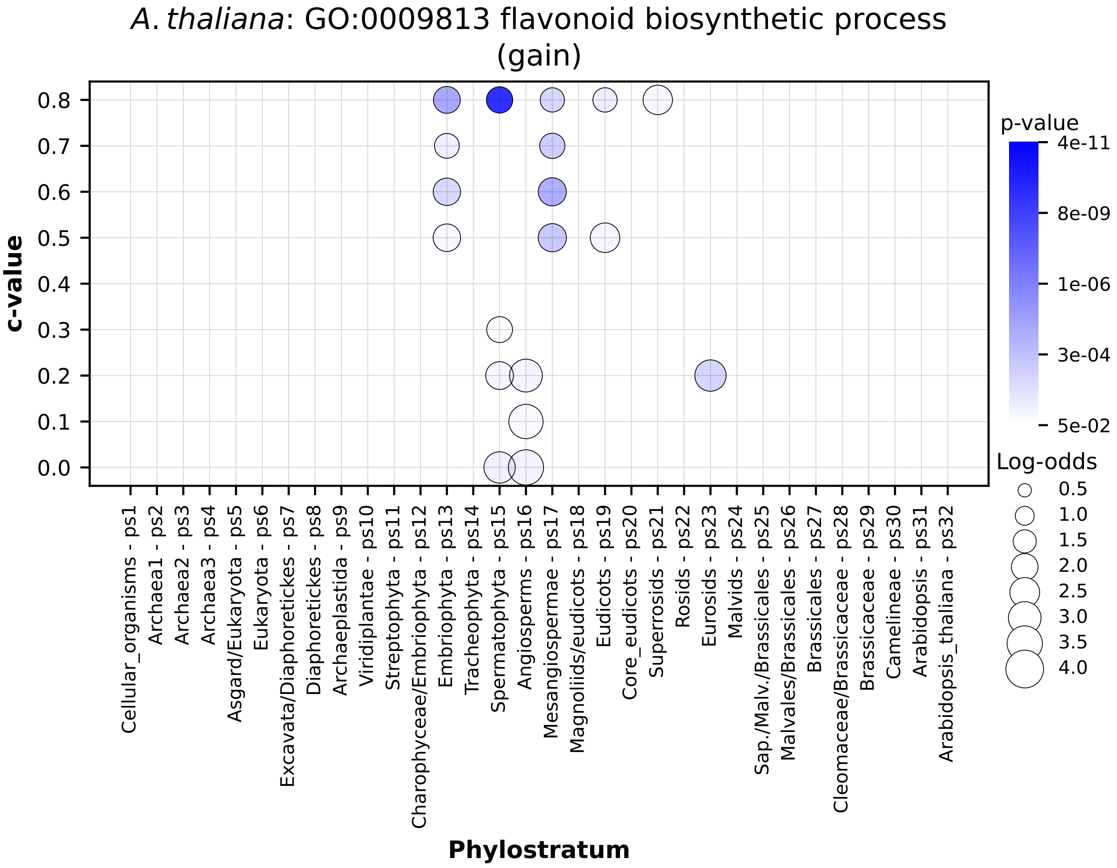

A. thaliana: GO:0009813 flavonoid biosynthetic process (gain)
c-value
0.8
0.7
0.6
0.5
0.4
0.3
0.2
0.1
0.0
p-value
4e-11
8e-09
1e-06
3e-04
5e-02
Log-odds
0.5
1.0
1.5
2.0
2.5
3.0
3.5
4.0
Cellular_organisms - ps1
Archaea1 - ps2
Archaea2 - ps3
Archaea3 - ps4
Asgard/Eukaryota - ps5
Eukaryota - ps6
Excavata/Diaphoretickes - ps7
Diaphoretickes - ps8
Archaeplastida - ps9
Viridiplantae - ps10
Streptophyta - ps11
Charophyceae/Embriophyta - ps12
Embriophyta - ps13
Tracheophyta - ps14
Spermatophyta - ps15
Angiosperms - ps16
Mesangiospermae - ps17
Magnoliids/eudicots - ps18
Eudicots - ps19
Core_eudicots - ps20
Superrosids - ps21
Rosids - ps22
Eurosids - ps23
Malvids - ps24
Sap./Malv./Brassicales - ps25
Malvales/Brassicales - ps26
Brassicales - ps27
Cleomaceae/Brassicaceae - ps28
Brassicaceae - ps29
Camelineae - ps30
Arabidopsis - ps31
Arabidopsis_thaliana - ps32
Phylostratum

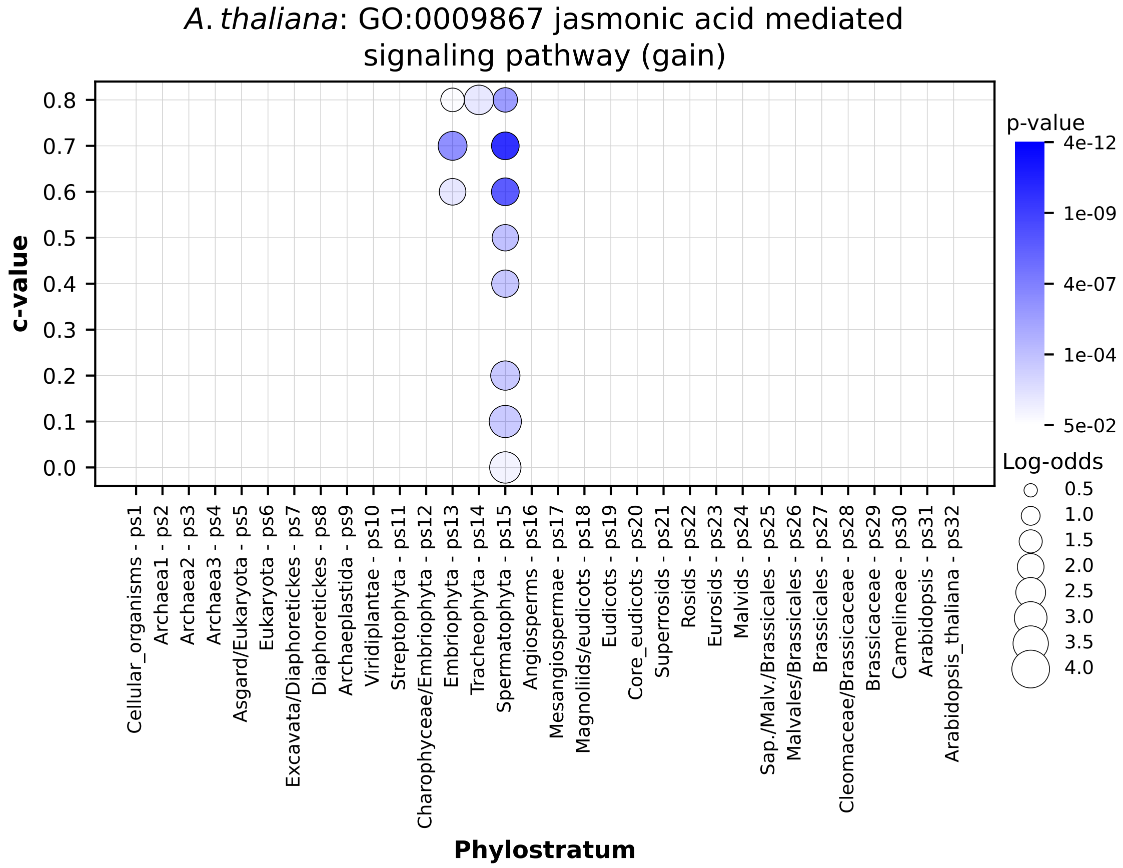
A. thaliana: GO:0009867 jasmonic acid mediated signaling pathway (gain)
c-value
0.8
0.7
0.6
0.5
0.4
0.3
0.2
0.1
0.0
p-value
4e-12
1e-09
4e-07
1e-04
5e-02
Log-odds
0.5
1.0
1.5
2.0
2.5
3.0
3.5
4.0
Cellular_organisms - ps1
Archaea1 - ps2
Archaea2 - ps3
Archaea3 - ps4
Asgard/Eukaryota - ps5
Eukaryota - ps6
Excavata/Diaphoretickes - ps7
Diaphoretickes - ps8
Archaeplastida - ps9
Viridiplantae - ps10
Streptophyta - ps11
Charophyceae/Embriophyta - ps12
Embriophyta - ps13
Tracheophyta - ps14
Spermatophyta - ps15
Angiosperms - ps16
Mesangiospermae - ps17
Magnoliids/eudicots - ps18
Eudicots - ps19
Core_eudicots - ps20
Superrosids - ps21
Rosids - ps22
Eurosids - ps23
Malvids - ps24
Sap./Malv./Brassicales - ps25
Malvales/Brassicales - ps26
Brassicales - ps27
Cleomaceae/Brassicaceae - ps28
Brassicaceae - ps29
Camelineae - ps30
Arabidopsis - ps31
Arabidopsis_thaliana - ps32
Phylostratum

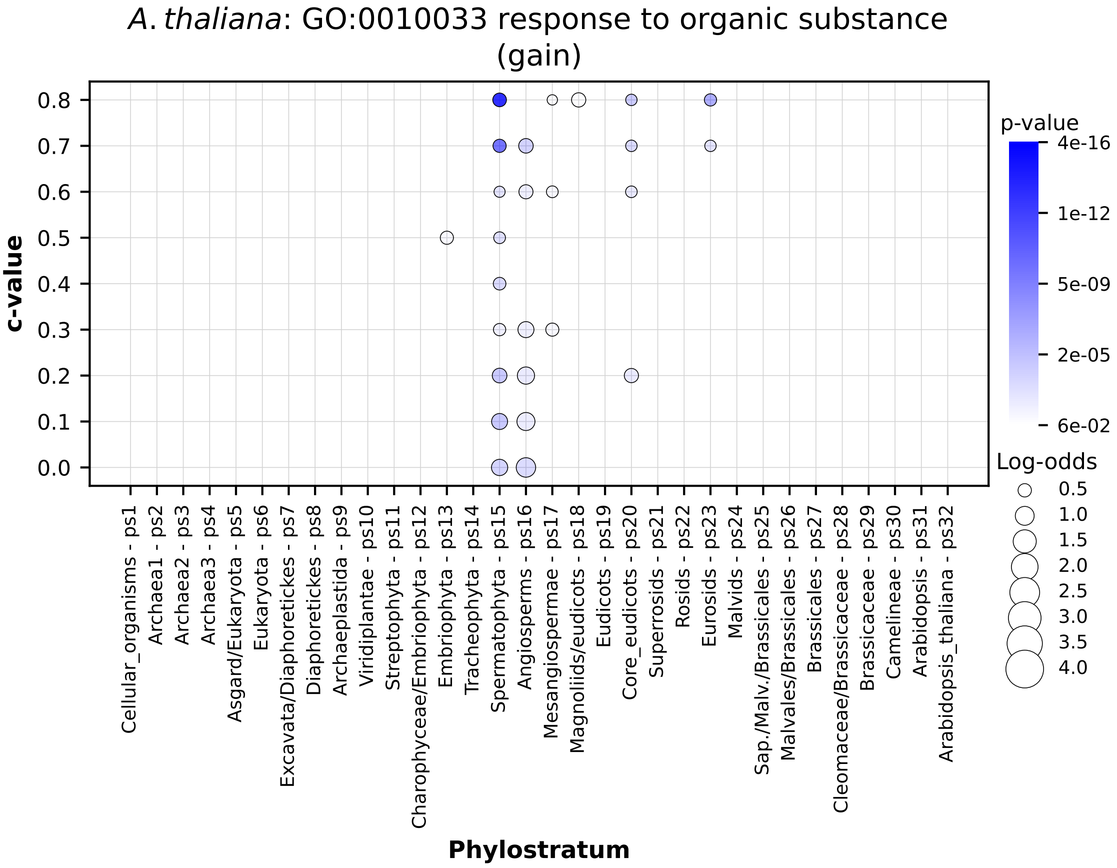
A. thaliana: GO:0010033 response to organic substance (gain)
c-value
0.8
0.7
0.6
0.5
0.4
0.3
0.2
0.1
0.0
p-value
4e-16
1e-12
5e-09
2e-05
6e-02
Log-odds
0.5
1.0
1.5
2.0
2.5
3.0
3.5
4.0
Cellular_organisms - ps1
Archaea1 - ps2
Archaea2 - ps3
Archaea3 - ps4
Asgard/Eukaryota - ps5
Eukaryota - ps6
Excavata/Diaphoretickes - ps7
Diaphoretickes - ps8
Archaeplastida - ps9
Viridiplantae - ps10
Streptophyta - ps11
Charophyceae/Embriophyta - ps12
Embriophyta - ps13
Tracheophyta - ps14
Spermatophyta - ps15
Angiosperms - ps16
Mesangiospermae - ps17
Magnoliids/eudicots - ps18
Eudicots - ps19
Core_eudicots - ps20
Superrosids - ps21
Rosids - ps22
Eurosids - ps23
Malvids - ps24
Sap./Malv./Brassicales - ps25
Malvales/Brassicales - ps26
Brassicales - ps27
Cleomaceae/Brassicaceae - ps28
Brassicaceae - ps29
Camelineae - ps30
Arabidopsis - ps31
Arabidopsis_thaliana - ps32
Phylostratum

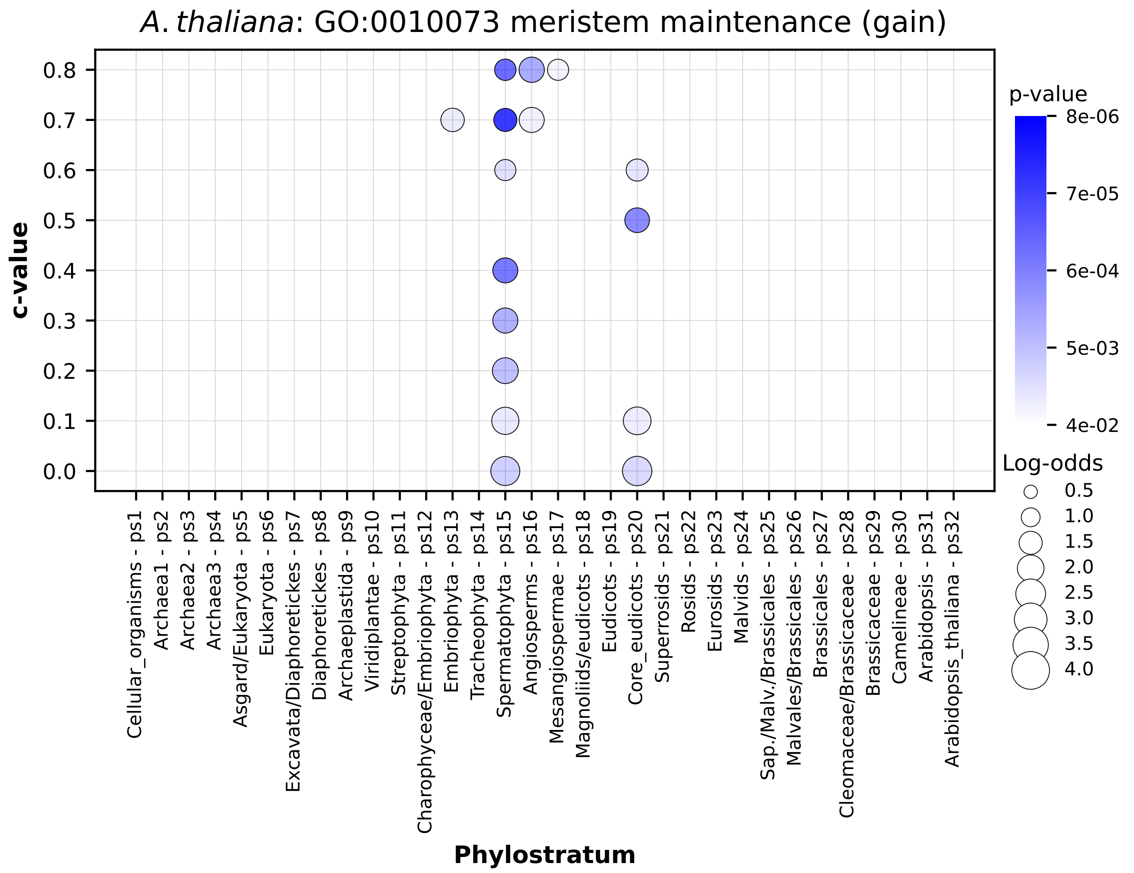

A. thaliana: GO:0010073 meristem maintenance (gain)
c-value
0.8
0.7
0.6
0.5
0.4
0.3
0.2
0.1
0.0
p-value
8e-06
7e-05
6e-04
5e-03
4e-02
Log-odds
0.5
1.0
1.5
2.0
2.5
3.0
3.5
4.0
Cellular_organisms - ps1
Archaea1 - ps2
Archaea2 - ps3
Archaea3 - ps4
Asgard/Eukaryota - ps5
Eukaryota - ps6
Excavata/Diaphoretickes - ps7
Diaphoretickes - ps8
Archaeplastida - ps9
Viridiplantae - ps10
Streptophyta - ps11
Charophyceae/Embriophyta - ps12
Embriophyta - ps13
Tracheophyta - ps14
Spermatophyta - ps15
Angiosperms - ps16
Mesangiospermae - ps17
Magnoliids/eudicots - ps18
Eudicots - ps19
Core_eudicots - ps20
Superrosids - ps21
Rosids - ps22
Eurosids - ps23
Malvids - ps24
Sap./Malv./Brassicales - ps25
Malvales/Brassicales - ps26
Brassicales - ps27
Cleomaceae/Brassicaceae - ps28
Brassicaceae - ps29
Camelineae - ps30
Arabidopsis - ps31
Arabidopsis_thaliana - ps32
Phylostratum

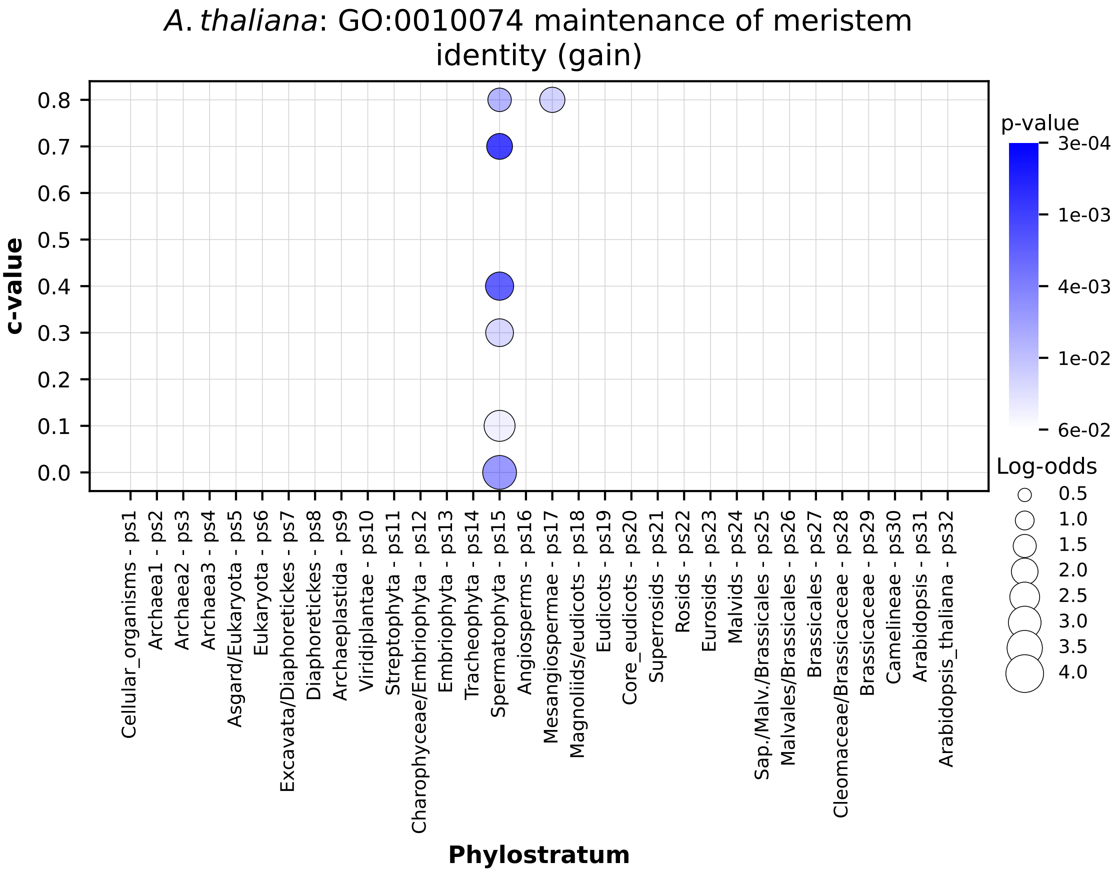

A. thaliana: GO:0010074 maintenance of meristem identity (gain)
c-value
0.8
0.7
0.6
0.5
0.4
0.3
0.2
0.1
0.0
p-value
3e-04
1e-03
4e-03
1e-02
6e-02
Log-odds
0.5
1.0
1.5
2.0
2.5
3.0
3.5
4.0
Cellular_organisms - ps1
Archaea1 - ps2
Archaea2 - ps3
Archaea3 - ps4
Asgard/Eukaryota - ps5
Eukaryota - ps6
Excavata/Diaphoretickes - ps7
Diaphoretickes - ps8
Archaeplastida - ps9
Viridiplantae - ps10
Streptophyta - ps11
Charophyceae/Embriophyta - ps12
Embriophyta - ps13
Tracheophyta - ps14
Spermatophyta - ps15
Angiosperms - ps16
Mesangiospermae - ps17
Magnoliids/eudicots - ps18
Eudicots - ps19
Core_eudicots - ps20
Superrosids - ps21
Rosids - ps22
Eurosids - ps23
Malvids - ps24
Sap./Malv./Brassicales - ps25
Malvales/Brassicales - ps26
Brassicales - ps27
Cleomaceae/Brassicaceae - ps28
Brassicaceae - ps29
Camelineae - ps30
Arabidopsis - ps31
Arabidopsis_thaliana - ps32
Phylostratum

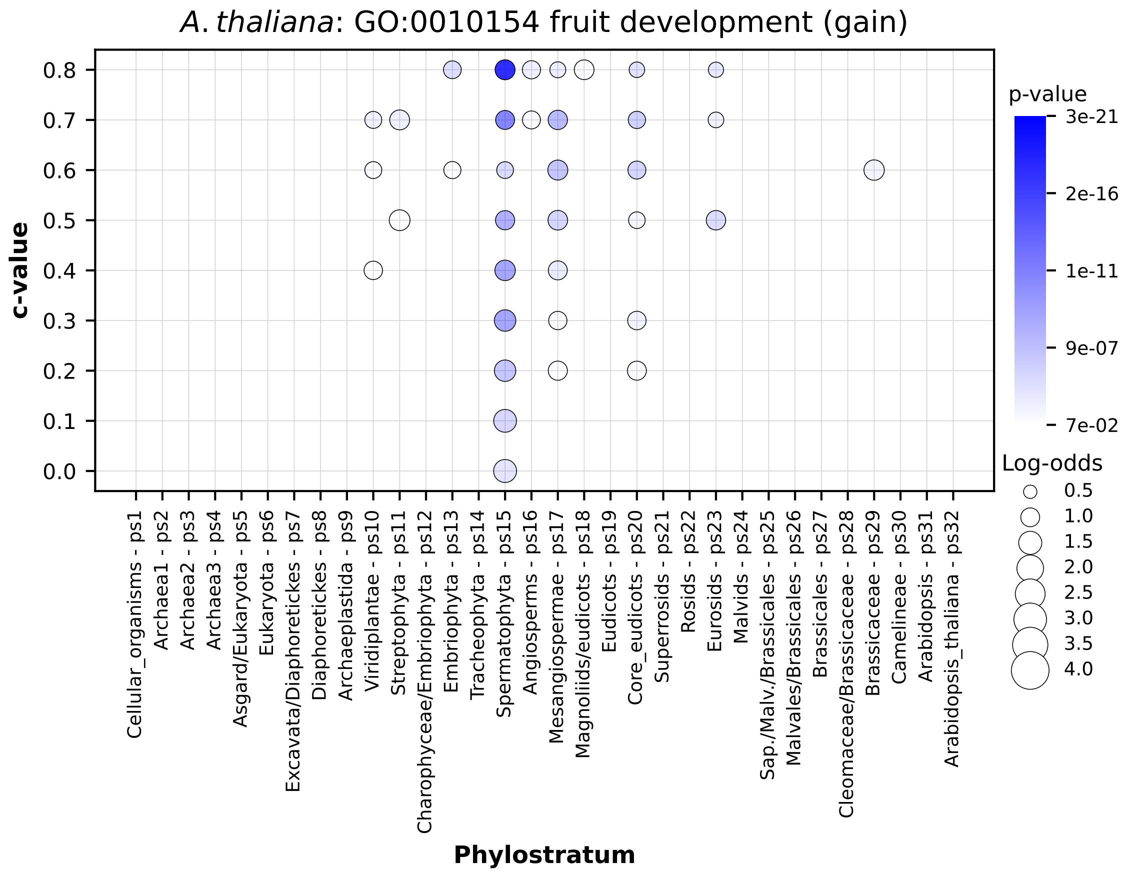

A. thaliana: GO:0010154 fruit development (gain)
c-value
0.8
0.7
0.6
0.5
0.4
0.3
0.2
0.1
0.0
p-value
3e-21
2e-16
1e-11
9e-07
7e-02
Log-odds
0.5
1.0
1.5
2.0
2.5
3.0
3.5
4.0
Cellular_organisms - ps1
Archaea1 - ps2
Archaea2 - ps3
Archaea3 - ps4
Asgard/Eukaryota - ps5
Eukaryota - ps6
Excavata/Diaphoretickes - ps7
Diaphoretickes - ps8
Archaeplastida - ps9
Viridiplantae - ps10
Streptophyta - ps11
Charophyceae/Embriophyta - ps12
Embriophyta - ps13
Tracheophyta - ps14
Spermatophyta - ps15
Angiosperms - ps16
Mesangiospermae - ps17
Magnoliids/eudicots - ps18
Eudicots - ps19
Core_eudicots - ps20
Superrosids - ps21
Rosids - ps22
Eurosids - ps23
Malvids - ps24
Sap./Malv./Brassicales - ps25
Malvales/Brassicales - ps26
Brassicales - ps27
Cleomaceae/Brassicaceae - ps28
Brassicaceae - ps29
Camelineae - ps30
Arabidopsis - ps31
Arabidopsis_thaliana - ps32
Phylostratum

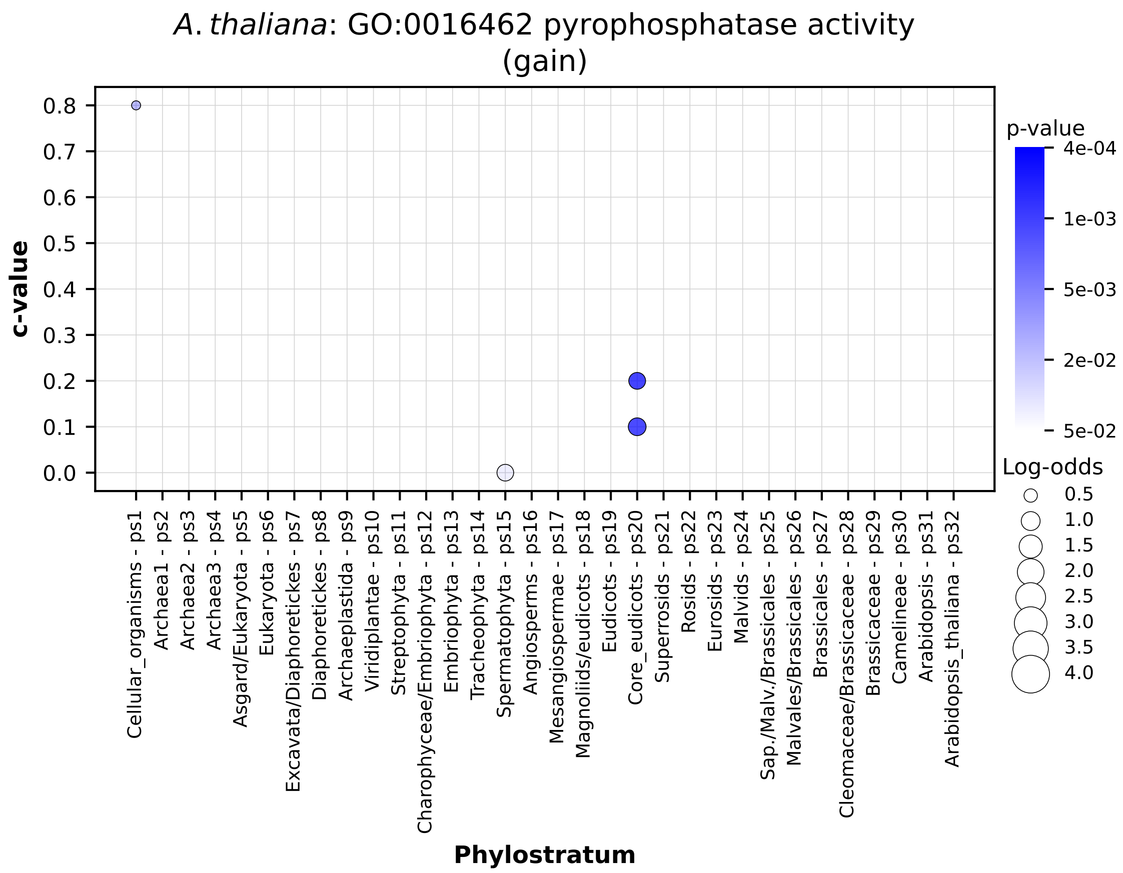

A. thaliana: GO:0016462 pyrophosphatase activity (gain)
c-value
0.0
0.1
0.2
0.3
0.4
0.5
0.6
0.7
0.8
Phylostratum
Cellular_organisms - ps1
Archaea1 - ps2
Archaea2 - ps3
Archaea3 - ps4
Asgard/Eukaryota - ps5
Eukaryota - ps6
Excavata/Diaphoretickes - ps7
Diaphoretickes - ps8
Archaeplastida - ps9
Viridiplantae - ps10
Streptophyta - ps11
Charophyceae/Embriophyta - ps12
Embriophyta - ps13
Tracheophyta - ps14
Spermatophyta - ps15
Angiosperms - ps16
Mesangiospermae - ps17
Magnoliids/eudicots - ps18
Eudicots - ps19
Core_eudicots - ps20
Superrosids - ps21
Rosids - ps22
Eurosids - ps23
Malvids - ps24
Sap./Malv./Brassicales - ps25
Malvales/Brassicales - ps26
Brassicales - ps27
Cleomaceae/Brassicaceae - ps28
Brassicaceae - ps29
Camelineae - ps30
Arabidopsis - ps31
Arabidopsis_thaliana - ps32
p-value
4e-04
1e-03
5e-03
2e-02
5e-02
Log-odds
0.5
1.0
1.5
2.0
2.5
3.0
3.5
4.0

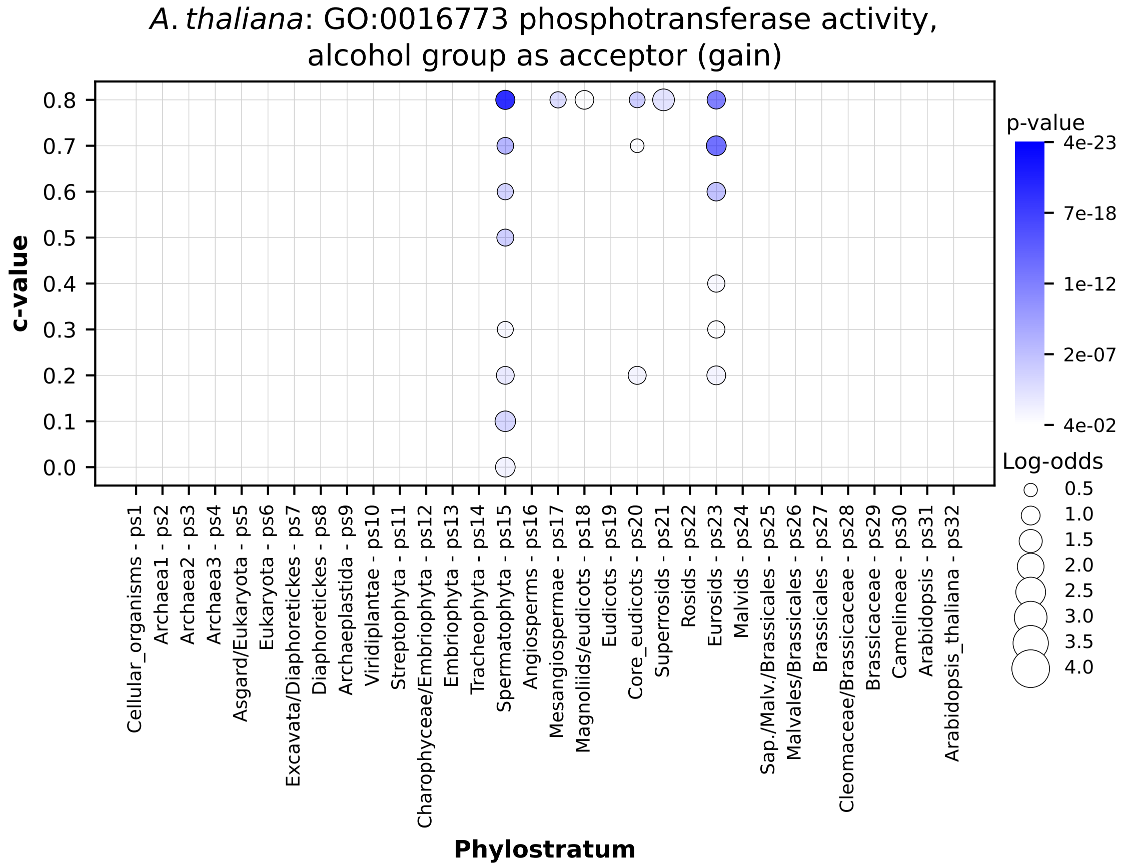
A. thaliana: GO:0016773 phosphotransferase activity, alcohol group as acceptor (gain)
c-value
0.8
0.7
0.6
0.5
0.4
0.3
0.2
0.1
0.0
p-value
4e-23
7e-18
1e-12
2e-07
4e-02
Log-odds
0.5
1.0
1.5
2.0
2.5
3.0
3.5
4.0
Cellular_organisms - ps1
Archaea1 - ps2
Archaea2 - ps3
Archaea3 - ps4
Asgard/Eukaryota - ps5
Eukaryota - ps6
Excavata/Diaphoretickes - ps7
Diaphoretickes - ps8
Archaeplastida - ps9
Viridiplantae - ps10
Streptophyta - ps11
Charophyceae/Embriophyta - ps12
Embriophyta - ps13
Tracheophyta - ps14
Spermatophyta - ps15
Angiosperms - ps16
Mesangiospermae - ps17
Magnoliids/eudicots - ps18
Eudicots - ps19
Core_eudicots - ps20
Superrosids - ps21
Rosids - ps22
Eurosids - ps23
Malvids - ps24
Sap./Malv./Brassicales - ps25
Malvales/Brassicales - ps26
Brassicales - ps27
Cleomaceae/Brassicaceae - ps28
Brassicaceae - ps29
Camelineae - ps30
Arabidopsis - ps31
Arabidopsis_thaliana - ps32
Phylostratum

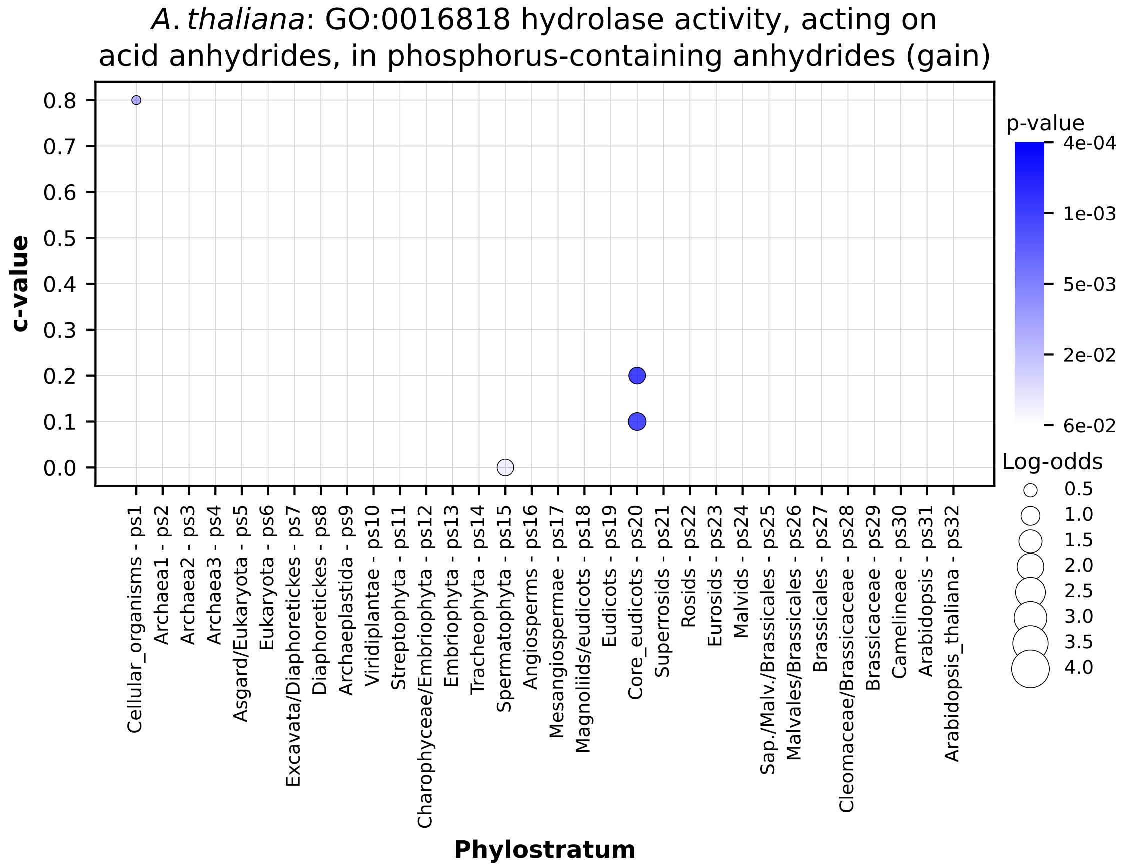

A. thaliana: GO:0016818 hydrolase activity, acting on acid anhydrides, in phosphorus-containing anhydrides (gain)
c-value
0.8
0.7
0.6
0.5
0.4
0.3
0.2
0.1
0.0
p-value
4e-04
1e-03
5e-03
2e-02
6e-02
Log-odds
0.5
1.0
1.5
2.0
2.5
3.0
3.5
4.0
Cellular_organisms - ps1
Archaea1 - ps2
Archaea2 - ps3
Archaea3 - ps4
Asgard/Eukaryota - ps5
Eukaryota - ps6
Excavata/Diaphoretickes - ps7
Diaphoretickes - ps8
Archaeplastida - ps9
Viridiplantae - ps10
Streptophyta - ps11
Charophyceae/Embriophyta - ps12
Embriophyta - ps13
Tracheophyta - ps14
Spermatophyta - ps15
Angiosperms - ps16
Mesangiospermae - ps17
Magnoliids/eudicots - ps18
Eudicots - ps19
Core_eudicots - ps20
Superrosids - ps21
Rosids - ps22
Eurosids - ps23
Malvids - ps24
Sap./Malv./Brassicales - ps25
Malvales/Brassicales - ps26
Brassicales - ps27
Cleomaceae/Brassicaceae - ps28
Brassicaceae - ps29
Camelineae - ps30
Arabidopsis - ps31
Arabidopsis_thaliana - ps32
Phylostratum

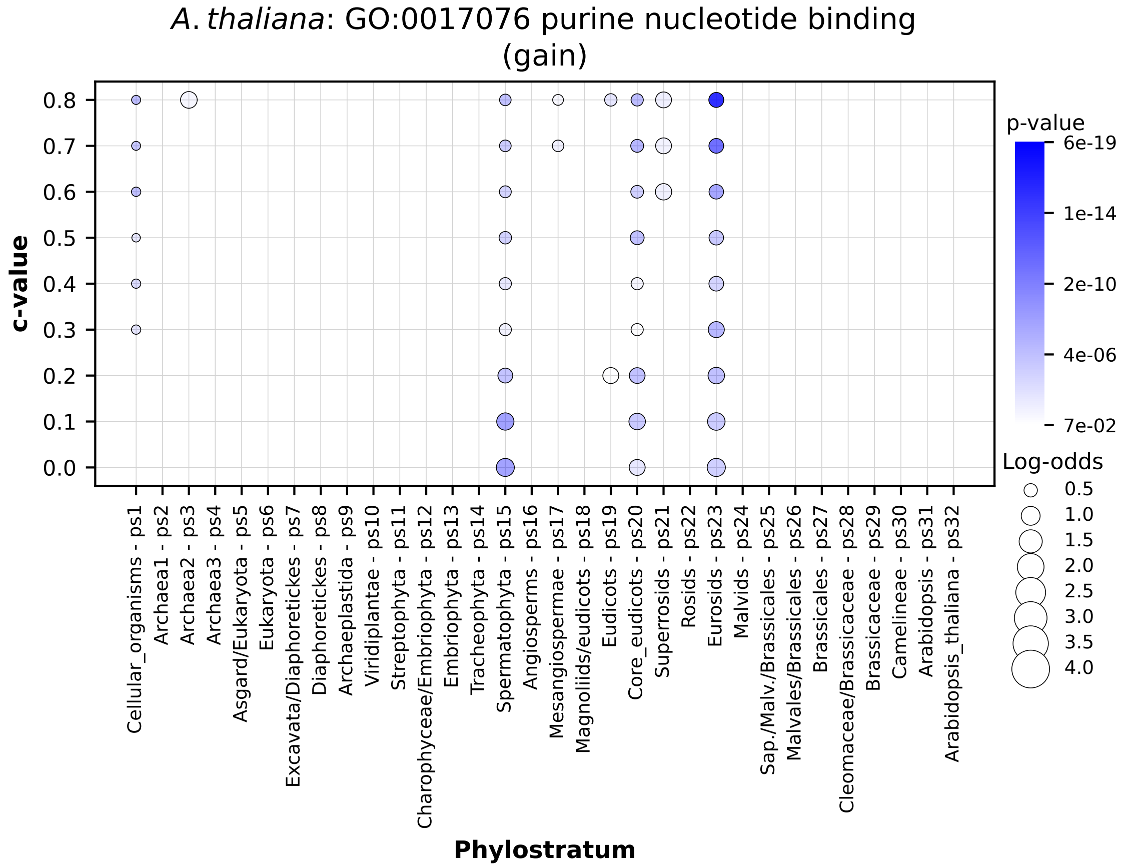
A. thaliana: GO:0017076 purine nucleotide binding
(gain)
c-value
0.8
0.7
0.6
0.5
0.4
0.3
0.2
0.1
0.0
p-value
6e-19
1e-14
2e-10
4e-06
7e-02
Log-odds
0.5
1.0
1.5
2.0
2.5
3.0
3.5
4.0
Cellular_organisms - ps1
Archaea1 - ps2
Archaea2 - ps3
Archaea3 - ps4
Asgard/Eukaryota - ps5
Eukaryota - ps6
Excavata/Diaphoretickes - ps7
Diaphoretickes - ps8
Archaeplastida - ps9
Viridiplantae - ps10
Streptophyta - ps11
Charophyceae/Embriophyta - ps12
Embriophyta - ps13
Tracheophyta - ps14
Spermatophyta - ps15
Angiosperms - ps16
Mesangiospermae - ps17
Magnoliids/eudicots - ps18
Eudicots - ps19
Core_eudicots - ps20
Superrosids - ps21
Rosids - ps22
Eurosids - ps23
Malvids - ps24
Sap./Malv./Brassicales - ps25
Malvales/Brassicales - ps26
Brassicales - ps27
Cleomaceae/Brassicaceae - ps28
Brassicaceae - ps29
Camelineae - ps30
Arabidopsis - ps31
Arabidopsis_thaliana - ps32
Phylostratum

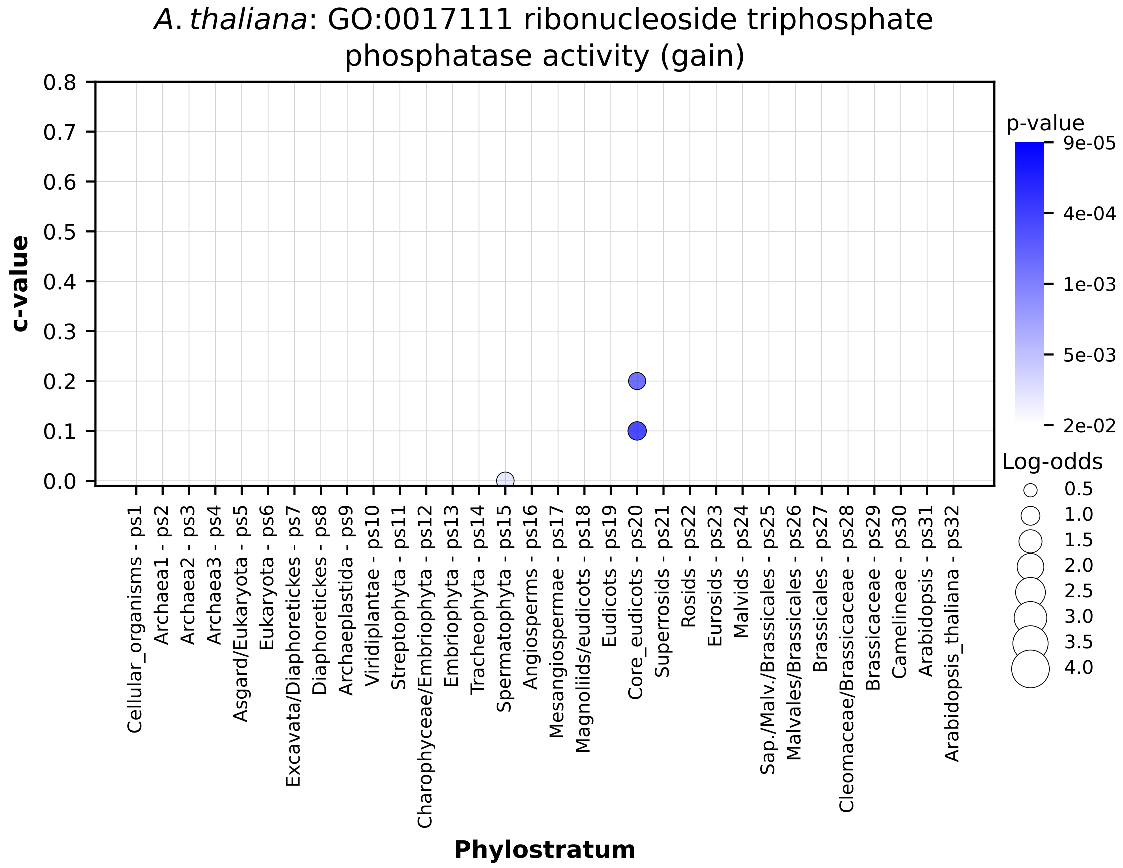

A. thaliana: GO:0017111 ribonucleoside triphosphate phosphatase activity (gain)
c-value
0.0
0.1
0.2
0.3
0.4
0.5
0.6
0.7
0.8
Phylostratum
Cellular_organisms - ps1
Archaea1 - ps2
Archaea2 - ps3
Archaea3 - ps4
Asgard/Eukaryota - ps5
Eukaryota - ps6
Excavata/Diaphoretickes - ps7
Diaphoretickes - ps8
Archaeplastida - ps9
Viridiplantae - ps10
Streptophyta - ps11
Charophyceae/Embriophyta - ps12
Embriophyta - ps13
Tracheophyta - ps14
Spermatophyta - ps15
Angiosperms - ps16
Mesangiospermae - ps17
Magnoliids/eudicots - ps18
Eudicots - ps19
Core_eudicots - ps20
Superrosids - ps21
Rosids - ps22
Eurosids - ps23
Malvids - ps24
Sap./Malv./Brassicales - ps25
Malvales/Brassicales - ps26
Brassicales - ps27
Cleomaceae/Brassicaceae - ps28
Brassicaceae - ps29
Camelineae - ps30
Arabidopsis - ps31
Arabidopsis_thaliana - ps32
p-value
9e-05
4e-04
1e-03
5e-03
2e-02
Log-odds
0.5
1.0
1.5
2.0
2.5
3.0
3.5
4.0

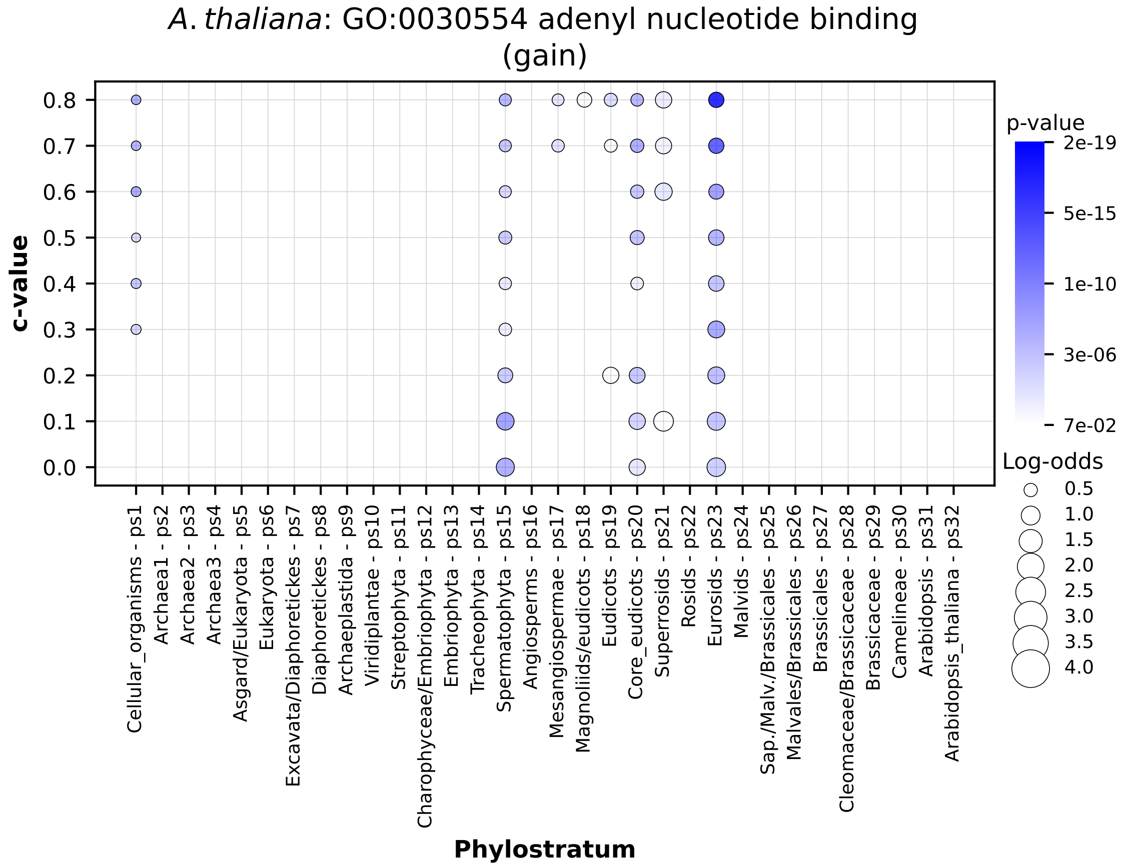

A. thaliana: GO:0030554 adenyl nucleotide binding
(gain)
c-value
0.8
0.7
0.6
0.5
0.4
0.3
0.2
0.1
0.0
Phylostratum
Cellular_organisms - ps1
Archaea1 - ps2
Archaea2 - ps3
Archaea3 - ps4
Asgard/Eukaryota - ps5
Eukaryota - ps6
Excavata/Diaphoretickes - ps7
Diaphoretickes - ps8
Archaeplastida - ps9
Viridiplantae - ps10
Streptophyta - ps11
Charophyceae/Embriophyta - ps12
Embriophyta - ps13
Tracheophyta - ps14
Spermatophyta - ps15
Angiosperms - ps16
Mesangiospermae - ps17
Magnoliids/eudicots - ps18
Eudicots - ps19
Core_eudicots - ps20
Superrosids - ps21
Rosids - ps22
Eurosids - ps23
Malvids - ps24
Sap./Malv./Brassicales - ps25
Malvales/Brassicales - ps26
Brassicales - ps27
Cleomaceae/Brassicaceae - ps28
Brassicaceae - ps29
Camelineae - ps30
Arabidopsis - ps31
Arabidopsis_thaliana - ps32
p-value
2e-19
5e-15
1e-10
3e-06
7e-02
Log-odds
0.5
1.0
1.5
2.0
2.5
3.0
3.5
4.0

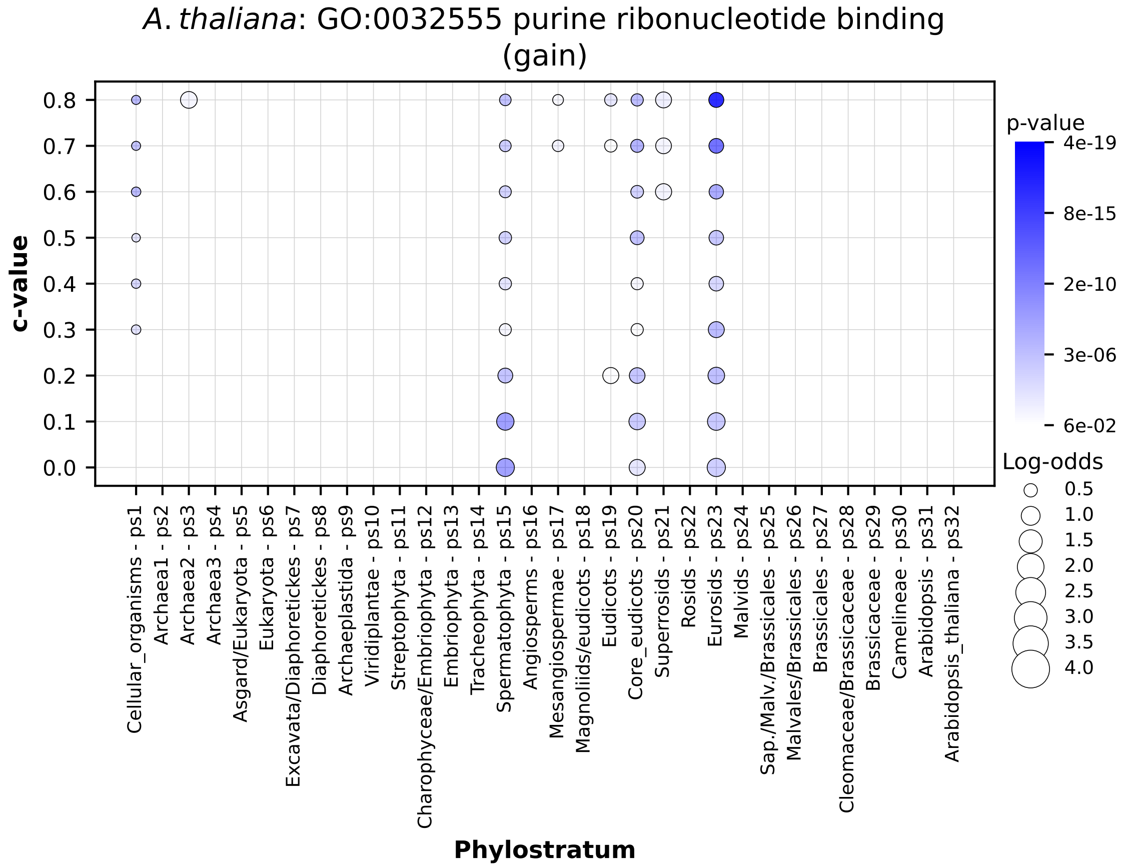
A. thaliana: GO:0032555 purine ribonucleotide binding (gain)
c-value
0.8
0.7
0.6
0.5
0.4
0.3
0.2
0.1
0.0
Phylostratum
Cellular_organisms - ps1
Archaea1 - ps2
Archaea2 - ps3
Archaea3 - ps4
Asgard/Eukaryota - ps5
Eukaryota - ps6
Excavata/Diaphoretickes - ps7
Diaphoretickes - ps8
Archaeplastida - ps9
Viridiplantae - ps10
Streptophyta - ps11
Charophyceae/Embriophyta - ps12
Embriophyta - ps13
Tracheophyta - ps14
Spermatophyta - ps15
Angiosperms - ps16
Mesangiospermae - ps17
Magnoliids/eudicots - ps18
Eudicots - ps19
Core_eudicots - ps20
Superrosids - ps21
Rosids - ps22
Eurosids - ps23
Malvids - ps24
Sap./Malv./Brassicales - ps25
Malvales/Brassicales - ps26
Brassicales - ps27
Cleomaceae/Brassicaceae - ps28
Brassicaceae - ps29
Camelineae - ps30
Arabidopsis - ps31
Arabidopsis_thaliana - ps32
p-value
4e-19
8e-15
2e-10
3e-06
6e-02
Log-odds
0.5
1.0
1.5
2.0
2.5
3.0
3.5
4.0

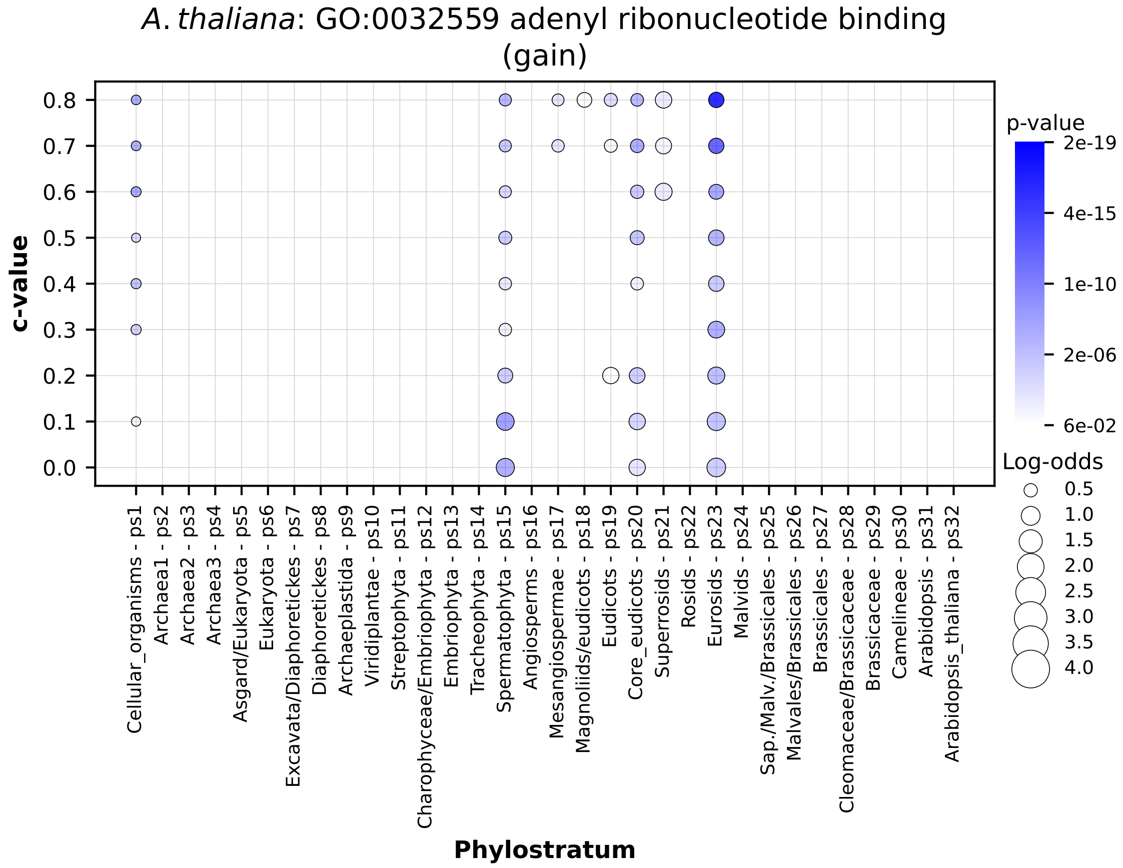

A. thaliana: GO:0032559 adenyl ribonucleotide binding (gain)
c-value
0.8
0.7
0.6
0.5
0.4
0.3
0.2
0.1
0.0
p-value
2e-19
4e-15
1e-10
2e-06
6e-02
Log-odds
0.5
1.0
1.5
2.0
2.5
3.0
3.5
4.0
Cellular_organisms - ps1
Archaea1 - ps2
Archaea2 - ps3
Archaea3 - ps4
Asgard/Eukaryota - ps5
Eukaryota - ps6
Excavata/Diaphoretickes - ps7
Diaphoretickes - ps8
Archaeplastida - ps9
Viridiplantae - ps10
Streptophyta - ps11
Charophyceae/Embriophyta - ps12
Embriophyta - ps13
Tracheophyta - ps14
Spermatophyta - ps15
Angiosperms - ps16
Mesangiospermae - ps17
Magnoliids/eudicots - ps18
Eudicots - ps19
Core_eudicots - ps20
Superrosids - ps21
Rosids - ps22
Eurosids - ps23
Malvids - ps24
Sap./Malv./Brassicales - ps25
Malvales/Brassicales - ps26
Brassicales - ps27
Cleomaceae/Brassicaceae - ps28
Brassicaceae - ps29
Camelineae - ps30
Arabidopsis - ps31
Arabidopsis_thaliana - ps32
Phylostratum

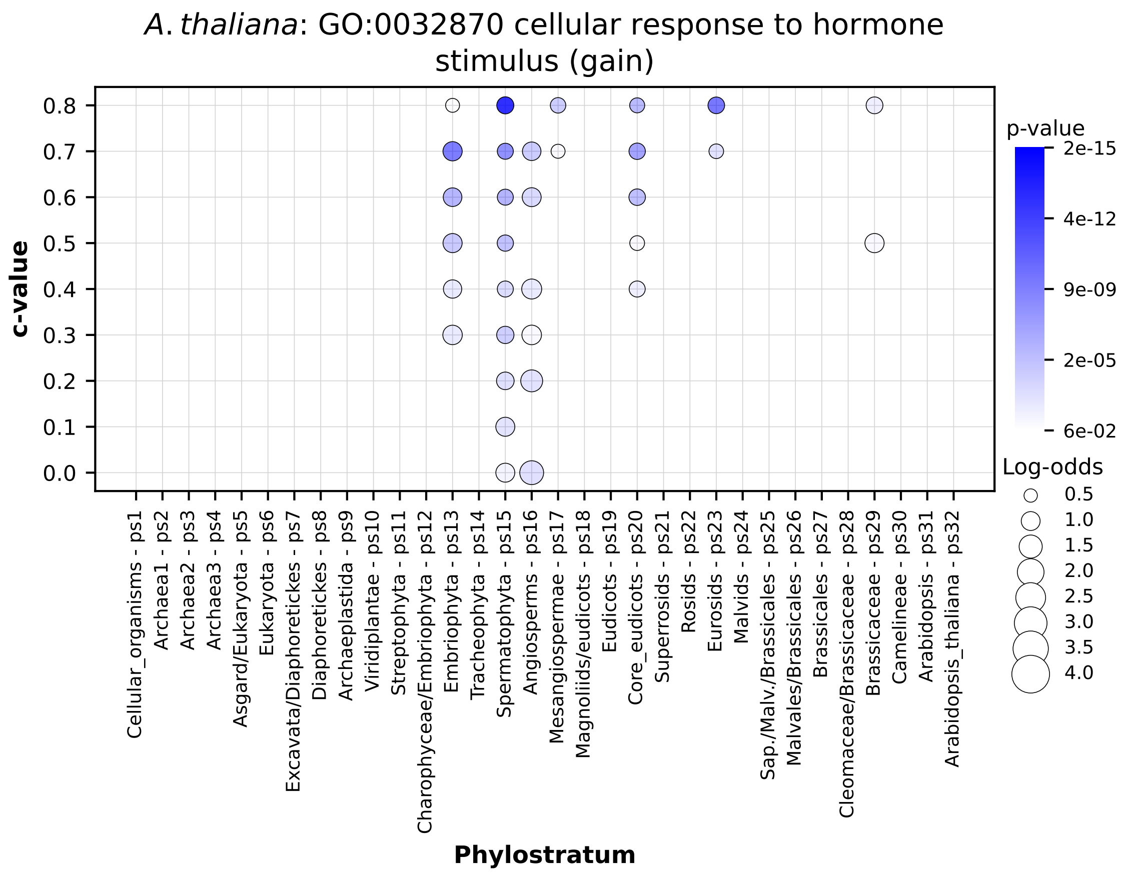

A. thaliana: GO:0032870 cellular response to hormone stimulus (gain)
c-value
0.8
0.7
0.6
0.5
0.4
0.3
0.2
0.1
0.0
p-value
2e-15
4e-12
9e-09
2e-05
6e-02
Log-odds
0.5
1.0
1.5
2.0
2.5
3.0
3.5
4.0
Cellular_organisms - ps1
Archaea1 - ps2
Archaea2 - ps3
Archaea3 - ps4
Asgard/Eukaryota - ps5
Eukaryota - ps6
Excavata/Diaphoretickes - ps7
Diaphoretickes - ps8
Archaeplastida - ps9
Viridiplantae - ps10
Streptophyta - ps11
Charophyceae/Embriophyta - ps12
Embriophyta - ps13
Tracheophyta - ps14
Spermatophyta - ps15
Angiosperms - ps16
Mesangiospermae - ps17
Magnoliids/eudicots - ps18
Eudicots - ps19
Core_eudicots - ps20
Superrosids - ps21
Rosids - ps22
Eurosids - ps23
Malvids - ps24
Sap./Malv./Brassicales - ps25
Malvales/Brassicales - ps26
Brassicales - ps27
Cleomaceae/Brassicaceae - ps28
Brassicaceae - ps29
Camelineae - ps30
Arabidopsis - ps31
Arabidopsis_thaliana - ps32
Phylostratum

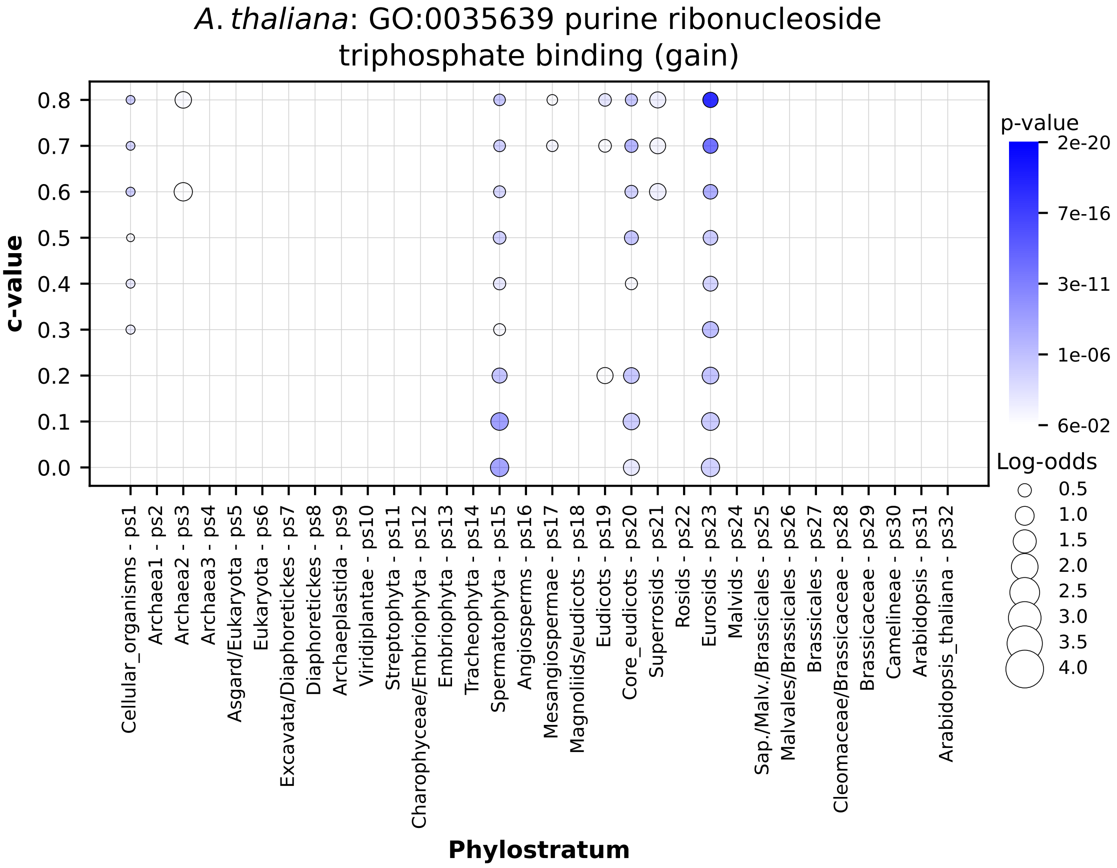

A. thaliana: GO:0035639 purine ribonucleoside triphosphate binding (gain)
c-value
0.8
0.7
0.6
0.5
0.4
0.3
0.2
0.1
0.0
Phylostratum
Cellular_organisms - ps1
Archaea1 - ps2
Archaea2 - ps3
Archaea3 - ps4
Asgard/Eukaryota - ps5
Eukaryota - ps6
Excavata/Diaphoretickes - ps7
Diaphoretickes - ps8
Archaeplastida - ps9
Viridiplantae - ps10
Streptophyta - ps11
Charophyceae/Embriophyta - ps12
Embriophyta - ps13
Tracheophyta - ps14
Spermatophyta - ps15
Angiosperms - ps16
Mesangiospermae - ps17
Magnoliids/eudicots - ps18
Eudicots - ps19
Core_eudicots - ps20
Superrosids - ps21
Rosids - ps22
Eurosids - ps23
Malvids - ps24
Sap./Malv./Brassicales - ps25
Malvales/Brassicales - ps26
Brassicales - ps27
Cleomaceae/Brassicaceae - ps28
Brassicaceae - ps29
Camelineae - ps30
Arabidopsis - ps31
Arabidopsis_thaliana - ps32
p-value
2e-20
7e-16
3e-11
1e-06
6e-02
Log-odds
0.5
1.0
1.5
2.0
2.5
3.0
3.5
4.0

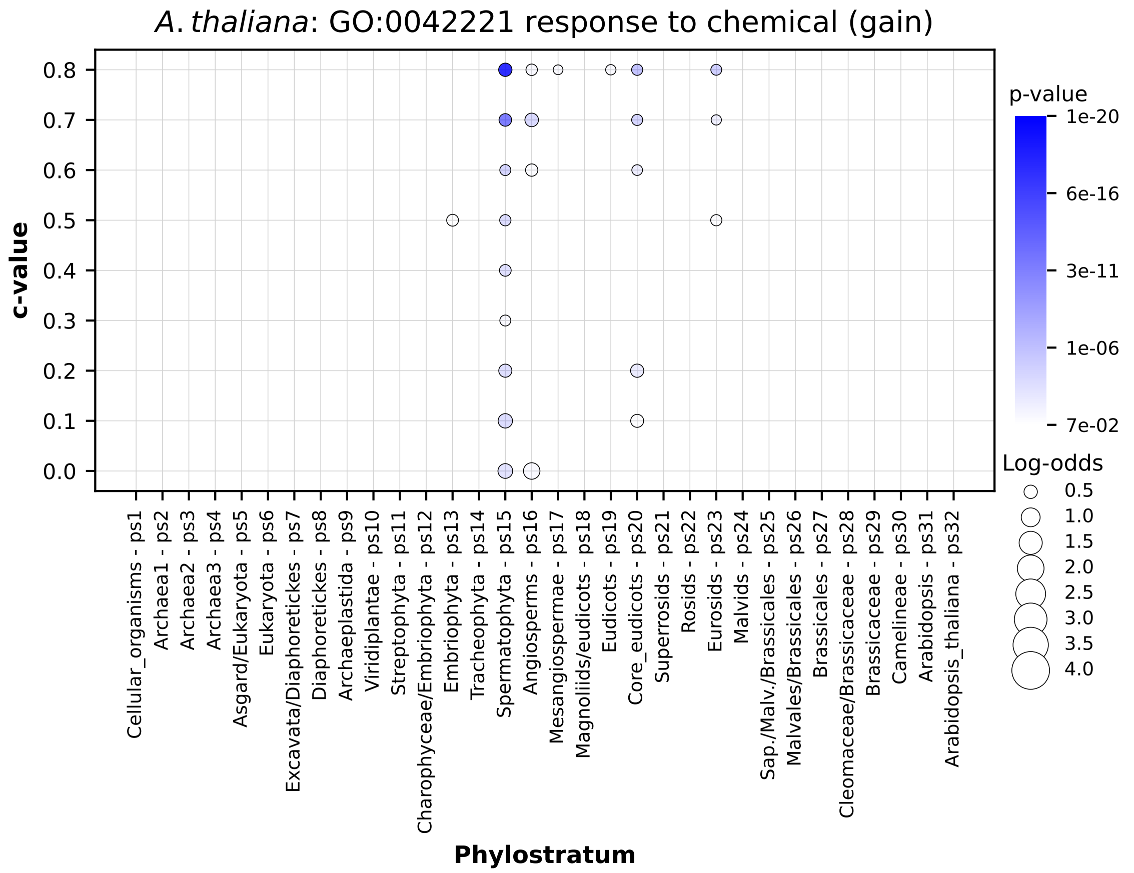
A. thaliana: GO:0042221 response to chemical (gain)
c-value
0.8
0.7
0.6
0.5
0.4
0.3
0.2
0.1
0.0
Phylostratum
Cellular_organisms - ps1
Archaea1 - ps2
Archaea2 - ps3
Archaea3 - ps4
Asgard/Eukaryota - ps5
Eukaryota - ps6
Excavata/Diaphoretickes - ps7
Diaphoretickes - ps8
Archaeplastida - ps9
Viridiplantae - ps10
Streptophyta - ps11
Charophyceae/Embriophyta - ps12
Embriophyta - ps13
Tracheophyta - ps14
Spermatophyta - ps15
Angiosperms - ps16
Mesangiospermae - ps17
Magnoliids/eudicots - ps18
Eudicots - ps19
Core_eudicots - ps20
Superrosids - ps21
Rosids - ps22
Eurosids - ps23
Malvids - ps24
Sap./Malv./Brassicales - ps25
Malvales/Brassicales - ps26
Brassicales - ps27
Cleomaceae/Brassicaceae - ps28
Brassicaceae - ps29
Camelineae - ps30
Arabidopsis - ps31
Arabidopsis_thaliana - ps32
p-value
1e-20
6e-16
3e-11
1e-06
7e-02
Log-odds
0.5
1.0
1.5
2.0
2.5
3.0
3.5
4.0

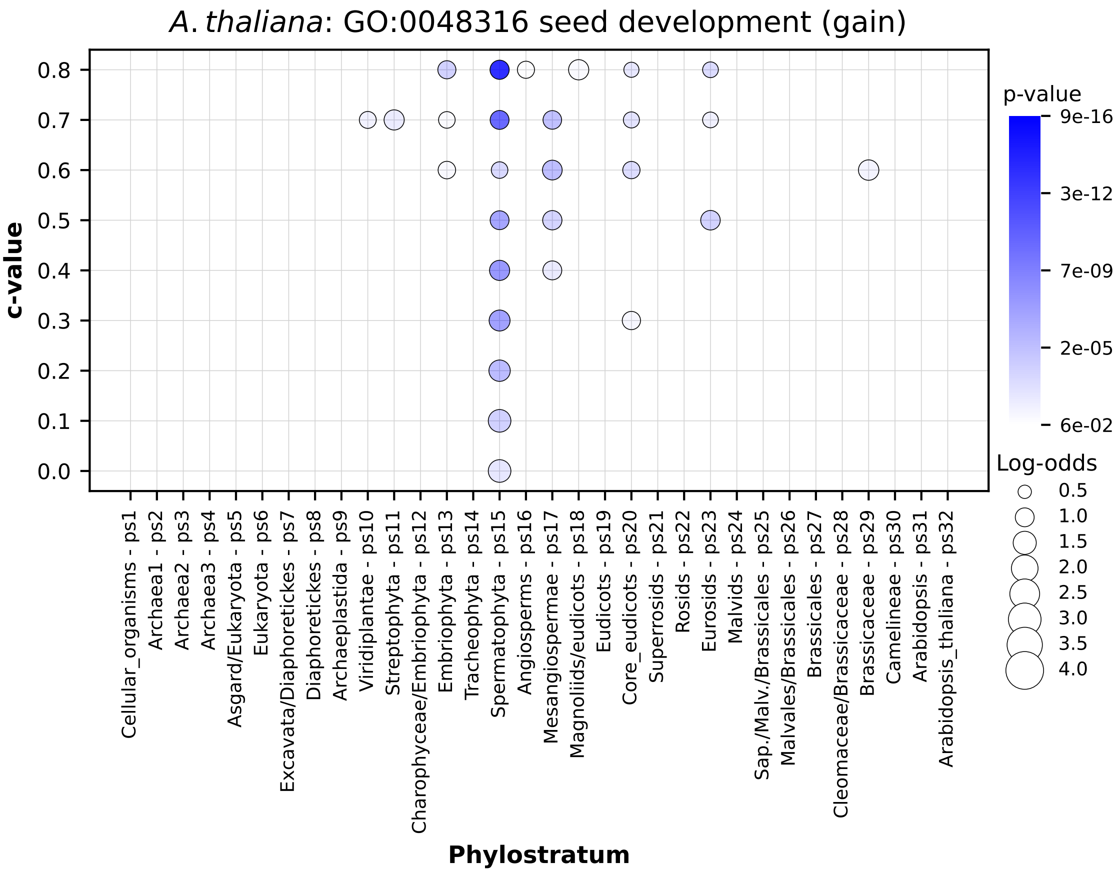

A. thaliana: GO:0048316 seed development (gain)
c-value
0.8
0.7
0.6
0.5
0.4
0.3
0.2
0.1
0.0
p-value
9e-16
3e-12
7e-09
2e-05
6e-02
Log-odds
0.5
1.0
1.5
2.0
2.5
3.0
3.5
4.0
Cellular_organisms - ps1
Archaea1 - ps2
Archaea2 - ps3
Archaea3 - ps4
Asgard/Eukaryota - ps5
Eukaryota - ps6
Excavata/Diaphoretickes - ps7
Diaphoretickes - ps8
Archaeplastida - ps9
Viridiplantae - ps10
Streptophyta - ps11
Charophyceae/Embriophyta - ps12
Embriophyta - ps13
Tracheophyta - ps14
Spermatophyta - ps15
Angiosperms - ps16
Mesangiospermae - ps17
Magnoliids/eudicots - ps18
Eudicots - ps19
Core_eudicots - ps20
Superrosids - ps21
Rosids - ps22
Eurosids - ps23
Malvids - ps24
Sap./Malv./Brassicales - ps25
Malvales/Brassicales - ps26
Brassicales - ps27
Cleomaceae/Brassicaceae - ps28
Brassicaceae - ps29
Camelineae - ps30
Arabidopsis - ps31
Arabidopsis_thaliana - ps32
Phylostratum

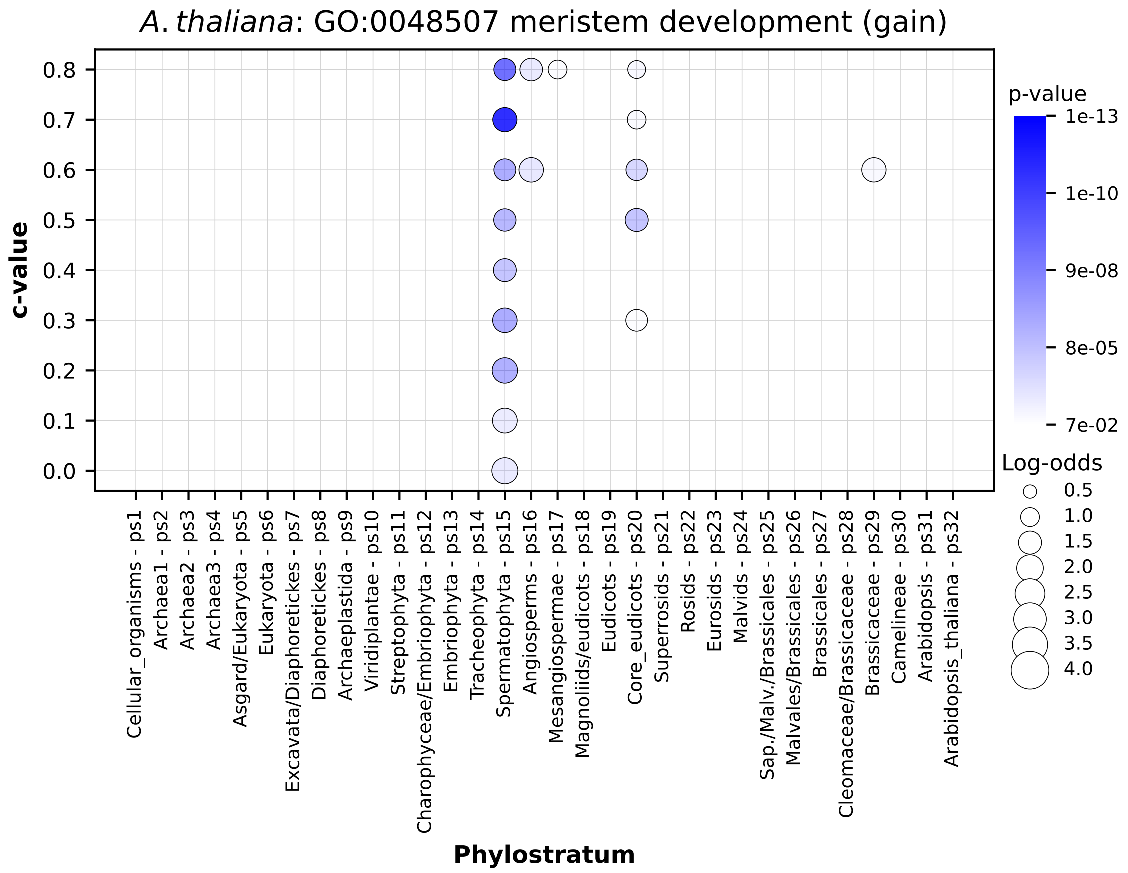

A. thaliana: GO:0048507 meristem development (gain)
c-value
0.8
0.7
0.6
0.5
0.4
0.3
0.2
0.1
0.0
p-value
1e-13
1e-10
9e-08
8e-05
7e-02
Log-odds
0.5
1.0
1.5
2.0
2.5
3.0
3.5
4.0
Cellular_organisms - ps1
Archaea1 - ps2
Archaea2 - ps3
Archaea3 - ps4
Asgard/Eukaryota - ps5
Eukaryota - ps6
Excavata/Diaphoretickes - ps7
Diaphoretickes - ps8
Archaeplastida - ps9
Viridiplantae - ps10
Streptophyta - ps11
Charophyceae/Embriophyta - ps12
Embriophyta - ps13
Tracheophyta - ps14
Spermatophyta - ps15
Angiosperms - ps16
Mesangiospermae - ps17
Magnoliids/eudicots - ps18
Eudicots - ps19
Core_eudicots - ps20
Superrosids - ps21
Rosids - ps22
Eurosids - ps23
Malvids - ps24
Sap./Malv./Brassicales - ps25
Malvales/Brassicales - ps26
Brassicales - ps27
Cleomaceae/Brassicaceae - ps28
Brassicaceae - ps29
Camelineae - ps30
Arabidopsis - ps31
Arabidopsis_thaliana - ps32
Phylostratum

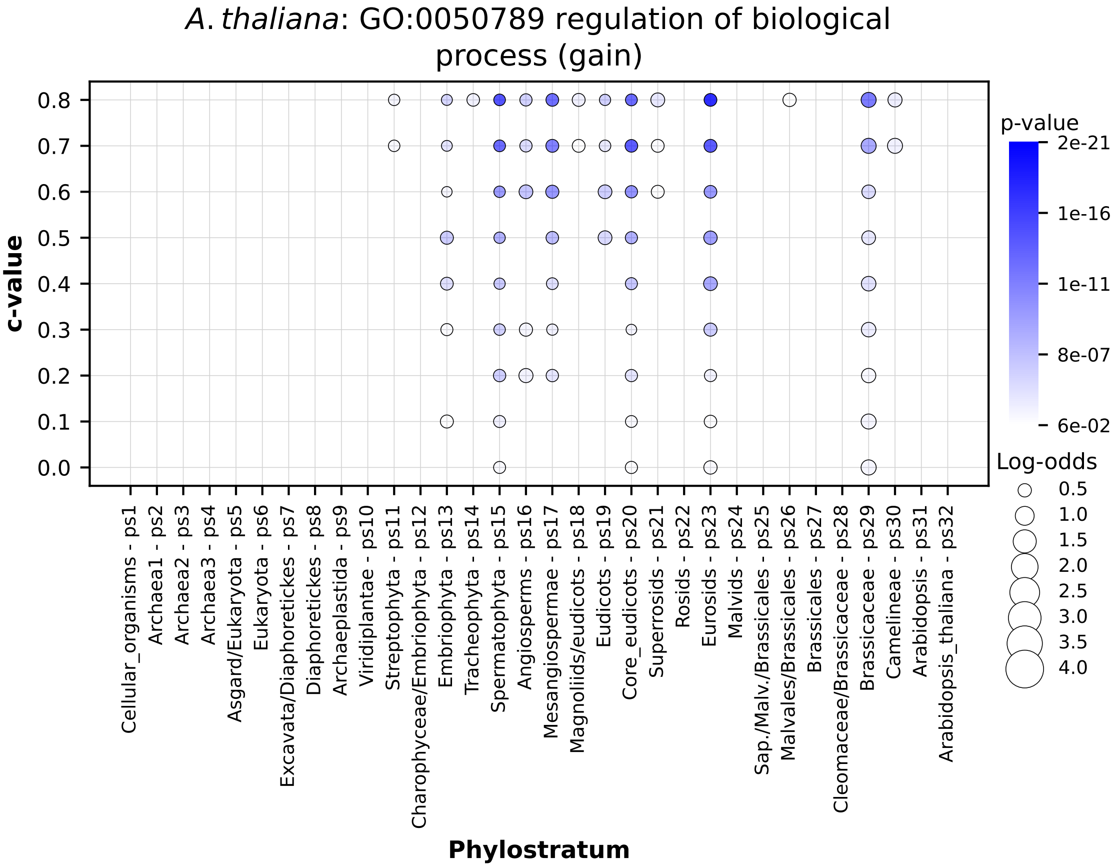
A. thaliana: GO:0050789 regulation of biological process (gain)
c-value
0.8
0.7
0.6
0.5
0.4
0.3
0.2
0.1
0.0
p-value
2e-21
1e-16
1e-11
8e-07
6e-02
Log-odds
0.5
1.0
1.5
2.0
2.5
3.0
3.5
4.0
Cellular_organisms - ps1
Archaea1 - ps2
Archaea2 - ps3
Archaea3 - ps4
Asgard/Eukaryota - ps5
Eukaryota - ps6
Excavata/Diaphoretickes - ps7
Diaphoretickes - ps8
Archaeplastida - ps9
Viridiplantae - ps10
Streptophyta - ps11
Charophyceae/Embriophyta - ps12
Embriophyta - ps13
Tracheophyta - ps14
Spermatophyta - ps15
Angiosperms - ps16
Mesangiospermae - ps17
Magnoliids/eudicots - ps18
Eudicots - ps19
Core_eudicots - ps20
Superrosids - ps21
Rosids - ps22
Eurosids - ps23
Malvids - ps24
Sap./Malv./Brassicales - ps25
Malvales/Brassicales - ps26
Brassicales - ps27
Cleomaceae/Brassicaceae - ps28
Brassicaceae - ps29
Camelineae - ps30
Arabidopsis - ps31
Arabidopsis_thaliana - ps32
Phylostratum

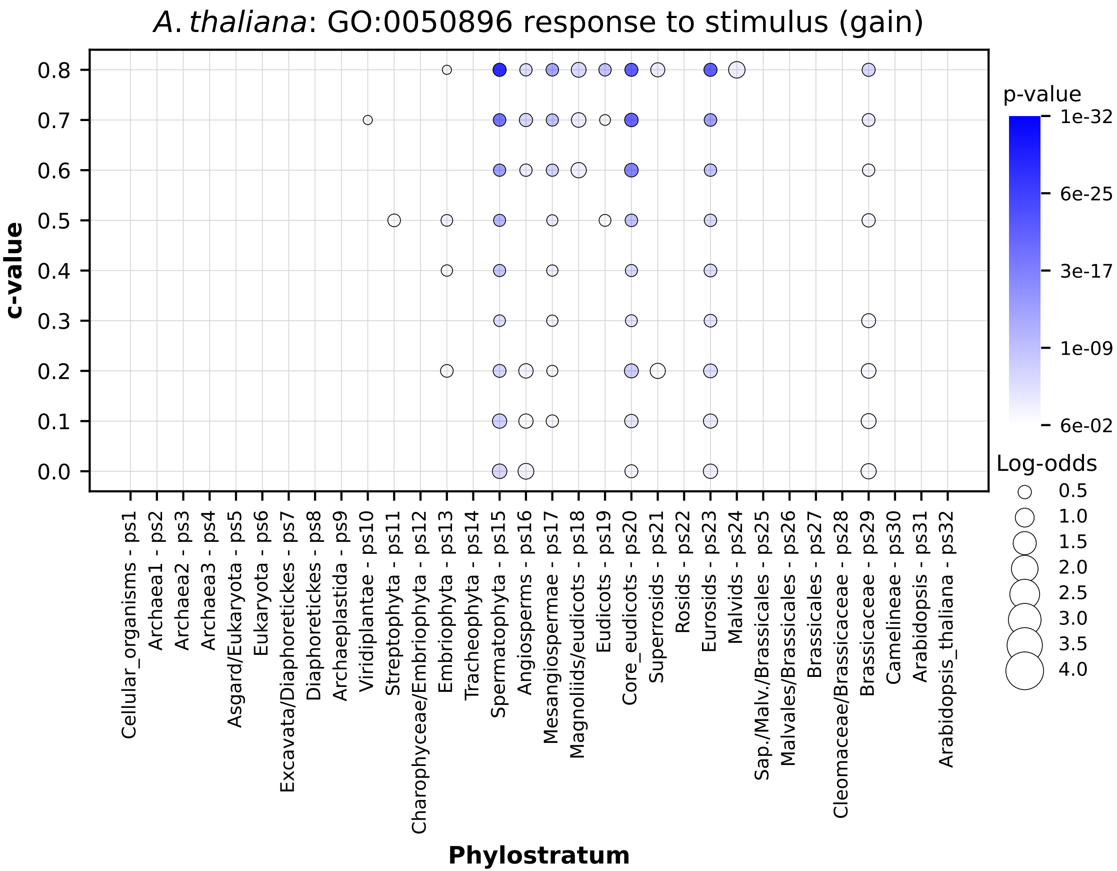
A. thaliana: GO:0050896 response to stimulus (gain)
c-value
0.0
0.1
0.2
0.3
0.4
0.5
0.6
0.7
0.8
Phylostratum
Cellular_organisms - ps1
Archaea1 - ps2
Archaea2 - ps3
Archaea3 - ps4
Asgard/Eukaryota - ps5
Eukaryota - ps6
Excavata/Diaphoretickes - ps7
Diaphoretickes - ps8
Archaeplastida - ps9
Viridiplantae - ps10
Streptophyta - ps11
Charophyceae/Embriophyta - ps12
Embriophyta - ps13
Tracheophyta - ps14
Spermatophyta - ps15
Angiosperms - ps16
Mesangiospermae - ps17
Magnoliids/eudicots - ps18
Eudicots - ps19
Core_eudicots - ps20
Superrosids - ps21
Rosids - ps22
Eurosids - ps23
Malvids - ps24
Sap./Malv./Brassicales - ps25
Malvales/Brassicales - ps26
Brassicales - ps27
Cleomaceae/Brassicaceae - ps28
Brassicaceae - ps29
Camelineae - ps30
Arabidopsis - ps31
Arabidopsis_thaliana - ps32
p-value
1e-32
6e-25
3e-17
1e-09
6e-02
Log-odds
0.5
1.0
1.5
2.0
2.5
3.0
3.5
4.0

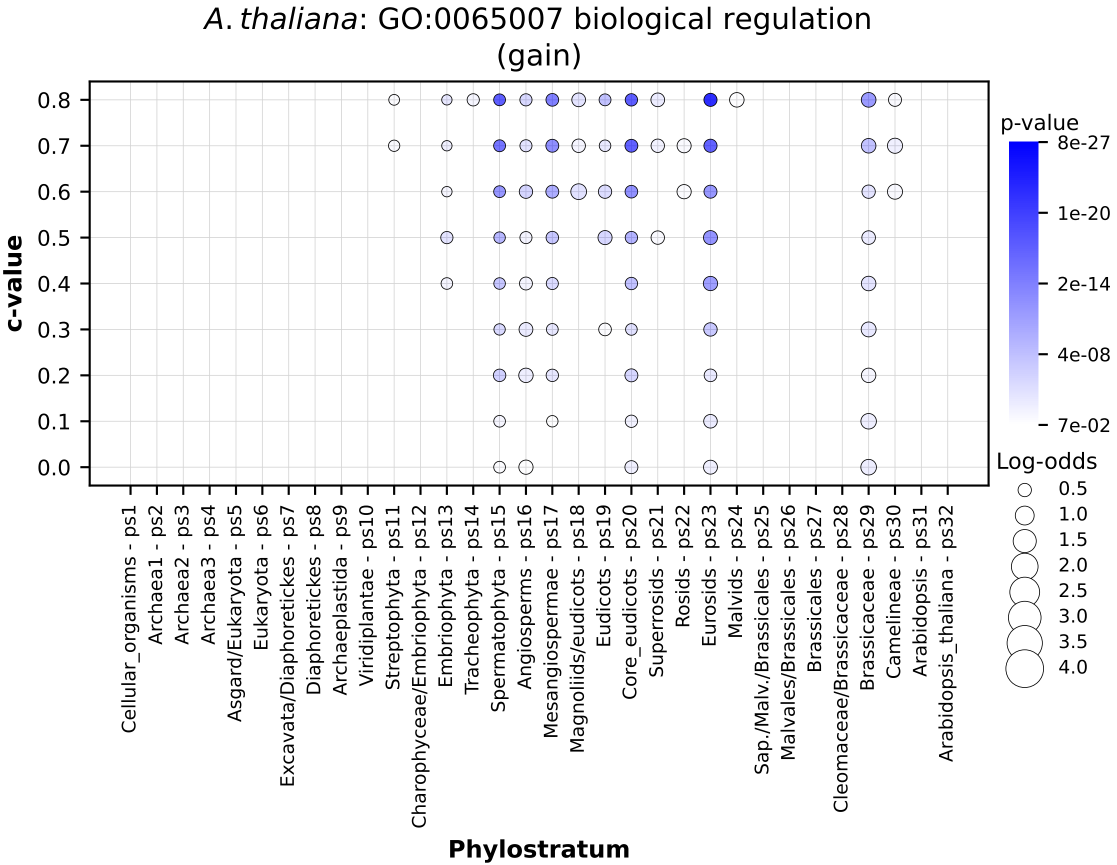

$A. thaliana$: GO:0065007 biological regulation
(gain)
c-value
0.8
0.7
0.6
0.5
0.4
0.3
0.2
0.1
0.0
p-value
8e-27
1e-20
2e-14
4e-08
7e-02
Log-odds
0.5
1.0
1.5
2.0
2.5
3.0
3.5
4.0
Cellular_organisms - ps1
Archaea1 - ps2
Archaea2 - ps3
Archaea3 - ps4
Asgard/Eukaryota - ps5
Eukaryota - ps6
Excavata/Diaphoretickes - ps7
Diaphoretickes - ps8
Archaeplastida - ps9
Viridiplantae - ps10
Streptophyta - ps11
Charophyceae/Embriophyta - ps12
Embriophyta - ps13
Tracheophyta - ps14
Spermatophyta - ps15
Angiosperms - ps16
Mesangiospermae - ps17
Magnoliids/eudicots - ps18
Eudicots - ps19
Core_eudicots - ps20
Superrosids - ps21
Rosids - ps22
Eurosids - ps23
Malvids - ps24
Sap./Malv./Brassicales - ps25
Malvales/Brassicales - ps26
Brassicales - ps27
Cleomaceae/Brassicaceae - ps28
Brassicaceae - ps29
Camelineae - ps30
Arabidopsis - ps31
Arabidopsis_thaliana - ps32
Phylostratum

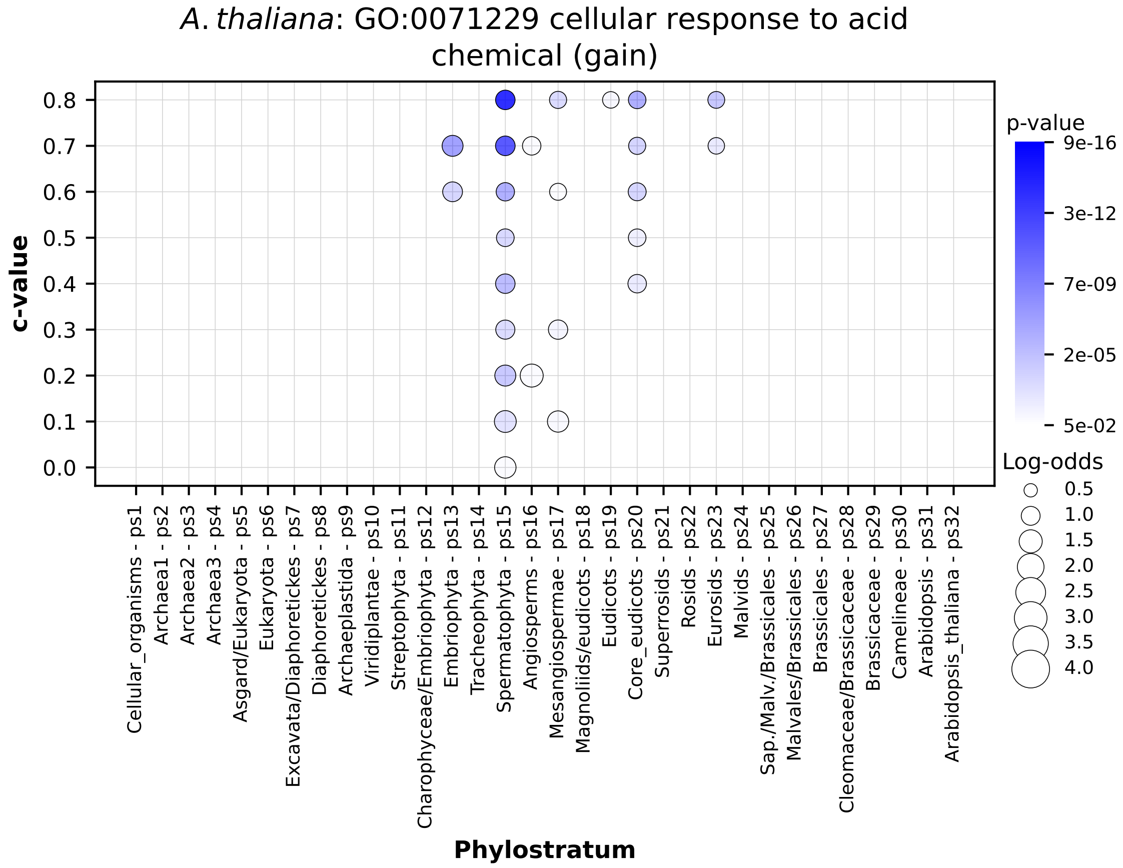

A. thaliana: GO:0071229 cellular response to acid chemical (gain)
c-value
0.8
0.7
0.6
0.5
0.4
0.3
0.2
0.1
0.0
Phylostratum
Cellular_organisms - ps1
Archaea1 - ps2
Archaea2 - ps3
Archaea3 - ps4
Asgard/Eukaryota - ps5
Eukaryota - ps6
Excavata/Diaphoretickes - ps7
Diaphoretickes - ps8
Archaeplastida - ps9
Viridiplantae - ps10
Streptophyta - ps11
Charophyceae/Embriophyta - ps12
Embriophyta - ps13
Tracheophyta - ps14
Spermatophyta - ps15
Angiosperms - ps16
Mesangiospermae - ps17
Magnoliids/eudicots - ps18
Eudicots - ps19
Core_eudicots - ps20
Superrosids - ps21
Rosids - ps22
Eurosids - ps23
Malvids - ps24
Sap./Malv./Brassicales - ps25
Malvales/Brassicales - ps26
Brassicales - ps27
Cleomaceae/Brassicaceae - ps28
Brassicaceae - ps29
Camelineae - ps30
Arabidopsis - ps31
Arabidopsis_thaliana - ps32
p-value
9e-16
3e-12
7e-09
2e-05
5e-02
Log-odds
0.5
1.0
1.5
2.0
2.5
3.0
3.5
4.0

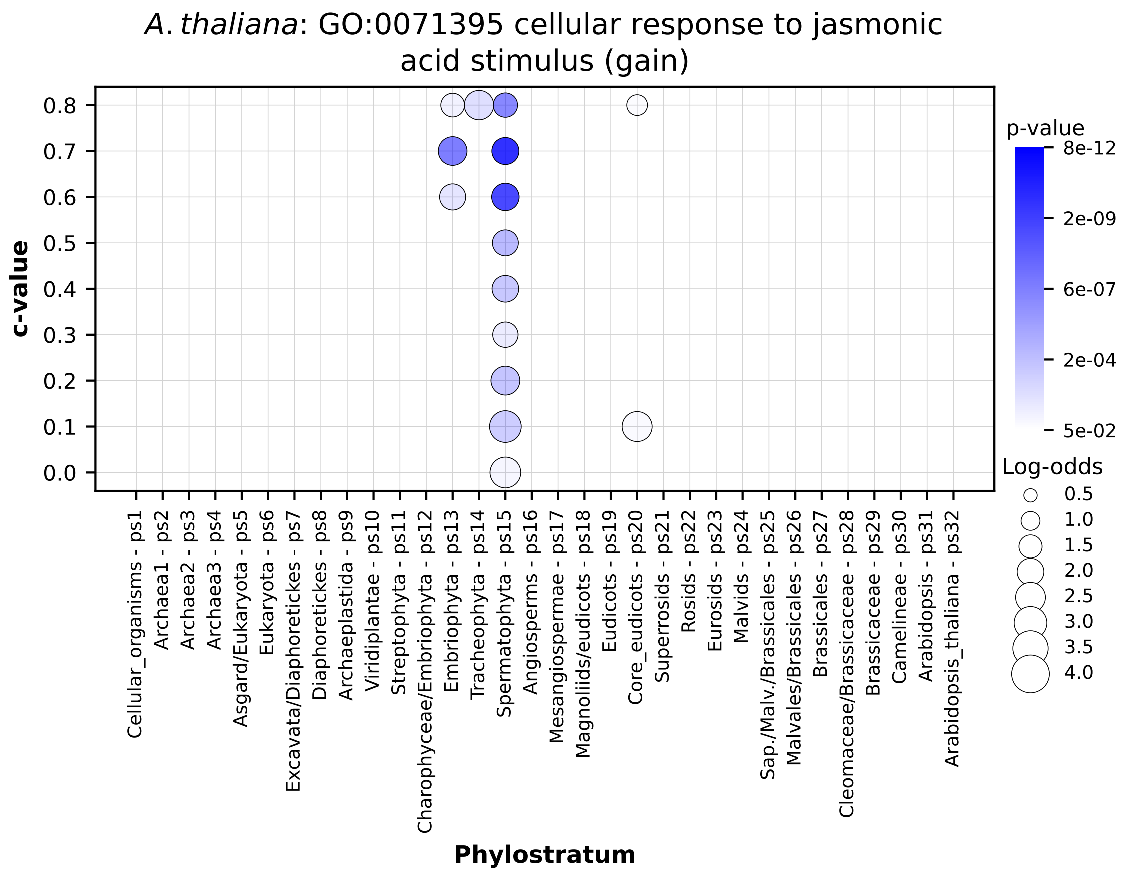

A. thaliana: GO:0071395 cellular response to jasmonic acid stimulus (gain)
c-value
0.8
0.7
0.6
0.5
0.4
0.3
0.2
0.1
0.0
p-value
8e-12
2e-09
6e-07
2e-04
5e-02
Log-odds
0.5
1.0
1.5
2.0
2.5
3.0
3.5
4.0
Cellular_organisms - ps1
Archaea1 - ps2
Archaea2 - ps3
Archaea3 - ps4
Asgard/Eukaryota - ps5
Eukaryota - ps6
Excavata/Diaphoretickes - ps7
Diaphoretickes - ps8
Archaeplastida - ps9
Viridiplantae - ps10
Streptophyta - ps11
Charophyceae/Embriophyta - ps12
Embriophyta - ps13
Tracheophyta - ps14
Spermatophyta - ps15
Angiosperms - ps16
Mesangiospermae - ps17
Magnoliids/eudicots - ps18
Eudicots - ps19
Core_eudicots - ps20
Superrosids - ps21
Rosids - ps22
Eurosids - ps23
Malvids - ps24
Sap./Malv./Brassicales - ps25
Malvales/Brassicales - ps26
Brassicales - ps27
Cleomaceae/Brassicaceae - ps28
Brassicaceae - ps29
Camelineae - ps30
Arabidopsis - ps31
Arabidopsis_thaliana - ps32
Phylostratum

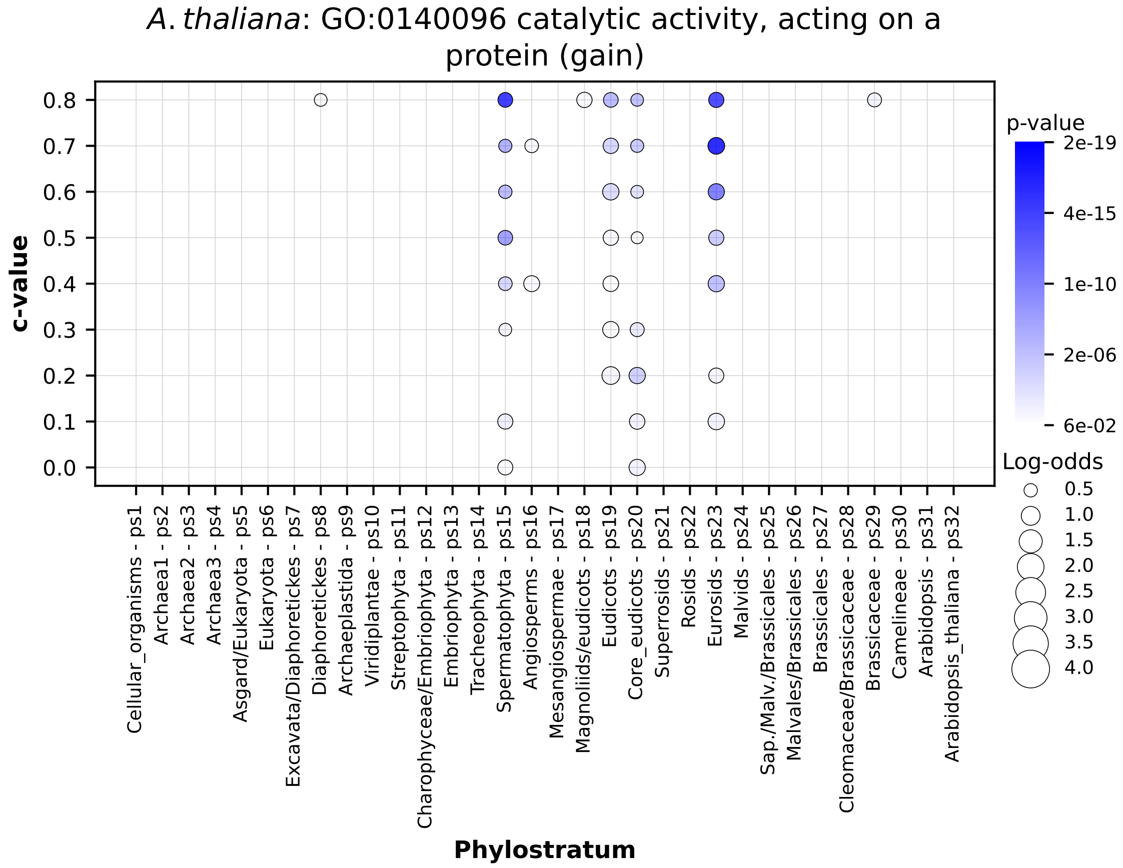

A. thaliana: GO:0140096 catalytic activity, acting on a protein (gain)
c-value
0.8
0.7
0.6
0.5
0.4
0.3
0.2
0.1
0.0
p-value
2e-19
4e-15
1e-10
2e-06
6e-02
Log-odds
0.5
1.0
1.5
2.0
2.5
3.0
3.5
4.0
Cellular_organisms - ps1
Archaea1 - ps2
Archaea2 - ps3
Archaea3 - ps4
Asgard/Eukaryota - ps5
Eukaryota - ps6
Excavata/Diaphoretickes - ps7
Diaphoretickes - ps8
Archaeplastida - ps9
Viridiplantae - ps10
Streptophyta - ps11
Charophyceae/Embriophyta - ps12
Embriophyta - ps13
Tracheophyta - ps14
Spermatophyta - ps15
Angiosperms - ps16
Mesangiospermae - ps17
Magnoliids/eudicots - ps18
Eudicots - ps19
Core_eudicots - ps20
Superrosids - ps21
Rosids - ps22
Eurosids - ps23
Malvids - ps24
Sap./Malv./Brassicales - ps25
Malvales/Brassicales - ps26
Brassicales - ps27
Cleomaceae/Brassicaceae - ps28
Brassicaceae - ps29
Camelineae - ps30
Arabidopsis - ps31
Arabidopsis_thaliana - ps32
Phylostratum

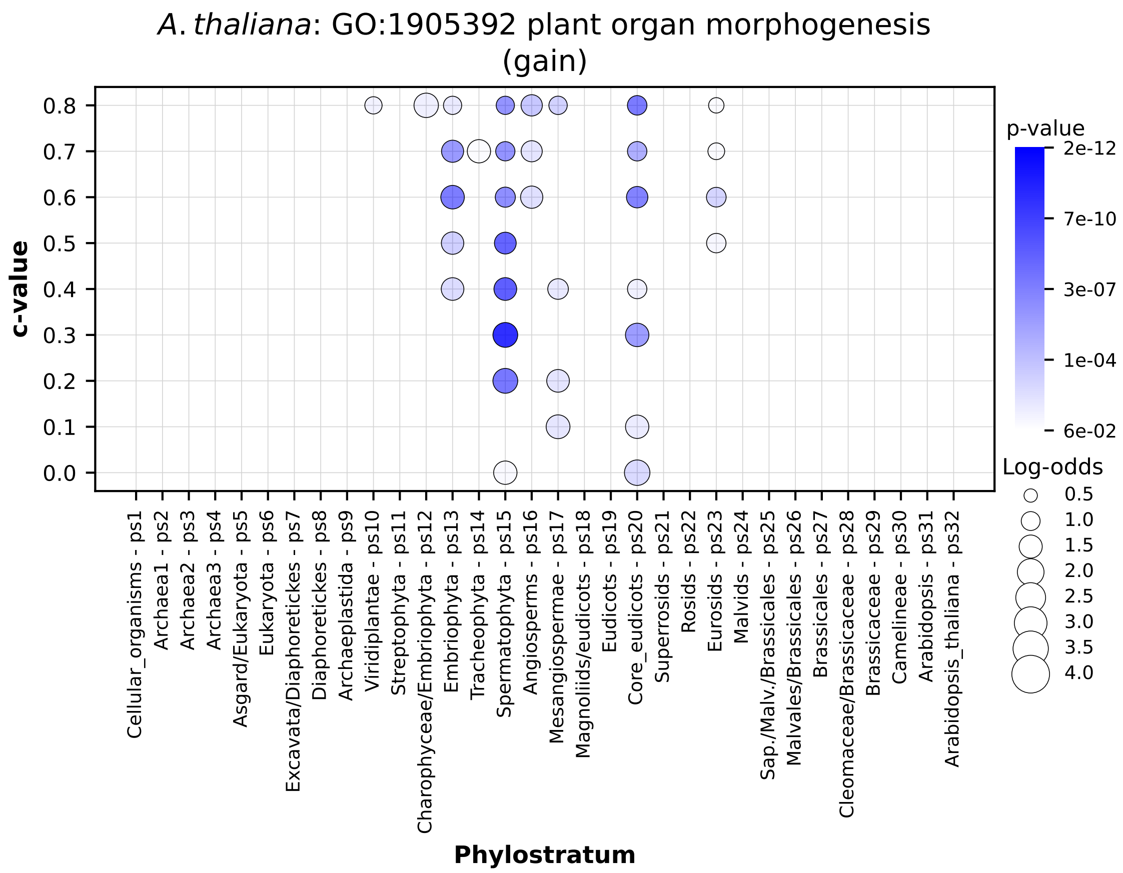
A. thaliana: GO:1905392 plant organ morphogenesis
(gain)
c-value
0.8
0.7
0.6
0.5
0.4
0.3
0.2
0.1
0.0
p-value
2e-12
7e-10
3e-07
1e-04
6e-02
Log-odds
0.5
1.0
1.5
2.0
2.5
3.0
3.5
4.0
Cellular_organisms - ps1
Archaea1 - ps2
Archaea2 - ps3
Archaea3 - ps4
Asgard/Eukaryota - ps5
Eukaryota - ps6
Excavata/Diaphoretickes - ps7
Diaphoretickes - ps8
Archaeplastida - ps9
Viridiplantae - ps10
Streptophyta - ps11
Charophyceae/Embriophyta - ps12
Embriophyta - ps13
Tracheophyta - ps14
Spermatophyta - ps15
Angiosperms - ps16
Mesangiospermae - ps17
Magnoliids/eudicots - ps18
Eudicots - ps19
Core_eudicots - ps20
Superrosids - ps21
Rosids - ps22
Eurosids - ps23
Malvids - ps24
Sap./Malv./Brassicales - ps25
Malvales/Brassicales - ps26
Brassicales - ps27
Cleomaceae/Brassicaceae - ps28
Brassicaceae - ps29
Camelineae - ps30
Arabidopsis - ps31
Arabidopsis_thaliana - ps32
Phylostratum

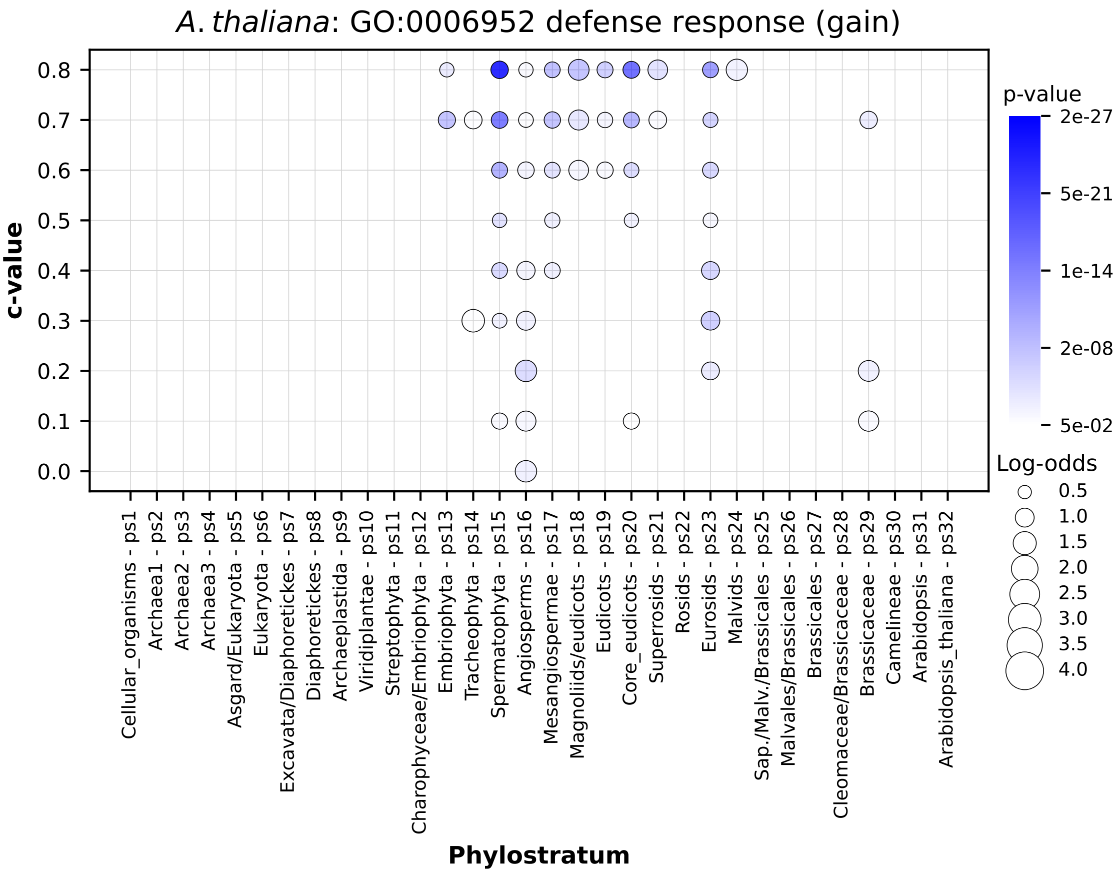

A. thaliana: GO:0006952 defense response (gain)
c-value
0.8
0.7
0.6
0.5
0.4
0.3
0.2
0.1
0.0
Phylostratum
Cellular_organisms - ps1
Archaea1 - ps2
Archaea2 - ps3
Archaea3 - ps4
Asgard/Eukaryota - ps5
Eukaryota - ps6
Excavata/Diaphoretickes - ps7
Diaphoretickes - ps8
Archaeplastida - ps9
Viridiplantae - ps10
Streptophyta - ps11
Charophyceae/Embriophyta - ps12
Embriophyta - ps13
Tracheophyta - ps14
Spermatophyta - ps15
Angiosperms - ps16
Mesangiospermae - ps17
Magnoliids/eudicots - ps18
Eudicots - ps19
Core_eudicots - ps20
Superrosids - ps21
Rosids - ps22
Eurosids - ps23
Malvids - ps24
Sap./Malv./Brassicales - ps25
Malvales/Brassicales - ps26
Brassicales - ps27
Cleomaceae/Brassicaceae - ps28
Brassicaceae - ps29
Camelineae - ps30
Arabidopsis - ps31
Arabidopsis_thaliana - ps32
p-value
2e-27
5e-21
1e-14
2e-08
5e-02
Log-odds
0.5
1.0
1.5
2.0
2.5
3.0
3.5
4.0

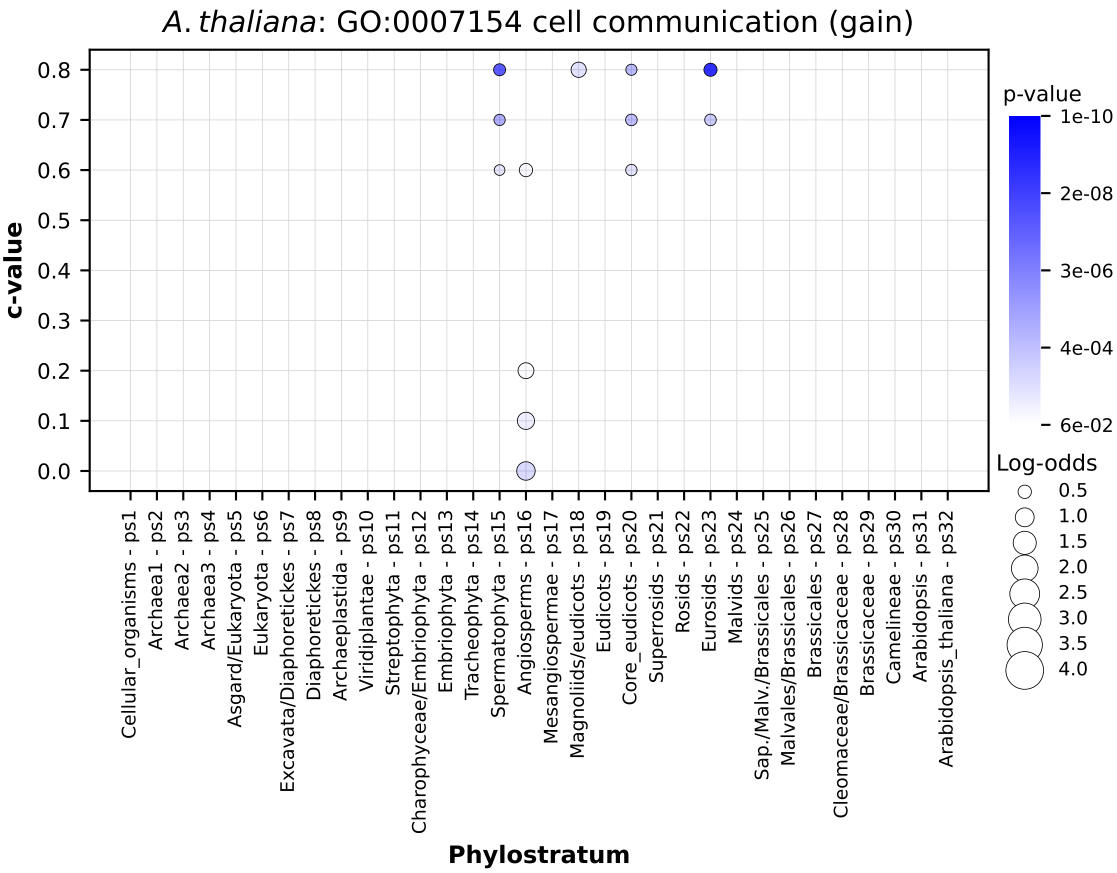
A. thaliana: GO:0007154 cell communication (gain)
c-value
0.8
0.7
0.6
0.5
0.4
0.3
0.2
0.1
0.0
p-value
1e-10
2e-08
3e-06
4e-04
6e-02
Log-odds
0.5
1.0
1.5
2.0
2.5
3.0
3.5
4.0
Cellular_organisms - ps1
Archaea1 - ps2
Archaea2 - ps3
Archaea3 - ps4
Asgard/Eukaryota - ps5
Eukaryota - ps6
Excavata/Diaphoretickes - ps7
Diaphoretickes - ps8
Archaeplastida - ps9
Viridiplantae - ps10
Streptophyta - ps11
Charophyceae/Embriophyta - ps12
Embriophyta - ps13
Tracheophyta - ps14
Spermatophyta - ps15
Angiosperms - ps16
Mesangiospermae - ps17
Magnoliids/eudicots - ps18
Eudicots - ps19
Core_eudicots - ps20
Superrosids - ps21
Rosids - ps22
Eurosids - ps23
Malvids - ps24
Sap./Malv./Brassicales - ps25
Malvales/Brassicales - ps26
Brassicales - ps27
Cleomaceae/Brassicaceae - ps28
Brassicaceae - ps29
Camelineae - ps30
Arabidopsis - ps31
Arabidopsis_thaliana - ps32
Phylostratum

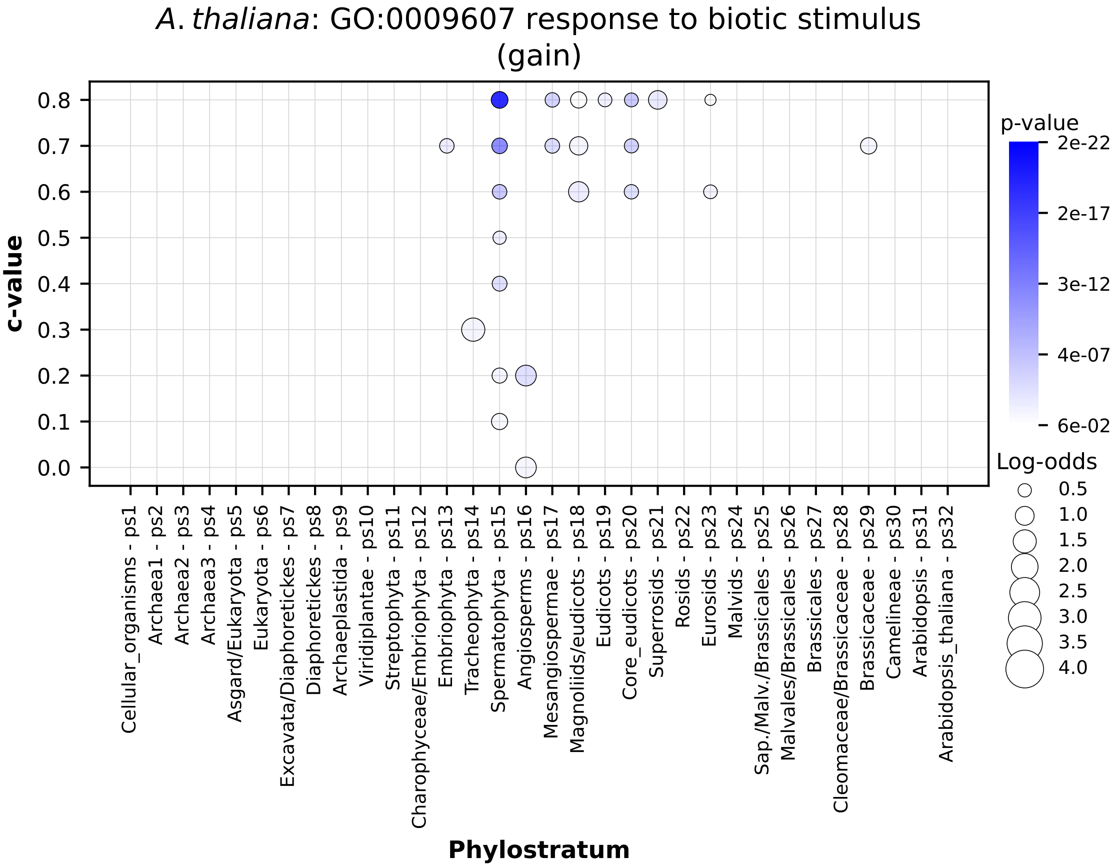
A. thaliana: GO:0009607 response to biotic stimulus
(gain)
c-value
0.8
0.7
0.6
0.5
0.4
0.3
0.2
0.1
0.0
Phylostratum
Cellular_organisms - ps1
Archaea1 - ps2
Archaea2 - ps3
Archaea3 - ps4
Asgard/Eukaryota - ps5
Eukaryota - ps6
Excavata/Diaphoretickes - ps7
Diaphoretickes - ps8
Archaeplastida - ps9
Viridiplantae - ps10
Streptophyta - ps11
Charophyceae/Embriophyta - ps12
Embriophyta - ps13
Tracheophyta - ps14
Spermatophyta - ps15
Angiosperms - ps16
Mesangiospermae - ps17
Magnoliids/eudicots - ps18
Eudicots - ps19
Core_eudicots - ps20
Superrosids - ps21
Rosids - ps22
Eurosids - ps23
Malvids - ps24
Sap./Malv./Brassicales - ps25
Malvales/Brassicales - ps26
Brassicales - ps27
Cleomaceae/Brassicaceae - ps28
Brassicaceae - ps29
Camelineae - ps30
Arabidopsis - ps31
Arabidopsis_thaliana - ps32
p-value
2e-22
2e-17
3e-12
4e-07
6e-02
Log-odds
0.5
1.0
1.5
2.0
2.5
3.0
3.5
4.0

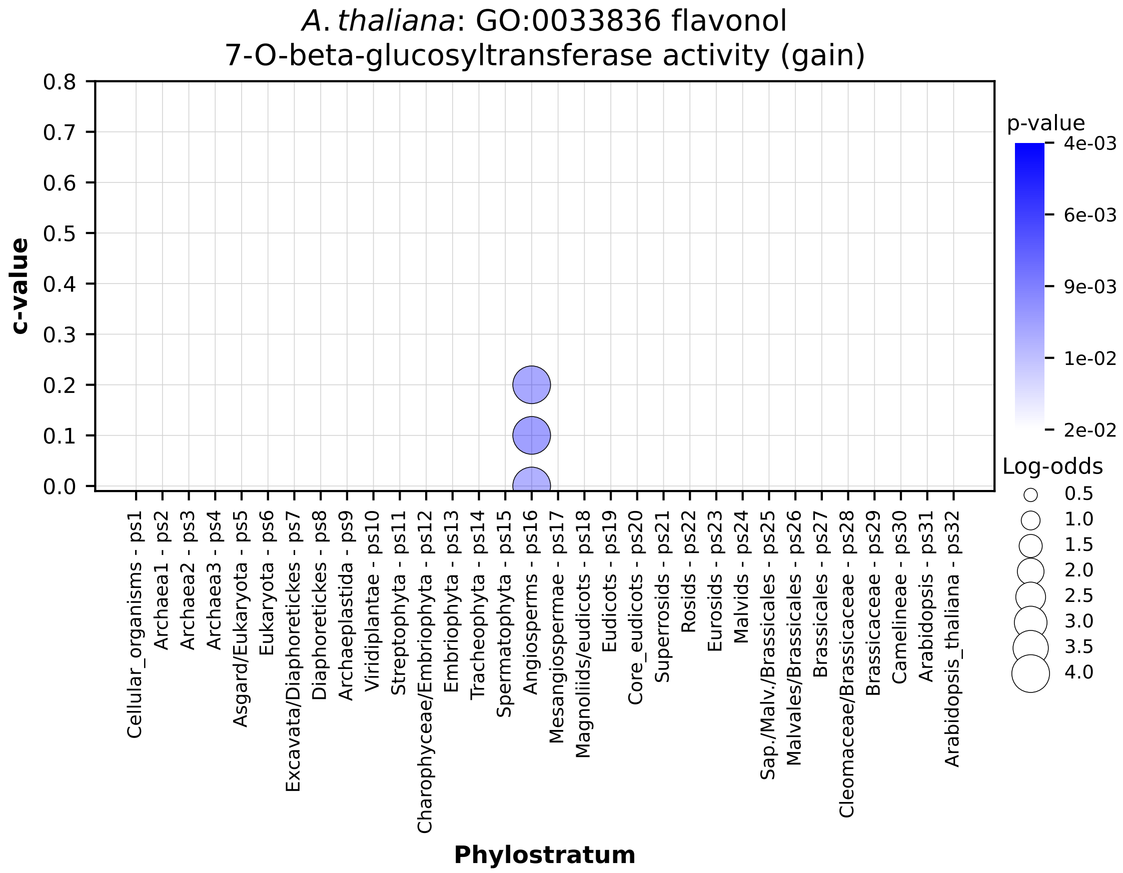
A. thaliana: GO:0033836 flavonol
7-O-beta-glucosyltransferase activity (gain)
c-value
0.0
0.1
0.2
0.3
0.4
0.5
0.6
0.7
0.8
Phylostratum
Cellular_organisms - ps1
Archaea1 - ps2
Archaea2 - ps3
Archaea3 - ps4
Asgard/Eukaryota - ps5
Eukaryota - ps6
Excavata/Diaphoretickes - ps7
Diaphoretickes - ps8
Archaeplastida - ps9
Viridiplantae - ps10
Streptophyta - ps11
Charophyceae/Embriophyta - ps12
Embriophyta - ps13
Tracheophyta - ps14
Spermatophyta - ps15
Angiosperms - ps16
Mesangiospermae - ps17
Magnoliids/eudicots - ps18
Eudicots - ps19
Core_eudicots - ps20
Superrosids - ps21
Rosids - ps22
Eurosids - ps23
Malvids - ps24
Sap./Malv./Brassicales - ps25
Malvales/Brassicales - ps26
Brassicales - ps27
Cleomaceae/Brassicaceae - ps28
Brassicaceae - ps29
Camelineae - ps30
Arabidopsis - ps31
Arabidopsis_thaliana - ps32
p-value
4e-03
6e-03
9e-03
1e-02
2e-02
Log-odds
0.5
1.0
1.5
2.0
2.5
3.0
3.5
4.0

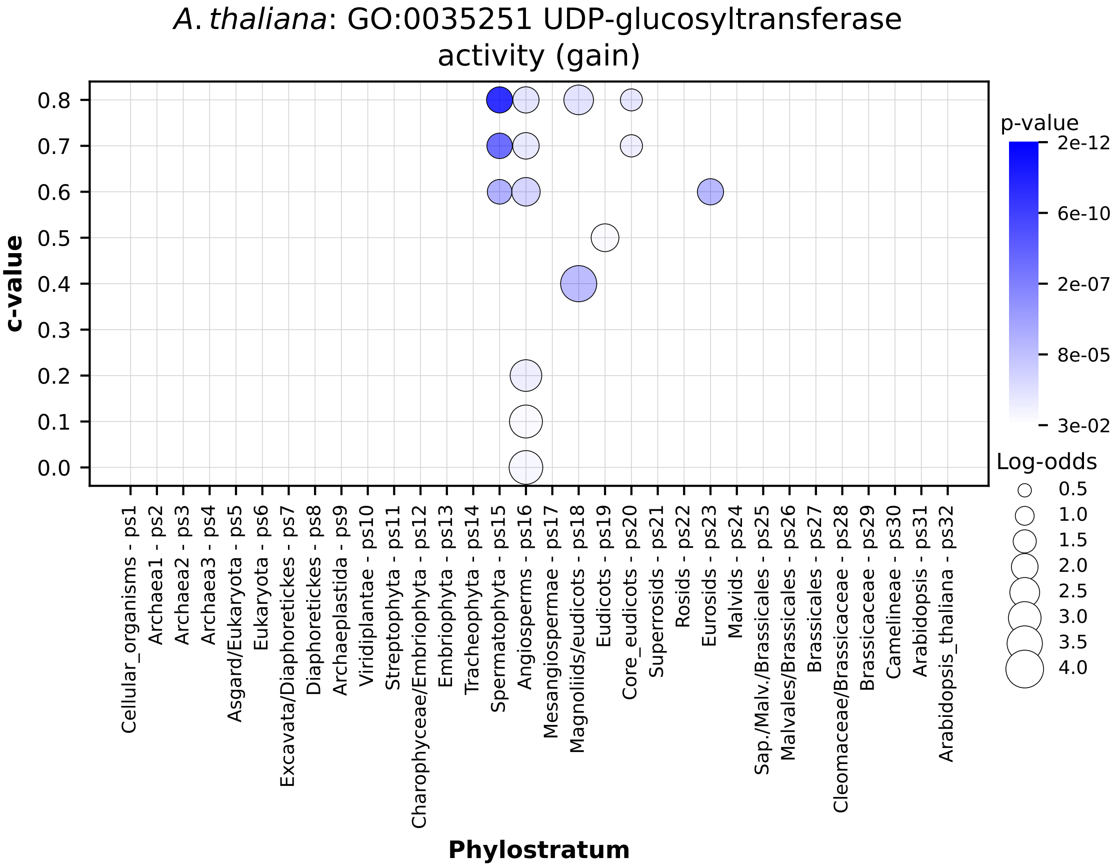
A. thaliana: GO:0035251 UDP-glucosyltransferase activity (gain)
c-value
0.8
0.7
0.6
0.5
0.4
0.3
0.2
0.1
0.0
Phylostratum
Cellular_organisms - ps1
Archaea1 - ps2
Archaea2 - ps3
Archaea3 - ps4
Asgard/Eukaryota - ps5
Eukaryota - ps6
Excavata/Diaphoretickes - ps7
Diaphoretickes - ps8
Archaeplastida - ps9
Viridiplantae - ps10
Streptophyta - ps11
Charophyceae/Embriophyta - ps12
Embriophyta - ps13
Tracheophyta - ps14
Spermatophyta - ps15
Angiosperms - ps16
Mesangiospermae - ps17
Magnoliids/eudicots - ps18
Eudicots - ps19
Core_eudicots - ps20
Superrosids - ps21
Rosids - ps22
Eurosids - ps23
Malvids - ps24
Sap./Malv./Brassicales - ps25
Malvales/Brassicales - ps26
Brassicales - ps27
Cleomaceae/Brassicaceae - ps28
Brassicaceae - ps29
Camelineae - ps30
Arabidopsis - ps31
Arabidopsis_thaliana - ps32
p-value
2e-12
6e-10
2e-07
8e-05
3e-02
Log-odds
0.5
1.0
1.5
2.0
2.5
3.0
3.5
4.0

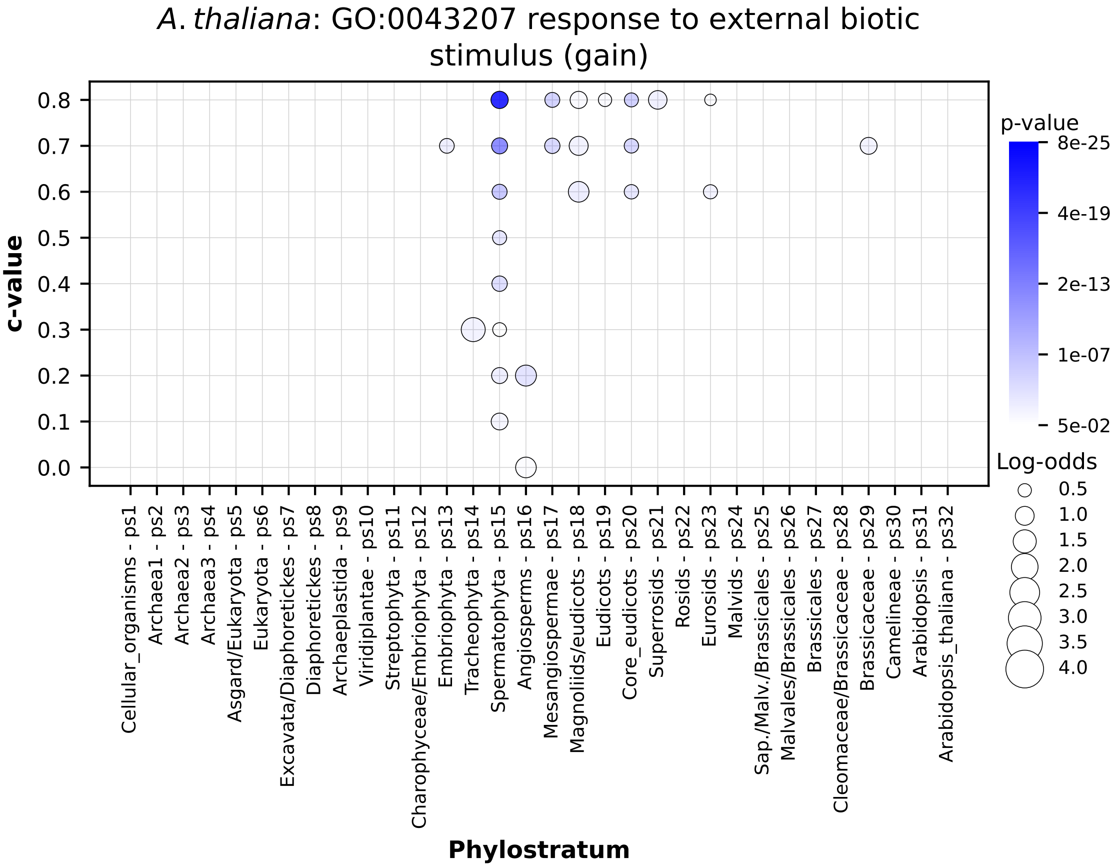
A. thaliana: GO:0043207 response to external biotic stimulus (gain)
c-value
0.8
0.7
0.6
0.5
0.4
0.3
0.2
0.1
0.0
Phylostratum
Cellular_organisms - ps1
Archaea1 - ps2
Archaea2 - ps3
Archaea3 - ps4
Asgard/Eukaryota - ps5
Eukaryota - ps6
Excavata/Diaphoretickes - ps7
Diaphoretickes - ps8
Archaeplastida - ps9
Viridiplantae - ps10
Streptophyta - ps11
Charophyceae/Embriophyta - ps12
Embriophyta - ps13
Tracheophyta - ps14
Spermatophyta - ps15
Angiosperms - ps16
Mesangiospermae - ps17
Magnoliids/eudicots - ps18
Eudicots - ps19
Core_eudicots - ps20
Superrosids - ps21
Rosids - ps22
Eurosids - ps23
Malvids - ps24
Sap./Malv./Brassicales - ps25
Malvales/Brassicales - ps26
Brassicales - ps27
Cleomaceae/Brassicaceae - ps28
Brassicaceae - ps29
Camelineae - ps30
Arabidopsis - ps31
Arabidopsis_thaliana - ps32
p-value
8e-25
4e-19
2e-13
1e-07
5e-02
Log-odds
0.5
1.0
1.5
2.0
2.5
3.0
3.5
4.0

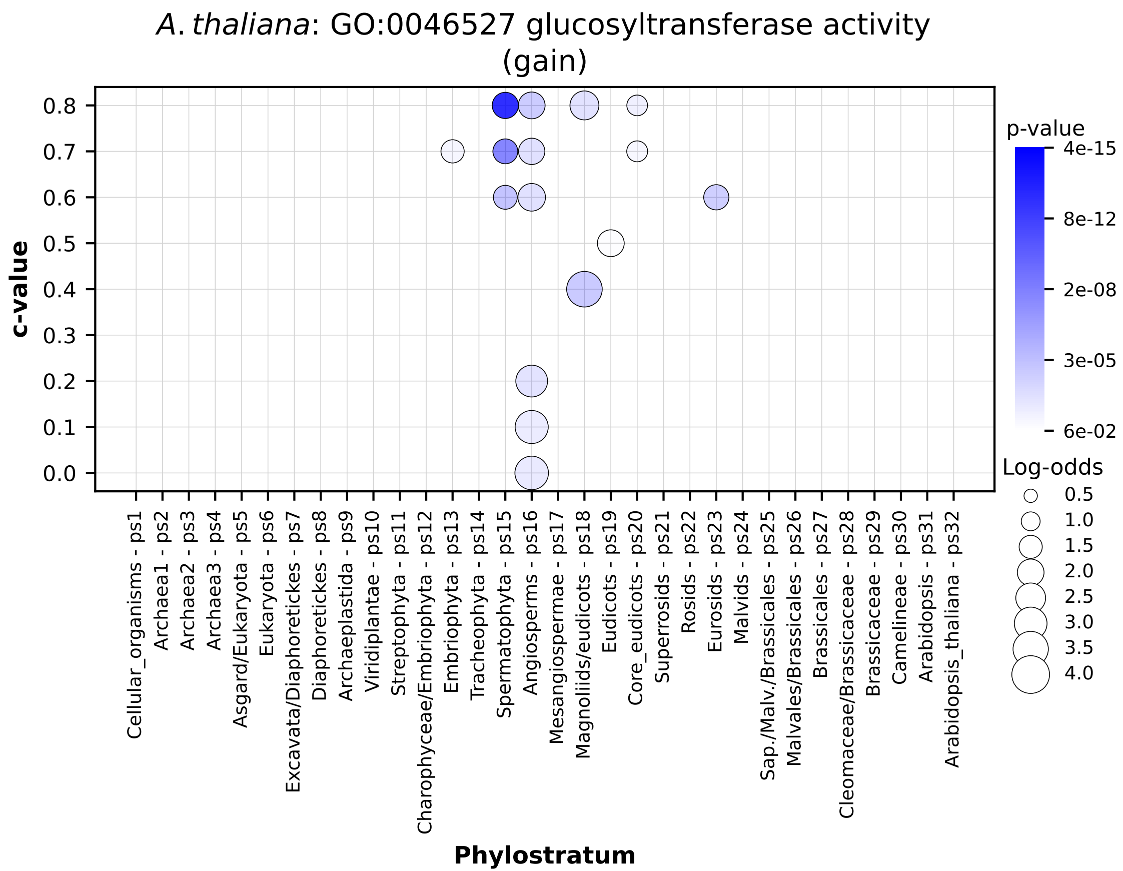
A. thaliana: GO:0046527 glucosyltransferase activity
(gain)
c-value
0.8
0.7
0.6
0.5
0.4
0.3
0.2
0.1
0.0
p-value
4e-15
8e-12
2e-08
3e-05
6e-02
Log-odds
0.5
1.0
1.5
2.0
2.5
3.0
3.5
4.0
Cellular_organisms - ps1
Archaea1 - ps2
Archaea2 - ps3
Archaea3 - ps4
Asgard/Eukaryota - ps5
Eukaryota - ps6
Excavata/Diaphoretickes - ps7
Diaphoretickes - ps8
Archaeplastida - ps9
Viridiplantae - ps10
Streptophyta - ps11
Charophyceae/Embriophyta - ps12
Embriophyta - ps13
Tracheophyta - ps14
Spermatophyta - ps15
Angiosperms - ps16
Mesangiospermae - ps17
Magnoliids/eudicots - ps18
Eudicots - ps19
Core_eudicots - ps20
Superrosids - ps21
Rosids - ps22
Eurosids - ps23
Malvids - ps24
Sap./Malv./Brassicales - ps25
Malvales/Brassicales - ps26
Brassicales - ps27
Cleomaceae/Brassicaceae - ps28
Brassicaceae - ps29
Camelineae - ps30
Arabidopsis - ps31
Arabidopsis_thaliana - ps32
Phylostratum

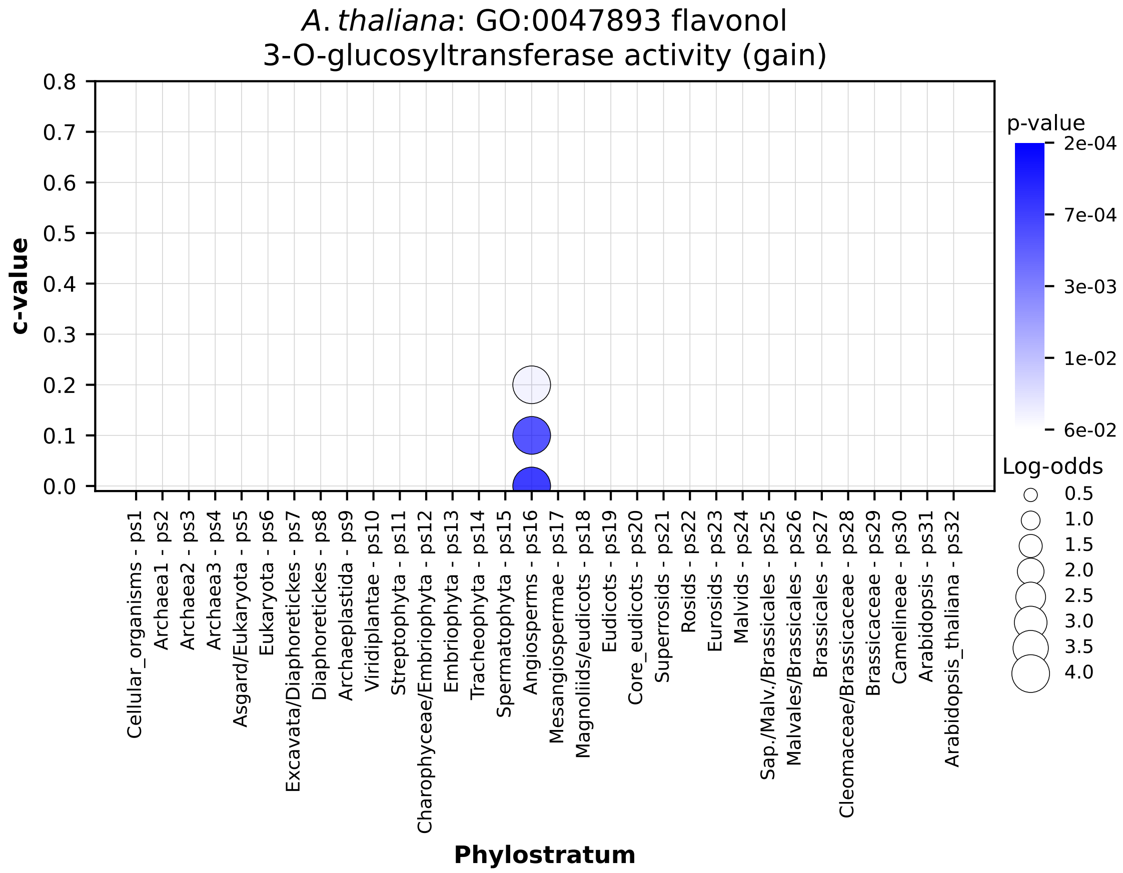

A. thaliana: GO:0047893 flavonol
3-O-glucosyltransferase activity (gain)
c-value
0.8
0.7
0.6
0.5
0.4
0.3
0.2
0.1
0.0
p-value
2e-04
7e-04
3e-03
1e-02
6e-02
Log-odds
0.5
1.0
1.5
2.0
2.5
3.0
3.5
4.0
Cellular_organisms - ps1
Archaea1 - ps2
Archaea2 - ps3
Archaea3 - ps4
Asgard/Eukaryota - ps5
Eukaryota - ps6
Excavata/Diaphoretickes - ps7
Diaphoretickes - ps8
Archaeplastida - ps9
Viridiplantae - ps10
Streptophyta - ps11
Charophyceae/Embriophyta - ps12
Embriophyta - ps13
Tracheophyta - ps14
Spermatophyta - ps15
Angiosperms - ps16
Mesangiospermae - ps17
Magnoliids/eudicots - ps18
Eudicots - ps19
Core_eudicots - ps20
Superrosids - ps21
Rosids - ps22
Eurosids - ps23
Malvids - ps24
Sap./Malv./Brassicales - ps25
Malvales/Brassicales - ps26
Brassicales - ps27
Cleomaceae/Brassicaceae - ps28
Brassicaceae - ps29
Camelineae - ps30
Arabidopsis - ps31
Arabidopsis_thaliana - ps32
Phylostratum

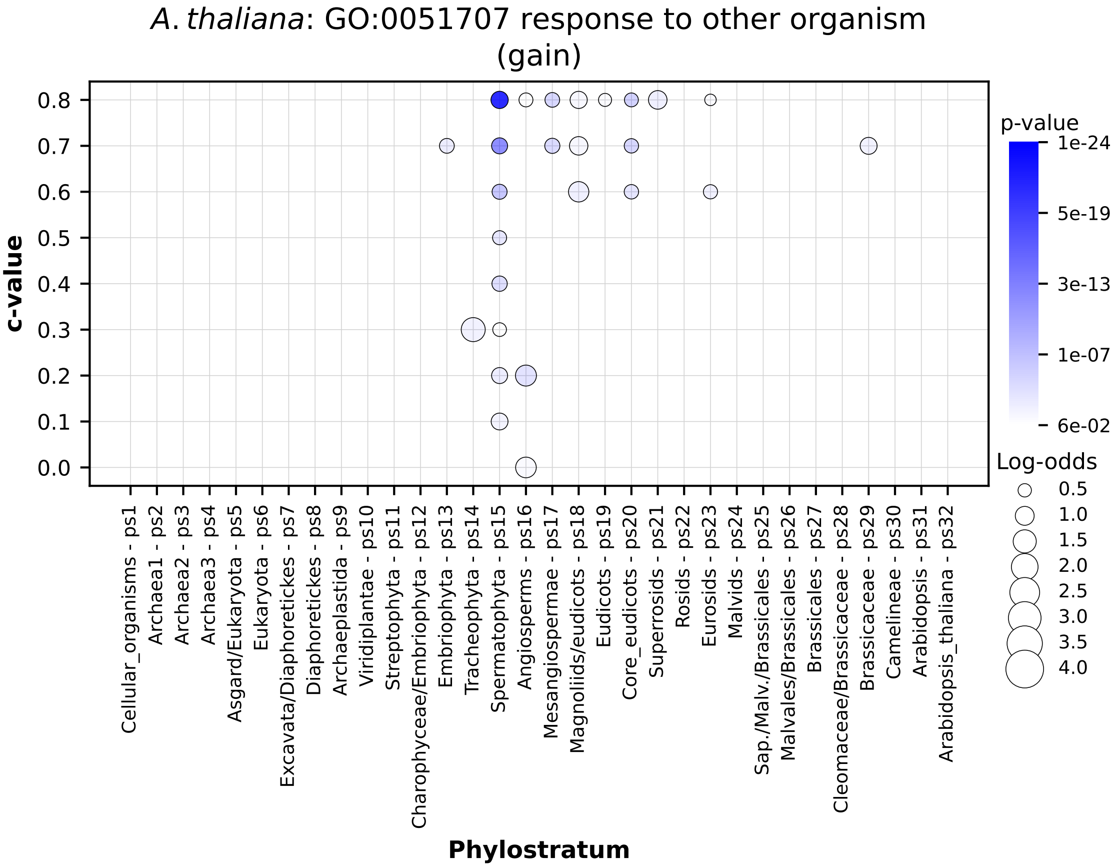

A. thaliana: GO:0051707 response to other organism
(gain)
c-value
0.8
0.7
0.6
0.5
0.4
0.3
0.2
0.1
0.0
p-value
1e-24
5e-19
3e-13
1e-07
6e-02
Log-odds
0.5
1.0
1.5
2.0
2.5
3.0
3.5
4.0
Cellular_organisms - ps1
Archaea1 - ps2
Archaea2 - ps3
Archaea3 - ps4
Asgard/Eukaryota - ps5
Eukaryota - ps6
Excavata/Diaphoretickes - ps7
Diaphoretickes - ps8
Archaeplastida - ps9
Viridiplantae - ps10
Streptophyta - ps11
Charophyceae/Embriophyta - ps12
Embriophyta - ps13
Tracheophyta - ps14
Spermatophyta - ps15
Angiosperms - ps16
Mesangiospermae - ps17
Magnoliids/eudicots - ps18
Eudicots - ps19
Core_eudicots - ps20
Superrosids - ps21
Rosids - ps22
Eurosids - ps23
Malvids - ps24
Sap./Malv./Brassicales - ps25
Malvales/Brassicales - ps26
Brassicales - ps27
Cleomaceae/Brassicaceae - ps28
Brassicaceae - ps29
Camelineae - ps30
Arabidopsis - ps31
Arabidopsis_thaliana - ps32
Phylostratum

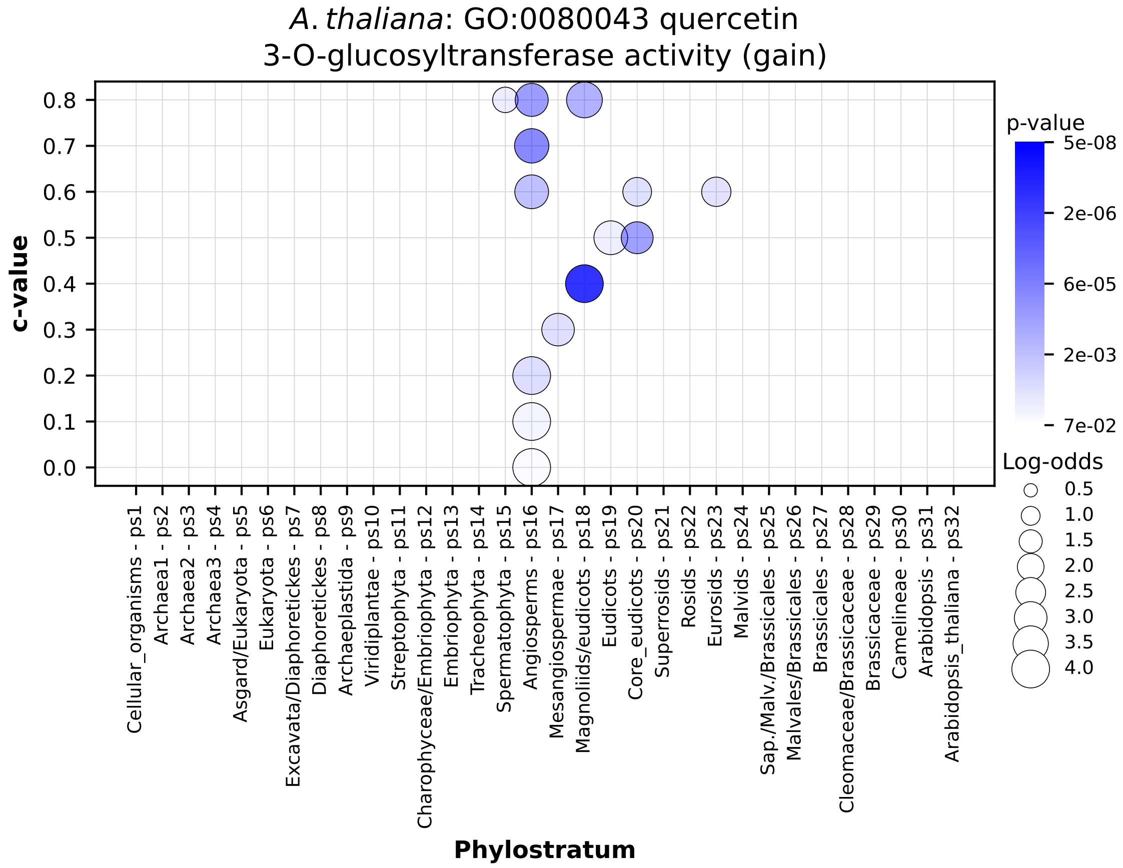

A. thaliana: GO:0080043 quercetin
3-O-glucosyltransferase activity (gain)
c-value
0.8
0.7
0.6
0.5
0.4
0.3
0.2
0.1
0.0
Phylostratum
Cellular_organisms - ps1
Archaea1 - ps2
Archaea2 - ps3
Archaea3 - ps4
Asgard/Eukaryota - ps5
Eukaryota - ps6
Excavata/Diaphoretickes - ps7
Diaphoretickes - ps8
Archaeplastida - ps9
Viridiplantae - ps10
Streptophyta - ps11
Charophyceae/Embriophyta - ps12
Embriophyta - ps13
Tracheophyta - ps14
Spermatophyta - ps15
Angiosperms - ps16
Mesangiospermae - ps17
Magnoliids/eudicots - ps18
Eudicots - ps19
Core_eudicots - ps20
Superrosids - ps21
Rosids - ps22
Eurosids - ps23
Malvids - ps24
Sap./Malv./Brassicales - ps25
Malvales/Brassicales - ps26
Brassicales - ps27
Cleomaceae/Brassicaceae - ps28
Brassicaceae - ps29
Camelineae - ps30
Arabidopsis - ps31
Arabidopsis_thaliana - ps32
p-value
5e-08
2e-06
6e-05
2e-03
7e-02
Log-odds
0.5
1.0
1.5
2.0
2.5
3.0
3.5
4.0

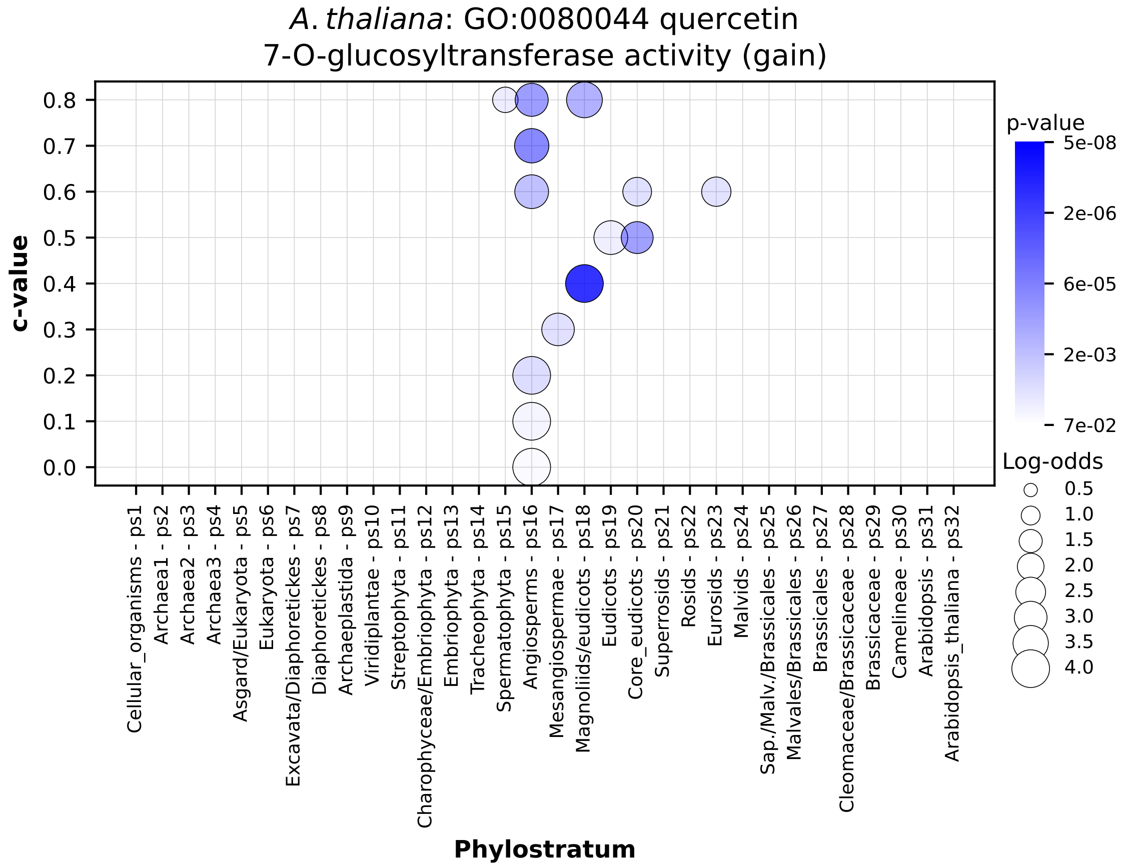

A. thaliana: GO:0080044 quercetin
7-O-glucosyltransferase activity (gain)
c-value
0.8
0.7
0.6
0.5
0.4
0.3
0.2
0.1
0.0
p-value
5e-08
2e-06
6e-05
2e-03
7e-02
Log-odds
0.5
1.0
1.5
2.0
2.5
3.0
3.5
4.0
Cellular_organisms - ps1
Archaea1 - ps2
Archaea2 - ps3
Archaea3 - ps4
Asgard/Eukaryota - ps5
Eukaryota - ps6
Excavata/Diaphoretickes - ps7
Diaphoretickes - ps8
Archaeplastida - ps9
Viridiplantae - ps10
Streptophyta - ps11
Charophyceae/Embriophyta - ps12
Embriophyta - ps13
Tracheophyta - ps14
Spermatophyta - ps15
Angiosperms - ps16
Mesangiospermae - ps17
Magnoliids/eudicots - ps18
Eudicots - ps19
Core_eudicots - ps20
Superrosids - ps21
Rosids - ps22
Eurosids - ps23
Malvids - ps24
Sap./Malv./Brassicales - ps25
Malvales/Brassicales - ps26
Brassicales - ps27
Cleomaceae/Brassicaceae - ps28
Brassicaceae - ps29
Camelineae - ps30
Arabidopsis - ps31
Arabidopsis_thaliana - ps32
Phylostratum

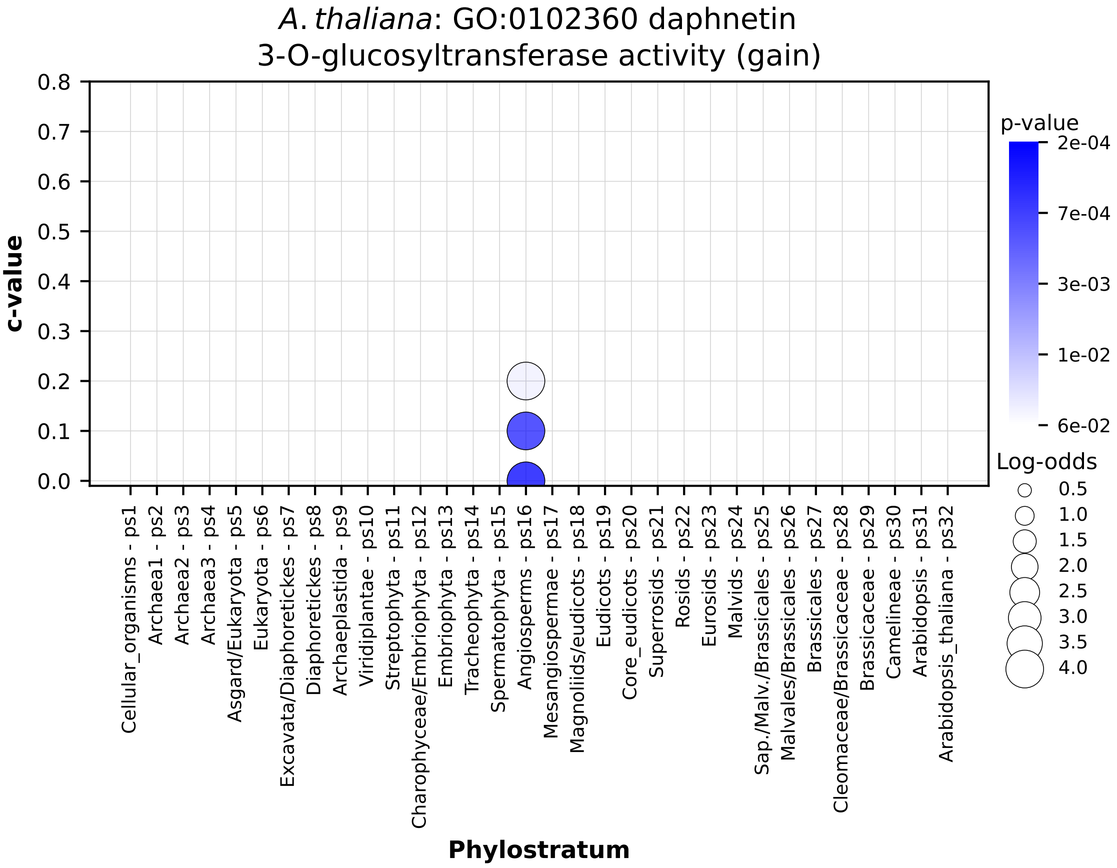
A. thaliana: GO:0102360 daphnetin
3-O-glucosyltransferase activity (gain)
c-value
0.8
0.7
0.6
0.5
0.4
0.3
0.2
0.1
0.0
p-value
2e-04
7e-04
3e-03
1e-02
6e-02
Log-odds
0.5
1.0
1.5
2.0
2.5
3.0
3.5
4.0
Cellular_organisms - ps1
Archaea1 - ps2
Archaea2 - ps3
Archaea3 - ps4
Asgard/Eukaryota - ps5
Eukaryota - ps6
Excavata/Diaphoretickes - ps7
Diaphoretickes - ps8
Archaeplastida - ps9
Viridiplantae - ps10
Streptophyta - ps11
Charophyceae/Embriophyta - ps12
Embriophyta - ps13
Tracheophyta - ps14
Spermatophyta - ps15
Angiosperms - ps16
Mesangiospermae - ps17
Magnoliids/eudicots - ps18
Eudicots - ps19
Core_eudicots - ps20
Superrosids - ps21
Rosids - ps22
Eurosids - ps23
Malvids - ps24
Sap./Malv./Brassicales - ps25
Malvales/Brassicales - ps26
Brassicales - ps27
Cleomaceae/Brassicaceae - ps28
Brassicaceae - ps29
Camelineae - ps30
Arabidopsis - ps31
Arabidopsis_thaliana - ps32
Phylostratum

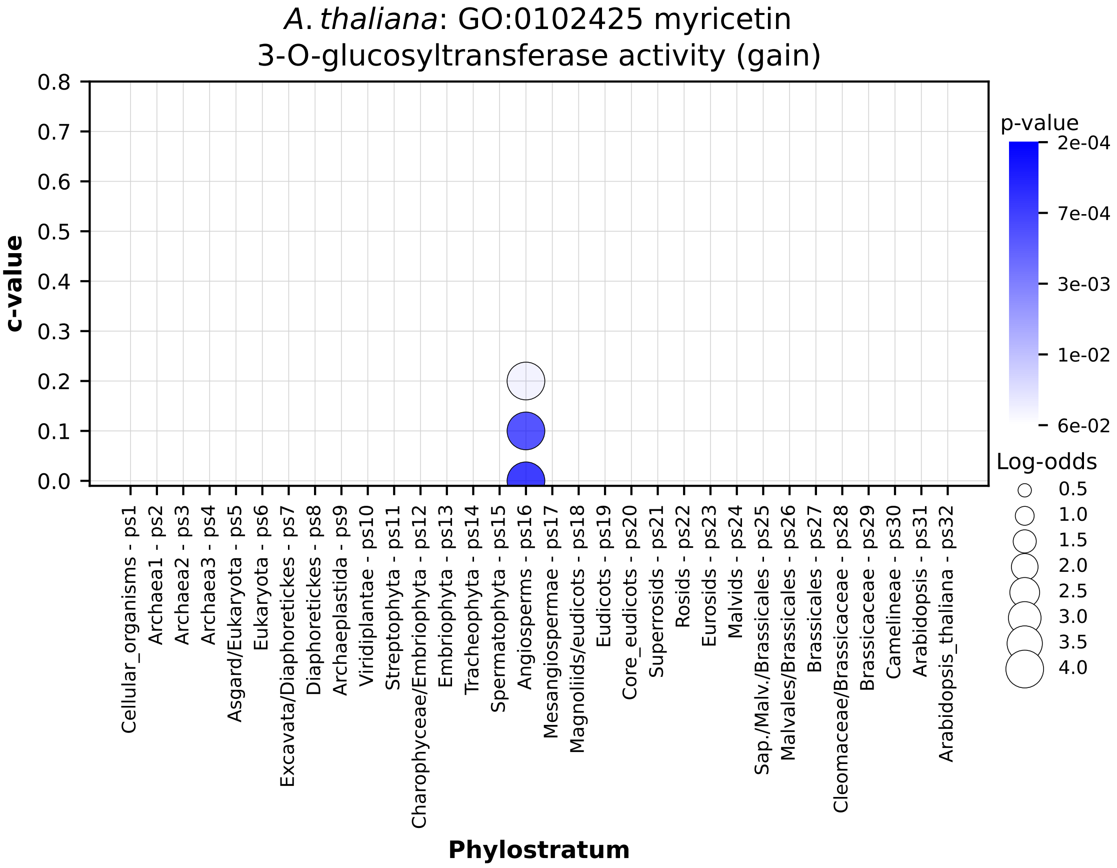
A. thaliana: GO:0102425 myricetin 3-O-glucosyltransferase activity (gain)
c-value
0.8
0.7
0.6
0.5
0.4
0.3
0.2
0.1
0.0
p-value
2e-04
7e-04
3e-03
1e-02
6e-02
Log-odds
0.5
1.0
1.5
2.0
2.5
3.0
3.5
4.0
Cellular_organisms - ps1
Archaea1 - ps2
Archaea2 - ps3
Archaea3 - ps4
Asgard/Eukaryota - ps5
Eukaryota - ps6
Excavata/Diaphoretickes - ps7
Diaphoretickes - ps8
Archaeplastida - ps9
Viridiplantae - ps10
Streptophyta - ps11
Charophyceae/Embriophyta - ps12
Embriophyta - ps13
Tracheophyta - ps14
Spermatophyta - ps15
Angiosperms - ps16
Mesangiospermae - ps17
Magnoliids/eudicots - ps18
Eudicots - ps19
Core_eudicots - ps20
Superrosids - ps21
Rosids - ps22
Eurosids - ps23
Malvids - ps24
Sap./Malv./Brassicales - ps25
Malvales/Brassicales - ps26
Brassicales - ps27
Cleomaceae/Brassicaceae - ps28
Brassicaceae - ps29
Camelineae - ps30
Arabidopsis - ps31
Arabidopsis_thaliana - ps32
Phylostratum

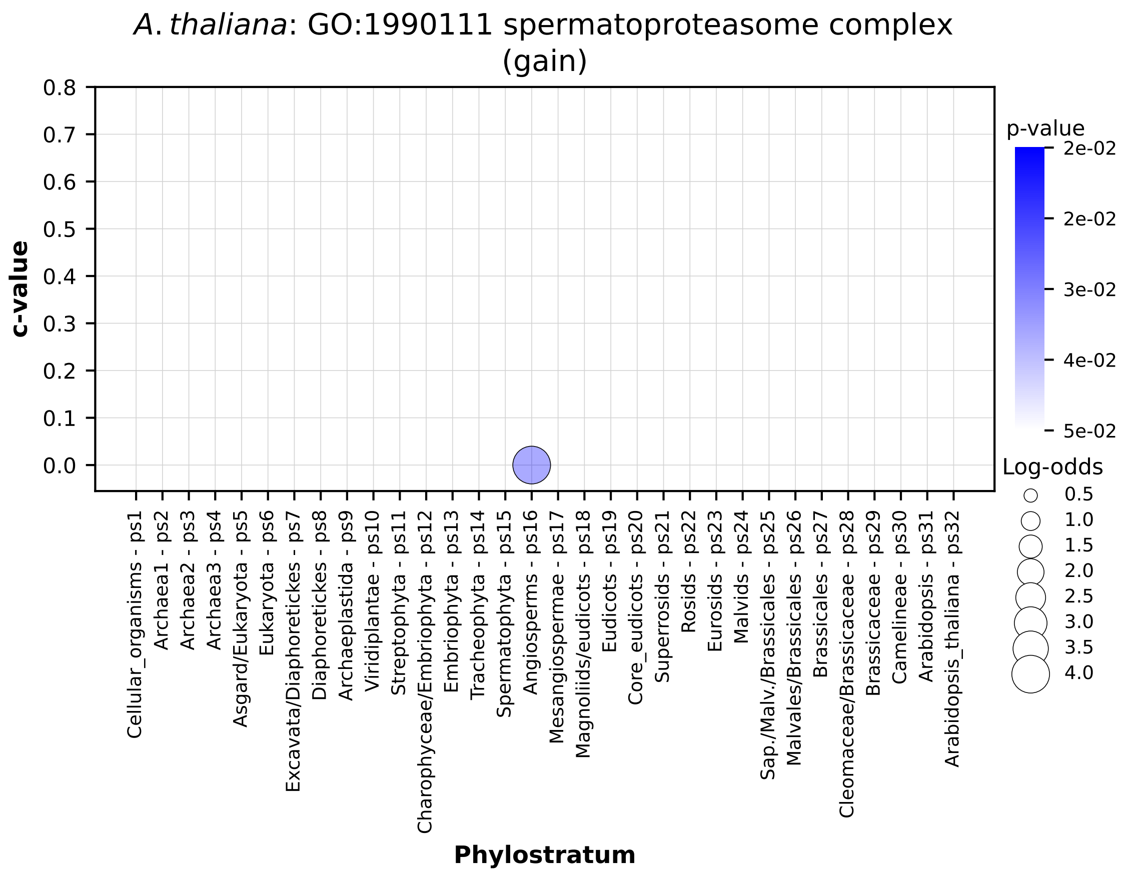

A. thaliana: GO:1990111 spermatoproteasome complex
(gain)
c-value
0.8
0.7
0.6
0.5
0.4
0.3
0.2
0.1
0.0
p-value
2e-02
2e-02
3e-02
4e-02
5e-02
Log-odds
0.5
1.0
1.5
2.0
2.5
3.0
3.5
4.0
Cellular_organisms - ps1
Archaea1 - ps2
Archaea2 - ps3
Archaea3 - ps4
Asgard/Eukaryota - ps5
Eukaryota - ps6
Excavata/Diaphoretickes - ps7
Diaphoretickes - ps8
Archaeplastida - ps9
Viridiplantae - ps10
Streptophyta - ps11
Charophyceae/Embriophyta - ps12
Embriophyta - ps13
Tracheophyta - ps14
Spermatophyta - ps15
Angiosperms - ps16
Mesangiospermae - ps17
Magnoliids/eudicots - ps18
Eudicots - ps19
Core_eudicots - ps20
Superrosids - ps21
Rosids - ps22
Eurosids - ps23
Malvids - ps24
Sap./Malv./Brassicales - ps25
Malvales/Brassicales - ps26
Brassicales - ps27
Cleomaceae/Brassicaceae - ps28
Brassicaceae - ps29
Camelineae - ps30
Arabidopsis - ps31
Arabidopsis_thaliana - ps32
Phylostratum

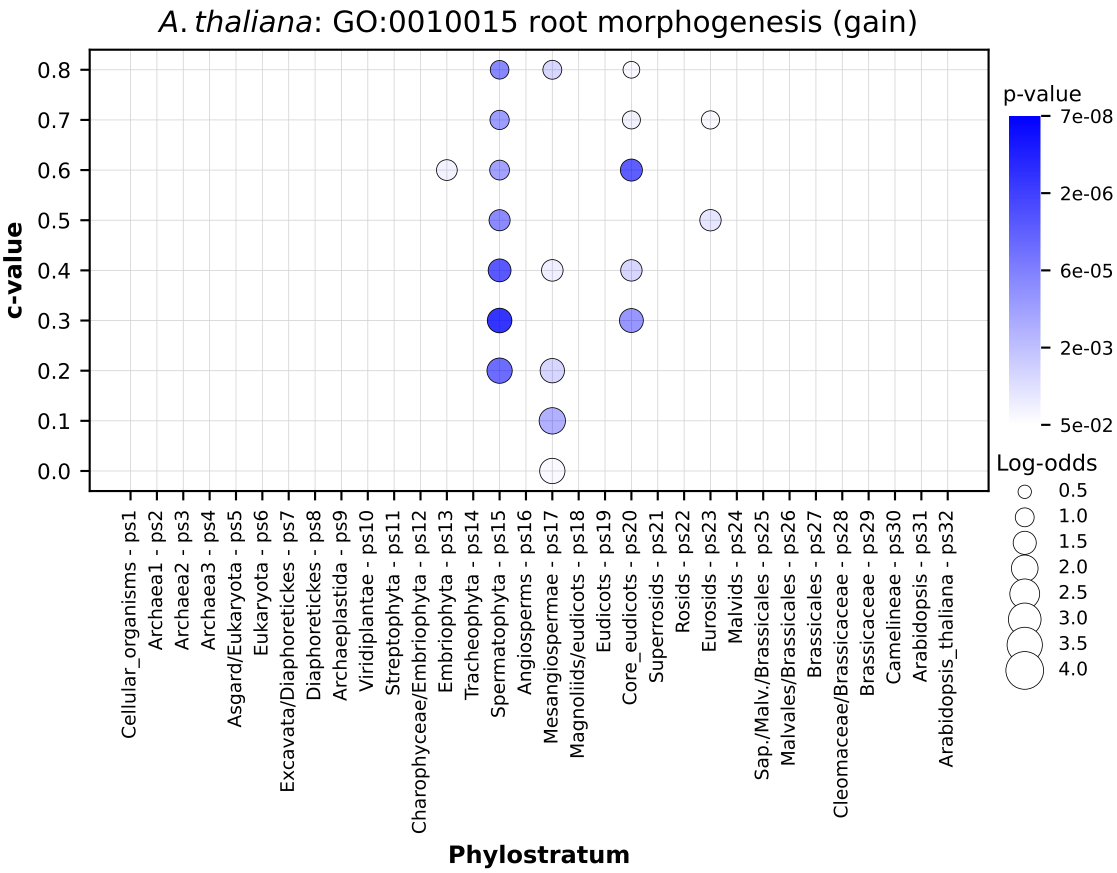

A. thaliana: GO:0010015 root morphogenesis (gain)
c-value
0.8
0.7
0.6
0.5
0.4
0.3
0.2
0.1
0.0
p-value
7e-08
2e-06
6e-05
2e-03
5e-02
Log-odds
0.5
1.0
1.5
2.0
2.5
3.0
3.5
4.0
Cellular_organisms - ps1
Archaea1 - ps2
Archaea2 - ps3
Archaea3 - ps4
Asgard/Eukaryota - ps5
Eukaryota - ps6
Excavata/Diaphoretickes - ps7
Diaphoretickes - ps8
Archaeplastida - ps9
Viridiplantae - ps10
Streptophyta - ps11
Charophyceae/Embriophyta - ps12
Embriophyta - ps13
Tracheophyta - ps14
Spermatophyta - ps15
Angiosperms - ps16
Mesangiospermae - ps17
Magnoliids/eudicots - ps18
Eudicots - ps19
Core_eudicots - ps20
Superrosids - ps21
Rosids - ps22
Eurosids - ps23
Malvids - ps24
Sap./Malv./Brassicales - ps25
Malvales/Brassicales - ps26
Brassicales - ps27
Cleomaceae/Brassicaceae - ps28
Brassicaceae - ps29
Camelineae - ps30
Arabidopsis - ps31
Arabidopsis_thaliana - ps32
Phylostratum

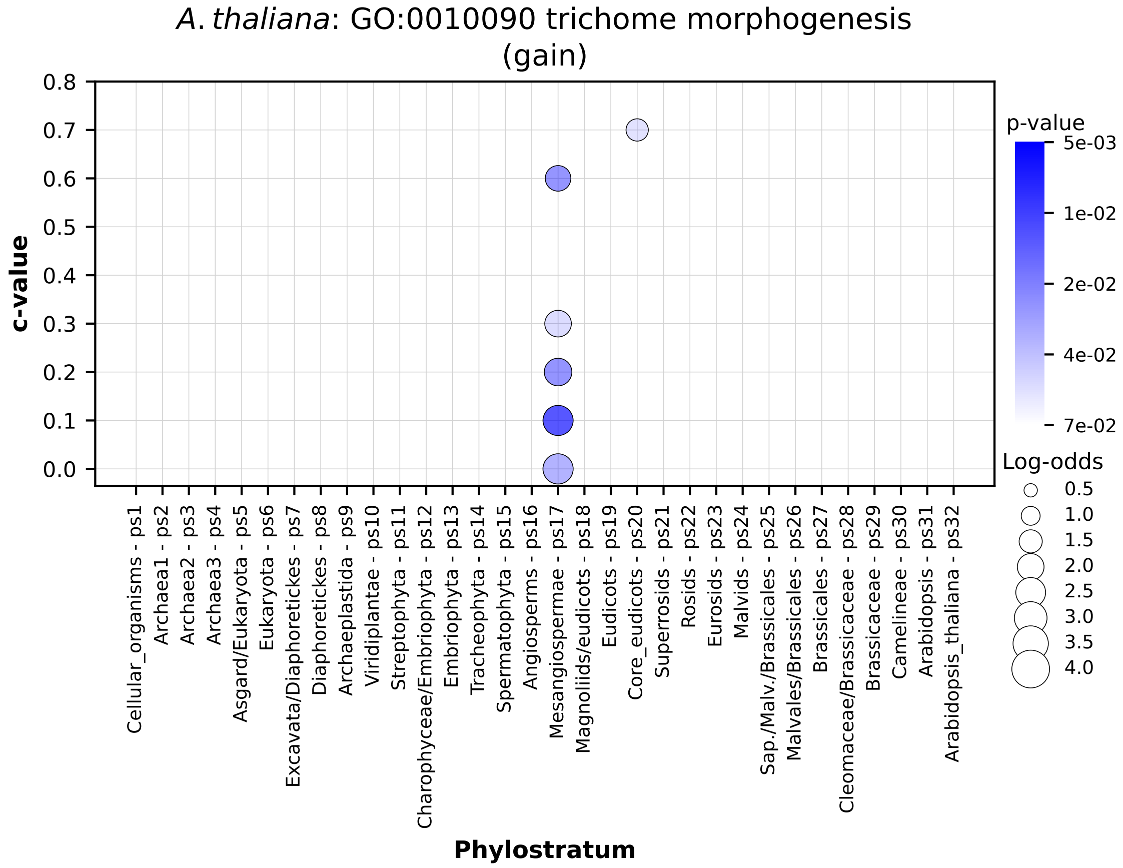
A. thaliana: GO:0010090 trichome morphogenesis
(gain)
c-value
0.8
0.7
0.6
0.5
0.4
0.3
0.2
0.1
0.0
p-value
5e-03
1e-02
2e-02
4e-02
7e-02
Log-odds
0.5
1.0
1.5
2.0
2.5
3.0
3.5
4.0
Cellular_organisms - ps1
Archaea1 - ps2
Archaea2 - ps3
Archaea3 - ps4
Asgard/Eukaryota - ps5
Eukaryota - ps6
Excavata/Diaphoretickes - ps7
Diaphoretickes - ps8
Archaeplastida - ps9
Viridiplantae - ps10
Streptophyta - ps11
Charophyceae/Embriophyta - ps12
Embriophyta - ps13
Tracheophyta - ps14
Spermatophyta - ps15
Angiosperms - ps16
Mesangiospermae - ps17
Magnoliids/eudicots - ps18
Eudicots - ps19
Core_eudicots - ps20
Superrosids - ps21
Rosids - ps22
Eurosids - ps23
Malvids - ps24
Sap./Malv./Brassicales - ps25
Malvales/Brassicales - ps26
Brassicales - ps27
Cleomaceae/Brassicaceae - ps28
Brassicaceae - ps29
Camelineae - ps30
Arabidopsis - ps31
Arabidopsis_thaliana - ps32
Phylostratum

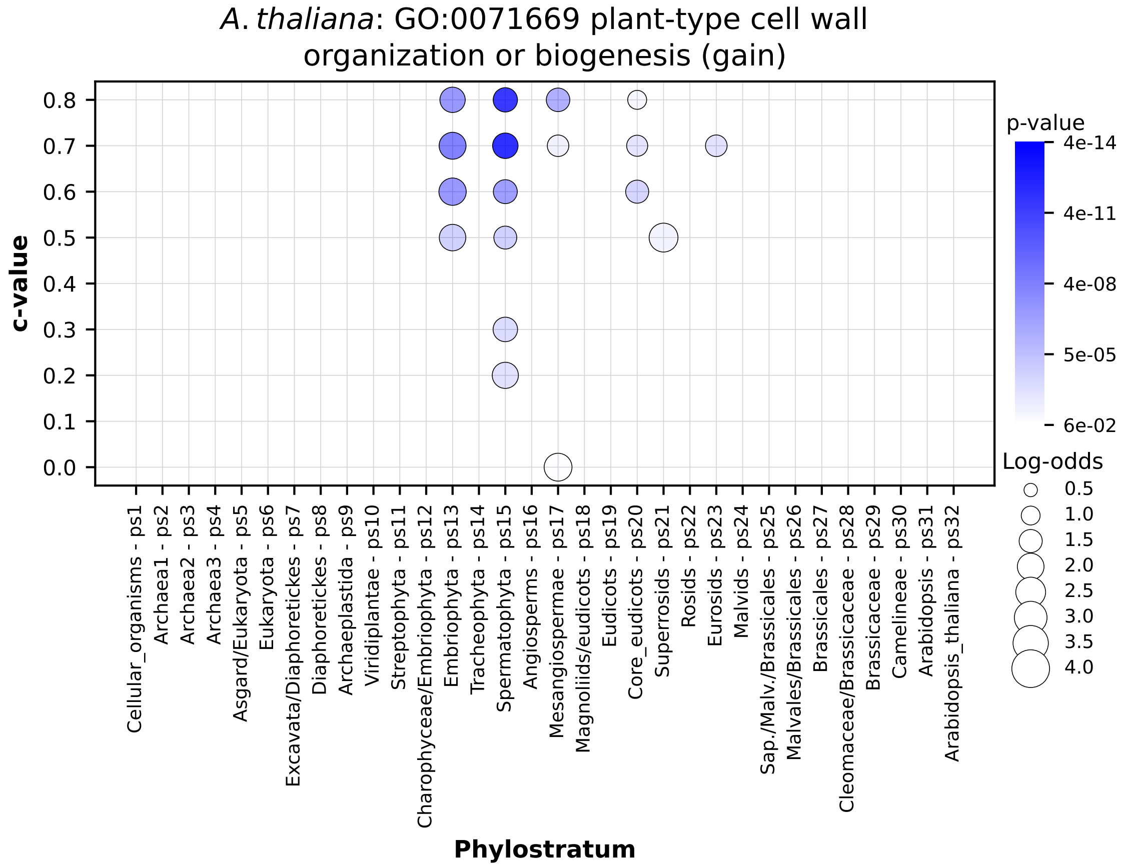

A. thaliana: GO:0071669 plant-type cell wall organization or biogenesis (gain)
c-value
0.8
0.7
0.6
0.5
0.4
0.3
0.2
0.1
0.0
Phylostratum
Cellular_organisms - ps1
Archaea1 - ps2
Archaea2 - ps3
Archaea3 - ps4
Asgard/Eukaryota - ps5
Eukaryota - ps6
Excavata/Diaphoretickes - ps7
Diaphoretickes - ps8
Archaeplastida - ps9
Viridiplantae - ps10
Streptophyta - ps11
Charophyceae/Embriophyta - ps12
Embriophyta - ps13
Tracheophyta - ps14
Spermatophyta - ps15
Angiosperms - ps16
Mesangiospermae - ps17
Magnoliids/eudicots - ps18
Eudicots - ps19
Core_eudicots - ps20
Superrosids - ps21
Rosids - ps22
Eurosids - ps23
Malvids - ps24
Sap./Malv./Brassicales - ps25
Malvales/Brassicales - ps26
Brassicales - ps27
Cleomaceae/Brassicaceae - ps28
Brassicaceae - ps29
Camelineae - ps30
Arabidopsis - ps31
Arabidopsis_thaliana - ps32
p-value
4e-14
4e-11
4e-08
5e-05
6e-02
Log-odds
0.5
1.0
1.5
2.0
2.5
3.0
3.5
4.0

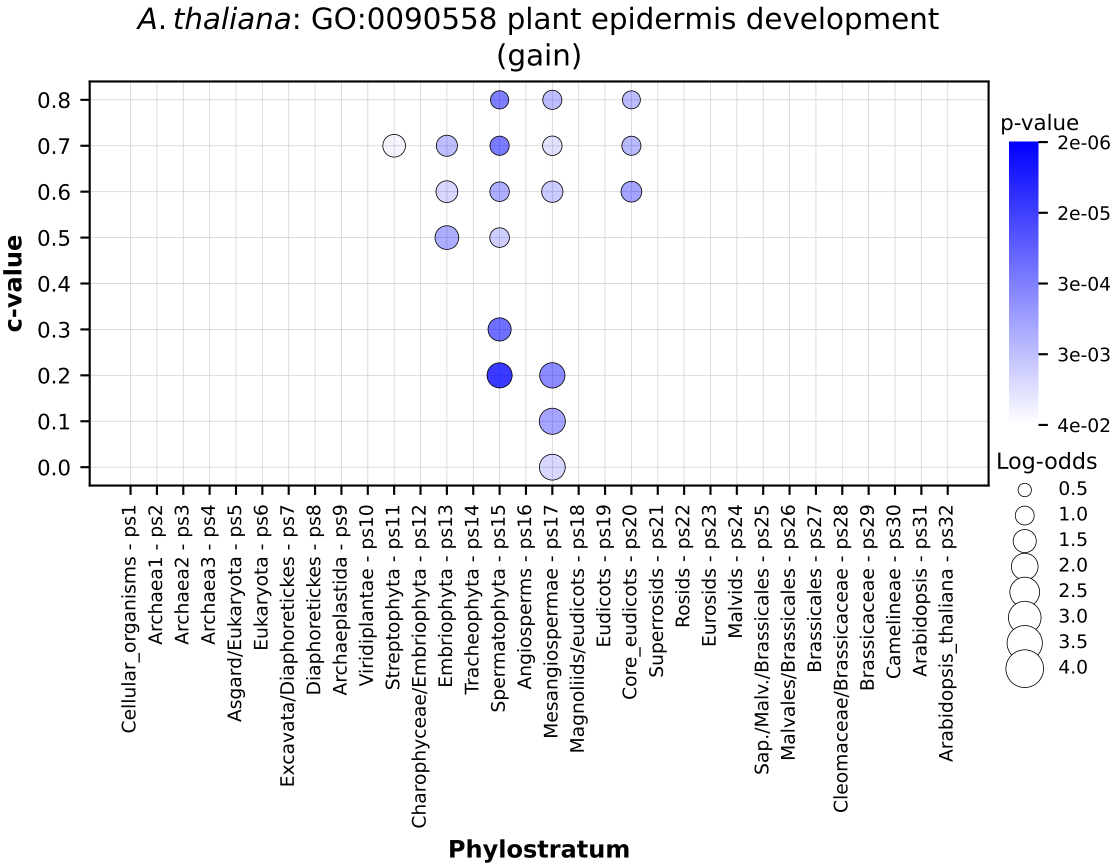
A. thaliana: GO:0090558 plant epidermis development
(gain)
c-value
0.8
0.7
0.6
0.5
0.4
0.3
0.2
0.1
0.0
p-value
2e-06
2e-05
3e-04
3e-03
4e-02
Log-odds
0.5
1.0
1.5
2.0
2.5
3.0
3.5
4.0
Cellular_organisms - ps1
Archaea1 - ps2
Archaea2 - ps3
Archaea3 - ps4
Asgard/Eukaryota - ps5
Eukaryota - ps6
Excavata/Diaphoretickes - ps7
Diaphoretickes - ps8
Archaeplastida - ps9
Viridiplantae - ps10
Streptophyta - ps11
Charophyceae/Embriophyta - ps12
Embriophyta - ps13
Tracheophyta - ps14
Spermatophyta - ps15
Angiosperms - ps16
Mesangiospermae - ps17
Magnoliids/eudicots - ps18
Eudicots - ps19
Core_eudicots - ps20
Superrosids - ps21
Rosids - ps22
Eurosids - ps23
Malvids - ps24
Sap./Malv./Brassicales - ps25
Malvales/Brassicales - ps26
Brassicales - ps27
Cleomaceae/Brassicaceae - ps28
Brassicaceae - ps29
Camelineae - ps30
Arabidopsis - ps31
Arabidopsis_thaliana - ps32
Phylostratum

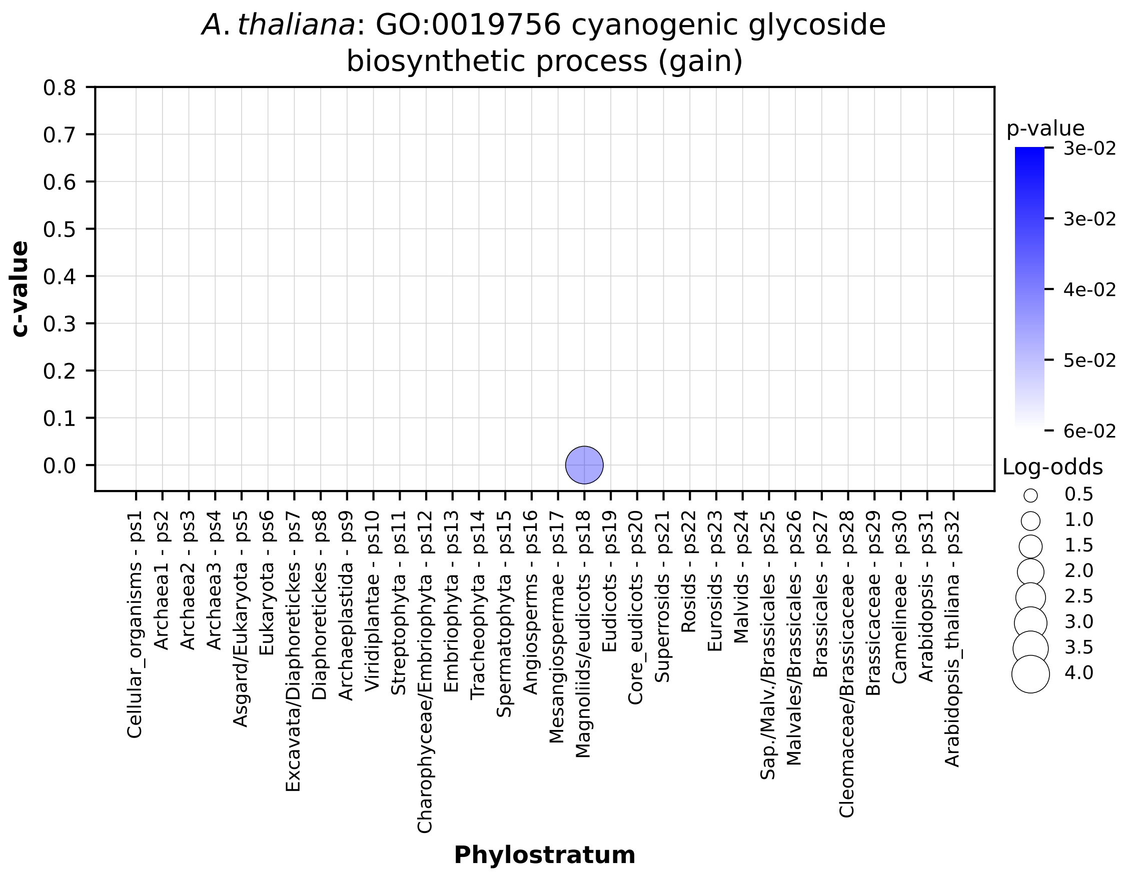

$A. thaliana$: GO:0019756 cyanogenic glycoside biosynthetic process (gain)
c-value
0.8
0.7
0.6
0.5
0.4
0.3
0.2
0.1
0.0
p-value
3e-02
3e-02
4e-02
5e-02
6e-02
Log-odds
0.5
1.0
1.5
2.0
2.5
3.0
3.5
4.0
Cellular_organisms - ps1
Archaea1 - ps2
Archaea2 - ps3
Archaea3 - ps4
Asgard/Eukaryota - ps5
Eukaryota - ps6
Excavata/Diaphoretickes - ps7
Diaphoretickes - ps8
Archaeplastida - ps9
Viridiplantae - ps10
Streptophyta - ps11
Charophyceae/Embriophyta - ps12
Embriophyta - ps13
Tracheophyta - ps14
Spermatophyta - ps15
Angiosperms - ps16
Mesangiospermae - ps17
Magnoliids/eudicots - ps18
Eudicots - ps19
Core_eudicots - ps20
Superrosids - ps21
Rosids - ps22
Eurosids - ps23
Malvids - ps24
Sap./Malv./Brassicales - ps25
Malvales/Brassicales - ps26
Brassicales - ps27
Cleomaceae/Brassicaceae - ps28
Brassicaceae - ps29
Camelineae - ps30
Arabidopsis - ps31
Arabidopsis_thaliana - ps32
Phylostratum

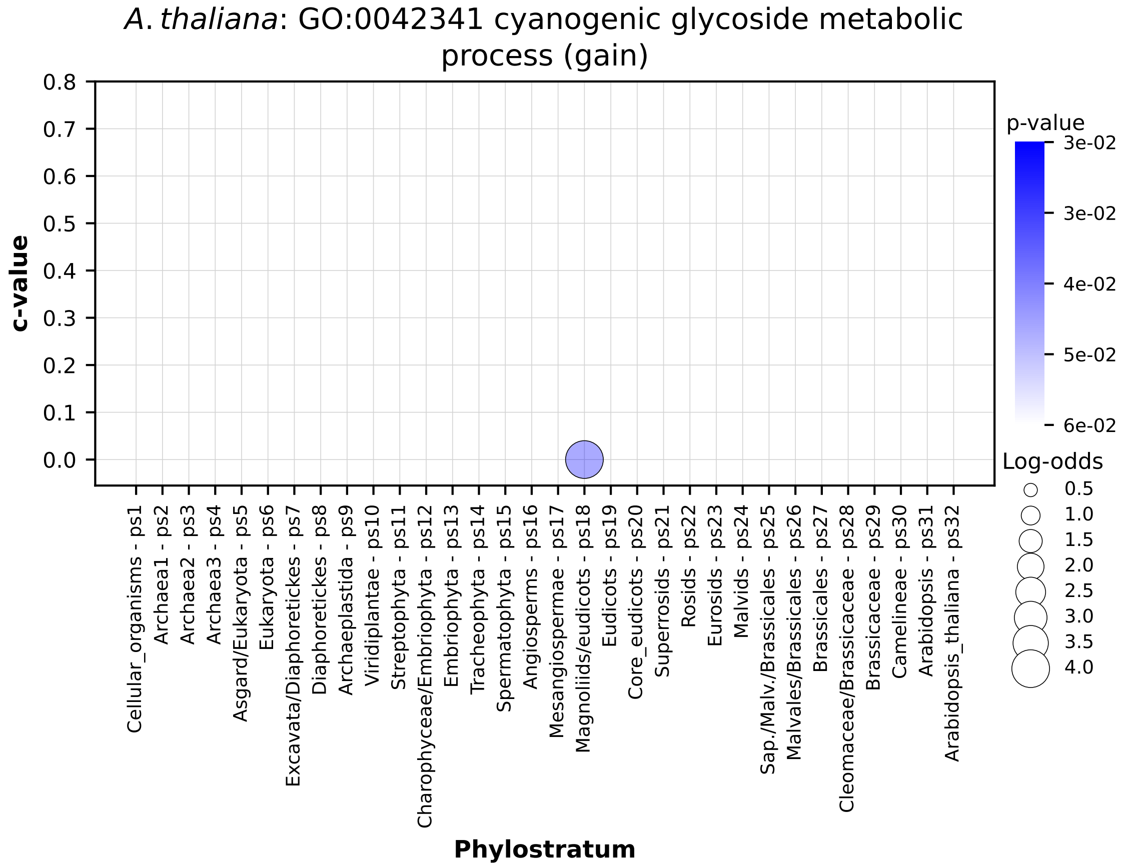

A. thaliana: GO:0042341 cyanogenic glycoside metabolic process (gain)
c-value
0.8
0.7
0.6
0.5
0.4
0.3
0.2
0.1
0.0
p-value
3e-02
3e-02
4e-02
5e-02
6e-02
Log-odds
0.5
1.0
1.5
2.0
2.5
3.0
3.5
4.0
Cellular_organisms - ps1
Archaea1 - ps2
Archaea2 - ps3
Archaea3 - ps4
Asgard/Eukaryota - ps5
Eukaryota - ps6
Excavata/Diaphoretickes - ps7
Diaphoretickes - ps8
Archaeplastida - ps9
Viridiplantae - ps10
Streptophyta - ps11
Charophyceae/Embriophyta - ps12
Embriophyta - ps13
Tracheophyta - ps14
Spermatophyta - ps15
Angiosperms - ps16
Mesangiospermae - ps17
Magnoliids/eudicots - ps18
Eudicots - ps19
Core_eudicots - ps20
Superrosids - ps21
Rosids - ps22
Eurosids - ps23
Malvids - ps24
Sap./Malv./Brassicales - ps25
Malvales/Brassicales - ps26
Brassicales - ps27
Cleomaceae/Brassicaceae - ps28
Brassicaceae - ps29
Camelineae - ps30
Arabidopsis - ps31
Arabidopsis_thaliana - ps32
Phylostratum

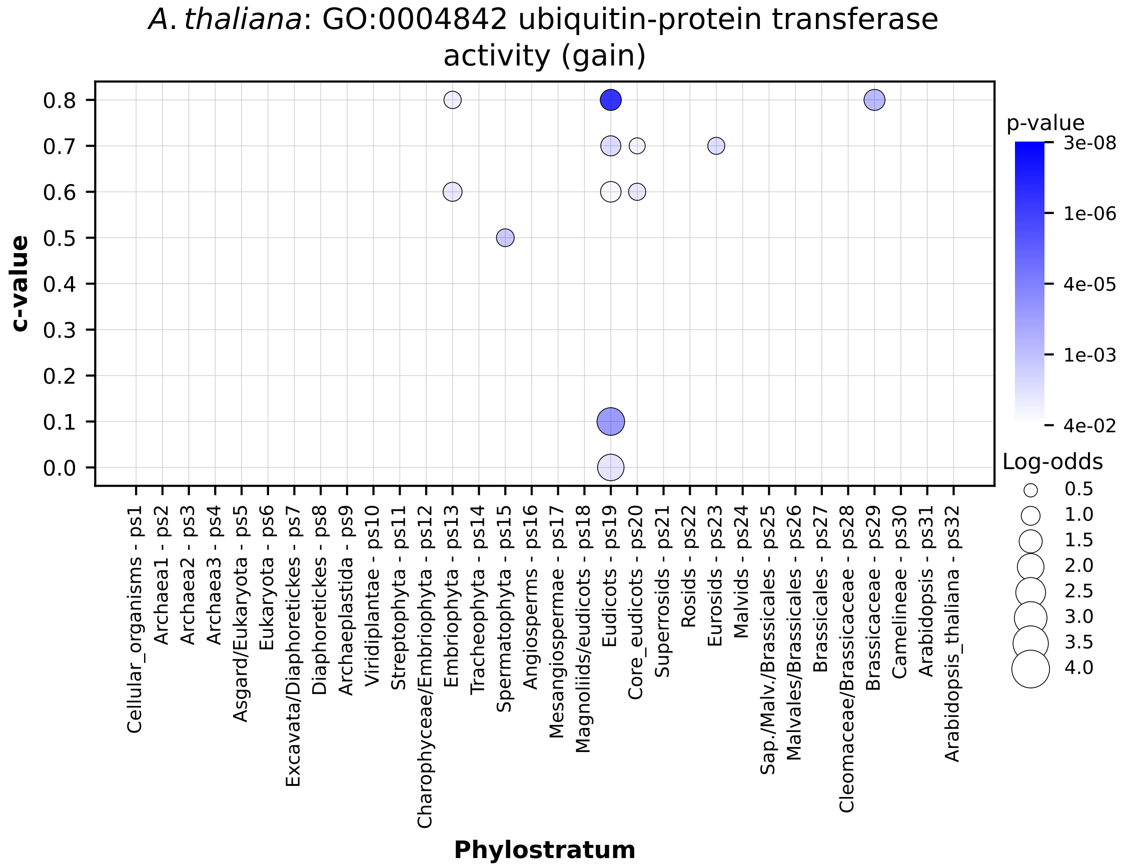
A. thaliana: GO:0004842 ubiquitin-protein transferase activity (gain)
c-value
0.8
0.7
0.6
0.5
0.4
0.3
0.2
0.1
0.0
p-value
3e-08
1e-06
4e-05
1e-03
4e-02
Log-odds
0.5
1.0
1.5
2.0
2.5
3.0
3.5
4.0
Cellular_organisms - ps1
Archaea1 - ps2
Archaea2 - ps3
Archaea3 - ps4
Asgard/Eukaryota - ps5
Eukaryota - ps6
Excavata/Diaphoretickes - ps7
Diaphoretickes - ps8
Archaeplastida - ps9
Viridiplantae - ps10
Streptophyta - ps11
Charophyceae/Embriophyta - ps12
Embriophyta - ps13
Tracheophyta - ps14
Spermatophyta - ps15
Angiosperms - ps16
Mesangiospermae - ps17
Magnoliids/eudicots - ps18
Eudicots - ps19
Core_eudicots - ps20
Superrosids - ps21
Rosids - ps22
Eurosids - ps23
Malvids - ps24
Sap./Malv./Brassicales - ps25
Malvales/Brassicales - ps26
Brassicales - ps27
Cleomaceae/Brassicaceae - ps28
Brassicaceae - ps29
Camelineae - ps30
Arabidopsis - ps31
Arabidopsis_thaliana - ps32
Phylostratum

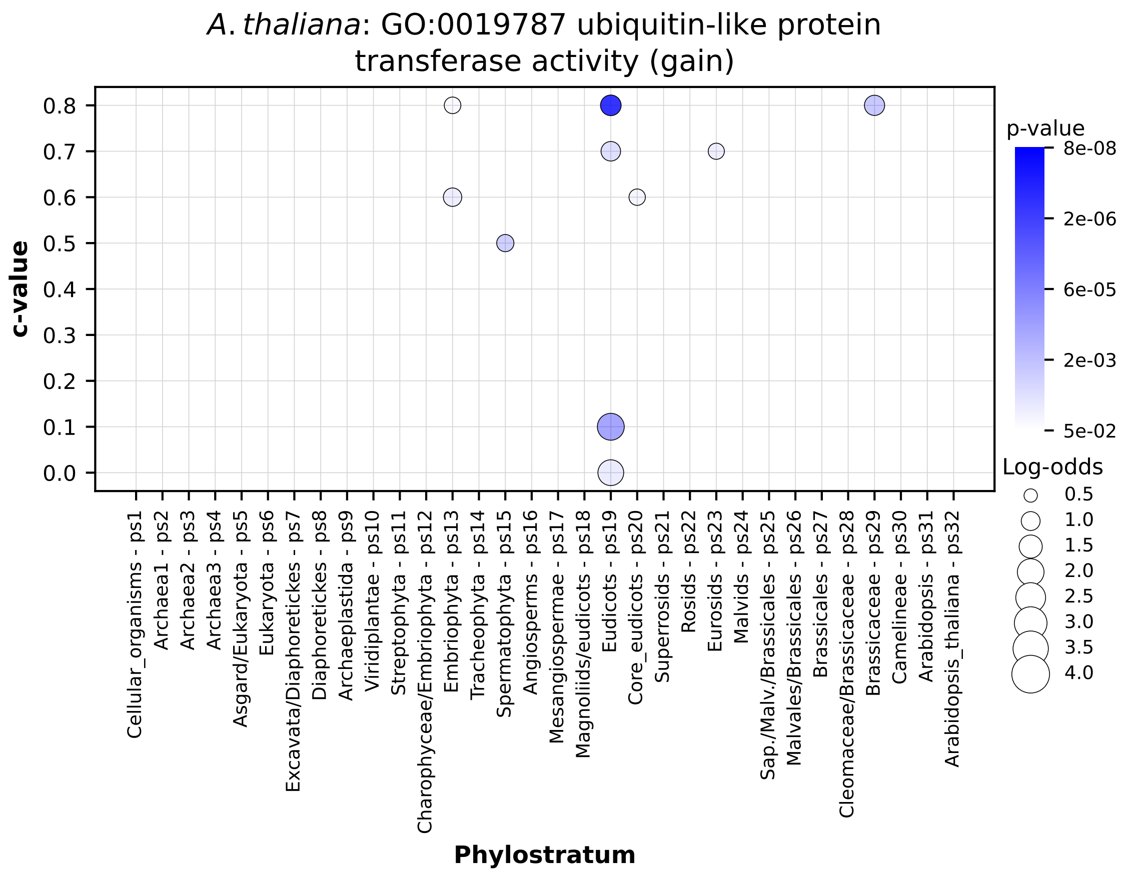

A. thaliana: GO:0019787 ubiquitin-like protein transferase activity (gain)
c-value
0.8
0.7
0.6
0.5
0.4
0.3
0.2
0.1
0.0
p-value
8e-08
2e-06
6e-05
2e-03
5e-02
Log-odds
0.5
1.0
1.5
2.0
2.5
3.0
3.5
4.0
Cellular_organisms - ps1
Archaea1 - ps2
Archaea2 - ps3
Archaea3 - ps4
Asgard/Eukaryota - ps5
Eukaryota - ps6
Excavata/Diaphoretickes - ps7
Diaphoretickes - ps8
Archaeplastida - ps9
Viridiplantae - ps10
Streptophyta - ps11
Charophyceae/Embriophyta - ps12
Embriophyta - ps13
Tracheophyta - ps14
Spermatophyta - ps15
Angiosperms - ps16
Mesangiospermae - ps17
Magnoliids/eudicots - ps18
Eudicots - ps19
Core_eudicots - ps20
Superrosids - ps21
Rosids - ps22
Eurosids - ps23
Malvids - ps24
Sap./Malv./Brassicales - ps25
Malvales/Brassicales - ps26
Brassicales - ps27
Cleomaceae/Brassicaceae - ps28
Brassicaceae - ps29
Camelineae - ps30
Arabidopsis - ps31
Arabidopsis_thaliana - ps32
Phylostratum

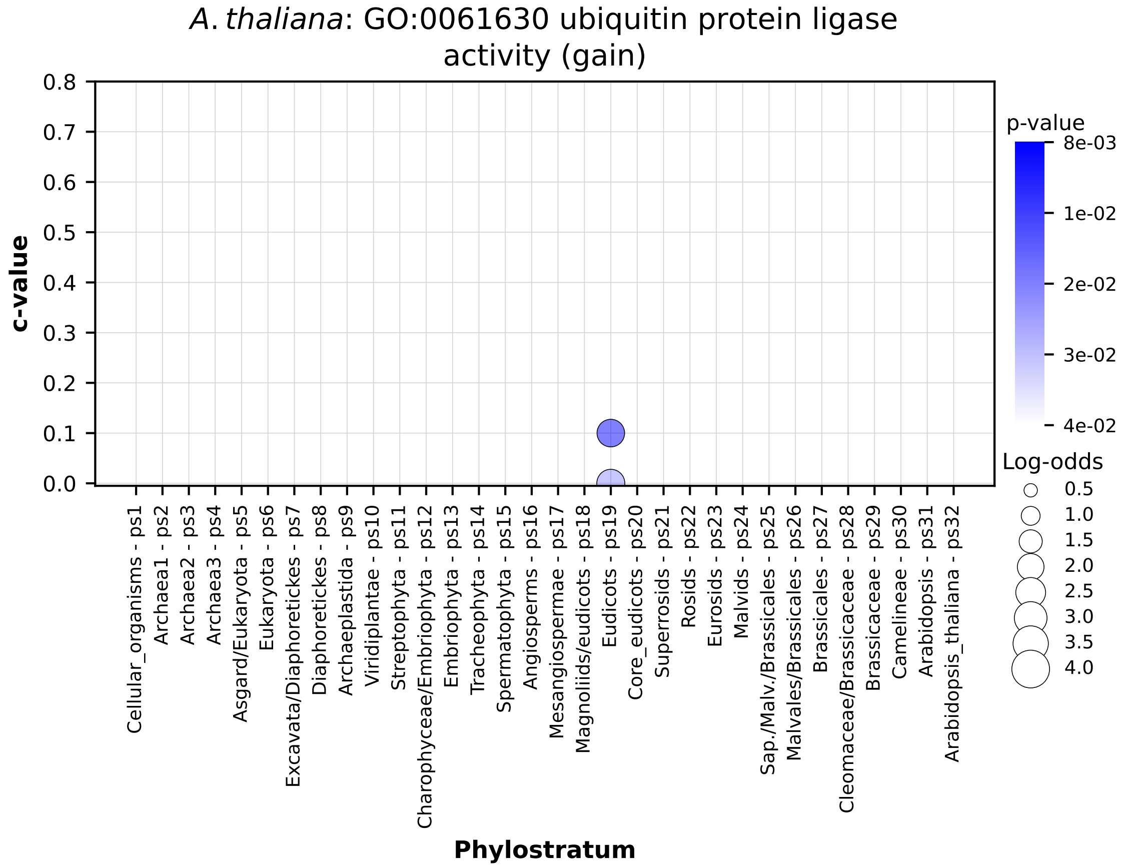

A. thaliana: GO:0061630 ubiquitin protein ligase activity (gain)
c-value
0.8
0.7
0.6
0.5
0.4
0.3
0.2
0.1
0.0
p-value
8e-03
1e-02
2e-02
3e-02
4e-02
Log-odds
0.5
1.0
1.5
2.0
2.5
3.0
3.5
4.0
Cellular_organisms - ps1
Archaea1 - ps2
Archaea2 - ps3
Archaea3 - ps4
Asgard/Eukaryota - ps5
Eukaryota - ps6
Excavata/Diaphoretickes - ps7
Diaphoretickes - ps8
Archaeplastida - ps9
Viridiplantae - ps10
Streptophyta - ps11
Charophyceae/Embriophyta - ps12
Embriophyta - ps13
Tracheophyta - ps14
Spermatophyta - ps15
Angiosperms - ps16
Mesangiospermae - ps17
Magnoliids/eudicots - ps18
Eudicots - ps19
Core_eudicots - ps20
Superrosids - ps21
Rosids - ps22
Eurosids - ps23
Malvids - ps24
Sap./Malv./Brassicales - ps25
Malvales/Brassicales - ps26
Brassicales - ps27
Cleomaceae/Brassicaceae - ps28
Brassicaceae - ps29
Camelineae - ps30
Arabidopsis - ps31
Arabidopsis_thaliana - ps32
Phylostratum

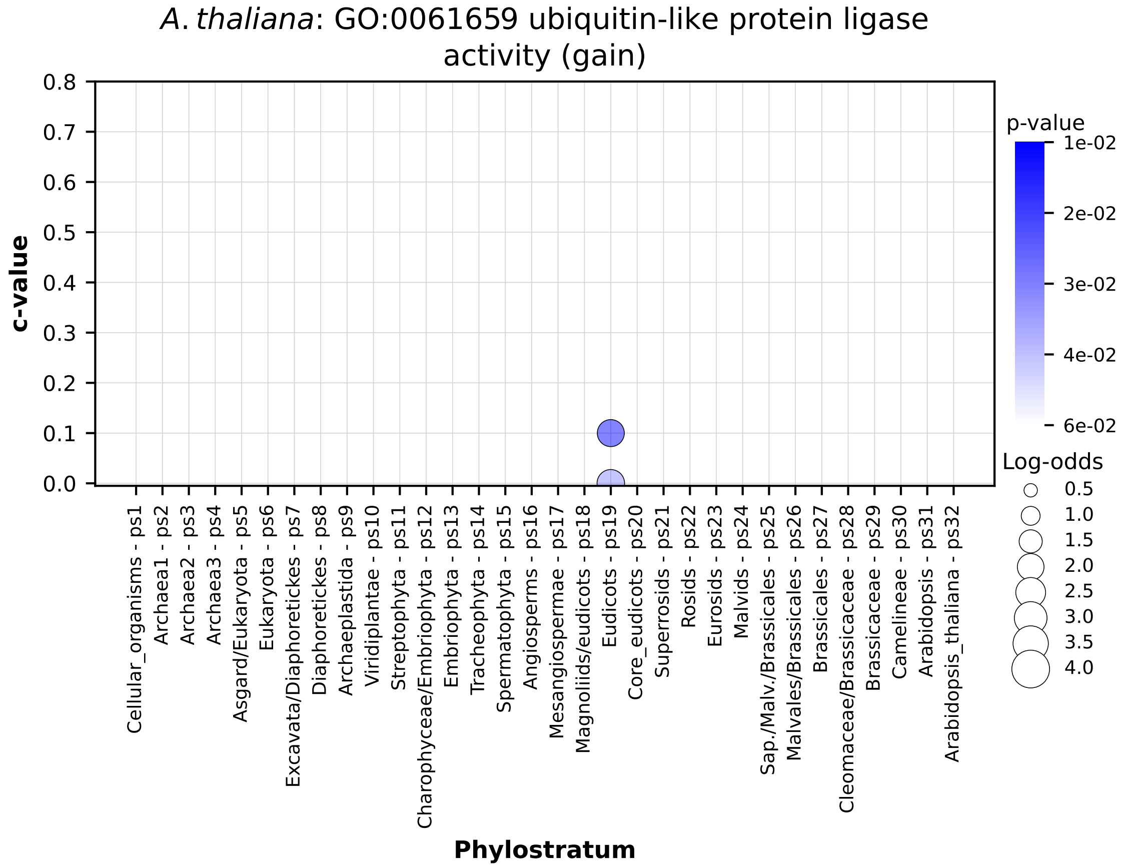
A. thaliana: GO:0061659 ubiquitin-like protein ligase activity (gain)
c-value
0.8
0.7
0.6
0.5
0.4
0.3
0.2
0.1
0.0
p-value
1e-02
2e-02
3e-02
4e-02
6e-02
Log-odds
0.5
1.0
1.5
2.0
2.5
3.0
3.5
4.0
Cellular_organisms - ps1
Archaea1 - ps2
Archaea2 - ps3
Archaea3 - ps4
Asgard/Eukaryota - ps5
Eukaryota - ps6
Excavata/Diaphoretickes - ps7
Diaphoretickes - ps8
Archaeplastida - ps9
Viridiplantae - ps10
Streptophyta - ps11
Charophyceae/Embriophyta - ps12
Embriophyta - ps13
Tracheophyta - ps14
Spermatophyta - ps15
Angiosperms - ps16
Mesangiospermae - ps17
Magnoliids/eudicots - ps18
Eudicots - ps19
Core_eudicots - ps20
Superrosids - ps21
Rosids - ps22
Eurosids - ps23
Malvids - ps24
Sap./Malv./Brassicales - ps25
Malvales/Brassicales - ps26
Brassicales - ps27
Cleomaceae/Brassicaceae - ps28
Brassicaceae - ps29
Camelineae - ps30
Arabidopsis - ps31
Arabidopsis_thaliana - ps32
Phylostratum

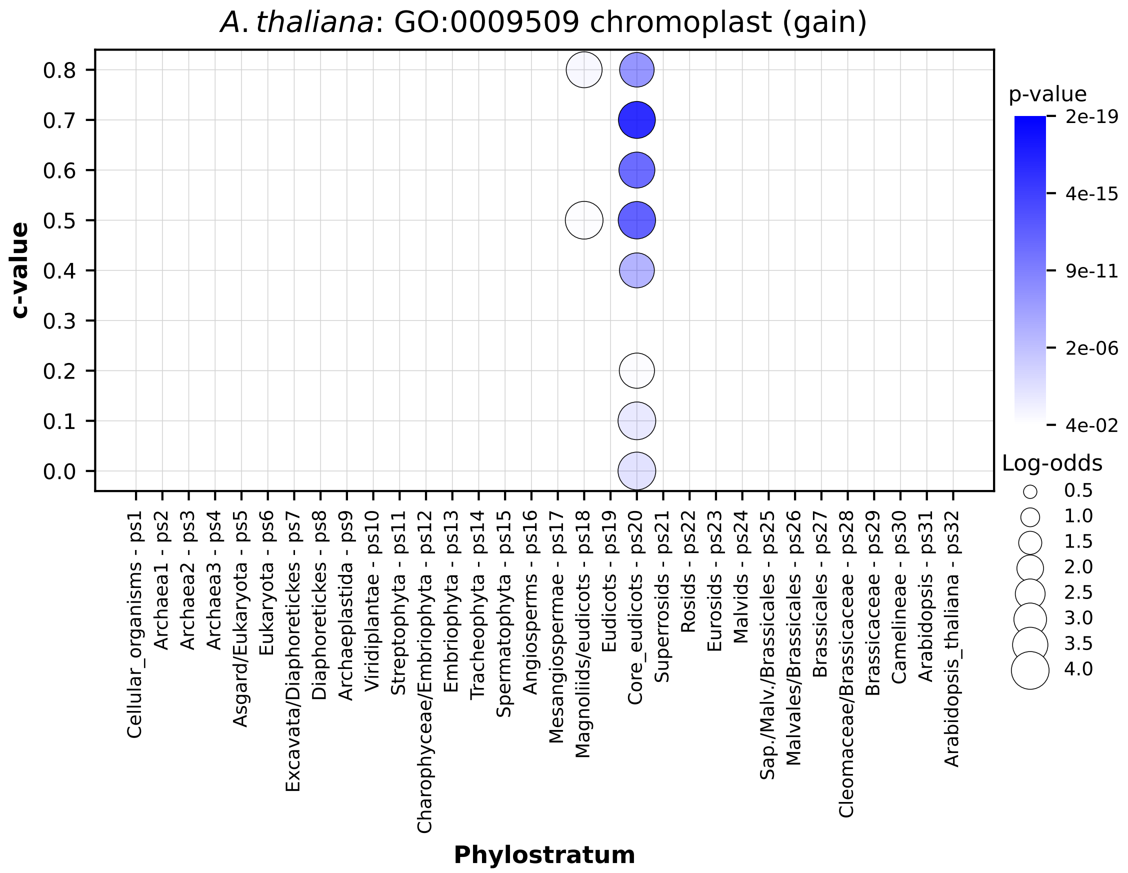

A. thaliana: GO:0009509 chromoplast (gain)
c-value
0.8
0.7
0.6
0.5
0.4
0.3
0.2
0.1
0.0
p-value
2e-19
4e-15
9e-11
2e-06
4e-02
Log-odds
0.5
1.0
1.5
2.0
2.5
3.0
3.5
4.0
Cellular_organisms - ps1
Archaea1 - ps2
Archaea2 - ps3
Archaea3 - ps4
Asgard/Eukaryota - ps5
Eukaryota - ps6
Excavata/Diaphoretickes - ps7
Diaphoretickes - ps8
Archaeplastida - ps9
Viridiplantae - ps10
Streptophyta - ps11
Charophyceae/Embriophyta - ps12
Embriophyta - ps13
Tracheophyta - ps14
Spermatophyta - ps15
Angiosperms - ps16
Mesangiospermae - ps17
Magnoliids/eudicots - ps18
Eudicots - ps19
Core_eudicots - ps20
Superrosids - ps21
Rosids - ps22
Eurosids - ps23
Malvids - ps24
Sap./Malv./Brassicales - ps25
Malvales/Brassicales - ps26
Brassicales - ps27
Cleomaceae/Brassicaceae - ps28
Brassicaceae - ps29
Camelineae - ps30
Arabidopsis - ps31
Arabidopsis_thaliana - ps32
Phylostratum

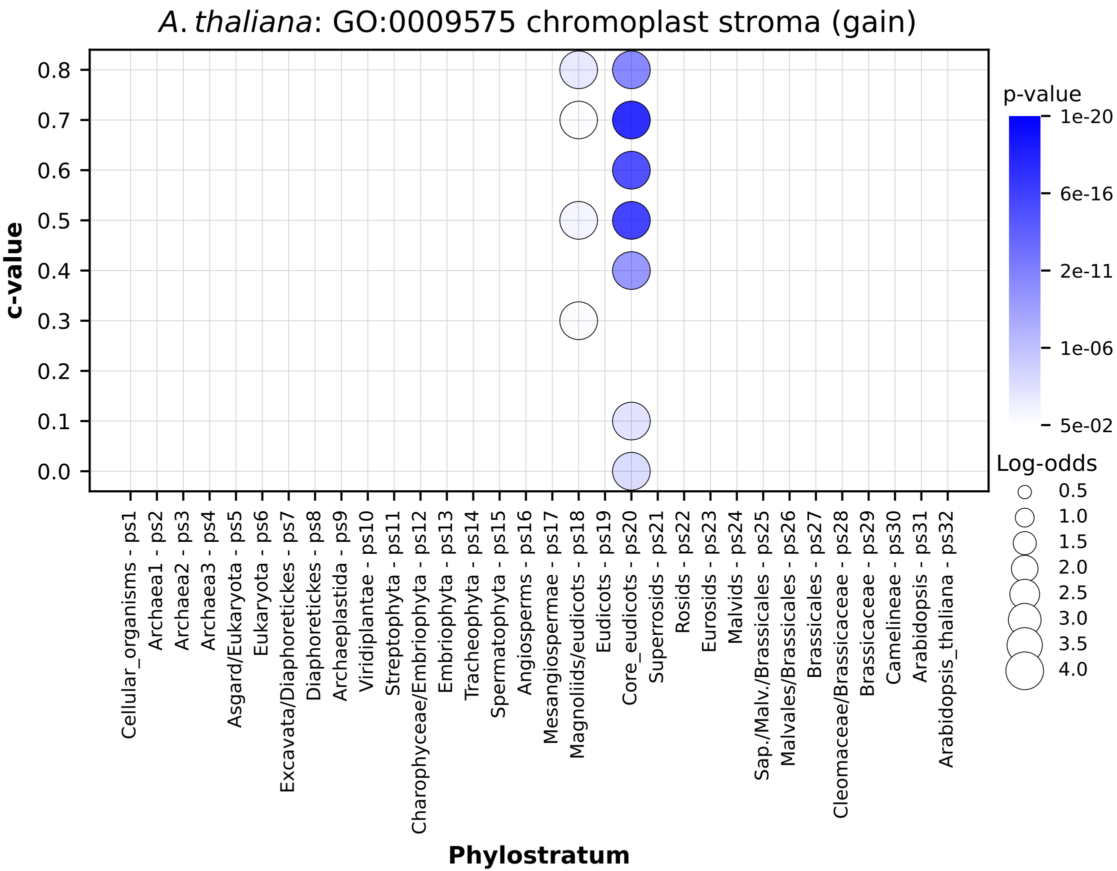

A. thaliana: GO:0009575 chromoplast stroma (gain)
c-value
0.8
0.7
0.6
0.5
0.4
0.3
0.2
0.1
0.0
p-value
1e-20
6e-16
2e-11
1e-06
5e-02
Log-odds
0.5
1.0
1.5
2.0
2.5
3.0
3.5
4.0
Cellular_organisms - ps1
Archaea1 - ps2
Archaea2 - ps3
Archaea3 - ps4
Asgard/Eukaryota - ps5
Eukaryota - ps6
Excavata/Diaphoretickes - ps7
Diaphoretickes - ps8
Archaeplastida - ps9
Viridiplantae - ps10
Streptophyta - ps11
Charophyceae/Embriophyta - ps12
Embriophyta - ps13
Tracheophyta - ps14
Spermatophyta - ps15
Angiosperms - ps16
Mesangiospermae - ps17
Magnoliids/eudicots - ps18
Eudicots - ps19
Core_eudicots - ps20
Superrosids - ps21
Rosids - ps22
Eurosids - ps23
Malvids - ps24
Sap./Malv./Brassicales - ps25
Malvales/Brassicales - ps26
Brassicales - ps27
Cleomaceae/Brassicaceae - ps28
Brassicaceae - ps29
Camelineae - ps30
Arabidopsis - ps31
Arabidopsis_thaliana - ps32
Phylostratum

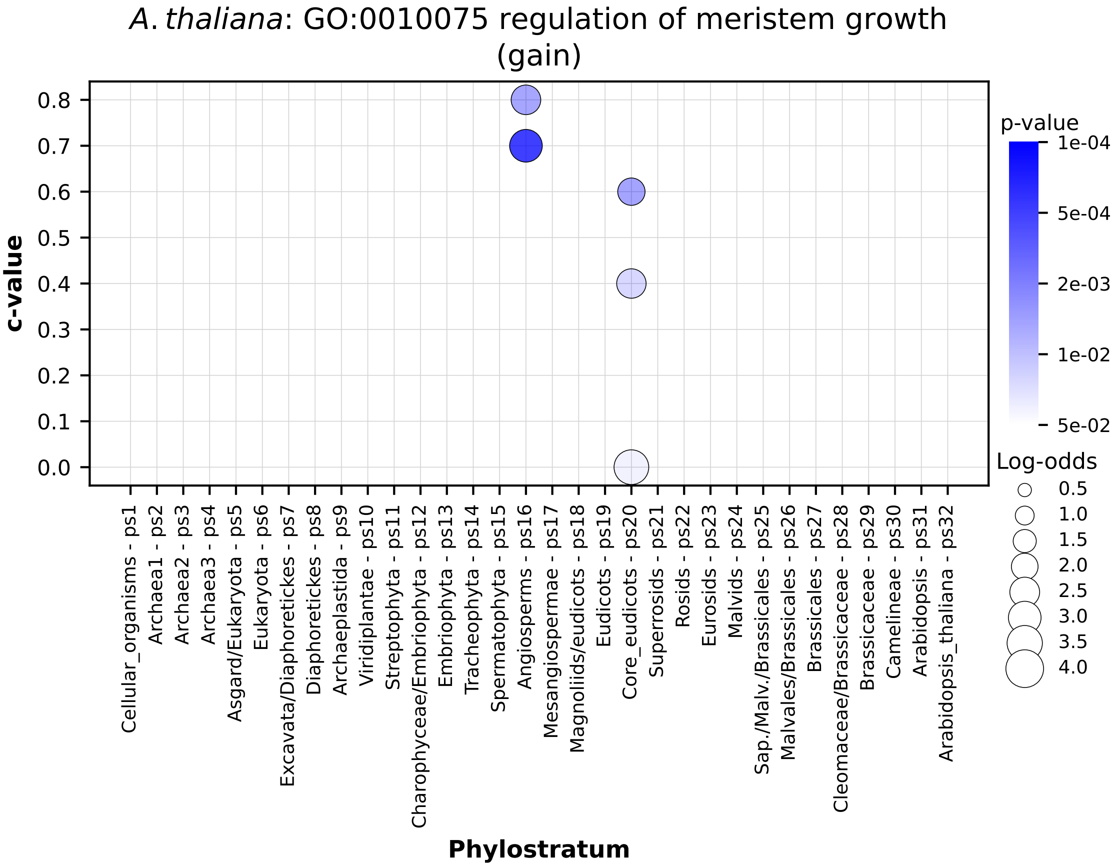

A. thaliana: GO:0010075 regulation of meristem growth
(gain)
c-value
0.8
0.7
0.6
0.5
0.4
0.3
0.2
0.1
0.0
p-value
1e-04
5e-04
2e-03
1e-02
5e-02
Log-odds
0.5
1.0
1.5
2.0
2.5
3.0
3.5
4.0
Cellular_organisms - ps1
Archaea1 - ps2
Archaea2 - ps3
Archaea3 - ps4
Asgard/Eukaryota - ps5
Eukaryota - ps6
Excavata/Diaphoretickes - ps7
Diaphoretickes - ps8
Archaeplastida - ps9
Viridiplantae - ps10
Streptophyta - ps11
Charophyceae/Embriophyta - ps12
Embriophyta - ps13
Tracheophyta - ps14
Spermatophyta - ps15
Angiosperms - ps16
Mesangiospermae - ps17
Magnoliids/eudicots - ps18
Eudicots - ps19
Core_eudicots - ps20
Superrosids - ps21
Rosids - ps22
Eurosids - ps23
Malvids - ps24
Sap./Malv./Brassicales - ps25
Malvales/Brassicales - ps26
Brassicales - ps27
Cleomaceae/Brassicaceae - ps28
Brassicaceae - ps29
Camelineae - ps30
Arabidopsis - ps31
Arabidopsis_thaliana - ps32
Phylostratum

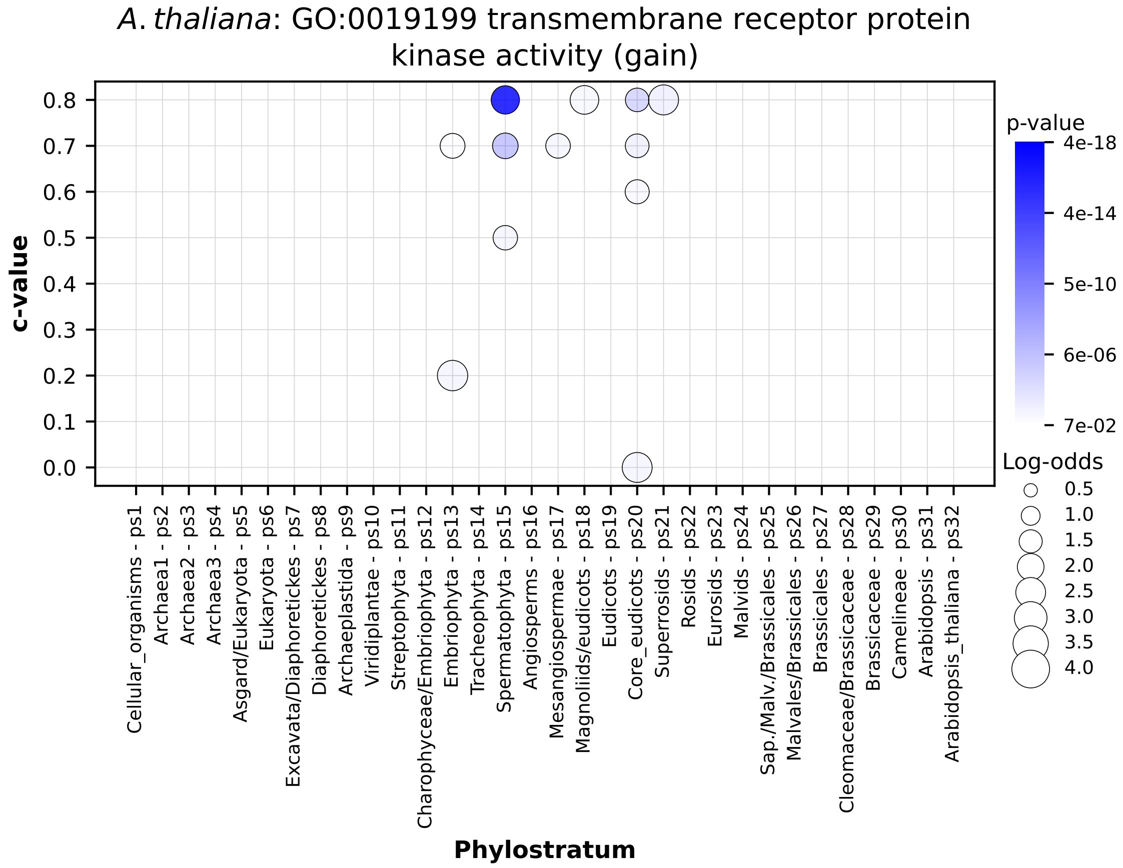
A. thaliana: GO:0019199 transmembrane receptor protein kinase activity (gain)
c-value
0.8
0.7
0.6
0.5
0.4
0.3
0.2
0.1
0.0
Phylostratum
Cellular_organisms - ps1
Archaea1 - ps2
Archaea2 - ps3
Archaea3 - ps4
Asgard/Eukaryota - ps5
Eukaryota - ps6
Excavata/Diaphoretickes - ps7
Diaphoretickes - ps8
Archaeplastida - ps9
Viridiplantae - ps10
Streptophyta - ps11
Charophyceae/Embriophyta - ps12
Embriophyta - ps13
Tracheophyta - ps14
Spermatophyta - ps15
Angiosperms - ps16
Mesangiospermae - ps17
Magnoliids/eudicots - ps18
Eudicots - ps19
Core_eudicots - ps20
Superrosids - ps21
Rosids - ps22
Eurosids - ps23
Malvids - ps24
Sap./Malv./Brassicales - ps25
Malvales/Brassicales - ps26
Brassicales - ps27
Cleomaceae/Brassicaceae - ps28
Brassicaceae - ps29
Camelineae - ps30
Arabidopsis - ps31
Arabidopsis_thaliana - ps32
p-value
4e-18
4e-14
5e-10
6e-06
7e-02
Log-odds
0.5
1.0
1.5
2.0
2.5
3.0
3.5
4.0

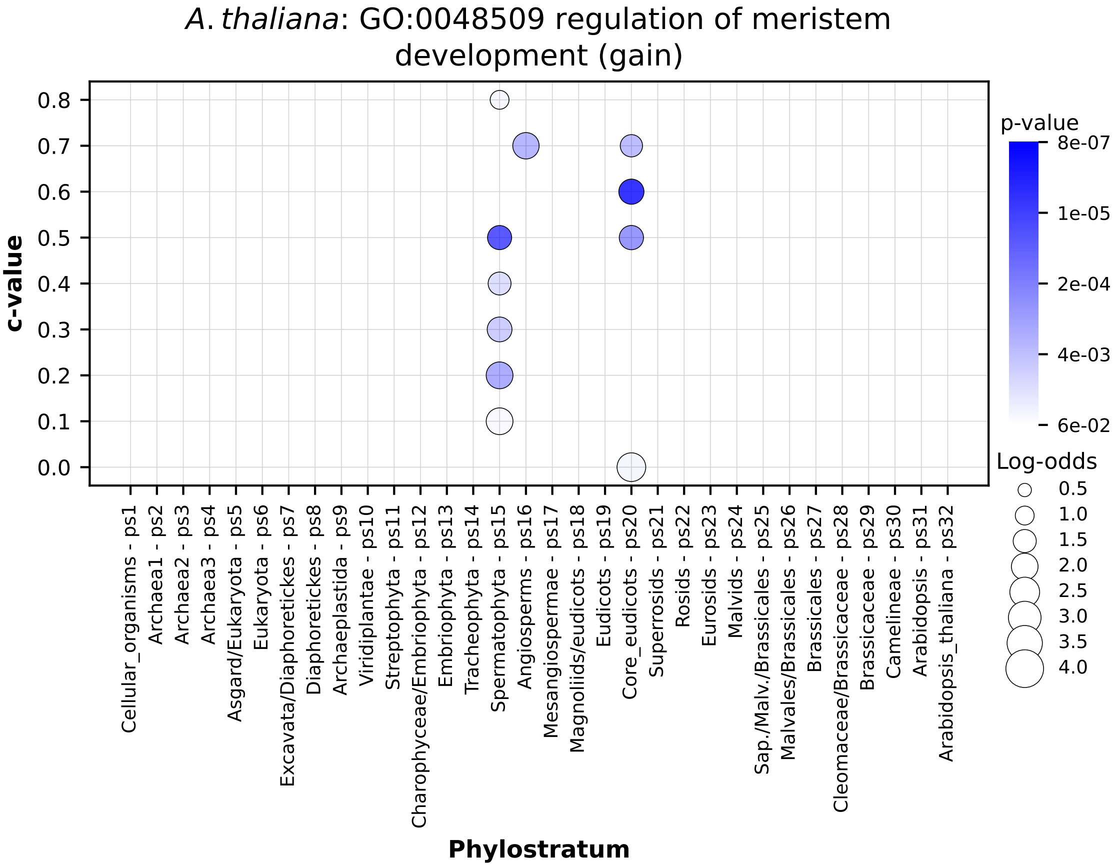
A. thaliana: GO:0048509 regulation of meristem development (gain)
c-value
0.8
0.7
0.6
0.5
0.4
0.3
0.2
0.1
0.0
p-value
8e-07
1e-05
2e-04
4e-03
6e-02
Log-odds
0.5
1.0
1.5
2.0
2.5
3.0
3.5
4.0
Cellular_organisms - ps1
Archaea1 - ps2
Archaea2 - ps3
Archaea3 - ps4
Asgard/Eukaryota - ps5
Eukaryota - ps6
Excavata/Diaphoretickes - ps7
Diaphoretickes - ps8
Archaeplastida - ps9
Viridiplantae - ps10
Streptophyta - ps11
Charophyceae/Embriophyta - ps12
Embriophyta - ps13
Tracheophyta - ps14
Spermatophyta - ps15
Angiosperms - ps16
Mesangiospermae - ps17
Magnoliids/eudicots - ps18
Eudicots - ps19
Core_eudicots - ps20
Superrosids - ps21
Rosids - ps22
Eurosids - ps23
Malvids - ps24
Sap./Malv./Brassicales - ps25
Malvales/Brassicales - ps26
Brassicales - ps27
Cleomaceae/Brassicaceae - ps28
Brassicaceae - ps29
Camelineae - ps30
Arabidopsis - ps31
Arabidopsis_thaliana - ps32
Phylostratum

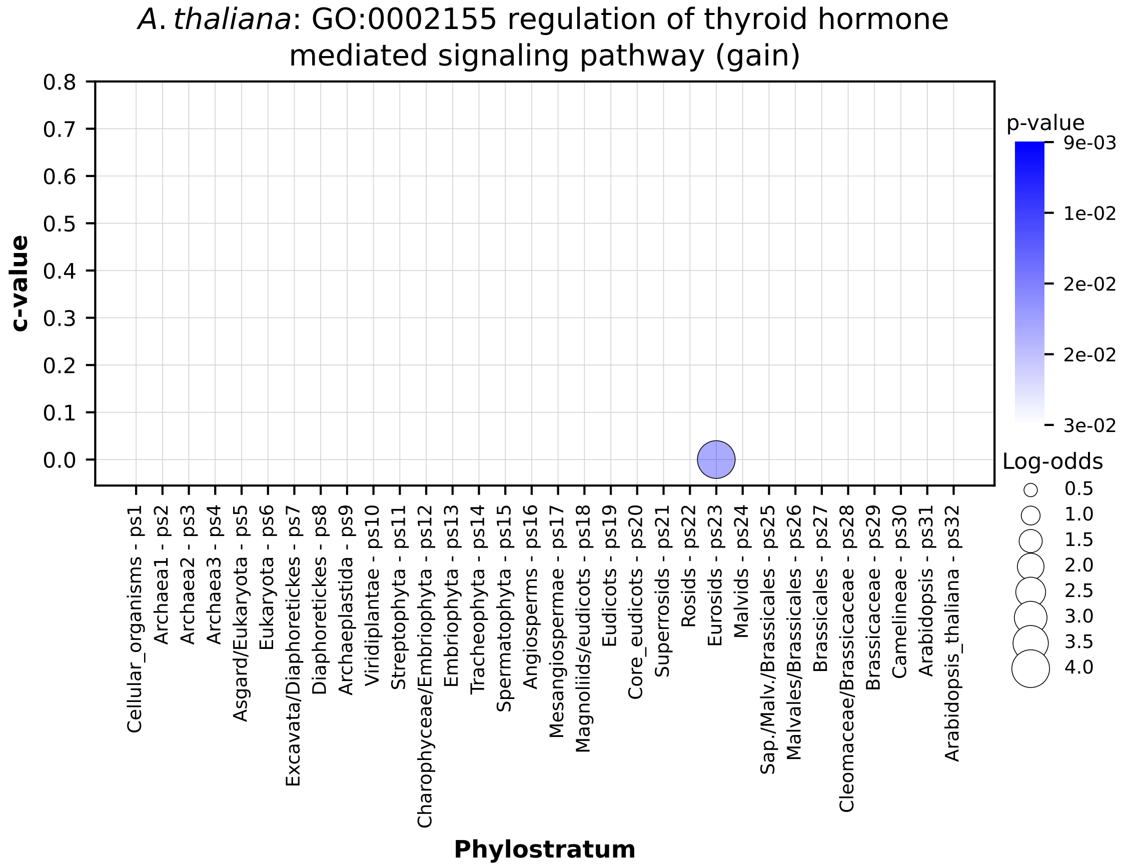

A. thaliana: GO:0002155 regulation of thyroid hormone mediated signaling pathway (gain)
c-value
0.8
0.7
0.6
0.5
0.4
0.3
0.2
0.1
0.0
p-value
9e-03
1e-02
2e-02
2e-02
3e-02
Log-odds
0.5
1.0
1.5
2.0
2.5
3.0
3.5
4.0
Cellular_organisms - ps1
Archaea1 - ps2
Archaea2 - ps3
Archaea3 - ps4
Asgard/Eukaryota - ps5
Eukaryota - ps6
Excavata/Diaphoretickes - ps7
Diaphoretickes - ps8
Archaeplastida - ps9
Viridiplantae - ps10
Streptophyta - ps11
Charophyceae/Embriophyta - ps12
Embriophyta - ps13
Tracheophyta - ps14
Spermatophyta - ps15
Angiosperms - ps16
Mesangiospermae - ps17
Magnoliids/eudicots - ps18
Eudicots - ps19
Core_eudicots - ps20
Superrosids - ps21
Rosids - ps22
Eurosids - ps23
Malvids - ps24
Sap./Malv./Brassicales - ps25
Malvales/Brassicales - ps26
Brassicales - ps27
Cleomaceae/Brassicaceae - ps28
Brassicaceae - ps29
Camelineae - ps30
Arabidopsis - ps31
Arabidopsis_thaliana - ps32
Phylostratum

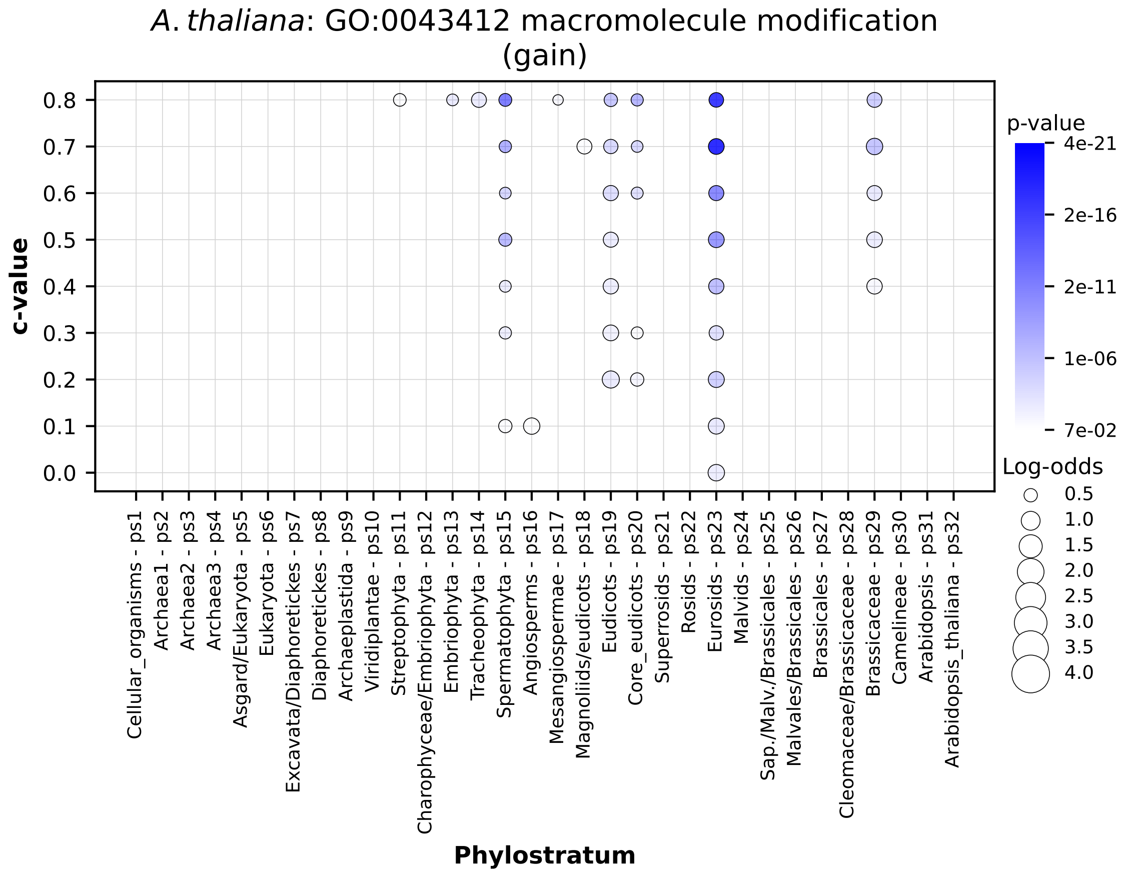

A. thaliana: GO:0043412 macromolecule modification (gain)
c-value
0.8
0.7
0.6
0.5
0.4
0.3
0.2
0.1
0.0
Phylostratum
Cellular_organisms - ps1
Archaea1 - ps2
Archaea2 - ps3
Archaea3 - ps4
Asgard/Eukaryota - ps5
Eukaryota - ps6
Excavata/Diaphoretickes - ps7
Diaphoretickes - ps8
Archaeplastida - ps9
Viridiplantae - ps10
Streptophyta - ps11
Charophyceae/Embriophyta - ps12
Embriophyta - ps13
Tracheophyta - ps14
Spermatophyta - ps15
Angiosperms - ps16
Mesangiospermae - ps17
Magnoliids/eudicots - ps18
Eudicots - ps19
Core_eudicots - ps20
Superrosids - ps21
Rosids - ps22
Eurosids - ps23
Malvids - ps24
Sap./Malv./Brassicales - ps25
Malvales/Brassicales - ps26
Brassicales - ps27
Cleomaceae/Brassicaceae - ps28
Brassicaceae - ps29
Camelineae - ps30
Arabidopsis - ps31
Arabidopsis_thaliana - ps32
p-value
4e-21
2e-16
2e-11
1e-06
7e-02
Log-odds
0.5
1.0
1.5
2.0
2.5
3.0
3.5
4.0

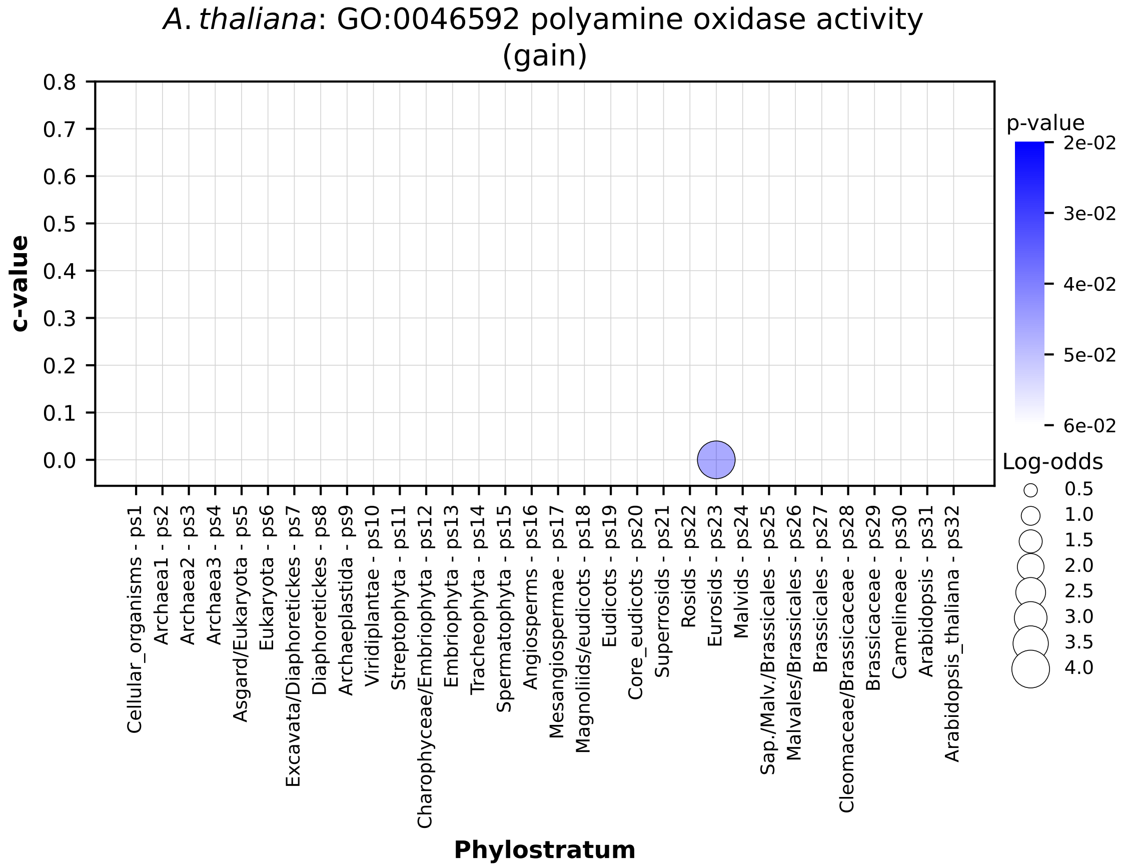

A. thaliana: GO:0046592 polyamine oxidase activity
(gain)
c-value
0.8
0.7
0.6
0.5
0.4
0.3
0.2
0.1
0.0
p-value
2e-02
3e-02
4e-02
5e-02
6e-02
Log-odds
0.5
1.0
1.5
2.0
2.5
3.0
3.5
4.0
Cellular_organisms - ps1
Archaea1 - ps2
Archaea2 - ps3
Archaea3 - ps4
Asgard/Eukaryota - ps5
Eukaryota - ps6
Excavata/Diaphoretickes - ps7
Diaphoretickes - ps8
Archaeplastida - ps9
Viridiplantae - ps10
Streptophyta - ps11
Charophyceae/Embriophyta - ps12
Embriophyta - ps13
Tracheophyta - ps14
Spermatophyta - ps15
Angiosperms - ps16
Mesangiospermae - ps17
Magnoliids/eudicots - ps18
Eudicots - ps19
Core_eudicots - ps20
Superrosids - ps21
Rosids - ps22
Eurosids - ps23
Malvids - ps24
Sap./Malv./Brassicales - ps25
Malvales/Brassicales - ps26
Brassicales - ps27
Cleomaceae/Brassicaceae - ps28
Brassicaceae - ps29
Camelineae - ps30
Arabidopsis - ps31
Arabidopsis_thaliana - ps32
Phylostratum

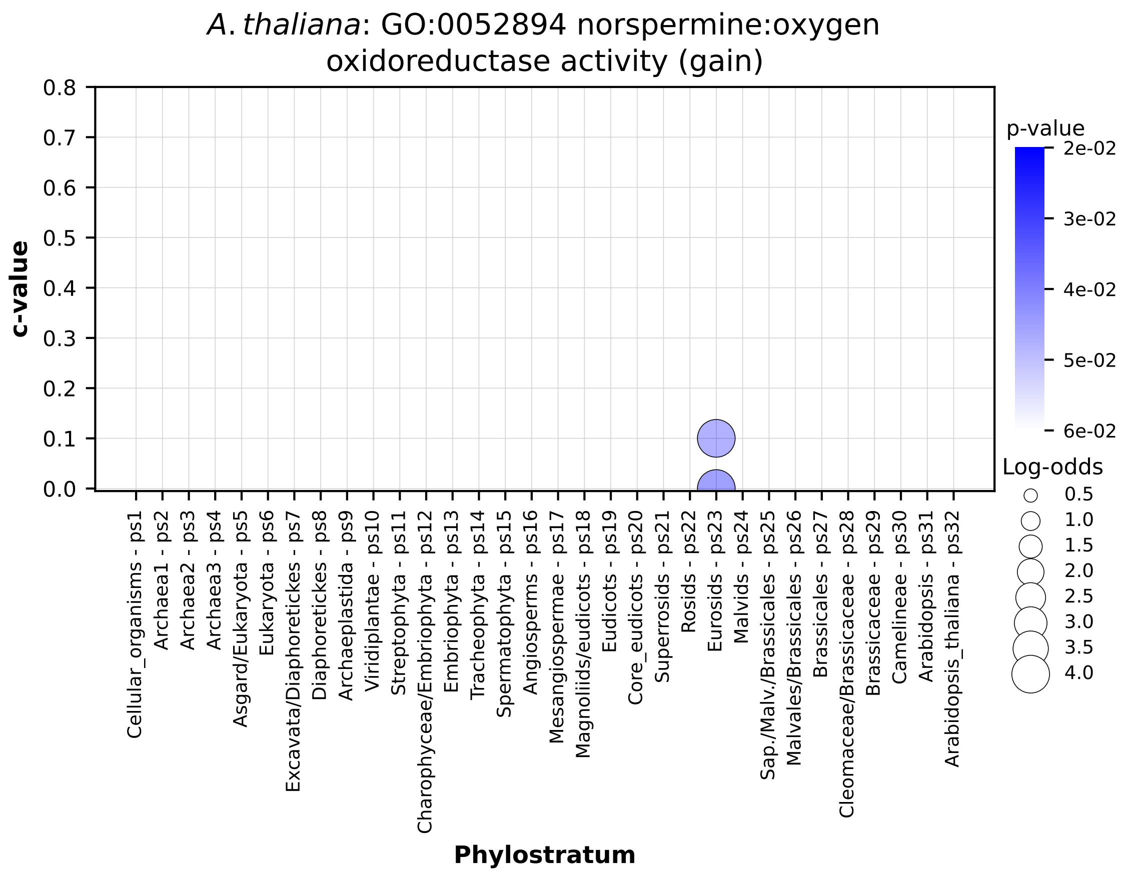

A. thaliana: GO:0052894 norspermine:oxygen oxidoreductase activity (gain)
c-value
0.8
0.7
0.6
0.5
0.4
0.3
0.2
0.1
0.0
p-value
2e-02
3e-02
4e-02
5e-02
6e-02
Log-odds
0.5
1.0
1.5
2.0
2.5
3.0
3.5
4.0
Cellular_organisms - ps1
Archaea1 - ps2
Archaea2 - ps3
Archaea3 - ps4
Asgard/Eukaryota - ps5
Eukaryota - ps6
Excavata/Diaphoretickes - ps7
Diaphoretickes - ps8
Archaeplastida - ps9
Viridiplantae - ps10
Streptophyta - ps11
Charophyceae/Embriophyta - ps12
Embriophyta - ps13
Tracheophyta - ps14
Spermatophyta - ps15
Angiosperms - ps16
Mesangiospermae - ps17
Magnoliids/eudicots - ps18
Eudicots - ps19
Core_eudicots - ps20
Superrosids - ps21
Rosids - ps22
Eurosids - ps23
Malvids - ps24
Sap./Malv./Brassicales - ps25
Malvales/Brassicales - ps26
Brassicales - ps27
Cleomaceae/Brassicaceae - ps28
Brassicaceae - ps29
Camelineae - ps30
Arabidopsis - ps31
Arabidopsis_thaliana - ps32
Phylostratum

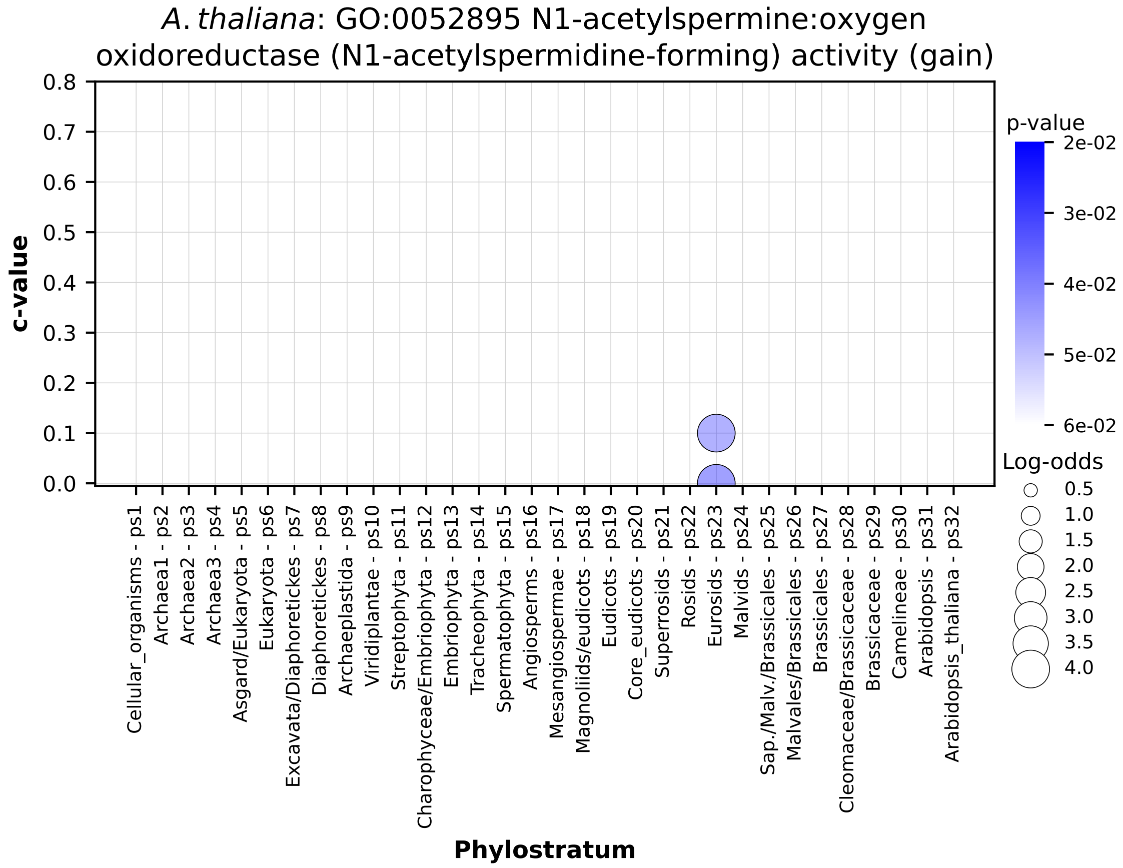

A. thaliana: GO:0052895 N1-acetylspermine:oxygen oxidoreductase (N1-acetylspermidine-forming) activity (gain)
c-value
0.8
0.7
0.6
0.5
0.4
0.3
0.2
0.1
0.0
p-value
2e-02
3e-02
4e-02
5e-02
6e-02
Log-odds
0.5
1.0
1.5
2.0
2.5
3.0
3.5
4.0
Cellular_organisms - ps1
Archaea1 - ps2
Archaea2 - ps3
Archaea3 - ps4
Asgard/Eukaryota - ps5
Eukaryota - ps6
Excavata/Diaphoretickes - ps7
Diaphoretickes - ps8
Archaeplastida - ps9
Viridiplantae - ps10
Streptophyta - ps11
Charophyceae/Embriophyta - ps12
Embriophyta - ps13
Tracheophyta - ps14
Spermatophyta - ps15
Angiosperms - ps16
Mesangiospermae - ps17
Magnoliids/eudicots - ps18
Eudicots - ps19
Core_eudicots - ps20
Superrosids - ps21
Rosids - ps22
Eurosids - ps23
Malvids - ps24
Sap./Malv./Brassicales - ps25
Malvales/Brassicales - ps26
Brassicales - ps27
Cleomaceae/Brassicaceae - ps28
Brassicaceae - ps29
Camelineae - ps30
Arabidopsis - ps31
Arabidopsis_thaliana - ps32
Phylostratum

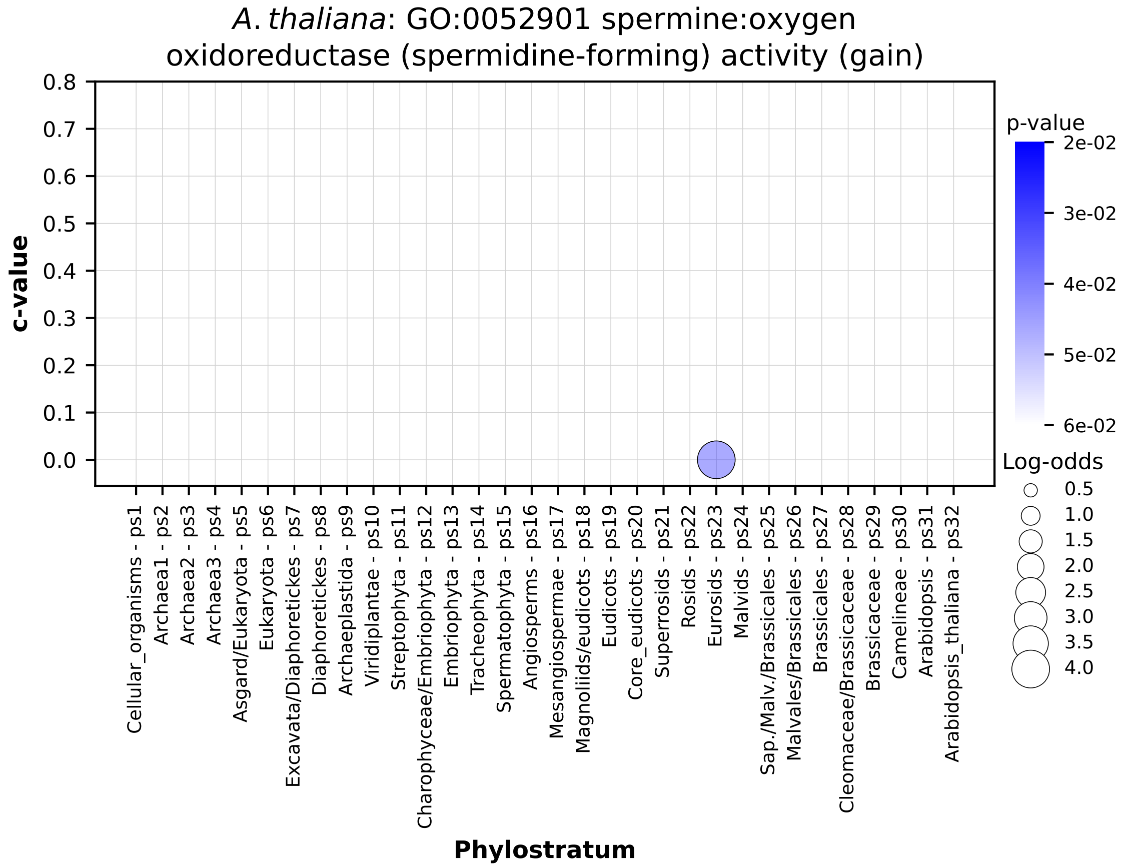

A. thaliana: GO:0052901 spermine:oxygen oxidoreductase (spermidine-forming) activity (gain)
c-value
0.8
0.7
0.6
0.5
0.4
0.3
0.2
0.1
0.0
p-value
2e-02
3e-02
4e-02
5e-02
6e-02
Log-odds
0.5
1.0
1.5
2.0
2.5
3.0
3.5
4.0
Cellular_organisms - ps1
Archaea1 - ps2
Archaea2 - ps3
Archaea3 - ps4
Asgard/Eukaryota - ps5
Eukaryota - ps6
Excavata/Diaphoretickes - ps7
Diaphoretickes - ps8
Archaeplastida - ps9
Viridiplantae - ps10
Streptophyta - ps11
Charophyceae/Embriophyta - ps12
Embriophyta - ps13
Tracheophyta - ps14
Spermatophyta - ps15
Angiosperms - ps16
Mesangiospermae - ps17
Magnoliids/eudicots - ps18
Eudicots - ps19
Core_eudicots - ps20
Superrosids - ps21
Rosids - ps22
Eurosids - ps23
Malvids - ps24
Sap./Malv./Brassicales - ps25
Malvales/Brassicales - ps26
Brassicales - ps27
Cleomaceae/Brassicaceae - ps28
Brassicaceae - ps29
Camelineae - ps30
Arabidopsis - ps31
Arabidopsis_thaliana - ps32
Phylostratum

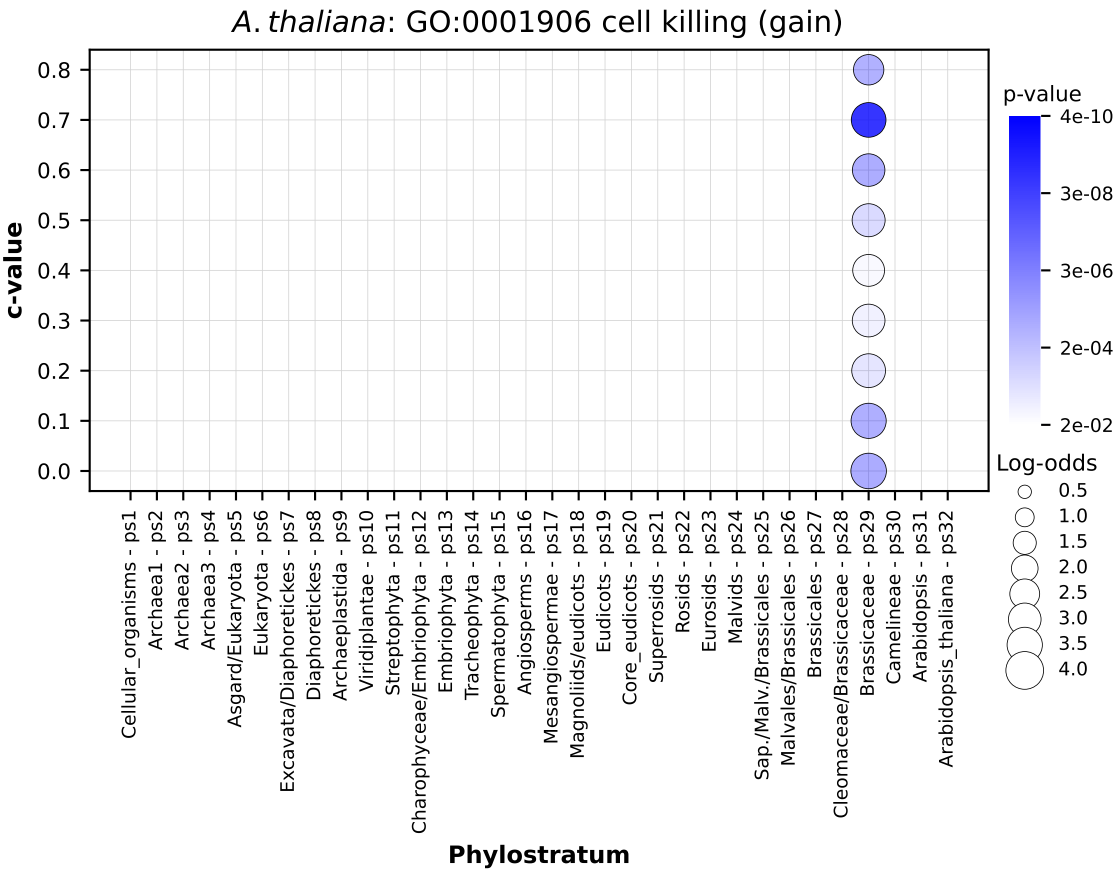

A. thaliana: GO:0001906 cell killing (gain)
c-value
0.0
0.1
0.2
0.3
0.4
0.5
0.6
0.7
0.8
p-value
4e-10
3e-08
3e-06
2e-04
2e-02
Log-odds
0.5
1.0
1.5
2.0
2.5
3.0
3.5
4.0
Cellular_organisms - ps1
Archaea1 - ps2
Archaea2 - ps3
Archaea3 - ps4
Asgard/Eukaryota - ps5
Eukaryota - ps6
Excavata/Diaphoretickes - ps7
Diaphoretickes - ps8
Archaeplastida - ps9
Viridiplantae - ps10
Streptophyta - ps11
Charophyceae/Embriophyta - ps12
Embriophyta - ps13
Tracheophyta - ps14
Spermatophyta - ps15
Angiosperms - ps16
Mesangiospermae - ps17
Magnoliids/eudicots - ps18
Eudicots - ps19
Core_eudicots - ps20
Superrosids - ps21
Rosids - ps22
Eurosids - ps23
Malvids - ps24
Sap./Malv./Brassicales - ps25
Malvales/Brassicales - ps26
Brassicales - ps27
Cleomaceae/Brassicaceae - ps28
Brassicaceae - ps29
Camelineae - ps30
Arabidopsis - ps31
Arabidopsis_thaliana - ps32
Phylostratum

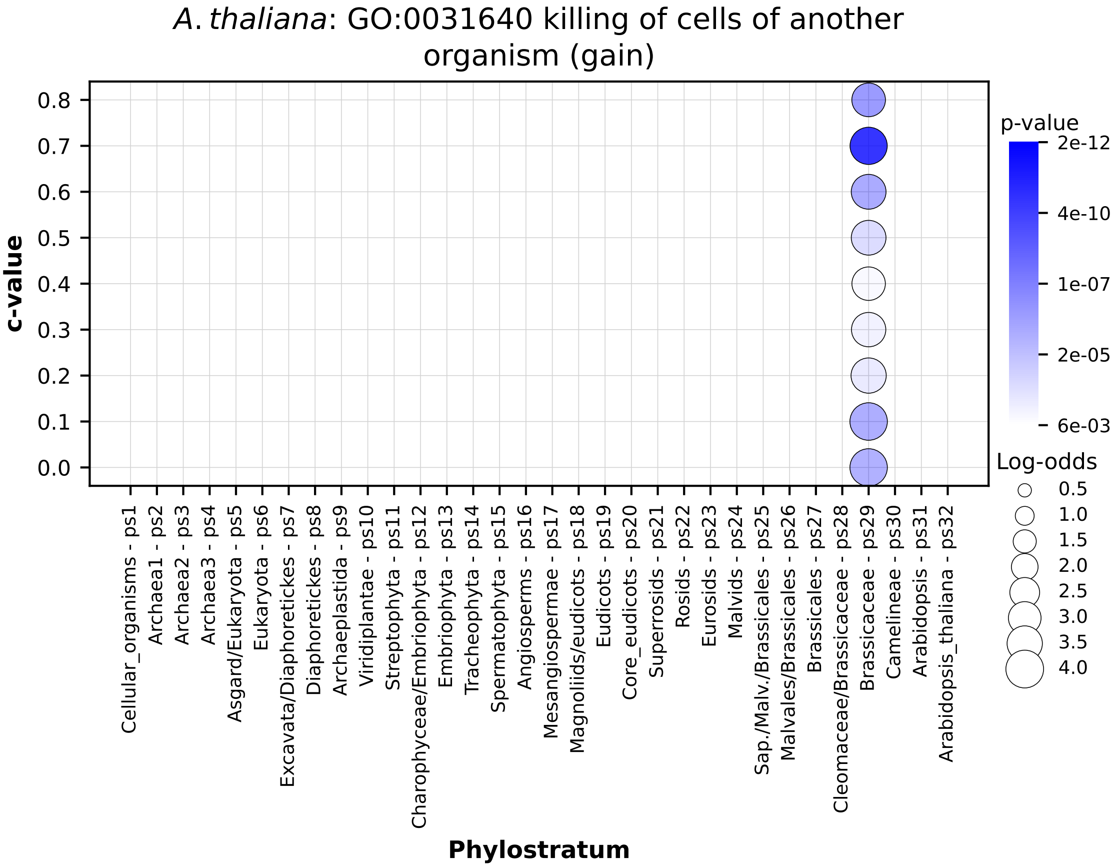
A. thaliana: GO:0031640 killing of cells of another organism (gain)
c-value
0.8
0.7
0.6
0.5
0.4
0.3
0.2
0.1
0.0
p-value
2e-12
4e-10
1e-07
2e-05
6e-03
Log-odds
0.5
1.0
1.5
2.0
2.5
3.0
3.5
4.0
Cellular_organisms - ps1
Archaea1 - ps2
Archaea2 - ps3
Archaea3 - ps4
Asgard/Eukaryota - ps5
Eukaryota - ps6
Excavata/Diaphoretickes - ps7
Diaphoretickes - ps8
Archaeplastida - ps9
Viridiplantae - ps10
Streptophyta - ps11
Charophyceae/Embriophyta - ps12
Embriophyta - ps13
Tracheophyta - ps14
Spermatophyta - ps15
Angiosperms - ps16
Mesangiospermae - ps17
Magnoliids/eudicots - ps18
Eudicots - ps19
Core_eudicots - ps20
Superrosids - ps21
Rosids - ps22
Eurosids - ps23
Malvids - ps24
Sap./Malv./Brassicales - ps25
Malvales/Brassicales - ps26
Brassicales - ps27
Cleomaceae/Brassicaceae - ps28
Brassicaceae - ps29
Camelineae - ps30
Arabidopsis - ps31
Arabidopsis_thaliana - ps32
Phylostratum

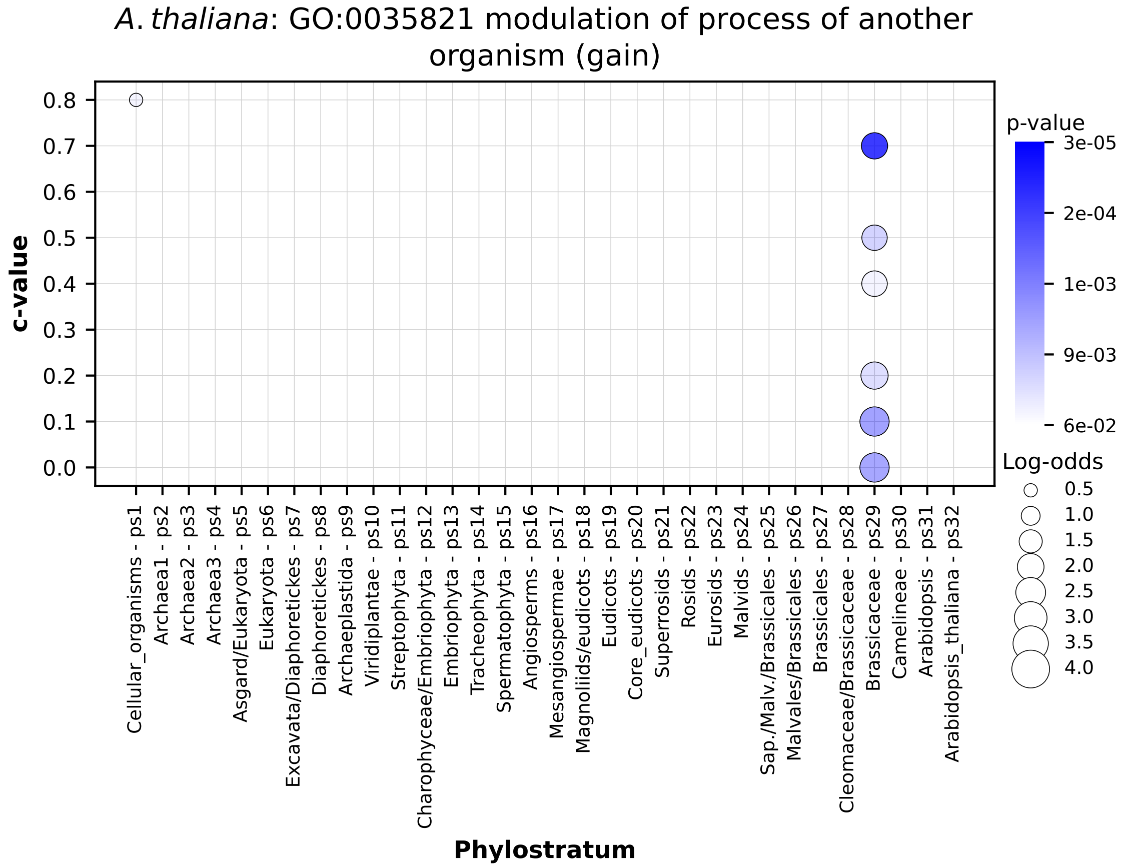

A. thaliana: GO:0035821 modulation of process of another organism (gain)
c-value
0.8
0.7
0.6
0.5
0.4
0.3
0.2
0.1
0.0
p-value
3e-05
2e-04
1e-03
9e-03
6e-02
Log-odds
0.5
1.0
1.5
2.0
2.5
3.0
3.5
4.0
Cellular_organisms - ps1
Archaea1 - ps2
Archaea2 - ps3
Archaea3 - ps4
Asgard/Eukaryota - ps5
Eukaryota - ps6
Excavata/Diaphoretickes - ps7
Diaphoretickes - ps8
Archaeplastida - ps9
Viridiplantae - ps10
Streptophyta - ps11
Charophyceae/Embriophyta - ps12
Embriophyta - ps13
Tracheophyta - ps14
Spermatophyta - ps15
Angiosperms - ps16
Mesangiospermae - ps17
Magnoliids/eudicots - ps18
Eudicots - ps19
Core_eudicots - ps20
Superrosids - ps21
Rosids - ps22
Eurosids - ps23
Malvids - ps24
Sap./Malv./Brassicales - ps25
Malvales/Brassicales - ps26
Brassicales - ps27
Cleomaceae/Brassicaceae - ps28
Brassicaceae - ps29
Camelineae - ps30
Arabidopsis - ps31
Arabidopsis_thaliana - ps32
Phylostratum

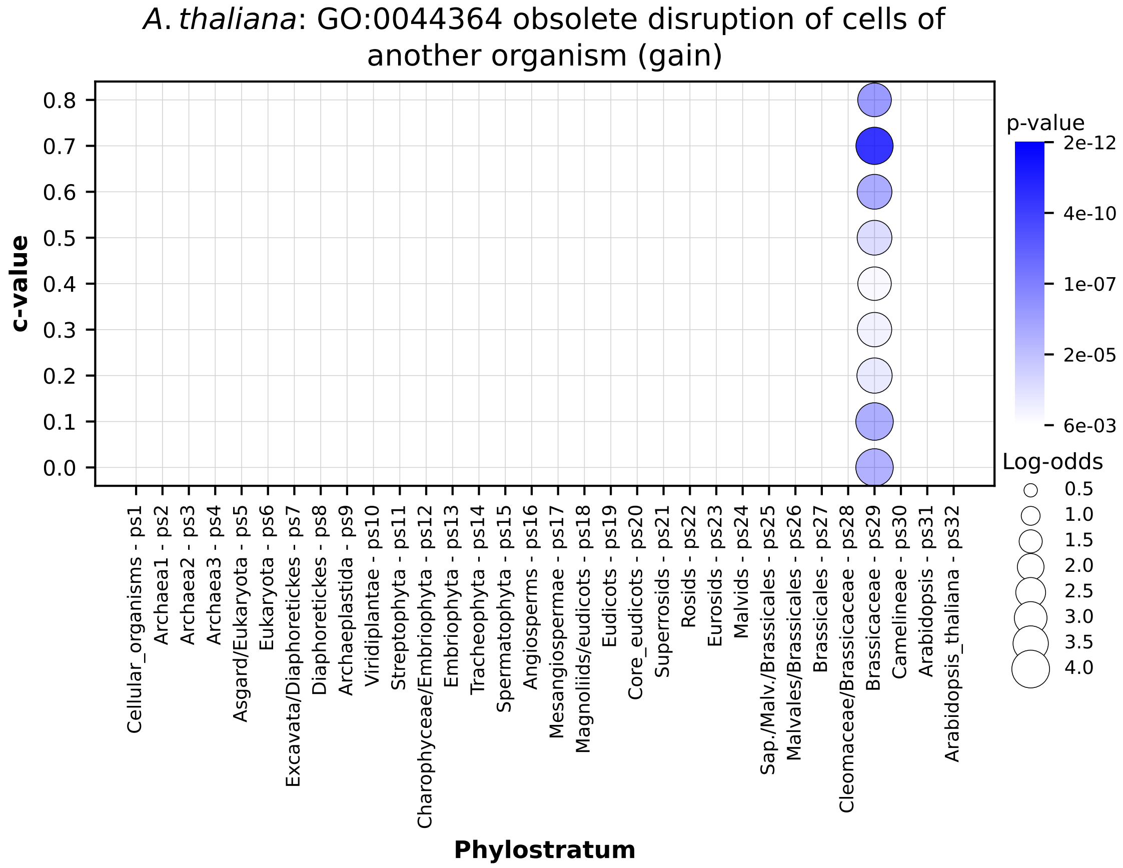
A. thaliana: GO:0044364 obsolete disruption of cells of another organism (gain)
c-value
0.8
0.7
0.6
0.5
0.4
0.3
0.2
0.1
0.0
Phylostratum
Cellular_organisms - ps1
Archaea1 - ps2
Archaea2 - ps3
Archaea3 - ps4
Asgard/Eukaryota - ps5
Eukaryota - ps6
Excavata/Diaphoretickes - ps7
Diaphoretickes - ps8
Archaeplastida - ps9
Viridiplantae - ps10
Streptophyta - ps11
Charophyceae/Embriophyta - ps12
Embriophyta - ps13
Tracheophyta - ps14
Spermatophyta - ps15
Angiosperms - ps16
Mesangiospermae - ps17
Magnoliids/eudicots - ps18
Eudicots - ps19
Core_eudicots - ps20
Superrosids - ps21
Rosids - ps22
Eurosids - ps23
Malvids - ps24
Sap./Malv./Brassicales - ps25
Malvales/Brassicales - ps26
Brassicales - ps27
Cleomaceae/Brassicaceae - ps28
Brassicaceae - ps29
Camelineae - ps30
Arabidopsis - ps31
Arabidopsis_thaliana - ps32
p-value
2e-12
4e-10
1e-07
2e-05
6e-03
Log-odds
0.5
1.0
1.5
2.0
2.5
3.0
3.5
4.0

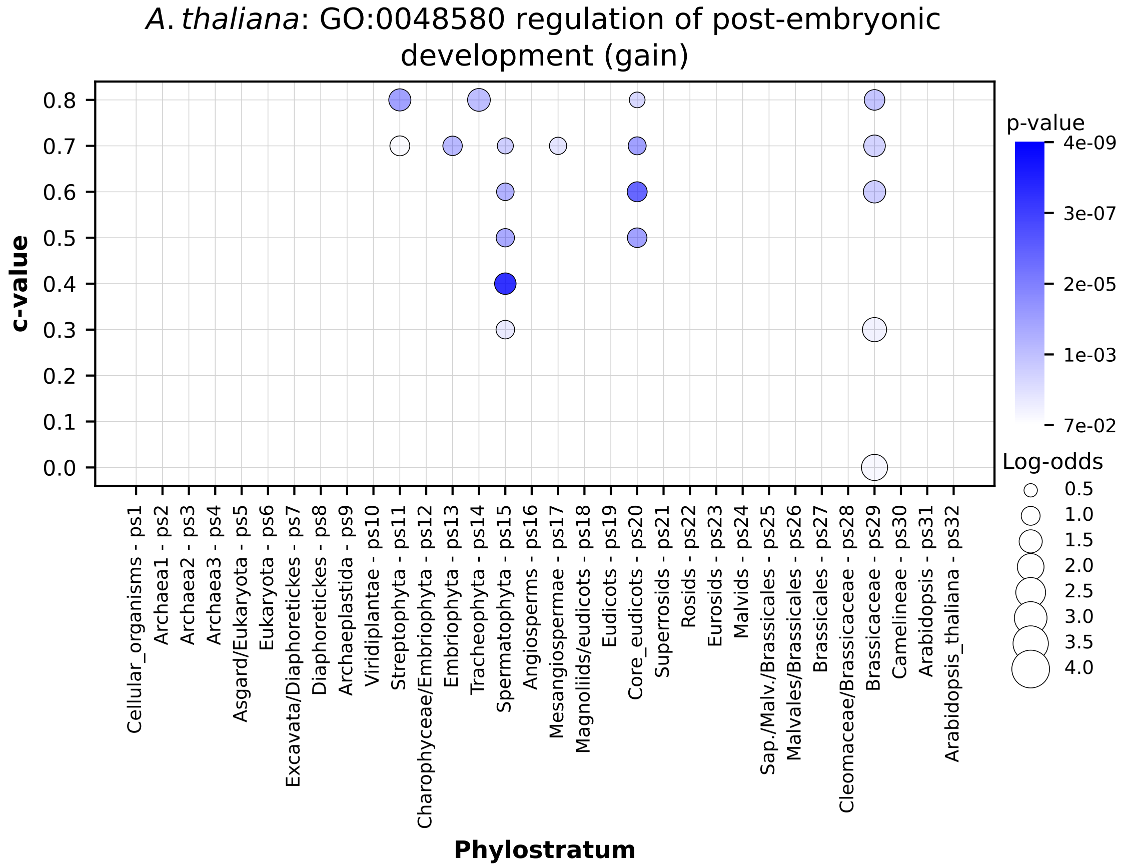
A. thaliana: GO:0048580 regulation of post-embryonic development (gain)
c-value
Phylostratum
p-value
4e-09
3e-07
2e-05
1e-03
7e-02
Log-odds
0.5
1.0
1.5
2.0
2.5
3.0
3.5
4.0
0.8
0.7
0.6
0.5
0.4
0.3
0.2
0.1
0.0
Cellular_organisms - ps1
Archaea1 - ps2
Archaea2 - ps3
Archaea3 - ps4
Asgard/Eukaryota - ps5
Eukaryota - ps6
Excavata/Diaphoretickes - ps7
Diaphoretickes - ps8
Archaeplastida - ps9
Viridiplantae - ps10
Streptophyta - ps11
Charophyceae/Embriophyta - ps12
Embriophyta - ps13
Tracheophyta - ps14
Spermatophyta - ps15
Angiosperms - ps16
Mesangiospermae - ps17
Magnoliids/eudicots - ps18
Eudicots - ps19
Core_eudicots - ps20
Superrosids - ps21
Rosids - ps22
Eurosids - ps23
Malvids - ps24
Sap./Malv./Brassicales - ps25
Malvales/Brassicales - ps26
Brassicales - ps27
Cleomaceae/Brassicaceae - ps28
Brassicaceae - ps29
Camelineae - ps30
Arabidopsis - ps31
Arabidopsis_thaliana - ps32

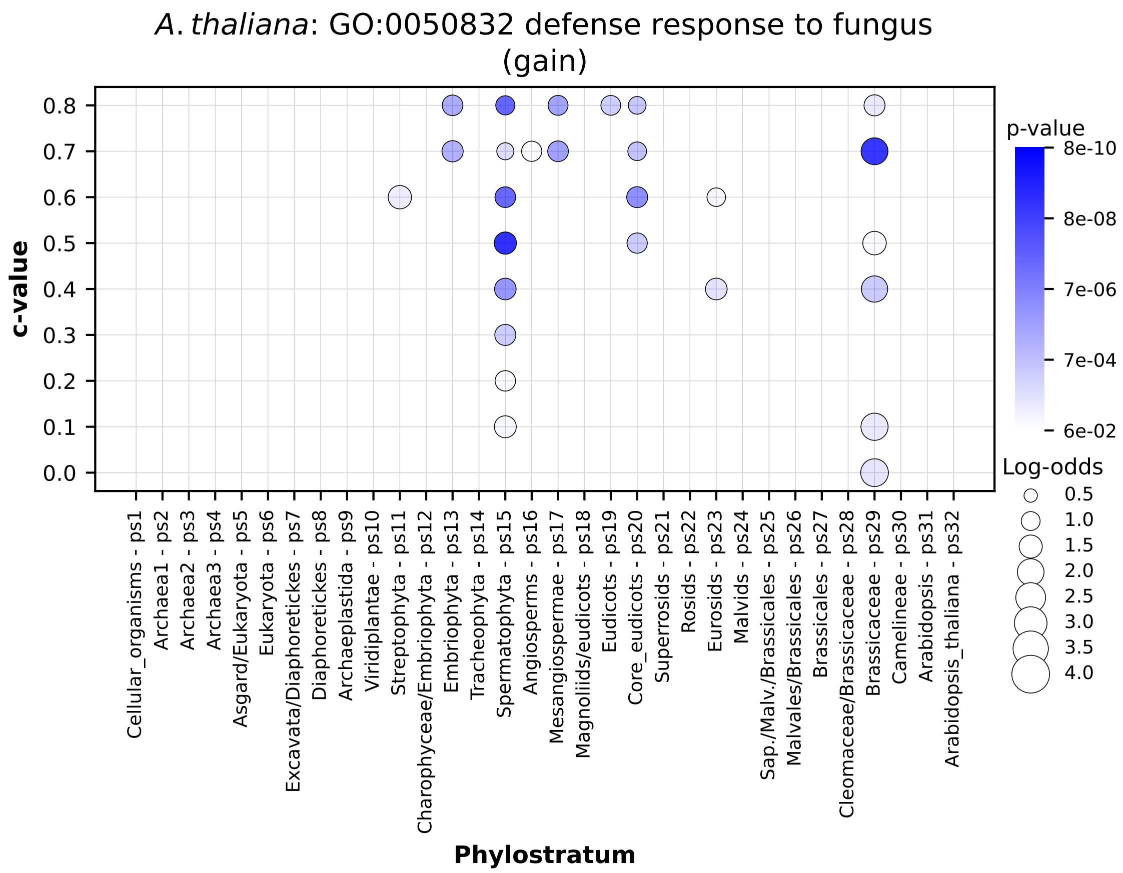
A. thaliana: GO:0050832 defense response to fungus
(gain)
c-value
Phylostratum
p-value
8e-10
8e-08
7e-06
7e-04
6e-02
Log-odds
0.5
1.0
1.5
2.0
2.5
3.0
3.5
4.0
0.8
0.7
0.6
0.5
0.4
0.3
0.2
0.1
0.0
Cellular_organisms - ps1
Archaea1 - ps2
Archaea2 - ps3
Archaea3 - ps4
Asgard/Eukaryota - ps5
Eukaryota - ps6
Excavata/Diaphoretickes - ps7
Diaphoretickes - ps8
Archaeplastida - ps9
Viridiplantae - ps10
Streptophyta - ps11
Charophyceae/Embriophyta - ps12
Embriophyta - ps13
Tracheophyta - ps14
Spermatophyta - ps15
Angiosperms - ps16
Mesangiospermae - ps17
Magnoliids/eudicots - ps18
Eudicots - ps19
Core_eudicots - ps20
Superrosids - ps21
Rosids - ps22
Eurosids - ps23
Malvids - ps24
Sap./Malv./Brassicales - ps25
Malvales/Brassicales - ps26
Brassicales - ps27
Cleomaceae/Brassicaceae - ps28
Brassicaceae - ps29
Camelineae - ps30
Arabidopsis - ps31
Arabidopsis_thaliana - ps32

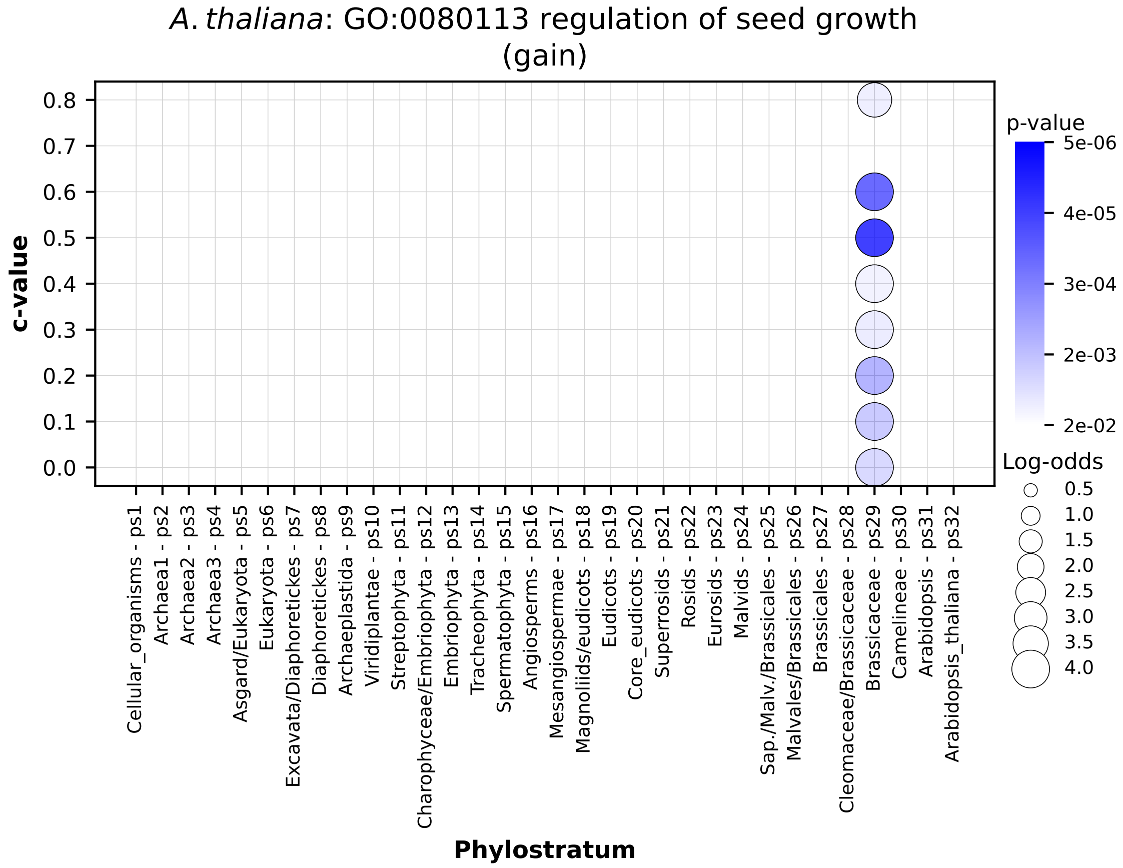

A. thaliana: GO:0080113 regulation of seed growth (gain)
c-value
0.8
0.7
0.6
0.5
0.4
0.3
0.2
0.1
0.0
p-value
5e-06
4e-05
3e-04
2e-03
2e-02
Log-odds
0.5
1.0
1.5
2.0
2.5
3.0
3.5
4.0
Cellular_organisms - ps1
Archaea1 - ps2
Archaea2 - ps3
Archaea3 - ps4
Asgard/Eukaryota - ps5
Eukaryota - ps6
Excavata/Diaphoretickes - ps7
Diaphoretickes - ps8
Archaeplastida - ps9
Viridiplantae - ps10
Streptophyta - ps11
Charophyceae/Embriophyta - ps12
Embriophyta - ps13
Tracheophyta - ps14
Spermatophyta - ps15
Angiosperms - ps16
Mesangiospermae - ps17
Magnoliids/eudicots - ps18
Eudicots - ps19
Core_eudicots - ps20
Superrosids - ps21
Rosids - ps22
Eurosids - ps23
Malvids - ps24
Sap./Malv./Brassicales - ps25
Malvales/Brassicales - ps26
Brassicales - ps27
Cleomaceae/Brassicaceae - ps28
Brassicaceae - ps29
Camelineae - ps30
Arabidopsis - ps31
Arabidopsis_thaliana - ps32
Phylostratum

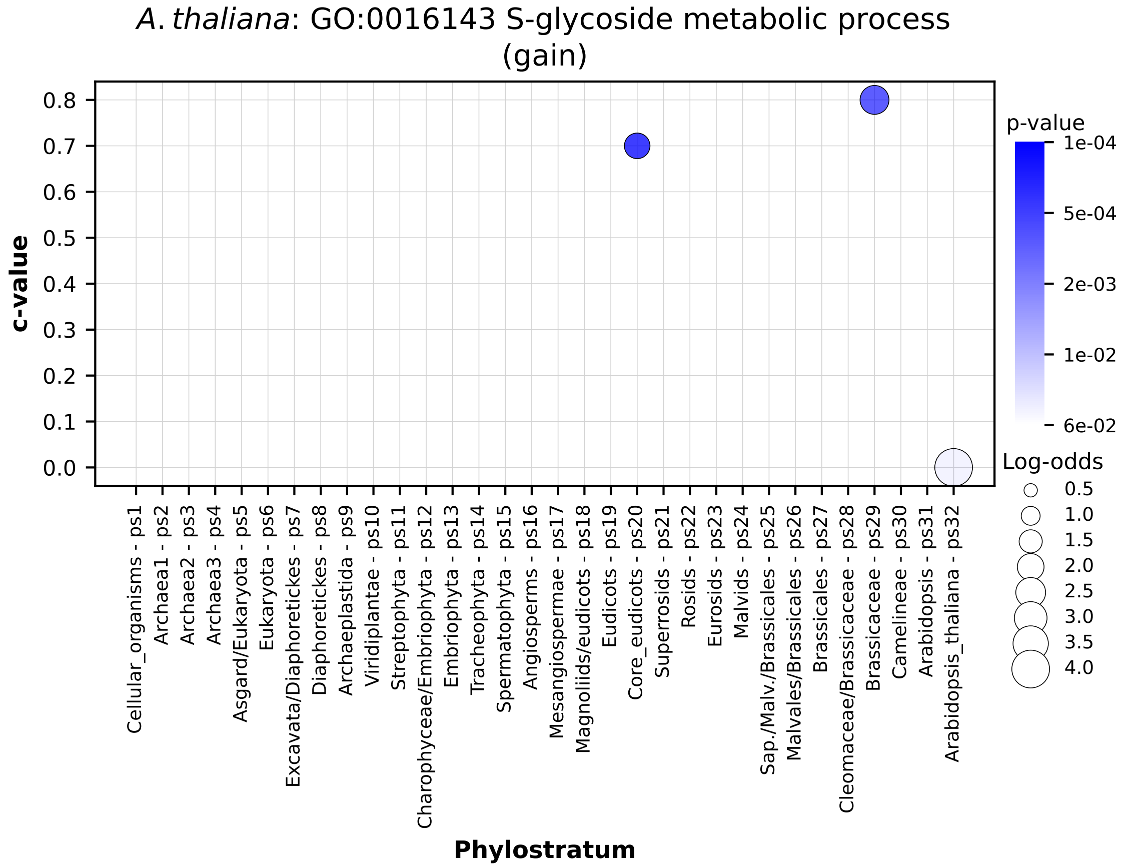

A. thaliana: GO:0016143 S-glycoside metabolic process
(gain)
c-value
0.0
0.1
0.2
0.3
0.4
0.5
0.6
0.7
0.8
p-value
1e-04
5e-04
2e-03
1e-02
6e-02
Log-odds
0.5
1.0
1.5
2.0
2.5
3.0
3.5
4.0
Cellular_organisms - ps1
Archaea1 - ps2
Archaea2 - ps3
Archaea3 - ps4
Asgard/Eukaryota - ps5
Eukaryota - ps6
Excavata/Diaphoretickes - ps7
Diaphoretickes - ps8
Archaeplastida - ps9
Viridiplantae - ps10
Streptophyta - ps11
Charophyceae/Embriophyta - ps12
Embriophyta - ps13
Tracheophyta - ps14
Spermatophyta - ps15
Angiosperms - ps16
Mesangiospermae - ps17
Magnoliids/eudicots - ps18
Eudicots - ps19
Core_eudicots - ps20
Superrosids - ps21
Rosids - ps22
Eurosids - ps23
Malvids - ps24
Sap./Malv./Brassicales - ps25
Malvales/Brassicales - ps26
Brassicales - ps27
Cleomaceae/Brassicaceae - ps28
Brassicaceae - ps29
Camelineae - ps30
Arabidopsis - ps31
Arabidopsis_thaliana - ps32
Phylostratum

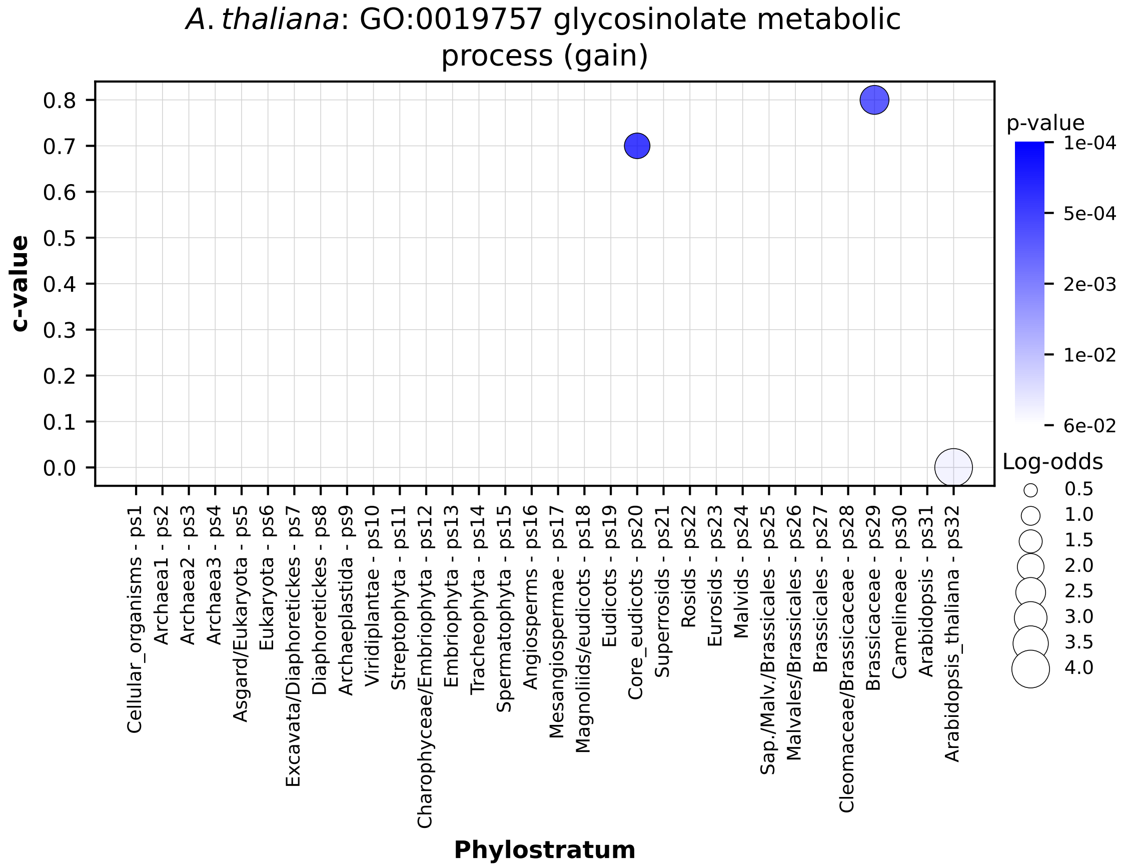

A. thaliana: GO:0019757 glycosinolate metabolic process (gain)
c-value
0.8
0.7
0.6
0.5
0.4
0.3
0.2
0.1
0.0
Phylostratum
Cellular_organisms - ps1
Archaea1 - ps2
Archaea2 - ps3
Archaea3 - ps4
Asgard/Eukaryota - ps5
Eukaryota - ps6
Excavata/Diaphoretickes - ps7
Diaphoretickes - ps8
Archaeplastida - ps9
Viridiplantae - ps10
Streptophyta - ps11
Charophyceae/Embriophyta - ps12
Embriophyta - ps13
Tracheophyta - ps14
Spermatophyta - ps15
Angiosperms - ps16
Mesangiospermae - ps17
Magnoliids/eudicots - ps18
Eudicots - ps19
Core_eudicots - ps20
Superrosids - ps21
Rosids - ps22
Eurosids - ps23
Malvids - ps24
Sap./Malv./Brassicales - ps25
Malvales/Brassicales - ps26
Brassicales - ps27
Cleomaceae/Brassicaceae - ps28
Brassicaceae - ps29
Camelineae - ps30
Arabidopsis - ps31
Arabidopsis_thaliana - ps32
p-value
1e-04
5e-04
2e-03
1e-02
6e-02
Log-odds
0.5
1.0
1.5
2.0
2.5
3.0
3.5
4.0

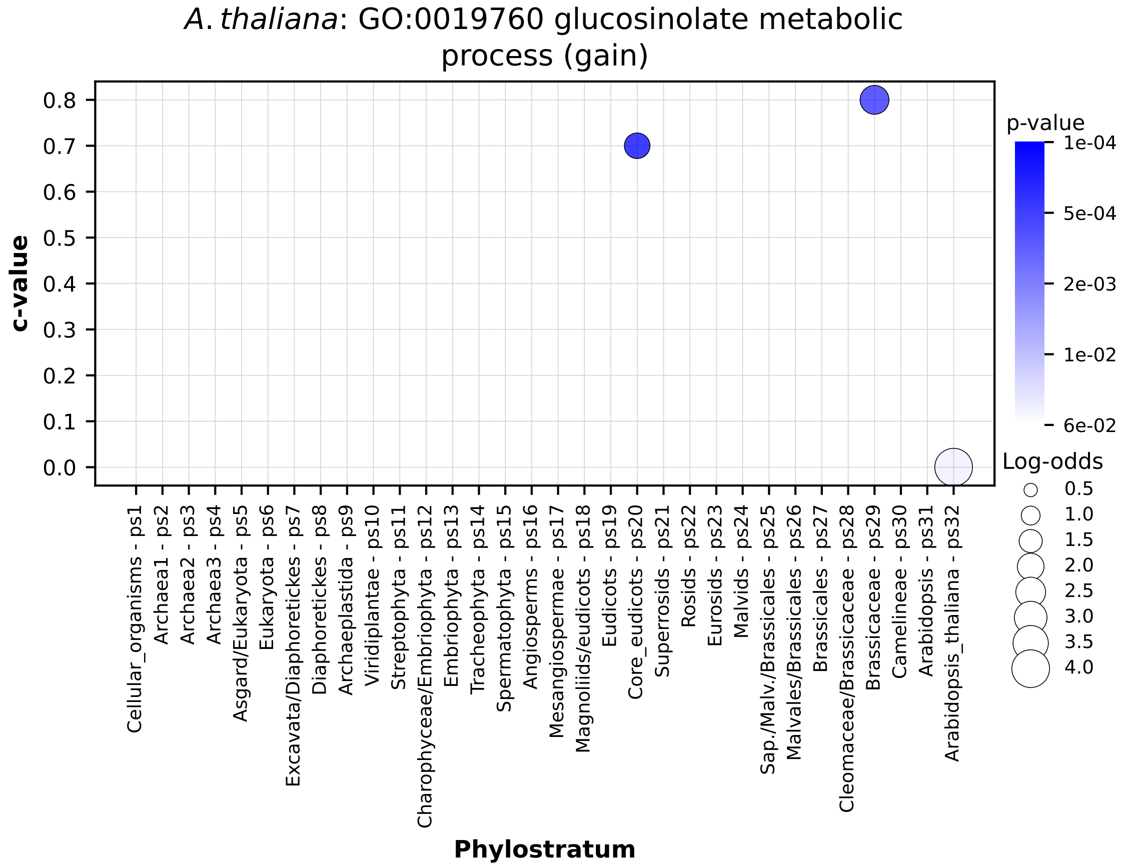

A. thaliana: GO:0019760 glucosinolate metabolic process (gain)
c-value
0.0
0.1
0.2
0.3
0.4
0.5
0.6
0.7
0.8
Phylostratum
Cellular_organisms - ps1
Archaea1 - ps2
Archaea2 - ps3
Archaea3 - ps4
Asgard/Eukaryota - ps5
Eukaryota - ps6
Excavata/Diaphoretickes - ps7
Diaphoretickes - ps8
Archaeplastida - ps9
Viridiplantae - ps10
Streptophyta - ps11
Charophyceae/Embriophyta - ps12
Embriophyta - ps13
Tracheophyta - ps14
Spermatophyta - ps15
Angiosperms - ps16
Mesangiospermae - ps17
Magnoliids/eudicots - ps18
Eudicots - ps19
Core_eudicots - ps20
Superrosids - ps21
Rosids - ps22
Eurosids - ps23
Malvids - ps24
Sap./Malv./Brassicales - ps25
Malvales/Brassicales - ps26
Brassicales - ps27
Cleomaceae/Brassicaceae - ps28
Brassicaceae - ps29
Camelineae - ps30
Arabidopsis - ps31
Arabidopsis_thaliana - ps32
p-value
1e-04
5e-04
2e-03
1e-02
6e-02
Log-odds
0.5
1.0
1.5
2.0
2.5
3.0
3.5
4.0

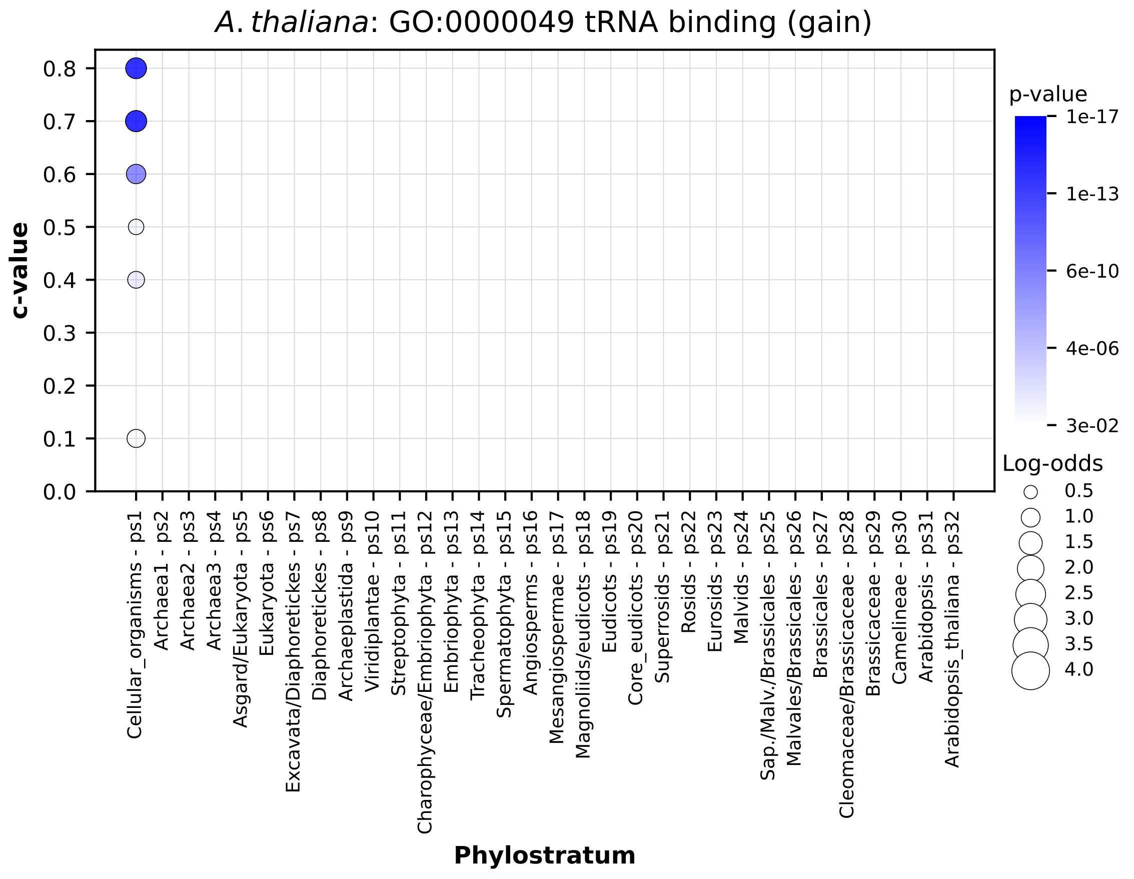

A. thaliana: GO:0000049 tRNA binding (gain)
c-value
0.0
0.1
0.2
0.3
0.4
0.5
0.6
0.7
0.8
p-value
1e-17
1e-13
6e-10
4e-06
3e-02
Log-odds
0.5
1.0
1.5
2.0
2.5
3.0
3.5
4.0
Cellular_organisms - ps1
Archaea1 - ps2
Archaea2 - ps3
Archaea3 - ps4
Asgard/Eukaryota - ps5
Eukaryota - ps6
Excavata/Diaphoretickes - ps7
Diaphoretickes - ps8
Archaeplastida - ps9
Viridiplantae - ps10
Streptophyta - ps11
Charophyceae/Embriophyta - ps12
Embriophyta - ps13
Tracheophyta - ps14
Spermatophyta - ps15
Angiosperms - ps16
Mesangiospermae - ps17
Magnoliids/eudicots - ps18
Eudicots - ps19
Core_eudicots - ps20
Superrosids - ps21
Rosids - ps22
Eurosids - ps23
Malvids - ps24
Sap./Malv./Brassicales - ps25
Malvales/Brassicales - ps26
Brassicales - ps27
Cleomaceae/Brassicaceae - ps28
Brassicaceae - ps29
Camelineae - ps30
Arabidopsis - ps31
Arabidopsis_thaliana - ps32
Phylostratum

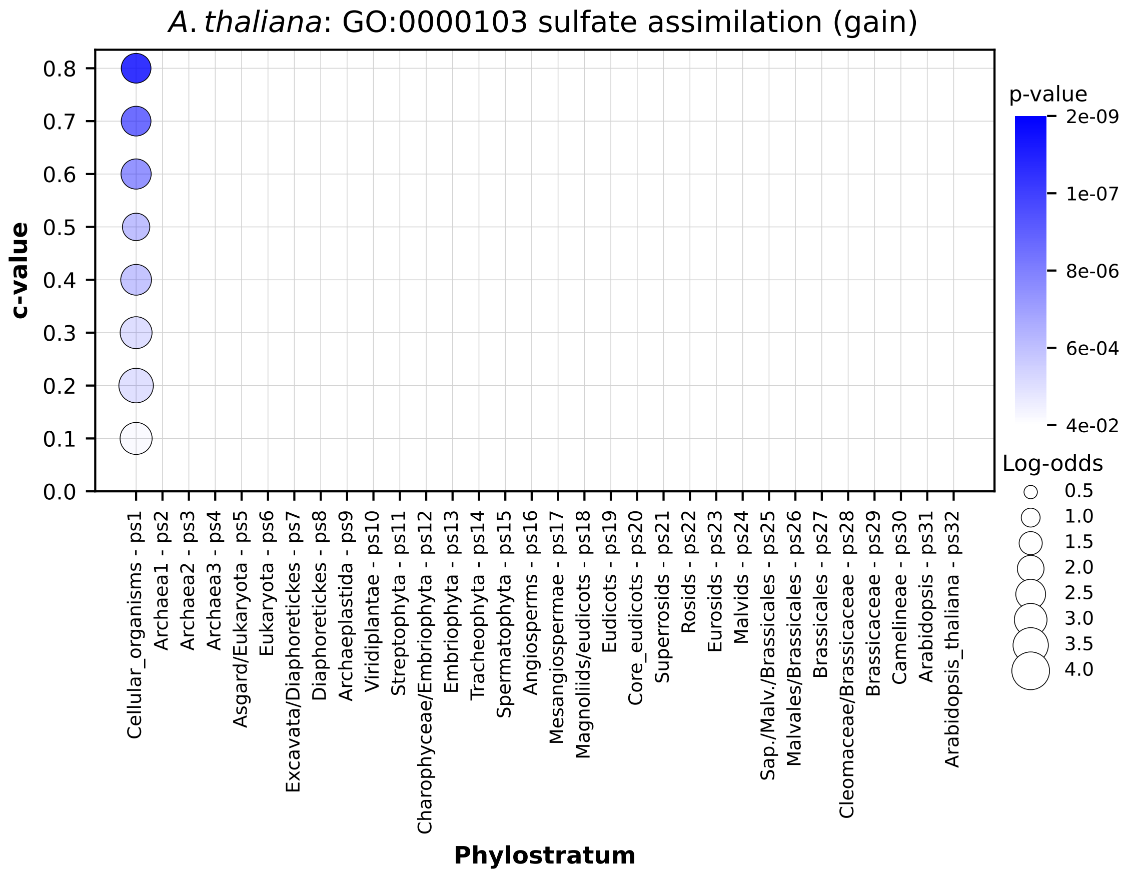
A. thaliana: GO:0000103 sulfate assimilation (gain)
c-value
0.0
0.1
0.2
0.3
0.4
0.5
0.6
0.7
0.8
p-value
2e-09
1e-07
8e-06
6e-04
4e-02
Log-odds
0.5
1.0
1.5
2.0
2.5
3.0
3.5
4.0
Cellular_organisms - ps1
Archaea1 - ps2
Archaea2 - ps3
Archaea3 - ps4
Asgard/Eukaryota - ps5
Eukaryota - ps6
Excavata/Diaphoretickes - ps7
Diaphoretickes - ps8
Archaeplastida - ps9
Viridiplantae - ps10
Streptophyta - ps11
Charophyceae/Embriophyta - ps12
Embriophyta - ps13
Tracheophyta - ps14
Spermatophyta - ps15
Angiosperms - ps16
Mesangiospermae - ps17
Magnoliids/eudicots - ps18
Eudicots - ps19
Core_eudicots - ps20
Superrosids - ps21
Rosids - ps22
Eurosids - ps23
Malvids - ps24
Sap./Malv./Brassicales - ps25
Malvales/Brassicales - ps26
Brassicales - ps27
Cleomaceae/Brassicaceae - ps28
Brassicaceae - ps29
Camelineae - ps30
Arabidopsis - ps31
Arabidopsis_thaliana - ps32
Phylostratum

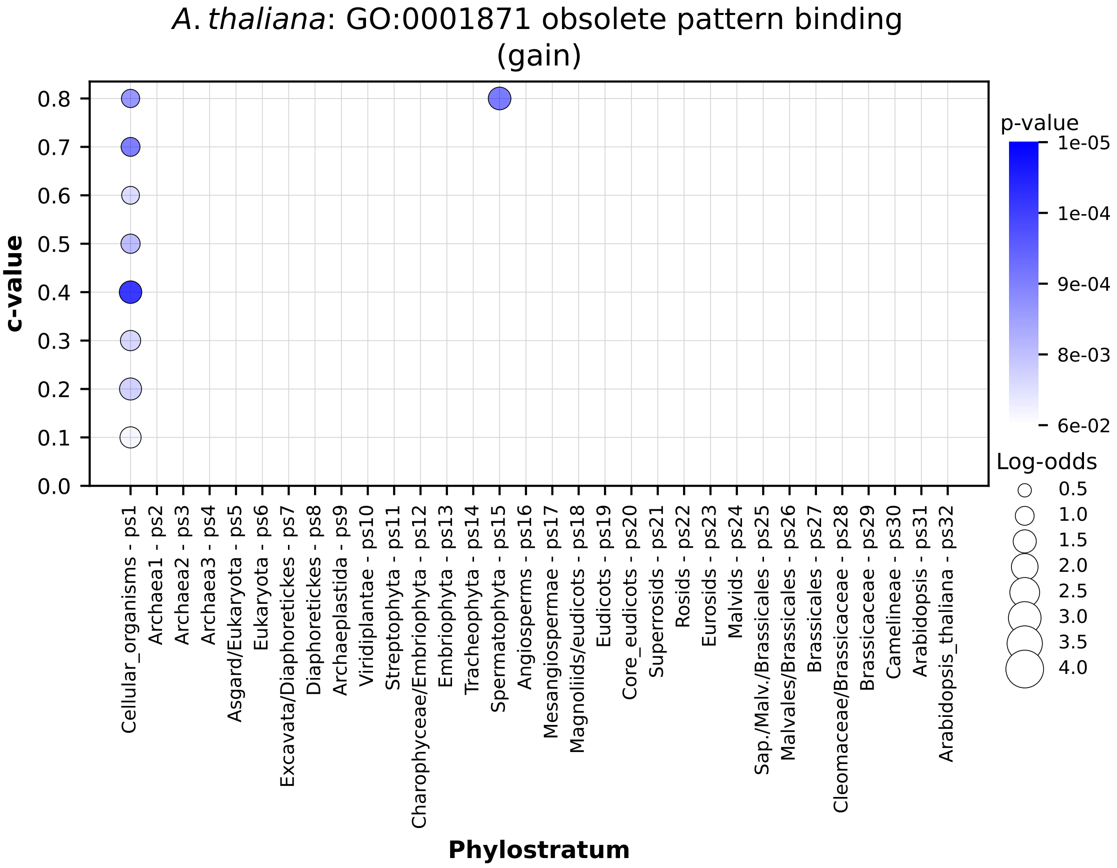

A. thaliana: GO:0001871 obsolete pattern binding (gain)
c-value
0.0
0.1
0.2
0.3
0.4
0.5
0.6
0.7
0.8
Phylostratum
Cellular_organisms - ps1
Archaea1 - ps2
Archaea2 - ps3
Archaea3 - ps4
Asgard/Eukaryota - ps5
Eukaryota - ps6
Excavata/Diaphoretickes - ps7
Diaphoretickes - ps8
Archaeplastida - ps9
Viridiplantae - ps10
Streptophyta - ps11
Charophyceae/Embriophyta - ps12
Embriophyta - ps13
Tracheophyta - ps14
Spermatophyta - ps15
Angiosperms - ps16
Mesangiospermae - ps17
Magnoliids/eudicots - ps18
Eudicots - ps19
Core_eudicots - ps20
Superrosids - ps21
Rosids - ps22
Eurosids - ps23
Malvids - ps24
Sap./Malv./Brassicales - ps25
Malvales/Brassicales - ps26
Brassicales - ps27
Cleomaceae/Brassicaceae - ps28
Brassicaceae - ps29
Camelineae - ps30
Arabidopsis - ps31
Arabidopsis_thaliana - ps32
p-value
1e-05
1e-04
9e-04
8e-03
6e-02
Log-odds
0.5
1.0
1.5
2.0
2.5
3.0
3.5
4.0

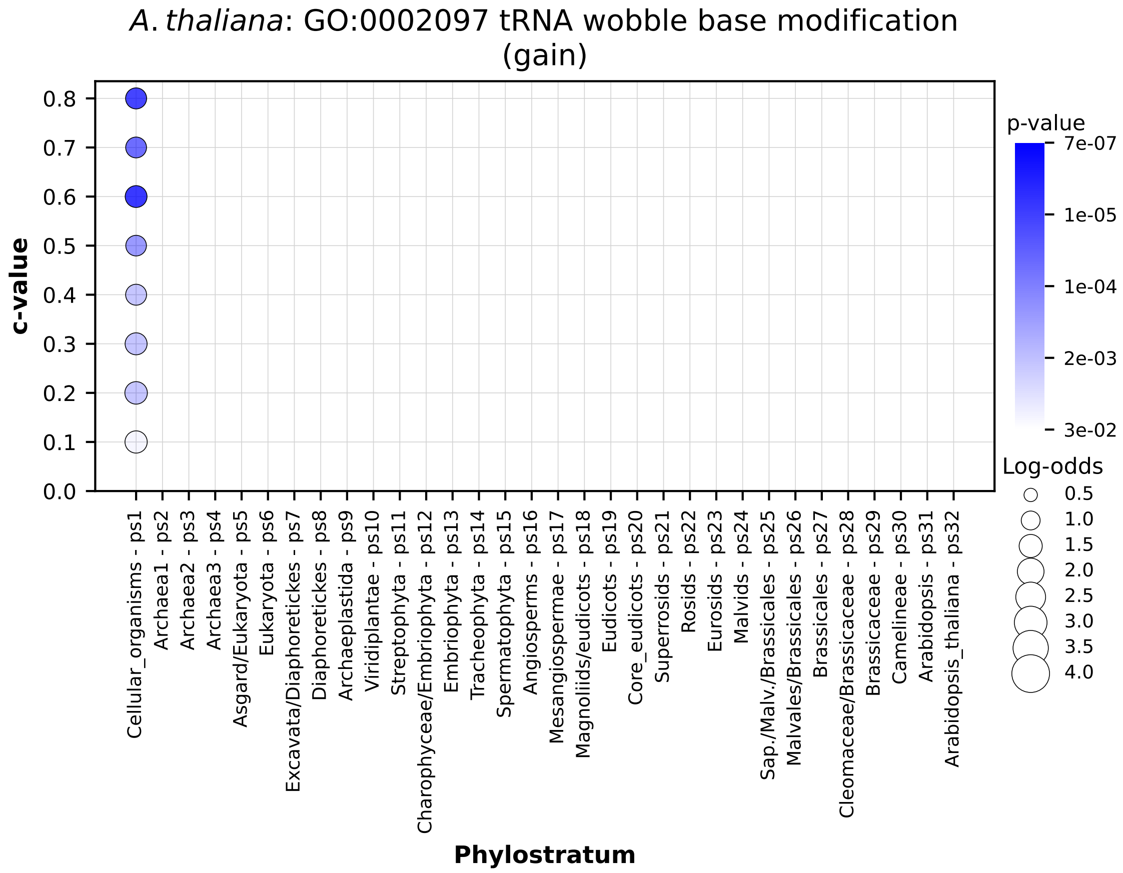

A. thaliana: GO:0002097 tRNA wobble base modification (gain)
c-value
0.0
0.1
0.2
0.3
0.4
0.5
0.6
0.7
0.8
Phylostratum
Cellular_organisms - ps1
Archaea1 - ps2
Archaea2 - ps3
Archaea3 - ps4
Asgard/Eukaryota - ps5
Eukaryota - ps6
Excavata/Diaphoretickes - ps7
Diaphoretickes - ps8
Archaeplastida - ps9
Viridiplantae - ps10
Streptophyta - ps11
Charophyceae/Embriophyta - ps12
Embriophyta - ps13
Tracheophyta - ps14
Spermatophyta - ps15
Angiosperms - ps16
Mesangiospermae - ps17
Magnoliids/eudicots - ps18
Eudicots - ps19
Core_eudicots - ps20
Superrosids - ps21
Rosids - ps22
Eurosids - ps23
Malvids - ps24
Sap./Malv./Brassicales - ps25
Malvales/Brassicales - ps26
Brassicales - ps27
Cleomaceae/Brassicaceae - ps28
Brassicaceae - ps29
Camelineae - ps30
Arabidopsis - ps31
Arabidopsis_thaliana - ps32
p-value
7e-07
1e-05
1e-04
2e-03
3e-02
Log-odds
0.5
1.0
1.5
2.0
2.5
3.0
3.5
4.0

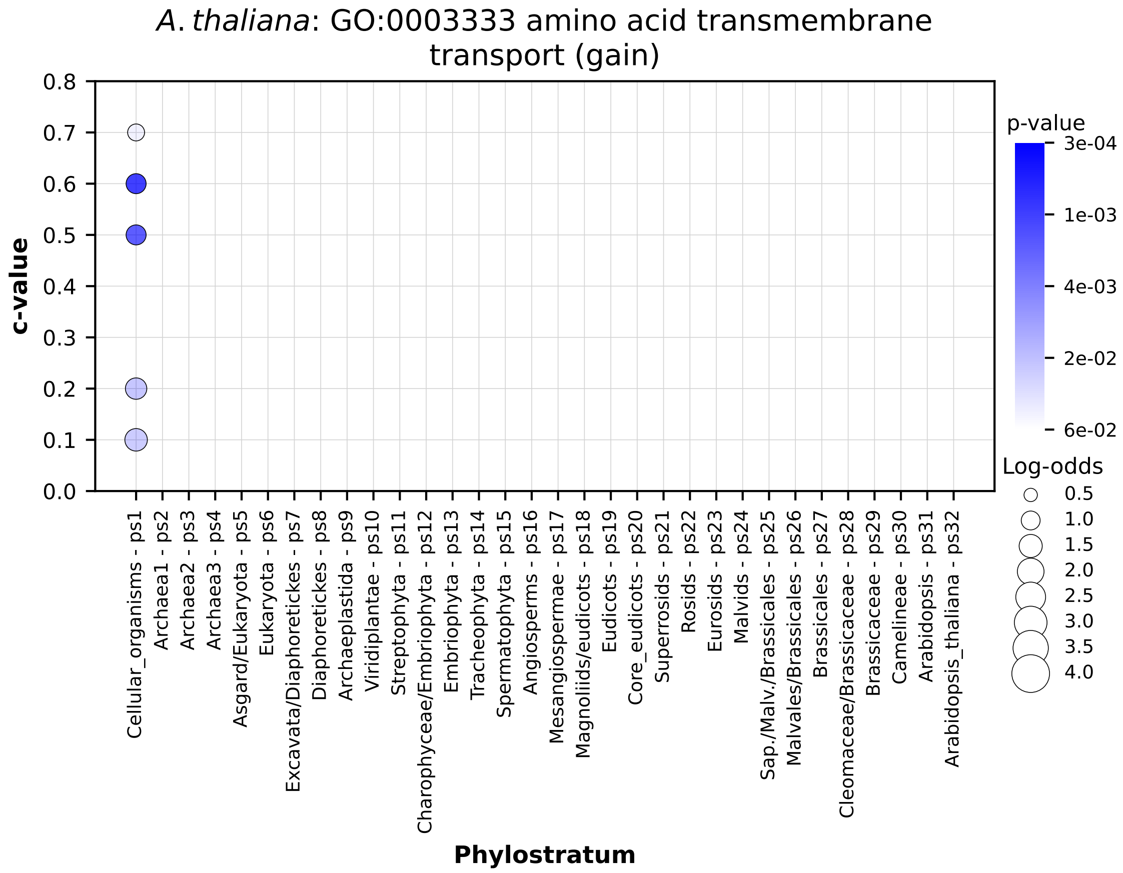

A. thaliana: GO:0003333 amino acid transmembrane transport (gain)
c-value
0.8
0.7
0.6
0.5
0.4
0.3
0.2
0.1
0.0
p-value
3e-04
1e-03
4e-03
2e-02
6e-02
Log-odds
0.5
1.0
1.5
2.0
2.5
3.0
3.5
4.0
Cellular_organisms - ps1
Archaea1 - ps2
Archaea2 - ps3
Archaea3 - ps4
Asgard/Eukaryota - ps5
Eukaryota - ps6
Excavata/Diaphoretickes - ps7
Diaphoretickes - ps8
Archaeplastida - ps9
Viridiplantae - ps10
Streptophyta - ps11
Charophyceae/Embriophyta - ps12
Embriophyta - ps13
Tracheophyta - ps14
Spermatophyta - ps15
Angiosperms - ps16
Mesangiospermae - ps17
Magnoliids/eudicots - ps18
Eudicots - ps19
Core_eudicots - ps20
Superrosids - ps21
Rosids - ps22
Eurosids - ps23
Malvids - ps24
Sap./Malv./Brassicales - ps25
Malvales/Brassicales - ps26
Brassicales - ps27
Cleomaceae/Brassicaceae - ps28
Brassicaceae - ps29
Camelineae - ps30
Arabidopsis - ps31
Arabidopsis_thaliana - ps32
Phylostratum

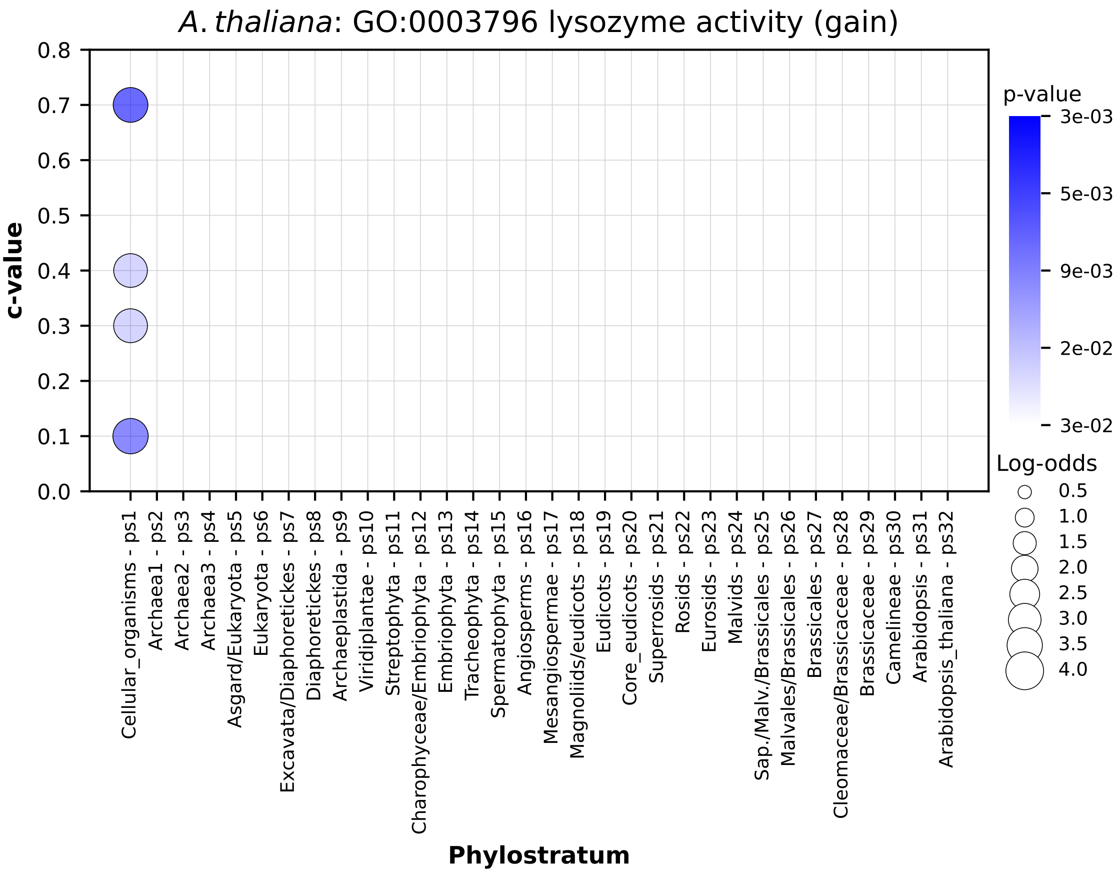

A. thaliana: GO:0003796 lysozyme activity (gain)
c-value
0.0
0.1
0.2
0.3
0.4
0.5
0.6
0.7
0.8
Phylostratum
Cellular_organisms - ps1
Archaea1 - ps2
Archaea2 - ps3
Archaea3 - ps4
Asgard/Eukaryota - ps5
Eukaryota - ps6
Excavata/Diaphoretickes - ps7
Diaphoretickes - ps8
Archaeplastida - ps9
Viridiplantae - ps10
Streptophyta - ps11
Charophyceae/Embriophyta - ps12
Embriophyta - ps13
Tracheophyta - ps14
Spermatophyta - ps15
Angiosperms - ps16
Mesangiospermae - ps17
Magnoliids/eudicots - ps18
Eudicots - ps19
Core_eudicots - ps20
Superrosids - ps21
Rosids - ps22
Eurosids - ps23
Malvids - ps24
Sap./Malv./Brassicales - ps25
Malvales/Brassicales - ps26
Brassicales - ps27
Cleomaceae/Brassicaceae - ps28
Brassicaceae - ps29
Camelineae - ps30
Arabidopsis - ps31
Arabidopsis_thaliana - ps32
p-value
3e-03
5e-03
9e-03
2e-02
3e-02
Log-odds
0.5
1.0
1.5
2.0
2.5
3.0
3.5
4.0

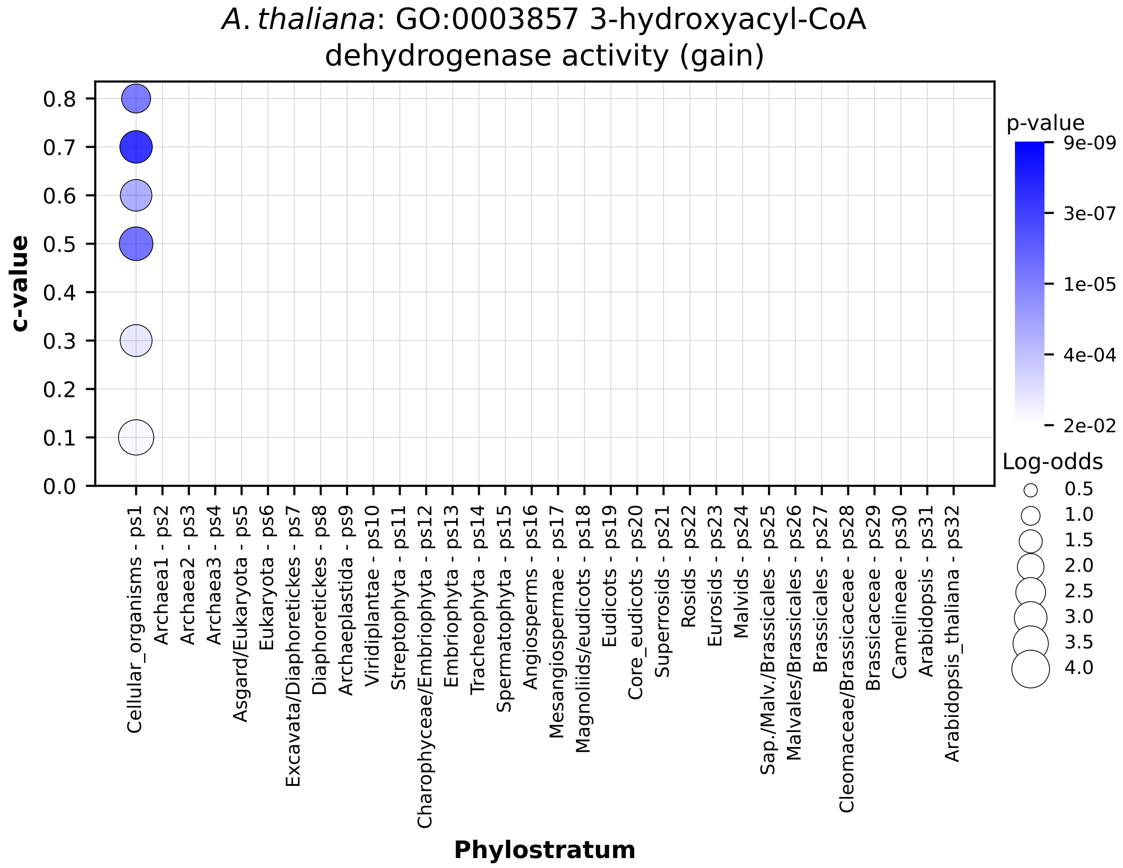
A. thaliana: GO:0003857 3-hydroxyacyl-CoA dehydrogenase activity (gain)
c-value
0.0
0.1
0.2
0.3
0.4
0.5
0.6
0.7
0.8
Phylostratum
Cellular_organisms - ps1
Archaea1 - ps2
Archaea2 - ps3
Archaea3 - ps4
Asgard/Eukaryota - ps5
Eukaryota - ps6
Excavata/Diaphoretickes - ps7
Diaphoretickes - ps8
Archaeplastida - ps9
Viridiplantae - ps10
Streptophyta - ps11
Charophyceae/Embriophyta - ps12
Embriophyta - ps13
Tracheophyta - ps14
Spermatophyta - ps15
Angiosperms - ps16
Mesangiospermae - ps17
Magnoliids/eudicots - ps18
Eudicots - ps19
Core_eudicots - ps20
Superrosids - ps21
Rosids - ps22
Eurosids - ps23
Malvids - ps24
Sap./Malv./Brassicales - ps25
Malvales/Brassicales - ps26
Brassicales - ps27
Cleomaceae/Brassicaceae - ps28
Brassicaceae - ps29
Camelineae - ps30
Arabidopsis - ps31
Arabidopsis_thaliana - ps32
p-value
9e-09
3e-07
1e-05
4e-04
2e-02
Log-odds
0.5
1.0
1.5
2.0
2.5
3.0
3.5
4.0

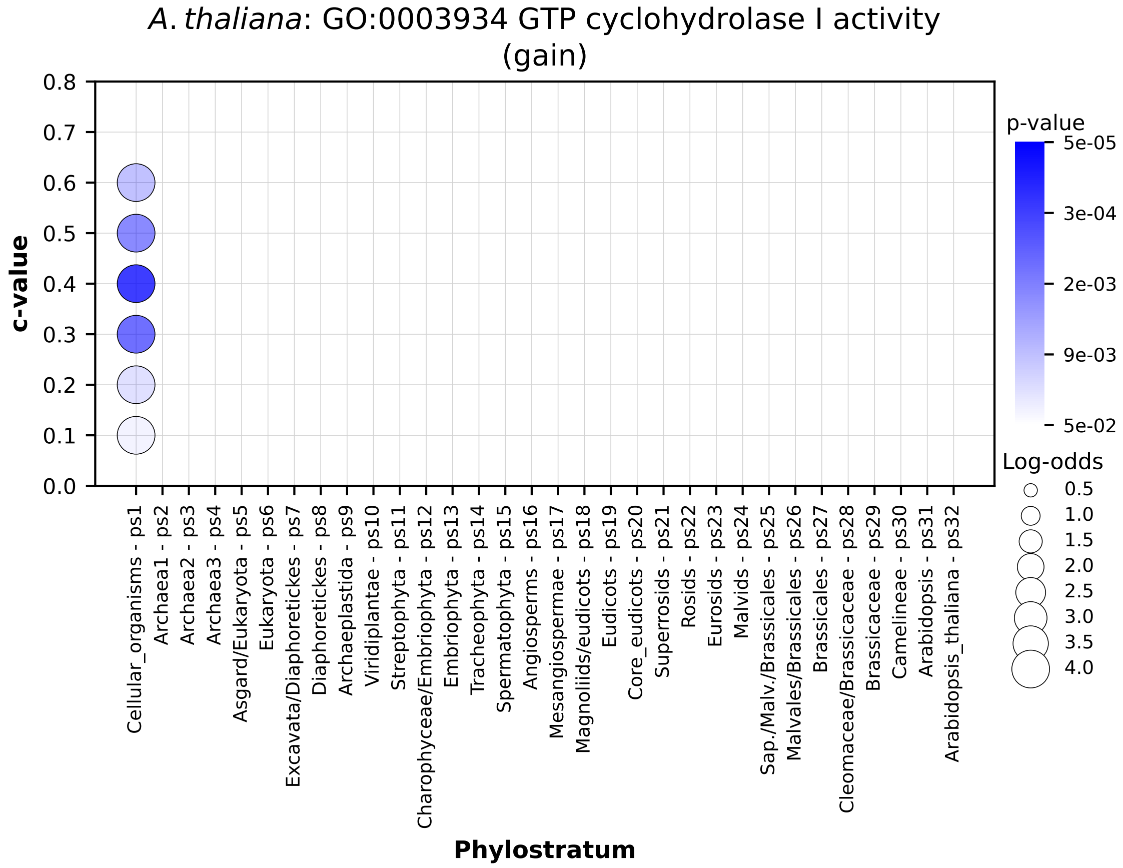

A. thaliana: GO:0003934 GTP cyclohydrolase I activity (gain)
c-value
0.0
0.1
0.2
0.3
0.4
0.5
0.6
0.7
0.8
Phylostratum
Cellular_organisms - ps1
Archaea1 - ps2
Archaea2 - ps3
Archaea3 - ps4
Asgard/Eukaryota - ps5
Eukaryota - ps6
Excavata/Diaphoretickes - ps7
Diaphoretickes - ps8
Archaeplastida - ps9
Viridiplantae - ps10
Streptophyta - ps11
Charophyceae/Embriophyta - ps12
Embriophyta - ps13
Tracheophyta - ps14
Spermatophyta - ps15
Angiosperms - ps16
Mesangiospermae - ps17
Magnoliids/eudicots - ps18
Eudicots - ps19
Core_eudicots - ps20
Superrosids - ps21
Rosids - ps22
Eurosids - ps23
Malvids - ps24
Sap./Malv./Brassicales - ps25
Malvales/Brassicales - ps26
Brassicales - ps27
Cleomaceae/Brassicaceae - ps28
Brassicaceae - ps29
Camelineae - ps30
Arabidopsis - ps31
Arabidopsis_thaliana - ps32
p-value
5e-05
3e-04
2e-03
9e-03
5e-02
Log-odds
0.5
1.0
1.5
2.0
2.5
3.0
3.5
4.0

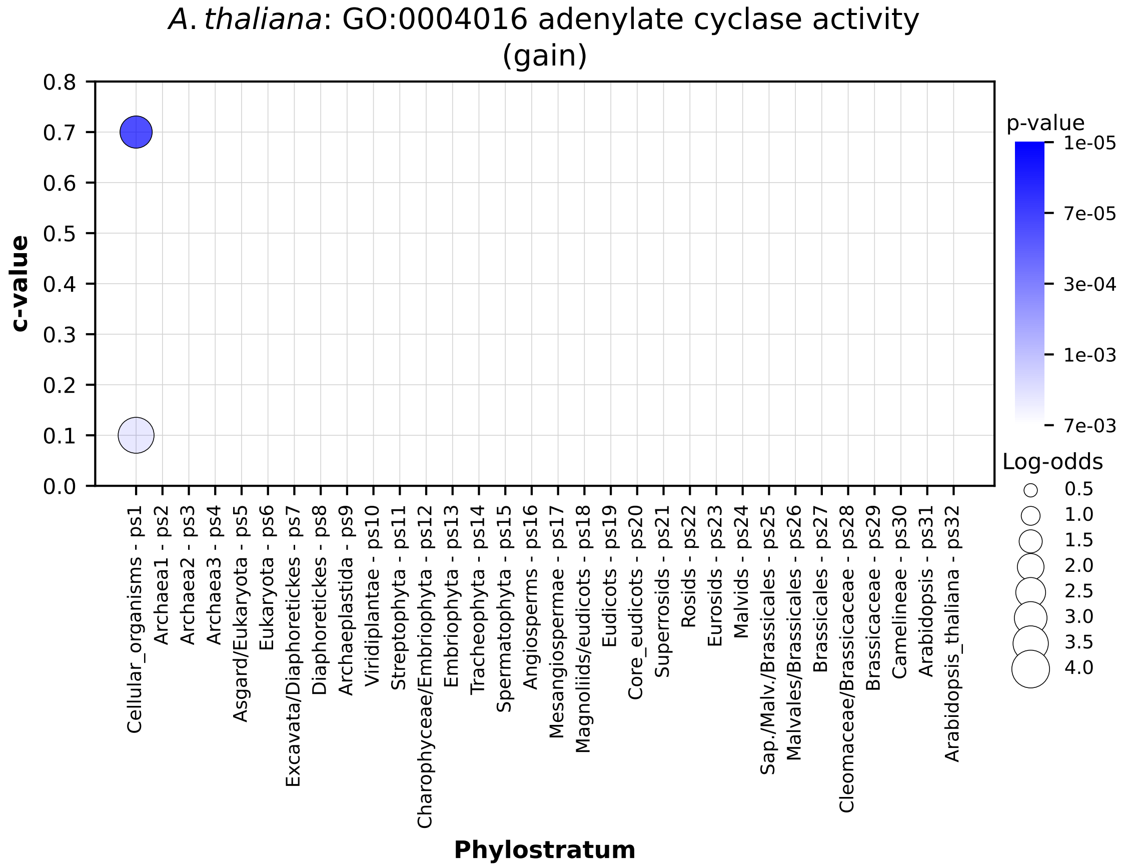

A. thaliana: GO:0004016 adenylate cyclase activity
(gain)
c-value
0.8
0.7
0.6
0.5
0.4
0.3
0.2
0.1
0.0
p-value
1e-05
7e-05
3e-04
1e-03
7e-03
Log-odds
0.5
1.0
1.5
2.0
2.5
3.0
3.5
4.0
Cellular_organisms - ps1
Archaea1 - ps2
Archaea2 - ps3
Archaea3 - ps4
Asgard/Eukaryota - ps5
Eukaryota - ps6
Excavata/Diaphoretickes - ps7
Diaphoretickes - ps8
Archaeplastida - ps9
Viridiplantae - ps10
Streptophyta - ps11
Charophyceae/Embriophyta - ps12
Embriophyta - ps13
Tracheophyta - ps14
Spermatophyta - ps15
Angiosperms - ps16
Mesangiospermae - ps17
Magnoliids/eudicots - ps18
Eudicots - ps19
Core_eudicots - ps20
Superrosids - ps21
Rosids - ps22
Eurosids - ps23
Malvids - ps24
Sap./Malv./Brassicales - ps25
Malvales/Brassicales - ps26
Brassicales - ps27
Cleomaceae/Brassicaceae - ps28
Brassicaceae - ps29
Camelineae - ps30
Arabidopsis - ps31
Arabidopsis_thaliana - ps32
Phylostratum

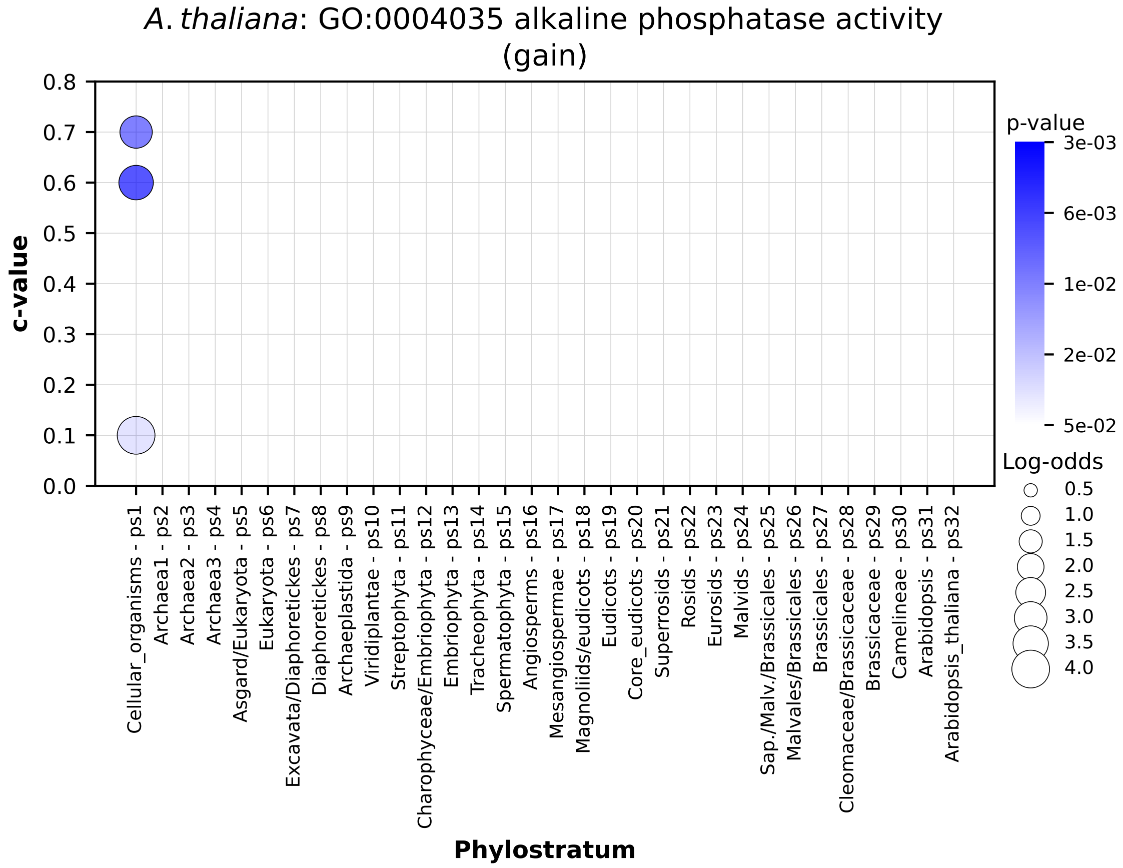

A. thaliana: GO:0004035 alkaline phosphatase activity (gain)
c-value
0.8
0.7
0.6
0.5
0.4
0.3
0.2
0.1
0.0
p-value
3e-03
6e-03
1e-02
2e-02
5e-02
Log-odds
0.5
1.0
1.5
2.0
2.5
3.0
3.5
4.0
Cellular_organisms - ps1
Archaea1 - ps2
Archaea2 - ps3
Archaea3 - ps4
Asgard/Eukaryota - ps5
Eukaryota - ps6
Excavata/Diaphoretickes - ps7
Diaphoretickes - ps8
Archaeplastida - ps9
Viridiplantae - ps10
Streptophyta - ps11
Charophyceae/Embriophyta - ps12
Embriophyta - ps13
Tracheophyta - ps14
Spermatophyta - ps15
Angiosperms - ps16
Mesangiospermae - ps17
Magnoliids/eudicots - ps18
Eudicots - ps19
Core_eudicots - ps20
Superrosids - ps21
Rosids - ps22
Eurosids - ps23
Malvids - ps24
Sap./Malv./Brassicales - ps25
Malvales/Brassicales - ps26
Brassicales - ps27
Cleomaceae/Brassicaceae - ps28
Brassicaceae - ps29
Camelineae - ps30
Arabidopsis - ps31
Arabidopsis_thaliana - ps32
Phylostratum

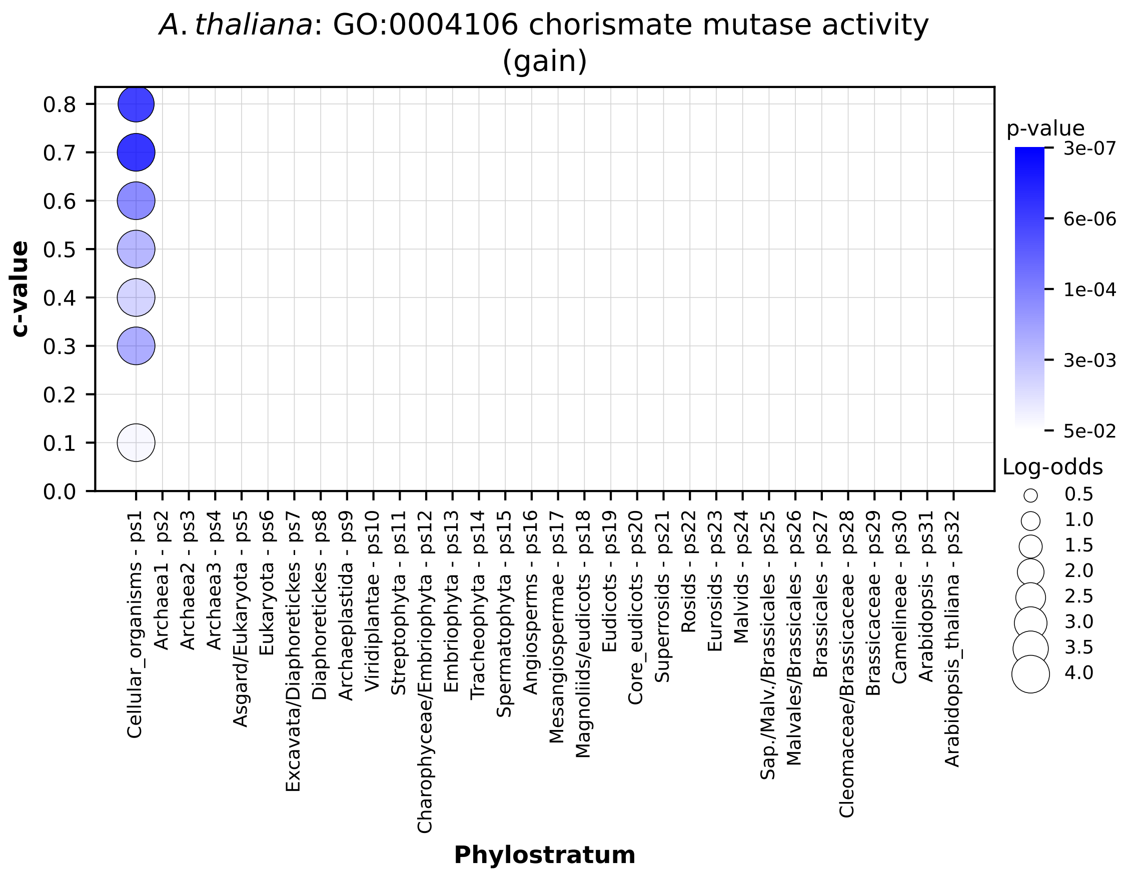
A. thaliana: GO:0004106 chorismate mutase activity (gain)
c-value
0.0
0.1
0.2
0.3
0.4
0.5
0.6
0.7
0.8
Phylostratum
Cellular_organisms - ps1
Archaea1 - ps2
Archaea2 - ps3
Archaea3 - ps4
Asgard/Eukaryota - ps5
Eukaryota - ps6
Excavata/Diaphoretickes - ps7
Diaphoretickes - ps8
Archaeplastida - ps9
Viridiplantae - ps10
Streptophyta - ps11
Charophyceae/Embriophyta - ps12
Embriophyta - ps13
Tracheophyta - ps14
Spermatophyta - ps15
Angiosperms - ps16
Mesangiospermae - ps17
Magnoliids/eudicots - ps18
Eudicots - ps19
Core_eudicots - ps20
Superrosids - ps21
Rosids - ps22
Eurosids - ps23
Malvids - ps24
Sap./Malv./Brassicales - ps25
Malvales/Brassicales - ps26
Brassicales - ps27
Cleomaceae/Brassicaceae - ps28
Brassicaceae - ps29
Camelineae - ps30
Arabidopsis - ps31
Arabidopsis_thaliana - ps32
p-value
3e-07
6e-06
1e-04
3e-03
5e-02
Log-odds
0.5
1.0
1.5
2.0
2.5
3.0
3.5
4.0

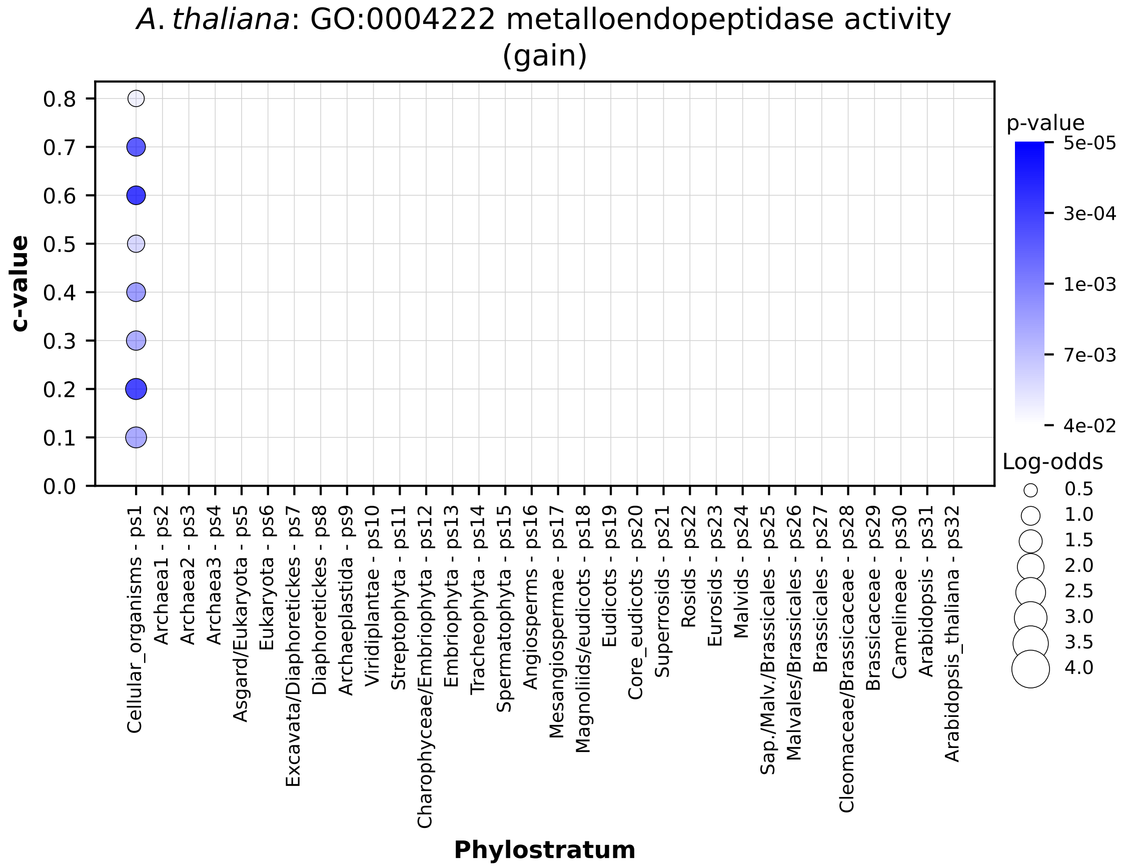

A. thaliana: GO:0004222 metalloendopeptidase activity
(gain)
c-value
0.0
0.1
0.2
0.3
0.4
0.5
0.6
0.7
0.8
Phylostratum
Cellular_organisms - ps1
Archaea1 - ps2
Archaea2 - ps3
Archaea3 - ps4
Asgard/Eukaryota - ps5
Eukaryota - ps6
Excavata/Diaphoretickes - ps7
Diaphoretickes - ps8
Archaeplastida - ps9
Viridiplantae - ps10
Streptophyta - ps11
Charophyceae/Embriophyta - ps12
Embriophyta - ps13
Tracheophyta - ps14
Spermatophyta - ps15
Angiosperms - ps16
Mesangiospermae - ps17
Magnoliids/eudicots - ps18
Eudicots - ps19
Core_eudicots - ps20
Superrosids - ps21
Rosids - ps22
Eurosids - ps23
Malvids - ps24
Sap./Malv./Brassicales - ps25
Malvales/Brassicales - ps26
Brassicales - ps27
Cleomaceae/Brassicaceae - ps28
Brassicaceae - ps29
Camelineae - ps30
Arabidopsis - ps31
Arabidopsis_thaliana - ps32
p-value
5e-05
3e-04
1e-03
7e-03
4e-02
Log-odds
0.5
1.0
1.5
2.0
2.5
3.0
3.5
4.0

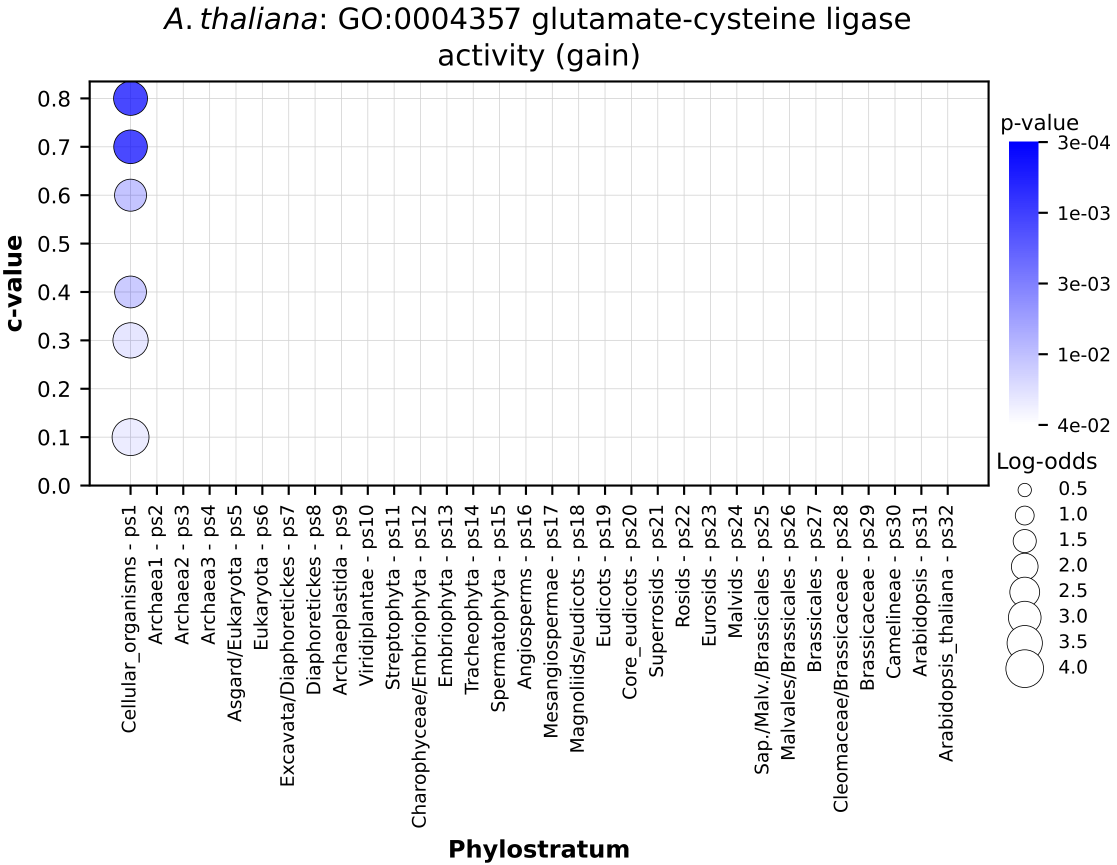

A. thaliana: GO:0004357 glutamate-cysteine ligase activity (gain)
c-value
0.0
0.1
0.2
0.3
0.4
0.5
0.6
0.7
0.8
Phylostratum
Cellular_organisms - ps1
Archaea1 - ps2
Archaea2 - ps3
Archaea3 - ps4
Asgard/Eukaryota - ps5
Eukaryota - ps6
Excavata/Diaphoretickes - ps7
Diaphoretickes - ps8
Archaeplastida - ps9
Viridiplantae - ps10
Streptophyta - ps11
Charophyceae/Embriophyta - ps12
Embriophyta - ps13
Tracheophyta - ps14
Spermatophyta - ps15
Angiosperms - ps16
Mesangiospermae - ps17
Magnoliids/eudicots - ps18
Eudicots - ps19
Core_eudicots - ps20
Superrosids - ps21
Rosids - ps22
Eurosids - ps23
Malvids - ps24
Sap./Malv./Brassicales - ps25
Malvales/Brassicales - ps26
Brassicales - ps27
Cleomaceae/Brassicaceae - ps28
Brassicaceae - ps29
Camelineae - ps30
Arabidopsis - ps31
Arabidopsis_thaliana - ps32
p-value
3e-04
1e-03
3e-03
1e-02
4e-02
Log-odds
0.5
1.0
1.5
2.0
2.5
3.0
3.5
4.0

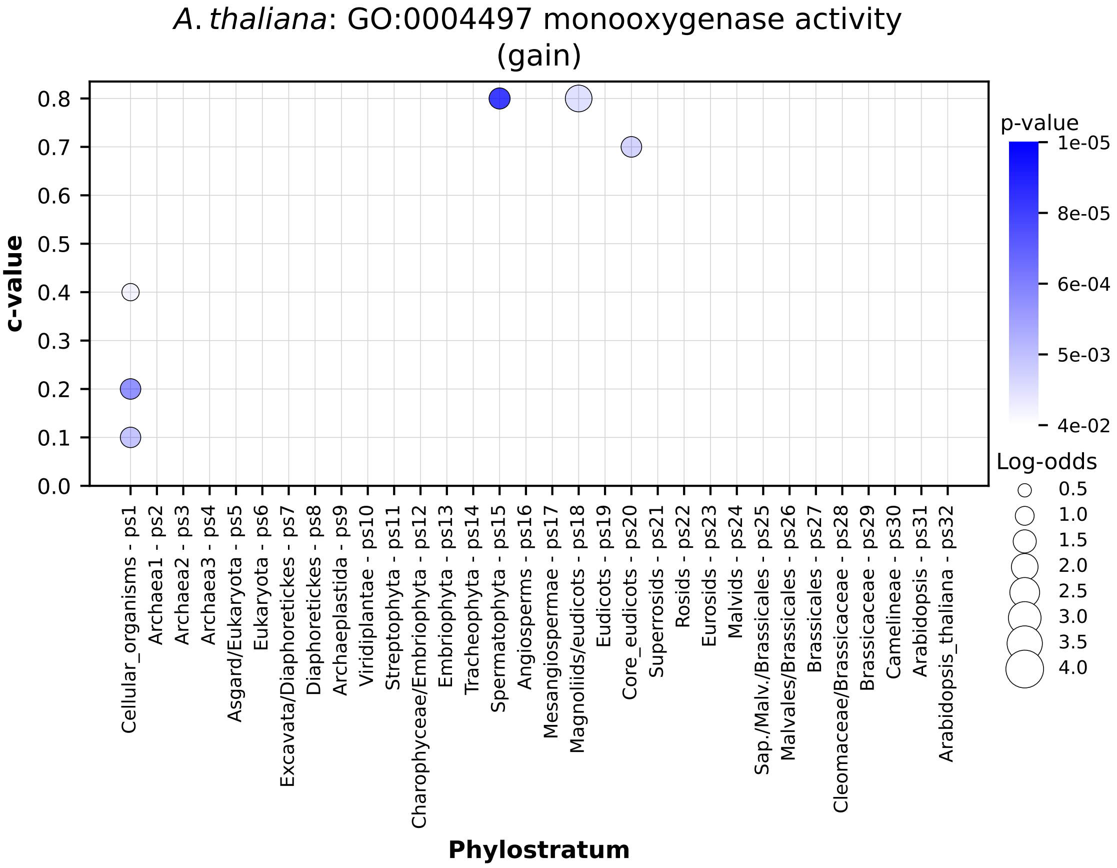

A. thaliana: GO:0004497 monooxygenase activity
(gain)
c-value
0.8
0.7
0.6
0.5
0.4
0.3
0.2
0.1
0.0
p-value
1e-05
8e-05
6e-04
5e-03
4e-02
Log-odds
0.5
1.0
1.5
2.0
2.5
3.0
3.5
4.0
Cellular_organisms - ps1
Archaea1 - ps2
Archaea2 - ps3
Archaea3 - ps4
Asgard/Eukaryota - ps5
Eukaryota - ps6
Excavata/Diaphoretickes - ps7
Diaphoretickes - ps8
Archaeplastida - ps9
Viridiplantae - ps10
Streptophyta - ps11
Charophyceae/Embriophyta - ps12
Embriophyta - ps13
Tracheophyta - ps14
Spermatophyta - ps15
Angiosperms - ps16
Mesangiospermae - ps17
Magnoliids/eudicots - ps18
Eudicots - ps19
Core_eudicots - ps20
Superrosids - ps21
Rosids - ps22
Eurosids - ps23
Malvids - ps24
Sap./Malv./Brassicales - ps25
Malvales/Brassicales - ps26
Brassicales - ps27
Cleomaceae/Brassicaceae - ps28
Brassicaceae - ps29
Camelineae - ps30
Arabidopsis - ps31
Arabidopsis_thaliana - ps32
Phylostratum

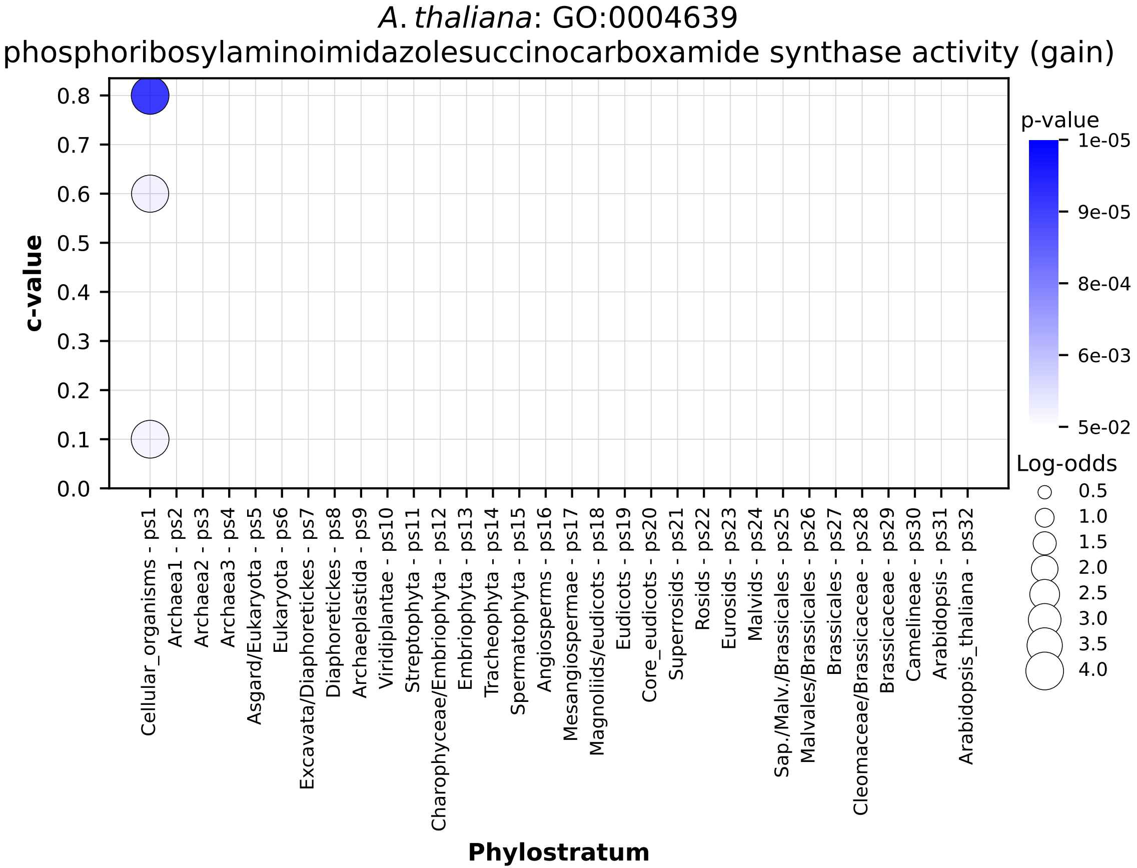

A. thaliana: GO:0004639
phosphoribosylaminoimidazolesuccinocarboxamide synthase activity (gain)
c-value
0.0
0.1
0.2
0.3
0.4
0.5
0.6
0.7
0.8
p-value
1e-05
9e-05
8e-04
6e-03
5e-02
Log-odds
0.5
1.0
1.5
2.0
2.5
3.0
3.5
4.0
Cellular_organisms - ps1
Archaea1 - ps2
Archaea2 - ps3
Archaea3 - ps4
Asgard/Eukaryota - ps5
Eukaryota - ps6
Excavata/Diaphoretickes - ps7
Diaphoretickes - ps8
Archaeplastida - ps9
Viridiplantae - ps10
Streptophyta - ps11
Charophyceae/Embriophyta - ps12
Embriophyta - ps13
Tracheophyta - ps14
Spermatophyta - ps15
Angiosperms - ps16
Mesangiospermae - ps17
Magnoliids/eudicots - ps18
Eudicots - ps19
Core_eudicots - ps20
Superrosids - ps21
Rosids - ps22
Eurosids - ps23
Malvids - ps24
Sap./Malv./Brassicales - ps25
Malvales/Brassicales - ps26
Brassicales - ps27
Cleomaceae/Brassicaceae - ps28
Brassicaceae - ps29
Camelineae - ps30
Arabidopsis - ps31
Arabidopsis_thaliana - ps32
Phylostratum

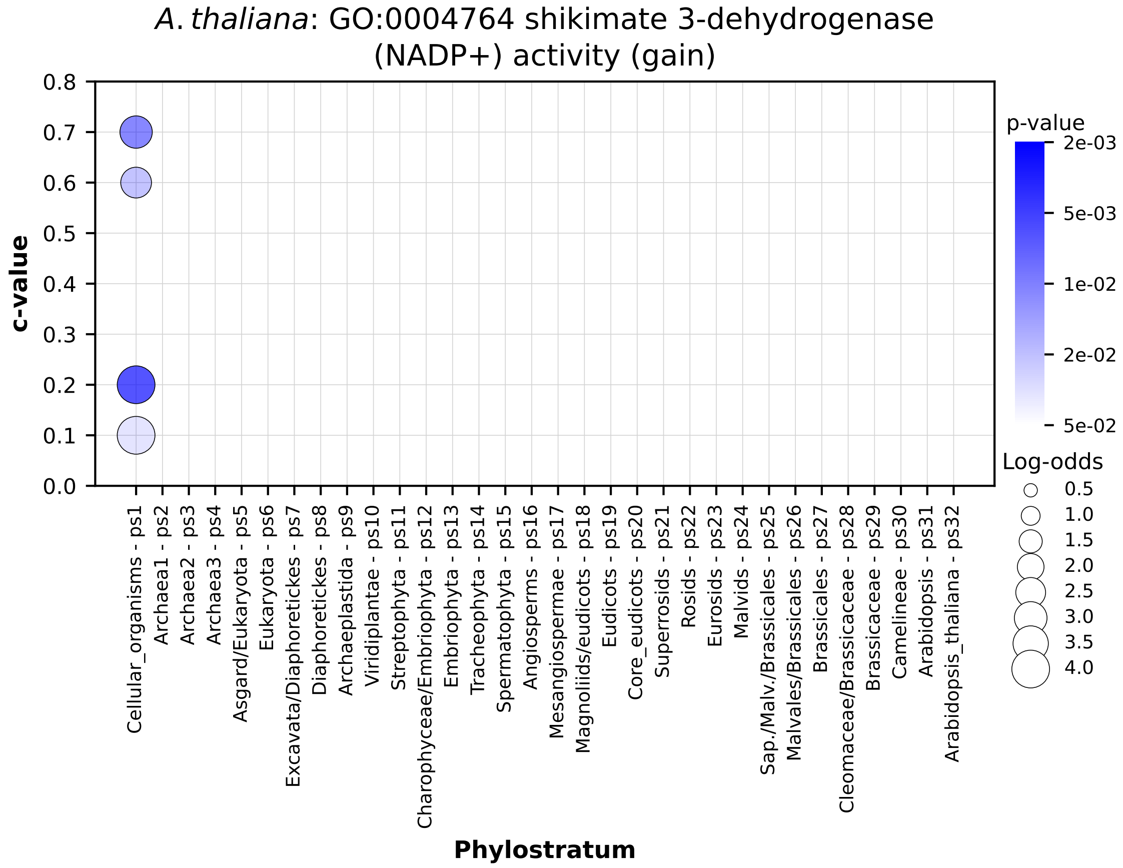
A. thaliana: GO:0004764 shikimate 3-dehydrogenase (NADP+) activity (gain)
c-value
0.8
0.7
0.6
0.5
0.4
0.3
0.2
0.1
0.0
p-value
2e-03
5e-03
1e-02
2e-02
5e-02
Log-odds
0.5
1.0
1.5
2.0
2.5
3.0
3.5
4.0
Cellular_organisms - ps1
Archaea1 - ps2
Archaea2 - ps3
Archaea3 - ps4
Asgard/Eukaryota - ps5
Eukaryota - ps6
Excavata/Diaphoretickes - ps7
Diaphoretickes - ps8
Archaeplastida - ps9
Viridiplantae - ps10
Streptophyta - ps11
Charophyceae/Embriophyta - ps12
Embriophyta - ps13
Tracheophyta - ps14
Spermatophyta - ps15
Angiosperms - ps16
Mesangiospermae - ps17
Magnoliids/eudicots - ps18
Eudicots - ps19
Core_eudicots - ps20
Superrosids - ps21
Rosids - ps22
Eurosids - ps23
Malvids - ps24
Sap./Malv./Brassicales - ps25
Malvales/Brassicales - ps26
Brassicales - ps27
Cleomaceae/Brassicaceae - ps28
Brassicaceae - ps29
Camelineae - ps30
Arabidopsis - ps31
Arabidopsis_thaliana - ps32
Phylostratum

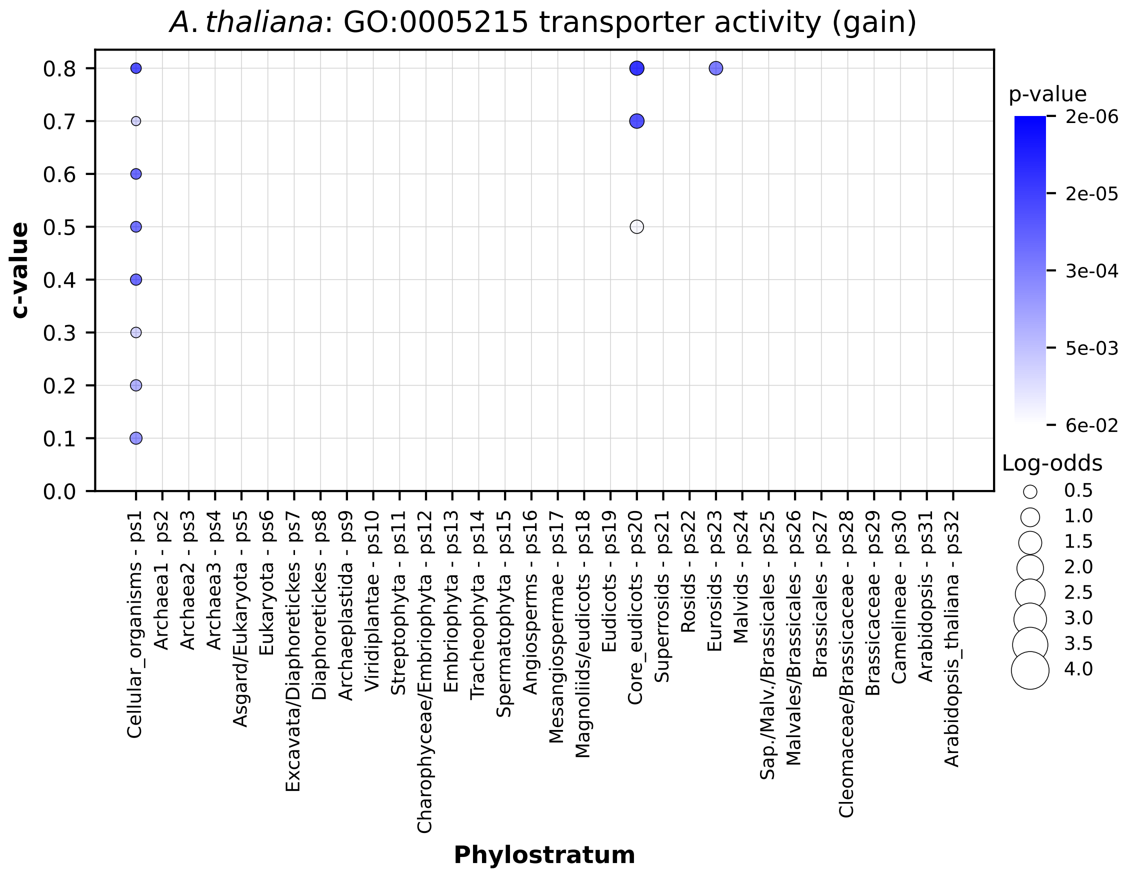

A. thaliana: GO:0005215 transporter activity (gain)
c-value
0.8
0.7
0.6
0.5
0.4
0.3
0.2
0.1
0.0
p-value
2e-06
2e-05
3e-04
5e-03
6e-02
Log-odds
0.5
1.0
1.5
2.0
2.5
3.0
3.5
4.0
Cellular_organisms - ps1
Archaea1 - ps2
Archaea2 - ps3
Archaea3 - ps4
Asgard/Eukaryota - ps5
Eukaryota - ps6
Excavata/Diaphoretickes - ps7
Diaphoretickes - ps8
Archaeplastida - ps9
Viridiplantae - ps10
Streptophyta - ps11
Charophyceae/Embriophyta - ps12
Embriophyta - ps13
Tracheophyta - ps14
Spermatophyta - ps15
Angiosperms - ps16
Mesangiospermae - ps17
Magnoliids/eudicots - ps18
Eudicots - ps19
Core_eudicots - ps20
Superrosids - ps21
Rosids - ps22
Eurosids - ps23
Malvids - ps24
Sap./Malv./Brassicales - ps25
Malvales/Brassicales - ps26
Brassicales - ps27
Cleomaceae/Brassicaceae - ps28
Brassicaceae - ps29
Camelineae - ps30
Arabidopsis - ps31
Arabidopsis_thaliana - ps32
Phylostratum

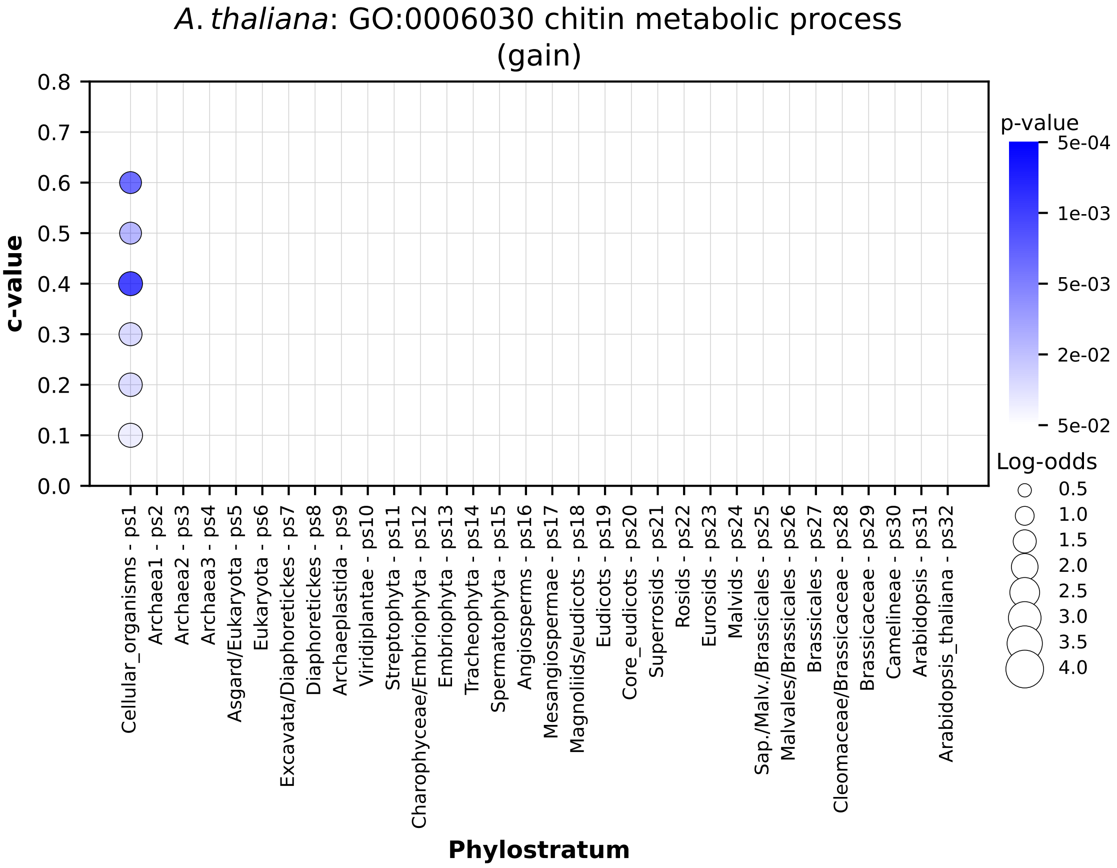
A. thaliana: GO:0006030 chitin metabolic process
(gain)
c-value
0.0
0.1
0.2
0.3
0.4
0.5
0.6
0.7
0.8
Phylostratum
Cellular_organisms - ps1
Archaea1 - ps2
Archaea2 - ps3
Archaea3 - ps4
Asgard/Eukaryota - ps5
Eukaryota - ps6
Excavata/Diaphoretickes - ps7
Diaphoretickes - ps8
Archaeplastida - ps9
Viridiplantae - ps10
Streptophyta - ps11
Charophyceae/Embriophyta - ps12
Embriophyta - ps13
Tracheophyta - ps14
Spermatophyta - ps15
Angiosperms - ps16
Mesangiospermae - ps17
Magnoliids/eudicots - ps18
Eudicots - ps19
Core_eudicots - ps20
Superrosids - ps21
Rosids - ps22
Eurosids - ps23
Malvids - ps24
Sap./Malv./Brassicales - ps25
Malvales/Brassicales - ps26
Brassicales - ps27
Cleomaceae/Brassicaceae - ps28
Brassicaceae - ps29
Camelineae - ps30
Arabidopsis - ps31
Arabidopsis_thaliana - ps32
p-value
5e-04
1e-03
5e-03
2e-02
5e-02
Log-odds
0.5
1.0
1.5
2.0
2.5
3.0
3.5
4.0

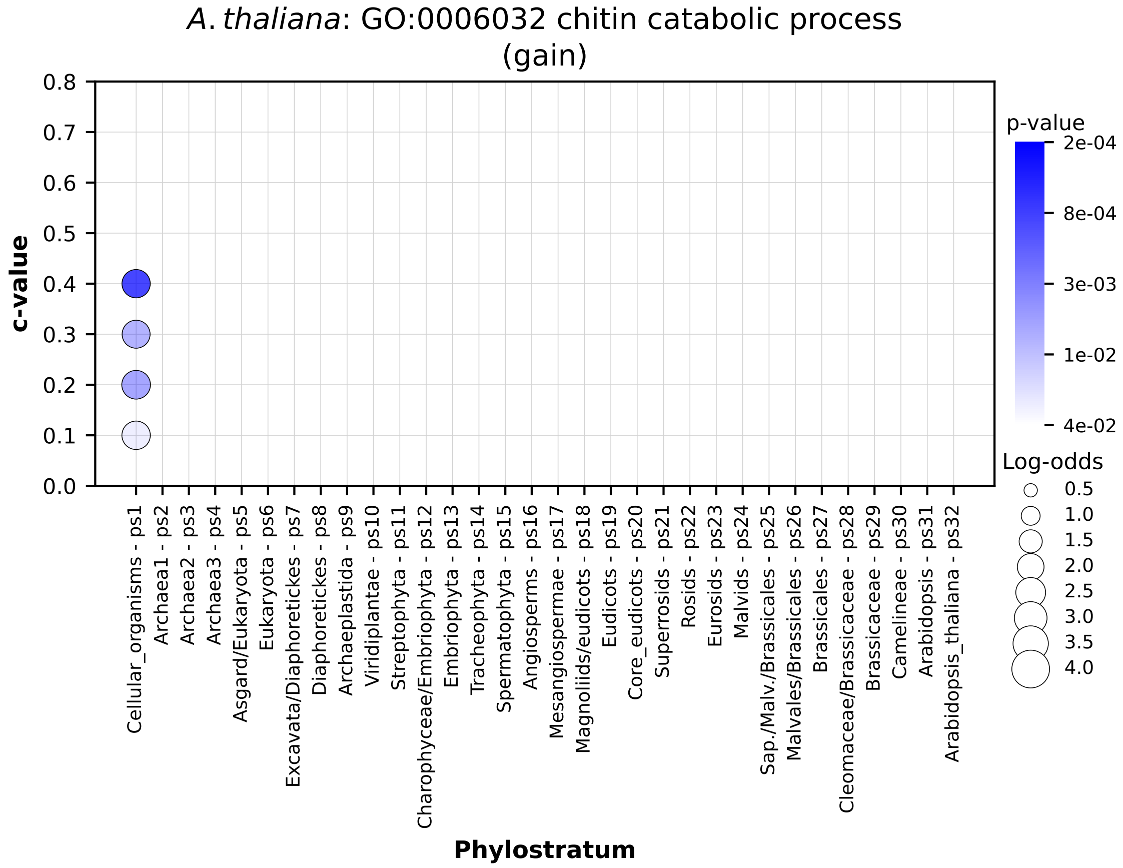

A. thaliana: GO:0006032 chitin catabolic process
(gain)
c-value
0.8
0.7
0.6
0.5
0.4
0.3
0.2
0.1
0.0
p-value
2e-04
8e-04
3e-03
1e-02
4e-02
Log-odds
0.5
1.0
1.5
2.0
2.5
3.0
3.5
4.0
Cellular_organisms - ps1
Archaea1 - ps2
Archaea2 - ps3
Archaea3 - ps4
Asgard/Eukaryota - ps5
Eukaryota - ps6
Excavata/Diaphoretickes - ps7
Diaphoretickes - ps8
Archaeplastida - ps9
Viridiplantae - ps10
Streptophyta - ps11
Charophyceae/Embriophyta - ps12
Embriophyta - ps13
Tracheophyta - ps14
Spermatophyta - ps15
Angiosperms - ps16
Mesangiospermae - ps17
Magnoliids/eudicots - ps18
Eudicots - ps19
Core_eudicots - ps20
Superrosids - ps21
Rosids - ps22
Eurosids - ps23
Malvids - ps24
Sap./Malv./Brassicales - ps25
Malvales/Brassicales - ps26
Brassicales - ps27
Cleomaceae/Brassicaceae - ps28
Brassicaceae - ps29
Camelineae - ps30
Arabidopsis - ps31
Arabidopsis_thaliana - ps32
Phylostratum

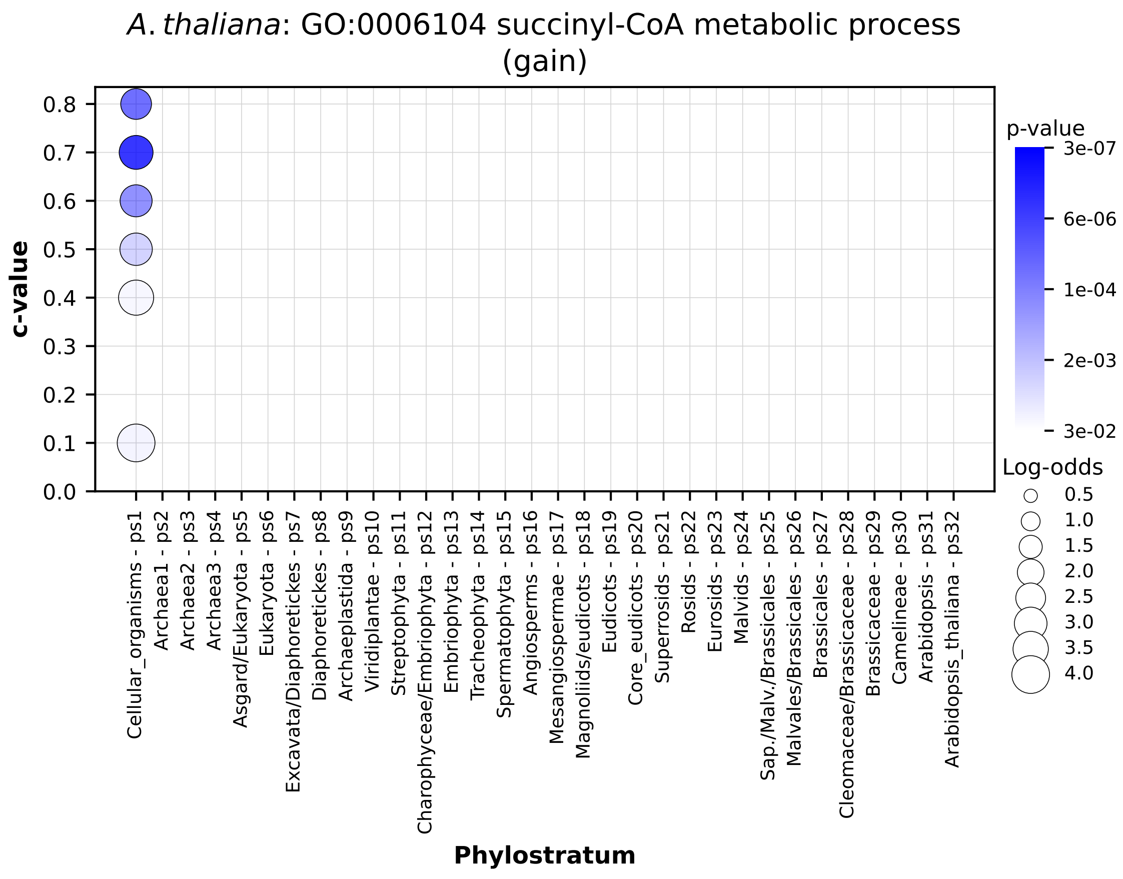

A. thaliana: GO:0006104 succinyl-CoA metabolic process
(gain)
c-value
0.0
0.1
0.2
0.3
0.4
0.5
0.6
0.7
0.8
Phylostratum
Cellular_organisms - ps1
Archaea1 - ps2
Archaea2 - ps3
Archaea3 - ps4
Asgard/Eukaryota - ps5
Eukaryota - ps6
Excavata/Diaphoretickes - ps7
Diaphoretickes - ps8
Archaeplastida - ps9
Viridiplantae - ps10
Streptophyta - ps11
Charophyceae/Embriophyta - ps12
Embriophyta - ps13
Tracheophyta - ps14
Spermatophyta - ps15
Angiosperms - ps16
Mesangiospermae - ps17
Magnoliids/eudicots - ps18
Eudicots - ps19
Core_eudicots - ps20
Superrosids - ps21
Rosids - ps22
Eurosids - ps23
Malvids - ps24
Sap./Malv./Brassicales - ps25
Malvales/Brassicales - ps26
Brassicales - ps27
Cleomaceae/Brassicaceae - ps28
Brassicaceae - ps29
Camelineae - ps30
Arabidopsis - ps31
Arabidopsis_thaliana - ps32
p-value
3e-07
6e-06
1e-04
2e-03
3e-02
Log-odds
0.5
1.0
1.5
2.0
2.5
3.0
3.5
4.0

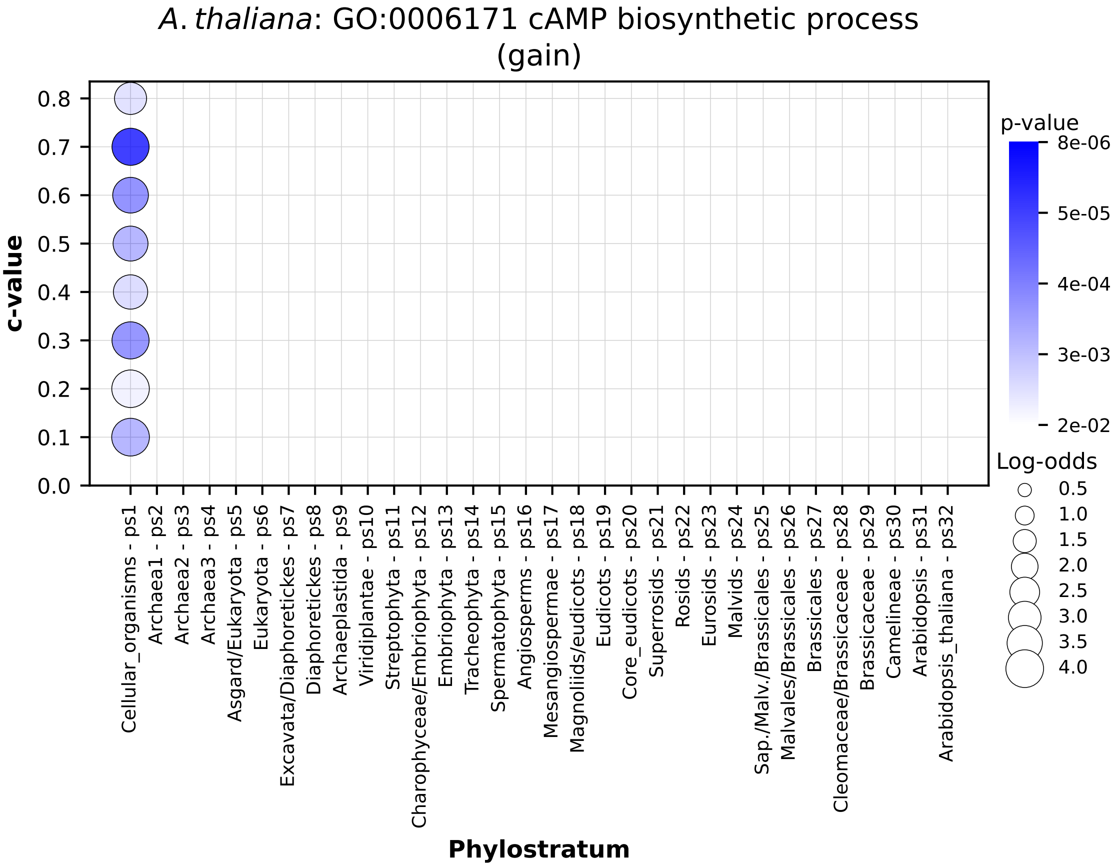

A. thaliana: GO:0006171 cAMP biosynthetic process
(gain)
c-value
0.0
0.1
0.2
0.3
0.4
0.5
0.6
0.7
0.8
p-value
8e-06
5e-05
4e-04
3e-03
2e-02
Log-odds
0.5
1.0
1.5
2.0
2.5
3.0
3.5
4.0
Cellular_organisms - ps1
Archaea1 - ps2
Archaea2 - ps3
Archaea3 - ps4
Asgard/Eukaryota - ps5
Eukaryota - ps6
Excavata/Diaphoretickes - ps7
Diaphoretickes - ps8
Archaeplastida - ps9
Viridiplantae - ps10
Streptophyta - ps11
Charophyceae/Embriophyta - ps12
Embriophyta - ps13
Tracheophyta - ps14
Spermatophyta - ps15
Angiosperms - ps16
Mesangiospermae - ps17
Magnoliids/eudicots - ps18
Eudicots - ps19
Core_eudicots - ps20
Superrosids - ps21
Rosids - ps22
Eurosids - ps23
Malvids - ps24
Sap./Malv./Brassicales - ps25
Malvales/Brassicales - ps26
Brassicales - ps27
Cleomaceae/Brassicaceae - ps28
Brassicaceae - ps29
Camelineae - ps30
Arabidopsis - ps31
Arabidopsis_thaliana - ps32
Phylostratum

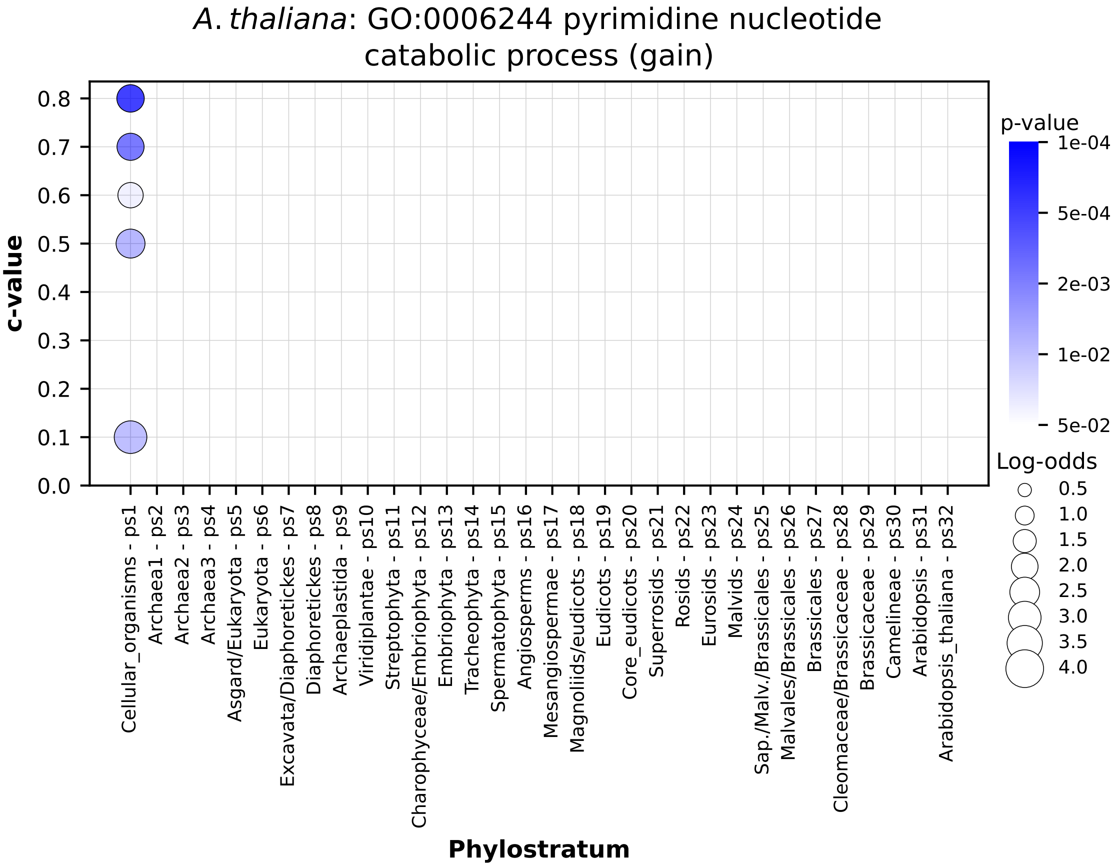

A. thaliana: GO:0006244 pyrimidine nucleotide catabolic process (gain)
c-value
0.0
0.1
0.2
0.3
0.4
0.5
0.6
0.7
0.8
Phylostratum
Cellular_organisms - ps1
Archaea1 - ps2
Archaea2 - ps3
Archaea3 - ps4
Asgard/Eukaryota - ps5
Eukaryota - ps6
Excavata/Diaphoretickes - ps7
Diaphoretickes - ps8
Archaeplastida - ps9
Viridiplantae - ps10
Streptophyta - ps11
Charophyceae/Embriophyta - ps12
Embriophyta - ps13
Tracheophyta - ps14
Spermatophyta - ps15
Angiosperms - ps16
Mesangiospermae - ps17
Magnoliids/eudicots - ps18
Eudicots - ps19
Core_eudicots - ps20
Superrosids - ps21
Rosids - ps22
Eurosids - ps23
Malvids - ps24
Sap./Malv./Brassicales - ps25
Malvales/Brassicales - ps26
Brassicales - ps27
Cleomaceae/Brassicaceae - ps28
Brassicaceae - ps29
Camelineae - ps30
Arabidopsis - ps31
Arabidopsis_thaliana - ps32
p-value
1e-04
5e-04
2e-03
1e-02
5e-02
Log-odds
0.5
1.0
1.5
2.0
2.5
3.0
3.5
4.0

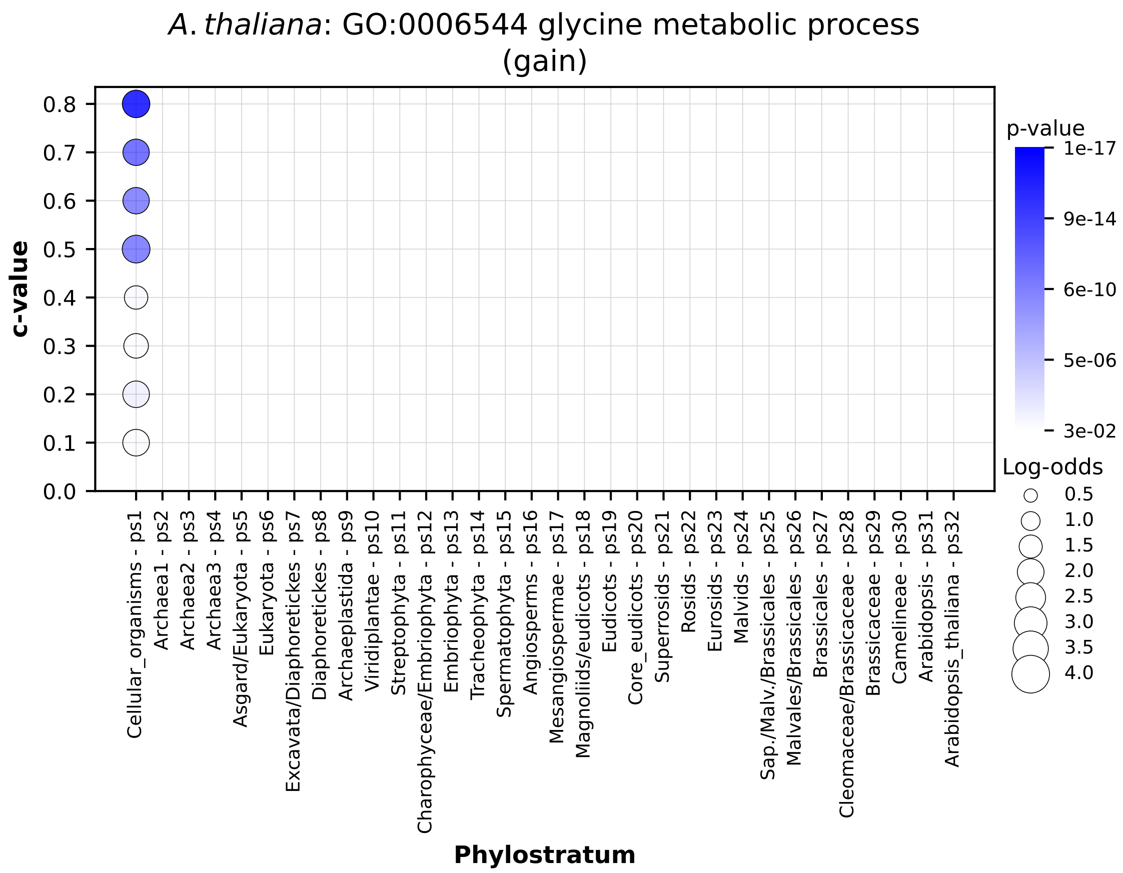

A. thaliana: GO:0006544 glycine metabolic process
(gain)
c-value
0.0
0.1
0.2
0.3
0.4
0.5
0.6
0.7
0.8
p-value
1e-17
9e-14
6e-10
5e-06
3e-02
Log-odds
0.5
1.0
1.5
2.0
2.5
3.0
3.5
4.0
Cellular_organisms - ps1
Archaea1 - ps2
Archaea2 - ps3
Archaea3 - ps4
Asgard/Eukaryota - ps5
Eukaryota - ps6
Excavata/Diaphoretickes - ps7
Diaphoretickes - ps8
Archaeplastida - ps9
Viridiplantae - ps10
Streptophyta - ps11
Charophyceae/Embriophyta - ps12
Embriophyta - ps13
Tracheophyta - ps14
Spermatophyta - ps15
Angiosperms - ps16
Mesangiospermae - ps17
Magnoliids/eudicots - ps18
Eudicots - ps19
Core_eudicots - ps20
Superrosids - ps21
Rosids - ps22
Eurosids - ps23
Malvids - ps24
Sap./Malv./Brassicales - ps25
Malvales/Brassicales - ps26
Brassicales - ps27
Cleomaceae/Brassicaceae - ps28
Brassicaceae - ps29
Camelineae - ps30
Arabidopsis - ps31
Arabidopsis_thaliana - ps32
Phylostratum

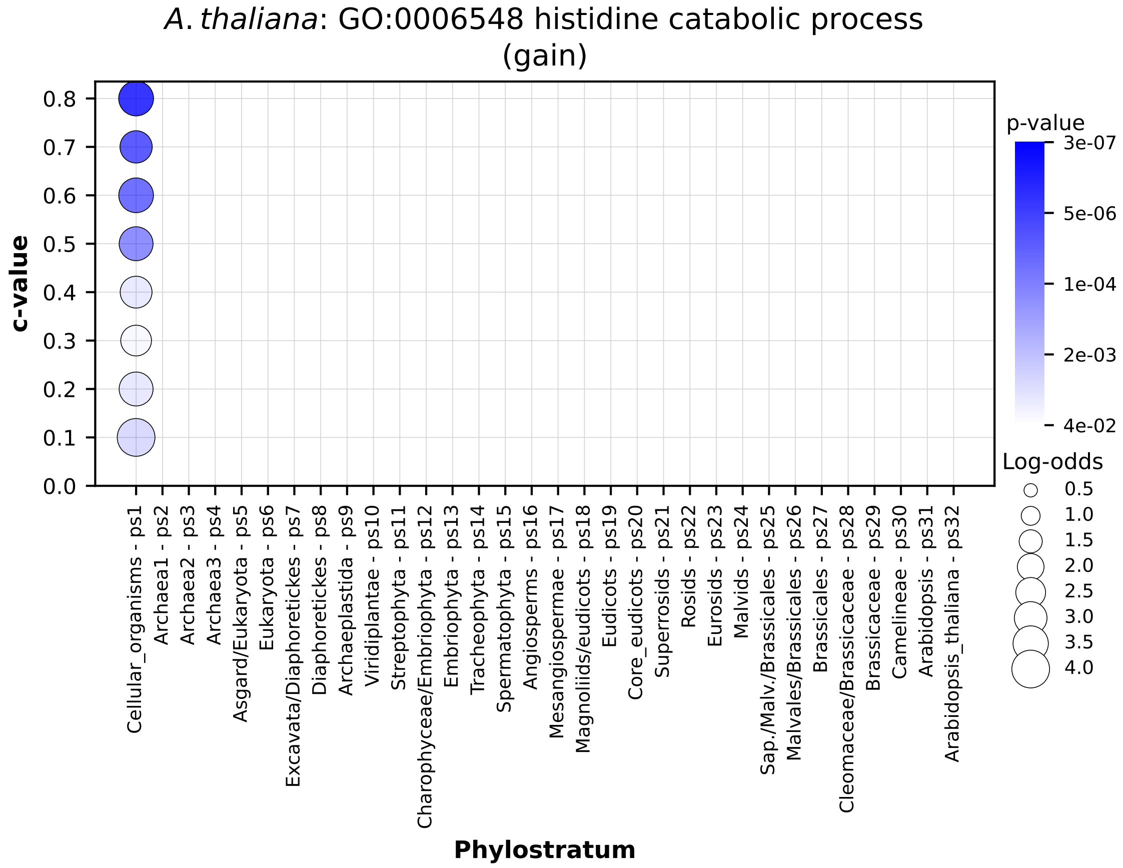

A. thaliana: GO:0006548 histidine catabolic process
(gain)
c-value
0.0
0.1
0.2
0.3
0.4
0.5
0.6
0.7
0.8
p-value
3e-07
5e-06
1e-04
2e-03
4e-02
Log-odds
0.5
1.0
1.5
2.0
2.5
3.0
3.5
4.0
Cellular_organisms - ps1
Archaea1 - ps2
Archaea2 - ps3
Archaea3 - ps4
Asgard/Eukaryota - ps5
Eukaryota - ps6
Excavata/Diaphoretickes - ps7
Diaphoretickes - ps8
Archaeplastida - ps9
Viridiplantae - ps10
Streptophyta - ps11
Charophyceae/Embriophyta - ps12
Embriophyta - ps13
Tracheophyta - ps14
Spermatophyta - ps15
Angiosperms - ps16
Mesangiospermae - ps17
Magnoliids/eudicots - ps18
Eudicots - ps19
Core_eudicots - ps20
Superrosids - ps21
Rosids - ps22
Eurosids - ps23
Malvids - ps24
Sap./Malv./Brassicales - ps25
Malvales/Brassicales - ps26
Brassicales - ps27
Cleomaceae/Brassicaceae - ps28
Brassicaceae - ps29
Camelineae - ps30
Arabidopsis - ps31
Arabidopsis_thaliana - ps32
Phylostratum

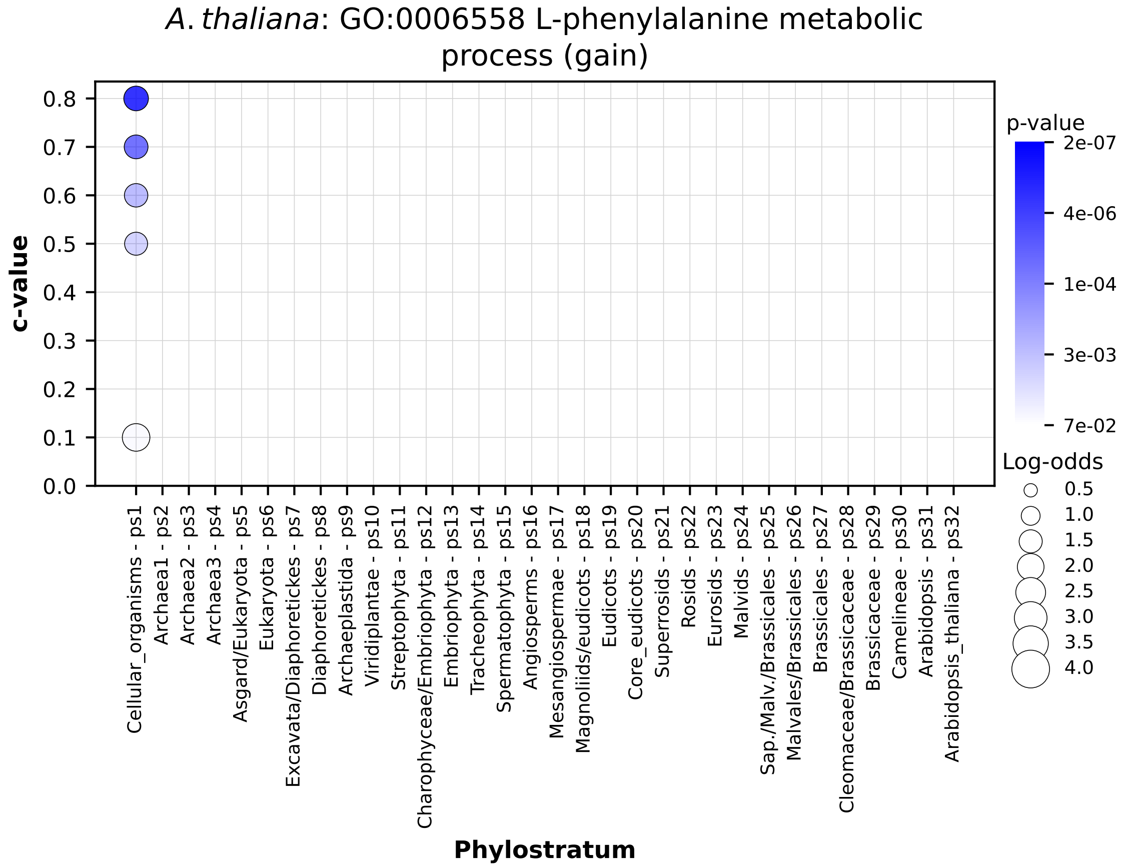
A. thaliana: GO:0006558 L-phenylalanine metabolic process (gain)
c-value
Phylostratum
p-value
2e-07
4e-06
1e-04
3e-03
7e-02
Log-odds
0.5
1.0
1.5
2.0
2.5
3.0
3.5
4.0
Cellular_organisms - ps1
Archaea1 - ps2
Archaea2 - ps3
Archaea3 - ps4
Asgard/Eukaryota - ps5
Eukaryota - ps6
Excavata/Diaphoretickes - ps7
Diaphoretickes - ps8
Archaeplastida - ps9
Viridiplantae - ps10
Streptophyta - ps11
Charophyceae/Embriophyta - ps12
Embriophyta - ps13
Tracheophyta - ps14
Spermatophyta - ps15
Angiosperms - ps16
Mesangiospermae - ps17
Magnoliids/eudicots - ps18
Eudicots - ps19
Core_eudicots - ps20
Superrosids - ps21
Rosids - ps22
Eurosids - ps23
Malvids - ps24
Sap./Malv./Brassicales - ps25
Malvales/Brassicales - ps26
Brassicales - ps27
Cleomaceae/Brassicaceae - ps28
Brassicaceae - ps29
Camelineae - ps30
Arabidopsis - ps31
Arabidopsis_thaliana - ps32

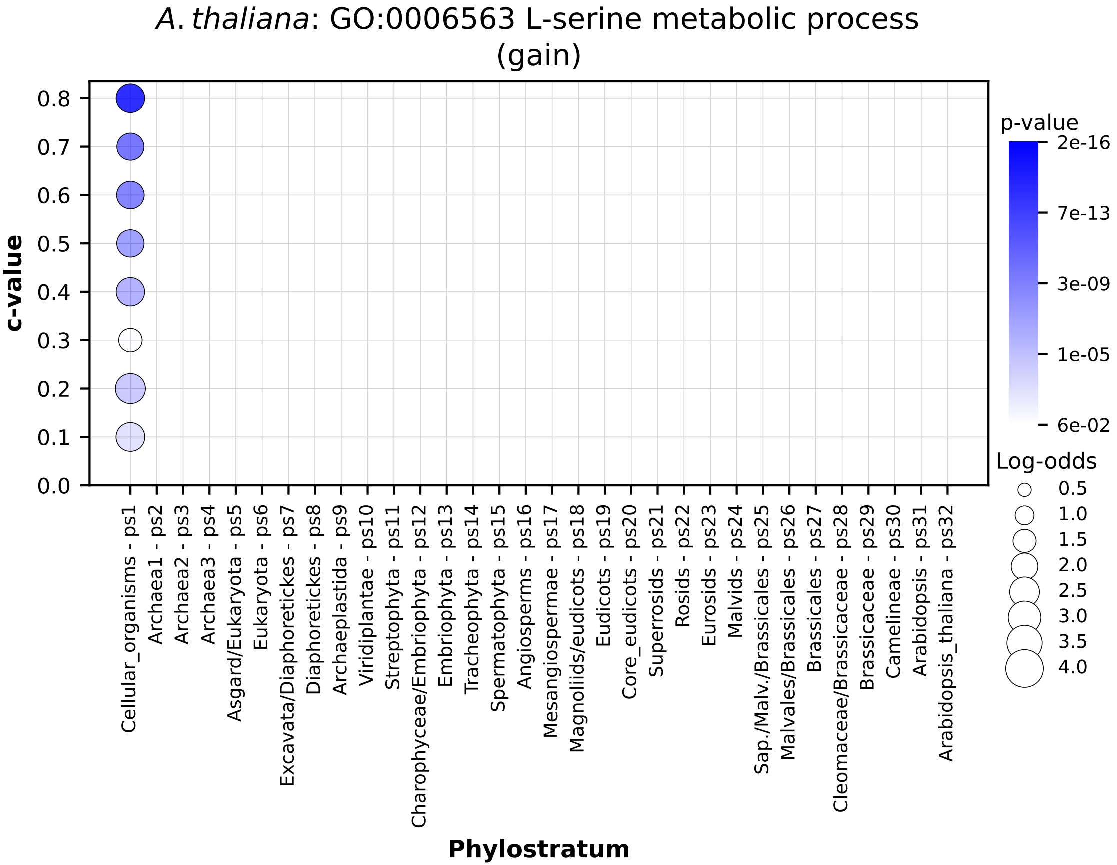

A. thaliana: GO:0006563 L-serine metabolic process (gain)
c-value
0.0
0.1
0.2
0.3
0.4
0.5
0.6
0.7
0.8
p-value
2e-16
7e-13
3e-09
1e-05
6e-02
Log-odds
0.5
1.0
1.5
2.0
2.5
3.0
3.5
4.0
Cellular_organisms - ps1
Archaea1 - ps2
Archaea2 - ps3
Archaea3 - ps4
Asgard/Eukaryota - ps5
Eukaryota - ps6
Excavata/Diaphoretickes - ps7
Diaphoretickes - ps8
Archaeplastida - ps9
Viridiplantae - ps10
Streptophyta - ps11
Charophyceae/Embriophyta - ps12
Embriophyta - ps13
Tracheophyta - ps14
Spermatophyta - ps15
Angiosperms - ps16
Mesangiospermae - ps17
Magnoliids/eudicots - ps18
Eudicots - ps19
Core_eudicots - ps20
Superrosids - ps21
Rosids - ps22
Eurosids - ps23
Malvids - ps24
Sap./Malv./Brassicales - ps25
Malvales/Brassicales - ps26
Brassicales - ps27
Cleomaceae/Brassicaceae - ps28
Brassicaceae - ps29
Camelineae - ps30
Arabidopsis - ps31
Arabidopsis_thaliana - ps32
Phylostratum

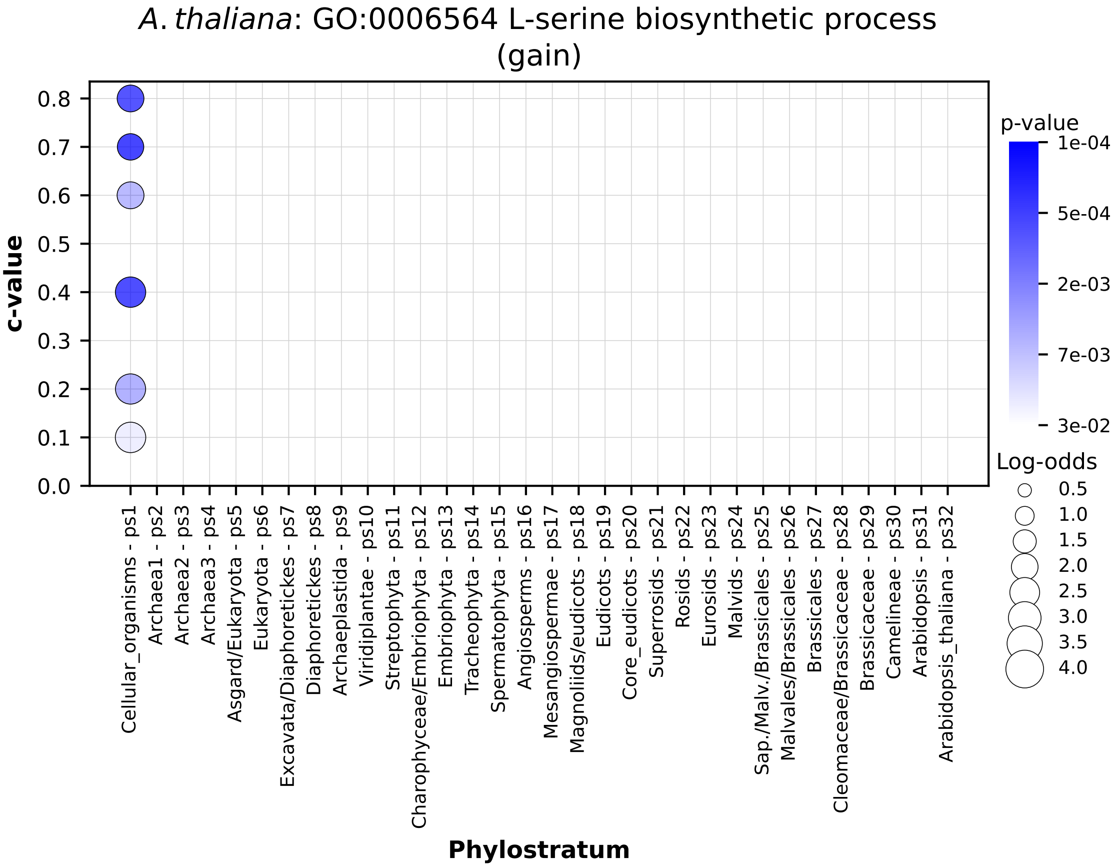

A. thaliana: GO:0006564 L-serine biosynthetic process
(gain)
c-value
0.8
0.7
0.6
0.5
0.4
0.3
0.2
0.1
0.0
p-value
1e-04
5e-04
2e-03
7e-03
3e-02
Log-odds
0.5
1.0
1.5
2.0
2.5
3.0
3.5
4.0
Cellular_organisms - ps1
Archaea1 - ps2
Archaea2 - ps3
Archaea3 - ps4
Asgard/Eukaryota - ps5
Eukaryota - ps6
Excavata/Diaphoretickes - ps7
Diaphoretickes - ps8
Archaeplastida - ps9
Viridiplantae - ps10
Streptophyta - ps11
Charophyceae/Embriophyta - ps12
Embriophyta - ps13
Tracheophyta - ps14
Spermatophyta - ps15
Angiosperms - ps16
Mesangiospermae - ps17
Magnoliids/eudicots - ps18
Eudicots - ps19
Core_eudicots - ps20
Superrosids - ps21
Rosids - ps22
Eurosids - ps23
Malvids - ps24
Sap./Malv./Brassicales - ps25
Malvales/Brassicales - ps26
Brassicales - ps27
Cleomaceae/Brassicaceae - ps28
Brassicaceae - ps29
Camelineae - ps30
Arabidopsis - ps31
Arabidopsis_thaliana - ps32
Phylostratum

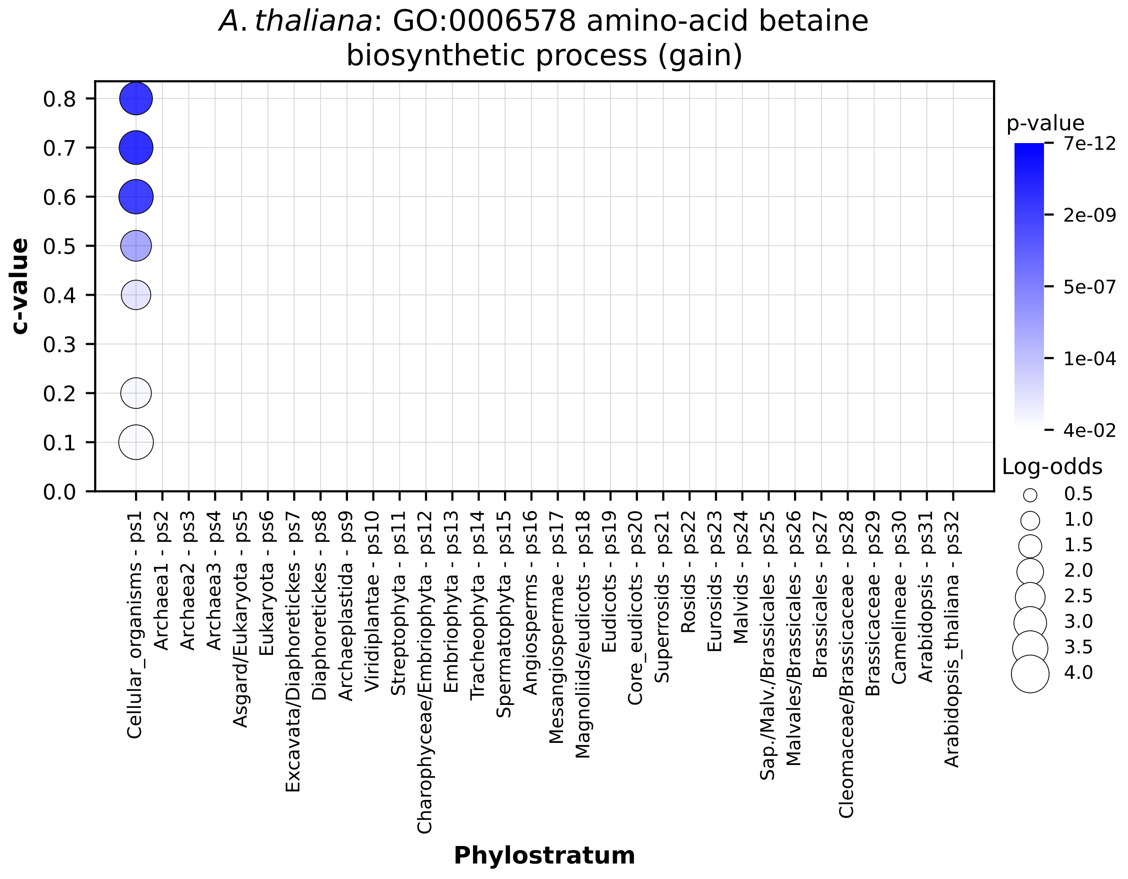

A. thaliana: GO:0006578 amino-acid betaine biosynthetic process (gain)
c-value
0.0
0.1
0.2
0.3
0.4
0.5
0.6
0.7
0.8
Phylostratum
Cellular_organisms - ps1
Archaea1 - ps2
Archaea2 - ps3
Archaea3 - ps4
Asgard/Eukaryota - ps5
Eukaryota - ps6
Excavata/Diaphoretickes - ps7
Diaphoretickes - ps8
Archaeplastida - ps9
Viridiplantae - ps10
Streptophyta - ps11
Charophyceae/Embriophyta - ps12
Embriophyta - ps13
Tracheophyta - ps14
Spermatophyta - ps15
Angiosperms - ps16
Mesangiospermae - ps17
Magnoliids/eudicots - ps18
Eudicots - ps19
Core_eudicots - ps20
Superrosids - ps21
Rosids - ps22
Eurosids - ps23
Malvids - ps24
Sap./Malv./Brassicales - ps25
Malvales/Brassicales - ps26
Brassicales - ps27
Cleomaceae/Brassicaceae - ps28
Brassicaceae - ps29
Camelineae - ps30
Arabidopsis - ps31
Arabidopsis_thaliana - ps32
p-value
7e-12
2e-09
5e-07
1e-04
4e-02
Log-odds
0.5
1.0
1.5
2.0
2.5
3.0
3.5
4.0

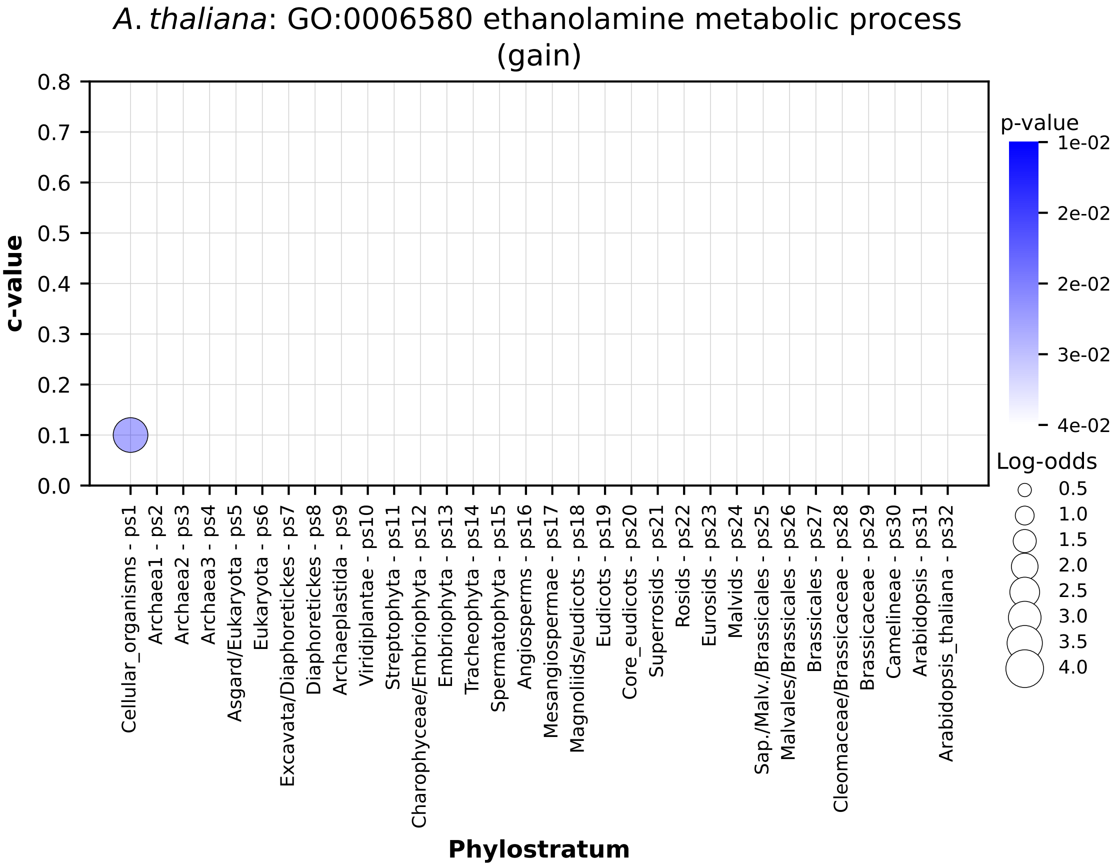

A. thaliana: GO:0006580 ethanolamine metabolic process (gain)
c-value
0.8
0.7
0.6
0.5
0.4
0.3
0.2
0.1
0.0
Phylostratum
Cellular_organisms - ps1
Archaea1 - ps2
Archaea2 - ps3
Archaea3 - ps4
Asgard/Eukaryota - ps5
Eukaryota - ps6
Excavata/Diaphoretickes - ps7
Diaphoretickes - ps8
Archaeplastida - ps9
Viridiplantae - ps10
Streptophyta - ps11
Charophyceae/Embriophyta - ps12
Embriophyta - ps13
Tracheophyta - ps14
Spermatophyta - ps15
Angiosperms - ps16
Mesangiospermae - ps17
Magnoliids/eudicots - ps18
Eudicots - ps19
Core_eudicots - ps20
Superrosids - ps21
Rosids - ps22
Eurosids - ps23
Malvids - ps24
Sap./Malv./Brassicales - ps25
Malvales/Brassicales - ps26
Brassicales - ps27
Cleomaceae/Brassicaceae - ps28
Brassicaceae - ps29
Camelineae - ps30
Arabidopsis - ps31
Arabidopsis_thaliana - ps32
p-value
1e-02
2e-02
2e-02
3e-02
4e-02
Log-odds
0.5
1.0
1.5
2.0
2.5
3.0
3.5
4.0

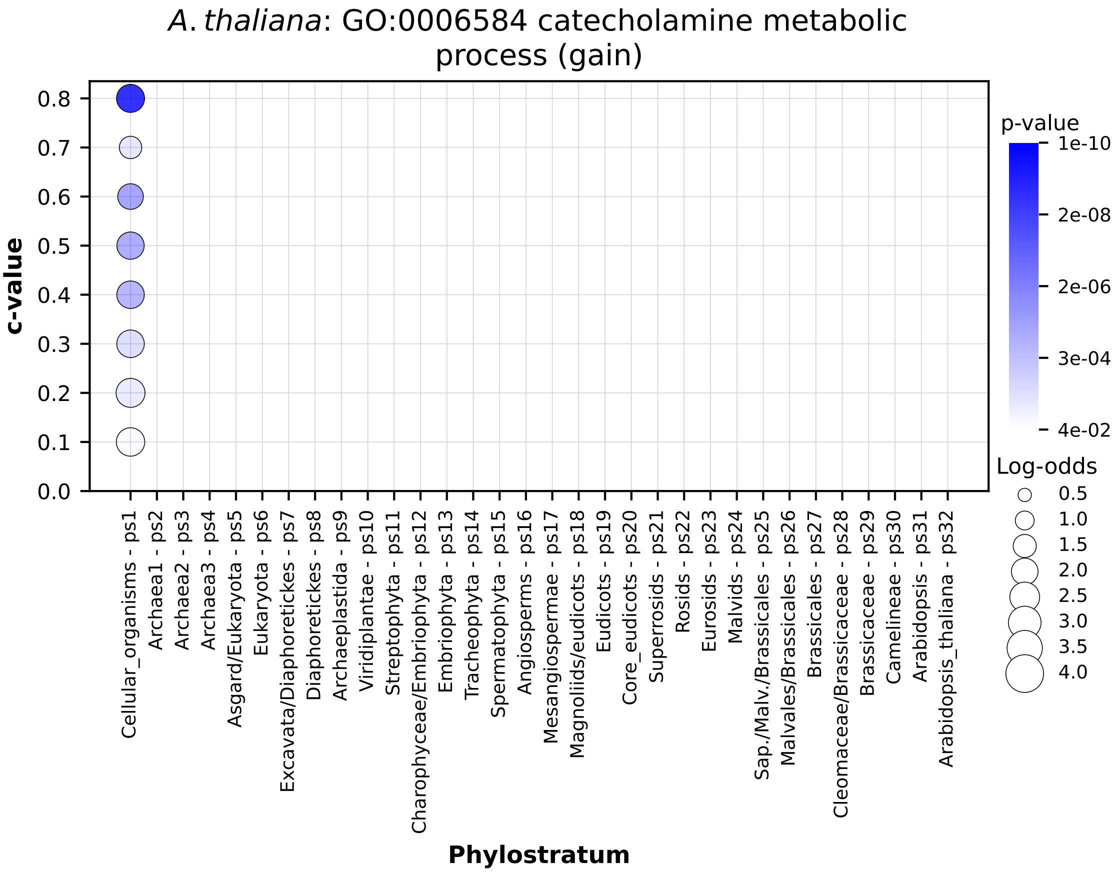

A. thaliana: GO:0006584 catecholamine metabolic process (gain)
c-value
0.8
0.7
0.6
0.5
0.4
0.3
0.2
0.1
0.0
p-value
1e-10
2e-08
2e-06
3e-04
4e-02
Log-odds
0.5
1.0
1.5
2.0
2.5
3.0
3.5
4.0
Cellular_organisms - ps1
Archaea1 - ps2
Archaea2 - ps3
Archaea3 - ps4
Asgard/Eukaryota - ps5
Eukaryota - ps6
Excavata/Diaphoretickes - ps7
Diaphoretickes - ps8
Archaeplastida - ps9
Viridiplantae - ps10
Streptophyta - ps11
Charophyceae/Embriophyta - ps12
Embriophyta - ps13
Tracheophyta - ps14
Spermatophyta - ps15
Angiosperms - ps16
Mesangiospermae - ps17
Magnoliids/eudicots - ps18
Eudicots - ps19
Core_eudicots - ps20
Superrosids - ps21
Rosids - ps22
Eurosids - ps23
Malvids - ps24
Sap./Malv./Brassicales - ps25
Malvales/Brassicales - ps26
Brassicales - ps27
Cleomaceae/Brassicaceae - ps28
Brassicaceae - ps29
Camelineae - ps30
Arabidopsis - ps31
Arabidopsis_thaliana - ps32
Phylostratum

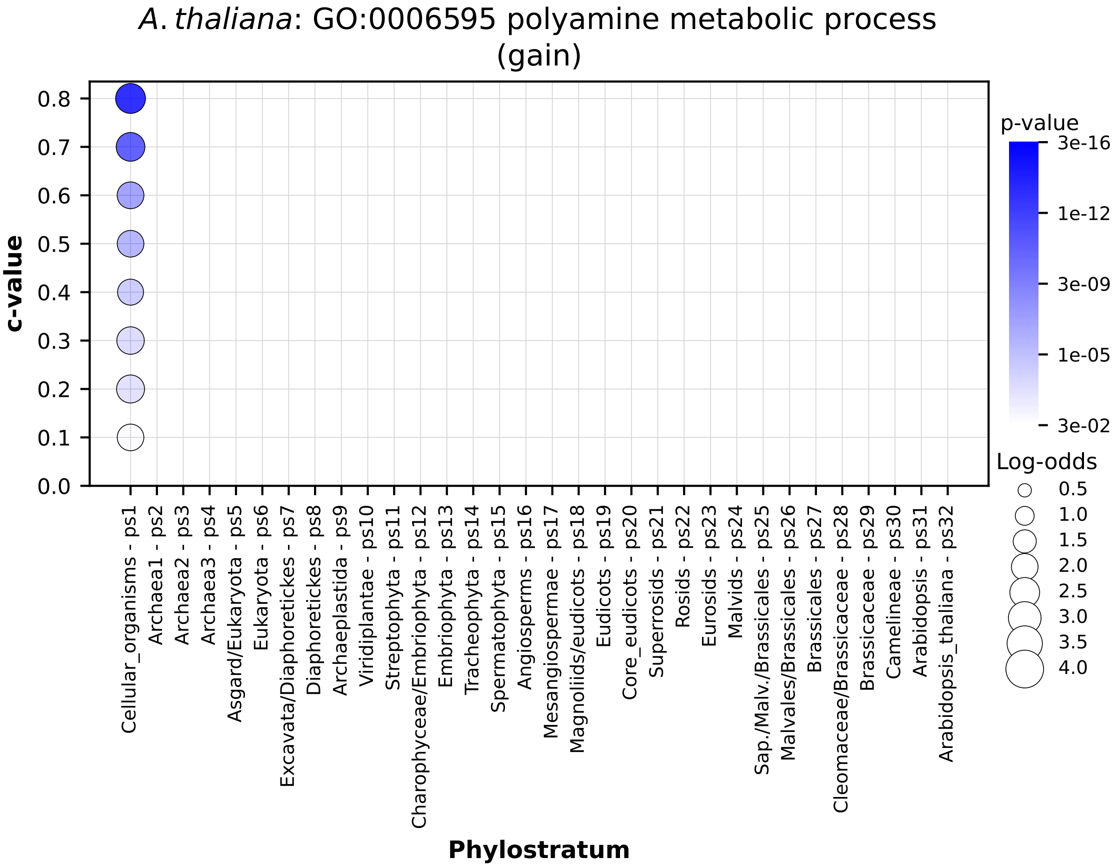

A. thaliana: GO:0006595 polyamine metabolic process
(gain)
c-value
0.0
0.1
0.2
0.3
0.4
0.5
0.6
0.7
0.8
p-value
3e-16
1e-12
3e-09
1e-05
3e-02
Log-odds
0.5
1.0
1.5
2.0
2.5
3.0
3.5
4.0
Cellular_organisms - ps1
Archaea1 - ps2
Archaea2 - ps3
Archaea3 - ps4
Asgard/Eukaryota - ps5
Eukaryota - ps6
Excavata/Diaphoretickes - ps7
Diaphoretickes - ps8
Archaeplastida - ps9
Viridiplantae - ps10
Streptophyta - ps11
Charophyceae/Embriophyta - ps12
Embriophyta - ps13
Tracheophyta - ps14
Spermatophyta - ps15
Angiosperms - ps16
Mesangiospermae - ps17
Magnoliids/eudicots - ps18
Eudicots - ps19
Core_eudicots - ps20
Superrosids - ps21
Rosids - ps22
Eurosids - ps23
Malvids - ps24
Sap./Malv./Brassicales - ps25
Malvales/Brassicales - ps26
Brassicales - ps27
Cleomaceae/Brassicaceae - ps28
Brassicaceae - ps29
Camelineae - ps30
Arabidopsis - ps31
Arabidopsis_thaliana - ps32
Phylostratum

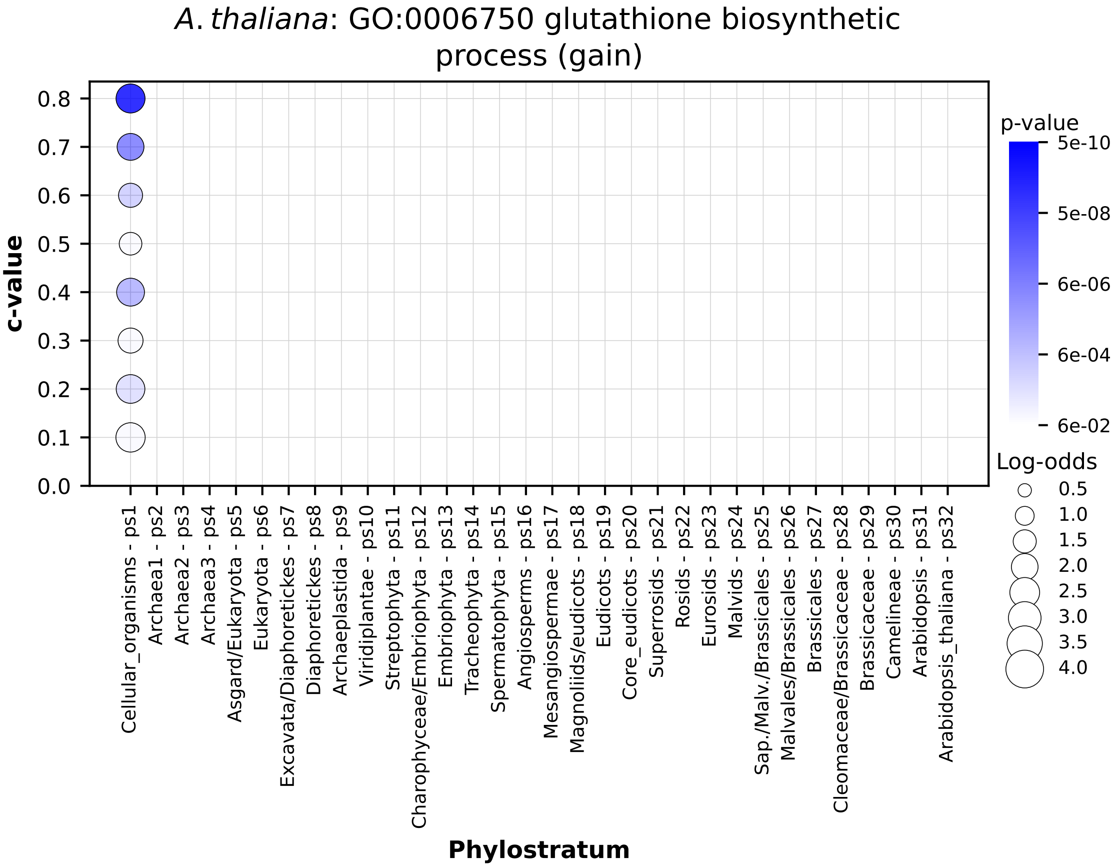

A. thaliana: GO:0006750 glutathione biosynthetic process (gain)
c-value
0.0
0.1
0.2
0.3
0.4
0.5
0.6
0.7
0.8
p-value
5e-10
5e-08
6e-06
6e-04
6e-02
Log-odds
0.5
1.0
1.5
2.0
2.5
3.0
3.5
4.0
Cellular_organisms - ps1
Archaea1 - ps2
Archaea2 - ps3
Archaea3 - ps4
Asgard/Eukaryota - ps5
Eukaryota - ps6
Excavata/Diaphoretickes - ps7
Diaphoretickes - ps8
Archaeplastida - ps9
Viridiplantae - ps10
Streptophyta - ps11
Charophyceae/Embriophyta - ps12
Embriophyta - ps13
Tracheophyta - ps14
Spermatophyta - ps15
Angiosperms - ps16
Mesangiospermae - ps17
Magnoliids/eudicots - ps18
Eudicots - ps19
Core_eudicots - ps20
Superrosids - ps21
Rosids - ps22
Eurosids - ps23
Malvids - ps24
Sap./Malv./Brassicales - ps25
Malvales/Brassicales - ps26
Brassicales - ps27
Cleomaceae/Brassicaceae - ps28
Brassicaceae - ps29
Camelineae - ps30
Arabidopsis - ps31
Arabidopsis_thaliana - ps32
Phylostratum

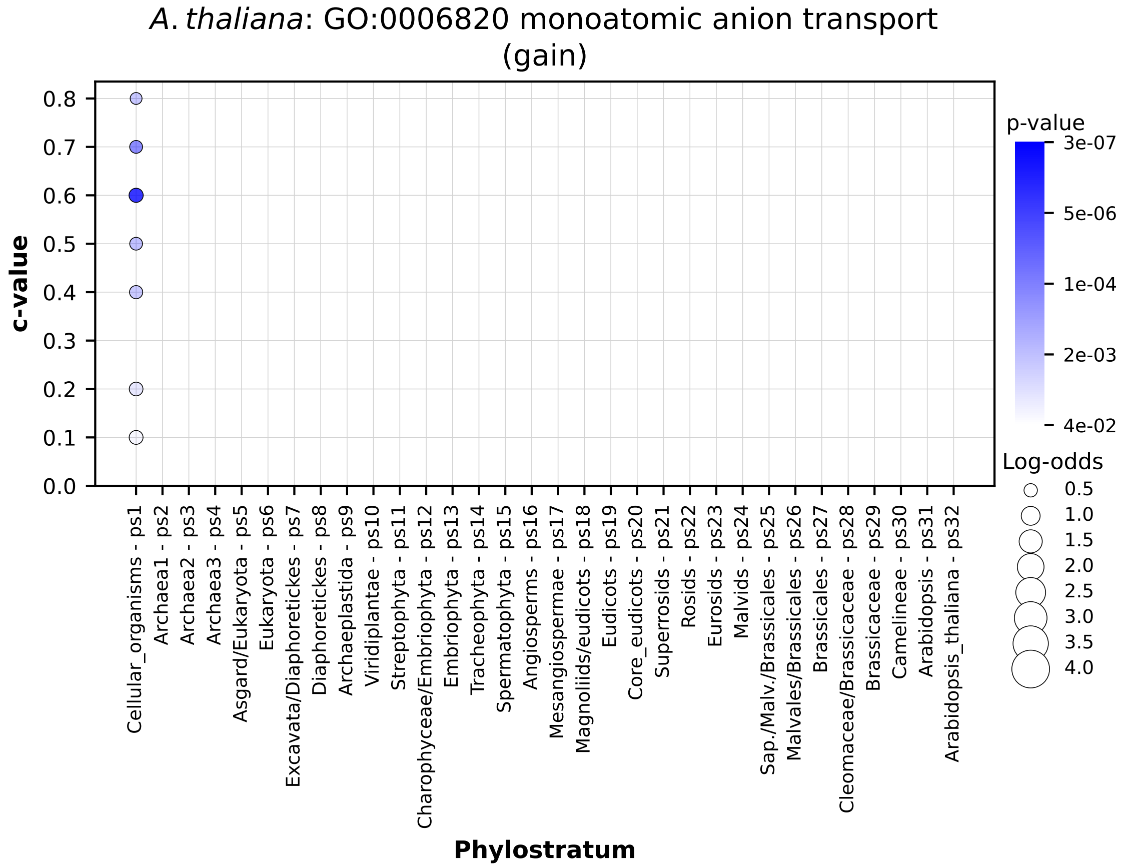

A. thaliana: GO:0006820 monoatomic anion transport (gain)
c-value
0.0
0.1
0.2
0.3
0.4
0.5
0.6
0.7
0.8
p-value
3e-07
5e-06
1e-04
2e-03
4e-02
Log-odds
0.5
1.0
1.5
2.0
2.5
3.0
3.5
4.0
Cellular_organisms - ps1
Archaea1 - ps2
Archaea2 - ps3
Archaea3 - ps4
Asgard/Eukaryota - ps5
Eukaryota - ps6
Excavata/Diaphoretickes - ps7
Diaphoretickes - ps8
Archaeplastida - ps9
Viridiplantae - ps10
Streptophyta - ps11
Charophyceae/Embriophyta - ps12
Embriophyta - ps13
Tracheophyta - ps14
Spermatophyta - ps15
Angiosperms - ps16
Mesangiospermae - ps17
Magnoliids/eudicots - ps18
Eudicots - ps19
Core_eudicots - ps20
Superrosids - ps21
Rosids - ps22
Eurosids - ps23
Malvids - ps24
Sap./Malv./Brassicales - ps25
Malvales/Brassicales - ps26
Brassicales - ps27
Cleomaceae/Brassicaceae - ps28
Brassicaceae - ps29
Camelineae - ps30
Arabidopsis - ps31
Arabidopsis_thaliana - ps32
Phylostratum

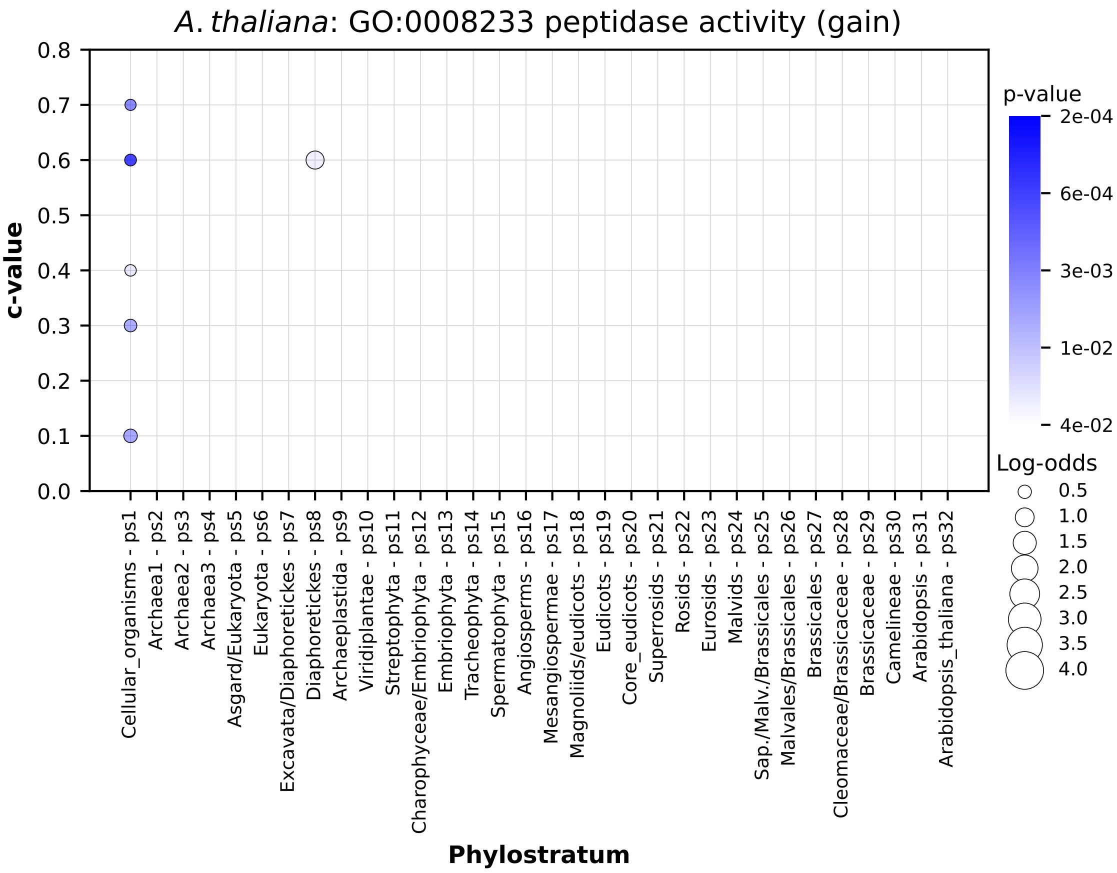

A. thaliana: GO:0008233 peptidase activity (gain)
c-value
0.8
0.7
0.6
0.5
0.4
0.3
0.2
0.1
0.0
p-value
2e-04
6e-04
3e-03
1e-02
4e-02
Log-odds
0.5
1.0
1.5
2.0
2.5
3.0
3.5
4.0
Cellular_organisms - ps1
Archaea1 - ps2
Archaea2 - ps3
Archaea3 - ps4
Asgard/Eukaryota - ps5
Eukaryota - ps6
Excavata/Diaphoretickes - ps7
Diaphoretickes - ps8
Archaeplastida - ps9
Viridiplantae - ps10
Streptophyta - ps11
Charophyceae/Embriophyta - ps12
Embriophyta - ps13
Tracheophyta - ps14
Spermatophyta - ps15
Angiosperms - ps16
Mesangiospermae - ps17
Magnoliids/eudicots - ps18
Eudicots - ps19
Core_eudicots - ps20
Superrosids - ps21
Rosids - ps22
Eurosids - ps23
Malvids - ps24
Sap./Malv./Brassicales - ps25
Malvales/Brassicales - ps26
Brassicales - ps27
Cleomaceae/Brassicaceae - ps28
Brassicaceae - ps29
Camelineae - ps30
Arabidopsis - ps31
Arabidopsis_thaliana - ps32
Phylostratum

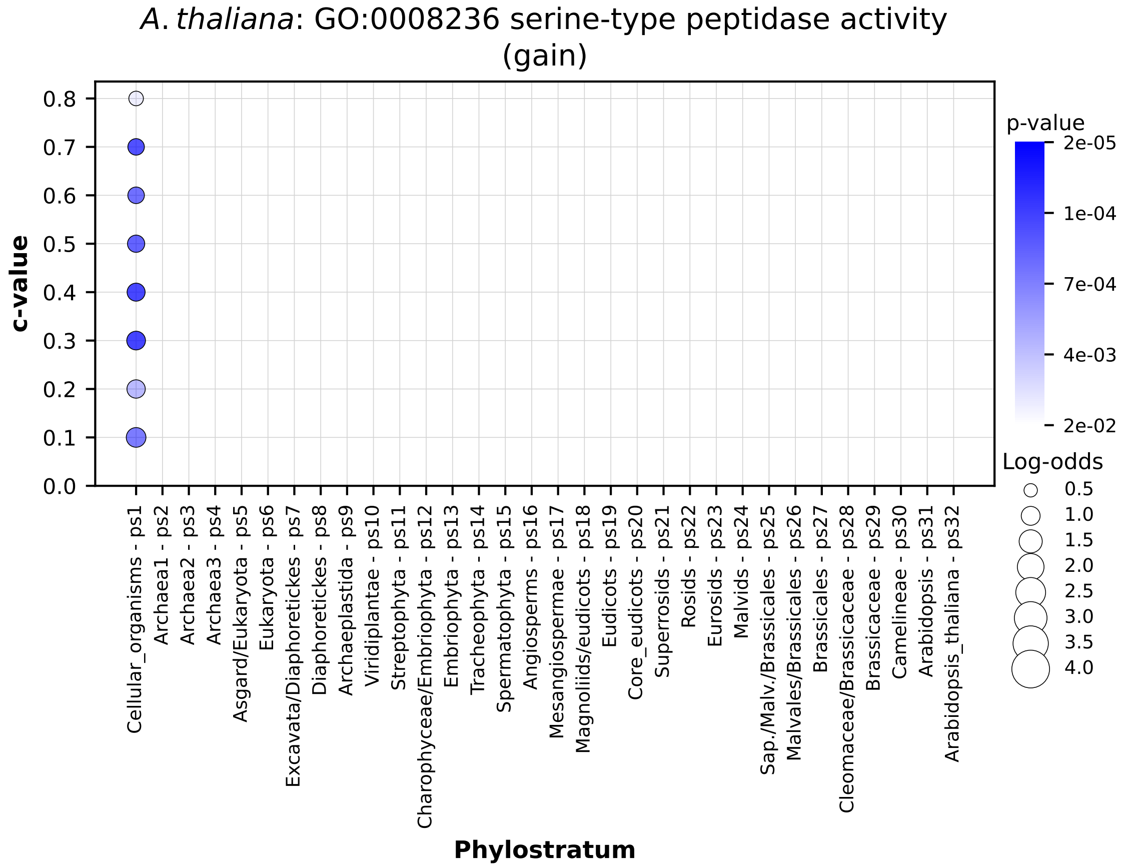

A. thaliana: GO:0008236 serine-type peptidase activity (gain)
c-value
0.0
0.1
0.2
0.3
0.4
0.5
0.6
0.7
0.8
p-value
2e-05
1e-04
7e-04
4e-03
2e-02
Log-odds
0.5
1.0
1.5
2.0
2.5
3.0
3.5
4.0
Cellular_organisms - ps1
Archaea1 - ps2
Archaea2 - ps3
Archaea3 - ps4
Asgard/Eukaryota - ps5
Eukaryota - ps6
Excavata/Diaphoretickes - ps7
Diaphoretickes - ps8
Archaeplastida - ps9
Viridiplantae - ps10
Streptophyta - ps11
Charophyceae/Embriophyta - ps12
Embriophyta - ps13
Tracheophyta - ps14
Spermatophyta - ps15
Angiosperms - ps16
Mesangiospermae - ps17
Magnoliids/eudicots - ps18
Eudicots - ps19
Core_eudicots - ps20
Superrosids - ps21
Rosids - ps22
Eurosids - ps23
Malvids - ps24
Sap./Malv./Brassicales - ps25
Malvales/Brassicales - ps26
Brassicales - ps27
Cleomaceae/Brassicaceae - ps28
Brassicaceae - ps29
Camelineae - ps30
Arabidopsis - ps31
Arabidopsis_thaliana - ps32
Phylostratum

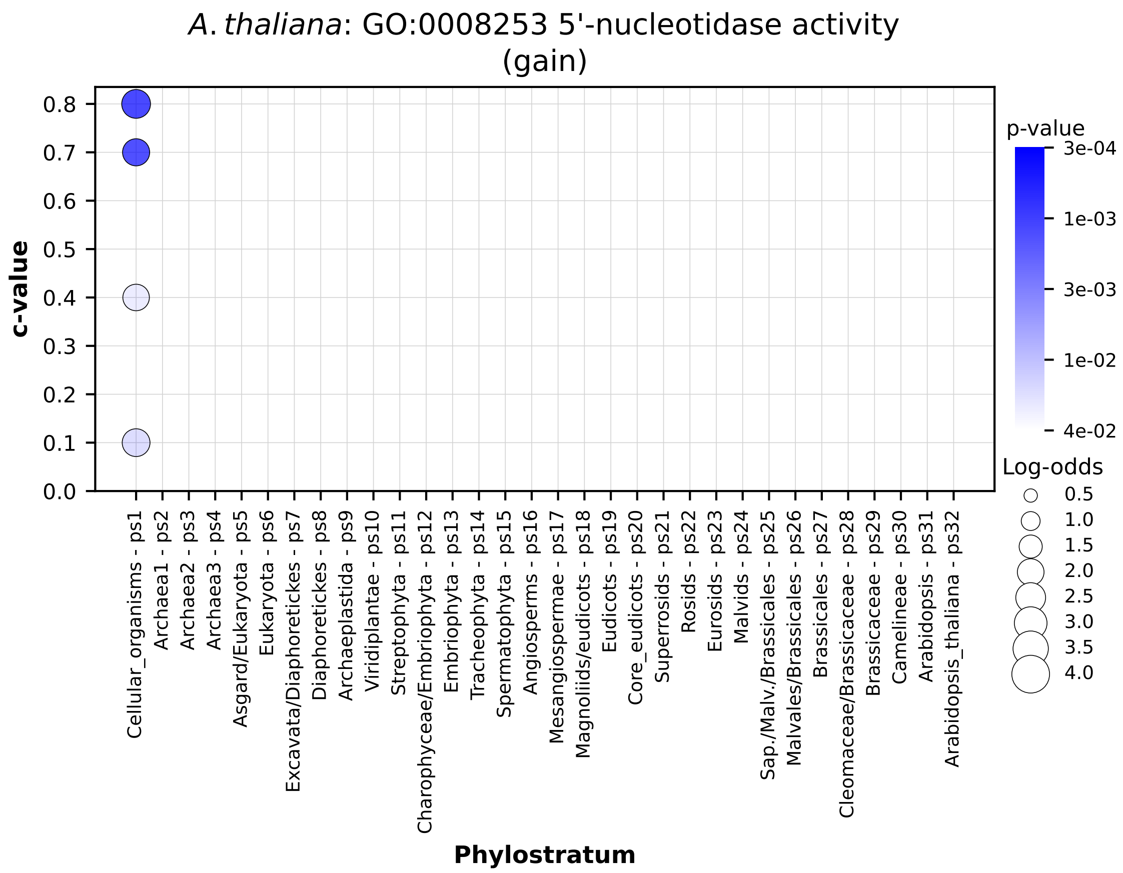

A. thaliana: GO:0008253 5'-nucleotidase activity
(gain)
c-value
0.0
0.1
0.2
0.3
0.4
0.5
0.6
0.7
0.8
p-value
3e-04
1e-03
3e-03
1e-02
4e-02
Log-odds
0.5
1.0
1.5
2.0
2.5
3.0
3.5
4.0
Cellular_organisms - ps1
Archaea1 - ps2
Archaea2 - ps3
Archaea3 - ps4
Asgard/Eukaryota - ps5
Eukaryota - ps6
Excavata/Diaphoretickes - ps7
Diaphoretickes - ps8
Archaeplastida - ps9
Viridiplantae - ps10
Streptophyta - ps11
Charophyceae/Embriophyta - ps12
Embriophyta - ps13
Tracheophyta - ps14
Spermatophyta - ps15
Angiosperms - ps16
Mesangiospermae - ps17
Magnoliids/eudicots - ps18
Eudicots - ps19
Core_eudicots - ps20
Superrosids - ps21
Rosids - ps22
Eurosids - ps23
Malvids - ps24
Sap./Malv./Brassicales - ps25
Malvales/Brassicales - ps26
Brassicales - ps27
Cleomaceae/Brassicaceae - ps28
Brassicaceae - ps29
Camelineae - ps30
Arabidopsis - ps31
Arabidopsis_thaliana - ps32
Phylostratum

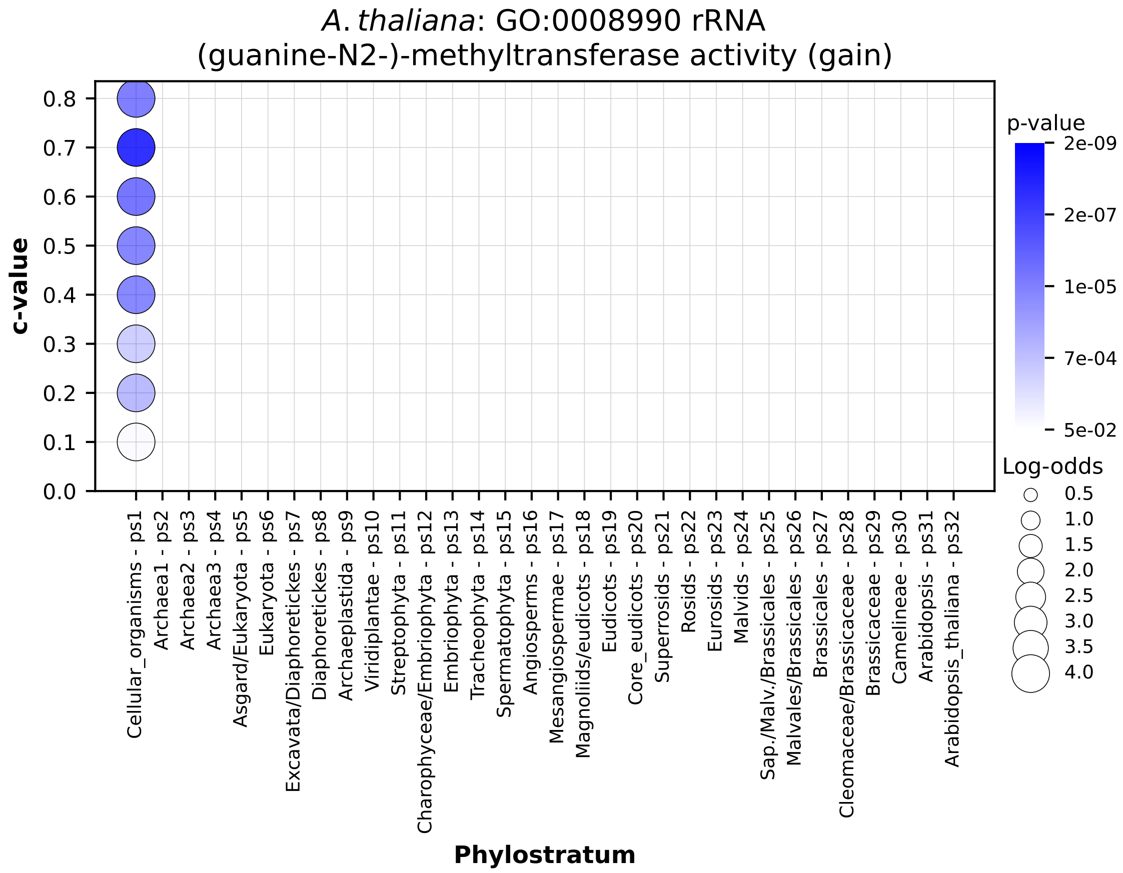
A. thaliana: GO:0008990 rRNA (guanine-N2-)-methyltransferase activity (gain)
c-value
0.8
0.7
0.6
0.5
0.4
0.3
0.2
0.1
0.0
p-value
2e-09
2e-07
1e-05
7e-04
5e-02
Log-odds
0.5
1.0
1.5
2.0
2.5
3.0
3.5
4.0
Cellular_organisms - ps1
Archaea1 - ps2
Archaea2 - ps3
Archaea3 - ps4
Asgard/Eukaryota - ps5
Eukaryota - ps6
Excavata/Diaphoretickes - ps7
Diaphoretickes - ps8
Archaeplastida - ps9
Viridiplantae - ps10
Streptophyta - ps11
Charophyceae/Embriophyta - ps12
Embriophyta - ps13
Tracheophyta - ps14
Spermatophyta - ps15
Angiosperms - ps16
Mesangiospermae - ps17
Magnoliids/eudicots - ps18
Eudicots - ps19
Core_eudicots - ps20
Superrosids - ps21
Rosids - ps22
Eurosids - ps23
Malvids - ps24
Sap./Malv./Brassicales - ps25
Malvales/Brassicales - ps26
Brassicales - ps27
Cleomaceae/Brassicaceae - ps28
Brassicaceae - ps29
Camelineae - ps30
Arabidopsis - ps31
Arabidopsis_thaliana - ps32
Phylostratum

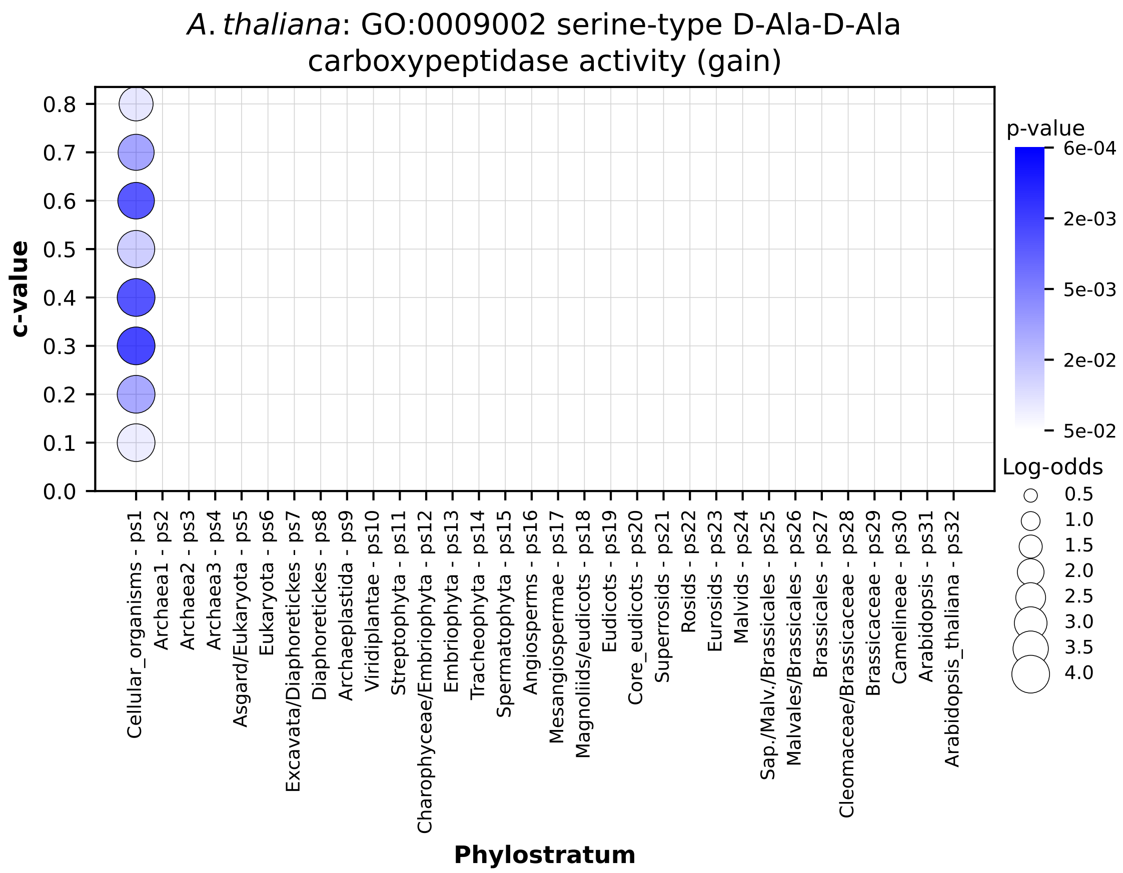
$A. thaliana$: GO:0009002 serine-type D-Ala-D-Ala carboxypeptidase activity (gain)
c-value
0.0
0.1
0.2
0.3
0.4
0.5
0.6
0.7
0.8
Phylostratum
Cellular_organisms - ps1
Archaea1 - ps2
Archaea2 - ps3
Archaea3 - ps4
Asgard/Eukaryota - ps5
Eukaryota - ps6
Excavata/Diaphoretickes - ps7
Diaphoretickes - ps8
Archaeplastida - ps9
Viridiplantae - ps10
Streptophyta - ps11
Charophyceae/Embriophyta - ps12
Embriophyta - ps13
Tracheophyta - ps14
Spermatophyta - ps15
Angiosperms - ps16
Mesangiospermae - ps17
Magnoliids/eudicots - ps18
Eudicots - ps19
Core_eudicots - ps20
Superrosids - ps21
Rosids - ps22
Eurosids - ps23
Malvids - ps24
Sap./Malv./Brassicales - ps25
Malvales/Brassicales - ps26
Brassicales - ps27
Cleomaceae/Brassicaceae - ps28
Brassicaceae - ps29
Camelineae - ps30
Arabidopsis - ps31
Arabidopsis_thaliana - ps32
p-value
6e-04
2e-03
5e-03
2e-02
5e-02
Log-odds
0.5
1.0
1.5
2.0
2.5
3.0
3.5
4.0

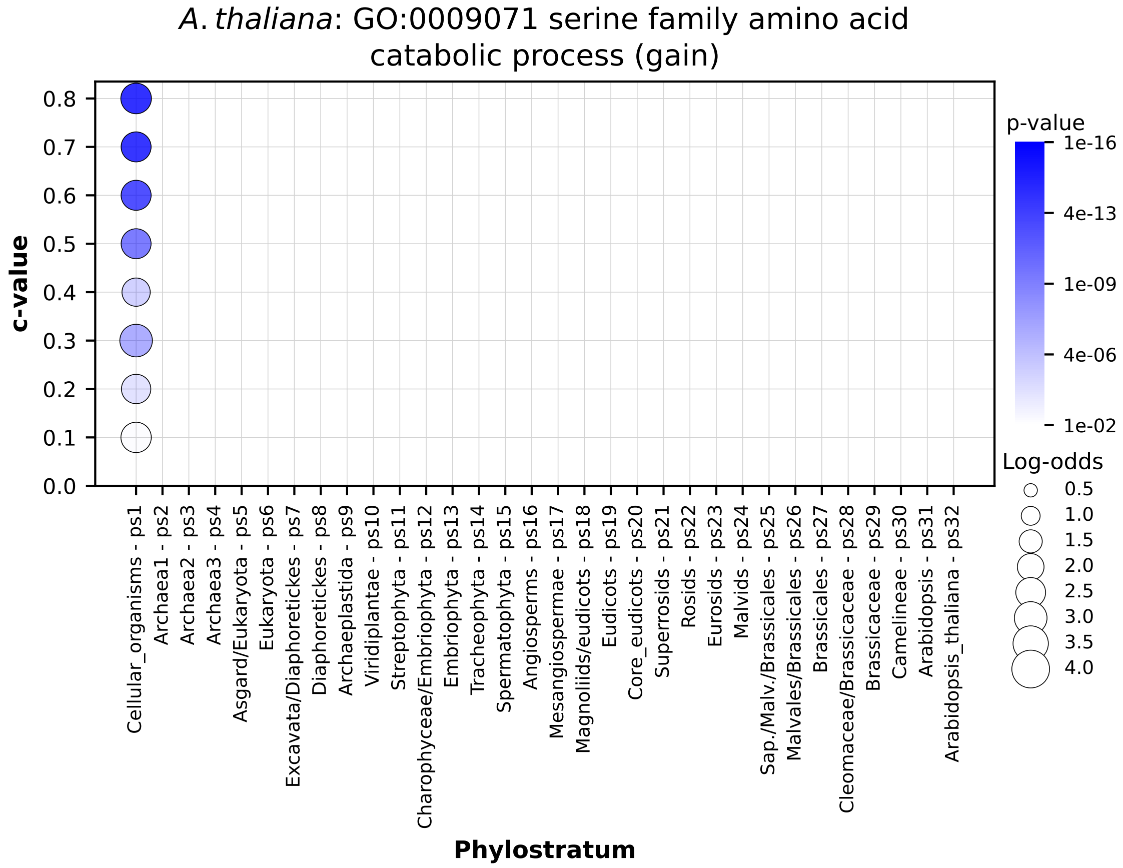

A. thaliana: GO:0009071 serine family amino acid catabolic process (gain)
c-value
0.0
0.1
0.2
0.3
0.4
0.5
0.6
0.7
0.8
p-value
1e-16
4e-13
1e-09
4e-06
1e-02
Log-odds
0.5
1.0
1.5
2.0
2.5
3.0
3.5
4.0
Cellular_organisms - ps1
Archaea1 - ps2
Archaea2 - ps3
Archaea3 - ps4
Asgard/Eukaryota - ps5
Eukaryota - ps6
Excavata/Diaphoretickes - ps7
Diaphoretickes - ps8
Archaeplastida - ps9
Viridiplantae - ps10
Streptophyta - ps11
Charophyceae/Embriophyta - ps12
Embriophyta - ps13
Tracheophyta - ps14
Spermatophyta - ps15
Angiosperms - ps16
Mesangiospermae - ps17
Magnoliids/eudicots - ps18
Eudicots - ps19
Core_eudicots - ps20
Superrosids - ps21
Rosids - ps22
Eurosids - ps23
Malvids - ps24
Sap./Malv./Brassicales - ps25
Malvales/Brassicales - ps26
Brassicales - ps27
Cleomaceae/Brassicaceae - ps28
Brassicaceae - ps29
Camelineae - ps30
Arabidopsis - ps31
Arabidopsis_thaliana - ps32
Phylostratum

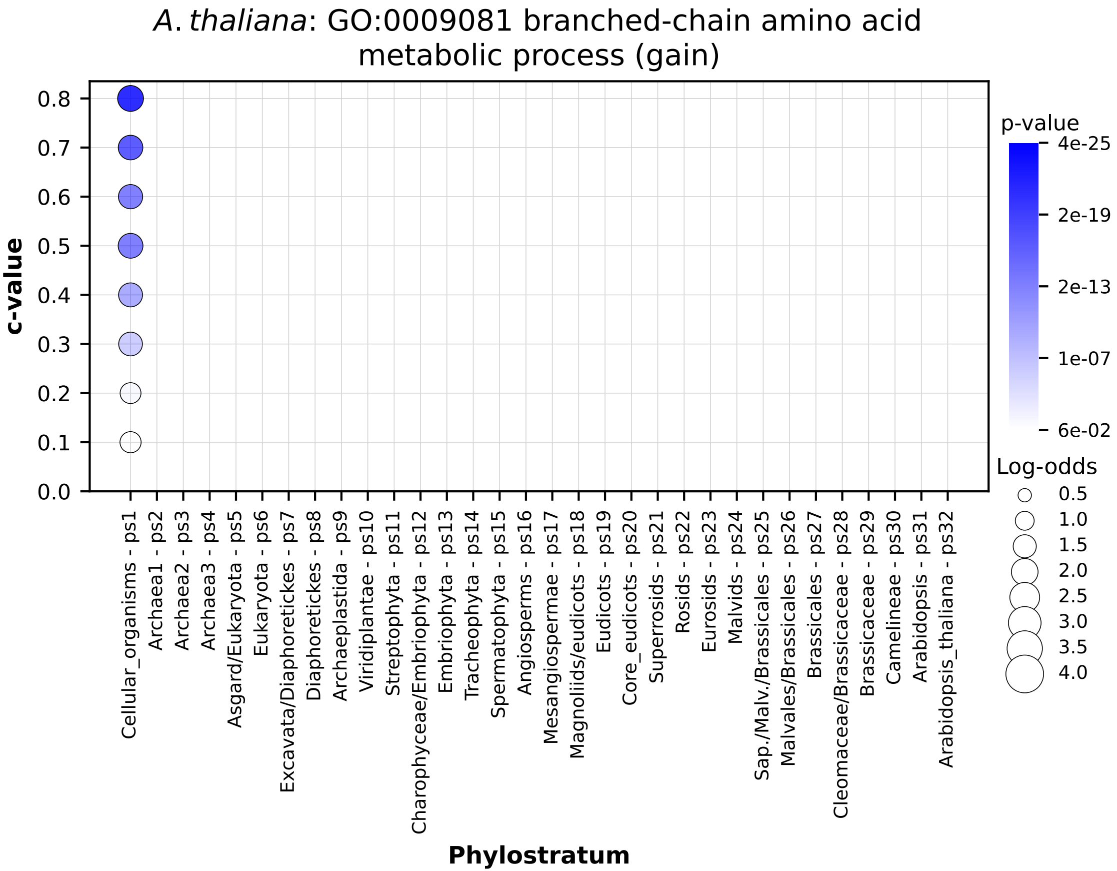

A. thaliana: GO:0009081 branched-chain amino acid metabolic process (gain)
c-value
0.0
0.1
0.2
0.3
0.4
0.5
0.6
0.7
0.8
Phylostratum
Cellular_organisms - ps1
Archaea1 - ps2
Archaea2 - ps3
Archaea3 - ps4
Asgard/Eukaryota - ps5
Eukaryota - ps6
Excavata/Diaphoretickes - ps7
Diaphoretickes - ps8
Archaeplastida - ps9
Viridiplantae - ps10
Streptophyta - ps11
Charophyceae/Embriophyta - ps12
Embriophyta - ps13
Tracheophyta - ps14
Spermatophyta - ps15
Angiosperms - ps16
Mesangiospermae - ps17
Magnoliids/eudicots - ps18
Eudicots - ps19
Core_eudicots - ps20
Superrosids - ps21
Rosids - ps22
Eurosids - ps23
Malvids - ps24
Sap./Malv./Brassicales - ps25
Malvales/Brassicales - ps26
Brassicales - ps27
Cleomaceae/Brassicaceae - ps28
Brassicaceae - ps29
Camelineae - ps30
Arabidopsis - ps31
Arabidopsis_thaliana - ps32
p-value
4e-25
2e-19
2e-13
1e-07
6e-02
Log-odds
0.5
1.0
1.5
2.0
2.5
3.0
3.5
4.0

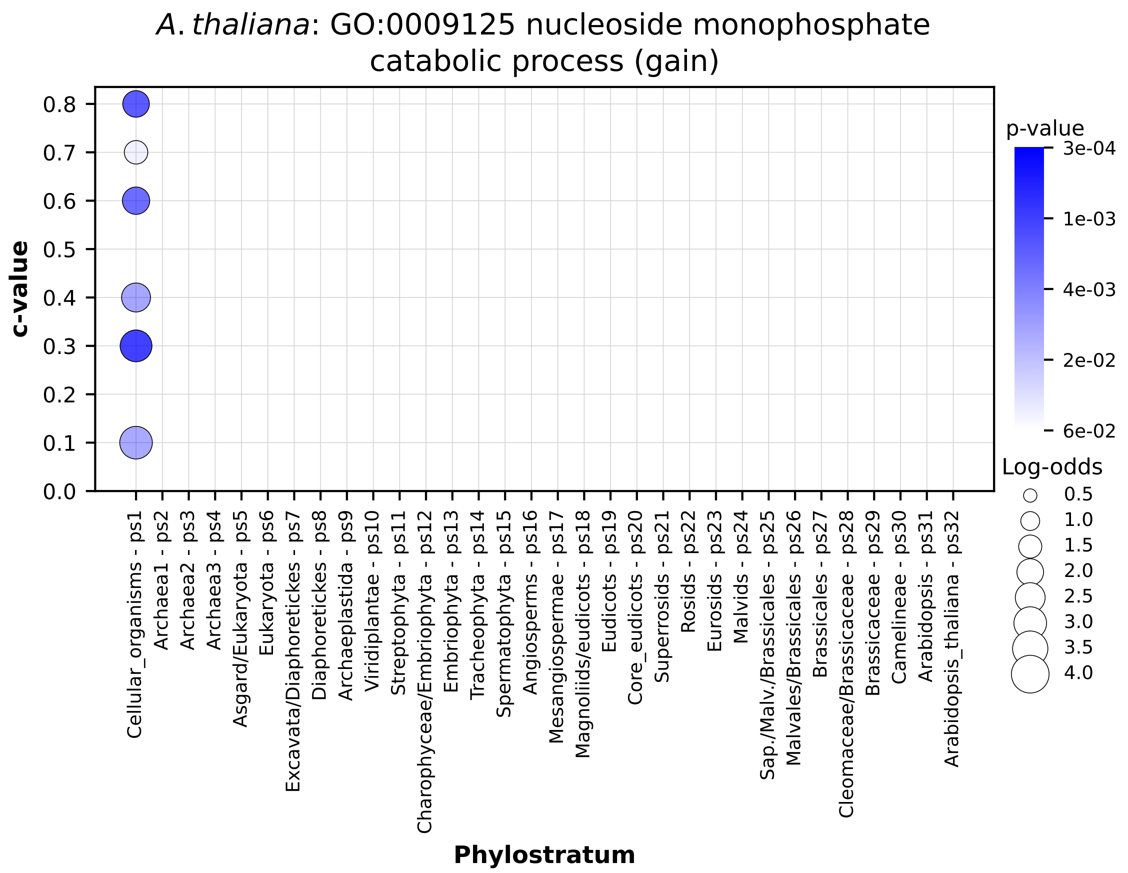
A. thaliana: GO:0009125 nucleoside monophosphate catabolic process (gain)
c-value
0.0
0.1
0.2
0.3
0.4
0.5
0.6
0.7
0.8
Phylostratum
Cellular_organisms - ps1
Archaea1 - ps2
Archaea2 - ps3
Archaea3 - ps4
Asgard/Eukaryota - ps5
Eukaryota - ps6
Excavata/Diaphoretickes - ps7
Diaphoretickes - ps8
Archaeplastida - ps9
Viridiplantae - ps10
Streptophyta - ps11
Charophyceae/Embriophyta - ps12
Embriophyta - ps13
Tracheophyta - ps14
Spermatophyta - ps15
Angiosperms - ps16
Mesangiospermae - ps17
Magnoliids/eudicots - ps18
Eudicots - ps19
Core_eudicots - ps20
Superrosids - ps21
Rosids - ps22
Eurosids - ps23
Malvids - ps24
Sap./Malv./Brassicales - ps25
Malvales/Brassicales - ps26
Brassicales - ps27
Cleomaceae/Brassicaceae - ps28
Brassicaceae - ps29
Camelineae - ps30
Arabidopsis - ps31
Arabidopsis_thaliana - ps32
p-value
3e-04
1e-03
4e-03
2e-02
6e-02
Log-odds
0.5
1.0
1.5
2.0
2.5
3.0
3.5
4.0

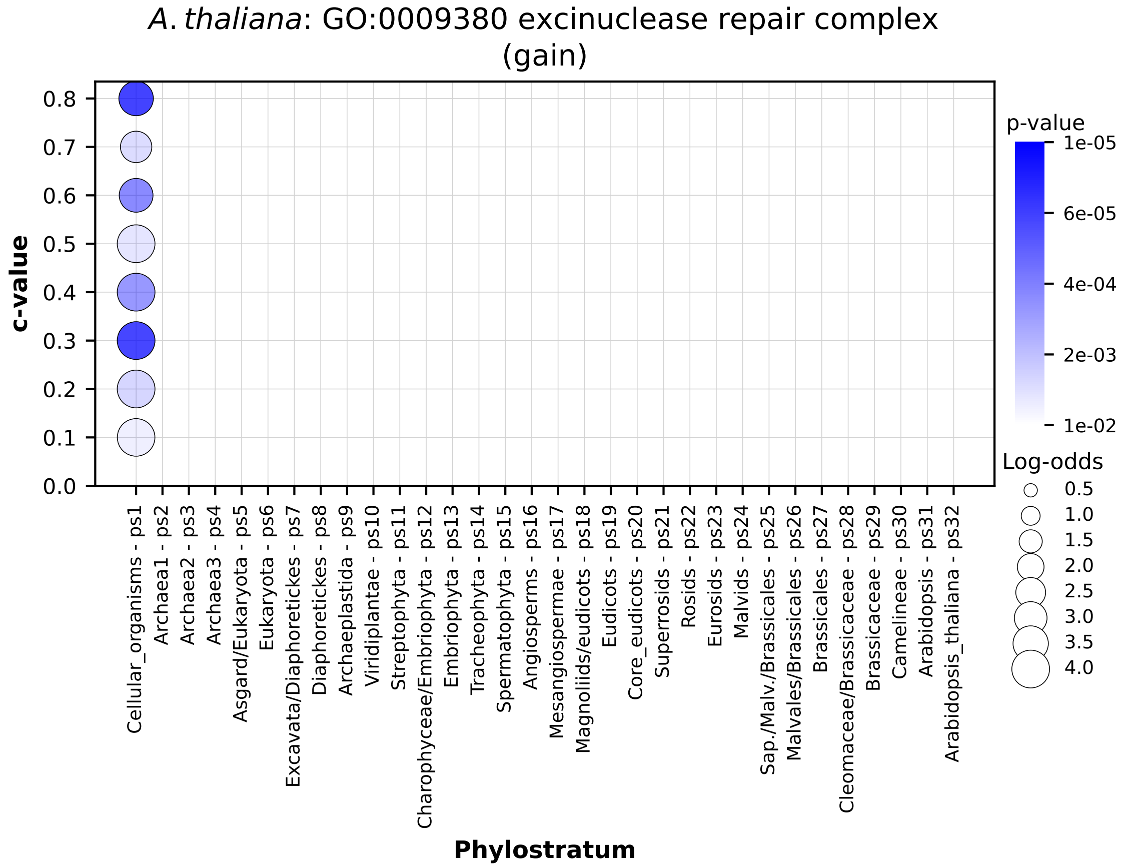

A. thaliana: GO:0009380 excinuclease repair complex
(gain)
c-value
0.0
0.1
0.2
0.3
0.4
0.5
0.6
0.7
0.8
p-value
1e-05
6e-05
4e-04
2e-03
1e-02
Log-odds
0.5
1.0
1.5
2.0
2.5
3.0
3.5
4.0
Cellular_organisms - ps1
Archaea1 - ps2
Archaea2 - ps3
Archaea3 - ps4
Asgard/Eukaryota - ps5
Eukaryota - ps6
Excavata/Diaphoretickes - ps7
Diaphoretickes - ps8
Archaeplastida - ps9
Viridiplantae - ps10
Streptophyta - ps11
Charophyceae/Embriophyta - ps12
Embriophyta - ps13
Tracheophyta - ps14
Spermatophyta - ps15
Angiosperms - ps16
Mesangiospermae - ps17
Magnoliids/eudicots - ps18
Eudicots - ps19
Core_eudicots - ps20
Superrosids - ps21
Rosids - ps22
Eurosids - ps23
Malvids - ps24
Sap./Malv./Brassicales - ps25
Malvales/Brassicales - ps26
Brassicales - ps27
Cleomaceae/Brassicaceae - ps28
Brassicaceae - ps29
Camelineae - ps30
Arabidopsis - ps31
Arabidopsis_thaliana - ps32
Phylostratum

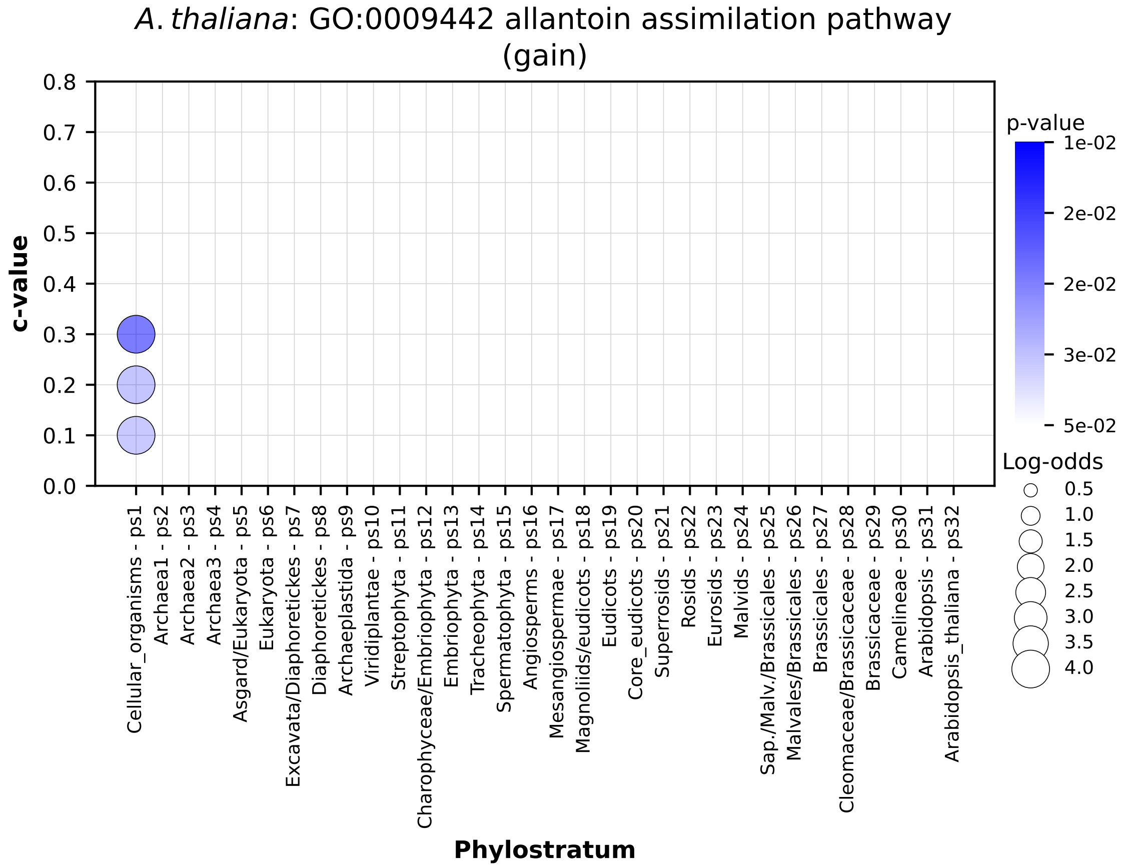

A. thaliana: GO:0009442 allantoin assimilation pathway (gain)
c-value
0.8
0.7
0.6
0.5
0.4
0.3
0.2
0.1
0.0
p-value
1e-02
2e-02
2e-02
3e-02
5e-02
Log-odds
0.5
1.0
1.5
2.0
2.5
3.0
3.5
4.0
Cellular_organisms - ps1
Archaea1 - ps2
Archaea2 - ps3
Archaea3 - ps4
Asgard/Eukaryota - ps5
Eukaryota - ps6
Excavata/Diaphoretickes - ps7
Diaphoretickes - ps8
Archaeplastida - ps9
Viridiplantae - ps10
Streptophyta - ps11
Charophyceae/Embriophyta - ps12
Embriophyta - ps13
Tracheophyta - ps14
Spermatophyta - ps15
Angiosperms - ps16
Mesangiospermae - ps17
Magnoliids/eudicots - ps18
Eudicots - ps19
Core_eudicots - ps20
Superrosids - ps21
Rosids - ps22
Eurosids - ps23
Malvids - ps24
Sap./Malv./Brassicales - ps25
Malvales/Brassicales - ps26
Brassicales - ps27
Cleomaceae/Brassicaceae - ps28
Brassicaceae - ps29
Camelineae - ps30
Arabidopsis - ps31
Arabidopsis_thaliana - ps32
Phylostratum

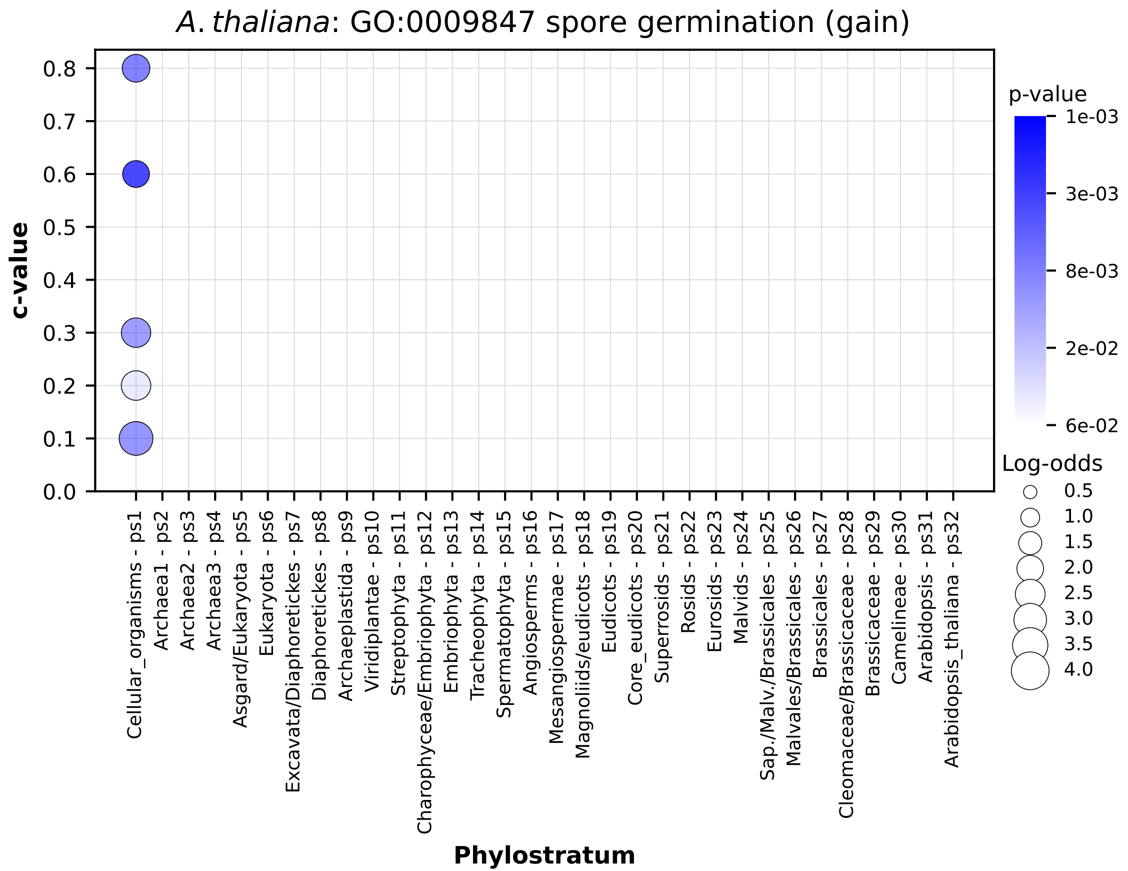

A. thaliana: GO:0009847 spore germination (gain)
c-value
0.0
0.1
0.2
0.3
0.4
0.5
0.6
0.7
0.8
Phylostratum
Cellular_organisms - ps1
Archaea1 - ps2
Archaea2 - ps3
Archaea3 - ps4
Asgard/Eukaryota - ps5
Eukaryota - ps6
Excavata/Diaphoretickes - ps7
Diaphoretickes - ps8
Archaeplastida - ps9
Viridiplantae - ps10
Streptophyta - ps11
Charophyceae/Embriophyta - ps12
Embriophyta - ps13
Tracheophyta - ps14
Spermatophyta - ps15
Angiosperms - ps16
Mesangiospermae - ps17
Magnoliids/eudicots - ps18
Eudicots - ps19
Core_eudicots - ps20
Superrosids - ps21
Rosids - ps22
Eurosids - ps23
Malvids - ps24
Sap./Malv./Brassicales - ps25
Malvales/Brassicales - ps26
Brassicales - ps27
Cleomaceae/Brassicaceae - ps28
Brassicaceae - ps29
Camelineae - ps30
Arabidopsis - ps31
Arabidopsis_thaliana - ps32
p-value
1e-03
3e-03
8e-03
2e-02
6e-02
Log-odds
0.5
1.0
1.5
2.0
2.5
3.0
3.5
4.0

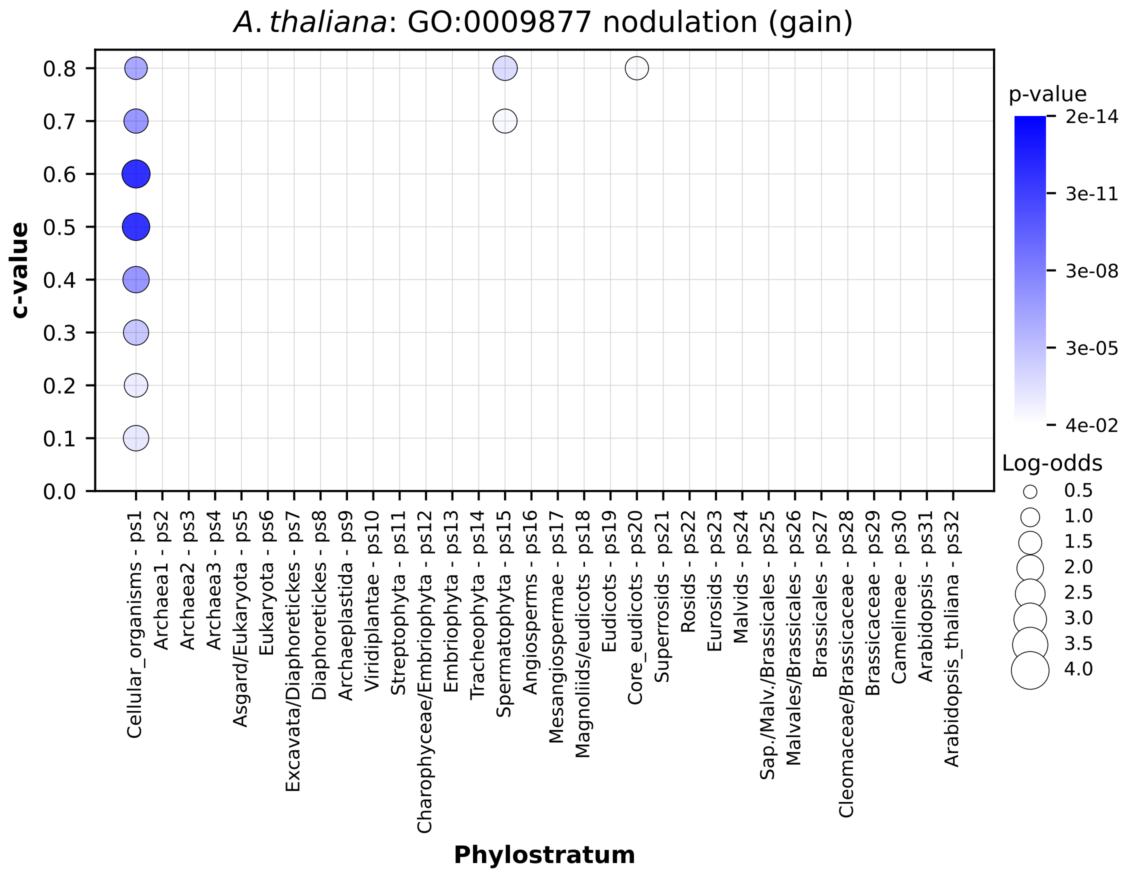

A. thaliana: GO:0009877 nodulation (gain)
c-value
0.0
0.1
0.2
0.3
0.4
0.5
0.6
0.7
0.8
Phylostratum
Cellular_organisms - ps1
Archaea1 - ps2
Archaea2 - ps3
Archaea3 - ps4
Asgard/Eukaryota - ps5
Eukaryota - ps6
Excavata/Diaphoretickes - ps7
Diaphoretickes - ps8
Archaeplastida - ps9
Viridiplantae - ps10
Streptophyta - ps11
Charophyceae/Embriophyta - ps12
Embriophyta - ps13
Tracheophyta - ps14
Spermatophyta - ps15
Angiosperms - ps16
Mesangiospermae - ps17
Magnoliids/eudicots - ps18
Eudicots - ps19
Core_eudicots - ps20
Superrosids - ps21
Rosids - ps22
Eurosids - ps23
Malvids - ps24
Sap./Malv./Brassicales - ps25
Malvales/Brassicales - ps26
Brassicales - ps27
Cleomaceae/Brassicaceae - ps28
Brassicaceae - ps29
Camelineae - ps30
Arabidopsis - ps31
Arabidopsis_thaliana - ps32
p-value
2e-14
3e-11
3e-08
3e-05
4e-02
Log-odds
0.5
1.0
1.5
2.0
2.5
3.0
3.5
4.0

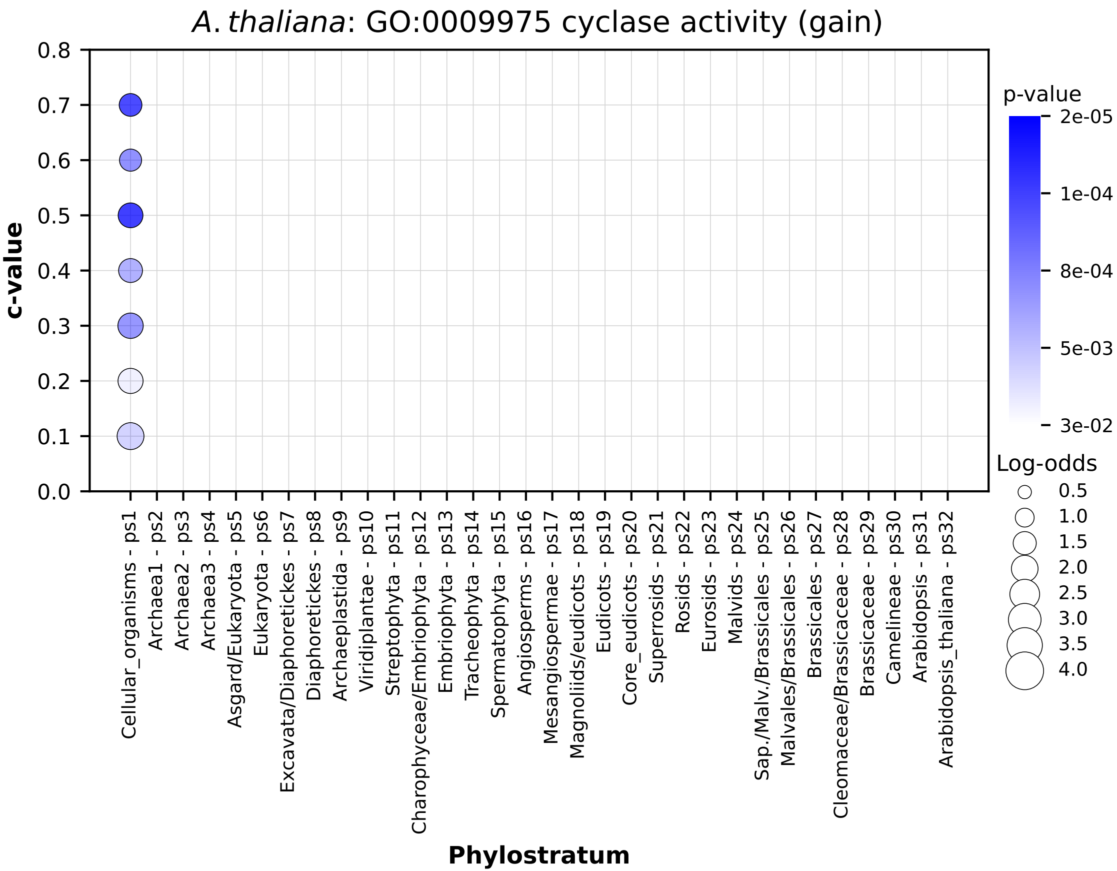

A. thaliana: GO:0009975 cyclase activity (gain)
c-value
0.0
0.1
0.2
0.3
0.4
0.5
0.6
0.7
0.8
p-value
2e-05
1e-04
8e-04
5e-03
3e-02
Log-odds
0.5
1.0
1.5
2.0
2.5
3.0
3.5
4.0
Cellular_organisms - ps1
Archaea1 - ps2
Archaea2 - ps3
Archaea3 - ps4
Asgard/Eukaryota - ps5
Eukaryota - ps6
Excavata/Diaphoretickes - ps7
Diaphoretickes - ps8
Archaeplastida - ps9
Viridiplantae - ps10
Streptophyta - ps11
Charophyceae/Embriophyta - ps12
Embriophyta - ps13
Tracheophyta - ps14
Spermatophyta - ps15
Angiosperms - ps16
Mesangiospermae - ps17
Magnoliids/eudicots - ps18
Eudicots - ps19
Core_eudicots - ps20
Superrosids - ps21
Rosids - ps22
Eurosids - ps23
Malvids - ps24
Sap./Malv./Brassicales - ps25
Malvales/Brassicales - ps26
Brassicales - ps27
Cleomaceae/Brassicaceae - ps28
Brassicaceae - ps29
Camelineae - ps30
Arabidopsis - ps31
Arabidopsis_thaliana - ps32
Phylostratum

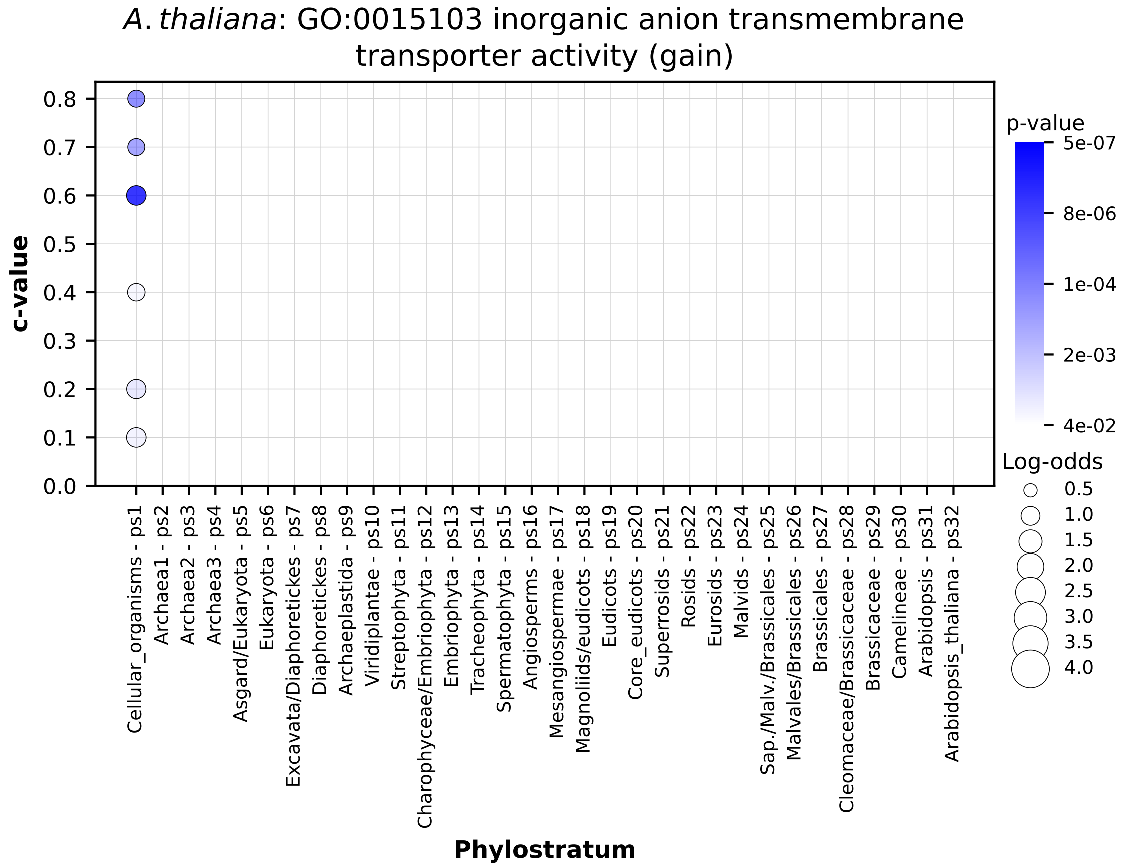
A. thaliana: GO:0015103 inorganic anion transmembrane transporter activity (gain)
c-value
0.0
0.1
0.2
0.3
0.4
0.5
0.6
0.7
0.8
Phylostratum
Cellular_organisms - ps1
Archaea1 - ps2
Archaea2 - ps3
Archaea3 - ps4
Asgard/Eukaryota - ps5
Eukaryota - ps6
Excavata/Diaphoretickes - ps7
Diaphoretickes - ps8
Archaeplastida - ps9
Viridiplantae - ps10
Streptophyta - ps11
Charophyceae/Embriophyta - ps12
Embriophyta - ps13
Tracheophyta - ps14
Spermatophyta - ps15
Angiosperms - ps16
Mesangiospermae - ps17
Magnoliids/eudicots - ps18
Eudicots - ps19
Core_eudicots - ps20
Superrosids - ps21
Rosids - ps22
Eurosids - ps23
Malvids - ps24
Sap./Malv./Brassicales - ps25
Malvales/Brassicales - ps26
Brassicales - ps27
Cleomaceae/Brassicaceae - ps28
Brassicaceae - ps29
Camelineae - ps30
Arabidopsis - ps31
Arabidopsis_thaliana - ps32
p-value
5e-07
8e-06
1e-04
2e-03
4e-02
Log-odds
0.5
1.0
1.5
2.0
2.5
3.0
3.5
4.0

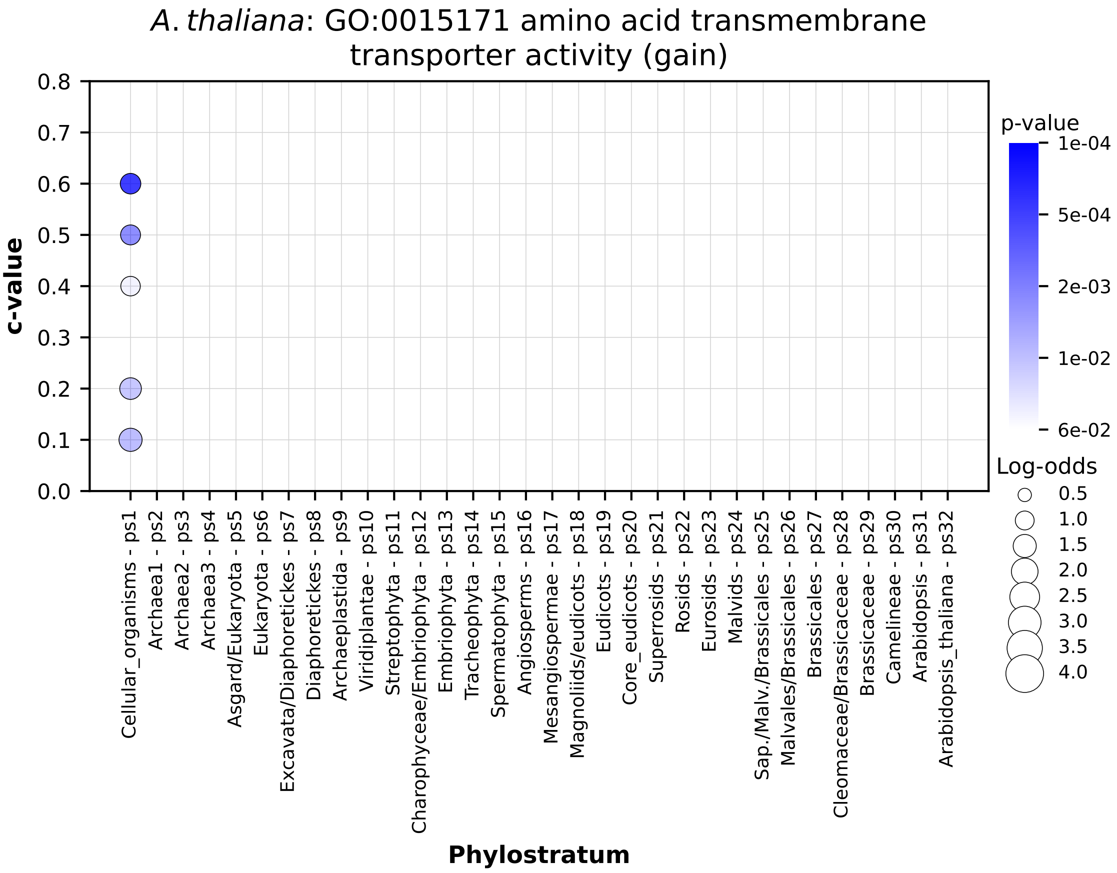

A. thaliana: GO:0015171 amino acid transmembrane transporter activity (gain)
c-value
0.0
0.1
0.2
0.3
0.4
0.5
0.6
0.7
0.8
p-value
1e-04
5e-04
2e-03
1e-02
6e-02
Log-odds
0.5
1.0
1.5
2.0
2.5
3.0
3.5
4.0
Cellular_organisms - ps1
Archaea1 - ps2
Archaea2 - ps3
Archaea3 - ps4
Asgard/Eukaryota - ps5
Eukaryota - ps6
Excavata/Diaphoretickes - ps7
Diaphoretickes - ps8
Archaeplastida - ps9
Viridiplantae - ps10
Streptophyta - ps11
Charophyceae/Embriophyta - ps12
Embriophyta - ps13
Tracheophyta - ps14
Spermatophyta - ps15
Angiosperms - ps16
Mesangiospermae - ps17
Magnoliids/eudicots - ps18
Eudicots - ps19
Core_eudicots - ps20
Superrosids - ps21
Rosids - ps22
Eurosids - ps23
Malvids - ps24
Sap./Malv./Brassicales - ps25
Malvales/Brassicales - ps26
Brassicales - ps27
Cleomaceae/Brassicaceae - ps28
Brassicaceae - ps29
Camelineae - ps30
Arabidopsis - ps31
Arabidopsis_thaliana - ps32
Phylostratum

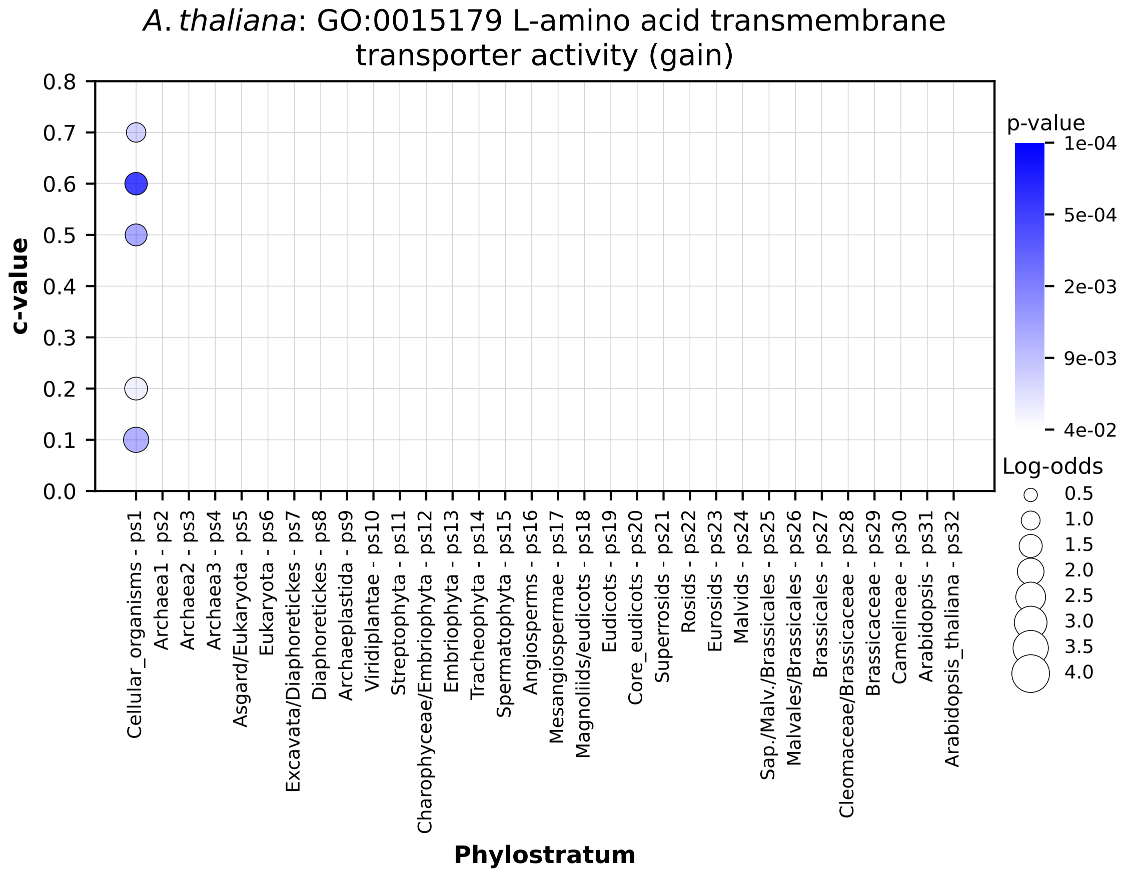

A. thaliana: GO:0015179 L-amino acid transmembrane transporter activity (gain)
c-value
0.8
0.7
0.6
0.5
0.4
0.3
0.2
0.1
0.0
p-value
1e-04
5e-04
2e-03
9e-03
4e-02
Log-odds
0.5
1.0
1.5
2.0
2.5
3.0
3.5
4.0
Cellular_organisms - ps1
Archaea1 - ps2
Archaea2 - ps3
Archaea3 - ps4
Asgard/Eukaryota - ps5
Eukaryota - ps6
Excavata/Diaphoretickes - ps7
Diaphoretickes - ps8
Archaeplastida - ps9
Viridiplantae - ps10
Streptophyta - ps11
Charophyceae/Embriophyta - ps12
Embriophyta - ps13
Tracheophyta - ps14
Spermatophyta - ps15
Angiosperms - ps16
Mesangiospermae - ps17
Magnoliids/eudicots - ps18
Eudicots - ps19
Core_eudicots - ps20
Superrosids - ps21
Rosids - ps22
Eurosids - ps23
Malvids - ps24
Sap./Malv./Brassicales - ps25
Malvales/Brassicales - ps26
Brassicales - ps27
Cleomaceae/Brassicaceae - ps28
Brassicaceae - ps29
Camelineae - ps30
Arabidopsis - ps31
Arabidopsis_thaliana - ps32
Phylostratum

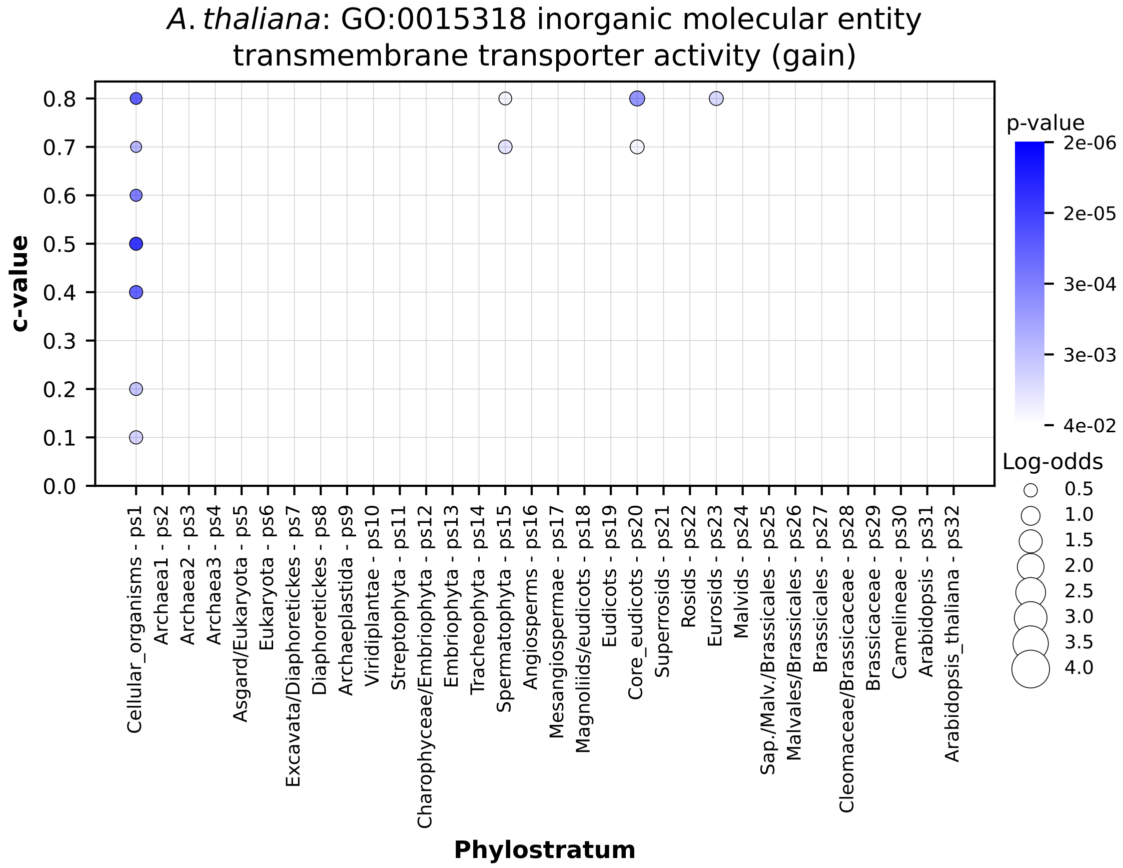

A. thaliana: GO:0015318 inorganic molecular entity transmembrane transporter activity (gain)
c-value
0.0
0.1
0.2
0.3
0.4
0.5
0.6
0.7
0.8
Phylostratum
Cellular_organisms - ps1
Archaea1 - ps2
Archaea2 - ps3
Archaea3 - ps4
Asgard/Eukaryota - ps5
Eukaryota - ps6
Excavata/Diaphoretickes - ps7
Diaphoretickes - ps8
Archaeplastida - ps9
Viridiplantae - ps10
Streptophyta - ps11
Charophyceae/Embriophyta - ps12
Embriophyta - ps13
Tracheophyta - ps14
Spermatophyta - ps15
Angiosperms - ps16
Mesangiospermae - ps17
Magnoliids/eudicots - ps18
Eudicots - ps19
Core_eudicots - ps20
Superrosids - ps21
Rosids - ps22
Eurosids - ps23
Malvids - ps24
Sap./Malv./Brassicales - ps25
Malvales/Brassicales - ps26
Brassicales - ps27
Cleomaceae/Brassicaceae - ps28
Brassicaceae - ps29
Camelineae - ps30
Arabidopsis - ps31
Arabidopsis_thaliana - ps32
p-value
2e-06
2e-05
3e-04
3e-03
4e-02
Log-odds
0.5
1.0
1.5
2.0
2.5
3.0
3.5
4.0

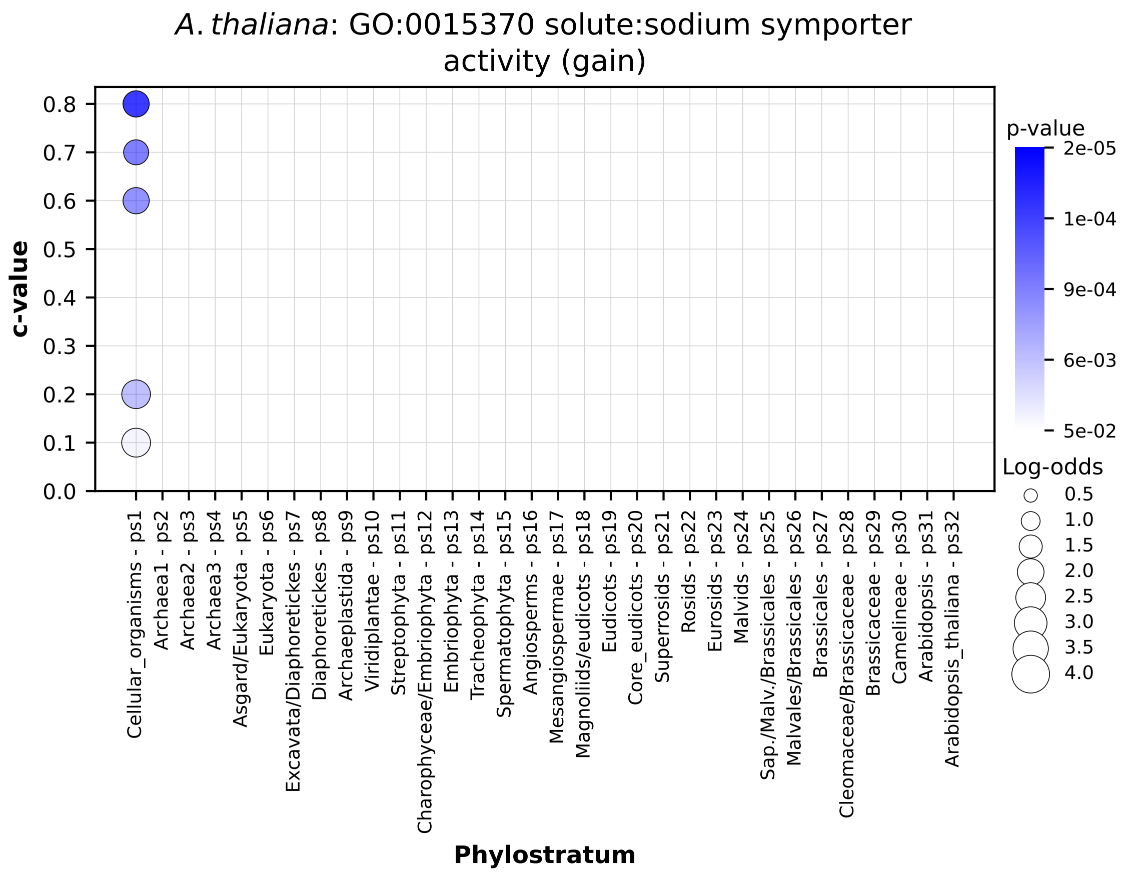
A. thaliana: GO:0015370 solute:sodium symporter activity (gain)
c-value
0.8
0.7
0.6
0.5
0.4
0.3
0.2
0.1
0.0
p-value
2e-05
1e-04
9e-04
6e-03
5e-02
Log-odds
0.5
1.0
1.5
2.0
2.5
3.0
3.5
4.0
Cellular_organisms - ps1
Archaea1 - ps2
Archaea2 - ps3
Archaea3 - ps4
Asgard/Eukaryota - ps5
Eukaryota - ps6
Excavata/Diaphoretickes - ps7
Diaphoretickes - ps8
Archaeplastida - ps9
Viridiplantae - ps10
Streptophyta - ps11
Charophyceae/Embriophyta - ps12
Embriophyta - ps13
Tracheophyta - ps14
Spermatophyta - ps15
Angiosperms - ps16
Mesangiospermae - ps17
Magnoliids/eudicots - ps18
Eudicots - ps19
Core_eudicots - ps20
Superrosids - ps21
Rosids - ps22
Eurosids - ps23
Malvids - ps24
Sap./Malv./Brassicales - ps25
Malvales/Brassicales - ps26
Brassicales - ps27
Cleomaceae/Brassicaceae - ps28
Brassicaceae - ps29
Camelineae - ps30
Arabidopsis - ps31
Arabidopsis_thaliana - ps32
Phylostratum

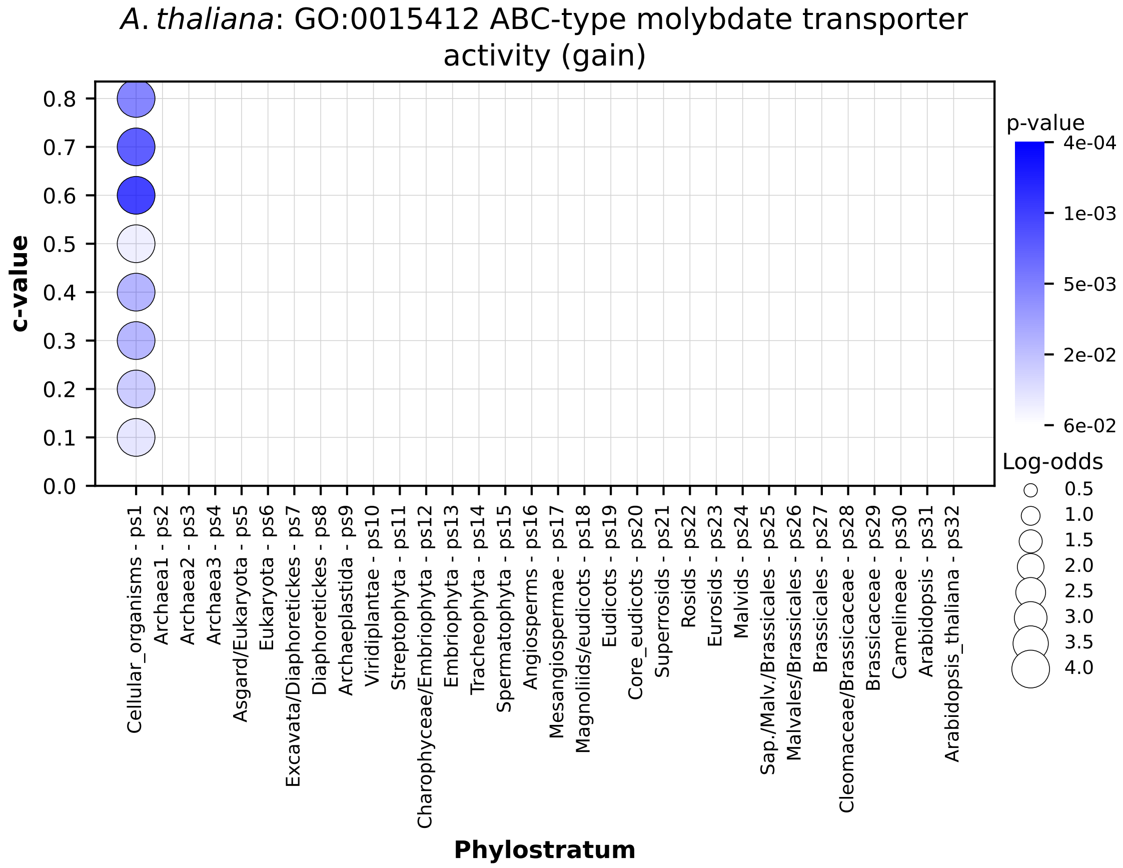

A. thaliana: GO:0015412 ABC-type molybdate transporter activity (gain)
c-value
0.8
0.7
0.6
0.5
0.4
0.3
0.2
0.1
0.0
p-value
4e-04
1e-03
5e-03
2e-02
6e-02
Log-odds
0.5
1.0
1.5
2.0
2.5
3.0
3.5
4.0
Cellular_organisms - ps1
Archaea1 - ps2
Archaea2 - ps3
Archaea3 - ps4
Asgard/Eukaryota - ps5
Eukaryota - ps6
Excavata/Diaphoretickes - ps7
Diaphoretickes - ps8
Archaeplastida - ps9
Viridiplantae - ps10
Streptophyta - ps11
Charophyceae/Embriophyta - ps12
Embriophyta - ps13
Tracheophyta - ps14
Spermatophyta - ps15
Angiosperms - ps16
Mesangiospermae - ps17
Magnoliids/eudicots - ps18
Eudicots - ps19
Core_eudicots - ps20
Superrosids - ps21
Rosids - ps22
Eurosids - ps23
Malvids - ps24
Sap./Malv./Brassicales - ps25
Malvales/Brassicales - ps26
Brassicales - ps27
Cleomaceae/Brassicaceae - ps28
Brassicaceae - ps29
Camelineae - ps30
Arabidopsis - ps31
Arabidopsis_thaliana - ps32
Phylostratum

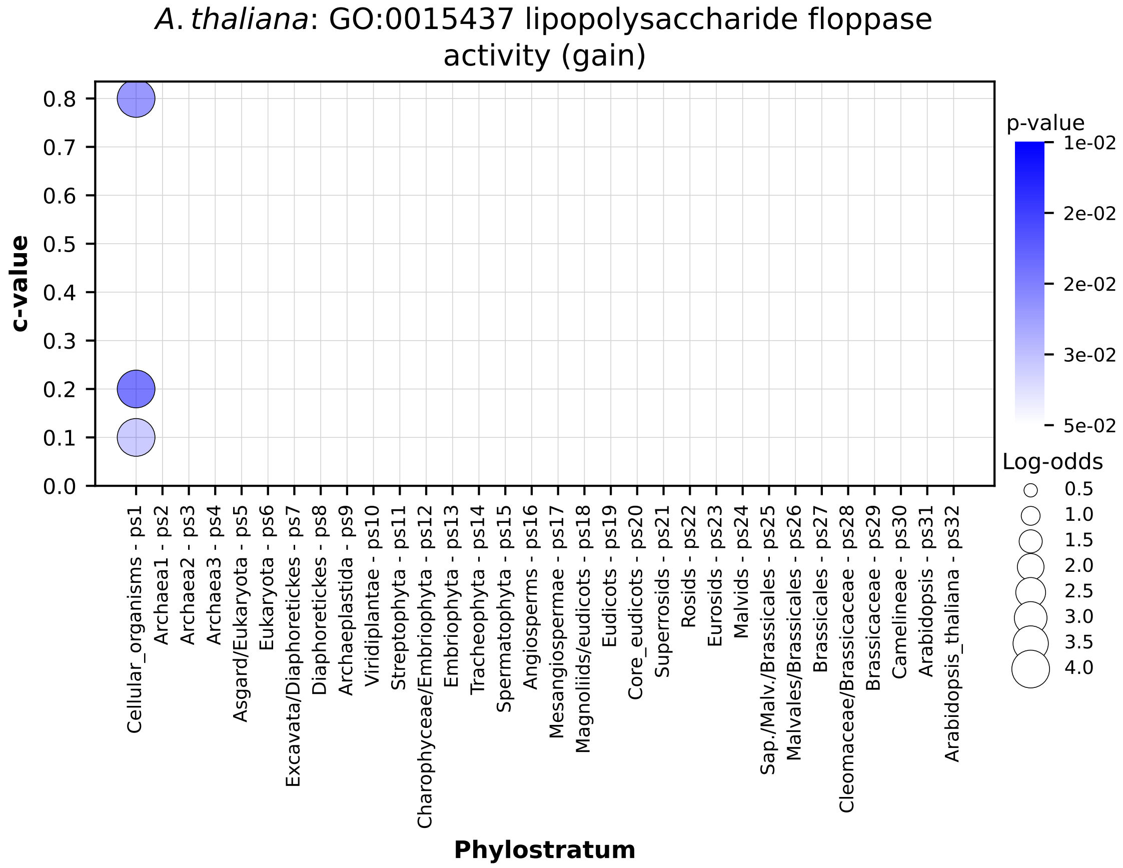

A. thaliana: GO:0015437 lipopolysaccharide floppase activity (gain)
c-value
0.8
0.7
0.6
0.5
0.4
0.3
0.2
0.1
0.0
Phylostratum
Cellular_organisms - ps1
Archaea1 - ps2
Archaea2 - ps3
Archaea3 - ps4
Asgard/Eukaryota - ps5
Eukaryota - ps6
Excavata/Diaphoretickes - ps7
Diaphoretickes - ps8
Archaeplastida - ps9
Viridiplantae - ps10
Streptophyta - ps11
Charophyceae/Embriophyta - ps12
Embriophyta - ps13
Tracheophyta - ps14
Spermatophyta - ps15
Angiosperms - ps16
Mesangiospermae - ps17
Magnoliids/eudicots - ps18
Eudicots - ps19
Core_eudicots - ps20
Superrosids - ps21
Rosids - ps22
Eurosids - ps23
Malvids - ps24
Sap./Malv./Brassicales - ps25
Malvales/Brassicales - ps26
Brassicales - ps27
Cleomaceae/Brassicaceae - ps28
Brassicaceae - ps29
Camelineae - ps30
Arabidopsis - ps31
Arabidopsis_thaliana - ps32
p-value
1e-02
2e-02
2e-02
3e-02
5e-02
Log-odds
0.5
1.0
1.5
2.0
2.5
3.0
3.5
4.0

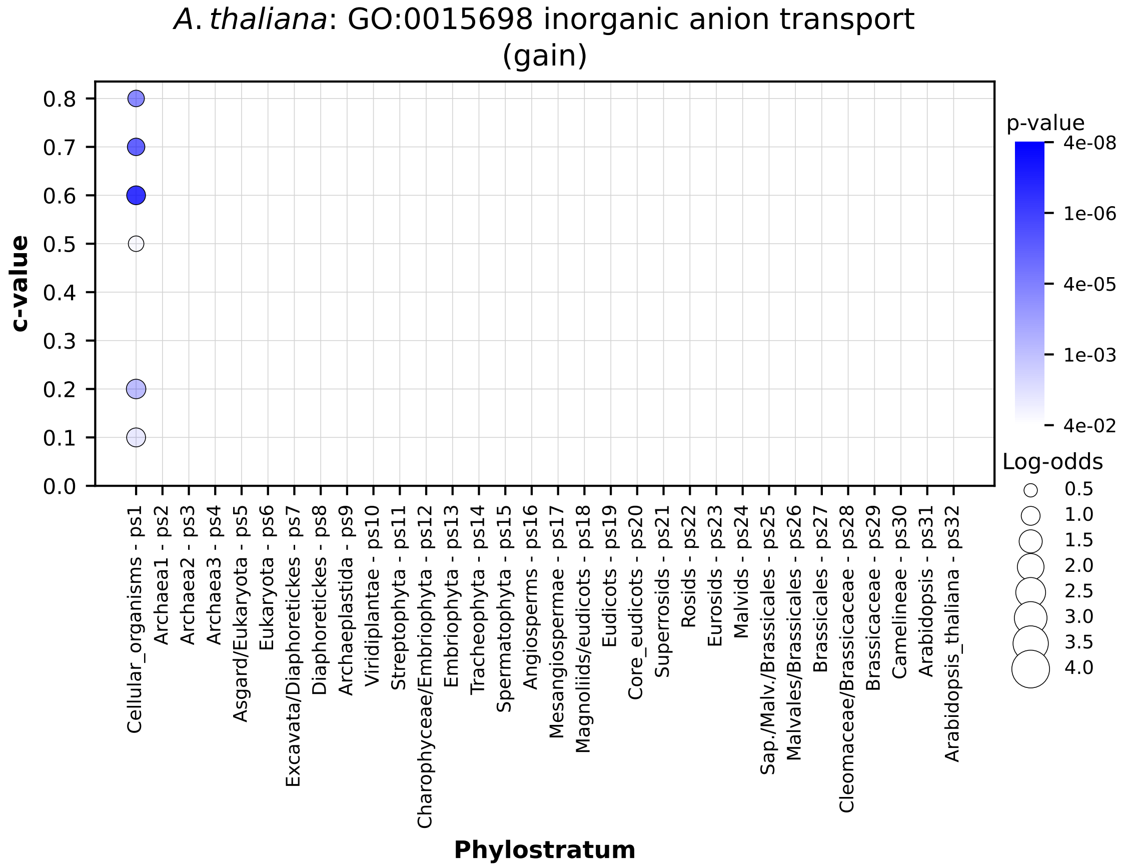

A. thaliana: GO:0015698 inorganic anion transport (gain)
c-value
0.0
0.1
0.2
0.3
0.4
0.5
0.6
0.7
0.8
Phylostratum
Cellular_organisms - ps1
Archaea1 - ps2
Archaea2 - ps3
Archaea3 - ps4
Asgard/Eukaryota - ps5
Eukaryota - ps6
Excavata/Diaphoretickes - ps7
Diaphoretickes - ps8
Archaeplastida - ps9
Viridiplantae - ps10
Streptophyta - ps11
Charophyceae/Embriophyta - ps12
Embriophyta - ps13
Tracheophyta - ps14
Spermatophyta - ps15
Angiosperms - ps16
Mesangiospermae - ps17
Magnoliids/eudicots - ps18
Eudicots - ps19
Core_eudicots - ps20
Superrosids - ps21
Rosids - ps22
Eurosids - ps23
Malvids - ps24
Sap./Malv./Brassicales - ps25
Malvales/Brassicales - ps26
Brassicales - ps27
Cleomaceae/Brassicaceae - ps28
Brassicaceae - ps29
Camelineae - ps30
Arabidopsis - ps31
Arabidopsis_thaliana - ps32
p-value
4e-08
1e-06
4e-05
1e-03
4e-02
Log-odds
0.5
1.0
1.5
2.0
2.5
3.0
3.5
4.0

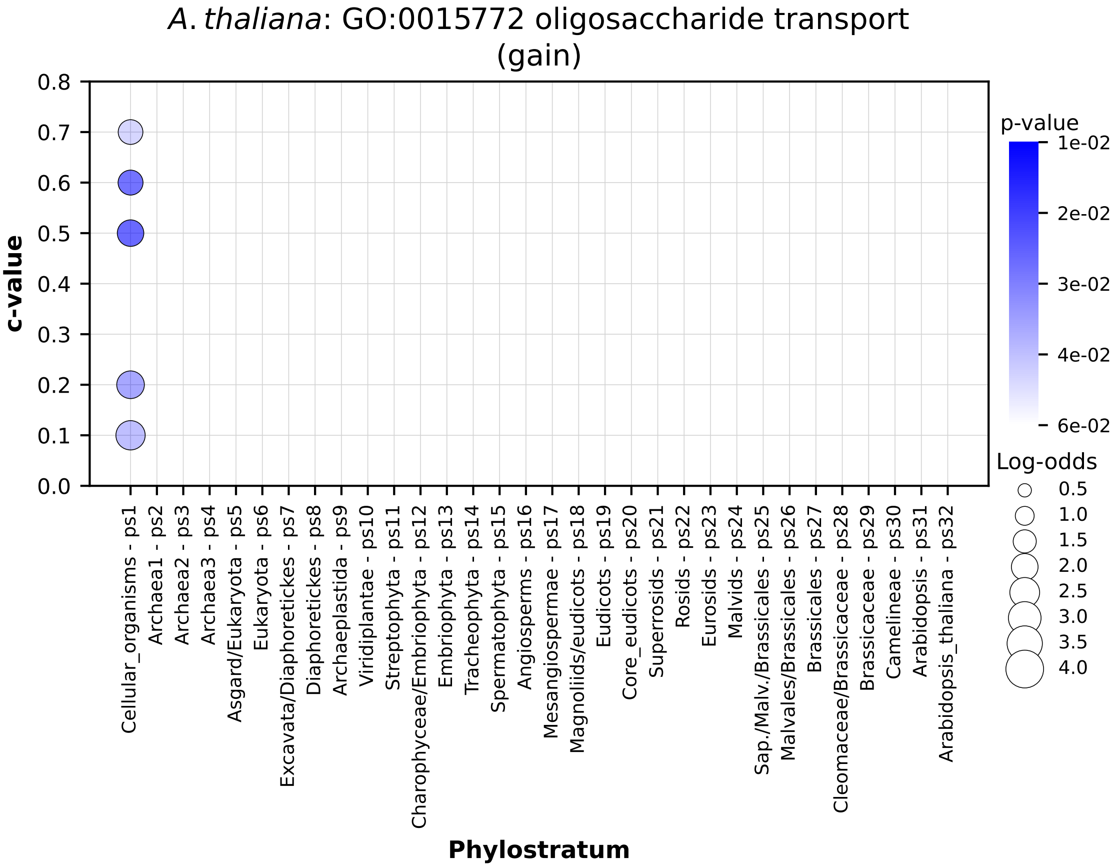
A. thaliana: GO:0015772 oligosaccharide transport
(gain)
c-value
0.0
0.1
0.2
0.3
0.4
0.5
0.6
0.7
0.8
Phylostratum
Cellular_organisms - ps1
Archaea1 - ps2
Archaea2 - ps3
Archaea3 - ps4
Asgard/Eukaryota - ps5
Eukaryota - ps6
Excavata/Diaphoretickes - ps7
Diaphoretickes - ps8
Archaeplastida - ps9
Viridiplantae - ps10
Streptophyta - ps11
Charophyceae/Embriophyta - ps12
Embriophyta - ps13
Tracheophyta - ps14
Spermatophyta - ps15
Angiosperms - ps16
Mesangiospermae - ps17
Magnoliids/eudicots - ps18
Eudicots - ps19
Core_eudicots - ps20
Superrosids - ps21
Rosids - ps22
Eurosids - ps23
Malvids - ps24
Sap./Malv./Brassicales - ps25
Malvales/Brassicales - ps26
Brassicales - ps27
Cleomaceae/Brassicaceae - ps28
Brassicaceae - ps29
Camelineae - ps30
Arabidopsis - ps31
Arabidopsis_thaliana - ps32
p-value
1e-02
2e-02
3e-02
4e-02
6e-02
Log-odds
0.5
1.0
1.5
2.0
2.5
3.0
3.5
4.0

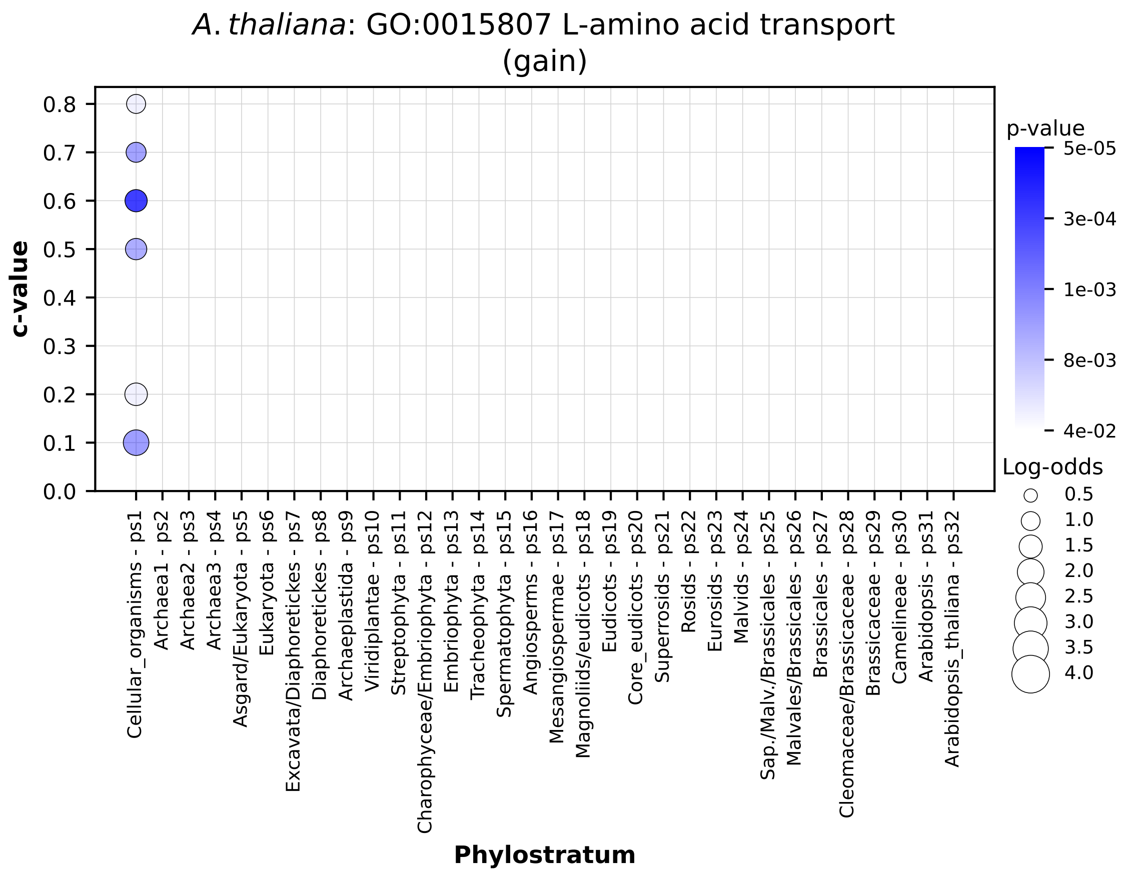

A. thaliana: GO:0015807 L-amino acid transport
(gain)
c-value
0.0
0.1
0.2
0.3
0.4
0.5
0.6
0.7
0.8
p-value
5e-05
3e-04
1e-03
8e-03
4e-02
Log-odds
0.5
1.0
1.5
2.0
2.5
3.0
3.5
4.0
Cellular_organisms - ps1
Archaea1 - ps2
Archaea2 - ps3
Archaea3 - ps4
Asgard/Eukaryota - ps5
Eukaryota - ps6
Excavata/Diaphoretickes - ps7
Diaphoretickes - ps8
Archaeplastida - ps9
Viridiplantae - ps10
Streptophyta - ps11
Charophyceae/Embriophyta - ps12
Embriophyta - ps13
Tracheophyta - ps14
Spermatophyta - ps15
Angiosperms - ps16
Mesangiospermae - ps17
Magnoliids/eudicots - ps18
Eudicots - ps19
Core_eudicots - ps20
Superrosids - ps21
Rosids - ps22
Eurosids - ps23
Malvids - ps24
Sap./Malv./Brassicales - ps25
Malvales/Brassicales - ps26
Brassicales - ps27
Cleomaceae/Brassicaceae - ps28
Brassicaceae - ps29
Camelineae - ps30
Arabidopsis - ps31
Arabidopsis_thaliana - ps32
Phylostratum

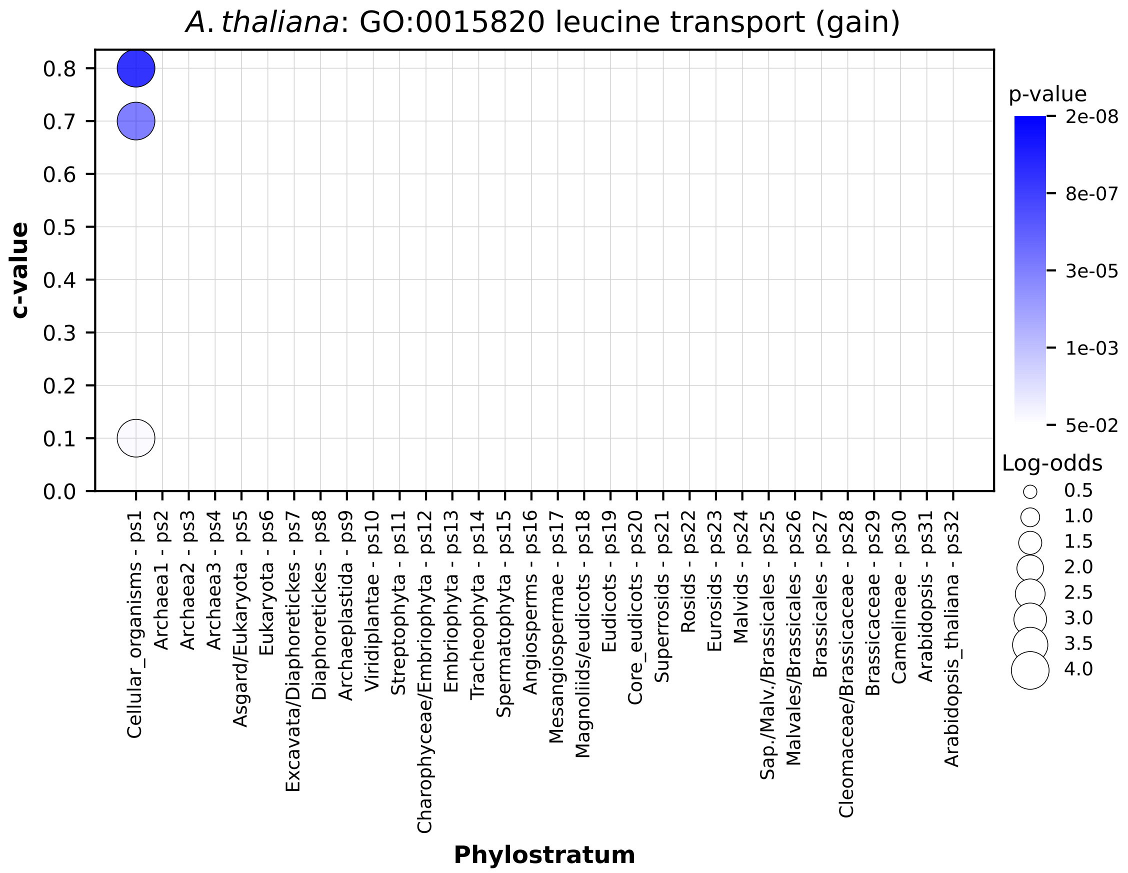

A. thaliana: GO:0015820 leucine transport (gain)
c-value
0.0
0.1
0.2
0.3
0.4
0.5
0.6
0.7
0.8
p-value
2e-08
8e-07
3e-05
1e-03
5e-02
Log-odds
0.5
1.0
1.5
2.0
2.5
3.0
3.5
4.0
Cellular_organisms - ps1
Archaea1 - ps2
Archaea2 - ps3
Archaea3 - ps4
Asgard/Eukaryota - ps5
Eukaryota - ps6
Excavata/Diaphoretickes - ps7
Diaphoretickes - ps8
Archaeplastida - ps9
Viridiplantae - ps10
Streptophyta - ps11
Charophyceae/Embriophyta - ps12
Embriophyta - ps13
Tracheophyta - ps14
Spermatophyta - ps15
Angiosperms - ps16
Mesangiospermae - ps17
Magnoliids/eudicots - ps18
Eudicots - ps19
Core_eudicots - ps20
Superrosids - ps21
Rosids - ps22
Eurosids - ps23
Malvids - ps24
Sap./Malv./Brassicales - ps25
Malvales/Brassicales - ps26
Brassicales - ps27
Cleomaceae/Brassicaceae - ps28
Brassicaceae - ps29
Camelineae - ps30
Arabidopsis - ps31
Arabidopsis_thaliana - ps32
Phylostratum

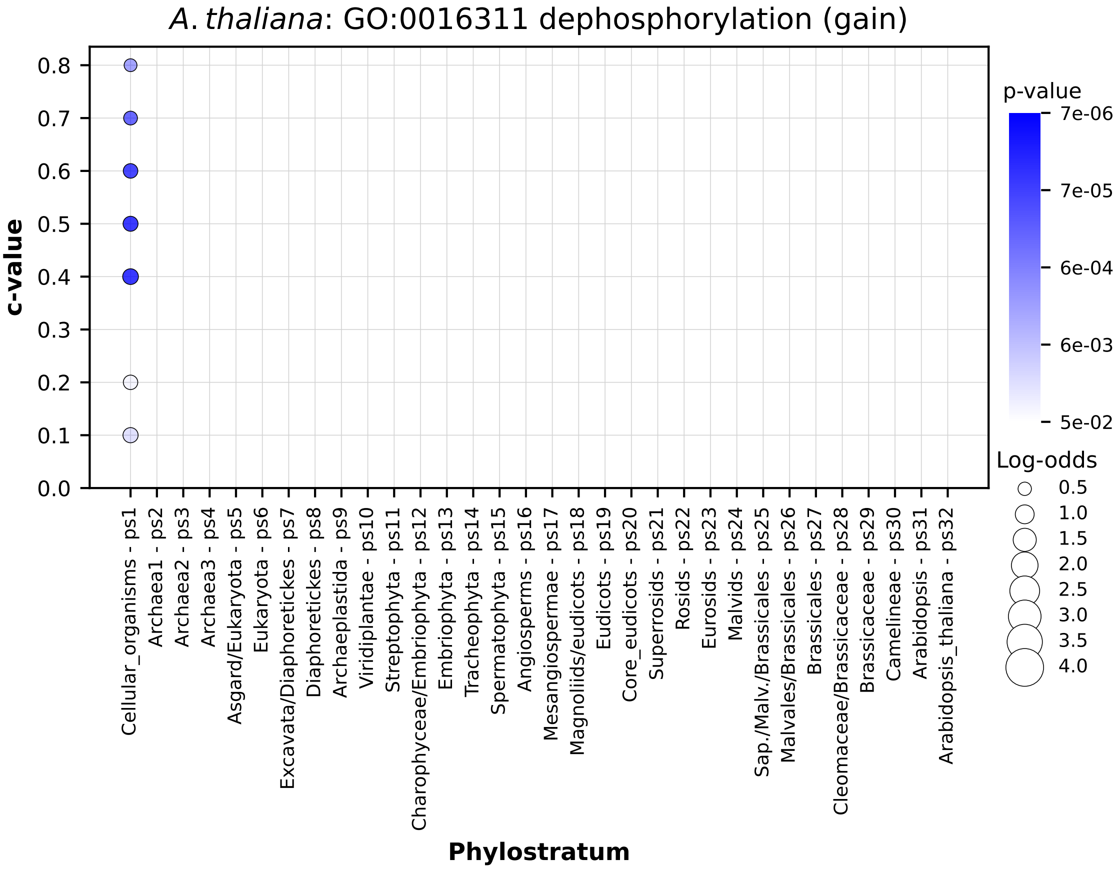

A. thaliana: GO:0016311 dephosphorylation (gain)
c-value
0.0
0.1
0.2
0.3
0.4
0.5
0.6
0.7
0.8
Phylostratum
Cellular_organisms - ps1
Archaea1 - ps2
Archaea2 - ps3
Archaea3 - ps4
Asgard/Eukaryota - ps5
Eukaryota - ps6
Excavata/Diaphoretickes - ps7
Diaphoretickes - ps8
Archaeplastida - ps9
Viridiplantae - ps10
Streptophyta - ps11
Charophyceae/Embriophyta - ps12
Embriophyta - ps13
Tracheophyta - ps14
Spermatophyta - ps15
Angiosperms - ps16
Mesangiospermae - ps17
Magnoliids/eudicots - ps18
Eudicots - ps19
Core_eudicots - ps20
Superrosids - ps21
Rosids - ps22
Eurosids - ps23
Malvids - ps24
Sap./Malv./Brassicales - ps25
Malvales/Brassicales - ps26
Brassicales - ps27
Cleomaceae/Brassicaceae - ps28
Brassicaceae - ps29
Camelineae - ps30
Arabidopsis - ps31
Arabidopsis_thaliana - ps32
p-value
7e-06
7e-05
6e-04
6e-03
5e-02
Log-odds
0.5
1.0
1.5
2.0
2.5
3.0
3.5
4.0

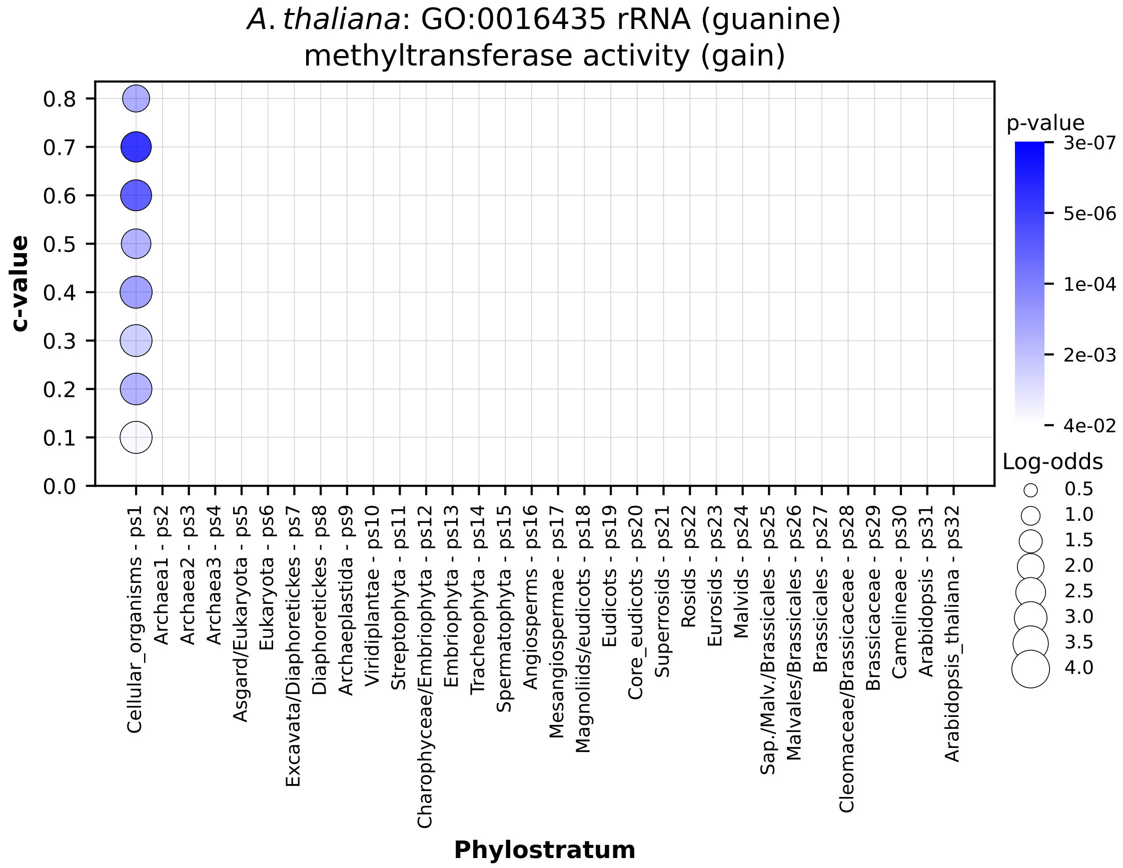

A. thaliana: GO:0016435 rRNA (guanine) methyltransferase activity (gain)
c-value
0.8
0.7
0.6
0.5
0.4
0.3
0.2
0.1
0.0
Phylostratum
Cellular_organisms - ps1
Archaea1 - ps2
Archaea2 - ps3
Archaea3 - ps4
Asgard/Eukaryota - ps5
Eukaryota - ps6
Excavata/Diaphoretickes - ps7
Diaphoretickes - ps8
Archaeplastida - ps9
Viridiplantae - ps10
Streptophyta - ps11
Charophyceae/Embriophyta - ps12
Embriophyta - ps13
Tracheophyta - ps14
Spermatophyta - ps15
Angiosperms - ps16
Mesangiospermae - ps17
Magnoliids/eudicots - ps18
Eudicots - ps19
Core_eudicots - ps20
Superrosids - ps21
Rosids - ps22
Eurosids - ps23
Malvids - ps24
Sap./Malv./Brassicales - ps25
Malvales/Brassicales - ps26
Brassicales - ps27
Cleomaceae/Brassicaceae - ps28
Brassicaceae - ps29
Camelineae - ps30
Arabidopsis - ps31
Arabidopsis_thaliana - ps32
p-value
3e-07
5e-06
1e-04
2e-03
4e-02
Log-odds
0.5
1.0
1.5
2.0
2.5
3.0
3.5
4.0

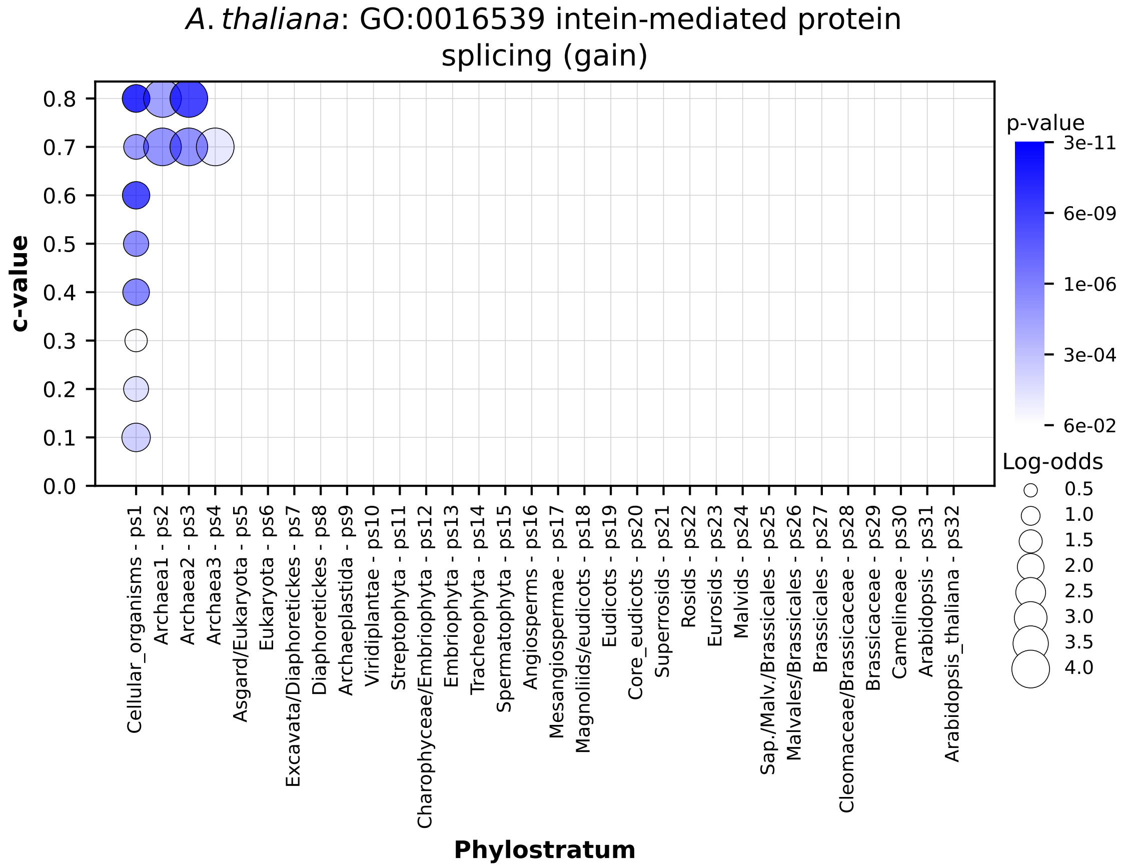

A. thaliana: GO:0016539 intein-mediated protein splicing (gain)
c-value
0.0
0.1
0.2
0.3
0.4
0.5
0.6
0.7
0.8
p-value
3e-11
6e-09
1e-06
3e-04
6e-02
Log-odds
0.5
1.0
1.5
2.0
2.5
3.0
3.5
4.0
Cellular_organisms - ps1
Archaea1 - ps2
Archaea2 - ps3
Archaea3 - ps4
Asgard/Eukaryota - ps5
Eukaryota - ps6
Excavata/Diaphoretickes - ps7
Diaphoretickes - ps8
Archaeplastida - ps9
Viridiplantae - ps10
Streptophyta - ps11
Charophyceae/Embriophyta - ps12
Embriophyta - ps13
Tracheophyta - ps14
Spermatophyta - ps15
Angiosperms - ps16
Mesangiospermae - ps17
Magnoliids/eudicots - ps18
Eudicots - ps19
Core_eudicots - ps20
Superrosids - ps21
Rosids - ps22
Eurosids - ps23
Malvids - ps24
Sap./Malv./Brassicales - ps25
Malvales/Brassicales - ps26
Brassicales - ps27
Cleomaceae/Brassicaceae - ps28
Brassicaceae - ps29
Camelineae - ps30
Arabidopsis - ps31
Arabidopsis_thaliana - ps32
Phylostratum

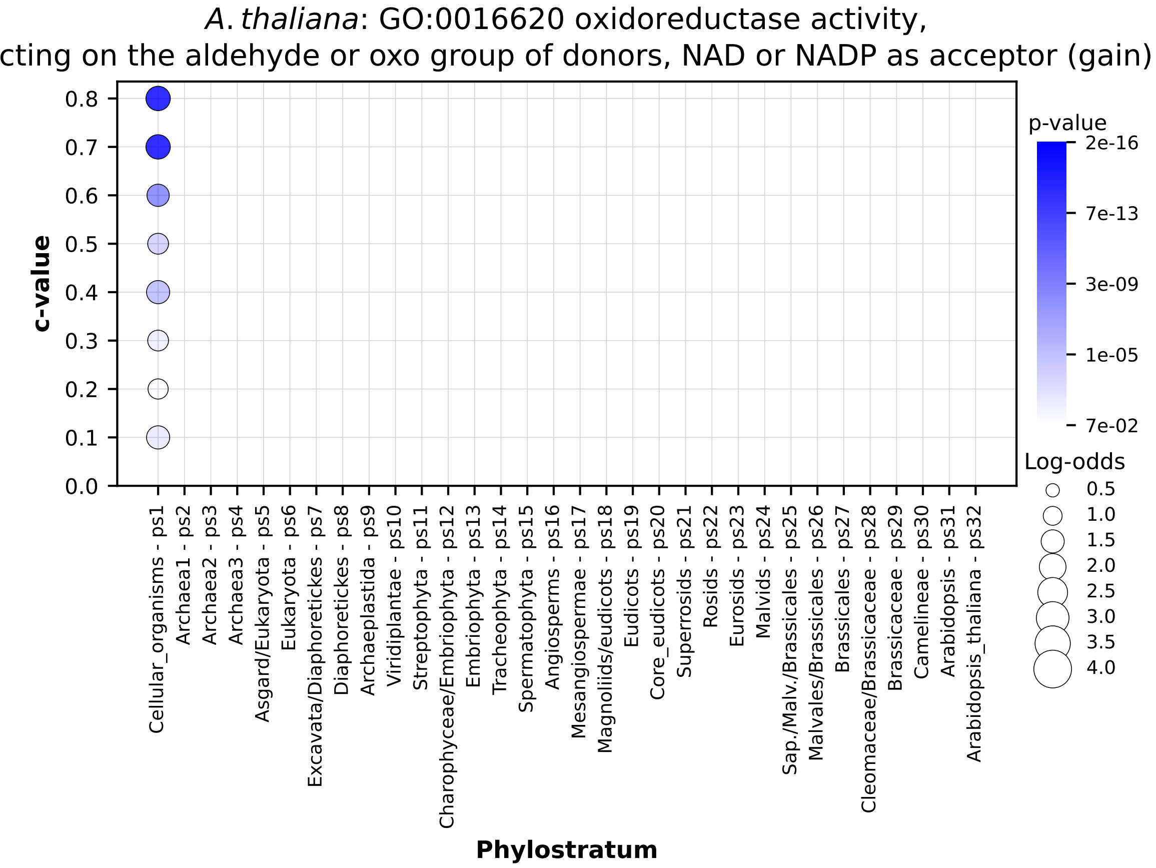
A. thaliana: GO:0016620 oxidoreductase activity,
cting on the aldehyde or oxo group of donors, NAD or NADP as acceptor (gain)
c-value
0.0
0.1
0.2
0.3
0.4
0.5
0.6
0.7
0.8
p-value
2e-16
7e-13
3e-09
1e-05
7e-02
Log-odds
0.5
1.0
1.5
2.0
2.5
3.0
3.5
4.0
Cellular_organisms - ps1
Archaea1 - ps2
Archaea2 - ps3
Archaea3 - ps4
Asgard/Eukaryota - ps5
Eukaryota - ps6
Excavata/Diaphoretickes - ps7
Diaphoretickes - ps8
Archaeplastida - ps9
Viridiplantae - ps10
Streptophyta - ps11
Charophyceae/Embriophyta - ps12
Embriophyta - ps13
Tracheophyta - ps14
Spermatophyta - ps15
Angiosperms - ps16
Mesangiospermae - ps17
Magnoliids/eudicots - ps18
Eudicots - ps19
Core_eudicots - ps20
Superrosids - ps21
Rosids - ps22
Eurosids - ps23
Malvids - ps24
Sap./Malv./Brassicales - ps25
Malvales/Brassicales - ps26
Brassicales - ps27
Cleomaceae/Brassicaceae - ps28
Brassicaceae - ps29
Camelineae - ps30
Arabidopsis - ps31
Arabidopsis_thaliana - ps32
Phylostratum

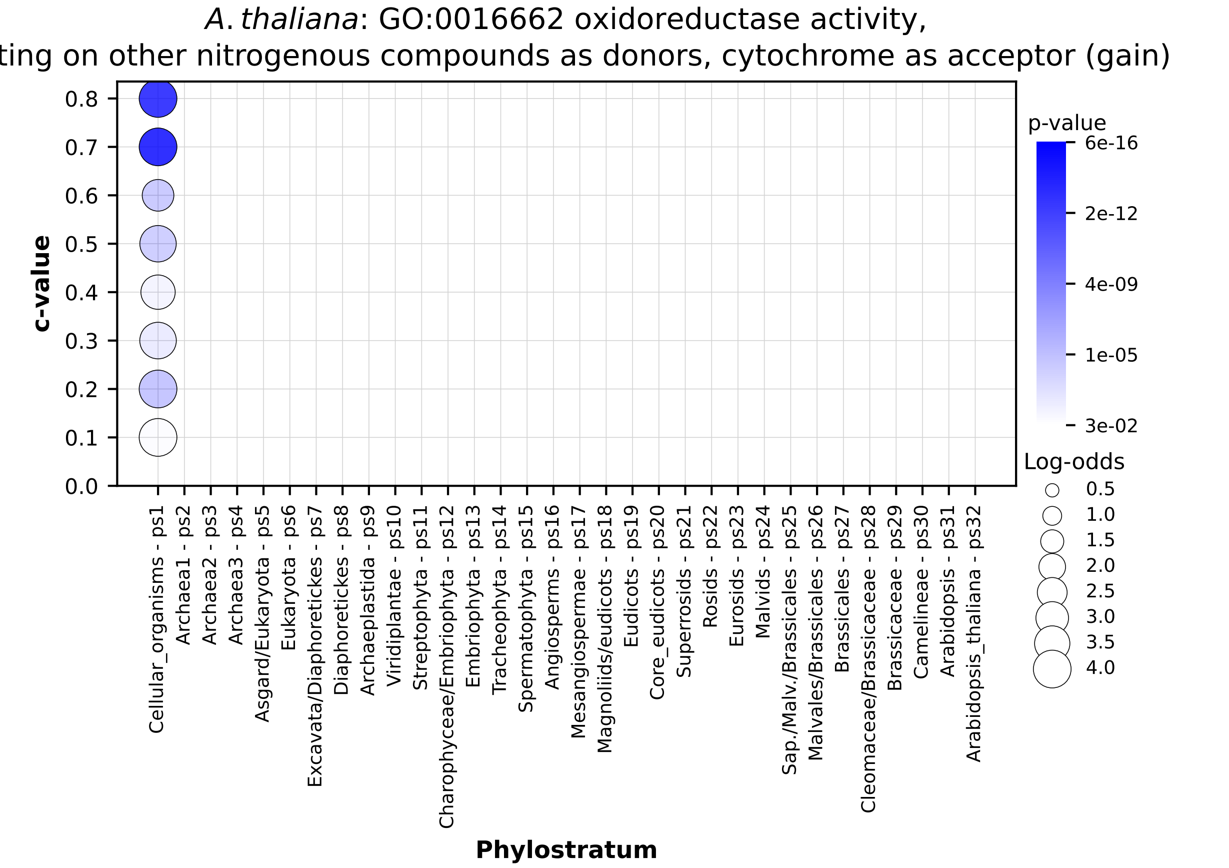

A. thaliana: GO:0016662 oxidoreductase activity,
ting on other nitrogenous compounds as donors, cytochrome as acceptor (gain)
c-value
0.0
0.1
0.2
0.3
0.4
0.5
0.6
0.7
0.8
p-value
6e-16
2e-12
4e-09
1e-05
3e-02
Log-odds
0.5
1.0
1.5
2.0
2.5
3.0
3.5
4.0
Cellular_organisms - ps1
Archaea1 - ps2
Archaea2 - ps3
Archaea3 - ps4
Asgard/Eukaryota - ps5
Eukaryota - ps6
Excavata/Diaphoretickes - ps7
Diaphoretickes - ps8
Archaeplastida - ps9
Viridiplantae - ps10
Streptophyta - ps11
Charophyceae/Embriophyta - ps12
Embriophyta - ps13
Tracheophyta - ps14
Spermatophyta - ps15
Angiosperms - ps16
Mesangiospermae - ps17
Magnoliids/eudicots - ps18
Eudicots - ps19
Core_eudicots - ps20
Superrosids - ps21
Rosids - ps22
Eurosids - ps23
Malvids - ps24
Sap./Malv./Brassicales - ps25
Malvales/Brassicales - ps26
Brassicales - ps27
Cleomaceae/Brassicaceae - ps28
Brassicaceae - ps29
Camelineae - ps30
Arabidopsis - ps31
Arabidopsis_thaliana - ps32
Phylostratum

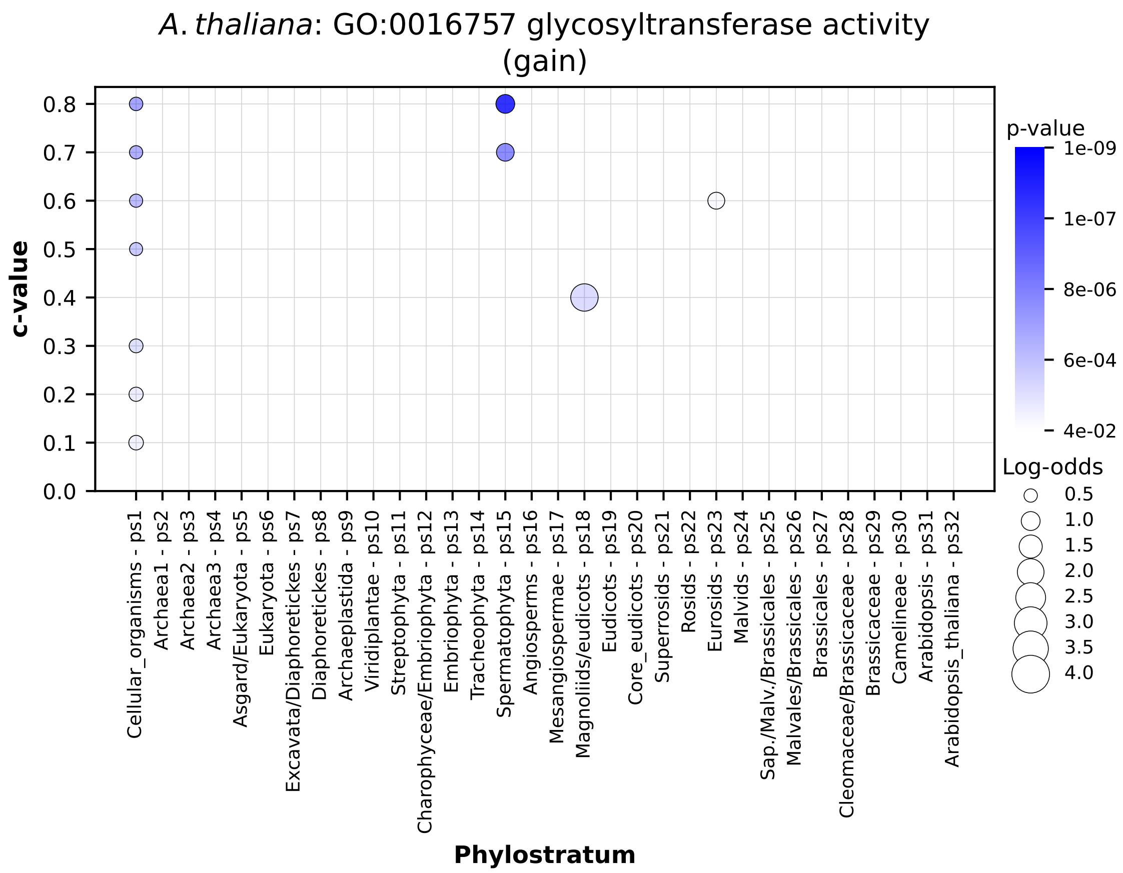

A. thaliana: GO:0016757 glycosyltransferase activity
(gain)
c-value
0.0
0.1
0.2
0.3
0.4
0.5
0.6
0.7
0.8
p-value
1e-09
1e-07
8e-06
6e-04
4e-02
Log-odds
0.5
1.0
1.5
2.0
2.5
3.0
3.5
4.0
Cellular_organisms - ps1
Archaea1 - ps2
Archaea2 - ps3
Archaea3 - ps4
Asgard/Eukaryota - ps5
Eukaryota - ps6
Excavata/Diaphoretickes - ps7
Diaphoretickes - ps8
Archaeplastida - ps9
Viridiplantae - ps10
Streptophyta - ps11
Charophyceae/Embriophyta - ps12
Embriophyta - ps13
Tracheophyta - ps14
Spermatophyta - ps15
Angiosperms - ps16
Mesangiospermae - ps17
Magnoliids/eudicots - ps18
Eudicots - ps19
Core_eudicots - ps20
Superrosids - ps21
Rosids - ps22
Eurosids - ps23
Malvids - ps24
Sap./Malv./Brassicales - ps25
Malvales/Brassicales - ps26
Brassicales - ps27
Cleomaceae/Brassicaceae - ps28
Brassicaceae - ps29
Camelineae - ps30
Arabidopsis - ps31
Arabidopsis_thaliana - ps32
Phylostratum

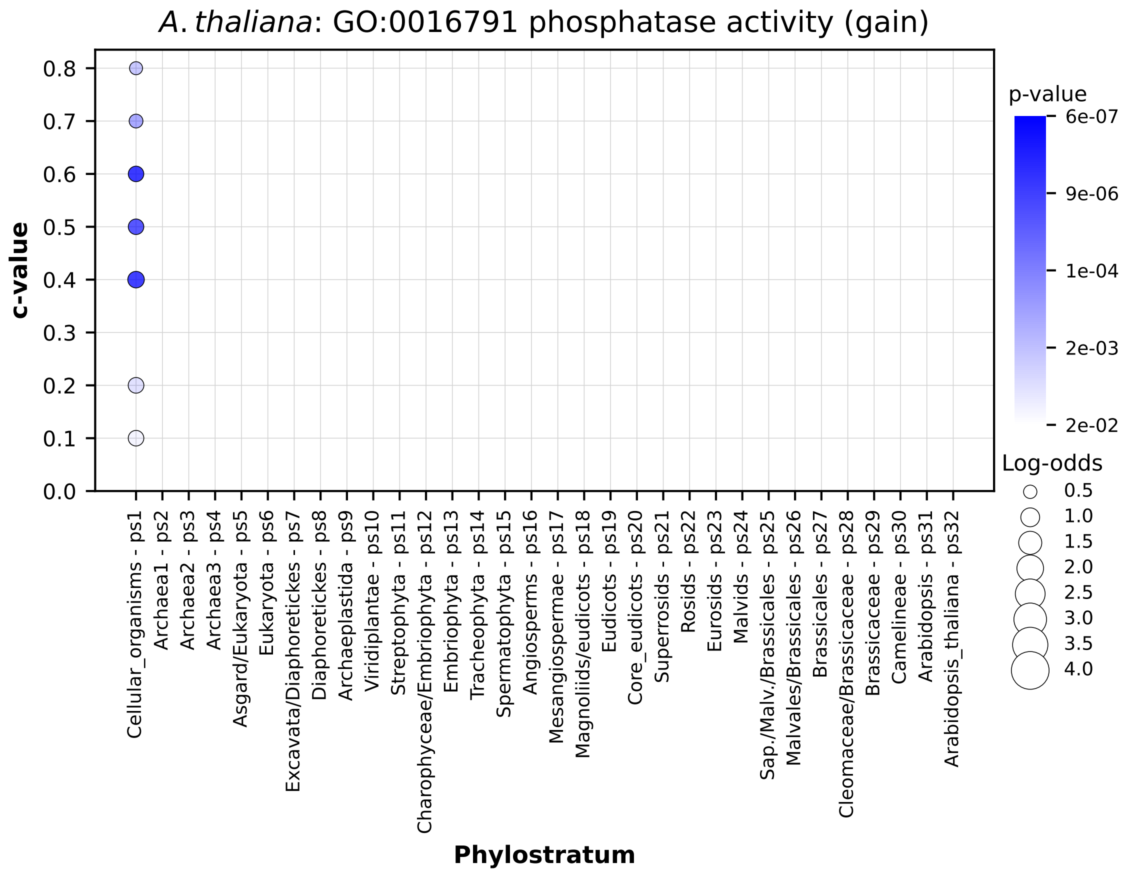
A. thaliana: GO:0016791 phosphatase activity (gain)
c-value
0.0
0.1
0.2
0.3
0.4
0.5
0.6
0.7
0.8
Phylostratum
Cellular_organisms - ps1
Archaea1 - ps2
Archaea2 - ps3
Archaea3 - ps4
Asgard/Eukaryota - ps5
Eukaryota - ps6
Excavata/Diaphoretickes - ps7
Diaphoretickes - ps8
Archaeplastida - ps9
Viridiplantae - ps10
Streptophyta - ps11
Charophyceae/Embriophyta - ps12
Embriophyta - ps13
Tracheophyta - ps14
Spermatophyta - ps15
Angiosperms - ps16
Mesangiospermae - ps17
Magnoliids/eudicots - ps18
Eudicots - ps19
Core_eudicots - ps20
Superrosids - ps21
Rosids - ps22
Eurosids - ps23
Malvids - ps24
Sap./Malv./Brassicales - ps25
Malvales/Brassicales - ps26
Brassicales - ps27
Cleomaceae/Brassicaceae - ps28
Brassicaceae - ps29
Camelineae - ps30
Arabidopsis - ps31
Arabidopsis_thaliana - ps32
p-value
6e-07
9e-06
1e-04
2e-03
2e-02
Log-odds
0.5
1.0
1.5
2.0
2.5
3.0
3.5
4.0

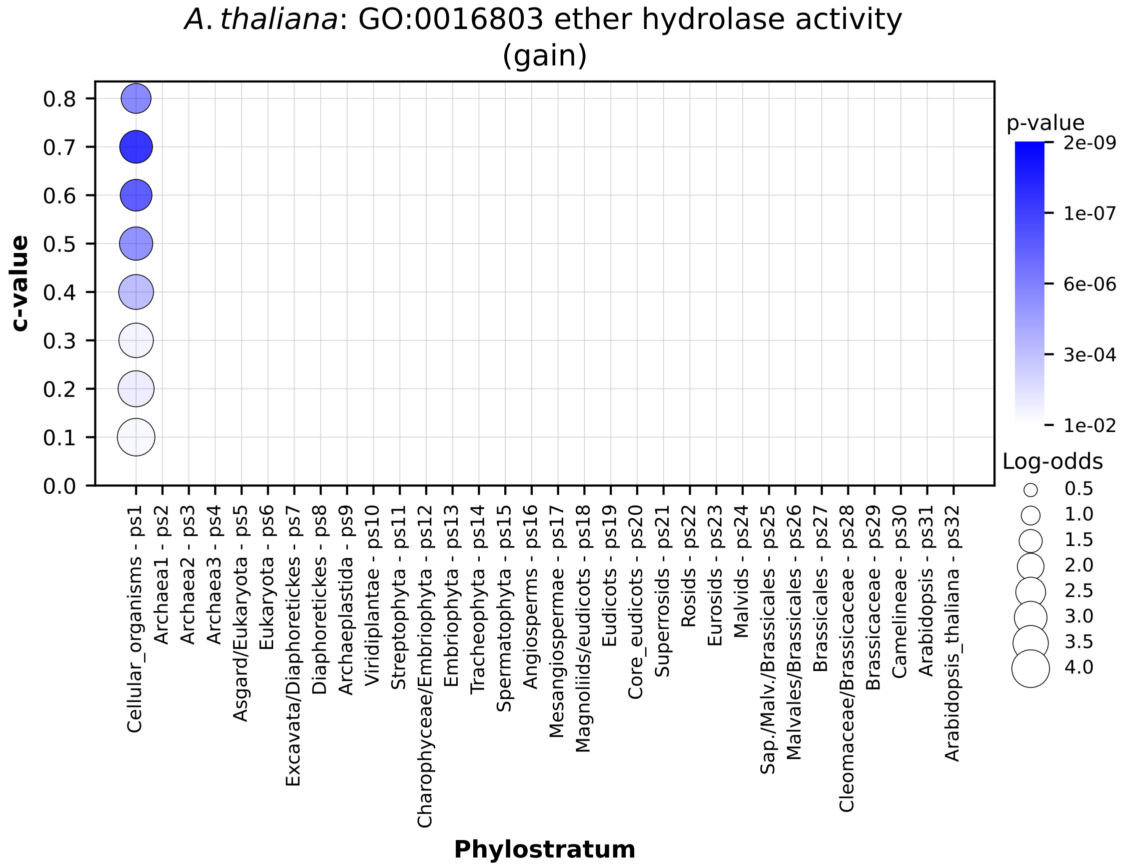
A. thaliana: GO:0016803 ether hydrolase activity
(gain)
c-value
0.0
0.1
0.2
0.3
0.4
0.5
0.6
0.7
0.8
p-value
2e-09
1e-07
6e-06
3e-04
1e-02
Log-odds
0.5
1.0
1.5
2.0
2.5
3.0
3.5
4.0
Cellular_organisms - ps1
Archaea1 - ps2
Archaea2 - ps3
Archaea3 - ps4
Asgard/Eukaryota - ps5
Eukaryota - ps6
Excavata/Diaphoretickes - ps7
Diaphoretickes - ps8
Archaeplastida - ps9
Viridiplantae - ps10
Streptophyta - ps11
Charophyceae/Embriophyta - ps12
Embriophyta - ps13
Tracheophyta - ps14
Spermatophyta - ps15
Angiosperms - ps16
Mesangiospermae - ps17
Magnoliids/eudicots - ps18
Eudicots - ps19
Core_eudicots - ps20
Superrosids - ps21
Rosids - ps22
Eurosids - ps23
Malvids - ps24
Sap./Malv./Brassicales - ps25
Malvales/Brassicales - ps26
Brassicales - ps27
Cleomaceae/Brassicaceae - ps28
Brassicaceae - ps29
Camelineae - ps30
Arabidopsis - ps31
Arabidopsis_thaliana - ps32
Phylostratum

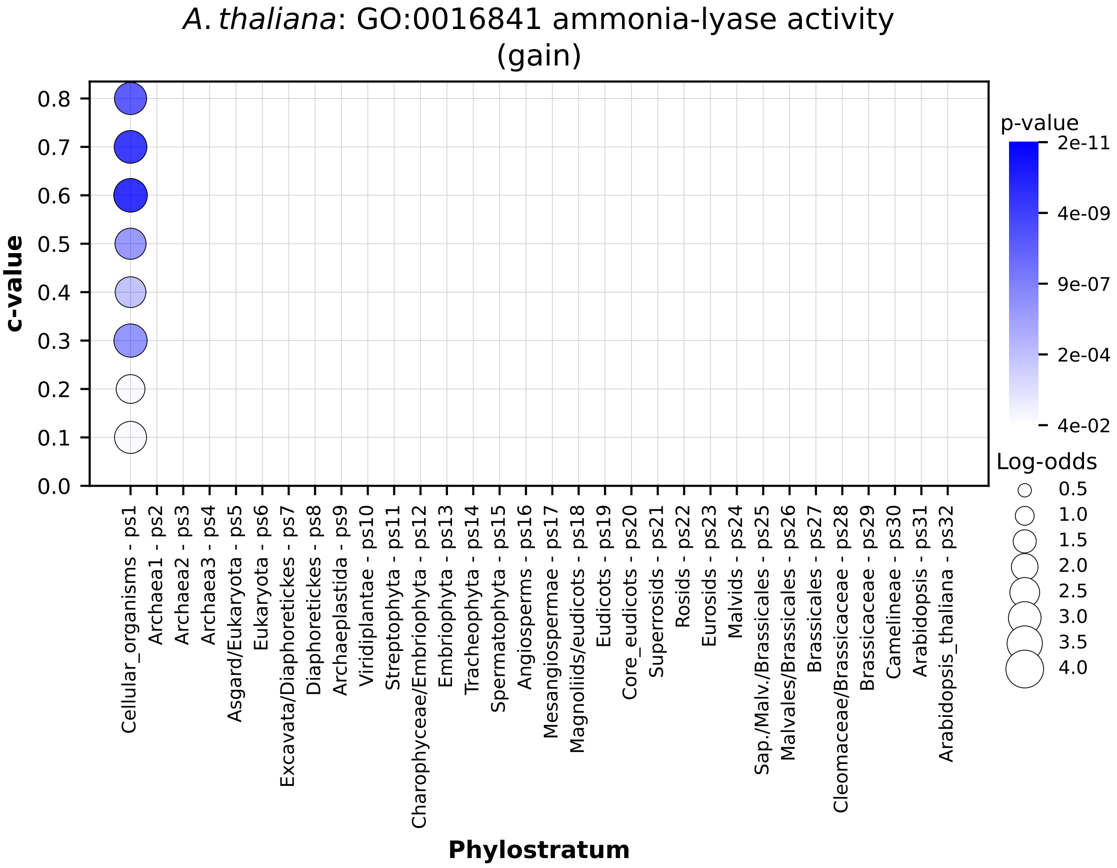

A. thaliana: GO:0016841 ammonia-lyase activity
(gain)
c-value
0.0
0.1
0.2
0.3
0.4
0.5
0.6
0.7
0.8
Phylostratum
Cellular_organisms - ps1
Archaea1 - ps2
Archaea2 - ps3
Archaea3 - ps4
Asgard/Eukaryota - ps5
Eukaryota - ps6
Excavata/Diaphoretickes - ps7
Diaphoretickes - ps8
Archaeplastida - ps9
Viridiplantae - ps10
Streptophyta - ps11
Charophyceae/Embriophyta - ps12
Embriophyta - ps13
Tracheophyta - ps14
Spermatophyta - ps15
Angiosperms - ps16
Mesangiospermae - ps17
Magnoliids/eudicots - ps18
Eudicots - ps19
Core_eudicots - ps20
Superrosids - ps21
Rosids - ps22
Eurosids - ps23
Malvids - ps24
Sap./Malv./Brassicales - ps25
Malvales/Brassicales - ps26
Brassicales - ps27
Cleomaceae/Brassicaceae - ps28
Brassicaceae - ps29
Camelineae - ps30
Arabidopsis - ps31
Arabidopsis_thaliana - ps32
p-value
2e-11
4e-09
9e-07
2e-04
4e-02
Log-odds
0.5
1.0
1.5
2.0
2.5
3.0
3.5
4.0

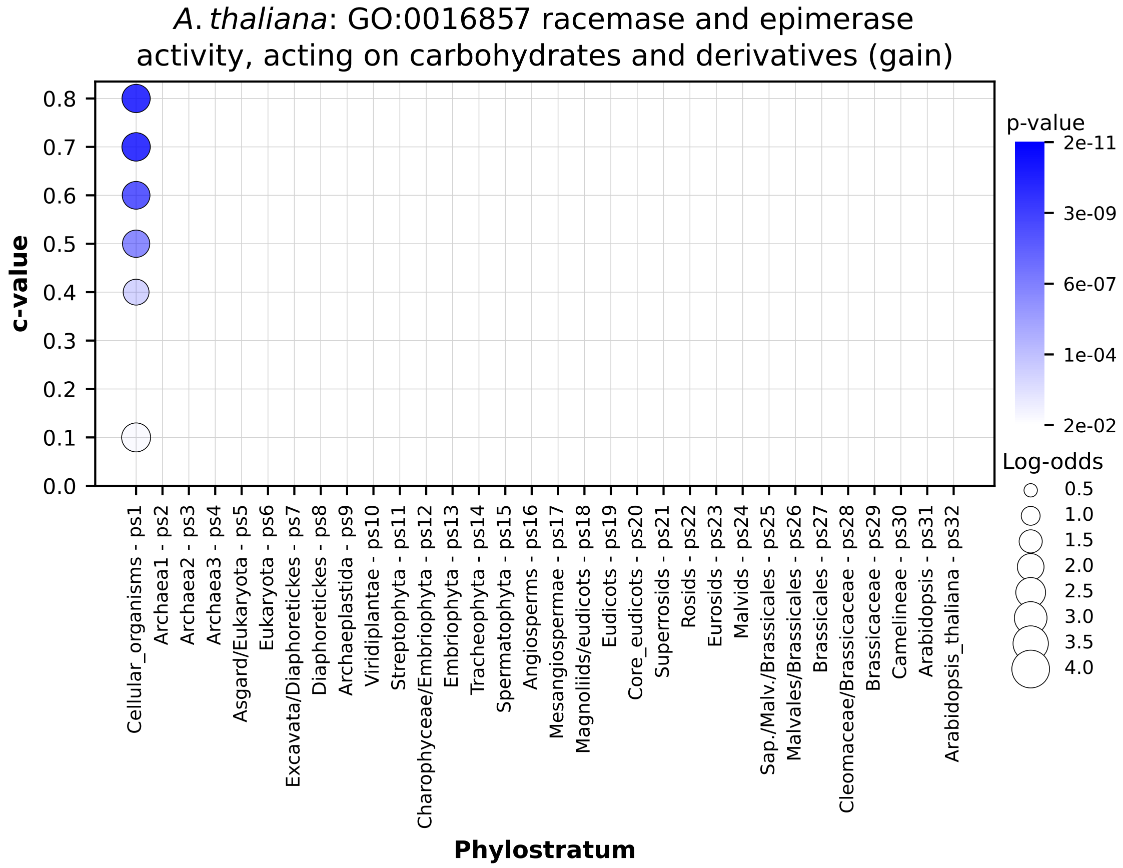
A. thaliana: GO:0016857 racemase and epimerase activity, acting on carbohydrates and derivatives (gain)
c-value
0.0
0.1
0.2
0.3
0.4
0.5
0.6
0.7
0.8
Phylostratum
Cellular_organisms - ps1
Archaea1 - ps2
Archaea2 - ps3
Archaea3 - ps4
Asgard/Eukaryota - ps5
Eukaryota - ps6
Excavata/Diaphoretickes - ps7
Diaphoretickes - ps8
Archaeplastida - ps9
Viridiplantae - ps10
Streptophyta - ps11
Charophyceae/Embriophyta - ps12
Embriophyta - ps13
Tracheophyta - ps14
Spermatophyta - ps15
Angiosperms - ps16
Mesangiospermae - ps17
Magnoliids/eudicots - ps18
Eudicots - ps19
Core_eudicots - ps20
Superrosids - ps21
Rosids - ps22
Eurosids - ps23
Malvids - ps24
Sap./Malv./Brassicales - ps25
Malvales/Brassicales - ps26
Brassicales - ps27
Cleomaceae/Brassicaceae - ps28
Brassicaceae - ps29
Camelineae - ps30
Arabidopsis - ps31
Arabidopsis_thaliana - ps32
p-value
2e-11
3e-09
6e-07
1e-04
2e-02
Log-odds
0.5
1.0
1.5
2.0
2.5
3.0
3.5
4.0

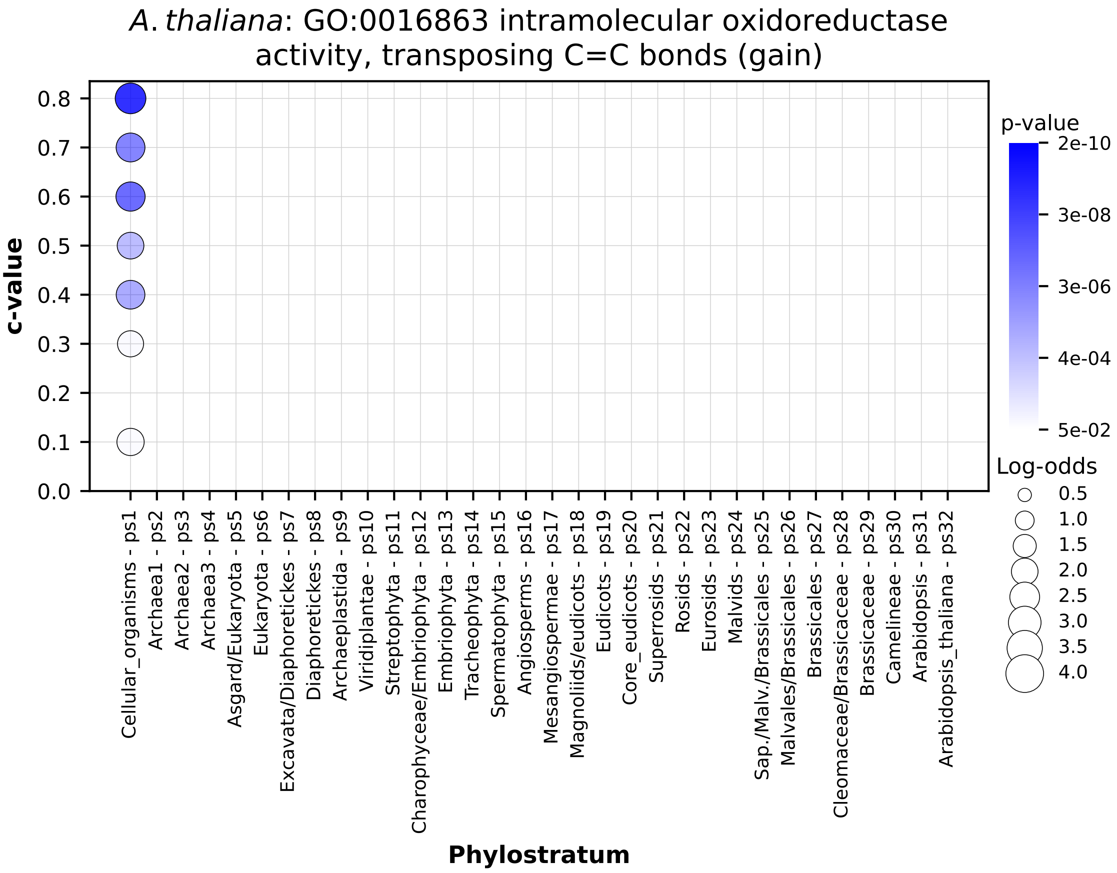

A. thaliana: GO:0016863 intramolecular oxidoreductase activity, transposing C=C bonds (gain)
c-value
0.0
0.1
0.2
0.3
0.4
0.5
0.6
0.7
0.8
p-value
2e-10
3e-08
3e-06
4e-04
5e-02
Log-odds
0.5
1.0
1.5
2.0
2.5
3.0
3.5
4.0
Cellular_organisms - ps1
Archaea1 - ps2
Archaea2 - ps3
Archaea3 - ps4
Asgard/Eukaryota - ps5
Eukaryota - ps6
Excavata/Diaphoretickes - ps7
Diaphoretickes - ps8
Archaeplastida - ps9
Viridiplantae - ps10
Streptophyta - ps11
Charophyceae/Embriophyta - ps12
Embriophyta - ps13
Tracheophyta - ps14
Spermatophyta - ps15
Angiosperms - ps16
Mesangiospermae - ps17
Magnoliids/eudicots - ps18
Eudicots - ps19
Core_eudicots - ps20
Superrosids - ps21
Rosids - ps22
Eurosids - ps23
Malvids - ps24
Sap./Malv./Brassicales - ps25
Malvales/Brassicales - ps26
Brassicales - ps27
Cleomaceae/Brassicaceae - ps28
Brassicaceae - ps29
Camelineae - ps30
Arabidopsis - ps31
Arabidopsis_thaliana - ps32
Phylostratum

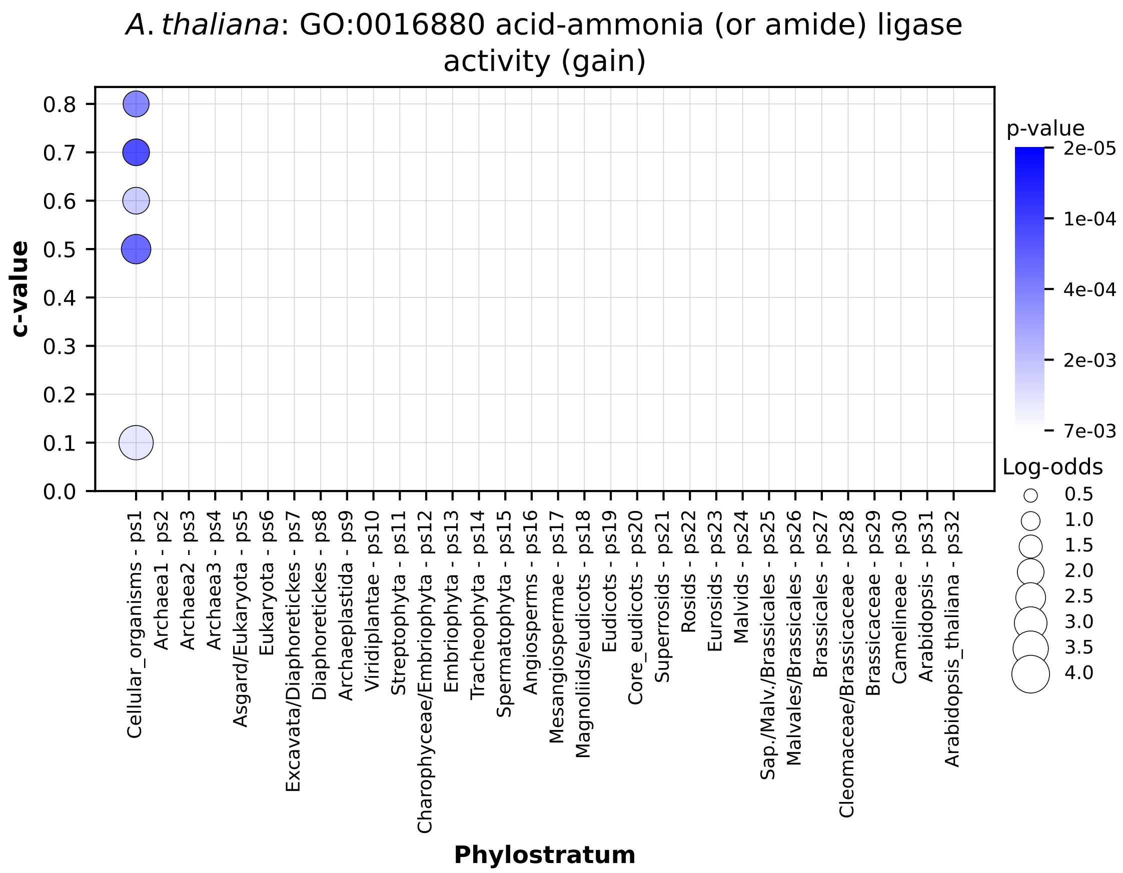

A. thaliana: GO:0016880 acid-ammonia (or amide) ligase activity (gain)
c-value
0.0
0.1
0.2
0.3
0.4
0.5
0.6
0.7
0.8
Phylostratum
Cellular_organisms - ps1
Archaea1 - ps2
Archaea2 - ps3
Archaea3 - ps4
Asgard/Eukaryota - ps5
Eukaryota - ps6
Excavata/Diaphoretickes - ps7
Diaphoretickes - ps8
Archaeplastida - ps9
Viridiplantae - ps10
Streptophyta - ps11
Charophyceae/Embriophyta - ps12
Embriophyta - ps13
Tracheophyta - ps14
Spermatophyta - ps15
Angiosperms - ps16
Mesangiospermae - ps17
Magnoliids/eudicots - ps18
Eudicots - ps19
Core_eudicots - ps20
Superrosids - ps21
Rosids - ps22
Eurosids - ps23
Malvids - ps24
Sap./Malv./Brassicales - ps25
Malvales/Brassicales - ps26
Brassicales - ps27
Cleomaceae/Brassicaceae - ps28
Brassicaceae - ps29
Camelineae - ps30
Arabidopsis - ps31
Arabidopsis_thaliana - ps32
p-value
2e-05
1e-04
4e-04
2e-03
7e-03
Log-odds
0.5
1.0
1.5
2.0
2.5
3.0
3.5
4.0

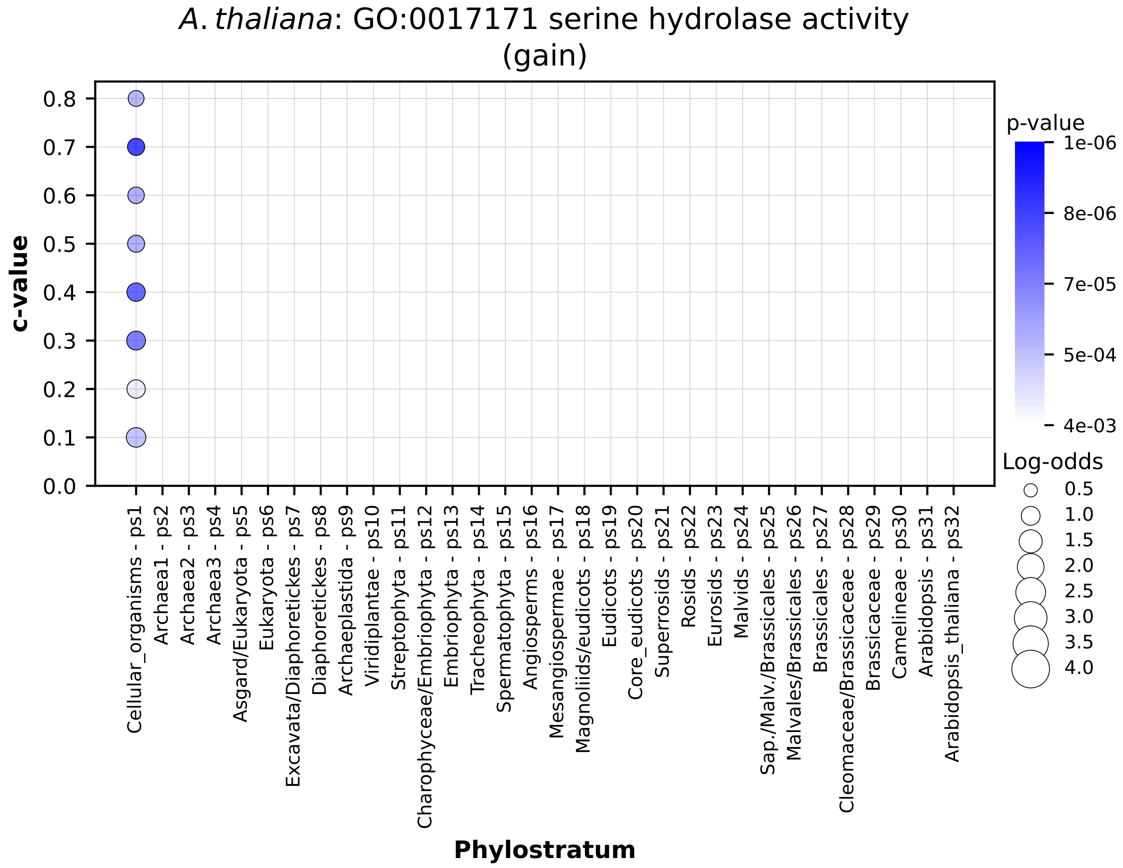
A. thaliana: GO:0017171 serine hydrolase activity
(gain)
c-value
0.0
0.1
0.2
0.3
0.4
0.5
0.6
0.7
0.8
Phylostratum
Cellular_organisms - ps1
Archaea1 - ps2
Archaea2 - ps3
Archaea3 - ps4
Asgard/Eukaryota - ps5
Eukaryota - ps6
Excavata/Diaphoretickes - ps7
Diaphoretickes - ps8
Archaeplastida - ps9
Viridiplantae - ps10
Streptophyta - ps11
Charophyceae/Embriophyta - ps12
Embriophyta - ps13
Tracheophyta - ps14
Spermatophyta - ps15
Angiosperms - ps16
Mesangiospermae - ps17
Magnoliids/eudicots - ps18
Eudicots - ps19
Core_eudicots - ps20
Superrosids - ps21
Rosids - ps22
Eurosids - ps23
Malvids - ps24
Sap./Malv./Brassicales - ps25
Malvales/Brassicales - ps26
Brassicales - ps27
Cleomaceae/Brassicaceae - ps28
Brassicaceae - ps29
Camelineae - ps30
Arabidopsis - ps31
Arabidopsis_thaliana - ps32
p-value
1e-06
8e-06
7e-05
5e-04
4e-03
Log-odds
0.5
1.0
1.5
2.0
2.5
3.0
3.5
4.0

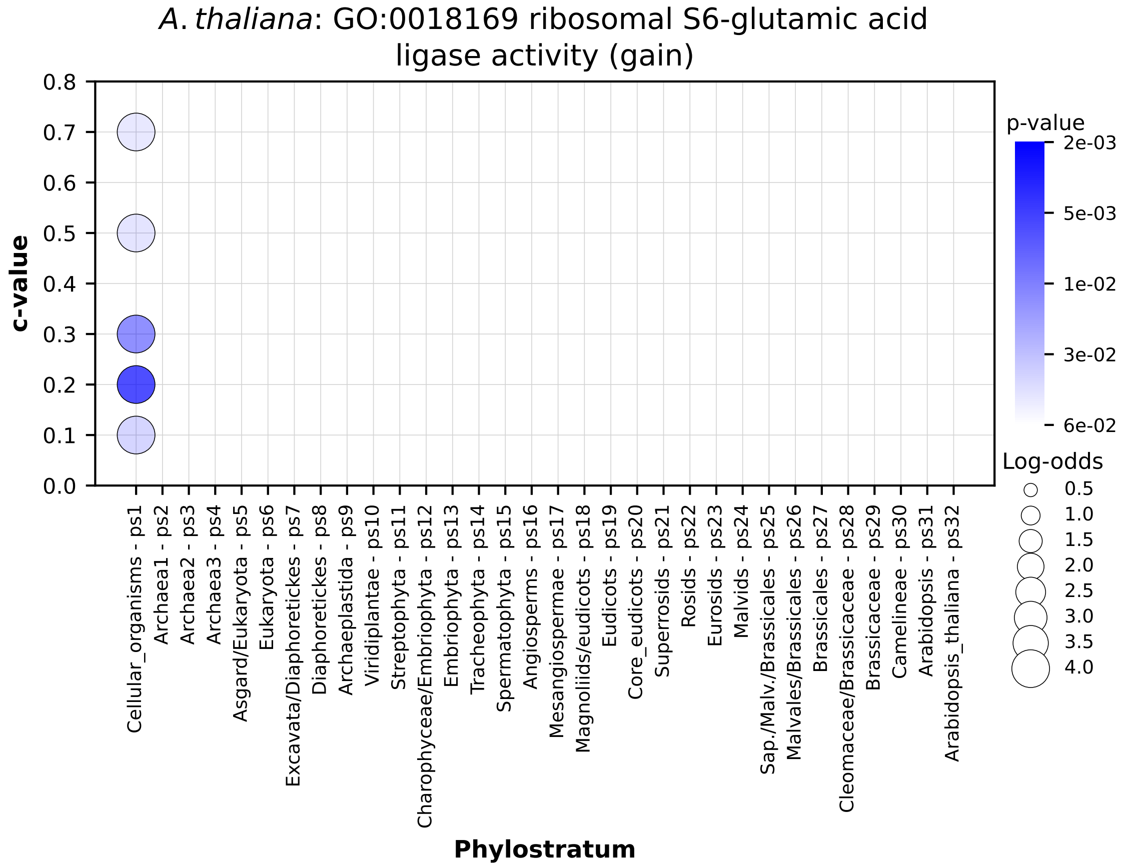

A. thaliana: GO:0018169 ribosomal S6-glutamic acid ligase activity (gain)
c-value
0.8
0.7
0.6
0.5
0.4
0.3
0.2
0.1
0.0
p-value
2e-03
5e-03
1e-02
3e-02
6e-02
Log-odds
0.5
1.0
1.5
2.0
2.5
3.0
3.5
4.0
Cellular_organisms - ps1
Archaea1 - ps2
Archaea2 - ps3
Archaea3 - ps4
Asgard/Eukaryota - ps5
Eukaryota - ps6
Excavata/Diaphoretickes - ps7
Diaphoretickes - ps8
Archaeplastida - ps9
Viridiplantae - ps10
Streptophyta - ps11
Charophyceae/Embriophyta - ps12
Embriophyta - ps13
Tracheophyta - ps14
Spermatophyta - ps15
Angiosperms - ps16
Mesangiospermae - ps17
Magnoliids/eudicots - ps18
Eudicots - ps19
Core_eudicots - ps20
Superrosids - ps21
Rosids - ps22
Eurosids - ps23
Malvids - ps24
Sap./Malv./Brassicales - ps25
Malvales/Brassicales - ps26
Brassicales - ps27
Cleomaceae/Brassicaceae - ps28
Brassicaceae - ps29
Camelineae - ps30
Arabidopsis - ps31
Arabidopsis_thaliana - ps32
Phylostratum

A. thaliana: GO:0019295 coenzyme M biosynthetic process (gain)

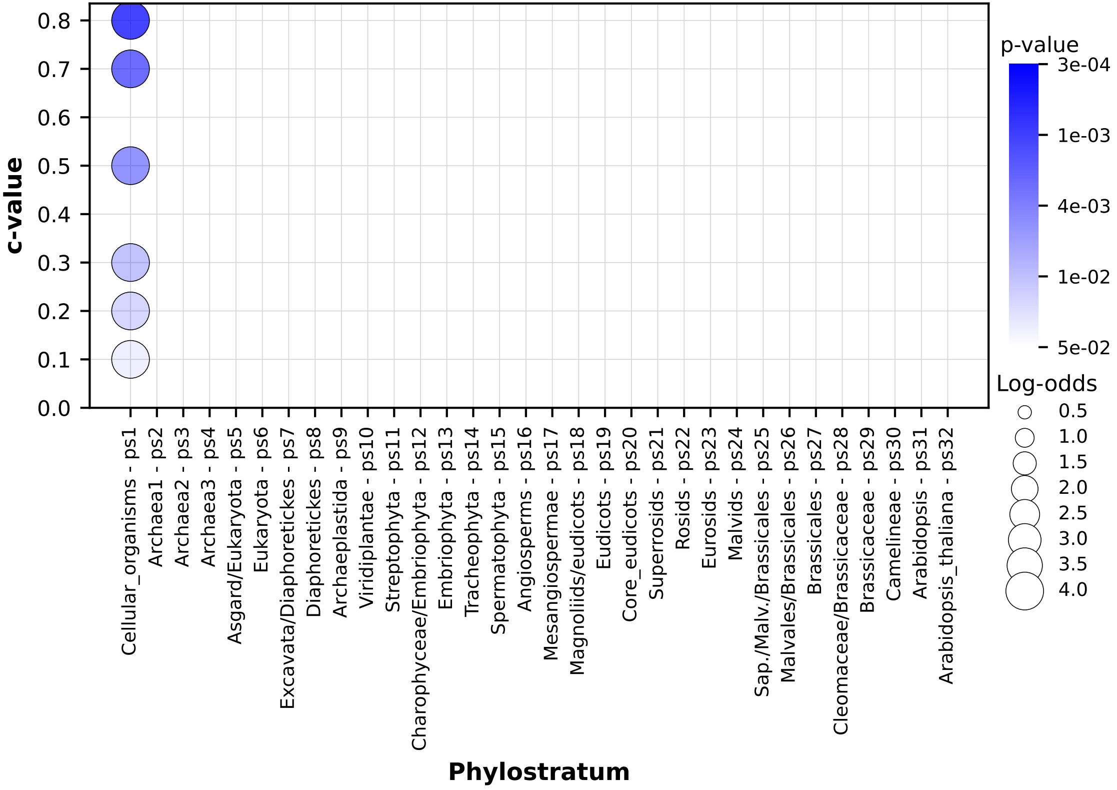

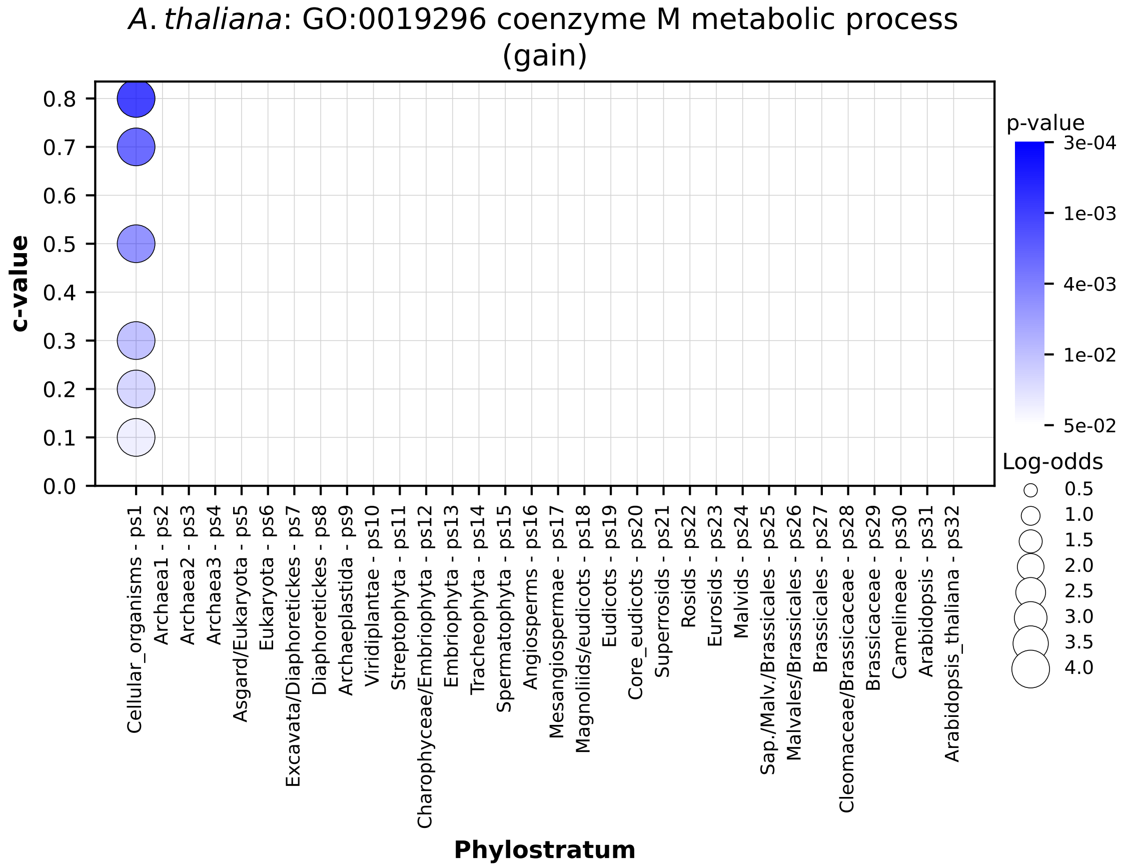

A. thaliana: GO:0019296 coenzyme M metabolic process (gain)
c-value
0.0
0.1
0.2
0.3
0.4
0.5
0.6
0.7
0.8
p-value
3e-04
1e-03
4e-03
1e-02
5e-02
Log-odds
0.5
1.0
1.5
2.0
2.5
3.0
3.5
4.0
Cellular_organisms - ps1
Archaea1 - ps2
Archaea2 - ps3
Archaea3 - ps4
Asgard/Eukaryota - ps5
Eukaryota - ps6
Excavata/Diaphoretickes - ps7
Diaphoretickes - ps8
Archaeplastida - ps9
Viridiplantae - ps10
Streptophyta - ps11
Charophyceae/Embriophyta - ps12
Embriophyta - ps13
Tracheophyta - ps14
Spermatophyta - ps15
Angiosperms - ps16
Mesangiospermae - ps17
Magnoliids/eudicots - ps18
Eudicots - ps19
Core_eudicots - ps20
Superrosids - ps21
Rosids - ps22
Eurosids - ps23
Malvids - ps24
Sap./Malv./Brassicales - ps25
Malvales/Brassicales - ps26
Brassicales - ps27
Cleomaceae/Brassicaceae - ps28
Brassicaceae - ps29
Camelineae - ps30
Arabidopsis - ps31
Arabidopsis_thaliana - ps32
Phylostratum

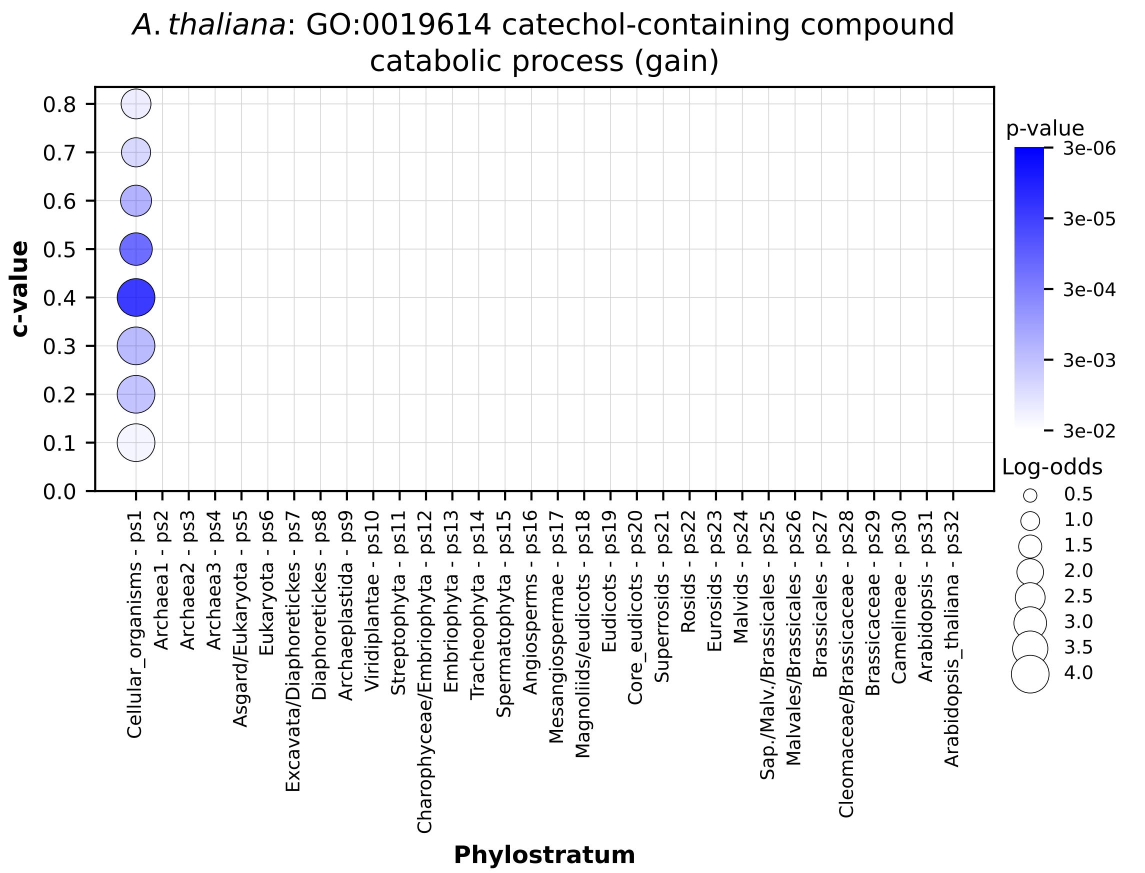
A. thaliana: GO:0019614 catechol-containing compound catabolic process (gain)
c-value
0.0
0.1
0.2
0.3
0.4
0.5
0.6
0.7
0.8
p-value
3e-06
3e-05
3e-04
3e-03
3e-02
Log-odds
0.5
1.0
1.5
2.0
2.5
3.0
3.5
4.0
Cellular_organisms - ps1
Archaea1 - ps2
Archaea2 - ps3
Archaea3 - ps4
Asgard/Eukaryota - ps5
Eukaryota - ps6
Excavata/Diaphoretickes - ps7
Diaphoretickes - ps8
Archaeplastida - ps9
Viridiplantae - ps10
Streptophyta - ps11
Charophyceae/Embriophyta - ps12
Embriophyta - ps13
Tracheophyta - ps14
Spermatophyta - ps15
Angiosperms - ps16
Mesangiospermae - ps17
Magnoliids/eudicots - ps18
Eudicots - ps19
Core_eudicots - ps20
Superrosids - ps21
Rosids - ps22
Eurosids - ps23
Malvids - ps24
Sap./Malv./Brassicales - ps25
Malvales/Brassicales - ps26
Brassicales - ps27
Cleomaceae/Brassicaceae - ps28
Brassicaceae - ps29
Camelineae - ps30
Arabidopsis - ps31
Arabidopsis_thaliana - ps32
Phylostratum

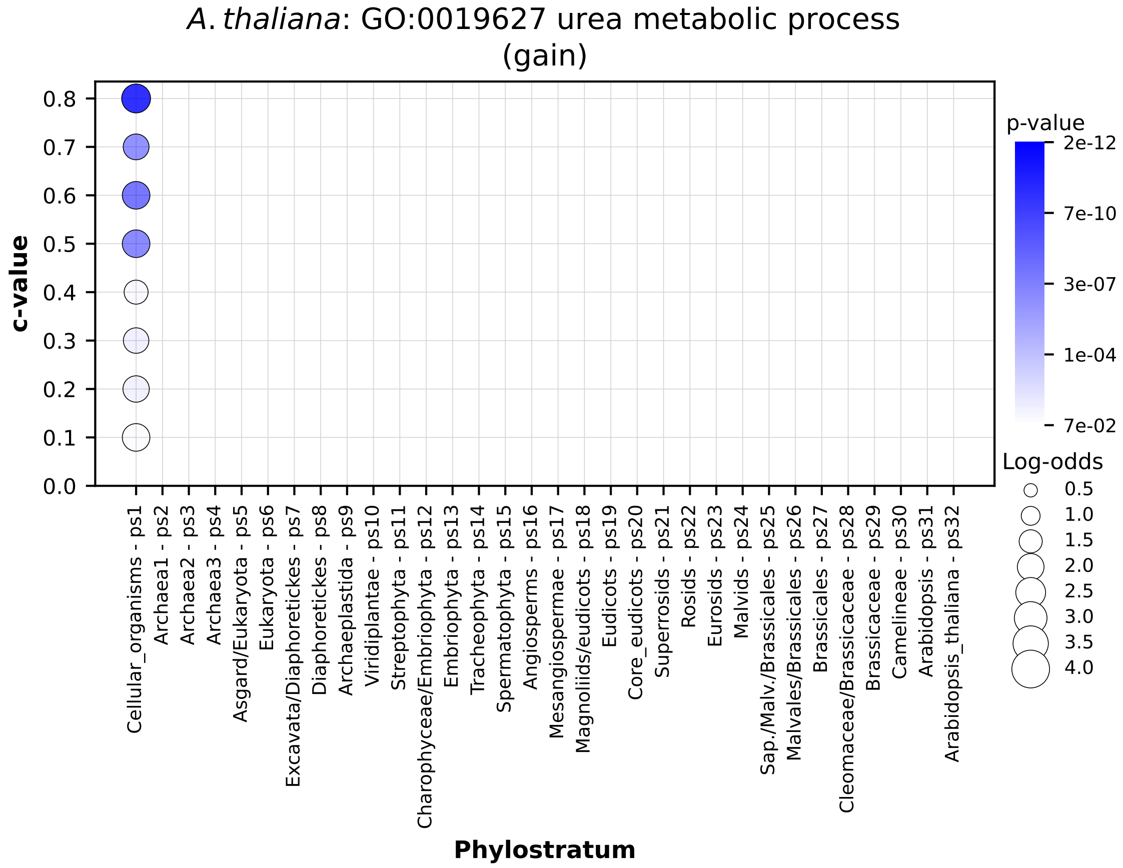
A. thaliana: GO:0019627 urea metabolic process
(gain)
c-value
0.0
0.1
0.2
0.3
0.4
0.5
0.6
0.7
0.8
p-value
2e-12
7e-10
3e-07
1e-04
7e-02
Log-odds
0.5
1.0
1.5
2.0
2.5
3.0
3.5
4.0
Cellular_organisms - ps1
Archaea1 - ps2
Archaea2 - ps3
Archaea3 - ps4
Asgard/Eukaryota - ps5
Eukaryota - ps6
Excavata/Diaphoretickes - ps7
Diaphoretickes - ps8
Archaeplastida - ps9
Viridiplantae - ps10
Streptophyta - ps11
Charophyceae/Embriophyta - ps12
Embriophyta - ps13
Tracheophyta - ps14
Spermatophyta - ps15
Angiosperms - ps16
Mesangiospermae - ps17
Magnoliids/eudicots - ps18
Eudicots - ps19
Core_eudicots - ps20
Superrosids - ps21
Rosids - ps22
Eurosids - ps23
Malvids - ps24
Sap./Malv./Brassicales - ps25
Malvales/Brassicales - ps26
Brassicales - ps27
Cleomaceae/Brassicaceae - ps28
Brassicaceae - ps29
Camelineae - ps30
Arabidopsis - ps31
Arabidopsis_thaliana - ps32
Phylostratum

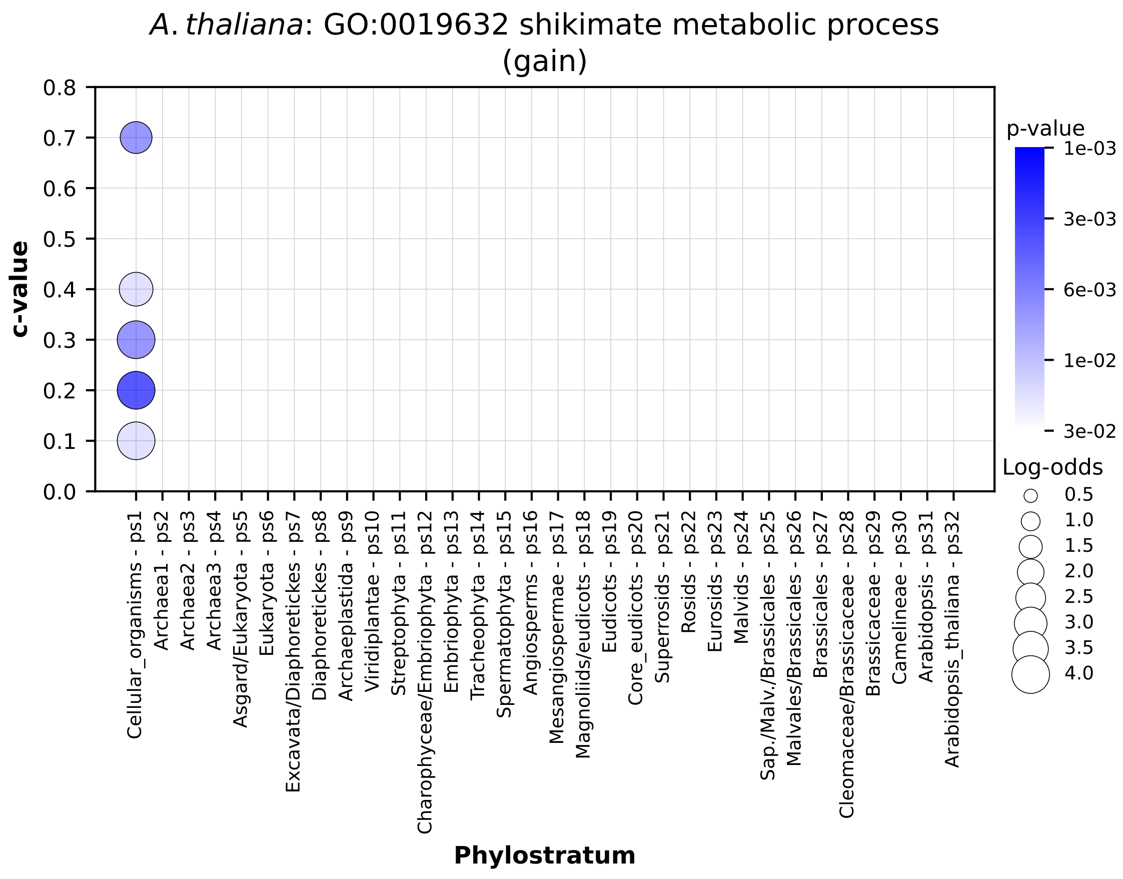
A. thaliana: GO:0019632 shikimate metabolic process
(gain)
c-value
0.8
0.7
0.6
0.5
0.4
0.3
0.2
0.1
0.0
p-value
1e-03
3e-03
6e-03
1e-02
3e-02
Log-odds
0.5
1.0
1.5
2.0
2.5
3.0
3.5
4.0
Cellular_organisms - ps1
Archaea1 - ps2
Archaea2 - ps3
Archaea3 - ps4
Asgard/Eukaryota - ps5
Eukaryota - ps6
Excavata/Diaphoretickes - ps7
Diaphoretickes - ps8
Archaeplastida - ps9
Viridiplantae - ps10
Streptophyta - ps11
Charophyceae/Embriophyta - ps12
Embriophyta - ps13
Tracheophyta - ps14
Spermatophyta - ps15
Angiosperms - ps16
Mesangiospermae - ps17
Magnoliids/eudicots - ps18
Eudicots - ps19
Core_eudicots - ps20
Superrosids - ps21
Rosids - ps22
Eurosids - ps23
Malvids - ps24
Sap./Malv./Brassicales - ps25
Malvales/Brassicales - ps26
Brassicales - ps27
Cleomaceae/Brassicaceae - ps28
Brassicaceae - ps29
Camelineae - ps30
Arabidopsis - ps31
Arabidopsis_thaliana - ps32
Phylostratum

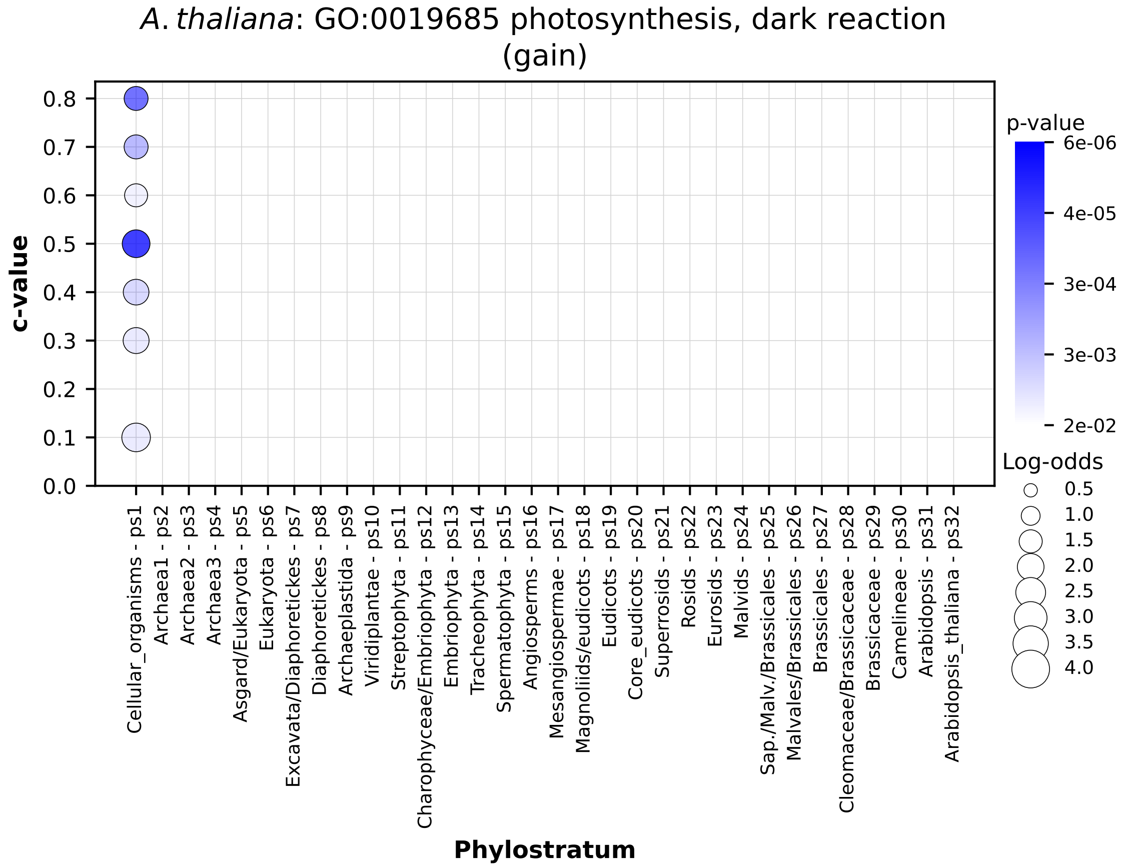
A. thaliana: GO:0019685 photosynthesis, dark reaction (gain)
c-value
0.0
0.1
0.2
0.3
0.4
0.5
0.6
0.7
0.8
p-value
6e-06
4e-05
3e-04
3e-03
2e-02
Log-odds
0.5
1.0
1.5
2.0
2.5
3.0
3.5
4.0
Cellular_organisms - ps1
Archaea1 - ps2
Archaea2 - ps3
Archaea3 - ps4
Asgard/Eukaryota - ps5
Eukaryota - ps6
Excavata/Diaphoretickes - ps7
Diaphoretickes - ps8
Archaeplastida - ps9
Viridiplantae - ps10
Streptophyta - ps11
Charophyceae/Embriophyta - ps12
Embriophyta - ps13
Tracheophyta - ps14
Spermatophyta - ps15
Angiosperms - ps16
Mesangiospermae - ps17
Magnoliids/eudicots - ps18
Eudicots - ps19
Core_eudicots - ps20
Superrosids - ps21
Rosids - ps22
Eurosids - ps23
Malvids - ps24
Sap./Malv./Brassicales - ps25
Malvales/Brassicales - ps26
Brassicales - ps27
Cleomaceae/Brassicaceae - ps28
Brassicaceae - ps29
Camelineae - ps30
Arabidopsis - ps31
Arabidopsis_thaliana - ps32
Phylostratum

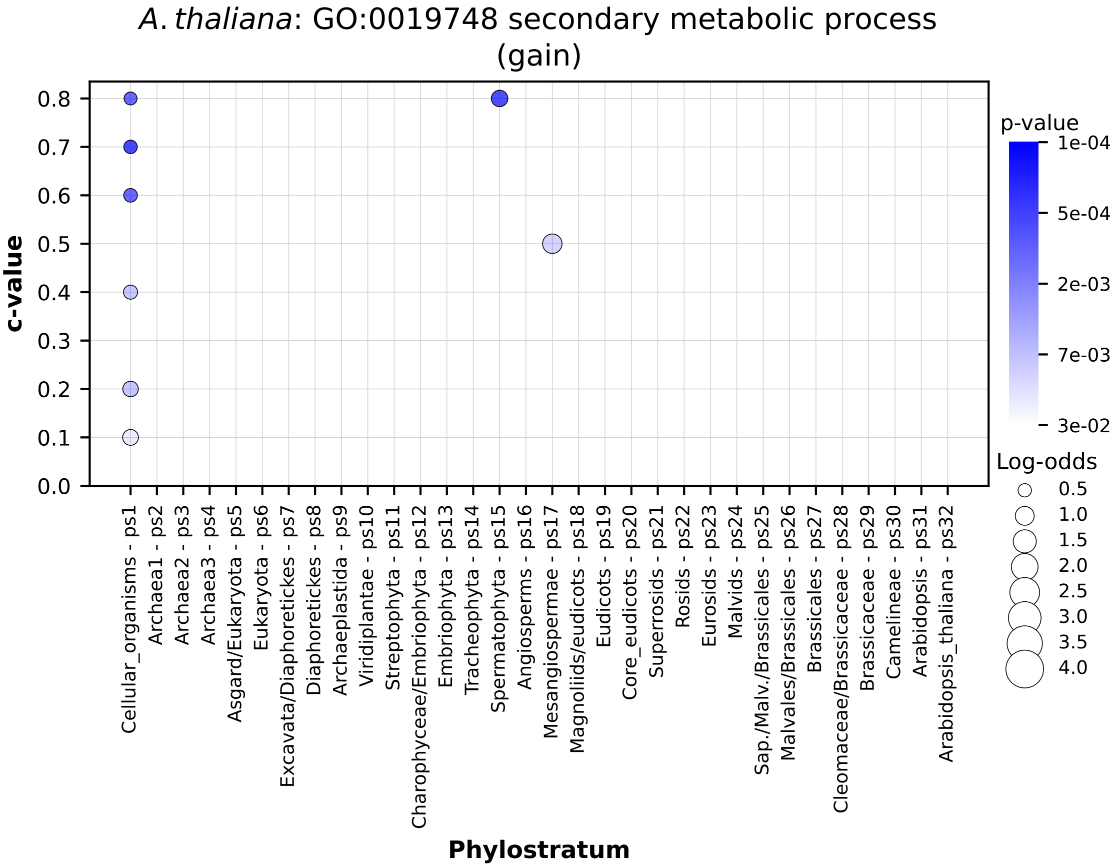

A. thaliana: GO:0019748 secondary metabolic process
(gain)
c-value
0.8
0.7
0.6
0.5
0.4
0.3
0.2
0.1
0.0
Phylostratum
Cellular_organisms - ps1
Archaea1 - ps2
Archaea2 - ps3
Archaea3 - ps4
Asgard/Eukaryota - ps5
Eukaryota - ps6
Excavata/Diaphoretickes - ps7
Diaphoretickes - ps8
Archaeplastida - ps9
Viridiplantae - ps10
Streptophyta - ps11
Charophyceae/Embriophyta - ps12
Embriophyta - ps13
Tracheophyta - ps14
Spermatophyta - ps15
Angiosperms - ps16
Mesangiospermae - ps17
Magnoliids/eudicots - ps18
Eudicots - ps19
Core_eudicots - ps20
Superrosids - ps21
Rosids - ps22
Eurosids - ps23
Malvids - ps24
Sap./Malv./Brassicales - ps25
Malvales/Brassicales - ps26
Brassicales - ps27
Cleomaceae/Brassicaceae - ps28
Brassicaceae - ps29
Camelineae - ps30
Arabidopsis - ps31
Arabidopsis_thaliana - ps32
p-value
1e-04
5e-04
2e-03
7e-03
3e-02
Log-odds
0.5
1.0
1.5
2.0
2.5
3.0
3.5
4.0

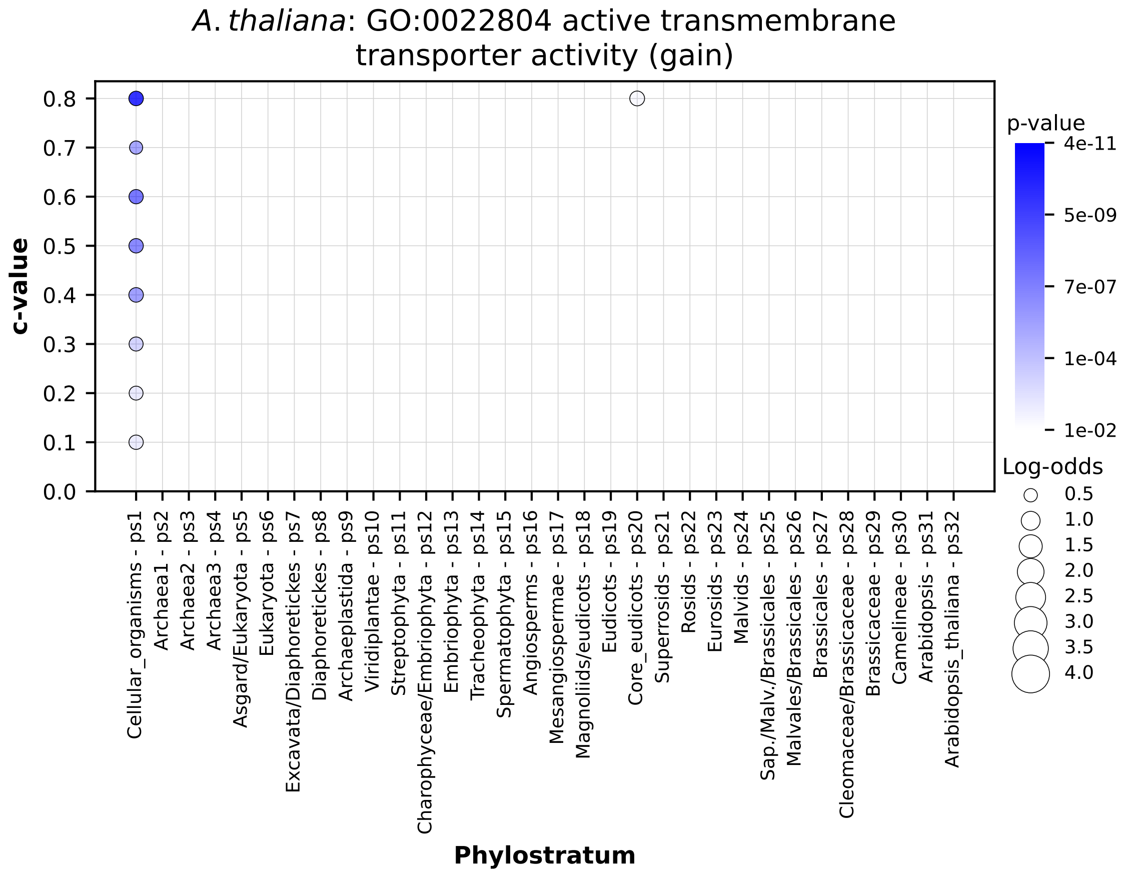

A. thaliana: GO:0022804 active transmembrane transporter activity (gain)
c-value
0.0
0.1
0.2
0.3
0.4
0.5
0.6
0.7
0.8
Phylostratum
Cellular_organisms - ps1
Archaea1 - ps2
Archaea2 - ps3
Archaea3 - ps4
Asgard/Eukaryota - ps5
Eukaryota - ps6
Excavata/Diaphoretickes - ps7
Diaphoretickes - ps8
Archaeplastida - ps9
Viridiplantae - ps10
Streptophyta - ps11
Charophyceae/Embriophyta - ps12
Embriophyta - ps13
Tracheophyta - ps14
Spermatophyta - ps15
Angiosperms - ps16
Mesangiospermae - ps17
Magnoliids/eudicots - ps18
Eudicots - ps19
Core_eudicots - ps20
Superrosids - ps21
Rosids - ps22
Eurosids - ps23
Malvids - ps24
Sap./Malv./Brassicales - ps25
Malvales/Brassicales - ps26
Brassicales - ps27
Cleomaceae/Brassicaceae - ps28
Brassicaceae - ps29
Camelineae - ps30
Arabidopsis - ps31
Arabidopsis_thaliana - ps32
p-value
4e-11
5e-09
7e-07
1e-04
1e-02
Log-odds
0.5
1.0
1.5
2.0
2.5
3.0
3.5
4.0

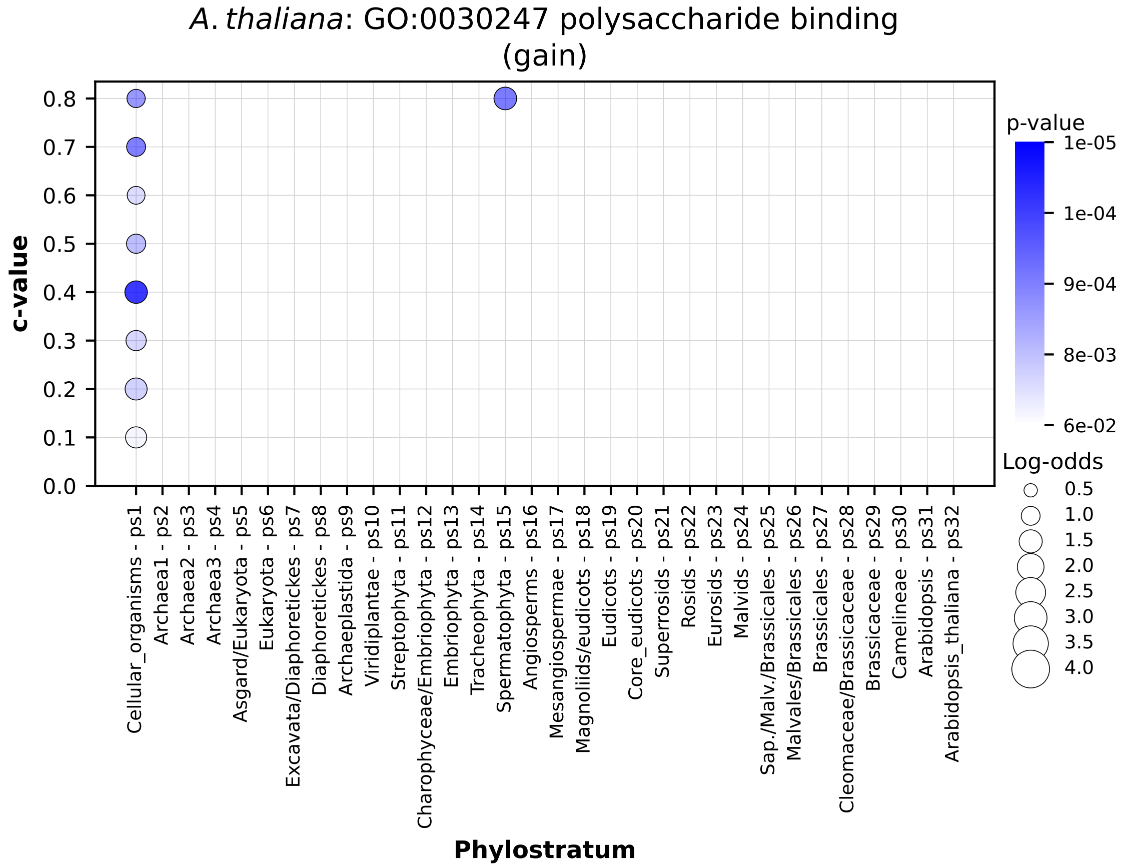
A. thaliana: GO:0030247 polysaccharide binding
(gain)
c-value
0.0
0.1
0.2
0.3
0.4
0.5
0.6
0.7
0.8
p-value
1e-05
1e-04
9e-04
8e-03
6e-02
Log-odds
0.5
1.0
1.5
2.0
2.5
3.0
3.5
4.0
Cellular_organisms - ps1
Archaea1 - ps2
Archaea2 - ps3
Archaea3 - ps4
Asgard/Eukaryota - ps5
Eukaryota - ps6
Excavata/Diaphoretickes - ps7
Diaphoretickes - ps8
Archaeplastida - ps9
Viridiplantae - ps10
Streptophyta - ps11
Charophyceae/Embriophyta - ps12
Embriophyta - ps13
Tracheophyta - ps14
Spermatophyta - ps15
Angiosperms - ps16
Mesangiospermae - ps17
Magnoliids/eudicots - ps18
Eudicots - ps19
Core_eudicots - ps20
Superrosids - ps21
Rosids - ps22
Eurosids - ps23
Malvids - ps24
Sap./Malv./Brassicales - ps25
Malvales/Brassicales - ps26
Brassicales - ps27
Cleomaceae/Brassicaceae - ps28
Brassicaceae - ps29
Camelineae - ps30
Arabidopsis - ps31
Arabidopsis_thaliana - ps32
Phylostratum

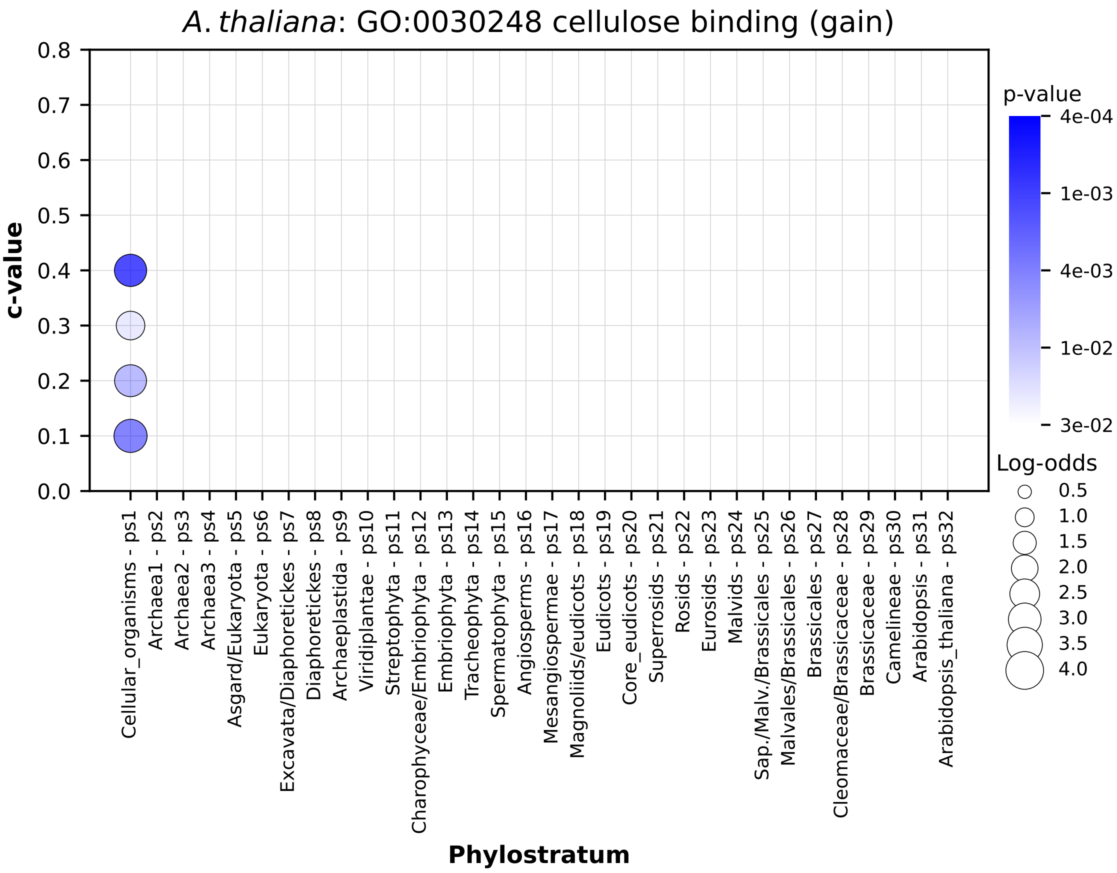

A. thaliana: GO:0030248 cellulose binding (gain)
c-value
0.0
0.1
0.2
0.3
0.4
0.5
0.6
0.7
0.8
p-value
4e-04
1e-03
4e-03
1e-02
3e-02
Log-odds
0.5
1.0
1.5
2.0
2.5
3.0
3.5
4.0
Cellular_organisms - ps1
Archaea1 - ps2
Archaea2 - ps3
Archaea3 - ps4
Asgard/Eukaryota - ps5
Eukaryota - ps6
Excavata/Diaphoretickes - ps7
Diaphoretickes - ps8
Archaeplastida - ps9
Viridiplantae - ps10
Streptophyta - ps11
Charophyceae/Embriophyta - ps12
Embriophyta - ps13
Tracheophyta - ps14
Spermatophyta - ps15
Angiosperms - ps16
Mesangiospermae - ps17
Magnoliids/eudicots - ps18
Eudicots - ps19
Core_eudicots - ps20
Superrosids - ps21
Rosids - ps22
Eurosids - ps23
Malvids - ps24
Sap./Malv./Brassicales - ps25
Malvales/Brassicales - ps26
Brassicales - ps27
Cleomaceae/Brassicaceae - ps28
Brassicaceae - ps29
Camelineae - ps30
Arabidopsis - ps31
Arabidopsis_thaliana - ps32
Phylostratum

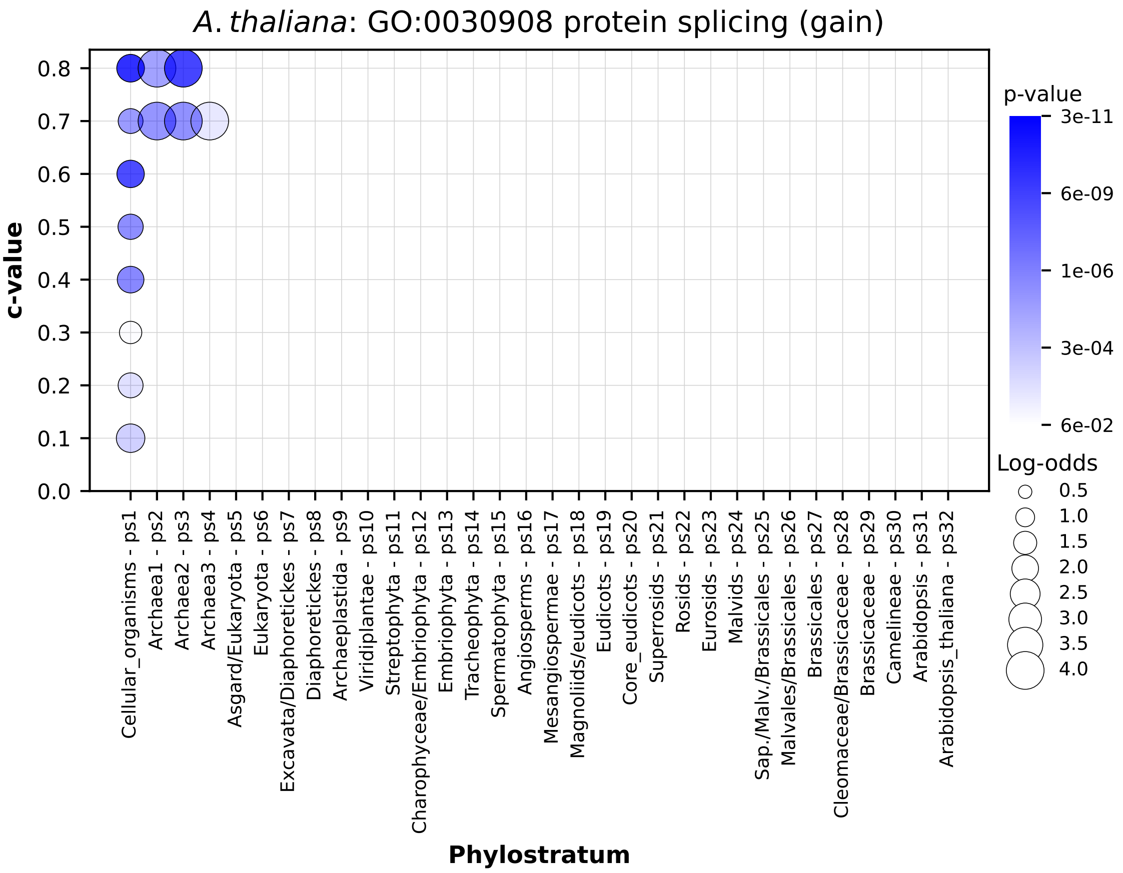

A. thaliana: GO:0030908 protein splicing (gain)
c-value
0.0
0.1
0.2
0.3
0.4
0.5
0.6
0.7
0.8
Phylostratum
Cellular_organisms - ps1
Archaea1 - ps2
Archaea2 - ps3
Archaea3 - ps4
Asgard/Eukaryota - ps5
Eukaryota - ps6
Excavata/Diaphoretickes - ps7
Diaphoretickes - ps8
Archaeplastida - ps9
Viridiplantae - ps10
Streptophyta - ps11
Charophyceae/Embriophyta - ps12
Embriophyta - ps13
Tracheophyta - ps14
Spermatophyta - ps15
Angiosperms - ps16
Mesangiospermae - ps17
Magnoliids/eudicots - ps18
Eudicots - ps19
Core_eudicots - ps20
Superrosids - ps21
Rosids - ps22
Eurosids - ps23
Malvids - ps24
Sap./Malv./Brassicales - ps25
Malvales/Brassicales - ps26
Brassicales - ps27
Cleomaceae/Brassicaceae - ps28
Brassicaceae - ps29
Camelineae - ps30
Arabidopsis - ps31
Arabidopsis_thaliana - ps32
p-value
3e-11
6e-09
1e-06
3e-04
6e-02
Log-odds
0.5
1.0
1.5
2.0
2.5
3.0
3.5
4.0

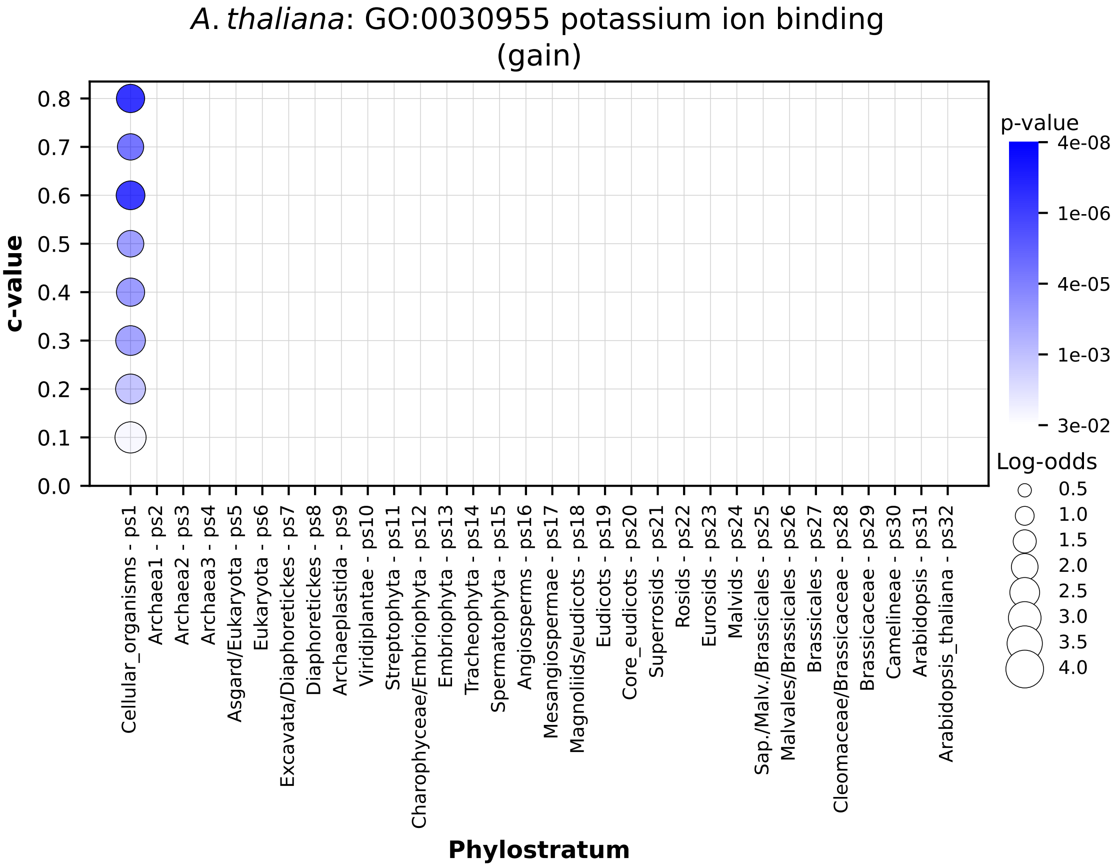

A. thaliana: GO:0030955 potassium ion binding
(gain)
c-value
0.0
0.1
0.2
0.3
0.4
0.5
0.6
0.7
0.8
p-value
4e-08
1e-06
4e-05
1e-03
3e-02
Log-odds
0.5
1.0
1.5
2.0
2.5
3.0
3.5
4.0
Cellular_organisms - ps1
Archaea1 - ps2
Archaea2 - ps3
Archaea3 - ps4
Asgard/Eukaryota - ps5
Eukaryota - ps6
Excavata/Diaphoretickes - ps7
Diaphoretickes - ps8
Archaeplastida - ps9
Viridiplantae - ps10
Streptophyta - ps11
Charophyceae/Embriophyta - ps12
Embriophyta - ps13
Tracheophyta - ps14
Spermatophyta - ps15
Angiosperms - ps16
Mesangiospermae - ps17
Magnoliids/eudicots - ps18
Eudicots - ps19
Core_eudicots - ps20
Superrosids - ps21
Rosids - ps22
Eurosids - ps23
Malvids - ps24
Sap./Malv./Brassicales - ps25
Malvales/Brassicales - ps26
Brassicales - ps27
Cleomaceae/Brassicaceae - ps28
Brassicaceae - ps29
Camelineae - ps30
Arabidopsis - ps31
Arabidopsis_thaliana - ps32
Phylostratum

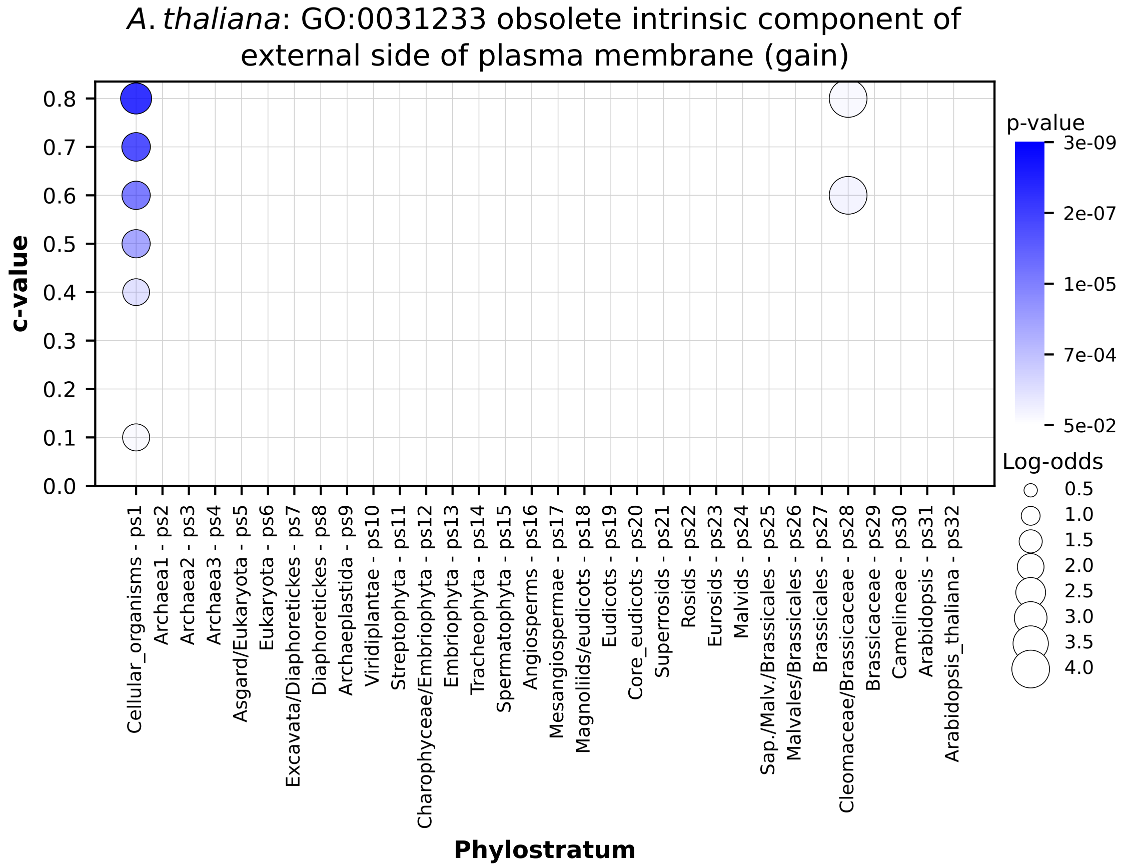

A. thaliana: GO:0031233 obsolete intrinsic component of external side of plasma membrane (gain)
c-value
0.0
0.1
0.2
0.3
0.4
0.5
0.6
0.7
0.8
Phylostratum
Cellular_organisms - ps1
Archaea1 - ps2
Archaea2 - ps3
Archaea3 - ps4
Asgard/Eukaryota - ps5
Eukaryota - ps6
Excavata/Diaphoretickes - ps7
Diaphoretickes - ps8
Archaeplastida - ps9
Viridiplantae - ps10
Streptophyta - ps11
Charophyceae/Embriophyta - ps12
Embriophyta - ps13
Tracheophyta - ps14
Spermatophyta - ps15
Angiosperms - ps16
Mesangiospermae - ps17
Magnoliids/eudicots - ps18
Eudicots - ps19
Core_eudicots - ps20
Superrosids - ps21
Rosids - ps22
Eurosids - ps23
Malvids - ps24
Sap./Malv./Brassicales - ps25
Malvales/Brassicales - ps26
Brassicales - ps27
Cleomaceae/Brassicaceae - ps28
Brassicaceae - ps29
Camelineae - ps30
Arabidopsis - ps31
Arabidopsis_thaliana - ps32
p-value
3e-09
2e-07
1e-05
7e-04
5e-02
Log-odds
0.5
1.0
1.5
2.0
2.5
3.0
3.5
4.0

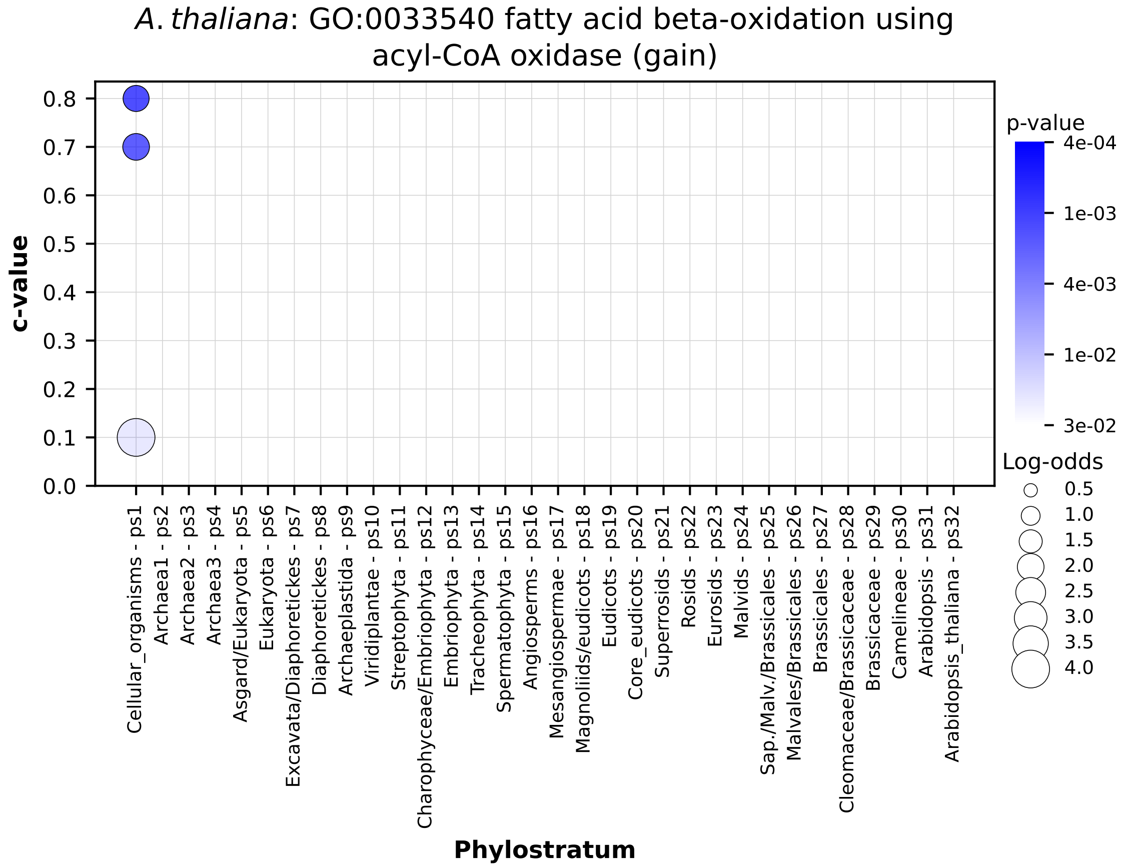

A. thaliana: GO:0033540 fatty acid beta-oxidation using acyl-CoA oxidase (gain)
c-value
0.8
0.7
0.6
0.5
0.4
0.3
0.2
0.1
0.0
p-value
4e-04
1e-03
4e-03
1e-02
3e-02
Log-odds
0.5
1.0
1.5
2.0
2.5
3.0
3.5
4.0
Cellular_organisms - ps1
Archaea1 - ps2
Archaea2 - ps3
Archaea3 - ps4
Asgard/Eukaryota - ps5
Eukaryota - ps6
Excavata/Diaphoretickes - ps7
Diaphoretickes - ps8
Archaeplastida - ps9
Viridiplantae - ps10
Streptophyta - ps11
Charophyceae/Embriophyta - ps12
Embriophyta - ps13
Tracheophyta - ps14
Spermatophyta - ps15
Angiosperms - ps16
Mesangiospermae - ps17
Magnoliids/eudicots - ps18
Eudicots - ps19
Core_eudicots - ps20
Superrosids - ps21
Rosids - ps22
Eurosids - ps23
Malvids - ps24
Sap./Malv./Brassicales - ps25
Malvales/Brassicales - ps26
Brassicales - ps27
Cleomaceae/Brassicaceae - ps28
Brassicaceae - ps29
Camelineae - ps30
Arabidopsis - ps31
Arabidopsis_thaliana - ps32
Phylostratum

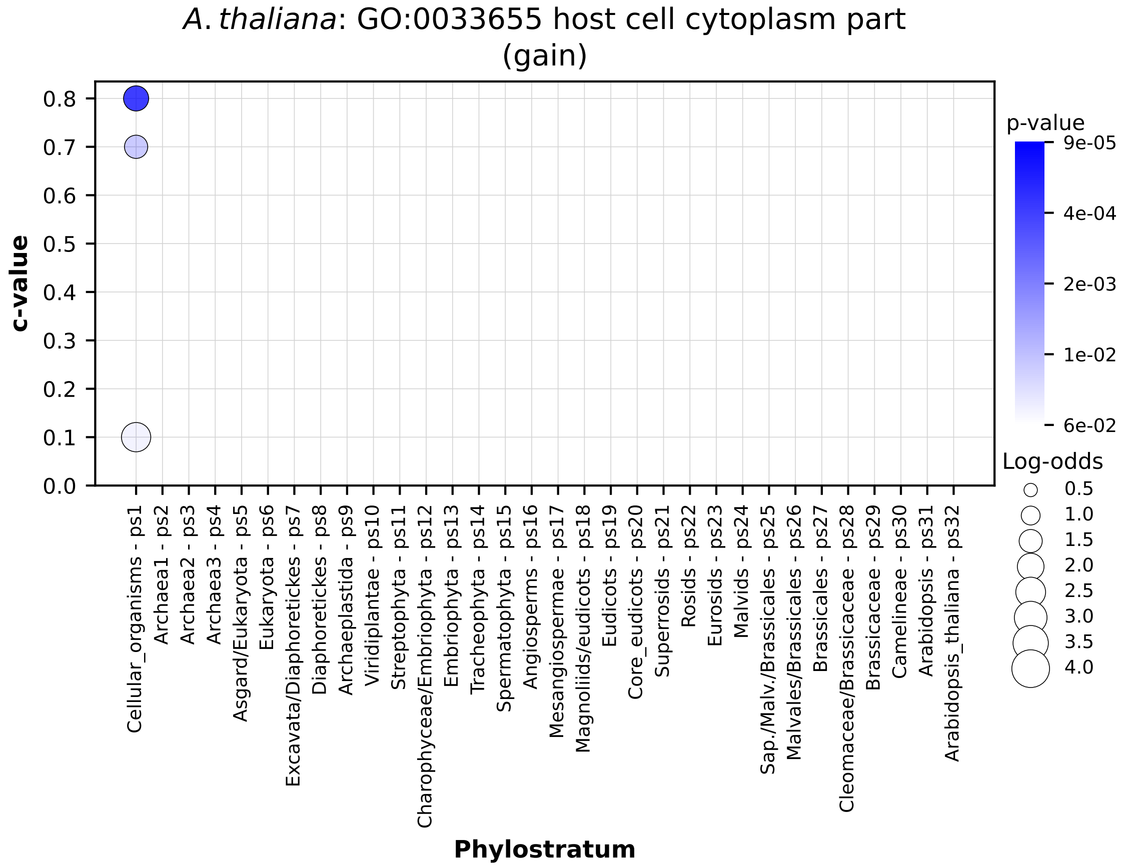

A. thaliana: GO:0033655 host cell cytoplasm part (gain)
c-value
0.8
0.7
0.6
0.5
0.4
0.3
0.2
0.1
0.0
p-value
9e-05
4e-04
2e-03
1e-02
6e-02
Log-odds
0.5
1.0
1.5
2.0
2.5
3.0
3.5
4.0
Cellular_organisms - ps1
Archaea1 - ps2
Archaea2 - ps3
Archaea3 - ps4
Asgard/Eukaryota - ps5
Eukaryota - ps6
Excavata/Diaphoretickes - ps7
Diaphoretickes - ps8
Archaeplastida - ps9
Viridiplantae - ps10
Streptophyta - ps11
Charophyceae/Embriophyta - ps12
Embriophyta - ps13
Tracheophyta - ps14
Spermatophyta - ps15
Angiosperms - ps16
Mesangiospermae - ps17
Magnoliids/eudicots - ps18
Eudicots - ps19
Core_eudicots - ps20
Superrosids - ps21
Rosids - ps22
Eurosids - ps23
Malvids - ps24
Sap./Malv./Brassicales - ps25
Malvales/Brassicales - ps26
Brassicales - ps27
Cleomaceae/Brassicaceae - ps28
Brassicaceae - ps29
Camelineae - ps30
Arabidopsis - ps31
Arabidopsis_thaliana - ps32
Phylostratum

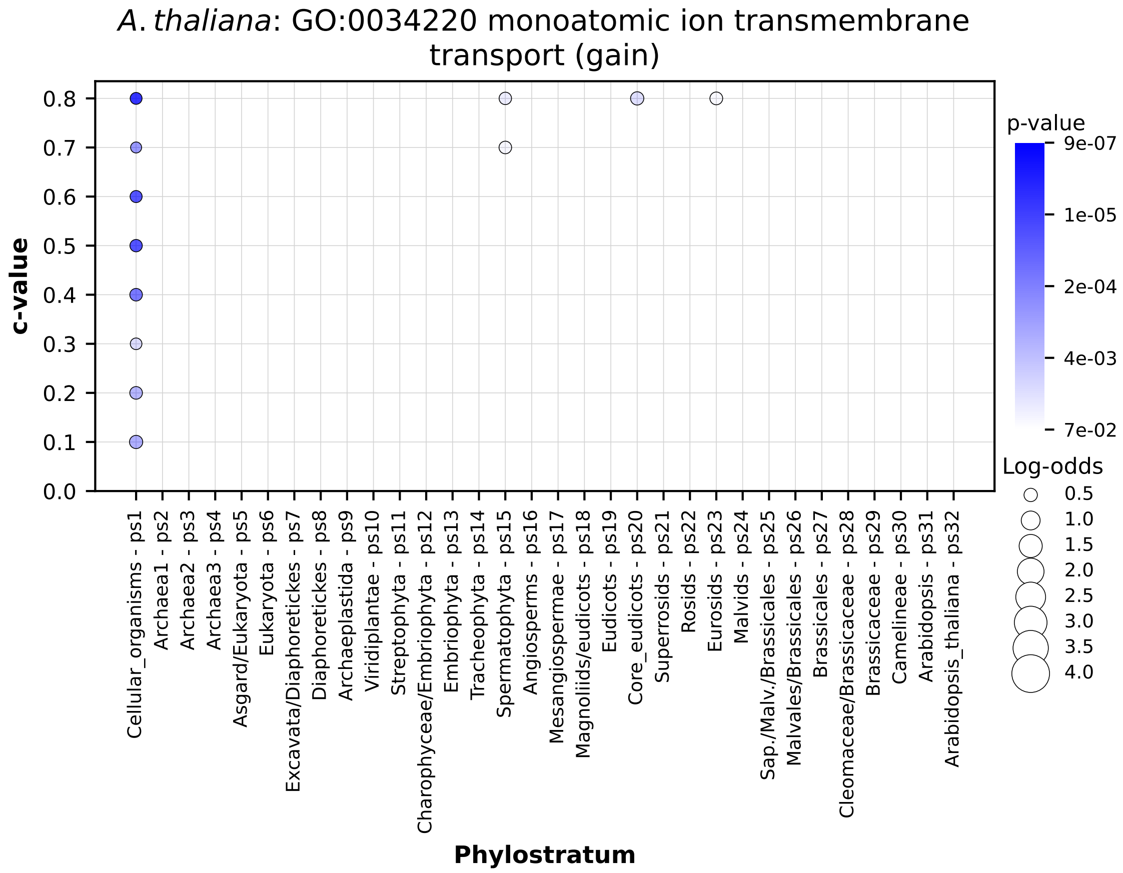

A. thaliana: GO:0034220 monoatomic ion transmembrane transport (gain)
c-value
0.0
0.1
0.2
0.3
0.4
0.5
0.6
0.7
0.8
Phylostratum
Cellular_organisms - ps1
Archaea1 - ps2
Archaea2 - ps3
Archaea3 - ps4
Asgard/Eukaryota - ps5
Eukaryota - ps6
Excavata/Diaphoretickes - ps7
Diaphoretickes - ps8
Archaeplastida - ps9
Viridiplantae - ps10
Streptophyta - ps11
Charophyceae/Embriophyta - ps12
Embriophyta - ps13
Tracheophyta - ps14
Spermatophyta - ps15
Angiosperms - ps16
Mesangiospermae - ps17
Magnoliids/eudicots - ps18
Eudicots - ps19
Core_eudicots - ps20
Superrosids - ps21
Rosids - ps22
Eurosids - ps23
Malvids - ps24
Sap./Malv./Brassicales - ps25
Malvales/Brassicales - ps26
Brassicales - ps27
Cleomaceae/Brassicaceae - ps28
Brassicaceae - ps29
Camelineae - ps30
Arabidopsis - ps31
Arabidopsis_thaliana - ps32
p-value
9e-07
1e-05
2e-04
4e-03
7e-02
Log-odds
0.5
1.0
1.5
2.0
2.5
3.0
3.5
4.0

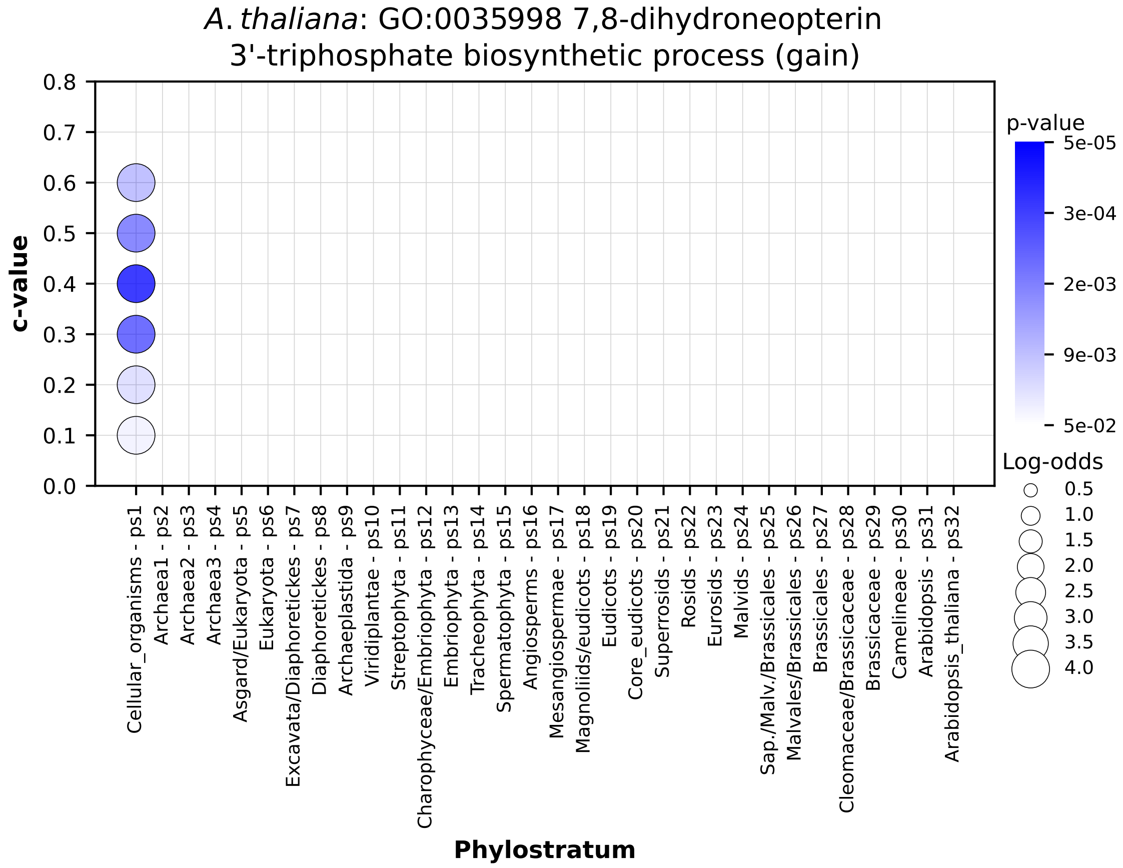

A. thaliana: GO:0035998 7,8-dihydroneopterin 3'-triphosphate biosynthetic process (gain)
c-value
0.0
0.1
0.2
0.3
0.4
0.5
0.6
0.7
0.8
Phylostratum
Cellular_organisms - ps1
Archaea1 - ps2
Archaea2 - ps3
Archaea3 - ps4
Asgard/Eukaryota - ps5
Eukaryota - ps6
Excavata/Diaphoretickes - ps7
Diaphoretickes - ps8
Archaeplastida - ps9
Viridiplantae - ps10
Streptophyta - ps11
Charophyceae/Embriophyta - ps12
Embriophyta - ps13
Tracheophyta - ps14
Spermatophyta - ps15
Angiosperms - ps16
Mesangiospermae - ps17
Magnoliids/eudicots - ps18
Eudicots - ps19
Core_eudicots - ps20
Superrosids - ps21
Rosids - ps22
Eurosids - ps23
Malvids - ps24
Sap./Malv./Brassicales - ps25
Malvales/Brassicales - ps26
Brassicales - ps27
Cleomaceae/Brassicaceae - ps28
Brassicaceae - ps29
Camelineae - ps30
Arabidopsis - ps31
Arabidopsis_thaliana - ps32
p-value
5e-05
3e-04
2e-03
9e-03
5e-02
Log-odds
0.5
1.0
1.5
2.0
2.5
3.0
3.5
4.0

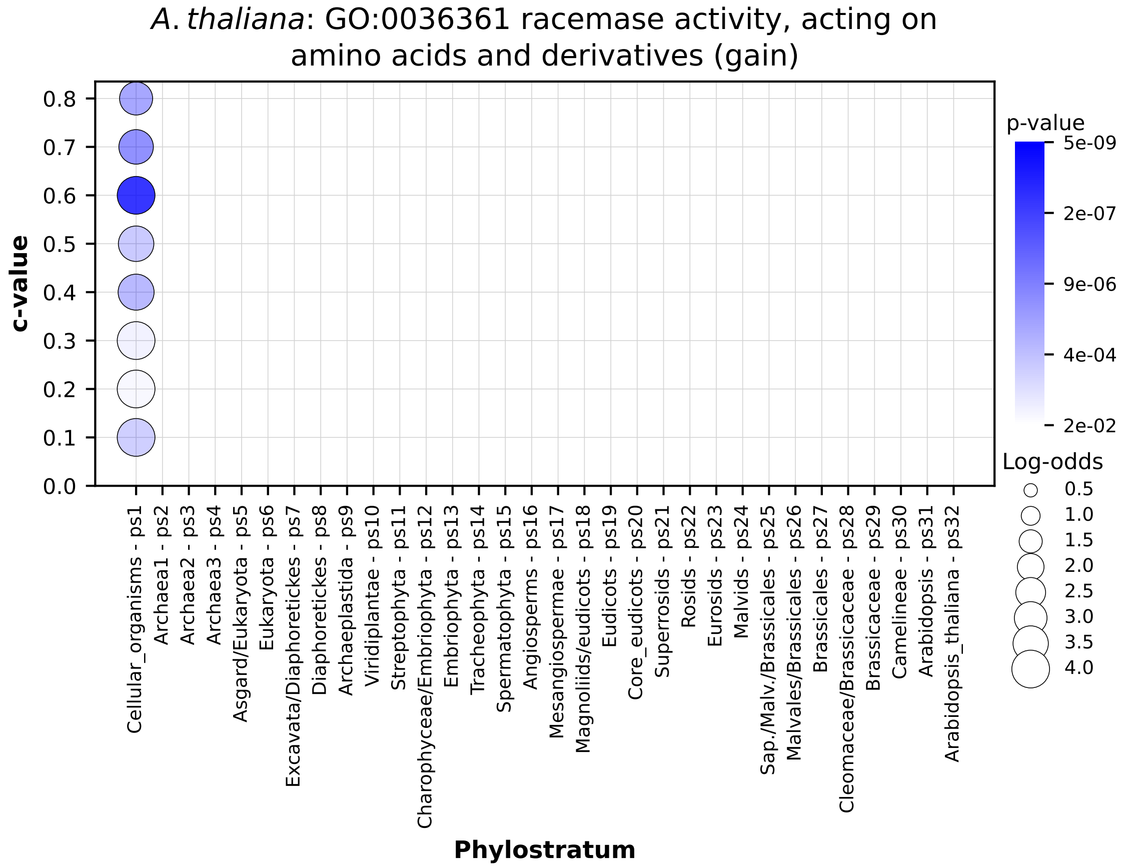
A. thaliana: GO:0036361 racemase activity, acting on amino acids and derivatives (gain)
c-value
0.8
0.7
0.6
0.5
0.4
0.3
0.2
0.1
0.0
p-value
5e-09
2e-07
9e-06
4e-04
2e-02
Log-odds
0.5
1.0
1.5
2.0
2.5
3.0
3.5
4.0
Cellular_organisms - ps1
Archaea1 - ps2
Archaea2 - ps3
Archaea3 - ps4
Asgard/Eukaryota - ps5
Eukaryota - ps6
Excavata/Diaphoretickes - ps7
Diaphoretickes - ps8
Archaeplastida - ps9
Viridiplantae - ps10
Streptophyta - ps11
Charophyceae/Embriophyta - ps12
Embriophyta - ps13
Tracheophyta - ps14
Spermatophyta - ps15
Angiosperms - ps16
Mesangiospermae - ps17
Magnoliids/eudicots - ps18
Eudicots - ps19
Core_eudicots - ps20
Superrosids - ps21
Rosids - ps22
Eurosids - ps23
Malvids - ps24
Sap./Malv./Brassicales - ps25
Malvales/Brassicales - ps26
Brassicales - ps27
Cleomaceae/Brassicaceae - ps28
Brassicaceae - ps29
Camelineae - ps30
Arabidopsis - ps31
Arabidopsis_thaliana - ps32
Phylostratum

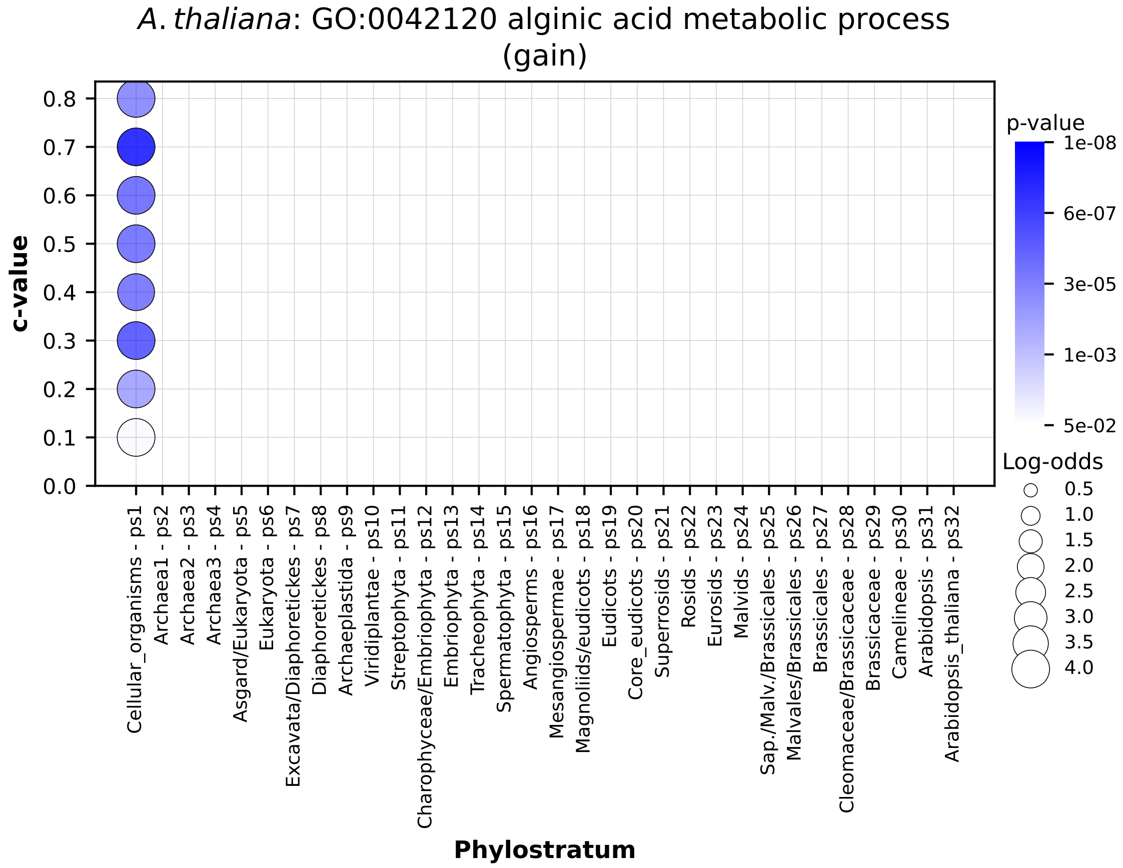
A. thaliana: GO:0042120 alginic acid metabolic process
(gain)
c-value
0.0
0.1
0.2
0.3
0.4
0.5
0.6
0.7
0.8
p-value
1e-08
6e-07
3e-05
1e-03
5e-02
Log-odds
0.5
1.0
1.5
2.0
2.5
3.0
3.5
4.0
Cellular_organisms - ps1
Archaea1 - ps2
Archaea2 - ps3
Archaea3 - ps4
Asgard/Eukaryota - ps5
Eukaryota - ps6
Excavata/Diaphoretickes - ps7
Diaphoretickes - ps8
Archaeplastida - ps9
Viridiplantae - ps10
Streptophyta - ps11
Charophyceae/Embriophyta - ps12
Embriophyta - ps13
Tracheophyta - ps14
Spermatophyta - ps15
Angiosperms - ps16
Mesangiospermae - ps17
Magnoliids/eudicots - ps18
Eudicots - ps19
Core_eudicots - ps20
Superrosids - ps21
Rosids - ps22
Eurosids - ps23
Malvids - ps24
Sap./Malv./Brassicales - ps25
Malvales/Brassicales - ps26
Brassicales - ps27
Cleomaceae/Brassicaceae - ps28
Brassicaceae - ps29
Camelineae - ps30
Arabidopsis - ps31
Arabidopsis_thaliana - ps32
Phylostratum

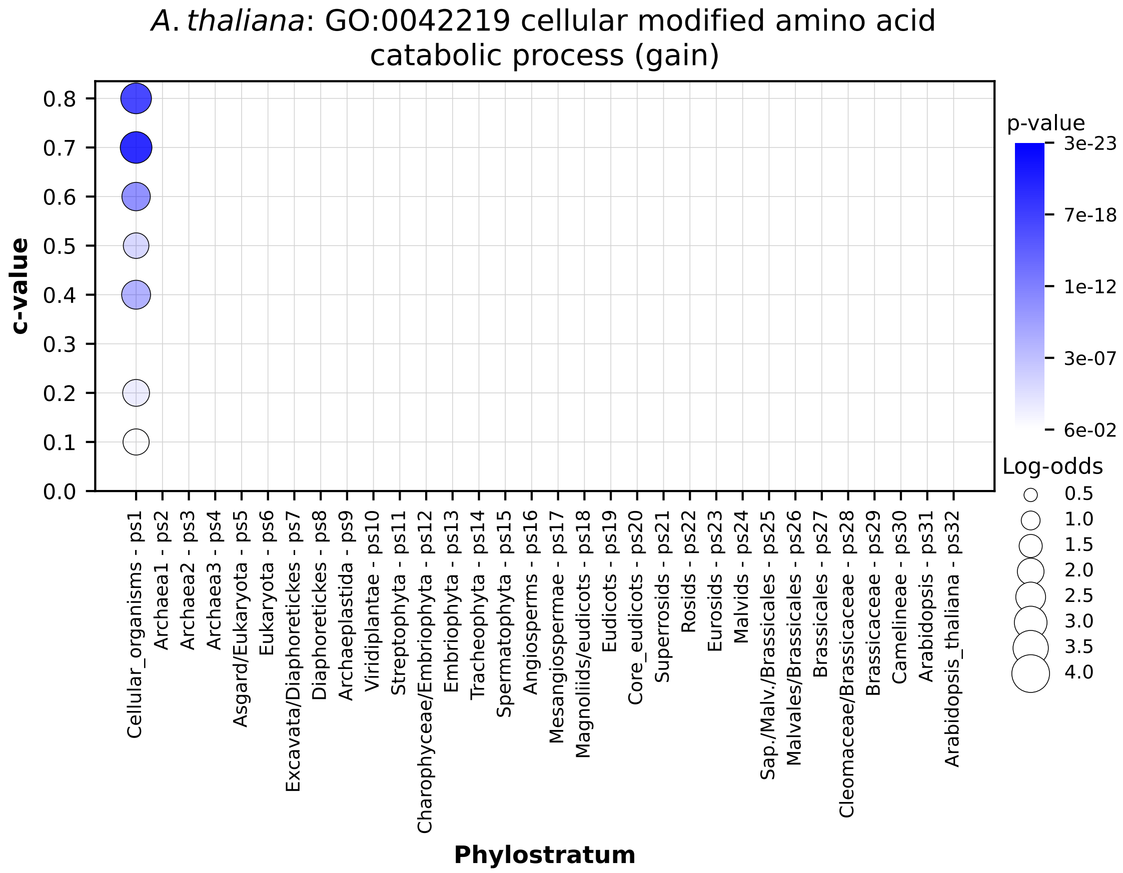

A. thaliana: GO:0042219 cellular modified amino acid catabolic process (gain)
c-value
0.0
0.1
0.2
0.3
0.4
0.5
0.6
0.7
0.8
p-value
3e-23
7e-18
1e-12
3e-07
6e-02
Log-odds
0.5
1.0
1.5
2.0
2.5
3.0
3.5
4.0
Cellular_organisms - ps1
Archaea1 - ps2
Archaea2 - ps3
Archaea3 - ps4
Asgard/Eukaryota - ps5
Eukaryota - ps6
Excavata/Diaphoretickes - ps7
Diaphoretickes - ps8
Archaeplastida - ps9
Viridiplantae - ps10
Streptophyta - ps11
Charophyceae/Embriophyta - ps12
Embriophyta - ps13
Tracheophyta - ps14
Spermatophyta - ps15
Angiosperms - ps16
Mesangiospermae - ps17
Magnoliids/eudicots - ps18
Eudicots - ps19
Core_eudicots - ps20
Superrosids - ps21
Rosids - ps22
Eurosids - ps23
Malvids - ps24
Sap./Malv./Brassicales - ps25
Malvales/Brassicales - ps26
Brassicales - ps27
Cleomaceae/Brassicaceae - ps28
Brassicaceae - ps29
Camelineae - ps30
Arabidopsis - ps31
Arabidopsis_thaliana - ps32
Phylostratum

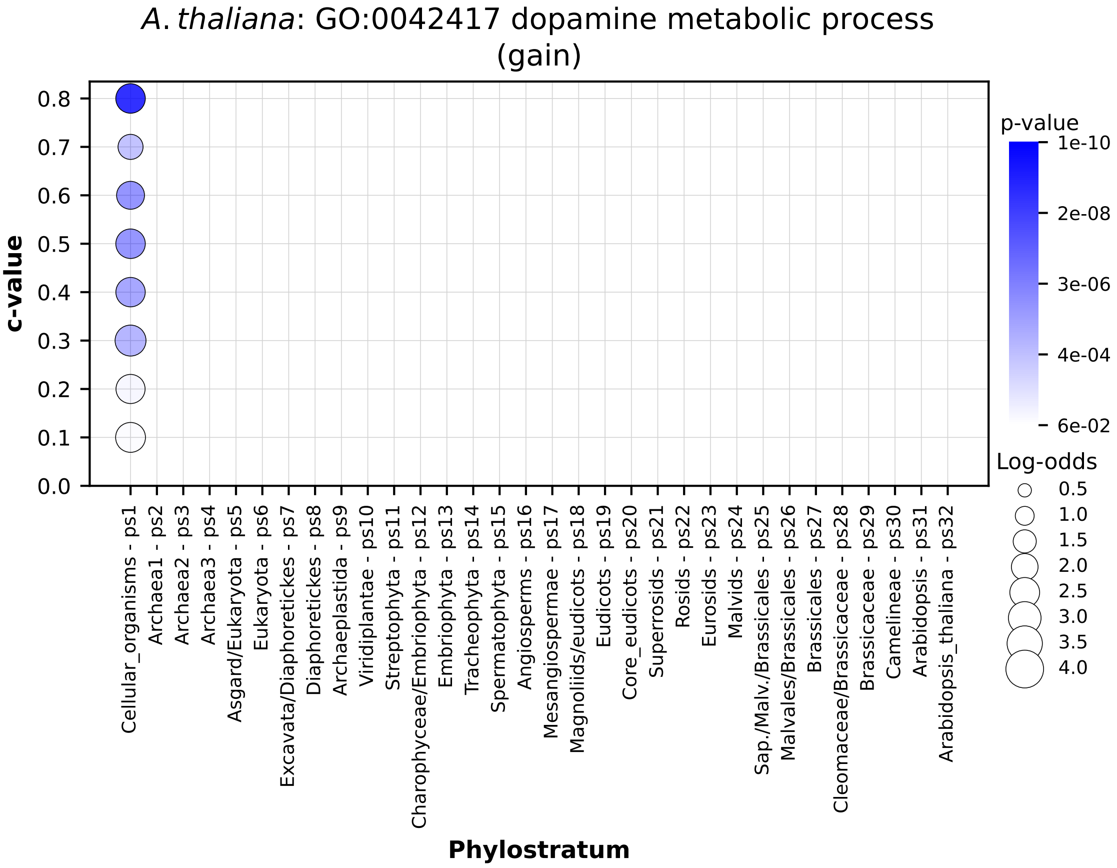

A. thaliana: GO:0042417 dopamine metabolic process
(gain)
c-value
0.0
0.1
0.2
0.3
0.4
0.5
0.6
0.7
0.8
p-value
1e-10
2e-08
3e-06
4e-04
6e-02
Log-odds
0.5
1.0
1.5
2.0
2.5
3.0
3.5
4.0
Cellular_organisms - ps1
Archaea1 - ps2
Archaea2 - ps3
Archaea3 - ps4
Asgard/Eukaryota - ps5
Eukaryota - ps6
Excavata/Diaphoretickes - ps7
Diaphoretickes - ps8
Archaeplastida - ps9
Viridiplantae - ps10
Streptophyta - ps11
Charophyceae/Embriophyta - ps12
Embriophyta - ps13
Tracheophyta - ps14
Spermatophyta - ps15
Angiosperms - ps16
Mesangiospermae - ps17
Magnoliids/eudicots - ps18
Eudicots - ps19
Core_eudicots - ps20
Superrosids - ps21
Rosids - ps22
Eurosids - ps23
Malvids - ps24
Sap./Malv./Brassicales - ps25
Malvales/Brassicales - ps26
Brassicales - ps27
Cleomaceae/Brassicaceae - ps28
Brassicaceae - ps29
Camelineae - ps30
Arabidopsis - ps31
Arabidopsis_thaliana - ps32
Phylostratum

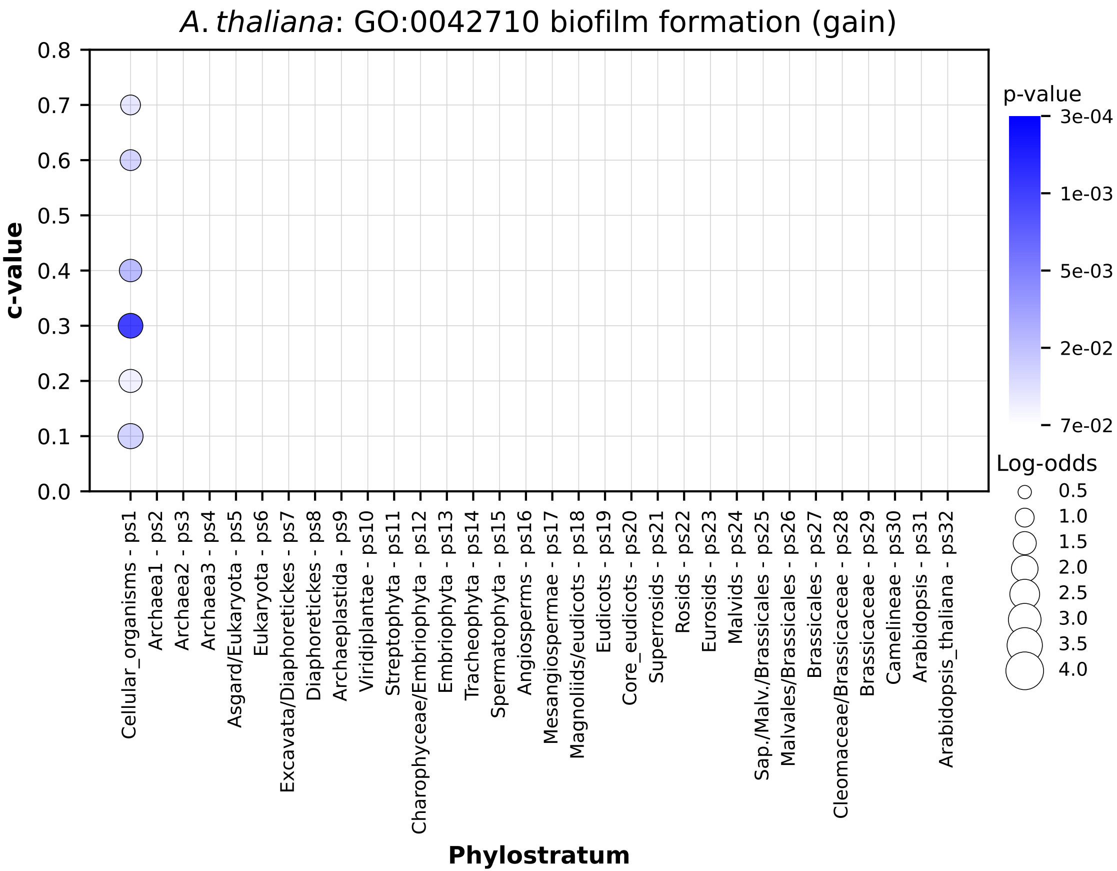
A. thaliana: GO:0042710 biofilm formation (gain)
c-value
0.0
0.1
0.2
0.3
0.4
0.5
0.6
0.7
0.8
Phylostratum
Cellular_organisms - ps1
Archaea1 - ps2
Archaea2 - ps3
Archaea3 - ps4
Asgard/Eukaryota - ps5
Eukaryota - ps6
Excavata/Diaphoretickes - ps7
Diaphoretickes - ps8
Archaeplastida - ps9
Viridiplantae - ps10
Streptophyta - ps11
Charophyceae/Embriophyta - ps12
Embriophyta - ps13
Tracheophyta - ps14
Spermatophyta - ps15
Angiosperms - ps16
Mesangiospermae - ps17
Magnoliids/eudicots - ps18
Eudicots - ps19
Core_eudicots - ps20
Superrosids - ps21
Rosids - ps22
Eurosids - ps23
Malvids - ps24
Sap./Malv./Brassicales - ps25
Malvales/Brassicales - ps26
Brassicales - ps27
Cleomaceae/Brassicaceae - ps28
Brassicaceae - ps29
Camelineae - ps30
Arabidopsis - ps31
Arabidopsis_thaliana - ps32
p-value
3e-04
1e-03
5e-03
2e-02
7e-02
Log-odds
0.5
1.0
1.5
2.0
2.5
3.0
3.5
4.0

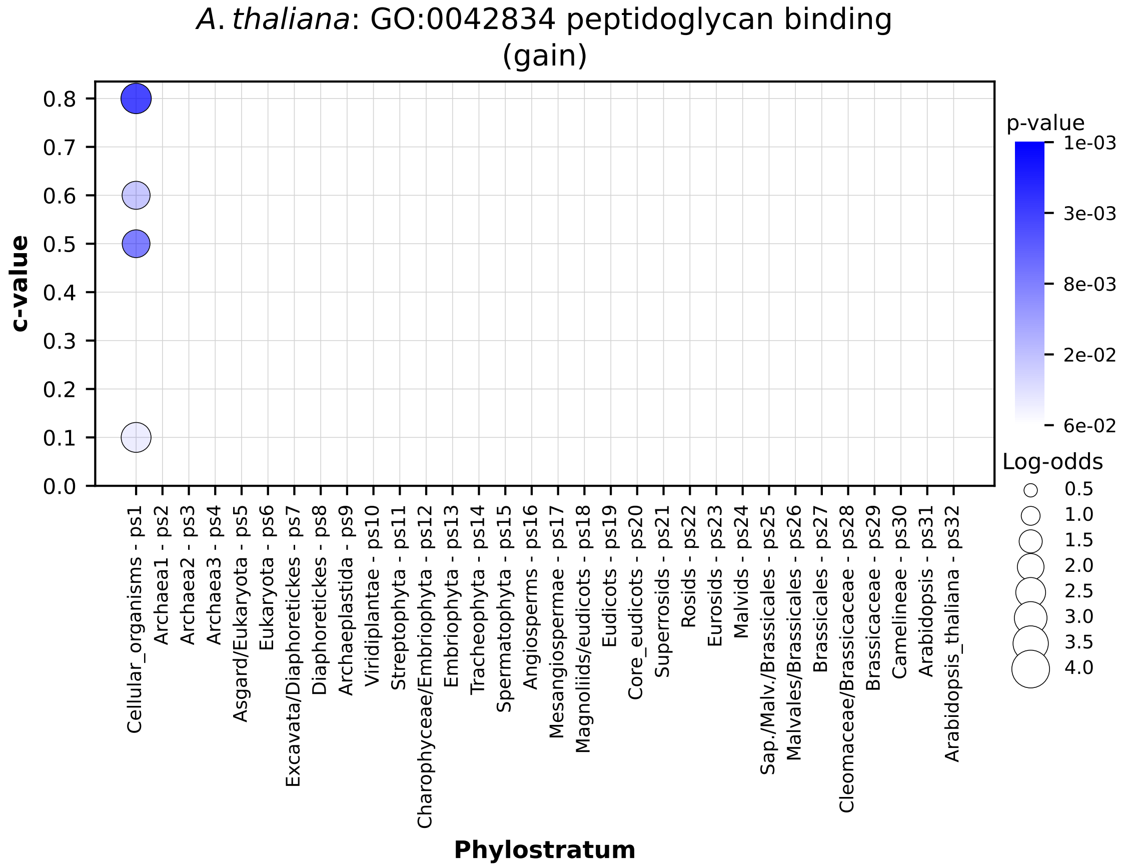

A. thaliana: GO:0042834 peptidoglycan binding
(gain)
c-value
0.0
0.1
0.2
0.3
0.4
0.5
0.6
0.7
0.8
p-value
1e-03
3e-03
8e-03
2e-02
6e-02
Log-odds
0.5
1.0
1.5
2.0
2.5
3.0
3.5
4.0
Cellular_organisms - ps1
Archaea1 - ps2
Archaea2 - ps3
Archaea3 - ps4
Asgard/Eukaryota - ps5
Eukaryota - ps6
Excavata/Diaphoretickes - ps7
Diaphoretickes - ps8
Archaeplastida - ps9
Viridiplantae - ps10
Streptophyta - ps11
Charophyceae/Embriophyta - ps12
Embriophyta - ps13
Tracheophyta - ps14
Spermatophyta - ps15
Angiosperms - ps16
Mesangiospermae - ps17
Magnoliids/eudicots - ps18
Eudicots - ps19
Core_eudicots - ps20
Superrosids - ps21
Rosids - ps22
Eurosids - ps23
Malvids - ps24
Sap./Malv./Brassicales - ps25
Malvales/Brassicales - ps26
Brassicales - ps27
Cleomaceae/Brassicaceae - ps28
Brassicaceae - ps29
Camelineae - ps30
Arabidopsis - ps31
Arabidopsis_thaliana - ps32
Phylostratum

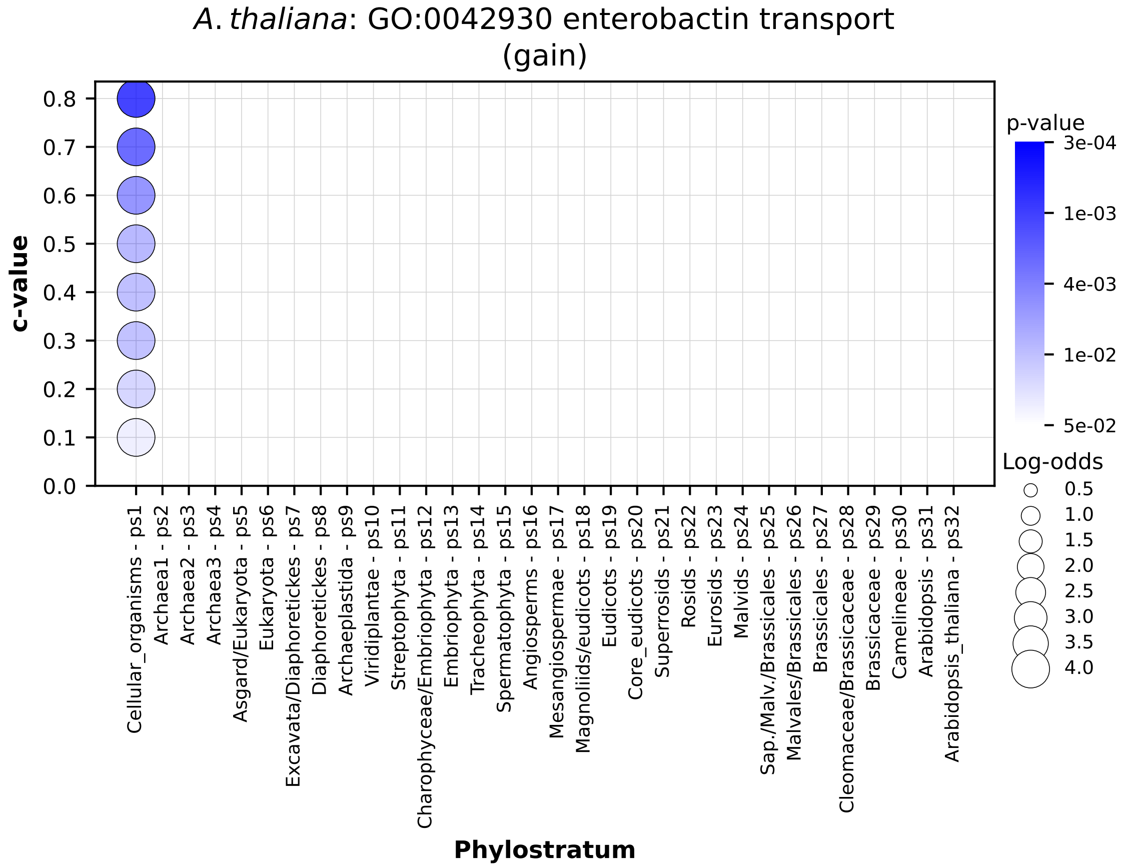

A. thaliana: GO:0042930 enterobactin transport
(gain)
c-value
0.8
0.7
0.6
0.5
0.4
0.3
0.2
0.1
0.0
p-value
3e-04
1e-03
4e-03
1e-02
5e-02
Log-odds
0.5
1.0
1.5
2.0
2.5
3.0
3.5
4.0
Cellular_organisms - ps1
Archaea1 - ps2
Archaea2 - ps3
Archaea3 - ps4
Asgard/Eukaryota - ps5
Eukaryota - ps6
Excavata/Diaphoretickes - ps7
Diaphoretickes - ps8
Archaeplastida - ps9
Viridiplantae - ps10
Streptophyta - ps11
Charophyceae/Embriophyta - ps12
Embriophyta - ps13
Tracheophyta - ps14
Spermatophyta - ps15
Angiosperms - ps16
Mesangiospermae - ps17
Magnoliids/eudicots - ps18
Eudicots - ps19
Core_eudicots - ps20
Superrosids - ps21
Rosids - ps22
Eurosids - ps23
Malvids - ps24
Sap./Malv./Brassicales - ps25
Malvales/Brassicales - ps26
Brassicales - ps27
Cleomaceae/Brassicaceae - ps28
Brassicaceae - ps29
Camelineae - ps30
Arabidopsis - ps31
Arabidopsis_thaliana - ps32
Phylostratum

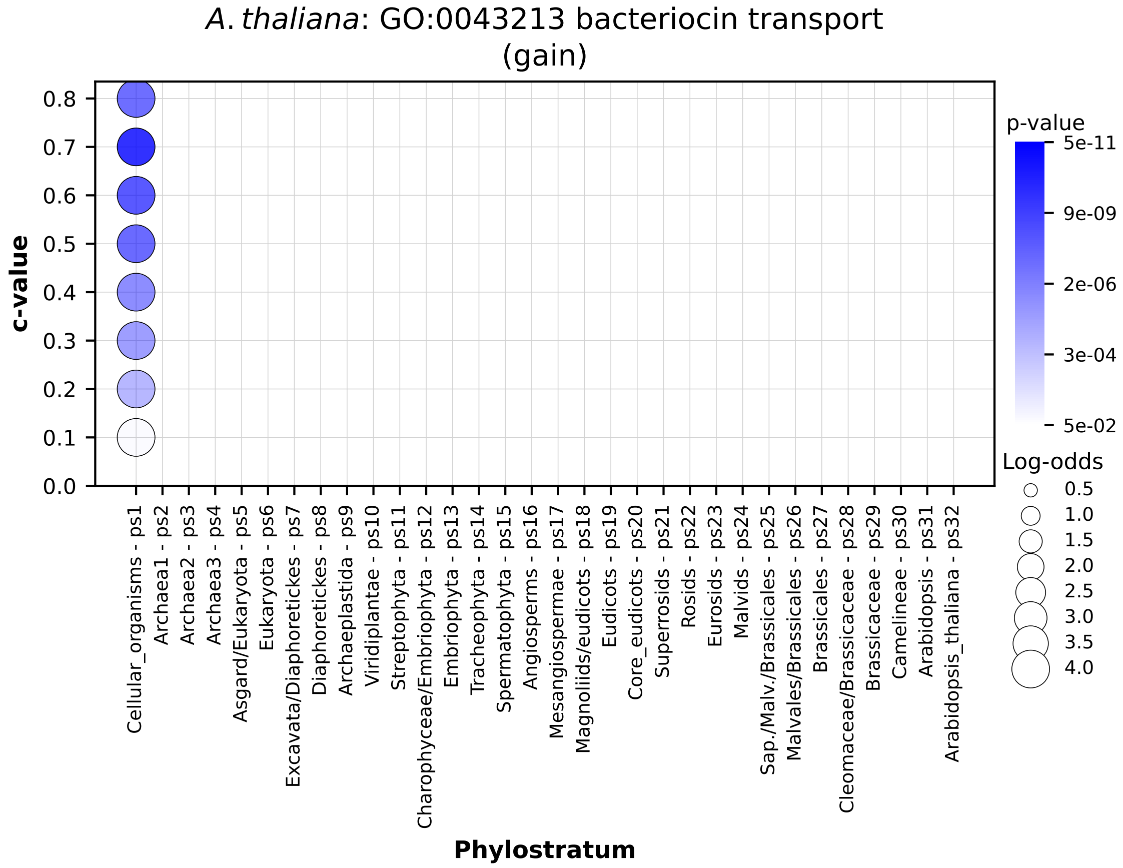

A. thaliana: GO:0043213 bacteriocin transport
(gain)
c-value
0.0
0.1
0.2
0.3
0.4
0.5
0.6
0.7
0.8
Phylostratum
Cellular_organisms - ps1
Archaea1 - ps2
Archaea2 - ps3
Archaea3 - ps4
Asgard/Eukaryota - ps5
Eukaryota - ps6
Excavata/Diaphoretickes - ps7
Diaphoretickes - ps8
Archaeplastida - ps9
Viridiplantae - ps10
Streptophyta - ps11
Charophyceae/Embriophyta - ps12
Embriophyta - ps13
Tracheophyta - ps14
Spermatophyta - ps15
Angiosperms - ps16
Mesangiospermae - ps17
Magnoliids/eudicots - ps18
Eudicots - ps19
Core_eudicots - ps20
Superrosids - ps21
Rosids - ps22
Eurosids - ps23
Malvids - ps24
Sap./Malv./Brassicales - ps25
Malvales/Brassicales - ps26
Brassicales - ps27
Cleomaceae/Brassicaceae - ps28
Brassicaceae - ps29
Camelineae - ps30
Arabidopsis - ps31
Arabidopsis_thaliana - ps32
p-value
5e-11
9e-09
2e-06
3e-04
5e-02
Log-odds
0.5
1.0
1.5
2.0
2.5
3.0
3.5
4.0

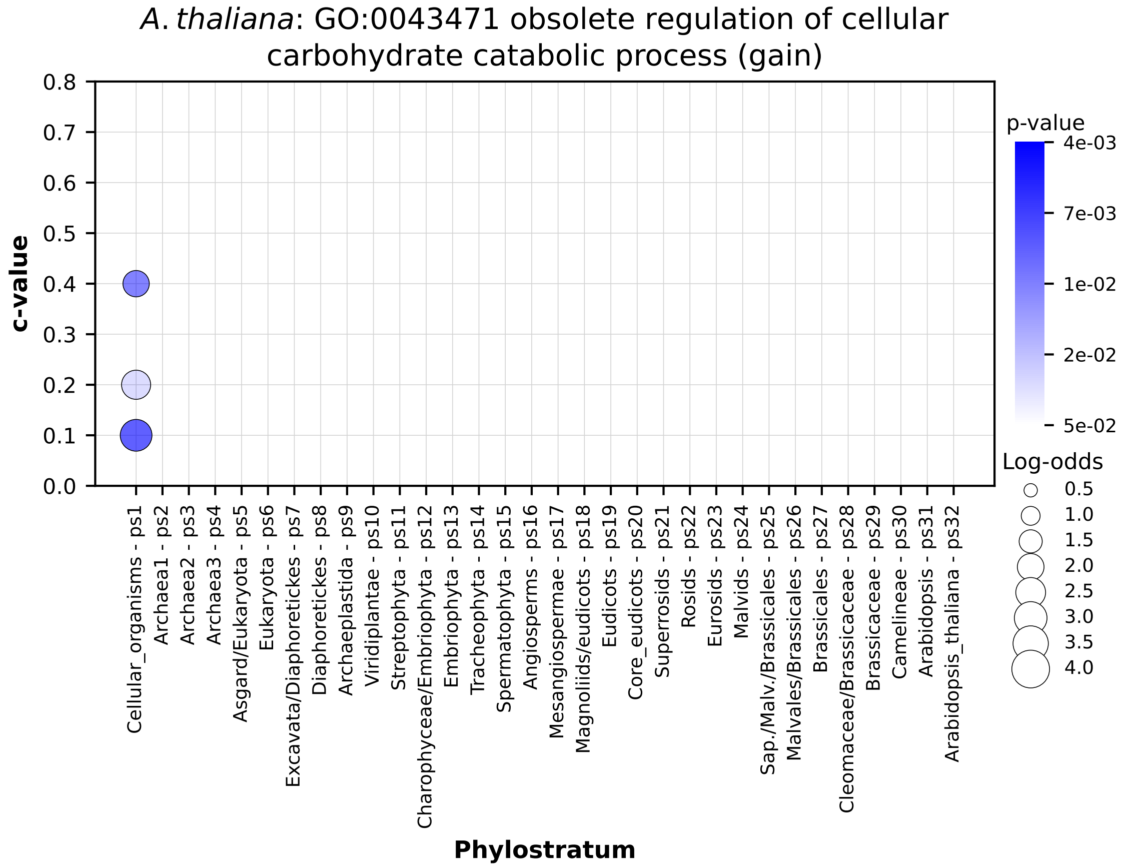

A. thaliana: GO:0043471 obsolete regulation of cellular carbohydrate catabolic process (gain)
c-value
0.0
0.1
0.2
0.3
0.4
0.5
0.6
0.7
0.8
Phylostratum
Cellular_organisms - ps1
Archaea1 - ps2
Archaea2 - ps3
Archaea3 - ps4
Asgard/Eukaryota - ps5
Eukaryota - ps6
Excavata/Diaphoretickes - ps7
Diaphoretickes - ps8
Archaeplastida - ps9
Viridiplantae - ps10
Streptophyta - ps11
Charophyceae/Embriophyta - ps12
Embriophyta - ps13
Tracheophyta - ps14
Spermatophyta - ps15
Angiosperms - ps16
Mesangiospermae - ps17
Magnoliids/eudicots - ps18
Eudicots - ps19
Core_eudicots - ps20
Superrosids - ps21
Rosids - ps22
Eurosids - ps23
Malvids - ps24
Sap./Malv./Brassicales - ps25
Malvales/Brassicales - ps26
Brassicales - ps27
Cleomaceae/Brassicaceae - ps28
Brassicaceae - ps29
Camelineae - ps30
Arabidopsis - ps31
Arabidopsis_thaliana - ps32
p-value
4e-03
7e-03
1e-02
2e-02
5e-02
Log-odds
0.5
1.0
1.5
2.0
2.5
3.0
3.5
4.0

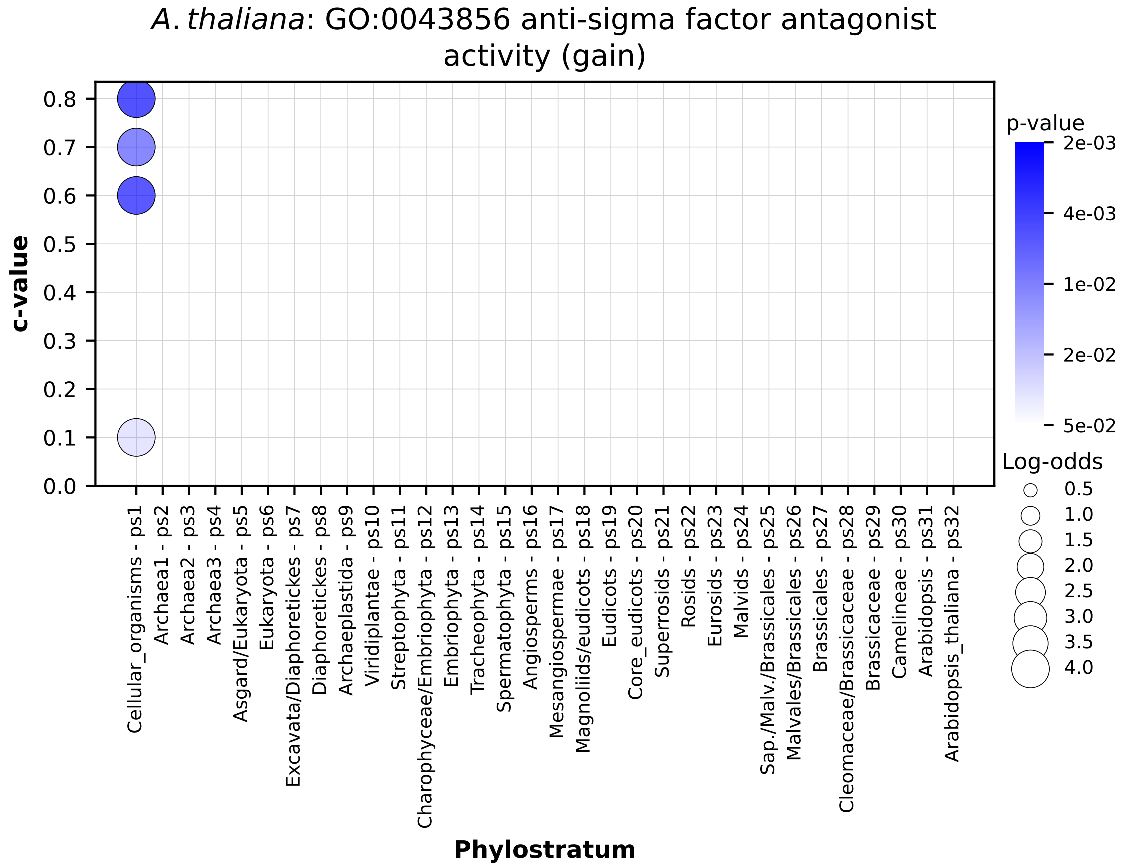

A. thaliana: GO:0043856 anti-sigma factor antagonist activity (gain)
c-value
Phylostratum
p-value
2e-03
4e-03
1e-02
2e-02
5e-02
Log-odds
0.5
1.0
1.5
2.0
2.5
3.0
3.5
4.0
Cellular_organisms - ps1
Archaea1 - ps2
Archaea2 - ps3
Archaea3 - ps4
Asgard/Eukaryota - ps5
Eukaryota - ps6
Excavata/Diaphoretickes - ps7
Diaphoretickes - ps8
Archaeplastida - ps9
Viridiplantae - ps10
Streptophyta - ps11
Charophyceae/Embriophyta - ps12
Embriophyta - ps13
Tracheophyta - ps14
Spermatophyta - ps15
Angiosperms - ps16
Mesangiospermae - ps17
Magnoliids/eudicots - ps18
Eudicots - ps19
Core_eudicots - ps20
Superrosids - ps21
Rosids - ps22
Eurosids - ps23
Malvids - ps24
Sap./Malv./Brassicales - ps25
Malvales/Brassicales - ps26
Brassicales - ps27
Cleomaceae/Brassicaceae - ps28
Brassicaceae - ps29
Camelineae - ps30
Arabidopsis - ps31
Arabidopsis_thaliana - ps32

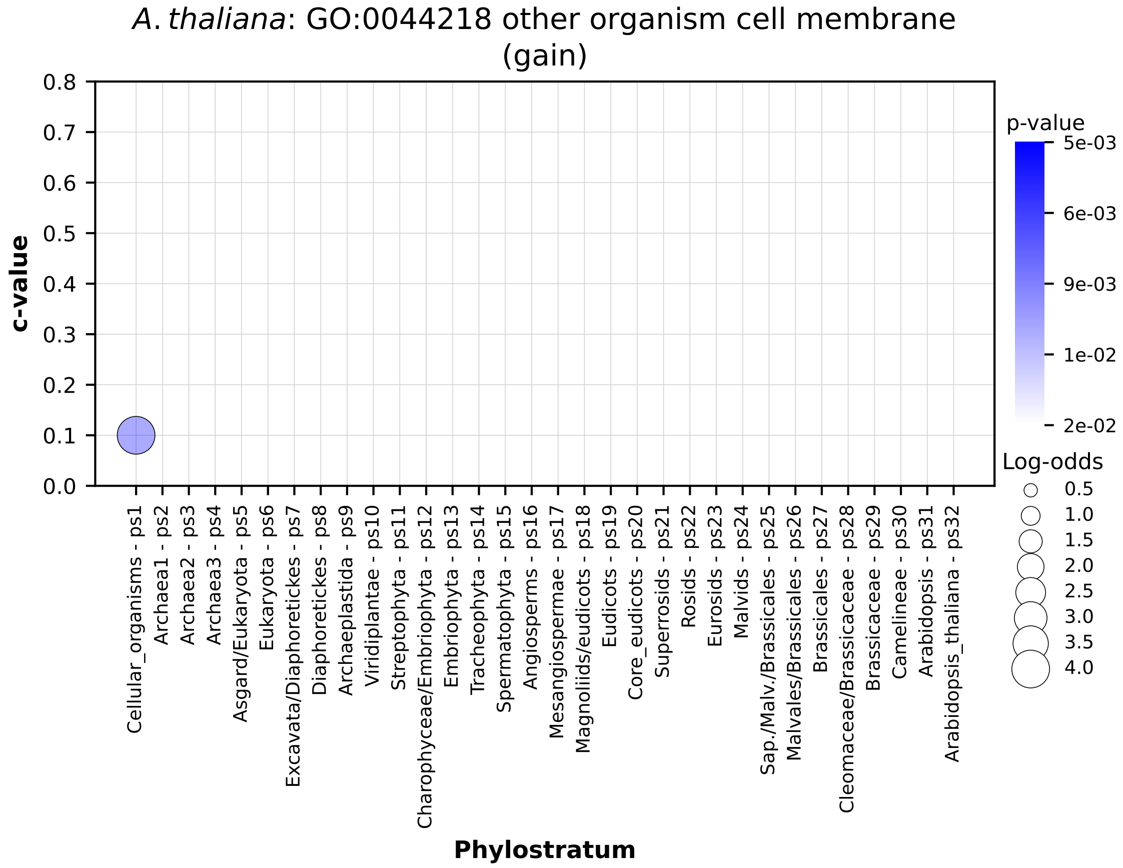

$A. thaliana$: GO:0044218 other organism cell membrane
(gain)
c-value
0.0
0.1
0.2
0.3
0.4
0.5
0.6
0.7
0.8
p-value
5e-03
6e-03
9e-03
1e-02
2e-02
Log-odds
0.5
1.0
1.5
2.0
2.5
3.0
3.5
4.0
Cellular_organisms - ps1
Archaea1 - ps2
Archaea2 - ps3
Archaea3 - ps4
Asgard/Eukaryota - ps5
Eukaryota - ps6
Excavata/Diaphoretickes - ps7
Diaphoretickes - ps8
Archaeplastida - ps9
Viridiplantae - ps10
Streptophyta - ps11
Charophyceae/Embriophyta - ps12
Embriophyta - ps13
Tracheophyta - ps14
Spermatophyta - ps15
Angiosperms - ps16
Mesangiospermae - ps17
Magnoliids/eudicots - ps18
Eudicots - ps19
Core_eudicots - ps20
Superrosids - ps21
Rosids - ps22
Eurosids - ps23
Malvids - ps24
Sap./Malv./Brassicales - ps25
Malvales/Brassicales - ps26
Brassicales - ps27
Cleomaceae/Brassicaceae - ps28
Brassicaceae - ps29
Camelineae - ps30
Arabidopsis - ps31
Arabidopsis_thaliana - ps32
Phylostratum

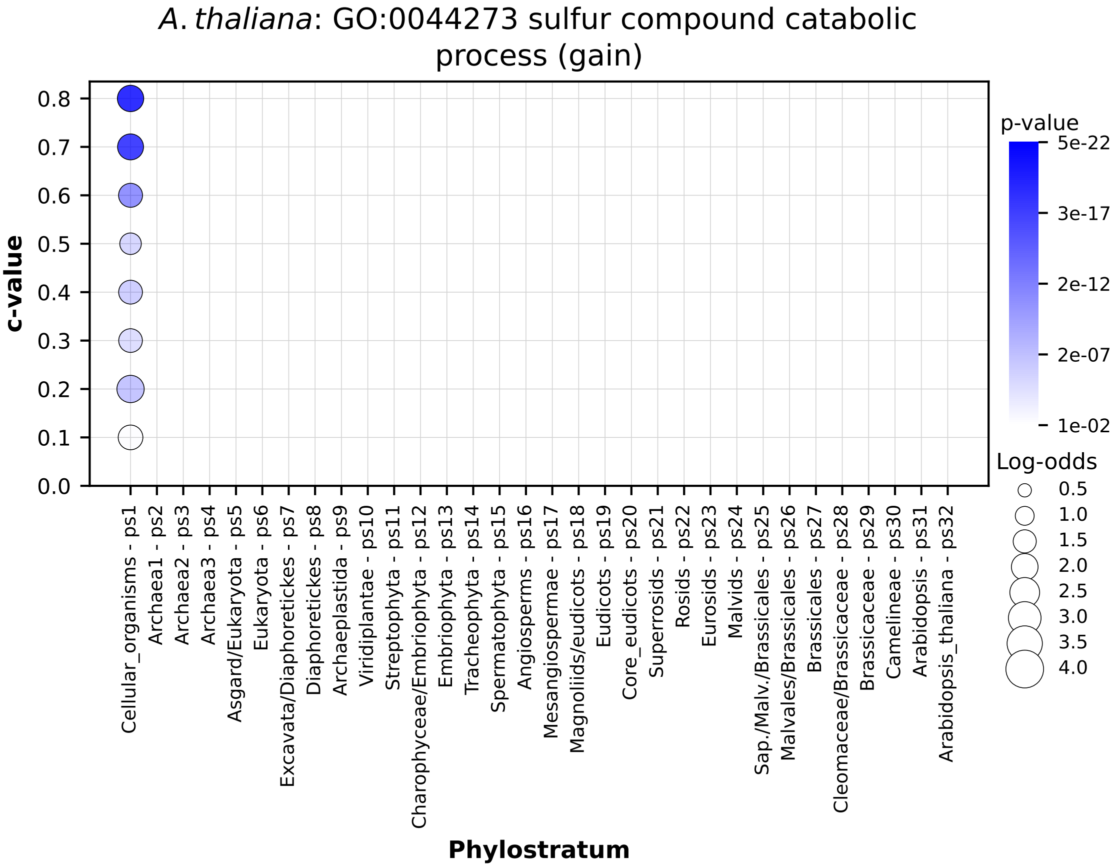

A. thaliana: GO:0044273 sulfur compound catabolic process (gain)
c-value
0.0
0.1
0.2
0.3
0.4
0.5
0.6
0.7
0.8
p-value
5e-22
3e-17
2e-12
2e-07
1e-02
Log-odds
0.5
1.0
1.5
2.0
2.5
3.0
3.5
4.0
Cellular_organisms - ps1
Archaea1 - ps2
Archaea2 - ps3
Archaea3 - ps4
Asgard/Eukaryota - ps5
Eukaryota - ps6
Excavata/Diaphoretickes - ps7
Diaphoretickes - ps8
Archaeplastida - ps9
Viridiplantae - ps10
Streptophyta - ps11
Charophyceae/Embriophyta - ps12
Embriophyta - ps13
Tracheophyta - ps14
Spermatophyta - ps15
Angiosperms - ps16
Mesangiospermae - ps17
Magnoliids/eudicots - ps18
Eudicots - ps19
Core_eudicots - ps20
Superrosids - ps21
Rosids - ps22
Eurosids - ps23
Malvids - ps24
Sap./Malv./Brassicales - ps25
Malvales/Brassicales - ps26
Brassicales - ps27
Cleomaceae/Brassicaceae - ps28
Brassicaceae - ps29
Camelineae - ps30
Arabidopsis - ps31
Arabidopsis_thaliana - ps32
Phylostratum

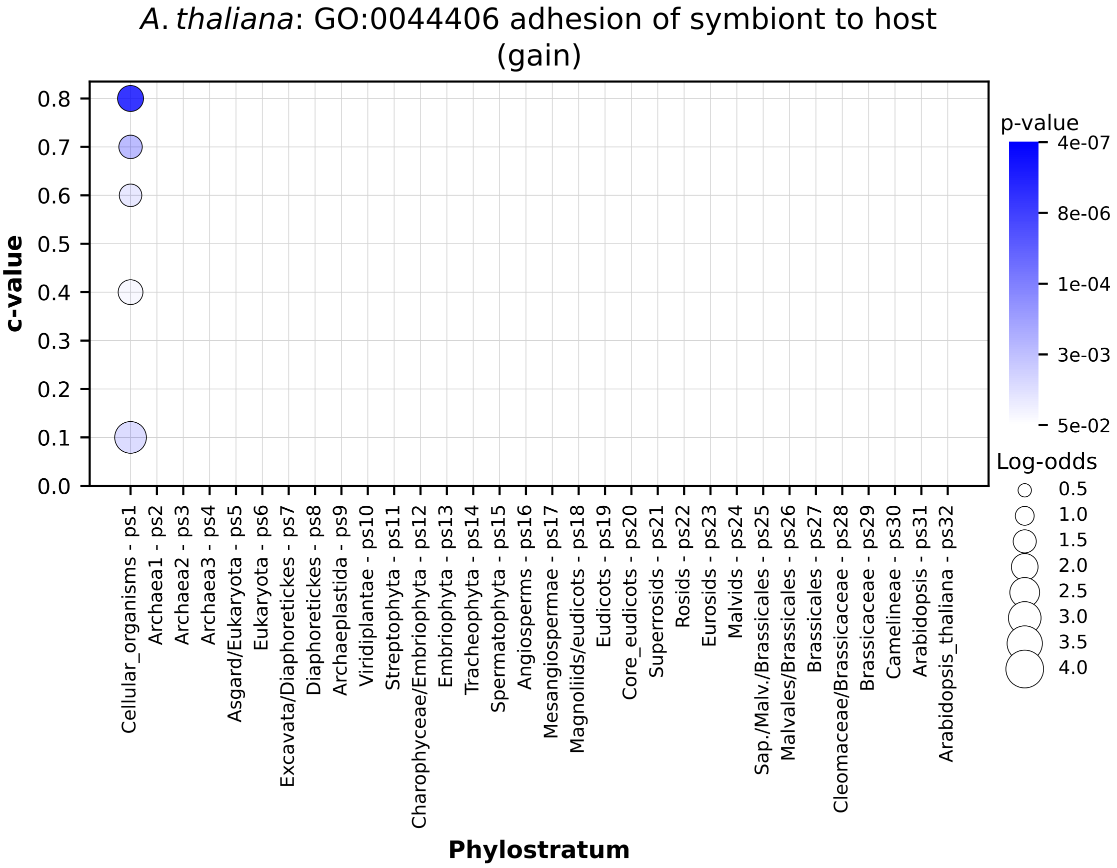

A. thaliana: GO:0044406 adhesion of symbiont to host (gain)
c-value
0.0
0.1
0.2
0.3
0.4
0.5
0.6
0.7
0.8
Phylostratum
Cellular_organisms - ps1
Archaea1 - ps2
Archaea2 - ps3
Archaea3 - ps4
Asgard/Eukaryota - ps5
Eukaryota - ps6
Excavata/Diaphoretickes - ps7
Diaphoretickes - ps8
Archaeplastida - ps9
Viridiplantae - ps10
Streptophyta - ps11
Charophyceae/Embriophyta - ps12
Embriophyta - ps13
Tracheophyta - ps14
Spermatophyta - ps15
Angiosperms - ps16
Mesangiospermae - ps17
Magnoliids/eudicots - ps18
Eudicots - ps19
Core_eudicots - ps20
Superrosids - ps21
Rosids - ps22
Eurosids - ps23
Malvids - ps24
Sap./Malv./Brassicales - ps25
Malvales/Brassicales - ps26
Brassicales - ps27
Cleomaceae/Brassicaceae - ps28
Brassicaceae - ps29
Camelineae - ps30
Arabidopsis - ps31
Arabidopsis_thaliana - ps32
p-value
4e-07
8e-06
1e-04
3e-03
5e-02
Log-odds
0.5
1.0
1.5
2.0
2.5
3.0
3.5
4.0

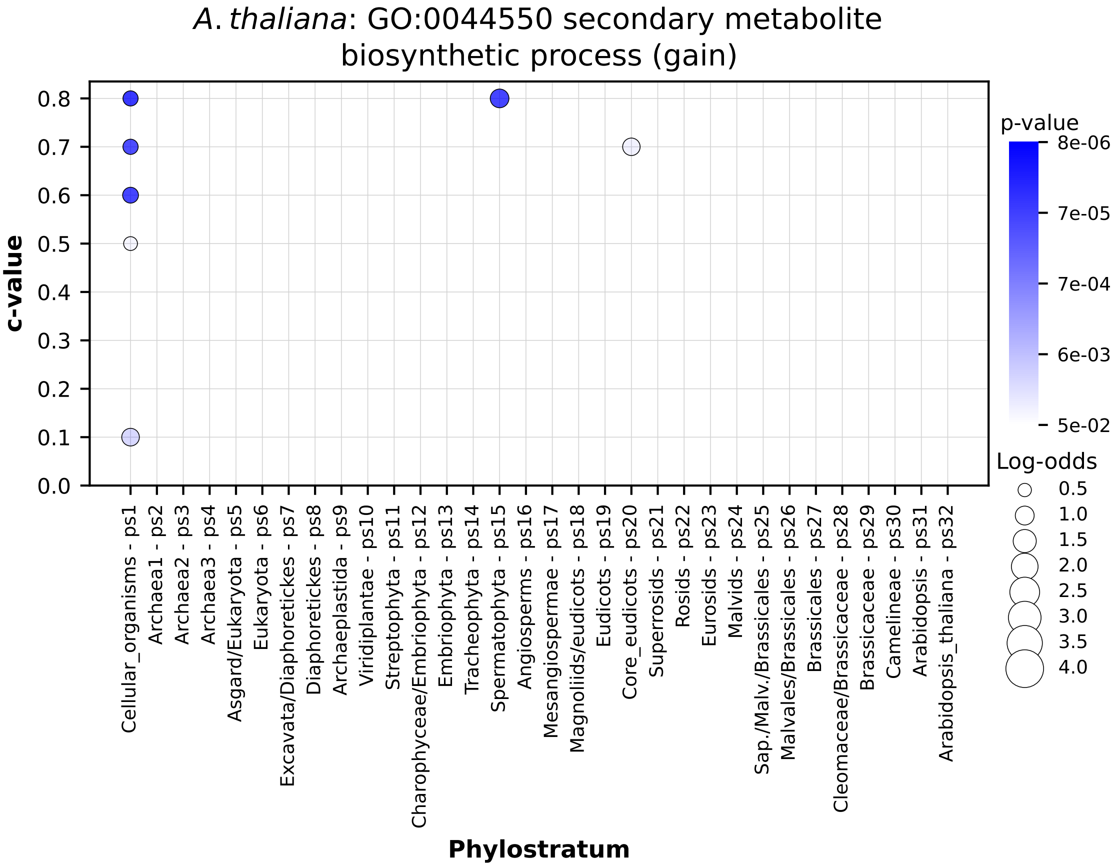

A. thaliana: GO:0044550 secondary metabolite biosynthetic process (gain)
c-value
0.0
0.1
0.2
0.3
0.4
0.5
0.6
0.7
0.8
Phylostratum
Cellular_organisms - ps1
Archaea1 - ps2
Archaea2 - ps3
Archaea3 - ps4
Asgard/Eukaryota - ps5
Eukaryota - ps6
Excavata/Diaphoretickes - ps7
Diaphoretickes - ps8
Archaeplastida - ps9
Viridiplantae - ps10
Streptophyta - ps11
Charophyceae/Embriophyta - ps12
Embriophyta - ps13
Tracheophyta - ps14
Spermatophyta - ps15
Angiosperms - ps16
Mesangiospermae - ps17
Magnoliids/eudicots - ps18
Eudicots - ps19
Core_eudicots - ps20
Superrosids - ps21
Rosids - ps22
Eurosids - ps23
Malvids - ps24
Sap./Malv./Brassicales - ps25
Malvales/Brassicales - ps26
Brassicales - ps27
Cleomaceae/Brassicaceae - ps28
Brassicaceae - ps29
Camelineae - ps30
Arabidopsis - ps31
Arabidopsis_thaliana - ps32
p-value
8e-06
7e-05
7e-04
6e-03
5e-02
Log-odds
0.5
1.0
1.5
2.0
2.5
3.0
3.5
4.0

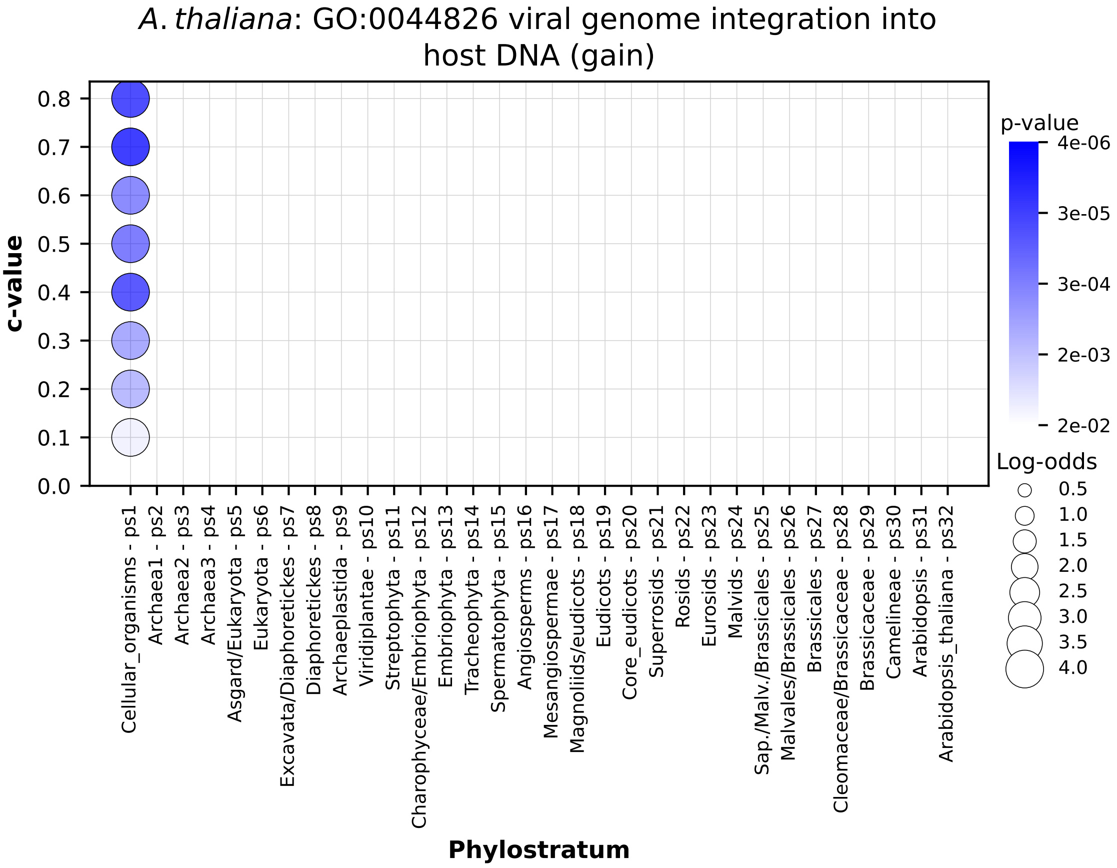

A. thaliana: GO:0044826 viral genome integration into host DNA (gain)
c-value
0.0
0.1
0.2
0.3
0.4
0.5
0.6
0.7
0.8
Phylostratum
Cellular_organisms - ps1
Archaea1 - ps2
Archaea2 - ps3
Archaea3 - ps4
Asgard/Eukaryota - ps5
Eukaryota - ps6
Excavata/Diaphoretickes - ps7
Diaphoretickes - ps8
Archaeplastida - ps9
Viridiplantae - ps10
Streptophyta - ps11
Charophyceae/Embriophyta - ps12
Embriophyta - ps13
Tracheophyta - ps14
Spermatophyta - ps15
Angiosperms - ps16
Mesangiospermae - ps17
Magnoliids/eudicots - ps18
Eudicots - ps19
Core_eudicots - ps20
Superrosids - ps21
Rosids - ps22
Eurosids - ps23
Malvids - ps24
Sap./Malv./Brassicales - ps25
Malvales/Brassicales - ps26
Brassicales - ps27
Cleomaceae/Brassicaceae - ps28
Brassicaceae - ps29
Camelineae - ps30
Arabidopsis - ps31
Arabidopsis_thaliana - ps32
p-value
4e-06
3e-05
3e-04
2e-03
2e-02
Log-odds
0.5
1.0
1.5
2.0
2.5
3.0
3.5
4.0

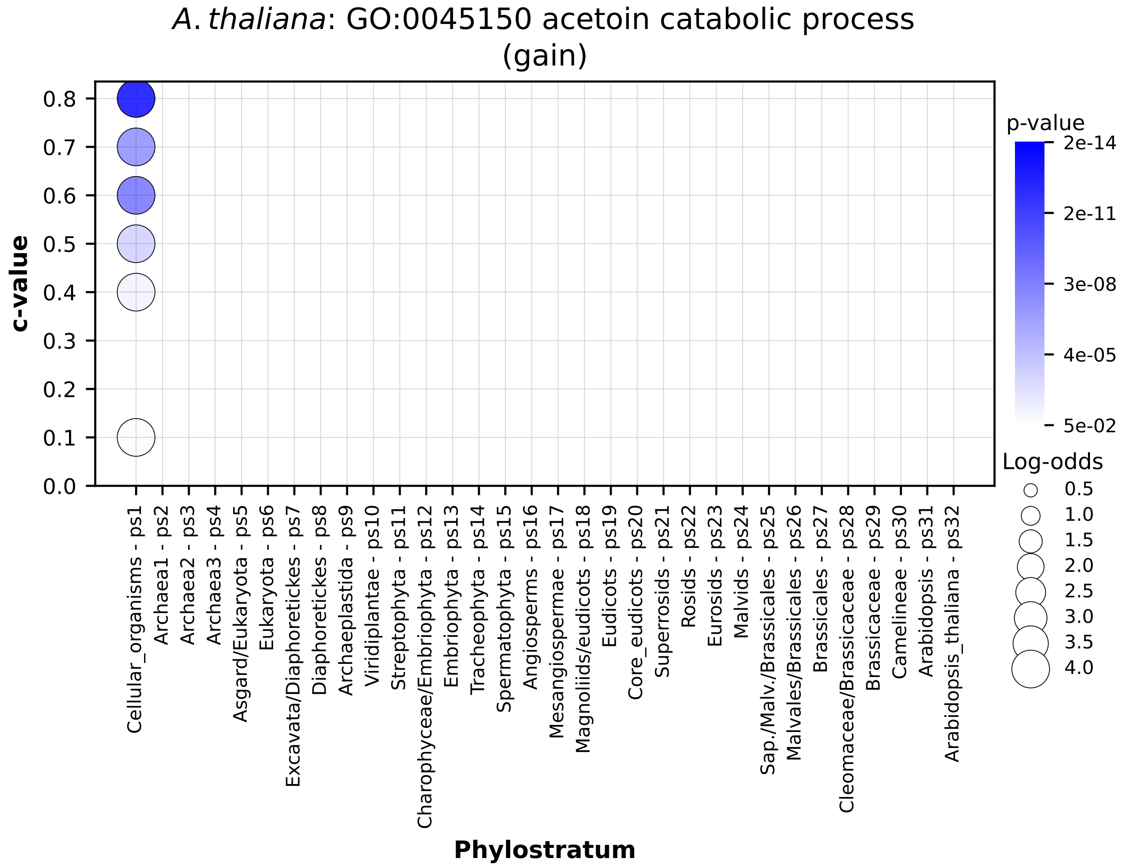
A. thaliana: GO:0045150 acetoin catabolic process
(gain)
c-value
0.0
0.1
0.2
0.3
0.4
0.5
0.6
0.7
0.8
p-value
2e-14
2e-11
3e-08
4e-05
5e-02
Log-odds
0.5
1.0
1.5
2.0
2.5
3.0
3.5
4.0
Cellular_organisms - ps1
Archaea1 - ps2
Archaea2 - ps3
Archaea3 - ps4
Asgard/Eukaryota - ps5
Eukaryota - ps6
Excavata/Diaphoretickes - ps7
Diaphoretickes - ps8
Archaeplastida - ps9
Viridiplantae - ps10
Streptophyta - ps11
Charophyceae/Embriophyta - ps12
Embriophyta - ps13
Tracheophyta - ps14
Spermatophyta - ps15
Angiosperms - ps16
Mesangiospermae - ps17
Magnoliids/eudicots - ps18
Eudicots - ps19
Core_eudicots - ps20
Superrosids - ps21
Rosids - ps22
Eurosids - ps23
Malvids - ps24
Sap./Malv./Brassicales - ps25
Malvales/Brassicales - ps26
Brassicales - ps27
Cleomaceae/Brassicaceae - ps28
Brassicaceae - ps29
Camelineae - ps30
Arabidopsis - ps31
Arabidopsis_thaliana - ps32
Phylostratum

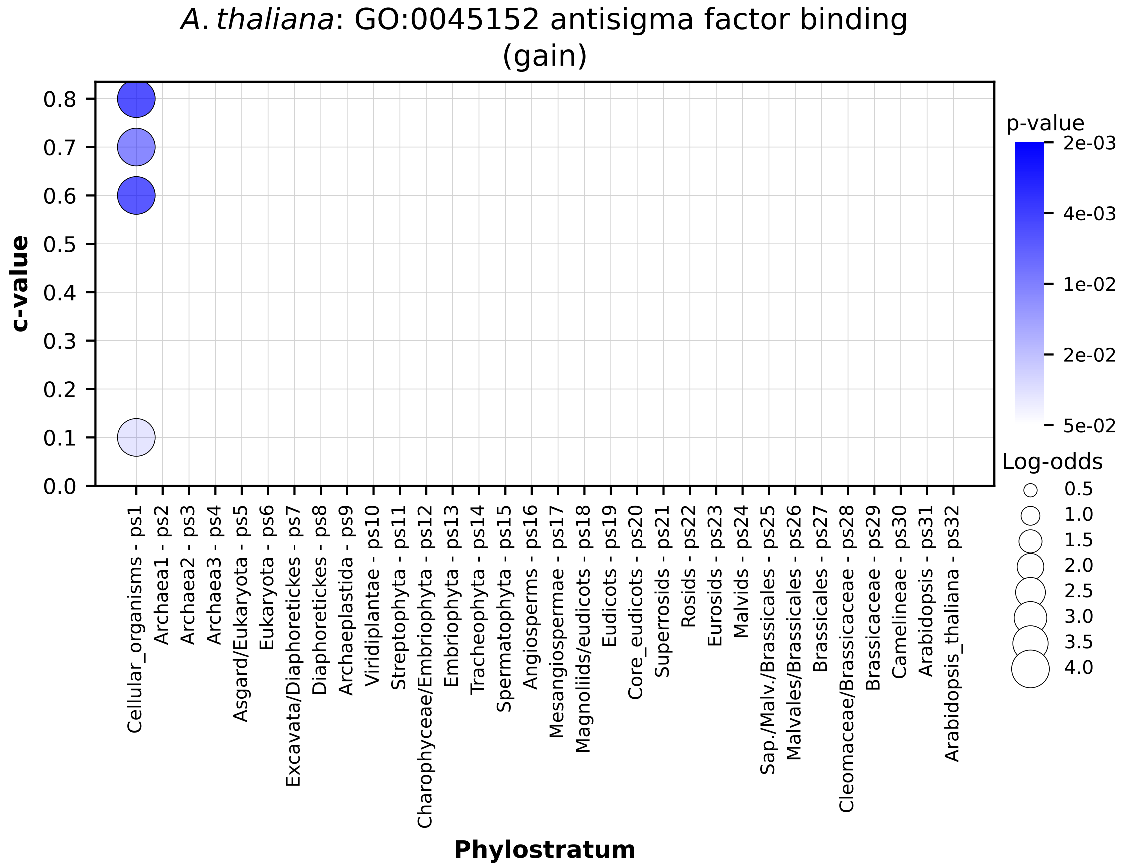
A. thaliana: GO:0045152 antisigma factor binding
(gain)
c-value
0.0
0.1
0.2
0.3
0.4
0.5
0.6
0.7
0.8
Phylostratum
Cellular_organisms - ps1
Archaea1 - ps2
Archaea2 - ps3
Archaea3 - ps4
Asgard/Eukaryota - ps5
Eukaryota - ps6
Excavata/Diaphoretickes - ps7
Diaphoretickes - ps8
Archaeplastida - ps9
Viridiplantae - ps10
Streptophyta - ps11
Charophyceae/Embriophyta - ps12
Embriophyta - ps13
Tracheophyta - ps14
Spermatophyta - ps15
Angiosperms - ps16
Mesangiospermae - ps17
Magnoliids/eudicots - ps18
Eudicots - ps19
Core_eudicots - ps20
Superrosids - ps21
Rosids - ps22
Eurosids - ps23
Malvids - ps24
Sap./Malv./Brassicales - ps25
Malvales/Brassicales - ps26
Brassicales - ps27
Cleomaceae/Brassicaceae - ps28
Brassicaceae - ps29
Camelineae - ps30
Arabidopsis - ps31
Arabidopsis_thaliana - ps32
p-value
2e-03
4e-03
1e-02
2e-02
5e-02
Log-odds
0.5
1.0
1.5
2.0
2.5
3.0
3.5
4.0

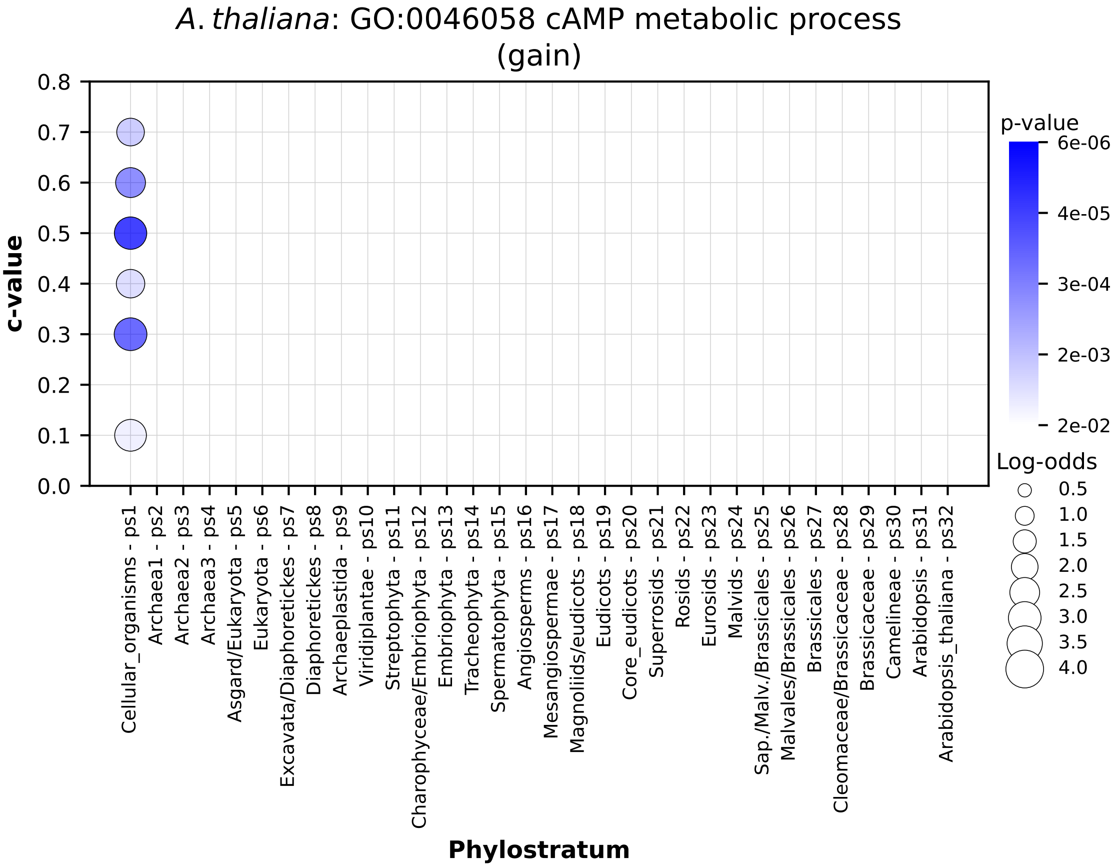

A. thaliana: GO:0046058 cAMP metabolic process
(gain)
c-value
0.0
0.1
0.2
0.3
0.4
0.5
0.6
0.7
0.8
Phylostratum
Cellular_organisms - ps1
Archaea1 - ps2
Archaea2 - ps3
Archaea3 - ps4
Asgard/Eukaryota - ps5
Eukaryota - ps6
Excavata/Diaphoretickes - ps7
Diaphoretickes - ps8
Archaeplastida - ps9
Viridiplantae - ps10
Streptophyta - ps11
Charophyceae/Embriophyta - ps12
Embriophyta - ps13
Tracheophyta - ps14
Spermatophyta - ps15
Angiosperms - ps16
Mesangiospermae - ps17
Magnoliids/eudicots - ps18
Eudicots - ps19
Core_eudicots - ps20
Superrosids - ps21
Rosids - ps22
Eurosids - ps23
Malvids - ps24
Sap./Malv./Brassicales - ps25
Malvales/Brassicales - ps26
Brassicales - ps27
Cleomaceae/Brassicaceae - ps28
Brassicaceae - ps29
Camelineae - ps30
Arabidopsis - ps31
Arabidopsis_thaliana - ps32
p-value
6e-06
4e-05
3e-04
2e-03
2e-02
Log-odds
0.5
1.0
1.5
2.0
2.5
3.0
3.5
4.0

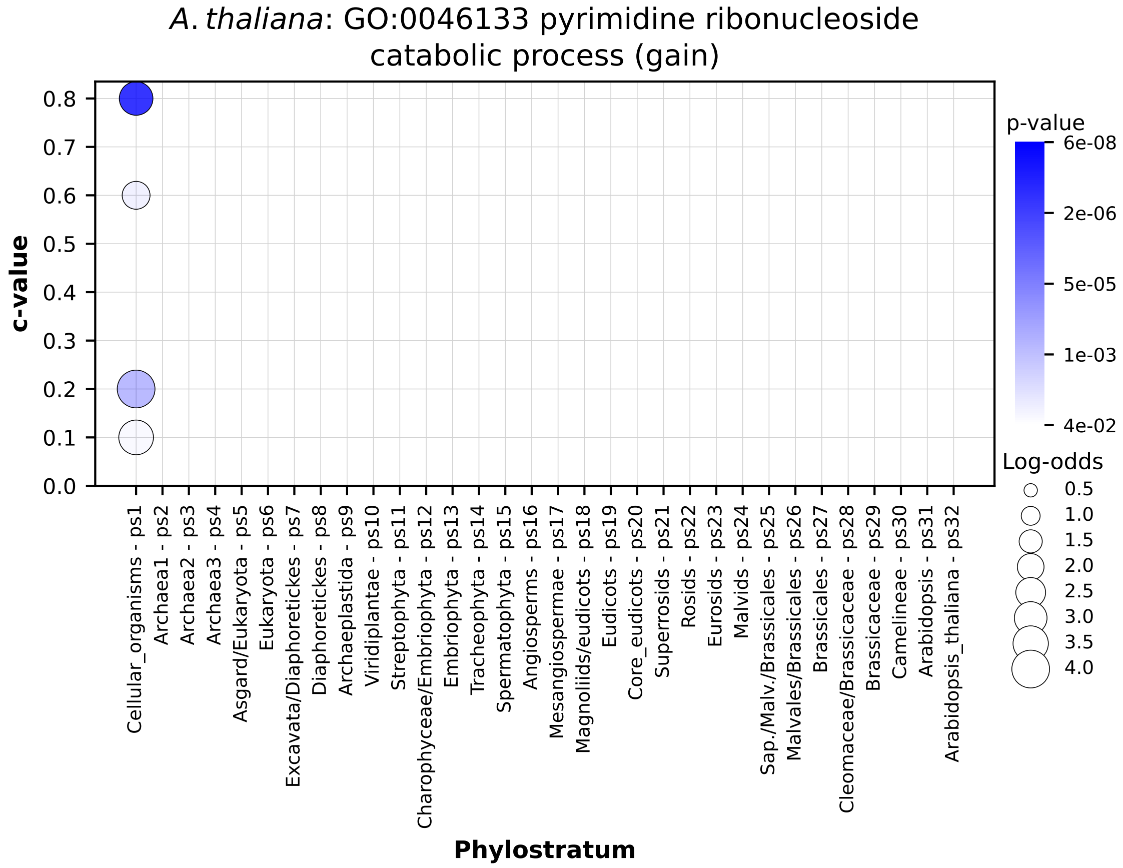
A. thaliana: GO:0046133 pyrimidine ribonucleoside catabolic process (gain)
c-value
0.0
0.1
0.2
0.3
0.4
0.5
0.6
0.7
0.8
Phylostratum
Cellular_organisms - ps1
Archaea1 - ps2
Archaea2 - ps3
Archaea3 - ps4
Asgard/Eukaryota - ps5
Eukaryota - ps6
Excavata/Diaphoretickes - ps7
Diaphoretickes - ps8
Archaeplastida - ps9
Viridiplantae - ps10
Streptophyta - ps11
Charophyceae/Embriophyta - ps12
Embriophyta - ps13
Tracheophyta - ps14
Spermatophyta - ps15
Angiosperms - ps16
Mesangiospermae - ps17
Magnoliids/eudicots - ps18
Eudicots - ps19
Core_eudicots - ps20
Superrosids - ps21
Rosids - ps22
Eurosids - ps23
Malvids - ps24
Sap./Malv./Brassicales - ps25
Malvales/Brassicales - ps26
Brassicales - ps27
Cleomaceae/Brassicaceae - ps28
Brassicaceae - ps29
Camelineae - ps30
Arabidopsis - ps31
Arabidopsis_thaliana - ps32
p-value
6e-08
2e-06
5e-05
1e-03
4e-02
Log-odds
0.5
1.0
1.5
2.0
2.5
3.0
3.5
4.0

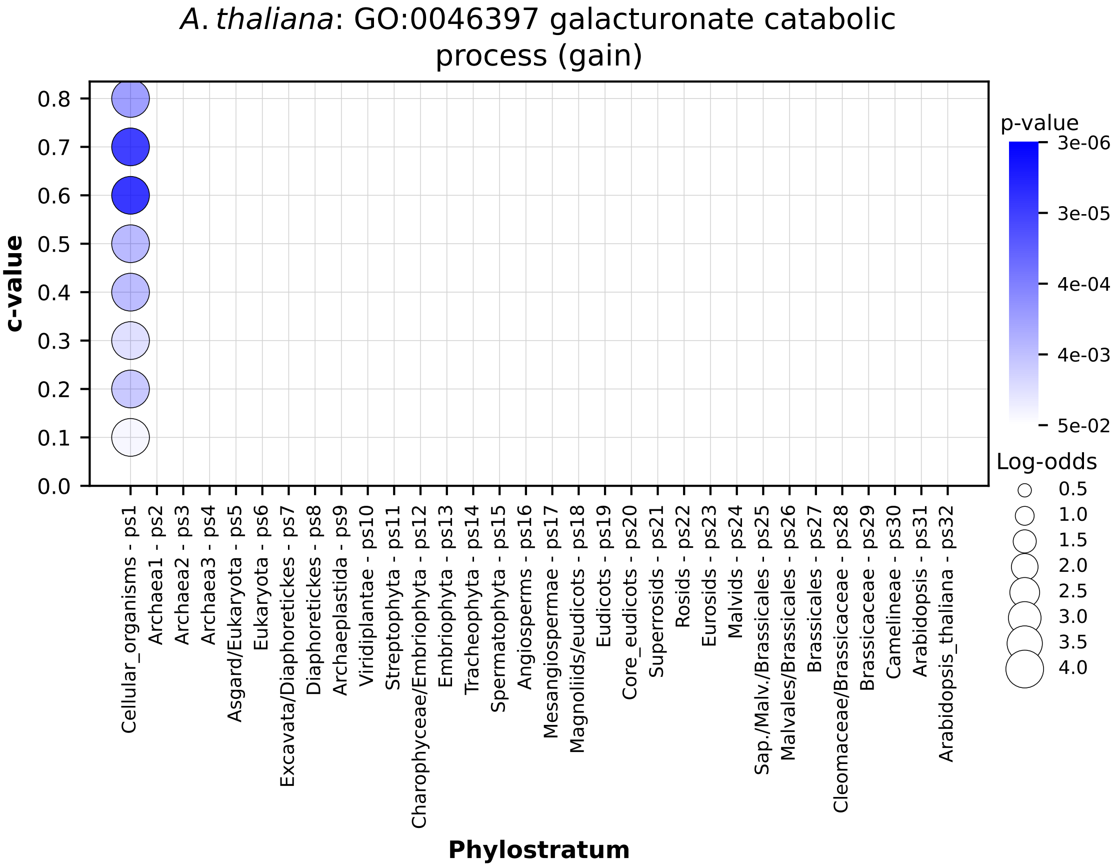

A. thaliana: GO:0046397 galacturonate catabolic process (gain)
c-value
0.0
0.1
0.2
0.3
0.4
0.5
0.6
0.7
0.8
Phylostratum
Cellular_organisms - ps1
Archaea1 - ps2
Archaea2 - ps3
Archaea3 - ps4
Asgard/Eukaryota - ps5
Eukaryota - ps6
Excavata/Diaphoretickes - ps7
Diaphoretickes - ps8
Archaeplastida - ps9
Viridiplantae - ps10
Streptophyta - ps11
Charophyceae/Embriophyta - ps12
Embriophyta - ps13
Tracheophyta - ps14
Spermatophyta - ps15
Angiosperms - ps16
Mesangiospermae - ps17
Magnoliids/eudicots - ps18
Eudicots - ps19
Core_eudicots - ps20
Superrosids - ps21
Rosids - ps22
Eurosids - ps23
Malvids - ps24
Sap./Malv./Brassicales - ps25
Malvales/Brassicales - ps26
Brassicales - ps27
Cleomaceae/Brassicaceae - ps28
Brassicaceae - ps29
Camelineae - ps30
Arabidopsis - ps31
Arabidopsis_thaliana - ps32
p-value
3e-06
3e-05
4e-04
4e-03
5e-02
Log-odds
0.5
1.0
1.5
2.0
2.5
3.0
3.5
4.0

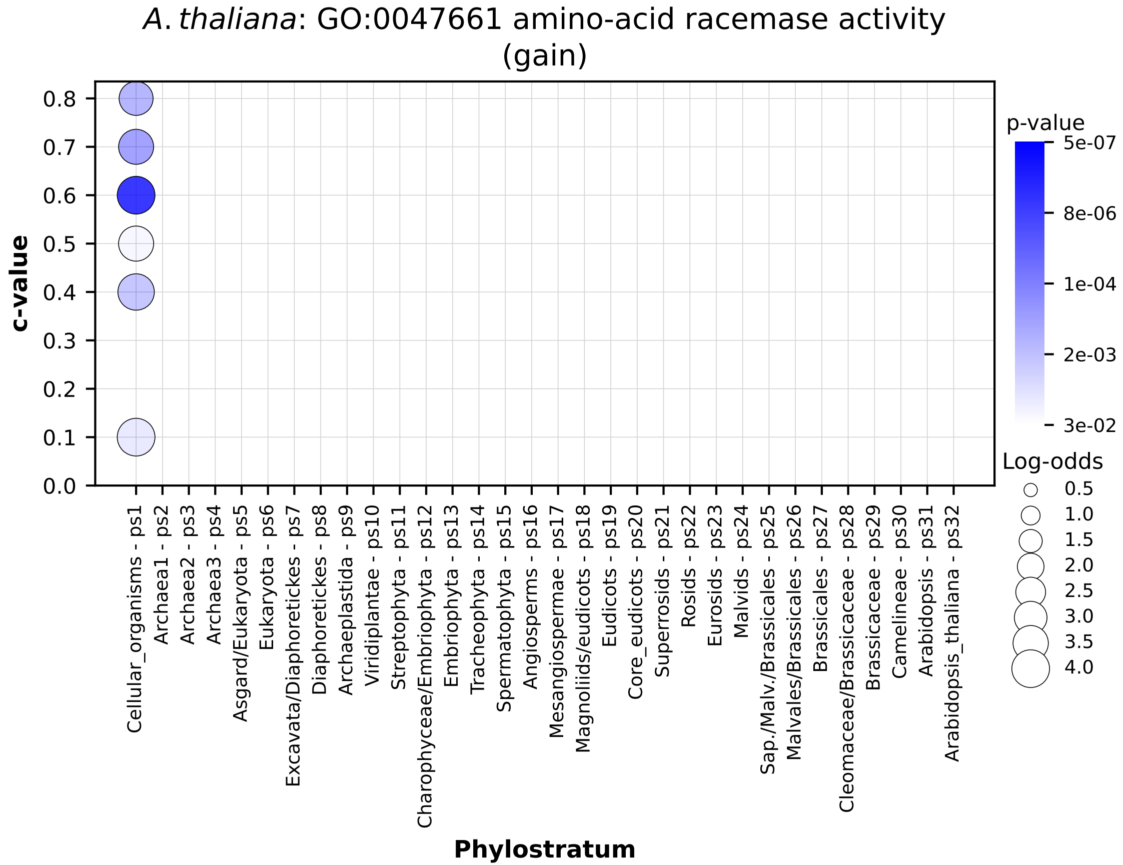

A. thaliana: GO:0047661 amino-acid racemase activity
(gain)
c-value
0.0
0.1
0.2
0.3
0.4
0.5
0.6
0.7
0.8
Phylostratum
Cellular_organisms - ps1
Archaea1 - ps2
Archaea2 - ps3
Archaea3 - ps4
Asgard/Eukaryota - ps5
Eukaryota - ps6
Excavata/Diaphoretickes - ps7
Diaphoretickes - ps8
Archaeplastida - ps9
Viridiplantae - ps10
Streptophyta - ps11
Charophyceae/Embriophyta - ps12
Embriophyta - ps13
Tracheophyta - ps14
Spermatophyta - ps15
Angiosperms - ps16
Mesangiospermae - ps17
Magnoliids/eudicots - ps18
Eudicots - ps19
Core_eudicots - ps20
Superrosids - ps21
Rosids - ps22
Eurosids - ps23
Malvids - ps24
Sap./Malv./Brassicales - ps25
Malvales/Brassicales - ps26
Brassicales - ps27
Cleomaceae/Brassicaceae - ps28
Brassicaceae - ps29
Camelineae - ps30
Arabidopsis - ps31
Arabidopsis_thaliana - ps32
p-value
5e-07
8e-06
1e-04
2e-03
3e-02
Log-odds
0.5
1.0
1.5
2.0
2.5
3.0
3.5
4.0

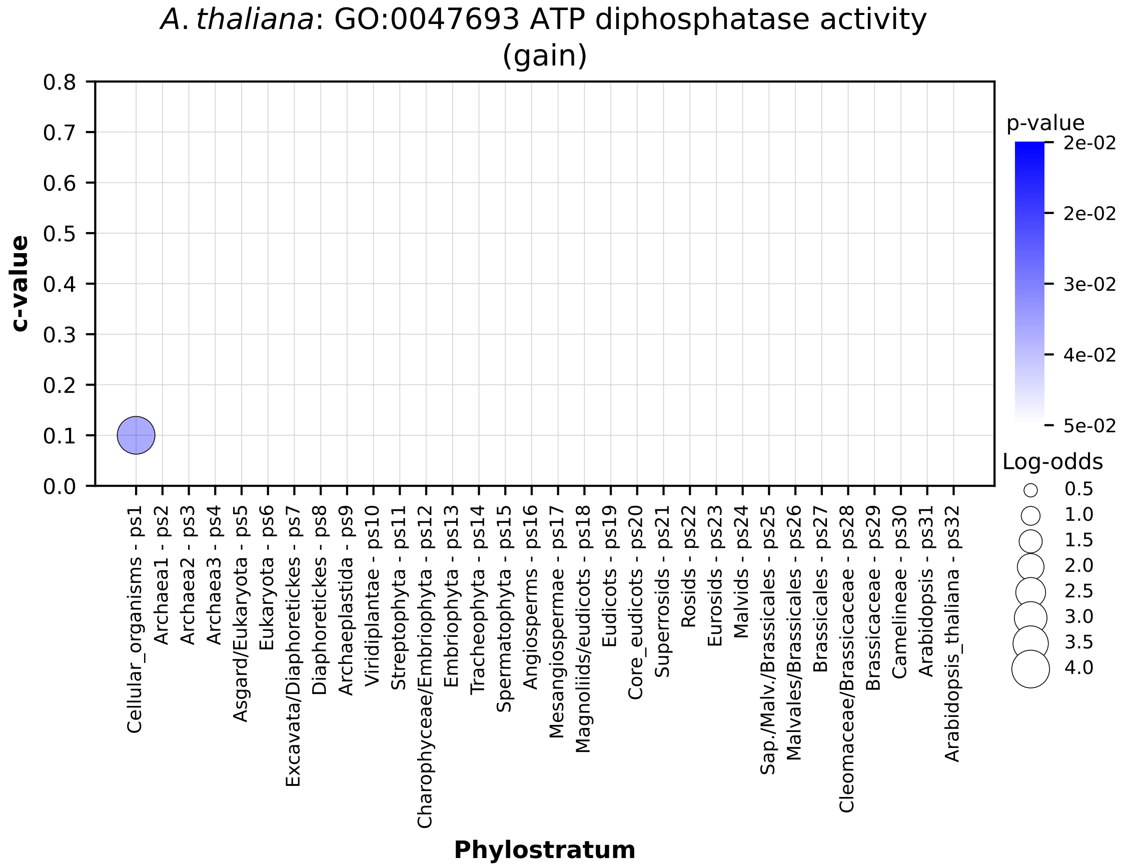
A. thaliana: GO:0047693 ATP diphosphatase activity (gain)
c-value
0.8
0.7
0.6
0.5
0.4
0.3
0.2
0.1
0.0
Phylostratum
Cellular_organisms - ps1
Archaea1 - ps2
Archaea2 - ps3
Archaea3 - ps4
Asgard/Eukaryota - ps5
Eukaryota - ps6
Excavata/Diaphoretickes - ps7
Diaphoretickes - ps8
Archaeplastida - ps9
Viridiplantae - ps10
Streptophyta - ps11
Charophyceae/Embriophyta - ps12
Embriophyta - ps13
Tracheophyta - ps14
Spermatophyta - ps15
Angiosperms - ps16
Mesangiospermae - ps17
Magnoliids/eudicots - ps18
Eudicots - ps19
Core_eudicots - ps20
Superrosids - ps21
Rosids - ps22
Eurosids - ps23
Malvids - ps24
Sap./Malv./Brassicales - ps25
Malvales/Brassicales - ps26
Brassicales - ps27
Cleomaceae/Brassicaceae - ps28
Brassicaceae - ps29
Camelineae - ps30
Arabidopsis - ps31
Arabidopsis_thaliana - ps32
p-value
2e-02
2e-02
3e-02
4e-02
5e-02
Log-odds
0.5
1.0
1.5
2.0
2.5
3.0
3.5
4.0

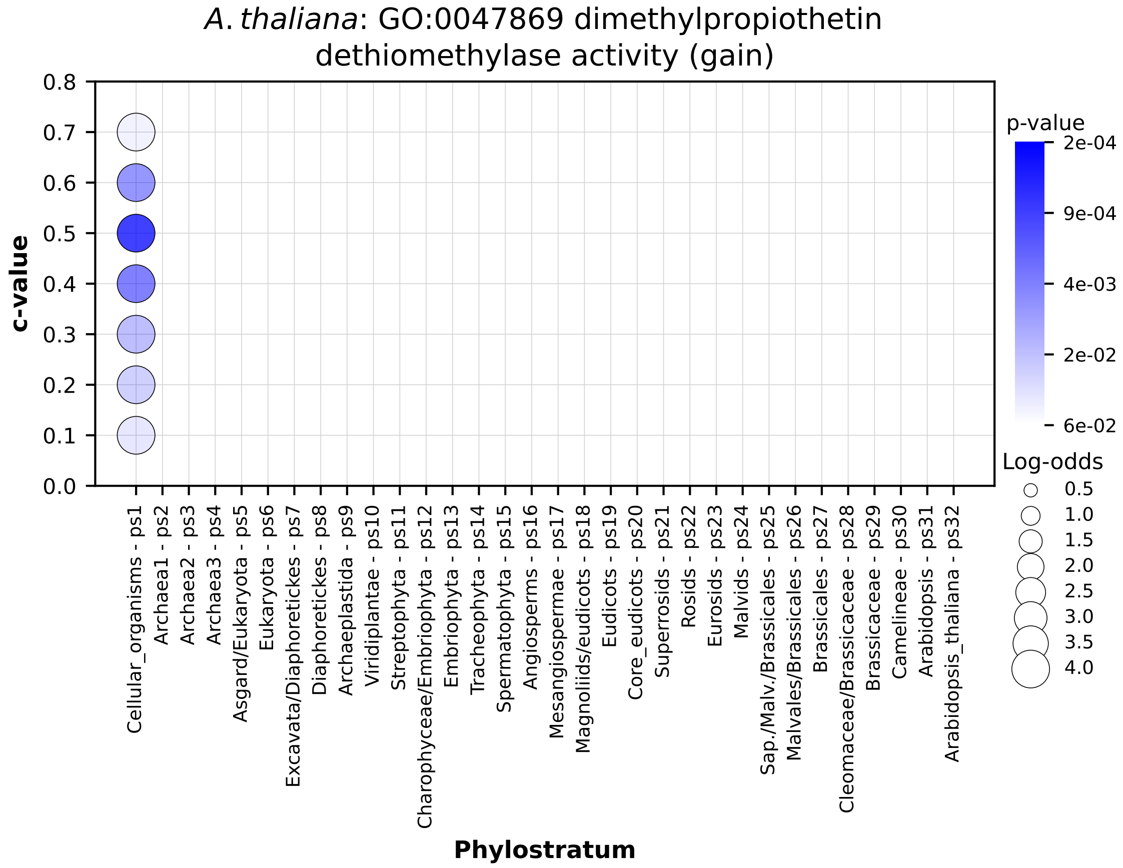

A. thaliana: GO:0047869 dimethylpropiothetin dethiomethylase activity (gain)
c-value
0.8
0.7
0.6
0.5
0.4
0.3
0.2
0.1
0.0
p-value
2e-04
9e-04
4e-03
2e-02
6e-02
Log-odds
0.5
1.0
1.5
2.0
2.5
3.0
3.5
4.0
Cellular_organisms - ps1
Archaea1 - ps2
Archaea2 - ps3
Archaea3 - ps4
Asgard/Eukaryota - ps5
Eukaryota - ps6
Excavata/Diaphoretickes - ps7
Diaphoretickes - ps8
Archaeplastida - ps9
Viridiplantae - ps10
Streptophyta - ps11
Charophyceae/Embriophyta - ps12
Embriophyta - ps13
Tracheophyta - ps14
Spermatophyta - ps15
Angiosperms - ps16
Mesangiospermae - ps17
Magnoliids/eudicots - ps18
Eudicots - ps19
Core_eudicots - ps20
Superrosids - ps21
Rosids - ps22
Eurosids - ps23
Malvids - ps24
Sap./Malv./Brassicales - ps25
Malvales/Brassicales - ps26
Brassicales - ps27
Cleomaceae/Brassicaceae - ps28
Brassicaceae - ps29
Camelineae - ps30
Arabidopsis - ps31
Arabidopsis_thaliana - ps32
Phylostratum

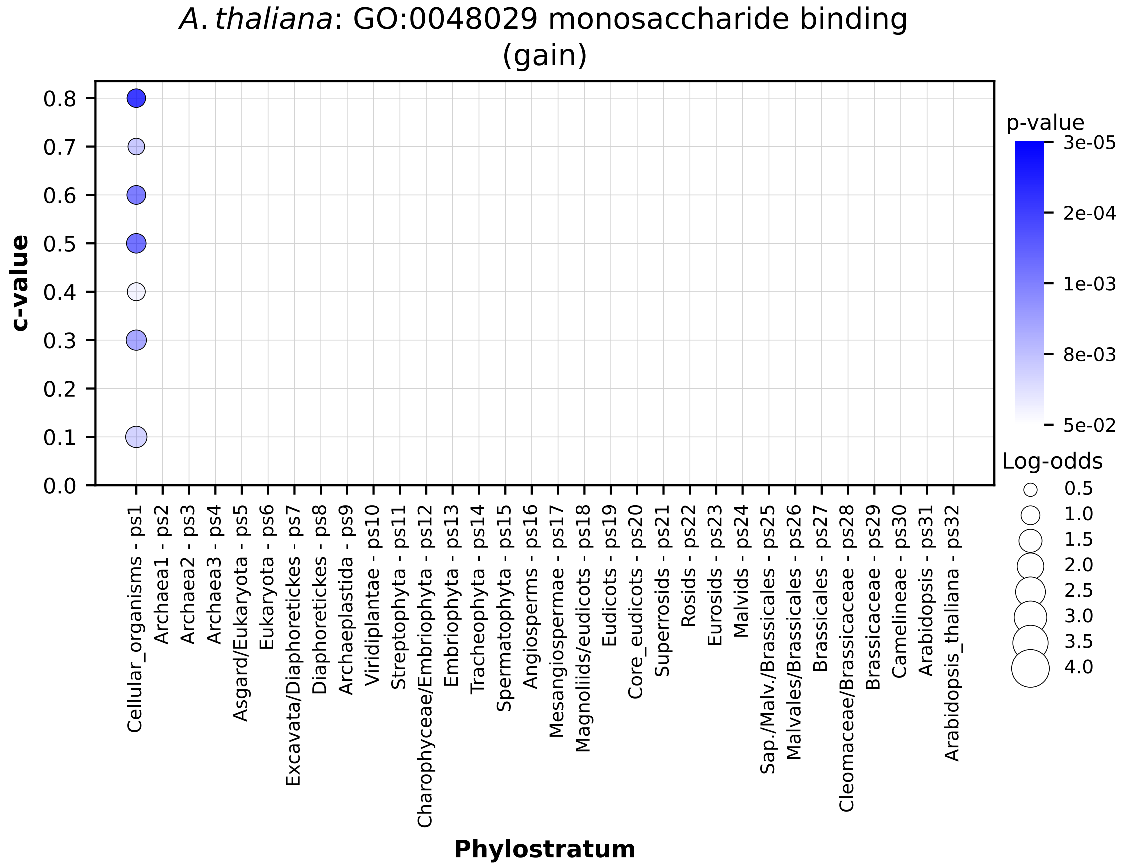

A. thaliana: GO:0048029 monosaccharide binding
(gain)
c-value
0.0
0.1
0.2
0.3
0.4
0.5
0.6
0.7
0.8
Phylostratum
Cellular_organisms - ps1
Archaea1 - ps2
Archaea2 - ps3
Archaea3 - ps4
Asgard/Eukaryota - ps5
Eukaryota - ps6
Excavata/Diaphoretickes - ps7
Diaphoretickes - ps8
Archaeplastida - ps9
Viridiplantae - ps10
Streptophyta - ps11
Charophyceae/Embriophyta - ps12
Embriophyta - ps13
Tracheophyta - ps14
Spermatophyta - ps15
Angiosperms - ps16
Mesangiospermae - ps17
Magnoliids/eudicots - ps18
Eudicots - ps19
Core_eudicots - ps20
Superrosids - ps21
Rosids - ps22
Eurosids - ps23
Malvids - ps24
Sap./Malv./Brassicales - ps25
Malvales/Brassicales - ps26
Brassicales - ps27
Cleomaceae/Brassicaceae - ps28
Brassicaceae - ps29
Camelineae - ps30
Arabidopsis - ps31
Arabidopsis_thaliana - ps32
p-value
3e-05
2e-04
1e-03
8e-03
5e-02
Log-odds
0.5
1.0
1.5
2.0
2.5
3.0
3.5
4.0

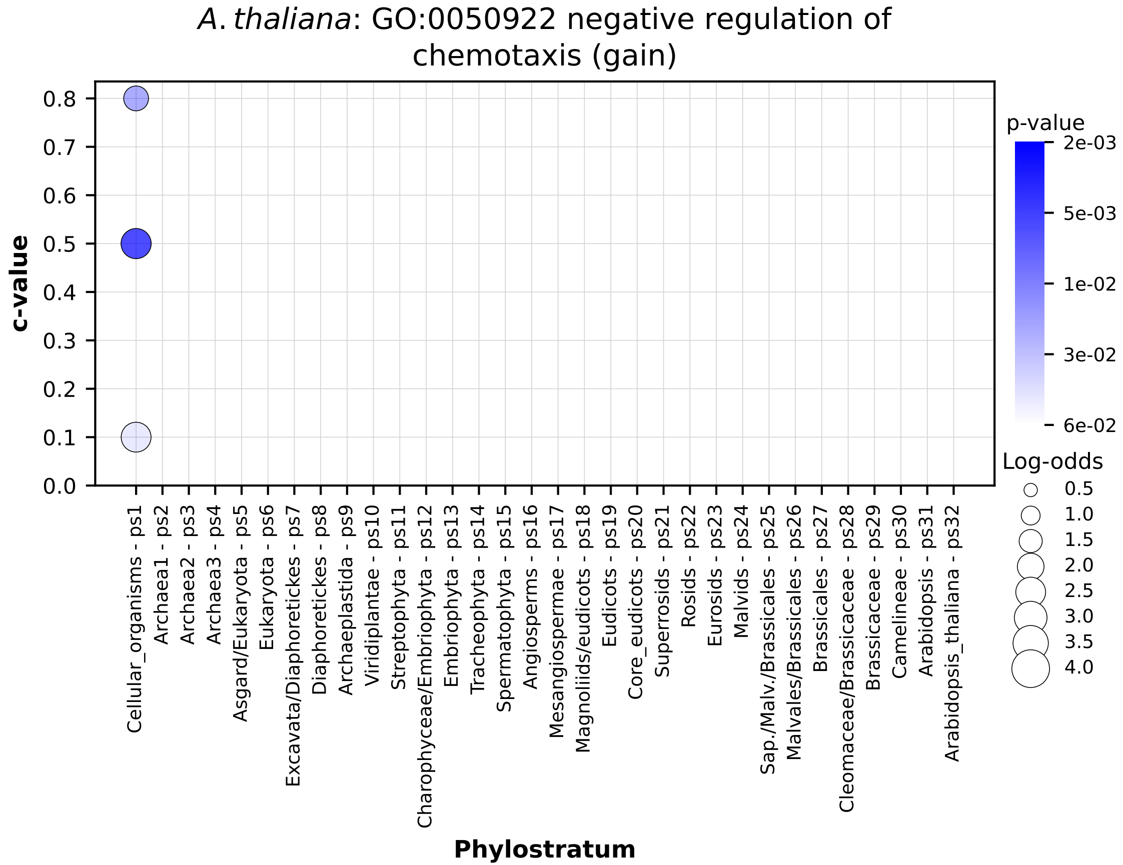

A. thaliana: GO:0050922 negative regulation of chemotaxis (gain)
c-value
0.0
0.1
0.2
0.3
0.4
0.5
0.6
0.7
0.8
Phylostratum
Cellular_organisms - ps1
Archaea1 - ps2
Archaea2 - ps3
Archaea3 - ps4
Asgard/Eukaryota - ps5
Eukaryota - ps6
Excavata/Diaphoretickes - ps7
Diaphoretickes - ps8
Archaeplastida - ps9
Viridiplantae - ps10
Streptophyta - ps11
Charophyceae/Embriophyta - ps12
Embriophyta - ps13
Tracheophyta - ps14
Spermatophyta - ps15
Angiosperms - ps16
Mesangiospermae - ps17
Magnoliids/eudicots - ps18
Eudicots - ps19
Core_eudicots - ps20
Superrosids - ps21
Rosids - ps22
Eurosids - ps23
Malvids - ps24
Sap./Malv./Brassicales - ps25
Malvales/Brassicales - ps26
Brassicales - ps27
Cleomaceae/Brassicaceae - ps28
Brassicaceae - ps29
Camelineae - ps30
Arabidopsis - ps31
Arabidopsis_thaliana - ps32
p-value
2e-03
5e-03
1e-02
3e-02
6e-02
Log-odds
0.5
1.0
1.5
2.0
2.5
3.0
3.5
4.0

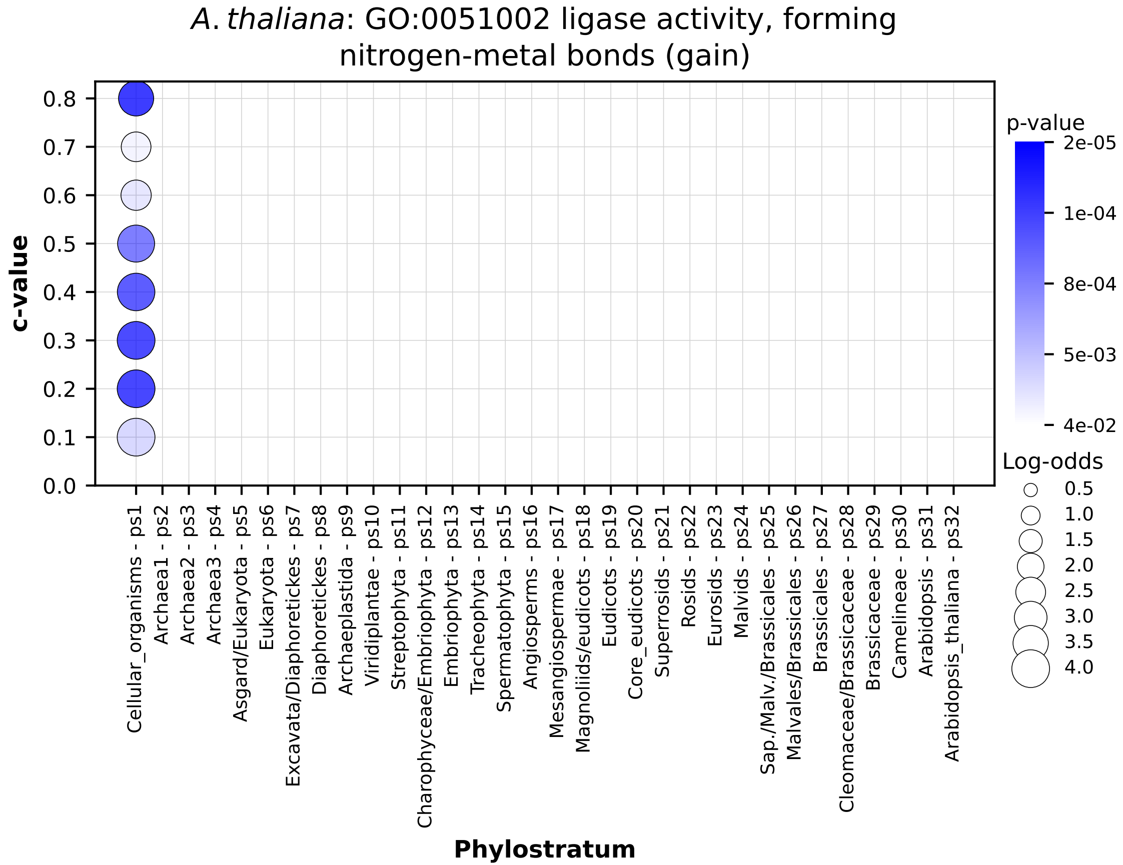
A. thaliana: GO:0051002 ligase activity, forming nitrogen-metal bonds (gain)
c-value
0.0
0.1
0.2
0.3
0.4
0.5
0.6
0.7
0.8
Phylostratum
Cellular_organisms - ps1
Archaea1 - ps2
Archaea2 - ps3
Archaea3 - ps4
Asgard/Eukaryota - ps5
Eukaryota - ps6
Excavata/Diaphoretickes - ps7
Diaphoretickes - ps8
Archaeplastida - ps9
Viridiplantae - ps10
Streptophyta - ps11
Charophyceae/Embriophyta - ps12
Embriophyta - ps13
Tracheophyta - ps14
Spermatophyta - ps15
Angiosperms - ps16
Mesangiospermae - ps17
Magnoliids/eudicots - ps18
Eudicots - ps19
Core_eudicots - ps20
Superrosids - ps21
Rosids - ps22
Eurosids - ps23
Malvids - ps24
Sap./Malv./Brassicales - ps25
Malvales/Brassicales - ps26
Brassicales - ps27
Cleomaceae/Brassicaceae - ps28
Brassicaceae - ps29
Camelineae - ps30
Arabidopsis - ps31
Arabidopsis_thaliana - ps32
p-value
2e-05
1e-04
8e-04
5e-03
4e-02
Log-odds
0.5
1.0
1.5
2.0
2.5
3.0
3.5
4.0

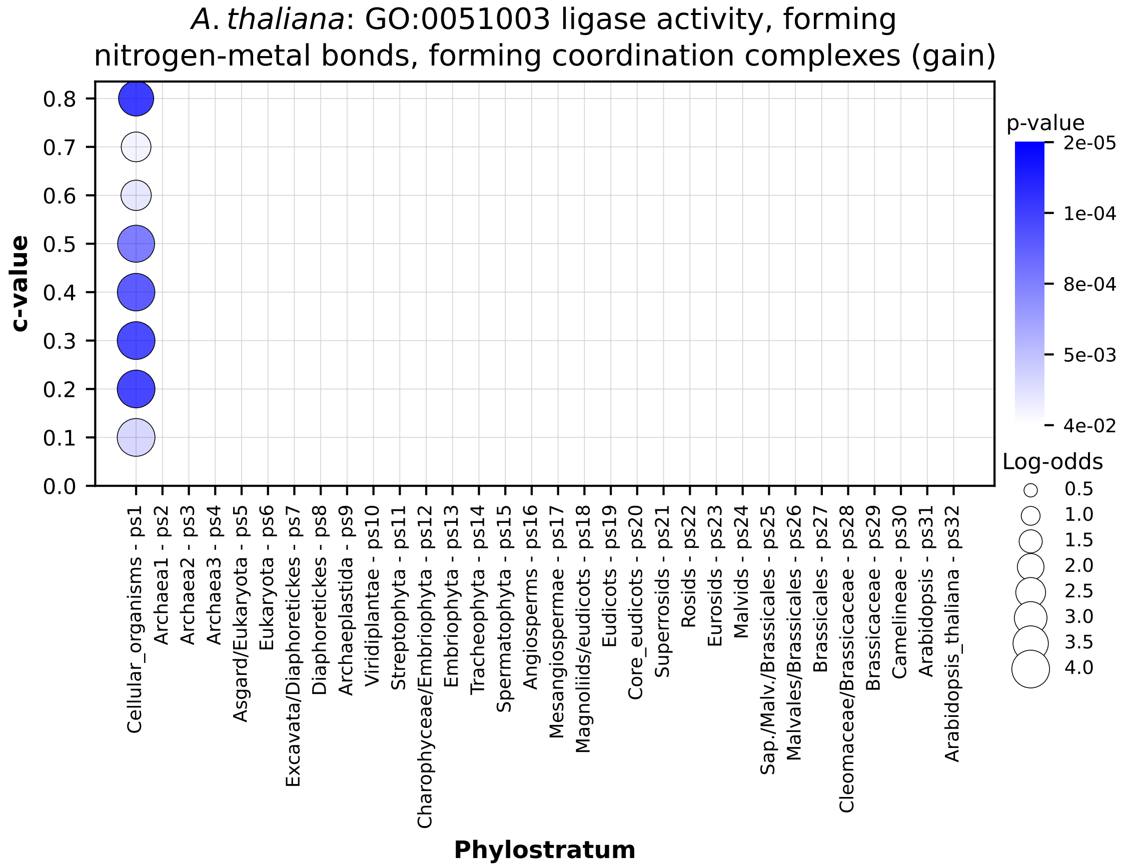
A. thaliana: GO:0051003 ligase activity, forming nitrogen-metal bonds, forming coordination complexes (gain)
c-value
0.0
0.1
0.2
0.3
0.4
0.5
0.6
0.7
0.8
p-value
2e-05
1e-04
8e-04
5e-03
4e-02
Log-odds
0.5
1.0
1.5
2.0
2.5
3.0
3.5
4.0
Cellular_organisms - ps1
Archaea1 - ps2
Archaea2 - ps3
Archaea3 - ps4
Asgard/Eukaryota - ps5
Eukaryota - ps6
Excavata/Diaphoretickes - ps7
Diaphoretickes - ps8
Archaeplastida - ps9
Viridiplantae - ps10
Streptophyta - ps11
Charophyceae/Embriophyta - ps12
Embriophyta - ps13
Tracheophyta - ps14
Spermatophyta - ps15
Angiosperms - ps16
Mesangiospermae - ps17
Magnoliids/eudicots - ps18
Eudicots - ps19
Core_eudicots - ps20
Superrosids - ps21
Rosids - ps22
Eurosids - ps23
Malvids - ps24
Sap./Malv./Brassicales - ps25
Malvales/Brassicales - ps26
Brassicales - ps27
Cleomaceae/Brassicaceae - ps28
Brassicaceae - ps29
Camelineae - ps30
Arabidopsis - ps31
Arabidopsis_thaliana - ps32
Phylostratum

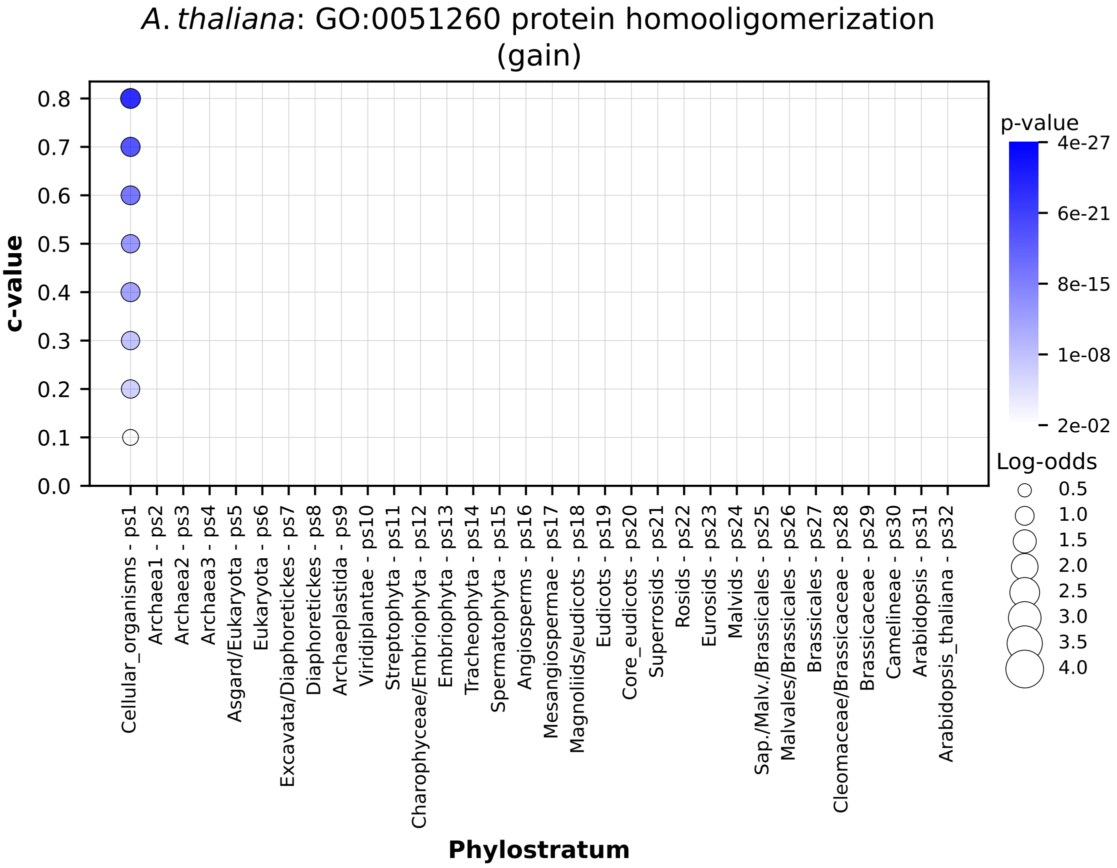

A. thaliana: GO:0051260 protein homooligomerization
(gain)
c-value
0.8
0.7
0.6
0.5
0.4
0.3
0.2
0.1
0.0
Phylostratum
Cellular_organisms - ps1
Archaea1 - ps2
Archaea2 - ps3
Archaea3 - ps4
Asgard/Eukaryota - ps5
Eukaryota - ps6
Excavata/Diaphoretickes - ps7
Diaphoretickes - ps8
Archaeplastida - ps9
Viridiplantae - ps10
Streptophyta - ps11
Charophyceae/Embriophyta - ps12
Embriophyta - ps13
Tracheophyta - ps14
Spermatophyta - ps15
Angiosperms - ps16
Mesangiospermae - ps17
Magnoliids/eudicots - ps18
Eudicots - ps19
Core_eudicots - ps20
Superrosids - ps21
Rosids - ps22
Eurosids - ps23
Malvids - ps24
Sap./Malv./Brassicales - ps25
Malvales/Brassicales - ps26
Brassicales - ps27
Cleomaceae/Brassicaceae - ps28
Brassicaceae - ps29
Camelineae - ps30
Arabidopsis - ps31
Arabidopsis_thaliana - ps32
p-value
4e-27
6e-21
8e-15
1e-08
2e-02
Log-odds
0.5
1.0
1.5
2.0
2.5
3.0
3.5
4.0

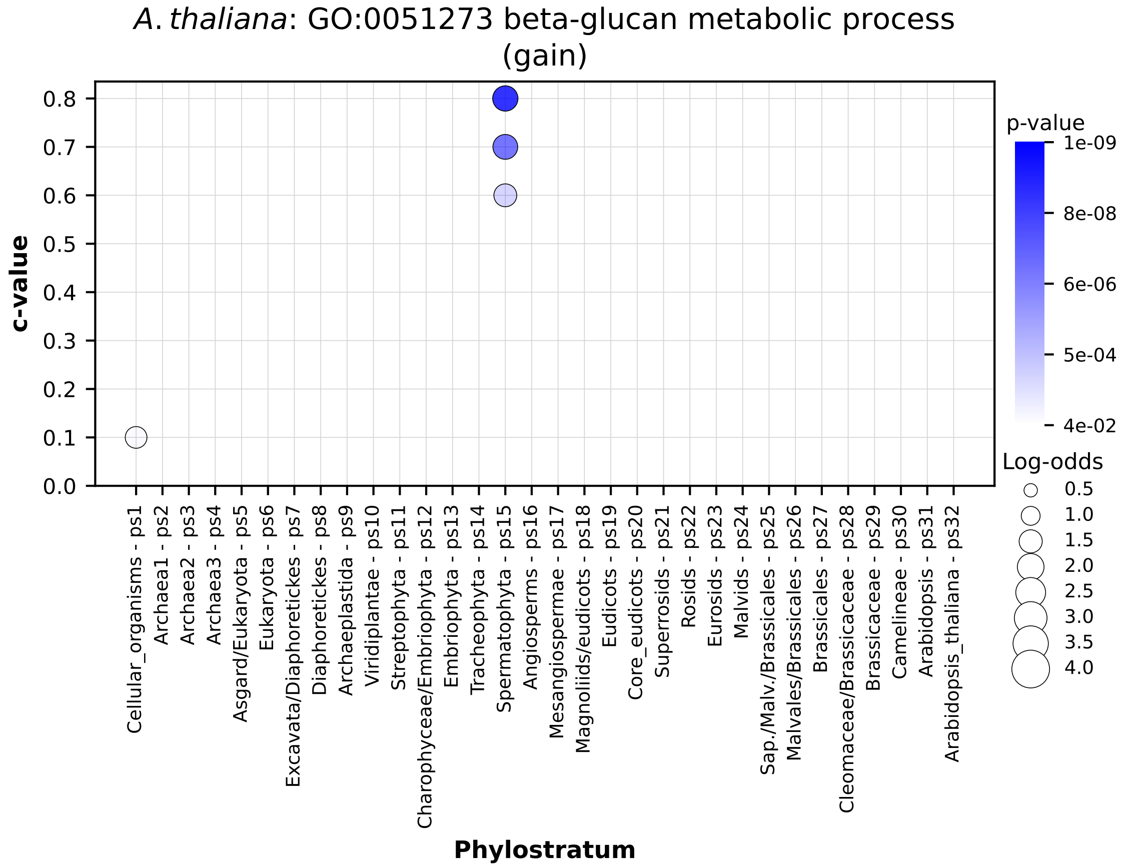
A. thaliana: GO:0051273 beta-glucan metabolic process
(gain)
c-value
0.0
0.1
0.2
0.3
0.4
0.5
0.6
0.7
0.8
Phylostratum
Cellular_organisms - ps1
Archaea1 - ps2
Archaea2 - ps3
Archaea3 - ps4
Asgard/Eukaryota - ps5
Eukaryota - ps6
Excavata/Diaphoretickes - ps7
Diaphoretickes - ps8
Archaeplastida - ps9
Viridiplantae - ps10
Streptophyta - ps11
Charophyceae/Embriophyta - ps12
Embriophyta - ps13
Tracheophyta - ps14
Spermatophyta - ps15
Angiosperms - ps16
Mesangiospermae - ps17
Magnoliids/eudicots - ps18
Eudicots - ps19
Core_eudicots - ps20
Superrosids - ps21
Rosids - ps22
Eurosids - ps23
Malvids - ps24
Sap./Malv./Brassicales - ps25
Malvales/Brassicales - ps26
Brassicales - ps27
Cleomaceae/Brassicaceae - ps28
Brassicaceae - ps29
Camelineae - ps30
Arabidopsis - ps31
Arabidopsis_thaliana - ps32
p-value
1e-09
8e-08
6e-06
5e-04
4e-02
Log-odds
0.5
1.0
1.5
2.0
2.5
3.0
3.5
4.0

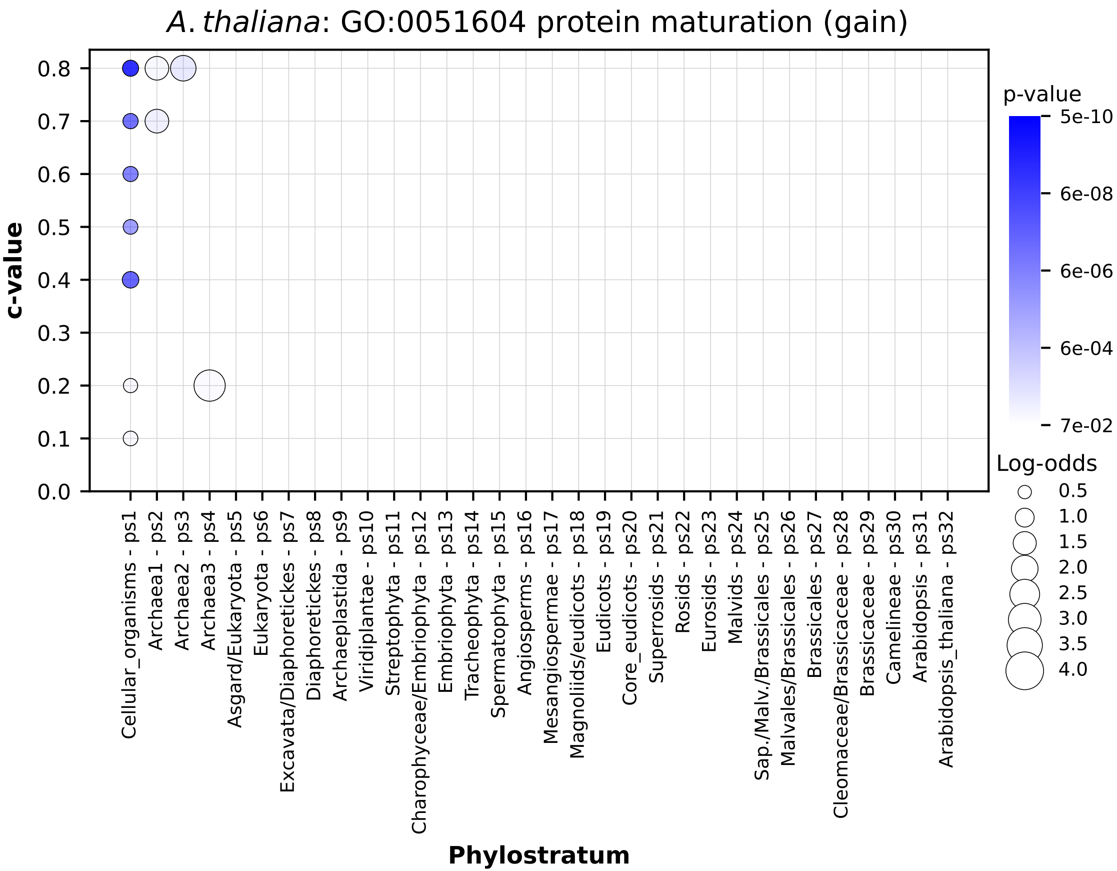

A. thaliana: GO:0051604 protein maturation (gain)
c-value
0.0
0.1
0.2
0.3
0.4
0.5
0.6
0.7
0.8
Phylostratum
Cellular_organisms - ps1
Archaea1 - ps2
Archaea2 - ps3
Archaea3 - ps4
Asgard/Eukaryota - ps5
Eukaryota - ps6
Excavata/Diaphoretickes - ps7
Diaphoretickes - ps8
Archaeplastida - ps9
Viridiplantae - ps10
Streptophyta - ps11
Charophyceae/Embriophyta - ps12
Embriophyta - ps13
Tracheophyta - ps14
Spermatophyta - ps15
Angiosperms - ps16
Mesangiospermae - ps17
Magnoliids/eudicots - ps18
Eudicots - ps19
Core_eudicots - ps20
Superrosids - ps21
Rosids - ps22
Eurosids - ps23
Malvids - ps24
Sap./Malv./Brassicales - ps25
Malvales/Brassicales - ps26
Brassicales - ps27
Cleomaceae/Brassicaceae - ps28
Brassicaceae - ps29
Camelineae - ps30
Arabidopsis - ps31
Arabidopsis_thaliana - ps32
p-value
5e-10
6e-08
6e-06
6e-04
7e-02
Log-odds
0.5
1.0
1.5
2.0
2.5
3.0
3.5
4.0

A. thaliana: GO:0051818 obsolete disruption of cells of other organism involved in symbiotic interaction (gain)

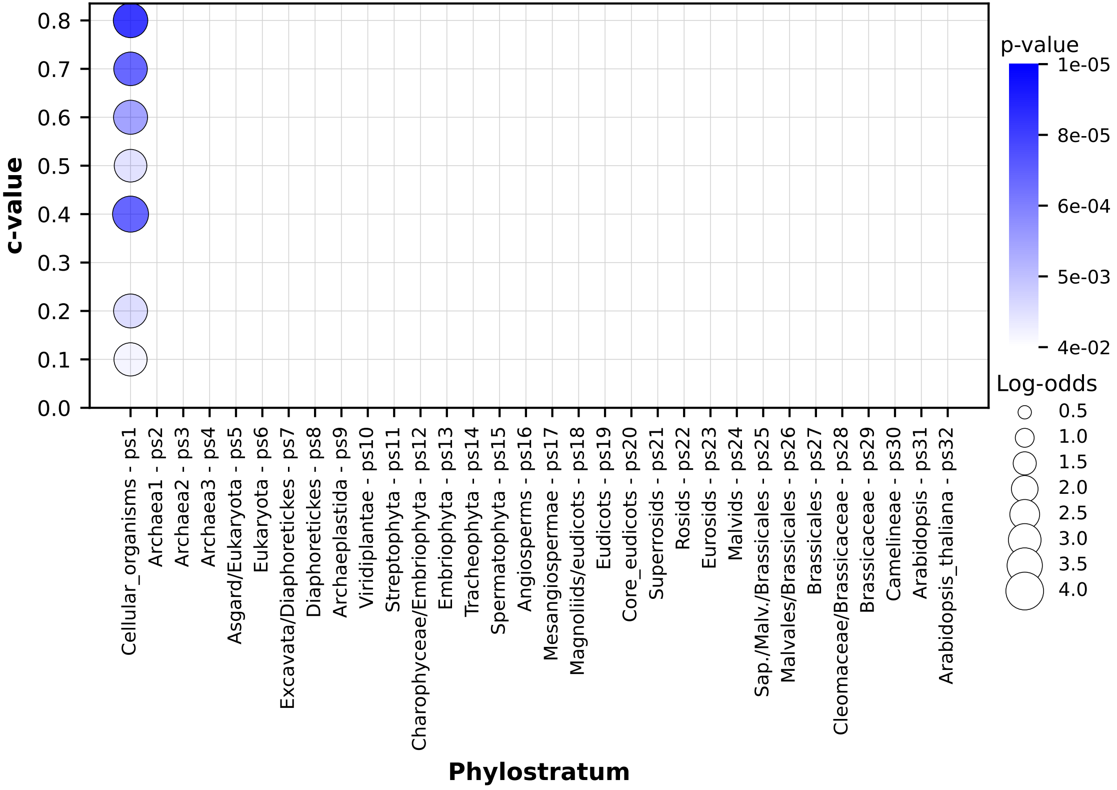

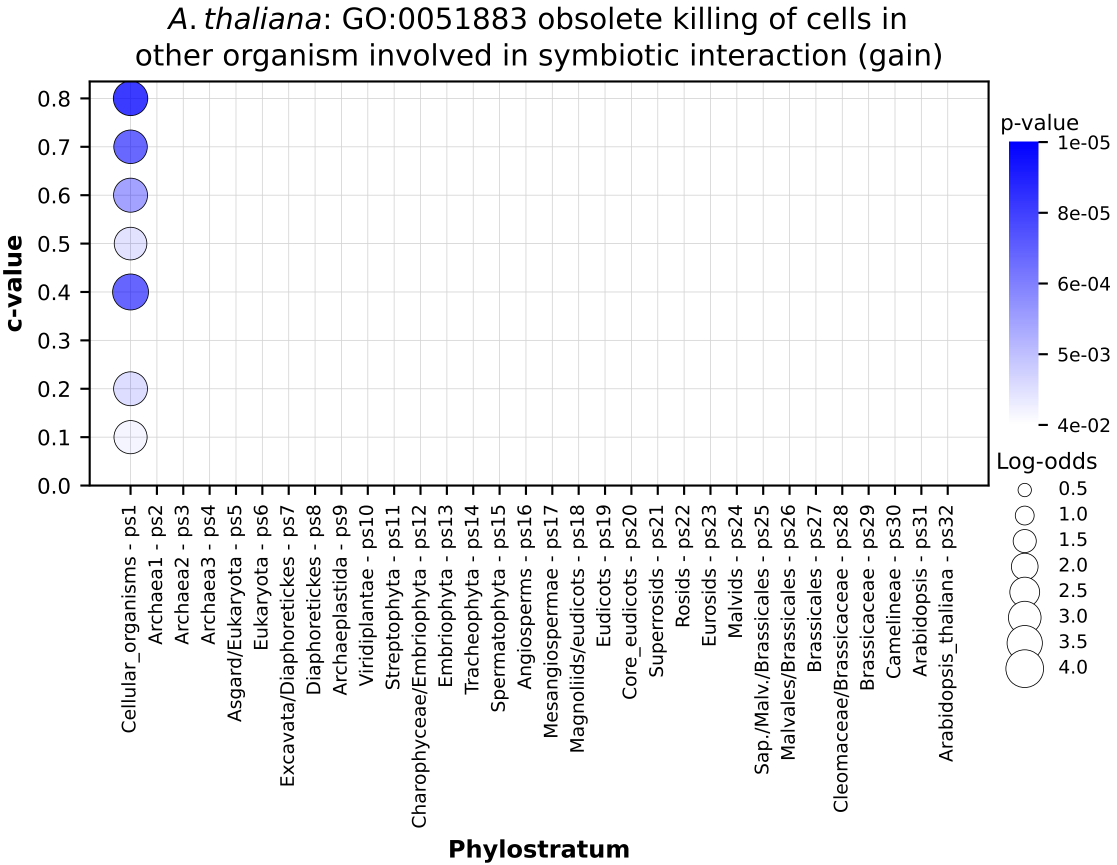

A. thaliana: GO:0051883 obsolete killing of cells in other organism involved in symbiotic interaction (gain)
c-value
0.0
0.1
0.2
0.3
0.4
0.5
0.6
0.7
0.8
Phylostratum
Cellular_organisms - ps1
Archaea1 - ps2
Archaea2 - ps3
Archaea3 - ps4
Asgard/Eukaryota - ps5
Eukaryota - ps6
Excavata/Diaphoretickes - ps7
Diaphoretickes - ps8
Archaeplastida - ps9
Viridiplantae - ps10
Streptophyta - ps11
Charophyceae/Embriophyta - ps12
Embriophyta - ps13
Tracheophyta - ps14
Spermatophyta - ps15
Angiosperms - ps16
Mesangiospermae - ps17
Magnoliids/eudicots - ps18
Eudicots - ps19
Core_eudicots - ps20
Superrosids - ps21
Rosids - ps22
Eurosids - ps23
Malvids - ps24
Sap./Malv./Brassicales - ps25
Malvales/Brassicales - ps26
Brassicales - ps27
Cleomaceae/Brassicaceae - ps28
Brassicaceae - ps29
Camelineae - ps30
Arabidopsis - ps31
Arabidopsis_thaliana - ps32
p-value
1e-05
8e-05
6e-04
5e-03
4e-02
Log-odds
0.5
1.0
1.5
2.0
2.5
3.0
3.5
4.0

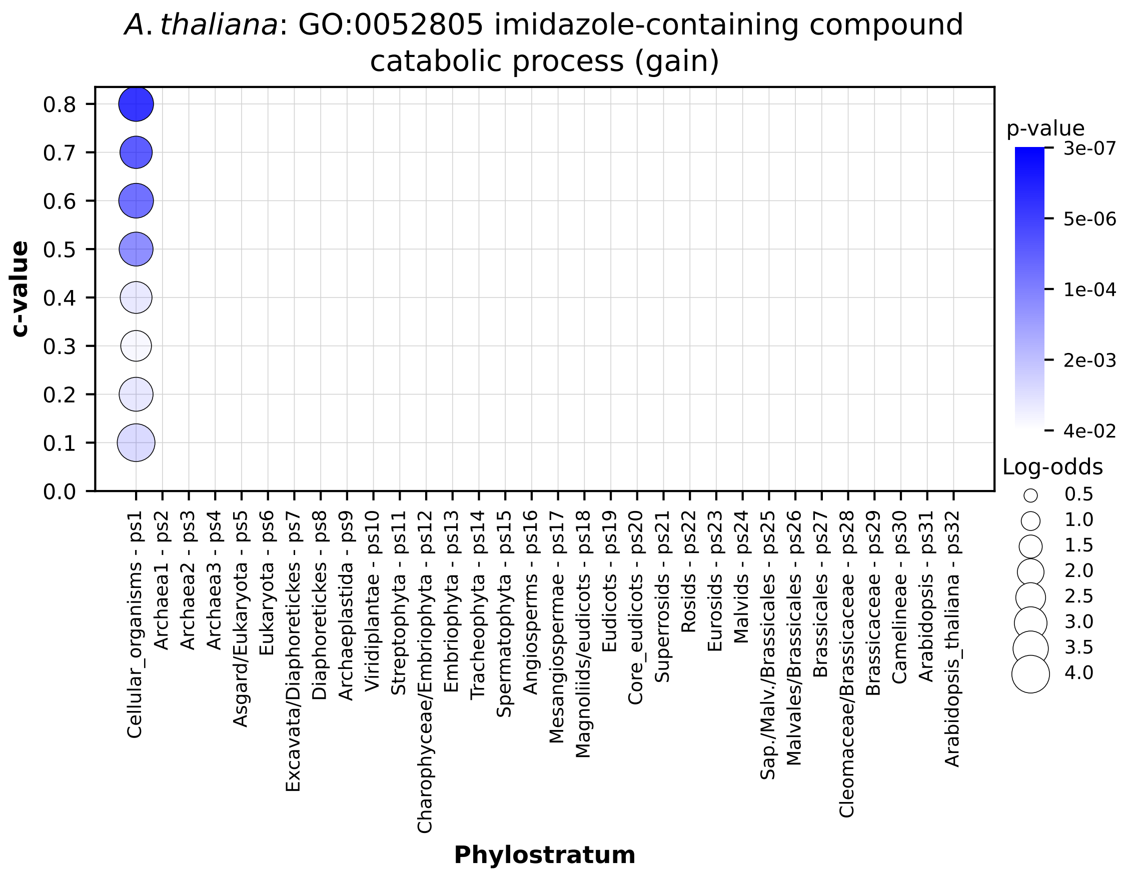

A. thaliana: GO:0052805 imidazole-containing compound catabolic process (gain)
c-value
0.0
0.1
0.2
0.3
0.4
0.5
0.6
0.7
0.8
Phylostratum
Cellular_organisms - ps1
Archaea1 - ps2
Archaea2 - ps3
Archaea3 - ps4
Asgard/Eukaryota - ps5
Eukaryota - ps6
Excavata/Diaphoretickes - ps7
Diaphoretickes - ps8
Archaeplastida - ps9
Viridiplantae - ps10
Streptophyta - ps11
Charophyceae/Embriophyta - ps12
Embriophyta - ps13
Tracheophyta - ps14
Spermatophyta - ps15
Angiosperms - ps16
Mesangiospermae - ps17
Magnoliids/eudicots - ps18
Eudicots - ps19
Core_eudicots - ps20
Superrosids - ps21
Rosids - ps22
Eurosids - ps23
Malvids - ps24
Sap./Malv./Brassicales - ps25
Malvales/Brassicales - ps26
Brassicales - ps27
Cleomaceae/Brassicaceae - ps28
Brassicaceae - ps29
Camelineae - ps30
Arabidopsis - ps31
Arabidopsis_thaliana - ps32
p-value
3e-07
5e-06
1e-04
2e-03
4e-02
Log-odds
0.5
1.0
1.5
2.0
2.5
3.0
3.5
4.0

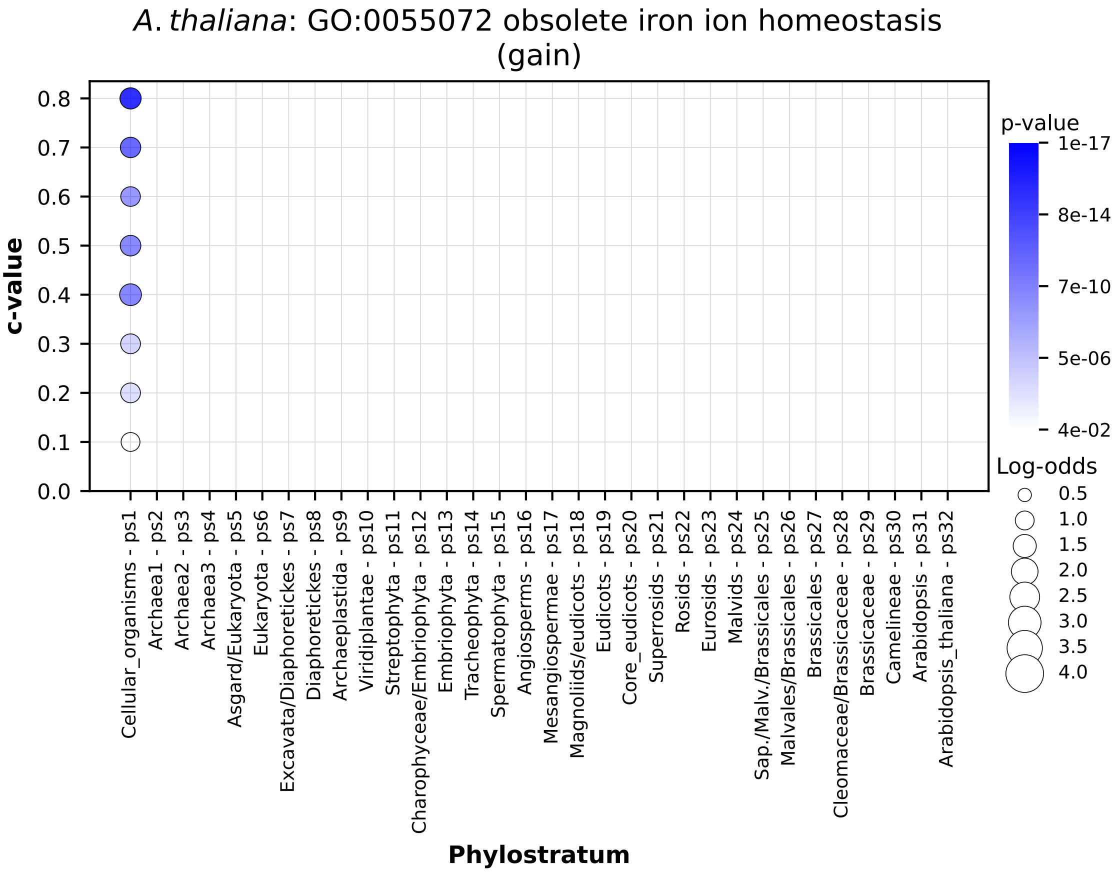

A. thaliana: GO:0055072 obsolete iron ion homeostasis (gain)
c-value
0.0
0.1
0.2
0.3
0.4
0.5
0.6
0.7
0.8
Phylostratum
Cellular_organisms - ps1
Archaea1 - ps2
Archaea2 - ps3
Archaea3 - ps4
Asgard/Eukaryota - ps5
Eukaryota - ps6
Excavata/Diaphoretickes - ps7
Diaphoretickes - ps8
Archaeplastida - ps9
Viridiplantae - ps10
Streptophyta - ps11
Charophyceae/Embriophyta - ps12
Embriophyta - ps13
Tracheophyta - ps14
Spermatophyta - ps15
Angiosperms - ps16
Mesangiospermae - ps17
Magnoliids/eudicots - ps18
Eudicots - ps19
Core_eudicots - ps20
Superrosids - ps21
Rosids - ps22
Eurosids - ps23
Malvids - ps24
Sap./Malv./Brassicales - ps25
Malvales/Brassicales - ps26
Brassicales - ps27
Cleomaceae/Brassicaceae - ps28
Brassicaceae - ps29
Camelineae - ps30
Arabidopsis - ps31
Arabidopsis_thaliana - ps32
p-value
1e-17
8e-14
7e-10
5e-06
4e-02
Log-odds
0.5
1.0
1.5
2.0
2.5
3.0
3.5
4.0

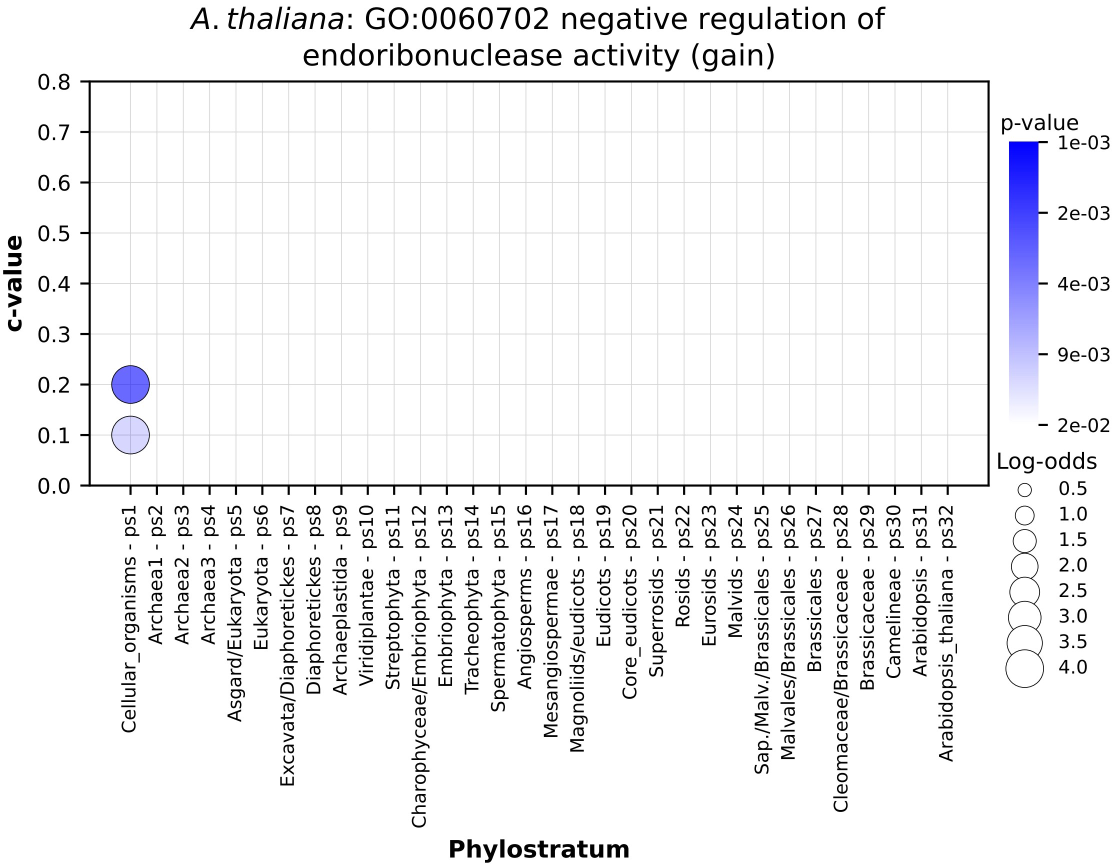
A. thaliana: GO:0060702 negative regulation of endoribonuclease activity (gain)
c-value
0.8
0.7
0.6
0.5
0.4
0.3
0.2
0.1
0.0
p-value
1e-03
2e-03
4e-03
9e-03
2e-02
Log-odds
0.5
1.0
1.5
2.0
2.5
3.0
3.5
4.0
Cellular_organisms - ps1
Archaea1 - ps2
Archaea2 - ps3
Archaea3 - ps4
Asgard/Eukaryota - ps5
Eukaryota - ps6
Excavata/Diaphoretickes - ps7
Diaphoretickes - ps8
Archaeplastida - ps9
Viridiplantae - ps10
Streptophyta - ps11
Charophyceae/Embriophyta - ps12
Embriophyta - ps13
Tracheophyta - ps14
Spermatophyta - ps15
Angiosperms - ps16
Mesangiospermae - ps17
Magnoliids/eudicots - ps18
Eudicots - ps19
Core_eudicots - ps20
Superrosids - ps21
Rosids - ps22
Eurosids - ps23
Malvids - ps24
Sap./Malv./Brassicales - ps25
Malvales/Brassicales - ps26
Brassicales - ps27
Cleomaceae/Brassicaceae - ps28
Brassicaceae - ps29
Camelineae - ps30
Arabidopsis - ps31
Arabidopsis_thaliana - ps32
Phylostratum

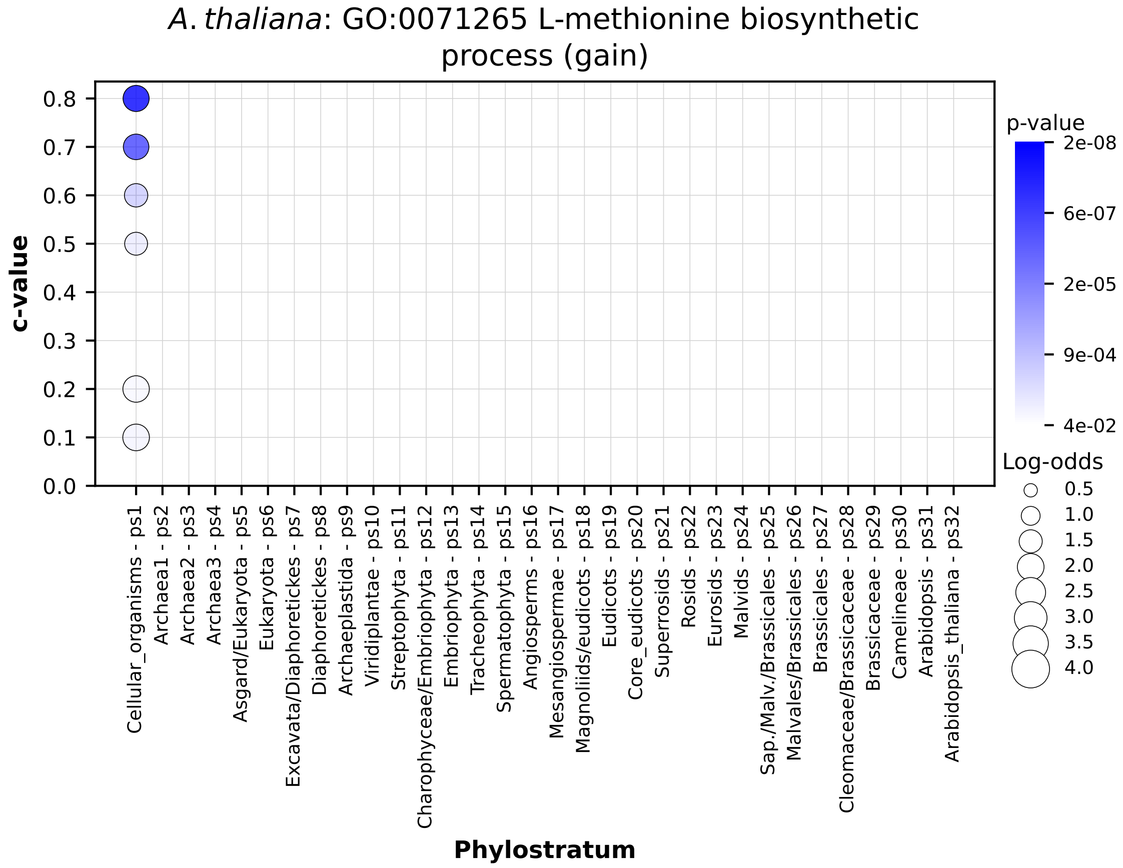

A. thaliana: GO:0071265 L-methionine biosynthetic process (gain)
c-value
0.0
0.1
0.2
0.3
0.4
0.5
0.6
0.7
0.8
p-value
2e-08
6e-07
2e-05
9e-04
4e-02
Log-odds
0.5
1.0
1.5
2.0
2.5
3.0
3.5
4.0
Cellular_organisms - ps1
Archaea1 - ps2
Archaea2 - ps3
Archaea3 - ps4
Asgard/Eukaryota - ps5
Eukaryota - ps6
Excavata/Diaphoretickes - ps7
Diaphoretickes - ps8
Archaeplastida - ps9
Viridiplantae - ps10
Streptophyta - ps11
Charophyceae/Embriophyta - ps12
Embriophyta - ps13
Tracheophyta - ps14
Spermatophyta - ps15
Angiosperms - ps16
Mesangiospermae - ps17
Magnoliids/eudicots - ps18
Eudicots - ps19
Core_eudicots - ps20
Superrosids - ps21
Rosids - ps22
Eurosids - ps23
Malvids - ps24
Sap./Malv./Brassicales - ps25
Malvales/Brassicales - ps26
Brassicales - ps27
Cleomaceae/Brassicaceae - ps28
Brassicaceae - ps29
Camelineae - ps30
Arabidopsis - ps31
Arabidopsis_thaliana - ps32
Phylostratum

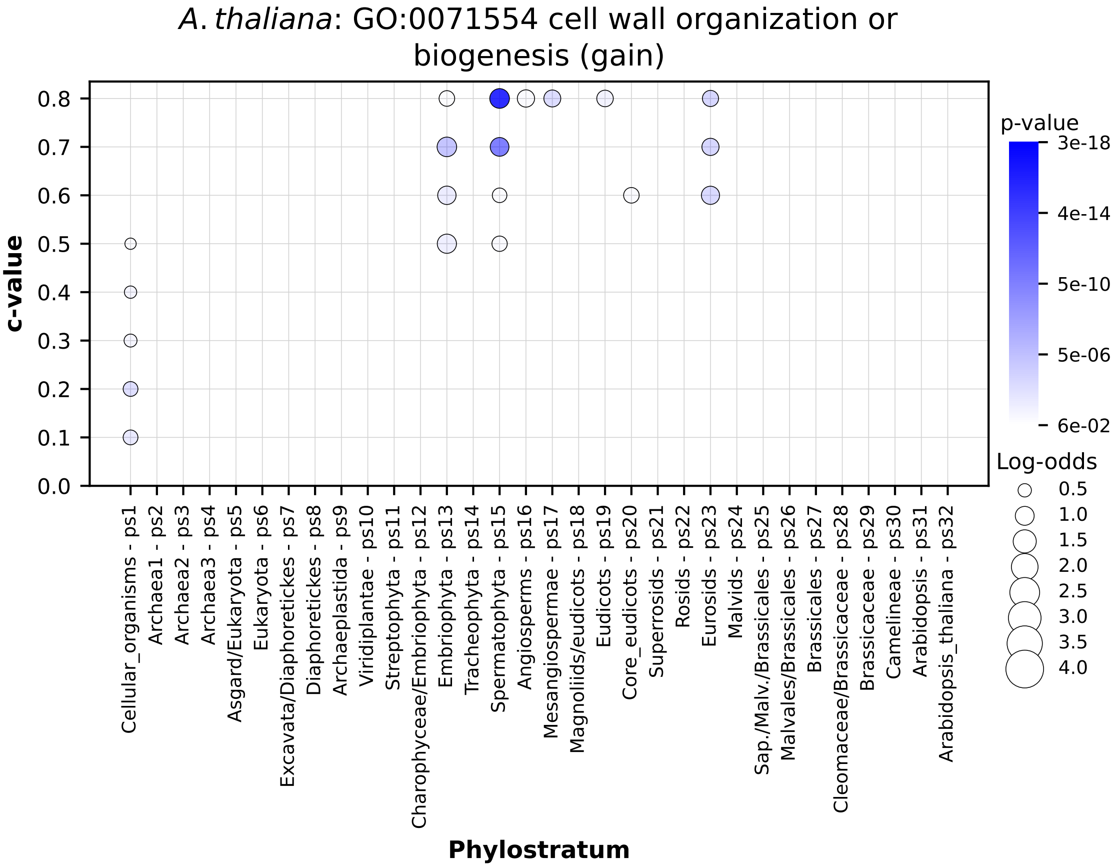

A. thaliana: GO:0071554 cell wall organization or biogenesis (gain)
c-value
0.8
0.7
0.6
0.5
0.4
0.3
0.2
0.1
0.0
p-value
3e-18
4e-14
5e-10
5e-06
6e-02
Log-odds
0.5
1.0
1.5
2.0
2.5
3.0
3.5
4.0
Cellular_organisms - ps1
Archaea1 - ps2
Archaea2 - ps3
Archaea3 - ps4
Asgard/Eukaryota - ps5
Eukaryota - ps6
Excavata/Diaphoretickes - ps7
Diaphoretickes - ps8
Archaeplastida - ps9
Viridiplantae - ps10
Streptophyta - ps11
Charophyceae/Embriophyta - ps12
Embriophyta - ps13
Tracheophyta - ps14
Spermatophyta - ps15
Angiosperms - ps16
Mesangiospermae - ps17
Magnoliids/eudicots - ps18
Eudicots - ps19
Core_eudicots - ps20
Superrosids - ps21
Rosids - ps22
Eurosids - ps23
Malvids - ps24
Sap./Malv./Brassicales - ps25
Malvales/Brassicales - ps26
Brassicales - ps27
Cleomaceae/Brassicaceae - ps28
Brassicaceae - ps29
Camelineae - ps30
Arabidopsis - ps31
Arabidopsis_thaliana - ps32
Phylostratum

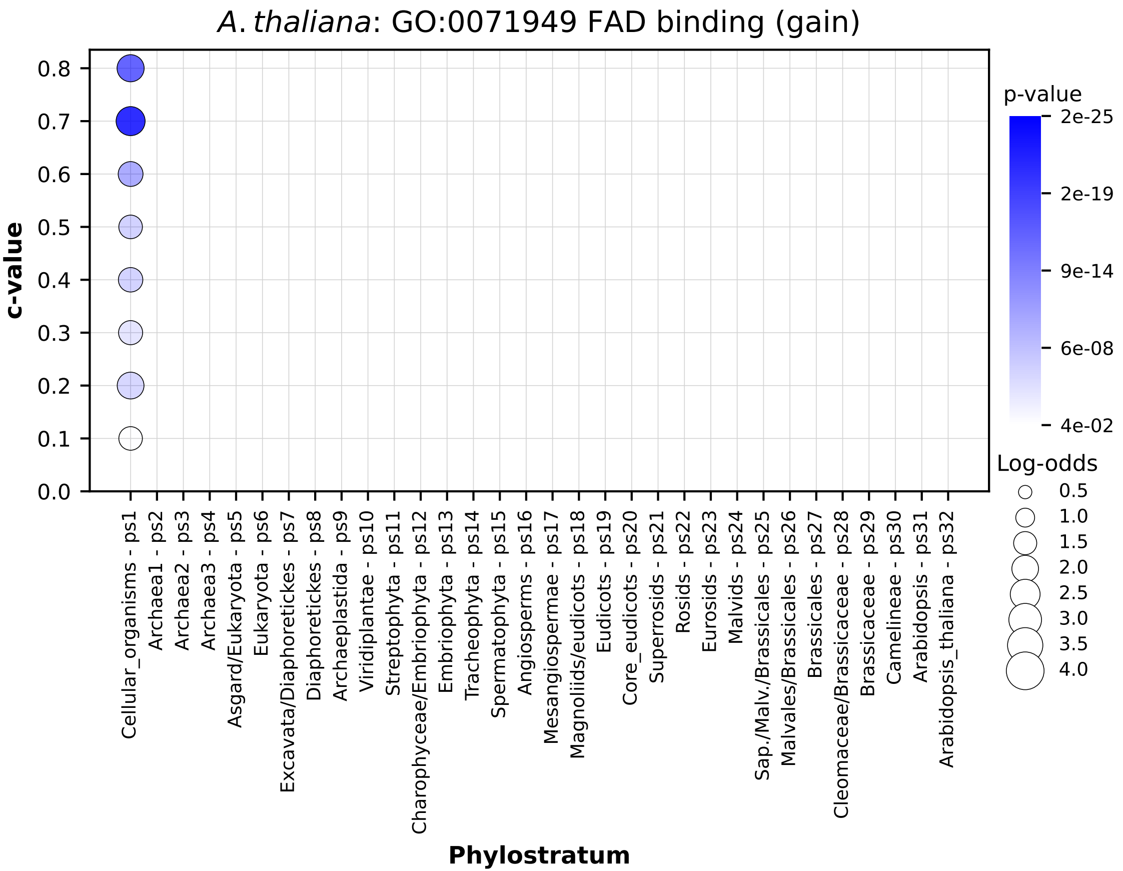

A. thaliana: GO:0071949 FAD binding (gain)
c-value
0.0
0.1
0.2
0.3
0.4
0.5
0.6
0.7
0.8
Phylostratum
Cellular_organisms - ps1
Archaea1 - ps2
Archaea2 - ps3
Archaea3 - ps4
Asgard/Eukaryota - ps5
Eukaryota - ps6
Excavata/Diaphoretickes - ps7
Diaphoretickes - ps8
Archaeplastida - ps9
Viridiplantae - ps10
Streptophyta - ps11
Charophyceae/Embriophyta - ps12
Embriophyta - ps13
Tracheophyta - ps14
Spermatophyta - ps15
Angiosperms - ps16
Mesangiospermae - ps17
Magnoliids/eudicots - ps18
Eudicots - ps19
Core_eudicots - ps20
Superrosids - ps21
Rosids - ps22
Eurosids - ps23
Malvids - ps24
Sap./Malv./Brassicales - ps25
Malvales/Brassicales - ps26
Brassicales - ps27
Cleomaceae/Brassicaceae - ps28
Brassicaceae - ps29
Camelineae - ps30
Arabidopsis - ps31
Arabidopsis_thaliana - ps32
p-value
2e-25
2e-19
9e-14
6e-08
4e-02
Log-odds
0.5
1.0
1.5
2.0
2.5
3.0
3.5
4.0

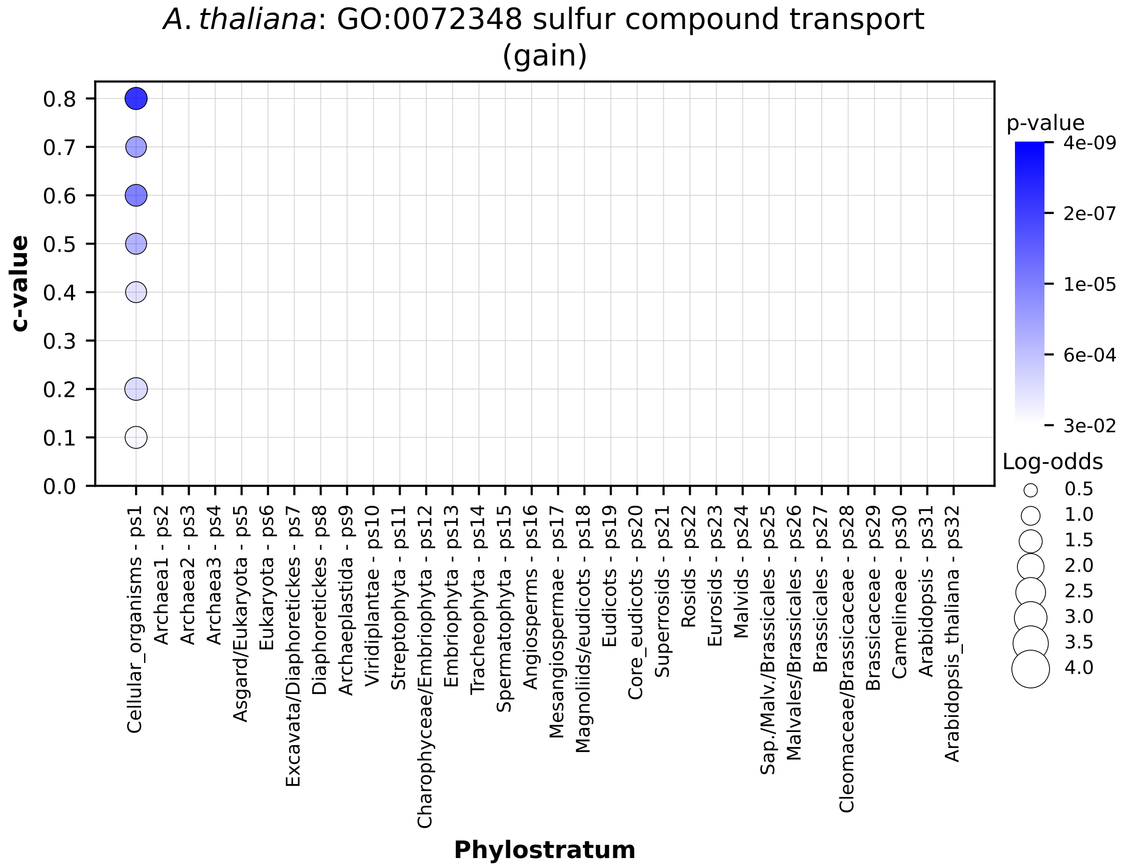

A. thaliana: GO:0072348 sulfur compound transport
(gain)
c-value
0.0
0.1
0.2
0.3
0.4
0.5
0.6
0.7
0.8
p-value
4e-09
2e-07
1e-05
6e-04
3e-02
Log-odds
0.5
1.0
1.5
2.0
2.5
3.0
3.5
4.0
Cellular_organisms - ps1
Archaea1 - ps2
Archaea2 - ps3
Archaea3 - ps4
Asgard/Eukaryota - ps5
Eukaryota - ps6
Excavata/Diaphoretickes - ps7
Diaphoretickes - ps8
Archaeplastida - ps9
Viridiplantae - ps10
Streptophyta - ps11
Charophyceae/Embriophyta - ps12
Embriophyta - ps13
Tracheophyta - ps14
Spermatophyta - ps15
Angiosperms - ps16
Mesangiospermae - ps17
Magnoliids/eudicots - ps18
Eudicots - ps19
Core_eudicots - ps20
Superrosids - ps21
Rosids - ps22
Eurosids - ps23
Malvids - ps24
Sap./Malv./Brassicales - ps25
Malvales/Brassicales - ps26
Brassicales - ps27
Cleomaceae/Brassicaceae - ps28
Brassicaceae - ps29
Camelineae - ps30
Arabidopsis - ps31
Arabidopsis_thaliana - ps32
Phylostratum

A. thaliana: GO:0086080 protein binding involved in heterotypic cell-cell adhesion (gain)

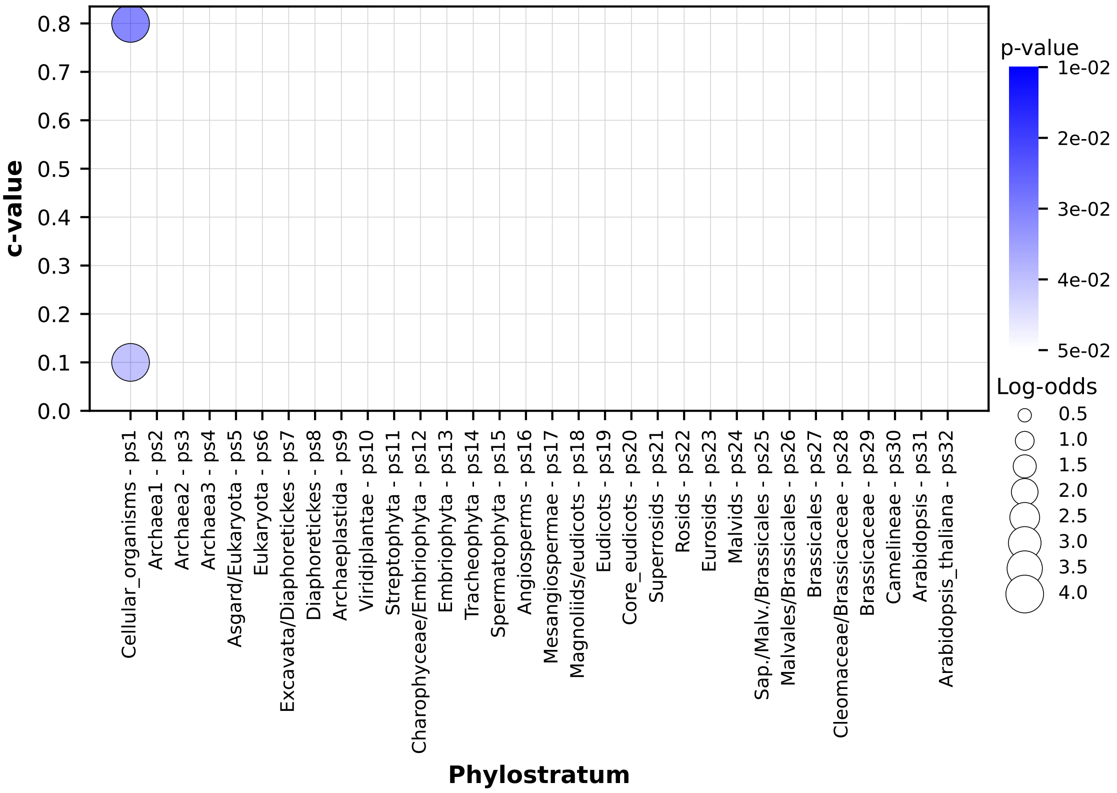

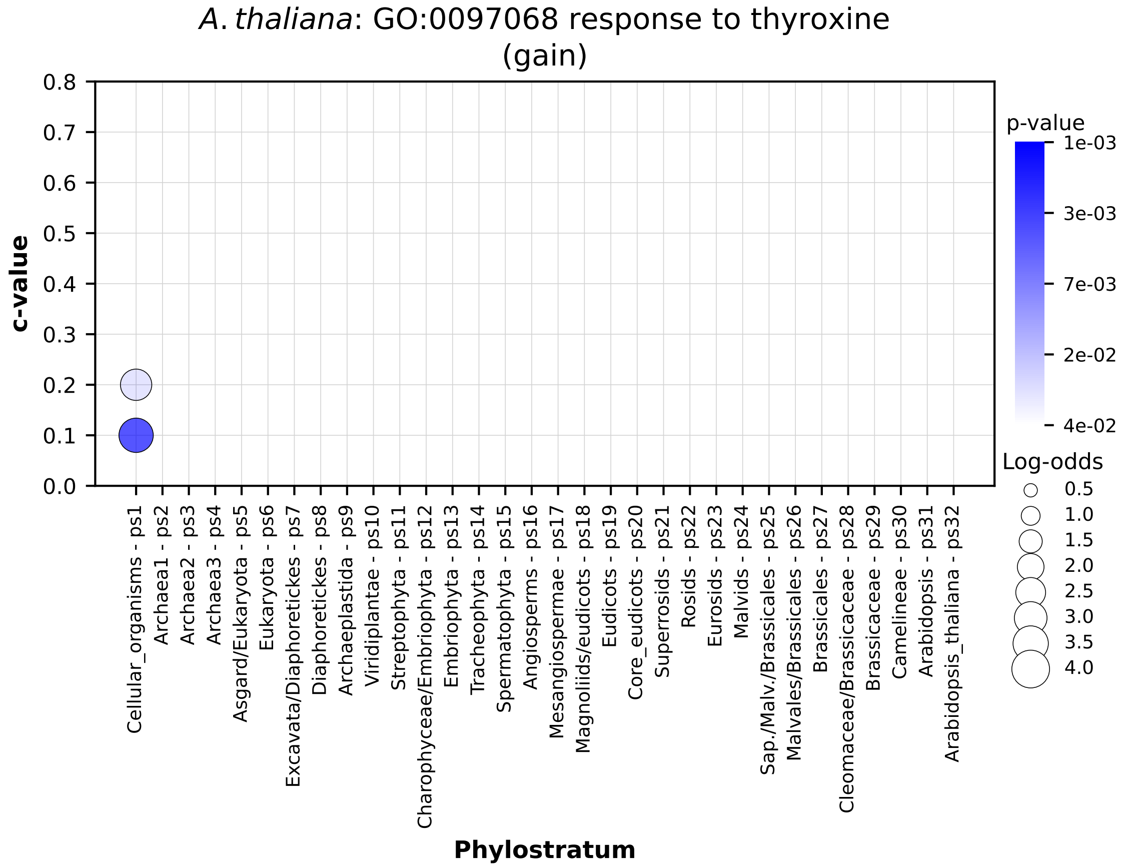

A. thaliana: GO:0097068 response to thyroxine
(gain)
c-value
0.8
0.7
0.6
0.5
0.4
0.3
0.2
0.1
0.0
p-value
1e-03
3e-03
7e-03
2e-02
4e-02
Log-odds
0.5
1.0
1.5
2.0
2.5
3.0
3.5
4.0
Cellular_organisms - ps1
Archaea1 - ps2
Archaea2 - ps3
Archaea3 - ps4
Asgard/Eukaryota - ps5
Eukaryota - ps6
Excavata/Diaphoretickes - ps7
Diaphoretickes - ps8
Archaeplastida - ps9
Viridiplantae - ps10
Streptophyta - ps11
Charophyceae/Embriophyta - ps12
Embriophyta - ps13
Tracheophyta - ps14
Spermatophyta - ps15
Angiosperms - ps16
Mesangiospermae - ps17
Magnoliids/eudicots - ps18
Eudicots - ps19
Core_eudicots - ps20
Superrosids - ps21
Rosids - ps22
Eurosids - ps23
Malvids - ps24
Sap./Malv./Brassicales - ps25
Malvales/Brassicales - ps26
Brassicales - ps27
Cleomaceae/Brassicaceae - ps28
Brassicaceae - ps29
Camelineae - ps30
Arabidopsis - ps31
Arabidopsis_thaliana - ps32
Phylostratum

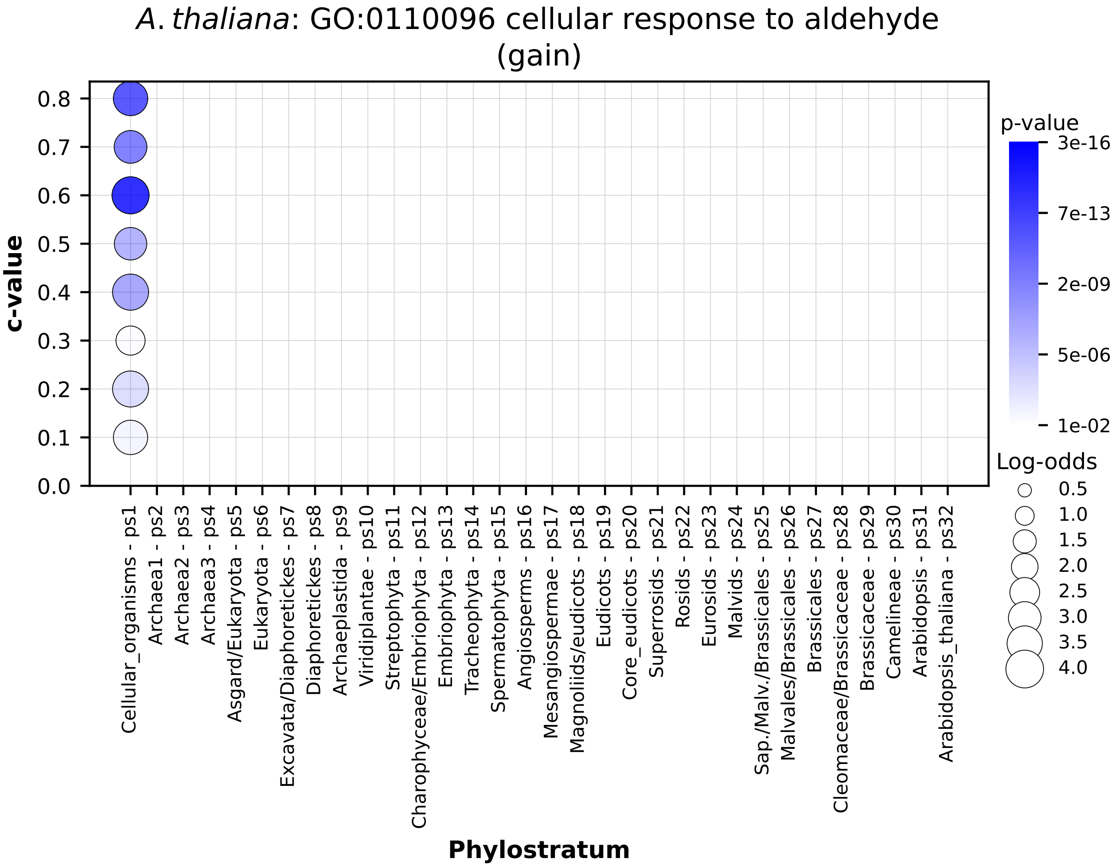
A. thaliana: GO:0110096 cellular response to aldehyde
(gain)
c-value
0.0
0.1
0.2
0.3
0.4
0.5
0.6
0.7
0.8
Phylostratum
Cellular_organisms - ps1
Archaea1 - ps2
Archaea2 - ps3
Archaea3 - ps4
Asgard/Eukaryota - ps5
Eukaryota - ps6
Excavata/Diaphoretickes - ps7
Diaphoretickes - ps8
Archaeplastida - ps9
Viridiplantae - ps10
Streptophyta - ps11
Charophyceae/Embriophyta - ps12
Embriophyta - ps13
Tracheophyta - ps14
Spermatophyta - ps15
Angiosperms - ps16
Mesangiospermae - ps17
Magnoliids/eudicots - ps18
Eudicots - ps19
Core_eudicots - ps20
Superrosids - ps21
Rosids - ps22
Eurosids - ps23
Malvids - ps24
Sap./Malv./Brassicales - ps25
Malvales/Brassicales - ps26
Brassicales - ps27
Cleomaceae/Brassicaceae - ps28
Brassicaceae - ps29
Camelineae - ps30
Arabidopsis - ps31
Arabidopsis_thaliana - ps32
p-value
3e-16
7e-13
2e-09
5e-06
1e-02
Log-odds
0.5
1.0
1.5
2.0
2.5
3.0
3.5
4.0

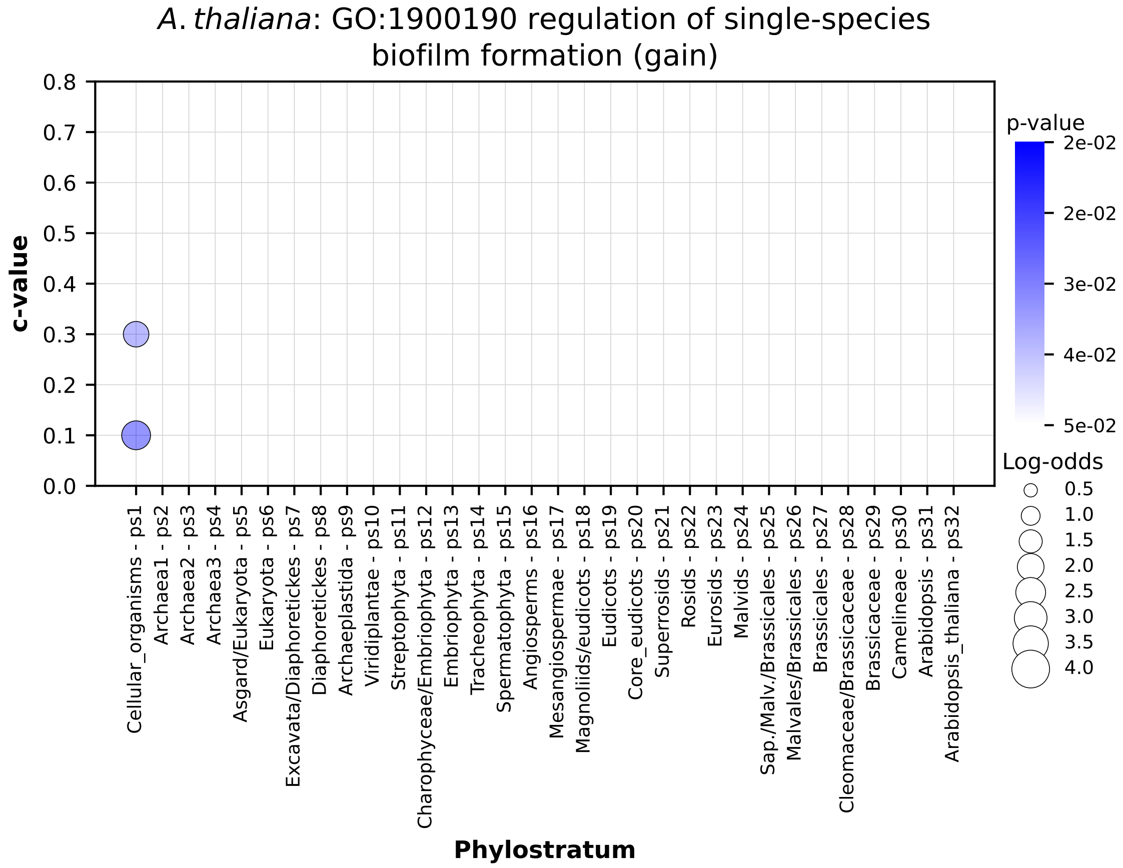

A. thaliana: GO:1900190 regulation of single-species biofilm formation (gain)
c-value
0.8
0.7
0.6
0.5
0.4
0.3
0.2
0.1
0.0
Phylostratum
Cellular_organisms - ps1
Archaea1 - ps2
Archaea2 - ps3
Archaea3 - ps4
Asgard/Eukaryota - ps5
Eukaryota - ps6
Excavata/Diaphoretickes - ps7
Diaphoretickes - ps8
Archaeplastida - ps9
Viridiplantae - ps10
Streptophyta - ps11
Charophyceae/Embriophyta - ps12
Embriophyta - ps13
Tracheophyta - ps14
Spermatophyta - ps15
Angiosperms - ps16
Mesangiospermae - ps17
Magnoliids/eudicots - ps18
Eudicots - ps19
Core_eudicots - ps20
Superrosids - ps21
Rosids - ps22
Eurosids - ps23
Malvids - ps24
Sap./Malv./Brassicales - ps25
Malvales/Brassicales - ps26
Brassicales - ps27
Cleomaceae/Brassicaceae - ps28
Brassicaceae - ps29
Camelineae - ps30
Arabidopsis - ps31
Arabidopsis_thaliana - ps32
p-value
2e-02
2e-02
3e-02
4e-02
5e-02
Log-odds
0.5
1.0
1.5
2.0
2.5
3.0
3.5
4.0

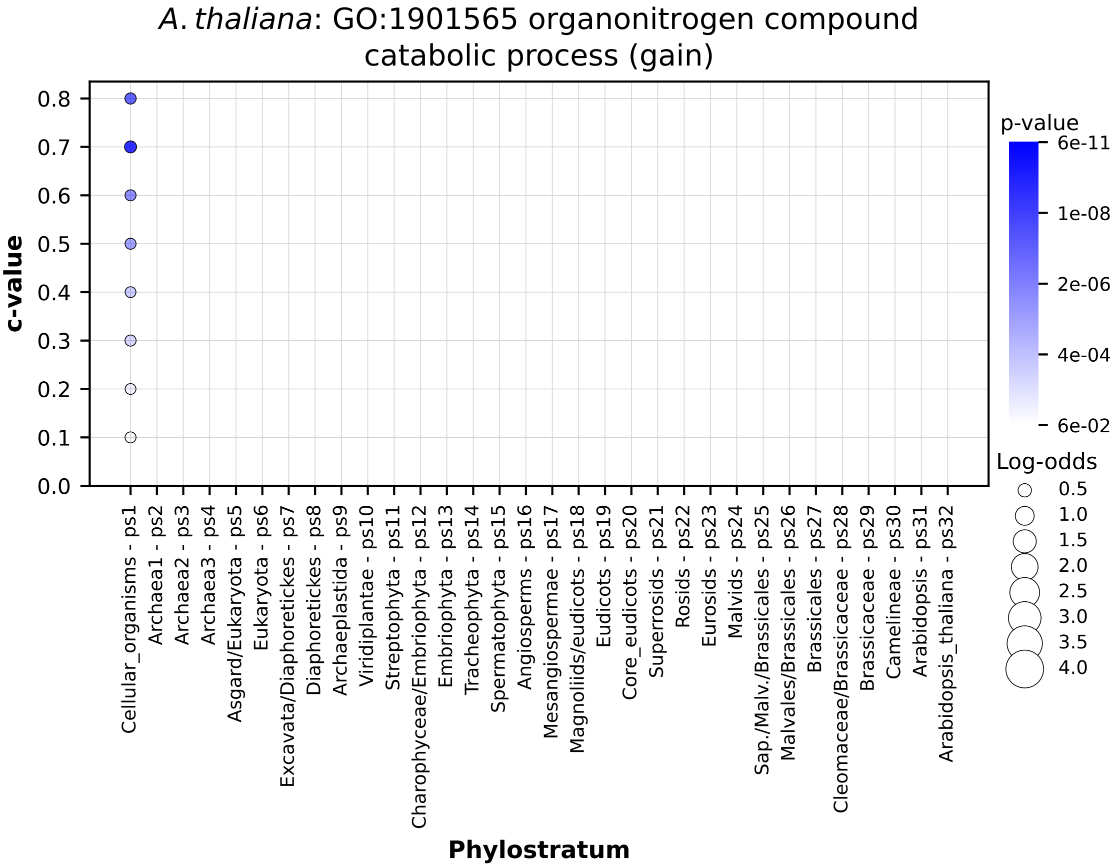

A. thaliana: GO:1901565 organonitrogen compound catabolic process (gain)
c-value
0.0
0.1
0.2
0.3
0.4
0.5
0.6
0.7
0.8
Phylostratum
Cellular_organisms - ps1
Archaea1 - ps2
Archaea2 - ps3
Archaea3 - ps4
Asgard/Eukaryota - ps5
Eukaryota - ps6
Excavata/Diaphoretickes - ps7
Diaphoretickes - ps8
Archaeplastida - ps9
Viridiplantae - ps10
Streptophyta - ps11
Charophyceae/Embriophyta - ps12
Embriophyta - ps13
Tracheophyta - ps14
Spermatophyta - ps15
Angiosperms - ps16
Mesangiospermae - ps17
Magnoliids/eudicots - ps18
Eudicots - ps19
Core_eudicots - ps20
Superrosids - ps21
Rosids - ps22
Eurosids - ps23
Malvids - ps24
Sap./Malv./Brassicales - ps25
Malvales/Brassicales - ps26
Brassicales - ps27
Cleomaceae/Brassicaceae - ps28
Brassicaceae - ps29
Camelineae - ps30
Arabidopsis - ps31
Arabidopsis_thaliana - ps32
p-value
6e-11
1e-08
2e-06
4e-04
6e-02
Log-odds
0.5
1.0
1.5
2.0
2.5
3.0
3.5
4.0

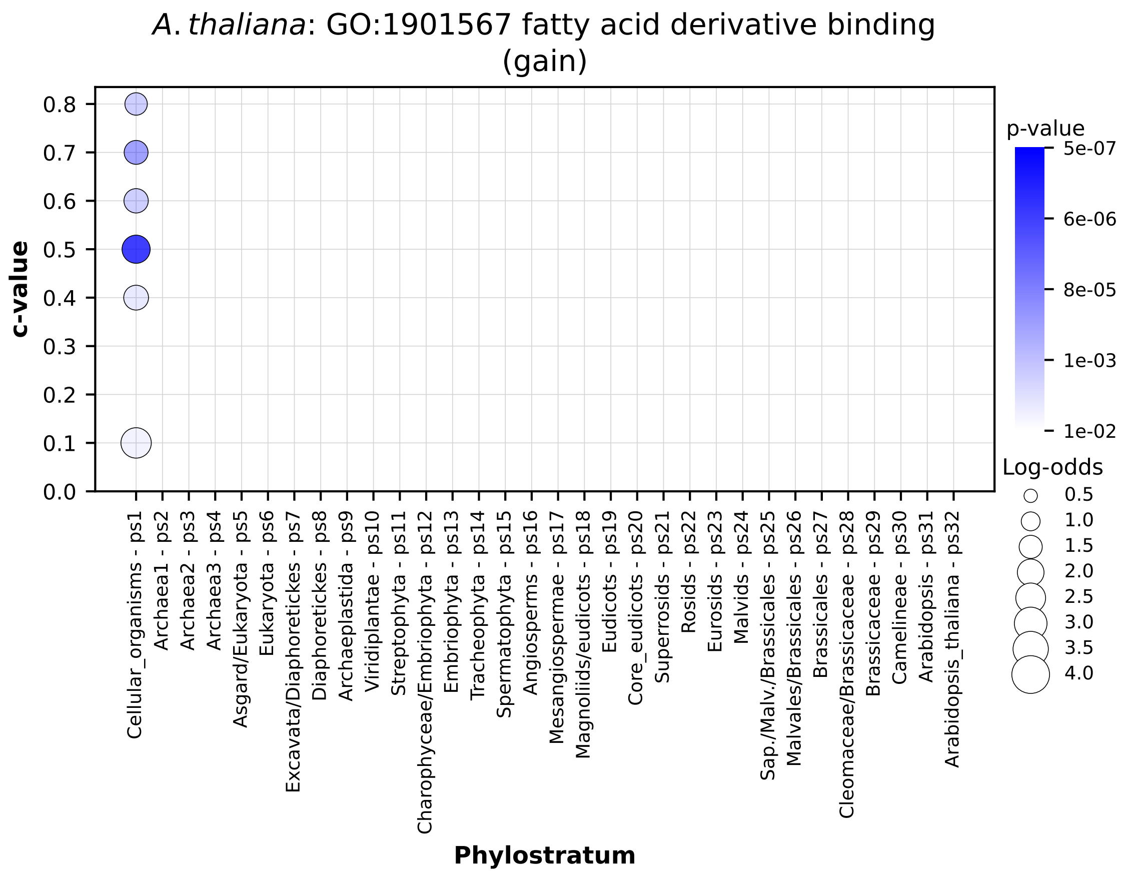
A. thaliana: GO:1901567 fatty acid derivative binding
(gain)
c-value
0.0
0.1
0.2
0.3
0.4
0.5
0.6
0.7
0.8
p-value
5e-07
6e-06
8e-05
1e-03
1e-02
Log-odds
0.5
1.0
1.5
2.0
2.5
3.0
3.5
4.0
Cellular_organisms - ps1
Archaea1 - ps2
Archaea2 - ps3
Archaea3 - ps4
Asgard/Eukaryota - ps5
Eukaryota - ps6
Excavata/Diaphoretickes - ps7
Diaphoretickes - ps8
Archaeplastida - ps9
Viridiplantae - ps10
Streptophyta - ps11
Charophyceae/Embriophyta - ps12
Embriophyta - ps13
Tracheophyta - ps14
Spermatophyta - ps15
Angiosperms - ps16
Mesangiospermae - ps17
Magnoliids/eudicots - ps18
Eudicots - ps19
Core_eudicots - ps20
Superrosids - ps21
Rosids - ps22
Eurosids - ps23
Malvids - ps24
Sap./Malv./Brassicales - ps25
Malvales/Brassicales - ps26
Brassicales - ps27
Cleomaceae/Brassicaceae - ps28
Brassicaceae - ps29
Camelineae - ps30
Arabidopsis - ps31
Arabidopsis_thaliana - ps32
Phylostratum

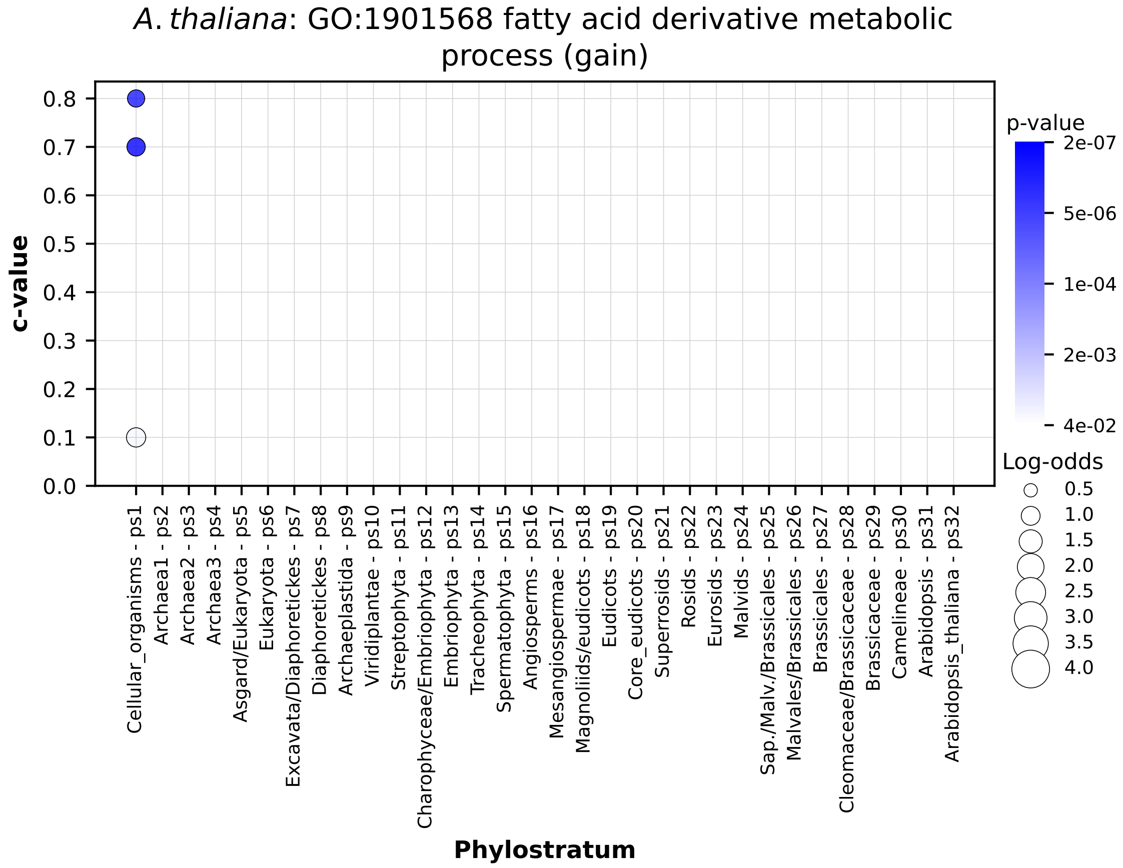

A. thaliana: GO:1901568 fatty acid derivative metabolic process (gain)
c-value
0.0
0.1
0.2
0.3
0.4
0.5
0.6
0.7
0.8
Phylostratum
Cellular_organisms - ps1
Archaea1 - ps2
Archaea2 - ps3
Archaea3 - ps4
Asgard/Eukaryota - ps5
Eukaryota - ps6
Excavata/Diaphoretickes - ps7
Diaphoretickes - ps8
Archaeplastida - ps9
Viridiplantae - ps10
Streptophyta - ps11
Charophyceae/Embriophyta - ps12
Embriophyta - ps13
Tracheophyta - ps14
Spermatophyta - ps15
Angiosperms - ps16
Mesangiospermae - ps17
Magnoliids/eudicots - ps18
Eudicots - ps19
Core_eudicots - ps20
Superrosids - ps21
Rosids - ps22
Eurosids - ps23
Malvids - ps24
Sap./Malv./Brassicales - ps25
Malvales/Brassicales - ps26
Brassicales - ps27
Cleomaceae/Brassicaceae - ps28
Brassicaceae - ps29
Camelineae - ps30
Arabidopsis - ps31
Arabidopsis_thaliana - ps32
p-value
2e-07
5e-06
1e-04
2e-03
4e-02
Log-odds
0.5
1.0
1.5
2.0
2.5
3.0
3.5
4.0

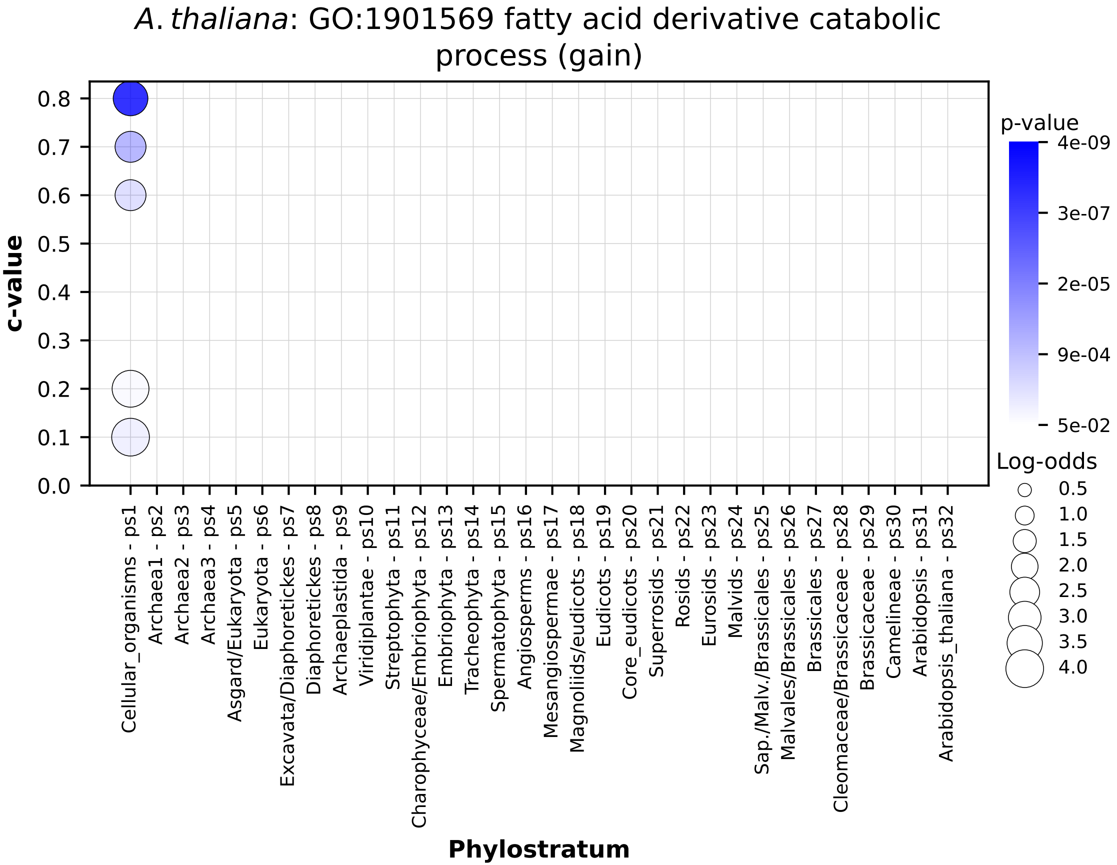

A. thaliana: GO:1901569 fatty acid derivative catabolic process (gain)
c-value
0.0
0.1
0.2
0.3
0.4
0.5
0.6
0.7
0.8
Phylostratum
Cellular_organisms - ps1
Archaea1 - ps2
Archaea2 - ps3
Archaea3 - ps4
Asgard/Eukaryota - ps5
Eukaryota - ps6
Excavata/Diaphoretickes - ps7
Diaphoretickes - ps8
Archaeplastida - ps9
Viridiplantae - ps10
Streptophyta - ps11
Charophyceae/Embriophyta - ps12
Embriophyta - ps13
Tracheophyta - ps14
Spermatophyta - ps15
Angiosperms - ps16
Mesangiospermae - ps17
Magnoliids/eudicots - ps18
Eudicots - ps19
Core_eudicots - ps20
Superrosids - ps21
Rosids - ps22
Eurosids - ps23
Malvids - ps24
Sap./Malv./Brassicales - ps25
Malvales/Brassicales - ps26
Brassicales - ps27
Cleomaceae/Brassicaceae - ps28
Brassicaceae - ps29
Camelineae - ps30
Arabidopsis - ps31
Arabidopsis_thaliana - ps32
p-value
4e-09
3e-07
2e-05
9e-04
5e-02
Log-odds
0.5
1.0
1.5
2.0
2.5
3.0
3.5
4.0

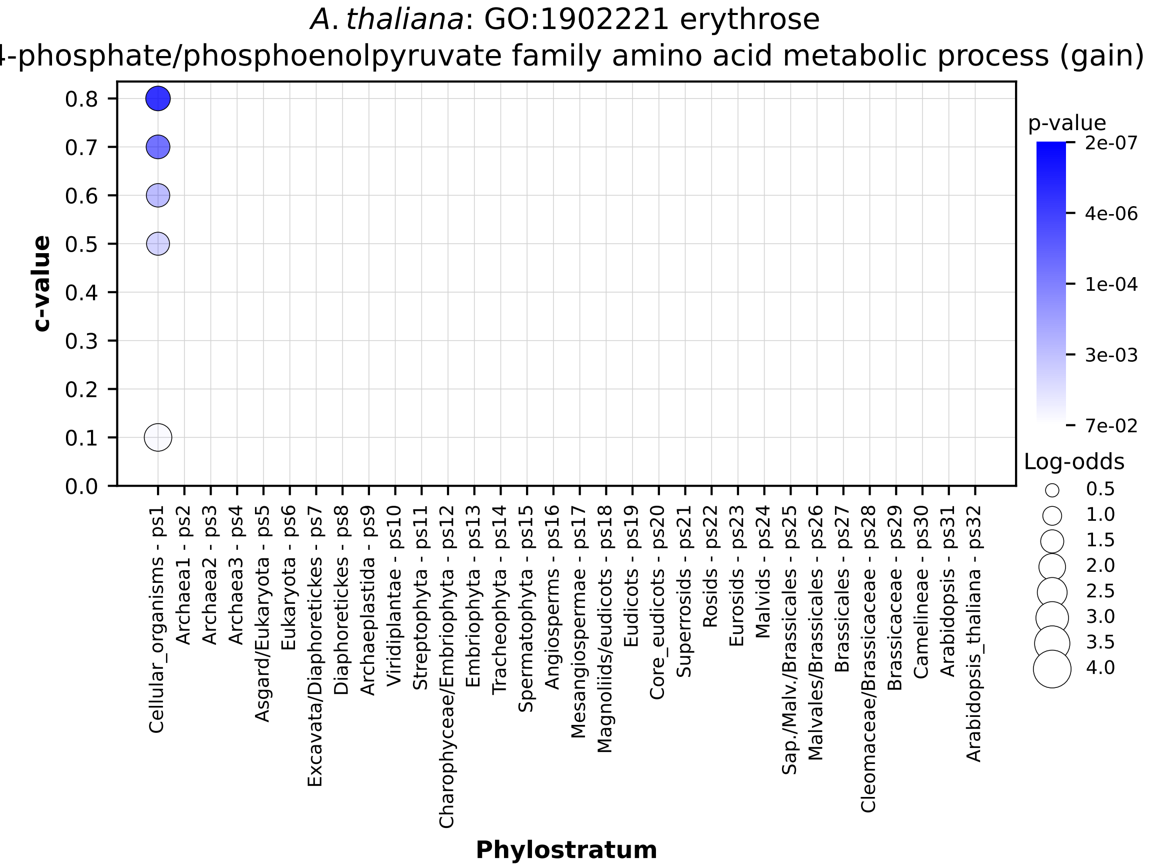

A. thaliana: GO:1902221 erythrose
4-phosphate/phosphoenolpyruvate family amino acid metabolic process (gain)
c-value
0.0
0.1
0.2
0.3
0.4
0.5
0.6
0.7
0.8
p-value
2e-07
4e-06
1e-04
3e-03
7e-02
Log-odds
0.5
1.0
1.5
2.0
2.5
3.0
3.5
4.0
Cellular_organisms - ps1
Archaea1 - ps2
Archaea2 - ps3
Archaea3 - ps4
Asgard/Eukaryota - ps5
Eukaryota - ps6
Excavata/Diaphoretickes - ps7
Diaphoretickes - ps8
Archaeplastida - ps9
Viridiplantae - ps10
Streptophyta - ps11
Charophyceae/Embriophyta - ps12
Embriophyta - ps13
Tracheophyta - ps14
Spermatophyta - ps15
Angiosperms - ps16
Mesangiospermae - ps17
Magnoliids/eudicots - ps18
Eudicots - ps19
Core_eudicots - ps20
Superrosids - ps21
Rosids - ps22
Eurosids - ps23
Malvids - ps24
Sap./Malv./Brassicales - ps25
Malvales/Brassicales - ps26
Brassicales - ps27
Cleomaceae/Brassicaceae - ps28
Brassicaceae - ps29
Camelineae - ps30
Arabidopsis - ps31
Arabidopsis_thaliana - ps32
Phylostratum

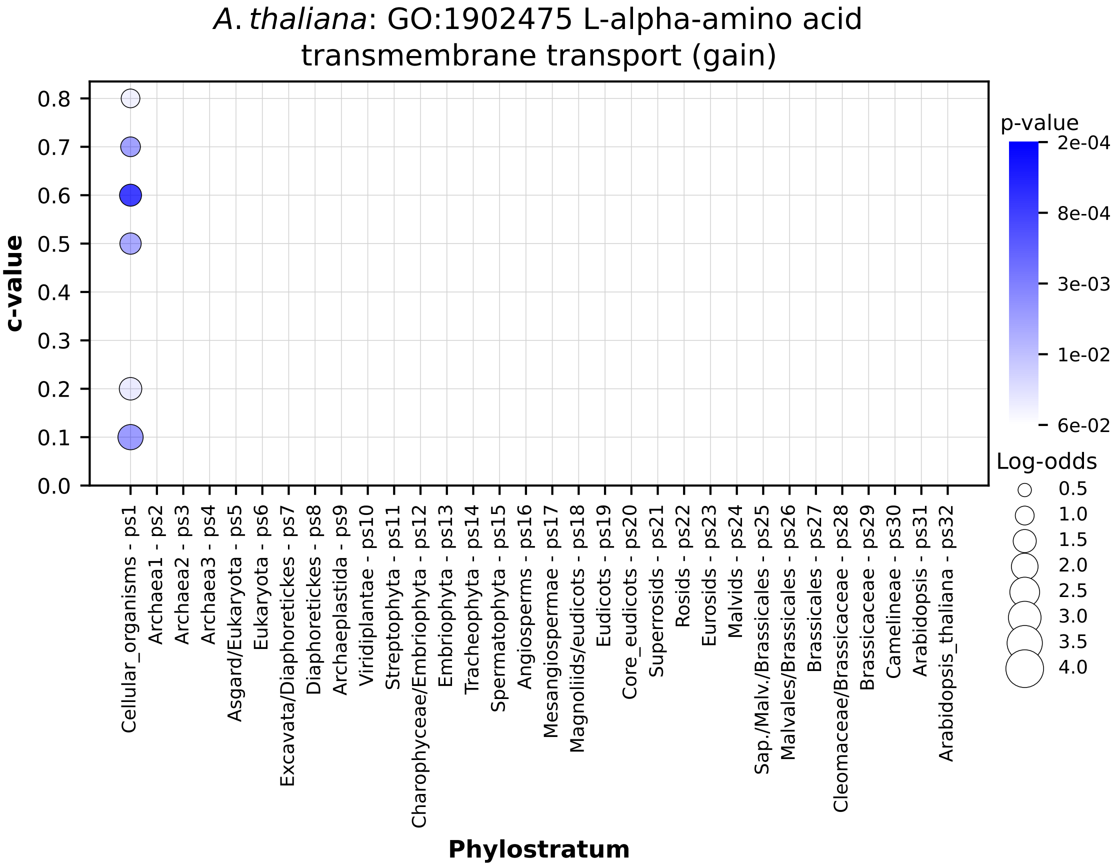
A. thaliana: GO:1902475 L-alpha-amino acid transmembrane transport (gain)
c-value
0.0
0.1
0.2
0.3
0.4
0.5
0.6
0.7
0.8
Phylostratum
Cellular_organisms - ps1
Archaea1 - ps2
Archaea2 - ps3
Archaea3 - ps4
Asgard/Eukaryota - ps5
Eukaryota - ps6
Excavata/Diaphoretickes - ps7
Diaphoretickes - ps8
Archaeplastida - ps9
Viridiplantae - ps10
Streptophyta - ps11
Charophyceae/Embriophyta - ps12
Embriophyta - ps13
Tracheophyta - ps14
Spermatophyta - ps15
Angiosperms - ps16
Mesangiospermae - ps17
Magnoliids/eudicots - ps18
Eudicots - ps19
Core_eudicots - ps20
Superrosids - ps21
Rosids - ps22
Eurosids - ps23
Malvids - ps24
Sap./Malv./Brassicales - ps25
Malvales/Brassicales - ps26
Brassicales - ps27
Cleomaceae/Brassicaceae - ps28
Brassicaceae - ps29
Camelineae - ps30
Arabidopsis - ps31
Arabidopsis_thaliana - ps32
p-value
2e-04
8e-04
3e-03
1e-02
6e-02
Log-odds
0.5
1.0
1.5
2.0
2.5
3.0
3.5
4.0

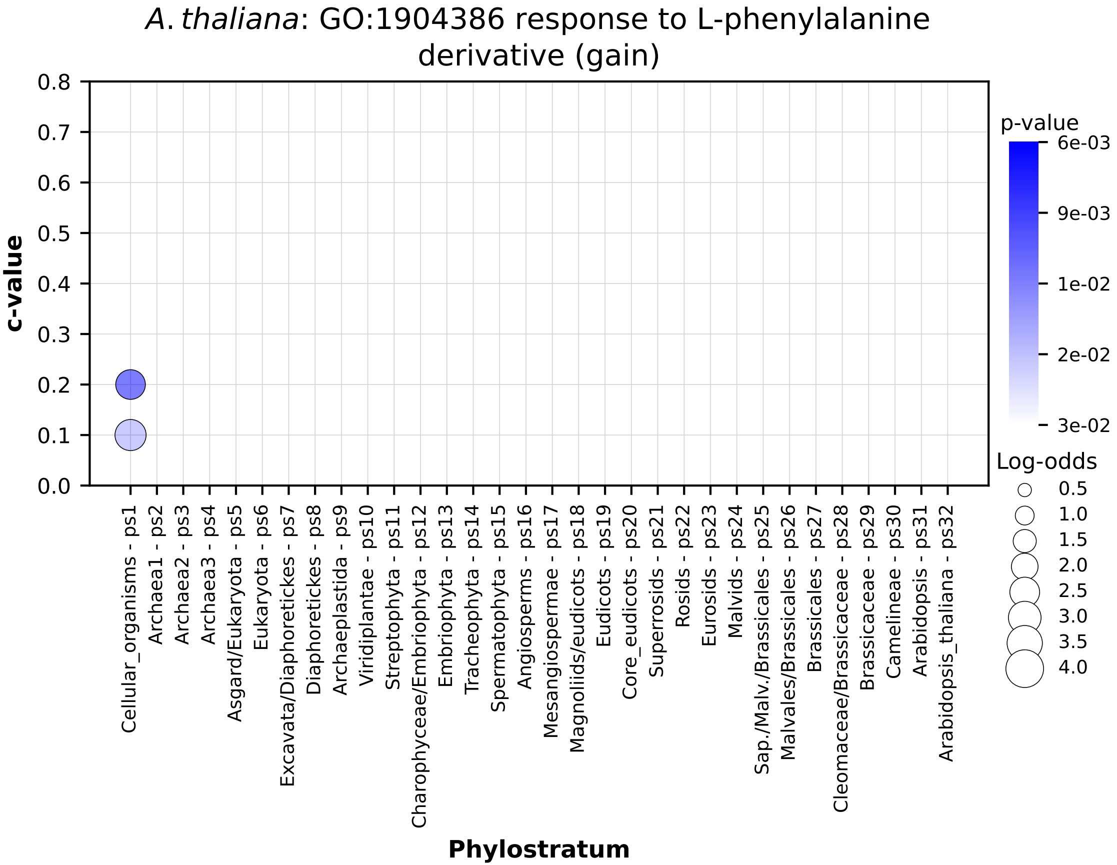
A. thaliana: GO:1904386 response to L-phenylalanine derivative (gain)
c-value
0.8
0.7
0.6
0.5
0.4
0.3
0.2
0.1
0.0
Phylostratum
Cellular_organisms - ps1
Archaea1 - ps2
Archaea2 - ps3
Archaea3 - ps4
Asgard/Eukaryota - ps5
Eukaryota - ps6
Excavata/Diaphoretickes - ps7
Diaphoretickes - ps8
Archaeplastida - ps9
Viridiplantae - ps10
Streptophyta - ps11
Charophyceae/Embriophyta - ps12
Embriophyta - ps13
Tracheophyta - ps14
Spermatophyta - ps15
Angiosperms - ps16
Mesangiospermae - ps17
Magnoliids/eudicots - ps18
Eudicots - ps19
Core_eudicots - ps20
Superrosids - ps21
Rosids - ps22
Eurosids - ps23
Malvids - ps24
Sap./Malv./Brassicales - ps25
Malvales/Brassicales - ps26
Brassicales - ps27
Cleomaceae/Brassicaceae - ps28
Brassicaceae - ps29
Camelineae - ps30
Arabidopsis - ps31
Arabidopsis_thaliana - ps32
p-value
6e-03
9e-03
1e-02
2e-02
3e-02
Log-odds
0.5
1.0
1.5
2.0
2.5
3.0
3.5
4.0

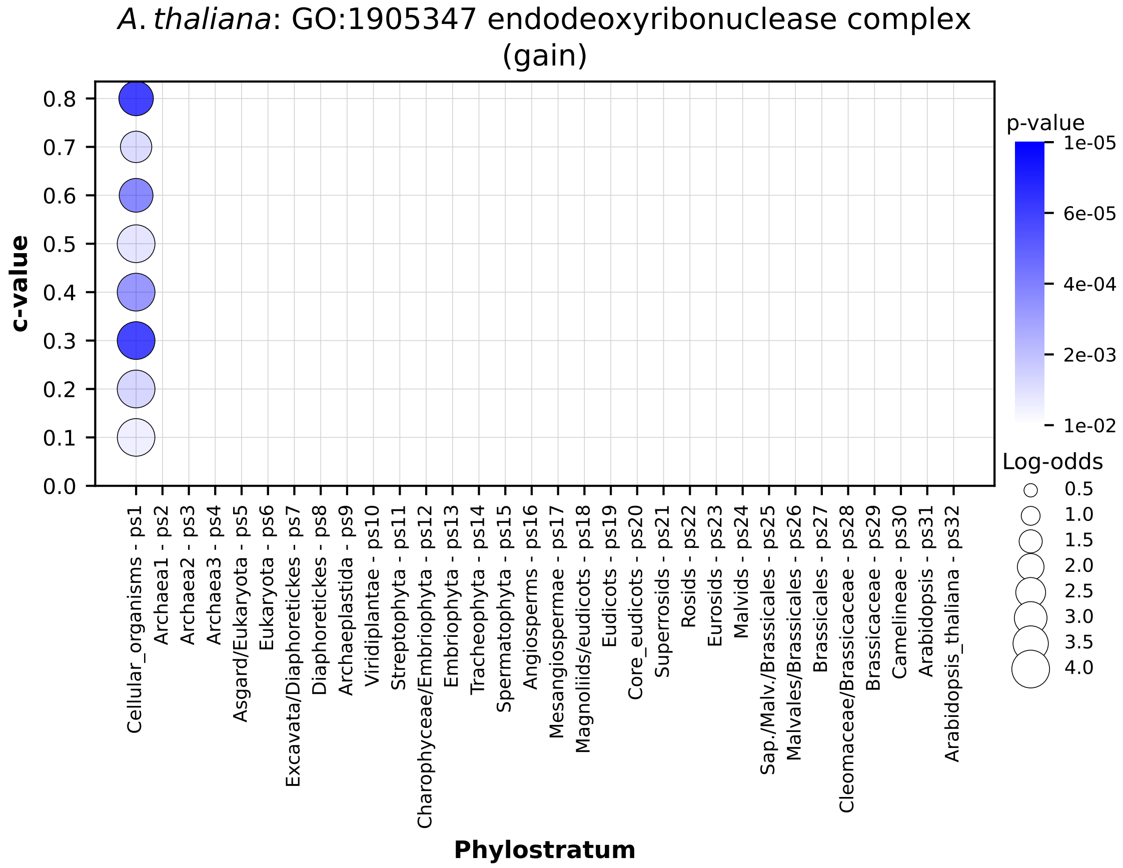

A. thaliana: GO:1905347 endodeoxyribonuclease complex
(gain)
c-value
0.0
0.1
0.2
0.3
0.4
0.5
0.6
0.7
0.8
p-value
1e-05
6e-05
4e-04
2e-03
1e-02
Log-odds
0.5
1.0
1.5
2.0
2.5
3.0
3.5
4.0
Cellular_organisms - ps1
Archaea1 - ps2
Archaea2 - ps3
Archaea3 - ps4
Asgard/Eukaryota - ps5
Eukaryota - ps6
Excavata/Diaphoretickes - ps7
Diaphoretickes - ps8
Archaeplastida - ps9
Viridiplantae - ps10
Streptophyta - ps11
Charophyceae/Embriophyta - ps12
Embriophyta - ps13
Tracheophyta - ps14
Spermatophyta - ps15
Angiosperms - ps16
Mesangiospermae - ps17
Magnoliids/eudicots - ps18
Eudicots - ps19
Core_eudicots - ps20
Superrosids - ps21
Rosids - ps22
Eurosids - ps23
Malvids - ps24
Sap./Malv./Brassicales - ps25
Malvales/Brassicales - ps26
Brassicales - ps27
Cleomaceae/Brassicaceae - ps28
Brassicaceae - ps29
Camelineae - ps30
Arabidopsis - ps31
Arabidopsis_thaliana - ps32
Phylostratum

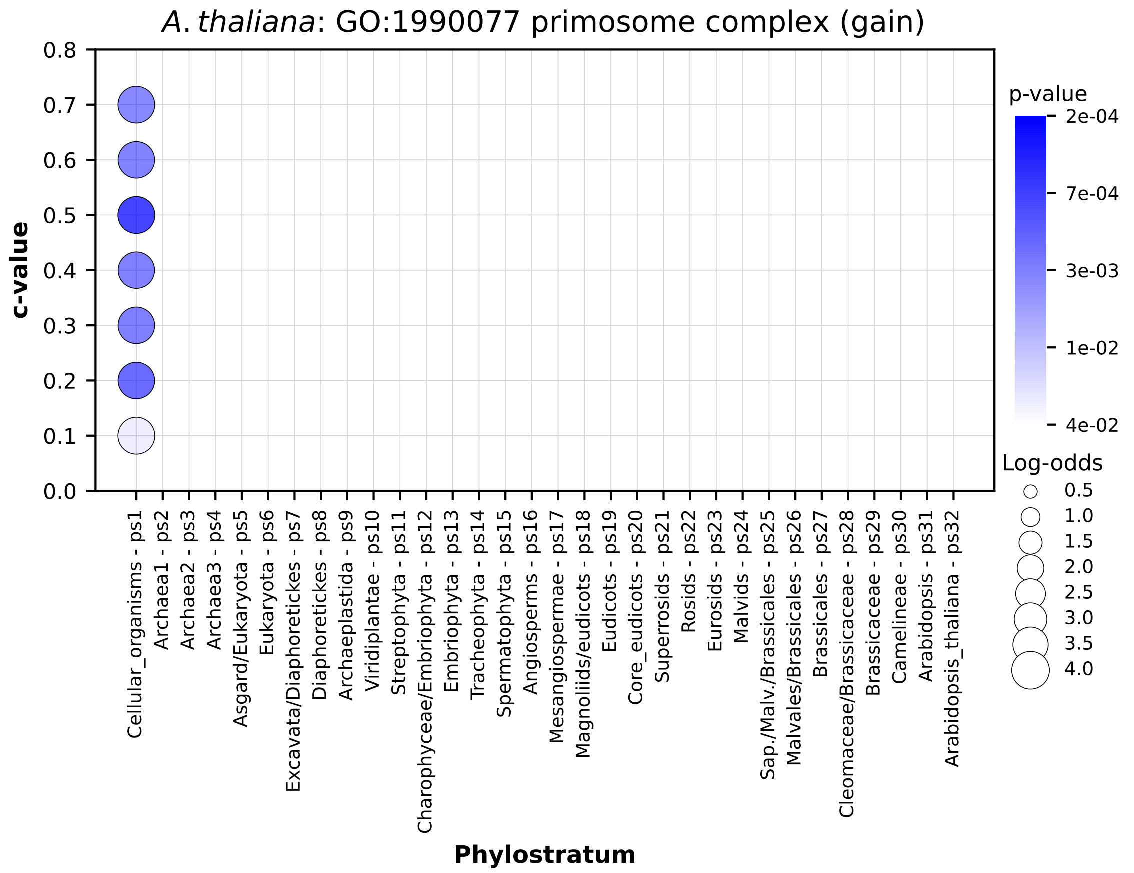

A. thaliana: GO:1990077 primosome complex (gain)
c-value
0.0
0.1
0.2
0.3
0.4
0.5
0.6
0.7
0.8
Phylostratum
Cellular_organisms - ps1
Archaea1 - ps2
Archaea2 - ps3
Archaea3 - ps4
Asgard/Eukaryota - ps5
Eukaryota - ps6
Excavata/Diaphoretickes - ps7
Diaphoretickes - ps8
Archaeplastida - ps9
Viridiplantae - ps10
Streptophyta - ps11
Charophyceae/Embriophyta - ps12
Embriophyta - ps13
Tracheophyta - ps14
Spermatophyta - ps15
Angiosperms - ps16
Mesangiospermae - ps17
Magnoliids/eudicots - ps18
Eudicots - ps19
Core_eudicots - ps20
Superrosids - ps21
Rosids - ps22
Eurosids - ps23
Malvids - ps24
Sap./Malv./Brassicales - ps25
Malvales/Brassicales - ps26
Brassicales - ps27
Cleomaceae/Brassicaceae - ps28
Brassicaceae - ps29
Camelineae - ps30
Arabidopsis - ps31
Arabidopsis_thaliana - ps32
p-value
2e-04
7e-04
3e-03
1e-02
4e-02
Log-odds
0.5
1.0
1.5
2.0
2.5
3.0
3.5
4.0

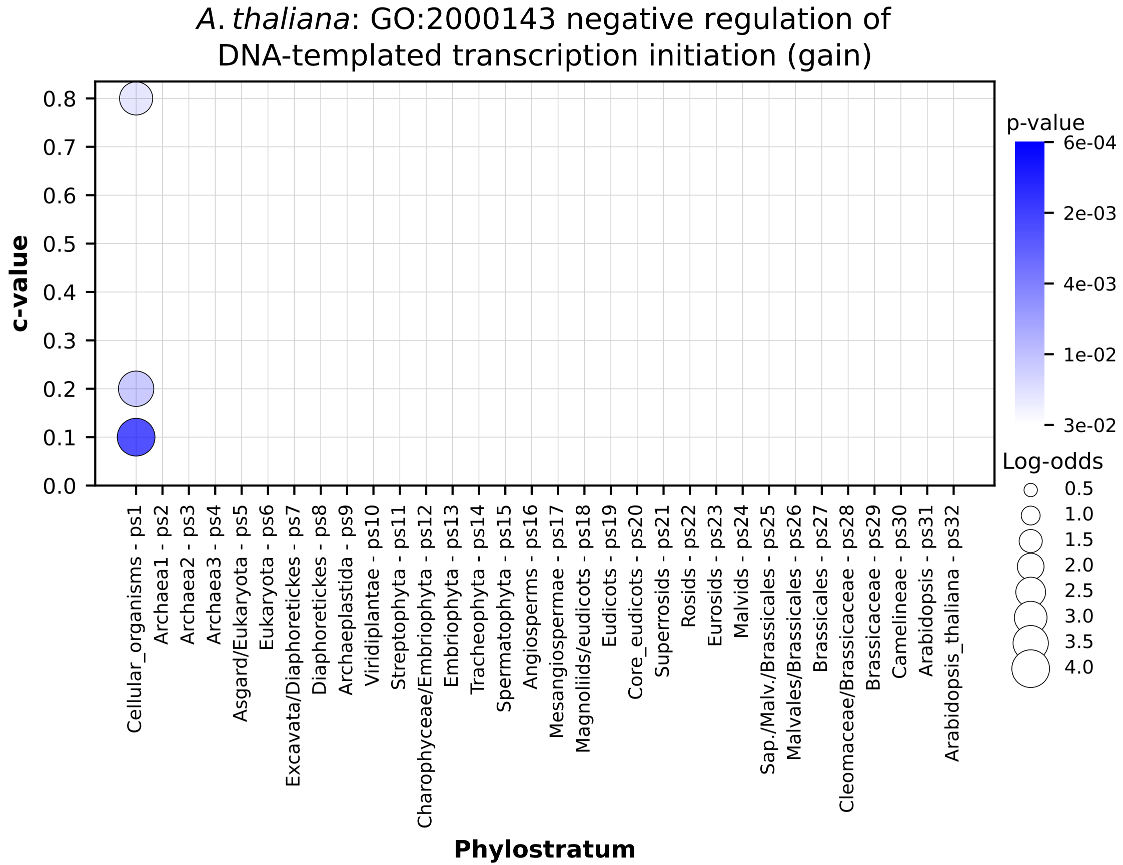

A. thaliana: GO:2000143 negative regulation of DNA-templated transcription initiation (gain)
c-value
0.8
0.7
0.6
0.5
0.4
0.3
0.2
0.1
0.0
Phylostratum
Cellular_organisms - ps1
Archaea1 - ps2
Archaea2 - ps3
Archaea3 - ps4
Asgard/Eukaryota - ps5
Eukaryota - ps6
Excavata/Diaphoretickes - ps7
Diaphoretickes - ps8
Archaeplastida - ps9
Viridiplantae - ps10
Streptophyta - ps11
Charophyceae/Embriophyta - ps12
Embriophyta - ps13
Tracheophyta - ps14
Spermatophyta - ps15
Angiosperms - ps16
Mesangiospermae - ps17
Magnoliids/eudicots - ps18
Eudicots - ps19
Core_eudicots - ps20
Superrosids - ps21
Rosids - ps22
Eurosids - ps23
Malvids - ps24
Sap./Malv./Brassicales - ps25
Malvales/Brassicales - ps26
Brassicales - ps27
Cleomaceae/Brassicaceae - ps28
Brassicaceae - ps29
Camelineae - ps30
Arabidopsis - ps31
Arabidopsis_thaliana - ps32
p-value
6e-04
2e-03
4e-03
1e-02
3e-02
Log-odds
0.5
1.0
1.5
2.0
2.5
3.0
3.5
4.0

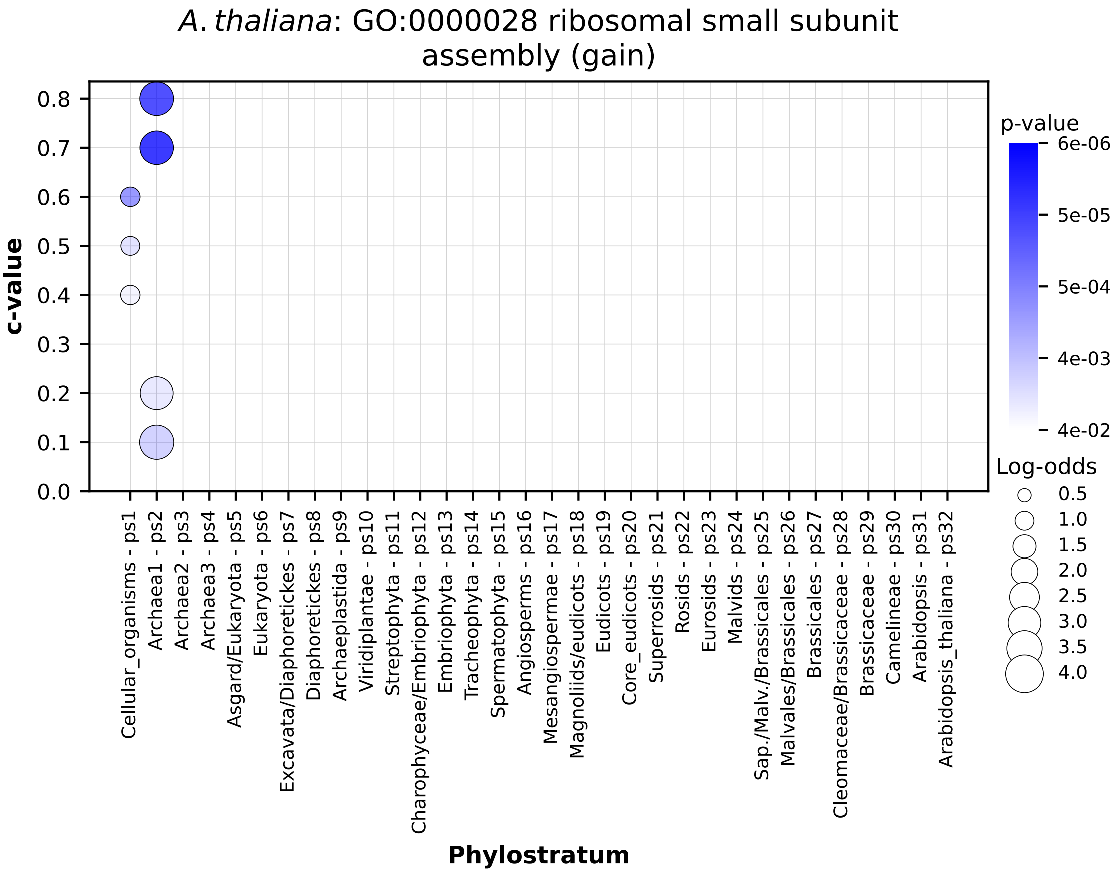

A. thaliana: GO:0000028 ribosomal small subunit assembly (gain)
c-value
0.0
0.1
0.2
0.3
0.4
0.5
0.6
0.7
0.8
Phylostratum
Cellular_organisms - ps1
Archaea1 - ps2
Archaea2 - ps3
Archaea3 - ps4
Asgard/Eukaryota - ps5
Eukaryota - ps6
Excavata/Diaphoretickes - ps7
Diaphoretickes - ps8
Archaeplastida - ps9
Viridiplantae - ps10
Streptophyta - ps11
Charophyceae/Embriophyta - ps12
Embriophyta - ps13
Tracheophyta - ps14
Spermatophyta - ps15
Angiosperms - ps16
Mesangiospermae - ps17
Magnoliids/eudicots - ps18
Eudicots - ps19
Core_eudicots - ps20
Superrosids - ps21
Rosids - ps22
Eurosids - ps23
Malvids - ps24
Sap./Malv./Brassicales - ps25
Malvales/Brassicales - ps26
Brassicales - ps27
Cleomaceae/Brassicaceae - ps28
Brassicaceae - ps29
Camelineae - ps30
Arabidopsis - ps31
Arabidopsis_thaliana - ps32
p-value
6e-06
5e-05
5e-04
4e-03
4e-02
Log-odds
0.5
1.0
1.5
2.0
2.5
3.0
3.5
4.0

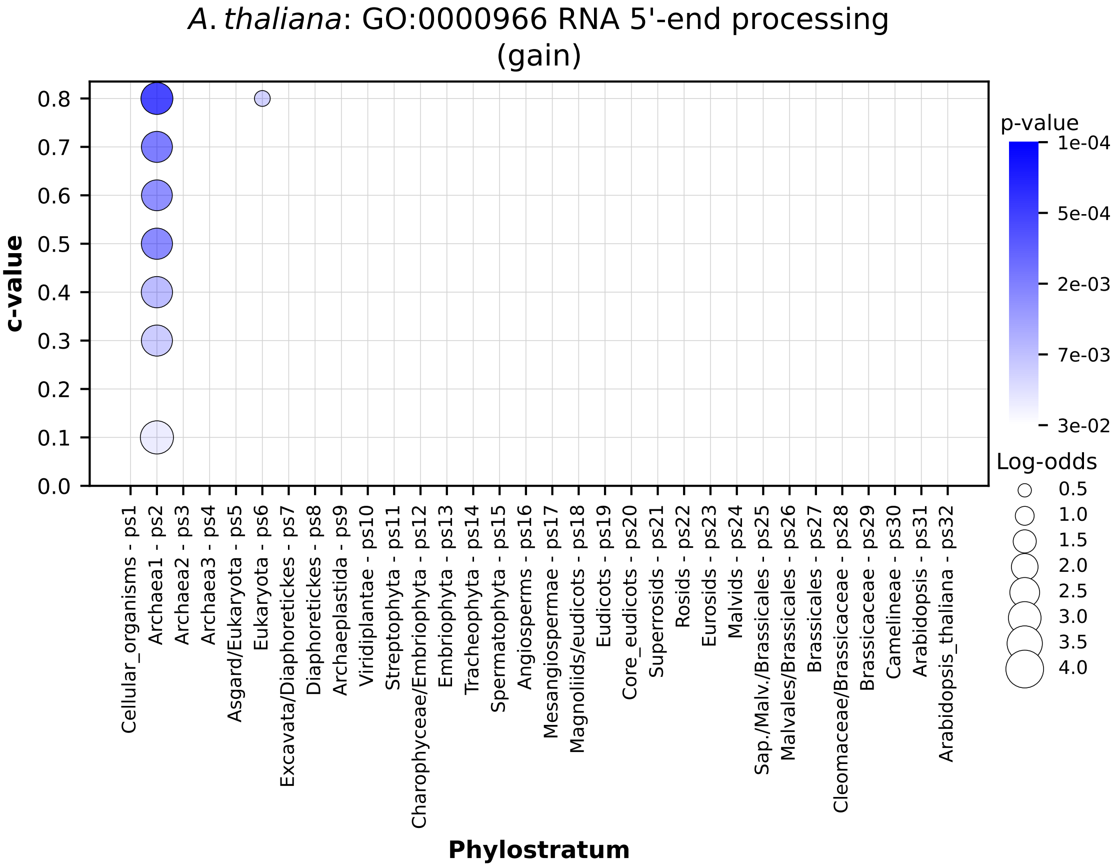

A. thaliana: GO:0000966 RNA 5'-end processing
(gain)
c-value
0.0
0.1
0.2
0.3
0.4
0.5
0.6
0.7
0.8
Phylostratum
Cellular_organisms - ps1
Archaea1 - ps2
Archaea2 - ps3
Archaea3 - ps4
Asgard/Eukaryota - ps5
Eukaryota - ps6
Excavata/Diaphoretickes - ps7
Diaphoretickes - ps8
Archaeplastida - ps9
Viridiplantae - ps10
Streptophyta - ps11
Charophyceae/Embriophyta - ps12
Embriophyta - ps13
Tracheophyta - ps14
Spermatophyta - ps15
Angiosperms - ps16
Mesangiospermae - ps17
Magnoliids/eudicots - ps18
Eudicots - ps19
Core_eudicots - ps20
Superrosids - ps21
Rosids - ps22
Eurosids - ps23
Malvids - ps24
Sap./Malv./Brassicales - ps25
Malvales/Brassicales - ps26
Brassicales - ps27
Cleomaceae/Brassicaceae - ps28
Brassicaceae - ps29
Camelineae - ps30
Arabidopsis - ps31
Arabidopsis_thaliana - ps32
p-value
1e-04
5e-04
2e-03
7e-03
3e-02
Log-odds
0.5
1.0
1.5
2.0
2.5
3.0
3.5
4.0

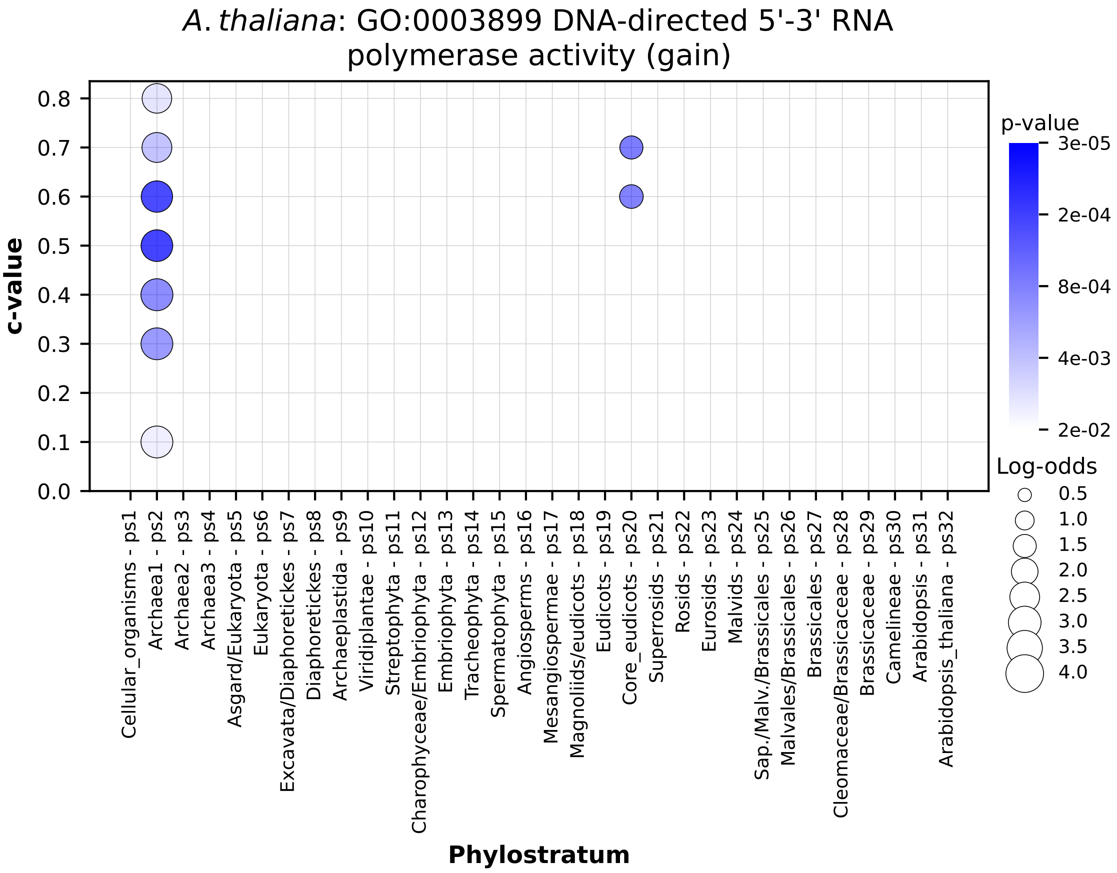

A. thaliana: GO:0003899 DNA-directed 5'-3' RNA polymerase activity (gain)
c-value
0.0
0.1
0.2
0.3
0.4
0.5
0.6
0.7
0.8
p-value
3e-05
2e-04
8e-04
4e-03
2e-02
Log-odds
0.5
1.0
1.5
2.0
2.5
3.0
3.5
4.0
Cellular_organisms - ps1
Archaea1 - ps2
Archaea2 - ps3
Archaea3 - ps4
Asgard/Eukaryota - ps5
Eukaryota - ps6
Excavata/Diaphoretickes - ps7
Diaphoretickes - ps8
Archaeplastida - ps9
Viridiplantae - ps10
Streptophyta - ps11
Charophyceae/Embriophyta - ps12
Embriophyta - ps13
Tracheophyta - ps14
Spermatophyta - ps15
Angiosperms - ps16
Mesangiospermae - ps17
Magnoliids/eudicots - ps18
Eudicots - ps19
Core_eudicots - ps20
Superrosids - ps21
Rosids - ps22
Eurosids - ps23
Malvids - ps24
Sap./Malv./Brassicales - ps25
Malvales/Brassicales - ps26
Brassicales - ps27
Cleomaceae/Brassicaceae - ps28
Brassicaceae - ps29
Camelineae - ps30
Arabidopsis - ps31
Arabidopsis_thaliana - ps32
Phylostratum

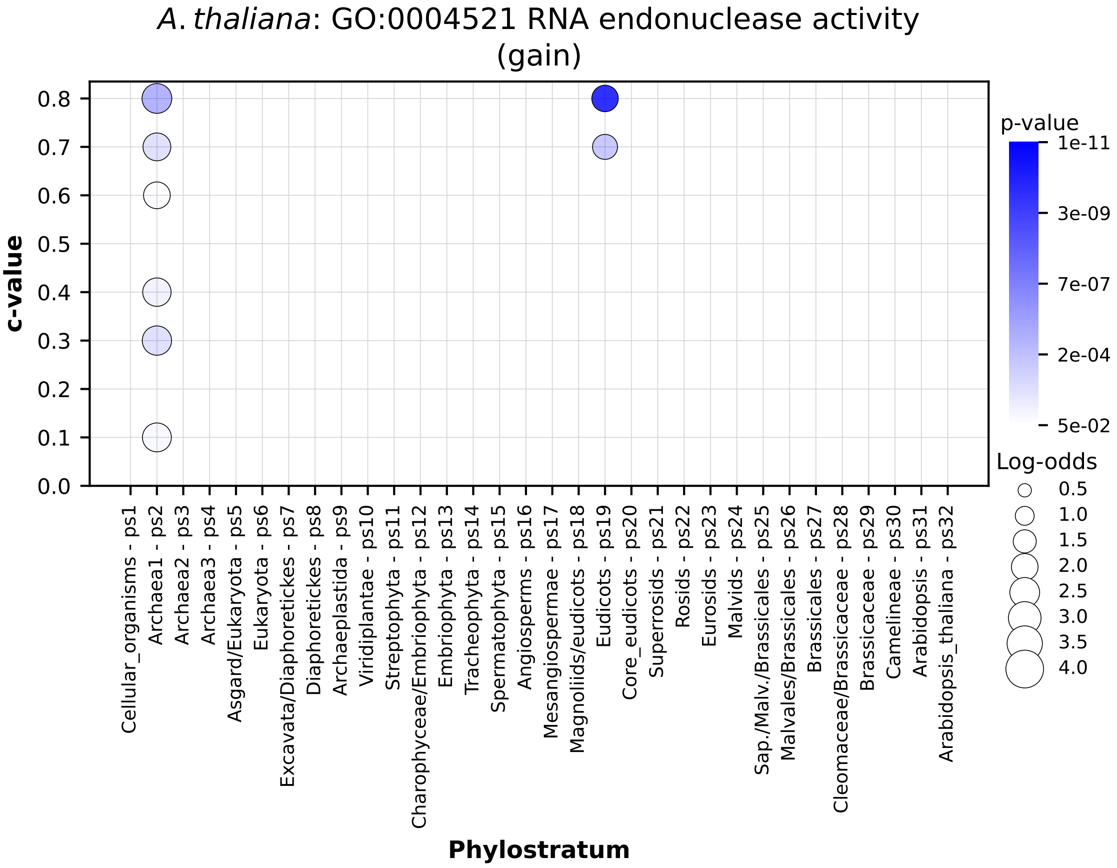

A. thaliana: GO:0004521 RNA endonuclease activity
(gain)
c-value
0.0
0.1
0.2
0.3
0.4
0.5
0.6
0.7
0.8
Phylostratum
Cellular_organisms - ps1
Archaea1 - ps2
Archaea2 - ps3
Archaea3 - ps4
Asgard/Eukaryota - ps5
Eukaryota - ps6
Excavata/Diaphoretickes - ps7
Diaphoretickes - ps8
Archaeplastida - ps9
Viridiplantae - ps10
Streptophyta - ps11
Charophyceae/Embriophyta - ps12
Embriophyta - ps13
Tracheophyta - ps14
Spermatophyta - ps15
Angiosperms - ps16
Mesangiospermae - ps17
Magnoliids/eudicots - ps18
Eudicots - ps19
Core_eudicots - ps20
Superrosids - ps21
Rosids - ps22
Eurosids - ps23
Malvids - ps24
Sap./Malv./Brassicales - ps25
Malvales/Brassicales - ps26
Brassicales - ps27
Cleomaceae/Brassicaceae - ps28
Brassicaceae - ps29
Camelineae - ps30
Arabidopsis - ps31
Arabidopsis_thaliana - ps32
p-value
1e-11
3e-09
7e-07
2e-04
5e-02
Log-odds
0.5
1.0
1.5
2.0
2.5
3.0
3.5
4.0

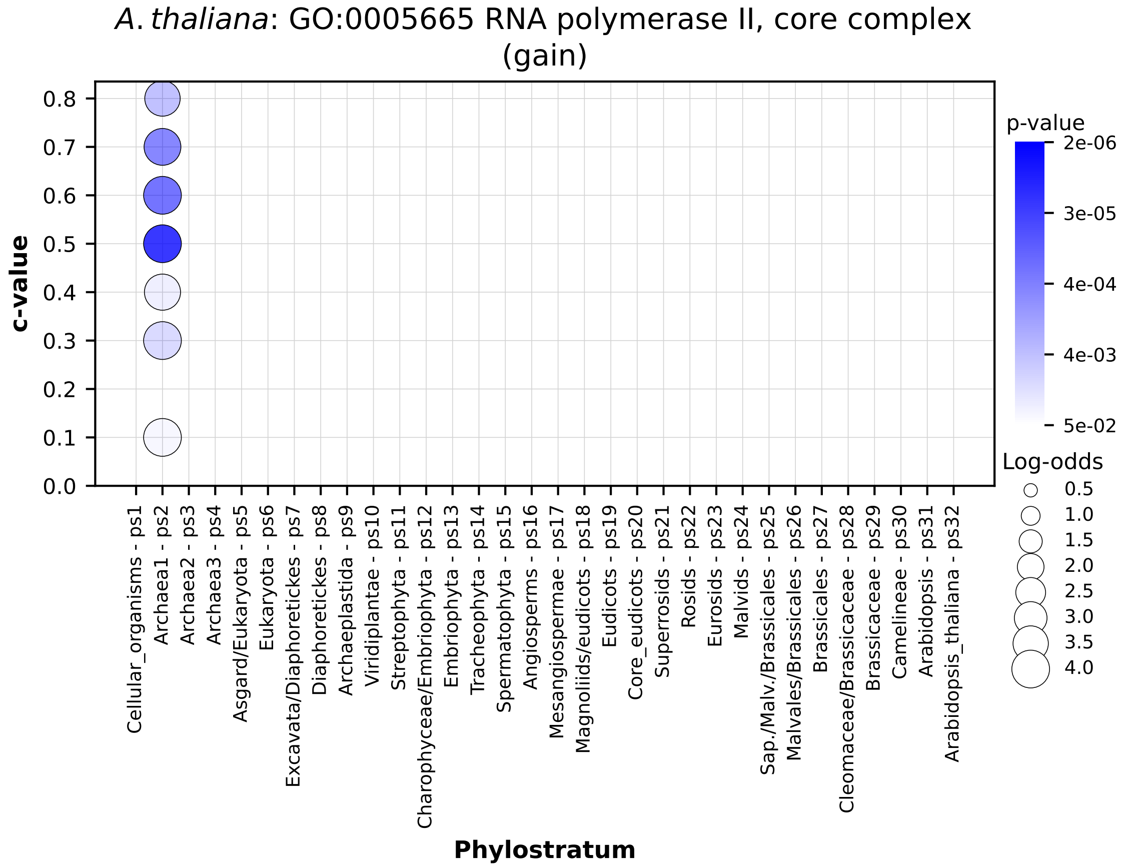

A. thaliana: GO:0005665 RNA polymerase II, core complex
(gain)
c-value
0.0
0.1
0.2
0.3
0.4
0.5
0.6
0.7
0.8
p-value
2e-06
3e-05
4e-04
4e-03
5e-02
Log-odds
0.5
1.0
1.5
2.0
2.5
3.0
3.5
4.0
Cellular_organisms - ps1
Archaea1 - ps2
Archaea2 - ps3
Archaea3 - ps4
Asgard/Eukaryota - ps5
Eukaryota - ps6
Excavata/Diaphoretickes - ps7
Diaphoretickes - ps8
Archaeplastida - ps9
Viridiplantae - ps10
Streptophyta - ps11
Charophyceae/Embriophyta - ps12
Embriophyta - ps13
Tracheophyta - ps14
Spermatophyta - ps15
Angiosperms - ps16
Mesangiospermae - ps17
Magnoliids/eudicots - ps18
Eudicots - ps19
Core_eudicots - ps20
Superrosids - ps21
Rosids - ps22
Eurosids - ps23
Malvids - ps24
Sap./Malv./Brassicales - ps25
Malvales/Brassicales - ps26
Brassicales - ps27
Cleomaceae/Brassicaceae - ps28
Brassicaceae - ps29
Camelineae - ps30
Arabidopsis - ps31
Arabidopsis_thaliana - ps32
Phylostratum

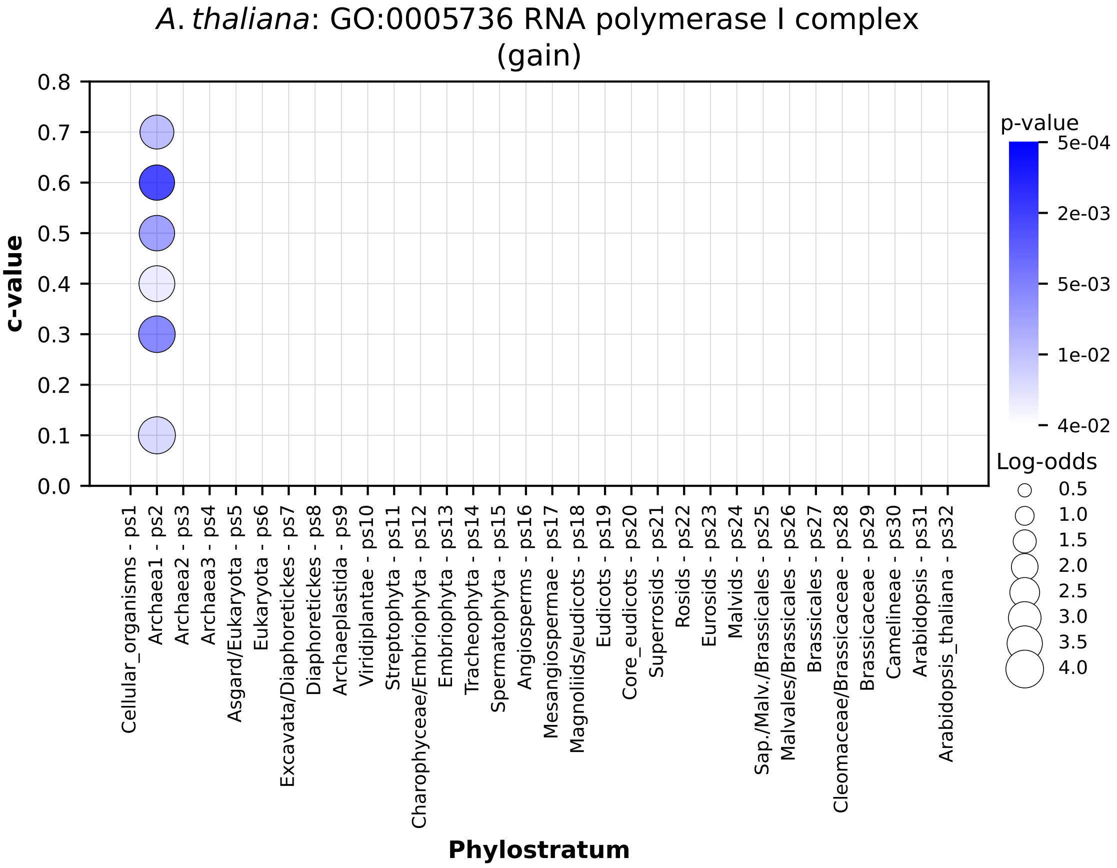

A. thaliana: GO:0005736 RNA polymerase I complex
(gain)
c-value
0.0
0.1
0.2
0.3
0.4
0.5
0.6
0.7
0.8
Phylostratum
Cellular_organisms - ps1
Archaea1 - ps2
Archaea2 - ps3
Archaea3 - ps4
Asgard/Eukaryota - ps5
Eukaryota - ps6
Excavata/Diaphoretickes - ps7
Diaphoretickes - ps8
Archaeplastida - ps9
Viridiplantae - ps10
Streptophyta - ps11
Charophyceae/Embriophyta - ps12
Embriophyta - ps13
Tracheophyta - ps14
Spermatophyta - ps15
Angiosperms - ps16
Mesangiospermae - ps17
Magnoliids/eudicots - ps18
Eudicots - ps19
Core_eudicots - ps20
Superrosids - ps21
Rosids - ps22
Eurosids - ps23
Malvids - ps24
Sap./Malv./Brassicales - ps25
Malvales/Brassicales - ps26
Brassicales - ps27
Cleomaceae/Brassicaceae - ps28
Brassicaceae - ps29
Camelineae - ps30
Arabidopsis - ps31
Arabidopsis_thaliana - ps32
p-value
5e-04
2e-03
5e-03
1e-02
4e-02
Log-odds
0.5
1.0
1.5
2.0
2.5
3.0
3.5
4.0

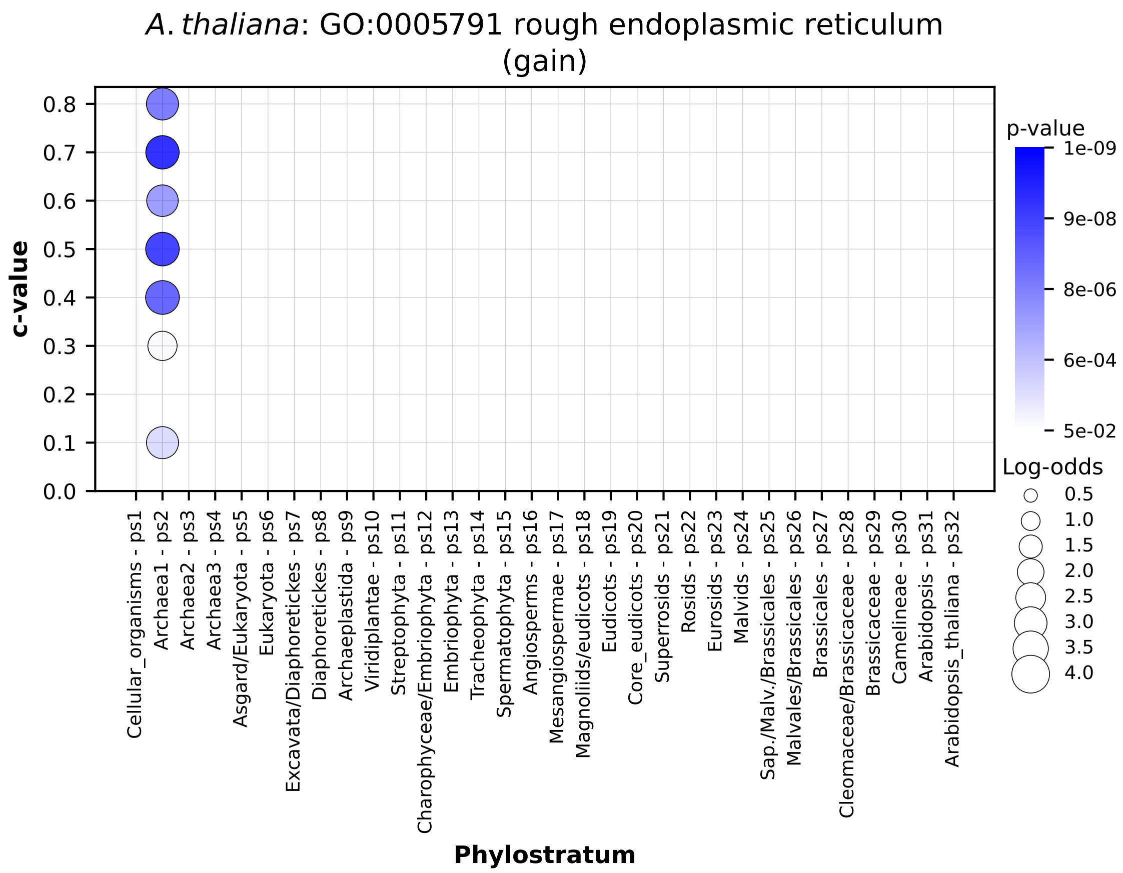
A. thaliana: GO:0005791 rough endoplasmic reticulum (gain)
c-value
0.0
0.1
0.2
0.3
0.4
0.5
0.6
0.7
0.8
Phylostratum
Cellular_organisms - ps1
Archaea1 - ps2
Archaea2 - ps3
Archaea3 - ps4
Asgard/Eukaryota - ps5
Eukaryota - ps6
Excavata/Diaphoretickes - ps7
Diaphoretickes - ps8
Archaeplastida - ps9
Viridiplantae - ps10
Streptophyta - ps11
Charophyceae/Embriophyta - ps12
Embriophyta - ps13
Tracheophyta - ps14
Spermatophyta - ps15
Angiosperms - ps16
Mesangiospermae - ps17
Magnoliids/eudicots - ps18
Eudicots - ps19
Core_eudicots - ps20
Superrosids - ps21
Rosids - ps22
Eurosids - ps23
Malvids - ps24
Sap./Malv./Brassicales - ps25
Malvales/Brassicales - ps26
Brassicales - ps27
Cleomaceae/Brassicaceae - ps28
Brassicaceae - ps29
Camelineae - ps30
Arabidopsis - ps31
Arabidopsis_thaliana - ps32
p-value
1e-09
9e-08
8e-06
6e-04
5e-02
Log-odds
0.5
1.0
1.5
2.0
2.5
3.0
3.5
4.0

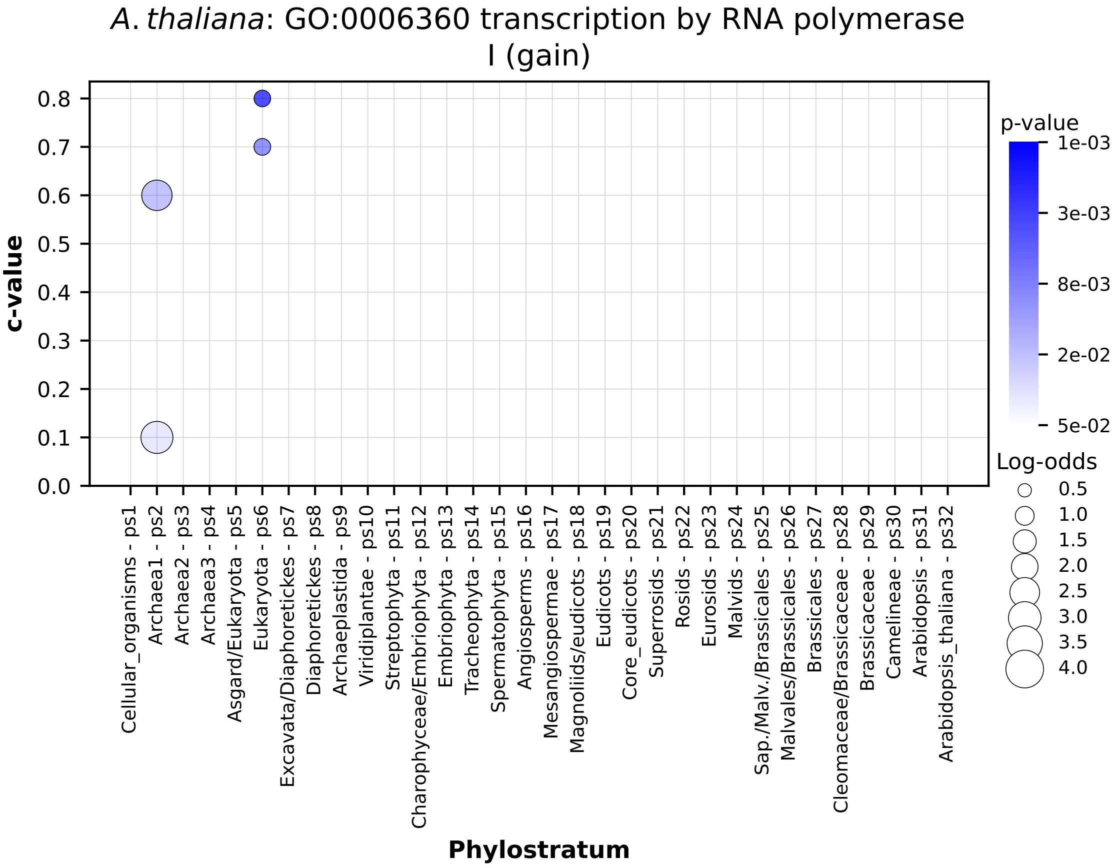

A. thaliana: GO:0006360 transcription by RNA polymerase I (gain)
c-value
0.0
0.1
0.2
0.3
0.4
0.5
0.6
0.7
0.8
Phylostratum
Cellular_organisms - ps1
Archaea1 - ps2
Archaea2 - ps3
Archaea3 - ps4
Asgard/Eukaryota - ps5
Eukaryota - ps6
Excavata/Diaphoretickes - ps7
Diaphoretickes - ps8
Archaeplastida - ps9
Viridiplantae - ps10
Streptophyta - ps11
Charophyceae/Embriophyta - ps12
Embriophyta - ps13
Tracheophyta - ps14
Spermatophyta - ps15
Angiosperms - ps16
Mesangiospermae - ps17
Magnoliids/eudicots - ps18
Eudicots - ps19
Core_eudicots - ps20
Superrosids - ps21
Rosids - ps22
Eurosids - ps23
Malvids - ps24
Sap./Malv./Brassicales - ps25
Malvales/Brassicales - ps26
Brassicales - ps27
Cleomaceae/Brassicaceae - ps28
Brassicaceae - ps29
Camelineae - ps30
Arabidopsis - ps31
Arabidopsis_thaliana - ps32
p-value
1e-03
3e-03
8e-03
2e-02
5e-02
Log-odds
0.5
1.0
1.5
2.0
2.5
3.0
3.5
4.0

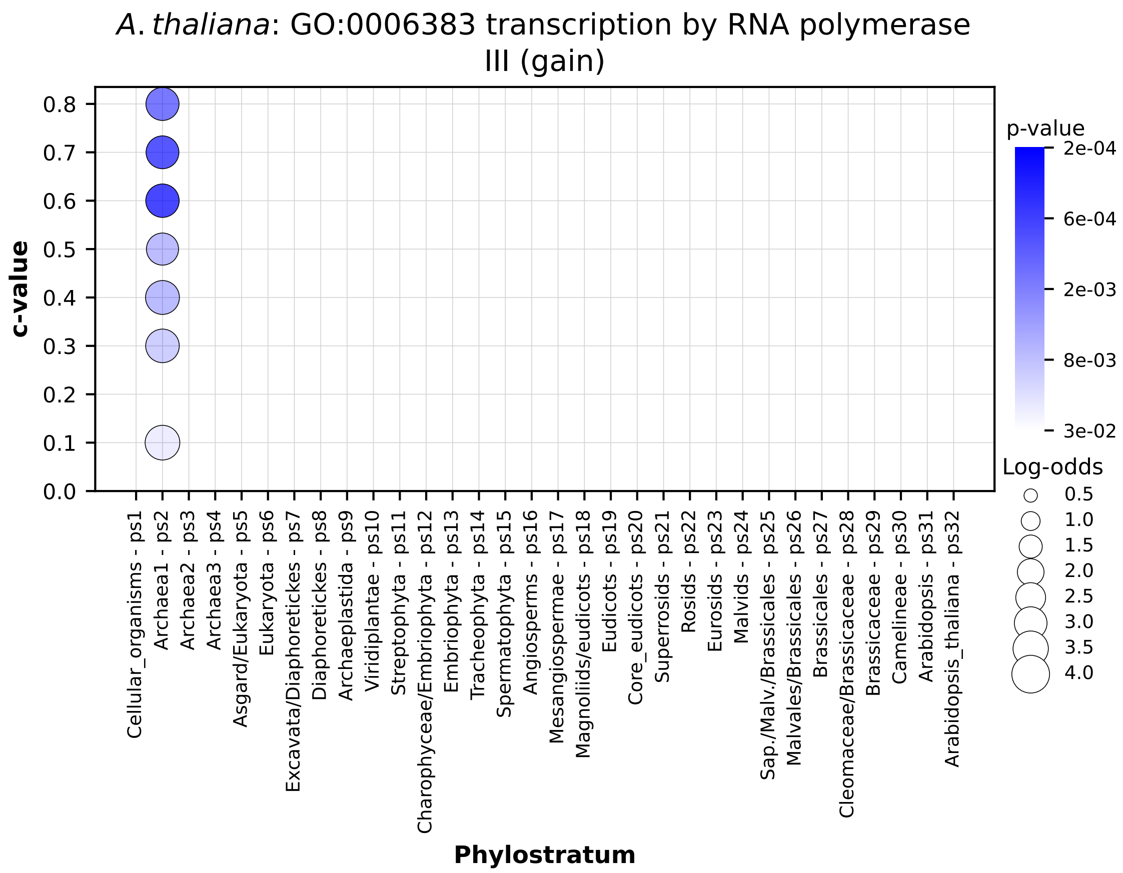

A. thaliana: GO:0006383 transcription by RNA polymerase III (gain)
c-value
0.0
0.1
0.2
0.3
0.4
0.5
0.6
0.7
0.8
Phylostratum
Cellular_organisms - ps1
Archaea1 - ps2
Archaea2 - ps3
Archaea3 - ps4
Asgard/Eukaryota - ps5
Eukaryota - ps6
Excavata/Diaphoretickes - ps7
Diaphoretickes - ps8
Archaeplastida - ps9
Viridiplantae - ps10
Streptophyta - ps11
Charophyceae/Embriophyta - ps12
Embriophyta - ps13
Tracheophyta - ps14
Spermatophyta - ps15
Angiosperms - ps16
Mesangiospermae - ps17
Magnoliids/eudicots - ps18
Eudicots - ps19
Core_eudicots - ps20
Superrosids - ps21
Rosids - ps22
Eurosids - ps23
Malvids - ps24
Sap./Malv./Brassicales - ps25
Malvales/Brassicales - ps26
Brassicales - ps27
Cleomaceae/Brassicaceae - ps28
Brassicaceae - ps29
Camelineae - ps30
Arabidopsis - ps31
Arabidopsis_thaliana - ps32
p-value
2e-04
6e-04
2e-03
8e-03
3e-02
Log-odds
0.5
1.0
1.5
2.0
2.5
3.0
3.5
4.0

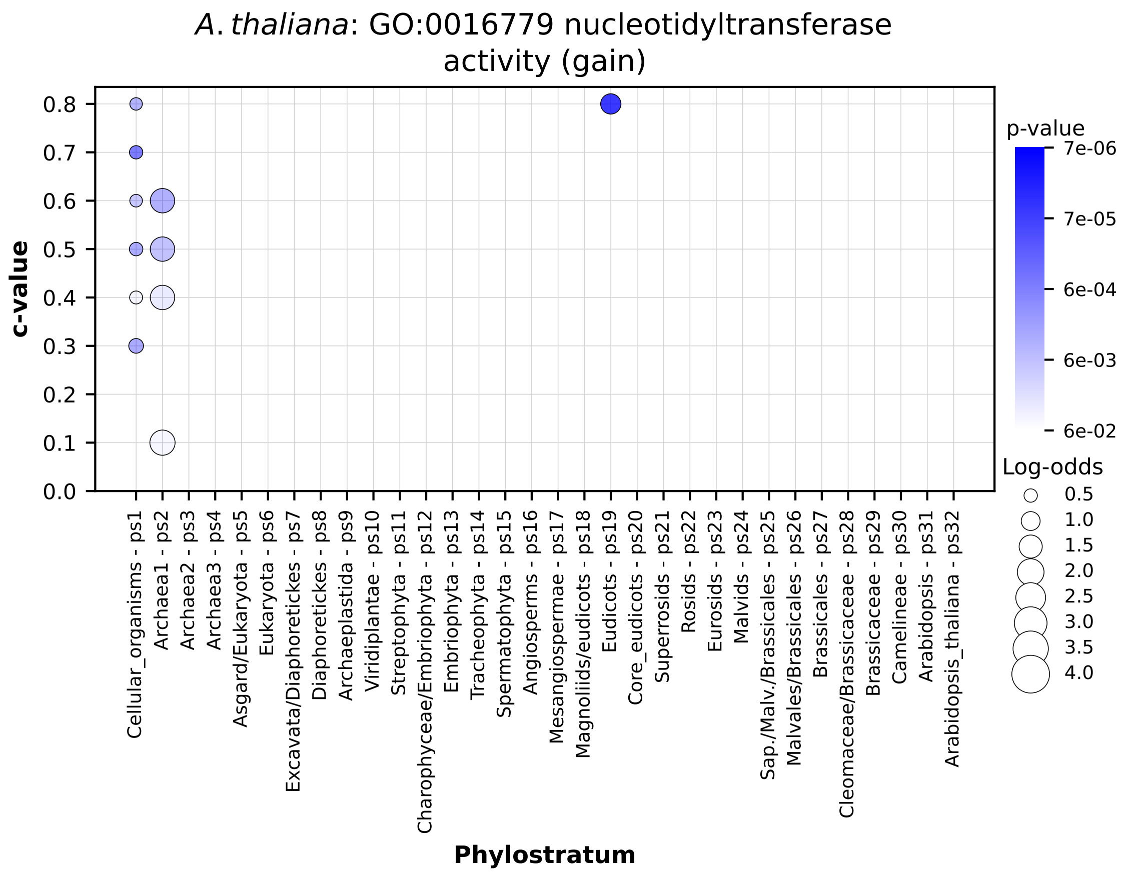
A. thaliana: GO:0016779 nucleotidyltransferase activity (gain)
c-value
0.0
0.1
0.2
0.3
0.4
0.5
0.6
0.7
0.8
Phylostratum
Cellular_organisms - ps1
Archaea1 - ps2
Archaea2 - ps3
Archaea3 - ps4
Asgard/Eukaryota - ps5
Eukaryota - ps6
Excavata/Diaphoretickes - ps7
Diaphoretickes - ps8
Archaeplastida - ps9
Viridiplantae - ps10
Streptophyta - ps11
Charophyceae/Embriophyta - ps12
Embriophyta - ps13
Tracheophyta - ps14
Spermatophyta - ps15
Angiosperms - ps16
Mesangiospermae - ps17
Magnoliids/eudicots - ps18
Eudicots - ps19
Core_eudicots - ps20
Superrosids - ps21
Rosids - ps22
Eurosids - ps23
Malvids - ps24
Sap./Malv./Brassicales - ps25
Malvales/Brassicales - ps26
Brassicales - ps27
Cleomaceae/Brassicaceae - ps28
Brassicaceae - ps29
Camelineae - ps30
Arabidopsis - ps31
Arabidopsis_thaliana - ps32
p-value
7e-06
7e-05
6e-04
6e-03
6e-02
Log-odds
0.5
1.0
1.5
2.0
2.5
3.0
3.5
4.0

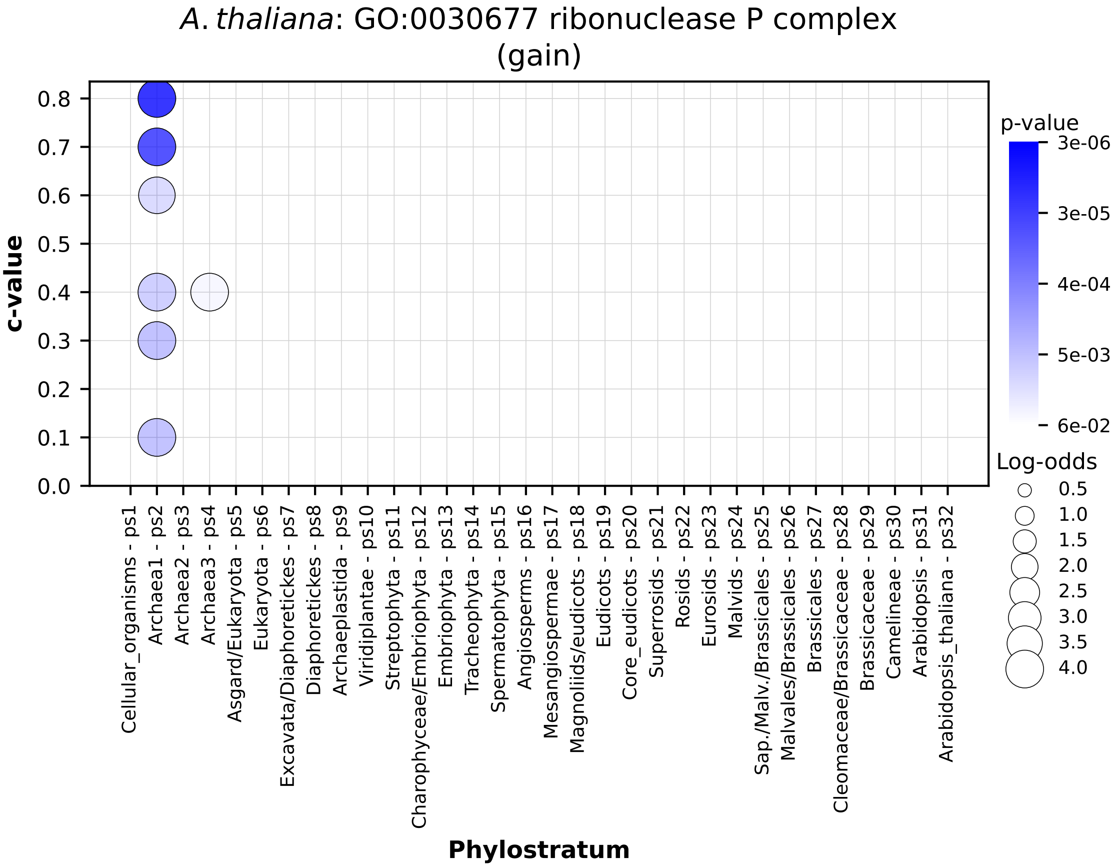

A. thaliana: GO:0030677 ribonuclease P complex
(gain)
c-value
0.0
0.1
0.2
0.3
0.4
0.5
0.6
0.7
0.8
Phylostratum
Cellular_organisms - ps1
Archaea1 - ps2
Archaea2 - ps3
Archaea3 - ps4
Asgard/Eukaryota - ps5
Eukaryota - ps6
Excavata/Diaphoretickes - ps7
Diaphoretickes - ps8
Archaeplastida - ps9
Viridiplantae - ps10
Streptophyta - ps11
Charophyceae/Embriophyta - ps12
Embriophyta - ps13
Tracheophyta - ps14
Spermatophyta - ps15
Angiosperms - ps16
Mesangiospermae - ps17
Magnoliids/eudicots - ps18
Eudicots - ps19
Core_eudicots - ps20
Superrosids - ps21
Rosids - ps22
Eurosids - ps23
Malvids - ps24
Sap./Malv./Brassicales - ps25
Malvales/Brassicales - ps26
Brassicales - ps27
Cleomaceae/Brassicaceae - ps28
Brassicaceae - ps29
Camelineae - ps30
Arabidopsis - ps31
Arabidopsis_thaliana - ps32
p-value
3e-06
3e-05
4e-04
5e-03
6e-02
Log-odds
0.5
1.0
1.5
2.0
2.5
3.0
3.5
4.0

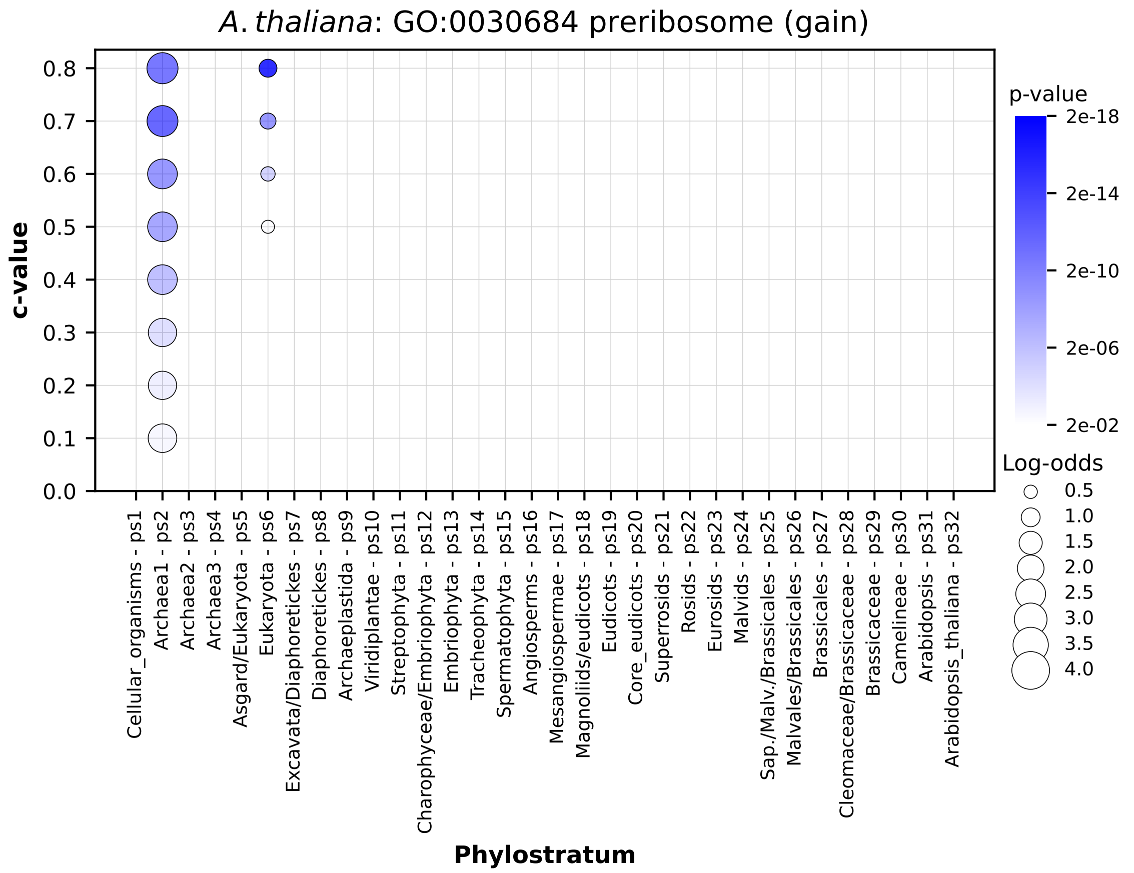
A. thaliana: GO:0030684 preribosome (gain)
c-value
0.0
0.1
0.2
0.3
0.4
0.5
0.6
0.7
0.8
Phylostratum
Cellular_organisms - ps1
Archaea1 - ps2
Archaea2 - ps3
Archaea3 - ps4
Asgard/Eukaryota - ps5
Eukaryota - ps6
Excavata/Diaphoretickes - ps7
Diaphoretickes - ps8
Archaeplastida - ps9
Viridiplantae - ps10
Streptophyta - ps11
Charophyceae/Embriophyta - ps12
Embriophyta - ps13
Tracheophyta - ps14
Spermatophyta - ps15
Angiosperms - ps16
Mesangiospermae - ps17
Magnoliids/eudicots - ps18
Eudicots - ps19
Core_eudicots - ps20
Superrosids - ps21
Rosids - ps22
Eurosids - ps23
Malvids - ps24
Sap./Malv./Brassicales - ps25
Malvales/Brassicales - ps26
Brassicales - ps27
Cleomaceae/Brassicaceae - ps28
Brassicaceae - ps29
Camelineae - ps30
Arabidopsis - ps31
Arabidopsis_thaliana - ps32
p-value
2e-18
2e-14
2e-10
2e-06
2e-02
Log-odds
0.5
1.0
1.5
2.0
2.5
3.0
3.5
4.0

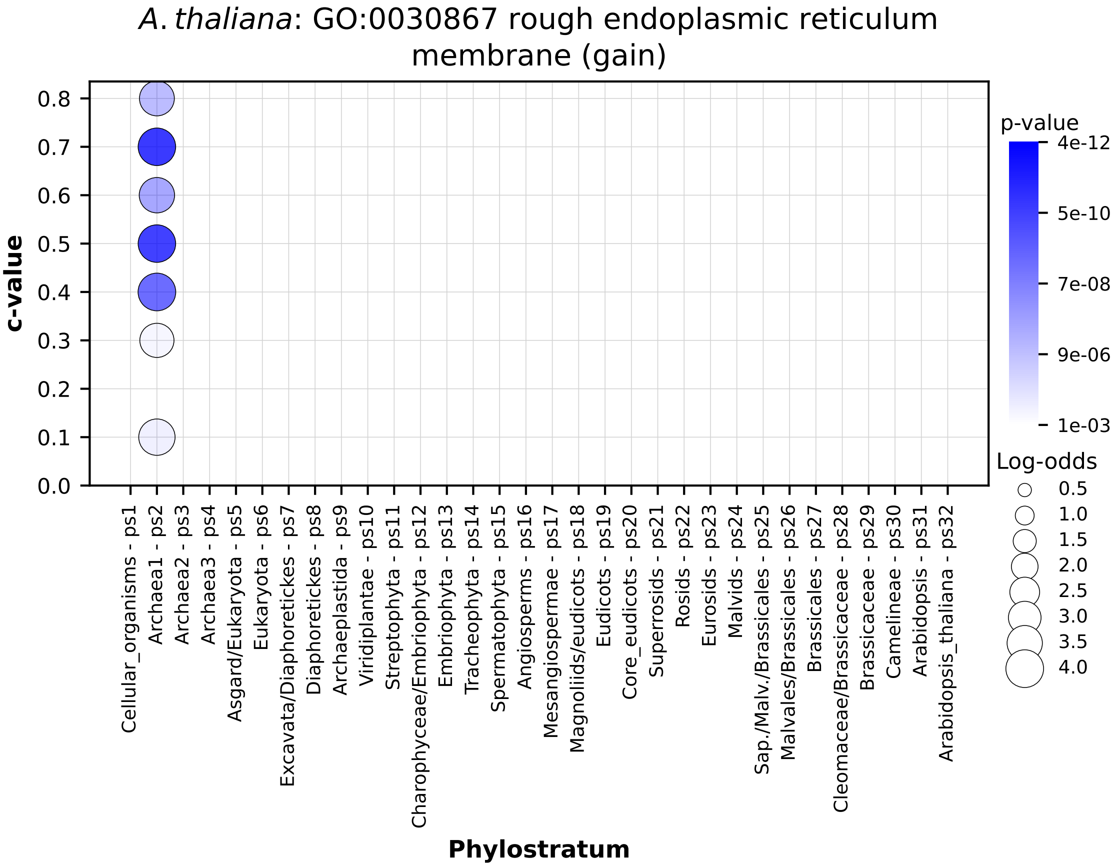

A. thaliana: GO:0030867 rough endoplasmic reticulum membrane (gain)
c-value
0.0
0.1
0.2
0.3
0.4
0.5
0.6
0.7
0.8
Phylostratum
Cellular_organisms - ps1
Archaea1 - ps2
Archaea2 - ps3
Archaea3 - ps4
Asgard/Eukaryota - ps5
Eukaryota - ps6
Excavata/Diaphoretickes - ps7
Diaphoretickes - ps8
Archaeplastida - ps9
Viridiplantae - ps10
Streptophyta - ps11
Charophyceae/Embriophyta - ps12
Embriophyta - ps13
Tracheophyta - ps14
Spermatophyta - ps15
Angiosperms - ps16
Mesangiospermae - ps17
Magnoliids/eudicots - ps18
Eudicots - ps19
Core_eudicots - ps20
Superrosids - ps21
Rosids - ps22
Eurosids - ps23
Malvids - ps24
Sap./Malv./Brassicales - ps25
Malvales/Brassicales - ps26
Brassicales - ps27
Cleomaceae/Brassicaceae - ps28
Brassicaceae - ps29
Camelineae - ps30
Arabidopsis - ps31
Arabidopsis_thaliana - ps32
p-value
4e-12
5e-10
7e-08
9e-06
1e-03
Log-odds
0.5
1.0
1.5
2.0
2.5
3.0
3.5
4.0

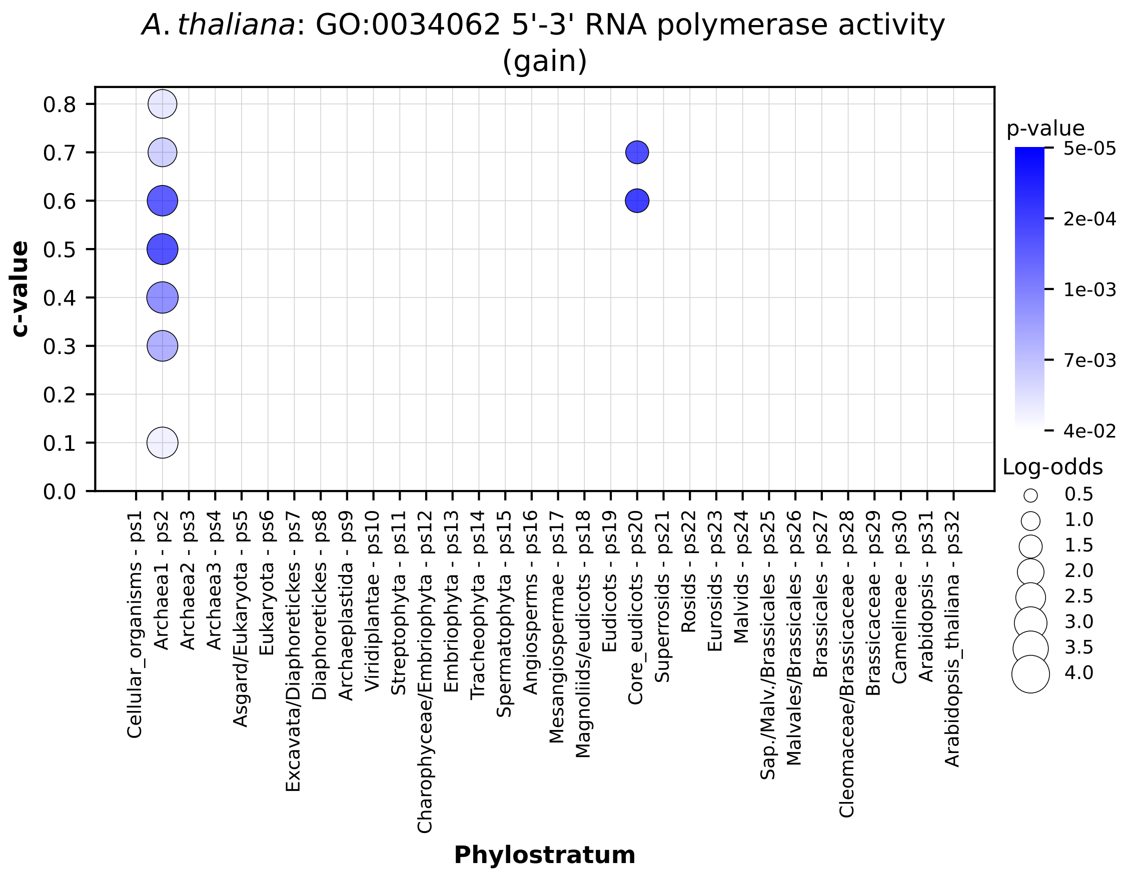

A. thaliana: GO:0034062 5'-3' RNA polymerase activity
(gain)
c-value
0.0
0.1
0.2
0.3
0.4
0.5
0.6
0.7
0.8
p-value
5e-05
2e-04
1e-03
7e-03
4e-02
Log-odds
0.5
1.0
1.5
2.0
2.5
3.0
3.5
4.0
Cellular_organisms - ps1
Archaea1 - ps2
Archaea2 - ps3
Archaea3 - ps4
Asgard/Eukaryota - ps5
Eukaryota - ps6
Excavata/Diaphoretickes - ps7
Diaphoretickes - ps8
Archaeplastida - ps9
Viridiplantae - ps10
Streptophyta - ps11
Charophyceae/Embriophyta - ps12
Embriophyta - ps13
Tracheophyta - ps14
Spermatophyta - ps15
Angiosperms - ps16
Mesangiospermae - ps17
Magnoliids/eudicots - ps18
Eudicots - ps19
Core_eudicots - ps20
Superrosids - ps21
Rosids - ps22
Eurosids - ps23
Malvids - ps24
Sap./Malv./Brassicales - ps25
Malvales/Brassicales - ps26
Brassicales - ps27
Cleomaceae/Brassicaceae - ps28
Brassicaceae - ps29
Camelineae - ps30
Arabidopsis - ps31
Arabidopsis_thaliana - ps32
Phylostratum

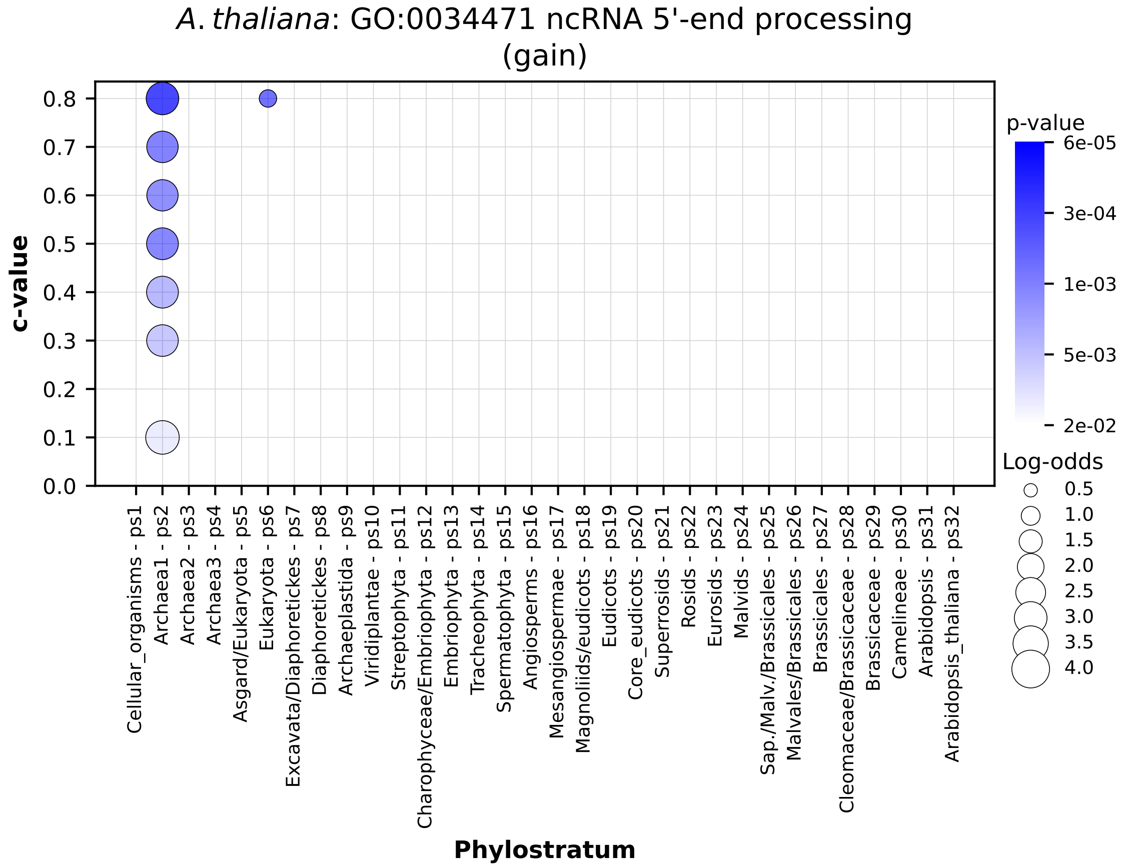

A. thaliana: GO:0034471 ncRNA 5'-end processing
(gain)
c-value
0.8
0.7
0.6
0.5
0.4
0.3
0.2
0.1
0.0
p-value
6e-05
3e-04
1e-03
5e-03
2e-02
Log-odds
0.5
1.0
1.5
2.0
2.5
3.0
3.5
4.0
Cellular_organisms - ps1
Archaea1 - ps2
Archaea2 - ps3
Archaea3 - ps4
Asgard/Eukaryota - ps5
Eukaryota - ps6
Excavata/Diaphoretickes - ps7
Diaphoretickes - ps8
Archaeplastida - ps9
Viridiplantae - ps10
Streptophyta - ps11
Charophyceae/Embriophyta - ps12
Embriophyta - ps13
Tracheophyta - ps14
Spermatophyta - ps15
Angiosperms - ps16
Mesangiospermae - ps17
Magnoliids/eudicots - ps18
Eudicots - ps19
Core_eudicots - ps20
Superrosids - ps21
Rosids - ps22
Eurosids - ps23
Malvids - ps24
Sap./Malv./Brassicales - ps25
Malvales/Brassicales - ps26
Brassicales - ps27
Cleomaceae/Brassicaceae - ps28
Brassicaceae - ps29
Camelineae - ps30
Arabidopsis - ps31
Arabidopsis_thaliana - ps32
Phylostratum

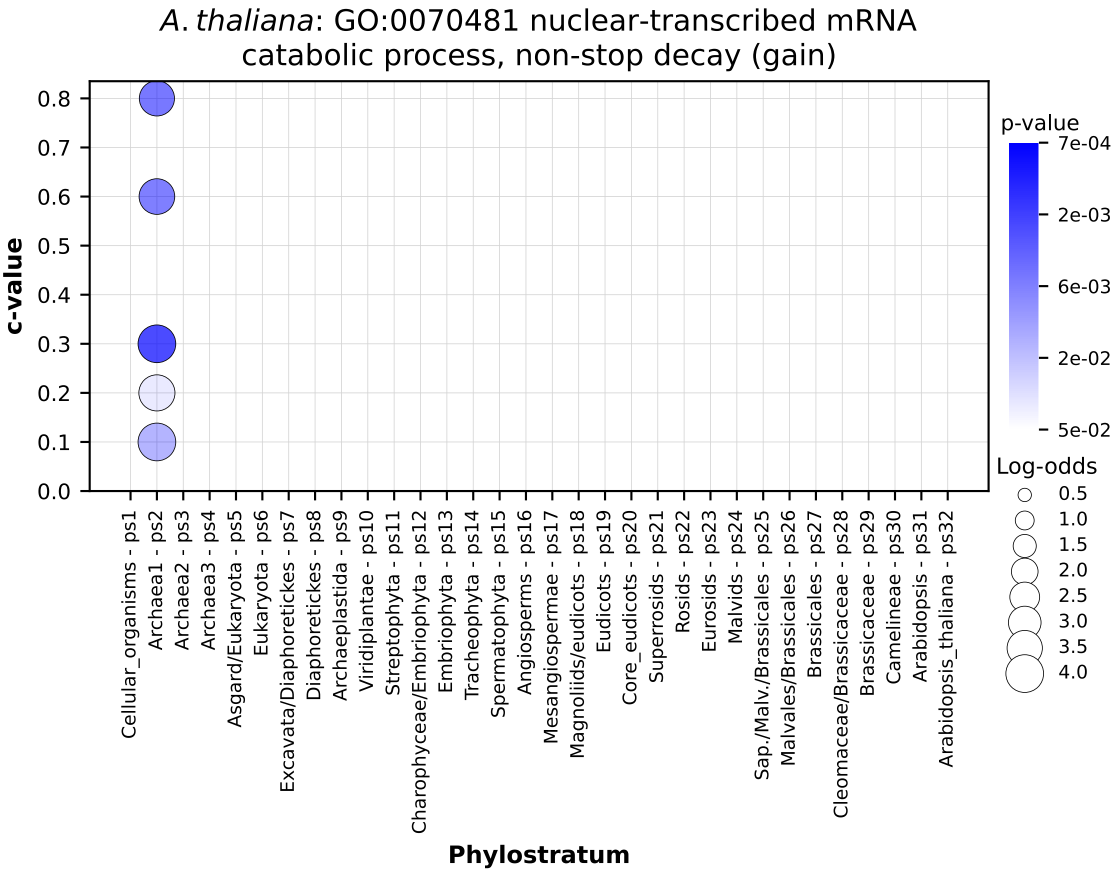

A. thaliana: GO:0070481 nuclear-transcribed mRNA catabolic process, non-stop decay (gain)
c-value
0.0
0.1
0.2
0.3
0.4
0.5
0.6
0.7
0.8
Phylostratum
Cellular_organisms - ps1
Archaea1 - ps2
Archaea2 - ps3
Archaea3 - ps4
Asgard/Eukaryota - ps5
Eukaryota - ps6
Excavata/Diaphoretickes - ps7
Diaphoretickes - ps8
Archaeplastida - ps9
Viridiplantae - ps10
Streptophyta - ps11
Charophyceae/Embriophyta - ps12
Embriophyta - ps13
Tracheophyta - ps14
Spermatophyta - ps15
Angiosperms - ps16
Mesangiospermae - ps17
Magnoliids/eudicots - ps18
Eudicots - ps19
Core_eudicots - ps20
Superrosids - ps21
Rosids - ps22
Eurosids - ps23
Malvids - ps24
Sap./Malv./Brassicales - ps25
Malvales/Brassicales - ps26
Brassicales - ps27
Cleomaceae/Brassicaceae - ps28
Brassicaceae - ps29
Camelineae - ps30
Arabidopsis - ps31
Arabidopsis_thaliana - ps32
p-value
7e-04
2e-03
6e-03
2e-02
5e-02
Log-odds
0.5
1.0
1.5
2.0
2.5
3.0
3.5
4.0

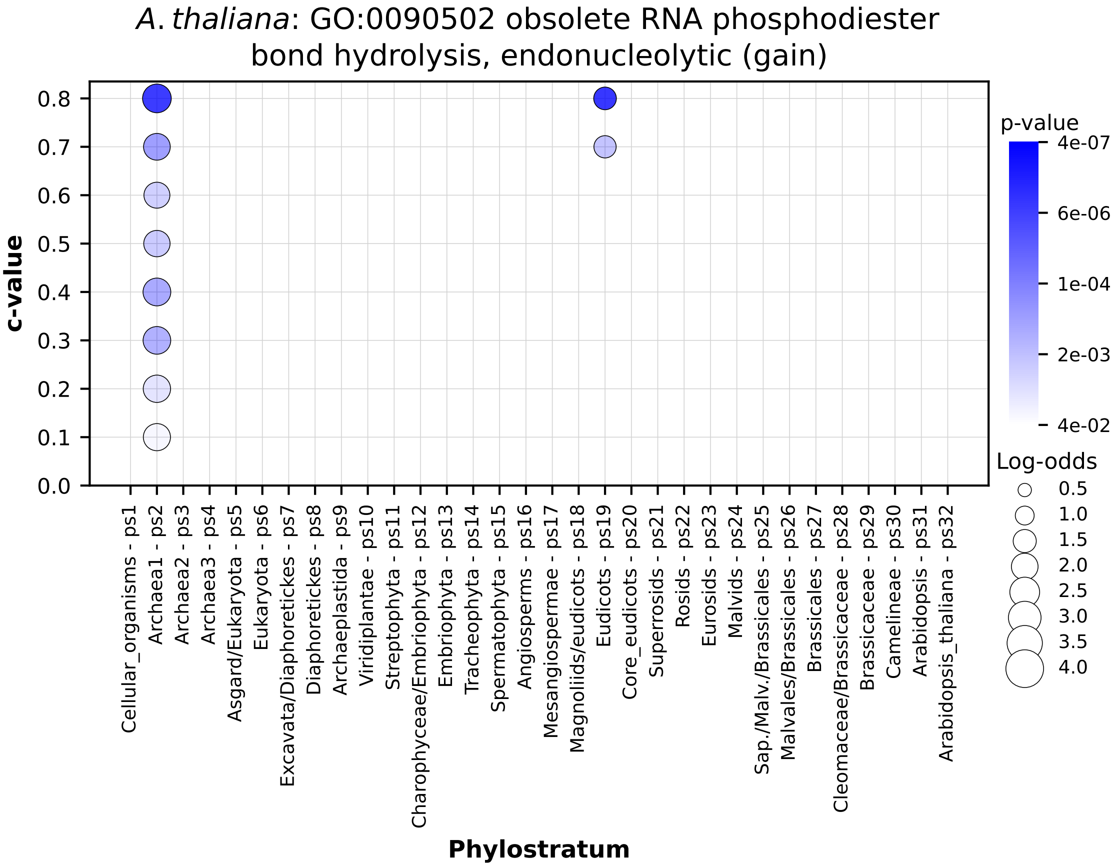

A. thaliana: GO:0090502 obsolete RNA phosphodiester bond hydrolysis, endonucleolytic (gain)
c-value
0.0
0.1
0.2
0.3
0.4
0.5
0.6
0.7
0.8
p-value
4e-07
6e-06
1e-04
2e-03
4e-02
Log-odds
0.5
1.0
1.5
2.0
2.5
3.0
3.5
4.0
Cellular_organisms - ps1
Archaea1 - ps2
Archaea2 - ps3
Archaea3 - ps4
Asgard/Eukaryota - ps5
Eukaryota - ps6
Excavata/Diaphoretickes - ps7
Diaphoretickes - ps8
Archaeplastida - ps9
Viridiplantae - ps10
Streptophyta - ps11
Charophyceae/Embriophyta - ps12
Embriophyta - ps13
Tracheophyta - ps14
Spermatophyta - ps15
Angiosperms - ps16
Mesangiospermae - ps17
Magnoliids/eudicots - ps18
Eudicots - ps19
Core_eudicots - ps20
Superrosids - ps21
Rosids - ps22
Eurosids - ps23
Malvids - ps24
Sap./Malv./Brassicales - ps25
Malvales/Brassicales - ps26
Brassicales - ps27
Cleomaceae/Brassicaceae - ps28
Brassicaceae - ps29
Camelineae - ps30
Arabidopsis - ps31
Arabidopsis_thaliana - ps32
Phylostratum

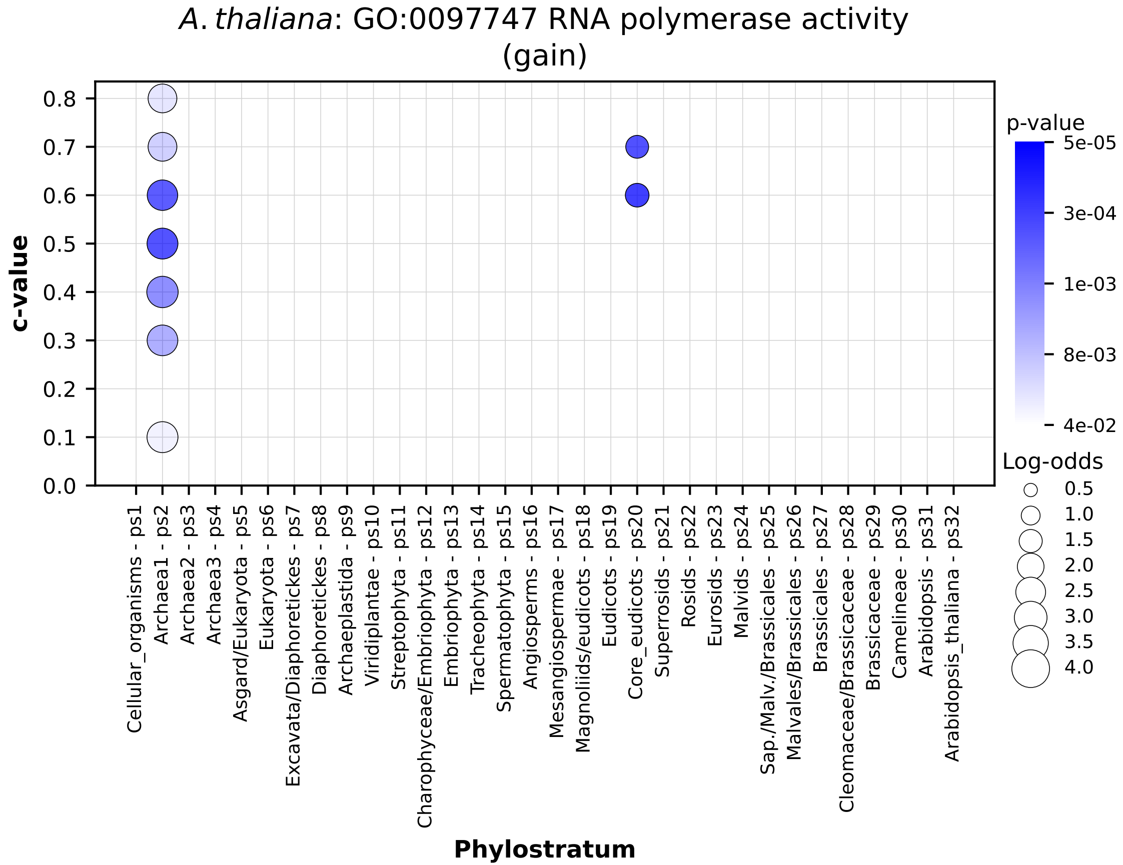

$A. thaliana$: GO:0097747 RNA polymerase activity (gain)
c-value
0.0
0.1
0.2
0.3
0.4
0.5
0.6
0.7
0.8
Phylostratum
Cellular_organisms - ps1
Archaea1 - ps2
Archaea2 - ps3
Archaea3 - ps4
Asgard/Eukaryota - ps5
Eukaryota - ps6
Excavata/Diaphoretickes - ps7
Diaphoretickes - ps8
Archaeplastida - ps9
Viridiplantae - ps10
Streptophyta - ps11
Charophyceae/Embriophyta - ps12
Embriophyta - ps13
Tracheophyta - ps14
Spermatophyta - ps15
Angiosperms - ps16
Mesangiospermae - ps17
Magnoliids/eudicots - ps18
Eudicots - ps19
Core_eudicots - ps20
Superrosids - ps21
Rosids - ps22
Eurosids - ps23
Malvids - ps24
Sap./Malv./Brassicales - ps25
Malvales/Brassicales - ps26
Brassicales - ps27
Cleomaceae/Brassicaceae - ps28
Brassicaceae - ps29
Camelineae - ps30
Arabidopsis - ps31
Arabidopsis_thaliana - ps32
p-value
5e-05
3e-04
1e-03
8e-03
4e-02
Log-odds
0.5
1.0
1.5
2.0
2.5
3.0
3.5
4.0

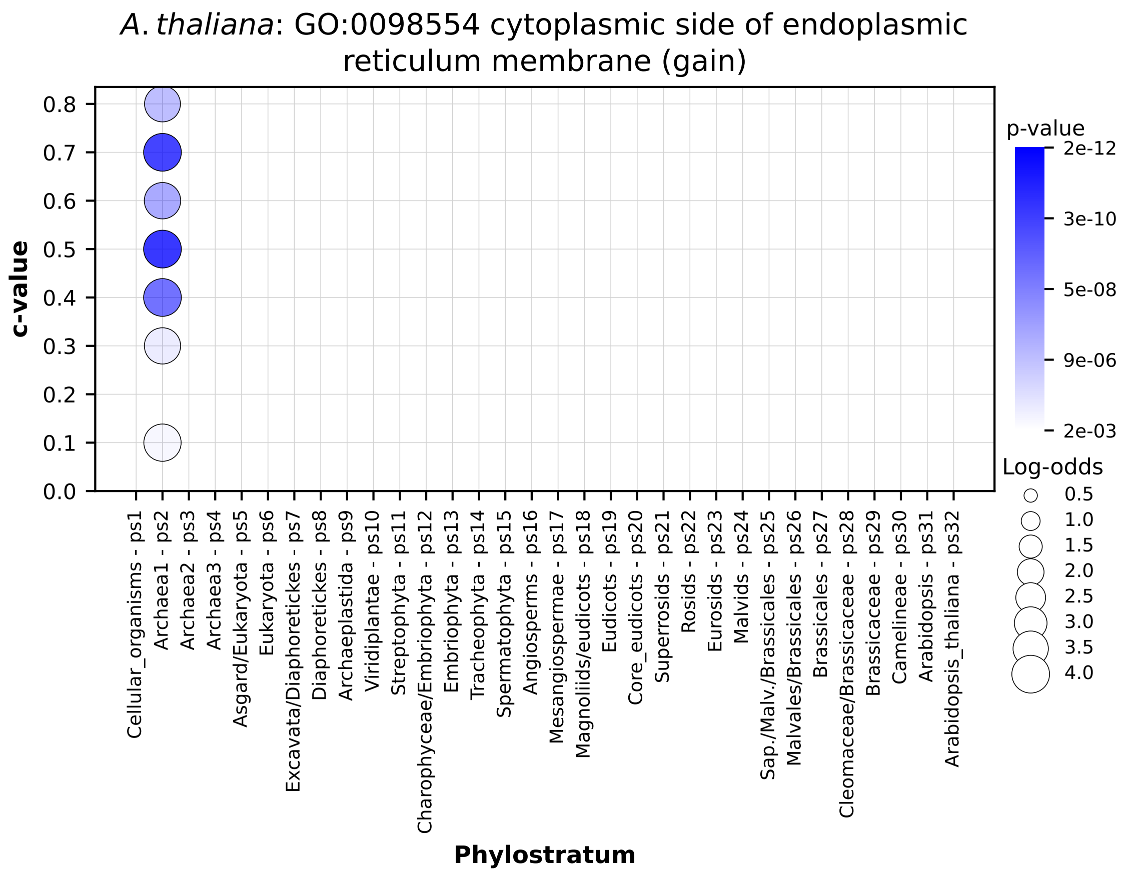
A. thaliana: GO:0098554 cytoplasmic side of endoplasmic reticulum membrane (gain)
c-value
0.0
0.1
0.2
0.3
0.4
0.5
0.6
0.7
0.8
Phylostratum
Cellular_organisms - ps1
Archaea1 - ps2
Archaea2 - ps3
Archaea3 - ps4
Asgard/Eukaryota - ps5
Eukaryota - ps6
Excavata/Diaphoretickes - ps7
Diaphoretickes - ps8
Archaeplastida - ps9
Viridiplantae - ps10
Streptophyta - ps11
Charophyceae/Embriophyta - ps12
Embriophyta - ps13
Tracheophyta - ps14
Spermatophyta - ps15
Angiosperms - ps16
Mesangiospermae - ps17
Magnoliids/eudicots - ps18
Eudicots - ps19
Core_eudicots - ps20
Superrosids - ps21
Rosids - ps22
Eurosids - ps23
Malvids - ps24
Sap./Malv./Brassicales - ps25
Malvales/Brassicales - ps26
Brassicales - ps27
Cleomaceae/Brassicaceae - ps28
Brassicaceae - ps29
Camelineae - ps30
Arabidopsis - ps31
Arabidopsis_thaliana - ps32
p-value
2e-12
3e-10
5e-08
9e-06
2e-03
Log-odds
0.5
1.0
1.5
2.0
2.5
3.0
3.5
4.0

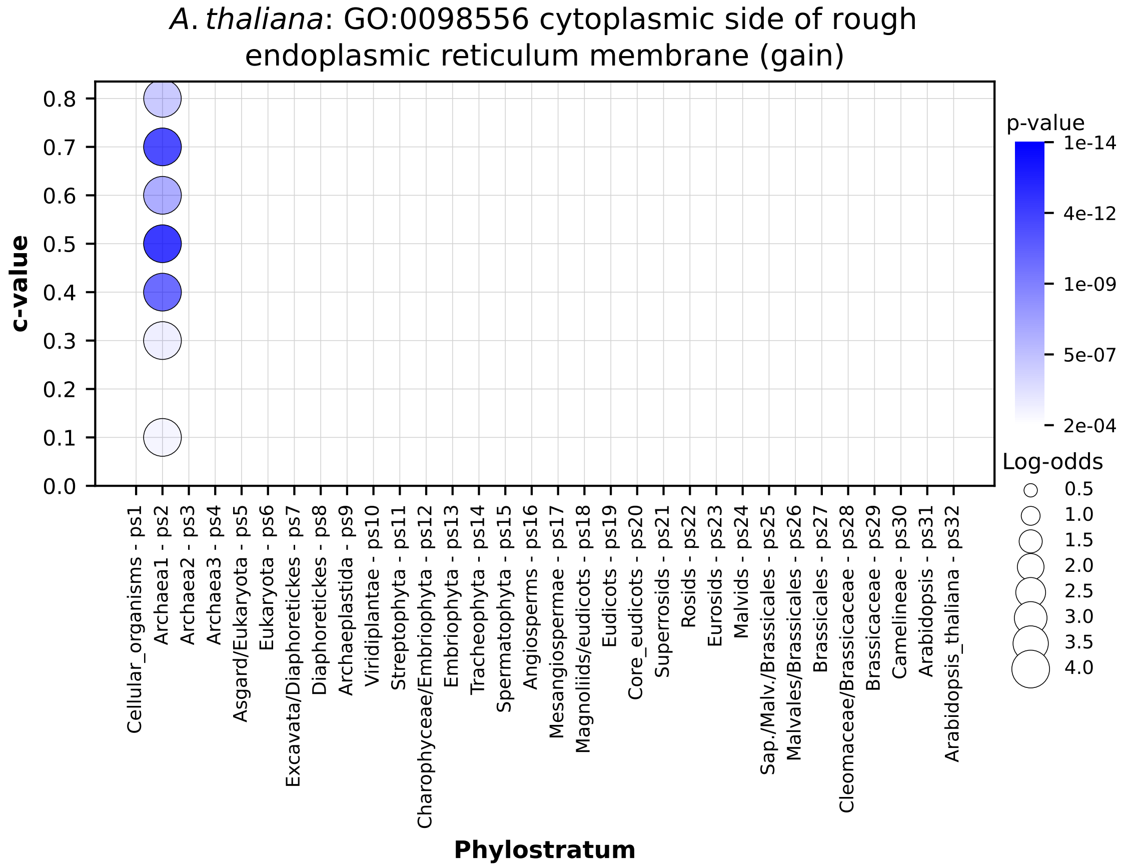

A. thaliana: GO:0098556 cytoplasmic side of rough endoplasmic reticulum membrane (gain)
c-value
0.0
0.1
0.2
0.3
0.4
0.5
0.6
0.7
0.8
p-value
1e-14
4e-12
1e-09
5e-07
2e-04
Log-odds
0.5
1.0
1.5
2.0
2.5
3.0
3.5
4.0
Cellular_organisms - ps1
Archaea1 - ps2
Archaea2 - ps3
Archaea3 - ps4
Asgard/Eukaryota - ps5
Eukaryota - ps6
Excavata/Diaphoretickes - ps7
Diaphoretickes - ps8
Archaeplastida - ps9
Viridiplantae - ps10
Streptophyta - ps11
Charophyceae/Embriophyta - ps12
Embriophyta - ps13
Tracheophyta - ps14
Spermatophyta - ps15
Angiosperms - ps16
Mesangiospermae - ps17
Magnoliids/eudicots - ps18
Eudicots - ps19
Core_eudicots - ps20
Superrosids - ps21
Rosids - ps22
Eurosids - ps23
Malvids - ps24
Sap./Malv./Brassicales - ps25
Malvales/Brassicales - ps26
Brassicales - ps27
Cleomaceae/Brassicaceae - ps28
Brassicaceae - ps29
Camelineae - ps30
Arabidopsis - ps31
Arabidopsis_thaliana - ps32
Phylostratum

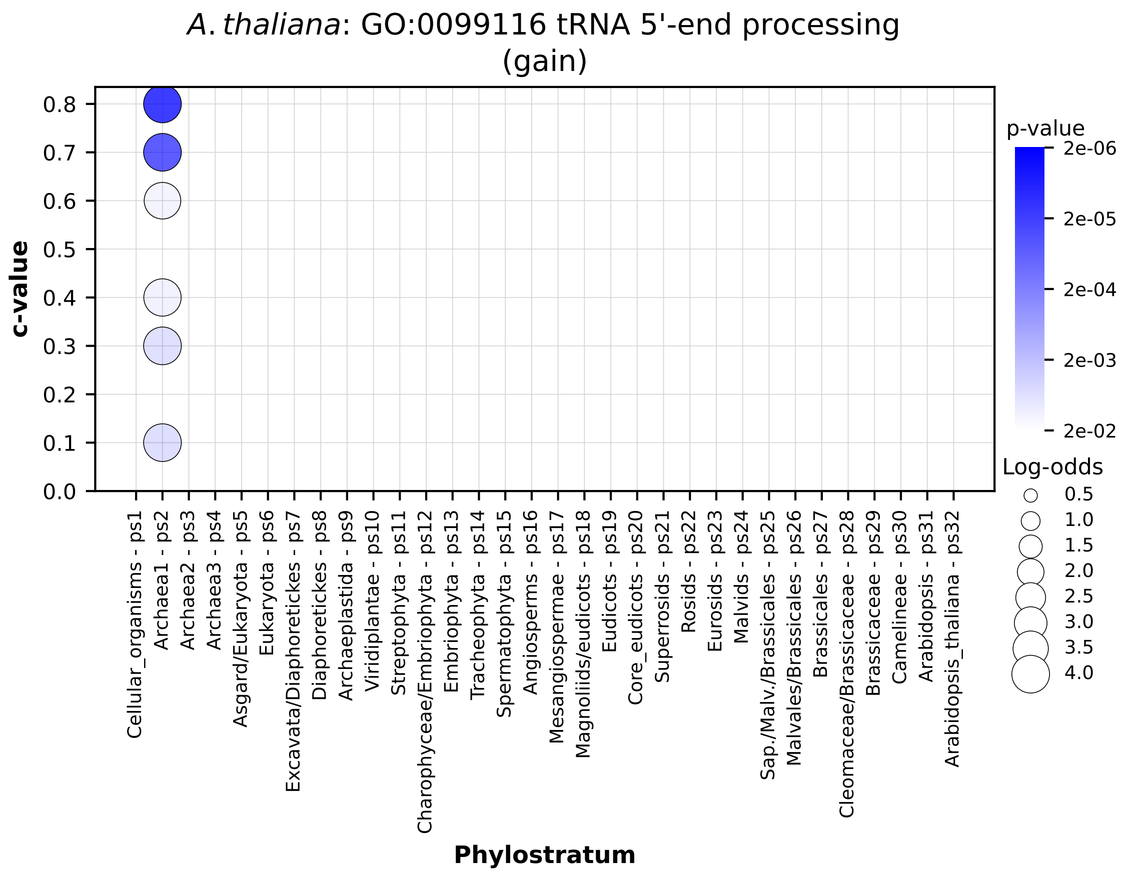

A. thaliana: GO:0099116 tRNA 5'-end processing
(gain)
c-value
0.0
0.1
0.2
0.3
0.4
0.5
0.6
0.7
0.8
Phylostratum
Cellular_organisms - ps1
Archaea1 - ps2
Archaea2 - ps3
Archaea3 - ps4
Asgard/Eukaryota - ps5
Eukaryota - ps6
Excavata/Diaphoretickes - ps7
Diaphoretickes - ps8
Archaeplastida - ps9
Viridiplantae - ps10
Streptophyta - ps11
Charophyceae/Embriophyta - ps12
Embriophyta - ps13
Tracheophyta - ps14
Spermatophyta - ps15
Angiosperms - ps16
Mesangiospermae - ps17
Magnoliids/eudicots - ps18
Eudicots - ps19
Core_eudicots - ps20
Superrosids - ps21
Rosids - ps22
Eurosids - ps23
Malvids - ps24
Sap./Malv./Brassicales - ps25
Malvales/Brassicales - ps26
Brassicales - ps27
Cleomaceae/Brassicaceae - ps28
Brassicaceae - ps29
Camelineae - ps30
Arabidopsis - ps31
Arabidopsis_thaliana - ps32
p-value
2e-06
2e-05
2e-04
2e-03
2e-02
Log-odds
0.5
1.0
1.5
2.0
2.5
3.0
3.5
4.0

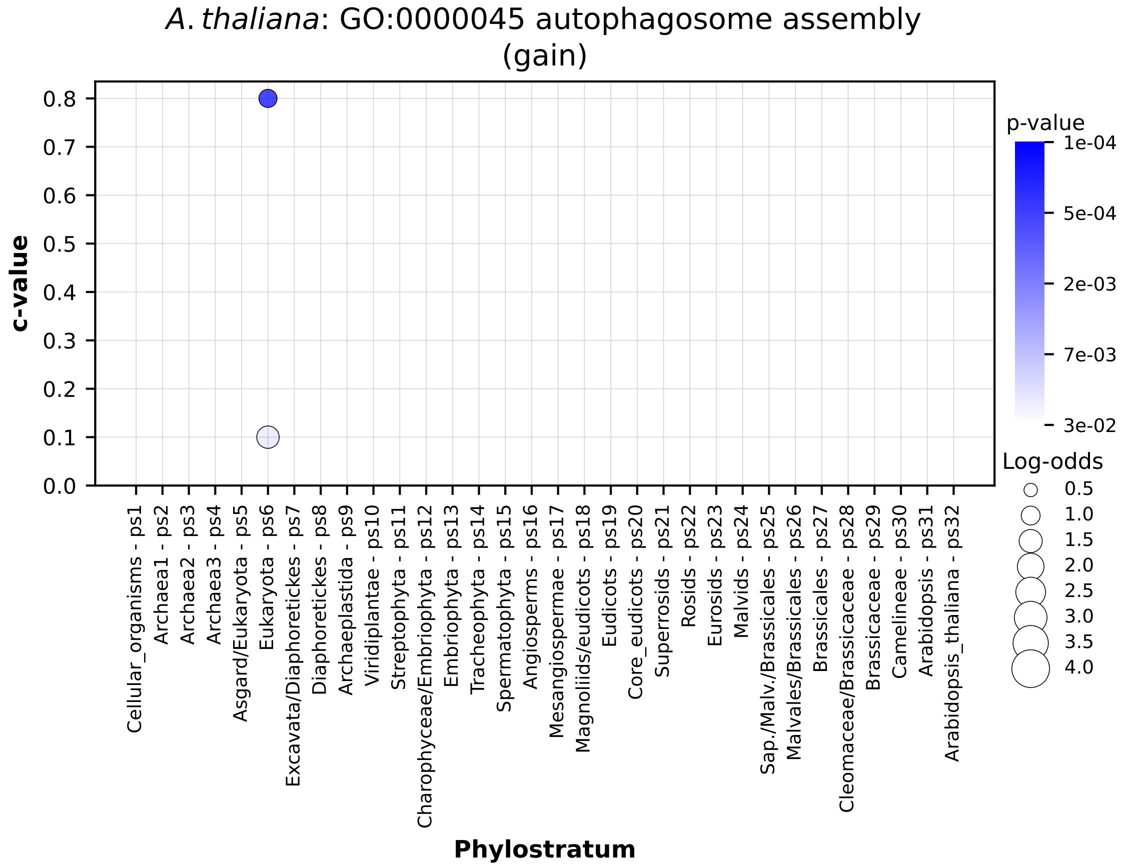

A. thaliana: GO:0000045 autophagosome assembly (gain)
c-value
0.0
0.1
0.2
0.3
0.4
0.5
0.6
0.7
0.8
Phylostratum
Cellular_organisms - ps1
Archaea1 - ps2
Archaea2 - ps3
Archaea3 - ps4
Asgard/Eukaryota - ps5
Eukaryota - ps6
Excavata/Diaphoretickes - ps7
Diaphoretickes - ps8
Archaeplastida - ps9
Viridiplantae - ps10
Streptophyta - ps11
Charophyceae/Embriophyta - ps12
Embriophyta - ps13
Tracheophyta - ps14
Spermatophyta - ps15
Angiosperms - ps16
Mesangiospermae - ps17
Magnoliids/eudicots - ps18
Eudicots - ps19
Core_eudicots - ps20
Superrosids - ps21
Rosids - ps22
Eurosids - ps23
Malvids - ps24
Sap./Malv./Brassicales - ps25
Malvales/Brassicales - ps26
Brassicales - ps27
Cleomaceae/Brassicaceae - ps28
Brassicaceae - ps29
Camelineae - ps30
Arabidopsis - ps31
Arabidopsis_thaliana - ps32
p-value
1e-04
5e-04
2e-03
7e-03
3e-02
Log-odds
0.5
1.0
1.5
2.0
2.5
3.0
3.5
4.0

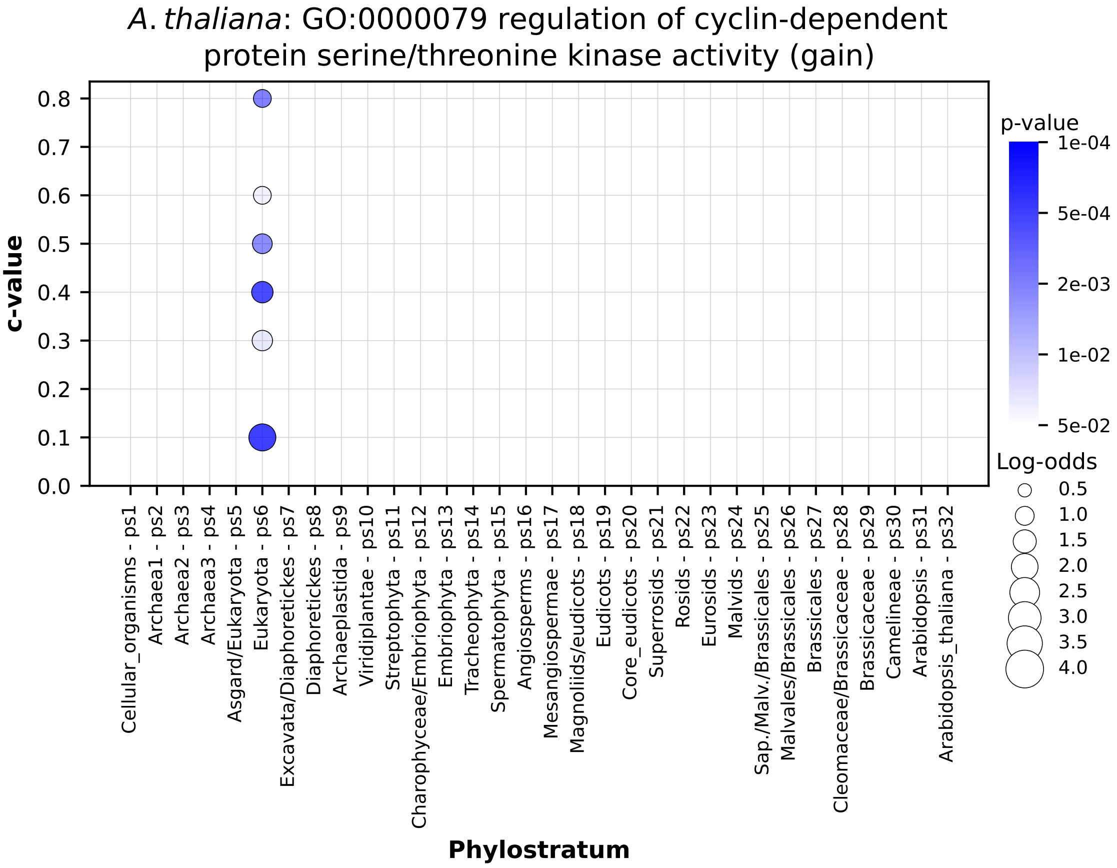

A. thaliana: GO:0000079 regulation of cyclin-dependent protein serine/threonine kinase activity (gain)
c-value
0.0
0.1
0.2
0.3
0.4
0.5
0.6
0.7
0.8
Phylostratum
Cellular_organisms - ps1
Archaea1 - ps2
Archaea2 - ps3
Archaea3 - ps4
Asgard/Eukaryota - ps5
Eukaryota - ps6
Excavata/Diaphoretickes - ps7
Diaphoretickes - ps8
Archaeplastida - ps9
Viridiplantae - ps10
Streptophyta - ps11
Charophyceae/Embriophyta - ps12
Embriophyta - ps13
Tracheophyta - ps14
Spermatophyta - ps15
Angiosperms - ps16
Mesangiospermae - ps17
Magnoliids/eudicots - ps18
Eudicots - ps19
Core_eudicots - ps20
Superrosids - ps21
Rosids - ps22
Eurosids - ps23
Malvids - ps24
Sap./Malv./Brassicales - ps25
Malvales/Brassicales - ps26
Brassicales - ps27
Cleomaceae/Brassicaceae - ps28
Brassicaceae - ps29
Camelineae - ps30
Arabidopsis - ps31
Arabidopsis_thaliana - ps32
p-value
1e-04
5e-04
2e-03
1e-02
5e-02
Log-odds
0.5
1.0
1.5
2.0
2.5
3.0
3.5
4.0

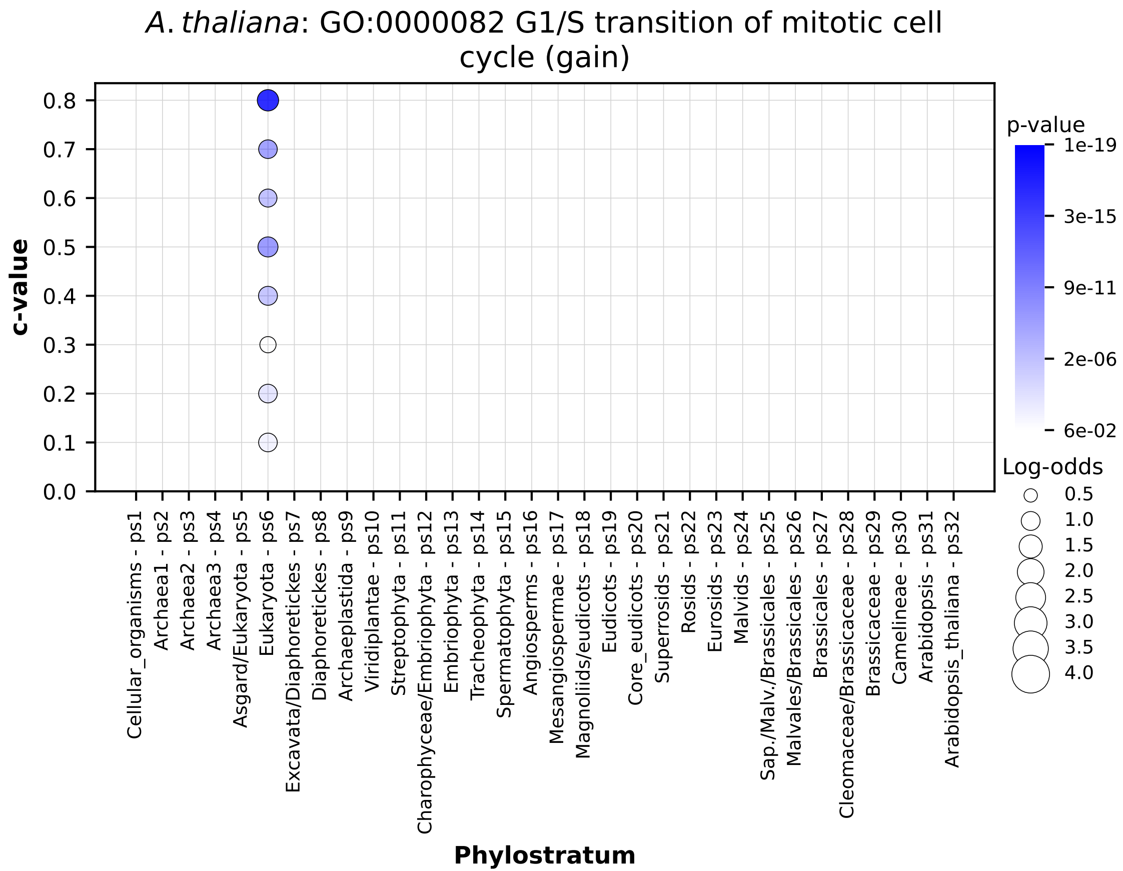

A. thaliana: GO:0000082 G1/S transition of mitotic cell cycle (gain)
c-value
0.0
0.1
0.2
0.3
0.4
0.5
0.6
0.7
0.8
p-value
1e-19
3e-15
9e-11
2e-06
6e-02
Log-odds
0.5
1.0
1.5
2.0
2.5
3.0
3.5
4.0
Cellular_organisms - ps1
Archaea1 - ps2
Archaea2 - ps3
Archaea3 - ps4
Asgard/Eukaryota - ps5
Eukaryota - ps6
Excavata/Diaphoretickes - ps7
Diaphoretickes - ps8
Archaeplastida - ps9
Viridiplantae - ps10
Streptophyta - ps11
Charophyceae/Embriophyta - ps12
Embriophyta - ps13
Tracheophyta - ps14
Spermatophyta - ps15
Angiosperms - ps16
Mesangiospermae - ps17
Magnoliids/eudicots - ps18
Eudicots - ps19
Core_eudicots - ps20
Superrosids - ps21
Rosids - ps22
Eurosids - ps23
Malvids - ps24
Sap./Malv./Brassicales - ps25
Malvales/Brassicales - ps26
Brassicales - ps27
Cleomaceae/Brassicaceae - ps28
Brassicaceae - ps29
Camelineae - ps30
Arabidopsis - ps31
Arabidopsis_thaliana - ps32
Phylostratum

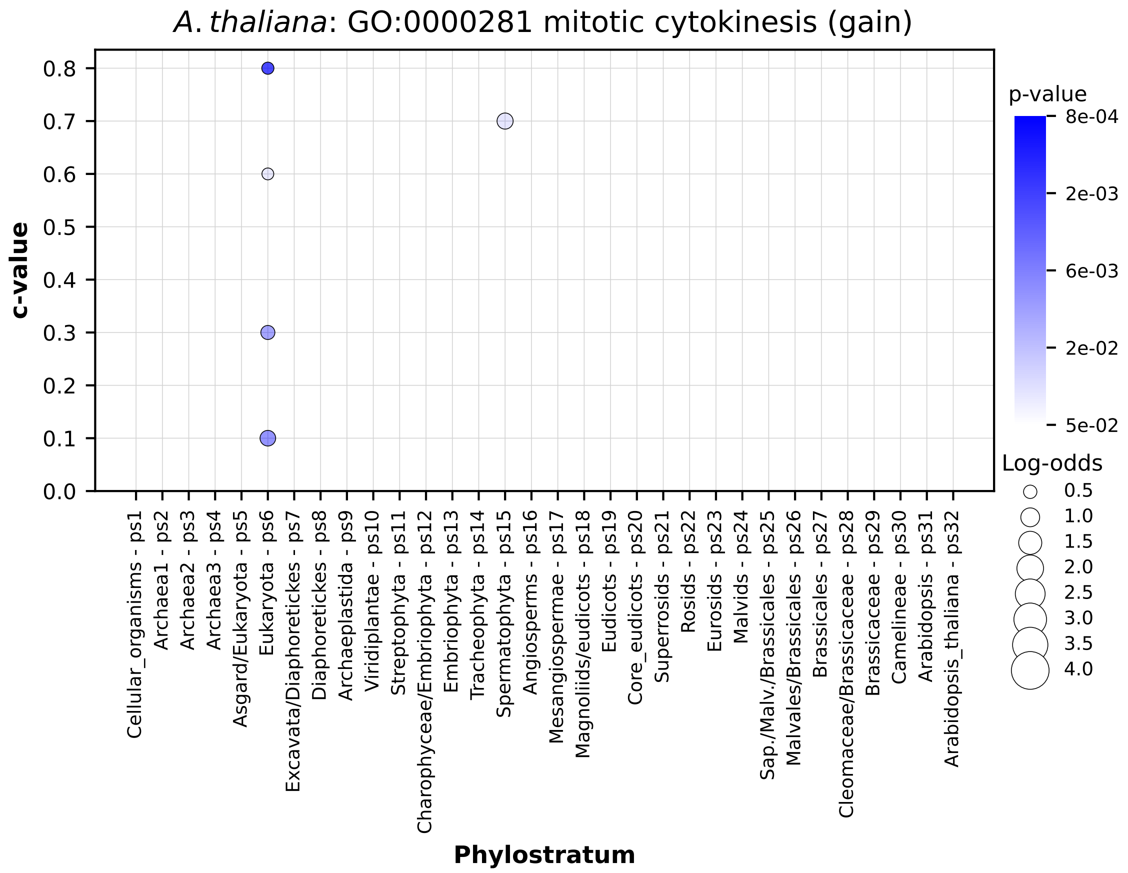
A. thaliana: GO:0000281 mitotic cytokinesis (gain)
c-value
0.0
0.1
0.2
0.3
0.4
0.5
0.6
0.7
0.8
Phylostratum
Cellular_organisms - ps1
Archaea1 - ps2
Archaea2 - ps3
Archaea3 - ps4
Asgard/Eukaryota - ps5
Eukaryota - ps6
Excavata/Diaphoretickes - ps7
Diaphoretickes - ps8
Archaeplastida - ps9
Viridiplantae - ps10
Streptophyta - ps11
Charophyceae/Embriophyta - ps12
Embriophyta - ps13
Tracheophyta - ps14
Spermatophyta - ps15
Angiosperms - ps16
Mesangiospermae - ps17
Magnoliids/eudicots - ps18
Eudicots - ps19
Core_eudicots - ps20
Superrosids - ps21
Rosids - ps22
Eurosids - ps23
Malvids - ps24
Sap./Malv./Brassicales - ps25
Malvales/Brassicales - ps26
Brassicales - ps27
Cleomaceae/Brassicaceae - ps28
Brassicaceae - ps29
Camelineae - ps30
Arabidopsis - ps31
Arabidopsis_thaliana - ps32
p-value
8e-04
2e-03
6e-03
2e-02
5e-02
Log-odds
0.5
1.0
1.5
2.0
2.5
3.0
3.5
4.0

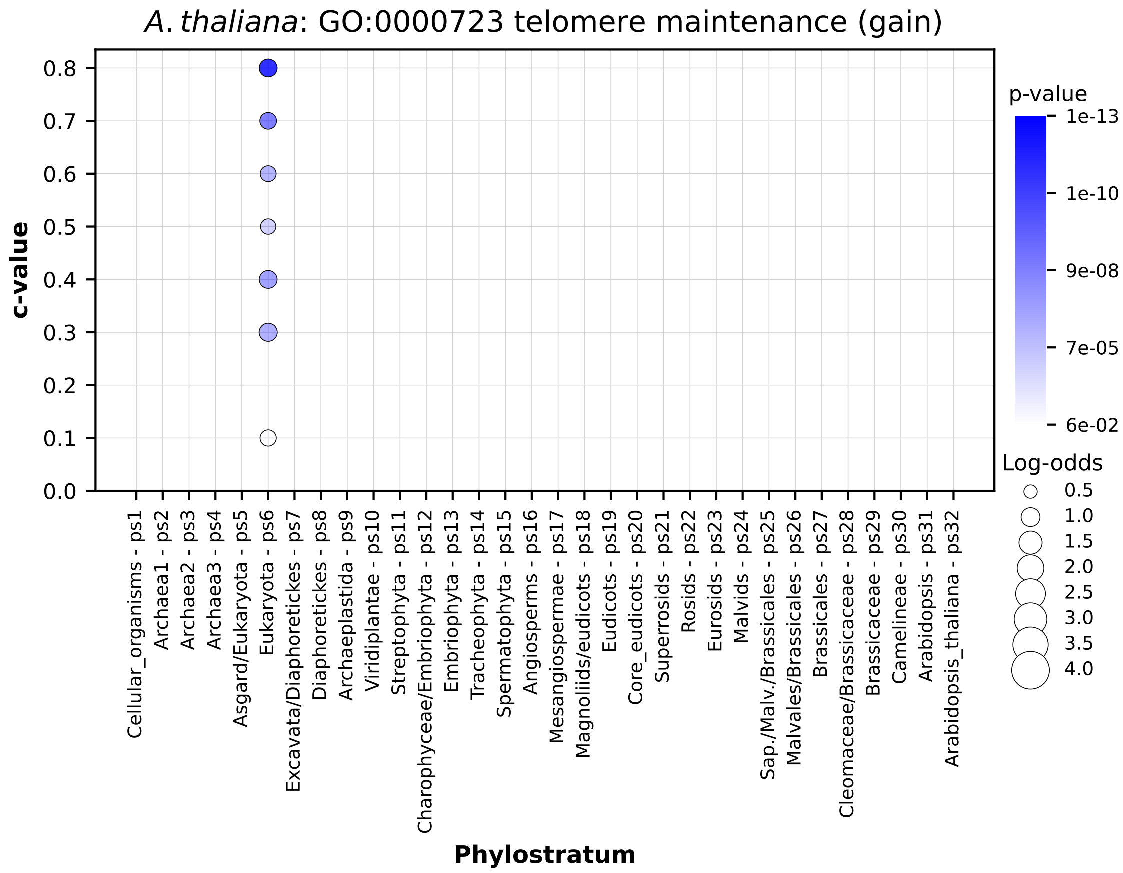
A. thaliana: GO:0000723 telomere maintenance (gain)
c-value
0.0
0.1
0.2
0.3
0.4
0.5
0.6
0.7
0.8
Phylostratum
Cellular_organisms - ps1
Archaea1 - ps2
Archaea2 - ps3
Archaea3 - ps4
Asgard/Eukaryota - ps5
Eukaryota - ps6
Excavata/Diaphoretickes - ps7
Diaphoretickes - ps8
Archaeplastida - ps9
Viridiplantae - ps10
Streptophyta - ps11
Charophyceae/Embriophyta - ps12
Embriophyta - ps13
Tracheophyta - ps14
Spermatophyta - ps15
Angiosperms - ps16
Mesangiospermae - ps17
Magnoliids/eudicots - ps18
Eudicots - ps19
Core_eudicots - ps20
Superrosids - ps21
Rosids - ps22
Eurosids - ps23
Malvids - ps24
Sap./Malv./Brassicales - ps25
Malvales/Brassicales - ps26
Brassicales - ps27
Cleomaceae/Brassicaceae - ps28
Brassicaceae - ps29
Camelineae - ps30
Arabidopsis - ps31
Arabidopsis_thaliana - ps32
p-value
1e-13
1e-10
9e-08
7e-05
6e-02
Log-odds
0.5
1.0
1.5
2.0
2.5
3.0
3.5
4.0

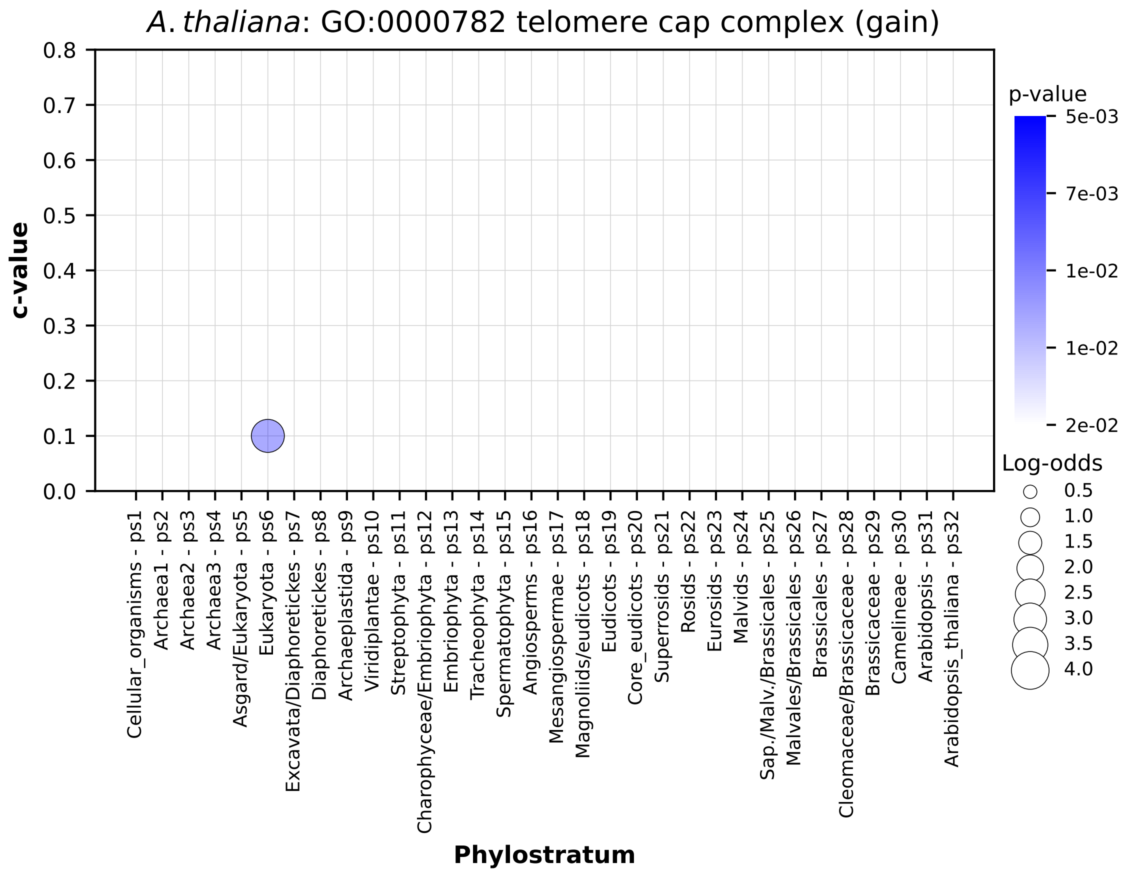
A. thaliana: GO:0000782 telomere cap complex (gain)
c-value
0.8
0.7
0.6
0.5
0.4
0.3
0.2
0.1
0.0
Phylostratum
Cellular_organisms - ps1
Archaea1 - ps2
Archaea2 - ps3
Archaea3 - ps4
Asgard/Eukaryota - ps5
Eukaryota - ps6
Excavata/Diaphoretickes - ps7
Diaphoretickes - ps8
Archaeplastida - ps9
Viridiplantae - ps10
Streptophyta - ps11
Charophyceae/Embriophyta - ps12
Embriophyta - ps13
Tracheophyta - ps14
Spermatophyta - ps15
Angiosperms - ps16
Mesangiospermae - ps17
Magnoliids/eudicots - ps18
Eudicots - ps19
Core_eudicots - ps20
Superrosids - ps21
Rosids - ps22
Eurosids - ps23
Malvids - ps24
Sap./Malv./Brassicales - ps25
Malvales/Brassicales - ps26
Brassicales - ps27
Cleomaceae/Brassicaceae - ps28
Brassicaceae - ps29
Camelineae - ps30
Arabidopsis - ps31
Arabidopsis_thaliana - ps32
p-value
5e-03
7e-03
1e-02
1e-02
2e-02
Log-odds
0.5
1.0
1.5
2.0
2.5
3.0
3.5
4.0

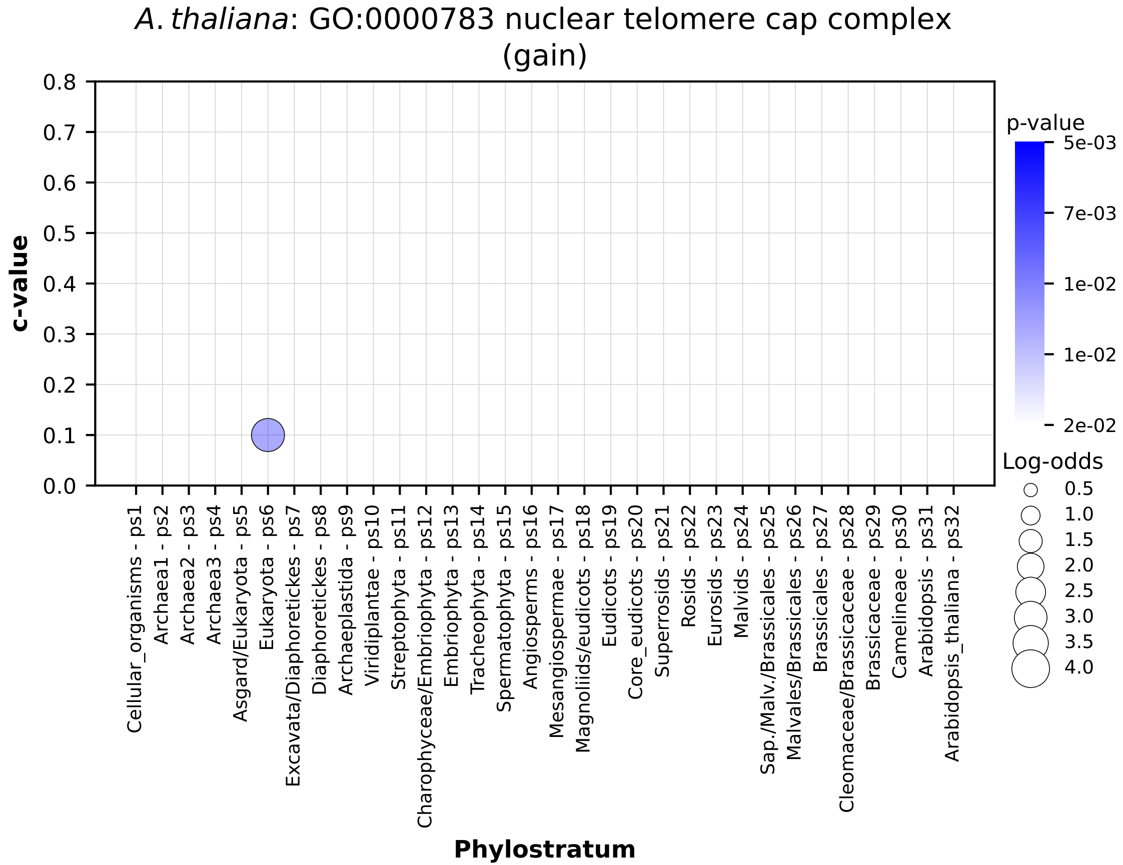

A. thaliana: GO:0000783 nuclear telomere cap complex
(gain)
c-value
0.0
0.1
0.2
0.3
0.4
0.5
0.6
0.7
0.8
Phylostratum
Cellular_organisms - ps1
Archaea1 - ps2
Archaea2 - ps3
Archaea3 - ps4
Asgard/Eukaryota - ps5
Eukaryota - ps6
Excavata/Diaphoretickes - ps7
Diaphoretickes - ps8
Archaeplastida - ps9
Viridiplantae - ps10
Streptophyta - ps11
Charophyceae/Embriophyta - ps12
Embriophyta - ps13
Tracheophyta - ps14
Spermatophyta - ps15
Angiosperms - ps16
Mesangiospermae - ps17
Magnoliids/eudicots - ps18
Eudicots - ps19
Core_eudicots - ps20
Superrosids - ps21
Rosids - ps22
Eurosids - ps23
Malvids - ps24
Sap./Malv./Brassicales - ps25
Malvales/Brassicales - ps26
Brassicales - ps27
Cleomaceae/Brassicaceae - ps28
Brassicaceae - ps29
Camelineae - ps30
Arabidopsis - ps31
Arabidopsis_thaliana - ps32
p-value
5e-03
7e-03
1e-02
1e-02
2e-02
Log-odds
0.5
1.0
1.5
2.0
2.5
3.0
3.5
4.0

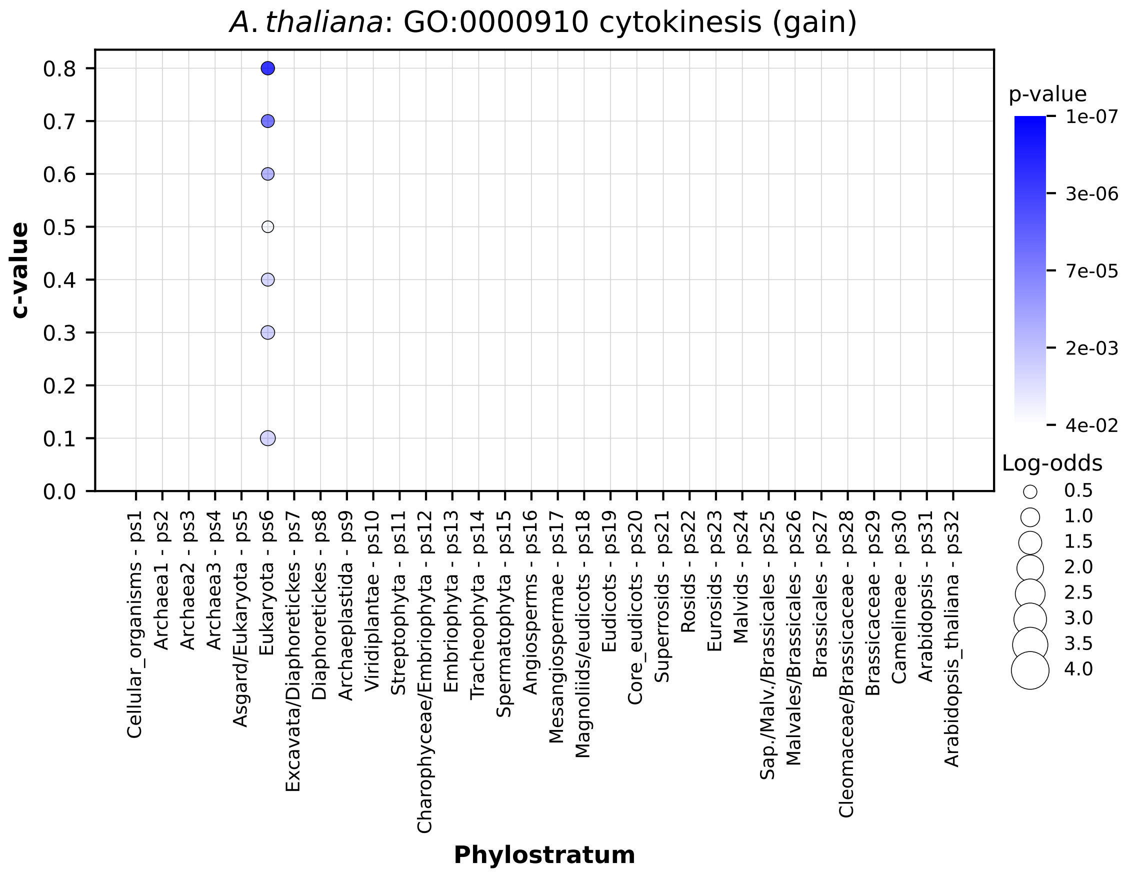

A. thaliana: GO:0000910 cytokinesis (gain)
c-value
0.0
0.1
0.2
0.3
0.4
0.5
0.6
0.7
0.8
Phylostratum
Cellular_organisms - ps1
Archaea1 - ps2
Archaea2 - ps3
Archaea3 - ps4
Asgard/Eukaryota - ps5
Eukaryota - ps6
Excavata/Diaphoretickes - ps7
Diaphoretickes - ps8
Archaeplastida - ps9
Viridiplantae - ps10
Streptophyta - ps11
Charophyceae/Embriophyta - ps12
Embriophyta - ps13
Tracheophyta - ps14
Spermatophyta - ps15
Angiosperms - ps16
Mesangiospermae - ps17
Magnoliids/eudicots - ps18
Eudicots - ps19
Core_eudicots - ps20
Superrosids - ps21
Rosids - ps22
Eurosids - ps23
Malvids - ps24
Sap./Malv./Brassicales - ps25
Malvales/Brassicales - ps26
Brassicales - ps27
Cleomaceae/Brassicaceae - ps28
Brassicaceae - ps29
Camelineae - ps30
Arabidopsis - ps31
Arabidopsis_thaliana - ps32
p-value
1e-07
3e-06
7e-05
2e-03
4e-02
Log-odds
0.5
1.0
1.5
2.0
2.5
3.0
3.5
4.0

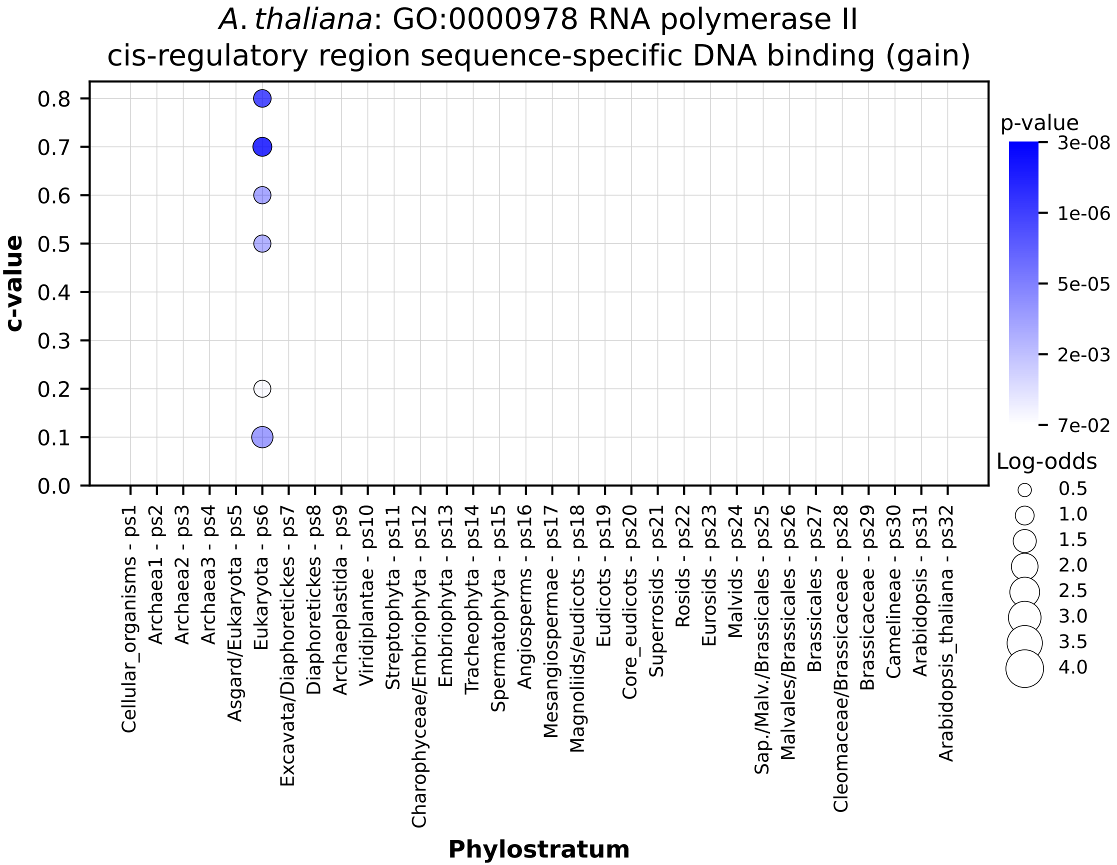

A. thaliana: GO:0000978 RNA polymerase II
cis-regulatory region sequence-specific DNA binding (gain)
c-value
0.8
0.7
0.6
0.5
0.4
0.3
0.2
0.1
0.0
p-value
3e-08
1e-06
5e-05
2e-03
7e-02
Log-odds
0.5
1.0
1.5
2.0
2.5
3.0
3.5
4.0
Cellular_organisms - ps1
Archaea1 - ps2
Archaea2 - ps3
Archaea3 - ps4
Asgard/Eukaryota - ps5
Eukaryota - ps6
Excavata/Diaphoretickes - ps7
Diaphoretickes - ps8
Archaeplastida - ps9
Viridiplantae - ps10
Streptophyta - ps11
Charophyceae/Embriophyta - ps12
Embriophyta - ps13
Tracheophyta - ps14
Spermatophyta - ps15
Angiosperms - ps16
Mesangiospermae - ps17
Magnoliids/eudicots - ps18
Eudicots - ps19
Core_eudicots - ps20
Superrosids - ps21
Rosids - ps22
Eurosids - ps23
Malvids - ps24
Sap./Malv./Brassicales - ps25
Malvales/Brassicales - ps26
Brassicales - ps27
Cleomaceae/Brassicaceae - ps28
Brassicaceae - ps29
Camelineae - ps30
Arabidopsis - ps31
Arabidopsis_thaliana - ps32
Phylostratum

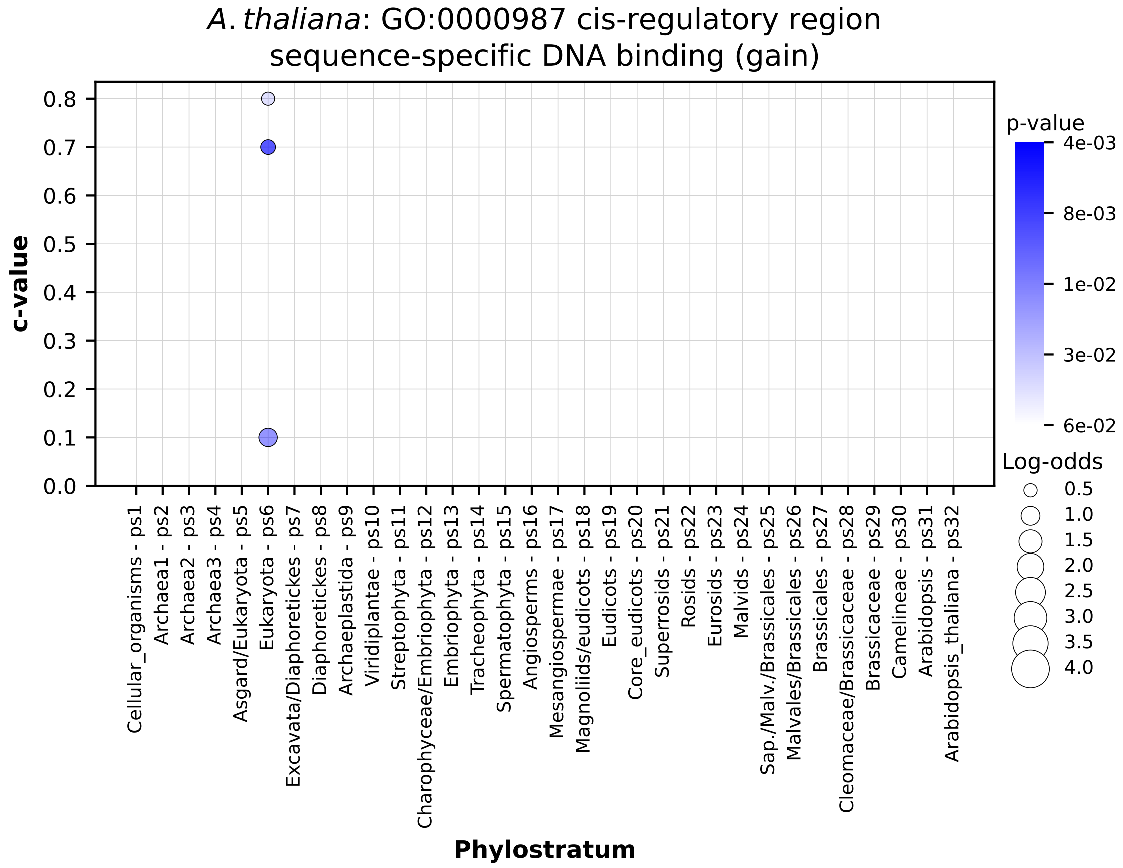

A. thaliana: GO:0000987 cis-regulatory region sequence-specific DNA binding (gain)
c-value
0.0
0.1
0.2
0.3
0.4
0.5
0.6
0.7
0.8
p-value
4e-03
8e-03
1e-02
3e-02
6e-02
Log-odds
0.5
1.0
1.5
2.0
2.5
3.0
3.5
4.0
Cellular_organisms - ps1
Archaea1 - ps2
Archaea2 - ps3
Archaea3 - ps4
Asgard/Eukaryota - ps5
Eukaryota - ps6
Excavata/Diaphoretickes - ps7
Diaphoretickes - ps8
Archaeplastida - ps9
Viridiplantae - ps10
Streptophyta - ps11
Charophyceae/Embriophyta - ps12
Embriophyta - ps13
Tracheophyta - ps14
Spermatophyta - ps15
Angiosperms - ps16
Mesangiospermae - ps17
Magnoliids/eudicots - ps18
Eudicots - ps19
Core_eudicots - ps20
Superrosids - ps21
Rosids - ps22
Eurosids - ps23
Malvids - ps24
Sap./Malv./Brassicales - ps25
Malvales/Brassicales - ps26
Brassicales - ps27
Cleomaceae/Brassicaceae - ps28
Brassicaceae - ps29
Camelineae - ps30
Arabidopsis - ps31
Arabidopsis_thaliana - ps32
Phylostratum

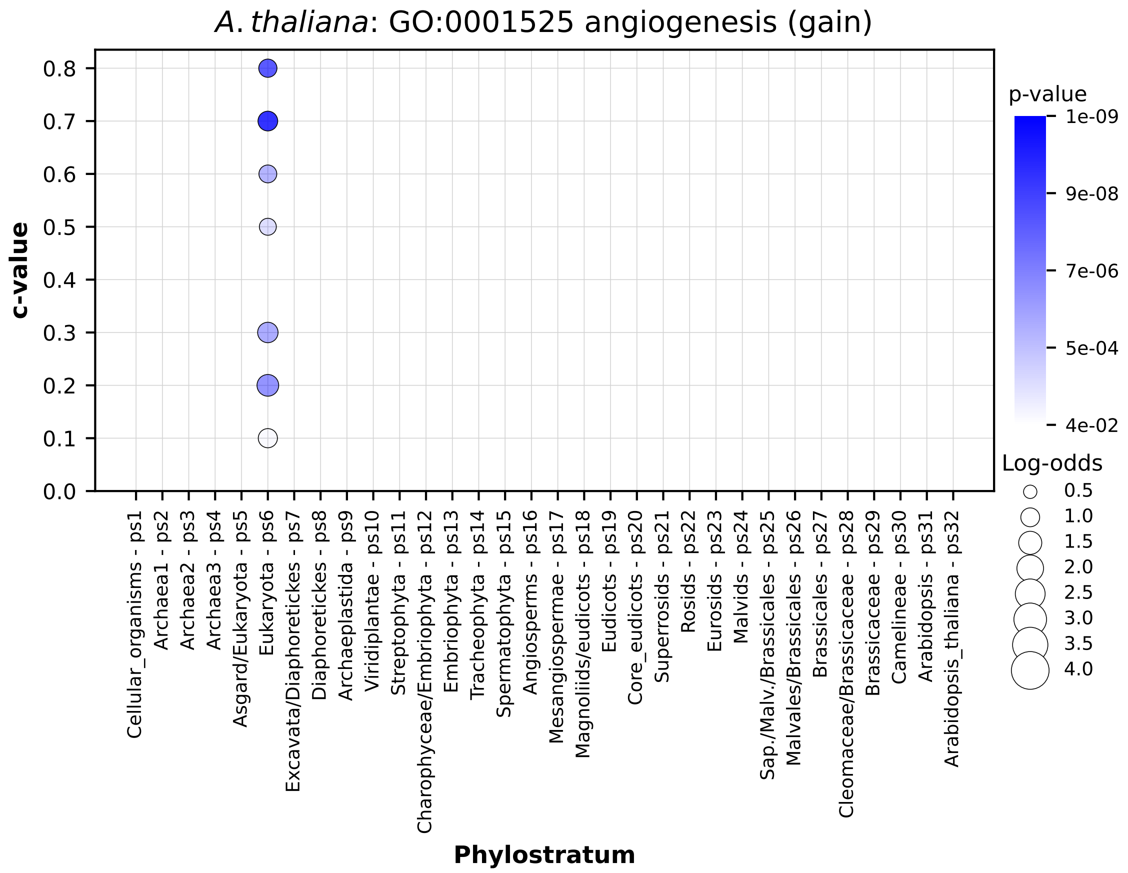

A. thaliana: GO:0001525 angiogenesis (gain)
c-value
0.0
0.1
0.2
0.3
0.4
0.5
0.6
0.7
0.8
p-value
1e-09
9e-08
7e-06
5e-04
4e-02
Log-odds
0.5
1.0
1.5
2.0
2.5
3.0
3.5
4.0
Cellular_organisms - ps1
Archaea1 - ps2
Archaea2 - ps3
Archaea3 - ps4
Asgard/Eukaryota - ps5
Eukaryota - ps6
Excavata/Diaphoretickes - ps7
Diaphoretickes - ps8
Archaeplastida - ps9
Viridiplantae - ps10
Streptophyta - ps11
Charophyceae/Embriophyta - ps12
Embriophyta - ps13
Tracheophyta - ps14
Spermatophyta - ps15
Angiosperms - ps16
Mesangiospermae - ps17
Magnoliids/eudicots - ps18
Eudicots - ps19
Core_eudicots - ps20
Superrosids - ps21
Rosids - ps22
Eurosids - ps23
Malvids - ps24
Sap./Malv./Brassicales - ps25
Malvales/Brassicales - ps26
Brassicales - ps27
Cleomaceae/Brassicaceae - ps28
Brassicaceae - ps29
Camelineae - ps30
Arabidopsis - ps31
Arabidopsis_thaliana - ps32
Phylostratum

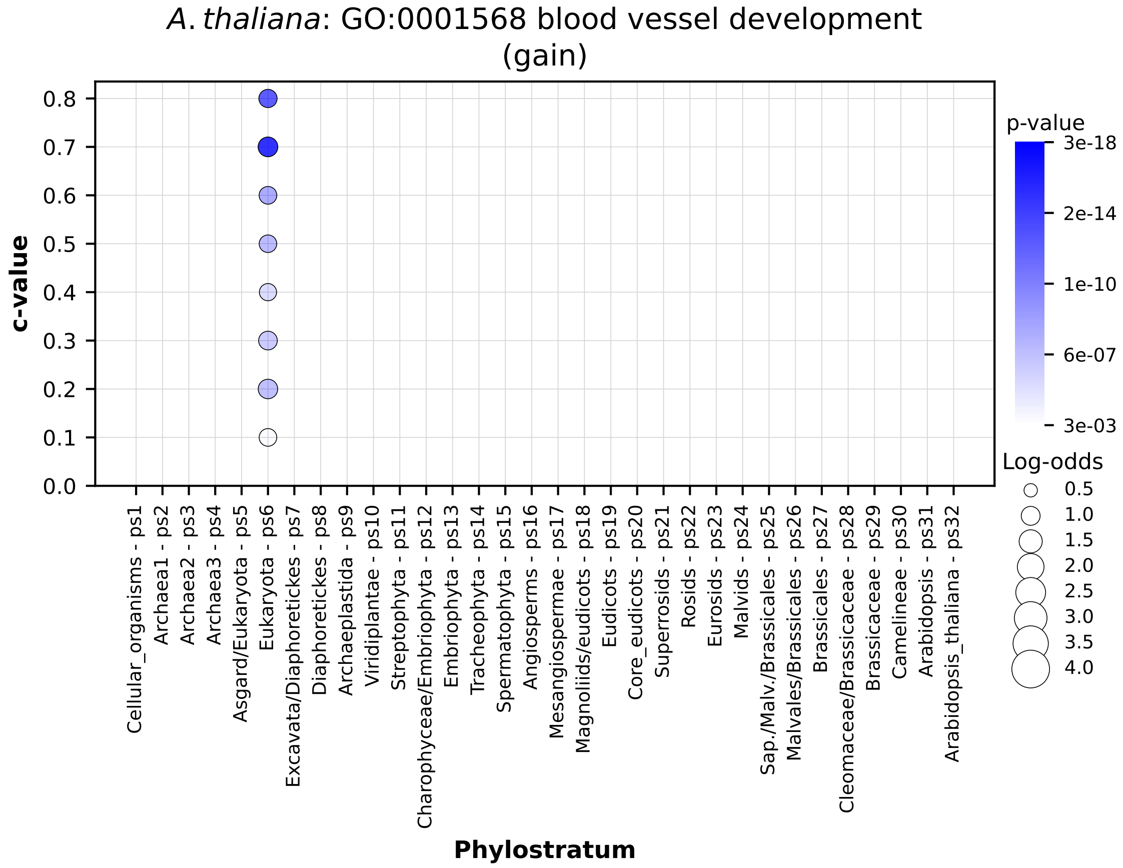

A. thaliana: GO:0001568 blood vessel development
(gain)
c-value
0.0
0.1
0.2
0.3
0.4
0.5
0.6
0.7
0.8
p-value
3e-18
2e-14
1e-10
6e-07
3e-03
Log-odds
0.5
1.0
1.5
2.0
2.5
3.0
3.5
4.0
Cellular_organisms - ps1
Archaea1 - ps2
Archaea2 - ps3
Archaea3 - ps4
Asgard/Eukaryota - ps5
Eukaryota - ps6
Excavata/Diaphoretickes - ps7
Diaphoretickes - ps8
Archaeplastida - ps9
Viridiplantae - ps10
Streptophyta - ps11
Charophyceae/Embriophyta - ps12
Embriophyta - ps13
Tracheophyta - ps14
Spermatophyta - ps15
Angiosperms - ps16
Mesangiospermae - ps17
Magnoliids/eudicots - ps18
Eudicots - ps19
Core_eudicots - ps20
Superrosids - ps21
Rosids - ps22
Eurosids - ps23
Malvids - ps24
Sap./Malv./Brassicales - ps25
Malvales/Brassicales - ps26
Brassicales - ps27
Cleomaceae/Brassicaceae - ps28
Brassicaceae - ps29
Camelineae - ps30
Arabidopsis - ps31
Arabidopsis_thaliana - ps32
Phylostratum

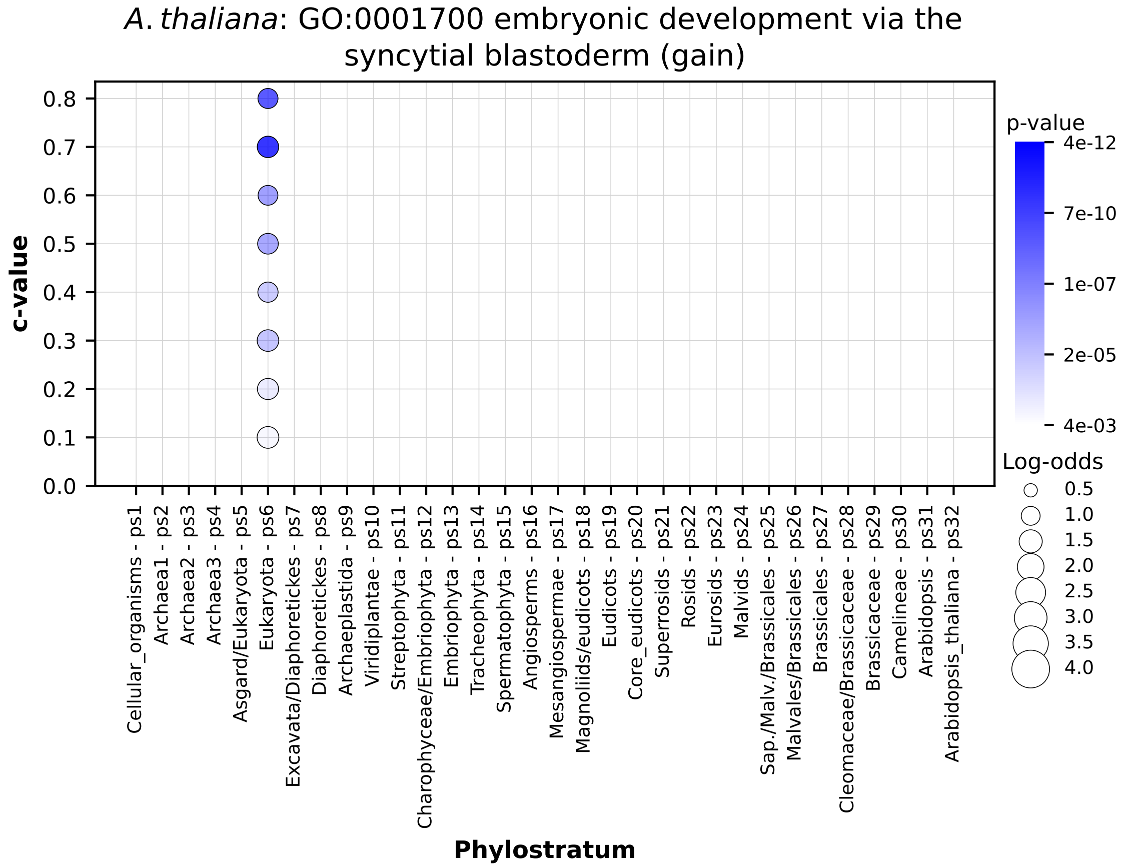

A. thaliana: GO:0001700 embryonic development via the syncytial blastoderm (gain)
c-value
0.0
0.1
0.2
0.3
0.4
0.5
0.6
0.7
0.8
Phylostratum
Cellular_organisms - ps1
Archaea1 - ps2
Archaea2 - ps3
Archaea3 - ps4
Asgard/Eukaryota - ps5
Eukaryota - ps6
Excavata/Diaphoretickes - ps7
Diaphoretickes - ps8
Archaeplastida - ps9
Viridiplantae - ps10
Streptophyta - ps11
Charophyceae/Embriophyta - ps12
Embriophyta - ps13
Tracheophyta - ps14
Spermatophyta - ps15
Angiosperms - ps16
Mesangiospermae - ps17
Magnoliids/eudicots - ps18
Eudicots - ps19
Core_eudicots - ps20
Superrosids - ps21
Rosids - ps22
Eurosids - ps23
Malvids - ps24
Sap./Malv./Brassicales - ps25
Malvales/Brassicales - ps26
Brassicales - ps27
Cleomaceae/Brassicaceae - ps28
Brassicaceae - ps29
Camelineae - ps30
Arabidopsis - ps31
Arabidopsis_thaliana - ps32
p-value
4e-12
7e-10
1e-07
2e-05
4e-03
Log-odds
0.5
1.0
1.5
2.0
2.5
3.0
3.5
4.0

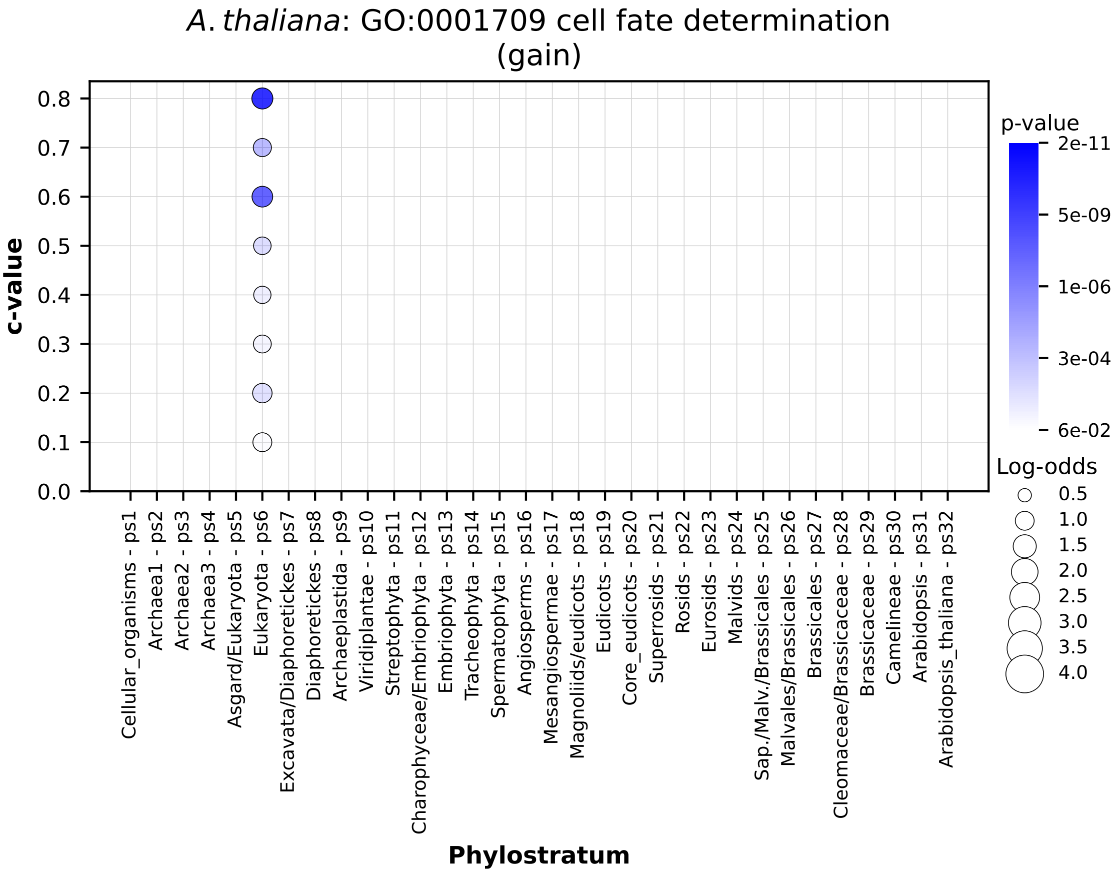

A. thaliana: GO:0001709 cell fate determination
(gain)
c-value
0.0
0.1
0.2
0.3
0.4
0.5
0.6
0.7
0.8
Phylostratum
Cellular_organisms - ps1
Archaea1 - ps2
Archaea2 - ps3
Archaea3 - ps4
Asgard/Eukaryota - ps5
Eukaryota - ps6
Excavata/Diaphoretickes - ps7
Diaphoretickes - ps8
Archaeplastida - ps9
Viridiplantae - ps10
Streptophyta - ps11
Charophyceae/Embriophyta - ps12
Embriophyta - ps13
Tracheophyta - ps14
Spermatophyta - ps15
Angiosperms - ps16
Mesangiospermae - ps17
Magnoliids/eudicots - ps18
Eudicots - ps19
Core_eudicots - ps20
Superrosids - ps21
Rosids - ps22
Eurosids - ps23
Malvids - ps24
Sap./Malv./Brassicales - ps25
Malvales/Brassicales - ps26
Brassicales - ps27
Cleomaceae/Brassicaceae - ps28
Brassicaceae - ps29
Camelineae - ps30
Arabidopsis - ps31
Arabidopsis_thaliana - ps32
p-value
2e-11
5e-09
1e-06
3e-04
6e-02
Log-odds
0.5
1.0
1.5
2.0
2.5
3.0
3.5
4.0

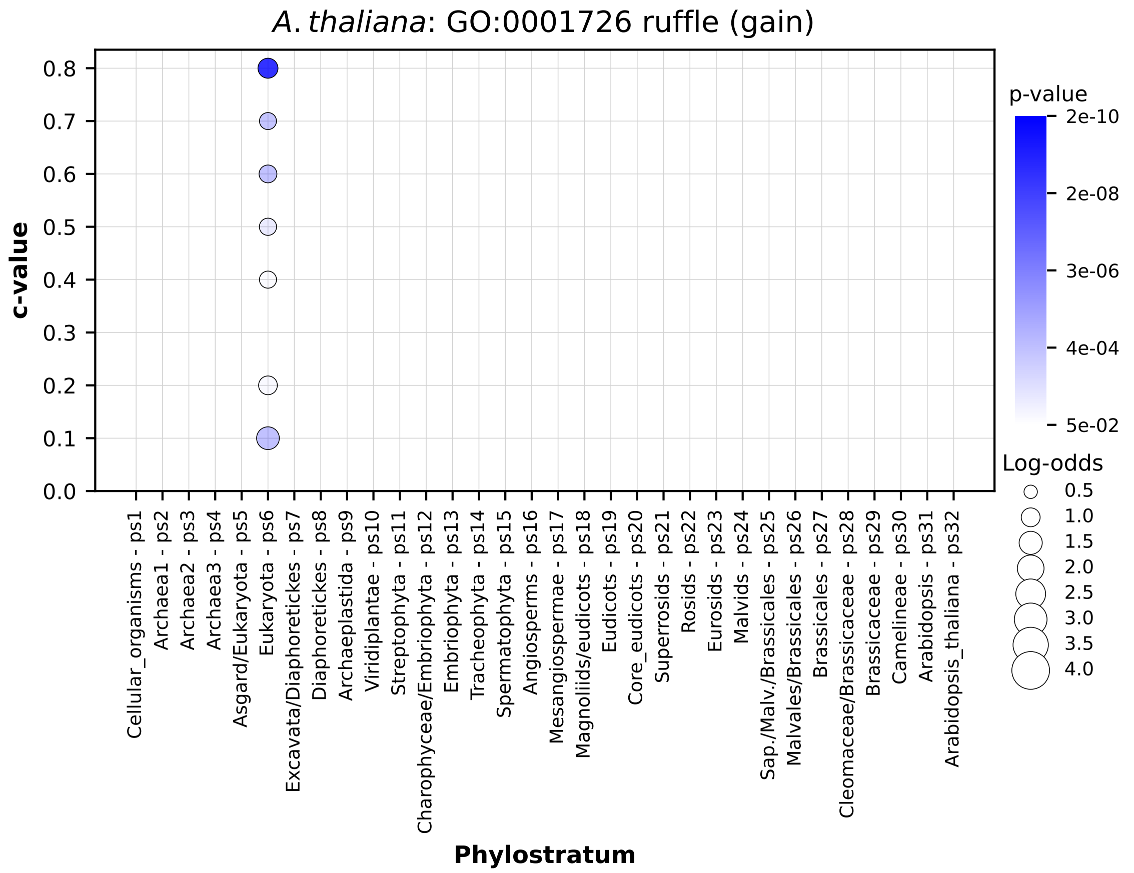

A. thaliana: GO:0001726 ruffle (gain)
c-value
0.0
0.1
0.2
0.3
0.4
0.5
0.6
0.7
0.8
Phylostratum
Cellular_organisms - ps1
Archaea1 - ps2
Archaea2 - ps3
Archaea3 - ps4
Asgard/Eukaryota - ps5
Eukaryota - ps6
Excavata/Diaphoretickes - ps7
Diaphoretickes - ps8
Archaeplastida - ps9
Viridiplantae - ps10
Streptophyta - ps11
Charophyceae/Embriophyta - ps12
Embriophyta - ps13
Tracheophyta - ps14
Spermatophyta - ps15
Angiosperms - ps16
Mesangiospermae - ps17
Magnoliids/eudicots - ps18
Eudicots - ps19
Core_eudicots - ps20
Superrosids - ps21
Rosids - ps22
Eurosids - ps23
Malvids - ps24
Sap./Malv./Brassicales - ps25
Malvales/Brassicales - ps26
Brassicales - ps27
Cleomaceae/Brassicaceae - ps28
Brassicaceae - ps29
Camelineae - ps30
Arabidopsis - ps31
Arabidopsis_thaliana - ps32
p-value
2e-10
2e-08
3e-06
4e-04
5e-02
Log-odds
0.5
1.0
1.5
2.0
2.5
3.0
3.5
4.0

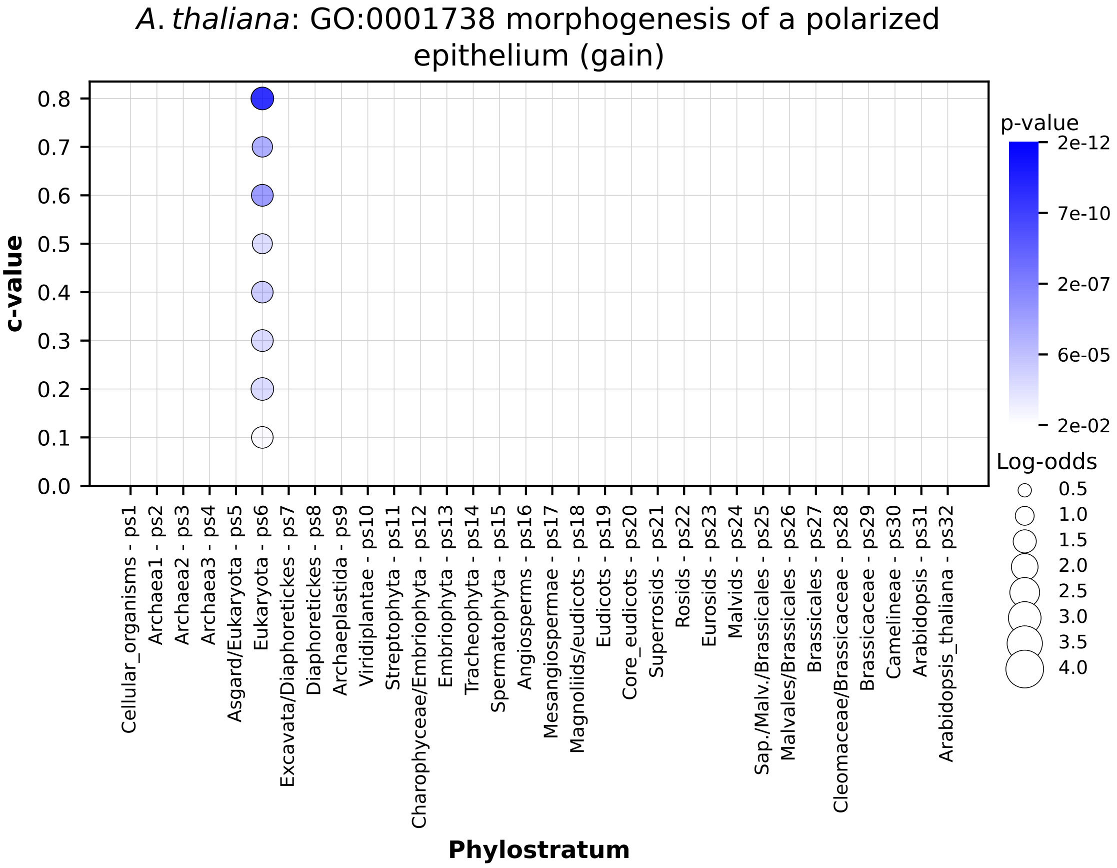
A. thaliana: GO:0001738 morphogenesis of a polarized epithelium (gain)
c-value
0.0
0.1
0.2
0.3
0.4
0.5
0.6
0.7
0.8
p-value
2e-12
7e-10
2e-07
6e-05
2e-02
Log-odds
0.5
1.0
1.5
2.0
2.5
3.0
3.5
4.0
Cellular_organisms - ps1
Archaea1 - ps2
Archaea2 - ps3
Archaea3 - ps4
Asgard/Eukaryota - ps5
Eukaryota - ps6
Excavata/Diaphoretickes - ps7
Diaphoretickes - ps8
Archaeplastida - ps9
Viridiplantae - ps10
Streptophyta - ps11
Charophyceae/Embriophyta - ps12
Embriophyta - ps13
Tracheophyta - ps14
Spermatophyta - ps15
Angiosperms - ps16
Mesangiospermae - ps17
Magnoliids/eudicots - ps18
Eudicots - ps19
Core_eudicots - ps20
Superrosids - ps21
Rosids - ps22
Eurosids - ps23
Malvids - ps24
Sap./Malv./Brassicales - ps25
Malvales/Brassicales - ps26
Brassicales - ps27
Cleomaceae/Brassicaceae - ps28
Brassicaceae - ps29
Camelineae - ps30
Arabidopsis - ps31
Arabidopsis_thaliana - ps32
Phylostratum

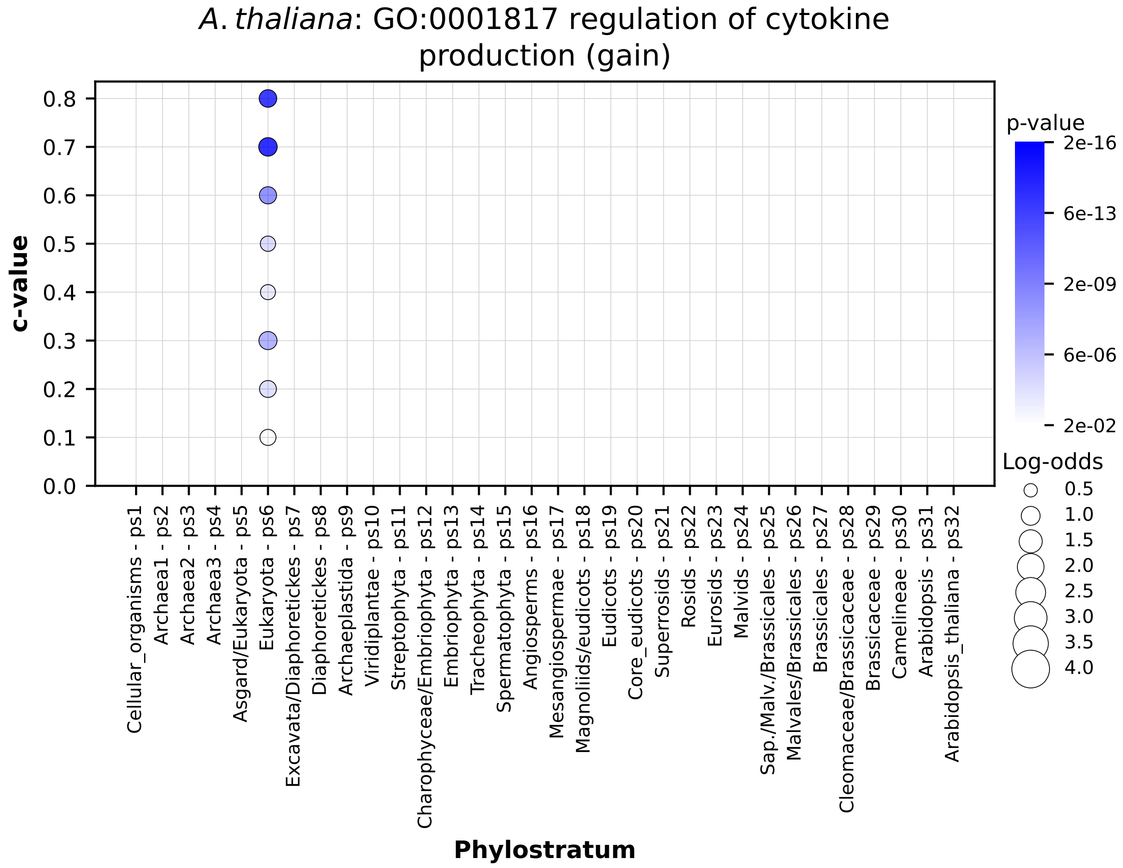

A. thaliana: GO:0001817 regulation of cytokine production (gain)
c-value
0.8
0.7
0.6
0.5
0.4
0.3
0.2
0.1
0.0
p-value
2e-16
6e-13
2e-09
6e-06
2e-02
Log-odds
0.5
1.0
1.5
2.0
2.5
3.0
3.5
4.0
Cellular_organisms - ps1
Archaea1 - ps2
Archaea2 - ps3
Archaea3 - ps4
Asgard/Eukaryota - ps5
Eukaryota - ps6
Excavata/Diaphoretickes - ps7
Diaphoretickes - ps8
Archaeplastida - ps9
Viridiplantae - ps10
Streptophyta - ps11
Charophyceae/Embriophyta - ps12
Embriophyta - ps13
Tracheophyta - ps14
Spermatophyta - ps15
Angiosperms - ps16
Mesangiospermae - ps17
Magnoliids/eudicots - ps18
Eudicots - ps19
Core_eudicots - ps20
Superrosids - ps21
Rosids - ps22
Eurosids - ps23
Malvids - ps24
Sap./Malv./Brassicales - ps25
Malvales/Brassicales - ps26
Brassicales - ps27
Cleomaceae/Brassicaceae - ps28
Brassicaceae - ps29
Camelineae - ps30
Arabidopsis - ps31
Arabidopsis_thaliana - ps32
Phylostratum

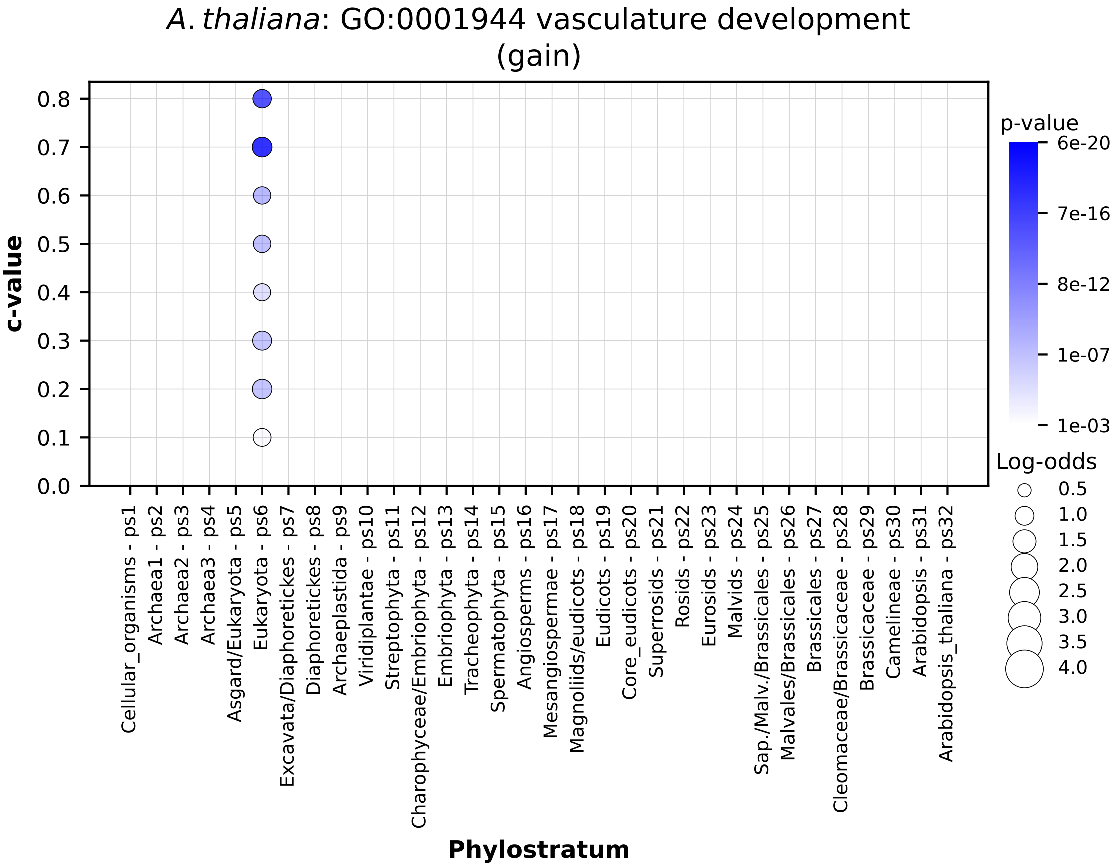
A. thaliana: GO:0001944 vasculature development
(gain)
c-value
0.8
0.7
0.6
0.5
0.4
0.3
0.2
0.1
0.0
p-value
6e-20
7e-16
8e-12
1e-07
1e-03
Log-odds
0.5
1.0
1.5
2.0
2.5
3.0
3.5
4.0
Cellular_organisms - ps1
Archaea1 - ps2
Archaea2 - ps3
Archaea3 - ps4
Asgard/Eukaryota - ps5
Eukaryota - ps6
Excavata/Diaphoretickes - ps7
Diaphoretickes - ps8
Archaeplastida - ps9
Viridiplantae - ps10
Streptophyta - ps11
Charophyceae/Embriophyta - ps12
Embriophyta - ps13
Tracheophyta - ps14
Spermatophyta - ps15
Angiosperms - ps16
Mesangiospermae - ps17
Magnoliids/eudicots - ps18
Eudicots - ps19
Core_eudicots - ps20
Superrosids - ps21
Rosids - ps22
Eurosids - ps23
Malvids - ps24
Sap./Malv./Brassicales - ps25
Malvales/Brassicales - ps26
Brassicales - ps27
Cleomaceae/Brassicaceae - ps28
Brassicaceae - ps29
Camelineae - ps30
Arabidopsis - ps31
Arabidopsis_thaliana - ps32
Phylostratum

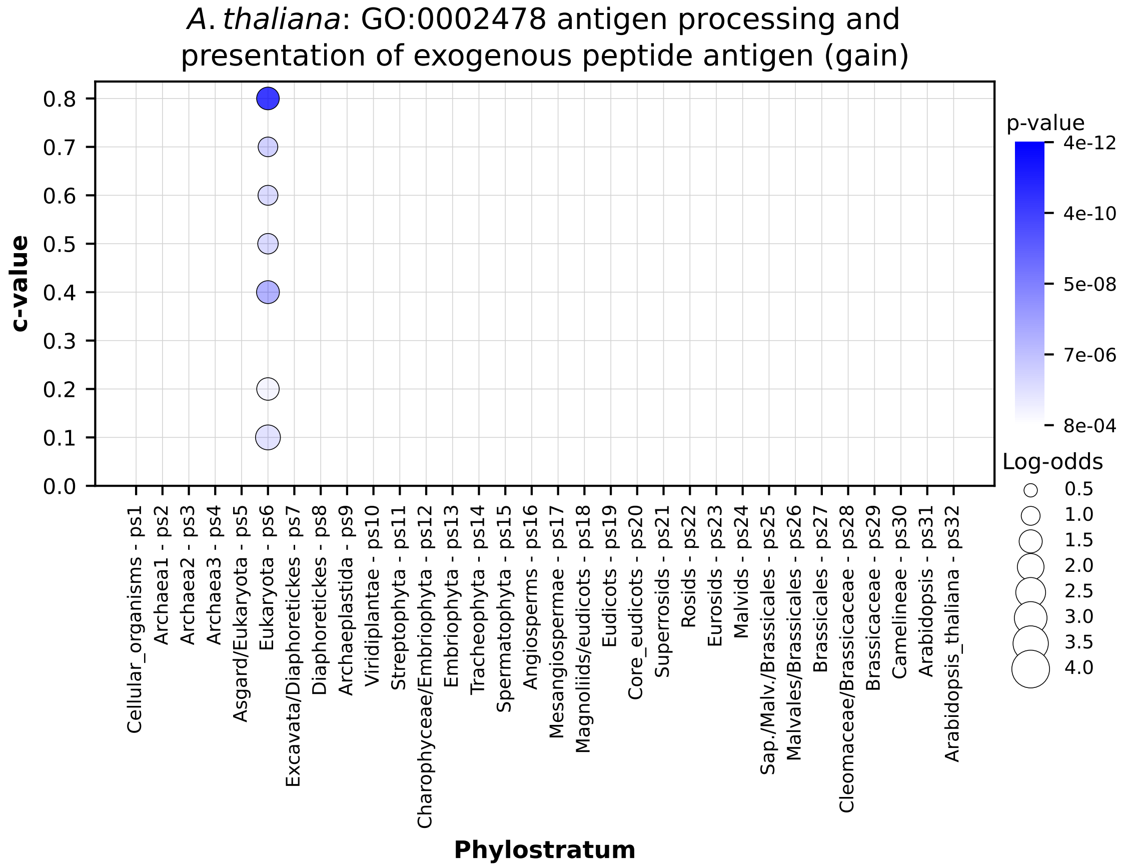

A. thaliana: GO:0002478 antigen processing and presentation of exogenous peptide antigen (gain)
c-value
0.0
0.1
0.2
0.3
0.4
0.5
0.6
0.7
0.8
Phylostratum
Cellular_organisms - ps1
Archaea1 - ps2
Archaea2 - ps3
Archaea3 - ps4
Asgard/Eukaryota - ps5
Eukaryota - ps6
Excavata/Diaphoretickes - ps7
Diaphoretickes - ps8
Archaeplastida - ps9
Viridiplantae - ps10
Streptophyta - ps11
Charophyceae/Embriophyta - ps12
Embriophyta - ps13
Tracheophyta - ps14
Spermatophyta - ps15
Angiosperms - ps16
Mesangiospermae - ps17
Magnoliids/eudicots - ps18
Eudicots - ps19
Core_eudicots - ps20
Superrosids - ps21
Rosids - ps22
Eurosids - ps23
Malvids - ps24
Sap./Malv./Brassicales - ps25
Malvales/Brassicales - ps26
Brassicales - ps27
Cleomaceae/Brassicaceae - ps28
Brassicaceae - ps29
Camelineae - ps30
Arabidopsis - ps31
Arabidopsis_thaliana - ps32
p-value
4e-12
4e-10
5e-08
7e-06
8e-04
Log-odds
0.5
1.0
1.5
2.0
2.5
3.0
3.5
4.0

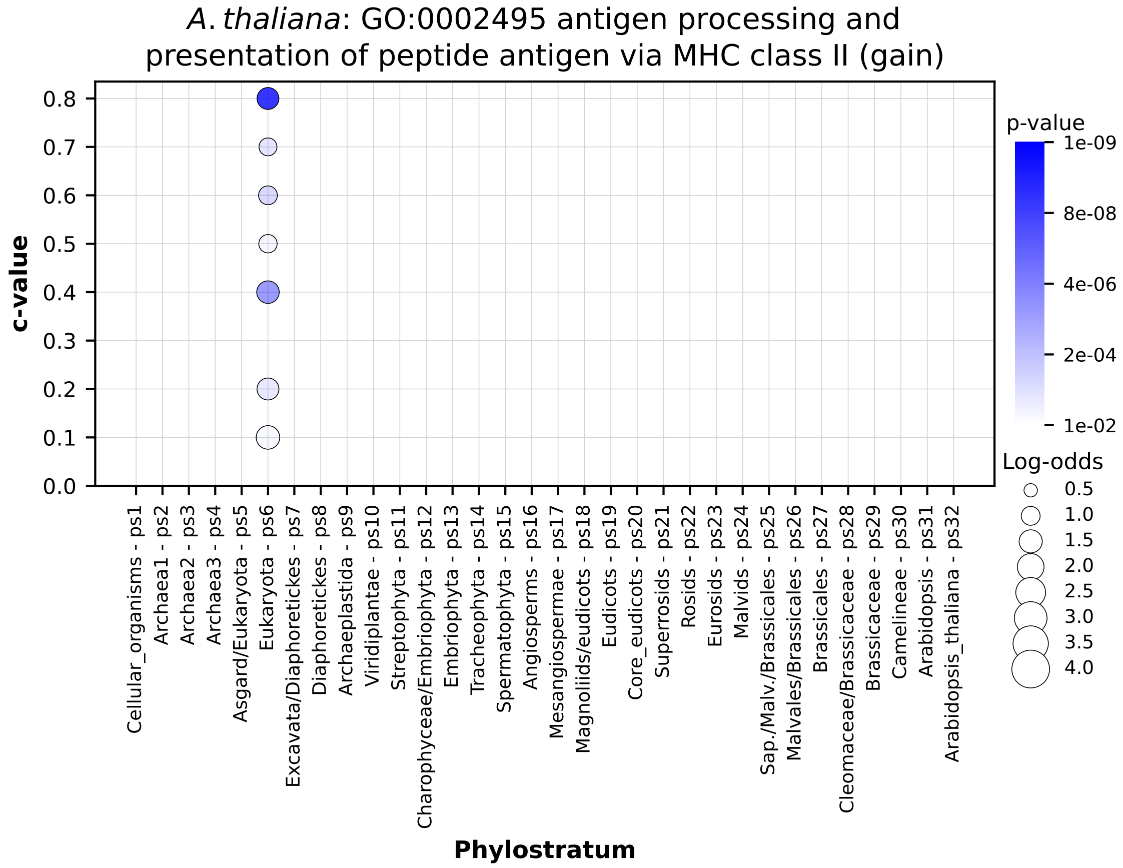

A. thaliana: GO:0002495 antigen processing and presentation of peptide antigen via MHC class II (gain)
c-value
0.0
0.1
0.2
0.3
0.4
0.5
0.6
0.7
0.8
Phylostratum
Cellular_organisms - ps1
Archaea1 - ps2
Archaea2 - ps3
Archaea3 - ps4
Asgard/Eukaryota - ps5
Eukaryota - ps6
Excavata/Diaphoretickes - ps7
Diaphoretickes - ps8
Archaeplastida - ps9
Viridiplantae - ps10
Streptophyta - ps11
Charophyceae/Embriophyta - ps12
Embriophyta - ps13
Tracheophyta - ps14
Spermatophyta - ps15
Angiosperms - ps16
Mesangiospermae - ps17
Magnoliids/eudicots - ps18
Eudicots - ps19
Core_eudicots - ps20
Superrosids - ps21
Rosids - ps22
Eurosids - ps23
Malvids - ps24
Sap./Malv./Brassicales - ps25
Malvales/Brassicales - ps26
Brassicales - ps27
Cleomaceae/Brassicaceae - ps28
Brassicaceae - ps29
Camelineae - ps30
Arabidopsis - ps31
Arabidopsis_thaliana - ps32
p-value
1e-09
8e-08
4e-06
2e-04
1e-02
Log-odds
0.5
1.0
1.5
2.0
2.5
3.0
3.5
4.0

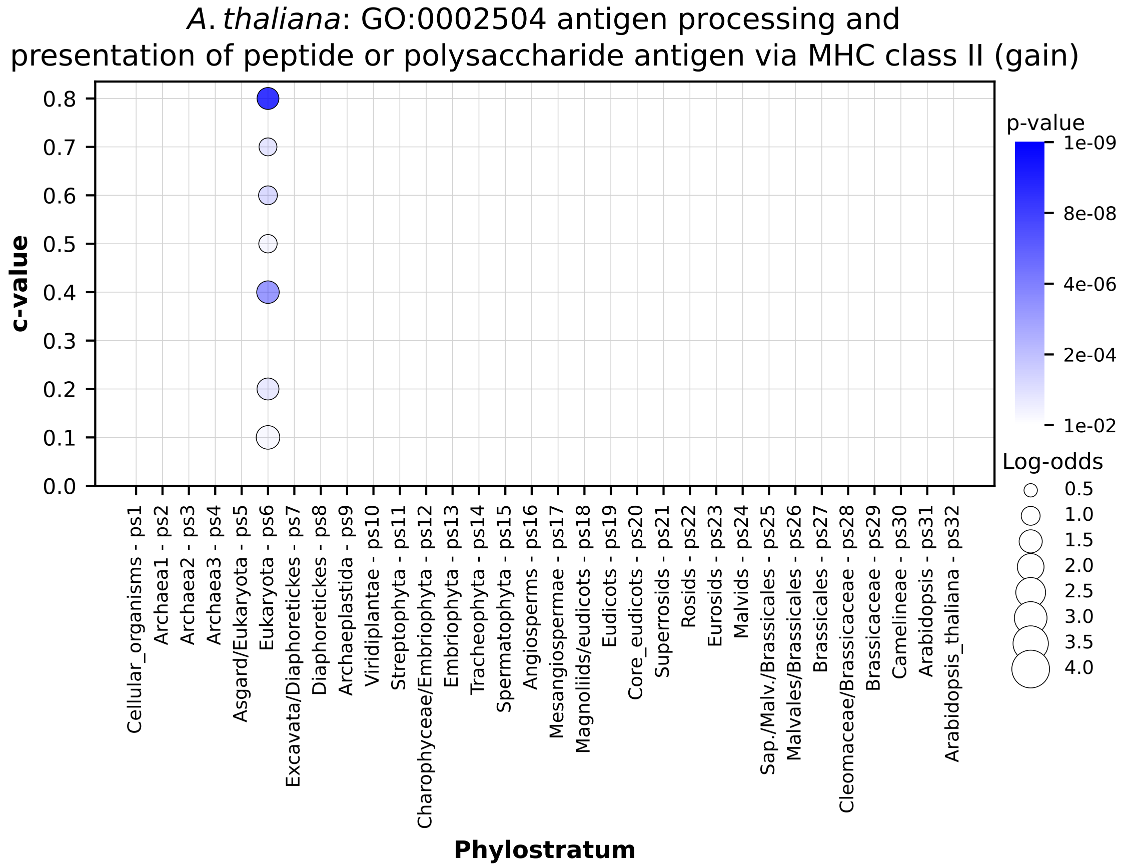

A. thaliana: GO:0002504 antigen processing and presentation of peptide or polysaccharide antigen via MHC class II (gain)
c-value
0.0
0.1
0.2
0.3
0.4
0.5
0.6
0.7
0.8
Phylostratum
Cellular_organisms - ps1
Archaea1 - ps2
Archaea2 - ps3
Archaea3 - ps4
Asgard/Eukaryota - ps5
Eukaryota - ps6
Excavata/Diaphoretickes - ps7
Diaphoretickes - ps8
Archaeplastida - ps9
Viridiplantae - ps10
Streptophyta - ps11
Charophyceae/Embriophyta - ps12
Embriophyta - ps13
Tracheophyta - ps14
Spermatophyta - ps15
Angiosperms - ps16
Mesangiospermae - ps17
Magnoliids/eudicots - ps18
Eudicots - ps19
Core_eudicots - ps20
Superrosids - ps21
Rosids - ps22
Eurosids - ps23
Malvids - ps24
Sap./Malv./Brassicales - ps25
Malvales/Brassicales - ps26
Brassicales - ps27
Cleomaceae/Brassicaceae - ps28
Brassicaceae - ps29
Camelineae - ps30
Arabidopsis - ps31
Arabidopsis_thaliana - ps32
p-value
1e-09
8e-08
4e-06
2e-04
1e-02
Log-odds
0.5
1.0
1.5
2.0
2.5
3.0
3.5
4.0

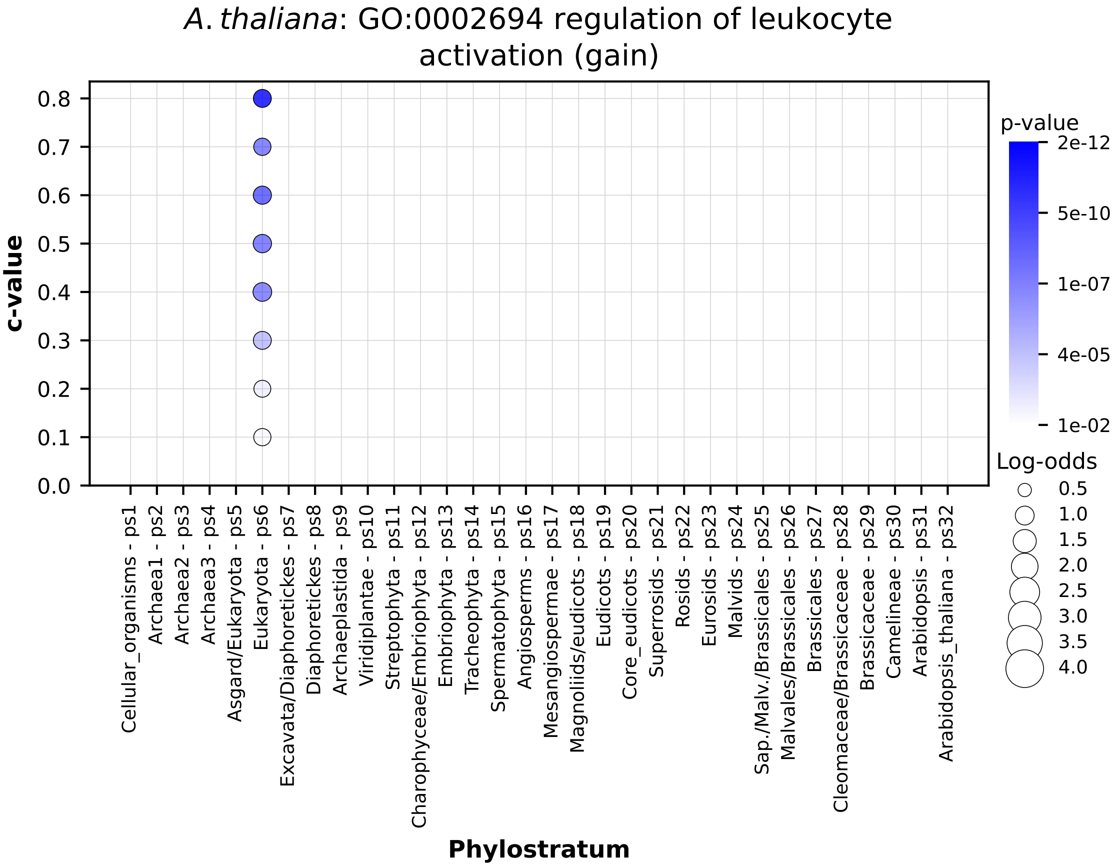

A. thaliana: GO:0002694 regulation of leukocyte activation (gain)
c-value
0.0
0.1
0.2
0.3
0.4
0.5
0.6
0.7
0.8
Phylostratum
Cellular_organisms - ps1
Archaea1 - ps2
Archaea2 - ps3
Archaea3 - ps4
Asgard/Eukaryota - ps5
Eukaryota - ps6
Excavata/Diaphoretickes - ps7
Diaphoretickes - ps8
Archaeplastida - ps9
Viridiplantae - ps10
Streptophyta - ps11
Charophyceae/Embriophyta - ps12
Embriophyta - ps13
Tracheophyta - ps14
Spermatophyta - ps15
Angiosperms - ps16
Mesangiospermae - ps17
Magnoliids/eudicots - ps18
Eudicots - ps19
Core_eudicots - ps20
Superrosids - ps21
Rosids - ps22
Eurosids - ps23
Malvids - ps24
Sap./Malv./Brassicales - ps25
Malvales/Brassicales - ps26
Brassicales - ps27
Cleomaceae/Brassicaceae - ps28
Brassicaceae - ps29
Camelineae - ps30
Arabidopsis - ps31
Arabidopsis_thaliana - ps32
p-value
2e-12
5e-10
1e-07
4e-05
1e-02
Log-odds
0.5
1.0
1.5
2.0
2.5
3.0
3.5
4.0

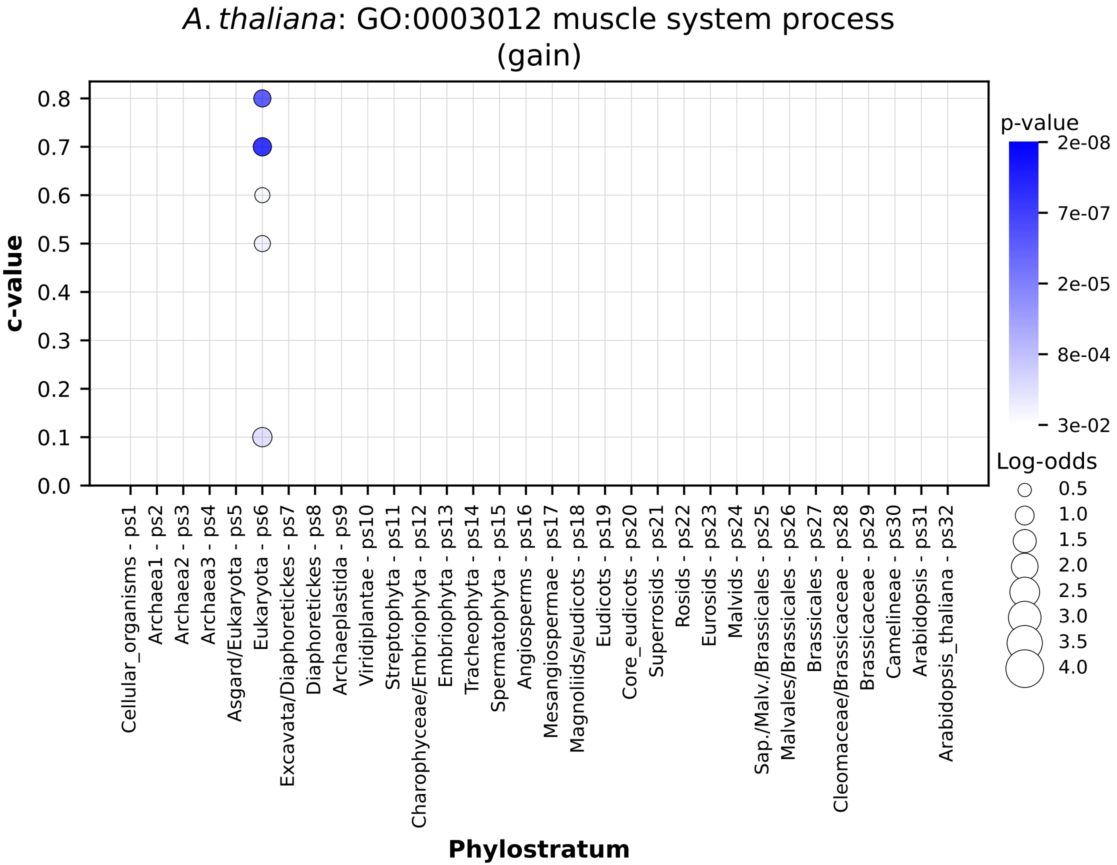

A. thaliana: GO:0003012 muscle system process
(gain)
c-value
0.0
0.1
0.2
0.3
0.4
0.5
0.6
0.7
0.8
Phylostratum
Cellular_organisms - ps1
Archaea1 - ps2
Archaea2 - ps3
Archaea3 - ps4
Asgard/Eukaryota - ps5
Eukaryota - ps6
Excavata/Diaphoretickes - ps7
Diaphoretickes - ps8
Archaeplastida - ps9
Viridiplantae - ps10
Streptophyta - ps11
Charophyceae/Embriophyta - ps12
Embriophyta - ps13
Tracheophyta - ps14
Spermatophyta - ps15
Angiosperms - ps16
Mesangiospermae - ps17
Magnoliids/eudicots - ps18
Eudicots - ps19
Core_eudicots - ps20
Superrosids - ps21
Rosids - ps22
Eurosids - ps23
Malvids - ps24
Sap./Malv./Brassicales - ps25
Malvales/Brassicales - ps26
Brassicales - ps27
Cleomaceae/Brassicaceae - ps28
Brassicaceae - ps29
Camelineae - ps30
Arabidopsis - ps31
Arabidopsis_thaliana - ps32
p-value
2e-08
7e-07
2e-05
8e-04
3e-02
Log-odds
0.5
1.0
1.5
2.0
2.5
3.0
3.5
4.0

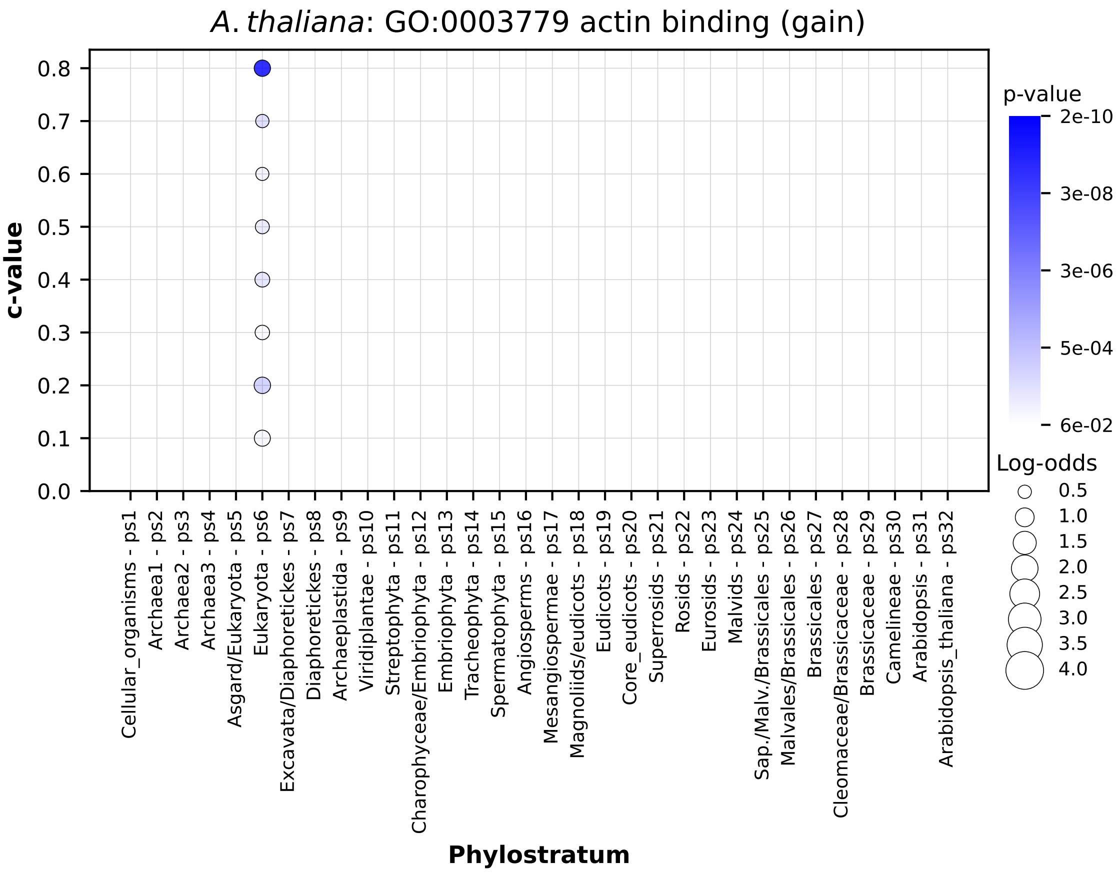

A. thaliana: GO:0003779 actin binding (gain)
c-value
0.0
0.1
0.2
0.3
0.4
0.5
0.6
0.7
0.8
Phylostratum
Cellular_organisms - ps1
Archaea1 - ps2
Archaea2 - ps3
Archaea3 - ps4
Asgard/Eukaryota - ps5
Eukaryota - ps6
Excavata/Diaphoretickes - ps7
Diaphoretickes - ps8
Archaeplastida - ps9
Viridiplantae - ps10
Streptophyta - ps11
Charophyceae/Embriophyta - ps12
Embriophyta - ps13
Tracheophyta - ps14
Spermatophyta - ps15
Angiosperms - ps16
Mesangiospermae - ps17
Magnoliids/eudicots - ps18
Eudicots - ps19
Core_eudicots - ps20
Superrosids - ps21
Rosids - ps22
Eurosids - ps23
Malvids - ps24
Sap./Malv./Brassicales - ps25
Malvales/Brassicales - ps26
Brassicales - ps27
Cleomaceae/Brassicaceae - ps28
Brassicaceae - ps29
Camelineae - ps30
Arabidopsis - ps31
Arabidopsis_thaliana - ps32
p-value
2e-10
3e-08
3e-06
5e-04
6e-02
Log-odds
0.5
1.0
1.5
2.0
2.5
3.0
3.5
4.0

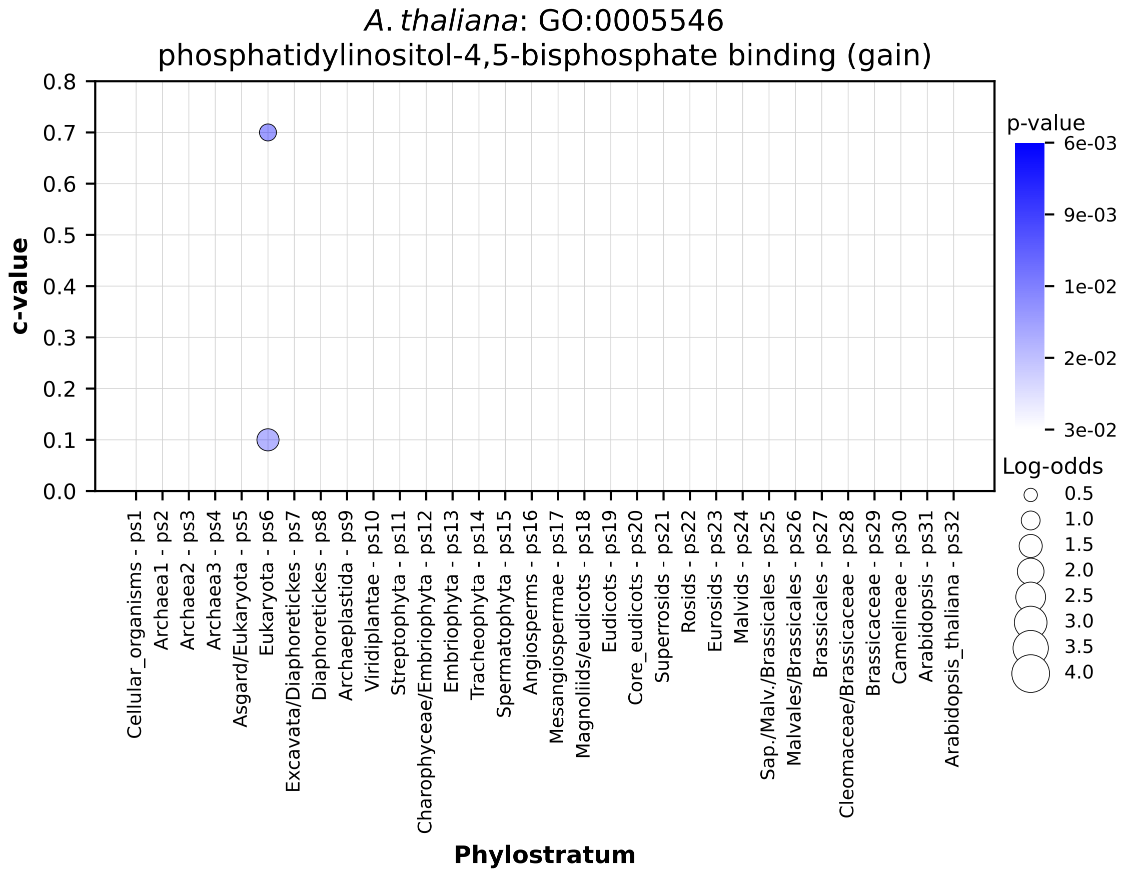

A. thaliana: GO:0005546
phosphatidylinositol-4,5-bisphosphate binding (gain)
c-value
0.0
0.1
0.2
0.3
0.4
0.5
0.6
0.7
0.8
Phylostratum
Cellular_organisms - ps1
Archaea1 - ps2
Archaea2 - ps3
Archaea3 - ps4
Asgard/Eukaryota - ps5
Eukaryota - ps6
Excavata/Diaphoretickes - ps7
Diaphoretickes - ps8
Archaeplastida - ps9
Viridiplantae - ps10
Streptophyta - ps11
Charophyceae/Embriophyta - ps12
Embriophyta - ps13
Tracheophyta - ps14
Spermatophyta - ps15
Angiosperms - ps16
Mesangiospermae - ps17
Magnoliids/eudicots - ps18
Eudicots - ps19
Core_eudicots - ps20
Superrosids - ps21
Rosids - ps22
Eurosids - ps23
Malvids - ps24
Sap./Malv./Brassicales - ps25
Malvales/Brassicales - ps26
Brassicales - ps27
Cleomaceae/Brassicaceae - ps28
Brassicaceae - ps29
Camelineae - ps30
Arabidopsis - ps31
Arabidopsis_thaliana - ps32
p-value
6e-03
9e-03
1e-02
2e-02
3e-02
Log-odds
0.5
1.0
1.5
2.0
2.5
3.0
3.5
4.0

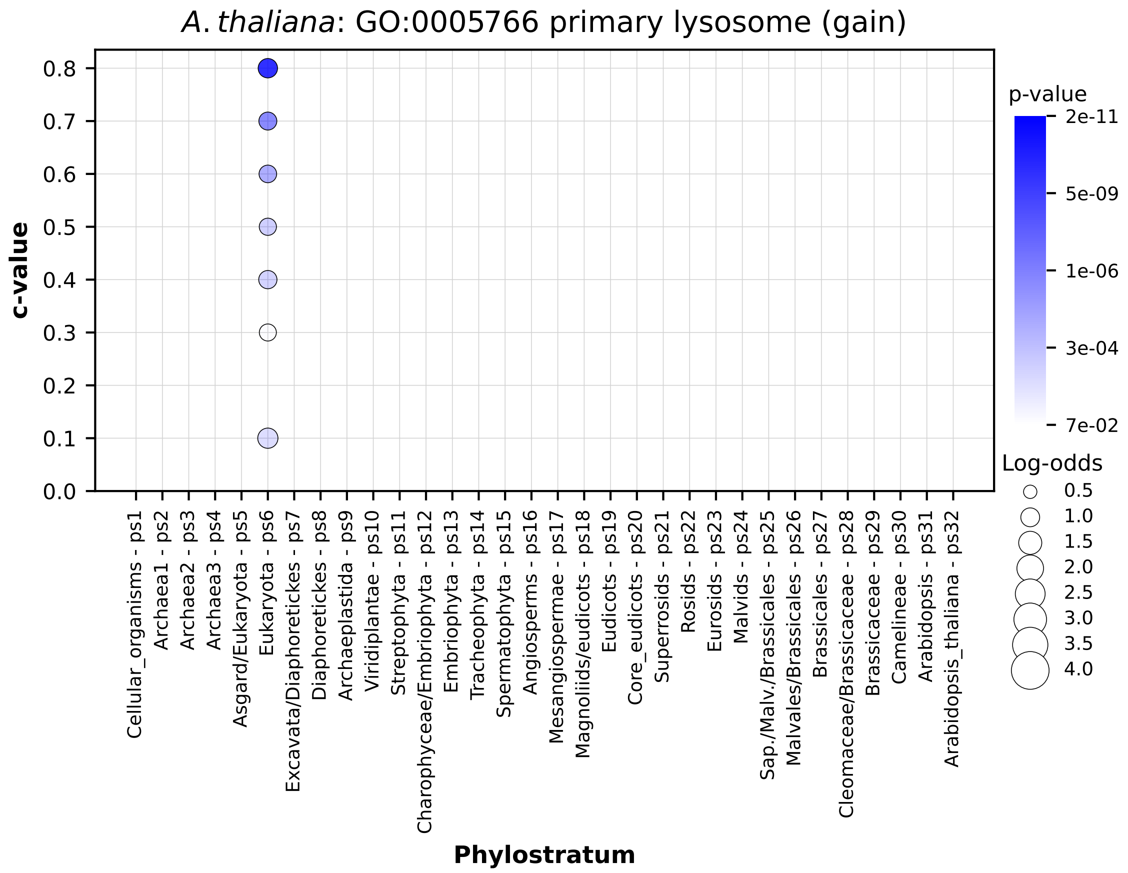
A. thaliana: GO:0005766 primary lysosome (gain)
c-value
0.8
0.7
0.6
0.5
0.4
0.3
0.2
0.1
0.0
p-value
2e-11
5e-09
1e-06
3e-04
7e-02
Log-odds
0.5
1.0
1.5
2.0
2.5
3.0
3.5
4.0
Cellular_organisms - ps1
Archaea1 - ps2
Archaea2 - ps3
Archaea3 - ps4
Asgard/Eukaryota - ps5
Eukaryota - ps6
Excavata/Diaphoretickes - ps7
Diaphoretickes - ps8
Archaeplastida - ps9
Viridiplantae - ps10
Streptophyta - ps11
Charophyceae/Embriophyta - ps12
Embriophyta - ps13
Tracheophyta - ps14
Spermatophyta - ps15
Angiosperms - ps16
Mesangiospermae - ps17
Magnoliids/eudicots - ps18
Eudicots - ps19
Core_eudicots - ps20
Superrosids - ps21
Rosids - ps22
Eurosids - ps23
Malvids - ps24
Sap./Malv./Brassicales - ps25
Malvales/Brassicales - ps26
Brassicales - ps27
Cleomaceae/Brassicaceae - ps28
Brassicaceae - ps29
Camelineae - ps30
Arabidopsis - ps31
Arabidopsis_thaliana - ps32
Phylostratum

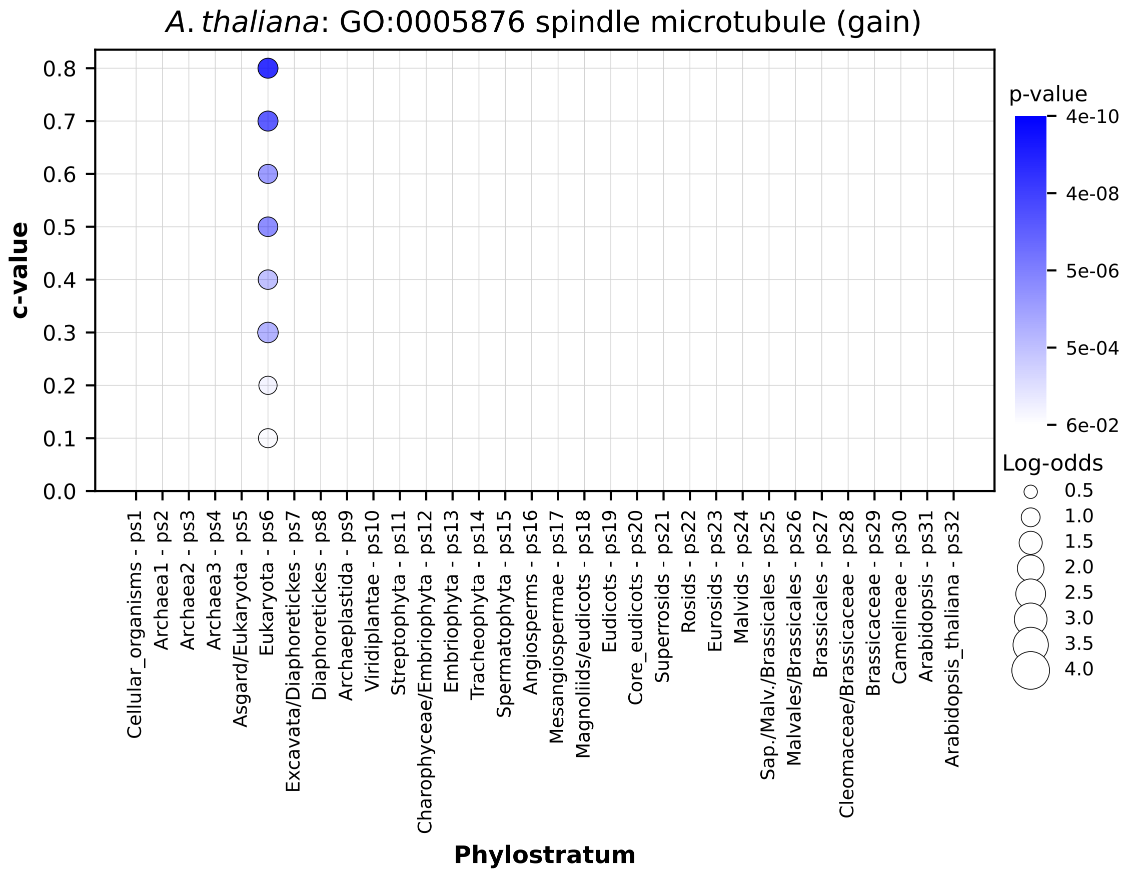
A. thaliana: GO:0005876 spindle microtubule (gain)
c-value
0.0
0.1
0.2
0.3
0.4
0.5
0.6
0.7
0.8
Phylostratum
Cellular_organisms - ps1
Archaea1 - ps2
Archaea2 - ps3
Archaea3 - ps4
Asgard/Eukaryota - ps5
Eukaryota - ps6
Excavata/Diaphoretickes - ps7
Diaphoretickes - ps8
Archaeplastida - ps9
Viridiplantae - ps10
Streptophyta - ps11
Charophyceae/Embriophyta - ps12
Embriophyta - ps13
Tracheophyta - ps14
Spermatophyta - ps15
Angiosperms - ps16
Mesangiospermae - ps17
Magnoliids/eudicots - ps18
Eudicots - ps19
Core_eudicots - ps20
Superrosids - ps21
Rosids - ps22
Eurosids - ps23
Malvids - ps24
Sap./Malv./Brassicales - ps25
Malvales/Brassicales - ps26
Brassicales - ps27
Cleomaceae/Brassicaceae - ps28
Brassicaceae - ps29
Camelineae - ps30
Arabidopsis - ps31
Arabidopsis_thaliana - ps32
p-value
4e-10
4e-08
5e-06
5e-04
6e-02
Log-odds
0.5
1.0
1.5
2.0
2.5
3.0
3.5
4.0

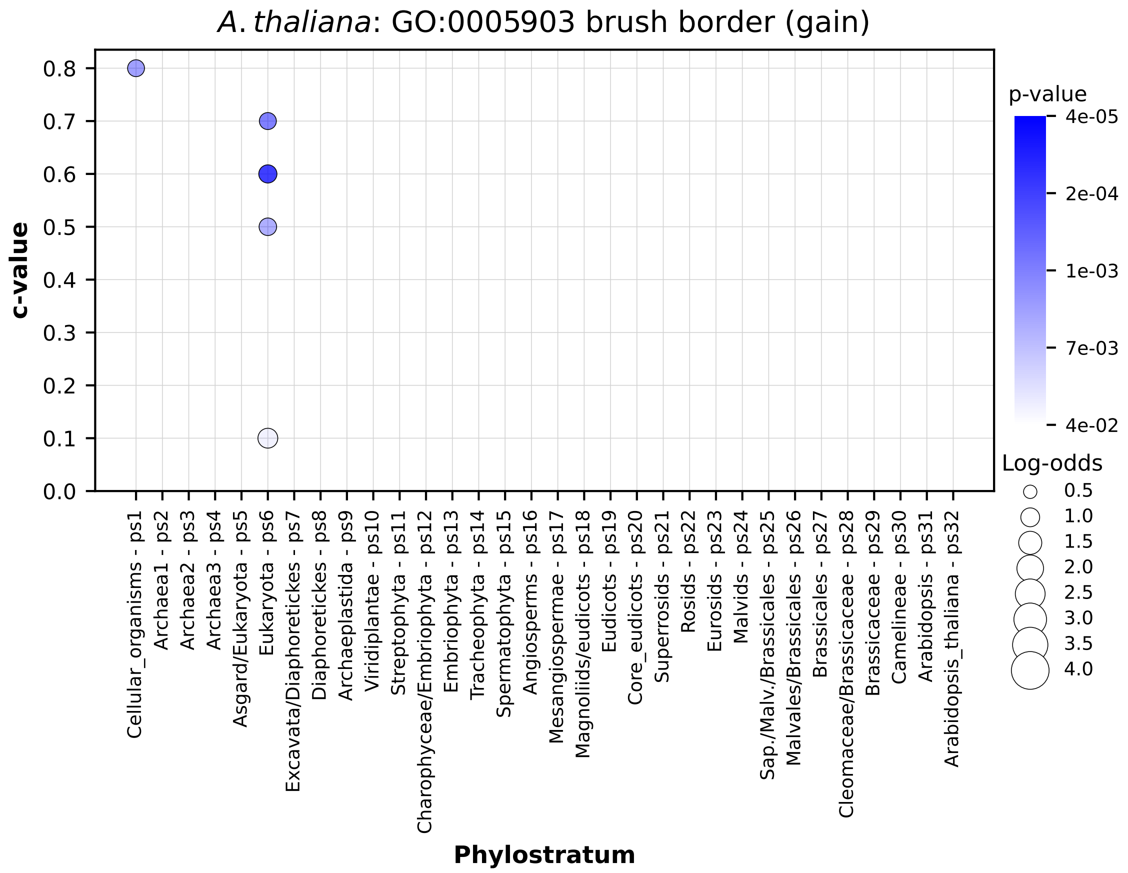

A. thaliana: GO:0005903 brush border (gain)
c-value
0.0
0.1
0.2
0.3
0.4
0.5
0.6
0.7
0.8
p-value
4e-05
2e-04
1e-03
7e-03
4e-02
Log-odds
0.5
1.0
1.5
2.0
2.5
3.0
3.5
4.0
Cellular_organisms - ps1
Archaea1 - ps2
Archaea2 - ps3
Archaea3 - ps4
Asgard/Eukaryota - ps5
Eukaryota - ps6
Excavata/Diaphoretickes - ps7
Diaphoretickes - ps8
Archaeplastida - ps9
Viridiplantae - ps10
Streptophyta - ps11
Charophyceae/Embriophyta - ps12
Embriophyta - ps13
Tracheophyta - ps14
Spermatophyta - ps15
Angiosperms - ps16
Mesangiospermae - ps17
Magnoliids/eudicots - ps18
Eudicots - ps19
Core_eudicots - ps20
Superrosids - ps21
Rosids - ps22
Eurosids - ps23
Malvids - ps24
Sap./Malv./Brassicales - ps25
Malvales/Brassicales - ps26
Brassicales - ps27
Cleomaceae/Brassicaceae - ps28
Brassicaceae - ps29
Camelineae - ps30
Arabidopsis - ps31
Arabidopsis_thaliana - ps32
Phylostratum

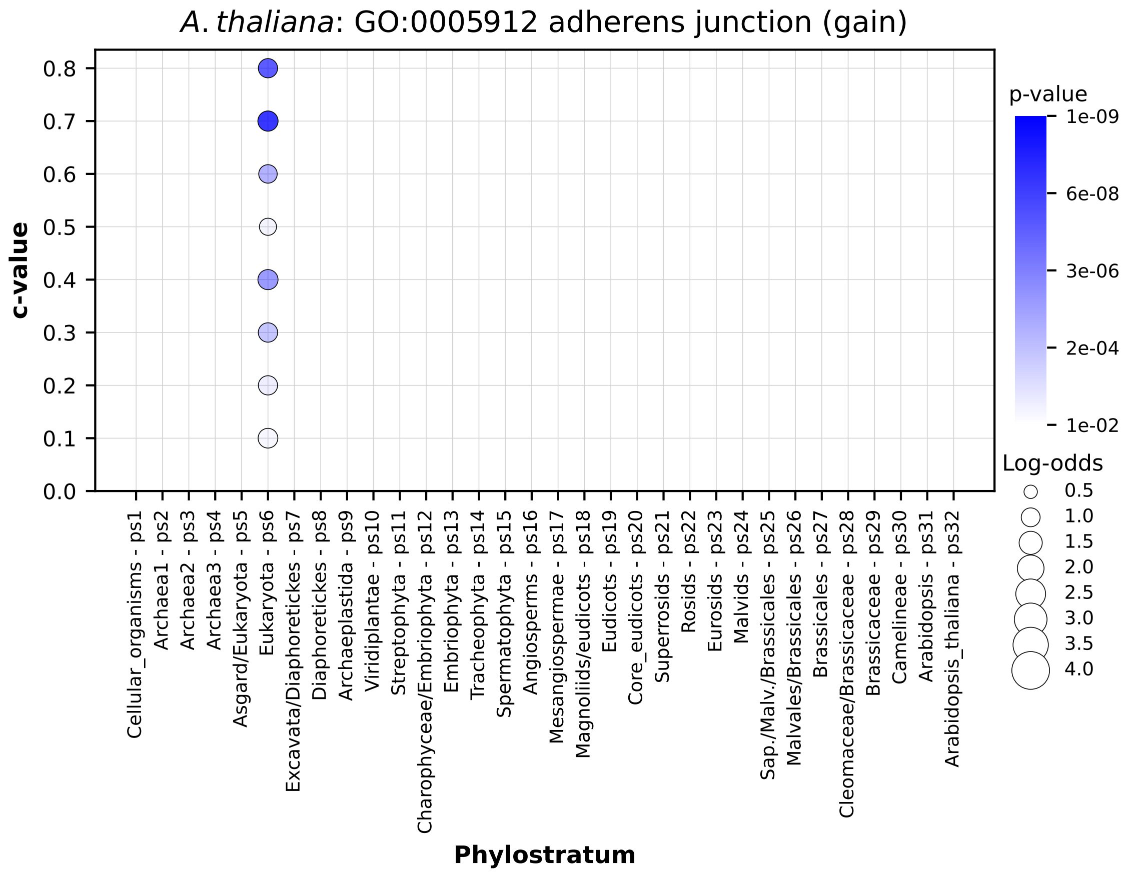

A. thaliana: GO:0005912 adherens junction (gain)
c-value
0.0
0.1
0.2
0.3
0.4
0.5
0.6
0.7
0.8
Phylostratum
Cellular_organisms - ps1
Archaea1 - ps2
Archaea2 - ps3
Archaea3 - ps4
Asgard/Eukaryota - ps5
Eukaryota - ps6
Excavata/Diaphoretickes - ps7
Diaphoretickes - ps8
Archaeplastida - ps9
Viridiplantae - ps10
Streptophyta - ps11
Charophyceae/Embriophyta - ps12
Embriophyta - ps13
Tracheophyta - ps14
Spermatophyta - ps15
Angiosperms - ps16
Mesangiospermae - ps17
Magnoliids/eudicots - ps18
Eudicots - ps19
Core_eudicots - ps20
Superrosids - ps21
Rosids - ps22
Eurosids - ps23
Malvids - ps24
Sap./Malv./Brassicales - ps25
Malvales/Brassicales - ps26
Brassicales - ps27
Cleomaceae/Brassicaceae - ps28
Brassicaceae - ps29
Camelineae - ps30
Arabidopsis - ps31
Arabidopsis_thaliana - ps32
p-value
1e-09
6e-08
3e-06
2e-04
1e-02
Log-odds
0.5
1.0
1.5
2.0
2.5
3.0
3.5
4.0

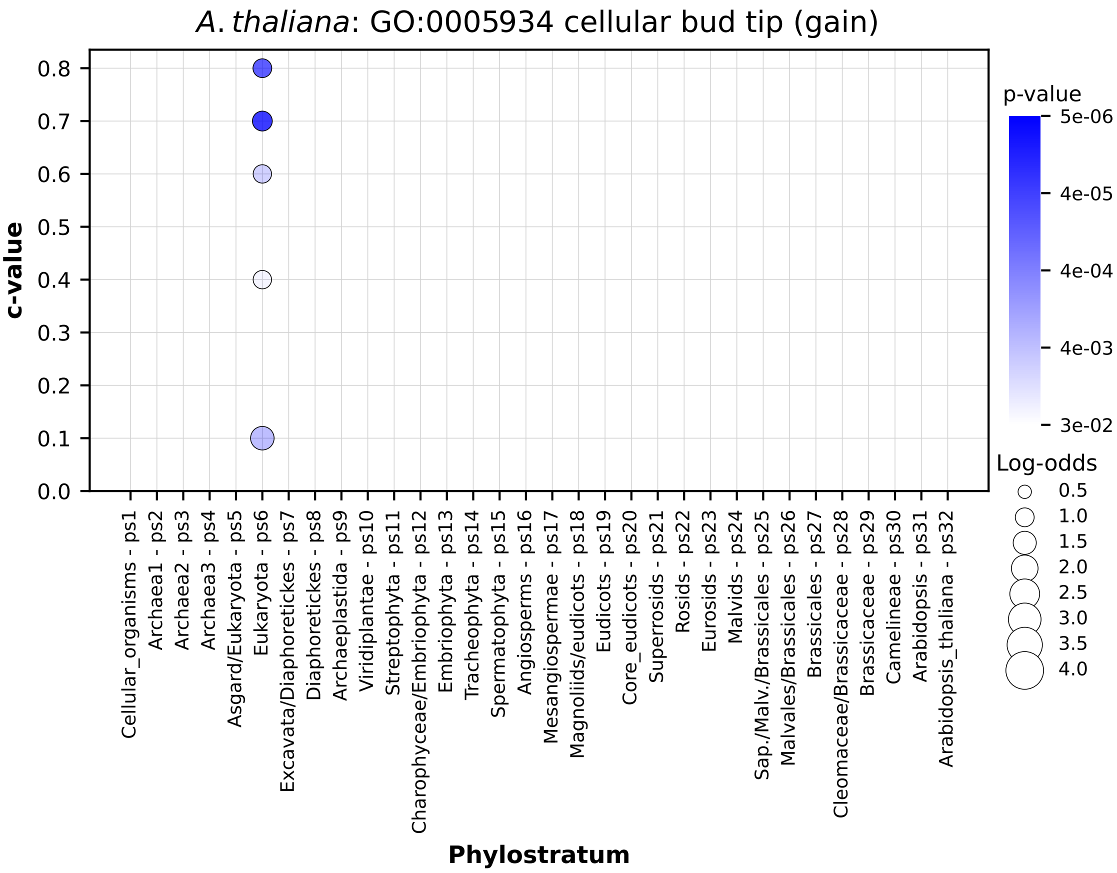

A. thaliana: GO:0005934 cellular bud tip (gain)
c-value
0.0
0.1
0.2
0.3
0.4
0.5
0.6
0.7
0.8
Phylostratum
Cellular_organisms - ps1
Archaea1 - ps2
Archaea2 - ps3
Archaea3 - ps4
Asgard/Eukaryota - ps5
Eukaryota - ps6
Excavata/Diaphoretickes - ps7
Diaphoretickes - ps8
Archaeplastida - ps9
Viridiplantae - ps10
Streptophyta - ps11
Charophyceae/Embriophyta - ps12
Embriophyta - ps13
Tracheophyta - ps14
Spermatophyta - ps15
Angiosperms - ps16
Mesangiospermae - ps17
Magnoliids/eudicots - ps18
Eudicots - ps19
Core_eudicots - ps20
Superrosids - ps21
Rosids - ps22
Eurosids - ps23
Malvids - ps24
Sap./Malv./Brassicales - ps25
Malvales/Brassicales - ps26
Brassicales - ps27
Cleomaceae/Brassicaceae - ps28
Brassicaceae - ps29
Camelineae - ps30
Arabidopsis - ps31
Arabidopsis_thaliana - ps32
p-value
5e-06
4e-05
4e-04
4e-03
3e-02
Log-odds
0.5
1.0
1.5
2.0
2.5
3.0
3.5
4.0

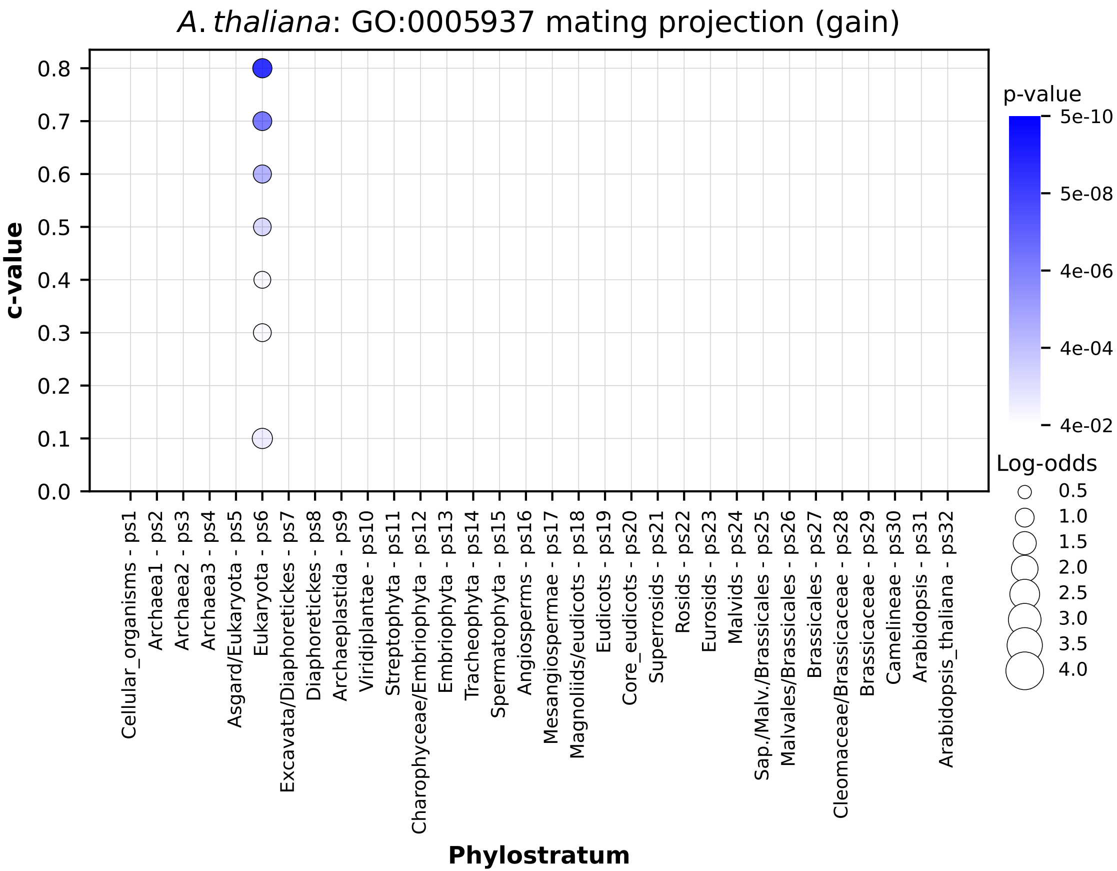

A. thaliana: GO:0005937 mating projection (gain)
c-value
0.0
0.1
0.2
0.3
0.4
0.5
0.6
0.7
0.8
Phylostratum
Cellular_organisms - ps1
Archaea1 - ps2
Archaea2 - ps3
Archaea3 - ps4
Asgard/Eukaryota - ps5
Eukaryota - ps6
Excavata/Diaphoretickes - ps7
Diaphoretickes - ps8
Archaeplastida - ps9
Viridiplantae - ps10
Streptophyta - ps11
Charophyceae/Embriophyta - ps12
Embriophyta - ps13
Tracheophyta - ps14
Spermatophyta - ps15
Angiosperms - ps16
Mesangiospermae - ps17
Magnoliids/eudicots - ps18
Eudicots - ps19
Core_eudicots - ps20
Superrosids - ps21
Rosids - ps22
Eurosids - ps23
Malvids - ps24
Sap./Malv./Brassicales - ps25
Malvales/Brassicales - ps26
Brassicales - ps27
Cleomaceae/Brassicaceae - ps28
Brassicaceae - ps29
Camelineae - ps30
Arabidopsis - ps31
Arabidopsis_thaliana - ps32
p-value
5e-10
5e-08
4e-06
4e-04
4e-02
Log-odds
0.5
1.0
1.5
2.0
2.5
3.0
3.5
4.0

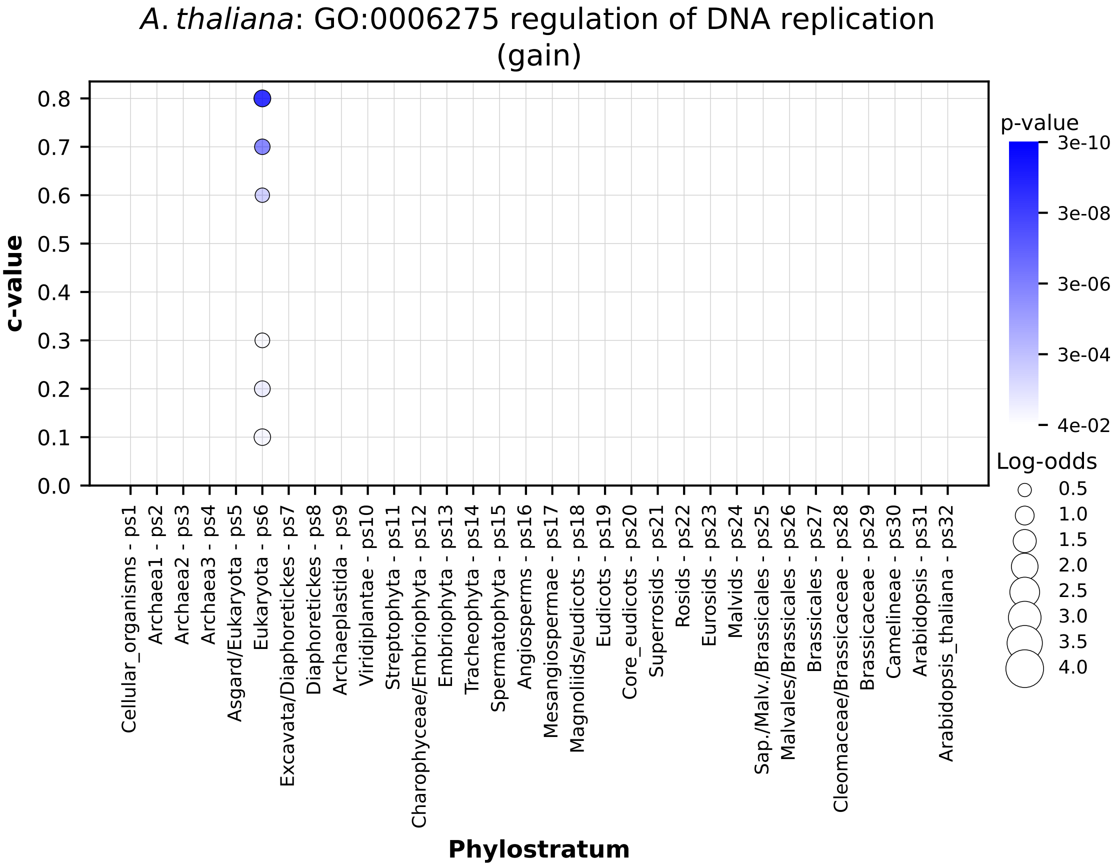
A. thaliana: GO:0006275 regulation of DNA replication (gain)
c-value
0.0
0.1
0.2
0.3
0.4
0.5
0.6
0.7
0.8
Phylostratum
Cellular_organisms - ps1
Archaea1 - ps2
Archaea2 - ps3
Archaea3 - ps4
Asgard/Eukaryota - ps5
Eukaryota - ps6
Excavata/Diaphoretickes - ps7
Diaphoretickes - ps8
Archaeplastida - ps9
Viridiplantae - ps10
Streptophyta - ps11
Charophyceae/Embriophyta - ps12
Embriophyta - ps13
Tracheophyta - ps14
Spermatophyta - ps15
Angiosperms - ps16
Mesangiospermae - ps17
Magnoliids/eudicots - ps18
Eudicots - ps19
Core_eudicots - ps20
Superrosids - ps21
Rosids - ps22
Eurosids - ps23
Malvids - ps24
Sap./Malv./Brassicales - ps25
Malvales/Brassicales - ps26
Brassicales - ps27
Cleomaceae/Brassicaceae - ps28
Brassicaceae - ps29
Camelineae - ps30
Arabidopsis - ps31
Arabidopsis_thaliana - ps32
p-value
3e-10
3e-08
3e-06
3e-04
4e-02
Log-odds
0.5
1.0
1.5
2.0
2.5
3.0
3.5
4.0

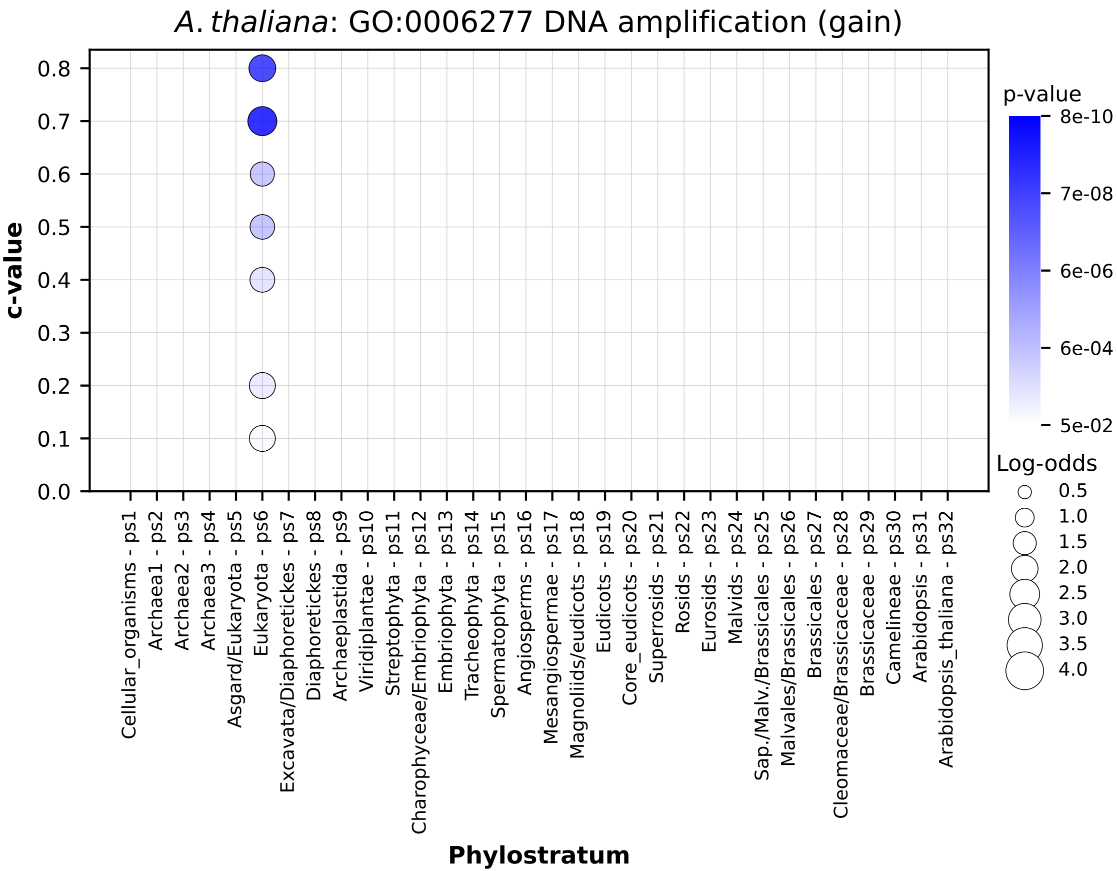

A. thaliana: GO:0006277 DNA amplification (gain)
c-value
0.0
0.1
0.2
0.3
0.4
0.5
0.6
0.7
0.8
Phylostratum
Cellular_organisms - ps1
Archaea1 - ps2
Archaea2 - ps3
Archaea3 - ps4
Asgard/Eukaryota - ps5
Eukaryota - ps6
Excavata/Diaphoretickes - ps7
Diaphoretickes - ps8
Archaeplastida - ps9
Viridiplantae - ps10
Streptophyta - ps11
Charophyceae/Embriophyta - ps12
Embriophyta - ps13
Tracheophyta - ps14
Spermatophyta - ps15
Angiosperms - ps16
Mesangiospermae - ps17
Magnoliids/eudicots - ps18
Eudicots - ps19
Core_eudicots - ps20
Superrosids - ps21
Rosids - ps22
Eurosids - ps23
Malvids - ps24
Sap./Malv./Brassicales - ps25
Malvales/Brassicales - ps26
Brassicales - ps27
Cleomaceae/Brassicaceae - ps28
Brassicaceae - ps29
Camelineae - ps30
Arabidopsis - ps31
Arabidopsis_thaliana - ps32
p-value
8e-10
7e-08
6e-06
6e-04
5e-02
Log-odds
0.5
1.0
1.5
2.0
2.5
3.0
3.5
4.0

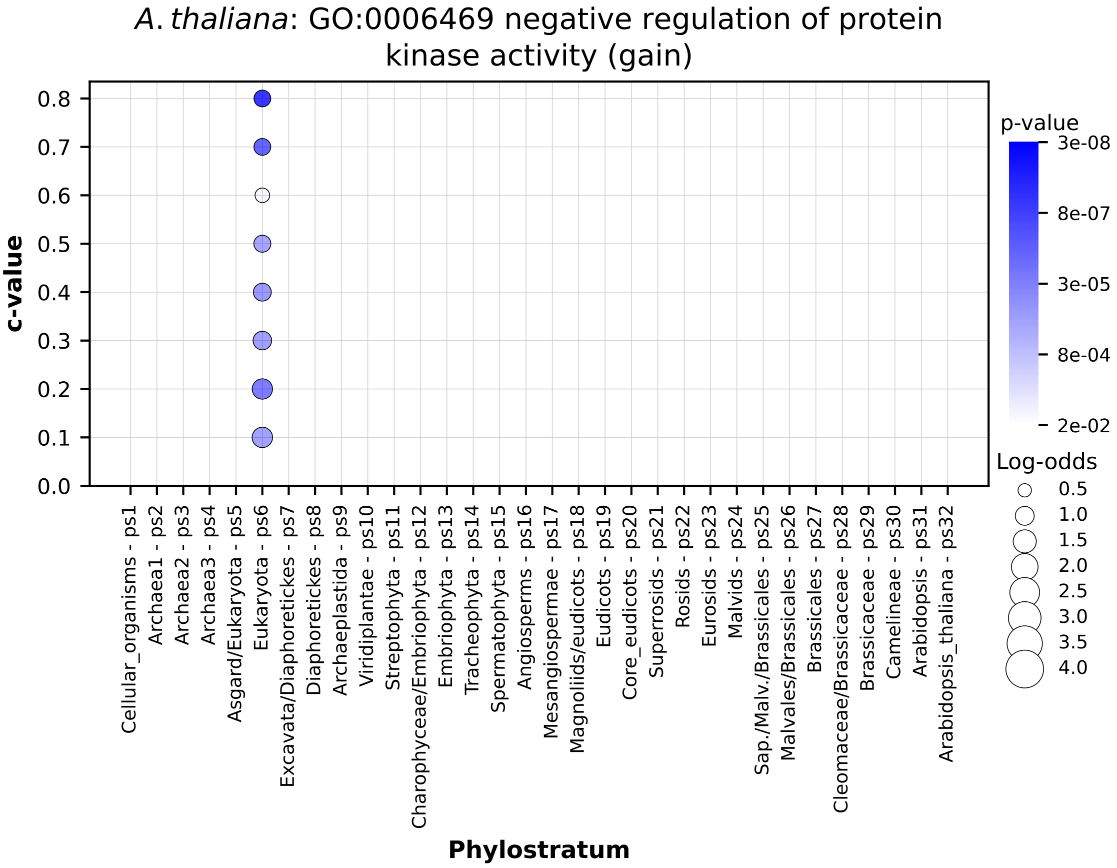

A. thaliana: GO:0006469 negative regulation of protein kinase activity (gain)
c-value
0.8
0.7
0.6
0.5
0.4
0.3
0.2
0.1
0.0
Phylostratum
Cellular_organisms - ps1
Archaea1 - ps2
Archaea2 - ps3
Archaea3 - ps4
Asgard/Eukaryota - ps5
Eukaryota - ps6
Excavata/Diaphoretickes - ps7
Diaphoretickes - ps8
Archaeplastida - ps9
Viridiplantae - ps10
Streptophyta - ps11
Charophyceae/Embriophyta - ps12
Embriophyta - ps13
Tracheophyta - ps14
Spermatophyta - ps15
Angiosperms - ps16
Mesangiospermae - ps17
Magnoliids/eudicots - ps18
Eudicots - ps19
Core_eudicots - ps20
Superrosids - ps21
Rosids - ps22
Eurosids - ps23
Malvids - ps24
Sap./Malv./Brassicales - ps25
Malvales/Brassicales - ps26
Brassicales - ps27
Cleomaceae/Brassicaceae - ps28
Brassicaceae - ps29
Camelineae - ps30
Arabidopsis - ps31
Arabidopsis_thaliana - ps32
p-value
3e-08
8e-07
3e-05
8e-04
2e-02
Log-odds
0.5
1.0
1.5
2.0
2.5
3.0
3.5
4.0

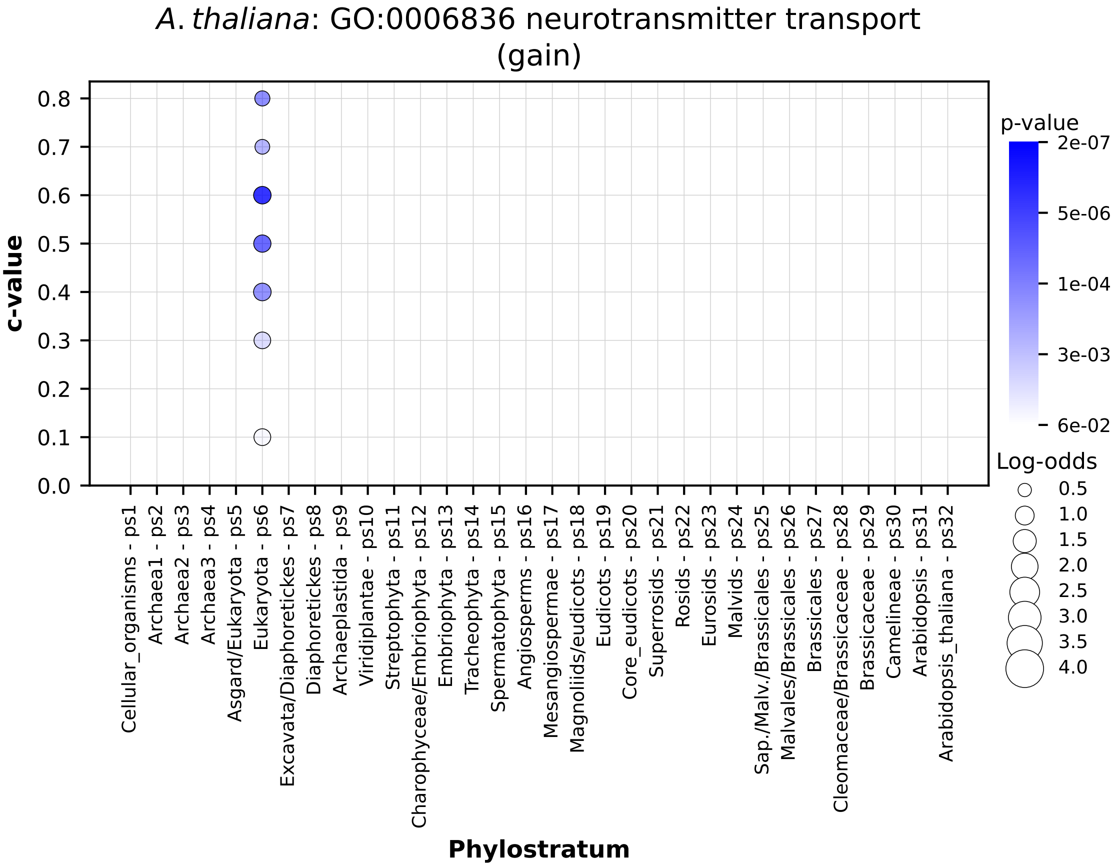
A. thaliana: GO:0006836 neurotransmitter transport
(gain)
c-value
0.0
0.1
0.2
0.3
0.4
0.5
0.6
0.7
0.8
p-value
2e-07
5e-06
1e-04
3e-03
6e-02
Log-odds
0.5
1.0
1.5
2.0
2.5
3.0
3.5
4.0
Cellular_organisms - ps1
Archaea1 - ps2
Archaea2 - ps3
Archaea3 - ps4
Asgard/Eukaryota - ps5
Eukaryota - ps6
Excavata/Diaphoretickes - ps7
Diaphoretickes - ps8
Archaeplastida - ps9
Viridiplantae - ps10
Streptophyta - ps11
Charophyceae/Embriophyta - ps12
Embriophyta - ps13
Tracheophyta - ps14
Spermatophyta - ps15
Angiosperms - ps16
Mesangiospermae - ps17
Magnoliids/eudicots - ps18
Eudicots - ps19
Core_eudicots - ps20
Superrosids - ps21
Rosids - ps22
Eurosids - ps23
Malvids - ps24
Sap./Malv./Brassicales - ps25
Malvales/Brassicales - ps26
Brassicales - ps27
Cleomaceae/Brassicaceae - ps28
Brassicaceae - ps29
Camelineae - ps30
Arabidopsis - ps31
Arabidopsis_thaliana - ps32
Phylostratum

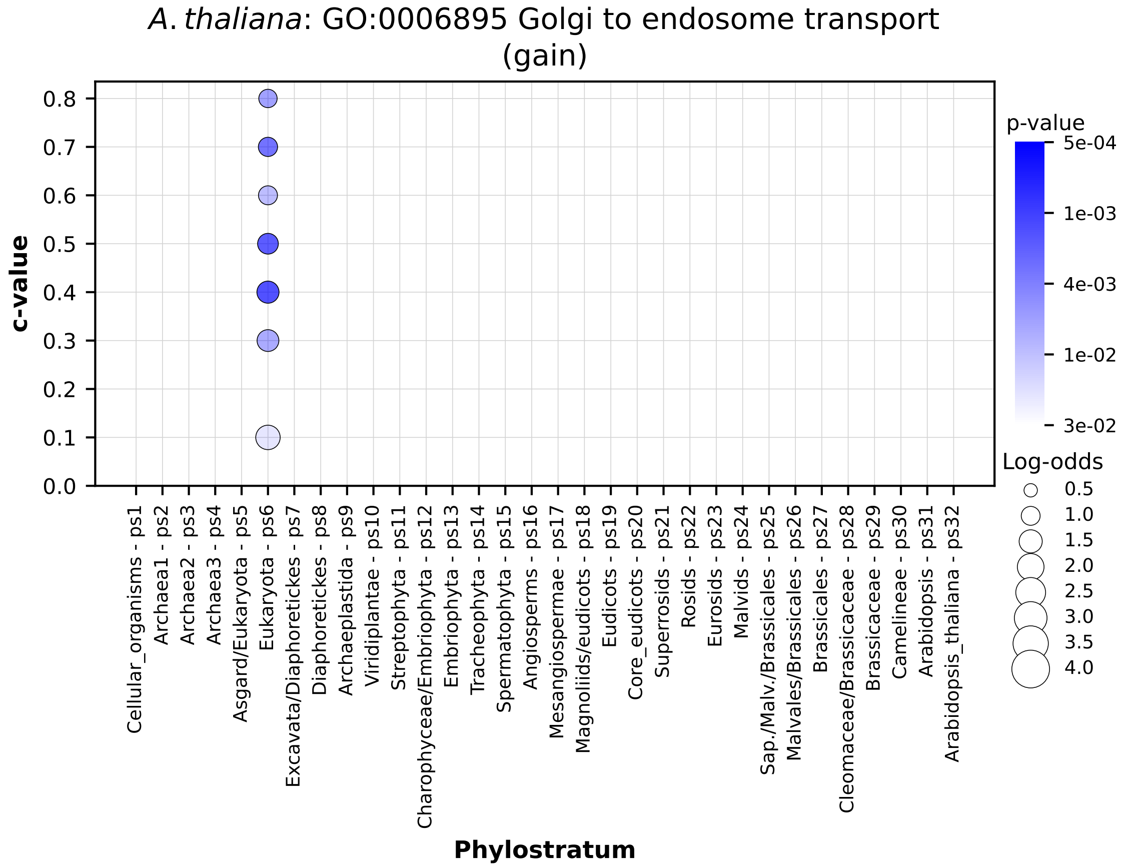
A. thaliana: GO:0006895 Golgi to endosome transport
(gain)
c-value
0.0
0.1
0.2
0.3
0.4
0.5
0.6
0.7
0.8
Phylostratum
Cellular_organisms - ps1
Archaea1 - ps2
Archaea2 - ps3
Archaea3 - ps4
Asgard/Eukaryota - ps5
Eukaryota - ps6
Excavata/Diaphoretickes - ps7
Diaphoretickes - ps8
Archaeplastida - ps9
Viridiplantae - ps10
Streptophyta - ps11
Charophyceae/Embriophyta - ps12
Embriophyta - ps13
Tracheophyta - ps14
Spermatophyta - ps15
Angiosperms - ps16
Mesangiospermae - ps17
Magnoliids/eudicots - ps18
Eudicots - ps19
Core_eudicots - ps20
Superrosids - ps21
Rosids - ps22
Eurosids - ps23
Malvids - ps24
Sap./Malv./Brassicales - ps25
Malvales/Brassicales - ps26
Brassicales - ps27
Cleomaceae/Brassicaceae - ps28
Brassicaceae - ps29
Camelineae - ps30
Arabidopsis - ps31
Arabidopsis_thaliana - ps32
p-value
5e-04
1e-03
4e-03
1e-02
3e-02
Log-odds
0.5
1.0
1.5
2.0
2.5
3.0
3.5
4.0

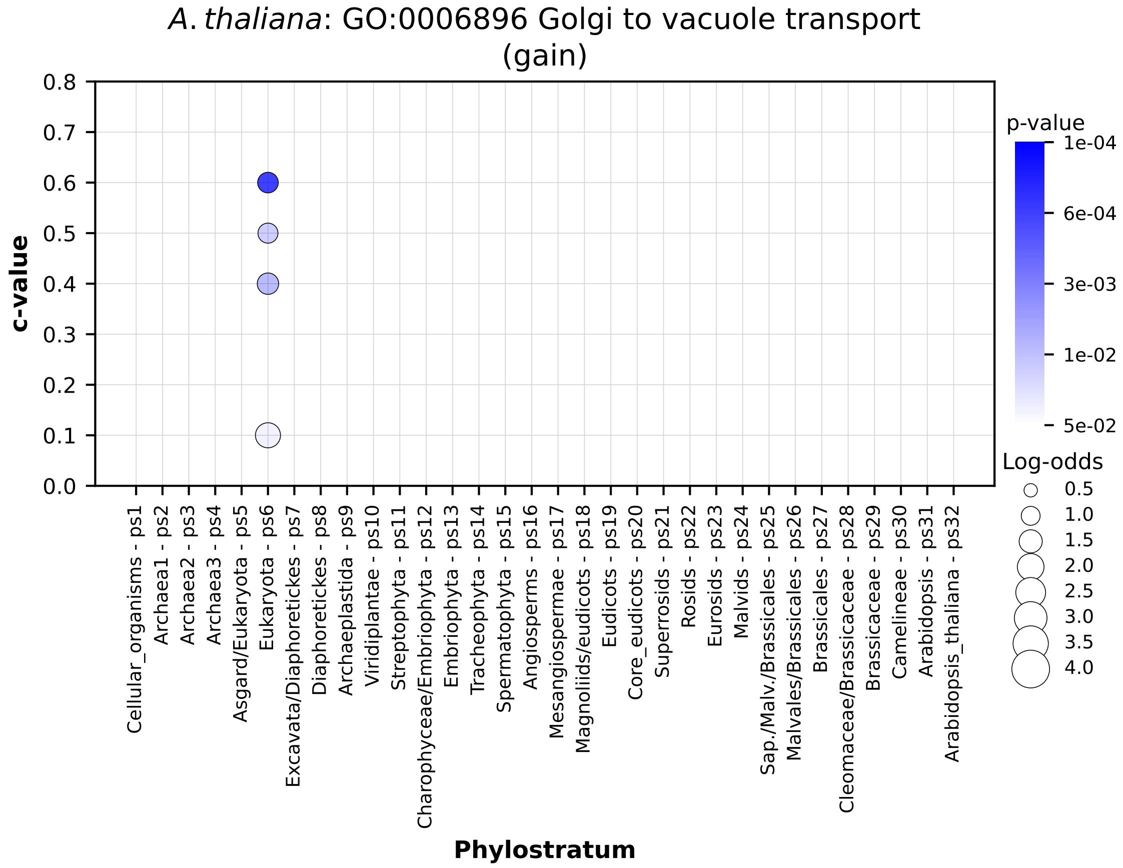

A. thaliana: GO:0006896 Golgi to vacuole transport
(gain)
c-value
0.8
0.7
0.6
0.5
0.4
0.3
0.2
0.1
0.0
Phylostratum
Cellular_organisms - ps1
Archaea1 - ps2
Archaea2 - ps3
Archaea3 - ps4
Asgard/Eukaryota - ps5
Eukaryota - ps6
Excavata/Diaphoretickes - ps7
Diaphoretickes - ps8
Archaeplastida - ps9
Viridiplantae - ps10
Streptophyta - ps11
Charophyceae/Embriophyta - ps12
Embriophyta - ps13
Tracheophyta - ps14
Spermatophyta - ps15
Angiosperms - ps16
Mesangiospermae - ps17
Magnoliids/eudicots - ps18
Eudicots - ps19
Core_eudicots - ps20
Superrosids - ps21
Rosids - ps22
Eurosids - ps23
Malvids - ps24
Sap./Malv./Brassicales - ps25
Malvales/Brassicales - ps26
Brassicales - ps27
Cleomaceae/Brassicaceae - ps28
Brassicaceae - ps29
Camelineae - ps30
Arabidopsis - ps31
Arabidopsis_thaliana - ps32
p-value
1e-04
6e-04
3e-03
1e-02
5e-02
Log-odds
0.5
1.0
1.5
2.0
2.5
3.0
3.5
4.0

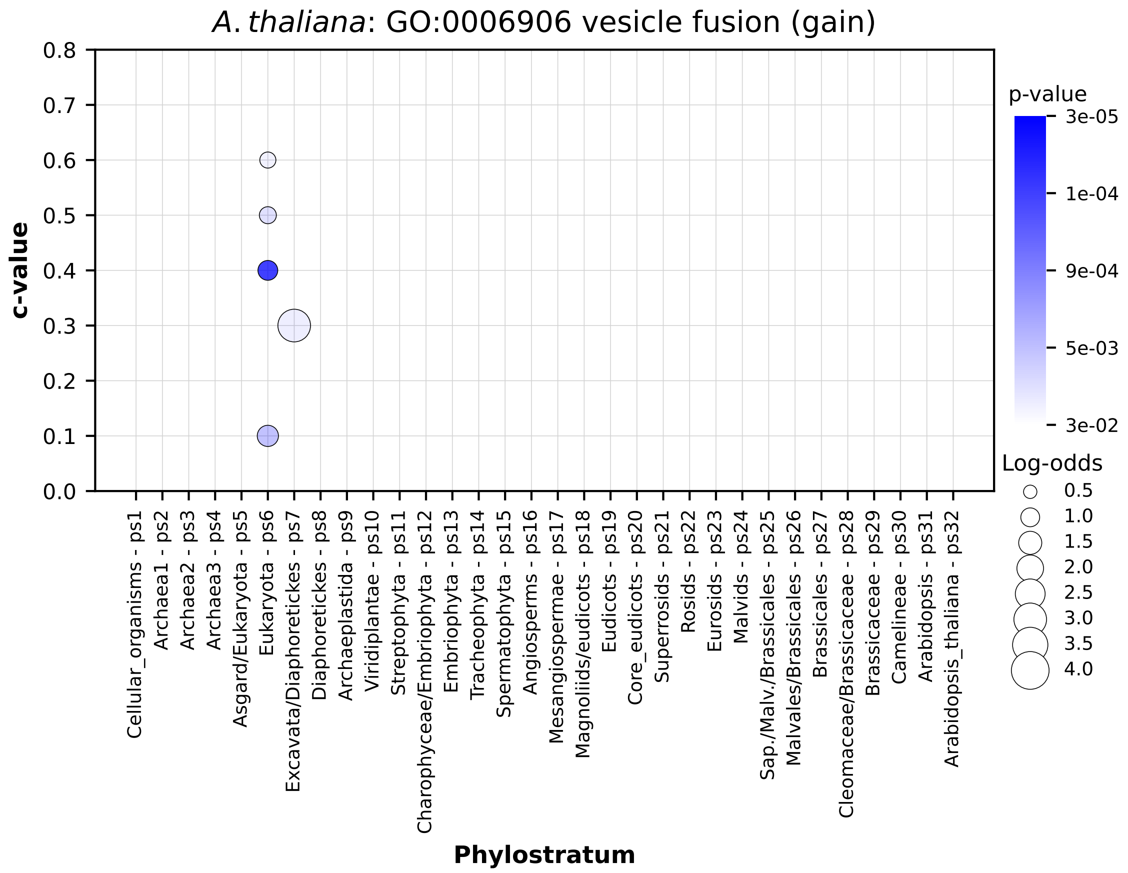
A. thaliana: GO:0006906 vesicle fusion (gain)
c-value
0.0
0.1
0.2
0.3
0.4
0.5
0.6
0.7
0.8
p-value
3e-05
1e-04
9e-04
5e-03
3e-02
Log-odds
0.5
1.0
1.5
2.0
2.5
3.0
3.5
4.0
Cellular_organisms - ps1
Archaea1 - ps2
Archaea2 - ps3
Archaea3 - ps4
Asgard/Eukaryota - ps5
Eukaryota - ps6
Excavata/Diaphoretickes - ps7
Diaphoretickes - ps8
Archaeplastida - ps9
Viridiplantae - ps10
Streptophyta - ps11
Charophyceae/Embriophyta - ps12
Embriophyta - ps13
Tracheophyta - ps14
Spermatophyta - ps15
Angiosperms - ps16
Mesangiospermae - ps17
Magnoliids/eudicots - ps18
Eudicots - ps19
Core_eudicots - ps20
Superrosids - ps21
Rosids - ps22
Eurosids - ps23
Malvids - ps24
Sap./Malv./Brassicales - ps25
Malvales/Brassicales - ps26
Brassicales - ps27
Cleomaceae/Brassicaceae - ps28
Brassicaceae - ps29
Camelineae - ps30
Arabidopsis - ps31
Arabidopsis_thaliana - ps32
Phylostratum

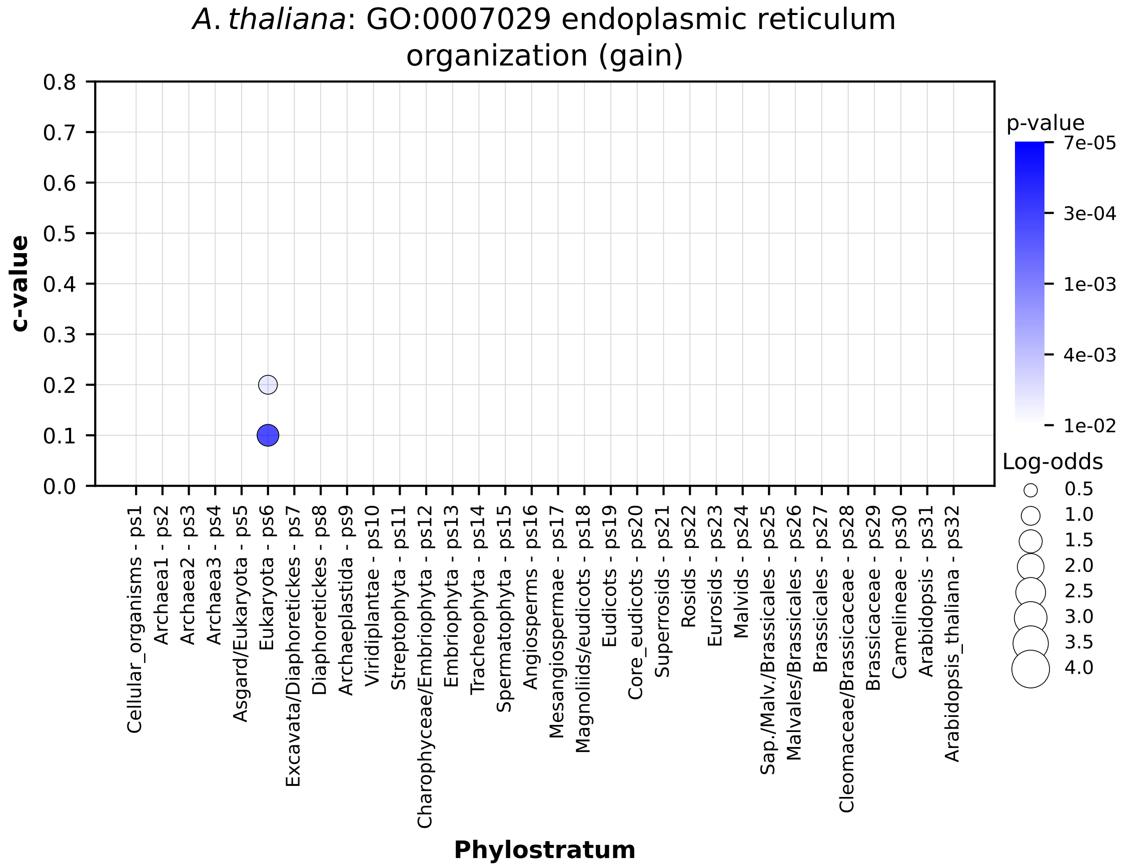

A. thaliana: GO:0007029 endoplasmic reticulum organization (gain)
c-value
0.0
0.1
0.2
0.3
0.4
0.5
0.6
0.7
0.8
p-value
7e-05
3e-04
1e-03
4e-03
1e-02
Log-odds
0.5
1.0
1.5
2.0
2.5
3.0
3.5
4.0
Cellular_organisms - ps1
Archaea1 - ps2
Archaea2 - ps3
Archaea3 - ps4
Asgard/Eukaryota - ps5
Eukaryota - ps6
Excavata/Diaphoretickes - ps7
Diaphoretickes - ps8
Archaeplastida - ps9
Viridiplantae - ps10
Streptophyta - ps11
Charophyceae/Embriophyta - ps12
Embriophyta - ps13
Tracheophyta - ps14
Spermatophyta - ps15
Angiosperms - ps16
Mesangiospermae - ps17
Magnoliids/eudicots - ps18
Eudicots - ps19
Core_eudicots - ps20
Superrosids - ps21
Rosids - ps22
Eurosids - ps23
Malvids - ps24
Sap./Malv./Brassicales - ps25
Malvales/Brassicales - ps26
Brassicales - ps27
Cleomaceae/Brassicaceae - ps28
Brassicaceae - ps29
Camelineae - ps30
Arabidopsis - ps31
Arabidopsis_thaliana - ps32
Phylostratum

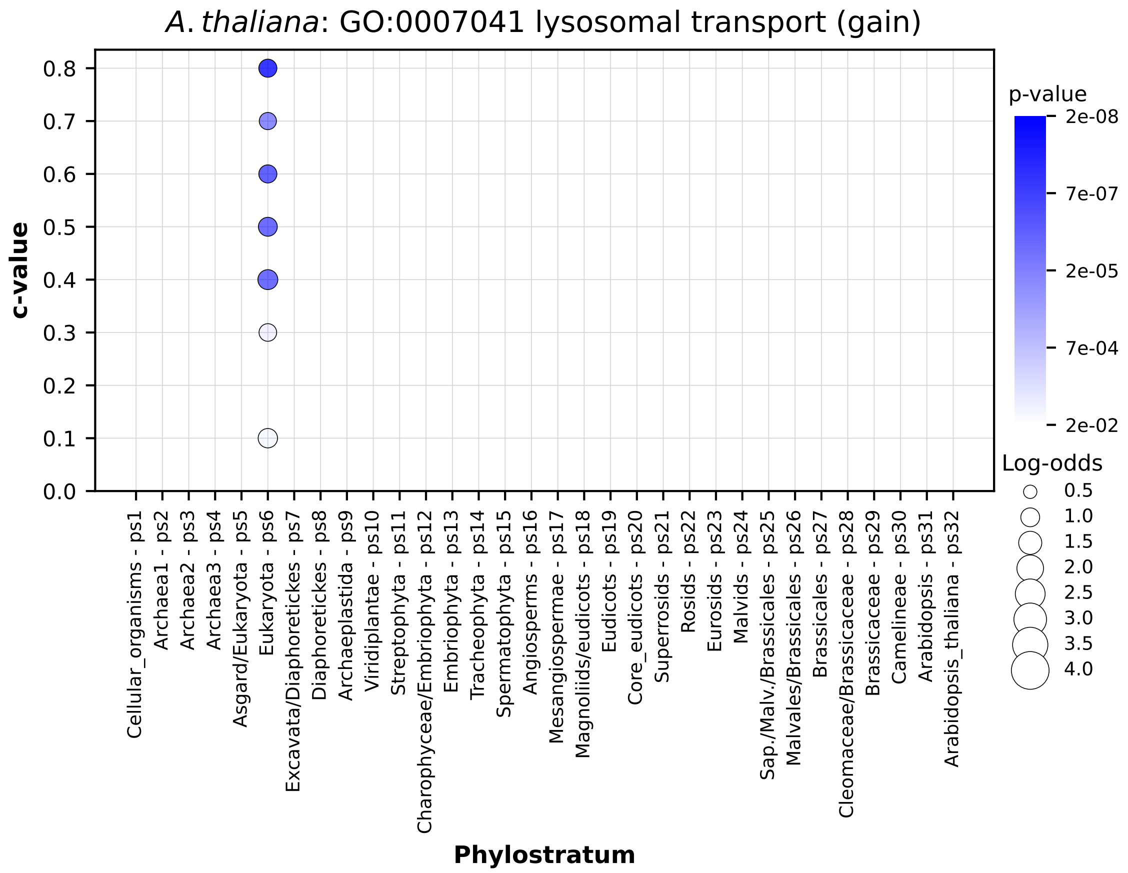

A. thaliana: GO:0007041 lysosomal transport (gain)
c-value
0.0
0.1
0.2
0.3
0.4
0.5
0.6
0.7
0.8
Phylostratum
Cellular_organisms - ps1
Archaea1 - ps2
Archaea2 - ps3
Archaea3 - ps4
Asgard/Eukaryota - ps5
Eukaryota - ps6
Excavata/Diaphoretickes - ps7
Diaphoretickes - ps8
Archaeplastida - ps9
Viridiplantae - ps10
Streptophyta - ps11
Charophyceae/Embriophyta - ps12
Embriophyta - ps13
Tracheophyta - ps14
Spermatophyta - ps15
Angiosperms - ps16
Mesangiospermae - ps17
Magnoliids/eudicots - ps18
Eudicots - ps19
Core_eudicots - ps20
Superrosids - ps21
Rosids - ps22
Eurosids - ps23
Malvids - ps24
Sap./Malv./Brassicales - ps25
Malvales/Brassicales - ps26
Brassicales - ps27
Cleomaceae/Brassicaceae - ps28
Brassicaceae - ps29
Camelineae - ps30
Arabidopsis - ps31
Arabidopsis_thaliana - ps32
p-value
2e-08
7e-07
2e-05
7e-04
2e-02
Log-odds
0.5
1.0
1.5
2.0
2.5
3.0
3.5
4.0

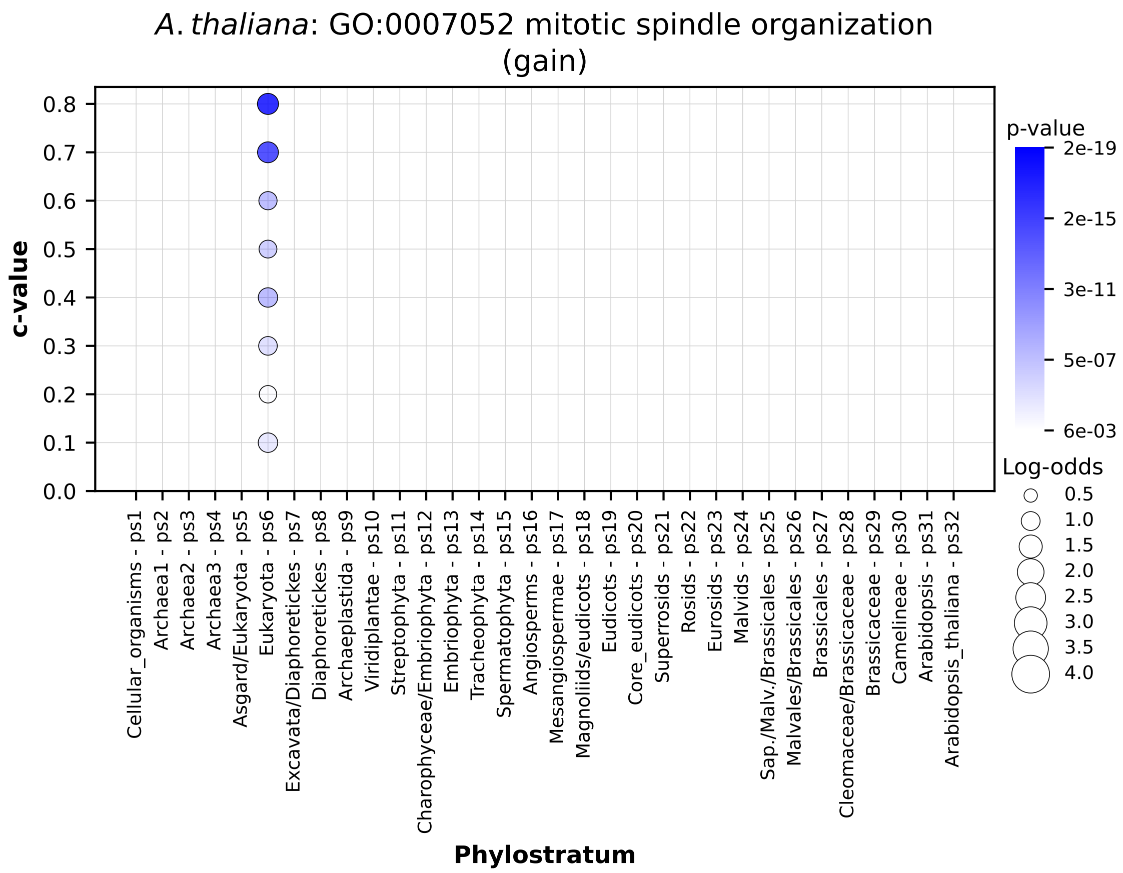
A. thaliana: GO:0007052 mitotic spindle organization (gain)
c-value
0.0
0.1
0.2
0.3
0.4
0.5
0.6
0.7
0.8
Phylostratum
Cellular_organisms - ps1
Archaea1 - ps2
Archaea2 - ps3
Archaea3 - ps4
Asgard/Eukaryota - ps5
Eukaryota - ps6
Excavata/Diaphoretickes - ps7
Diaphoretickes - ps8
Archaeplastida - ps9
Viridiplantae - ps10
Streptophyta - ps11
Charophyceae/Embriophyta - ps12
Embriophyta - ps13
Tracheophyta - ps14
Spermatophyta - ps15
Angiosperms - ps16
Mesangiospermae - ps17
Magnoliids/eudicots - ps18
Eudicots - ps19
Core_eudicots - ps20
Superrosids - ps21
Rosids - ps22
Eurosids - ps23
Malvids - ps24
Sap./Malv./Brassicales - ps25
Malvales/Brassicales - ps26
Brassicales - ps27
Cleomaceae/Brassicaceae - ps28
Brassicaceae - ps29
Camelineae - ps30
Arabidopsis - ps31
Arabidopsis_thaliana - ps32
p-value
2e-19
2e-15
3e-11
5e-07
6e-03
Log-odds
0.5
1.0
1.5
2.0
2.5
3.0
3.5
4.0

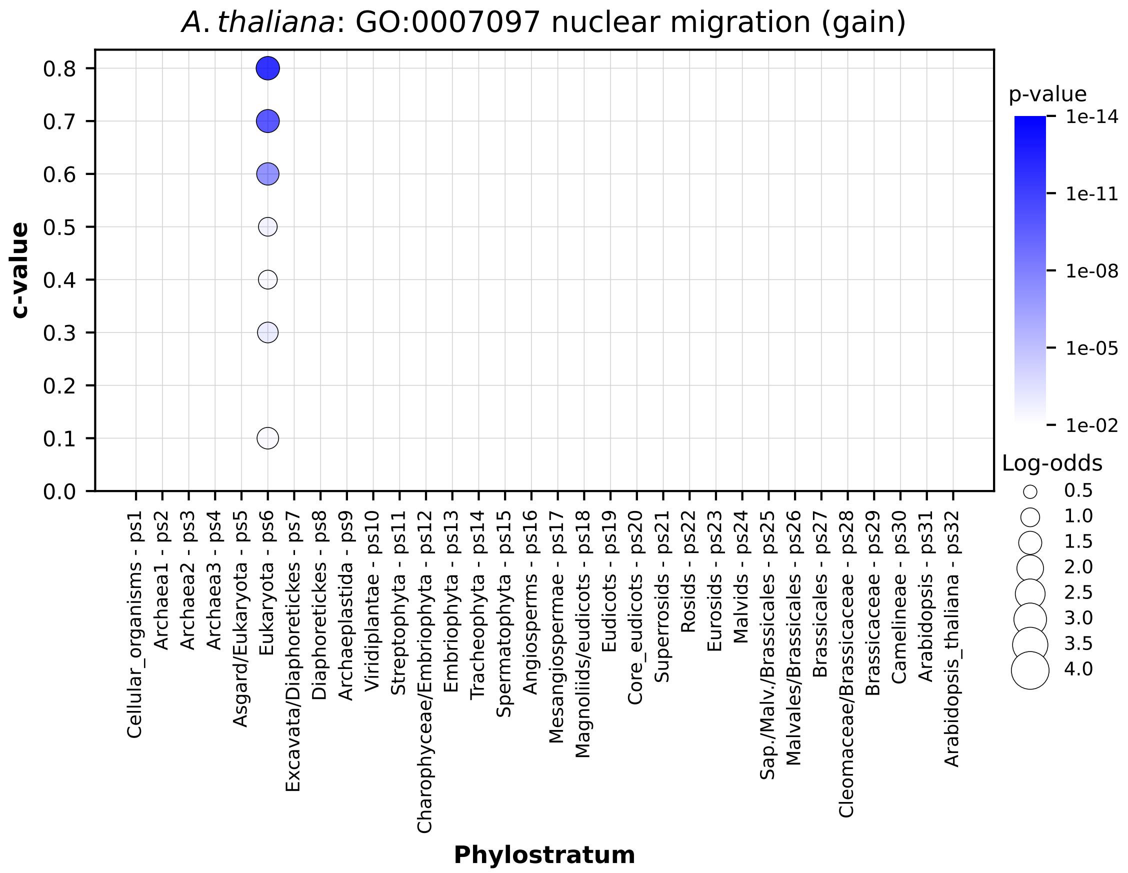

A. thaliana: GO:0007097 nuclear migration (gain)
c-value
0.0
0.1
0.2
0.3
0.4
0.5
0.6
0.7
0.8
Phylostratum
Cellular_organisms - ps1
Archaea1 - ps2
Archaea2 - ps3
Archaea3 - ps4
Asgard/Eukaryota - ps5
Eukaryota - ps6
Excavata/Diaphoretickes - ps7
Diaphoretickes - ps8
Archaeplastida - ps9
Viridiplantae - ps10
Streptophyta - ps11
Charophyceae/Embriophyta - ps12
Embriophyta - ps13
Tracheophyta - ps14
Spermatophyta - ps15
Angiosperms - ps16
Mesangiospermae - ps17
Magnoliids/eudicots - ps18
Eudicots - ps19
Core_eudicots - ps20
Superrosids - ps21
Rosids - ps22
Eurosids - ps23
Malvids - ps24
Sap./Malv./Brassicales - ps25
Malvales/Brassicales - ps26
Brassicales - ps27
Cleomaceae/Brassicaceae - ps28
Brassicaceae - ps29
Camelineae - ps30
Arabidopsis - ps31
Arabidopsis_thaliana - ps32
p-value
1e-14
1e-11
1e-08
1e-05
1e-02
Log-odds
0.5
1.0
1.5
2.0
2.5
3.0
3.5
4.0

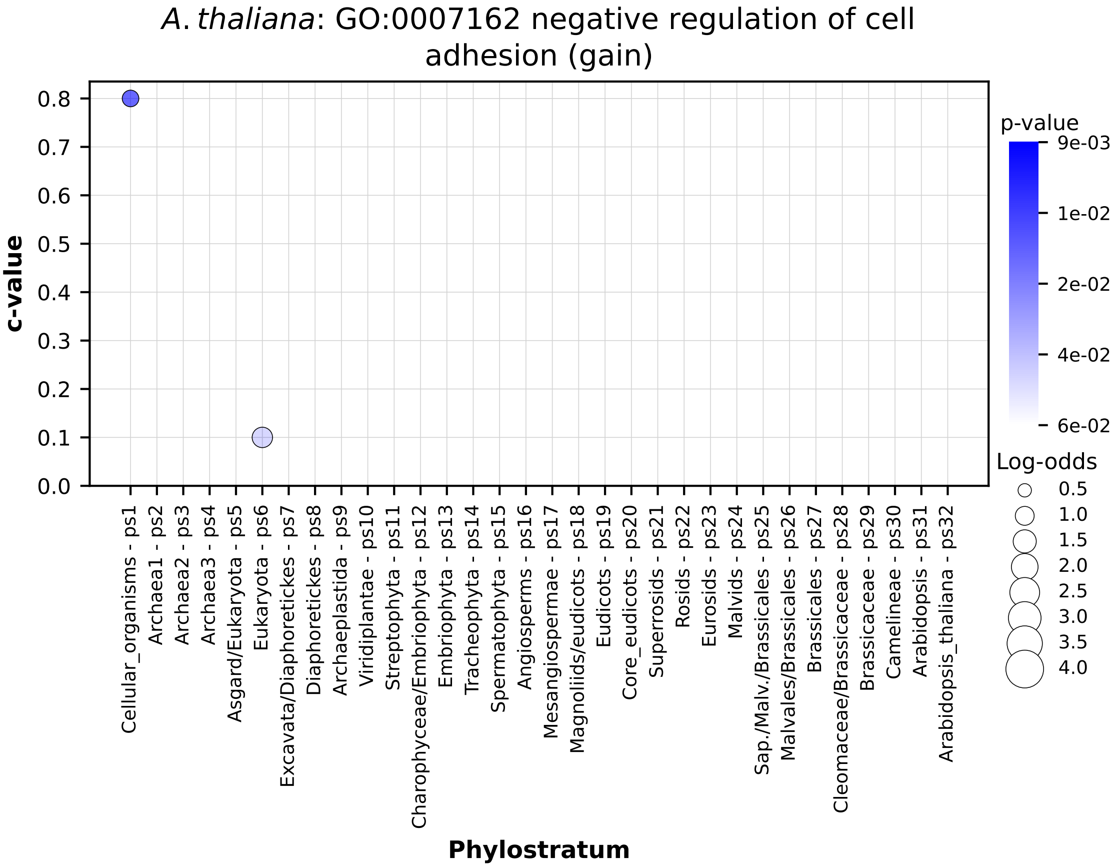

A. thaliana: GO:0007162 negative regulation of cell adhesion (gain)
c-value
0.0
0.1
0.2
0.3
0.4
0.5
0.6
0.7
0.8
Phylostratum
Cellular_organisms - ps1
Archaea1 - ps2
Archaea2 - ps3
Archaea3 - ps4
Asgard/Eukaryota - ps5
Eukaryota - ps6
Excavata/Diaphoretickes - ps7
Diaphoretickes - ps8
Archaeplastida - ps9
Viridiplantae - ps10
Streptophyta - ps11
Charophyceae/Embriophyta - ps12
Embriophyta - ps13
Tracheophyta - ps14
Spermatophyta - ps15
Angiosperms - ps16
Mesangiospermae - ps17
Magnoliids/eudicots - ps18
Eudicots - ps19
Core_eudicots - ps20
Superrosids - ps21
Rosids - ps22
Eurosids - ps23
Malvids - ps24
Sap./Malv./Brassicales - ps25
Malvales/Brassicales - ps26
Brassicales - ps27
Cleomaceae/Brassicaceae - ps28
Brassicaceae - ps29
Camelineae - ps30
Arabidopsis - ps31
Arabidopsis_thaliana - ps32
p-value
9e-03
1e-02
2e-02
4e-02
6e-02
Log-odds
0.5
1.0
1.5
2.0
2.5
3.0
3.5
4.0

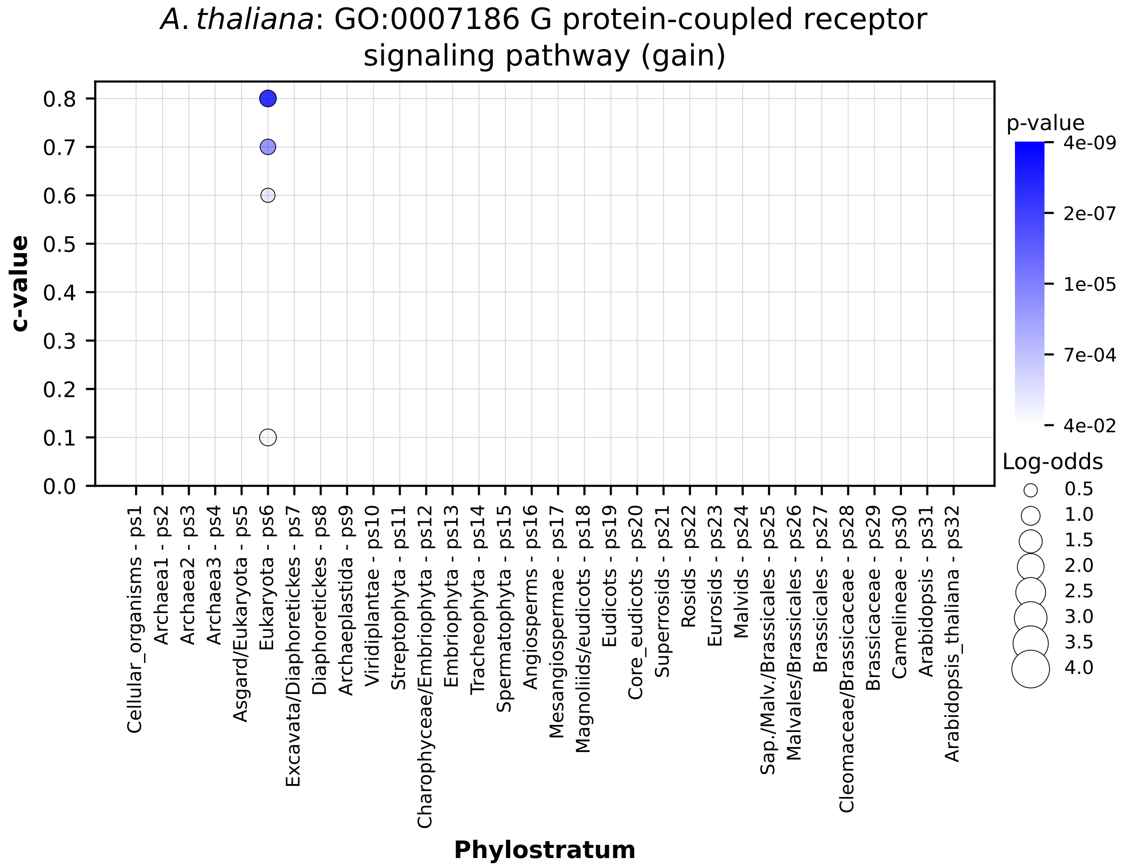

A. thaliana: GO:0007186 G protein-coupled receptor signaling pathway (gain)
c-value
0.0
0.1
0.2
0.3
0.4
0.5
0.6
0.7
0.8
Phylostratum
Cellular_organisms - ps1
Archaea1 - ps2
Archaea2 - ps3
Archaea3 - ps4
Asgard/Eukaryota - ps5
Eukaryota - ps6
Excavata/Diaphoretickes - ps7
Diaphoretickes - ps8
Archaeplastida - ps9
Viridiplantae - ps10
Streptophyta - ps11
Charophyceae/Embriophyta - ps12
Embriophyta - ps13
Tracheophyta - ps14
Spermatophyta - ps15
Angiosperms - ps16
Mesangiospermae - ps17
Magnoliids/eudicots - ps18
Eudicots - ps19
Core_eudicots - ps20
Superrosids - ps21
Rosids - ps22
Eurosids - ps23
Malvids - ps24
Sap./Malv./Brassicales - ps25
Malvales/Brassicales - ps26
Brassicales - ps27
Cleomaceae/Brassicaceae - ps28
Brassicaceae - ps29
Camelineae - ps30
Arabidopsis - ps31
Arabidopsis_thaliana - ps32
p-value
4e-09
2e-07
1e-05
7e-04
4e-02
Log-odds
0.5
1.0
1.5
2.0
2.5
3.0
3.5
4.0

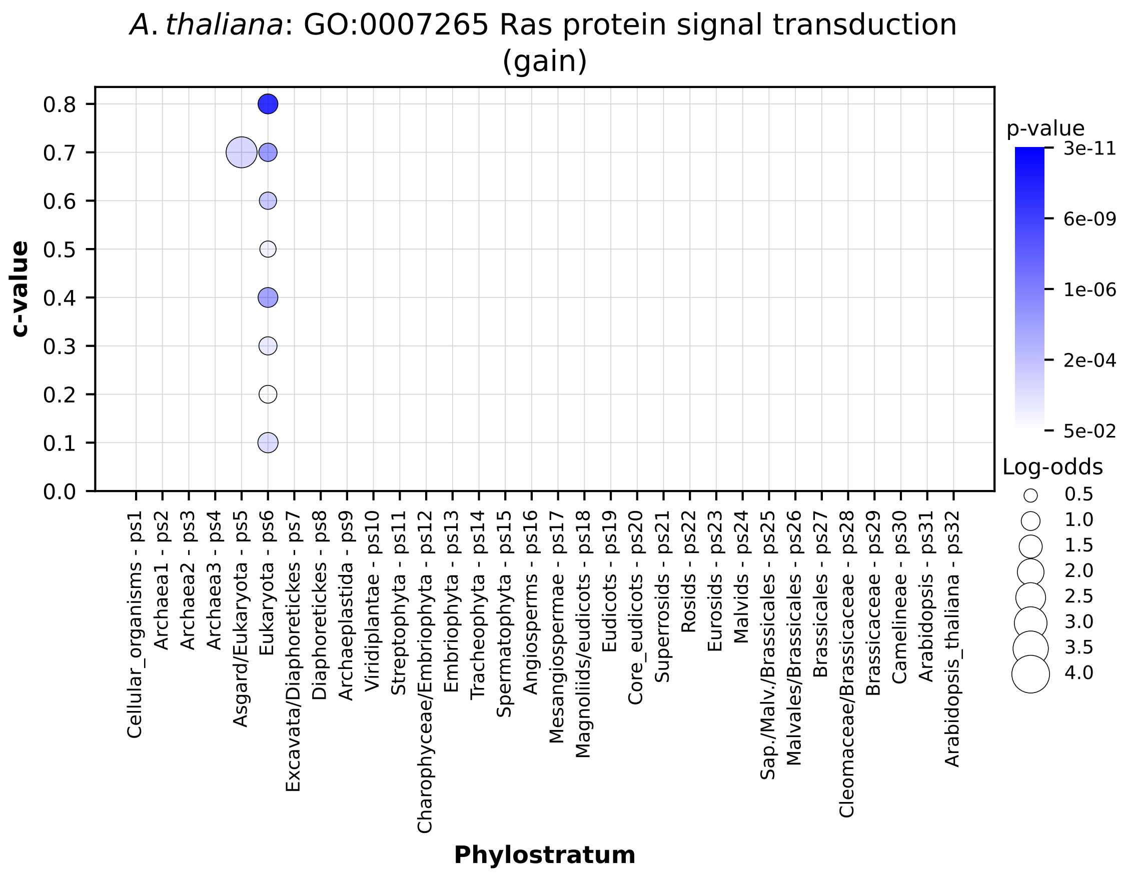

A. thaliana: GO:0007265 Ras protein signal transduction (gain)
c-value
0.0
0.1
0.2
0.3
0.4
0.5
0.6
0.7
0.8
Phylostratum
Cellular_organisms - ps1
Archaea1 - ps2
Archaea2 - ps3
Archaea3 - ps4
Asgard/Eukaryota - ps5
Eukaryota - ps6
Excavata/Diaphoretickes - ps7
Diaphoretickes - ps8
Archaeplastida - ps9
Viridiplantae - ps10
Streptophyta - ps11
Charophyceae/Embriophyta - ps12
Embriophyta - ps13
Tracheophyta - ps14
Spermatophyta - ps15
Angiosperms - ps16
Mesangiospermae - ps17
Magnoliids/eudicots - ps18
Eudicots - ps19
Core_eudicots - ps20
Superrosids - ps21
Rosids - ps22
Eurosids - ps23
Malvids - ps24
Sap./Malv./Brassicales - ps25
Malvales/Brassicales - ps26
Brassicales - ps27
Cleomaceae/Brassicaceae - ps28
Brassicaceae - ps29
Camelineae - ps30
Arabidopsis - ps31
Arabidopsis_thaliana - ps32
p-value
3e-11
6e-09
1e-06
2e-04
5e-02
Log-odds
0.5
1.0
1.5
2.0
2.5
3.0
3.5
4.0

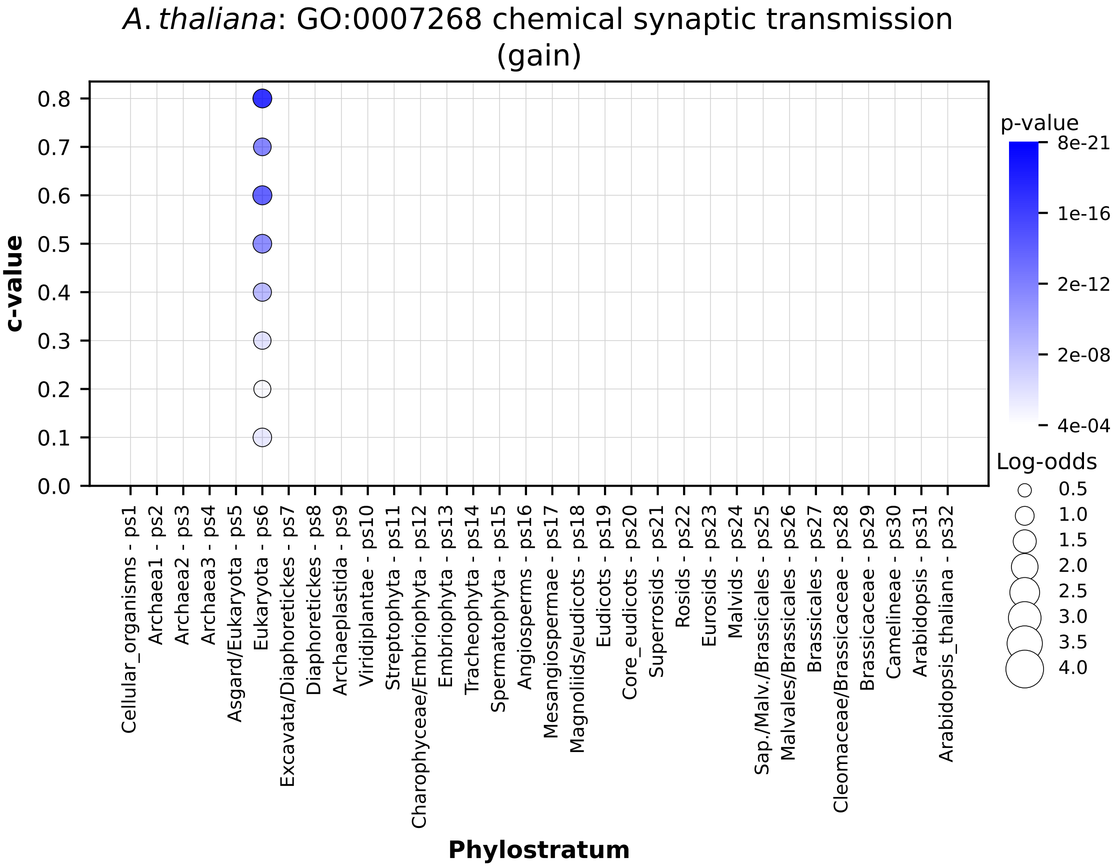

A. thaliana: GO:0007268 chemical synaptic transmission (gain)
c-value
0.0
0.1
0.2
0.3
0.4
0.5
0.6
0.7
0.8
p-value
8e-21
1e-16
2e-12
2e-08
4e-04
Log-odds
0.5
1.0
1.5
2.0
2.5
3.0
3.5
4.0
Cellular_organisms - ps1
Archaea1 - ps2
Archaea2 - ps3
Archaea3 - ps4
Asgard/Eukaryota - ps5
Eukaryota - ps6
Excavata/Diaphoretickes - ps7
Diaphoretickes - ps8
Archaeplastida - ps9
Viridiplantae - ps10
Streptophyta - ps11
Charophyceae/Embriophyta - ps12
Embriophyta - ps13
Tracheophyta - ps14
Spermatophyta - ps15
Angiosperms - ps16
Mesangiospermae - ps17
Magnoliids/eudicots - ps18
Eudicots - ps19
Core_eudicots - ps20
Superrosids - ps21
Rosids - ps22
Eurosids - ps23
Malvids - ps24
Sap./Malv./Brassicales - ps25
Malvales/Brassicales - ps26
Brassicales - ps27
Cleomaceae/Brassicaceae - ps28
Brassicaceae - ps29
Camelineae - ps30
Arabidopsis - ps31
Arabidopsis_thaliana - ps32
Phylostratum

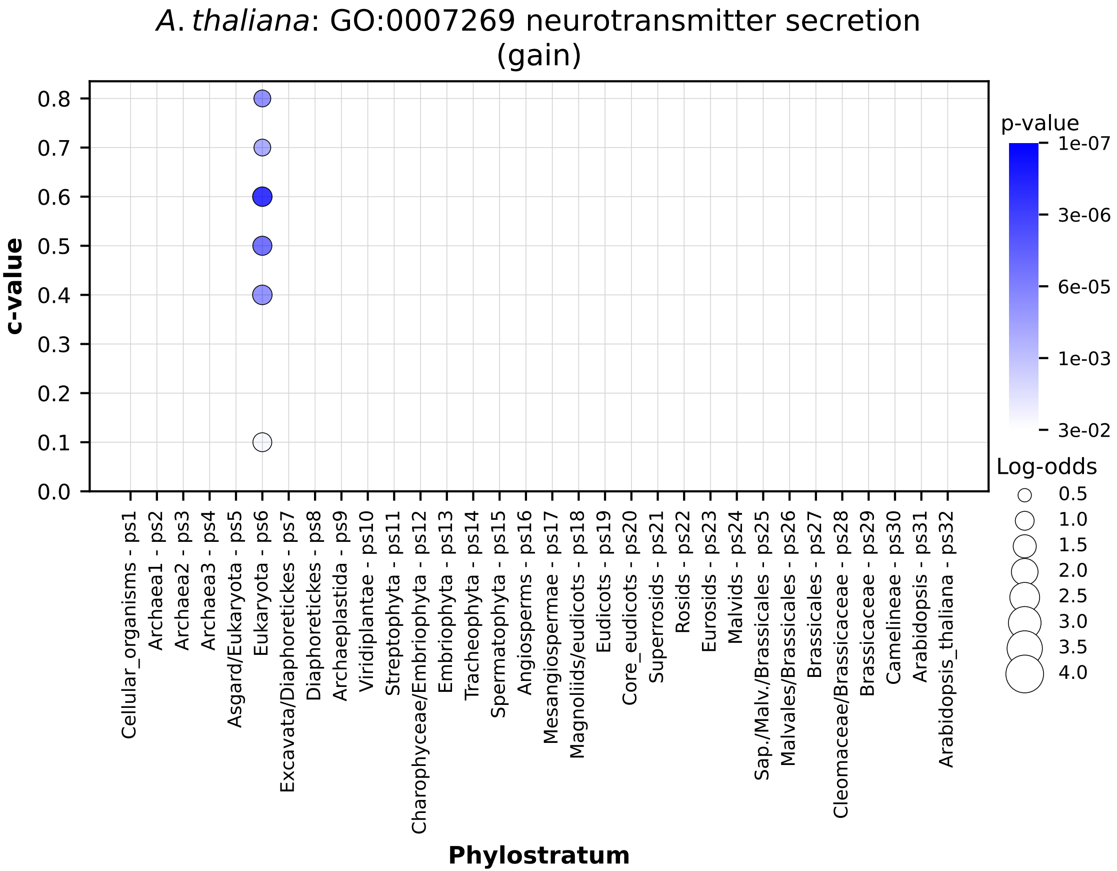

A. thaliana: GO:0007269 neurotransmitter secretion (gain)
c-value
0.8
0.7
0.6
0.5
0.4
0.3
0.2
0.1
0.0
p-value
1e-07
3e-06
6e-05
1e-03
3e-02
Log-odds
0.5
1.0
1.5
2.0
2.5
3.0
3.5
4.0
Cellular_organisms - ps1
Archaea1 - ps2
Archaea2 - ps3
Archaea3 - ps4
Asgard/Eukaryota - ps5
Eukaryota - ps6
Excavata/Diaphoretickes - ps7
Diaphoretickes - ps8
Archaeplastida - ps9
Viridiplantae - ps10
Streptophyta - ps11
Charophyceae/Embriophyta - ps12
Embriophyta - ps13
Tracheophyta - ps14
Spermatophyta - ps15
Angiosperms - ps16
Mesangiospermae - ps17
Magnoliids/eudicots - ps18
Eudicots - ps19
Core_eudicots - ps20
Superrosids - ps21
Rosids - ps22
Eurosids - ps23
Malvids - ps24
Sap./Malv./Brassicales - ps25
Malvales/Brassicales - ps26
Brassicales - ps27
Cleomaceae/Brassicaceae - ps28
Brassicaceae - ps29
Camelineae - ps30
Arabidopsis - ps31
Arabidopsis_thaliana - ps32
Phylostratum

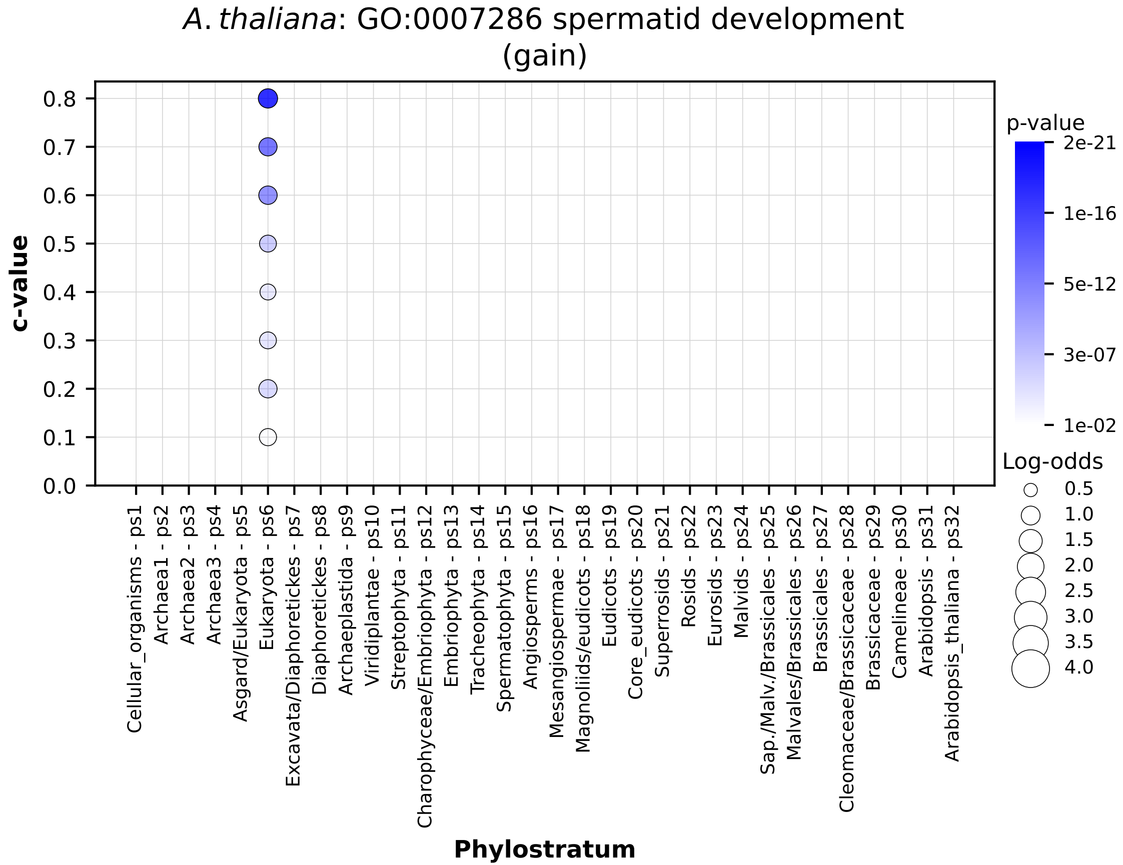

A. thaliana: GO:0007286 spermatid development
(gain)
c-value
0.0
0.1
0.2
0.3
0.4
0.5
0.6
0.7
0.8
Phylostratum
Cellular_organisms - ps1
Archaea1 - ps2
Archaea2 - ps3
Archaea3 - ps4
Asgard/Eukaryota - ps5
Eukaryota - ps6
Excavata/Diaphoretickes - ps7
Diaphoretickes - ps8
Archaeplastida - ps9
Viridiplantae - ps10
Streptophyta - ps11
Charophyceae/Embriophyta - ps12
Embriophyta - ps13
Tracheophyta - ps14
Spermatophyta - ps15
Angiosperms - ps16
Mesangiospermae - ps17
Magnoliids/eudicots - ps18
Eudicots - ps19
Core_eudicots - ps20
Superrosids - ps21
Rosids - ps22
Eurosids - ps23
Malvids - ps24
Sap./Malv./Brassicales - ps25
Malvales/Brassicales - ps26
Brassicales - ps27
Cleomaceae/Brassicaceae - ps28
Brassicaceae - ps29
Camelineae - ps30
Arabidopsis - ps31
Arabidopsis_thaliana - ps32
p-value
2e-21
1e-16
5e-12
3e-07
1e-02
Log-odds
0.5
1.0
1.5
2.0
2.5
3.0
3.5
4.0

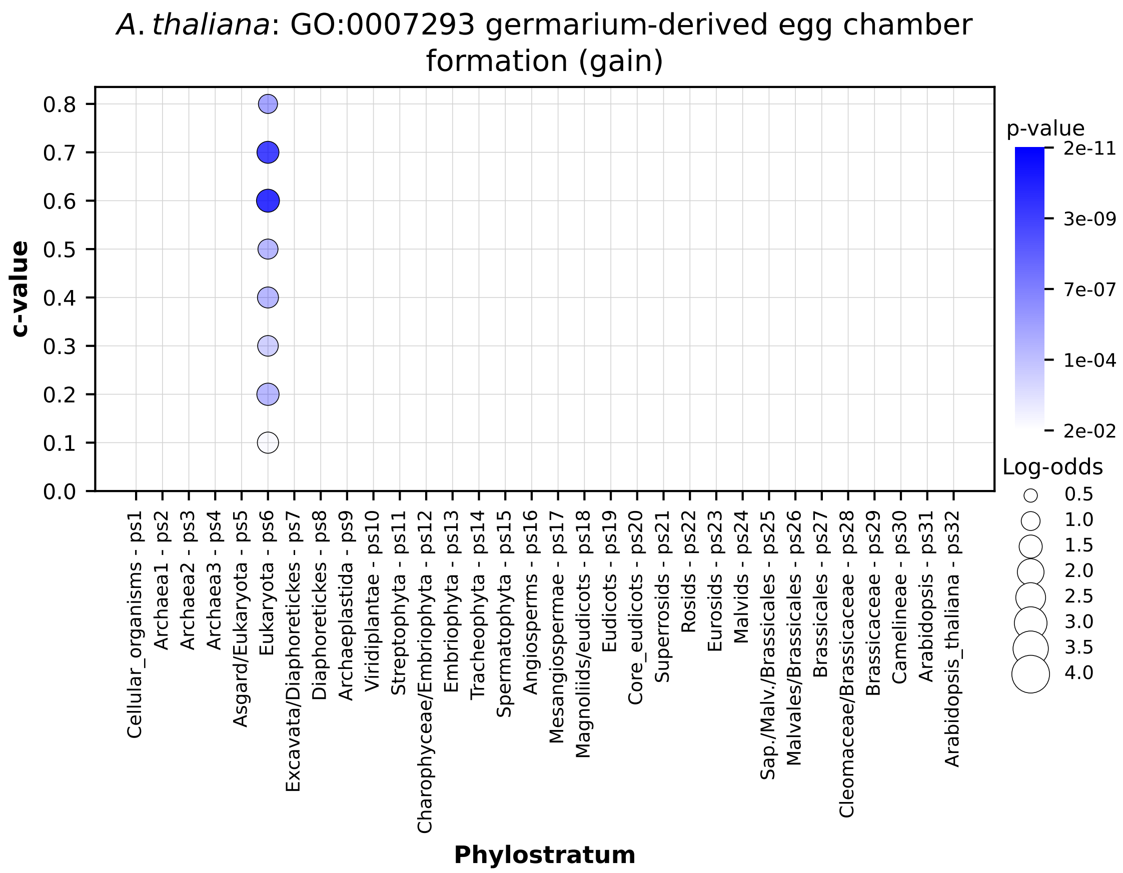

A. thaliana: GO:0007293 germarium-derived egg chamber formation (gain)
c-value
0.0
0.1
0.2
0.3
0.4
0.5
0.6
0.7
0.8
Phylostratum
Cellular_organisms - ps1
Archaea1 - ps2
Archaea2 - ps3
Archaea3 - ps4
Asgard/Eukaryota - ps5
Eukaryota - ps6
Excavata/Diaphoretickes - ps7
Diaphoretickes - ps8
Archaeplastida - ps9
Viridiplantae - ps10
Streptophyta - ps11
Charophyceae/Embriophyta - ps12
Embriophyta - ps13
Tracheophyta - ps14
Spermatophyta - ps15
Angiosperms - ps16
Mesangiospermae - ps17
Magnoliids/eudicots - ps18
Eudicots - ps19
Core_eudicots - ps20
Superrosids - ps21
Rosids - ps22
Eurosids - ps23
Malvids - ps24
Sap./Malv./Brassicales - ps25
Malvales/Brassicales - ps26
Brassicales - ps27
Cleomaceae/Brassicaceae - ps28
Brassicaceae - ps29
Camelineae - ps30
Arabidopsis - ps31
Arabidopsis_thaliana - ps32
p-value
2e-11
3e-09
7e-07
1e-04
2e-02
Log-odds
0.5
1.0
1.5
2.0
2.5
3.0
3.5
4.0

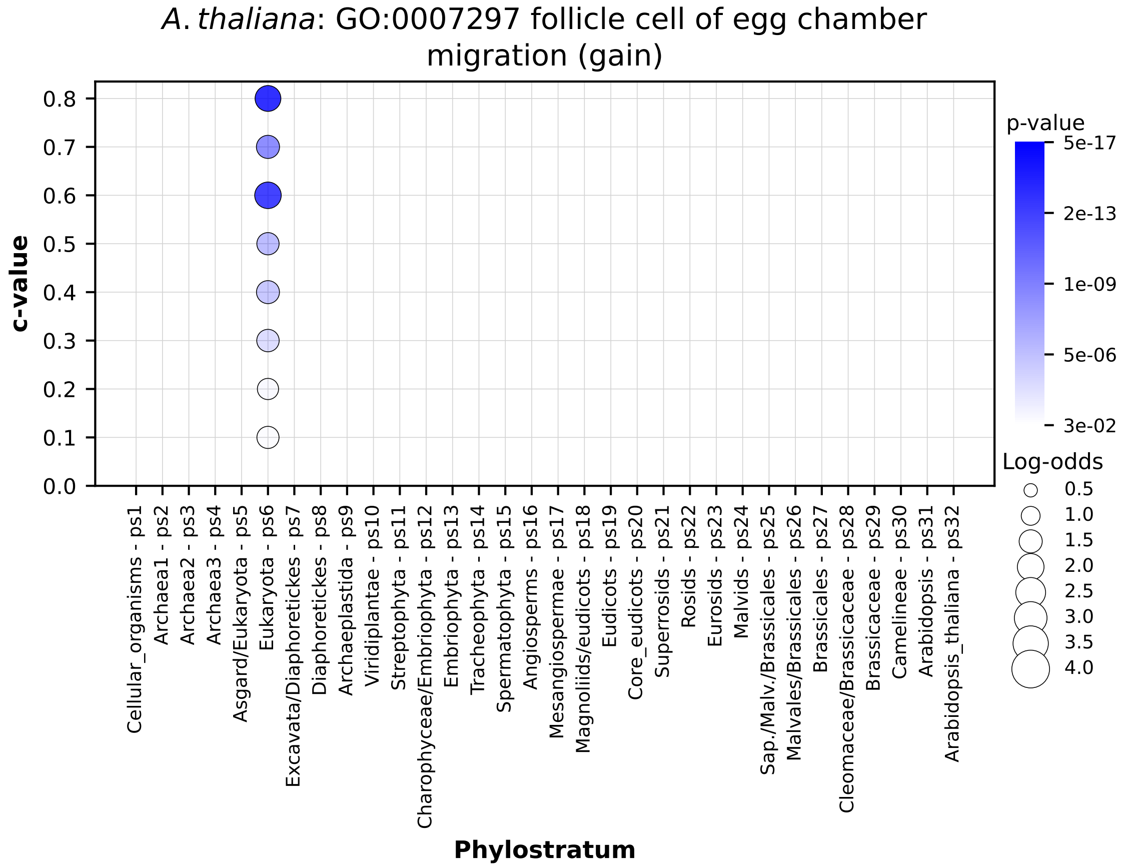
$A. thaliana$: GO:0007297 follicle cell of egg chamber migration (gain)
c-value
0.8
0.7
0.6
0.5
0.4
0.3
0.2
0.1
0.0
p-value
5e-17
2e-13
1e-09
5e-06
3e-02
Log-odds
0.5
1.0
1.5
2.0
2.5
3.0
3.5
4.0
Cellular_organisms - ps1
Archaea1 - ps2
Archaea2 - ps3
Archaea3 - ps4
Asgard/Eukaryota - ps5
Eukaryota - ps6
Excavata/Diaphoretickes - ps7
Diaphoretickes - ps8
Archaeplastida - ps9
Viridiplantae - ps10
Streptophyta - ps11
Charophyceae/Embriophyta - ps12
Embriophyta - ps13
Tracheophyta - ps14
Spermatophyta - ps15
Angiosperms - ps16
Mesangiospermae - ps17
Magnoliids/eudicots - ps18
Eudicots - ps19
Core_eudicots - ps20
Superrosids - ps21
Rosids - ps22
Eurosids - ps23
Malvids - ps24
Sap./Malv./Brassicales - ps25
Malvales/Brassicales - ps26
Brassicales - ps27
Cleomaceae/Brassicaceae - ps28
Brassicaceae - ps29
Camelineae - ps30
Arabidopsis - ps31
Arabidopsis_thaliana - ps32
Phylostratum

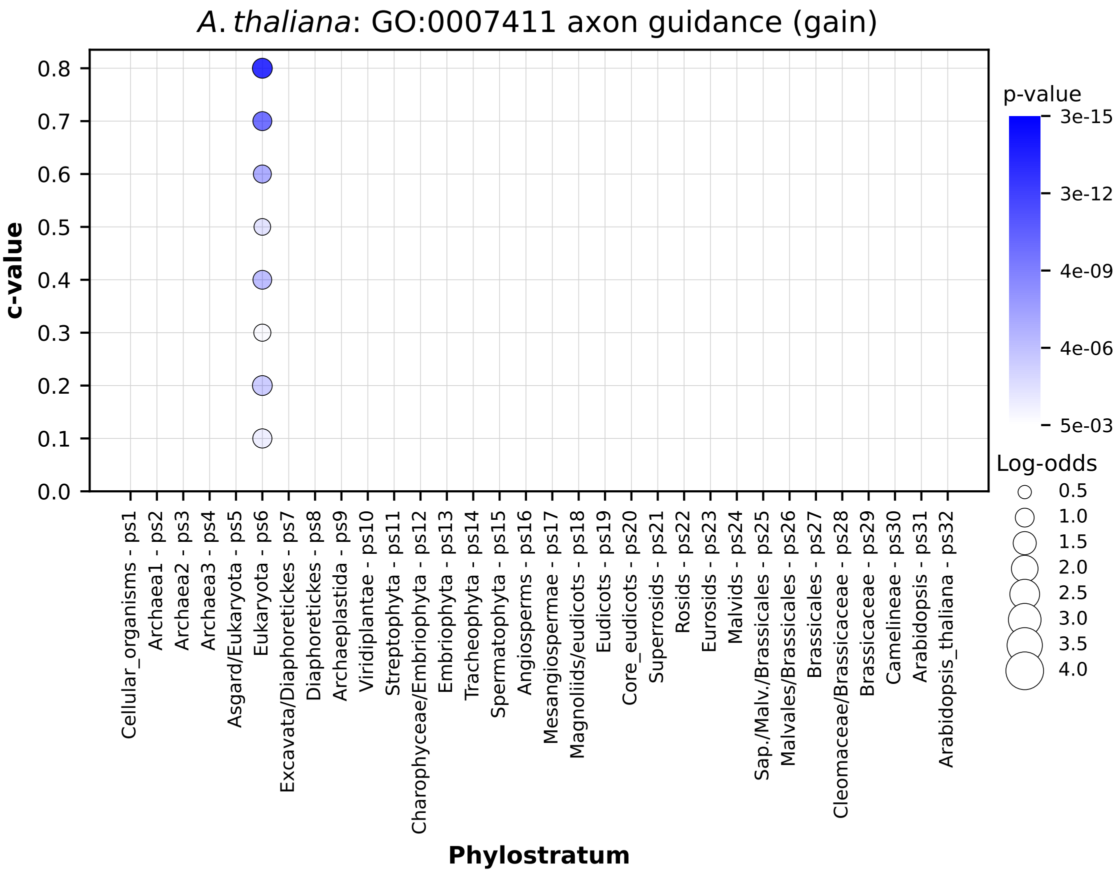

A. thaliana: GO:0007411 axon guidance (gain)
c-value
0.0
0.1
0.2
0.3
0.4
0.5
0.6
0.7
0.8
Phylostratum
Cellular_organisms - ps1
Archaea1 - ps2
Archaea2 - ps3
Archaea3 - ps4
Asgard/Eukaryota - ps5
Eukaryota - ps6
Excavata/Diaphoretickes - ps7
Diaphoretickes - ps8
Archaeplastida - ps9
Viridiplantae - ps10
Streptophyta - ps11
Charophyceae/Embriophyta - ps12
Embriophyta - ps13
Tracheophyta - ps14
Spermatophyta - ps15
Angiosperms - ps16
Mesangiospermae - ps17
Magnoliids/eudicots - ps18
Eudicots - ps19
Core_eudicots - ps20
Superrosids - ps21
Rosids - ps22
Eurosids - ps23
Malvids - ps24
Sap./Malv./Brassicales - ps25
Malvales/Brassicales - ps26
Brassicales - ps27
Cleomaceae/Brassicaceae - ps28
Brassicaceae - ps29
Camelineae - ps30
Arabidopsis - ps31
Arabidopsis_thaliana - ps32
p-value
3e-15
3e-12
4e-09
4e-06
5e-03
Log-odds
0.5
1.0
1.5
2.0
2.5
3.0
3.5
4.0

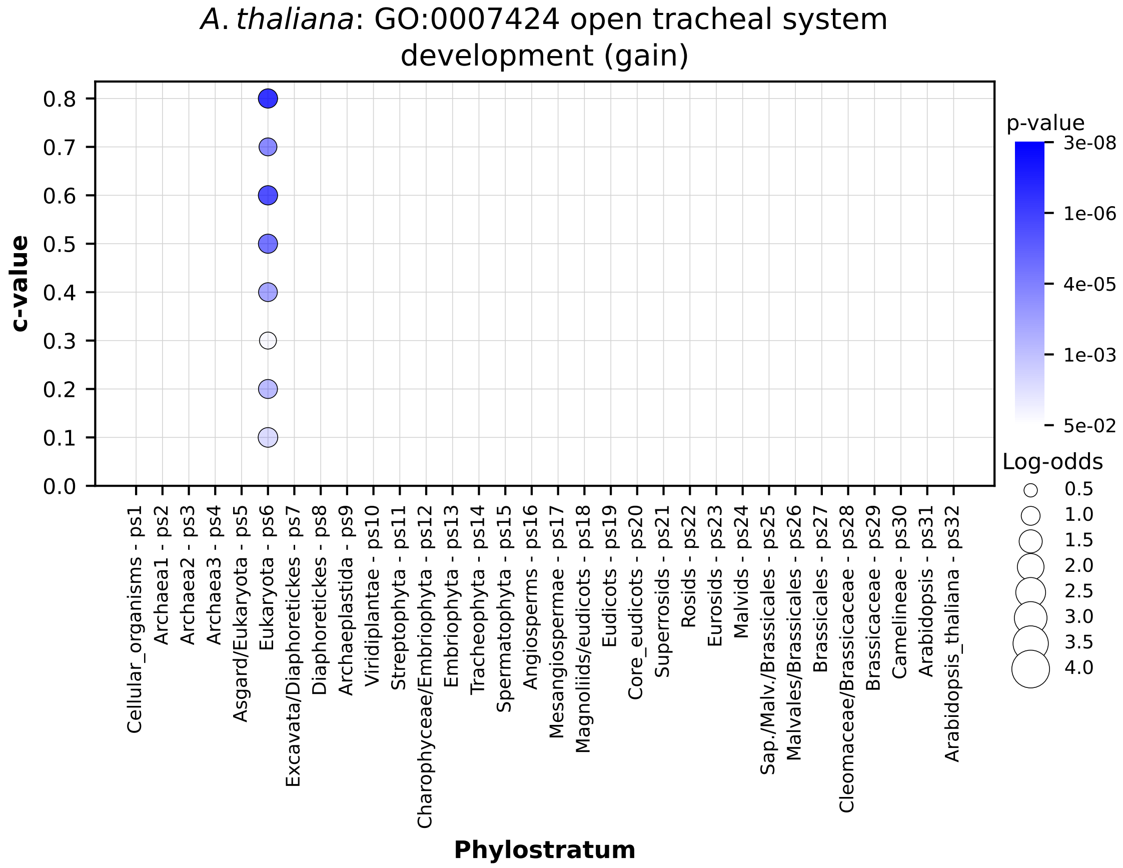

A. thaliana: GO:0007424 open tracheal system development (gain)
c-value
0.8
0.7
0.6
0.5
0.4
0.3
0.2
0.1
0.0
Phylostratum
Cellular_organisms - ps1
Archaea1 - ps2
Archaea2 - ps3
Archaea3 - ps4
Asgard/Eukaryota - ps5
Eukaryota - ps6
Excavata/Diaphoretickes - ps7
Diaphoretickes - ps8
Archaeplastida - ps9
Viridiplantae - ps10
Streptophyta - ps11
Charophyceae/Embriophyta - ps12
Embriophyta - ps13
Tracheophyta - ps14
Spermatophyta - ps15
Angiosperms - ps16
Mesangiospermae - ps17
Magnoliids/eudicots - ps18
Eudicots - ps19
Core_eudicots - ps20
Superrosids - ps21
Rosids - ps22
Eurosids - ps23
Malvids - ps24
Sap./Malv./Brassicales - ps25
Malvales/Brassicales - ps26
Brassicales - ps27
Cleomaceae/Brassicaceae - ps28
Brassicaceae - ps29
Camelineae - ps30
Arabidopsis - ps31
Arabidopsis_thaliana - ps32
p-value
3e-08
1e-06
4e-05
1e-03
5e-02
Log-odds
0.5
1.0
1.5
2.0
2.5
3.0
3.5
4.0

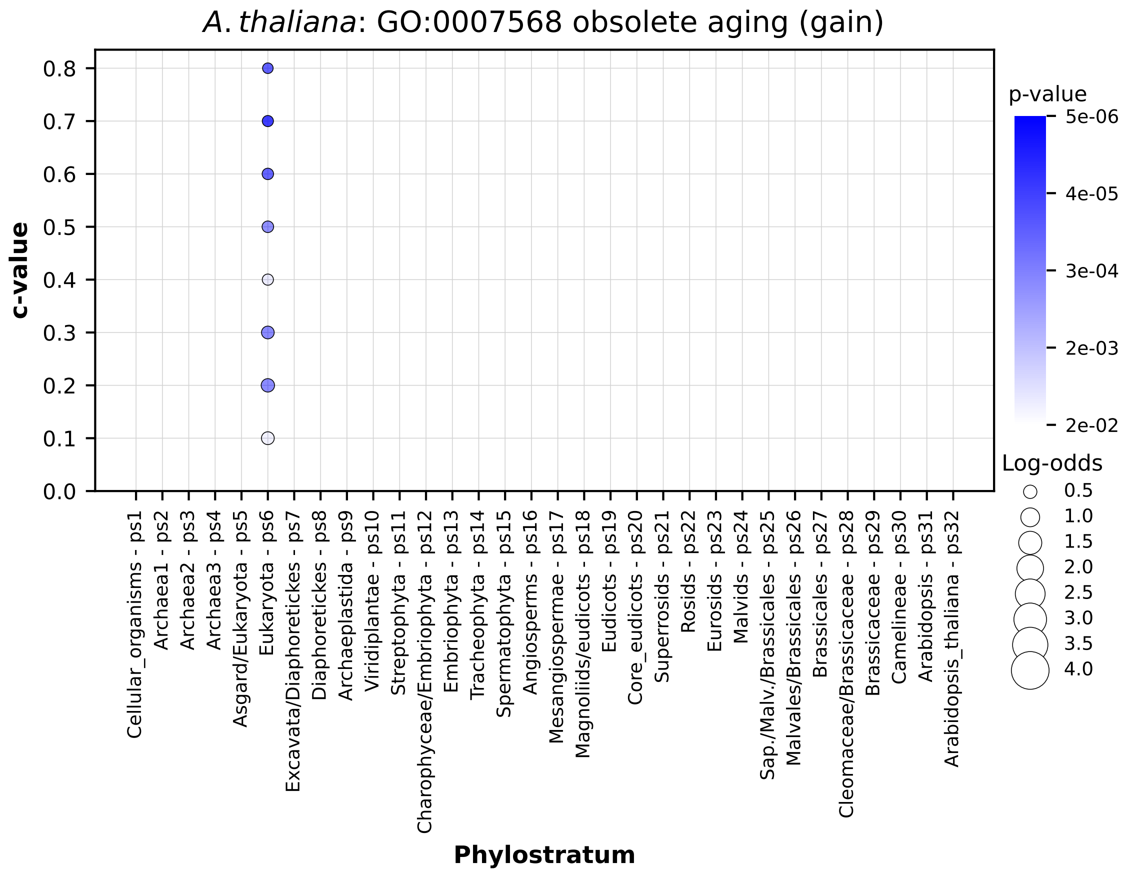

A. thaliana: GO:0007568 obsolete aging (gain)
c-value
0.0
0.1
0.2
0.3
0.4
0.5
0.6
0.7
0.8
p-value
5e-06
4e-05
3e-04
2e-03
2e-02
Log-odds
0.5
1.0
1.5
2.0
2.5
3.0
3.5
4.0
Cellular_organisms - ps1
Archaea1 - ps2
Archaea2 - ps3
Archaea3 - ps4
Asgard/Eukaryota - ps5
Eukaryota - ps6
Excavata/Diaphoretickes - ps7
Diaphoretickes - ps8
Archaeplastida - ps9
Viridiplantae - ps10
Streptophyta - ps11
Charophyceae/Embriophyta - ps12
Embriophyta - ps13
Tracheophyta - ps14
Spermatophyta - ps15
Angiosperms - ps16
Mesangiospermae - ps17
Magnoliids/eudicots - ps18
Eudicots - ps19
Core_eudicots - ps20
Superrosids - ps21
Rosids - ps22
Eurosids - ps23
Malvids - ps24
Sap./Malv./Brassicales - ps25
Malvales/Brassicales - ps26
Brassicales - ps27
Cleomaceae/Brassicaceae - ps28
Brassicaceae - ps29
Camelineae - ps30
Arabidopsis - ps31
Arabidopsis_thaliana - ps32
Phylostratum

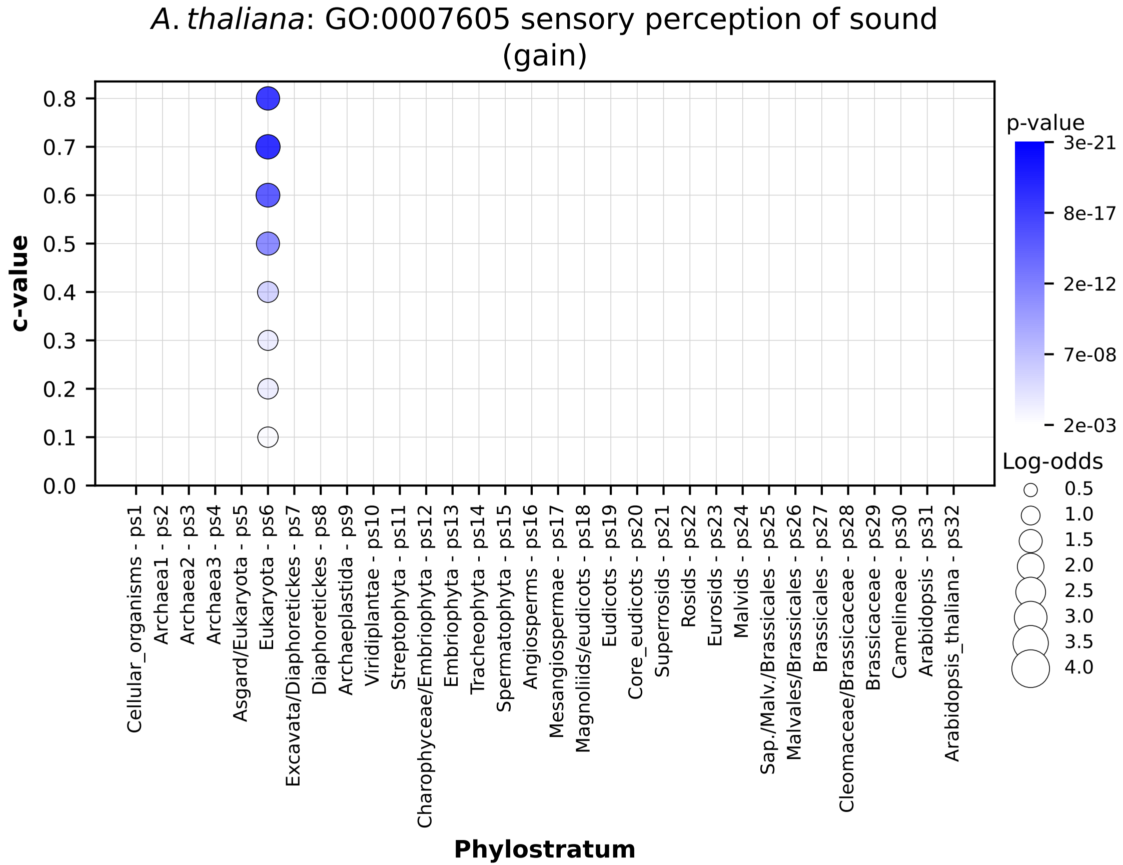

A. thaliana: GO:0007605 sensory perception of sound
(gain)
c-value
0.8
0.7
0.6
0.5
0.4
0.3
0.2
0.1
0.0
p-value
3e-21
8e-17
2e-12
7e-08
2e-03
Log-odds
0.5
1.0
1.5
2.0
2.5
3.0
3.5
4.0
Cellular_organisms - ps1
Archaea1 - ps2
Archaea2 - ps3
Archaea3 - ps4
Asgard/Eukaryota - ps5
Eukaryota - ps6
Excavata/Diaphoretickes - ps7
Diaphoretickes - ps8
Archaeplastida - ps9
Viridiplantae - ps10
Streptophyta - ps11
Charophyceae/Embriophyta - ps12
Embriophyta - ps13
Tracheophyta - ps14
Spermatophyta - ps15
Angiosperms - ps16
Mesangiospermae - ps17
Magnoliids/eudicots - ps18
Eudicots - ps19
Core_eudicots - ps20
Superrosids - ps21
Rosids - ps22
Eurosids - ps23
Malvids - ps24
Sap./Malv./Brassicales - ps25
Malvales/Brassicales - ps26
Brassicales - ps27
Cleomaceae/Brassicaceae - ps28
Brassicaceae - ps29
Camelineae - ps30
Arabidopsis - ps31
Arabidopsis_thaliana - ps32
Phylostratum

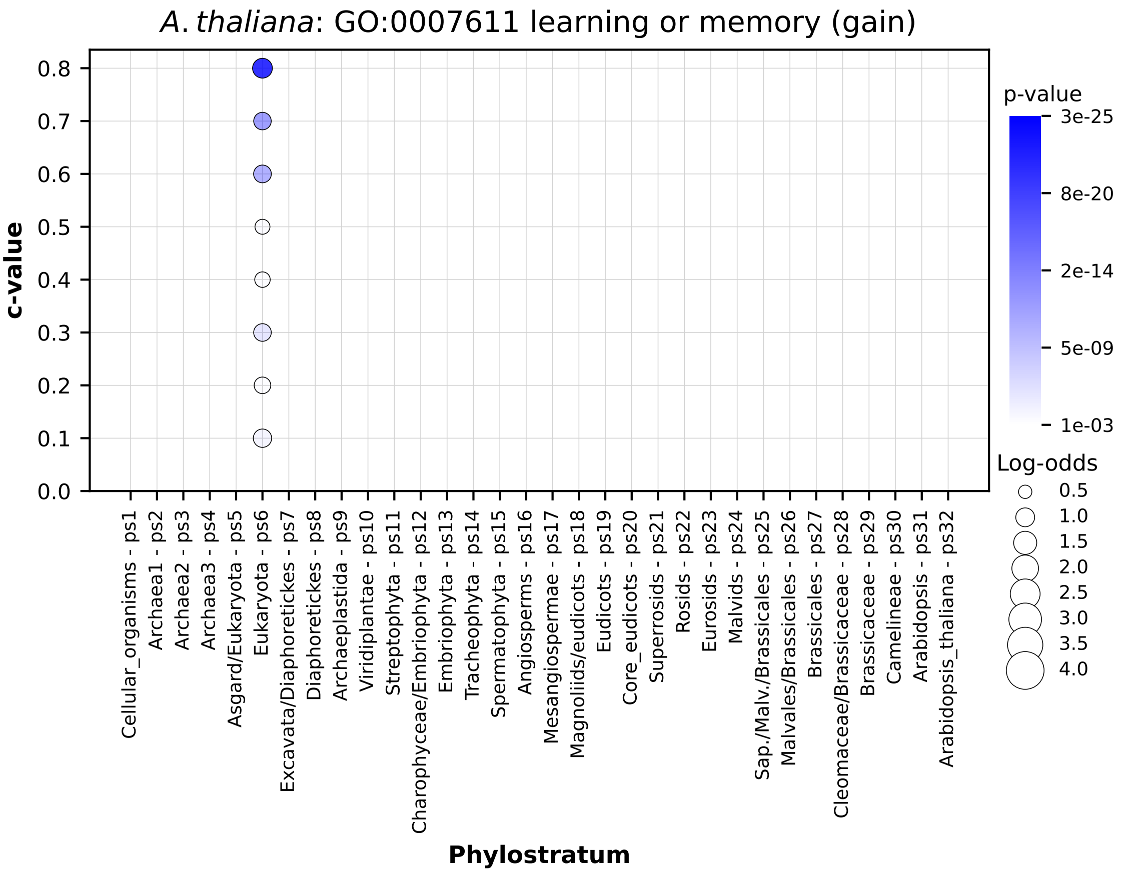

A. thaliana: GO:0007611 learning or memory (gain)
c-value
0.0
0.1
0.2
0.3
0.4
0.5
0.6
0.7
0.8
Phylostratum
Cellular_organisms - ps1
Archaea1 - ps2
Archaea2 - ps3
Archaea3 - ps4
Asgard/Eukaryota - ps5
Eukaryota - ps6
Excavata/Diaphoretickes - ps7
Diaphoretickes - ps8
Archaeplastida - ps9
Viridiplantae - ps10
Streptophyta - ps11
Charophyceae/Embriophyta - ps12
Embriophyta - ps13
Tracheophyta - ps14
Spermatophyta - ps15
Angiosperms - ps16
Mesangiospermae - ps17
Magnoliids/eudicots - ps18
Eudicots - ps19
Core_eudicots - ps20
Superrosids - ps21
Rosids - ps22
Eurosids - ps23
Malvids - ps24
Sap./Malv./Brassicales - ps25
Malvales/Brassicales - ps26
Brassicales - ps27
Cleomaceae/Brassicaceae - ps28
Brassicaceae - ps29
Camelineae - ps30
Arabidopsis - ps31
Arabidopsis_thaliana - ps32
p-value
3e-25
8e-20
2e-14
5e-09
1e-03
Log-odds
0.5
1.0
1.5
2.0
2.5
3.0
3.5
4.0

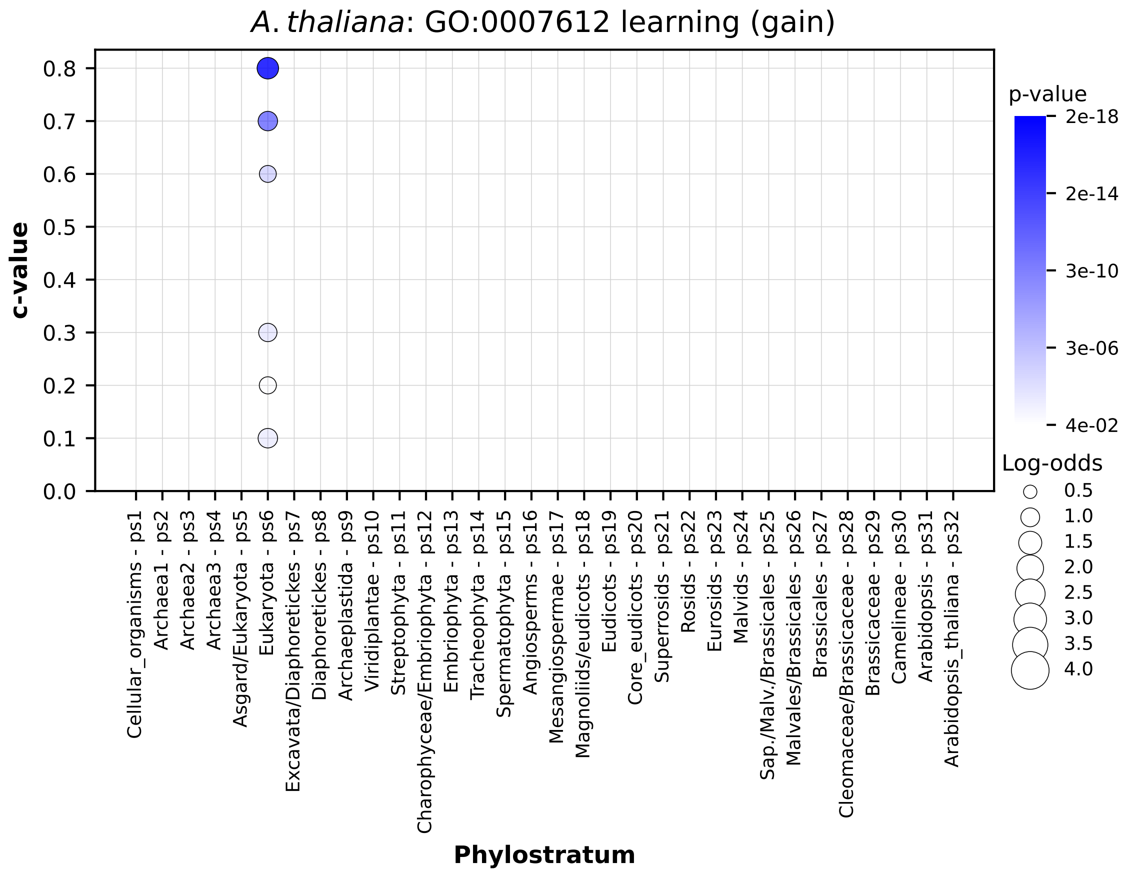

A. thaliana: GO:0007612 learning (gain)
c-value
0.0
0.1
0.2
0.3
0.4
0.5
0.6
0.7
0.8
Phylostratum
Cellular_organisms - ps1
Archaea1 - ps2
Archaea2 - ps3
Archaea3 - ps4
Asgard/Eukaryota - ps5
Eukaryota - ps6
Excavata/Diaphoretickes - ps7
Diaphoretickes - ps8
Archaeplastida - ps9
Viridiplantae - ps10
Streptophyta - ps11
Charophyceae/Embriophyta - ps12
Embriophyta - ps13
Tracheophyta - ps14
Spermatophyta - ps15
Angiosperms - ps16
Mesangiospermae - ps17
Magnoliids/eudicots - ps18
Eudicots - ps19
Core_eudicots - ps20
Superrosids - ps21
Rosids - ps22
Eurosids - ps23
Malvids - ps24
Sap./Malv./Brassicales - ps25
Malvales/Brassicales - ps26
Brassicales - ps27
Cleomaceae/Brassicaceae - ps28
Brassicaceae - ps29
Camelineae - ps30
Arabidopsis - ps31
Arabidopsis_thaliana - ps32
p-value
2e-18
2e-14
3e-10
3e-06
4e-02
Log-odds
0.5
1.0
1.5
2.0
2.5
3.0
3.5
4.0

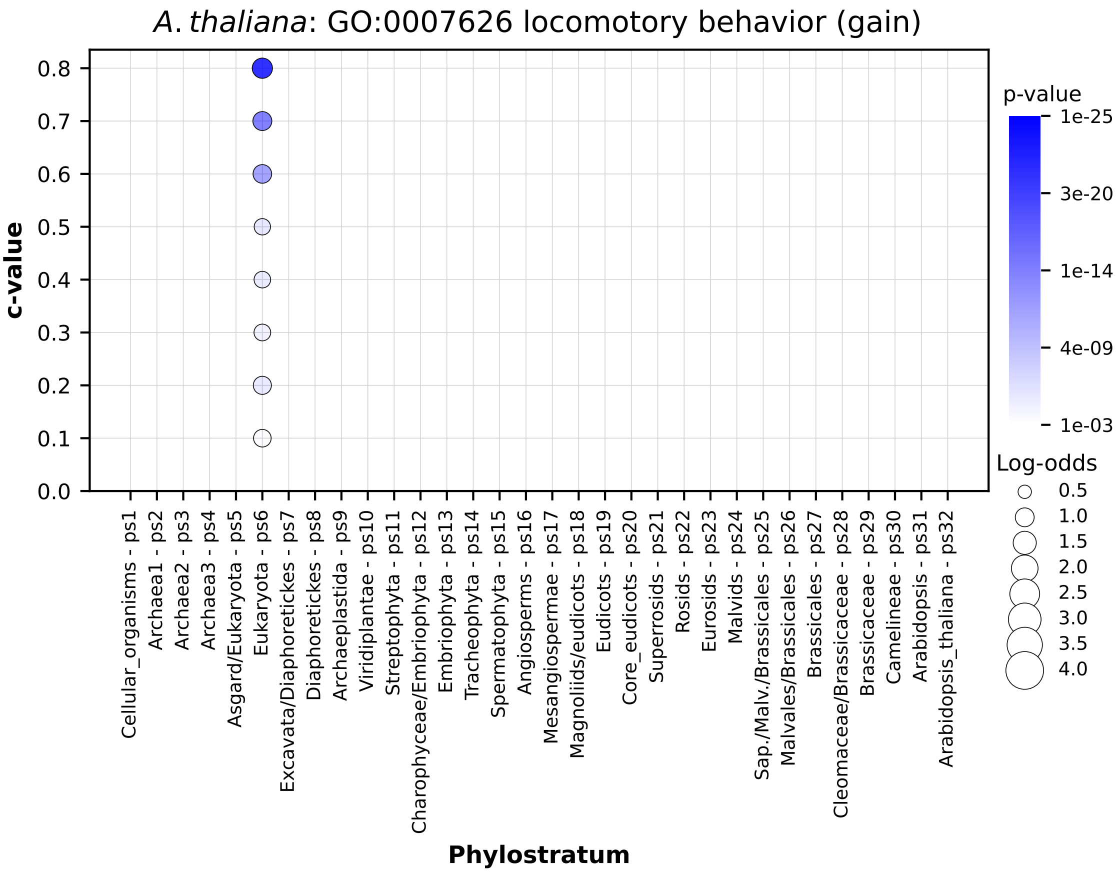

A. thaliana: GO:0007626 locomotory behavior (gain)
c-value
0.0
0.1
0.2
0.3
0.4
0.5
0.6
0.7
0.8
Phylostratum
Cellular_organisms - ps1
Archaea1 - ps2
Archaea2 - ps3
Archaea3 - ps4
Asgard/Eukaryota - ps5
Eukaryota - ps6
Excavata/Diaphoretickes - ps7
Diaphoretickes - ps8
Archaeplastida - ps9
Viridiplantae - ps10
Streptophyta - ps11
Charophyceae/Embriophyta - ps12
Embriophyta - ps13
Tracheophyta - ps14
Spermatophyta - ps15
Angiosperms - ps16
Mesangiospermae - ps17
Magnoliids/eudicots - ps18
Eudicots - ps19
Core_eudicots - ps20
Superrosids - ps21
Rosids - ps22
Eurosids - ps23
Malvids - ps24
Sap./Malv./Brassicales - ps25
Malvales/Brassicales - ps26
Brassicales - ps27
Cleomaceae/Brassicaceae - ps28
Brassicaceae - ps29
Camelineae - ps30
Arabidopsis - ps31
Arabidopsis_thaliana - ps32
p-value
1e-25
3e-20
1e-14
4e-09
1e-03
Log-odds
0.5
1.0
1.5
2.0
2.5
3.0
3.5
4.0

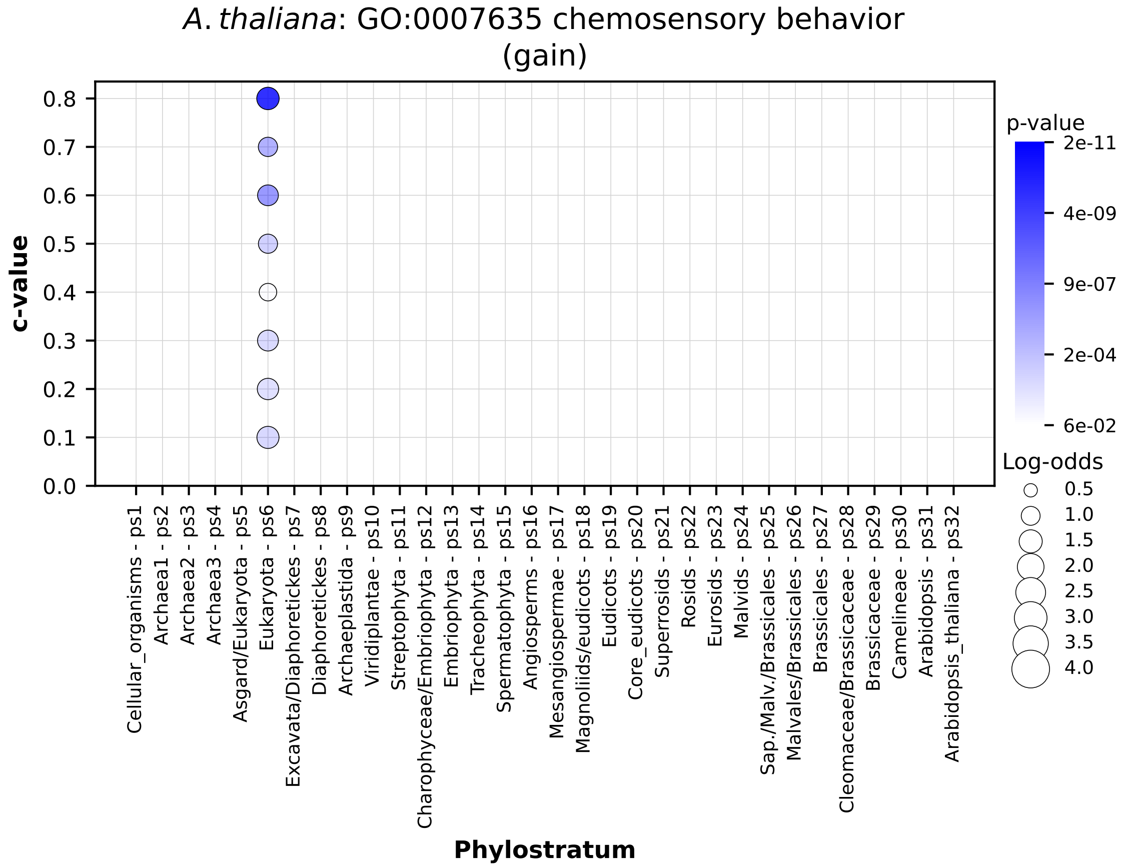

A. thaliana: GO:0007635 chemosensory behavior
(gain)
c-value
0.0
0.1
0.2
0.3
0.4
0.5
0.6
0.7
0.8
Phylostratum
Cellular_organisms - ps1
Archaea1 - ps2
Archaea2 - ps3
Archaea3 - ps4
Asgard/Eukaryota - ps5
Eukaryota - ps6
Excavata/Diaphoretickes - ps7
Diaphoretickes - ps8
Archaeplastida - ps9
Viridiplantae - ps10
Streptophyta - ps11
Charophyceae/Embriophyta - ps12
Embriophyta - ps13
Tracheophyta - ps14
Spermatophyta - ps15
Angiosperms - ps16
Mesangiospermae - ps17
Magnoliids/eudicots - ps18
Eudicots - ps19
Core_eudicots - ps20
Superrosids - ps21
Rosids - ps22
Eurosids - ps23
Malvids - ps24
Sap./Malv./Brassicales - ps25
Malvales/Brassicales - ps26
Brassicales - ps27
Cleomaceae/Brassicaceae - ps28
Brassicaceae - ps29
Camelineae - ps30
Arabidopsis - ps31
Arabidopsis_thaliana - ps32
p-value
2e-11
4e-09
9e-07
2e-04
6e-02
Log-odds
0.5
1.0
1.5
2.0
2.5
3.0
3.5
4.0

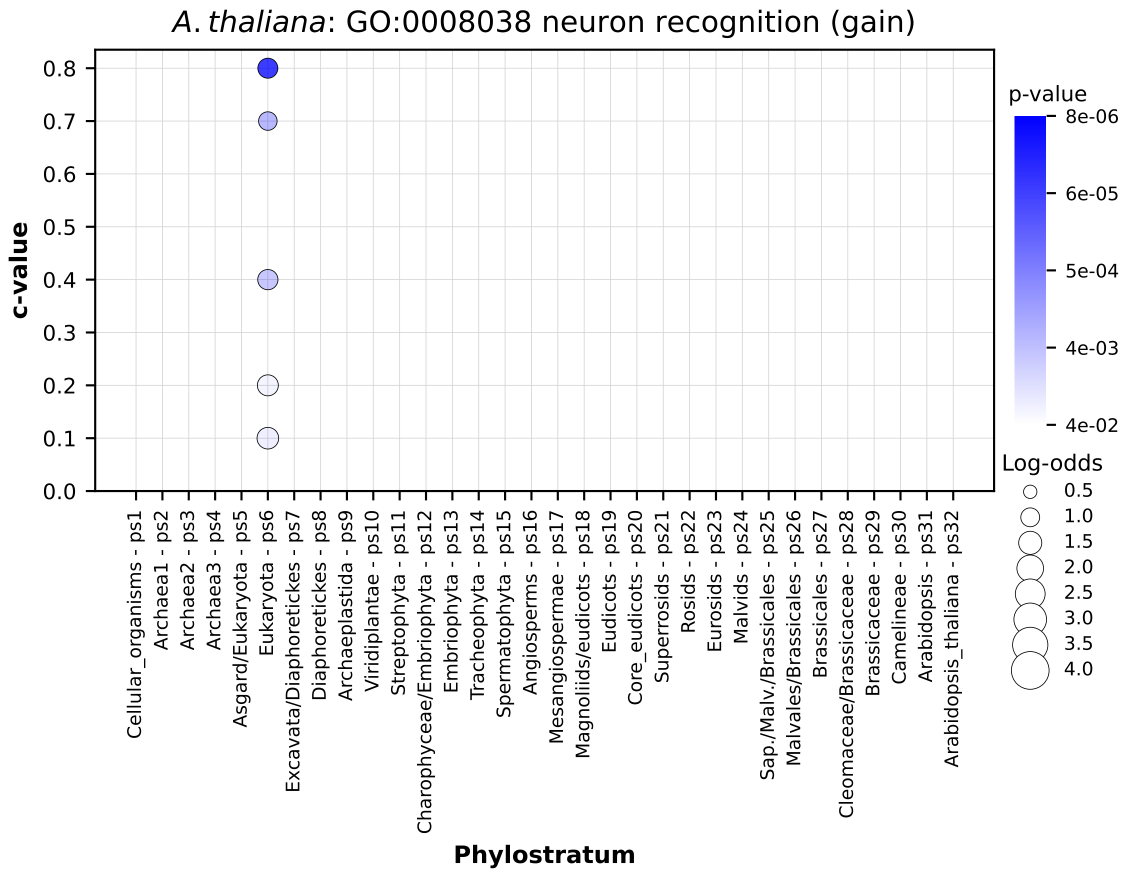

A. thaliana: GO:0008038 neuron recognition (gain)
c-value
0.0
0.1
0.2
0.3
0.4
0.5
0.6
0.7
0.8
Phylostratum
Cellular_organisms - ps1
Archaea1 - ps2
Archaea2 - ps3
Archaea3 - ps4
Asgard/Eukaryota - ps5
Eukaryota - ps6
Excavata/Diaphoretickes - ps7
Diaphoretickes - ps8
Archaeplastida - ps9
Viridiplantae - ps10
Streptophyta - ps11
Charophyceae/Embriophyta - ps12
Embriophyta - ps13
Tracheophyta - ps14
Spermatophyta - ps15
Angiosperms - ps16
Mesangiospermae - ps17
Magnoliids/eudicots - ps18
Eudicots - ps19
Core_eudicots - ps20
Superrosids - ps21
Rosids - ps22
Eurosids - ps23
Malvids - ps24
Sap./Malv./Brassicales - ps25
Malvales/Brassicales - ps26
Brassicales - ps27
Cleomaceae/Brassicaceae - ps28
Brassicaceae - ps29
Camelineae - ps30
Arabidopsis - ps31
Arabidopsis_thaliana - ps32
p-value
8e-06
6e-05
5e-04
4e-03
4e-02
Log-odds
0.5
1.0
1.5
2.0
2.5
3.0
3.5
4.0

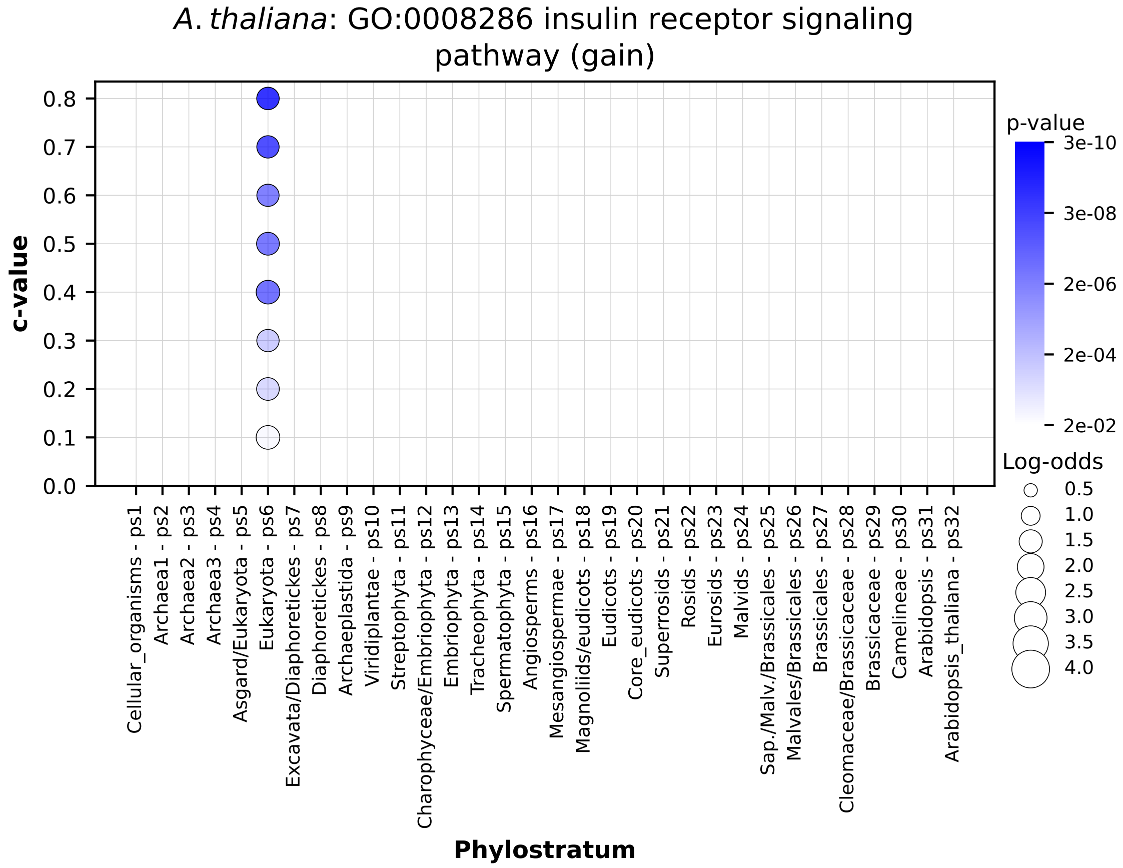

A. thaliana: GO:0008286 insulin receptor signaling pathway (gain)
c-value
0.0
0.1
0.2
0.3
0.4
0.5
0.6
0.7
0.8
Phylostratum
Cellular_organisms - ps1
Archaea1 - ps2
Archaea2 - ps3
Archaea3 - ps4
Asgard/Eukaryota - ps5
Eukaryota - ps6
Excavata/Diaphoretickes - ps7
Diaphoretickes - ps8
Archaeplastida - ps9
Viridiplantae - ps10
Streptophyta - ps11
Charophyceae/Embriophyta - ps12
Embriophyta - ps13
Tracheophyta - ps14
Spermatophyta - ps15
Angiosperms - ps16
Mesangiospermae - ps17
Magnoliids/eudicots - ps18
Eudicots - ps19
Core_eudicots - ps20
Superrosids - ps21
Rosids - ps22
Eurosids - ps23
Malvids - ps24
Sap./Malv./Brassicales - ps25
Malvales/Brassicales - ps26
Brassicales - ps27
Cleomaceae/Brassicaceae - ps28
Brassicaceae - ps29
Camelineae - ps30
Arabidopsis - ps31
Arabidopsis_thaliana - ps32
p-value
3e-10
3e-08
2e-06
2e-04
2e-02
Log-odds
0.5
1.0
1.5
2.0
2.5
3.0
3.5
4.0

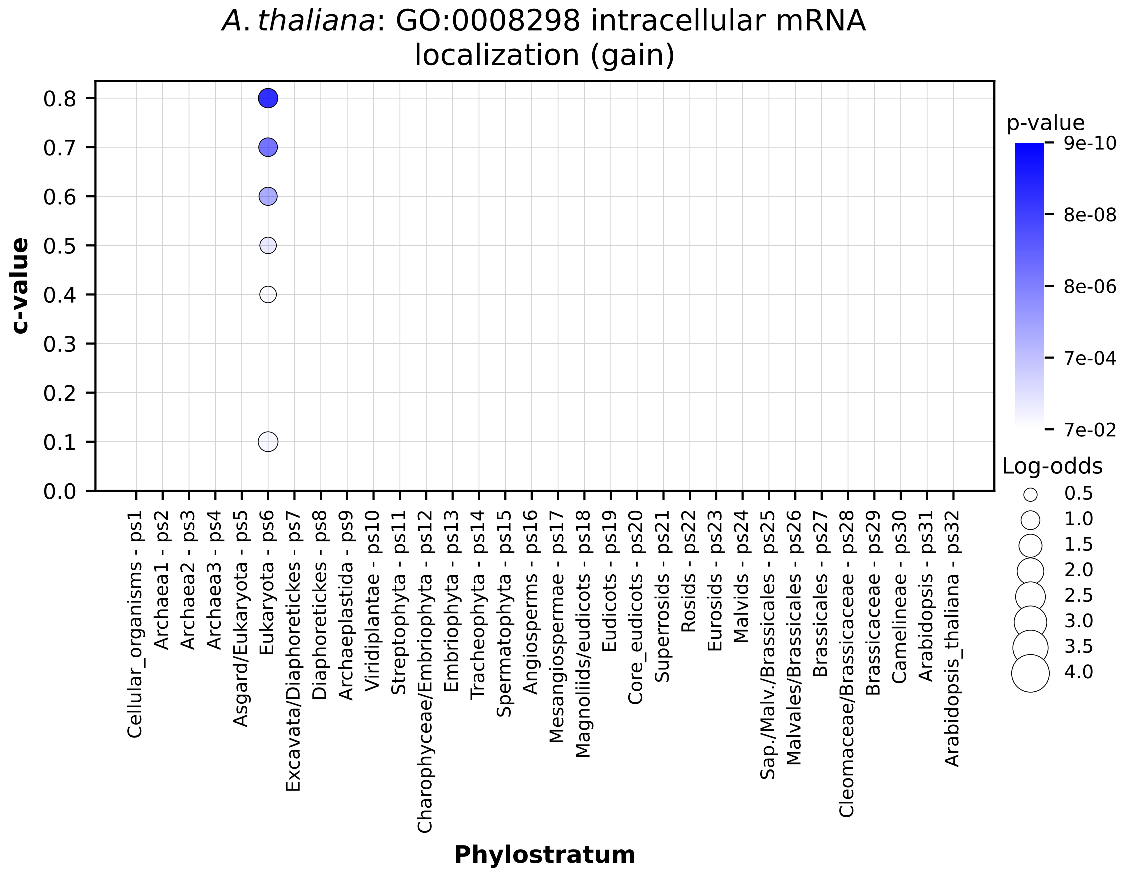

A. thaliana: GO:0008298 intracellular mRNA localization (gain)
c-value
0.8
0.7
0.6
0.5
0.4
0.3
0.2
0.1
0.0
p-value
9e-10
8e-08
8e-06
7e-04
7e-02
Log-odds
0.5
1.0
1.5
2.0
2.5
3.0
3.5
4.0
Cellular_organisms - ps1
Archaea1 - ps2
Archaea2 - ps3
Archaea3 - ps4
Asgard/Eukaryota - ps5
Eukaryota - ps6
Excavata/Diaphoretickes - ps7
Diaphoretickes - ps8
Archaeplastida - ps9
Viridiplantae - ps10
Streptophyta - ps11
Charophyceae/Embriophyta - ps12
Embriophyta - ps13
Tracheophyta - ps14
Spermatophyta - ps15
Angiosperms - ps16
Mesangiospermae - ps17
Magnoliids/eudicots - ps18
Eudicots - ps19
Core_eudicots - ps20
Superrosids - ps21
Rosids - ps22
Eurosids - ps23
Malvids - ps24
Sap./Malv./Brassicales - ps25
Malvales/Brassicales - ps26
Brassicales - ps27
Cleomaceae/Brassicaceae - ps28
Brassicaceae - ps29
Camelineae - ps30
Arabidopsis - ps31
Arabidopsis_thaliana - ps32
Phylostratum

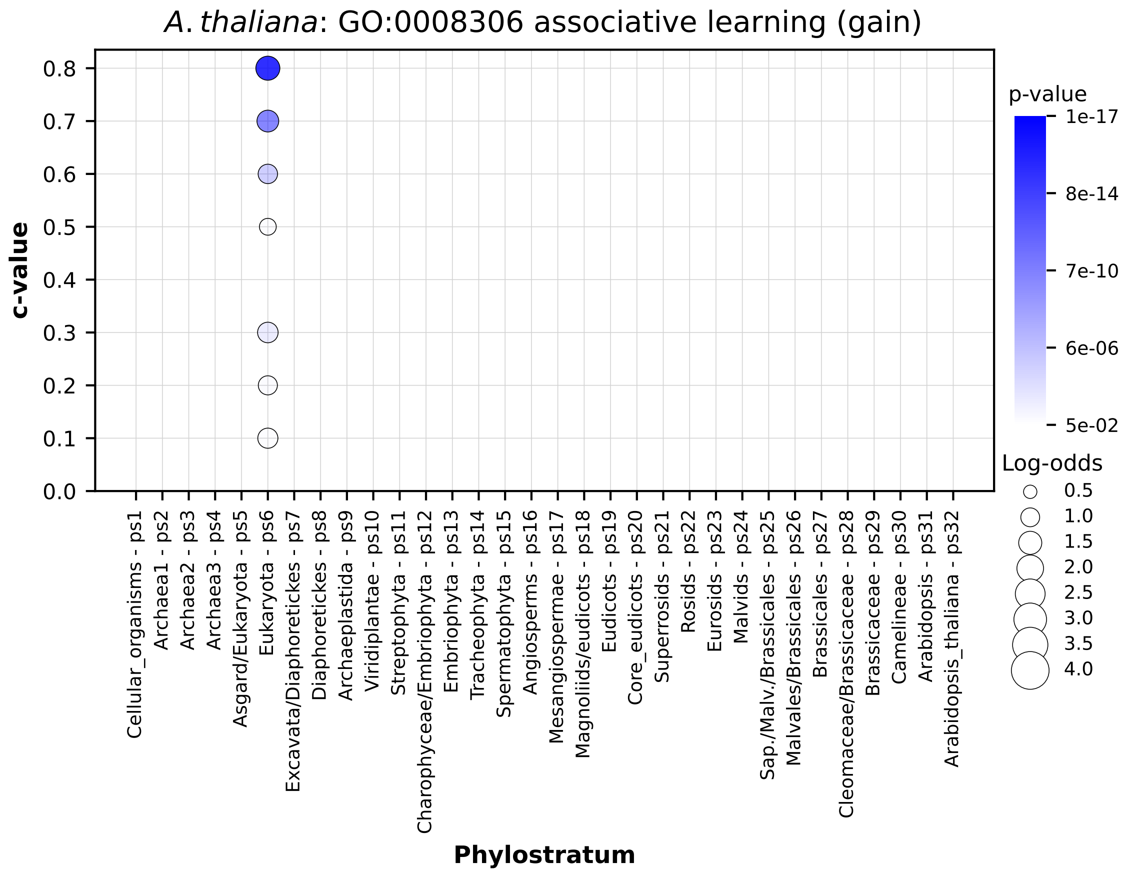

A. thaliana: GO:0008306 associative learning (gain)
c-value
0.0
0.1
0.2
0.3
0.4
0.5
0.6
0.7
0.8
Phylostratum
Cellular_organisms - ps1
Archaea1 - ps2
Archaea2 - ps3
Archaea3 - ps4
Asgard/Eukaryota - ps5
Eukaryota - ps6
Excavata/Diaphoretickes - ps7
Diaphoretickes - ps8
Archaeplastida - ps9
Viridiplantae - ps10
Streptophyta - ps11
Charophyceae/Embriophyta - ps12
Embriophyta - ps13
Tracheophyta - ps14
Spermatophyta - ps15
Angiosperms - ps16
Mesangiospermae - ps17
Magnoliids/eudicots - ps18
Eudicots - ps19
Core_eudicots - ps20
Superrosids - ps21
Rosids - ps22
Eurosids - ps23
Malvids - ps24
Sap./Malv./Brassicales - ps25
Malvales/Brassicales - ps26
Brassicales - ps27
Cleomaceae/Brassicaceae - ps28
Brassicaceae - ps29
Camelineae - ps30
Arabidopsis - ps31
Arabidopsis_thaliana - ps32
p-value
1e-17
8e-14
7e-10
6e-06
5e-02
Log-odds
0.5
1.0
1.5
2.0
2.5
3.0
3.5
4.0

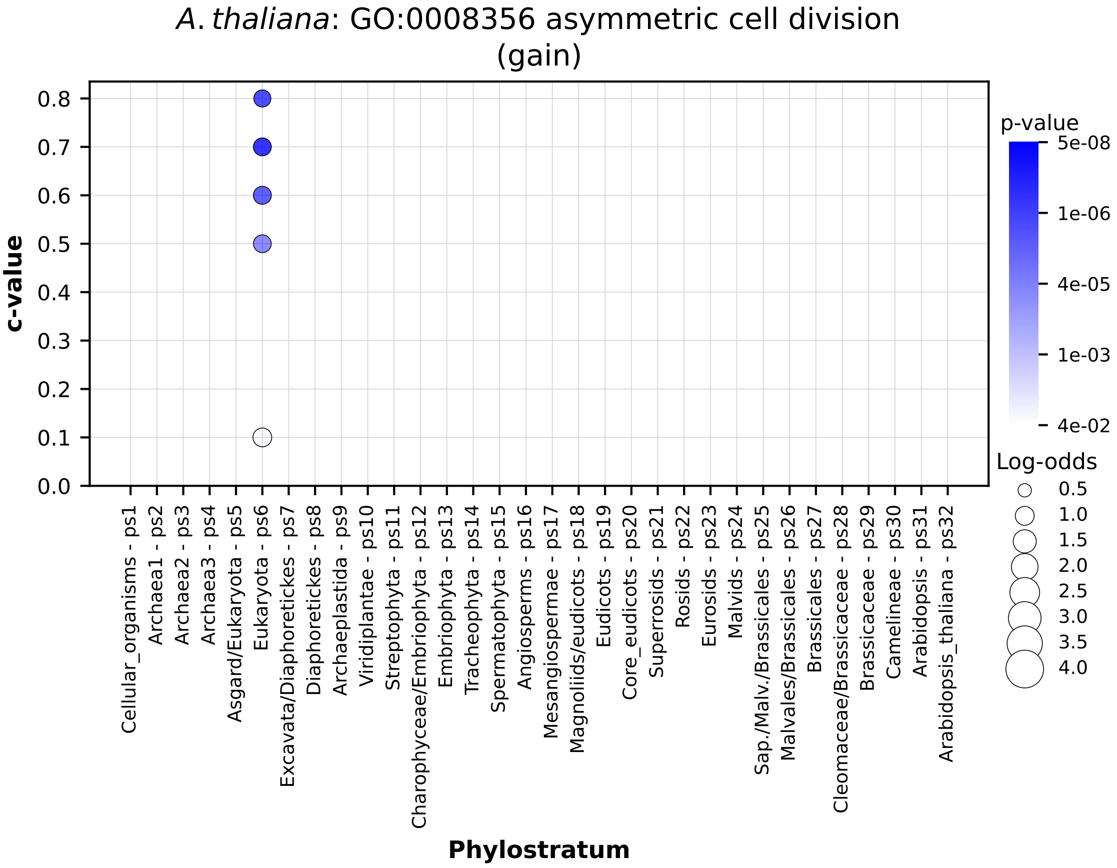
A. thaliana: GO:0008356 asymmetric cell division
(gain)
c-value
0.8
0.7
0.6
0.5
0.4
0.3
0.2
0.1
0.0
p-value
5e-08
1e-06
4e-05
1e-03
4e-02
Log-odds
0.5
1.0
1.5
2.0
2.5
3.0
3.5
4.0
Cellular_organisms - ps1
Archaea1 - ps2
Archaea2 - ps3
Archaea3 - ps4
Asgard/Eukaryota - ps5
Eukaryota - ps6
Excavata/Diaphoretickes - ps7
Diaphoretickes - ps8
Archaeplastida - ps9
Viridiplantae - ps10
Streptophyta - ps11
Charophyceae/Embriophyta - ps12
Embriophyta - ps13
Tracheophyta - ps14
Spermatophyta - ps15
Angiosperms - ps16
Mesangiospermae - ps17
Magnoliids/eudicots - ps18
Eudicots - ps19
Core_eudicots - ps20
Superrosids - ps21
Rosids - ps22
Eurosids - ps23
Malvids - ps24
Sap./Malv./Brassicales - ps25
Malvales/Brassicales - ps26
Brassicales - ps27
Cleomaceae/Brassicaceae - ps28
Brassicaceae - ps29
Camelineae - ps30
Arabidopsis - ps31
Arabidopsis_thaliana - ps32
Phylostratum

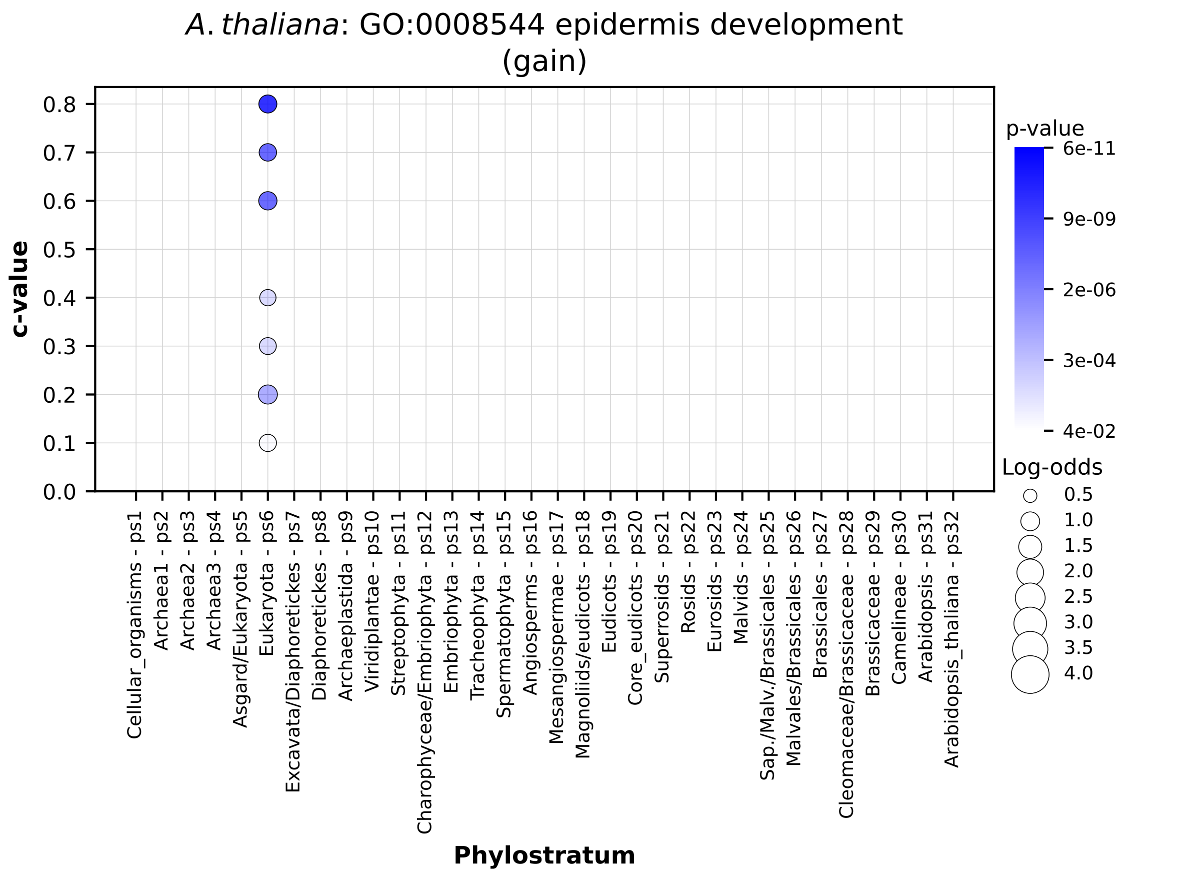
A. thaliana: GO:0008544 epidermis development
(gain)
c-value
0.8
0.7
0.6
0.5
0.4
0.3
0.2
0.1
0.0
p-value
6e-11
9e-09
2e-06
3e-04
4e-02
Log-odds
0.5
1.0
1.5
2.0
2.5
3.0
3.5
4.0
Cellular_organisms - ps1
Archaea1 - ps2
Archaea2 - ps3
Archaea3 - ps4
Asgard/Eukaryota - ps5
Eukaryota - ps6
Excavata/Diaphoretickes - ps7
Diaphoretickes - ps8
Archaeplastida - ps9
Viridiplantae - ps10
Streptophyta - ps11
Charophyceae/Embriophyta - ps12
Embriophyta - ps13
Tracheophyta - ps14
Spermatophyta - ps15
Angiosperms - ps16
Mesangiospermae - ps17
Magnoliids/eudicots - ps18
Eudicots - ps19
Core_eudicots - ps20
Superrosids - ps21
Rosids - ps22
Eurosids - ps23
Malvids - ps24
Sap./Malv./Brassicales - ps25
Malvales/Brassicales - ps26
Brassicales - ps27
Cleomaceae/Brassicaceae - ps28
Brassicaceae - ps29
Camelineae - ps30
Arabidopsis - ps31
Arabidopsis_thaliana - ps32
Phylostratum

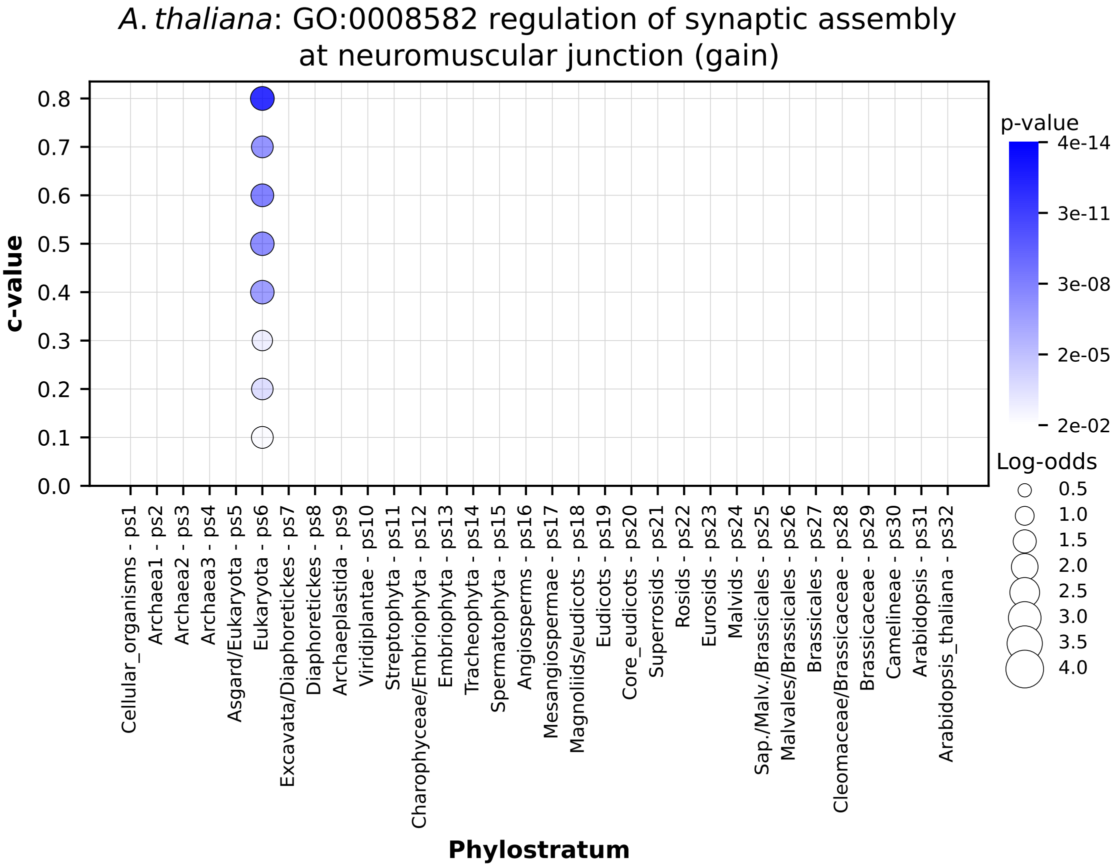
A. thaliana: GO:0008582 regulation of synaptic assembly at neuromuscular junction (gain)
c-value
0.0
0.1
0.2
0.3
0.4
0.5
0.6
0.7
0.8
Phylostratum
Cellular_organisms - ps1
Archaea1 - ps2
Archaea2 - ps3
Archaea3 - ps4
Asgard/Eukaryota - ps5
Eukaryota - ps6
Excavata/Diaphoretickes - ps7
Diaphoretickes - ps8
Archaeplastida - ps9
Viridiplantae - ps10
Streptophyta - ps11
Charophyceae/Embriophyta - ps12
Embriophyta - ps13
Tracheophyta - ps14
Spermatophyta - ps15
Angiosperms - ps16
Mesangiospermae - ps17
Magnoliids/eudicots - ps18
Eudicots - ps19
Core_eudicots - ps20
Superrosids - ps21
Rosids - ps22
Eurosids - ps23
Malvids - ps24
Sap./Malv./Brassicales - ps25
Malvales/Brassicales - ps26
Brassicales - ps27
Cleomaceae/Brassicaceae - ps28
Brassicaceae - ps29
Camelineae - ps30
Arabidopsis - ps31
Arabidopsis_thaliana - ps32
p-value
4e-14
3e-11
3e-08
2e-05
2e-02
Log-odds
0.5
1.0
1.5
2.0
2.5
3.0
3.5
4.0

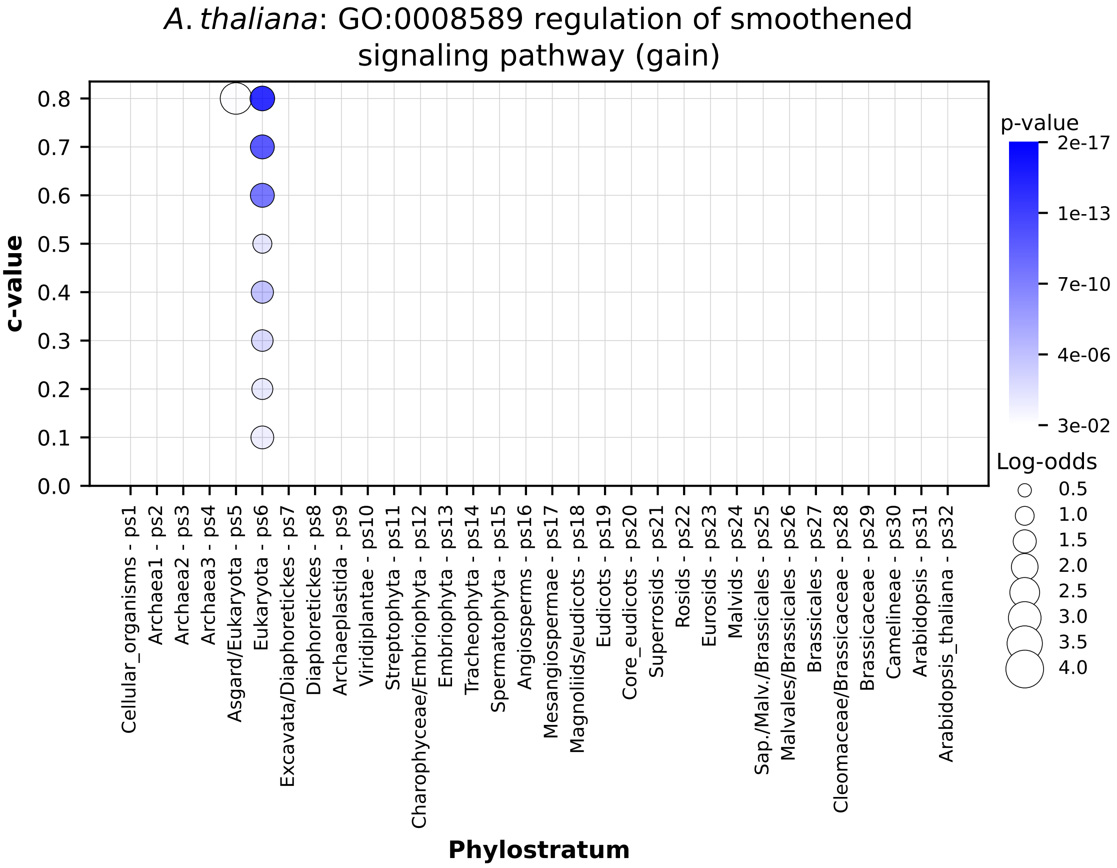

A. thaliana: GO:0008589 regulation of smoothened signaling pathway (gain)
c-value
0.0
0.1
0.2
0.3
0.4
0.5
0.6
0.7
0.8
Phylostratum
Cellular_organisms - ps1
Archaea1 - ps2
Archaea2 - ps3
Archaea3 - ps4
Asgard/Eukaryota - ps5
Eukaryota - ps6
Excavata/Diaphoretickes - ps7
Diaphoretickes - ps8
Archaeplastida - ps9
Viridiplantae - ps10
Streptophyta - ps11
Charophyceae/Embriophyta - ps12
Embriophyta - ps13
Tracheophyta - ps14
Spermatophyta - ps15
Angiosperms - ps16
Mesangiospermae - ps17
Magnoliids/eudicots - ps18
Eudicots - ps19
Core_eudicots - ps20
Superrosids - ps21
Rosids - ps22
Eurosids - ps23
Malvids - ps24
Sap./Malv./Brassicales - ps25
Malvales/Brassicales - ps26
Brassicales - ps27
Cleomaceae/Brassicaceae - ps28
Brassicaceae - ps29
Camelineae - ps30
Arabidopsis - ps31
Arabidopsis_thaliana - ps32
p-value
2e-17
1e-13
7e-10
4e-06
3e-02
Log-odds
0.5
1.0
1.5
2.0
2.5
3.0
3.5
4.0

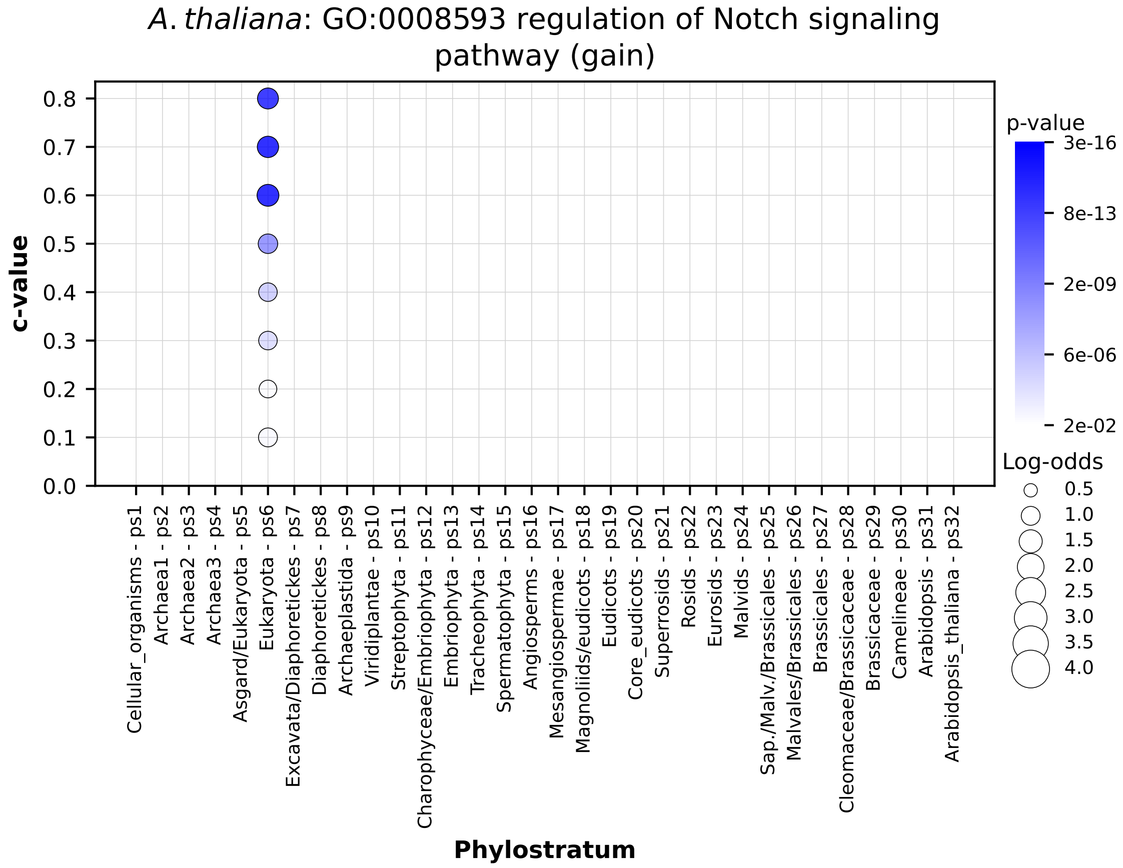
A. thaliana: GO:0008593 regulation of Notch signaling pathway (gain)
c-value
0.0
0.1
0.2
0.3
0.4
0.5
0.6
0.7
0.8
p-value
3e-16
8e-13
2e-09
6e-06
2e-02
Log-odds
0.5
1.0
1.5
2.0
2.5
3.0
3.5
4.0
Cellular_organisms - ps1
Archaea1 - ps2
Archaea2 - ps3
Archaea3 - ps4
Asgard/Eukaryota - ps5
Eukaryota - ps6
Excavata/Diaphoretickes - ps7
Diaphoretickes - ps8
Archaeplastida - ps9
Viridiplantae - ps10
Streptophyta - ps11
Charophyceae/Embriophyta - ps12
Embriophyta - ps13
Tracheophyta - ps14
Spermatophyta - ps15
Angiosperms - ps16
Mesangiospermae - ps17
Magnoliids/eudicots - ps18
Eudicots - ps19
Core_eudicots - ps20
Superrosids - ps21
Rosids - ps22
Eurosids - ps23
Malvids - ps24
Sap./Malv./Brassicales - ps25
Malvales/Brassicales - ps26
Brassicales - ps27
Cleomaceae/Brassicaceae - ps28
Brassicaceae - ps29
Camelineae - ps30
Arabidopsis - ps31
Arabidopsis_thaliana - ps32
Phylostratum

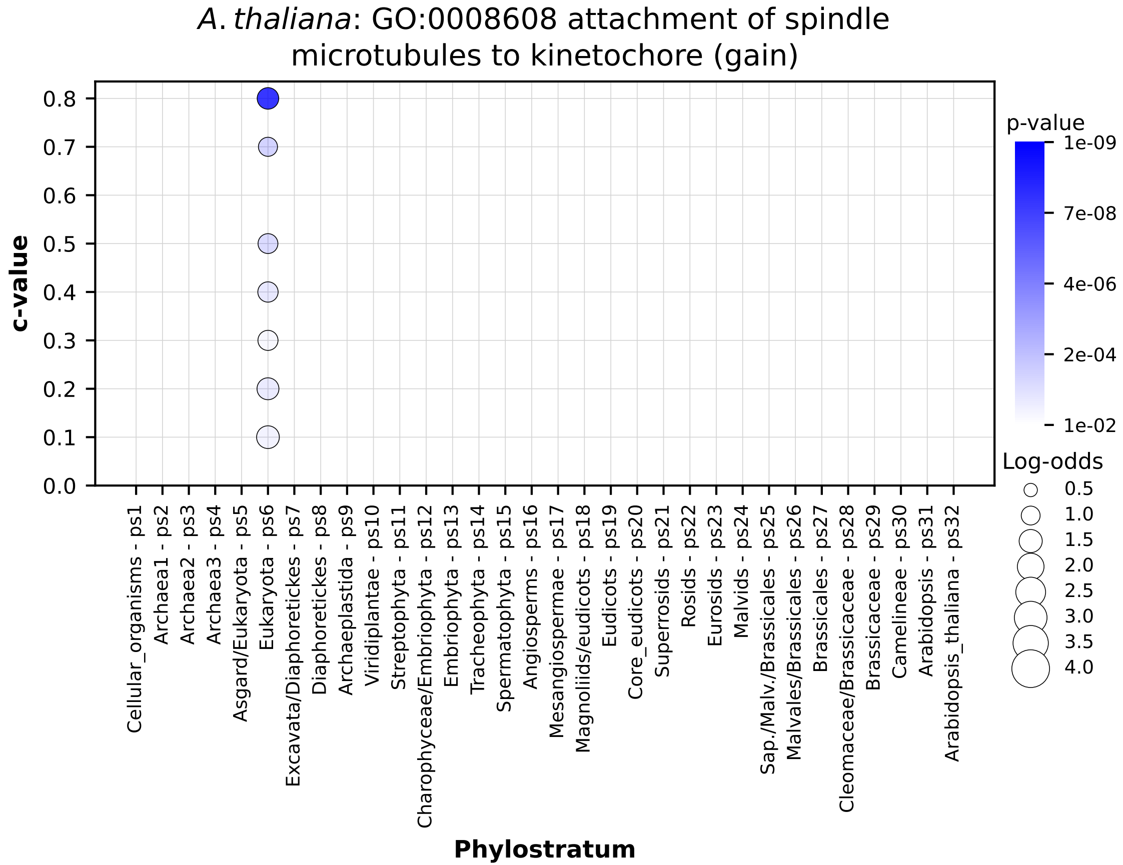

A. thaliana: GO:0008608 attachment of spindle microtubules to kinetochore (gain)
c-value
0.0
0.1
0.2
0.3
0.4
0.5
0.6
0.7
0.8
Phylostratum
Cellular_organisms - ps1
Archaea1 - ps2
Archaea2 - ps3
Archaea3 - ps4
Asgard/Eukaryota - ps5
Eukaryota - ps6
Excavata/Diaphoretickes - ps7
Diaphoretickes - ps8
Archaeplastida - ps9
Viridiplantae - ps10
Streptophyta - ps11
Charophyceae/Embriophyta - ps12
Embriophyta - ps13
Tracheophyta - ps14
Spermatophyta - ps15
Angiosperms - ps16
Mesangiospermae - ps17
Magnoliids/eudicots - ps18
Eudicots - ps19
Core_eudicots - ps20
Superrosids - ps21
Rosids - ps22
Eurosids - ps23
Malvids - ps24
Sap./Malv./Brassicales - ps25
Malvales/Brassicales - ps26
Brassicales - ps27
Cleomaceae/Brassicaceae - ps28
Brassicaceae - ps29
Camelineae - ps30
Arabidopsis - ps31
Arabidopsis_thaliana - ps32
p-value
1e-09
7e-08
4e-06
2e-04
1e-02
Log-odds
0.5
1.0
1.5
2.0
2.5
3.0
3.5
4.0

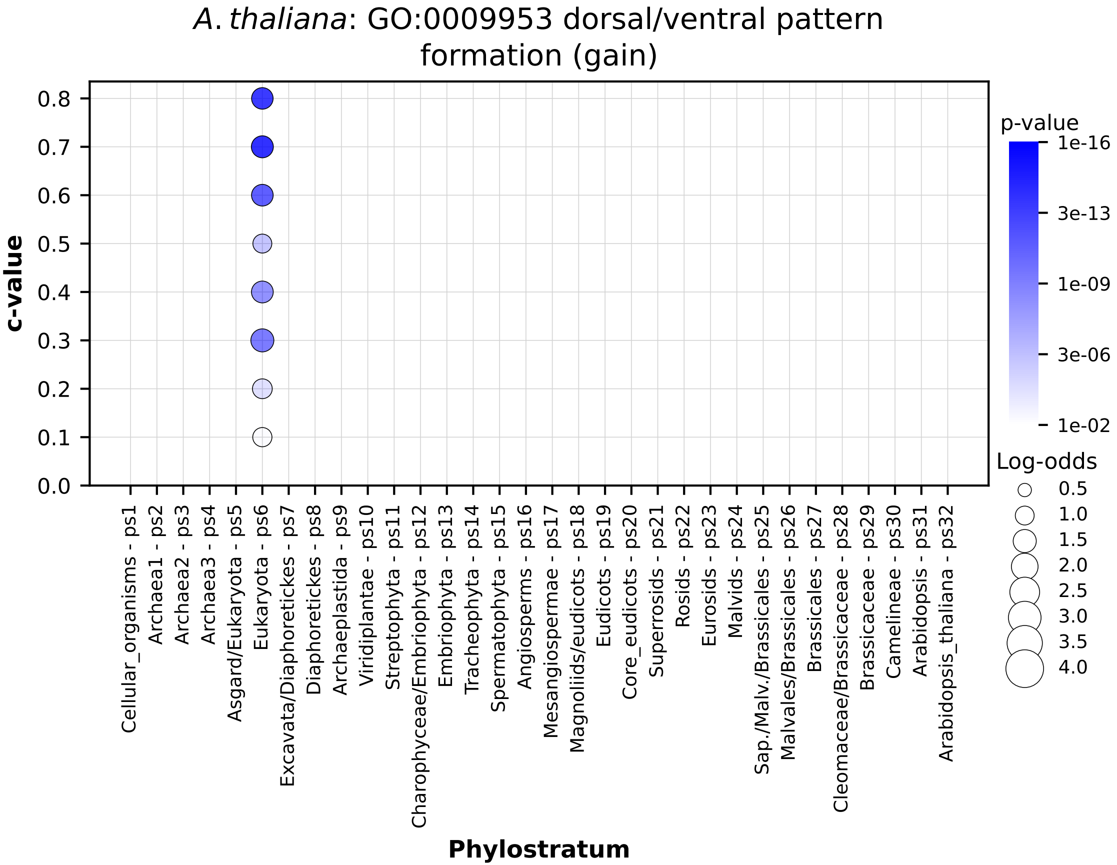

A. thaliana: GO:0009953 dorsal/ventral pattern formation (gain)
c-value
0.0
0.1
0.2
0.3
0.4
0.5
0.6
0.7
0.8
p-value
1e-16
3e-13
1e-09
3e-06
1e-02
Log-odds
0.5
1.0
1.5
2.0
2.5
3.0
3.5
4.0
Cellular_organisms - ps1
Archaea1 - ps2
Archaea2 - ps3
Archaea3 - ps4
Asgard/Eukaryota - ps5
Eukaryota - ps6
Excavata/Diaphoretickes - ps7
Diaphoretickes - ps8
Archaeplastida - ps9
Viridiplantae - ps10
Streptophyta - ps11
Charophyceae/Embriophyta - ps12
Embriophyta - ps13
Tracheophyta - ps14
Spermatophyta - ps15
Angiosperms - ps16
Mesangiospermae - ps17
Magnoliids/eudicots - ps18
Eudicots - ps19
Core_eudicots - ps20
Superrosids - ps21
Rosids - ps22
Eurosids - ps23
Malvids - ps24
Sap./Malv./Brassicales - ps25
Malvales/Brassicales - ps26
Brassicales - ps27
Cleomaceae/Brassicaceae - ps28
Brassicaceae - ps29
Camelineae - ps30
Arabidopsis - ps31
Arabidopsis_thaliana - ps32
Phylostratum

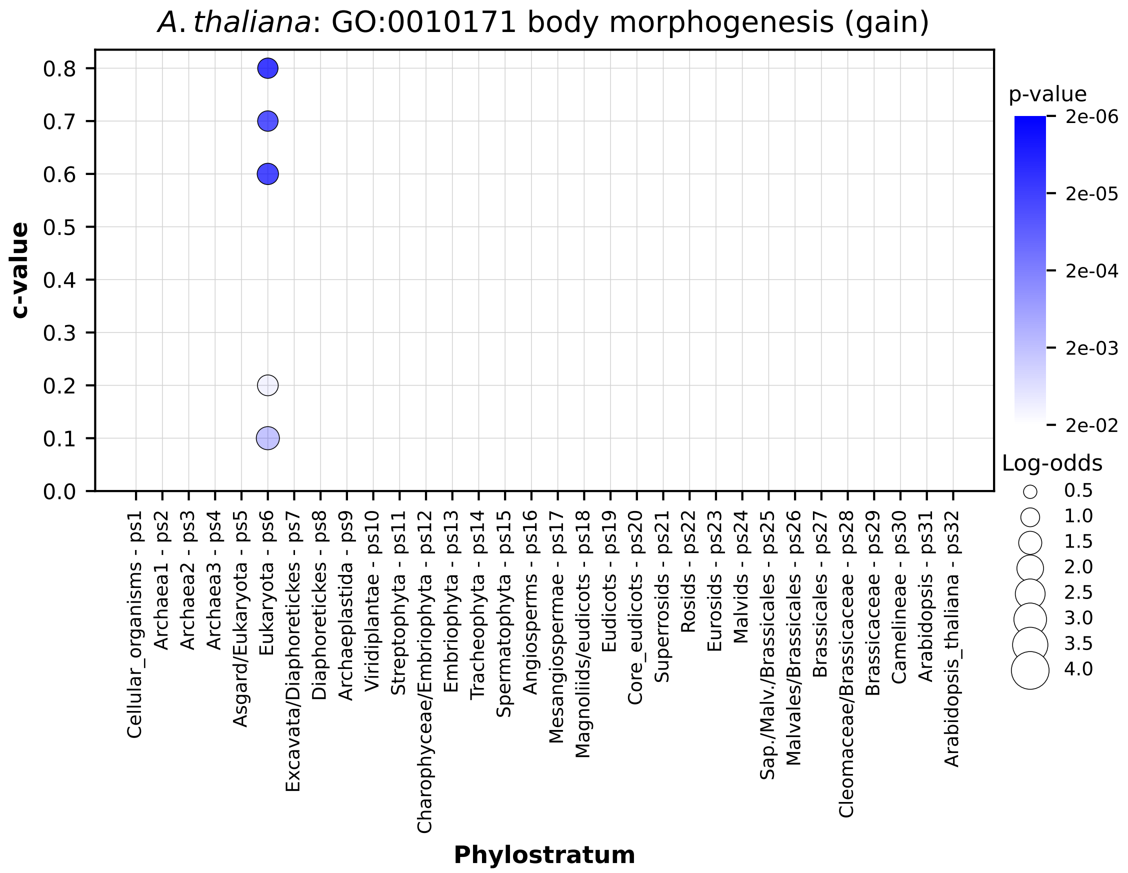

A. thaliana: GO:0010171 body morphogenesis (gain)
c-value
0.0
0.1
0.2
0.3
0.4
0.5
0.6
0.7
0.8
Phylostratum
Cellular_organisms - ps1
Archaea1 - ps2
Archaea2 - ps3
Archaea3 - ps4
Asgard/Eukaryota - ps5
Eukaryota - ps6
Excavata/Diaphoretickes - ps7
Diaphoretickes - ps8
Archaeplastida - ps9
Viridiplantae - ps10
Streptophyta - ps11
Charophyceae/Embriophyta - ps12
Embriophyta - ps13
Tracheophyta - ps14
Spermatophyta - ps15
Angiosperms - ps16
Mesangiospermae - ps17
Magnoliids/eudicots - ps18
Eudicots - ps19
Core_eudicots - ps20
Superrosids - ps21
Rosids - ps22
Eurosids - ps23
Malvids - ps24
Sap./Malv./Brassicales - ps25
Malvales/Brassicales - ps26
Brassicales - ps27
Cleomaceae/Brassicaceae - ps28
Brassicaceae - ps29
Camelineae - ps30
Arabidopsis - ps31
Arabidopsis_thaliana - ps32
p-value
2e-06
2e-05
2e-04
2e-03
2e-02
Log-odds
0.5
1.0
1.5
2.0
2.5
3.0
3.5
4.0

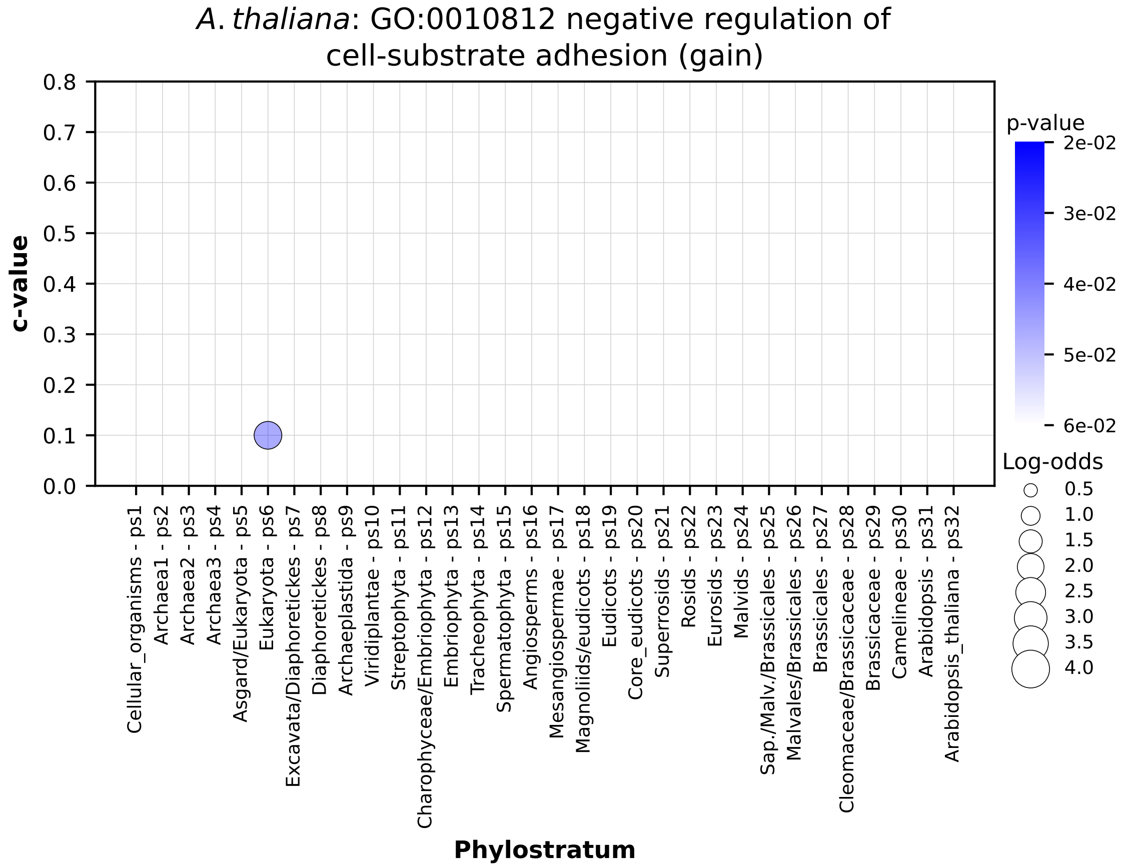

A. thaliana: GO:0010812 negative regulation of cell-substrate adhesion (gain)
c-value
0.8
0.7
0.6
0.5
0.4
0.3
0.2
0.1
0.0
Phylostratum
Cellular_organisms - ps1
Archaea1 - ps2
Archaea2 - ps3
Archaea3 - ps4
Asgard/Eukaryota - ps5
Eukaryota - ps6
Excavata/Diaphoretickes - ps7
Diaphoretickes - ps8
Archaeplastida - ps9
Viridiplantae - ps10
Streptophyta - ps11
Charophyceae/Embriophyta - ps12
Embriophyta - ps13
Tracheophyta - ps14
Spermatophyta - ps15
Angiosperms - ps16
Mesangiospermae - ps17
Magnoliids/eudicots - ps18
Eudicots - ps19
Core_eudicots - ps20
Superrosids - ps21
Rosids - ps22
Eurosids - ps23
Malvids - ps24
Sap./Malv./Brassicales - ps25
Malvales/Brassicales - ps26
Brassicales - ps27
Cleomaceae/Brassicaceae - ps28
Brassicaceae - ps29
Camelineae - ps30
Arabidopsis - ps31
Arabidopsis_thaliana - ps32
p-value
2e-02
3e-02
4e-02
5e-02
6e-02
Log-odds
0.5
1.0
1.5
2.0
2.5
3.0
3.5
4.0

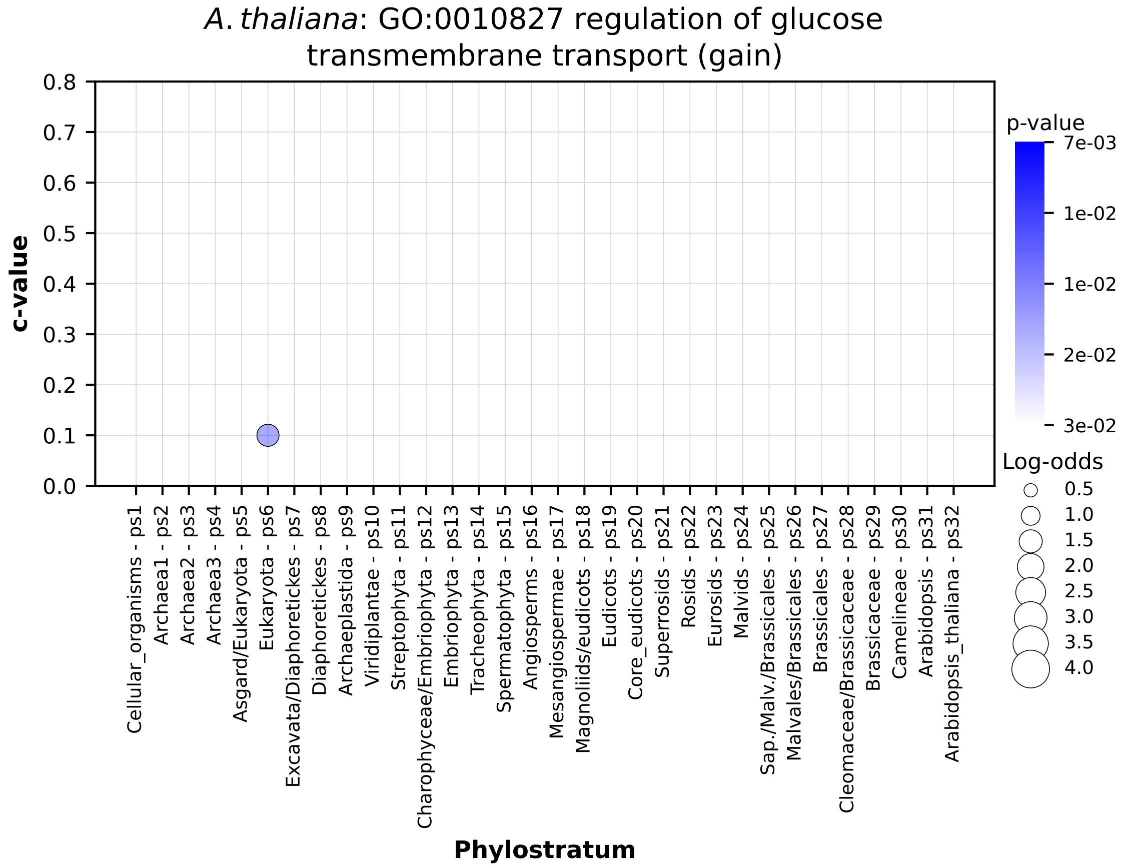

A. thaliana: GO:0010827 regulation of glucose transmembrane transport (gain)
c-value
0.8
0.7
0.6
0.5
0.4
0.3
0.2
0.1
0.0
p-value
7e-03
1e-02
1e-02
2e-02
3e-02
Log-odds
0.5
1.0
1.5
2.0
2.5
3.0
3.5
4.0
Cellular_organisms - ps1
Archaea1 - ps2
Archaea2 - ps3
Archaea3 - ps4
Asgard/Eukaryota - ps5
Eukaryota - ps6
Excavata/Diaphoretickes - ps7
Diaphoretickes - ps8
Archaeplastida - ps9
Viridiplantae - ps10
Streptophyta - ps11
Charophyceae/Embriophyta - ps12
Embriophyta - ps13
Tracheophyta - ps14
Spermatophyta - ps15
Angiosperms - ps16
Mesangiospermae - ps17
Magnoliids/eudicots - ps18
Eudicots - ps19
Core_eudicots - ps20
Superrosids - ps21
Rosids - ps22
Eurosids - ps23
Malvids - ps24
Sap./Malv./Brassicales - ps25
Malvales/Brassicales - ps26
Brassicales - ps27
Cleomaceae/Brassicaceae - ps28
Brassicaceae - ps29
Camelineae - ps30
Arabidopsis - ps31
Arabidopsis_thaliana - ps32
Phylostratum

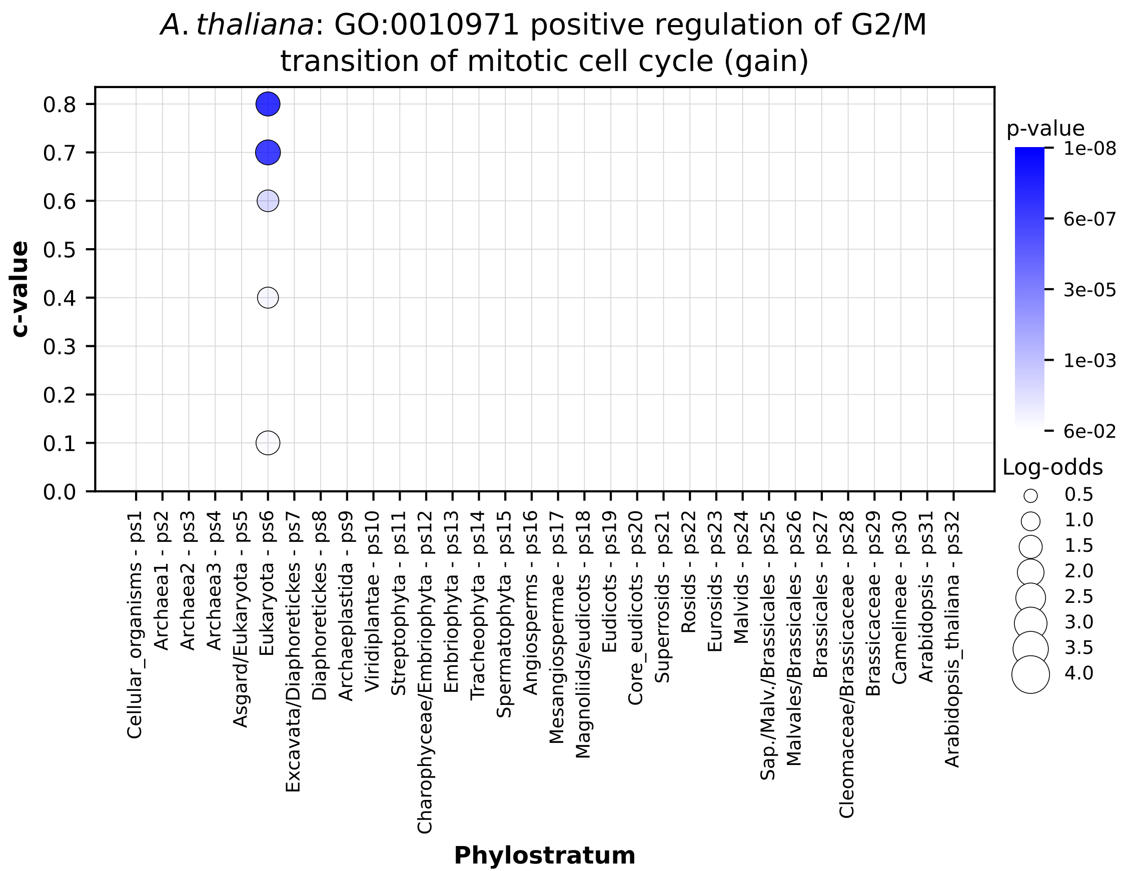

A. thaliana: GO:0010971 positive regulation of G2/M transition of mitotic cell cycle (gain)
c-value
0.0
0.1
0.2
0.3
0.4
0.5
0.6
0.7
0.8
p-value
1e-08
6e-07
3e-05
1e-03
6e-02
Log-odds
0.5
1.0
1.5
2.0
2.5
3.0
3.5
4.0
Cellular_organisms - ps1
Archaea1 - ps2
Archaea2 - ps3
Archaea3 - ps4
Asgard/Eukaryota - ps5
Eukaryota - ps6
Excavata/Diaphoretickes - ps7
Diaphoretickes - ps8
Archaeplastida - ps9
Viridiplantae - ps10
Streptophyta - ps11
Charophyceae/Embriophyta - ps12
Embriophyta - ps13
Tracheophyta - ps14
Spermatophyta - ps15
Angiosperms - ps16
Mesangiospermae - ps17
Magnoliids/eudicots - ps18
Eudicots - ps19
Core_eudicots - ps20
Superrosids - ps21
Rosids - ps22
Eurosids - ps23
Malvids - ps24
Sap./Malv./Brassicales - ps25
Malvales/Brassicales - ps26
Brassicales - ps27
Cleomaceae/Brassicaceae - ps28
Brassicaceae - ps29
Camelineae - ps30
Arabidopsis - ps31
Arabidopsis_thaliana - ps32
Phylostratum

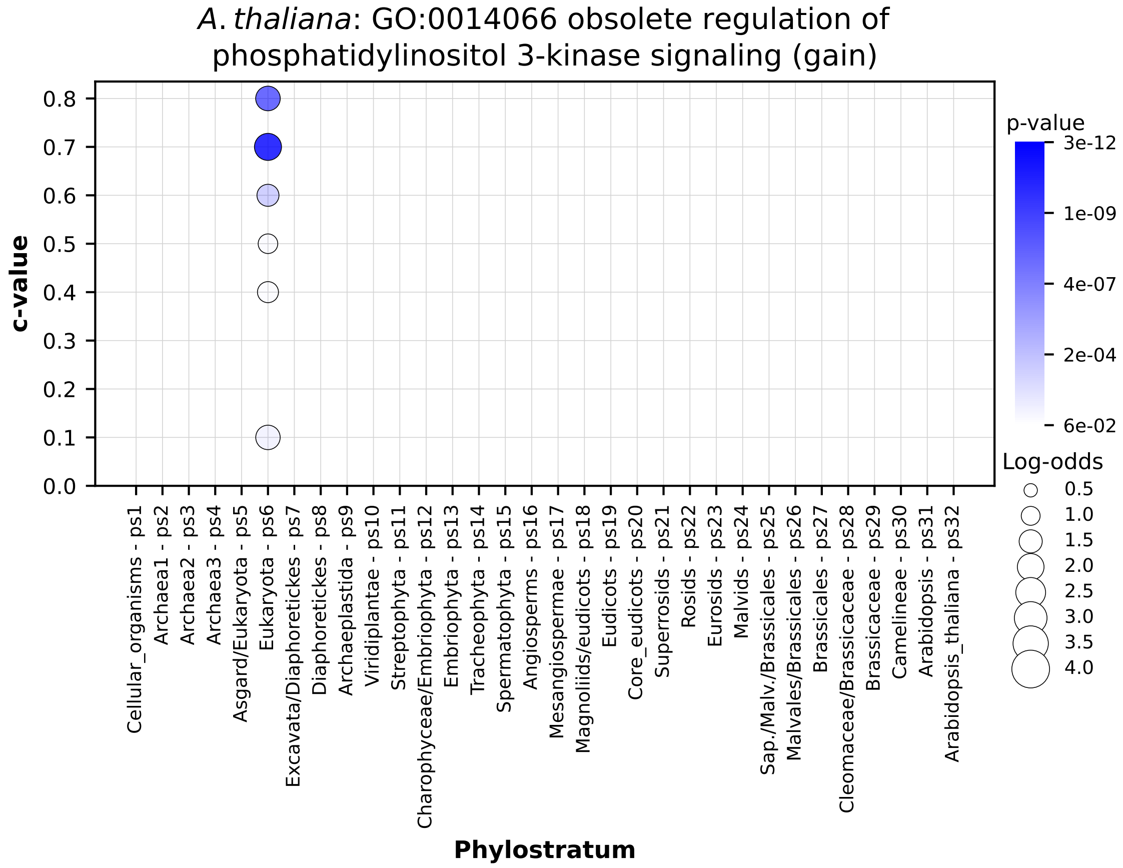

A. thaliana: GO:0014066 obsolete regulation of phosphatidylinositol 3-kinase signaling (gain)
c-value
0.0
0.1
0.2
0.3
0.4
0.5
0.6
0.7
0.8
Phylostratum
Cellular_organisms - ps1
Archaea1 - ps2
Archaea2 - ps3
Archaea3 - ps4
Asgard/Eukaryota - ps5
Eukaryota - ps6
Excavata/Diaphoretickes - ps7
Diaphoretickes - ps8
Archaeplastida - ps9
Viridiplantae - ps10
Streptophyta - ps11
Charophyceae/Embriophyta - ps12
Embriophyta - ps13
Tracheophyta - ps14
Spermatophyta - ps15
Angiosperms - ps16
Mesangiospermae - ps17
Magnoliids/eudicots - ps18
Eudicots - ps19
Core_eudicots - ps20
Superrosids - ps21
Rosids - ps22
Eurosids - ps23
Malvids - ps24
Sap./Malv./Brassicales - ps25
Malvales/Brassicales - ps26
Brassicales - ps27
Cleomaceae/Brassicaceae - ps28
Brassicaceae - ps29
Camelineae - ps30
Arabidopsis - ps31
Arabidopsis_thaliana - ps32
p-value
3e-12
1e-09
4e-07
2e-04
6e-02
Log-odds
0.5
1.0
1.5
2.0
2.5
3.0
3.5
4.0

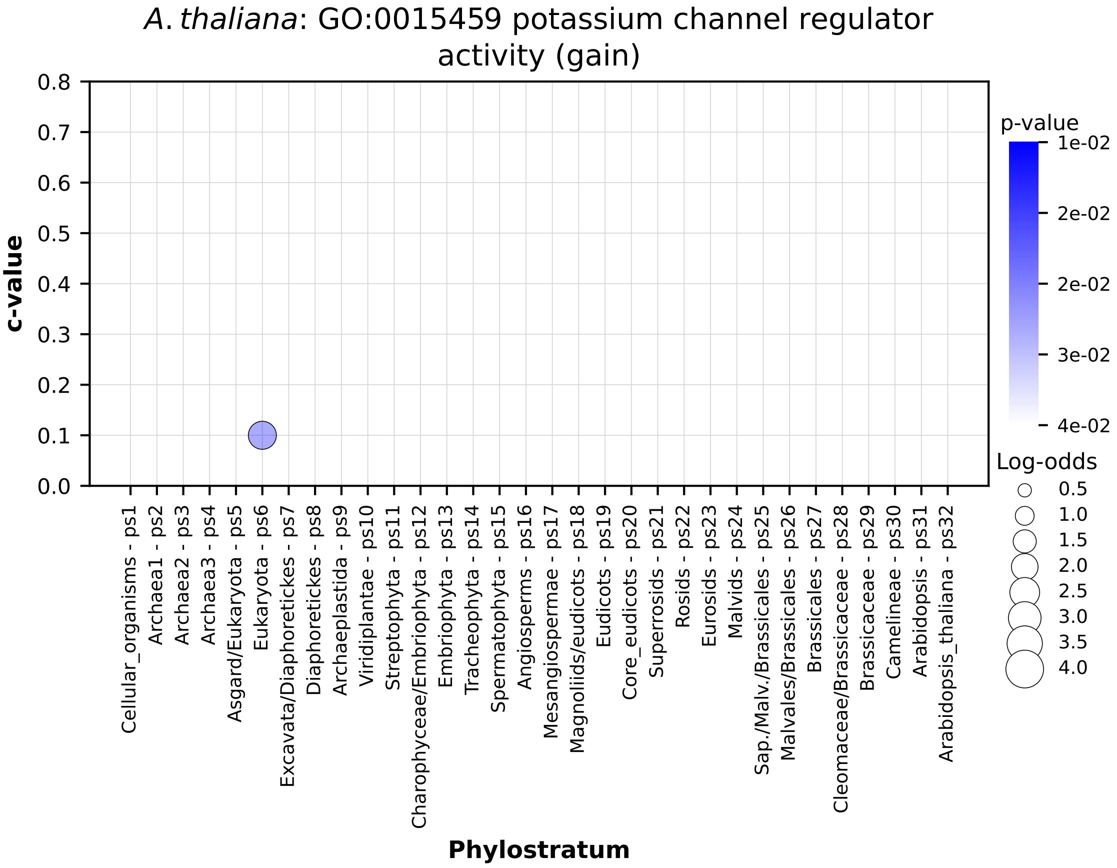
A. thaliana: GO:0015459 potassium channel regulator activity (gain)
c-value
0.0
0.1
0.2
0.3
0.4
0.5
0.6
0.7
0.8
Phylostratum
Cellular_organisms - ps1
Archaea1 - ps2
Archaea2 - ps3
Archaea3 - ps4
Asgard/Eukaryota - ps5
Eukaryota - ps6
Excavata/Diaphoretickes - ps7
Diaphoretickes - ps8
Archaeplastida - ps9
Viridiplantae - ps10
Streptophyta - ps11
Charophyceae/Embriophyta - ps12
Embriophyta - ps13
Tracheophyta - ps14
Spermatophyta - ps15
Angiosperms - ps16
Mesangiospermae - ps17
Magnoliids/eudicots - ps18
Eudicots - ps19
Core_eudicots - ps20
Superrosids - ps21
Rosids - ps22
Eurosids - ps23
Malvids - ps24
Sap./Malv./Brassicales - ps25
Malvales/Brassicales - ps26
Brassicales - ps27
Cleomaceae/Brassicaceae - ps28
Brassicaceae - ps29
Camelineae - ps30
Arabidopsis - ps31
Arabidopsis_thaliana - ps32
p-value
1e-02
2e-02
2e-02
3e-02
4e-02
Log-odds
0.5
1.0
1.5
2.0
2.5
3.0
3.5
4.0

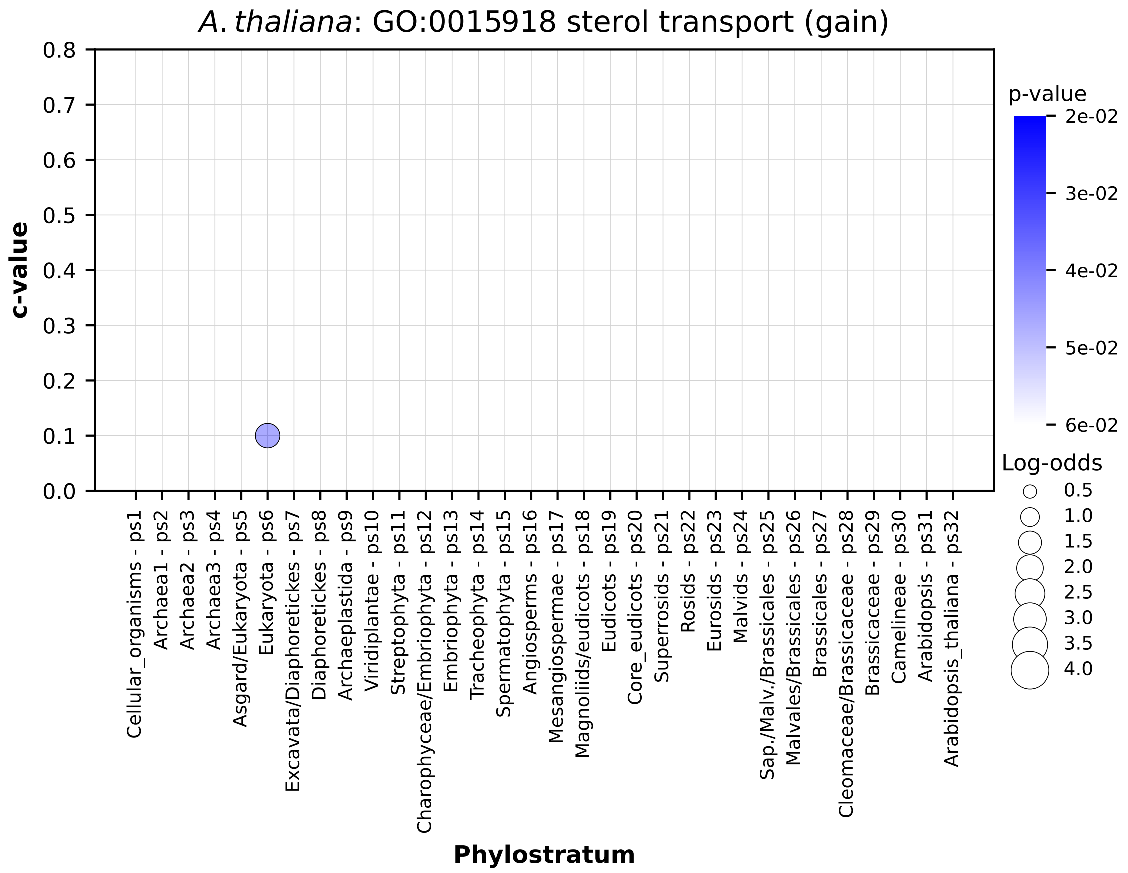
A. thaliana: GO:0015918 sterol transport (gain)
c-value
0.0
0.1
0.2
0.3
0.4
0.5
0.6
0.7
0.8
Phylostratum
Cellular_organisms - ps1
Archaea1 - ps2
Archaea2 - ps3
Archaea3 - ps4
Asgard/Eukaryota - ps5
Eukaryota - ps6
Excavata/Diaphoretickes - ps7
Diaphoretickes - ps8
Archaeplastida - ps9
Viridiplantae - ps10
Streptophyta - ps11
Charophyceae/Embriophyta - ps12
Embriophyta - ps13
Tracheophyta - ps14
Spermatophyta - ps15
Angiosperms - ps16
Mesangiospermae - ps17
Magnoliids/eudicots - ps18
Eudicots - ps19
Core_eudicots - ps20
Superrosids - ps21
Rosids - ps22
Eurosids - ps23
Malvids - ps24
Sap./Malv./Brassicales - ps25
Malvales/Brassicales - ps26
Brassicales - ps27
Cleomaceae/Brassicaceae - ps28
Brassicaceae - ps29
Camelineae - ps30
Arabidopsis - ps31
Arabidopsis_thaliana - ps32
p-value
2e-02
3e-02
4e-02
5e-02
6e-02
Log-odds
0.5
1.0
1.5
2.0
2.5
3.0
3.5
4.0

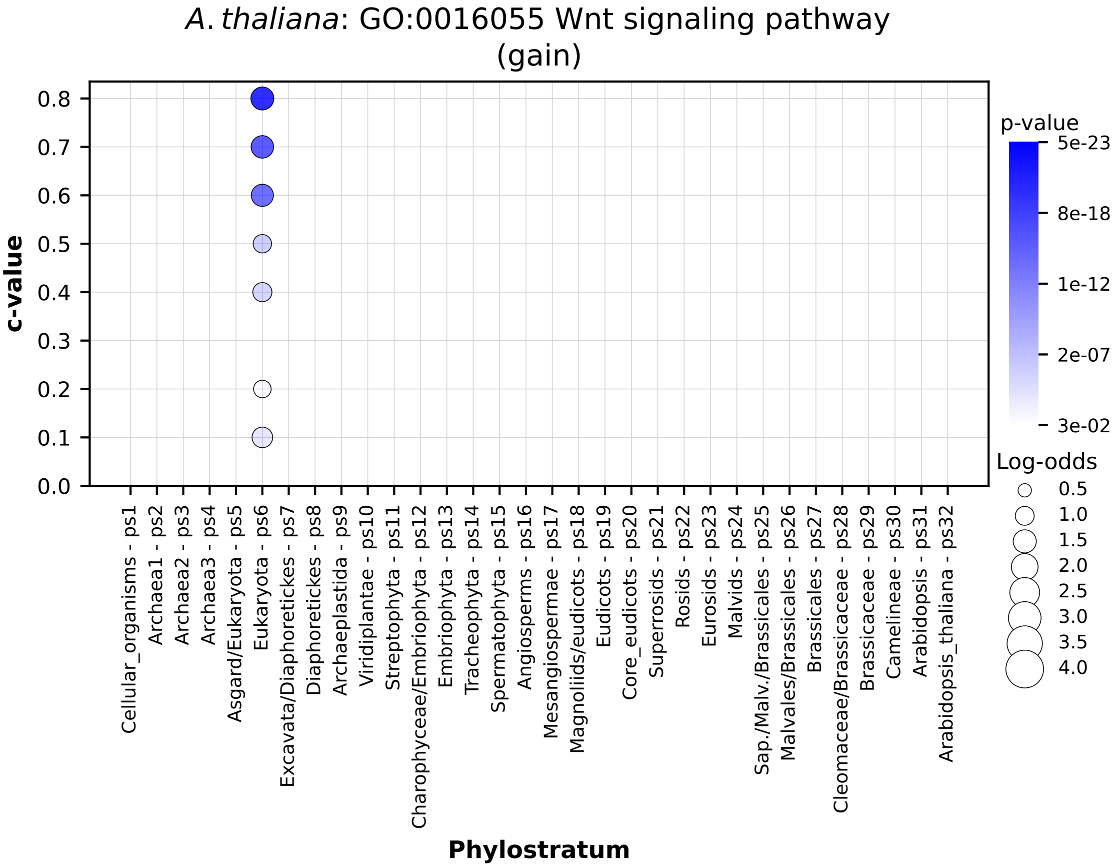
A. thaliana: GO:0016055 Wnt signaling pathway
(gain)
c-value
0.0
0.1
0.2
0.3
0.4
0.5
0.6
0.7
0.8
Phylostratum
Cellular_organisms - ps1
Archaea1 - ps2
Archaea2 - ps3
Archaea3 - ps4
Asgard/Eukaryota - ps5
Eukaryota - ps6
Excavata/Diaphoretickes - ps7
Diaphoretickes - ps8
Archaeplastida - ps9
Viridiplantae - ps10
Streptophyta - ps11
Charophyceae/Embriophyta - ps12
Embriophyta - ps13
Tracheophyta - ps14
Spermatophyta - ps15
Angiosperms - ps16
Mesangiospermae - ps17
Magnoliids/eudicots - ps18
Eudicots - ps19
Core_eudicots - ps20
Superrosids - ps21
Rosids - ps22
Eurosids - ps23
Malvids - ps24
Sap./Malv./Brassicales - ps25
Malvales/Brassicales - ps26
Brassicales - ps27
Cleomaceae/Brassicaceae - ps28
Brassicaceae - ps29
Camelineae - ps30
Arabidopsis - ps31
Arabidopsis_thaliana - ps32
p-value
5e-23
8e-18
1e-12
2e-07
3e-02
Log-odds
0.5
1.0
1.5
2.0
2.5
3.0
3.5
4.0

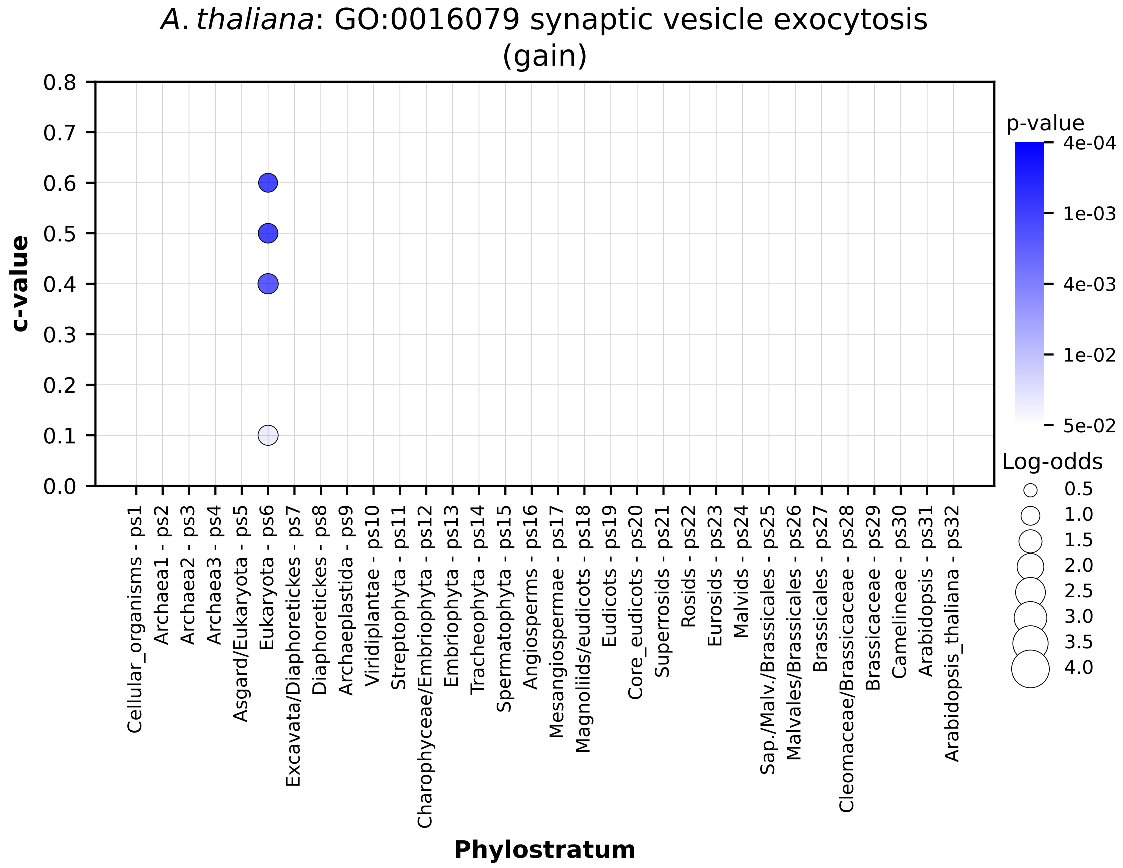

A. thaliana: GO:0016079 synaptic vesicle exocytosis (gain)
c-value
0.0
0.1
0.2
0.3
0.4
0.5
0.6
0.7
0.8
Phylostratum
Cellular_organisms - ps1
Archaea1 - ps2
Archaea2 - ps3
Archaea3 - ps4
Asgard/Eukaryota - ps5
Eukaryota - ps6
Excavata/Diaphoretickes - ps7
Diaphoretickes - ps8
Archaeplastida - ps9
Viridiplantae - ps10
Streptophyta - ps11
Charophyceae/Embriophyta - ps12
Embriophyta - ps13
Tracheophyta - ps14
Spermatophyta - ps15
Angiosperms - ps16
Mesangiospermae - ps17
Magnoliids/eudicots - ps18
Eudicots - ps19
Core_eudicots - ps20
Superrosids - ps21
Rosids - ps22
Eurosids - ps23
Malvids - ps24
Sap./Malv./Brassicales - ps25
Malvales/Brassicales - ps26
Brassicales - ps27
Cleomaceae/Brassicaceae - ps28
Brassicaceae - ps29
Camelineae - ps30
Arabidopsis - ps31
Arabidopsis_thaliana - ps32
p-value
4e-04
1e-03
4e-03
1e-02
5e-02
Log-odds
0.5
1.0
1.5
2.0
2.5
3.0
3.5
4.0

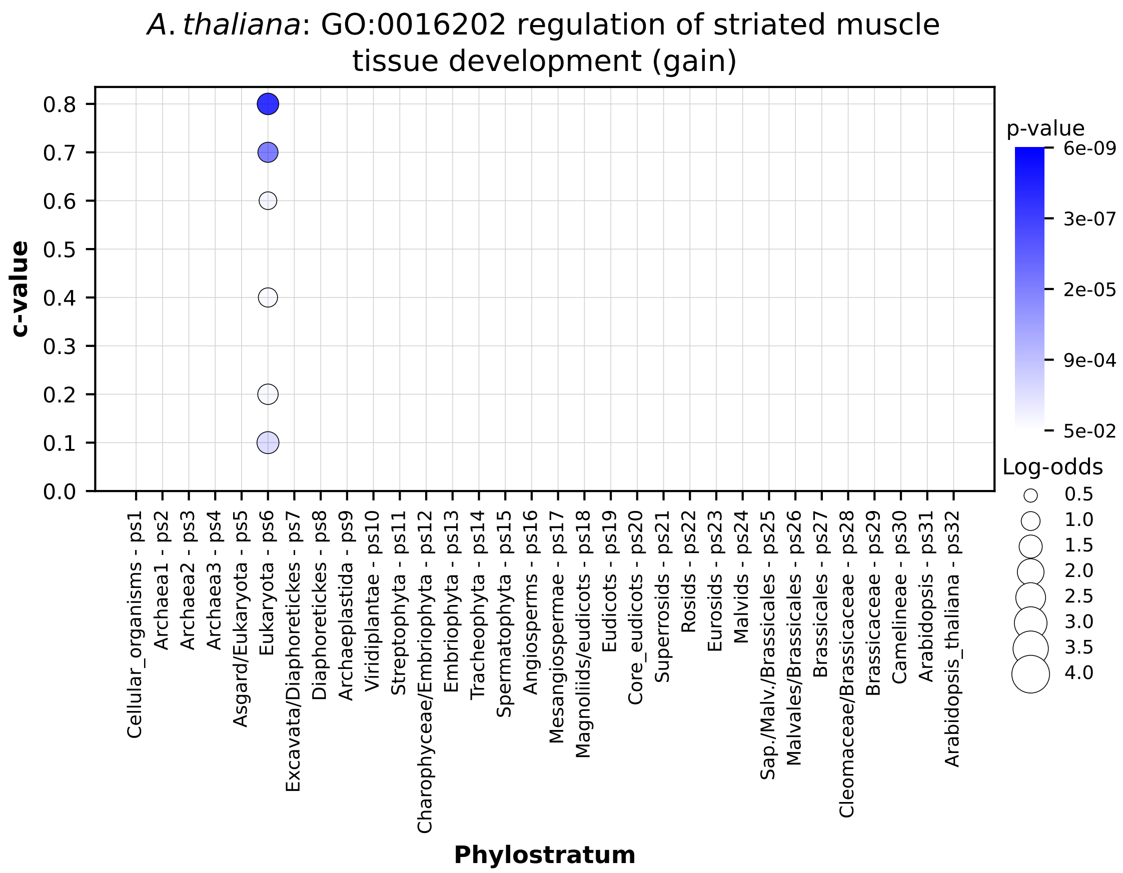
A. thaliana: GO:0016202 regulation of striated muscle tissue development (gain)
c-value
0.8
0.7
0.6
0.5
0.4
0.3
0.2
0.1
0.0
Phylostratum
Cellular_organisms - ps1
Archaea1 - ps2
Archaea2 - ps3
Archaea3 - ps4
Asgard/Eukaryota - ps5
Eukaryota - ps6
Excavata/Diaphoretickes - ps7
Diaphoretickes - ps8
Archaeplastida - ps9
Viridiplantae - ps10
Streptophyta - ps11
Charophyceae/Embriophyta - ps12
Embriophyta - ps13
Tracheophyta - ps14
Spermatophyta - ps15
Angiosperms - ps16
Mesangiospermae - ps17
Magnoliids/eudicots - ps18
Eudicots - ps19
Core_eudicots - ps20
Superrosids - ps21
Rosids - ps22
Eurosids - ps23
Malvids - ps24
Sap./Malv./Brassicales - ps25
Malvales/Brassicales - ps26
Brassicales - ps27
Cleomaceae/Brassicaceae - ps28
Brassicaceae - ps29
Camelineae - ps30
Arabidopsis - ps31
Arabidopsis_thaliana - ps32
p-value
6e-09
3e-07
2e-05
9e-04
5e-02
Log-odds
0.5
1.0
1.5
2.0
2.5
3.0
3.5
4.0

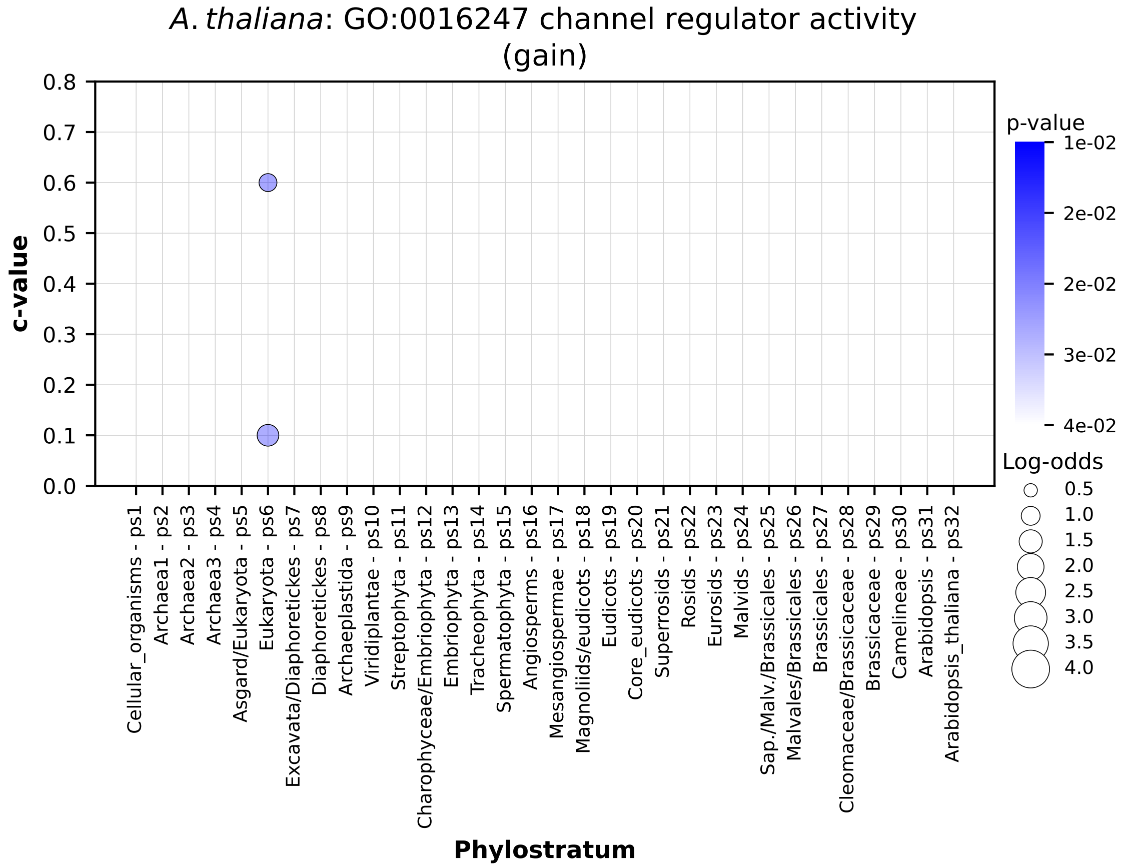

A. thaliana: GO:0016247 channel regulator activity
(gain)
c-value
0.0
0.1
0.2
0.3
0.4
0.5
0.6
0.7
0.8
Phylostratum
Cellular_organisms - ps1
Archaea1 - ps2
Archaea2 - ps3
Archaea3 - ps4
Asgard/Eukaryota - ps5
Eukaryota - ps6
Excavata/Diaphoretickes - ps7
Diaphoretickes - ps8
Archaeplastida - ps9
Viridiplantae - ps10
Streptophyta - ps11
Charophyceae/Embriophyta - ps12
Embriophyta - ps13
Tracheophyta - ps14
Spermatophyta - ps15
Angiosperms - ps16
Mesangiospermae - ps17
Magnoliids/eudicots - ps18
Eudicots - ps19
Core_eudicots - ps20
Superrosids - ps21
Rosids - ps22
Eurosids - ps23
Malvids - ps24
Sap./Malv./Brassicales - ps25
Malvales/Brassicales - ps26
Brassicales - ps27
Cleomaceae/Brassicaceae - ps28
Brassicaceae - ps29
Camelineae - ps30
Arabidopsis - ps31
Arabidopsis_thaliana - ps32
p-value
1e-02
2e-02
2e-02
3e-02
4e-02
Log-odds
0.5
1.0
1.5
2.0
2.5
3.0
3.5
4.0

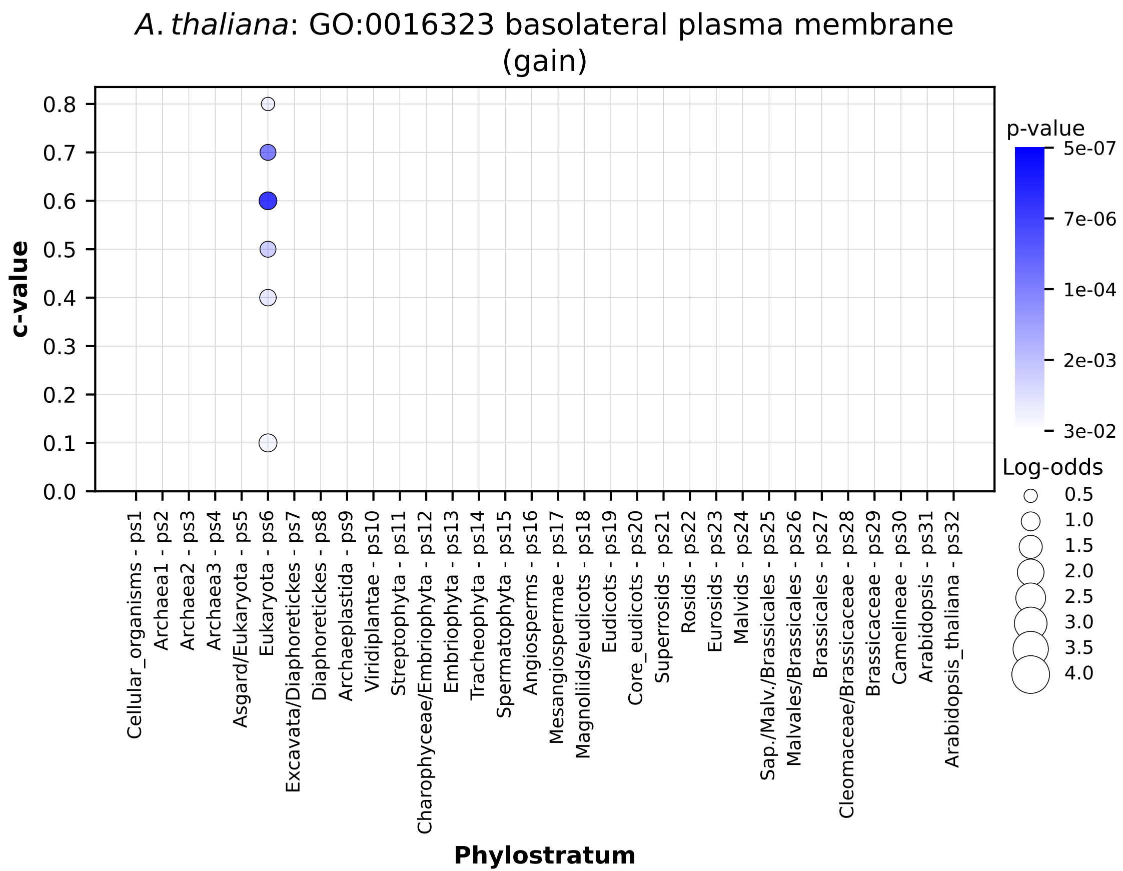

A. thaliana: GO:0016323 basolateral plasma membrane
(gain)
c-value
0.0
0.1
0.2
0.3
0.4
0.5
0.6
0.7
0.8
p-value
5e-07
7e-06
1e-04
2e-03
3e-02
Log-odds
0.5
1.0
1.5
2.0
2.5
3.0
3.5
4.0
Cellular_organisms - ps1
Archaea1 - ps2
Archaea2 - ps3
Archaea3 - ps4
Asgard/Eukaryota - ps5
Eukaryota - ps6
Excavata/Diaphoretickes - ps7
Diaphoretickes - ps8
Archaeplastida - ps9
Viridiplantae - ps10
Streptophyta - ps11
Charophyceae/Embriophyta - ps12
Embriophyta - ps13
Tracheophyta - ps14
Spermatophyta - ps15
Angiosperms - ps16
Mesangiospermae - ps17
Magnoliids/eudicots - ps18
Eudicots - ps19
Core_eudicots - ps20
Superrosids - ps21
Rosids - ps22
Eurosids - ps23
Malvids - ps24
Sap./Malv./Brassicales - ps25
Malvales/Brassicales - ps26
Brassicales - ps27
Cleomaceae/Brassicaceae - ps28
Brassicaceae - ps29
Camelineae - ps30
Arabidopsis - ps31
Arabidopsis_thaliana - ps32
Phylostratum

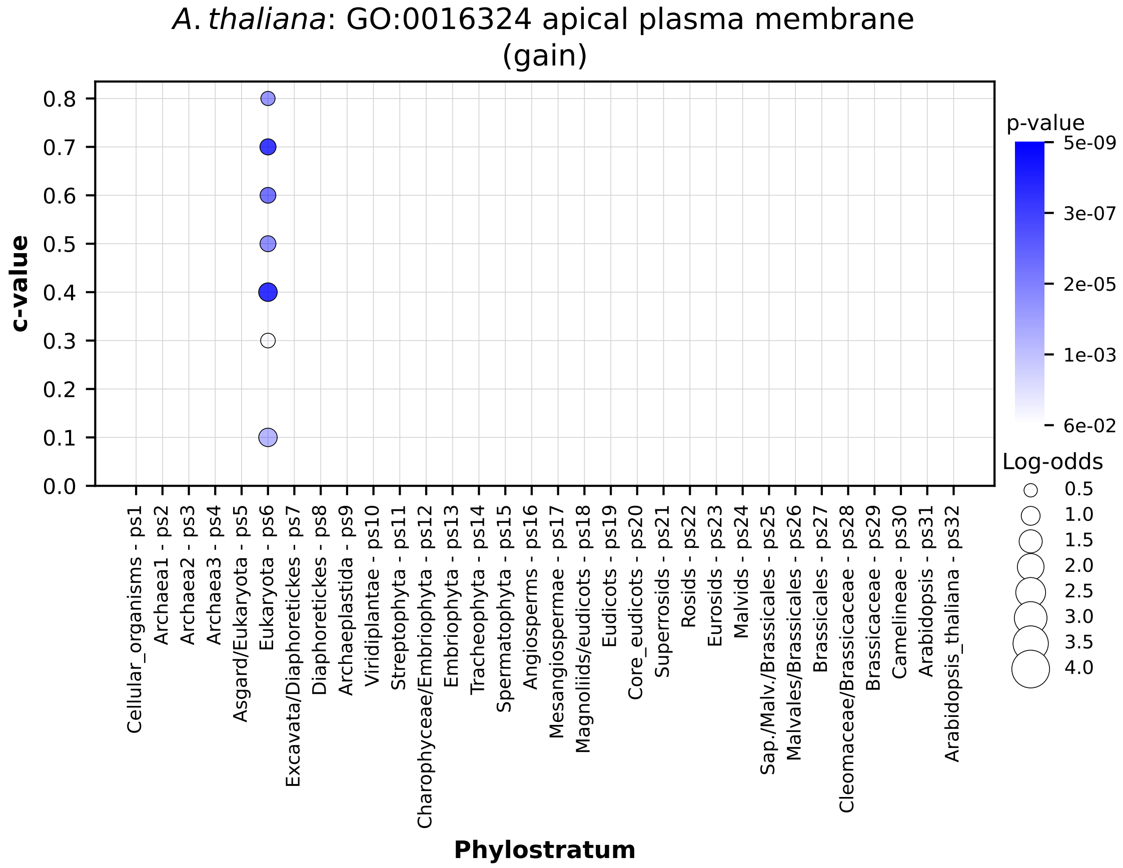

A. thaliana: GO:0016324 apical plasma membrane
(gain)
c-value
0.0
0.1
0.2
0.3
0.4
0.5
0.6
0.7
0.8
Phylostratum
Cellular_organisms - ps1
Archaea1 - ps2
Archaea2 - ps3
Archaea3 - ps4
Asgard/Eukaryota - ps5
Eukaryota - ps6
Excavata/Diaphoretickes - ps7
Diaphoretickes - ps8
Archaeplastida - ps9
Viridiplantae - ps10
Streptophyta - ps11
Charophyceae/Embriophyta - ps12
Embriophyta - ps13
Tracheophyta - ps14
Spermatophyta - ps15
Angiosperms - ps16
Mesangiospermae - ps17
Magnoliids/eudicots - ps18
Eudicots - ps19
Core_eudicots - ps20
Superrosids - ps21
Rosids - ps22
Eurosids - ps23
Malvids - ps24
Sap./Malv./Brassicales - ps25
Malvales/Brassicales - ps26
Brassicales - ps27
Cleomaceae/Brassicaceae - ps28
Brassicaceae - ps29
Camelineae - ps30
Arabidopsis - ps31
Arabidopsis_thaliana - ps32
p-value
5e-09
3e-07
2e-05
1e-03
6e-02
Log-odds
0.5
1.0
1.5
2.0
2.5
3.0
3.5
4.0

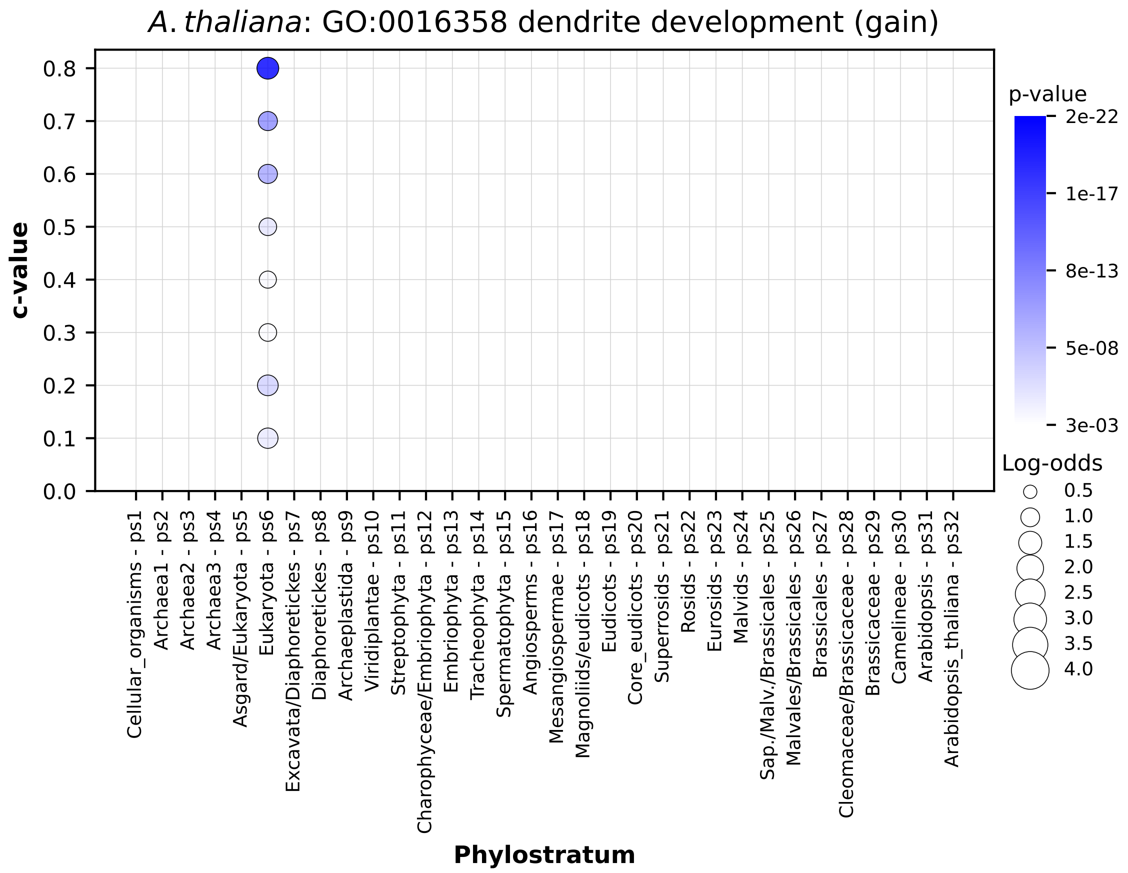

A. thaliana: GO:0016358 dendrite development (gain)
c-value
0.0
0.1
0.2
0.3
0.4
0.5
0.6
0.7
0.8
Phylostratum
Cellular_organisms - ps1
Archaea1 - ps2
Archaea2 - ps3
Archaea3 - ps4
Asgard/Eukaryota - ps5
Eukaryota - ps6
Excavata/Diaphoretickes - ps7
Diaphoretickes - ps8
Archaeplastida - ps9
Viridiplantae - ps10
Streptophyta - ps11
Charophyceae/Embriophyta - ps12
Embriophyta - ps13
Tracheophyta - ps14
Spermatophyta - ps15
Angiosperms - ps16
Mesangiospermae - ps17
Magnoliids/eudicots - ps18
Eudicots - ps19
Core_eudicots - ps20
Superrosids - ps21
Rosids - ps22
Eurosids - ps23
Malvids - ps24
Sap./Malv./Brassicales - ps25
Malvales/Brassicales - ps26
Brassicales - ps27
Cleomaceae/Brassicaceae - ps28
Brassicaceae - ps29
Camelineae - ps30
Arabidopsis - ps31
Arabidopsis_thaliana - ps32
p-value
2e-22
1e-17
8e-13
5e-08
3e-03
Log-odds
0.5
1.0
1.5
2.0
2.5
3.0
3.5
4.0

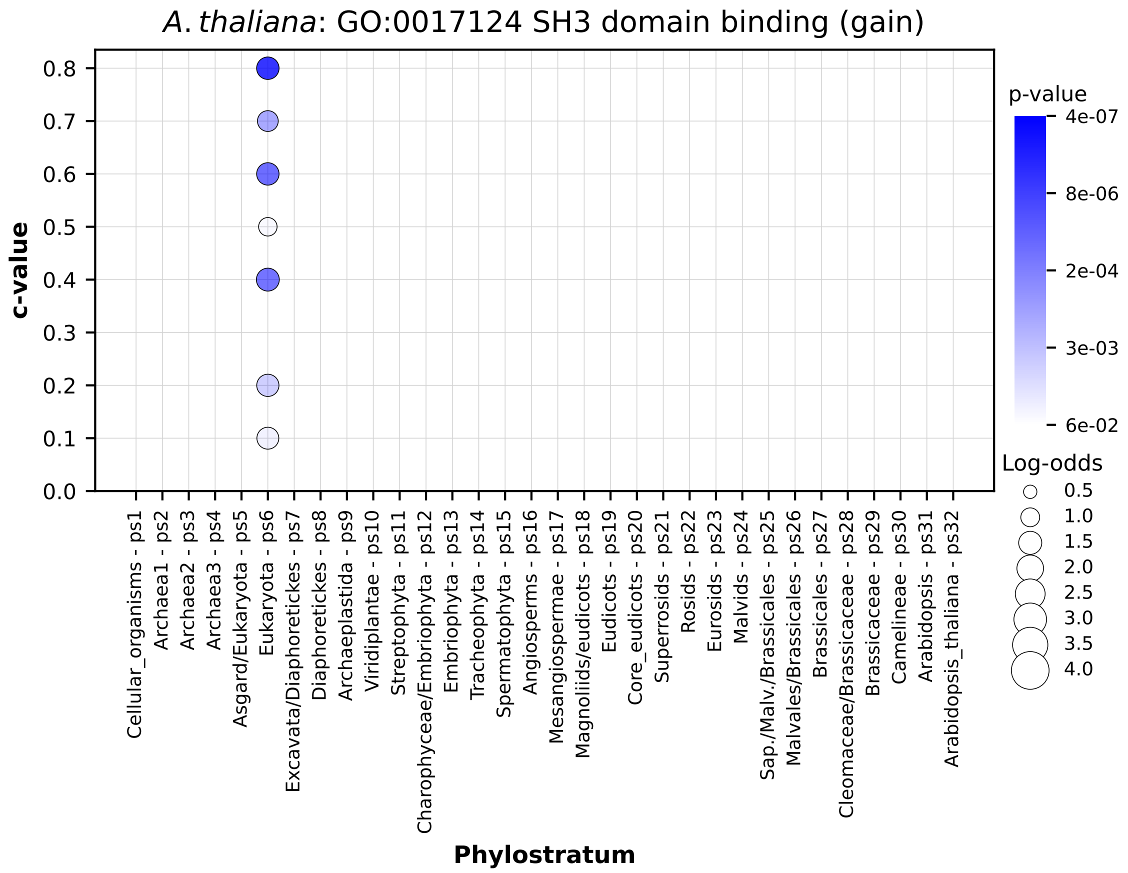

A. thaliana: GO:0017124 SH3 domain binding (gain)
c-value
0.8
0.7
0.6
0.5
0.4
0.3
0.2
0.1
0.0
Phylostratum
Cellular_organisms - ps1
Archaea1 - ps2
Archaea2 - ps3
Archaea3 - ps4
Asgard/Eukaryota - ps5
Eukaryota - ps6
Excavata/Diaphoretickes - ps7
Diaphoretickes - ps8
Archaeplastida - ps9
Viridiplantae - ps10
Streptophyta - ps11
Charophyceae/Embriophyta - ps12
Embriophyta - ps13
Tracheophyta - ps14
Spermatophyta - ps15
Angiosperms - ps16
Mesangiospermae - ps17
Magnoliids/eudicots - ps18
Eudicots - ps19
Core_eudicots - ps20
Superrosids - ps21
Rosids - ps22
Eurosids - ps23
Malvids - ps24
Sap./Malv./Brassicales - ps25
Malvales/Brassicales - ps26
Brassicales - ps27
Cleomaceae/Brassicaceae - ps28
Brassicaceae - ps29
Camelineae - ps30
Arabidopsis - ps31
Arabidopsis_thaliana - ps32
p-value
4e-07
8e-06
2e-04
3e-03
6e-02
Log-odds
0.5
1.0
1.5
2.0
2.5
3.0
3.5
4.0

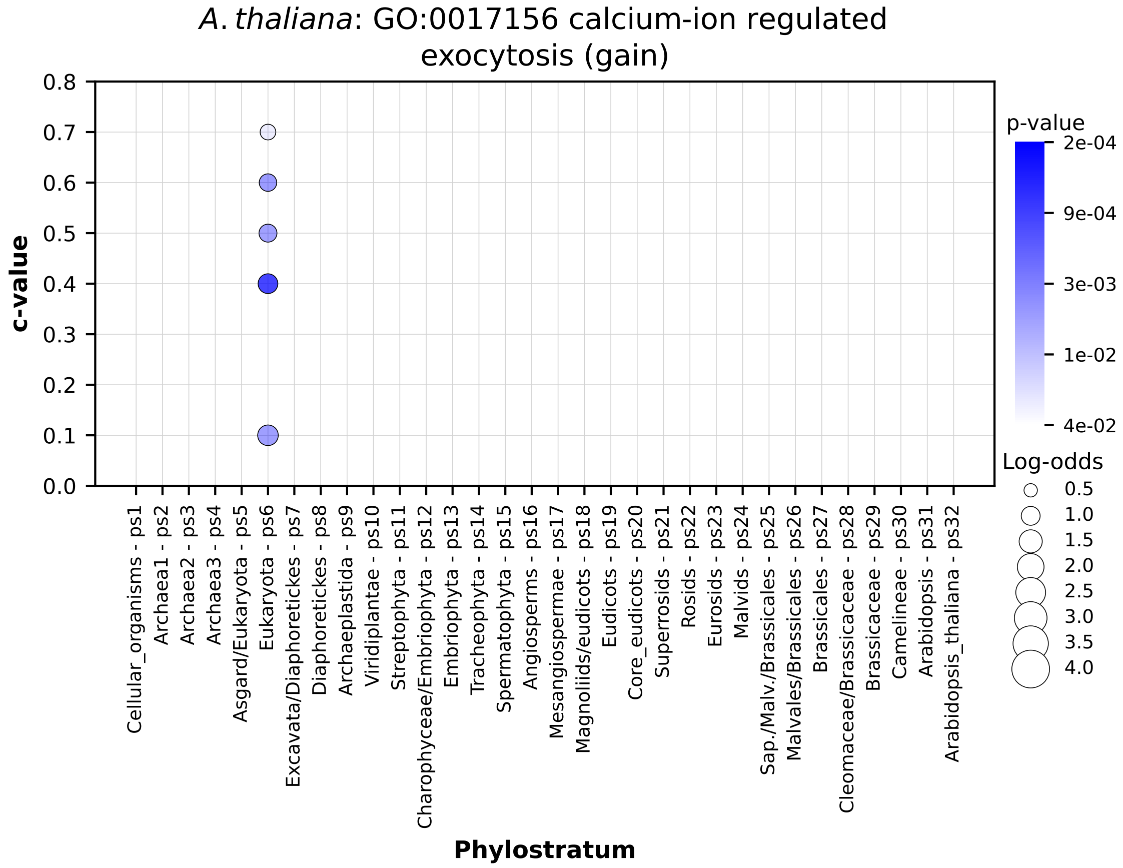

A. thaliana: GO:0017156 calcium-ion regulated exocytosis (gain)
c-value
0.8
0.7
0.6
0.5
0.4
0.3
0.2
0.1
0.0
p-value
2e-04
9e-04
3e-03
1e-02
4e-02
Log-odds
0.5
1.0
1.5
2.0
2.5
3.0
3.5
4.0
Cellular_organisms - ps1
Archaea1 - ps2
Archaea2 - ps3
Archaea3 - ps4
Asgard/Eukaryota - ps5
Eukaryota - ps6
Excavata/Diaphoretickes - ps7
Diaphoretickes - ps8
Archaeplastida - ps9
Viridiplantae - ps10
Streptophyta - ps11
Charophyceae/Embriophyta - ps12
Embriophyta - ps13
Tracheophyta - ps14
Spermatophyta - ps15
Angiosperms - ps16
Mesangiospermae - ps17
Magnoliids/eudicots - ps18
Eudicots - ps19
Core_eudicots - ps20
Superrosids - ps21
Rosids - ps22
Eurosids - ps23
Malvids - ps24
Sap./Malv./Brassicales - ps25
Malvales/Brassicales - ps26
Brassicales - ps27
Cleomaceae/Brassicaceae - ps28
Brassicaceae - ps29
Camelineae - ps30
Arabidopsis - ps31
Arabidopsis_thaliana - ps32
Phylostratum

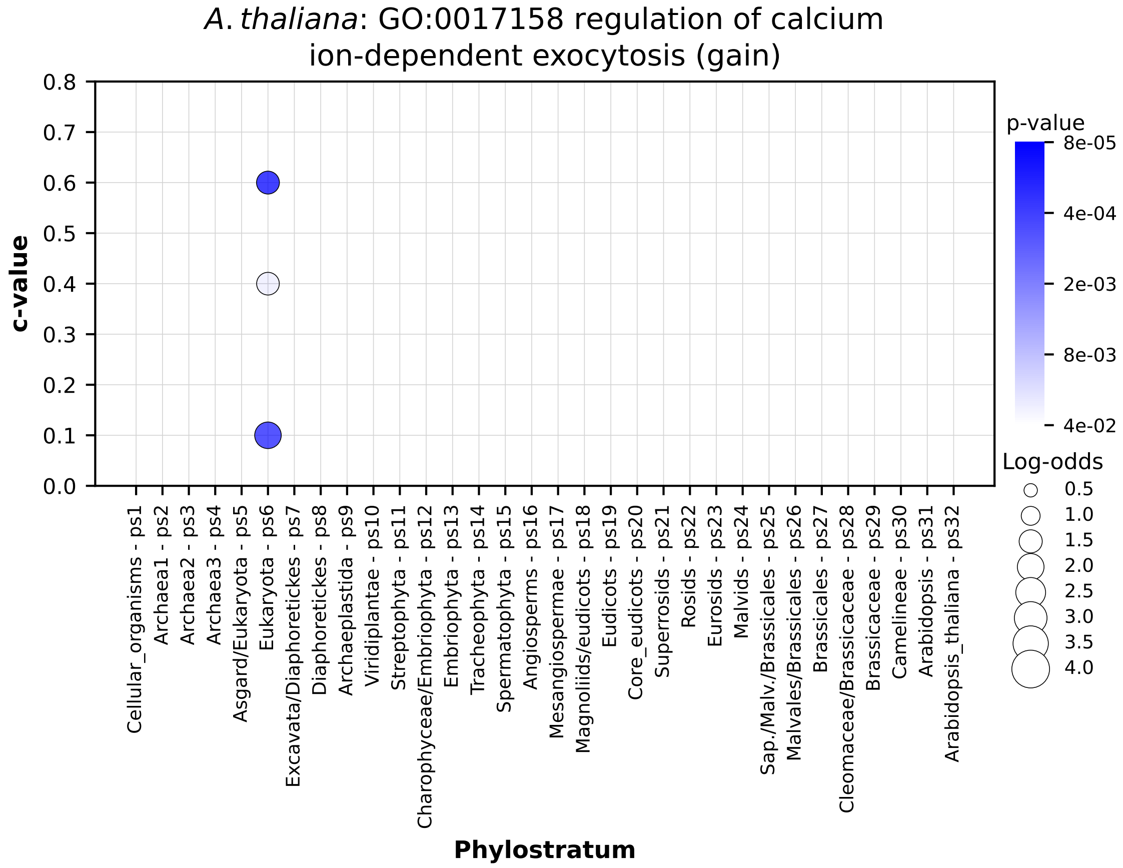
A. thaliana: GO:0017158 regulation of calcium ion-dependent exocytosis (gain)
c-value
0.8
0.7
0.6
0.5
0.4
0.3
0.2
0.1
0.0
p-value
8e-05
4e-04
2e-03
8e-03
4e-02
Log-odds
0.5
1.0
1.5
2.0
2.5
3.0
3.5
4.0
Cellular_organisms - ps1
Archaea1 - ps2
Archaea2 - ps3
Archaea3 - ps4
Asgard/Eukaryota - ps5
Eukaryota - ps6
Excavata/Diaphoretickes - ps7
Diaphoretickes - ps8
Archaeplastida - ps9
Viridiplantae - ps10
Streptophyta - ps11
Charophyceae/Embriophyta - ps12
Embriophyta - ps13
Tracheophyta - ps14
Spermatophyta - ps15
Angiosperms - ps16
Mesangiospermae - ps17
Magnoliids/eudicots - ps18
Eudicots - ps19
Core_eudicots - ps20
Superrosids - ps21
Rosids - ps22
Eurosids - ps23
Malvids - ps24
Sap./Malv./Brassicales - ps25
Malvales/Brassicales - ps26
Brassicales - ps27
Cleomaceae/Brassicaceae - ps28
Brassicaceae - ps29
Camelineae - ps30
Arabidopsis - ps31
Arabidopsis_thaliana - ps32
Phylostratum

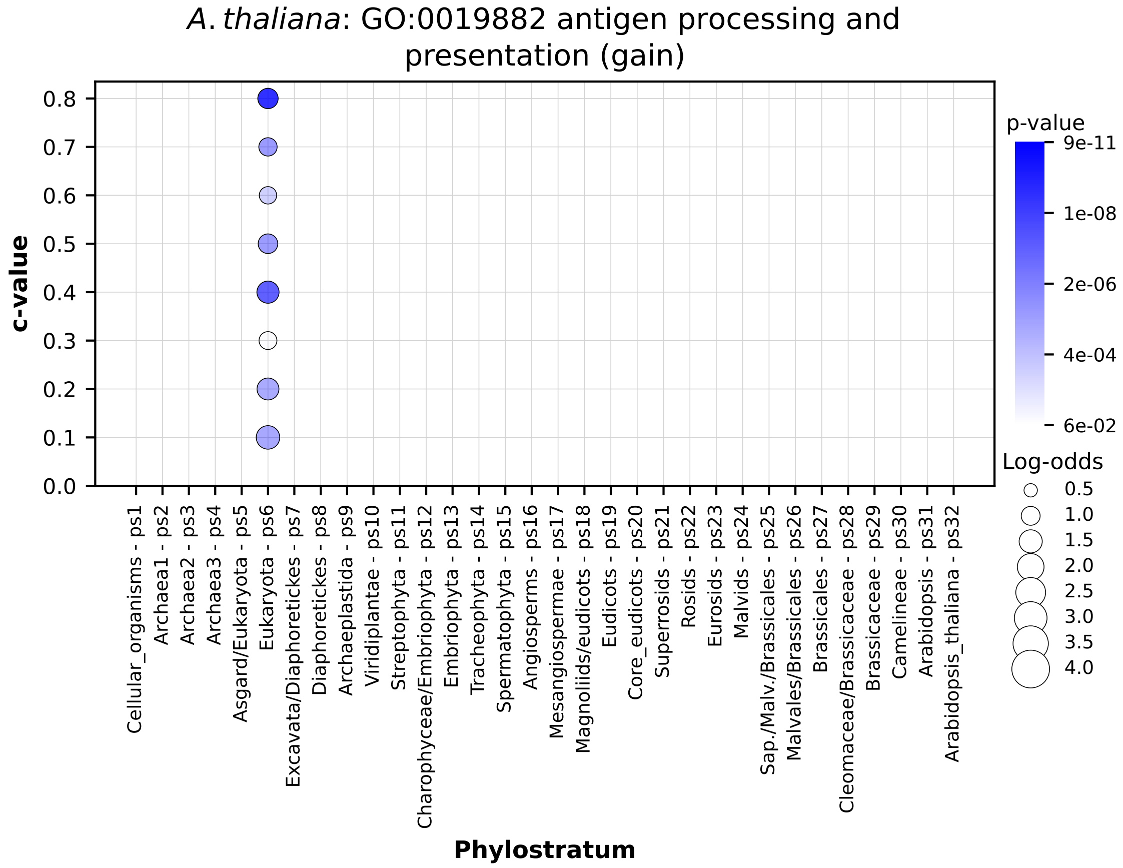

A. thaliana: GO:0019882 antigen processing and presentation (gain)
c-value
0.8
0.7
0.6
0.5
0.4
0.3
0.2
0.1
0.0
p-value
9e-11
1e-08
2e-06
4e-04
6e-02
Log-odds
0.5
1.0
1.5
2.0
2.5
3.0
3.5
4.0
Cellular_organisms - ps1
Archaea1 - ps2
Archaea2 - ps3
Archaea3 - ps4
Asgard/Eukaryota - ps5
Eukaryota - ps6
Excavata/Diaphoretickes - ps7
Diaphoretickes - ps8
Archaeplastida - ps9
Viridiplantae - ps10
Streptophyta - ps11
Charophyceae/Embriophyta - ps12
Embriophyta - ps13
Tracheophyta - ps14
Spermatophyta - ps15
Angiosperms - ps16
Mesangiospermae - ps17
Magnoliids/eudicots - ps18
Eudicots - ps19
Core_eudicots - ps20
Superrosids - ps21
Rosids - ps22
Eurosids - ps23
Malvids - ps24
Sap./Malv./Brassicales - ps25
Malvales/Brassicales - ps26
Brassicales - ps27
Cleomaceae/Brassicaceae - ps28
Brassicaceae - ps29
Camelineae - ps30
Arabidopsis - ps31
Arabidopsis_thaliana - ps32
Phylostratum

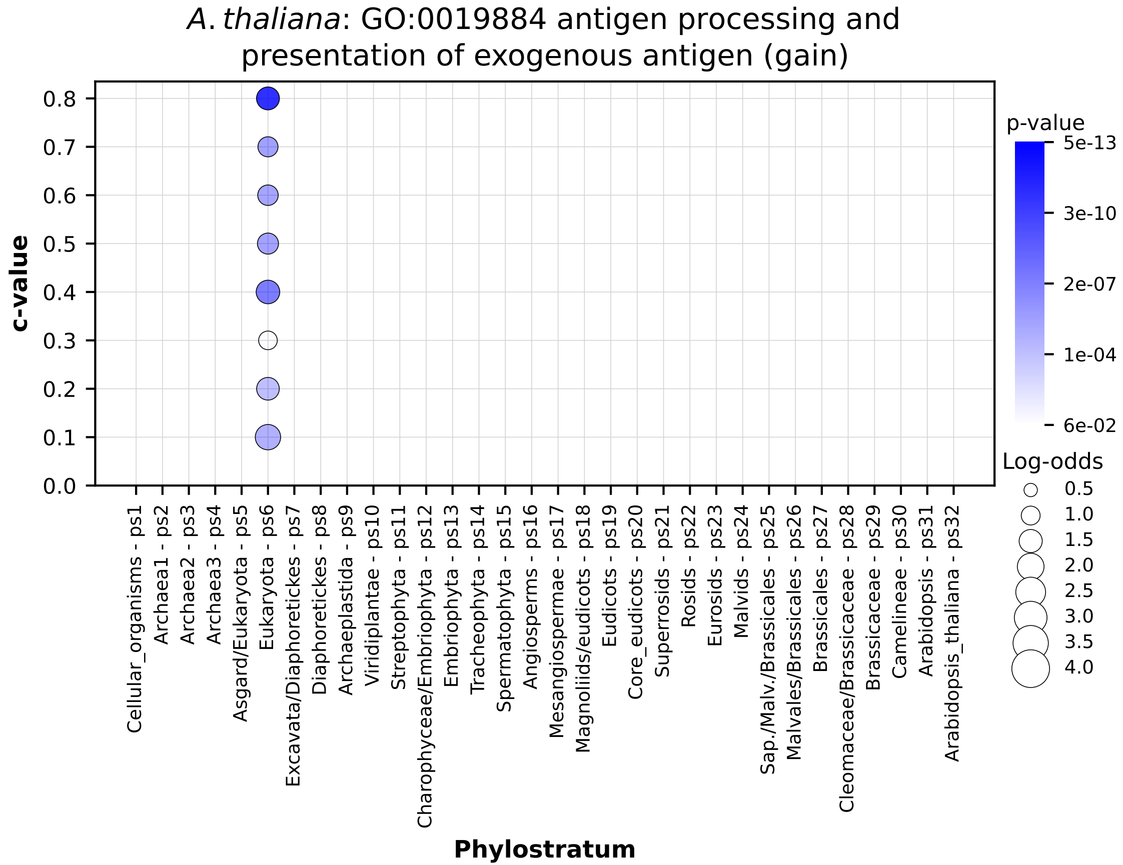

A. thaliana: GO:0019884 antigen processing and presentation of exogenous antigen (gain)
c-value
0.0
0.1
0.2
0.3
0.4
0.5
0.6
0.7
0.8
Phylostratum
Cellular_organisms - ps1
Archaea1 - ps2
Archaea2 - ps3
Archaea3 - ps4
Asgard/Eukaryota - ps5
Eukaryota - ps6
Excavata/Diaphoretickes - ps7
Diaphoretickes - ps8
Archaeplastida - ps9
Viridiplantae - ps10
Streptophyta - ps11
Charophyceae/Embriophyta - ps12
Embriophyta - ps13
Tracheophyta - ps14
Spermatophyta - ps15
Angiosperms - ps16
Mesangiospermae - ps17
Magnoliids/eudicots - ps18
Eudicots - ps19
Core_eudicots - ps20
Superrosids - ps21
Rosids - ps22
Eurosids - ps23
Malvids - ps24
Sap./Malv./Brassicales - ps25
Malvales/Brassicales - ps26
Brassicales - ps27
Cleomaceae/Brassicaceae - ps28
Brassicaceae - ps29
Camelineae - ps30
Arabidopsis - ps31
Arabidopsis_thaliana - ps32
p-value
5e-13
3e-10
2e-07
1e-04
6e-02
Log-odds
0.5
1.0
1.5
2.0
2.5
3.0
3.5
4.0

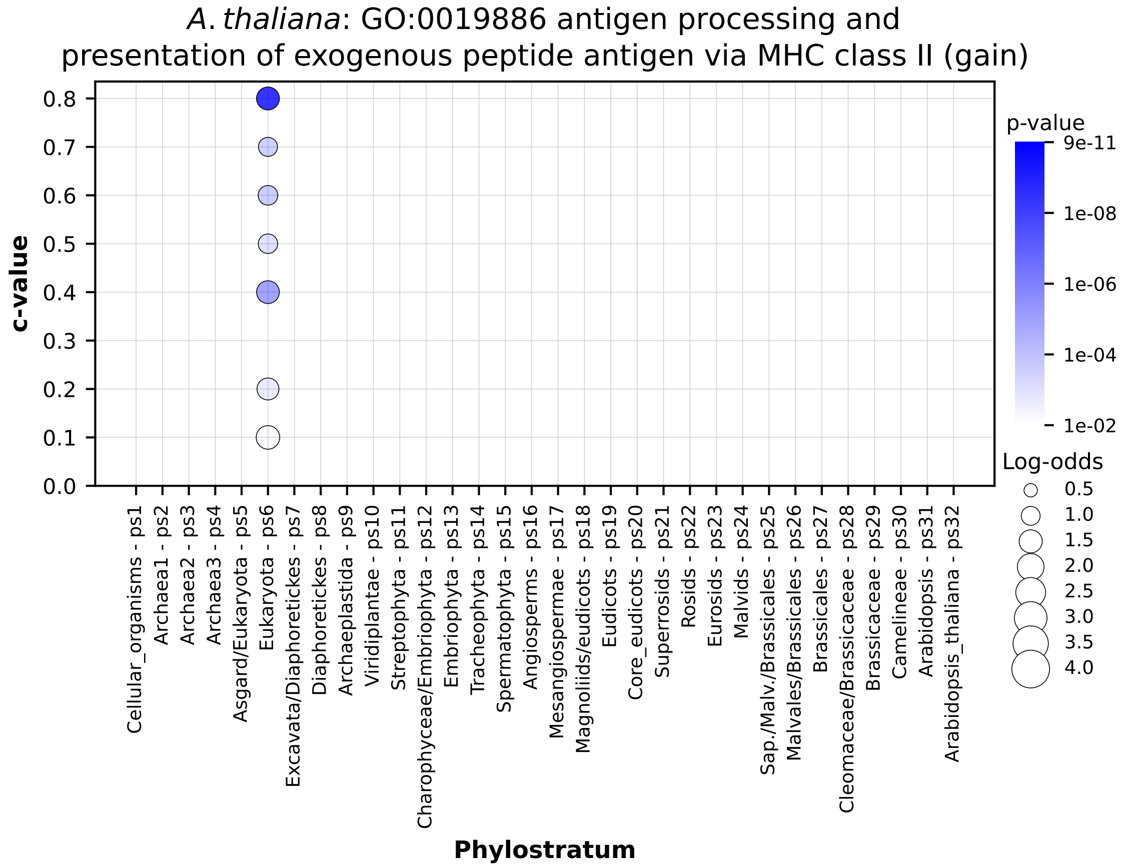

A. thaliana: GO:0019886 antigen processing and presentation of exogenous peptide antigen via MHC class II (gain)
c-value
0.8
0.7
0.6
0.5
0.4
0.3
0.2
0.1
0.0
p-value
9e-11
1e-08
1e-06
1e-04
1e-02
Log-odds
0.5
1.0
1.5
2.0
2.5
3.0
3.5
4.0
Cellular_organisms - ps1
Archaea1 - ps2
Archaea2 - ps3
Archaea3 - ps4
Asgard/Eukaryota - ps5
Eukaryota - ps6
Excavata/Diaphoretickes - ps7
Diaphoretickes - ps8
Archaeplastida - ps9
Viridiplantae - ps10
Streptophyta - ps11
Charophyceae/Embriophyta - ps12
Embriophyta - ps13
Tracheophyta - ps14
Spermatophyta - ps15
Angiosperms - ps16
Mesangiospermae - ps17
Magnoliids/eudicots - ps18
Eudicots - ps19
Core_eudicots - ps20
Superrosids - ps21
Rosids - ps22
Eurosids - ps23
Malvids - ps24
Sap./Malv./Brassicales - ps25
Malvales/Brassicales - ps26
Brassicales - ps27
Cleomaceae/Brassicaceae - ps28
Brassicaceae - ps29
Camelineae - ps30
Arabidopsis - ps31
Arabidopsis_thaliana - ps32
Phylostratum

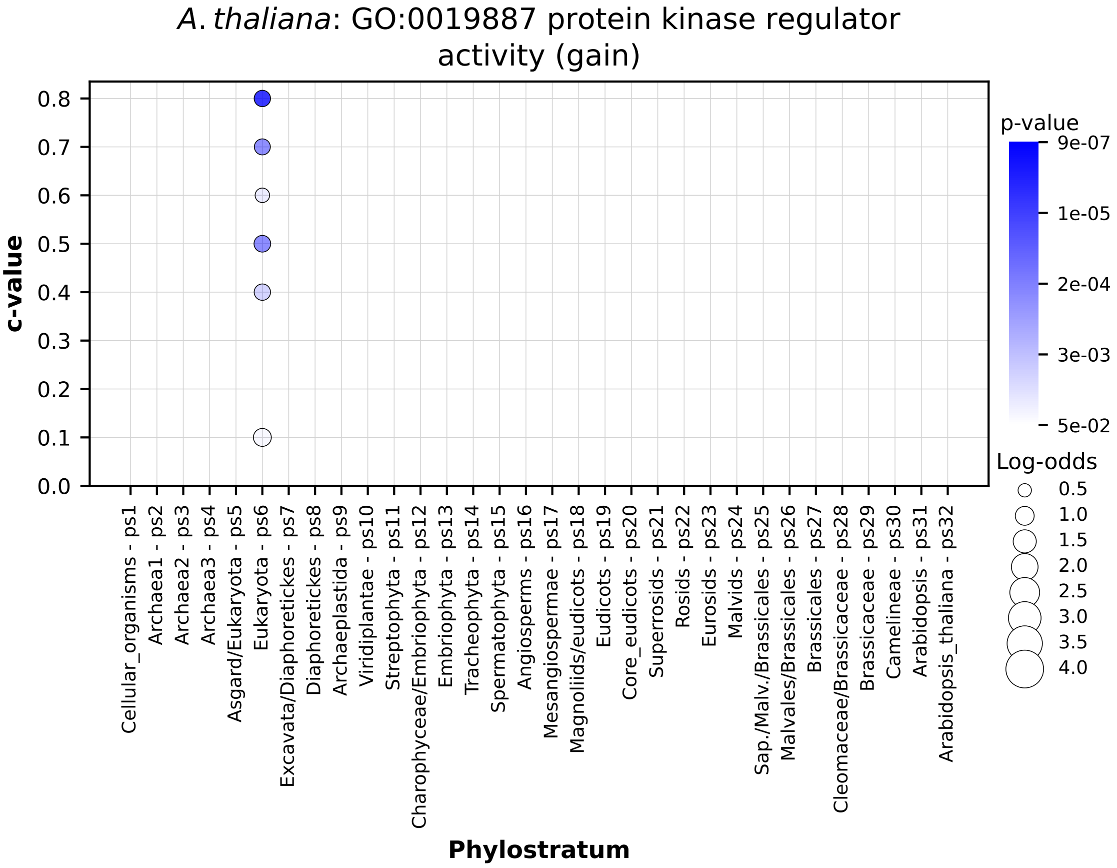

A. thaliana: GO:0019887 protein kinase regulator activity (gain)
c-value
0.8
0.7
0.6
0.5
0.4
0.3
0.2
0.1
0.0
p-value
9e-07
1e-05
2e-04
3e-03
5e-02
Log-odds
0.5
1.0
1.5
2.0
2.5
3.0
3.5
4.0
Cellular_organisms - ps1
Archaea1 - ps2
Archaea2 - ps3
Archaea3 - ps4
Asgard/Eukaryota - ps5
Eukaryota - ps6
Excavata/Diaphoretickes - ps7
Diaphoretickes - ps8
Archaeplastida - ps9
Viridiplantae - ps10
Streptophyta - ps11
Charophyceae/Embriophyta - ps12
Embriophyta - ps13
Tracheophyta - ps14
Spermatophyta - ps15
Angiosperms - ps16
Mesangiospermae - ps17
Magnoliids/eudicots - ps18
Eudicots - ps19
Core_eudicots - ps20
Superrosids - ps21
Rosids - ps22
Eurosids - ps23
Malvids - ps24
Sap./Malv./Brassicales - ps25
Malvales/Brassicales - ps26
Brassicales - ps27
Cleomaceae/Brassicaceae - ps28
Brassicaceae - ps29
Camelineae - ps30
Arabidopsis - ps31
Arabidopsis_thaliana - ps32
Phylostratum

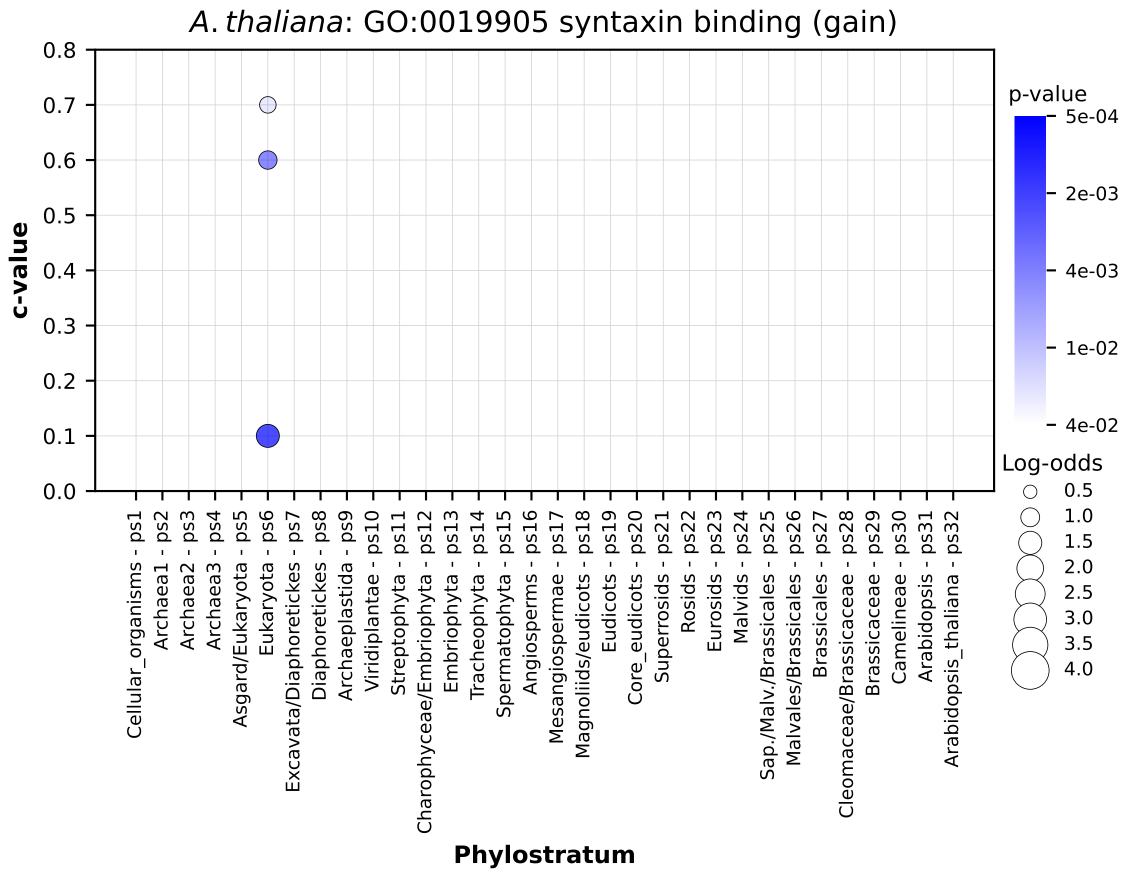

A. thaliana: GO:0019905 syntaxin binding (gain)
c-value
0.8
0.7
0.6
0.5
0.4
0.3
0.2
0.1
0.0
Phylostratum
Cellular_organisms - ps1
Archaea1 - ps2
Archaea2 - ps3
Archaea3 - ps4
Asgard/Eukaryota - ps5
Eukaryota - ps6
Excavata/Diaphoretickes - ps7
Diaphoretickes - ps8
Archaeplastida - ps9
Viridiplantae - ps10
Streptophyta - ps11
Charophyceae/Embriophyta - ps12
Embriophyta - ps13
Tracheophyta - ps14
Spermatophyta - ps15
Angiosperms - ps16
Mesangiospermae - ps17
Magnoliids/eudicots - ps18
Eudicots - ps19
Core_eudicots - ps20
Superrosids - ps21
Rosids - ps22
Eurosids - ps23
Malvids - ps24
Sap./Malv./Brassicales - ps25
Malvales/Brassicales - ps26
Brassicales - ps27
Cleomaceae/Brassicaceae - ps28
Brassicaceae - ps29
Camelineae - ps30
Arabidopsis - ps31
Arabidopsis_thaliana - ps32
p-value
5e-04
2e-03
4e-03
1e-02
4e-02
Log-odds
0.5
1.0
1.5
2.0
2.5
3.0
3.5
4.0

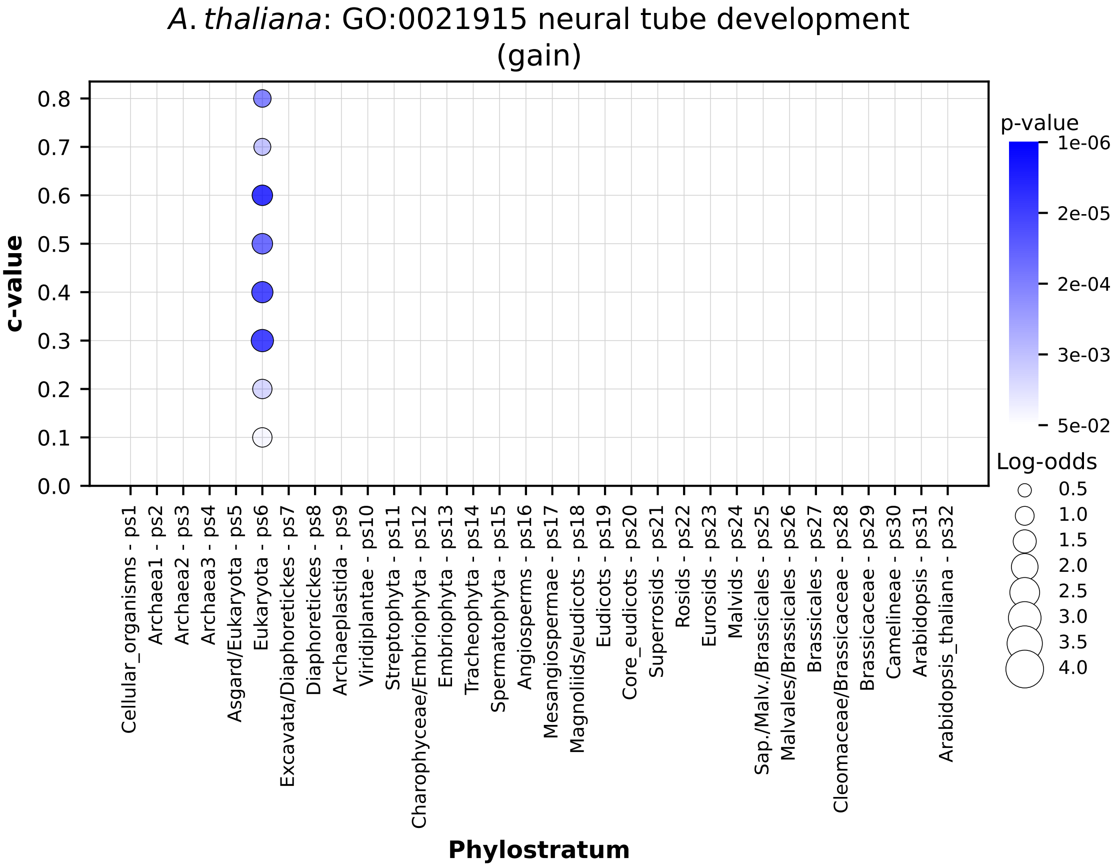
A. thaliana: GO:0021915 neural tube development
(gain)
c-value
0.0
0.1
0.2
0.3
0.4
0.5
0.6
0.7
0.8
Phylostratum
Cellular_organisms - ps1
Archaea1 - ps2
Archaea2 - ps3
Archaea3 - ps4
Asgard/Eukaryota - ps5
Eukaryota - ps6
Excavata/Diaphoretickes - ps7
Diaphoretickes - ps8
Archaeplastida - ps9
Viridiplantae - ps10
Streptophyta - ps11
Charophyceae/Embriophyta - ps12
Embriophyta - ps13
Tracheophyta - ps14
Spermatophyta - ps15
Angiosperms - ps16
Mesangiospermae - ps17
Magnoliids/eudicots - ps18
Eudicots - ps19
Core_eudicots - ps20
Superrosids - ps21
Rosids - ps22
Eurosids - ps23
Malvids - ps24
Sap./Malv./Brassicales - ps25
Malvales/Brassicales - ps26
Brassicales - ps27
Cleomaceae/Brassicaceae - ps28
Brassicaceae - ps29
Camelineae - ps30
Arabidopsis - ps31
Arabidopsis_thaliana - ps32
p-value
1e-06
2e-05
2e-04
3e-03
5e-02
Log-odds
0.5
1.0
1.5
2.0
2.5
3.0
3.5
4.0

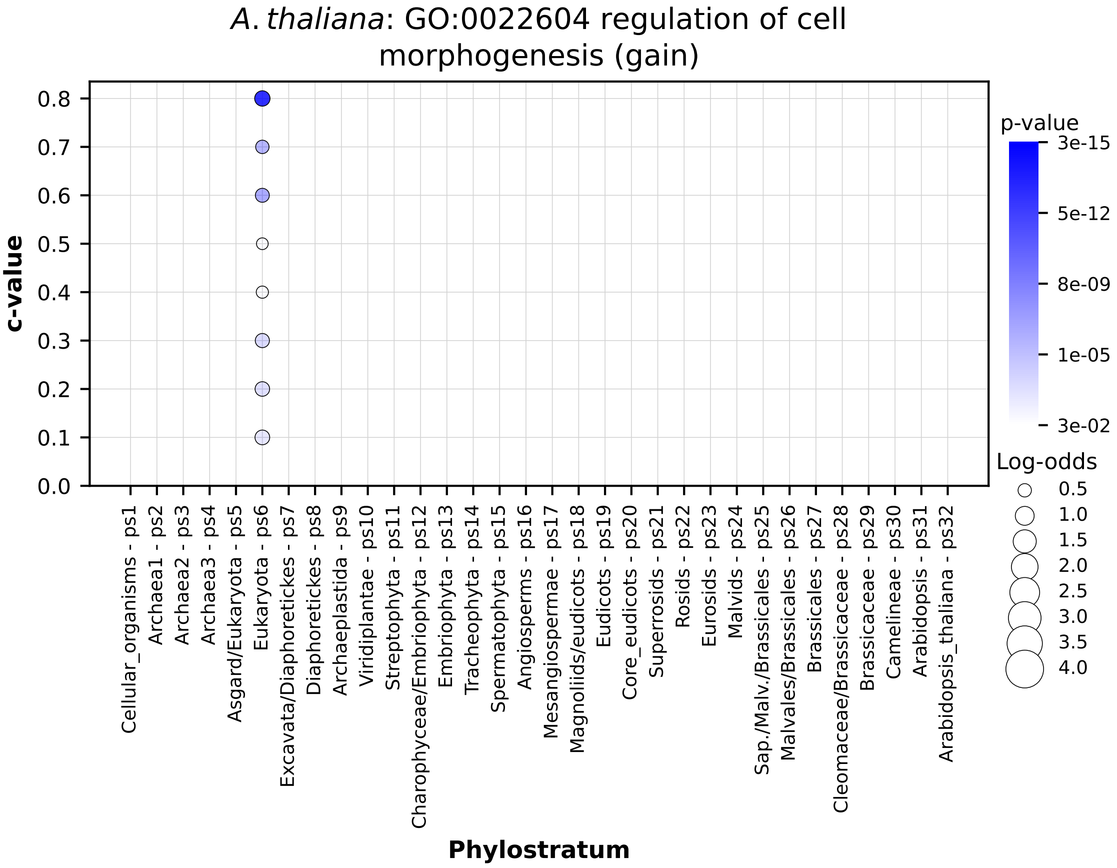

A. thaliana: GO:0022604 regulation of cell morphogenesis (gain)
c-value
0.0
0.1
0.2
0.3
0.4
0.5
0.6
0.7
0.8
Phylostratum
Cellular_organisms - ps1
Archaea1 - ps2
Archaea2 - ps3
Archaea3 - ps4
Asgard/Eukaryota - ps5
Eukaryota - ps6
Excavata/Diaphoretickes - ps7
Diaphoretickes - ps8
Archaeplastida - ps9
Viridiplantae - ps10
Streptophyta - ps11
Charophyceae/Embriophyta - ps12
Embriophyta - ps13
Tracheophyta - ps14
Spermatophyta - ps15
Angiosperms - ps16
Mesangiospermae - ps17
Magnoliids/eudicots - ps18
Eudicots - ps19
Core_eudicots - ps20
Superrosids - ps21
Rosids - ps22
Eurosids - ps23
Malvids - ps24
Sap./Malv./Brassicales - ps25
Malvales/Brassicales - ps26
Brassicales - ps27
Cleomaceae/Brassicaceae - ps28
Brassicaceae - ps29
Camelineae - ps30
Arabidopsis - ps31
Arabidopsis_thaliana - ps32
p-value
3e-15
5e-12
8e-09
1e-05
3e-02
Log-odds
0.5
1.0
1.5
2.0
2.5
3.0
3.5
4.0

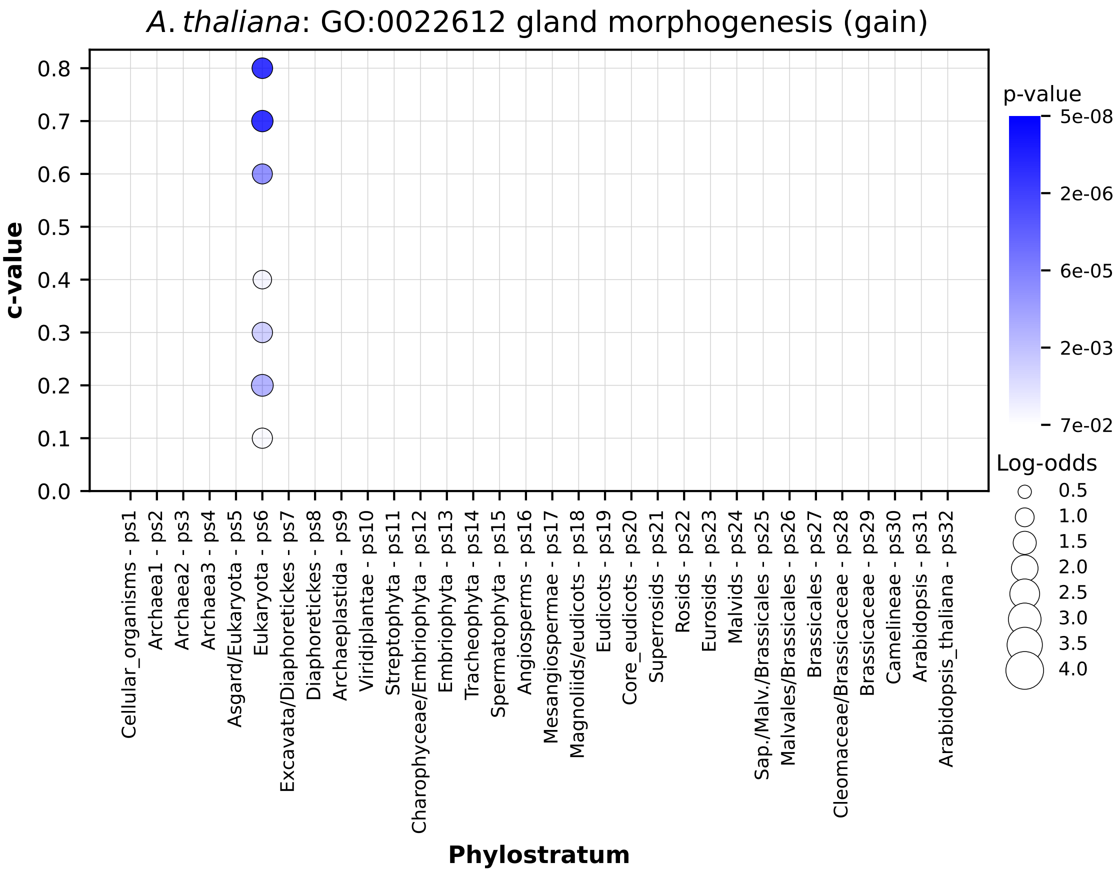

A. thaliana: GO:0022612 gland morphogenesis (gain)
c-value
0.0
0.1
0.2
0.3
0.4
0.5
0.6
0.7
0.8
p-value
5e-08
2e-06
6e-05
2e-03
7e-02
Log-odds
0.5
1.0
1.5
2.0
2.5
3.0
3.5
4.0
Cellular_organisms - ps1
Archaea1 - ps2
Archaea2 - ps3
Archaea3 - ps4
Asgard/Eukaryota - ps5
Eukaryota - ps6
Excavata/Diaphoretickes - ps7
Diaphoretickes - ps8
Archaeplastida - ps9
Viridiplantae - ps10
Streptophyta - ps11
Charophyceae/Embriophyta - ps12
Embriophyta - ps13
Tracheophyta - ps14
Spermatophyta - ps15
Angiosperms - ps16
Mesangiospermae - ps17
Magnoliids/eudicots - ps18
Eudicots - ps19
Core_eudicots - ps20
Superrosids - ps21
Rosids - ps22
Eurosids - ps23
Malvids - ps24
Sap./Malv./Brassicales - ps25
Malvales/Brassicales - ps26
Brassicales - ps27
Cleomaceae/Brassicaceae - ps28
Brassicaceae - ps29
Camelineae - ps30
Arabidopsis - ps31
Arabidopsis_thaliana - ps32
Phylostratum

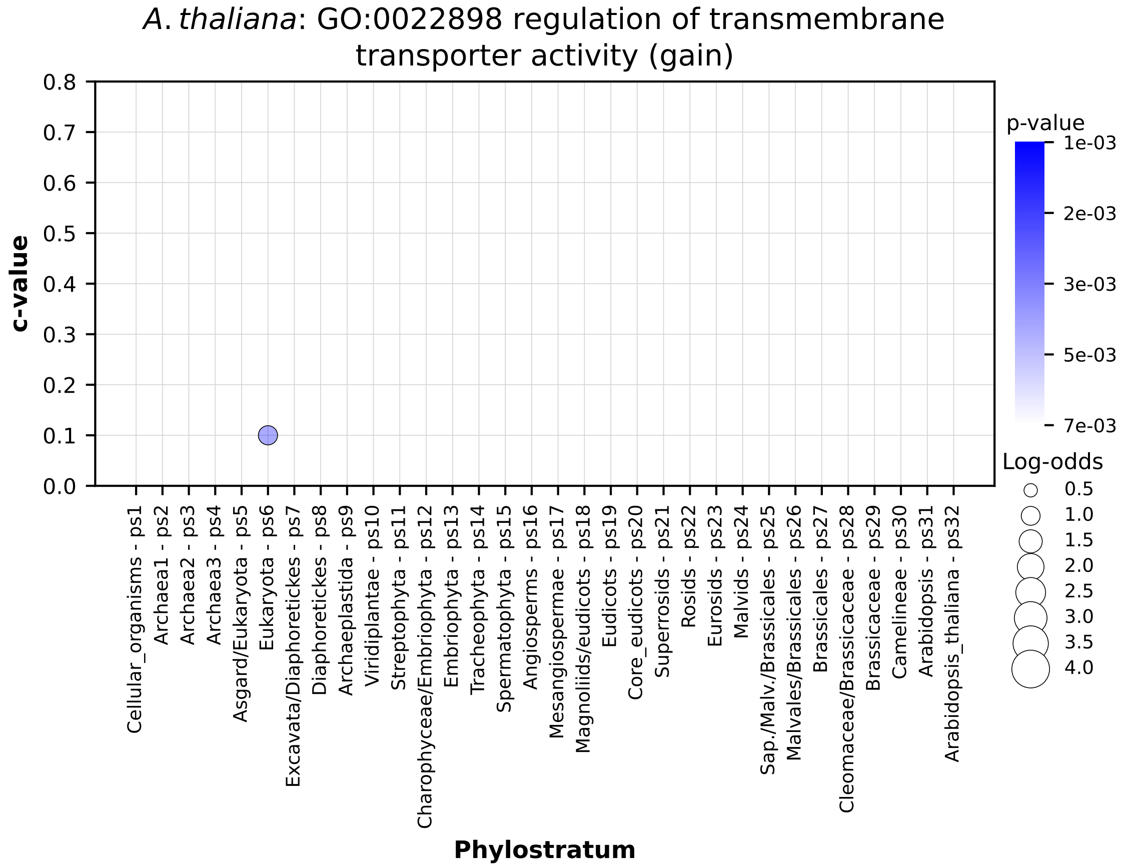

A. thaliana: GO:0022898 regulation of transmembrane transporter activity (gain)
c-value
0.0
0.1
0.2
0.3
0.4
0.5
0.6
0.7
0.8
Phylostratum
Cellular_organisms - ps1
Archaea1 - ps2
Archaea2 - ps3
Archaea3 - ps4
Asgard/Eukaryota - ps5
Eukaryota - ps6
Excavata/Diaphoretickes - ps7
Diaphoretickes - ps8
Archaeplastida - ps9
Viridiplantae - ps10
Streptophyta - ps11
Charophyceae/Embriophyta - ps12
Embriophyta - ps13
Tracheophyta - ps14
Spermatophyta - ps15
Angiosperms - ps16
Mesangiospermae - ps17
Magnoliids/eudicots - ps18
Eudicots - ps19
Core_eudicots - ps20
Superrosids - ps21
Rosids - ps22
Eurosids - ps23
Malvids - ps24
Sap./Malv./Brassicales - ps25
Malvales/Brassicales - ps26
Brassicales - ps27
Cleomaceae/Brassicaceae - ps28
Brassicaceae - ps29
Camelineae - ps30
Arabidopsis - ps31
Arabidopsis_thaliana - ps32
p-value
1e-03
2e-03
3e-03
5e-03
7e-03
Log-odds
0.5
1.0
1.5
2.0
2.5
3.0
3.5
4.0

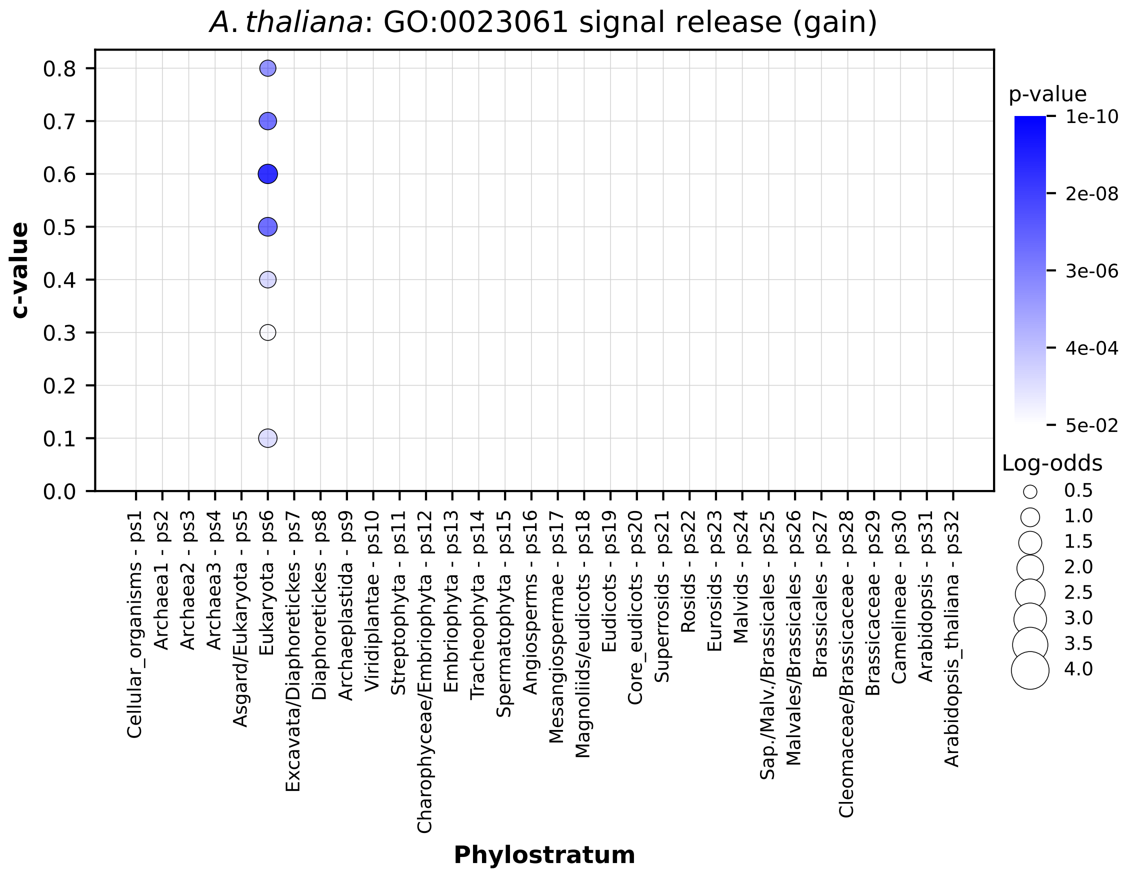

A. thaliana: GO:0023061 signal release (gain)
c-value
0.0
0.1
0.2
0.3
0.4
0.5
0.6
0.7
0.8
p-value
1e-10
2e-08
3e-06
4e-04
5e-02
Log-odds
0.5
1.0
1.5
2.0
2.5
3.0
3.5
4.0
Cellular_organisms - ps1
Archaea1 - ps2
Archaea2 - ps3
Archaea3 - ps4
Asgard/Eukaryota - ps5
Eukaryota - ps6
Excavata/Diaphoretickes - ps7
Diaphoretickes - ps8
Archaeplastida - ps9
Viridiplantae - ps10
Streptophyta - ps11
Charophyceae/Embriophyta - ps12
Embriophyta - ps13
Tracheophyta - ps14
Spermatophyta - ps15
Angiosperms - ps16
Mesangiospermae - ps17
Magnoliids/eudicots - ps18
Eudicots - ps19
Core_eudicots - ps20
Superrosids - ps21
Rosids - ps22
Eurosids - ps23
Malvids - ps24
Sap./Malv./Brassicales - ps25
Malvales/Brassicales - ps26
Brassicales - ps27
Cleomaceae/Brassicaceae - ps28
Brassicaceae - ps29
Camelineae - ps30
Arabidopsis - ps31
Arabidopsis_thaliana - ps32
Phylostratum

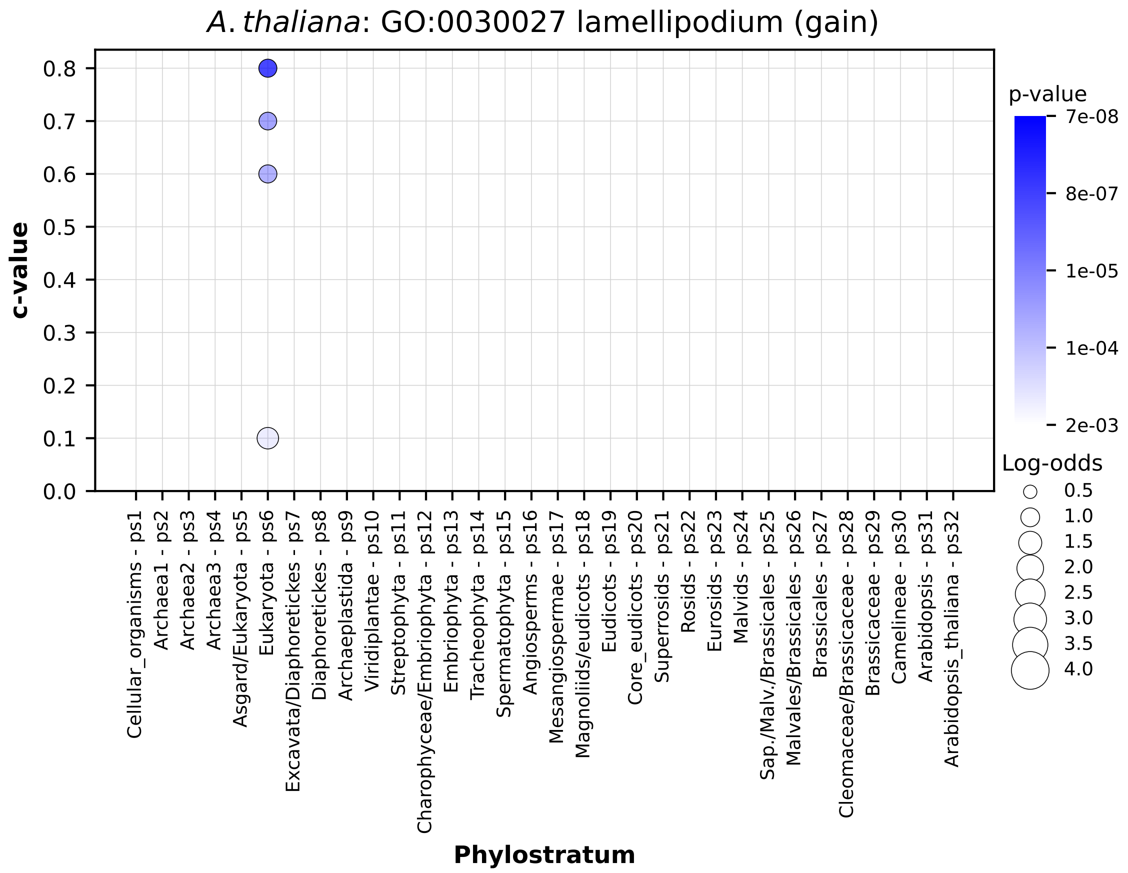
A. thaliana: GO:0030027 lamellipodium (gain)
c-value
0.0
0.1
0.2
0.3
0.4
0.5
0.6
0.7
0.8
Phylostratum
Cellular_organisms - ps1
Archaea1 - ps2
Archaea2 - ps3
Archaea3 - ps4
Asgard/Eukaryota - ps5
Eukaryota - ps6
Excavata/Diaphoretickes - ps7
Diaphoretickes - ps8
Archaeplastida - ps9
Viridiplantae - ps10
Streptophyta - ps11
Charophyceae/Embriophyta - ps12
Embriophyta - ps13
Tracheophyta - ps14
Spermatophyta - ps15
Angiosperms - ps16
Mesangiospermae - ps17
Magnoliids/eudicots - ps18
Eudicots - ps19
Core_eudicots - ps20
Superrosids - ps21
Rosids - ps22
Eurosids - ps23
Malvids - ps24
Sap./Malv./Brassicales - ps25
Malvales/Brassicales - ps26
Brassicales - ps27
Cleomaceae/Brassicaceae - ps28
Brassicaceae - ps29
Camelineae - ps30
Arabidopsis - ps31
Arabidopsis_thaliana - ps32
p-value
7e-08
8e-07
1e-05
1e-04
2e-03
Log-odds
0.5
1.0
1.5
2.0
2.5
3.0
3.5
4.0

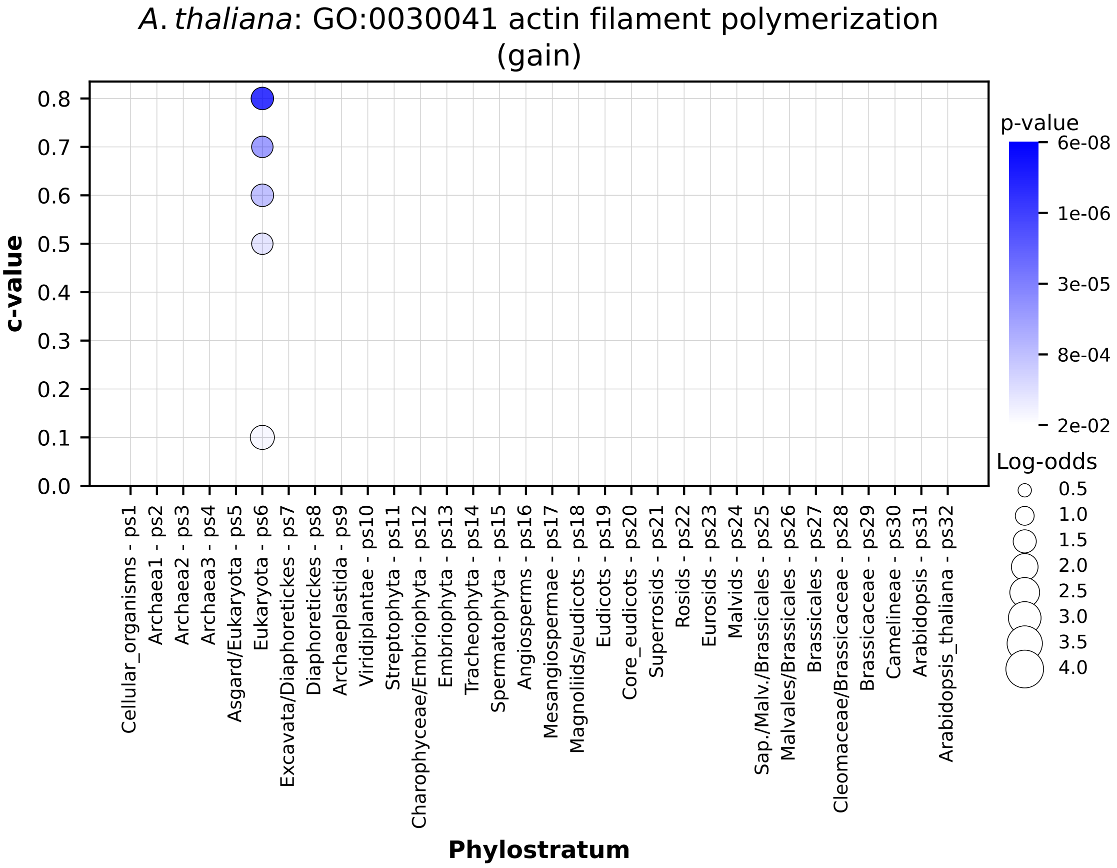

A. thaliana: GO:0030041 actin filament polymerization (gain)
c-value
0.0
0.1
0.2
0.3
0.4
0.5
0.6
0.7
0.8
Cellular_organisms - ps1
Archaea1 - ps2
Archaea2 - ps3
Archaea3 - ps4
Asgard/Eukaryota - ps5
Eukaryota - ps6
Excavata/Diaphoretickes - ps7
Diaphoretickes - ps8
Archaeplastida - ps9
Viridiplantae - ps10
Streptophyta - ps11
Charophyceae/Embriophyta - ps12
Embriophyta - ps13
Tracheophyta - ps14
Spermatophyta - ps15
Angiosperms - ps16
Mesangiospermae - ps17
Magnoliids/eudicots - ps18
Eudicots - ps19
Core_eudicots - ps20
Superrosids - ps21
Rosids - ps22
Eurosids - ps23
Malvids - ps24
Sap./Malv./Brassicales - ps25
Malvales/Brassicales - ps26
Brassicales - ps27
Cleomaceae/Brassicaceae - ps28
Brassicaceae - ps29
Camelineae - ps30
Arabidopsis - ps31
Arabidopsis_thaliana - ps32
Phylostratum
p-value
6e-08
1e-06
3e-05
8e-04
2e-02
Log-odds
0.5
1.0
1.5
2.0
2.5
3.0
3.5
4.0

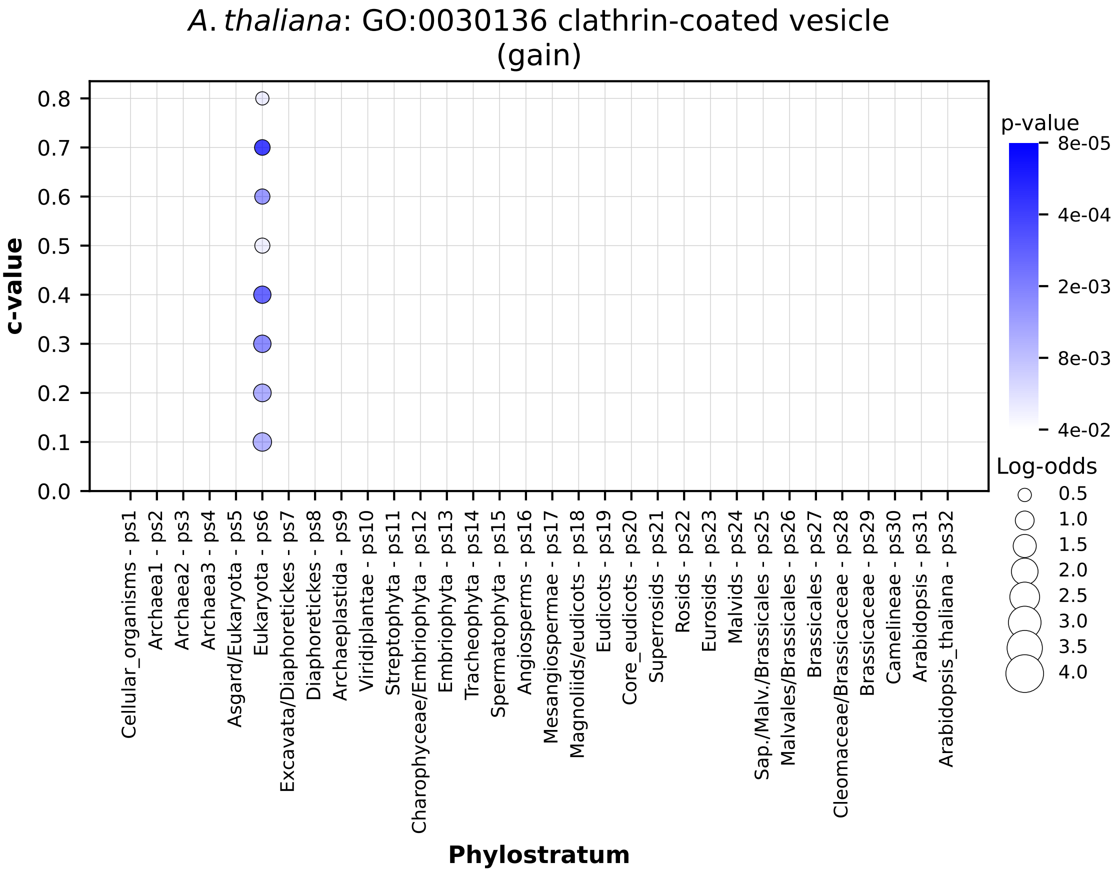

A. thaliana: GO:0030136 clathrin-coated vesicle
(gain)
c-value
0.8
0.7
0.6
0.5
0.4
0.3
0.2
0.1
0.0
p-value
8e-05
4e-04
2e-03
8e-03
4e-02
Log-odds
0.5
1.0
1.5
2.0
2.5
3.0
3.5
4.0
Cellular_organisms - ps1
Archaea1 - ps2
Archaea2 - ps3
Archaea3 - ps4
Asgard/Eukaryota - ps5
Eukaryota - ps6
Excavata/Diaphoretickes - ps7
Diaphoretickes - ps8
Archaeplastida - ps9
Viridiplantae - ps10
Streptophyta - ps11
Charophyceae/Embriophyta - ps12
Embriophyta - ps13
Tracheophyta - ps14
Spermatophyta - ps15
Angiosperms - ps16
Mesangiospermae - ps17
Magnoliids/eudicots - ps18
Eudicots - ps19
Core_eudicots - ps20
Superrosids - ps21
Rosids - ps22
Eurosids - ps23
Malvids - ps24
Sap./Malv./Brassicales - ps25
Malvales/Brassicales - ps26
Brassicales - ps27
Cleomaceae/Brassicaceae - ps28
Brassicaceae - ps29
Camelineae - ps30
Arabidopsis - ps31
Arabidopsis_thaliana - ps32
Phylostratum

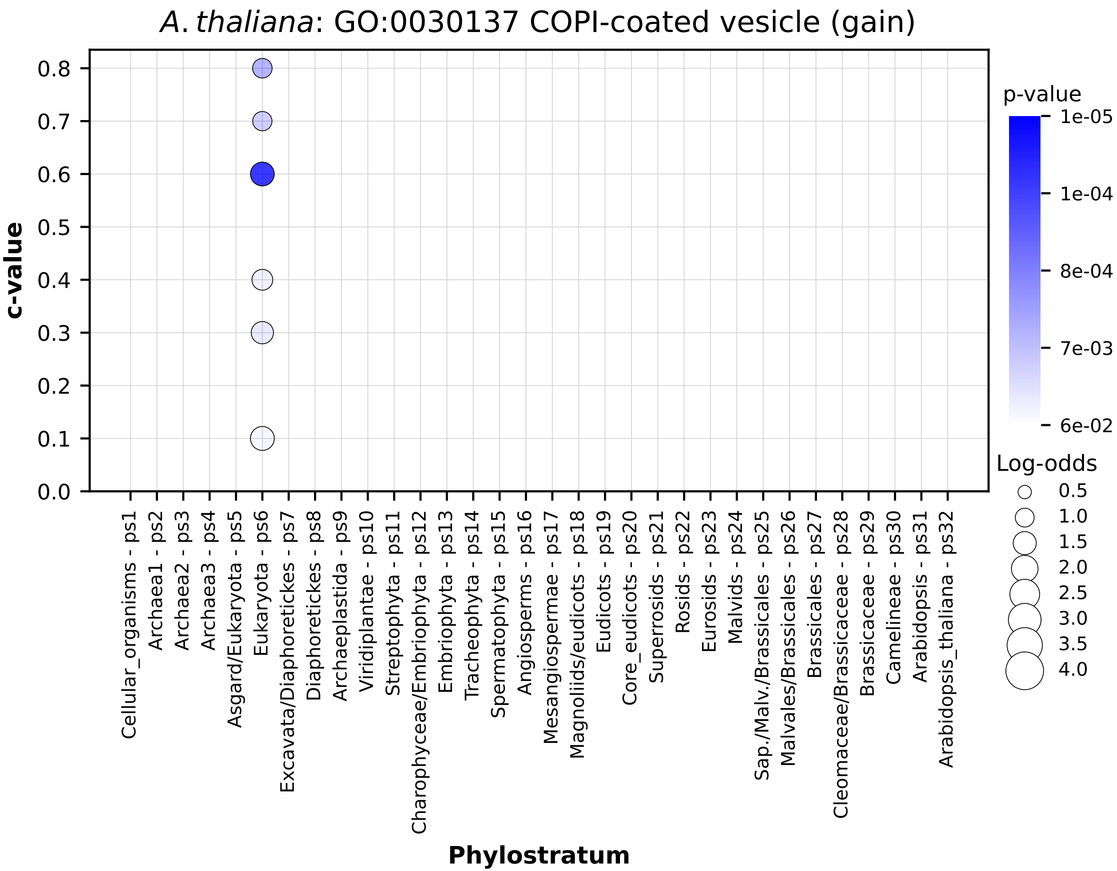
A. thaliana: GO:0030137 COPI-coated vesicle (gain)
c-value
0.0
0.1
0.2
0.3
0.4
0.5
0.6
0.7
0.8
Phylostratum
Cellular_organisms - ps1
Archaea1 - ps2
Archaea2 - ps3
Archaea3 - ps4
Asgard/Eukaryota - ps5
Eukaryota - ps6
Excavata/Diaphoretickes - ps7
Diaphoretickes - ps8
Archaeplastida - ps9
Viridiplantae - ps10
Streptophyta - ps11
Charophyceae/Embriophyta - ps12
Embriophyta - ps13
Tracheophyta - ps14
Spermatophyta - ps15
Angiosperms - ps16
Mesangiospermae - ps17
Magnoliids/eudicots - ps18
Eudicots - ps19
Core_eudicots - ps20
Superrosids - ps21
Rosids - ps22
Eurosids - ps23
Malvids - ps24
Sap./Malv./Brassicales - ps25
Malvales/Brassicales - ps26
Brassicales - ps27
Cleomaceae/Brassicaceae - ps28
Brassicaceae - ps29
Camelineae - ps30
Arabidopsis - ps31
Arabidopsis_thaliana - ps32
p-value
1e-05
1e-04
8e-04
7e-03
6e-02
Log-odds
0.5
1.0
1.5
2.0
2.5
3.0
3.5
4.0

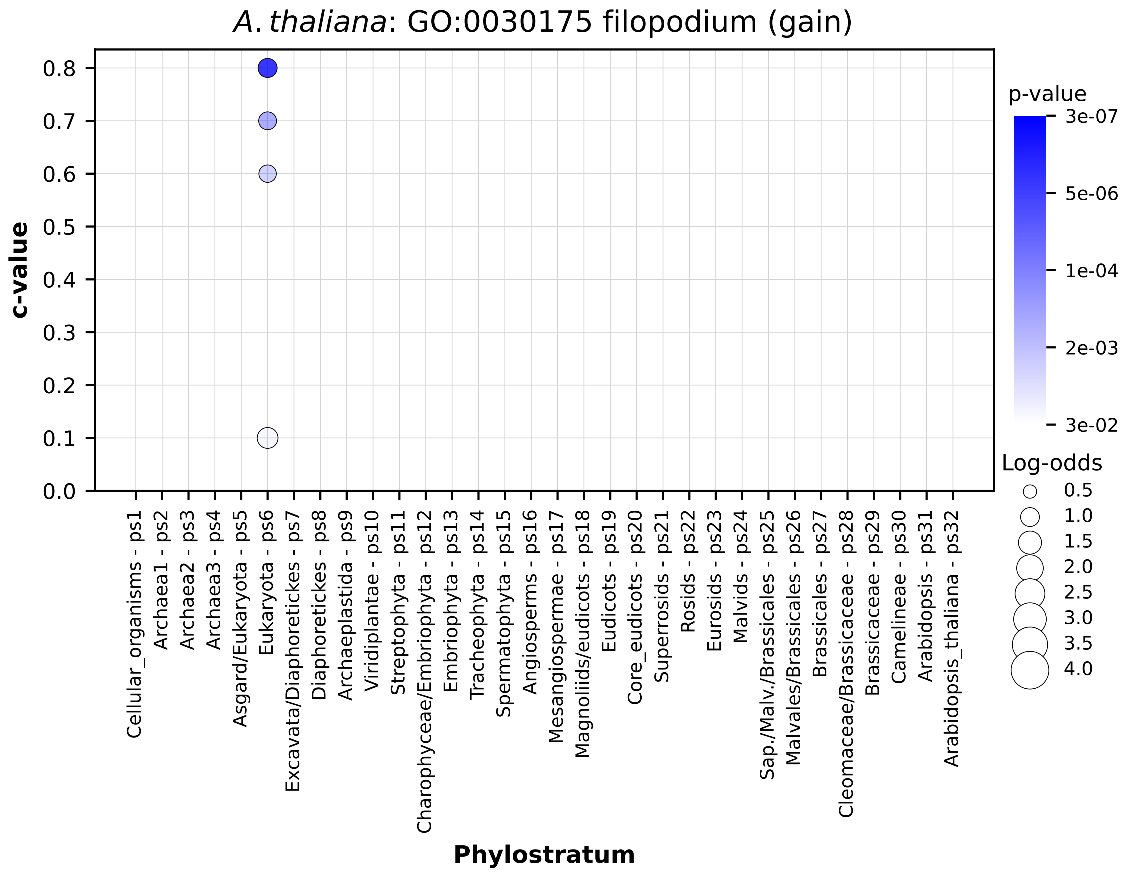

A. thaliana: GO:0030175 filopodium (gain)
c-value
0.0
0.1
0.2
0.3
0.4
0.5
0.6
0.7
0.8
Phylostratum
Cellular_organisms - ps1
Archaea1 - ps2
[truncated: 493,093 more chars]
