# Supplementary material for: Macroevolutionary dynamics of gene family gain and loss along multicellular eukaryotic lineages
Source: Nat Commun. 2024 Mar 26;15:2663. doi: 10.1038/s41467-024-47017-w (PMC10966110; doi:10.1038/s41467-024-47017-w)
Supplement: Supplementary file 15 — Supplementary Dataset 10 [file 41467_2024_47017_MOESM15_ESM.pdf]

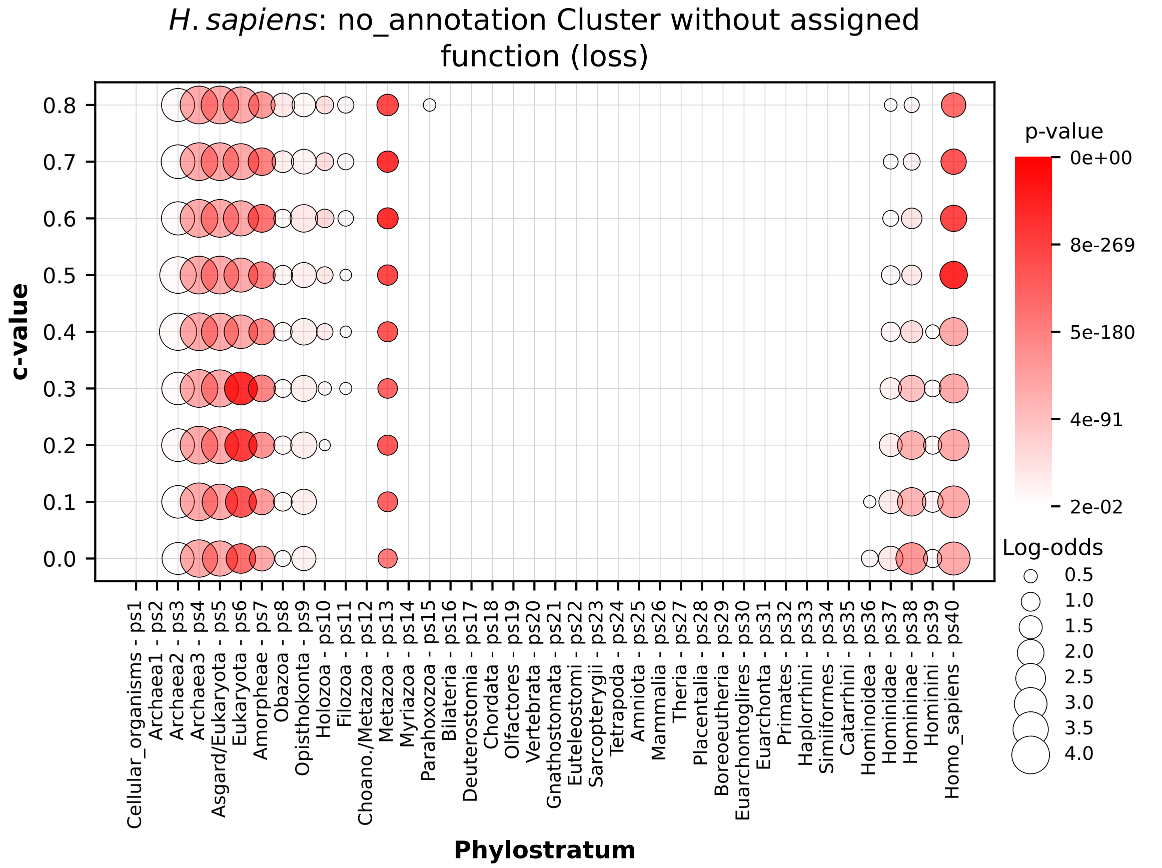

H. sapiens: no_annotation Cluster without assigned function (loss)
c-value
0.8
0.7
0.6
0.5
0.4
0.3
0.2
0.1
0.0
p-value
0e+00
8e-269
5e-180
4e-91
2e-02
Log-odds
0.5
1.0
1.5
2.0
2.5
3.0
3.5
4.0
Cellular_organisms - ps1
Archaea1 - ps2
Archaea2 - ps3
Archaea3 - ps4
Asgard/Eukaryota - ps5
Eukaryota - ps6
Amorpheae - ps7
Obazoa - ps8
Opisthokonta - ps9
Holozoa - ps10
Filozoa - ps11
Choano./Metazoa - ps12
Metazoa - ps13
Myriazoa - ps14
Parahoxozoa - ps15
Bilateria - ps16
Deuterostomia - ps17
Chordata - ps18
Olfactores - ps19
Vertebrata - ps20
Gnathostomata - ps21
Euteleostomi - ps22
Sarcopterygii - ps23
Tetrapoda - ps24
Amniota - ps25
Mammalia - ps26
Theria - ps27
Placentalia - ps28
Boreoeutheria - ps29
Euarchontoglires - ps30
Euarchonta - ps31
Primates - ps32
Haplorrhini - ps33
Simiiformes - ps34
Catarrhini - ps35
Hominoidea - ps36
Hominidae - ps37
Homininae - ps38
Hominini - ps39
Homo_sapiens - ps40
Phylostratum

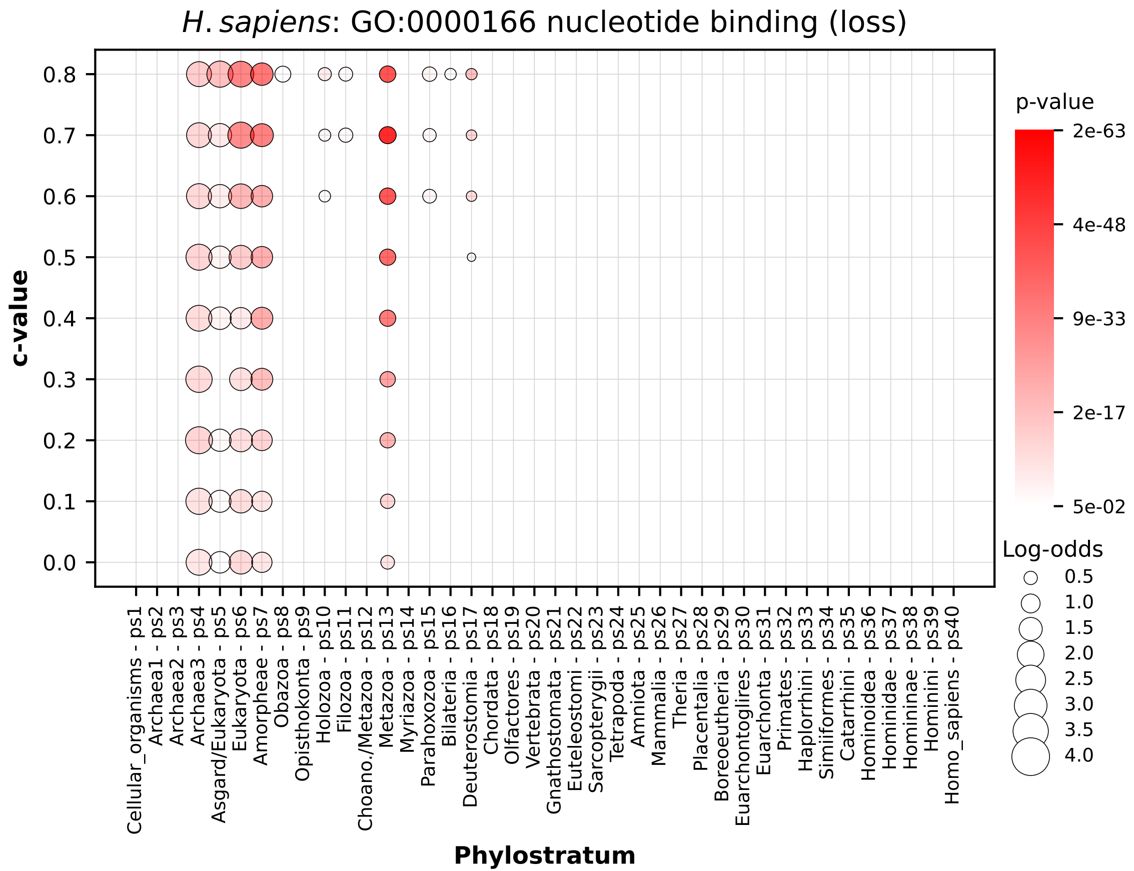

H. sapiens: GO:0000166 nucleotide binding (loss)
c-value
0.8
0.7
0.6
0.5
0.4
0.3
0.2
0.1
0.0
p-value
2e-63
4e-48
9e-33
2e-17
5e-02
Log-odds
0.5
1.0
1.5
2.0
2.5
3.0
3.5
4.0
Cellular_organisms - ps1
Archaea1 - ps2
Archaea2 - ps3
Archaea3 - ps4
Asgard/Eukaryota - ps5
Eukaryota - ps6
Amorpheae - ps7
Obazoa - ps8
Opisthokonta - ps9
Holozoa - ps10
Filozoa - ps11
Choano./Metazoa - ps12
Metazoa - ps13
Myriazoa - ps14
Parahoxozoa - ps15
Bilateria - ps16
Deuterostomia - ps17
Chordata - ps18
Olfactores - ps19
Vertebrata - ps20
Gnathostomata - ps21
Euteleostomi - ps22
Sarcopterygii - ps23
Tetrapoda - ps24
Amniota - ps25
Mammalia - ps26
Theria - ps27
Placentalia - ps28
Boreoeutheria - ps29
Euarchontoglires - ps30
Euarchonta - ps31
Primates - ps32
Haplorrhini - ps33
Simiiformes - ps34
Catarrhini - ps35
Hominoidea - ps36
Hominidae - ps37
Homininae - ps38
Hominini - ps39
Homo_sapiens - ps40
Phylostratum

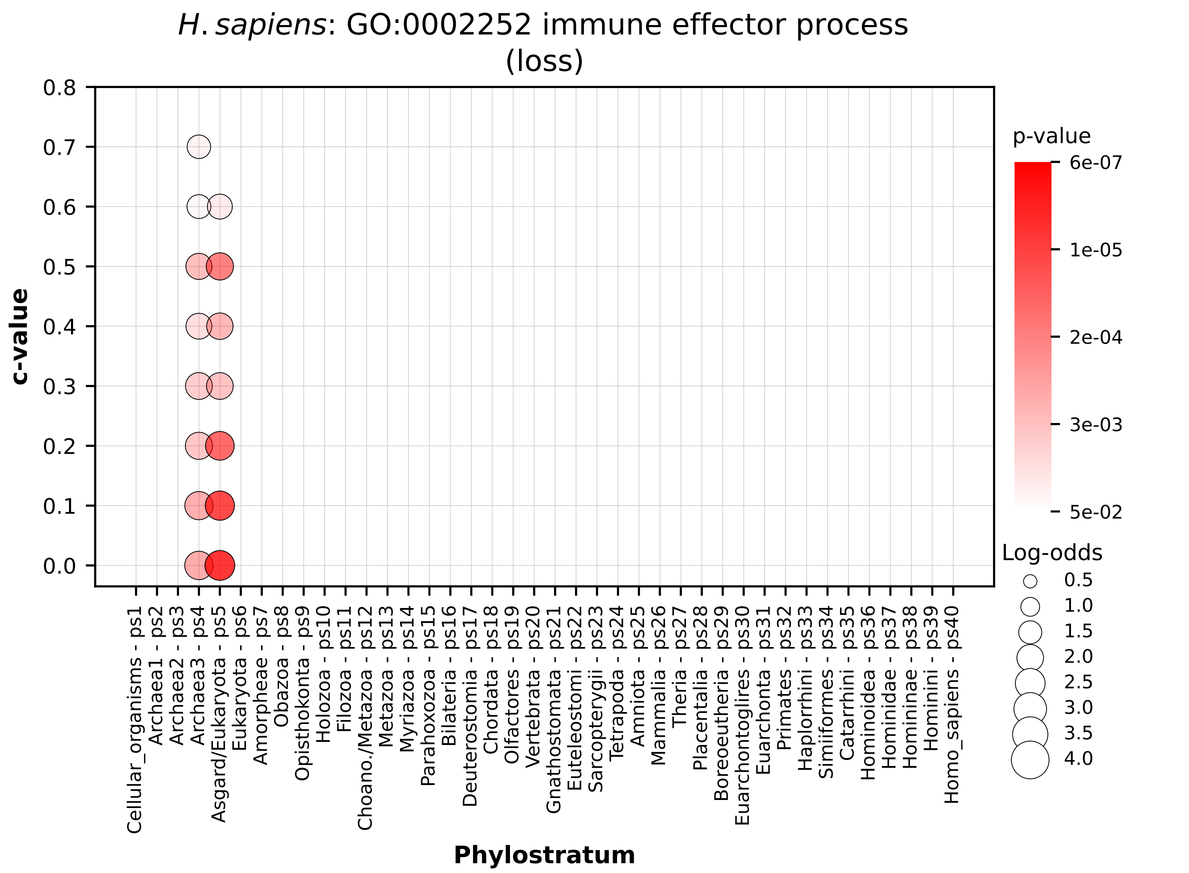

H. sapiens: GO:0002252 immune effector process
(loss)
c-value
0.8
0.7
0.6
0.5
0.4
0.3
0.2
0.1
0.0
p-value
6e-07
1e-05
2e-04
3e-03
5e-02
Log-odds
0.5
1.0
1.5
2.0
2.5
3.0
3.5
4.0
Cellular_organisms - ps1
Archaea1 - ps2
Archaea2 - ps3
Archaea3 - ps4
Asgard/Eukaryota - ps5
Eukaryota - ps6
Amorpheae - ps7
Obazoa - ps8
Opisthokonta - ps9
Holozoa - ps10
Filozoa - ps11
Choano./Metazoa - ps12
Metazoa - ps13
Myriazoa - ps14
Parahoxozoa - ps15
Bilateria - ps16
Deuterostomia - ps17
Chordata - ps18
Olfactores - ps19
Vertebrata - ps20
Gnathostomata - ps21
Euteleostomi - ps22
Sarcopterygii - ps23
Tetrapoda - ps24
Amniota - ps25
Mammalia - ps26
Theria - ps27
Placentalia - ps28
Boreoeutheria - ps29
Euarchontoglires - ps30
Euarchonta - ps31
Primates - ps32
Haplorrhini - ps33
Simiiformes - ps34
Catarrhini - ps35
Hominoidea - ps36
Hominidae - ps37
Homininae - ps38
Hominini - ps39
Homo_sapiens - ps40
Phylostratum

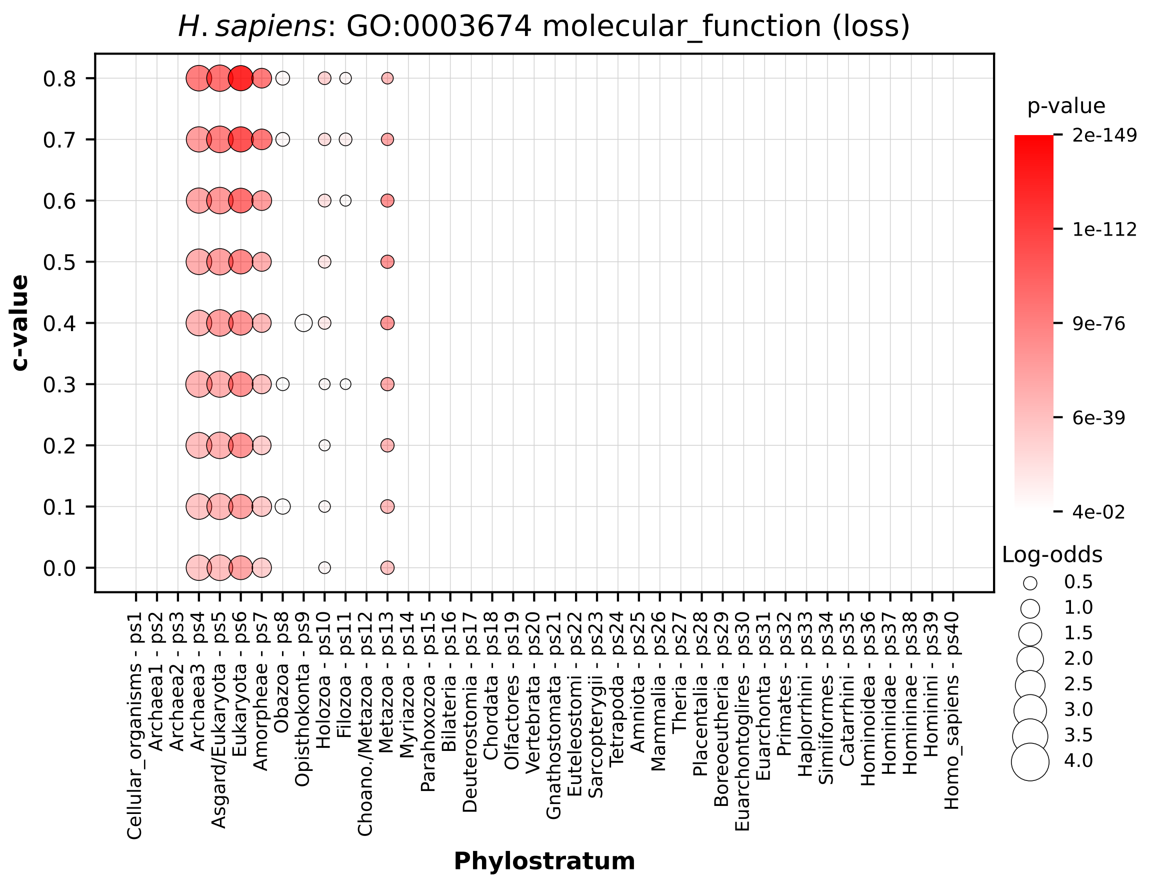

H. sapiens: GO:0003674 molecular_function (loss)
c-value
0.0
0.1
0.2
0.3
0.4
0.5
0.6
0.7
0.8
Phylostratum
Cellular_organisms - ps1
Archaea1 - ps2
Archaea2 - ps3
Archaea3 - ps4
Asgard/Eukaryota - ps5
Eukaryota - ps6
Amorpheae - ps7
Obazoa - ps8
Opisthokonta - ps9
Holozoa - ps10
Filozoa - ps11
Choano./Metazoa - ps12
Metazoa - ps13
Myriazoa - ps14
Parahoxozoa - ps15
Bilateria - ps16
Deuterostomia - ps17
Chordata - ps18
Olfactores - ps19
Vertebrata - ps20
Gnathostomata - ps21
Euteleostomi - ps22
Sarcopterygii - ps23
Tetrapoda - ps24
Amniota - ps25
Mammalia - ps26
Theria - ps27
Placentalia - ps28
Boreoeutheria - ps29
Euarchontoglires - ps30
Euarchonta - ps31
Primates - ps32
Haplorrhini - ps33
Simiiformes - ps34
Catarrhini - ps35
Hominoidea - ps36
Hominidae - ps37
Homininae - ps38
Hominini - ps39
Homo_sapiens - ps40
p-value
2e-149
1e-112
9e-76
6e-39
4e-02
Log-odds
0.5
1.0
1.5
2.0
2.5
3.0
3.5
4.0

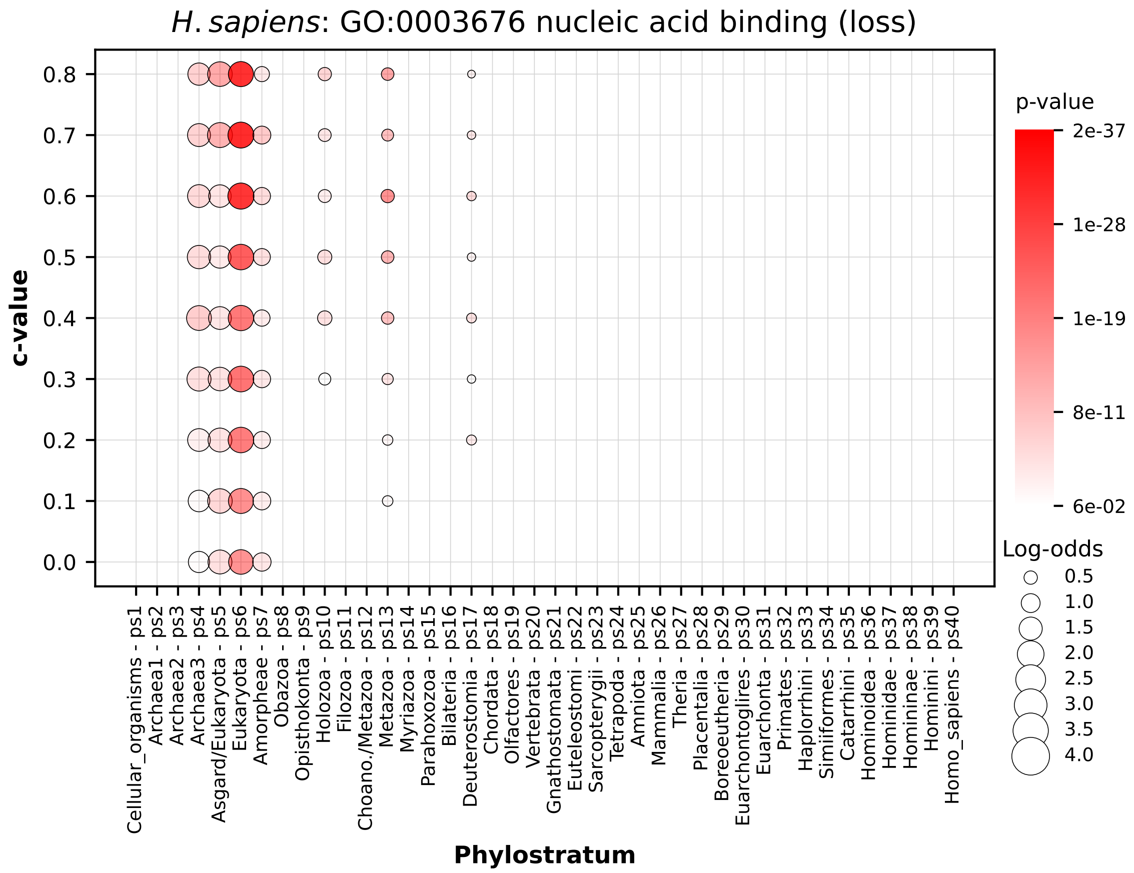

H. sapiens: GO:0003676 nucleic acid binding (loss)
c-value
0.8
0.7
0.6
0.5
0.4
0.3
0.2
0.1
0.0
p-value
2e-37
1e-28
1e-19
8e-11
6e-02
Log-odds
0.5
1.0
1.5
2.0
2.5
3.0
3.5
4.0
Cellular_organisms - ps1
Archaea1 - ps2
Archaea2 - ps3
Archaea3 - ps4
Asgard/Eukaryota - ps5
Eukaryota - ps6
Amorpheae - ps7
Obazoa - ps8
Opisthokonta - ps9
Holozoa - ps10
Filozoa - ps11
Choano./Metazoa - ps12
Metazoa - ps13
Myriazoa - ps14
Parahoxozoa - ps15
Bilateria - ps16
Deuterostomia - ps17
Chordata - ps18
Olfactores - ps19
Vertebrata - ps20
Gnathostomata - ps21
Euteleostomi - ps22
Sarcopterygii - ps23
Tetrapoda - ps24
Amniota - ps25
Mammalia - ps26
Theria - ps27
Placentalia - ps28
Boreoeutheria - ps29
Euarchontoglires - ps30
Euarchonta - ps31
Primates - ps32
Haplorrhini - ps33
Simiiformes - ps34
Catarrhini - ps35
Hominoidea - ps36
Hominidae - ps37
Homininae - ps38
Hominini - ps39
Homo_sapiens - ps40
Phylostratum

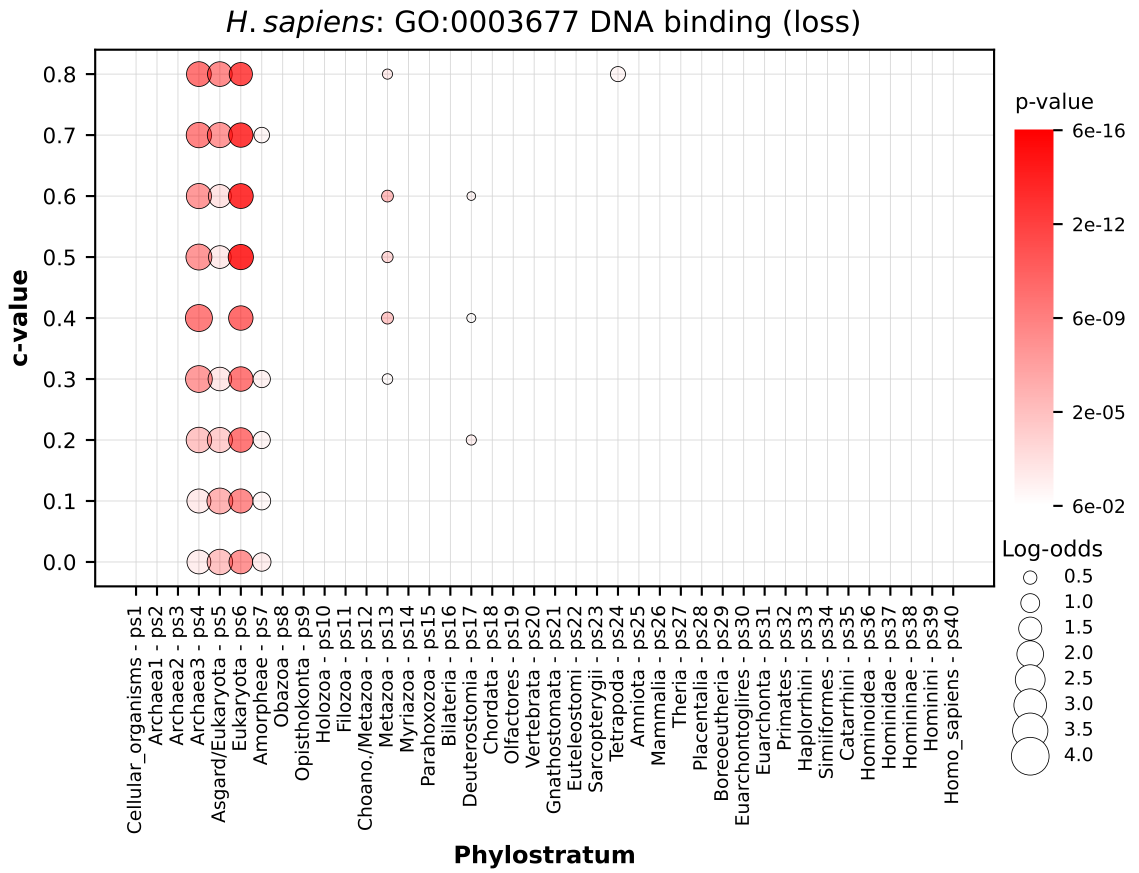

H. sapiens: GO:0003677 DNA binding (loss)
c-value
0.8
0.7
0.6
0.5
0.4
0.3
0.2
0.1
0.0
p-value
6e-16
2e-12
6e-09
2e-05
6e-02
Log-odds
0.5
1.0
1.5
2.0
2.5
3.0
3.5
4.0
Cellular_organisms - ps1
Archaea1 - ps2
Archaea2 - ps3
Archaea3 - ps4
Asgard/Eukaryota - ps5
Eukaryota - ps6
Amorpheae - ps7
Obazoa - ps8
Opisthokonta - ps9
Holozoa - ps10
Filozoa - ps11
Choano./Metazoa - ps12
Metazoa - ps13
Myriazoa - ps14
Parahoxozoa - ps15
Bilateria - ps16
Deuterostomia - ps17
Chordata - ps18
Olfactores - ps19
Vertebrata - ps20
Gnathostomata - ps21
Euteleostomi - ps22
Sarcopterygii - ps23
Tetrapoda - ps24
Amniota - ps25
Mammalia - ps26
Theria - ps27
Placentalia - ps28
Boreoeutheria - ps29
Euarchontoglires - ps30
Euarchonta - ps31
Primates - ps32
Haplorrhini - ps33
Simiiformes - ps34
Catarrhini - ps35
Hominoidea - ps36
Hominidae - ps37
Homininae - ps38
Hominini - ps39
Homo_sapiens - ps40
Phylostratum

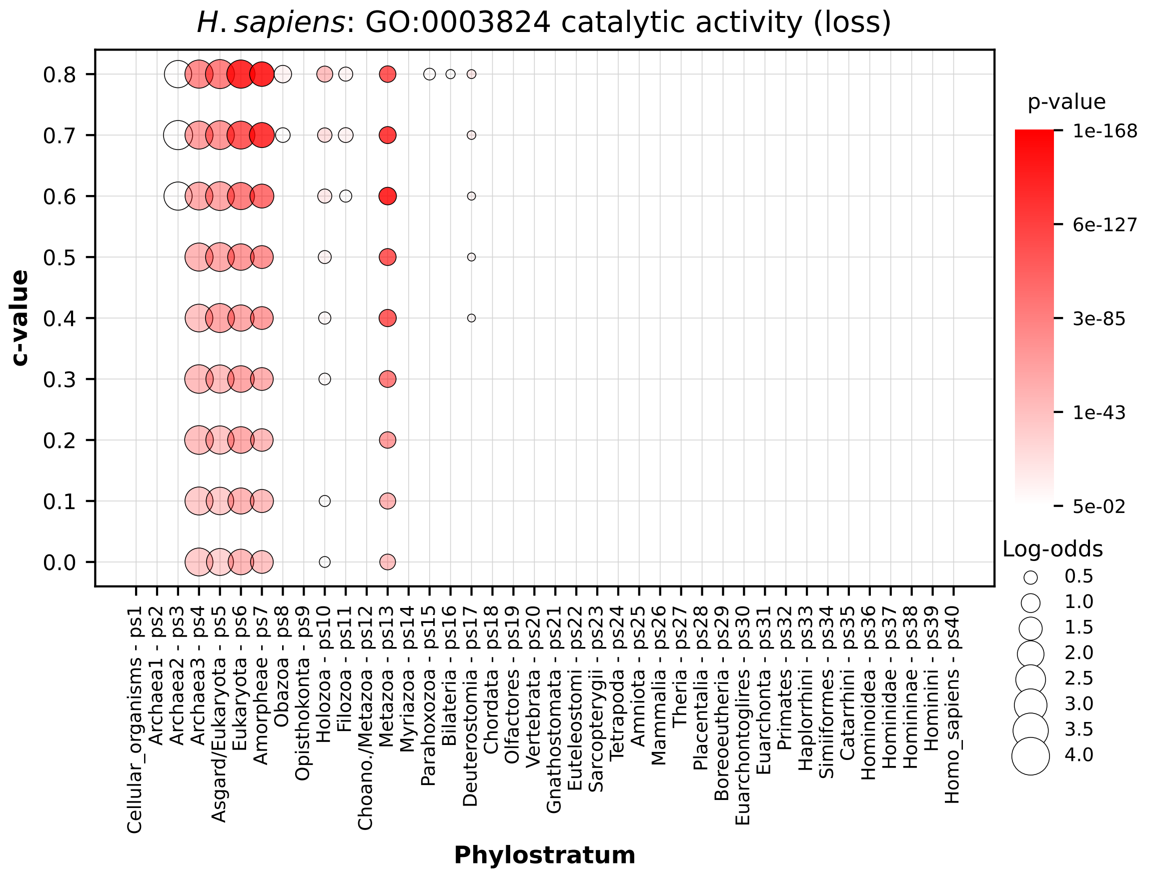
H. sapiens: GO:0003824 catalytic activity (loss)
c-value
0.8
0.7
0.6
0.5
0.4
0.3
0.2
0.1
0.0
p-value
1e-168
6e-127
3e-85
1e-43
5e-02
Log-odds
0.5
1.0
1.5
2.0
2.5
3.0
3.5
4.0
Cellular_organisms - ps1
Archaea1 - ps2
Archaea2 - ps3
Archaea3 - ps4
Asgard/Eukaryota - ps5
Eukaryota - ps6
Amorpheae - ps7
Obazoa - ps8
Opisthokonta - ps9
Holozoa - ps10
Filozoa - ps11
Choano./Metazoa - ps12
Metazoa - ps13
Myriazoa - ps14
Parahoxozoa - ps15
Bilateria - ps16
Deuterostomia - ps17
Chordata - ps18
Olfactores - ps19
Vertebrata - ps20
Gnathostomata - ps21
Euteleostomi - ps22
Sarcopterygii - ps23
Tetrapoda - ps24
Amniota - ps25
Mammalia - ps26
Theria - ps27
Placentalia - ps28
Boreoeutheria - ps29
Euarchontoglires - ps30
Euarchonta - ps31
Primates - ps32
Haplorrhini - ps33
Simiiformes - ps34
Catarrhini - ps35
Hominoidea - ps36
Hominidae - ps37
Homininae - ps38
Hominini - ps39
Homo_sapiens - ps40
Phylostratum

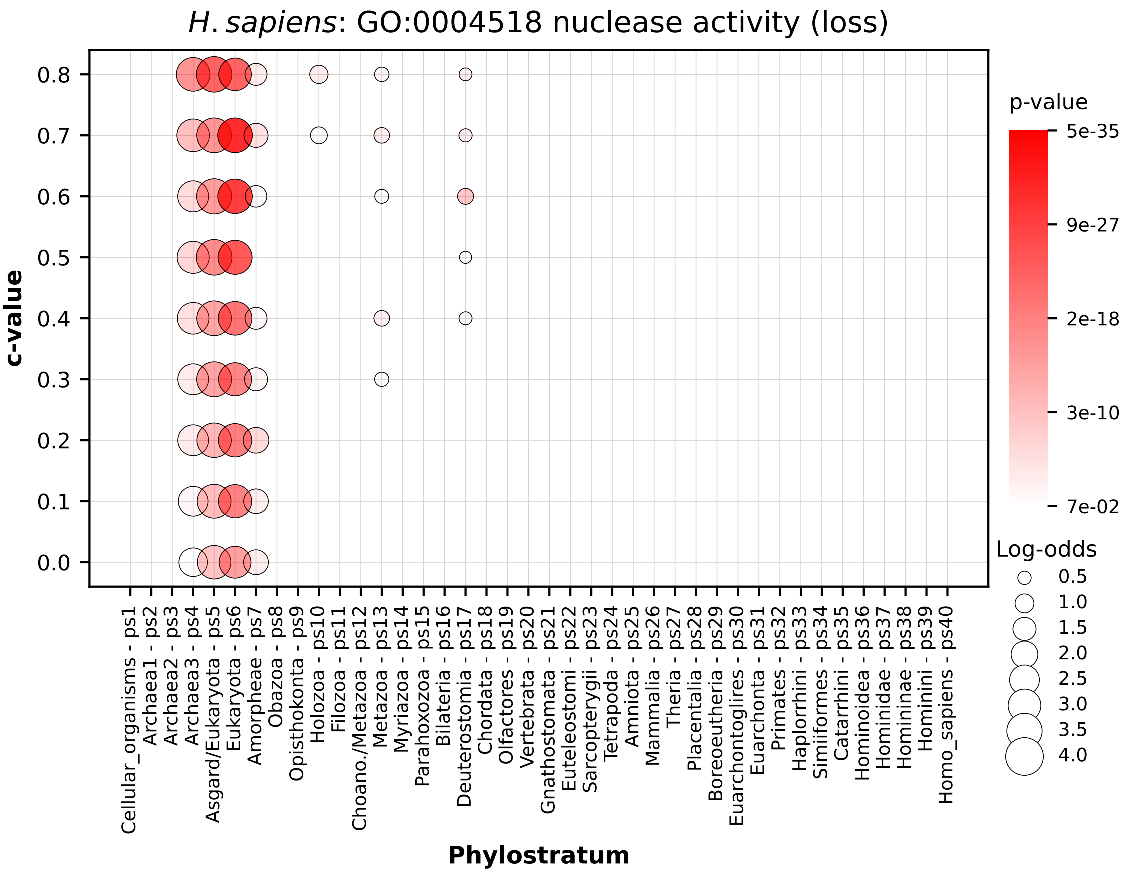

H. sapiens: GO:0004518 nuclease activity (loss)
c-value
0.8
0.7
0.6
0.5
0.4
0.3
0.2
0.1
0.0
p-value
5e-35
9e-27
2e-18
3e-10
7e-02
Log-odds
0.5
1.0
1.5
2.0
2.5
3.0
3.5
4.0
Cellular_organisms - ps1
Archaea1 - ps2
Archaea2 - ps3
Archaea3 - ps4
Asgard/Eukaryota - ps5
Eukaryota - ps6
Amorpheae - ps7
Obazoa - ps8
Opisthokonta - ps9
Holozoa - ps10
Filozoa - ps11
Choano./Metazoa - ps12
Metazoa - ps13
Myriazoa - ps14
Parahoxozoa - ps15
Bilateria - ps16
Deuterostomia - ps17
Chordata - ps18
Olfactores - ps19
Vertebrata - ps20
Gnathostomata - ps21
Euteleostomi - ps22
Sarcopterygii - ps23
Tetrapoda - ps24
Amniota - ps25
Mammalia - ps26
Theria - ps27
Placentalia - ps28
Boreoeutheria - ps29
Euarchontoglires - ps30
Euarchonta - ps31
Primates - ps32
Haplorrhini - ps33
Simiiformes - ps34
Catarrhini - ps35
Hominoidea - ps36
Hominidae - ps37
Homininae - ps38
Hominini - ps39
Homo_sapiens - ps40
Phylostratum

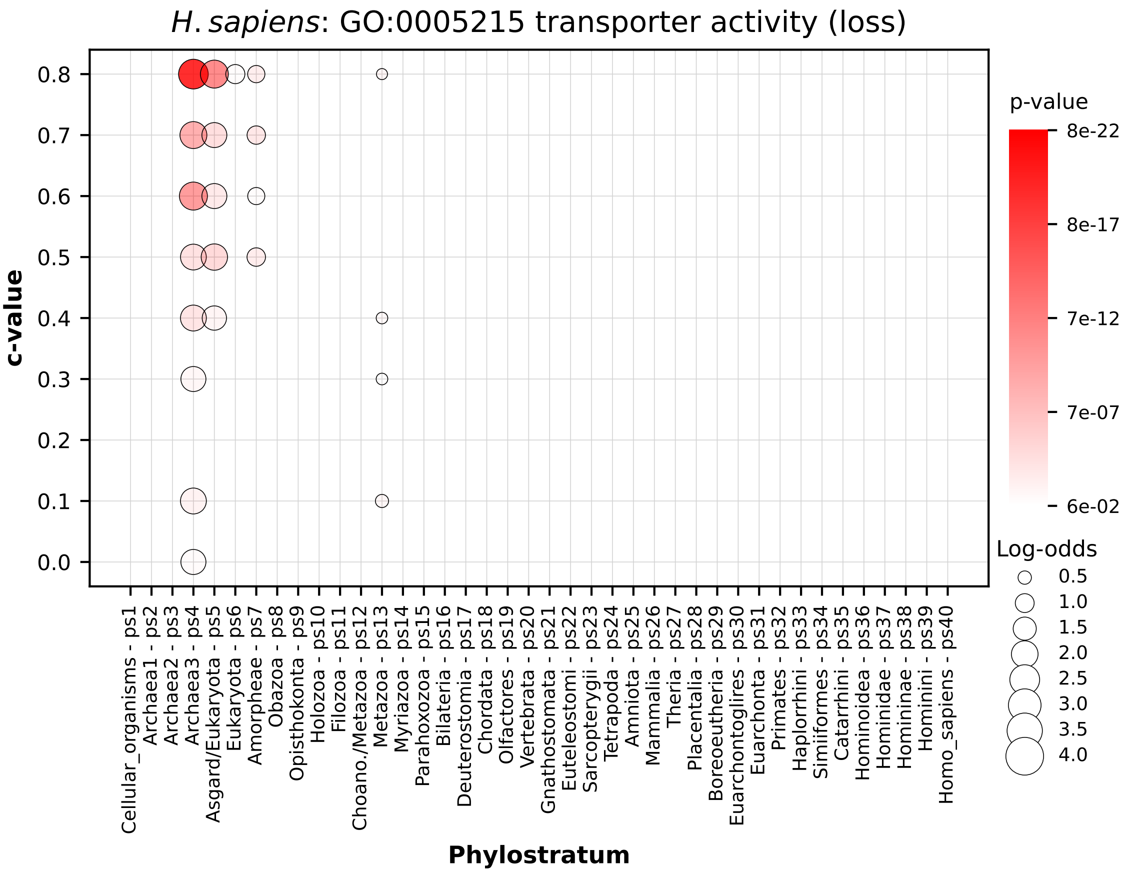

H. sapiens: GO:0005215 transporter activity (loss)
c-value
0.8
0.7
0.6
0.5
0.4
0.3
0.2
0.1
0.0
p-value
8e-22
8e-17
7e-12
7e-07
6e-02
Log-odds
0.5
1.0
1.5
2.0
2.5
3.0
3.5
4.0
Cellular_organisms - ps1
Archaea1 - ps2
Archaea2 - ps3
Archaea3 - ps4
Asgard/Eukaryota - ps5
Eukaryota - ps6
Amorpheae - ps7
Obazoa - ps8
Opisthokonta - ps9
Holozoa - ps10
Filozoa - ps11
Choano./Metazoa - ps12
Metazoa - ps13
Myriazoa - ps14
Parahoxozoa - ps15
Bilateria - ps16
Deuterostomia - ps17
Chordata - ps18
Olfactores - ps19
Vertebrata - ps20
Gnathostomata - ps21
Euteleostomi - ps22
Sarcopterygii - ps23
Tetrapoda - ps24
Amniota - ps25
Mammalia - ps26
Theria - ps27
Placentalia - ps28
Boreoeutheria - ps29
Euarchontoglires - ps30
Euarchonta - ps31
Primates - ps32
Haplorrhini - ps33
Simiiformes - ps34
Catarrhini - ps35
Hominoidea - ps36
Hominidae - ps37
Homininae - ps38
Hominini - ps39
Homo_sapiens - ps40
Phylostratum

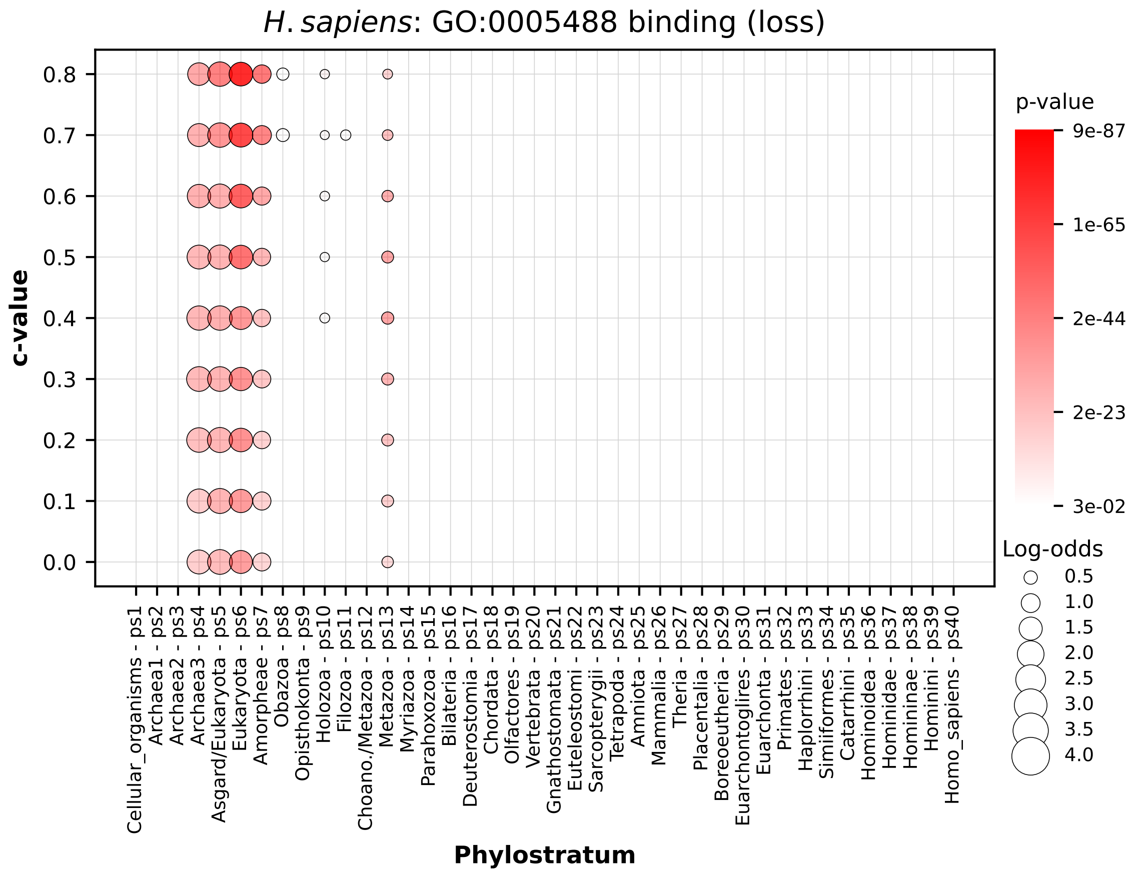

H. sapiens: GO:0005488 binding (loss)
c-value
0.0
0.1
0.2
0.3
0.4
0.5
0.6
0.7
0.8
Phylostratum
Cellular_organisms - ps1
Archaea1 - ps2
Archaea2 - ps3
Archaea3 - ps4
Asgard/Eukaryota - ps5
Eukaryota - ps6
Amorpheae - ps7
Obazoa - ps8
Opisthokonta - ps9
Holozoa - ps10
Filozoa - ps11
Choano./Metazoa - ps12
Metazoa - ps13
Myriazoa - ps14
Parahoxozoa - ps15
Bilateria - ps16
Deuterostomia - ps17
Chordata - ps18
Olfactores - ps19
Vertebrata - ps20
Gnathostomata - ps21
Euteleostomi - ps22
Sarcopterygii - ps23
Tetrapoda - ps24
Amniota - ps25
Mammalia - ps26
Theria - ps27
Placentalia - ps28
Boreoeutheria - ps29
Euarchontoglires - ps30
Euarchonta - ps31
Primates - ps32
Haplorrhini - ps33
Simiiformes - ps34
Catarrhini - ps35
Hominoidea - ps36
Hominidae - ps37
Homininae - ps38
Hominini - ps39
Homo_sapiens - ps40
p-value
9e-87
1e-65
2e-44
2e-23
3e-02
Log-odds
0.5
1.0
1.5
2.0
2.5
3.0
3.5
4.0

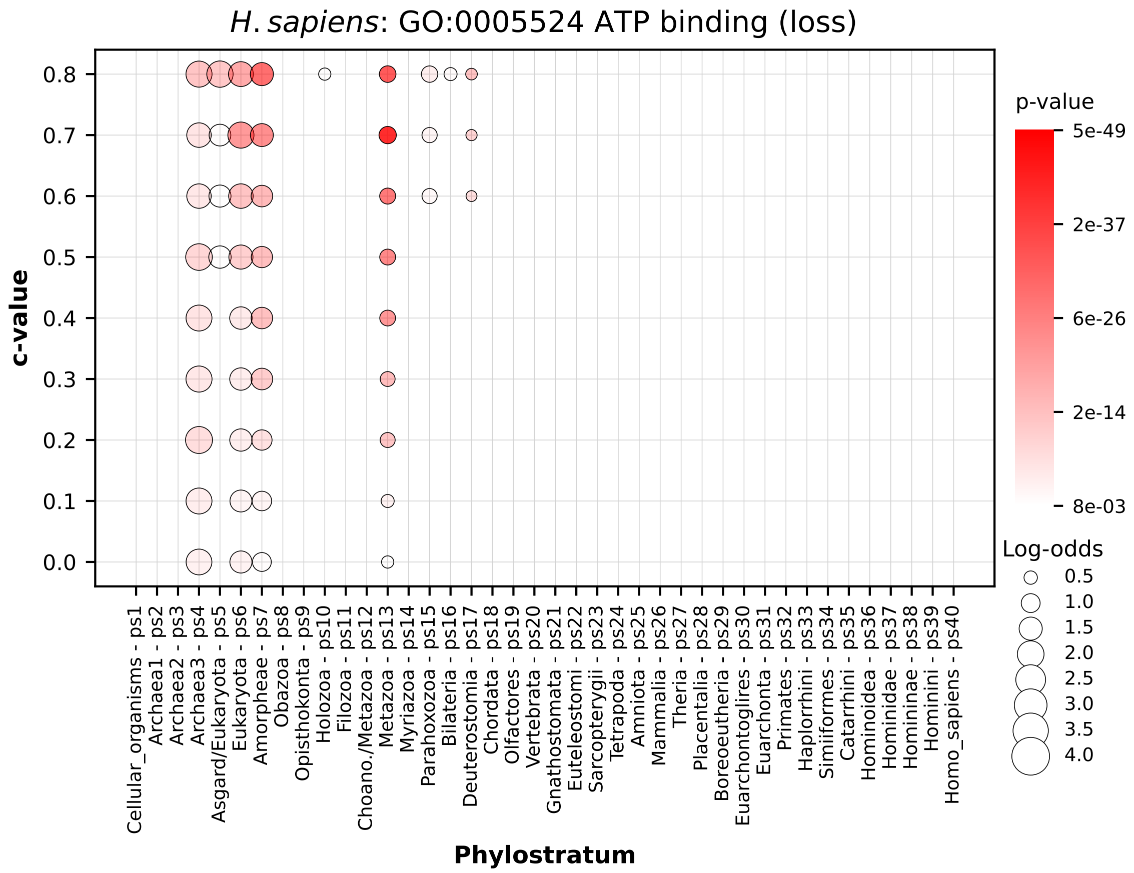
H. sapiens: GO:0005524 ATP binding (loss)
c-value
0.8
0.7
0.6
0.5
0.4
0.3
0.2
0.1
0.0
Phylostratum
Cellular_organisms - ps1
Archaea1 - ps2
Archaea2 - ps3
Archaea3 - ps4
Asgard/Eukaryota - ps5
Eukaryota - ps6
Amorpheae - ps7
Obazoa - ps8
Opisthokonta - ps9
Holozoa - ps10
Filozoa - ps11
Choano./Metazoa - ps12
Metazoa - ps13
Myriazoa - ps14
Parahoxozoa - ps15
Bilateria - ps16
Deuterostomia - ps17
Chordata - ps18
Olfactores - ps19
Vertebrata - ps20
Gnathostomata - ps21
Euteleostomi - ps22
Sarcopterygii - ps23
Tetrapoda - ps24
Amniota - ps25
Mammalia - ps26
Theria - ps27
Placentalia - ps28
Boreoeutheria - ps29
Euarchontoglires - ps30
Euarchonta - ps31
Primates - ps32
Haplorrhini - ps33
Simiiformes - ps34
Catarrhini - ps35
Hominoidea - ps36
Hominidae - ps37
Homininae - ps38
Hominini - ps39
Homo_sapiens - ps40
p-value
5e-49
2e-37
6e-26
2e-14
8e-03
Log-odds
0.5
1.0
1.5
2.0
2.5
3.0
3.5
4.0

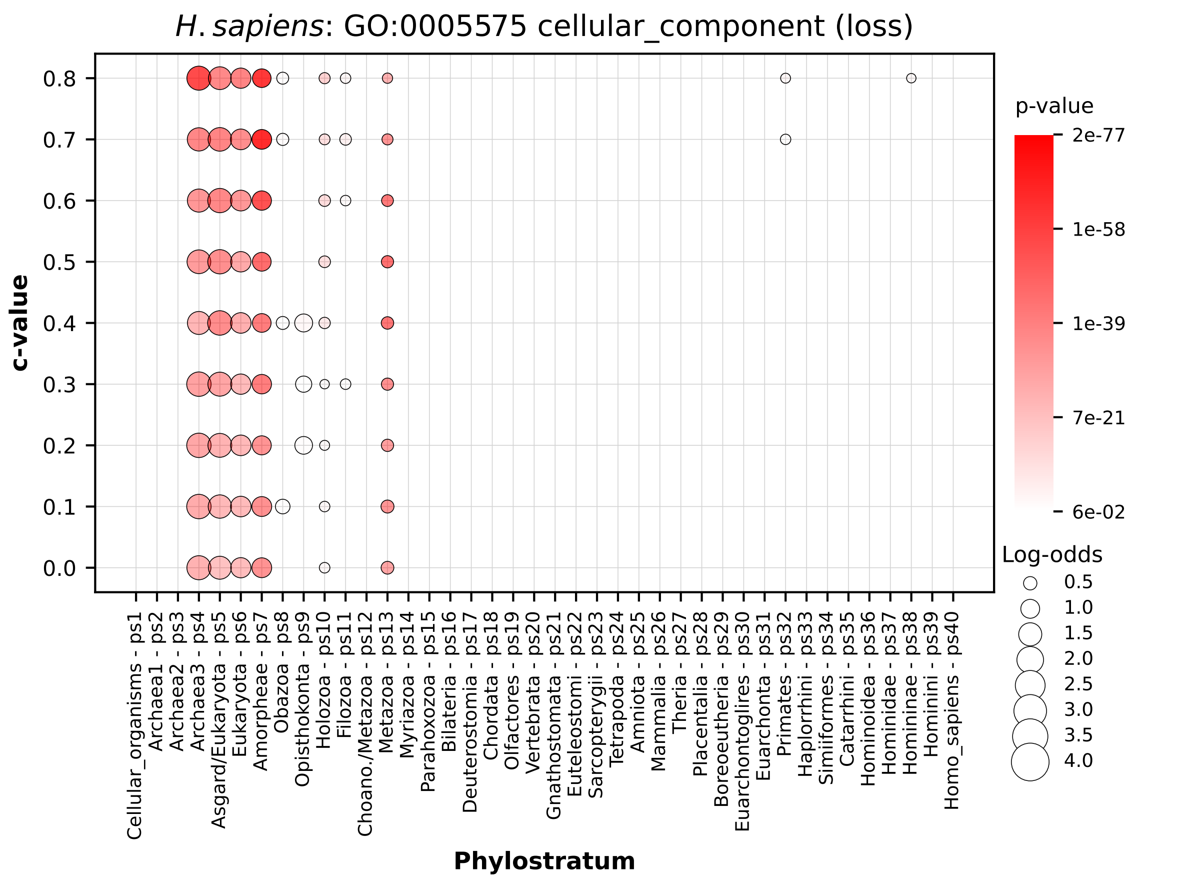

H. sapiens: GO:0005575 cellular_component (loss)
c-value
0.0
0.1
0.2
0.3
0.4
0.5
0.6
0.7
0.8
Phylostratum
Cellular_organisms - ps1
Archaea1 - ps2
Archaea2 - ps3
Archaea3 - ps4
Asgard/Eukaryota - ps5
Eukaryota - ps6
Amorpheae - ps7
Obazoa - ps8
Opisthokonta - ps9
Holozoa - ps10
Filozoa - ps11
Choano./Metazoa - ps12
Metazoa - ps13
Myriazoa - ps14
Parahoxozoa - ps15
Bilateria - ps16
Deuterostomia - ps17
Chordata - ps18
Olfactores - ps19
Vertebrata - ps20
Gnathostomata - ps21
Euteleostomi - ps22
Sarcopterygii - ps23
Tetrapoda - ps24
Amniota - ps25
Mammalia - ps26
Theria - ps27
Placentalia - ps28
Boreoeutheria - ps29
Euarchontoglires - ps30
Euarchonta - ps31
Primates - ps32
Haplorrhini - ps33
Simiiformes - ps34
Catarrhini - ps35
Hominoidea - ps36
Hominidae - ps37
Homininae - ps38
Hominini - ps39
Homo_sapiens - ps40
p-value
2e-77
1e-58
1e-39
7e-21
6e-02
Log-odds
0.5
1.0
1.5
2.0
2.5
3.0
3.5
4.0

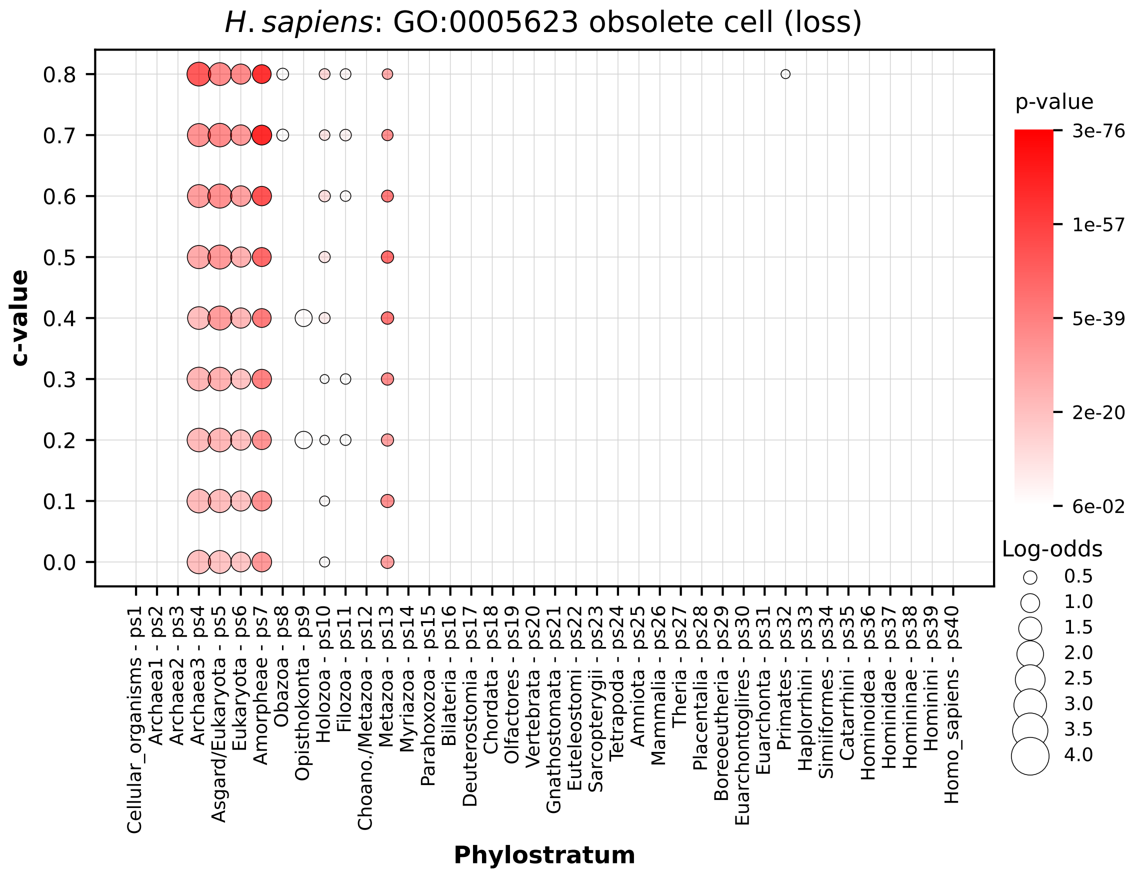

H. sapiens: GO:0005623 obsolete cell (loss)
c-value
0.8
0.7
0.6
0.5
0.4
0.3
0.2
0.1
0.0
Phylostratum
Cellular_organisms - ps1
Archaea1 - ps2
Archaea2 - ps3
Archaea3 - ps4
Asgard/Eukaryota - ps5
Eukaryota - ps6
Amorpheae - ps7
Obazoa - ps8
Opisthokonta - ps9
Holozoa - ps10
Filozoa - ps11
Choano./Metazoa - ps12
Metazoa - ps13
Myriazoa - ps14
Parahoxozoa - ps15
Bilateria - ps16
Deuterostomia - ps17
Chordata - ps18
Olfactores - ps19
Vertebrata - ps20
Gnathostomata - ps21
Euteleostomi - ps22
Sarcopterygii - ps23
Tetrapoda - ps24
Amniota - ps25
Mammalia - ps26
Theria - ps27
Placentalia - ps28
Boreoeutheria - ps29
Euarchontoglires - ps30
Euarchonta - ps31
Primates - ps32
Haplorrhini - ps33
Simiiformes - ps34
Catarrhini - ps35
Hominoidea - ps36
Hominidae - ps37
Homininae - ps38
Hominini - ps39
Homo_sapiens - ps40
p-value
3e-76
1e-57
5e-39
2e-20
6e-02
Log-odds
0.5
1.0
1.5
2.0
2.5
3.0
3.5
4.0

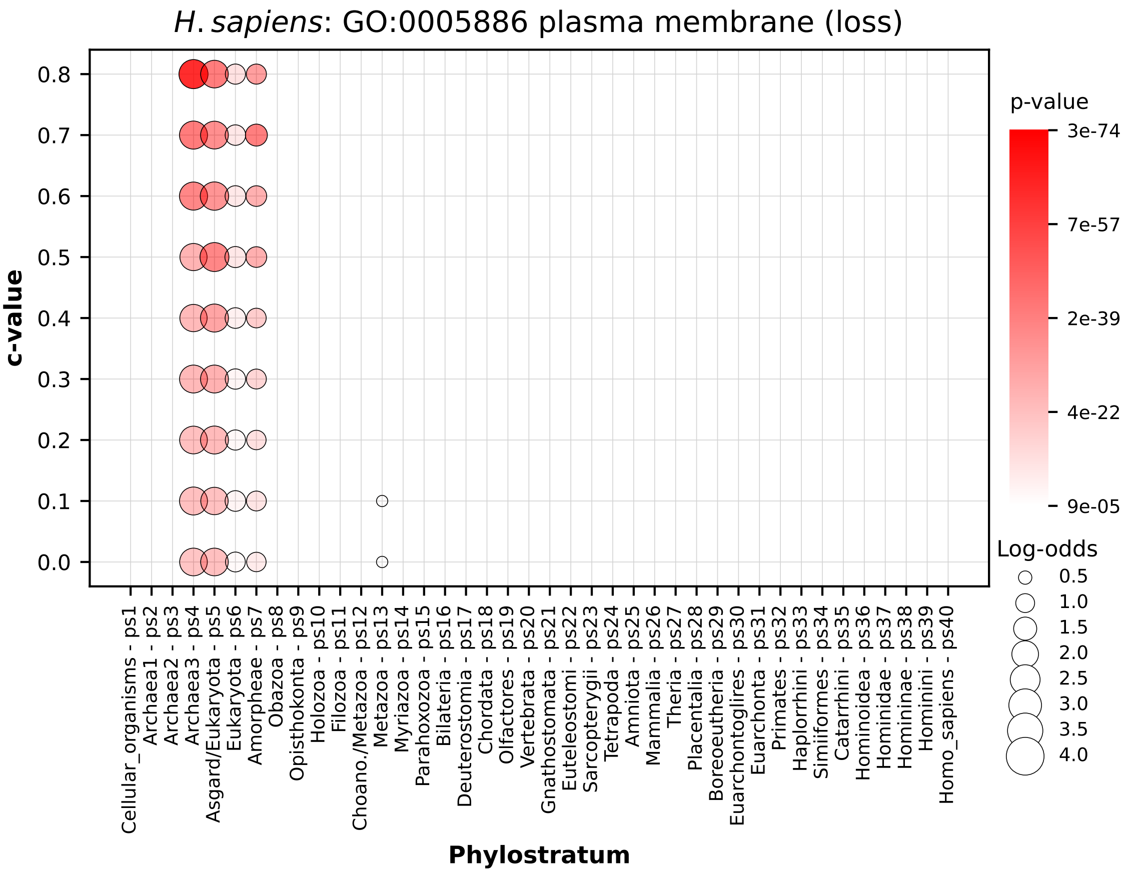
H. sapiens: GO:0005886 plasma membrane (loss)
c-value
0.8
0.7
0.6
0.5
0.4
0.3
0.2
0.1
0.0
p-value
3e-74
7e-57
2e-39
4e-22
9e-05
Log-odds
0.5
1.0
1.5
2.0
2.5
3.0
3.5
4.0
Cellular_organisms - ps1
Archaea1 - ps2
Archaea2 - ps3
Archaea3 - ps4
Asgard/Eukaryota - ps5
Eukaryota - ps6
Amorpheae - ps7
Obazoa - ps8
Opisthokonta - ps9
Holozoa - ps10
Filozoa - ps11
Choano./Metazoa - ps12
Metazoa - ps13
Myriazoa - ps14
Parahoxozoa - ps15
Bilateria - ps16
Deuterostomia - ps17
Chordata - ps18
Olfactores - ps19
Vertebrata - ps20
Gnathostomata - ps21
Euteleostomi - ps22
Sarcopterygii - ps23
Tetrapoda - ps24
Amniota - ps25
Mammalia - ps26
Theria - ps27
Placentalia - ps28
Boreoeutheria - ps29
Euarchontoglires - ps30
Euarchonta - ps31
Primates - ps32
Haplorrhini - ps33
Simiiformes - ps34
Catarrhini - ps35
Hominoidea - ps36
Hominidae - ps37
Homininae - ps38
Hominini - ps39
Homo_sapiens - ps40
Phylostratum

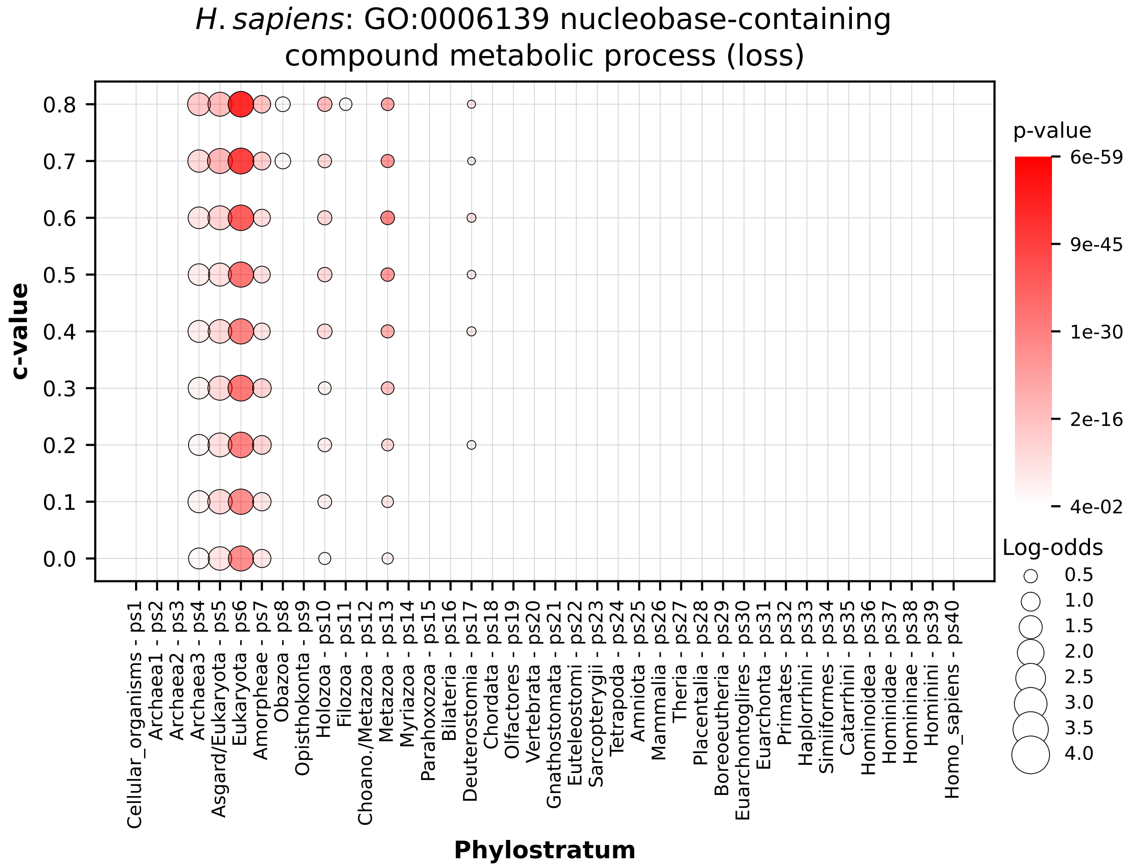
H. sapiens: GO:0006139 nucleobase-containing compound metabolic process (loss)
c-value
0.8
0.7
0.6
0.5
0.4
0.3
0.2
0.1
0.0
Phylostratum
Cellular_organisms - ps1
Archaea1 - ps2
Archaea2 - ps3
Archaea3 - ps4
Asgard/Eukaryota - ps5
Eukaryota - ps6
Amorpheae - ps7
Obazoa - ps8
Opisthokonta - ps9
Holozoa - ps10
Filozoa - ps11
Choano./Metazoa - ps12
Metazoa - ps13
Myriazoa - ps14
Parahoxozoa - ps15
Bilateria - ps16
Deuterostomia - ps17
Chordata - ps18
Olfactores - ps19
Vertebrata - ps20
Gnathostomata - ps21
Euteleostomi - ps22
Sarcopterygii - ps23
Tetrapoda - ps24
Amniota - ps25
Mammalia - ps26
Theria - ps27
Placentalia - ps28
Boreoeutheria - ps29
Euarchontoglires - ps30
Euarchonta - ps31
Primates - ps32
Haplorrhini - ps33
Simiiformes - ps34
Catarrhini - ps35
Hominoidea - ps36
Hominidae - ps37
Homininae - ps38
Hominini - ps39
Homo_sapiens - ps40
p-value
6e-59
9e-45
1e-30
2e-16
4e-02
Log-odds
0.5
1.0
1.5
2.0
2.5
3.0
3.5
4.0

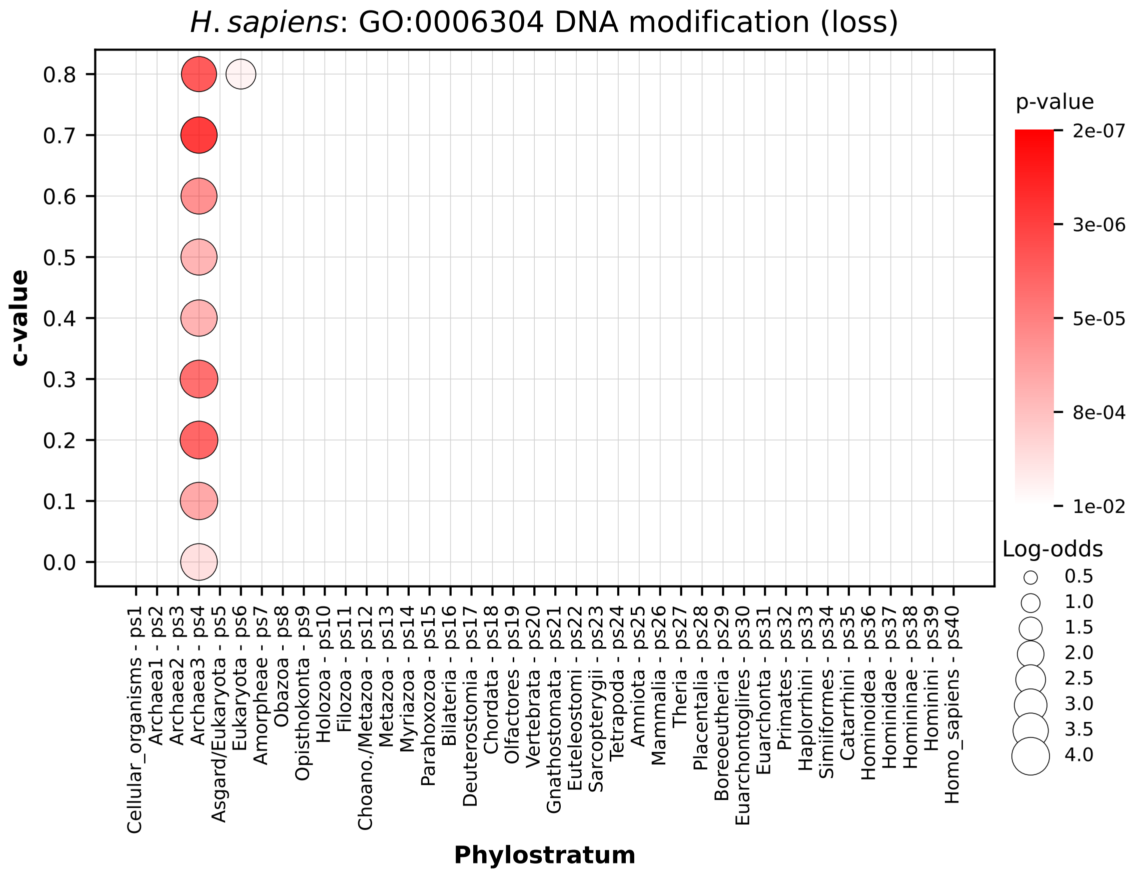

H. sapiens: GO:0006304 DNA modification (loss)
c-value
0.0
0.1
0.2
0.3
0.4
0.5
0.6
0.7
0.8
Phylostratum
Cellular_organisms - ps1
Archaea1 - ps2
Archaea2 - ps3
Archaea3 - ps4
Asgard/Eukaryota - ps5
Eukaryota - ps6
Amorpheae - ps7
Obazoa - ps8
Opisthokonta - ps9
Holozoa - ps10
Filozoa - ps11
Choano./Metazoa - ps12
Metazoa - ps13
Myriazoa - ps14
Parahoxozoa - ps15
Bilateria - ps16
Deuterostomia - ps17
Chordata - ps18
Olfactores - ps19
Vertebrata - ps20
Gnathostomata - ps21
Euteleostomi - ps22
Sarcopterygii - ps23
Tetrapoda - ps24
Amniota - ps25
Mammalia - ps26
Theria - ps27
Placentalia - ps28
Boreoeutheria - ps29
Euarchontoglires - ps30
Euarchonta - ps31
Primates - ps32
Haplorrhini - ps33
Simiiformes - ps34
Catarrhini - ps35
Hominoidea - ps36
Hominidae - ps37
Homininae - ps38
Hominini - ps39
Homo_sapiens - ps40
p-value
2e-07
3e-06
5e-05
8e-04
1e-02
Log-odds
0.5
1.0
1.5
2.0
2.5
3.0
3.5
4.0

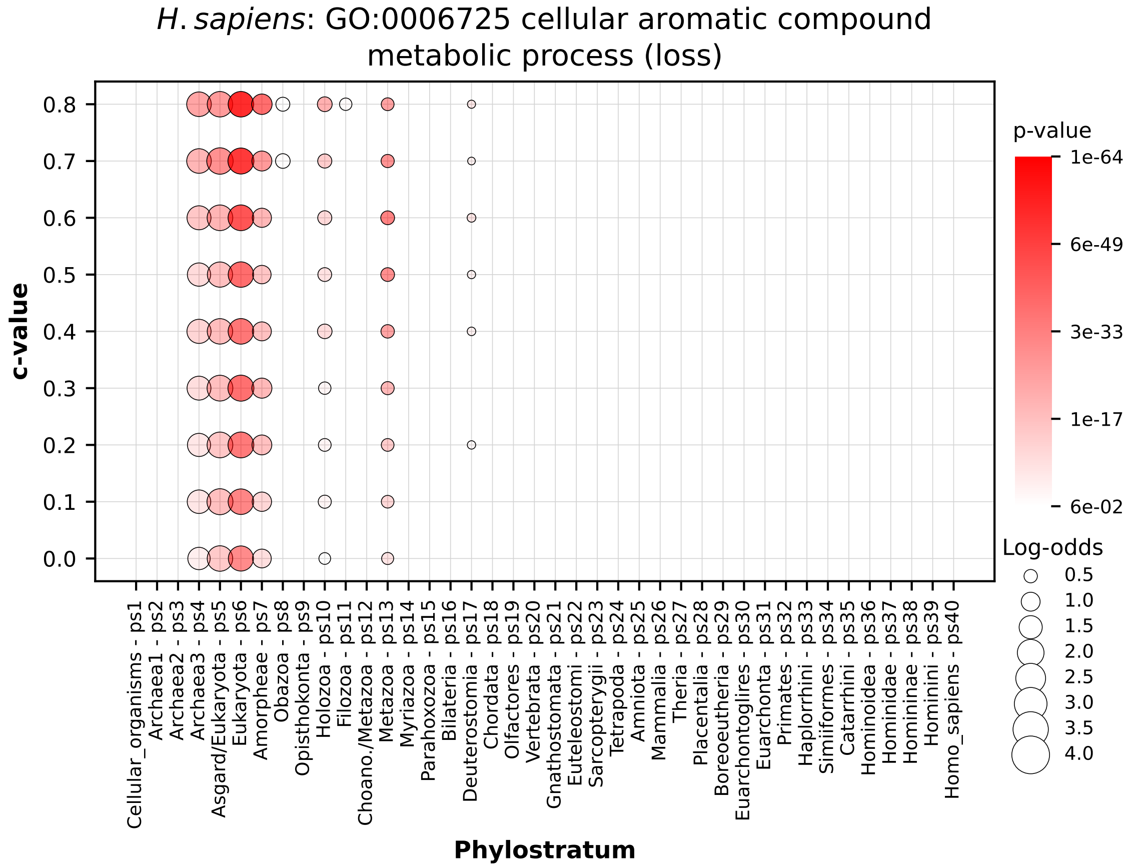
H. sapiens: GO:0006725 cellular aromatic compound metabolic process (loss)
c-value
0.8
0.7
0.6
0.5
0.4
0.3
0.2
0.1
0.0
Phylostratum
Cellular_organisms - ps1
Archaea1 - ps2
Archaea2 - ps3
Archaea3 - ps4
Asgard/Eukaryota - ps5
Eukaryota - ps6
Amorpheae - ps7
Obazoa - ps8
Opisthokonta - ps9
Holozoa - ps10
Filozoa - ps11
Choano./Metazoa - ps12
Metazoa - ps13
Myriazoa - ps14
Parahoxozoa - ps15
Bilateria - ps16
Deuterostomia - ps17
Chordata - ps18
Olfactores - ps19
Vertebrata - ps20
Gnathostomata - ps21
Euteleostomi - ps22
Sarcopterygii - ps23
Tetrapoda - ps24
Amniota - ps25
Mammalia - ps26
Theria - ps27
Placentalia - ps28
Boreoeutheria - ps29
Euarchontoglires - ps30
Euarchonta - ps31
Primates - ps32
Haplorrhini - ps33
Simiiformes - ps34
Catarrhini - ps35
Hominoidea - ps36
Hominidae - ps37
Homininae - ps38
Hominini - ps39
Homo_sapiens - ps40
p-value
1e-64
6e-49
3e-33
1e-17
6e-02
Log-odds
0.5
1.0
1.5
2.0
2.5
3.0
3.5
4.0

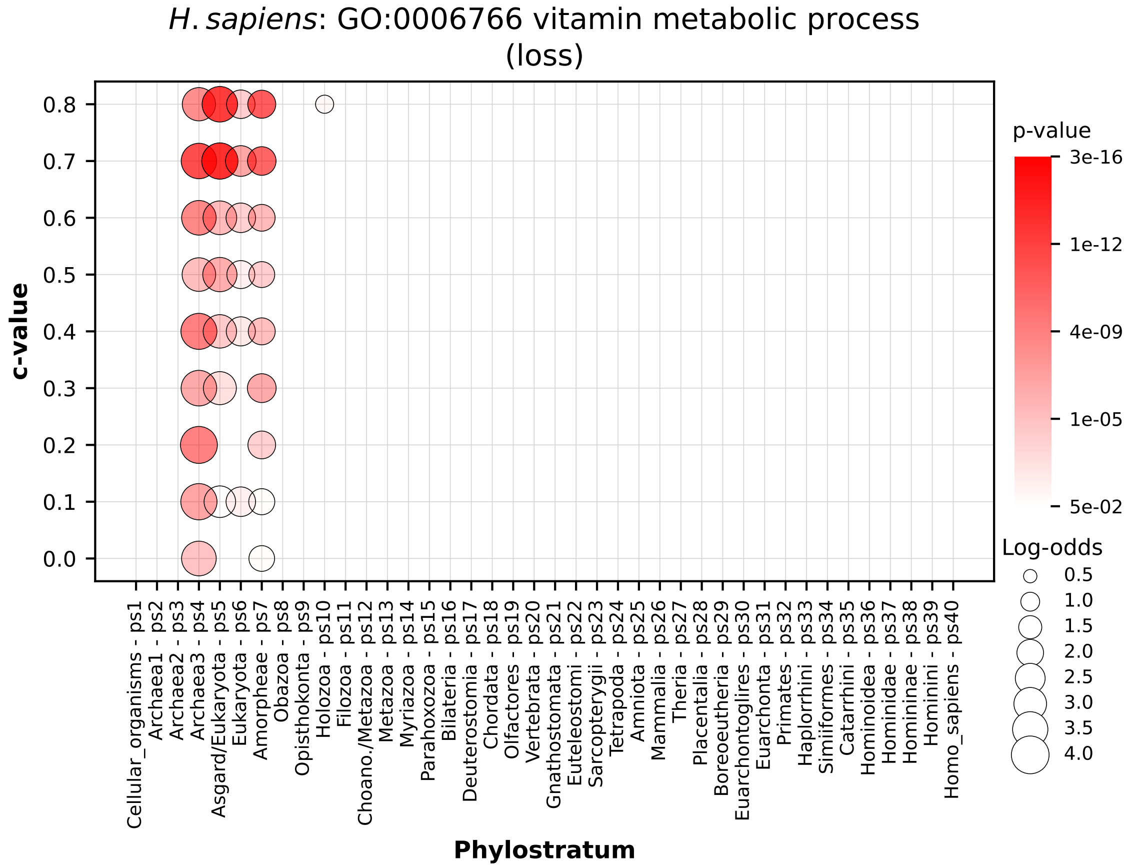

H. sapiens: GO:0006766 vitamin metabolic process
(loss)
c-value
0.8
0.7
0.6
0.5
0.4
0.3
0.2
0.1
0.0
p-value
3e-16
1e-12
4e-09
1e-05
5e-02
Log-odds
0.5
1.0
1.5
2.0
2.5
3.0
3.5
4.0
Cellular_organisms - ps1
Archaea1 - ps2
Archaea2 - ps3
Archaea3 - ps4
Asgard/Eukaryota - ps5
Eukaryota - ps6
Amorpheae - ps7
Obazoa - ps8
Opisthokonta - ps9
Holozoa - ps10
Filozoa - ps11
Choano./Metazoa - ps12
Metazoa - ps13
Myriazoa - ps14
Parahoxozoa - ps15
Bilateria - ps16
Deuterostomia - ps17
Chordata - ps18
Olfactores - ps19
Vertebrata - ps20
Gnathostomata - ps21
Euteleostomi - ps22
Sarcopterygii - ps23
Tetrapoda - ps24
Amniota - ps25
Mammalia - ps26
Theria - ps27
Placentalia - ps28
Boreoeutheria - ps29
Euarchontoglires - ps30
Euarchonta - ps31
Primates - ps32
Haplorrhini - ps33
Simiiformes - ps34
Catarrhini - ps35
Hominoidea - ps36
Hominidae - ps37
Homininae - ps38
Hominini - ps39
Homo_sapiens - ps40
Phylostratum

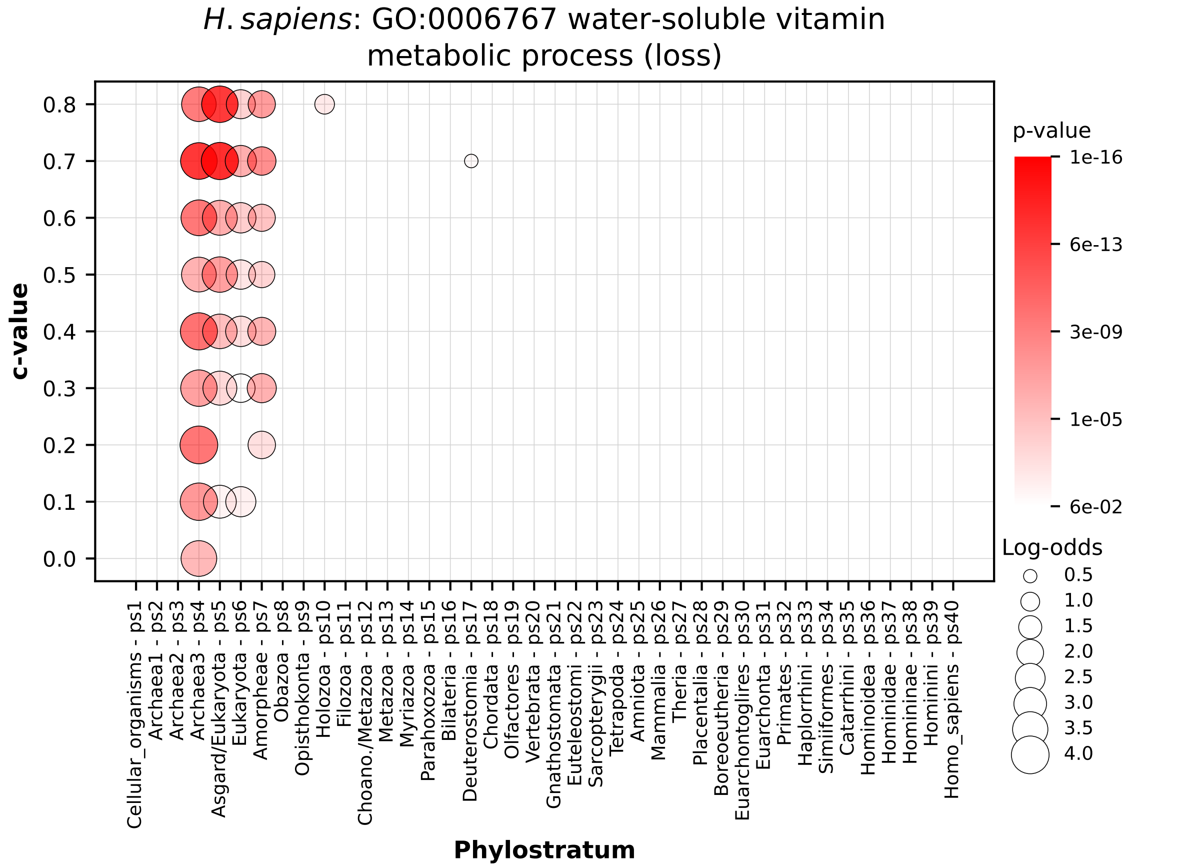

H. sapiens: GO:0006767 water-soluble vitamin metabolic process (loss)
c-value
0.8
0.7
0.6
0.5
0.4
0.3
0.2
0.1
0.0
p-value
1e-16
6e-13
3e-09
1e-05
6e-02
Log-odds
0.5
1.0
1.5
2.0
2.5
3.0
3.5
4.0
Cellular_organisms - ps1
Archaea1 - ps2
Archaea2 - ps3
Archaea3 - ps4
Asgard/Eukaryota - ps5
Eukaryota - ps6
Amorpheae - ps7
Obazoa - ps8
Opisthokonta - ps9
Holozoa - ps10
Filozoa - ps11
Choano./Metazoa - ps12
Metazoa - ps13
Myriazoa - ps14
Parahoxozoa - ps15
Bilateria - ps16
Deuterostomia - ps17
Chordata - ps18
Olfactores - ps19
Vertebrata - ps20
Gnathostomata - ps21
Euteleostomi - ps22
Sarcopterygii - ps23
Tetrapoda - ps24
Amniota - ps25
Mammalia - ps26
Theria - ps27
Placentalia - ps28
Boreoeutheria - ps29
Euarchontoglires - ps30
Euarchonta - ps31
Primates - ps32
Haplorrhini - ps33
Simiiformes - ps34
Catarrhini - ps35
Hominoidea - ps36
Hominidae - ps37
Homininae - ps38
Hominini - ps39
Homo_sapiens - ps40
Phylostratum

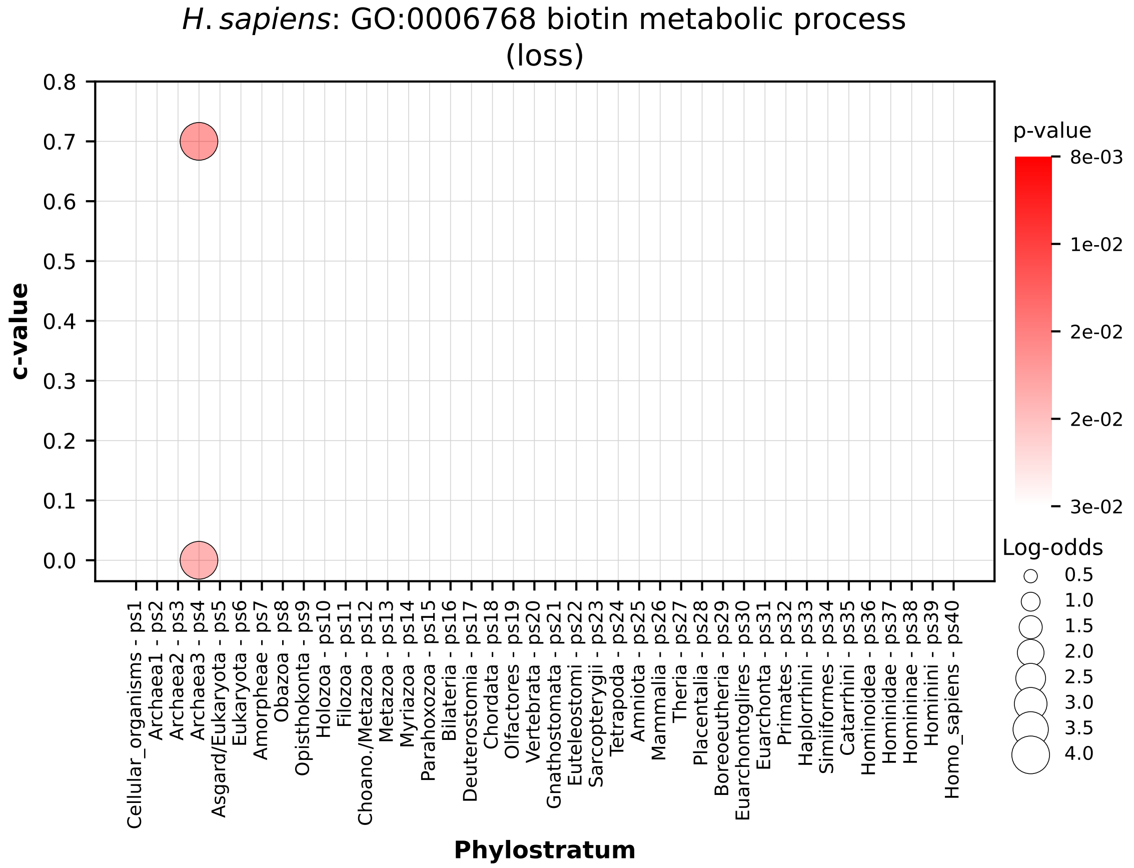

H. sapiens: GO:0006768 biotin metabolic process
(loss)
c-value
0.8
0.7
0.6
0.5
0.4
0.3
0.2
0.1
0.0
p-value
8e-03
1e-02
2e-02
2e-02
3e-02
Log-odds
0.5
1.0
1.5
2.0
2.5
3.0
3.5
4.0
Cellular_organisms - ps1
Archaea1 - ps2
Archaea2 - ps3
Archaea3 - ps4
Asgard/Eukaryota - ps5
Eukaryota - ps6
Amorpheae - ps7
Obazoa - ps8
Opisthokonta - ps9
Holozoa - ps10
Filozoa - ps11
Choano./Metazoa - ps12
Metazoa - ps13
Myriazoa - ps14
Parahoxozoa - ps15
Bilateria - ps16
Deuterostomia - ps17
Chordata - ps18
Olfactores - ps19
Vertebrata - ps20
Gnathostomata - ps21
Euteleostomi - ps22
Sarcopterygii - ps23
Tetrapoda - ps24
Amniota - ps25
Mammalia - ps26
Theria - ps27
Placentalia - ps28
Boreoeutheria - ps29
Euarchontoglires - ps30
Euarchonta - ps31
Primates - ps32
Haplorrhini - ps33
Simiiformes - ps34
Catarrhini - ps35
Hominoidea - ps36
Hominidae - ps37
Homininae - ps38
Hominini - ps39
Homo_sapiens - ps40
Phylostratum

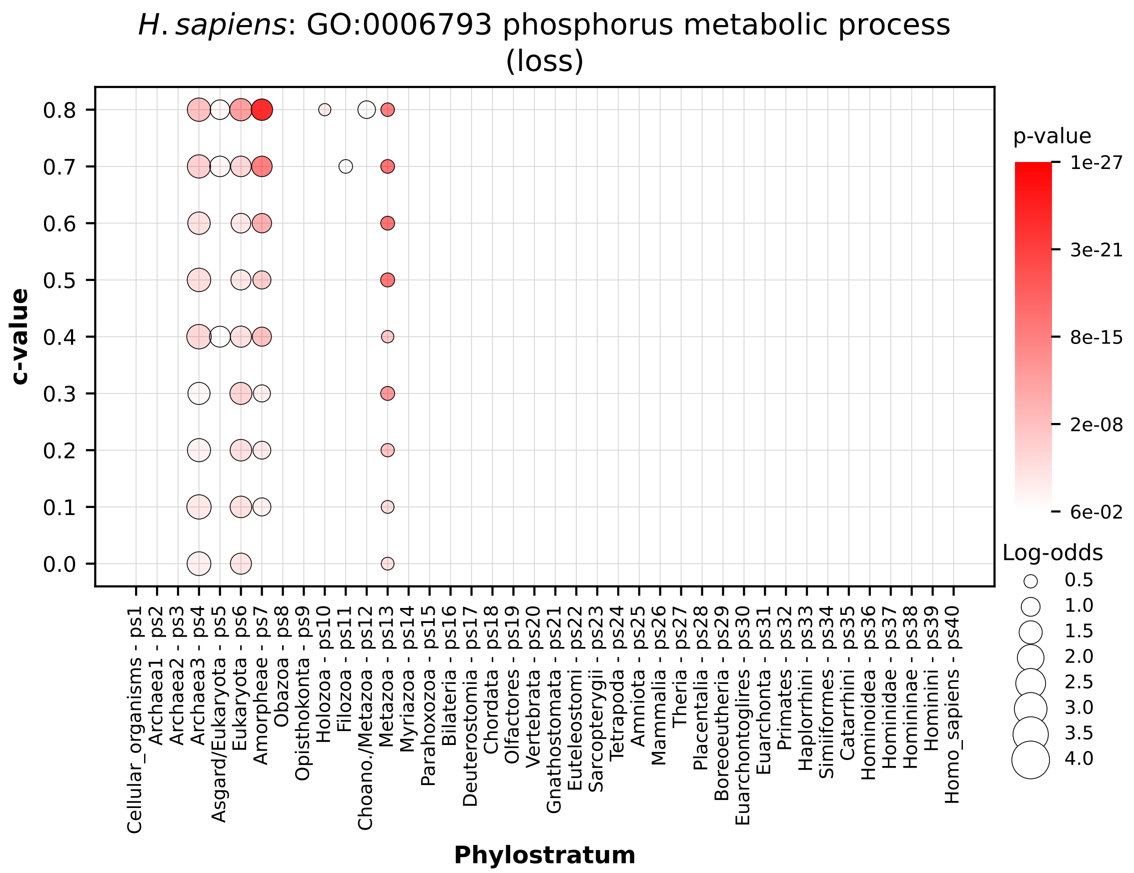

H. sapiens: GO:0006793 phosphorus metabolic process
(loss)
c-value
0.8
0.7
0.6
0.5
0.4
0.3
0.2
0.1
0.0
Phylostratum
Cellular_organisms - ps1
Archaea1 - ps2
Archaea2 - ps3
Archaea3 - ps4
Asgard/Eukaryota - ps5
Eukaryota - ps6
Amorpheae - ps7
Obazoa - ps8
Opisthokonta - ps9
Holozoa - ps10
Filozoa - ps11
Choano./Metazoa - ps12
Metazoa - ps13
Myriazoa - ps14
Parahoxozoa - ps15
Bilateria - ps16
Deuterostomia - ps17
Chordata - ps18
Olfactores - ps19
Vertebrata - ps20
Gnathostomata - ps21
Euteleostomi - ps22
Sarcopterygii - ps23
Tetrapoda - ps24
Amniota - ps25
Mammalia - ps26
Theria - ps27
Placentalia - ps28
Boreoeutheria - ps29
Euarchontoglires - ps30
Euarchonta - ps31
Primates - ps32
Haplorrhini - ps33
Simiiformes - ps34
Catarrhini - ps35
Hominoidea - ps36
Hominidae - ps37
Homininae - ps38
Hominini - ps39
Homo_sapiens - ps40
p-value
1e-27
3e-21
8e-15
2e-08
6e-02
Log-odds
0.5
1.0
1.5
2.0
2.5
3.0
3.5
4.0

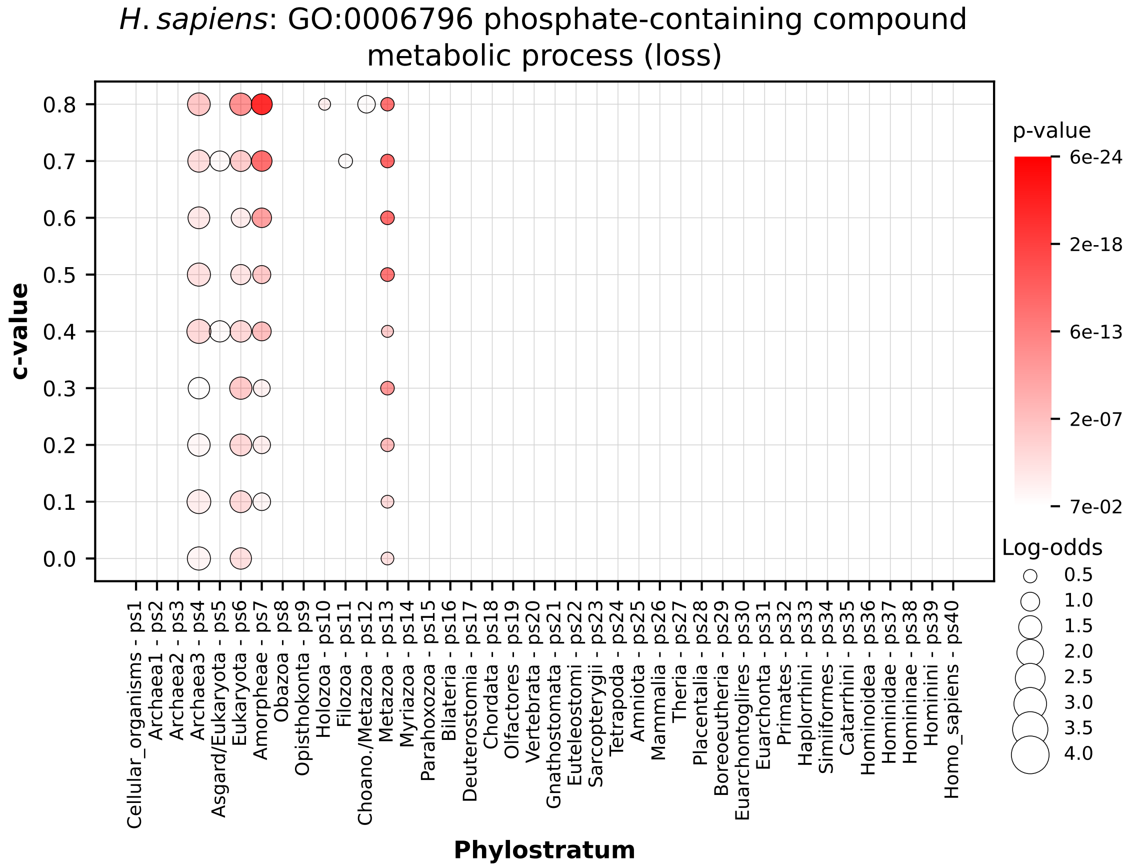
H. sapiens: GO:0006796 phosphate-containing compound metabolic process (loss)
c-value
0.8
0.7
0.6
0.5
0.4
0.3
0.2
0.1
0.0
Phylostratum
Cellular_organisms - ps1
Archaea1 - ps2
Archaea2 - ps3
Archaea3 - ps4
Asgard/Eukaryota - ps5
Eukaryota - ps6
Amorpheae - ps7
Obazoa - ps8
Opisthokonta - ps9
Holozoa - ps10
Filozoa - ps11
Choano./Metazoa - ps12
Metazoa - ps13
Myriazoa - ps14
Parahoxozoa - ps15
Bilateria - ps16
Deuterostomia - ps17
Chordata - ps18
Olfactores - ps19
Vertebrata - ps20
Gnathostomata - ps21
Euteleostomi - ps22
Sarcopterygii - ps23
Tetrapoda - ps24
Amniota - ps25
Mammalia - ps26
Theria - ps27
Placentalia - ps28
Boreoeutheria - ps29
Euarchontoglires - ps30
Euarchonta - ps31
Primates - ps32
Haplorrhini - ps33
Simiiformes - ps34
Catarrhini - ps35
Hominoidea - ps36
Hominidae - ps37
Homininae - ps38
Hominini - ps39
Homo_sapiens - ps40
p-value
6e-24
2e-18
6e-13
2e-07
7e-02
Log-odds
0.5
1.0
1.5
2.0
2.5
3.0
3.5
4.0

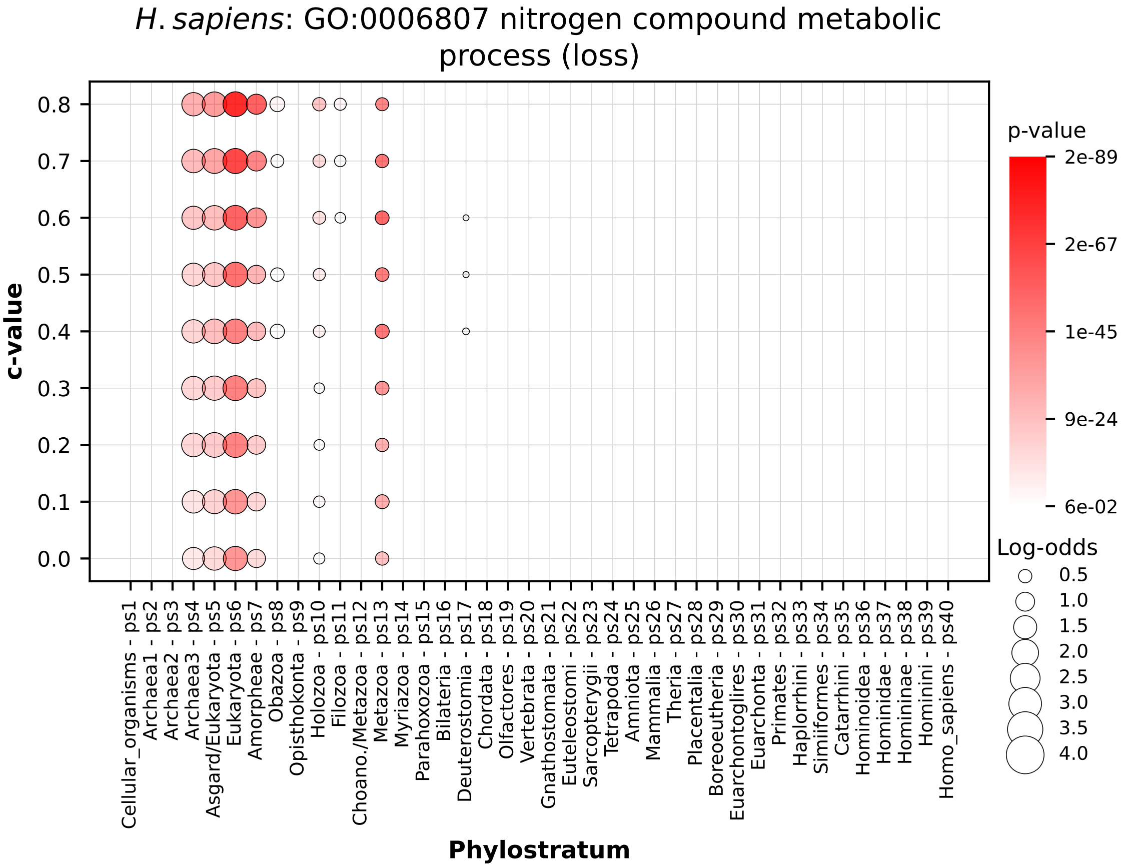

H. sapiens: GO:0006807 nitrogen compound metabolic process (loss)
c-value
0.8
0.7
0.6
0.5
0.4
0.3
0.2
0.1
0.0
p-value
2e-89
2e-67
1e-45
9e-24
6e-02
Log-odds
0.5
1.0
1.5
2.0
2.5
3.0
3.5
4.0
Cellular_organisms - ps1
Archaea1 - ps2
Archaea2 - ps3
Archaea3 - ps4
Asgard/Eukaryota - ps5
Eukaryota - ps6
Amorpheae - ps7
Obazoa - ps8
Opisthokonta - ps9
Holozoa - ps10
Filozoa - ps11
Choano./Metazoa - ps12
Metazoa - ps13
Myriazoa - ps14
Parahoxozoa - ps15
Bilateria - ps16
Deuterostomia - ps17
Chordata - ps18
Olfactores - ps19
Vertebrata - ps20
Gnathostomata - ps21
Euteleostomi - ps22
Sarcopterygii - ps23
Tetrapoda - ps24
Amniota - ps25
Mammalia - ps26
Theria - ps27
Placentalia - ps28
Boreoeutheria - ps29
Euarchontoglires - ps30
Euarchonta - ps31
Primates - ps32
Haplorrhini - ps33
Simiiformes - ps34
Catarrhini - ps35
Hominoidea - ps36
Hominidae - ps37
Homininae - ps38
Hominini - ps39
Homo_sapiens - ps40
Phylostratum

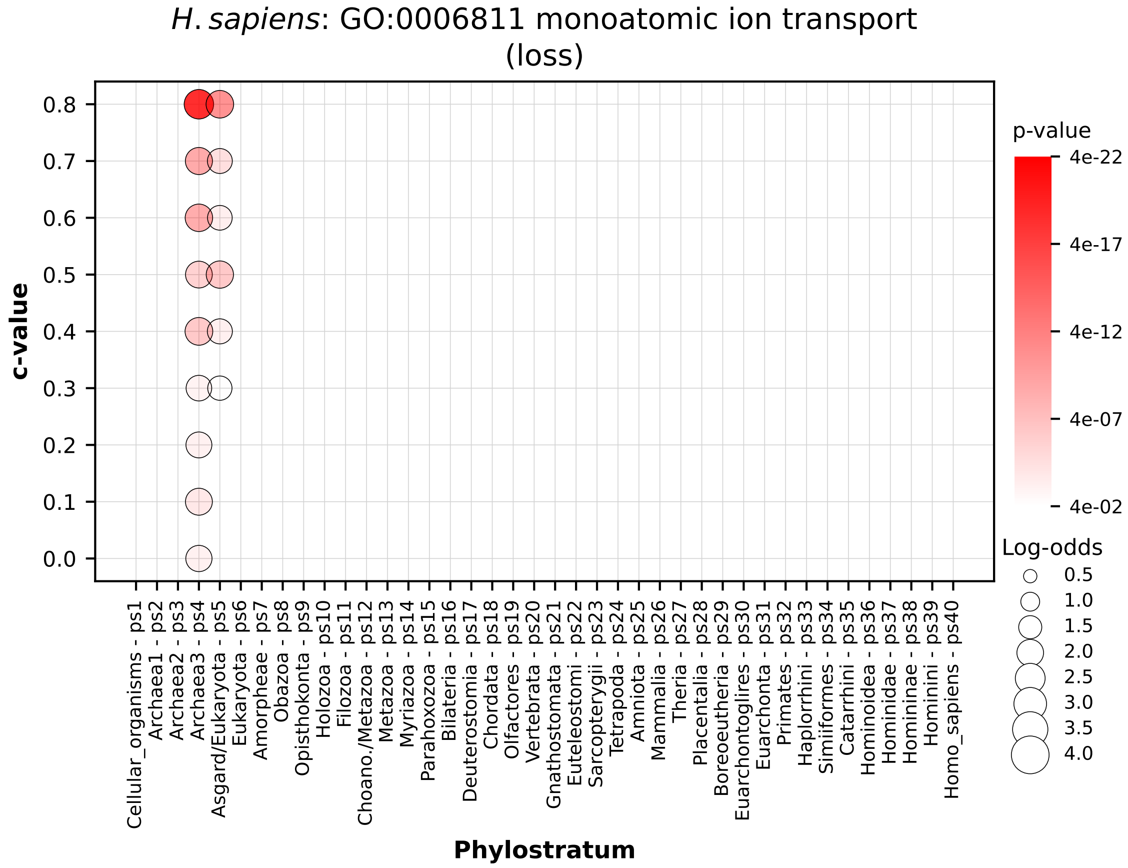

H. sapiens: GO:0006811 monoatomic ion transport
(loss)
c-value
0.0
0.1
0.2
0.3
0.4
0.5
0.6
0.7
0.8
Phylostratum
Cellular_organisms - ps1
Archaea1 - ps2
Archaea2 - ps3
Archaea3 - ps4
Asgard/Eukaryota - ps5
Eukaryota - ps6
Amorpheae - ps7
Obazoa - ps8
Opisthokonta - ps9
Holozoa - ps10
Filozoa - ps11
Choano./Metazoa - ps12
Metazoa - ps13
Myriazoa - ps14
Parahoxozoa - ps15
Bilateria - ps16
Deuterostomia - ps17
Chordata - ps18
Olfactores - ps19
Vertebrata - ps20
Gnathostomata - ps21
Euteleostomi - ps22
Sarcopterygii - ps23
Tetrapoda - ps24
Amniota - ps25
Mammalia - ps26
Theria - ps27
Placentalia - ps28
Boreoeutheria - ps29
Euarchontoglires - ps30
Euarchonta - ps31
Primates - ps32
Haplorrhini - ps33
Simiiformes - ps34
Catarrhini - ps35
Hominoidea - ps36
Hominidae - ps37
Homininae - ps38
Hominini - ps39
Homo_sapiens - ps40
p-value
4e-22
4e-17
4e-12
4e-07
4e-02
Log-odds
0.5
1.0
1.5
2.0
2.5
3.0
3.5
4.0

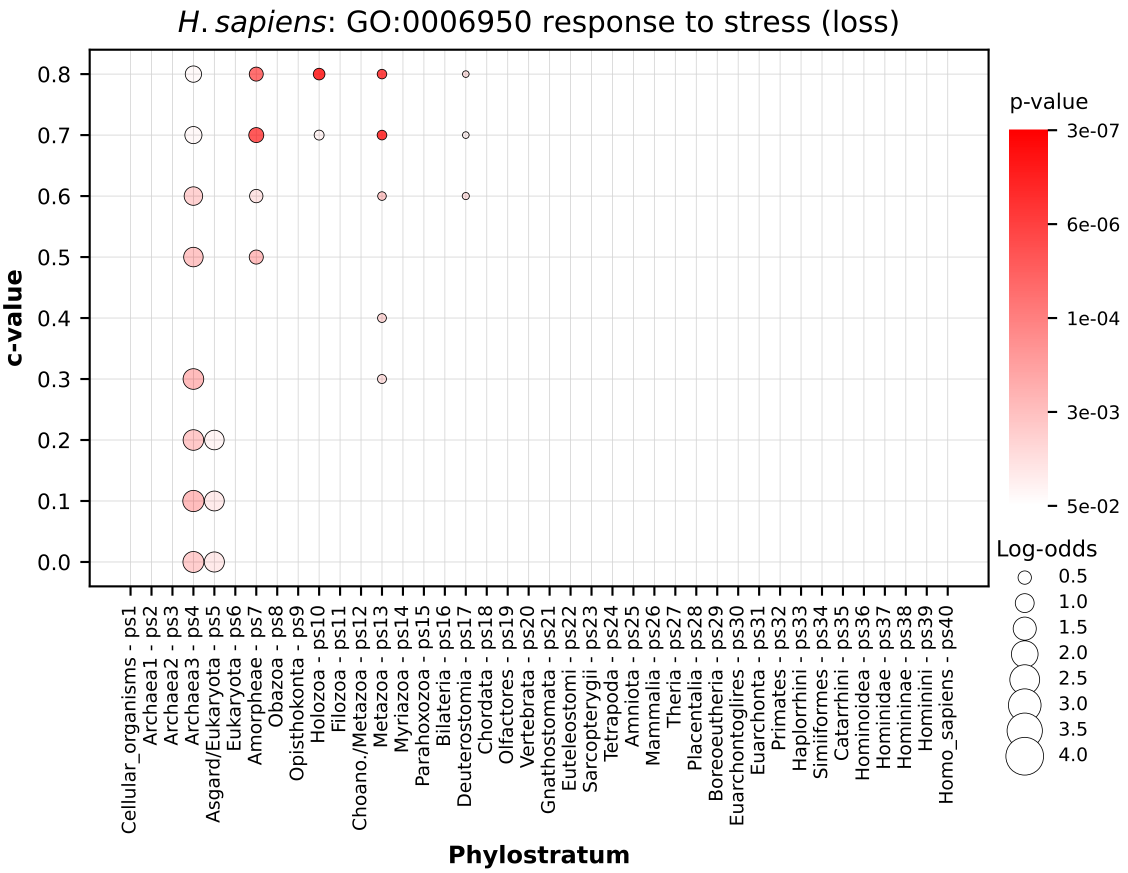

H. sapiens: GO:0006950 response to stress (loss)
c-value
0.8
0.7
0.6
0.5
0.4
0.3
0.2
0.1
0.0
Phylostratum
Cellular_organisms - ps1
Archaea1 - ps2
Archaea2 - ps3
Archaea3 - ps4
Asgard/Eukaryota - ps5
Eukaryota - ps6
Amorpheae - ps7
Obazoa - ps8
Opisthokonta - ps9
Holozoa - ps10
Filozoa - ps11
Choano./Metazoa - ps12
Metazoa - ps13
Myriazoa - ps14
Parahoxozoa - ps15
Bilateria - ps16
Deuterostomia - ps17
Chordata - ps18
Olfactores - ps19
Vertebrata - ps20
Gnathostomata - ps21
Euteleostomi - ps22
Sarcopterygii - ps23
Tetrapoda - ps24
Amniota - ps25
Mammalia - ps26
Theria - ps27
Placentalia - ps28
Boreoeutheria - ps29
Euarchontoglires - ps30
Euarchonta - ps31
Primates - ps32
Haplorrhini - ps33
Simiiformes - ps34
Catarrhini - ps35
Hominoidea - ps36
Hominidae - ps37
Homininae - ps38
Hominini - ps39
Homo_sapiens - ps40
p-value
3e-07
6e-06
1e-04
3e-03
5e-02
Log-odds
0.5
1.0
1.5
2.0
2.5
3.0
3.5
4.0

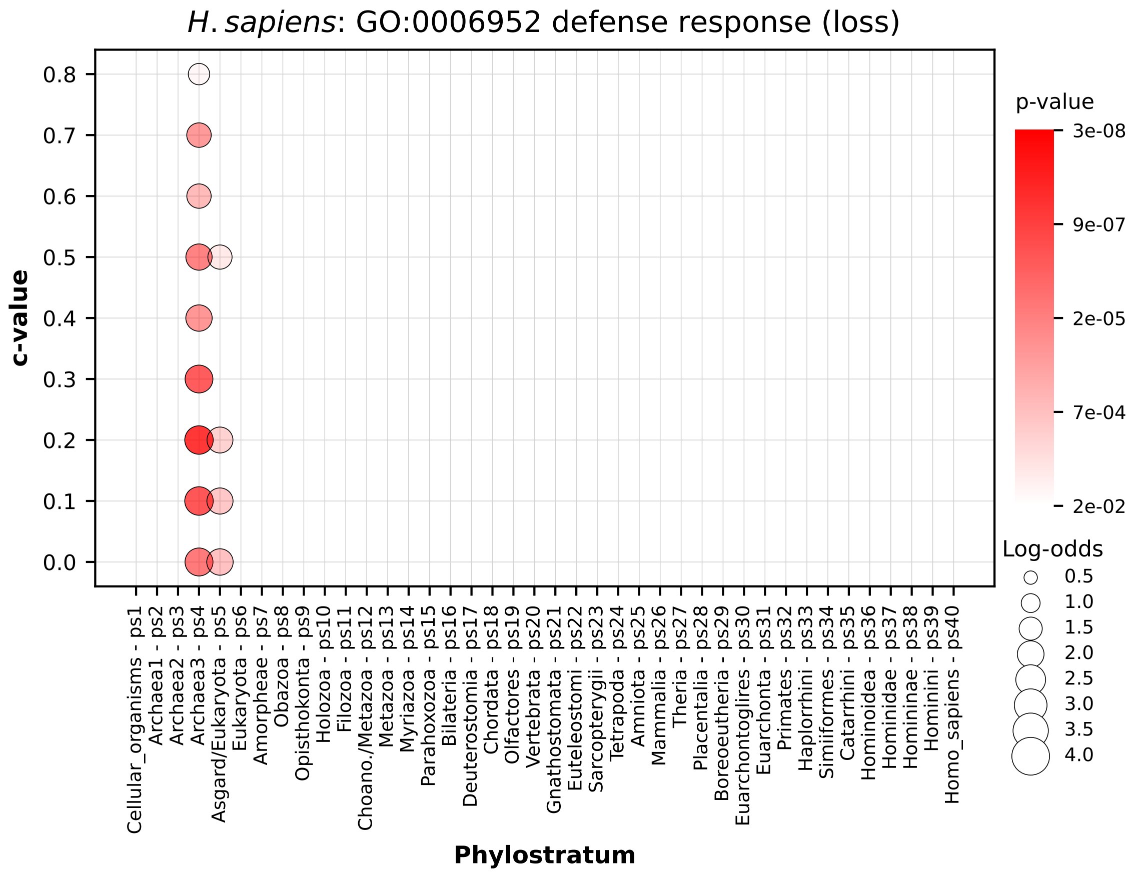

H. sapiens: GO:0006952 defense response (loss)
c-value
0.8
0.7
0.6
0.5
0.4
0.3
0.2
0.1
0.0
Phylostratum
Cellular_organisms - ps1
Archaea1 - ps2
Archaea2 - ps3
Archaea3 - ps4
Asgard/Eukaryota - ps5
Eukaryota - ps6
Amorpheae - ps7
Obazoa - ps8
Opisthokonta - ps9
Holozoa - ps10
Filozoa - ps11
Choano./Metazoa - ps12
Metazoa - ps13
Myriazoa - ps14
Parahoxozoa - ps15
Bilateria - ps16
Deuterostomia - ps17
Chordata - ps18
Olfactores - ps19
Vertebrata - ps20
Gnathostomata - ps21
Euteleostomi - ps22
Sarcopterygii - ps23
Tetrapoda - ps24
Amniota - ps25
Mammalia - ps26
Theria - ps27
Placentalia - ps28
Boreoeutheria - ps29
Euarchontoglires - ps30
Euarchonta - ps31
Primates - ps32
Haplorrhini - ps33
Simiiformes - ps34
Catarrhini - ps35
Hominoidea - ps36
Hominidae - ps37
Homininae - ps38
Hominini - ps39
Homo_sapiens - ps40
p-value
3e-08
9e-07
2e-05
7e-04
2e-02
Log-odds
0.5
1.0
1.5
2.0
2.5
3.0
3.5
4.0

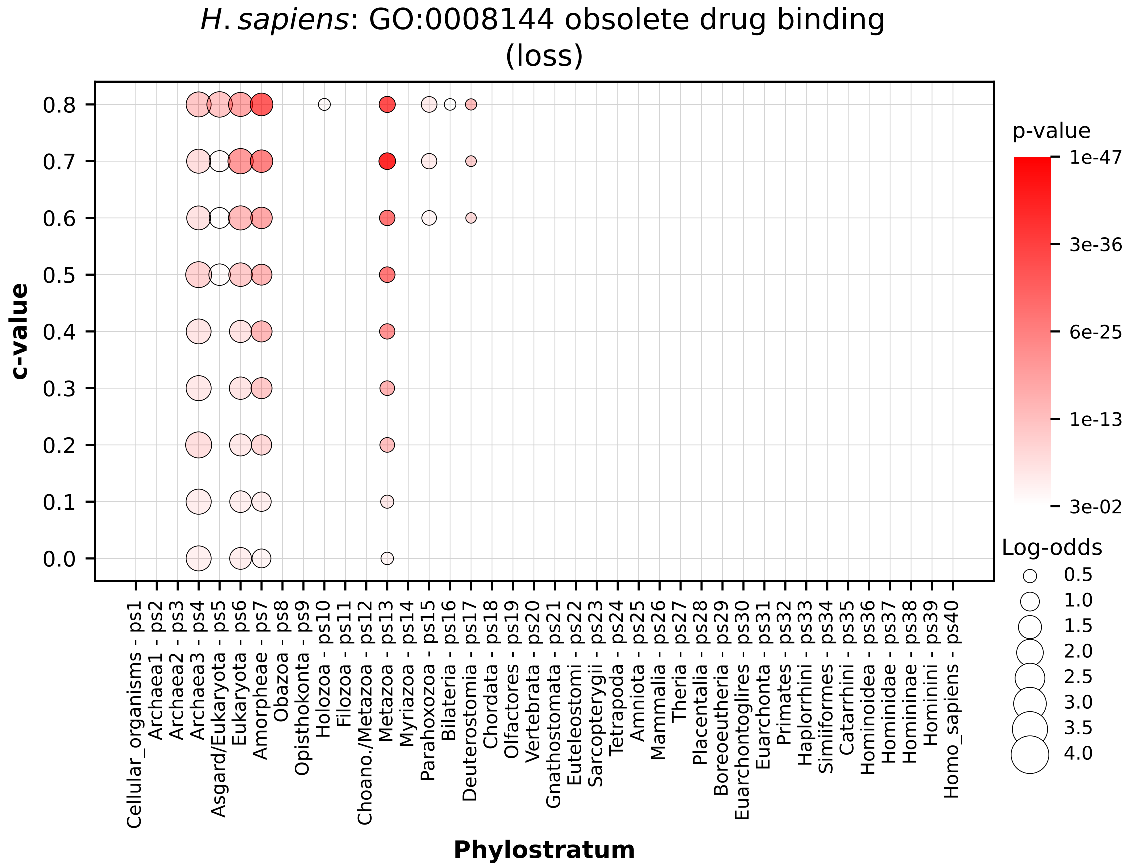

H. sapiens: GO:0008144 obsolete drug binding
(loss)
c-value
0.8
0.7
0.6
0.5
0.4
0.3
0.2
0.1
0.0
Phylostratum
Cellular_organisms - ps1
Archaea1 - ps2
Archaea2 - ps3
Archaea3 - ps4
Asgard/Eukaryota - ps5
Eukaryota - ps6
Amorpheae - ps7
Obazoa - ps8
Opisthokonta - ps9
Holozoa - ps10
Filozoa - ps11
Choano./Metazoa - ps12
Metazoa - ps13
Myriazoa - ps14
Parahoxozoa - ps15
Bilateria - ps16
Deuterostomia - ps17
Chordata - ps18
Olfactores - ps19
Vertebrata - ps20
Gnathostomata - ps21
Euteleostomi - ps22
Sarcopterygii - ps23
Tetrapoda - ps24
Amniota - ps25
Mammalia - ps26
Theria - ps27
Placentalia - ps28
Boreoeutheria - ps29
Euarchontoglires - ps30
Euarchonta - ps31
Primates - ps32
Haplorrhini - ps33
Simiiformes - ps34
Catarrhini - ps35
Hominoidea - ps36
Hominidae - ps37
Homininae - ps38
Hominini - ps39
Homo_sapiens - ps40
p-value
1e-47
3e-36
6e-25
1e-13
3e-02
Log-odds
0.5
1.0
1.5
2.0
2.5
3.0
3.5
4.0

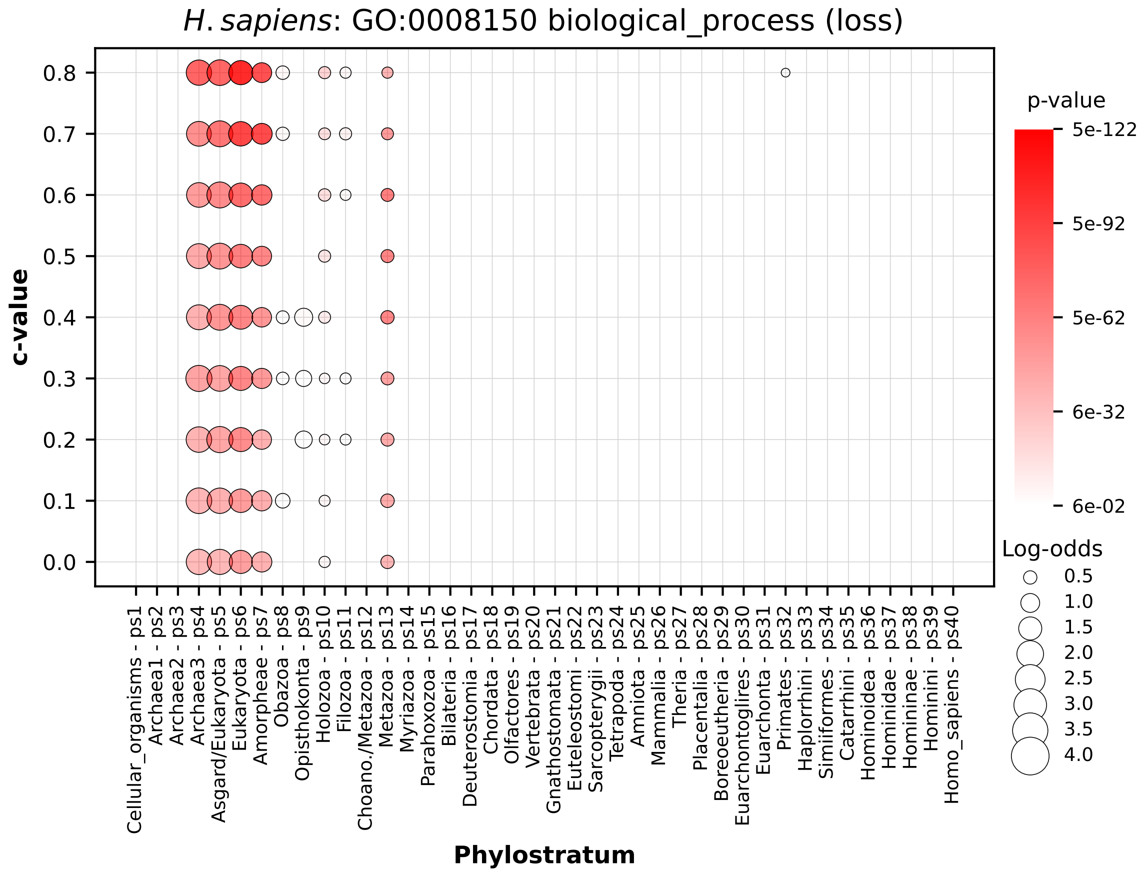

H. sapiens: GO:0008150 biological_process (loss)
c-value
0.8
0.7
0.6
0.5
0.4
0.3
0.2
0.1
0.0
Phylostratum
Cellular_organisms - ps1
Archaea1 - ps2
Archaea2 - ps3
Archaea3 - ps4
Asgard/Eukaryota - ps5
Eukaryota - ps6
Amorpheae - ps7
Obazoa - ps8
Opisthokonta - ps9
Holozoa - ps10
Filozoa - ps11
Choano./Metazoa - ps12
Metazoa - ps13
Myriazoa - ps14
Parahoxozoa - ps15
Bilateria - ps16
Deuterostomia - ps17
Chordata - ps18
Olfactores - ps19
Vertebrata - ps20
Gnathostomata - ps21
Euteleostomi - ps22
Sarcopterygii - ps23
Tetrapoda - ps24
Amniota - ps25
Mammalia - ps26
Theria - ps27
Placentalia - ps28
Boreoeutheria - ps29
Euarchontoglires - ps30
Euarchonta - ps31
Primates - ps32
Haplorrhini - ps33
Simiiformes - ps34
Catarrhini - ps35
Hominoidea - ps36
Hominidae - ps37
Homininae - ps38
Hominini - ps39
Homo_sapiens - ps40
p-value
5e-122
5e-92
5e-62
6e-32
6e-02
Log-odds
0.5
1.0
1.5
2.0
2.5
3.0
3.5
4.0

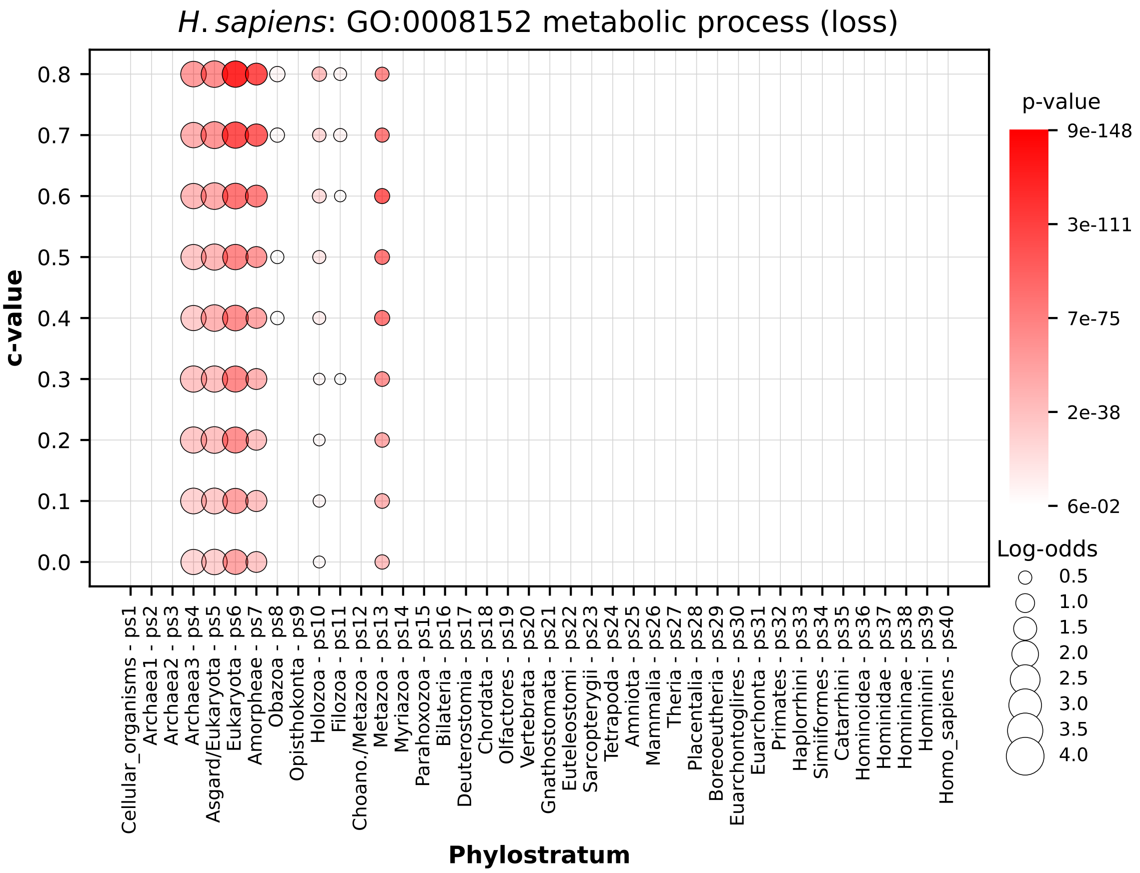
H. sapiens: GO:0008152 metabolic process (loss)
c-value
0.8
0.7
0.6
0.5
0.4
0.3
0.2
0.1
0.0
p-value
9e-148
3e-111
7e-75
2e-38
6e-02
Log-odds
0.5
1.0
1.5
2.0
2.5
3.0
3.5
4.0
Cellular_organisms - ps1
Archaea1 - ps2
Archaea2 - ps3
Archaea3 - ps4
Asgard/Eukaryota - ps5
Eukaryota - ps6
Amorpheae - ps7
Obazoa - ps8
Opisthokonta - ps9
Holozoa - ps10
Filozoa - ps11
Choano./Metazoa - ps12
Metazoa - ps13
Myriazoa - ps14
Parahoxozoa - ps15
Bilateria - ps16
Deuterostomia - ps17
Chordata - ps18
Olfactores - ps19
Vertebrata - ps20
Gnathostomata - ps21
Euteleostomi - ps22
Sarcopterygii - ps23
Tetrapoda - ps24
Amniota - ps25
Mammalia - ps26
Theria - ps27
Placentalia - ps28
Boreoeutheria - ps29
Euarchontoglires - ps30
Euarchonta - ps31
Primates - ps32
Haplorrhini - ps33
Simiiformes - ps34
Catarrhini - ps35
Hominoidea - ps36
Hominidae - ps37
Homininae - ps38
Hominini - ps39
Homo_sapiens - ps40
Phylostratum

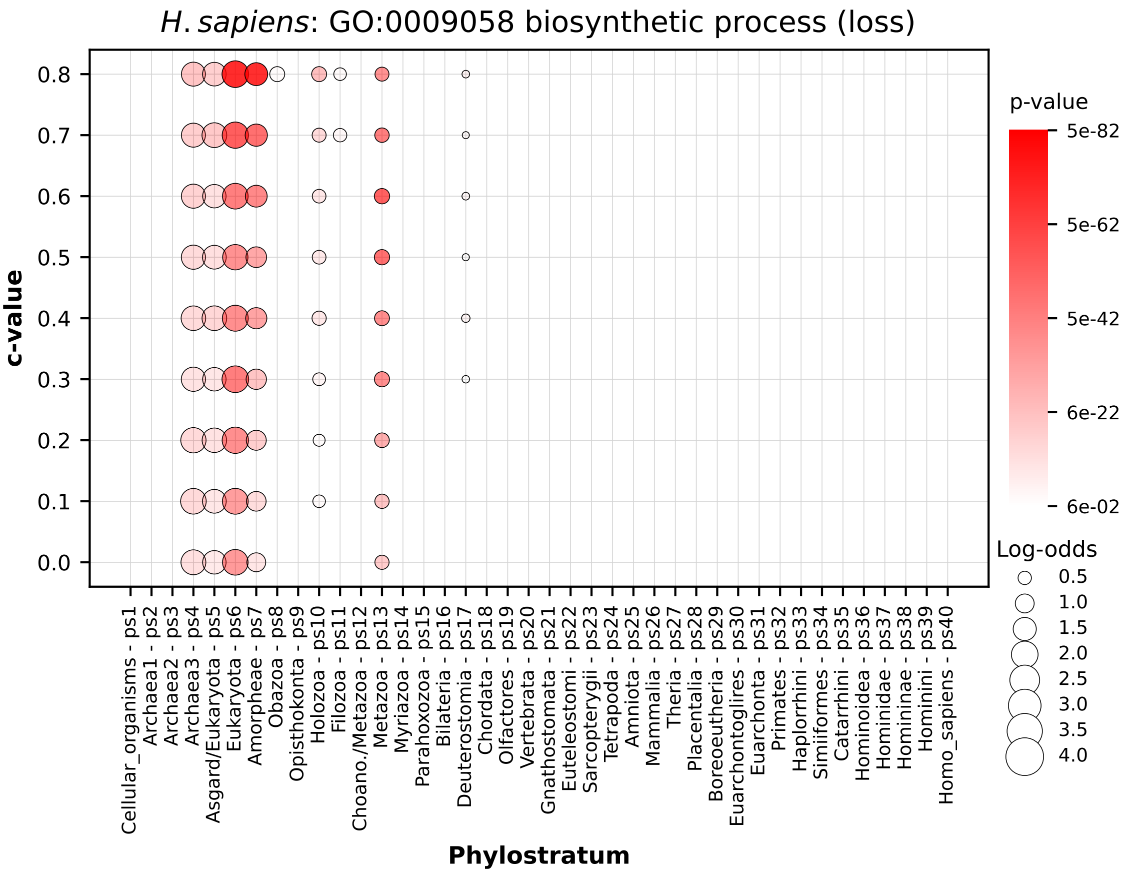
H. sapiens: GO:0009058 biosynthetic process (loss)
c-value
0.0
0.1
0.2
0.3
0.4
0.5
0.6
0.7
0.8
Phylostratum
Cellular_organisms - ps1
Archaea1 - ps2
Archaea2 - ps3
Archaea3 - ps4
Asgard/Eukaryota - ps5
Eukaryota - ps6
Amorpheae - ps7
Obazoa - ps8
Opisthokonta - ps9
Holozoa - ps10
Filozoa - ps11
Choano./Metazoa - ps12
Metazoa - ps13
Myriazoa - ps14
Parahoxozoa - ps15
Bilateria - ps16
Deuterostomia - ps17
Chordata - ps18
Olfactores - ps19
Vertebrata - ps20
Gnathostomata - ps21
Euteleostomi - ps22
Sarcopterygii - ps23
Tetrapoda - ps24
Amniota - ps25
Mammalia - ps26
Theria - ps27
Placentalia - ps28
Boreoeutheria - ps29
Euarchontoglires - ps30
Euarchonta - ps31
Primates - ps32
Haplorrhini - ps33
Simiiformes - ps34
Catarrhini - ps35
Hominoidea - ps36
Hominidae - ps37
Homininae - ps38
Hominini - ps39
Homo_sapiens - ps40
p-value
5e-82
5e-62
5e-42
6e-22
6e-02
Log-odds
0.5
1.0
1.5
2.0
2.5
3.0
3.5
4.0

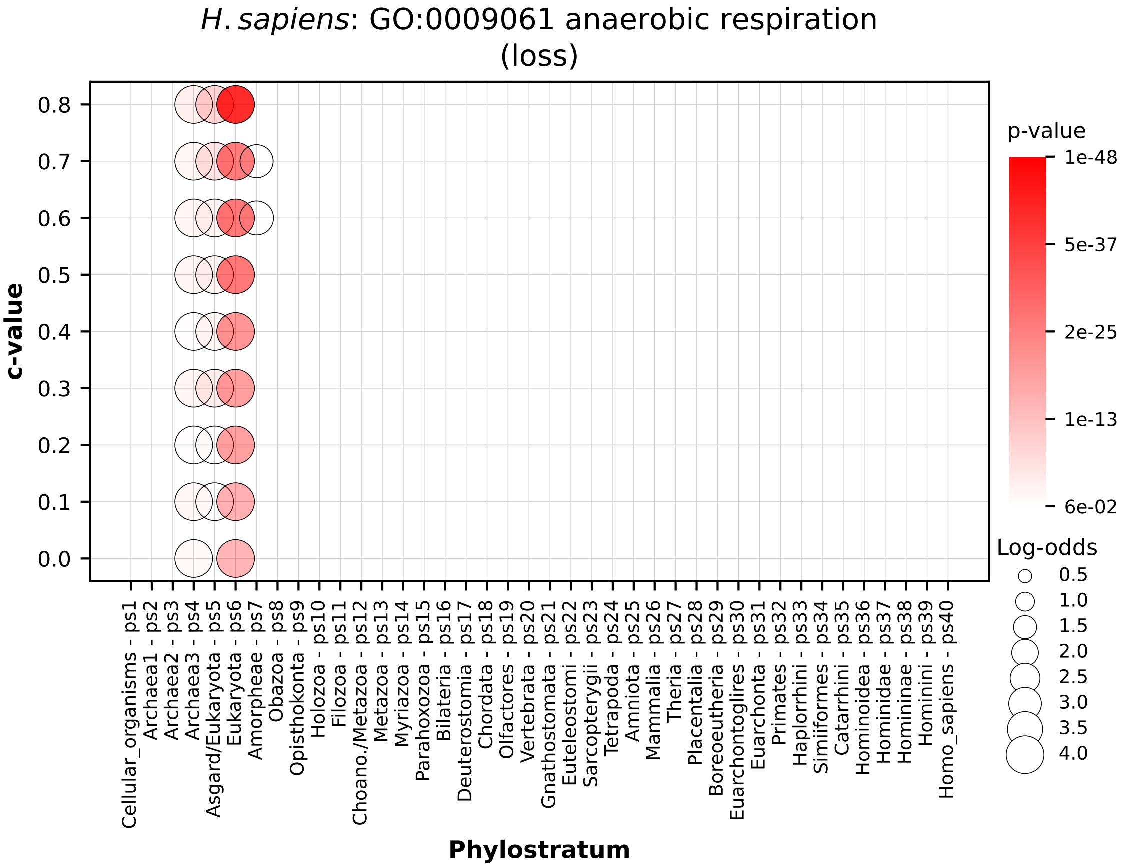

H. sapiens: GO:0009061 anaerobic respiration
(loss)
c-value
0.0
0.1
0.2
0.3
0.4
0.5
0.6
0.7
0.8
Phylostratum
Cellular_organisms - ps1
Archaea1 - ps2
Archaea2 - ps3
Archaea3 - ps4
Asgard/Eukaryota - ps5
Eukaryota - ps6
Amorpheae - ps7
Obazoa - ps8
Opisthokonta - ps9
Holozoa - ps10
Filozoa - ps11
Choano./Metazoa - ps12
Metazoa - ps13
Myriazoa - ps14
Parahoxozoa - ps15
Bilateria - ps16
Deuterostomia - ps17
Chordata - ps18
Olfactores - ps19
Vertebrata - ps20
Gnathostomata - ps21
Euteleostomi - ps22
Sarcopterygii - ps23
Tetrapoda - ps24
Amniota - ps25
Mammalia - ps26
Theria - ps27
Placentalia - ps28
Boreoeutheria - ps29
Euarchontoglires - ps30
Euarchonta - ps31
Primates - ps32
Haplorrhini - ps33
Simiiformes - ps34
Catarrhini - ps35
Hominoidea - ps36
Hominidae - ps37
Homininae - ps38
Hominini - ps39
Homo_sapiens - ps40
p-value
1e-48
5e-37
2e-25
1e-13
6e-02
Log-odds
0.5
1.0
1.5
2.0
2.5
3.0
3.5
4.0

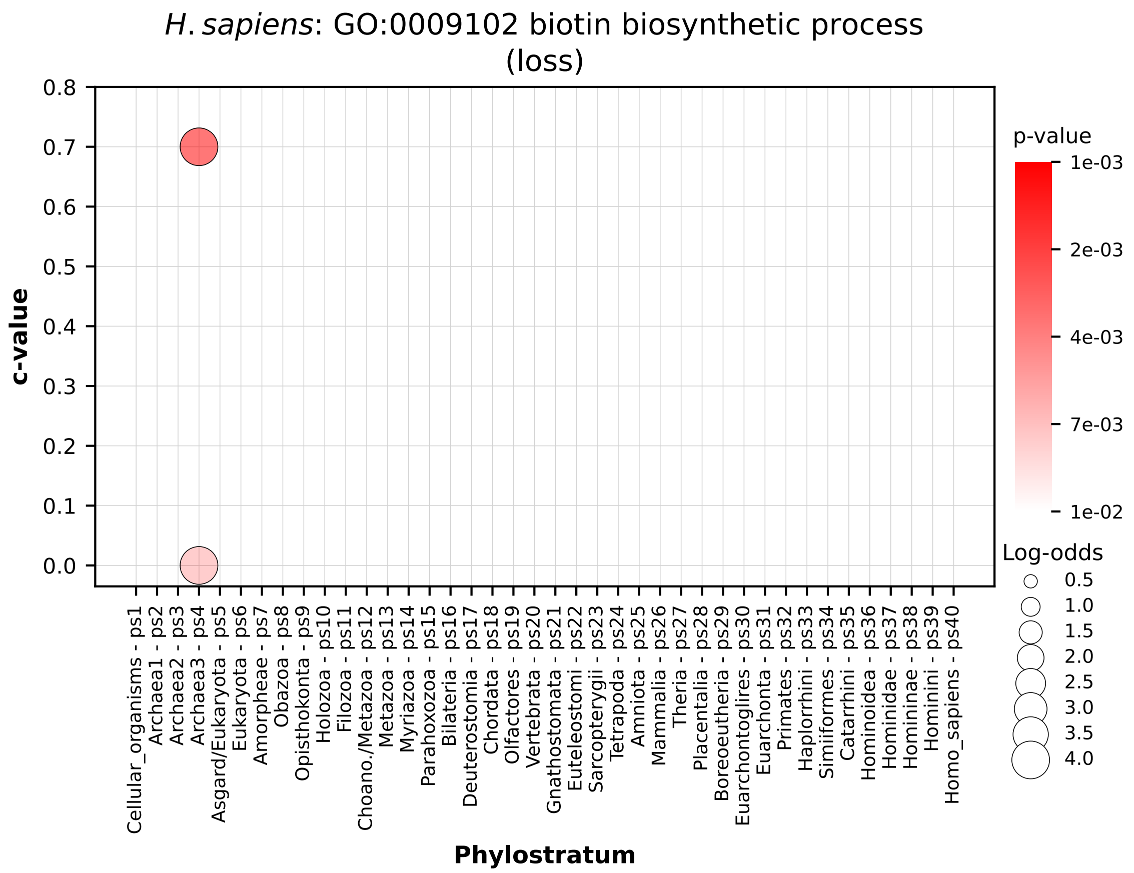

H. sapiens: GO:0009102 biotin biosynthetic process
(loss)
c-value
0.8
0.7
0.6
0.5
0.4
0.3
0.2
0.1
0.0
p-value
1e-03
2e-03
4e-03
7e-03
1e-02
Log-odds
0.5
1.0
1.5
2.0
2.5
3.0
3.5
4.0
Cellular_organisms - ps1
Archaea1 - ps2
Archaea2 - ps3
Archaea3 - ps4
Asgard/Eukaryota - ps5
Eukaryota - ps6
Amorpheae - ps7
Obazoa - ps8
Opisthokonta - ps9
Holozoa - ps10
Filozoa - ps11
Choano./Metazoa - ps12
Metazoa - ps13
Myriazoa - ps14
Parahoxozoa - ps15
Bilateria - ps16
Deuterostomia - ps17
Chordata - ps18
Olfactores - ps19
Vertebrata - ps20
Gnathostomata - ps21
Euteleostomi - ps22
Sarcopterygii - ps23
Tetrapoda - ps24
Amniota - ps25
Mammalia - ps26
Theria - ps27
Placentalia - ps28
Boreoeutheria - ps29
Euarchontoglires - ps30
Euarchonta - ps31
Primates - ps32
Haplorrhini - ps33
Simiiformes - ps34
Catarrhini - ps35
Hominoidea - ps36
Hominidae - ps37
Homininae - ps38
Hominini - ps39
Homo_sapiens - ps40
Phylostratum

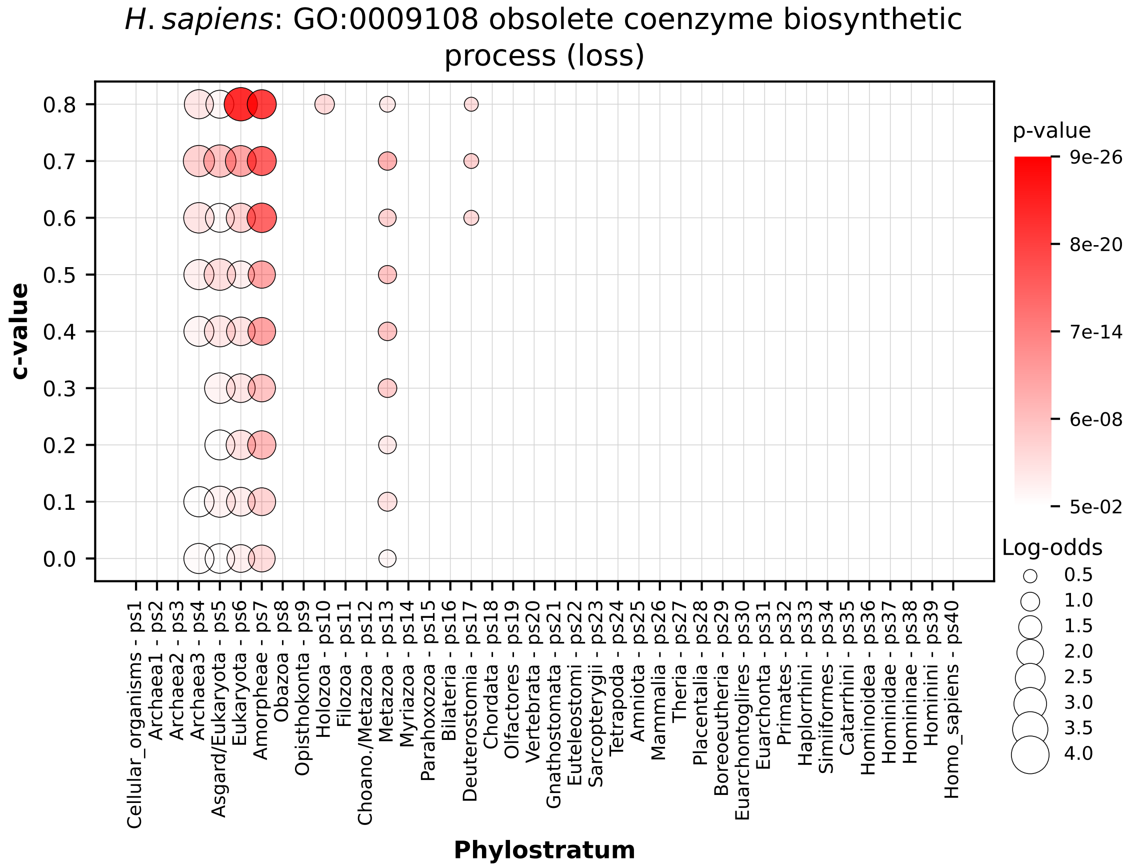

H. sapiens: GO:0009108 obsolete coenzyme biosynthetic process (loss)
c-value
0.0
0.1
0.2
0.3
0.4
0.5
0.6
0.7
0.8
p-value
9e-26
8e-20
7e-14
6e-08
5e-02
Log-odds
0.5
1.0
1.5
2.0
2.5
3.0
3.5
4.0
Cellular_organisms - ps1
Archaea1 - ps2
Archaea2 - ps3
Archaea3 - ps4
Asgard/Eukaryota - ps5
Eukaryota - ps6
Amorpheae - ps7
Obazoa - ps8
Opisthokonta - ps9
Holozoa - ps10
Filozoa - ps11
Choano./Metazoa - ps12
Metazoa - ps13
Myriazoa - ps14
Parahoxozoa - ps15
Bilateria - ps16
Deuterostomia - ps17
Chordata - ps18
Olfactores - ps19
Vertebrata - ps20
Gnathostomata - ps21
Euteleostomi - ps22
Sarcopterygii - ps23
Tetrapoda - ps24
Amniota - ps25
Mammalia - ps26
Theria - ps27
Placentalia - ps28
Boreoeutheria - ps29
Euarchontoglires - ps30
Euarchonta - ps31
Primates - ps32
Haplorrhini - ps33
Simiiformes - ps34
Catarrhini - ps35
Hominoidea - ps36
Hominidae - ps37
Homininae - ps38
Hominini - ps39
Homo_sapiens - ps40
Phylostratum

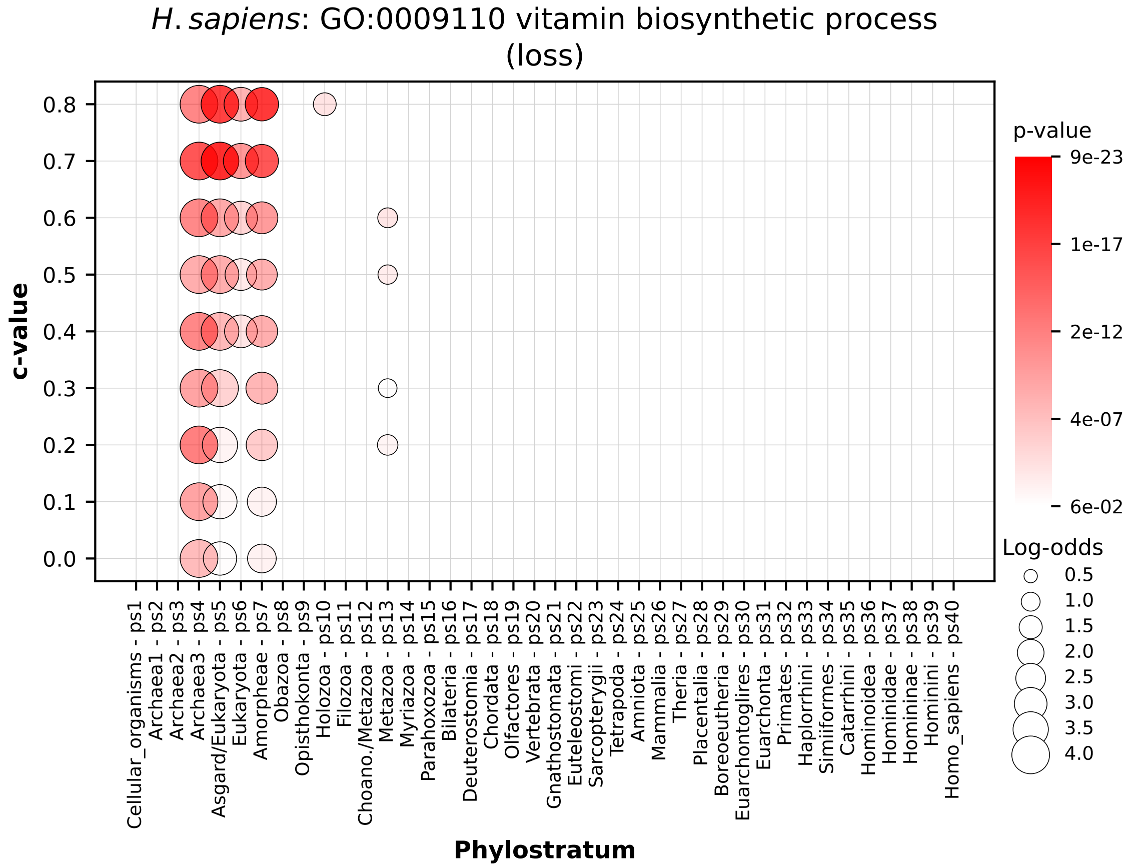

H. sapiens: GO:0009110 vitamin biosynthetic process
(loss)
c-value
0.8
0.7
0.6
0.5
0.4
0.3
0.2
0.1
0.0
Phylostratum
Cellular_organisms - ps1
Archaea1 - ps2
Archaea2 - ps3
Archaea3 - ps4
Asgard/Eukaryota - ps5
Eukaryota - ps6
Amorpheae - ps7
Obazoa - ps8
Opisthokonta - ps9
Holozoa - ps10
Filozoa - ps11
Choano./Metazoa - ps12
Metazoa - ps13
Myriazoa - ps14
Parahoxozoa - ps15
Bilateria - ps16
Deuterostomia - ps17
Chordata - ps18
Olfactores - ps19
Vertebrata - ps20
Gnathostomata - ps21
Euteleostomi - ps22
Sarcopterygii - ps23
Tetrapoda - ps24
Amniota - ps25
Mammalia - ps26
Theria - ps27
Placentalia - ps28
Boreoeutheria - ps29
Euarchontoglires - ps30
Euarchonta - ps31
Primates - ps32
Haplorrhini - ps33
Simiiformes - ps34
Catarrhini - ps35
Hominoidea - ps36
Hominidae - ps37
Homininae - ps38
Hominini - ps39
Homo_sapiens - ps40
p-value
9e-23
1e-17
2e-12
4e-07
6e-02
Log-odds
0.5
1.0
1.5
2.0
2.5
3.0
3.5
4.0

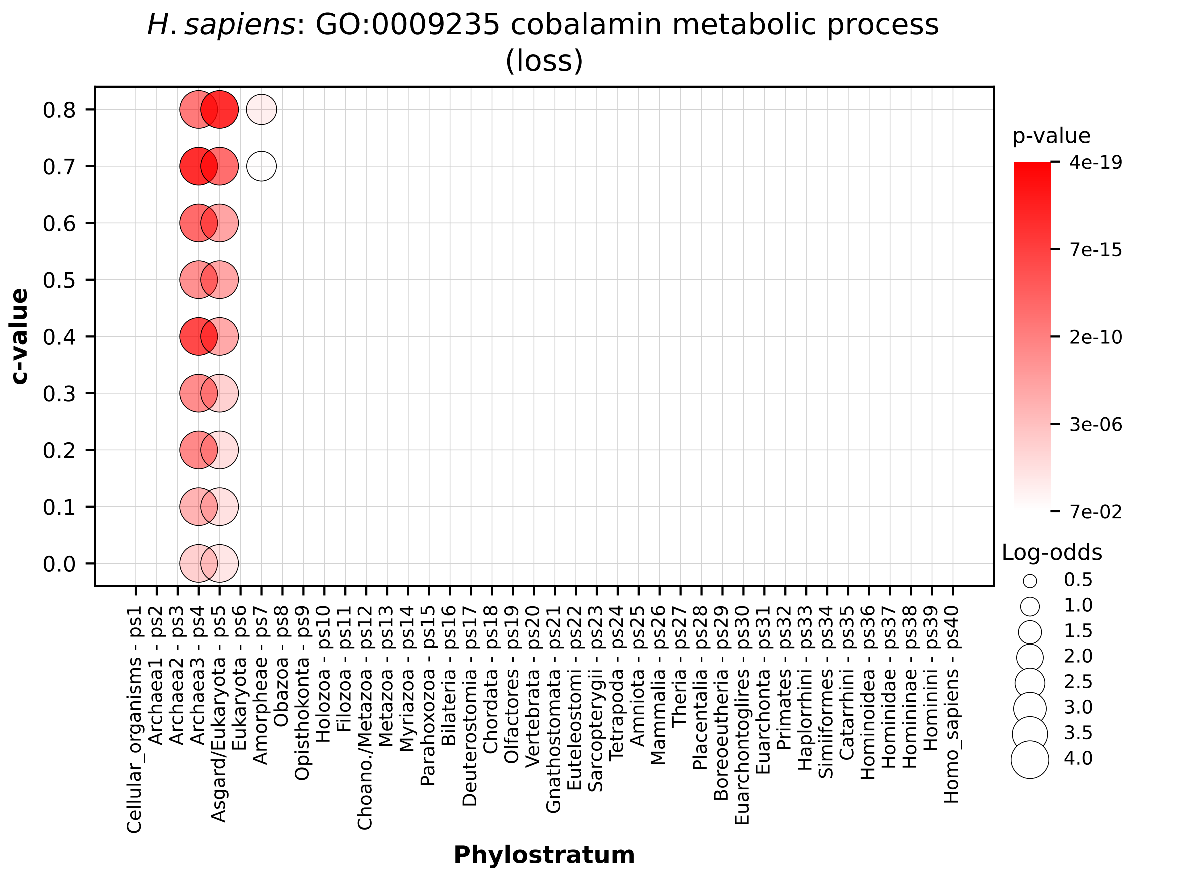

H. sapiens: GO:0009235 cobalamin metabolic process
(loss)
c-value
0.8
0.7
0.6
0.5
0.4
0.3
0.2
0.1
0.0
p-value
4e-19
7e-15
2e-10
3e-06
7e-02
Log-odds
0.5
1.0
1.5
2.0
2.5
3.0
3.5
4.0
Cellular_organisms - ps1
Archaea1 - ps2
Archaea2 - ps3
Archaea3 - ps4
Asgard/Eukaryota - ps5
Eukaryota - ps6
Amorpheae - ps7
Obazoa - ps8
Opisthokonta - ps9
Holozoa - ps10
Filozoa - ps11
Choano./Metazoa - ps12
Metazoa - ps13
Myriazoa - ps14
Parahoxozoa - ps15
Bilateria - ps16
Deuterostomia - ps17
Chordata - ps18
Olfactores - ps19
Vertebrata - ps20
Gnathostomata - ps21
Euteleostomi - ps22
Sarcopterygii - ps23
Tetrapoda - ps24
Amniota - ps25
Mammalia - ps26
Theria - ps27
Placentalia - ps28
Boreoeutheria - ps29
Euarchontoglires - ps30
Euarchonta - ps31
Primates - ps32
Haplorrhini - ps33
Simiiformes - ps34
Catarrhini - ps35
Hominoidea - ps36
Hominidae - ps37
Homininae - ps38
Hominini - ps39
Homo_sapiens - ps40
Phylostratum

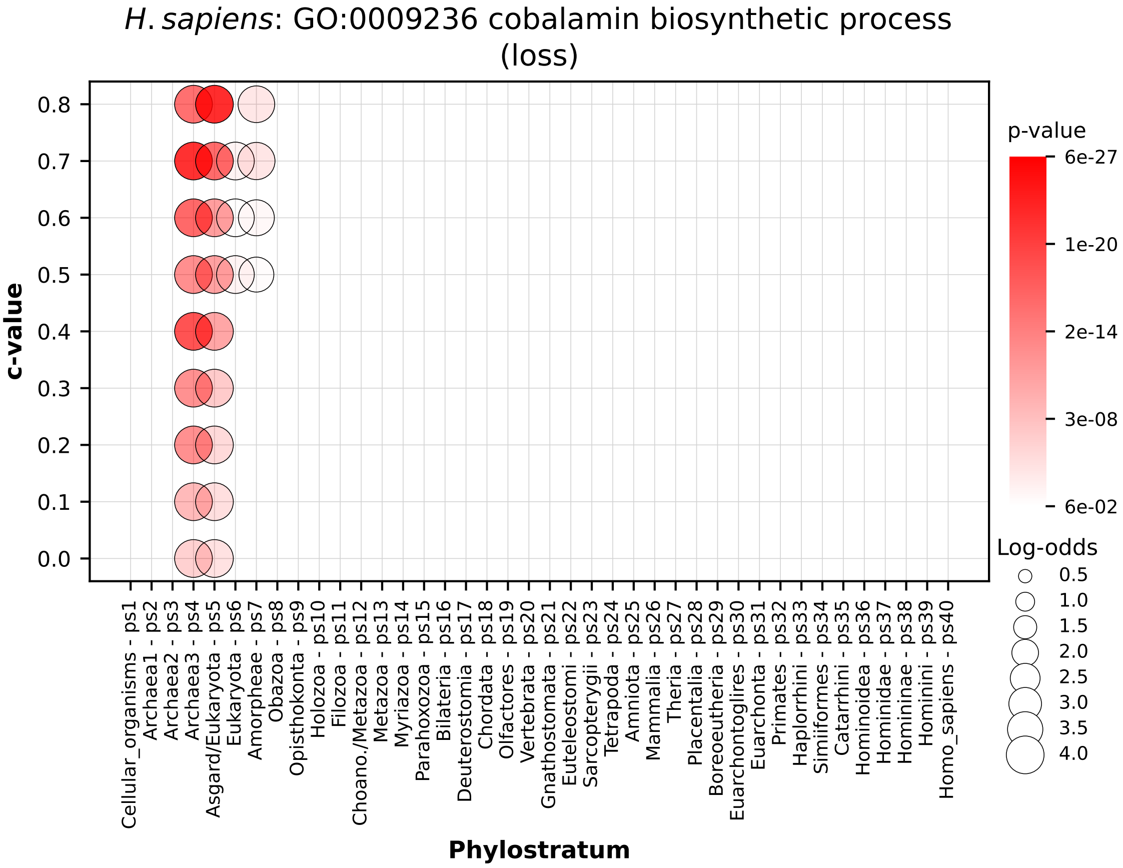
H. sapiens: GO:0009236 cobalamin biosynthetic process
(loss)
c-value
0.8
0.7
0.6
0.5
0.4
0.3
0.2
0.1
0.0
p-value
6e-27
1e-20
2e-14
3e-08
6e-02
Log-odds
0.5
1.0
1.5
2.0
2.5
3.0
3.5
4.0
Cellular_organisms - ps1
Archaea1 - ps2
Archaea2 - ps3
Archaea3 - ps4
Asgard/Eukaryota - ps5
Eukaryota - ps6
Amorpheae - ps7
Obazoa - ps8
Opisthokonta - ps9
Holozoa - ps10
Filozoa - ps11
Choano./Metazoa - ps12
Metazoa - ps13
Myriazoa - ps14
Parahoxozoa - ps15
Bilateria - ps16
Deuterostomia - ps17
Chordata - ps18
Olfactores - ps19
Vertebrata - ps20
Gnathostomata - ps21
Euteleostomi - ps22
Sarcopterygii - ps23
Tetrapoda - ps24
Amniota - ps25
Mammalia - ps26
Theria - ps27
Placentalia - ps28
Boreoeutheria - ps29
Euarchontoglires - ps30
Euarchonta - ps31
Primates - ps32
Haplorrhini - ps33
Simiiformes - ps34
Catarrhini - ps35
Hominoidea - ps36
Hominidae - ps37
Homininae - ps38
Hominini - ps39
Homo_sapiens - ps40
Phylostratum

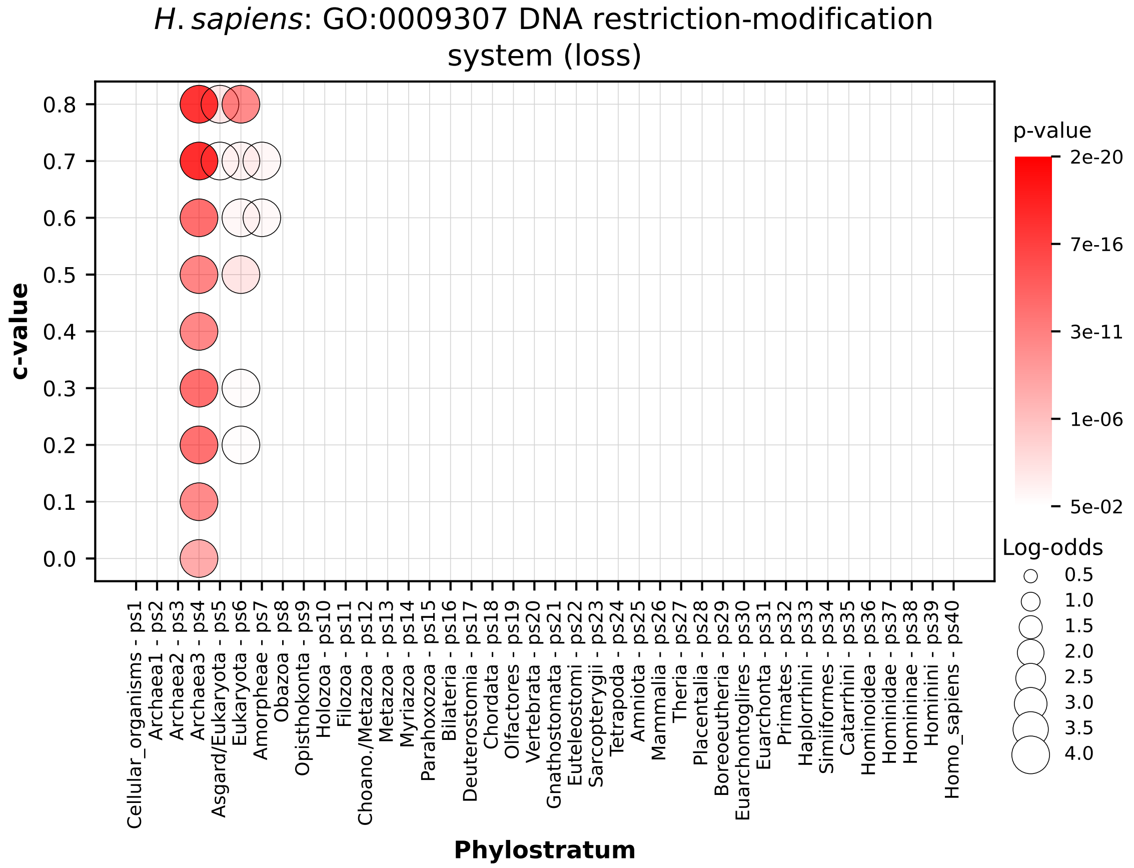

H. sapiens: GO:0009307 DNA restriction-modification system (loss)
c-value
0.8
0.7
0.6
0.5
0.4
0.3
0.2
0.1
0.0
p-value
2e-20
7e-16
3e-11
1e-06
5e-02
Log-odds
0.5
1.0
1.5
2.0
2.5
3.0
3.5
4.0
Cellular_organisms - ps1
Archaea1 - ps2
Archaea2 - ps3
Archaea3 - ps4
Asgard/Eukaryota - ps5
Eukaryota - ps6
Amorpheae - ps7
Obazoa - ps8
Opisthokonta - ps9
Holozoa - ps10
Filozoa - ps11
Choano./Metazoa - ps12
Metazoa - ps13
Myriazoa - ps14
Parahoxozoa - ps15
Bilateria - ps16
Deuterostomia - ps17
Chordata - ps18
Olfactores - ps19
Vertebrata - ps20
Gnathostomata - ps21
Euteleostomi - ps22
Sarcopterygii - ps23
Tetrapoda - ps24
Amniota - ps25
Mammalia - ps26
Theria - ps27
Placentalia - ps28
Boreoeutheria - ps29
Euarchontoglires - ps30
Euarchonta - ps31
Primates - ps32
Haplorrhini - ps33
Simiiformes - ps34
Catarrhini - ps35
Hominoidea - ps36
Hominidae - ps37
Homininae - ps38
Hominini - ps39
Homo_sapiens - ps40
Phylostratum

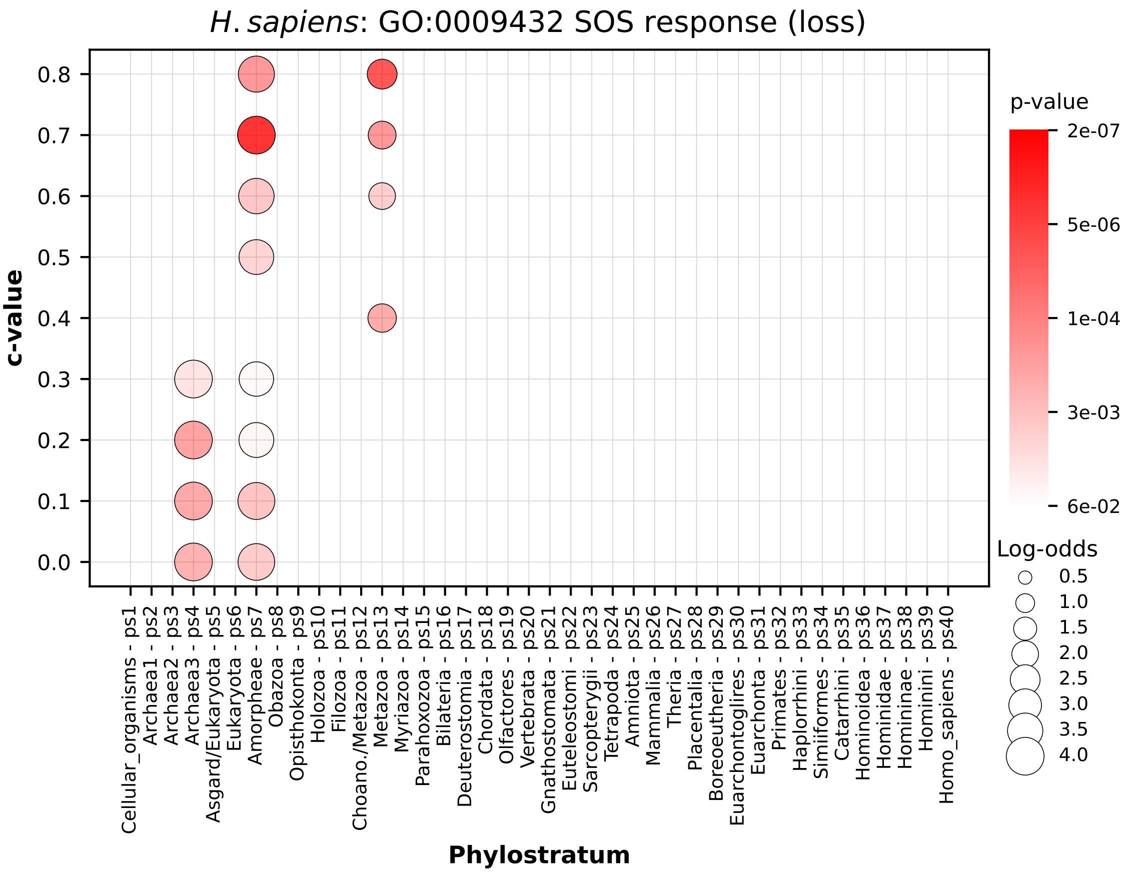

H. sapiens: GO:0009432 SOS response (loss)
c-value
0.0
0.1
0.2
0.3
0.4
0.5
0.6
0.7
0.8
p-value
2e-07
5e-06
1e-04
3e-03
6e-02
Log-odds
0.5
1.0
1.5
2.0
2.5
3.0
3.5
4.0
Cellular_organisms - ps1
Archaea1 - ps2
Archaea2 - ps3
Archaea3 - ps4
Asgard/Eukaryota - ps5
Eukaryota - ps6
Amorpheae - ps7
Obazoa - ps8
Opisthokonta - ps9
Holozoa - ps10
Filozoa - ps11
Choano./Metazoa - ps12
Metazoa - ps13
Myriazoa - ps14
Parahoxozoa - ps15
Bilateria - ps16
Deuterostomia - ps17
Chordata - ps18
Olfactores - ps19
Vertebrata - ps20
Gnathostomata - ps21
Euteleostomi - ps22
Sarcopterygii - ps23
Tetrapoda - ps24
Amniota - ps25
Mammalia - ps26
Theria - ps27
Placentalia - ps28
Boreoeutheria - ps29
Euarchontoglires - ps30
Euarchonta - ps31
Primates - ps32
Haplorrhini - ps33
Simiiformes - ps34
Catarrhini - ps35
Hominoidea - ps36
Hominidae - ps37
Homininae - ps38
Hominini - ps39
Homo_sapiens - ps40
Phylostratum

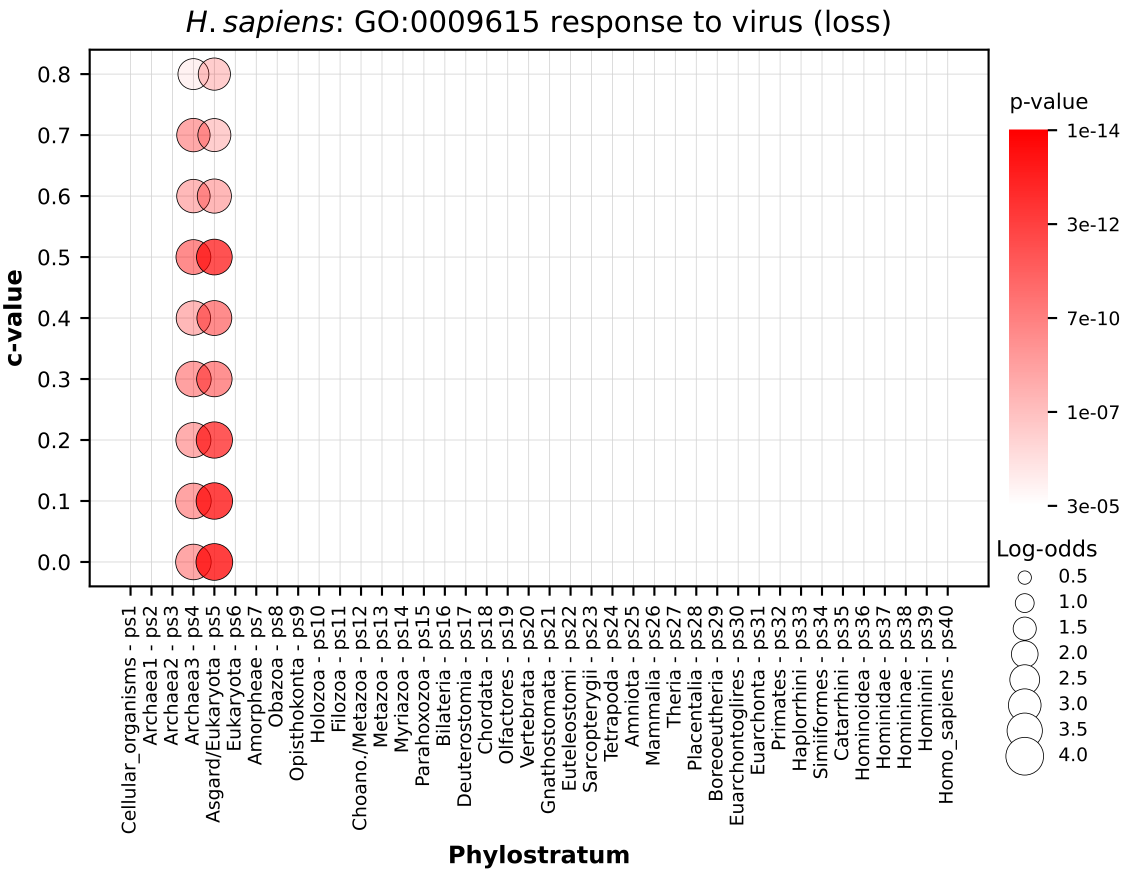
H. sapiens: GO:0009615 response to virus (loss)
c-value
0.8
0.7
0.6
0.5
0.4
0.3
0.2
0.1
0.0
Phylostratum
Cellular_organisms - ps1
Archaea1 - ps2
Archaea2 - ps3
Archaea3 - ps4
Asgard/Eukaryota - ps5
Eukaryota - ps6
Amorpheae - ps7
Obazoa - ps8
Opisthokonta - ps9
Holozoa - ps10
Filozoa - ps11
Choano./Metazoa - ps12
Metazoa - ps13
Myriazoa - ps14
Parahoxozoa - ps15
Bilateria - ps16
Deuterostomia - ps17
Chordata - ps18
Olfactores - ps19
Vertebrata - ps20
Gnathostomata - ps21
Euteleostomi - ps22
Sarcopterygii - ps23
Tetrapoda - ps24
Amniota - ps25
Mammalia - ps26
Theria - ps27
Placentalia - ps28
Boreoeutheria - ps29
Euarchontoglires - ps30
Euarchonta - ps31
Primates - ps32
Haplorrhini - ps33
Simiiformes - ps34
Catarrhini - ps35
Hominoidea - ps36
Hominidae - ps37
Homininae - ps38
Hominini - ps39
Homo_sapiens - ps40
p-value
1e-14
3e-12
7e-10
1e-07
3e-05
Log-odds
0.5
1.0
1.5
2.0
2.5
3.0
3.5
4.0

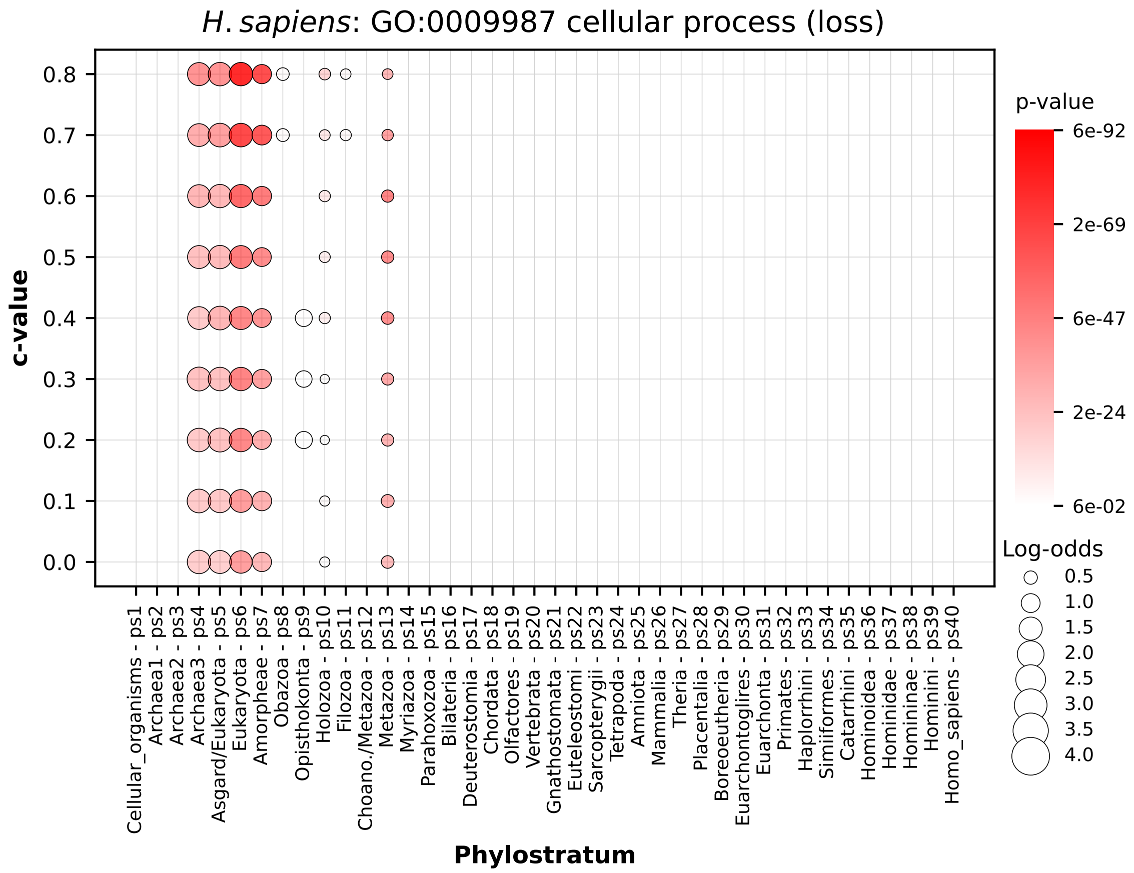

H. sapiens: GO:0009987 cellular process (loss)
c-value
0.0
0.1
0.2
0.3
0.4
0.5
0.6
0.7
0.8
p-value
6e-92
2e-69
6e-47
2e-24
6e-02
Log-odds
0.5
1.0
1.5
2.0
2.5
3.0
3.5
4.0
Cellular_organisms - ps1
Archaea1 - ps2
Archaea2 - ps3
Archaea3 - ps4
Asgard/Eukaryota - ps5
Eukaryota - ps6
Amorpheae - ps7
Obazoa - ps8
Opisthokonta - ps9
Holozoa - ps10
Filozoa - ps11
Choano./Metazoa - ps12
Metazoa - ps13
Myriazoa - ps14
Parahoxozoa - ps15
Bilateria - ps16
Deuterostomia - ps17
Chordata - ps18
Olfactores - ps19
Vertebrata - ps20
Gnathostomata - ps21
Euteleostomi - ps22
Sarcopterygii - ps23
Tetrapoda - ps24
Amniota - ps25
Mammalia - ps26
Theria - ps27
Placentalia - ps28
Boreoeutheria - ps29
Euarchontoglires - ps30
Euarchonta - ps31
Primates - ps32
Haplorrhini - ps33
Simiiformes - ps34
Catarrhini - ps35
Hominoidea - ps36
Hominidae - ps37
Homininae - ps38
Hominini - ps39
Homo_sapiens - ps40
Phylostratum

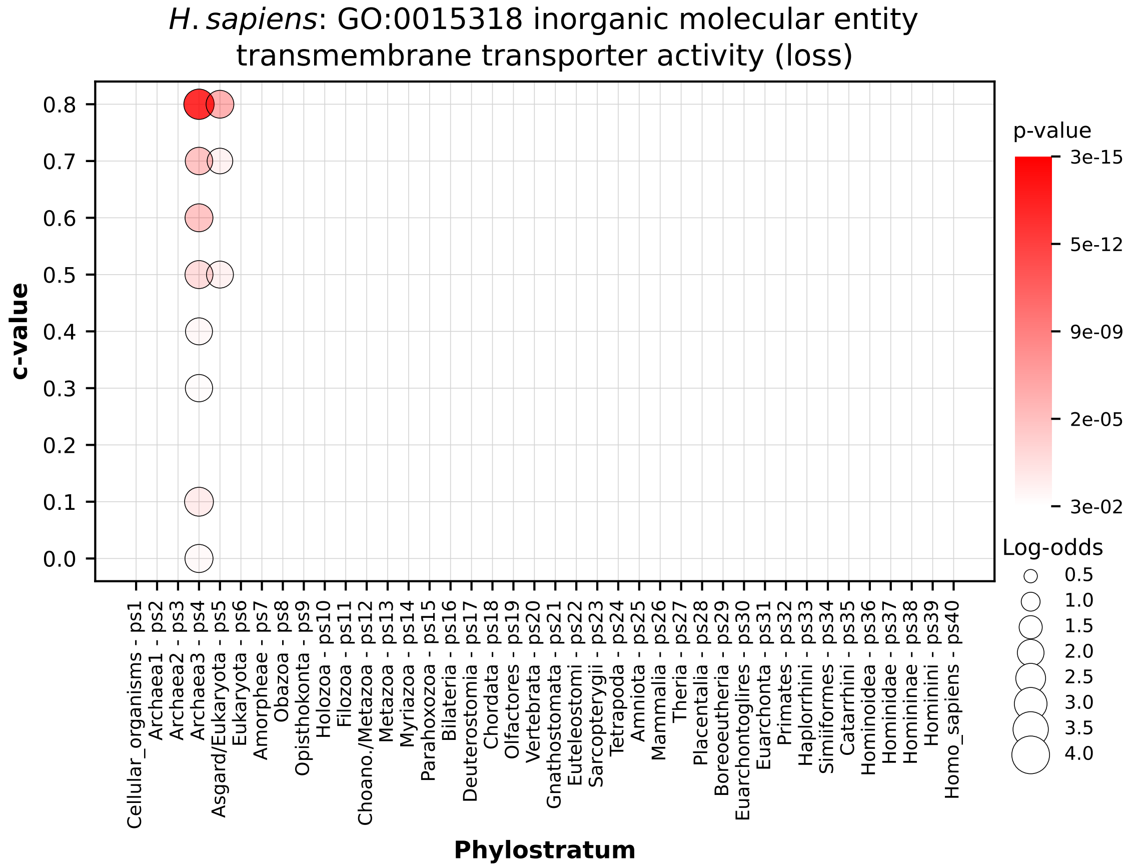

H. sapiens: GO:0015318 inorganic molecular entity transmembrane transporter activity (loss)
c-value
0.0
0.1
0.2
0.3
0.4
0.5
0.6
0.7
0.8
Phylostratum
Cellular_organisms - ps1
Archaea1 - ps2
Archaea2 - ps3
Archaea3 - ps4
Asgard/Eukaryota - ps5
Eukaryota - ps6
Amorpheae - ps7
Obazoa - ps8
Opisthokonta - ps9
Holozoa - ps10
Filozoa - ps11
Choano./Metazoa - ps12
Metazoa - ps13
Myriazoa - ps14
Parahoxozoa - ps15
Bilateria - ps16
Deuterostomia - ps17
Chordata - ps18
Olfactores - ps19
Vertebrata - ps20
Gnathostomata - ps21
Euteleostomi - ps22
Sarcopterygii - ps23
Tetrapoda - ps24
Amniota - ps25
Mammalia - ps26
Theria - ps27
Placentalia - ps28
Boreoeutheria - ps29
Euarchontoglires - ps30
Euarchonta - ps31
Primates - ps32
Haplorrhini - ps33
Simiiformes - ps34
Catarrhini - ps35
Hominoidea - ps36
Hominidae - ps37
Homininae - ps38
Hominini - ps39
Homo_sapiens - ps40
p-value
3e-15
5e-12
9e-09
2e-05
3e-02
Log-odds
0.5
1.0
1.5
2.0
2.5
3.0
3.5
4.0

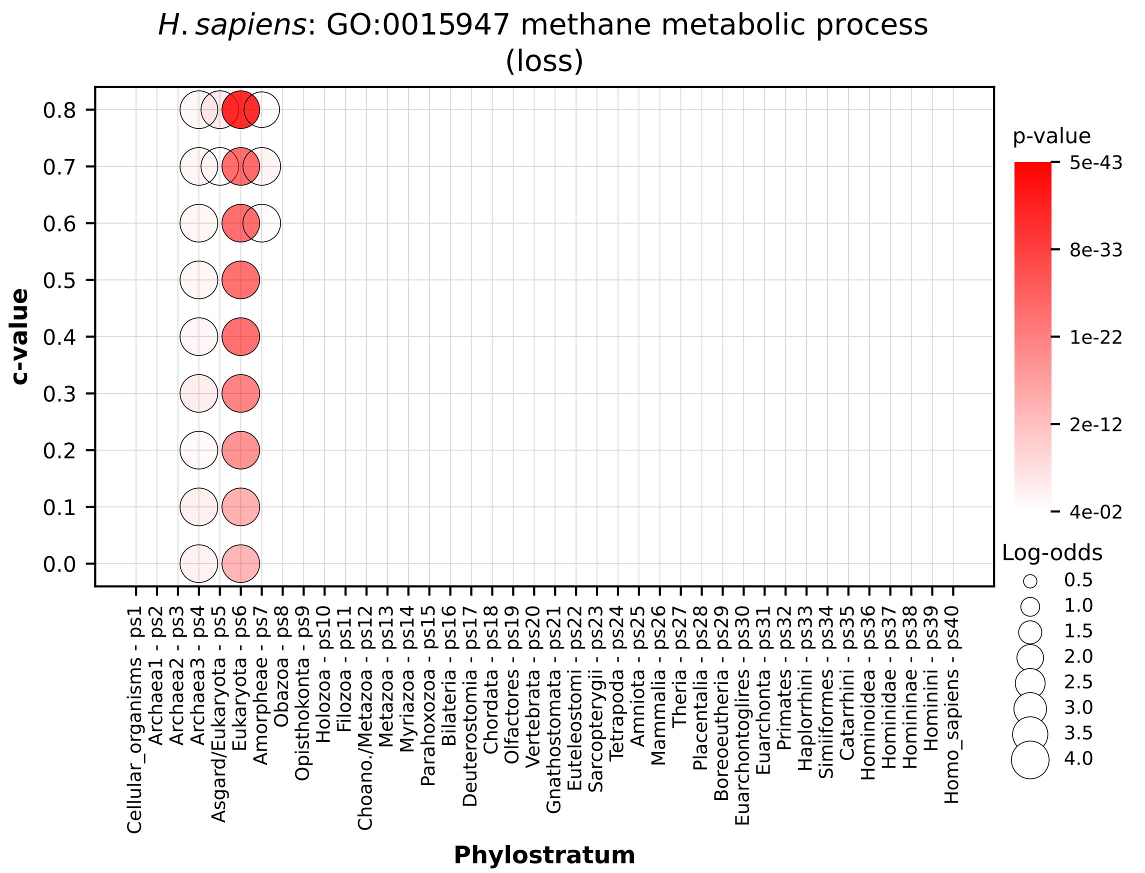
H. sapiens: GO:0015947 methane metabolic process
(loss)
c-value
0.8
0.7
0.6
0.5
0.4
0.3
0.2
0.1
0.0
p-value
5e-43
8e-33
1e-22
2e-12
4e-02
Log-odds
0.5
1.0
1.5
2.0
2.5
3.0
3.5
4.0
Cellular_organisms - ps1
Archaea1 - ps2
Archaea2 - ps3
Archaea3 - ps4
Asgard/Eukaryota - ps5
Eukaryota - ps6
Amorpheae - ps7
Obazoa - ps8
Opisthokonta - ps9
Holozoa - ps10
Filozoa - ps11
Choano./Metazoa - ps12
Metazoa - ps13
Myriazoa - ps14
Parahoxozoa - ps15
Bilateria - ps16
Deuterostomia - ps17
Chordata - ps18
Olfactores - ps19
Vertebrata - ps20
Gnathostomata - ps21
Euteleostomi - ps22
Sarcopterygii - ps23
Tetrapoda - ps24
Amniota - ps25
Mammalia - ps26
Theria - ps27
Placentalia - ps28
Boreoeutheria - ps29
Euarchontoglires - ps30
Euarchonta - ps31
Primates - ps32
Haplorrhini - ps33
Simiiformes - ps34
Catarrhini - ps35
Hominoidea - ps36
Hominidae - ps37
Homininae - ps38
Hominini - ps39
Homo_sapiens - ps40
Phylostratum

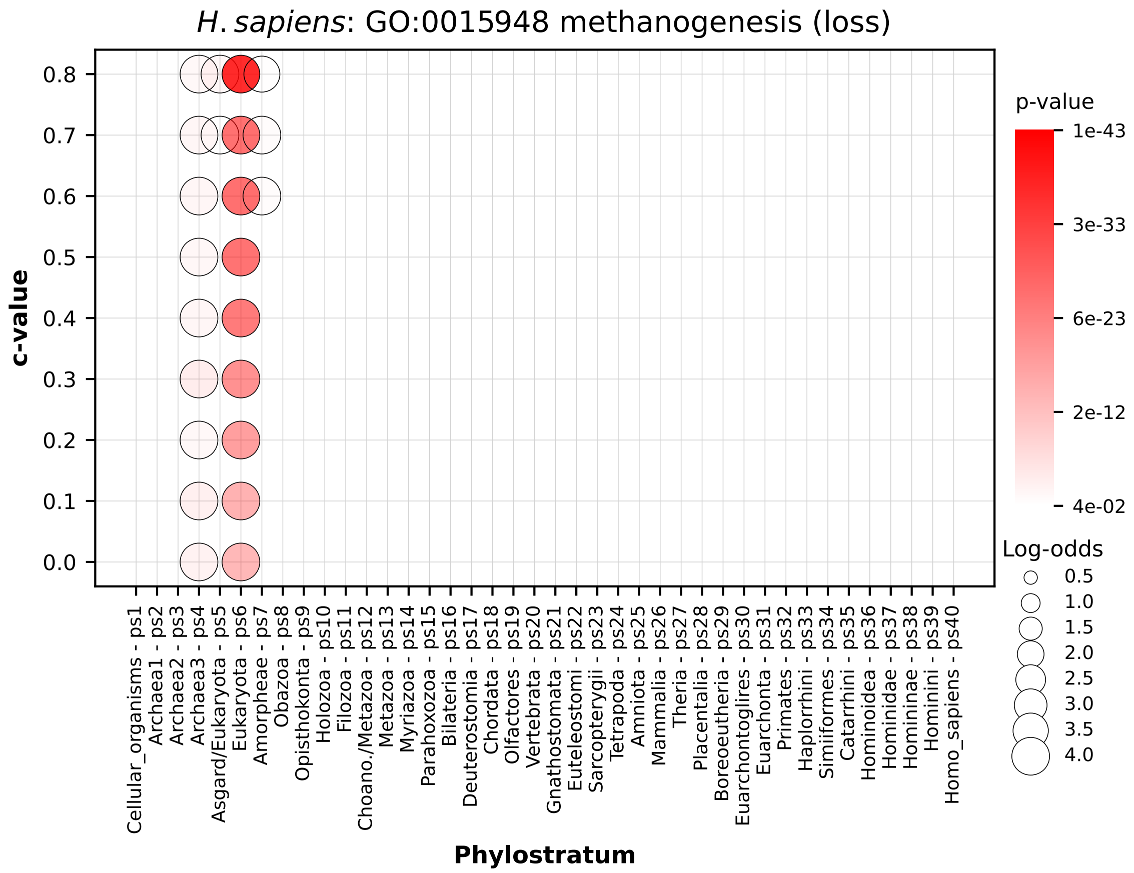

H. sapiens: GO:0015948 methanogenesis (loss)
c-value
0.0
0.1
0.2
0.3
0.4
0.5
0.6
0.7
0.8
Phylostratum
Cellular_organisms - ps1
Archaea1 - ps2
Archaea2 - ps3
Archaea3 - ps4
Asgard/Eukaryota - ps5
Eukaryota - ps6
Amorpheae - ps7
Obazoa - ps8
Opisthokonta - ps9
Holozoa - ps10
Filozoa - ps11
Choano./Metazoa - ps12
Metazoa - ps13
Myriazoa - ps14
Parahoxozoa - ps15
Bilateria - ps16
Deuterostomia - ps17
Chordata - ps18
Olfactores - ps19
Vertebrata - ps20
Gnathostomata - ps21
Euteleostomi - ps22
Sarcopterygii - ps23
Tetrapoda - ps24
Amniota - ps25
Mammalia - ps26
Theria - ps27
Placentalia - ps28
Boreoeutheria - ps29
Euarchontoglires - ps30
Euarchonta - ps31
Primates - ps32
Haplorrhini - ps33
Simiiformes - ps34
Catarrhini - ps35
Hominoidea - ps36
Hominidae - ps37
Homininae - ps38
Hominini - ps39
Homo_sapiens - ps40
p-value
1e-43
3e-33
6e-23
2e-12
4e-02
Log-odds
0.5
1.0
1.5
2.0
2.5
3.0
3.5
4.0

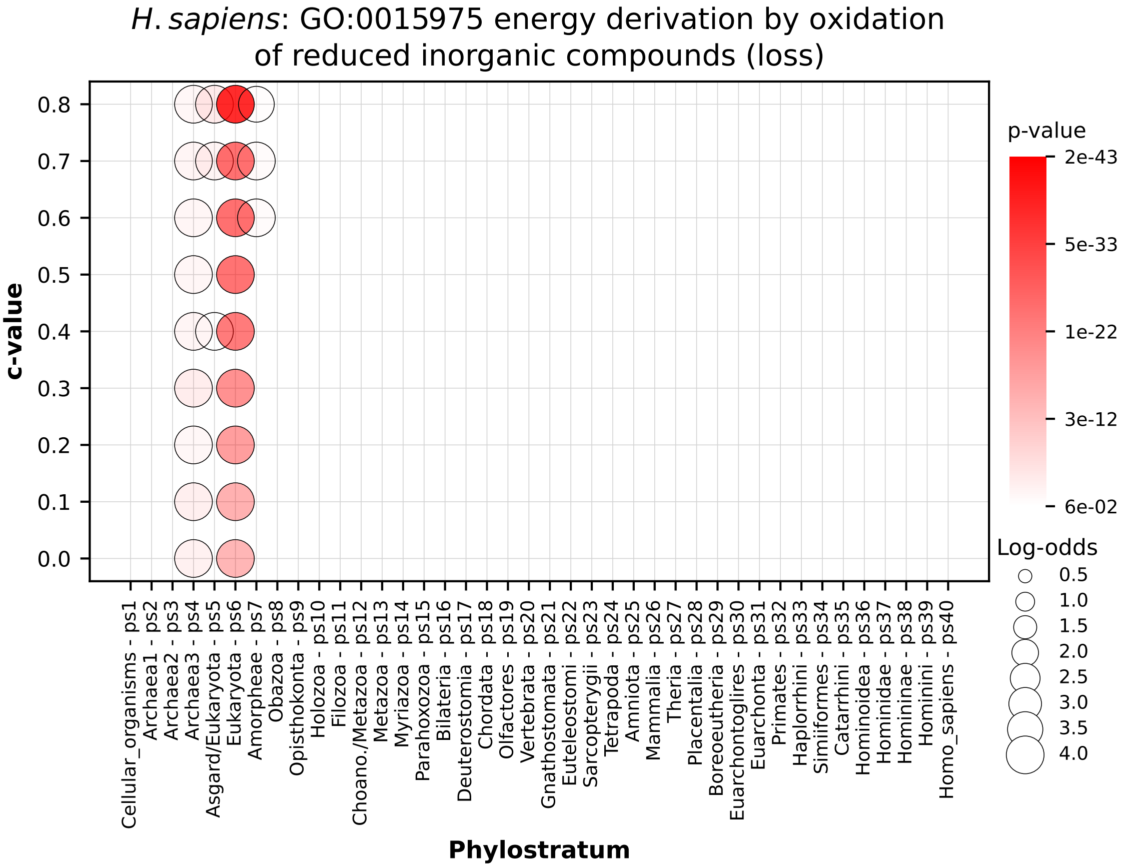

H. sapiens: GO:0015975 energy derivation by oxidation of reduced inorganic compounds (loss)
c-value
0.8
0.7
0.6
0.5
0.4
0.3
0.2
0.1
0.0
Phylostratum
Cellular_organisms - ps1
Archaea1 - ps2
Archaea2 - ps3
Archaea3 - ps4
Asgard/Eukaryota - ps5
Eukaryota - ps6
Amorpheae - ps7
Obazoa - ps8
Opisthokonta - ps9
Holozoa - ps10
Filozoa - ps11
Choano./Metazoa - ps12
Metazoa - ps13
Myriazoa - ps14
Parahoxozoa - ps15
Bilateria - ps16
Deuterostomia - ps17
Chordata - ps18
Olfactores - ps19
Vertebrata - ps20
Gnathostomata - ps21
Euteleostomi - ps22
Sarcopterygii - ps23
Tetrapoda - ps24
Amniota - ps25
Mammalia - ps26
Theria - ps27
Placentalia - ps28
Boreoeutheria - ps29
Euarchontoglires - ps30
Euarchonta - ps31
Primates - ps32
Haplorrhini - ps33
Simiiformes - ps34
Catarrhini - ps35
Hominoidea - ps36
Hominidae - ps37
Homininae - ps38
Hominini - ps39
Homo_sapiens - ps40
p-value
2e-43
5e-33
1e-22
3e-12
6e-02
Log-odds
0.5
1.0
1.5
2.0
2.5
3.0
3.5
4.0

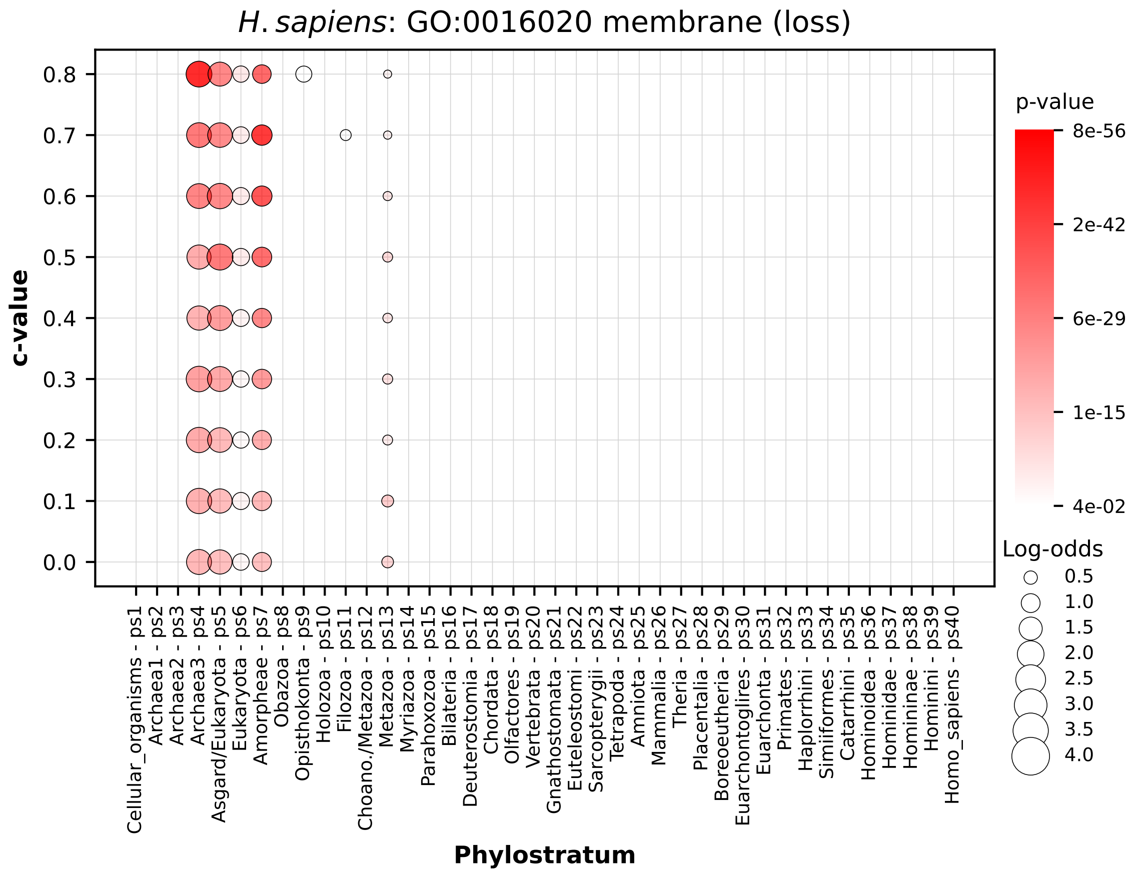

H. sapiens: GO:0016020 membrane (loss)
c-value
0.8
0.7
0.6
0.5
0.4
0.3
0.2
0.1
0.0
Phylostratum
Cellular_organisms - ps1
Archaea1 - ps2
Archaea2 - ps3
Archaea3 - ps4
Asgard/Eukaryota - ps5
Eukaryota - ps6
Amorpheae - ps7
Obazoa - ps8
Opisthokonta - ps9
Holozoa - ps10
Filozoa - ps11
Choano./Metazoa - ps12
Metazoa - ps13
Myriazoa - ps14
Parahoxozoa - ps15
Bilateria - ps16
Deuterostomia - ps17
Chordata - ps18
Olfactores - ps19
Vertebrata - ps20
Gnathostomata - ps21
Euteleostomi - ps22
Sarcopterygii - ps23
Tetrapoda - ps24
Amniota - ps25
Mammalia - ps26
Theria - ps27
Placentalia - ps28
Boreoeutheria - ps29
Euarchontoglires - ps30
Euarchonta - ps31
Primates - ps32
Haplorrhini - ps33
Simiiformes - ps34
Catarrhini - ps35
Hominoidea - ps36
Hominidae - ps37
Homininae - ps38
Hominini - ps39
Homo_sapiens - ps40
p-value
8e-56
2e-42
6e-29
1e-15
4e-02
Log-odds
0.5
1.0
1.5
2.0
2.5
3.0
3.5
4.0

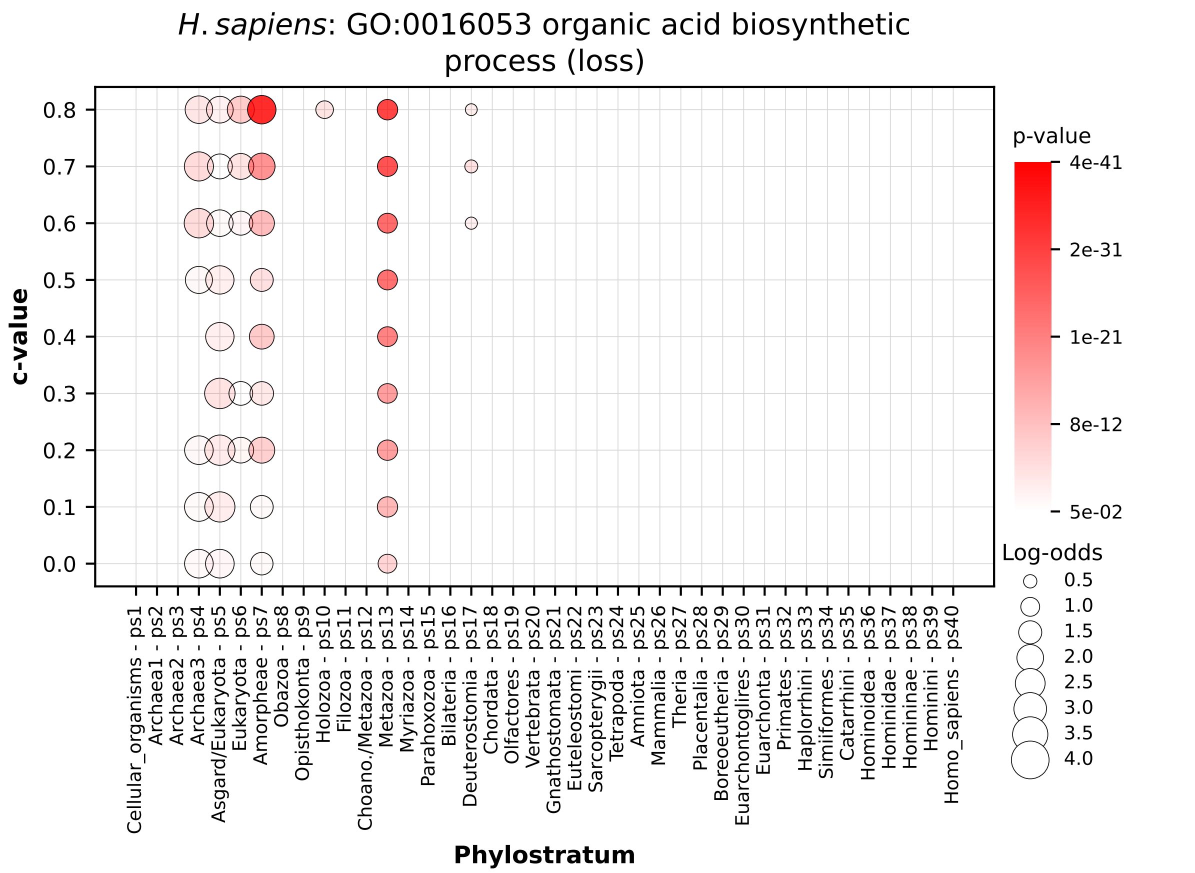
H. sapiens: GO:0016053 organic acid biosynthetic process (loss)
c-value
0.8
0.7
0.6
0.5
0.4
0.3
0.2
0.1
0.0
Phylostratum
Cellular_organisms - ps1
Archaea1 - ps2
Archaea2 - ps3
Archaea3 - ps4
Asgard/Eukaryota - ps5
Eukaryota - ps6
Amorpheae - ps7
Obazoa - ps8
Opisthokonta - ps9
Holozoa - ps10
Filozoa - ps11
Choano./Metazoa - ps12
Metazoa - ps13
Myriazoa - ps14
Parahoxozoa - ps15
Bilateria - ps16
Deuterostomia - ps17
Chordata - ps18
Olfactores - ps19
Vertebrata - ps20
Gnathostomata - ps21
Euteleostomi - ps22
Sarcopterygii - ps23
Tetrapoda - ps24
Amniota - ps25
Mammalia - ps26
Theria - ps27
Placentalia - ps28
Boreoeutheria - ps29
Euarchontoglires - ps30
Euarchonta - ps31
Primates - ps32
Haplorrhini - ps33
Simiiformes - ps34
Catarrhini - ps35
Hominoidea - ps36
Hominidae - ps37
Homininae - ps38
Hominini - ps39
Homo_sapiens - ps40
p-value
4e-41
2e-31
1e-21
8e-12
5e-02
Log-odds
0.5
1.0
1.5
2.0
2.5
3.0
3.5
4.0

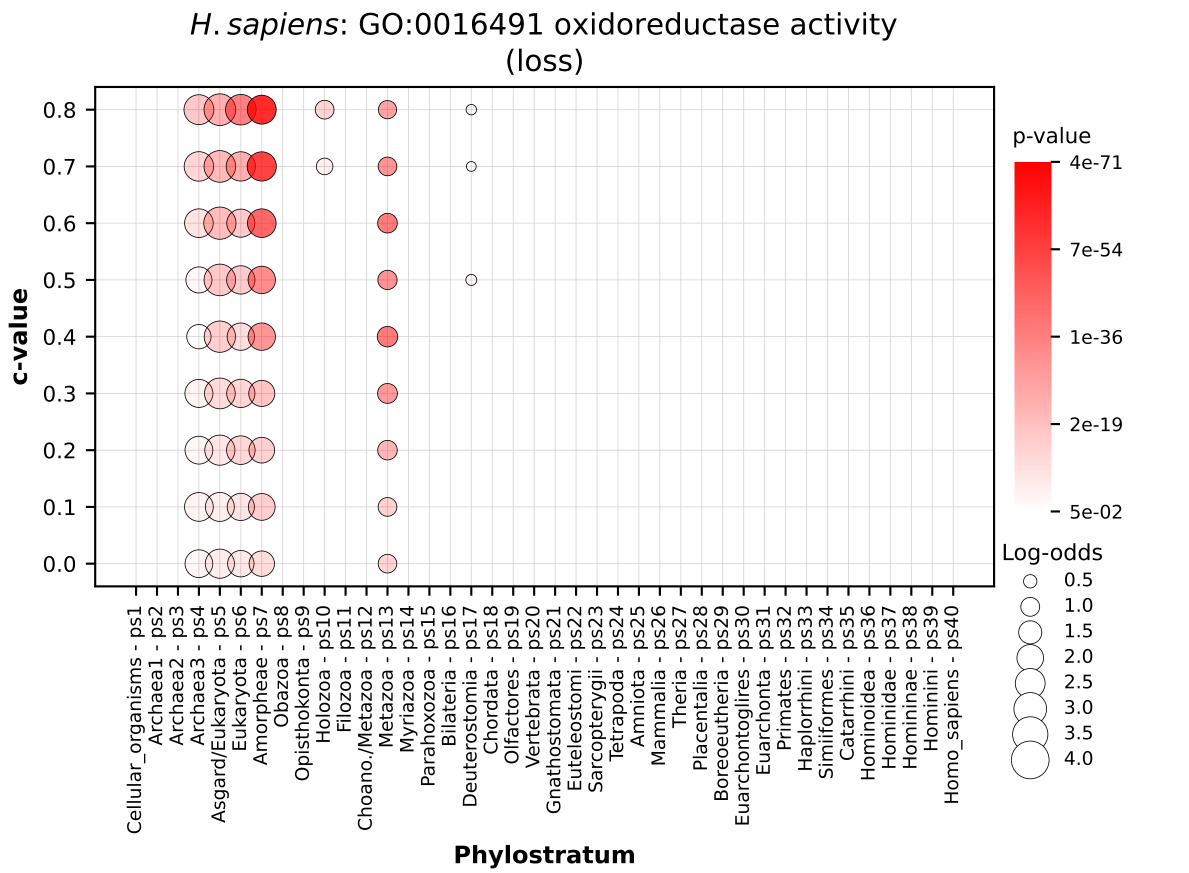

H. sapiens: GO:0016491 oxidoreductase activity
(loss)
c-value
0.8
0.7
0.6
0.5
0.4
0.3
0.2
0.1
0.0
p-value
4e-71
7e-54
1e-36
2e-19
5e-02
Log-odds
0.5
1.0
1.5
2.0
2.5
3.0
3.5
4.0
Cellular_organisms - ps1
Archaea1 - ps2
Archaea2 - ps3
Archaea3 - ps4
Asgard/Eukaryota - ps5
Eukaryota - ps6
Amorpheae - ps7
Obazoa - ps8
Opisthokonta - ps9
Holozoa - ps10
Filozoa - ps11
Choano./Metazoa - ps12
Metazoa - ps13
Myriazoa - ps14
Parahoxozoa - ps15
Bilateria - ps16
Deuterostomia - ps17
Chordata - ps18
Olfactores - ps19
Vertebrata - ps20
Gnathostomata - ps21
Euteleostomi - ps22
Sarcopterygii - ps23
Tetrapoda - ps24
Amniota - ps25
Mammalia - ps26
Theria - ps27
Placentalia - ps28
Boreoeutheria - ps29
Euarchontoglires - ps30
Euarchonta - ps31
Primates - ps32
Haplorrhini - ps33
Simiiformes - ps34
Catarrhini - ps35
Hominoidea - ps36
Hominidae - ps37
Homininae - ps38
Hominini - ps39
Homo_sapiens - ps40
Phylostratum

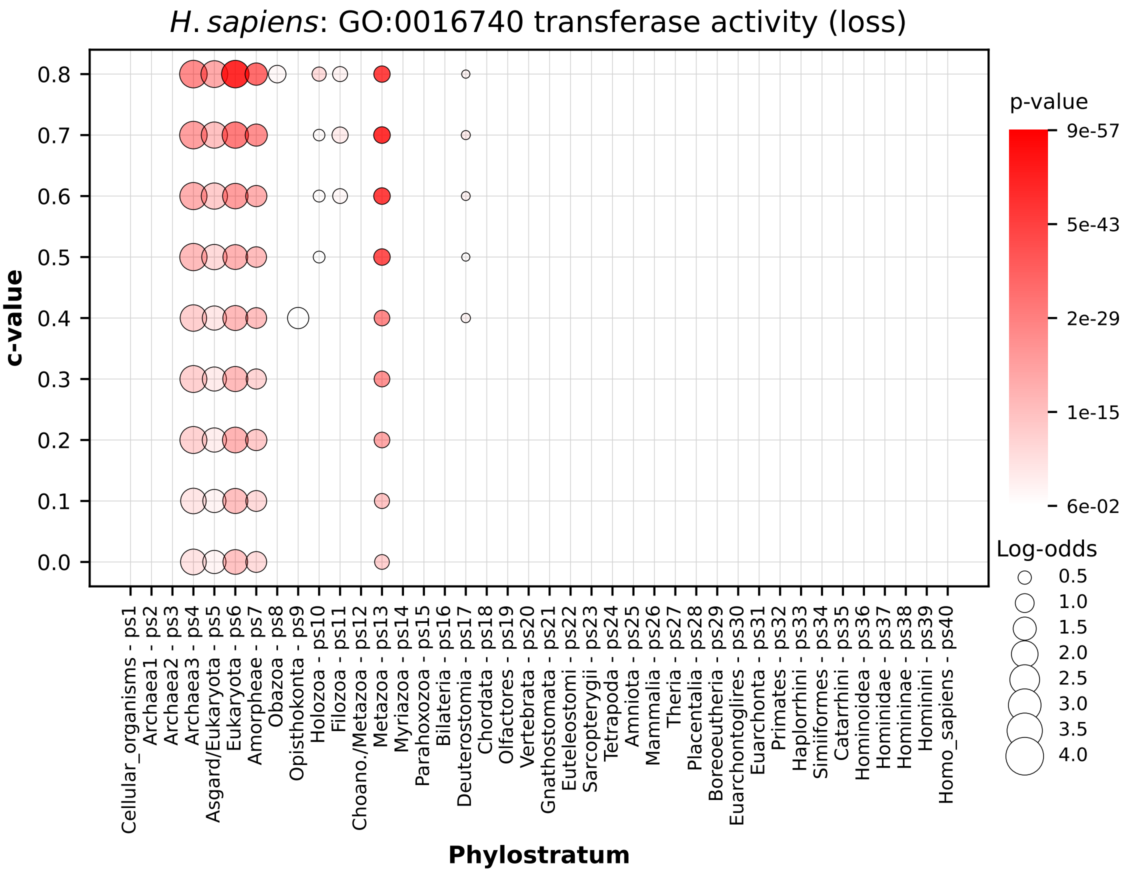
H. sapiens: GO:0016740 transferase activity (loss)
c-value
0.8
0.7
0.6
0.5
0.4
0.3
0.2
0.1
0.0
Phylostratum
Cellular_organisms - ps1
Archaea1 - ps2
Archaea2 - ps3
Archaea3 - ps4
Asgard/Eukaryota - ps5
Eukaryota - ps6
Amorpheae - ps7
Obazoa - ps8
Opisthokonta - ps9
Holozoa - ps10
Filozoa - ps11
Choano./Metazoa - ps12
Metazoa - ps13
Myriazoa - ps14
Parahoxozoa - ps15
Bilateria - ps16
Deuterostomia - ps17
Chordata - ps18
Olfactores - ps19
Vertebrata - ps20
Gnathostomata - ps21
Euteleostomi - ps22
Sarcopterygii - ps23
Tetrapoda - ps24
Amniota - ps25
Mammalia - ps26
Theria - ps27
Placentalia - ps28
Boreoeutheria - ps29
Euarchontoglires - ps30
Euarchonta - ps31
Primates - ps32
Haplorrhini - ps33
Simiiformes - ps34
Catarrhini - ps35
Hominoidea - ps36
Hominidae - ps37
Homininae - ps38
Hominini - ps39
Homo_sapiens - ps40
p-value
9e-57
5e-43
2e-29
1e-15
6e-02
Log-odds
0.5
1.0
1.5
2.0
2.5
3.0
3.5
4.0

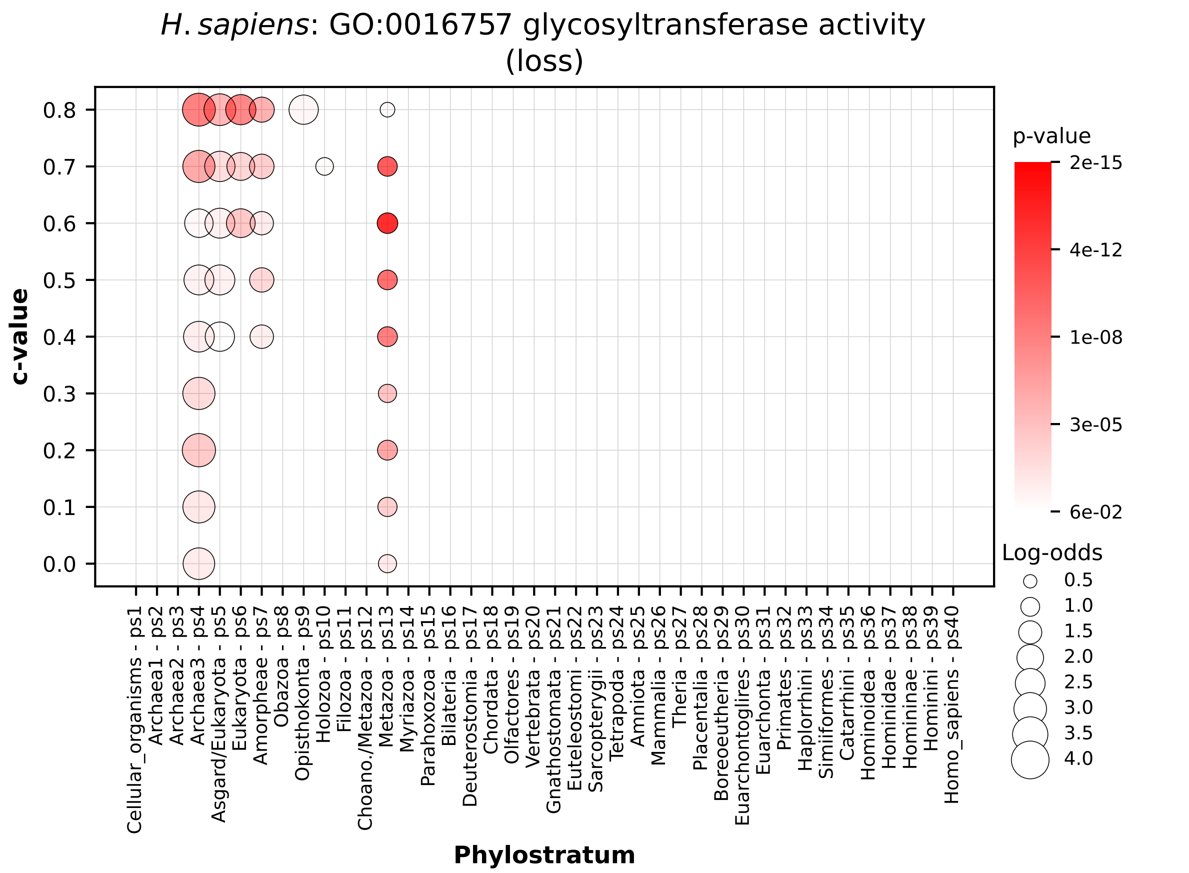

H. sapiens: GO:0016757 glycosyltransferase activity
(loss)
c-value
0.8
0.7
0.6
0.5
0.4
0.3
0.2
0.1
0.0
p-value
2e-15
4e-12
1e-08
3e-05
6e-02
Log-odds
0.5
1.0
1.5
2.0
2.5
3.0
3.5
4.0
Cellular_organisms - ps1
Archaea1 - ps2
Archaea2 - ps3
Archaea3 - ps4
Asgard/Eukaryota - ps5
Eukaryota - ps6
Amorpheae - ps7
Obazoa - ps8
Opisthokonta - ps9
Holozoa - ps10
Filozoa - ps11
Choano./Metazoa - ps12
Metazoa - ps13
Myriazoa - ps14
Parahoxozoa - ps15
Bilateria - ps16
Deuterostomia - ps17
Chordata - ps18
Olfactores - ps19
Vertebrata - ps20
Gnathostomata - ps21
Euteleostomi - ps22
Sarcopterygii - ps23
Tetrapoda - ps24
Amniota - ps25
Mammalia - ps26
Theria - ps27
Placentalia - ps28
Boreoeutheria - ps29
Euarchontoglires - ps30
Euarchonta - ps31
Primates - ps32
Haplorrhini - ps33
Simiiformes - ps34
Catarrhini - ps35
Hominoidea - ps36
Hominidae - ps37
Homininae - ps38
Hominini - ps39
Homo_sapiens - ps40
Phylostratum

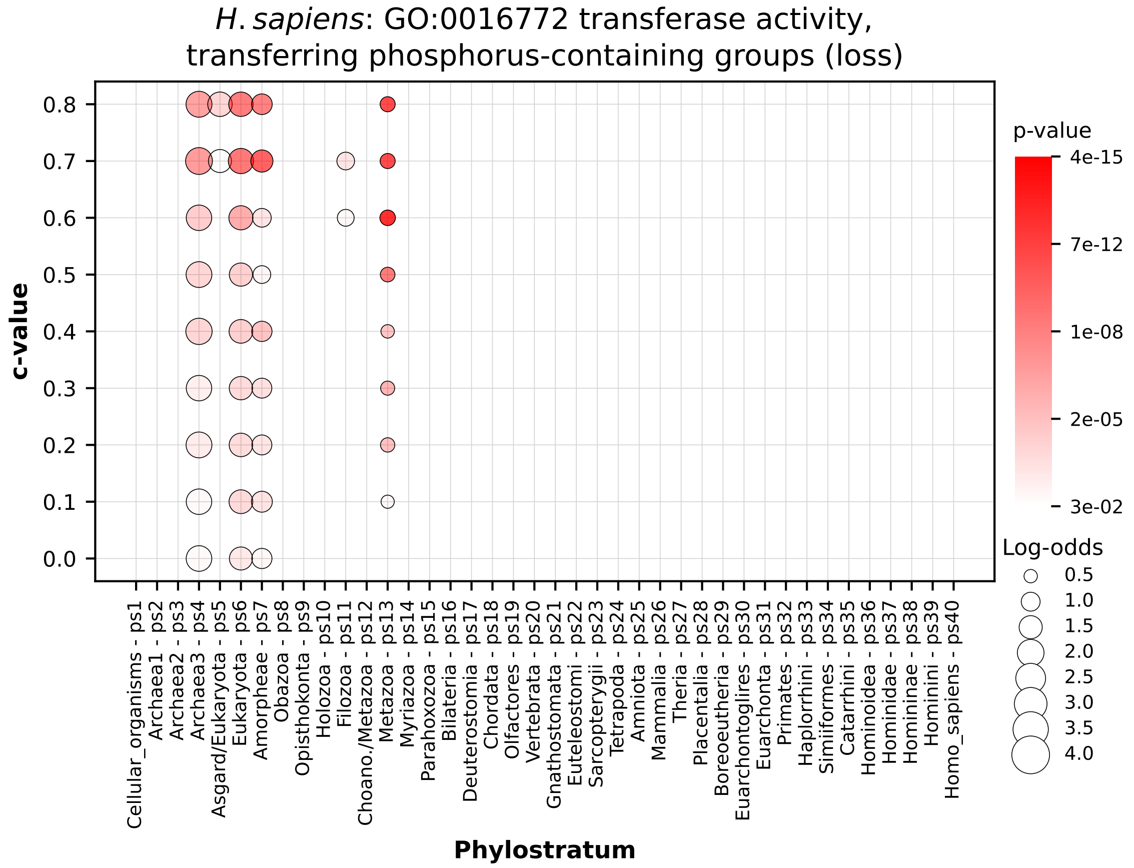

H. sapiens: GO:0016772 transferase activity, transferring phosphorus-containing groups (loss)
c-value
Phylostratum
p-value
4e-15
7e-12
1e-08
2e-05
3e-02
Log-odds
0.5
1.0
1.5
2.0
2.5
3.0
3.5
4.0
Cellular_organisms - ps1
Archaea1 - ps2
Archaea2 - ps3
Archaea3 - ps4
Asgard/Eukaryota - ps5
Eukaryota - ps6
Amorpheae - ps7
Obazoa - ps8
Opisthokonta - ps9
Holozoa - ps10
Filozoa - ps11
Choano./Metazoa - ps12
Metazoa - ps13
Myriazoa - ps14
Parahoxozoa - ps15
Bilateria - ps16
Deuterostomia - ps17
Chordata - ps18
Olfactores - ps19
Vertebrata - ps20
Gnathostomata - ps21
Euteleostomi - ps22
Sarcopterygii - ps23
Tetrapoda - ps24
Amniota - ps25
Mammalia - ps26
Theria - ps27
Placentalia - ps28
Boreoeutheria - ps29
Euarchontoglires - ps30
Euarchonta - ps31
Primates - ps32
Haplorrhini - ps33
Simiiformes - ps34
Catarrhini - ps35
Hominoidea - ps36
Hominidae - ps37
Homininae - ps38
Hominini - ps39
Homo_sapiens - ps40

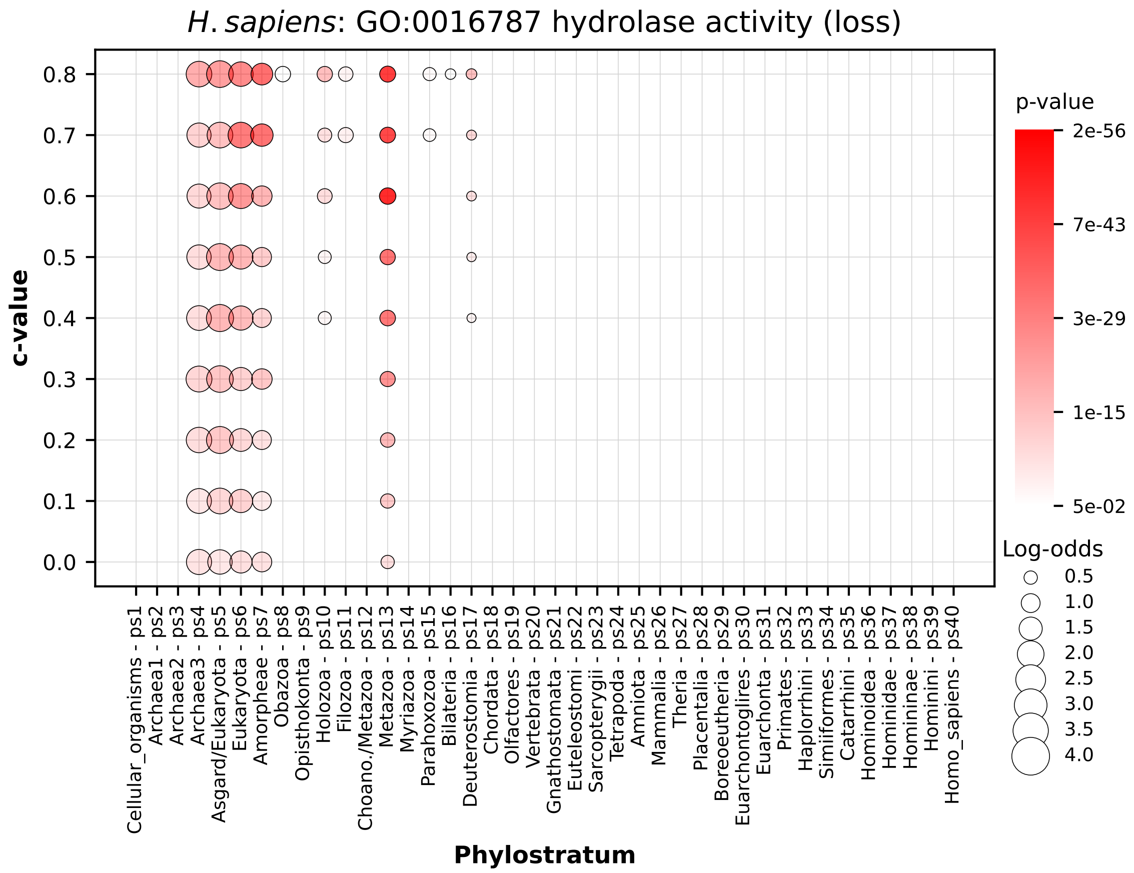

H. sapiens: GO:0016787 hydrolase activity (loss)
c-value
0.8
0.7
0.6
0.5
0.4
0.3
0.2
0.1
0.0
Phylostratum
Cellular_organisms - ps1
Archaea1 - ps2
Archaea2 - ps3
Archaea3 - ps4
Asgard/Eukaryota - ps5
Eukaryota - ps6
Amorpheae - ps7
Obazoa - ps8
Opisthokonta - ps9
Holozoa - ps10
Filozoa - ps11
Choano./Metazoa - ps12
Metazoa - ps13
Myriazoa - ps14
Parahoxozoa - ps15
Bilateria - ps16
Deuterostomia - ps17
Chordata - ps18
Olfactores - ps19
Vertebrata - ps20
Gnathostomata - ps21
Euteleostomi - ps22
Sarcopterygii - ps23
Tetrapoda - ps24
Amniota - ps25
Mammalia - ps26
Theria - ps27
Placentalia - ps28
Boreoeutheria - ps29
Euarchontoglires - ps30
Euarchonta - ps31
Primates - ps32
Haplorrhini - ps33
Simiiformes - ps34
Catarrhini - ps35
Hominoidea - ps36
Hominidae - ps37
Homininae - ps38
Hominini - ps39
Homo_sapiens - ps40
p-value
2e-56
7e-43
3e-29
1e-15
5e-02
Log-odds
0.5
1.0
1.5
2.0
2.5
3.0
3.5
4.0

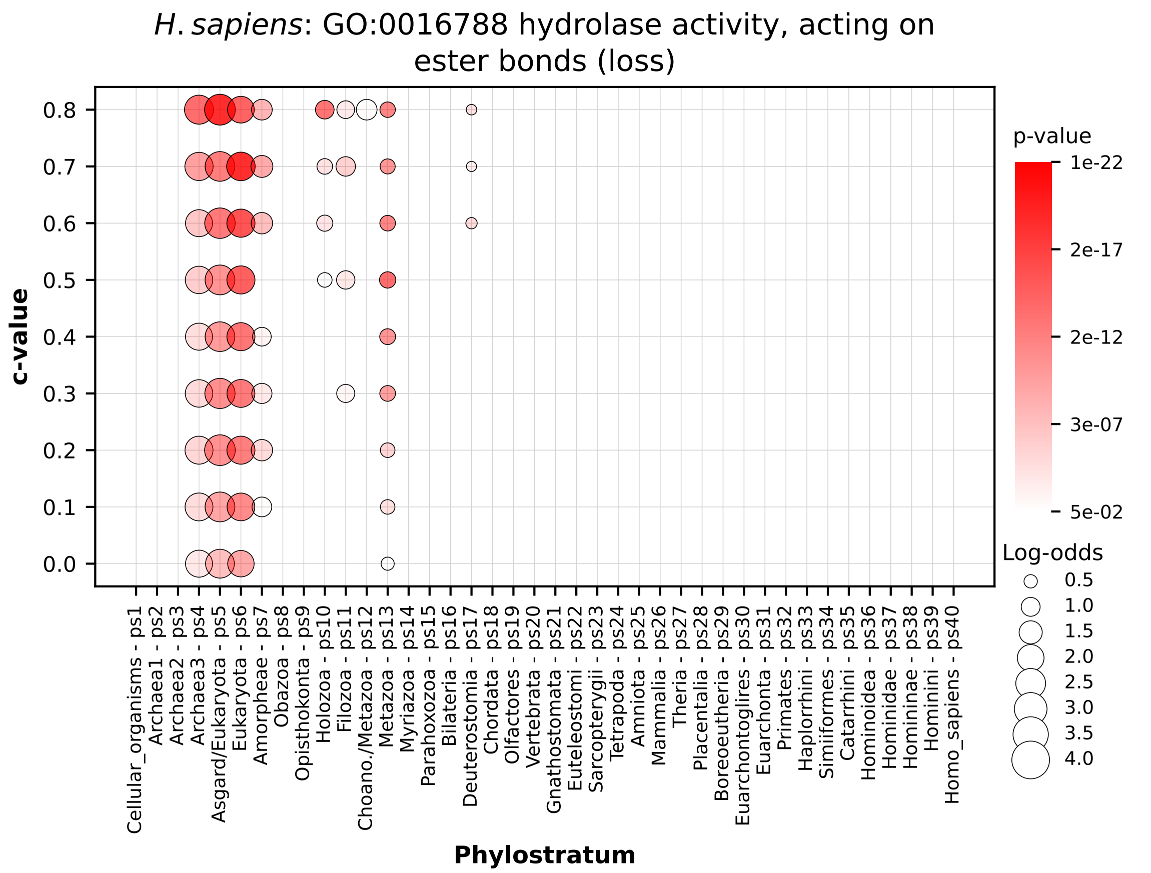

H. sapiens: GO:0016788 hydrolase activity, acting on ester bonds (loss)
c-value
0.8
0.7
0.6
0.5
0.4
0.3
0.2
0.1
0.0
Phylostratum
Cellular_organisms - ps1
Archaea1 - ps2
Archaea2 - ps3
Archaea3 - ps4
Asgard/Eukaryota - ps5
Eukaryota - ps6
Amorpheae - ps7
Obazoa - ps8
Opisthokonta - ps9
Holozoa - ps10
Filozoa - ps11
Choano./Metazoa - ps12
Metazoa - ps13
Myriazoa - ps14
Parahoxozoa - ps15
Bilateria - ps16
Deuterostomia - ps17
Chordata - ps18
Olfactores - ps19
Vertebrata - ps20
Gnathostomata - ps21
Euteleostomi - ps22
Sarcopterygii - ps23
Tetrapoda - ps24
Amniota - ps25
Mammalia - ps26
Theria - ps27
Placentalia - ps28
Boreoeutheria - ps29
Euarchontoglires - ps30
Euarchonta - ps31
Primates - ps32
Haplorrhini - ps33
Simiiformes - ps34
Catarrhini - ps35
Hominoidea - ps36
Hominidae - ps37
Homininae - ps38
Hominini - ps39
Homo_sapiens - ps40
p-value
1e-22
2e-17
2e-12
3e-07
5e-02
Log-odds
0.5
1.0
1.5
2.0
2.5
3.0
3.5
4.0

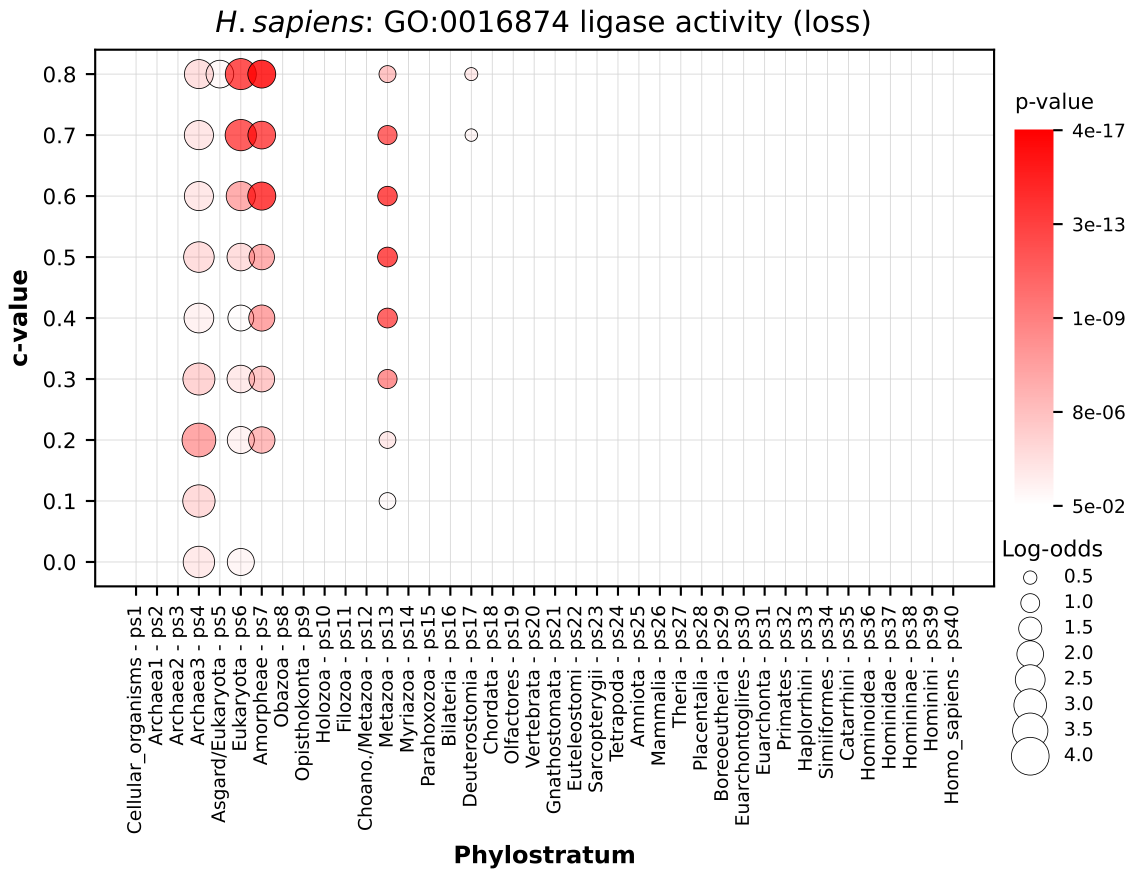
H. sapiens: GO:0016874 ligase activity (loss)
c-value
0.8
0.7
0.6
0.5
0.4
0.3
0.2
0.1
0.0
Phylostratum
Cellular_organisms - ps1
Archaea1 - ps2
Archaea2 - ps3
Archaea3 - ps4
Asgard/Eukaryota - ps5
Eukaryota - ps6
Amorpheae - ps7
Obazoa - ps8
Opisthokonta - ps9
Holozoa - ps10
Filozoa - ps11
Choano./Metazoa - ps12
Metazoa - ps13
Myriazoa - ps14
Parahoxozoa - ps15
Bilateria - ps16
Deuterostomia - ps17
Chordata - ps18
Olfactores - ps19
Vertebrata - ps20
Gnathostomata - ps21
Euteleostomi - ps22
Sarcopterygii - ps23
Tetrapoda - ps24
Amniota - ps25
Mammalia - ps26
Theria - ps27
Placentalia - ps28
Boreoeutheria - ps29
Euarchontoglires - ps30
Euarchonta - ps31
Primates - ps32
Haplorrhini - ps33
Simiiformes - ps34
Catarrhini - ps35
Hominoidea - ps36
Hominidae - ps37
Homininae - ps38
Hominini - ps39
Homo_sapiens - ps40
p-value
4e-17
3e-13
1e-09
8e-06
5e-02
Log-odds
0.5
1.0
1.5
2.0
2.5
3.0
3.5
4.0

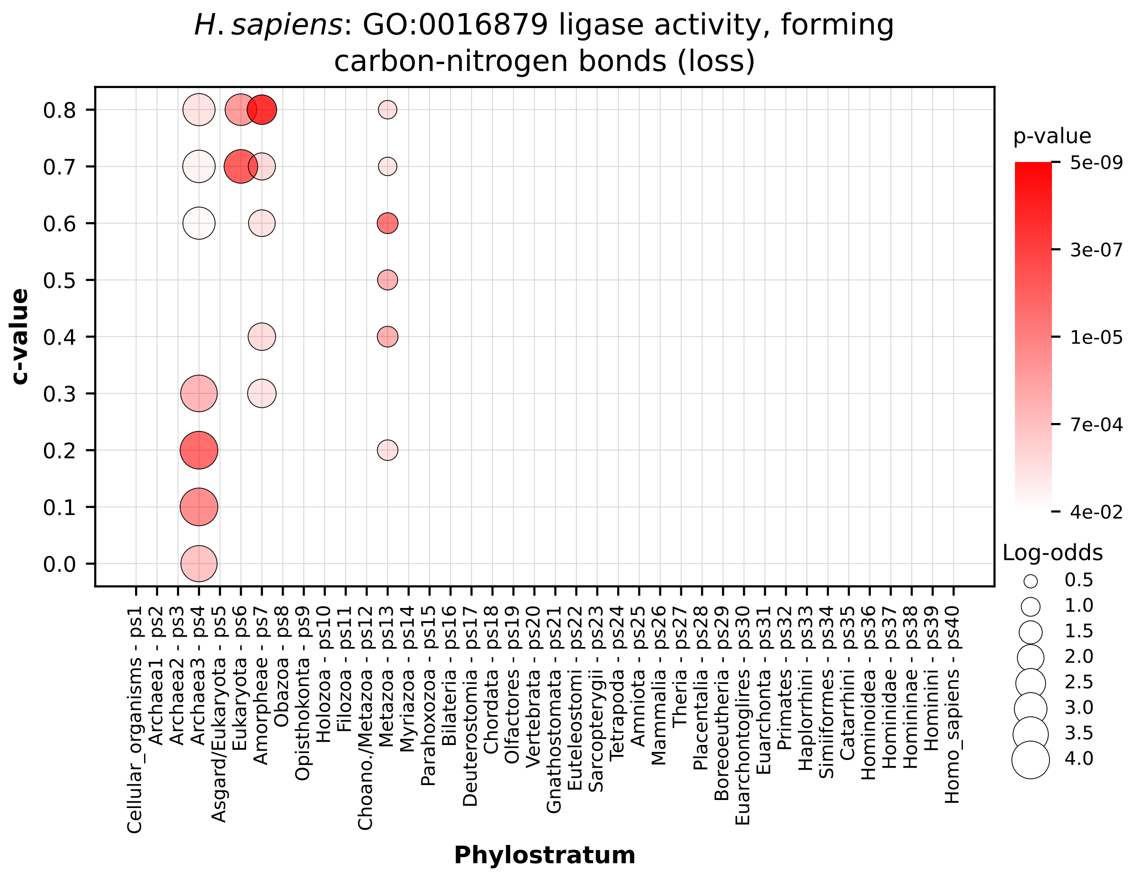

H. sapiens: GO:0016879 ligase activity, forming carbon-nitrogen bonds (loss)
c-value
0.8
0.7
0.6
0.5
0.4
0.3
0.2
0.1
0.0
p-value
5e-09
3e-07
1e-05
7e-04
4e-02
Log-odds
0.5
1.0
1.5
2.0
2.5
3.0
3.5
4.0
Cellular_organisms - ps1
Archaea1 - ps2
Archaea2 - ps3
Archaea3 - ps4
Asgard/Eukaryota - ps5
Eukaryota - ps6
Amorpheae - ps7
Obazoa - ps8
Opisthokonta - ps9
Holozoa - ps10
Filozoa - ps11
Choano./Metazoa - ps12
Metazoa - ps13
Myriazoa - ps14
Parahoxozoa - ps15
Bilateria - ps16
Deuterostomia - ps17
Chordata - ps18
Olfactores - ps19
Vertebrata - ps20
Gnathostomata - ps21
Euteleostomi - ps22
Sarcopterygii - ps23
Tetrapoda - ps24
Amniota - ps25
Mammalia - ps26
Theria - ps27
Placentalia - ps28
Boreoeutheria - ps29
Euarchontoglires - ps30
Euarchonta - ps31
Primates - ps32
Haplorrhini - ps33
Simiiformes - ps34
Catarrhini - ps35
Hominoidea - ps36
Hominidae - ps37
Homininae - ps38
Hominini - ps39
Homo_sapiens - ps40
Phylostratum

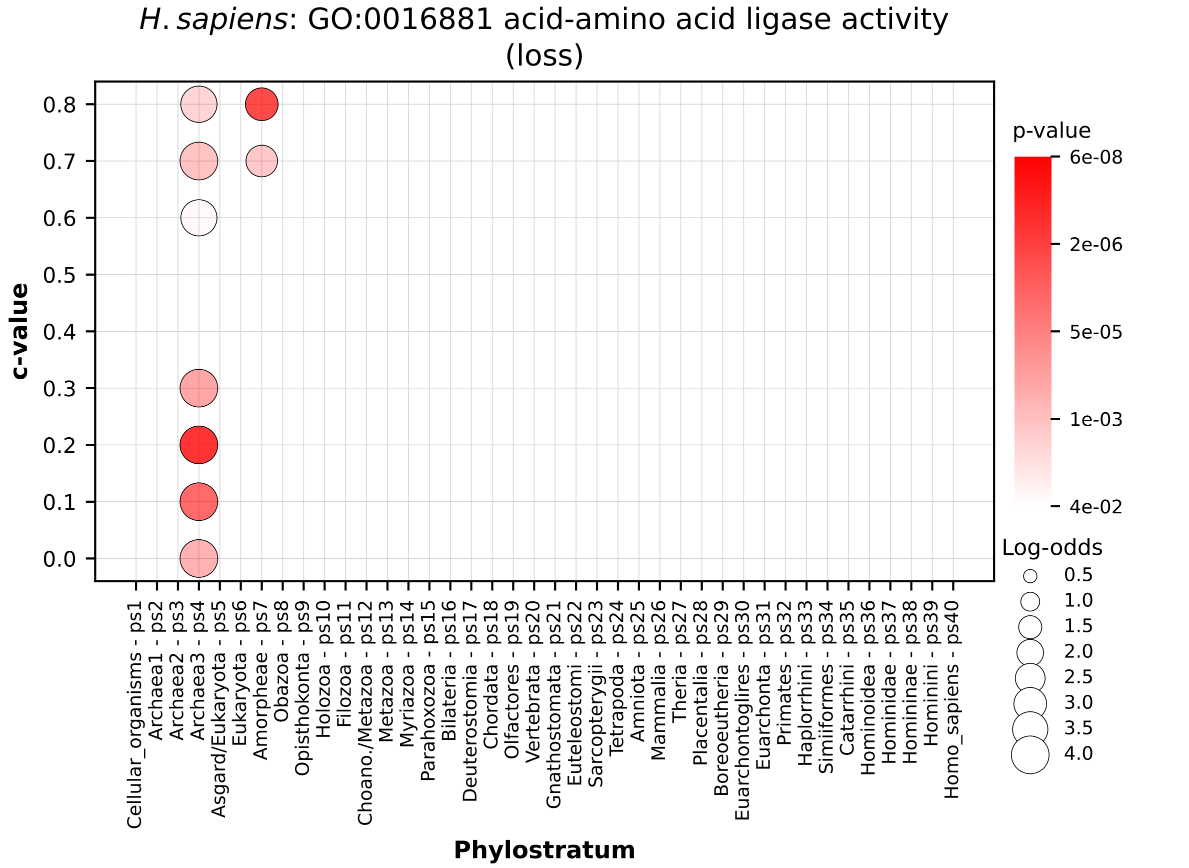
H. sapiens: GO:0016881 acid-amino acid ligase activity
(loss)
c-value
0.8
0.7
0.6
0.5
0.4
0.3
0.2
0.1
0.0
Phylostratum
Cellular_organisms - ps1
Archaea1 - ps2
Archaea2 - ps3
Archaea3 - ps4
Asgard/Eukaryota - ps5
Eukaryota - ps6
Amorpheae - ps7
Obazoa - ps8
Opisthokonta - ps9
Holozoa - ps10
Filozoa - ps11
Choano./Metazoa - ps12
Metazoa - ps13
Myriazoa - ps14
Parahoxozoa - ps15
Bilateria - ps16
Deuterostomia - ps17
Chordata - ps18
Olfactores - ps19
Vertebrata - ps20
Gnathostomata - ps21
Euteleostomi - ps22
Sarcopterygii - ps23
Tetrapoda - ps24
Amniota - ps25
Mammalia - ps26
Theria - ps27
Placentalia - ps28
Boreoeutheria - ps29
Euarchontoglires - ps30
Euarchonta - ps31
Primates - ps32
Haplorrhini - ps33
Simiiformes - ps34
Catarrhini - ps35
Hominoidea - ps36
Hominidae - ps37
Homininae - ps38
Hominini - ps39
Homo_sapiens - ps40
p-value
6e-08
2e-06
5e-05
1e-03
4e-02
Log-odds
0.5
1.0
1.5
2.0
2.5
3.0
3.5
4.0

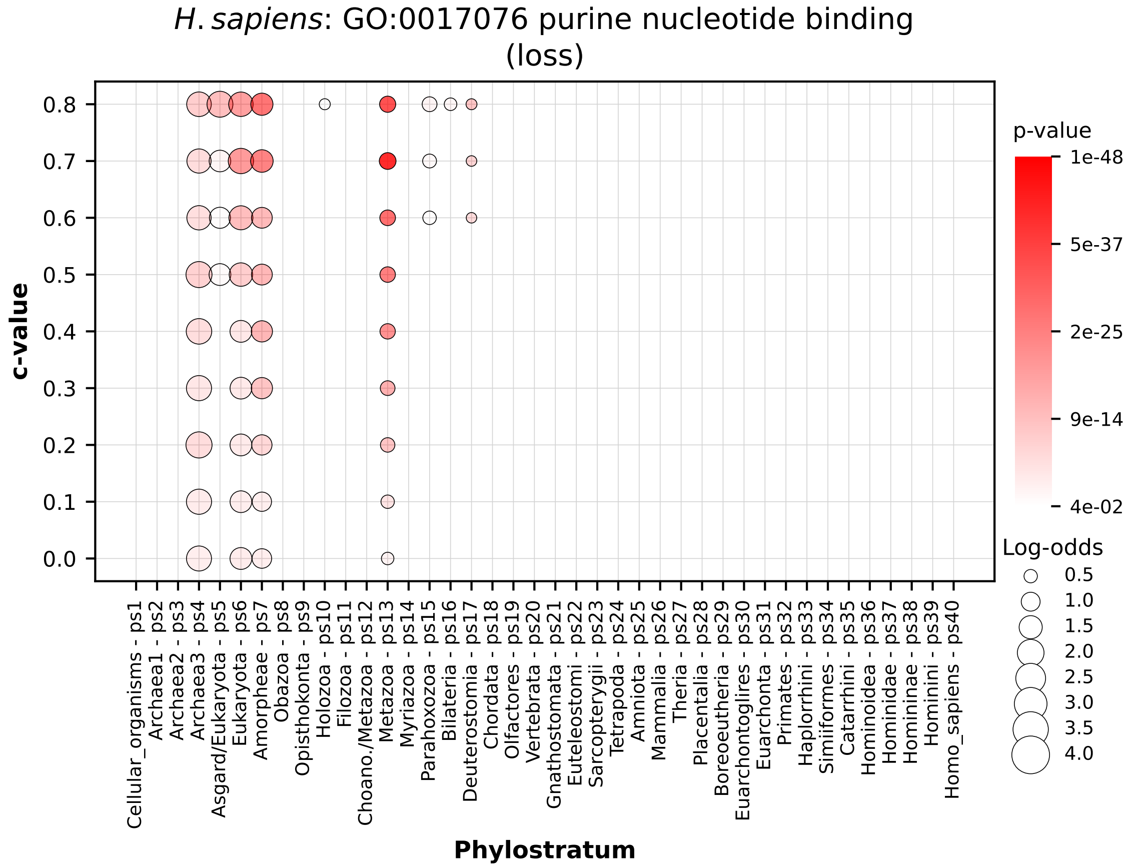

H. sapiens: GO:0017076 purine nucleotide binding
(loss)
c-value
0.8
0.7
0.6
0.5
0.4
0.3
0.2
0.1
0.0
p-value
1e-48
5e-37
2e-25
9e-14
4e-02
Log-odds
0.5
1.0
1.5
2.0
2.5
3.0
3.5
4.0
Cellular_organisms - ps1
Archaea1 - ps2
Archaea2 - ps3
Archaea3 - ps4
Asgard/Eukaryota - ps5
Eukaryota - ps6
Amorpheae - ps7
Obazoa - ps8
Opisthokonta - ps9
Holozoa - ps10
Filozoa - ps11
Choano./Metazoa - ps12
Metazoa - ps13
Myriazoa - ps14
Parahoxozoa - ps15
Bilateria - ps16
Deuterostomia - ps17
Chordata - ps18
Olfactores - ps19
Vertebrata - ps20
Gnathostomata - ps21
Euteleostomi - ps22
Sarcopterygii - ps23
Tetrapoda - ps24
Amniota - ps25
Mammalia - ps26
Theria - ps27
Placentalia - ps28
Boreoeutheria - ps29
Euarchontoglires - ps30
Euarchonta - ps31
Primates - ps32
Haplorrhini - ps33
Simiiformes - ps34
Catarrhini - ps35
Hominoidea - ps36
Hominidae - ps37
Homininae - ps38
Hominini - ps39
Homo_sapiens - ps40
Phylostratum

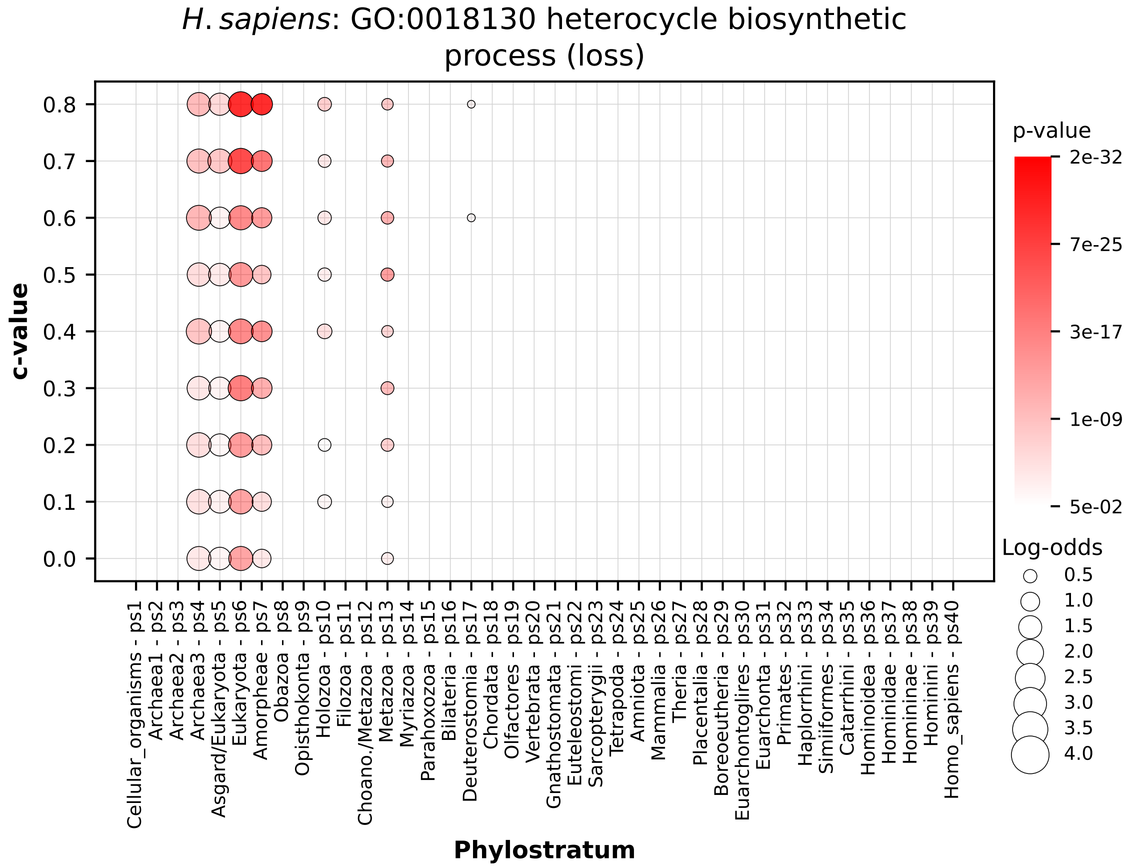

H. sapiens: GO:0018130 heterocycle biosynthetic process (loss)
c-value
0.8
0.7
0.6
0.5
0.4
0.3
0.2
0.1
0.0
Phylostratum
Cellular_organisms - ps1
Archaea1 - ps2
Archaea2 - ps3
Archaea3 - ps4
Asgard/Eukaryota - ps5
Eukaryota - ps6
Amorpheae - ps7
Obazoa - ps8
Opisthokonta - ps9
Holozoa - ps10
Filozoa - ps11
Choano./Metazoa - ps12
Metazoa - ps13
Myriazoa - ps14
Parahoxozoa - ps15
Bilateria - ps16
Deuterostomia - ps17
Chordata - ps18
Olfactores - ps19
Vertebrata - ps20
Gnathostomata - ps21
Euteleostomi - ps22
Sarcopterygii - ps23
Tetrapoda - ps24
Amniota - ps25
Mammalia - ps26
Theria - ps27
Placentalia - ps28
Boreoeutheria - ps29
Euarchontoglires - ps30
Euarchonta - ps31
Primates - ps32
Haplorrhini - ps33
Simiiformes - ps34
Catarrhini - ps35
Hominoidea - ps36
Hominidae - ps37
Homininae - ps38
Hominini - ps39
Homo_sapiens - ps40
p-value
2e-32
7e-25
3e-17
1e-09
5e-02
Log-odds
0.5
1.0
1.5
2.0
2.5
3.0
3.5
4.0

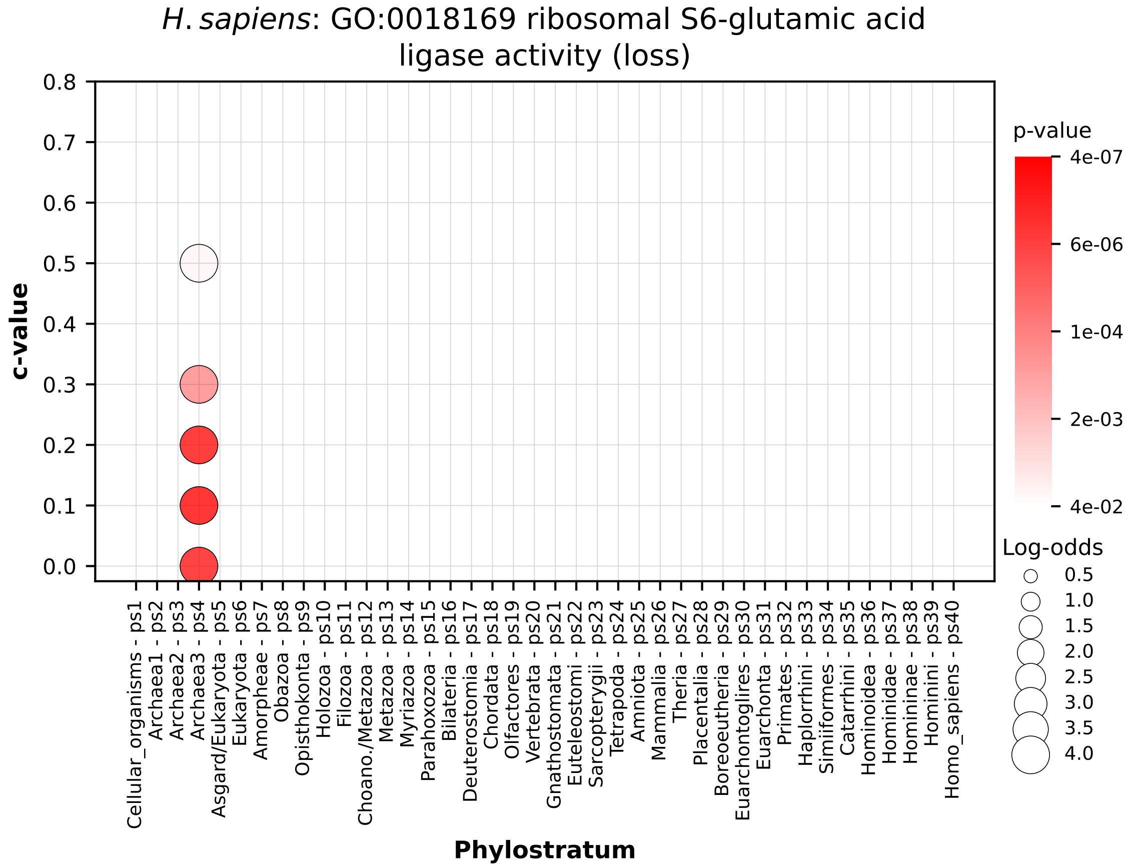

H. sapiens: GO:0018169 ribosomal S6-glutamic acid ligase activity (loss)
c-value
0.8
0.7
0.6
0.5
0.4
0.3
0.2
0.1
0.0
p-value
4e-07
6e-06
1e-04
2e-03
4e-02
Log-odds
0.5
1.0
1.5
2.0
2.5
3.0
3.5
4.0
Cellular_organisms - ps1
Archaea1 - ps2
Archaea2 - ps3
Archaea3 - ps4
Asgard/Eukaryota - ps5
Eukaryota - ps6
Amorpheae - ps7
Obazoa - ps8
Opisthokonta - ps9
Holozoa - ps10
Filozoa - ps11
Choano./Metazoa - ps12
Metazoa - ps13
Myriazoa - ps14
Parahoxozoa - ps15
Bilateria - ps16
Deuterostomia - ps17
Chordata - ps18
Olfactores - ps19
Vertebrata - ps20
Gnathostomata - ps21
Euteleostomi - ps22
Sarcopterygii - ps23
Tetrapoda - ps24
Amniota - ps25
Mammalia - ps26
Theria - ps27
Placentalia - ps28
Boreoeutheria - ps29
Euarchontoglires - ps30
Euarchonta - ps31
Primates - ps32
Haplorrhini - ps33
Simiiformes - ps34
Catarrhini - ps35
Hominoidea - ps36
Hominidae - ps37
Homininae - ps38
Hominini - ps39
Homo_sapiens - ps40
Phylostratum

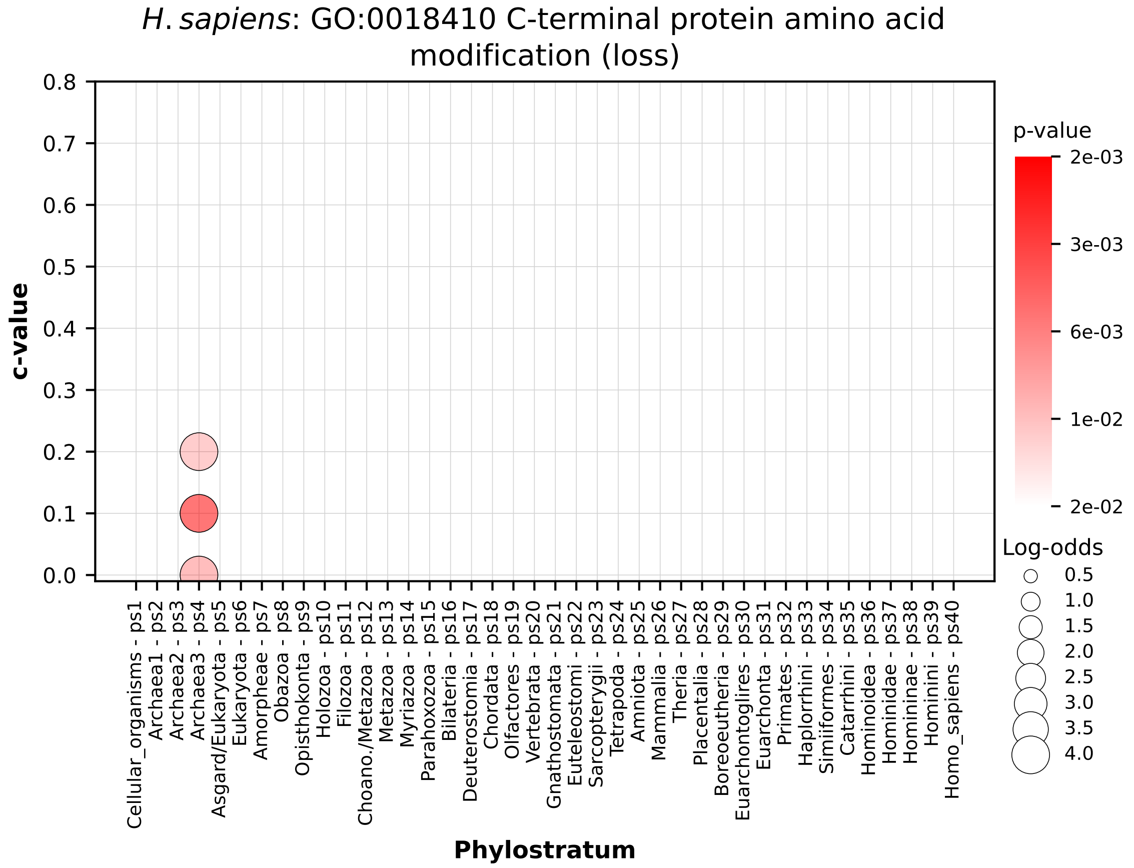

H. sapiens: GO:0018410 C-terminal protein amino acid modification (loss)
c-value
0.8
0.7
0.6
0.5
0.4
0.3
0.2
0.1
0.0
p-value
2e-03
3e-03
6e-03
1e-02
2e-02
Log-odds
0.5
1.0
1.5
2.0
2.5
3.0
3.5
4.0
Cellular_organisms - ps1
Archaea1 - ps2
Archaea2 - ps3
Archaea3 - ps4
Asgard/Eukaryota - ps5
Eukaryota - ps6
Amorpheae - ps7
Obazoa - ps8
Opisthokonta - ps9
Holozoa - ps10
Filozoa - ps11
Choano./Metazoa - ps12
Metazoa - ps13
Myriazoa - ps14
Parahoxozoa - ps15
Bilateria - ps16
Deuterostomia - ps17
Chordata - ps18
Olfactores - ps19
Vertebrata - ps20
Gnathostomata - ps21
Euteleostomi - ps22
Sarcopterygii - ps23
Tetrapoda - ps24
Amniota - ps25
Mammalia - ps26
Theria - ps27
Placentalia - ps28
Boreoeutheria - ps29
Euarchontoglires - ps30
Euarchonta - ps31
Primates - ps32
Haplorrhini - ps33
Simiiformes - ps34
Catarrhini - ps35
Hominoidea - ps36
Hominidae - ps37
Homininae - ps38
Hominini - ps39
Homo_sapiens - ps40
Phylostratum

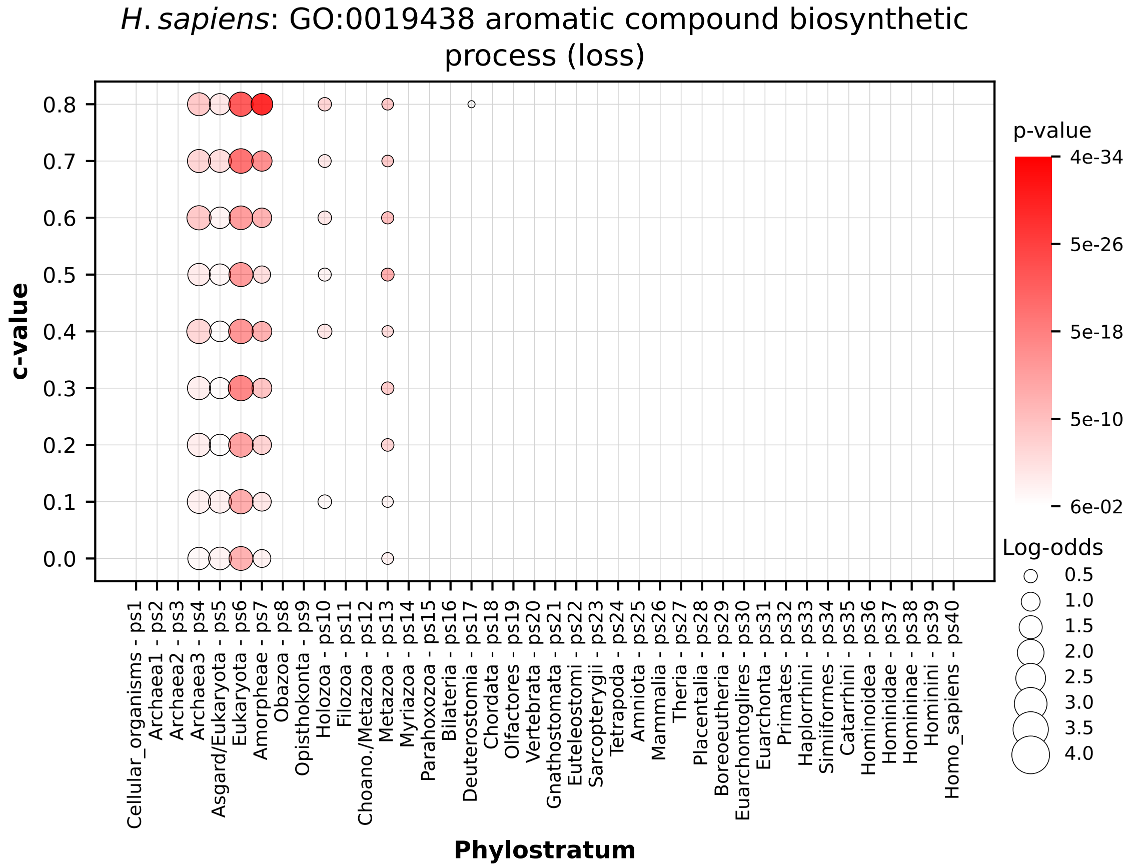

H. sapiens: GO:0019438 aromatic compound biosynthetic process (loss)
c-value
0.8
0.7
0.6
0.5
0.4
0.3
0.2
0.1
0.0
p-value
4e-34
5e-26
5e-18
5e-10
6e-02
Log-odds
0.5
1.0
1.5
2.0
2.5
3.0
3.5
4.0
Cellular_organisms - ps1
Archaea1 - ps2
Archaea2 - ps3
Archaea3 - ps4
Asgard/Eukaryota - ps5
Eukaryota - ps6
Amorpheae - ps7
Obazoa - ps8
Opisthokonta - ps9
Holozoa - ps10
Filozoa - ps11
Choano./Metazoa - ps12
Metazoa - ps13
Myriazoa - ps14
Parahoxozoa - ps15
Bilateria - ps16
Deuterostomia - ps17
Chordata - ps18
Olfactores - ps19
Vertebrata - ps20
Gnathostomata - ps21
Euteleostomi - ps22
Sarcopterygii - ps23
Tetrapoda - ps24
Amniota - ps25
Mammalia - ps26
Theria - ps27
Placentalia - ps28
Boreoeutheria - ps29
Euarchontoglires - ps30
Euarchonta - ps31
Primates - ps32
Haplorrhini - ps33
Simiiformes - ps34
Catarrhini - ps35
Hominoidea - ps36
Hominidae - ps37
Homininae - ps38
Hominini - ps39
Homo_sapiens - ps40
Phylostratum

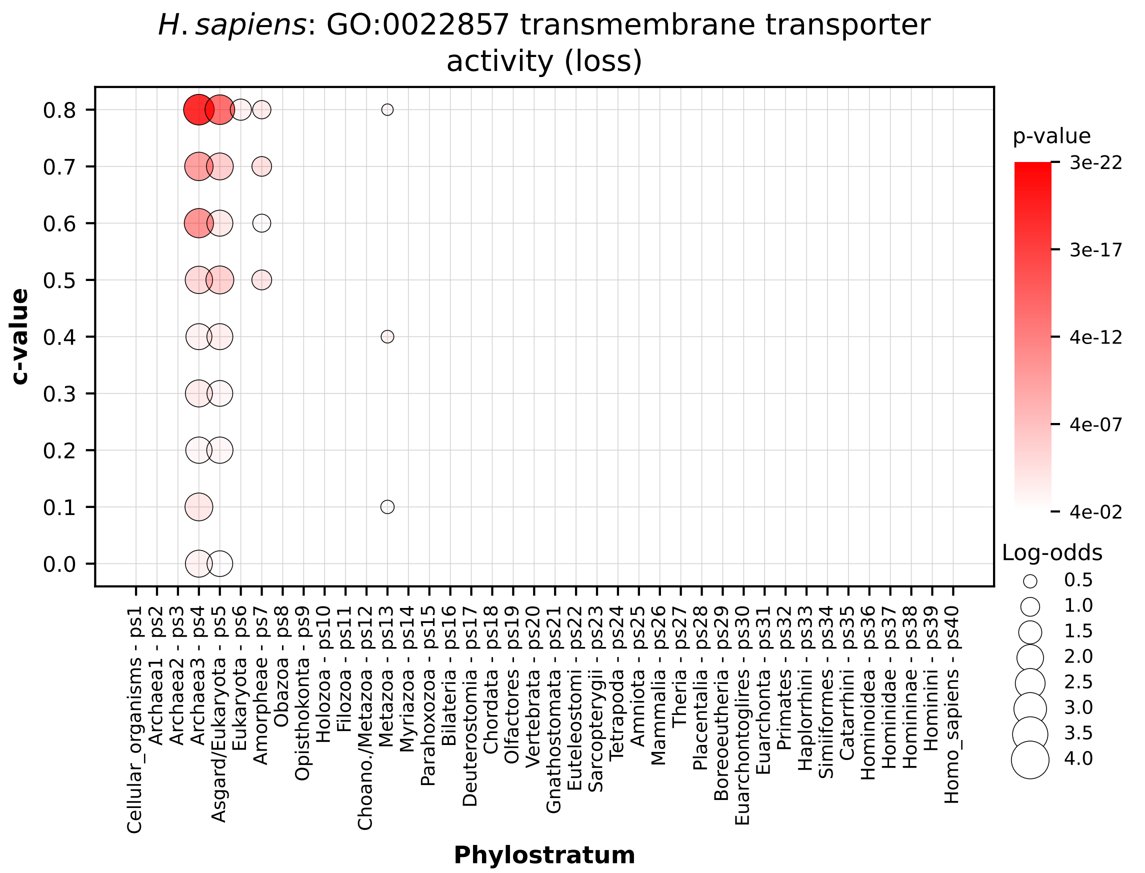
H. sapiens: GO:0022857 transmembrane transporter activity (loss)
c-value
0.8
0.7
0.6
0.5
0.4
0.3
0.2
0.1
0.0
p-value
3e-22
3e-17
4e-12
4e-07
4e-02
Log-odds
0.5
1.0
1.5
2.0
2.5
3.0
3.5
4.0
Cellular_organisms - ps1
Archaea1 - ps2
Archaea2 - ps3
Archaea3 - ps4
Asgard/Eukaryota - ps5
Eukaryota - ps6
Amorpheae - ps7
Obazoa - ps8
Opisthokonta - ps9
Holozoa - ps10
Filozoa - ps11
Choano./Metazoa - ps12
Metazoa - ps13
Myriazoa - ps14
Parahoxozoa - ps15
Bilateria - ps16
Deuterostomia - ps17
Chordata - ps18
Olfactores - ps19
Vertebrata - ps20
Gnathostomata - ps21
Euteleostomi - ps22
Sarcopterygii - ps23
Tetrapoda - ps24
Amniota - ps25
Mammalia - ps26
Theria - ps27
Placentalia - ps28
Boreoeutheria - ps29
Euarchontoglires - ps30
Euarchonta - ps31
Primates - ps32
Haplorrhini - ps33
Simiiformes - ps34
Catarrhini - ps35
Hominoidea - ps36
Hominidae - ps37
Homininae - ps38
Hominini - ps39
Homo_sapiens - ps40
Phylostratum

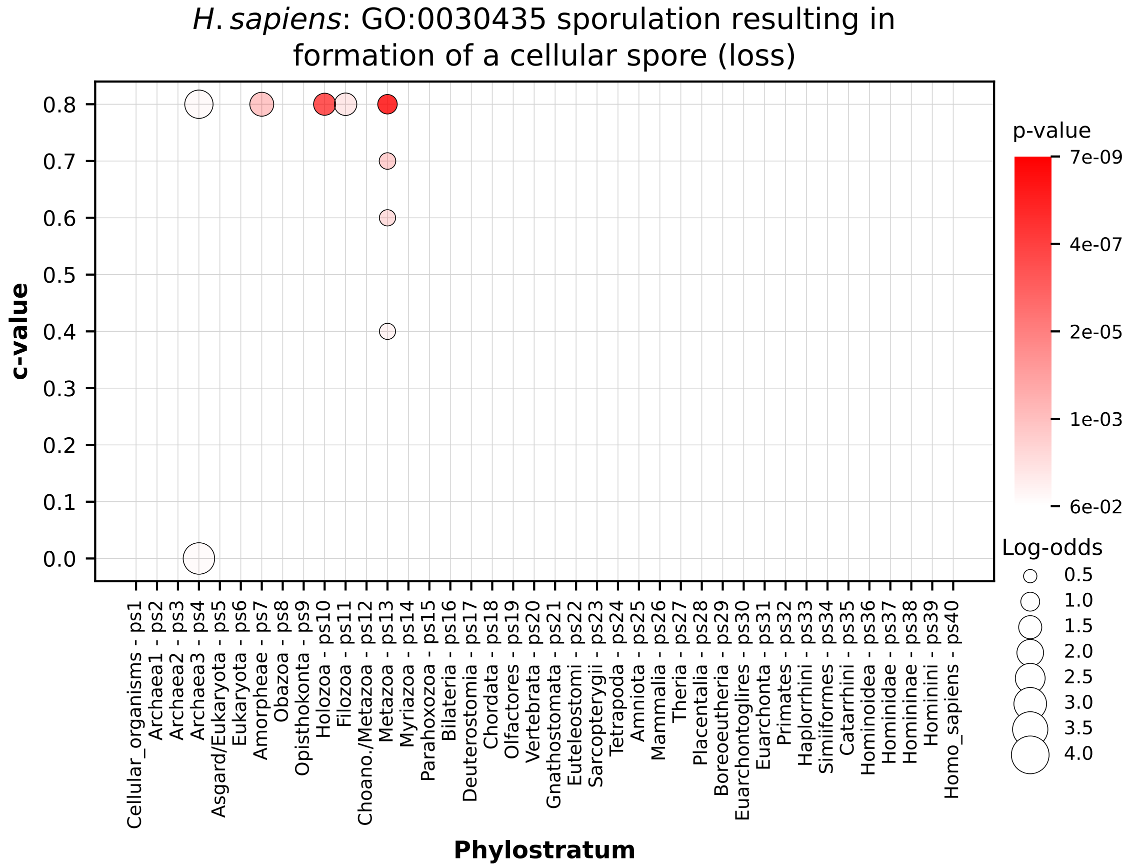

H. sapiens: GO:0030435 sporulation resulting in formation of a cellular spore (loss)
c-value
0.0
0.1
0.2
0.3
0.4
0.5
0.6
0.7
0.8
Phylostratum
Cellular_organisms - ps1
Archaea1 - ps2
Archaea2 - ps3
Archaea3 - ps4
Asgard/Eukaryota - ps5
Eukaryota - ps6
Amorpheae - ps7
Obazoa - ps8
Opisthokonta - ps9
Holozoa - ps10
Filozoa - ps11
Choano./Metazoa - ps12
Metazoa - ps13
Myriazoa - ps14
Parahoxozoa - ps15
Bilateria - ps16
Deuterostomia - ps17
Chordata - ps18
Olfactores - ps19
Vertebrata - ps20
Gnathostomata - ps21
Euteleostomi - ps22
Sarcopterygii - ps23
Tetrapoda - ps24
Amniota - ps25
Mammalia - ps26
Theria - ps27
Placentalia - ps28
Boreoeutheria - ps29
Euarchontoglires - ps30
Euarchonta - ps31
Primates - ps32
Haplorrhini - ps33
Simiiformes - ps34
Catarrhini - ps35
Hominoidea - ps36
Hominidae - ps37
Homininae - ps38
Hominini - ps39
Homo_sapiens - ps40
p-value
7e-09
4e-07
2e-05
1e-03
6e-02
Log-odds
0.5
1.0
1.5
2.0
2.5
3.0
3.5
4.0

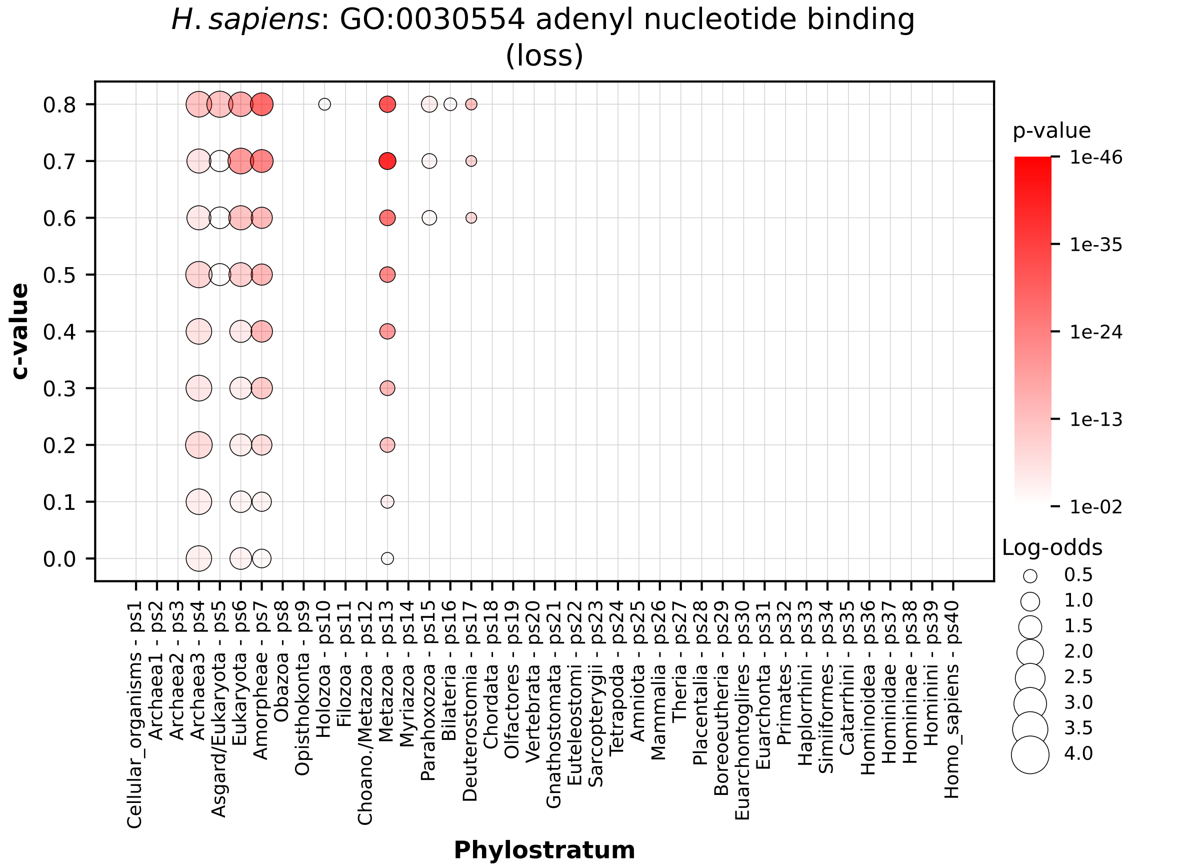

H. sapiens: GO:0030554 adenyl nucleotide binding
(loss)
c-value
0.0
0.1
0.2
0.3
0.4
0.5
0.6
0.7
0.8
p-value
1e-46
1e-35
1e-24
1e-13
1e-02
Log-odds
0.5
1.0
1.5
2.0
2.5
3.0
3.5
4.0
Cellular_organisms - ps1
Archaea1 - ps2
Archaea2 - ps3
Archaea3 - ps4
Asgard/Eukaryota - ps5
Eukaryota - ps6
Amorpheae - ps7
Obazoa - ps8
Opisthokonta - ps9
Holozoa - ps10
Filozoa - ps11
Choano./Metazoa - ps12
Metazoa - ps13
Myriazoa - ps14
Parahoxozoa - ps15
Bilateria - ps16
Deuterostomia - ps17
Chordata - ps18
Olfactores - ps19
Vertebrata - ps20
Gnathostomata - ps21
Euteleostomi - ps22
Sarcopterygii - ps23
Tetrapoda - ps24
Amniota - ps25
Mammalia - ps26
Theria - ps27
Placentalia - ps28
Boreoeutheria - ps29
Euarchontoglires - ps30
Euarchonta - ps31
Primates - ps32
Haplorrhini - ps33
Simiiformes - ps34
Catarrhini - ps35
Hominoidea - ps36
Hominidae - ps37
Homininae - ps38
Hominini - ps39
Homo_sapiens - ps40
Phylostratum

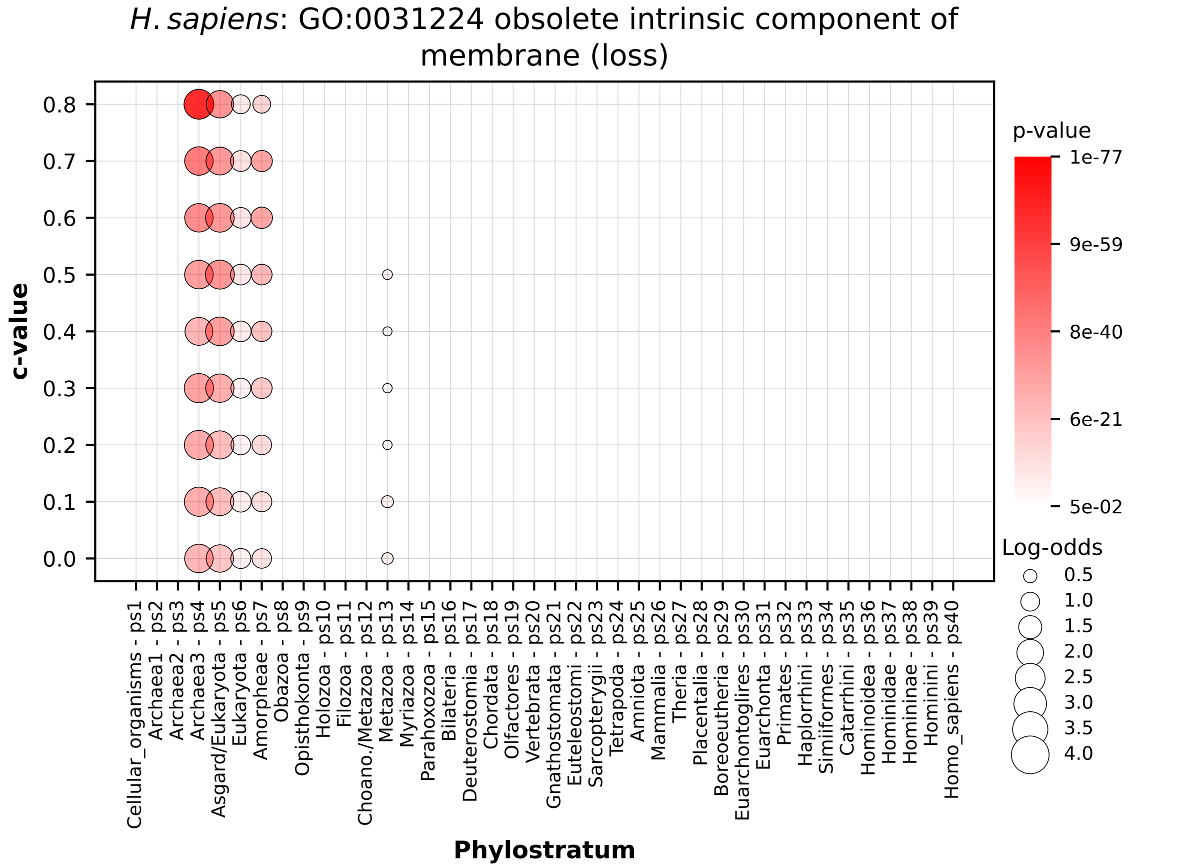

H. sapiens: GO:0031224 obsolete intrinsic component of membrane (loss)
c-value
0.8
0.7
0.6
0.5
0.4
0.3
0.2
0.1
0.0
p-value
1e-77
9e-59
8e-40
6e-21
5e-02
Log-odds
0.5
1.0
1.5
2.0
2.5
3.0
3.5
4.0
Cellular_organisms - ps1
Archaea1 - ps2
Archaea2 - ps3
Archaea3 - ps4
Asgard/Eukaryota - ps5
Eukaryota - ps6
Amorpheae - ps7
Obazoa - ps8
Opisthokonta - ps9
Holozoa - ps10
Filozoa - ps11
Choano./Metazoa - ps12
Metazoa - ps13
Myriazoa - ps14
Parahoxozoa - ps15
Bilateria - ps16
Deuterostomia - ps17
Chordata - ps18
Olfactores - ps19
Vertebrata - ps20
Gnathostomata - ps21
Euteleostomi - ps22
Sarcopterygii - ps23
Tetrapoda - ps24
Amniota - ps25
Mammalia - ps26
Theria - ps27
Placentalia - ps28
Boreoeutheria - ps29
Euarchontoglires - ps30
Euarchonta - ps31
Primates - ps32
Haplorrhini - ps33
Simiiformes - ps34
Catarrhini - ps35
Hominoidea - ps36
Hominidae - ps37
Homininae - ps38
Hominini - ps39
Homo_sapiens - ps40
Phylostratum

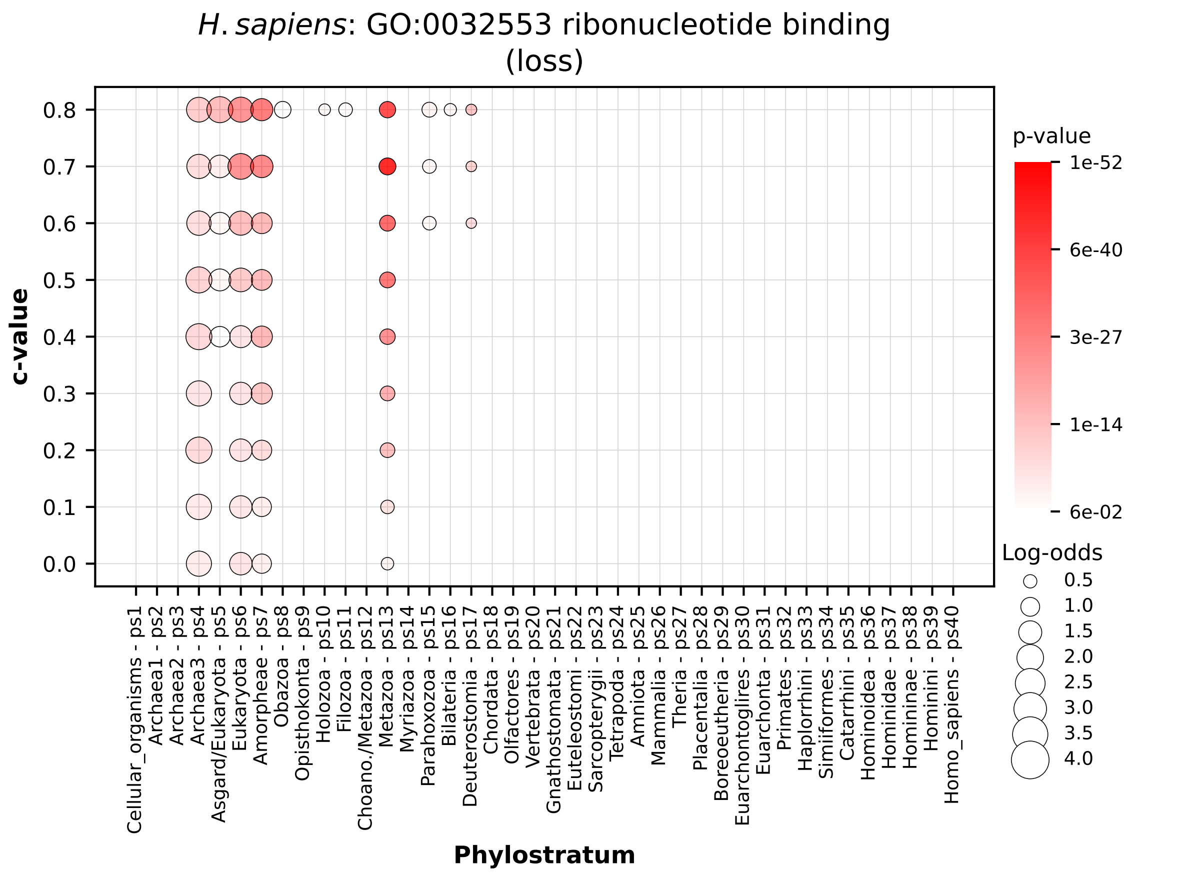

H. sapiens: GO:0032553 ribonucleotide binding
(loss)
c-value
0.8
0.7
0.6
0.5
0.4
0.3
0.2
0.1
0.0
p-value
1e-52
6e-40
3e-27
1e-14
6e-02
Log-odds
0.5
1.0
1.5
2.0
2.5
3.0
3.5
4.0
Cellular_organisms - ps1
Archaea1 - ps2
Archaea2 - ps3
Archaea3 - ps4
Asgard/Eukaryota - ps5
Eukaryota - ps6
Amorpheae - ps7
Obazoa - ps8
Opisthokonta - ps9
Holozoa - ps10
Filozoa - ps11
Choano./Metazoa - ps12
Metazoa - ps13
Myriazoa - ps14
Parahoxozoa - ps15
Bilateria - ps16
Deuterostomia - ps17
Chordata - ps18
Olfactores - ps19
Vertebrata - ps20
Gnathostomata - ps21
Euteleostomi - ps22
Sarcopterygii - ps23
Tetrapoda - ps24
Amniota - ps25
Mammalia - ps26
Theria - ps27
Placentalia - ps28
Boreoeutheria - ps29
Euarchontoglires - ps30
Euarchonta - ps31
Primates - ps32
Haplorrhini - ps33
Simiiformes - ps34
Catarrhini - ps35
Hominoidea - ps36
Hominidae - ps37
Homininae - ps38
Hominini - ps39
Homo_sapiens - ps40
Phylostratum

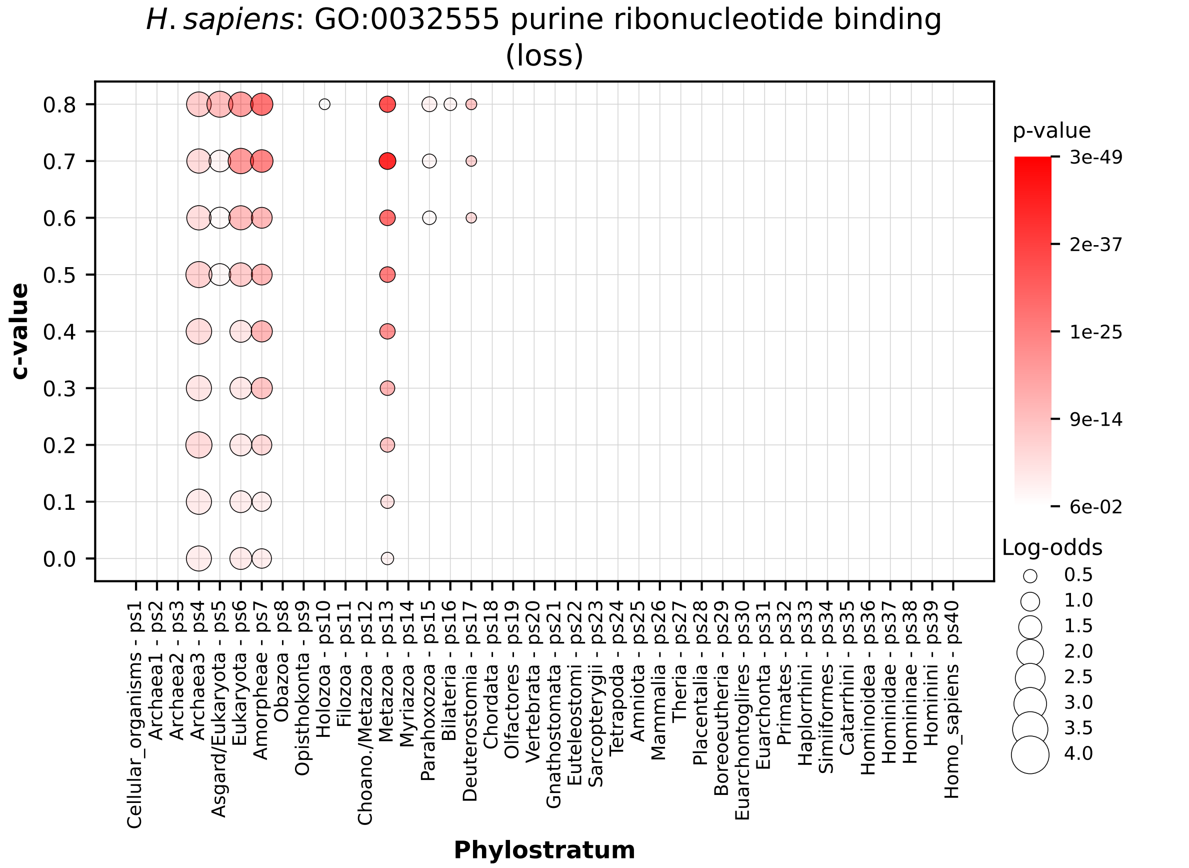

H. sapiens: GO:0032555 purine ribonucleotide binding (loss)
c-value
0.8
0.7
0.6
0.5
0.4
0.3
0.2
0.1
0.0
p-value
3e-49
2e-37
1e-25
9e-14
6e-02
Log-odds
0.5
1.0
1.5
2.0
2.5
3.0
3.5
4.0
Cellular_organisms - ps1
Archaea1 - ps2
Archaea2 - ps3
Archaea3 - ps4
Asgard/Eukaryota - ps5
Eukaryota - ps6
Amorpheae - ps7
Obazoa - ps8
Opisthokonta - ps9
Holozoa - ps10
Filozoa - ps11
Choano./Metazoa - ps12
Metazoa - ps13
Myriazoa - ps14
Parahoxozoa - ps15
Bilateria - ps16
Deuterostomia - ps17
Chordata - ps18
Olfactores - ps19
Vertebrata - ps20
Gnathostomata - ps21
Euteleostomi - ps22
Sarcopterygii - ps23
Tetrapoda - ps24
Amniota - ps25
Mammalia - ps26
Theria - ps27
Placentalia - ps28
Boreoeutheria - ps29
Euarchontoglires - ps30
Euarchonta - ps31
Primates - ps32
Haplorrhini - ps33
Simiiformes - ps34
Catarrhini - ps35
Hominoidea - ps36
Hominidae - ps37
Homininae - ps38
Hominini - ps39
Homo_sapiens - ps40
Phylostratum

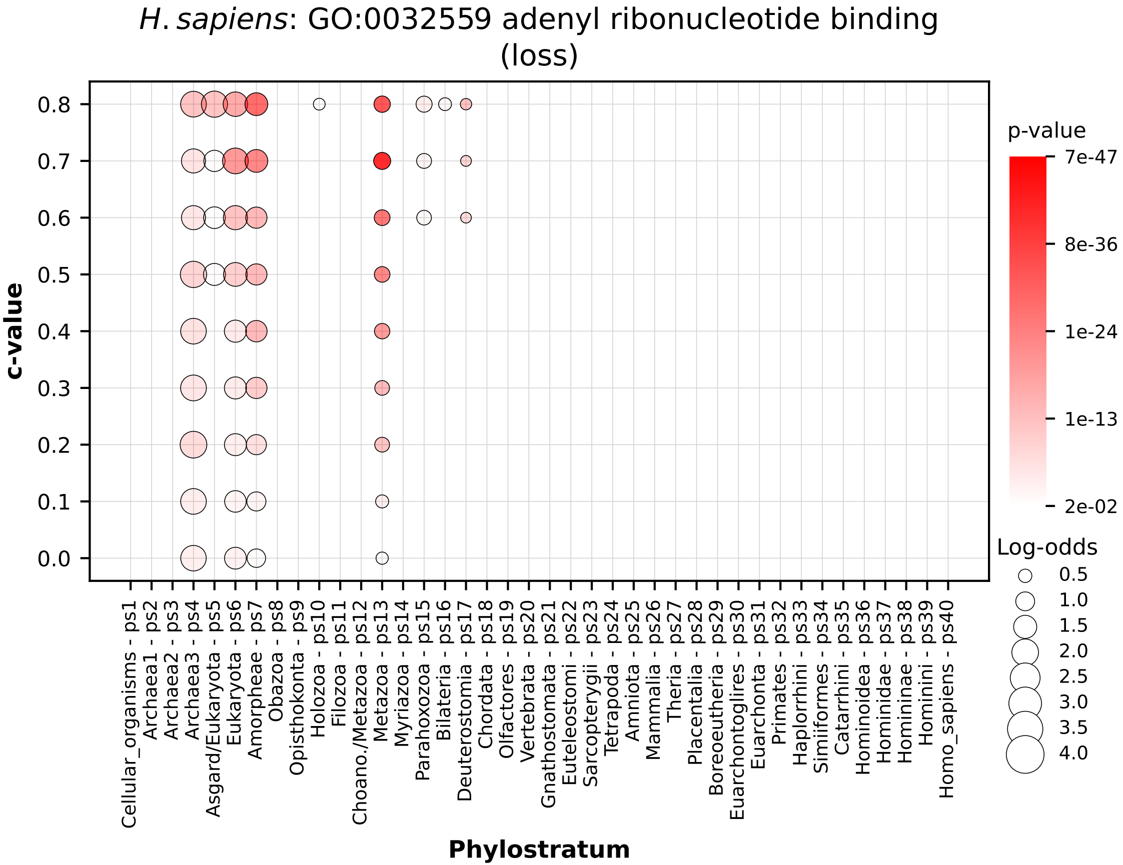

H. sapiens: GO:0032559 adenyl ribonucleotide binding
(loss)
c-value
0.8
0.7
0.6
0.5
0.4
0.3
0.2
0.1
0.0
Phylostratum
Cellular_organisms - ps1
Archaea1 - ps2
Archaea2 - ps3
Archaea3 - ps4
Asgard/Eukaryota - ps5
Eukaryota - ps6
Amorpheae - ps7
Obazoa - ps8
Opisthokonta - ps9
Holozoa - ps10
Filozoa - ps11
Choano./Metazoa - ps12
Metazoa - ps13
Myriazoa - ps14
Parahoxozoa - ps15
Bilateria - ps16
Deuterostomia - ps17
Chordata - ps18
Olfactores - ps19
Vertebrata - ps20
Gnathostomata - ps21
Euteleostomi - ps22
Sarcopterygii - ps23
Tetrapoda - ps24
Amniota - ps25
Mammalia - ps26
Theria - ps27
Placentalia - ps28
Boreoeutheria - ps29
Euarchontoglires - ps30
Euarchonta - ps31
Primates - ps32
Haplorrhini - ps33
Simiiformes - ps34
Catarrhini - ps35
Hominoidea - ps36
Hominidae - ps37
Homininae - ps38
Hominini - ps39
Homo_sapiens - ps40
p-value
7e-47
8e-36
1e-24
1e-13
2e-02
Log-odds
0.5
1.0
1.5
2.0
2.5
3.0
3.5
4.0

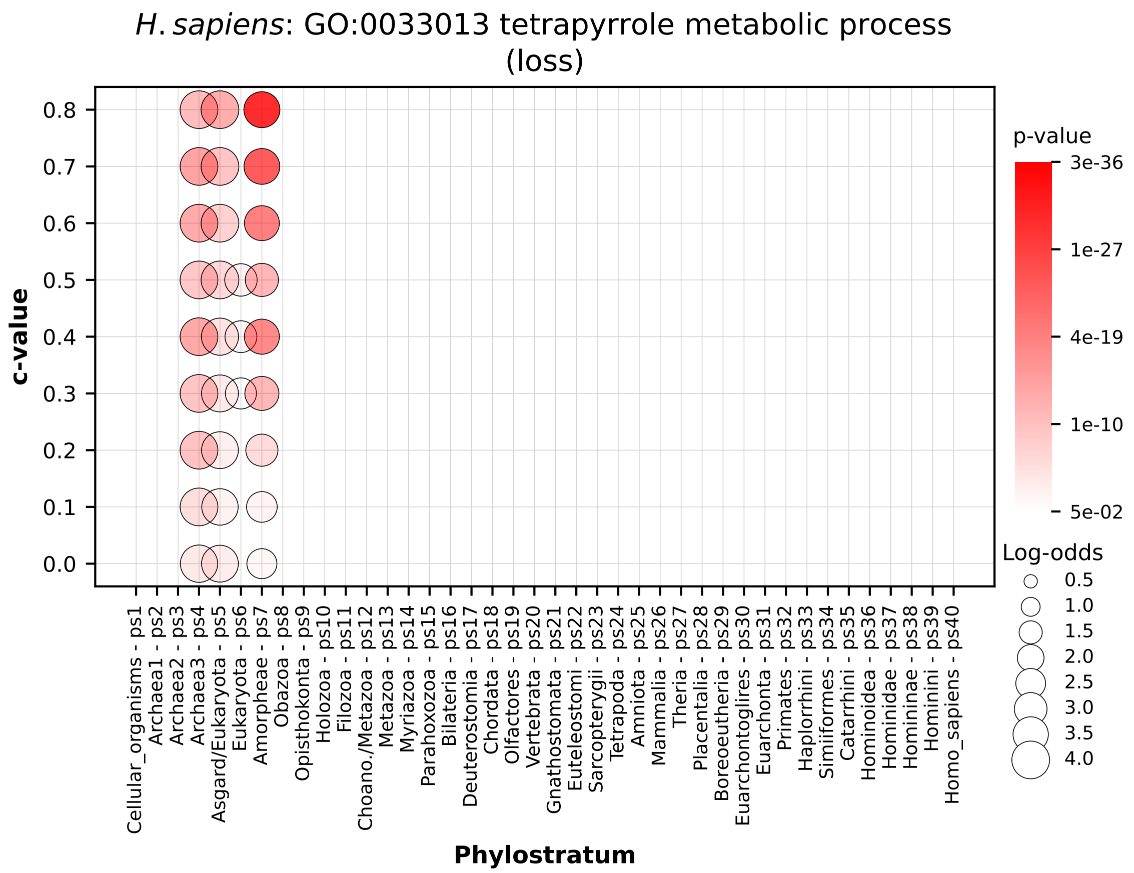

H. sapiens: GO:0033013 tetrapyrrole metabolic process
(loss)
c-value
0.0
0.1
0.2
0.3
0.4
0.5
0.6
0.7
0.8
Phylostratum
Cellular_organisms - ps1
Archaea1 - ps2
Archaea2 - ps3
Archaea3 - ps4
Asgard/Eukaryota - ps5
Eukaryota - ps6
Amorpheae - ps7
Obazoa - ps8
Opisthokonta - ps9
Holozoa - ps10
Filozoa - ps11
Choano./Metazoa - ps12
Metazoa - ps13
Myriazoa - ps14
Parahoxozoa - ps15
Bilateria - ps16
Deuterostomia - ps17
Chordata - ps18
Olfactores - ps19
Vertebrata - ps20
Gnathostomata - ps21
Euteleostomi - ps22
Sarcopterygii - ps23
Tetrapoda - ps24
Amniota - ps25
Mammalia - ps26
Theria - ps27
Placentalia - ps28
Boreoeutheria - ps29
Euarchontoglires - ps30
Euarchonta - ps31
Primates - ps32
Haplorrhini - ps33
Simiiformes - ps34
Catarrhini - ps35
Hominoidea - ps36
Hominidae - ps37
Homininae - ps38
Hominini - ps39
Homo_sapiens - ps40
p-value
3e-36
1e-27
4e-19
1e-10
5e-02
Log-odds
0.5
1.0
1.5
2.0
2.5
3.0
3.5
4.0

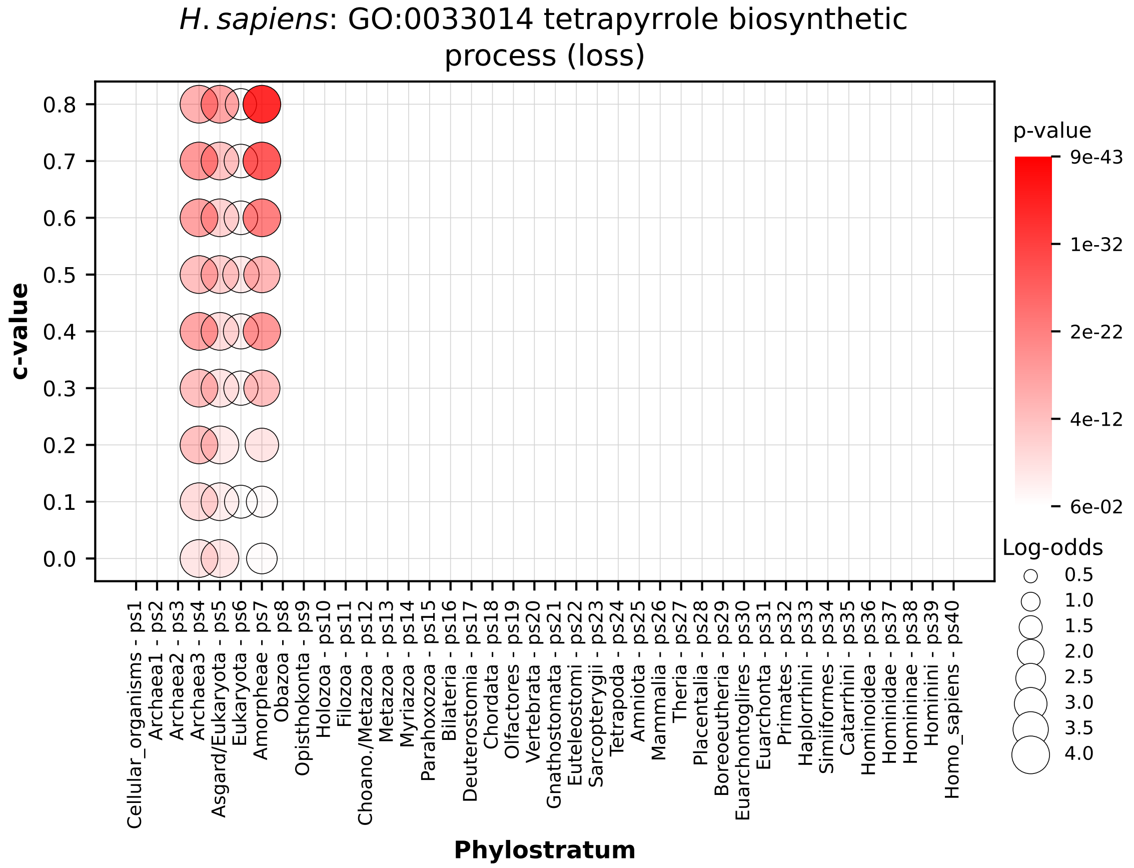

H. sapiens: GO:0033014 tetrapyrrole biosynthetic process (loss)
c-value
0.0
0.1
0.2
0.3
0.4
0.5
0.6
0.7
0.8
p-value
9e-43
1e-32
2e-22
4e-12
6e-02
Log-odds
0.5
1.0
1.5
2.0
2.5
3.0
3.5
4.0
Cellular_organisms - ps1
Archaea1 - ps2
Archaea2 - ps3
Archaea3 - ps4
Asgard/Eukaryota - ps5
Eukaryota - ps6
Amorpheae - ps7
Obazoa - ps8
Opisthokonta - ps9
Holozoa - ps10
Filozoa - ps11
Choano./Metazoa - ps12
Metazoa - ps13
Myriazoa - ps14
Parahoxozoa - ps15
Bilateria - ps16
Deuterostomia - ps17
Chordata - ps18
Olfactores - ps19
Vertebrata - ps20
Gnathostomata - ps21
Euteleostomi - ps22
Sarcopterygii - ps23
Tetrapoda - ps24
Amniota - ps25
Mammalia - ps26
Theria - ps27
Placentalia - ps28
Boreoeutheria - ps29
Euarchontoglires - ps30
Euarchonta - ps31
Primates - ps32
Haplorrhini - ps33
Simiiformes - ps34
Catarrhini - ps35
Hominoidea - ps36
Hominidae - ps37
Homininae - ps38
Hominini - ps39
Homo_sapiens - ps40
Phylostratum

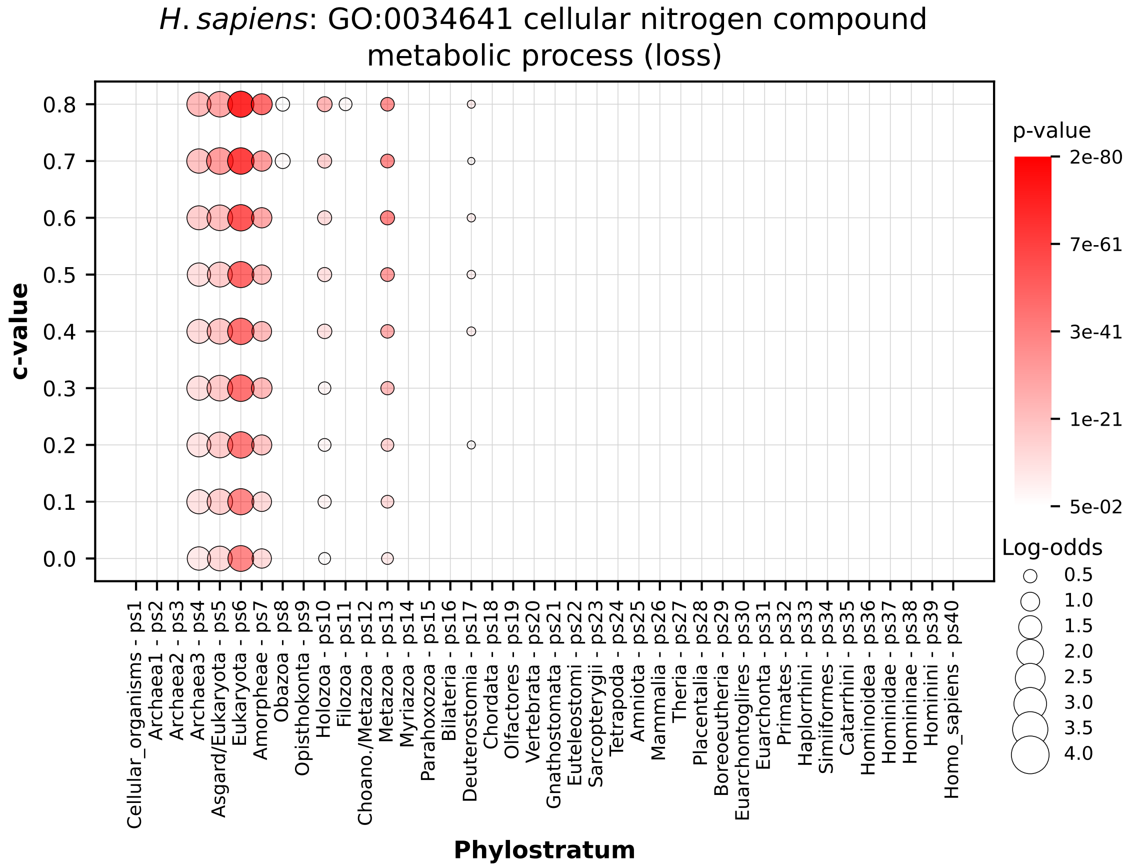

H. sapiens: GO:0034641 cellular nitrogen compound metabolic process (loss)
c-value
0.0
0.1
0.2
0.3
0.4
0.5
0.6
0.7
0.8
Phylostratum
Cellular_organisms - ps1
Archaea1 - ps2
Archaea2 - ps3
Archaea3 - ps4
Asgard/Eukaryota - ps5
Eukaryota - ps6
Amorpheae - ps7
Obazoa - ps8
Opisthokonta - ps9
Holozoa - ps10
Filozoa - ps11
Choano./Metazoa - ps12
Metazoa - ps13
Myriazoa - ps14
Parahoxozoa - ps15
Bilateria - ps16
Deuterostomia - ps17
Chordata - ps18
Olfactores - ps19
Vertebrata - ps20
Gnathostomata - ps21
Euteleostomi - ps22
Sarcopterygii - ps23
Tetrapoda - ps24
Amniota - ps25
Mammalia - ps26
Theria - ps27
Placentalia - ps28
Boreoeutheria - ps29
Euarchontoglires - ps30
Euarchonta - ps31
Primates - ps32
Haplorrhini - ps33
Simiiformes - ps34
Catarrhini - ps35
Hominoidea - ps36
Hominidae - ps37
Homininae - ps38
Hominini - ps39
Homo_sapiens - ps40
p-value
2e-80
7e-61
3e-41
1e-21
5e-02
Log-odds
0.5
1.0
1.5
2.0
2.5
3.0
3.5
4.0

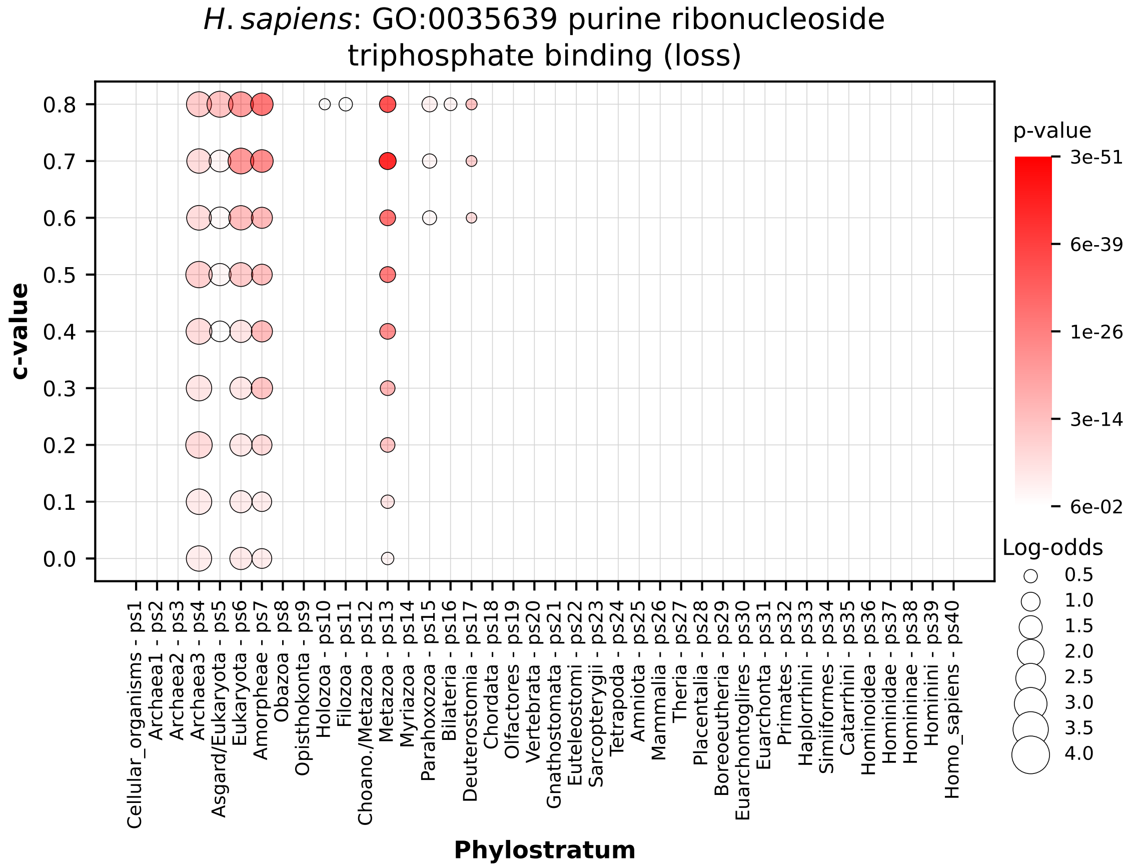

H. sapiens: GO:0035639 purine ribonucleoside triphosphate binding (loss)
c-value
0.8
0.7
0.6
0.5
0.4
0.3
0.2
0.1
0.0
Phylostratum
Cellular_organisms - ps1
Archaea1 - ps2
Archaea2 - ps3
Archaea3 - ps4
Asgard/Eukaryota - ps5
Eukaryota - ps6
Amorpheae - ps7
Obazoa - ps8
Opisthokonta - ps9
Holozoa - ps10
Filozoa - ps11
Choano./Metazoa - ps12
Metazoa - ps13
Myriazoa - ps14
Parahoxozoa - ps15
Bilateria - ps16
Deuterostomia - ps17
Chordata - ps18
Olfactores - ps19
Vertebrata - ps20
Gnathostomata - ps21
Euteleostomi - ps22
Sarcopterygii - ps23
Tetrapoda - ps24
Amniota - ps25
Mammalia - ps26
Theria - ps27
Placentalia - ps28
Boreoeutheria - ps29
Euarchontoglires - ps30
Euarchonta - ps31
Primates - ps32
Haplorrhini - ps33
Simiiformes - ps34
Catarrhini - ps35
Hominoidea - ps36
Hominidae - ps37
Homininae - ps38
Hominini - ps39
Homo_sapiens - ps40
p-value
3e-51
6e-39
1e-26
3e-14
6e-02
Log-odds
0.5
1.0
1.5
2.0
2.5
3.0
3.5
4.0

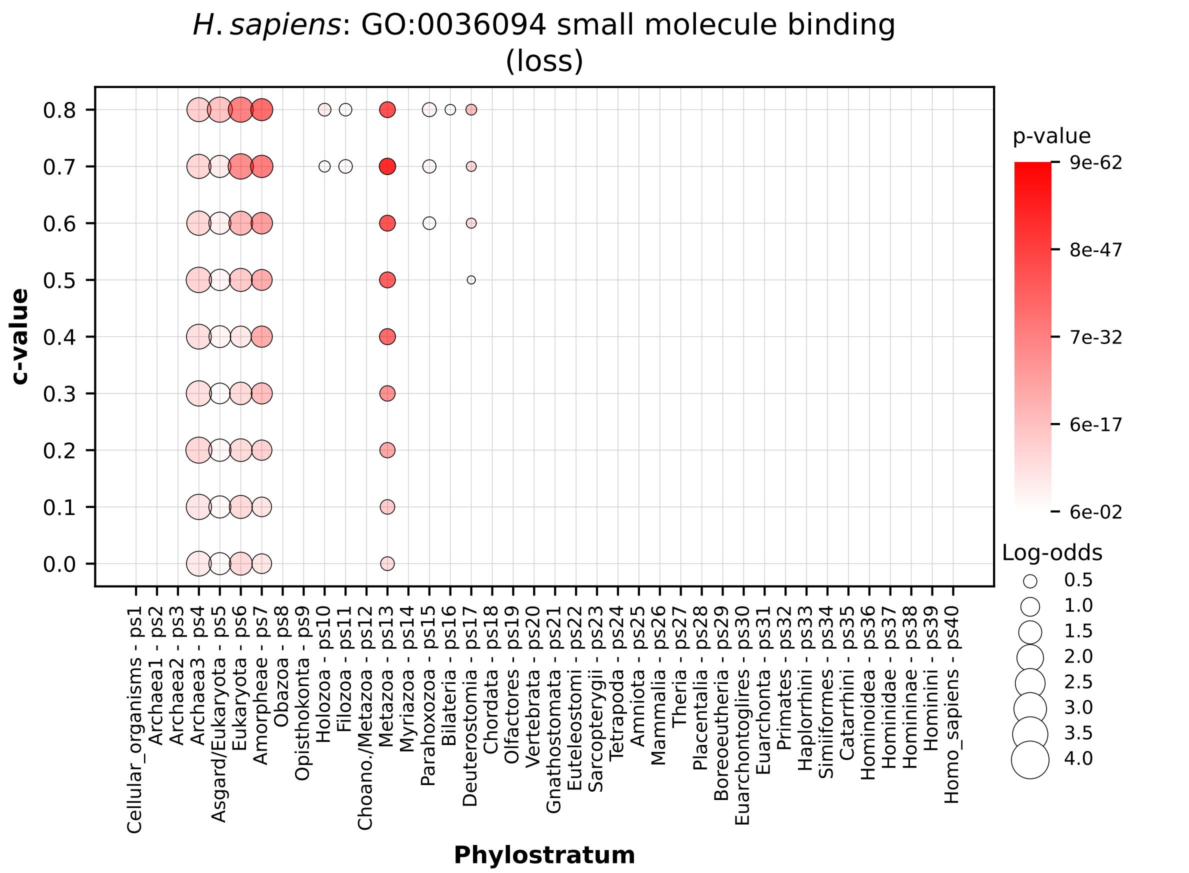

H. sapiens: GO:0036094 small molecule binding
(loss)
c-value
0.0
0.1
0.2
0.3
0.4
0.5
0.6
0.7
0.8
Phylostratum
Cellular_organisms - ps1
Archaea1 - ps2
Archaea2 - ps3
Archaea3 - ps4
Asgard/Eukaryota - ps5
Eukaryota - ps6
Amorpheae - ps7
Obazoa - ps8
Opisthokonta - ps9
Holozoa - ps10
Filozoa - ps11
Choano./Metazoa - ps12
Metazoa - ps13
Myriazoa - ps14
Parahoxozoa - ps15
Bilateria - ps16
Deuterostomia - ps17
Chordata - ps18
Olfactores - ps19
Vertebrata - ps20
Gnathostomata - ps21
Euteleostomi - ps22
Sarcopterygii - ps23
Tetrapoda - ps24
Amniota - ps25
Mammalia - ps26
Theria - ps27
Placentalia - ps28
Boreoeutheria - ps29
Euarchontoglires - ps30
Euarchonta - ps31
Primates - ps32
Haplorrhini - ps33
Simiiformes - ps34
Catarrhini - ps35
Hominoidea - ps36
Hominidae - ps37
Homininae - ps38
Hominini - ps39
Homo_sapiens - ps40
p-value
9e-62
8e-47
7e-32
6e-17
6e-02
Log-odds
0.5
1.0
1.5
2.0
2.5
3.0
3.5
4.0

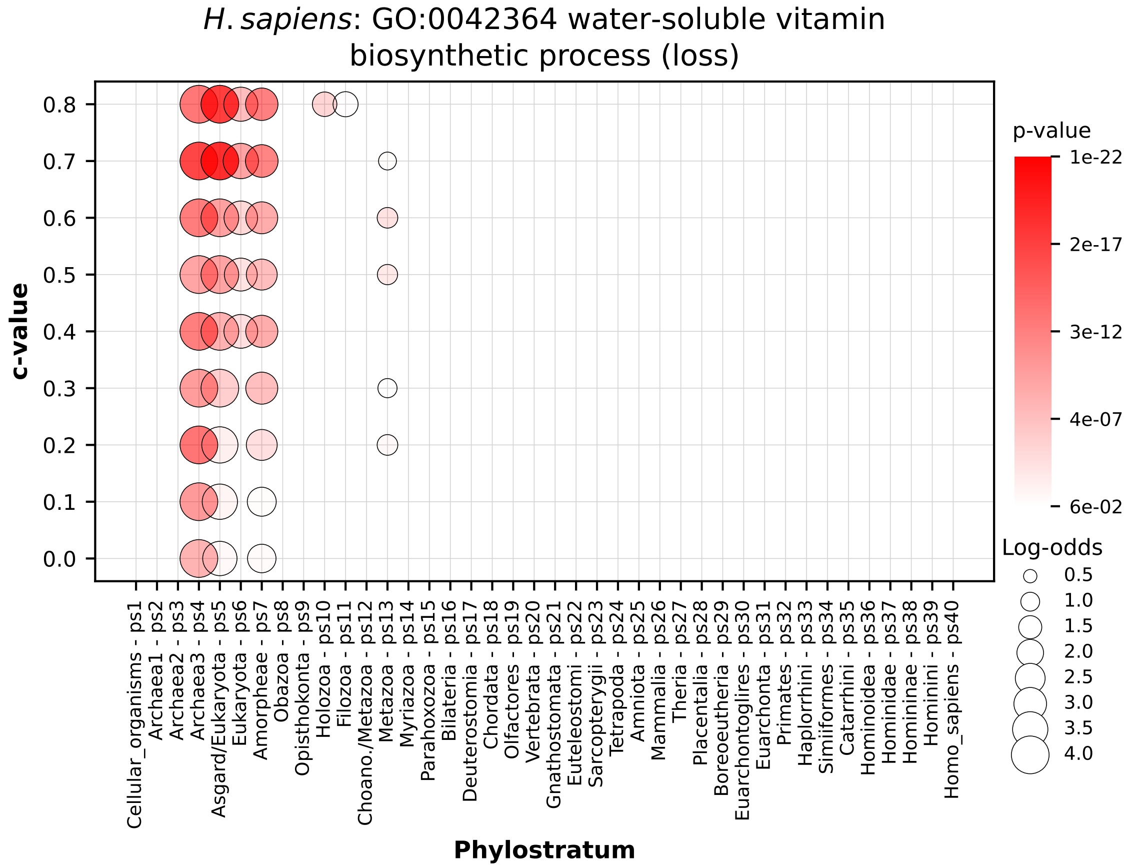

H. sapiens: GO:0042364 water-soluble vitamin biosynthetic process (loss)
c-value
0.8
0.7
0.6
0.5
0.4
0.3
0.2
0.1
0.0
p-value
1e-22
2e-17
3e-12
4e-07
6e-02
Log-odds
0.5
1.0
1.5
2.0
2.5
3.0
3.5
4.0
Cellular_organisms - ps1
Archaea1 - ps2
Archaea2 - ps3
Archaea3 - ps4
Asgard/Eukaryota - ps5
Eukaryota - ps6
Amorpheae - ps7
Obazoa - ps8
Opisthokonta - ps9
Holozoa - ps10
Filozoa - ps11
Choano./Metazoa - ps12
Metazoa - ps13
Myriazoa - ps14
Parahoxozoa - ps15
Bilateria - ps16
Deuterostomia - ps17
Chordata - ps18
Olfactores - ps19
Vertebrata - ps20
Gnathostomata - ps21
Euteleostomi - ps22
Sarcopterygii - ps23
Tetrapoda - ps24
Amniota - ps25
Mammalia - ps26
Theria - ps27
Placentalia - ps28
Boreoeutheria - ps29
Euarchontoglires - ps30
Euarchonta - ps31
Primates - ps32
Haplorrhini - ps33
Simiiformes - ps34
Catarrhini - ps35
Hominoidea - ps36
Hominidae - ps37
Homininae - ps38
Hominini - ps39
Homo_sapiens - ps40
Phylostratum

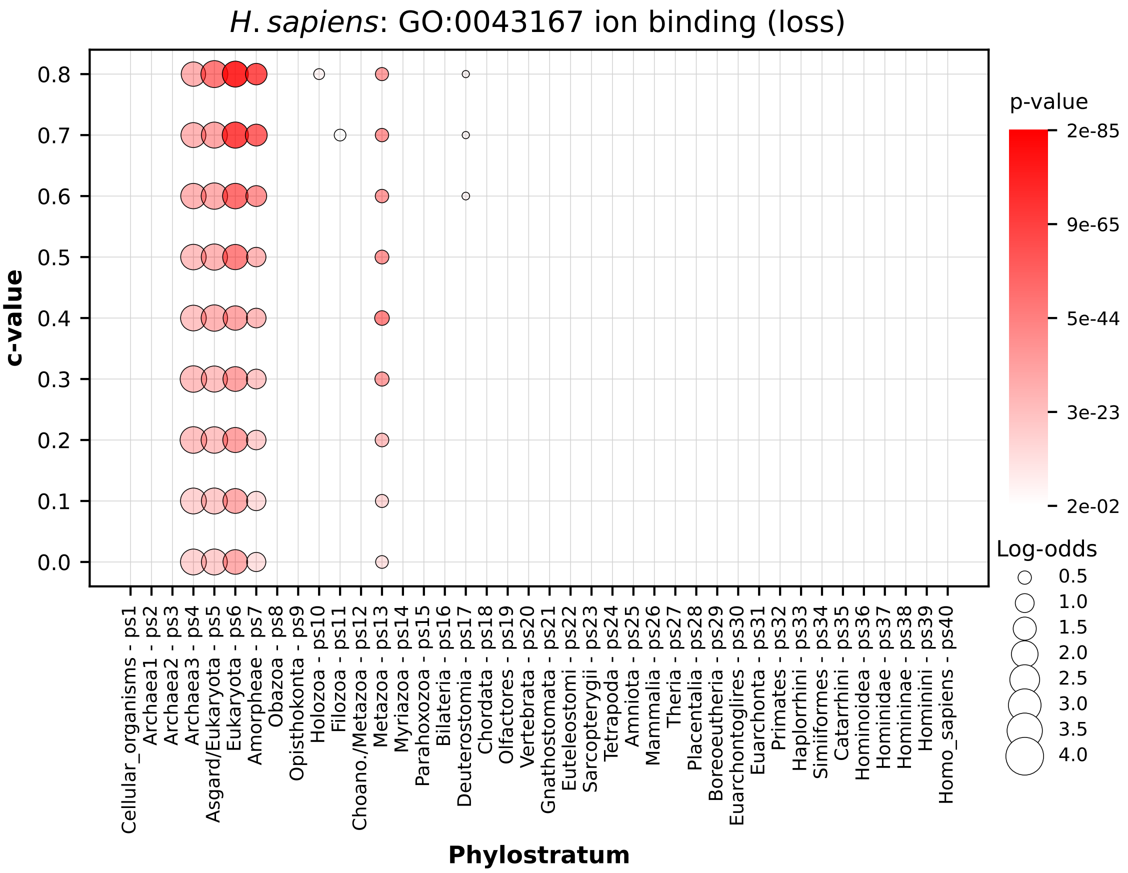
H. sapiens: GO:0043167 ion binding (loss)
c-value
0.8
0.7
0.6
0.5
0.4
0.3
0.2
0.1
0.0
Phylostratum
Cellular_organisms - ps1
Archaea1 - ps2
Archaea2 - ps3
Archaea3 - ps4
Asgard/Eukaryota - ps5
Eukaryota - ps6
Amorpheae - ps7
Obazoa - ps8
Opisthokonta - ps9
Holozoa - ps10
Filozoa - ps11
Choano./Metazoa - ps12
Metazoa - ps13
Myriazoa - ps14
Parahoxozoa - ps15
Bilateria - ps16
Deuterostomia - ps17
Chordata - ps18
Olfactores - ps19
Vertebrata - ps20
Gnathostomata - ps21
Euteleostomi - ps22
Sarcopterygii - ps23
Tetrapoda - ps24
Amniota - ps25
Mammalia - ps26
Theria - ps27
Placentalia - ps28
Boreoeutheria - ps29
Euarchontoglires - ps30
Euarchonta - ps31
Primates - ps32
Haplorrhini - ps33
Simiiformes - ps34
Catarrhini - ps35
Hominoidea - ps36
Hominidae - ps37
Homininae - ps38
Hominini - ps39
Homo_sapiens - ps40
p-value
2e-85
9e-65
5e-44
3e-23
2e-02
Log-odds
0.5
1.0
1.5
2.0
2.5
3.0
3.5
4.0

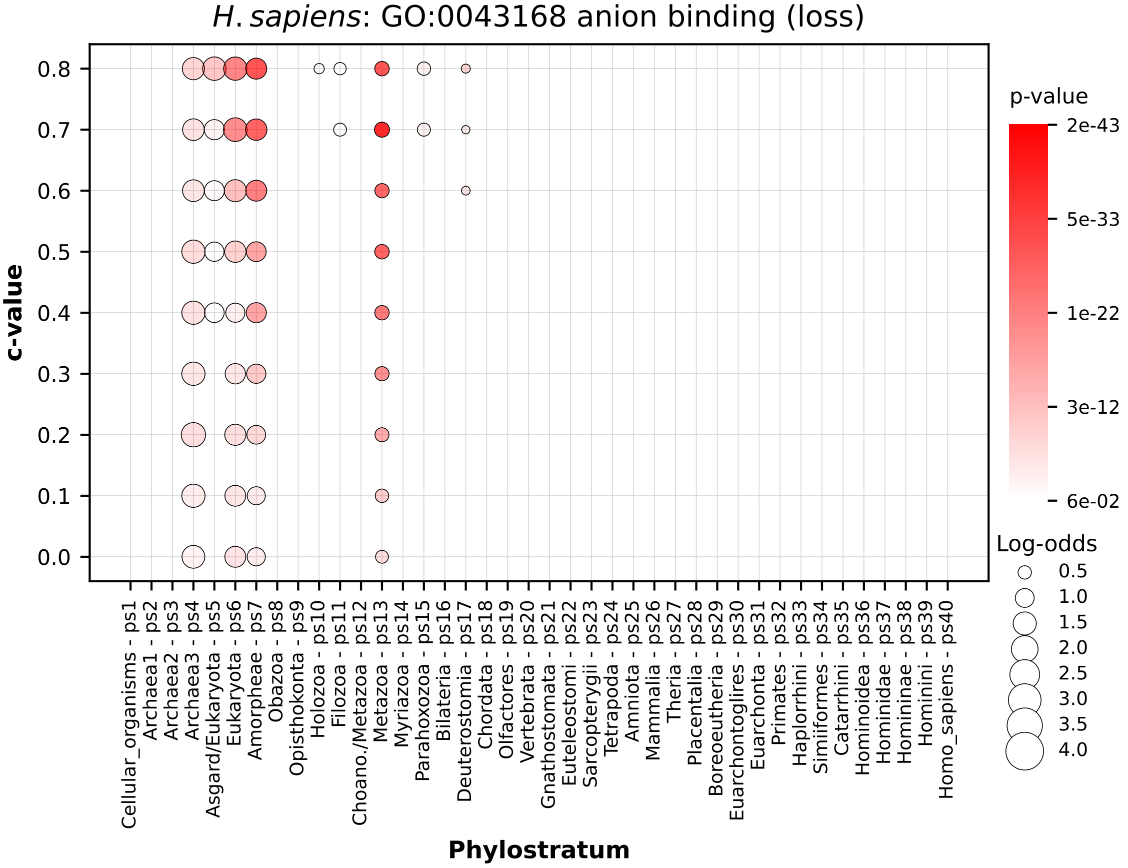

H. sapiens: GO:0043168 anion binding (loss)
c-value
0.8
0.7
0.6
0.5
0.4
0.3
0.2
0.1
0.0
Phylostratum
Cellular_organisms - ps1
Archaea1 - ps2
Archaea2 - ps3
Archaea3 - ps4
Asgard/Eukaryota - ps5
Eukaryota - ps6
Amorpheae - ps7
Obazoa - ps8
Opisthokonta - ps9
Holozoa - ps10
Filozoa - ps11
Choano./Metazoa - ps12
Metazoa - ps13
Myriazoa - ps14
Parahoxozoa - ps15
Bilateria - ps16
Deuterostomia - ps17
Chordata - ps18
Olfactores - ps19
Vertebrata - ps20
Gnathostomata - ps21
Euteleostomi - ps22
Sarcopterygii - ps23
Tetrapoda - ps24
Amniota - ps25
Mammalia - ps26
Theria - ps27
Placentalia - ps28
Boreoeutheria - ps29
Euarchontoglires - ps30
Euarchonta - ps31
Primates - ps32
Haplorrhini - ps33
Simiiformes - ps34
Catarrhini - ps35
Hominoidea - ps36
Hominidae - ps37
Homininae - ps38
Hominini - ps39
Homo_sapiens - ps40
p-value
2e-43
5e-33
1e-22
3e-12
6e-02
Log-odds
0.5
1.0
1.5
2.0
2.5
3.0
3.5
4.0

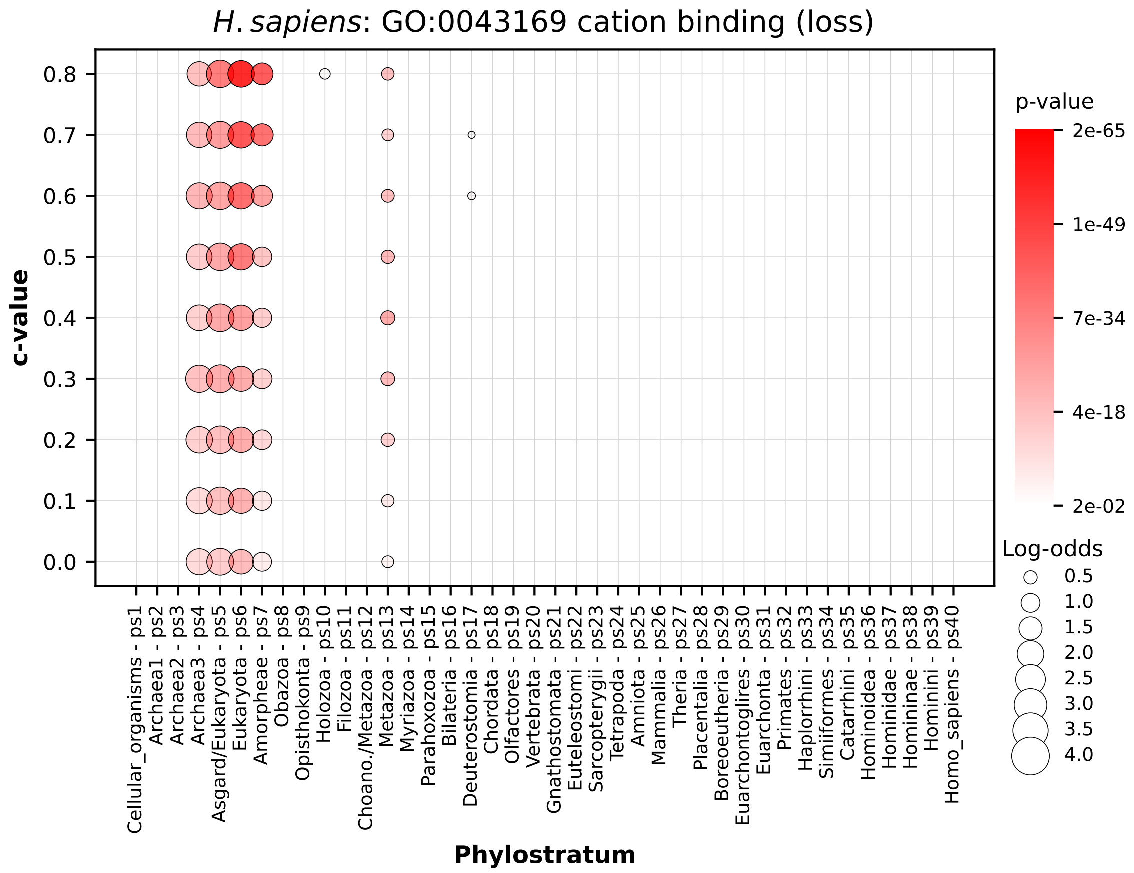

H. sapiens: GO:0043169 cation binding (loss)
c-value
0.8
0.7
0.6
0.5
0.4
0.3
0.2
0.1
0.0
Phylostratum
Cellular_organisms - ps1
Archaea1 - ps2
Archaea2 - ps3
Archaea3 - ps4
Asgard/Eukaryota - ps5
Eukaryota - ps6
Amorpheae - ps7
Obazoa - ps8
Opisthokonta - ps9
Holozoa - ps10
Filozoa - ps11
Choano./Metazoa - ps12
Metazoa - ps13
Myriazoa - ps14
Parahoxozoa - ps15
Bilateria - ps16
Deuterostomia - ps17
Chordata - ps18
Olfactores - ps19
Vertebrata - ps20
Gnathostomata - ps21
Euteleostomi - ps22
Sarcopterygii - ps23
Tetrapoda - ps24
Amniota - ps25
Mammalia - ps26
Theria - ps27
Placentalia - ps28
Boreoeutheria - ps29
Euarchontoglires - ps30
Euarchonta - ps31
Primates - ps32
Haplorrhini - ps33
Simiiformes - ps34
Catarrhini - ps35
Hominoidea - ps36
Hominidae - ps37
Homininae - ps38
Hominini - ps39
Homo_sapiens - ps40
p-value
2e-65
1e-49
7e-34
4e-18
2e-02
Log-odds
0.5
1.0
1.5
2.0
2.5
3.0
3.5
4.0

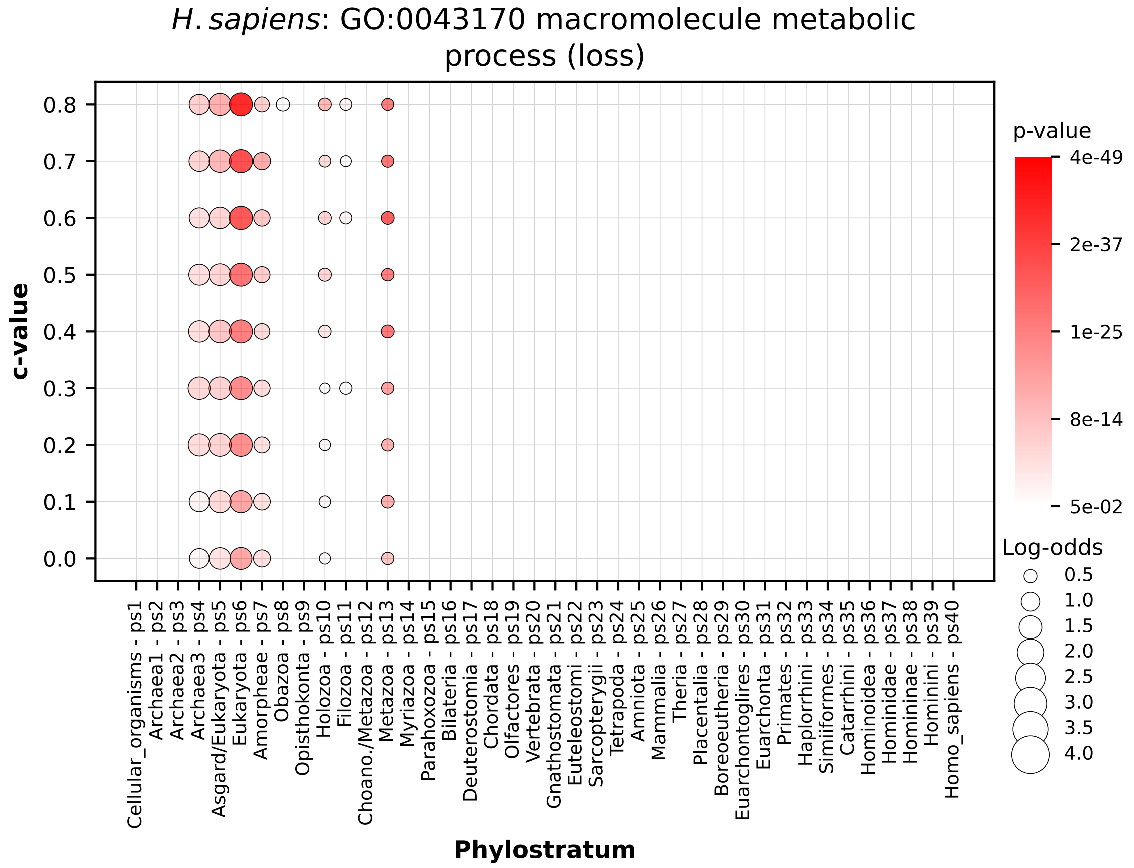

H. sapiens: GO:0043170 macromolecule metabolic process (loss)
c-value
0.8
0.7
0.6
0.5
0.4
0.3
0.2
0.1
0.0
Phylostratum
Cellular_organisms - ps1
Archaea1 - ps2
Archaea2 - ps3
Archaea3 - ps4
Asgard/Eukaryota - ps5
Eukaryota - ps6
Amorpheae - ps7
Obazoa - ps8
Opisthokonta - ps9
Holozoa - ps10
Filozoa - ps11
Choano./Metazoa - ps12
Metazoa - ps13
Myriazoa - ps14
Parahoxozoa - ps15
Bilateria - ps16
Deuterostomia - ps17
Chordata - ps18
Olfactores - ps19
Vertebrata - ps20
Gnathostomata - ps21
Euteleostomi - ps22
Sarcopterygii - ps23
Tetrapoda - ps24
Amniota - ps25
Mammalia - ps26
Theria - ps27
Placentalia - ps28
Boreoeutheria - ps29
Euarchontoglires - ps30
Euarchonta - ps31
Primates - ps32
Haplorrhini - ps33
Simiiformes - ps34
Catarrhini - ps35
Hominoidea - ps36
Hominidae - ps37
Homininae - ps38
Hominini - ps39
Homo_sapiens - ps40
p-value
4e-49
2e-37
1e-25
8e-14
5e-02
Log-odds
0.5
1.0
1.5
2.0
2.5
3.0
3.5
4.0

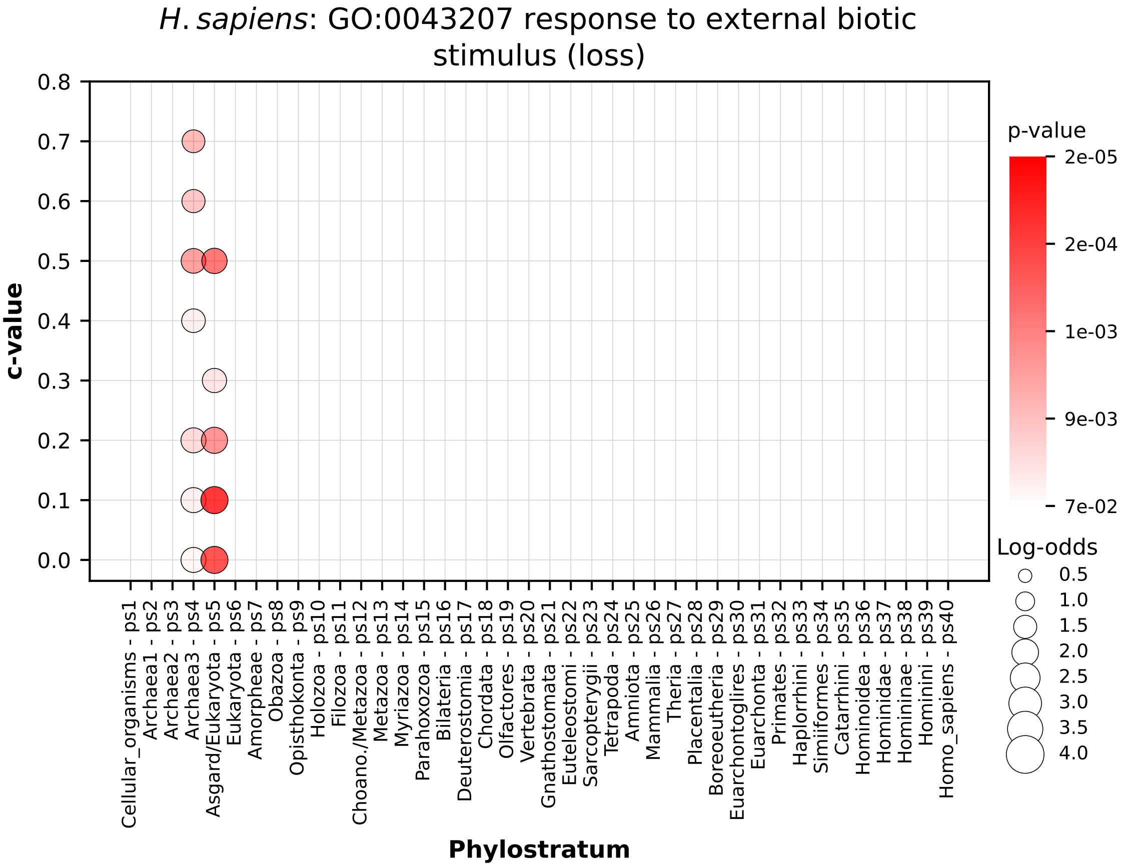

H. sapiens: GO:0043207 response to external biotic stimulus (loss)
c-value
0.0
0.1
0.2
0.3
0.4
0.5
0.6
0.7
0.8
Phylostratum
Cellular_organisms - ps1
Archaea1 - ps2
Archaea2 - ps3
Archaea3 - ps4
Asgard/Eukaryota - ps5
Eukaryota - ps6
Amorpheae - ps7
Obazoa - ps8
Opisthokonta - ps9
Holozoa - ps10
Filozoa - ps11
Choano./Metazoa - ps12
Metazoa - ps13
Myriazoa - ps14
Parahoxozoa - ps15
Bilateria - ps16
Deuterostomia - ps17
Chordata - ps18
Olfactores - ps19
Vertebrata - ps20
Gnathostomata - ps21
Euteleostomi - ps22
Sarcopterygii - ps23
Tetrapoda - ps24
Amniota - ps25
Mammalia - ps26
Theria - ps27
Placentalia - ps28
Boreoeutheria - ps29
Euarchontoglires - ps30
Euarchonta - ps31
Primates - ps32
Haplorrhini - ps33
Simiiformes - ps34
Catarrhini - ps35
Hominoidea - ps36
Hominidae - ps37
Homininae - ps38
Hominini - ps39
Homo_sapiens - ps40
p-value
2e-05
2e-04
1e-03
9e-03
7e-02
Log-odds
0.5
1.0
1.5
2.0
2.5
3.0
3.5
4.0

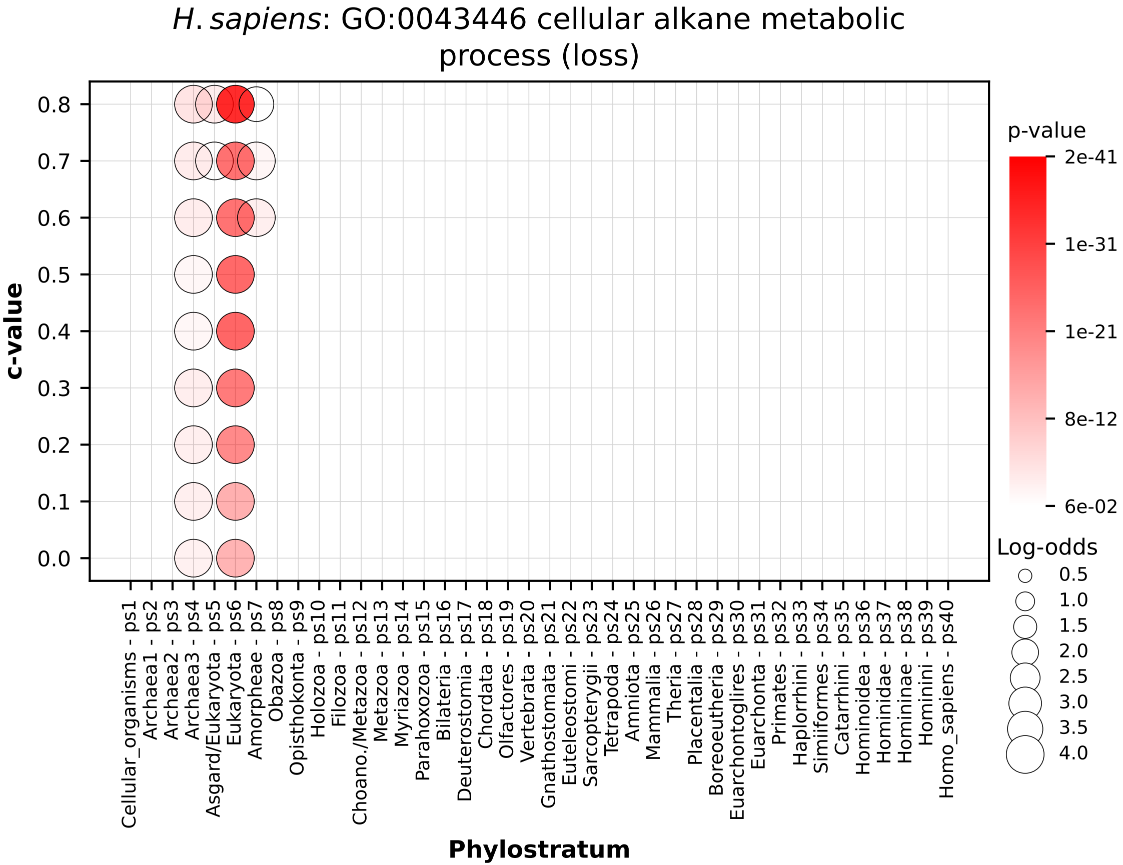

H. sapiens: GO:0043446 cellular alkane metabolic process (loss)
c-value
0.0
0.1
0.2
0.3
0.4
0.5
0.6
0.7
0.8
Phylostratum
Cellular_organisms - ps1
Archaea1 - ps2
Archaea2 - ps3
Archaea3 - ps4
Asgard/Eukaryota - ps5
Eukaryota - ps6
Amorpheae - ps7
Obazoa - ps8
Opisthokonta - ps9
Holozoa - ps10
Filozoa - ps11
Choano./Metazoa - ps12
Metazoa - ps13
Myriazoa - ps14
Parahoxozoa - ps15
Bilateria - ps16
Deuterostomia - ps17
Chordata - ps18
Olfactores - ps19
Vertebrata - ps20
Gnathostomata - ps21
Euteleostomi - ps22
Sarcopterygii - ps23
Tetrapoda - ps24
Amniota - ps25
Mammalia - ps26
Theria - ps27
Placentalia - ps28
Boreoeutheria - ps29
Euarchontoglires - ps30
Euarchonta - ps31
Primates - ps32
Haplorrhini - ps33
Simiiformes - ps34
Catarrhini - ps35
Hominoidea - ps36
Hominidae - ps37
Homininae - ps38
Hominini - ps39
Homo_sapiens - ps40
p-value
2e-41
1e-31
1e-21
8e-12
6e-02
Log-odds
0.5
1.0
1.5
2.0
2.5
3.0
3.5
4.0

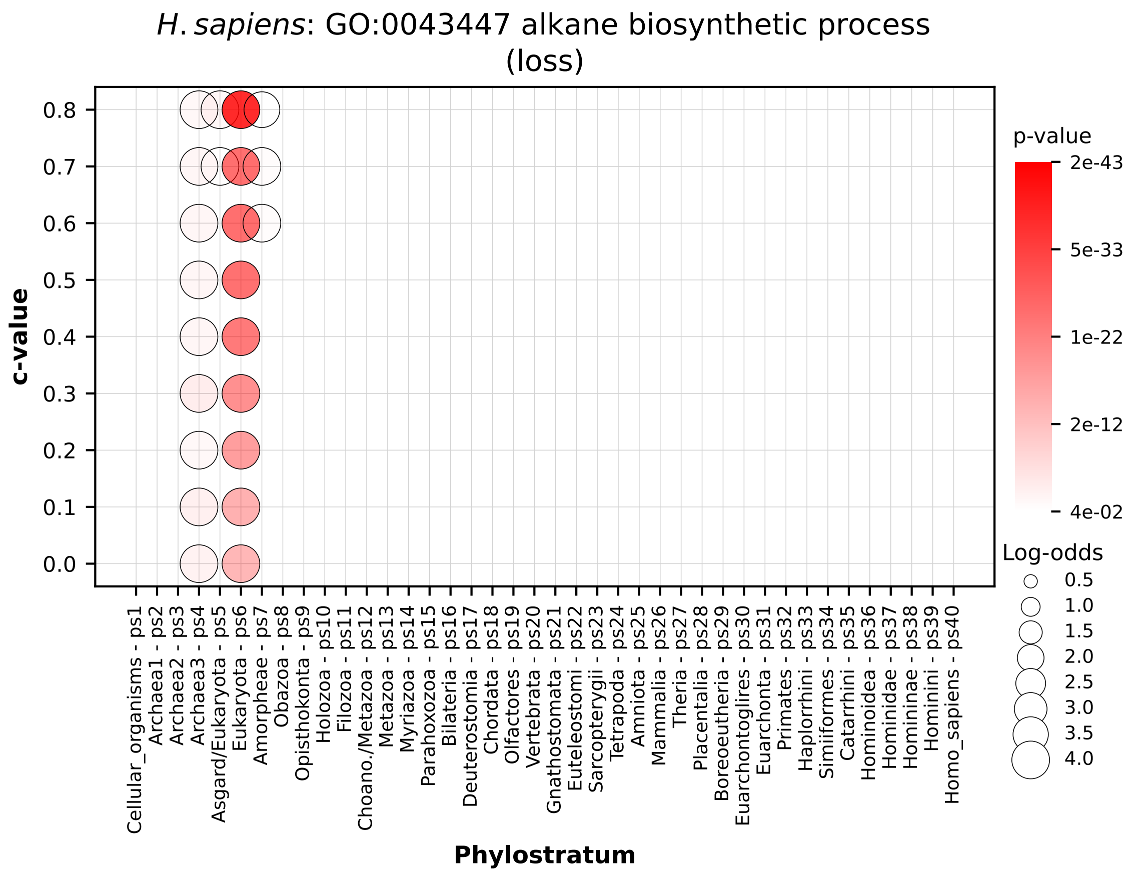

H. sapiens: GO:0043447 alkane biosynthetic process
(loss)
c-value
0.8
0.7
0.6
0.5
0.4
0.3
0.2
0.1
0.0
Phylostratum
Cellular_organisms - ps1
Archaea1 - ps2
Archaea2 - ps3
Archaea3 - ps4
Asgard/Eukaryota - ps5
Eukaryota - ps6
Amorpheae - ps7
Obazoa - ps8
Opisthokonta - ps9
Holozoa - ps10
Filozoa - ps11
Choano./Metazoa - ps12
Metazoa - ps13
Myriazoa - ps14
Parahoxozoa - ps15
Bilateria - ps16
Deuterostomia - ps17
Chordata - ps18
Olfactores - ps19
Vertebrata - ps20
Gnathostomata - ps21
Euteleostomi - ps22
Sarcopterygii - ps23
Tetrapoda - ps24
Amniota - ps25
Mammalia - ps26
Theria - ps27
Placentalia - ps28
Boreoeutheria - ps29
Euarchontoglires - ps30
Euarchonta - ps31
Primates - ps32
Haplorrhini - ps33
Simiiformes - ps34
Catarrhini - ps35
Hominoidea - ps36
Hominidae - ps37
Homininae - ps38
Hominini - ps39
Homo_sapiens - ps40
p-value
2e-43
5e-33
1e-22
2e-12
4e-02
Log-odds
0.5
1.0
1.5
2.0
2.5
3.0
3.5
4.0

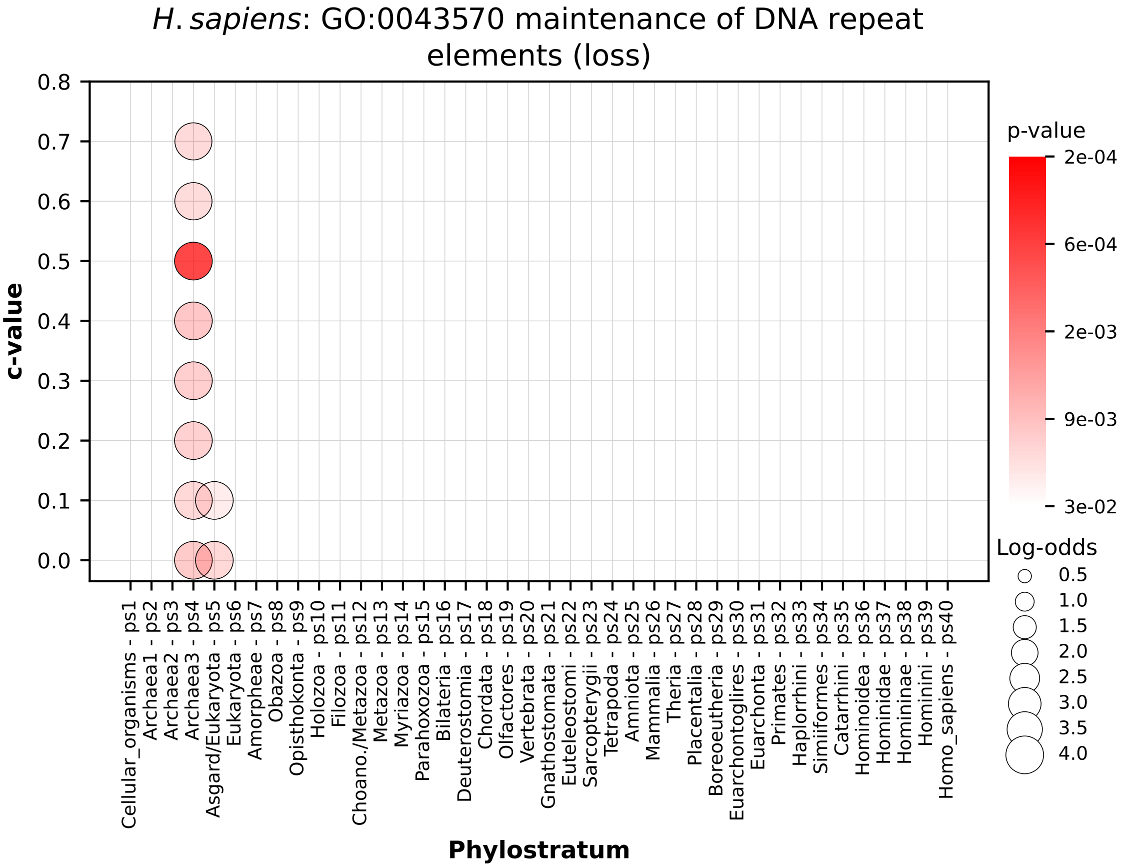

H. sapiens: GO:0043570 maintenance of DNA repeat elements (loss)
c-value
0.8
0.7
0.6
0.5
0.4
0.3
0.2
0.1
0.0
p-value
2e-04
6e-04
2e-03
9e-03
3e-02
Log-odds
0.5
1.0
1.5
2.0
2.5
3.0
3.5
4.0
Cellular_organisms - ps1
Archaea1 - ps2
Archaea2 - ps3
Archaea3 - ps4
Asgard/Eukaryota - ps5
Eukaryota - ps6
Amorpheae - ps7
Obazoa - ps8
Opisthokonta - ps9
Holozoa - ps10
Filozoa - ps11
Choano./Metazoa - ps12
Metazoa - ps13
Myriazoa - ps14
Parahoxozoa - ps15
Bilateria - ps16
Deuterostomia - ps17
Chordata - ps18
Olfactores - ps19
Vertebrata - ps20
Gnathostomata - ps21
Euteleostomi - ps22
Sarcopterygii - ps23
Tetrapoda - ps24
Amniota - ps25
Mammalia - ps26
Theria - ps27
Placentalia - ps28
Boreoeutheria - ps29
Euarchontoglires - ps30
Euarchonta - ps31
Primates - ps32
Haplorrhini - ps33
Simiiformes - ps34
Catarrhini - ps35
Hominoidea - ps36
Hominidae - ps37
Homininae - ps38
Hominini - ps39
Homo_sapiens - ps40
Phylostratum

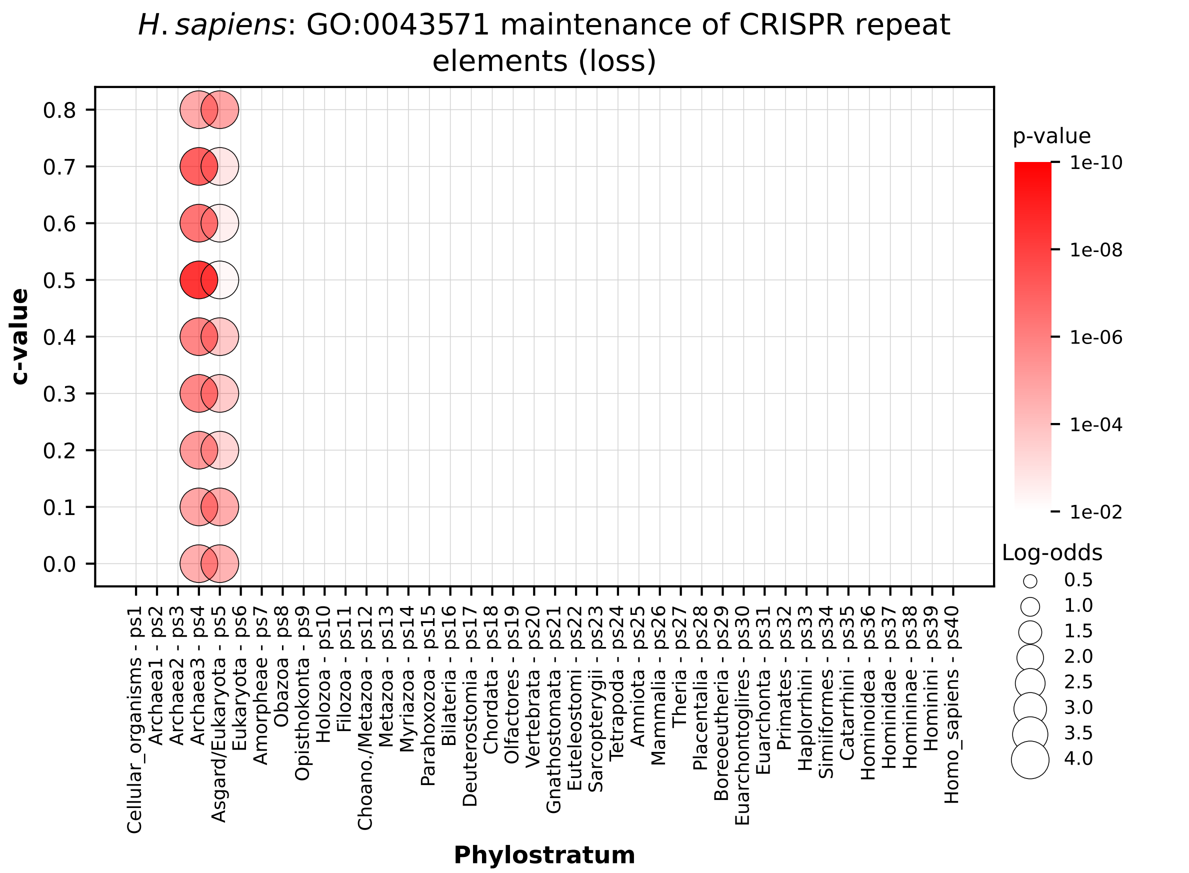

H. sapiens: GO:0043571 maintenance of CRISPR repeat elements (loss)
c-value
0.8
0.7
0.6
0.5
0.4
0.3
0.2
0.1
0.0
p-value
1e-10
1e-08
1e-06
1e-04
1e-02
Log-odds
0.5
1.0
1.5
2.0
2.5
3.0
3.5
4.0
Cellular_organisms - ps1
Archaea1 - ps2
Archaea2 - ps3
Archaea3 - ps4
Asgard/Eukaryota - ps5
Eukaryota - ps6
Amorpheae - ps7
Obazoa - ps8
Opisthokonta - ps9
Holozoa - ps10
Filozoa - ps11
Choano./Metazoa - ps12
Metazoa - ps13
Myriazoa - ps14
Parahoxozoa - ps15
Bilateria - ps16
Deuterostomia - ps17
Chordata - ps18
Olfactores - ps19
Vertebrata - ps20
Gnathostomata - ps21
Euteleostomi - ps22
Sarcopterygii - ps23
Tetrapoda - ps24
Amniota - ps25
Mammalia - ps26
Theria - ps27
Placentalia - ps28
Boreoeutheria - ps29
Euarchontoglires - ps30
Euarchonta - ps31
Primates - ps32
Haplorrhini - ps33
Simiiformes - ps34
Catarrhini - ps35
Hominoidea - ps36
Hominidae - ps37
Homininae - ps38
Hominini - ps39
Homo_sapiens - ps40
Phylostratum

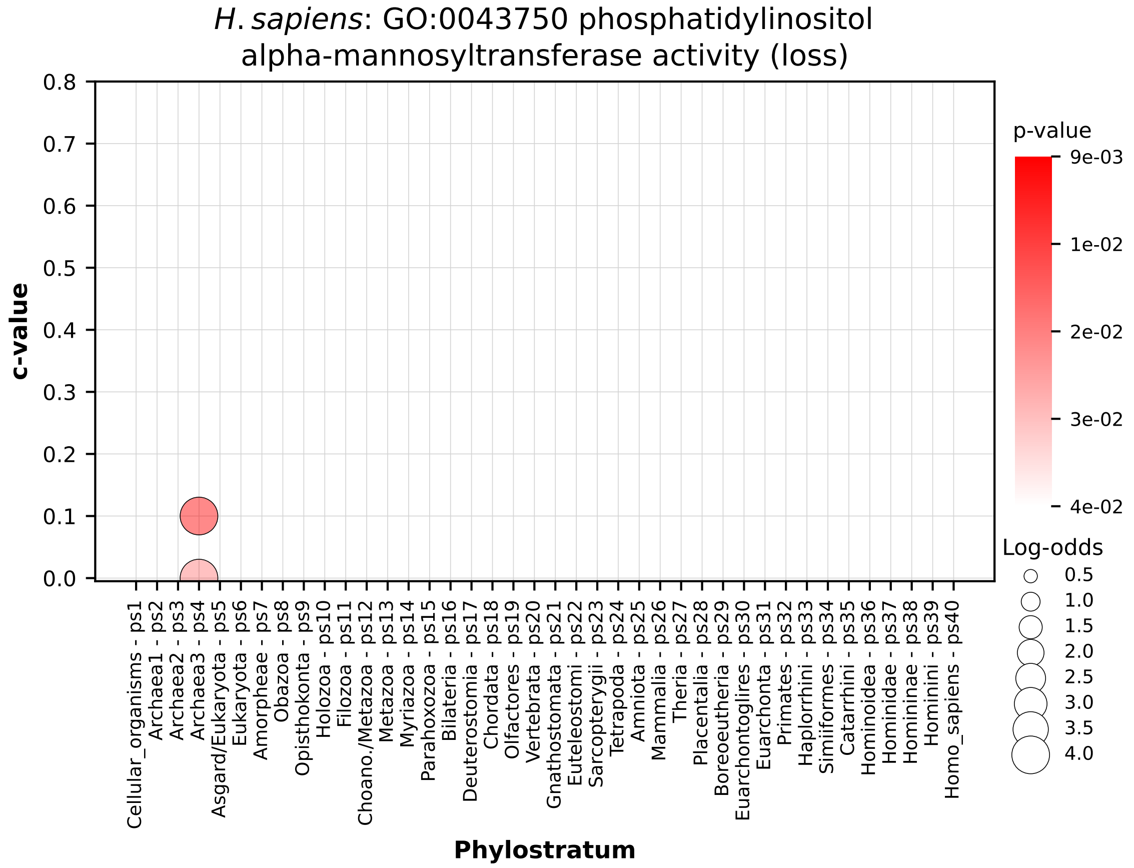

H. sapiens: GO:0043750 phosphatidylinositol alpha-mannosyltransferase activity (loss)
c-value
0.8
0.7
0.6
0.5
0.4
0.3
0.2
0.1
0.0
Phylostratum
Cellular_organisms - ps1
Archaea1 - ps2
Archaea2 - ps3
Archaea3 - ps4
Asgard/Eukaryota - ps5
Eukaryota - ps6
Amorpheae - ps7
Obazoa - ps8
Opisthokonta - ps9
Holozoa - ps10
Filozoa - ps11
Choano./Metazoa - ps12
Metazoa - ps13
Myriazoa - ps14
Parahoxozoa - ps15
Bilateria - ps16
Deuterostomia - ps17
Chordata - ps18
Olfactores - ps19
Vertebrata - ps20
Gnathostomata - ps21
Euteleostomi - ps22
Sarcopterygii - ps23
Tetrapoda - ps24
Amniota - ps25
Mammalia - ps26
Theria - ps27
Placentalia - ps28
Boreoeutheria - ps29
Euarchontoglires - ps30
Euarchonta - ps31
Primates - ps32
Haplorrhini - ps33
Simiiformes - ps34
Catarrhini - ps35
Hominoidea - ps36
Hominidae - ps37
Homininae - ps38
Hominini - ps39
Homo_sapiens - ps40
p-value
9e-03
1e-02
2e-02
3e-02
4e-02
Log-odds
0.5
1.0
1.5
2.0
2.5
3.0
3.5
4.0

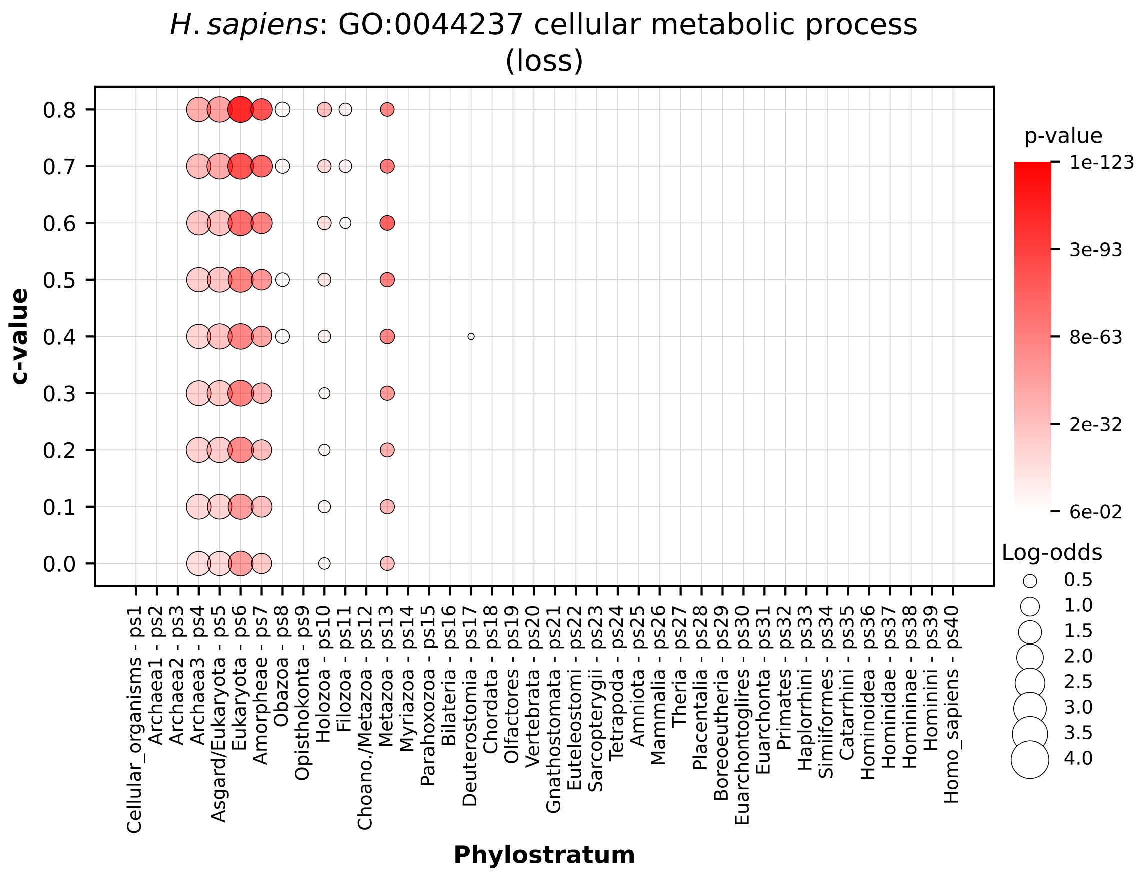

H. sapiens: GO:0044237 cellular metabolic process
(loss)
c-value
0.8
0.7
0.6
0.5
0.4
0.3
0.2
0.1
0.0
Phylostratum
Cellular_organisms - ps1
Archaea1 - ps2
Archaea2 - ps3
Archaea3 - ps4
Asgard/Eukaryota - ps5
Eukaryota - ps6
Amorpheae - ps7
Obazoa - ps8
Opisthokonta - ps9
Holozoa - ps10
Filozoa - ps11
Choano./Metazoa - ps12
Metazoa - ps13
Myriazoa - ps14
Parahoxozoa - ps15
Bilateria - ps16
Deuterostomia - ps17
Chordata - ps18
Olfactores - ps19
Vertebrata - ps20
Gnathostomata - ps21
Euteleostomi - ps22
Sarcopterygii - ps23
Tetrapoda - ps24
Amniota - ps25
Mammalia - ps26
Theria - ps27
Placentalia - ps28
Boreoeutheria - ps29
Euarchontoglires - ps30
Euarchonta - ps31
Primates - ps32
Haplorrhini - ps33
Simiiformes - ps34
Catarrhini - ps35
Hominoidea - ps36
Hominidae - ps37
Homininae - ps38
Hominini - ps39
Homo_sapiens - ps40
p-value
1e-123
3e-93
8e-63
2e-32
6e-02
Log-odds
0.5
1.0
1.5
2.0
2.5
3.0
3.5
4.0

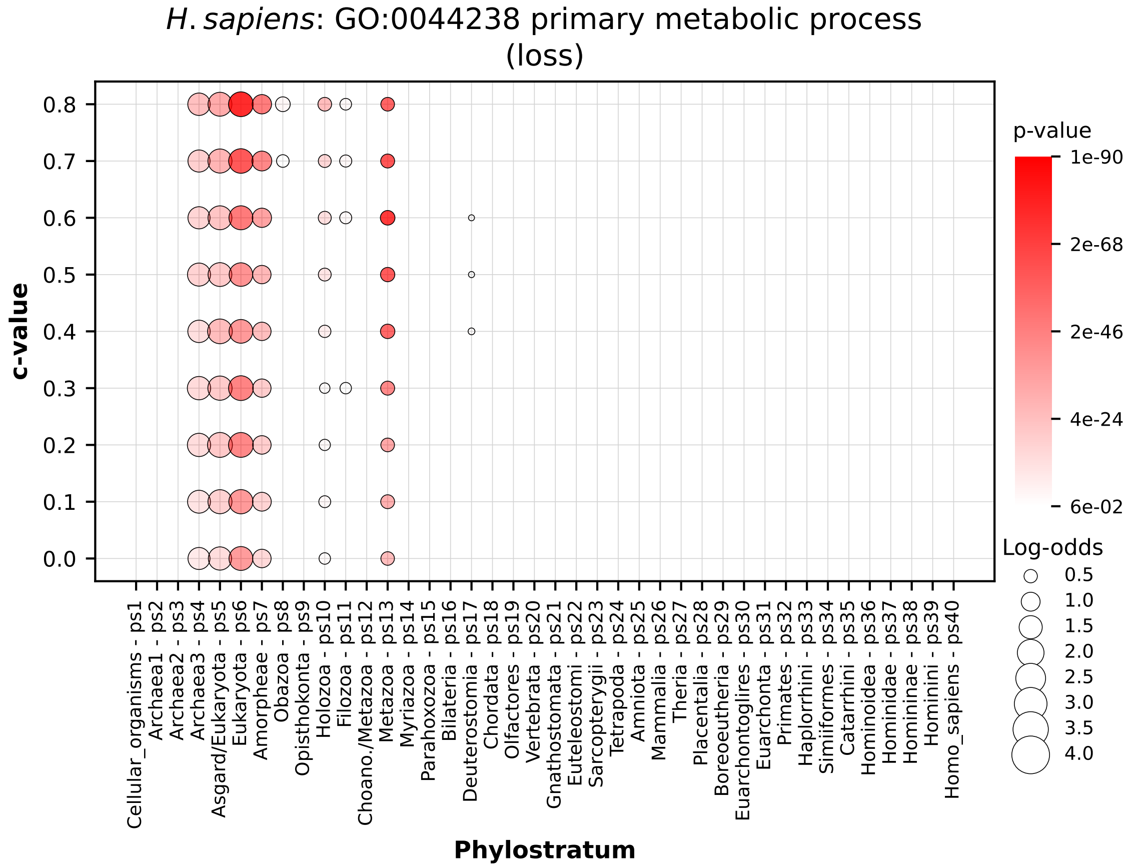

H. sapiens: GO:0044238 primary metabolic process
(loss)
c-value
0.8
0.7
0.6
0.5
0.4
0.3
0.2
0.1
0.0
Phylostratum
Cellular_organisms - ps1
Archaea1 - ps2
Archaea2 - ps3
Archaea3 - ps4
Asgard/Eukaryota - ps5
Eukaryota - ps6
Amorpheae - ps7
Obazoa - ps8
Opisthokonta - ps9
Holozoa - ps10
Filozoa - ps11
Choano./Metazoa - ps12
Metazoa - ps13
Myriazoa - ps14
Parahoxozoa - ps15
Bilateria - ps16
Deuterostomia - ps17
Chordata - ps18
Olfactores - ps19
Vertebrata - ps20
Gnathostomata - ps21
Euteleostomi - ps22
Sarcopterygii - ps23
Tetrapoda - ps24
Amniota - ps25
Mammalia - ps26
Theria - ps27
Placentalia - ps28
Boreoeutheria - ps29
Euarchontoglires - ps30
Euarchonta - ps31
Primates - ps32
Haplorrhini - ps33
Simiiformes - ps34
Catarrhini - ps35
Hominoidea - ps36
Hominidae - ps37
Homininae - ps38
Hominini - ps39
Homo_sapiens - ps40
p-value
1e-90
2e-68
2e-46
4e-24
6e-02
Log-odds
0.5
1.0
1.5
2.0
2.5
3.0
3.5
4.0

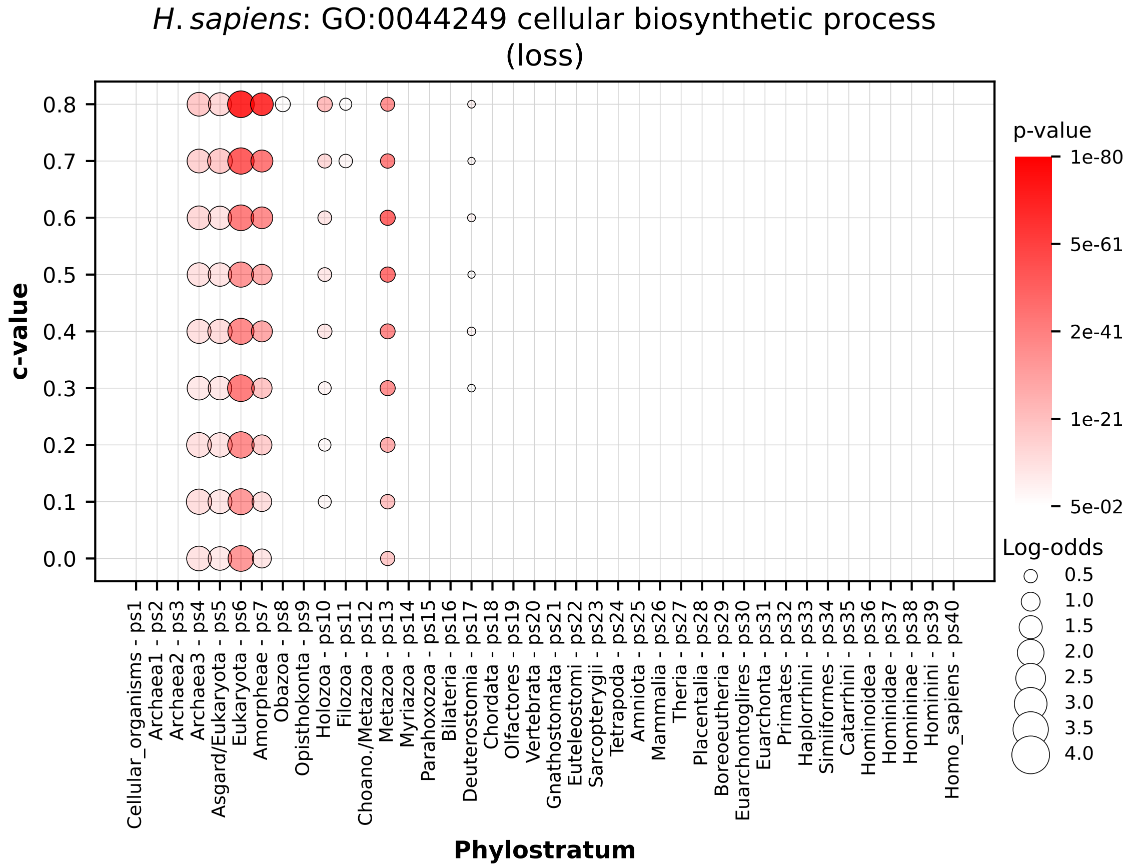

H. sapiens: GO:0044249 cellular biosynthetic process
(loss)
c-value
0.8
0.7
0.6
0.5
0.4
0.3
0.2
0.1
0.0
p-value
1e-80
5e-61
2e-41
1e-21
5e-02
Log-odds
0.5
1.0
1.5
2.0
2.5
3.0
3.5
4.0
Cellular_organisms - ps1
Archaea1 - ps2
Archaea2 - ps3
Archaea3 - ps4
Asgard/Eukaryota - ps5
Eukaryota - ps6
Amorpheae - ps7
Obazoa - ps8
Opisthokonta - ps9
Holozoa - ps10
Filozoa - ps11
Choano./Metazoa - ps12
Metazoa - ps13
Myriazoa - ps14
Parahoxozoa - ps15
Bilateria - ps16
Deuterostomia - ps17
Chordata - ps18
Olfactores - ps19
Vertebrata - ps20
Gnathostomata - ps21
Euteleostomi - ps22
Sarcopterygii - ps23
Tetrapoda - ps24
Amniota - ps25
Mammalia - ps26
Theria - ps27
Placentalia - ps28
Boreoeutheria - ps29
Euarchontoglires - ps30
Euarchonta - ps31
Primates - ps32
Haplorrhini - ps33
Simiiformes - ps34
Catarrhini - ps35
Hominoidea - ps36
Hominidae - ps37
Homininae - ps38
Hominini - ps39
Homo_sapiens - ps40
Phylostratum

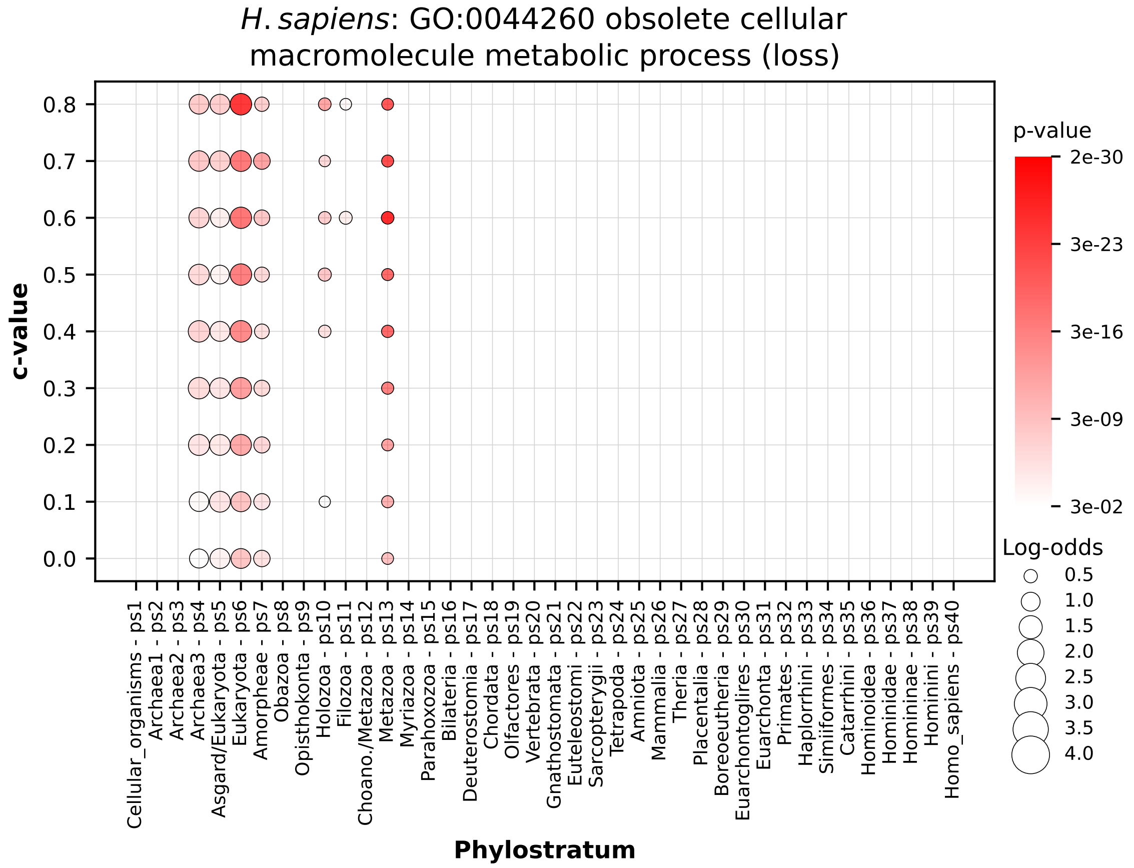

H. sapiens: GO:0044260 obsolete cellular macromolecule metabolic process (loss)
c-value
0.8
0.7
0.6
0.5
0.4
0.3
0.2
0.1
0.0
Phylostratum
Cellular_organisms - ps1
Archaea1 - ps2
Archaea2 - ps3
Archaea3 - ps4
Asgard/Eukaryota - ps5
Eukaryota - ps6
Amorpheae - ps7
Obazoa - ps8
Opisthokonta - ps9
Holozoa - ps10
Filozoa - ps11
Choano./Metazoa - ps12
Metazoa - ps13
Myriazoa - ps14
Parahoxozoa - ps15
Bilateria - ps16
Deuterostomia - ps17
Chordata - ps18
Olfactores - ps19
Vertebrata - ps20
Gnathostomata - ps21
Euteleostomi - ps22
Sarcopterygii - ps23
Tetrapoda - ps24
Amniota - ps25
Mammalia - ps26
Theria - ps27
Placentalia - ps28
Boreoeutheria - ps29
Euarchontoglires - ps30
Euarchonta - ps31
Primates - ps32
Haplorrhini - ps33
Simiiformes - ps34
Catarrhini - ps35
Hominoidea - ps36
Hominidae - ps37
Homininae - ps38
Hominini - ps39
Homo_sapiens - ps40
p-value
2e-30
3e-23
3e-16
3e-09
3e-02
Log-odds
0.5
1.0
1.5
2.0
2.5
3.0
3.5
4.0

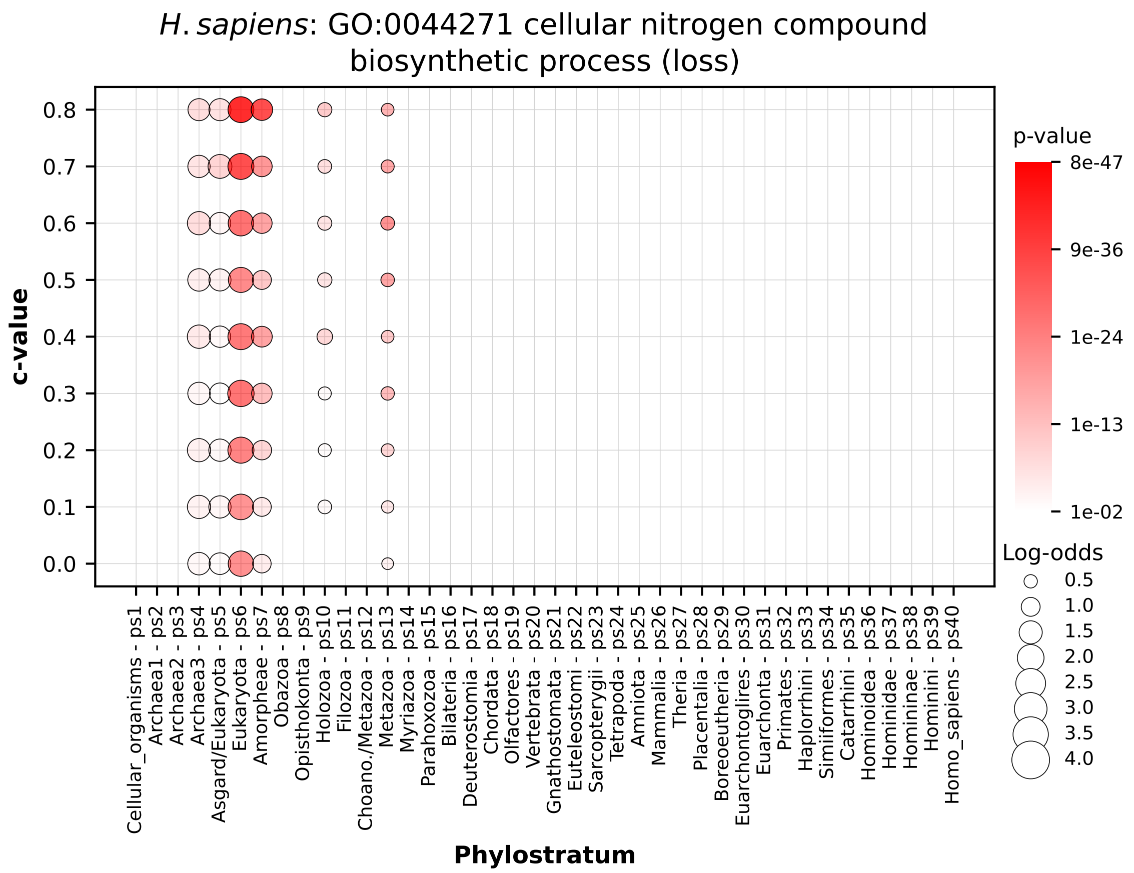

H. sapiens: GO:0044271 cellular nitrogen compound biosynthetic process (loss)
c-value
0.8
0.7
0.6
0.5
0.4
0.3
0.2
0.1
0.0
Phylostratum
Cellular_organisms - ps1
Archaea1 - ps2
Archaea2 - ps3
Archaea3 - ps4
Asgard/Eukaryota - ps5
Eukaryota - ps6
Amorpheae - ps7
Obazoa - ps8
Opisthokonta - ps9
Holozoa - ps10
Filozoa - ps11
Choano./Metazoa - ps12
Metazoa - ps13
Myriazoa - ps14
Parahoxozoa - ps15
Bilateria - ps16
Deuterostomia - ps17
Chordata - ps18
Olfactores - ps19
Vertebrata - ps20
Gnathostomata - ps21
Euteleostomi - ps22
Sarcopterygii - ps23
Tetrapoda - ps24
Amniota - ps25
Mammalia - ps26
Theria - ps27
Placentalia - ps28
Boreoeutheria - ps29
Euarchontoglires - ps30
Euarchonta - ps31
Primates - ps32
Haplorrhini - ps33
Simiiformes - ps34
Catarrhini - ps35
Hominoidea - ps36
Hominidae - ps37
Homininae - ps38
Hominini - ps39
Homo_sapiens - ps40
p-value
8e-47
9e-36
1e-24
1e-13
1e-02
Log-odds
0.5
1.0
1.5
2.0
2.5
3.0
3.5
4.0

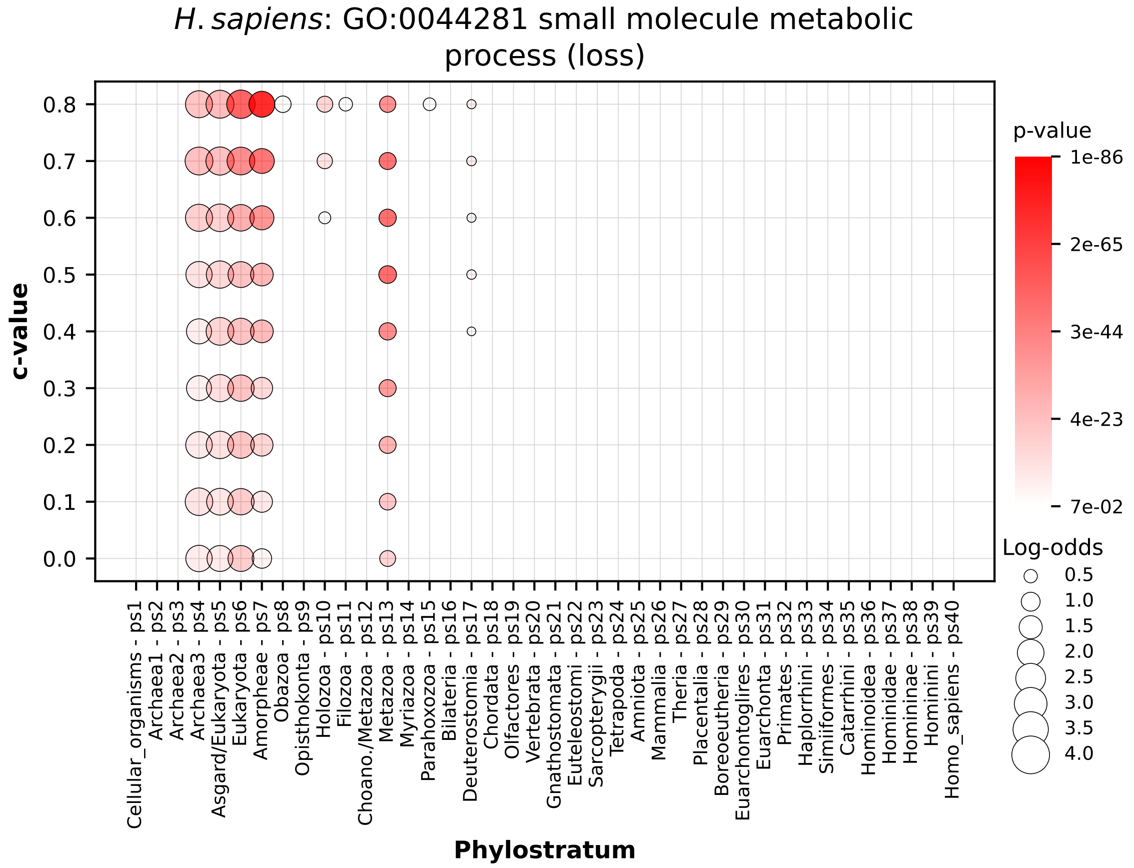

H. sapiens: GO:0044281 small molecule metabolic process (loss)
c-value
0.8
0.7
0.6
0.5
0.4
0.3
0.2
0.1
0.0
p-value
1e-86
2e-65
3e-44
4e-23
7e-02
Log-odds
0.5
1.0
1.5
2.0
2.5
3.0
3.5
4.0
Cellular_organisms - ps1
Archaea1 - ps2
Archaea2 - ps3
Archaea3 - ps4
Asgard/Eukaryota - ps5
Eukaryota - ps6
Amorpheae - ps7
Obazoa - ps8
Opisthokonta - ps9
Holozoa - ps10
Filozoa - ps11
Choano./Metazoa - ps12
Metazoa - ps13
Myriazoa - ps14
Parahoxozoa - ps15
Bilateria - ps16
Deuterostomia - ps17
Chordata - ps18
Olfactores - ps19
Vertebrata - ps20
Gnathostomata - ps21
Euteleostomi - ps22
Sarcopterygii - ps23
Tetrapoda - ps24
Amniota - ps25
Mammalia - ps26
Theria - ps27
Placentalia - ps28
Boreoeutheria - ps29
Euarchontoglires - ps30
Euarchonta - ps31
Primates - ps32
Haplorrhini - ps33
Simiiformes - ps34
Catarrhini - ps35
Hominoidea - ps36
Hominidae - ps37
Homininae - ps38
Hominini - ps39
Homo_sapiens - ps40
Phylostratum

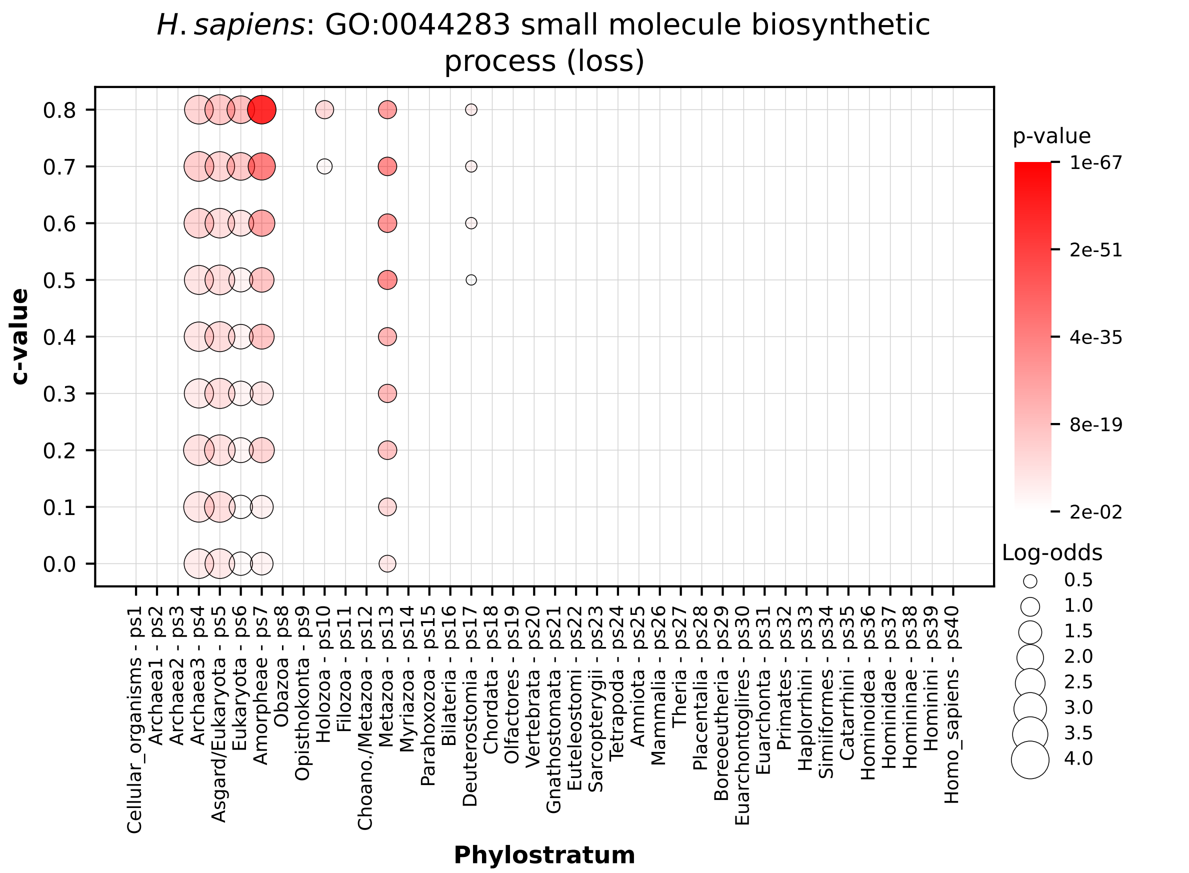

H. sapiens: GO:0044283 small molecule biosynthetic process (loss)
c-value
0.8
0.7
0.6
0.5
0.4
0.3
0.2
0.1
0.0
p-value
1e-67
2e-51
4e-35
8e-19
2e-02
Log-odds
0.5
1.0
1.5
2.0
2.5
3.0
3.5
4.0
Cellular_organisms - ps1
Archaea1 - ps2
Archaea2 - ps3
Archaea3 - ps4
Asgard/Eukaryota - ps5
Eukaryota - ps6
Amorpheae - ps7
Obazoa - ps8
Opisthokonta - ps9
Holozoa - ps10
Filozoa - ps11
Choano./Metazoa - ps12
Metazoa - ps13
Myriazoa - ps14
Parahoxozoa - ps15
Bilateria - ps16
Deuterostomia - ps17
Chordata - ps18
Olfactores - ps19
Vertebrata - ps20
Gnathostomata - ps21
Euteleostomi - ps22
Sarcopterygii - ps23
Tetrapoda - ps24
Amniota - ps25
Mammalia - ps26
Theria - ps27
Placentalia - ps28
Boreoeutheria - ps29
Euarchontoglires - ps30
Euarchonta - ps31
Primates - ps32
Haplorrhini - ps33
Simiiformes - ps34
Catarrhini - ps35
Hominoidea - ps36
Hominidae - ps37
Homininae - ps38
Hominini - ps39
Homo_sapiens - ps40
Phylostratum

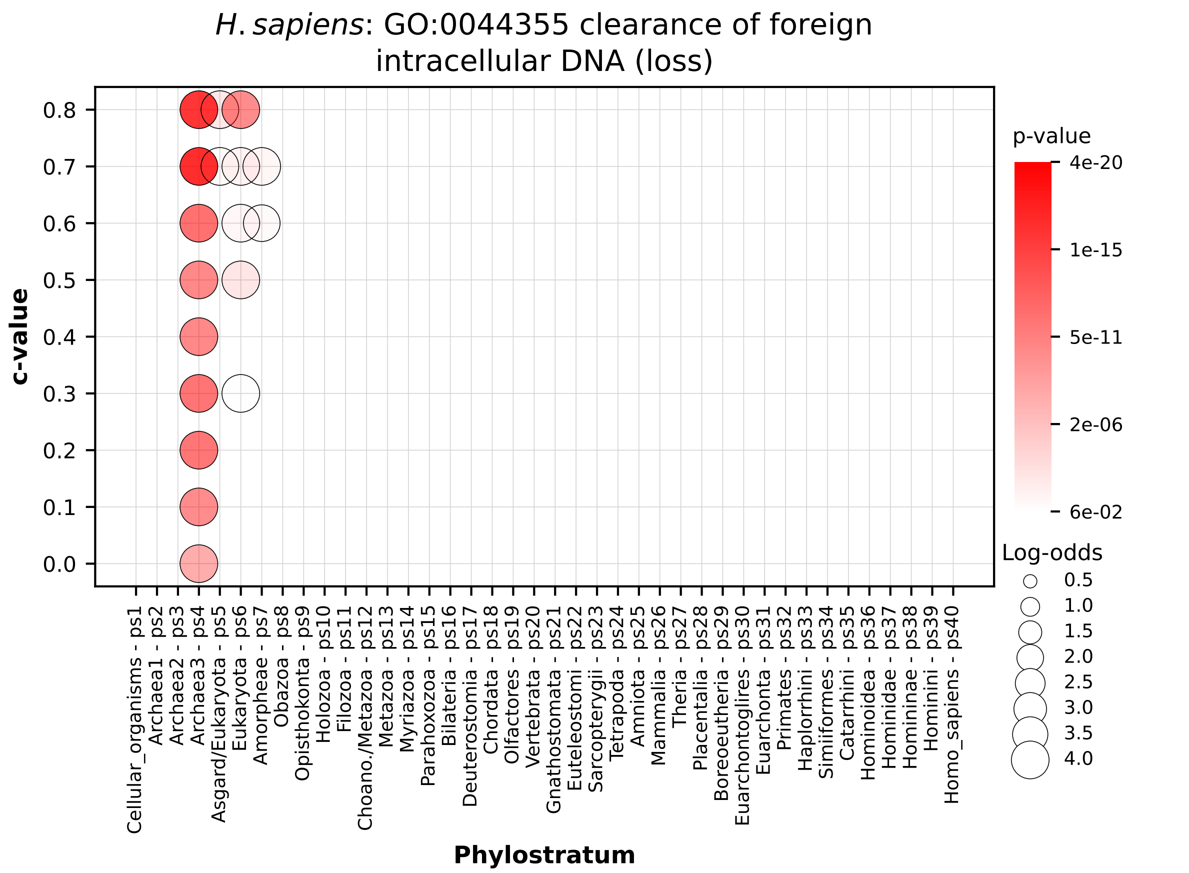

$H. sapiens$: GO:0044355 clearance of foreign intracellular DNA (loss)
c-value
0.8
0.7
0.6
0.5
0.4
0.3
0.2
0.1
0.0
Phylostratum
Cellular_organisms - ps1
Archaea1 - ps2
Archaea2 - ps3
Archaea3 - ps4
Asgard/Eukaryota - ps5
Eukaryota - ps6
Amorpheae - ps7
Obazoa - ps8
Opisthokonta - ps9
Holozoa - ps10
Filozoa - ps11
Choano./Metazoa - ps12
Metazoa - ps13
Myriazoa - ps14
Parahoxozoa - ps15
Bilateria - ps16
Deuterostomia - ps17
Chordata - ps18
Olfactores - ps19
Vertebrata - ps20
Gnathostomata - ps21
Euteleostomi - ps22
Sarcopterygii - ps23
Tetrapoda - ps24
Amniota - ps25
Mammalia - ps26
Theria - ps27
Placentalia - ps28
Boreoeutheria - ps29
Euarchontoglires - ps30
Euarchonta - ps31
Primates - ps32
Haplorrhini - ps33
Simiiformes - ps34
Catarrhini - ps35
Hominoidea - ps36
Hominidae - ps37
Homininae - ps38
Hominini - ps39
Homo_sapiens - ps40
p-value
4e-20
1e-15
5e-11
2e-06
6e-02
Log-odds
0.5
1.0
1.5
2.0
2.5
3.0
3.5
4.0

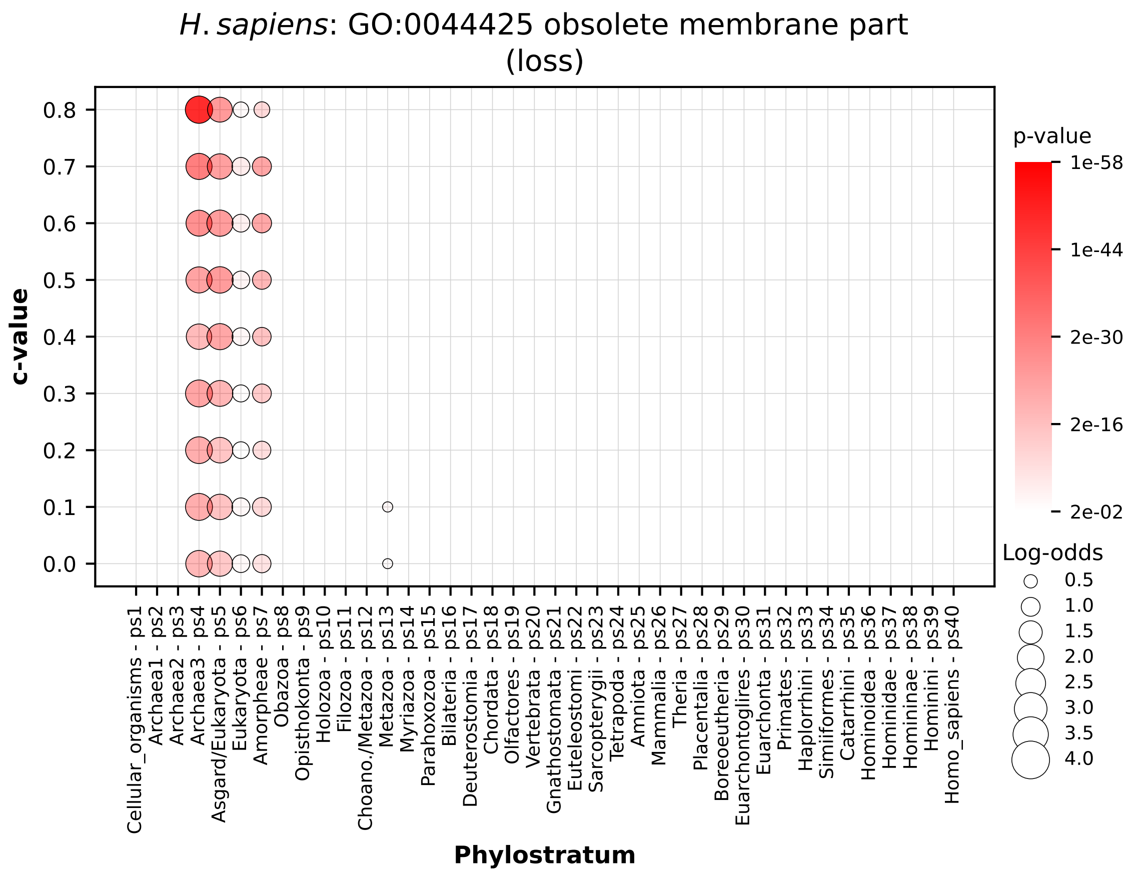

H. sapiens: GO:0044425 obsolete membrane part
(loss)
c-value
0.8
0.7
0.6
0.5
0.4
0.3
0.2
0.1
0.0
p-value
1e-58
1e-44
2e-30
2e-16
2e-02
Log-odds
0.5
1.0
1.5
2.0
2.5
3.0
3.5
4.0
Cellular_organisms - ps1
Archaea1 - ps2
Archaea2 - ps3
Archaea3 - ps4
Asgard/Eukaryota - ps5
Eukaryota - ps6
Amorpheae - ps7
Obazoa - ps8
Opisthokonta - ps9
Holozoa - ps10
Filozoa - ps11
Choano./Metazoa - ps12
Metazoa - ps13
Myriazoa - ps14
Parahoxozoa - ps15
Bilateria - ps16
Deuterostomia - ps17
Chordata - ps18
Olfactores - ps19
Vertebrata - ps20
Gnathostomata - ps21
Euteleostomi - ps22
Sarcopterygii - ps23
Tetrapoda - ps24
Amniota - ps25
Mammalia - ps26
Theria - ps27
Placentalia - ps28
Boreoeutheria - ps29
Euarchontoglires - ps30
Euarchonta - ps31
Primates - ps32
Haplorrhini - ps33
Simiiformes - ps34
Catarrhini - ps35
Hominoidea - ps36
Hominidae - ps37
Homininae - ps38
Hominini - ps39
Homo_sapiens - ps40
Phylostratum

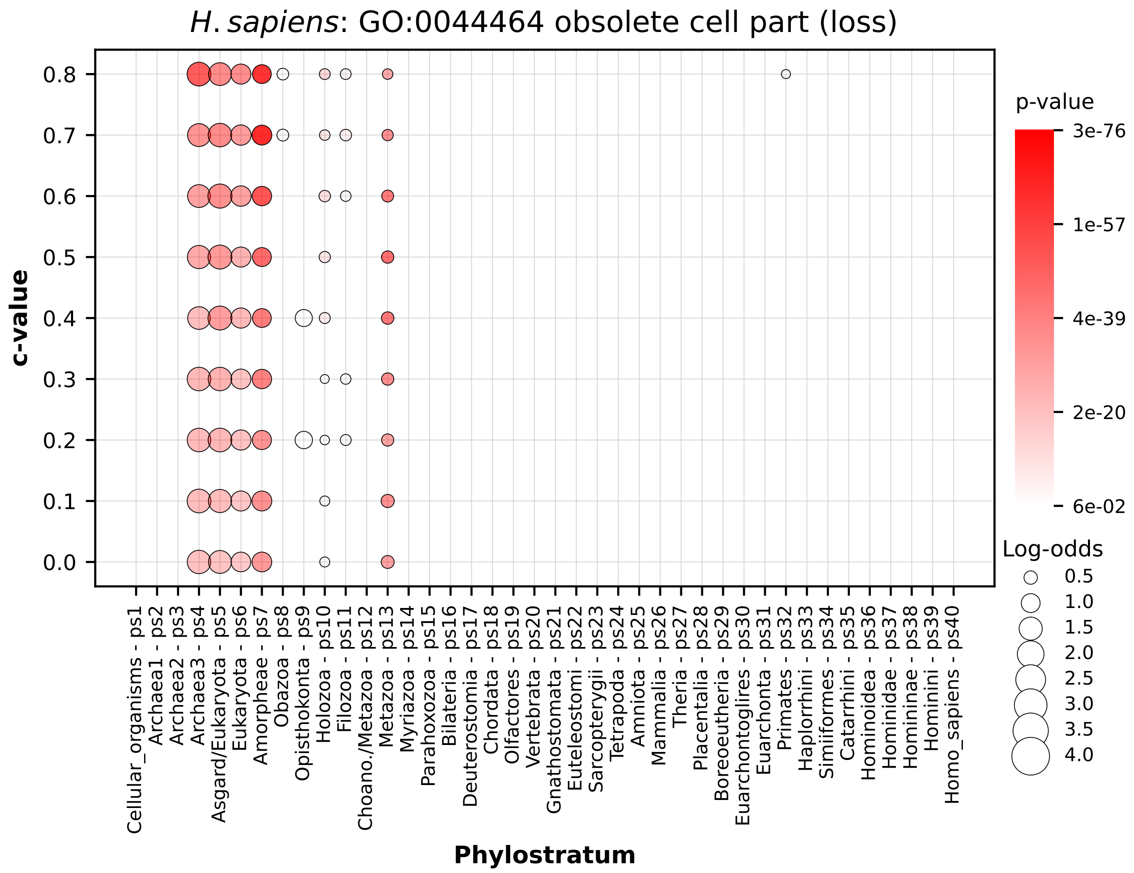
H. sapiens: GO:0044464 obsolete cell part (loss)
c-value
0.8
0.7
0.6
0.5
0.4
0.3
0.2
0.1
0.0
Phylostratum
Cellular_organisms - ps1
Archaea1 - ps2
Archaea2 - ps3
Archaea3 - ps4
Asgard/Eukaryota - ps5
Eukaryota - ps6
Amorpheae - ps7
Obazoa - ps8
Opisthokonta - ps9
Holozoa - ps10
Filozoa - ps11
Choano./Metazoa - ps12
Metazoa - ps13
Myriazoa - ps14
Parahoxozoa - ps15
Bilateria - ps16
Deuterostomia - ps17
Chordata - ps18
Olfactores - ps19
Vertebrata - ps20
Gnathostomata - ps21
Euteleostomi - ps22
Sarcopterygii - ps23
Tetrapoda - ps24
Amniota - ps25
Mammalia - ps26
Theria - ps27
Placentalia - ps28
Boreoeutheria - ps29
Euarchontoglires - ps30
Euarchonta - ps31
Primates - ps32
Haplorrhini - ps33
Simiiformes - ps34
Catarrhini - ps35
Hominoidea - ps36
Hominidae - ps37
Homininae - ps38
Hominini - ps39
Homo_sapiens - ps40
p-value
3e-76
1e-57
4e-39
2e-20
6e-02
Log-odds
0.5
1.0
1.5
2.0
2.5
3.0
3.5
4.0

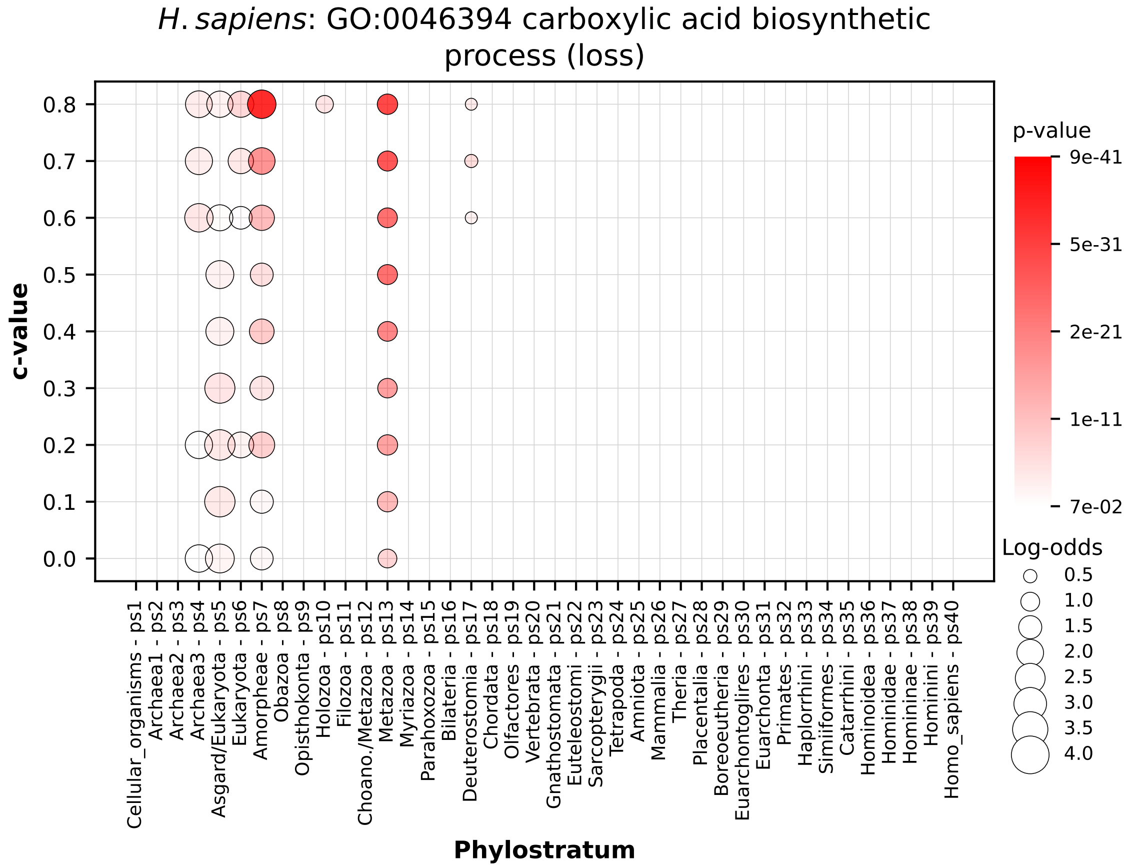
H. sapiens: GO:0046394 carboxylic acid biosynthetic process (loss)
c-value
0.8
0.7
0.6
0.5
0.4
0.3
0.2
0.1
0.0
p-value
9e-41
5e-31
2e-21
1e-11
7e-02
Log-odds
0.5
1.0
1.5
2.0
2.5
3.0
3.5
4.0
Cellular_organisms - ps1
Archaea1 - ps2
Archaea2 - ps3
Archaea3 - ps4
Asgard/Eukaryota - ps5
Eukaryota - ps6
Amorpheae - ps7
Obazoa - ps8
Opisthokonta - ps9
Holozoa - ps10
Filozoa - ps11
Choano./Metazoa - ps12
Metazoa - ps13
Myriazoa - ps14
Parahoxozoa - ps15
Bilateria - ps16
Deuterostomia - ps17
Chordata - ps18
Olfactores - ps19
Vertebrata - ps20
Gnathostomata - ps21
Euteleostomi - ps22
Sarcopterygii - ps23
Tetrapoda - ps24
Amniota - ps25
Mammalia - ps26
Theria - ps27
Placentalia - ps28
Boreoeutheria - ps29
Euarchontoglires - ps30
Euarchonta - ps31
Primates - ps32
Haplorrhini - ps33
Simiiformes - ps34
Catarrhini - ps35
Hominoidea - ps36
Hominidae - ps37
Homininae - ps38
Hominini - ps39
Homo_sapiens - ps40
Phylostratum

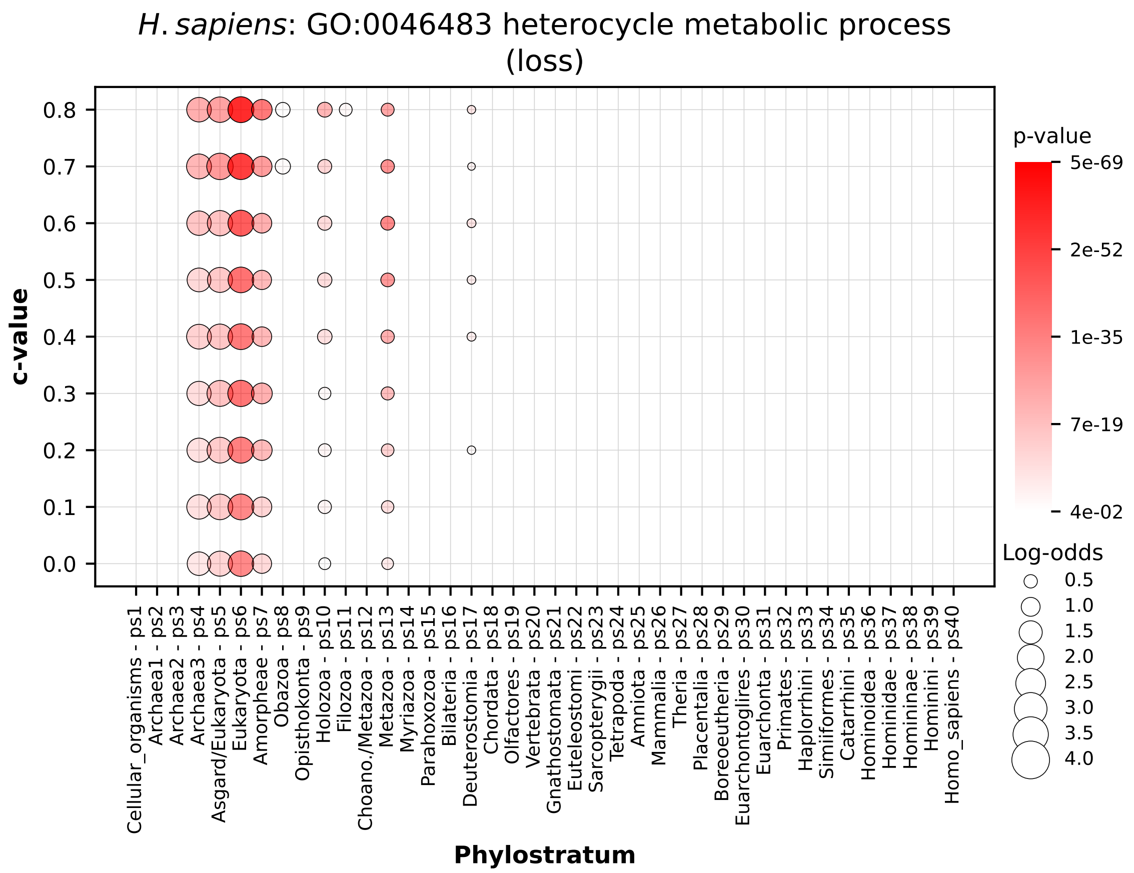

H. sapiens: GO:0046483 heterocycle metabolic process
(loss)
c-value
0.0
0.1
0.2
0.3
0.4
0.5
0.6
0.7
0.8
p-value
5e-69
2e-52
1e-35
7e-19
4e-02
Log-odds
0.5
1.0
1.5
2.0
2.5
3.0
3.5
4.0
Cellular_organisms - ps1
Archaea1 - ps2
Archaea2 - ps3
Archaea3 - ps4
Asgard/Eukaryota - ps5
Eukaryota - ps6
Amorpheae - ps7
Obazoa - ps8
Opisthokonta - ps9
Holozoa - ps10
Filozoa - ps11
Choano./Metazoa - ps12
Metazoa - ps13
Myriazoa - ps14
Parahoxozoa - ps15
Bilateria - ps16
Deuterostomia - ps17
Chordata - ps18
Olfactores - ps19
Vertebrata - ps20
Gnathostomata - ps21
Euteleostomi - ps22
Sarcopterygii - ps23
Tetrapoda - ps24
Amniota - ps25
Mammalia - ps26
Theria - ps27
Placentalia - ps28
Boreoeutheria - ps29
Euarchontoglires - ps30
Euarchonta - ps31
Primates - ps32
Haplorrhini - ps33
Simiiformes - ps34
Catarrhini - ps35
Hominoidea - ps36
Hominidae - ps37
Homininae - ps38
Hominini - ps39
Homo_sapiens - ps40
Phylostratum

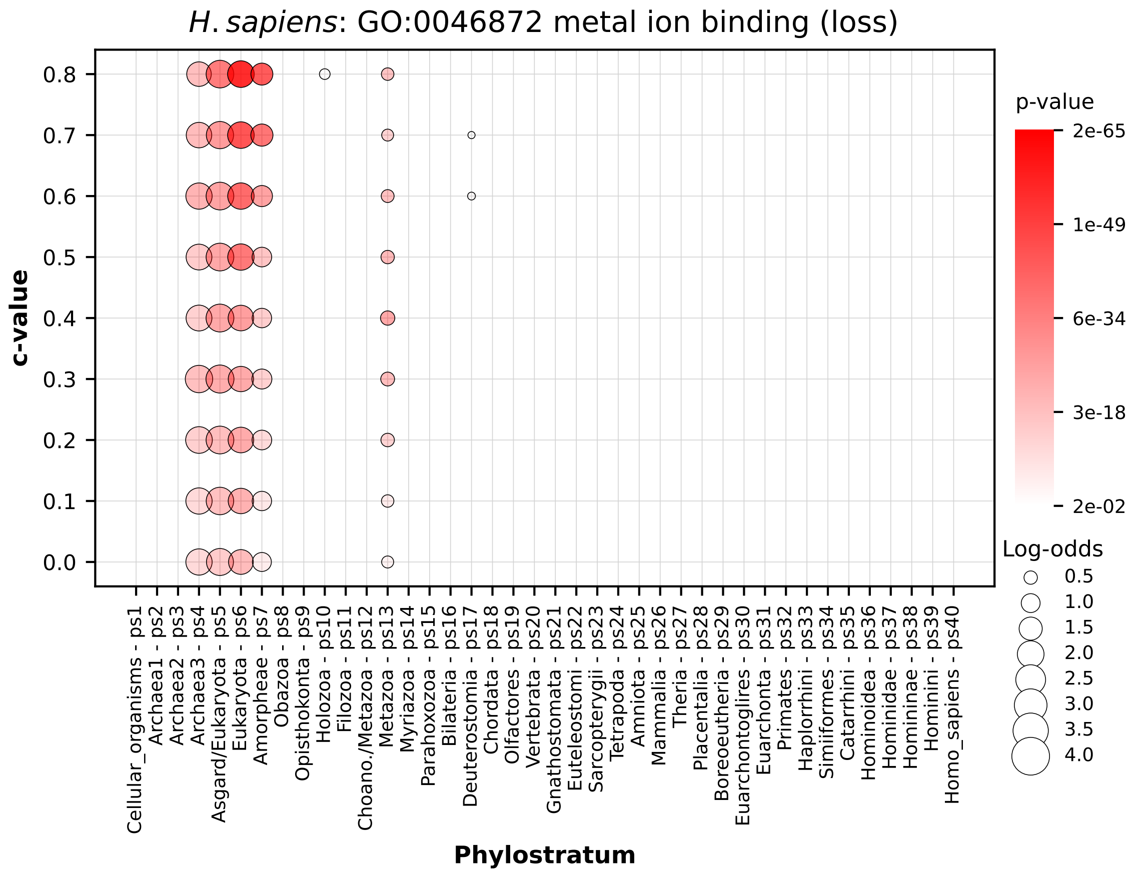

H. sapiens: GO:0046872 metal ion binding (loss)
c-value
0.8
0.7
0.6
0.5
0.4
0.3
0.2
0.1
0.0
Phylostratum
Cellular_organisms - ps1
Archaea1 - ps2
Archaea2 - ps3
Archaea3 - ps4
Asgard/Eukaryota - ps5
Eukaryota - ps6
Amorpheae - ps7
Obazoa - ps8
Opisthokonta - ps9
Holozoa - ps10
Filozoa - ps11
Choano./Metazoa - ps12
Metazoa - ps13
Myriazoa - ps14
Parahoxozoa - ps15
Bilateria - ps16
Deuterostomia - ps17
Chordata - ps18
Olfactores - ps19
Vertebrata - ps20
Gnathostomata - ps21
Euteleostomi - ps22
Sarcopterygii - ps23
Tetrapoda - ps24
Amniota - ps25
Mammalia - ps26
Theria - ps27
Placentalia - ps28
Boreoeutheria - ps29
Euarchontoglires - ps30
Euarchonta - ps31
Primates - ps32
Haplorrhini - ps33
Simiiformes - ps34
Catarrhini - ps35
Hominoidea - ps36
Hominidae - ps37
Homininae - ps38
Hominini - ps39
Homo_sapiens - ps40
p-value
2e-65
1e-49
6e-34
3e-18
2e-02
Log-odds
0.5
1.0
1.5
2.0
2.5
3.0
3.5
4.0

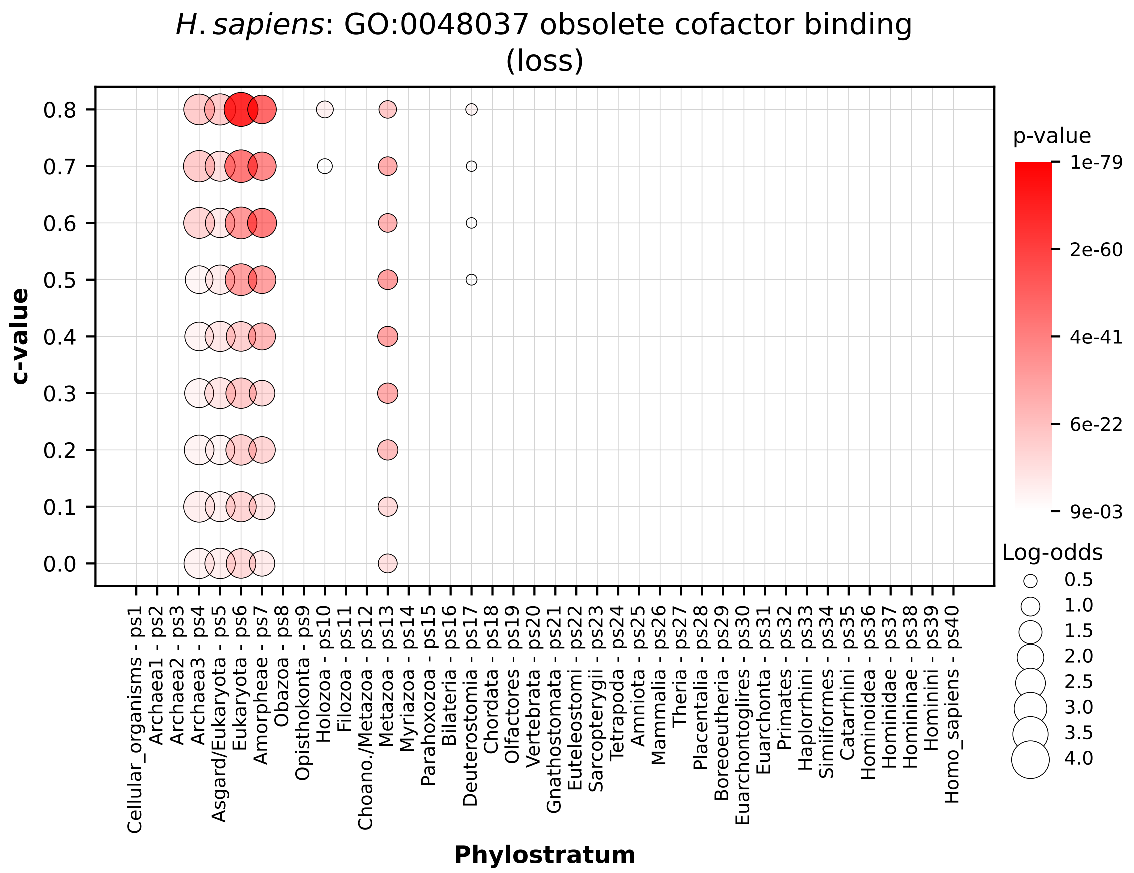

H. sapiens: GO:0048037 obsolete cofactor binding
(loss)
c-value
0.8
0.7
0.6
0.5
0.4
0.3
0.2
0.1
0.0
p-value
1e-79
2e-60
4e-41
6e-22
9e-03
Log-odds
0.5
1.0
1.5
2.0
2.5
3.0
3.5
4.0
Cellular_organisms - ps1
Archaea1 - ps2
Archaea2 - ps3
Archaea3 - ps4
Asgard/Eukaryota - ps5
Eukaryota - ps6
Amorpheae - ps7
Obazoa - ps8
Opisthokonta - ps9
Holozoa - ps10
Filozoa - ps11
Choano./Metazoa - ps12
Metazoa - ps13
Myriazoa - ps14
Parahoxozoa - ps15
Bilateria - ps16
Deuterostomia - ps17
Chordata - ps18
Olfactores - ps19
Vertebrata - ps20
Gnathostomata - ps21
Euteleostomi - ps22
Sarcopterygii - ps23
Tetrapoda - ps24
Amniota - ps25
Mammalia - ps26
Theria - ps27
Placentalia - ps28
Boreoeutheria - ps29
Euarchontoglires - ps30
Euarchonta - ps31
Primates - ps32
Haplorrhini - ps33
Simiiformes - ps34
Catarrhini - ps35
Hominoidea - ps36
Hominidae - ps37
Homininae - ps38
Hominini - ps39
Homo_sapiens - ps40
Phylostratum

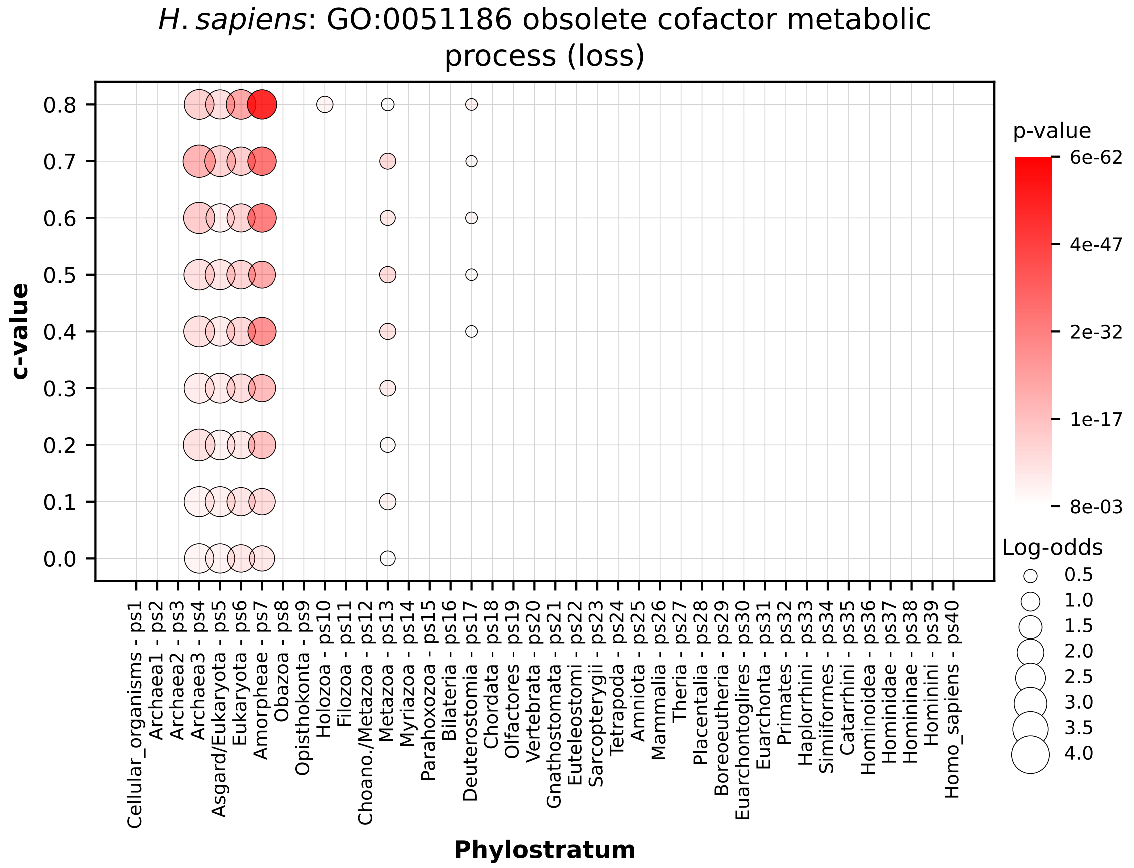
H. sapiens: GO:0051186 obsolete cofactor metabolic process (loss)
c-value
0.0
0.1
0.2
0.3
0.4
0.5
0.6
0.7
0.8
p-value
6e-62
4e-47
2e-32
1e-17
8e-03
Log-odds
0.5
1.0
1.5
2.0
2.5
3.0
3.5
4.0
Cellular_organisms - ps1
Archaea1 - ps2
Archaea2 - ps3
Archaea3 - ps4
Asgard/Eukaryota - ps5
Eukaryota - ps6
Amorpheae - ps7
Obazoa - ps8
Opisthokonta - ps9
Holozoa - ps10
Filozoa - ps11
Choano./Metazoa - ps12
Metazoa - ps13
Myriazoa - ps14
Parahoxozoa - ps15
Bilateria - ps16
Deuterostomia - ps17
Chordata - ps18
Olfactores - ps19
Vertebrata - ps20
Gnathostomata - ps21
Euteleostomi - ps22
Sarcopterygii - ps23
Tetrapoda - ps24
Amniota - ps25
Mammalia - ps26
Theria - ps27
Placentalia - ps28
Boreoeutheria - ps29
Euarchontoglires - ps30
Euarchonta - ps31
Primates - ps32
Haplorrhini - ps33
Simiiformes - ps34
Catarrhini - ps35
Hominoidea - ps36
Hominidae - ps37
Homininae - ps38
Hominini - ps39
Homo_sapiens - ps40
Phylostratum

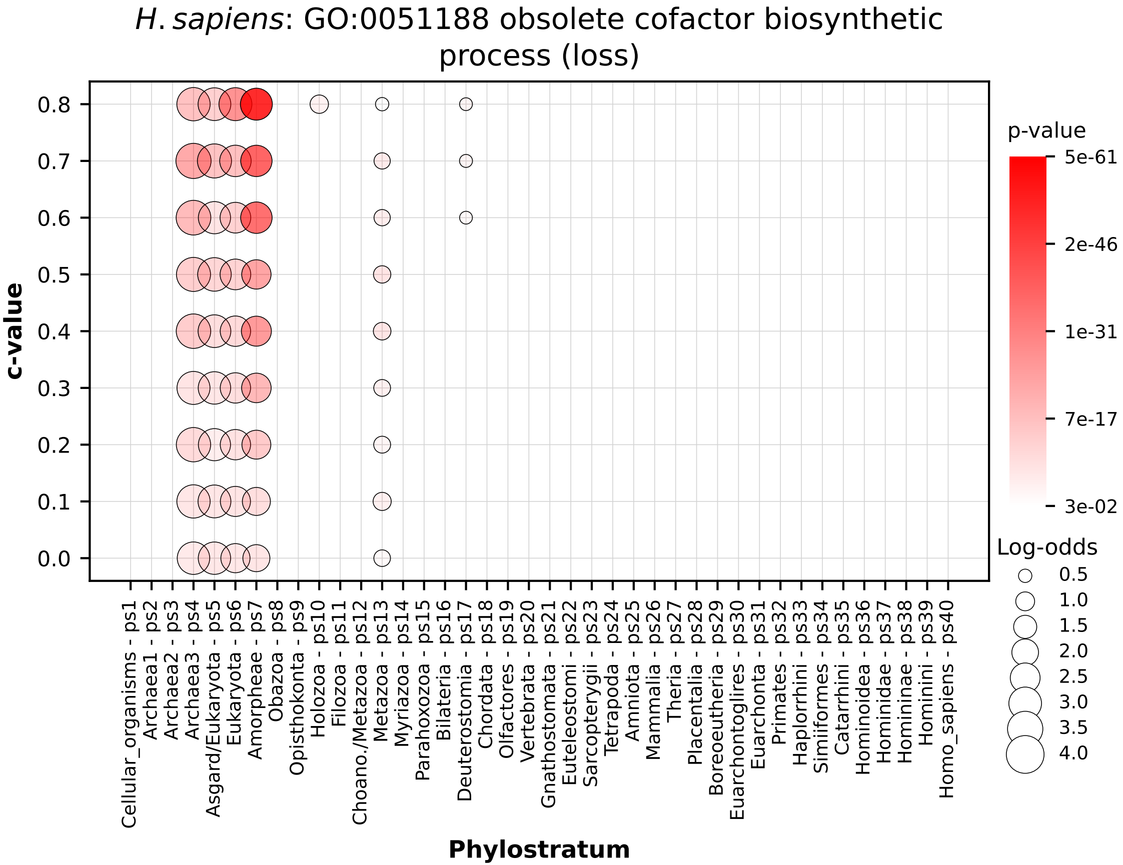

H. sapiens: GO:0051188 obsolete cofactor biosynthetic process (loss)
c-value
0.8
0.7
0.6
0.5
0.4
0.3
0.2
0.1
0.0
Phylostratum
Cellular_organisms - ps1
Archaea1 - ps2
Archaea2 - ps3
Archaea3 - ps4
Asgard/Eukaryota - ps5
Eukaryota - ps6
Amorpheae - ps7
Obazoa - ps8
Opisthokonta - ps9
Holozoa - ps10
Filozoa - ps11
Choano./Metazoa - ps12
Metazoa - ps13
Myriazoa - ps14
Parahoxozoa - ps15
Bilateria - ps16
Deuterostomia - ps17
Chordata - ps18
Olfactores - ps19
Vertebrata - ps20
Gnathostomata - ps21
Euteleostomi - ps22
Sarcopterygii - ps23
Tetrapoda - ps24
Amniota - ps25
Mammalia - ps26
Theria - ps27
Placentalia - ps28
Boreoeutheria - ps29
Euarchontoglires - ps30
Euarchonta - ps31
Primates - ps32
Haplorrhini - ps33
Simiiformes - ps34
Catarrhini - ps35
Hominoidea - ps36
Hominidae - ps37
Homininae - ps38
Hominini - ps39
Homo_sapiens - ps40
p-value
5e-61
2e-46
1e-31
7e-17
3e-02
Log-odds
0.5
1.0
1.5
2.0
2.5
3.0
3.5
4.0

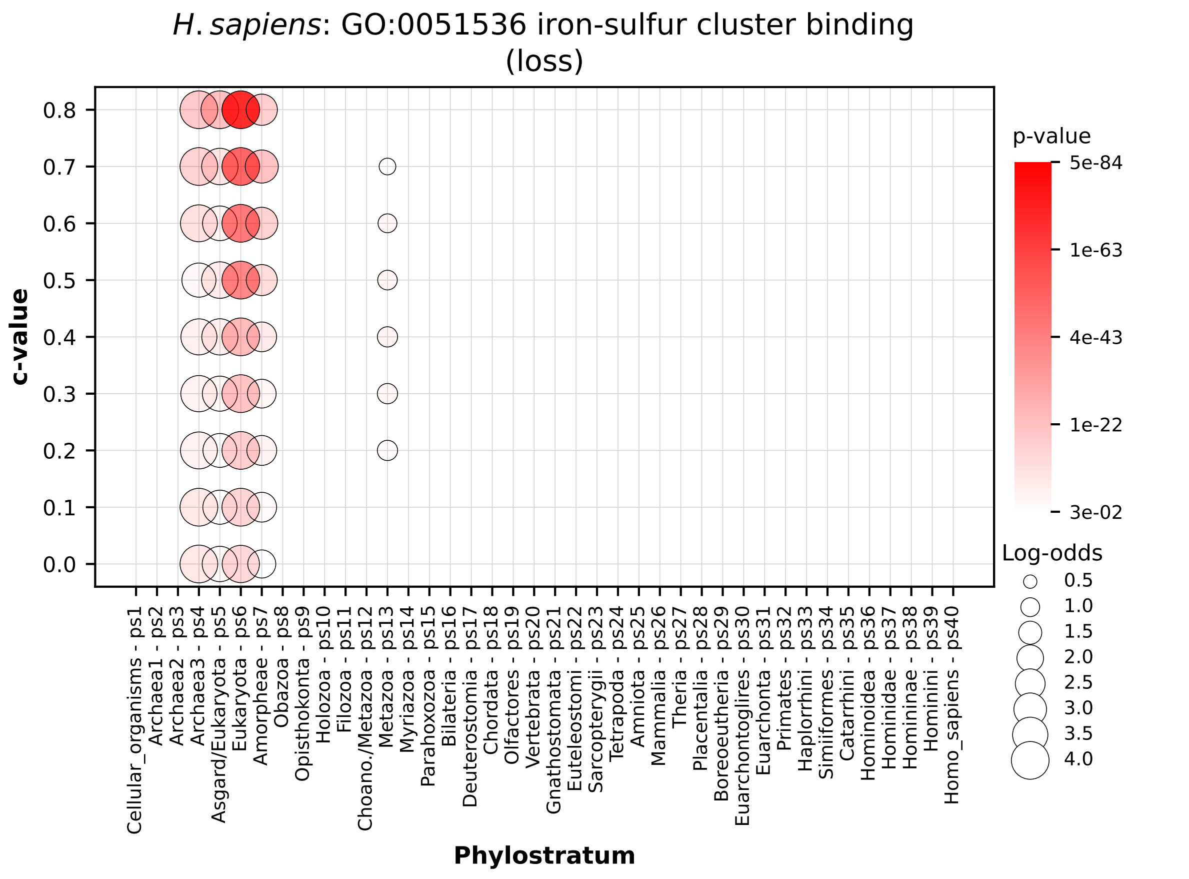

H. sapiens: GO:0051536 iron-sulfur cluster binding
(loss)
c-value
0.8
0.7
0.6
0.5
0.4
0.3
0.2
0.1
0.0
p-value
5e-84
1e-63
4e-43
1e-22
3e-02
Log-odds
0.5
1.0
1.5
2.0
2.5
3.0
3.5
4.0
Cellular_organisms - ps1
Archaea1 - ps2
Archaea2 - ps3
Archaea3 - ps4
Asgard/Eukaryota - ps5
Eukaryota - ps6
Amorpheae - ps7
Obazoa - ps8
Opisthokonta - ps9
Holozoa - ps10
Filozoa - ps11
Choano./Metazoa - ps12
Metazoa - ps13
Myriazoa - ps14
Parahoxozoa - ps15
Bilateria - ps16
Deuterostomia - ps17
Chordata - ps18
Olfactores - ps19
Vertebrata - ps20
Gnathostomata - ps21
Euteleostomi - ps22
Sarcopterygii - ps23
Tetrapoda - ps24
Amniota - ps25
Mammalia - ps26
Theria - ps27
Placentalia - ps28
Boreoeutheria - ps29
Euarchontoglires - ps30
Euarchonta - ps31
Primates - ps32
Haplorrhini - ps33
Simiiformes - ps34
Catarrhini - ps35
Hominoidea - ps36
Hominidae - ps37
Homininae - ps38
Hominini - ps39
Homo_sapiens - ps40
Phylostratum

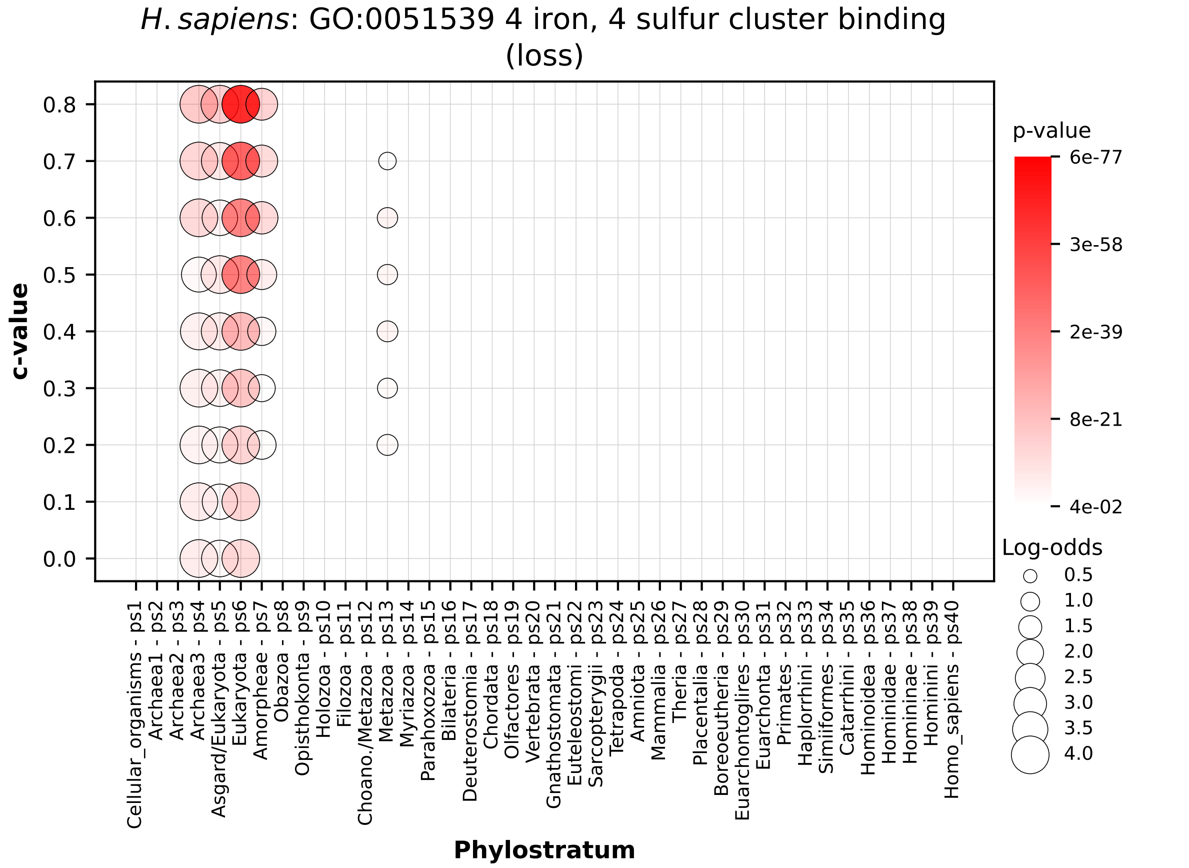

H. sapiens: GO:0051539 4 iron, 4 sulfur cluster binding (loss)
c-value
0.0
0.1
0.2
0.3
0.4
0.5
0.6
0.7
0.8
Phylostratum
Cellular_organisms - ps1
Archaea1 - ps2
Archaea2 - ps3
Archaea3 - ps4
Asgard/Eukaryota - ps5
Eukaryota - ps6
Amorpheae - ps7
Obazoa - ps8
Opisthokonta - ps9
Holozoa - ps10
Filozoa - ps11
Choano./Metazoa - ps12
Metazoa - ps13
Myriazoa - ps14
Parahoxozoa - ps15
Bilateria - ps16
Deuterostomia - ps17
Chordata - ps18
Olfactores - ps19
Vertebrata - ps20
Gnathostomata - ps21
Euteleostomi - ps22
Sarcopterygii - ps23
Tetrapoda - ps24
Amniota - ps25
Mammalia - ps26
Theria - ps27
Placentalia - ps28
Boreoeutheria - ps29
Euarchontoglires - ps30
Euarchonta - ps31
Primates - ps32
Haplorrhini - ps33
Simiiformes - ps34
Catarrhini - ps35
Hominoidea - ps36
Hominidae - ps37
Homininae - ps38
Hominini - ps39
Homo_sapiens - ps40
p-value
6e-77
3e-58
2e-39
8e-21
4e-02
Log-odds
0.5
1.0
1.5
2.0
2.5
3.0
3.5
4.0

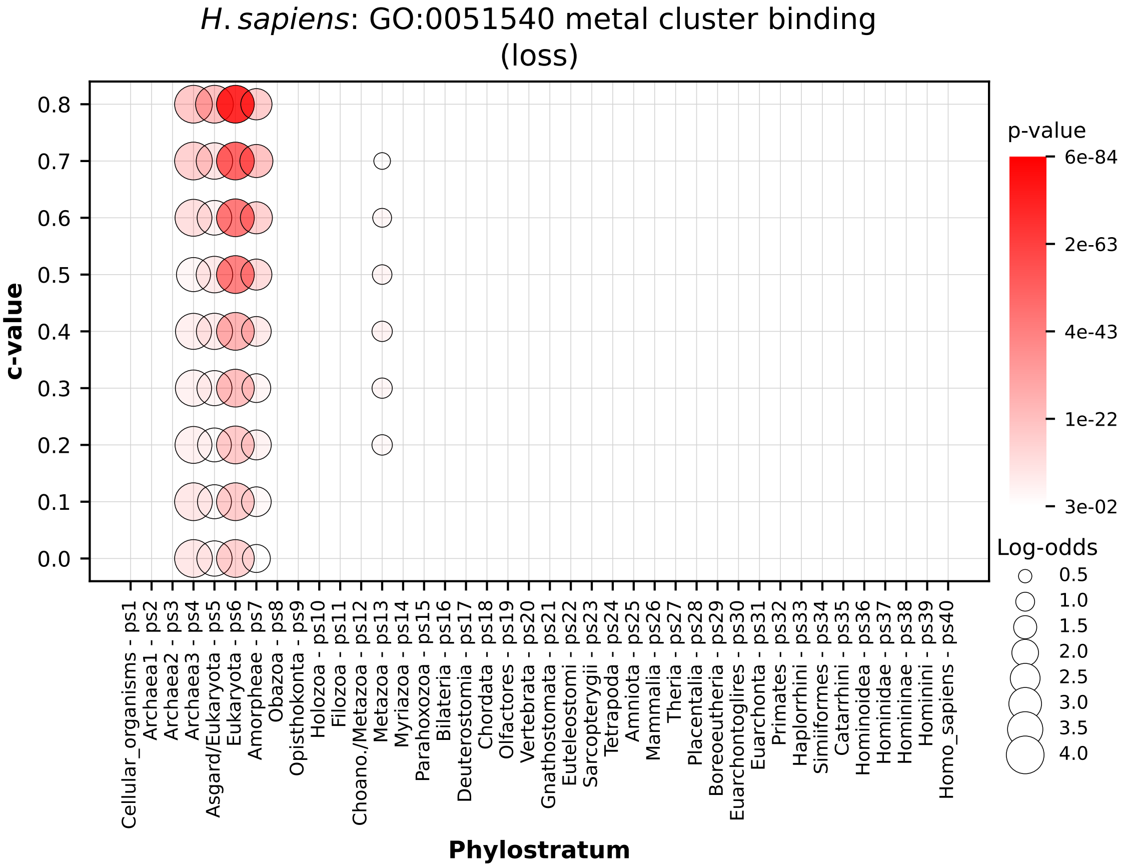

H. sapiens: GO:0051540 metal cluster binding
(loss)
c-value
0.8
0.7
0.6
0.5
0.4
0.3
0.2
0.1
0.0
Phylostratum
Cellular_organisms - ps1
Archaea1 - ps2
Archaea2 - ps3
Archaea3 - ps4
Asgard/Eukaryota - ps5
Eukaryota - ps6
Amorpheae - ps7
Obazoa - ps8
Opisthokonta - ps9
Holozoa - ps10
Filozoa - ps11
Choano./Metazoa - ps12
Metazoa - ps13
Myriazoa - ps14
Parahoxozoa - ps15
Bilateria - ps16
Deuterostomia - ps17
Chordata - ps18
Olfactores - ps19
Vertebrata - ps20
Gnathostomata - ps21
Euteleostomi - ps22
Sarcopterygii - ps23
Tetrapoda - ps24
Amniota - ps25
Mammalia - ps26
Theria - ps27
Placentalia - ps28
Boreoeutheria - ps29
Euarchontoglires - ps30
Euarchonta - ps31
Primates - ps32
Haplorrhini - ps33
Simiiformes - ps34
Catarrhini - ps35
Hominoidea - ps36
Hominidae - ps37
Homininae - ps38
Hominini - ps39
Homo_sapiens - ps40
p-value
6e-84
2e-63
4e-43
1e-22
3e-02
Log-odds
0.5
1.0
1.5
2.0
2.5
3.0
3.5
4.0

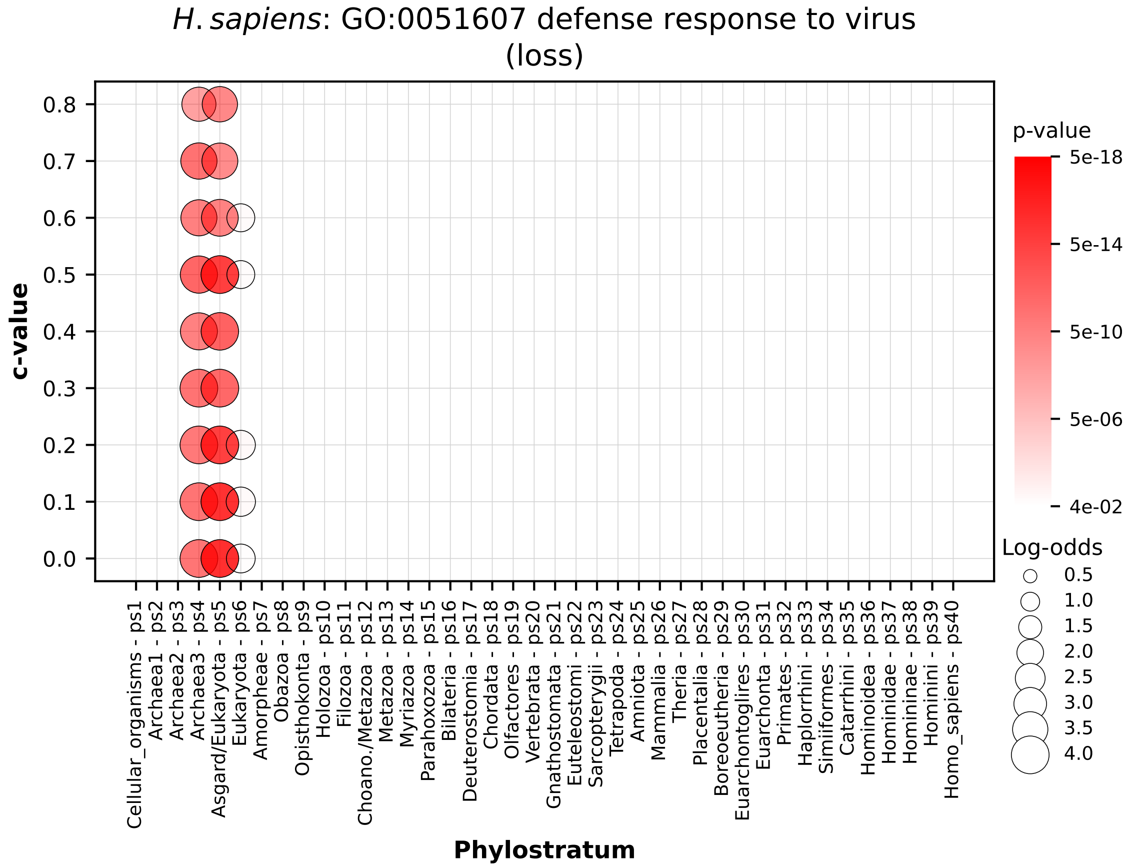

H. sapiens: GO:0051607 defense response to virus
(loss)
c-value
0.8
0.7
0.6
0.5
0.4
0.3
0.2
0.1
0.0
p-value
5e-18
5e-14
5e-10
5e-06
4e-02
Log-odds
0.5
1.0
1.5
2.0
2.5
3.0
3.5
4.0
Cellular_organisms - ps1
Archaea1 - ps2
Archaea2 - ps3
Archaea3 - ps4
Asgard/Eukaryota - ps5
Eukaryota - ps6
Amorpheae - ps7
Obazoa - ps8
Opisthokonta - ps9
Holozoa - ps10
Filozoa - ps11
Choano./Metazoa - ps12
Metazoa - ps13
Myriazoa - ps14
Parahoxozoa - ps15
Bilateria - ps16
Deuterostomia - ps17
Chordata - ps18
Olfactores - ps19
Vertebrata - ps20
Gnathostomata - ps21
Euteleostomi - ps22
Sarcopterygii - ps23
Tetrapoda - ps24
Amniota - ps25
Mammalia - ps26
Theria - ps27
Placentalia - ps28
Boreoeutheria - ps29
Euarchontoglires - ps30
Euarchonta - ps31
Primates - ps32
Haplorrhini - ps33
Simiiformes - ps34
Catarrhini - ps35
Hominoidea - ps36
Hominidae - ps37
Homininae - ps38
Hominini - ps39
Homo_sapiens - ps40
Phylostratum

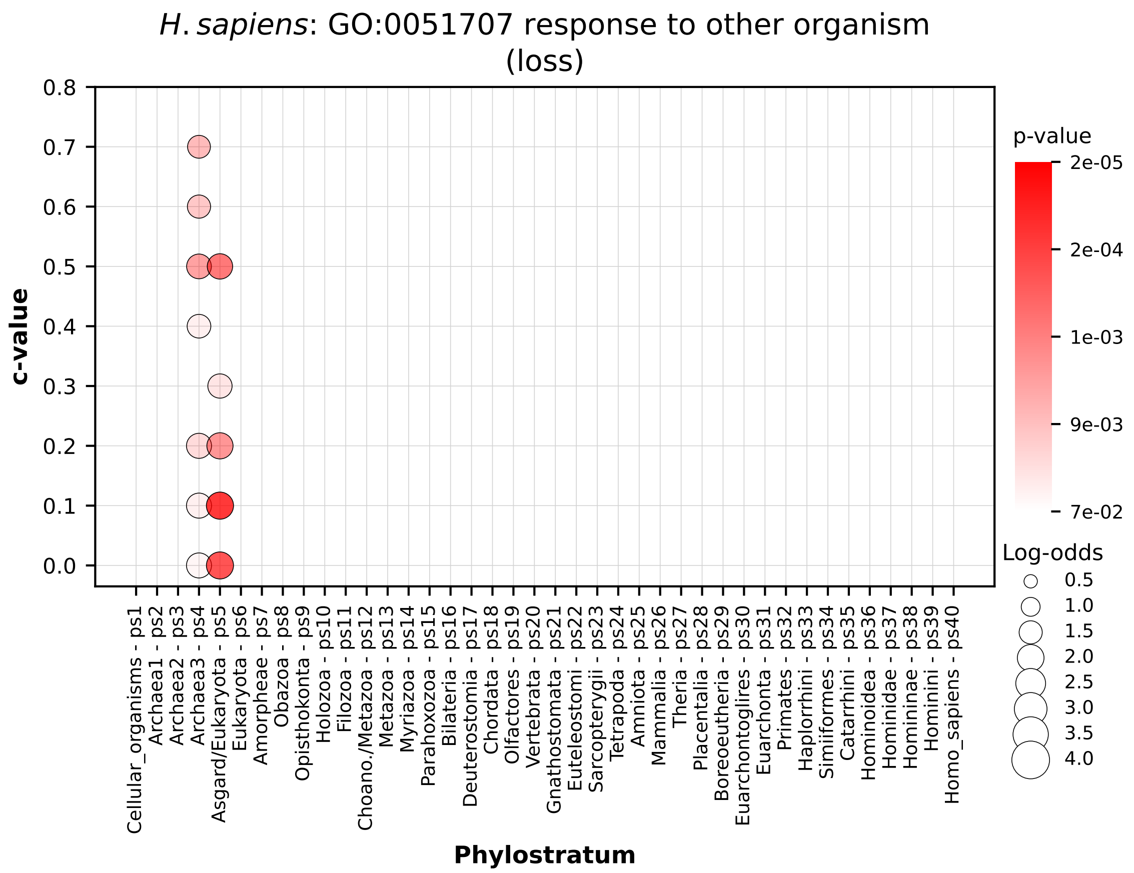
H. sapiens: GO:0051707 response to other organism
(loss)
c-value
0.8
0.7
0.6
0.5
0.4
0.3
0.2
0.1
0.0
p-value
2e-05
2e-04
1e-03
9e-03
7e-02
Log-odds
0.5
1.0
1.5
2.0
2.5
3.0
3.5
4.0
Cellular_organisms - ps1
Archaea1 - ps2
Archaea2 - ps3
Archaea3 - ps4
Asgard/Eukaryota - ps5
Eukaryota - ps6
Amorpheae - ps7
Obazoa - ps8
Opisthokonta - ps9
Holozoa - ps10
Filozoa - ps11
Choano./Metazoa - ps12
Metazoa - ps13
Myriazoa - ps14
Parahoxozoa - ps15
Bilateria - ps16
Deuterostomia - ps17
Chordata - ps18
Olfactores - ps19
Vertebrata - ps20
Gnathostomata - ps21
Euteleostomi - ps22
Sarcopterygii - ps23
Tetrapoda - ps24
Amniota - ps25
Mammalia - ps26
Theria - ps27
Placentalia - ps28
Boreoeutheria - ps29
Euarchontoglires - ps30
Euarchonta - ps31
Primates - ps32
Haplorrhini - ps33
Simiiformes - ps34
Catarrhini - ps35
Hominoidea - ps36
Hominidae - ps37
Homininae - ps38
Hominini - ps39
Homo_sapiens - ps40
Phylostratum

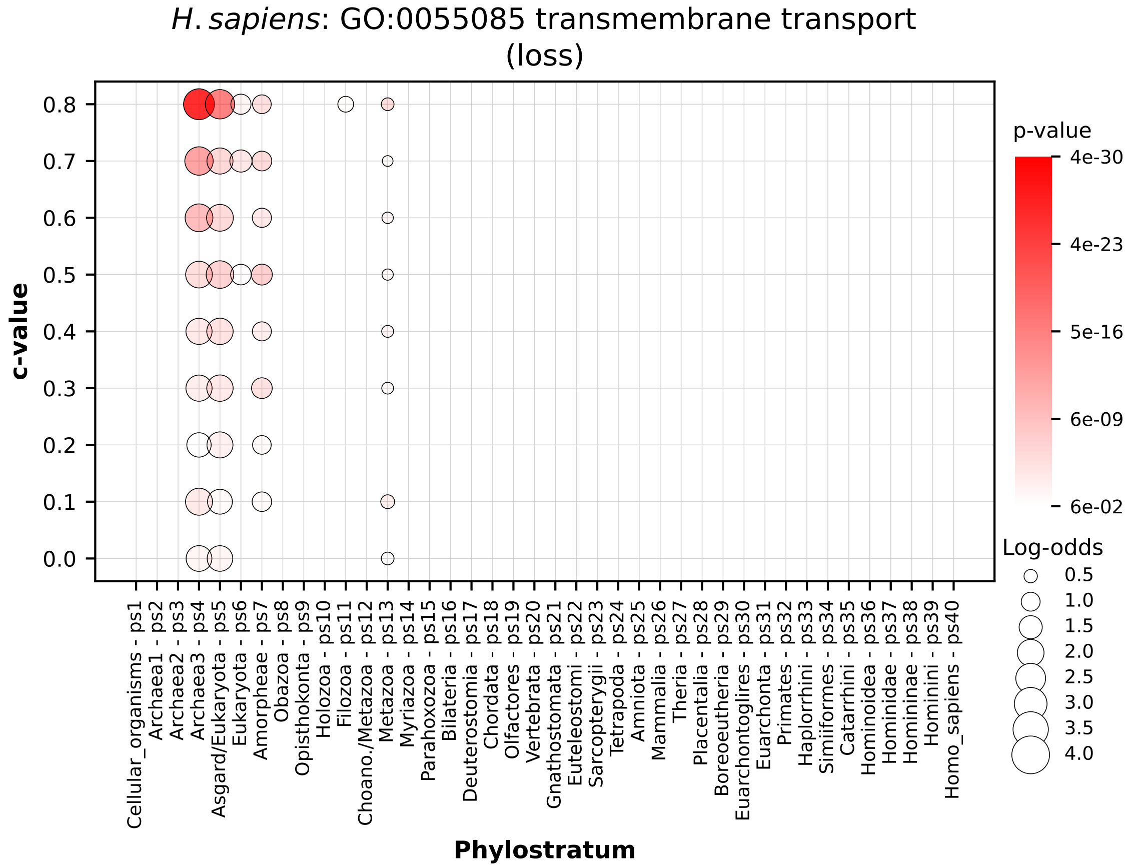

H. sapiens: GO:0055085 transmembrane transport
(loss)
c-value
0.8
0.7
0.6
0.5
0.4
0.3
0.2
0.1
0.0
p-value
4e-30
4e-23
5e-16
6e-09
6e-02
Log-odds
0.5
1.0
1.5
2.0
2.5
3.0
3.5
4.0
Cellular_organisms - ps1
Archaea1 - ps2
Archaea2 - ps3
Archaea3 - ps4
Asgard/Eukaryota - ps5
Eukaryota - ps6
Amorpheae - ps7
Obazoa - ps8
Opisthokonta - ps9
Holozoa - ps10
Filozoa - ps11
Choano./Metazoa - ps12
Metazoa - ps13
Myriazoa - ps14
Parahoxozoa - ps15
Bilateria - ps16
Deuterostomia - ps17
Chordata - ps18
Olfactores - ps19
Vertebrata - ps20
Gnathostomata - ps21
Euteleostomi - ps22
Sarcopterygii - ps23
Tetrapoda - ps24
Amniota - ps25
Mammalia - ps26
Theria - ps27
Placentalia - ps28
Boreoeutheria - ps29
Euarchontoglires - ps30
Euarchonta - ps31
Primates - ps32
Haplorrhini - ps33
Simiiformes - ps34
Catarrhini - ps35
Hominoidea - ps36
Hominidae - ps37
Homininae - ps38
Hominini - ps39
Homo_sapiens - ps40
Phylostratum

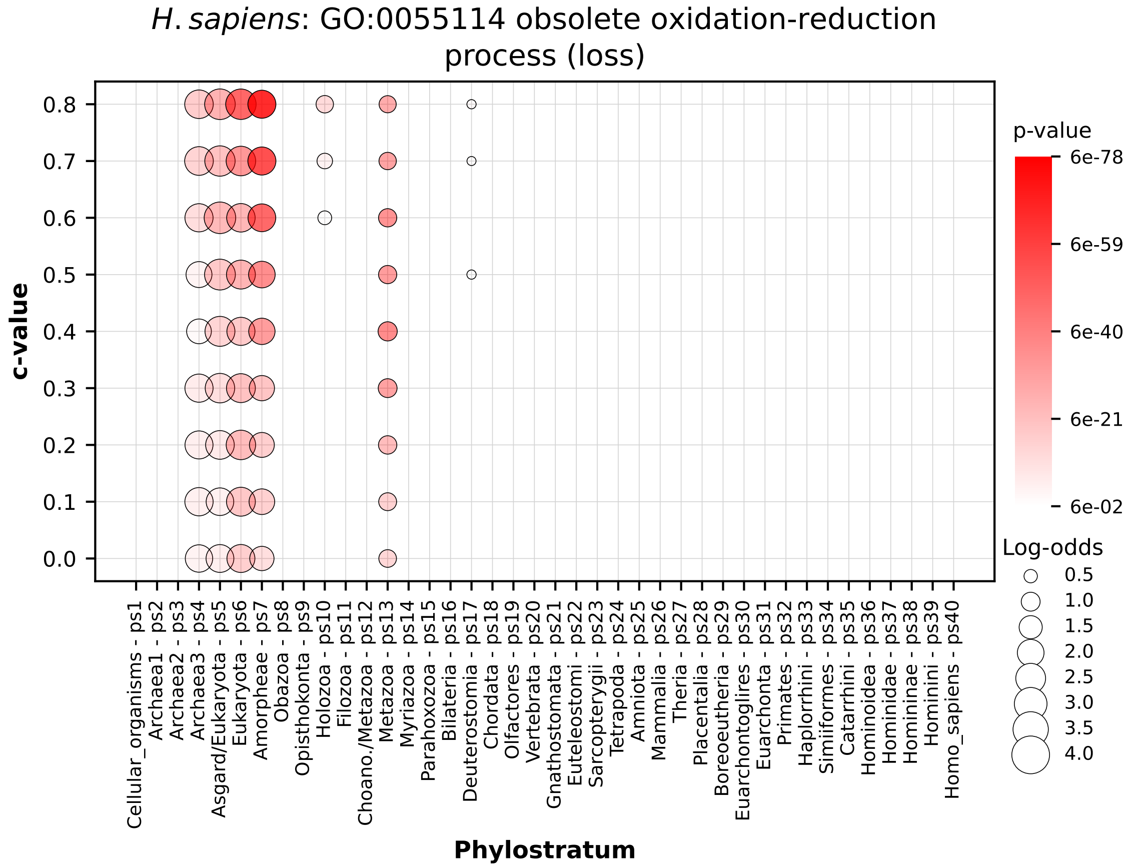

H. sapiens: GO:0055114 obsolete oxidation-reduction process (loss)
c-value
0.8
0.7
0.6
0.5
0.4
0.3
0.2
0.1
0.0
Phylostratum
Cellular_organisms - ps1
Archaea1 - ps2
Archaea2 - ps3
Archaea3 - ps4
Asgard/Eukaryota - ps5
Eukaryota - ps6
Amorpheae - ps7
Obazoa - ps8
Opisthokonta - ps9
Holozoa - ps10
Filozoa - ps11
Choano./Metazoa - ps12
Metazoa - ps13
Myriazoa - ps14
Parahoxozoa - ps15
Bilateria - ps16
Deuterostomia - ps17
Chordata - ps18
Olfactores - ps19
Vertebrata - ps20
Gnathostomata - ps21
Euteleostomi - ps22
Sarcopterygii - ps23
Tetrapoda - ps24
Amniota - ps25
Mammalia - ps26
Theria - ps27
Placentalia - ps28
Boreoeutheria - ps29
Euarchontoglires - ps30
Euarchonta - ps31
Primates - ps32
Haplorrhini - ps33
Simiiformes - ps34
Catarrhini - ps35
Hominoidea - ps36
Hominidae - ps37
Homininae - ps38
Hominini - ps39
Homo_sapiens - ps40
p-value
6e-78
6e-59
6e-40
6e-21
6e-02
Log-odds
0.5
1.0
1.5
2.0
2.5
3.0
3.5
4.0

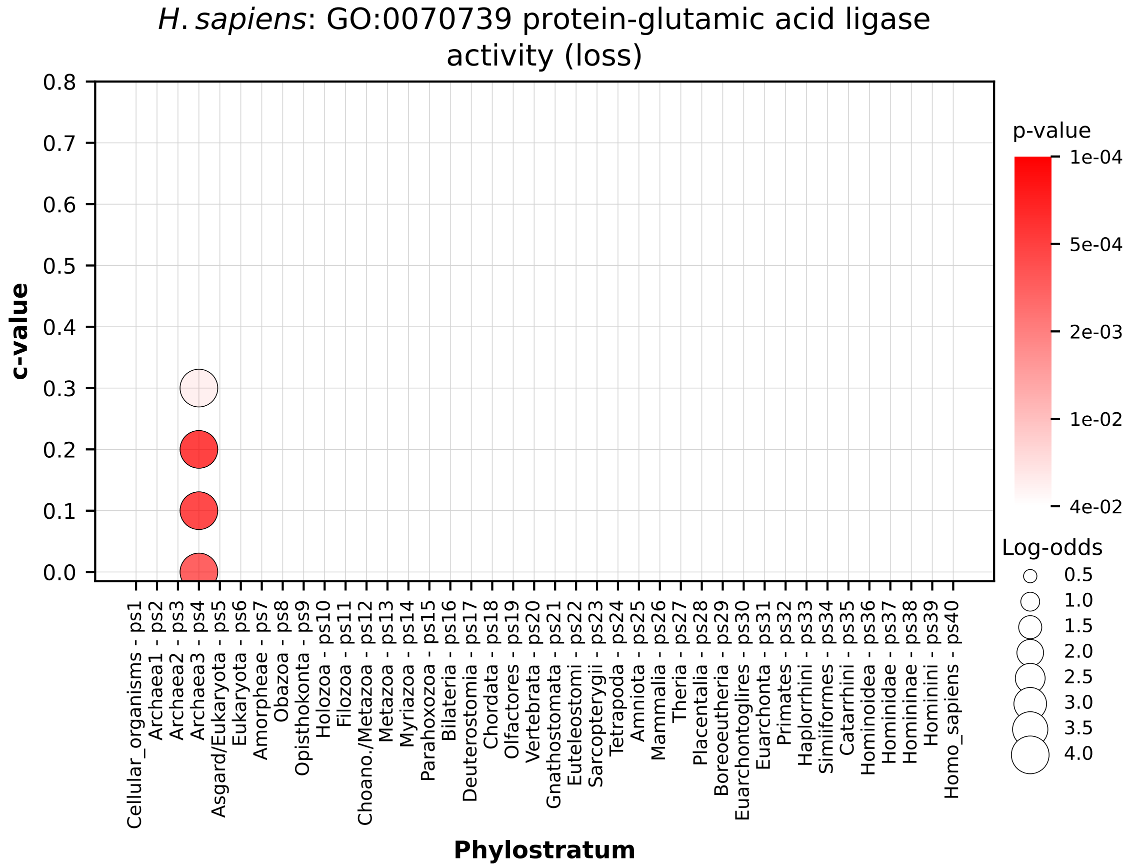

H. sapiens: GO:0070739 protein-glutamic acid ligase activity (loss)
c-value
0.0
0.1
0.2
0.3
0.4
0.5
0.6
0.7
0.8
Phylostratum
Cellular_organisms - ps1
Archaea1 - ps2
Archaea2 - ps3
Archaea3 - ps4
Asgard/Eukaryota - ps5
Eukaryota - ps6
Amorpheae - ps7
Obazoa - ps8
Opisthokonta - ps9
Holozoa - ps10
Filozoa - ps11
Choano./Metazoa - ps12
Metazoa - ps13
Myriazoa - ps14
Parahoxozoa - ps15
Bilateria - ps16
Deuterostomia - ps17
Chordata - ps18
Olfactores - ps19
Vertebrata - ps20
Gnathostomata - ps21
Euteleostomi - ps22
Sarcopterygii - ps23
Tetrapoda - ps24
Amniota - ps25
Mammalia - ps26
Theria - ps27
Placentalia - ps28
Boreoeutheria - ps29
Euarchontoglires - ps30
Euarchonta - ps31
Primates - ps32
Haplorrhini - ps33
Simiiformes - ps34
Catarrhini - ps35
Hominoidea - ps36
Hominidae - ps37
Homininae - ps38
Hominini - ps39
Homo_sapiens - ps40
p-value
1e-04
5e-04
2e-03
1e-02
4e-02
Log-odds
0.5
1.0
1.5
2.0
2.5
3.0
3.5
4.0

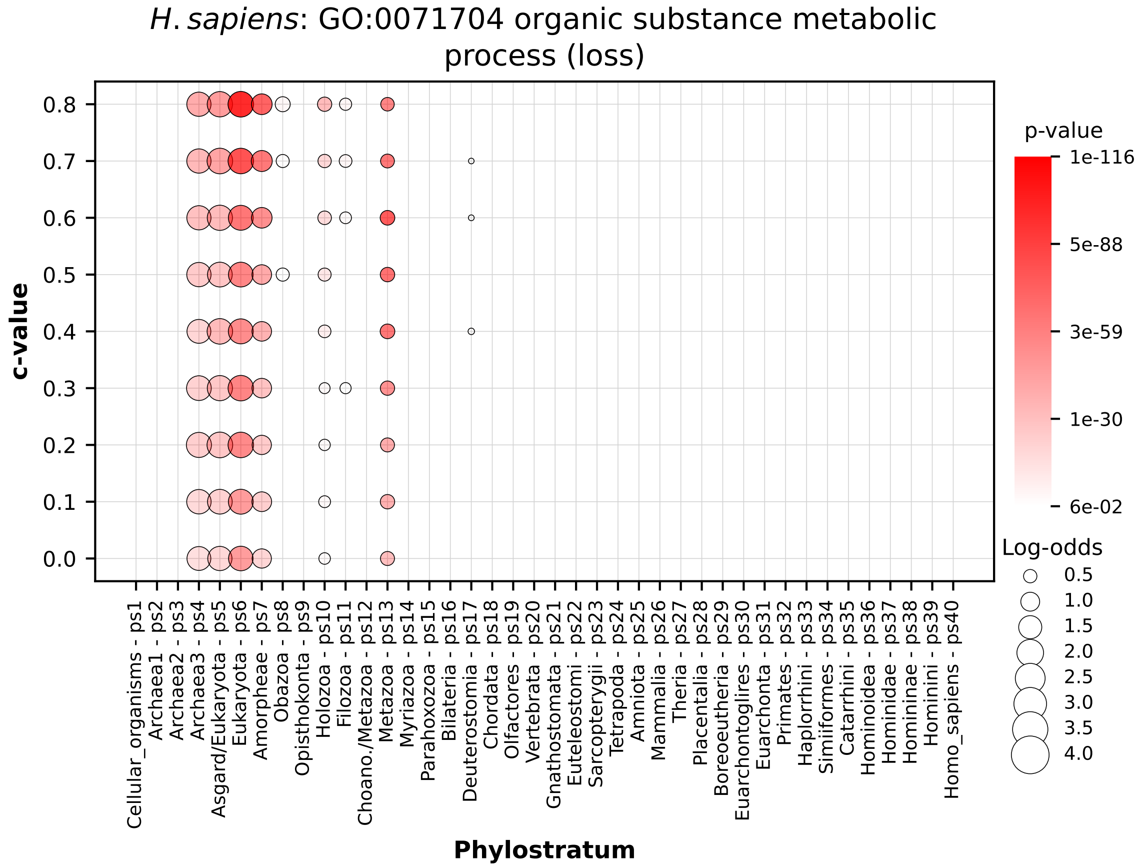

H. sapiens: GO:0071704 organic substance metabolic process (loss)
c-value
0.8
0.7
0.6
0.5
0.4
0.3
0.2
0.1
0.0
Phylostratum
Cellular_organisms - ps1
Archaea1 - ps2
Archaea2 - ps3
Archaea3 - ps4
Asgard/Eukaryota - ps5
Eukaryota - ps6
Amorpheae - ps7
Obazoa - ps8
Opisthokonta - ps9
Holozoa - ps10
Filozoa - ps11
Choano./Metazoa - ps12
Metazoa - ps13
Myriazoa - ps14
Parahoxozoa - ps15
Bilateria - ps16
Deuterostomia - ps17
Chordata - ps18
Olfactores - ps19
Vertebrata - ps20
Gnathostomata - ps21
Euteleostomi - ps22
Sarcopterygii - ps23
Tetrapoda - ps24
Amniota - ps25
Mammalia - ps26
Theria - ps27
Placentalia - ps28
Boreoeutheria - ps29
Euarchontoglires - ps30
Euarchonta - ps31
Primates - ps32
Haplorrhini - ps33
Simiiformes - ps34
Catarrhini - ps35
Hominoidea - ps36
Hominidae - ps37
Homininae - ps38
Hominini - ps39
Homo_sapiens - ps40
p-value
1e-116
5e-88
3e-59
1e-30
6e-02
Log-odds
0.5
1.0
1.5
2.0
2.5
3.0
3.5
4.0

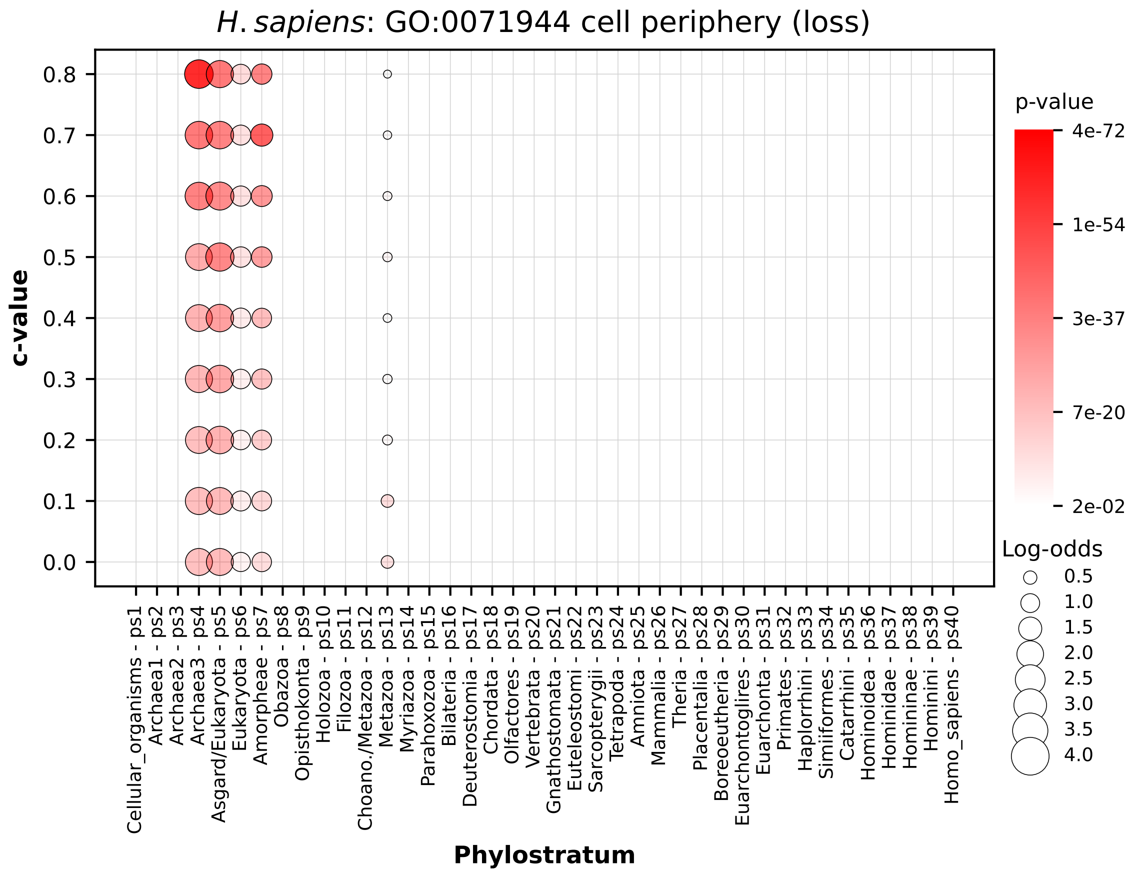

H. sapiens: GO:0071944 cell periphery (loss)
c-value
0.8
0.7
0.6
0.5
0.4
0.3
0.2
0.1
0.0
Phylostratum
Cellular_organisms - ps1
Archaea1 - ps2
Archaea2 - ps3
Archaea3 - ps4
Asgard/Eukaryota - ps5
Eukaryota - ps6
Amorpheae - ps7
Obazoa - ps8
Opisthokonta - ps9
Holozoa - ps10
Filozoa - ps11
Choano./Metazoa - ps12
Metazoa - ps13
Myriazoa - ps14
Parahoxozoa - ps15
Bilateria - ps16
Deuterostomia - ps17
Chordata - ps18
Olfactores - ps19
Vertebrata - ps20
Gnathostomata - ps21
Euteleostomi - ps22
Sarcopterygii - ps23
Tetrapoda - ps24
Amniota - ps25
Mammalia - ps26
Theria - ps27
Placentalia - ps28
Boreoeutheria - ps29
Euarchontoglires - ps30
Euarchonta - ps31
Primates - ps32
Haplorrhini - ps33
Simiiformes - ps34
Catarrhini - ps35
Hominoidea - ps36
Hominidae - ps37
Homininae - ps38
Hominini - ps39
Homo_sapiens - ps40
p-value
4e-72
1e-54
3e-37
7e-20
2e-02
Log-odds
0.5
1.0
1.5
2.0
2.5
3.0
3.5
4.0

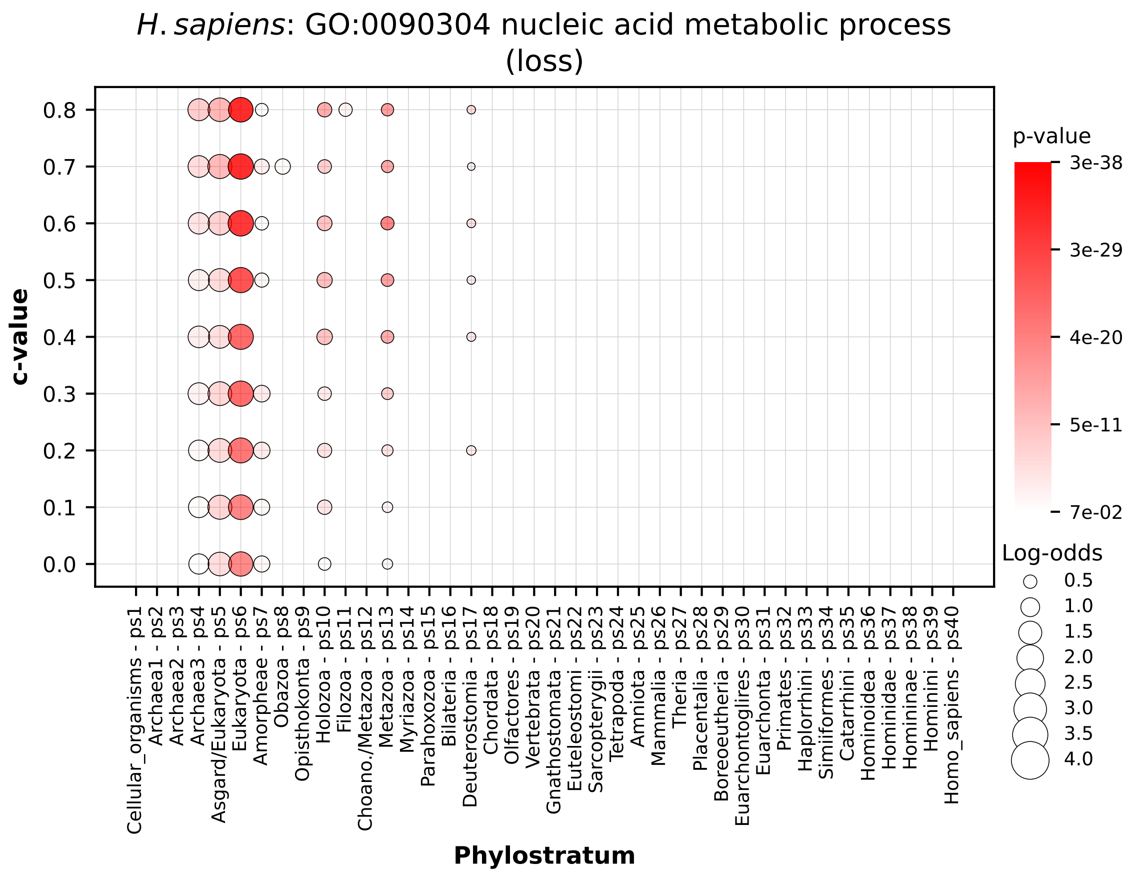

H. sapiens: GO:0090304 nucleic acid metabolic process
(loss)
c-value
0.8
0.7
0.6
0.5
0.4
0.3
0.2
0.1
0.0
p-value
3e-38
3e-29
4e-20
5e-11
7e-02
Log-odds
0.5
1.0
1.5
2.0
2.5
3.0
3.5
4.0
Cellular_organisms - ps1
Archaea1 - ps2
Archaea2 - ps3
Archaea3 - ps4
Asgard/Eukaryota - ps5
Eukaryota - ps6
Amorpheae - ps7
Obazoa - ps8
Opisthokonta - ps9
Holozoa - ps10
Filozoa - ps11
Choano./Metazoa - ps12
Metazoa - ps13
Myriazoa - ps14
Parahoxozoa - ps15
Bilateria - ps16
Deuterostomia - ps17
Chordata - ps18
Olfactores - ps19
Vertebrata - ps20
Gnathostomata - ps21
Euteleostomi - ps22
Sarcopterygii - ps23
Tetrapoda - ps24
Amniota - ps25
Mammalia - ps26
Theria - ps27
Placentalia - ps28
Boreoeutheria - ps29
Euarchontoglires - ps30
Euarchonta - ps31
Primates - ps32
Haplorrhini - ps33
Simiiformes - ps34
Catarrhini - ps35
Hominoidea - ps36
Hominidae - ps37
Homininae - ps38
Hominini - ps39
Homo_sapiens - ps40
Phylostratum

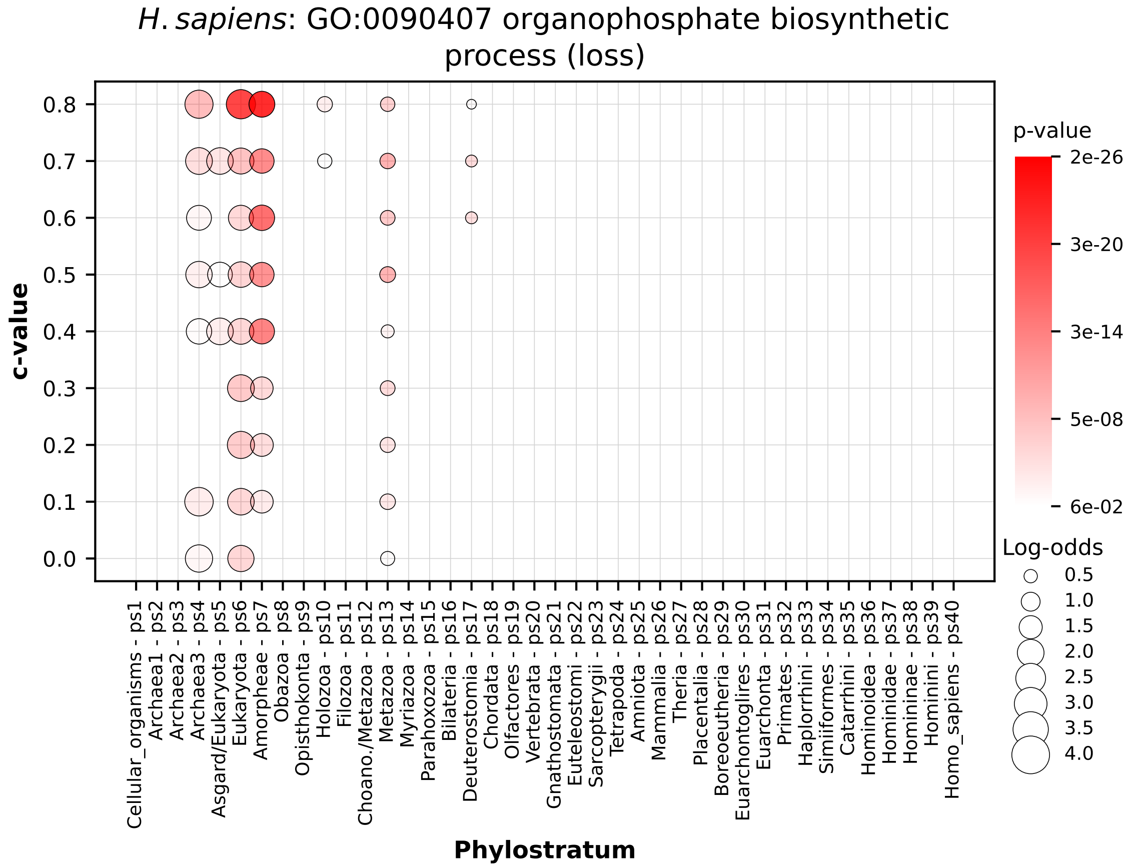

H. sapiens: GO:0090407 organophosphate biosynthetic process (loss)
c-value
0.8
0.7
0.6
0.5
0.4
0.3
0.2
0.1
0.0
p-value
2e-26
3e-20
3e-14
5e-08
6e-02
Log-odds
0.5
1.0
1.5
2.0
2.5
3.0
3.5
4.0
Cellular_organisms - ps1
Archaea1 - ps2
Archaea2 - ps3
Archaea3 - ps4
Asgard/Eukaryota - ps5
Eukaryota - ps6
Amorpheae - ps7
Obazoa - ps8
Opisthokonta - ps9
Holozoa - ps10
Filozoa - ps11
Choano./Metazoa - ps12
Metazoa - ps13
Myriazoa - ps14
Parahoxozoa - ps15
Bilateria - ps16
Deuterostomia - ps17
Chordata - ps18
Olfactores - ps19
Vertebrata - ps20
Gnathostomata - ps21
Euteleostomi - ps22
Sarcopterygii - ps23
Tetrapoda - ps24
Amniota - ps25
Mammalia - ps26
Theria - ps27
Placentalia - ps28
Boreoeutheria - ps29
Euarchontoglires - ps30
Euarchonta - ps31
Primates - ps32
Haplorrhini - ps33
Simiiformes - ps34
Catarrhini - ps35
Hominoidea - ps36
Hominidae - ps37
Homininae - ps38
Hominini - ps39
Homo_sapiens - ps40
Phylostratum

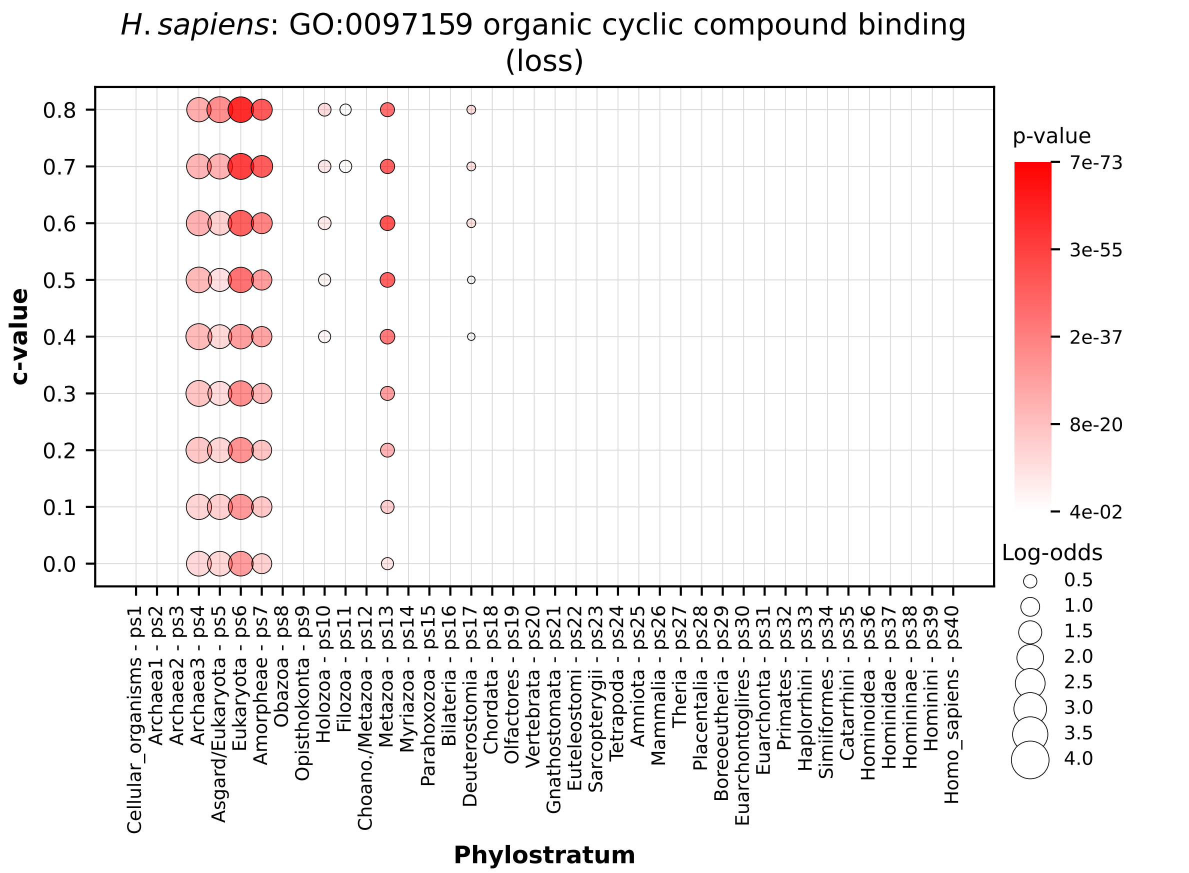

H. sapiens: GO:0097159 organic cyclic compound binding
(loss)
c-value
0.8
0.7
0.6
0.5
0.4
0.3
0.2
0.1
0.0
p-value
7e-73
3e-55
2e-37
8e-20
4e-02
Log-odds
0.5
1.0
1.5
2.0
2.5
3.0
3.5
4.0
Cellular_organisms - ps1
Archaea1 - ps2
Archaea2 - ps3
Archaea3 - ps4
Asgard/Eukaryota - ps5
Eukaryota - ps6
Amorpheae - ps7
Obazoa - ps8
Opisthokonta - ps9
Holozoa - ps10
Filozoa - ps11
Choano./Metazoa - ps12
Metazoa - ps13
Myriazoa - ps14
Parahoxozoa - ps15
Bilateria - ps16
Deuterostomia - ps17
Chordata - ps18
Olfactores - ps19
Vertebrata - ps20
Gnathostomata - ps21
Euteleostomi - ps22
Sarcopterygii - ps23
Tetrapoda - ps24
Amniota - ps25
Mammalia - ps26
Theria - ps27
Placentalia - ps28
Boreoeutheria - ps29
Euarchontoglires - ps30
Euarchonta - ps31
Primates - ps32
Haplorrhini - ps33
Simiiformes - ps34
Catarrhini - ps35
Hominoidea - ps36
Hominidae - ps37
Homininae - ps38
Hominini - ps39
Homo_sapiens - ps40
Phylostratum

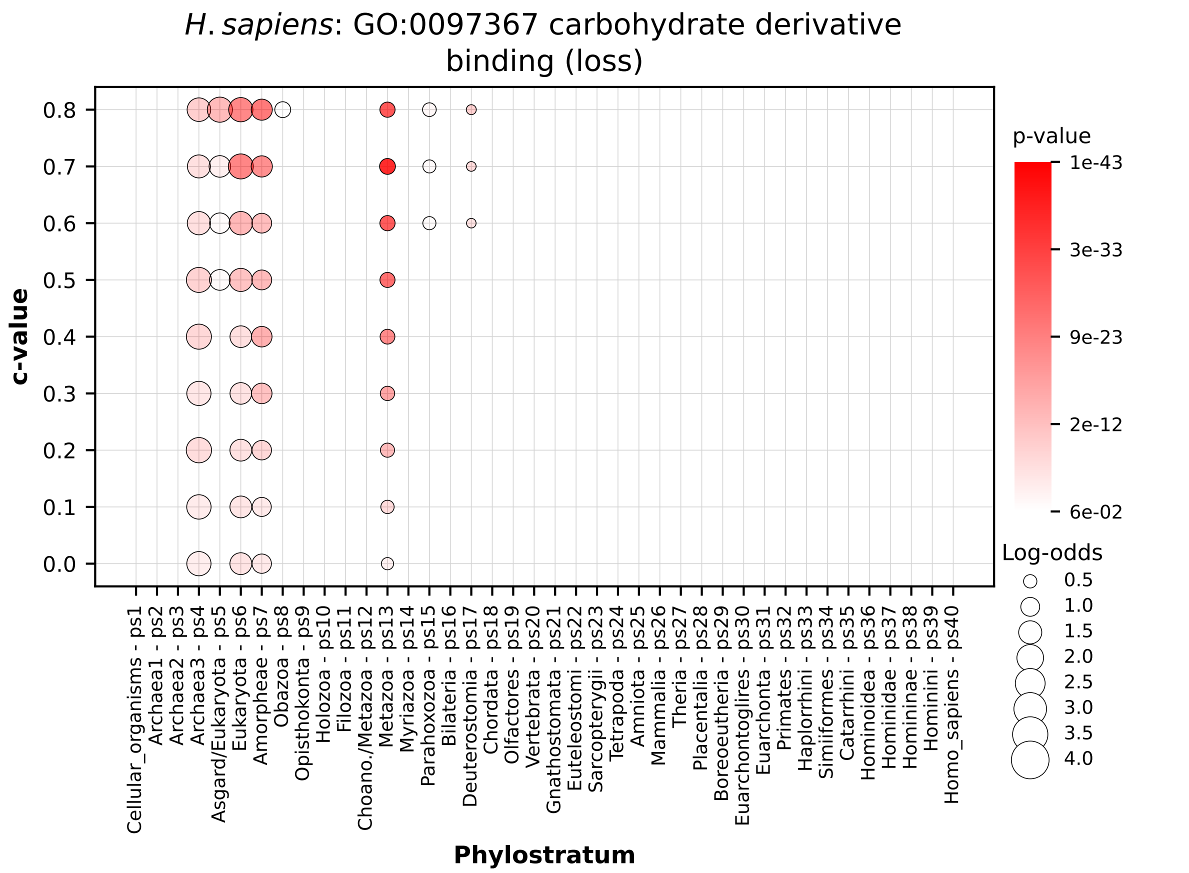

H. sapiens: GO:0097367 carbohydrate derivative binding (loss)
c-value
0.0
0.1
0.2
0.3
0.4
0.5
0.6
0.7
0.8
Phylostratum
Cellular_organisms - ps1
Archaea1 - ps2
Archaea2 - ps3
Archaea3 - ps4
Asgard/Eukaryota - ps5
Eukaryota - ps6
Amorpheae - ps7
Obazoa - ps8
Opisthokonta - ps9
Holozoa - ps10
Filozoa - ps11
Choano./Metazoa - ps12
Metazoa - ps13
Myriazoa - ps14
Parahoxozoa - ps15
Bilateria - ps16
Deuterostomia - ps17
Chordata - ps18
Olfactores - ps19
Vertebrata - ps20
Gnathostomata - ps21
Euteleostomi - ps22
Sarcopterygii - ps23
Tetrapoda - ps24
Amniota - ps25
Mammalia - ps26
Theria - ps27
Placentalia - ps28
Boreoeutheria - ps29
Euarchontoglires - ps30
Euarchonta - ps31
Primates - ps32
Haplorrhini - ps33
Simiiformes - ps34
Catarrhini - ps35
Hominoidea - ps36
Hominidae - ps37
Homininae - ps38
Hominini - ps39
Homo_sapiens - ps40
p-value
1e-43
3e-33
9e-23
2e-12
6e-02
Log-odds
0.5
1.0
1.5
2.0
2.5
3.0
3.5
4.0

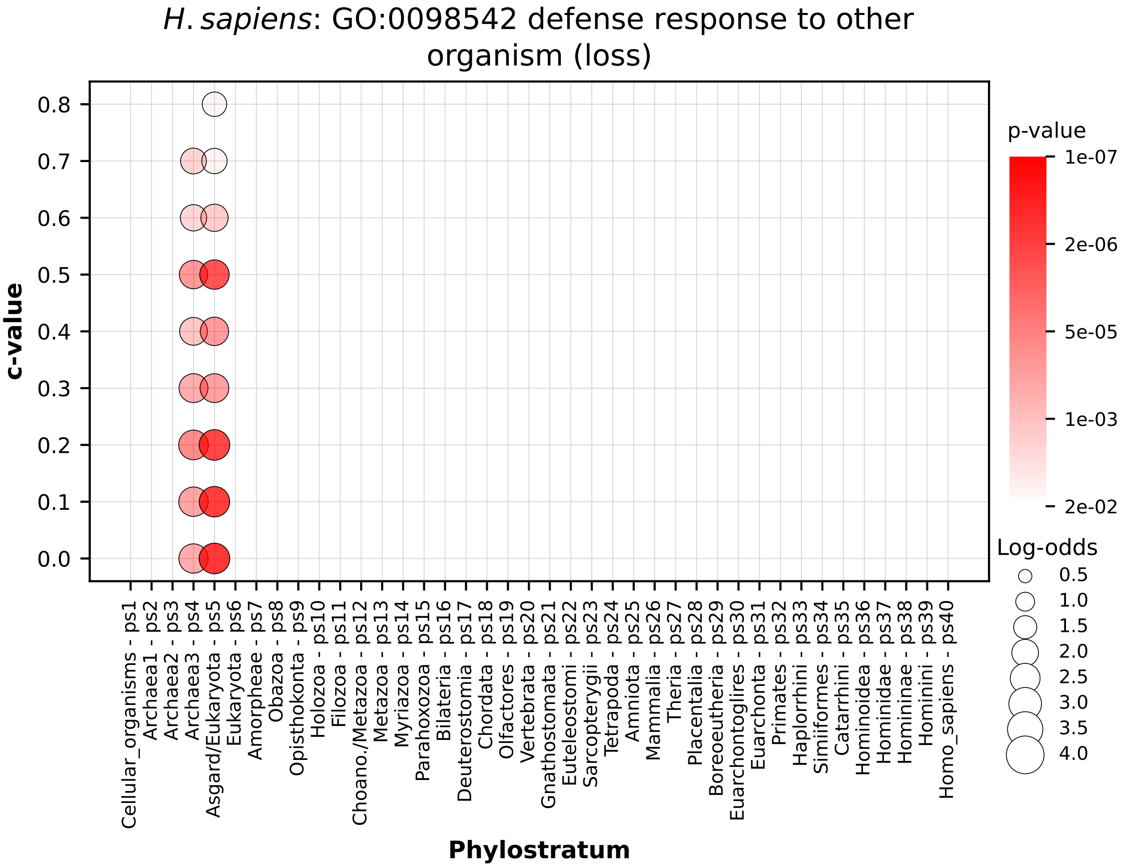

H. sapiens: GO:0098542 defense response to other organism (loss)
c-value
0.8
0.7
0.6
0.5
0.4
0.3
0.2
0.1
0.0
p-value
1e-07
2e-06
5e-05
1e-03
2e-02
Log-odds
0.5
1.0
1.5
2.0
2.5
3.0
3.5
4.0
Cellular_organisms - ps1
Archaea1 - ps2
Archaea2 - ps3
Archaea3 - ps4
Asgard/Eukaryota - ps5
Eukaryota - ps6
Amorpheae - ps7
Obazoa - ps8
Opisthokonta - ps9
Holozoa - ps10
Filozoa - ps11
Choano./Metazoa - ps12
Metazoa - ps13
Myriazoa - ps14
Parahoxozoa - ps15
Bilateria - ps16
Deuterostomia - ps17
Chordata - ps18
Olfactores - ps19
Vertebrata - ps20
Gnathostomata - ps21
Euteleostomi - ps22
Sarcopterygii - ps23
Tetrapoda - ps24
Amniota - ps25
Mammalia - ps26
Theria - ps27
Placentalia - ps28
Boreoeutheria - ps29
Euarchontoglires - ps30
Euarchonta - ps31
Primates - ps32
Haplorrhini - ps33
Simiiformes - ps34
Catarrhini - ps35
Hominoidea - ps36
Hominidae - ps37
Homininae - ps38
Hominini - ps39
Homo_sapiens - ps40
Phylostratum

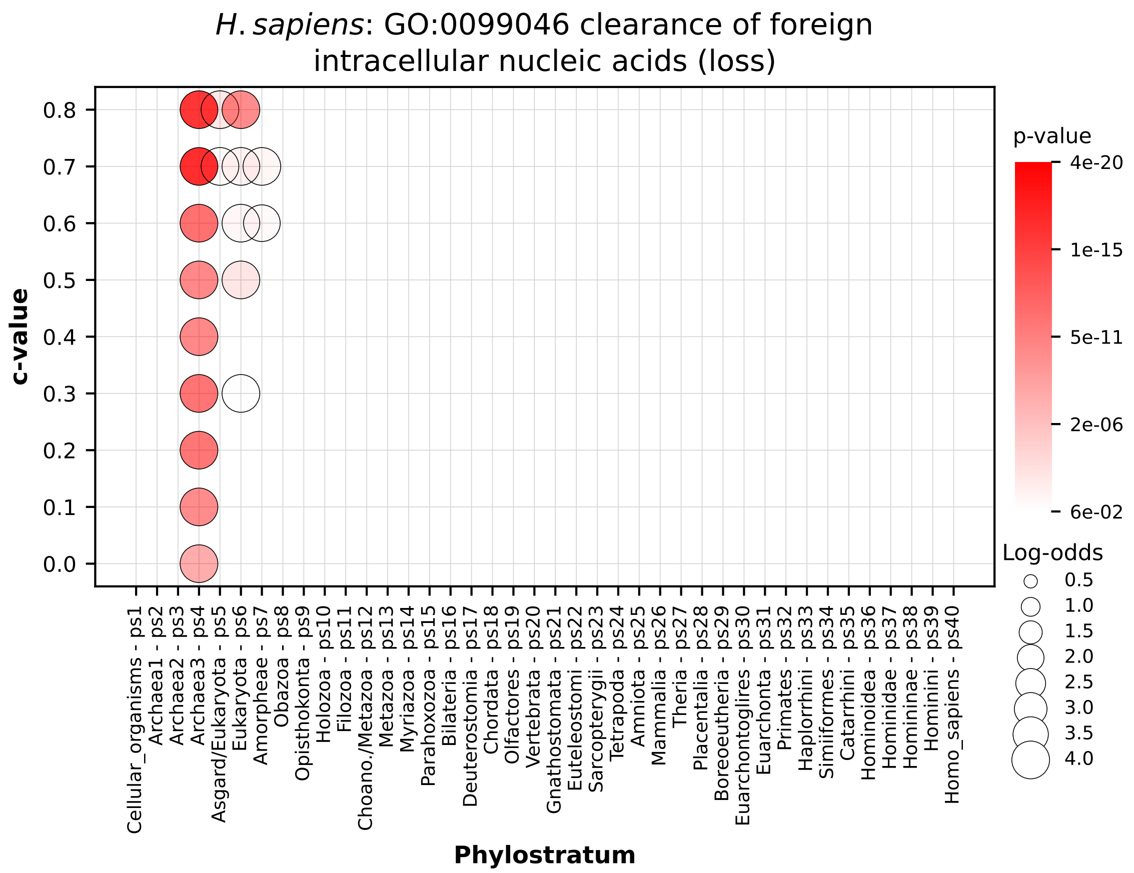

H. sapiens: GO:0099046 clearance of foreign intracellular nucleic acids (loss)
c-value
0.8
0.7
0.6
0.5
0.4
0.3
0.2
0.1
0.0
p-value
4e-20
1e-15
5e-11
2e-06
6e-02
Log-odds
0.5
1.0
1.5
2.0
2.5
3.0
3.5
4.0
Cellular_organisms - ps1
Archaea1 - ps2
Archaea2 - ps3
Archaea3 - ps4
Asgard/Eukaryota - ps5
Eukaryota - ps6
Amorpheae - ps7
Obazoa - ps8
Opisthokonta - ps9
Holozoa - ps10
Filozoa - ps11
Choano./Metazoa - ps12
Metazoa - ps13
Myriazoa - ps14
Parahoxozoa - ps15
Bilateria - ps16
Deuterostomia - ps17
Chordata - ps18
Olfactores - ps19
Vertebrata - ps20
Gnathostomata - ps21
Euteleostomi - ps22
Sarcopterygii - ps23
Tetrapoda - ps24
Amniota - ps25
Mammalia - ps26
Theria - ps27
Placentalia - ps28
Boreoeutheria - ps29
Euarchontoglires - ps30
Euarchonta - ps31
Primates - ps32
Haplorrhini - ps33
Simiiformes - ps34
Catarrhini - ps35
Hominoidea - ps36
Hominidae - ps37
Homininae - ps38
Hominini - ps39
Homo_sapiens - ps40
Phylostratum

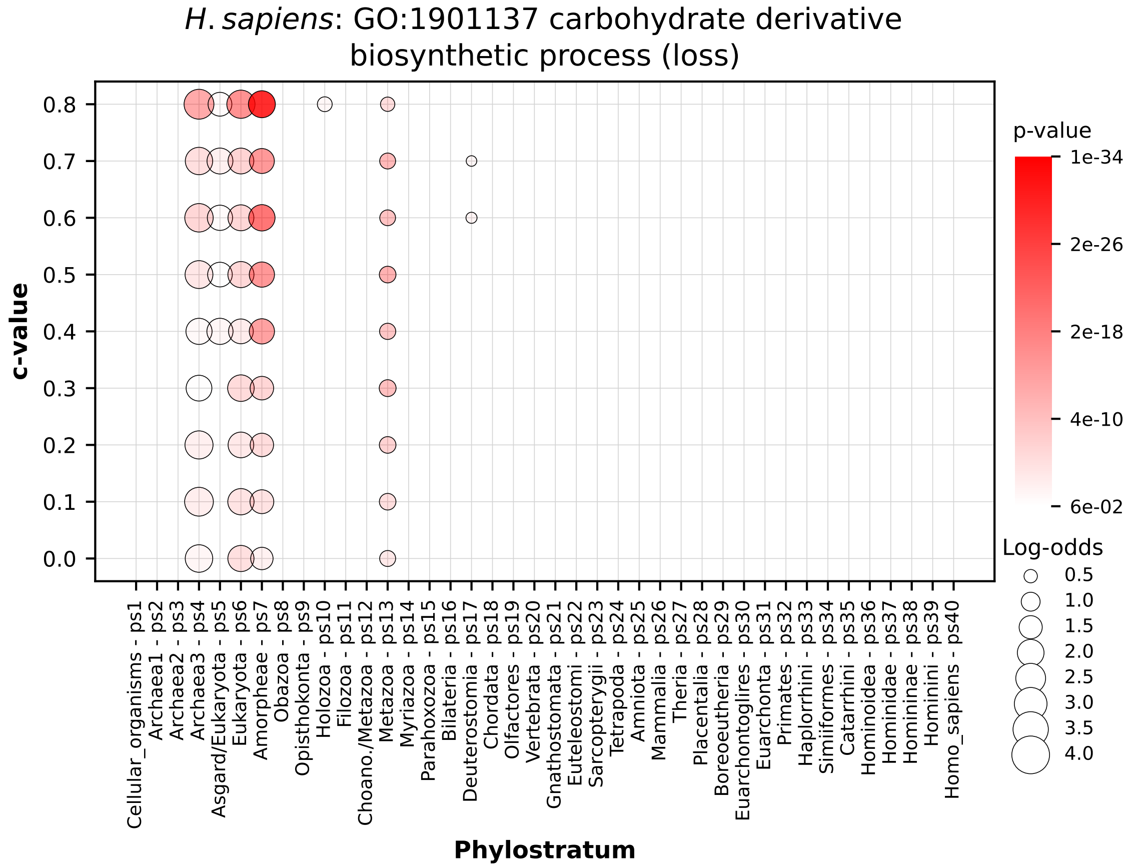
H. sapiens: GO:1901137 carbohydrate derivative biosynthetic process (loss)
c-value
Phylostratum
p-value
1e-34
2e-26
2e-18
4e-10
6e-02
Log-odds
0.5
1.0
1.5
2.0
2.5
3.0
3.5
4.0
Cellular_organisms - ps1
Archaea1 - ps2
Archaea2 - ps3
Archaea3 - ps4
Asgard/Eukaryota - ps5
Eukaryota - ps6
Amorpheae - ps7
Obazoa - ps8
Opisthokonta - ps9
Holozoa - ps10
Filozoa - ps11
Choano./Metazoa - ps12
Metazoa - ps13
Myriazoa - ps14
Parahoxozoa - ps15
Bilateria - ps16
Deuterostomia - ps17
Chordata - ps18
Olfactores - ps19
Vertebrata - ps20
Gnathostomata - ps21
Euteleostomi - ps22
Sarcopterygii - ps23
Tetrapoda - ps24
Amniota - ps25
Mammalia - ps26
Theria - ps27
Placentalia - ps28
Boreoeutheria - ps29
Euarchontoglires - ps30
Euarchonta - ps31
Primates - ps32
Haplorrhini - ps33
Simiiformes - ps34
Catarrhini - ps35
Hominoidea - ps36
Hominidae - ps37
Homininae - ps38
Hominini - ps39
Homo_sapiens - ps40

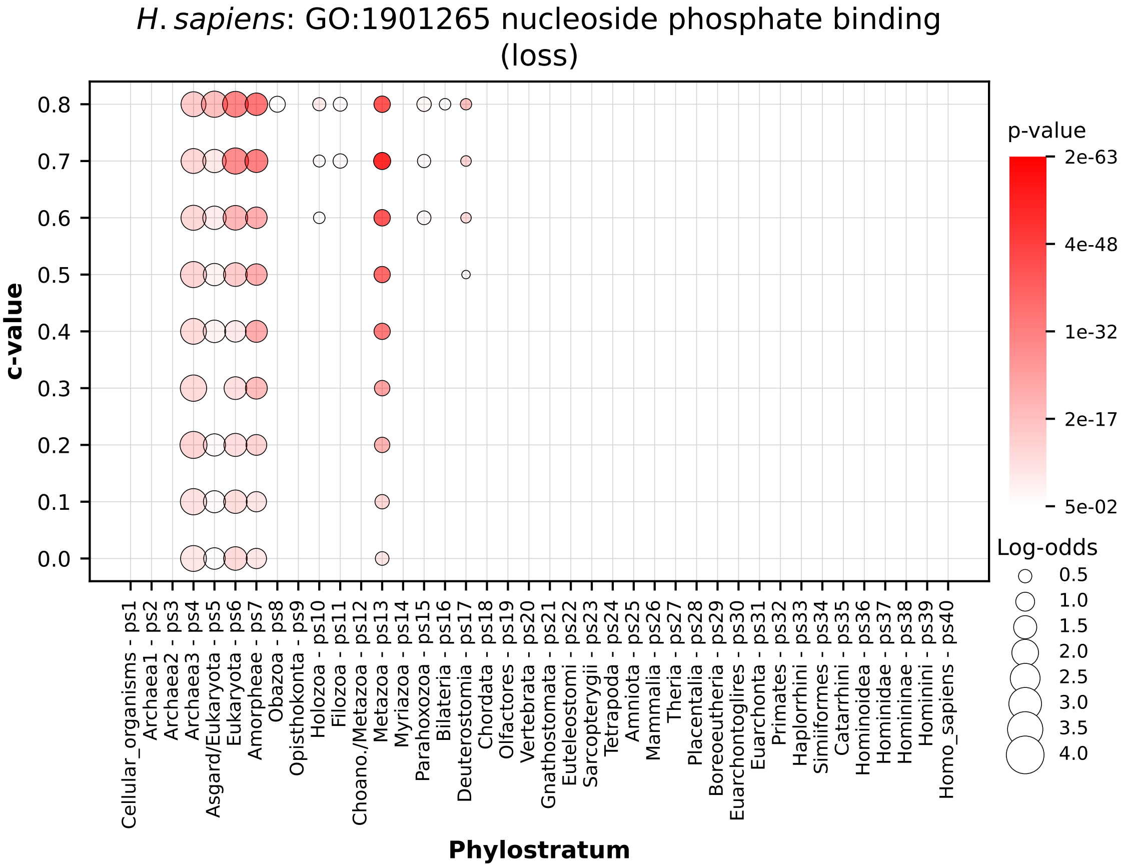

H. sapiens: GO:1901265 nucleoside phosphate binding
(loss)
c-value
0.8
0.7
0.6
0.5
0.4
0.3
0.2
0.1
0.0
p-value
2e-63
4e-48
1e-32
2e-17
5e-02
Log-odds
0.5
1.0
1.5
2.0
2.5
3.0
3.5
4.0
Cellular_organisms - ps1
Archaea1 - ps2
Archaea2 - ps3
Archaea3 - ps4
Asgard/Eukaryota - ps5
Eukaryota - ps6
Amorpheae - ps7
Obazoa - ps8
Opisthokonta - ps9
Holozoa - ps10
Filozoa - ps11
Choano./Metazoa - ps12
Metazoa - ps13
Myriazoa - ps14
Parahoxozoa - ps15
Bilateria - ps16
Deuterostomia - ps17
Chordata - ps18
Olfactores - ps19
Vertebrata - ps20
Gnathostomata - ps21
Euteleostomi - ps22
Sarcopterygii - ps23
Tetrapoda - ps24
Amniota - ps25
Mammalia - ps26
Theria - ps27
Placentalia - ps28
Boreoeutheria - ps29
Euarchontoglires - ps30
Euarchonta - ps31
Primates - ps32
Haplorrhini - ps33
Simiiformes - ps34
Catarrhini - ps35
Hominoidea - ps36
Hominidae - ps37
Homininae - ps38
Hominini - ps39
Homo_sapiens - ps40
Phylostratum

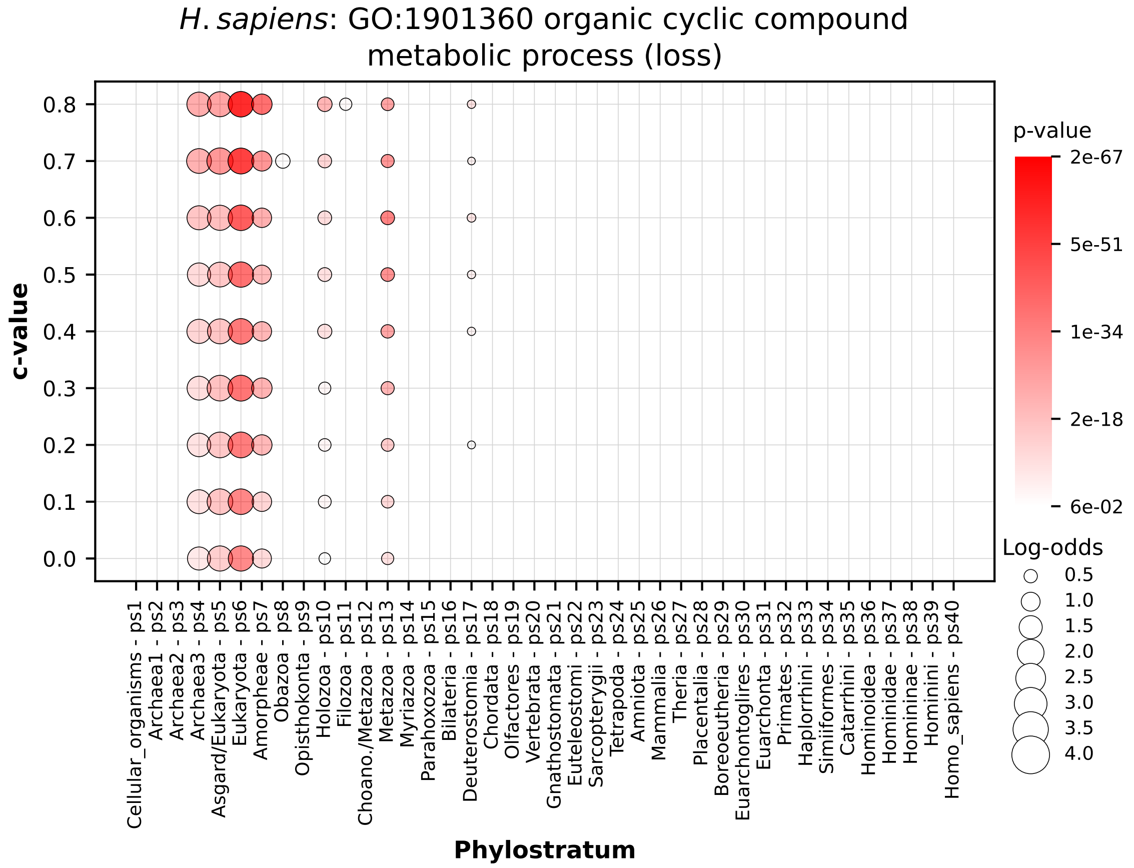

H. sapiens: GO:1901360 organic cyclic compound metabolic process (loss)
c-value
0.8
0.7
0.6
0.5
0.4
0.3
0.2
0.1
0.0
Phylostratum
Cellular_organisms - ps1
Archaea1 - ps2
Archaea2 - ps3
Archaea3 - ps4
Asgard/Eukaryota - ps5
Eukaryota - ps6
Amorpheae - ps7
Obazoa - ps8
Opisthokonta - ps9
Holozoa - ps10
Filozoa - ps11
Choano./Metazoa - ps12
Metazoa - ps13
Myriazoa - ps14
Parahoxozoa - ps15
Bilateria - ps16
Deuterostomia - ps17
Chordata - ps18
Olfactores - ps19
Vertebrata - ps20
Gnathostomata - ps21
Euteleostomi - ps22
Sarcopterygii - ps23
Tetrapoda - ps24
Amniota - ps25
Mammalia - ps26
Theria - ps27
Placentalia - ps28
Boreoeutheria - ps29
Euarchontoglires - ps30
Euarchonta - ps31
Primates - ps32
Haplorrhini - ps33
Simiiformes - ps34
Catarrhini - ps35
Hominoidea - ps36
Hominidae - ps37
Homininae - ps38
Hominini - ps39
Homo_sapiens - ps40
p-value
2e-67
5e-51
1e-34
2e-18
6e-02
Log-odds
0.5
1.0
1.5
2.0
2.5
3.0
3.5
4.0

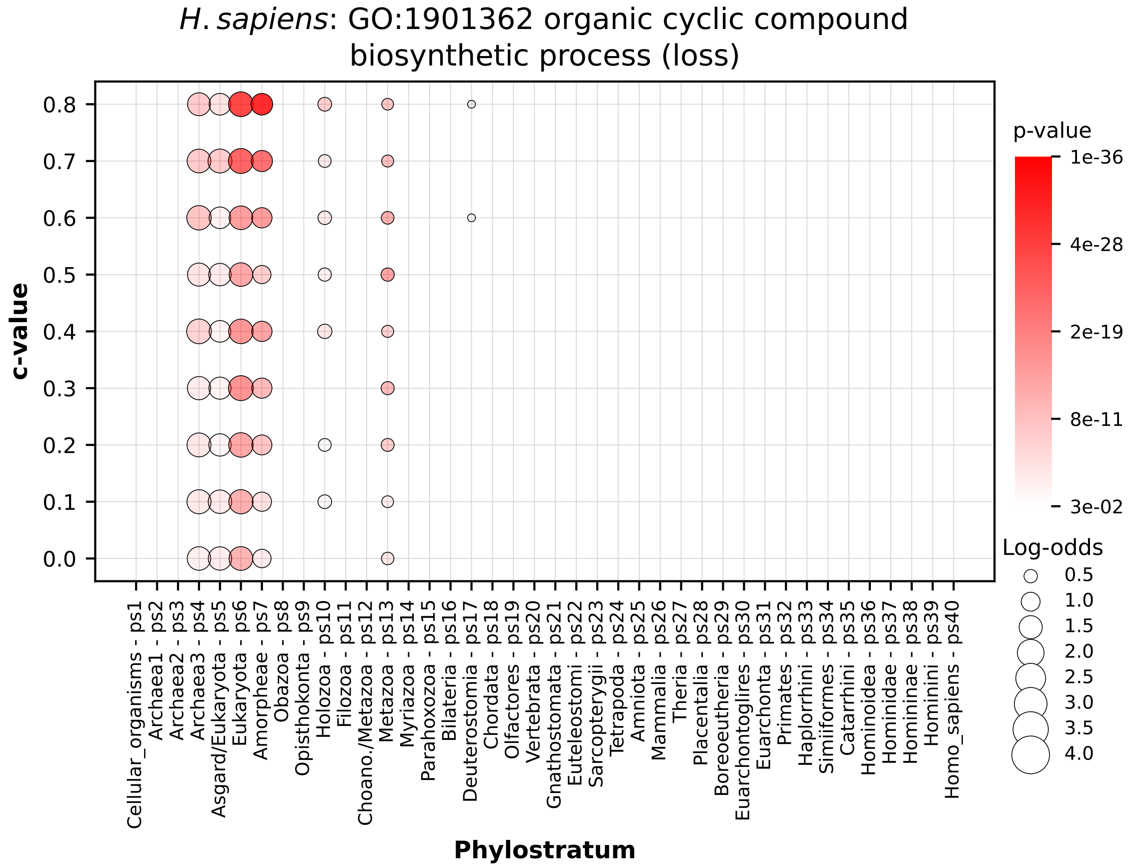

H. sapiens: GO:1901362 organic cyclic compound biosynthetic process (loss)
c-value
0.0
0.1
0.2
0.3
0.4
0.5
0.6
0.7
0.8
Phylostratum
Cellular_organisms - ps1
Archaea1 - ps2
Archaea2 - ps3
Archaea3 - ps4
Asgard/Eukaryota - ps5
Eukaryota - ps6
Amorpheae - ps7
Obazoa - ps8
Opisthokonta - ps9
Holozoa - ps10
Filozoa - ps11
Choano./Metazoa - ps12
Metazoa - ps13
Myriazoa - ps14
Parahoxozoa - ps15
Bilateria - ps16
Deuterostomia - ps17
Chordata - ps18
Olfactores - ps19
Vertebrata - ps20
Gnathostomata - ps21
Euteleostomi - ps22
Sarcopterygii - ps23
Tetrapoda - ps24
Amniota - ps25
Mammalia - ps26
Theria - ps27
Placentalia - ps28
Boreoeutheria - ps29
Euarchontoglires - ps30
Euarchonta - ps31
Primates - ps32
Haplorrhini - ps33
Simiiformes - ps34
Catarrhini - ps35
Hominoidea - ps36
Hominidae - ps37
Homininae - ps38
Hominini - ps39
Homo_sapiens - ps40
p-value
1e-36
4e-28
2e-19
8e-11
3e-02
Log-odds
0.5
1.0
1.5
2.0
2.5
3.0
3.5
4.0

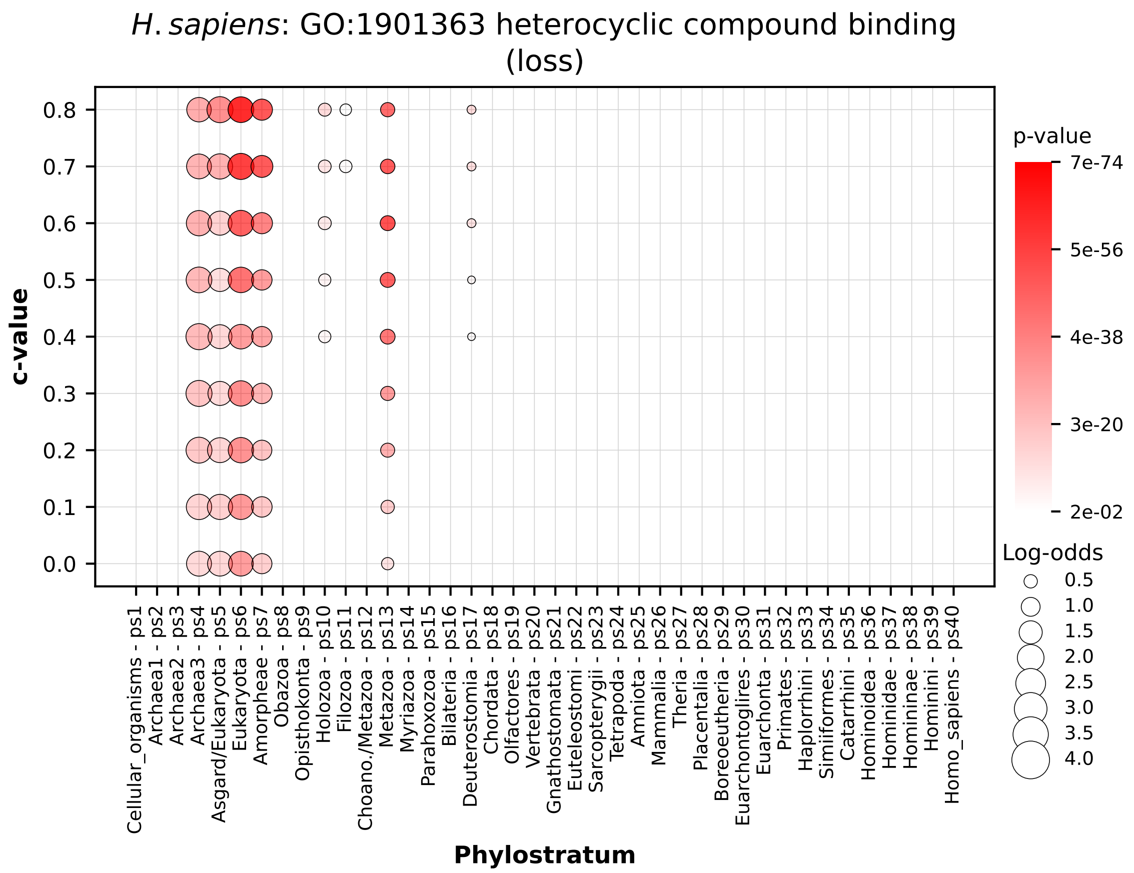

H. sapiens: GO:1901363 heterocyclic compound binding
(loss)
c-value
0.8
0.7
0.6
0.5
0.4
0.3
0.2
0.1
0.0
p-value
7e-74
5e-56
4e-38
3e-20
2e-02
Log-odds
0.5
1.0
1.5
2.0
2.5
3.0
3.5
4.0
Cellular_organisms - ps1
Archaea1 - ps2
Archaea2 - ps3
Archaea3 - ps4
Asgard/Eukaryota - ps5
Eukaryota - ps6
Amorpheae - ps7
Obazoa - ps8
Opisthokonta - ps9
Holozoa - ps10
Filozoa - ps11
Choano./Metazoa - ps12
Metazoa - ps13
Myriazoa - ps14
Parahoxozoa - ps15
Bilateria - ps16
Deuterostomia - ps17
Chordata - ps18
Olfactores - ps19
Vertebrata - ps20
Gnathostomata - ps21
Euteleostomi - ps22
Sarcopterygii - ps23
Tetrapoda - ps24
Amniota - ps25
Mammalia - ps26
Theria - ps27
Placentalia - ps28
Boreoeutheria - ps29
Euarchontoglires - ps30
Euarchonta - ps31
Primates - ps32
Haplorrhini - ps33
Simiiformes - ps34
Catarrhini - ps35
Hominoidea - ps36
Hominidae - ps37
Homininae - ps38
Hominini - ps39
Homo_sapiens - ps40
Phylostratum

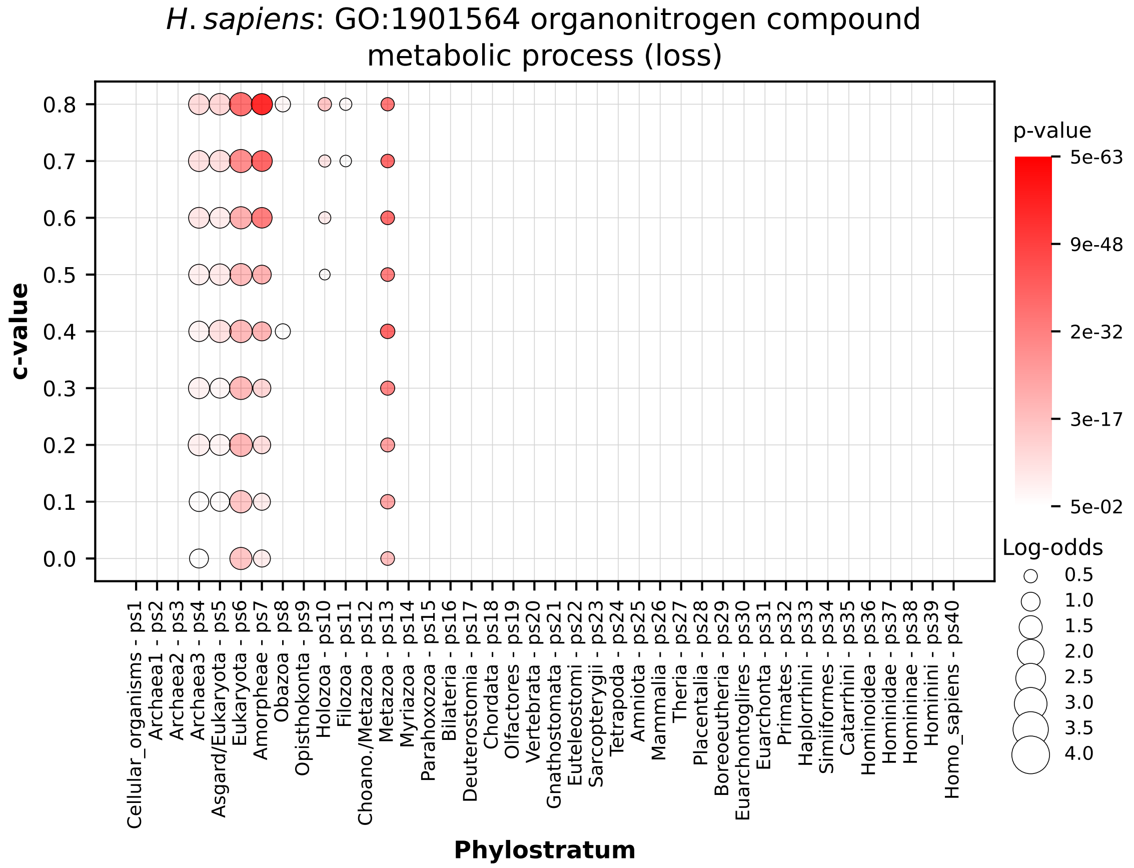
H. sapiens: GO:1901564 organonitrogen compound metabolic process (loss)
c-value
0.8
0.7
0.6
0.5
0.4
0.3
0.2
0.1
0.0
Phylostratum
Cellular_organisms - ps1
Archaea1 - ps2
Archaea2 - ps3
Archaea3 - ps4
Asgard/Eukaryota - ps5
Eukaryota - ps6
Amorpheae - ps7
Obazoa - ps8
Opisthokonta - ps9
Holozoa - ps10
Filozoa - ps11
Choano./Metazoa - ps12
Metazoa - ps13
Myriazoa - ps14
Parahoxozoa - ps15
Bilateria - ps16
Deuterostomia - ps17
Chordata - ps18
Olfactores - ps19
Vertebrata - ps20
Gnathostomata - ps21
Euteleostomi - ps22
Sarcopterygii - ps23
Tetrapoda - ps24
Amniota - ps25
Mammalia - ps26
Theria - ps27
Placentalia - ps28
Boreoeutheria - ps29
Euarchontoglires - ps30
Euarchonta - ps31
Primates - ps32
Haplorrhini - ps33
Simiiformes - ps34
Catarrhini - ps35
Hominoidea - ps36
Hominidae - ps37
Homininae - ps38
Hominini - ps39
Homo_sapiens - ps40
p-value
5e-63
9e-48
2e-32
3e-17
5e-02
Log-odds
0.5
1.0
1.5
2.0
2.5
3.0
3.5
4.0

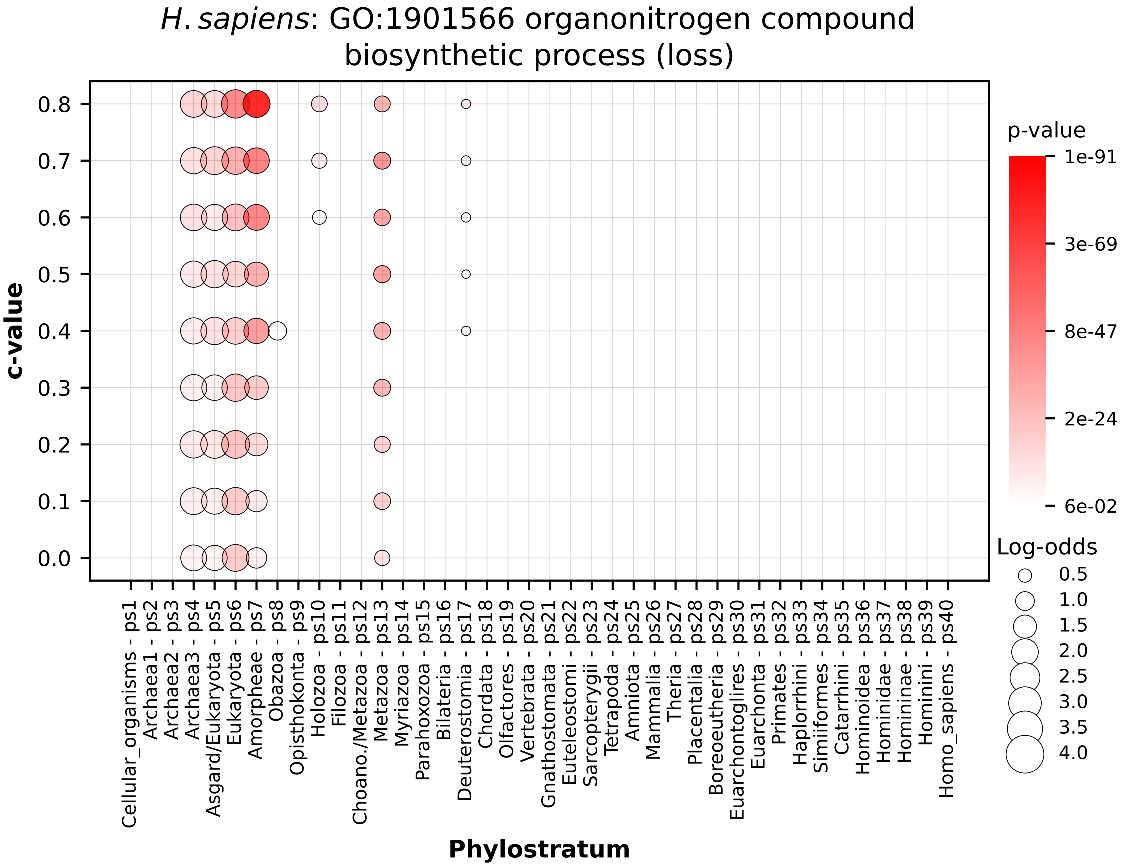

H. sapiens: GO:1901566 organonitrogen compound biosynthetic process (loss)
c-value
0.0
0.1
0.2
0.3
0.4
0.5
0.6
0.7
0.8
Phylostratum
Cellular_organisms - ps1
Archaea1 - ps2
Archaea2 - ps3
Archaea3 - ps4
Asgard/Eukaryota - ps5
Eukaryota - ps6
Amorpheae - ps7
Obazoa - ps8
Opisthokonta - ps9
Holozoa - ps10
Filozoa - ps11
Choano./Metazoa - ps12
Metazoa - ps13
Myriazoa - ps14
Parahoxozoa - ps15
Bilateria - ps16
Deuterostomia - ps17
Chordata - ps18
Olfactores - ps19
Vertebrata - ps20
Gnathostomata - ps21
Euteleostomi - ps22
Sarcopterygii - ps23
Tetrapoda - ps24
Amniota - ps25
Mammalia - ps26
Theria - ps27
Placentalia - ps28
Boreoeutheria - ps29
Euarchontoglires - ps30
Euarchonta - ps31
Primates - ps32
Haplorrhini - ps33
Simiiformes - ps34
Catarrhini - ps35
Hominoidea - ps36
Hominidae - ps37
Homininae - ps38
Hominini - ps39
Homo_sapiens - ps40
p-value
1e-91
3e-69
8e-47
2e-24
6e-02
Log-odds
0.5
1.0
1.5
2.0
2.5
3.0
3.5
4.0

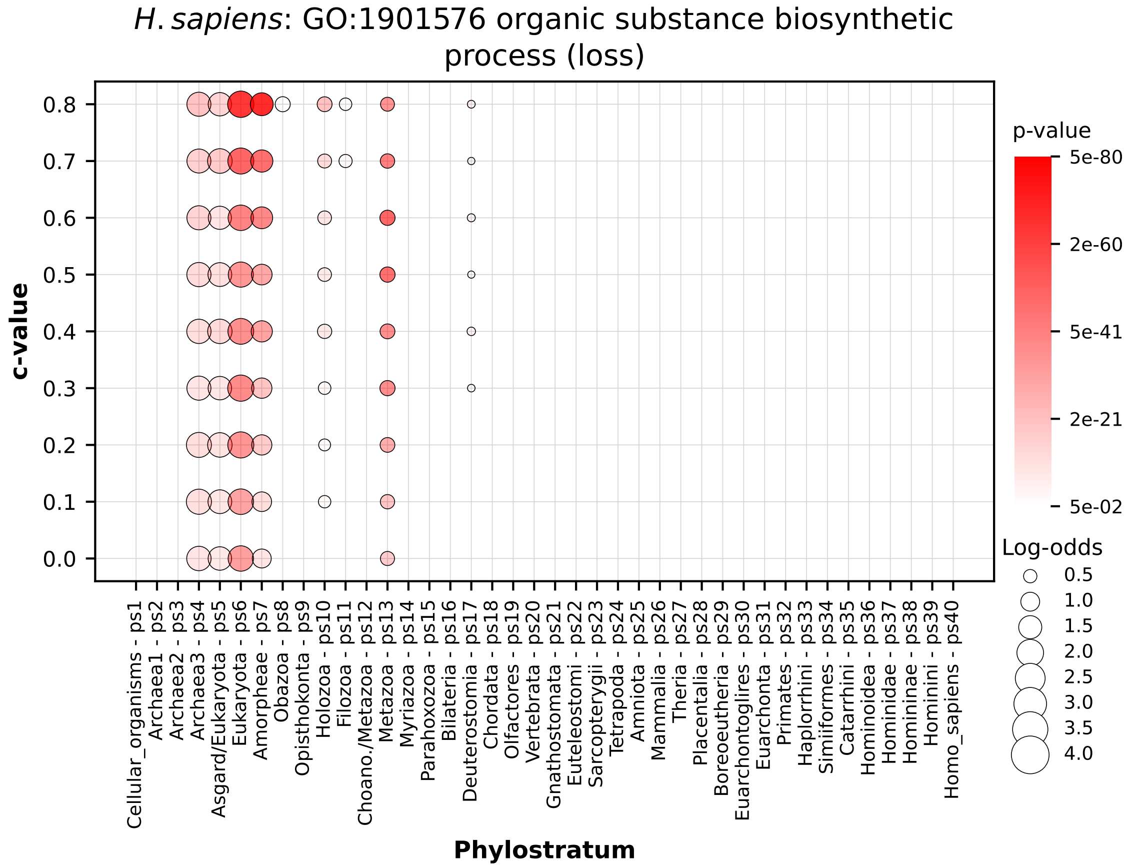

H. sapiens: GO:1901576 organic substance biosynthetic process (loss)
c-value
0.8
0.7
0.6
0.5
0.4
0.3
0.2
0.1
0.0
Phylostratum
Cellular_organisms - ps1
Archaea1 - ps2
Archaea2 - ps3
Archaea3 - ps4
Asgard/Eukaryota - ps5
Eukaryota - ps6
Amorpheae - ps7
Obazoa - ps8
Opisthokonta - ps9
Holozoa - ps10
Filozoa - ps11
Choano./Metazoa - ps12
Metazoa - ps13
Myriazoa - ps14
Parahoxozoa - ps15
Bilateria - ps16
Deuterostomia - ps17
Chordata - ps18
Olfactores - ps19
Vertebrata - ps20
Gnathostomata - ps21
Euteleostomi - ps22
Sarcopterygii - ps23
Tetrapoda - ps24
Amniota - ps25
Mammalia - ps26
Theria - ps27
Placentalia - ps28
Boreoeutheria - ps29
Euarchontoglires - ps30
Euarchonta - ps31
Primates - ps32
Haplorrhini - ps33
Simiiformes - ps34
Catarrhini - ps35
Hominoidea - ps36
Hominidae - ps37
Homininae - ps38
Hominini - ps39
Homo_sapiens - ps40
p-value
5e-80
2e-60
5e-41
2e-21
5e-02
Log-odds
0.5
1.0
1.5
2.0
2.5
3.0
3.5
4.0

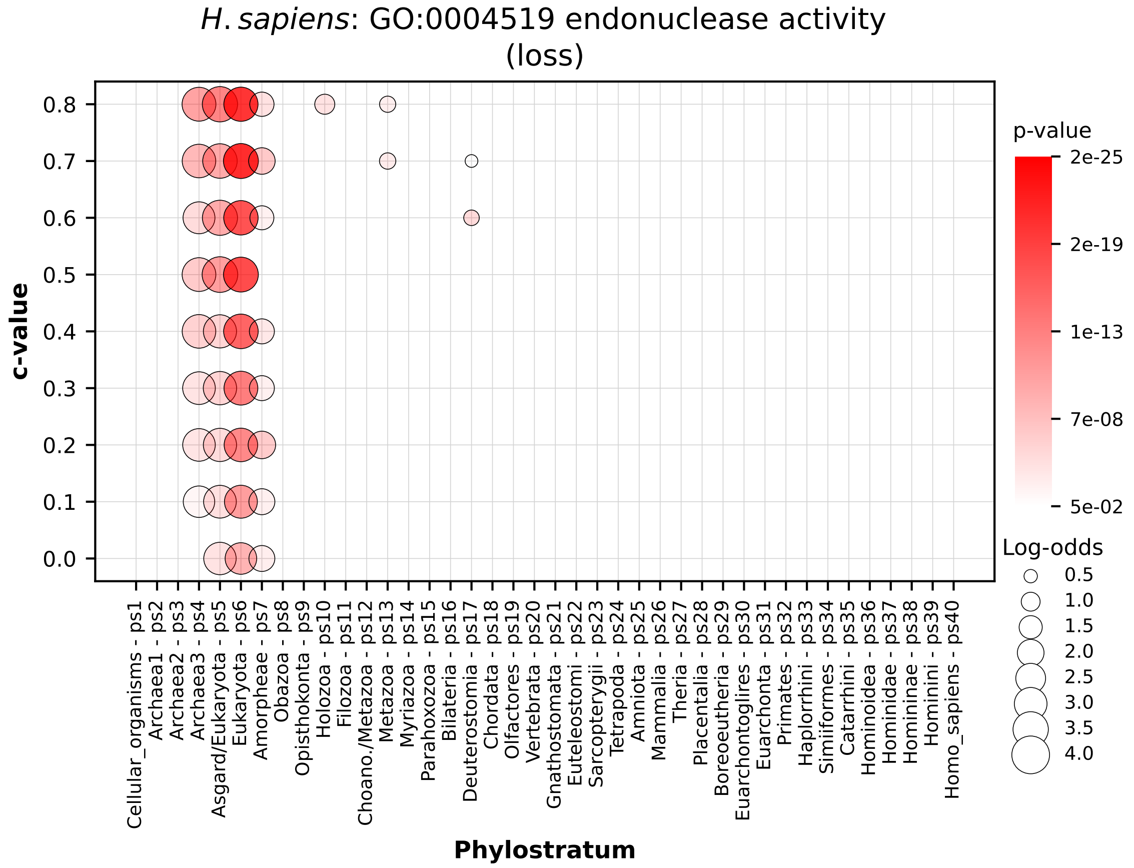
H. sapiens: GO:0004519 endonuclease activity
(loss)
c-value
0.8
0.7
0.6
0.5
0.4
0.3
0.2
0.1
0.0
p-value
2e-25
2e-19
1e-13
7e-08
5e-02
Log-odds
0.5
1.0
1.5
2.0
2.5
3.0
3.5
4.0
Cellular_organisms - ps1
Archaea1 - ps2
Archaea2 - ps3
Archaea3 - ps4
Asgard/Eukaryota - ps5
Eukaryota - ps6
Amorpheae - ps7
Obazoa - ps8
Opisthokonta - ps9
Holozoa - ps10
Filozoa - ps11
Choano./Metazoa - ps12
Metazoa - ps13
Myriazoa - ps14
Parahoxozoa - ps15
Bilateria - ps16
Deuterostomia - ps17
Chordata - ps18
Olfactores - ps19
Vertebrata - ps20
Gnathostomata - ps21
Euteleostomi - ps22
Sarcopterygii - ps23
Tetrapoda - ps24
Amniota - ps25
Mammalia - ps26
Theria - ps27
Placentalia - ps28
Boreoeutheria - ps29
Euarchontoglires - ps30
Euarchonta - ps31
Primates - ps32
Haplorrhini - ps33
Simiiformes - ps34
Catarrhini - ps35
Hominoidea - ps36
Hominidae - ps37
Homininae - ps38
Hominini - ps39
Homo_sapiens - ps40
Phylostratum

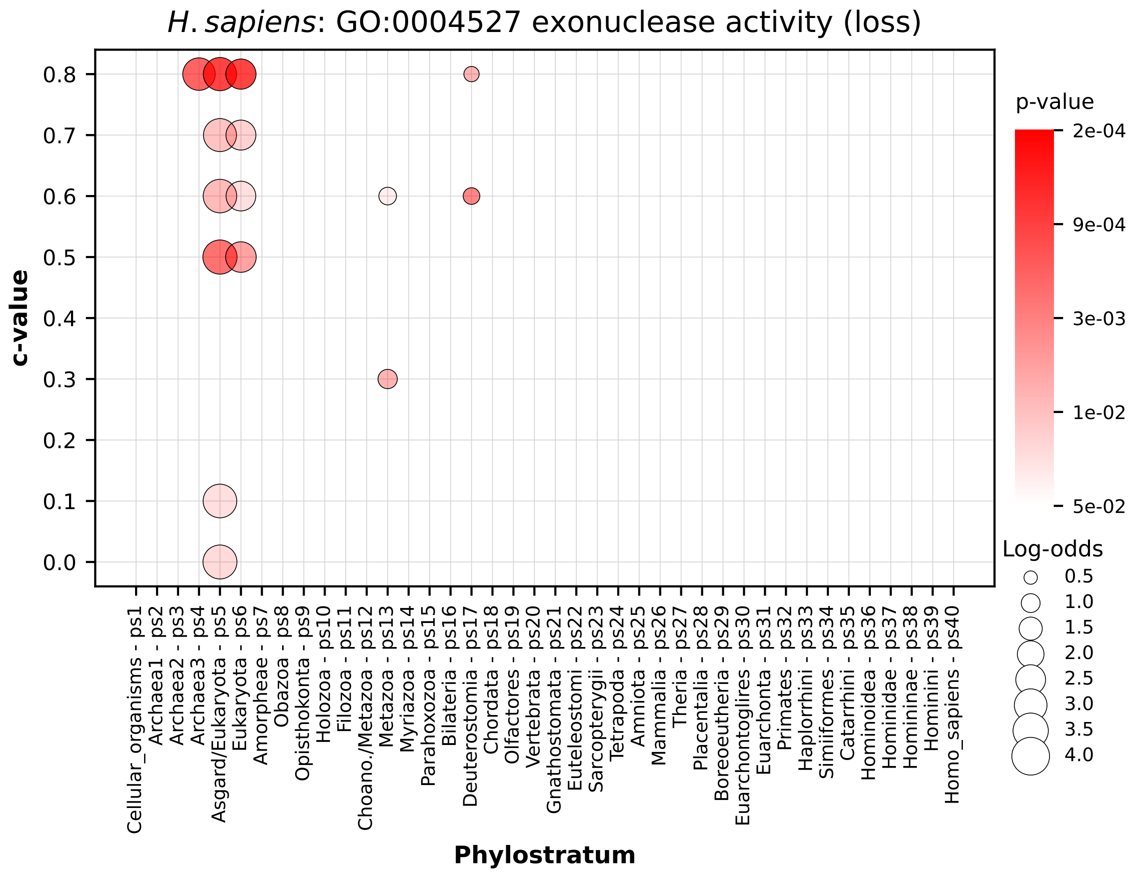

H. sapiens: GO:0004527 exonuclease activity (loss)
c-value
0.8
0.7
0.6
0.5
0.4
0.3
0.2
0.1
0.0
Phylostratum
Cellular_organisms - ps1
Archaea1 - ps2
Archaea2 - ps3
Archaea3 - ps4
Asgard/Eukaryota - ps5
Eukaryota - ps6
Amorpheae - ps7
Obazoa - ps8
Opisthokonta - ps9
Holozoa - ps10
Filozoa - ps11
Choano./Metazoa - ps12
Metazoa - ps13
Myriazoa - ps14
Parahoxozoa - ps15
Bilateria - ps16
Deuterostomia - ps17
Chordata - ps18
Olfactores - ps19
Vertebrata - ps20
Gnathostomata - ps21
Euteleostomi - ps22
Sarcopterygii - ps23
Tetrapoda - ps24
Amniota - ps25
Mammalia - ps26
Theria - ps27
Placentalia - ps28
Boreoeutheria - ps29
Euarchontoglires - ps30
Euarchonta - ps31
Primates - ps32
Haplorrhini - ps33
Simiiformes - ps34
Catarrhini - ps35
Hominoidea - ps36
Hominidae - ps37
Homininae - ps38
Hominini - ps39
Homo_sapiens - ps40
p-value
2e-04
9e-04
3e-03
1e-02
5e-02
Log-odds
0.5
1.0
1.5
2.0
2.5
3.0
3.5
4.0

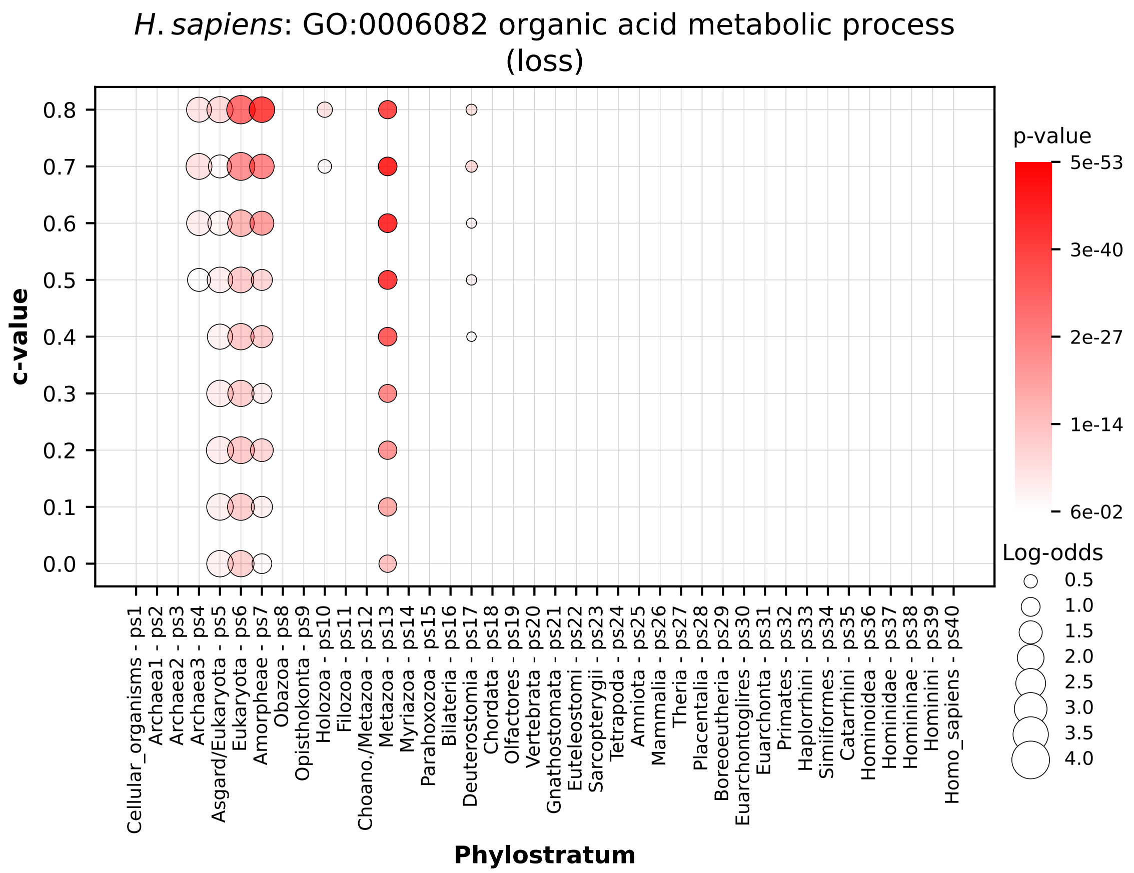

H. sapiens: GO:0006082 organic acid metabolic process (loss)
c-value
0.8
0.7
0.6
0.5
0.4
0.3
0.2
0.1
0.0
p-value
5e-53
3e-40
2e-27
1e-14
6e-02
Log-odds
0.5
1.0
1.5
2.0
2.5
3.0
3.5
4.0
Cellular_organisms - ps1
Archaea1 - ps2
Archaea2 - ps3
Archaea3 - ps4
Asgard/Eukaryota - ps5
Eukaryota - ps6
Amorpheae - ps7
Obazoa - ps8
Opisthokonta - ps9
Holozoa - ps10
Filozoa - ps11
Choano./Metazoa - ps12
Metazoa - ps13
Myriazoa - ps14
Parahoxozoa - ps15
Bilateria - ps16
Deuterostomia - ps17
Chordata - ps18
Olfactores - ps19
Vertebrata - ps20
Gnathostomata - ps21
Euteleostomi - ps22
Sarcopterygii - ps23
Tetrapoda - ps24
Amniota - ps25
Mammalia - ps26
Theria - ps27
Placentalia - ps28
Boreoeutheria - ps29
Euarchontoglires - ps30
Euarchonta - ps31
Primates - ps32
Haplorrhini - ps33
Simiiformes - ps34
Catarrhini - ps35
Hominoidea - ps36
Hominidae - ps37
Homininae - ps38
Hominini - ps39
Homo_sapiens - ps40
Phylostratum

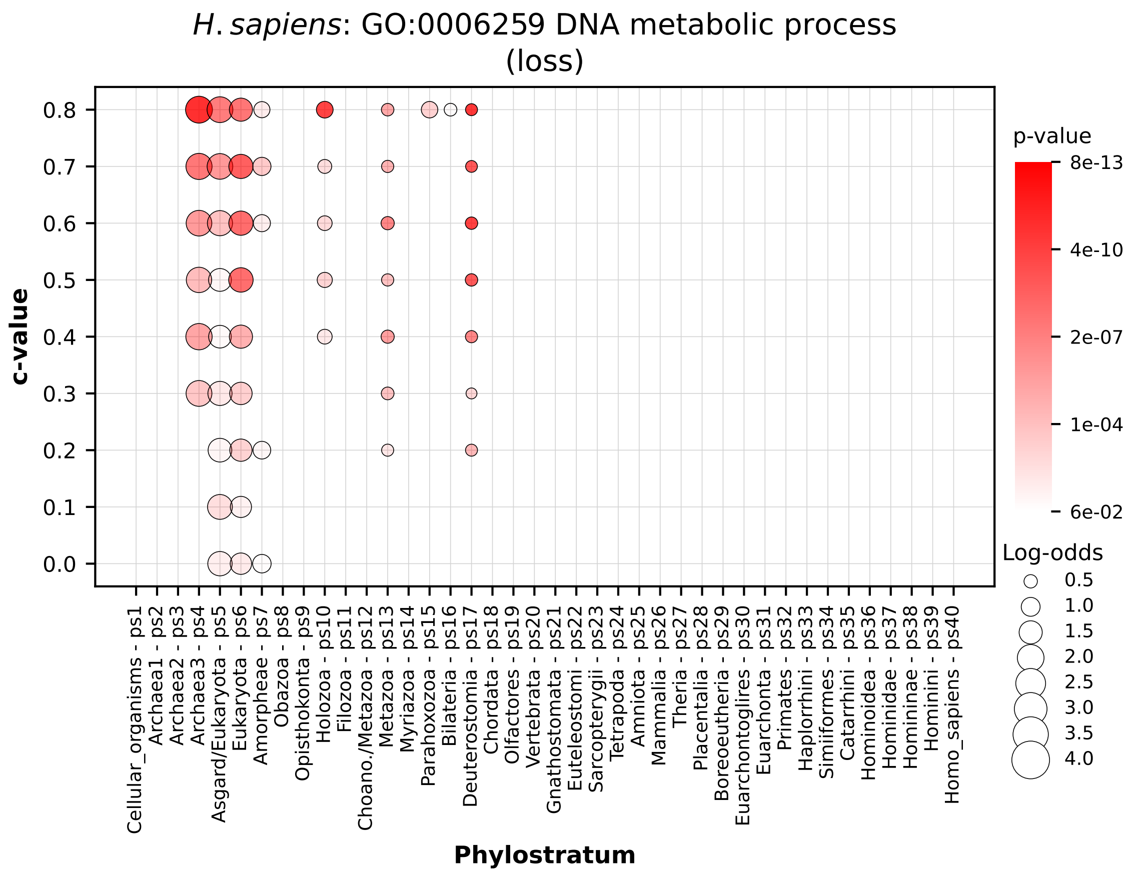

H. sapiens: GO:0006259 DNA metabolic process
(loss)
c-value
0.0
0.1
0.2
0.3
0.4
0.5
0.6
0.7
0.8
Phylostratum
Cellular_organisms - ps1
Archaea1 - ps2
Archaea2 - ps3
Archaea3 - ps4
Asgard/Eukaryota - ps5
Eukaryota - ps6
Amorpheae - ps7
Obazoa - ps8
Opisthokonta - ps9
Holozoa - ps10
Filozoa - ps11
Choano./Metazoa - ps12
Metazoa - ps13
Myriazoa - ps14
Parahoxozoa - ps15
Bilateria - ps16
Deuterostomia - ps17
Chordata - ps18
Olfactores - ps19
Vertebrata - ps20
Gnathostomata - ps21
Euteleostomi - ps22
Sarcopterygii - ps23
Tetrapoda - ps24
Amniota - ps25
Mammalia - ps26
Theria - ps27
Placentalia - ps28
Boreoeutheria - ps29
Euarchontoglires - ps30
Euarchonta - ps31
Primates - ps32
Haplorrhini - ps33
Simiiformes - ps34
Catarrhini - ps35
Hominoidea - ps36
Hominidae - ps37
Homininae - ps38
Hominini - ps39
Homo_sapiens - ps40
p-value
8e-13
4e-10
2e-07
1e-04
6e-02
Log-odds
0.5
1.0
1.5
2.0
2.5
3.0
3.5
4.0

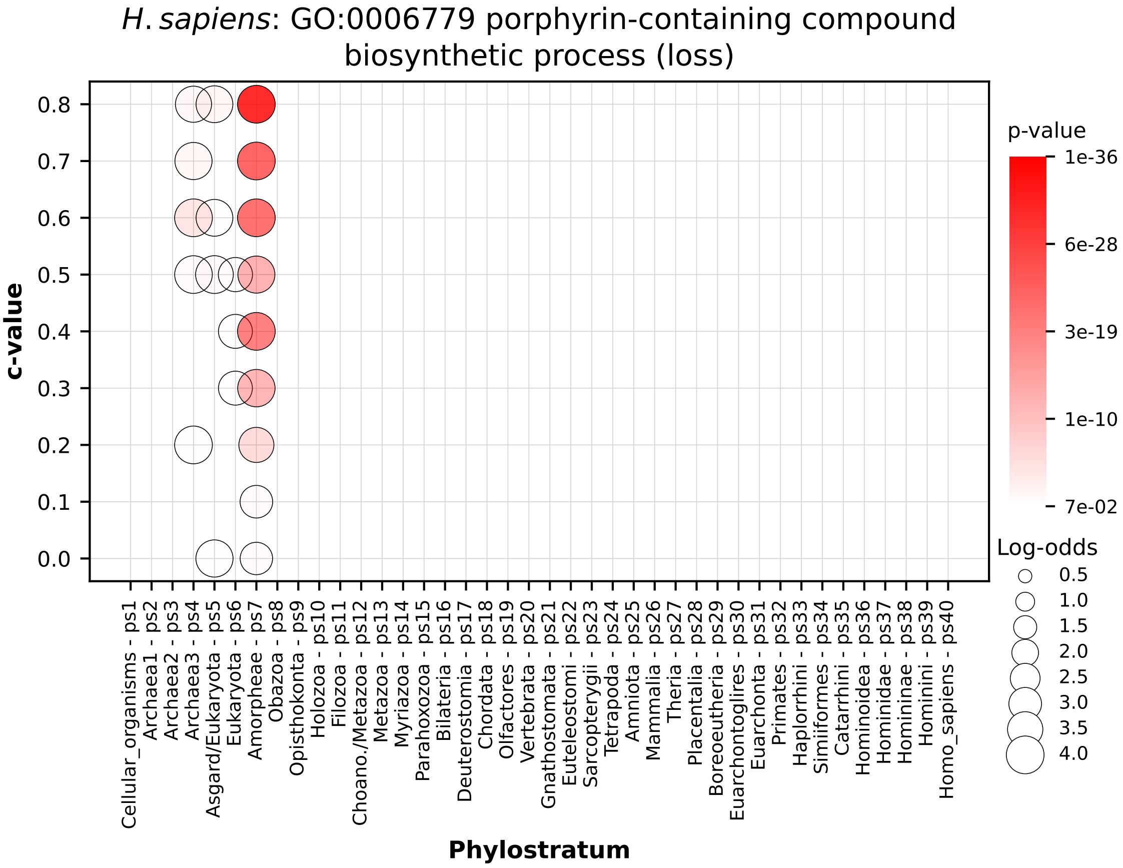
H. sapiens: GO:0006779 porphyrin-containing compound biosynthetic process (loss)
c-value
0.8
0.7
0.6
0.5
0.4
0.3
0.2
0.1
0.0
Phylostratum
Cellular_organisms - ps1
Archaea1 - ps2
Archaea2 - ps3
Archaea3 - ps4
Asgard/Eukaryota - ps5
Eukaryota - ps6
Amorpheae - ps7
Obazoa - ps8
Opisthokonta - ps9
Holozoa - ps10
Filozoa - ps11
Choano./Metazoa - ps12
Metazoa - ps13
Myriazoa - ps14
Parahoxozoa - ps15
Bilateria - ps16
Deuterostomia - ps17
Chordata - ps18
Olfactores - ps19
Vertebrata - ps20
Gnathostomata - ps21
Euteleostomi - ps22
Sarcopterygii - ps23
Tetrapoda - ps24
Amniota - ps25
Mammalia - ps26
Theria - ps27
Placentalia - ps28
Boreoeutheria - ps29
Euarchontoglires - ps30
Euarchonta - ps31
Primates - ps32
Haplorrhini - ps33
Simiiformes - ps34
Catarrhini - ps35
Hominoidea - ps36
Hominidae - ps37
Homininae - ps38
Hominini - ps39
Homo_sapiens - ps40
p-value
1e-36
6e-28
3e-19
1e-10
7e-02
Log-odds
0.5
1.0
1.5
2.0
2.5
3.0
3.5
4.0

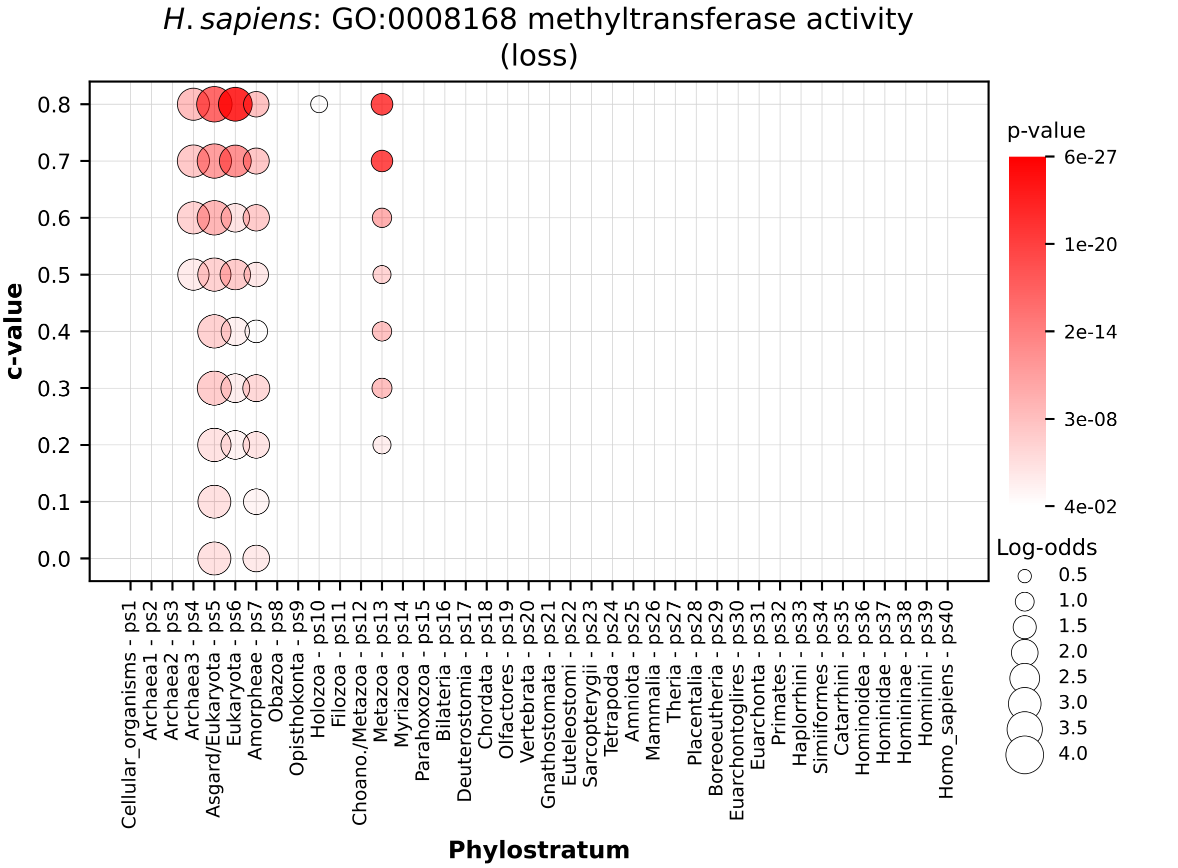

H. sapiens: GO:0008168 methyltransferase activity
(loss)
c-value
0.8
0.7
0.6
0.5
0.4
0.3
0.2
0.1
0.0
p-value
6e-27
1e-20
2e-14
3e-08
4e-02
Log-odds
0.5
1.0
1.5
2.0
2.5
3.0
3.5
4.0
Cellular_organisms - ps1
Archaea1 - ps2
Archaea2 - ps3
Archaea3 - ps4
Asgard/Eukaryota - ps5
Eukaryota - ps6
Amorpheae - ps7
Obazoa - ps8
Opisthokonta - ps9
Holozoa - ps10
Filozoa - ps11
Choano./Metazoa - ps12
Metazoa - ps13
Myriazoa - ps14
Parahoxozoa - ps15
Bilateria - ps16
Deuterostomia - ps17
Chordata - ps18
Olfactores - ps19
Vertebrata - ps20
Gnathostomata - ps21
Euteleostomi - ps22
Sarcopterygii - ps23
Tetrapoda - ps24
Amniota - ps25
Mammalia - ps26
Theria - ps27
Placentalia - ps28
Boreoeutheria - ps29
Euarchontoglires - ps30
Euarchonta - ps31
Primates - ps32
Haplorrhini - ps33
Simiiformes - ps34
Catarrhini - ps35
Hominoidea - ps36
Hominidae - ps37
Homininae - ps38
Hominini - ps39
Homo_sapiens - ps40
Phylostratum

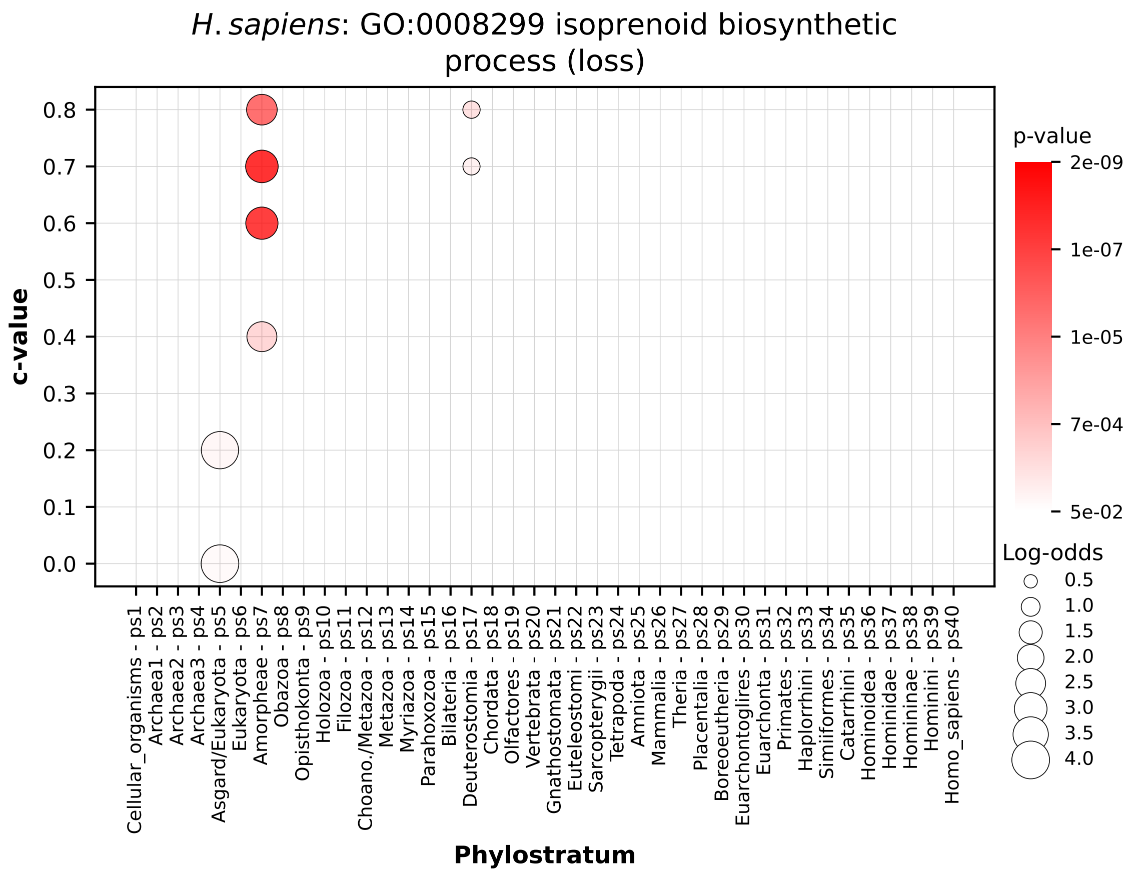

H. sapiens: GO:0008299 isoprenoid biosynthetic process (loss)
c-value
0.8
0.7
0.6
0.5
0.4
0.3
0.2
0.1
0.0
p-value
2e-09
1e-07
1e-05
7e-04
5e-02
Log-odds
0.5
1.0
1.5
2.0
2.5
3.0
3.5
4.0
Cellular_organisms - ps1
Archaea1 - ps2
Archaea2 - ps3
Archaea3 - ps4
Asgard/Eukaryota - ps5
Eukaryota - ps6
Amorpheae - ps7
Obazoa - ps8
Opisthokonta - ps9
Holozoa - ps10
Filozoa - ps11
Choano./Metazoa - ps12
Metazoa - ps13
Myriazoa - ps14
Parahoxozoa - ps15
Bilateria - ps16
Deuterostomia - ps17
Chordata - ps18
Olfactores - ps19
Vertebrata - ps20
Gnathostomata - ps21
Euteleostomi - ps22
Sarcopterygii - ps23
Tetrapoda - ps24
Amniota - ps25
Mammalia - ps26
Theria - ps27
Placentalia - ps28
Boreoeutheria - ps29
Euarchontoglires - ps30
Euarchonta - ps31
Primates - ps32
Haplorrhini - ps33
Simiiformes - ps34
Catarrhini - ps35
Hominoidea - ps36
Hominidae - ps37
Homininae - ps38
Hominini - ps39
Homo_sapiens - ps40
Phylostratum

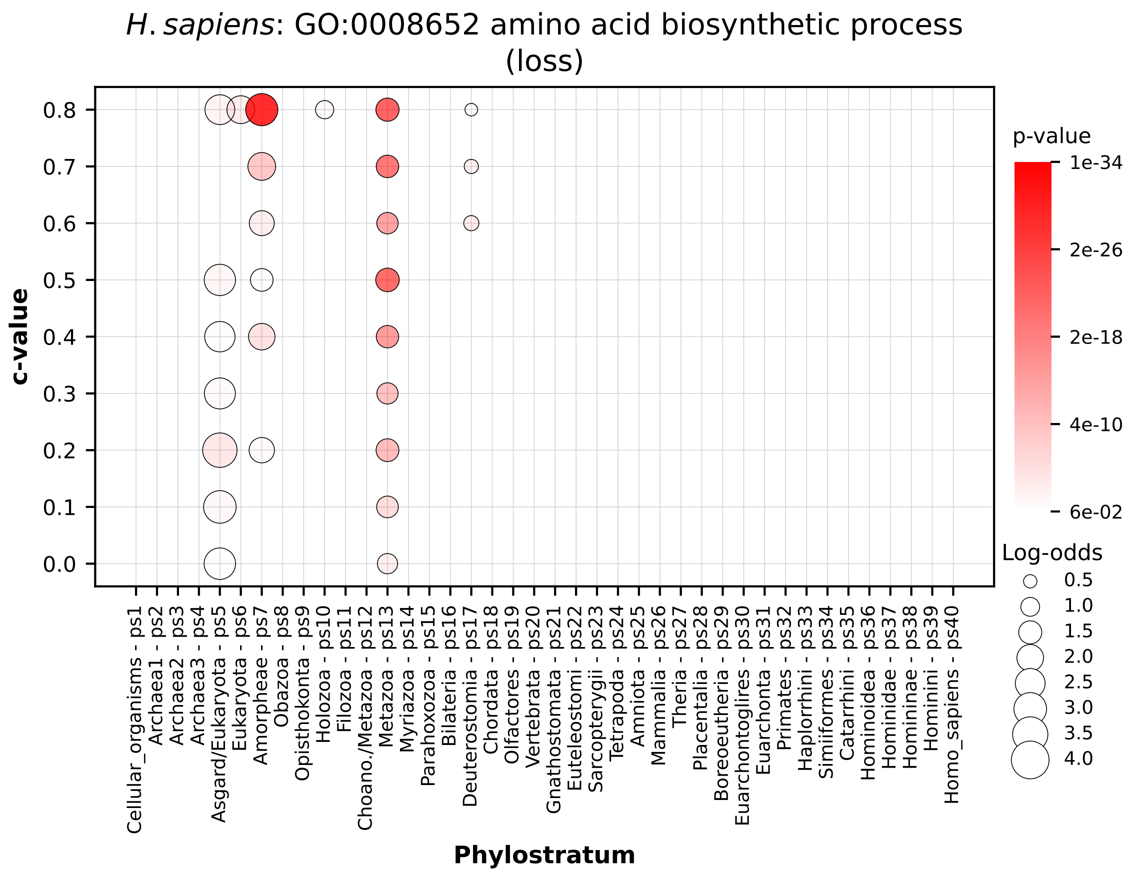
H. sapiens: GO:0008652 amino acid biosynthetic process
(loss)
c-value
0.0
0.1
0.2
0.3
0.4
0.5
0.6
0.7
0.8
Phylostratum
Cellular_organisms - ps1
Archaea1 - ps2
Archaea2 - ps3
Archaea3 - ps4
Asgard/Eukaryota - ps5
Eukaryota - ps6
Amorpheae - ps7
Obazoa - ps8
Opisthokonta - ps9
Holozoa - ps10
Filozoa - ps11
Choano./Metazoa - ps12
Metazoa - ps13
Myriazoa - ps14
Parahoxozoa - ps15
Bilateria - ps16
Deuterostomia - ps17
Chordata - ps18
Olfactores - ps19
Vertebrata - ps20
Gnathostomata - ps21
Euteleostomi - ps22
Sarcopterygii - ps23
Tetrapoda - ps24
Amniota - ps25
Mammalia - ps26
Theria - ps27
Placentalia - ps28
Boreoeutheria - ps29
Euarchontoglires - ps30
Euarchonta - ps31
Primates - ps32
Haplorrhini - ps33
Simiiformes - ps34
Catarrhini - ps35
Hominoidea - ps36
Hominidae - ps37
Homininae - ps38
Hominini - ps39
Homo_sapiens - ps40
p-value
1e-34
2e-26
2e-18
4e-10
6e-02
Log-odds
0.5
1.0
1.5
2.0
2.5
3.0
3.5
4.0

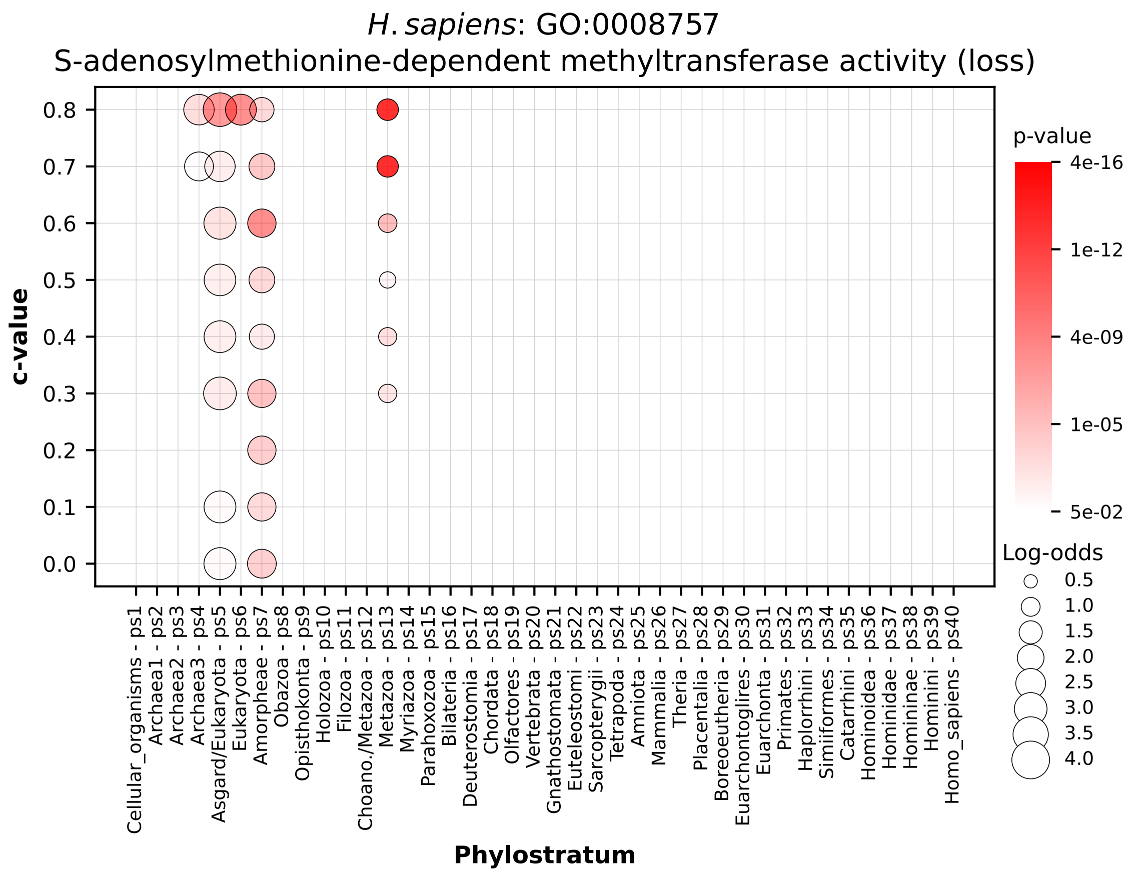

H. sapiens: GO:0008757
S-adenosylmethionine-dependent methyltransferase activity (loss)
c-value
0.8
0.7
0.6
0.5
0.4
0.3
0.2
0.1
0.0
p-value
4e-16
1e-12
4e-09
1e-05
5e-02
Log-odds
0.5
1.0
1.5
2.0
2.5
3.0
3.5
4.0
Cellular_organisms - ps1
Archaea1 - ps2
Archaea2 - ps3
Archaea3 - ps4
Asgard/Eukaryota - ps5
Eukaryota - ps6
Amorpheae - ps7
Obazoa - ps8
Opisthokonta - ps9
Holozoa - ps10
Filozoa - ps11
Choano./Metazoa - ps12
Metazoa - ps13
Myriazoa - ps14
Parahoxozoa - ps15
Bilateria - ps16
Deuterostomia - ps17
Chordata - ps18
Olfactores - ps19
Vertebrata - ps20
Gnathostomata - ps21
Euteleostomi - ps22
Sarcopterygii - ps23
Tetrapoda - ps24
Amniota - ps25
Mammalia - ps26
Theria - ps27
Placentalia - ps28
Boreoeutheria - ps29
Euarchontoglires - ps30
Euarchonta - ps31
Primates - ps32
Haplorrhini - ps33
Simiiformes - ps34
Catarrhini - ps35
Hominoidea - ps36
Hominidae - ps37
Homininae - ps38
Hominini - ps39
Homo_sapiens - ps40
Phylostratum

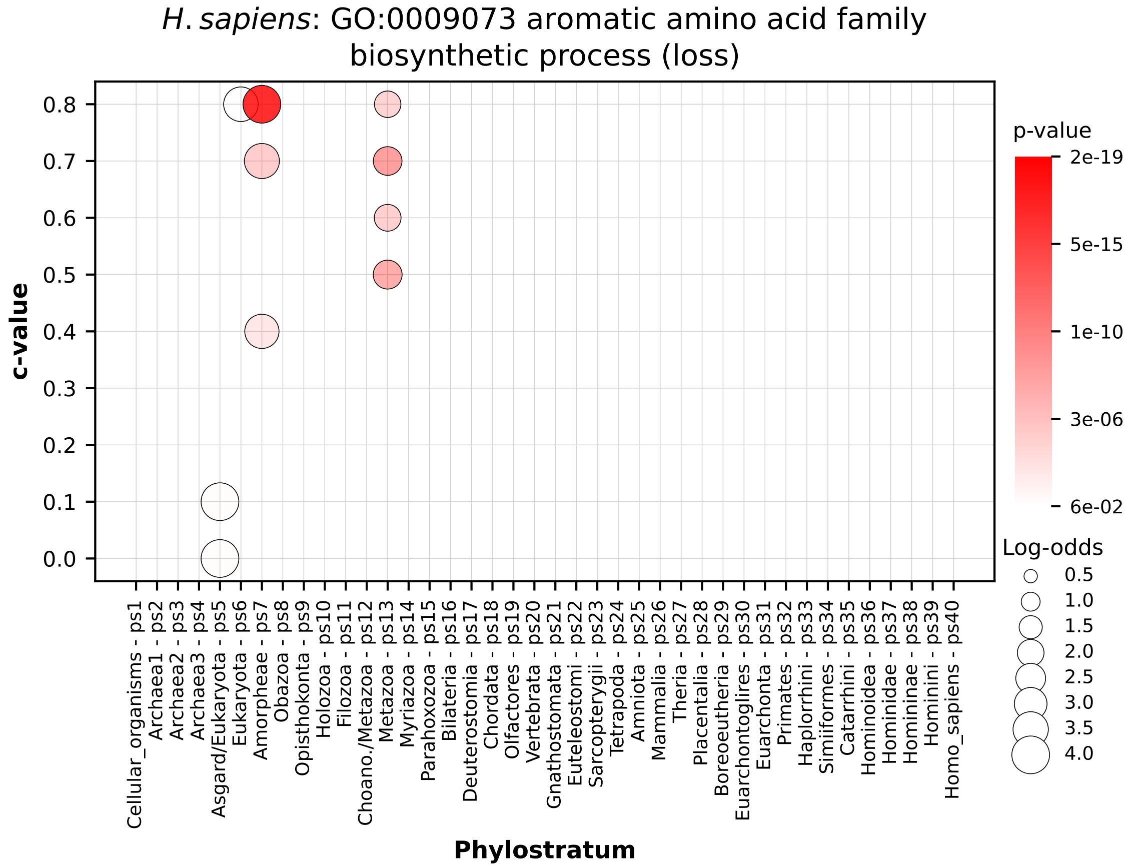

H. sapiens: GO:0009073 aromatic amino acid family biosynthetic process (loss)
c-value
0.8
0.7
0.6
0.5
0.4
0.3
0.2
0.1
0.0
Phylostratum
Cellular_organisms - ps1
Archaea1 - ps2
Archaea2 - ps3
Archaea3 - ps4
Asgard/Eukaryota - ps5
Eukaryota - ps6
Amorpheae - ps7
Obazoa - ps8
Opisthokonta - ps9
Holozoa - ps10
Filozoa - ps11
Choano./Metazoa - ps12
Metazoa - ps13
Myriazoa - ps14
Parahoxozoa - ps15
Bilateria - ps16
Deuterostomia - ps17
Chordata - ps18
Olfactores - ps19
Vertebrata - ps20
Gnathostomata - ps21
Euteleostomi - ps22
Sarcopterygii - ps23
Tetrapoda - ps24
Amniota - ps25
Mammalia - ps26
Theria - ps27
Placentalia - ps28
Boreoeutheria - ps29
Euarchontoglires - ps30
Euarchonta - ps31
Primates - ps32
Haplorrhini - ps33
Simiiformes - ps34
Catarrhini - ps35
Hominoidea - ps36
Hominidae - ps37
Homininae - ps38
Hominini - ps39
Homo_sapiens - ps40
p-value
2e-19
5e-15
1e-10
3e-06
6e-02
Log-odds
0.5
1.0
1.5
2.0
2.5
3.0
3.5
4.0

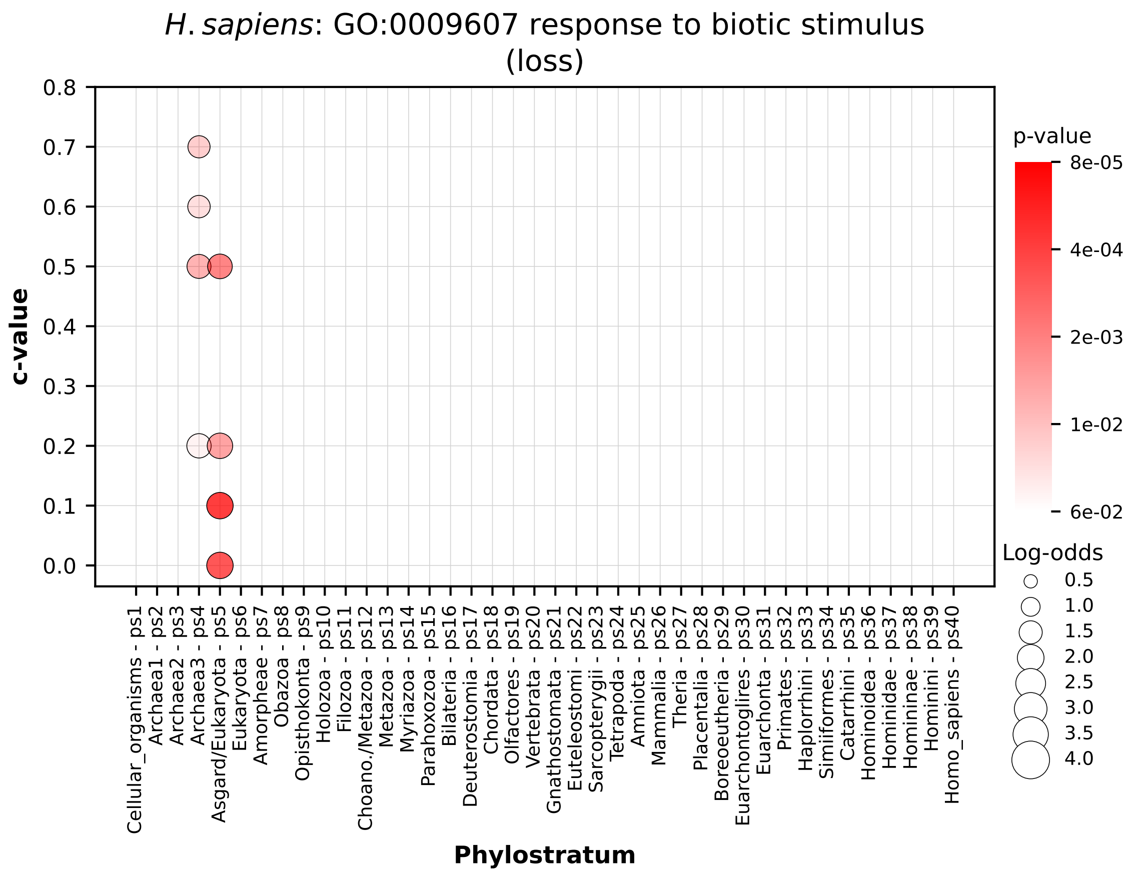

H. sapiens: GO:0009607 response to biotic stimulus
(loss)
c-value
0.8
0.7
0.6
0.5
0.4
0.3
0.2
0.1
0.0
p-value
8e-05
4e-04
2e-03
1e-02
6e-02
Log-odds
0.5
1.0
1.5
2.0
2.5
3.0
3.5
4.0
Cellular_organisms - ps1
Archaea1 - ps2
Archaea2 - ps3
Archaea3 - ps4
Asgard/Eukaryota - ps5
Eukaryota - ps6
Amorpheae - ps7
Obazoa - ps8
Opisthokonta - ps9
Holozoa - ps10
Filozoa - ps11
Choano./Metazoa - ps12
Metazoa - ps13
Myriazoa - ps14
Parahoxozoa - ps15
Bilateria - ps16
Deuterostomia - ps17
Chordata - ps18
Olfactores - ps19
Vertebrata - ps20
Gnathostomata - ps21
Euteleostomi - ps22
Sarcopterygii - ps23
Tetrapoda - ps24
Amniota - ps25
Mammalia - ps26
Theria - ps27
Placentalia - ps28
Boreoeutheria - ps29
Euarchontoglires - ps30
Euarchonta - ps31
Primates - ps32
Haplorrhini - ps33
Simiiformes - ps34
Catarrhini - ps35
Hominoidea - ps36
Hominidae - ps37
Homininae - ps38
Hominini - ps39
Homo_sapiens - ps40
Phylostratum

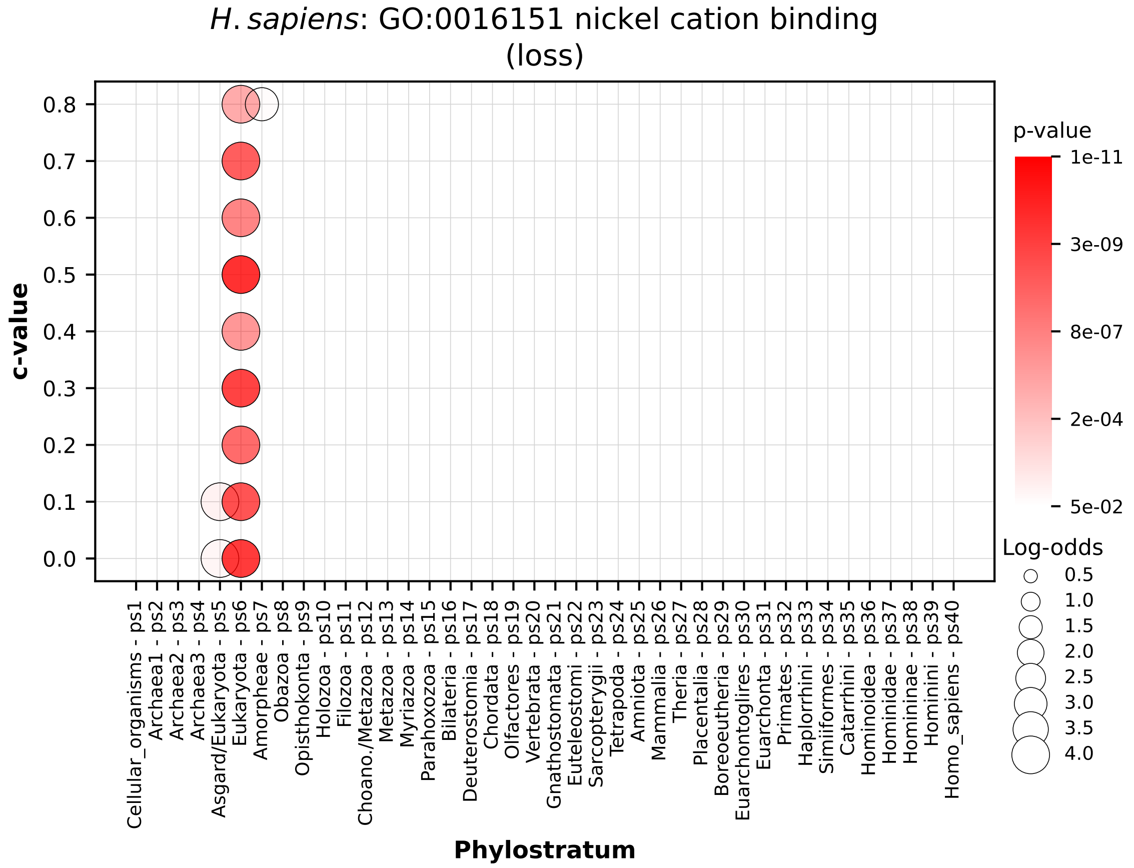
H. sapiens: GO:0016151 nickel cation binding
(loss)
c-value
0.0
0.1
0.2
0.3
0.4
0.5
0.6
0.7
0.8
p-value
1e-11
3e-09
8e-07
2e-04
5e-02
Log-odds
0.5
1.0
1.5
2.0
2.5
3.0
3.5
4.0
Cellular_organisms - ps1
Archaea1 - ps2
Archaea2 - ps3
Archaea3 - ps4
Asgard/Eukaryota - ps5
Eukaryota - ps6
Amorpheae - ps7
Obazoa - ps8
Opisthokonta - ps9
Holozoa - ps10
Filozoa - ps11
Choano./Metazoa - ps12
Metazoa - ps13
Myriazoa - ps14
Parahoxozoa - ps15
Bilateria - ps16
Deuterostomia - ps17
Chordata - ps18
Olfactores - ps19
Vertebrata - ps20
Gnathostomata - ps21
Euteleostomi - ps22
Sarcopterygii - ps23
Tetrapoda - ps24
Amniota - ps25
Mammalia - ps26
Theria - ps27
Placentalia - ps28
Boreoeutheria - ps29
Euarchontoglires - ps30
Euarchonta - ps31
Primates - ps32
Haplorrhini - ps33
Simiiformes - ps34
Catarrhini - ps35
Hominoidea - ps36
Hominidae - ps37
Homininae - ps38
Hominini - ps39
Homo_sapiens - ps40
Phylostratum

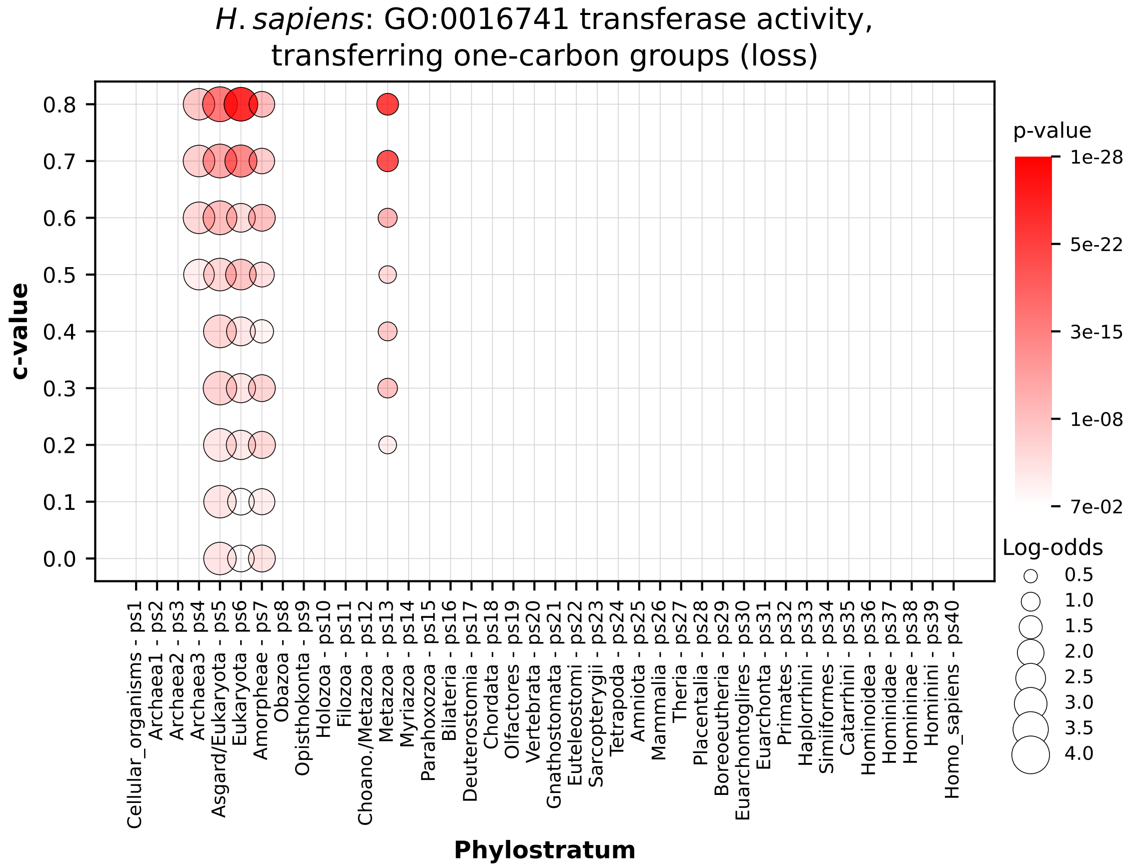

H. sapiens: GO:0016741 transferase activity,
transferring one-carbon groups (loss)
c-value
0.0
0.1
0.2
0.3
0.4
0.5
0.6
0.7
0.8
Phylostratum
Cellular_organisms - ps1
Archaea1 - ps2
Archaea2 - ps3
Archaea3 - ps4
Asgard/Eukaryota - ps5
Eukaryota - ps6
Amorpheae - ps7
Obazoa - ps8
Opisthokonta - ps9
Holozoa - ps10
Filozoa - ps11
Choano./Metazoa - ps12
Metazoa - ps13
Myriazoa - ps14
Parahoxozoa - ps15
Bilateria - ps16
Deuterostomia - ps17
Chordata - ps18
Olfactores - ps19
Vertebrata - ps20
Gnathostomata - ps21
Euteleostomi - ps22
Sarcopterygii - ps23
Tetrapoda - ps24
Amniota - ps25
Mammalia - ps26
Theria - ps27
Placentalia - ps28
Boreoeutheria - ps29
Euarchontoglires - ps30
Euarchonta - ps31
Primates - ps32
Haplorrhini - ps33
Simiiformes - ps34
Catarrhini - ps35
Hominoidea - ps36
Hominidae - ps37
Homininae - ps38
Hominini - ps39
Homo_sapiens - ps40
p-value
1e-28
5e-22
3e-15
1e-08
7e-02
Log-odds
0.5
1.0
1.5
2.0
2.5
3.0
3.5
4.0

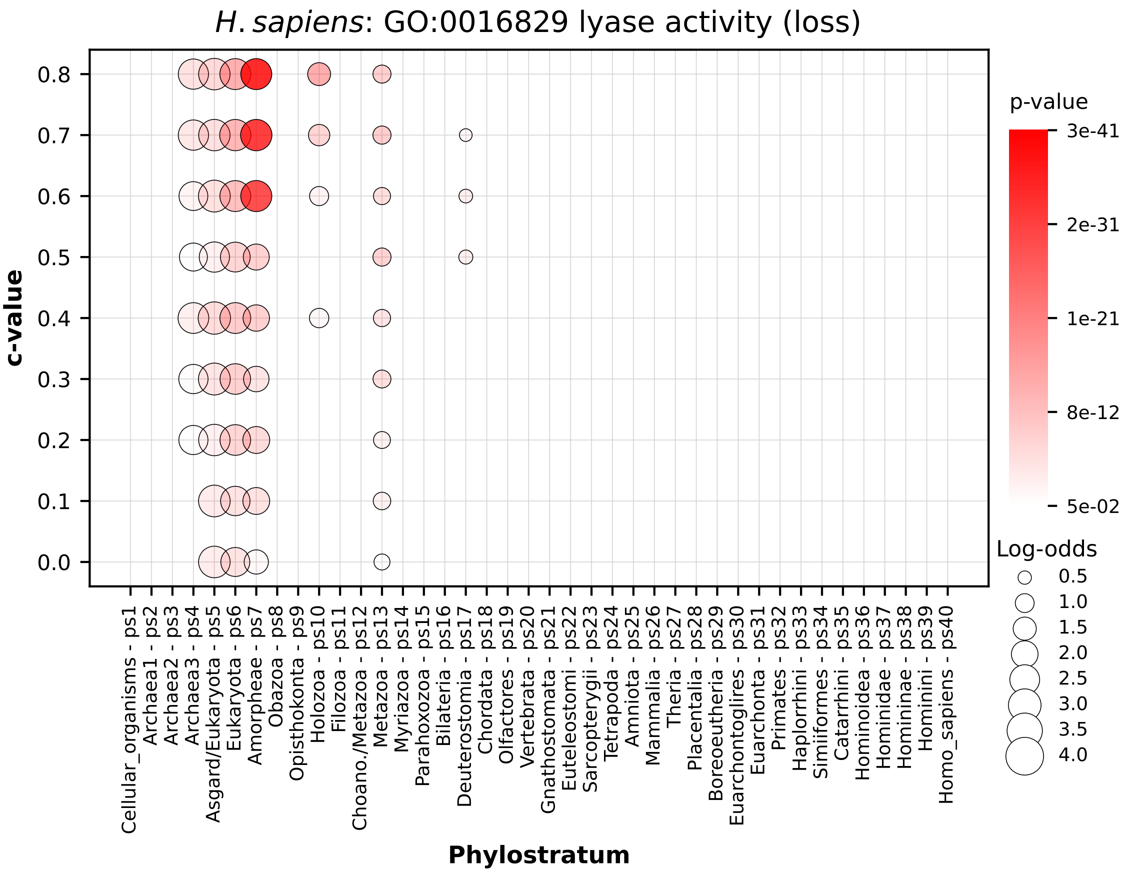

H. sapiens: GO:0016829 lyase activity (loss)
c-value
0.8
0.7
0.6
0.5
0.4
0.3
0.2
0.1
0.0
p-value
3e-41
2e-31
1e-21
8e-12
5e-02
Log-odds
0.5
1.0
1.5
2.0
2.5
3.0
3.5
4.0
Cellular_organisms - ps1
Archaea1 - ps2
Archaea2 - ps3
Archaea3 - ps4
Asgard/Eukaryota - ps5
Eukaryota - ps6
Amorpheae - ps7
Obazoa - ps8
Opisthokonta - ps9
Holozoa - ps10
Filozoa - ps11
Choano./Metazoa - ps12
Metazoa - ps13
Myriazoa - ps14
Parahoxozoa - ps15
Bilateria - ps16
Deuterostomia - ps17
Chordata - ps18
Olfactores - ps19
Vertebrata - ps20
Gnathostomata - ps21
Euteleostomi - ps22
Sarcopterygii - ps23
Tetrapoda - ps24
Amniota - ps25
Mammalia - ps26
Theria - ps27
Placentalia - ps28
Boreoeutheria - ps29
Euarchontoglires - ps30
Euarchonta - ps31
Primates - ps32
Haplorrhini - ps33
Simiiformes - ps34
Catarrhini - ps35
Hominoidea - ps36
Hominidae - ps37
Homininae - ps38
Hominini - ps39
Homo_sapiens - ps40
Phylostratum

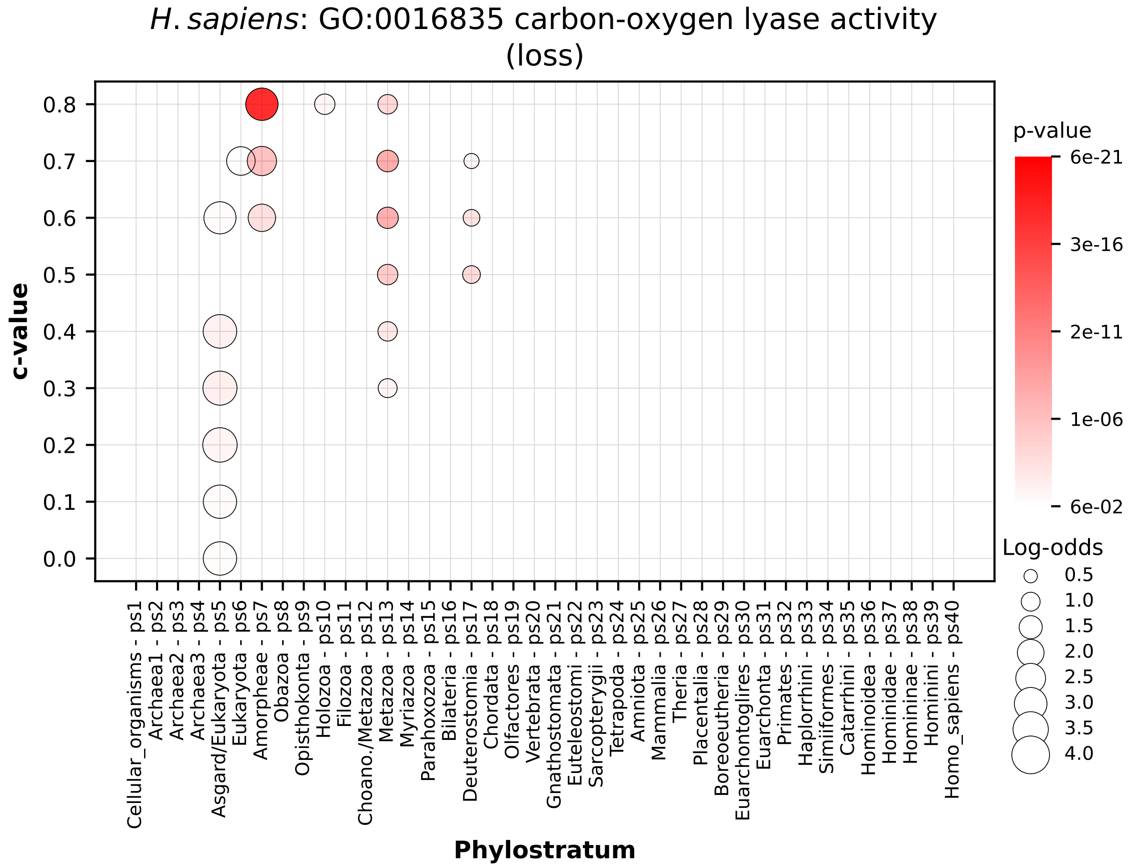

H. sapiens: GO:0016835 carbon-oxygen lyase activity (loss)
c-value
0.0
0.1
0.2
0.3
0.4
0.5
0.6
0.7
0.8
Phylostratum
Cellular_organisms - ps1
Archaea1 - ps2
Archaea2 - ps3
Archaea3 - ps4
Asgard/Eukaryota - ps5
Eukaryota - ps6
Amorpheae - ps7
Obazoa - ps8
Opisthokonta - ps9
Holozoa - ps10
Filozoa - ps11
Choano./Metazoa - ps12
Metazoa - ps13
Myriazoa - ps14
Parahoxozoa - ps15
Bilateria - ps16
Deuterostomia - ps17
Chordata - ps18
Olfactores - ps19
Vertebrata - ps20
Gnathostomata - ps21
Euteleostomi - ps22
Sarcopterygii - ps23
Tetrapoda - ps24
Amniota - ps25
Mammalia - ps26
Theria - ps27
Placentalia - ps28
Boreoeutheria - ps29
Euarchontoglires - ps30
Euarchonta - ps31
Primates - ps32
Haplorrhini - ps33
Simiiformes - ps34
Catarrhini - ps35
Hominoidea - ps36
Hominidae - ps37
Homininae - ps38
Hominini - ps39
Homo_sapiens - ps40
p-value
6e-21
3e-16
2e-11
1e-06
6e-02
Log-odds
0.5
1.0
1.5
2.0
2.5
3.0
3.5
4.0

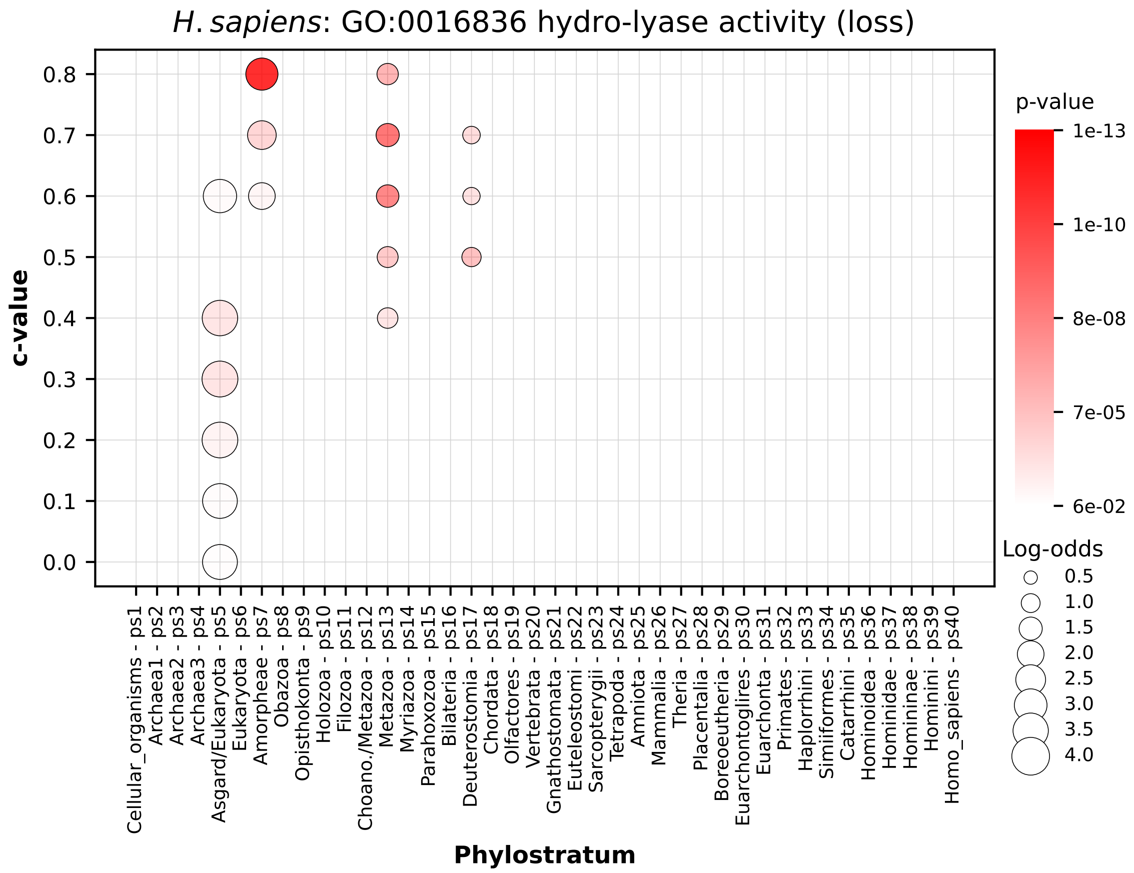

H. sapiens: GO:0016836 hydro-lyase activity (loss)
c-value
0.8
0.7
0.6
0.5
0.4
0.3
0.2
0.1
0.0
Phylostratum
Cellular_organisms - ps1
Archaea1 - ps2
Archaea2 - ps3
Archaea3 - ps4
Asgard/Eukaryota - ps5
Eukaryota - ps6
Amorpheae - ps7
Obazoa - ps8
Opisthokonta - ps9
Holozoa - ps10
Filozoa - ps11
Choano./Metazoa - ps12
Metazoa - ps13
Myriazoa - ps14
Parahoxozoa - ps15
Bilateria - ps16
Deuterostomia - ps17
Chordata - ps18
Olfactores - ps19
Vertebrata - ps20
Gnathostomata - ps21
Euteleostomi - ps22
Sarcopterygii - ps23
Tetrapoda - ps24
Amniota - ps25
Mammalia - ps26
Theria - ps27
Placentalia - ps28
Boreoeutheria - ps29
Euarchontoglires - ps30
Euarchonta - ps31
Primates - ps32
Haplorrhini - ps33
Simiiformes - ps34
Catarrhini - ps35
Hominoidea - ps36
Hominidae - ps37
Homininae - ps38
Hominini - ps39
Homo_sapiens - ps40
p-value
1e-13
1e-10
8e-08
7e-05
6e-02
Log-odds
0.5
1.0
1.5
2.0
2.5
3.0
3.5
4.0

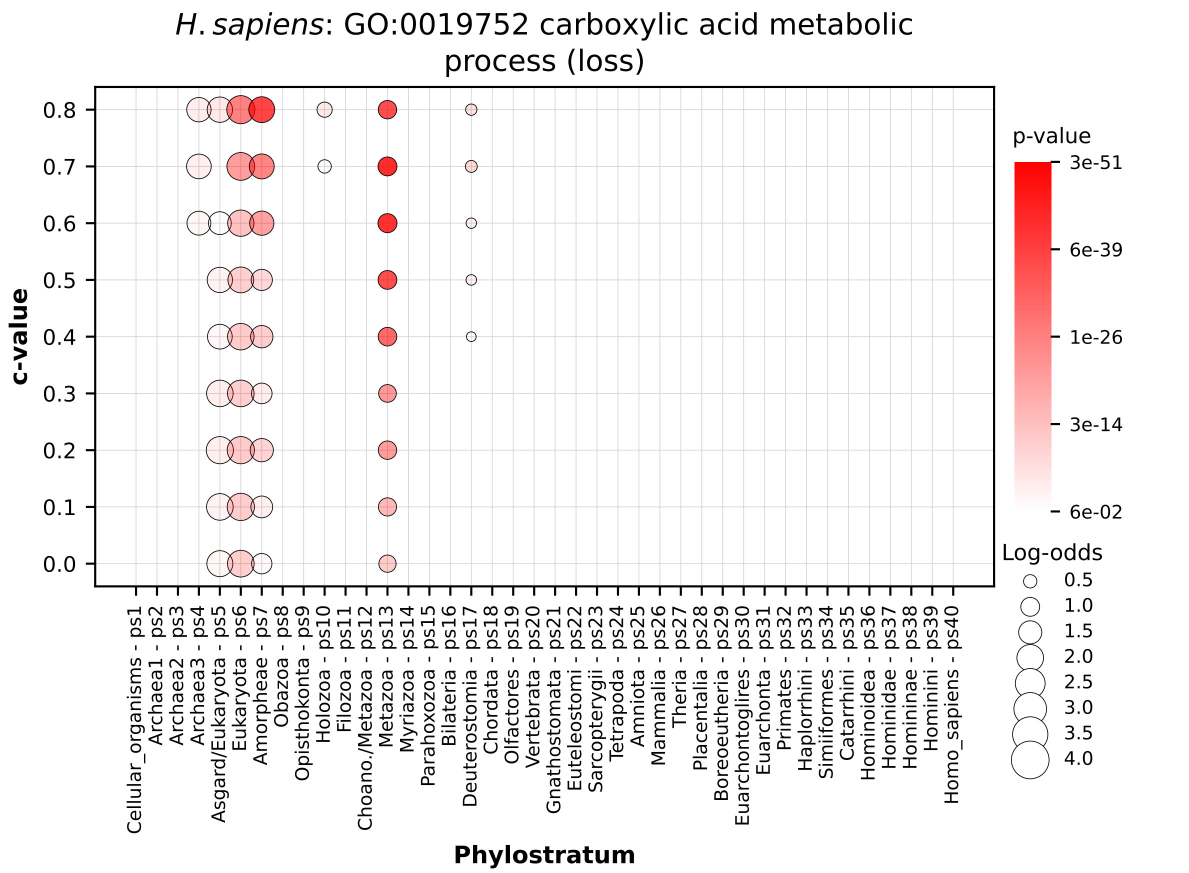

H. sapiens: GO:0019752 carboxylic acid metabolic process (loss)
c-value
0.8
0.7
0.6
0.5
0.4
0.3
0.2
0.1
0.0
Phylostratum
Cellular_organisms - ps1
Archaea1 - ps2
Archaea2 - ps3
Archaea3 - ps4
Asgard/Eukaryota - ps5
Eukaryota - ps6
Amorpheae - ps7
Obazoa - ps8
Opisthokonta - ps9
Holozoa - ps10
Filozoa - ps11
Choano./Metazoa - ps12
Metazoa - ps13
Myriazoa - ps14
Parahoxozoa - ps15
Bilateria - ps16
Deuterostomia - ps17
Chordata - ps18
Olfactores - ps19
Vertebrata - ps20
Gnathostomata - ps21
Euteleostomi - ps22
Sarcopterygii - ps23
Tetrapoda - ps24
Amniota - ps25
Mammalia - ps26
Theria - ps27
Placentalia - ps28
Boreoeutheria - ps29
Euarchontoglires - ps30
Euarchonta - ps31
Primates - ps32
Haplorrhini - ps33
Simiiformes - ps34
Catarrhini - ps35
Hominoidea - ps36
Hominidae - ps37
Homininae - ps38
Hominini - ps39
Homo_sapiens - ps40
p-value
3e-51
6e-39
1e-26
3e-14
6e-02
Log-odds
0.5
1.0
1.5
2.0
2.5
3.0
3.5
4.0

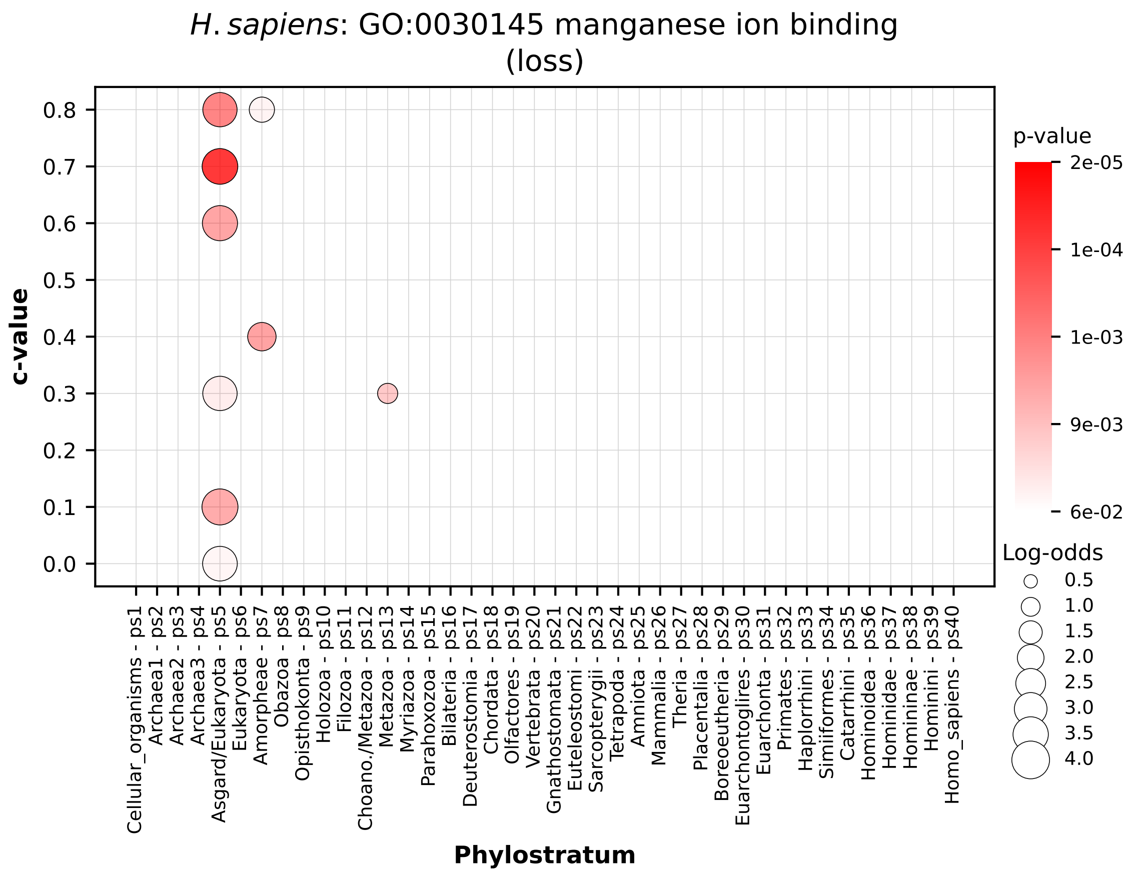

H. sapiens: GO:0030145 manganese ion binding
(loss)
c-value
0.8
0.7
0.6
0.5
0.4
0.3
0.2
0.1
0.0
p-value
2e-05
1e-04
1e-03
9e-03
6e-02
Log-odds
0.5
1.0
1.5
2.0
2.5
3.0
3.5
4.0
Cellular_organisms - ps1
Archaea1 - ps2
Archaea2 - ps3
Archaea3 - ps4
Asgard/Eukaryota - ps5
Eukaryota - ps6
Amorpheae - ps7
Obazoa - ps8
Opisthokonta - ps9
Holozoa - ps10
Filozoa - ps11
Choano./Metazoa - ps12
Metazoa - ps13
Myriazoa - ps14
Parahoxozoa - ps15
Bilateria - ps16
Deuterostomia - ps17
Chordata - ps18
Olfactores - ps19
Vertebrata - ps20
Gnathostomata - ps21
Euteleostomi - ps22
Sarcopterygii - ps23
Tetrapoda - ps24
Amniota - ps25
Mammalia - ps26
Theria - ps27
Placentalia - ps28
Boreoeutheria - ps29
Euarchontoglires - ps30
Euarchonta - ps31
Primates - ps32
Haplorrhini - ps33
Simiiformes - ps34
Catarrhini - ps35
Hominoidea - ps36
Hominidae - ps37
Homininae - ps38
Hominini - ps39
Homo_sapiens - ps40
Phylostratum

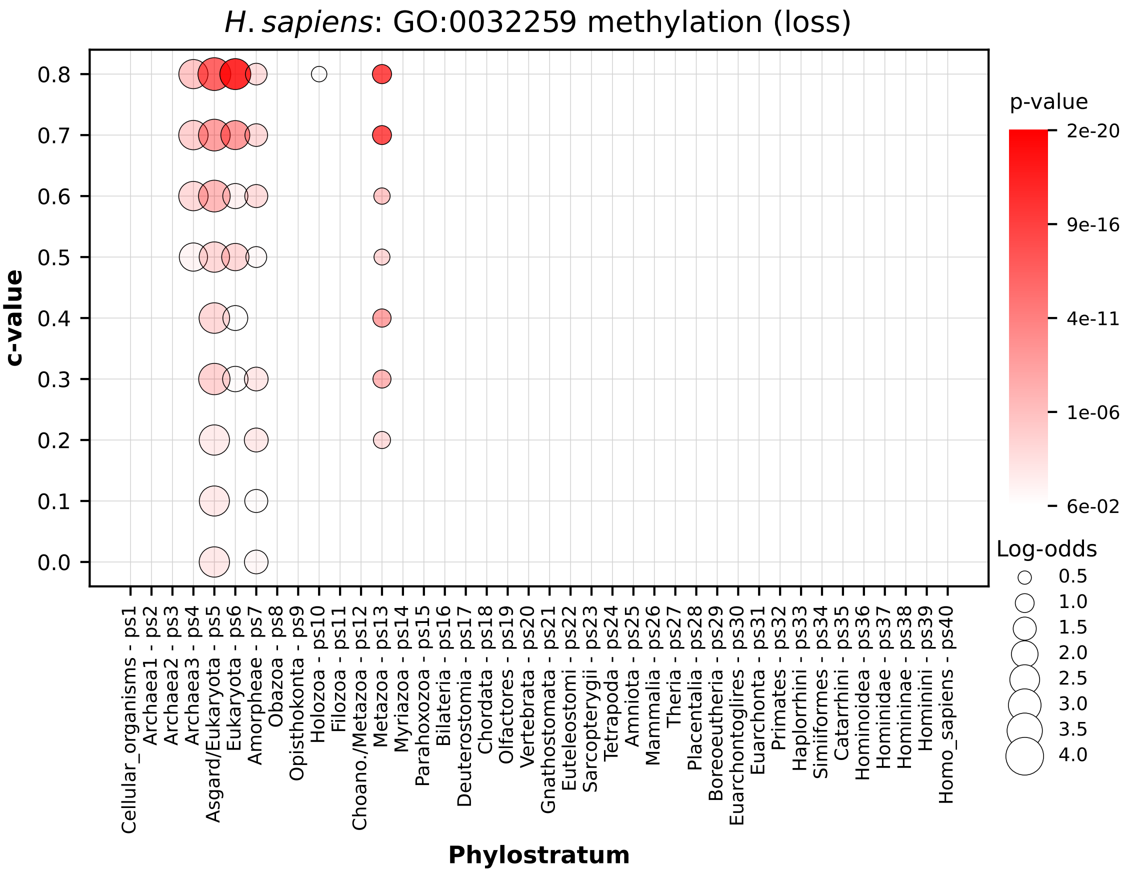

H. sapiens: GO:0032259 methylation (loss)
c-value
0.0
0.1
0.2
0.3
0.4
0.5
0.6
0.7
0.8
Phylostratum
Cellular_organisms - ps1
Archaea1 - ps2
Archaea2 - ps3
Archaea3 - ps4
Asgard/Eukaryota - ps5
Eukaryota - ps6
Amorpheae - ps7
Obazoa - ps8
Opisthokonta - ps9
Holozoa - ps10
Filozoa - ps11
Choano./Metazoa - ps12
Metazoa - ps13
Myriazoa - ps14
Parahoxozoa - ps15
Bilateria - ps16
Deuterostomia - ps17
Chordata - ps18
Olfactores - ps19
Vertebrata - ps20
Gnathostomata - ps21
Euteleostomi - ps22
Sarcopterygii - ps23
Tetrapoda - ps24
Amniota - ps25
Mammalia - ps26
Theria - ps27
Placentalia - ps28
Boreoeutheria - ps29
Euarchontoglires - ps30
Euarchonta - ps31
Primates - ps32
Haplorrhini - ps33
Simiiformes - ps34
Catarrhini - ps35
Hominoidea - ps36
Hominidae - ps37
Homininae - ps38
Hominini - ps39
Homo_sapiens - ps40
p-value
2e-20
9e-16
4e-11
1e-06
6e-02
Log-odds
0.5
1.0
1.5
2.0
2.5
3.0
3.5
4.0

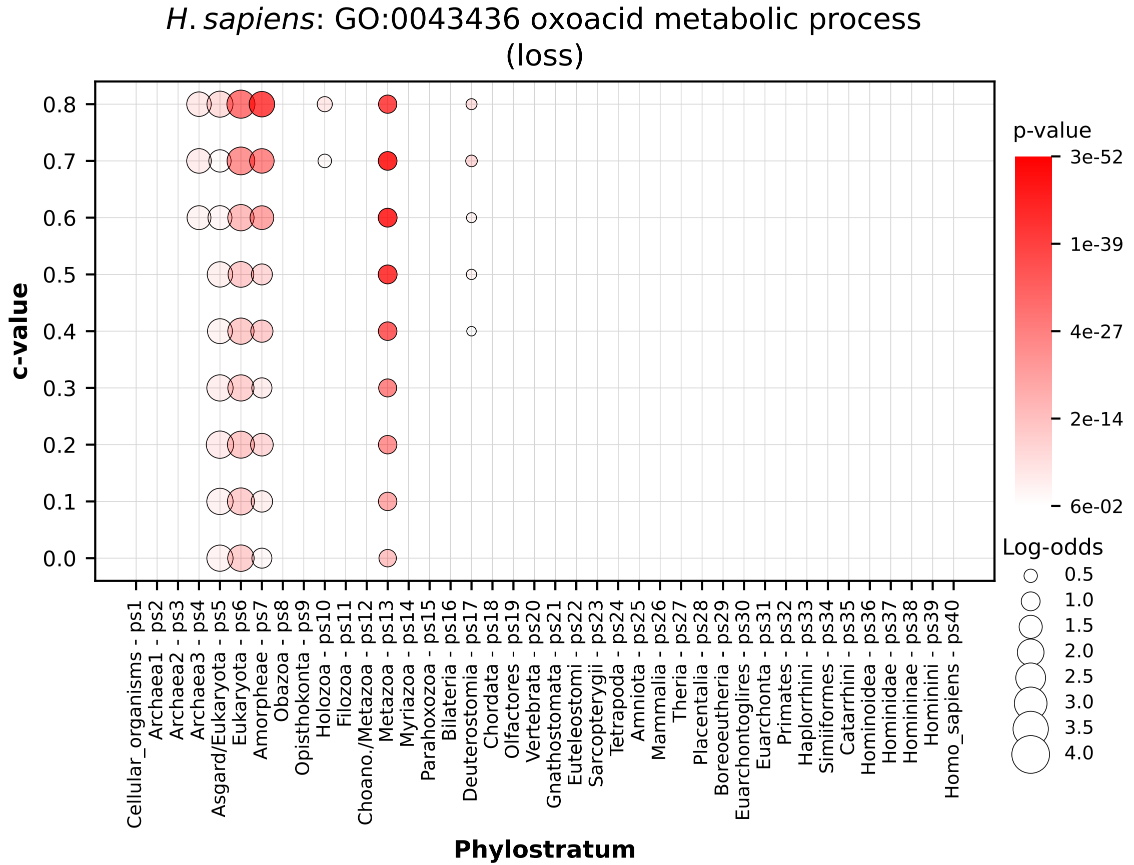

H. sapiens: GO:0043436 oxoacid metabolic process (loss)
c-value
0.8
0.7
0.6
0.5
0.4
0.3
0.2
0.1
0.0
Phylostratum
Cellular_organisms - ps1
Archaea1 - ps2
Archaea2 - ps3
Archaea3 - ps4
Asgard/Eukaryota - ps5
Eukaryota - ps6
Amorpheae - ps7
Obazoa - ps8
Opisthokonta - ps9
Holozoa - ps10
Filozoa - ps11
Choano./Metazoa - ps12
Metazoa - ps13
Myriazoa - ps14
Parahoxozoa - ps15
Bilateria - ps16
Deuterostomia - ps17
Chordata - ps18
Olfactores - ps19
Vertebrata - ps20
Gnathostomata - ps21
Euteleostomi - ps22
Sarcopterygii - ps23
Tetrapoda - ps24
Amniota - ps25
Mammalia - ps26
Theria - ps27
Placentalia - ps28
Boreoeutheria - ps29
Euarchontoglires - ps30
Euarchonta - ps31
Primates - ps32
Haplorrhini - ps33
Simiiformes - ps34
Catarrhini - ps35
Hominoidea - ps36
Hominidae - ps37
Homininae - ps38
Hominini - ps39
Homo_sapiens - ps40
p-value
3e-52
1e-39
4e-27
2e-14
6e-02
Log-odds
0.5
1.0
1.5
2.0
2.5
3.0
3.5
4.0

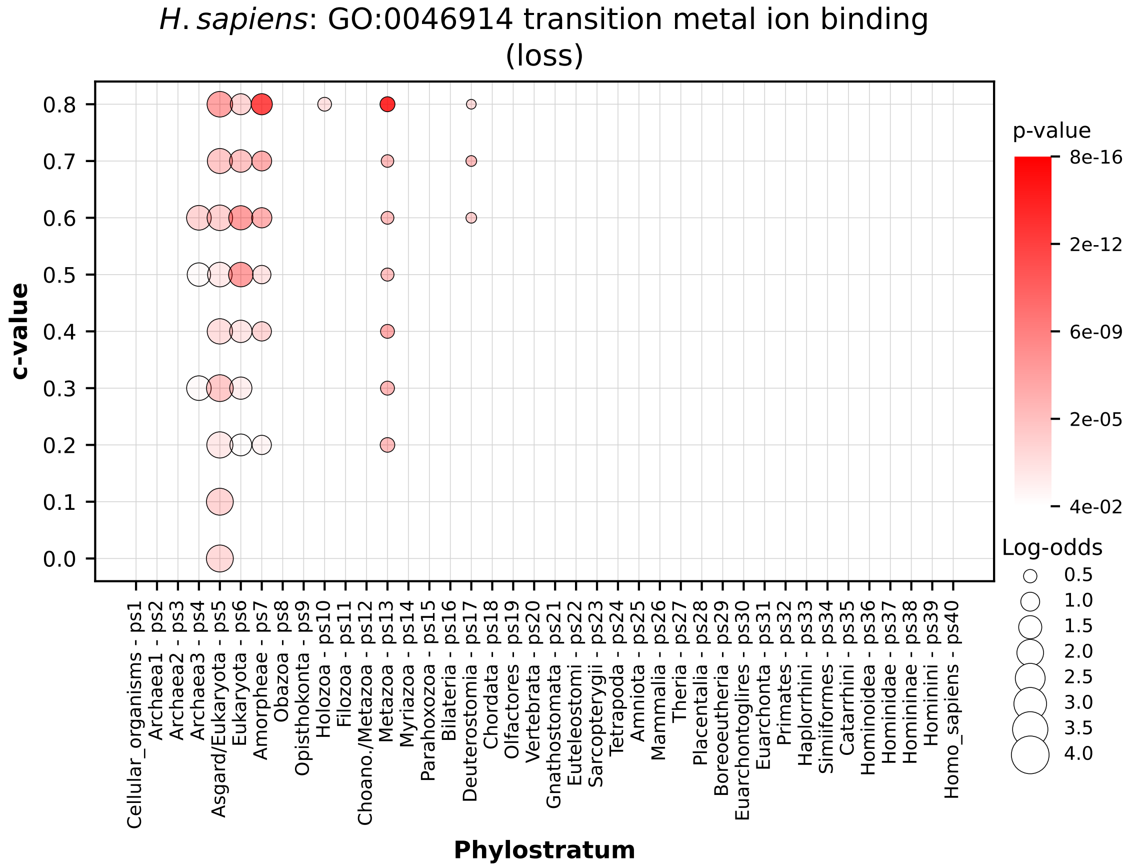

H. sapiens: GO:0046914 transition metal ion binding
(loss)
c-value
0.8
0.7
0.6
0.5
0.4
0.3
0.2
0.1
0.0
p-value
8e-16
2e-12
6e-09
2e-05
4e-02
Log-odds
0.5
1.0
1.5
2.0
2.5
3.0
3.5
4.0
Cellular_organisms - ps1
Archaea1 - ps2
Archaea2 - ps3
Archaea3 - ps4
Asgard/Eukaryota - ps5
Eukaryota - ps6
Amorpheae - ps7
Obazoa - ps8
Opisthokonta - ps9
Holozoa - ps10
Filozoa - ps11
Choano./Metazoa - ps12
Metazoa - ps13
Myriazoa - ps14
Parahoxozoa - ps15
Bilateria - ps16
Deuterostomia - ps17
Chordata - ps18
Olfactores - ps19
Vertebrata - ps20
Gnathostomata - ps21
Euteleostomi - ps22
Sarcopterygii - ps23
Tetrapoda - ps24
Amniota - ps25
Mammalia - ps26
Theria - ps27
Placentalia - ps28
Boreoeutheria - ps29
Euarchontoglires - ps30
Euarchonta - ps31
Primates - ps32
Haplorrhini - ps33
Simiiformes - ps34
Catarrhini - ps35
Hominoidea - ps36
Hominidae - ps37
Homininae - ps38
Hominini - ps39
Homo_sapiens - ps40
Phylostratum

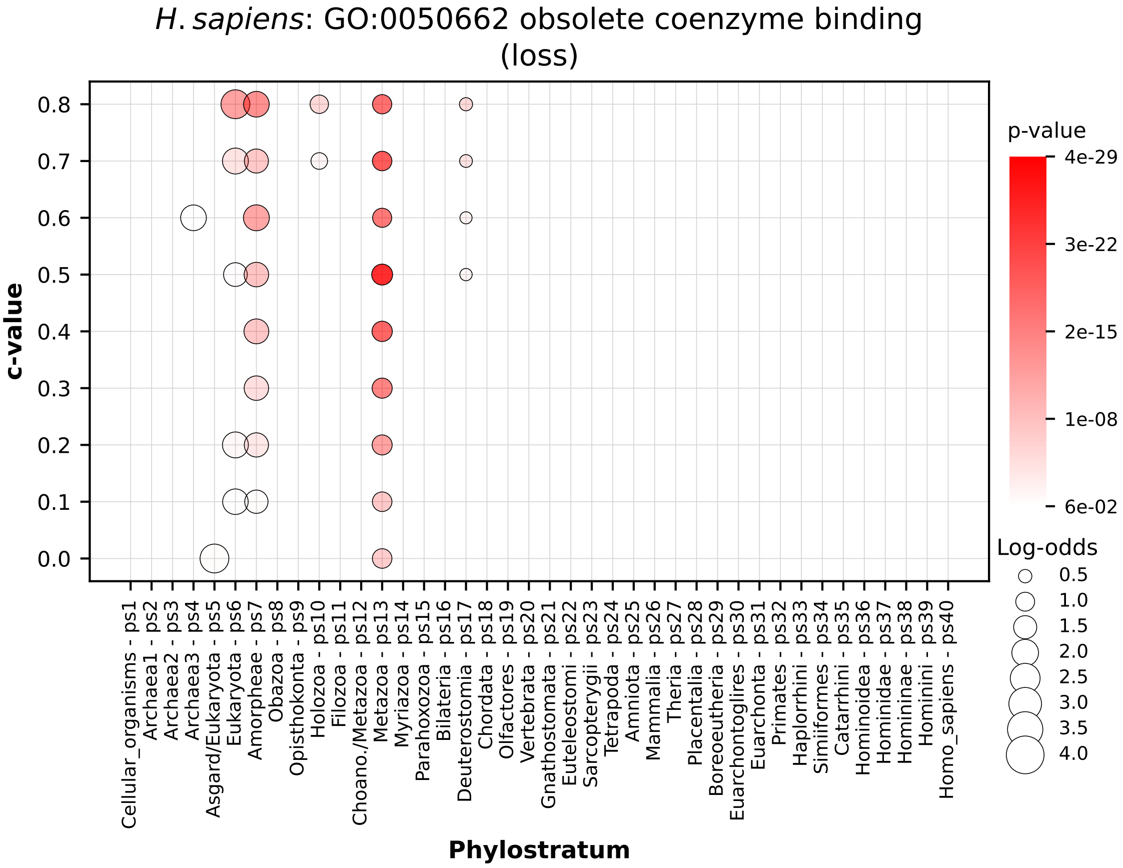

H. sapiens: GO:0050662 obsolete coenzyme binding
(loss)
c-value
0.8
0.7
0.6
0.5
0.4
0.3
0.2
0.1
0.0
Phylostratum
Cellular_organisms - ps1
Archaea1 - ps2
Archaea2 - ps3
Archaea3 - ps4
Asgard/Eukaryota - ps5
Eukaryota - ps6
Amorpheae - ps7
Obazoa - ps8
Opisthokonta - ps9
Holozoa - ps10
Filozoa - ps11
Choano./Metazoa - ps12
Metazoa - ps13
Myriazoa - ps14
Parahoxozoa - ps15
Bilateria - ps16
Deuterostomia - ps17
Chordata - ps18
Olfactores - ps19
Vertebrata - ps20
Gnathostomata - ps21
Euteleostomi - ps22
Sarcopterygii - ps23
Tetrapoda - ps24
Amniota - ps25
Mammalia - ps26
Theria - ps27
Placentalia - ps28
Boreoeutheria - ps29
Euarchontoglires - ps30
Euarchonta - ps31
Primates - ps32
Haplorrhini - ps33
Simiiformes - ps34
Catarrhini - ps35
Hominoidea - ps36
Hominidae - ps37
Homininae - ps38
Hominini - ps39
Homo_sapiens - ps40
p-value
4e-29
3e-22
2e-15
1e-08
6e-02
Log-odds
0.5
1.0
1.5
2.0
2.5
3.0
3.5
4.0

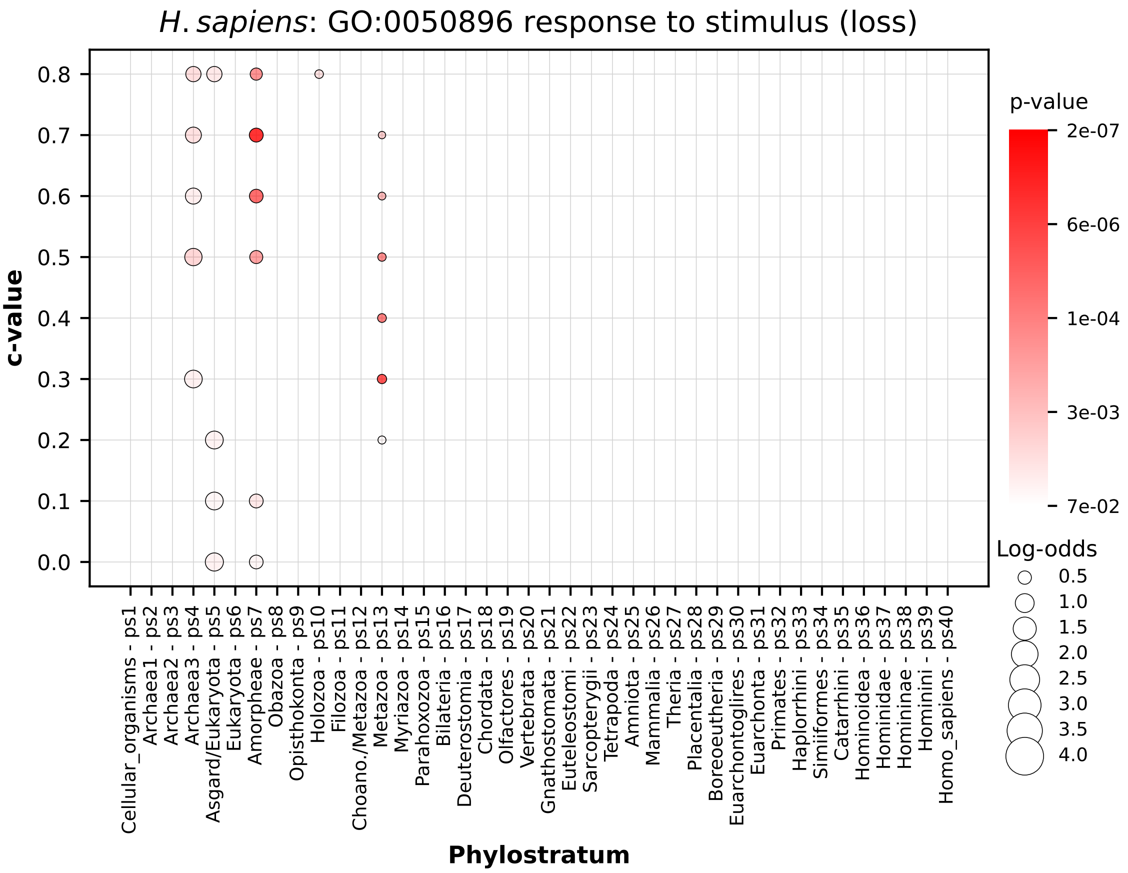

H. sapiens: GO:0050896 response to stimulus (loss)
c-value
0.0
0.1
0.2
0.3
0.4
0.5
0.6
0.7
0.8
Phylostratum
Cellular_organisms - ps1
Archaea1 - ps2
Archaea2 - ps3
Archaea3 - ps4
Asgard/Eukaryota - ps5
Eukaryota - ps6
Amorpheae - ps7
Obazoa - ps8
Opisthokonta - ps9
Holozoa - ps10
Filozoa - ps11
Choano./Metazoa - ps12
Metazoa - ps13
Myriazoa - ps14
Parahoxozoa - ps15
Bilateria - ps16
Deuterostomia - ps17
Chordata - ps18
Olfactores - ps19
Vertebrata - ps20
Gnathostomata - ps21
Euteleostomi - ps22
Sarcopterygii - ps23
Tetrapoda - ps24
Amniota - ps25
Mammalia - ps26
Theria - ps27
Placentalia - ps28
Boreoeutheria - ps29
Euarchontoglires - ps30
Euarchonta - ps31
Primates - ps32
Haplorrhini - ps33
Simiiformes - ps34
Catarrhini - ps35
Hominoidea - ps36
Hominidae - ps37
Homininae - ps38
Hominini - ps39
Homo_sapiens - ps40
p-value
2e-07
6e-06
1e-04
3e-03
7e-02
Log-odds
0.5
1.0
1.5
2.0
2.5
3.0
3.5
4.0

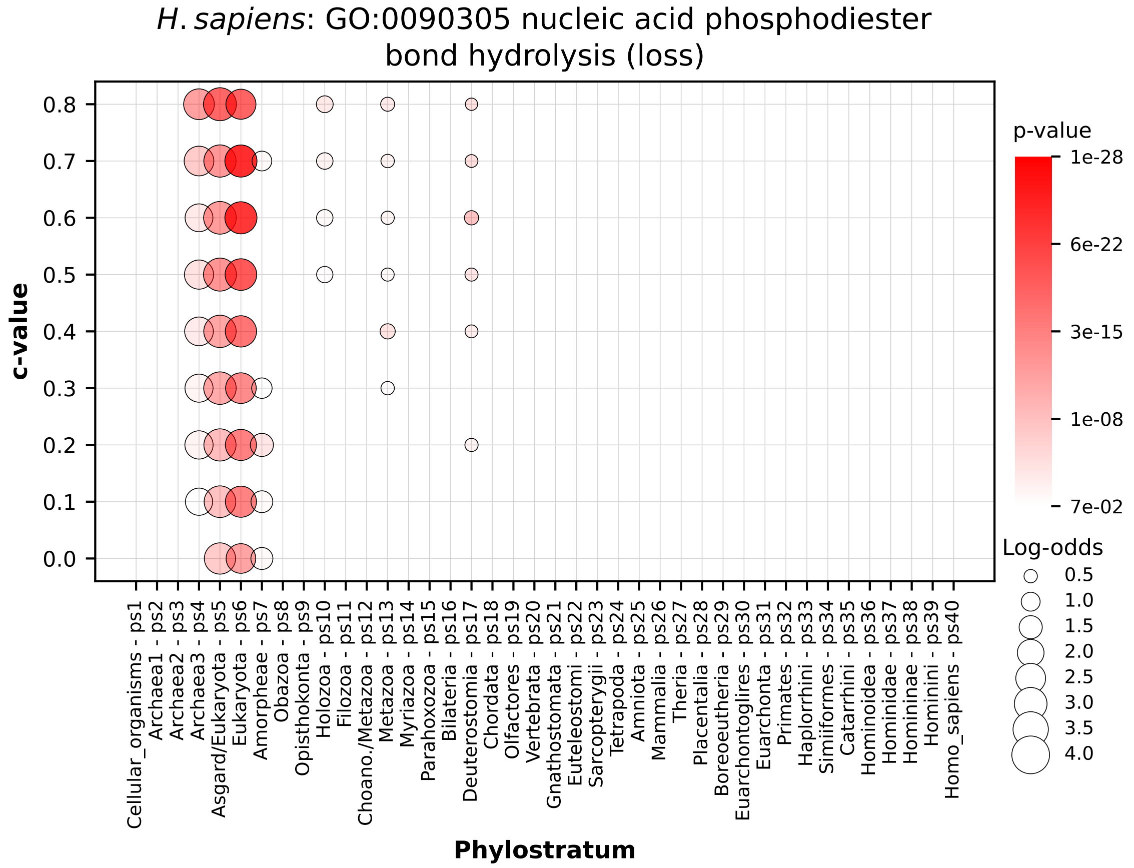

H. sapiens: GO:0090305 nucleic acid phosphodiester bond hydrolysis (loss)
c-value
0.8
0.7
0.6
0.5
0.4
0.3
0.2
0.1
0.0
p-value
1e-28
6e-22
3e-15
1e-08
7e-02
Log-odds
0.5
1.0
1.5
2.0
2.5
3.0
3.5
4.0
Cellular_organisms - ps1
Archaea1 - ps2
Archaea2 - ps3
Archaea3 - ps4
Asgard/Eukaryota - ps5
Eukaryota - ps6
Amorpheae - ps7
Obazoa - ps8
Opisthokonta - ps9
Holozoa - ps10
Filozoa - ps11
Choano./Metazoa - ps12
Metazoa - ps13
Myriazoa - ps14
Parahoxozoa - ps15
Bilateria - ps16
Deuterostomia - ps17
Chordata - ps18
Olfactores - ps19
Vertebrata - ps20
Gnathostomata - ps21
Euteleostomi - ps22
Sarcopterygii - ps23
Tetrapoda - ps24
Amniota - ps25
Mammalia - ps26
Theria - ps27
Placentalia - ps28
Boreoeutheria - ps29
Euarchontoglires - ps30
Euarchonta - ps31
Primates - ps32
Haplorrhini - ps33
Simiiformes - ps34
Catarrhini - ps35
Hominoidea - ps36
Hominidae - ps37
Homininae - ps38
Hominini - ps39
Homo_sapiens - ps40
Phylostratum

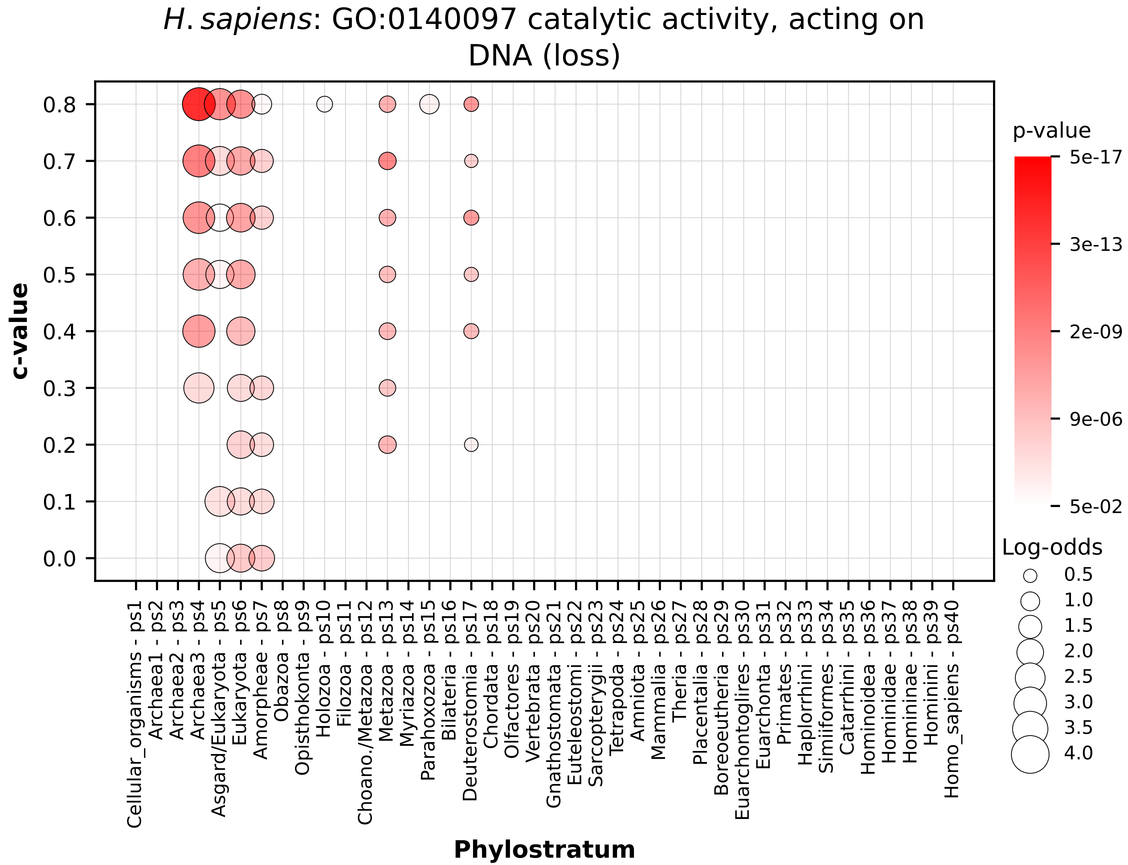
H. sapiens: GO:0140097 catalytic activity, acting on DNA (loss)
c-value
0.8
0.7
0.6
0.5
0.4
0.3
0.2
0.1
0.0
Phylostratum
Cellular_organisms - ps1
Archaea1 - ps2
Archaea2 - ps3
Archaea3 - ps4
Asgard/Eukaryota - ps5
Eukaryota - ps6
Amorpheae - ps7
Obazoa - ps8
Opisthokonta - ps9
Holozoa - ps10
Filozoa - ps11
Choano./Metazoa - ps12
Metazoa - ps13
Myriazoa - ps14
Parahoxozoa - ps15
Bilateria - ps16
Deuterostomia - ps17
Chordata - ps18
Olfactores - ps19
Vertebrata - ps20
Gnathostomata - ps21
Euteleostomi - ps22
Sarcopterygii - ps23
Tetrapoda - ps24
Amniota - ps25
Mammalia - ps26
Theria - ps27
Placentalia - ps28
Boreoeutheria - ps29
Euarchontoglires - ps30
Euarchonta - ps31
Primates - ps32
Haplorrhini - ps33
Simiiformes - ps34
Catarrhini - ps35
Hominoidea - ps36
Hominidae - ps37
Homininae - ps38
Hominini - ps39
Homo_sapiens - ps40
p-value
5e-17
3e-13
2e-09
9e-06
5e-02
Log-odds
0.5
1.0
1.5
2.0
2.5
3.0
3.5
4.0

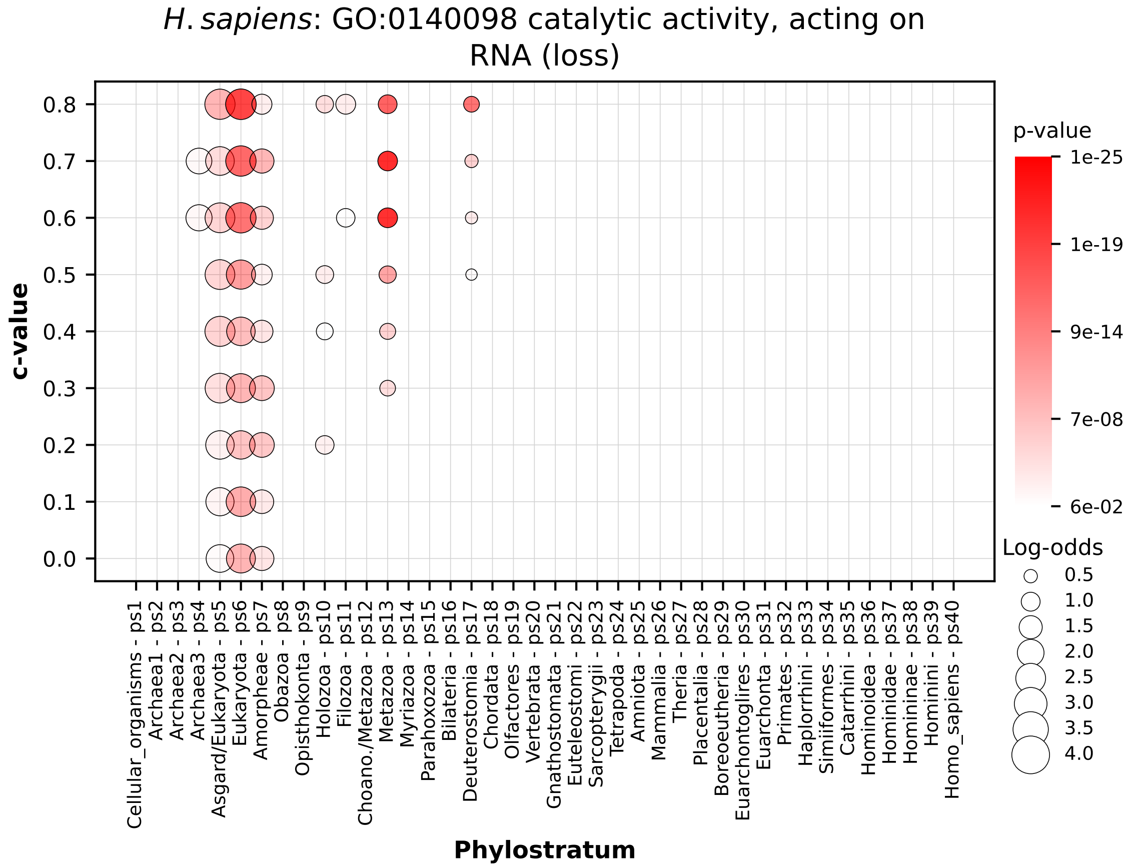

H. sapiens: GO:0140098 catalytic activity, acting on RNA (loss)
c-value
0.8
0.7
0.6
0.5
0.4
0.3
0.2
0.1
0.0
p-value
1e-25
1e-19
9e-14
7e-08
6e-02
Log-odds
0.5
1.0
1.5
2.0
2.5
3.0
3.5
4.0
Cellular_organisms - ps1
Archaea1 - ps2
Archaea2 - ps3
Archaea3 - ps4
Asgard/Eukaryota - ps5
Eukaryota - ps6
Amorpheae - ps7
Obazoa - ps8
Opisthokonta - ps9
Holozoa - ps10
Filozoa - ps11
Choano./Metazoa - ps12
Metazoa - ps13
Myriazoa - ps14
Parahoxozoa - ps15
Bilateria - ps16
Deuterostomia - ps17
Chordata - ps18
Olfactores - ps19
Vertebrata - ps20
Gnathostomata - ps21
Euteleostomi - ps22
Sarcopterygii - ps23
Tetrapoda - ps24
Amniota - ps25
Mammalia - ps26
Theria - ps27
Placentalia - ps28
Boreoeutheria - ps29
Euarchontoglires - ps30
Euarchonta - ps31
Primates - ps32
Haplorrhini - ps33
Simiiformes - ps34
Catarrhini - ps35
Hominoidea - ps36
Hominidae - ps37
Homininae - ps38
Hominini - ps39
Homo_sapiens - ps40
Phylostratum

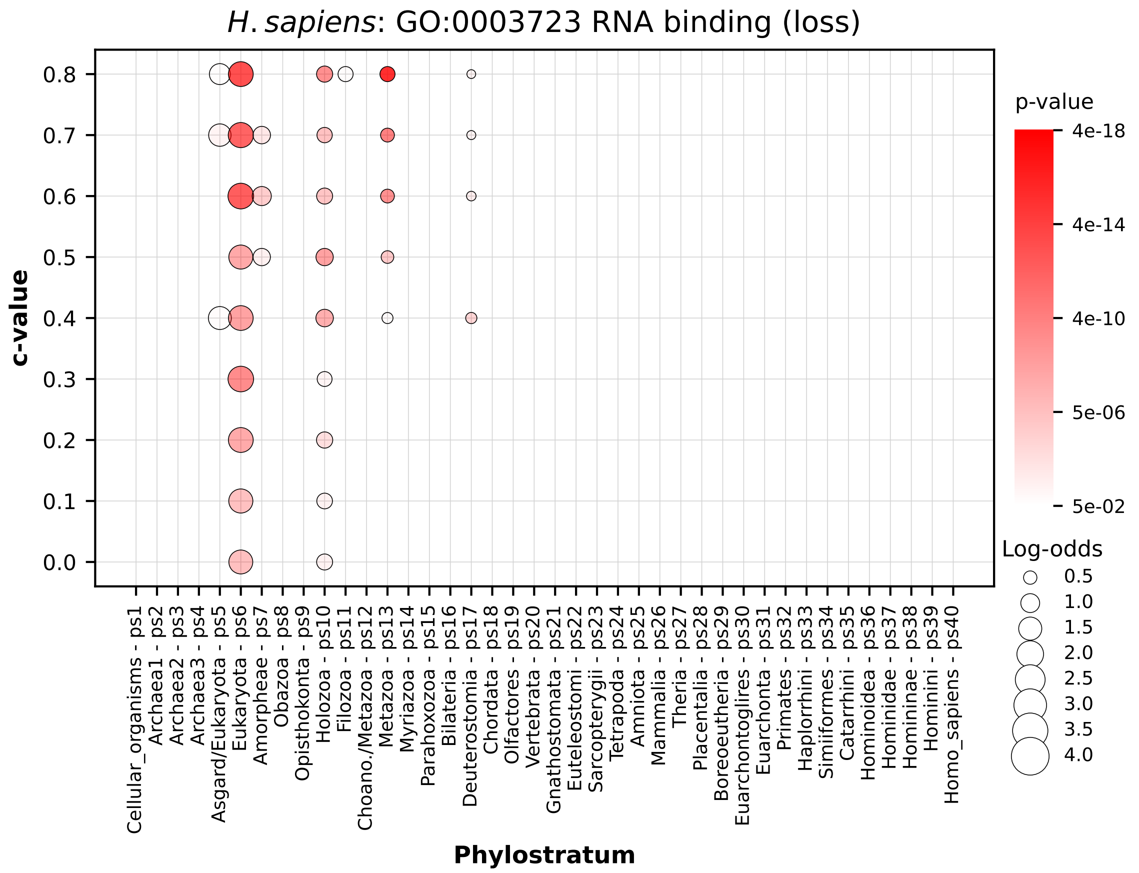

H. sapiens: GO:0003723 RNA binding (loss)
c-value
0.8
0.7
0.6
0.5
0.4
0.3
0.2
0.1
0.0
p-value
4e-18
4e-14
4e-10
5e-06
5e-02
Log-odds
0.5
1.0
1.5
2.0
2.5
3.0
3.5
4.0
Cellular_organisms - ps1
Archaea1 - ps2
Archaea2 - ps3
Archaea3 - ps4
Asgard/Eukaryota - ps5
Eukaryota - ps6
Amorpheae - ps7
Obazoa - ps8
Opisthokonta - ps9
Holozoa - ps10
Filozoa - ps11
Choano./Metazoa - ps12
Metazoa - ps13
Myriazoa - ps14
Parahoxozoa - ps15
Bilateria - ps16
Deuterostomia - ps17
Chordata - ps18
Olfactores - ps19
Vertebrata - ps20
Gnathostomata - ps21
Euteleostomi - ps22
Sarcopterygii - ps23
Tetrapoda - ps24
Amniota - ps25
Mammalia - ps26
Theria - ps27
Placentalia - ps28
Boreoeutheria - ps29
Euarchontoglires - ps30
Euarchonta - ps31
Primates - ps32
Haplorrhini - ps33
Simiiformes - ps34
Catarrhini - ps35
Hominoidea - ps36
Hominidae - ps37
Homininae - ps38
Hominini - ps39
Homo_sapiens - ps40
Phylostratum

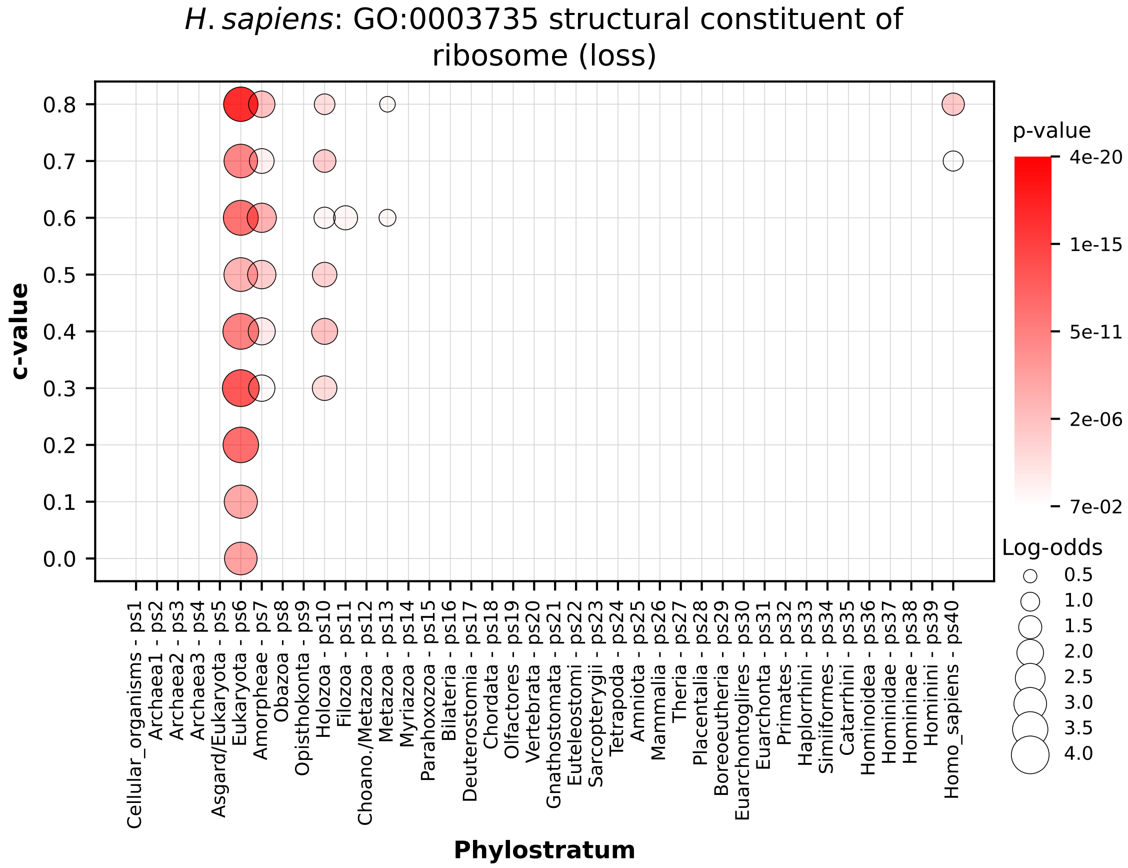

H. sapiens: GO:0003735 structural constituent of ribosome (loss)
c-value
0.8
0.7
0.6
0.5
0.4
0.3
0.2
0.1
0.0
p-value
4e-20
1e-15
5e-11
2e-06
7e-02
Log-odds
0.5
1.0
1.5
2.0
2.5
3.0
3.5
4.0
Cellular_organisms - ps1
Archaea1 - ps2
Archaea2 - ps3
Archaea3 - ps4
Asgard/Eukaryota - ps5
Eukaryota - ps6
Amorpheae - ps7
Obazoa - ps8
Opisthokonta - ps9
Holozoa - ps10
Filozoa - ps11
Choano./Metazoa - ps12
Metazoa - ps13
Myriazoa - ps14
Parahoxozoa - ps15
Bilateria - ps16
Deuterostomia - ps17
Chordata - ps18
Olfactores - ps19
Vertebrata - ps20
Gnathostomata - ps21
Euteleostomi - ps22
Sarcopterygii - ps23
Tetrapoda - ps24
Amniota - ps25
Mammalia - ps26
Theria - ps27
Placentalia - ps28
Boreoeutheria - ps29
Euarchontoglires - ps30
Euarchonta - ps31
Primates - ps32
Haplorrhini - ps33
Simiiformes - ps34
Catarrhini - ps35
Hominoidea - ps36
Hominidae - ps37
Homininae - ps38
Hominini - ps39
Homo_sapiens - ps40
Phylostratum

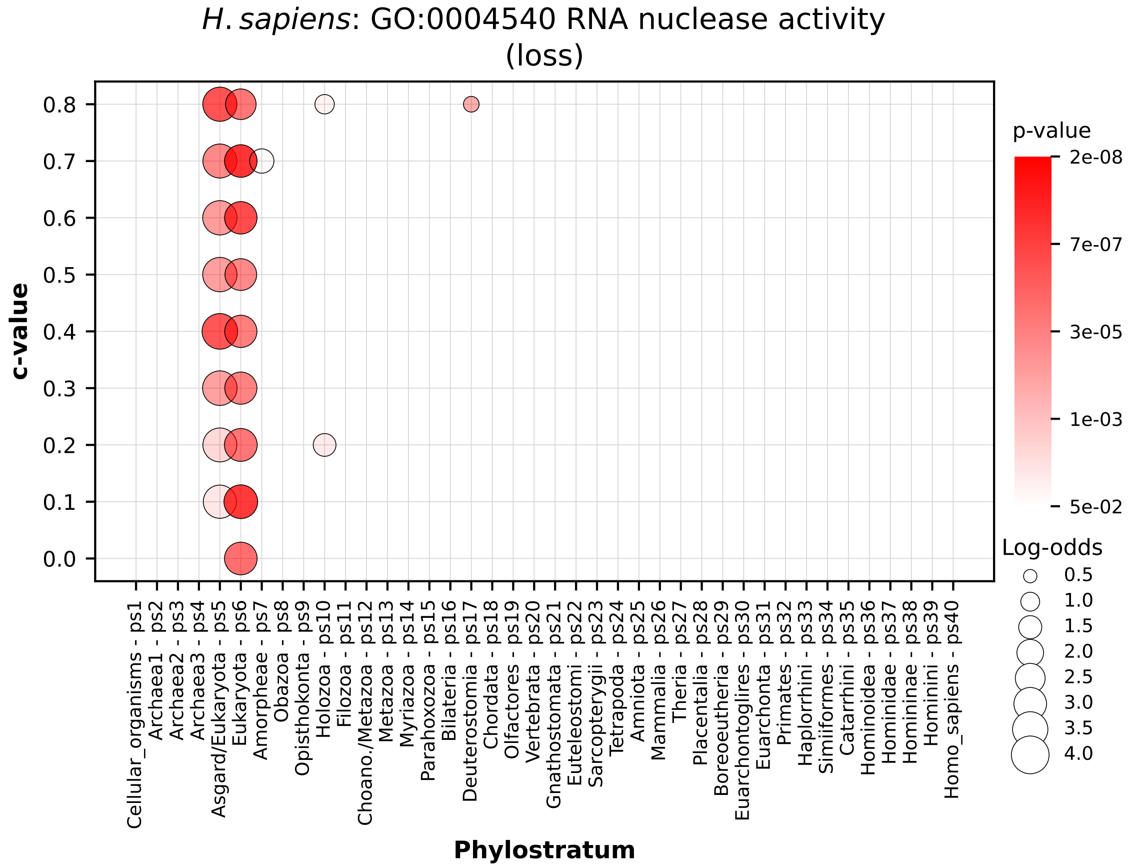

H. sapiens: GO:0004540 RNA nuclease activity
(loss)
c-value
0.8
0.7
0.6
0.5
0.4
0.3
0.2
0.1
0.0
p-value
2e-08
7e-07
3e-05
1e-03
5e-02
Log-odds
0.5
1.0
1.5
2.0
2.5
3.0
3.5
4.0
Cellular_organisms - ps1
Archaea1 - ps2
Archaea2 - ps3
Archaea3 - ps4
Asgard/Eukaryota - ps5
Eukaryota - ps6
Amorpheae - ps7
Obazoa - ps8
Opisthokonta - ps9
Holozoa - ps10
Filozoa - ps11
Choano./Metazoa - ps12
Metazoa - ps13
Myriazoa - ps14
Parahoxozoa - ps15
Bilateria - ps16
Deuterostomia - ps17
Chordata - ps18
Olfactores - ps19
Vertebrata - ps20
Gnathostomata - ps21
Euteleostomi - ps22
Sarcopterygii - ps23
Tetrapoda - ps24
Amniota - ps25
Mammalia - ps26
Theria - ps27
Placentalia - ps28
Boreoeutheria - ps29
Euarchontoglires - ps30
Euarchonta - ps31
Primates - ps32
Haplorrhini - ps33
Simiiformes - ps34
Catarrhini - ps35
Hominoidea - ps36
Hominidae - ps37
Homininae - ps38
Hominini - ps39
Homo_sapiens - ps40
Phylostratum

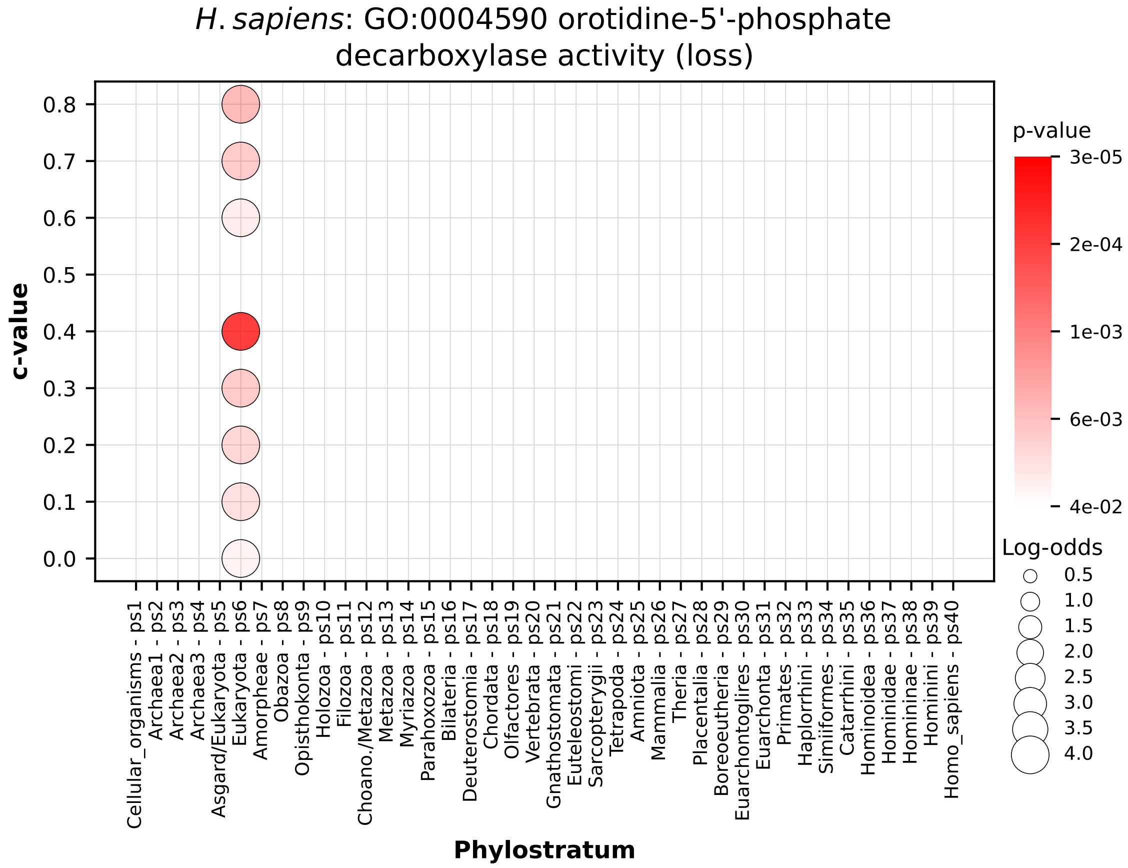

H. sapiens: GO:0004590 orotidine-5'-phosphate decarboxylase activity (loss)
c-value
0.8
0.7
0.6
0.5
0.4
0.3
0.2
0.1
0.0
Phylostratum
Cellular_organisms - ps1
Archaea1 - ps2
Archaea2 - ps3
Archaea3 - ps4
Asgard/Eukaryota - ps5
Eukaryota - ps6
Amorpheae - ps7
Obazoa - ps8
Opisthokonta - ps9
Holozoa - ps10
Filozoa - ps11
Choano./Metazoa - ps12
Metazoa - ps13
Myriazoa - ps14
Parahoxozoa - ps15
Bilateria - ps16
Deuterostomia - ps17
Chordata - ps18
Olfactores - ps19
Vertebrata - ps20
Gnathostomata - ps21
Euteleostomi - ps22
Sarcopterygii - ps23
Tetrapoda - ps24
Amniota - ps25
Mammalia - ps26
Theria - ps27
Placentalia - ps28
Boreoeutheria - ps29
Euarchontoglires - ps30
Euarchonta - ps31
Primates - ps32
Haplorrhini - ps33
Simiiformes - ps34
Catarrhini - ps35
Hominoidea - ps36
Hominidae - ps37
Homininae - ps38
Hominini - ps39
Homo_sapiens - ps40
p-value
3e-05
2e-04
1e-03
6e-03
4e-02
Log-odds
0.5
1.0
1.5
2.0
2.5
3.0
3.5
4.0

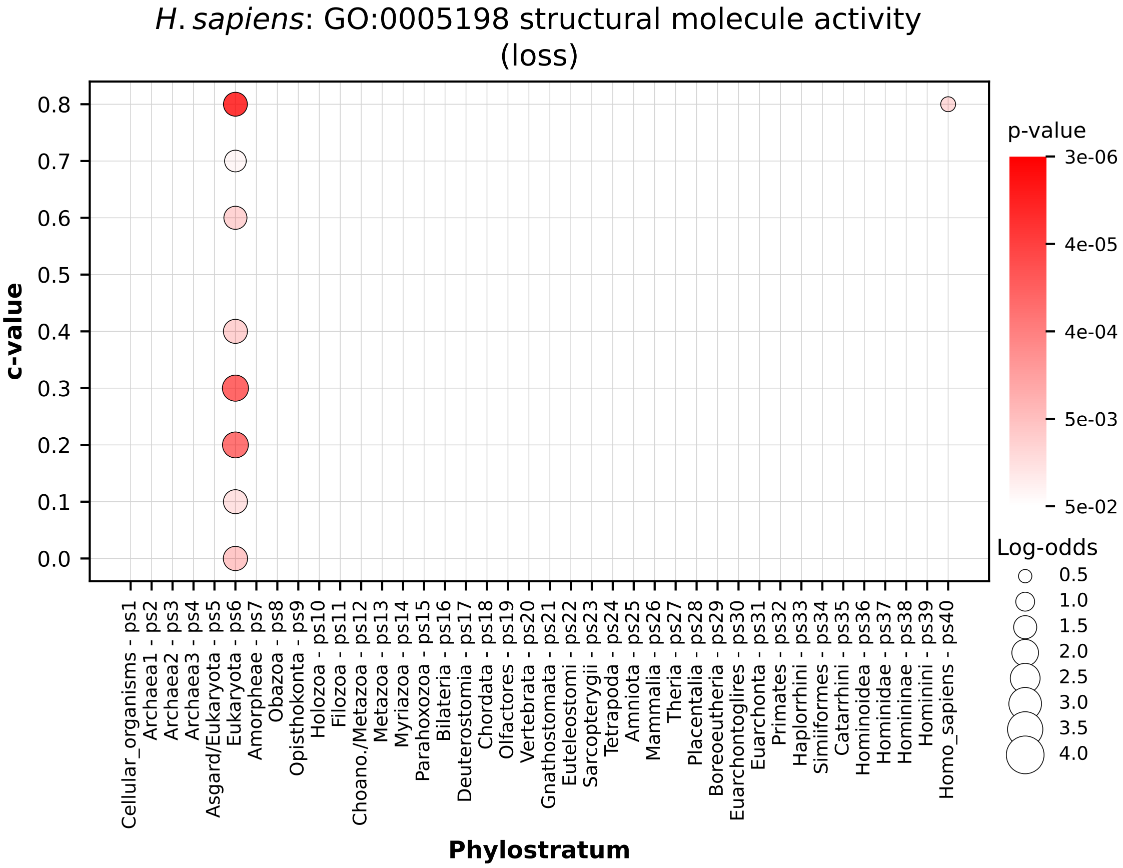

H. sapiens: GO:0005198 structural molecule activity
(loss)
c-value
0.8
0.7
0.6
0.5
0.4
0.3
0.2
0.1
0.0
p-value
3e-06
4e-05
4e-04
5e-03
5e-02
Log-odds
0.5
1.0
1.5
2.0
2.5
3.0
3.5
4.0
Cellular_organisms - ps1
Archaea1 - ps2
Archaea2 - ps3
Archaea3 - ps4
Asgard/Eukaryota - ps5
Eukaryota - ps6
Amorpheae - ps7
Obazoa - ps8
Opisthokonta - ps9
Holozoa - ps10
Filozoa - ps11
Choano./Metazoa - ps12
Metazoa - ps13
Myriazoa - ps14
Parahoxozoa - ps15
Bilateria - ps16
Deuterostomia - ps17
Chordata - ps18
Olfactores - ps19
Vertebrata - ps20
Gnathostomata - ps21
Euteleostomi - ps22
Sarcopterygii - ps23
Tetrapoda - ps24
Amniota - ps25
Mammalia - ps26
Theria - ps27
Placentalia - ps28
Boreoeutheria - ps29
Euarchontoglires - ps30
Euarchonta - ps31
Primates - ps32
Haplorrhini - ps33
Simiiformes - ps34
Catarrhini - ps35
Hominoidea - ps36
Hominidae - ps37
Homininae - ps38
Hominini - ps39
Homo_sapiens - ps40
Phylostratum

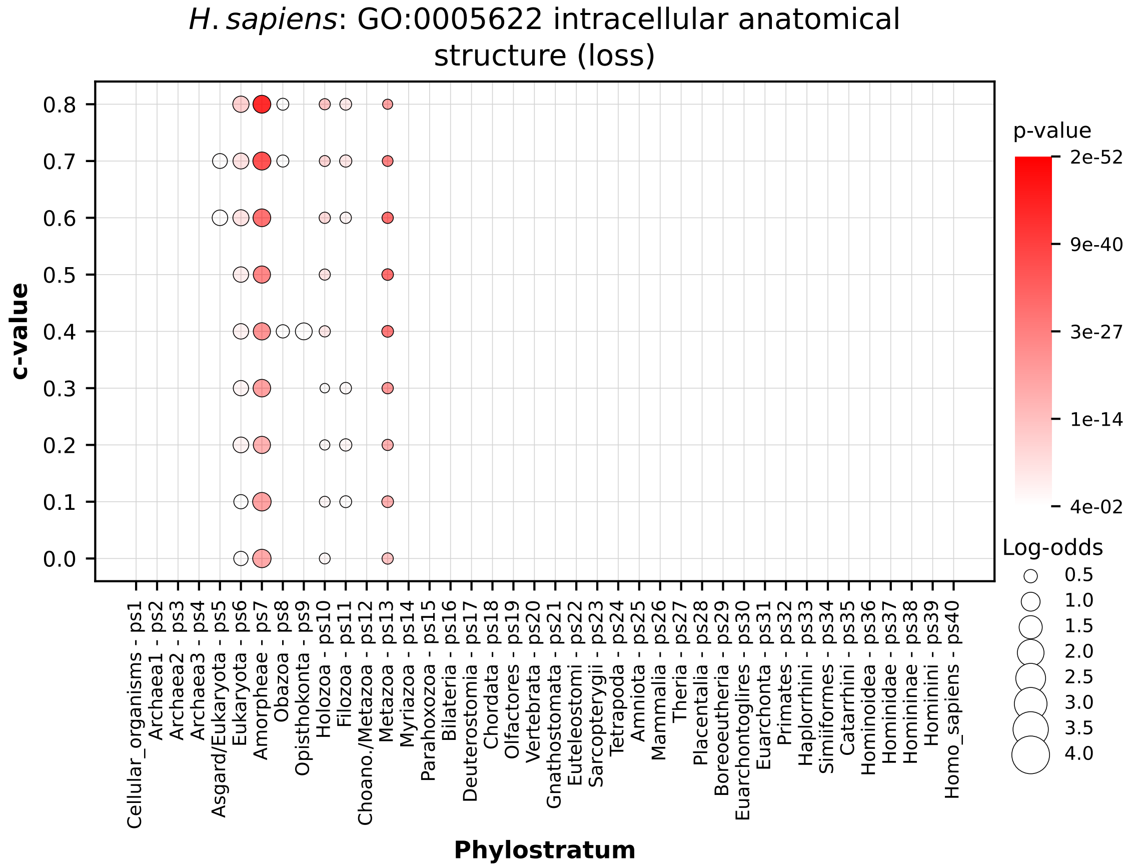

H. sapiens: GO:0005622 intracellular anatomical structure (loss)
c-value
0.8
0.7
0.6
0.5
0.4
0.3
0.2
0.1
0.0
Phylostratum
Cellular_organisms - ps1
Archaea1 - ps2
Archaea2 - ps3
Archaea3 - ps4
Asgard/Eukaryota - ps5
Eukaryota - ps6
Amorpheae - ps7
Obazoa - ps8
Opisthokonta - ps9
Holozoa - ps10
Filozoa - ps11
Choano./Metazoa - ps12
Metazoa - ps13
Myriazoa - ps14
Parahoxozoa - ps15
Bilateria - ps16
Deuterostomia - ps17
Chordata - ps18
Olfactores - ps19
Vertebrata - ps20
Gnathostomata - ps21
Euteleostomi - ps22
Sarcopterygii - ps23
Tetrapoda - ps24
Amniota - ps25
Mammalia - ps26
Theria - ps27
Placentalia - ps28
Boreoeutheria - ps29
Euarchontoglires - ps30
Euarchonta - ps31
Primates - ps32
Haplorrhini - ps33
Simiiformes - ps34
Catarrhini - ps35
Hominoidea - ps36
Hominidae - ps37
Homininae - ps38
Hominini - ps39
Homo_sapiens - ps40
p-value
2e-52
9e-40
3e-27
1e-14
4e-02
Log-odds
0.5
1.0
1.5
2.0
2.5
3.0
3.5
4.0

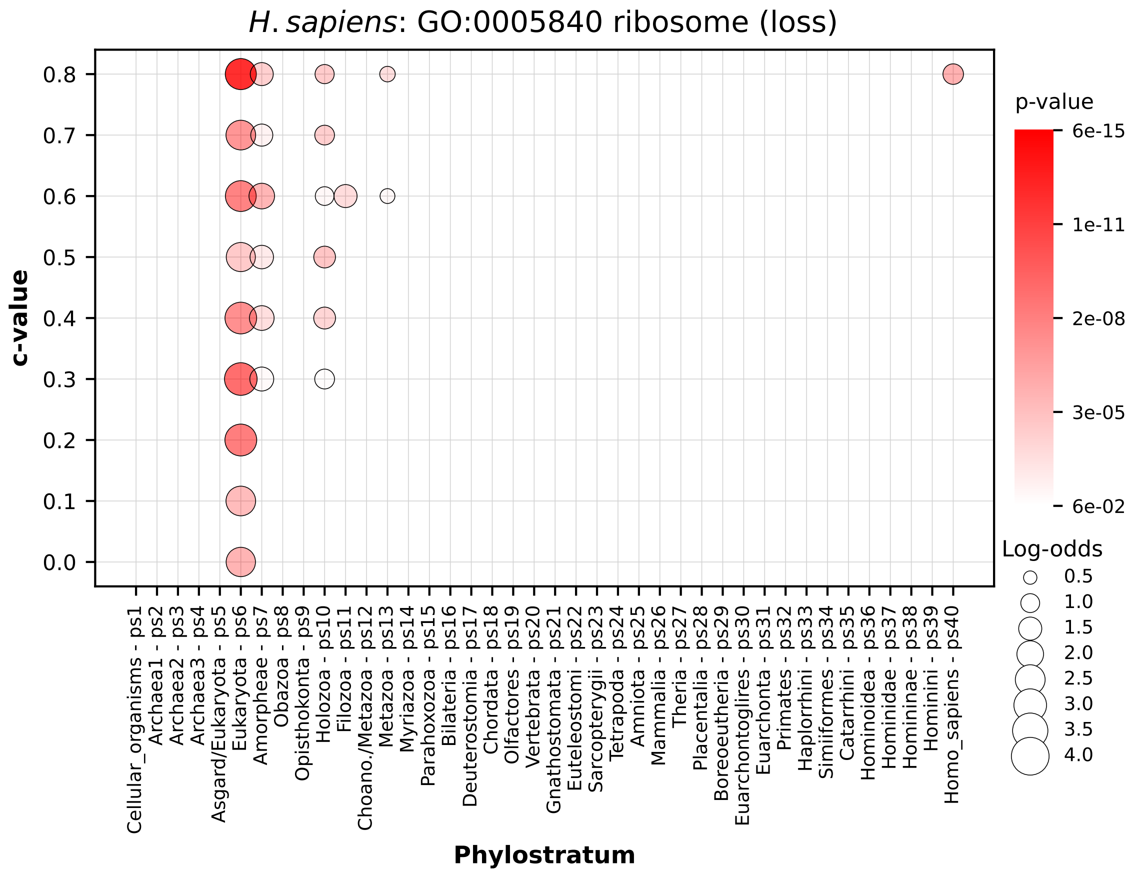

H. sapiens: GO:0005840 ribosome (loss)
c-value
0.8
0.7
0.6
0.5
0.4
0.3
0.2
0.1
0.0
p-value
6e-15
1e-11
2e-08
3e-05
6e-02
Log-odds
0.5
1.0
1.5
2.0
2.5
3.0
3.5
4.0
Cellular_organisms - ps1
Archaea1 - ps2
Archaea2 - ps3
Archaea3 - ps4
Asgard/Eukaryota - ps5
Eukaryota - ps6
Amorpheae - ps7
Obazoa - ps8
Opisthokonta - ps9
Holozoa - ps10
Filozoa - ps11
Choano./Metazoa - ps12
Metazoa - ps13
Myriazoa - ps14
Parahoxozoa - ps15
Bilateria - ps16
Deuterostomia - ps17
Chordata - ps18
Olfactores - ps19
Vertebrata - ps20
Gnathostomata - ps21
Euteleostomi - ps22
Sarcopterygii - ps23
Tetrapoda - ps24
Amniota - ps25
Mammalia - ps26
Theria - ps27
Placentalia - ps28
Boreoeutheria - ps29
Euarchontoglires - ps30
Euarchonta - ps31
Primates - ps32
Haplorrhini - ps33
Simiiformes - ps34
Catarrhini - ps35
Hominoidea - ps36
Hominidae - ps37
Homininae - ps38
Hominini - ps39
Homo_sapiens - ps40
Phylostratum

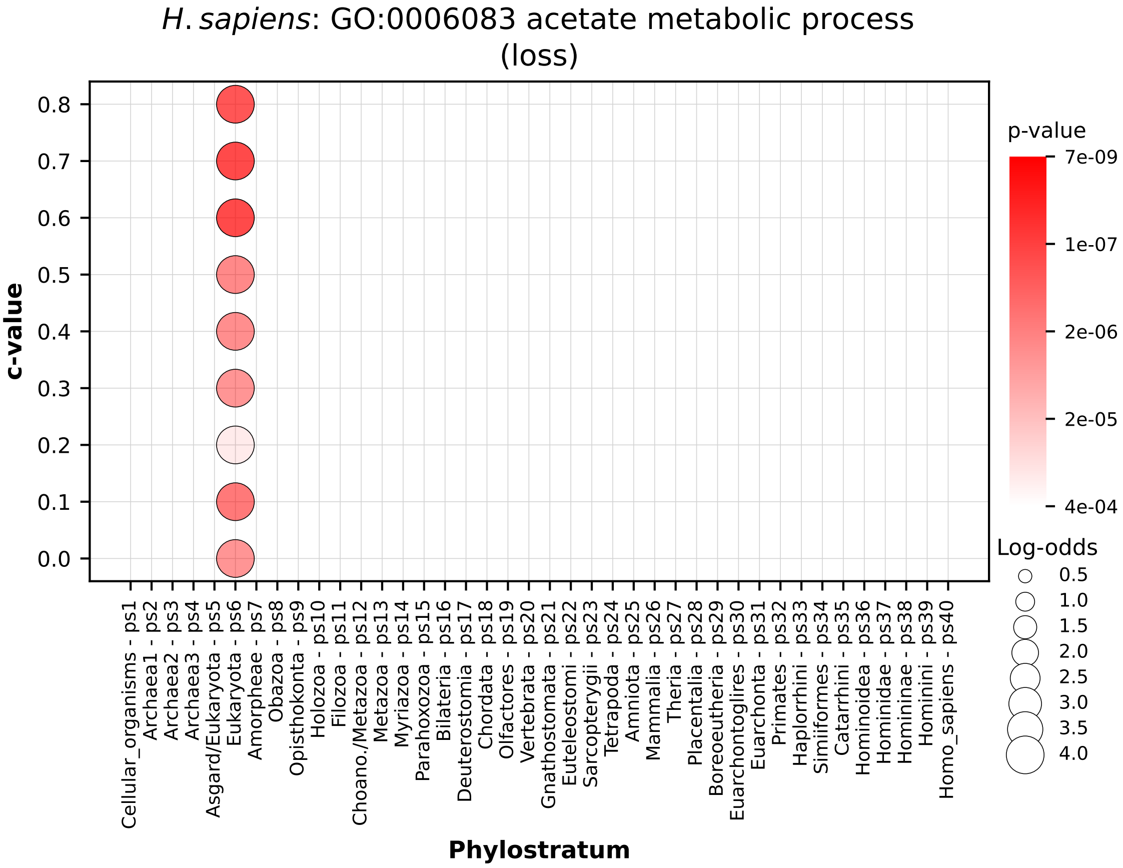

H. sapiens: GO:0006083 acetate metabolic process
(loss)
c-value
0.8
0.7
0.6
0.5
0.4
0.3
0.2
0.1
0.0
Phylostratum
Cellular_organisms - ps1
Archaea1 - ps2
Archaea2 - ps3
Archaea3 - ps4
Asgard/Eukaryota - ps5
Eukaryota - ps6
Amorpheae - ps7
Obazoa - ps8
Opisthokonta - ps9
Holozoa - ps10
Filozoa - ps11
Choano./Metazoa - ps12
Metazoa - ps13
Myriazoa - ps14
Parahoxozoa - ps15
Bilateria - ps16
Deuterostomia - ps17
Chordata - ps18
Olfactores - ps19
Vertebrata - ps20
Gnathostomata - ps21
Euteleostomi - ps22
Sarcopterygii - ps23
Tetrapoda - ps24
Amniota - ps25
Mammalia - ps26
Theria - ps27
Placentalia - ps28
Boreoeutheria - ps29
Euarchontoglires - ps30
Euarchonta - ps31
Primates - ps32
Haplorrhini - ps33
Simiiformes - ps34
Catarrhini - ps35
Hominoidea - ps36
Hominidae - ps37
Homininae - ps38
Hominini - ps39
Homo_sapiens - ps40
p-value
7e-09
1e-07
2e-06
2e-05
4e-04
Log-odds
0.5
1.0
1.5
2.0
2.5
3.0
3.5
4.0

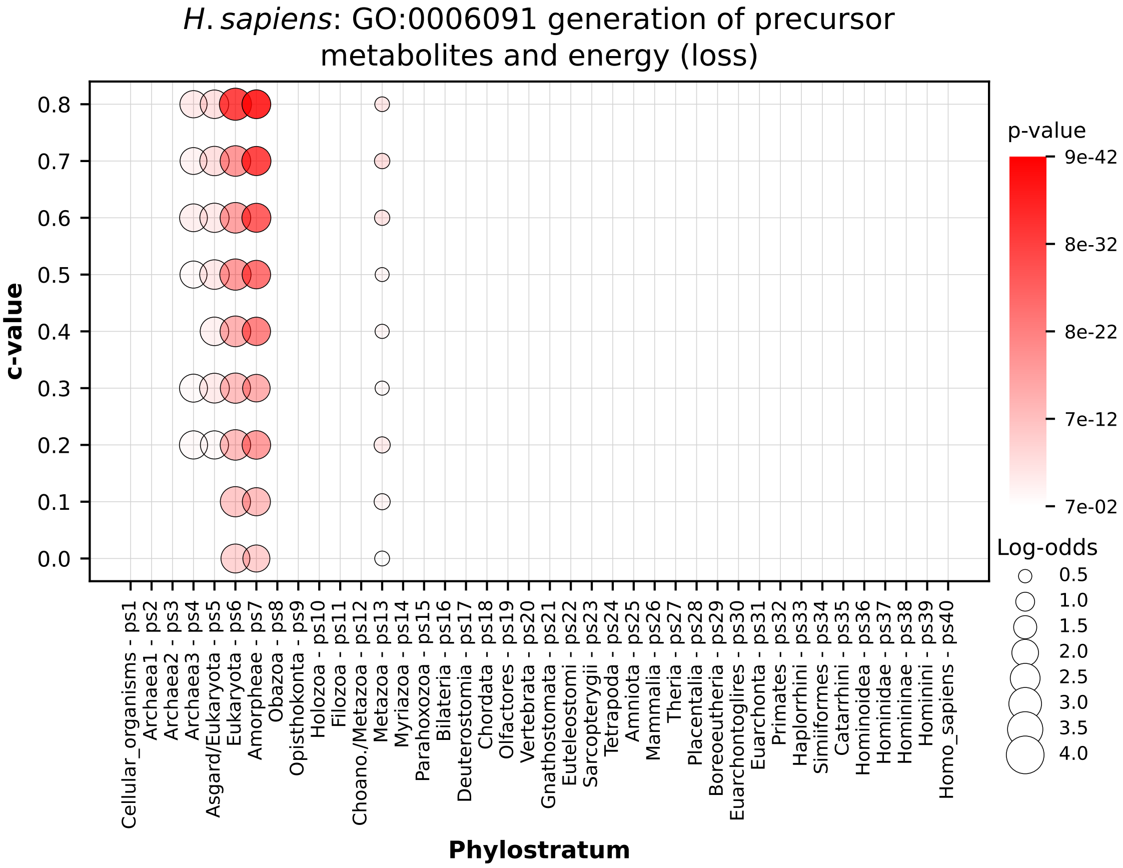

H. sapiens: GO:0006091 generation of precursor metabolites and energy (loss)
c-value
0.8
0.7
0.6
0.5
0.4
0.3
0.2
0.1
0.0
p-value
9e-42
8e-32
8e-22
7e-12
7e-02
Log-odds
0.5
1.0
1.5
2.0
2.5
3.0
3.5
4.0
Cellular_organisms - ps1
Archaea1 - ps2
Archaea2 - ps3
Archaea3 - ps4
Asgard/Eukaryota - ps5
Eukaryota - ps6
Amorpheae - ps7
Obazoa - ps8
Opisthokonta - ps9
Holozoa - ps10
Filozoa - ps11
Choano./Metazoa - ps12
Metazoa - ps13
Myriazoa - ps14
Parahoxozoa - ps15
Bilateria - ps16
Deuterostomia - ps17
Chordata - ps18
Olfactores - ps19
Vertebrata - ps20
Gnathostomata - ps21
Euteleostomi - ps22
Sarcopterygii - ps23
Tetrapoda - ps24
Amniota - ps25
Mammalia - ps26
Theria - ps27
Placentalia - ps28
Boreoeutheria - ps29
Euarchontoglires - ps30
Euarchonta - ps31
Primates - ps32
Haplorrhini - ps33
Simiiformes - ps34
Catarrhini - ps35
Hominoidea - ps36
Hominidae - ps37
Homininae - ps38
Hominini - ps39
Homo_sapiens - ps40
Phylostratum

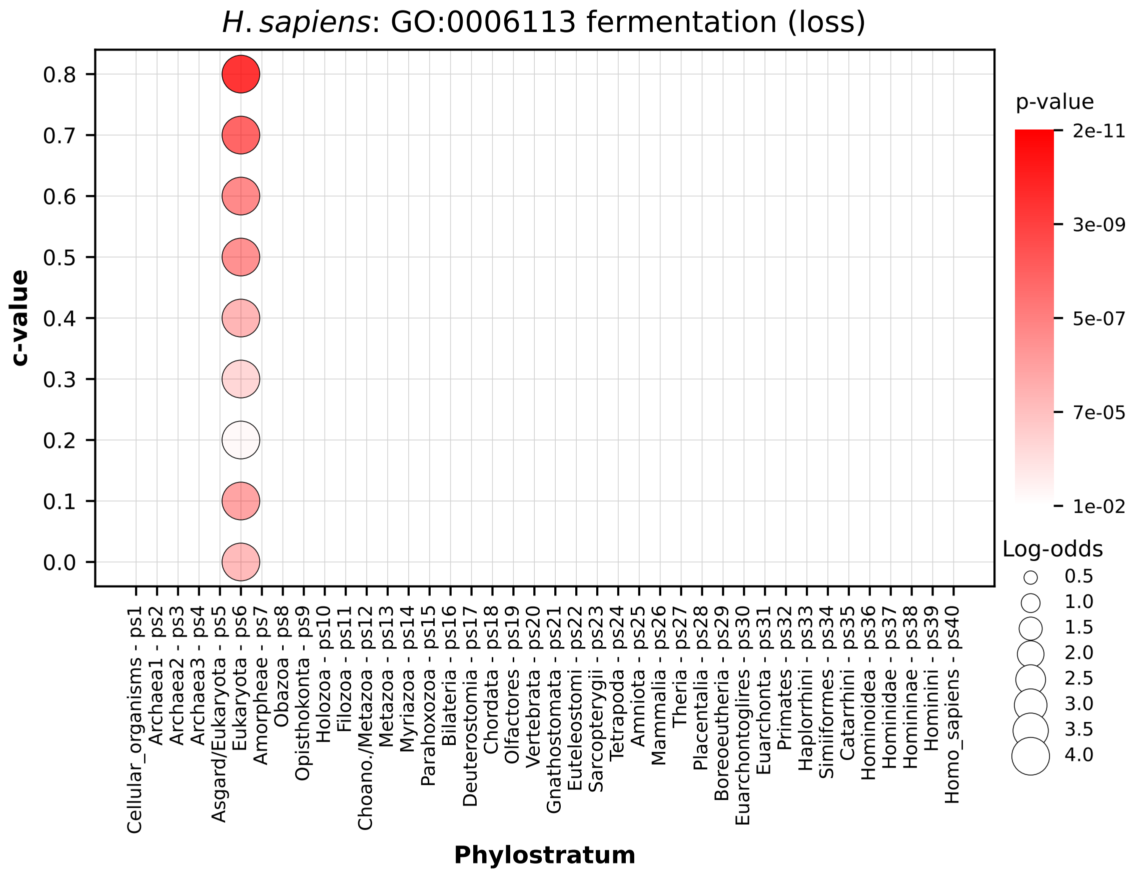

H. sapiens: GO:0006113 fermentation (loss)
c-value
0.8
0.7
0.6
0.5
0.4
0.3
0.2
0.1
0.0
p-value
2e-11
3e-09
5e-07
7e-05
1e-02
Log-odds
0.5
1.0
1.5
2.0
2.5
3.0
3.5
4.0
Cellular_organisms - ps1
Archaea1 - ps2
Archaea2 - ps3
Archaea3 - ps4
Asgard/Eukaryota - ps5
Eukaryota - ps6
Amorpheae - ps7
Obazoa - ps8
Opisthokonta - ps9
Holozoa - ps10
Filozoa - ps11
Choano./Metazoa - ps12
Metazoa - ps13
Myriazoa - ps14
Parahoxozoa - ps15
Bilateria - ps16
Deuterostomia - ps17
Chordata - ps18
Olfactores - ps19
Vertebrata - ps20
Gnathostomata - ps21
Euteleostomi - ps22
Sarcopterygii - ps23
Tetrapoda - ps24
Amniota - ps25
Mammalia - ps26
Theria - ps27
Placentalia - ps28
Boreoeutheria - ps29
Euarchontoglires - ps30
Euarchonta - ps31
Primates - ps32
Haplorrhini - ps33
Simiiformes - ps34
Catarrhini - ps35
Hominoidea - ps36
Hominidae - ps37
Homininae - ps38
Hominini - ps39
Homo_sapiens - ps40
Phylostratum

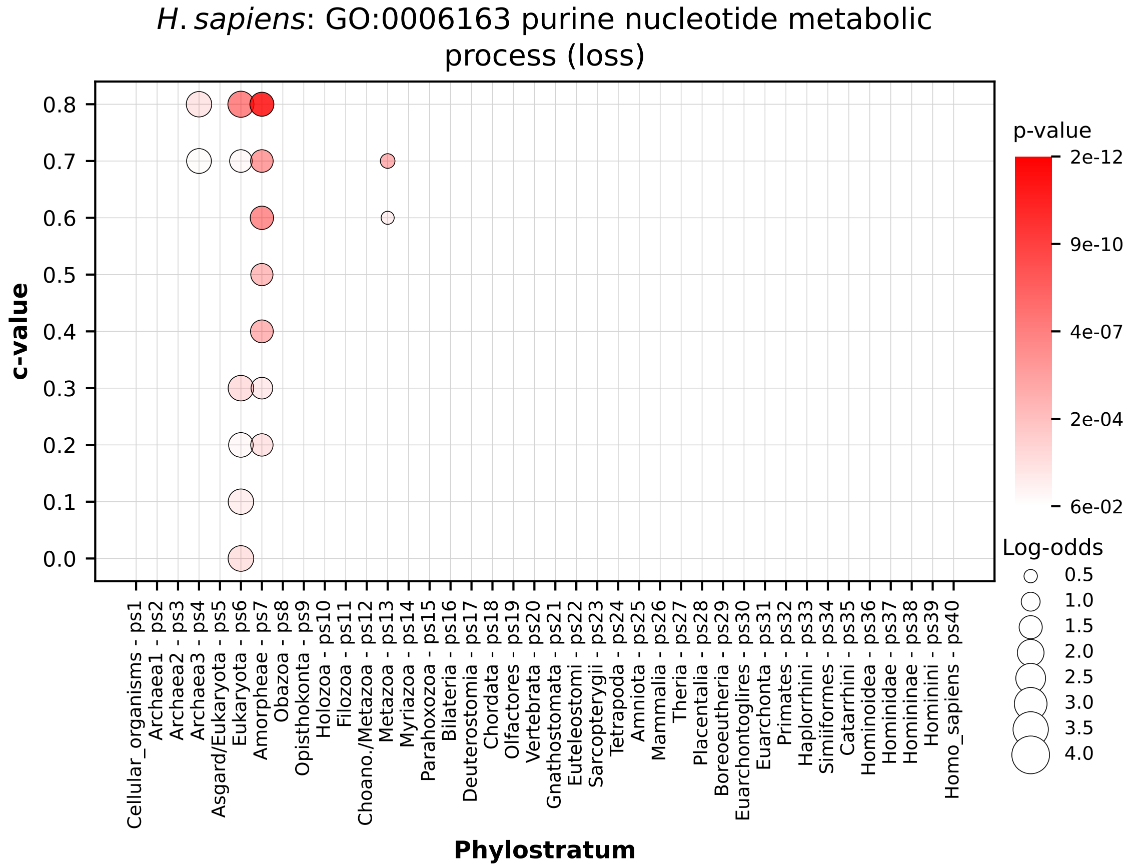

H. sapiens: GO:0006163 purine nucleotide metabolic process (loss)
c-value
0.8
0.7
0.6
0.5
0.4
0.3
0.2
0.1
0.0
p-value
2e-12
9e-10
4e-07
2e-04
6e-02
Log-odds
0.5
1.0
1.5
2.0
2.5
3.0
3.5
4.0
Cellular_organisms - ps1
Archaea1 - ps2
Archaea2 - ps3
Archaea3 - ps4
Asgard/Eukaryota - ps5
Eukaryota - ps6
Amorpheae - ps7
Obazoa - ps8
Opisthokonta - ps9
Holozoa - ps10
Filozoa - ps11
Choano./Metazoa - ps12
Metazoa - ps13
Myriazoa - ps14
Parahoxozoa - ps15
Bilateria - ps16
Deuterostomia - ps17
Chordata - ps18
Olfactores - ps19
Vertebrata - ps20
Gnathostomata - ps21
Euteleostomi - ps22
Sarcopterygii - ps23
Tetrapoda - ps24
Amniota - ps25
Mammalia - ps26
Theria - ps27
Placentalia - ps28
Boreoeutheria - ps29
Euarchontoglires - ps30
Euarchonta - ps31
Primates - ps32
Haplorrhini - ps33
Simiiformes - ps34
Catarrhini - ps35
Hominoidea - ps36
Hominidae - ps37
Homininae - ps38
Hominini - ps39
Homo_sapiens - ps40
Phylostratum

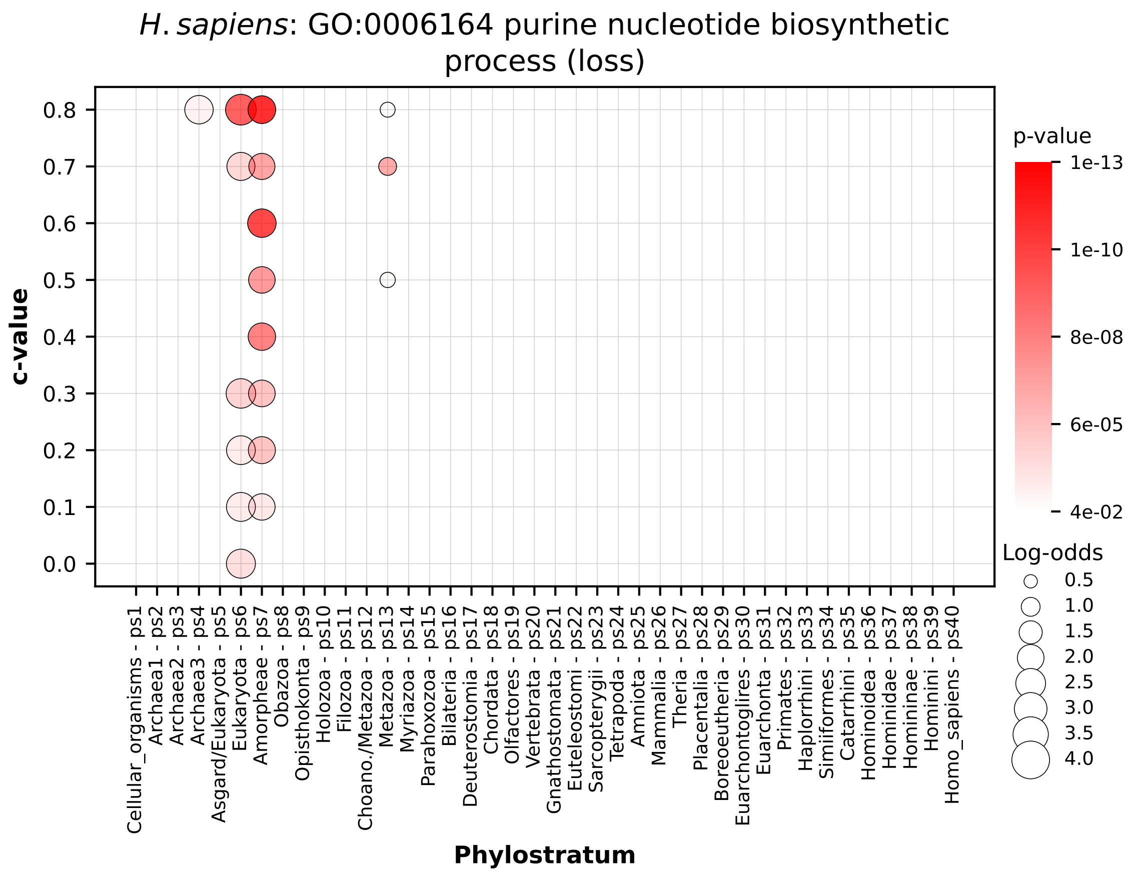

H. sapiens: GO:0006164 purine nucleotide biosynthetic process (loss)
c-value
0.8
0.7
0.6
0.5
0.4
0.3
0.2
0.1
0.0
p-value
1e-13
1e-10
8e-08
6e-05
4e-02
Log-odds
0.5
1.0
1.5
2.0
2.5
3.0
3.5
4.0
Cellular_organisms - ps1
Archaea1 - ps2
Archaea2 - ps3
Archaea3 - ps4
Asgard/Eukaryota - ps5
Eukaryota - ps6
Amorpheae - ps7
Obazoa - ps8
Opisthokonta - ps9
Holozoa - ps10
Filozoa - ps11
Choano./Metazoa - ps12
Metazoa - ps13
Myriazoa - ps14
Parahoxozoa - ps15
Bilateria - ps16
Deuterostomia - ps17
Chordata - ps18
Olfactores - ps19
Vertebrata - ps20
Gnathostomata - ps21
Euteleostomi - ps22
Sarcopterygii - ps23
Tetrapoda - ps24
Amniota - ps25
Mammalia - ps26
Theria - ps27
Placentalia - ps28
Boreoeutheria - ps29
Euarchontoglires - ps30
Euarchonta - ps31
Primates - ps32
Haplorrhini - ps33
Simiiformes - ps34
Catarrhini - ps35
Hominoidea - ps36
Hominidae - ps37
Homininae - ps38
Hominini - ps39
Homo_sapiens - ps40
Phylostratum

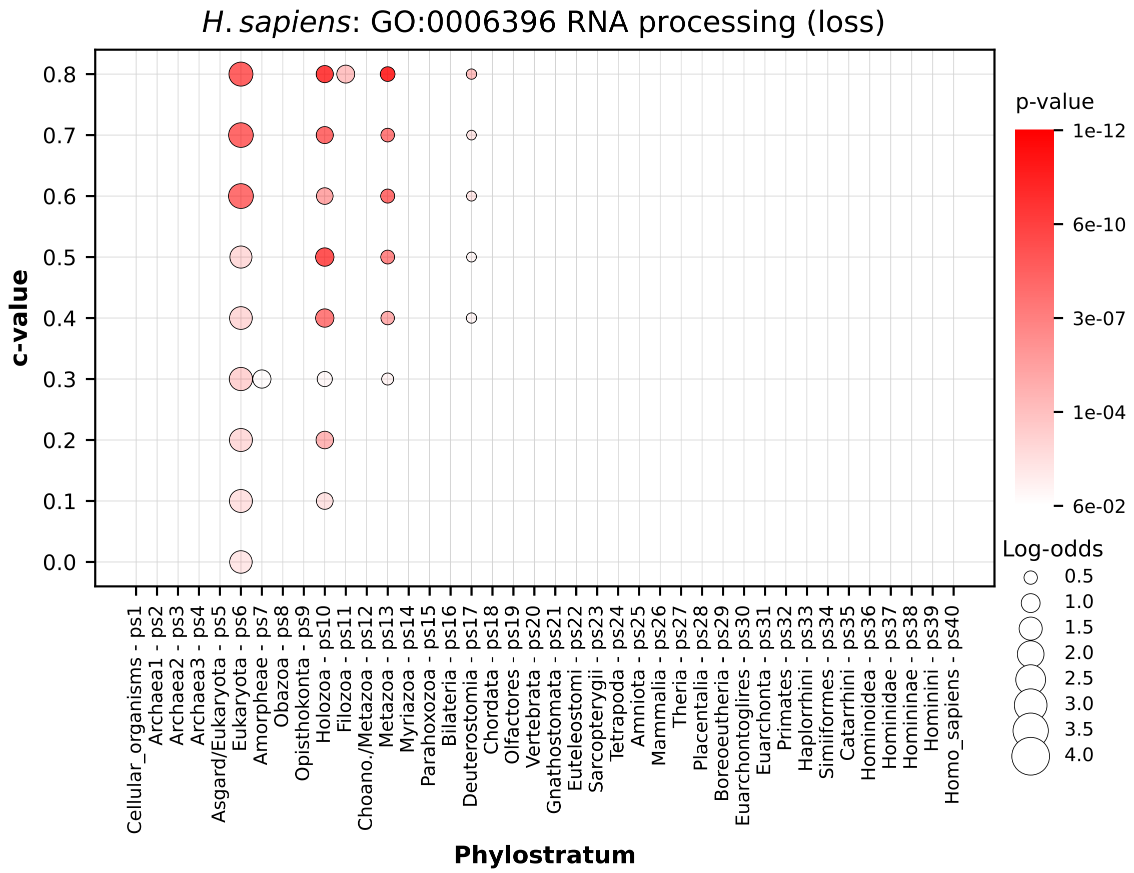
H. sapiens: GO:0006396 RNA processing (loss)
c-value
0.8
0.7
0.6
0.5
0.4
0.3
0.2
0.1
0.0
Phylostratum
Cellular_organisms - ps1
Archaea1 - ps2
Archaea2 - ps3
Archaea3 - ps4
Asgard/Eukaryota - ps5
Eukaryota - ps6
Amorpheae - ps7
Obazoa - ps8
Opisthokonta - ps9
Holozoa - ps10
Filozoa - ps11
Choano./Metazoa - ps12
Metazoa - ps13
Myriazoa - ps14
Parahoxozoa - ps15
Bilateria - ps16
Deuterostomia - ps17
Chordata - ps18
Olfactores - ps19
Vertebrata - ps20
Gnathostomata - ps21
Euteleostomi - ps22
Sarcopterygii - ps23
Tetrapoda - ps24
Amniota - ps25
Mammalia - ps26
Theria - ps27
Placentalia - ps28
Boreoeutheria - ps29
Euarchontoglires - ps30
Euarchonta - ps31
Primates - ps32
Haplorrhini - ps33
Simiiformes - ps34
Catarrhini - ps35
Hominoidea - ps36
Hominidae - ps37
Homininae - ps38
Hominini - ps39
Homo_sapiens - ps40
p-value
1e-12
6e-10
3e-07
1e-04
6e-02
Log-odds
0.5
1.0
1.5
2.0
2.5
3.0
3.5
4.0

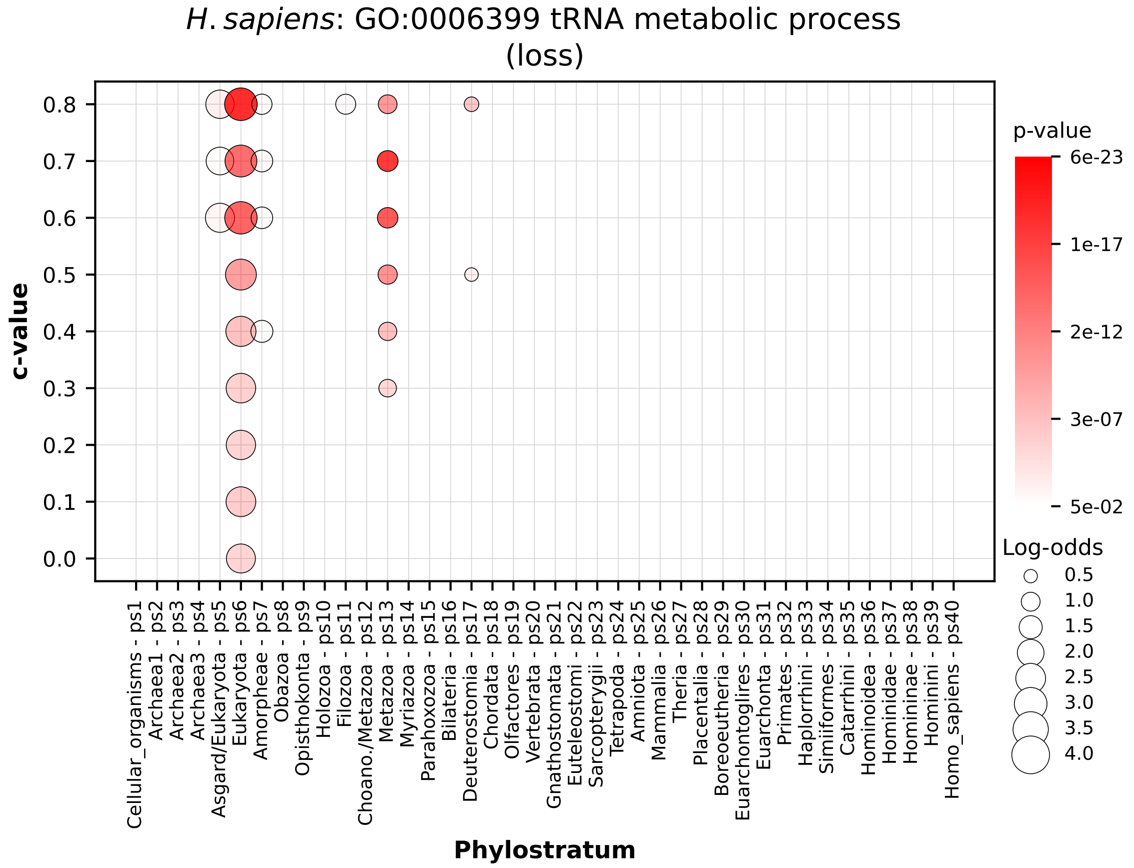

H. sapiens: GO:0006399 tRNA metabolic process
(loss)
c-value
0.8
0.7
0.6
0.5
0.4
0.3
0.2
0.1
0.0
p-value
6e-23
1e-17
2e-12
3e-07
5e-02
Log-odds
0.5
1.0
1.5
2.0
2.5
3.0
3.5
4.0
Cellular_organisms - ps1
Archaea1 - ps2
Archaea2 - ps3
Archaea3 - ps4
Asgard/Eukaryota - ps5
Eukaryota - ps6
Amorpheae - ps7
Obazoa - ps8
Opisthokonta - ps9
Holozoa - ps10
Filozoa - ps11
Choano./Metazoa - ps12
Metazoa - ps13
Myriazoa - ps14
Parahoxozoa - ps15
Bilateria - ps16
Deuterostomia - ps17
Chordata - ps18
Olfactores - ps19
Vertebrata - ps20
Gnathostomata - ps21
Euteleostomi - ps22
Sarcopterygii - ps23
Tetrapoda - ps24
Amniota - ps25
Mammalia - ps26
Theria - ps27
Placentalia - ps28
Boreoeutheria - ps29
Euarchontoglires - ps30
Euarchonta - ps31
Primates - ps32
Haplorrhini - ps33
Simiiformes - ps34
Catarrhini - ps35
Hominoidea - ps36
Hominidae - ps37
Homininae - ps38
Hominini - ps39
Homo_sapiens - ps40
Phylostratum

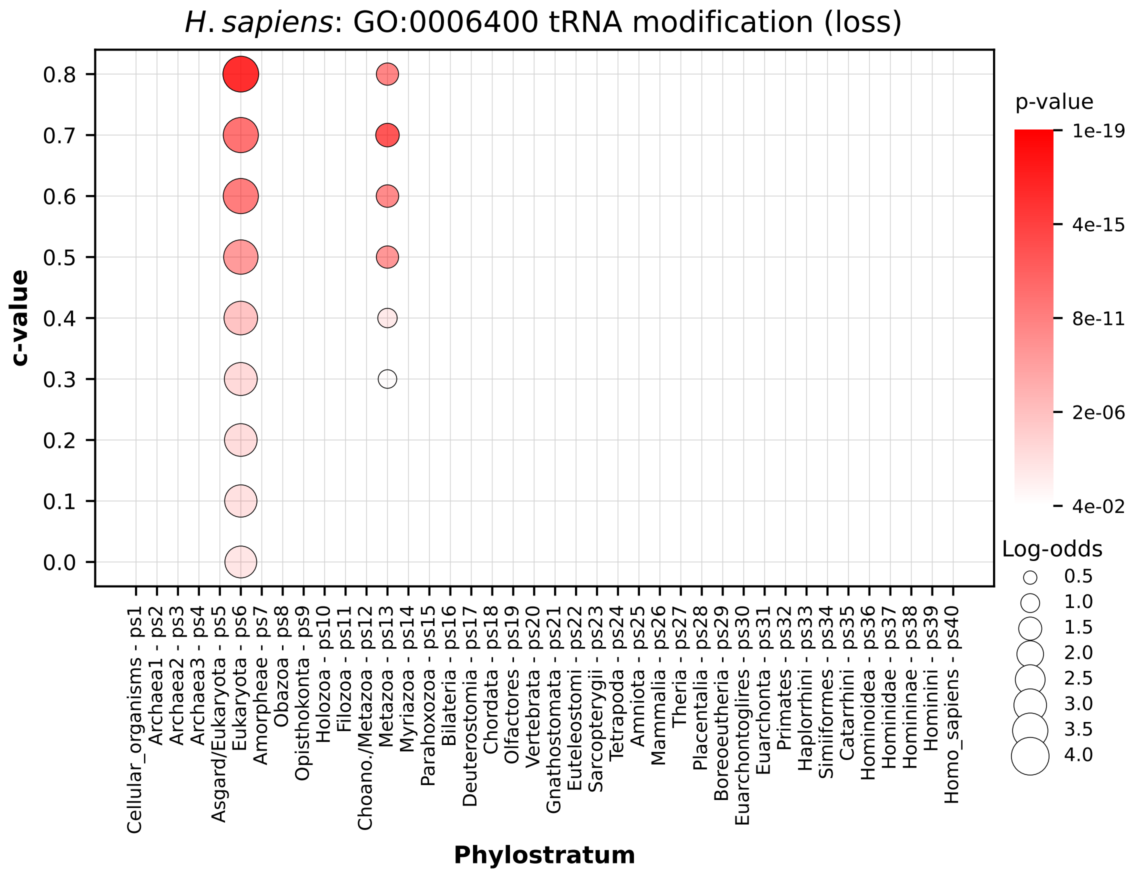

H. sapiens: GO:0006400 tRNA modification (loss)
c-value
0.0
0.1
0.2
0.3
0.4
0.5
0.6
0.7
0.8
Phylostratum
Cellular_organisms - ps1
Archaea1 - ps2
Archaea2 - ps3
Archaea3 - ps4
Asgard/Eukaryota - ps5
Eukaryota - ps6
Amorpheae - ps7
Obazoa - ps8
Opisthokonta - ps9
Holozoa - ps10
Filozoa - ps11
Choano./Metazoa - ps12
Metazoa - ps13
Myriazoa - ps14
Parahoxozoa - ps15
Bilateria - ps16
Deuterostomia - ps17
Chordata - ps18
Olfactores - ps19
Vertebrata - ps20
Gnathostomata - ps21
Euteleostomi - ps22
Sarcopterygii - ps23
Tetrapoda - ps24
Amniota - ps25
Mammalia - ps26
Theria - ps27
Placentalia - ps28
Boreoeutheria - ps29
Euarchontoglires - ps30
Euarchonta - ps31
Primates - ps32
Haplorrhini - ps33
Simiiformes - ps34
Catarrhini - ps35
Hominoidea - ps36
Hominidae - ps37
Homininae - ps38
Hominini - ps39
Homo_sapiens - ps40
p-value
1e-19
4e-15
8e-11
2e-06
4e-02
Log-odds
0.5
1.0
1.5
2.0
2.5
3.0
3.5
4.0

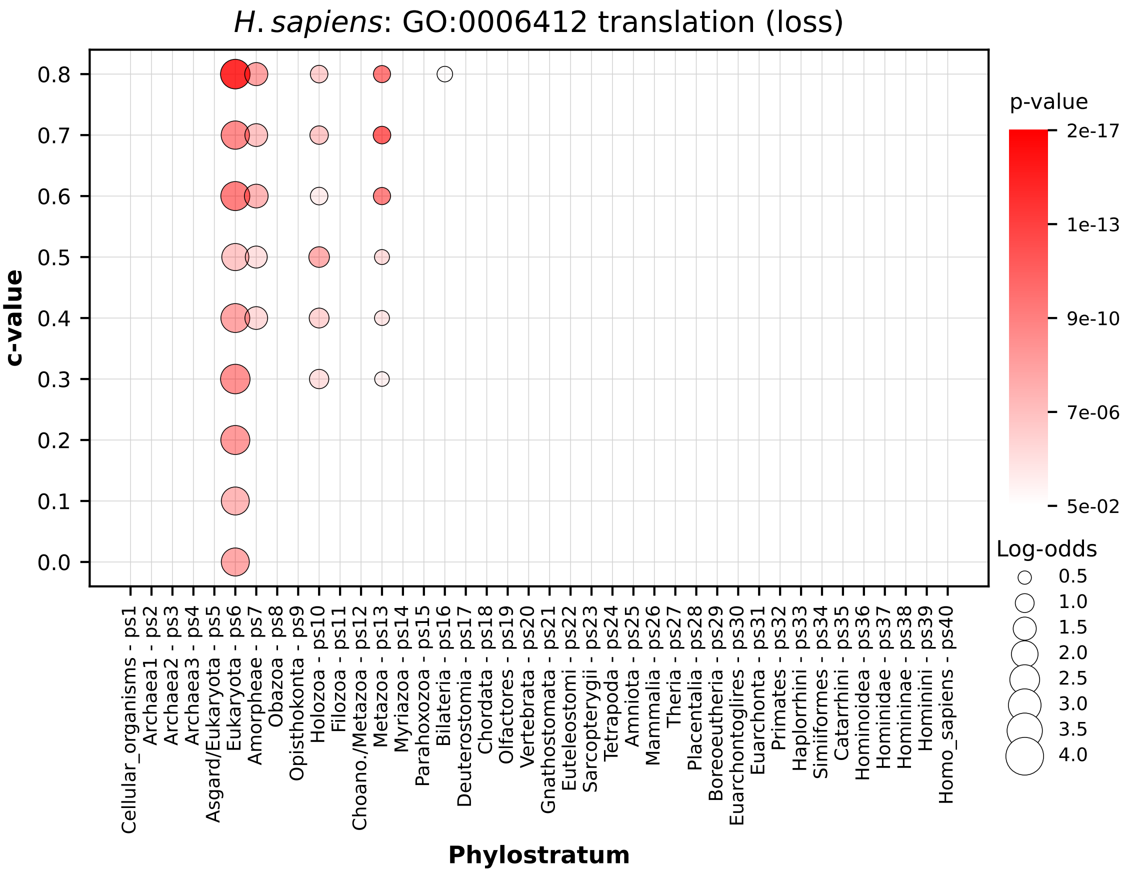
H. sapiens: GO:0006412 translation (loss)
c-value
0.8
0.7
0.6
0.5
0.4
0.3
0.2
0.1
0.0
Phylostratum
Cellular_organisms - ps1
Archaea1 - ps2
Archaea2 - ps3
Archaea3 - ps4
Asgard/Eukaryota - ps5
Eukaryota - ps6
Amorpheae - ps7
Obazoa - ps8
Opisthokonta - ps9
Holozoa - ps10
Filozoa - ps11
Choano./Metazoa - ps12
Metazoa - ps13
Myriazoa - ps14
Parahoxozoa - ps15
Bilateria - ps16
Deuterostomia - ps17
Chordata - ps18
Olfactores - ps19
Vertebrata - ps20
Gnathostomata - ps21
Euteleostomi - ps22
Sarcopterygii - ps23
Tetrapoda - ps24
Amniota - ps25
Mammalia - ps26
Theria - ps27
Placentalia - ps28
Boreoeutheria - ps29
Euarchontoglires - ps30
Euarchonta - ps31
Primates - ps32
Haplorrhini - ps33
Simiiformes - ps34
Catarrhini - ps35
Hominoidea - ps36
Hominidae - ps37
Homininae - ps38
Hominini - ps39
Homo_sapiens - ps40
p-value
2e-17
1e-13
9e-10
7e-06
5e-02
Log-odds
0.5
1.0
1.5
2.0
2.5
3.0
3.5
4.0

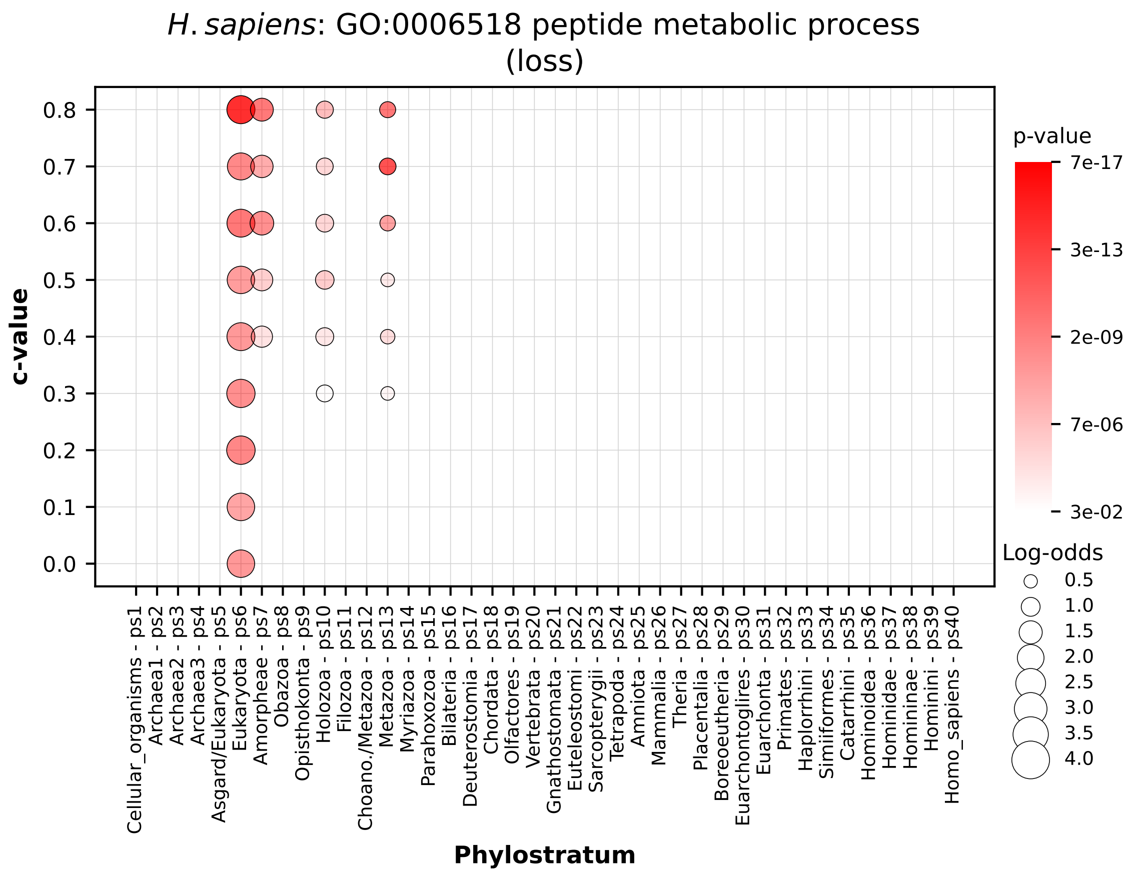

H. sapiens: GO:0006518 peptide metabolic process
(loss)
c-value
0.8
0.7
0.6
0.5
0.4
0.3
0.2
0.1
0.0
Phylostratum
Cellular_organisms - ps1
Archaea1 - ps2
Archaea2 - ps3
Archaea3 - ps4
Asgard/Eukaryota - ps5
Eukaryota - ps6
Amorpheae - ps7
Obazoa - ps8
Opisthokonta - ps9
Holozoa - ps10
Filozoa - ps11
Choano./Metazoa - ps12
Metazoa - ps13
Myriazoa - ps14
Parahoxozoa - ps15
Bilateria - ps16
Deuterostomia - ps17
Chordata - ps18
Olfactores - ps19
Vertebrata - ps20
Gnathostomata - ps21
Euteleostomi - ps22
Sarcopterygii - ps23
Tetrapoda - ps24
Amniota - ps25
Mammalia - ps26
Theria - ps27
Placentalia - ps28
Boreoeutheria - ps29
Euarchontoglires - ps30
Euarchonta - ps31
Primates - ps32
Haplorrhini - ps33
Simiiformes - ps34
Catarrhini - ps35
Hominoidea - ps36
Hominidae - ps37
Homininae - ps38
Hominini - ps39
Homo_sapiens - ps40
p-value
7e-17
3e-13
2e-09
7e-06
3e-02
Log-odds
0.5
1.0
1.5
2.0
2.5
3.0
3.5
4.0

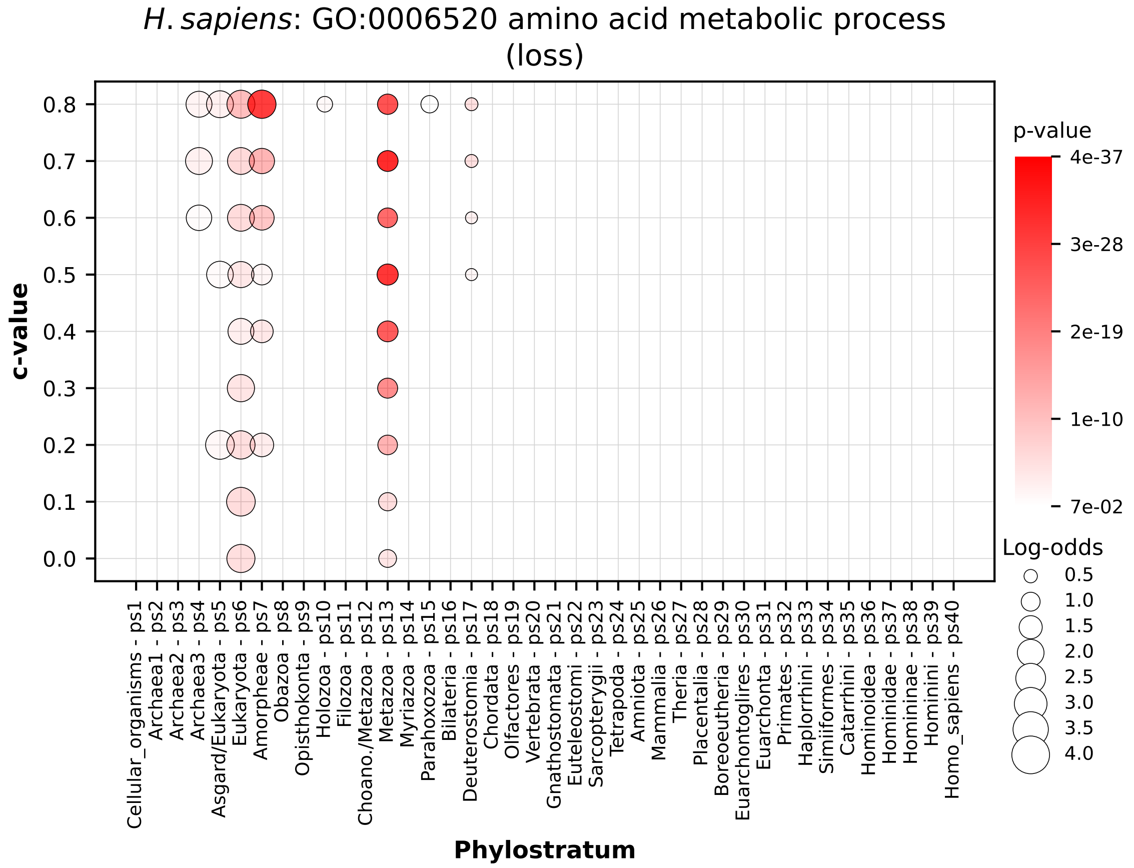

H. sapiens: GO:0006520 amino acid metabolic process
(loss)
c-value
0.0
0.1
0.2
0.3
0.4
0.5
0.6
0.7
0.8
Phylostratum
Cellular_organisms - ps1
Archaea1 - ps2
Archaea2 - ps3
Archaea3 - ps4
Asgard/Eukaryota - ps5
Eukaryota - ps6
Amorpheae - ps7
Obazoa - ps8
Opisthokonta - ps9
Holozoa - ps10
Filozoa - ps11
Choano./Metazoa - ps12
Metazoa - ps13
Myriazoa - ps14
Parahoxozoa - ps15
Bilateria - ps16
Deuterostomia - ps17
Chordata - ps18
Olfactores - ps19
Vertebrata - ps20
Gnathostomata - ps21
Euteleostomi - ps22
Sarcopterygii - ps23
Tetrapoda - ps24
Amniota - ps25
Mammalia - ps26
Theria - ps27
Placentalia - ps28
Boreoeutheria - ps29
Euarchontoglires - ps30
Euarchonta - ps31
Primates - ps32
Haplorrhini - ps33
Simiiformes - ps34
Catarrhini - ps35
Hominoidea - ps36
Hominidae - ps37
Homininae - ps38
Hominini - ps39
Homo_sapiens - ps40
p-value
4e-37
3e-28
2e-19
1e-10
7e-02
Log-odds
0.5
1.0
1.5
2.0
2.5
3.0
3.5
4.0

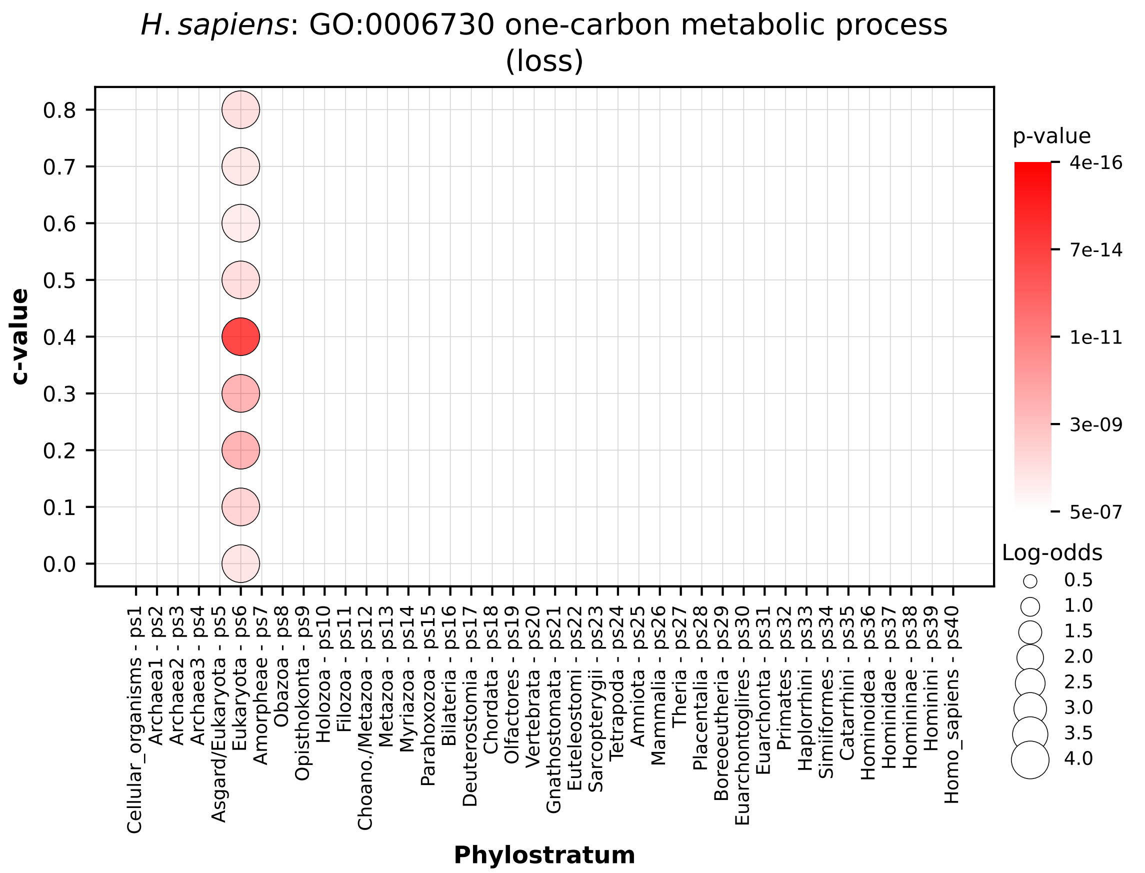

H. sapiens: GO:0006730 one-carbon metabolic process
(loss)
c-value
0.0
0.1
0.2
0.3
0.4
0.5
0.6
0.7
0.8
Phylostratum
Cellular_organisms - ps1
Archaea1 - ps2
Archaea2 - ps3
Archaea3 - ps4
Asgard/Eukaryota - ps5
Eukaryota - ps6
Amorpheae - ps7
Obazoa - ps8
Opisthokonta - ps9
Holozoa - ps10
Filozoa - ps11
Choano./Metazoa - ps12
Metazoa - ps13
Myriazoa - ps14
Parahoxozoa - ps15
Bilateria - ps16
Deuterostomia - ps17
Chordata - ps18
Olfactores - ps19
Vertebrata - ps20
Gnathostomata - ps21
Euteleostomi - ps22
Sarcopterygii - ps23
Tetrapoda - ps24
Amniota - ps25
Mammalia - ps26
Theria - ps27
Placentalia - ps28
Boreoeutheria - ps29
Euarchontoglires - ps30
Euarchonta - ps31
Primates - ps32
Haplorrhini - ps33
Simiiformes - ps34
Catarrhini - ps35
Hominoidea - ps36
Hominidae - ps37
Homininae - ps38
Hominini - ps39
Homo_sapiens - ps40
p-value
4e-16
7e-14
1e-11
3e-09
5e-07
Log-odds
0.5
1.0
1.5
2.0
2.5
3.0
3.5
4.0

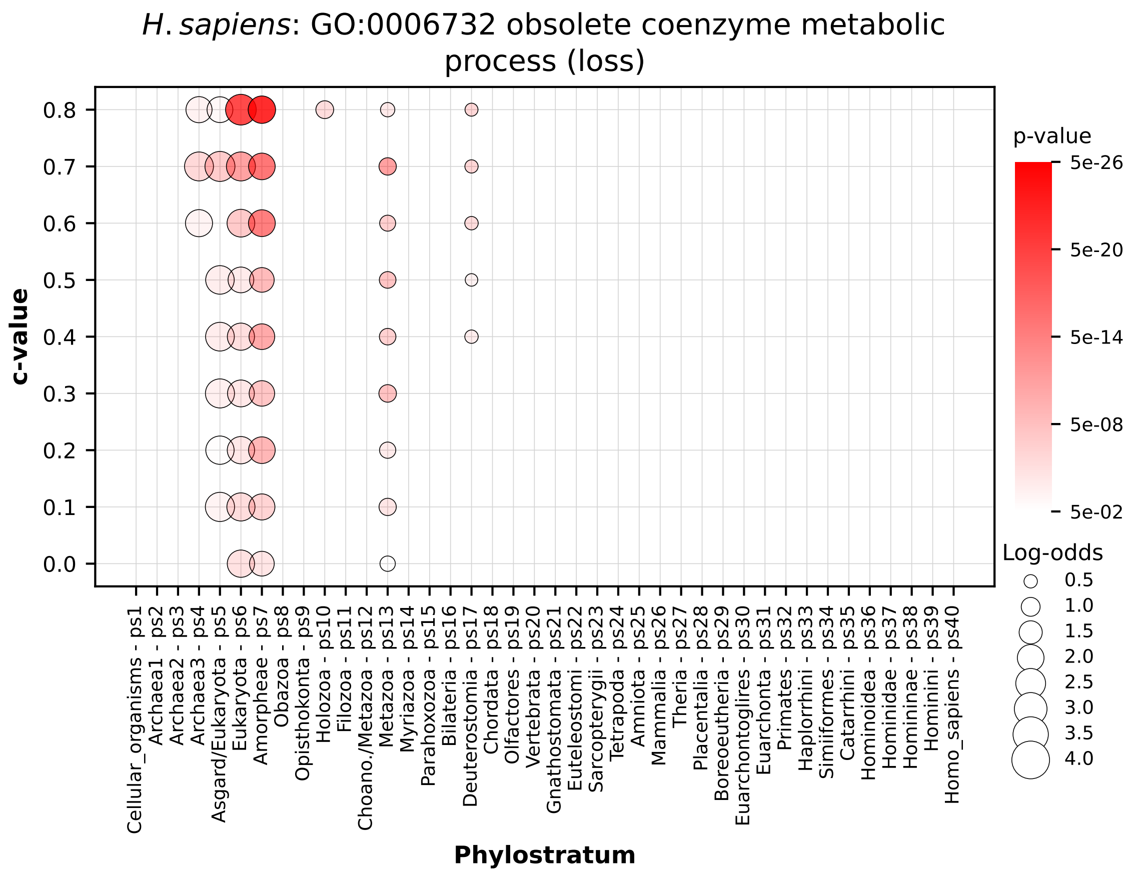

H. sapiens: GO:0006732 obsolete coenzyme metabolic process (loss)
c-value
0.8
0.7
0.6
0.5
0.4
0.3
0.2
0.1
0.0
Phylostratum
Cellular_organisms - ps1
Archaea1 - ps2
Archaea2 - ps3
Archaea3 - ps4
Asgard/Eukaryota - ps5
Eukaryota - ps6
Amorpheae - ps7
Obazoa - ps8
Opisthokonta - ps9
Holozoa - ps10
Filozoa - ps11
Choano./Metazoa - ps12
Metazoa - ps13
Myriazoa - ps14
Parahoxozoa - ps15
Bilateria - ps16
Deuterostomia - ps17
Chordata - ps18
Olfactores - ps19
Vertebrata - ps20
Gnathostomata - ps21
Euteleostomi - ps22
Sarcopterygii - ps23
Tetrapoda - ps24
Amniota - ps25
Mammalia - ps26
Theria - ps27
Placentalia - ps28
Boreoeutheria - ps29
Euarchontoglires - ps30
Euarchonta - ps31
Primates - ps32
Haplorrhini - ps33
Simiiformes - ps34
Catarrhini - ps35
Hominoidea - ps36
Hominidae - ps37
Homininae - ps38
Hominini - ps39
Homo_sapiens - ps40
p-value
5e-26
5e-20
5e-14
5e-08
5e-02
Log-odds
0.5
1.0
1.5
2.0
2.5
3.0
3.5
4.0

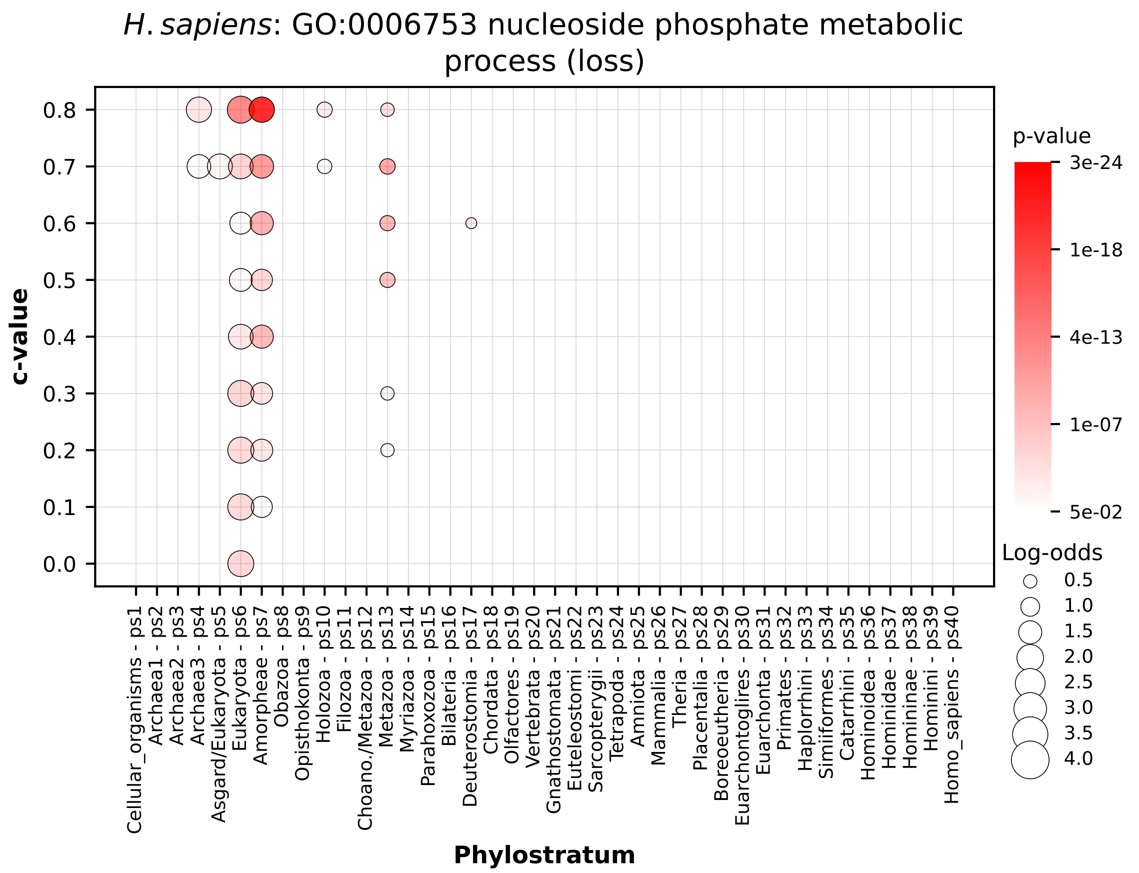

H. sapiens: GO:0006753 nucleoside phosphate metabolic process (loss)
c-value
0.8
0.7
0.6
0.5
0.4
0.3
0.2
0.1
0.0
Phylostratum
Cellular_organisms - ps1
Archaea1 - ps2
Archaea2 - ps3
Archaea3 - ps4
Asgard/Eukaryota - ps5
Eukaryota - ps6
Amorpheae - ps7
Obazoa - ps8
Opisthokonta - ps9
Holozoa - ps10
Filozoa - ps11
Choano./Metazoa - ps12
Metazoa - ps13
Myriazoa - ps14
Parahoxozoa - ps15
Bilateria - ps16
Deuterostomia - ps17
Chordata - ps18
Olfactores - ps19
Vertebrata - ps20
Gnathostomata - ps21
Euteleostomi - ps22
Sarcopterygii - ps23
Tetrapoda - ps24
Amniota - ps25
Mammalia - ps26
Theria - ps27
Placentalia - ps28
Boreoeutheria - ps29
Euarchontoglires - ps30
Euarchonta - ps31
Primates - ps32
Haplorrhini - ps33
Simiiformes - ps34
Catarrhini - ps35
Hominoidea - ps36
Hominidae - ps37
Homininae - ps38
Hominini - ps39
Homo_sapiens - ps40
p-value
3e-24
1e-18
4e-13
1e-07
5e-02
Log-odds
0.5
1.0
1.5
2.0
2.5
3.0
3.5
4.0

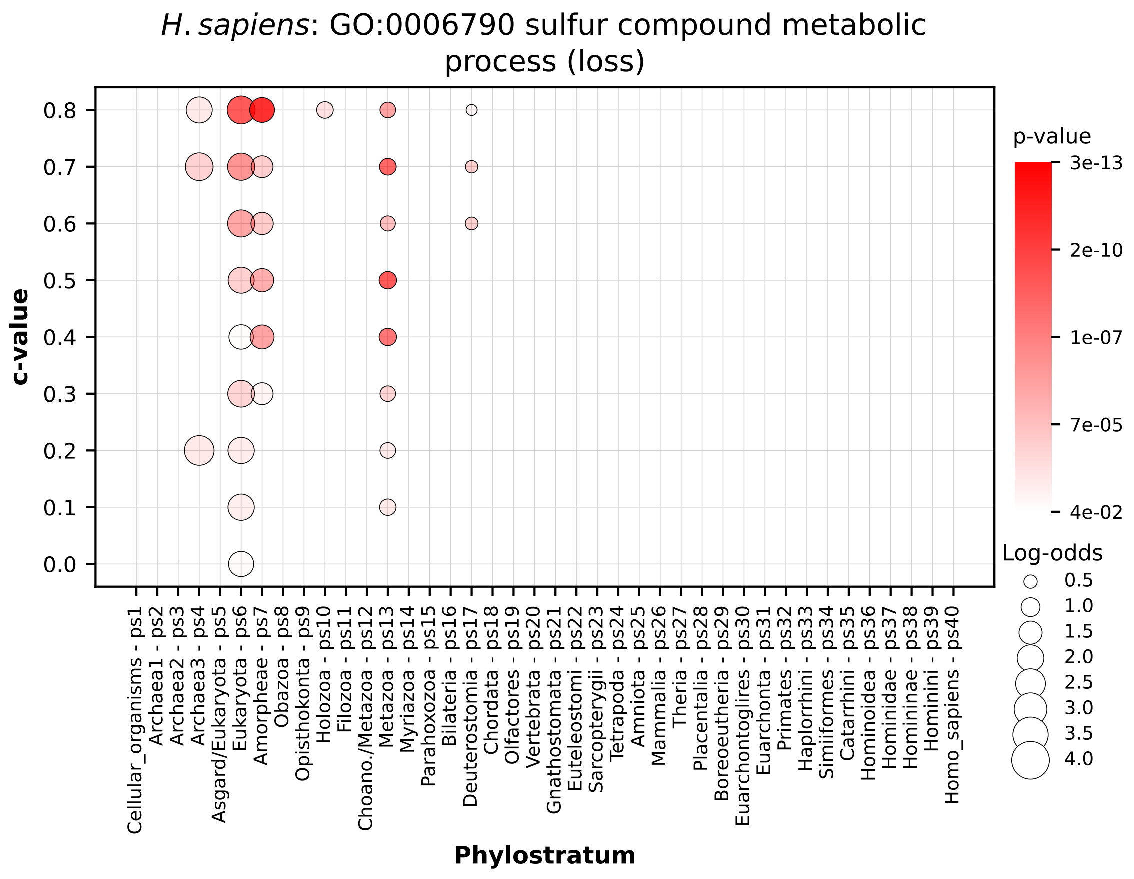

H. sapiens: GO:0006790 sulfur compound metabolic process (loss)
c-value
0.8
0.7
0.6
0.5
0.4
0.3
0.2
0.1
0.0
p-value
3e-13
2e-10
1e-07
7e-05
4e-02
Log-odds
0.5
1.0
1.5
2.0
2.5
3.0
3.5
4.0
Cellular_organisms - ps1
Archaea1 - ps2
Archaea2 - ps3
Archaea3 - ps4
Asgard/Eukaryota - ps5
Eukaryota - ps6
Amorpheae - ps7
Obazoa - ps8
Opisthokonta - ps9
Holozoa - ps10
Filozoa - ps11
Choano./Metazoa - ps12
Metazoa - ps13
Myriazoa - ps14
Parahoxozoa - ps15
Bilateria - ps16
Deuterostomia - ps17
Chordata - ps18
Olfactores - ps19
Vertebrata - ps20
Gnathostomata - ps21
Euteleostomi - ps22
Sarcopterygii - ps23
Tetrapoda - ps24
Amniota - ps25
Mammalia - ps26
Theria - ps27
Placentalia - ps28
Boreoeutheria - ps29
Euarchontoglires - ps30
Euarchonta - ps31
Primates - ps32
Haplorrhini - ps33
Simiiformes - ps34
Catarrhini - ps35
Hominoidea - ps36
Hominidae - ps37
Homininae - ps38
Hominini - ps39
Homo_sapiens - ps40
Phylostratum

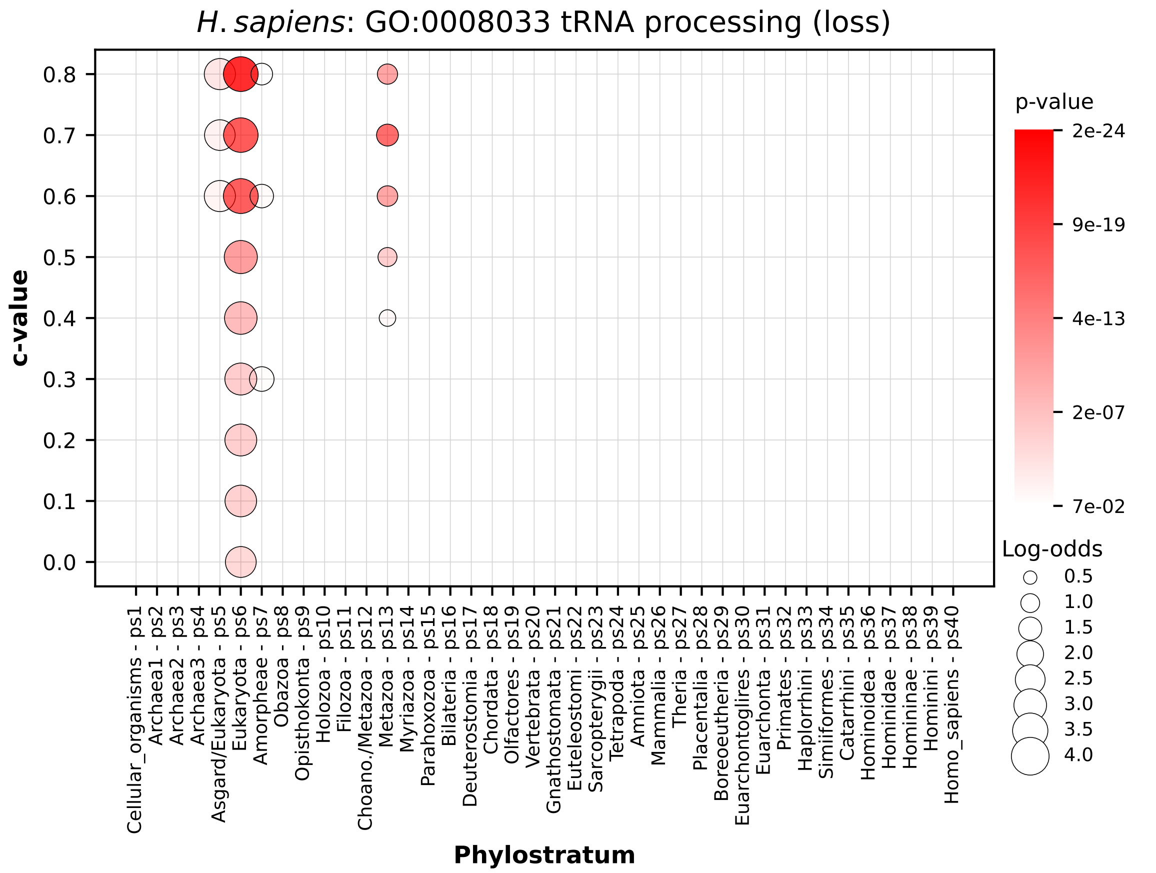

H. sapiens: GO:0008033 tRNA processing (loss)
c-value
0.0
0.1
0.2
0.3
0.4
0.5
0.6
0.7
0.8
Phylostratum
Cellular_organisms - ps1
Archaea1 - ps2
Archaea2 - ps3
Archaea3 - ps4
Asgard/Eukaryota - ps5
Eukaryota - ps6
Amorpheae - ps7
Obazoa - ps8
Opisthokonta - ps9
Holozoa - ps10
Filozoa - ps11
Choano./Metazoa - ps12
Metazoa - ps13
Myriazoa - ps14
Parahoxozoa - ps15
Bilateria - ps16
Deuterostomia - ps17
Chordata - ps18
Olfactores - ps19
Vertebrata - ps20
Gnathostomata - ps21
Euteleostomi - ps22
Sarcopterygii - ps23
Tetrapoda - ps24
Amniota - ps25
Mammalia - ps26
Theria - ps27
Placentalia - ps28
Boreoeutheria - ps29
Euarchontoglires - ps30
Euarchonta - ps31
Primates - ps32
Haplorrhini - ps33
Simiiformes - ps34
Catarrhini - ps35
Hominoidea - ps36
Hominidae - ps37
Homininae - ps38
Hominini - ps39
Homo_sapiens - ps40
p-value
2e-24
9e-19
4e-13
2e-07
7e-02
Log-odds
0.5
1.0
1.5
2.0
2.5
3.0
3.5
4.0

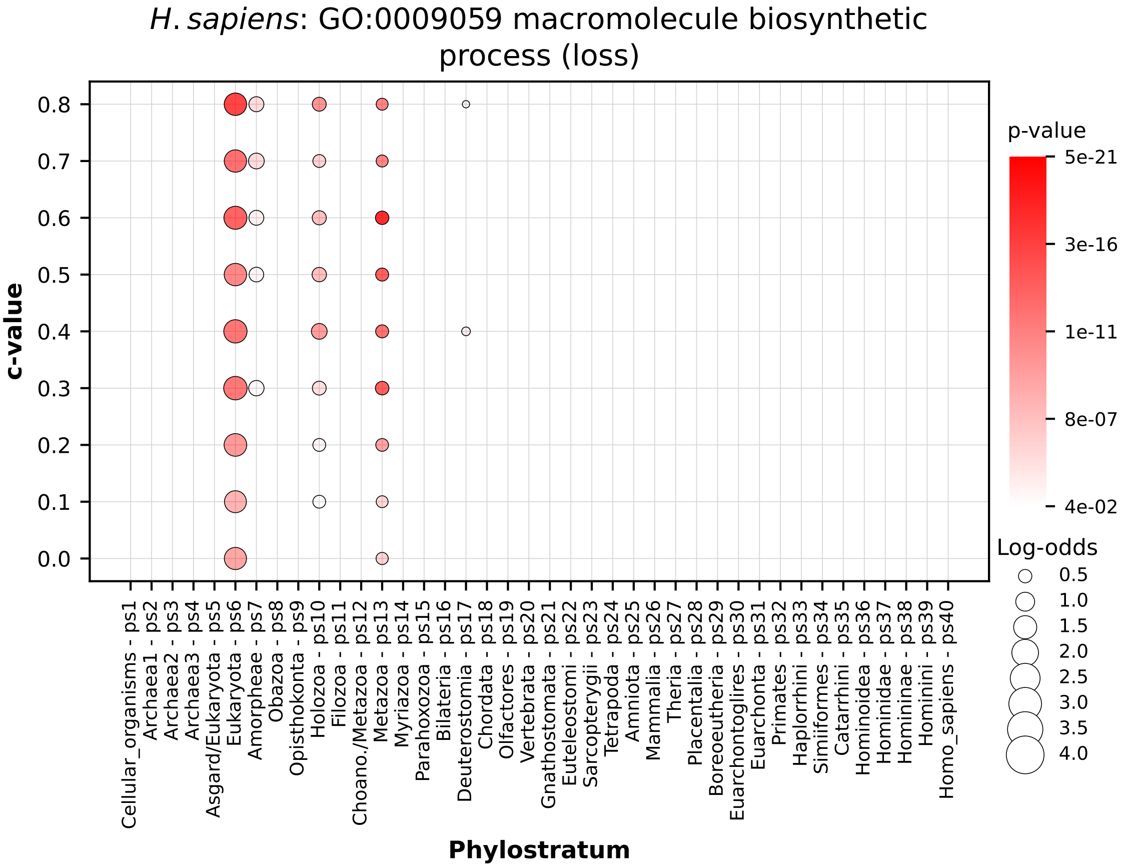

H. sapiens: GO:0009059 macromolecule biosynthetic process (loss)
c-value
0.0
0.1
0.2
0.3
0.4
0.5
0.6
0.7
0.8
Phylostratum
Cellular_organisms - ps1
Archaea1 - ps2
Archaea2 - ps3
Archaea3 - ps4
Asgard/Eukaryota - ps5
Eukaryota - ps6
Amorpheae - ps7
Obazoa - ps8
Opisthokonta - ps9
Holozoa - ps10
Filozoa - ps11
Choano./Metazoa - ps12
Metazoa - ps13
Myriazoa - ps14
Parahoxozoa - ps15
Bilateria - ps16
Deuterostomia - ps17
Chordata - ps18
Olfactores - ps19
Vertebrata - ps20
Gnathostomata - ps21
Euteleostomi - ps22
Sarcopterygii - ps23
Tetrapoda - ps24
Amniota - ps25
Mammalia - ps26
Theria - ps27
Placentalia - ps28
Boreoeutheria - ps29
Euarchontoglires - ps30
Euarchonta - ps31
Primates - ps32
Haplorrhini - ps33
Simiiformes - ps34
Catarrhini - ps35
Hominoidea - ps36
Hominidae - ps37
Homininae - ps38
Hominini - ps39
Homo_sapiens - ps40
p-value
5e-21
3e-16
1e-11
8e-07
4e-02
Log-odds
0.5
1.0
1.5
2.0
2.5
3.0
3.5
4.0

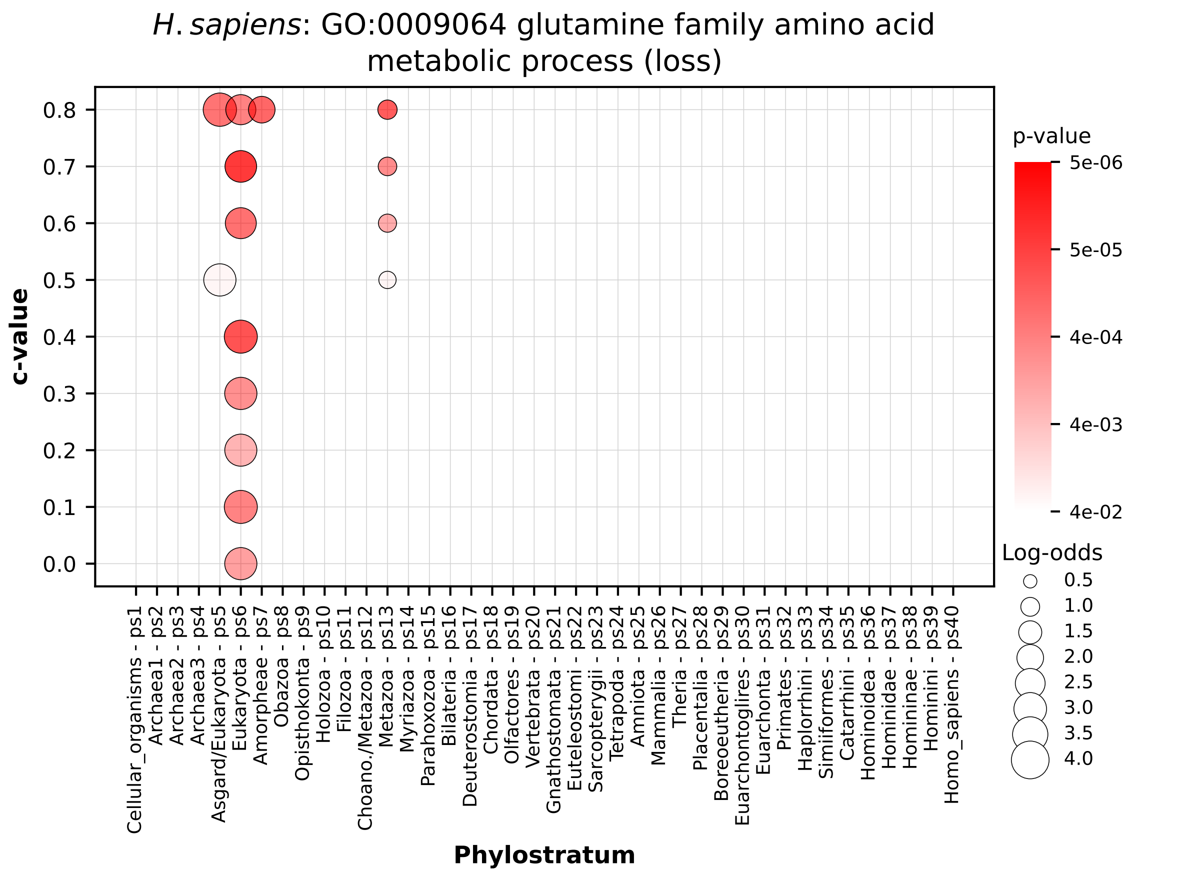

H. sapiens: GO:0009064 glutamine family amino acid metabolic process (loss)
c-value
0.8
0.7
0.6
0.5
0.4
0.3
0.2
0.1
0.0
p-value
5e-06
5e-05
4e-04
4e-03
4e-02
Log-odds
0.5
1.0
1.5
2.0
2.5
3.0
3.5
4.0
Cellular_organisms - ps1
Archaea1 - ps2
Archaea2 - ps3
Archaea3 - ps4
Asgard/Eukaryota - ps5
Eukaryota - ps6
Amorpheae - ps7
Obazoa - ps8
Opisthokonta - ps9
Holozoa - ps10
Filozoa - ps11
Choano./Metazoa - ps12
Metazoa - ps13
Myriazoa - ps14
Parahoxozoa - ps15
Bilateria - ps16
Deuterostomia - ps17
Chordata - ps18
Olfactores - ps19
Vertebrata - ps20
Gnathostomata - ps21
Euteleostomi - ps22
Sarcopterygii - ps23
Tetrapoda - ps24
Amniota - ps25
Mammalia - ps26
Theria - ps27
Placentalia - ps28
Boreoeutheria - ps29
Euarchontoglires - ps30
Euarchonta - ps31
Primates - ps32
Haplorrhini - ps33
Simiiformes - ps34
Catarrhini - ps35
Hominoidea - ps36
Hominidae - ps37
Homininae - ps38
Hominini - ps39
Homo_sapiens - ps40
Phylostratum

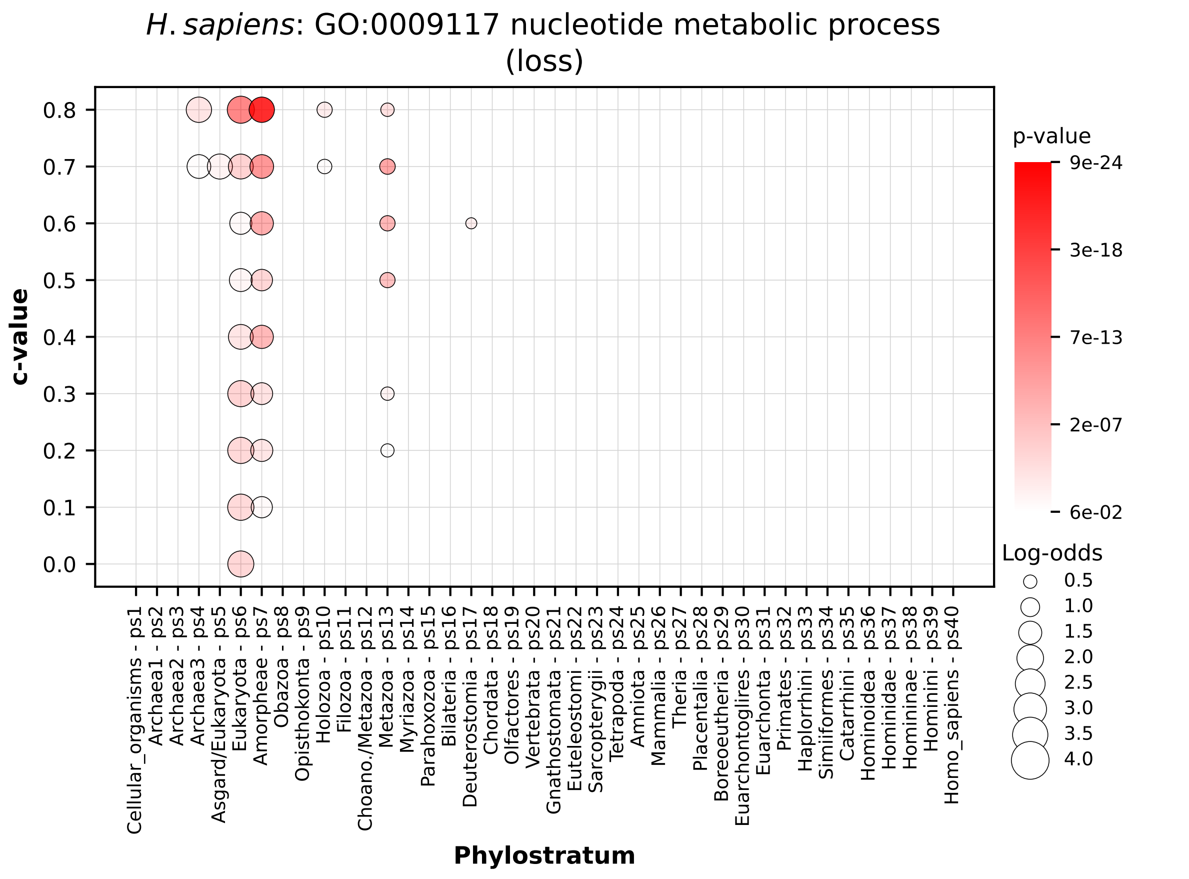

H. sapiens: GO:0009117 nucleotide metabolic process
(loss)
c-value
0.8
0.7
0.6
0.5
0.4
0.3
0.2
0.1
0.0
Phylostratum
Cellular_organisms - ps1
Archaea1 - ps2
Archaea2 - ps3
Archaea3 - ps4
Asgard/Eukaryota - ps5
Eukaryota - ps6
Amorpheae - ps7
Obazoa - ps8
Opisthokonta - ps9
Holozoa - ps10
Filozoa - ps11
Choano./Metazoa - ps12
Metazoa - ps13
Myriazoa - ps14
Parahoxozoa - ps15
Bilateria - ps16
Deuterostomia - ps17
Chordata - ps18
Olfactores - ps19
Vertebrata - ps20
Gnathostomata - ps21
Euteleostomi - ps22
Sarcopterygii - ps23
Tetrapoda - ps24
Amniota - ps25
Mammalia - ps26
Theria - ps27
Placentalia - ps28
Boreoeutheria - ps29
Euarchontoglires - ps30
Euarchonta - ps31
Primates - ps32
Haplorrhini - ps33
Simiiformes - ps34
Catarrhini - ps35
Hominoidea - ps36
Hominidae - ps37
Homininae - ps38
Hominini - ps39
Homo_sapiens - ps40
p-value
9e-24
3e-18
7e-13
2e-07
6e-02
Log-odds
0.5
1.0
1.5
2.0
2.5
3.0
3.5
4.0

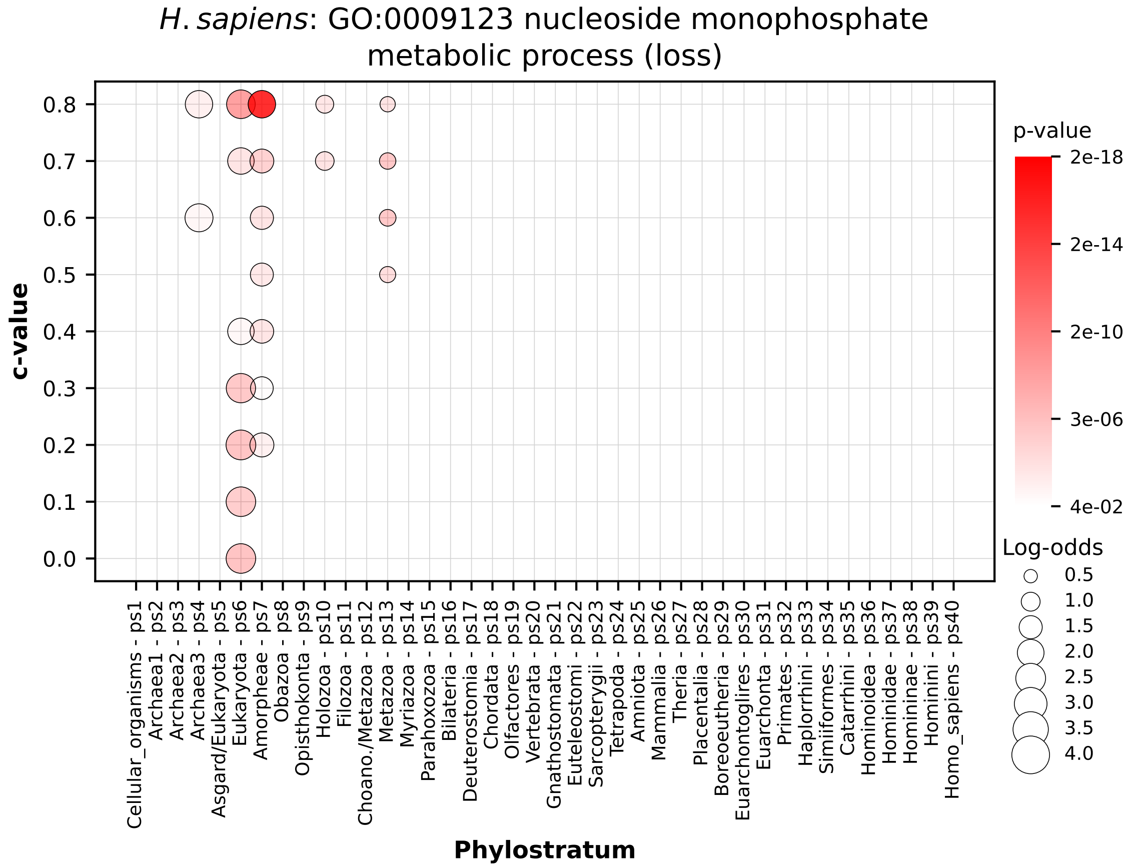

H. sapiens: GO:0009123 nucleoside monophosphate metabolic process (loss)
c-value
0.8
0.7
0.6
0.5
0.4
0.3
0.2
0.1
0.0
p-value
2e-18
2e-14
2e-10
3e-06
4e-02
Log-odds
0.5
1.0
1.5
2.0
2.5
3.0
3.5
4.0
Cellular_organisms - ps1
Archaea1 - ps2
Archaea2 - ps3
Archaea3 - ps4
Asgard/Eukaryota - ps5
Eukaryota - ps6
Amorpheae - ps7
Obazoa - ps8
Opisthokonta - ps9
Holozoa - ps10
Filozoa - ps11
Choano./Metazoa - ps12
Metazoa - ps13
Myriazoa - ps14
Parahoxozoa - ps15
Bilateria - ps16
Deuterostomia - ps17
Chordata - ps18
Olfactores - ps19
Vertebrata - ps20
Gnathostomata - ps21
Euteleostomi - ps22
Sarcopterygii - ps23
Tetrapoda - ps24
Amniota - ps25
Mammalia - ps26
Theria - ps27
Placentalia - ps28
Boreoeutheria - ps29
Euarchontoglires - ps30
Euarchonta - ps31
Primates - ps32
Haplorrhini - ps33
Simiiformes - ps34
Catarrhini - ps35
Hominoidea - ps36
Hominidae - ps37
Homininae - ps38
Hominini - ps39
Homo_sapiens - ps40
Phylostratum

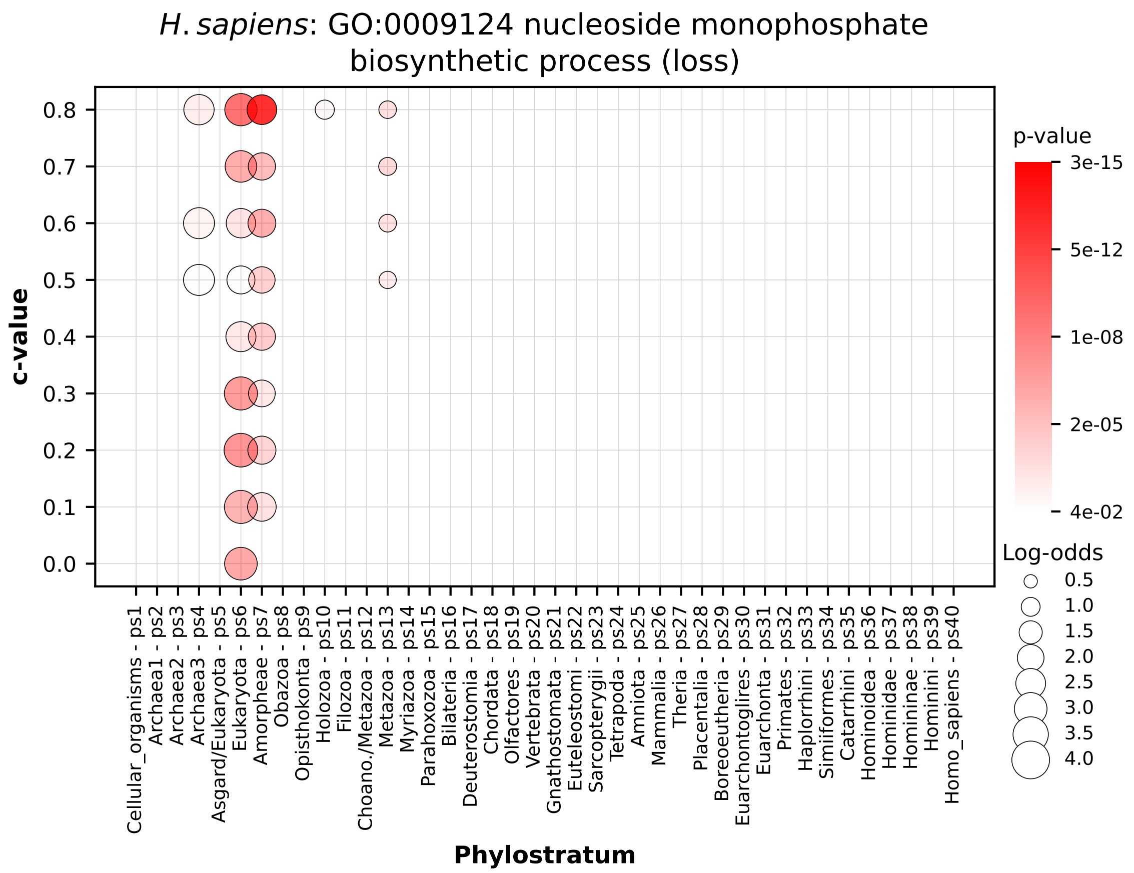

H. sapiens: GO:0009124 nucleoside monophosphate biosynthetic process (loss)
c-value
0.8
0.7
0.6
0.5
0.4
0.3
0.2
0.1
0.0
p-value
3e-15
5e-12
1e-08
2e-05
4e-02
Log-odds
0.5
1.0
1.5
2.0
2.5
3.0
3.5
4.0
Cellular_organisms - ps1
Archaea1 - ps2
Archaea2 - ps3
Archaea3 - ps4
Asgard/Eukaryota - ps5
Eukaryota - ps6
Amorpheae - ps7
Obazoa - ps8
Opisthokonta - ps9
Holozoa - ps10
Filozoa - ps11
Choano./Metazoa - ps12
Metazoa - ps13
Myriazoa - ps14
Parahoxozoa - ps15
Bilateria - ps16
Deuterostomia - ps17
Chordata - ps18
Olfactores - ps19
Vertebrata - ps20
Gnathostomata - ps21
Euteleostomi - ps22
Sarcopterygii - ps23
Tetrapoda - ps24
Amniota - ps25
Mammalia - ps26
Theria - ps27
Placentalia - ps28
Boreoeutheria - ps29
Euarchontoglires - ps30
Euarchonta - ps31
Primates - ps32
Haplorrhini - ps33
Simiiformes - ps34
Catarrhini - ps35
Hominoidea - ps36
Hominidae - ps37
Homininae - ps38
Hominini - ps39
Homo_sapiens - ps40
Phylostratum

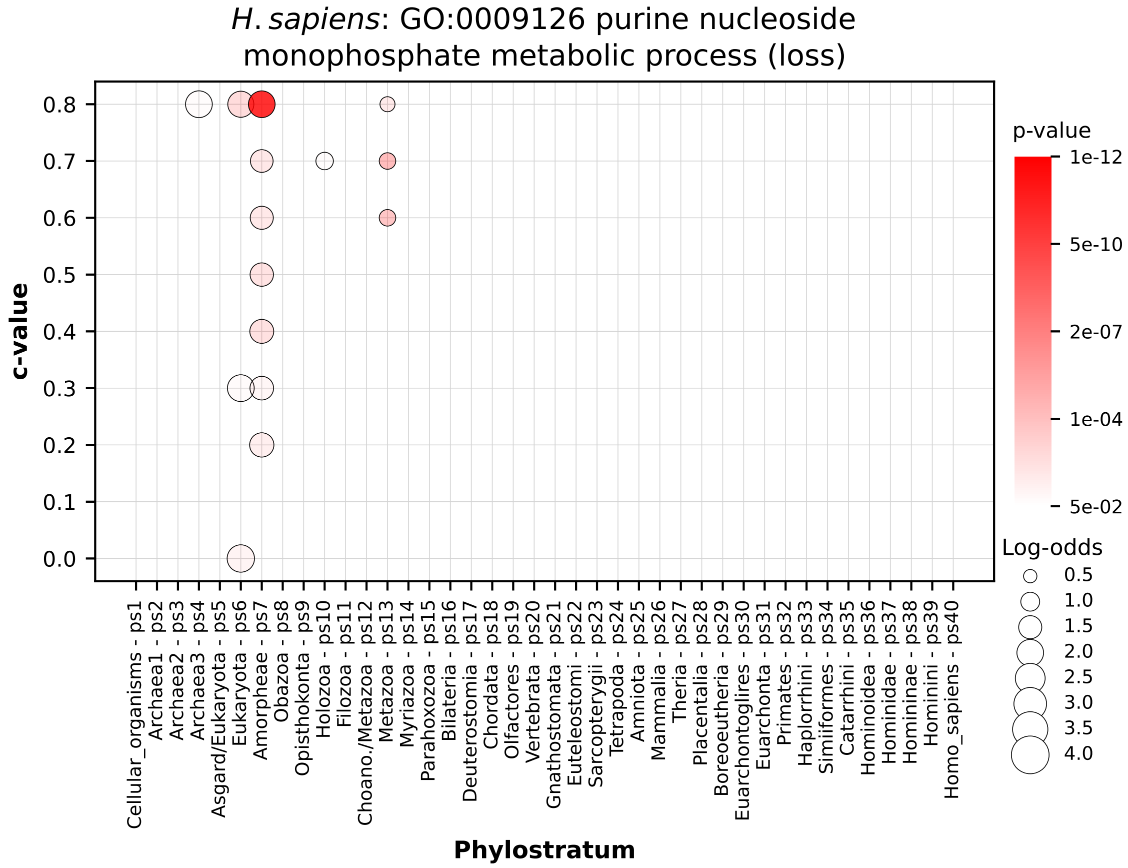

H. sapiens: GO:0009126 purine nucleoside monophosphate metabolic process (loss)
c-value
0.8
0.7
0.6
0.5
0.4
0.3
0.2
0.1
0.0
Phylostratum
Cellular_organisms - ps1
Archaea1 - ps2
Archaea2 - ps3
Archaea3 - ps4
Asgard/Eukaryota - ps5
Eukaryota - ps6
Amorpheae - ps7
Obazoa - ps8
Opisthokonta - ps9
Holozoa - ps10
Filozoa - ps11
Choano./Metazoa - ps12
Metazoa - ps13
Myriazoa - ps14
Parahoxozoa - ps15
Bilateria - ps16
Deuterostomia - ps17
Chordata - ps18
Olfactores - ps19
Vertebrata - ps20
Gnathostomata - ps21
Euteleostomi - ps22
Sarcopterygii - ps23
Tetrapoda - ps24
Amniota - ps25
Mammalia - ps26
Theria - ps27
Placentalia - ps28
Boreoeutheria - ps29
Euarchontoglires - ps30
Euarchonta - ps31
Primates - ps32
Haplorrhini - ps33
Simiiformes - ps34
Catarrhini - ps35
Hominoidea - ps36
Hominidae - ps37
Homininae - ps38
Hominini - ps39
Homo_sapiens - ps40
p-value
1e-12
5e-10
2e-07
1e-04
5e-02
Log-odds
0.5
1.0
1.5
2.0
2.5
3.0
3.5
4.0

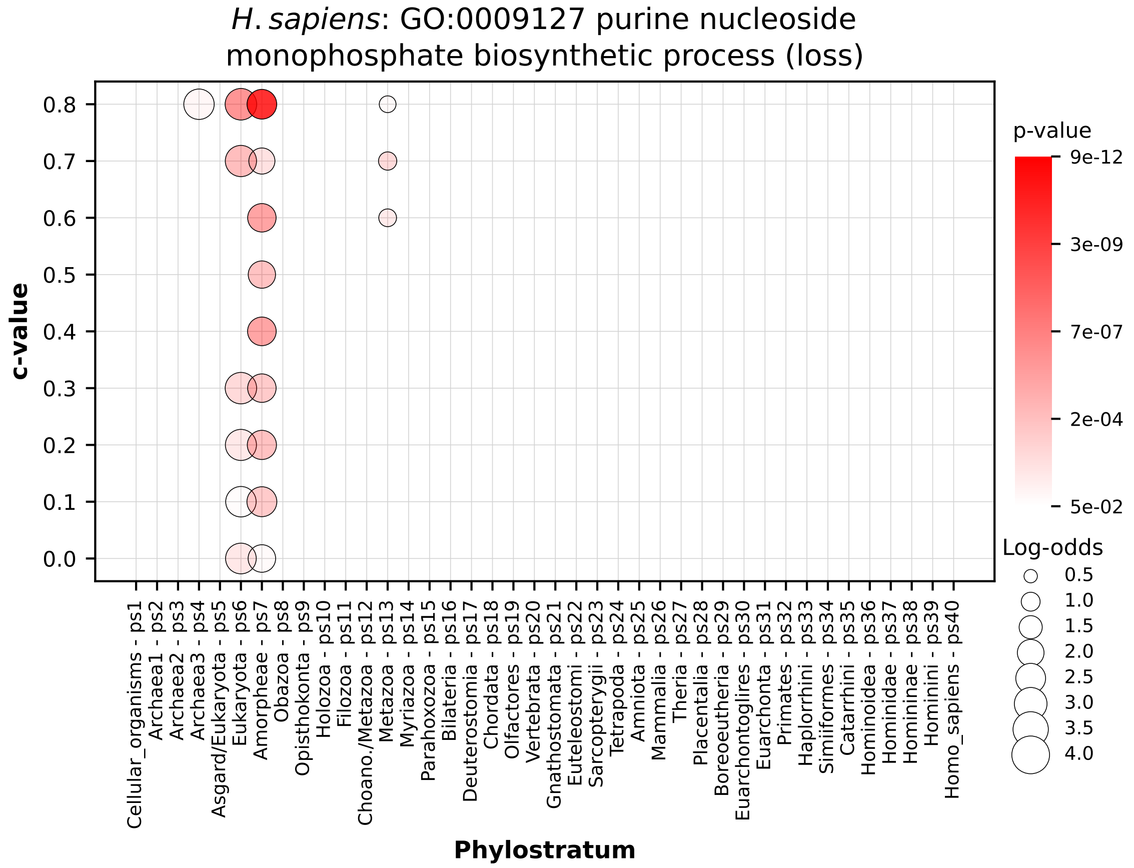

H. sapiens: GO:0009127 purine nucleoside monophosphate biosynthetic process (loss)
c-value
0.8
0.7
0.6
0.5
0.4
0.3
0.2
0.1
0.0
p-value
9e-12
3e-09
7e-07
2e-04
5e-02
Log-odds
0.5
1.0
1.5
2.0
2.5
3.0
3.5
4.0
Cellular_organisms - ps1
Archaea1 - ps2
Archaea2 - ps3
Archaea3 - ps4
Asgard/Eukaryota - ps5
Eukaryota - ps6
Amorpheae - ps7
Obazoa - ps8
Opisthokonta - ps9
Holozoa - ps10
Filozoa - ps11
Choano./Metazoa - ps12
Metazoa - ps13
Myriazoa - ps14
Parahoxozoa - ps15
Bilateria - ps16
Deuterostomia - ps17
Chordata - ps18
Olfactores - ps19
Vertebrata - ps20
Gnathostomata - ps21
Euteleostomi - ps22
Sarcopterygii - ps23
Tetrapoda - ps24
Amniota - ps25
Mammalia - ps26
Theria - ps27
Placentalia - ps28
Boreoeutheria - ps29
Euarchontoglires - ps30
Euarchonta - ps31
Primates - ps32
Haplorrhini - ps33
Simiiformes - ps34
Catarrhini - ps35
Hominoidea - ps36
Hominidae - ps37
Homininae - ps38
Hominini - ps39
Homo_sapiens - ps40
Phylostratum

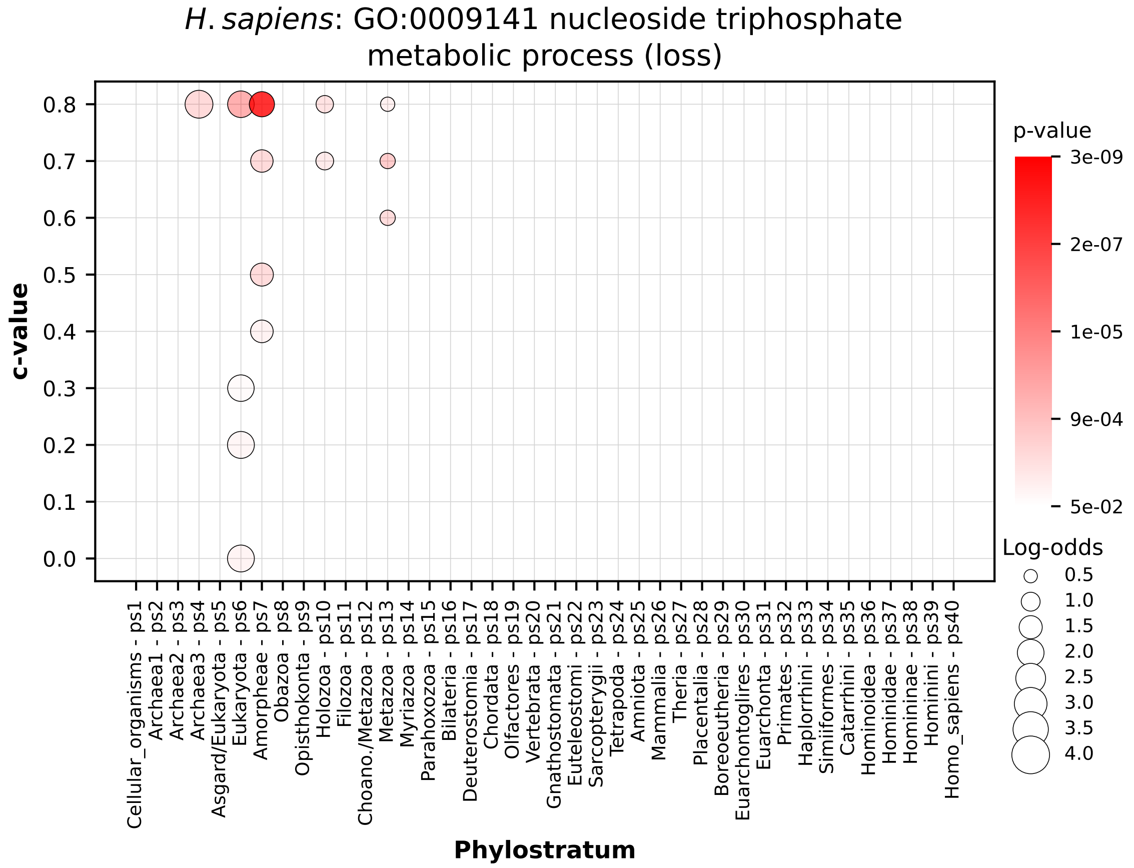

H. sapiens: GO:0009141 nucleoside triphosphate metabolic process (loss)
c-value
0.8
0.7
0.6
0.5
0.4
0.3
0.2
0.1
0.0
Phylostratum
Cellular_organisms - ps1
Archaea1 - ps2
Archaea2 - ps3
Archaea3 - ps4
Asgard/Eukaryota - ps5
Eukaryota - ps6
Amorpheae - ps7
Obazoa - ps8
Opisthokonta - ps9
Holozoa - ps10
Filozoa - ps11
Choano./Metazoa - ps12
Metazoa - ps13
Myriazoa - ps14
Parahoxozoa - ps15
Bilateria - ps16
Deuterostomia - ps17
Chordata - ps18
Olfactores - ps19
Vertebrata - ps20
Gnathostomata - ps21
Euteleostomi - ps22
Sarcopterygii - ps23
Tetrapoda - ps24
Amniota - ps25
Mammalia - ps26
Theria - ps27
Placentalia - ps28
Boreoeutheria - ps29
Euarchontoglires - ps30
Euarchonta - ps31
Primates - ps32
Haplorrhini - ps33
Simiiformes - ps34
Catarrhini - ps35
Hominoidea - ps36
Hominidae - ps37
Homininae - ps38
Hominini - ps39
Homo_sapiens - ps40
p-value
3e-09
2e-07
1e-05
9e-04
5e-02
Log-odds
0.5
1.0
1.5
2.0
2.5
3.0
3.5
4.0

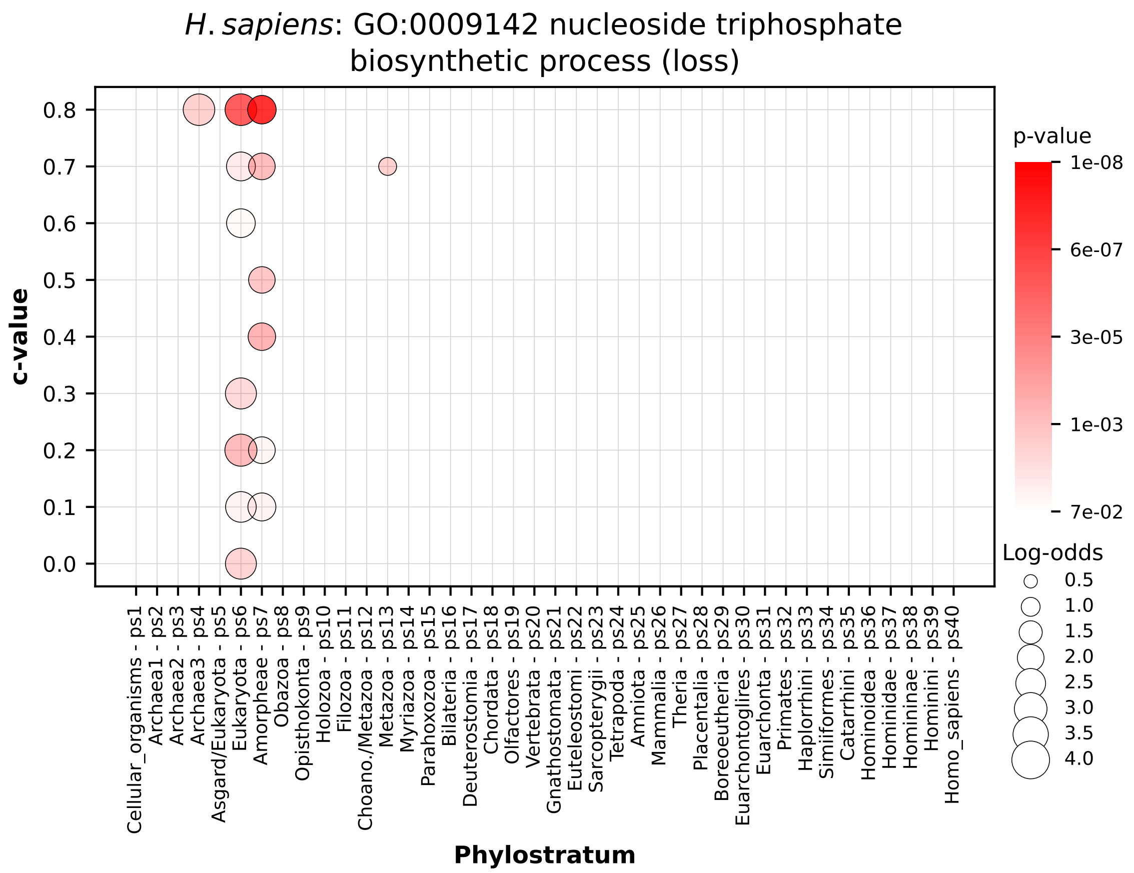

H. sapiens: GO:0009142 nucleoside triphosphate biosynthetic process (loss)
c-value
0.8
0.7
0.6
0.5
0.4
0.3
0.2
0.1
0.0
p-value
1e-08
6e-07
3e-05
1e-03
7e-02
Log-odds
0.5
1.0
1.5
2.0
2.5
3.0
3.5
4.0
Cellular_organisms - ps1
Archaea1 - ps2
Archaea2 - ps3
Archaea3 - ps4
Asgard/Eukaryota - ps5
Eukaryota - ps6
Amorpheae - ps7
Obazoa - ps8
Opisthokonta - ps9
Holozoa - ps10
Filozoa - ps11
Choano./Metazoa - ps12
Metazoa - ps13
Myriazoa - ps14
Parahoxozoa - ps15
Bilateria - ps16
Deuterostomia - ps17
Chordata - ps18
Olfactores - ps19
Vertebrata - ps20
Gnathostomata - ps21
Euteleostomi - ps22
Sarcopterygii - ps23
Tetrapoda - ps24
Amniota - ps25
Mammalia - ps26
Theria - ps27
Placentalia - ps28
Boreoeutheria - ps29
Euarchontoglires - ps30
Euarchonta - ps31
Primates - ps32
Haplorrhini - ps33
Simiiformes - ps34
Catarrhini - ps35
Hominoidea - ps36
Hominidae - ps37
Homininae - ps38
Hominini - ps39
Homo_sapiens - ps40
Phylostratum

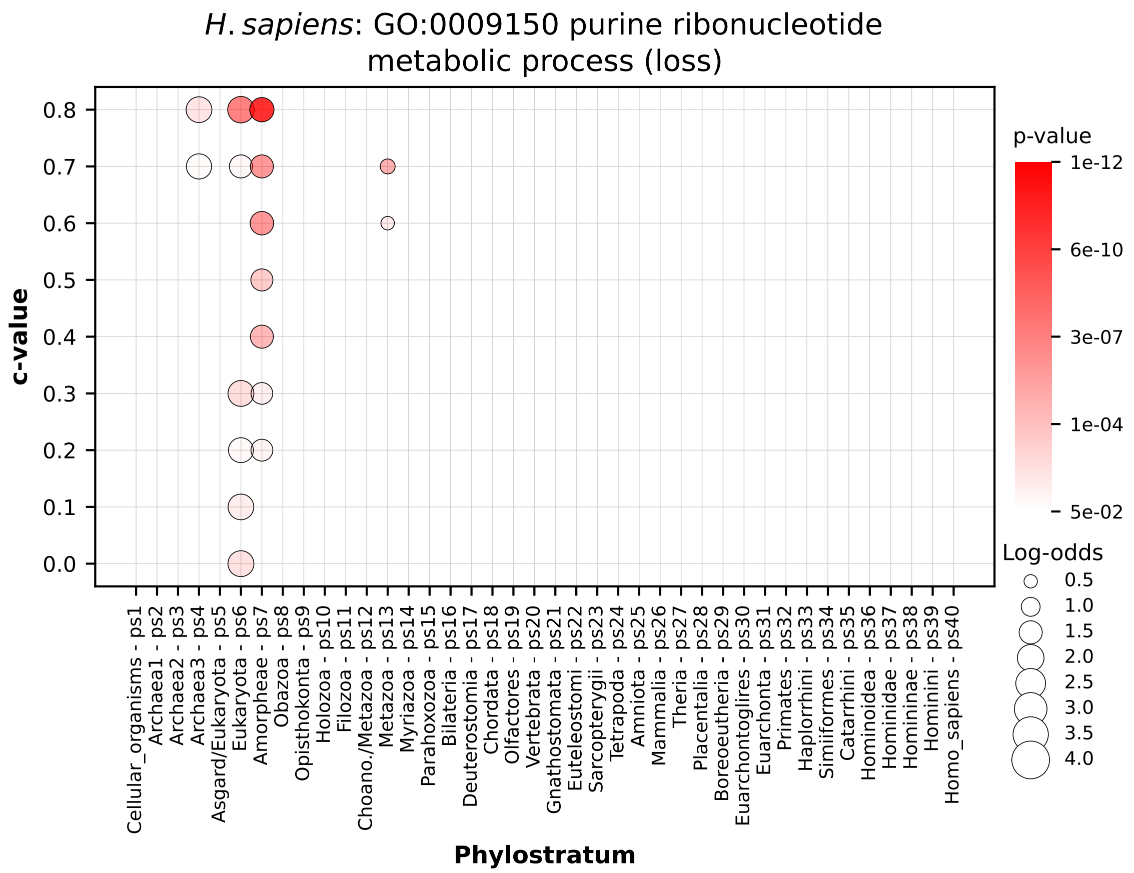

H. sapiens: GO:0009150 purine ribonucleotide metabolic process (loss)
c-value
0.8
0.7
0.6
0.5
0.4
0.3
0.2
0.1
0.0
Phylostratum
Cellular_organisms - ps1
Archaea1 - ps2
Archaea2 - ps3
Archaea3 - ps4
Asgard/Eukaryota - ps5
Eukaryota - ps6
Amorpheae - ps7
Obazoa - ps8
Opisthokonta - ps9
Holozoa - ps10
Filozoa - ps11
Choano./Metazoa - ps12
Metazoa - ps13
Myriazoa - ps14
Parahoxozoa - ps15
Bilateria - ps16
Deuterostomia - ps17
Chordata - ps18
Olfactores - ps19
Vertebrata - ps20
Gnathostomata - ps21
Euteleostomi - ps22
Sarcopterygii - ps23
Tetrapoda - ps24
Amniota - ps25
Mammalia - ps26
Theria - ps27
Placentalia - ps28
Boreoeutheria - ps29
Euarchontoglires - ps30
Euarchonta - ps31
Primates - ps32
Haplorrhini - ps33
Simiiformes - ps34
Catarrhini - ps35
Hominoidea - ps36
Hominidae - ps37
Homininae - ps38
Hominini - ps39
Homo_sapiens - ps40
p-value
1e-12
6e-10
3e-07
1e-04
5e-02
Log-odds
0.5
1.0
1.5
2.0
2.5
3.0
3.5
4.0

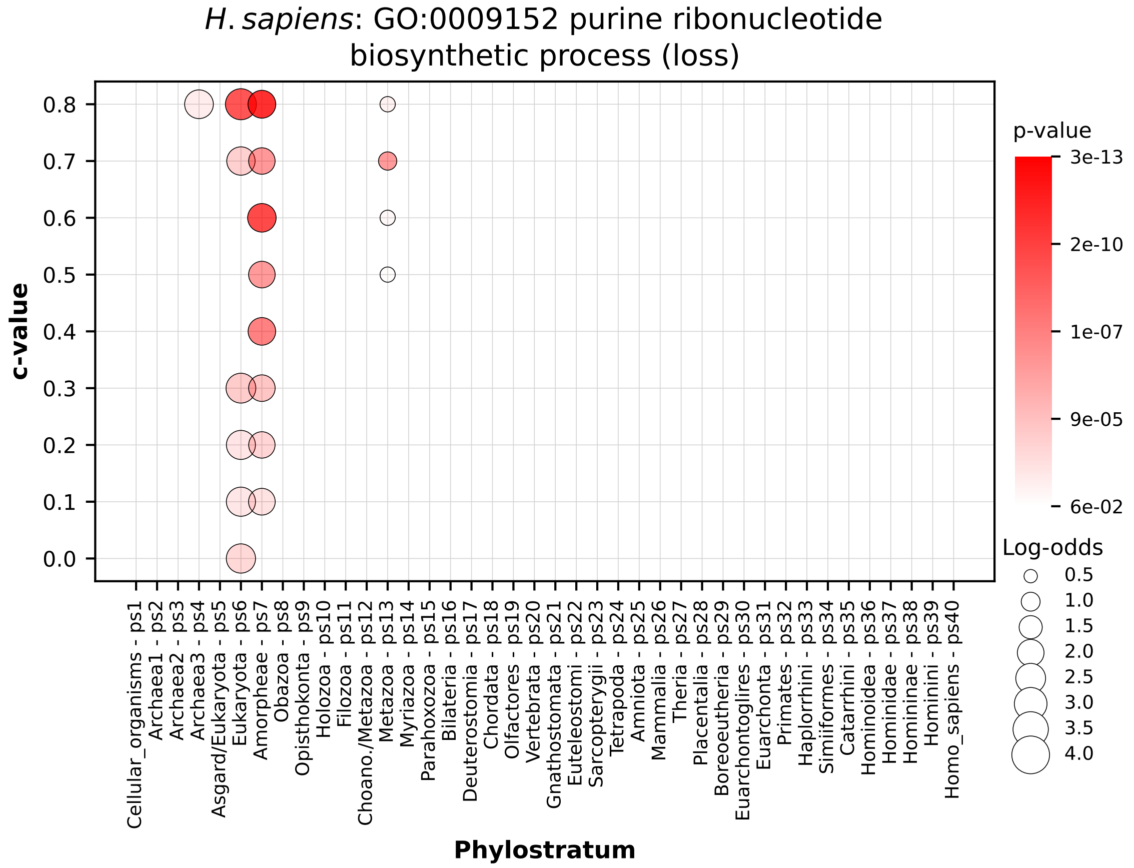

H. sapiens: GO:0009152 purine ribonucleotide biosynthetic process (loss)
c-value
0.0
0.1
0.2
0.3
0.4
0.5
0.6
0.7
0.8
Phylostratum
Cellular_organisms - ps1
Archaea1 - ps2
Archaea2 - ps3
Archaea3 - ps4
Asgard/Eukaryota - ps5
Eukaryota - ps6
Amorpheae - ps7
Obazoa - ps8
Opisthokonta - ps9
Holozoa - ps10
Filozoa - ps11
Choano./Metazoa - ps12
Metazoa - ps13
Myriazoa - ps14
Parahoxozoa - ps15
Bilateria - ps16
Deuterostomia - ps17
Chordata - ps18
Olfactores - ps19
Vertebrata - ps20
Gnathostomata - ps21
Euteleostomi - ps22
Sarcopterygii - ps23
Tetrapoda - ps24
Amniota - ps25
Mammalia - ps26
Theria - ps27
Placentalia - ps28
Boreoeutheria - ps29
Euarchontoglires - ps30
Euarchonta - ps31
Primates - ps32
Haplorrhini - ps33
Simiiformes - ps34
Catarrhini - ps35
Hominoidea - ps36
Hominidae - ps37
Homininae - ps38
Hominini - ps39
Homo_sapiens - ps40
p-value
3e-13
2e-10
1e-07
9e-05
6e-02
Log-odds
0.5
1.0
1.5
2.0
2.5
3.0
3.5
4.0

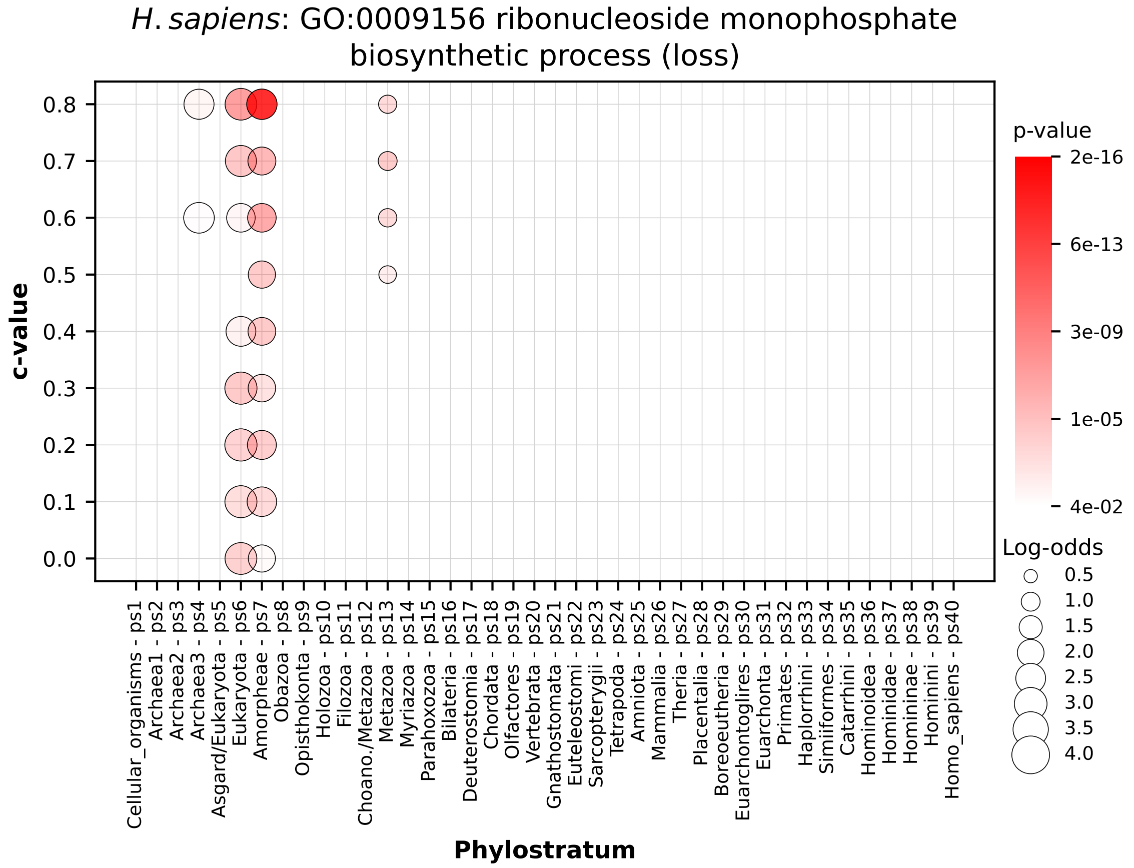

H. sapiens: GO:0009156 ribonucleoside monophosphate biosynthetic process (loss)
c-value
0.8
0.7
0.6
0.5
0.4
0.3
0.2
0.1
0.0
p-value
2e-16
6e-13
3e-09
1e-05
4e-02
Log-odds
0.5
1.0
1.5
2.0
2.5
3.0
3.5
4.0
Cellular_organisms - ps1
Archaea1 - ps2
Archaea2 - ps3
Archaea3 - ps4
Asgard/Eukaryota - ps5
Eukaryota - ps6
Amorpheae - ps7
Obazoa - ps8
Opisthokonta - ps9
Holozoa - ps10
Filozoa - ps11
Choano./Metazoa - ps12
Metazoa - ps13
Myriazoa - ps14
Parahoxozoa - ps15
Bilateria - ps16
Deuterostomia - ps17
Chordata - ps18
Olfactores - ps19
Vertebrata - ps20
Gnathostomata - ps21
Euteleostomi - ps22
Sarcopterygii - ps23
Tetrapoda - ps24
Amniota - ps25
Mammalia - ps26
Theria - ps27
Placentalia - ps28
Boreoeutheria - ps29
Euarchontoglires - ps30
Euarchonta - ps31
Primates - ps32
Haplorrhini - ps33
Simiiformes - ps34
Catarrhini - ps35
Hominoidea - ps36
Hominidae - ps37
Homininae - ps38
Hominini - ps39
Homo_sapiens - ps40
Phylostratum

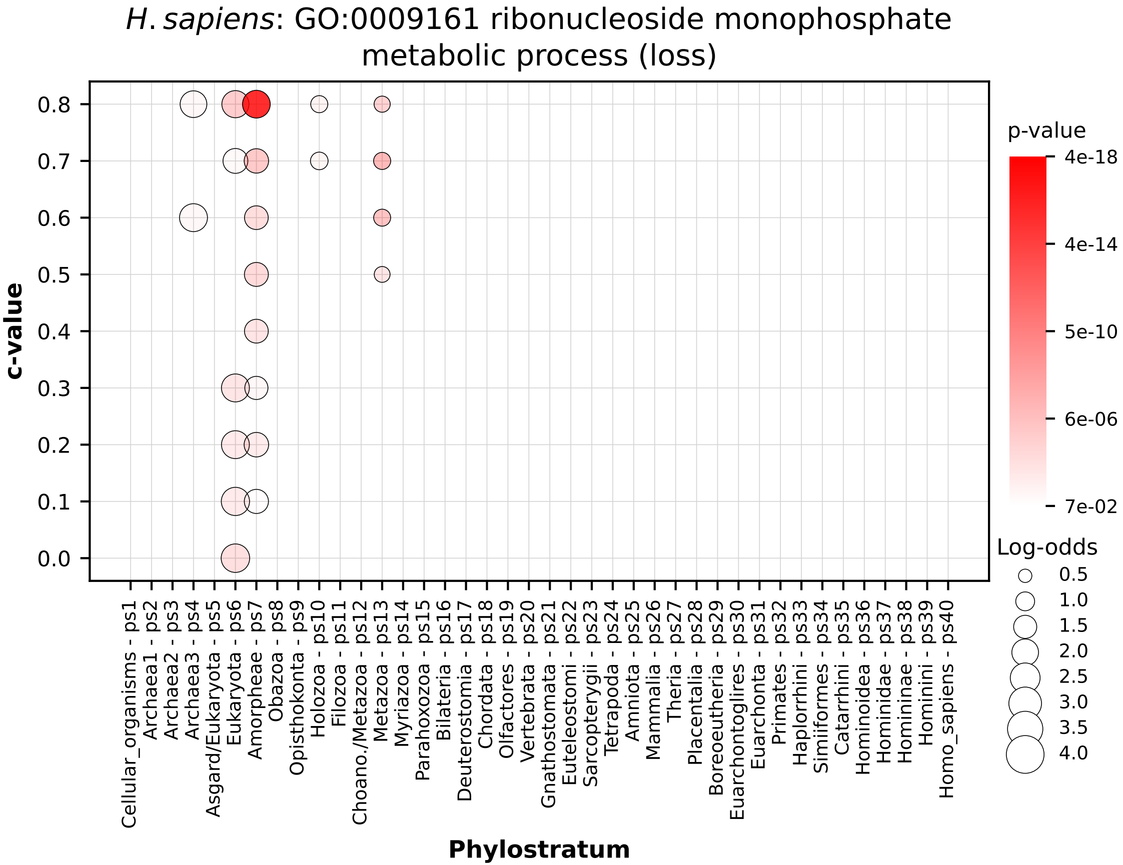

H. sapiens: GO:0009161 ribonucleoside monophosphate metabolic process (loss)
c-value
0.8
0.7
0.6
0.5
0.4
0.3
0.2
0.1
0.0
Phylostratum
Cellular_organisms - ps1
Archaea1 - ps2
Archaea2 - ps3
Archaea3 - ps4
Asgard/Eukaryota - ps5
Eukaryota - ps6
Amorpheae - ps7
Obazoa - ps8
Opisthokonta - ps9
Holozoa - ps10
Filozoa - ps11
Choano./Metazoa - ps12
Metazoa - ps13
Myriazoa - ps14
Parahoxozoa - ps15
Bilateria - ps16
Deuterostomia - ps17
Chordata - ps18
Olfactores - ps19
Vertebrata - ps20
Gnathostomata - ps21
Euteleostomi - ps22
Sarcopterygii - ps23
Tetrapoda - ps24
Amniota - ps25
Mammalia - ps26
Theria - ps27
Placentalia - ps28
Boreoeutheria - ps29
Euarchontoglires - ps30
Euarchonta - ps31
Primates - ps32
Haplorrhini - ps33
Simiiformes - ps34
Catarrhini - ps35
Hominoidea - ps36
Hominidae - ps37
Homininae - ps38
Hominini - ps39
Homo_sapiens - ps40
p-value
4e-18
4e-14
5e-10
6e-06
7e-02
Log-odds
0.5
1.0
1.5
2.0
2.5
3.0
3.5
4.0

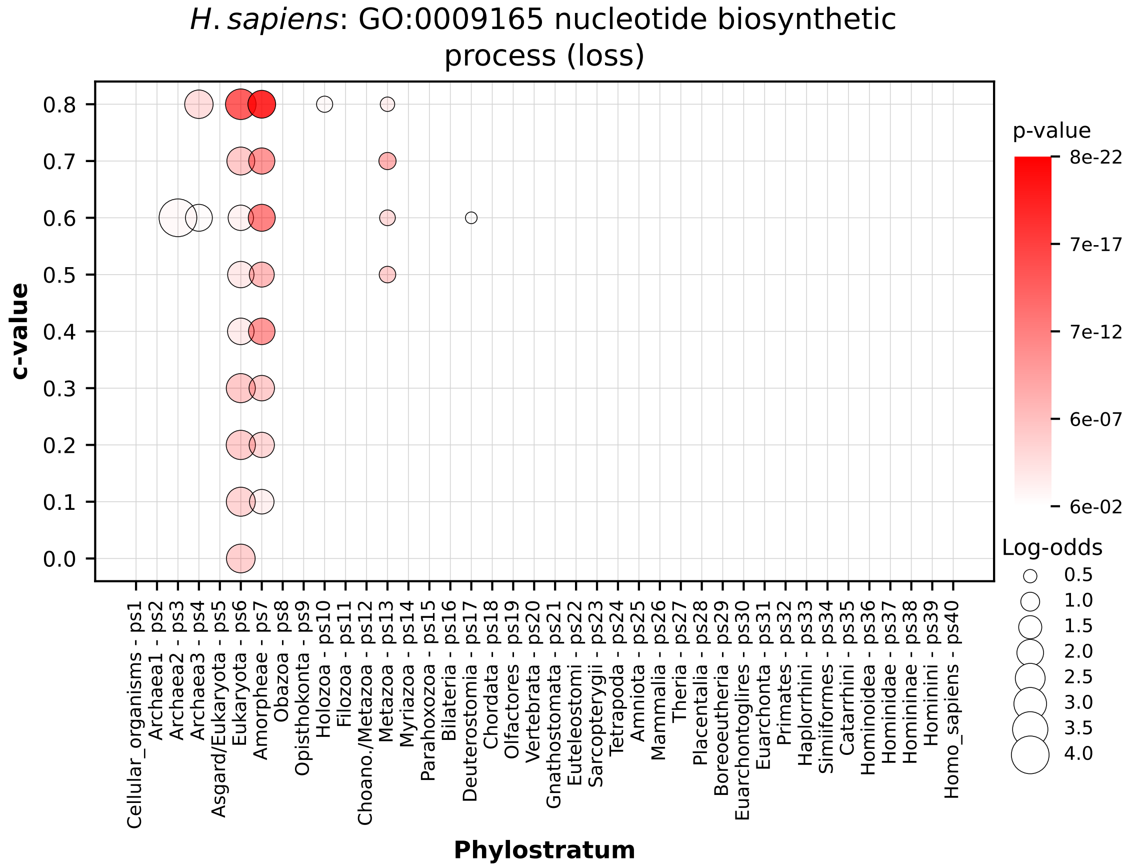

H. sapiens: GO:0009165 nucleotide biosynthetic process (loss)
c-value
0.8
0.7
0.6
0.5
0.4
0.3
0.2
0.1
0.0
p-value
8e-22
7e-17
7e-12
6e-07
6e-02
Log-odds
0.5
1.0
1.5
2.0
2.5
3.0
3.5
4.0
Cellular_organisms - ps1
Archaea1 - ps2
Archaea2 - ps3
Archaea3 - ps4
Asgard/Eukaryota - ps5
Eukaryota - ps6
Amorpheae - ps7
Obazoa - ps8
Opisthokonta - ps9
Holozoa - ps10
Filozoa - ps11
Choano./Metazoa - ps12
Metazoa - ps13
Myriazoa - ps14
Parahoxozoa - ps15
Bilateria - ps16
Deuterostomia - ps17
Chordata - ps18
Olfactores - ps19
Vertebrata - ps20
Gnathostomata - ps21
Euteleostomi - ps22
Sarcopterygii - ps23
Tetrapoda - ps24
Amniota - ps25
Mammalia - ps26
Theria - ps27
Placentalia - ps28
Boreoeutheria - ps29
Euarchontoglires - ps30
Euarchonta - ps31
Primates - ps32
Haplorrhini - ps33
Simiiformes - ps34
Catarrhini - ps35
Hominoidea - ps36
Hominidae - ps37
Homininae - ps38
Hominini - ps39
Homo_sapiens - ps40
Phylostratum

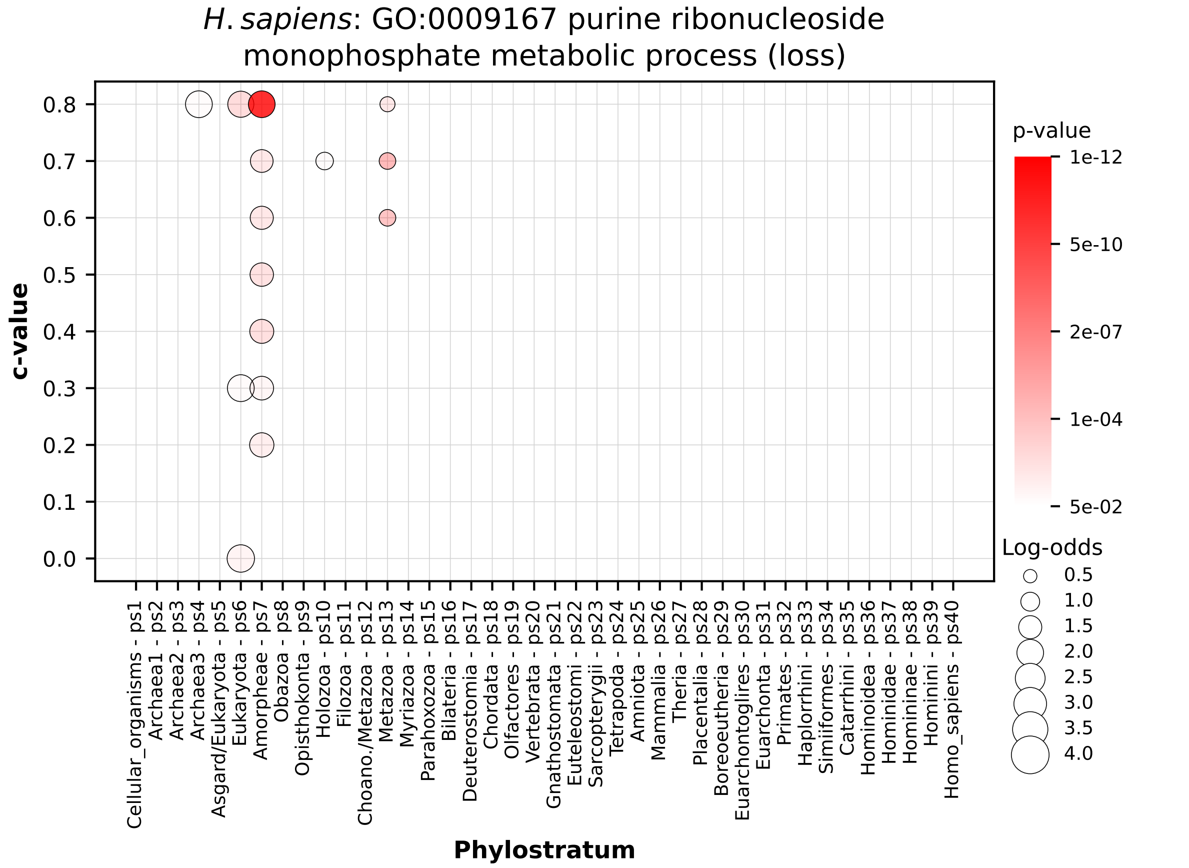

H. sapiens: GO:0009167 purine ribonucleoside monophosphate metabolic process (loss)
c-value
0.8
0.7
0.6
0.5
0.4
0.3
0.2
0.1
0.0
Phylostratum
Cellular_organisms - ps1
Archaea1 - ps2
Archaea2 - ps3
Archaea3 - ps4
Asgard/Eukaryota - ps5
Eukaryota - ps6
Amorpheae - ps7
Obazoa - ps8
Opisthokonta - ps9
Holozoa - ps10
Filozoa - ps11
Choano./Metazoa - ps12
Metazoa - ps13
Myriazoa - ps14
Parahoxozoa - ps15
Bilateria - ps16
Deuterostomia - ps17
Chordata - ps18
Olfactores - ps19
Vertebrata - ps20
Gnathostomata - ps21
Euteleostomi - ps22
Sarcopterygii - ps23
Tetrapoda - ps24
Amniota - ps25
Mammalia - ps26
Theria - ps27
Placentalia - ps28
Boreoeutheria - ps29
Euarchontoglires - ps30
Euarchonta - ps31
Primates - ps32
Haplorrhini - ps33
Simiiformes - ps34
Catarrhini - ps35
Hominoidea - ps36
Hominidae - ps37
Homininae - ps38
Hominini - ps39
Homo_sapiens - ps40
p-value
1e-12
5e-10
2e-07
1e-04
5e-02
Log-odds
0.5
1.0
1.5
2.0
2.5
3.0
3.5
4.0

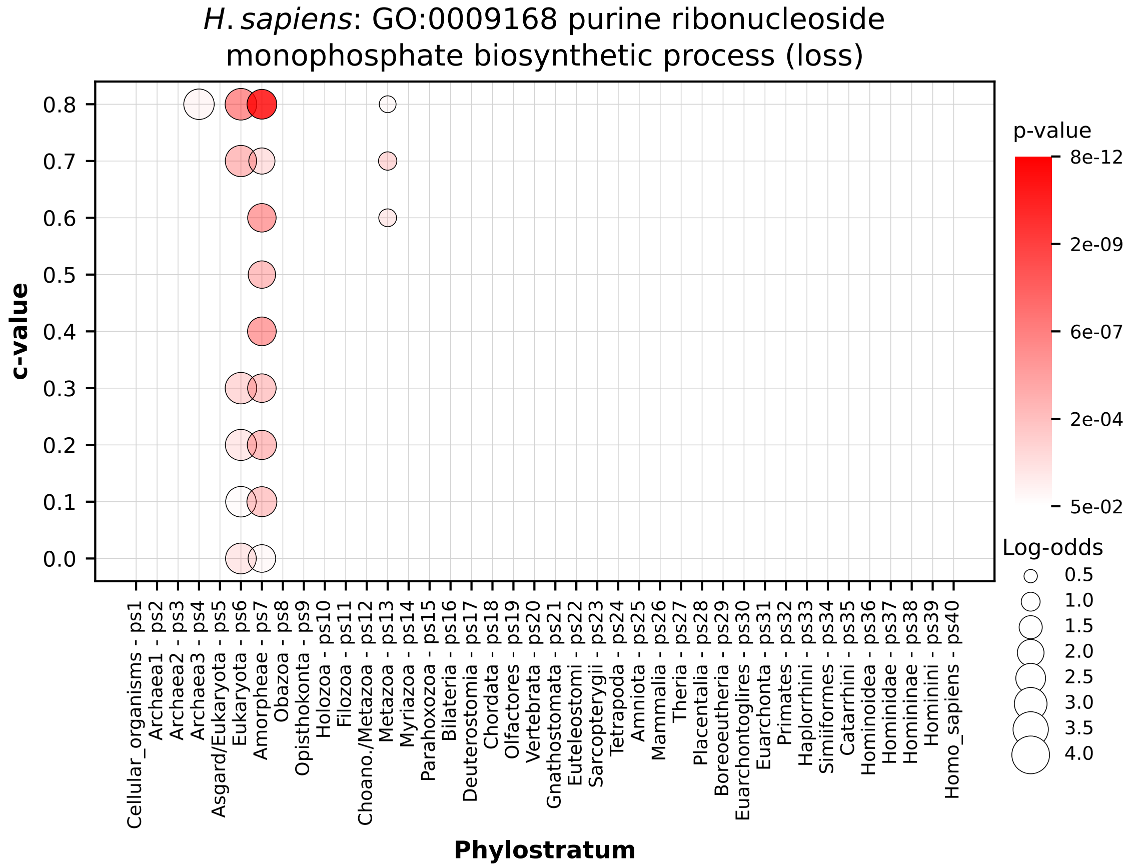

H. sapiens: GO:0009168 purine ribonucleoside monophosphate biosynthetic process (loss)
c-value
0.0
0.1
0.2
0.3
0.4
0.5
0.6
0.7
0.8
p-value
8e-12
2e-09
6e-07
2e-04
5e-02
Log-odds
0.5
1.0
1.5
2.0
2.5
3.0
3.5
4.0
Cellular_organisms - ps1
Archaea1 - ps2
Archaea2 - ps3
Archaea3 - ps4
Asgard/Eukaryota - ps5
Eukaryota - ps6
Amorpheae - ps7
Obazoa - ps8
Opisthokonta - ps9
Holozoa - ps10
Filozoa - ps11
Choano./Metazoa - ps12
Metazoa - ps13
Myriazoa - ps14
Parahoxozoa - ps15
Bilateria - ps16
Deuterostomia - ps17
Chordata - ps18
Olfactores - ps19
Vertebrata - ps20
Gnathostomata - ps21
Euteleostomi - ps22
Sarcopterygii - ps23
Tetrapoda - ps24
Amniota - ps25
Mammalia - ps26
Theria - ps27
Placentalia - ps28
Boreoeutheria - ps29
Euarchontoglires - ps30
Euarchonta - ps31
Primates - ps32
Haplorrhini - ps33
Simiiformes - ps34
Catarrhini - ps35
Hominoidea - ps36
Hominidae - ps37
Homininae - ps38
Hominini - ps39
Homo_sapiens - ps40
Phylostratum

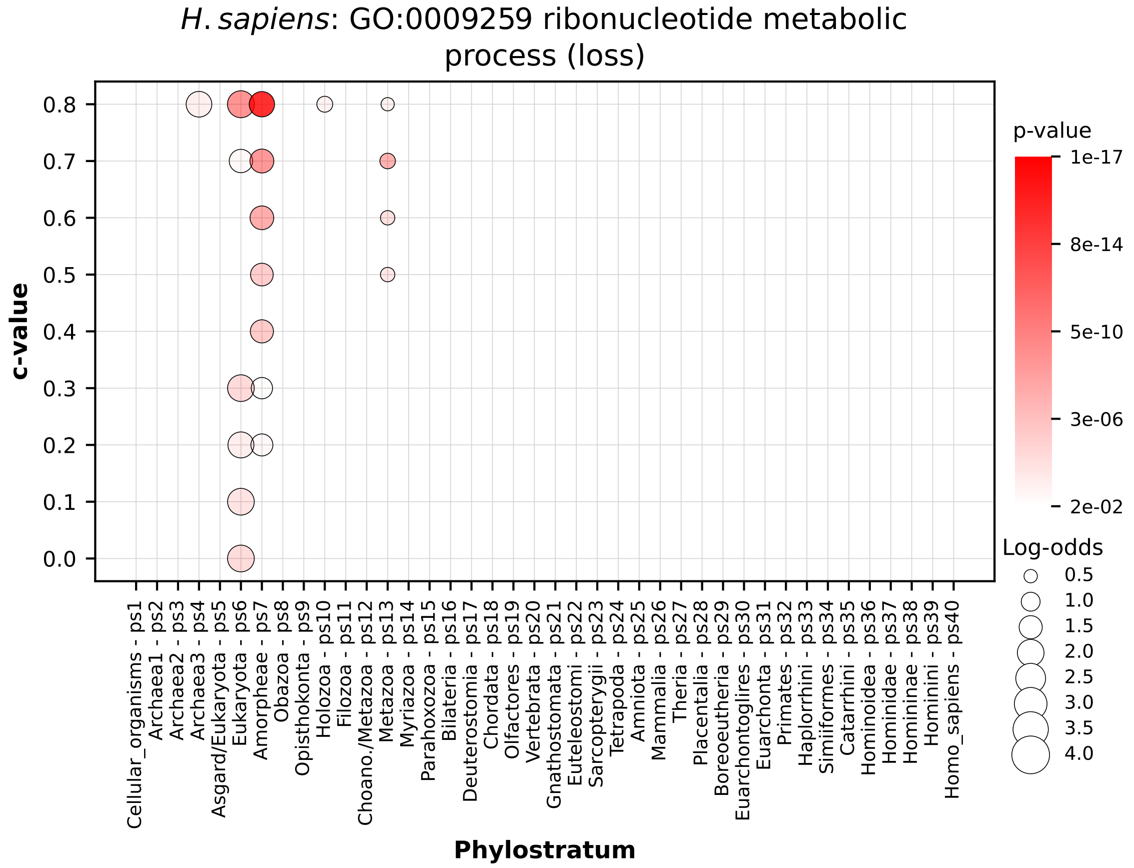

H. sapiens: GO:0009259 ribonucleotide metabolic process (loss)
c-value
0.0
0.1
0.2
0.3
0.4
0.5
0.6
0.7
0.8
Phylostratum
Cellular_organisms - ps1
Archaea1 - ps2
Archaea2 - ps3
Archaea3 - ps4
Asgard/Eukaryota - ps5
Eukaryota - ps6
Amorpheae - ps7
Obazoa - ps8
Opisthokonta - ps9
Holozoa - ps10
Filozoa - ps11
Choano./Metazoa - ps12
Metazoa - ps13
Myriazoa - ps14
Parahoxozoa - ps15
Bilateria - ps16
Deuterostomia - ps17
Chordata - ps18
Olfactores - ps19
Vertebrata - ps20
Gnathostomata - ps21
Euteleostomi - ps22
Sarcopterygii - ps23
Tetrapoda - ps24
Amniota - ps25
Mammalia - ps26
Theria - ps27
Placentalia - ps28
Boreoeutheria - ps29
Euarchontoglires - ps30
Euarchonta - ps31
Primates - ps32
Haplorrhini - ps33
Simiiformes - ps34
Catarrhini - ps35
Hominoidea - ps36
Hominidae - ps37
Homininae - ps38
Hominini - ps39
Homo_sapiens - ps40
p-value
1e-17
8e-14
5e-10
3e-06
2e-02
Log-odds
0.5
1.0
1.5
2.0
2.5
3.0
3.5
4.0

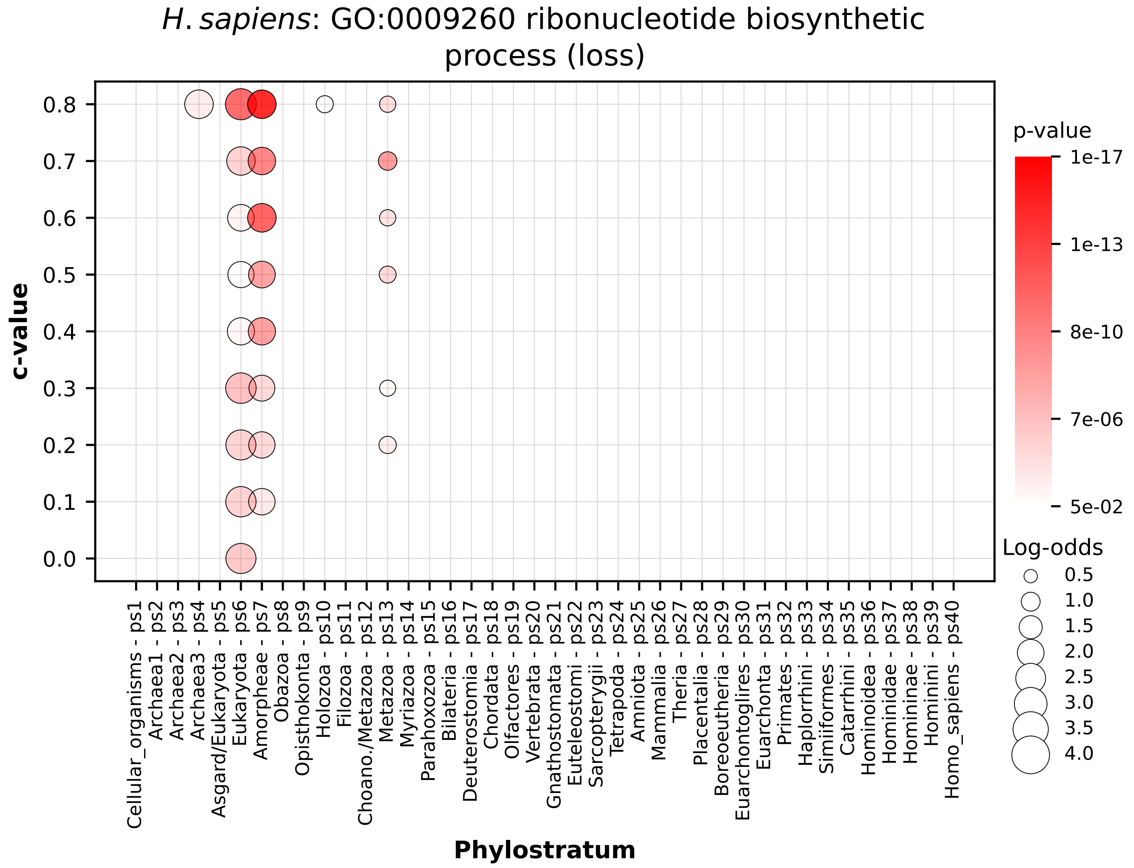

H. sapiens: GO:0009260 ribonucleotide biosynthetic process (loss)
c-value
0.0
0.1
0.2
0.3
0.4
0.5
0.6
0.7
0.8
Phylostratum
Cellular_organisms - ps1
Archaea1 - ps2
Archaea2 - ps3
Archaea3 - ps4
Asgard/Eukaryota - ps5
Eukaryota - ps6
Amorpheae - ps7
Obazoa - ps8
Opisthokonta - ps9
Holozoa - ps10
Filozoa - ps11
Choano./Metazoa - ps12
Metazoa - ps13
Myriazoa - ps14
Parahoxozoa - ps15
Bilateria - ps16
Deuterostomia - ps17
Chordata - ps18
Olfactores - ps19
Vertebrata - ps20
Gnathostomata - ps21
Euteleostomi - ps22
Sarcopterygii - ps23
Tetrapoda - ps24
Amniota - ps25
Mammalia - ps26
Theria - ps27
Placentalia - ps28
Boreoeutheria - ps29
Euarchontoglires - ps30
Euarchonta - ps31
Primates - ps32
Haplorrhini - ps33
Simiiformes - ps34
Catarrhini - ps35
Hominoidea - ps36
Hominidae - ps37
Homininae - ps38
Hominini - ps39
Homo_sapiens - ps40
p-value
1e-17
1e-13
8e-10
7e-06
5e-02
Log-odds
0.5
1.0
1.5
2.0
2.5
3.0
3.5
4.0

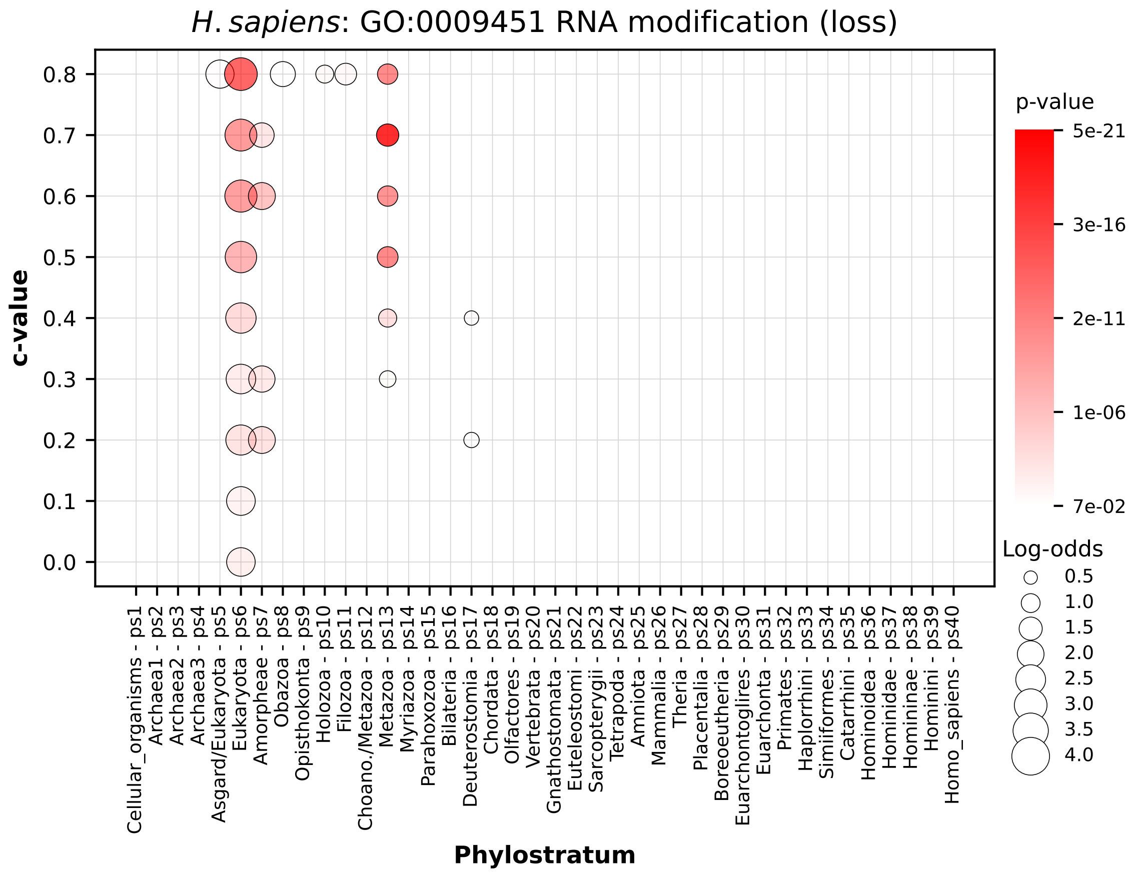
H. sapiens: GO:0009451 RNA modification (loss)
c-value
0.8
0.7
0.6
0.5
0.4
0.3
0.2
0.1
0.0
Phylostratum
Cellular_organisms - ps1
Archaea1 - ps2
Archaea2 - ps3
Archaea3 - ps4
Asgard/Eukaryota - ps5
Eukaryota - ps6
Amorpheae - ps7
Obazoa - ps8
Opisthokonta - ps9
Holozoa - ps10
Filozoa - ps11
Choano./Metazoa - ps12
Metazoa - ps13
Myriazoa - ps14
Parahoxozoa - ps15
Bilateria - ps16
Deuterostomia - ps17
Chordata - ps18
Olfactores - ps19
Vertebrata - ps20
Gnathostomata - ps21
Euteleostomi - ps22
Sarcopterygii - ps23
Tetrapoda - ps24
Amniota - ps25
Mammalia - ps26
Theria - ps27
Placentalia - ps28
Boreoeutheria - ps29
Euarchontoglires - ps30
Euarchonta - ps31
Primates - ps32
Haplorrhini - ps33
Simiiformes - ps34
Catarrhini - ps35
Hominoidea - ps36
Hominidae - ps37
Homininae - ps38
Hominini - ps39
Homo_sapiens - ps40
p-value
5e-21
3e-16
2e-11
1e-06
7e-02
Log-odds
0.5
1.0
1.5
2.0
2.5
3.0
3.5
4.0

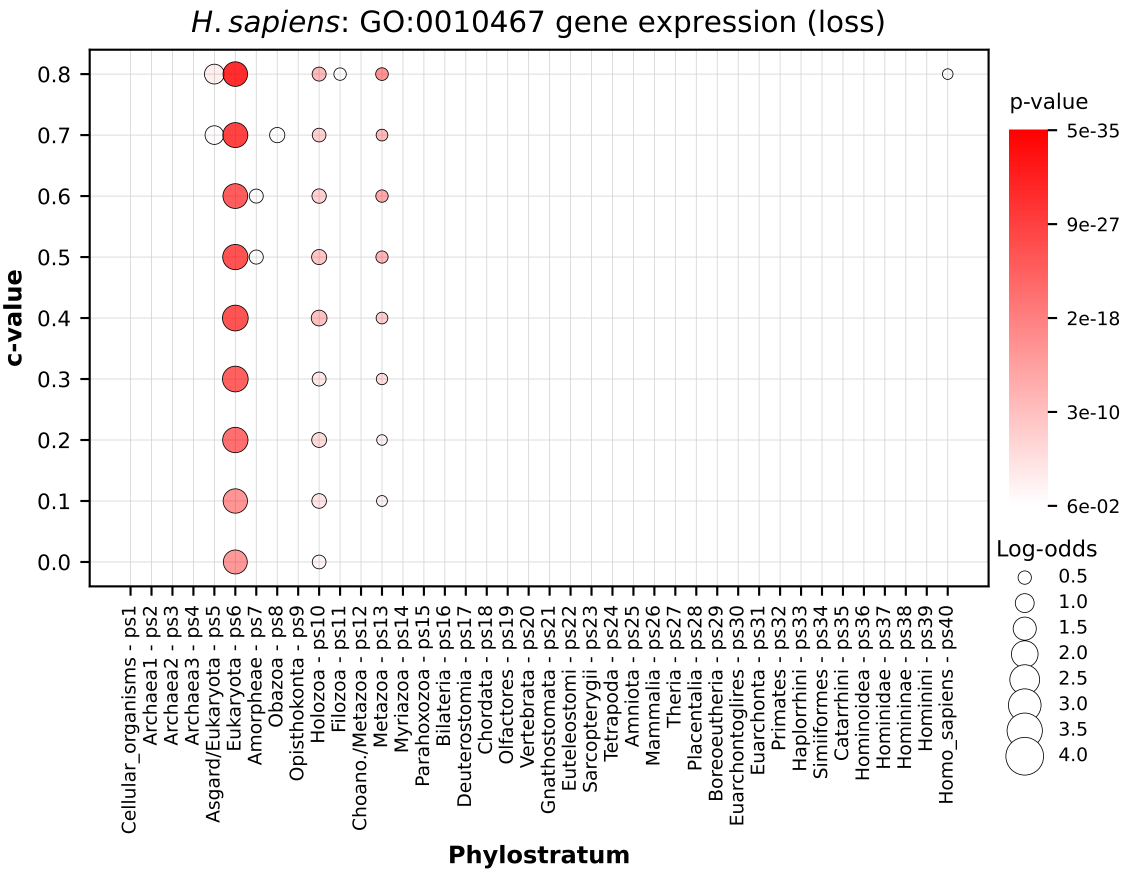

H. sapiens: GO:0010467 gene expression (loss)
c-value
0.0
0.1
0.2
0.3
0.4
0.5
0.6
0.7
0.8
Phylostratum
Cellular_organisms - ps1
Archaea1 - ps2
Archaea2 - ps3
Archaea3 - ps4
Asgard/Eukaryota - ps5
Eukaryota - ps6
Amorpheae - ps7
Obazoa - ps8
Opisthokonta - ps9
Holozoa - ps10
Filozoa - ps11
Choano./Metazoa - ps12
Metazoa - ps13
Myriazoa - ps14
Parahoxozoa - ps15
Bilateria - ps16
Deuterostomia - ps17
Chordata - ps18
Olfactores - ps19
Vertebrata - ps20
Gnathostomata - ps21
Euteleostomi - ps22
Sarcopterygii - ps23
Tetrapoda - ps24
Amniota - ps25
Mammalia - ps26
Theria - ps27
Placentalia - ps28
Boreoeutheria - ps29
Euarchontoglires - ps30
Euarchonta - ps31
Primates - ps32
Haplorrhini - ps33
Simiiformes - ps34
Catarrhini - ps35
Hominoidea - ps36
Hominidae - ps37
Homininae - ps38
Hominini - ps39
Homo_sapiens - ps40
p-value
5e-35
9e-27
2e-18
3e-10
6e-02
Log-odds
0.5
1.0
1.5
2.0
2.5
3.0
3.5
4.0

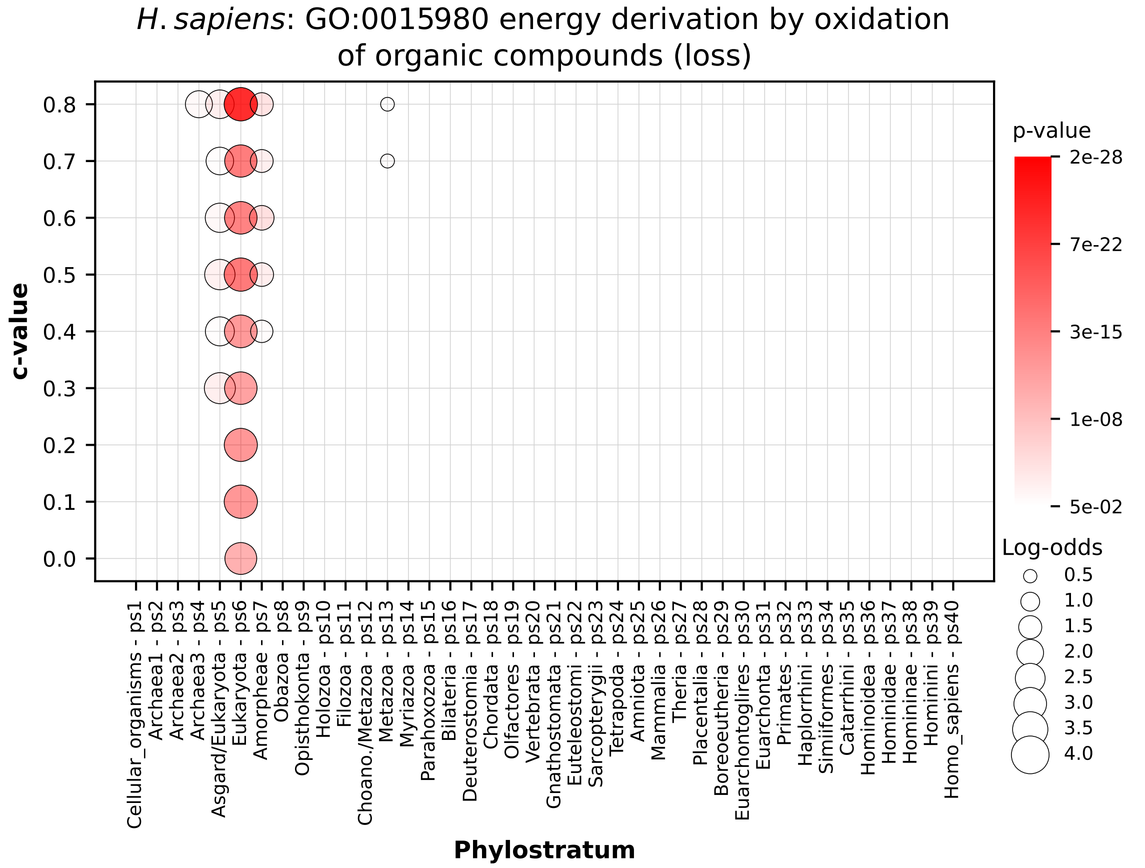

H. sapiens: GO:0015980 energy derivation by oxidation of organic compounds (loss)
c-value
0.8
0.7
0.6
0.5
0.4
0.3
0.2
0.1
0.0
Phylostratum
Cellular_organisms - ps1
Archaea1 - ps2
Archaea2 - ps3
Archaea3 - ps4
Asgard/Eukaryota - ps5
Eukaryota - ps6
Amorpheae - ps7
Obazoa - ps8
Opisthokonta - ps9
Holozoa - ps10
Filozoa - ps11
Choano./Metazoa - ps12
Metazoa - ps13
Myriazoa - ps14
Parahoxozoa - ps15
Bilateria - ps16
Deuterostomia - ps17
Chordata - ps18
Olfactores - ps19
Vertebrata - ps20
Gnathostomata - ps21
Euteleostomi - ps22
Sarcopterygii - ps23
Tetrapoda - ps24
Amniota - ps25
Mammalia - ps26
Theria - ps27
Placentalia - ps28
Boreoeutheria - ps29
Euarchontoglires - ps30
Euarchonta - ps31
Primates - ps32
Haplorrhini - ps33
Simiiformes - ps34
Catarrhini - ps35
Hominoidea - ps36
Hominidae - ps37
Homininae - ps38
Hominini - ps39
Homo_sapiens - ps40
p-value
2e-28
7e-22
3e-15
1e-08
5e-02
Log-odds
0.5
1.0
1.5
2.0
2.5
3.0
3.5
4.0

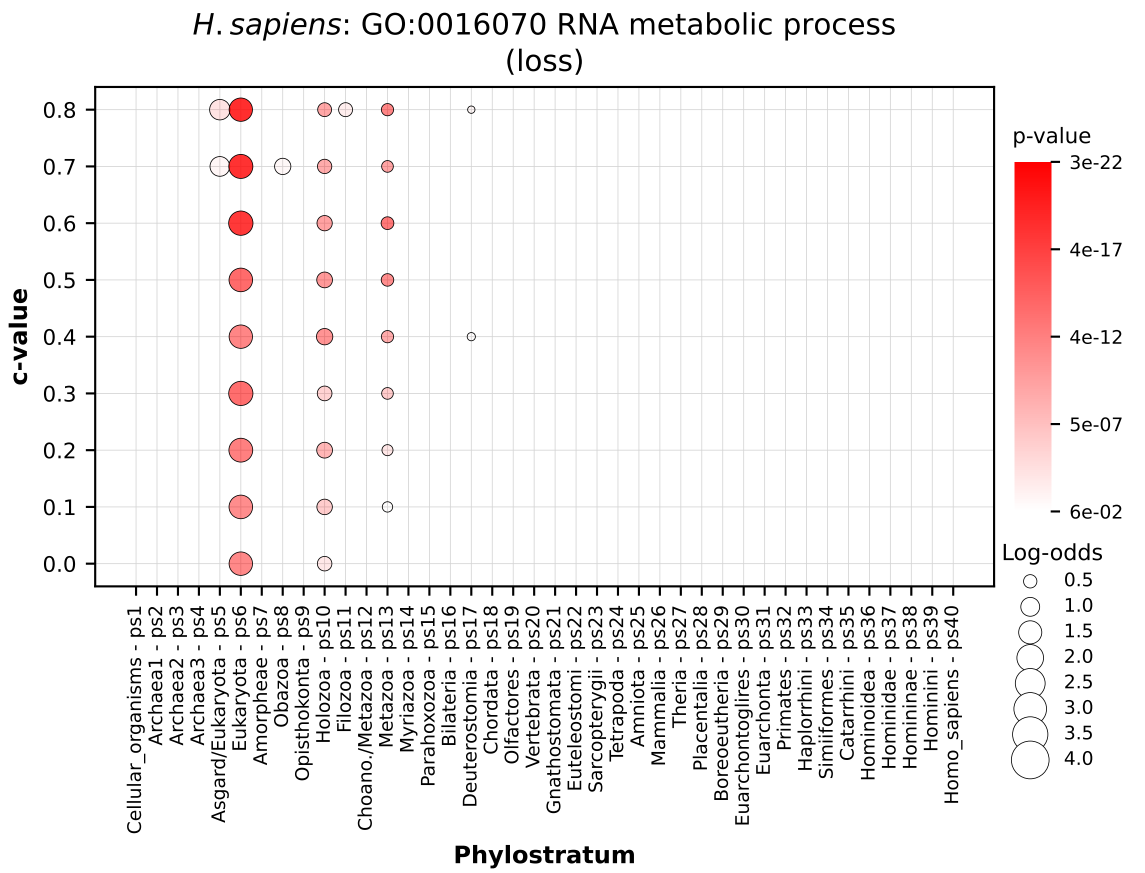

H. sapiens: GO:0016070 RNA metabolic process
(loss)
c-value
0.8
0.7
0.6
0.5
0.4
0.3
0.2
0.1
0.0
Phylostratum
Cellular_organisms - ps1
Archaea1 - ps2
Archaea2 - ps3
Archaea3 - ps4
Asgard/Eukaryota - ps5
Eukaryota - ps6
Amorpheae - ps7
Obazoa - ps8
Opisthokonta - ps9
Holozoa - ps10
Filozoa - ps11
Choano./Metazoa - ps12
Metazoa - ps13
Myriazoa - ps14
Parahoxozoa - ps15
Bilateria - ps16
Deuterostomia - ps17
Chordata - ps18
Olfactores - ps19
Vertebrata - ps20
Gnathostomata - ps21
Euteleostomi - ps22
Sarcopterygii - ps23
Tetrapoda - ps24
Amniota - ps25
Mammalia - ps26
Theria - ps27
Placentalia - ps28
Boreoeutheria - ps29
Euarchontoglires - ps30
Euarchonta - ps31
Primates - ps32
Haplorrhini - ps33
Simiiformes - ps34
Catarrhini - ps35
Hominoidea - ps36
Hominidae - ps37
Homininae - ps38
Hominini - ps39
Homo_sapiens - ps40
p-value
3e-22
4e-17
4e-12
5e-07
6e-02
Log-odds
0.5
1.0
1.5
2.0
2.5
3.0
3.5
4.0

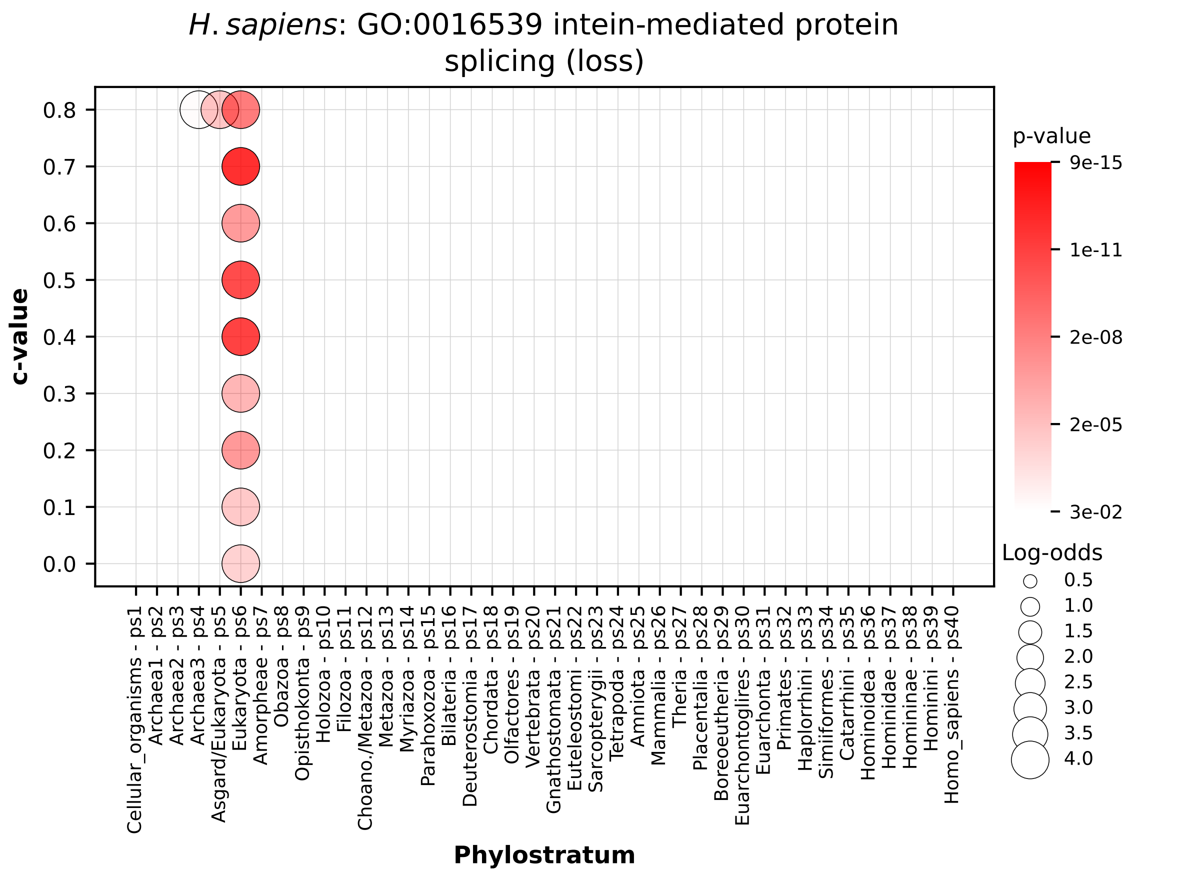

H. sapiens: GO:0016539 intein-mediated protein splicing (loss)
c-value
0.8
0.7
0.6
0.5
0.4
0.3
0.2
0.1
0.0
p-value
9e-15
1e-11
2e-08
2e-05
3e-02
Log-odds
0.5
1.0
1.5
2.0
2.5
3.0
3.5
4.0
Cellular_organisms - ps1
Archaea1 - ps2
Archaea2 - ps3
Archaea3 - ps4
Asgard/Eukaryota - ps5
Eukaryota - ps6
Amorpheae - ps7
Obazoa - ps8
Opisthokonta - ps9
Holozoa - ps10
Filozoa - ps11
Choano./Metazoa - ps12
Metazoa - ps13
Myriazoa - ps14
Parahoxozoa - ps15
Bilateria - ps16
Deuterostomia - ps17
Chordata - ps18
Olfactores - ps19
Vertebrata - ps20
Gnathostomata - ps21
Euteleostomi - ps22
Sarcopterygii - ps23
Tetrapoda - ps24
Amniota - ps25
Mammalia - ps26
Theria - ps27
Placentalia - ps28
Boreoeutheria - ps29
Euarchontoglires - ps30
Euarchonta - ps31
Primates - ps32
Haplorrhini - ps33
Simiiformes - ps34
Catarrhini - ps35
Hominoidea - ps36
Hominidae - ps37
Homininae - ps38
Hominini - ps39
Homo_sapiens - ps40
Phylostratum

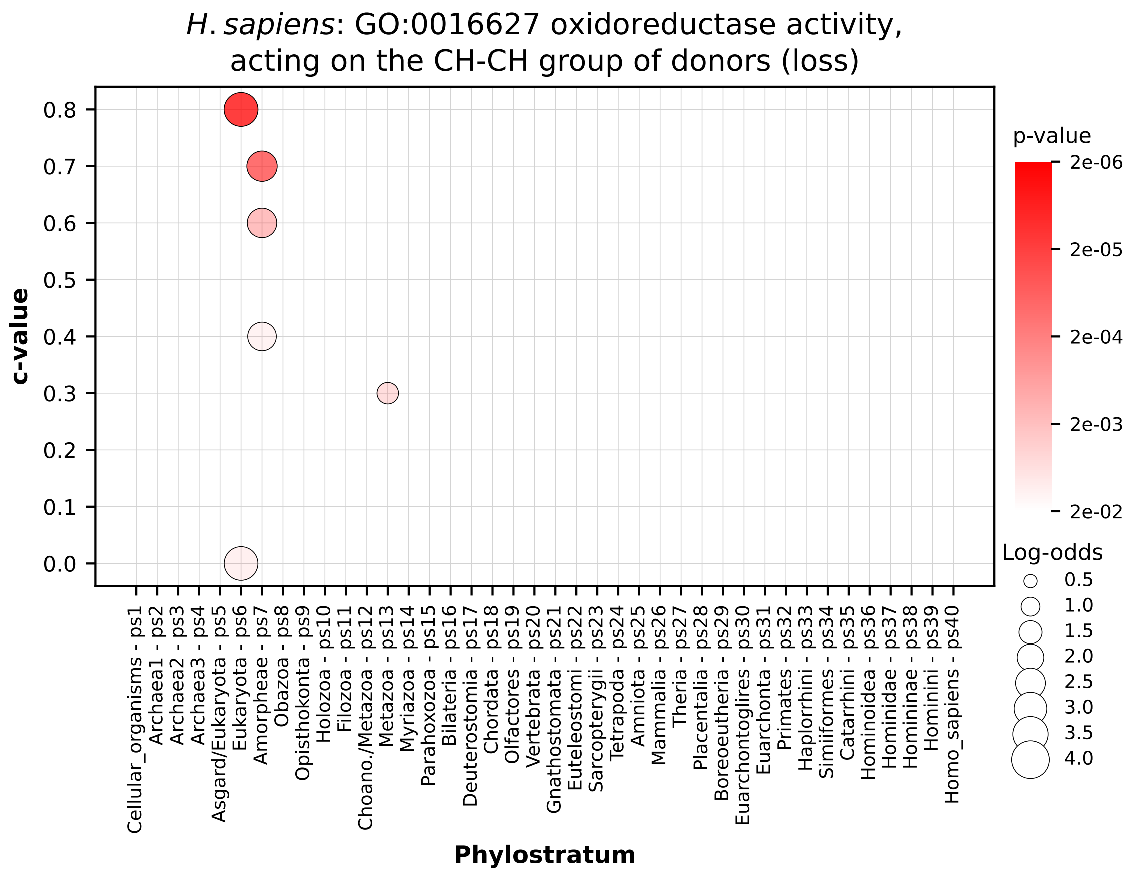

H. sapiens: GO:0016627 oxidoreductase activity, acting on the CH-CH group of donors (loss)
c-value
0.0
0.1
0.2
0.3
0.4
0.5
0.6
0.7
0.8
Phylostratum
Cellular_organisms - ps1
Archaea1 - ps2
Archaea2 - ps3
Archaea3 - ps4
Asgard/Eukaryota - ps5
Eukaryota - ps6
Amorpheae - ps7
Obazoa - ps8
Opisthokonta - ps9
Holozoa - ps10
Filozoa - ps11
Choano./Metazoa - ps12
Metazoa - ps13
Myriazoa - ps14
Parahoxozoa - ps15
Bilateria - ps16
Deuterostomia - ps17
Chordata - ps18
Olfactores - ps19
Vertebrata - ps20
Gnathostomata - ps21
Euteleostomi - ps22
Sarcopterygii - ps23
Tetrapoda - ps24
Amniota - ps25
Mammalia - ps26
Theria - ps27
Placentalia - ps28
Boreoeutheria - ps29
Euarchontoglires - ps30
Euarchonta - ps31
Primates - ps32
Haplorrhini - ps33
Simiiformes - ps34
Catarrhini - ps35
Hominoidea - ps36
Hominidae - ps37
Homininae - ps38
Hominini - ps39
Homo_sapiens - ps40
p-value
2e-06
2e-05
2e-04
2e-03
2e-02
Log-odds
0.5
1.0
1.5
2.0
2.5
3.0
3.5
4.0

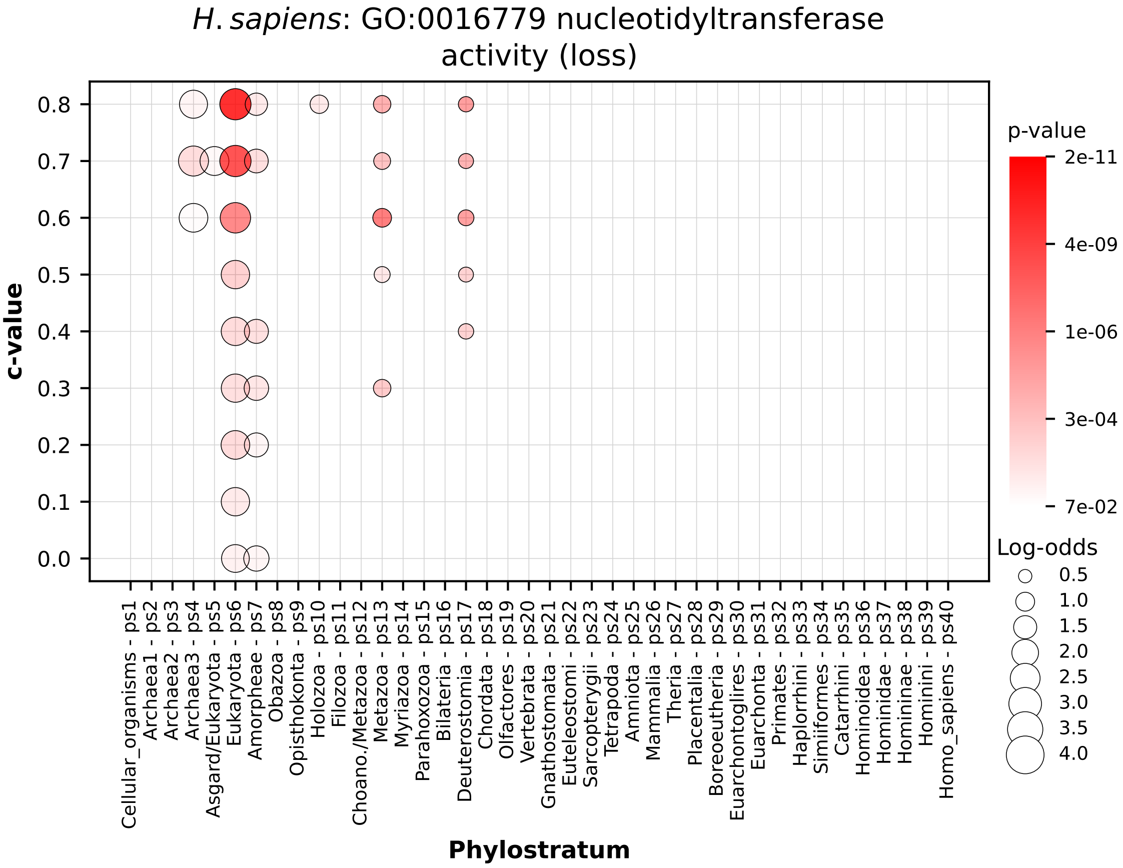

H. sapiens: GO:0016779 nucleotidyltransferase activity (loss)
c-value
0.8
0.7
0.6
0.5
0.4
0.3
0.2
0.1
0.0
Phylostratum
Cellular_organisms - ps1
Archaea1 - ps2
Archaea2 - ps3
Archaea3 - ps4
Asgard/Eukaryota - ps5
Eukaryota - ps6
Amorpheae - ps7
Obazoa - ps8
Opisthokonta - ps9
Holozoa - ps10
Filozoa - ps11
Choano./Metazoa - ps12
Metazoa - ps13
Myriazoa - ps14
Parahoxozoa - ps15
Bilateria - ps16
Deuterostomia - ps17
Chordata - ps18
Olfactores - ps19
Vertebrata - ps20
Gnathostomata - ps21
Euteleostomi - ps22
Sarcopterygii - ps23
Tetrapoda - ps24
Amniota - ps25
Mammalia - ps26
Theria - ps27
Placentalia - ps28
Boreoeutheria - ps29
Euarchontoglires - ps30
Euarchonta - ps31
Primates - ps32
Haplorrhini - ps33
Simiiformes - ps34
Catarrhini - ps35
Hominoidea - ps36
Hominidae - ps37
Homininae - ps38
Hominini - ps39
Homo_sapiens - ps40
p-value
2e-11
4e-09
1e-06
3e-04
7e-02
Log-odds
0.5
1.0
1.5
2.0
2.5
3.0
3.5
4.0

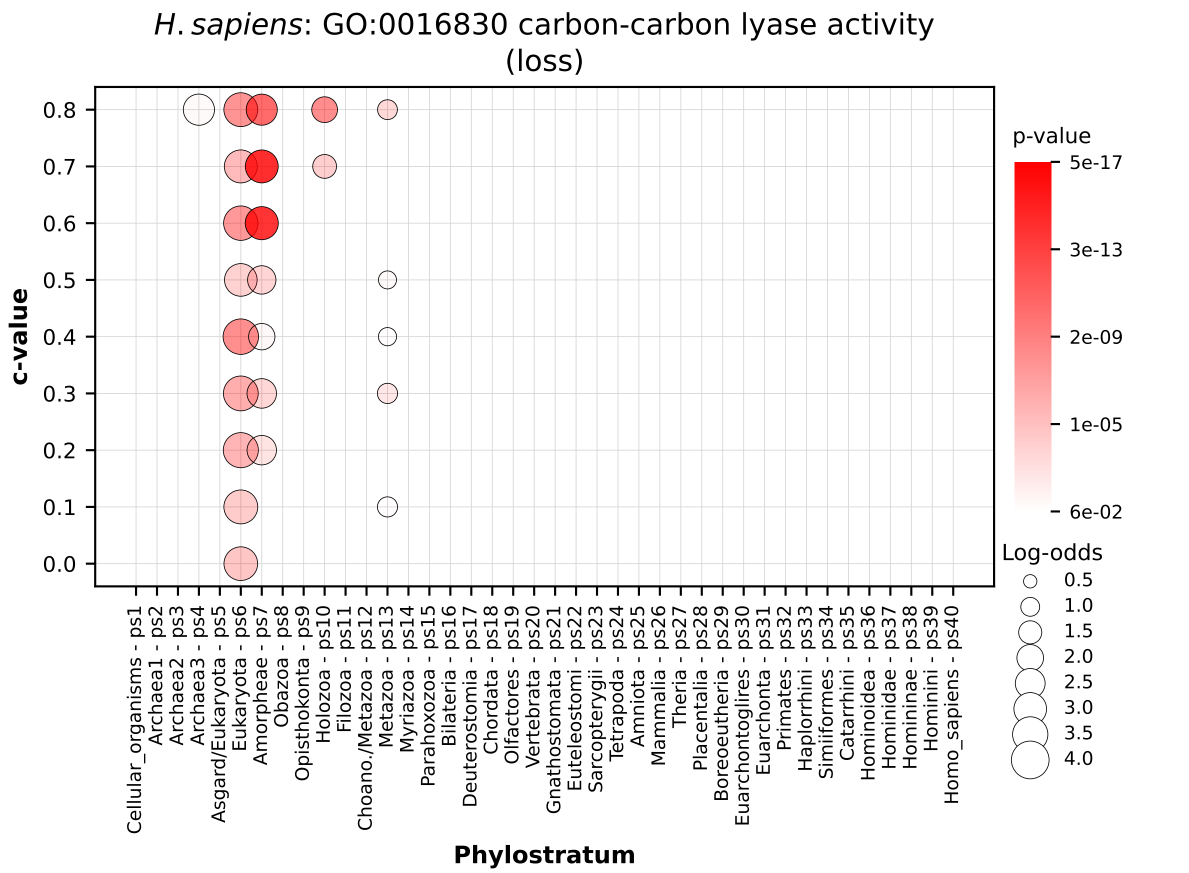

H. sapiens: GO:0016830 carbon-carbon lyase activity (loss)
c-value
0.8
0.7
0.6
0.5
0.4
0.3
0.2
0.1
0.0
Phylostratum
Cellular_organisms - ps1
Archaea1 - ps2
Archaea2 - ps3
Archaea3 - ps4
Asgard/Eukaryota - ps5
Eukaryota - ps6
Amorpheae - ps7
Obazoa - ps8
Opisthokonta - ps9
Holozoa - ps10
Filozoa - ps11
Choano./Metazoa - ps12
Metazoa - ps13
Myriazoa - ps14
Parahoxozoa - ps15
Bilateria - ps16
Deuterostomia - ps17
Chordata - ps18
Olfactores - ps19
Vertebrata - ps20
Gnathostomata - ps21
Euteleostomi - ps22
Sarcopterygii - ps23
Tetrapoda - ps24
Amniota - ps25
Mammalia - ps26
Theria - ps27
Placentalia - ps28
Boreoeutheria - ps29
Euarchontoglires - ps30
Euarchonta - ps31
Primates - ps32
Haplorrhini - ps33
Simiiformes - ps34
Catarrhini - ps35
Hominoidea - ps36
Hominidae - ps37
Homininae - ps38
Hominini - ps39
Homo_sapiens - ps40
p-value
5e-17
3e-13
2e-09
1e-05
6e-02
Log-odds
0.5
1.0
1.5
2.0
2.5
3.0
3.5
4.0

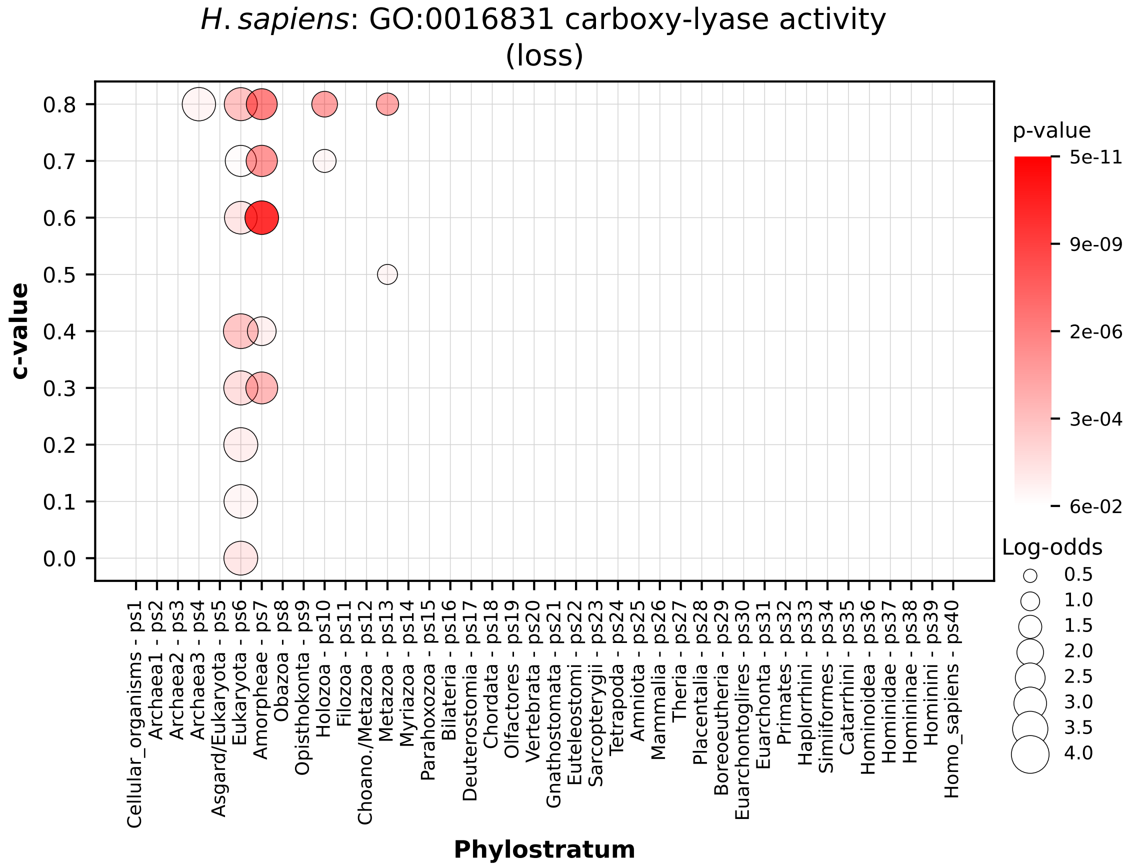

H. sapiens: GO:0016831 carboxy-lyase activity
(loss)
c-value
0.8
0.7
0.6
0.5
0.4
0.3
0.2
0.1
0.0
Phylostratum
Cellular_organisms - ps1
Archaea1 - ps2
Archaea2 - ps3
Archaea3 - ps4
Asgard/Eukaryota - ps5
Eukaryota - ps6
Amorpheae - ps7
Obazoa - ps8
Opisthokonta - ps9
Holozoa - ps10
Filozoa - ps11
Choano./Metazoa - ps12
Metazoa - ps13
Myriazoa - ps14
Parahoxozoa - ps15
Bilateria - ps16
Deuterostomia - ps17
Chordata - ps18
Olfactores - ps19
Vertebrata - ps20
Gnathostomata - ps21
Euteleostomi - ps22
Sarcopterygii - ps23
Tetrapoda - ps24
Amniota - ps25
Mammalia - ps26
Theria - ps27
Placentalia - ps28
Boreoeutheria - ps29
Euarchontoglires - ps30
Euarchonta - ps31
Primates - ps32
Haplorrhini - ps33
Simiiformes - ps34
Catarrhini - ps35
Hominoidea - ps36
Hominidae - ps37
Homininae - ps38
Hominini - ps39
Homo_sapiens - ps40
p-value
5e-11
9e-09
2e-06
3e-04
6e-02
Log-odds
0.5
1.0
1.5
2.0
2.5
3.0
3.5
4.0

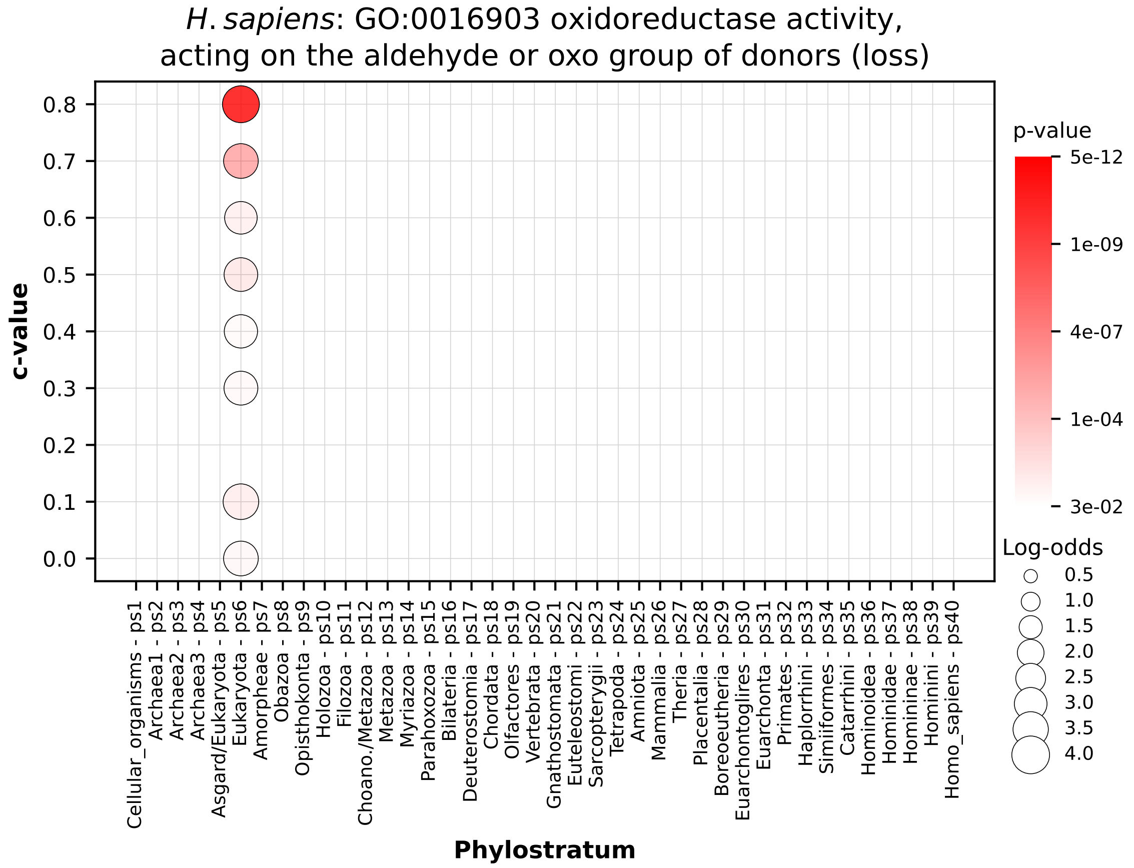

H. sapiens: GO:0016903 oxidoreductase activity, acting on the aldehyde or oxo group of donors (loss)
c-value
0.0
0.1
0.2
0.3
0.4
0.5
0.6
0.7
0.8
Phylostratum
Cellular_organisms - ps1
Archaea1 - ps2
Archaea2 - ps3
Archaea3 - ps4
Asgard/Eukaryota - ps5
Eukaryota - ps6
Amorpheae - ps7
Obazoa - ps8
Opisthokonta - ps9
Holozoa - ps10
Filozoa - ps11
Choano./Metazoa - ps12
Metazoa - ps13
Myriazoa - ps14
Parahoxozoa - ps15
Bilateria - ps16
Deuterostomia - ps17
Chordata - ps18
Olfactores - ps19
Vertebrata - ps20
Gnathostomata - ps21
Euteleostomi - ps22
Sarcopterygii - ps23
Tetrapoda - ps24
Amniota - ps25
Mammalia - ps26
Theria - ps27
Placentalia - ps28
Boreoeutheria - ps29
Euarchontoglires - ps30
Euarchonta - ps31
Primates - ps32
Haplorrhini - ps33
Simiiformes - ps34
Catarrhini - ps35
Hominoidea - ps36
Hominidae - ps37
Homininae - ps38
Hominini - ps39
Homo_sapiens - ps40
p-value
5e-12
1e-09
4e-07
1e-04
3e-02
Log-odds
0.5
1.0
1.5
2.0
2.5
3.0
3.5
4.0

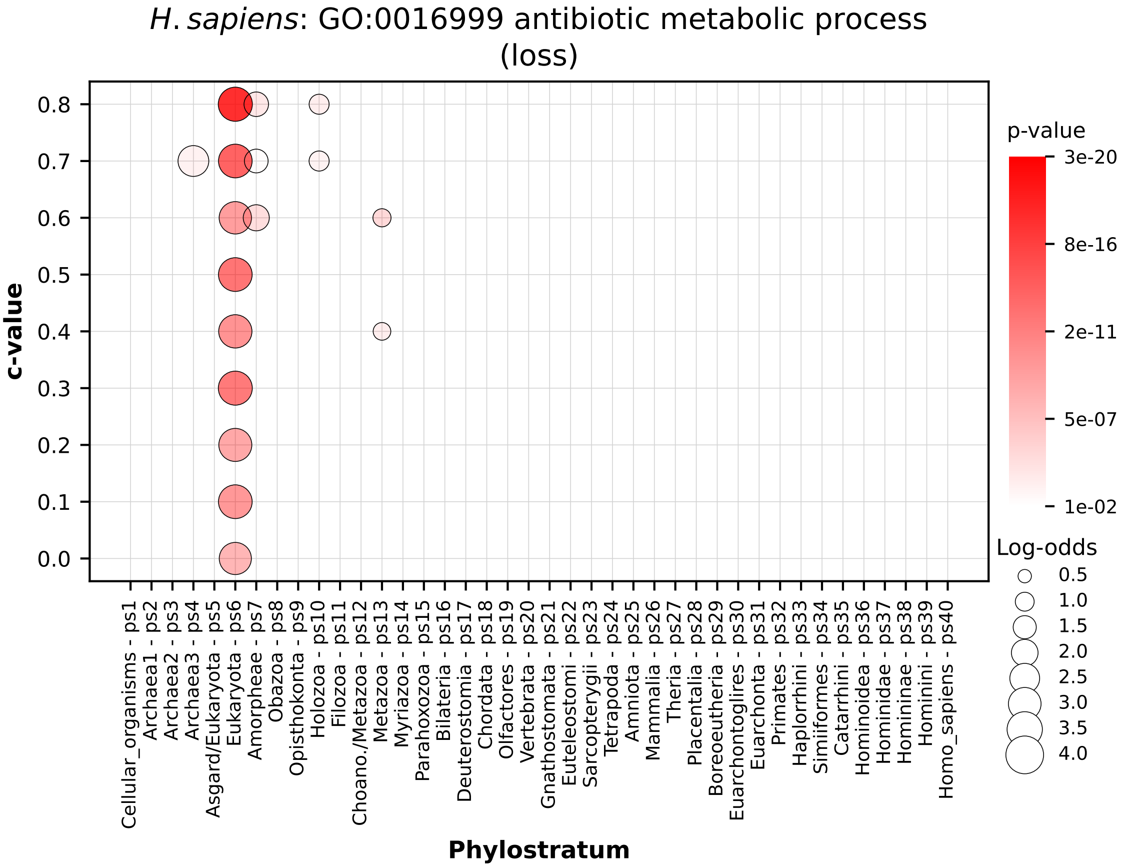
H. sapiens: GO:0016999 antibiotic metabolic process (loss)
c-value
0.0
0.1
0.2
0.3
0.4
0.5
0.6
0.7
0.8
p-value
3e-20
8e-16
2e-11
5e-07
1e-02
Log-odds
0.5
1.0
1.5
2.0
2.5
3.0
3.5
4.0
Cellular_organisms - ps1
Archaea1 - ps2
Archaea2 - ps3
Archaea3 - ps4
Asgard/Eukaryota - ps5
Eukaryota - ps6
Amorpheae - ps7
Obazoa - ps8
Opisthokonta - ps9
Holozoa - ps10
Filozoa - ps11
Choano./Metazoa - ps12
Metazoa - ps13
Myriazoa - ps14
Parahoxozoa - ps15
Bilateria - ps16
Deuterostomia - ps17
Chordata - ps18
Olfactores - ps19
Vertebrata - ps20
Gnathostomata - ps21
Euteleostomi - ps22
Sarcopterygii - ps23
Tetrapoda - ps24
Amniota - ps25
Mammalia - ps26
Theria - ps27
Placentalia - ps28
Boreoeutheria - ps29
Euarchontoglires - ps30
Euarchonta - ps31
Primates - ps32
Haplorrhini - ps33
Simiiformes - ps34
Catarrhini - ps35
Hominoidea - ps36
Hominidae - ps37
Homininae - ps38
Hominini - ps39
Homo_sapiens - ps40
Phylostratum

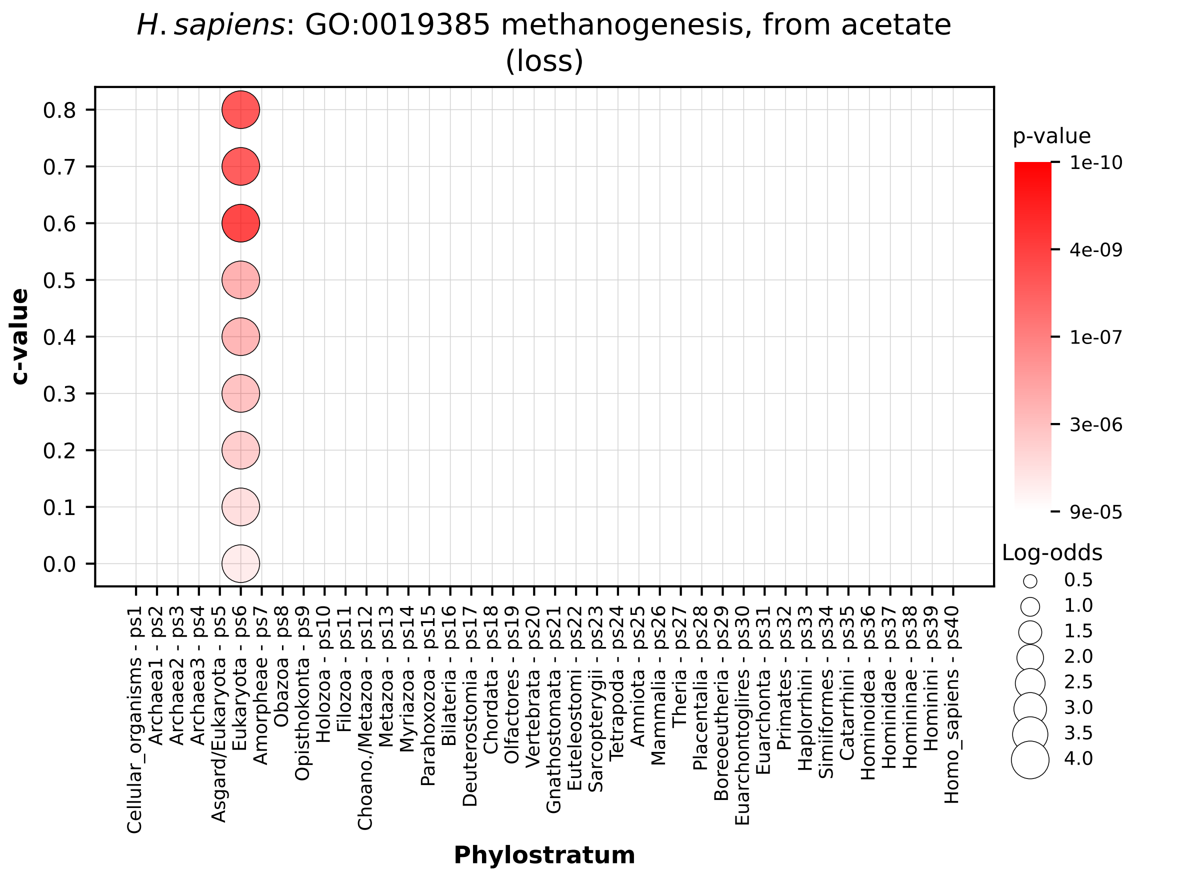
H. sapiens: GO:0019385 methanogenesis, from acetate (loss)
c-value
0.8
0.7
0.6
0.5
0.4
0.3
0.2
0.1
0.0
p-value
1e-10
4e-09
1e-07
3e-06
9e-05
Log-odds
0.5
1.0
1.5
2.0
2.5
3.0
3.5
4.0
Cellular_organisms - ps1
Archaea1 - ps2
Archaea2 - ps3
Archaea3 - ps4
Asgard/Eukaryota - ps5
Eukaryota - ps6
Amorpheae - ps7
Obazoa - ps8
Opisthokonta - ps9
Holozoa - ps10
Filozoa - ps11
Choano./Metazoa - ps12
Metazoa - ps13
Myriazoa - ps14
Parahoxozoa - ps15
Bilateria - ps16
Deuterostomia - ps17
Chordata - ps18
Olfactores - ps19
Vertebrata - ps20
Gnathostomata - ps21
Euteleostomi - ps22
Sarcopterygii - ps23
Tetrapoda - ps24
Amniota - ps25
Mammalia - ps26
Theria - ps27
Placentalia - ps28
Boreoeutheria - ps29
Euarchontoglires - ps30
Euarchonta - ps31
Primates - ps32
Haplorrhini - ps33
Simiiformes - ps34
Catarrhini - ps35
Hominoidea - ps36
Hominidae - ps37
Homininae - ps38
Hominini - ps39
Homo_sapiens - ps40
Phylostratum

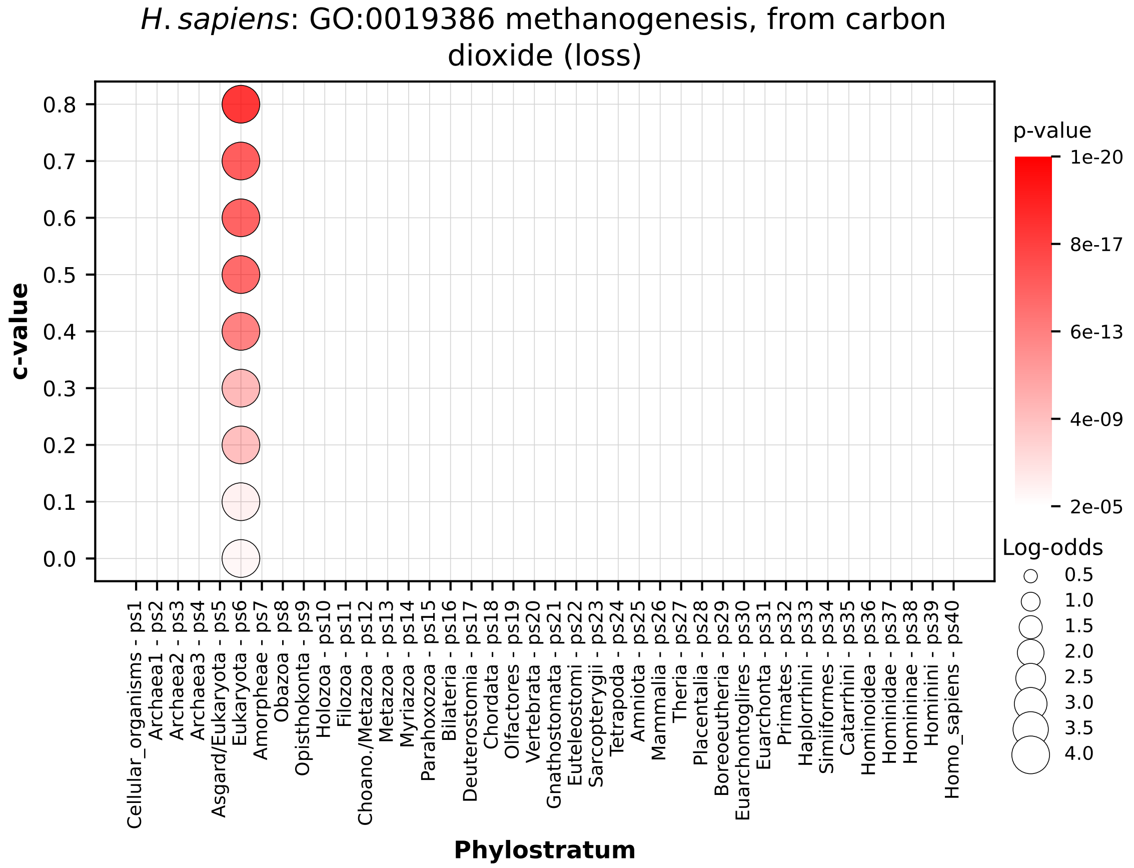

H. sapiens: GO:0019386 methanogenesis, from carbon dioxide (loss)
c-value
0.8
0.7
0.6
0.5
0.4
0.3
0.2
0.1
0.0
p-value
1e-20
8e-17
6e-13
4e-09
2e-05
Log-odds
0.5
1.0
1.5
2.0
2.5
3.0
3.5
4.0
Cellular_organisms - ps1
Archaea1 - ps2
Archaea2 - ps3
Archaea3 - ps4
Asgard/Eukaryota - ps5
Eukaryota - ps6
Amorpheae - ps7
Obazoa - ps8
Opisthokonta - ps9
Holozoa - ps10
Filozoa - ps11
Choano./Metazoa - ps12
Metazoa - ps13
Myriazoa - ps14
Parahoxozoa - ps15
Bilateria - ps16
Deuterostomia - ps17
Chordata - ps18
Olfactores - ps19
Vertebrata - ps20
Gnathostomata - ps21
Euteleostomi - ps22
Sarcopterygii - ps23
Tetrapoda - ps24
Amniota - ps25
Mammalia - ps26
Theria - ps27
Placentalia - ps28
Boreoeutheria - ps29
Euarchontoglires - ps30
Euarchonta - ps31
Primates - ps32
Haplorrhini - ps33
Simiiformes - ps34
Catarrhini - ps35
Hominoidea - ps36
Hominidae - ps37
Homininae - ps38
Hominini - ps39
Homo_sapiens - ps40
Phylostratum

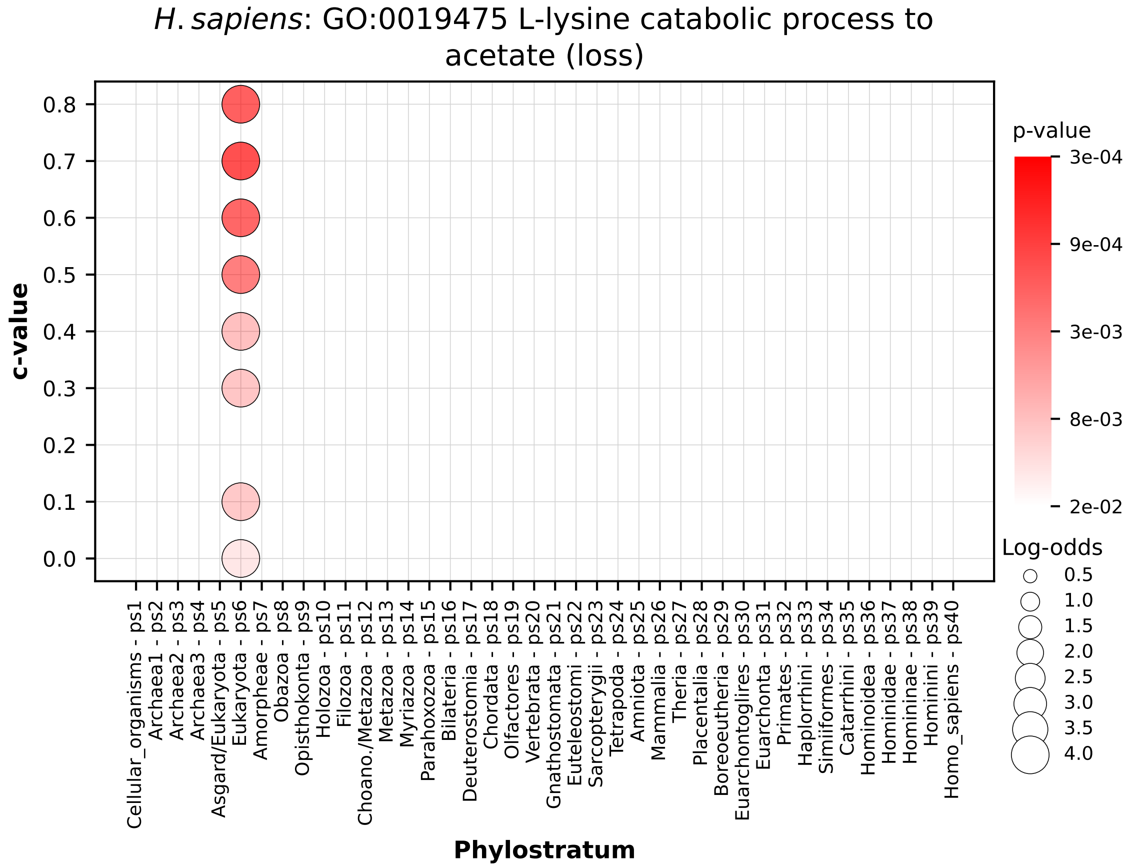

H. sapiens: GO:0019475 L-lysine catabolic process to acetate (loss)
c-value
0.8
0.7
0.6
0.5
0.4
0.3
0.2
0.1
0.0
Phylostratum
Cellular_organisms - ps1
Archaea1 - ps2
Archaea2 - ps3
Archaea3 - ps4
Asgard/Eukaryota - ps5
Eukaryota - ps6
Amorpheae - ps7
Obazoa - ps8
Opisthokonta - ps9
Holozoa - ps10
Filozoa - ps11
Choano./Metazoa - ps12
Metazoa - ps13
Myriazoa - ps14
Parahoxozoa - ps15
Bilateria - ps16
Deuterostomia - ps17
Chordata - ps18
Olfactores - ps19
Vertebrata - ps20
Gnathostomata - ps21
Euteleostomi - ps22
Sarcopterygii - ps23
Tetrapoda - ps24
Amniota - ps25
Mammalia - ps26
Theria - ps27
Placentalia - ps28
Boreoeutheria - ps29
Euarchontoglires - ps30
Euarchonta - ps31
Primates - ps32
Haplorrhini - ps33
Simiiformes - ps34
Catarrhini - ps35
Hominoidea - ps36
Hominidae - ps37
Homininae - ps38
Hominini - ps39
Homo_sapiens - ps40
p-value
3e-04
9e-04
3e-03
8e-03
2e-02
Log-odds
0.5
1.0
1.5
2.0
2.5
3.0
3.5
4.0

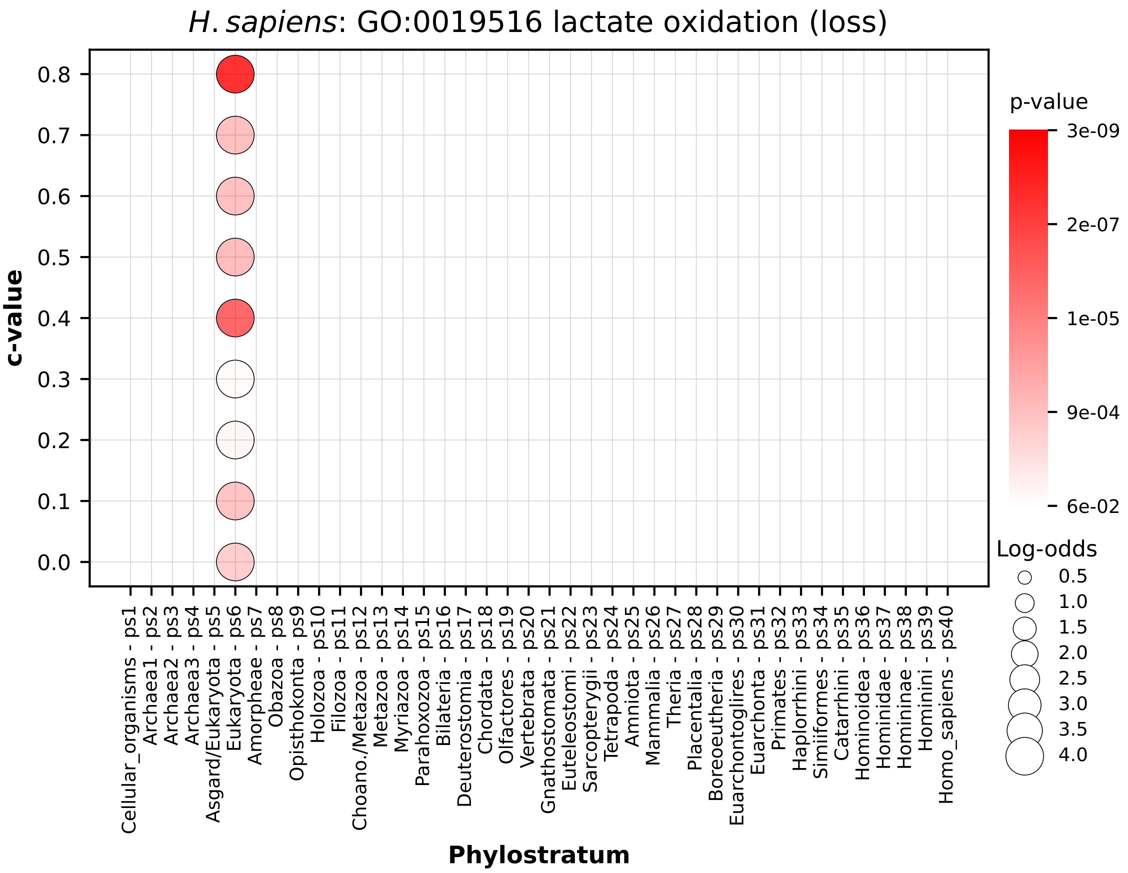

H. sapiens: GO:0019516 lactate oxidation (loss)
c-value
0.0
0.1
0.2
0.3
0.4
0.5
0.6
0.7
0.8
Phylostratum
Cellular_organisms - ps1
Archaea1 - ps2
Archaea2 - ps3
Archaea3 - ps4
Asgard/Eukaryota - ps5
Eukaryota - ps6
Amorpheae - ps7
Obazoa - ps8
Opisthokonta - ps9
Holozoa - ps10
Filozoa - ps11
Choano./Metazoa - ps12
Metazoa - ps13
Myriazoa - ps14
Parahoxozoa - ps15
Bilateria - ps16
Deuterostomia - ps17
Chordata - ps18
Olfactores - ps19
Vertebrata - ps20
Gnathostomata - ps21
Euteleostomi - ps22
Sarcopterygii - ps23
Tetrapoda - ps24
Amniota - ps25
Mammalia - ps26
Theria - ps27
Placentalia - ps28
Boreoeutheria - ps29
Euarchontoglires - ps30
Euarchonta - ps31
Primates - ps32
Haplorrhini - ps33
Simiiformes - ps34
Catarrhini - ps35
Hominoidea - ps36
Hominidae - ps37
Homininae - ps38
Hominini - ps39
Homo_sapiens - ps40
p-value
3e-09
2e-07
1e-05
9e-04
6e-02
Log-odds
0.5
1.0
1.5
2.0
2.5
3.0
3.5
4.0

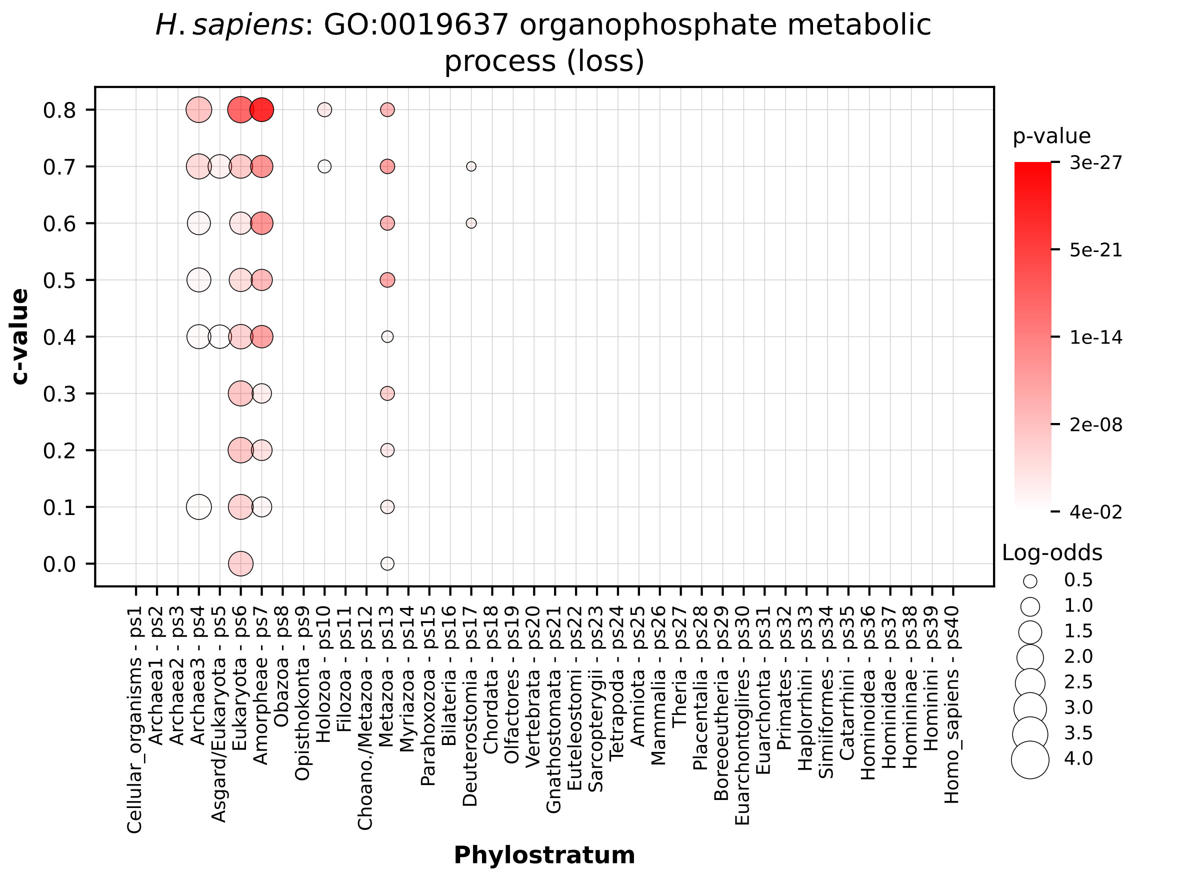

H. sapiens: GO:0019637 organophosphate metabolic process (loss)
c-value
0.8
0.7
0.6
0.5
0.4
0.3
0.2
0.1
0.0
p-value
3e-27
5e-21
1e-14
2e-08
4e-02
Log-odds
0.5
1.0
1.5
2.0
2.5
3.0
3.5
4.0
Cellular_organisms - ps1
Archaea1 - ps2
Archaea2 - ps3
Archaea3 - ps4
Asgard/Eukaryota - ps5
Eukaryota - ps6
Amorpheae - ps7
Obazoa - ps8
Opisthokonta - ps9
Holozoa - ps10
Filozoa - ps11
Choano./Metazoa - ps12
Metazoa - ps13
Myriazoa - ps14
Parahoxozoa - ps15
Bilateria - ps16
Deuterostomia - ps17
Chordata - ps18
Olfactores - ps19
Vertebrata - ps20
Gnathostomata - ps21
Euteleostomi - ps22
Sarcopterygii - ps23
Tetrapoda - ps24
Amniota - ps25
Mammalia - ps26
Theria - ps27
Placentalia - ps28
Boreoeutheria - ps29
Euarchontoglires - ps30
Euarchonta - ps31
Primates - ps32
Haplorrhini - ps33
Simiiformes - ps34
Catarrhini - ps35
Hominoidea - ps36
Hominidae - ps37
Homininae - ps38
Hominini - ps39
Homo_sapiens - ps40
Phylostratum

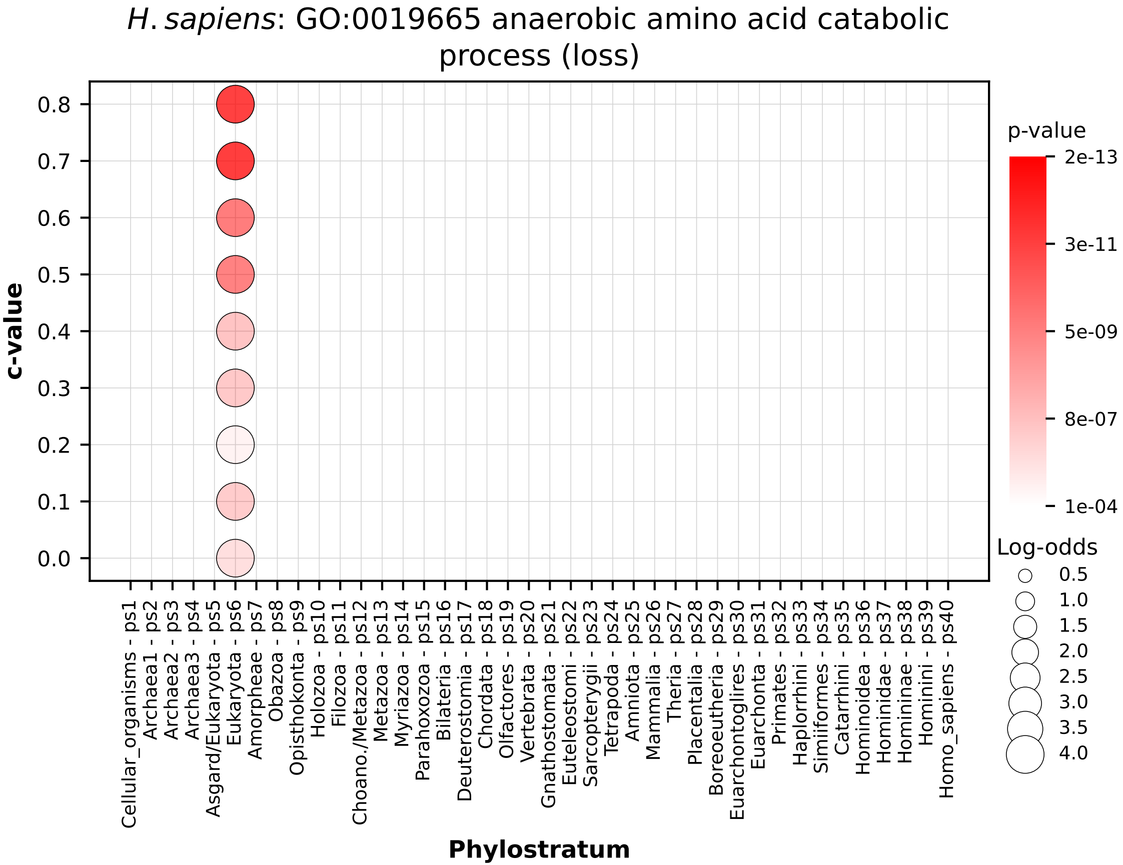

H. sapiens: GO:0019665 anaerobic amino acid catabolic process (loss)
c-value
0.0
0.1
0.2
0.3
0.4
0.5
0.6
0.7
0.8
Phylostratum
Cellular_organisms - ps1
Archaea1 - ps2
Archaea2 - ps3
Archaea3 - ps4
Asgard/Eukaryota - ps5
Eukaryota - ps6
Amorpheae - ps7
Obazoa - ps8
Opisthokonta - ps9
Holozoa - ps10
Filozoa - ps11
Choano./Metazoa - ps12
Metazoa - ps13
Myriazoa - ps14
Parahoxozoa - ps15
Bilateria - ps16
Deuterostomia - ps17
Chordata - ps18
Olfactores - ps19
Vertebrata - ps20
Gnathostomata - ps21
Euteleostomi - ps22
Sarcopterygii - ps23
Tetrapoda - ps24
Amniota - ps25
Mammalia - ps26
Theria - ps27
Placentalia - ps28
Boreoeutheria - ps29
Euarchontoglires - ps30
Euarchonta - ps31
Primates - ps32
Haplorrhini - ps33
Simiiformes - ps34
Catarrhini - ps35
Hominoidea - ps36
Hominidae - ps37
Homininae - ps38
Hominini - ps39
Homo_sapiens - ps40
p-value
2e-13
3e-11
5e-09
8e-07
1e-04
Log-odds
0.5
1.0
1.5
2.0
2.5
3.0
3.5
4.0

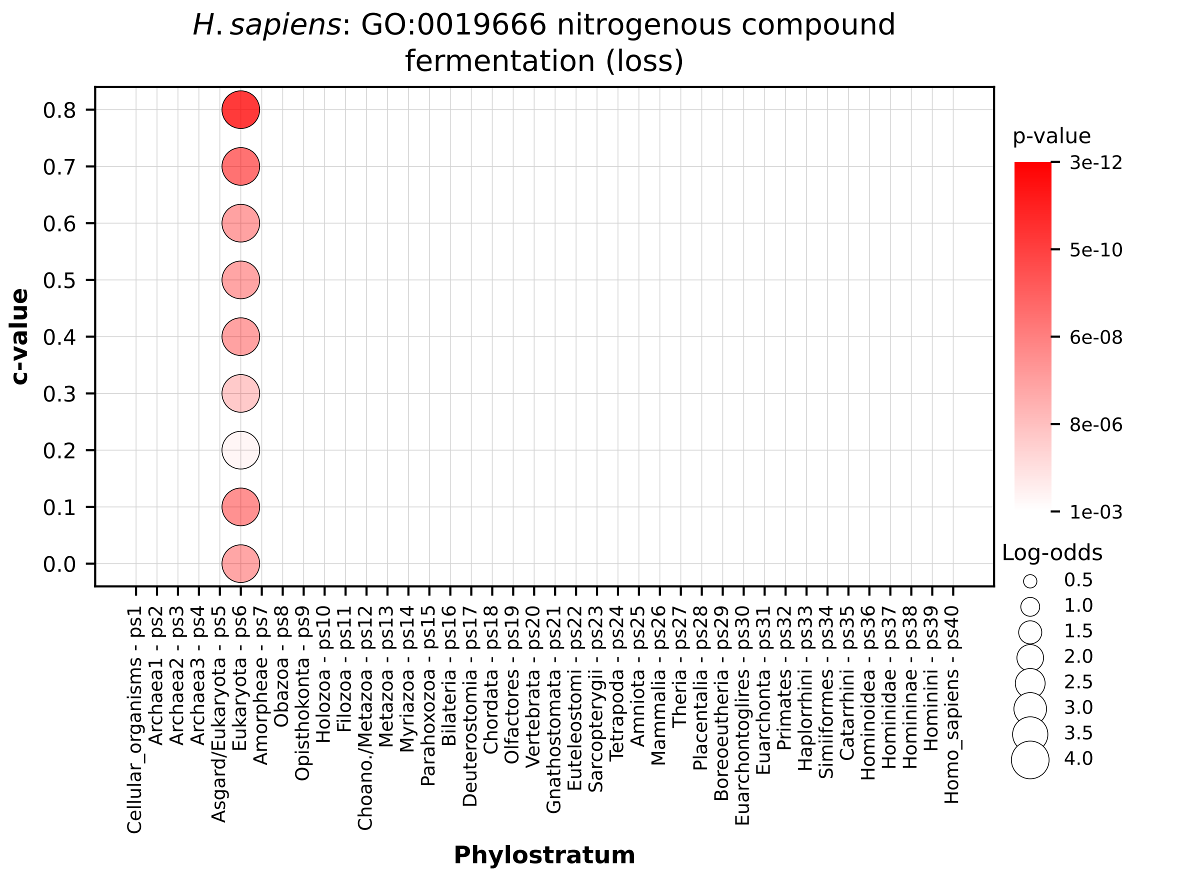

H. sapiens: GO:0019666 nitrogenous compound fermentation (loss)
c-value
0.8
0.7
0.6
0.5
0.4
0.3
0.2
0.1
0.0
Phylostratum
Cellular_organisms - ps1
Archaea1 - ps2
Archaea2 - ps3
Archaea3 - ps4
Asgard/Eukaryota - ps5
Eukaryota - ps6
Amorpheae - ps7
Obazoa - ps8
Opisthokonta - ps9
Holozoa - ps10
Filozoa - ps11
Choano./Metazoa - ps12
Metazoa - ps13
Myriazoa - ps14
Parahoxozoa - ps15
Bilateria - ps16
Deuterostomia - ps17
Chordata - ps18
Olfactores - ps19
Vertebrata - ps20
Gnathostomata - ps21
Euteleostomi - ps22
Sarcopterygii - ps23
Tetrapoda - ps24
Amniota - ps25
Mammalia - ps26
Theria - ps27
Placentalia - ps28
Boreoeutheria - ps29
Euarchontoglires - ps30
Euarchonta - ps31
Primates - ps32
Haplorrhini - ps33
Simiiformes - ps34
Catarrhini - ps35
Hominoidea - ps36
Hominidae - ps37
Homininae - ps38
Hominini - ps39
Homo_sapiens - ps40
p-value
3e-12
5e-10
6e-08
8e-06
1e-03
Log-odds
0.5
1.0
1.5
2.0
2.5
3.0
3.5
4.0

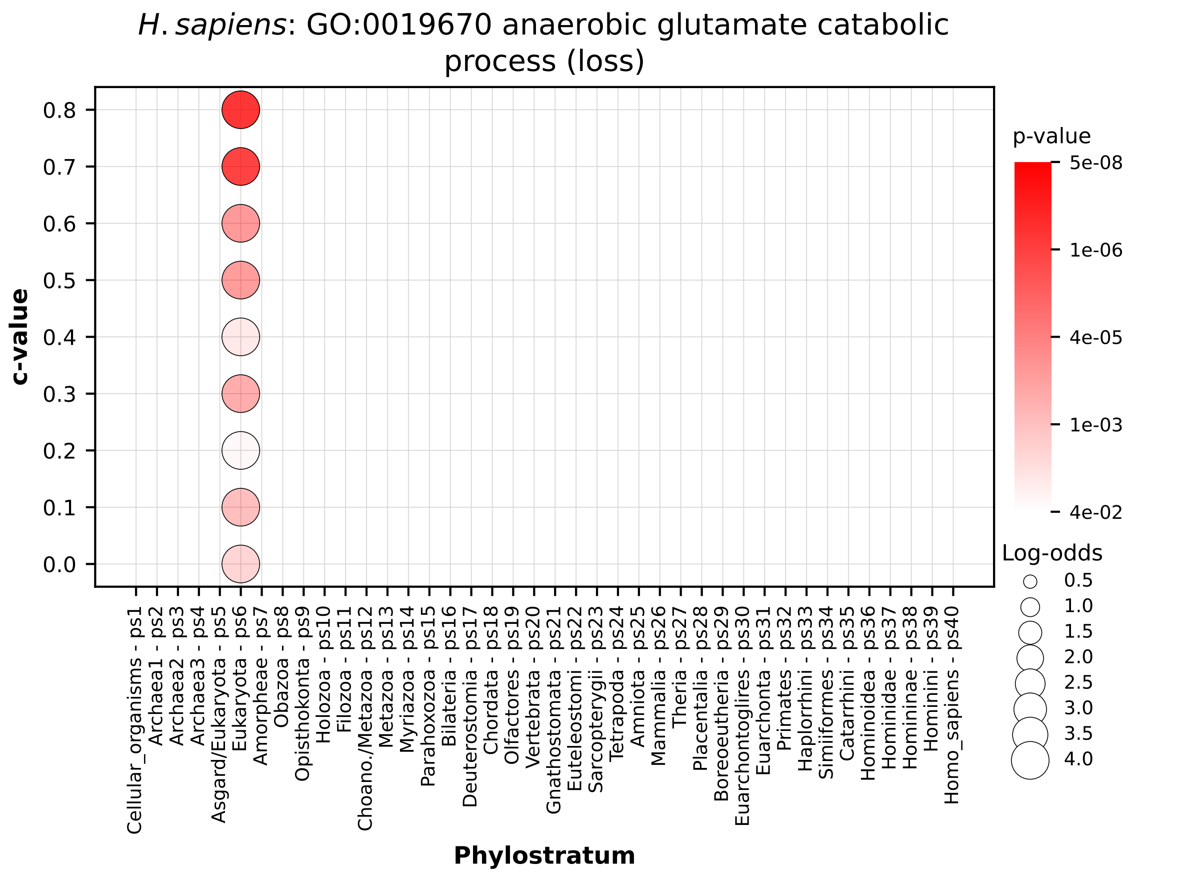

H. sapiens: GO:0019670 anaerobic glutamate catabolic process (loss)
c-value
0.0
0.1
0.2
0.3
0.4
0.5
0.6
0.7
0.8
Phylostratum
Cellular_organisms - ps1
Archaea1 - ps2
Archaea2 - ps3
Archaea3 - ps4
Asgard/Eukaryota - ps5
Eukaryota - ps6
Amorpheae - ps7
Obazoa - ps8
Opisthokonta - ps9
Holozoa - ps10
Filozoa - ps11
Choano./Metazoa - ps12
Metazoa - ps13
Myriazoa - ps14
Parahoxozoa - ps15
Bilateria - ps16
Deuterostomia - ps17
Chordata - ps18
Olfactores - ps19
Vertebrata - ps20
Gnathostomata - ps21
Euteleostomi - ps22
Sarcopterygii - ps23
Tetrapoda - ps24
Amniota - ps25
Mammalia - ps26
Theria - ps27
Placentalia - ps28
Boreoeutheria - ps29
Euarchontoglires - ps30
Euarchonta - ps31
Primates - ps32
Haplorrhini - ps33
Simiiformes - ps34
Catarrhini - ps35
Hominoidea - ps36
Hominidae - ps37
Homininae - ps38
Hominini - ps39
Homo_sapiens - ps40
p-value
5e-08
1e-06
4e-05
1e-03
4e-02
Log-odds
0.5
1.0
1.5
2.0
2.5
3.0
3.5
4.0

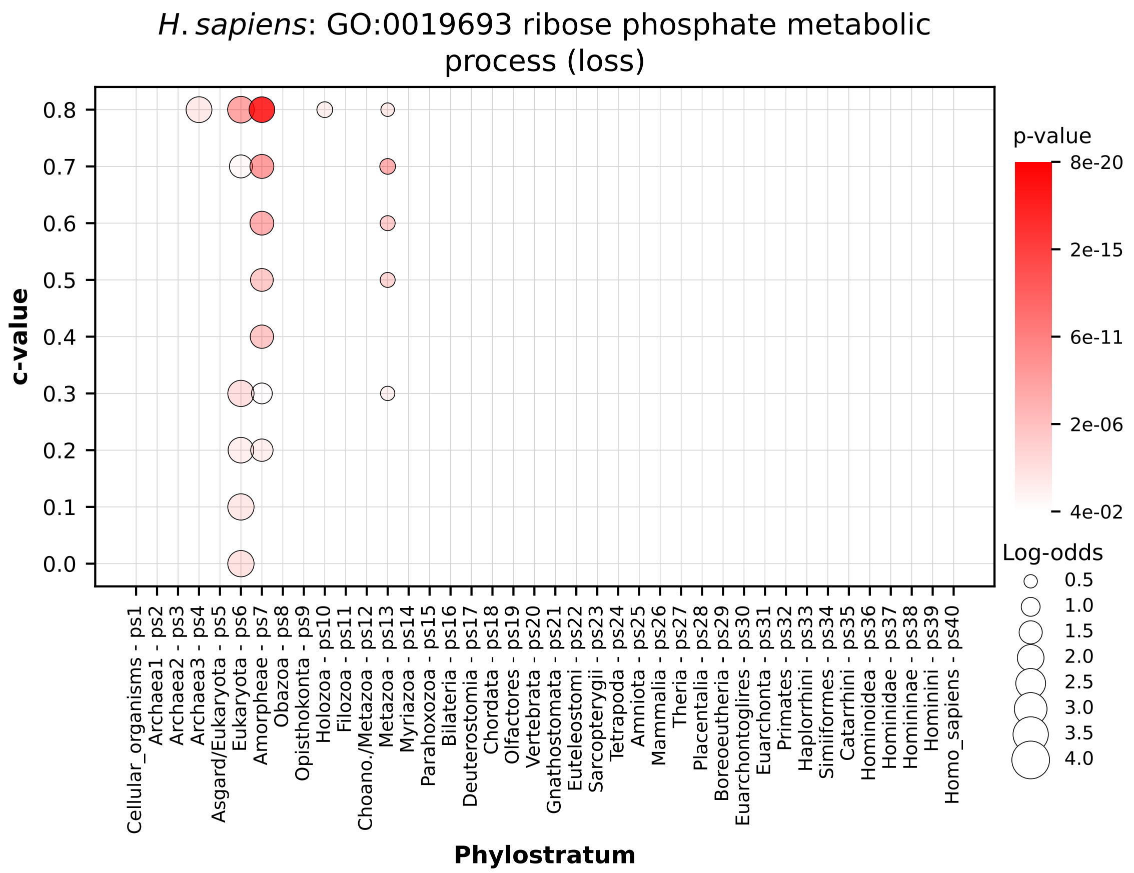

H. sapiens: GO:0019693 ribose phosphate metabolic process (loss)
c-value
0.8
0.7
0.6
0.5
0.4
0.3
0.2
0.1
0.0
p-value
8e-20
2e-15
6e-11
2e-06
4e-02
Log-odds
0.5
1.0
1.5
2.0
2.5
3.0
3.5
4.0
Cellular_organisms - ps1
Archaea1 - ps2
Archaea2 - ps3
Archaea3 - ps4
Asgard/Eukaryota - ps5
Eukaryota - ps6
Amorpheae - ps7
Obazoa - ps8
Opisthokonta - ps9
Holozoa - ps10
Filozoa - ps11
Choano./Metazoa - ps12
Metazoa - ps13
Myriazoa - ps14
Parahoxozoa - ps15
Bilateria - ps16
Deuterostomia - ps17
Chordata - ps18
Olfactores - ps19
Vertebrata - ps20
Gnathostomata - ps21
Euteleostomi - ps22
Sarcopterygii - ps23
Tetrapoda - ps24
Amniota - ps25
Mammalia - ps26
Theria - ps27
Placentalia - ps28
Boreoeutheria - ps29
Euarchontoglires - ps30
Euarchonta - ps31
Primates - ps32
Haplorrhini - ps33
Simiiformes - ps34
Catarrhini - ps35
Hominoidea - ps36
Hominidae - ps37
Homininae - ps38
Hominini - ps39
Homo_sapiens - ps40
Phylostratum

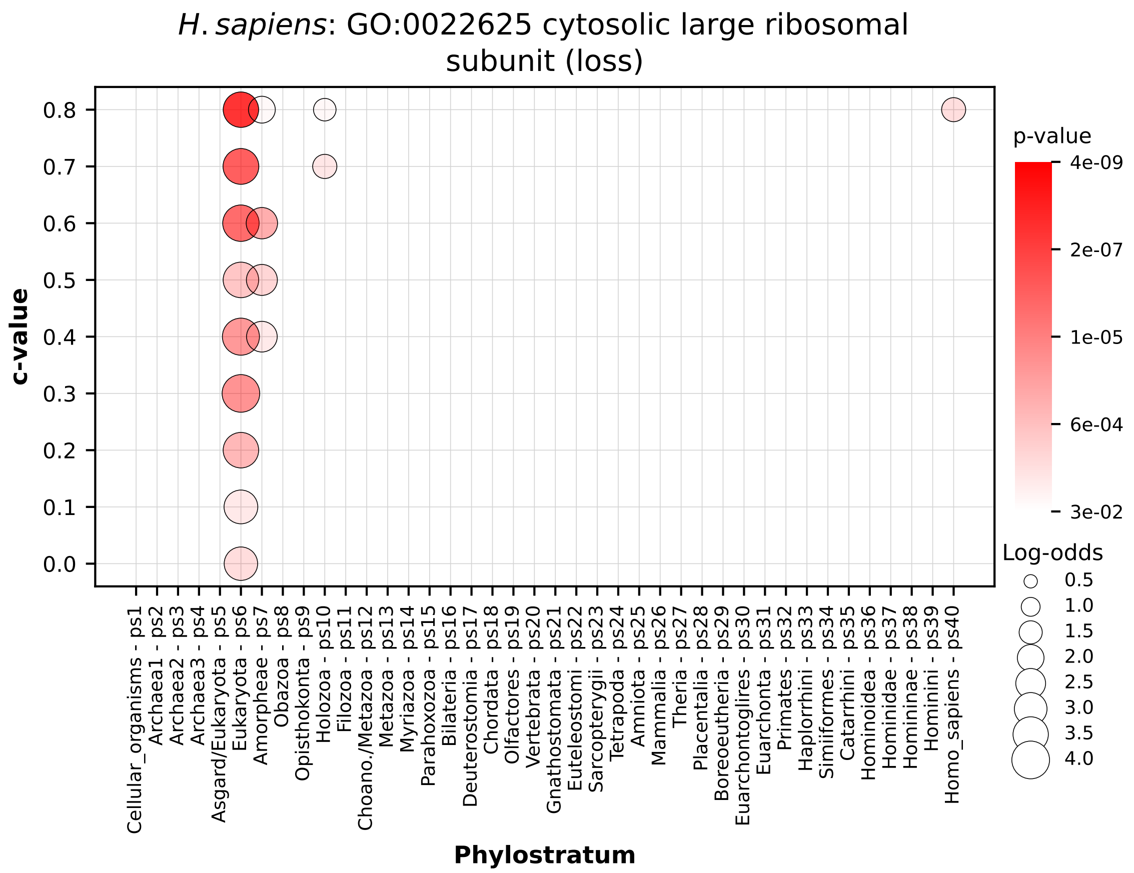

H. sapiens: GO:0022625 cytosolic large ribosomal subunit (loss)
c-value
0.0
0.1
0.2
0.3
0.4
0.5
0.6
0.7
0.8
p-value
4e-09
2e-07
1e-05
6e-04
3e-02
Log-odds
0.5
1.0
1.5
2.0
2.5
3.0
3.5
4.0
Cellular_organisms - ps1
Archaea1 - ps2
Archaea2 - ps3
Archaea3 - ps4
Asgard/Eukaryota - ps5
Eukaryota - ps6
Amorpheae - ps7
Obazoa - ps8
Opisthokonta - ps9
Holozoa - ps10
Filozoa - ps11
Choano./Metazoa - ps12
Metazoa - ps13
Myriazoa - ps14
Parahoxozoa - ps15
Bilateria - ps16
Deuterostomia - ps17
Chordata - ps18
Olfactores - ps19
Vertebrata - ps20
Gnathostomata - ps21
Euteleostomi - ps22
Sarcopterygii - ps23
Tetrapoda - ps24
Amniota - ps25
Mammalia - ps26
Theria - ps27
Placentalia - ps28
Boreoeutheria - ps29
Euarchontoglires - ps30
Euarchonta - ps31
Primates - ps32
Haplorrhini - ps33
Simiiformes - ps34
Catarrhini - ps35
Hominoidea - ps36
Hominidae - ps37
Homininae - ps38
Hominini - ps39
Homo_sapiens - ps40
Phylostratum

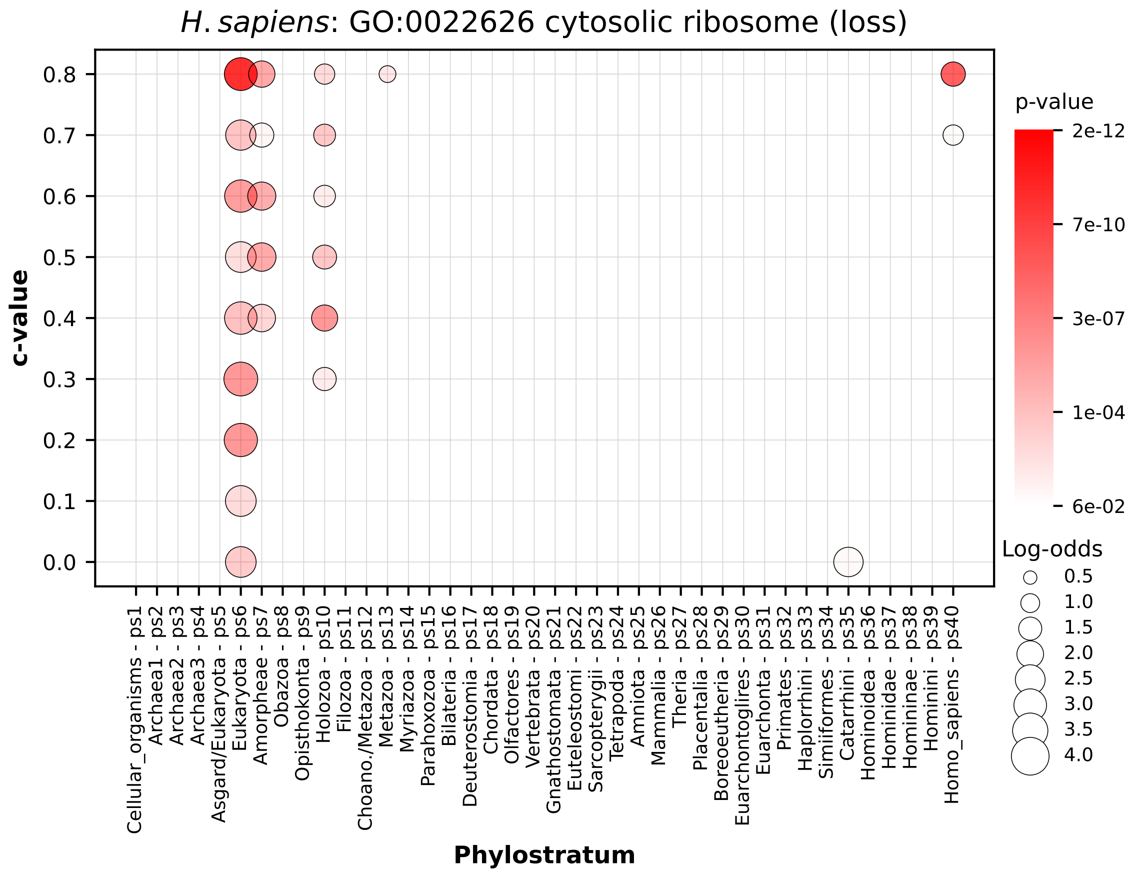
H. sapiens: GO:0022626 cytosolic ribosome (loss)
c-value
0.8
0.7
0.6
0.5
0.4
0.3
0.2
0.1
0.0
p-value
2e-12
7e-10
3e-07
1e-04
6e-02
Log-odds
0.5
1.0
1.5
2.0
2.5
3.0
3.5
4.0
Cellular_organisms - ps1
Archaea1 - ps2
Archaea2 - ps3
Archaea3 - ps4
Asgard/Eukaryota - ps5
Eukaryota - ps6
Amorpheae - ps7
Obazoa - ps8
Opisthokonta - ps9
Holozoa - ps10
Filozoa - ps11
Choano./Metazoa - ps12
Metazoa - ps13
Myriazoa - ps14
Parahoxozoa - ps15
Bilateria - ps16
Deuterostomia - ps17
Chordata - ps18
Olfactores - ps19
Vertebrata - ps20
Gnathostomata - ps21
Euteleostomi - ps22
Sarcopterygii - ps23
Tetrapoda - ps24
Amniota - ps25
Mammalia - ps26
Theria - ps27
Placentalia - ps28
Boreoeutheria - ps29
Euarchontoglires - ps30
Euarchonta - ps31
Primates - ps32
Haplorrhini - ps33
Simiiformes - ps34
Catarrhini - ps35
Hominoidea - ps36
Hominidae - ps37
Homininae - ps38
Hominini - ps39
Homo_sapiens - ps40
Phylostratum

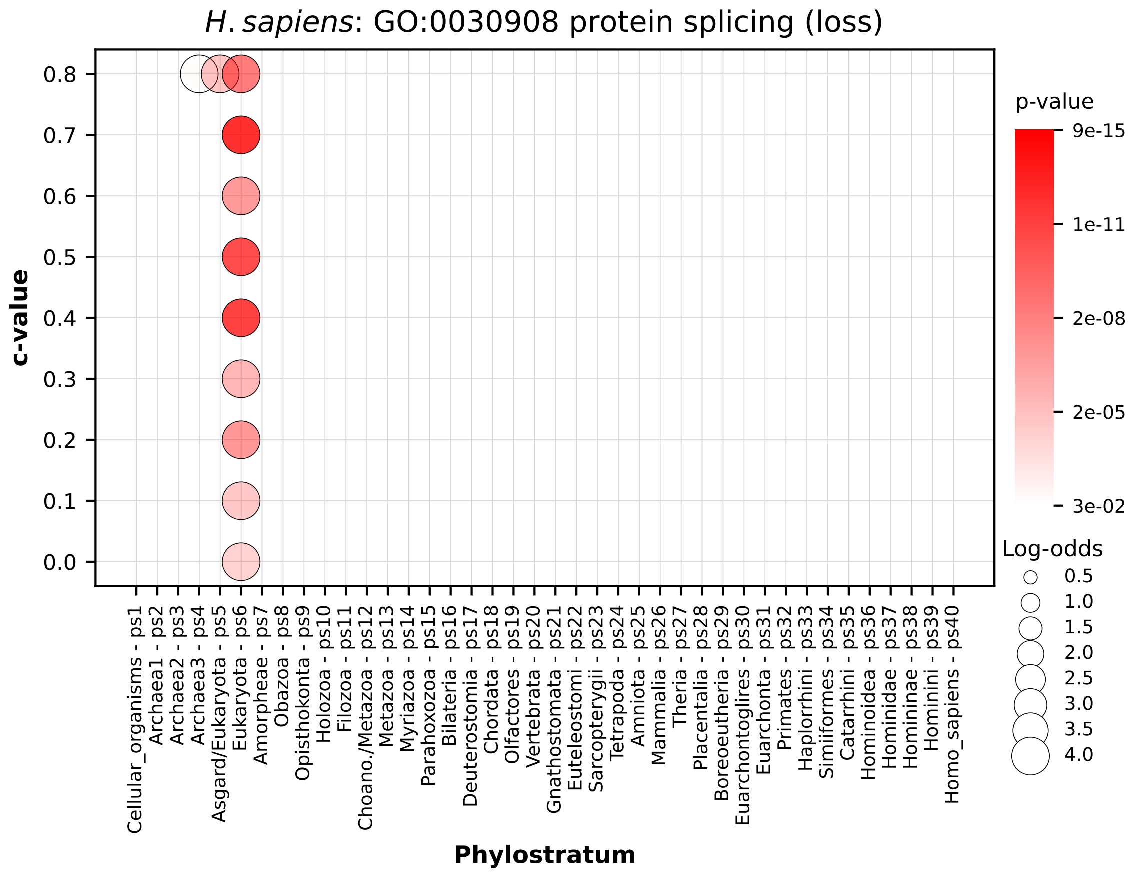

H. sapiens: GO:0030908 protein splicing (loss)
c-value
0.0
0.1
0.2
0.3
0.4
0.5
0.6
0.7
0.8
Phylostratum
Cellular_organisms - ps1
Archaea1 - ps2
Archaea2 - ps3
Archaea3 - ps4
Asgard/Eukaryota - ps5
Eukaryota - ps6
Amorpheae - ps7
Obazoa - ps8
Opisthokonta - ps9
Holozoa - ps10
Filozoa - ps11
Choano./Metazoa - ps12
Metazoa - ps13
Myriazoa - ps14
Parahoxozoa - ps15
Bilateria - ps16
Deuterostomia - ps17
Chordata - ps18
Olfactores - ps19
Vertebrata - ps20
Gnathostomata - ps21
Euteleostomi - ps22
Sarcopterygii - ps23
Tetrapoda - ps24
Amniota - ps25
Mammalia - ps26
Theria - ps27
Placentalia - ps28
Boreoeutheria - ps29
Euarchontoglires - ps30
Euarchonta - ps31
Primates - ps32
Haplorrhini - ps33
Simiiformes - ps34
Catarrhini - ps35
Hominoidea - ps36
Hominidae - ps37
Homininae - ps38
Hominini - ps39
Homo_sapiens - ps40
p-value
9e-15
1e-11
2e-08
2e-05
3e-02
Log-odds
0.5
1.0
1.5
2.0
2.5
3.0
3.5
4.0

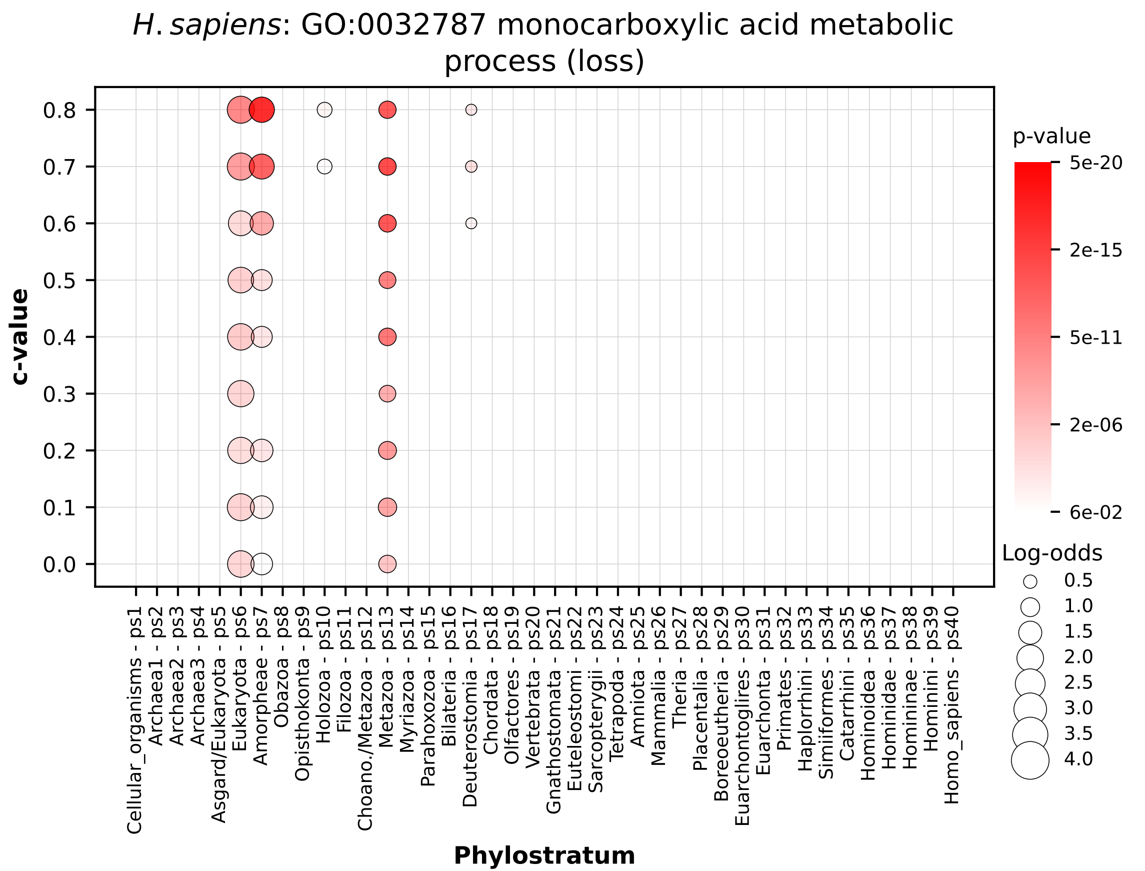
H. sapiens: GO:0032787 monocarboxylic acid metabolic process (loss)
c-value
0.0
0.1
0.2
0.3
0.4
0.5
0.6
0.7
0.8
Phylostratum
Cellular_organisms - ps1
Archaea1 - ps2
Archaea2 - ps3
Archaea3 - ps4
Asgard/Eukaryota - ps5
Eukaryota - ps6
Amorpheae - ps7
Obazoa - ps8
Opisthokonta - ps9
Holozoa - ps10
Filozoa - ps11
Choano./Metazoa - ps12
Metazoa - ps13
Myriazoa - ps14
Parahoxozoa - ps15
Bilateria - ps16
Deuterostomia - ps17
Chordata - ps18
Olfactores - ps19
Vertebrata - ps20
Gnathostomata - ps21
Euteleostomi - ps22
Sarcopterygii - ps23
Tetrapoda - ps24
Amniota - ps25
Mammalia - ps26
Theria - ps27
Placentalia - ps28
Boreoeutheria - ps29
Euarchontoglires - ps30
Euarchonta - ps31
Primates - ps32
Haplorrhini - ps33
Simiiformes - ps34
Catarrhini - ps35
Hominoidea - ps36
Hominidae - ps37
Homininae - ps38
Hominini - ps39
Homo_sapiens - ps40
p-value
5e-20
2e-15
5e-11
2e-06
6e-02
Log-odds
0.5
1.0
1.5
2.0
2.5
3.0
3.5
4.0

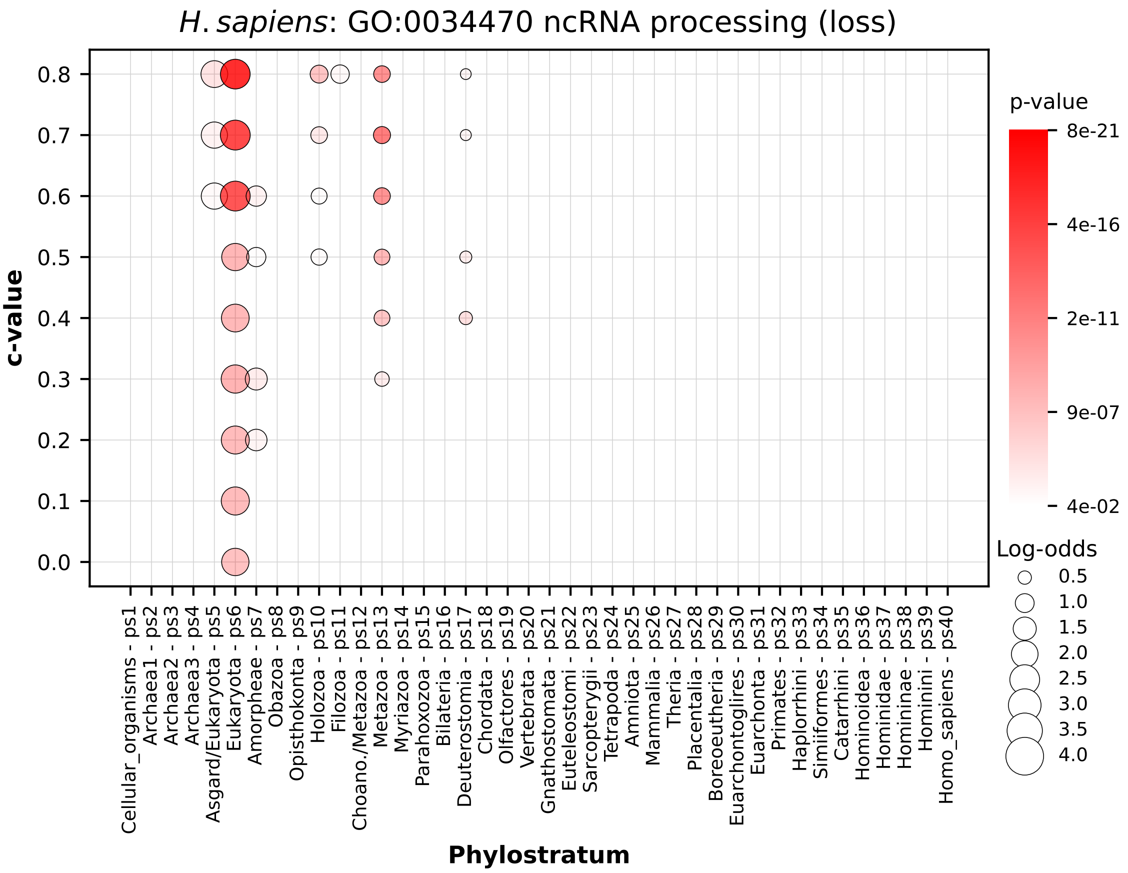

H. sapiens: GO:0034470 ncRNA processing (loss)
c-value
0.8
0.7
0.6
0.5
0.4
0.3
0.2
0.1
0.0
Phylostratum
Cellular_organisms - ps1
Archaea1 - ps2
Archaea2 - ps3
Archaea3 - ps4
Asgard/Eukaryota - ps5
Eukaryota - ps6
Amorpheae - ps7
Obazoa - ps8
Opisthokonta - ps9
Holozoa - ps10
Filozoa - ps11
Choano./Metazoa - ps12
Metazoa - ps13
Myriazoa - ps14
Parahoxozoa - ps15
Bilateria - ps16
Deuterostomia - ps17
Chordata - ps18
Olfactores - ps19
Vertebrata - ps20
Gnathostomata - ps21
Euteleostomi - ps22
Sarcopterygii - ps23
Tetrapoda - ps24
Amniota - ps25
Mammalia - ps26
Theria - ps27
Placentalia - ps28
Boreoeutheria - ps29
Euarchontoglires - ps30
Euarchonta - ps31
Primates - ps32
Haplorrhini - ps33
Simiiformes - ps34
Catarrhini - ps35
Hominoidea - ps36
Hominidae - ps37
Homininae - ps38
Hominini - ps39
Homo_sapiens - ps40
p-value
8e-21
4e-16
2e-11
9e-07
4e-02
Log-odds
0.5
1.0
1.5
2.0
2.5
3.0
3.5
4.0

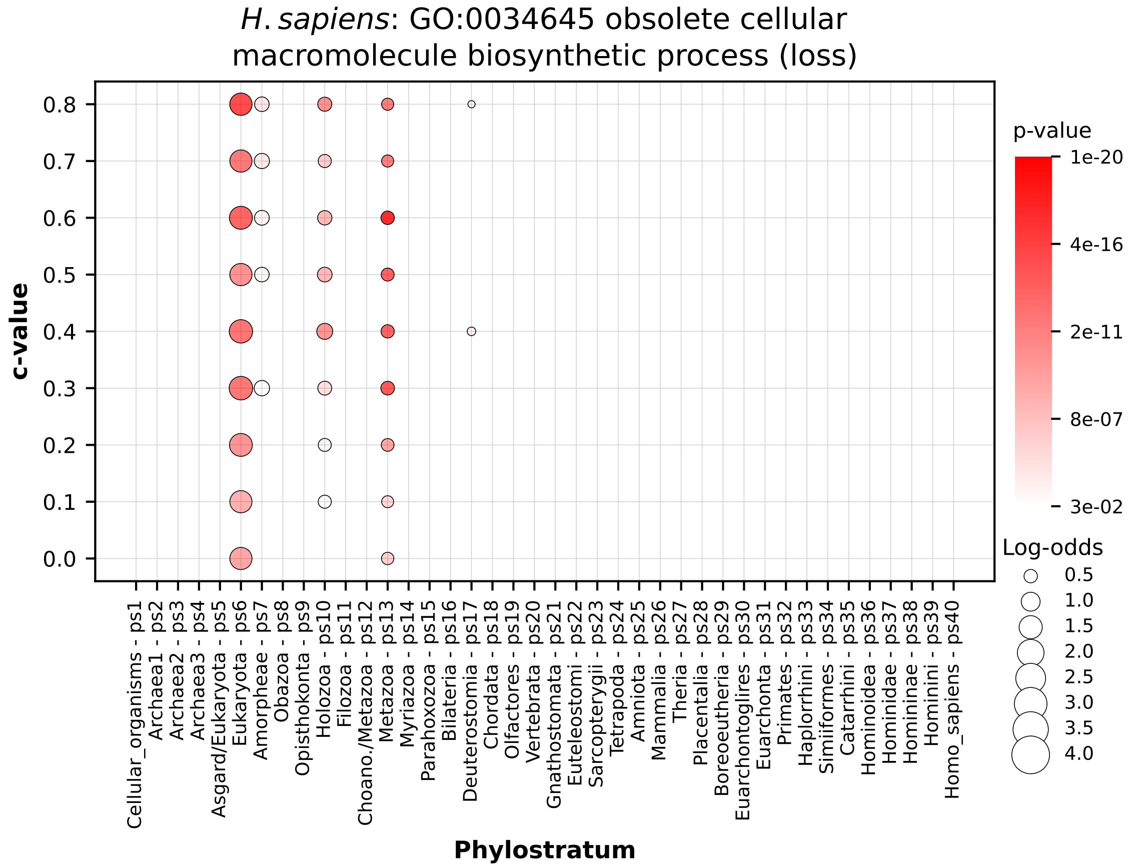

H. sapiens: GO:0034645 obsolete cellular macromolecule biosynthetic process (loss)
c-value
0.8
0.7
0.6
0.5
0.4
0.3
0.2
0.1
0.0
Phylostratum
Cellular_organisms - ps1
Archaea1 - ps2
Archaea2 - ps3
Archaea3 - ps4
Asgard/Eukaryota - ps5
Eukaryota - ps6
Amorpheae - ps7
Obazoa - ps8
Opisthokonta - ps9
Holozoa - ps10
Filozoa - ps11
Choano./Metazoa - ps12
Metazoa - ps13
Myriazoa - ps14
Parahoxozoa - ps15
Bilateria - ps16
Deuterostomia - ps17
Chordata - ps18
Olfactores - ps19
Vertebrata - ps20
Gnathostomata - ps21
Euteleostomi - ps22
Sarcopterygii - ps23
Tetrapoda - ps24
Amniota - ps25
Mammalia - ps26
Theria - ps27
Placentalia - ps28
Boreoeutheria - ps29
Euarchontoglires - ps30
Euarchonta - ps31
Primates - ps32
Haplorrhini - ps33
Simiiformes - ps34
Catarrhini - ps35
Hominoidea - ps36
Hominidae - ps37
Homininae - ps38
Hominini - ps39
Homo_sapiens - ps40
p-value
1e-20
4e-16
2e-11
8e-07
3e-02
Log-odds
0.5
1.0
1.5
2.0
2.5
3.0
3.5
4.0

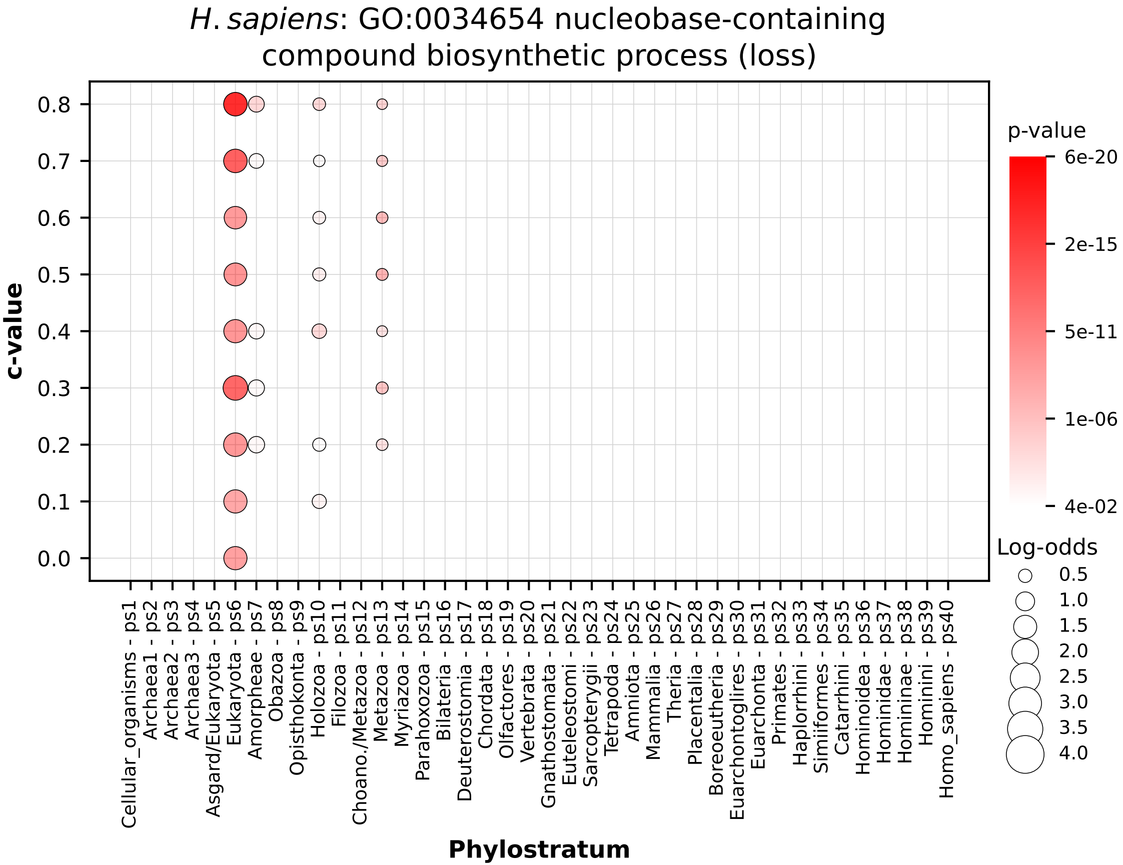

H. sapiens: GO:0034654 nucleobase-containing compound biosynthetic process (loss)
c-value
0.0
0.1
0.2
0.3
0.4
0.5
0.6
0.7
0.8
Phylostratum
Cellular_organisms - ps1
Archaea1 - ps2
Archaea2 - ps3
Archaea3 - ps4
Asgard/Eukaryota - ps5
Eukaryota - ps6
Amorpheae - ps7
Obazoa - ps8
Opisthokonta - ps9
Holozoa - ps10
Filozoa - ps11
Choano./Metazoa - ps12
Metazoa - ps13
Myriazoa - ps14
Parahoxozoa - ps15
Bilateria - ps16
Deuterostomia - ps17
Chordata - ps18
Olfactores - ps19
Vertebrata - ps20
Gnathostomata - ps21
Euteleostomi - ps22
Sarcopterygii - ps23
Tetrapoda - ps24
Amniota - ps25
Mammalia - ps26
Theria - ps27
Placentalia - ps28
Boreoeutheria - ps29
Euarchontoglires - ps30
Euarchonta - ps31
Primates - ps32
Haplorrhini - ps33
Simiiformes - ps34
Catarrhini - ps35
Hominoidea - ps36
Hominidae - ps37
Homininae - ps38
Hominini - ps39
Homo_sapiens - ps40
p-value
6e-20
2e-15
5e-11
1e-06
4e-02
Log-odds
0.5
1.0
1.5
2.0
2.5
3.0
3.5
4.0

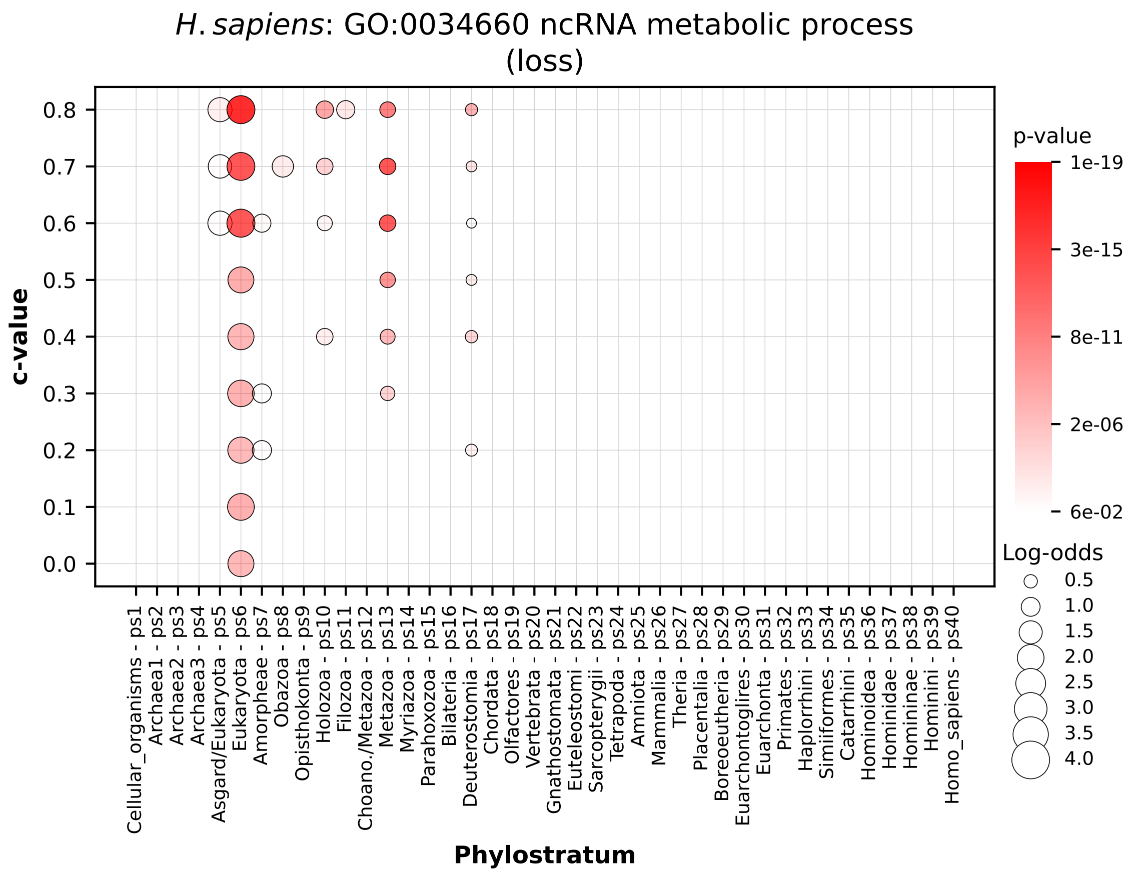

H. sapiens: GO:0034660 ncRNA metabolic process
(loss)
c-value
0.8
0.7
0.6
0.5
0.4
0.3
0.2
0.1
0.0
p-value
1e-19
3e-15
8e-11
2e-06
6e-02
Log-odds
0.5
1.0
1.5
2.0
2.5
3.0
3.5
4.0
Cellular_organisms - ps1
Archaea1 - ps2
Archaea2 - ps3
Archaea3 - ps4
Asgard/Eukaryota - ps5
Eukaryota - ps6
Amorpheae - ps7
Obazoa - ps8
Opisthokonta - ps9
Holozoa - ps10
Filozoa - ps11
Choano./Metazoa - ps12
Metazoa - ps13
Myriazoa - ps14
Parahoxozoa - ps15
Bilateria - ps16
Deuterostomia - ps17
Chordata - ps18
Olfactores - ps19
Vertebrata - ps20
Gnathostomata - ps21
Euteleostomi - ps22
Sarcopterygii - ps23
Tetrapoda - ps24
Amniota - ps25
Mammalia - ps26
Theria - ps27
Placentalia - ps28
Boreoeutheria - ps29
Euarchontoglires - ps30
Euarchonta - ps31
Primates - ps32
Haplorrhini - ps33
Simiiformes - ps34
Catarrhini - ps35
Hominoidea - ps36
Hominidae - ps37
Homininae - ps38
Hominini - ps39
Homo_sapiens - ps40
Phylostratum

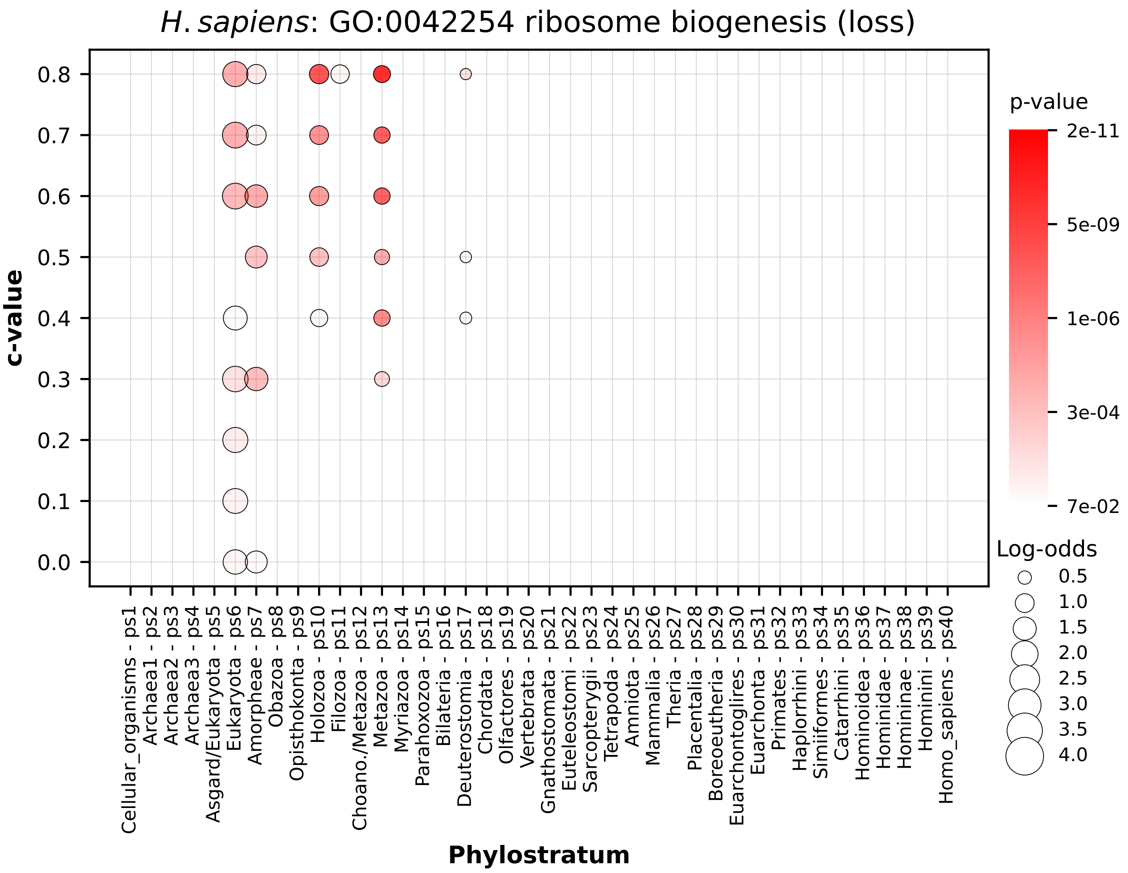

H. sapiens: GO:0042254 ribosome biogenesis (loss)
c-value
0.0
0.1
0.2
0.3
0.4
0.5
0.6
0.7
0.8
Phylostratum
Cellular_organisms - ps1
Archaea1 - ps2
Archaea2 - ps3
Archaea3 - ps4
Asgard/Eukaryota - ps5
Eukaryota - ps6
Amorpheae - ps7
Obazoa - ps8
Opisthokonta - ps9
Holozoa - ps10
Filozoa - ps11
Choano./Metazoa - ps12
Metazoa - ps13
Myriazoa - ps14
Parahoxozoa - ps15
Bilateria - ps16
Deuterostomia - ps17
Chordata - ps18
Olfactores - ps19
Vertebrata - ps20
Gnathostomata - ps21
Euteleostomi - ps22
Sarcopterygii - ps23
Tetrapoda - ps24
Amniota - ps25
Mammalia - ps26
Theria - ps27
Placentalia - ps28
Boreoeutheria - ps29
Euarchontoglires - ps30
Euarchonta - ps31
Primates - ps32
Haplorrhini - ps33
Simiiformes - ps34
Catarrhini - ps35
Hominoidea - ps36
Hominidae - ps37
Homininae - ps38
Hominini - ps39
Homo_sapiens - ps40
p-value
2e-11
5e-09
1e-06
3e-04
7e-02
Log-odds
0.5
1.0
1.5
2.0
2.5
3.0
3.5
4.0

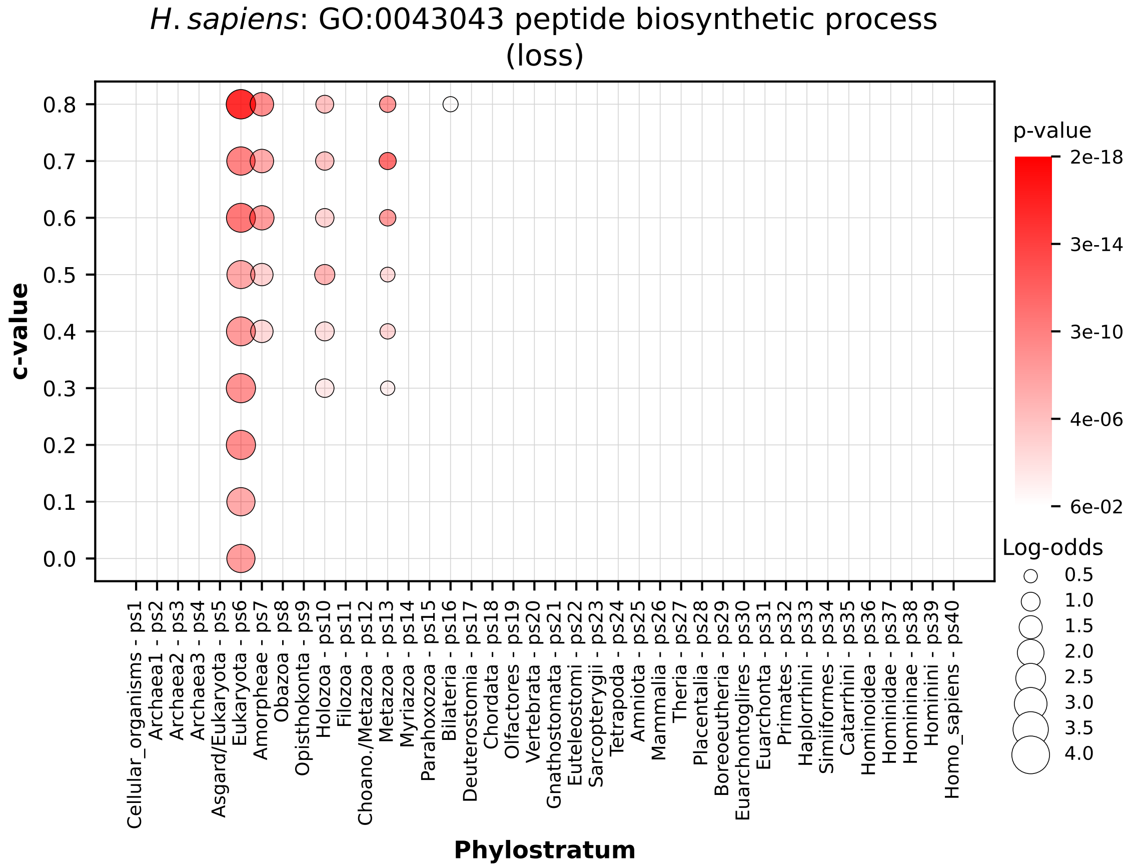

H. sapiens: GO:0043043 peptide biosynthetic process (loss)
c-value
0.8
0.7
0.6
0.5
0.4
0.3
0.2
0.1
0.0
Phylostratum
Cellular_organisms - ps1
Archaea1 - ps2
Archaea2 - ps3
Archaea3 - ps4
Asgard/Eukaryota - ps5
Eukaryota - ps6
Amorpheae - ps7
Obazoa - ps8
Opisthokonta - ps9
Holozoa - ps10
Filozoa - ps11
Choano./Metazoa - ps12
Metazoa - ps13
Myriazoa - ps14
Parahoxozoa - ps15
Bilateria - ps16
Deuterostomia - ps17
Chordata - ps18
Olfactores - ps19
Vertebrata - ps20
Gnathostomata - ps21
Euteleostomi - ps22
Sarcopterygii - ps23
Tetrapoda - ps24
Amniota - ps25
Mammalia - ps26
Theria - ps27
Placentalia - ps28
Boreoeutheria - ps29
Euarchontoglires - ps30
Euarchonta - ps31
Primates - ps32
Haplorrhini - ps33
Simiiformes - ps34
Catarrhini - ps35
Hominoidea - ps36
Hominidae - ps37
Homininae - ps38
Hominini - ps39
Homo_sapiens - ps40
p-value
2e-18
3e-14
3e-10
4e-06
6e-02
Log-odds
0.5
1.0
1.5
2.0
2.5
3.0
3.5
4.0

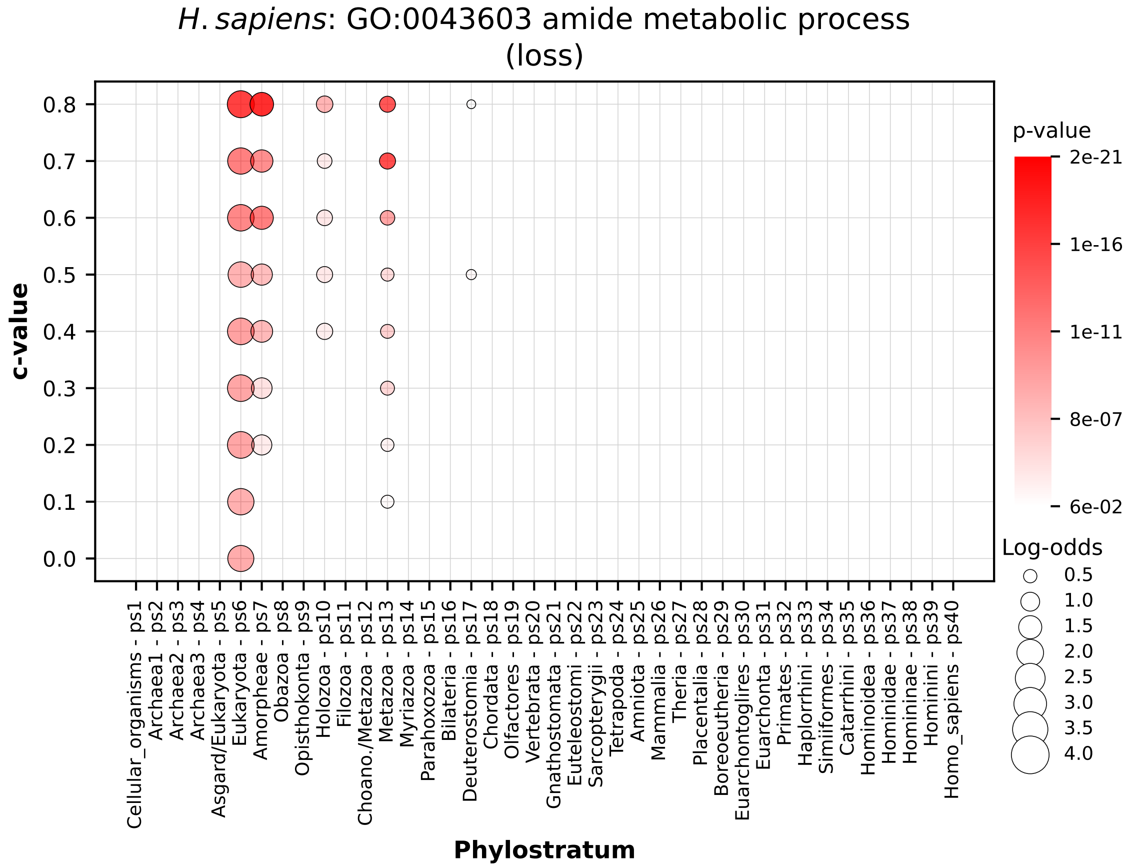

$H. sapiens$: GO:0043603 amide metabolic process
(loss)
c-value
0.8
0.7
0.6
0.5
0.4
0.3
0.2
0.1
0.0
p-value
2e-21
1e-16
1e-11
8e-07
6e-02
Log-odds
0.5
1.0
1.5
2.0
2.5
3.0
3.5
4.0
Cellular_organisms - ps1
Archaea1 - ps2
Archaea2 - ps3
Archaea3 - ps4
Asgard/Eukaryota - ps5
Eukaryota - ps6
Amorpheae - ps7
Obazoa - ps8
Opisthokonta - ps9
Holozoa - ps10
Filozoa - ps11
Choano./Metazoa - ps12
Metazoa - ps13
Myriazoa - ps14
Parahoxozoa - ps15
Bilateria - ps16
Deuterostomia - ps17
Chordata - ps18
Olfactores - ps19
Vertebrata - ps20
Gnathostomata - ps21
Euteleostomi - ps22
Sarcopterygii - ps23
Tetrapoda - ps24
Amniota - ps25
Mammalia - ps26
Theria - ps27
Placentalia - ps28
Boreoeutheria - ps29
Euarchontoglires - ps30
Euarchonta - ps31
Primates - ps32
Haplorrhini - ps33
Simiiformes - ps34
Catarrhini - ps35
Hominoidea - ps36
Hominidae - ps37
Homininae - ps38
Hominini - ps39
Homo_sapiens - ps40
Phylostratum

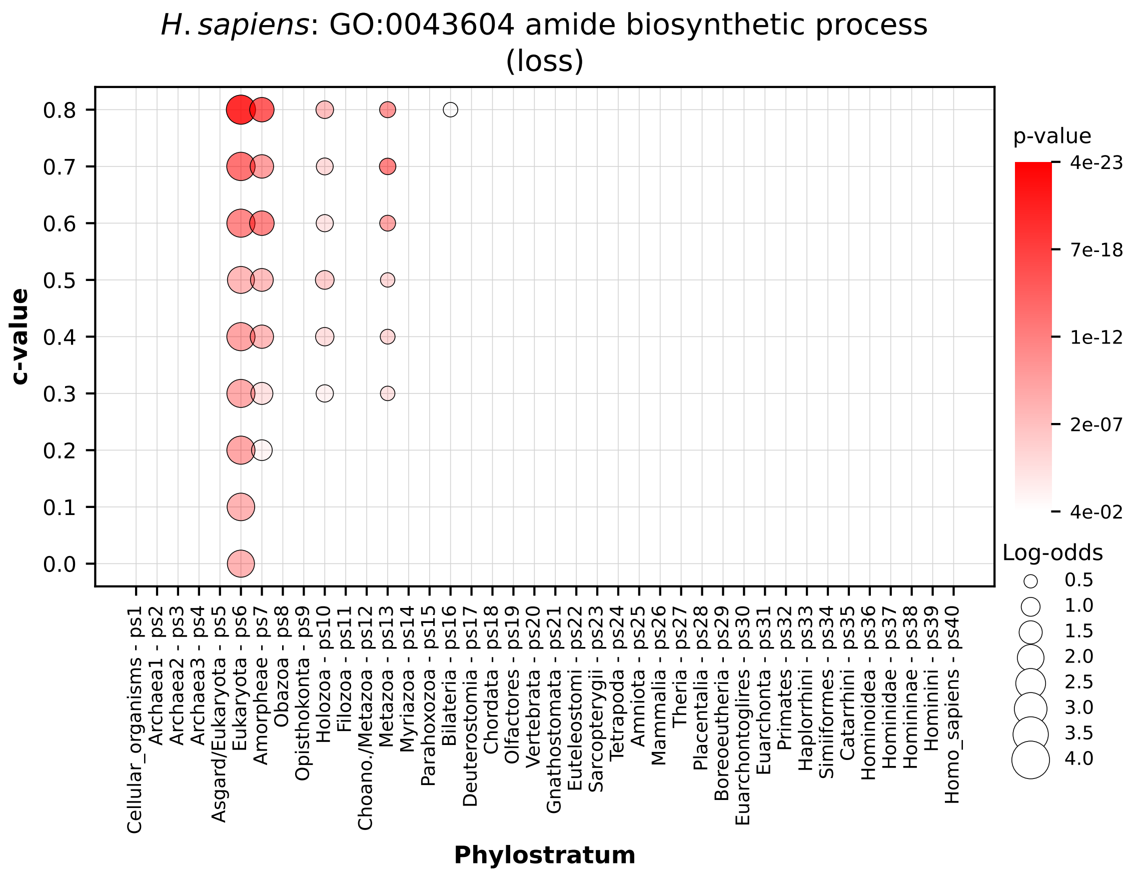

H. sapiens: GO:0043604 amide biosynthetic process
(loss)
c-value
0.0
0.1
0.2
0.3
0.4
0.5
0.6
0.7
0.8
Phylostratum
Cellular_organisms - ps1
Archaea1 - ps2
Archaea2 - ps3
Archaea3 - ps4
Asgard/Eukaryota - ps5
Eukaryota - ps6
Amorpheae - ps7
Obazoa - ps8
Opisthokonta - ps9
Holozoa - ps10
Filozoa - ps11
Choano./Metazoa - ps12
Metazoa - ps13
Myriazoa - ps14
Parahoxozoa - ps15
Bilateria - ps16
Deuterostomia - ps17
Chordata - ps18
Olfactores - ps19
Vertebrata - ps20
Gnathostomata - ps21
Euteleostomi - ps22
Sarcopterygii - ps23
Tetrapoda - ps24
Amniota - ps25
Mammalia - ps26
Theria - ps27
Placentalia - ps28
Boreoeutheria - ps29
Euarchontoglires - ps30
Euarchonta - ps31
Primates - ps32
Haplorrhini - ps33
Simiiformes - ps34
Catarrhini - ps35
Hominoidea - ps36
Hominidae - ps37
Homininae - ps38
Hominini - ps39
Homo_sapiens - ps40
p-value
4e-23
7e-18
1e-12
2e-07
4e-02
Log-odds
0.5
1.0
1.5
2.0
2.5
3.0
3.5
4.0

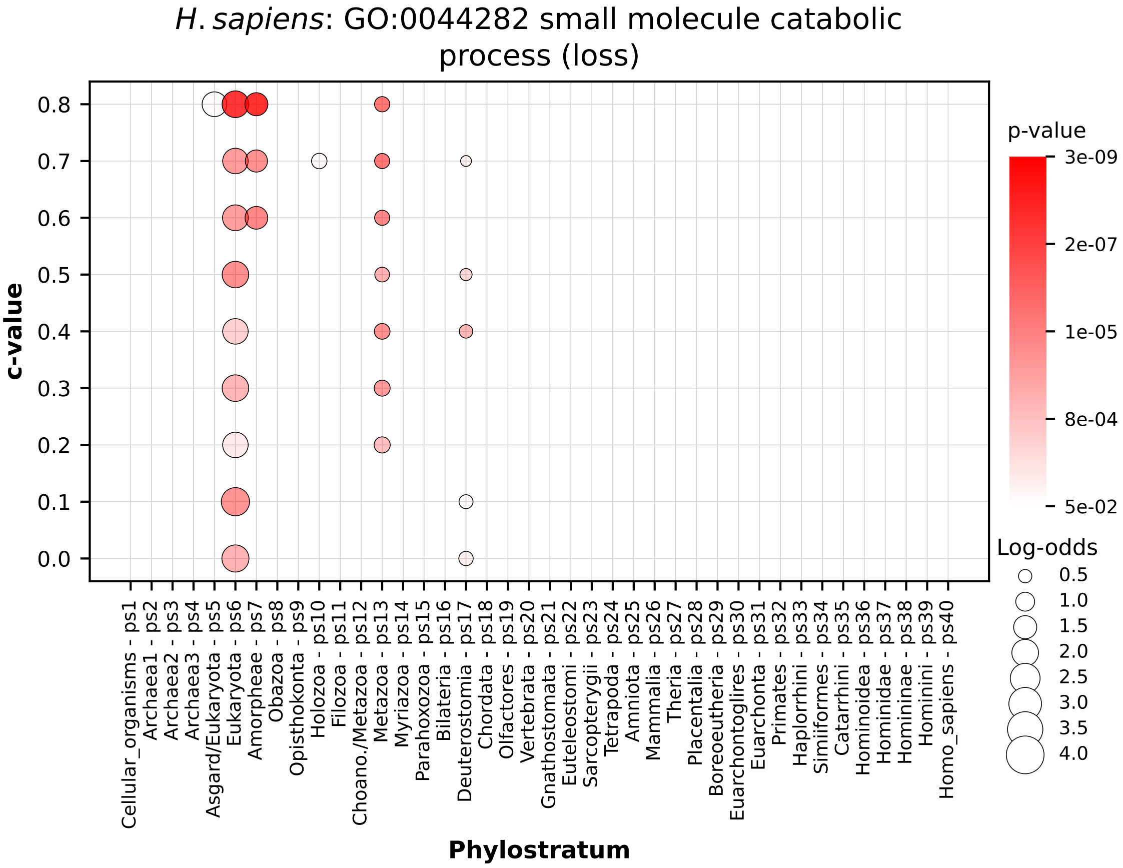

H. sapiens: GO:0044282 small molecule catabolic process (loss)
c-value
0.8
0.7
0.6
0.5
0.4
0.3
0.2
0.1
0.0
p-value
3e-09
2e-07
1e-05
8e-04
5e-02
Log-odds
0.5
1.0
1.5
2.0
2.5
3.0
3.5
4.0
Cellular_organisms - ps1
Archaea1 - ps2
Archaea2 - ps3
Archaea3 - ps4
Asgard/Eukaryota - ps5
Eukaryota - ps6
Amorpheae - ps7
Obazoa - ps8
Opisthokonta - ps9
Holozoa - ps10
Filozoa - ps11
Choano./Metazoa - ps12
Metazoa - ps13
Myriazoa - ps14
Parahoxozoa - ps15
Bilateria - ps16
Deuterostomia - ps17
Chordata - ps18
Olfactores - ps19
Vertebrata - ps20
Gnathostomata - ps21
Euteleostomi - ps22
Sarcopterygii - ps23
Tetrapoda - ps24
Amniota - ps25
Mammalia - ps26
Theria - ps27
Placentalia - ps28
Boreoeutheria - ps29
Euarchontoglires - ps30
Euarchonta - ps31
Primates - ps32
Haplorrhini - ps33
Simiiformes - ps34
Catarrhini - ps35
Hominoidea - ps36
Hominidae - ps37
Homininae - ps38
Hominini - ps39
Homo_sapiens - ps40
Phylostratum

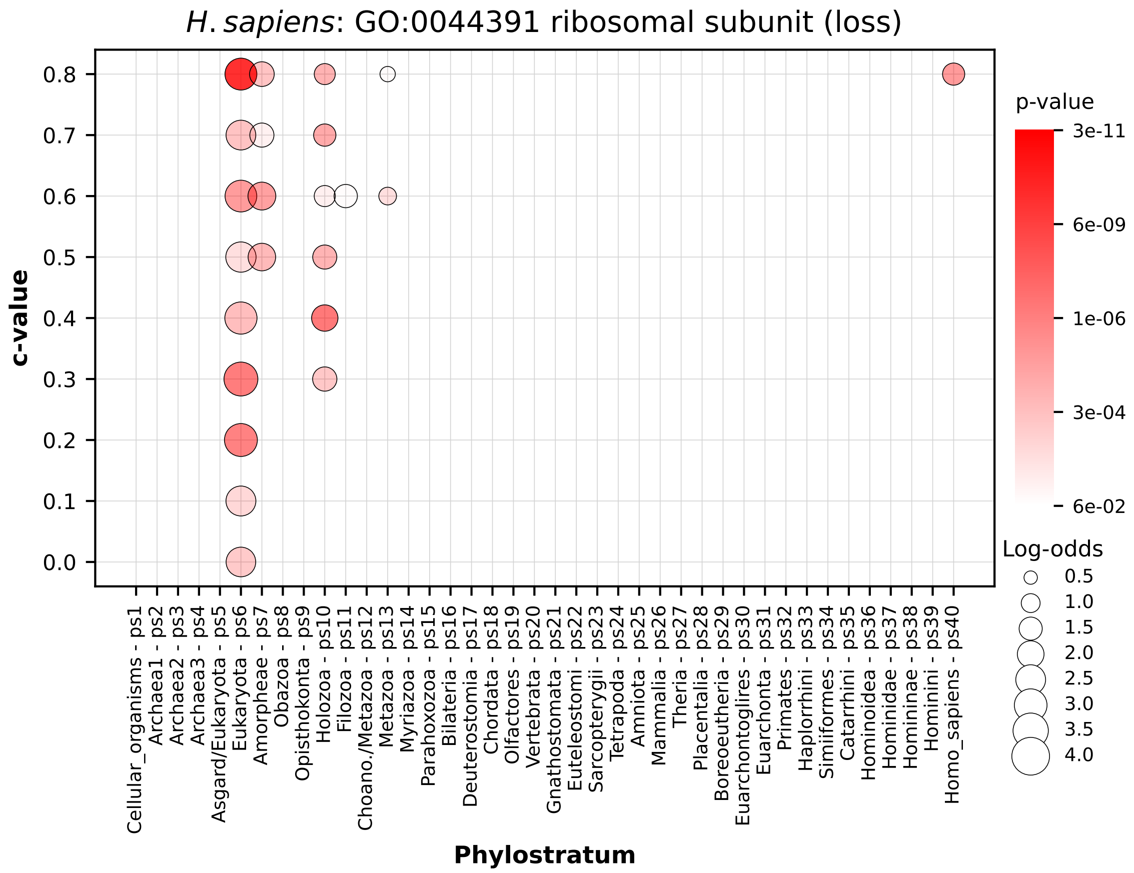

H. sapiens: GO:0044391 ribosomal subunit (loss)
c-value
0.0
0.1
0.2
0.3
0.4
0.5
0.6
0.7
0.8
Phylostratum
Cellular_organisms - ps1
Archaea1 - ps2
Archaea2 - ps3
Archaea3 - ps4
Asgard/Eukaryota - ps5
Eukaryota - ps6
Amorpheae - ps7
Obazoa - ps8
Opisthokonta - ps9
Holozoa - ps10
Filozoa - ps11
Choano./Metazoa - ps12
Metazoa - ps13
Myriazoa - ps14
Parahoxozoa - ps15
Bilateria - ps16
Deuterostomia - ps17
Chordata - ps18
Olfactores - ps19
Vertebrata - ps20
Gnathostomata - ps21
Euteleostomi - ps22
Sarcopterygii - ps23
Tetrapoda - ps24
Amniota - ps25
Mammalia - ps26
Theria - ps27
Placentalia - ps28
Boreoeutheria - ps29
Euarchontoglires - ps30
Euarchonta - ps31
Primates - ps32
Haplorrhini - ps33
Simiiformes - ps34
Catarrhini - ps35
Hominoidea - ps36
Hominidae - ps37
Homininae - ps38
Hominini - ps39
Homo_sapiens - ps40
p-value
3e-11
6e-09
1e-06
3e-04
6e-02
Log-odds
0.5
1.0
1.5
2.0
2.5
3.0
3.5
4.0

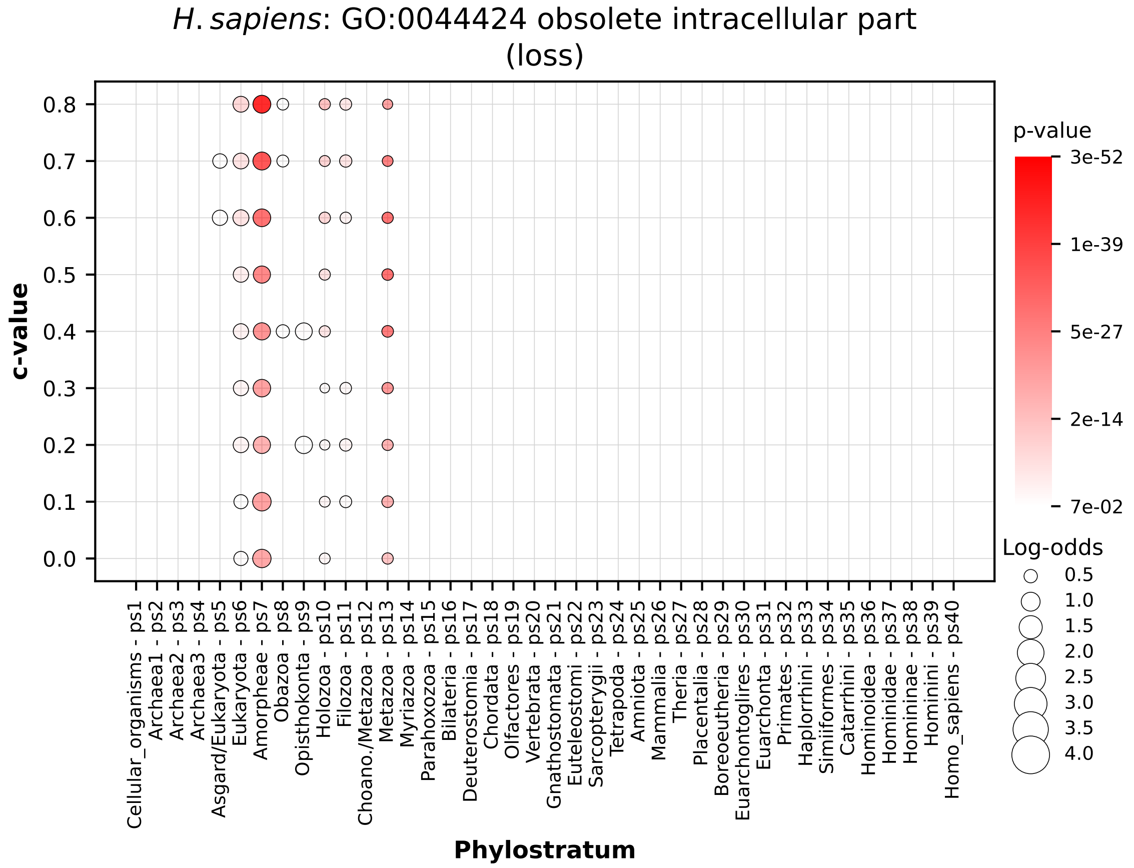

H. sapiens: GO:0044424 obsolete intracellular part
(loss)
c-value
0.8
0.7
0.6
0.5
0.4
0.3
0.2
0.1
0.0
p-value
3e-52
1e-39
5e-27
2e-14
7e-02
Log-odds
0.5
1.0
1.5
2.0
2.5
3.0
3.5
4.0
Cellular_organisms - ps1
Archaea1 - ps2
Archaea2 - ps3
Archaea3 - ps4
Asgard/Eukaryota - ps5
Eukaryota - ps6
Amorpheae - ps7
Obazoa - ps8
Opisthokonta - ps9
Holozoa - ps10
Filozoa - ps11
Choano./Metazoa - ps12
Metazoa - ps13
Myriazoa - ps14
Parahoxozoa - ps15
Bilateria - ps16
Deuterostomia - ps17
Chordata - ps18
Olfactores - ps19
Vertebrata - ps20
Gnathostomata - ps21
Euteleostomi - ps22
Sarcopterygii - ps23
Tetrapoda - ps24
Amniota - ps25
Mammalia - ps26
Theria - ps27
Placentalia - ps28
Boreoeutheria - ps29
Euarchontoglires - ps30
Euarchonta - ps31
Primates - ps32
Haplorrhini - ps33
Simiiformes - ps34
Catarrhini - ps35
Hominoidea - ps36
Hominidae - ps37
Homininae - ps38
Hominini - ps39
Homo_sapiens - ps40
Phylostratum

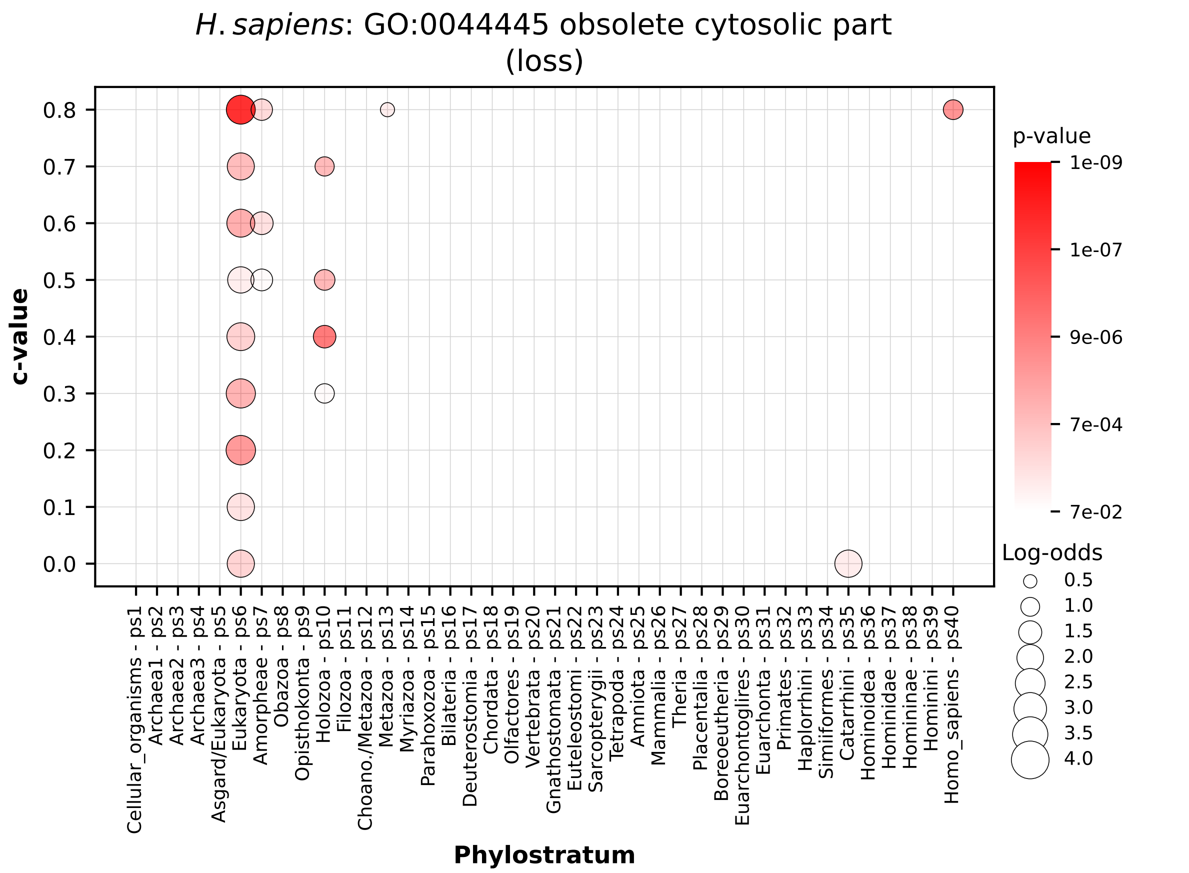

H. sapiens: GO:0044445 obsolete cytosolic part
(loss)
c-value
0.8
0.7
0.6
0.5
0.4
0.3
0.2
0.1
0.0
p-value
1e-09
1e-07
9e-06
7e-04
7e-02
Log-odds
0.5
1.0
1.5
2.0
2.5
3.0
3.5
4.0
Cellular_organisms - ps1
Archaea1 - ps2
Archaea2 - ps3
Archaea3 - ps4
Asgard/Eukaryota - ps5
Eukaryota - ps6
Amorpheae - ps7
Obazoa - ps8
Opisthokonta - ps9
Holozoa - ps10
Filozoa - ps11
Choano./Metazoa - ps12
Metazoa - ps13
Myriazoa - ps14
Parahoxozoa - ps15
Bilateria - ps16
Deuterostomia - ps17
Chordata - ps18
Olfactores - ps19
Vertebrata - ps20
Gnathostomata - ps21
Euteleostomi - ps22
Sarcopterygii - ps23
Tetrapoda - ps24
Amniota - ps25
Mammalia - ps26
Theria - ps27
Placentalia - ps28
Boreoeutheria - ps29
Euarchontoglires - ps30
Euarchonta - ps31
Primates - ps32
Haplorrhini - ps33
Simiiformes - ps34
Catarrhini - ps35
Hominoidea - ps36
Hominidae - ps37
Homininae - ps38
Hominini - ps39
Homo_sapiens - ps40
Phylostratum

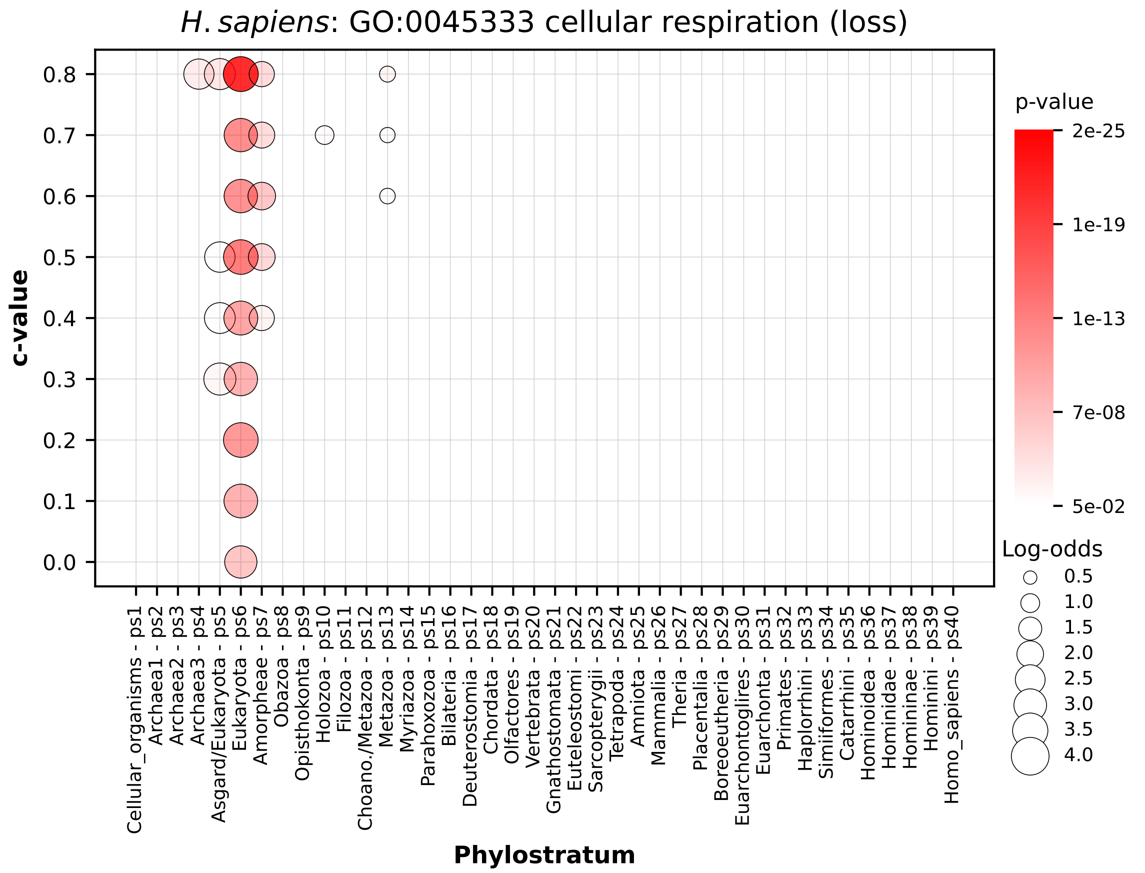

H. sapiens: GO:0045333 cellular respiration (loss)
c-value
0.8
0.7
0.6
0.5
0.4
0.3
0.2
0.1
0.0
Phylostratum
Cellular_organisms - ps1
Archaea1 - ps2
Archaea2 - ps3
Archaea3 - ps4
Asgard/Eukaryota - ps5
Eukaryota - ps6
Amorpheae - ps7
Obazoa - ps8
Opisthokonta - ps9
Holozoa - ps10
Filozoa - ps11
Choano./Metazoa - ps12
Metazoa - ps13
Myriazoa - ps14
Parahoxozoa - ps15
Bilateria - ps16
Deuterostomia - ps17
Chordata - ps18
Olfactores - ps19
Vertebrata - ps20
Gnathostomata - ps21
Euteleostomi - ps22
Sarcopterygii - ps23
Tetrapoda - ps24
Amniota - ps25
Mammalia - ps26
Theria - ps27
Placentalia - ps28
Boreoeutheria - ps29
Euarchontoglires - ps30
Euarchonta - ps31
Primates - ps32
Haplorrhini - ps33
Simiiformes - ps34
Catarrhini - ps35
Hominoidea - ps36
Hominidae - ps37
Homininae - ps38
Hominini - ps39
Homo_sapiens - ps40
p-value
2e-25
1e-19
1e-13
7e-08
5e-02
Log-odds
0.5
1.0
1.5
2.0
2.5
3.0
3.5
4.0

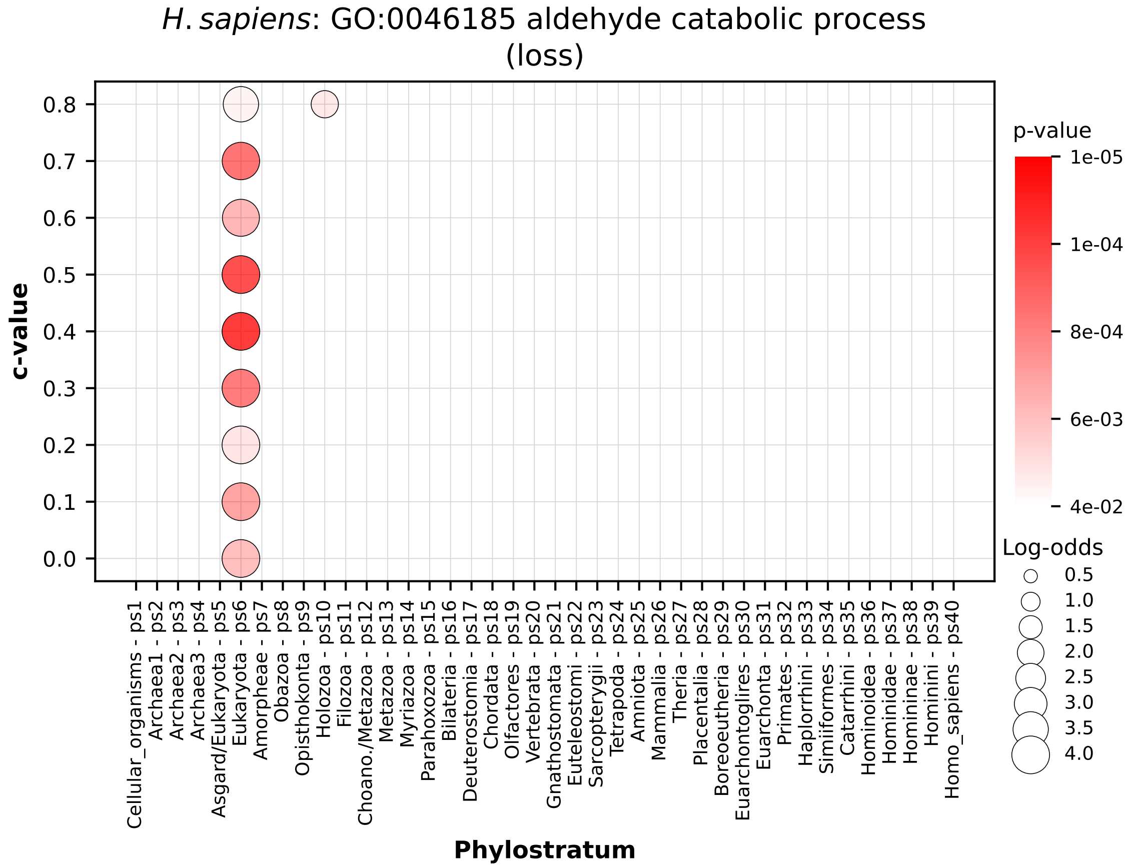

H. sapiens: GO:0046185 aldehyde catabolic process
(loss)
c-value
0.0
0.1
0.2
0.3
0.4
0.5
0.6
0.7
0.8
Phylostratum
Cellular_organisms - ps1
Archaea1 - ps2
Archaea2 - ps3
Archaea3 - ps4
Asgard/Eukaryota - ps5
Eukaryota - ps6
Amorpheae - ps7
Obazoa - ps8
Opisthokonta - ps9
Holozoa - ps10
Filozoa - ps11
Choano./Metazoa - ps12
Metazoa - ps13
Myriazoa - ps14
Parahoxozoa - ps15
Bilateria - ps16
Deuterostomia - ps17
Chordata - ps18
Olfactores - ps19
Vertebrata - ps20
Gnathostomata - ps21
Euteleostomi - ps22
Sarcopterygii - ps23
Tetrapoda - ps24
Amniota - ps25
Mammalia - ps26
Theria - ps27
Placentalia - ps28
Boreoeutheria - ps29
Euarchontoglires - ps30
Euarchonta - ps31
Primates - ps32
Haplorrhini - ps33
Simiiformes - ps34
Catarrhini - ps35
Hominoidea - ps36
Hominidae - ps37
Homininae - ps38
Hominini - ps39
Homo_sapiens - ps40
p-value
1e-05
1e-04
8e-04
6e-03
4e-02
Log-odds
0.5
1.0
1.5
2.0
2.5
3.0
3.5
4.0

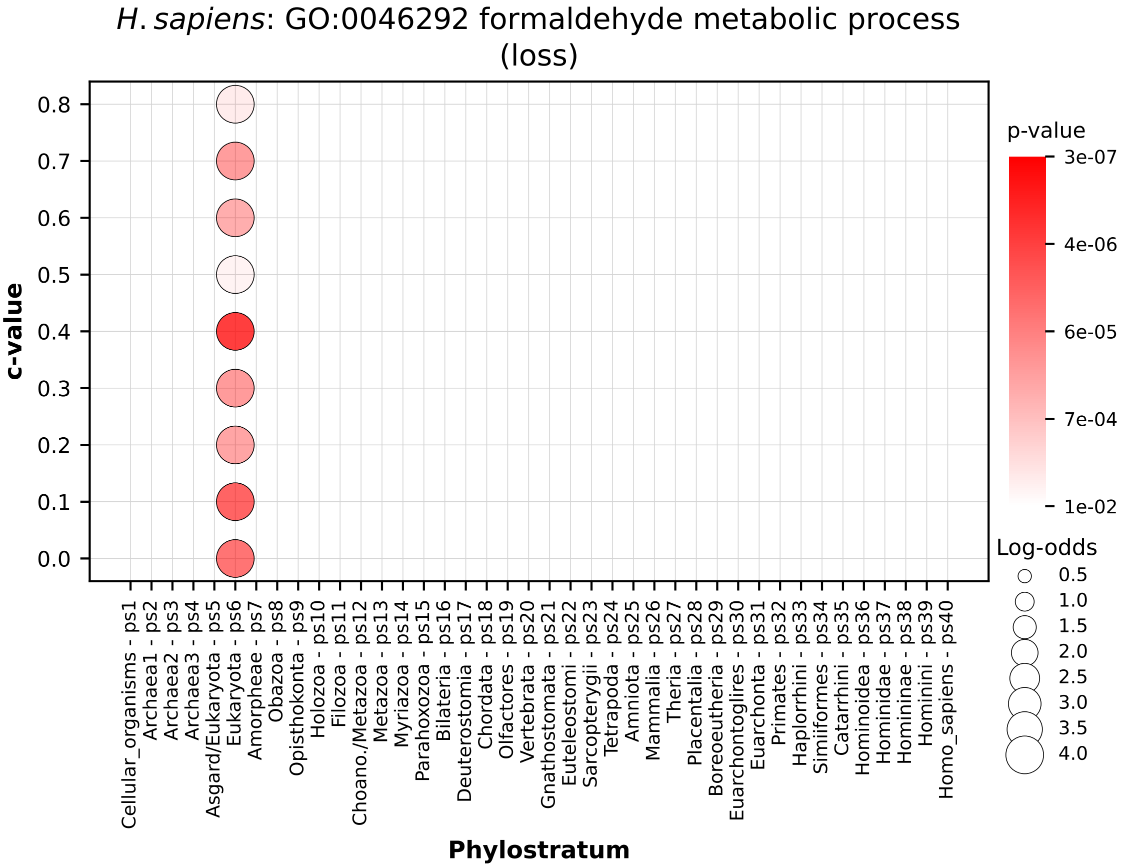
H. sapiens: GO:0046292 formaldehyde metabolic process
(loss)
c-value
0.8
0.7
0.6
0.5
0.4
0.3
0.2
0.1
0.0
p-value
3e-07
4e-06
6e-05
7e-04
1e-02
Log-odds
0.5
1.0
1.5
2.0
2.5
3.0
3.5
4.0
Cellular_organisms - ps1
Archaea1 - ps2
Archaea2 - ps3
Archaea3 - ps4
Asgard/Eukaryota - ps5
Eukaryota - ps6
Amorpheae - ps7
Obazoa - ps8
Opisthokonta - ps9
Holozoa - ps10
Filozoa - ps11
Choano./Metazoa - ps12
Metazoa - ps13
Myriazoa - ps14
Parahoxozoa - ps15
Bilateria - ps16
Deuterostomia - ps17
Chordata - ps18
Olfactores - ps19
Vertebrata - ps20
Gnathostomata - ps21
Euteleostomi - ps22
Sarcopterygii - ps23
Tetrapoda - ps24
Amniota - ps25
Mammalia - ps26
Theria - ps27
Placentalia - ps28
Boreoeutheria - ps29
Euarchontoglires - ps30
Euarchonta - ps31
Primates - ps32
Haplorrhini - ps33
Simiiformes - ps34
Catarrhini - ps35
Hominoidea - ps36
Hominidae - ps37
Homininae - ps38
Hominini - ps39
Homo_sapiens - ps40
Phylostratum

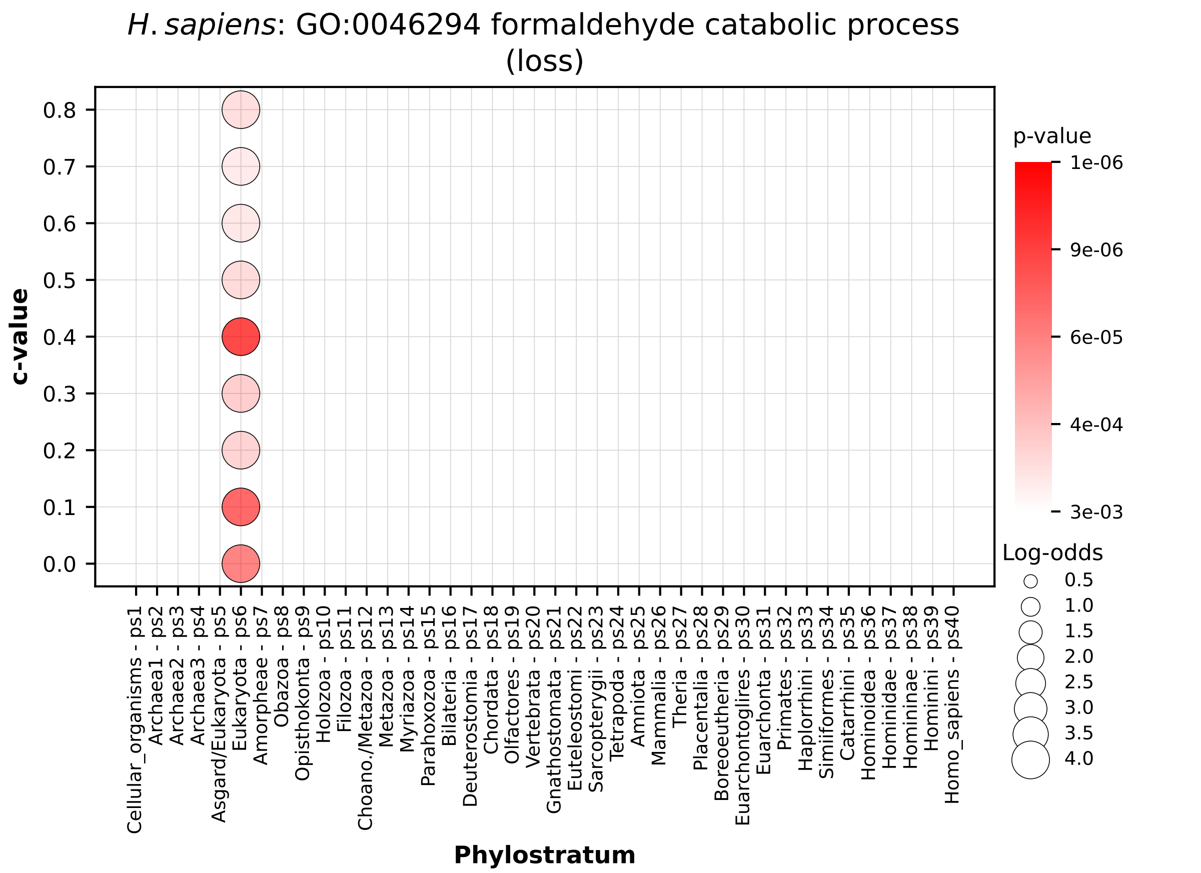

H. sapiens: GO:0046294 formaldehyde catabolic process (loss)
c-value
0.8
0.7
0.6
0.5
0.4
0.3
0.2
0.1
0.0
Phylostratum
Cellular_organisms - ps1
Archaea1 - ps2
Archaea2 - ps3
Archaea3 - ps4
Asgard/Eukaryota - ps5
Eukaryota - ps6
Amorpheae - ps7
Obazoa - ps8
Opisthokonta - ps9
Holozoa - ps10
Filozoa - ps11
Choano./Metazoa - ps12
Metazoa - ps13
Myriazoa - ps14
Parahoxozoa - ps15
Bilateria - ps16
Deuterostomia - ps17
Chordata - ps18
Olfactores - ps19
Vertebrata - ps20
Gnathostomata - ps21
Euteleostomi - ps22
Sarcopterygii - ps23
Tetrapoda - ps24
Amniota - ps25
Mammalia - ps26
Theria - ps27
Placentalia - ps28
Boreoeutheria - ps29
Euarchontoglires - ps30
Euarchonta - ps31
Primates - ps32
Haplorrhini - ps33
Simiiformes - ps34
Catarrhini - ps35
Hominoidea - ps36
Hominidae - ps37
Homininae - ps38
Hominini - ps39
Homo_sapiens - ps40
p-value
1e-06
9e-06
6e-05
4e-04
3e-03
Log-odds
0.5
1.0
1.5
2.0
2.5
3.0
3.5
4.0

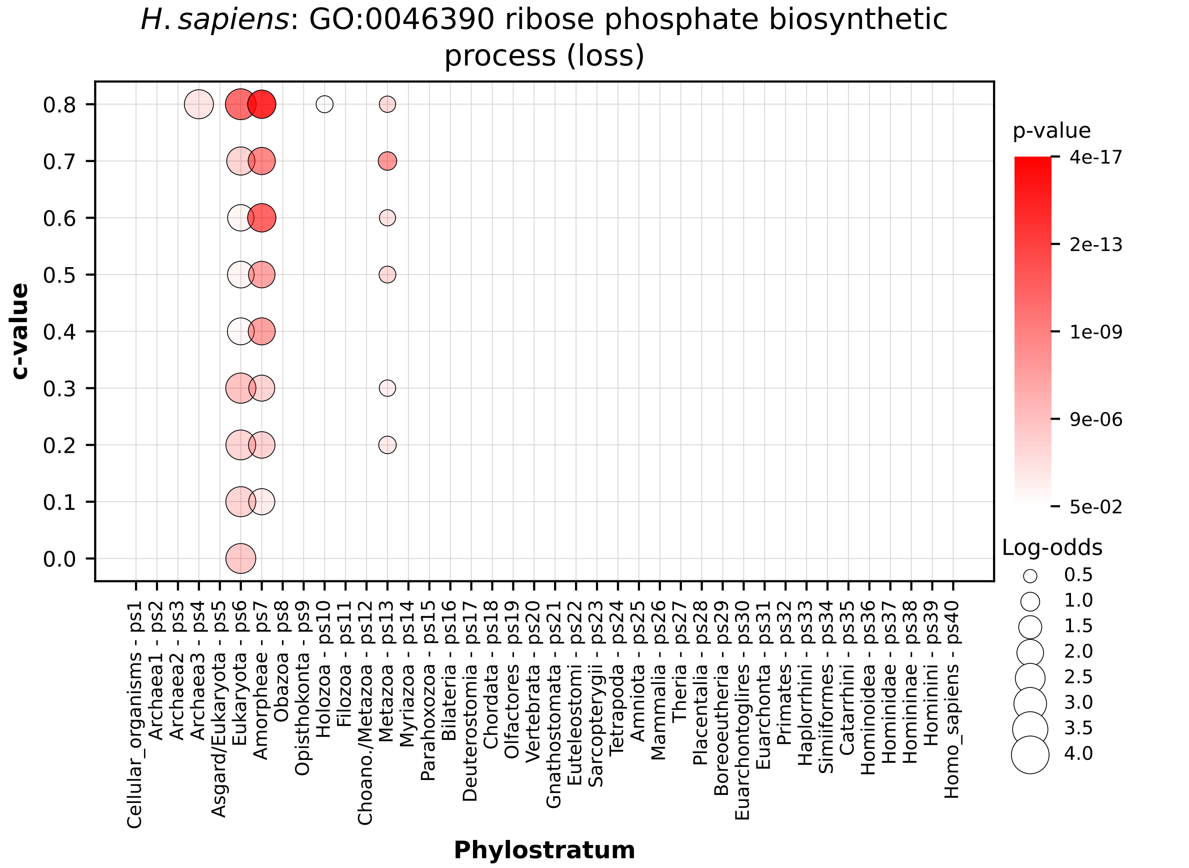

H. sapiens: GO:0046390 ribose phosphate biosynthetic process (loss)
c-value
0.8
0.7
0.6
0.5
0.4
0.3
0.2
0.1
0.0
Phylostratum
Cellular_organisms - ps1
Archaea1 - ps2
Archaea2 - ps3
Archaea3 - ps4
Asgard/Eukaryota - ps5
Eukaryota - ps6
Amorpheae - ps7
Obazoa - ps8
Opisthokonta - ps9
Holozoa - ps10
Filozoa - ps11
Choano./Metazoa - ps12
Metazoa - ps13
Myriazoa - ps14
Parahoxozoa - ps15
Bilateria - ps16
Deuterostomia - ps17
Chordata - ps18
Olfactores - ps19
Vertebrata - ps20
Gnathostomata - ps21
Euteleostomi - ps22
Sarcopterygii - ps23
Tetrapoda - ps24
Amniota - ps25
Mammalia - ps26
Theria - ps27
Placentalia - ps28
Boreoeutheria - ps29
Euarchontoglires - ps30
Euarchonta - ps31
Primates - ps32
Haplorrhini - ps33
Simiiformes - ps34
Catarrhini - ps35
Hominoidea - ps36
Hominidae - ps37
Homininae - ps38
Hominini - ps39
Homo_sapiens - ps40
p-value
4e-17
2e-13
1e-09
9e-06
5e-02
Log-odds
0.5
1.0
1.5
2.0
2.5
3.0
3.5
4.0

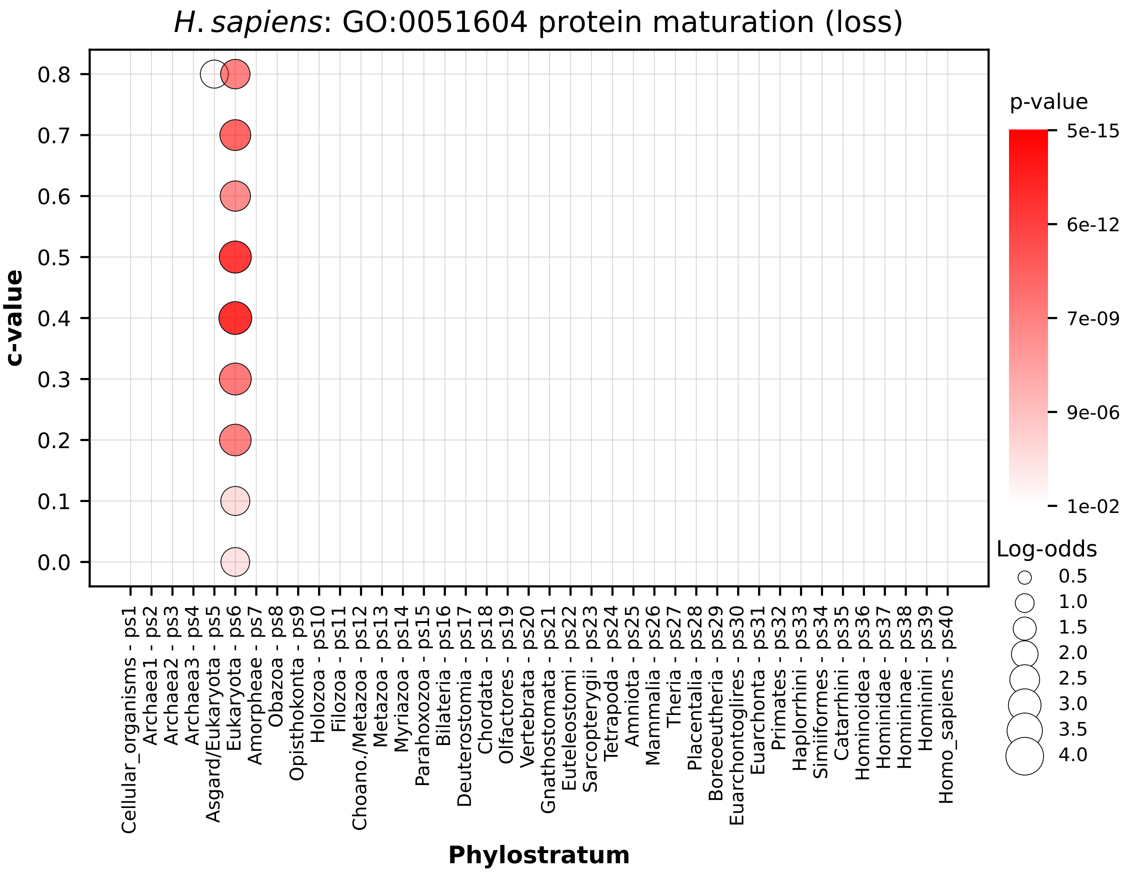

H. sapiens: GO:0051604 protein maturation (loss)
c-value
0.0
0.1
0.2
0.3
0.4
0.5
0.6
0.7
0.8
p-value
5e-15
6e-12
7e-09
9e-06
1e-02
Log-odds
0.5
1.0
1.5
2.0
2.5
3.0
3.5
4.0
Cellular_organisms - ps1
Archaea1 - ps2
Archaea2 - ps3
Archaea3 - ps4
Asgard/Eukaryota - ps5
Eukaryota - ps6
Amorpheae - ps7
Obazoa - ps8
Opisthokonta - ps9
Holozoa - ps10
Filozoa - ps11
Choano./Metazoa - ps12
Metazoa - ps13
Myriazoa - ps14
Parahoxozoa - ps15
Bilateria - ps16
Deuterostomia - ps17
Chordata - ps18
Olfactores - ps19
Vertebrata - ps20
Gnathostomata - ps21
Euteleostomi - ps22
Sarcopterygii - ps23
Tetrapoda - ps24
Amniota - ps25
Mammalia - ps26
Theria - ps27
Placentalia - ps28
Boreoeutheria - ps29
Euarchontoglires - ps30
Euarchonta - ps31
Primates - ps32
Haplorrhini - ps33
Simiiformes - ps34
Catarrhini - ps35
Hominoidea - ps36
Hominidae - ps37
Homininae - ps38
Hominini - ps39
Homo_sapiens - ps40
Phylostratum

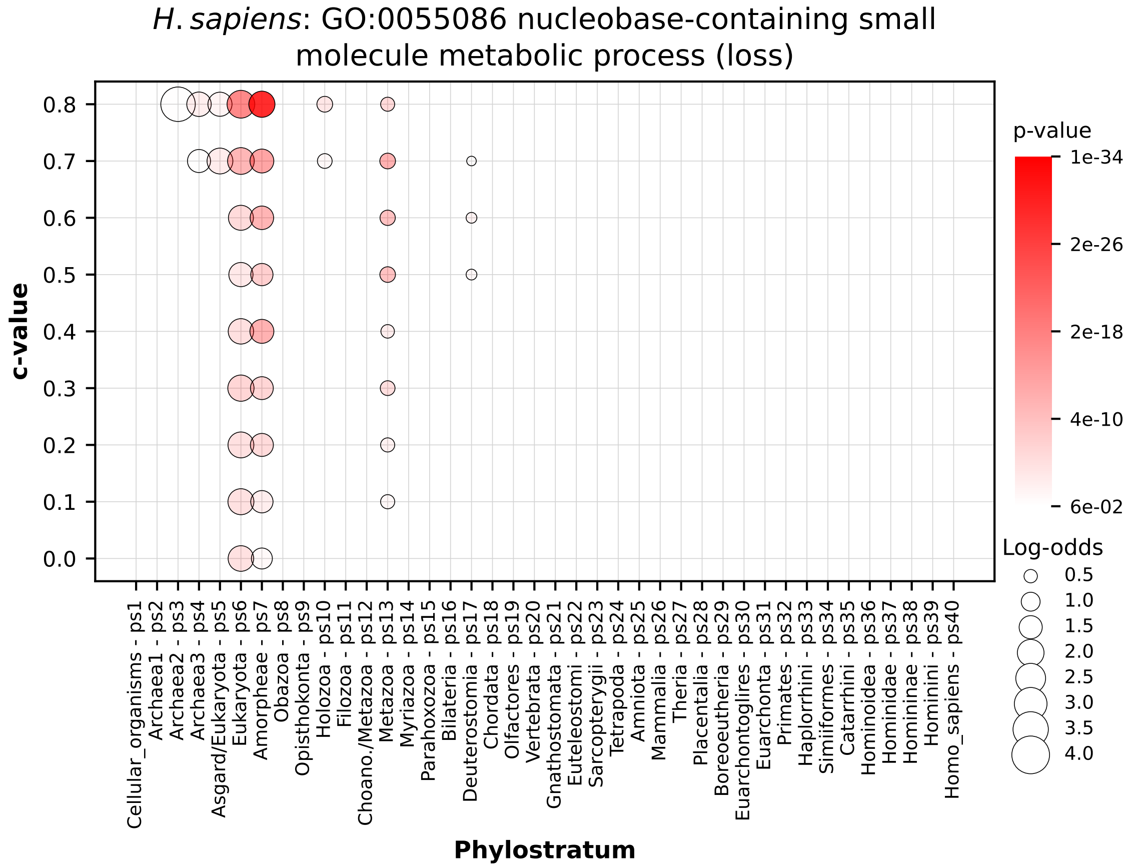

H. sapiens: GO:0055086 nucleobase-containing small molecule metabolic process (loss)
c-value
0.8
0.7
0.6
0.5
0.4
0.3
0.2
0.1
0.0
p-value
1e-34
2e-26
2e-18
4e-10
6e-02
Log-odds
0.5
1.0
1.5
2.0
2.5
3.0
3.5
4.0
Cellular_organisms - ps1
Archaea1 - ps2
Archaea2 - ps3
Archaea3 - ps4
Asgard/Eukaryota - ps5
Eukaryota - ps6
Amorpheae - ps7
Obazoa - ps8
Opisthokonta - ps9
Holozoa - ps10
Filozoa - ps11
Choano./Metazoa - ps12
Metazoa - ps13
Myriazoa - ps14
Parahoxozoa - ps15
Bilateria - ps16
Deuterostomia - ps17
Chordata - ps18
Olfactores - ps19
Vertebrata - ps20
Gnathostomata - ps21
Euteleostomi - ps22
Sarcopterygii - ps23
Tetrapoda - ps24
Amniota - ps25
Mammalia - ps26
Theria - ps27
Placentalia - ps28
Boreoeutheria - ps29
Euarchontoglires - ps30
Euarchonta - ps31
Primates - ps32
Haplorrhini - ps33
Simiiformes - ps34
Catarrhini - ps35
Hominoidea - ps36
Hominidae - ps37
Homininae - ps38
Hominini - ps39
Homo_sapiens - ps40
Phylostratum

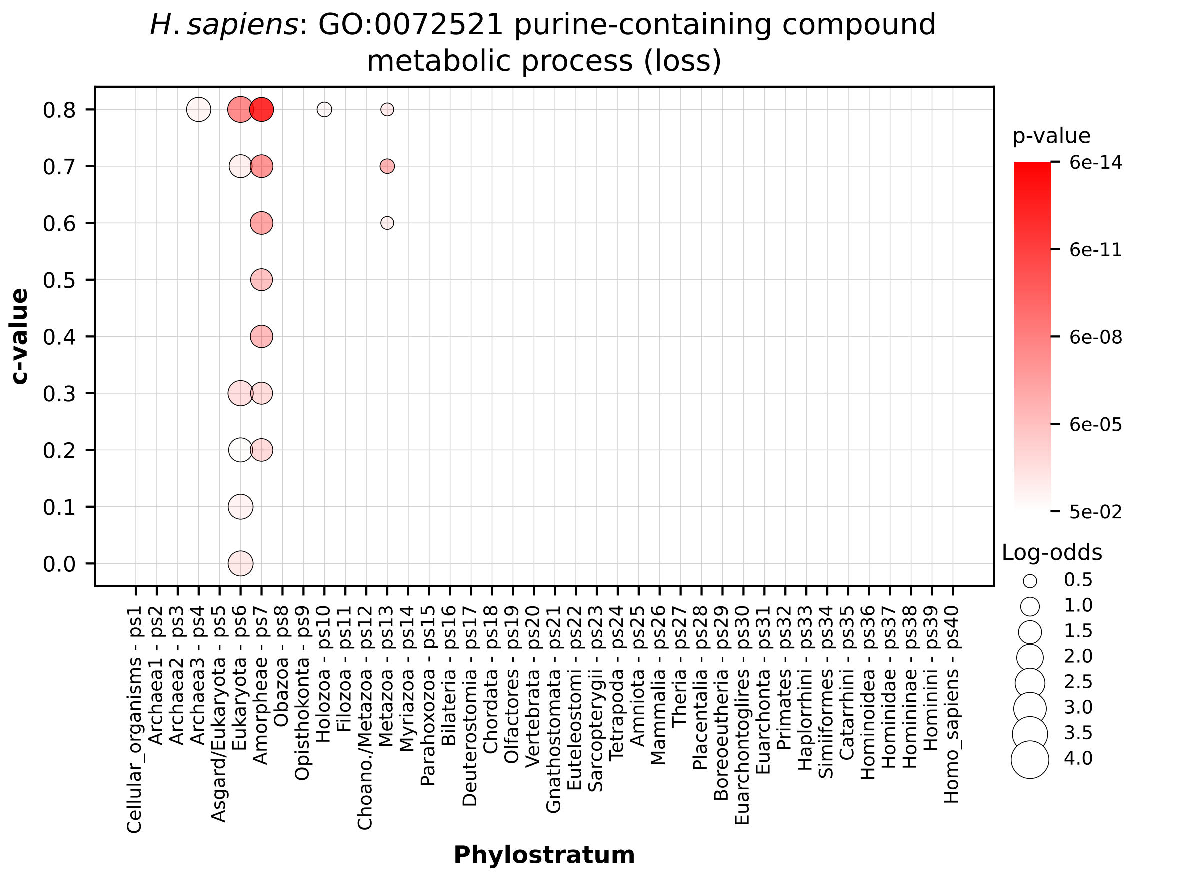

H. sapiens: GO:0072521 purine-containing compound metabolic process (loss)
c-value
0.8
0.7
0.6
0.5
0.4
0.3
0.2
0.1
0.0
Phylostratum
Cellular_organisms - ps1
Archaea1 - ps2
Archaea2 - ps3
Archaea3 - ps4
Asgard/Eukaryota - ps5
Eukaryota - ps6
Amorpheae - ps7
Obazoa - ps8
Opisthokonta - ps9
Holozoa - ps10
Filozoa - ps11
Choano./Metazoa - ps12
Metazoa - ps13
Myriazoa - ps14
Parahoxozoa - ps15
Bilateria - ps16
Deuterostomia - ps17
Chordata - ps18
Olfactores - ps19
Vertebrata - ps20
Gnathostomata - ps21
Euteleostomi - ps22
Sarcopterygii - ps23
Tetrapoda - ps24
Amniota - ps25
Mammalia - ps26
Theria - ps27
Placentalia - ps28
Boreoeutheria - ps29
Euarchontoglires - ps30
Euarchonta - ps31
Primates - ps32
Haplorrhini - ps33
Simiiformes - ps34
Catarrhini - ps35
Hominoidea - ps36
Hominidae - ps37
Homininae - ps38
Hominini - ps39
Homo_sapiens - ps40
p-value
6e-14
6e-11
6e-08
6e-05
5e-02
Log-odds
0.5
1.0
1.5
2.0
2.5
3.0
3.5
4.0

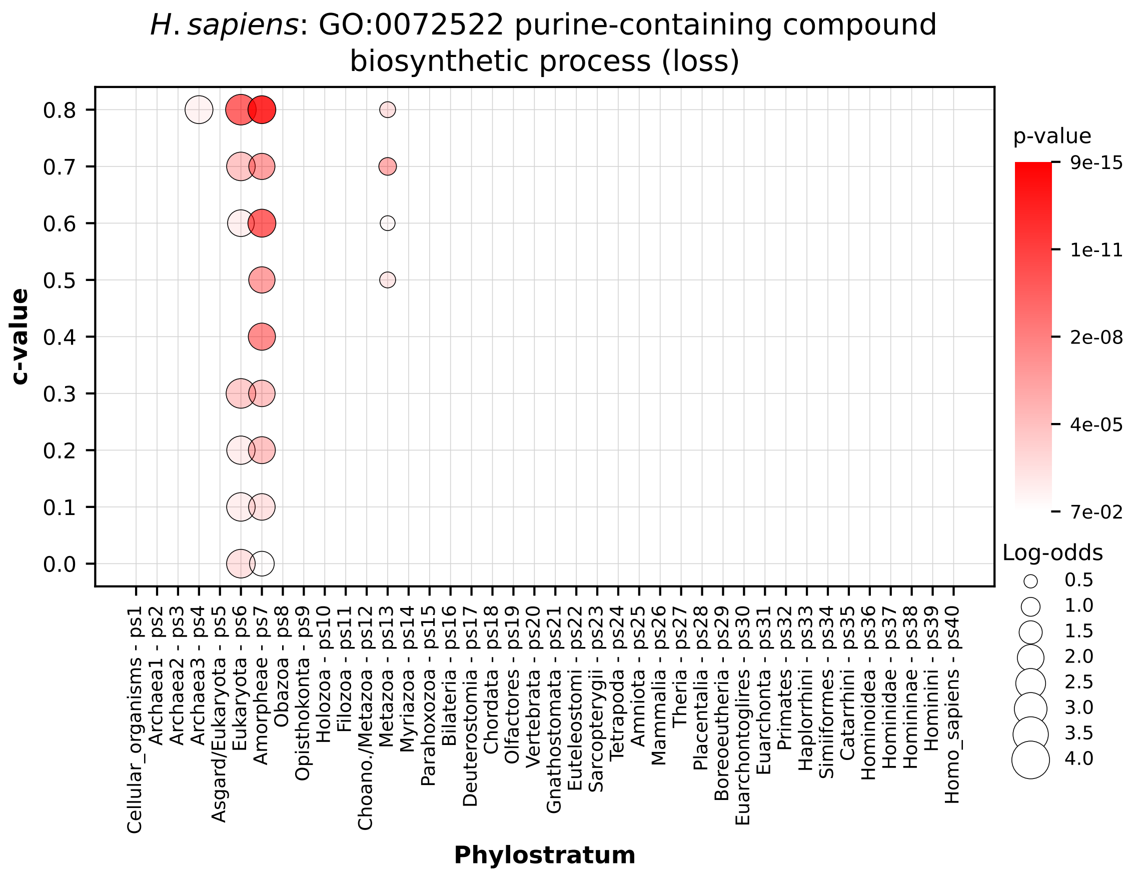

H. sapiens: GO:0072522 purine-containing compound biosynthetic process (loss)
c-value
0.8
0.7
0.6
0.5
0.4
0.3
0.2
0.1
0.0
p-value
9e-15
1e-11
2e-08
4e-05
7e-02
Log-odds
0.5
1.0
1.5
2.0
2.5
3.0
3.5
4.0
Cellular_organisms - ps1
Archaea1 - ps2
Archaea2 - ps3
Archaea3 - ps4
Asgard/Eukaryota - ps5
Eukaryota - ps6
Amorpheae - ps7
Obazoa - ps8
Opisthokonta - ps9
Holozoa - ps10
Filozoa - ps11
Choano./Metazoa - ps12
Metazoa - ps13
Myriazoa - ps14
Parahoxozoa - ps15
Bilateria - ps16
Deuterostomia - ps17
Chordata - ps18
Olfactores - ps19
Vertebrata - ps20
Gnathostomata - ps21
Euteleostomi - ps22
Sarcopterygii - ps23
Tetrapoda - ps24
Amniota - ps25
Mammalia - ps26
Theria - ps27
Placentalia - ps28
Boreoeutheria - ps29
Euarchontoglires - ps30
Euarchonta - ps31
Primates - ps32
Haplorrhini - ps33
Simiiformes - ps34
Catarrhini - ps35
Hominoidea - ps36
Hominidae - ps37
Homininae - ps38
Hominini - ps39
Homo_sapiens - ps40
Phylostratum

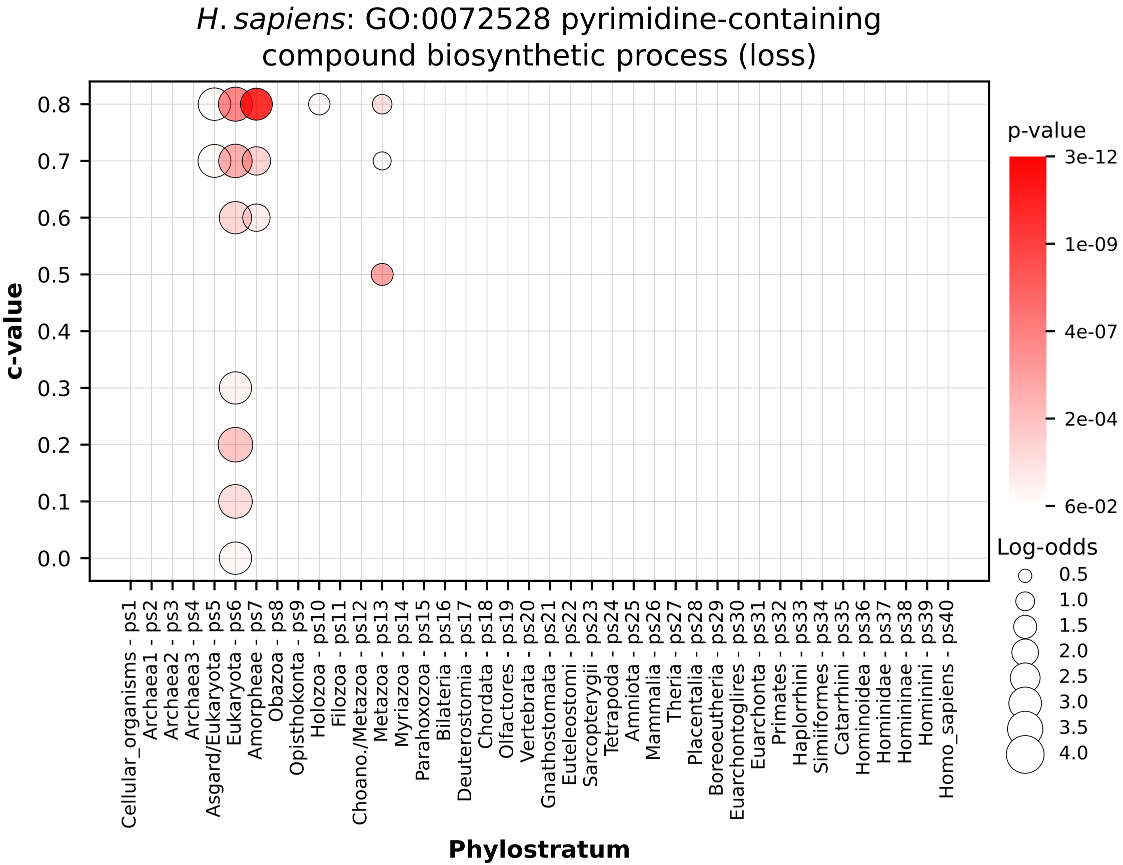

H. sapiens: GO:0072528 pyrimidine-containing compound biosynthetic process (loss)
c-value
0.8
0.7
0.6
0.5
0.4
0.3
0.2
0.1
0.0
Phylostratum
Cellular_organisms - ps1
Archaea1 - ps2
Archaea2 - ps3
Archaea3 - ps4
Asgard/Eukaryota - ps5
Eukaryota - ps6
Amorpheae - ps7
Obazoa - ps8
Opisthokonta - ps9
Holozoa - ps10
Filozoa - ps11
Choano./Metazoa - ps12
Metazoa - ps13
Myriazoa - ps14
Parahoxozoa - ps15
Bilateria - ps16
Deuterostomia - ps17
Chordata - ps18
Olfactores - ps19
Vertebrata - ps20
Gnathostomata - ps21
Euteleostomi - ps22
Sarcopterygii - ps23
Tetrapoda - ps24
Amniota - ps25
Mammalia - ps26
Theria - ps27
Placentalia - ps28
Boreoeutheria - ps29
Euarchontoglires - ps30
Euarchonta - ps31
Primates - ps32
Haplorrhini - ps33
Simiiformes - ps34
Catarrhini - ps35
Hominoidea - ps36
Hominidae - ps37
Homininae - ps38
Hominini - ps39
Homo_sapiens - ps40
p-value
3e-12
1e-09
4e-07
2e-04
6e-02
Log-odds
0.5
1.0
1.5
2.0
2.5
3.0
3.5
4.0

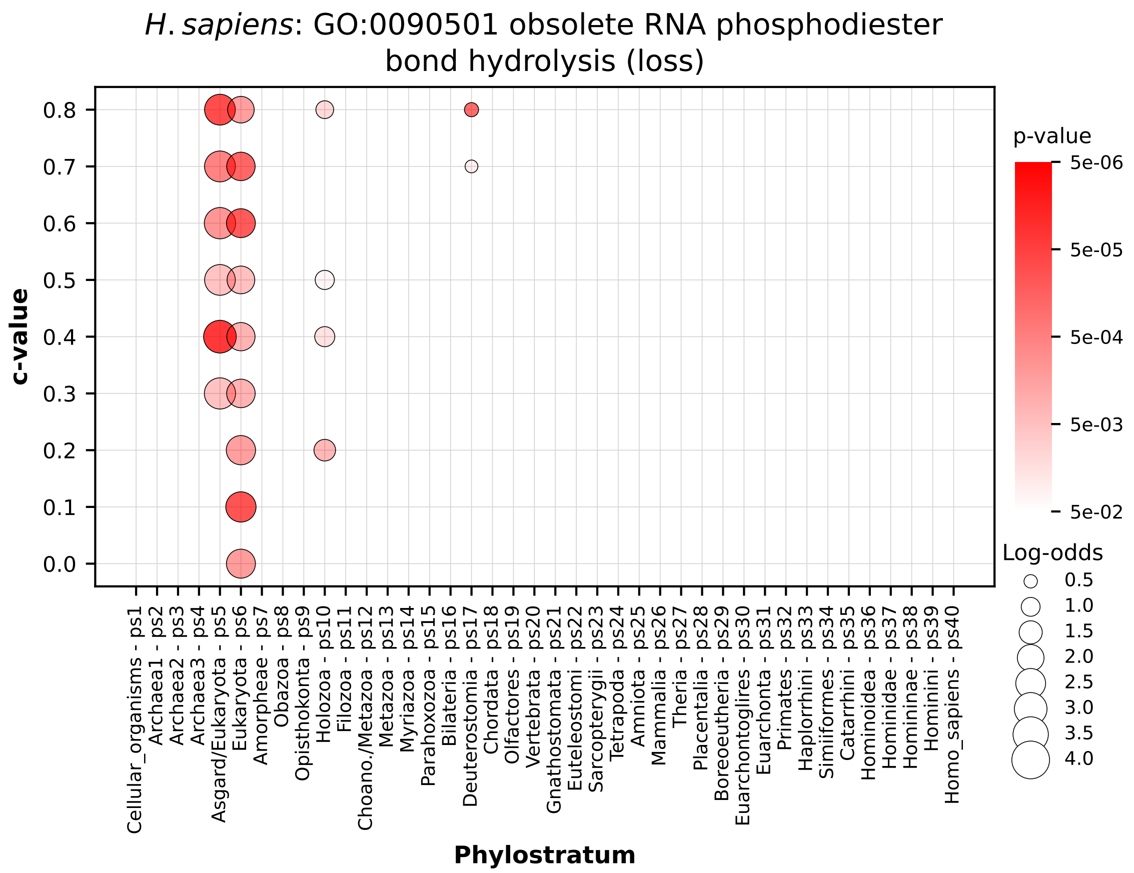

H. sapiens: GO:0090501 obsolete RNA phosphodiester bond hydrolysis (loss)
c-value
0.8
0.7
0.6
0.5
0.4
0.3
0.2
0.1
0.0
p-value
5e-06
5e-05
5e-04
5e-03
5e-02
Log-odds
0.5
1.0
1.5
2.0
2.5
3.0
3.5
4.0
Cellular_organisms - ps1
Archaea1 - ps2
Archaea2 - ps3
Archaea3 - ps4
Asgard/Eukaryota - ps5
Eukaryota - ps6
Amorpheae - ps7
Obazoa - ps8
Opisthokonta - ps9
Holozoa - ps10
Filozoa - ps11
Choano./Metazoa - ps12
Metazoa - ps13
Myriazoa - ps14
Parahoxozoa - ps15
Bilateria - ps16
Deuterostomia - ps17
Chordata - ps18
Olfactores - ps19
Vertebrata - ps20
Gnathostomata - ps21
Euteleostomi - ps22
Sarcopterygii - ps23
Tetrapoda - ps24
Amniota - ps25
Mammalia - ps26
Theria - ps27
Placentalia - ps28
Boreoeutheria - ps29
Euarchontoglires - ps30
Euarchonta - ps31
Primates - ps32
Haplorrhini - ps33
Simiiformes - ps34
Catarrhini - ps35
Hominoidea - ps36
Hominidae - ps37
Homininae - ps38
Hominini - ps39
Homo_sapiens - ps40
Phylostratum

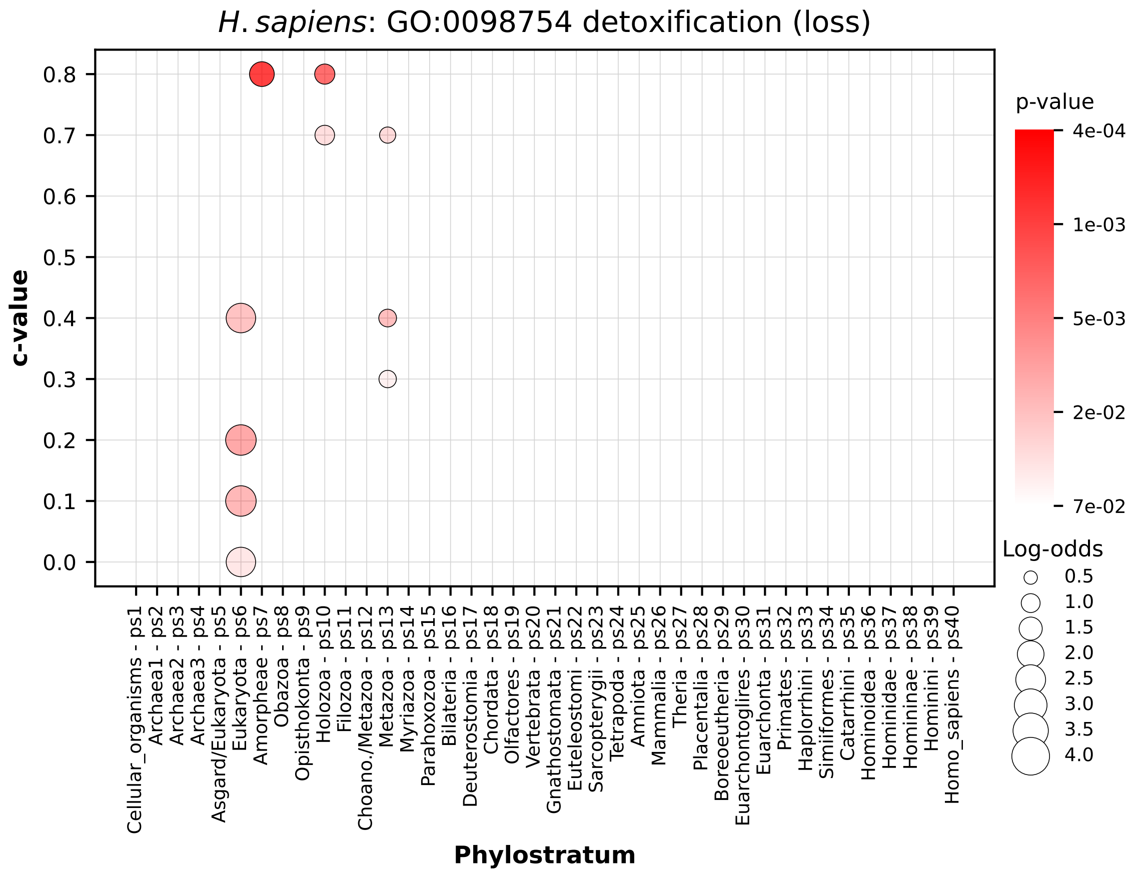

H. sapiens: GO:0098754 detoxification (loss)
c-value
0.8
0.7
0.6
0.5
0.4
0.3
0.2
0.1
0.0
Phylostratum
Cellular_organisms - ps1
Archaea1 - ps2
Archaea2 - ps3
Archaea3 - ps4
Asgard/Eukaryota - ps5
Eukaryota - ps6
Amorpheae - ps7
Obazoa - ps8
Opisthokonta - ps9
Holozoa - ps10
Filozoa - ps11
Choano./Metazoa - ps12
Metazoa - ps13
Myriazoa - ps14
Parahoxozoa - ps15
Bilateria - ps16
Deuterostomia - ps17
Chordata - ps18
Olfactores - ps19
Vertebrata - ps20
Gnathostomata - ps21
Euteleostomi - ps22
Sarcopterygii - ps23
Tetrapoda - ps24
Amniota - ps25
Mammalia - ps26
Theria - ps27
Placentalia - ps28
Boreoeutheria - ps29
Euarchontoglires - ps30
Euarchonta - ps31
Primates - ps32
Haplorrhini - ps33
Simiiformes - ps34
Catarrhini - ps35
Hominoidea - ps36
Hominidae - ps37
Homininae - ps38
Hominini - ps39
Homo_sapiens - ps40
p-value
4e-04
1e-03
5e-03
2e-02
7e-02
Log-odds
0.5
1.0
1.5
2.0
2.5
3.0
3.5
4.0

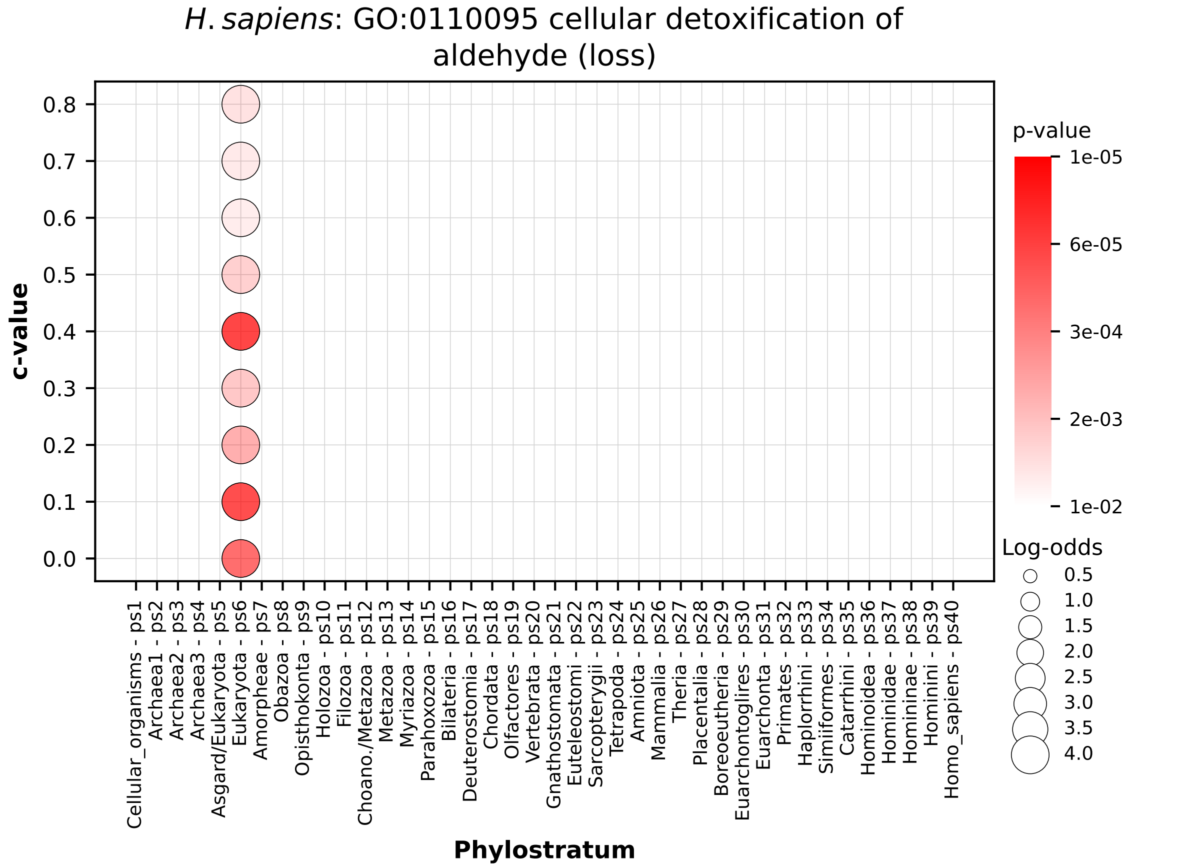

H. sapiens: GO:0110095 cellular detoxification of aldehyde (loss)
c-value
0.8
0.7
0.6
0.5
0.4
0.3
0.2
0.1
0.0
p-value
1e-05
6e-05
3e-04
2e-03
1e-02
Log-odds
0.5
1.0
1.5
2.0
2.5
3.0
3.5
4.0
Cellular_organisms - ps1
Archaea1 - ps2
Archaea2 - ps3
Archaea3 - ps4
Asgard/Eukaryota - ps5
Eukaryota - ps6
Amorpheae - ps7
Obazoa - ps8
Opisthokonta - ps9
Holozoa - ps10
Filozoa - ps11
Choano./Metazoa - ps12
Metazoa - ps13
Myriazoa - ps14
Parahoxozoa - ps15
Bilateria - ps16
Deuterostomia - ps17
Chordata - ps18
Olfactores - ps19
Vertebrata - ps20
Gnathostomata - ps21
Euteleostomi - ps22
Sarcopterygii - ps23
Tetrapoda - ps24
Amniota - ps25
Mammalia - ps26
Theria - ps27
Placentalia - ps28
Boreoeutheria - ps29
Euarchontoglires - ps30
Euarchonta - ps31
Primates - ps32
Haplorrhini - ps33
Simiiformes - ps34
Catarrhini - ps35
Hominoidea - ps36
Hominidae - ps37
Homininae - ps38
Hominini - ps39
Homo_sapiens - ps40
Phylostratum

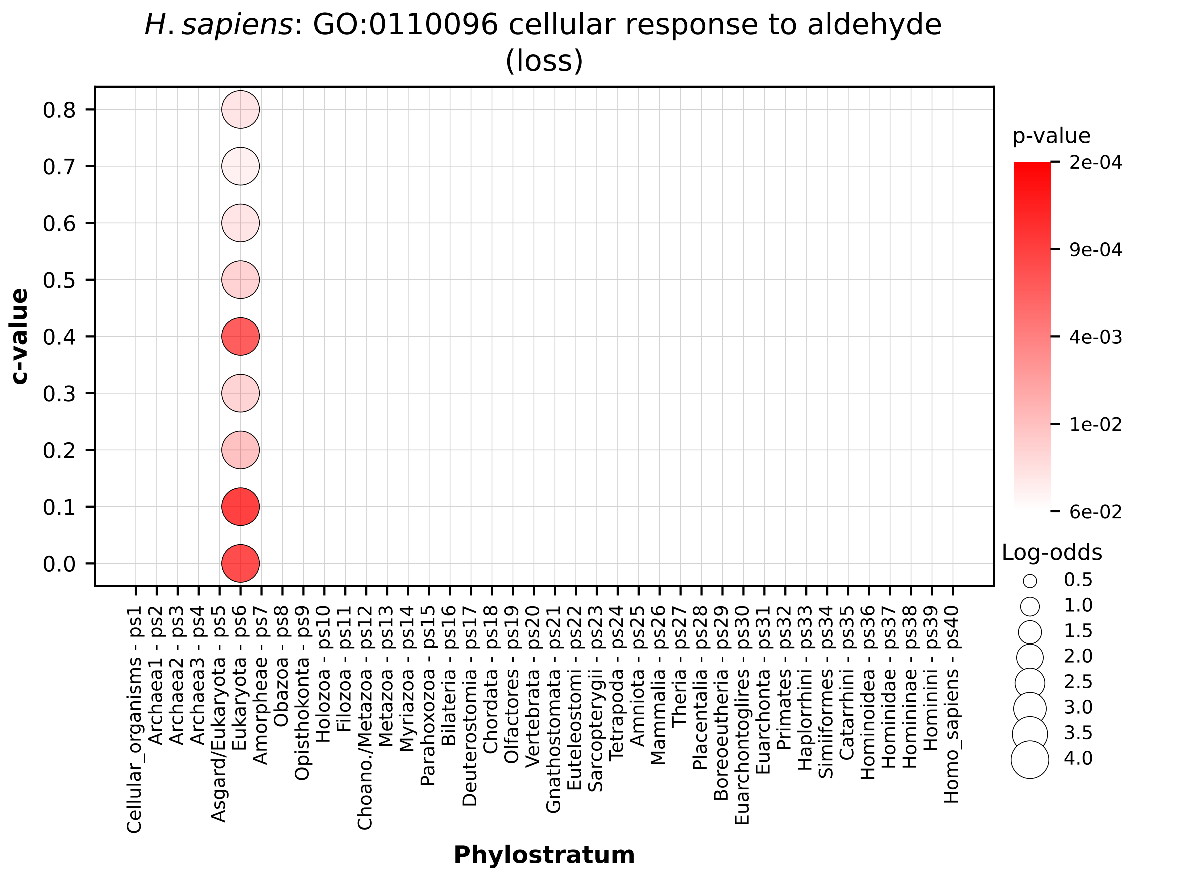

H. sapiens: GO:0110096 cellular response to aldehyde
(loss)
c-value
0.8
0.7
0.6
0.5
0.4
0.3
0.2
0.1
0.0
p-value
2e-04
9e-04
4e-03
1e-02
6e-02
Log-odds
0.5
1.0
1.5
2.0
2.5
3.0
3.5
4.0
Cellular_organisms - ps1
Archaea1 - ps2
Archaea2 - ps3
Archaea3 - ps4
Asgard/Eukaryota - ps5
Eukaryota - ps6
Amorpheae - ps7
Obazoa - ps8
Opisthokonta - ps9
Holozoa - ps10
Filozoa - ps11
Choano./Metazoa - ps12
Metazoa - ps13
Myriazoa - ps14
Parahoxozoa - ps15
Bilateria - ps16
Deuterostomia - ps17
Chordata - ps18
Olfactores - ps19
Vertebrata - ps20
Gnathostomata - ps21
Euteleostomi - ps22
Sarcopterygii - ps23
Tetrapoda - ps24
Amniota - ps25
Mammalia - ps26
Theria - ps27
Placentalia - ps28
Boreoeutheria - ps29
Euarchontoglires - ps30
Euarchonta - ps31
Primates - ps32
Haplorrhini - ps33
Simiiformes - ps34
Catarrhini - ps35
Hominoidea - ps36
Hominidae - ps37
Homininae - ps38
Hominini - ps39
Homo_sapiens - ps40
Phylostratum

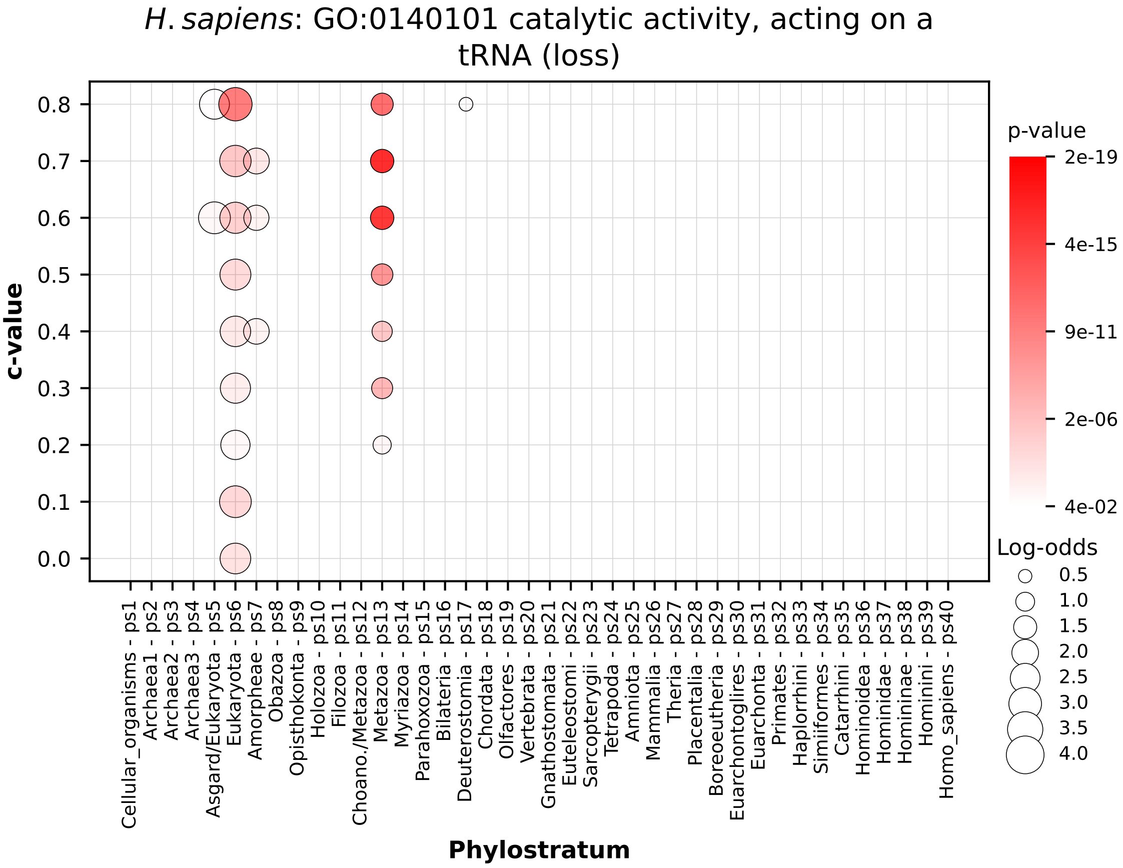

H. sapiens: GO:0140101 catalytic activity, acting on a tRNA (loss)
c-value
0.8
0.7
0.6
0.5
0.4
0.3
0.2
0.1
0.0
Phylostratum
Cellular_organisms - ps1
Archaea1 - ps2
Archaea2 - ps3
Archaea3 - ps4
Asgard/Eukaryota - ps5
Eukaryota - ps6
Amorpheae - ps7
Obazoa - ps8
Opisthokonta - ps9
Holozoa - ps10
Filozoa - ps11
Choano./Metazoa - ps12
Metazoa - ps13
Myriazoa - ps14
Parahoxozoa - ps15
Bilateria - ps16
Deuterostomia - ps17
Chordata - ps18
Olfactores - ps19
Vertebrata - ps20
Gnathostomata - ps21
Euteleostomi - ps22
Sarcopterygii - ps23
Tetrapoda - ps24
Amniota - ps25
Mammalia - ps26
Theria - ps27
Placentalia - ps28
Boreoeutheria - ps29
Euarchontoglires - ps30
Euarchonta - ps31
Primates - ps32
Haplorrhini - ps33
Simiiformes - ps34
Catarrhini - ps35
Hominoidea - ps36
Hominidae - ps37
Homininae - ps38
Hominini - ps39
Homo_sapiens - ps40
p-value
2e-19
4e-15
9e-11
2e-06
4e-02
Log-odds
0.5
1.0
1.5
2.0
2.5
3.0
3.5
4.0

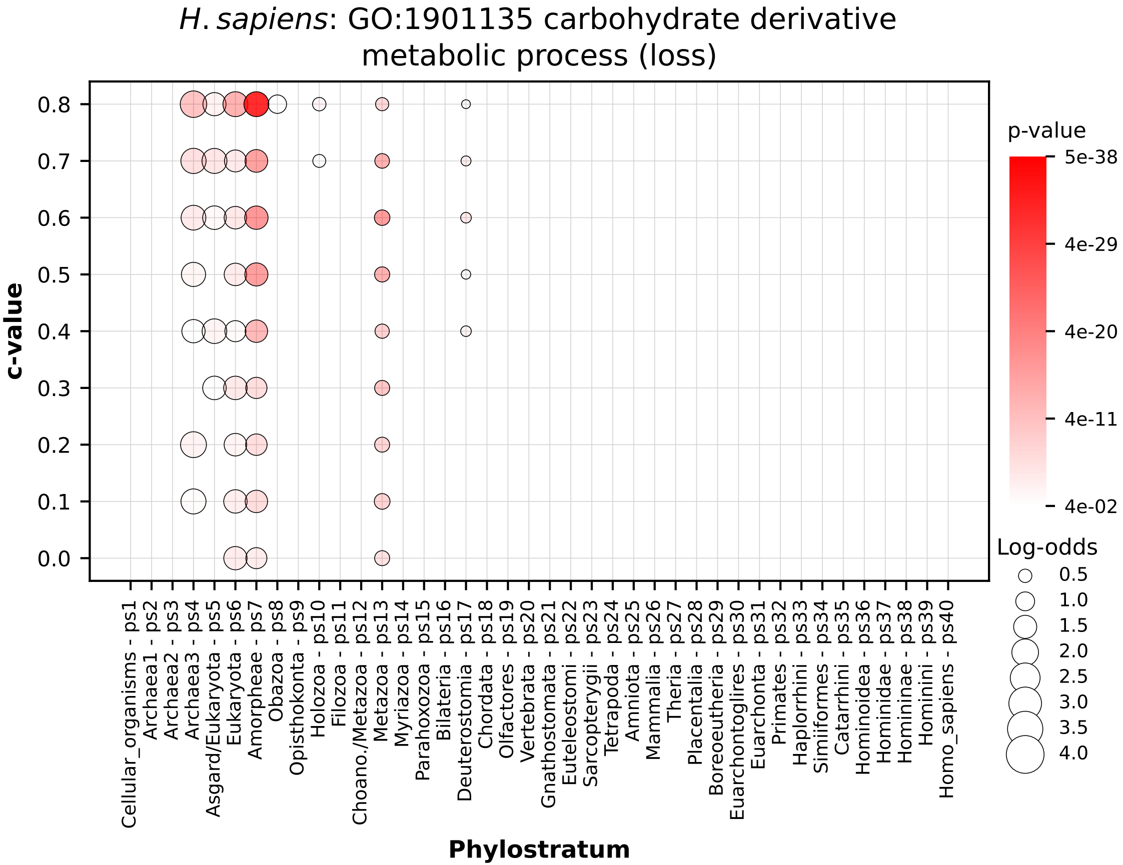

H. sapiens: GO:1901135 carbohydrate derivative metabolic process (loss)
c-value
0.8
0.7
0.6
0.5
0.4
0.3
0.2
0.1
0.0
Phylostratum
Cellular_organisms - ps1
Archaea1 - ps2
Archaea2 - ps3
Archaea3 - ps4
Asgard/Eukaryota - ps5
Eukaryota - ps6
Amorpheae - ps7
Obazoa - ps8
Opisthokonta - ps9
Holozoa - ps10
Filozoa - ps11
Choano./Metazoa - ps12
Metazoa - ps13
Myriazoa - ps14
Parahoxozoa - ps15
Bilateria - ps16
Deuterostomia - ps17
Chordata - ps18
Olfactores - ps19
Vertebrata - ps20
Gnathostomata - ps21
Euteleostomi - ps22
Sarcopterygii - ps23
Tetrapoda - ps24
Amniota - ps25
Mammalia - ps26
Theria - ps27
Placentalia - ps28
Boreoeutheria - ps29
Euarchontoglires - ps30
Euarchonta - ps31
Primates - ps32
Haplorrhini - ps33
Simiiformes - ps34
Catarrhini - ps35
Hominoidea - ps36
Hominidae - ps37
Homininae - ps38
Hominini - ps39
Homo_sapiens - ps40
p-value
5e-38
4e-29
4e-20
4e-11
4e-02
Log-odds
0.5
1.0
1.5
2.0
2.5
3.0
3.5
4.0

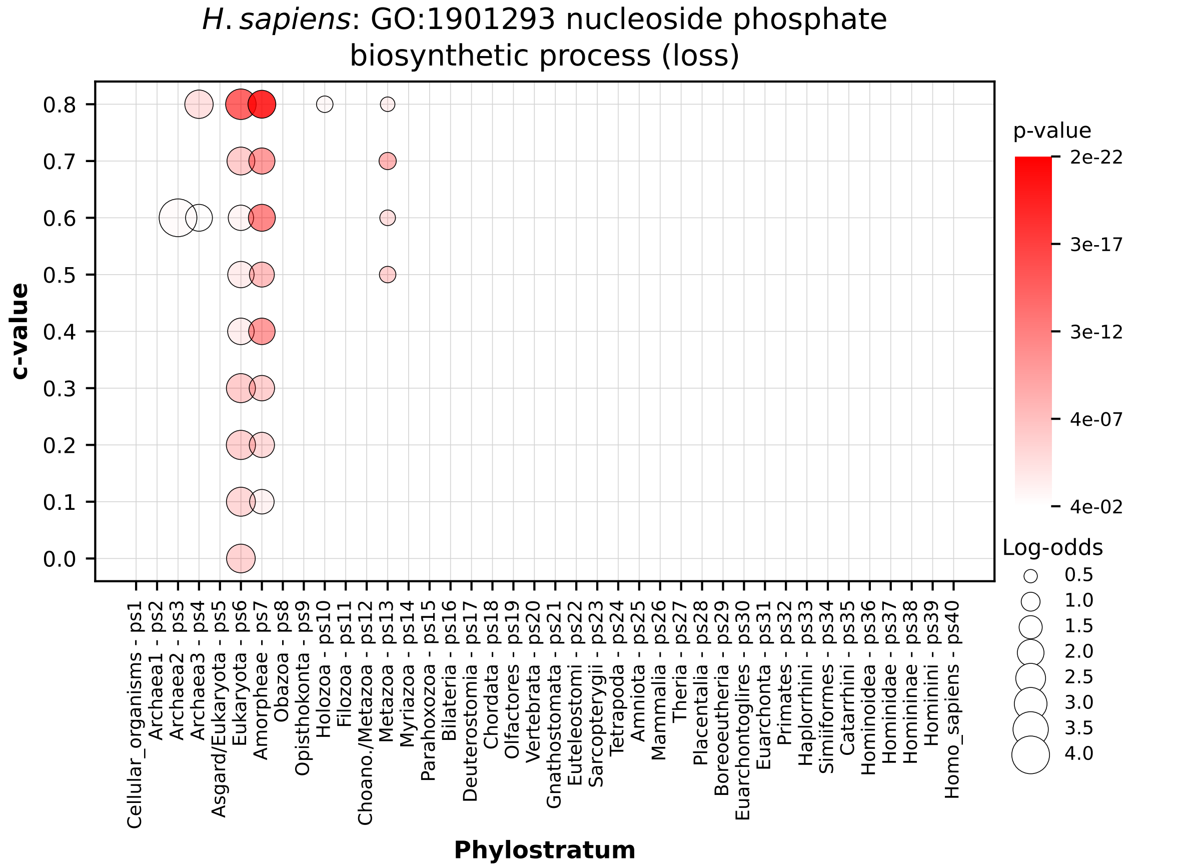

H. sapiens: GO:1901293 nucleoside phosphate biosynthetic process (loss)
c-value
0.8
0.7
0.6
0.5
0.4
0.3
0.2
0.1
0.0
Phylostratum
Cellular_organisms - ps1
Archaea1 - ps2
Archaea2 - ps3
Archaea3 - ps4
Asgard/Eukaryota - ps5
Eukaryota - ps6
Amorpheae - ps7
Obazoa - ps8
Opisthokonta - ps9
Holozoa - ps10
Filozoa - ps11
Choano./Metazoa - ps12
Metazoa - ps13
Myriazoa - ps14
Parahoxozoa - ps15
Bilateria - ps16
Deuterostomia - ps17
Chordata - ps18
Olfactores - ps19
Vertebrata - ps20
Gnathostomata - ps21
Euteleostomi - ps22
Sarcopterygii - ps23
Tetrapoda - ps24
Amniota - ps25
Mammalia - ps26
Theria - ps27
Placentalia - ps28
Boreoeutheria - ps29
Euarchontoglires - ps30
Euarchonta - ps31
Primates - ps32
Haplorrhini - ps33
Simiiformes - ps34
Catarrhini - ps35
Hominoidea - ps36
Hominidae - ps37
Homininae - ps38
Hominini - ps39
Homo_sapiens - ps40
p-value
2e-22
3e-17
3e-12
4e-07
4e-02
Log-odds
0.5
1.0
1.5
2.0
2.5
3.0
3.5
4.0

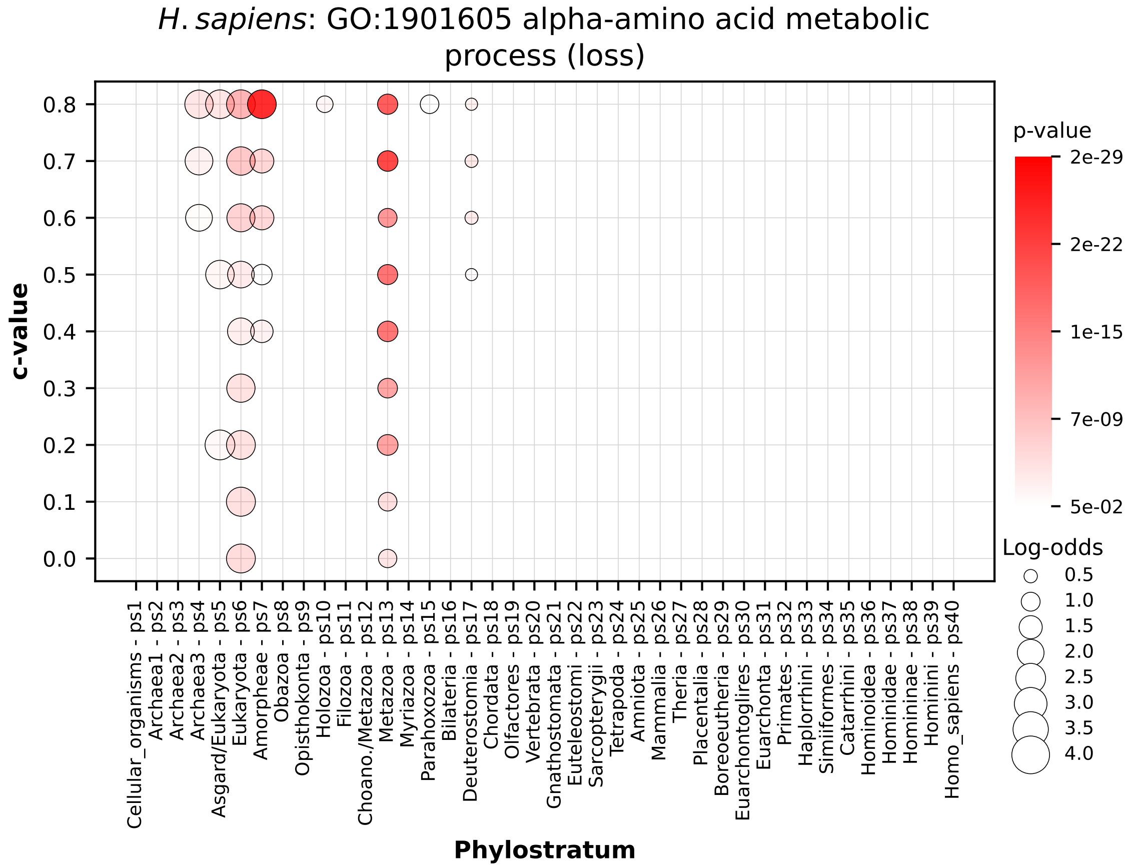

H. sapiens: GO:1901605 alpha-amino acid metabolic process (loss)
c-value
0.0
0.1
0.2
0.3
0.4
0.5
0.6
0.7
0.8
Phylostratum
Cellular_organisms - ps1
Archaea1 - ps2
Archaea2 - ps3
Archaea3 - ps4
Asgard/Eukaryota - ps5
Eukaryota - ps6
Amorpheae - ps7
Obazoa - ps8
Opisthokonta - ps9
Holozoa - ps10
Filozoa - ps11
Choano./Metazoa - ps12
Metazoa - ps13
Myriazoa - ps14
Parahoxozoa - ps15
Bilateria - ps16
Deuterostomia - ps17
Chordata - ps18
Olfactores - ps19
Vertebrata - ps20
Gnathostomata - ps21
Euteleostomi - ps22
Sarcopterygii - ps23
Tetrapoda - ps24
Amniota - ps25
Mammalia - ps26
Theria - ps27
Placentalia - ps28
Boreoeutheria - ps29
Euarchontoglires - ps30
Euarchonta - ps31
Primates - ps32
Haplorrhini - ps33
Simiiformes - ps34
Catarrhini - ps35
Hominoidea - ps36
Hominidae - ps37
Homininae - ps38
Hominini - ps39
Homo_sapiens - ps40
p-value
2e-29
2e-22
1e-15
7e-09
5e-02
Log-odds
0.5
1.0
1.5
2.0
2.5
3.0
3.5
4.0

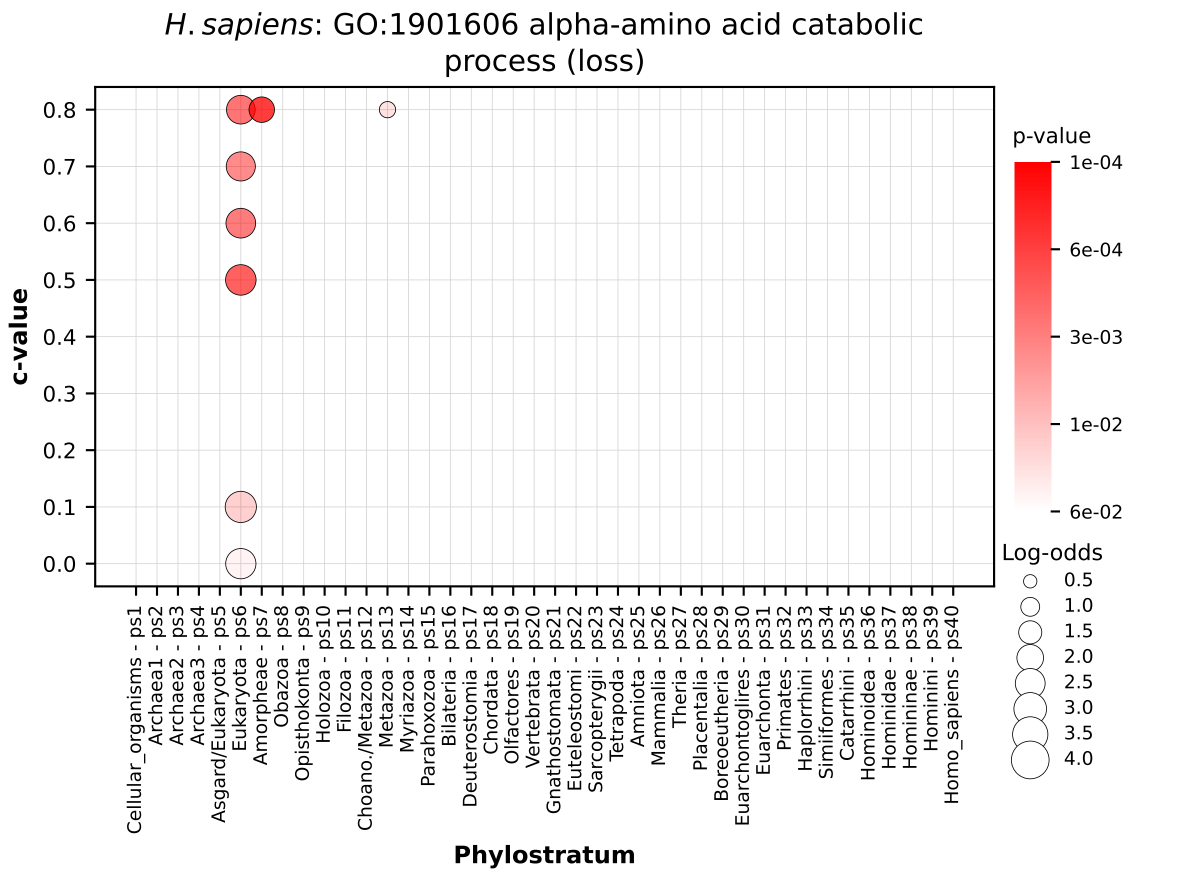

H. sapiens: GO:1901606 alpha-amino acid catabolic process (loss)
c-value
0.0
0.1
0.2
0.3
0.4
0.5
0.6
0.7
0.8
Phylostratum
Cellular_organisms - ps1
Archaea1 - ps2
Archaea2 - ps3
Archaea3 - ps4
Asgard/Eukaryota - ps5
Eukaryota - ps6
Amorpheae - ps7
Obazoa - ps8
Opisthokonta - ps9
Holozoa - ps10
Filozoa - ps11
Choano./Metazoa - ps12
Metazoa - ps13
Myriazoa - ps14
Parahoxozoa - ps15
Bilateria - ps16
Deuterostomia - ps17
Chordata - ps18
Olfactores - ps19
Vertebrata - ps20
Gnathostomata - ps21
Euteleostomi - ps22
Sarcopterygii - ps23
Tetrapoda - ps24
Amniota - ps25
Mammalia - ps26
Theria - ps27
Placentalia - ps28
Boreoeutheria - ps29
Euarchontoglires - ps30
Euarchonta - ps31
Primates - ps32
Haplorrhini - ps33
Simiiformes - ps34
Catarrhini - ps35
Hominoidea - ps36
Hominidae - ps37
Homininae - ps38
Hominini - ps39
Homo_sapiens - ps40
p-value
1e-04
6e-04
3e-03
1e-02
6e-02
Log-odds
0.5
1.0
1.5
2.0
2.5
3.0
3.5
4.0

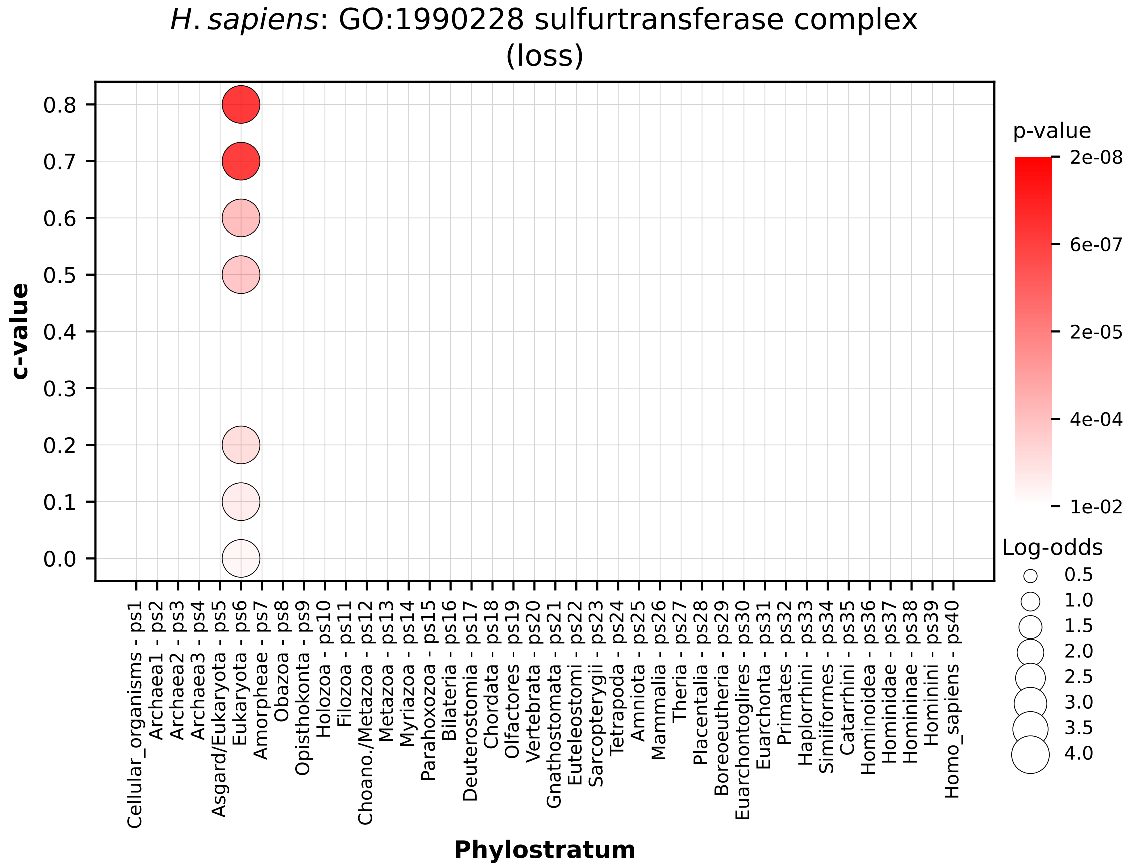

H. sapiens: GO:1990228 sulfurtransferase complex
(loss)
c-value
0.8
0.7
0.6
0.5
0.4
0.3
0.2
0.1
0.0
Phylostratum
Cellular_organisms - ps1
Archaea1 - ps2
Archaea2 - ps3
Archaea3 - ps4
Asgard/Eukaryota - ps5
Eukaryota - ps6
Amorpheae - ps7
Obazoa - ps8
Opisthokonta - ps9
Holozoa - ps10
Filozoa - ps11
Choano./Metazoa - ps12
Metazoa - ps13
Myriazoa - ps14
Parahoxozoa - ps15
Bilateria - ps16
Deuterostomia - ps17
Chordata - ps18
Olfactores - ps19
Vertebrata - ps20
Gnathostomata - ps21
Euteleostomi - ps22
Sarcopterygii - ps23
Tetrapoda - ps24
Amniota - ps25
Mammalia - ps26
Theria - ps27
Placentalia - ps28
Boreoeutheria - ps29
Euarchontoglires - ps30
Euarchonta - ps31
Primates - ps32
Haplorrhini - ps33
Simiiformes - ps34
Catarrhini - ps35
Hominoidea - ps36
Hominidae - ps37
Homininae - ps38
Hominini - ps39
Homo_sapiens - ps40
p-value
2e-08
6e-07
2e-05
4e-04
1e-02
Log-odds
0.5
1.0
1.5
2.0
2.5
3.0
3.5
4.0

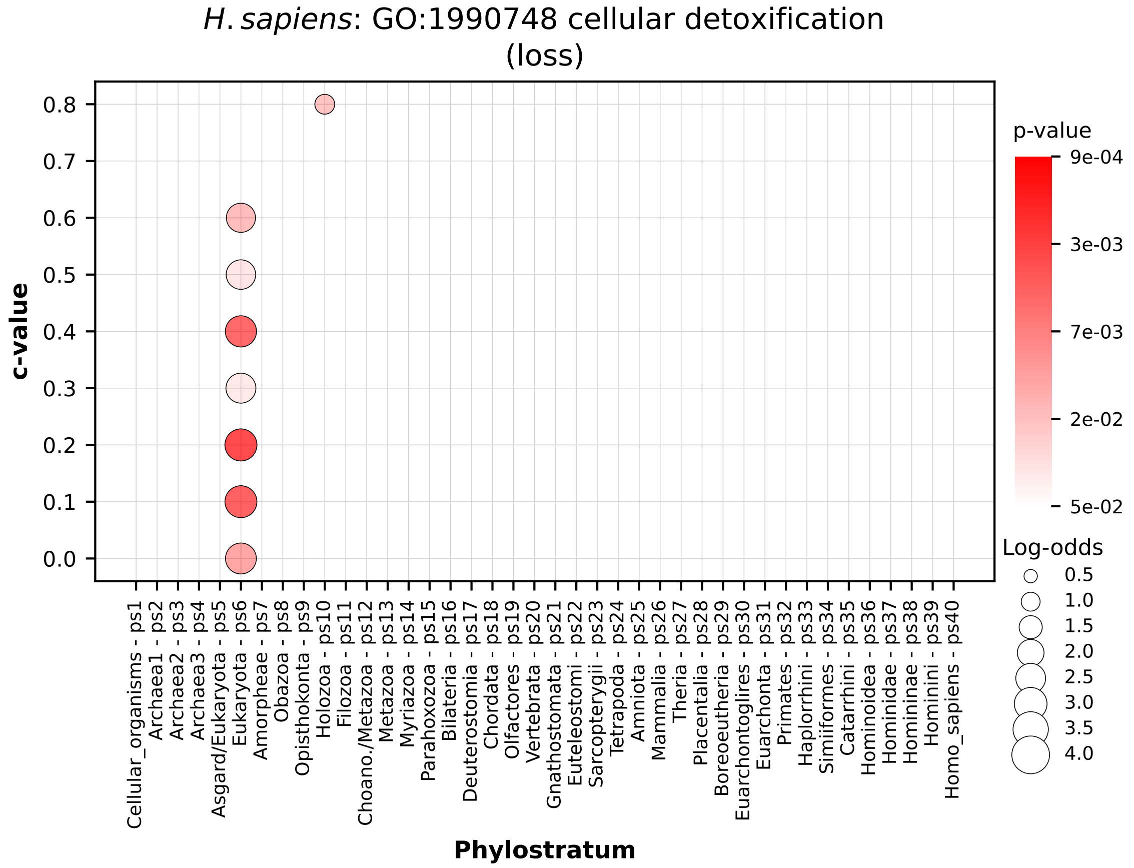

H. sapiens: GO:1990748 cellular detoxification
(loss)
c-value
0.8
0.7
0.6
0.5
0.4
0.3
0.2
0.1
0.0
p-value
9e-04
3e-03
7e-03
2e-02
5e-02
Log-odds
0.5
1.0
1.5
2.0
2.5
3.0
3.5
4.0
Cellular_organisms - ps1
Archaea1 - ps2
Archaea2 - ps3
Archaea3 - ps4
Asgard/Eukaryota - ps5
Eukaryota - ps6
Amorpheae - ps7
Obazoa - ps8
Opisthokonta - ps9
Holozoa - ps10
Filozoa - ps11
Choano./Metazoa - ps12
Metazoa - ps13
Myriazoa - ps14
Parahoxozoa - ps15
Bilateria - ps16
Deuterostomia - ps17
Chordata - ps18
Olfactores - ps19
Vertebrata - ps20
Gnathostomata - ps21
Euteleostomi - ps22
Sarcopterygii - ps23
Tetrapoda - ps24
Amniota - ps25
Mammalia - ps26
Theria - ps27
Placentalia - ps28
Boreoeutheria - ps29
Euarchontoglires - ps30
Euarchonta - ps31
Primates - ps32
Haplorrhini - ps33
Simiiformes - ps34
Catarrhini - ps35
Hominoidea - ps36
Hominidae - ps37
Homininae - ps38
Hominini - ps39
Homo_sapiens - ps40
Phylostratum

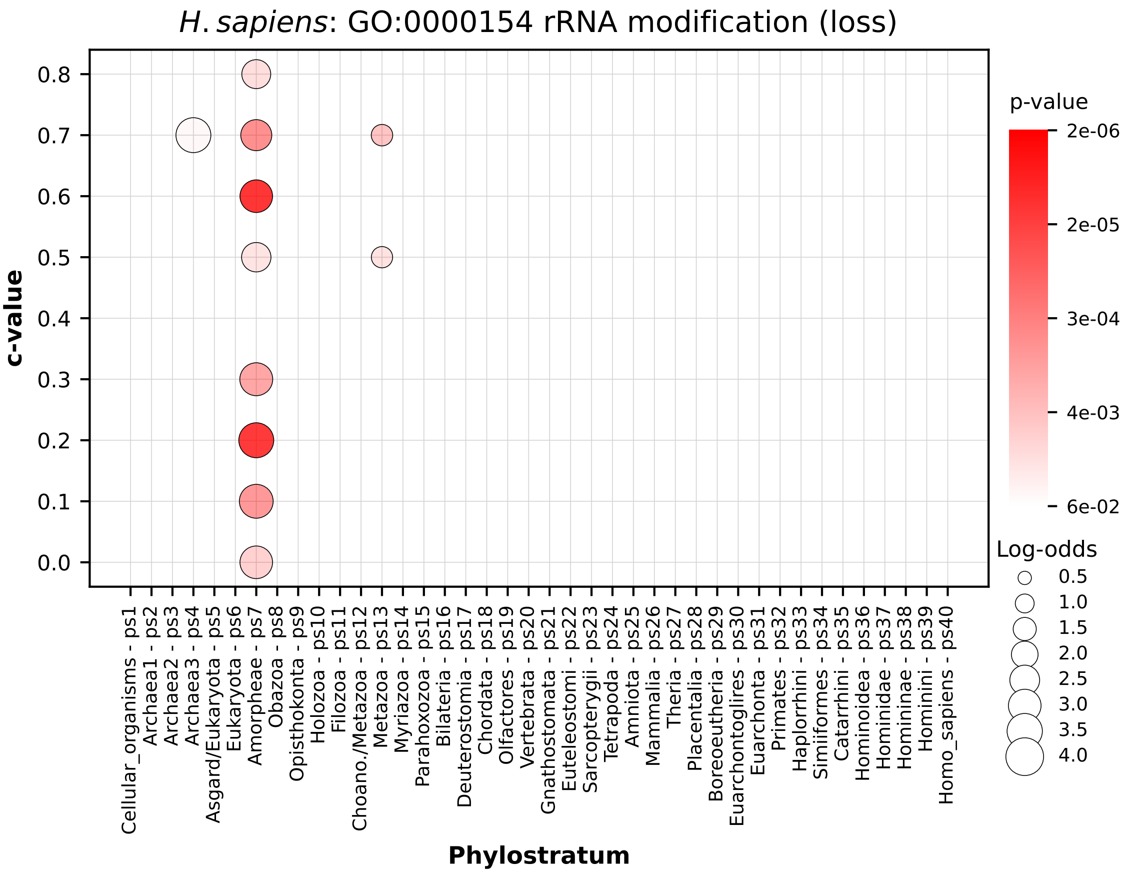

H. sapiens: GO:0000154 rRNA modification (loss)
c-value
0.0
0.1
0.2
0.3
0.4
0.5
0.6
0.7
0.8
Phylostratum
Cellular_organisms - ps1
Archaea1 - ps2
Archaea2 - ps3
Archaea3 - ps4
Asgard/Eukaryota - ps5
Eukaryota - ps6
Amorpheae - ps7
Obazoa - ps8
Opisthokonta - ps9
Holozoa - ps10
Filozoa - ps11
Choano./Metazoa - ps12
Metazoa - ps13
Myriazoa - ps14
Parahoxozoa - ps15
Bilateria - ps16
Deuterostomia - ps17
Chordata - ps18
Olfactores - ps19
Vertebrata - ps20
Gnathostomata - ps21
Euteleostomi - ps22
Sarcopterygii - ps23
Tetrapoda - ps24
Amniota - ps25
Mammalia - ps26
Theria - ps27
Placentalia - ps28
Boreoeutheria - ps29
Euarchontoglires - ps30
Euarchonta - ps31
Primates - ps32
Haplorrhini - ps33
Simiiformes - ps34
Catarrhini - ps35
Hominoidea - ps36
Hominidae - ps37
Homininae - ps38
Hominini - ps39
Homo_sapiens - ps40
p-value
2e-06
2e-05
3e-04
4e-03
6e-02
Log-odds
0.5
1.0
1.5
2.0
2.5
3.0
3.5
4.0

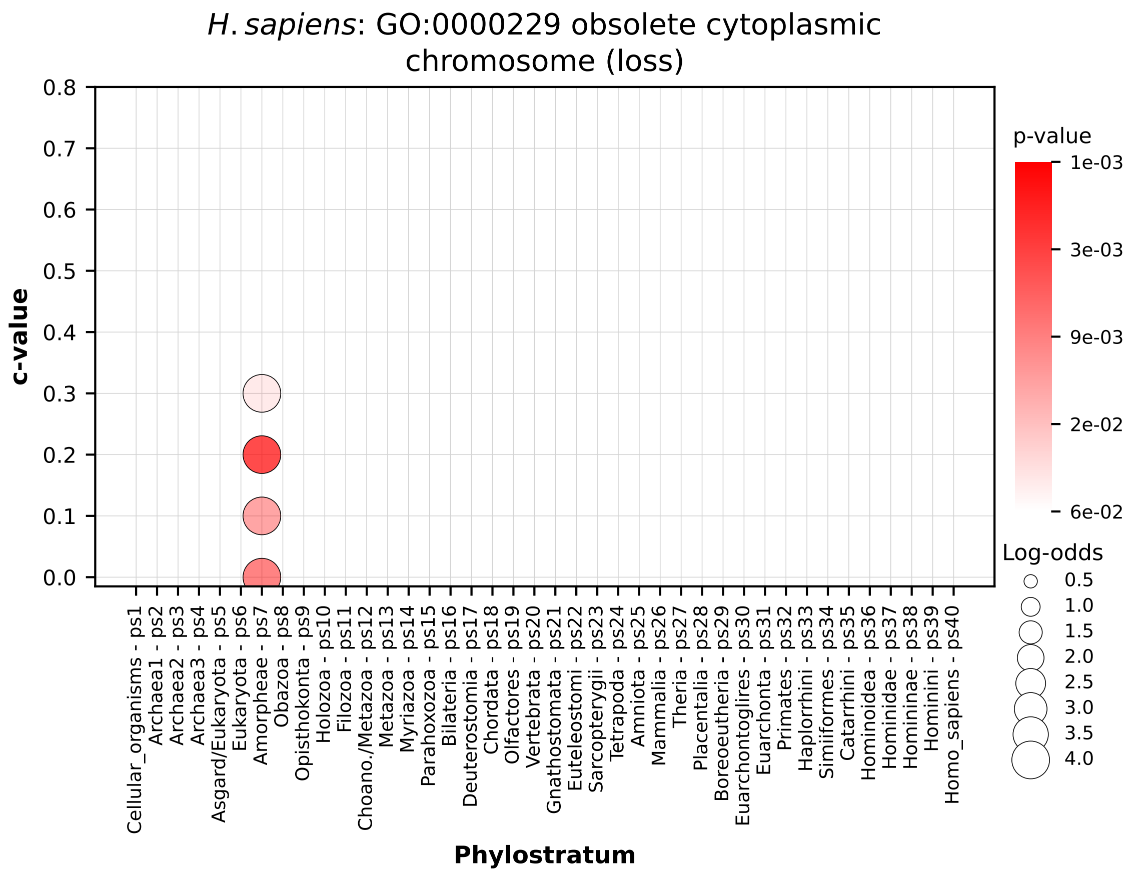

H. sapiens: GO:0000229 obsolete cytoplasmic chromosome (loss)
c-value
0.0
0.1
0.2
0.3
0.4
0.5
0.6
0.7
0.8
Phylostratum
Cellular_organisms - ps1
Archaea1 - ps2
Archaea2 - ps3
Archaea3 - ps4
Asgard/Eukaryota - ps5
Eukaryota - ps6
Amorpheae - ps7
Obazoa - ps8
Opisthokonta - ps9
Holozoa - ps10
Filozoa - ps11
Choano./Metazoa - ps12
Metazoa - ps13
Myriazoa - ps14
Parahoxozoa - ps15
Bilateria - ps16
Deuterostomia - ps17
Chordata - ps18
Olfactores - ps19
Vertebrata - ps20
Gnathostomata - ps21
Euteleostomi - ps22
Sarcopterygii - ps23
Tetrapoda - ps24
Amniota - ps25
Mammalia - ps26
Theria - ps27
Placentalia - ps28
Boreoeutheria - ps29
Euarchontoglires - ps30
Euarchonta - ps31
Primates - ps32
Haplorrhini - ps33
Simiiformes - ps34
Catarrhini - ps35
Hominoidea - ps36
Hominidae - ps37
Homininae - ps38
Hominini - ps39
Homo_sapiens - ps40
p-value
1e-03
3e-03
9e-03
2e-02
6e-02
Log-odds
0.5
1.0
1.5
2.0
2.5
3.0
3.5
4.0

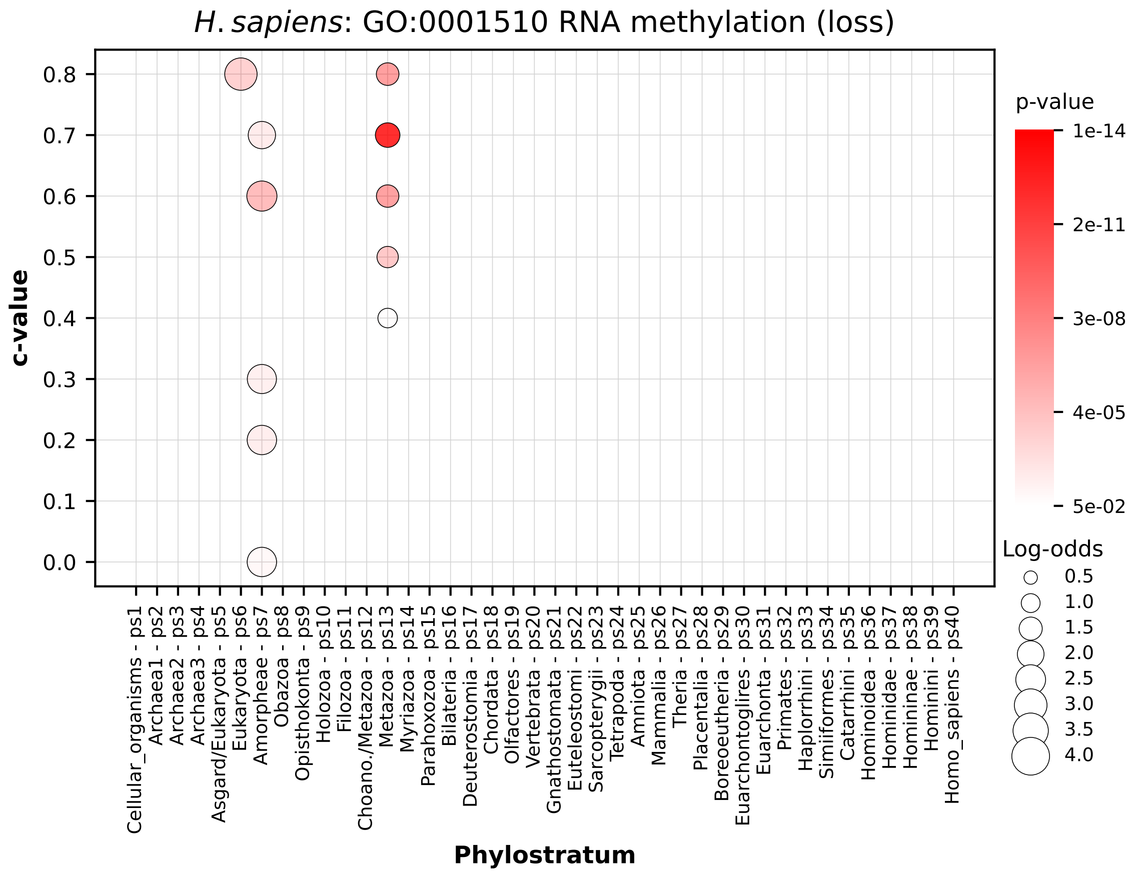

H. sapiens: GO:0001510 RNA methylation (loss)
c-value
0.8
0.7
0.6
0.5
0.4
0.3
0.2
0.1
0.0
Phylostratum
Cellular_organisms - ps1
Archaea1 - ps2
Archaea2 - ps3
Archaea3 - ps4
Asgard/Eukaryota - ps5
Eukaryota - ps6
Amorpheae - ps7
Obazoa - ps8
Opisthokonta - ps9
Holozoa - ps10
Filozoa - ps11
Choano./Metazoa - ps12
Metazoa - ps13
Myriazoa - ps14
Parahoxozoa - ps15
Bilateria - ps16
Deuterostomia - ps17
Chordata - ps18
Olfactores - ps19
Vertebrata - ps20
Gnathostomata - ps21
Euteleostomi - ps22
Sarcopterygii - ps23
Tetrapoda - ps24
Amniota - ps25
Mammalia - ps26
Theria - ps27
Placentalia - ps28
Boreoeutheria - ps29
Euarchontoglires - ps30
Euarchonta - ps31
Primates - ps32
Haplorrhini - ps33
Simiiformes - ps34
Catarrhini - ps35
Hominoidea - ps36
Hominidae - ps37
Homininae - ps38
Hominini - ps39
Homo_sapiens - ps40
p-value
1e-14
2e-11
3e-08
4e-05
5e-02
Log-odds
0.5
1.0
1.5
2.0
2.5
3.0
3.5
4.0

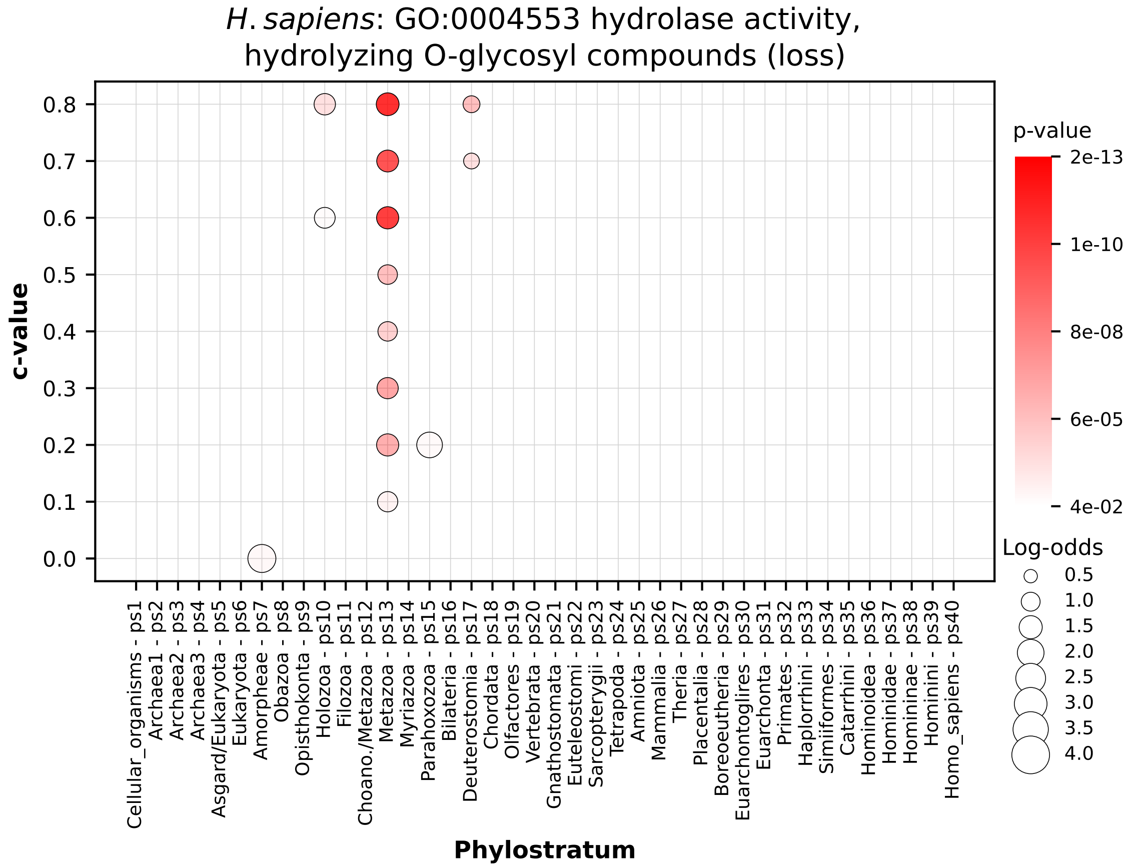

H. sapiens: GO:0004553 hydrolase activity, hydrolyzing O-glycosyl compounds (loss)
c-value
0.0
0.1
0.2
0.3
0.4
0.5
0.6
0.7
0.8
Phylostratum
Cellular_organisms - ps1
Archaea1 - ps2
Archaea2 - ps3
Archaea3 - ps4
Asgard/Eukaryota - ps5
Eukaryota - ps6
Amorpheae - ps7
Obazoa - ps8
Opisthokonta - ps9
Holozoa - ps10
Filozoa - ps11
Choano./Metazoa - ps12
Metazoa - ps13
Myriazoa - ps14
Parahoxozoa - ps15
Bilateria - ps16
Deuterostomia - ps17
Chordata - ps18
Olfactores - ps19
Vertebrata - ps20
Gnathostomata - ps21
Euteleostomi - ps22
Sarcopterygii - ps23
Tetrapoda - ps24
Amniota - ps25
Mammalia - ps26
Theria - ps27
Placentalia - ps28
Boreoeutheria - ps29
Euarchontoglires - ps30
Euarchonta - ps31
Primates - ps32
Haplorrhini - ps33
Simiiformes - ps34
Catarrhini - ps35
Hominoidea - ps36
Hominidae - ps37
Homininae - ps38
Hominini - ps39
Homo_sapiens - ps40
p-value
2e-13
1e-10
8e-08
6e-05
4e-02
Log-odds
0.5
1.0
1.5
2.0
2.5
3.0
3.5
4.0

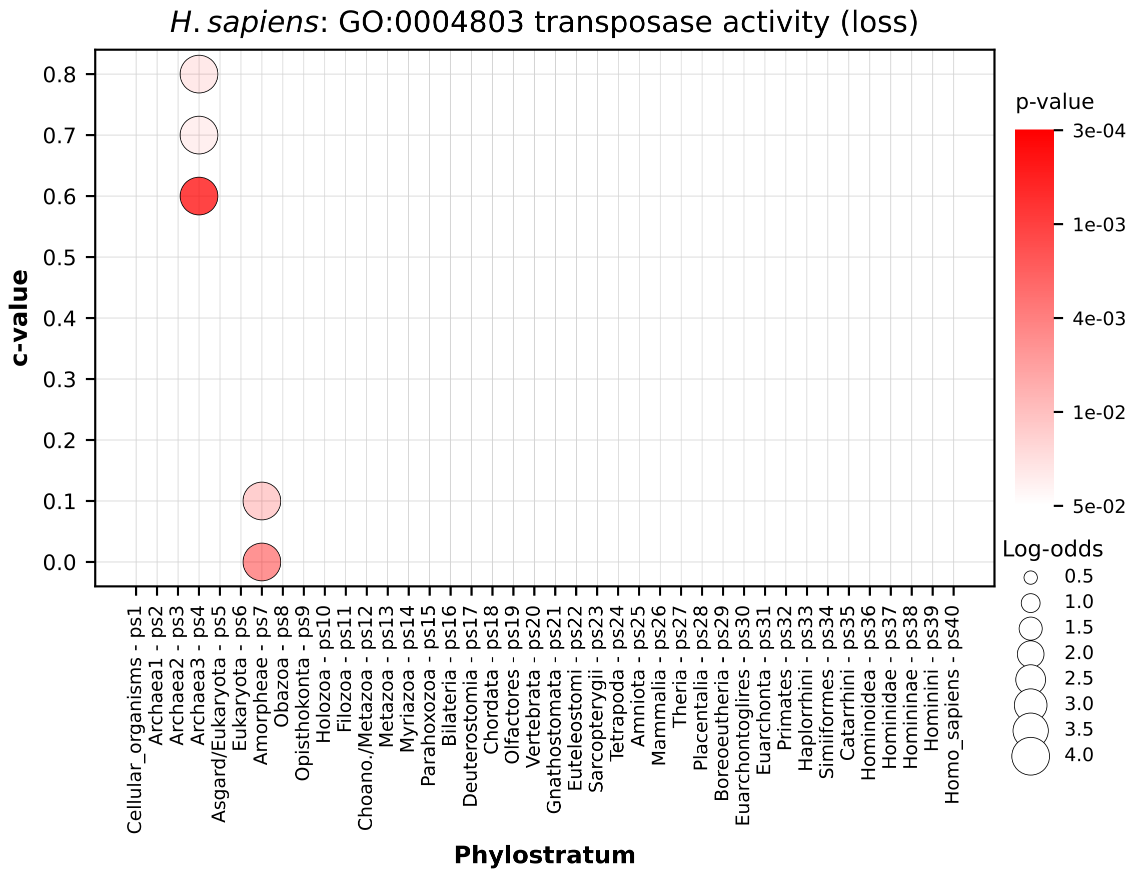

H. sapiens: GO:0004803 transposase activity (loss)
c-value
Phylostratum
p-value
3e-04
1e-03
4e-03
1e-02
5e-02
Log-odds
0.5
1.0
1.5
2.0
2.5
3.0
3.5
4.0
0.0
0.1
0.2
0.3
0.4
0.5
0.6
0.7
0.8
Cellular_organisms - ps1
Archaea1 - ps2
Archaea2 - ps3
Archaea3 - ps4
Asgard/Eukaryota - ps5
Eukaryota - ps6
Amorpheae - ps7
Obazoa - ps8
Opisthokonta - ps9
Holozoa - ps10
Filozoa - ps11
Choano./Metazoa - ps12
Metazoa - ps13
Myriazoa - ps14
Parahoxozoa - ps15
Bilateria - ps16
Deuterostomia - ps17
Chordata - ps18
Olfactores - ps19
Vertebrata - ps20
Gnathostomata - ps21
Euteleostomi - ps22
Sarcopterygii - ps23
Tetrapoda - ps24
Amniota - ps25
Mammalia - ps26
Theria - ps27
Placentalia - ps28
Boreoeutheria - ps29
Euarchontoglires - ps30
Euarchonta - ps31
Primates - ps32
Haplorrhini - ps33
Simiiformes - ps34
Catarrhini - ps35
Hominoidea - ps36
Hominidae - ps37
Homininae - ps38
Hominini - ps39
Homo_sapiens - ps40

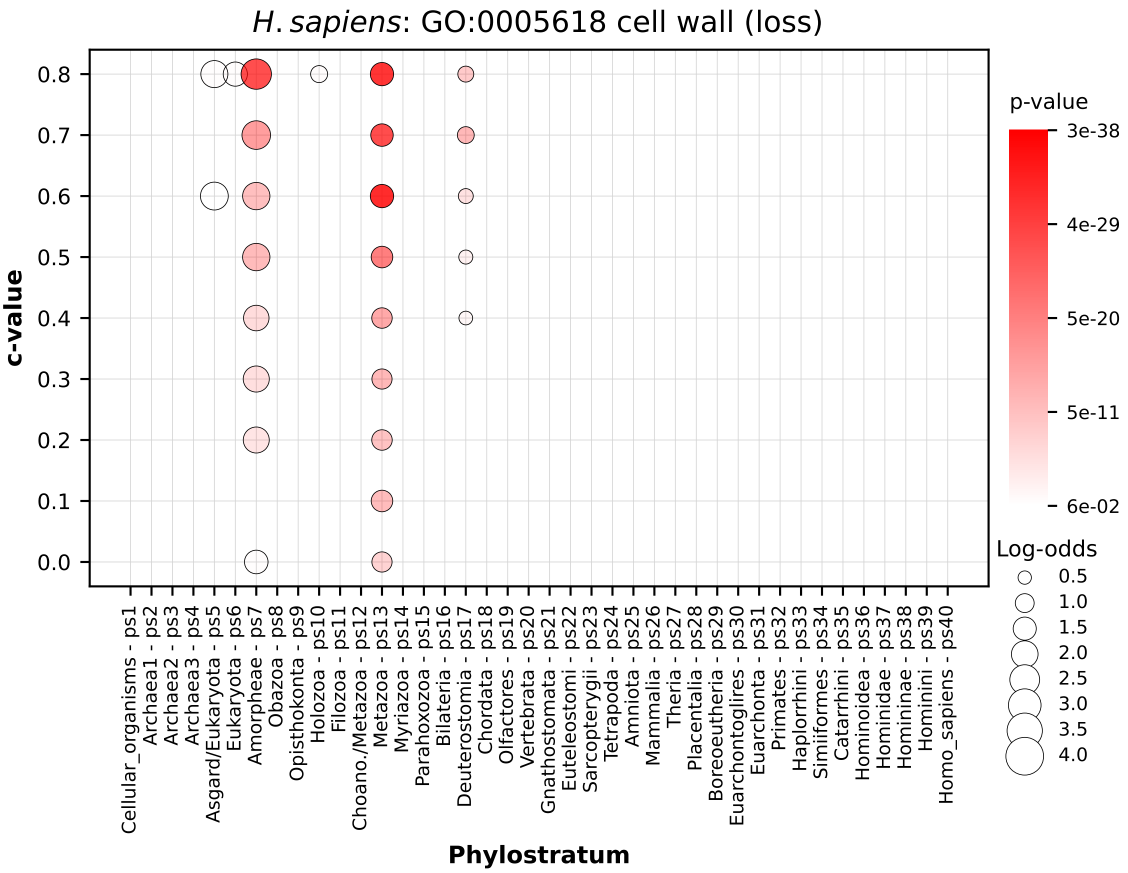

H. sapiens: GO:0005618 cell wall (loss)
c-value
0.8
0.7
0.6
0.5
0.4
0.3
0.2
0.1
0.0
Phylostratum
Cellular_organisms - ps1
Archaea1 - ps2
Archaea2 - ps3
Archaea3 - ps4
Asgard/Eukaryota - ps5
Eukaryota - ps6
Amorpheae - ps7
Obazoa - ps8
Opisthokonta - ps9
Holozoa - ps10
Filozoa - ps11
Choano./Metazoa - ps12
Metazoa - ps13
Myriazoa - ps14
Parahoxozoa - ps15
Bilateria - ps16
Deuterostomia - ps17
Chordata - ps18
Olfactores - ps19
Vertebrata - ps20
Gnathostomata - ps21
Euteleostomi - ps22
Sarcopterygii - ps23
Tetrapoda - ps24
Amniota - ps25
Mammalia - ps26
Theria - ps27
Placentalia - ps28
Boreoeutheria - ps29
Euarchontoglires - ps30
Euarchonta - ps31
Primates - ps32
Haplorrhini - ps33
Simiiformes - ps34
Catarrhini - ps35
Hominoidea - ps36
Hominidae - ps37
Homininae - ps38
Hominini - ps39
Homo_sapiens - ps40
p-value
3e-38
4e-29
5e-20
5e-11
6e-02
Log-odds
0.5
1.0
1.5
2.0
2.5
3.0
3.5
4.0

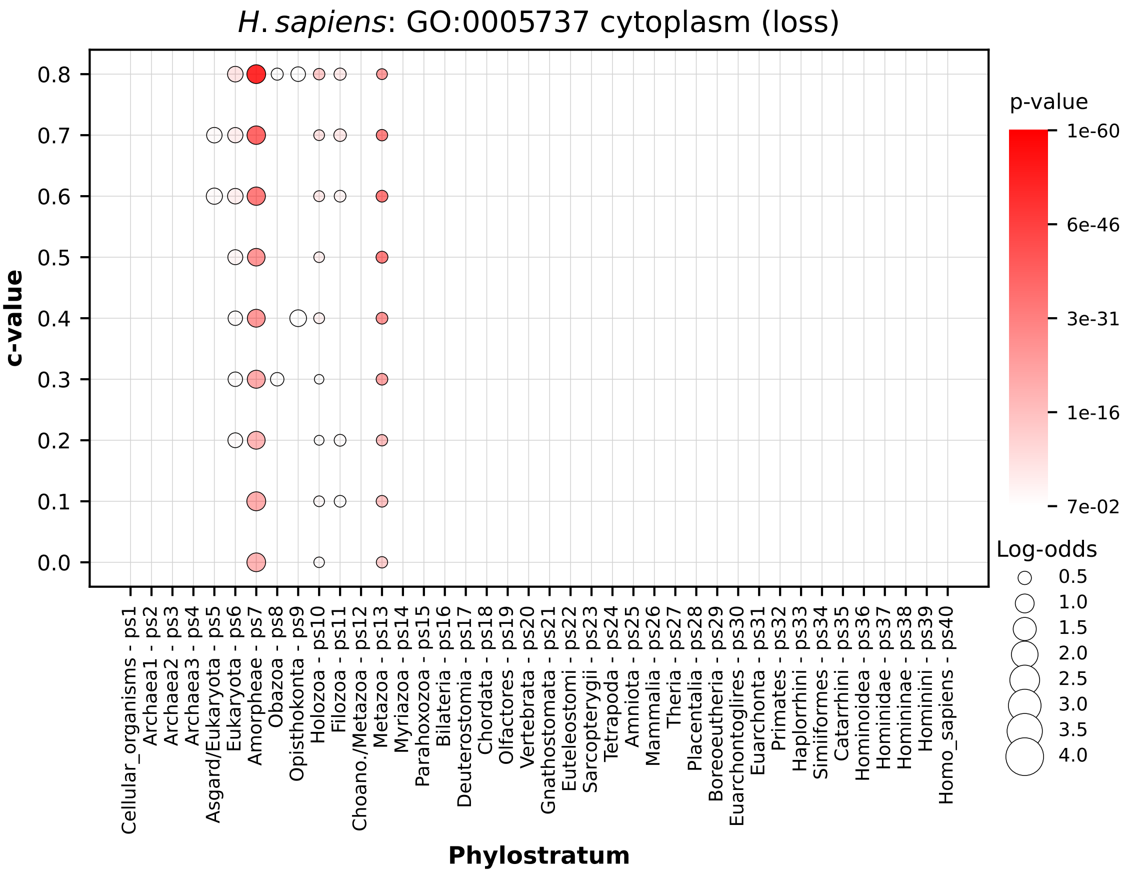

H. sapiens: GO:0005737 cytoplasm (loss)
c-value
0.0
0.1
0.2
0.3
0.4
0.5
0.6
0.7
0.8
Phylostratum
Cellular_organisms - ps1
Archaea1 - ps2
Archaea2 - ps3
Archaea3 - ps4
Asgard/Eukaryota - ps5
Eukaryota - ps6
Amorpheae - ps7
Obazoa - ps8
Opisthokonta - ps9
Holozoa - ps10
Filozoa - ps11
Choano./Metazoa - ps12
Metazoa - ps13
Myriazoa - ps14
Parahoxozoa - ps15
Bilateria - ps16
Deuterostomia - ps17
Chordata - ps18
Olfactores - ps19
Vertebrata - ps20
Gnathostomata - ps21
Euteleostomi - ps22
Sarcopterygii - ps23
Tetrapoda - ps24
Amniota - ps25
Mammalia - ps26
Theria - ps27
Placentalia - ps28
Boreoeutheria - ps29
Euarchontoglires - ps30
Euarchonta - ps31
Primates - ps32
Haplorrhini - ps33
Simiiformes - ps34
Catarrhini - ps35
Hominoidea - ps36
Hominidae - ps37
Homininae - ps38
Hominini - ps39
Homo_sapiens - ps40
p-value
1e-60
6e-46
3e-31
1e-16
7e-02
Log-odds
0.5
1.0
1.5
2.0
2.5
3.0
3.5
4.0

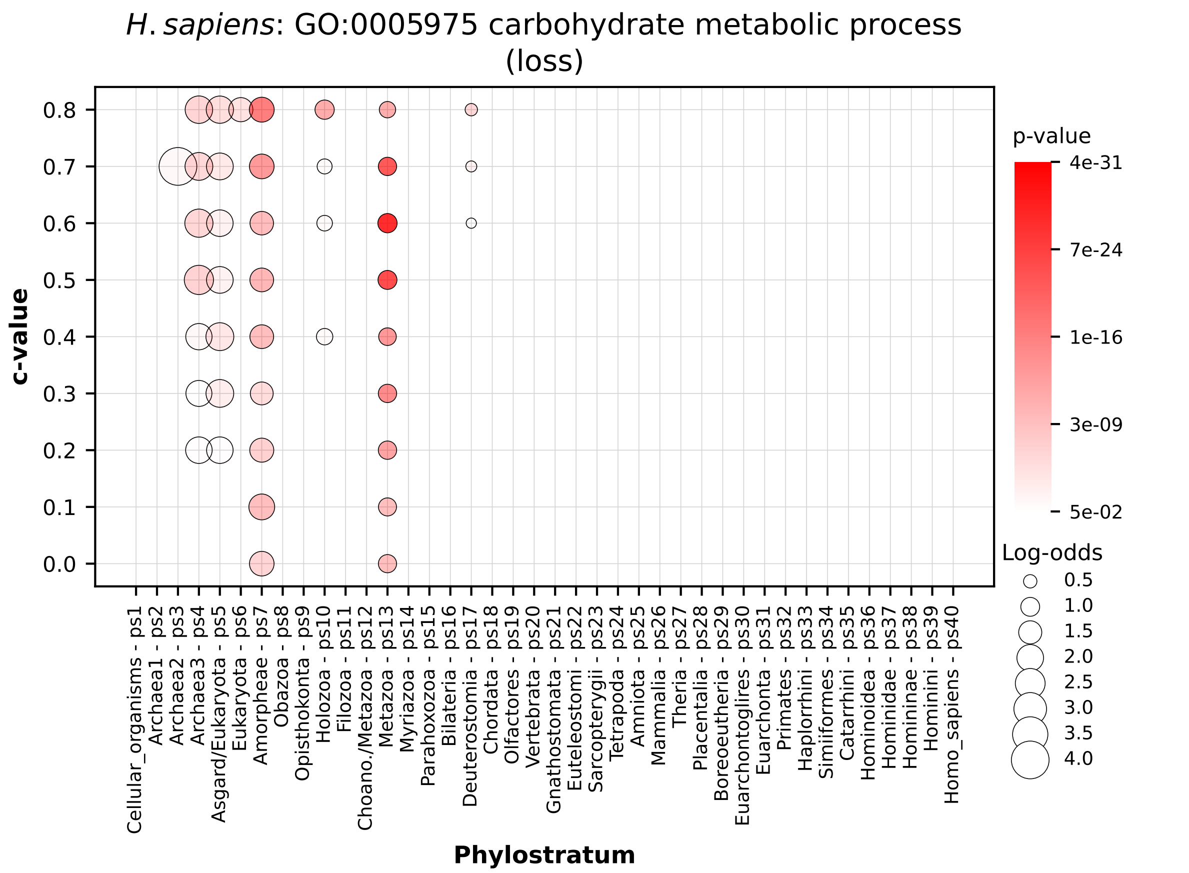
H. sapiens: GO:0005975 carbohydrate metabolic process
(loss)
c-value
0.8
0.7
0.6
0.5
0.4
0.3
0.2
0.1
0.0
Phylostratum
Cellular_organisms - ps1
Archaea1 - ps2
Archaea2 - ps3
Archaea3 - ps4
Asgard/Eukaryota - ps5
Eukaryota - ps6
Amorpheae - ps7
Obazoa - ps8
Opisthokonta - ps9
Holozoa - ps10
Filozoa - ps11
Choano./Metazoa - ps12
Metazoa - ps13
Myriazoa - ps14
Parahoxozoa - ps15
Bilateria - ps16
Deuterostomia - ps17
Chordata - ps18
Olfactores - ps19
Vertebrata - ps20
Gnathostomata - ps21
Euteleostomi - ps22
Sarcopterygii - ps23
Tetrapoda - ps24
Amniota - ps25
Mammalia - ps26
Theria - ps27
Placentalia - ps28
Boreoeutheria - ps29
Euarchontoglires - ps30
Euarchonta - ps31
Primates - ps32
Haplorrhini - ps33
Simiiformes - ps34
Catarrhini - ps35
Hominoidea - ps36
Hominidae - ps37
Homininae - ps38
Hominini - ps39
Homo_sapiens - ps40
p-value
4e-31
7e-24
1e-16
3e-09
5e-02
Log-odds
0.5
1.0
1.5
2.0
2.5
3.0
3.5
4.0

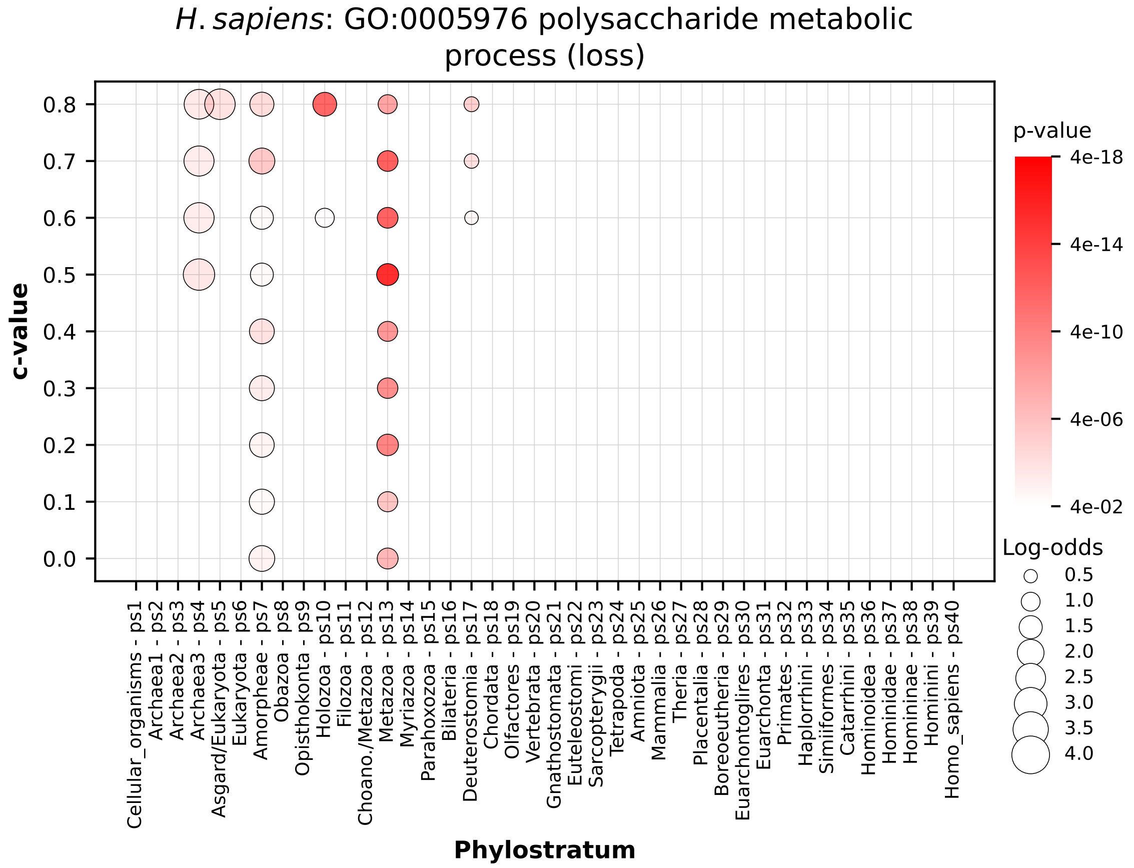

H. sapiens: GO:0005976 polysaccharide metabolic process (loss)
c-value
0.8
0.7
0.6
0.5
0.4
0.3
0.2
0.1
0.0
Phylostratum
Cellular_organisms - ps1
Archaea1 - ps2
Archaea2 - ps3
Archaea3 - ps4
Asgard/Eukaryota - ps5
Eukaryota - ps6
Amorpheae - ps7
Obazoa - ps8
Opisthokonta - ps9
Holozoa - ps10
Filozoa - ps11
Choano./Metazoa - ps12
Metazoa - ps13
Myriazoa - ps14
Parahoxozoa - ps15
Bilateria - ps16
Deuterostomia - ps17
Chordata - ps18
Olfactores - ps19
Vertebrata - ps20
Gnathostomata - ps21
Euteleostomi - ps22
Sarcopterygii - ps23
Tetrapoda - ps24
Amniota - ps25
Mammalia - ps26
Theria - ps27
Placentalia - ps28
Boreoeutheria - ps29
Euarchontoglires - ps30
Euarchonta - ps31
Primates - ps32
Haplorrhini - ps33
Simiiformes - ps34
Catarrhini - ps35
Hominoidea - ps36
Hominidae - ps37
Homininae - ps38
Hominini - ps39
Homo_sapiens - ps40
p-value
4e-18
4e-14
4e-10
4e-06
4e-02
Log-odds
0.5
1.0
1.5
2.0
2.5
3.0
3.5
4.0

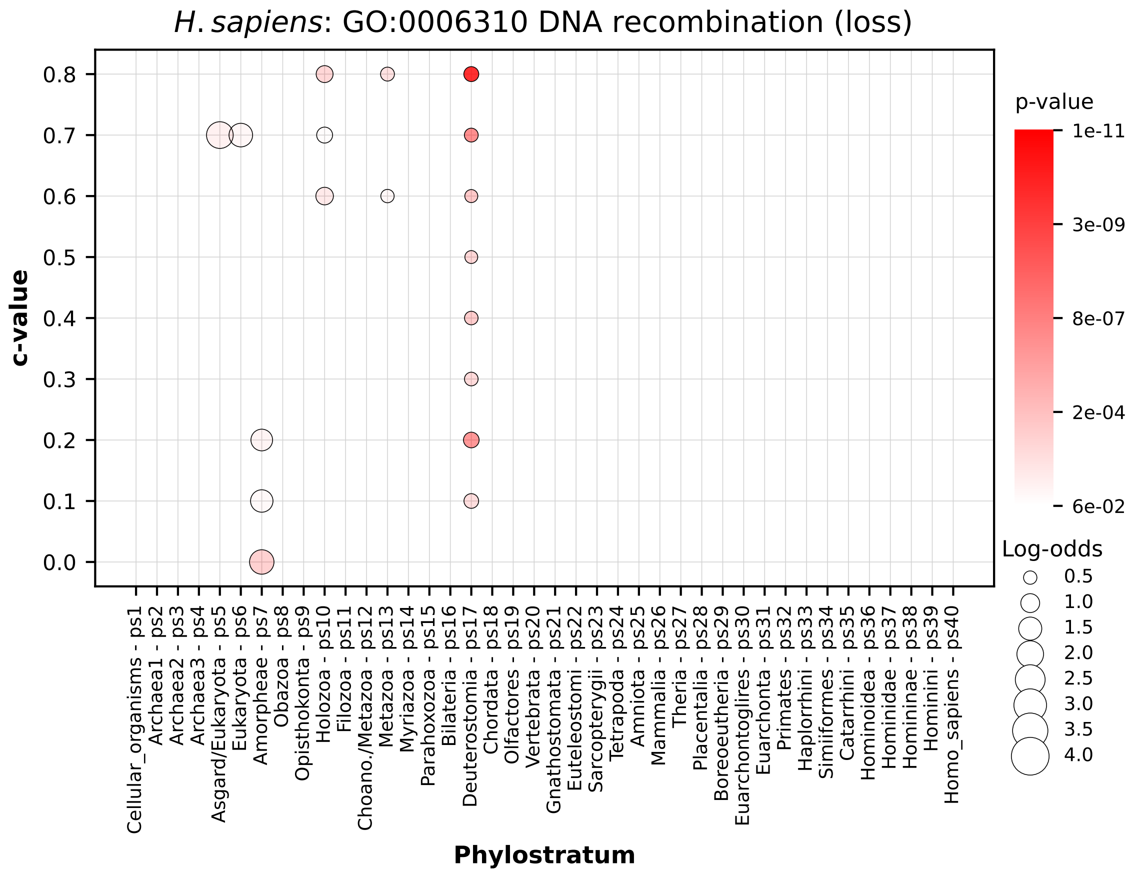

H. sapiens: GO:0006310 DNA recombination (loss)
c-value
0.8
0.7
0.6
0.5
0.4
0.3
0.2
0.1
0.0
Phylostratum
Cellular_organisms - ps1
Archaea1 - ps2
Archaea2 - ps3
Archaea3 - ps4
Asgard/Eukaryota - ps5
Eukaryota - ps6
Amorpheae - ps7
Obazoa - ps8
Opisthokonta - ps9
Holozoa - ps10
Filozoa - ps11
Choano./Metazoa - ps12
Metazoa - ps13
Myriazoa - ps14
Parahoxozoa - ps15
Bilateria - ps16
Deuterostomia - ps17
Chordata - ps18
Olfactores - ps19
Vertebrata - ps20
Gnathostomata - ps21
Euteleostomi - ps22
Sarcopterygii - ps23
Tetrapoda - ps24
Amniota - ps25
Mammalia - ps26
Theria - ps27
Placentalia - ps28
Boreoeutheria - ps29
Euarchontoglires - ps30
Euarchonta - ps31
Primates - ps32
Haplorrhini - ps33
Simiiformes - ps34
Catarrhini - ps35
Hominoidea - ps36
Hominidae - ps37
Homininae - ps38
Hominini - ps39
Homo_sapiens - ps40
p-value
1e-11
3e-09
8e-07
2e-04
6e-02
Log-odds
0.5
1.0
1.5
2.0
2.5
3.0
3.5
4.0

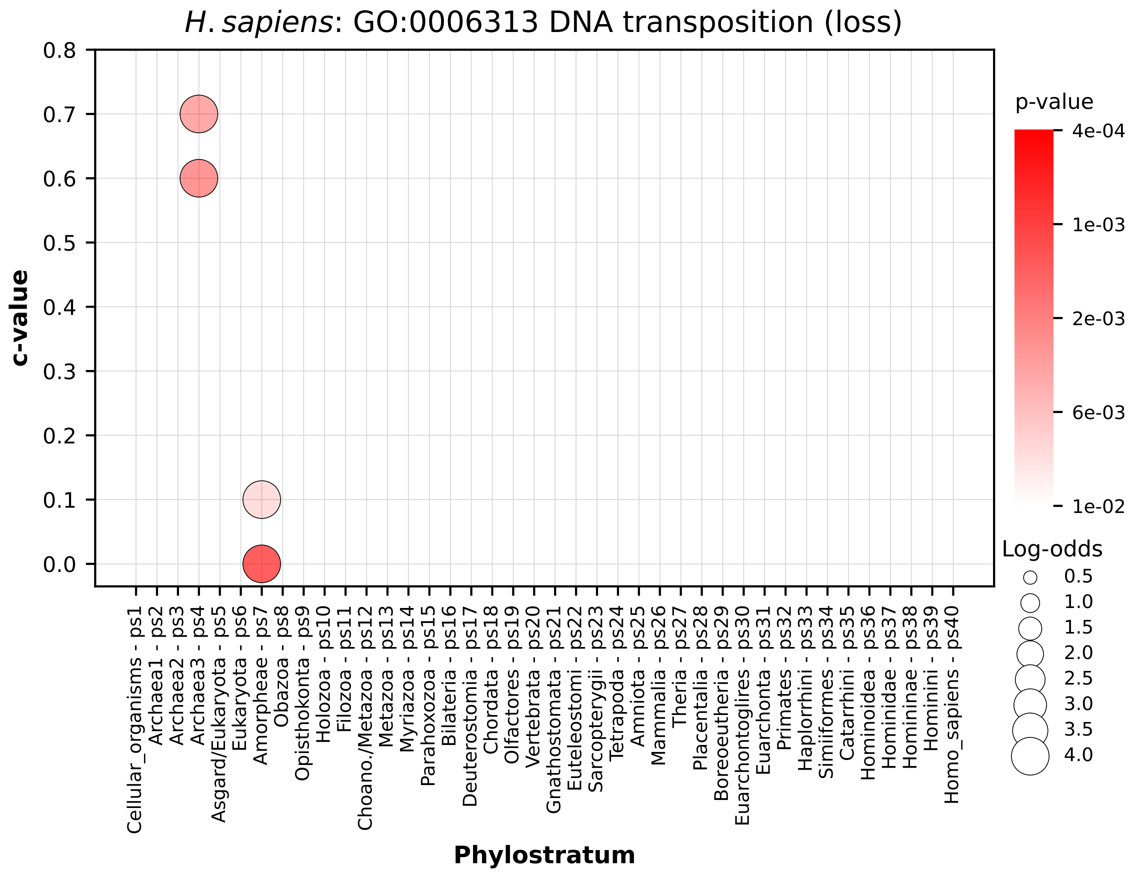

H. sapiens: GO:0006313 DNA transposition (loss)
c-value
0.8
0.7
0.6
0.5
0.4
0.3
0.2
0.1
0.0
p-value
4e-04
1e-03
2e-03
6e-03
1e-02
Log-odds
0.5
1.0
1.5
2.0
2.5
3.0
3.5
4.0
Cellular_organisms - ps1
Archaea1 - ps2
Archaea2 - ps3
Archaea3 - ps4
Asgard/Eukaryota - ps5
Eukaryota - ps6
Amorpheae - ps7
Obazoa - ps8
Opisthokonta - ps9
Holozoa - ps10
Filozoa - ps11
Choano./Metazoa - ps12
Metazoa - ps13
Myriazoa - ps14
Parahoxozoa - ps15
Bilateria - ps16
Deuterostomia - ps17
Chordata - ps18
Olfactores - ps19
Vertebrata - ps20
Gnathostomata - ps21
Euteleostomi - ps22
Sarcopterygii - ps23
Tetrapoda - ps24
Amniota - ps25
Mammalia - ps26
Theria - ps27
Placentalia - ps28
Boreoeutheria - ps29
Euarchontoglires - ps30
Euarchonta - ps31
Primates - ps32
Haplorrhini - ps33
Simiiformes - ps34
Catarrhini - ps35
Hominoidea - ps36
Hominidae - ps37
Homininae - ps38
Hominini - ps39
Homo_sapiens - ps40
Phylostratum

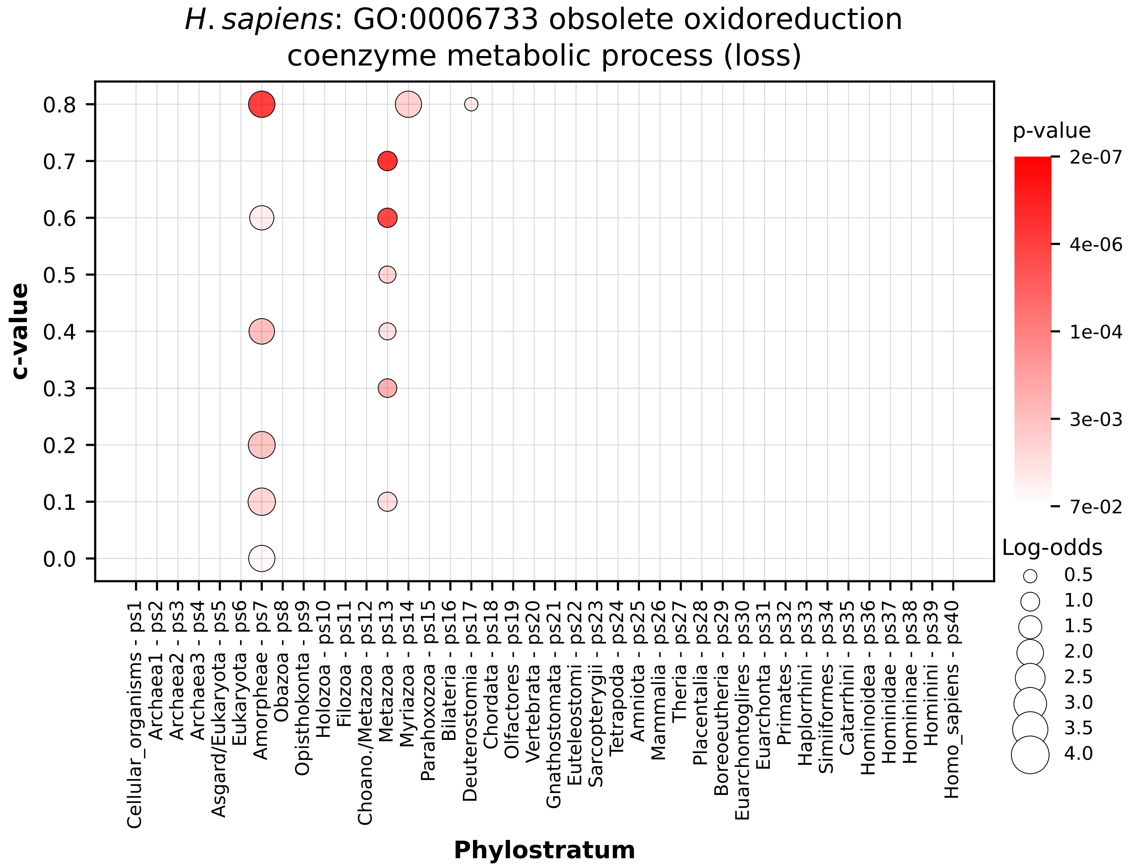

H. sapiens: GO:0006733 obsolete oxidoreduction coenzyme metabolic process (loss)
c-value
0.0
0.1
0.2
0.3
0.4
0.5
0.6
0.7
0.8
Phylostratum
Cellular_organisms - ps1
Archaea1 - ps2
Archaea2 - ps3
Archaea3 - ps4
Asgard/Eukaryota - ps5
Eukaryota - ps6
Amorpheae - ps7
Obazoa - ps8
Opisthokonta - ps9
Holozoa - ps10
Filozoa - ps11
Choano./Metazoa - ps12
Metazoa - ps13
Myriazoa - ps14
Parahoxozoa - ps15
Bilateria - ps16
Deuterostomia - ps17
Chordata - ps18
Olfactores - ps19
Vertebrata - ps20
Gnathostomata - ps21
Euteleostomi - ps22
Sarcopterygii - ps23
Tetrapoda - ps24
Amniota - ps25
Mammalia - ps26
Theria - ps27
Placentalia - ps28
Boreoeutheria - ps29
Euarchontoglires - ps30
Euarchonta - ps31
Primates - ps32
Haplorrhini - ps33
Simiiformes - ps34
Catarrhini - ps35
Hominoidea - ps36
Hominidae - ps37
Homininae - ps38
Hominini - ps39
Homo_sapiens - ps40
p-value
2e-07
4e-06
1e-04
3e-03
7e-02
Log-odds
0.5
1.0
1.5
2.0
2.5
3.0
3.5
4.0

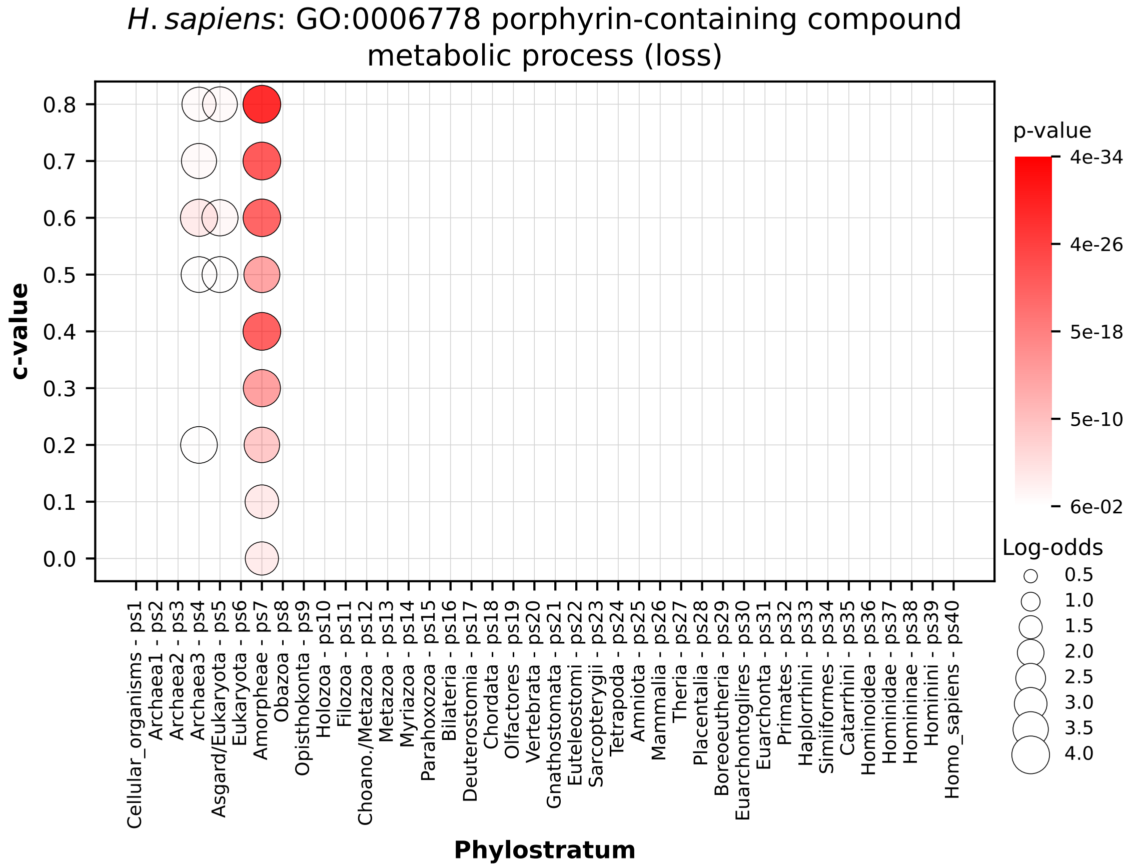

H. sapiens: GO:0006778 porphyrin-containing compound metabolic process (loss)
c-value
0.8
0.7
0.6
0.5
0.4
0.3
0.2
0.1
0.0
Phylostratum
Cellular_organisms - ps1
Archaea1 - ps2
Archaea2 - ps3
Archaea3 - ps4
Asgard/Eukaryota - ps5
Eukaryota - ps6
Amorpheae - ps7
Obazoa - ps8
Opisthokonta - ps9
Holozoa - ps10
Filozoa - ps11
Choano./Metazoa - ps12
Metazoa - ps13
Myriazoa - ps14
Parahoxozoa - ps15
Bilateria - ps16
Deuterostomia - ps17
Chordata - ps18
Olfactores - ps19
Vertebrata - ps20
Gnathostomata - ps21
Euteleostomi - ps22
Sarcopterygii - ps23
Tetrapoda - ps24
Amniota - ps25
Mammalia - ps26
Theria - ps27
Placentalia - ps28
Boreoeutheria - ps29
Euarchontoglires - ps30
Euarchonta - ps31
Primates - ps32
Haplorrhini - ps33
Simiiformes - ps34
Catarrhini - ps35
Hominoidea - ps36
Hominidae - ps37
Homininae - ps38
Hominini - ps39
Homo_sapiens - ps40
p-value
4e-34
4e-26
5e-18
5e-10
6e-02
Log-odds
0.5
1.0
1.5
2.0
2.5
3.0
3.5
4.0

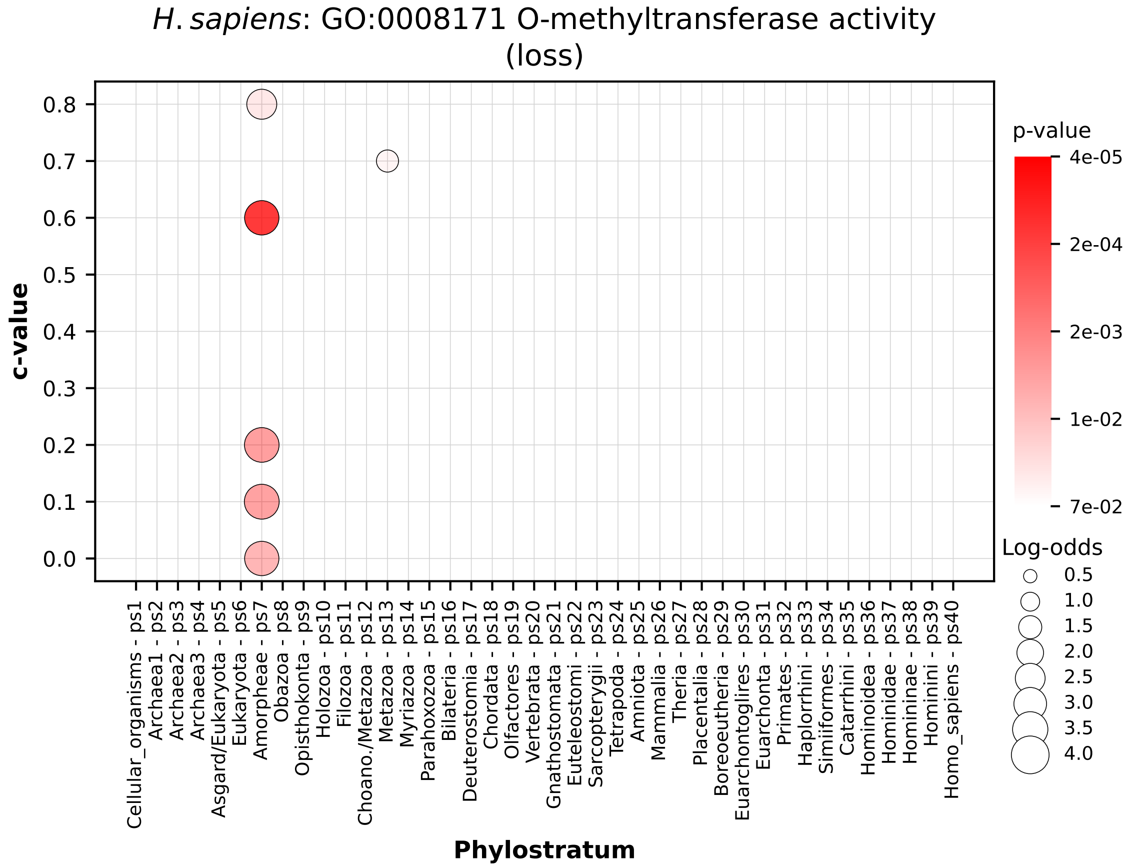

H. sapiens: GO:0008171 O-methyltransferase activity
(loss)
c-value
0.8
0.7
0.6
0.5
0.4
0.3
0.2
0.1
0.0
p-value
4e-05
2e-04
2e-03
1e-02
7e-02
Log-odds
0.5
1.0
1.5
2.0
2.5
3.0
3.5
4.0
Cellular_organisms - ps1
Archaea1 - ps2
Archaea2 - ps3
Archaea3 - ps4
Asgard/Eukaryota - ps5
Eukaryota - ps6
Amorpheae - ps7
Obazoa - ps8
Opisthokonta - ps9
Holozoa - ps10
Filozoa - ps11
Choano./Metazoa - ps12
Metazoa - ps13
Myriazoa - ps14
Parahoxozoa - ps15
Bilateria - ps16
Deuterostomia - ps17
Chordata - ps18
Olfactores - ps19
Vertebrata - ps20
Gnathostomata - ps21
Euteleostomi - ps22
Sarcopterygii - ps23
Tetrapoda - ps24
Amniota - ps25
Mammalia - ps26
Theria - ps27
Placentalia - ps28
Boreoeutheria - ps29
Euarchontoglires - ps30
Euarchonta - ps31
Primates - ps32
Haplorrhini - ps33
Simiiformes - ps34
Catarrhini - ps35
Hominoidea - ps36
Hominidae - ps37
Homininae - ps38
Hominini - ps39
Homo_sapiens - ps40
Phylostratum

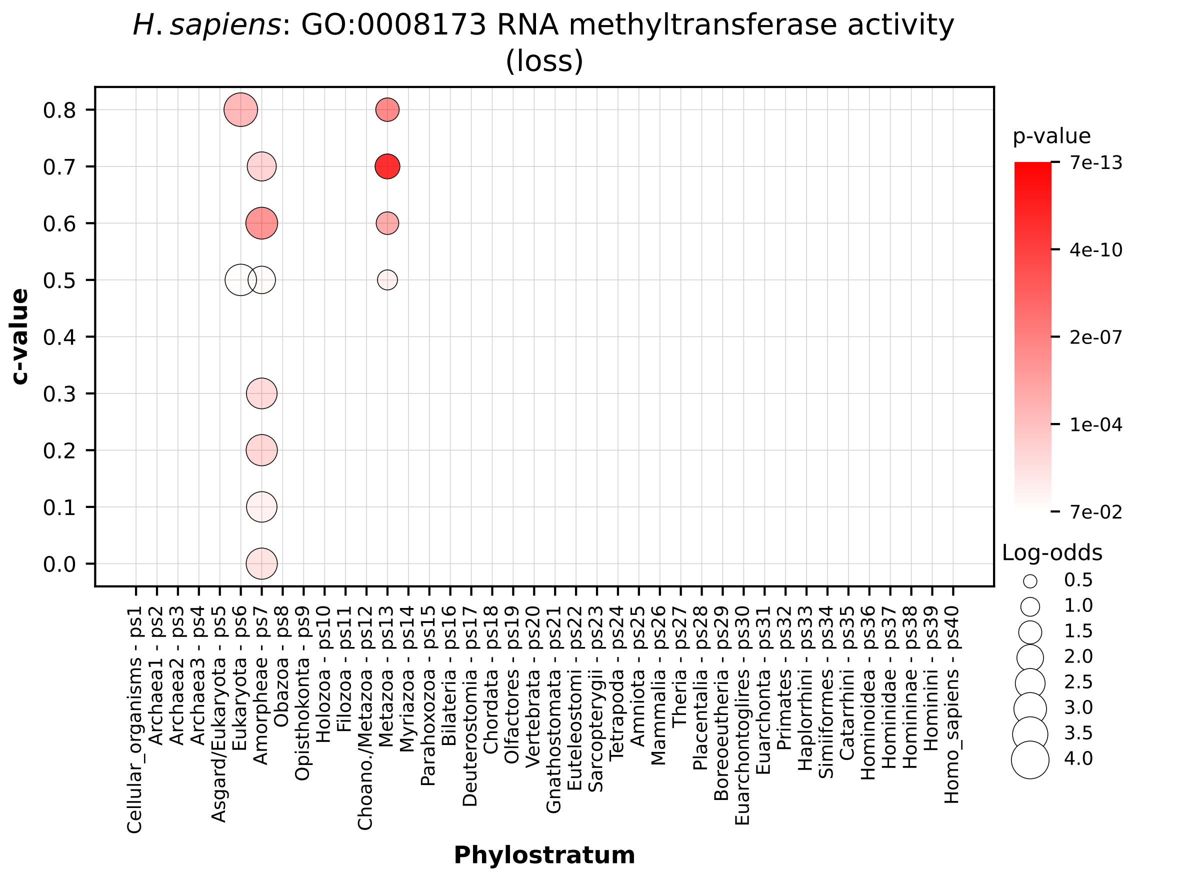

H. sapiens: GO:0008173 RNA methyltransferase activity
(loss)
c-value
0.8
0.7
0.6
0.5
0.4
0.3
0.2
0.1
0.0
p-value
7e-13
4e-10
2e-07
1e-04
7e-02
Log-odds
0.5
1.0
1.5
2.0
2.5
3.0
3.5
4.0
Cellular_organisms - ps1
Archaea1 - ps2
Archaea2 - ps3
Archaea3 - ps4
Asgard/Eukaryota - ps5
Eukaryota - ps6
Amorpheae - ps7
Obazoa - ps8
Opisthokonta - ps9
Holozoa - ps10
Filozoa - ps11
Choano./Metazoa - ps12
Metazoa - ps13
Myriazoa - ps14
Parahoxozoa - ps15
Bilateria - ps16
Deuterostomia - ps17
Chordata - ps18
Olfactores - ps19
Vertebrata - ps20
Gnathostomata - ps21
Euteleostomi - ps22
Sarcopterygii - ps23
Tetrapoda - ps24
Amniota - ps25
Mammalia - ps26
Theria - ps27
Placentalia - ps28
Boreoeutheria - ps29
Euarchontoglires - ps30
Euarchonta - ps31
Primates - ps32
Haplorrhini - ps33
Simiiformes - ps34
Catarrhini - ps35
Hominoidea - ps36
Hominidae - ps37
Homininae - ps38
Hominini - ps39
Homo_sapiens - ps40
Phylostratum

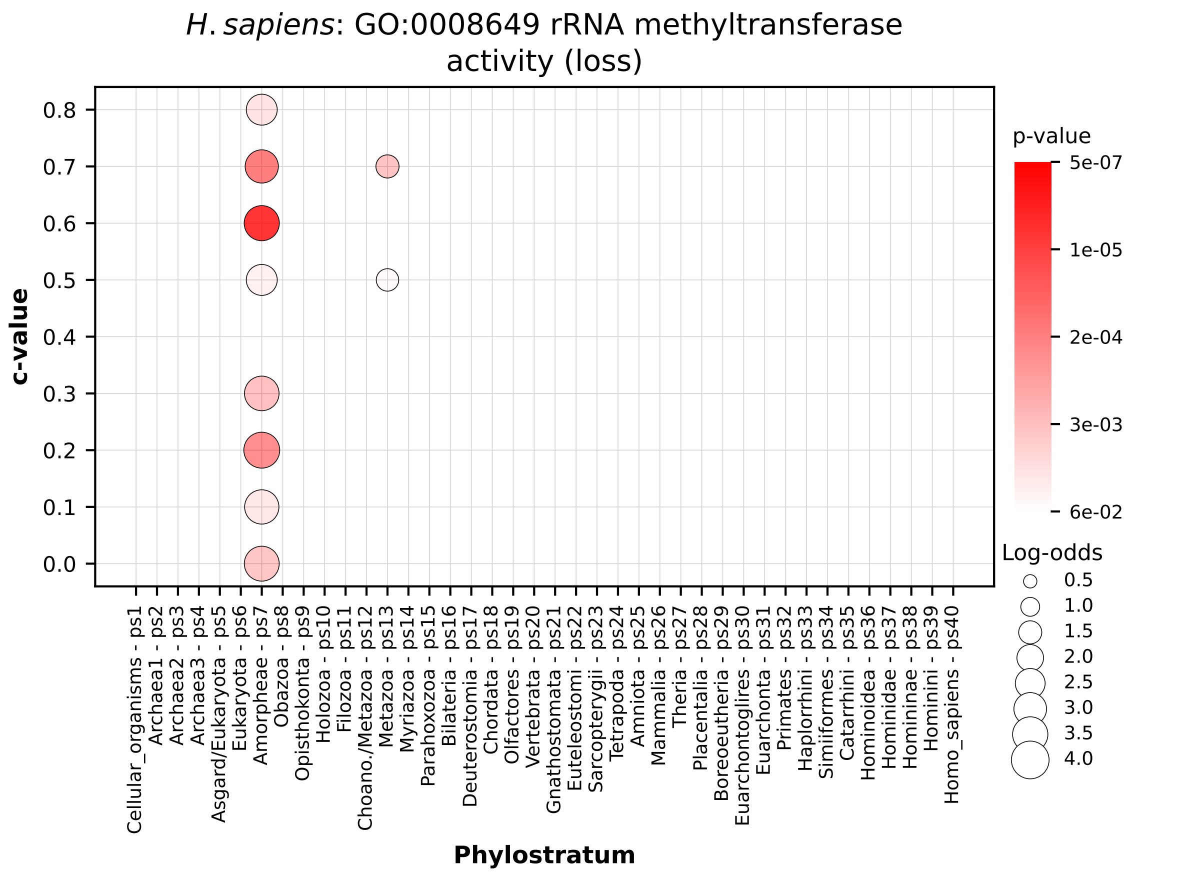

H. sapiens: GO:0008649 rRNA methyltransferase activity (loss)
c-value
0.8
0.7
0.6
0.5
0.4
0.3
0.2
0.1
0.0
Phylostratum
Cellular_organisms - ps1
Archaea1 - ps2
Archaea2 - ps3
Archaea3 - ps4
Asgard/Eukaryota - ps5
Eukaryota - ps6
Amorpheae - ps7
Obazoa - ps8
Opisthokonta - ps9
Holozoa - ps10
Filozoa - ps11
Choano./Metazoa - ps12
Metazoa - ps13
Myriazoa - ps14
Parahoxozoa - ps15
Bilateria - ps16
Deuterostomia - ps17
Chordata - ps18
Olfactores - ps19
Vertebrata - ps20
Gnathostomata - ps21
Euteleostomi - ps22
Sarcopterygii - ps23
Tetrapoda - ps24
Amniota - ps25
Mammalia - ps26
Theria - ps27
Placentalia - ps28
Boreoeutheria - ps29
Euarchontoglires - ps30
Euarchonta - ps31
Primates - ps32
Haplorrhini - ps33
Simiiformes - ps34
Catarrhini - ps35
Hominoidea - ps36
Hominidae - ps37
Homininae - ps38
Hominini - ps39
Homo_sapiens - ps40
p-value
5e-07
1e-05
2e-04
3e-03
6e-02
Log-odds
0.5
1.0
1.5
2.0
2.5
3.0
3.5
4.0

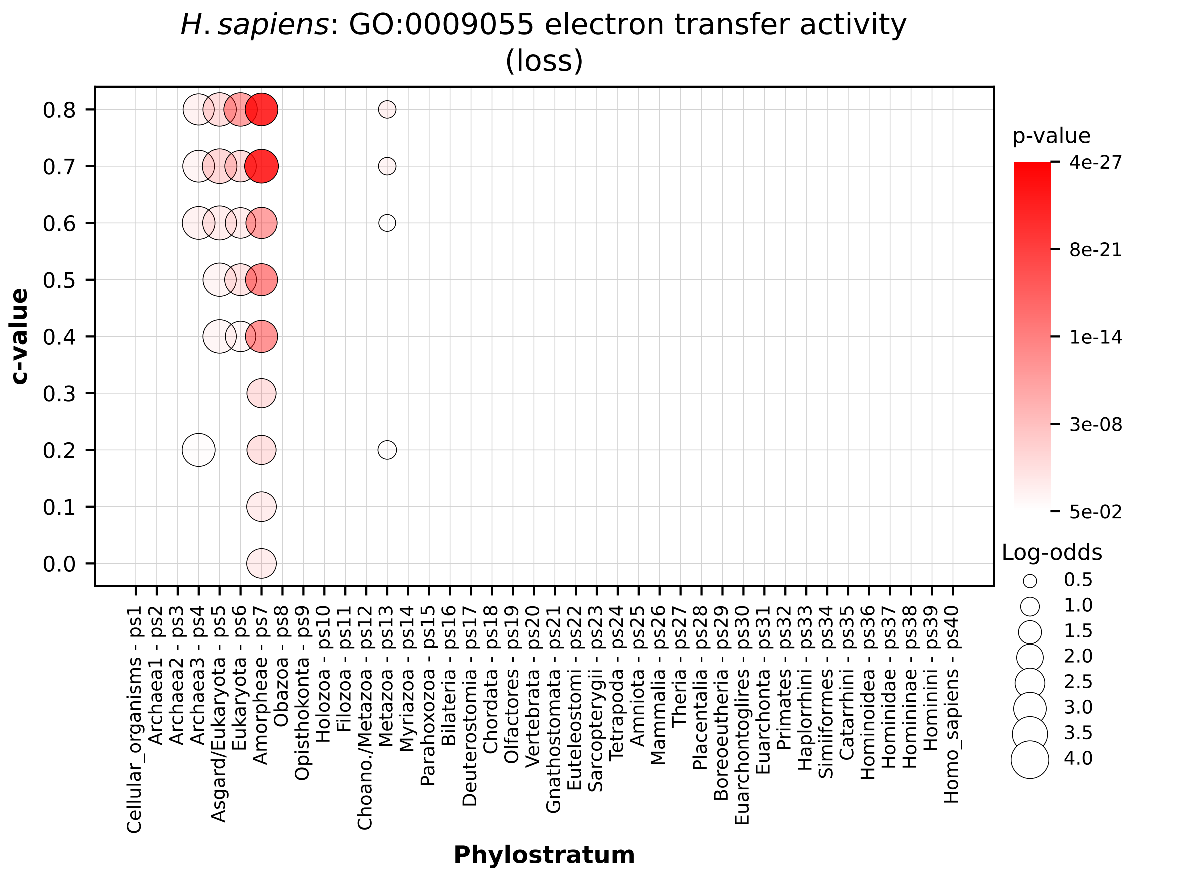

H. sapiens: GO:0009055 electron transfer activity (loss)
c-value
0.8
0.7
0.6
0.5
0.4
0.3
0.2
0.1
0.0
p-value
4e-27
8e-21
1e-14
3e-08
5e-02
Log-odds
0.5
1.0
1.5
2.0
2.5
3.0
3.5
4.0
Cellular_organisms - ps1
Archaea1 - ps2
Archaea2 - ps3
Archaea3 - ps4
Asgard/Eukaryota - ps5
Eukaryota - ps6
Amorpheae - ps7
Obazoa - ps8
Opisthokonta - ps9
Holozoa - ps10
Filozoa - ps11
Choano./Metazoa - ps12
Metazoa - ps13
Myriazoa - ps14
Parahoxozoa - ps15
Bilateria - ps16
Deuterostomia - ps17
Chordata - ps18
Olfactores - ps19
Vertebrata - ps20
Gnathostomata - ps21
Euteleostomi - ps22
Sarcopterygii - ps23
Tetrapoda - ps24
Amniota - ps25
Mammalia - ps26
Theria - ps27
Placentalia - ps28
Boreoeutheria - ps29
Euarchontoglires - ps30
Euarchonta - ps31
Primates - ps32
Haplorrhini - ps33
Simiiformes - ps34
Catarrhini - ps35
Hominoidea - ps36
Hominidae - ps37
Homininae - ps38
Hominini - ps39
Homo_sapiens - ps40
Phylostratum

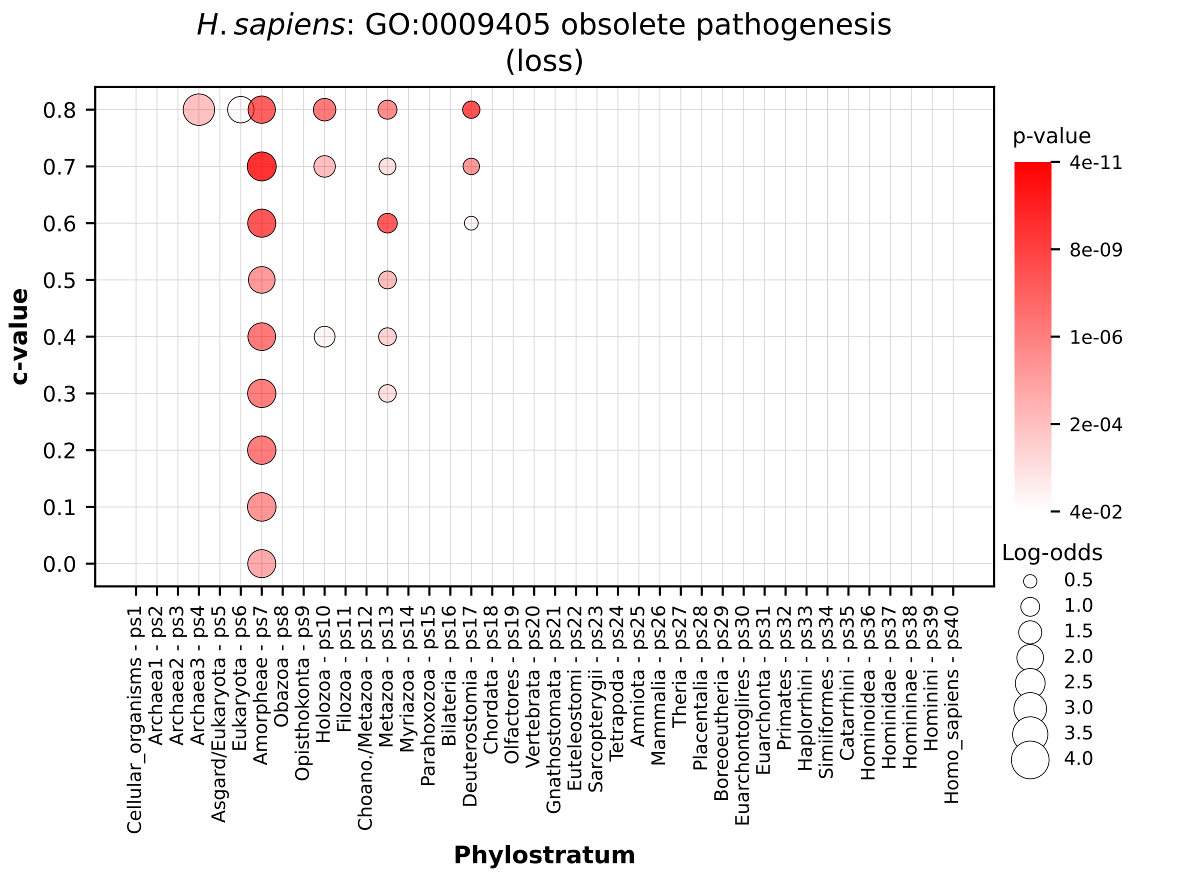

H. sapiens: GO:0009405 obsolete pathogenesis
(loss)
c-value
0.8
0.7
0.6
0.5
0.4
0.3
0.2
0.1
0.0
p-value
4e-11
8e-09
1e-06
2e-04
4e-02
Log-odds
0.5
1.0
1.5
2.0
2.5
3.0
3.5
4.0
Cellular_organisms - ps1
Archaea1 - ps2
Archaea2 - ps3
Archaea3 - ps4
Asgard/Eukaryota - ps5
Eukaryota - ps6
Amorpheae - ps7
Obazoa - ps8
Opisthokonta - ps9
Holozoa - ps10
Filozoa - ps11
Choano./Metazoa - ps12
Metazoa - ps13
Myriazoa - ps14
Parahoxozoa - ps15
Bilateria - ps16
Deuterostomia - ps17
Chordata - ps18
Olfactores - ps19
Vertebrata - ps20
Gnathostomata - ps21
Euteleostomi - ps22
Sarcopterygii - ps23
Tetrapoda - ps24
Amniota - ps25
Mammalia - ps26
Theria - ps27
Placentalia - ps28
Boreoeutheria - ps29
Euarchontoglires - ps30
Euarchonta - ps31
Primates - ps32
Haplorrhini - ps33
Simiiformes - ps34
Catarrhini - ps35
Hominoidea - ps36
Hominidae - ps37
Homininae - ps38
Hominini - ps39
Homo_sapiens - ps40
Phylostratum

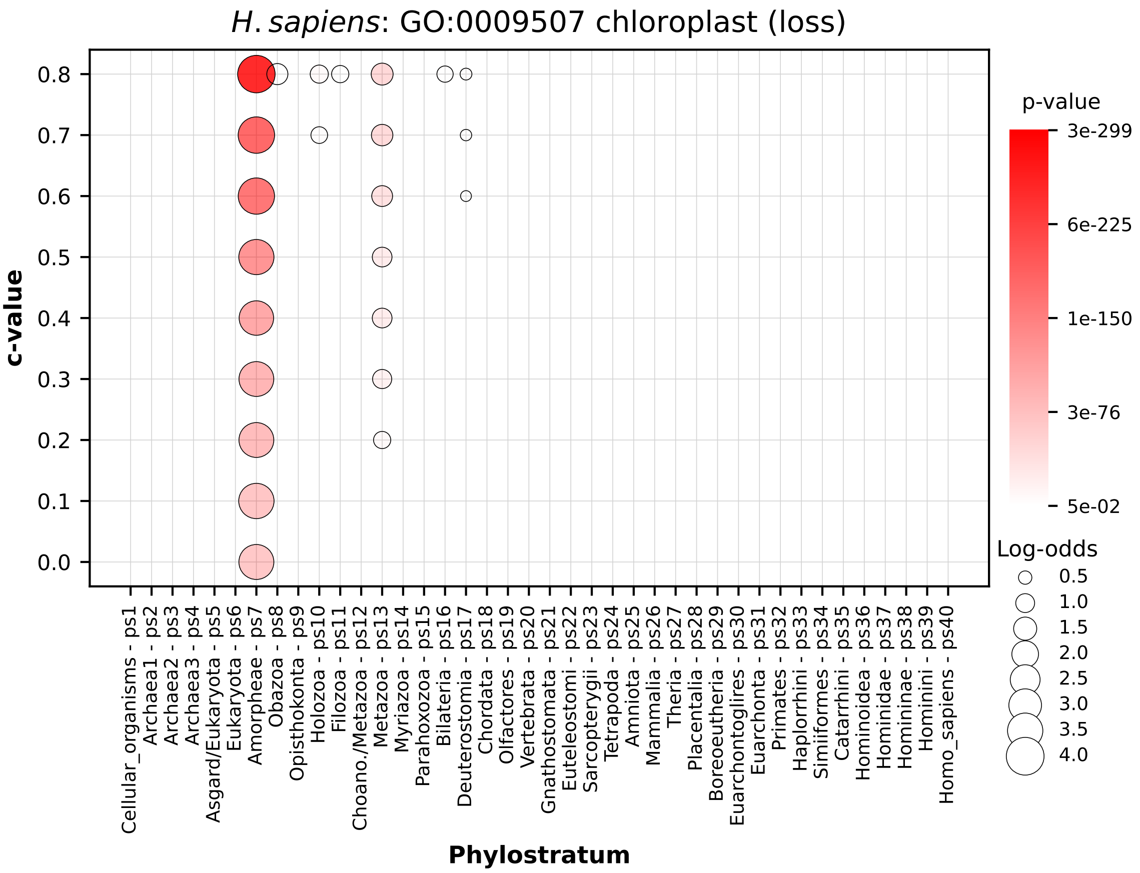

H. sapiens: GO:0009507 chloroplast (loss)
c-value
0.0
0.1
0.2
0.3
0.4
0.5
0.6
0.7
0.8
Phylostratum
Cellular_organisms - ps1
Archaea1 - ps2
Archaea2 - ps3
Archaea3 - ps4
Asgard/Eukaryota - ps5
Eukaryota - ps6
Amorpheae - ps7
Obazoa - ps8
Opisthokonta - ps9
Holozoa - ps10
Filozoa - ps11
Choano./Metazoa - ps12
Metazoa - ps13
Myriazoa - ps14
Parahoxozoa - ps15
Bilateria - ps16
Deuterostomia - ps17
Chordata - ps18
Olfactores - ps19
Vertebrata - ps20
Gnathostomata - ps21
Euteleostomi - ps22
Sarcopterygii - ps23
Tetrapoda - ps24
Amniota - ps25
Mammalia - ps26
Theria - ps27
Placentalia - ps28
Boreoeutheria - ps29
Euarchontoglires - ps30
Euarchonta - ps31
Primates - ps32
Haplorrhini - ps33
Simiiformes - ps34
Catarrhini - ps35
Hominoidea - ps36
Hominidae - ps37
Homininae - ps38
Hominini - ps39
Homo_sapiens - ps40
p-value
3e-299
6e-225
1e-150
3e-76
5e-02
Log-odds
0.5
1.0
1.5
2.0
2.5
3.0
3.5
4.0

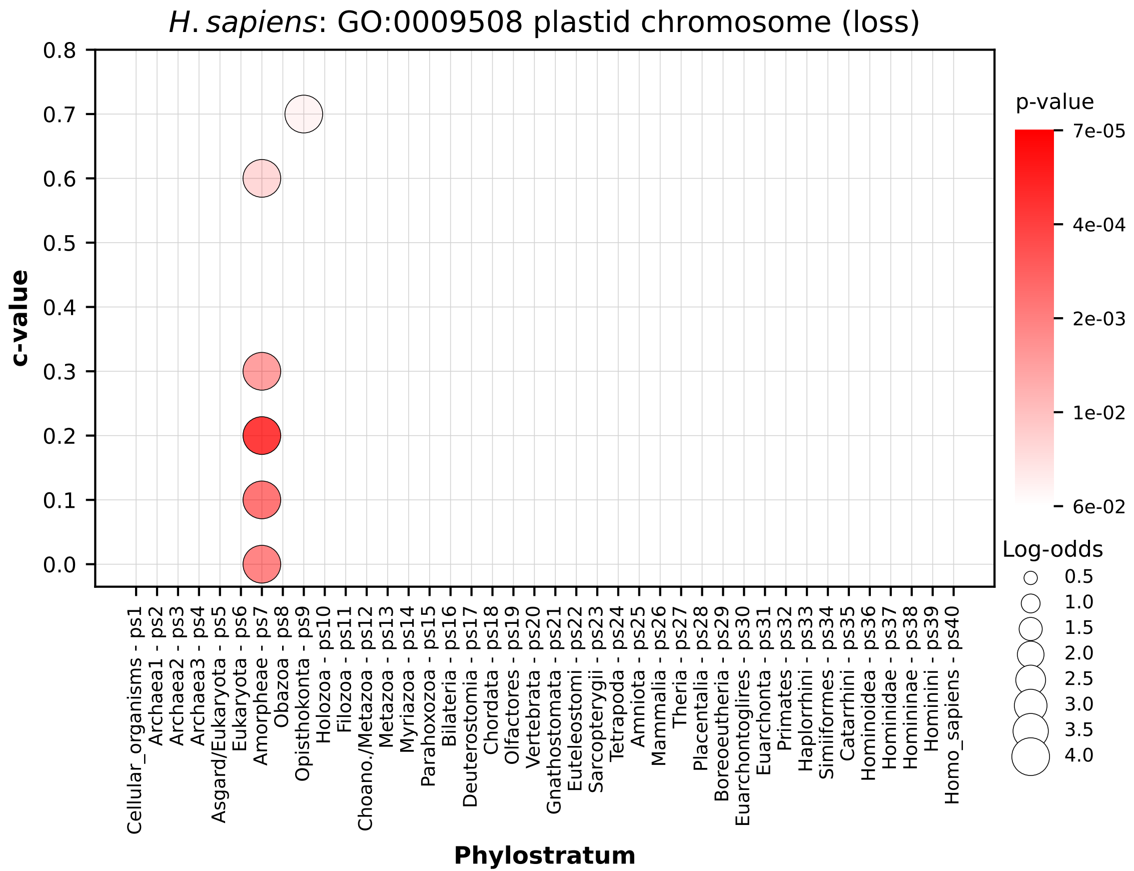

H. sapiens: GO:0009508 plastid chromosome (loss)
c-value
0.8
0.7
0.6
0.5
0.4
0.3
0.2
0.1
0.0
Phylostratum
Cellular_organisms - ps1
Archaea1 - ps2
Archaea2 - ps3
Archaea3 - ps4
Asgard/Eukaryota - ps5
Eukaryota - ps6
Amorpheae - ps7
Obazoa - ps8
Opisthokonta - ps9
Holozoa - ps10
Filozoa - ps11
Choano./Metazoa - ps12
Metazoa - ps13
Myriazoa - ps14
Parahoxozoa - ps15
Bilateria - ps16
Deuterostomia - ps17
Chordata - ps18
Olfactores - ps19
Vertebrata - ps20
Gnathostomata - ps21
Euteleostomi - ps22
Sarcopterygii - ps23
Tetrapoda - ps24
Amniota - ps25
Mammalia - ps26
Theria - ps27
Placentalia - ps28
Boreoeutheria - ps29
Euarchontoglires - ps30
Euarchonta - ps31
Primates - ps32
Haplorrhini - ps33
Simiiformes - ps34
Catarrhini - ps35
Hominoidea - ps36
Hominidae - ps37
Homininae - ps38
Hominini - ps39
Homo_sapiens - ps40
p-value
7e-05
4e-04
2e-03
1e-02
6e-02
Log-odds
0.5
1.0
1.5
2.0
2.5
3.0
3.5
4.0

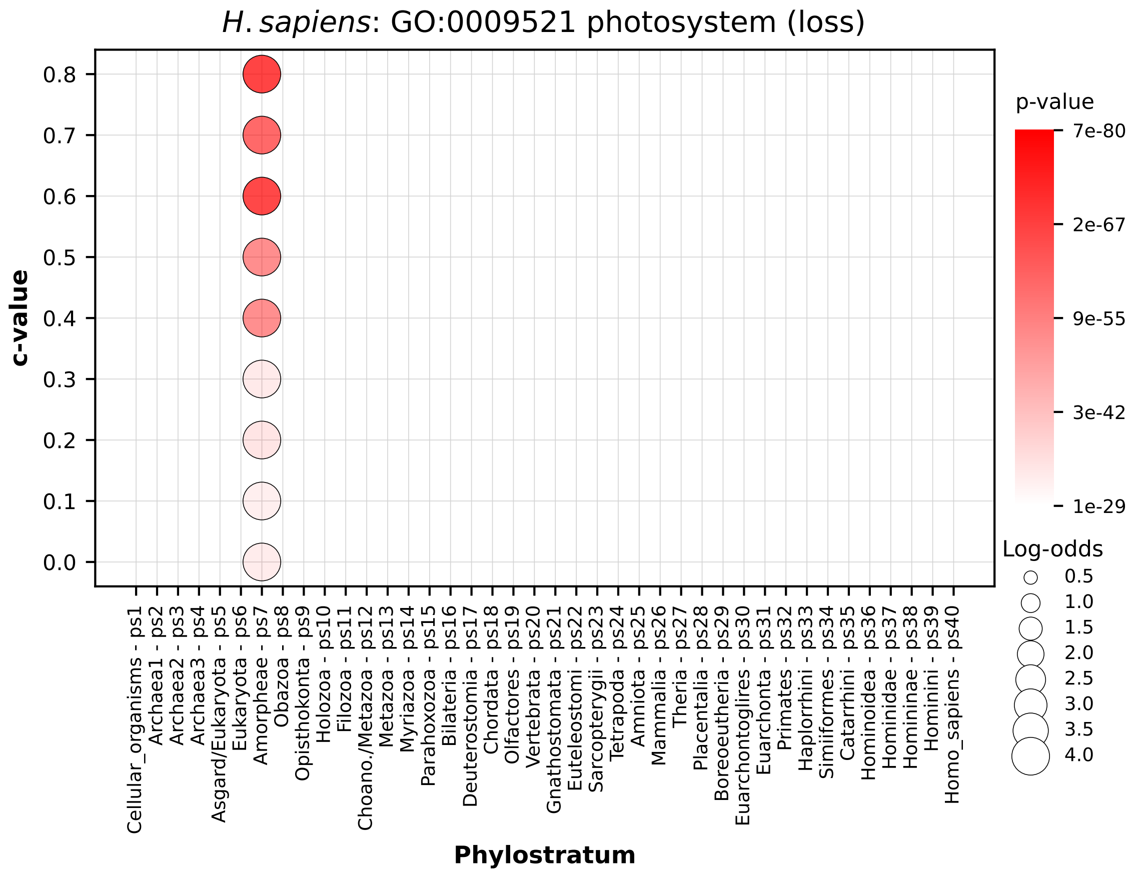

H. sapiens: GO:0009521 photosystem (loss)
c-value
0.8
0.7
0.6
0.5
0.4
0.3
0.2
0.1
0.0
p-value
7e-80
2e-67
9e-55
3e-42
1e-29
Log-odds
0.5
1.0
1.5
2.0
2.5
3.0
3.5
4.0
Cellular_organisms - ps1
Archaea1 - ps2
Archaea2 - ps3
Archaea3 - ps4
Asgard/Eukaryota - ps5
Eukaryota - ps6
Amorpheae - ps7
Obazoa - ps8
Opisthokonta - ps9
Holozoa - ps10
Filozoa - ps11
Choano./Metazoa - ps12
Metazoa - ps13
Myriazoa - ps14
Parahoxozoa - ps15
Bilateria - ps16
Deuterostomia - ps17
Chordata - ps18
Olfactores - ps19
Vertebrata - ps20
Gnathostomata - ps21
Euteleostomi - ps22
Sarcopterygii - ps23
Tetrapoda - ps24
Amniota - ps25
Mammalia - ps26
Theria - ps27
Placentalia - ps28
Boreoeutheria - ps29
Euarchontoglires - ps30
Euarchonta - ps31
Primates - ps32
Haplorrhini - ps33
Simiiformes - ps34
Catarrhini - ps35
Hominoidea - ps36
Hominidae - ps37
Homininae - ps38
Hominini - ps39
Homo_sapiens - ps40
Phylostratum

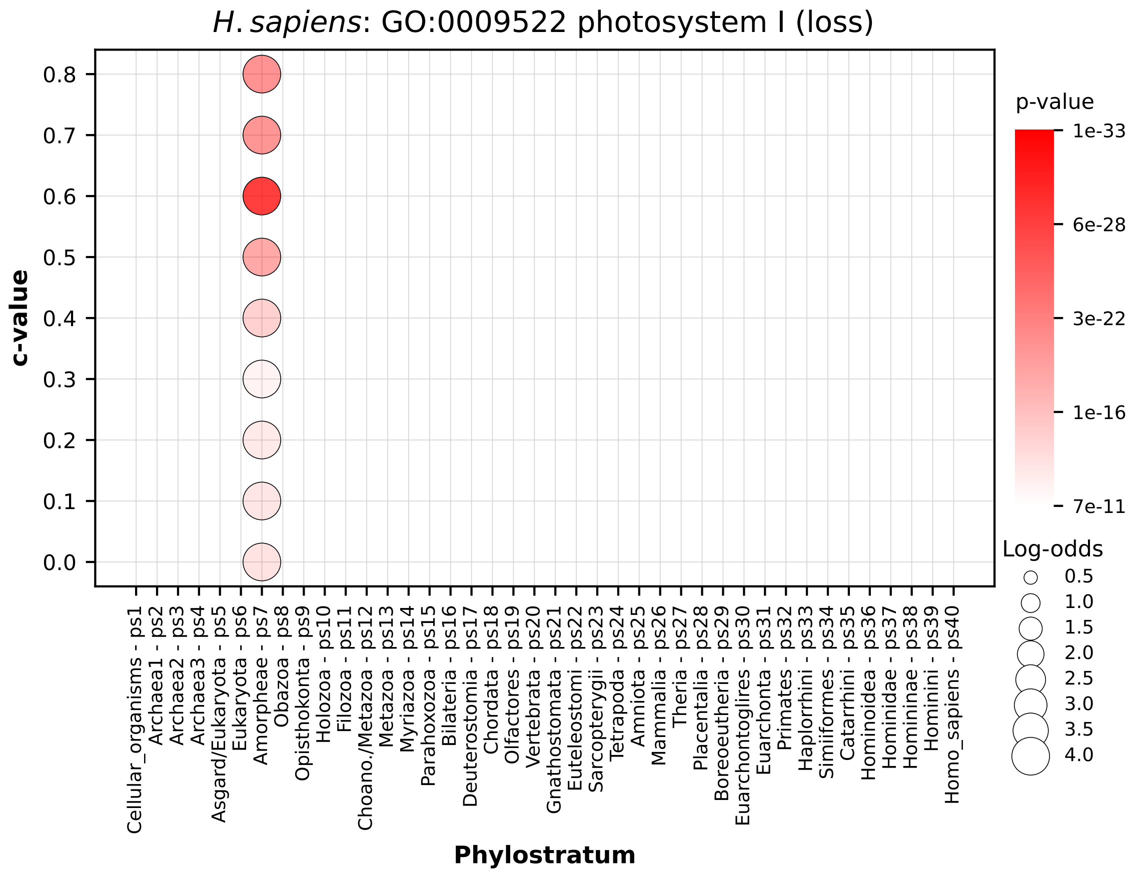

H. sapiens: GO:0009522 photosystem I (loss)
c-value
0.0
0.1
0.2
0.3
0.4
0.5
0.6
0.7
0.8
Phylostratum
Cellular_organisms - ps1
Archaea1 - ps2
Archaea2 - ps3
Archaea3 - ps4
Asgard/Eukaryota - ps5
Eukaryota - ps6
Amorpheae - ps7
Obazoa - ps8
Opisthokonta - ps9
Holozoa - ps10
Filozoa - ps11
Choano./Metazoa - ps12
Metazoa - ps13
Myriazoa - ps14
Parahoxozoa - ps15
Bilateria - ps16
Deuterostomia - ps17
Chordata - ps18
Olfactores - ps19
Vertebrata - ps20
Gnathostomata - ps21
Euteleostomi - ps22
Sarcopterygii - ps23
Tetrapoda - ps24
Amniota - ps25
Mammalia - ps26
Theria - ps27
Placentalia - ps28
Boreoeutheria - ps29
Euarchontoglires - ps30
Euarchonta - ps31
Primates - ps32
Haplorrhini - ps33
Simiiformes - ps34
Catarrhini - ps35
Hominoidea - ps36
Hominidae - ps37
Homininae - ps38
Hominini - ps39
Homo_sapiens - ps40
p-value
1e-33
6e-28
3e-22
1e-16
7e-11
Log-odds
0.5
1.0
1.5
2.0
2.5
3.0
3.5
4.0

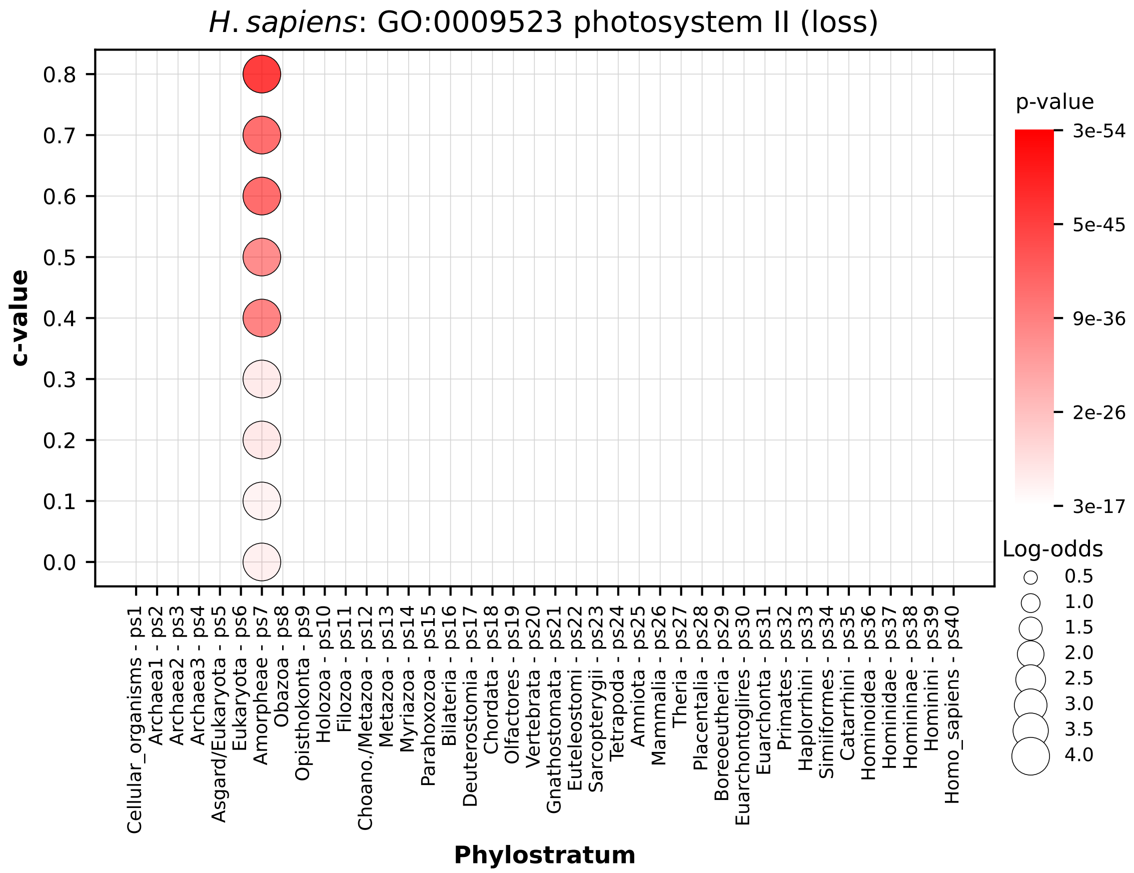

H. sapiens: GO:0009523 photosystem II (loss)
c-value
0.8
0.7
0.6
0.5
0.4
0.3
0.2
0.1
0.0
Phylostratum
Cellular_organisms - ps1
Archaea1 - ps2
Archaea2 - ps3
Archaea3 - ps4
Asgard/Eukaryota - ps5
Eukaryota - ps6
Amorpheae - ps7
Obazoa - ps8
Opisthokonta - ps9
Holozoa - ps10
Filozoa - ps11
Choano./Metazoa - ps12
Metazoa - ps13
Myriazoa - ps14
Parahoxozoa - ps15
Bilateria - ps16
Deuterostomia - ps17
Chordata - ps18
Olfactores - ps19
Vertebrata - ps20
Gnathostomata - ps21
Euteleostomi - ps22
Sarcopterygii - ps23
Tetrapoda - ps24
Amniota - ps25
Mammalia - ps26
Theria - ps27
Placentalia - ps28
Boreoeutheria - ps29
Euarchontoglires - ps30
Euarchonta - ps31
Primates - ps32
Haplorrhini - ps33
Simiiformes - ps34
Catarrhini - ps35
Hominoidea - ps36
Hominidae - ps37
Homininae - ps38
Hominini - ps39
Homo_sapiens - ps40
p-value
3e-54
5e-45
9e-36
2e-26
3e-17
Log-odds
0.5
1.0
1.5
2.0
2.5
3.0
3.5
4.0

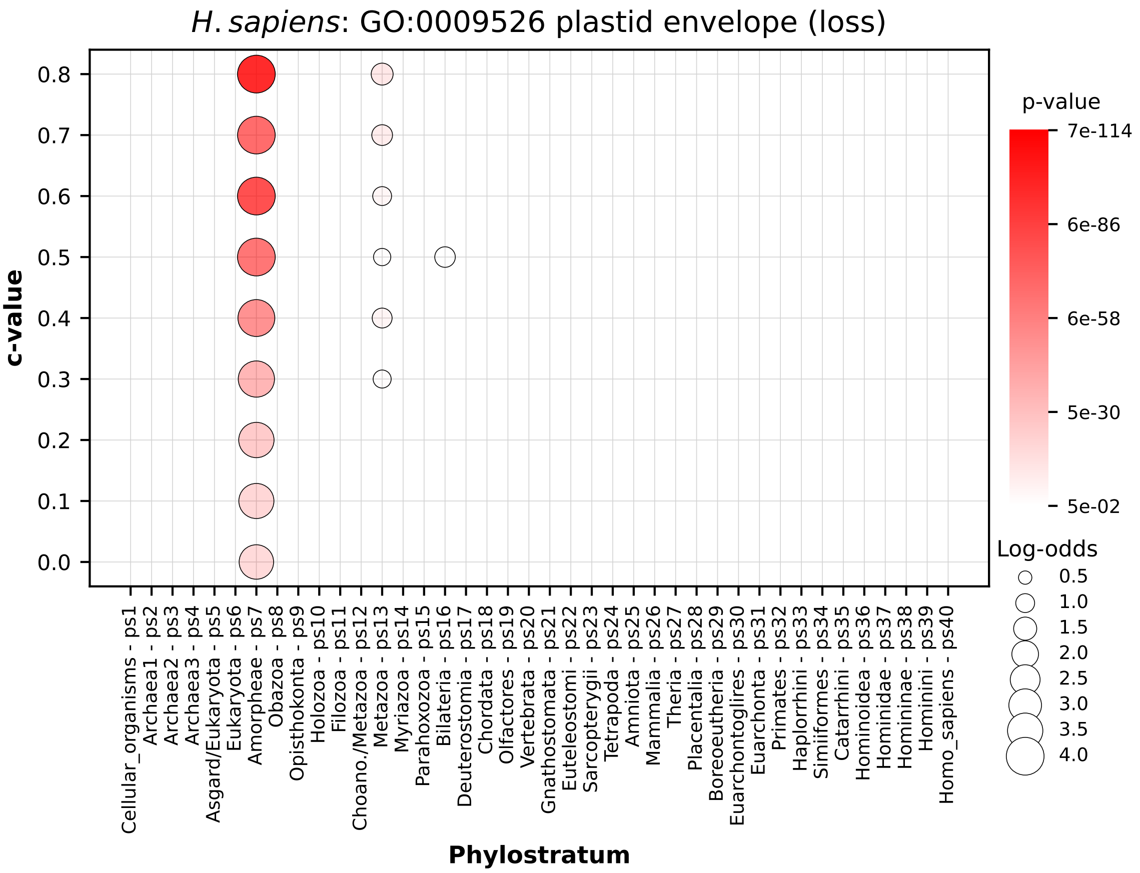
H. sapiens: GO:0009526 plastid envelope (loss)
c-value
0.8
0.7
0.6
0.5
0.4
0.3
0.2
0.1
0.0
p-value
7e-114
6e-86
6e-58
5e-30
5e-02
Log-odds
0.5
1.0
1.5
2.0
2.5
3.0
3.5
4.0
Cellular_organisms - ps1
Archaea1 - ps2
Archaea2 - ps3
Archaea3 - ps4
Asgard/Eukaryota - ps5
Eukaryota - ps6
Amorpheae - ps7
Obazoa - ps8
Opisthokonta - ps9
Holozoa - ps10
Filozoa - ps11
Choano./Metazoa - ps12
Metazoa - ps13
Myriazoa - ps14
Parahoxozoa - ps15
Bilateria - ps16
Deuterostomia - ps17
Chordata - ps18
Olfactores - ps19
Vertebrata - ps20
Gnathostomata - ps21
Euteleostomi - ps22
Sarcopterygii - ps23
Tetrapoda - ps24
Amniota - ps25
Mammalia - ps26
Theria - ps27
Placentalia - ps28
Boreoeutheria - ps29
Euarchontoglires - ps30
Euarchonta - ps31
Primates - ps32
Haplorrhini - ps33
Simiiformes - ps34
Catarrhini - ps35
Hominoidea - ps36
Hominidae - ps37
Homininae - ps38
Hominini - ps39
Homo_sapiens - ps40
Phylostratum

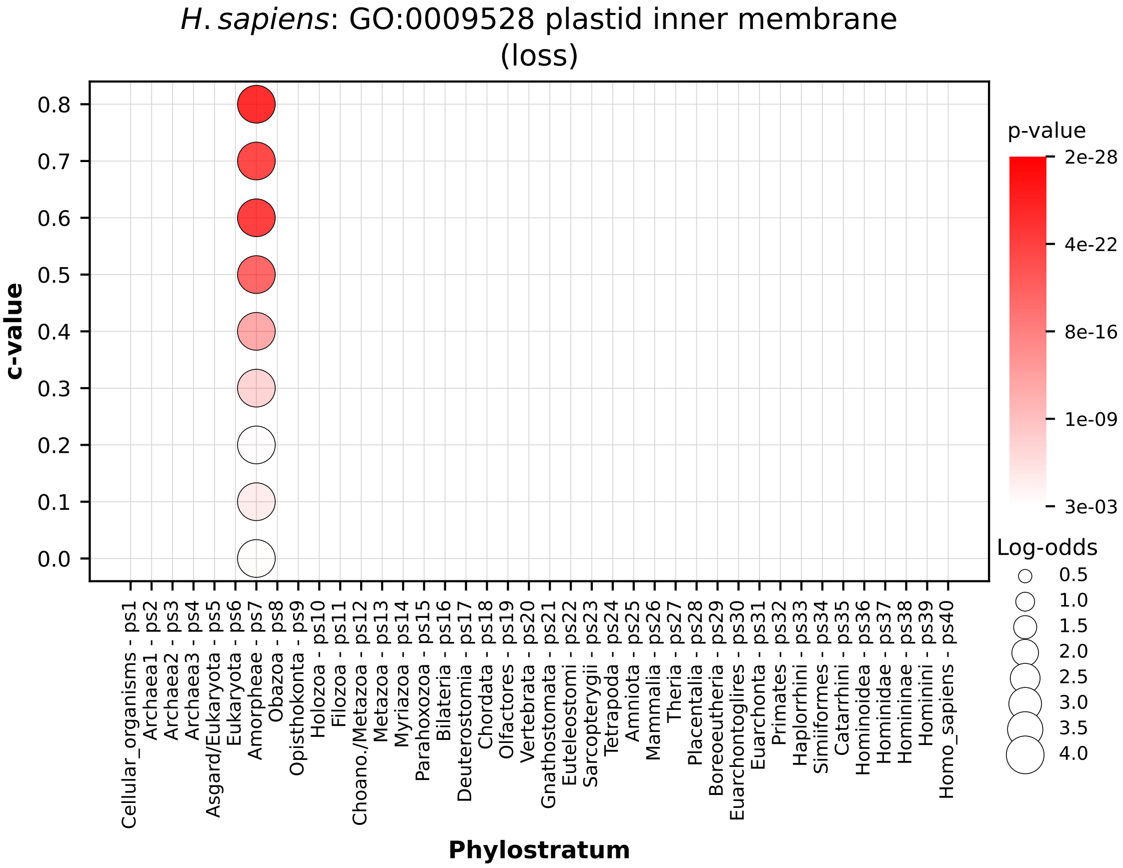

H. sapiens: GO:0009528 plastid inner membrane
(loss)
c-value
0.8
0.7
0.6
0.5
0.4
0.3
0.2
0.1
0.0
Phylostratum
Cellular_organisms - ps1
Archaea1 - ps2
Archaea2 - ps3
Archaea3 - ps4
Asgard/Eukaryota - ps5
Eukaryota - ps6
Amorpheae - ps7
Obazoa - ps8
Opisthokonta - ps9
Holozoa - ps10
Filozoa - ps11
Choano./Metazoa - ps12
Metazoa - ps13
Myriazoa - ps14
Parahoxozoa - ps15
Bilateria - ps16
Deuterostomia - ps17
Chordata - ps18
Olfactores - ps19
Vertebrata - ps20
Gnathostomata - ps21
Euteleostomi - ps22
Sarcopterygii - ps23
Tetrapoda - ps24
Amniota - ps25
Mammalia - ps26
Theria - ps27
Placentalia - ps28
Boreoeutheria - ps29
Euarchontoglires - ps30
Euarchonta - ps31
Primates - ps32
Haplorrhini - ps33
Simiiformes - ps34
Catarrhini - ps35
Hominoidea - ps36
Hominidae - ps37
Homininae - ps38
Hominini - ps39
Homo_sapiens - ps40
p-value
2e-28
4e-22
8e-16
1e-09
3e-03
Log-odds
0.5
1.0
1.5
2.0
2.5
3.0
3.5
4.0

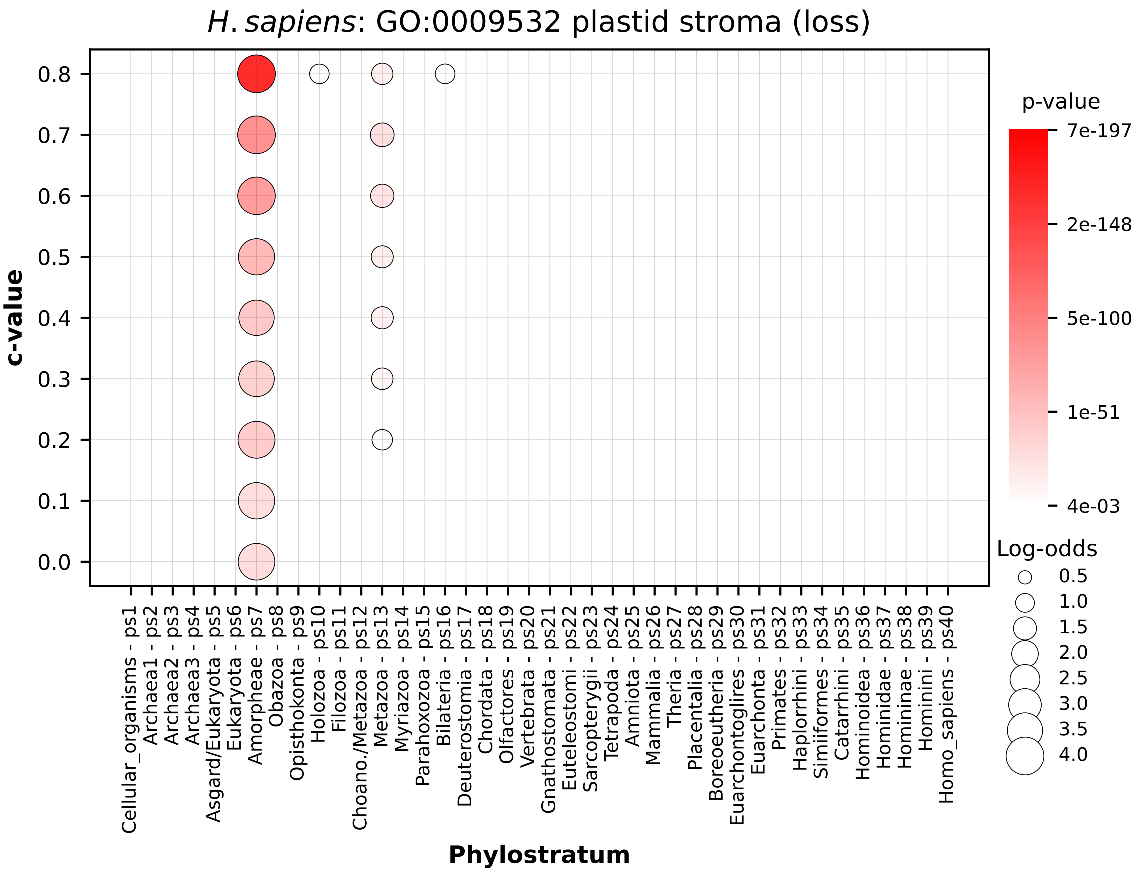
H. sapiens: GO:0009532 plastid stroma (loss)
c-value
0.0
0.1
0.2
0.3
0.4
0.5
0.6
0.7
0.8
Phylostratum
Cellular_organisms - ps1
Archaea1 - ps2
Archaea2 - ps3
Archaea3 - ps4
Asgard/Eukaryota - ps5
Eukaryota - ps6
Amorpheae - ps7
Obazoa - ps8
Opisthokonta - ps9
Holozoa - ps10
Filozoa - ps11
Choano./Metazoa - ps12
Metazoa - ps13
Myriazoa - ps14
Parahoxozoa - ps15
Bilateria - ps16
Deuterostomia - ps17
Chordata - ps18
Olfactores - ps19
Vertebrata - ps20
Gnathostomata - ps21
Euteleostomi - ps22
Sarcopterygii - ps23
Tetrapoda - ps24
Amniota - ps25
Mammalia - ps26
Theria - ps27
Placentalia - ps28
Boreoeutheria - ps29
Euarchontoglires - ps30
Euarchonta - ps31
Primates - ps32
Haplorrhini - ps33
Simiiformes - ps34
Catarrhini - ps35
Hominoidea - ps36
Hominidae - ps37
Homininae - ps38
Hominini - ps39
Homo_sapiens - ps40
p-value
7e-197
2e-148
5e-100
1e-51
4e-03
Log-odds
0.5
1.0
1.5
2.0
2.5
3.0
3.5
4.0

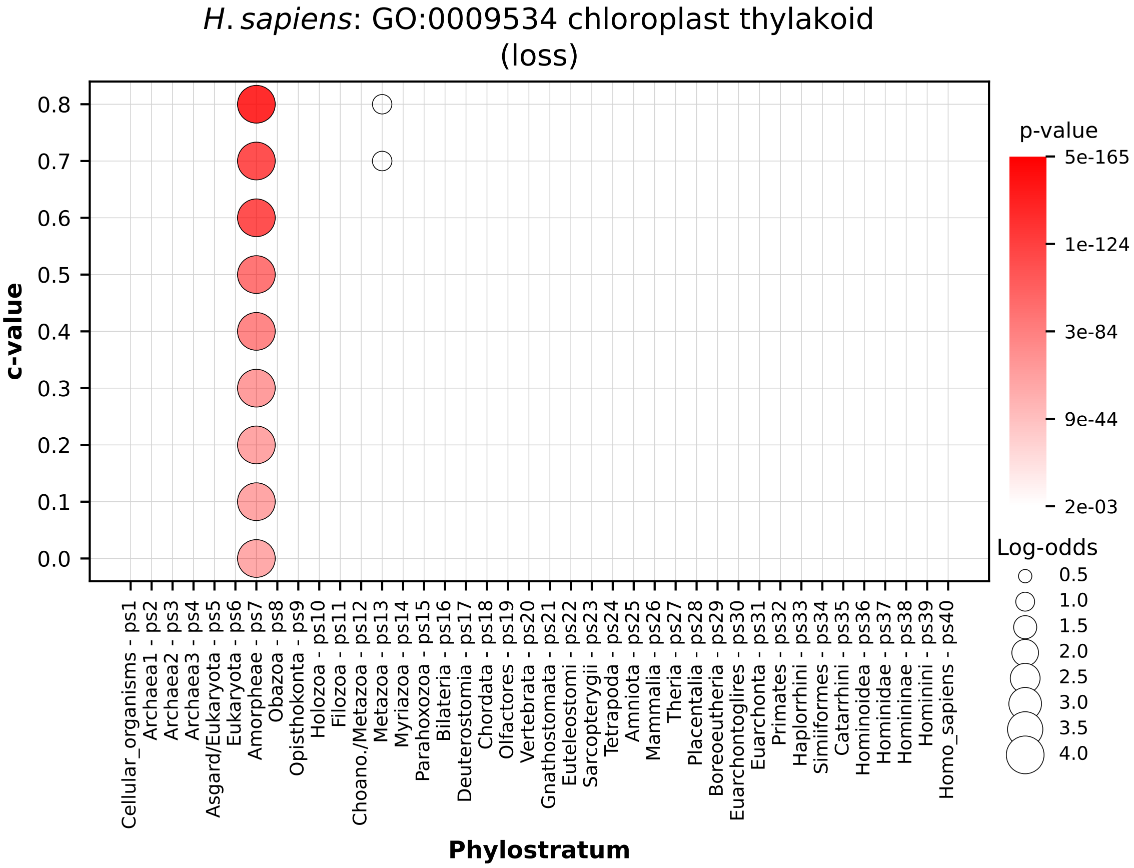

H. sapiens: GO:0009534 chloroplast thylakoid (loss)
c-value
0.8
0.7
0.6
0.5
0.4
0.3
0.2
0.1
0.0
p-value
5e-165
1e-124
3e-84
9e-44
2e-03
Log-odds
0.5
1.0
1.5
2.0
2.5
3.0
3.5
4.0
Cellular_organisms - ps1
Archaea1 - ps2
Archaea2 - ps3
Archaea3 - ps4
Asgard/Eukaryota - ps5
Eukaryota - ps6
Amorpheae - ps7
Obazoa - ps8
Opisthokonta - ps9
Holozoa - ps10
Filozoa - ps11
Choano./Metazoa - ps12
Metazoa - ps13
Myriazoa - ps14
Parahoxozoa - ps15
Bilateria - ps16
Deuterostomia - ps17
Chordata - ps18
Olfactores - ps19
Vertebrata - ps20
Gnathostomata - ps21
Euteleostomi - ps22
Sarcopterygii - ps23
Tetrapoda - ps24
Amniota - ps25
Mammalia - ps26
Theria - ps27
Placentalia - ps28
Boreoeutheria - ps29
Euarchontoglires - ps30
Euarchonta - ps31
Primates - ps32
Haplorrhini - ps33
Simiiformes - ps34
Catarrhini - ps35
Hominoidea - ps36
Hominidae - ps37
Homininae - ps38
Hominini - ps39
Homo_sapiens - ps40
Phylostratum

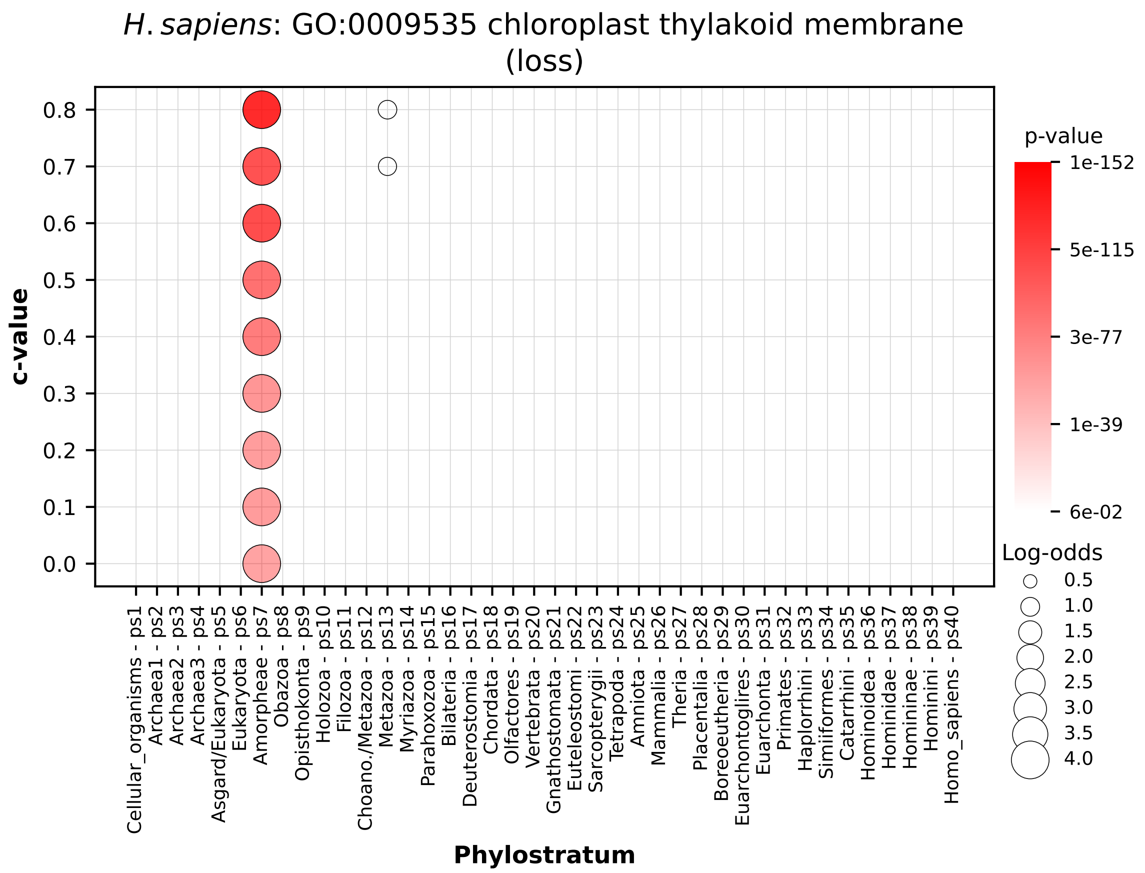

H. sapiens: GO:0009535 chloroplast thylakoid membrane
(loss)
c-value
0.8
0.7
0.6
0.5
0.4
0.3
0.2
0.1
0.0
Phylostratum
Cellular_organisms - ps1
Archaea1 - ps2
Archaea2 - ps3
Archaea3 - ps4
Asgard/Eukaryota - ps5
Eukaryota - ps6
Amorpheae - ps7
Obazoa - ps8
Opisthokonta - ps9
Holozoa - ps10
Filozoa - ps11
Choano./Metazoa - ps12
Metazoa - ps13
Myriazoa - ps14
Parahoxozoa - ps15
Bilateria - ps16
Deuterostomia - ps17
Chordata - ps18
Olfactores - ps19
Vertebrata - ps20
Gnathostomata - ps21
Euteleostomi - ps22
Sarcopterygii - ps23
Tetrapoda - ps24
Amniota - ps25
Mammalia - ps26
Theria - ps27
Placentalia - ps28
Boreoeutheria - ps29
Euarchontoglires - ps30
Euarchonta - ps31
Primates - ps32
Haplorrhini - ps33
Simiiformes - ps34
Catarrhini - ps35
Hominoidea - ps36
Hominidae - ps37
Homininae - ps38
Hominini - ps39
Homo_sapiens - ps40
p-value
1e-152
5e-115
3e-77
1e-39
6e-02
Log-odds
0.5
1.0
1.5
2.0
2.5
3.0
3.5
4.0

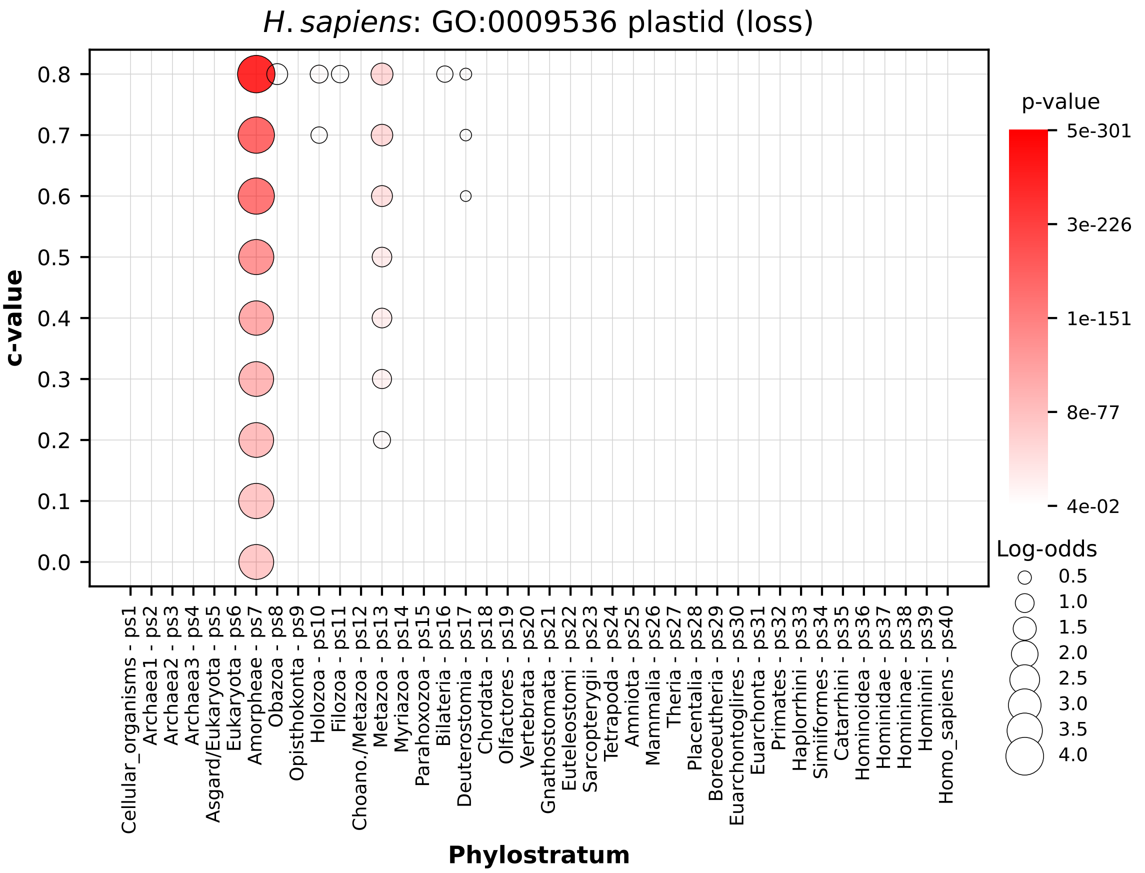

H. sapiens: GO:0009536 plastid (loss)
c-value
0.8
0.7
0.6
0.5
0.4
0.3
0.2
0.1
0.0
Phylostratum
Cellular_organisms - ps1
Archaea1 - ps2
Archaea2 - ps3
Archaea3 - ps4
Asgard/Eukaryota - ps5
Eukaryota - ps6
Amorpheae - ps7
Obazoa - ps8
Opisthokonta - ps9
Holozoa - ps10
Filozoa - ps11
Choano./Metazoa - ps12
Metazoa - ps13
Myriazoa - ps14
Parahoxozoa - ps15
Bilateria - ps16
Deuterostomia - ps17
Chordata - ps18
Olfactores - ps19
Vertebrata - ps20
Gnathostomata - ps21
Euteleostomi - ps22
Sarcopterygii - ps23
Tetrapoda - ps24
Amniota - ps25
Mammalia - ps26
Theria - ps27
Placentalia - ps28
Boreoeutheria - ps29
Euarchontoglires - ps30
Euarchonta - ps31
Primates - ps32
Haplorrhini - ps33
Simiiformes - ps34
Catarrhini - ps35
Hominoidea - ps36
Hominidae - ps37
Homininae - ps38
Hominini - ps39
Homo_sapiens - ps40
p-value
5e-301
3e-226
1e-151
8e-77
4e-02
Log-odds
0.5
1.0
1.5
2.0
2.5
3.0
3.5
4.0

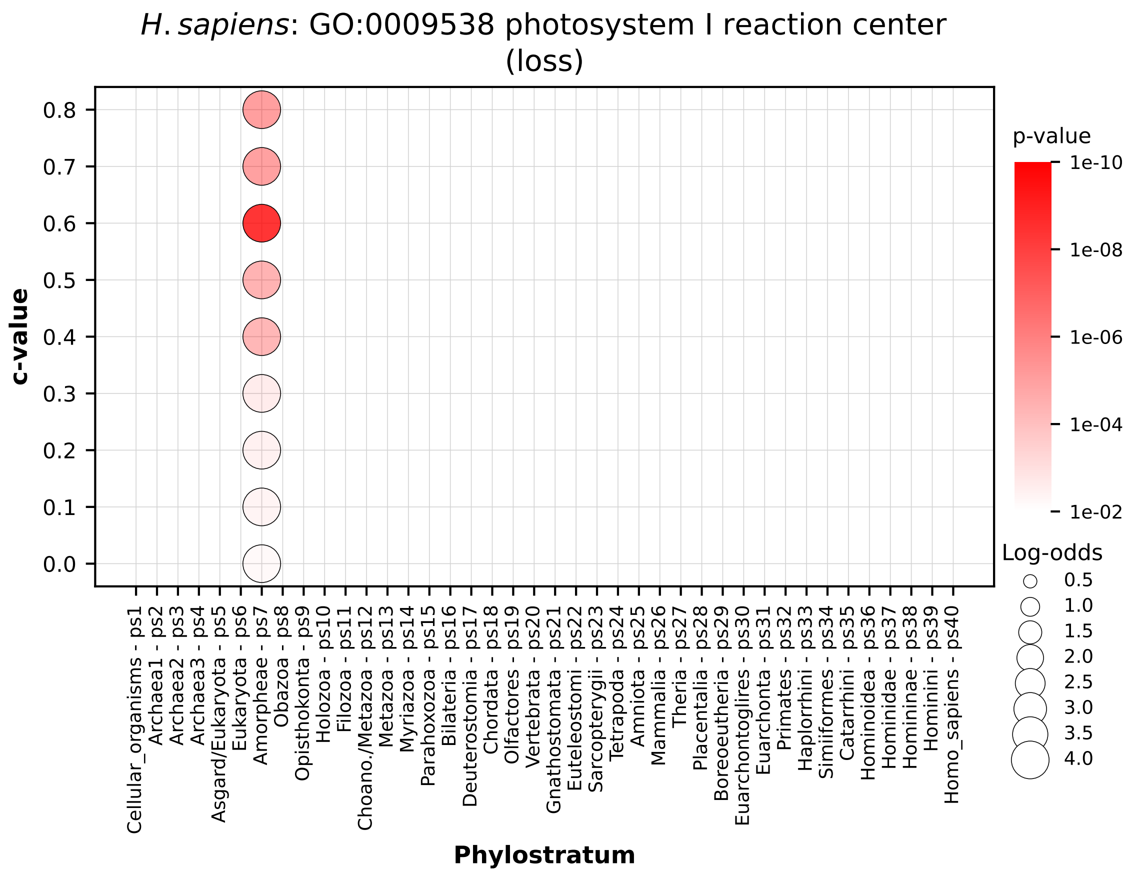

H. sapiens: GO:0009538 photosystem I reaction center
(loss)
c-value
0.8
0.7
0.6
0.5
0.4
0.3
0.2
0.1
0.0
p-value
1e-10
1e-08
1e-06
1e-04
1e-02
Log-odds
0.5
1.0
1.5
2.0
2.5
3.0
3.5
4.0
Cellular_organisms - ps1
Archaea1 - ps2
Archaea2 - ps3
Archaea3 - ps4
Asgard/Eukaryota - ps5
Eukaryota - ps6
Amorpheae - ps7
Obazoa - ps8
Opisthokonta - ps9
Holozoa - ps10
Filozoa - ps11
Choano./Metazoa - ps12
Metazoa - ps13
Myriazoa - ps14
Parahoxozoa - ps15
Bilateria - ps16
Deuterostomia - ps17
Chordata - ps18
Olfactores - ps19
Vertebrata - ps20
Gnathostomata - ps21
Euteleostomi - ps22
Sarcopterygii - ps23
Tetrapoda - ps24
Amniota - ps25
Mammalia - ps26
Theria - ps27
Placentalia - ps28
Boreoeutheria - ps29
Euarchontoglires - ps30
Euarchonta - ps31
Primates - ps32
Haplorrhini - ps33
Simiiformes - ps34
Catarrhini - ps35
Hominoidea - ps36
Hominidae - ps37
Homininae - ps38
Hominini - ps39
Homo_sapiens - ps40
Phylostratum

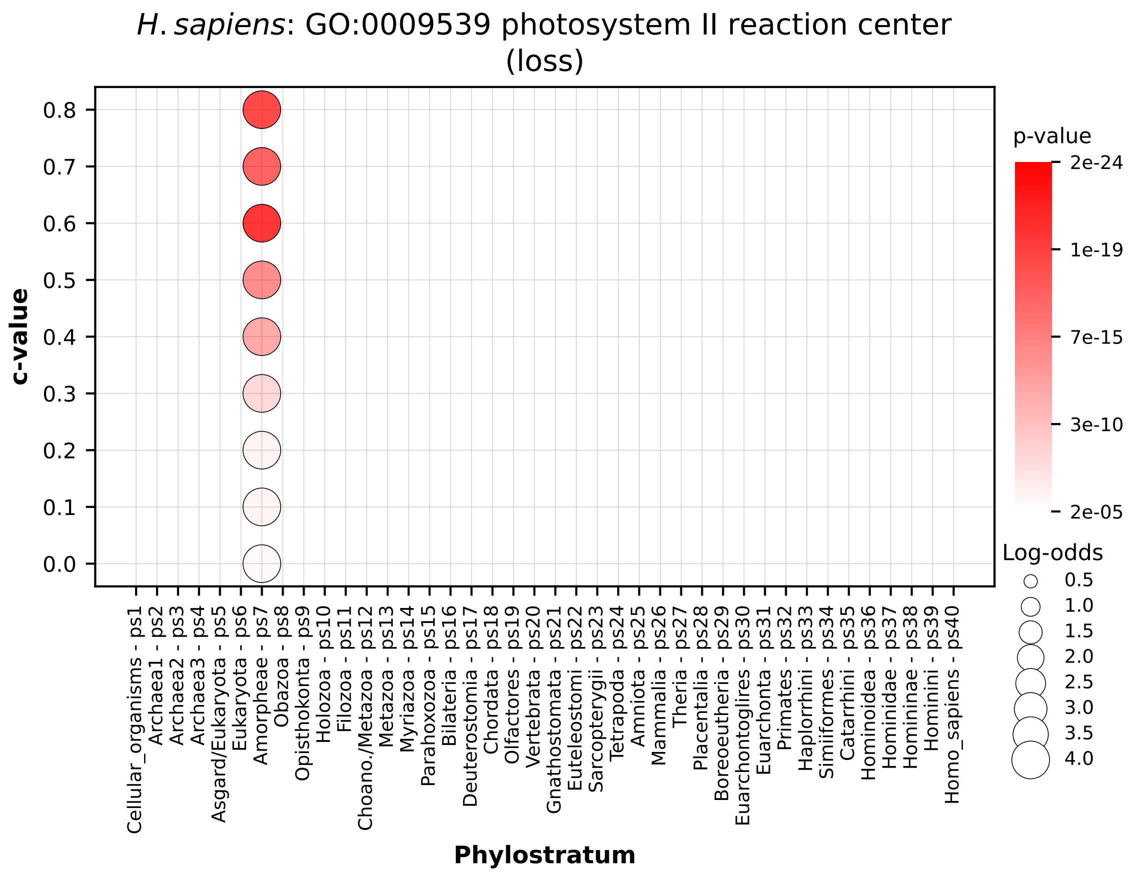

H. sapiens: GO:0009539 photosystem II reaction center (loss)
c-value
0.0
0.1
0.2
0.3
0.4
0.5
0.6
0.7
0.8
Phylostratum
Cellular_organisms - ps1
Archaea1 - ps2
Archaea2 - ps3
Archaea3 - ps4
Asgard/Eukaryota - ps5
Eukaryota - ps6
Amorpheae - ps7
Obazoa - ps8
Opisthokonta - ps9
Holozoa - ps10
Filozoa - ps11
Choano./Metazoa - ps12
Metazoa - ps13
Myriazoa - ps14
Parahoxozoa - ps15
Bilateria - ps16
Deuterostomia - ps17
Chordata - ps18
Olfactores - ps19
Vertebrata - ps20
Gnathostomata - ps21
Euteleostomi - ps22
Sarcopterygii - ps23
Tetrapoda - ps24
Amniota - ps25
Mammalia - ps26
Theria - ps27
Placentalia - ps28
Boreoeutheria - ps29
Euarchontoglires - ps30
Euarchonta - ps31
Primates - ps32
Haplorrhini - ps33
Simiiformes - ps34
Catarrhini - ps35
Hominoidea - ps36
Hominidae - ps37
Homininae - ps38
Hominini - ps39
Homo_sapiens - ps40
p-value
2e-24
1e-19
7e-15
3e-10
2e-05
Log-odds
0.5
1.0
1.5
2.0
2.5
3.0
3.5
4.0

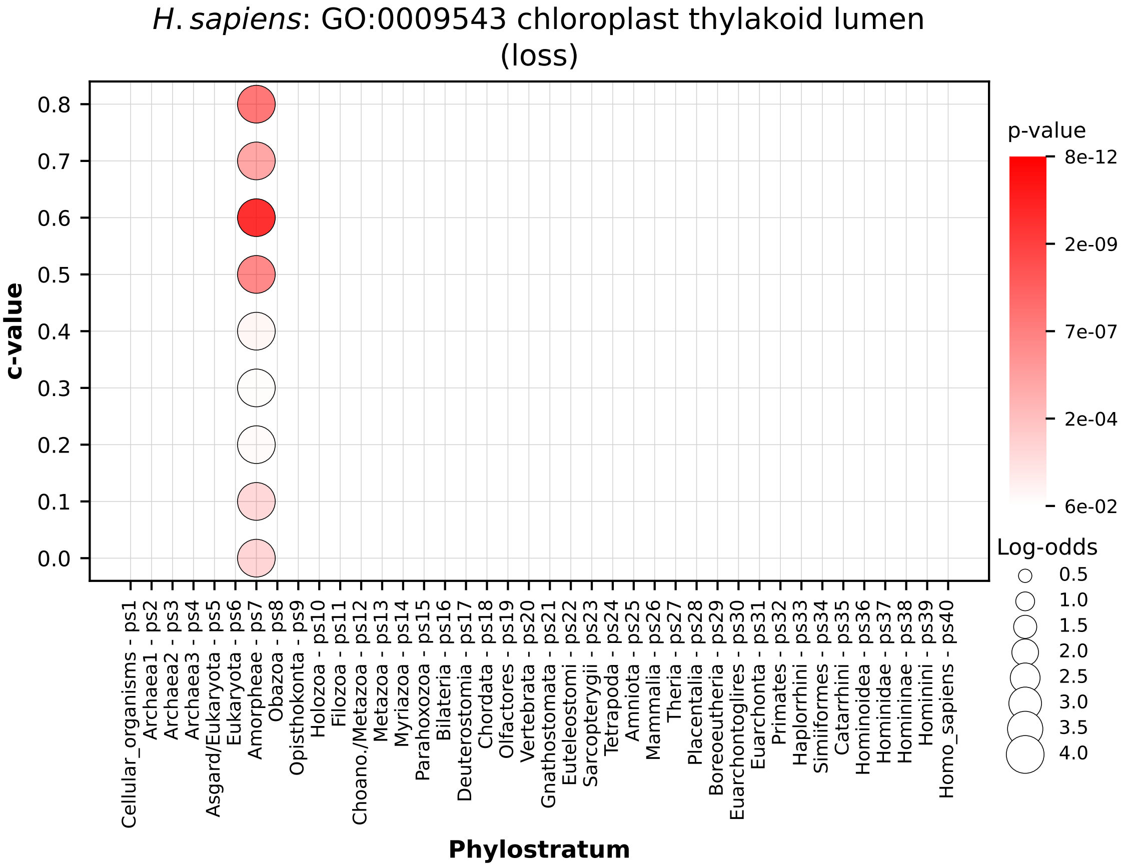

H. sapiens: GO:0009543 chloroplast thylakoid lumen
(loss)
c-value
0.8
0.7
0.6
0.5
0.4
0.3
0.2
0.1
0.0
Phylostratum
Cellular_organisms - ps1
Archaea1 - ps2
Archaea2 - ps3
Archaea3 - ps4
Asgard/Eukaryota - ps5
Eukaryota - ps6
Amorpheae - ps7
Obazoa - ps8
Opisthokonta - ps9
Holozoa - ps10
Filozoa - ps11
Choano./Metazoa - ps12
Metazoa - ps13
Myriazoa - ps14
Parahoxozoa - ps15
Bilateria - ps16
Deuterostomia - ps17
Chordata - ps18
Olfactores - ps19
Vertebrata - ps20
Gnathostomata - ps21
Euteleostomi - ps22
Sarcopterygii - ps23
Tetrapoda - ps24
Amniota - ps25
Mammalia - ps26
Theria - ps27
Placentalia - ps28
Boreoeutheria - ps29
Euarchontoglires - ps30
Euarchonta - ps31
Primates - ps32
Haplorrhini - ps33
Simiiformes - ps34
Catarrhini - ps35
Hominoidea - ps36
Hominidae - ps37
Homininae - ps38
Hominini - ps39
Homo_sapiens - ps40
p-value
8e-12
2e-09
7e-07
2e-04
6e-02
Log-odds
0.5
1.0
1.5
2.0
2.5
3.0
3.5
4.0

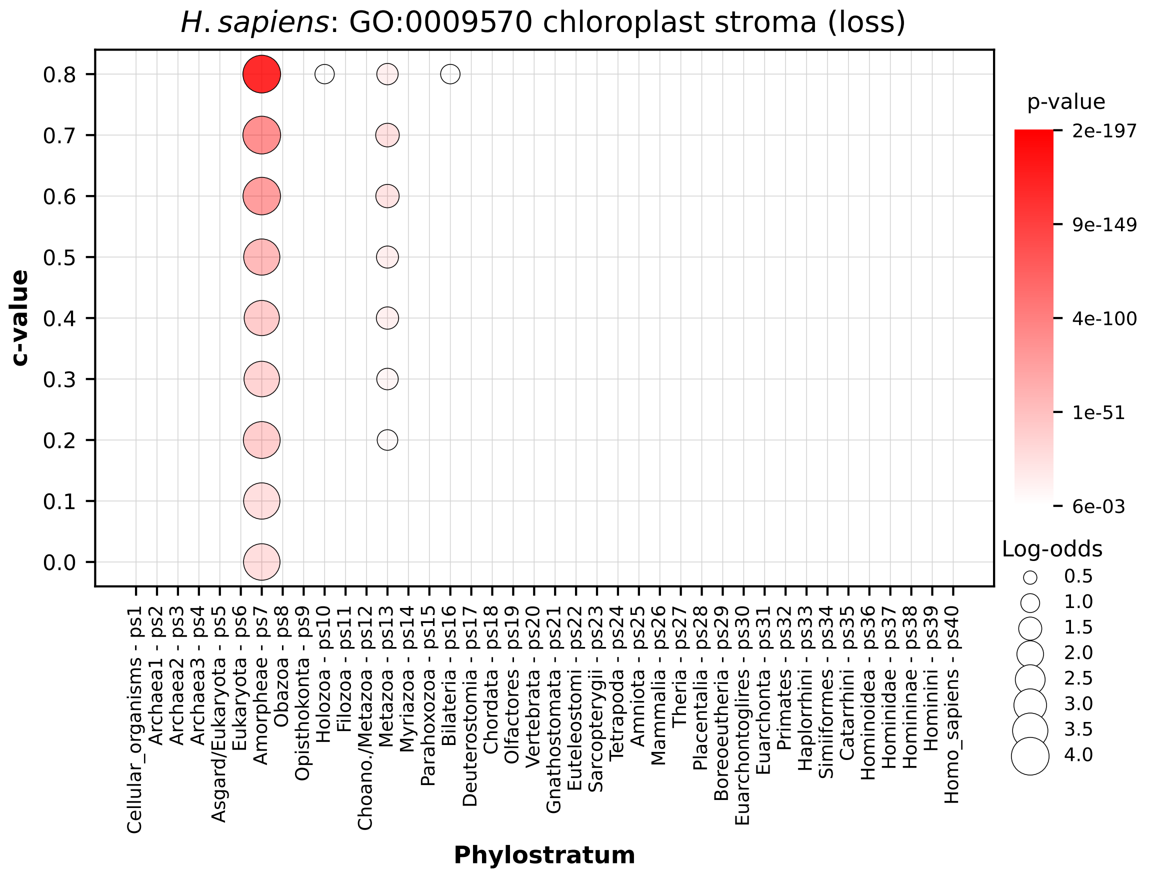

H. sapiens: GO:0009570 chloroplast stroma (loss)
c-value
0.0
0.1
0.2
0.3
0.4
0.5
0.6
0.7
0.8
Phylostratum
Cellular_organisms - ps1
Archaea1 - ps2
Archaea2 - ps3
Archaea3 - ps4
Asgard/Eukaryota - ps5
Eukaryota - ps6
Amorpheae - ps7
Obazoa - ps8
Opisthokonta - ps9
Holozoa - ps10
Filozoa - ps11
Choano./Metazoa - ps12
Metazoa - ps13
Myriazoa - ps14
Parahoxozoa - ps15
Bilateria - ps16
Deuterostomia - ps17
Chordata - ps18
Olfactores - ps19
Vertebrata - ps20
Gnathostomata - ps21
Euteleostomi - ps22
Sarcopterygii - ps23
Tetrapoda - ps24
Amniota - ps25
Mammalia - ps26
Theria - ps27
Placentalia - ps28
Boreoeutheria - ps29
Euarchontoglires - ps30
Euarchonta - ps31
Primates - ps32
Haplorrhini - ps33
Simiiformes - ps34
Catarrhini - ps35
Hominoidea - ps36
Hominidae - ps37
Homininae - ps38
Hominini - ps39
Homo_sapiens - ps40
p-value
2e-197
9e-149
4e-100
1e-51
6e-03
Log-odds
0.5
1.0
1.5
2.0
2.5
3.0
3.5
4.0

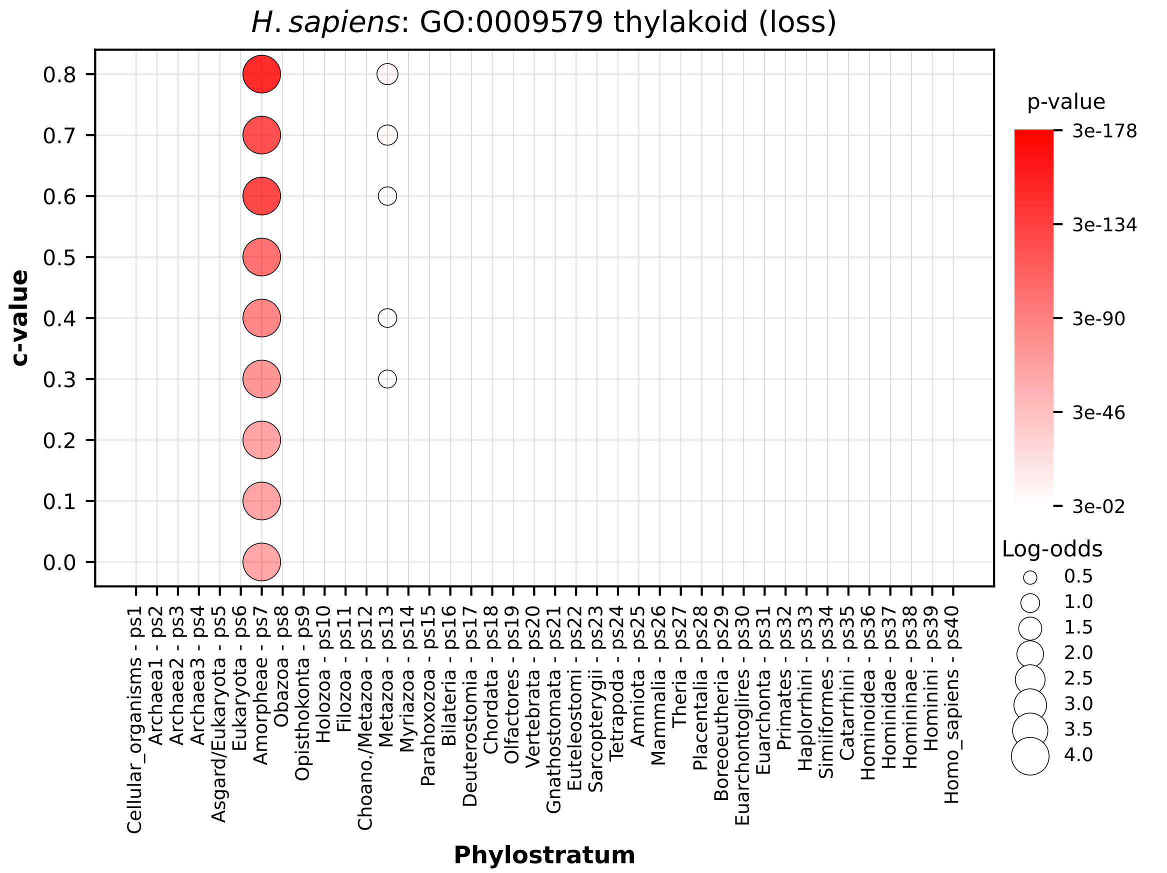
H. sapiens: GO:0009579 thylakoid (loss)
c-value
0.0
0.1
0.2
0.3
0.4
0.5
0.6
0.7
0.8
Phylostratum
Cellular_organisms - ps1
Archaea1 - ps2
Archaea2 - ps3
Archaea3 - ps4
Asgard/Eukaryota - ps5
Eukaryota - ps6
Amorpheae - ps7
Obazoa - ps8
Opisthokonta - ps9
Holozoa - ps10
Filozoa - ps11
Choano./Metazoa - ps12
Metazoa - ps13
Myriazoa - ps14
Parahoxozoa - ps15
Bilateria - ps16
Deuterostomia - ps17
Chordata - ps18
Olfactores - ps19
Vertebrata - ps20
Gnathostomata - ps21
Euteleostomi - ps22
Sarcopterygii - ps23
Tetrapoda - ps24
Amniota - ps25
Mammalia - ps26
Theria - ps27
Placentalia - ps28
Boreoeutheria - ps29
Euarchontoglires - ps30
Euarchonta - ps31
Primates - ps32
Haplorrhini - ps33
Simiiformes - ps34
Catarrhini - ps35
Hominoidea - ps36
Hominidae - ps37
Homininae - ps38
Hominini - ps39
Homo_sapiens - ps40
p-value
3e-178
3e-134
3e-90
3e-46
3e-02
Log-odds
0.5
1.0
1.5
2.0
2.5
3.0
3.5
4.0

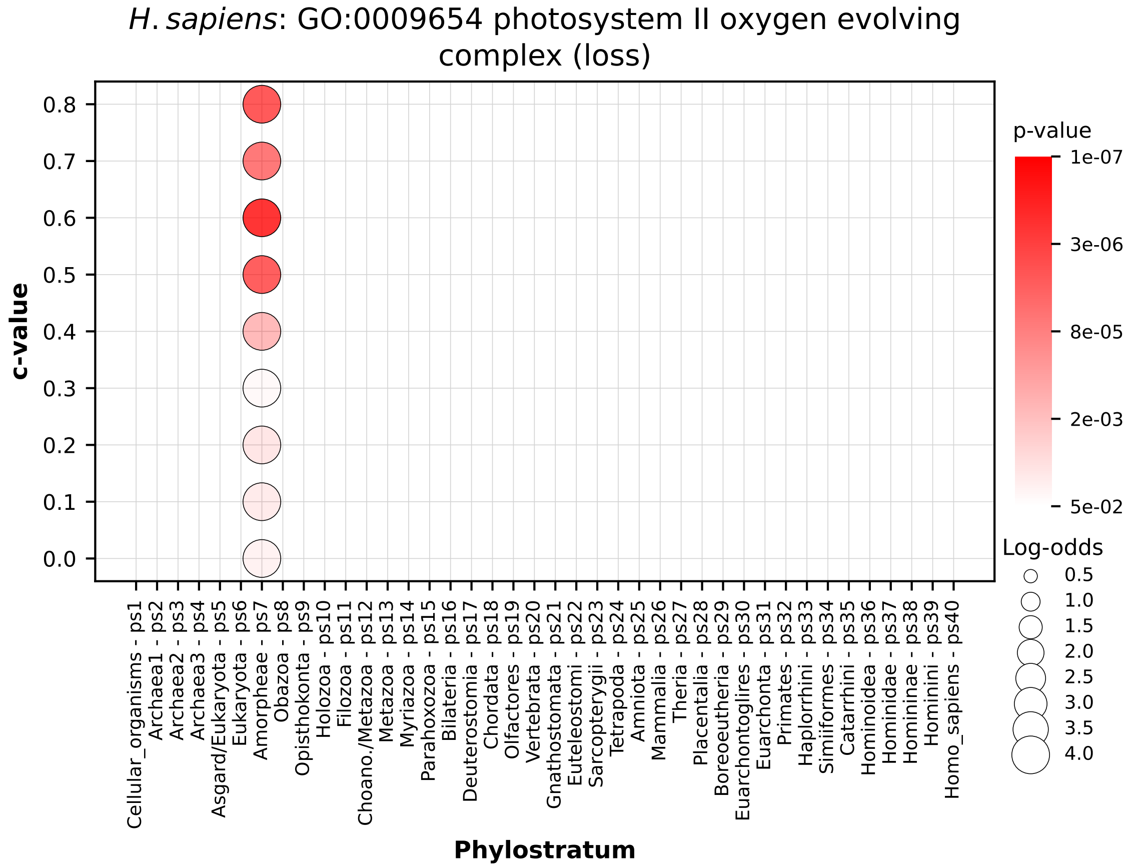
H. sapiens: GO:0009654 photosystem II oxygen evolving complex (loss)
c-value
0.0
0.1
0.2
0.3
0.4
0.5
0.6
0.7
0.8
p-value
1e-07
3e-06
8e-05
2e-03
5e-02
Log-odds
0.5
1.0
1.5
2.0
2.5
3.0
3.5
4.0
Cellular_organisms - ps1
Archaea1 - ps2
Archaea2 - ps3
Archaea3 - ps4
Asgard/Eukaryota - ps5
Eukaryota - ps6
Amorpheae - ps7
Obazoa - ps8
Opisthokonta - ps9
Holozoa - ps10
Filozoa - ps11
Choano./Metazoa - ps12
Metazoa - ps13
Myriazoa - ps14
Parahoxozoa - ps15
Bilateria - ps16
Deuterostomia - ps17
Chordata - ps18
Olfactores - ps19
Vertebrata - ps20
Gnathostomata - ps21
Euteleostomi - ps22
Sarcopterygii - ps23
Tetrapoda - ps24
Amniota - ps25
Mammalia - ps26
Theria - ps27
Placentalia - ps28
Boreoeutheria - ps29
Euarchontoglires - ps30
Euarchonta - ps31
Primates - ps32
Haplorrhini - ps33
Simiiformes - ps34
Catarrhini - ps35
Hominoidea - ps36
Hominidae - ps37
Homininae - ps38
Hominini - ps39
Homo_sapiens - ps40
Phylostratum

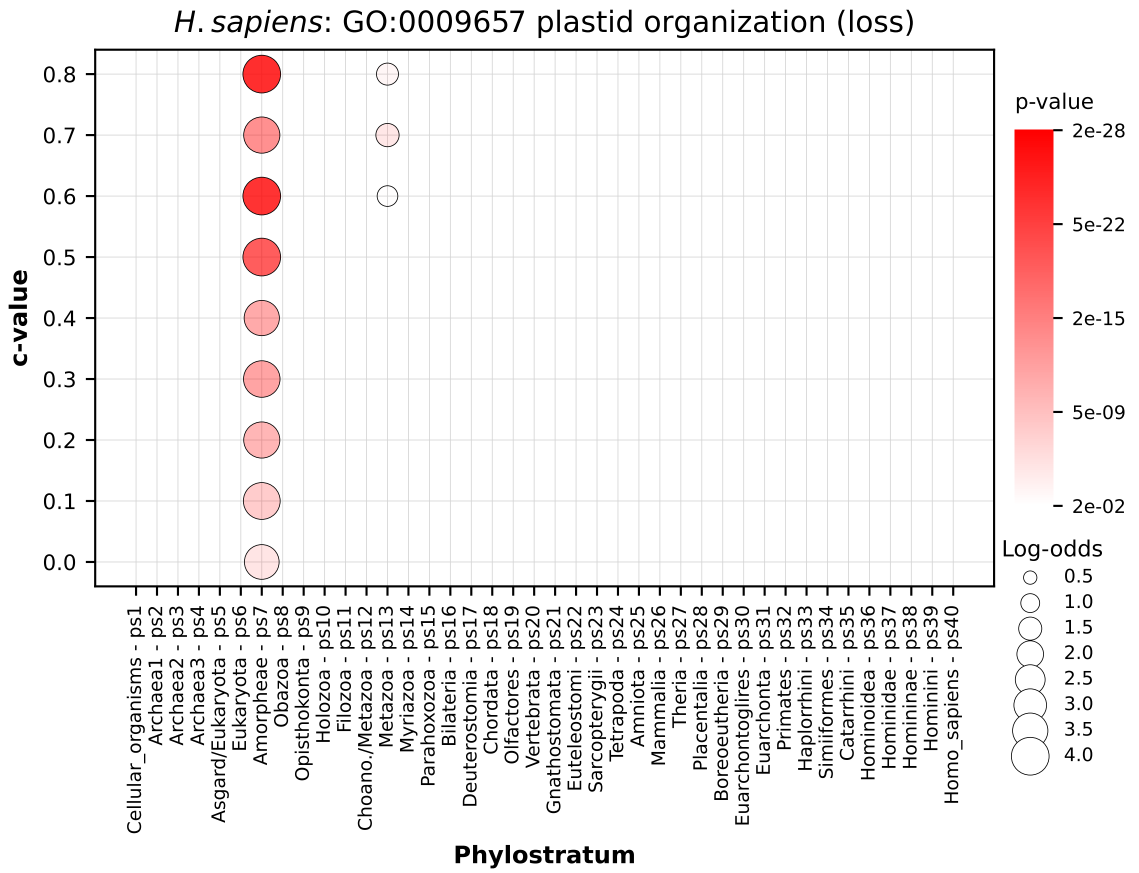

H. sapiens: GO:0009657 plastid organization (loss)
c-value
0.0
0.1
0.2
0.3
0.4
0.5
0.6
0.7
0.8
Phylostratum
Cellular_organisms - ps1
Archaea1 - ps2
Archaea2 - ps3
Archaea3 - ps4
Asgard/Eukaryota - ps5
Eukaryota - ps6
Amorpheae - ps7
Obazoa - ps8
Opisthokonta - ps9
Holozoa - ps10
Filozoa - ps11
Choano./Metazoa - ps12
Metazoa - ps13
Myriazoa - ps14
Parahoxozoa - ps15
Bilateria - ps16
Deuterostomia - ps17
Chordata - ps18
Olfactores - ps19
Vertebrata - ps20
Gnathostomata - ps21
Euteleostomi - ps22
Sarcopterygii - ps23
Tetrapoda - ps24
Amniota - ps25
Mammalia - ps26
Theria - ps27
Placentalia - ps28
Boreoeutheria - ps29
Euarchontoglires - ps30
Euarchonta - ps31
Primates - ps32
Haplorrhini - ps33
Simiiformes - ps34
Catarrhini - ps35
Hominoidea - ps36
Hominidae - ps37
Homininae - ps38
Hominini - ps39
Homo_sapiens - ps40
p-value
2e-28
5e-22
2e-15
5e-09
2e-02
Log-odds
0.5
1.0
1.5
2.0
2.5
3.0
3.5
4.0

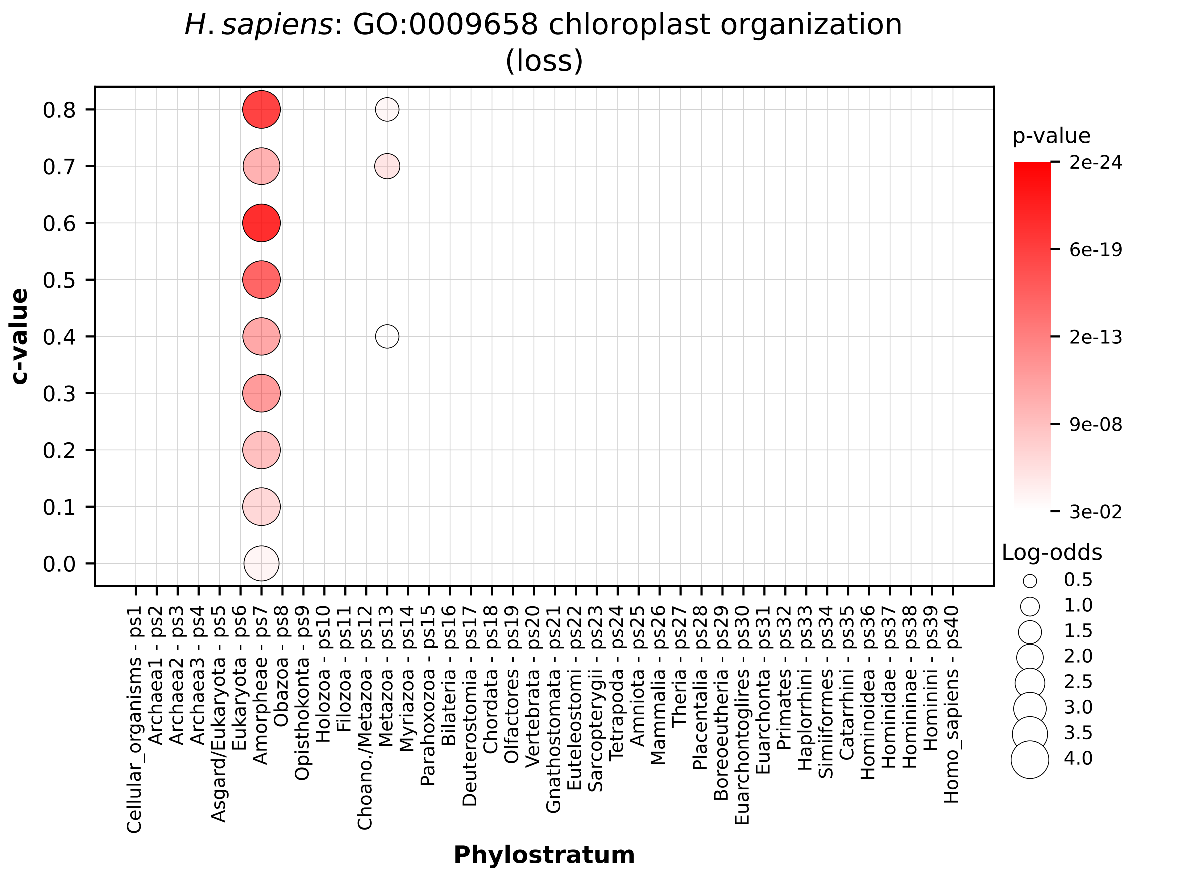

H. sapiens: GO:0009658 chloroplast organization
(loss)
c-value
0.8
0.7
0.6
0.5
0.4
0.3
0.2
0.1
0.0
p-value
2e-24
6e-19
2e-13
9e-08
3e-02
Log-odds
0.5
1.0
1.5
2.0
2.5
3.0
3.5
4.0
Cellular_organisms - ps1
Archaea1 - ps2
Archaea2 - ps3
Archaea3 - ps4
Asgard/Eukaryota - ps5
Eukaryota - ps6
Amorpheae - ps7
Obazoa - ps8
Opisthokonta - ps9
Holozoa - ps10
Filozoa - ps11
Choano./Metazoa - ps12
Metazoa - ps13
Myriazoa - ps14
Parahoxozoa - ps15
Bilateria - ps16
Deuterostomia - ps17
Chordata - ps18
Olfactores - ps19
Vertebrata - ps20
Gnathostomata - ps21
Euteleostomi - ps22
Sarcopterygii - ps23
Tetrapoda - ps24
Amniota - ps25
Mammalia - ps26
Theria - ps27
Placentalia - ps28
Boreoeutheria - ps29
Euarchontoglires - ps30
Euarchonta - ps31
Primates - ps32
Haplorrhini - ps33
Simiiformes - ps34
Catarrhini - ps35
Hominoidea - ps36
Hominidae - ps37
Homininae - ps38
Hominini - ps39
Homo_sapiens - ps40
Phylostratum

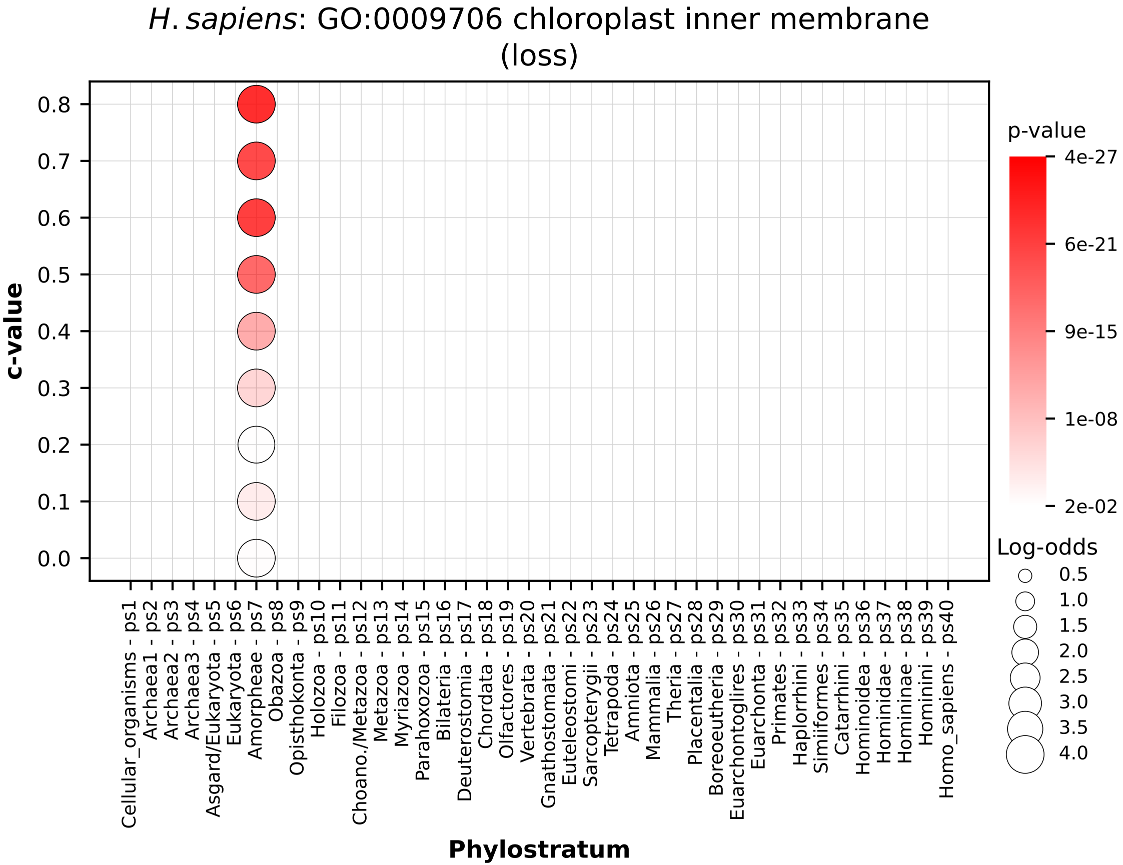
H. sapiens: GO:0009706 chloroplast inner membrane
(loss)
c-value
0.8
0.7
0.6
0.5
0.4
0.3
0.2
0.1
0.0
Phylostratum
Cellular_organisms - ps1
Archaea1 - ps2
Archaea2 - ps3
Archaea3 - ps4
Asgard/Eukaryota - ps5
Eukaryota - ps6
Amorpheae - ps7
Obazoa - ps8
Opisthokonta - ps9
Holozoa - ps10
Filozoa - ps11
Choano./Metazoa - ps12
Metazoa - ps13
Myriazoa - ps14
Parahoxozoa - ps15
Bilateria - ps16
Deuterostomia - ps17
Chordata - ps18
Olfactores - ps19
Vertebrata - ps20
Gnathostomata - ps21
Euteleostomi - ps22
Sarcopterygii - ps23
Tetrapoda - ps24
Amniota - ps25
Mammalia - ps26
Theria - ps27
Placentalia - ps28
Boreoeutheria - ps29
Euarchontoglires - ps30
Euarchonta - ps31
Primates - ps32
Haplorrhini - ps33
Simiiformes - ps34
Catarrhini - ps35
Hominoidea - ps36
Hominidae - ps37
Homininae - ps38
Hominini - ps39
Homo_sapiens - ps40
p-value
4e-27
6e-21
9e-15
1e-08
2e-02
Log-odds
0.5
1.0
1.5
2.0
2.5
3.0
3.5
4.0

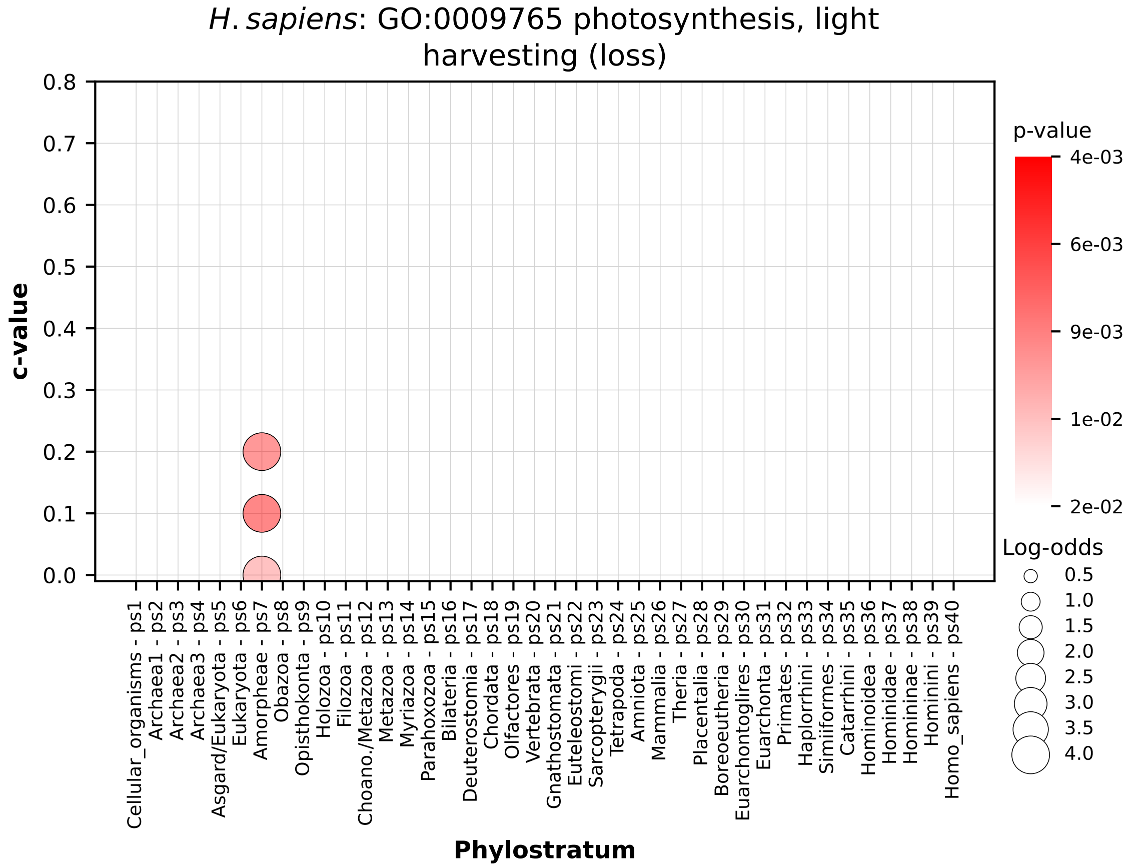

H. sapiens: GO:0009765 photosynthesis, light harvesting (loss)
c-value
0.0
0.1
0.2
0.3
0.4
0.5
0.6
0.7
0.8
Phylostratum
Cellular_organisms - ps1
Archaea1 - ps2
Archaea2 - ps3
Archaea3 - ps4
Asgard/Eukaryota - ps5
Eukaryota - ps6
Amorpheae - ps7
Obazoa - ps8
Opisthokonta - ps9
Holozoa - ps10
Filozoa - ps11
Choano./Metazoa - ps12
Metazoa - ps13
Myriazoa - ps14
Parahoxozoa - ps15
Bilateria - ps16
Deuterostomia - ps17
Chordata - ps18
Olfactores - ps19
Vertebrata - ps20
Gnathostomata - ps21
Euteleostomi - ps22
Sarcopterygii - ps23
Tetrapoda - ps24
Amniota - ps25
Mammalia - ps26
Theria - ps27
Placentalia - ps28
Boreoeutheria - ps29
Euarchontoglires - ps30
Euarchonta - ps31
Primates - ps32
Haplorrhini - ps33
Simiiformes - ps34
Catarrhini - ps35
Hominoidea - ps36
Hominidae - ps37
Homininae - ps38
Hominini - ps39
Homo_sapiens - ps40
p-value
4e-03
6e-03
9e-03
1e-02
2e-02
Log-odds
0.5
1.0
1.5
2.0
2.5
3.0
3.5
4.0

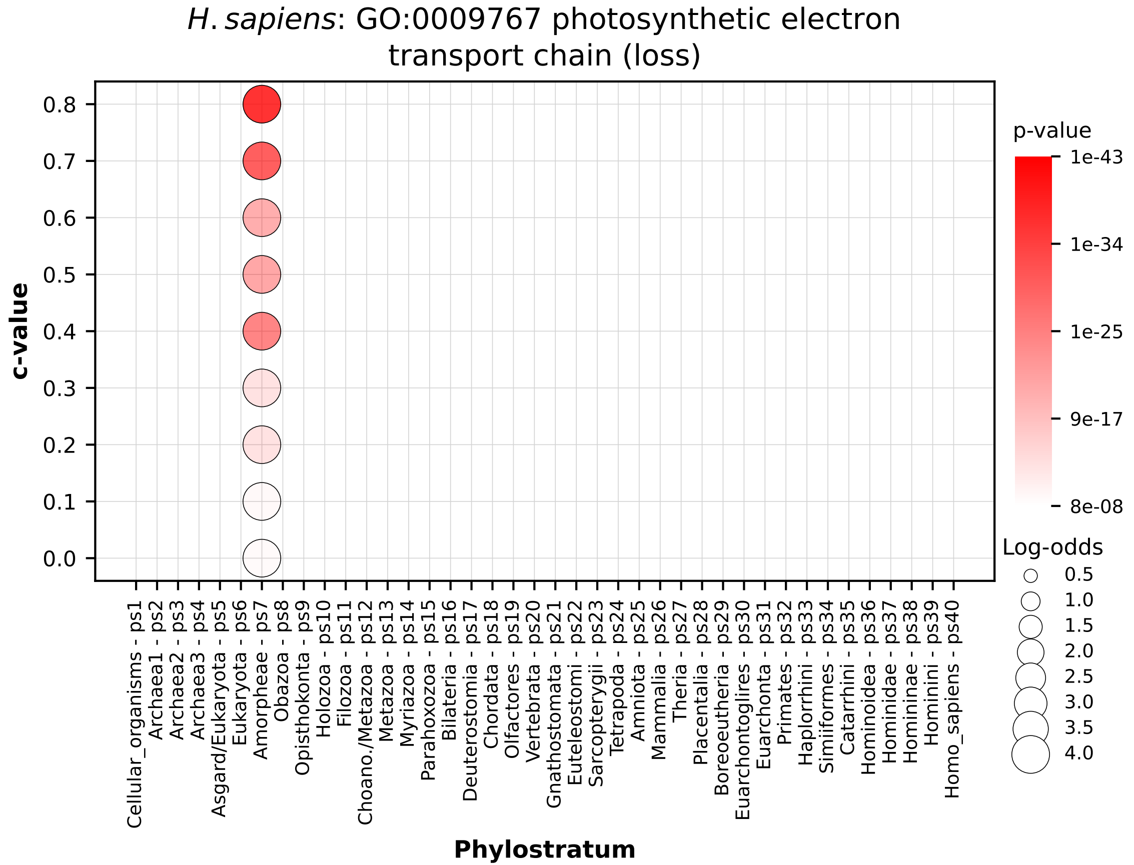
H. sapiens: GO:0009767 photosynthetic electron transport chain (loss)
c-value
0.8
0.7
0.6
0.5
0.4
0.3
0.2
0.1
0.0
p-value
1e-43
1e-34
1e-25
9e-17
8e-08
Log-odds
0.5
1.0
1.5
2.0
2.5
3.0
3.5
4.0
Cellular_organisms - ps1
Archaea1 - ps2
Archaea2 - ps3
Archaea3 - ps4
Asgard/Eukaryota - ps5
Eukaryota - ps6
Amorpheae - ps7
Obazoa - ps8
Opisthokonta - ps9
Holozoa - ps10
Filozoa - ps11
Choano./Metazoa - ps12
Metazoa - ps13
Myriazoa - ps14
Parahoxozoa - ps15
Bilateria - ps16
Deuterostomia - ps17
Chordata - ps18
Olfactores - ps19
Vertebrata - ps20
Gnathostomata - ps21
Euteleostomi - ps22
Sarcopterygii - ps23
Tetrapoda - ps24
Amniota - ps25
Mammalia - ps26
Theria - ps27
Placentalia - ps28
Boreoeutheria - ps29
Euarchontoglires - ps30
Euarchonta - ps31
Primates - ps32
Haplorrhini - ps33
Simiiformes - ps34
Catarrhini - ps35
Hominoidea - ps36
Hominidae - ps37
Homininae - ps38
Hominini - ps39
Homo_sapiens - ps40
Phylostratum

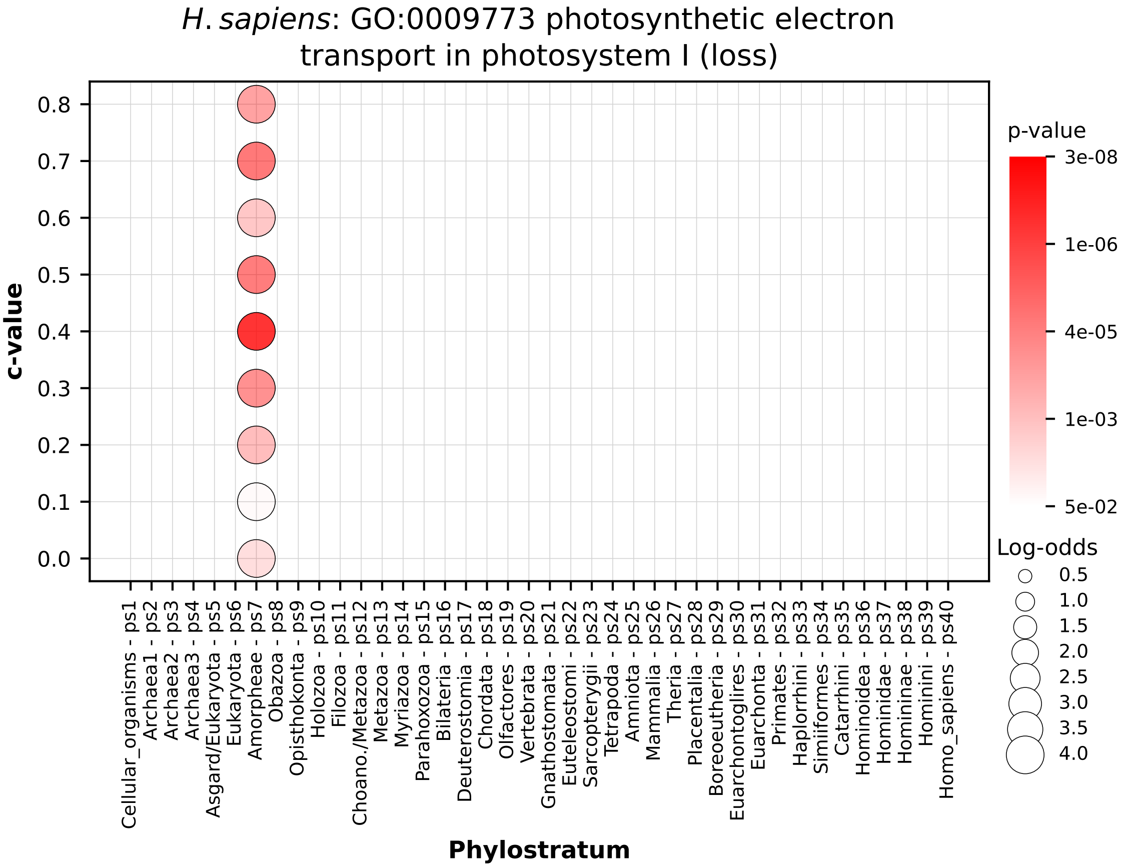

H. sapiens: GO:0009773 photosynthetic electron transport in photosystem I (loss)
c-value
0.0
0.1
0.2
0.3
0.4
0.5
0.6
0.7
0.8
Phylostratum
Cellular_organisms - ps1
Archaea1 - ps2
Archaea2 - ps3
Archaea3 - ps4
Asgard/Eukaryota - ps5
Eukaryota - ps6
Amorpheae - ps7
Obazoa - ps8
Opisthokonta - ps9
Holozoa - ps10
Filozoa - ps11
Choano./Metazoa - ps12
Metazoa - ps13
Myriazoa - ps14
Parahoxozoa - ps15
Bilateria - ps16
Deuterostomia - ps17
Chordata - ps18
Olfactores - ps19
Vertebrata - ps20
Gnathostomata - ps21
Euteleostomi - ps22
Sarcopterygii - ps23
Tetrapoda - ps24
Amniota - ps25
Mammalia - ps26
Theria - ps27
Placentalia - ps28
Boreoeutheria - ps29
Euarchontoglires - ps30
Euarchonta - ps31
Primates - ps32
Haplorrhini - ps33
Simiiformes - ps34
Catarrhini - ps35
Hominoidea - ps36
Hominidae - ps37
Homininae - ps38
Hominini - ps39
Homo_sapiens - ps40
p-value
3e-08
1e-06
4e-05
1e-03
5e-02
Log-odds
0.5
1.0
1.5
2.0
2.5
3.0
3.5
4.0

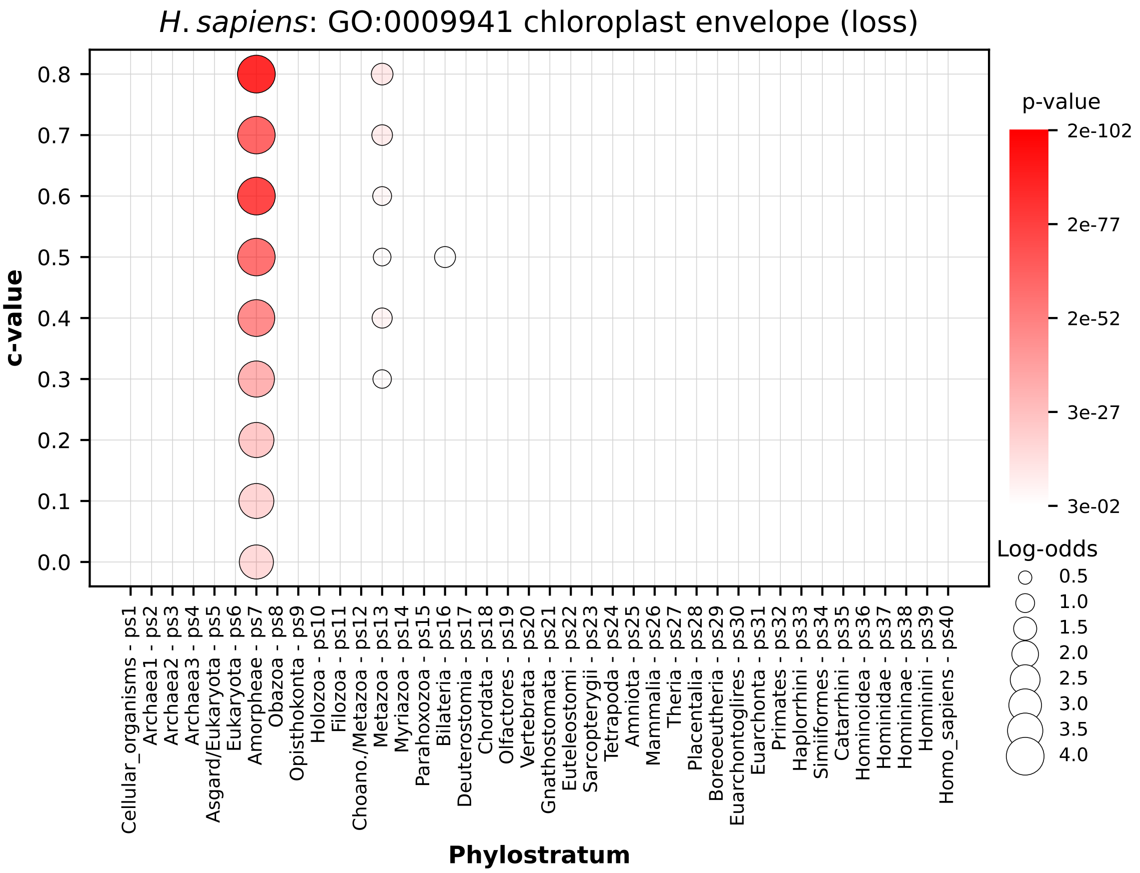

H. sapiens: GO:0009941 chloroplast envelope (loss)
c-value
0.8
0.7
0.6
0.5
0.4
0.3
0.2
0.1
0.0
Phylostratum
Cellular_organisms - ps1
Archaea1 - ps2
Archaea2 - ps3
Archaea3 - ps4
Asgard/Eukaryota - ps5
Eukaryota - ps6
Amorpheae - ps7
Obazoa - ps8
Opisthokonta - ps9
Holozoa - ps10
Filozoa - ps11
Choano./Metazoa - ps12
Metazoa - ps13
Myriazoa - ps14
Parahoxozoa - ps15
Bilateria - ps16
Deuterostomia - ps17
Chordata - ps18
Olfactores - ps19
Vertebrata - ps20
Gnathostomata - ps21
Euteleostomi - ps22
Sarcopterygii - ps23
Tetrapoda - ps24
Amniota - ps25
Mammalia - ps26
Theria - ps27
Placentalia - ps28
Boreoeutheria - ps29
Euarchontoglires - ps30
Euarchonta - ps31
Primates - ps32
Haplorrhini - ps33
Simiiformes - ps34
Catarrhini - ps35
Hominoidea - ps36
Hominidae - ps37
Homininae - ps38
Hominini - ps39
Homo_sapiens - ps40
p-value
2e-102
2e-77
2e-52
3e-27
3e-02
Log-odds
0.5
1.0
1.5
2.0
2.5
3.0
3.5
4.0

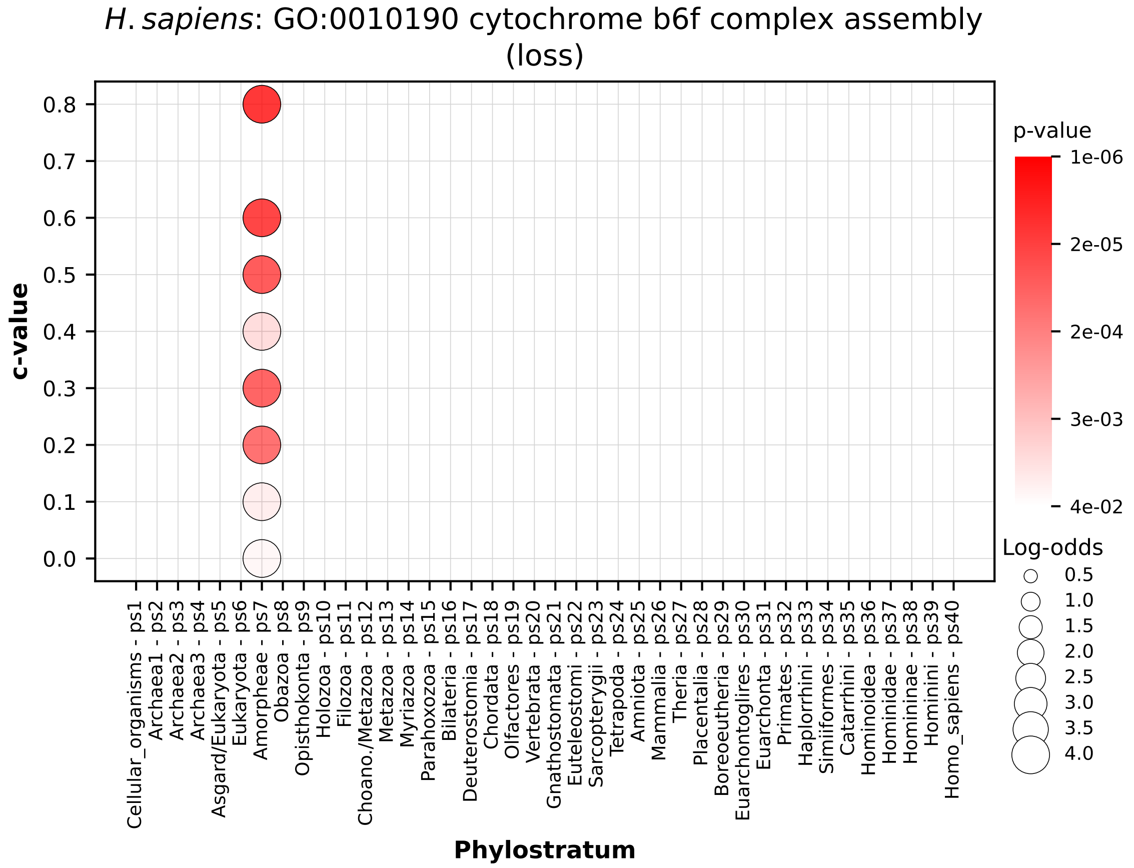

H. sapiens: GO:0010190 cytochrome b6f complex assembly
(loss)
c-value
0.8
0.7
0.6
0.5
0.4
0.3
0.2
0.1
0.0
p-value
1e-06
2e-05
2e-04
3e-03
4e-02
Log-odds
0.5
1.0
1.5
2.0
2.5
3.0
3.5
4.0
Cellular_organisms - ps1
Archaea1 - ps2
Archaea2 - ps3
Archaea3 - ps4
Asgard/Eukaryota - ps5
Eukaryota - ps6
Amorpheae - ps7
Obazoa - ps8
Opisthokonta - ps9
Holozoa - ps10
Filozoa - ps11
Choano./Metazoa - ps12
Metazoa - ps13
Myriazoa - ps14
Parahoxozoa - ps15
Bilateria - ps16
Deuterostomia - ps17
Chordata - ps18
Olfactores - ps19
Vertebrata - ps20
Gnathostomata - ps21
Euteleostomi - ps22
Sarcopterygii - ps23
Tetrapoda - ps24
Amniota - ps25
Mammalia - ps26
Theria - ps27
Placentalia - ps28
Boreoeutheria - ps29
Euarchontoglires - ps30
Euarchonta - ps31
Primates - ps32
Haplorrhini - ps33
Simiiformes - ps34
Catarrhini - ps35
Hominoidea - ps36
Hominidae - ps37
Homininae - ps38
Hominini - ps39
Homo_sapiens - ps40
Phylostratum

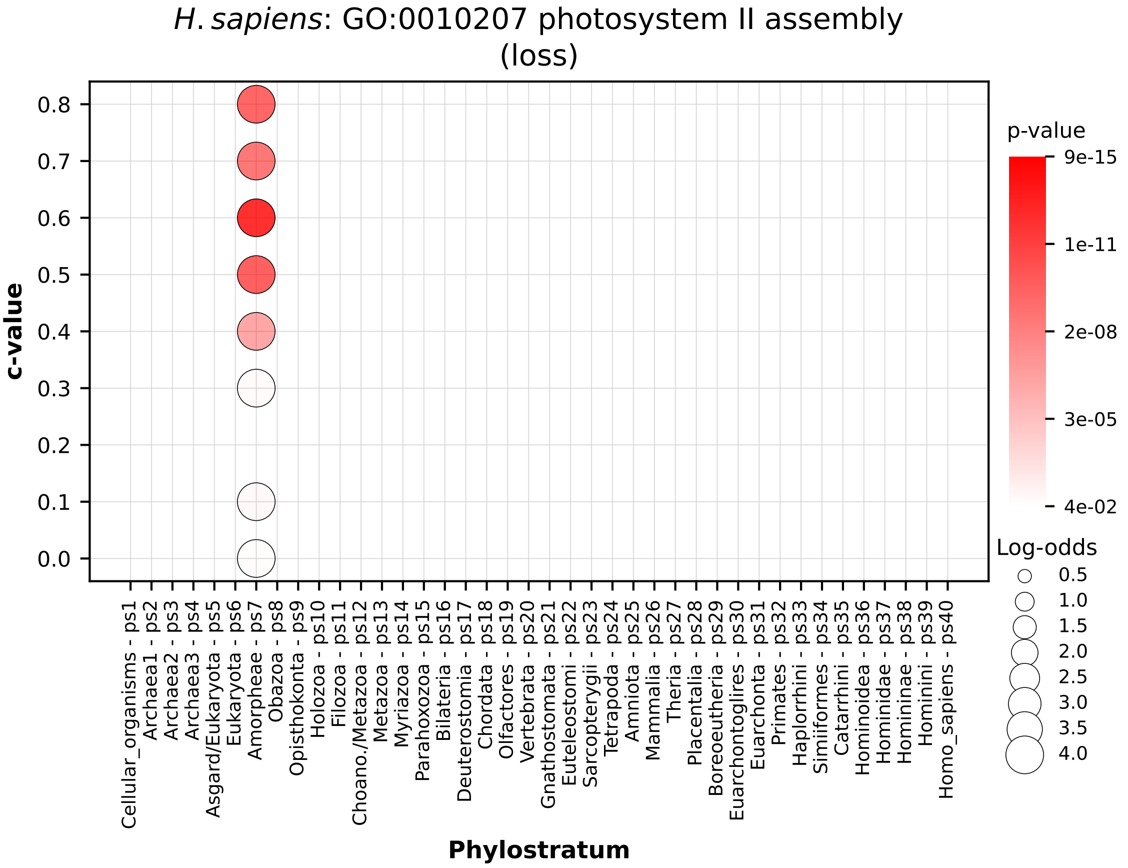

H. sapiens: GO:0010207 photosystem II assembly (loss)
c-value
0.8
0.7
0.6
0.5
0.4
0.3
0.2
0.1
0.0
p-value
9e-15
1e-11
2e-08
3e-05
4e-02
Log-odds
0.5
1.0
1.5
2.0
2.5
3.0
3.5
4.0
Cellular_organisms - ps1
Archaea1 - ps2
Archaea2 - ps3
Archaea3 - ps4
Asgard/Eukaryota - ps5
Eukaryota - ps6
Amorpheae - ps7
Obazoa - ps8
Opisthokonta - ps9
Holozoa - ps10
Filozoa - ps11
Choano./Metazoa - ps12
Metazoa - ps13
Myriazoa - ps14
Parahoxozoa - ps15
Bilateria - ps16
Deuterostomia - ps17
Chordata - ps18
Olfactores - ps19
Vertebrata - ps20
Gnathostomata - ps21
Euteleostomi - ps22
Sarcopterygii - ps23
Tetrapoda - ps24
Amniota - ps25
Mammalia - ps26
Theria - ps27
Placentalia - ps28
Boreoeutheria - ps29
Euarchontoglires - ps30
Euarchonta - ps31
Primates - ps32
Haplorrhini - ps33
Simiiformes - ps34
Catarrhini - ps35
Hominoidea - ps36
Hominidae - ps37
Homininae - ps38
Hominini - ps39
Homo_sapiens - ps40
Phylostratum

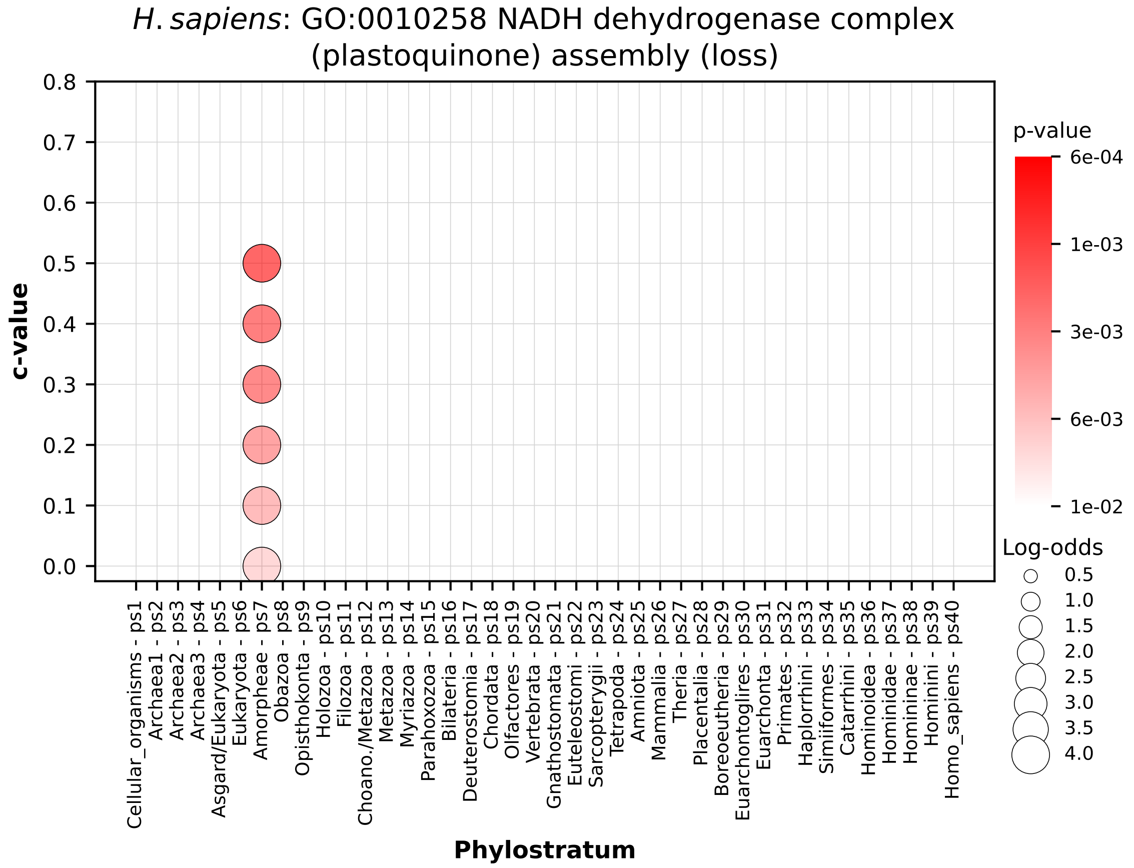

H. sapiens: GO:0010258 NADH dehydrogenase complex (plastoquinone) assembly (loss)
c-value
0.8
0.7
0.6
0.5
0.4
0.3
0.2
0.1
0.0
Phylostratum
Cellular_organisms - ps1
Archaea1 - ps2
Archaea2 - ps3
Archaea3 - ps4
Asgard/Eukaryota - ps5
Eukaryota - ps6
Amorpheae - ps7
Obazoa - ps8
Opisthokonta - ps9
Holozoa - ps10
Filozoa - ps11
Choano./Metazoa - ps12
Metazoa - ps13
Myriazoa - ps14
Parahoxozoa - ps15
Bilateria - ps16
Deuterostomia - ps17
Chordata - ps18
Olfactores - ps19
Vertebrata - ps20
Gnathostomata - ps21
Euteleostomi - ps22
Sarcopterygii - ps23
Tetrapoda - ps24
Amniota - ps25
Mammalia - ps26
Theria - ps27
Placentalia - ps28
Boreoeutheria - ps29
Euarchontoglires - ps30
Euarchonta - ps31
Primates - ps32
Haplorrhini - ps33
Simiiformes - ps34
Catarrhini - ps35
Hominoidea - ps36
Hominidae - ps37
Homininae - ps38
Hominini - ps39
Homo_sapiens - ps40
p-value
6e-04
1e-03
3e-03
6e-03
1e-02
Log-odds
0.5
1.0
1.5
2.0
2.5
3.0
3.5
4.0

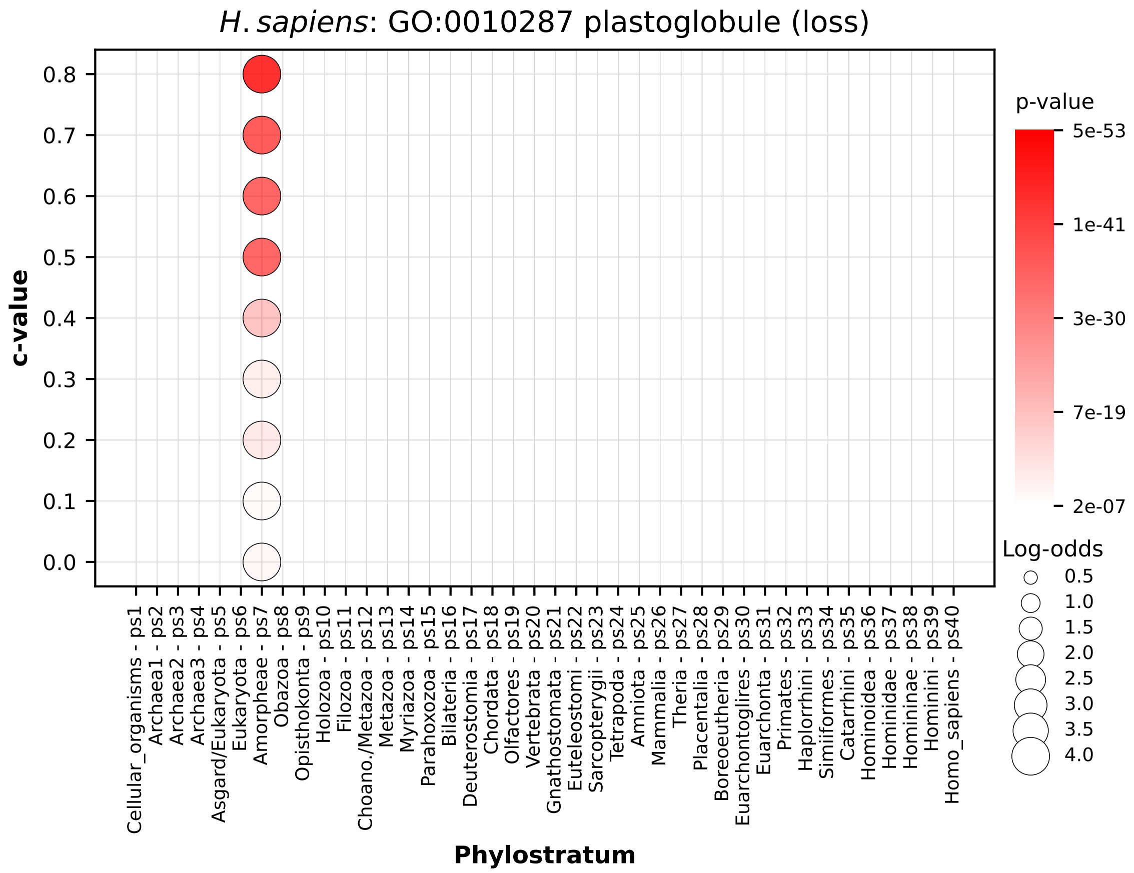

H. sapiens: GO:0010287 plastoglobule (loss)
c-value
0.8
0.7
0.6
0.5
0.4
0.3
0.2
0.1
0.0
Phylostratum
Cellular_organisms - ps1
Archaea1 - ps2
Archaea2 - ps3
Archaea3 - ps4
Asgard/Eukaryota - ps5
Eukaryota - ps6
Amorpheae - ps7
Obazoa - ps8
Opisthokonta - ps9
Holozoa - ps10
Filozoa - ps11
Choano./Metazoa - ps12
Metazoa - ps13
Myriazoa - ps14
Parahoxozoa - ps15
Bilateria - ps16
Deuterostomia - ps17
Chordata - ps18
Olfactores - ps19
Vertebrata - ps20
Gnathostomata - ps21
Euteleostomi - ps22
Sarcopterygii - ps23
Tetrapoda - ps24
Amniota - ps25
Mammalia - ps26
Theria - ps27
Placentalia - ps28
Boreoeutheria - ps29
Euarchontoglires - ps30
Euarchonta - ps31
Primates - ps32
Haplorrhini - ps33
Simiiformes - ps34
Catarrhini - ps35
Hominoidea - ps36
Hominidae - ps37
Homininae - ps38
Hominini - ps39
Homo_sapiens - ps40
p-value
5e-53
1e-41
3e-30
7e-19
2e-07
Log-odds
0.5
1.0
1.5
2.0
2.5
3.0
3.5
4.0

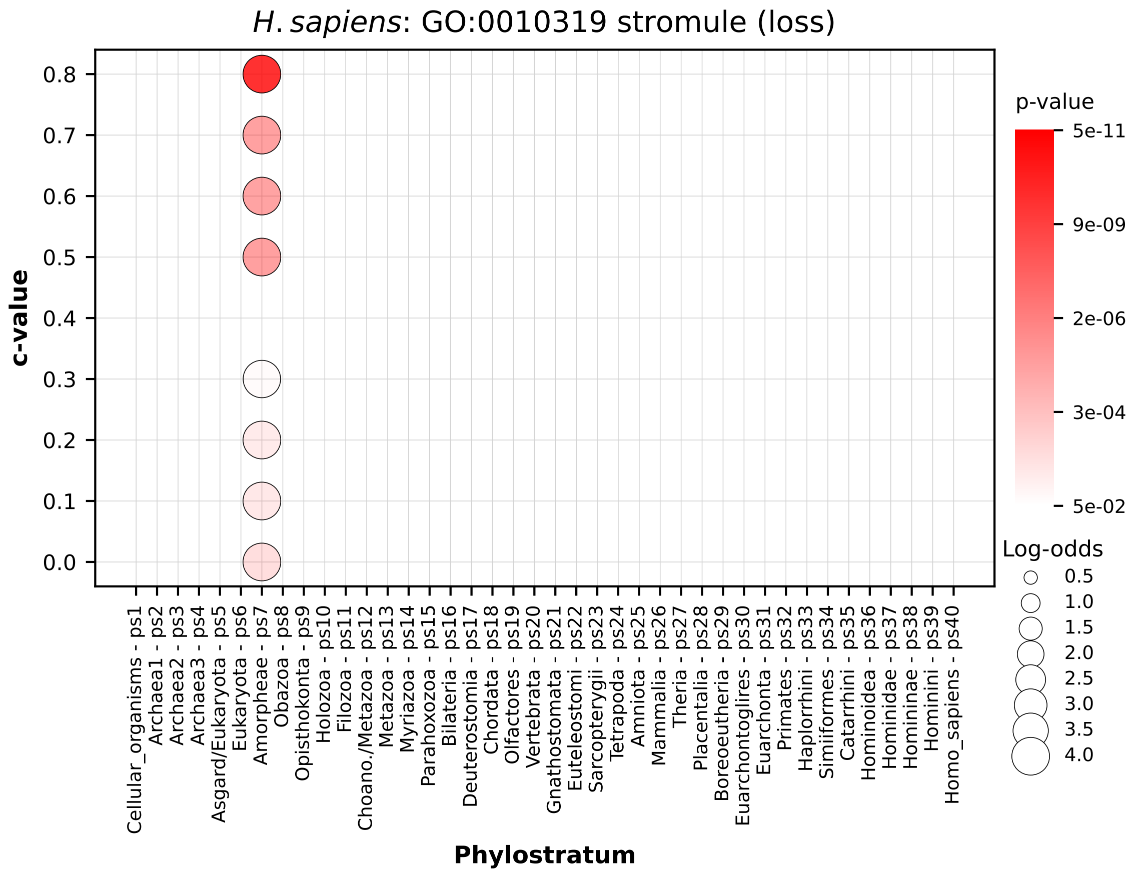

H. sapiens: GO:0010319 stromule (loss)
c-value
0.0
0.1
0.2
0.3
0.4
0.5
0.6
0.7
0.8
p-value
5e-11
9e-09
2e-06
3e-04
5e-02
Log-odds
0.5
1.0
1.5
2.0
2.5
3.0
3.5
4.0
Cellular_organisms - ps1
Archaea1 - ps2
Archaea2 - ps3
Archaea3 - ps4
Asgard/Eukaryota - ps5
Eukaryota - ps6
Amorpheae - ps7
Obazoa - ps8
Opisthokonta - ps9
Holozoa - ps10
Filozoa - ps11
Choano./Metazoa - ps12
Metazoa - ps13
Myriazoa - ps14
Parahoxozoa - ps15
Bilateria - ps16
Deuterostomia - ps17
Chordata - ps18
Olfactores - ps19
Vertebrata - ps20
Gnathostomata - ps21
Euteleostomi - ps22
Sarcopterygii - ps23
Tetrapoda - ps24
Amniota - ps25
Mammalia - ps26
Theria - ps27
Placentalia - ps28
Boreoeutheria - ps29
Euarchontoglires - ps30
Euarchonta - ps31
Primates - ps32
Haplorrhini - ps33
Simiiformes - ps34
Catarrhini - ps35
Hominoidea - ps36
Hominidae - ps37
Homininae - ps38
Hominini - ps39
Homo_sapiens - ps40
Phylostratum

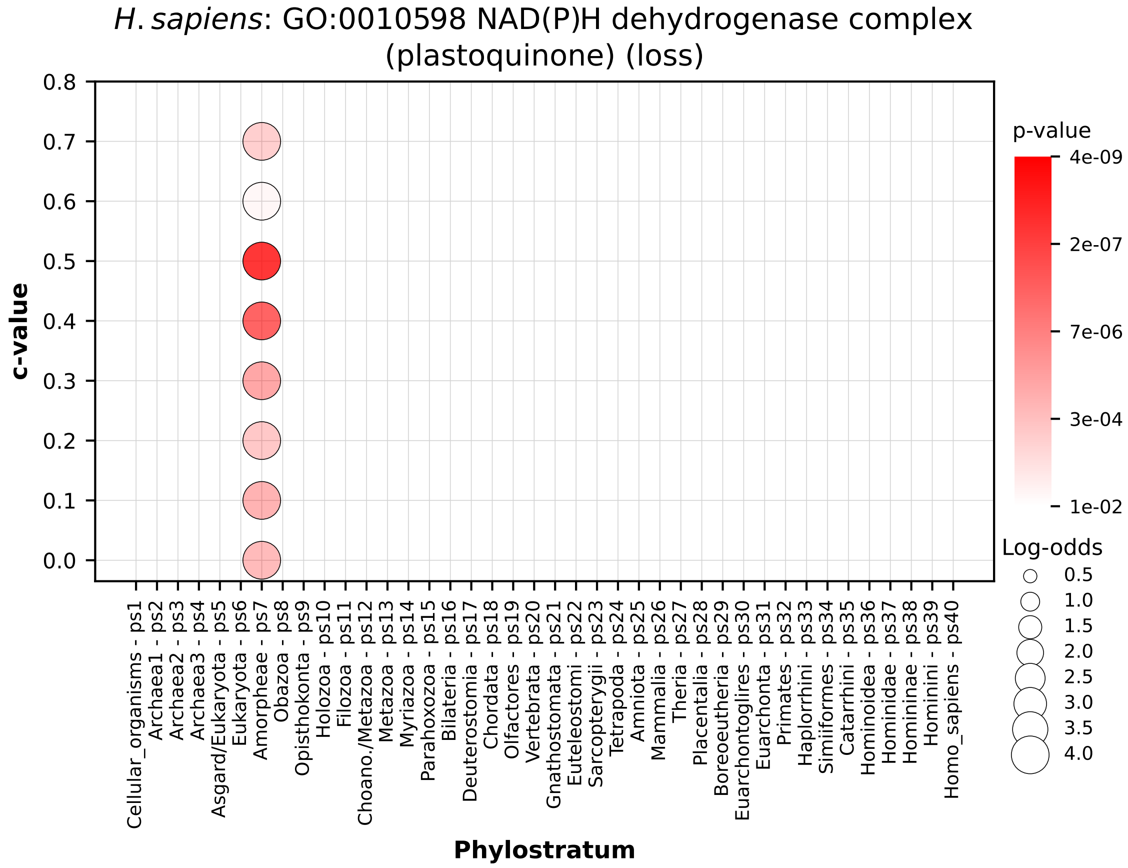

H. sapiens: GO:0010598 NAD(P)H dehydrogenase complex (plastoquinone) (loss)
c-value
0.0
0.1
0.2
0.3
0.4
0.5
0.6
0.7
0.8
Phylostratum
Cellular_organisms - ps1
Archaea1 - ps2
Archaea2 - ps3
Archaea3 - ps4
Asgard/Eukaryota - ps5
Eukaryota - ps6
Amorpheae - ps7
Obazoa - ps8
Opisthokonta - ps9
Holozoa - ps10
Filozoa - ps11
Choano./Metazoa - ps12
Metazoa - ps13
Myriazoa - ps14
Parahoxozoa - ps15
Bilateria - ps16
Deuterostomia - ps17
Chordata - ps18
Olfactores - ps19
Vertebrata - ps20
Gnathostomata - ps21
Euteleostomi - ps22
Sarcopterygii - ps23
Tetrapoda - ps24
Amniota - ps25
Mammalia - ps26
Theria - ps27
Placentalia - ps28
Boreoeutheria - ps29
Euarchontoglires - ps30
Euarchonta - ps31
Primates - ps32
Haplorrhini - ps33
Simiiformes - ps34
Catarrhini - ps35
Hominoidea - ps36
Hominidae - ps37
Homininae - ps38
Hominini - ps39
Homo_sapiens - ps40
p-value
4e-09
2e-07
7e-06
3e-04
1e-02
Log-odds
0.5
1.0
1.5
2.0
2.5
3.0
3.5
4.0

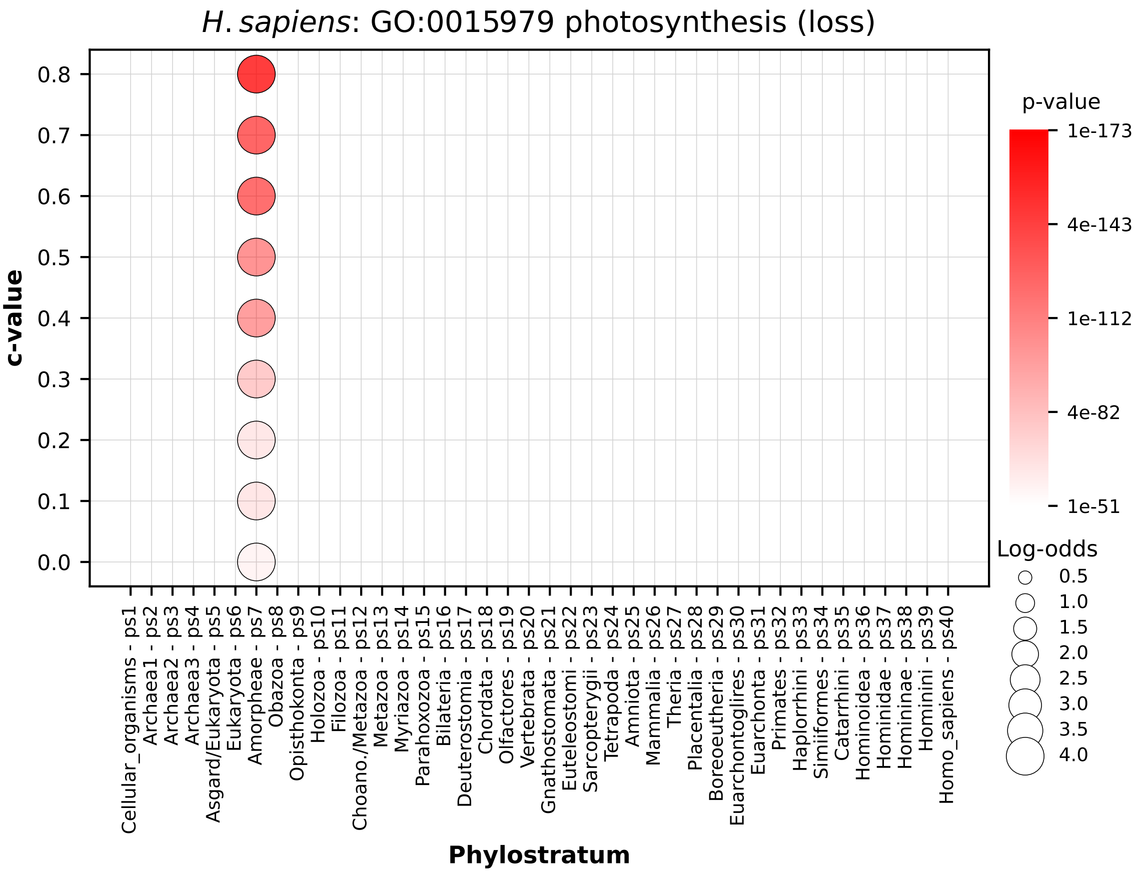

H. sapiens: GO:0015979 photosynthesis (loss)
c-value
0.8
0.7
0.6
0.5
0.4
0.3
0.2
0.1
0.0
p-value
1e-173
4e-143
1e-112
4e-82
1e-51
Log-odds
0.5
1.0
1.5
2.0
2.5
3.0
3.5
4.0
Cellular_organisms - ps1
Archaea1 - ps2
Archaea2 - ps3
Archaea3 - ps4
Asgard/Eukaryota - ps5
Eukaryota - ps6
Amorpheae - ps7
Obazoa - ps8
Opisthokonta - ps9
Holozoa - ps10
Filozoa - ps11
Choano./Metazoa - ps12
Metazoa - ps13
Myriazoa - ps14
Parahoxozoa - ps15
Bilateria - ps16
Deuterostomia - ps17
Chordata - ps18
Olfactores - ps19
Vertebrata - ps20
Gnathostomata - ps21
Euteleostomi - ps22
Sarcopterygii - ps23
Tetrapoda - ps24
Amniota - ps25
Mammalia - ps26
Theria - ps27
Placentalia - ps28
Boreoeutheria - ps29
Euarchontoglires - ps30
Euarchonta - ps31
Primates - ps32
Haplorrhini - ps33
Simiiformes - ps34
Catarrhini - ps35
Hominoidea - ps36
Hominidae - ps37
Homininae - ps38
Hominini - ps39
Homo_sapiens - ps40
Phylostratum

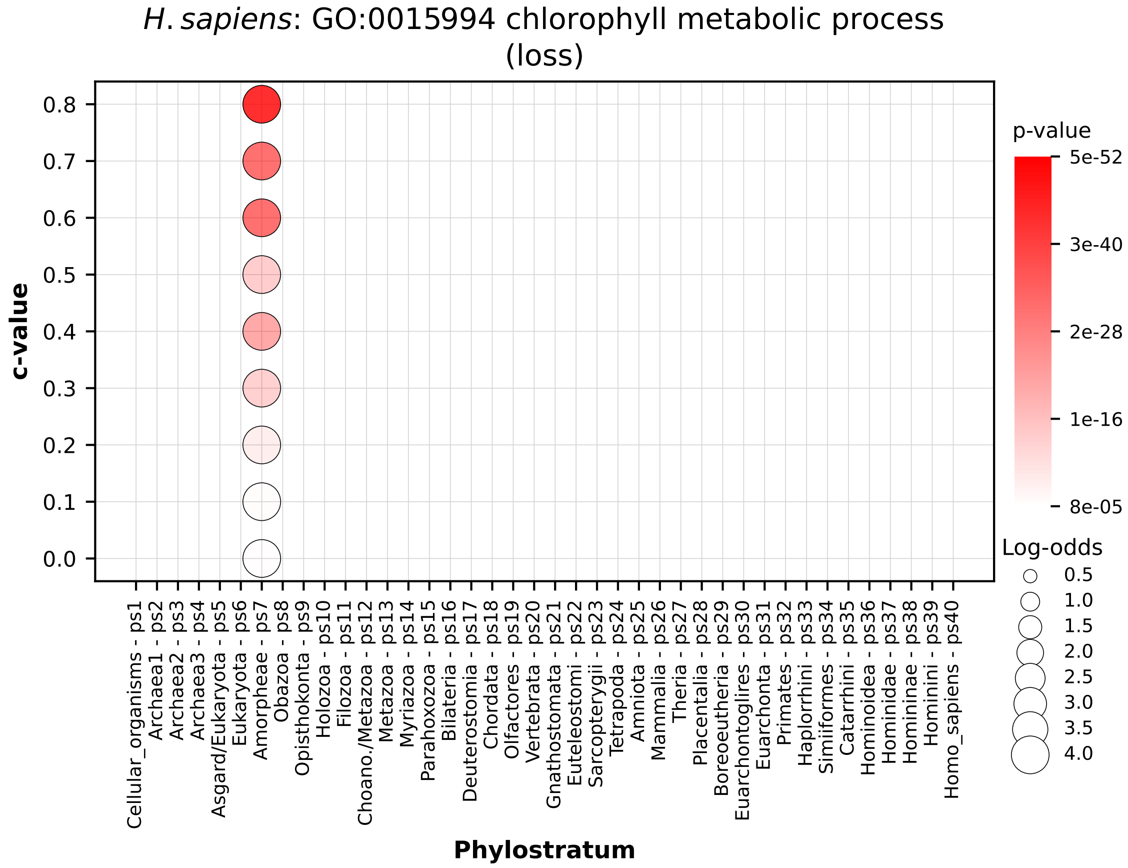

H. sapiens: GO:0015994 chlorophyll metabolic process (loss)
c-value
0.8
0.7
0.6
0.5
0.4
0.3
0.2
0.1
0.0
p-value
5e-52
3e-40
2e-28
1e-16
8e-05
Log-odds
0.5
1.0
1.5
2.0
2.5
3.0
3.5
4.0
Cellular_organisms - ps1
Archaea1 - ps2
Archaea2 - ps3
Archaea3 - ps4
Asgard/Eukaryota - ps5
Eukaryota - ps6
Amorpheae - ps7
Obazoa - ps8
Opisthokonta - ps9
Holozoa - ps10
Filozoa - ps11
Choano./Metazoa - ps12
Metazoa - ps13
Myriazoa - ps14
Parahoxozoa - ps15
Bilateria - ps16
Deuterostomia - ps17
Chordata - ps18
Olfactores - ps19
Vertebrata - ps20
Gnathostomata - ps21
Euteleostomi - ps22
Sarcopterygii - ps23
Tetrapoda - ps24
Amniota - ps25
Mammalia - ps26
Theria - ps27
Placentalia - ps28
Boreoeutheria - ps29
Euarchontoglires - ps30
Euarchonta - ps31
Primates - ps32
Haplorrhini - ps33
Simiiformes - ps34
Catarrhini - ps35
Hominoidea - ps36
Hominidae - ps37
Homininae - ps38
Hominini - ps39
Homo_sapiens - ps40
Phylostratum

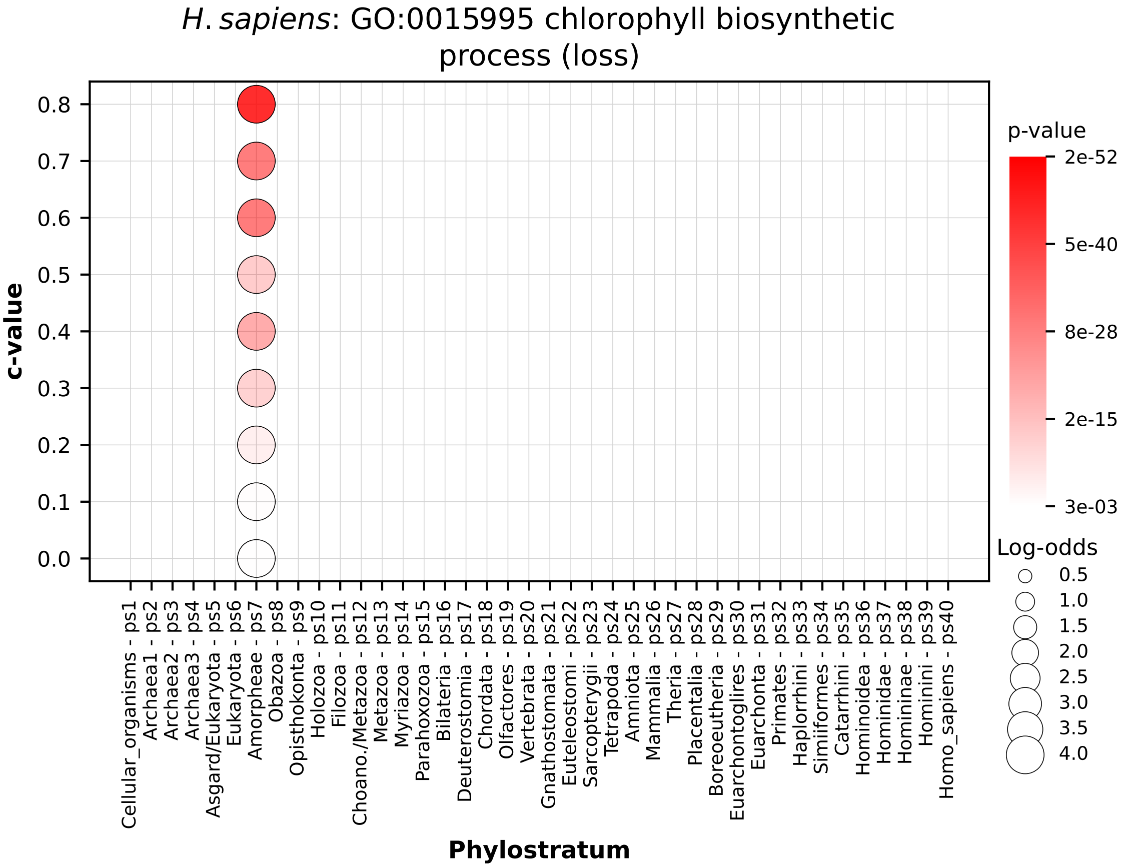

H. sapiens: GO:0015995 chlorophyll biosynthetic process (loss)
c-value
0.0
0.1
0.2
0.3
0.4
0.5
0.6
0.7
0.8
Phylostratum
Cellular_organisms - ps1
Archaea1 - ps2
Archaea2 - ps3
Archaea3 - ps4
Asgard/Eukaryota - ps5
Eukaryota - ps6
Amorpheae - ps7
Obazoa - ps8
Opisthokonta - ps9
Holozoa - ps10
Filozoa - ps11
Choano./Metazoa - ps12
Metazoa - ps13
Myriazoa - ps14
Parahoxozoa - ps15
Bilateria - ps16
Deuterostomia - ps17
Chordata - ps18
Olfactores - ps19
Vertebrata - ps20
Gnathostomata - ps21
Euteleostomi - ps22
Sarcopterygii - ps23
Tetrapoda - ps24
Amniota - ps25
Mammalia - ps26
Theria - ps27
Placentalia - ps28
Boreoeutheria - ps29
Euarchontoglires - ps30
Euarchonta - ps31
Primates - ps32
Haplorrhini - ps33
Simiiformes - ps34
Catarrhini - ps35
Hominoidea - ps36
Hominidae - ps37
Homininae - ps38
Hominini - ps39
Homo_sapiens - ps40
p-value
2e-52
5e-40
8e-28
2e-15
3e-03
Log-odds
0.5
1.0
1.5
2.0
2.5
3.0
3.5
4.0

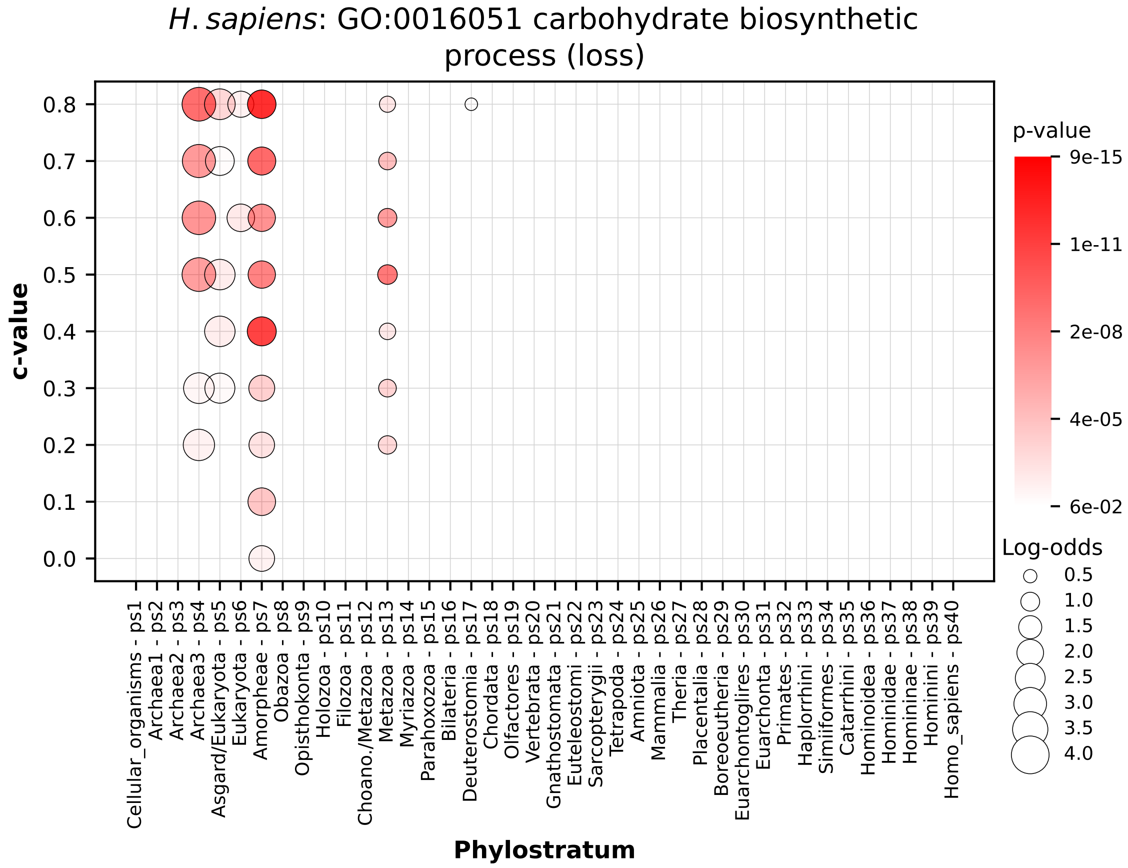

H. sapiens: GO:0016051 carbohydrate biosynthetic process (loss)
c-value
0.8
0.7
0.6
0.5
0.4
0.3
0.2
0.1
0.0
Phylostratum
Cellular_organisms - ps1
Archaea1 - ps2
Archaea2 - ps3
Archaea3 - ps4
Asgard/Eukaryota - ps5
Eukaryota - ps6
Amorpheae - ps7
Obazoa - ps8
Opisthokonta - ps9
Holozoa - ps10
Filozoa - ps11
Choano./Metazoa - ps12
Metazoa - ps13
Myriazoa - ps14
Parahoxozoa - ps15
Bilateria - ps16
Deuterostomia - ps17
Chordata - ps18
Olfactores - ps19
Vertebrata - ps20
Gnathostomata - ps21
Euteleostomi - ps22
Sarcopterygii - ps23
Tetrapoda - ps24
Amniota - ps25
Mammalia - ps26
Theria - ps27
Placentalia - ps28
Boreoeutheria - ps29
Euarchontoglires - ps30
Euarchonta - ps31
Primates - ps32
Haplorrhini - ps33
Simiiformes - ps34
Catarrhini - ps35
Hominoidea - ps36
Hominidae - ps37
Homininae - ps38
Hominini - ps39
Homo_sapiens - ps40
p-value
9e-15
1e-11
2e-08
4e-05
6e-02
Log-odds
0.5
1.0
1.5
2.0
2.5
3.0
3.5
4.0

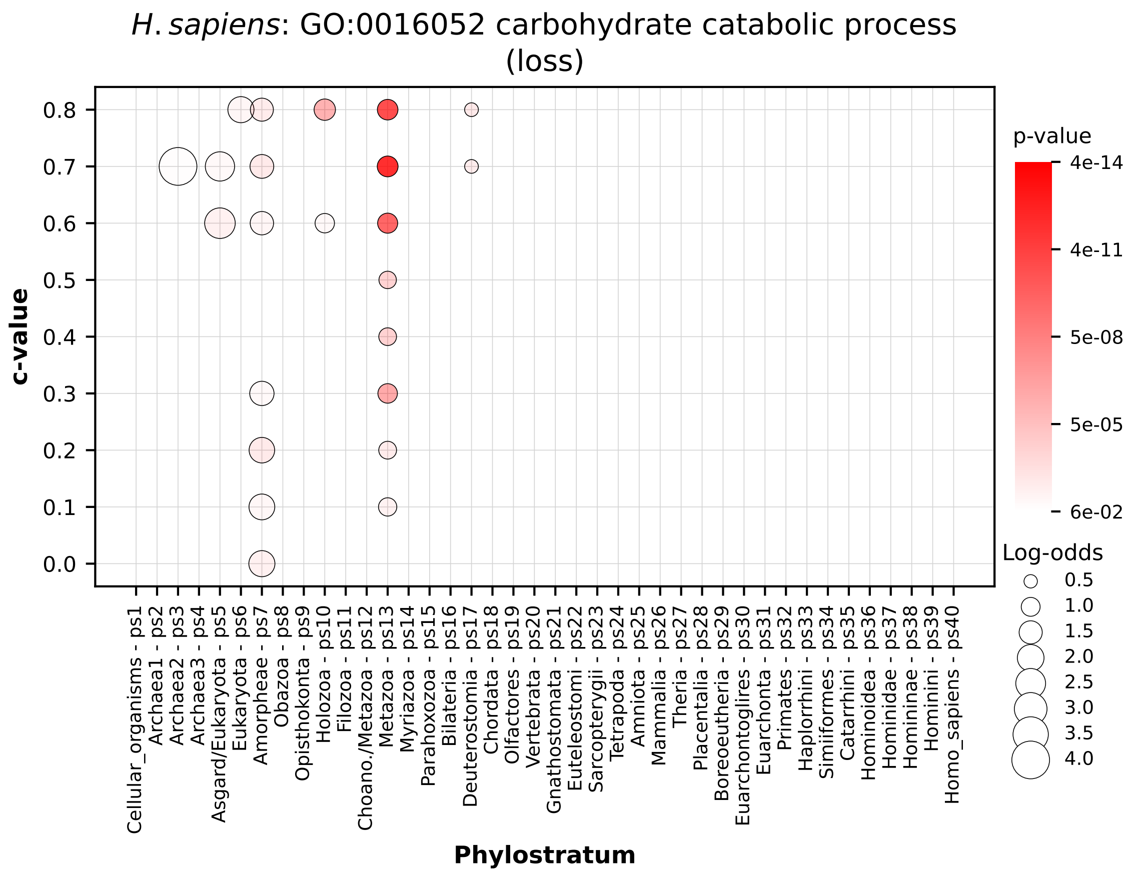
H. sapiens: GO:0016052 carbohydrate catabolic process
(loss)
c-value
0.8
0.7
0.6
0.5
0.4
0.3
0.2
0.1
0.0
p-value
4e-14
4e-11
5e-08
5e-05
6e-02
Log-odds
0.5
1.0
1.5
2.0
2.5
3.0
3.5
4.0
Cellular_organisms - ps1
Archaea1 - ps2
Archaea2 - ps3
Archaea3 - ps4
Asgard/Eukaryota - ps5
Eukaryota - ps6
Amorpheae - ps7
Obazoa - ps8
Opisthokonta - ps9
Holozoa - ps10
Filozoa - ps11
Choano./Metazoa - ps12
Metazoa - ps13
Myriazoa - ps14
Parahoxozoa - ps15
Bilateria - ps16
Deuterostomia - ps17
Chordata - ps18
Olfactores - ps19
Vertebrata - ps20
Gnathostomata - ps21
Euteleostomi - ps22
Sarcopterygii - ps23
Tetrapoda - ps24
Amniota - ps25
Mammalia - ps26
Theria - ps27
Placentalia - ps28
Boreoeutheria - ps29
Euarchontoglires - ps30
Euarchonta - ps31
Primates - ps32
Haplorrhini - ps33
Simiiformes - ps34
Catarrhini - ps35
Hominoidea - ps36
Hominidae - ps37
Homininae - ps38
Hominini - ps39
Homo_sapiens - ps40
Phylostratum

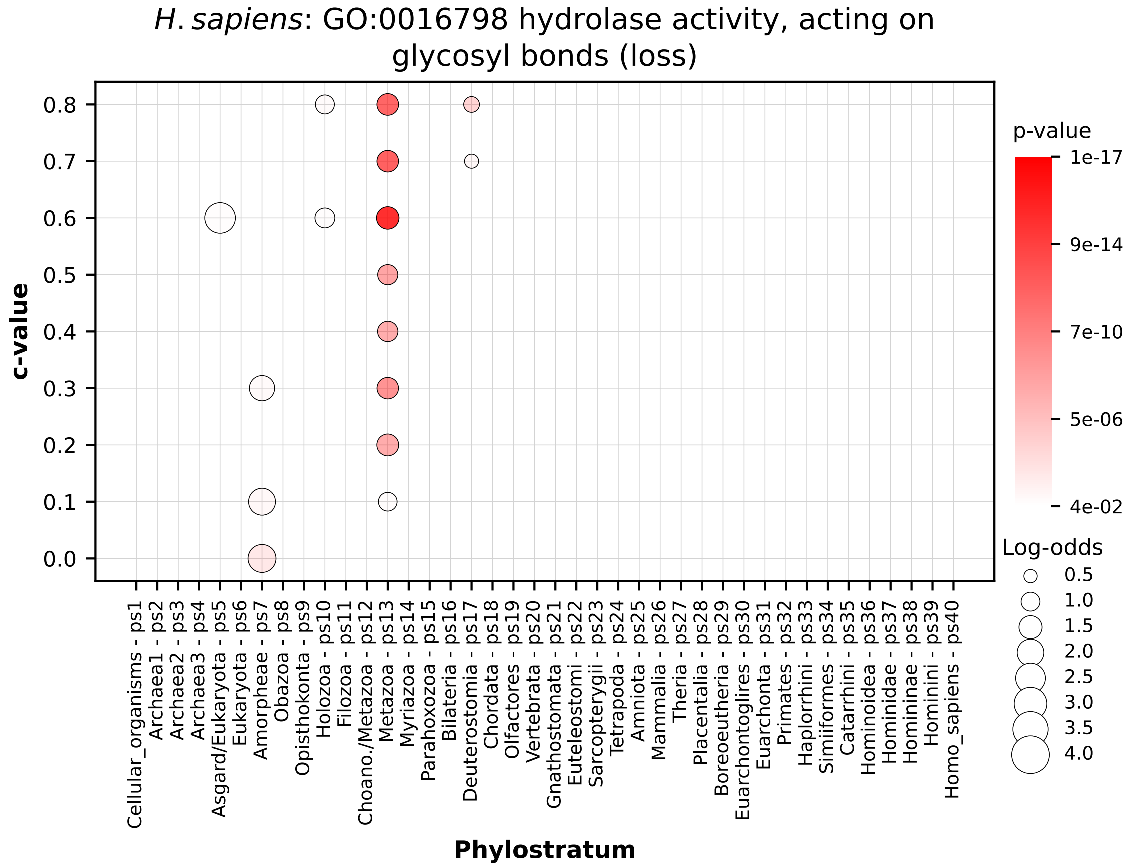

H. sapiens: GO:0016798 hydrolase activity, acting on glycosyl bonds (loss)
c-value
0.0
0.1
0.2
0.3
0.4
0.5
0.6
0.7
0.8
p-value
1e-17
9e-14
7e-10
5e-06
4e-02
Log-odds
0.5
1.0
1.5
2.0
2.5
3.0
3.5
4.0
Cellular_organisms - ps1
Archaea1 - ps2
Archaea2 - ps3
Archaea3 - ps4
Asgard/Eukaryota - ps5
Eukaryota - ps6
Amorpheae - ps7
Obazoa - ps8
Opisthokonta - ps9
Holozoa - ps10
Filozoa - ps11
Choano./Metazoa - ps12
Metazoa - ps13
Myriazoa - ps14
Parahoxozoa - ps15
Bilateria - ps16
Deuterostomia - ps17
Chordata - ps18
Olfactores - ps19
Vertebrata - ps20
Gnathostomata - ps21
Euteleostomi - ps22
Sarcopterygii - ps23
Tetrapoda - ps24
Amniota - ps25
Mammalia - ps26
Theria - ps27
Placentalia - ps28
Boreoeutheria - ps29
Euarchontoglires - ps30
Euarchonta - ps31
Primates - ps32
Haplorrhini - ps33
Simiiformes - ps34
Catarrhini - ps35
Hominoidea - ps36
Hominidae - ps37
Homininae - ps38
Hominini - ps39
Homo_sapiens - ps40
Phylostratum

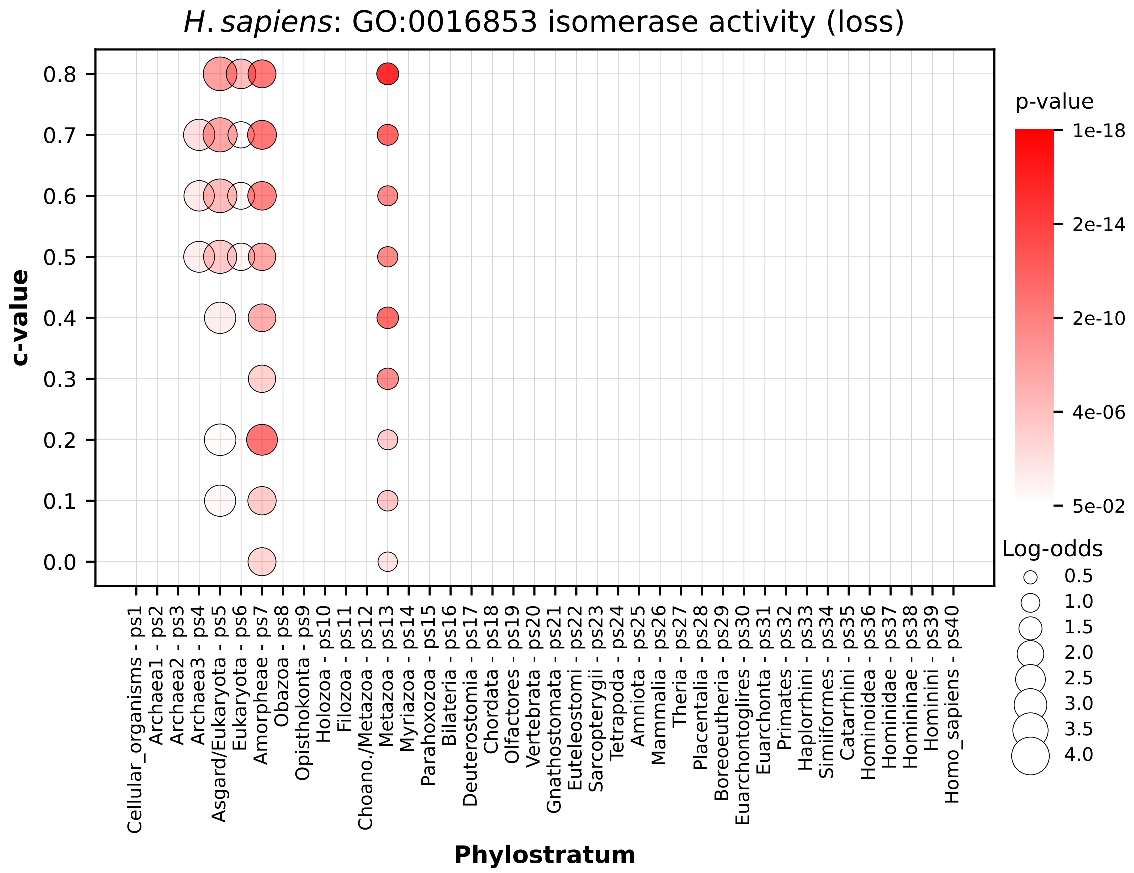
H. sapiens: GO:0016853 isomerase activity (loss)
c-value
0.8
0.7
0.6
0.5
0.4
0.3
0.2
0.1
0.0
Phylostratum
Cellular_organisms - ps1
Archaea1 - ps2
Archaea2 - ps3
Archaea3 - ps4
Asgard/Eukaryota - ps5
Eukaryota - ps6
Amorpheae - ps7
Obazoa - ps8
Opisthokonta - ps9
Holozoa - ps10
Filozoa - ps11
Choano./Metazoa - ps12
Metazoa - ps13
Myriazoa - ps14
Parahoxozoa - ps15
Bilateria - ps16
Deuterostomia - ps17
Chordata - ps18
Olfactores - ps19
Vertebrata - ps20
Gnathostomata - ps21
Euteleostomi - ps22
Sarcopterygii - ps23
Tetrapoda - ps24
Amniota - ps25
Mammalia - ps26
Theria - ps27
Placentalia - ps28
Boreoeutheria - ps29
Euarchontoglires - ps30
Euarchonta - ps31
Primates - ps32
Haplorrhini - ps33
Simiiformes - ps34
Catarrhini - ps35
Hominoidea - ps36
Hominidae - ps37
Homininae - ps38
Hominini - ps39
Homo_sapiens - ps40
p-value
1e-18
2e-14
2e-10
4e-06
5e-02
Log-odds
0.5
1.0
1.5
2.0
2.5
3.0
3.5
4.0

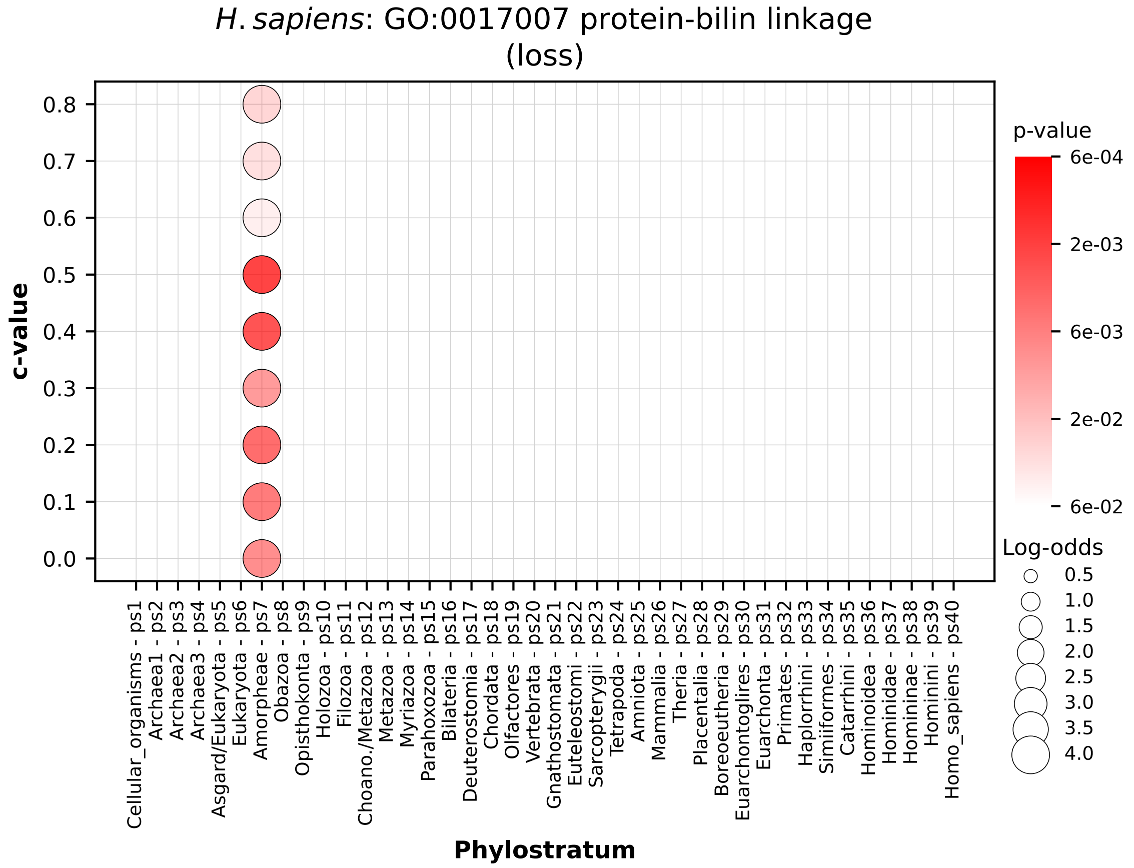

H. sapiens: GO:0017007 protein-bilin linkage
(loss)
c-value
0.0
0.1
0.2
0.3
0.4
0.5
0.6
0.7
0.8
p-value
6e-04
2e-03
6e-03
2e-02
6e-02
Log-odds
0.5
1.0
1.5
2.0
2.5
3.0
3.5
4.0
Cellular_organisms - ps1
Archaea1 - ps2
Archaea2 - ps3
Archaea3 - ps4
Asgard/Eukaryota - ps5
Eukaryota - ps6
Amorpheae - ps7
Obazoa - ps8
Opisthokonta - ps9
Holozoa - ps10
Filozoa - ps11
Choano./Metazoa - ps12
Metazoa - ps13
Myriazoa - ps14
Parahoxozoa - ps15
Bilateria - ps16
Deuterostomia - ps17
Chordata - ps18
Olfactores - ps19
Vertebrata - ps20
Gnathostomata - ps21
Euteleostomi - ps22
Sarcopterygii - ps23
Tetrapoda - ps24
Amniota - ps25
Mammalia - ps26
Theria - ps27
Placentalia - ps28
Boreoeutheria - ps29
Euarchontoglires - ps30
Euarchonta - ps31
Primates - ps32
Haplorrhini - ps33
Simiiformes - ps34
Catarrhini - ps35
Hominoidea - ps36
Hominidae - ps37
Homininae - ps38
Hominini - ps39
Homo_sapiens - ps40
Phylostratum

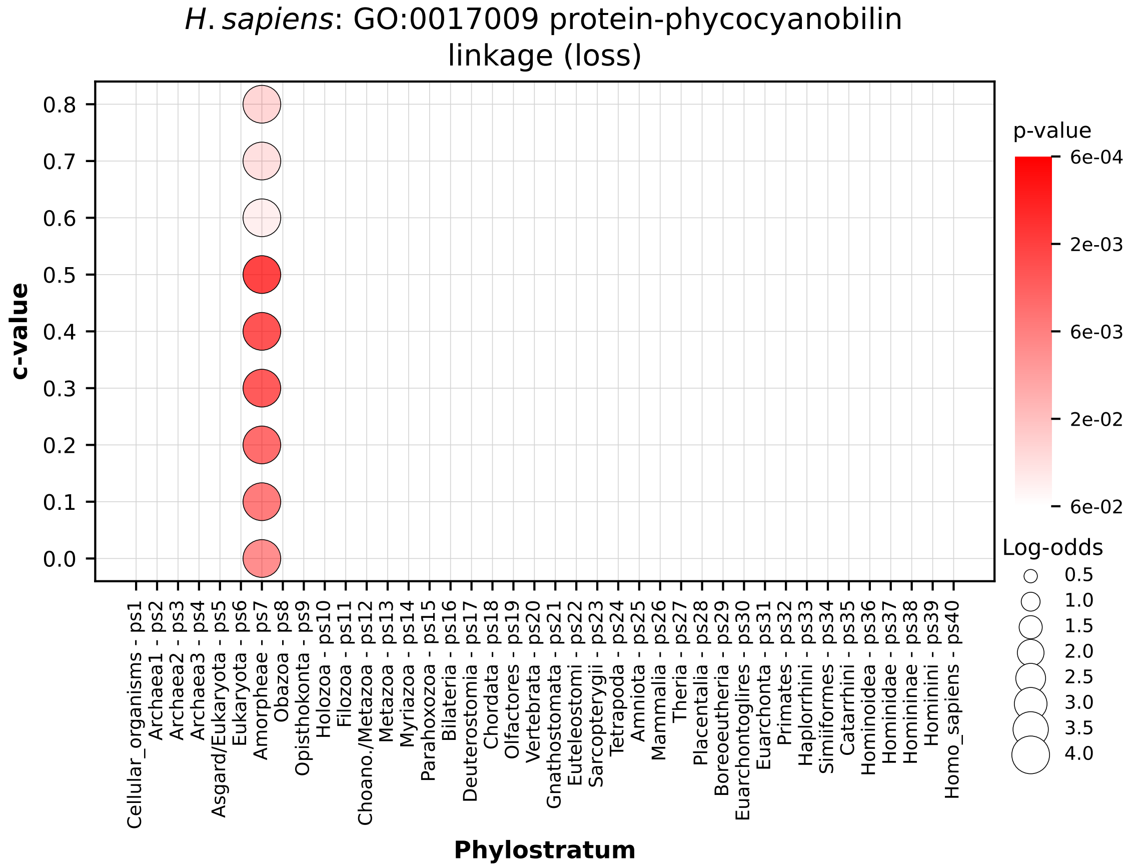

H. sapiens: GO:0017009 protein-phycocyanobilin linkage (loss)
c-value
0.8
0.7
0.6
0.5
0.4
0.3
0.2
0.1
0.0
p-value
6e-04
2e-03
6e-03
2e-02
6e-02
Log-odds
0.5
1.0
1.5
2.0
2.5
3.0
3.5
4.0
Cellular_organisms - ps1
Archaea1 - ps2
Archaea2 - ps3
Archaea3 - ps4
Asgard/Eukaryota - ps5
Eukaryota - ps6
Amorpheae - ps7
Obazoa - ps8
Opisthokonta - ps9
Holozoa - ps10
Filozoa - ps11
Choano./Metazoa - ps12
Metazoa - ps13
Myriazoa - ps14
Parahoxozoa - ps15
Bilateria - ps16
Deuterostomia - ps17
Chordata - ps18
Olfactores - ps19
Vertebrata - ps20
Gnathostomata - ps21
Euteleostomi - ps22
Sarcopterygii - ps23
Tetrapoda - ps24
Amniota - ps25
Mammalia - ps26
Theria - ps27
Placentalia - ps28
Boreoeutheria - ps29
Euarchontoglires - ps30
Euarchonta - ps31
Primates - ps32
Haplorrhini - ps33
Simiiformes - ps34
Catarrhini - ps35
Hominoidea - ps36
Hominidae - ps37
Homininae - ps38
Hominini - ps39
Homo_sapiens - ps40
Phylostratum

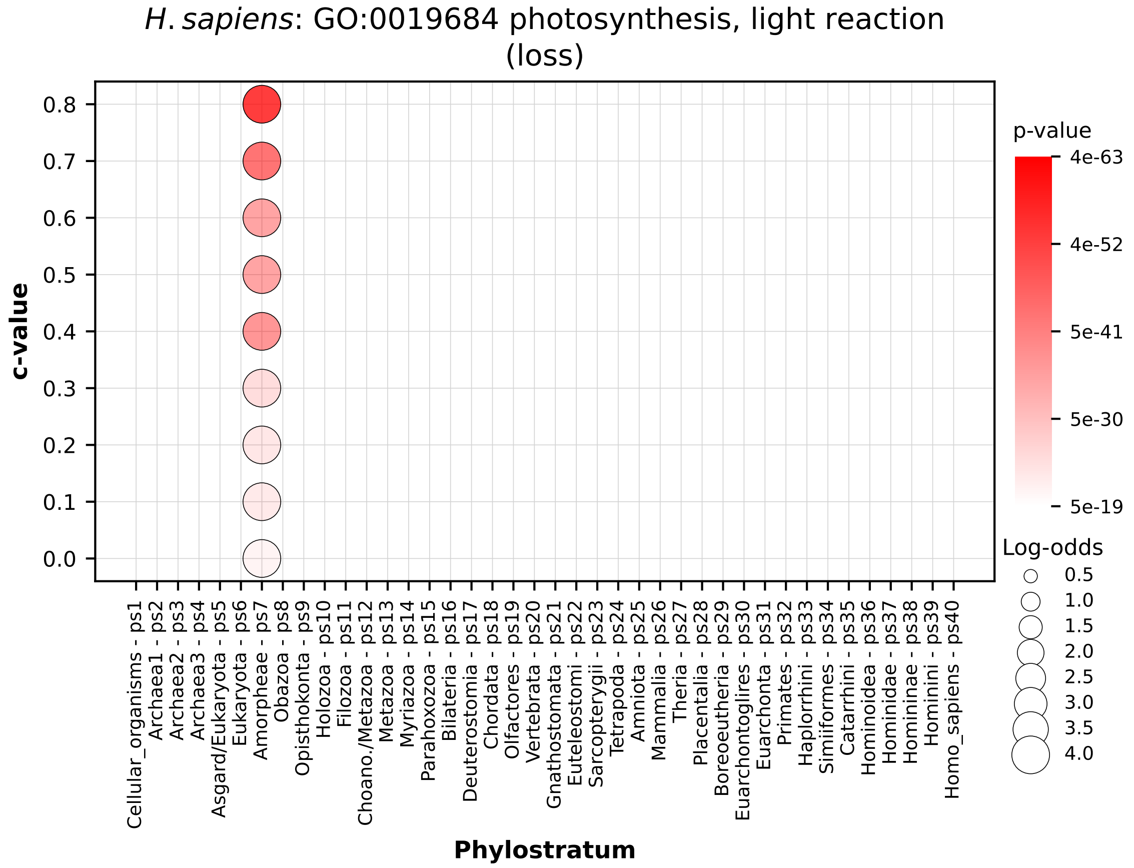

H. sapiens: GO:0019684 photosynthesis, light reaction (loss)
c-value
0.8
0.7
0.6
0.5
0.4
0.3
0.2
0.1
0.0
p-value
4e-63
4e-52
5e-41
5e-30
5e-19
Log-odds
0.5
1.0
1.5
2.0
2.5
3.0
3.5
4.0
Cellular_organisms - ps1
Archaea1 - ps2
Archaea2 - ps3
Archaea3 - ps4
Asgard/Eukaryota - ps5
Eukaryota - ps6
Amorpheae - ps7
Obazoa - ps8
Opisthokonta - ps9
Holozoa - ps10
Filozoa - ps11
Choano./Metazoa - ps12
Metazoa - ps13
Myriazoa - ps14
Parahoxozoa - ps15
Bilateria - ps16
Deuterostomia - ps17
Chordata - ps18
Olfactores - ps19
Vertebrata - ps20
Gnathostomata - ps21
Euteleostomi - ps22
Sarcopterygii - ps23
Tetrapoda - ps24
Amniota - ps25
Mammalia - ps26
Theria - ps27
Placentalia - ps28
Boreoeutheria - ps29
Euarchontoglires - ps30
Euarchonta - ps31
Primates - ps32
Haplorrhini - ps33
Simiiformes - ps34
Catarrhini - ps35
Hominoidea - ps36
Hominidae - ps37
Homininae - ps38
Hominini - ps39
Homo_sapiens - ps40
Phylostratum

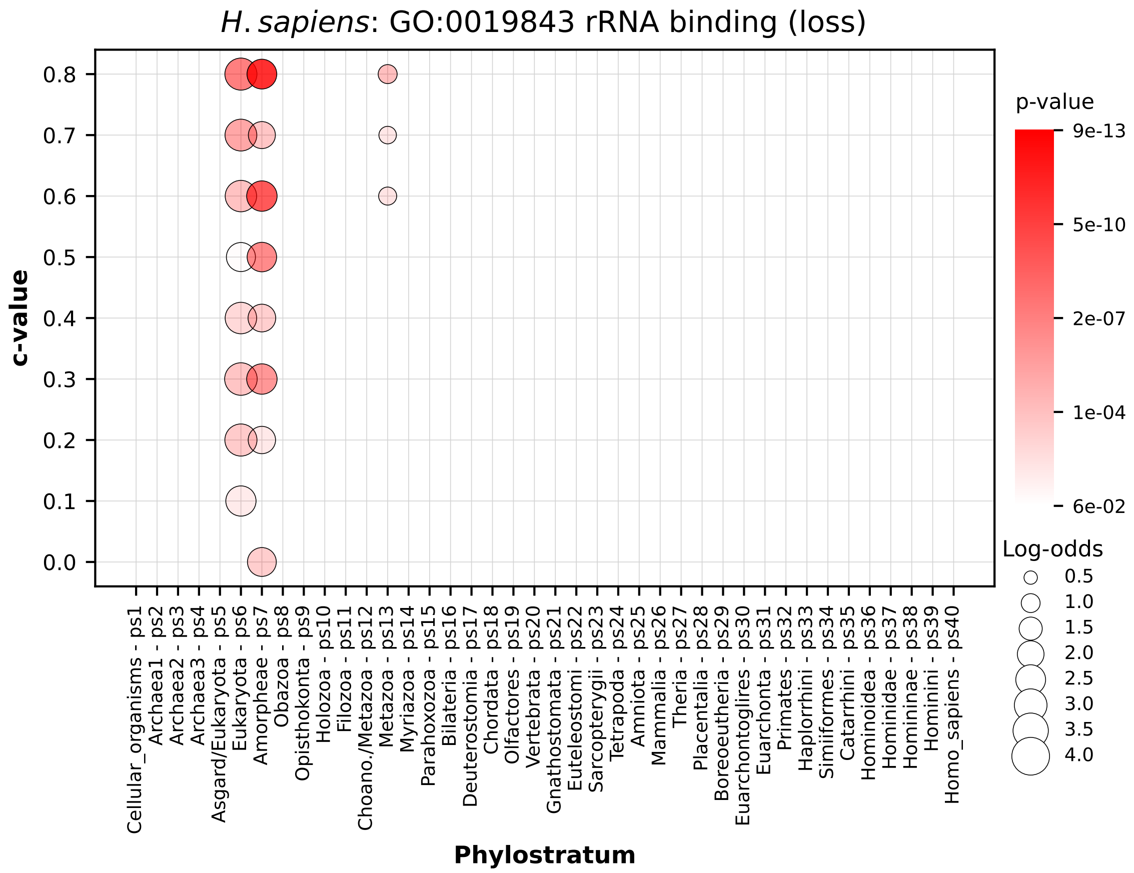

H. sapiens: GO:0019843 rRNA binding (loss)
c-value
0.0
0.1
0.2
0.3
0.4
0.5
0.6
0.7
0.8
Phylostratum
Cellular_organisms - ps1
Archaea1 - ps2
Archaea2 - ps3
Archaea3 - ps4
Asgard/Eukaryota - ps5
Eukaryota - ps6
Amorpheae - ps7
Obazoa - ps8
Opisthokonta - ps9
Holozoa - ps10
Filozoa - ps11
Choano./Metazoa - ps12
Metazoa - ps13
Myriazoa - ps14
Parahoxozoa - ps15
Bilateria - ps16
Deuterostomia - ps17
Chordata - ps18
Olfactores - ps19
Vertebrata - ps20
Gnathostomata - ps21
Euteleostomi - ps22
Sarcopterygii - ps23
Tetrapoda - ps24
Amniota - ps25
Mammalia - ps26
Theria - ps27
Placentalia - ps28
Boreoeutheria - ps29
Euarchontoglires - ps30
Euarchonta - ps31
Primates - ps32
Haplorrhini - ps33
Simiiformes - ps34
Catarrhini - ps35
Hominoidea - ps36
Hominidae - ps37
Homininae - ps38
Hominini - ps39
Homo_sapiens - ps40
p-value
9e-13
5e-10
2e-07
1e-04
6e-02
Log-odds
0.5
1.0
1.5
2.0
2.5
3.0
3.5
4.0

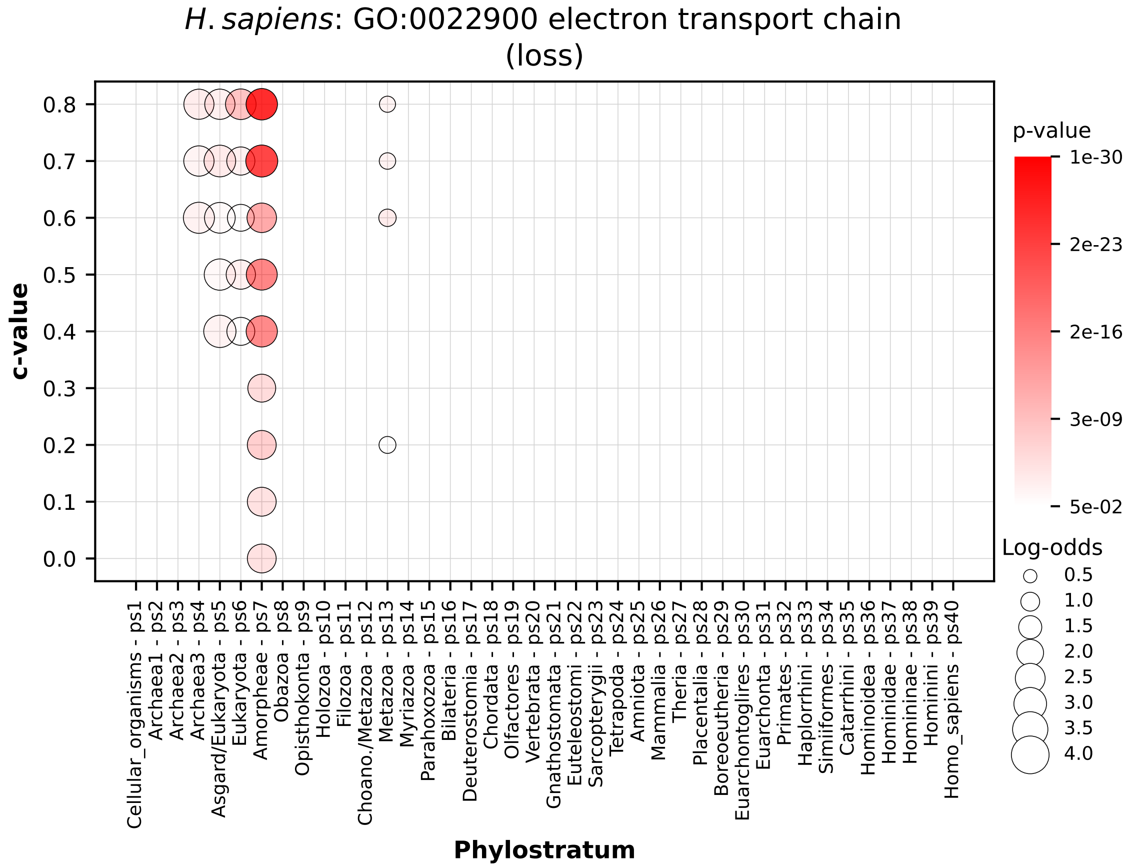

H. sapiens: GO:0022900 electron transport chain
(loss)
c-value
0.8
0.7
0.6
0.5
0.4
0.3
0.2
0.1
0.0
p-value
1e-30
2e-23
2e-16
3e-09
5e-02
Log-odds
0.5
1.0
1.5
2.0
2.5
3.0
3.5
4.0
Cellular_organisms - ps1
Archaea1 - ps2
Archaea2 - ps3
Archaea3 - ps4
Asgard/Eukaryota - ps5
Eukaryota - ps6
Amorpheae - ps7
Obazoa - ps8
Opisthokonta - ps9
Holozoa - ps10
Filozoa - ps11
Choano./Metazoa - ps12
Metazoa - ps13
Myriazoa - ps14
Parahoxozoa - ps15
Bilateria - ps16
Deuterostomia - ps17
Chordata - ps18
Olfactores - ps19
Vertebrata - ps20
Gnathostomata - ps21
Euteleostomi - ps22
Sarcopterygii - ps23
Tetrapoda - ps24
Amniota - ps25
Mammalia - ps26
Theria - ps27
Placentalia - ps28
Boreoeutheria - ps29
Euarchontoglires - ps30
Euarchonta - ps31
Primates - ps32
Haplorrhini - ps33
Simiiformes - ps34
Catarrhini - ps35
Hominoidea - ps36
Hominidae - ps37
Homininae - ps38
Hominini - ps39
Homo_sapiens - ps40
Phylostratum

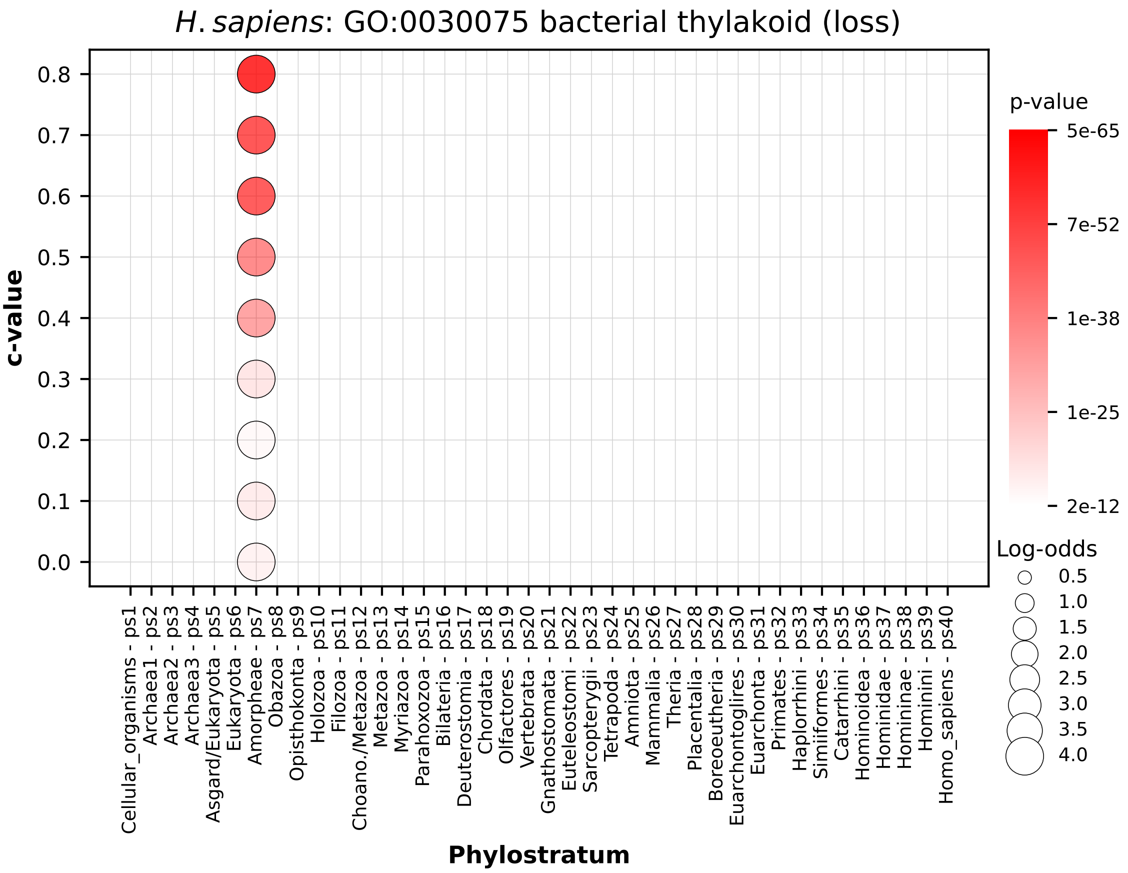

H. sapiens: GO:0030075 bacterial thylakoid (loss)
c-value
0.8
0.7
0.6
0.5
0.4
0.3
0.2
0.1
0.0
p-value
5e-65
7e-52
1e-38
1e-25
2e-12
Log-odds
0.5
1.0
1.5
2.0
2.5
3.0
3.5
4.0
Cellular_organisms - ps1
Archaea1 - ps2
Archaea2 - ps3
Archaea3 - ps4
Asgard/Eukaryota - ps5
Eukaryota - ps6
Amorpheae - ps7
Obazoa - ps8
Opisthokonta - ps9
Holozoa - ps10
Filozoa - ps11
Choano./Metazoa - ps12
Metazoa - ps13
Myriazoa - ps14
Parahoxozoa - ps15
Bilateria - ps16
Deuterostomia - ps17
Chordata - ps18
Olfactores - ps19
Vertebrata - ps20
Gnathostomata - ps21
Euteleostomi - ps22
Sarcopterygii - ps23
Tetrapoda - ps24
Amniota - ps25
Mammalia - ps26
Theria - ps27
Placentalia - ps28
Boreoeutheria - ps29
Euarchontoglires - ps30
Euarchonta - ps31
Primates - ps32
Haplorrhini - ps33
Simiiformes - ps34
Catarrhini - ps35
Hominoidea - ps36
Hominidae - ps37
Homininae - ps38
Hominini - ps39
Homo_sapiens - ps40
Phylostratum

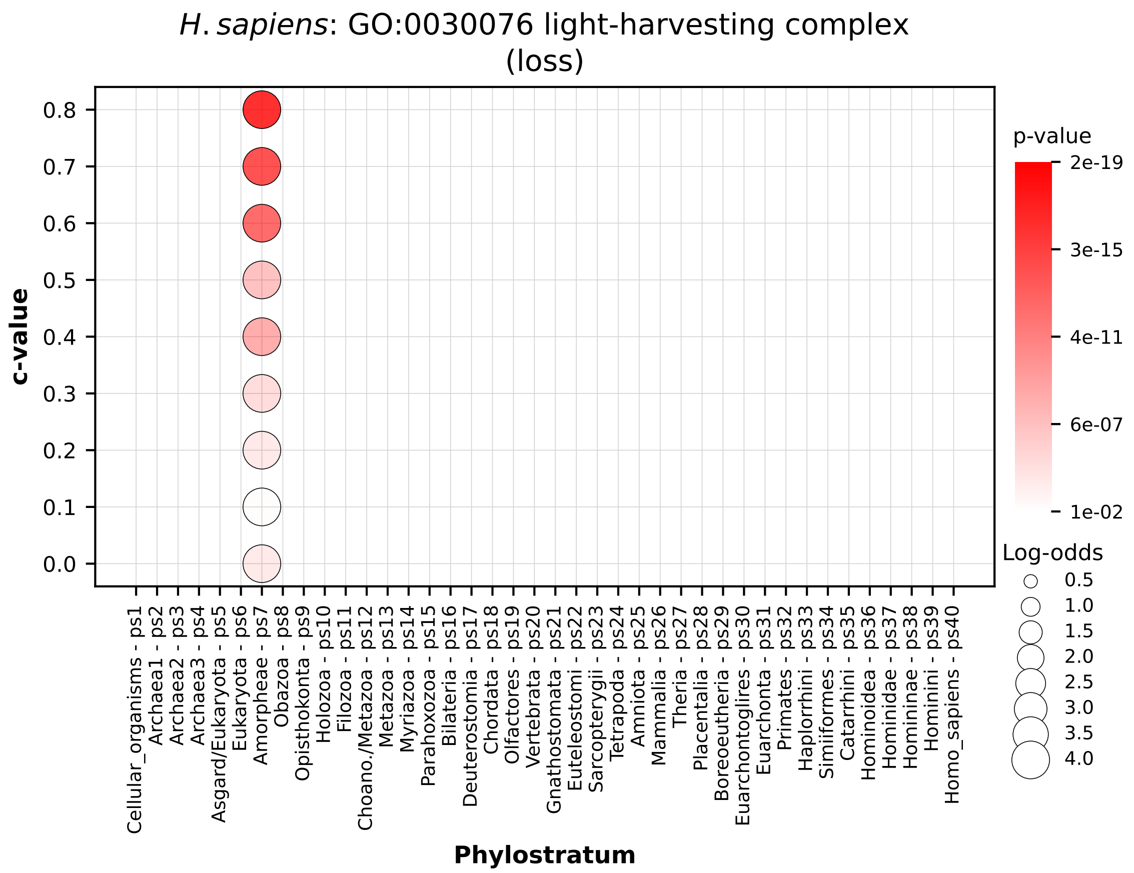

H. sapiens: GO:0030076 light-harvesting complex
(loss)
c-value
0.0
0.1
0.2
0.3
0.4
0.5
0.6
0.7
0.8
Phylostratum
Cellular_organisms - ps1
Archaea1 - ps2
Archaea2 - ps3
Archaea3 - ps4
Asgard/Eukaryota - ps5
Eukaryota - ps6
Amorpheae - ps7
Obazoa - ps8
Opisthokonta - ps9
Holozoa - ps10
Filozoa - ps11
Choano./Metazoa - ps12
Metazoa - ps13
Myriazoa - ps14
Parahoxozoa - ps15
Bilateria - ps16
Deuterostomia - ps17
Chordata - ps18
Olfactores - ps19
Vertebrata - ps20
Gnathostomata - ps21
Euteleostomi - ps22
Sarcopterygii - ps23
Tetrapoda - ps24
Amniota - ps25
Mammalia - ps26
Theria - ps27
Placentalia - ps28
Boreoeutheria - ps29
Euarchontoglires - ps30
Euarchonta - ps31
Primates - ps32
Haplorrhini - ps33
Simiiformes - ps34
Catarrhini - ps35
Hominoidea - ps36
Hominidae - ps37
Homininae - ps38
Hominini - ps39
Homo_sapiens - ps40
p-value
2e-19
3e-15
4e-11
6e-07
1e-02
Log-odds
0.5
1.0
1.5
2.0
2.5
3.0
3.5
4.0

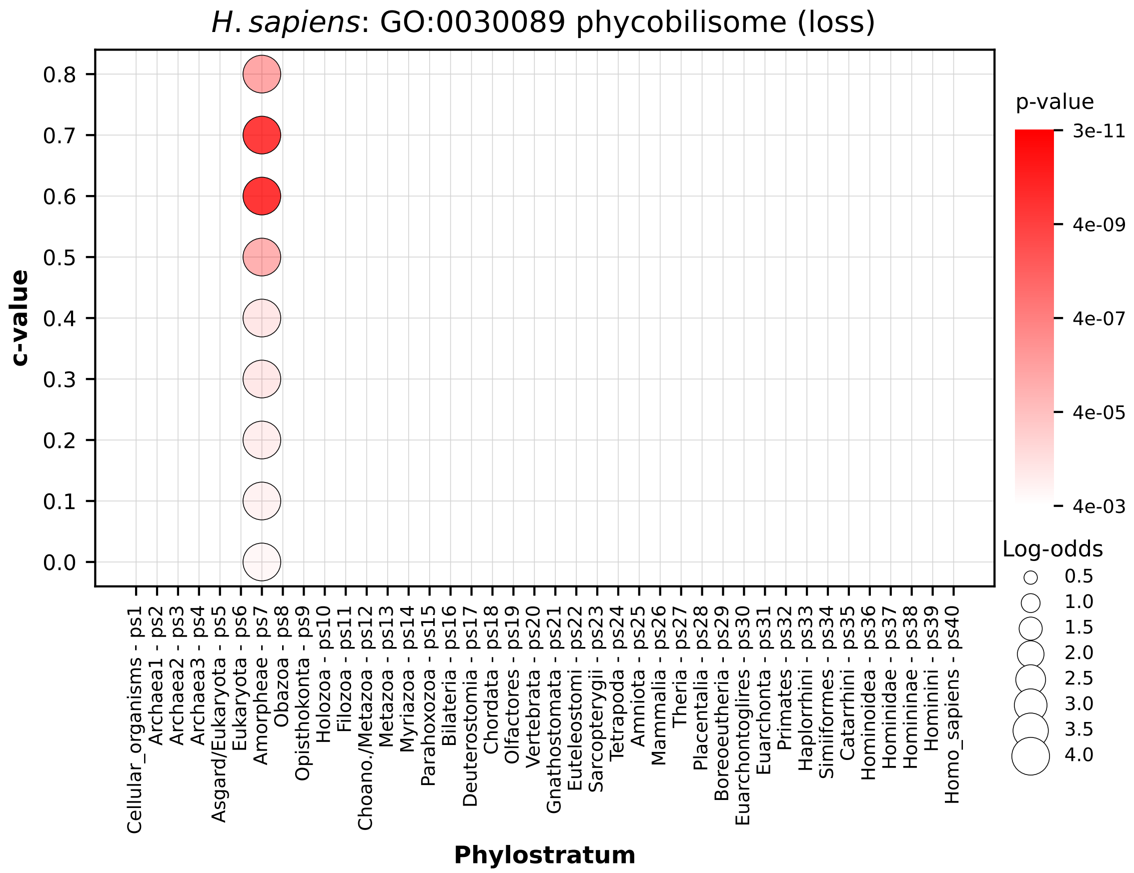

H. sapiens: GO:0030089 phycobilisome (loss)
c-value
0.0
0.1
0.2
0.3
0.4
0.5
0.6
0.7
0.8
Phylostratum
Cellular_organisms - ps1
Archaea1 - ps2
Archaea2 - ps3
Archaea3 - ps4
Asgard/Eukaryota - ps5
Eukaryota - ps6
Amorpheae - ps7
Obazoa - ps8
Opisthokonta - ps9
Holozoa - ps10
Filozoa - ps11
Choano./Metazoa - ps12
Metazoa - ps13
Myriazoa - ps14
Parahoxozoa - ps15
Bilateria - ps16
Deuterostomia - ps17
Chordata - ps18
Olfactores - ps19
Vertebrata - ps20
Gnathostomata - ps21
Euteleostomi - ps22
Sarcopterygii - ps23
Tetrapoda - ps24
Amniota - ps25
Mammalia - ps26
Theria - ps27
Placentalia - ps28
Boreoeutheria - ps29
Euarchontoglires - ps30
Euarchonta - ps31
Primates - ps32
Haplorrhini - ps33
Simiiformes - ps34
Catarrhini - ps35
Hominoidea - ps36
Hominidae - ps37
Homininae - ps38
Hominini - ps39
Homo_sapiens - ps40
p-value
3e-11
4e-09
4e-07
4e-05
4e-03
Log-odds
0.5
1.0
1.5
2.0
2.5
3.0
3.5
4.0

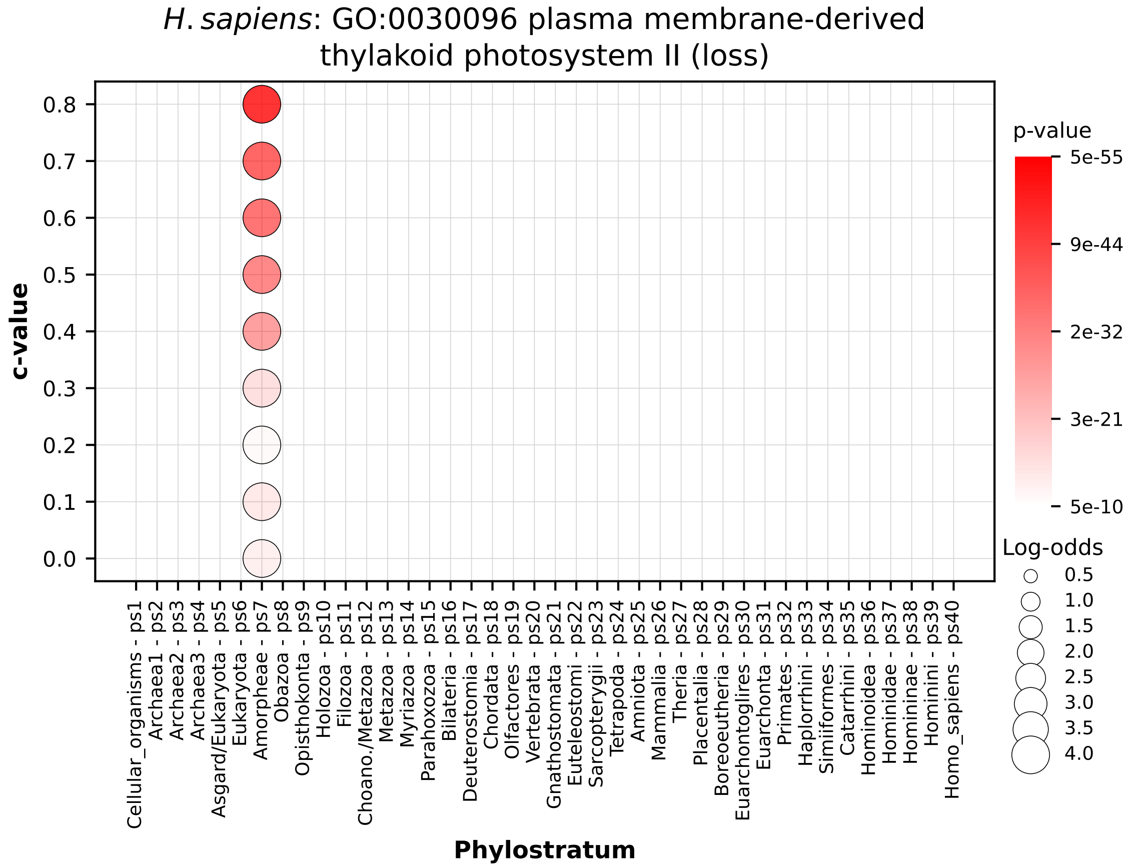

H. sapiens: GO:0030096 plasma membrane-derived thylakoid photosystem II (loss)
c-value
0.8
0.7
0.6
0.5
0.4
0.3
0.2
0.1
0.0
p-value
5e-55
9e-44
2e-32
3e-21
5e-10
Log-odds
0.5
1.0
1.5
2.0
2.5
3.0
3.5
4.0
Cellular_organisms - ps1
Archaea1 - ps2
Archaea2 - ps3
Archaea3 - ps4
Asgard/Eukaryota - ps5
Eukaryota - ps6
Amorpheae - ps7
Obazoa - ps8
Opisthokonta - ps9
Holozoa - ps10
Filozoa - ps11
Choano./Metazoa - ps12
Metazoa - ps13
Myriazoa - ps14
Parahoxozoa - ps15
Bilateria - ps16
Deuterostomia - ps17
Chordata - ps18
Olfactores - ps19
Vertebrata - ps20
Gnathostomata - ps21
Euteleostomi - ps22
Sarcopterygii - ps23
Tetrapoda - ps24
Amniota - ps25
Mammalia - ps26
Theria - ps27
Placentalia - ps28
Boreoeutheria - ps29
Euarchontoglires - ps30
Euarchonta - ps31
Primates - ps32
Haplorrhini - ps33
Simiiformes - ps34
Catarrhini - ps35
Hominoidea - ps36
Hominidae - ps37
Homininae - ps38
Hominini - ps39
Homo_sapiens - ps40
Phylostratum

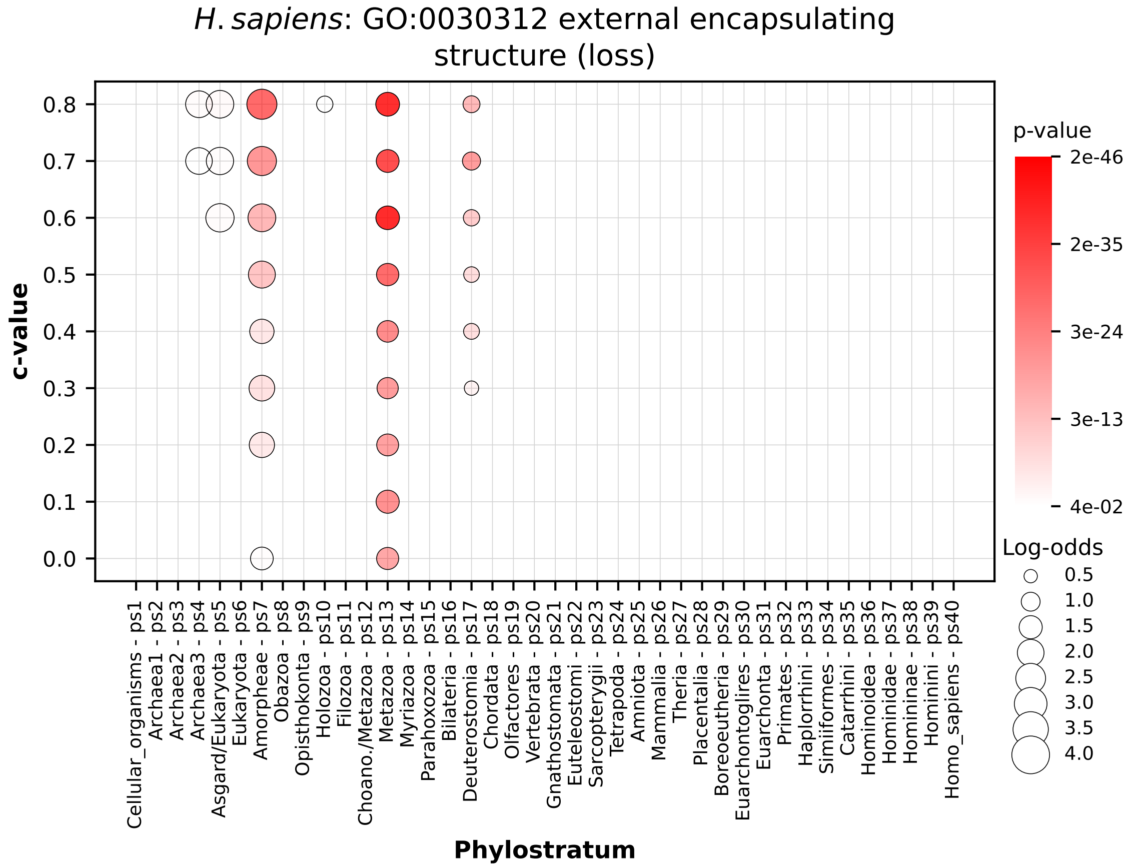

H. sapiens: GO:0030312 external encapsulating structure (loss)
c-value
0.8
0.7
0.6
0.5
0.4
0.3
0.2
0.1
0.0
Cellular_organisms - ps1
Archaea1 - ps2
Archaea2 - ps3
Archaea3 - ps4
Asgard/Eukaryota - ps5
Eukaryota - ps6
Amorpheae - ps7
Obazoa - ps8
Opisthokonta - ps9
Holozoa - ps10
Filozoa - ps11
Choano./Metazoa - ps12
Metazoa - ps13
Myriazoa - ps14
Parahoxozoa - ps15
Bilateria - ps16
Deuterostomia - ps17
Chordata - ps18
Olfactores - ps19
Vertebrata - ps20
Gnathostomata - ps21
Euteleostomi - ps22
Sarcopterygii - ps23
Tetrapoda - ps24
Amniota - ps25
Mammalia - ps26
Theria - ps27
Placentalia - ps28
Boreoeutheria - ps29
Euarchontoglires - ps30
Euarchonta - ps31
Primates - ps32
Haplorrhini - ps33
Simiiformes - ps34
Catarrhini - ps35
Hominoidea - ps36
Hominidae - ps37
Homininae - ps38
Hominini - ps39
Homo_sapiens - ps40
Phylostratum
p-value
2e-46
2e-35
3e-24
3e-13
4e-02
Log-odds
0.5
1.0
1.5
2.0
2.5
3.0
3.5
4.0

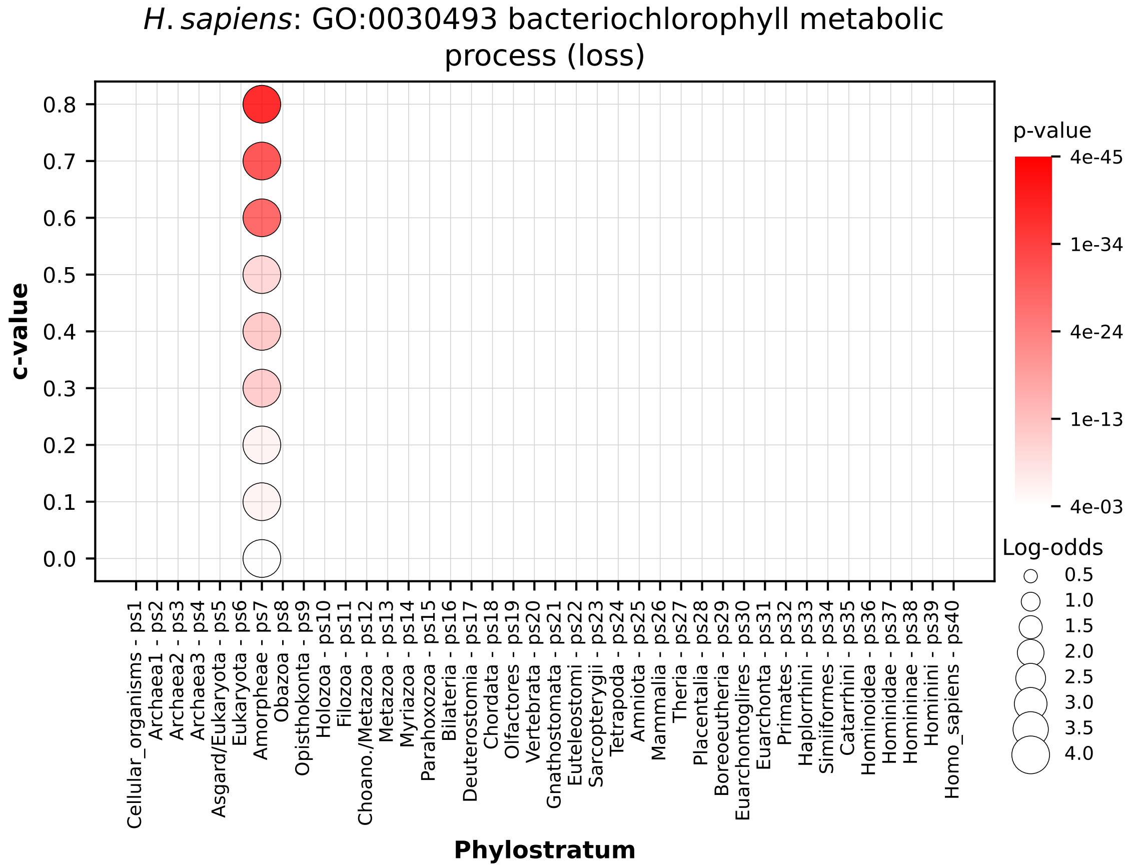
H. sapiens: GO:0030493 bacteriochlorophyll metabolic process (loss)
c-value
0.8
0.7
0.6
0.5
0.4
0.3
0.2
0.1
0.0
Phylostratum
Cellular_organisms - ps1
Archaea1 - ps2
Archaea2 - ps3
Archaea3 - ps4
Asgard/Eukaryota - ps5
Eukaryota - ps6
Amorpheae - ps7
Obazoa - ps8
Opisthokonta - ps9
Holozoa - ps10
Filozoa - ps11
Choano./Metazoa - ps12
Metazoa - ps13
Myriazoa - ps14
Parahoxozoa - ps15
Bilateria - ps16
Deuterostomia - ps17
Chordata - ps18
Olfactores - ps19
Vertebrata - ps20
Gnathostomata - ps21
Euteleostomi - ps22
Sarcopterygii - ps23
Tetrapoda - ps24
Amniota - ps25
Mammalia - ps26
Theria - ps27
Placentalia - ps28
Boreoeutheria - ps29
Euarchontoglires - ps30
Euarchonta - ps31
Primates - ps32
Haplorrhini - ps33
Simiiformes - ps34
Catarrhini - ps35
Hominoidea - ps36
Hominidae - ps37
Homininae - ps38
Hominini - ps39
Homo_sapiens - ps40
p-value
4e-45
1e-34
4e-24
1e-13
4e-03
Log-odds
0.5
1.0
1.5
2.0
2.5
3.0
3.5
4.0

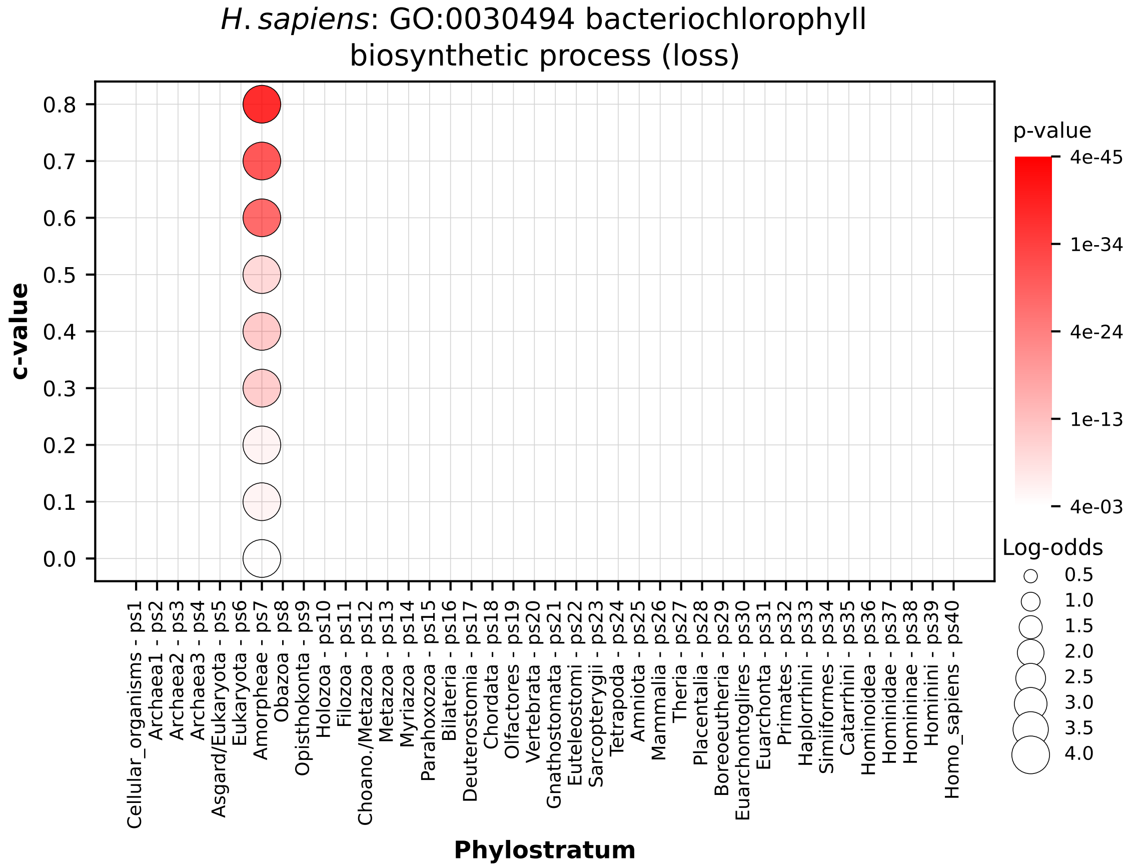

H. sapiens: GO:0030494 bacteriochlorophyll biosynthetic process (loss)
c-value
0.8
0.7
0.6
0.5
0.4
0.3
0.2
0.1
0.0
p-value
4e-45
1e-34
4e-24
1e-13
4e-03
Log-odds
0.5
1.0
1.5
2.0
2.5
3.0
3.5
4.0
Cellular_organisms - ps1
Archaea1 - ps2
Archaea2 - ps3
Archaea3 - ps4
Asgard/Eukaryota - ps5
Eukaryota - ps6
Amorpheae - ps7
Obazoa - ps8
Opisthokonta - ps9
Holozoa - ps10
Filozoa - ps11
Choano./Metazoa - ps12
Metazoa - ps13
Myriazoa - ps14
Parahoxozoa - ps15
Bilateria - ps16
Deuterostomia - ps17
Chordata - ps18
Olfactores - ps19
Vertebrata - ps20
Gnathostomata - ps21
Euteleostomi - ps22
Sarcopterygii - ps23
Tetrapoda - ps24
Amniota - ps25
Mammalia - ps26
Theria - ps27
Placentalia - ps28
Boreoeutheria - ps29
Euarchontoglires - ps30
Euarchonta - ps31
Primates - ps32
Haplorrhini - ps33
Simiiformes - ps34
Catarrhini - ps35
Hominoidea - ps36
Hominidae - ps37
Homininae - ps38
Hominini - ps39
Homo_sapiens - ps40
Phylostratum

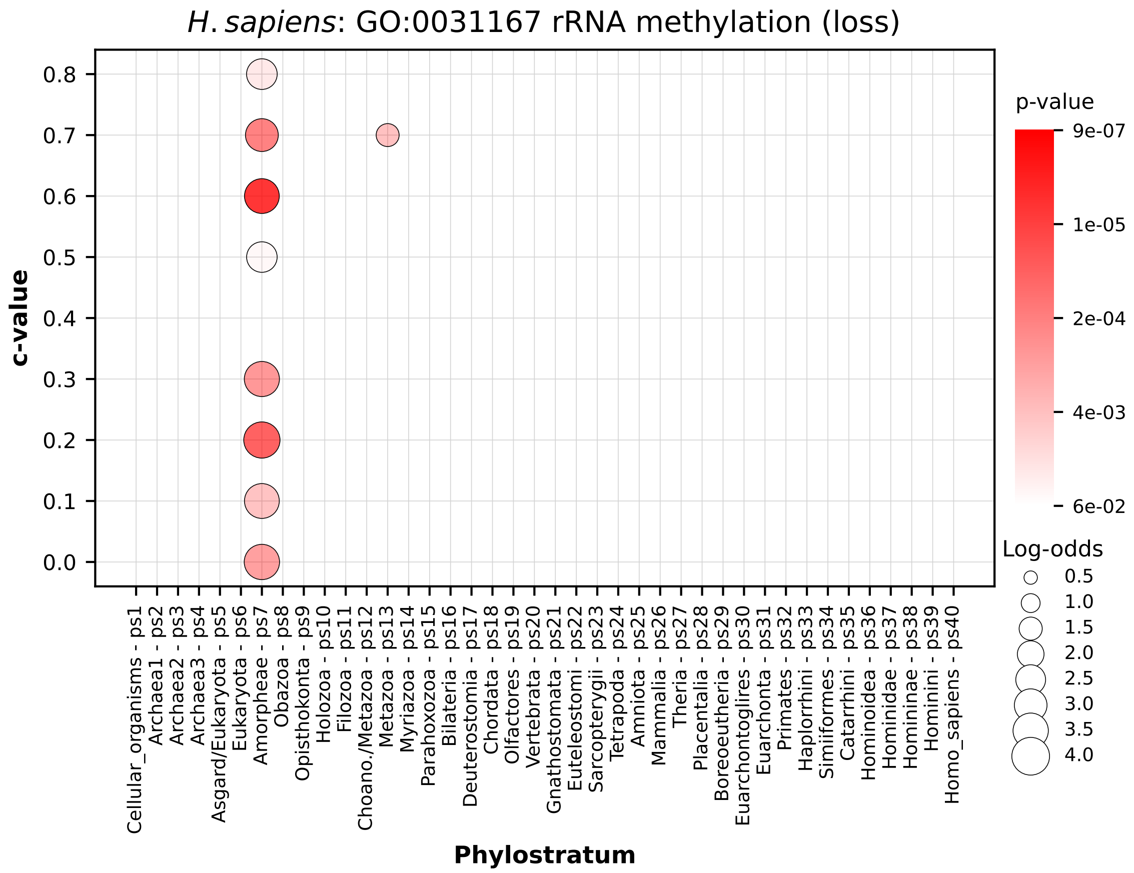

H. sapiens: GO:0031167 rRNA methylation (loss)
c-value
0.8
0.7
0.6
0.5
0.4
0.3
0.2
0.1
0.0
p-value
9e-07
1e-05
2e-04
4e-03
6e-02
Log-odds
0.5
1.0
1.5
2.0
2.5
3.0
3.5
4.0
Cellular_organisms - ps1
Archaea1 - ps2
Archaea2 - ps3
Archaea3 - ps4
Asgard/Eukaryota - ps5
Eukaryota - ps6
Amorpheae - ps7
Obazoa - ps8
Opisthokonta - ps9
Holozoa - ps10
Filozoa - ps11
Choano./Metazoa - ps12
Metazoa - ps13
Myriazoa - ps14
Parahoxozoa - ps15
Bilateria - ps16
Deuterostomia - ps17
Chordata - ps18
Olfactores - ps19
Vertebrata - ps20
Gnathostomata - ps21
Euteleostomi - ps22
Sarcopterygii - ps23
Tetrapoda - ps24
Amniota - ps25
Mammalia - ps26
Theria - ps27
Placentalia - ps28
Boreoeutheria - ps29
Euarchontoglires - ps30
Euarchonta - ps31
Primates - ps32
Haplorrhini - ps33
Simiiformes - ps34
Catarrhini - ps35
Hominoidea - ps36
Hominidae - ps37
Homininae - ps38
Hominini - ps39
Homo_sapiens - ps40
Phylostratum

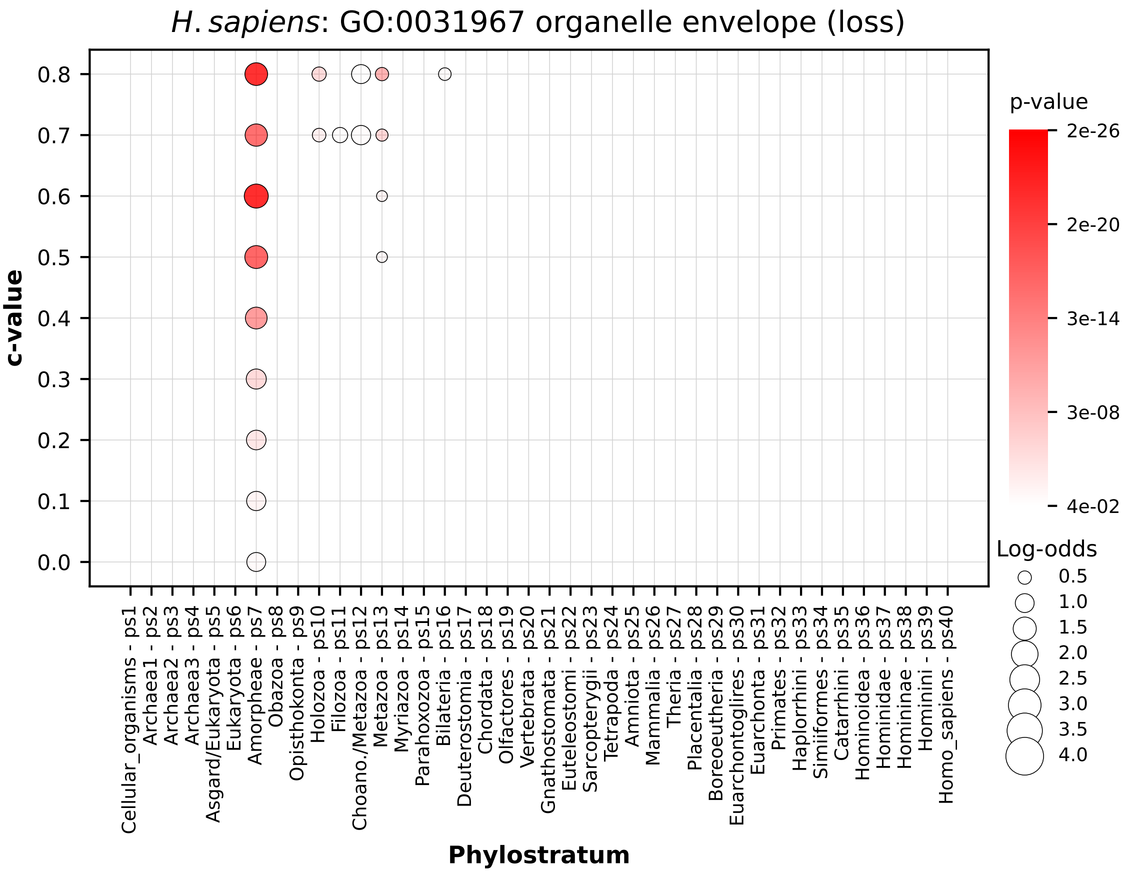
H. sapiens: GO:0031967 organelle envelope (loss)
c-value
0.8
0.7
0.6
0.5
0.4
0.3
0.2
0.1
0.0
p-value
2e-26
2e-20
3e-14
3e-08
4e-02
Log-odds
0.5
1.0
1.5
2.0
2.5
3.0
3.5
4.0
Cellular_organisms - ps1
Archaea1 - ps2
Archaea2 - ps3
Archaea3 - ps4
Asgard/Eukaryota - ps5
Eukaryota - ps6
Amorpheae - ps7
Obazoa - ps8
Opisthokonta - ps9
Holozoa - ps10
Filozoa - ps11
Choano./Metazoa - ps12
Metazoa - ps13
Myriazoa - ps14
Parahoxozoa - ps15
Bilateria - ps16
Deuterostomia - ps17
Chordata - ps18
Olfactores - ps19
Vertebrata - ps20
Gnathostomata - ps21
Euteleostomi - ps22
Sarcopterygii - ps23
Tetrapoda - ps24
Amniota - ps25
Mammalia - ps26
Theria - ps27
Placentalia - ps28
Boreoeutheria - ps29
Euarchontoglires - ps30
Euarchonta - ps31
Primates - ps32
Haplorrhini - ps33
Simiiformes - ps34
Catarrhini - ps35
Hominoidea - ps36
Hominidae - ps37
Homininae - ps38
Hominini - ps39
Homo_sapiens - ps40
Phylostratum

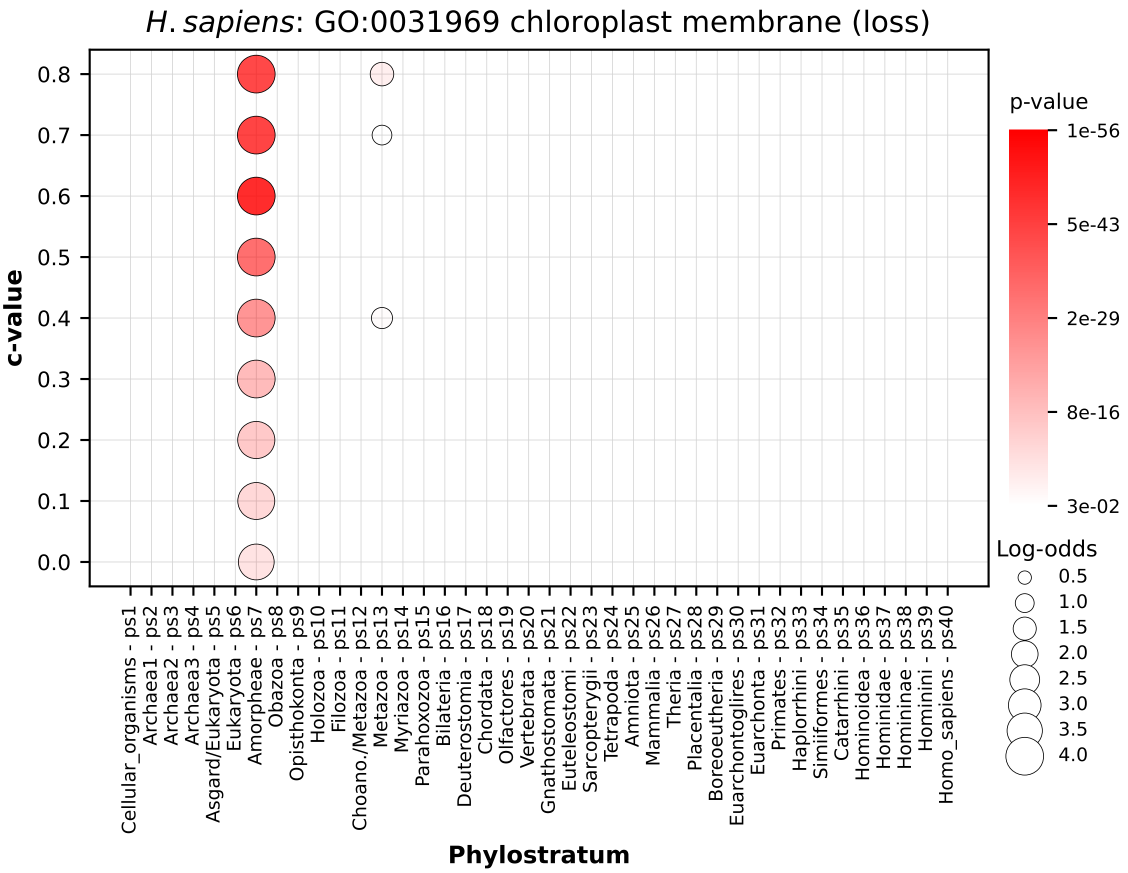

H. sapiens: GO:0031969 chloroplast membrane (loss)
c-value
0.0
0.1
0.2
0.3
0.4
0.5
0.6
0.7
0.8
Phylostratum
Cellular_organisms - ps1
Archaea1 - ps2
Archaea2 - ps3
Archaea3 - ps4
Asgard/Eukaryota - ps5
Eukaryota - ps6
Amorpheae - ps7
Obazoa - ps8
Opisthokonta - ps9
Holozoa - ps10
Filozoa - ps11
Choano./Metazoa - ps12
Metazoa - ps13
Myriazoa - ps14
Parahoxozoa - ps15
Bilateria - ps16
Deuterostomia - ps17
Chordata - ps18
Olfactores - ps19
Vertebrata - ps20
Gnathostomata - ps21
Euteleostomi - ps22
Sarcopterygii - ps23
Tetrapoda - ps24
Amniota - ps25
Mammalia - ps26
Theria - ps27
Placentalia - ps28
Boreoeutheria - ps29
Euarchontoglires - ps30
Euarchonta - ps31
Primates - ps32
Haplorrhini - ps33
Simiiformes - ps34
Catarrhini - ps35
Hominoidea - ps36
Hominidae - ps37
Homininae - ps38
Hominini - ps39
Homo_sapiens - ps40
p-value
1e-56
5e-43
2e-29
8e-16
3e-02
Log-odds
0.5
1.0
1.5
2.0
2.5
3.0
3.5
4.0

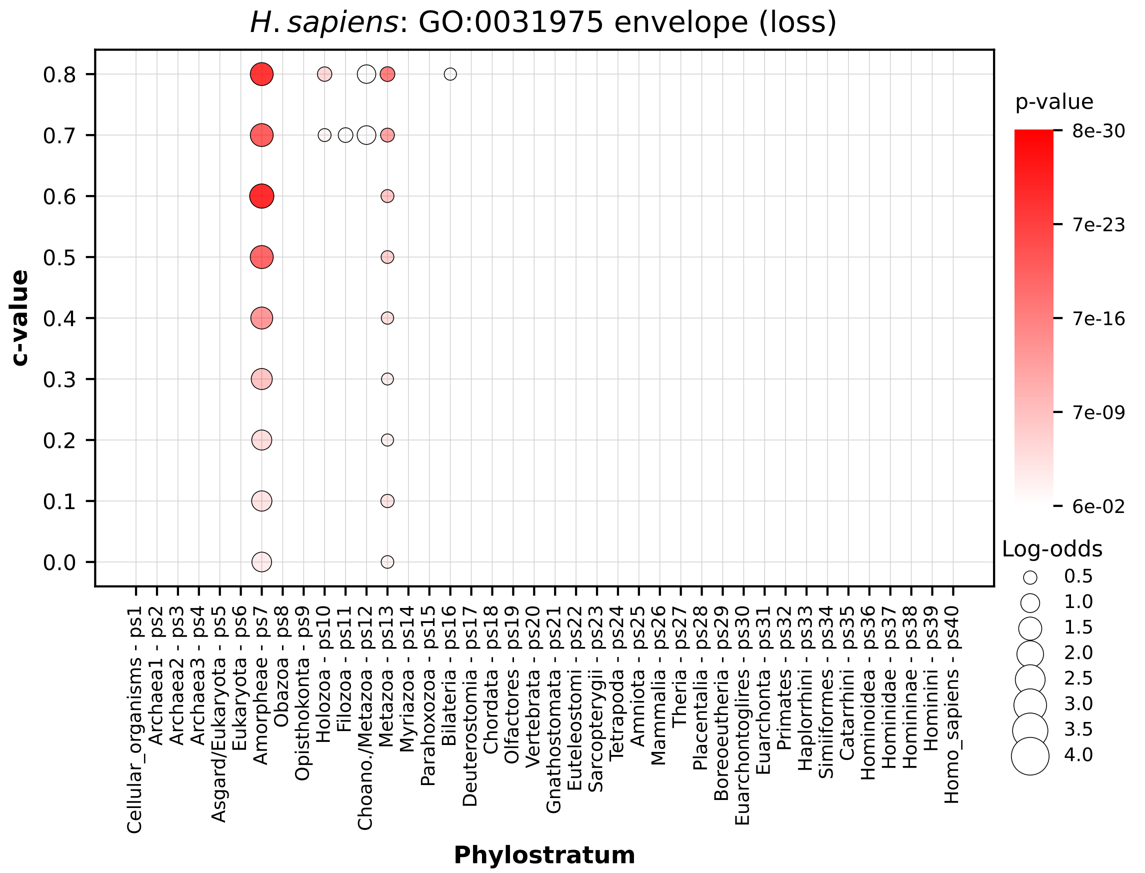
H. sapiens: GO:0031975 envelope (loss)
c-value
0.8
0.7
0.6
0.5
0.4
0.3
0.2
0.1
0.0
p-value
8e-30
7e-23
7e-16
7e-09
6e-02
Log-odds
0.5
1.0
1.5
2.0
2.5
3.0
3.5
4.0
Cellular_organisms - ps1
Archaea1 - ps2
Archaea2 - ps3
Archaea3 - ps4
Asgard/Eukaryota - ps5
Eukaryota - ps6
Amorpheae - ps7
Obazoa - ps8
Opisthokonta - ps9
Holozoa - ps10
Filozoa - ps11
Choano./Metazoa - ps12
Metazoa - ps13
Myriazoa - ps14
Parahoxozoa - ps15
Bilateria - ps16
Deuterostomia - ps17
Chordata - ps18
Olfactores - ps19
Vertebrata - ps20
Gnathostomata - ps21
Euteleostomi - ps22
Sarcopterygii - ps23
Tetrapoda - ps24
Amniota - ps25
Mammalia - ps26
Theria - ps27
Placentalia - ps28
Boreoeutheria - ps29
Euarchontoglires - ps30
Euarchonta - ps31
Primates - ps32
Haplorrhini - ps33
Simiiformes - ps34
Catarrhini - ps35
Hominoidea - ps36
Hominidae - ps37
Homininae - ps38
Hominini - ps39
Homo_sapiens - ps40
Phylostratum

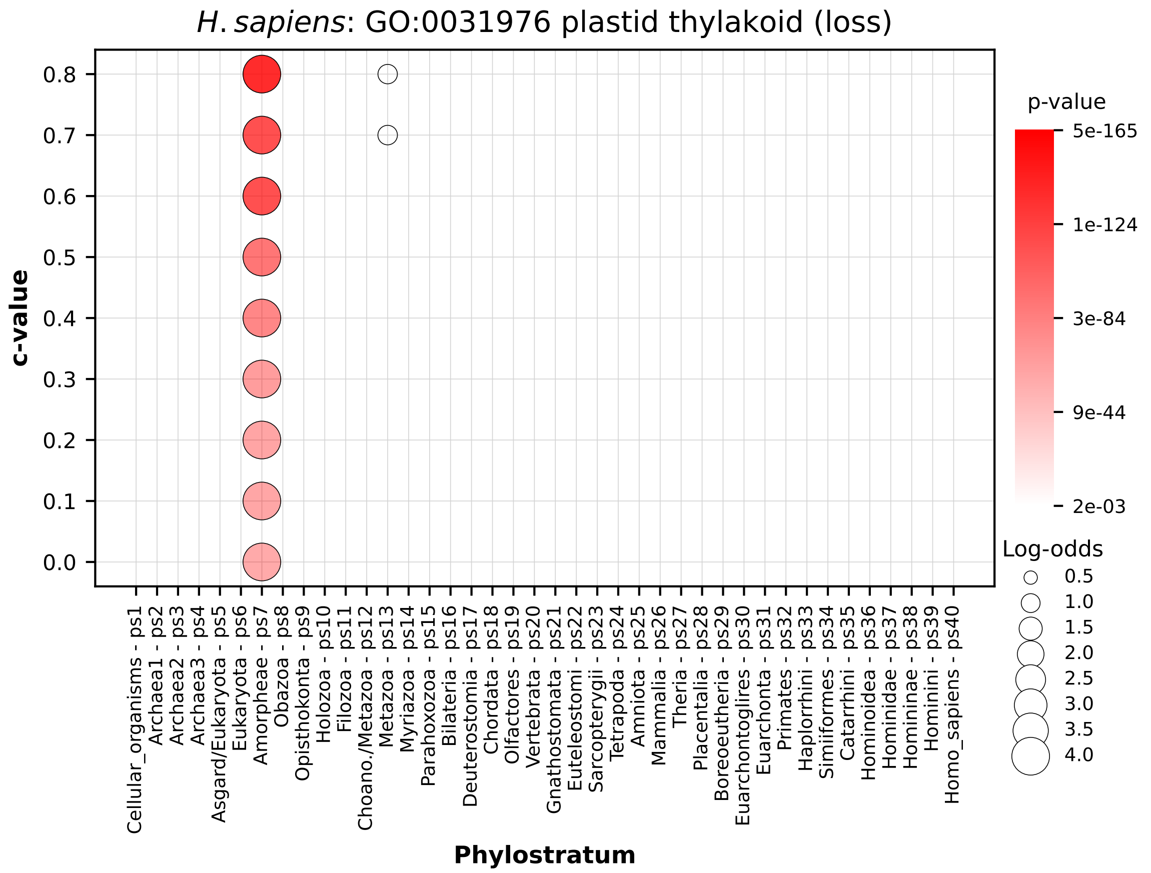

H. sapiens: GO:0031976 plastid thylakoid (loss)
c-value
0.8
0.7
0.6
0.5
0.4
0.3
0.2
0.1
0.0
p-value
5e-165
1e-124
3e-84
9e-44
2e-03
Log-odds
0.5
1.0
1.5
2.0
2.5
3.0
3.5
4.0
Cellular_organisms - ps1
Archaea1 - ps2
Archaea2 - ps3
Archaea3 - ps4
Asgard/Eukaryota - ps5
Eukaryota - ps6
Amorpheae - ps7
Obazoa - ps8
Opisthokonta - ps9
Holozoa - ps10
Filozoa - ps11
Choano./Metazoa - ps12
Metazoa - ps13
Myriazoa - ps14
Parahoxozoa - ps15
Bilateria - ps16
Deuterostomia - ps17
Chordata - ps18
Olfactores - ps19
Vertebrata - ps20
Gnathostomata - ps21
Euteleostomi - ps22
Sarcopterygii - ps23
Tetrapoda - ps24
Amniota - ps25
Mammalia - ps26
Theria - ps27
Placentalia - ps28
Boreoeutheria - ps29
Euarchontoglires - ps30
Euarchonta - ps31
Primates - ps32
Haplorrhini - ps33
Simiiformes - ps34
Catarrhini - ps35
Hominoidea - ps36
Hominidae - ps37
Homininae - ps38
Hominini - ps39
Homo_sapiens - ps40
Phylostratum

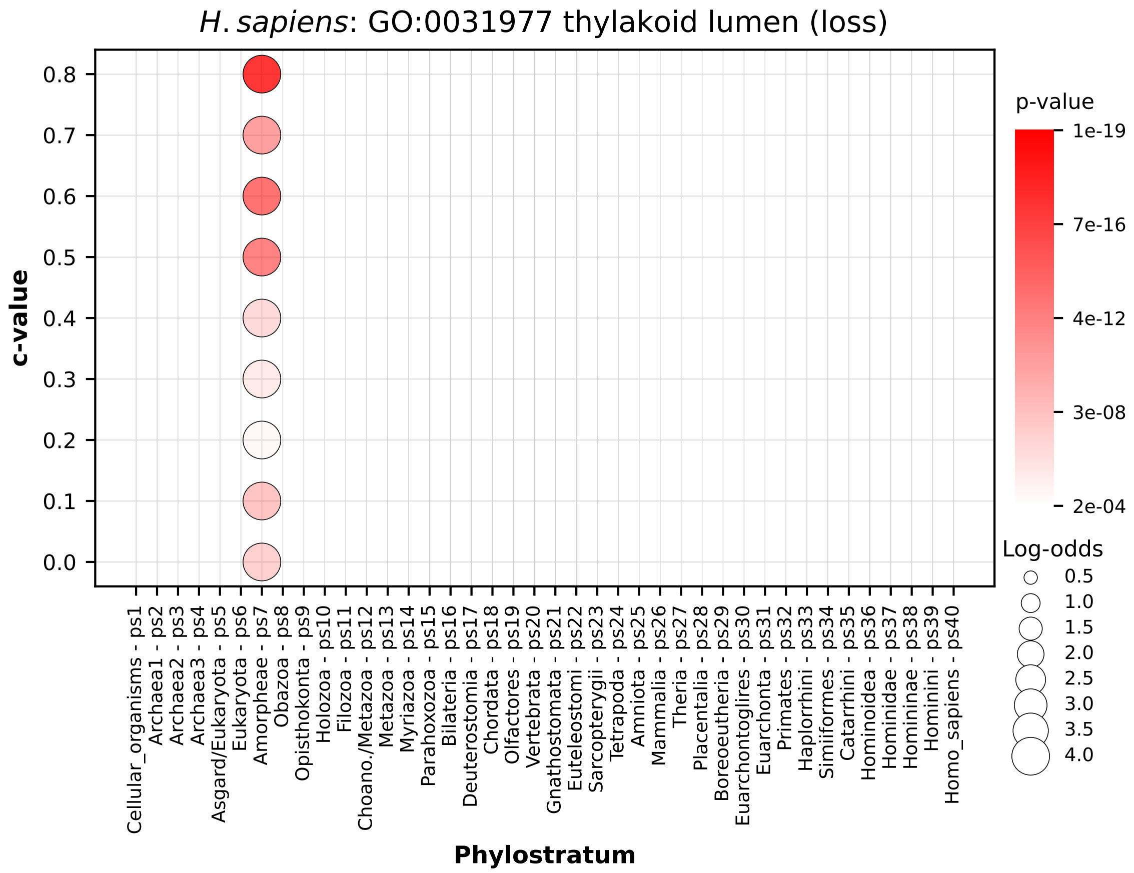

H. sapiens: GO:0031977 thylakoid lumen (loss)
c-value
0.8
0.7
0.6
0.5
0.4
0.3
0.2
0.1
0.0
p-value
1e-19
7e-16
4e-12
3e-08
2e-04
Log-odds
0.5
1.0
1.5
2.0
2.5
3.0
3.5
4.0
Cellular_organisms - ps1
Archaea1 - ps2
Archaea2 - ps3
Archaea3 - ps4
Asgard/Eukaryota - ps5
Eukaryota - ps6
Amorpheae - ps7
Obazoa - ps8
Opisthokonta - ps9
Holozoa - ps10
Filozoa - ps11
Choano./Metazoa - ps12
Metazoa - ps13
Myriazoa - ps14
Parahoxozoa - ps15
Bilateria - ps16
Deuterostomia - ps17
Chordata - ps18
Olfactores - ps19
Vertebrata - ps20
Gnathostomata - ps21
Euteleostomi - ps22
Sarcopterygii - ps23
Tetrapoda - ps24
Amniota - ps25
Mammalia - ps26
Theria - ps27
Placentalia - ps28
Boreoeutheria - ps29
Euarchontoglires - ps30
Euarchonta - ps31
Primates - ps32
Haplorrhini - ps33
Simiiformes - ps34
Catarrhini - ps35
Hominoidea - ps36
Hominidae - ps37
Homininae - ps38
Hominini - ps39
Homo_sapiens - ps40
Phylostratum

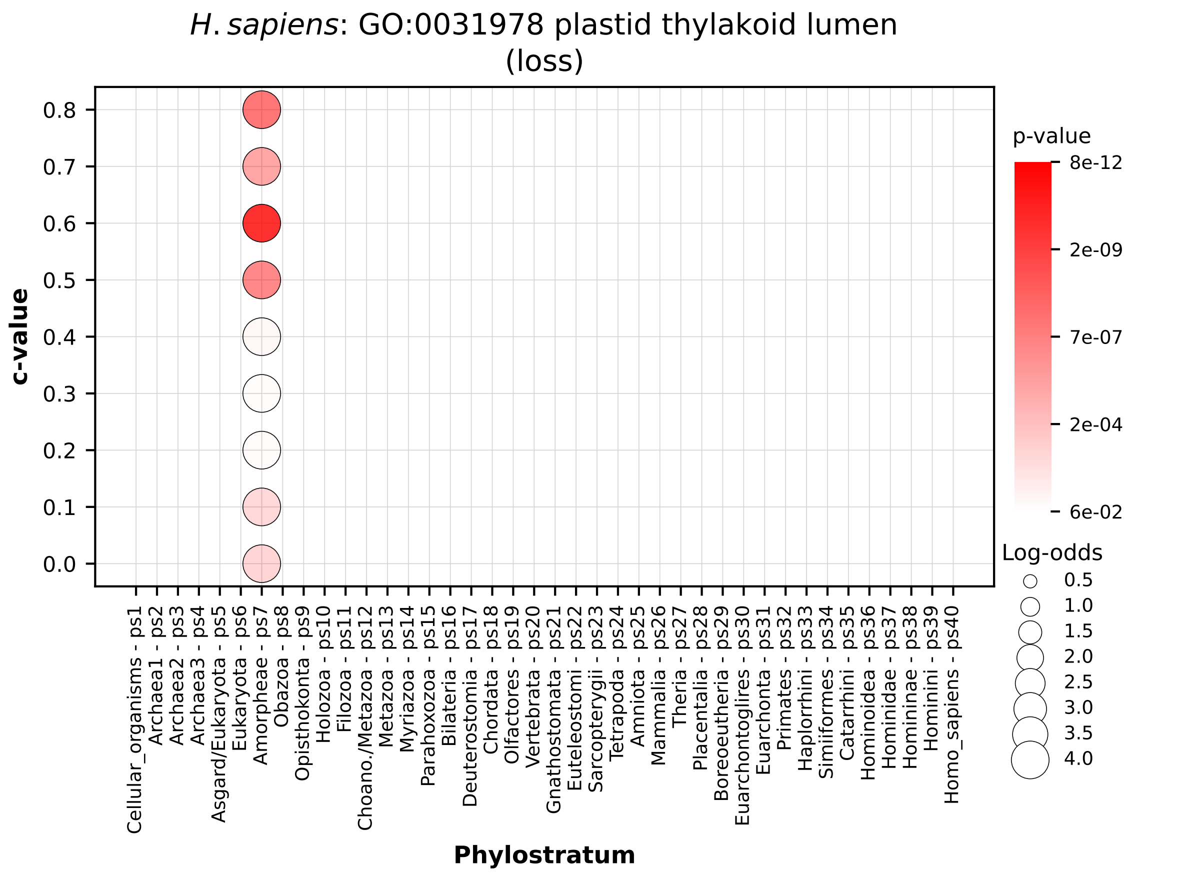

H. sapiens: GO:0031978 plastid thylakoid lumen
(loss)
c-value
0.8
0.7
0.6
0.5
0.4
0.3
0.2
0.1
0.0
p-value
8e-12
2e-09
7e-07
2e-04
6e-02
Log-odds
0.5
1.0
1.5
2.0
2.5
3.0
3.5
4.0
Cellular_organisms - ps1
Archaea1 - ps2
Archaea2 - ps3
Archaea3 - ps4
Asgard/Eukaryota - ps5
Eukaryota - ps6
Amorpheae - ps7
Obazoa - ps8
Opisthokonta - ps9
Holozoa - ps10
Filozoa - ps11
Choano./Metazoa - ps12
Metazoa - ps13
Myriazoa - ps14
Parahoxozoa - ps15
Bilateria - ps16
Deuterostomia - ps17
Chordata - ps18
Olfactores - ps19
Vertebrata - ps20
Gnathostomata - ps21
Euteleostomi - ps22
Sarcopterygii - ps23
Tetrapoda - ps24
Amniota - ps25
Mammalia - ps26
Theria - ps27
Placentalia - ps28
Boreoeutheria - ps29
Euarchontoglires - ps30
Euarchonta - ps31
Primates - ps32
Haplorrhini - ps33
Simiiformes - ps34
Catarrhini - ps35
Hominoidea - ps36
Hominidae - ps37
Homininae - ps38
Hominini - ps39
Homo_sapiens - ps40
Phylostratum

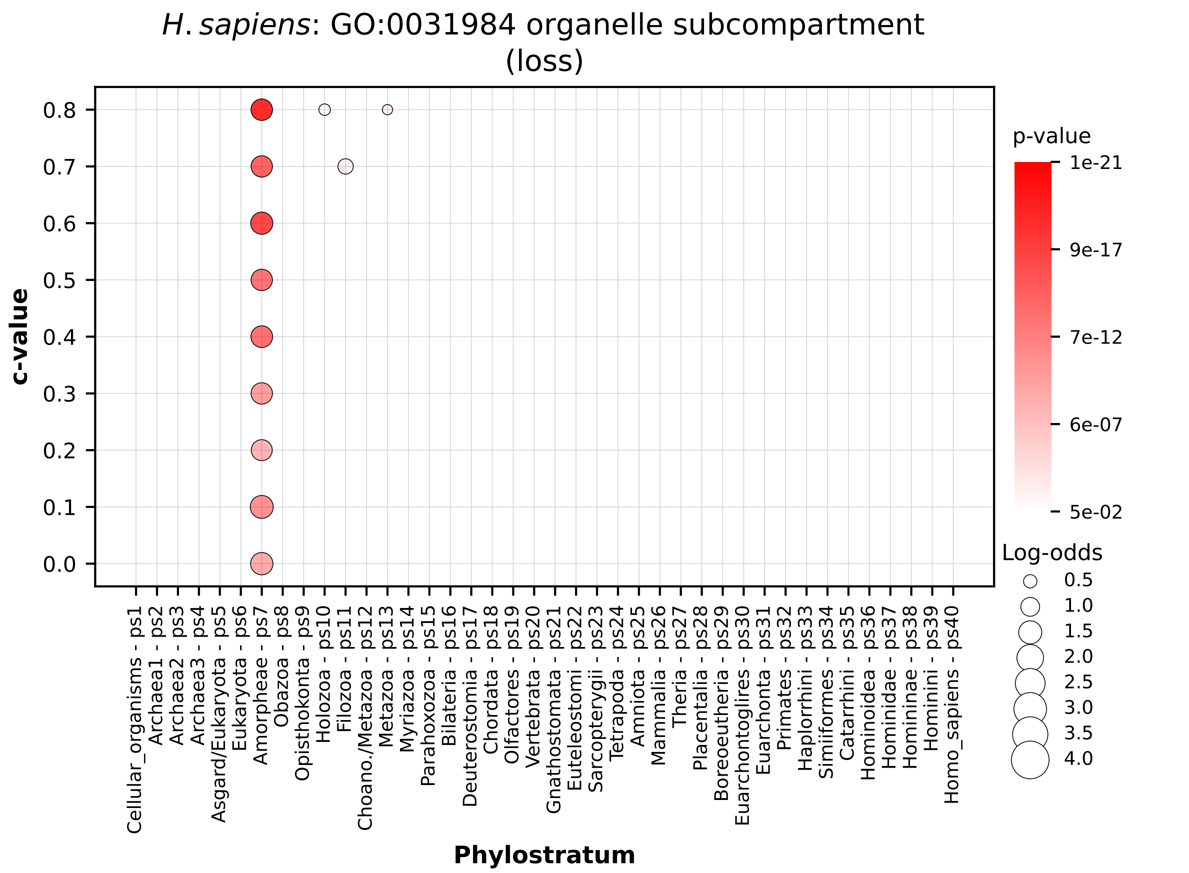

H. sapiens: GO:0031984 organelle subcompartment
(loss)
c-value
0.0
0.1
0.2
0.3
0.4
0.5
0.6
0.7
0.8
p-value
1e-21
9e-17
7e-12
6e-07
5e-02
Log-odds
0.5
1.0
1.5
2.0
2.5
3.0
3.5
4.0
Cellular_organisms - ps1
Archaea1 - ps2
Archaea2 - ps3
Archaea3 - ps4
Asgard/Eukaryota - ps5
Eukaryota - ps6
Amorpheae - ps7
Obazoa - ps8
Opisthokonta - ps9
Holozoa - ps10
Filozoa - ps11
Choano./Metazoa - ps12
Metazoa - ps13
Myriazoa - ps14
Parahoxozoa - ps15
Bilateria - ps16
Deuterostomia - ps17
Chordata - ps18
Olfactores - ps19
Vertebrata - ps20
Gnathostomata - ps21
Euteleostomi - ps22
Sarcopterygii - ps23
Tetrapoda - ps24
Amniota - ps25
Mammalia - ps26
Theria - ps27
Placentalia - ps28
Boreoeutheria - ps29
Euarchontoglires - ps30
Euarchonta - ps31
Primates - ps32
Haplorrhini - ps33
Simiiformes - ps34
Catarrhini - ps35
Hominoidea - ps36
Hominidae - ps37
Homininae - ps38
Hominini - ps39
Homo_sapiens - ps40
Phylostratum

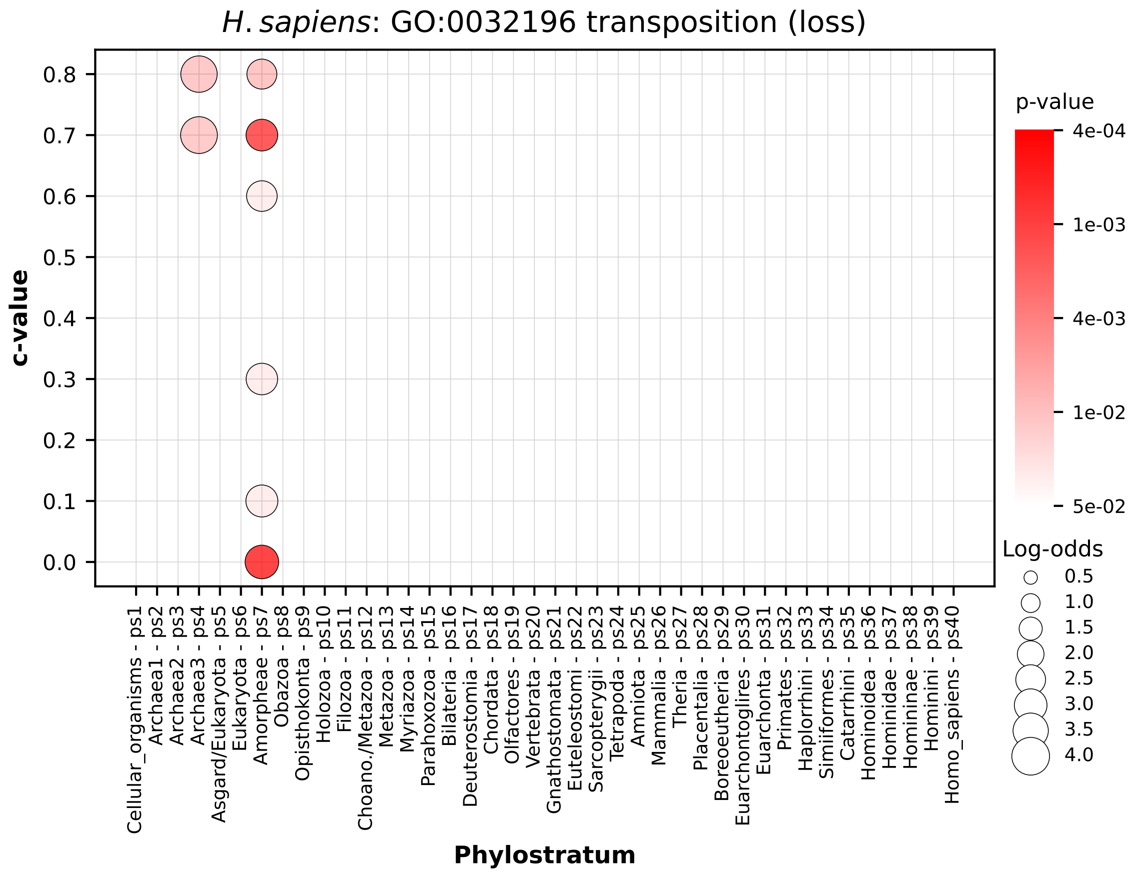

H. sapiens: GO:0032196 transposition (loss)
c-value
0.8
0.7
0.6
0.5
0.4
0.3
0.2
0.1
0.0
Cellular_organisms - ps1
Archaea1 - ps2
Archaea2 - ps3
Archaea3 - ps4
Asgard/Eukaryota - ps5
Eukaryota - ps6
Amorpheae - ps7
Obazoa - ps8
Opisthokonta - ps9
Holozoa - ps10
Filozoa - ps11
Choano./Metazoa - ps12
Metazoa - ps13
Myriazoa - ps14
Parahoxozoa - ps15
Bilateria - ps16
Deuterostomia - ps17
Chordata - ps18
Olfactores - ps19
Vertebrata - ps20
Gnathostomata - ps21
Euteleostomi - ps22
Sarcopterygii - ps23
Tetrapoda - ps24
Amniota - ps25
Mammalia - ps26
Theria - ps27
Placentalia - ps28
Boreoeutheria - ps29
Euarchontoglires - ps30
Euarchonta - ps31
Primates - ps32
Haplorrhini - ps33
Simiiformes - ps34
Catarrhini - ps35
Hominoidea - ps36
Hominidae - ps37
Homininae - ps38
Hominini - ps39
Homo_sapiens - ps40
Phylostratum
p-value
4e-04
1e-03
4e-03
1e-02
5e-02
Log-odds
0.5
1.0
1.5
2.0
2.5
3.0
3.5
4.0

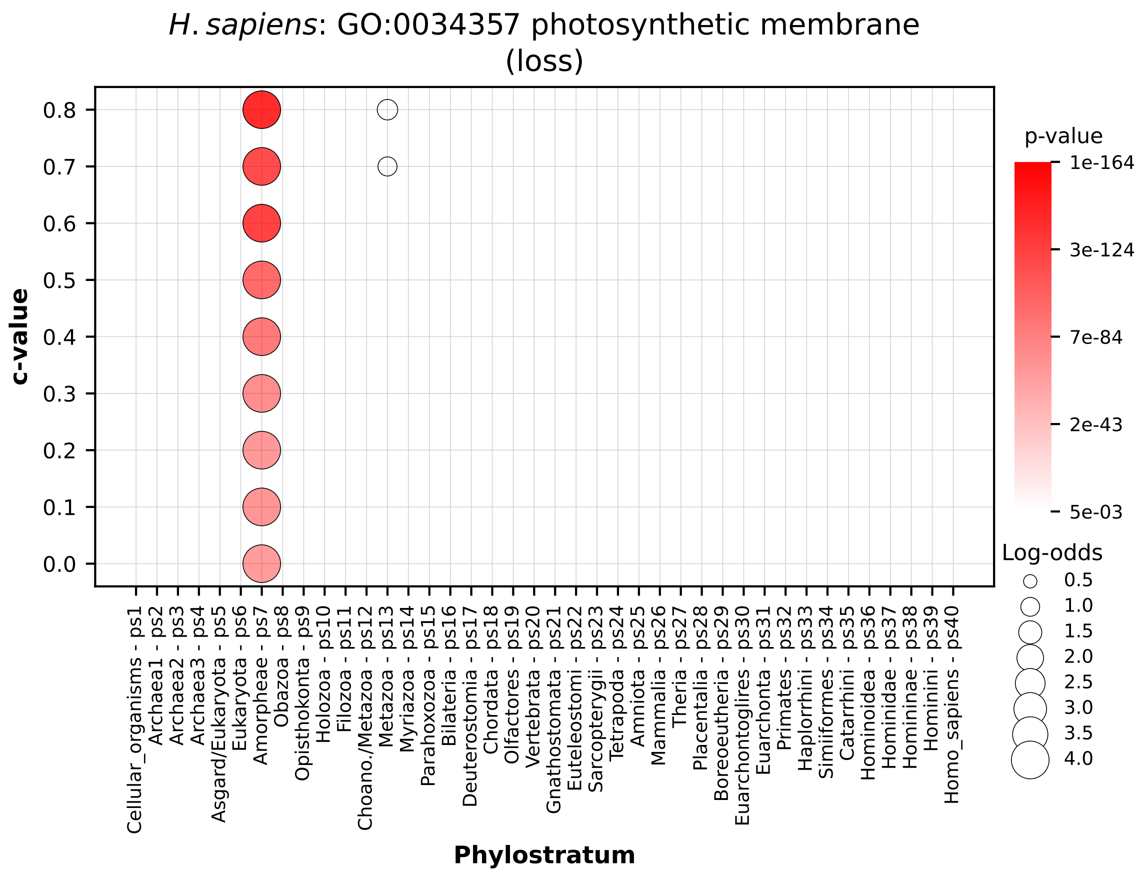

H. sapiens: GO:0034357 photosynthetic membrane
(loss)
c-value
0.8
0.7
0.6
0.5
0.4
0.3
0.2
0.1
0.0
p-value
1e-164
3e-124
7e-84
2e-43
5e-03
Log-odds
0.5
1.0
1.5
2.0
2.5
3.0
3.5
4.0
Cellular_organisms - ps1
Archaea1 - ps2
Archaea2 - ps3
Archaea3 - ps4
Asgard/Eukaryota - ps5
Eukaryota - ps6
Amorpheae - ps7
Obazoa - ps8
Opisthokonta - ps9
Holozoa - ps10
Filozoa - ps11
Choano./Metazoa - ps12
Metazoa - ps13
Myriazoa - ps14
Parahoxozoa - ps15
Bilateria - ps16
Deuterostomia - ps17
Chordata - ps18
Olfactores - ps19
Vertebrata - ps20
Gnathostomata - ps21
Euteleostomi - ps22
Sarcopterygii - ps23
Tetrapoda - ps24
Amniota - ps25
Mammalia - ps26
Theria - ps27
Placentalia - ps28
Boreoeutheria - ps29
Euarchontoglires - ps30
Euarchonta - ps31
Primates - ps32
Haplorrhini - ps33
Simiiformes - ps34
Catarrhini - ps35
Hominoidea - ps36
Hominidae - ps37
Homininae - ps38
Hominini - ps39
Homo_sapiens - ps40
Phylostratum

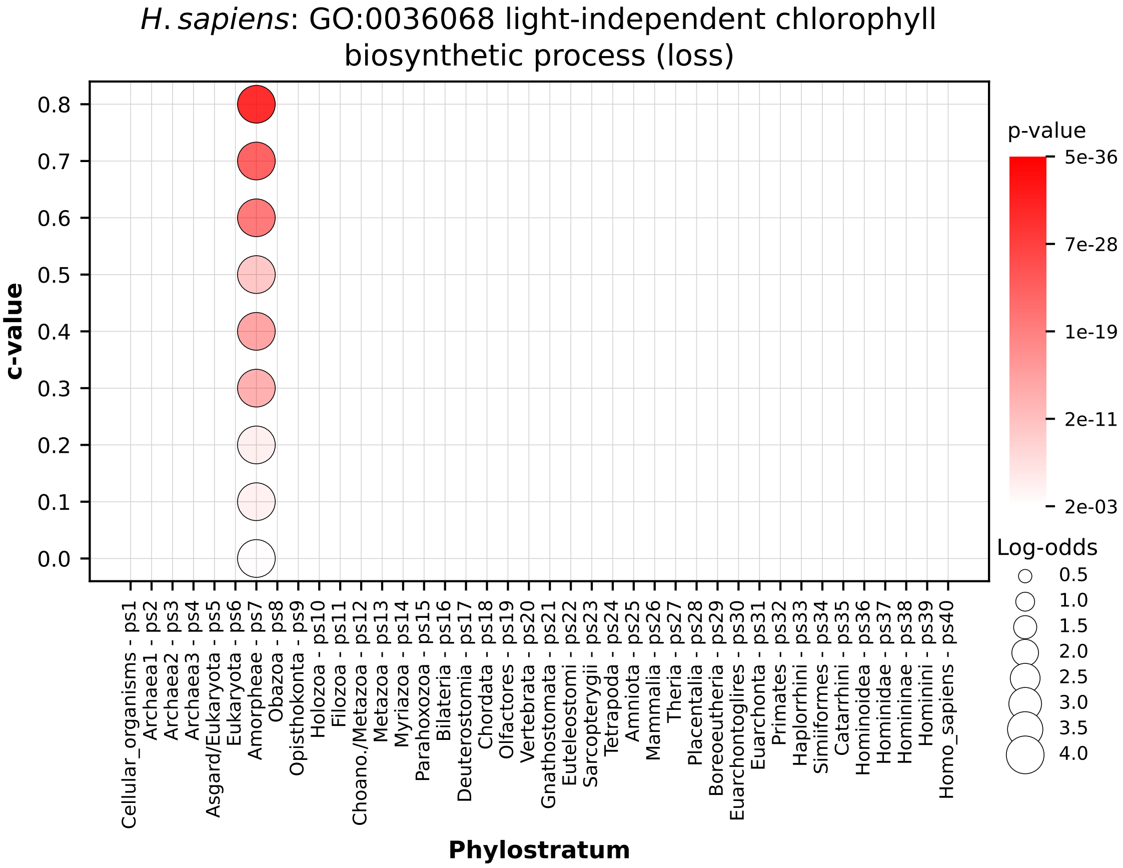

H. sapiens: GO:0036068 light-independent chlorophyll biosynthetic process (loss)
c-value
0.8
0.7
0.6
0.5
0.4
0.3
0.2
0.1
0.0
p-value
5e-36
7e-28
1e-19
2e-11
2e-03
Log-odds
0.5
1.0
1.5
2.0
2.5
3.0
3.5
4.0
Cellular_organisms - ps1
Archaea1 - ps2
Archaea2 - ps3
Archaea3 - ps4
Asgard/Eukaryota - ps5
Eukaryota - ps6
Amorpheae - ps7
Obazoa - ps8
Opisthokonta - ps9
Holozoa - ps10
Filozoa - ps11
Choano./Metazoa - ps12
Metazoa - ps13
Myriazoa - ps14
Parahoxozoa - ps15
Bilateria - ps16
Deuterostomia - ps17
Chordata - ps18
Olfactores - ps19
Vertebrata - ps20
Gnathostomata - ps21
Euteleostomi - ps22
Sarcopterygii - ps23
Tetrapoda - ps24
Amniota - ps25
Mammalia - ps26
Theria - ps27
Placentalia - ps28
Boreoeutheria - ps29
Euarchontoglires - ps30
Euarchonta - ps31
Primates - ps32
Haplorrhini - ps33
Simiiformes - ps34
Catarrhini - ps35
Hominoidea - ps36
Hominidae - ps37
Homininae - ps38
Hominini - ps39
Homo_sapiens - ps40
Phylostratum

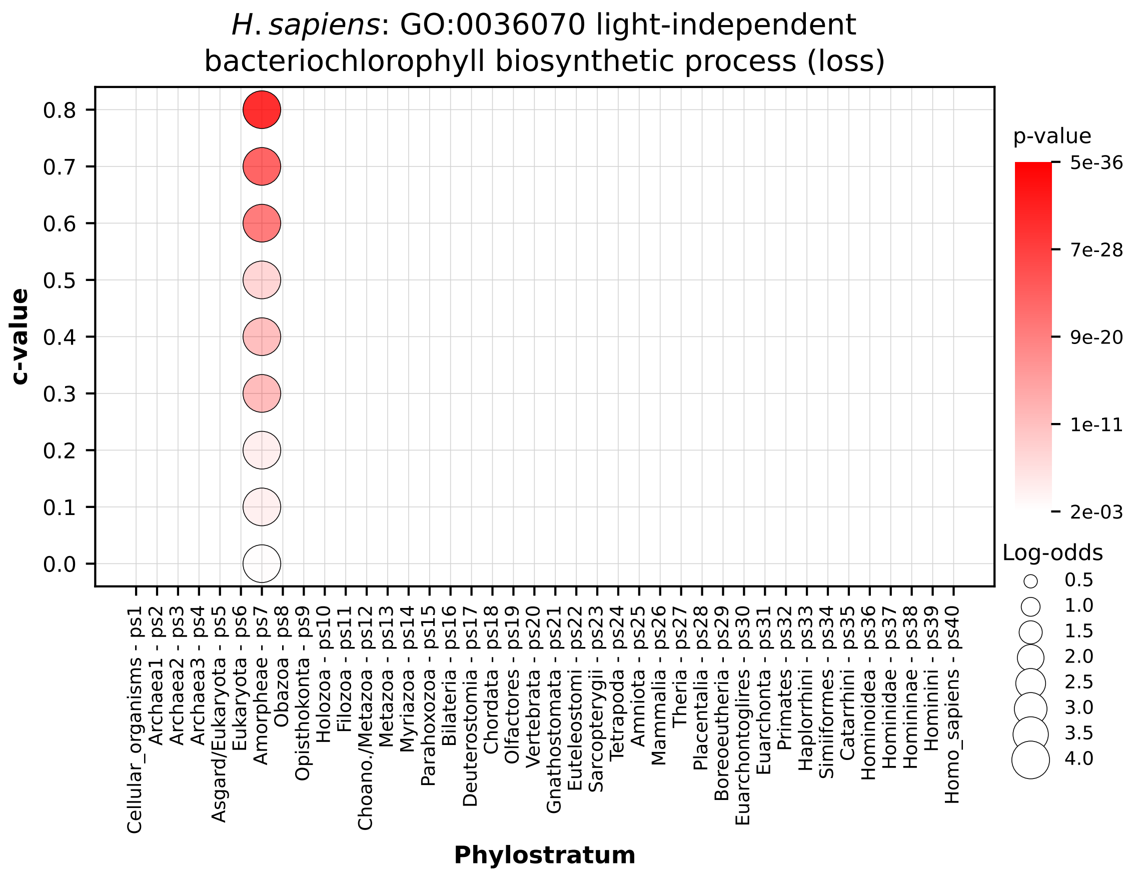

H. sapiens: GO:0036070 light-independent bacteriochlorophyll biosynthetic process (loss)
c-value
0.8
0.7
0.6
0.5
0.4
0.3
0.2
0.1
0.0
p-value
5e-36
7e-28
9e-20
1e-11
2e-03
Log-odds
0.5
1.0
1.5
2.0
2.5
3.0
3.5
4.0
Cellular_organisms - ps1
Archaea1 - ps2
Archaea2 - ps3
Archaea3 - ps4
Asgard/Eukaryota - ps5
Eukaryota - ps6
Amorpheae - ps7
Obazoa - ps8
Opisthokonta - ps9
Holozoa - ps10
Filozoa - ps11
Choano./Metazoa - ps12
Metazoa - ps13
Myriazoa - ps14
Parahoxozoa - ps15
Bilateria - ps16
Deuterostomia - ps17
Chordata - ps18
Olfactores - ps19
Vertebrata - ps20
Gnathostomata - ps21
Euteleostomi - ps22
Sarcopterygii - ps23
Tetrapoda - ps24
Amniota - ps25
Mammalia - ps26
Theria - ps27
Placentalia - ps28
Boreoeutheria - ps29
Euarchontoglires - ps30
Euarchonta - ps31
Primates - ps32
Haplorrhini - ps33
Simiiformes - ps34
Catarrhini - ps35
Hominoidea - ps36
Hominidae - ps37
Homininae - ps38
Hominini - ps39
Homo_sapiens - ps40
Phylostratum

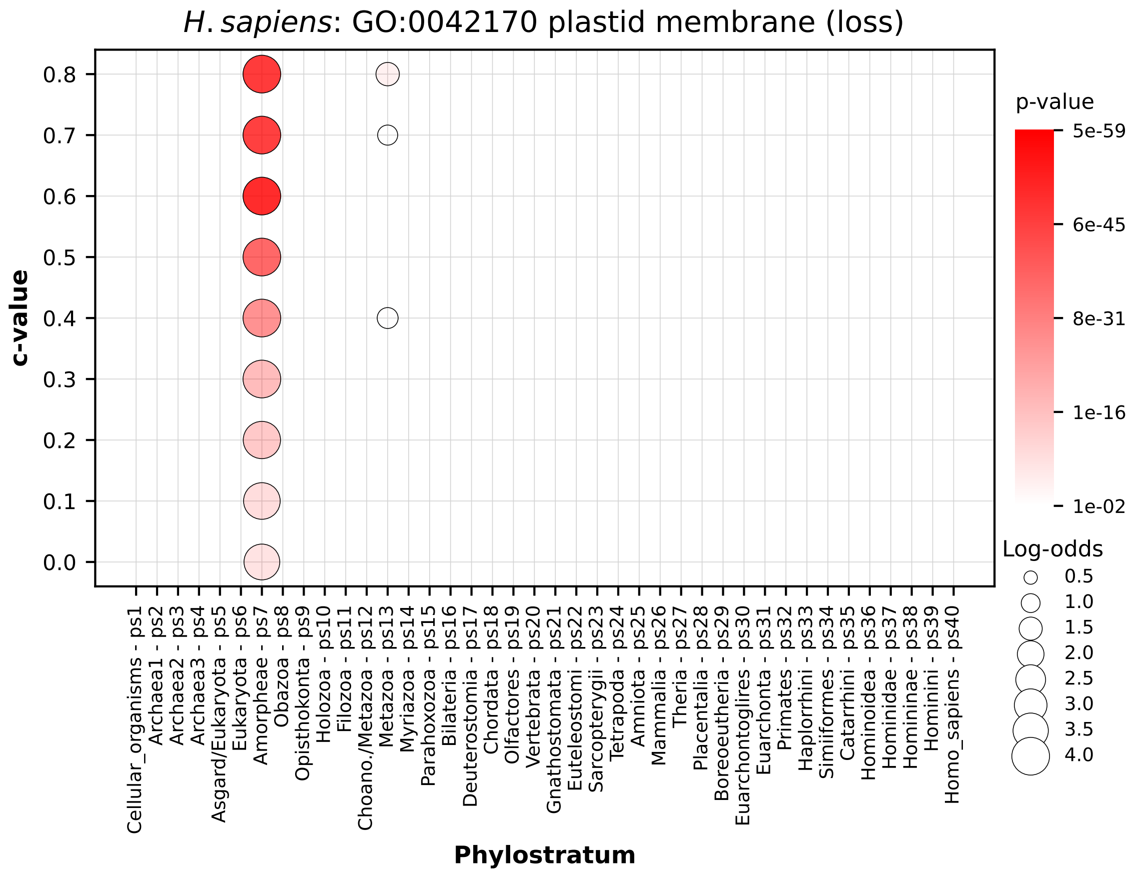

$H. sapiens$: GO:0042170 plastid membrane (loss)
c-value
0.0
0.1
0.2
0.3
0.4
0.5
0.6
0.7
0.8
p-value
5e-59
6e-45
8e-31
1e-16
1e-02
Log-odds
0.5
1.0
1.5
2.0
2.5
3.0
3.5
4.0
Cellular_organisms - ps1
Archaea1 - ps2
Archaea2 - ps3
Archaea3 - ps4
Asgard/Eukaryota - ps5
Eukaryota - ps6
Amorpheae - ps7
Obazoa - ps8
Opisthokonta - ps9
Holozoa - ps10
Filozoa - ps11
Choano./Metazoa - ps12
Metazoa - ps13
Myriazoa - ps14
Parahoxozoa - ps15
Bilateria - ps16
Deuterostomia - ps17
Chordata - ps18
Olfactores - ps19
Vertebrata - ps20
Gnathostomata - ps21
Euteleostomi - ps22
Sarcopterygii - ps23
Tetrapoda - ps24
Amniota - ps25
Mammalia - ps26
Theria - ps27
Placentalia - ps28
Boreoeutheria - ps29
Euarchontoglires - ps30
Euarchonta - ps31
Primates - ps32
Haplorrhini - ps33
Simiiformes - ps34
Catarrhini - ps35
Hominoidea - ps36
Hominidae - ps37
Homininae - ps38
Hominini - ps39
Homo_sapiens - ps40
Phylostratum

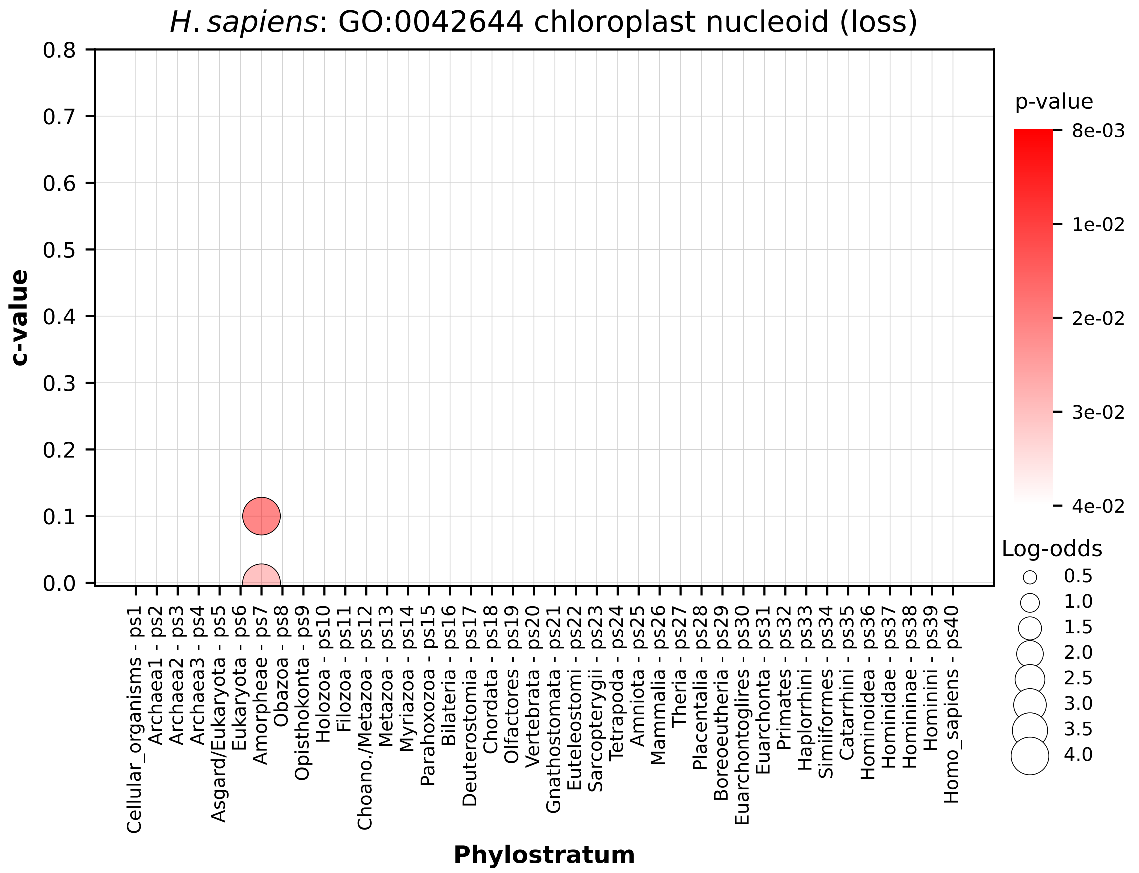

H. sapiens: GO:0042644 chloroplast nucleoid (loss)
c-value
0.0
0.1
0.2
0.3
0.4
0.5
0.6
0.7
0.8
Phylostratum
Cellular_organisms - ps1
Archaea1 - ps2
Archaea2 - ps3
Archaea3 - ps4
Asgard/Eukaryota - ps5
Eukaryota - ps6
Amorpheae - ps7
Obazoa - ps8
Opisthokonta - ps9
Holozoa - ps10
Filozoa - ps11
Choano./Metazoa - ps12
Metazoa - ps13
Myriazoa - ps14
Parahoxozoa - ps15
Bilateria - ps16
Deuterostomia - ps17
Chordata - ps18
Olfactores - ps19
Vertebrata - ps20
Gnathostomata - ps21
Euteleostomi - ps22
Sarcopterygii - ps23
Tetrapoda - ps24
Amniota - ps25
Mammalia - ps26
Theria - ps27
Placentalia - ps28
Boreoeutheria - ps29
Euarchontoglires - ps30
Euarchonta - ps31
Primates - ps32
Haplorrhini - ps33
Simiiformes - ps34
Catarrhini - ps35
Hominoidea - ps36
Hominidae - ps37
Homininae - ps38
Hominini - ps39
Homo_sapiens - ps40
p-value
8e-03
1e-02
2e-02
3e-02
4e-02
Log-odds
0.5
1.0
1.5
2.0
2.5
3.0
3.5
4.0

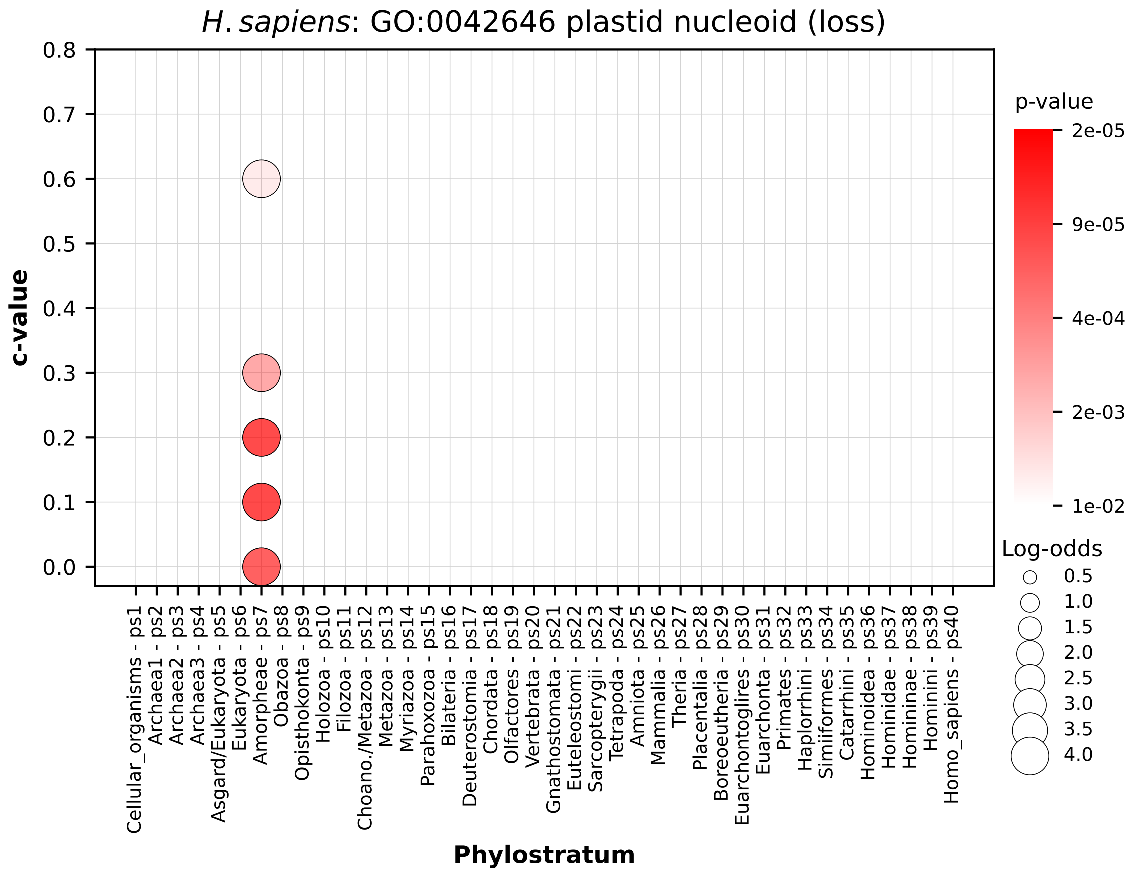

H. sapiens: GO:0042646 plastid nucleoid (loss)
c-value
0.8
0.7
0.6
0.5
0.4
0.3
0.2
0.1
0.0
Phylostratum
Cellular_organisms - ps1
Archaea1 - ps2
Archaea2 - ps3
Archaea3 - ps4
Asgard/Eukaryota - ps5
Eukaryota - ps6
Amorpheae - ps7
Obazoa - ps8
Opisthokonta - ps9
Holozoa - ps10
Filozoa - ps11
Choano./Metazoa - ps12
Metazoa - ps13
Myriazoa - ps14
Parahoxozoa - ps15
Bilateria - ps16
Deuterostomia - ps17
Chordata - ps18
Olfactores - ps19
Vertebrata - ps20
Gnathostomata - ps21
Euteleostomi - ps22
Sarcopterygii - ps23
Tetrapoda - ps24
Amniota - ps25
Mammalia - ps26
Theria - ps27
Placentalia - ps28
Boreoeutheria - ps29
Euarchontoglires - ps30
Euarchonta - ps31
Primates - ps32
Haplorrhini - ps33
Simiiformes - ps34
Catarrhini - ps35
Hominoidea - ps36
Hominidae - ps37
Homininae - ps38
Hominini - ps39
Homo_sapiens - ps40
p-value
2e-05
9e-05
4e-04
2e-03
1e-02
Log-odds
0.5
1.0
1.5
2.0
2.5
3.0
3.5
4.0

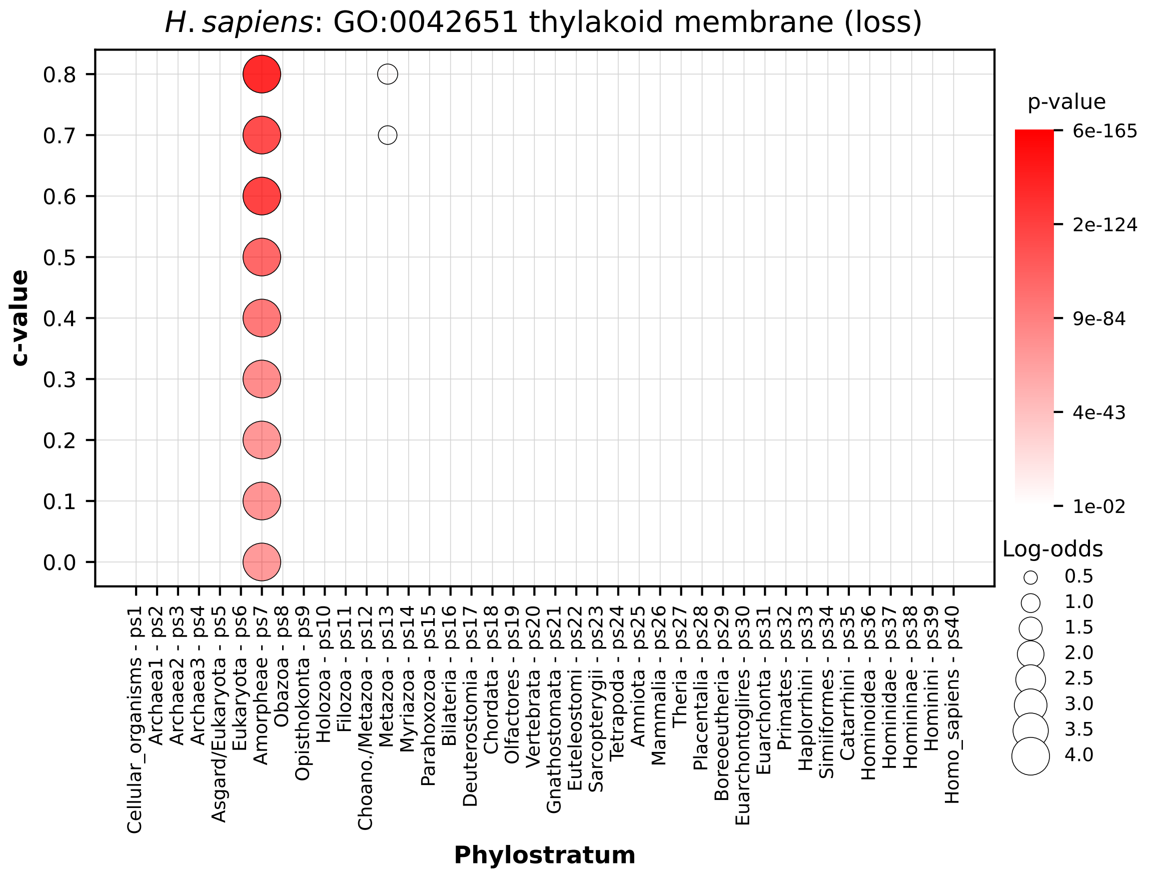

H. sapiens: GO:0042651 thylakoid membrane (loss)
c-value
0.8
0.7
0.6
0.5
0.4
0.3
0.2
0.1
0.0
p-value
6e-165
2e-124
9e-84
4e-43
1e-02
Log-odds
0.5
1.0
1.5
2.0
2.5
3.0
3.5
4.0
Cellular_organisms - ps1
Archaea1 - ps2
Archaea2 - ps3
Archaea3 - ps4
Asgard/Eukaryota - ps5
Eukaryota - ps6
Amorpheae - ps7
Obazoa - ps8
Opisthokonta - ps9
Holozoa - ps10
Filozoa - ps11
Choano./Metazoa - ps12
Metazoa - ps13
Myriazoa - ps14
Parahoxozoa - ps15
Bilateria - ps16
Deuterostomia - ps17
Chordata - ps18
Olfactores - ps19
Vertebrata - ps20
Gnathostomata - ps21
Euteleostomi - ps22
Sarcopterygii - ps23
Tetrapoda - ps24
Amniota - ps25
Mammalia - ps26
Theria - ps27
Placentalia - ps28
Boreoeutheria - ps29
Euarchontoglires - ps30
Euarchonta - ps31
Primates - ps32
Haplorrhini - ps33
Simiiformes - ps34
Catarrhini - ps35
Hominoidea - ps36
Hominidae - ps37
Homininae - ps38
Hominini - ps39
Homo_sapiens - ps40
Phylostratum

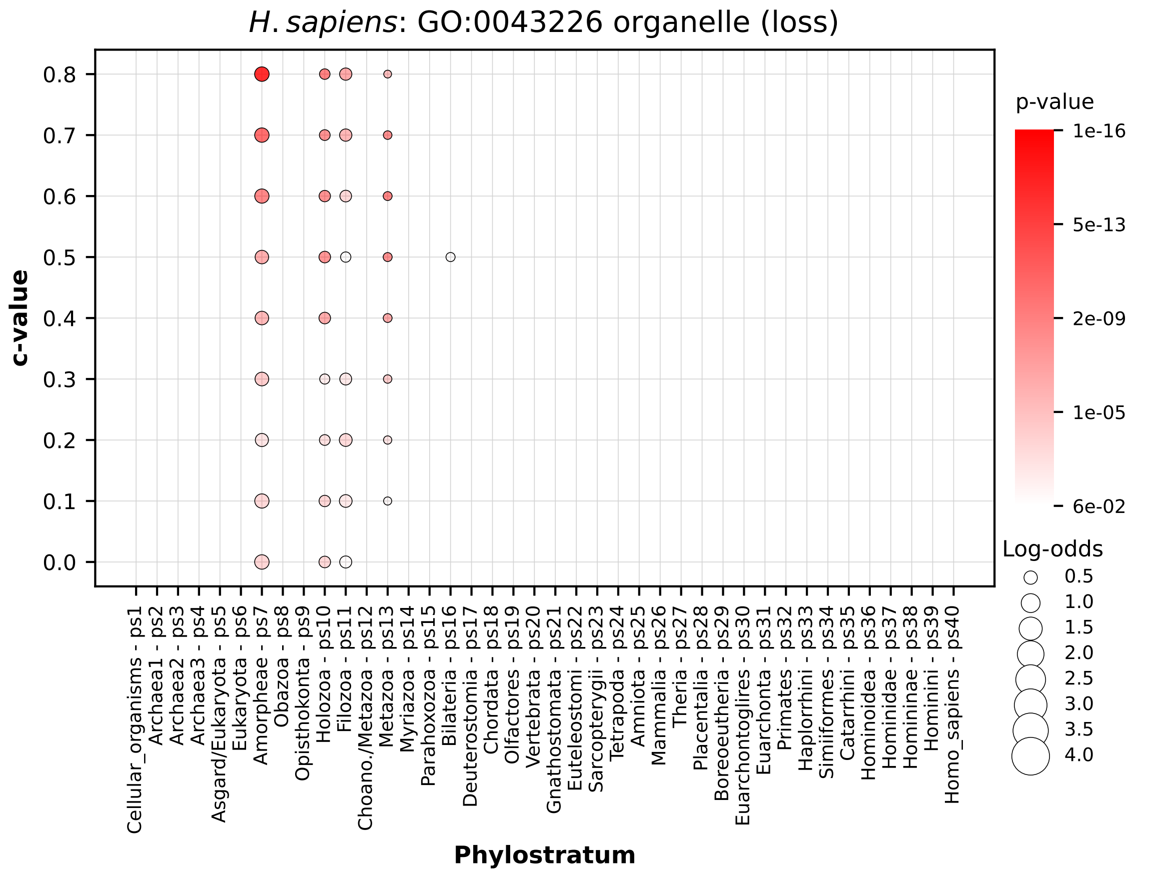

H. sapiens: GO:0043226 organelle (loss)
c-value
0.0
0.1
0.2
0.3
0.4
0.5
0.6
0.7
0.8
Phylostratum
Cellular_organisms - ps1
Archaea1 - ps2
Archaea2 - ps3
Archaea3 - ps4
Asgard/Eukaryota - ps5
Eukaryota - ps6
Amorpheae - ps7
Obazoa - ps8
Opisthokonta - ps9
Holozoa - ps10
Filozoa - ps11
Choano./Metazoa - ps12
Metazoa - ps13
Myriazoa - ps14
Parahoxozoa - ps15
Bilateria - ps16
Deuterostomia - ps17
Chordata - ps18
Olfactores - ps19
Vertebrata - ps20
Gnathostomata - ps21
Euteleostomi - ps22
Sarcopterygii - ps23
Tetrapoda - ps24
Amniota - ps25
Mammalia - ps26
Theria - ps27
Placentalia - ps28
Boreoeutheria - ps29
Euarchontoglires - ps30
Euarchonta - ps31
Primates - ps32
Haplorrhini - ps33
Simiiformes - ps34
Catarrhini - ps35
Hominoidea - ps36
Hominidae - ps37
Homininae - ps38
Hominini - ps39
Homo_sapiens - ps40
p-value
1e-16
5e-13
2e-09
1e-05
6e-02
Log-odds
0.5
1.0
1.5
2.0
2.5
3.0
3.5
4.0

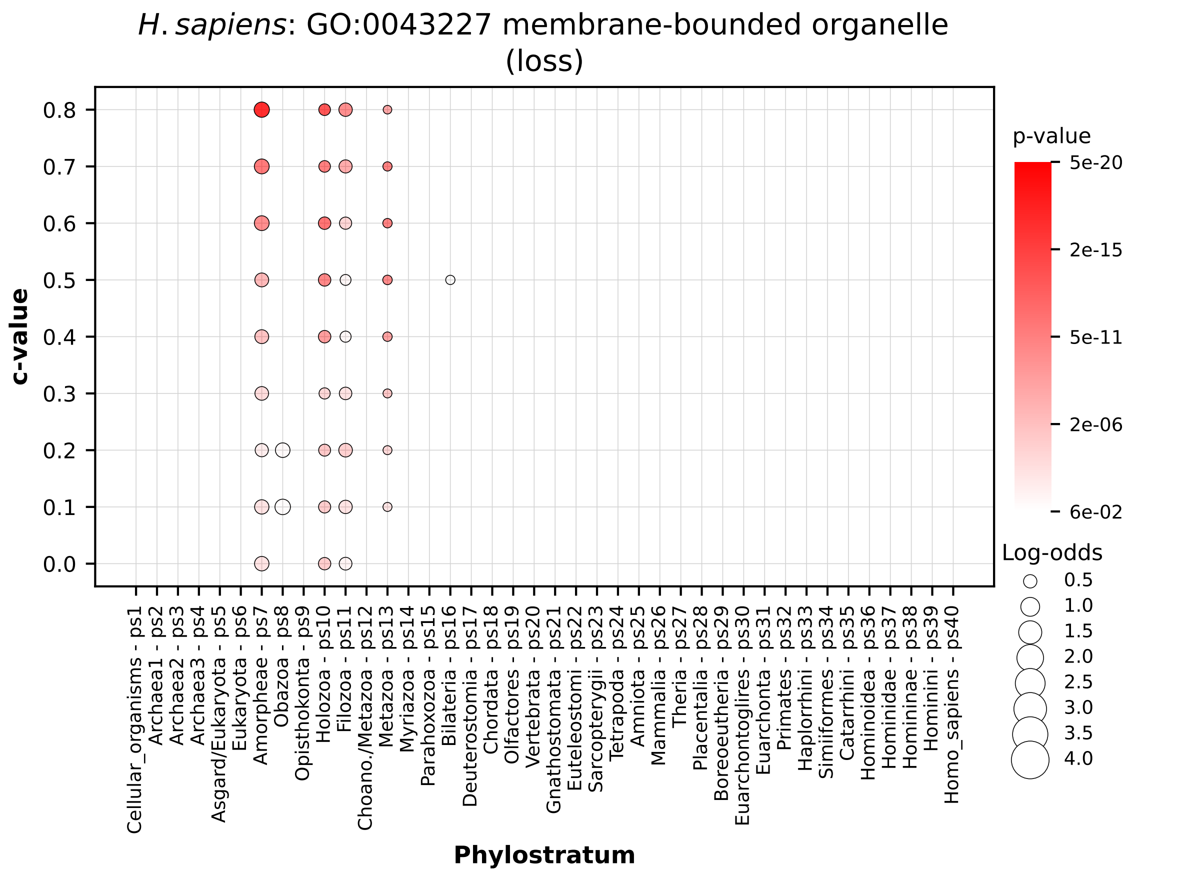
H. sapiens: GO:0043227 membrane-bounded organelle
(loss)
c-value
0.8
0.7
0.6
0.5
0.4
0.3
0.2
0.1
0.0
p-value
5e-20
2e-15
5e-11
2e-06
6e-02
Log-odds
0.5
1.0
1.5
2.0
2.5
3.0
3.5
4.0
Cellular_organisms - ps1
Archaea1 - ps2
Archaea2 - ps3
Archaea3 - ps4
Asgard/Eukaryota - ps5
Eukaryota - ps6
Amorpheae - ps7
Obazoa - ps8
Opisthokonta - ps9
Holozoa - ps10
Filozoa - ps11
Choano./Metazoa - ps12
Metazoa - ps13
Myriazoa - ps14
Parahoxozoa - ps15
Bilateria - ps16
Deuterostomia - ps17
Chordata - ps18
Olfactores - ps19
Vertebrata - ps20
Gnathostomata - ps21
Euteleostomi - ps22
Sarcopterygii - ps23
Tetrapoda - ps24
Amniota - ps25
Mammalia - ps26
Theria - ps27
Placentalia - ps28
Boreoeutheria - ps29
Euarchontoglires - ps30
Euarchonta - ps31
Primates - ps32
Haplorrhini - ps33
Simiiformes - ps34
Catarrhini - ps35
Hominoidea - ps36
Hominidae - ps37
Homininae - ps38
Hominini - ps39
Homo_sapiens - ps40
Phylostratum

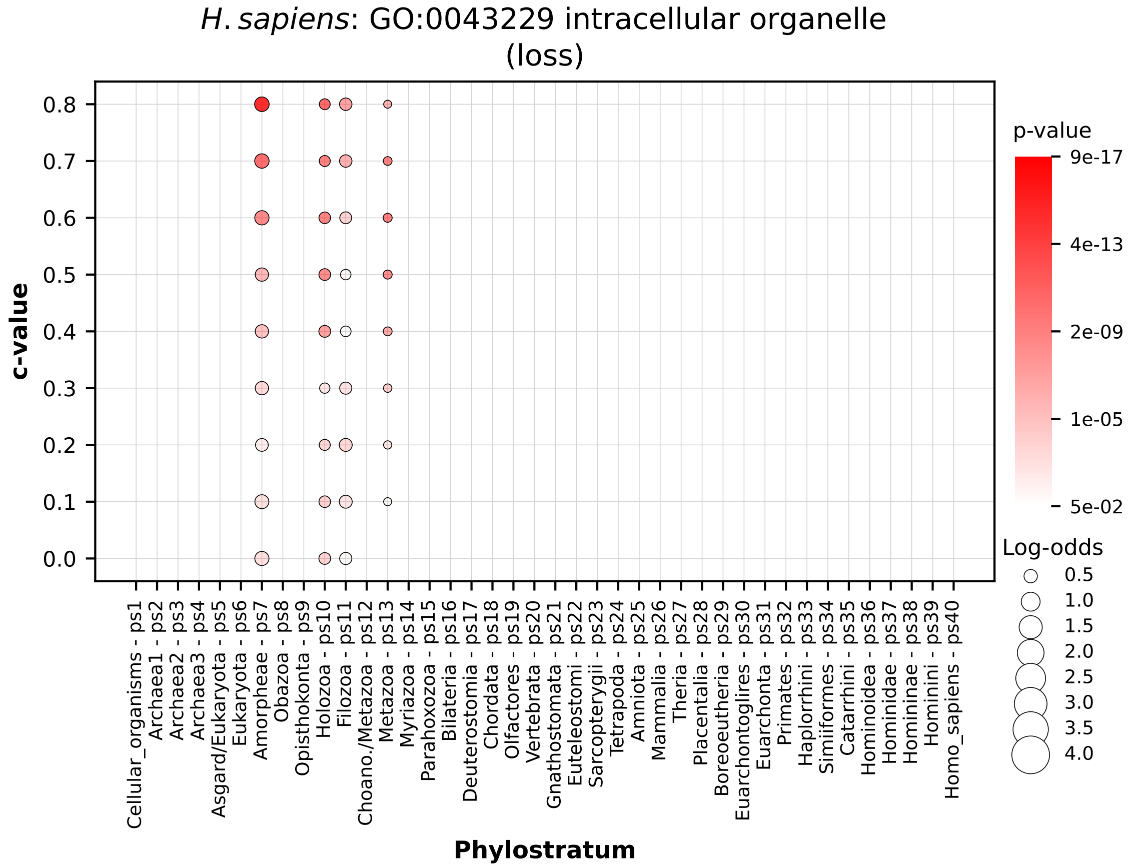

H. sapiens: GO:0043229 intracellular organelle
(loss)
c-value
0.8
0.7
0.6
0.5
0.4
0.3
0.2
0.1
0.0
Phylostratum
Cellular_organisms - ps1
Archaea1 - ps2
Archaea2 - ps3
Archaea3 - ps4
Asgard/Eukaryota - ps5
Eukaryota - ps6
Amorpheae - ps7
Obazoa - ps8
Opisthokonta - ps9
Holozoa - ps10
Filozoa - ps11
Choano./Metazoa - ps12
Metazoa - ps13
Myriazoa - ps14
Parahoxozoa - ps15
Bilateria - ps16
Deuterostomia - ps17
Chordata - ps18
Olfactores - ps19
Vertebrata - ps20
Gnathostomata - ps21
Euteleostomi - ps22
Sarcopterygii - ps23
Tetrapoda - ps24
Amniota - ps25
Mammalia - ps26
Theria - ps27
Placentalia - ps28
Boreoeutheria - ps29
Euarchontoglires - ps30
Euarchonta - ps31
Primates - ps32
Haplorrhini - ps33
Simiiformes - ps34
Catarrhini - ps35
Hominoidea - ps36
Hominidae - ps37
Homininae - ps38
Hominini - ps39
Homo_sapiens - ps40
p-value
9e-17
4e-13
2e-09
1e-05
5e-02
Log-odds
0.5
1.0
1.5
2.0
2.5
3.0
3.5
4.0

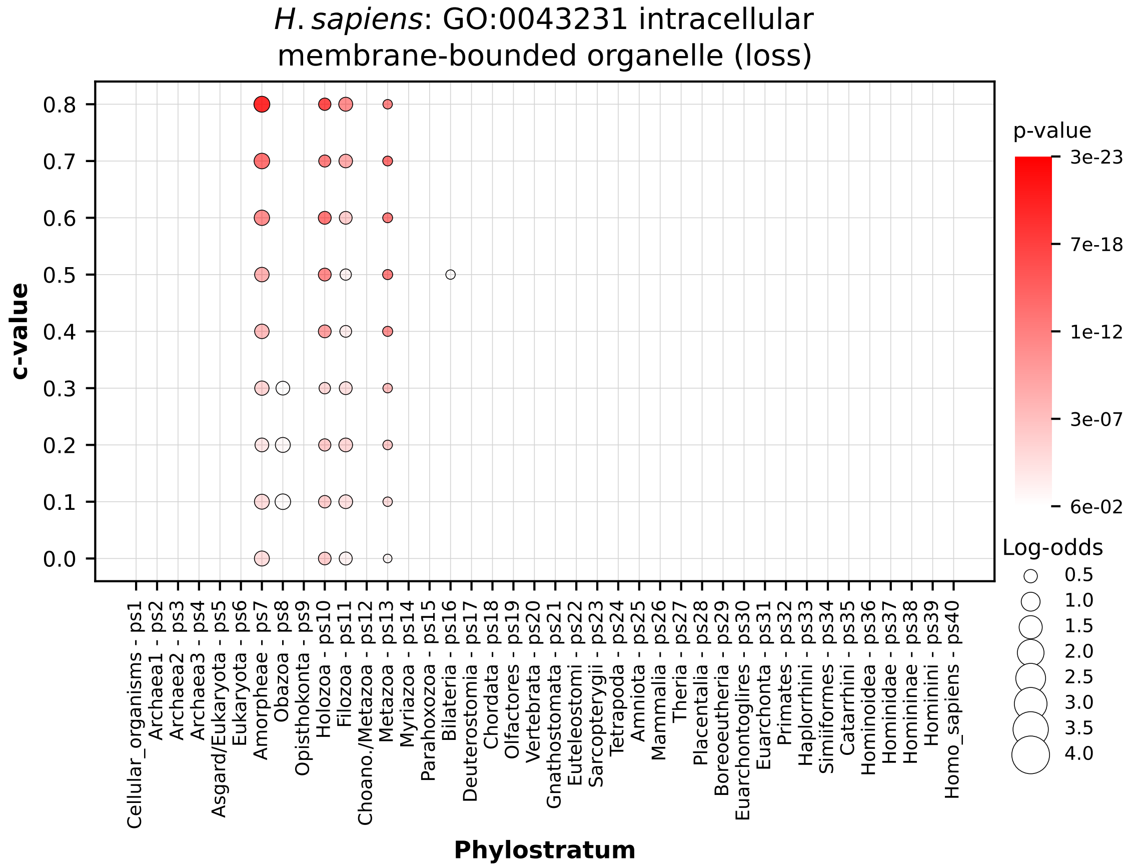

H. sapiens: GO:0043231 intracellular membrane-bounded organelle (loss)
c-value
0.8
0.7
0.6
0.5
0.4
0.3
0.2
0.1
0.0
Phylostratum
Cellular_organisms - ps1
Archaea1 - ps2
Archaea2 - ps3
Archaea3 - ps4
Asgard/Eukaryota - ps5
Eukaryota - ps6
Amorpheae - ps7
Obazoa - ps8
Opisthokonta - ps9
Holozoa - ps10
Filozoa - ps11
Choano./Metazoa - ps12
Metazoa - ps13
Myriazoa - ps14
Parahoxozoa - ps15
Bilateria - ps16
Deuterostomia - ps17
Chordata - ps18
Olfactores - ps19
Vertebrata - ps20
Gnathostomata - ps21
Euteleostomi - ps22
Sarcopterygii - ps23
Tetrapoda - ps24
Amniota - ps25
Mammalia - ps26
Theria - ps27
Placentalia - ps28
Boreoeutheria - ps29
Euarchontoglires - ps30
Euarchonta - ps31
Primates - ps32
Haplorrhini - ps33
Simiiformes - ps34
Catarrhini - ps35
Hominoidea - ps36
Hominidae - ps37
Homininae - ps38
Hominini - ps39
Homo_sapiens - ps40
p-value
3e-23
7e-18
1e-12
3e-07
6e-02
Log-odds
0.5
1.0
1.5
2.0
2.5
3.0
3.5
4.0

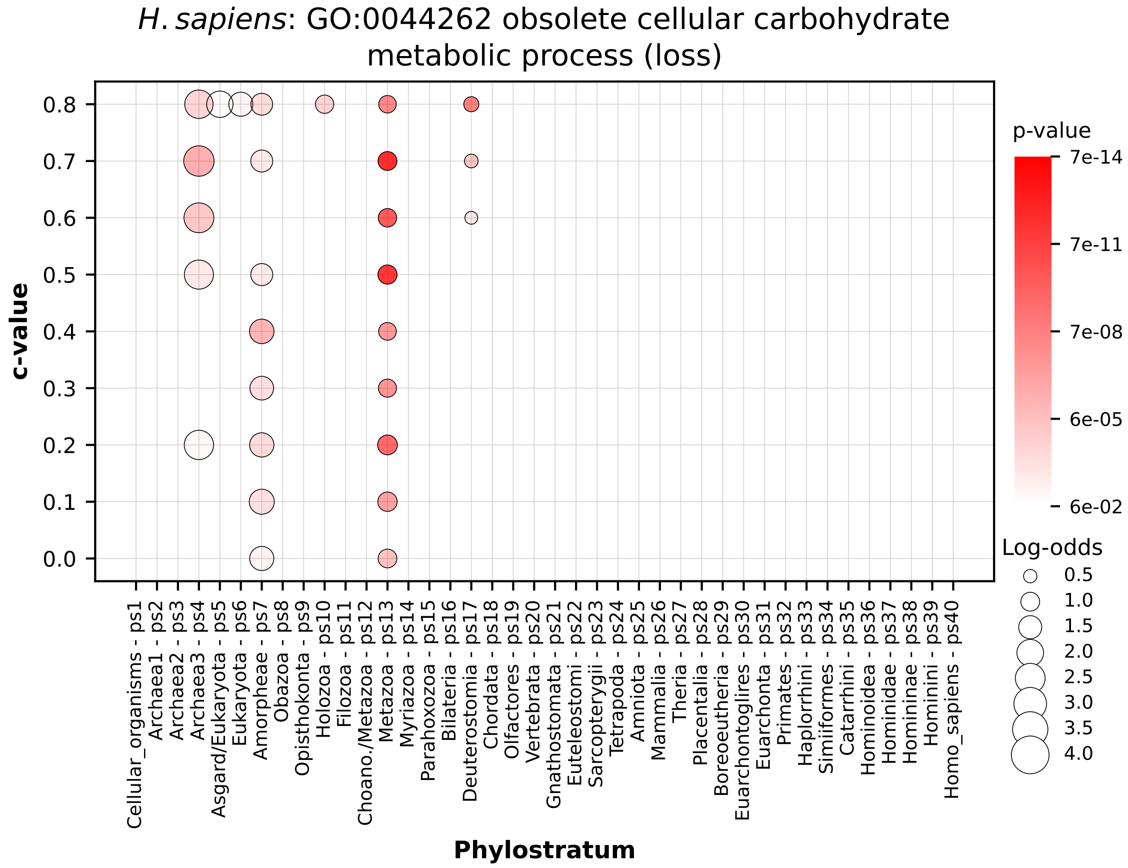

H. sapiens: GO:0044262 obsolete cellular carbohydrate metabolic process (loss)
c-value
0.8
0.7
0.6
0.5
0.4
0.3
0.2
0.1
0.0
Phylostratum
Cellular_organisms - ps1
Archaea1 - ps2
Archaea2 - ps3
Archaea3 - ps4
Asgard/Eukaryota - ps5
Eukaryota - ps6
Amorpheae - ps7
Obazoa - ps8
Opisthokonta - ps9
Holozoa - ps10
Filozoa - ps11
Choano./Metazoa - ps12
Metazoa - ps13
Myriazoa - ps14
Parahoxozoa - ps15
Bilateria - ps16
Deuterostomia - ps17
Chordata - ps18
Olfactores - ps19
Vertebrata - ps20
Gnathostomata - ps21
Euteleostomi - ps22
Sarcopterygii - ps23
Tetrapoda - ps24
Amniota - ps25
Mammalia - ps26
Theria - ps27
Placentalia - ps28
Boreoeutheria - ps29
Euarchontoglires - ps30
Euarchonta - ps31
Primates - ps32
Haplorrhini - ps33
Simiiformes - ps34
Catarrhini - ps35
Hominoidea - ps36
Hominidae - ps37
Homininae - ps38
Hominini - ps39
Homo_sapiens - ps40
p-value
7e-14
7e-11
7e-08
6e-05
6e-02
Log-odds
0.5
1.0
1.5
2.0
2.5
3.0
3.5
4.0

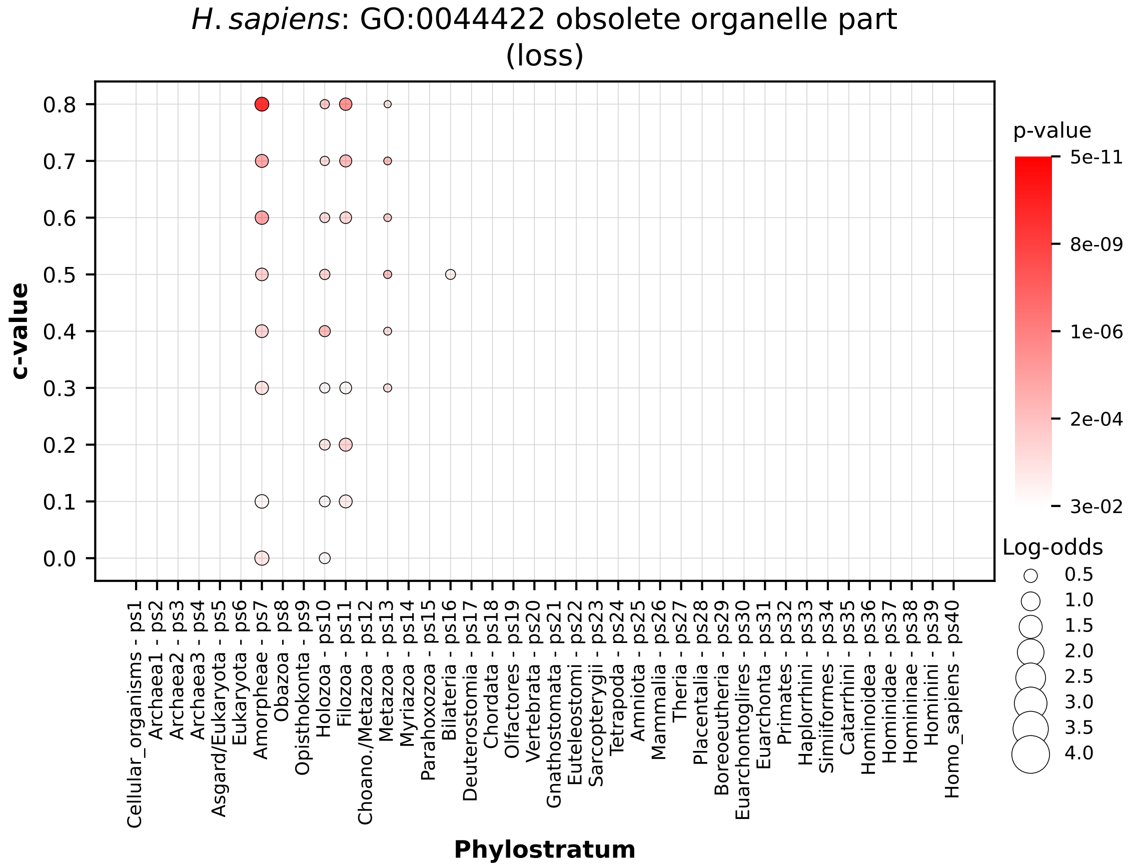

H. sapiens: GO:0044422 obsolete organelle part
(loss)
c-value
0.8
0.7
0.6
0.5
0.4
0.3
0.2
0.1
0.0
p-value
5e-11
8e-09
1e-06
2e-04
3e-02
Log-odds
0.5
1.0
1.5
2.0
2.5
3.0
3.5
4.0
Cellular_organisms - ps1
Archaea1 - ps2
Archaea2 - ps3
Archaea3 - ps4
Asgard/Eukaryota - ps5
Eukaryota - ps6
Amorpheae - ps7
Obazoa - ps8
Opisthokonta - ps9
Holozoa - ps10
Filozoa - ps11
Choano./Metazoa - ps12
Metazoa - ps13
Myriazoa - ps14
Parahoxozoa - ps15
Bilateria - ps16
Deuterostomia - ps17
Chordata - ps18
Olfactores - ps19
Vertebrata - ps20
Gnathostomata - ps21
Euteleostomi - ps22
Sarcopterygii - ps23
Tetrapoda - ps24
Amniota - ps25
Mammalia - ps26
Theria - ps27
Placentalia - ps28
Boreoeutheria - ps29
Euarchontoglires - ps30
Euarchonta - ps31
Primates - ps32
Haplorrhini - ps33
Simiiformes - ps34
Catarrhini - ps35
Hominoidea - ps36
Hominidae - ps37
Homininae - ps38
Hominini - ps39
Homo_sapiens - ps40
Phylostratum

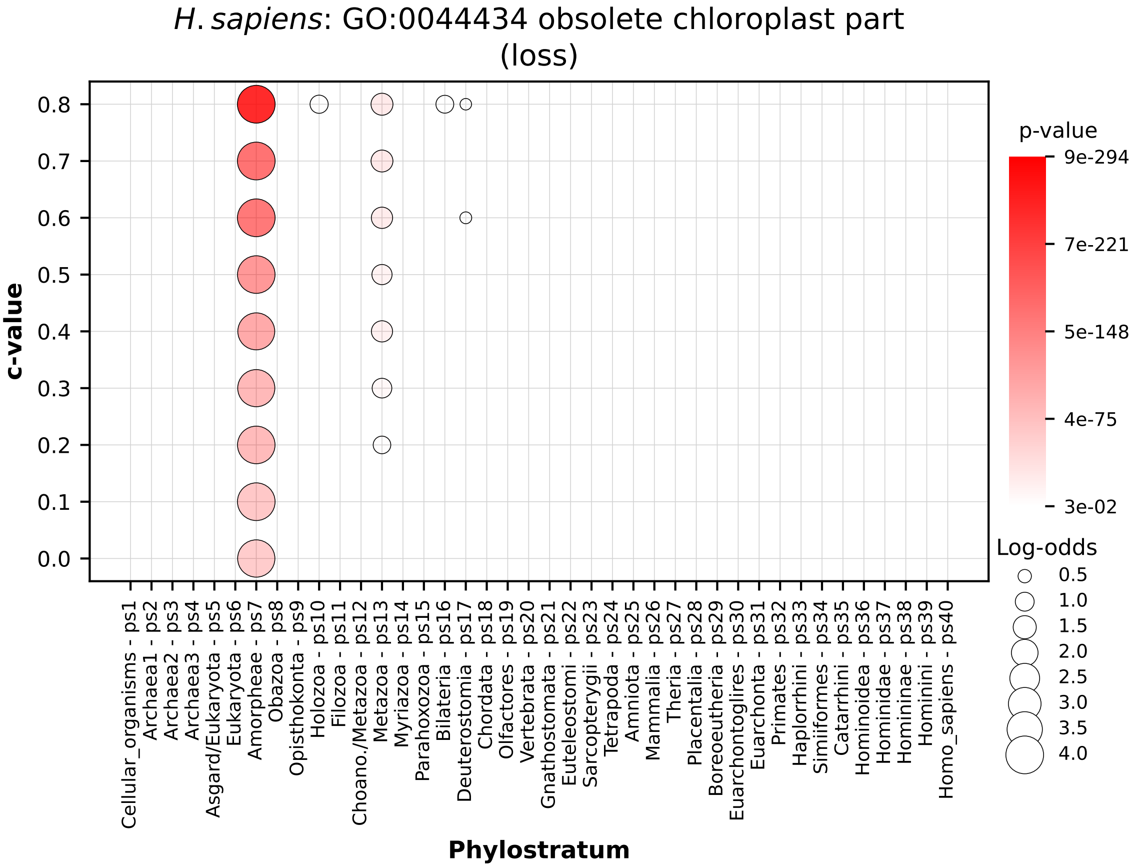

H. sapiens: GO:0044434 obsolete chloroplast part (loss)
c-value
0.0
0.1
0.2
0.3
0.4
0.5
0.6
0.7
0.8
Phylostratum
Cellular_organisms - ps1
Archaea1 - ps2
Archaea2 - ps3
Archaea3 - ps4
Asgard/Eukaryota - ps5
Eukaryota - ps6
Amorpheae - ps7
Obazoa - ps8
Opisthokonta - ps9
Holozoa - ps10
Filozoa - ps11
Choano./Metazoa - ps12
Metazoa - ps13
Myriazoa - ps14
Parahoxozoa - ps15
Bilateria - ps16
Deuterostomia - ps17
Chordata - ps18
Olfactores - ps19
Vertebrata - ps20
Gnathostomata - ps21
Euteleostomi - ps22
Sarcopterygii - ps23
Tetrapoda - ps24
Amniota - ps25
Mammalia - ps26
Theria - ps27
Placentalia - ps28
Boreoeutheria - ps29
Euarchontoglires - ps30
Euarchonta - ps31
Primates - ps32
Haplorrhini - ps33
Simiiformes - ps34
Catarrhini - ps35
Hominoidea - ps36
Hominidae - ps37
Homininae - ps38
Hominini - ps39
Homo_sapiens - ps40
p-value
9e-294
7e-221
5e-148
4e-75
3e-02
Log-odds
0.5
1.0
1.5
2.0
2.5
3.0
3.5
4.0

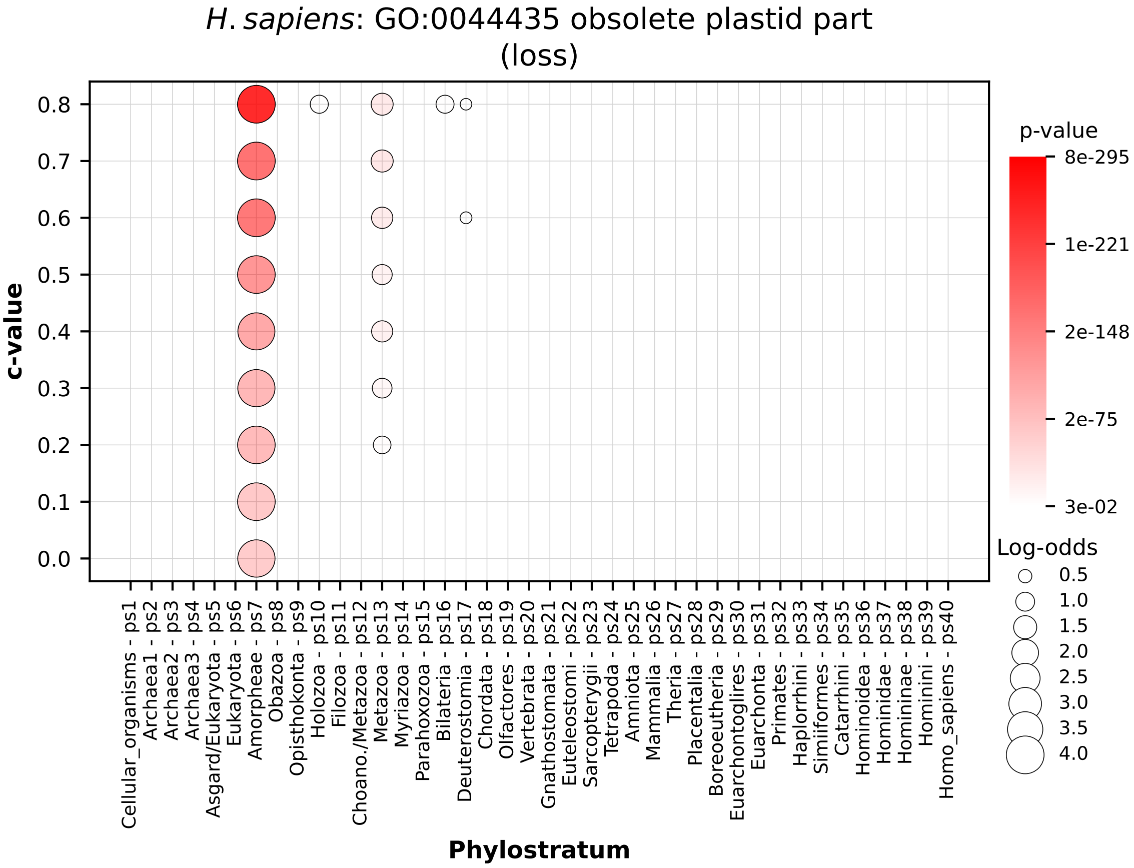

H. sapiens: GO:0044435 obsolete plastid part
(loss)
c-value
0.8
0.7
0.6
0.5
0.4
0.3
0.2
0.1
0.0
p-value
8e-295
1e-221
2e-148
2e-75
3e-02
Log-odds
0.5
1.0
1.5
2.0
2.5
3.0
3.5
4.0
Cellular_organisms - ps1
Archaea1 - ps2
Archaea2 - ps3
Archaea3 - ps4
Asgard/Eukaryota - ps5
Eukaryota - ps6
Amorpheae - ps7
Obazoa - ps8
Opisthokonta - ps9
Holozoa - ps10
Filozoa - ps11
Choano./Metazoa - ps12
Metazoa - ps13
Myriazoa - ps14
Parahoxozoa - ps15
Bilateria - ps16
Deuterostomia - ps17
Chordata - ps18
Olfactores - ps19
Vertebrata - ps20
Gnathostomata - ps21
Euteleostomi - ps22
Sarcopterygii - ps23
Tetrapoda - ps24
Amniota - ps25
Mammalia - ps26
Theria - ps27
Placentalia - ps28
Boreoeutheria - ps29
Euarchontoglires - ps30
Euarchonta - ps31
Primates - ps32
Haplorrhini - ps33
Simiiformes - ps34
Catarrhini - ps35
Hominoidea - ps36
Hominidae - ps37
Homininae - ps38
Hominini - ps39
Homo_sapiens - ps40
Phylostratum

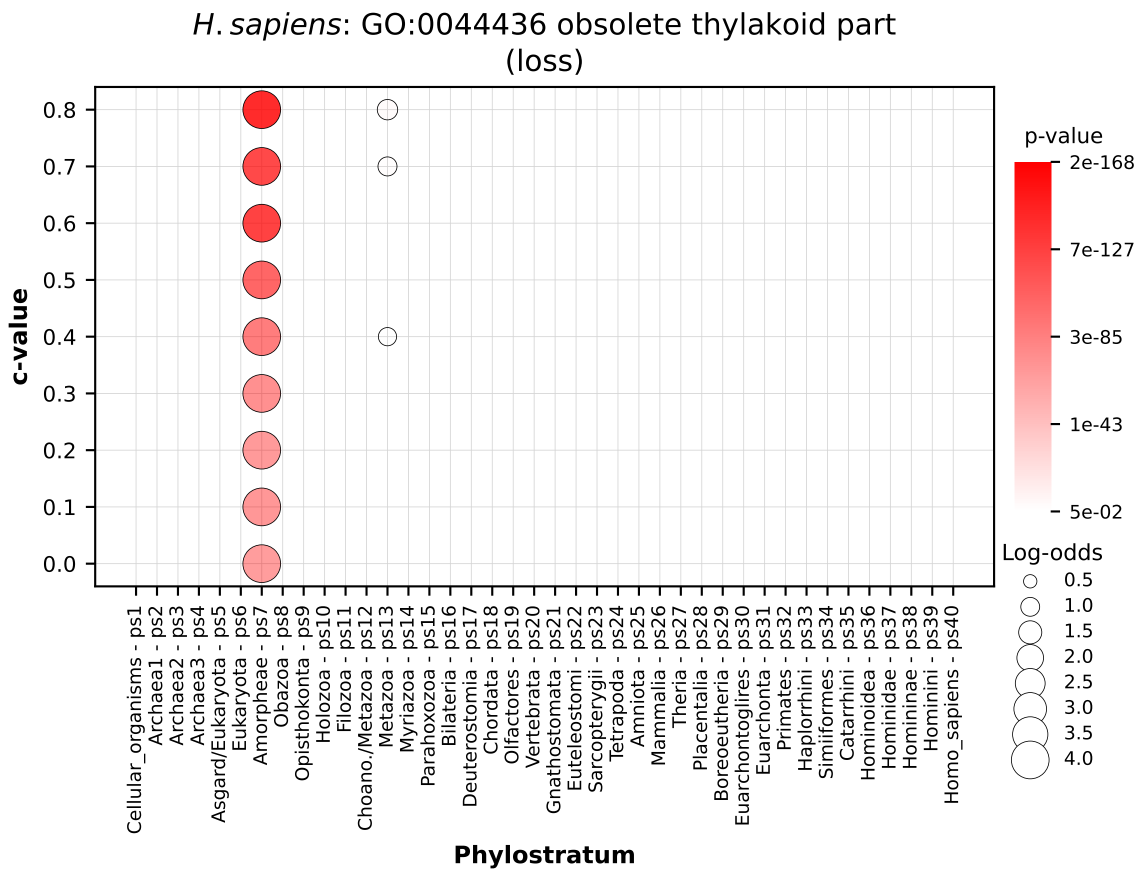

H. sapiens: GO:0044436 obsolete thylakoid part
(loss)
c-value
0.8
0.7
0.6
0.5
0.4
0.3
0.2
0.1
0.0
p-value
2e-168
7e-127
3e-85
1e-43
5e-02
Log-odds
0.5
1.0
1.5
2.0
2.5
3.0
3.5
4.0
Cellular_organisms - ps1
Archaea1 - ps2
Archaea2 - ps3
Archaea3 - ps4
Asgard/Eukaryota - ps5
Eukaryota - ps6
Amorpheae - ps7
Obazoa - ps8
Opisthokonta - ps9
Holozoa - ps10
Filozoa - ps11
Choano./Metazoa - ps12
Metazoa - ps13
Myriazoa - ps14
Parahoxozoa - ps15
Bilateria - ps16
Deuterostomia - ps17
Chordata - ps18
Olfactores - ps19
Vertebrata - ps20
Gnathostomata - ps21
Euteleostomi - ps22
Sarcopterygii - ps23
Tetrapoda - ps24
Amniota - ps25
Mammalia - ps26
Theria - ps27
Placentalia - ps28
Boreoeutheria - ps29
Euarchontoglires - ps30
Euarchonta - ps31
Primates - ps32
Haplorrhini - ps33
Simiiformes - ps34
Catarrhini - ps35
Hominoidea - ps36
Hominidae - ps37
Homininae - ps38
Hominini - ps39
Homo_sapiens - ps40
Phylostratum

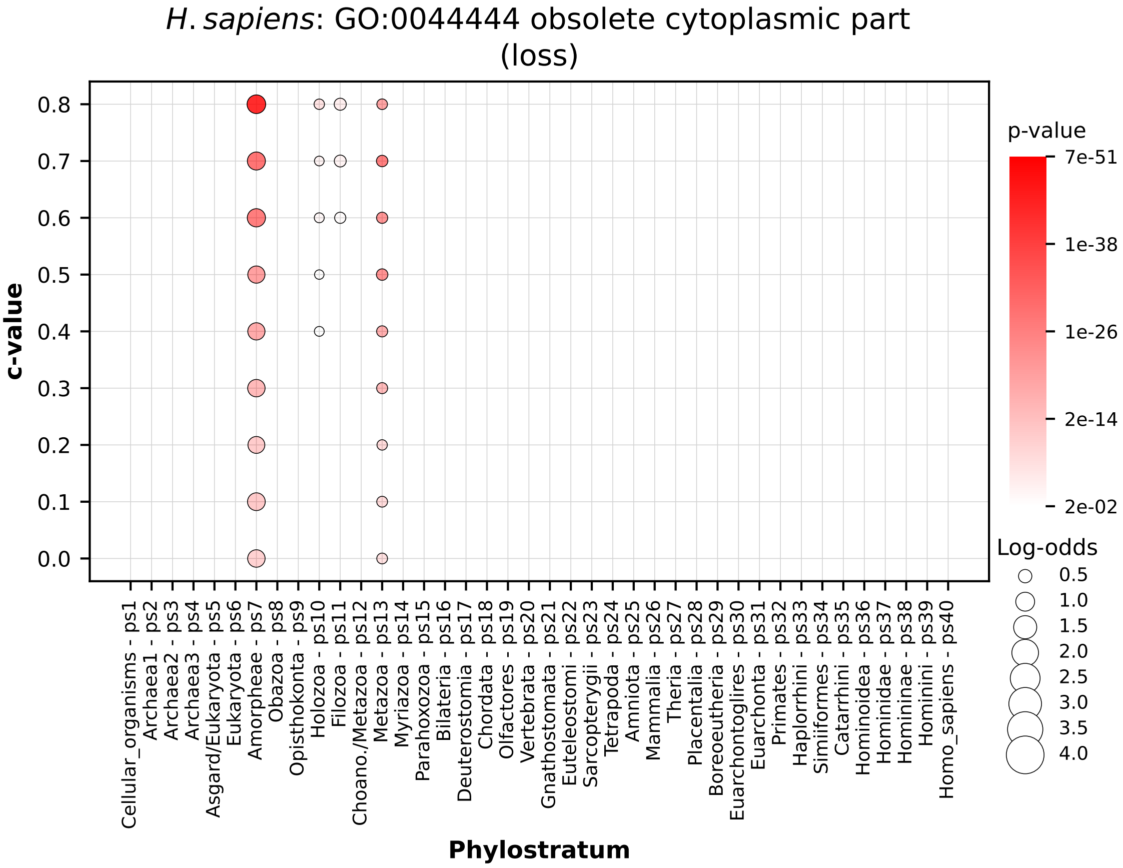

H. sapiens: GO:0044444 obsolete cytoplasmic part
(loss)
c-value
0.8
0.7
0.6
0.5
0.4
0.3
0.2
0.1
0.0
p-value
7e-51
1e-38
1e-26
2e-14
2e-02
Log-odds
0.5
1.0
1.5
2.0
2.5
3.0
3.5
4.0
Cellular_organisms - ps1
Archaea1 - ps2
Archaea2 - ps3
Archaea3 - ps4
Asgard/Eukaryota - ps5
Eukaryota - ps6
Amorpheae - ps7
Obazoa - ps8
Opisthokonta - ps9
Holozoa - ps10
Filozoa - ps11
Choano./Metazoa - ps12
Metazoa - ps13
Myriazoa - ps14
Parahoxozoa - ps15
Bilateria - ps16
Deuterostomia - ps17
Chordata - ps18
Olfactores - ps19
Vertebrata - ps20
Gnathostomata - ps21
Euteleostomi - ps22
Sarcopterygii - ps23
Tetrapoda - ps24
Amniota - ps25
Mammalia - ps26
Theria - ps27
Placentalia - ps28
Boreoeutheria - ps29
Euarchontoglires - ps30
Euarchonta - ps31
Primates - ps32
Haplorrhini - ps33
Simiiformes - ps34
Catarrhini - ps35
Hominoidea - ps36
Hominidae - ps37
Homininae - ps38
Hominini - ps39
Homo_sapiens - ps40
Phylostratum

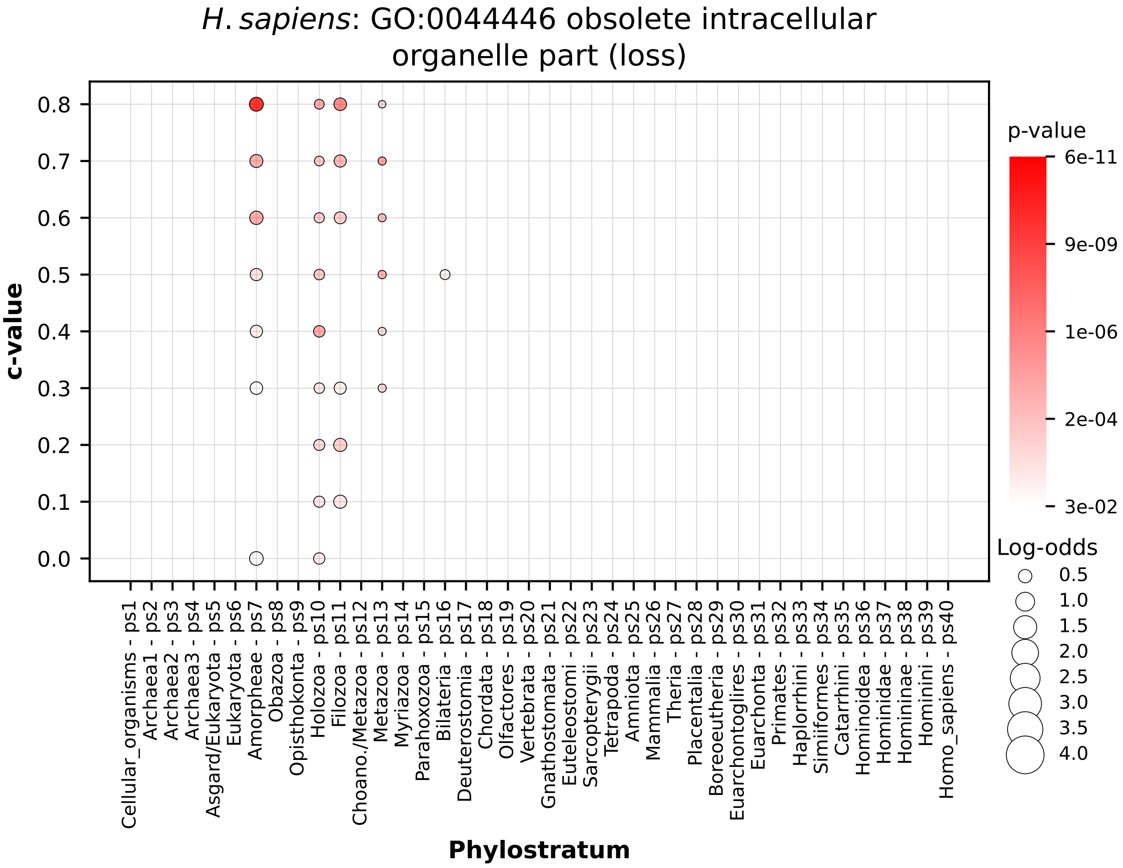

H. sapiens: GO:0044446 obsolete intracellular organelle part (loss)
c-value
0.8
0.7
0.6
0.5
0.4
0.3
0.2
0.1
0.0
p-value
6e-11
9e-09
1e-06
2e-04
3e-02
Log-odds
0.5
1.0
1.5
2.0
2.5
3.0
3.5
4.0
Cellular_organisms - ps1
Archaea1 - ps2
Archaea2 - ps3
Archaea3 - ps4
Asgard/Eukaryota - ps5
Eukaryota - ps6
Amorpheae - ps7
Obazoa - ps8
Opisthokonta - ps9
Holozoa - ps10
Filozoa - ps11
Choano./Metazoa - ps12
Metazoa - ps13
Myriazoa - ps14
Parahoxozoa - ps15
Bilateria - ps16
Deuterostomia - ps17
Chordata - ps18
Olfactores - ps19
Vertebrata - ps20
Gnathostomata - ps21
Euteleostomi - ps22
Sarcopterygii - ps23
Tetrapoda - ps24
Amniota - ps25
Mammalia - ps26
Theria - ps27
Placentalia - ps28
Boreoeutheria - ps29
Euarchontoglires - ps30
Euarchonta - ps31
Primates - ps32
Haplorrhini - ps33
Simiiformes - ps34
Catarrhini - ps35
Hominoidea - ps36
Hominidae - ps37
Homininae - ps38
Hominini - ps39
Homo_sapiens - ps40
Phylostratum

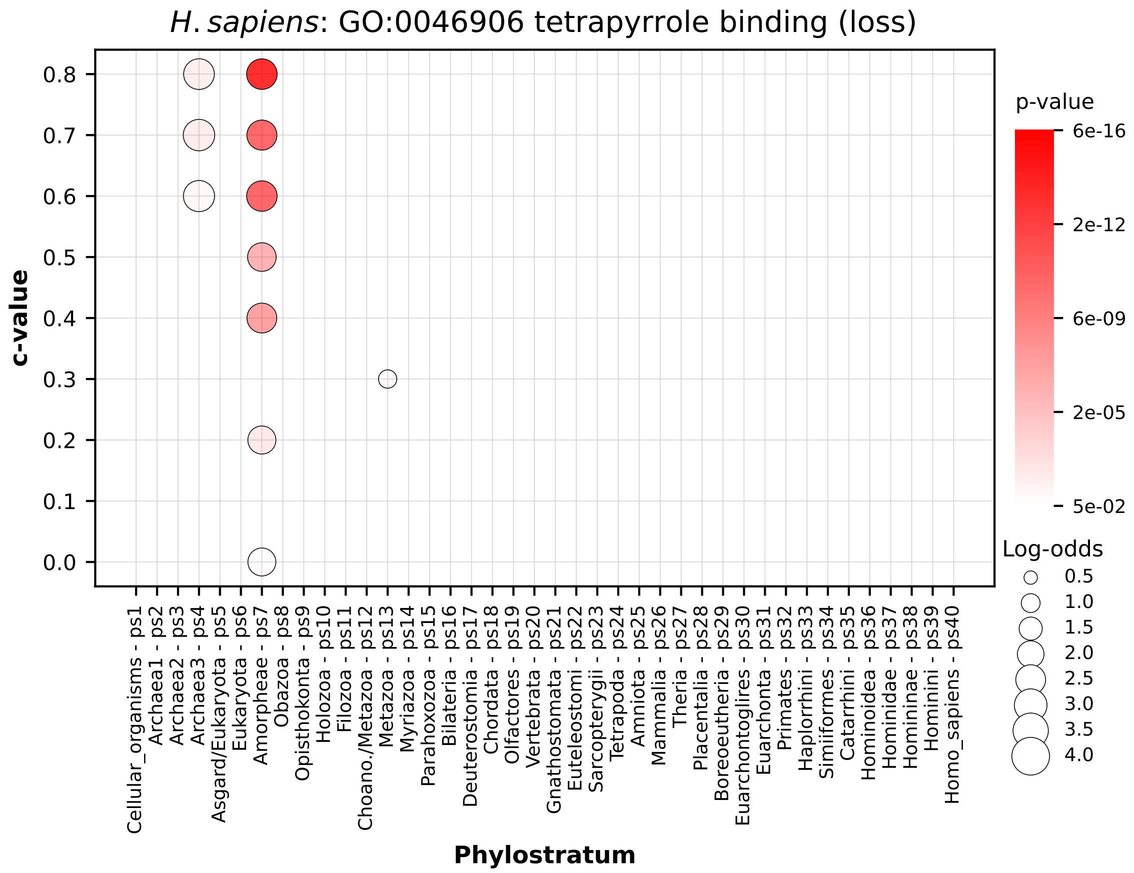

H. sapiens: GO:0046906 tetrapyrrole binding (loss)
c-value
0.8
0.7
0.6
0.5
0.4
0.3
0.2
0.1
0.0
Phylostratum
Cellular_organisms - ps1
Archaea1 - ps2
Archaea2 - ps3
Archaea3 - ps4
Asgard/Eukaryota - ps5
Eukaryota - ps6
Amorpheae - ps7
Obazoa - ps8
Opisthokonta - ps9
Holozoa - ps10
Filozoa - ps11
Choano./Metazoa - ps12
Metazoa - ps13
Myriazoa - ps14
Parahoxozoa - ps15
Bilateria - ps16
Deuterostomia - ps17
Chordata - ps18
Olfactores - ps19
Vertebrata - ps20
Gnathostomata - ps21
Euteleostomi - ps22
Sarcopterygii - ps23
Tetrapoda - ps24
Amniota - ps25
Mammalia - ps26
Theria - ps27
Placentalia - ps28
Boreoeutheria - ps29
Euarchontoglires - ps30
Euarchonta - ps31
Primates - ps32
Haplorrhini - ps33
Simiiformes - ps34
Catarrhini - ps35
Hominoidea - ps36
Hominidae - ps37
Homininae - ps38
Hominini - ps39
Homo_sapiens - ps40
p-value
6e-16
2e-12
6e-09
2e-05
5e-02
Log-odds
0.5
1.0
1.5
2.0
2.5
3.0
3.5
4.0

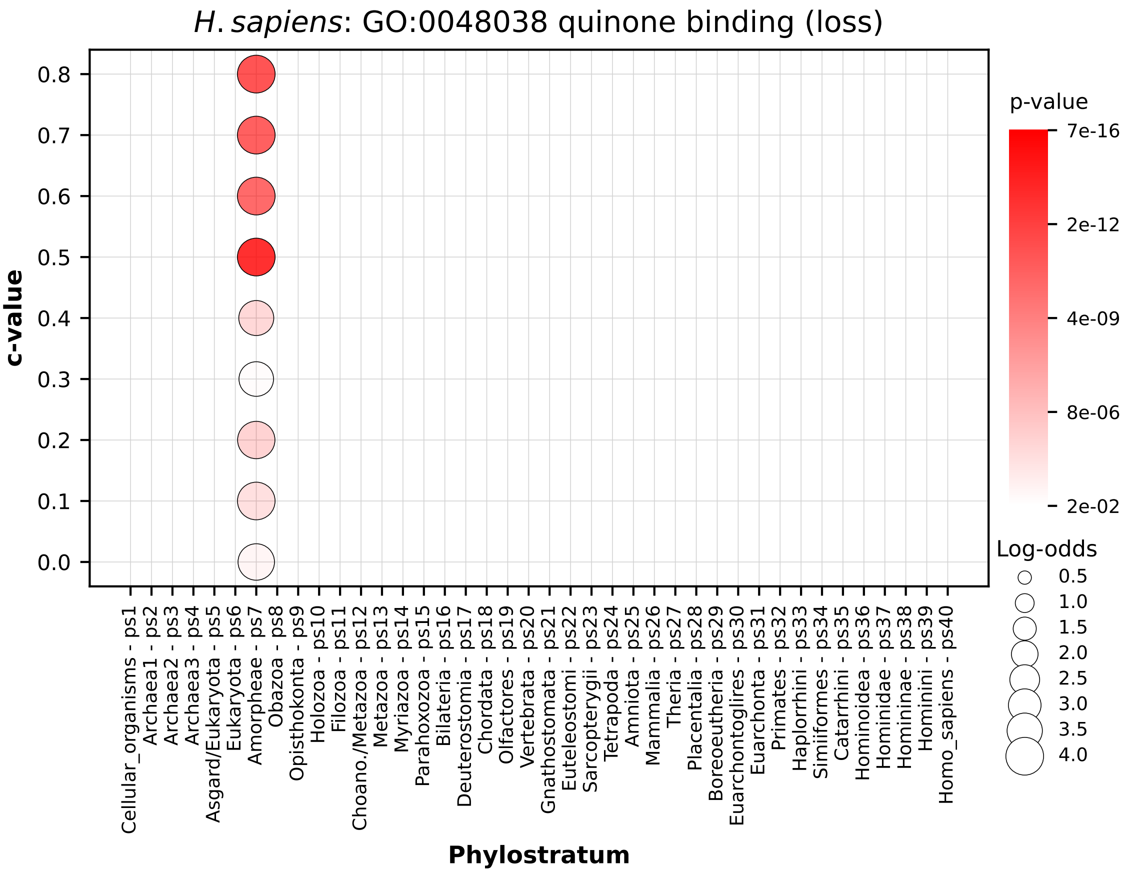

H. sapiens: GO:0048038 quinone binding (loss)
c-value
0.0
0.1
0.2
0.3
0.4
0.5
0.6
0.7
0.8
p-value
7e-16
2e-12
4e-09
8e-06
2e-02
Log-odds
0.5
1.0
1.5
2.0
2.5
3.0
3.5
4.0
Cellular_organisms - ps1
Archaea1 - ps2
Archaea2 - ps3
Archaea3 - ps4
Asgard/Eukaryota - ps5
Eukaryota - ps6
Amorpheae - ps7
Obazoa - ps8
Opisthokonta - ps9
Holozoa - ps10
Filozoa - ps11
Choano./Metazoa - ps12
Metazoa - ps13
Myriazoa - ps14
Parahoxozoa - ps15
Bilateria - ps16
Deuterostomia - ps17
Chordata - ps18
Olfactores - ps19
Vertebrata - ps20
Gnathostomata - ps21
Euteleostomi - ps22
Sarcopterygii - ps23
Tetrapoda - ps24
Amniota - ps25
Mammalia - ps26
Theria - ps27
Placentalia - ps28
Boreoeutheria - ps29
Euarchontoglires - ps30
Euarchonta - ps31
Primates - ps32
Haplorrhini - ps33
Simiiformes - ps34
Catarrhini - ps35
Hominoidea - ps36
Hominidae - ps37
Homininae - ps38
Hominini - ps39
Homo_sapiens - ps40
Phylostratum

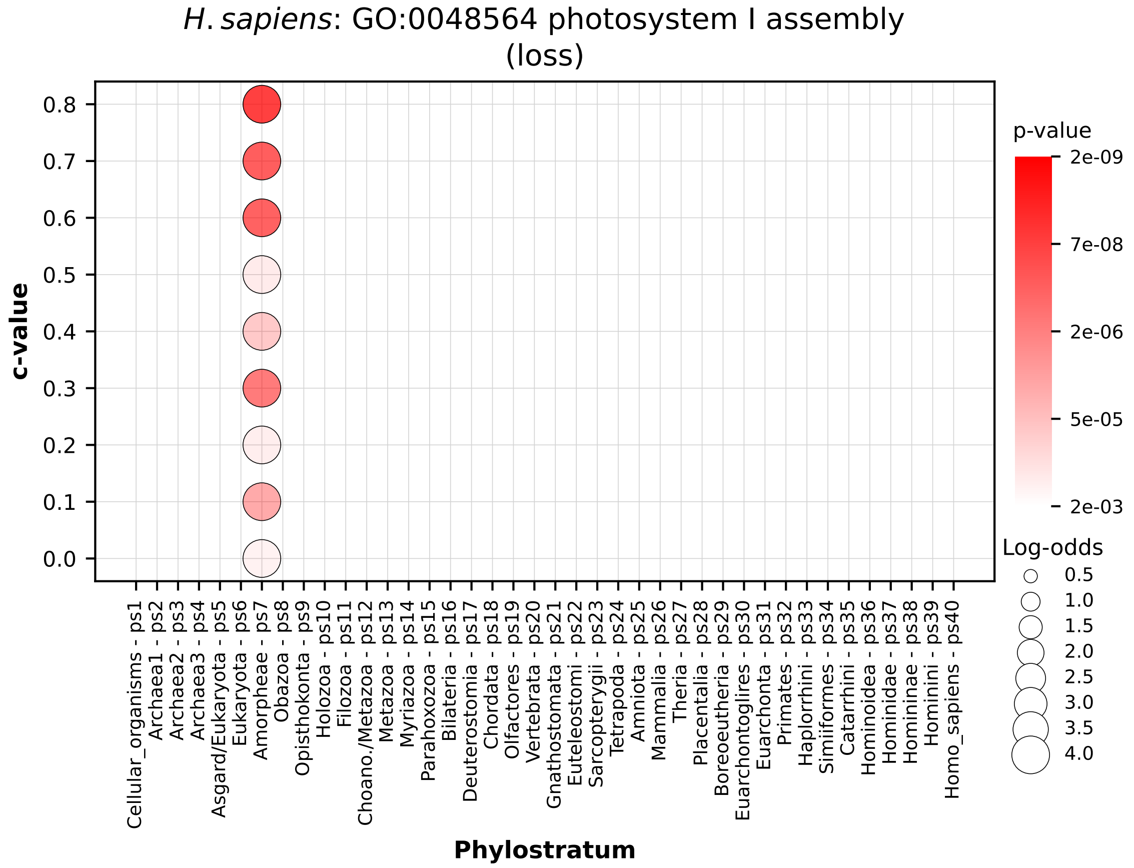

H. sapiens: GO:0048564 photosystem I assembly
(loss)
c-value
0.8
0.7
0.6
0.5
0.4
0.3
0.2
0.1
0.0
p-value
2e-09
7e-08
2e-06
5e-05
2e-03
Log-odds
0.5
1.0
1.5
2.0
2.5
3.0
3.5
4.0
Cellular_organisms - ps1
Archaea1 - ps2
Archaea2 - ps3
Archaea3 - ps4
Asgard/Eukaryota - ps5
Eukaryota - ps6
Amorpheae - ps7
Obazoa - ps8
Opisthokonta - ps9
Holozoa - ps10
Filozoa - ps11
Choano./Metazoa - ps12
Metazoa - ps13
Myriazoa - ps14
Parahoxozoa - ps15
Bilateria - ps16
Deuterostomia - ps17
Chordata - ps18
Olfactores - ps19
Vertebrata - ps20
Gnathostomata - ps21
Euteleostomi - ps22
Sarcopterygii - ps23
Tetrapoda - ps24
Amniota - ps25
Mammalia - ps26
Theria - ps27
Placentalia - ps28
Boreoeutheria - ps29
Euarchontoglires - ps30
Euarchonta - ps31
Primates - ps32
Haplorrhini - ps33
Simiiformes - ps34
Catarrhini - ps35
Hominoidea - ps36
Hominidae - ps37
Homininae - ps38
Hominini - ps39
Homo_sapiens - ps40
Phylostratum

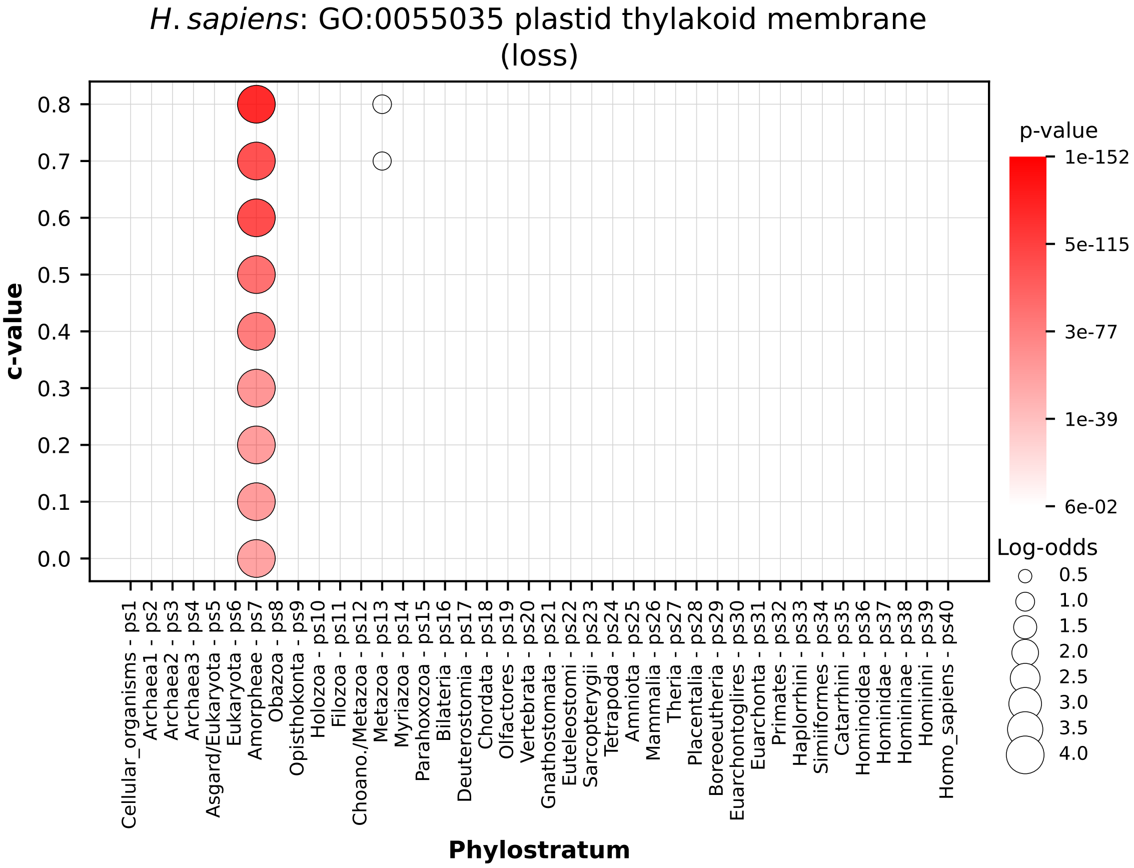

H. sapiens: GO:0055035 plastid thylakoid membrane
(loss)
c-value
0.0
0.1
0.2
0.3
0.4
0.5
0.6
0.7
0.8
p-value
1e-152
5e-115
3e-77
1e-39
6e-02
Log-odds
0.5
1.0
1.5
2.0
2.5
3.0
3.5
4.0
Cellular_organisms - ps1
Archaea1 - ps2
Archaea2 - ps3
Archaea3 - ps4
Asgard/Eukaryota - ps5
Eukaryota - ps6
Amorpheae - ps7
Obazoa - ps8
Opisthokonta - ps9
Holozoa - ps10
Filozoa - ps11
Choano./Metazoa - ps12
Metazoa - ps13
Myriazoa - ps14
Parahoxozoa - ps15
Bilateria - ps16
Deuterostomia - ps17
Chordata - ps18
Olfactores - ps19
Vertebrata - ps20
Gnathostomata - ps21
Euteleostomi - ps22
Sarcopterygii - ps23
Tetrapoda - ps24
Amniota - ps25
Mammalia - ps26
Theria - ps27
Placentalia - ps28
Boreoeutheria - ps29
Euarchontoglires - ps30
Euarchonta - ps31
Primates - ps32
Haplorrhini - ps33
Simiiformes - ps34
Catarrhini - ps35
Hominoidea - ps36
Hominidae - ps37
Homininae - ps38
Hominini - ps39
Homo_sapiens - ps40
Phylostratum

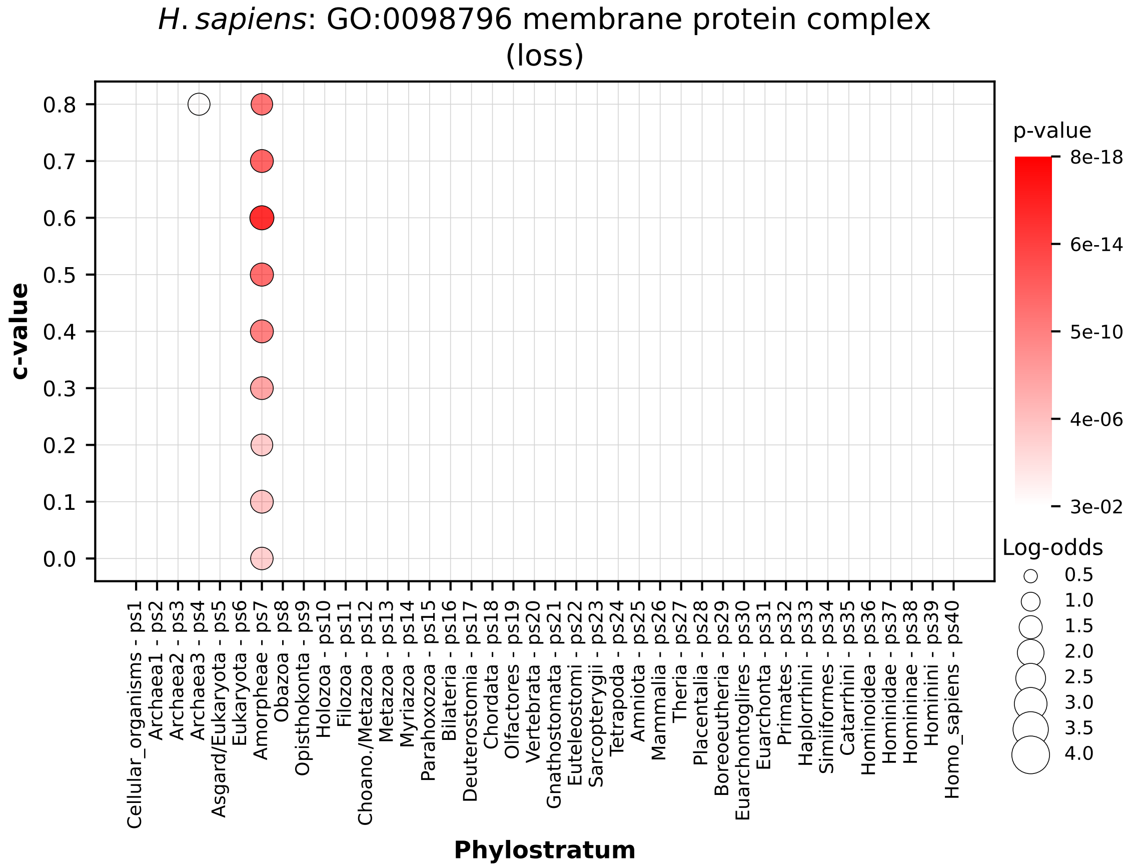

H. sapiens: GO:0098796 membrane protein complex (loss)
c-value
0.8
0.7
0.6
0.5
0.4
0.3
0.2
0.1
0.0
p-value
8e-18
6e-14
5e-10
4e-06
3e-02
Log-odds
0.5
1.0
1.5
2.0
2.5
3.0
3.5
4.0
Cellular_organisms - ps1
Archaea1 - ps2
Archaea2 - ps3
Archaea3 - ps4
Asgard/Eukaryota - ps5
Eukaryota - ps6
Amorpheae - ps7
Obazoa - ps8
Opisthokonta - ps9
Holozoa - ps10
Filozoa - ps11
Choano./Metazoa - ps12
Metazoa - ps13
Myriazoa - ps14
Parahoxozoa - ps15
Bilateria - ps16
Deuterostomia - ps17
Chordata - ps18
Olfactores - ps19
Vertebrata - ps20
Gnathostomata - ps21
Euteleostomi - ps22
Sarcopterygii - ps23
Tetrapoda - ps24
Amniota - ps25
Mammalia - ps26
Theria - ps27
Placentalia - ps28
Boreoeutheria - ps29
Euarchontoglires - ps30
Euarchonta - ps31
Primates - ps32
Haplorrhini - ps33
Simiiformes - ps34
Catarrhini - ps35
Hominoidea - ps36
Hominidae - ps37
Homininae - ps38
Hominini - ps39
Homo_sapiens - ps40
Phylostratum

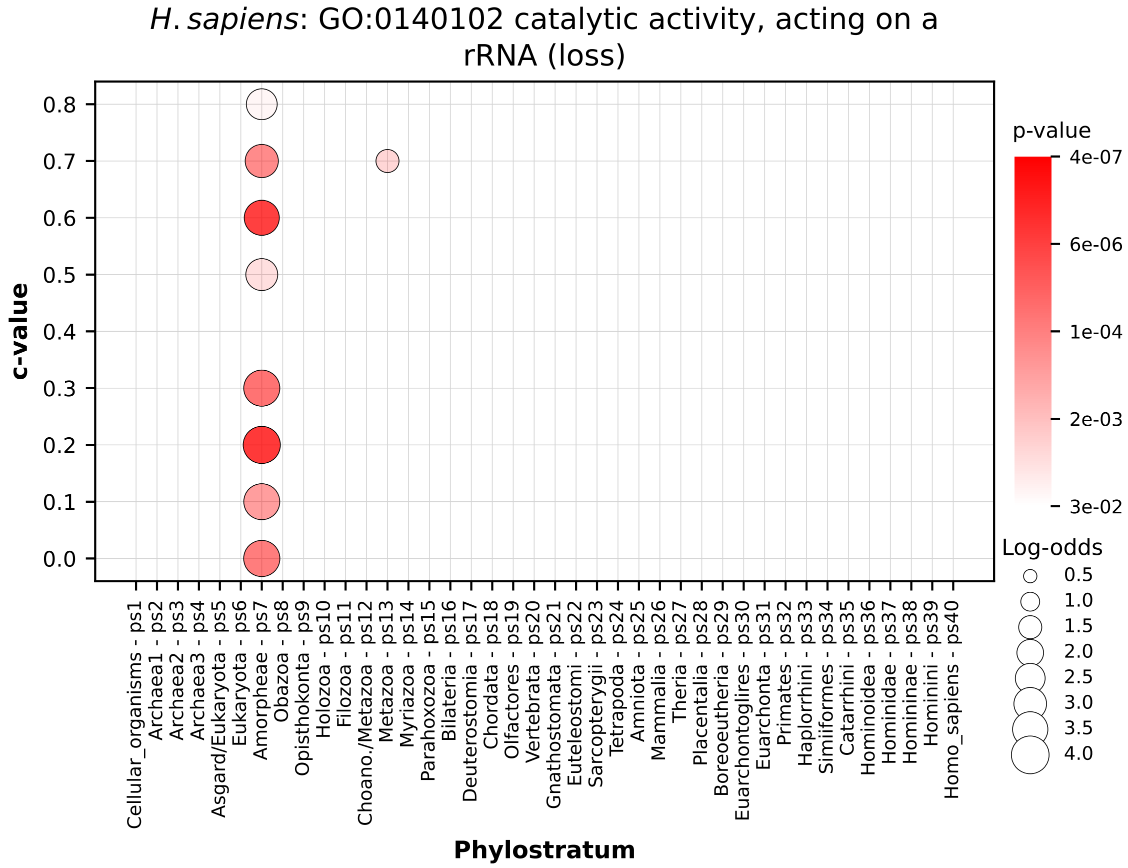

H. sapiens: GO:0140102 catalytic activity, acting on a rRNA (loss)
c-value
0.0
0.1
0.2
0.3
0.4
0.5
0.6
0.7
0.8
p-value
4e-07
6e-06
1e-04
2e-03
3e-02
Log-odds
0.5
1.0
1.5
2.0
2.5
3.0
3.5
4.0
Cellular_organisms - ps1
Archaea1 - ps2
Archaea2 - ps3
Archaea3 - ps4
Asgard/Eukaryota - ps5
Eukaryota - ps6
Amorpheae - ps7
Obazoa - ps8
Opisthokonta - ps9
Holozoa - ps10
Filozoa - ps11
Choano./Metazoa - ps12
Metazoa - ps13
Myriazoa - ps14
Parahoxozoa - ps15
Bilateria - ps16
Deuterostomia - ps17
Chordata - ps18
Olfactores - ps19
Vertebrata - ps20
Gnathostomata - ps21
Euteleostomi - ps22
Sarcopterygii - ps23
Tetrapoda - ps24
Amniota - ps25
Mammalia - ps26
Theria - ps27
Placentalia - ps28
Boreoeutheria - ps29
Euarchontoglires - ps30
Euarchonta - ps31
Primates - ps32
Haplorrhini - ps33
Simiiformes - ps34
Catarrhini - ps35
Hominoidea - ps36
Hominidae - ps37
Homininae - ps38
Hominini - ps39
Homo_sapiens - ps40
Phylostratum

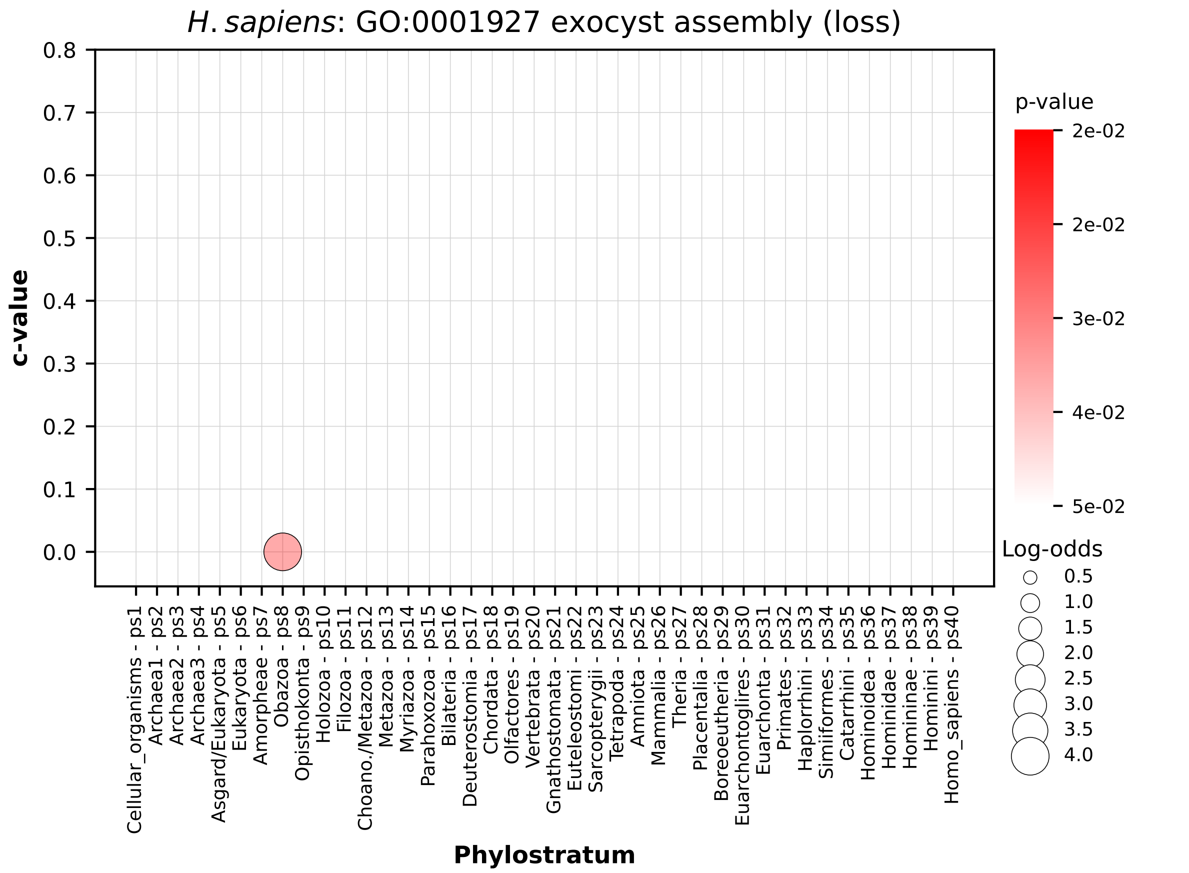

H. sapiens: GO:0001927 exocyst assembly (loss)
c-value
0.8
0.7
0.6
0.5
0.4
0.3
0.2
0.1
0.0
Phylostratum
Cellular_organisms - ps1
Archaea1 - ps2
Archaea2 - ps3
Archaea3 - ps4
Asgard/Eukaryota - ps5
Eukaryota - ps6
Amorpheae - ps7
Obazoa - ps8
Opisthokonta - ps9
Holozoa - ps10
Filozoa - ps11
Choano./Metazoa - ps12
Metazoa - ps13
Myriazoa - ps14
Parahoxozoa - ps15
Bilateria - ps16
Deuterostomia - ps17
Chordata - ps18
Olfactores - ps19
Vertebrata - ps20
Gnathostomata - ps21
Euteleostomi - ps22
Sarcopterygii - ps23
Tetrapoda - ps24
Amniota - ps25
Mammalia - ps26
Theria - ps27
Placentalia - ps28
Boreoeutheria - ps29
Euarchontoglires - ps30
Euarchonta - ps31
Primates - ps32
Haplorrhini - ps33
Simiiformes - ps34
Catarrhini - ps35
Hominoidea - ps36
Hominidae - ps37
Homininae - ps38
Hominini - ps39
Homo_sapiens - ps40
p-value
2e-02
2e-02
3e-02
4e-02
5e-02
Log-odds
0.5
1.0
1.5
2.0
2.5
3.0
3.5
4.0

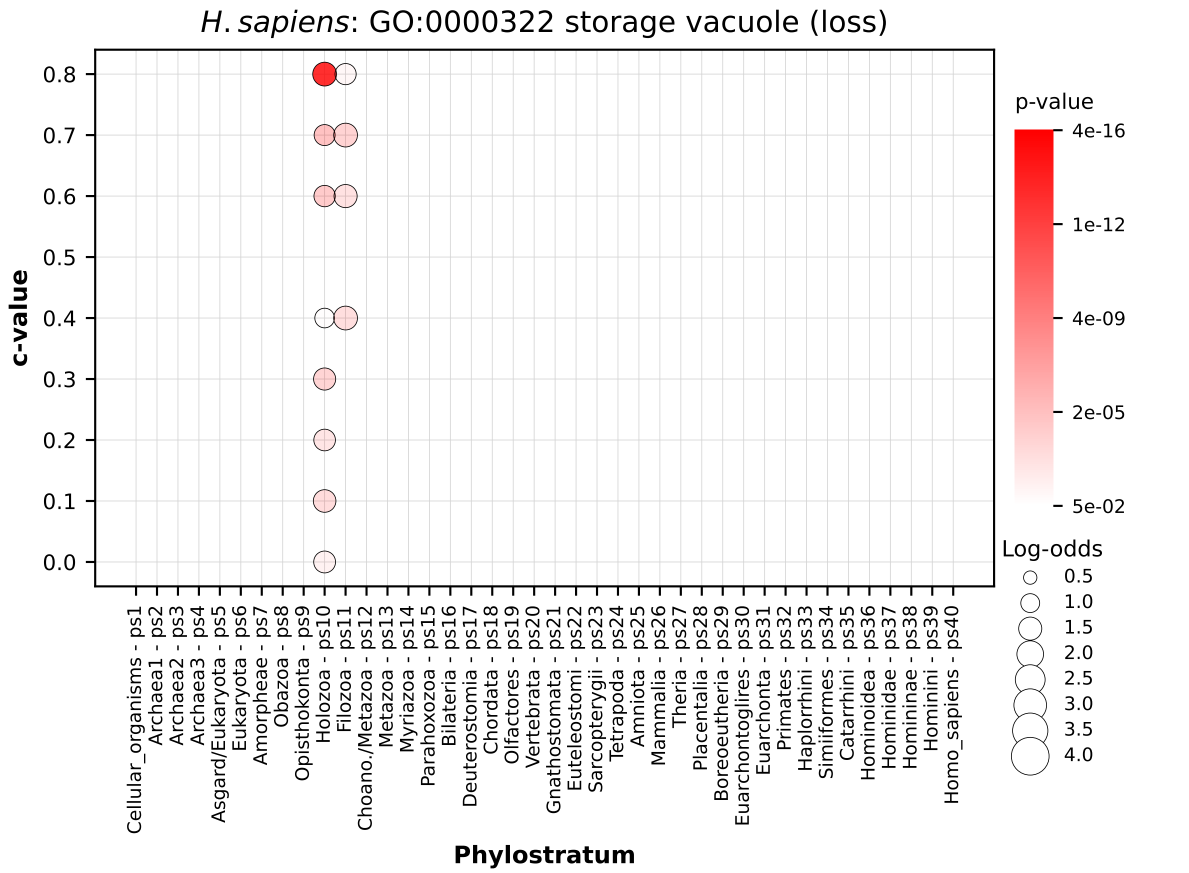
H. sapiens: GO:0000322 storage vacuole (loss)
c-value
0.0
0.1
0.2
0.3
0.4
0.5
0.6
0.7
0.8
Phylostratum
Cellular_organisms - ps1
Archaea1 - ps2
Archaea2 - ps3
Archaea3 - ps4
Asgard/Eukaryota - ps5
Eukaryota - ps6
Amorpheae - ps7
Obazoa - ps8
Opisthokonta - ps9
Holozoa - ps10
Filozoa - ps11
Choano./Metazoa - ps12
Metazoa - ps13
Myriazoa - ps14
Parahoxozoa - ps15
Bilateria - ps16
Deuterostomia - ps17
Chordata - ps18
Olfactores - ps19
Vertebrata - ps20
Gnathostomata - ps21
Euteleostomi - ps22
Sarcopterygii - ps23
Tetrapoda - ps24
Amniota - ps25
Mammalia - ps26
Theria - ps27
Placentalia - ps28
Boreoeutheria - ps29
Euarchontoglires - ps30
Euarchonta - ps31
Primates - ps32
Haplorrhini - ps33
Simiiformes - ps34
Catarrhini - ps35
Hominoidea - ps36
Hominidae - ps37
Homininae - ps38
Hominini - ps39
Homo_sapiens - ps40
p-value
4e-16
1e-12
4e-09
2e-05
5e-02
Log-odds
0.5
1.0
1.5
2.0
2.5
3.0
3.5
4.0

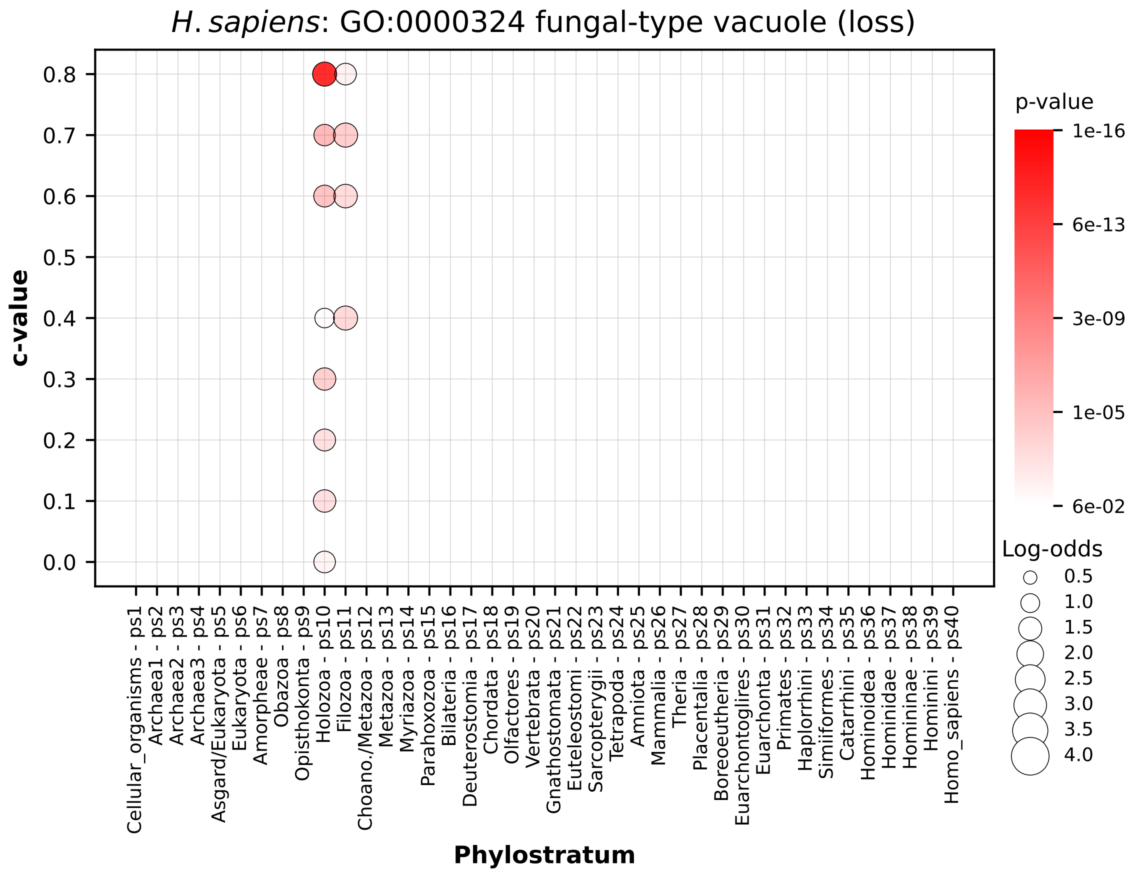

H. sapiens: GO:0000324 fungal-type vacuole (loss)
c-value
0.0
0.1
0.2
0.3
0.4
0.5
0.6
0.7
0.8
Phylostratum
Cellular_organisms - ps1
Archaea1 - ps2
Archaea2 - ps3
Archaea3 - ps4
Asgard/Eukaryota - ps5
Eukaryota - ps6
Amorpheae - ps7
Obazoa - ps8
Opisthokonta - ps9
Holozoa - ps10
Filozoa - ps11
Choano./Metazoa - ps12
Metazoa - ps13
Myriazoa - ps14
Parahoxozoa - ps15
Bilateria - ps16
Deuterostomia - ps17
Chordata - ps18
Olfactores - ps19
Vertebrata - ps20
Gnathostomata - ps21
Euteleostomi - ps22
Sarcopterygii - ps23
Tetrapoda - ps24
Amniota - ps25
Mammalia - ps26
Theria - ps27
Placentalia - ps28
Boreoeutheria - ps29
Euarchontoglires - ps30
Euarchonta - ps31
Primates - ps32
Haplorrhini - ps33
Simiiformes - ps34
Catarrhini - ps35
Hominoidea - ps36
Hominidae - ps37
Homininae - ps38
Hominini - ps39
Homo_sapiens - ps40
p-value
1e-16
6e-13
3e-09
1e-05
6e-02
Log-odds
0.5
1.0
1.5
2.0
2.5
3.0
3.5
4.0

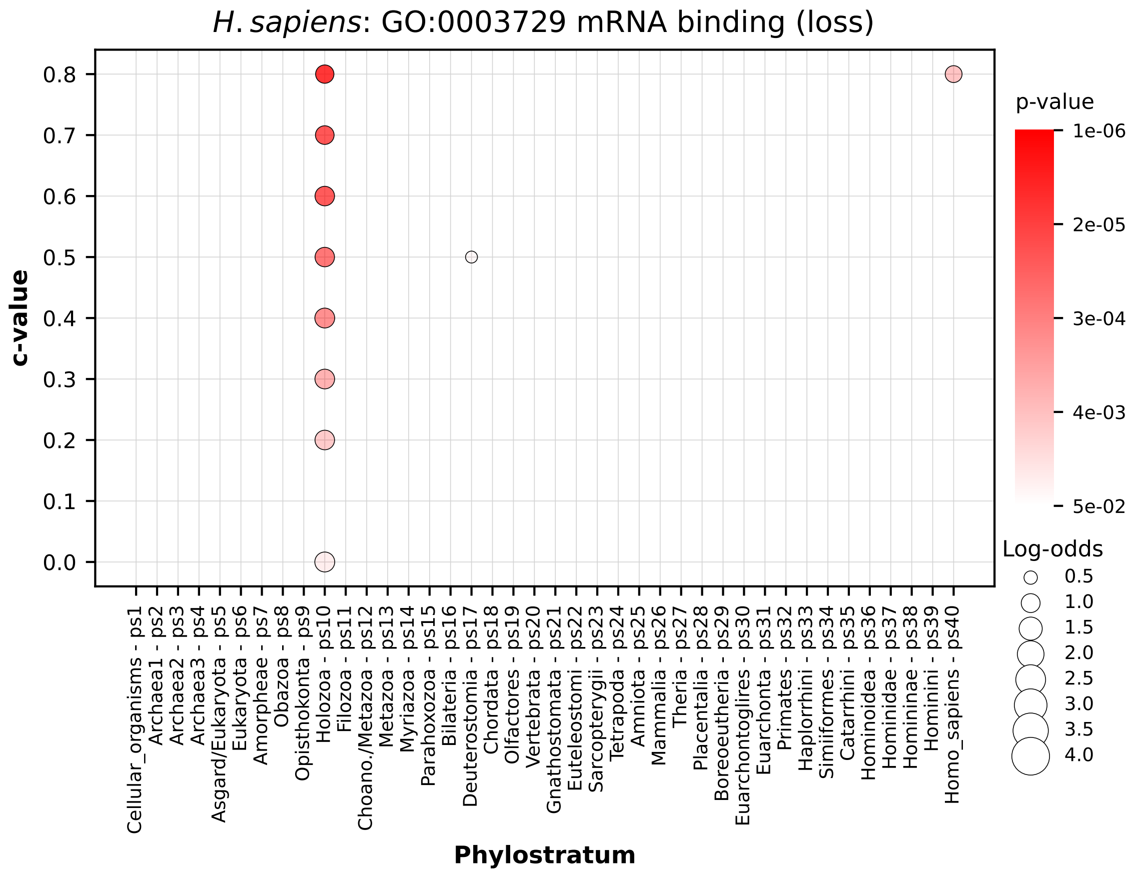
H. sapiens: GO:0003729 mRNA binding (loss)
c-value
0.8
0.7
0.6
0.5
0.4
0.3
0.2
0.1
0.0
Phylostratum
Cellular_organisms - ps1
Archaea1 - ps2
Archaea2 - ps3
Archaea3 - ps4
Asgard/Eukaryota - ps5
Eukaryota - ps6
Amorpheae - ps7
Obazoa - ps8
Opisthokonta - ps9
Holozoa - ps10
Filozoa - ps11
Choano./Metazoa - ps12
Metazoa - ps13
Myriazoa - ps14
Parahoxozoa - ps15
Bilateria - ps16
Deuterostomia - ps17
Chordata - ps18
Olfactores - ps19
Vertebrata - ps20
Gnathostomata - ps21
Euteleostomi - ps22
Sarcopterygii - ps23
Tetrapoda - ps24
Amniota - ps25
Mammalia - ps26
Theria - ps27
Placentalia - ps28
Boreoeutheria - ps29
Euarchontoglires - ps30
Euarchonta - ps31
Primates - ps32
Haplorrhini - ps33
Simiiformes - ps34
Catarrhini - ps35
Hominoidea - ps36
Hominidae - ps37
Homininae - ps38
Hominini - ps39
Homo_sapiens - ps40
p-value
1e-06
2e-05
3e-04
4e-03
5e-02
Log-odds
0.5
1.0
1.5
2.0
2.5
3.0
3.5
4.0

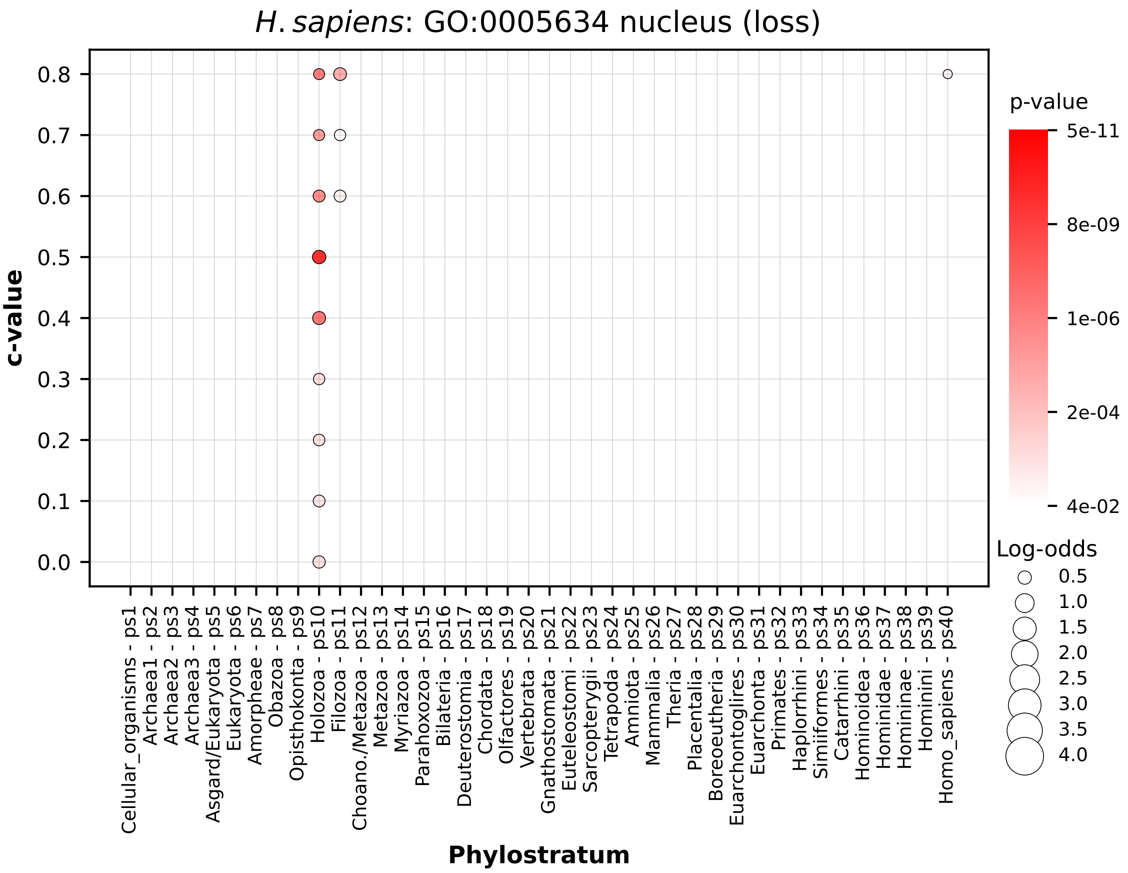
H. sapiens: GO:0005634 nucleus (loss)
c-value
0.8
0.7
0.6
0.5
0.4
0.3
0.2
0.1
0.0
Phylostratum
Cellular_organisms - ps1
Archaea1 - ps2
Archaea2 - ps3
Archaea3 - ps4
Asgard/Eukaryota - ps5
Eukaryota - ps6
Amorpheae - ps7
Obazoa - ps8
Opisthokonta - ps9
Holozoa - ps10
Filozoa - ps11
Choano./Metazoa - ps12
Metazoa - ps13
Myriazoa - ps14
Parahoxozoa - ps15
Bilateria - ps16
Deuterostomia - ps17
Chordata - ps18
Olfactores - ps19
Vertebrata - ps20
Gnathostomata - ps21
Euteleostomi - ps22
Sarcopterygii - ps23
Tetrapoda - ps24
Amniota - ps25
Mammalia - ps26
Theria - ps27
Placentalia - ps28
Boreoeutheria - ps29
Euarchontoglires - ps30
Euarchonta - ps31
Primates - ps32
Haplorrhini - ps33
Simiiformes - ps34
Catarrhini - ps35
Hominoidea - ps36
Hominidae - ps37
Homininae - ps38
Hominini - ps39
Homo_sapiens - ps40
p-value
5e-11
8e-09
1e-06
2e-04
4e-02
Log-odds
0.5
1.0
1.5
2.0
2.5
3.0
3.5
4.0

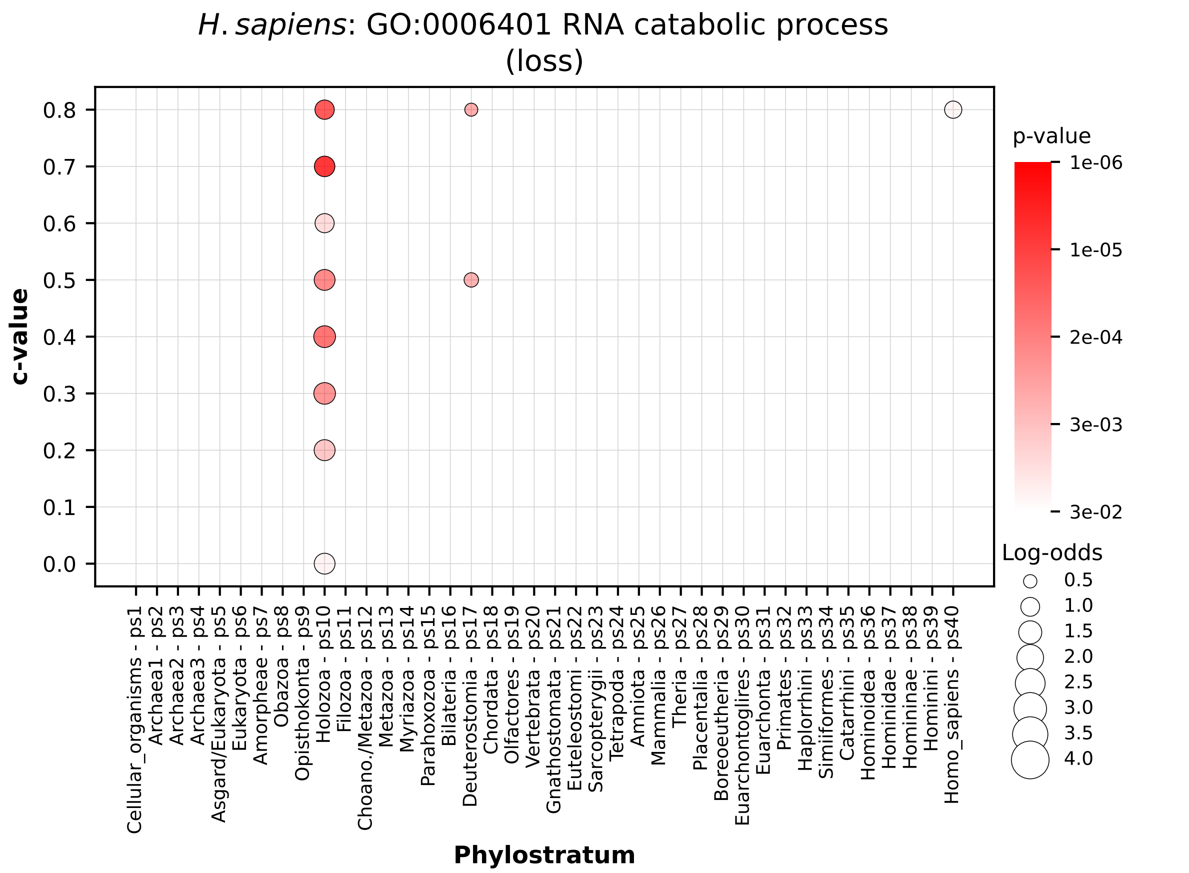

H. sapiens: GO:0006401 RNA catabolic process
(loss)
c-value
0.8
0.7
0.6
0.5
0.4
0.3
0.2
0.1
0.0
p-value
1e-06
1e-05
2e-04
3e-03
3e-02
Log-odds
0.5
1.0
1.5
2.0
2.5
3.0
3.5
4.0
Cellular_organisms - ps1
Archaea1 - ps2
Archaea2 - ps3
Archaea3 - ps4
Asgard/Eukaryota - ps5
Eukaryota - ps6
Amorpheae - ps7
Obazoa - ps8
Opisthokonta - ps9
Holozoa - ps10
Filozoa - ps11
Choano./Metazoa - ps12
Metazoa - ps13
Myriazoa - ps14
Parahoxozoa - ps15
Bilateria - ps16
Deuterostomia - ps17
Chordata - ps18
Olfactores - ps19
Vertebrata - ps20
Gnathostomata - ps21
Euteleostomi - ps22
Sarcopterygii - ps23
Tetrapoda - ps24
Amniota - ps25
Mammalia - ps26
Theria - ps27
Placentalia - ps28
Boreoeutheria - ps29
Euarchontoglires - ps30
Euarchonta - ps31
Primates - ps32
Haplorrhini - ps33
Simiiformes - ps34
Catarrhini - ps35
Hominoidea - ps36
Hominidae - ps37
Homininae - ps38
Hominini - ps39
Homo_sapiens - ps40
Phylostratum

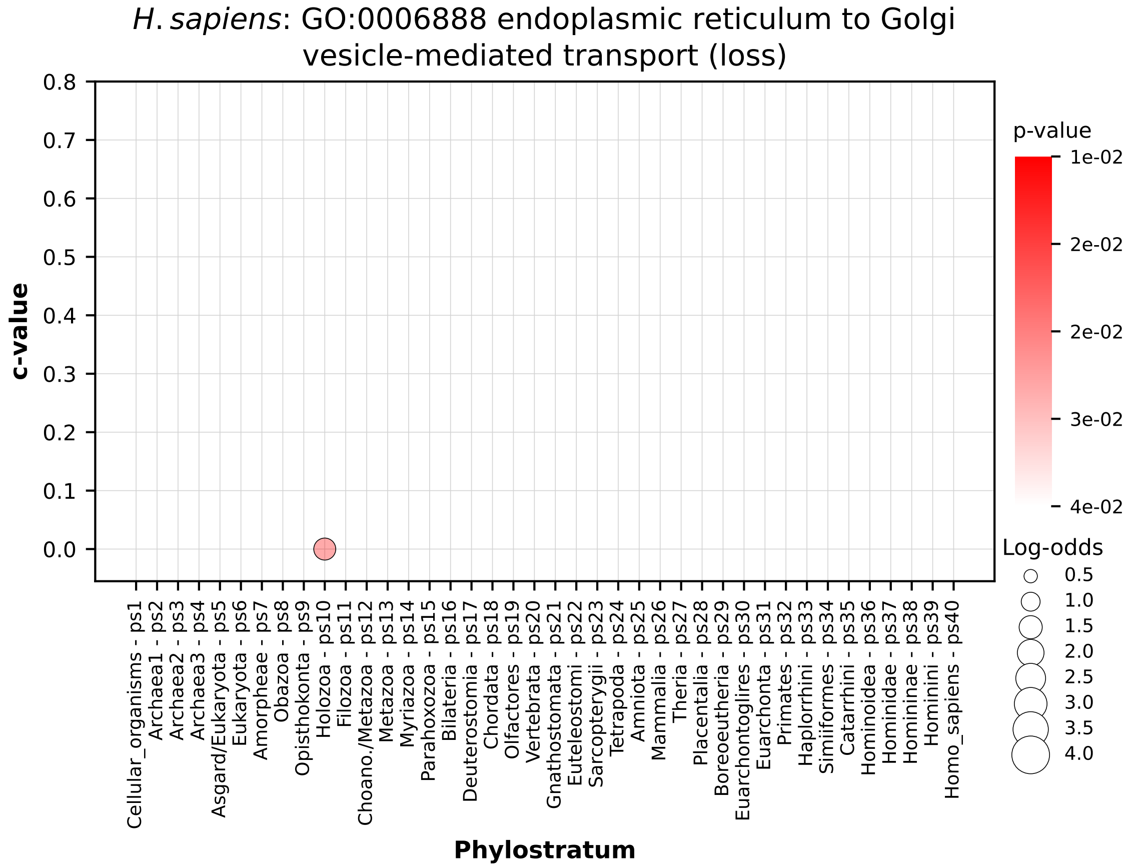

H. sapiens: GO:0006888 endoplasmic reticulum to Golgi vesicle-mediated transport (loss)
c-value
0.8
0.7
0.6
0.5
0.4
0.3
0.2
0.1
0.0
p-value
1e-02
2e-02
2e-02
3e-02
4e-02
Log-odds
0.5
1.0
1.5
2.0
2.5
3.0
3.5
4.0
Cellular_organisms - ps1
Archaea1 - ps2
Archaea2 - ps3
Archaea3 - ps4
Asgard/Eukaryota - ps5
Eukaryota - ps6
Amorpheae - ps7
Obazoa - ps8
Opisthokonta - ps9
Holozoa - ps10
Filozoa - ps11
Choano./Metazoa - ps12
Metazoa - ps13
Myriazoa - ps14
Parahoxozoa - ps15
Bilateria - ps16
Deuterostomia - ps17
Chordata - ps18
Olfactores - ps19
Vertebrata - ps20
Gnathostomata - ps21
Euteleostomi - ps22
Sarcopterygii - ps23
Tetrapoda - ps24
Amniota - ps25
Mammalia - ps26
Theria - ps27
Placentalia - ps28
Boreoeutheria - ps29
Euarchontoglires - ps30
Euarchonta - ps31
Primates - ps32
Haplorrhini - ps33
Simiiformes - ps34
Catarrhini - ps35
Hominoidea - ps36
Hominidae - ps37
Homininae - ps38
Hominini - ps39
Homo_sapiens - ps40
Phylostratum

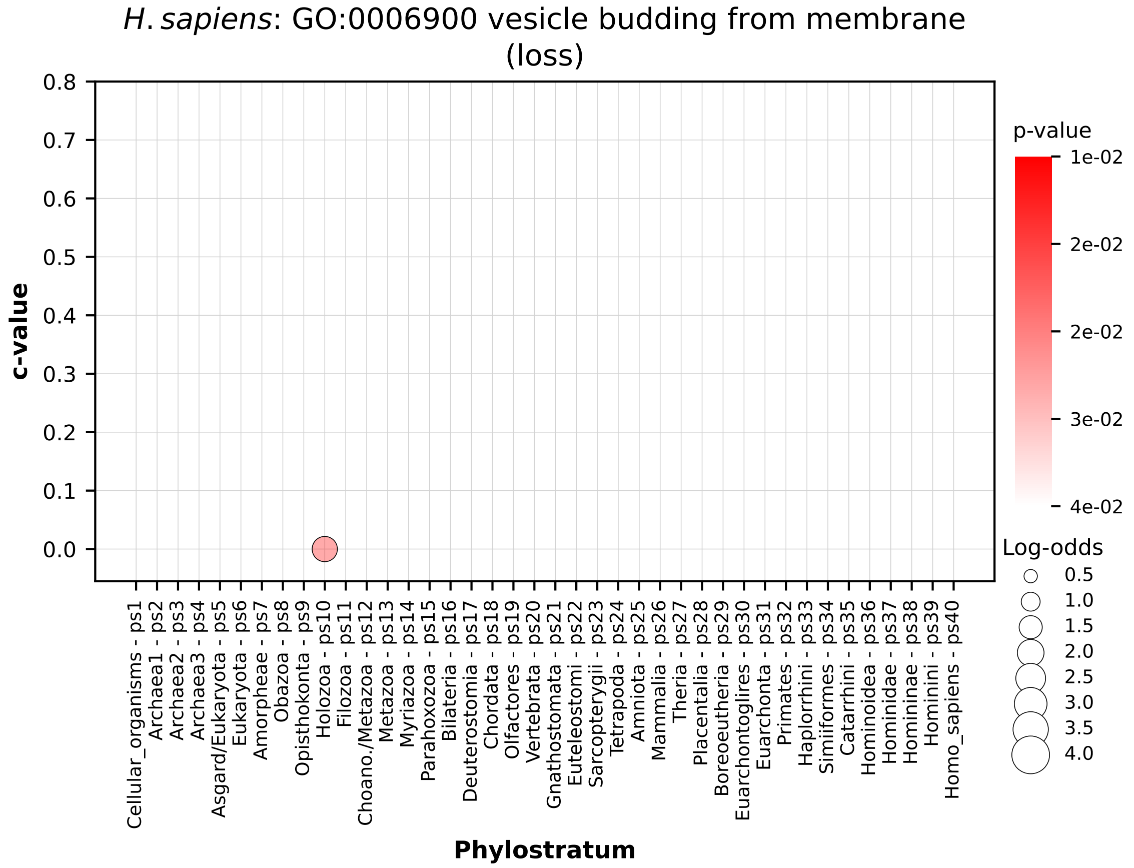

H. sapiens: GO:0006900 vesicle budding from membrane (loss)
c-value
0.8
0.7
0.6
0.5
0.4
0.3
0.2
0.1
0.0
p-value
1e-02
2e-02
2e-02
3e-02
4e-02
Log-odds
0.5
1.0
1.5
2.0
2.5
3.0
3.5
4.0
Cellular_organisms - ps1
Archaea1 - ps2
Archaea2 - ps3
Archaea3 - ps4
Asgard/Eukaryota - ps5
Eukaryota - ps6
Amorpheae - ps7
Obazoa - ps8
Opisthokonta - ps9
Holozoa - ps10
Filozoa - ps11
Choano./Metazoa - ps12
Metazoa - ps13
Myriazoa - ps14
Parahoxozoa - ps15
Bilateria - ps16
Deuterostomia - ps17
Chordata - ps18
Olfactores - ps19
Vertebrata - ps20
Gnathostomata - ps21
Euteleostomi - ps22
Sarcopterygii - ps23
Tetrapoda - ps24
Amniota - ps25
Mammalia - ps26
Theria - ps27
Placentalia - ps28
Boreoeutheria - ps29
Euarchontoglires - ps30
Euarchonta - ps31
Primates - ps32
Haplorrhini - ps33
Simiiformes - ps34
Catarrhini - ps35
Hominoidea - ps36
Hominidae - ps37
Homininae - ps38
Hominini - ps39
Homo_sapiens - ps40
Phylostratum

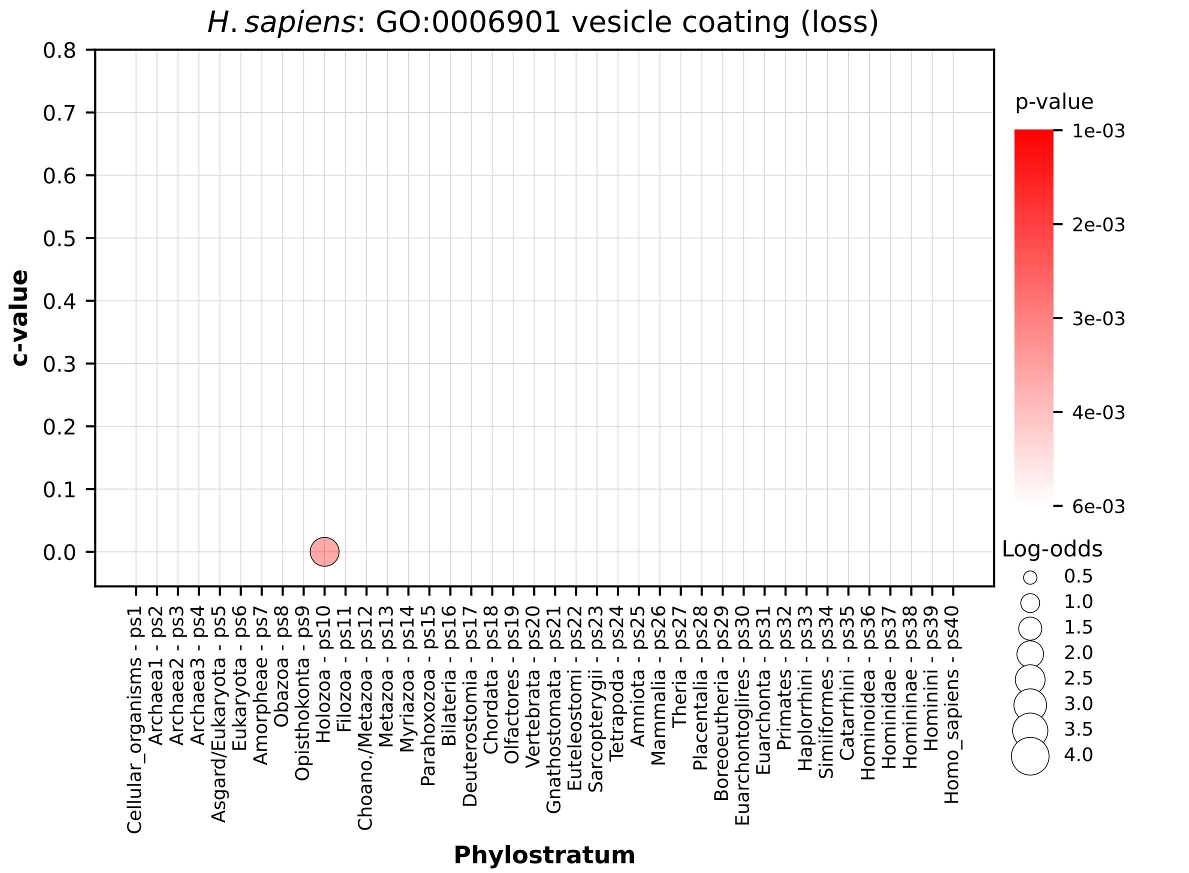

H. sapiens: GO:0006901 vesicle coating (loss)
c-value
0.8
0.7
0.6
0.5
0.4
0.3
0.2
0.1
0.0
p-value
1e-03
2e-03
3e-03
4e-03
6e-03
Log-odds
0.5
1.0
1.5
2.0
2.5
3.0
3.5
4.0
Cellular_organisms - ps1
Archaea1 - ps2
Archaea2 - ps3
Archaea3 - ps4
Asgard/Eukaryota - ps5
Eukaryota - ps6
Amorpheae - ps7
Obazoa - ps8
Opisthokonta - ps9
Holozoa - ps10
Filozoa - ps11
Choano./Metazoa - ps12
Metazoa - ps13
Myriazoa - ps14
Parahoxozoa - ps15
Bilateria - ps16
Deuterostomia - ps17
Chordata - ps18
Olfactores - ps19
Vertebrata - ps20
Gnathostomata - ps21
Euteleostomi - ps22
Sarcopterygii - ps23
Tetrapoda - ps24
Amniota - ps25
Mammalia - ps26
Theria - ps27
Placentalia - ps28
Boreoeutheria - ps29
Euarchontoglires - ps30
Euarchonta - ps31
Primates - ps32
Haplorrhini - ps33
Simiiformes - ps34
Catarrhini - ps35
Hominoidea - ps36
Hominidae - ps37
Homininae - ps38
Hominini - ps39
Homo_sapiens - ps40
Phylostratum

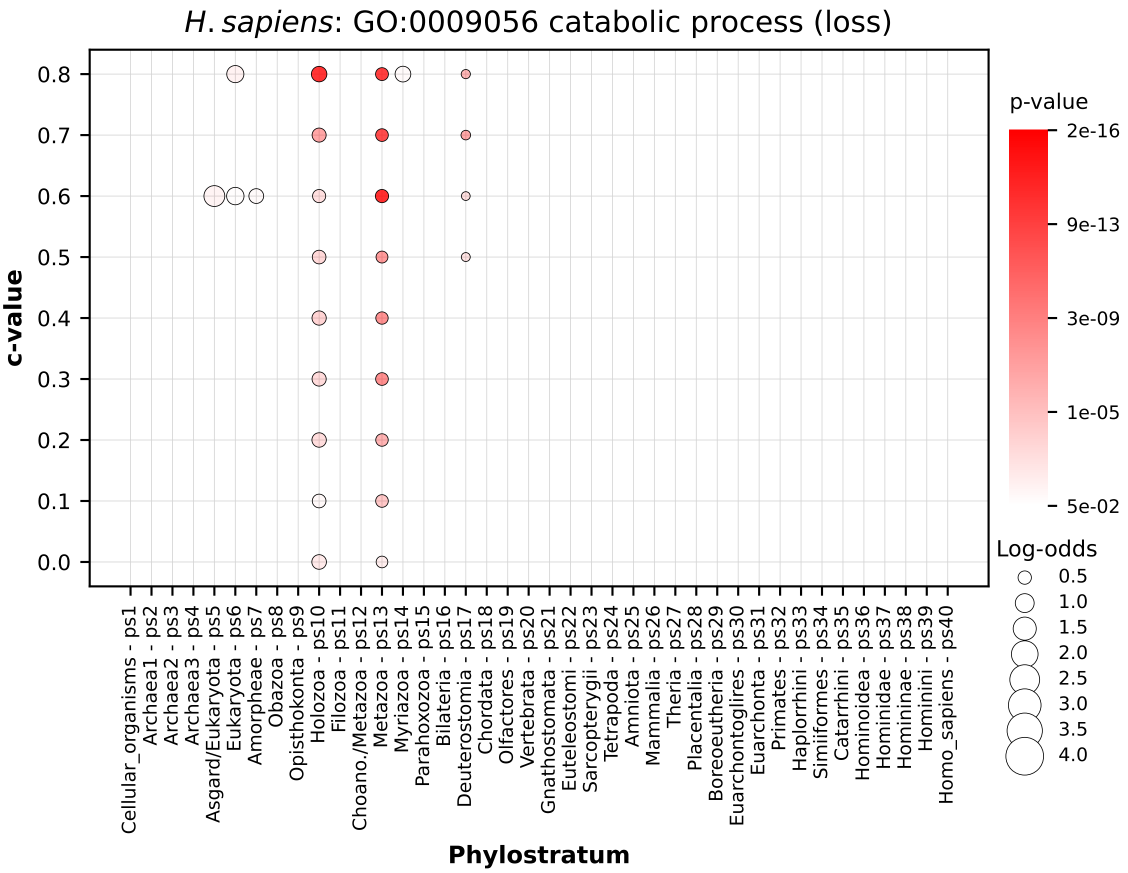

H. sapiens: GO:0009056 catabolic process (loss)
c-value
0.8
0.7
0.6
0.5
0.4
0.3
0.2
0.1
0.0
Phylostratum
Cellular_organisms - ps1
Archaea1 - ps2
Archaea2 - ps3
Archaea3 - ps4
Asgard/Eukaryota - ps5
Eukaryota - ps6
Amorpheae - ps7
Obazoa - ps8
Opisthokonta - ps9
Holozoa - ps10
Filozoa - ps11
Choano./Metazoa - ps12
Metazoa - ps13
Myriazoa - ps14
Parahoxozoa - ps15
Bilateria - ps16
Deuterostomia - ps17
Chordata - ps18
Olfactores - ps19
Vertebrata - ps20
Gnathostomata - ps21
Euteleostomi - ps22
Sarcopterygii - ps23
Tetrapoda - ps24
Amniota - ps25
Mammalia - ps26
Theria - ps27
Placentalia - ps28
Boreoeutheria - ps29
Euarchontoglires - ps30
Euarchonta - ps31
Primates - ps32
Haplorrhini - ps33
Simiiformes - ps34
Catarrhini - ps35
Hominoidea - ps36
Hominidae - ps37
Homininae - ps38
Hominini - ps39
Homo_sapiens - ps40
p-value
2e-16
9e-13
3e-09
1e-05
5e-02
Log-odds
0.5
1.0
1.5
2.0
2.5
3.0
3.5
4.0

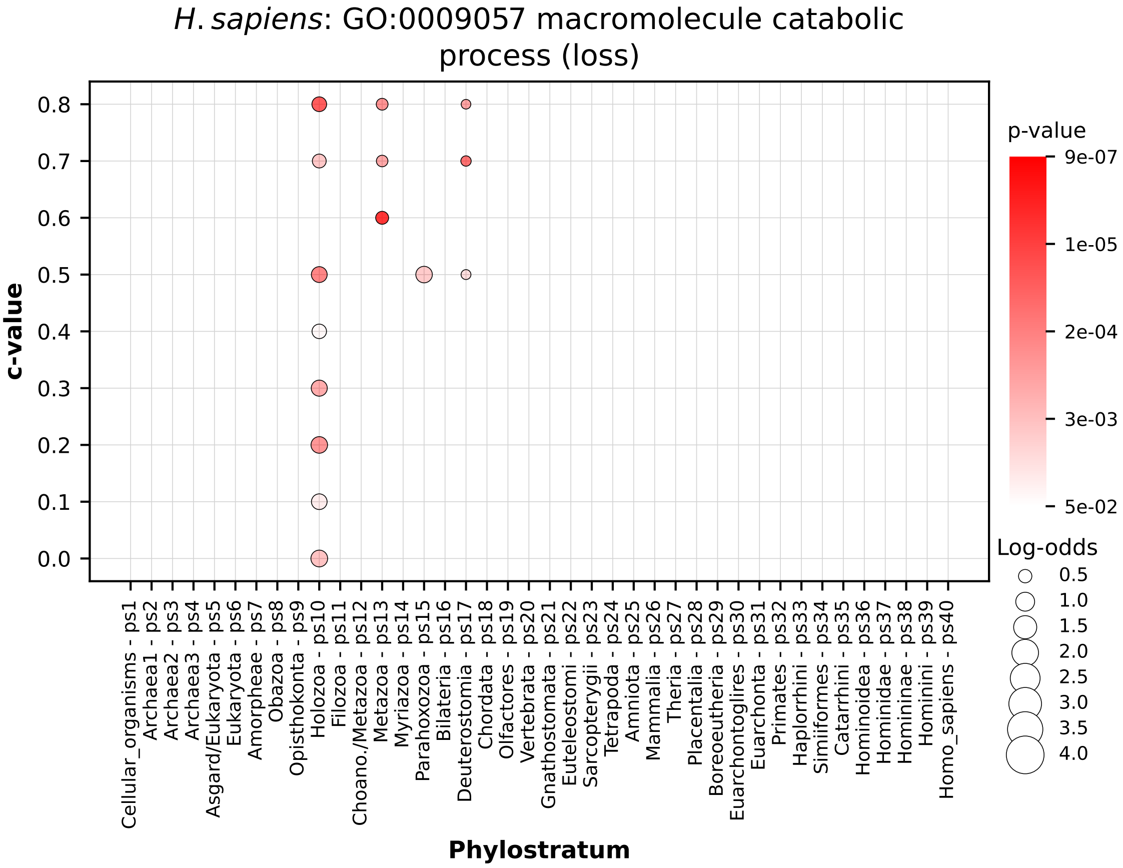

H. sapiens: GO:0009057 macromolecule catabolic process (loss)
c-value
0.8
0.7
0.6
0.5
0.4
0.3
0.2
0.1
0.0
Phylostratum
Cellular_organisms - ps1
Archaea1 - ps2
Archaea2 - ps3
Archaea3 - ps4
Asgard/Eukaryota - ps5
Eukaryota - ps6
Amorpheae - ps7
Obazoa - ps8
Opisthokonta - ps9
Holozoa - ps10
Filozoa - ps11
Choano./Metazoa - ps12
Metazoa - ps13
Myriazoa - ps14
Parahoxozoa - ps15
Bilateria - ps16
Deuterostomia - ps17
Chordata - ps18
Olfactores - ps19
Vertebrata - ps20
Gnathostomata - ps21
Euteleostomi - ps22
Sarcopterygii - ps23
Tetrapoda - ps24
Amniota - ps25
Mammalia - ps26
Theria - ps27
Placentalia - ps28
Boreoeutheria - ps29
Euarchontoglires - ps30
Euarchonta - ps31
Primates - ps32
Haplorrhini - ps33
Simiiformes - ps34
Catarrhini - ps35
Hominoidea - ps36
Hominidae - ps37
Homininae - ps38
Hominini - ps39
Homo_sapiens - ps40
p-value
9e-07
1e-05
2e-04
3e-03
5e-02
Log-odds
0.5
1.0
1.5
2.0
2.5
3.0
3.5
4.0

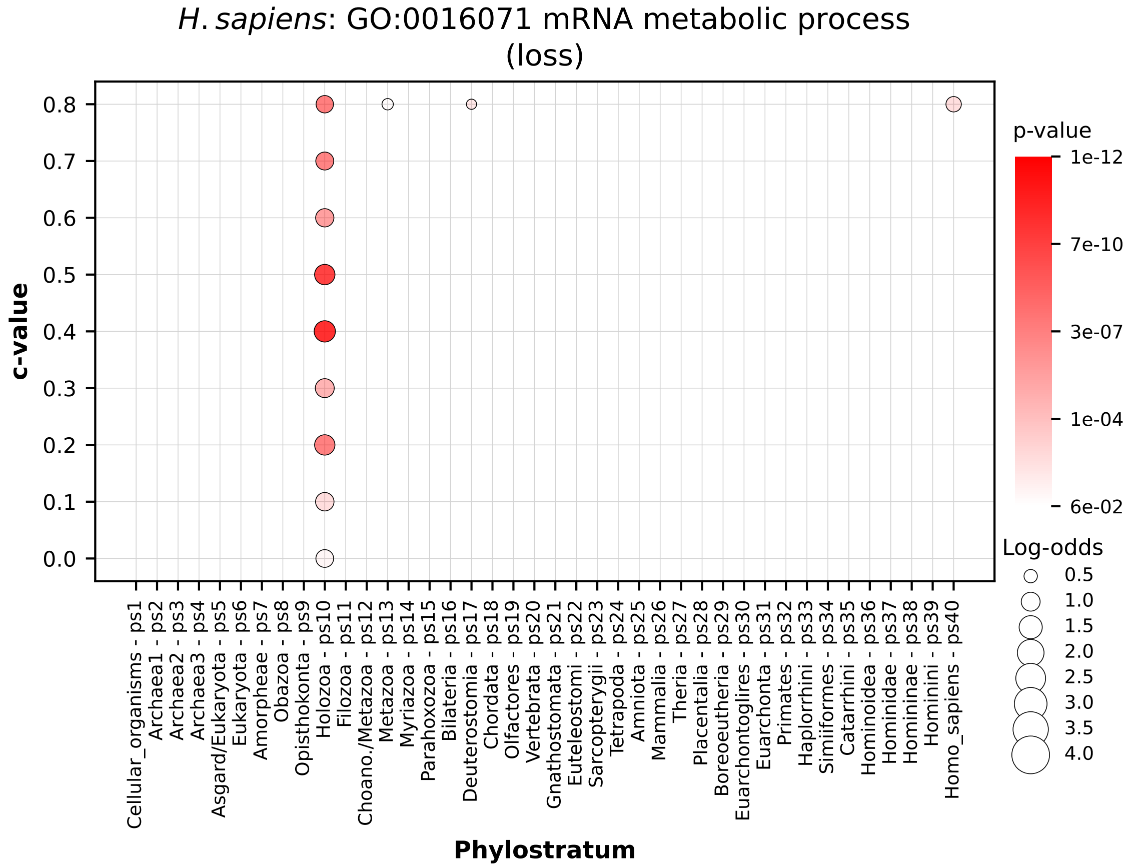

H. sapiens: GO:0016071 mRNA metabolic process
(loss)
c-value
0.8
0.7
0.6
0.5
0.4
0.3
0.2
0.1
0.0
p-value
1e-12
7e-10
3e-07
1e-04
6e-02
Log-odds
0.5
1.0
1.5
2.0
2.5
3.0
3.5
4.0
Cellular_organisms - ps1
Archaea1 - ps2
Archaea2 - ps3
Archaea3 - ps4
Asgard/Eukaryota - ps5
Eukaryota - ps6
Amorpheae - ps7
Obazoa - ps8
Opisthokonta - ps9
Holozoa - ps10
Filozoa - ps11
Choano./Metazoa - ps12
Metazoa - ps13
Myriazoa - ps14
Parahoxozoa - ps15
Bilateria - ps16
Deuterostomia - ps17
Chordata - ps18
Olfactores - ps19
Vertebrata - ps20
Gnathostomata - ps21
Euteleostomi - ps22
Sarcopterygii - ps23
Tetrapoda - ps24
Amniota - ps25
Mammalia - ps26
Theria - ps27
Placentalia - ps28
Boreoeutheria - ps29
Euarchontoglires - ps30
Euarchonta - ps31
Primates - ps32
Haplorrhini - ps33
Simiiformes - ps34
Catarrhini - ps35
Hominoidea - ps36
Hominidae - ps37
Homininae - ps38
Hominini - ps39
Homo_sapiens - ps40
Phylostratum

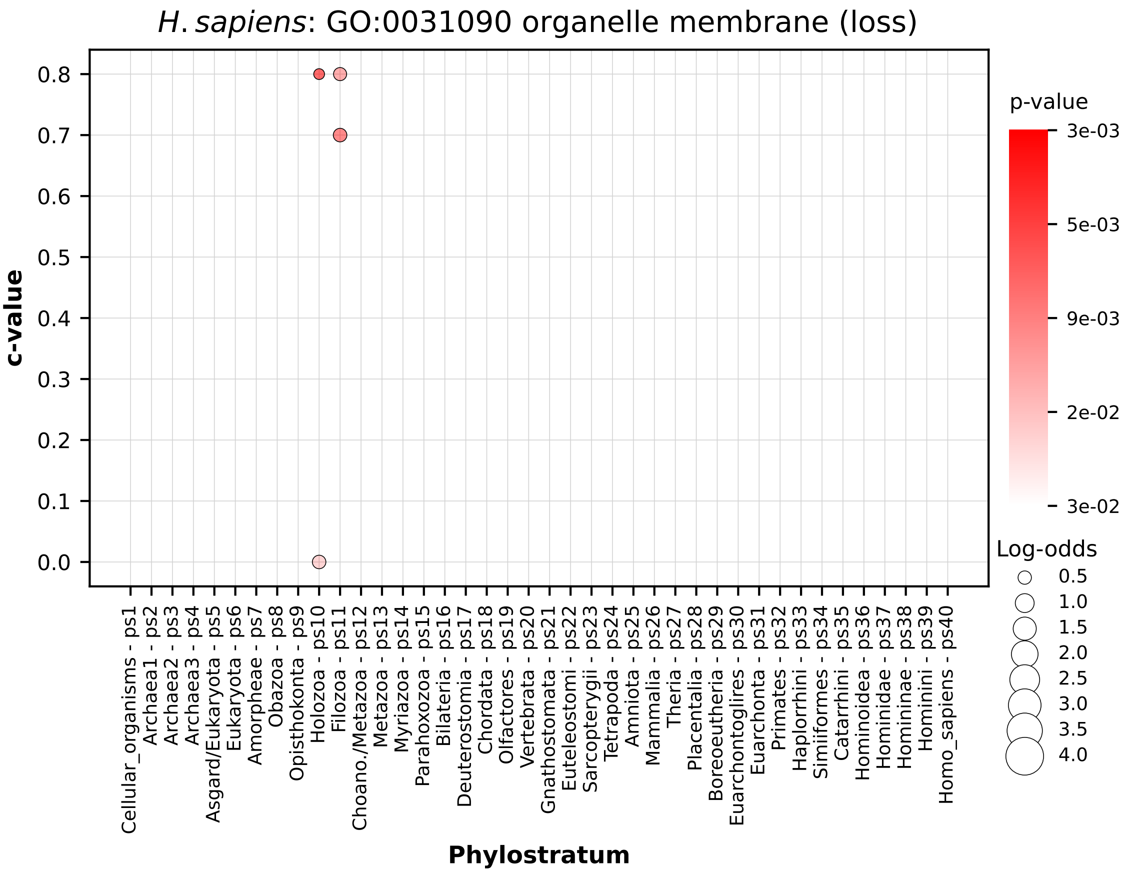

H. sapiens: GO:0031090 organelle membrane (loss)
c-value
0.8
0.7
0.6
0.5
0.4
0.3
0.2
0.1
0.0
p-value
3e-03
5e-03
9e-03
2e-02
3e-02
Log-odds
0.5
1.0
1.5
2.0
2.5
3.0
3.5
4.0
Cellular_organisms - ps1
Archaea1 - ps2
Archaea2 - ps3
Archaea3 - ps4
Asgard/Eukaryota - ps5
Eukaryota - ps6
Amorpheae - ps7
Obazoa - ps8
Opisthokonta - ps9
Holozoa - ps10
Filozoa - ps11
Choano./Metazoa - ps12
Metazoa - ps13
Myriazoa - ps14
Parahoxozoa - ps15
Bilateria - ps16
Deuterostomia - ps17
Chordata - ps18
Olfactores - ps19
Vertebrata - ps20
Gnathostomata - ps21
Euteleostomi - ps22
Sarcopterygii - ps23
Tetrapoda - ps24
Amniota - ps25
Mammalia - ps26
Theria - ps27
Placentalia - ps28
Boreoeutheria - ps29
Euarchontoglires - ps30
Euarchonta - ps31
Primates - ps32
Haplorrhini - ps33
Simiiformes - ps34
Catarrhini - ps35
Hominoidea - ps36
Hominidae - ps37
Homininae - ps38
Hominini - ps39
Homo_sapiens - ps40
Phylostratum

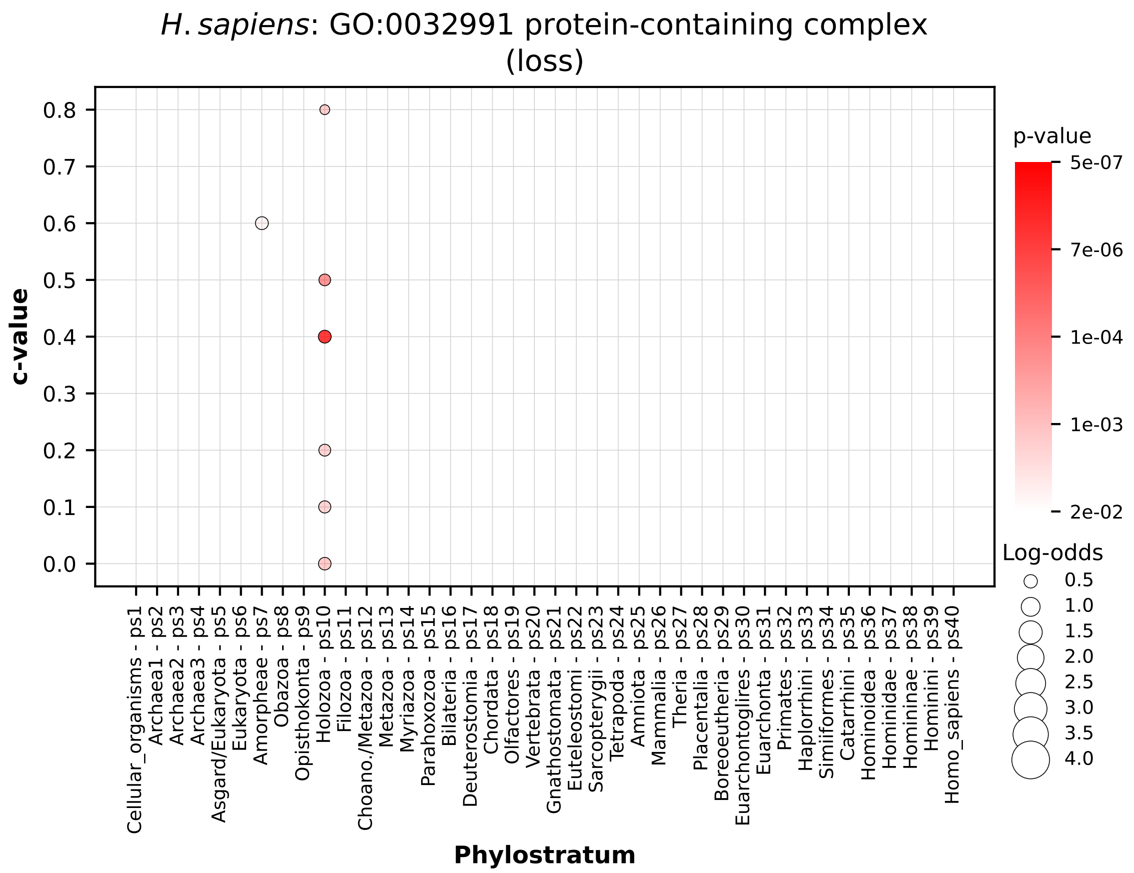

H. sapiens: GO:0032991 protein-containing complex
(loss)
c-value
0.8
0.7
0.6
0.5
0.4
0.3
0.2
0.1
0.0
p-value
5e-07
7e-06
1e-04
1e-03
2e-02
Log-odds
0.5
1.0
1.5
2.0
2.5
3.0
3.5
4.0
Cellular_organisms - ps1
Archaea1 - ps2
Archaea2 - ps3
Archaea3 - ps4
Asgard/Eukaryota - ps5
Eukaryota - ps6
Amorpheae - ps7
Obazoa - ps8
Opisthokonta - ps9
Holozoa - ps10
Filozoa - ps11
Choano./Metazoa - ps12
Metazoa - ps13
Myriazoa - ps14
Parahoxozoa - ps15
Bilateria - ps16
Deuterostomia - ps17
Chordata - ps18
Olfactores - ps19
Vertebrata - ps20
Gnathostomata - ps21
Euteleostomi - ps22
Sarcopterygii - ps23
Tetrapoda - ps24
Amniota - ps25
Mammalia - ps26
Theria - ps27
Placentalia - ps28
Boreoeutheria - ps29
Euarchontoglires - ps30
Euarchonta - ps31
Primates - ps32
Haplorrhini - ps33
Simiiformes - ps34
Catarrhini - ps35
Hominoidea - ps36
Hominidae - ps37
Homininae - ps38
Hominini - ps39
Homo_sapiens - ps40
Phylostratum

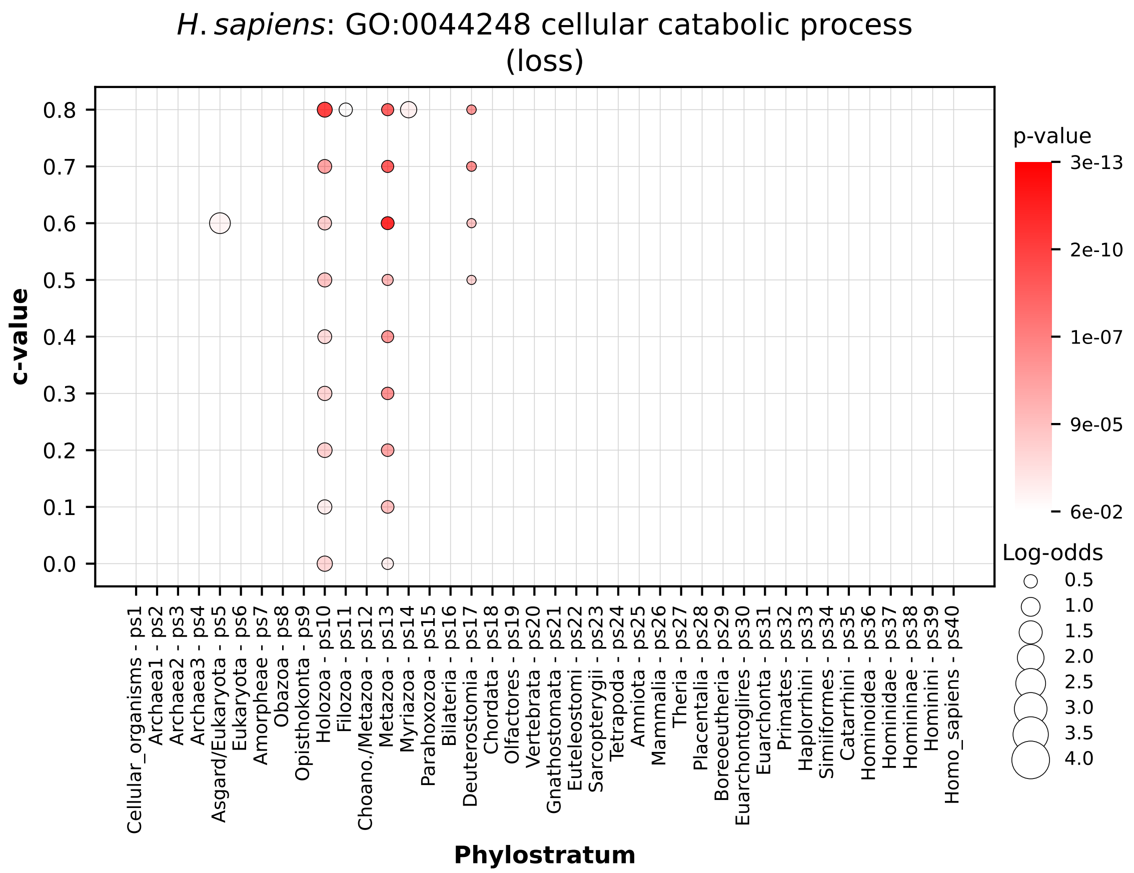

H. sapiens: GO:0044248 cellular catabolic process
(loss)
c-value
0.8
0.7
0.6
0.5
0.4
0.3
0.2
0.1
0.0
p-value
3e-13
2e-10
1e-07
9e-05
6e-02
Log-odds
0.5
1.0
1.5
2.0
2.5
3.0
3.5
4.0
Cellular_organisms - ps1
Archaea1 - ps2
Archaea2 - ps3
Archaea3 - ps4
Asgard/Eukaryota - ps5
Eukaryota - ps6
Amorpheae - ps7
Obazoa - ps8
Opisthokonta - ps9
Holozoa - ps10
Filozoa - ps11
Choano./Metazoa - ps12
Metazoa - ps13
Myriazoa - ps14
Parahoxozoa - ps15
Bilateria - ps16
Deuterostomia - ps17
Chordata - ps18
Olfactores - ps19
Vertebrata - ps20
Gnathostomata - ps21
Euteleostomi - ps22
Sarcopterygii - ps23
Tetrapoda - ps24
Amniota - ps25
Mammalia - ps26
Theria - ps27
Placentalia - ps28
Boreoeutheria - ps29
Euarchontoglires - ps30
Euarchonta - ps31
Primates - ps32
Haplorrhini - ps33
Simiiformes - ps34
Catarrhini - ps35
Hominoidea - ps36
Hominidae - ps37
Homininae - ps38
Hominini - ps39
Homo_sapiens - ps40
Phylostratum

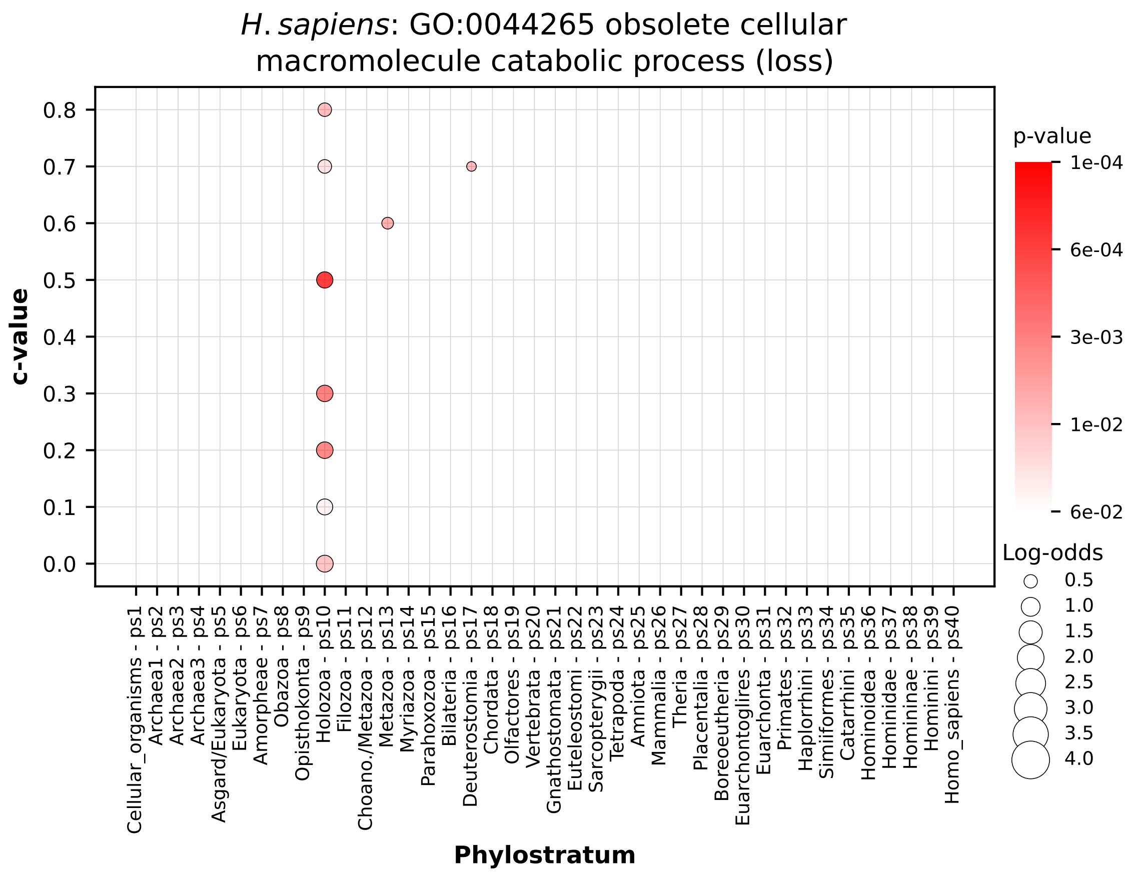
H. sapiens: GO:0044265 obsolete cellular macromolecule catabolic process (loss)
c-value
Phylostratum
p-value
1e-04
6e-04
3e-03
1e-02
6e-02
Log-odds
0.5
1.0
1.5
2.0
2.5
3.0
3.5
4.0
Cellular_organisms - ps1
Archaea1 - ps2
Archaea2 - ps3
Archaea3 - ps4
Asgard/Eukaryota - ps5
Eukaryota - ps6
Amorpheae - ps7
Obazoa - ps8
Opisthokonta - ps9
Holozoa - ps10
Filozoa - ps11
Choano./Metazoa - ps12
Metazoa - ps13
Myriazoa - ps14
Parahoxozoa - ps15
Bilateria - ps16
Deuterostomia - ps17
Chordata - ps18
Olfactores - ps19
Vertebrata - ps20
Gnathostomata - ps21
Euteleostomi - ps22
Sarcopterygii - ps23
Tetrapoda - ps24
Amniota - ps25
Mammalia - ps26
Theria - ps27
Placentalia - ps28
Boreoeutheria - ps29
Euarchontoglires - ps30
Euarchonta - ps31
Primates - ps32
Haplorrhini - ps33
Simiiformes - ps34
Catarrhini - ps35
Hominoidea - ps36
Hominidae - ps37
Homininae - ps38
Hominini - ps39
Homo_sapiens - ps40

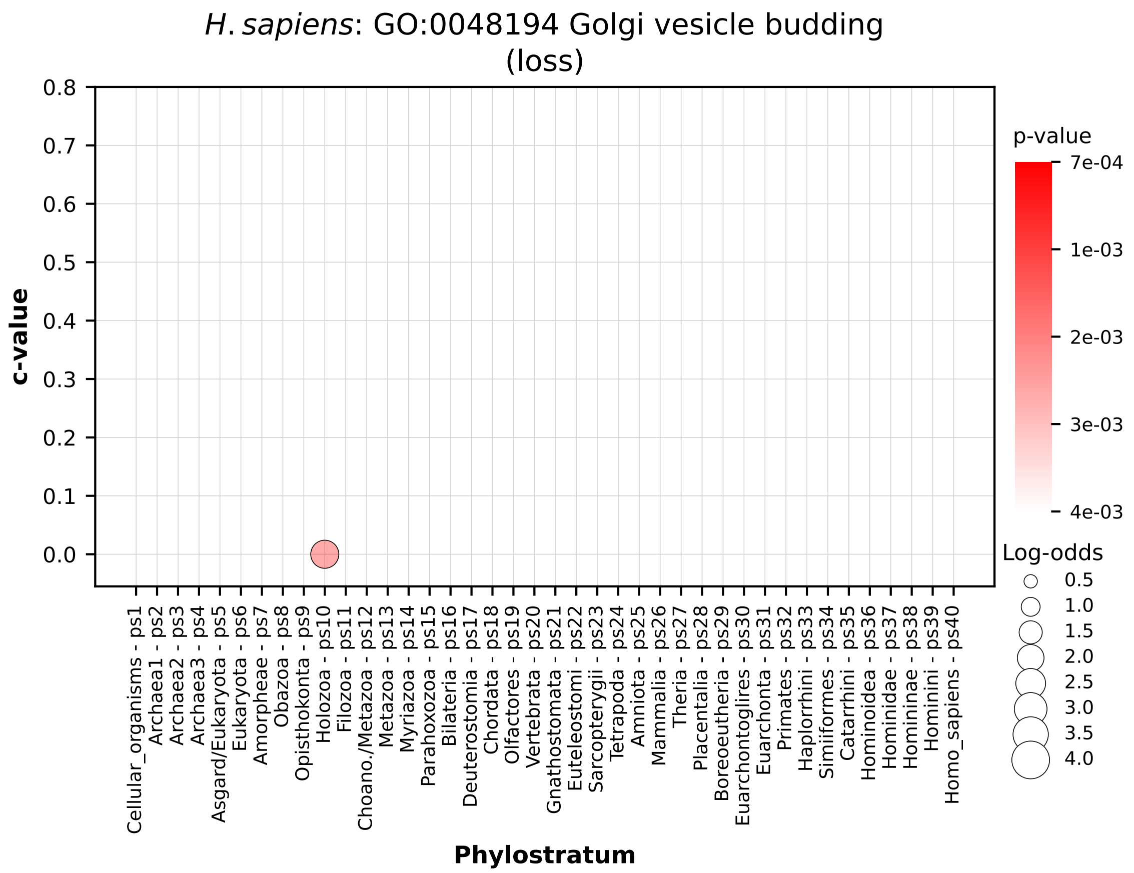

H. sapiens: GO:0048194 Golgi vesicle budding
(loss)
c-value
0.8
0.7
0.6
0.5
0.4
0.3
0.2
0.1
0.0
p-value
7e-04
1e-03
2e-03
3e-03
4e-03
Log-odds
0.5
1.0
1.5
2.0
2.5
3.0
3.5
4.0
Cellular_organisms - ps1
Archaea1 - ps2
Archaea2 - ps3
Archaea3 - ps4
Asgard/Eukaryota - ps5
Eukaryota - ps6
Amorpheae - ps7
Obazoa - ps8
Opisthokonta - ps9
Holozoa - ps10
Filozoa - ps11
Choano./Metazoa - ps12
Metazoa - ps13
Myriazoa - ps14
Parahoxozoa - ps15
Bilateria - ps16
Deuterostomia - ps17
Chordata - ps18
Olfactores - ps19
Vertebrata - ps20
Gnathostomata - ps21
Euteleostomi - ps22
Sarcopterygii - ps23
Tetrapoda - ps24
Amniota - ps25
Mammalia - ps26
Theria - ps27
Placentalia - ps28
Boreoeutheria - ps29
Euarchontoglires - ps30
Euarchonta - ps31
Primates - ps32
Haplorrhini - ps33
Simiiformes - ps34
Catarrhini - ps35
Hominoidea - ps36
Hominidae - ps37
Homininae - ps38
Hominini - ps39
Homo_sapiens - ps40
Phylostratum

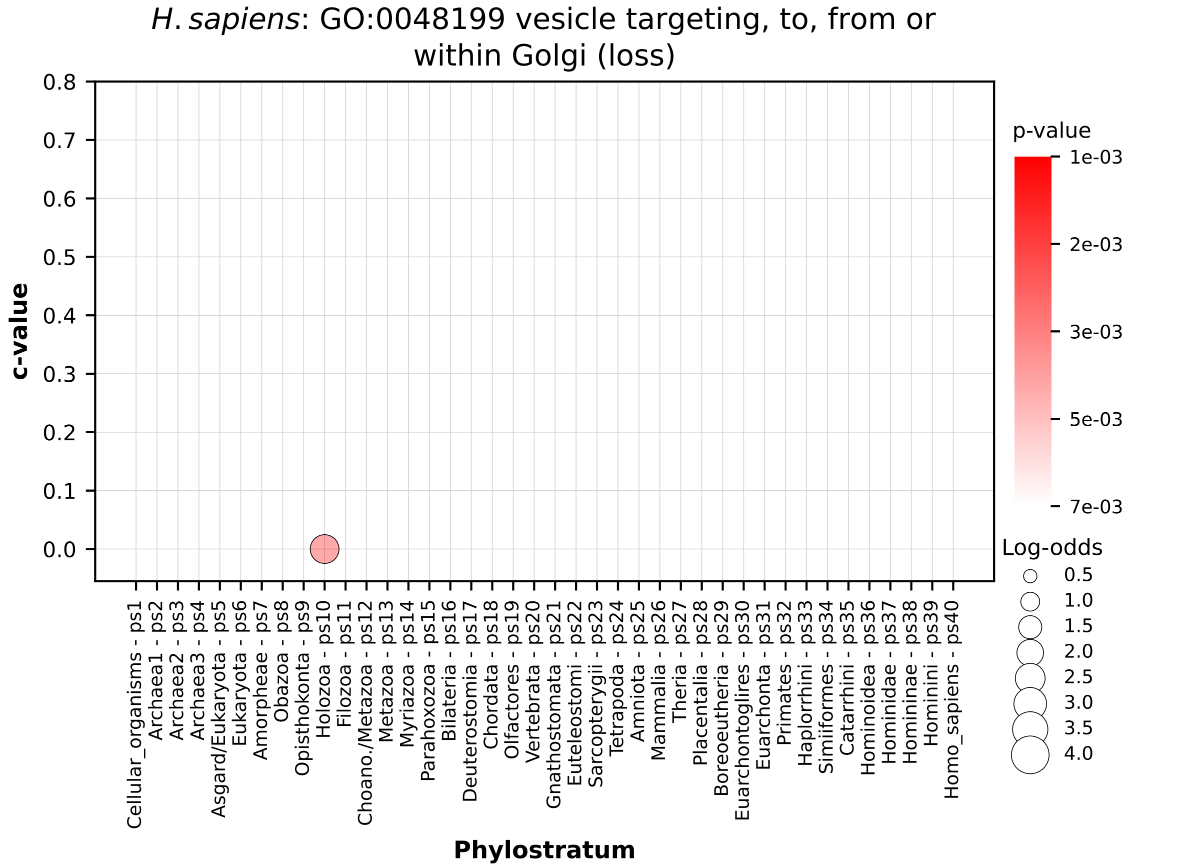

H. sapiens: GO:0048199 vesicle targeting, to, from or within Golgi (loss)
c-value
0.8
0.7
0.6
0.5
0.4
0.3
0.2
0.1
0.0
Phylostratum
Cellular_organisms - ps1
Archaea1 - ps2
Archaea2 - ps3
Archaea3 - ps4
Asgard/Eukaryota - ps5
Eukaryota - ps6
Amorpheae - ps7
Obazoa - ps8
Opisthokonta - ps9
Holozoa - ps10
Filozoa - ps11
Choano./Metazoa - ps12
Metazoa - ps13
Myriazoa - ps14
Parahoxozoa - ps15
Bilateria - ps16
Deuterostomia - ps17
Chordata - ps18
Olfactores - ps19
Vertebrata - ps20
Gnathostomata - ps21
Euteleostomi - ps22
Sarcopterygii - ps23
Tetrapoda - ps24
Amniota - ps25
Mammalia - ps26
Theria - ps27
Placentalia - ps28
Boreoeutheria - ps29
Euarchontoglires - ps30
Euarchonta - ps31
Primates - ps32
Haplorrhini - ps33
Simiiformes - ps34
Catarrhini - ps35
Hominoidea - ps36
Hominidae - ps37
Homininae - ps38
Hominini - ps39
Homo_sapiens - ps40
p-value
1e-03
2e-03
3e-03
5e-03
7e-03
Log-odds
0.5
1.0
1.5
2.0
2.5
3.0
3.5
4.0

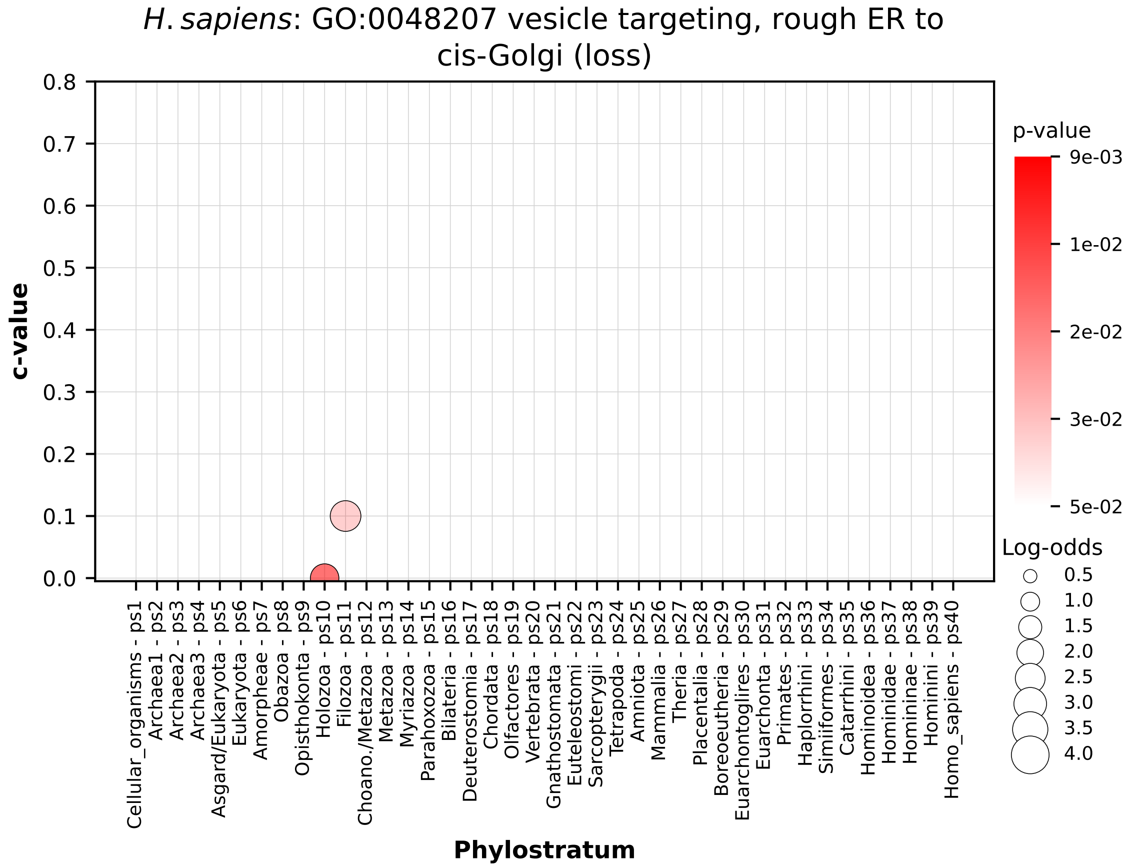

H. sapiens: GO:0048207 vesicle targeting, rough ER to cis-Golgi (loss)
c-value
0.0
0.1
0.2
0.3
0.4
0.5
0.6
0.7
0.8
Phylostratum
Cellular_organisms - ps1
Archaea1 - ps2
Archaea2 - ps3
Archaea3 - ps4
Asgard/Eukaryota - ps5
Eukaryota - ps6
Amorpheae - ps7
Obazoa - ps8
Opisthokonta - ps9
Holozoa - ps10
Filozoa - ps11
Choano./Metazoa - ps12
Metazoa - ps13
Myriazoa - ps14
Parahoxozoa - ps15
Bilateria - ps16
Deuterostomia - ps17
Chordata - ps18
Olfactores - ps19
Vertebrata - ps20
Gnathostomata - ps21
Euteleostomi - ps22
Sarcopterygii - ps23
Tetrapoda - ps24
Amniota - ps25
Mammalia - ps26
Theria - ps27
Placentalia - ps28
Boreoeutheria - ps29
Euarchontoglires - ps30
Euarchonta - ps31
Primates - ps32
Haplorrhini - ps33
Simiiformes - ps34
Catarrhini - ps35
Hominoidea - ps36
Hominidae - ps37
Homininae - ps38
Hominini - ps39
Homo_sapiens - ps40
p-value
9e-03
1e-02
2e-02
3e-02
5e-02
Log-odds
0.5
1.0
1.5
2.0
2.5
3.0
3.5
4.0

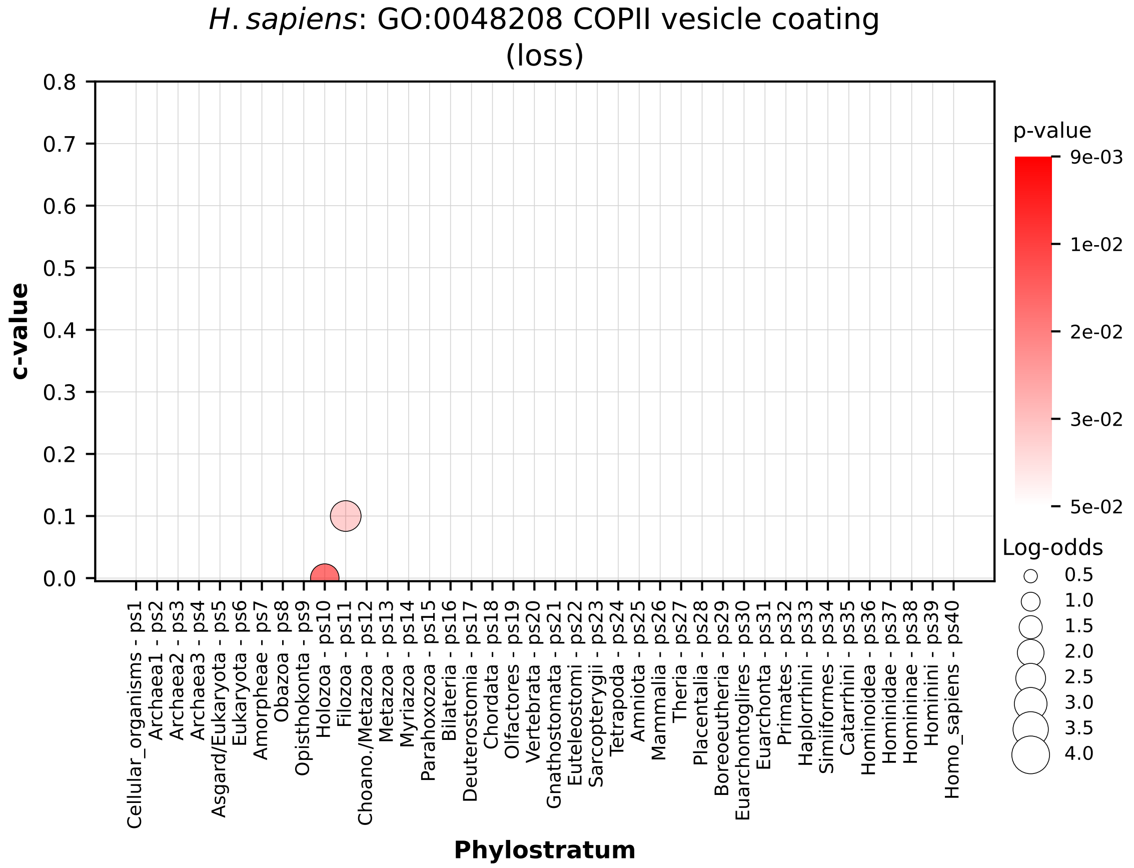
H. sapiens: GO:0048208 COPII vesicle coating
(loss)
c-value
0.8
0.7
0.6
0.5
0.4
0.3
0.2
0.1
0.0
Phylostratum
Cellular_organisms - ps1
Archaea1 - ps2
Archaea2 - ps3
Archaea3 - ps4
Asgard/Eukaryota - ps5
Eukaryota - ps6
Amorpheae - ps7
Obazoa - ps8
Opisthokonta - ps9
Holozoa - ps10
Filozoa - ps11
Choano./Metazoa - ps12
Metazoa - ps13
Myriazoa - ps14
Parahoxozoa - ps15
Bilateria - ps16
Deuterostomia - ps17
Chordata - ps18
Olfactores - ps19
Vertebrata - ps20
Gnathostomata - ps21
Euteleostomi - ps22
Sarcopterygii - ps23
Tetrapoda - ps24
Amniota - ps25
Mammalia - ps26
Theria - ps27
Placentalia - ps28
Boreoeutheria - ps29
Euarchontoglires - ps30
Euarchonta - ps31
Primates - ps32
Haplorrhini - ps33
Simiiformes - ps34
Catarrhini - ps35
Hominoidea - ps36
Hominidae - ps37
Homininae - ps38
Hominini - ps39
Homo_sapiens - ps40
p-value
9e-03
1e-02
2e-02
3e-02
5e-02
Log-odds
0.5
1.0
1.5
2.0
2.5
3.0
3.5
4.0

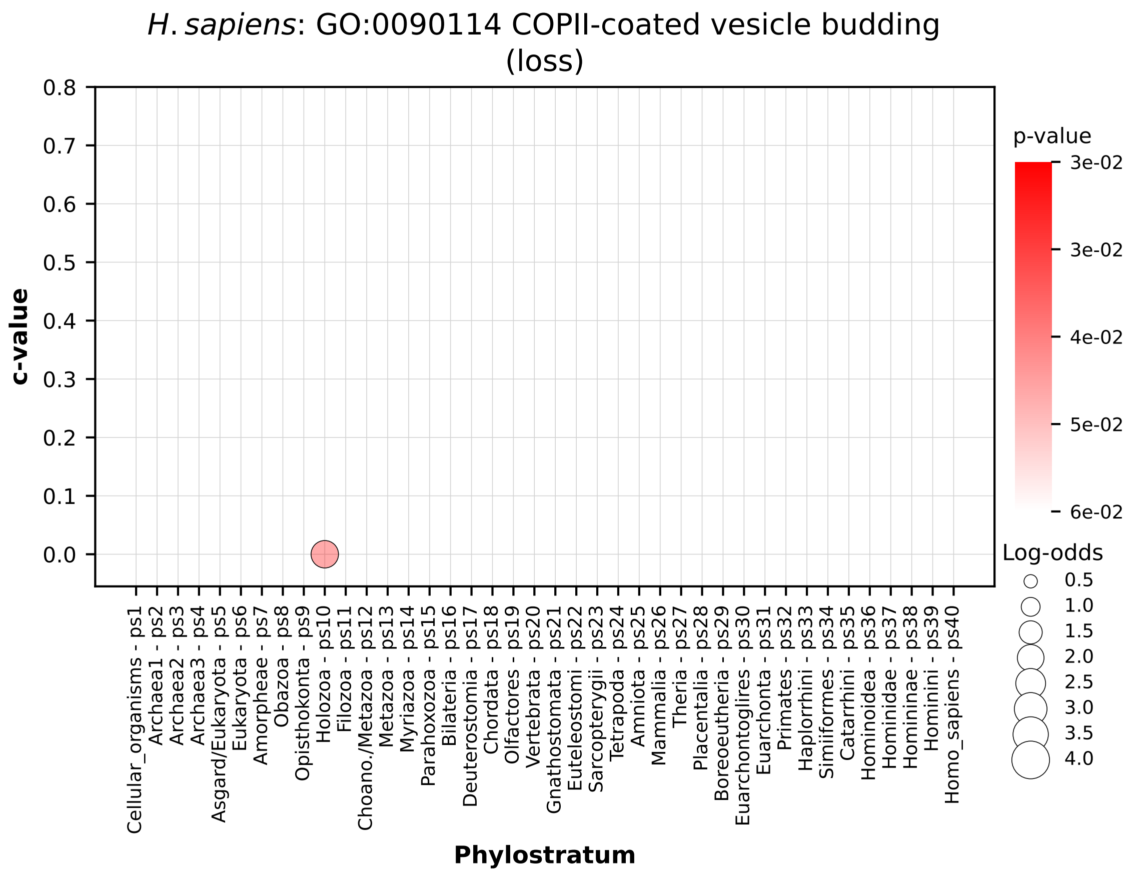

H. sapiens: GO:0090114 COPII-coated vesicle budding (loss)
c-value
0.8
0.7
0.6
0.5
0.4
0.3
0.2
0.1
0.0
p-value
3e-02
3e-02
4e-02
5e-02
6e-02
Log-odds
0.5
1.0
1.5
2.0
2.5
3.0
3.5
4.0
Cellular_organisms - ps1
Archaea1 - ps2
Archaea2 - ps3
Archaea3 - ps4
Asgard/Eukaryota - ps5
Eukaryota - ps6
Amorpheae - ps7
Obazoa - ps8
Opisthokonta - ps9
Holozoa - ps10
Filozoa - ps11
Choano./Metazoa - ps12
Metazoa - ps13
Myriazoa - ps14
Parahoxozoa - ps15
Bilateria - ps16
Deuterostomia - ps17
Chordata - ps18
Olfactores - ps19
Vertebrata - ps20
Gnathostomata - ps21
Euteleostomi - ps22
Sarcopterygii - ps23
Tetrapoda - ps24
Amniota - ps25
Mammalia - ps26
Theria - ps27
Placentalia - ps28
Boreoeutheria - ps29
Euarchontoglires - ps30
Euarchonta - ps31
Primates - ps32
Haplorrhini - ps33
Simiiformes - ps34
Catarrhini - ps35
Hominoidea - ps36
Hominidae - ps37
Homininae - ps38
Hominini - ps39
Homo_sapiens - ps40
Phylostratum

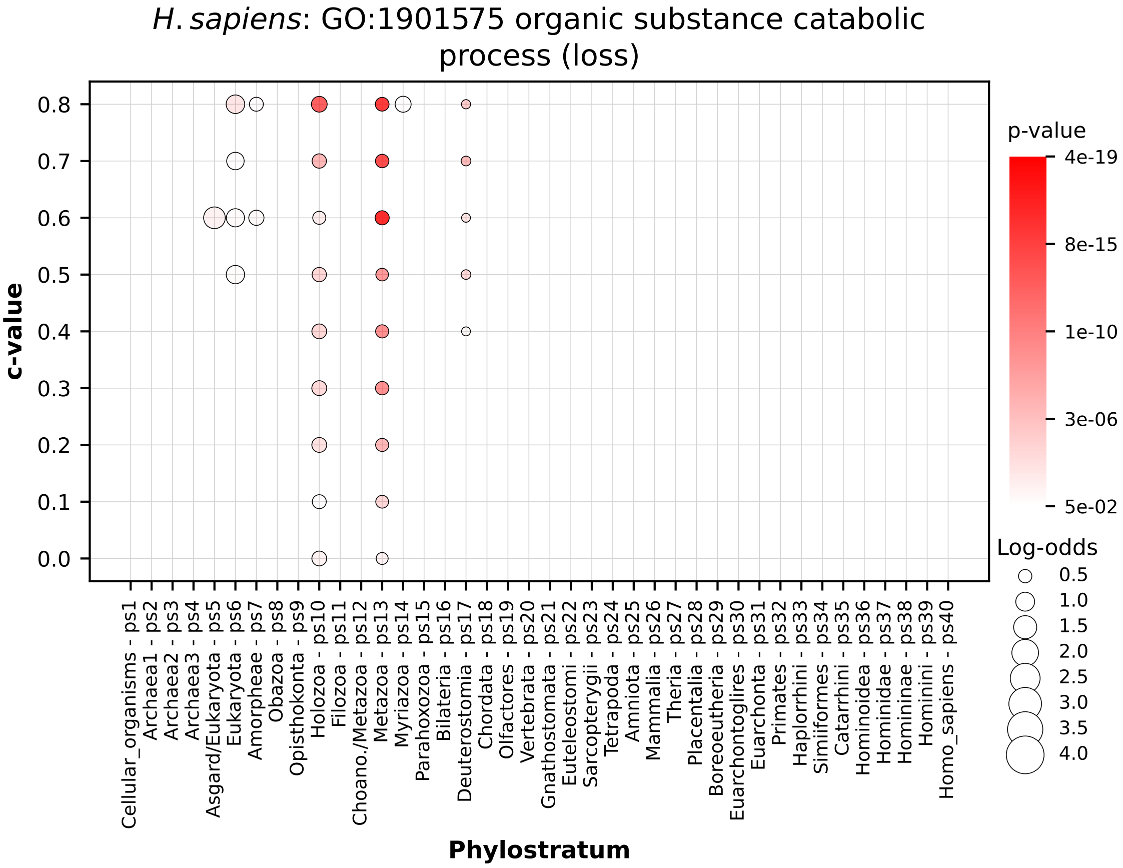
H. sapiens: GO:1901575 organic substance catabolic process (loss)
c-value
0.8
0.7
0.6
0.5
0.4
0.3
0.2
0.1
0.0
Phylostratum
Cellular_organisms - ps1
Archaea1 - ps2
Archaea2 - ps3
Archaea3 - ps4
Asgard/Eukaryota - ps5
Eukaryota - ps6
Amorpheae - ps7
Obazoa - ps8
Opisthokonta - ps9
Holozoa - ps10
Filozoa - ps11
Choano./Metazoa - ps12
Metazoa - ps13
Myriazoa - ps14
Parahoxozoa - ps15
Bilateria - ps16
Deuterostomia - ps17
Chordata - ps18
Olfactores - ps19
Vertebrata - ps20
Gnathostomata - ps21
Euteleostomi - ps22
Sarcopterygii - ps23
Tetrapoda - ps24
Amniota - ps25
Mammalia - ps26
Theria - ps27
Placentalia - ps28
Boreoeutheria - ps29
Euarchontoglires - ps30
Euarchonta - ps31
Primates - ps32
Haplorrhini - ps33
Simiiformes - ps34
Catarrhini - ps35
Hominoidea - ps36
Hominidae - ps37
Homininae - ps38
Hominini - ps39
Homo_sapiens - ps40
p-value
4e-19
8e-15
1e-10
3e-06
5e-02
Log-odds
0.5
1.0
1.5
2.0
2.5
3.0
3.5
4.0

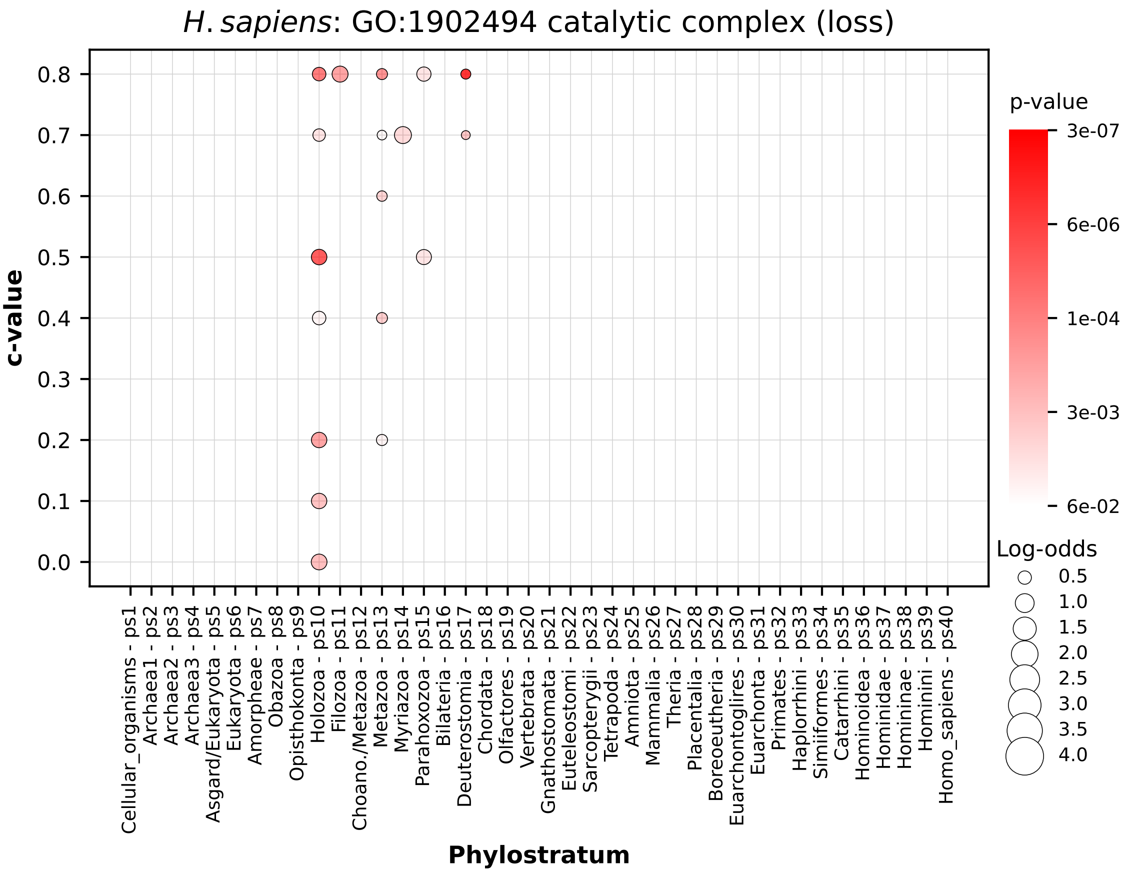

H. sapiens: GO:1902494 catalytic complex (loss)
c-value
0.0
0.1
0.2
0.3
0.4
0.5
0.6
0.7
0.8
Phylostratum
Cellular_organisms - ps1
Archaea1 - ps2
Archaea2 - ps3
Archaea3 - ps4
Asgard/Eukaryota - ps5
Eukaryota - ps6
Amorpheae - ps7
Obazoa - ps8
Opisthokonta - ps9
Holozoa - ps10
Filozoa - ps11
Choano./Metazoa - ps12
Metazoa - ps13
Myriazoa - ps14
Parahoxozoa - ps15
Bilateria - ps16
Deuterostomia - ps17
Chordata - ps18
Olfactores - ps19
Vertebrata - ps20
Gnathostomata - ps21
Euteleostomi - ps22
Sarcopterygii - ps23
Tetrapoda - ps24
Amniota - ps25
Mammalia - ps26
Theria - ps27
Placentalia - ps28
Boreoeutheria - ps29
Euarchontoglires - ps30
Euarchonta - ps31
Primates - ps32
Haplorrhini - ps33
Simiiformes - ps34
Catarrhini - ps35
Hominoidea - ps36
Hominidae - ps37
Homininae - ps38
Hominini - ps39
Homo_sapiens - ps40
p-value
3e-07
6e-06
1e-04
3e-03
6e-02
Log-odds
0.5
1.0
1.5
2.0
2.5
3.0
3.5
4.0

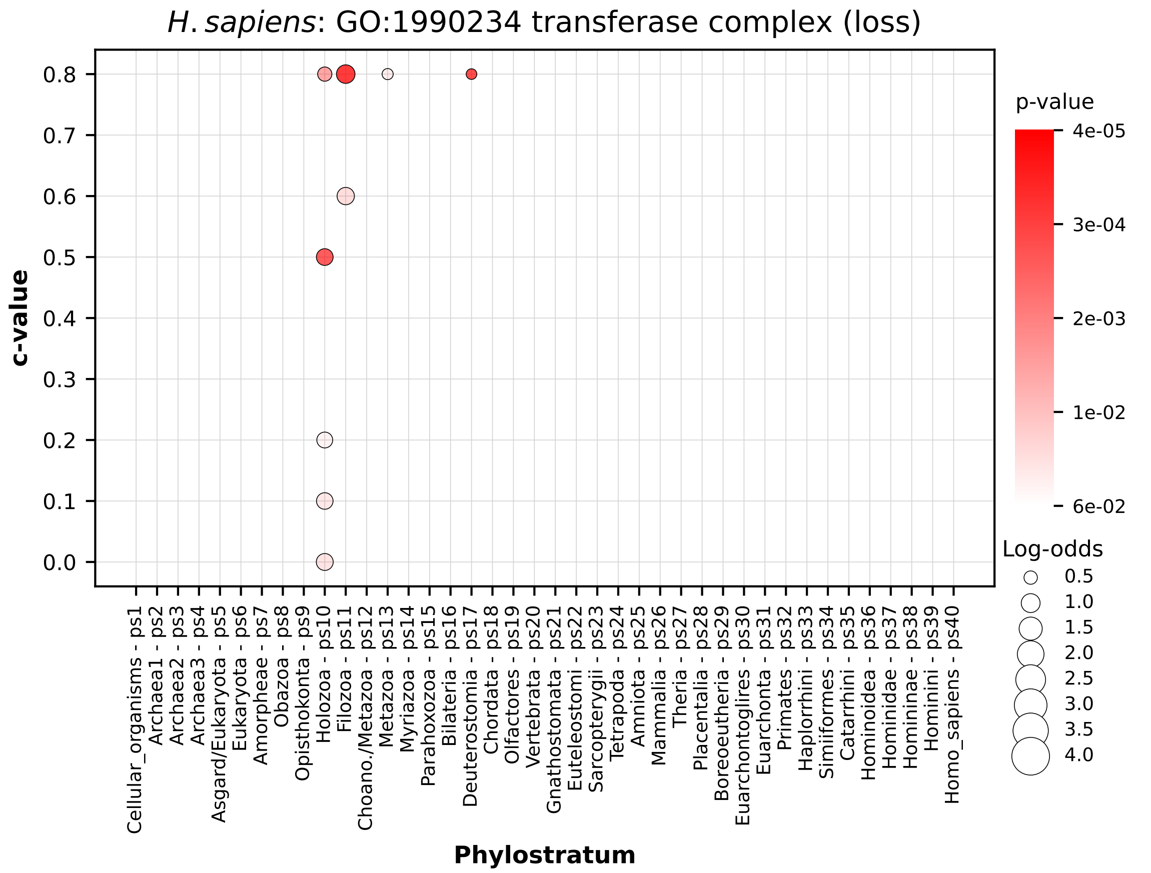

H. sapiens: GO:1990234 transferase complex (loss)
c-value
0.0
0.1
0.2
0.3
0.4
0.5
0.6
0.7
0.8
Phylostratum
Cellular_organisms - ps1
Archaea1 - ps2
Archaea2 - ps3
Archaea3 - ps4
Asgard/Eukaryota - ps5
Eukaryota - ps6
Amorpheae - ps7
Obazoa - ps8
Opisthokonta - ps9
Holozoa - ps10
Filozoa - ps11
Choano./Metazoa - ps12
Metazoa - ps13
Myriazoa - ps14
Parahoxozoa - ps15
Bilateria - ps16
Deuterostomia - ps17
Chordata - ps18
Olfactores - ps19
Vertebrata - ps20
Gnathostomata - ps21
Euteleostomi - ps22
Sarcopterygii - ps23
Tetrapoda - ps24
Amniota - ps25
Mammalia - ps26
Theria - ps27
Placentalia - ps28
Boreoeutheria - ps29
Euarchontoglires - ps30
Euarchonta - ps31
Primates - ps32
Haplorrhini - ps33
Simiiformes - ps34
Catarrhini - ps35
Hominoidea - ps36
Hominidae - ps37
Homininae - ps38
Hominini - ps39
Homo_sapiens - ps40
p-value
4e-05
3e-04
2e-03
1e-02
6e-02
Log-odds
0.5
1.0
1.5
2.0
2.5
3.0
3.5
4.0

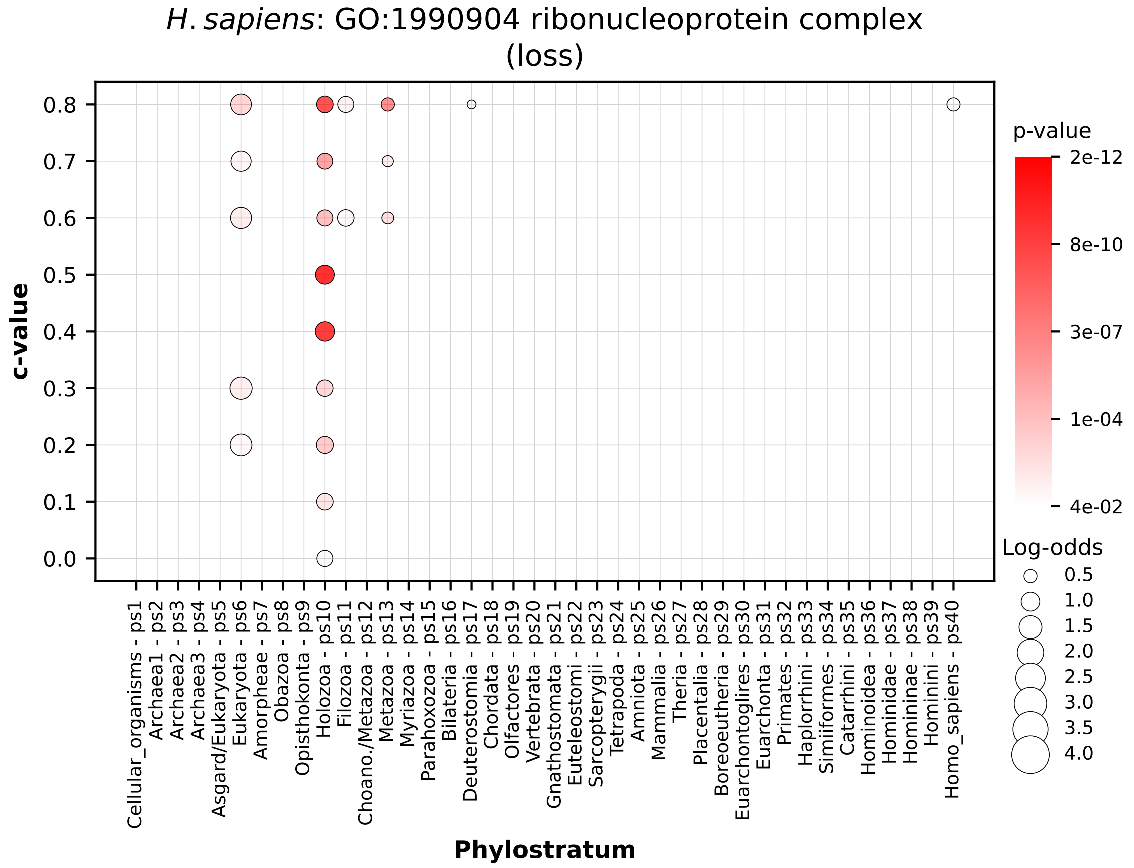

H. sapiens: GO:1990904 ribonucleoprotein complex
(loss)
c-value
0.8
0.7
0.6
0.5
0.4
0.3
0.2
0.1
0.0
Phylostratum
Cellular_organisms - ps1
Archaea1 - ps2
Archaea2 - ps3
Archaea3 - ps4
Asgard/Eukaryota - ps5
Eukaryota - ps6
Amorpheae - ps7
Obazoa - ps8
Opisthokonta - ps9
Holozoa - ps10
Filozoa - ps11
Choano./Metazoa - ps12
Metazoa - ps13
Myriazoa - ps14
Parahoxozoa - ps15
Bilateria - ps16
Deuterostomia - ps17
Chordata - ps18
Olfactores - ps19
Vertebrata - ps20
Gnathostomata - ps21
Euteleostomi - ps22
Sarcopterygii - ps23
Tetrapoda - ps24
Amniota - ps25
Mammalia - ps26
Theria - ps27
Placentalia - ps28
Boreoeutheria - ps29
Euarchontoglires - ps30
Euarchonta - ps31
Primates - ps32
Haplorrhini - ps33
Simiiformes - ps34
Catarrhini - ps35
Hominoidea - ps36
Hominidae - ps37
Homininae - ps38
Hominini - ps39
Homo_sapiens - ps40
p-value
2e-12
8e-10
3e-07
1e-04
4e-02
Log-odds
0.5
1.0
1.5
2.0
2.5
3.0
3.5
4.0

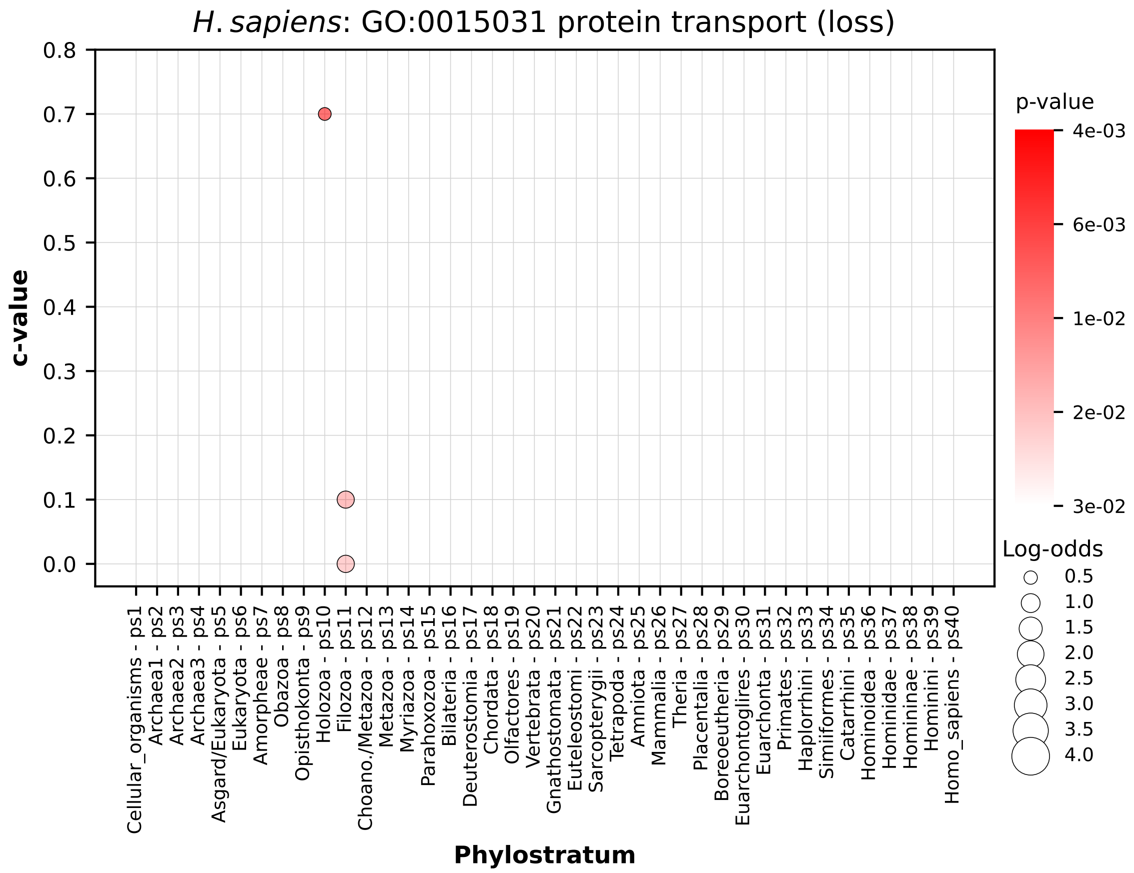

H. sapiens: GO:0015031 protein transport (loss)
c-value
0.8
0.7
0.6
0.5
0.4
0.3
0.2
0.1
0.0
Phylostratum
Cellular_organisms - ps1
Archaea1 - ps2
Archaea2 - ps3
Archaea3 - ps4
Asgard/Eukaryota - ps5
Eukaryota - ps6
Amorpheae - ps7
Obazoa - ps8
Opisthokonta - ps9
Holozoa - ps10
Filozoa - ps11
Choano./Metazoa - ps12
Metazoa - ps13
Myriazoa - ps14
Parahoxozoa - ps15
Bilateria - ps16
Deuterostomia - ps17
Chordata - ps18
Olfactores - ps19
Vertebrata - ps20
Gnathostomata - ps21
Euteleostomi - ps22
Sarcopterygii - ps23
Tetrapoda - ps24
Amniota - ps25
Mammalia - ps26
Theria - ps27
Placentalia - ps28
Boreoeutheria - ps29
Euarchontoglires - ps30
Euarchonta - ps31
Primates - ps32
Haplorrhini - ps33
Simiiformes - ps34
Catarrhini - ps35
Hominoidea - ps36
Hominidae - ps37
Homininae - ps38
Hominini - ps39
Homo_sapiens - ps40
p-value
4e-03
6e-03
1e-02
2e-02
3e-02
Log-odds
0.5
1.0
1.5
2.0
2.5
3.0
3.5
4.0

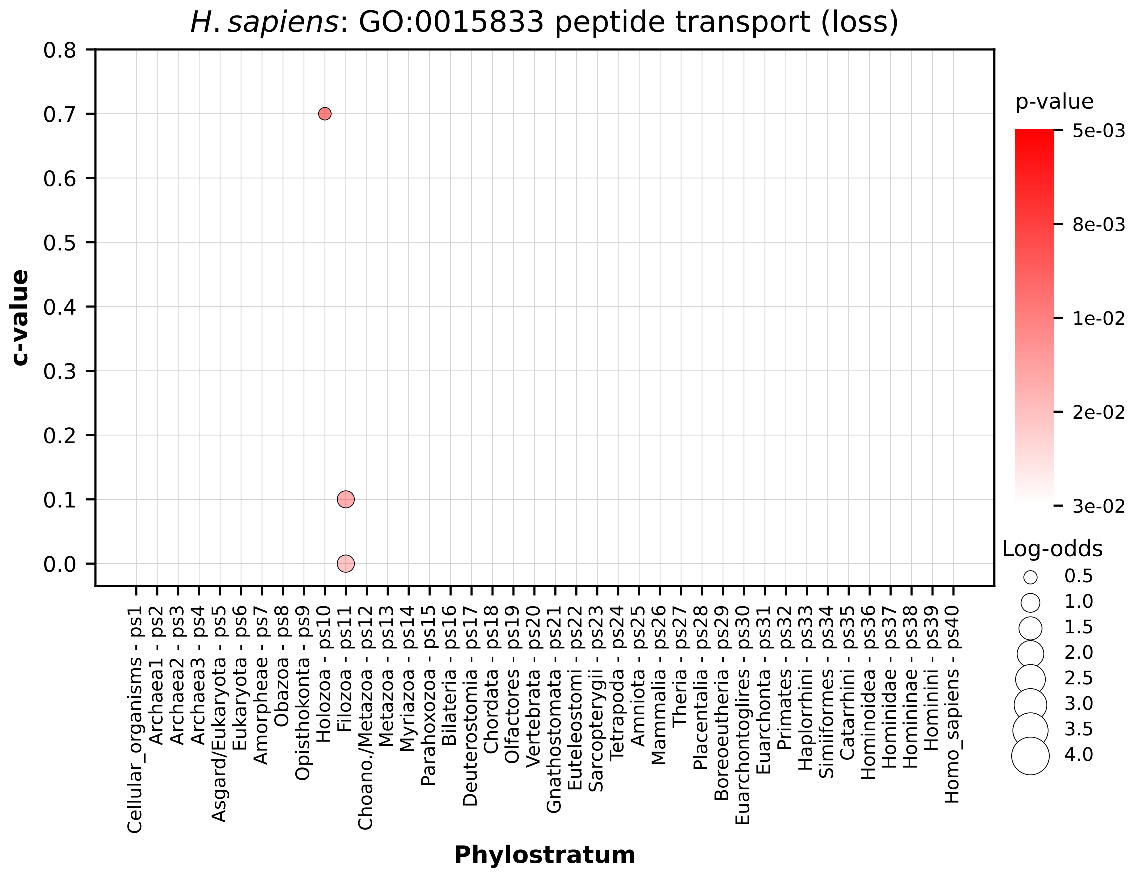

H. sapiens: GO:0015833 peptide transport (loss)
c-value
0.0
0.1
0.2
0.3
0.4
0.5
0.6
0.7
0.8
Phylostratum
Cellular_organisms - ps1
Archaea1 - ps2
Archaea2 - ps3
Archaea3 - ps4
Asgard/Eukaryota - ps5
Eukaryota - ps6
Amorpheae - ps7
Obazoa - ps8
Opisthokonta - ps9
Holozoa - ps10
Filozoa - ps11
Choano./Metazoa - ps12
Metazoa - ps13
Myriazoa - ps14
Parahoxozoa - ps15
Bilateria - ps16
Deuterostomia - ps17
Chordata - ps18
Olfactores - ps19
Vertebrata - ps20
Gnathostomata - ps21
Euteleostomi - ps22
Sarcopterygii - ps23
Tetrapoda - ps24
Amniota - ps25
Mammalia - ps26
Theria - ps27
Placentalia - ps28
Boreoeutheria - ps29
Euarchontoglires - ps30
Euarchonta - ps31
Primates - ps32
Haplorrhini - ps33
Simiiformes - ps34
Catarrhini - ps35
Hominoidea - ps36
Hominidae - ps37
Homininae - ps38
Hominini - ps39
Homo_sapiens - ps40
p-value
5e-03
8e-03
1e-02
2e-02
3e-02
Log-odds
0.5
1.0
1.5
2.0
2.5
3.0
3.5
4.0

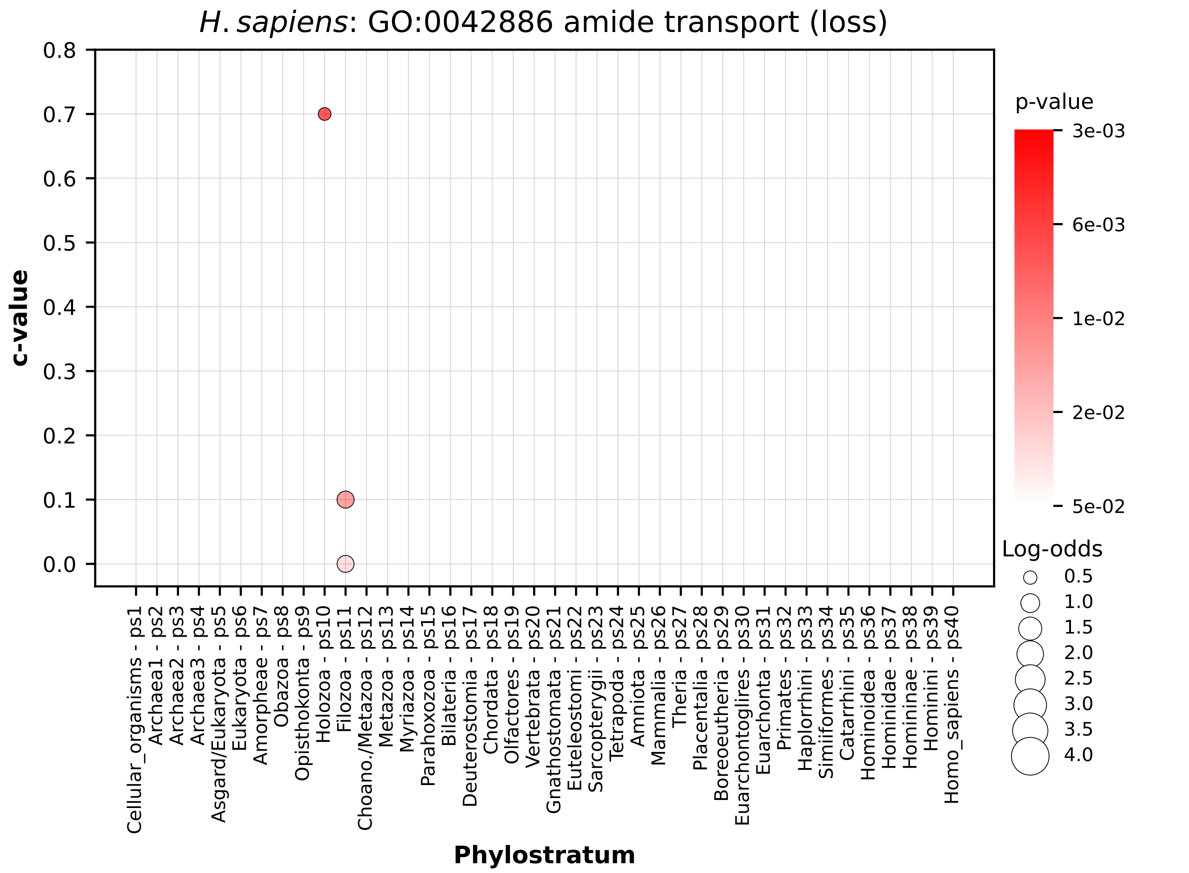

H. sapiens: GO:0042886 amide transport (loss)
c-value
0.8
0.7
0.6
0.5
0.4
0.3
0.2
0.1
0.0
Phylostratum
Cellular_organisms - ps1
Archaea1 - ps2
Archaea2 - ps3
Archaea3 - ps4
Asgard/Eukaryota - ps5
Eukaryota - ps6
Amorpheae - ps7
Obazoa - ps8
Opisthokonta - ps9
Holozoa - ps10
Filozoa - ps11
Choano./Metazoa - ps12
Metazoa - ps13
Myriazoa - ps14
Parahoxozoa - ps15
Bilateria - ps16
Deuterostomia - ps17
Chordata - ps18
Olfactores - ps19
Vertebrata - ps20
Gnathostomata - ps21
Euteleostomi - ps22
Sarcopterygii - ps23
Tetrapoda - ps24
Amniota - ps25
Mammalia - ps26
Theria - ps27
Placentalia - ps28
Boreoeutheria - ps29
Euarchontoglires - ps30
Euarchonta - ps31
Primates - ps32
Haplorrhini - ps33
Simiiformes - ps34
Catarrhini - ps35
Hominoidea - ps36
Hominidae - ps37
Homininae - ps38
Hominini - ps39
Homo_sapiens - ps40
p-value
3e-03
6e-03
1e-02
2e-02
5e-02
Log-odds
0.5
1.0
1.5
2.0
2.5
3.0
3.5
4.0

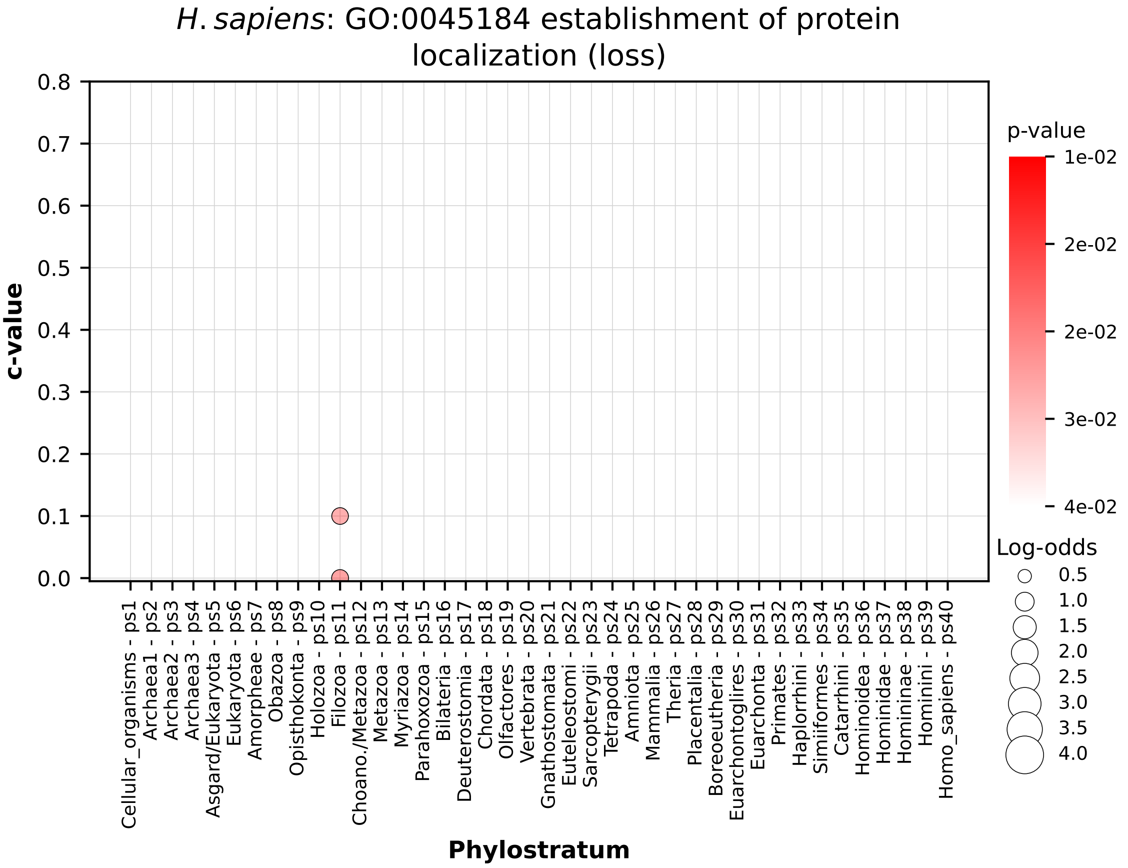

H. sapiens: GO:0045184 establishment of protein localization (loss)
c-value
0.0
0.1
0.2
0.3
0.4
0.5
0.6
0.7
0.8
Phylostratum
Cellular_organisms - ps1
Archaea1 - ps2
Archaea2 - ps3
Archaea3 - ps4
Asgard/Eukaryota - ps5
Eukaryota - ps6
Amorpheae - ps7
Obazoa - ps8
Opisthokonta - ps9
Holozoa - ps10
Filozoa - ps11
Choano./Metazoa - ps12
Metazoa - ps13
Myriazoa - ps14
Parahoxozoa - ps15
Bilateria - ps16
Deuterostomia - ps17
Chordata - ps18
Olfactores - ps19
Vertebrata - ps20
Gnathostomata - ps21
Euteleostomi - ps22
Sarcopterygii - ps23
Tetrapoda - ps24
Amniota - ps25
Mammalia - ps26
Theria - ps27
Placentalia - ps28
Boreoeutheria - ps29
Euarchontoglires - ps30
Euarchonta - ps31
Primates - ps32
Haplorrhini - ps33
Simiiformes - ps34
Catarrhini - ps35
Hominoidea - ps36
Hominidae - ps37
Homininae - ps38
Hominini - ps39
Homo_sapiens - ps40
p-value
1e-02
2e-02
2e-02
3e-02
4e-02
Log-odds
0.5
1.0
1.5
2.0
2.5
3.0
3.5
4.0

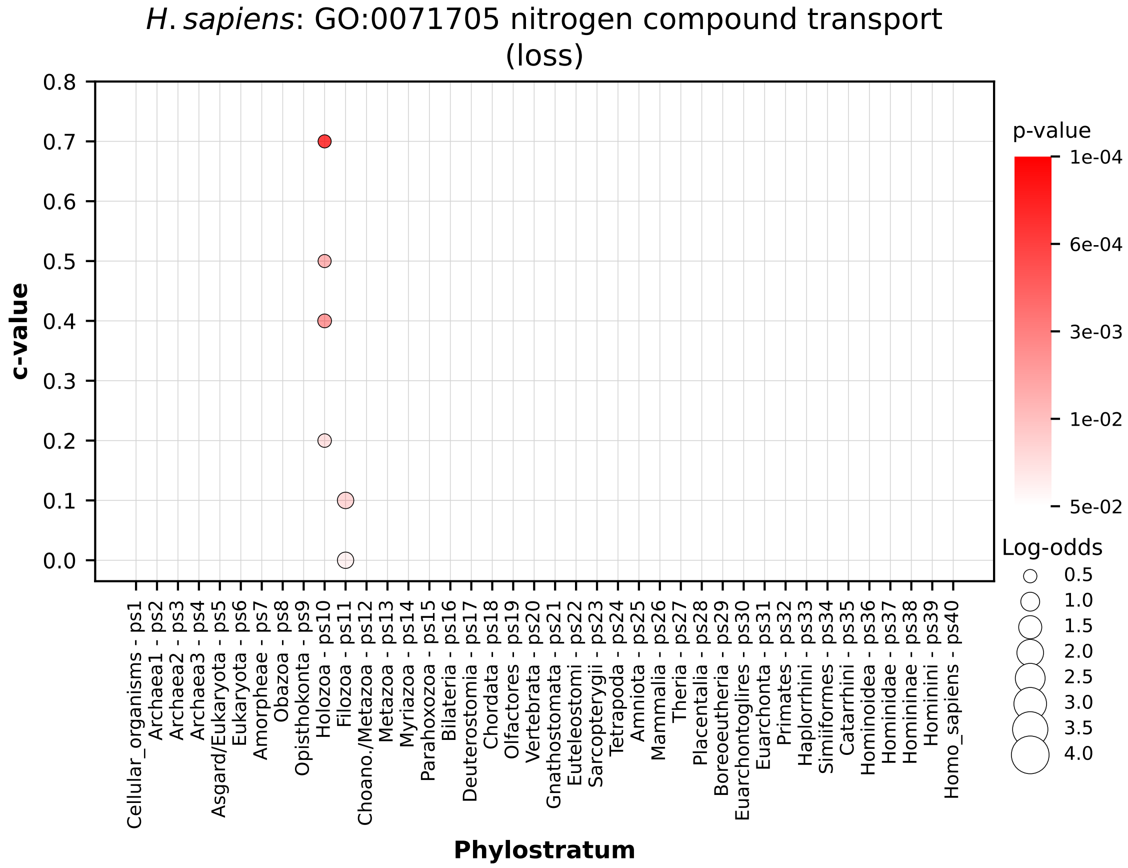

H. sapiens: GO:0071705 nitrogen compound transport
(loss)
c-value
0.8
0.7
0.6
0.5
0.4
0.3
0.2
0.1
0.0
p-value
1e-04
6e-04
3e-03
1e-02
5e-02
Log-odds
0.5
1.0
1.5
2.0
2.5
3.0
3.5
4.0
Cellular_organisms - ps1
Archaea1 - ps2
Archaea2 - ps3
Archaea3 - ps4
Asgard/Eukaryota - ps5
Eukaryota - ps6
Amorpheae - ps7
Obazoa - ps8
Opisthokonta - ps9
Holozoa - ps10
Filozoa - ps11
Choano./Metazoa - ps12
Metazoa - ps13
Myriazoa - ps14
Parahoxozoa - ps15
Bilateria - ps16
Deuterostomia - ps17
Chordata - ps18
Olfactores - ps19
Vertebrata - ps20
Gnathostomata - ps21
Euteleostomi - ps22
Sarcopterygii - ps23
Tetrapoda - ps24
Amniota - ps25
Mammalia - ps26
Theria - ps27
Placentalia - ps28
Boreoeutheria - ps29
Euarchontoglires - ps30
Euarchonta - ps31
Primates - ps32
Haplorrhini - ps33
Simiiformes - ps34
Catarrhini - ps35
Hominoidea - ps36
Hominidae - ps37
Homininae - ps38
Hominini - ps39
Homo_sapiens - ps40
Phylostratum

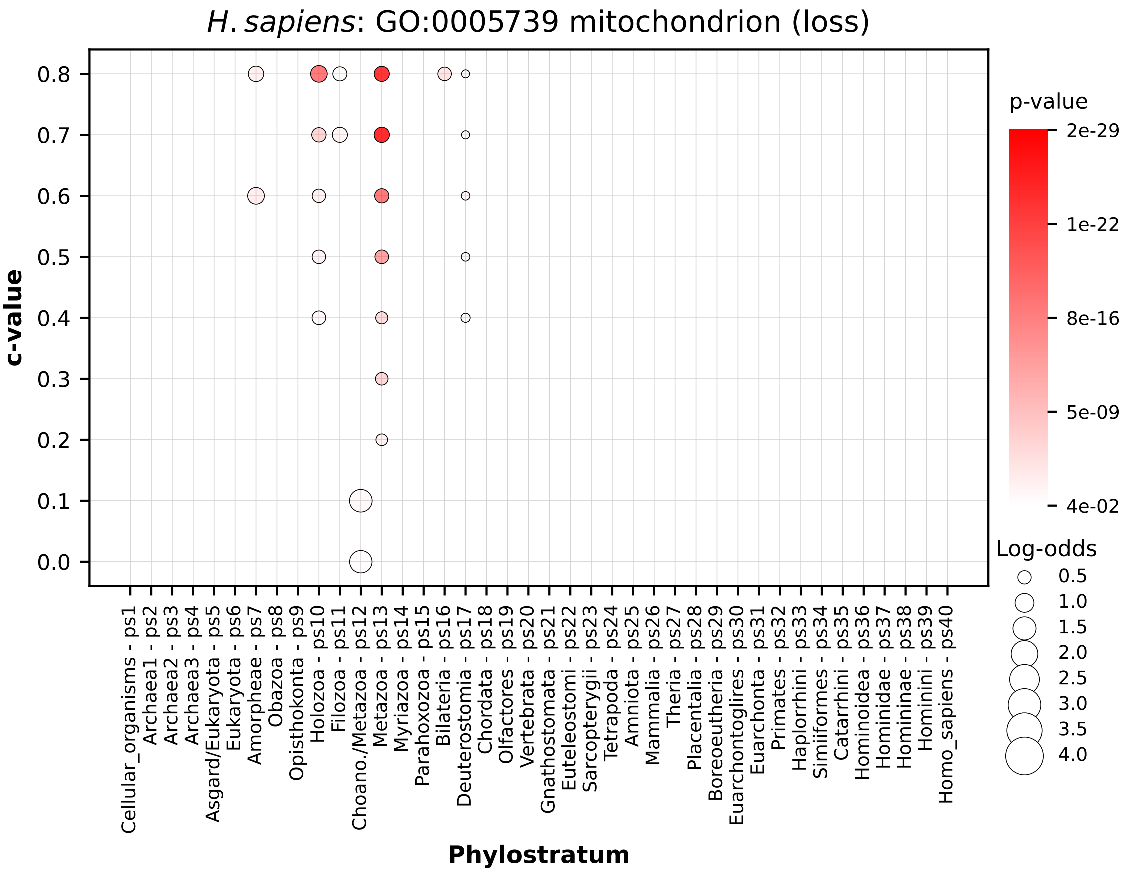

H. sapiens: GO:0005739 mitochondrion (loss)
c-value
0.8
0.7
0.6
0.5
0.4
0.3
0.2
0.1
0.0
Phylostratum
Cellular_organisms - ps1
Archaea1 - ps2
Archaea2 - ps3
Archaea3 - ps4
Asgard/Eukaryota - ps5
Eukaryota - ps6
Amorpheae - ps7
Obazoa - ps8
Opisthokonta - ps9
Holozoa - ps10
Filozoa - ps11
Choano./Metazoa - ps12
Metazoa - ps13
Myriazoa - ps14
Parahoxozoa - ps15
Bilateria - ps16
Deuterostomia - ps17
Chordata - ps18
Olfactores - ps19
Vertebrata - ps20
Gnathostomata - ps21
Euteleostomi - ps22
Sarcopterygii - ps23
Tetrapoda - ps24
Amniota - ps25
Mammalia - ps26
Theria - ps27
Placentalia - ps28
Boreoeutheria - ps29
Euarchontoglires - ps30
Euarchonta - ps31
Primates - ps32
Haplorrhini - ps33
Simiiformes - ps34
Catarrhini - ps35
Hominoidea - ps36
Hominidae - ps37
Homininae - ps38
Hominini - ps39
Homo_sapiens - ps40
p-value
2e-29
1e-22
8e-16
5e-09
4e-02
Log-odds
0.5
1.0
1.5
2.0
2.5
3.0
3.5
4.0

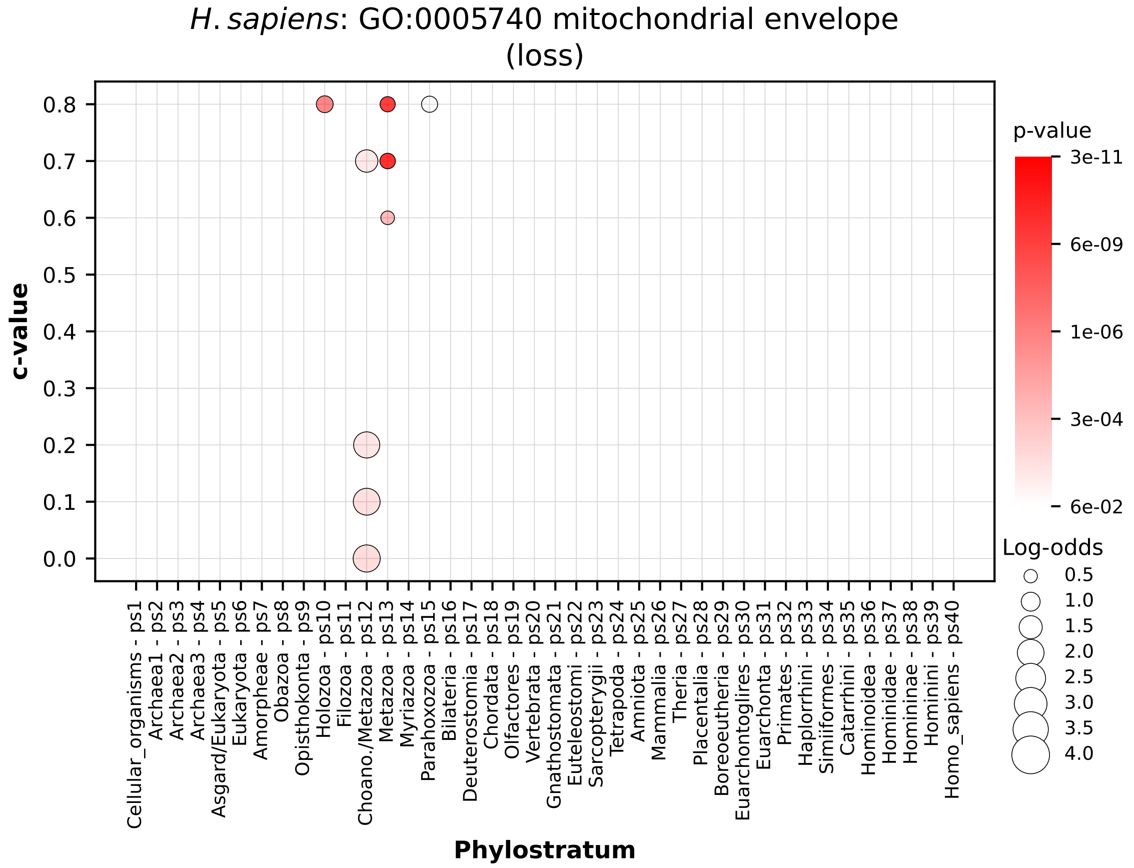

H. sapiens: GO:0005740 mitochondrial envelope
(loss)
c-value
0.8
0.7
0.6
0.5
0.4
0.3
0.2
0.1
0.0
Phylostratum
Cellular_organisms - ps1
Archaea1 - ps2
Archaea2 - ps3
Archaea3 - ps4
Asgard/Eukaryota - ps5
Eukaryota - ps6
Amorpheae - ps7
Obazoa - ps8
Opisthokonta - ps9
Holozoa - ps10
Filozoa - ps11
Choano./Metazoa - ps12
Metazoa - ps13
Myriazoa - ps14
Parahoxozoa - ps15
Bilateria - ps16
Deuterostomia - ps17
Chordata - ps18
Olfactores - ps19
Vertebrata - ps20
Gnathostomata - ps21
Euteleostomi - ps22
Sarcopterygii - ps23
Tetrapoda - ps24
Amniota - ps25
Mammalia - ps26
Theria - ps27
Placentalia - ps28
Boreoeutheria - ps29
Euarchontoglires - ps30
Euarchonta - ps31
Primates - ps32
Haplorrhini - ps33
Simiiformes - ps34
Catarrhini - ps35
Hominoidea - ps36
Hominidae - ps37
Homininae - ps38
Hominini - ps39
Homo_sapiens - ps40
p-value
3e-11
6e-09
1e-06
3e-04
6e-02
Log-odds
0.5
1.0
1.5
2.0
2.5
3.0
3.5
4.0

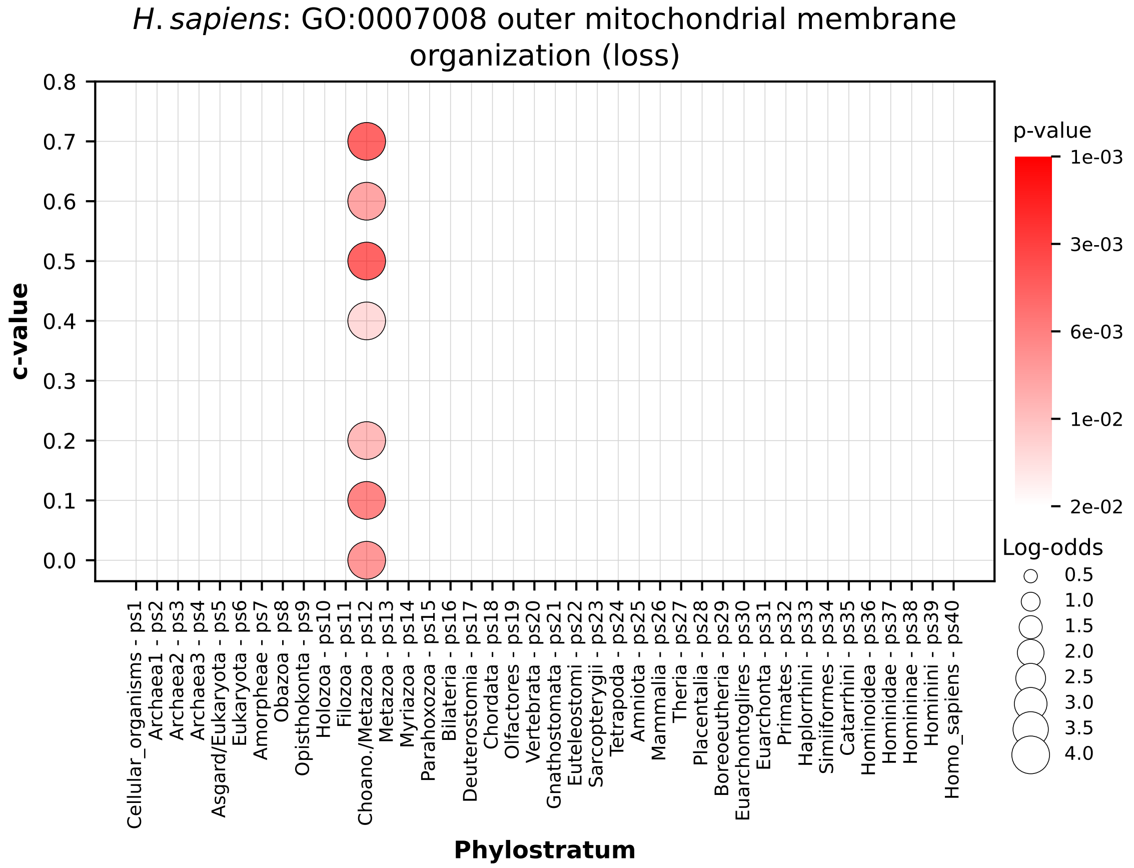

H. sapiens: GO:0007008 outer mitochondrial membrane organization (loss)
c-value
0.0
0.1
0.2
0.3
0.4
0.5
0.6
0.7
0.8
p-value
1e-03
3e-03
6e-03
1e-02
2e-02
Log-odds
0.5
1.0
1.5
2.0
2.5
3.0
3.5
4.0
Cellular_organisms - ps1
Archaea1 - ps2
Archaea2 - ps3
Archaea3 - ps4
Asgard/Eukaryota - ps5
Eukaryota - ps6
Amorpheae - ps7
Obazoa - ps8
Opisthokonta - ps9
Holozoa - ps10
Filozoa - ps11
Choano./Metazoa - ps12
Metazoa - ps13
Myriazoa - ps14
Parahoxozoa - ps15
Bilateria - ps16
Deuterostomia - ps17
Chordata - ps18
Olfactores - ps19
Vertebrata - ps20
Gnathostomata - ps21
Euteleostomi - ps22
Sarcopterygii - ps23
Tetrapoda - ps24
Amniota - ps25
Mammalia - ps26
Theria - ps27
Placentalia - ps28
Boreoeutheria - ps29
Euarchontoglires - ps30
Euarchonta - ps31
Primates - ps32
Haplorrhini - ps33
Simiiformes - ps34
Catarrhini - ps35
Hominoidea - ps36
Hominidae - ps37
Homininae - ps38
Hominini - ps39
Homo_sapiens - ps40
Phylostratum

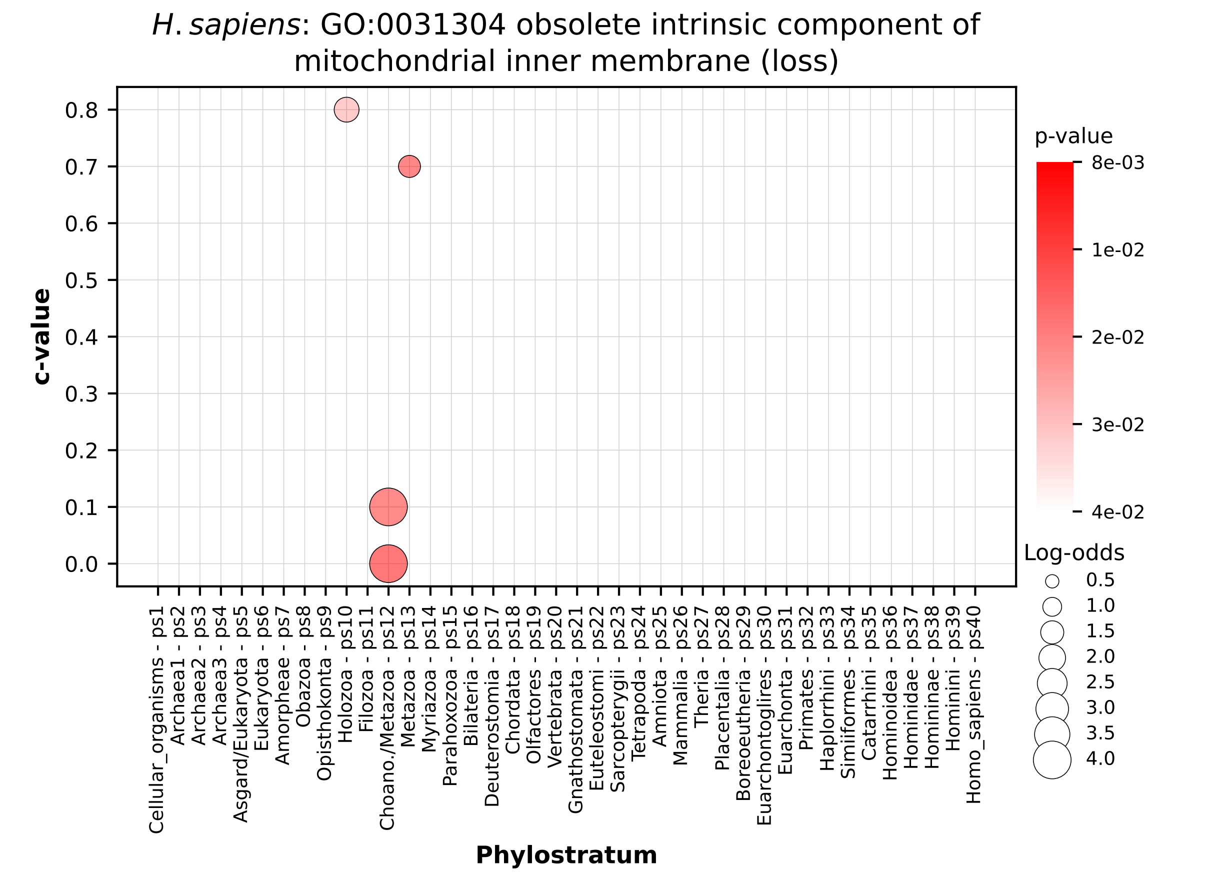

H. sapiens: GO:0031304 obsolete intrinsic component of mitochondrial inner membrane (loss)
c-value
0.8
0.7
0.6
0.5
0.4
0.3
0.2
0.1
0.0
Phylostratum
Cellular_organisms - ps1
Archaea1 - ps2
Archaea2 - ps3
Archaea3 - ps4
Asgard/Eukaryota - ps5
Eukaryota - ps6
Amorpheae - ps7
Obazoa - ps8
Opisthokonta - ps9
Holozoa - ps10
Filozoa - ps11
Choano./Metazoa - ps12
Metazoa - ps13
Myriazoa - ps14
Parahoxozoa - ps15
Bilateria - ps16
Deuterostomia - ps17
Chordata - ps18
Olfactores - ps19
Vertebrata - ps20
Gnathostomata - ps21
Euteleostomi - ps22
Sarcopterygii - ps23
Tetrapoda - ps24
Amniota - ps25
Mammalia - ps26
Theria - ps27
Placentalia - ps28
Boreoeutheria - ps29
Euarchontoglires - ps30
Euarchonta - ps31
Primates - ps32
Haplorrhini - ps33
Simiiformes - ps34
Catarrhini - ps35
Hominoidea - ps36
Hominidae - ps37
Homininae - ps38
Hominini - ps39
Homo_sapiens - ps40
p-value
8e-03
1e-02
2e-02
3e-02
4e-02
Log-odds
0.5
1.0
1.5
2.0
2.5
3.0
3.5
4.0

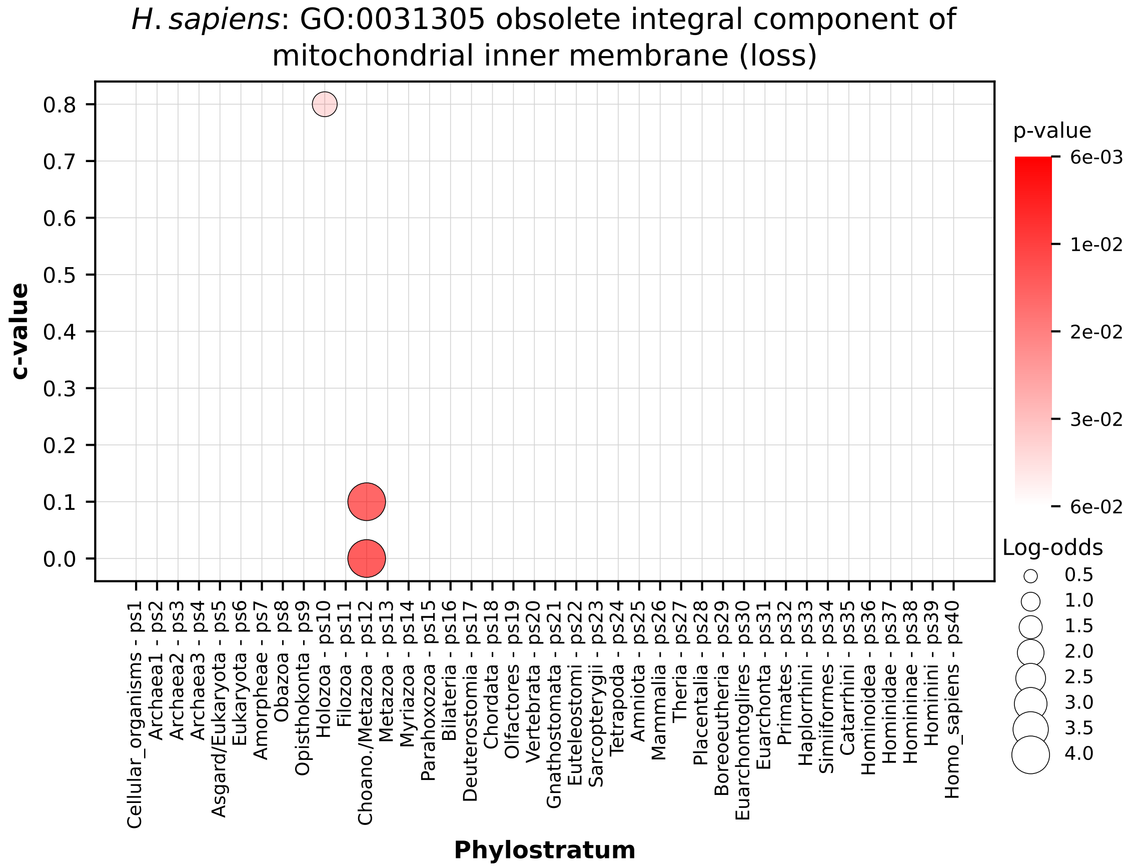
H. sapiens: GO:0031305 obsolete integral component of mitochondrial inner membrane (loss)
c-value
0.8
0.7
0.6
0.5
0.4
0.3
0.2
0.1
0.0
Phylostratum
Cellular_organisms - ps1
Archaea1 - ps2
Archaea2 - ps3
Archaea3 - ps4
Asgard/Eukaryota - ps5
Eukaryota - ps6
Amorpheae - ps7
Obazoa - ps8
Opisthokonta - ps9
Holozoa - ps10
Filozoa - ps11
Choano./Metazoa - ps12
Metazoa - ps13
Myriazoa - ps14
Parahoxozoa - ps15
Bilateria - ps16
Deuterostomia - ps17
Chordata - ps18
Olfactores - ps19
Vertebrata - ps20
Gnathostomata - ps21
Euteleostomi - ps22
Sarcopterygii - ps23
Tetrapoda - ps24
Amniota - ps25
Mammalia - ps26
Theria - ps27
Placentalia - ps28
Boreoeutheria - ps29
Euarchontoglires - ps30
Euarchonta - ps31
Primates - ps32
Haplorrhini - ps33
Simiiformes - ps34
Catarrhini - ps35
Hominoidea - ps36
Hominidae - ps37
Homininae - ps38
Hominini - ps39
Homo_sapiens - ps40
p-value
6e-03
1e-02
2e-02
3e-02
6e-02
Log-odds
0.5
1.0
1.5
2.0
2.5
3.0
3.5
4.0

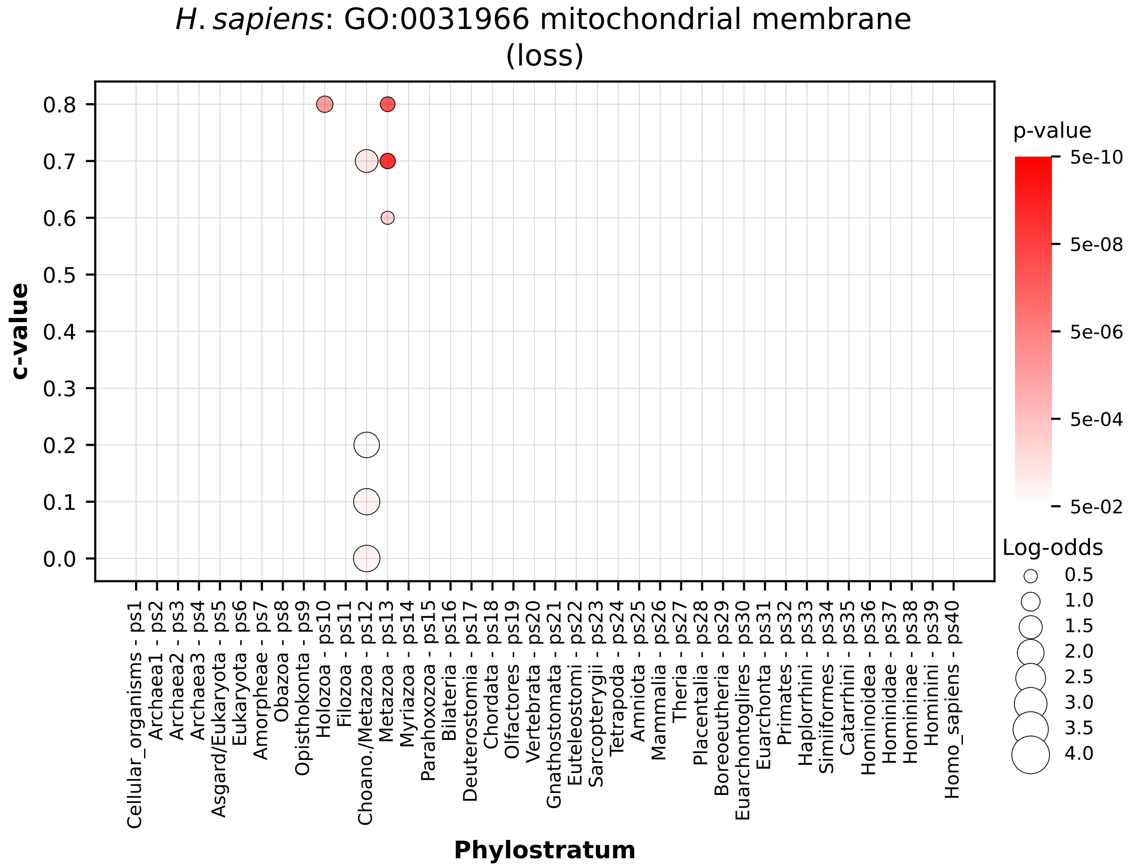
H. sapiens: GO:0031966 mitochondrial membrane
(loss)
c-value
0.8
0.7
0.6
0.5
0.4
0.3
0.2
0.1
0.0
p-value
5e-10
5e-08
5e-06
5e-04
5e-02
Log-odds
0.5
1.0
1.5
2.0
2.5
3.0
3.5
4.0
Cellular_organisms - ps1
Archaea1 - ps2
Archaea2 - ps3
Archaea3 - ps4
Asgard/Eukaryota - ps5
Eukaryota - ps6
Amorpheae - ps7
Obazoa - ps8
Opisthokonta - ps9
Holozoa - ps10
Filozoa - ps11
Choano./Metazoa - ps12
Metazoa - ps13
Myriazoa - ps14
Parahoxozoa - ps15
Bilateria - ps16
Deuterostomia - ps17
Chordata - ps18
Olfactores - ps19
Vertebrata - ps20
Gnathostomata - ps21
Euteleostomi - ps22
Sarcopterygii - ps23
Tetrapoda - ps24
Amniota - ps25
Mammalia - ps26
Theria - ps27
Placentalia - ps28
Boreoeutheria - ps29
Euarchontoglires - ps30
Euarchonta - ps31
Primates - ps32
Haplorrhini - ps33
Simiiformes - ps34
Catarrhini - ps35
Hominoidea - ps36
Hominidae - ps37
Homininae - ps38
Hominini - ps39
Homo_sapiens - ps40
Phylostratum

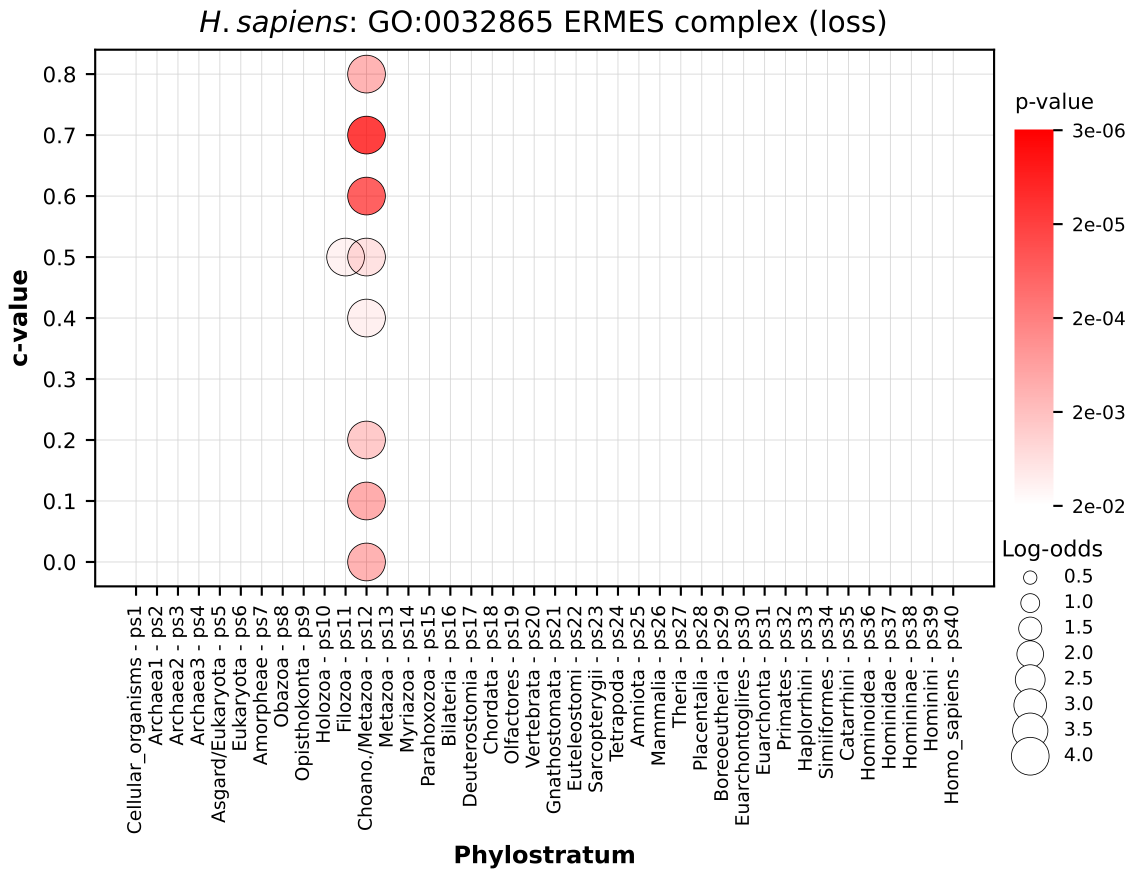

$H. sapiens$: GO:0032865 ERMES complex (loss)
c-value
0.8
0.7
0.6
0.5
0.4
0.3
0.2
0.1
0.0
Phylostratum
Cellular_organisms - ps1
Archaea1 - ps2
Archaea2 - ps3
Archaea3 - ps4
Asgard/Eukaryota - ps5
Eukaryota - ps6
Amorpheae - ps7
Obazoa - ps8
Opisthokonta - ps9
Holozoa - ps10
Filozoa - ps11
Choano./Metazoa - ps12
Metazoa - ps13
Myriazoa - ps14
Parahoxozoa - ps15
Bilateria - ps16
Deuterostomia - ps17
Chordata - ps18
Olfactores - ps19
Vertebrata - ps20
Gnathostomata - ps21
Euteleostomi - ps22
Sarcopterygii - ps23
Tetrapoda - ps24
Amniota - ps25
Mammalia - ps26
Theria - ps27
Placentalia - ps28
Boreoeutheria - ps29
Euarchontoglires - ps30
Euarchonta - ps31
Primates - ps32
Haplorrhini - ps33
Simiiformes - ps34
Catarrhini - ps35
Hominoidea - ps36
Hominidae - ps37
Homininae - ps38
Hominini - ps39
Homo_sapiens - ps40
p-value
3e-06
2e-05
2e-04
2e-03
2e-02
Log-odds
0.5
1.0
1.5
2.0
2.5
3.0
3.5
4.0

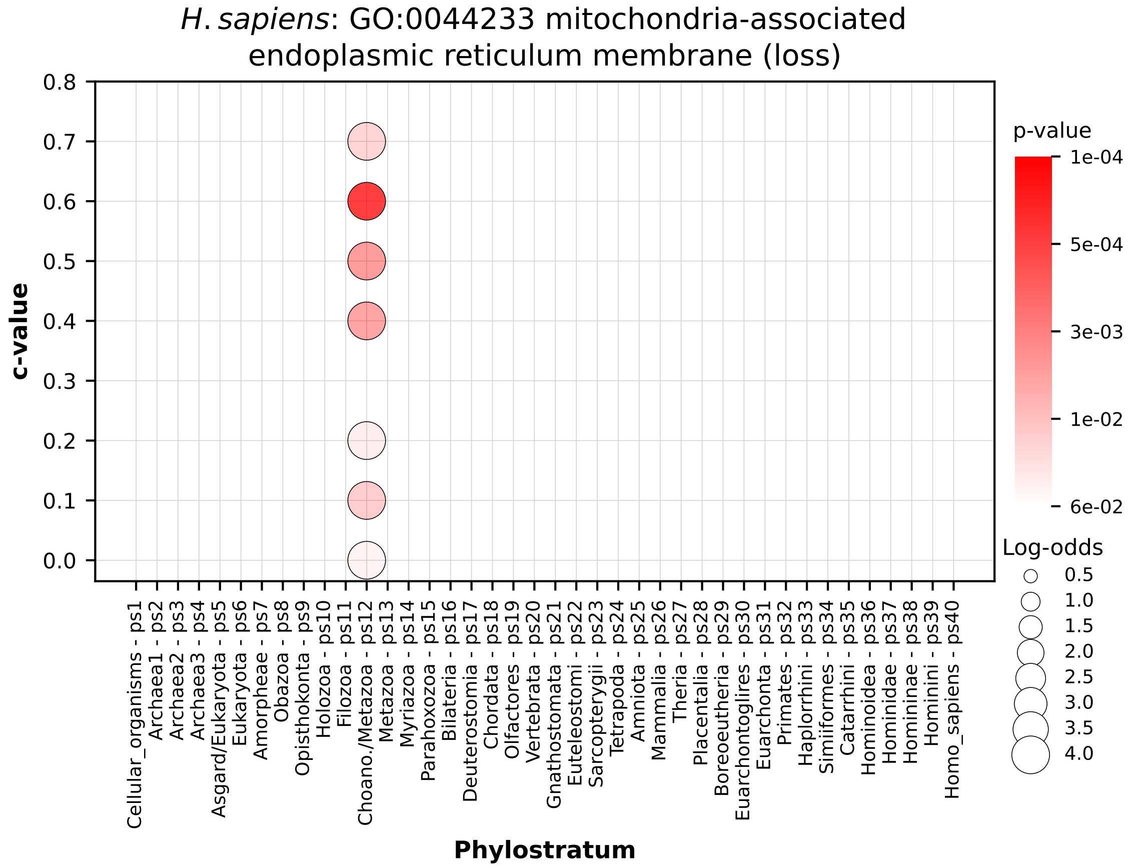

H. sapiens: GO:0044233 mitochondria-associated endoplasmic reticulum membrane (loss)
c-value
0.8
0.7
0.6
0.5
0.4
0.3
0.2
0.1
0.0
Phylostratum
Cellular_organisms - ps1
Archaea1 - ps2
Archaea2 - ps3
Archaea3 - ps4
Asgard/Eukaryota - ps5
Eukaryota - ps6
Amorpheae - ps7
Obazoa - ps8
Opisthokonta - ps9
Holozoa - ps10
Filozoa - ps11
Choano./Metazoa - ps12
Metazoa - ps13
Myriazoa - ps14
Parahoxozoa - ps15
Bilateria - ps16
Deuterostomia - ps17
Chordata - ps18
Olfactores - ps19
Vertebrata - ps20
Gnathostomata - ps21
Euteleostomi - ps22
Sarcopterygii - ps23
Tetrapoda - ps24
Amniota - ps25
Mammalia - ps26
Theria - ps27
Placentalia - ps28
Boreoeutheria - ps29
Euarchontoglires - ps30
Euarchonta - ps31
Primates - ps32
Haplorrhini - ps33
Simiiformes - ps34
Catarrhini - ps35
Hominoidea - ps36
Hominidae - ps37
Homininae - ps38
Hominini - ps39
Homo_sapiens - ps40
p-value
1e-04
5e-04
3e-03
1e-02
6e-02
Log-odds
0.5
1.0
1.5
2.0
2.5
3.0
3.5
4.0

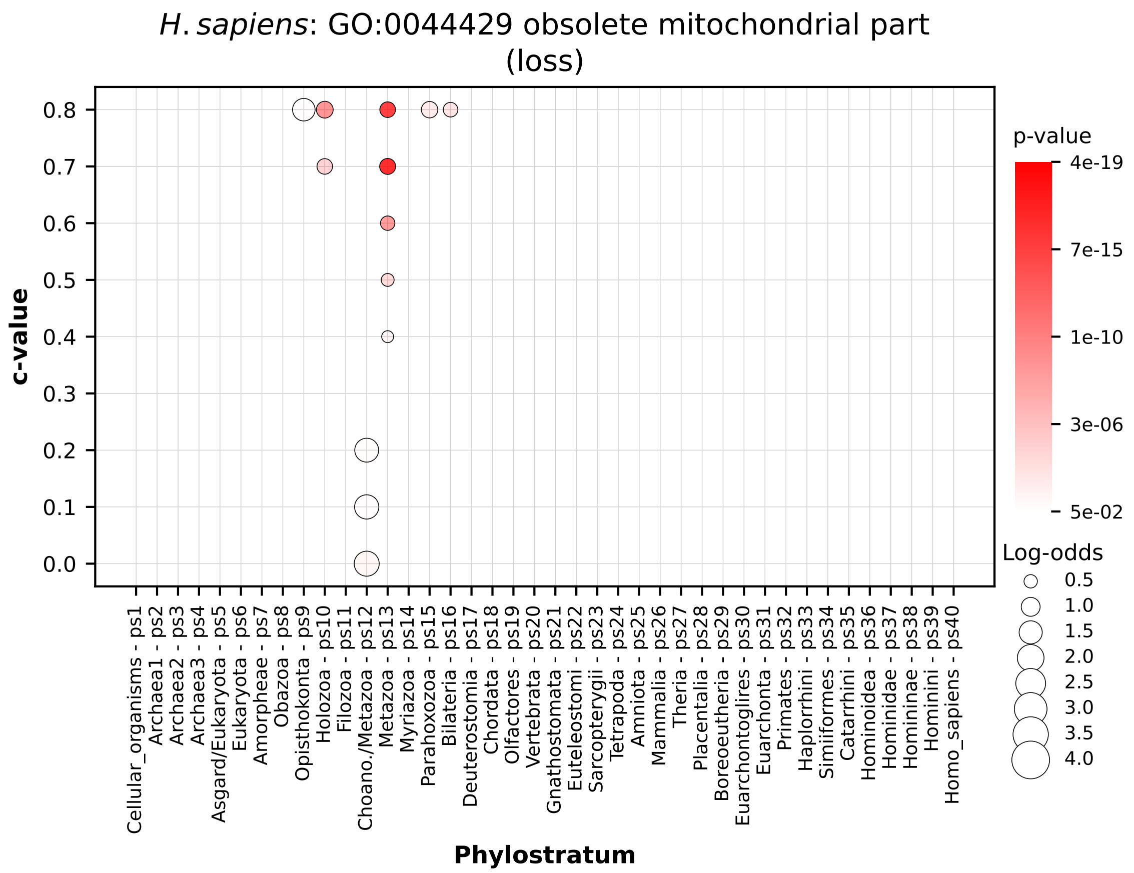

H. sapiens: GO:0044429 obsolete mitochondrial part
(loss)
c-value
0.0
0.1
0.2
0.3
0.4
0.5
0.6
0.7
0.8
Phylostratum
Cellular_organisms - ps1
Archaea1 - ps2
Archaea2 - ps3
Archaea3 - ps4
Asgard/Eukaryota - ps5
Eukaryota - ps6
Amorpheae - ps7
Obazoa - ps8
Opisthokonta - ps9
Holozoa - ps10
Filozoa - ps11
Choano./Metazoa - ps12
Metazoa - ps13
Myriazoa - ps14
Parahoxozoa - ps15
Bilateria - ps16
Deuterostomia - ps17
Chordata - ps18
Olfactores - ps19
Vertebrata - ps20
Gnathostomata - ps21
Euteleostomi - ps22
Sarcopterygii - ps23
Tetrapoda - ps24
Amniota - ps25
Mammalia - ps26
Theria - ps27
Placentalia - ps28
Boreoeutheria - ps29
Euarchontoglires - ps30
Euarchonta - ps31
Primates - ps32
Haplorrhini - ps33
Simiiformes - ps34
Catarrhini - ps35
Hominoidea - ps36
Hominidae - ps37
Homininae - ps38
Hominini - ps39
Homo_sapiens - ps40
p-value
4e-19
7e-15
1e-10
3e-06
5e-02
Log-odds
0.5
1.0
1.5
2.0
2.5
3.0
3.5
4.0

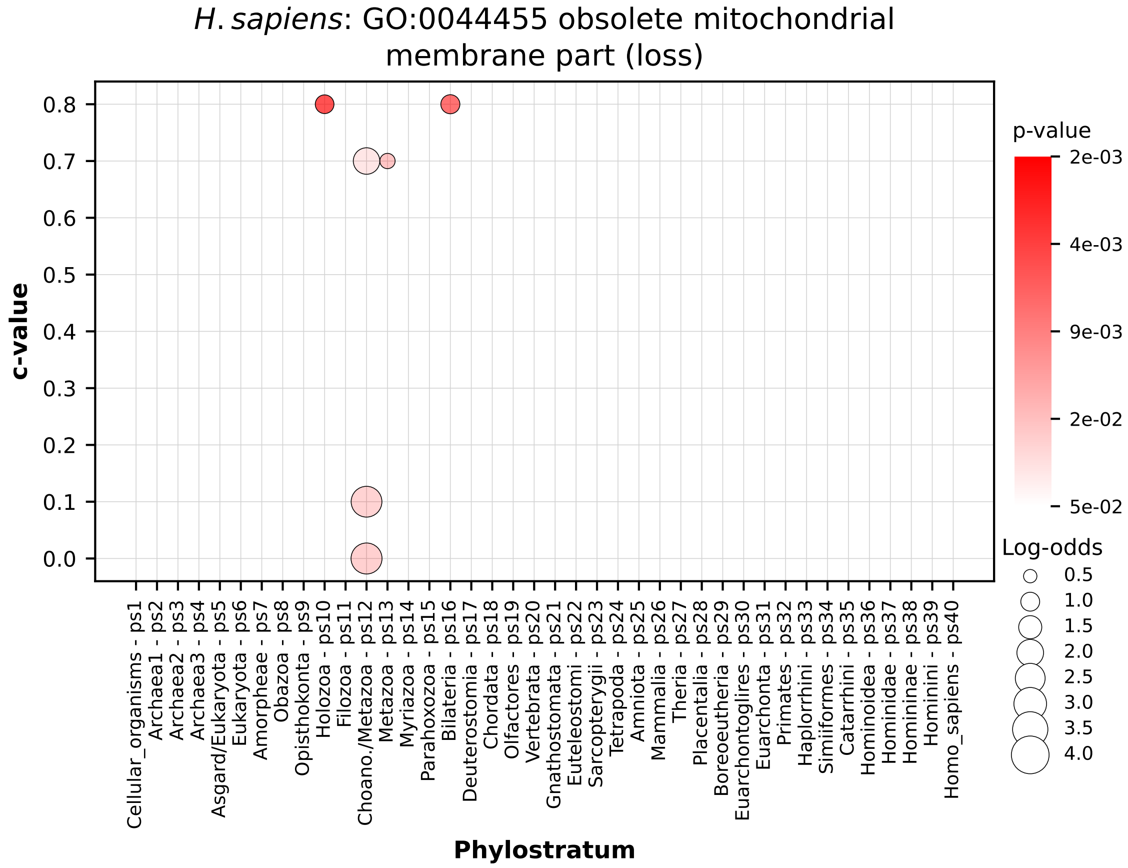

H. sapiens: GO:0044455 obsolete mitochondrial membrane part (loss)
c-value
0.8
0.7
0.6
0.5
0.4
0.3
0.2
0.1
0.0
p-value
2e-03
4e-03
9e-03
2e-02
5e-02
Log-odds
0.5
1.0
1.5
2.0
2.5
3.0
3.5
4.0
Cellular_organisms - ps1
Archaea1 - ps2
Archaea2 - ps3
Archaea3 - ps4
Asgard/Eukaryota - ps5
Eukaryota - ps6
Amorpheae - ps7
Obazoa - ps8
Opisthokonta - ps9
Holozoa - ps10
Filozoa - ps11
Choano./Metazoa - ps12
Metazoa - ps13
Myriazoa - ps14
Parahoxozoa - ps15
Bilateria - ps16
Deuterostomia - ps17
Chordata - ps18
Olfactores - ps19
Vertebrata - ps20
Gnathostomata - ps21
Euteleostomi - ps22
Sarcopterygii - ps23
Tetrapoda - ps24
Amniota - ps25
Mammalia - ps26
Theria - ps27
Placentalia - ps28
Boreoeutheria - ps29
Euarchontoglires - ps30
Euarchonta - ps31
Primates - ps32
Haplorrhini - ps33
Simiiformes - ps34
Catarrhini - ps35
Hominoidea - ps36
Hominidae - ps37
Homininae - ps38
Hominini - ps39
Homo_sapiens - ps40
Phylostratum

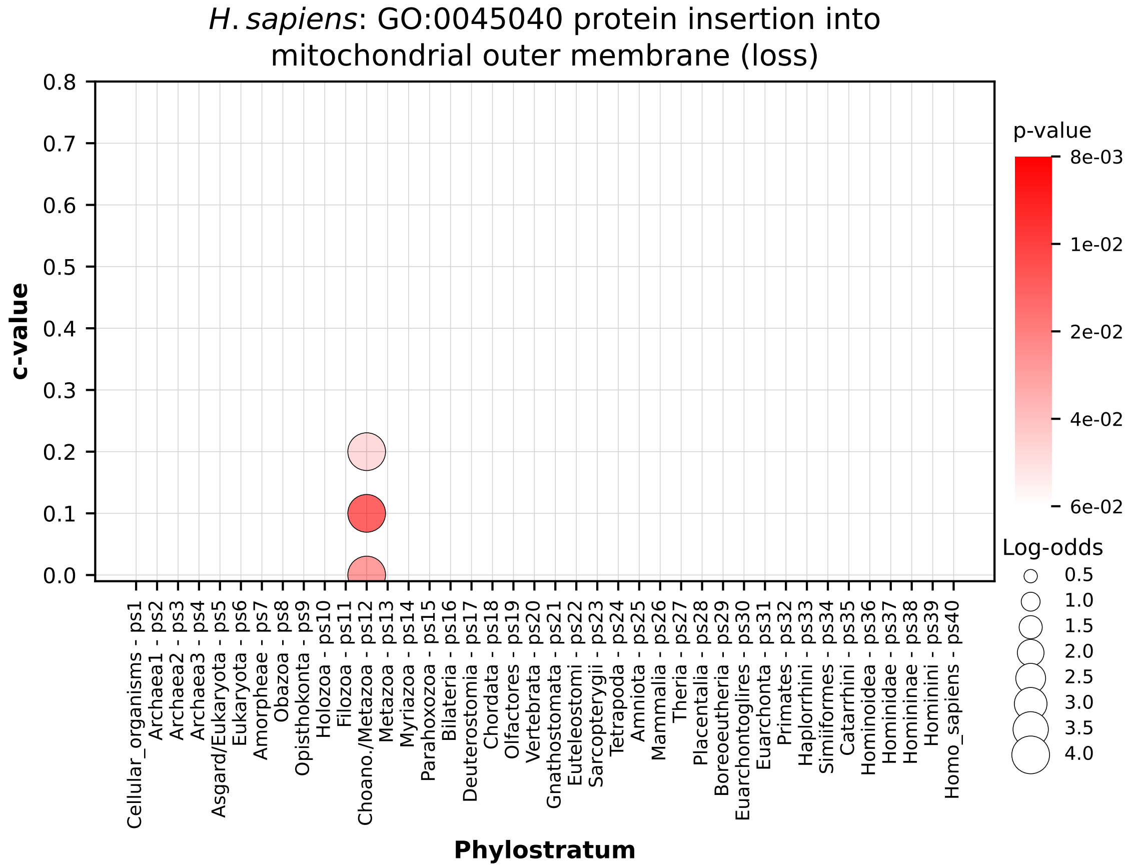

H. sapiens: GO:0045040 protein insertion into mitochondrial outer membrane (loss)
c-value
0.0
0.1
0.2
0.3
0.4
0.5
0.6
0.7
0.8
Phylostratum
Cellular_organisms - ps1
Archaea1 - ps2
Archaea2 - ps3
Archaea3 - ps4
Asgard/Eukaryota - ps5
Eukaryota - ps6
Amorpheae - ps7
Obazoa - ps8
Opisthokonta - ps9
Holozoa - ps10
Filozoa - ps11
Choano./Metazoa - ps12
Metazoa - ps13
Myriazoa - ps14
Parahoxozoa - ps15
Bilateria - ps16
Deuterostomia - ps17
Chordata - ps18
Olfactores - ps19
Vertebrata - ps20
Gnathostomata - ps21
Euteleostomi - ps22
Sarcopterygii - ps23
Tetrapoda - ps24
Amniota - ps25
Mammalia - ps26
Theria - ps27
Placentalia - ps28
Boreoeutheria - ps29
Euarchontoglires - ps30
Euarchonta - ps31
Primates - ps32
Haplorrhini - ps33
Simiiformes - ps34
Catarrhini - ps35
Hominoidea - ps36
Hominidae - ps37
Homininae - ps38
Hominini - ps39
Homo_sapiens - ps40
p-value
8e-03
1e-02
2e-02
4e-02
6e-02
Log-odds
0.5
1.0
1.5
2.0
2.5
3.0
3.5
4.0

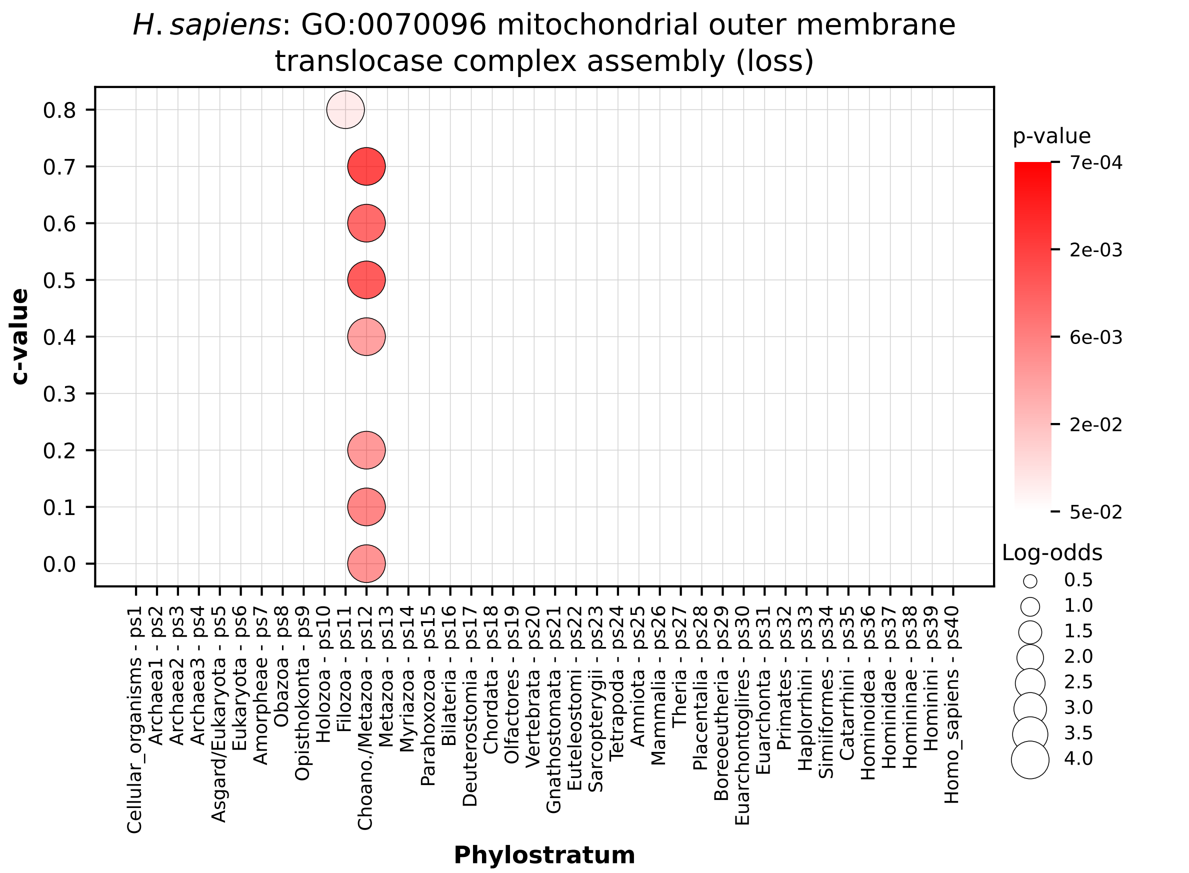

H. sapiens: GO:0070096 mitochondrial outer membrane translocase complex assembly (loss)
c-value
0.8
0.7
0.6
0.5
0.4
0.3
0.2
0.1
0.0
p-value
7e-04
2e-03
6e-03
2e-02
5e-02
Log-odds
0.5
1.0
1.5
2.0
2.5
3.0
3.5
4.0
Cellular_organisms - ps1
Archaea1 - ps2
Archaea2 - ps3
Archaea3 - ps4
Asgard/Eukaryota - ps5
Eukaryota - ps6
Amorpheae - ps7
Obazoa - ps8
Opisthokonta - ps9
Holozoa - ps10
Filozoa - ps11
Choano./Metazoa - ps12
Metazoa - ps13
Myriazoa - ps14
Parahoxozoa - ps15
Bilateria - ps16
Deuterostomia - ps17
Chordata - ps18
Olfactores - ps19
Vertebrata - ps20
Gnathostomata - ps21
Euteleostomi - ps22
Sarcopterygii - ps23
Tetrapoda - ps24
Amniota - ps25
Mammalia - ps26
Theria - ps27
Placentalia - ps28
Boreoeutheria - ps29
Euarchontoglires - ps30
Euarchonta - ps31
Primates - ps32
Haplorrhini - ps33
Simiiformes - ps34
Catarrhini - ps35
Hominoidea - ps36
Hominidae - ps37
Homininae - ps38
Hominini - ps39
Homo_sapiens - ps40
Phylostratum

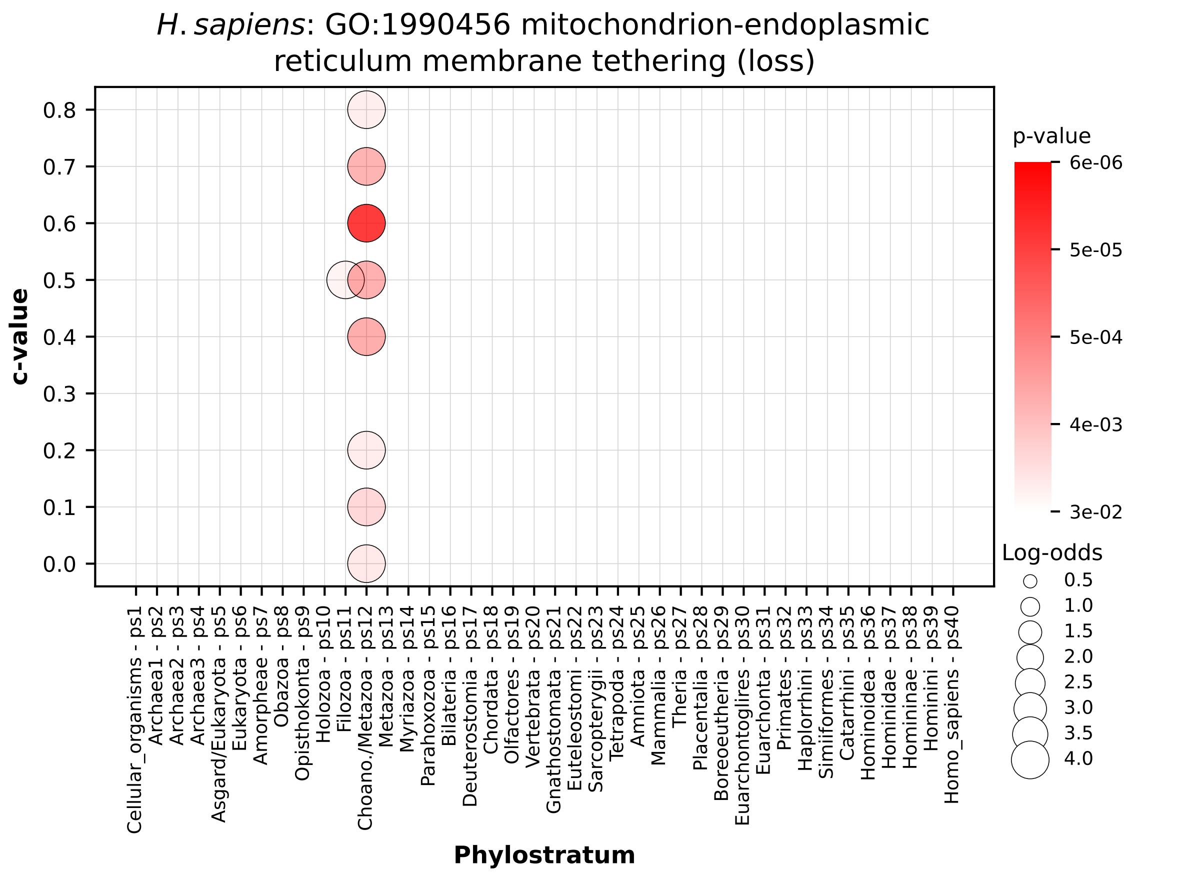

H. sapiens: GO:1990456 mitochondrion-endoplasmic reticulum membrane tethering (loss)
c-value
0.8
0.7
0.6
0.5
0.4
0.3
0.2
0.1
0.0
Phylostratum
Cellular_organisms - ps1
Archaea1 - ps2
Archaea2 - ps3
Archaea3 - ps4
Asgard/Eukaryota - ps5
Eukaryota - ps6
Amorpheae - ps7
Obazoa - ps8
Opisthokonta - ps9
Holozoa - ps10
Filozoa - ps11
Choano./Metazoa - ps12
Metazoa - ps13
Myriazoa - ps14
Parahoxozoa - ps15
Bilateria - ps16
Deuterostomia - ps17
Chordata - ps18
Olfactores - ps19
Vertebrata - ps20
Gnathostomata - ps21
Euteleostomi - ps22
Sarcopterygii - ps23
Tetrapoda - ps24
Amniota - ps25
Mammalia - ps26
Theria - ps27
Placentalia - ps28
Boreoeutheria - ps29
Euarchontoglires - ps30
Euarchonta - ps31
Primates - ps32
Haplorrhini - ps33
Simiiformes - ps34
Catarrhini - ps35
Hominoidea - ps36
Hominidae - ps37
Homininae - ps38
Hominini - ps39
Homo_sapiens - ps40
p-value
6e-06
5e-05
5e-04
4e-03
3e-02
Log-odds
0.5
1.0
1.5
2.0
2.5
3.0
3.5
4.0

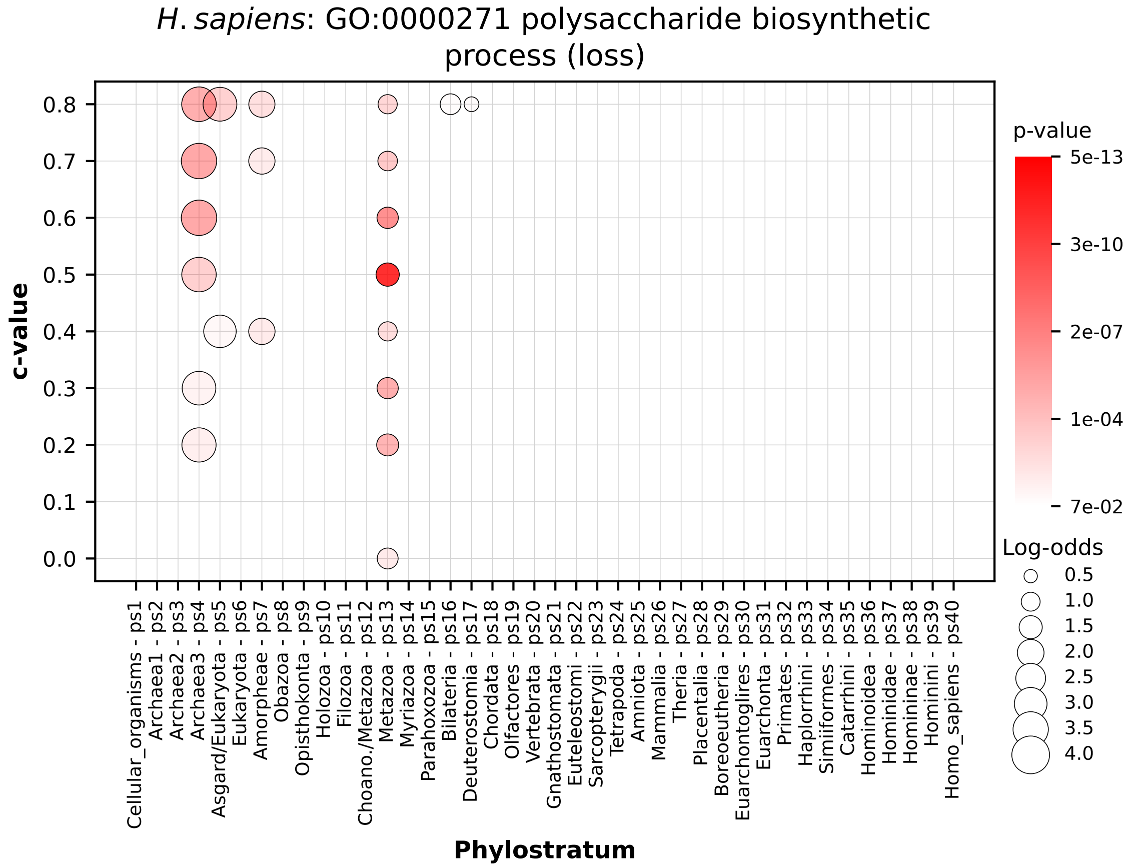

H. sapiens: GO:0000271 polysaccharide biosynthetic process (loss)
c-value
0.8
0.7
0.6
0.5
0.4
0.3
0.2
0.1
0.0
Phylostratum
Cellular_organisms - ps1
Archaea1 - ps2
Archaea2 - ps3
Archaea3 - ps4
Asgard/Eukaryota - ps5
Eukaryota - ps6
Amorpheae - ps7
Obazoa - ps8
Opisthokonta - ps9
Holozoa - ps10
Filozoa - ps11
Choano./Metazoa - ps12
Metazoa - ps13
Myriazoa - ps14
Parahoxozoa - ps15
Bilateria - ps16
Deuterostomia - ps17
Chordata - ps18
Olfactores - ps19
Vertebrata - ps20
Gnathostomata - ps21
Euteleostomi - ps22
Sarcopterygii - ps23
Tetrapoda - ps24
Amniota - ps25
Mammalia - ps26
Theria - ps27
Placentalia - ps28
Boreoeutheria - ps29
Euarchontoglires - ps30
Euarchonta - ps31
Primates - ps32
Haplorrhini - ps33
Simiiformes - ps34
Catarrhini - ps35
Hominoidea - ps36
Hominidae - ps37
Homininae - ps38
Hominini - ps39
Homo_sapiens - ps40
p-value
5e-13
3e-10
2e-07
1e-04
7e-02
Log-odds
0.5
1.0
1.5
2.0
2.5
3.0
3.5
4.0

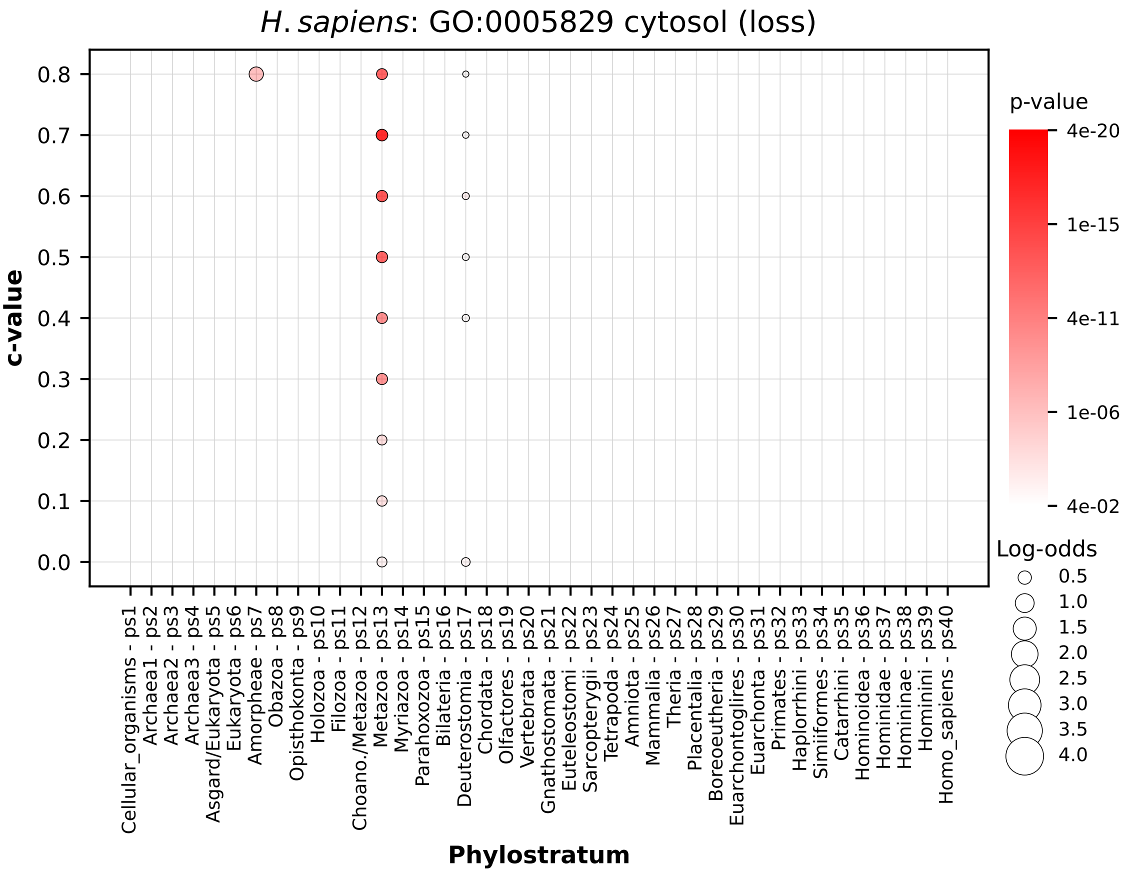
H. sapiens: GO:0005829 cytosol (loss)
c-value
0.8
0.7
0.6
0.5
0.4
0.3
0.2
0.1
0.0
p-value
4e-20
1e-15
4e-11
1e-06
4e-02
Log-odds
0.5
1.0
1.5
2.0
2.5
3.0
3.5
4.0
Cellular_organisms - ps1
Archaea1 - ps2
Archaea2 - ps3
Archaea3 - ps4
Asgard/Eukaryota - ps5
Eukaryota - ps6
Amorpheae - ps7
Obazoa - ps8
Opisthokonta - ps9
Holozoa - ps10
Filozoa - ps11
Choano./Metazoa - ps12
Metazoa - ps13
Myriazoa - ps14
Parahoxozoa - ps15
Bilateria - ps16
Deuterostomia - ps17
Chordata - ps18
Olfactores - ps19
Vertebrata - ps20
Gnathostomata - ps21
Euteleostomi - ps22
Sarcopterygii - ps23
Tetrapoda - ps24
Amniota - ps25
Mammalia - ps26
Theria - ps27
Placentalia - ps28
Boreoeutheria - ps29
Euarchontoglires - ps30
Euarchonta - ps31
Primates - ps32
Haplorrhini - ps33
Simiiformes - ps34
Catarrhini - ps35
Hominoidea - ps36
Hominidae - ps37
Homininae - ps38
Hominini - ps39
Homo_sapiens - ps40
Phylostratum

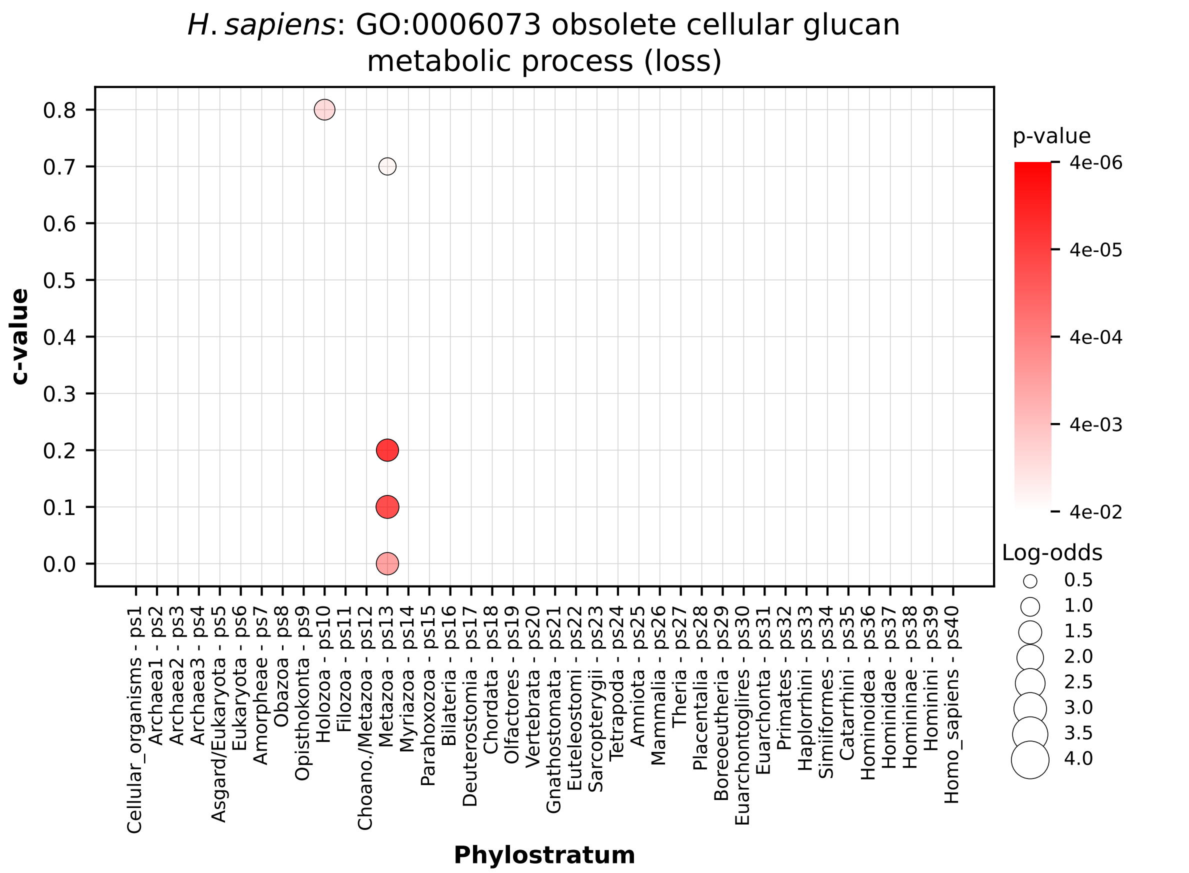
H. sapiens: GO:0006073 obsolete cellular glucan metabolic process (loss)
c-value
0.8
0.7
0.6
0.5
0.4
0.3
0.2
0.1
0.0
Phylostratum
Cellular_organisms - ps1
Archaea1 - ps2
Archaea2 - ps3
Archaea3 - ps4
Asgard/Eukaryota - ps5
Eukaryota - ps6
Amorpheae - ps7
Obazoa - ps8
Opisthokonta - ps9
Holozoa - ps10
Filozoa - ps11
Choano./Metazoa - ps12
Metazoa - ps13
Myriazoa - ps14
Parahoxozoa - ps15
Bilateria - ps16
Deuterostomia - ps17
Chordata - ps18
Olfactores - ps19
Vertebrata - ps20
Gnathostomata - ps21
Euteleostomi - ps22
Sarcopterygii - ps23
Tetrapoda - ps24
Amniota - ps25
Mammalia - ps26
Theria - ps27
Placentalia - ps28
Boreoeutheria - ps29
Euarchontoglires - ps30
Euarchonta - ps31
Primates - ps32
Haplorrhini - ps33
Simiiformes - ps34
Catarrhini - ps35
Hominoidea - ps36
Hominidae - ps37
Homininae - ps38
Hominini - ps39
Homo_sapiens - ps40
p-value
4e-06
4e-05
4e-04
4e-03
4e-02
Log-odds
0.5
1.0
1.5
2.0
2.5
3.0
3.5
4.0

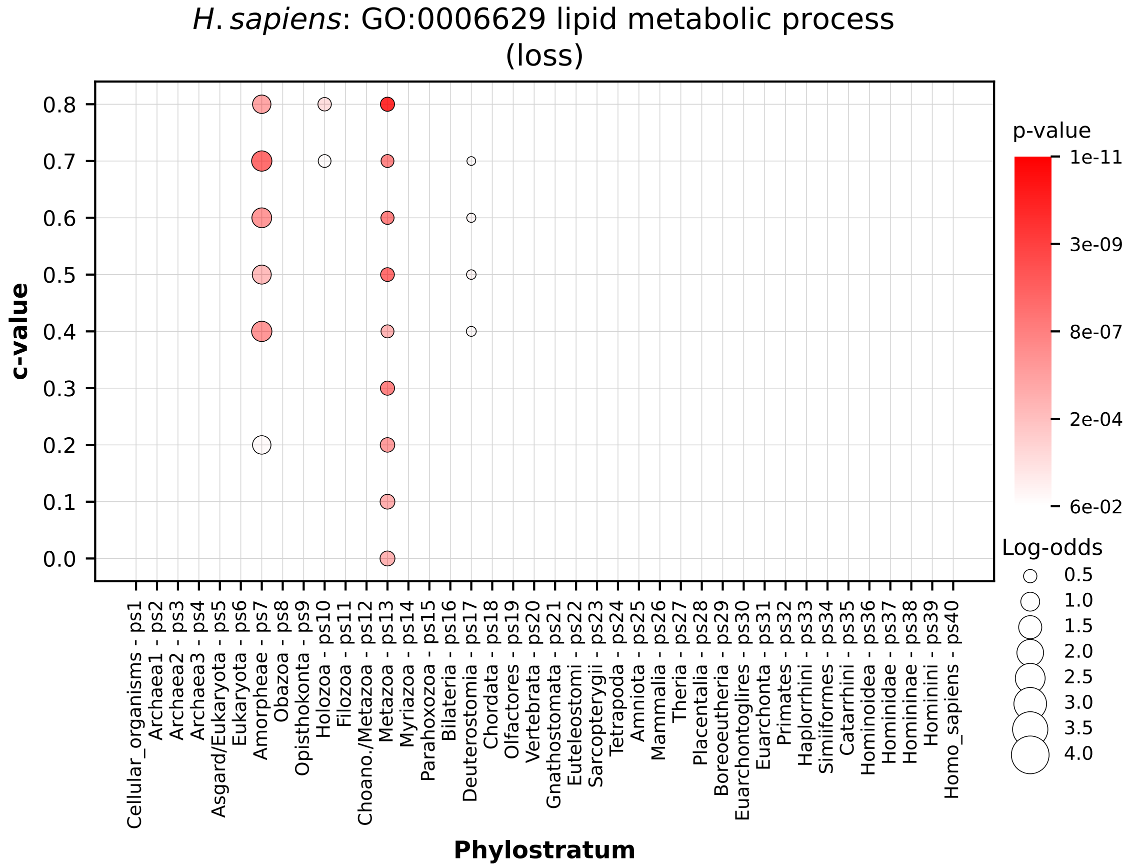

H. sapiens: GO:0006629 lipid metabolic process
(loss)
c-value
0.8
0.7
0.6
0.5
0.4
0.3
0.2
0.1
0.0
p-value
1e-11
3e-09
8e-07
2e-04
6e-02
Log-odds
0.5
1.0
1.5
2.0
2.5
3.0
3.5
4.0
Cellular_organisms - ps1
Archaea1 - ps2
Archaea2 - ps3
Archaea3 - ps4
Asgard/Eukaryota - ps5
Eukaryota - ps6
Amorpheae - ps7
Obazoa - ps8
Opisthokonta - ps9
Holozoa - ps10
Filozoa - ps11
Choano./Metazoa - ps12
Metazoa - ps13
Myriazoa - ps14
Parahoxozoa - ps15
Bilateria - ps16
Deuterostomia - ps17
Chordata - ps18
Olfactores - ps19
Vertebrata - ps20
Gnathostomata - ps21
Euteleostomi - ps22
Sarcopterygii - ps23
Tetrapoda - ps24
Amniota - ps25
Mammalia - ps26
Theria - ps27
Placentalia - ps28
Boreoeutheria - ps29
Euarchontoglires - ps30
Euarchonta - ps31
Primates - ps32
Haplorrhini - ps33
Simiiformes - ps34
Catarrhini - ps35
Hominoidea - ps36
Hominidae - ps37
Homininae - ps38
Hominini - ps39
Homo_sapiens - ps40
Phylostratum

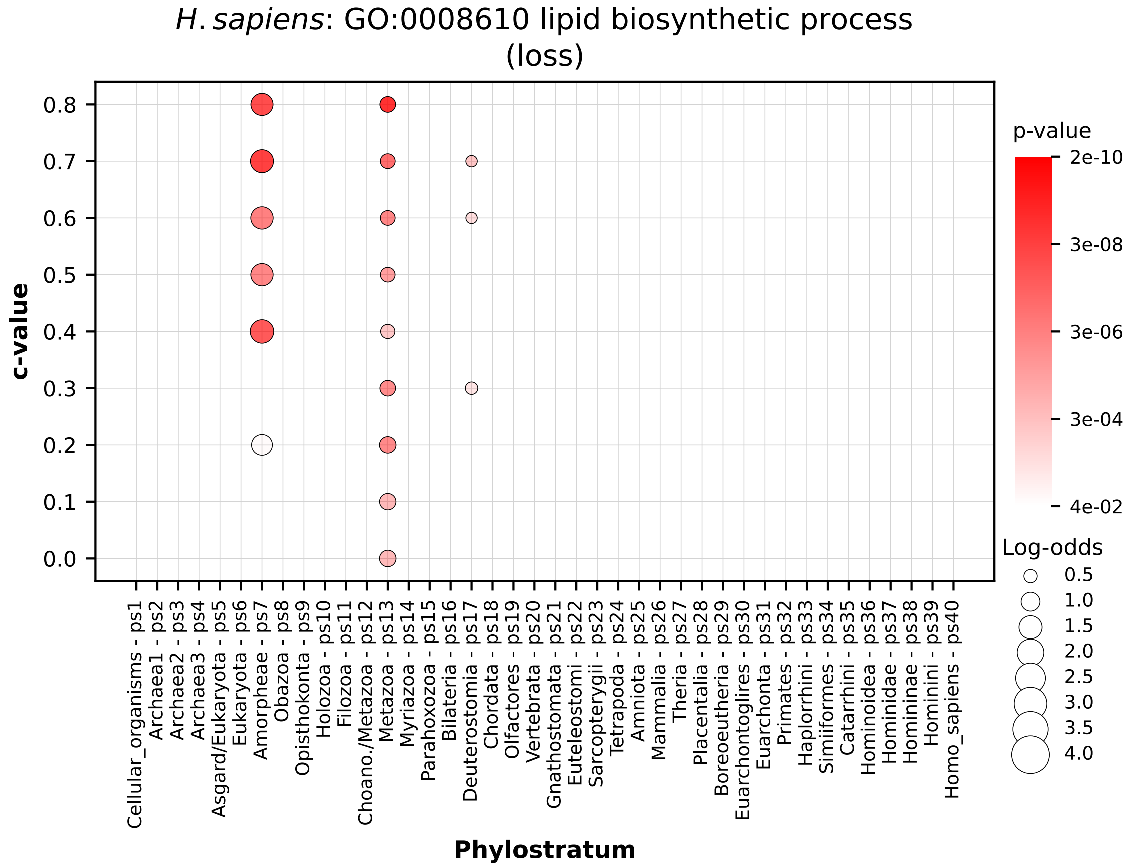
H. sapiens: GO:0008610 lipid biosynthetic process
(loss)
c-value
0.8
0.7
0.6
0.5
0.4
0.3
0.2
0.1
0.0
Phylostratum
Cellular_organisms - ps1
Archaea1 - ps2
Archaea2 - ps3
Archaea3 - ps4
Asgard/Eukaryota - ps5
Eukaryota - ps6
Amorpheae - ps7
Obazoa - ps8
Opisthokonta - ps9
Holozoa - ps10
Filozoa - ps11
Choano./Metazoa - ps12
Metazoa - ps13
Myriazoa - ps14
Parahoxozoa - ps15
Bilateria - ps16
Deuterostomia - ps17
Chordata - ps18
Olfactores - ps19
Vertebrata - ps20
Gnathostomata - ps21
Euteleostomi - ps22
Sarcopterygii - ps23
Tetrapoda - ps24
Amniota - ps25
Mammalia - ps26
Theria - ps27
Placentalia - ps28
Boreoeutheria - ps29
Euarchontoglires - ps30
Euarchonta - ps31
Primates - ps32
Haplorrhini - ps33
Simiiformes - ps34
Catarrhini - ps35
Hominoidea - ps36
Hominidae - ps37
Homininae - ps38
Hominini - ps39
Homo_sapiens - ps40
p-value
2e-10
3e-08
3e-06
3e-04
4e-02
Log-odds
0.5
1.0
1.5
2.0
2.5
3.0
3.5
4.0

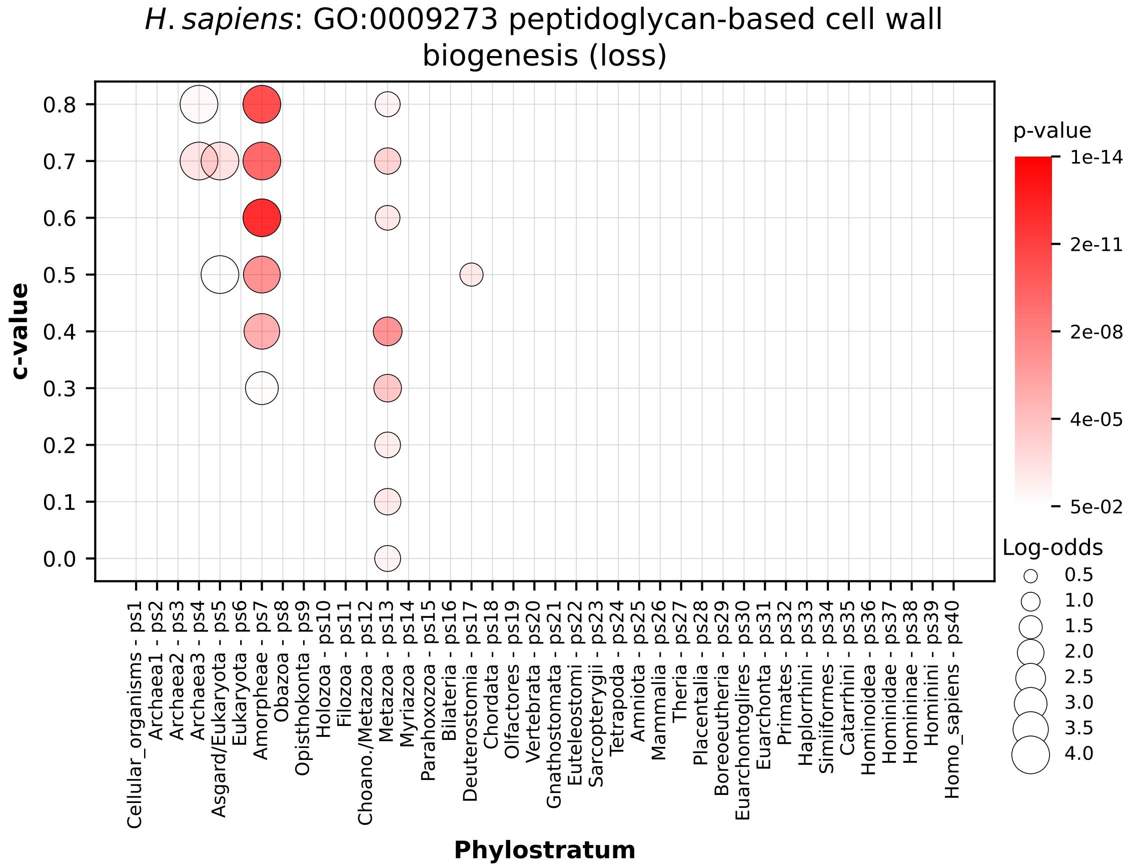
H. sapiens: GO:0009273 peptidoglycan-based cell wall biogenesis (loss)
c-value
0.8
0.7
0.6
0.5
0.4
0.3
0.2
0.1
0.0
Phylostratum
Cellular_organisms - ps1
Archaea1 - ps2
Archaea2 - ps3
Archaea3 - ps4
Asgard/Eukaryota - ps5
Eukaryota - ps6
Amorpheae - ps7
Obazoa - ps8
Opisthokonta - ps9
Holozoa - ps10
Filozoa - ps11
Choano./Metazoa - ps12
Metazoa - ps13
Myriazoa - ps14
Parahoxozoa - ps15
Bilateria - ps16
Deuterostomia - ps17
Chordata - ps18
Olfactores - ps19
Vertebrata - ps20
Gnathostomata - ps21
Euteleostomi - ps22
Sarcopterygii - ps23
Tetrapoda - ps24
Amniota - ps25
Mammalia - ps26
Theria - ps27
Placentalia - ps28
Boreoeutheria - ps29
Euarchontoglires - ps30
Euarchonta - ps31
Primates - ps32
Haplorrhini - ps33
Simiiformes - ps34
Catarrhini - ps35
Hominoidea - ps36
Hominidae - ps37
Homininae - ps38
Hominini - ps39
Homo_sapiens - ps40
p-value
1e-14
2e-11
2e-08
4e-05
5e-02
Log-odds
0.5
1.0
1.5
2.0
2.5
3.0
3.5
4.0

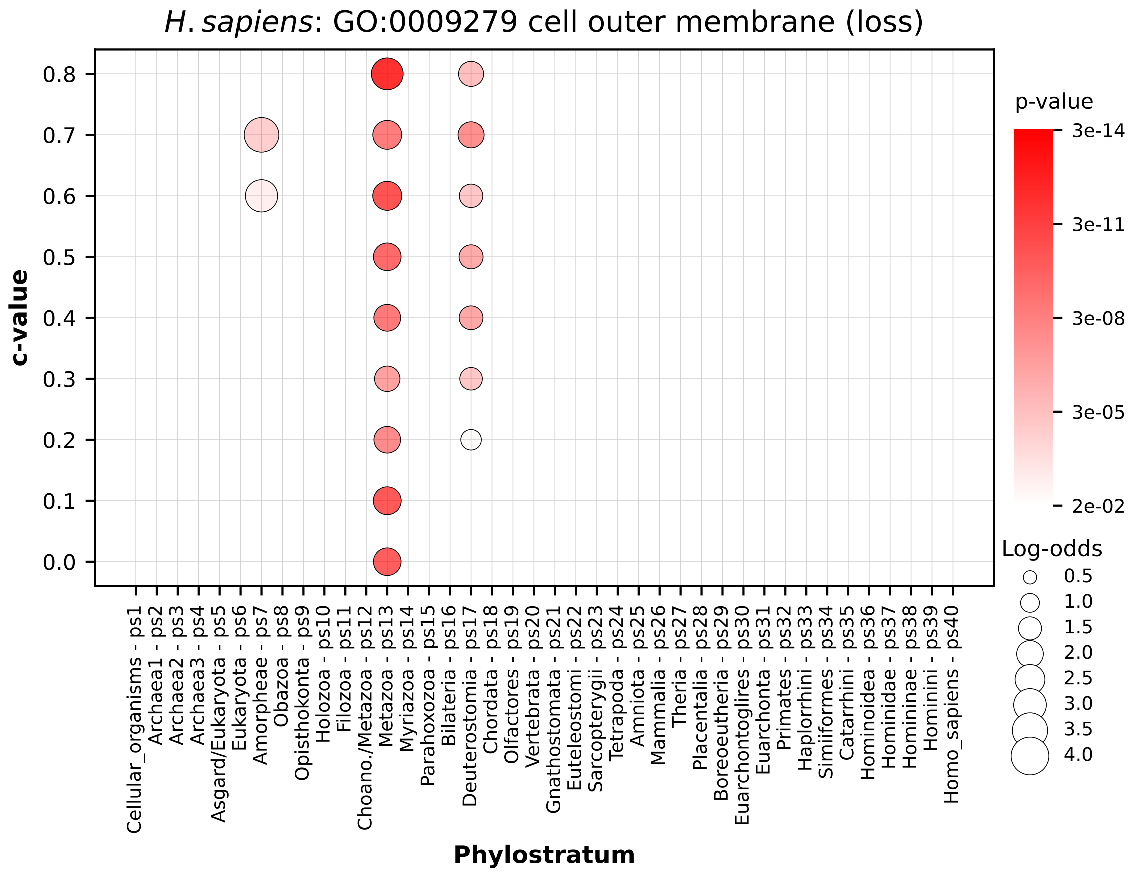

H. sapiens: GO:0009279 cell outer membrane (loss)
c-value
0.8
0.7
0.6
0.5
0.4
0.3
0.2
0.1
0.0
Phylostratum
Cellular_organisms - ps1
Archaea1 - ps2
Archaea2 - ps3
Archaea3 - ps4
Asgard/Eukaryota - ps5
Eukaryota - ps6
Amorpheae - ps7
Obazoa - ps8
Opisthokonta - ps9
Holozoa - ps10
Filozoa - ps11
Choano./Metazoa - ps12
Metazoa - ps13
Myriazoa - ps14
Parahoxozoa - ps15
Bilateria - ps16
Deuterostomia - ps17
Chordata - ps18
Olfactores - ps19
Vertebrata - ps20
Gnathostomata - ps21
Euteleostomi - ps22
Sarcopterygii - ps23
Tetrapoda - ps24
Amniota - ps25
Mammalia - ps26
Theria - ps27
Placentalia - ps28
Boreoeutheria - ps29
Euarchontoglires - ps30
Euarchonta - ps31
Primates - ps32
Haplorrhini - ps33
Simiiformes - ps34
Catarrhini - ps35
Hominoidea - ps36
Hominidae - ps37
Homininae - ps38
Hominini - ps39
Homo_sapiens - ps40
p-value
3e-14
3e-11
3e-08
3e-05
2e-02
Log-odds
0.5
1.0
1.5
2.0
2.5
3.0
3.5
4.0

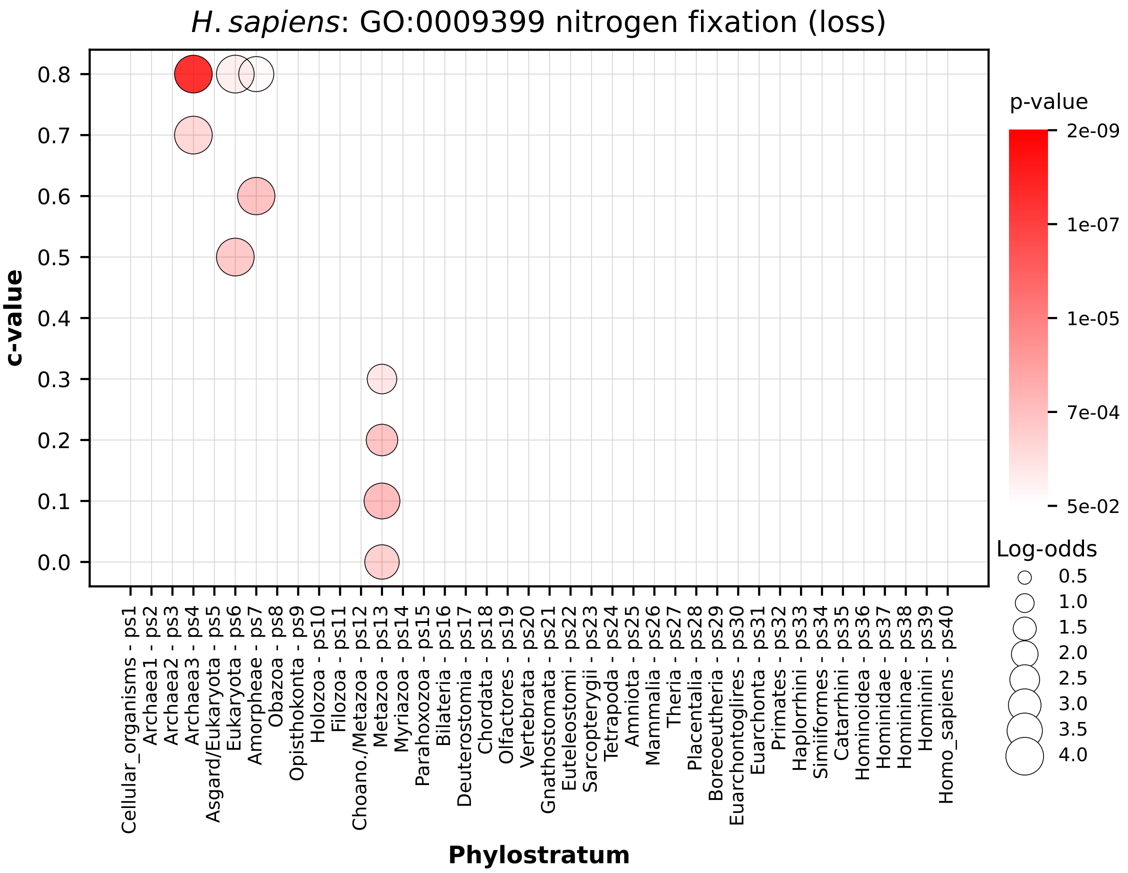

H. sapiens: GO:0009399 nitrogen fixation (loss)
c-value
0.8
0.7
0.6
0.5
0.4
0.3
0.2
0.1
0.0
Phylostratum
Cellular_organisms - ps1
Archaea1 - ps2
Archaea2 - ps3
Archaea3 - ps4
Asgard/Eukaryota - ps5
Eukaryota - ps6
Amorpheae - ps7
Obazoa - ps8
Opisthokonta - ps9
Holozoa - ps10
Filozoa - ps11
Choano./Metazoa - ps12
Metazoa - ps13
Myriazoa - ps14
Parahoxozoa - ps15
Bilateria - ps16
Deuterostomia - ps17
Chordata - ps18
Olfactores - ps19
Vertebrata - ps20
Gnathostomata - ps21
Euteleostomi - ps22
Sarcopterygii - ps23
Tetrapoda - ps24
Amniota - ps25
Mammalia - ps26
Theria - ps27
Placentalia - ps28
Boreoeutheria - ps29
Euarchontoglires - ps30
Euarchonta - ps31
Primates - ps32
Haplorrhini - ps33
Simiiformes - ps34
Catarrhini - ps35
Hominoidea - ps36
Hominidae - ps37
Homininae - ps38
Hominini - ps39
Homo_sapiens - ps40
p-value
2e-09
1e-07
1e-05
7e-04
5e-02
Log-odds
0.5
1.0
1.5
2.0
2.5
3.0
3.5
4.0

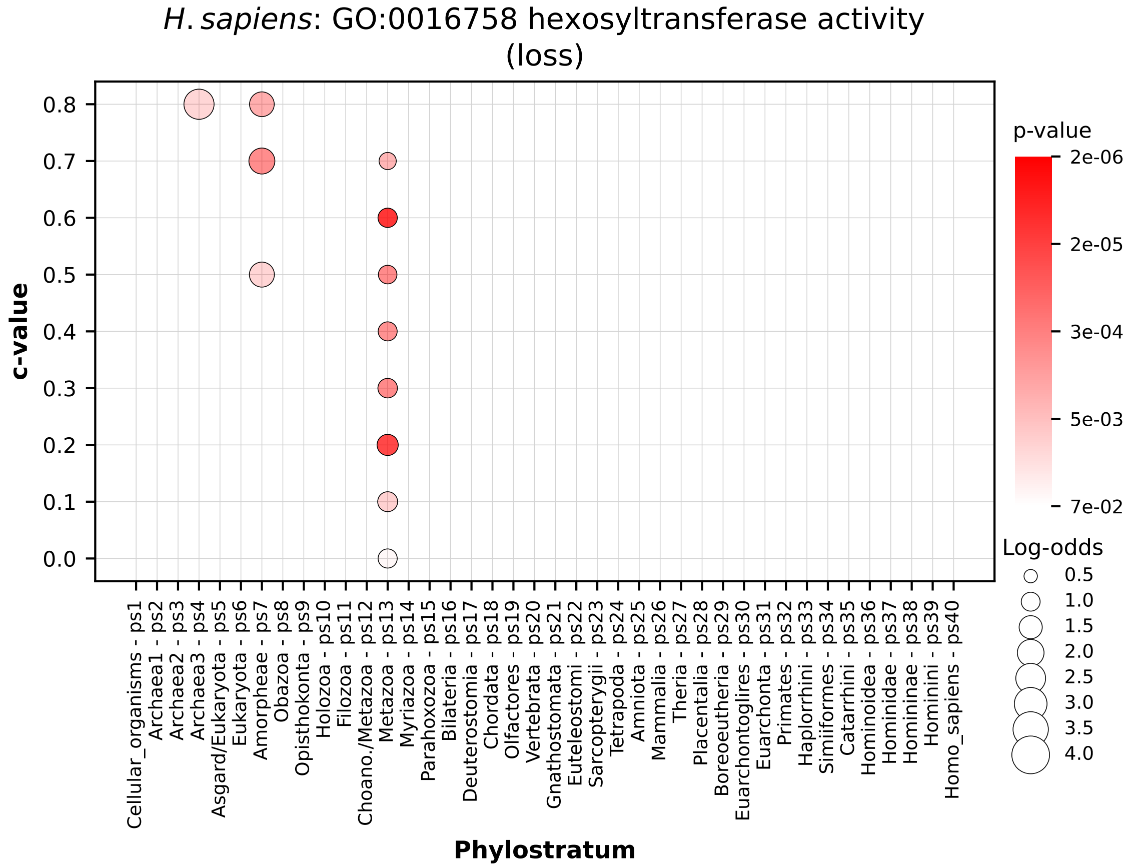

H. sapiens: GO:0016758 hexosyltransferase activity (loss)
c-value
0.8
0.7
0.6
0.5
0.4
0.3
0.2
0.1
0.0
Phylostratum
Cellular_organisms - ps1
Archaea1 - ps2
Archaea2 - ps3
Archaea3 - ps4
Asgard/Eukaryota - ps5
Eukaryota - ps6
Amorpheae - ps7
Obazoa - ps8
Opisthokonta - ps9
Holozoa - ps10
Filozoa - ps11
Choano./Metazoa - ps12
Metazoa - ps13
Myriazoa - ps14
Parahoxozoa - ps15
Bilateria - ps16
Deuterostomia - ps17
Chordata - ps18
Olfactores - ps19
Vertebrata - ps20
Gnathostomata - ps21
Euteleostomi - ps22
Sarcopterygii - ps23
Tetrapoda - ps24
Amniota - ps25
Mammalia - ps26
Theria - ps27
Placentalia - ps28
Boreoeutheria - ps29
Euarchontoglires - ps30
Euarchonta - ps31
Primates - ps32
Haplorrhini - ps33
Simiiformes - ps34
Catarrhini - ps35
Hominoidea - ps36
Hominidae - ps37
Homininae - ps38
Hominini - ps39
Homo_sapiens - ps40
p-value
2e-06
2e-05
3e-04
5e-03
7e-02
Log-odds
0.5
1.0
1.5
2.0
2.5
3.0
3.5
4.0

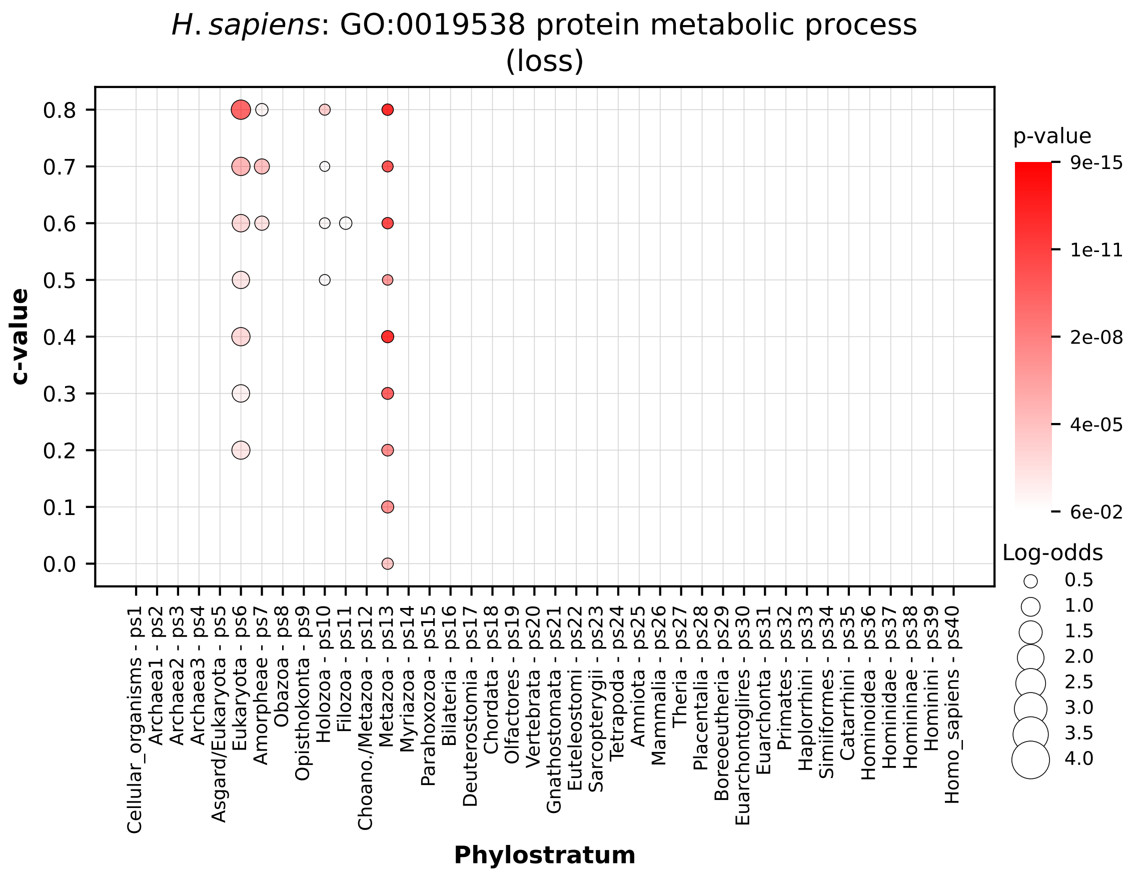

H. sapiens: GO:0019538 protein metabolic process
(loss)
c-value
0.8
0.7
0.6
0.5
0.4
0.3
0.2
0.1
0.0
Phylostratum
Cellular_organisms - ps1
Archaea1 - ps2
Archaea2 - ps3
Archaea3 - ps4
Asgard/Eukaryota - ps5
Eukaryota - ps6
Amorpheae - ps7
Obazoa - ps8
Opisthokonta - ps9
Holozoa - ps10
Filozoa - ps11
Choano./Metazoa - ps12
Metazoa - ps13
Myriazoa - ps14
Parahoxozoa - ps15
Bilateria - ps16
Deuterostomia - ps17
Chordata - ps18
Olfactores - ps19
Vertebrata - ps20
Gnathostomata - ps21
Euteleostomi - ps22
Sarcopterygii - ps23
Tetrapoda - ps24
Amniota - ps25
Mammalia - ps26
Theria - ps27
Placentalia - ps28
Boreoeutheria - ps29
Euarchontoglires - ps30
Euarchonta - ps31
Primates - ps32
Haplorrhini - ps33
Simiiformes - ps34
Catarrhini - ps35
Hominoidea - ps36
Hominidae - ps37
Homininae - ps38
Hominini - ps39
Homo_sapiens - ps40
p-value
9e-15
1e-11
2e-08
4e-05
6e-02
Log-odds
0.5
1.0
1.5
2.0
2.5
3.0
3.5
4.0

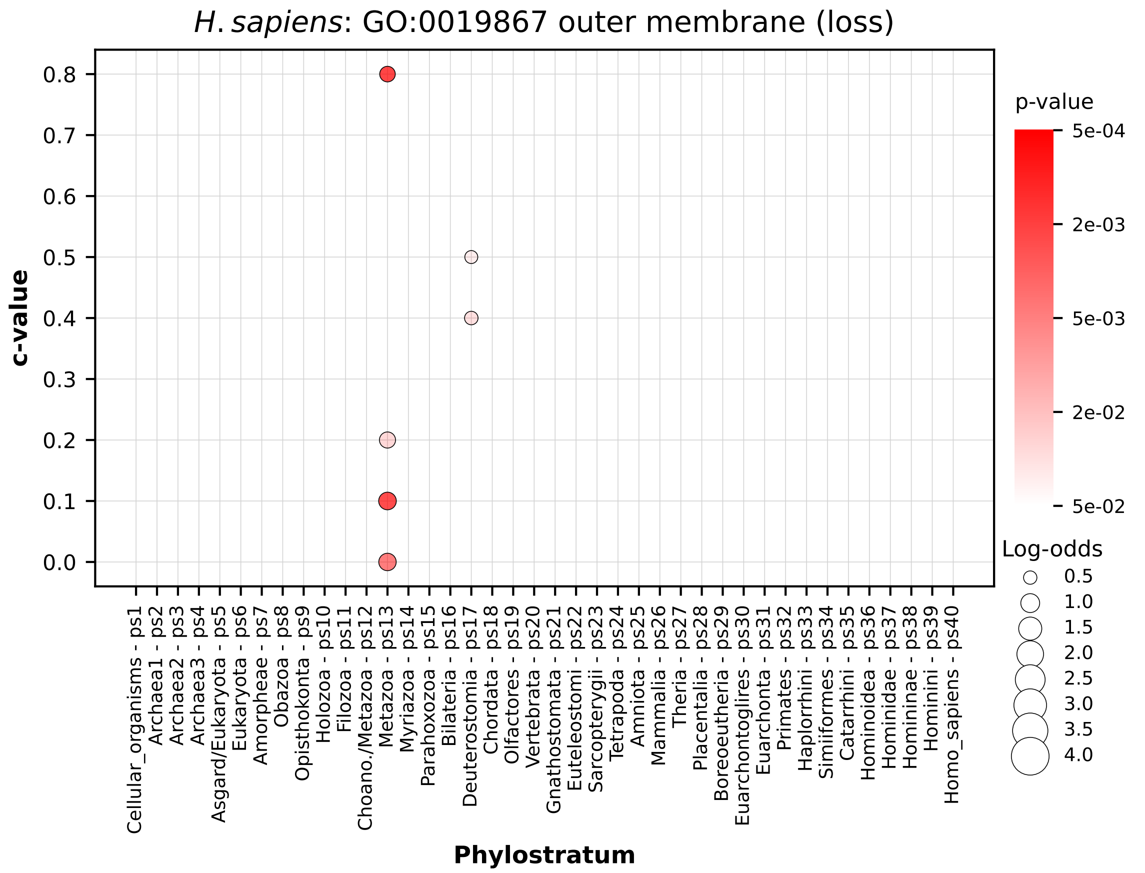

H. sapiens: GO:0019867 outer membrane (loss)
c-value
0.0
0.1
0.2
0.3
0.4
0.5
0.6
0.7
0.8
Phylostratum
Cellular_organisms - ps1
Archaea1 - ps2
Archaea2 - ps3
Archaea3 - ps4
Asgard/Eukaryota - ps5
Eukaryota - ps6
Amorpheae - ps7
Obazoa - ps8
Opisthokonta - ps9
Holozoa - ps10
Filozoa - ps11
Choano./Metazoa - ps12
Metazoa - ps13
Myriazoa - ps14
Parahoxozoa - ps15
Bilateria - ps16
Deuterostomia - ps17
Chordata - ps18
Olfactores - ps19
Vertebrata - ps20
Gnathostomata - ps21
Euteleostomi - ps22
Sarcopterygii - ps23
Tetrapoda - ps24
Amniota - ps25
Mammalia - ps26
Theria - ps27
Placentalia - ps28
Boreoeutheria - ps29
Euarchontoglires - ps30
Euarchonta - ps31
Primates - ps32
Haplorrhini - ps33
Simiiformes - ps34
Catarrhini - ps35
Hominoidea - ps36
Hominidae - ps37
Homininae - ps38
Hominini - ps39
Homo_sapiens - ps40
p-value
5e-04
2e-03
5e-03
2e-02
5e-02
Log-odds
0.5
1.0
1.5
2.0
2.5
3.0
3.5
4.0

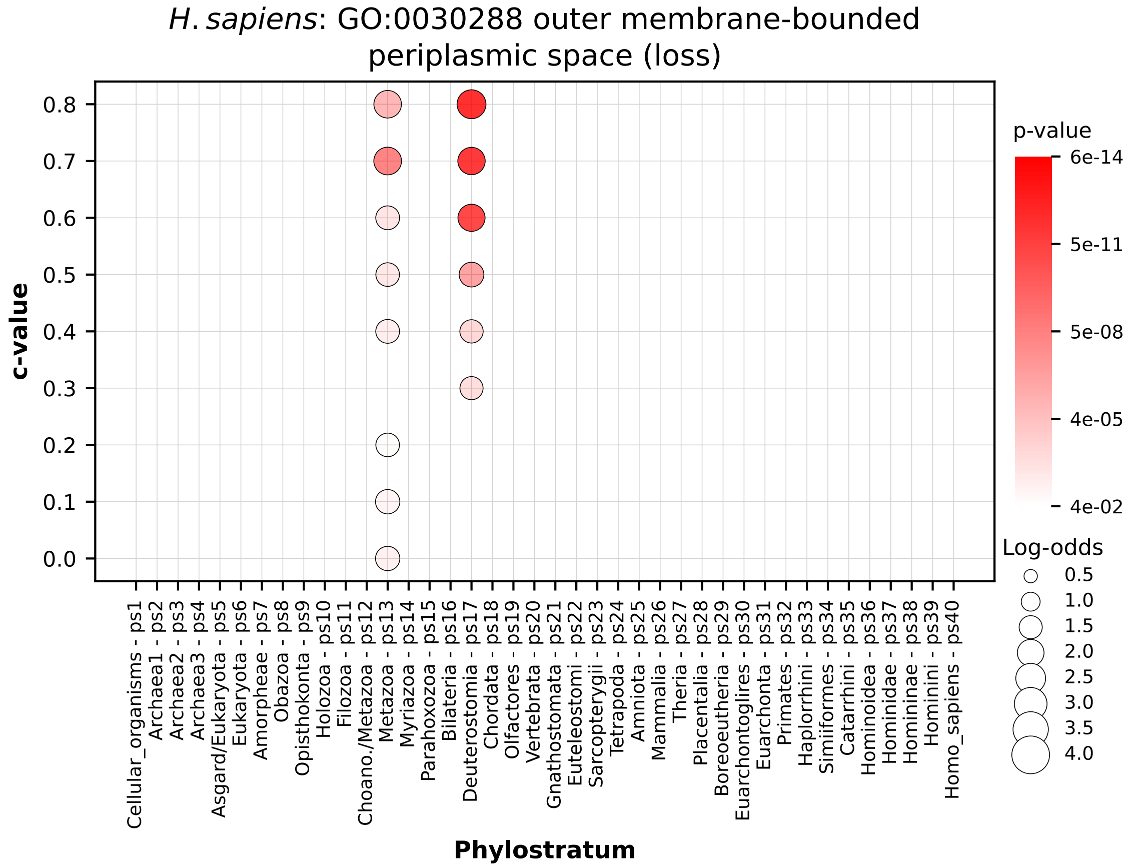

H. sapiens: GO:0030288 outer membrane-bounded periplasmic space (loss)
c-value
0.8
0.7
0.6
0.5
0.4
0.3
0.2
0.1
0.0
Phylostratum
Cellular_organisms - ps1
Archaea1 - ps2
Archaea2 - ps3
Archaea3 - ps4
Asgard/Eukaryota - ps5
Eukaryota - ps6
Amorpheae - ps7
Obazoa - ps8
Opisthokonta - ps9
Holozoa - ps10
Filozoa - ps11
Choano./Metazoa - ps12
Metazoa - ps13
Myriazoa - ps14
Parahoxozoa - ps15
Bilateria - ps16
Deuterostomia - ps17
Chordata - ps18
Olfactores - ps19
Vertebrata - ps20
Gnathostomata - ps21
Euteleostomi - ps22
Sarcopterygii - ps23
Tetrapoda - ps24
Amniota - ps25
Mammalia - ps26
Theria - ps27
Placentalia - ps28
Boreoeutheria - ps29
Euarchontoglires - ps30
Euarchonta - ps31
Primates - ps32
Haplorrhini - ps33
Simiiformes - ps34
Catarrhini - ps35
Hominoidea - ps36
Hominidae - ps37
Homininae - ps38
Hominini - ps39
Homo_sapiens - ps40
p-value
6e-14
5e-11
5e-08
4e-05
4e-02
Log-odds
0.5
1.0
1.5
2.0
2.5
3.0
3.5
4.0

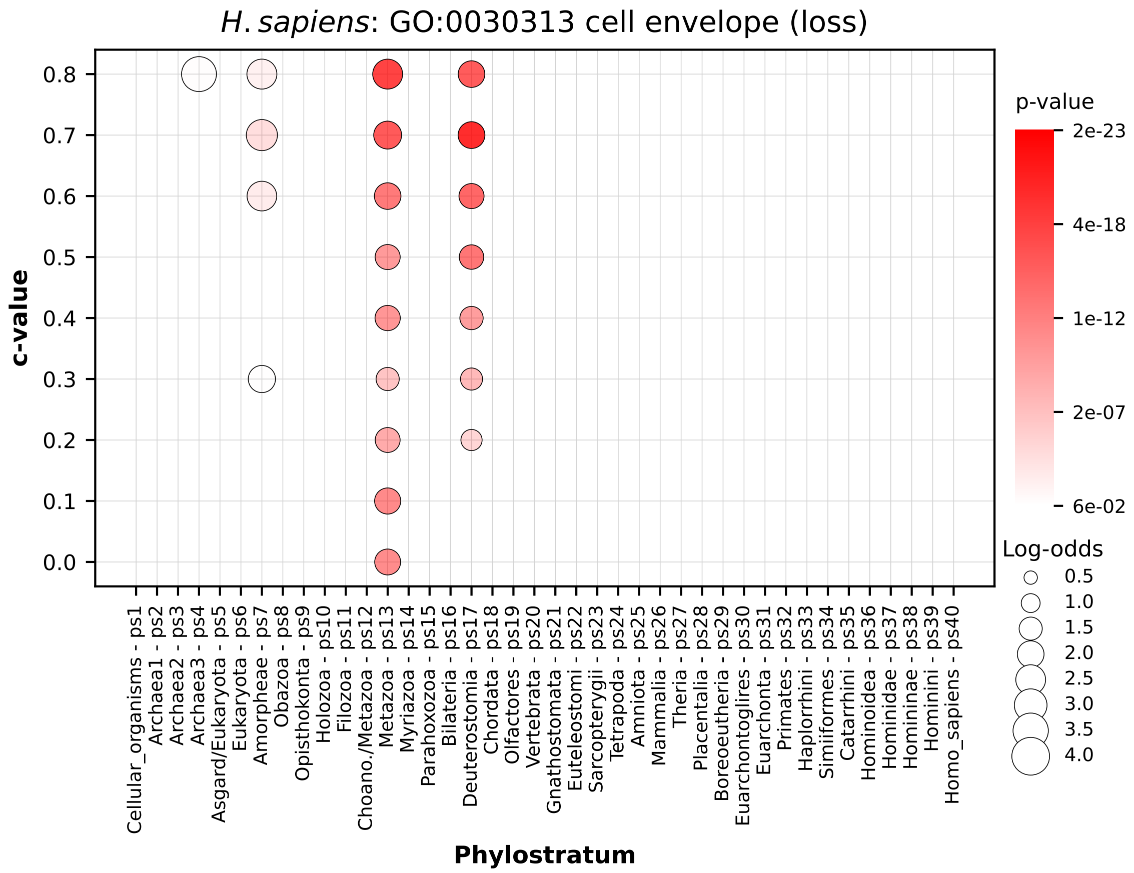

H. sapiens: GO:0030313 cell envelope (loss)
c-value
0.0
0.1
0.2
0.3
0.4
0.5
0.6
0.7
0.8
Phylostratum
Cellular_organisms - ps1
Archaea1 - ps2
Archaea2 - ps3
Archaea3 - ps4
Asgard/Eukaryota - ps5
Eukaryota - ps6
Amorpheae - ps7
Obazoa - ps8
Opisthokonta - ps9
Holozoa - ps10
Filozoa - ps11
Choano./Metazoa - ps12
Metazoa - ps13
Myriazoa - ps14
Parahoxozoa - ps15
Bilateria - ps16
Deuterostomia - ps17
Chordata - ps18
Olfactores - ps19
Vertebrata - ps20
Gnathostomata - ps21
Euteleostomi - ps22
Sarcopterygii - ps23
Tetrapoda - ps24
Amniota - ps25
Mammalia - ps26
Theria - ps27
Placentalia - ps28
Boreoeutheria - ps29
Euarchontoglires - ps30
Euarchonta - ps31
Primates - ps32
Haplorrhini - ps33
Simiiformes - ps34
Catarrhini - ps35
Hominoidea - ps36
Hominidae - ps37
Homininae - ps38
Hominini - ps39
Homo_sapiens - ps40
p-value
2e-23
4e-18
1e-12
2e-07
6e-02
Log-odds
0.5
1.0
1.5
2.0
2.5
3.0
3.5
4.0

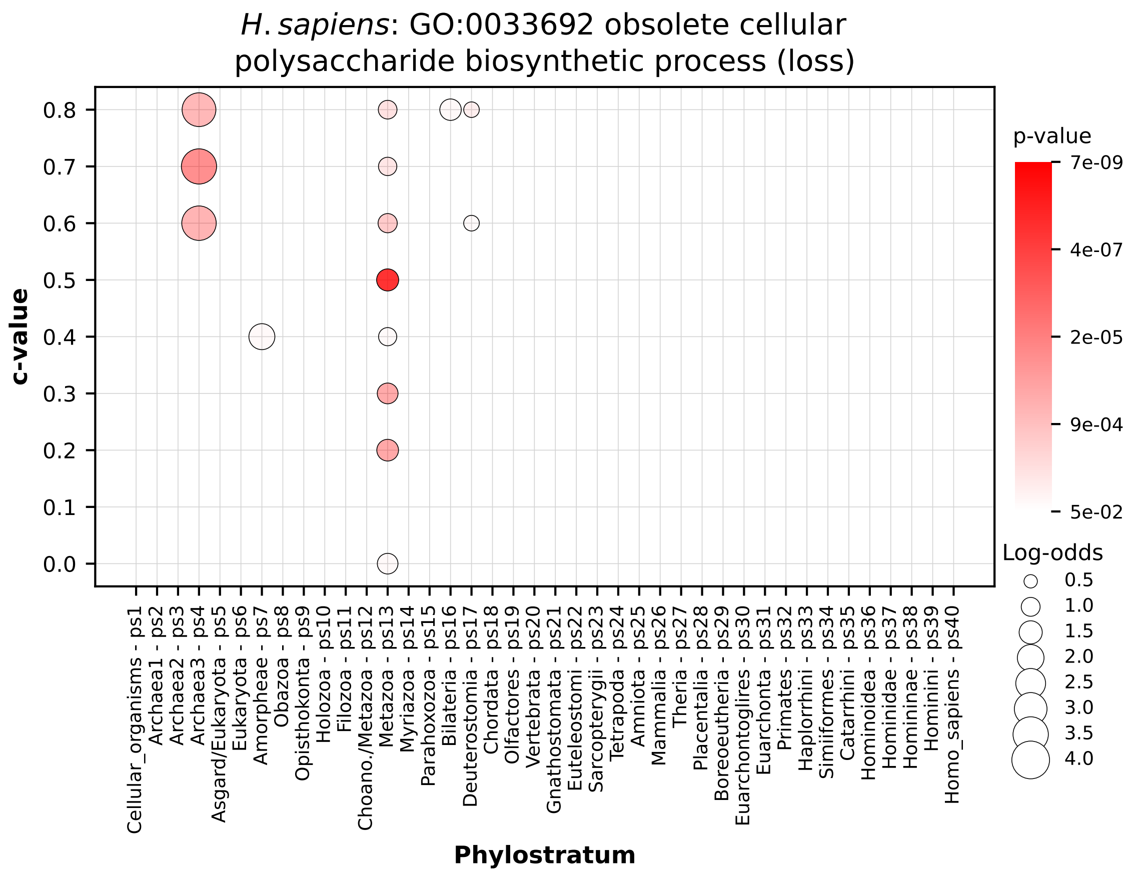

H. sapiens: GO:0033692 obsolete cellular polysaccharide biosynthetic process (loss)
c-value
0.8
0.7
0.6
0.5
0.4
0.3
0.2
0.1
0.0
Phylostratum
Cellular_organisms - ps1
Archaea1 - ps2
Archaea2 - ps3
Archaea3 - ps4
Asgard/Eukaryota - ps5
Eukaryota - ps6
Amorpheae - ps7
Obazoa - ps8
Opisthokonta - ps9
Holozoa - ps10
Filozoa - ps11
Choano./Metazoa - ps12
Metazoa - ps13
Myriazoa - ps14
Parahoxozoa - ps15
Bilateria - ps16
Deuterostomia - ps17
Chordata - ps18
Olfactores - ps19
Vertebrata - ps20
Gnathostomata - ps21
Euteleostomi - ps22
Sarcopterygii - ps23
Tetrapoda - ps24
Amniota - ps25
Mammalia - ps26
Theria - ps27
Placentalia - ps28
Boreoeutheria - ps29
Euarchontoglires - ps30
Euarchonta - ps31
Primates - ps32
Haplorrhini - ps33
Simiiformes - ps34
Catarrhini - ps35
Hominoidea - ps36
Hominidae - ps37
Homininae - ps38
Hominini - ps39
Homo_sapiens - ps40
p-value
7e-09
4e-07
2e-05
9e-04
5e-02
Log-odds
0.5
1.0
1.5
2.0
2.5
3.0
3.5
4.0

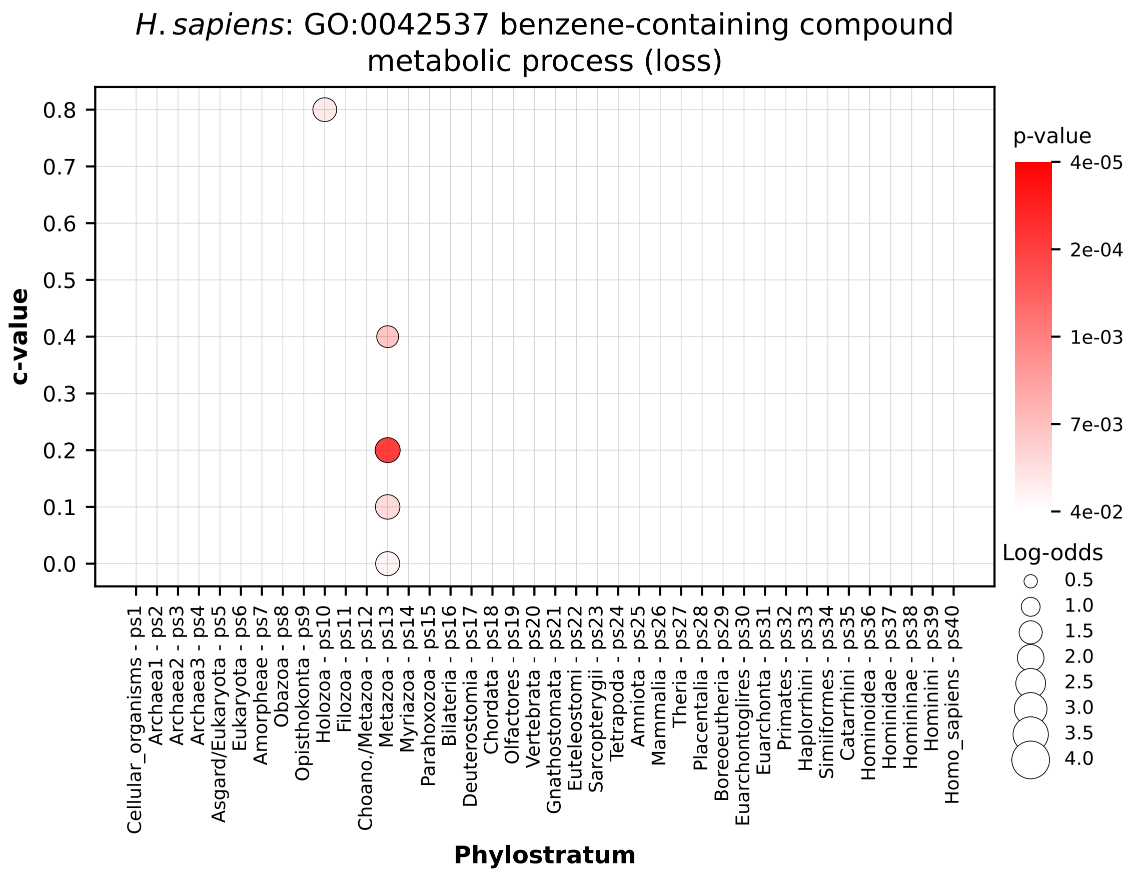
H. sapiens: GO:0042537 benzene-containing compound metabolic process (loss)
c-value
0.0
0.1
0.2
0.3
0.4
0.5
0.6
0.7
0.8
p-value
4e-05
2e-04
1e-03
7e-03
4e-02
Log-odds
0.5
1.0
1.5
2.0
2.5
3.0
3.5
4.0
Cellular_organisms - ps1
Archaea1 - ps2
Archaea2 - ps3
Archaea3 - ps4
Asgard/Eukaryota - ps5
Eukaryota - ps6
Amorpheae - ps7
Obazoa - ps8
Opisthokonta - ps9
Holozoa - ps10
Filozoa - ps11
Choano./Metazoa - ps12
Metazoa - ps13
Myriazoa - ps14
Parahoxozoa - ps15
Bilateria - ps16
Deuterostomia - ps17
Chordata - ps18
Olfactores - ps19
Vertebrata - ps20
Gnathostomata - ps21
Euteleostomi - ps22
Sarcopterygii - ps23
Tetrapoda - ps24
Amniota - ps25
Mammalia - ps26
Theria - ps27
Placentalia - ps28
Boreoeutheria - ps29
Euarchontoglires - ps30
Euarchonta - ps31
Primates - ps32
Haplorrhini - ps33
Simiiformes - ps34
Catarrhini - ps35
Hominoidea - ps36
Hominidae - ps37
Homininae - ps38
Hominini - ps39
Homo_sapiens - ps40
Phylostratum

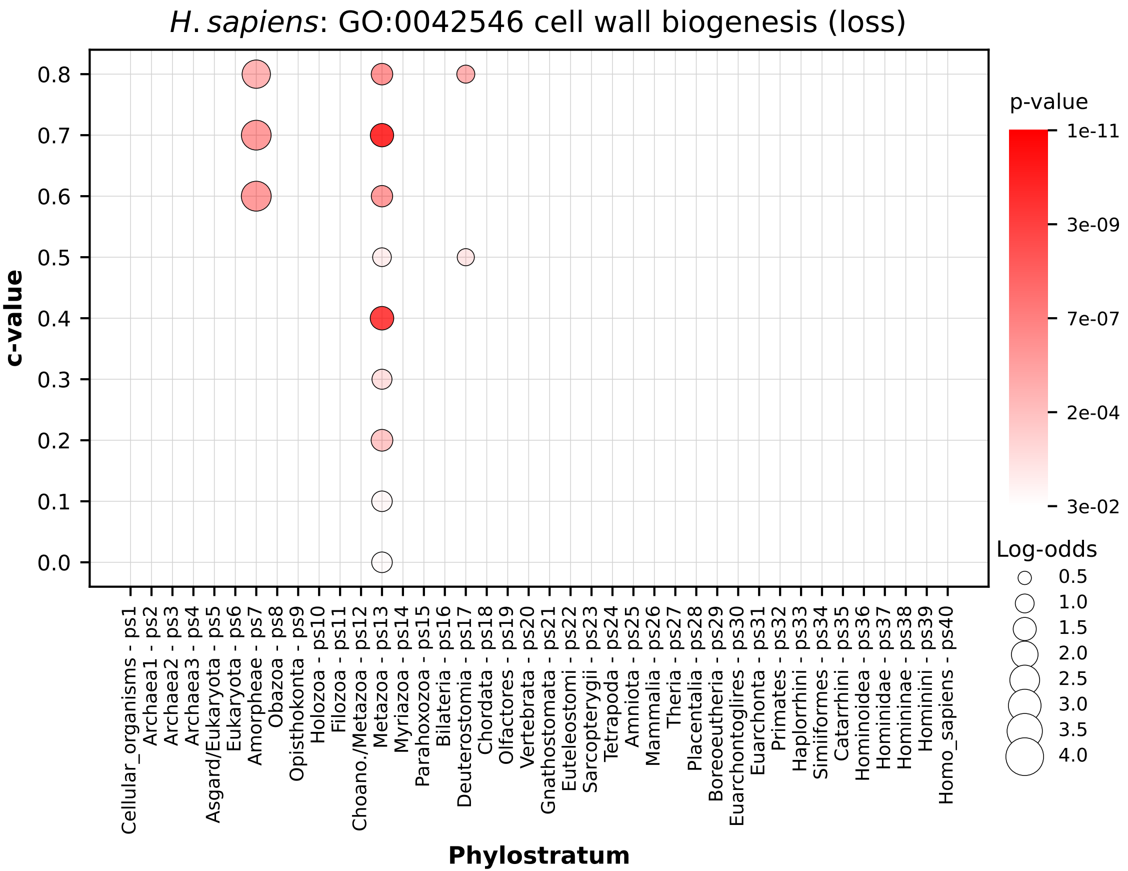

H. sapiens: GO:0042546 cell wall biogenesis (loss)
c-value
0.0
0.1
0.2
0.3
0.4
0.5
0.6
0.7
0.8
Phylostratum
Cellular_organisms - ps1
Archaea1 - ps2
Archaea2 - ps3
Archaea3 - ps4
Asgard/Eukaryota - ps5
Eukaryota - ps6
Amorpheae - ps7
Obazoa - ps8
Opisthokonta - ps9
Holozoa - ps10
Filozoa - ps11
Choano./Metazoa - ps12
Metazoa - ps13
Myriazoa - ps14
Parahoxozoa - ps15
Bilateria - ps16
Deuterostomia - ps17
Chordata - ps18
Olfactores - ps19
Vertebrata - ps20
Gnathostomata - ps21
Euteleostomi - ps22
Sarcopterygii - ps23
Tetrapoda - ps24
Amniota - ps25
Mammalia - ps26
Theria - ps27
Placentalia - ps28
Boreoeutheria - ps29
Euarchontoglires - ps30
Euarchonta - ps31
Primates - ps32
Haplorrhini - ps33
Simiiformes - ps34
Catarrhini - ps35
Hominoidea - ps36
Hominidae - ps37
Homininae - ps38
Hominini - ps39
Homo_sapiens - ps40
p-value
1e-11
3e-09
7e-07
2e-04
3e-02
Log-odds
0.5
1.0
1.5
2.0
2.5
3.0
3.5
4.0

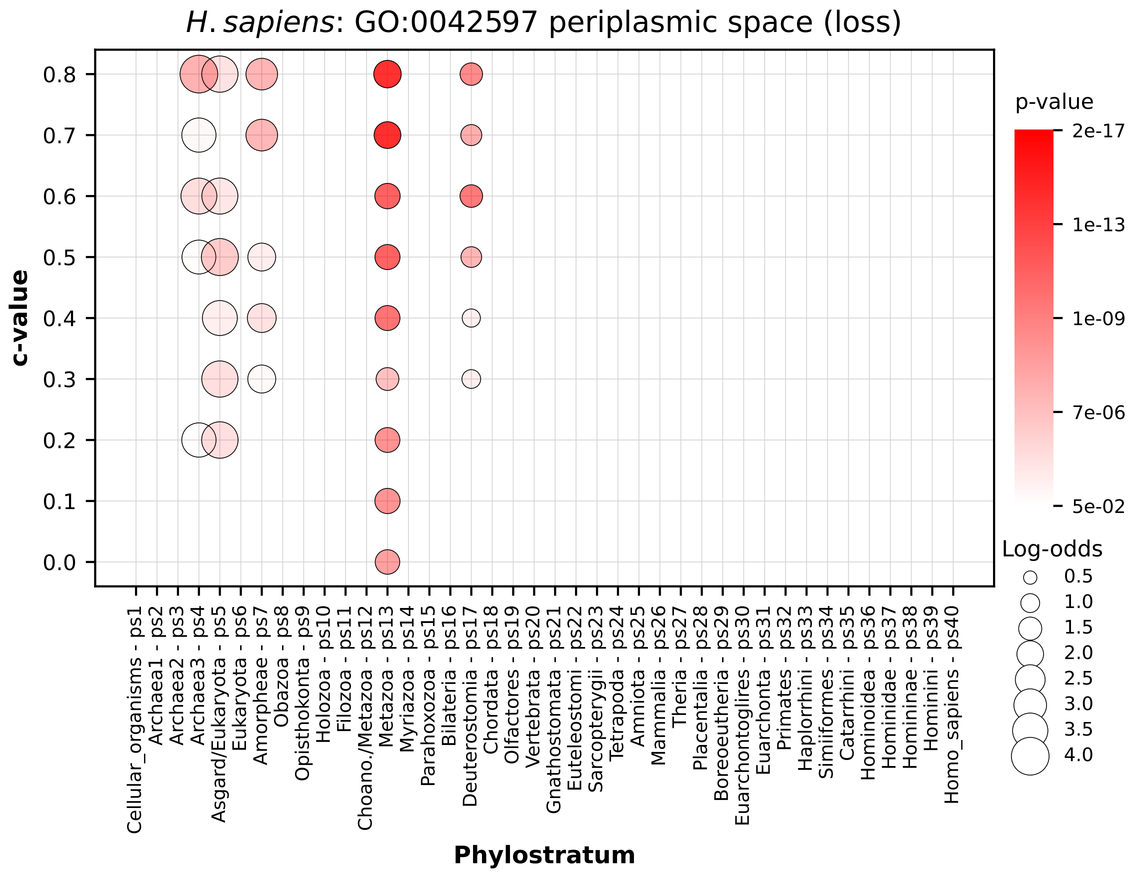

H. sapiens: GO:0042597 periplasmic space (loss)
c-value
0.8
0.7
0.6
0.5
0.4
0.3
0.2
0.1
0.0
Phylostratum
Cellular_organisms - ps1
Archaea1 - ps2
Archaea2 - ps3
Archaea3 - ps4
Asgard/Eukaryota - ps5
Eukaryota - ps6
Amorpheae - ps7
Obazoa - ps8
Opisthokonta - ps9
Holozoa - ps10
Filozoa - ps11
Choano./Metazoa - ps12
Metazoa - ps13
Myriazoa - ps14
Parahoxozoa - ps15
Bilateria - ps16
Deuterostomia - ps17
Chordata - ps18
Olfactores - ps19
Vertebrata - ps20
Gnathostomata - ps21
Euteleostomi - ps22
Sarcopterygii - ps23
Tetrapoda - ps24
Amniota - ps25
Mammalia - ps26
Theria - ps27
Placentalia - ps28
Boreoeutheria - ps29
Euarchontoglires - ps30
Euarchonta - ps31
Primates - ps32
Haplorrhini - ps33
Simiiformes - ps34
Catarrhini - ps35
Hominoidea - ps36
Hominidae - ps37
Homininae - ps38
Hominini - ps39
Homo_sapiens - ps40
p-value
2e-17
1e-13
1e-09
7e-06
5e-02
Log-odds
0.5
1.0
1.5
2.0
2.5
3.0
3.5
4.0

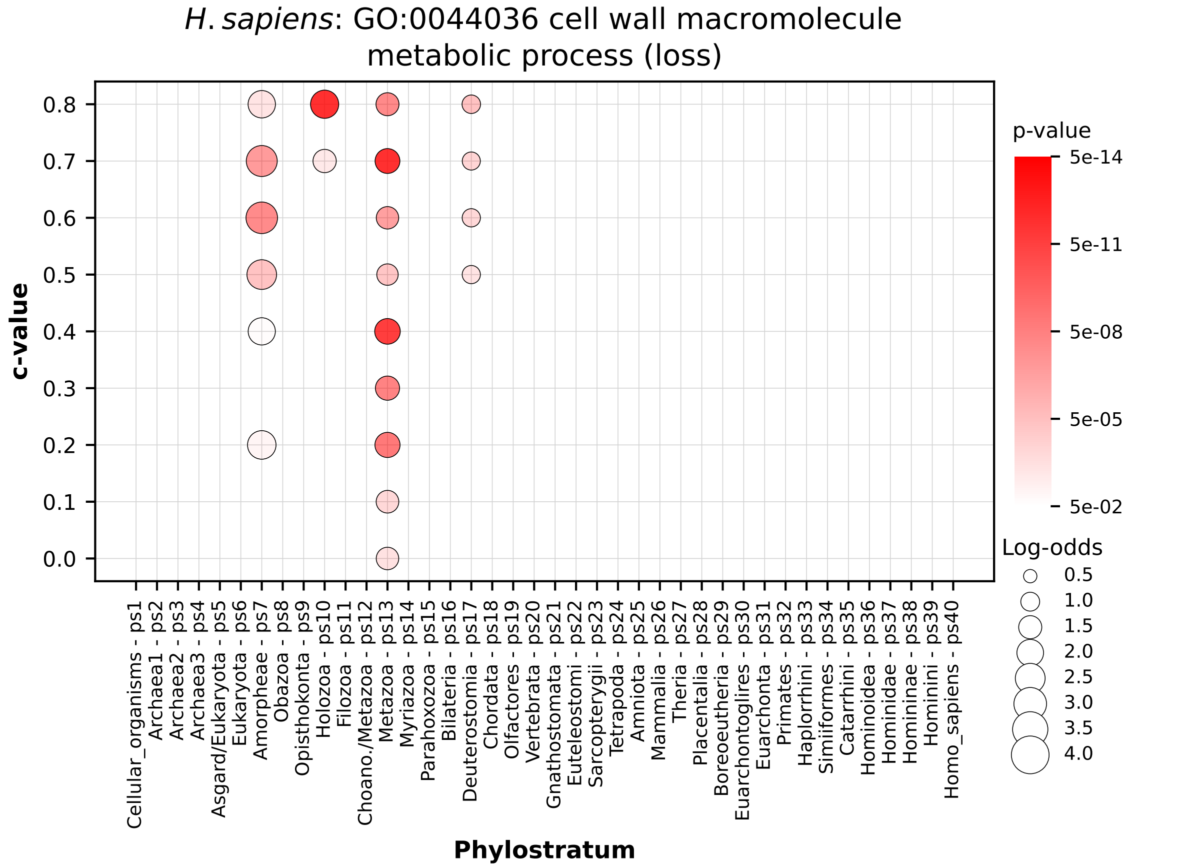

H. sapiens: GO:0044036 cell wall macromolecule metabolic process (loss)
c-value
0.8
0.7
0.6
0.5
0.4
0.3
0.2
0.1
0.0
p-value
5e-14
5e-11
5e-08
5e-05
5e-02
Log-odds
0.5
1.0
1.5
2.0
2.5
3.0
3.5
4.0
Cellular_organisms - ps1
Archaea1 - ps2
Archaea2 - ps3
Archaea3 - ps4
Asgard/Eukaryota - ps5
Eukaryota - ps6
Amorpheae - ps7
Obazoa - ps8
Opisthokonta - ps9
Holozoa - ps10
Filozoa - ps11
Choano./Metazoa - ps12
Metazoa - ps13
Myriazoa - ps14
Parahoxozoa - ps15
Bilateria - ps16
Deuterostomia - ps17
Chordata - ps18
Olfactores - ps19
Vertebrata - ps20
Gnathostomata - ps21
Euteleostomi - ps22
Sarcopterygii - ps23
Tetrapoda - ps24
Amniota - ps25
Mammalia - ps26
Theria - ps27
Placentalia - ps28
Boreoeutheria - ps29
Euarchontoglires - ps30
Euarchonta - ps31
Primates - ps32
Haplorrhini - ps33
Simiiformes - ps34
Catarrhini - ps35
Hominoidea - ps36
Hominidae - ps37
Homininae - ps38
Hominini - ps39
Homo_sapiens - ps40
Phylostratum

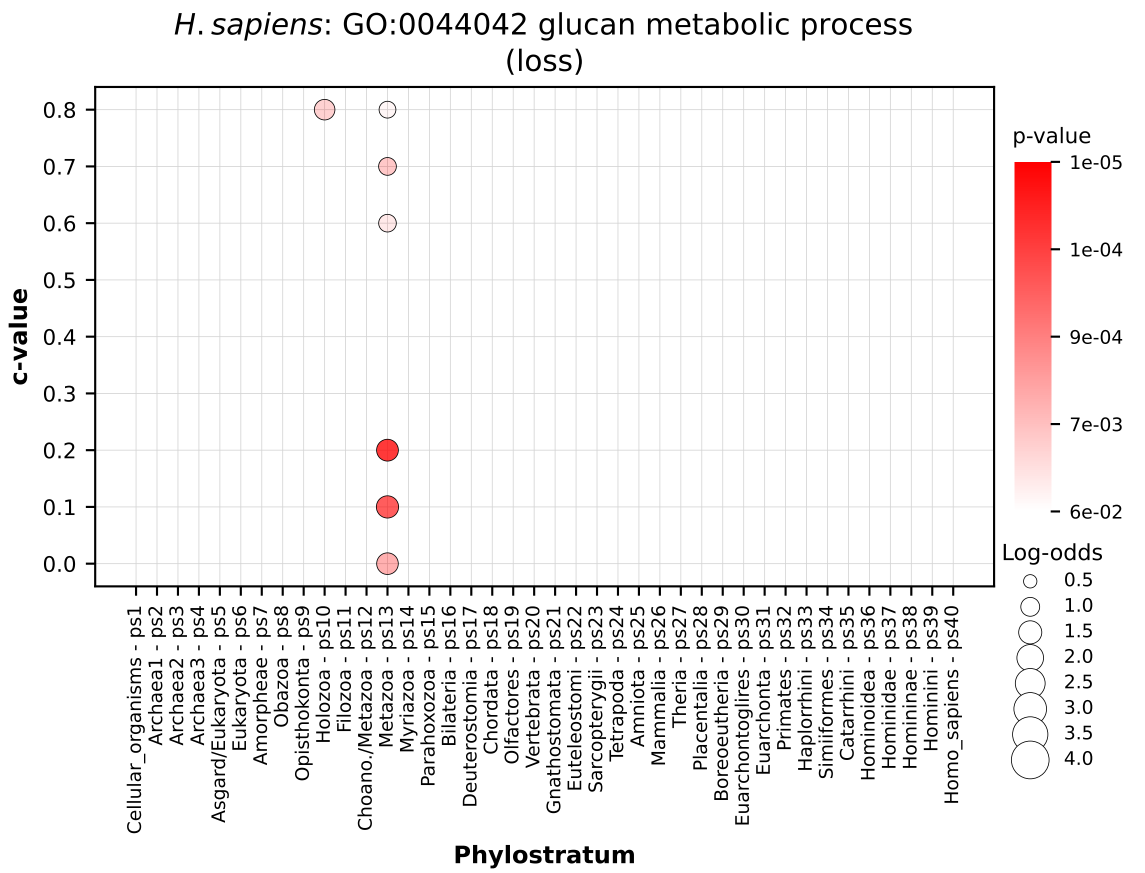

H. sapiens: GO:0044042 glucan metabolic process
(loss)
c-value
0.0
0.1
0.2
0.3
0.4
0.5
0.6
0.7
0.8
Phylostratum
Cellular_organisms - ps1
Archaea1 - ps2
Archaea2 - ps3
Archaea3 - ps4
Asgard/Eukaryota - ps5
Eukaryota - ps6
Amorpheae - ps7
Obazoa - ps8
Opisthokonta - ps9
Holozoa - ps10
Filozoa - ps11
Choano./Metazoa - ps12
Metazoa - ps13
Myriazoa - ps14
Parahoxozoa - ps15
Bilateria - ps16
Deuterostomia - ps17
Chordata - ps18
Olfactores - ps19
Vertebrata - ps20
Gnathostomata - ps21
Euteleostomi - ps22
Sarcopterygii - ps23
Tetrapoda - ps24
Amniota - ps25
Mammalia - ps26
Theria - ps27
Placentalia - ps28
Boreoeutheria - ps29
Euarchontoglires - ps30
Euarchonta - ps31
Primates - ps32
Haplorrhini - ps33
Simiiformes - ps34
Catarrhini - ps35
Hominoidea - ps36
Hominidae - ps37
Homininae - ps38
Hominini - ps39
Homo_sapiens - ps40
p-value
1e-05
1e-04
9e-04
7e-03
6e-02
Log-odds
0.5
1.0
1.5
2.0
2.5
3.0
3.5
4.0

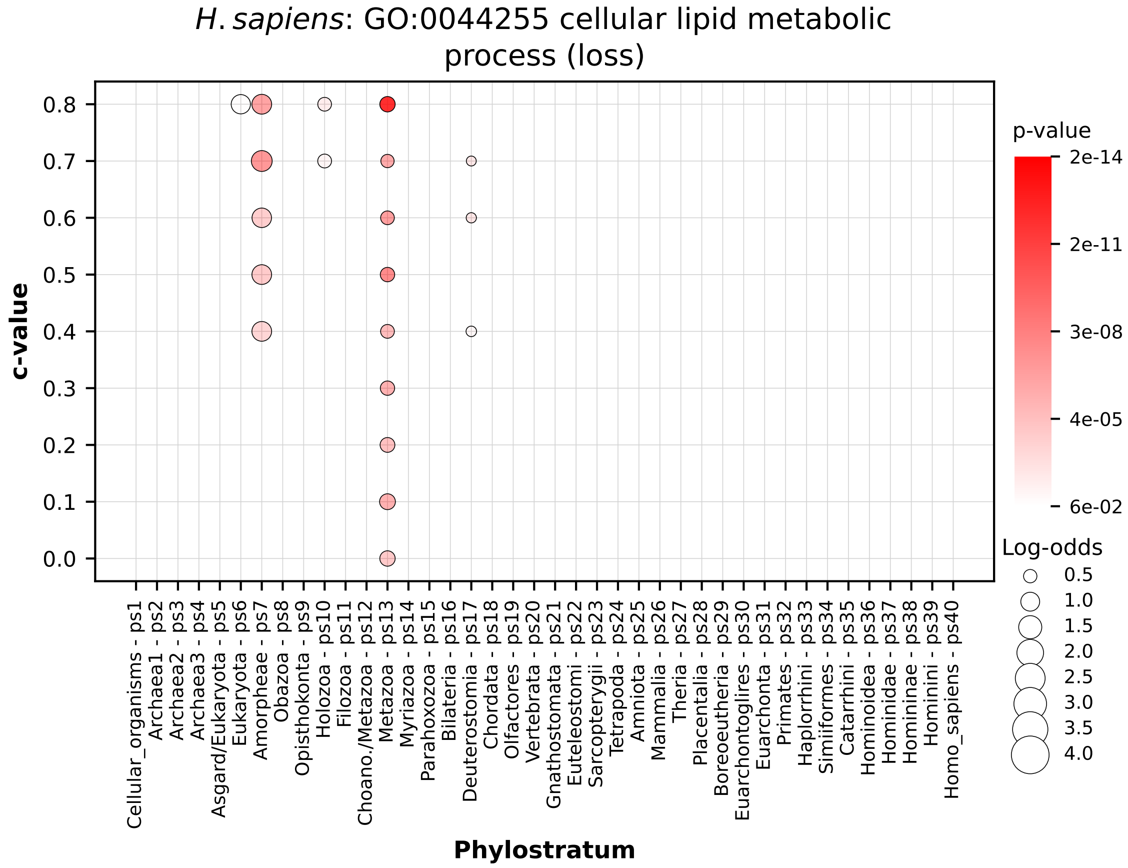

H. sapiens: GO:0044255 cellular lipid metabolic process (loss)
c-value
0.8
0.7
0.6
0.5
0.4
0.3
0.2
0.1
0.0
Phylostratum
Cellular_organisms - ps1
Archaea1 - ps2
Archaea2 - ps3
Archaea3 - ps4
Asgard/Eukaryota - ps5
Eukaryota - ps6
Amorpheae - ps7
Obazoa - ps8
Opisthokonta - ps9
Holozoa - ps10
Filozoa - ps11
Choano./Metazoa - ps12
Metazoa - ps13
Myriazoa - ps14
Parahoxozoa - ps15
Bilateria - ps16
Deuterostomia - ps17
Chordata - ps18
Olfactores - ps19
Vertebrata - ps20
Gnathostomata - ps21
Euteleostomi - ps22
Sarcopterygii - ps23
Tetrapoda - ps24
Amniota - ps25
Mammalia - ps26
Theria - ps27
Placentalia - ps28
Boreoeutheria - ps29
Euarchontoglires - ps30
Euarchonta - ps31
Primates - ps32
Haplorrhini - ps33
Simiiformes - ps34
Catarrhini - ps35
Hominoidea - ps36
Hominidae - ps37
Homininae - ps38
Hominini - ps39
Homo_sapiens - ps40
p-value
2e-14
2e-11
3e-08
4e-05
6e-02
Log-odds
0.5
1.0
1.5
2.0
2.5
3.0
3.5
4.0

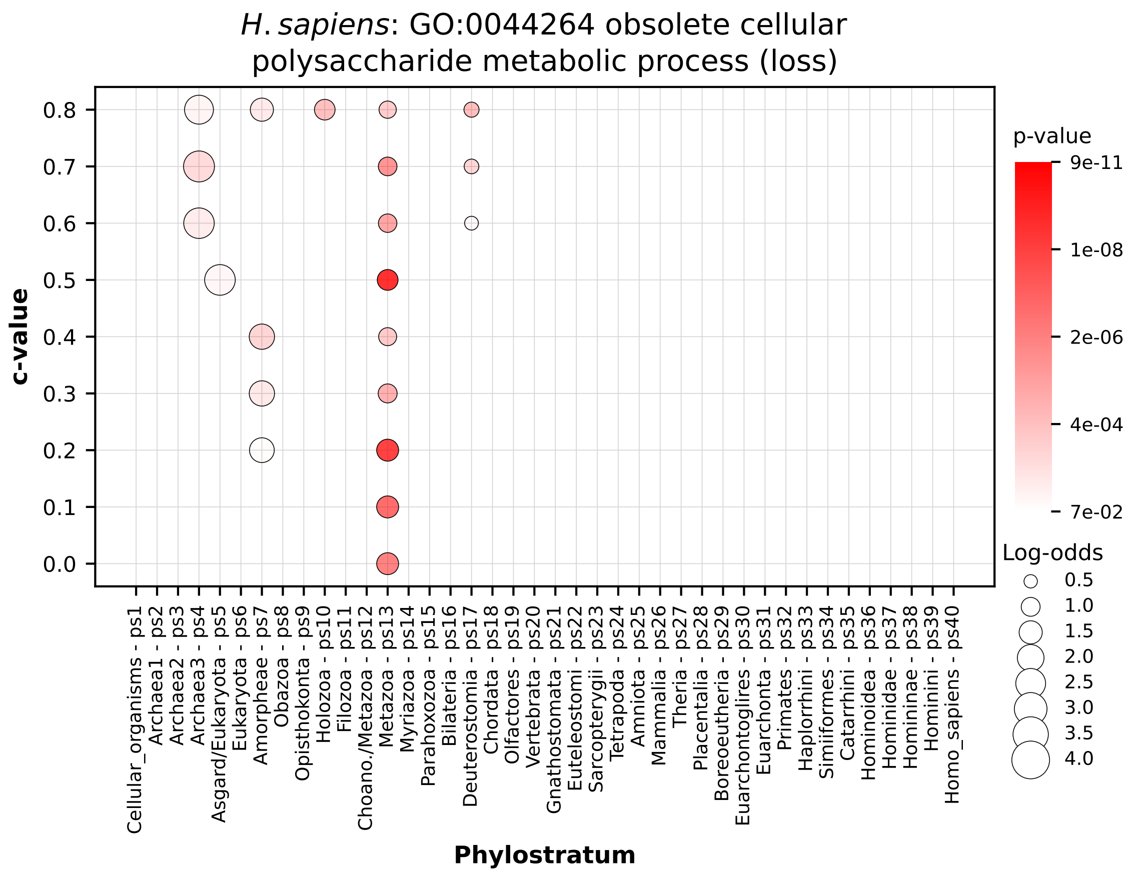

H. sapiens: GO:0044264 obsolete cellular polysaccharide metabolic process (loss)
c-value
Phylostratum
p-value
9e-11
1e-08
2e-06
4e-04
7e-02
Log-odds
0.5
1.0
1.5
2.0
2.5
3.0
3.5
4.0
0.8
0.7
0.6
0.5
0.4
0.3
0.2
0.1
0.0
Cellular_organisms - ps1
Archaea1 - ps2
Archaea2 - ps3
Archaea3 - ps4
Asgard/Eukaryota - ps5
Eukaryota - ps6
Amorpheae - ps7
Obazoa - ps8
Opisthokonta - ps9
Holozoa - ps10
Filozoa - ps11
Choano./Metazoa - ps12
Metazoa - ps13
Myriazoa - ps14
Parahoxozoa - ps15
Bilateria - ps16
Deuterostomia - ps17
Chordata - ps18
Olfactores - ps19
Vertebrata - ps20
Gnathostomata - ps21
Euteleostomi - ps22
Sarcopterygii - ps23
Tetrapoda - ps24
Amniota - ps25
Mammalia - ps26
Theria - ps27
Placentalia - ps28
Boreoeutheria - ps29
Euarchontoglires - ps30
Euarchonta - ps31
Primates - ps32
Haplorrhini - ps33
Simiiformes - ps34
Catarrhini - ps35
Hominoidea - ps36
Hominidae - ps37
Homininae - ps38
Hominini - ps39
Homo_sapiens - ps40

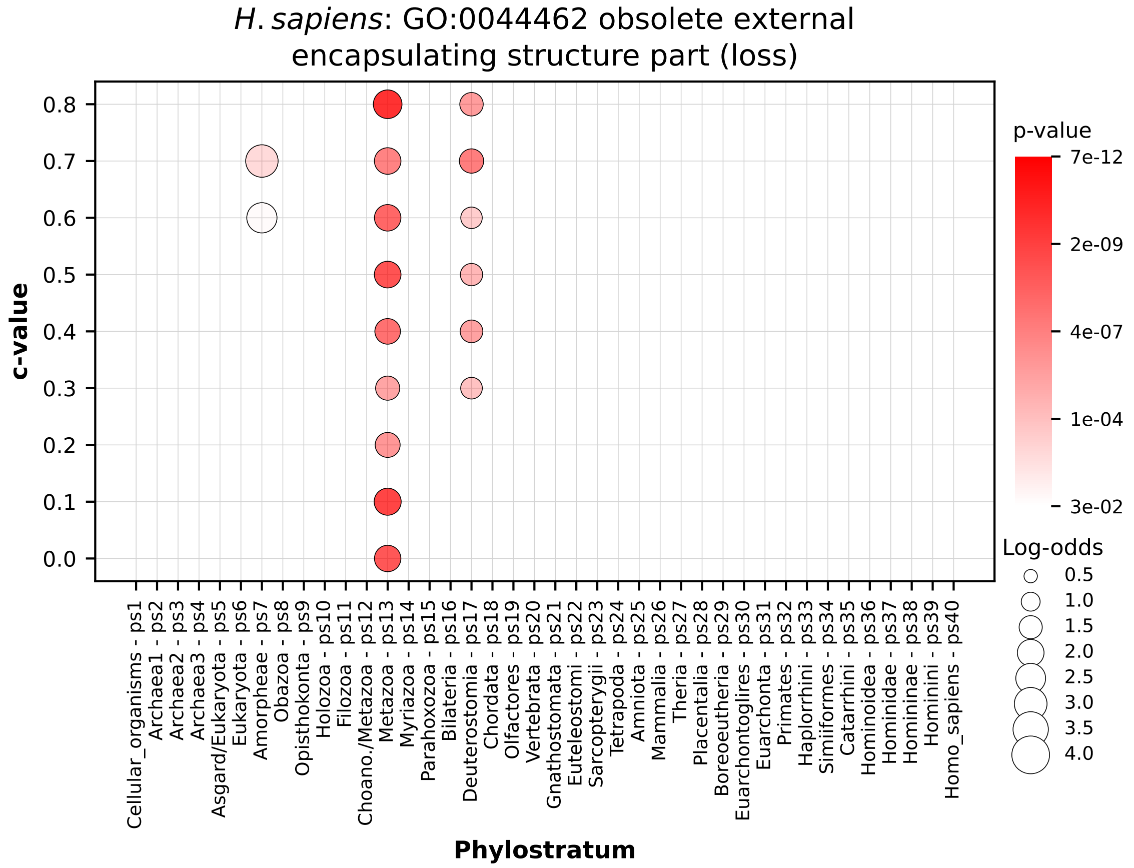

H. sapiens: GO:0044462 obsolete external encapsulating structure part (loss)
c-value
0.0
0.1
0.2
0.3
0.4
0.5
0.6
0.7
0.8
p-value
7e-12
2e-09
4e-07
1e-04
3e-02
Log-odds
0.5
1.0
1.5
2.0
2.5
3.0
3.5
4.0
Cellular_organisms - ps1
Archaea1 - ps2
Archaea2 - ps3
Archaea3 - ps4
Asgard/Eukaryota - ps5
Eukaryota - ps6
Amorpheae - ps7
Obazoa - ps8
Opisthokonta - ps9
Holozoa - ps10
Filozoa - ps11
Choano./Metazoa - ps12
Metazoa - ps13
Myriazoa - ps14
Parahoxozoa - ps15
Bilateria - ps16
Deuterostomia - ps17
Chordata - ps18
Olfactores - ps19
Vertebrata - ps20
Gnathostomata - ps21
Euteleostomi - ps22
Sarcopterygii - ps23
Tetrapoda - ps24
Amniota - ps25
Mammalia - ps26
Theria - ps27
Placentalia - ps28
Boreoeutheria - ps29
Euarchontoglires - ps30
Euarchonta - ps31
Primates - ps32
Haplorrhini - ps33
Simiiformes - ps34
Catarrhini - ps35
Hominoidea - ps36
Hominidae - ps37
Homininae - ps38
Hominini - ps39
Homo_sapiens - ps40
Phylostratum

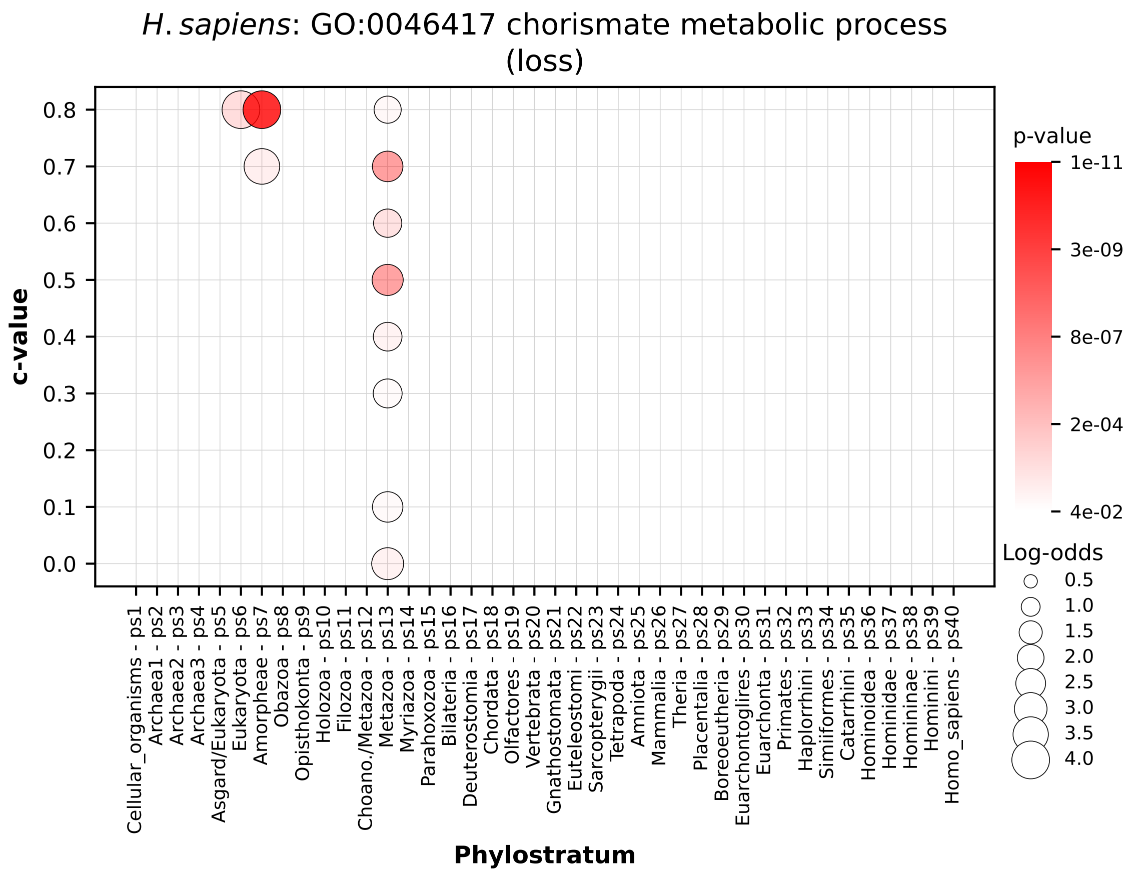

H. sapiens: GO:0046417 chorismate metabolic process
(loss)
c-value
0.8
0.7
0.6
0.5
0.4
0.3
0.2
0.1
0.0
p-value
1e-11
3e-09
8e-07
2e-04
4e-02
Log-odds
0.5
1.0
1.5
2.0
2.5
3.0
3.5
4.0
Cellular_organisms - ps1
Archaea1 - ps2
Archaea2 - ps3
Archaea3 - ps4
Asgard/Eukaryota - ps5
Eukaryota - ps6
Amorpheae - ps7
Obazoa - ps8
Opisthokonta - ps9
Holozoa - ps10
Filozoa - ps11
Choano./Metazoa - ps12
Metazoa - ps13
Myriazoa - ps14
Parahoxozoa - ps15
Bilateria - ps16
Deuterostomia - ps17
Chordata - ps18
Olfactores - ps19
Vertebrata - ps20
Gnathostomata - ps21
Euteleostomi - ps22
Sarcopterygii - ps23
Tetrapoda - ps24
Amniota - ps25
Mammalia - ps26
Theria - ps27
Placentalia - ps28
Boreoeutheria - ps29
Euarchontoglires - ps30
Euarchonta - ps31
Primates - ps32
Haplorrhini - ps33
Simiiformes - ps34
Catarrhini - ps35
Hominoidea - ps36
Hominidae - ps37
Homininae - ps38
Hominini - ps39
Homo_sapiens - ps40
Phylostratum

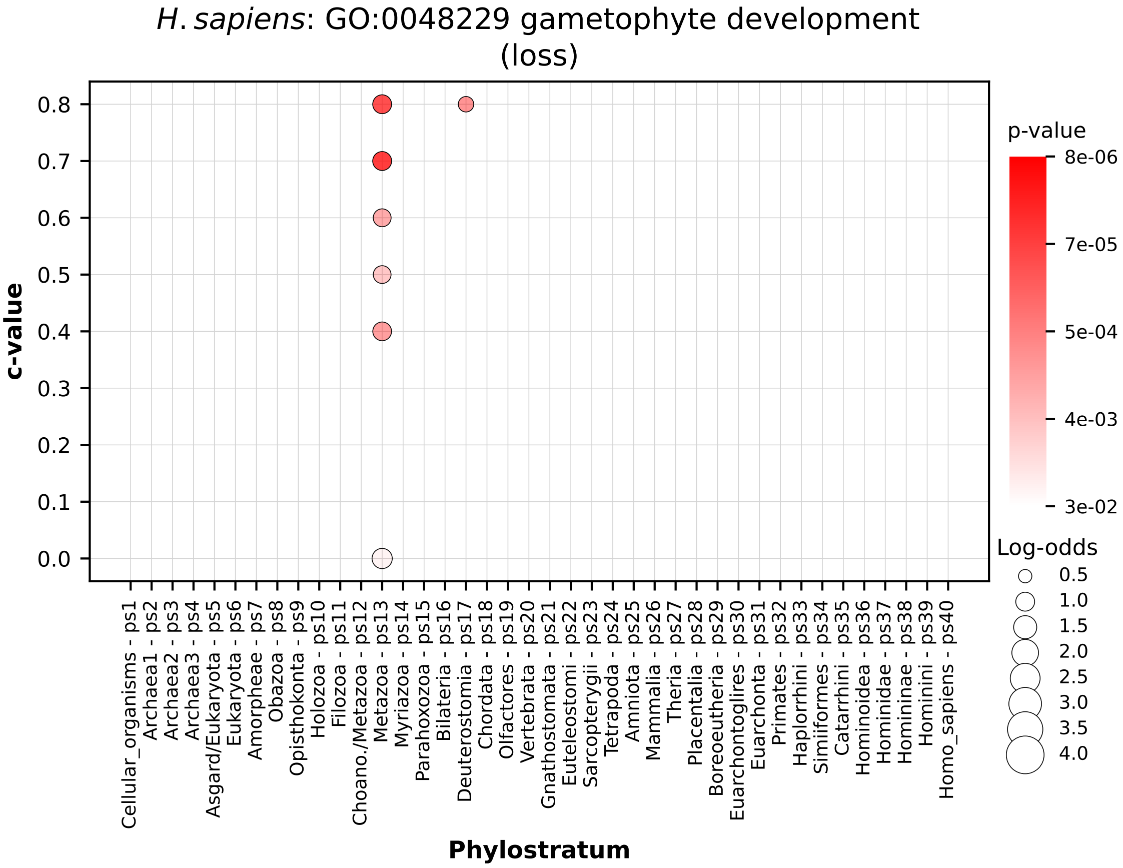

H. sapiens: GO:0048229 gametophyte development
(loss)
c-value
0.0
0.1
0.2
0.3
0.4
0.5
0.6
0.7
0.8
p-value
8e-06
7e-05
5e-04
4e-03
3e-02
Log-odds
0.5
1.0
1.5
2.0
2.5
3.0
3.5
4.0
Cellular_organisms - ps1
Archaea1 - ps2
Archaea2 - ps3
Archaea3 - ps4
Asgard/Eukaryota - ps5
Eukaryota - ps6
Amorpheae - ps7
Obazoa - ps8
Opisthokonta - ps9
Holozoa - ps10
Filozoa - ps11
Choano./Metazoa - ps12
Metazoa - ps13
Myriazoa - ps14
Parahoxozoa - ps15
Bilateria - ps16
Deuterostomia - ps17
Chordata - ps18
Olfactores - ps19
Vertebrata - ps20
Gnathostomata - ps21
Euteleostomi - ps22
Sarcopterygii - ps23
Tetrapoda - ps24
Amniota - ps25
Mammalia - ps26
Theria - ps27
Placentalia - ps28
Boreoeutheria - ps29
Euarchontoglires - ps30
Euarchonta - ps31
Primates - ps32
Haplorrhini - ps33
Simiiformes - ps34
Catarrhini - ps35
Hominoidea - ps36
Hominidae - ps37
Homininae - ps38
Hominini - ps39
Homo_sapiens - ps40
Phylostratum

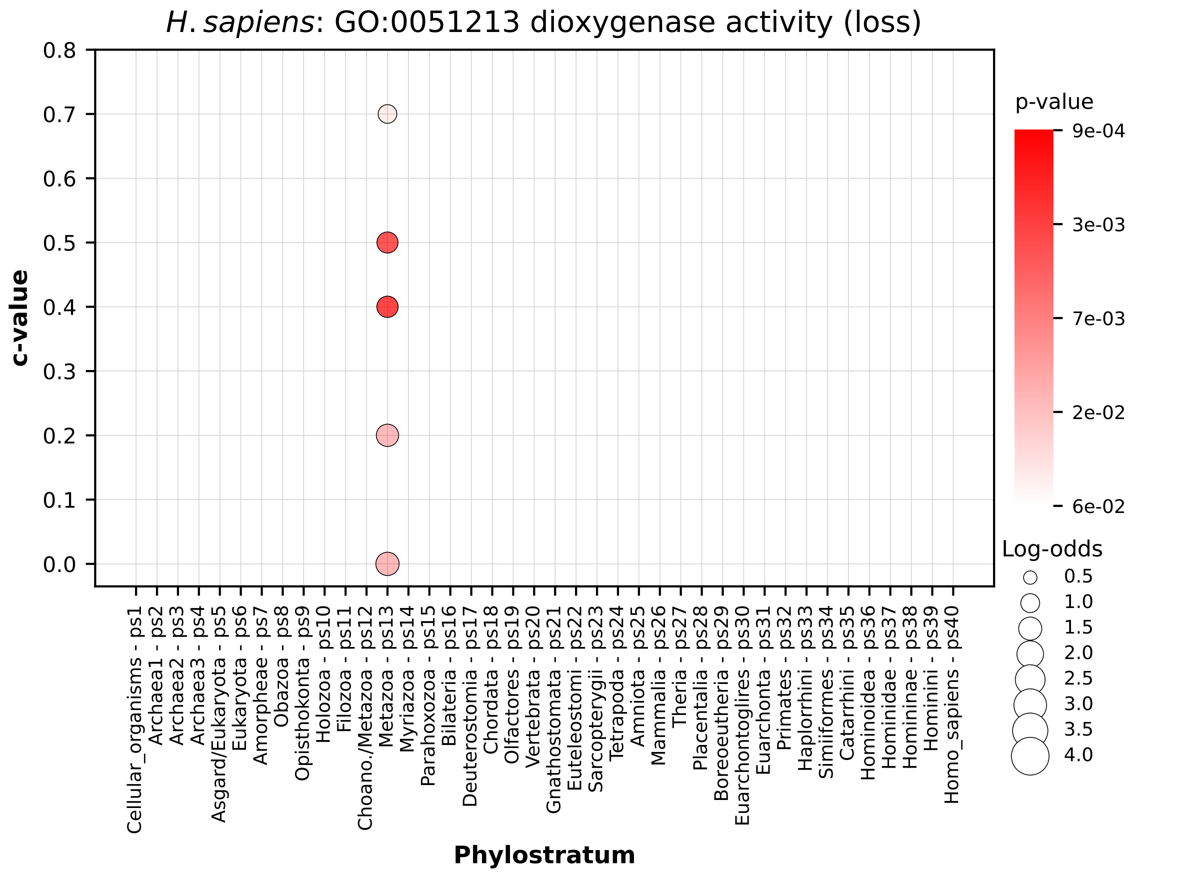

H. sapiens: GO:0051213 dioxygenase activity (loss)
c-value
0.8
0.7
0.6
0.5
0.4
0.3
0.2
0.1
0.0
Phylostratum
Cellular_organisms - ps1
Archaea1 - ps2
Archaea2 - ps3
Archaea3 - ps4
Asgard/Eukaryota - ps5
Eukaryota - ps6
Amorpheae - ps7
Obazoa - ps8
Opisthokonta - ps9
Holozoa - ps10
Filozoa - ps11
Choano./Metazoa - ps12
Metazoa - ps13
Myriazoa - ps14
Parahoxozoa - ps15
Bilateria - ps16
Deuterostomia - ps17
Chordata - ps18
Olfactores - ps19
Vertebrata - ps20
Gnathostomata - ps21
Euteleostomi - ps22
Sarcopterygii - ps23
Tetrapoda - ps24
Amniota - ps25
Mammalia - ps26
Theria - ps27
Placentalia - ps28
Boreoeutheria - ps29
Euarchontoglires - ps30
Euarchonta - ps31
Primates - ps32
Haplorrhini - ps33
Simiiformes - ps34
Catarrhini - ps35
Hominoidea - ps36
Hominidae - ps37
Homininae - ps38
Hominini - ps39
Homo_sapiens - ps40
p-value
9e-04
3e-03
7e-03
2e-02
6e-02
Log-odds
0.5
1.0
1.5
2.0
2.5
3.0
3.5
4.0

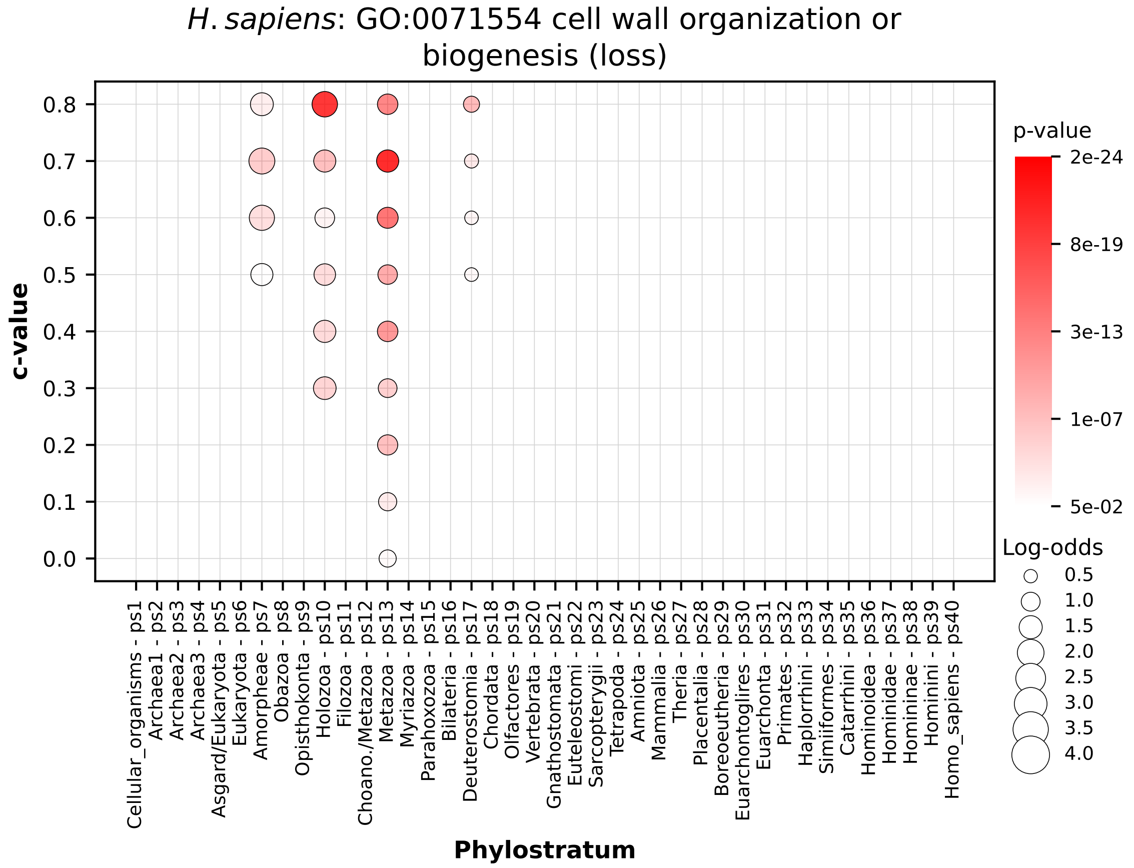

H. sapiens: GO:0071554 cell wall organization or biogenesis (loss)
c-value
0.8
0.7
0.6
0.5
0.4
0.3
0.2
0.1
0.0
Phylostratum
Cellular_organisms - ps1
Archaea1 - ps2
Archaea2 - ps3
Archaea3 - ps4
Asgard/Eukaryota - ps5
Eukaryota - ps6
Amorpheae - ps7
Obazoa - ps8
Opisthokonta - ps9
Holozoa - ps10
Filozoa - ps11
Choano./Metazoa - ps12
Metazoa - ps13
Myriazoa - ps14
Parahoxozoa - ps15
Bilateria - ps16
Deuterostomia - ps17
Chordata - ps18
Olfactores - ps19
Vertebrata - ps20
Gnathostomata - ps21
Euteleostomi - ps22
Sarcopterygii - ps23
Tetrapoda - ps24
Amniota - ps25
Mammalia - ps26
Theria - ps27
Placentalia - ps28
Boreoeutheria - ps29
Euarchontoglires - ps30
Euarchonta - ps31
Primates - ps32
Haplorrhini - ps33
Simiiformes - ps34
Catarrhini - ps35
Hominoidea - ps36
Hominidae - ps37
Homininae - ps38
Hominini - ps39
Homo_sapiens - ps40
p-value
2e-24
8e-19
3e-13
1e-07
5e-02
Log-odds
0.5
1.0
1.5
2.0
2.5
3.0
3.5
4.0

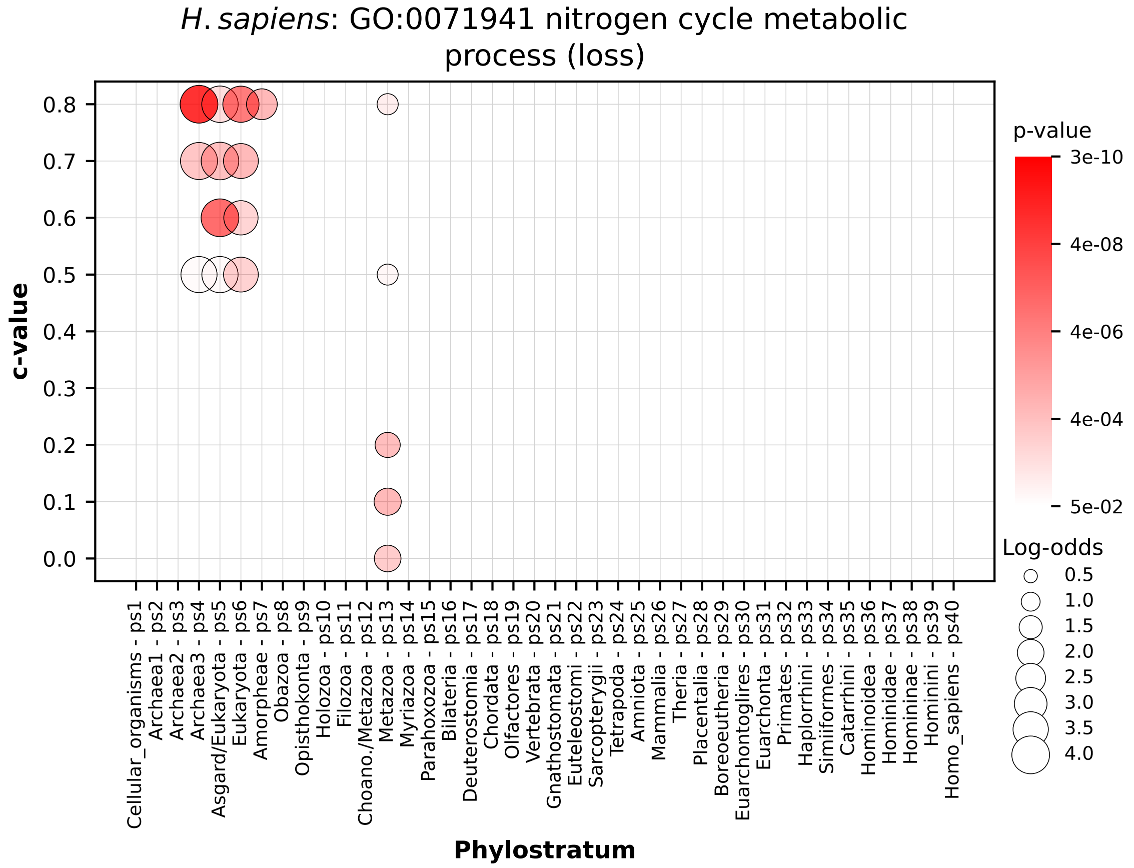

H. sapiens: GO:0071941 nitrogen cycle metabolic process (loss)
c-value
0.8
0.7
0.6
0.5
0.4
0.3
0.2
0.1
0.0
Phylostratum
Cellular_organisms - ps1
Archaea1 - ps2
Archaea2 - ps3
Archaea3 - ps4
Asgard/Eukaryota - ps5
Eukaryota - ps6
Amorpheae - ps7
Obazoa - ps8
Opisthokonta - ps9
Holozoa - ps10
Filozoa - ps11
Choano./Metazoa - ps12
Metazoa - ps13
Myriazoa - ps14
Parahoxozoa - ps15
Bilateria - ps16
Deuterostomia - ps17
Chordata - ps18
Olfactores - ps19
Vertebrata - ps20
Gnathostomata - ps21
Euteleostomi - ps22
Sarcopterygii - ps23
Tetrapoda - ps24
Amniota - ps25
Mammalia - ps26
Theria - ps27
Placentalia - ps28
Boreoeutheria - ps29
Euarchontoglires - ps30
Euarchonta - ps31
Primates - ps32
Haplorrhini - ps33
Simiiformes - ps34
Catarrhini - ps35
Hominoidea - ps36
Hominidae - ps37
Homininae - ps38
Hominini - ps39
Homo_sapiens - ps40
p-value
3e-10
4e-08
4e-06
4e-04
5e-02
Log-odds
0.5
1.0
1.5
2.0
2.5
3.0
3.5
4.0

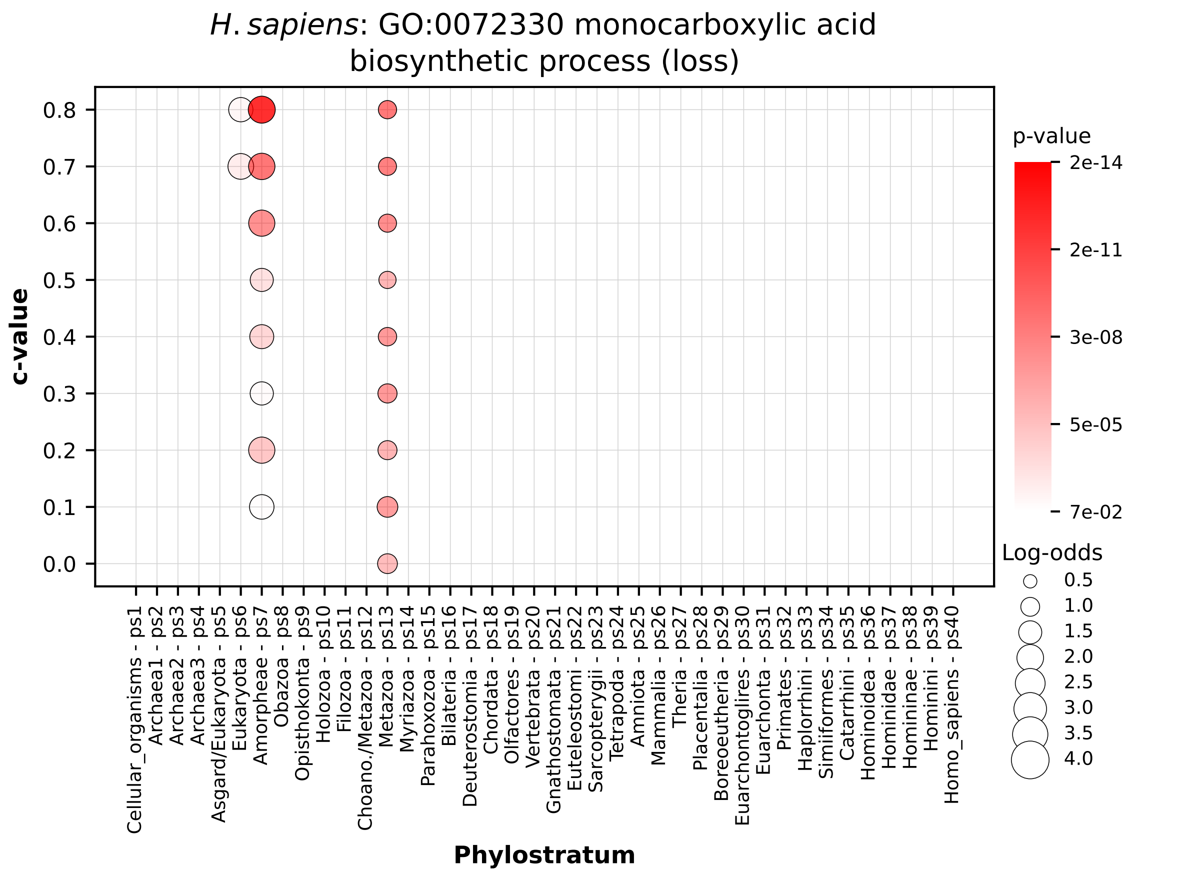

H. sapiens: GO:0072330 monocarboxylic acid biosynthetic process (loss)
c-value
0.8
0.7
0.6
0.5
0.4
0.3
0.2
0.1
0.0
p-value
2e-14
2e-11
3e-08
5e-05
7e-02
Log-odds
0.5
1.0
1.5
2.0
2.5
3.0
3.5
4.0
Cellular_organisms - ps1
Archaea1 - ps2
Archaea2 - ps3
Archaea3 - ps4
Asgard/Eukaryota - ps5
Eukaryota - ps6
Amorpheae - ps7
Obazoa - ps8
Opisthokonta - ps9
Holozoa - ps10
Filozoa - ps11
Choano./Metazoa - ps12
Metazoa - ps13
Myriazoa - ps14
Parahoxozoa - ps15
Bilateria - ps16
Deuterostomia - ps17
Chordata - ps18
Olfactores - ps19
Vertebrata - ps20
Gnathostomata - ps21
Euteleostomi - ps22
Sarcopterygii - ps23
Tetrapoda - ps24
Amniota - ps25
Mammalia - ps26
Theria - ps27
Placentalia - ps28
Boreoeutheria - ps29
Euarchontoglires - ps30
Euarchonta - ps31
Primates - ps32
Haplorrhini - ps33
Simiiformes - ps34
Catarrhini - ps35
Hominoidea - ps36
Hominidae - ps37
Homininae - ps38
Hominini - ps39
Homo_sapiens - ps40
Phylostratum

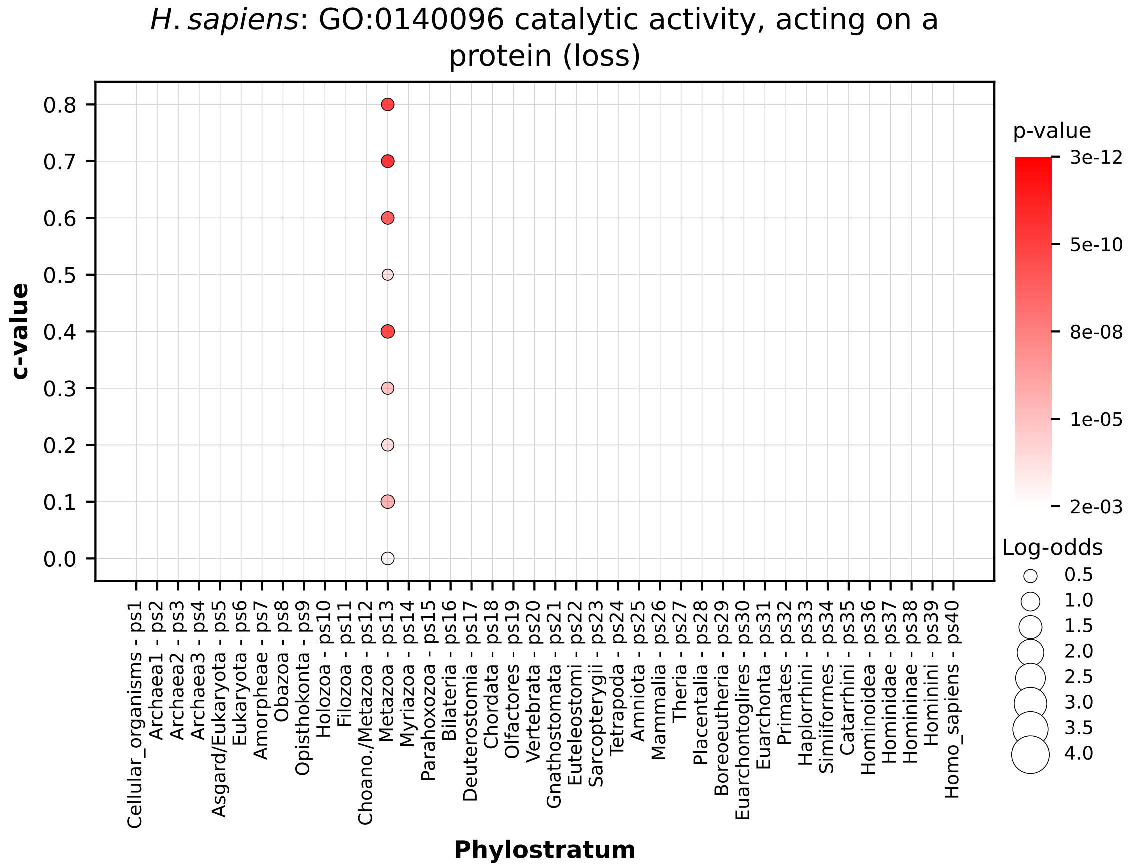

H. sapiens: GO:0140096 catalytic activity, acting on a protein (loss)
c-value
0.0
0.1
0.2
0.3
0.4
0.5
0.6
0.7
0.8
Phylostratum
Cellular_organisms - ps1
Archaea1 - ps2
Archaea2 - ps3
Archaea3 - ps4
Asgard/Eukaryota - ps5
Eukaryota - ps6
Amorpheae - ps7
Obazoa - ps8
Opisthokonta - ps9
Holozoa - ps10
Filozoa - ps11
Choano./Metazoa - ps12
Metazoa - ps13
Myriazoa - ps14
Parahoxozoa - ps15
Bilateria - ps16
Deuterostomia - ps17
Chordata - ps18
Olfactores - ps19
Vertebrata - ps20
Gnathostomata - ps21
Euteleostomi - ps22
Sarcopterygii - ps23
Tetrapoda - ps24
Amniota - ps25
Mammalia - ps26
Theria - ps27
Placentalia - ps28
Boreoeutheria - ps29
Euarchontoglires - ps30
Euarchonta - ps31
Primates - ps32
Haplorrhini - ps33
Simiiformes - ps34
Catarrhini - ps35
Hominoidea - ps36
Hominidae - ps37
Homininae - ps38
Hominini - ps39
Homo_sapiens - ps40
p-value
3e-12
5e-10
8e-08
1e-05
2e-03
Log-odds
0.5
1.0
1.5
2.0
2.5
3.0
3.5
4.0

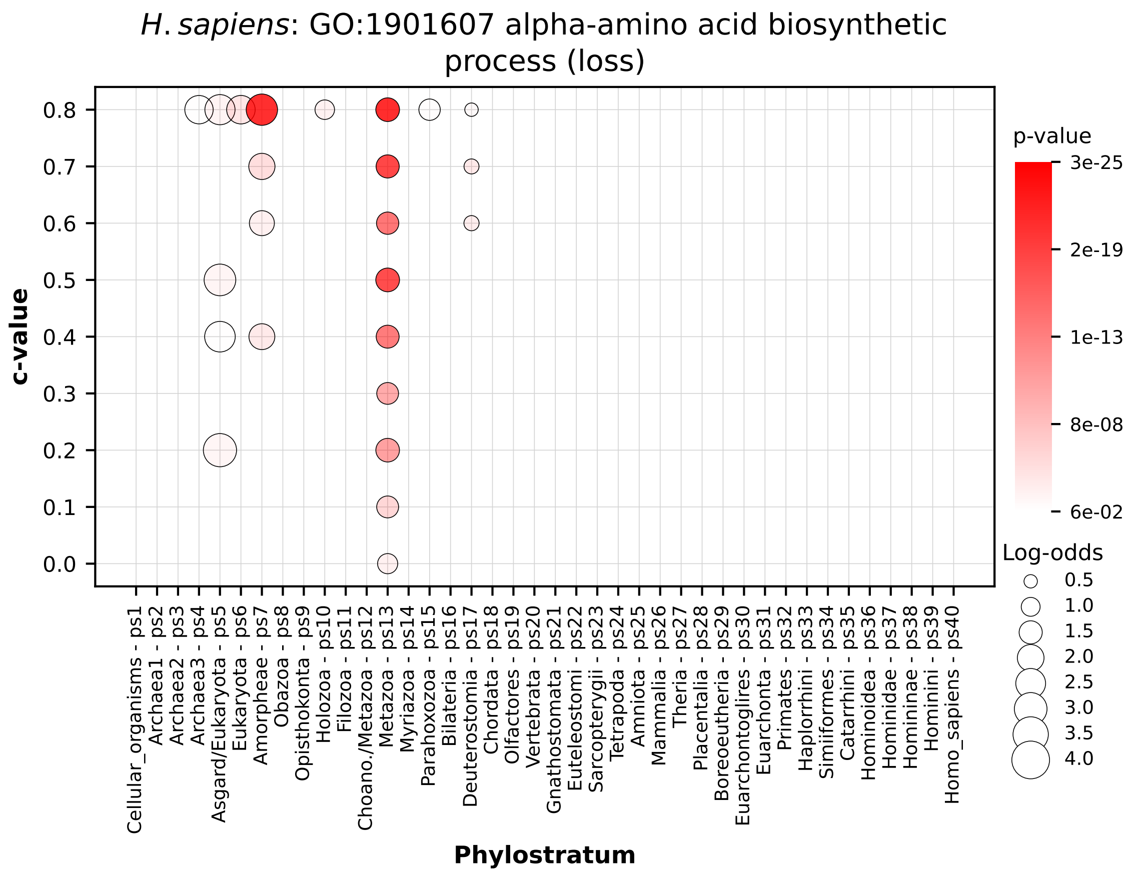

H. sapiens: GO:1901607 alpha-amino acid biosynthetic process (loss)
c-value
0.8
0.7
0.6
0.5
0.4
0.3
0.2
0.1
0.0
p-value
3e-25
2e-19
1e-13
8e-08
6e-02
Log-odds
0.5
1.0
1.5
2.0
2.5
3.0
3.5
4.0
Cellular_organisms - ps1
Archaea1 - ps2
Archaea2 - ps3
Archaea3 - ps4
Asgard/Eukaryota - ps5
Eukaryota - ps6
Amorpheae - ps7
Obazoa - ps8
Opisthokonta - ps9
Holozoa - ps10
Filozoa - ps11
Choano./Metazoa - ps12
Metazoa - ps13
Myriazoa - ps14
Parahoxozoa - ps15
Bilateria - ps16
Deuterostomia - ps17
Chordata - ps18
Olfactores - ps19
Vertebrata - ps20
Gnathostomata - ps21
Euteleostomi - ps22
Sarcopterygii - ps23
Tetrapoda - ps24
Amniota - ps25
Mammalia - ps26
Theria - ps27
Placentalia - ps28
Boreoeutheria - ps29
Euarchontoglires - ps30
Euarchonta - ps31
Primates - ps32
Haplorrhini - ps33
Simiiformes - ps34
Catarrhini - ps35
Hominoidea - ps36
Hominidae - ps37
Homininae - ps38
Hominini - ps39
Homo_sapiens - ps40
Phylostratum

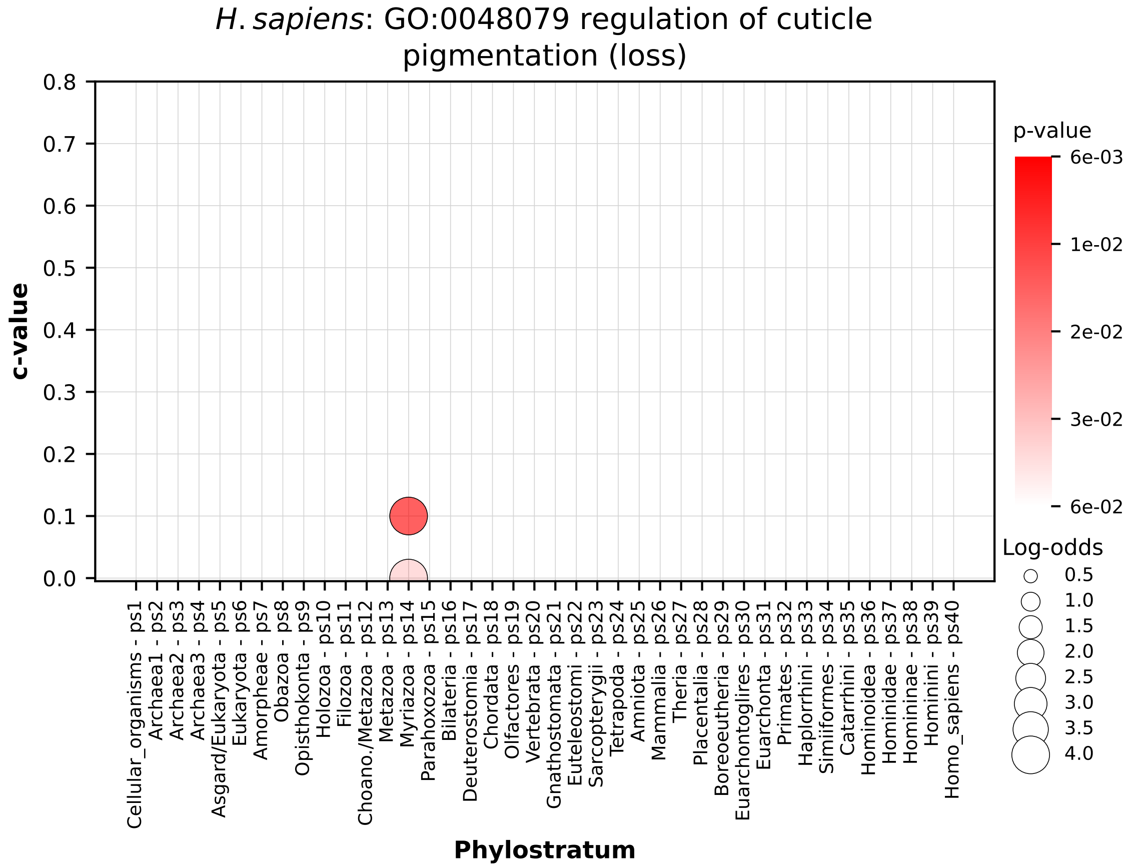

H. sapiens: GO:0048079 regulation of cuticle pigmentation (loss)
c-value
0.0
0.1
0.2
0.3
0.4
0.5
0.6
0.7
0.8
Phylostratum
Cellular_organisms - ps1
Archaea1 - ps2
Archaea2 - ps3
Archaea3 - ps4
Asgard/Eukaryota - ps5
Eukaryota - ps6
Amorpheae - ps7
Obazoa - ps8
Opisthokonta - ps9
Holozoa - ps10
Filozoa - ps11
Choano./Metazoa - ps12
Metazoa - ps13
Myriazoa - ps14
Parahoxozoa - ps15
Bilateria - ps16
Deuterostomia - ps17
Chordata - ps18
Olfactores - ps19
Vertebrata - ps20
Gnathostomata - ps21
Euteleostomi - ps22
Sarcopterygii - ps23
Tetrapoda - ps24
Amniota - ps25
Mammalia - ps26
Theria - ps27
Placentalia - ps28
Boreoeutheria - ps29
Euarchontoglires - ps30
Euarchonta - ps31
Primates - ps32
Haplorrhini - ps33
Simiiformes - ps34
Catarrhini - ps35
Hominoidea - ps36
Hominidae - ps37
Homininae - ps38
Hominini - ps39
Homo_sapiens - ps40
p-value
6e-03
1e-02
2e-02
3e-02
6e-02
Log-odds
0.5
1.0
1.5
2.0
2.5
3.0
3.5
4.0

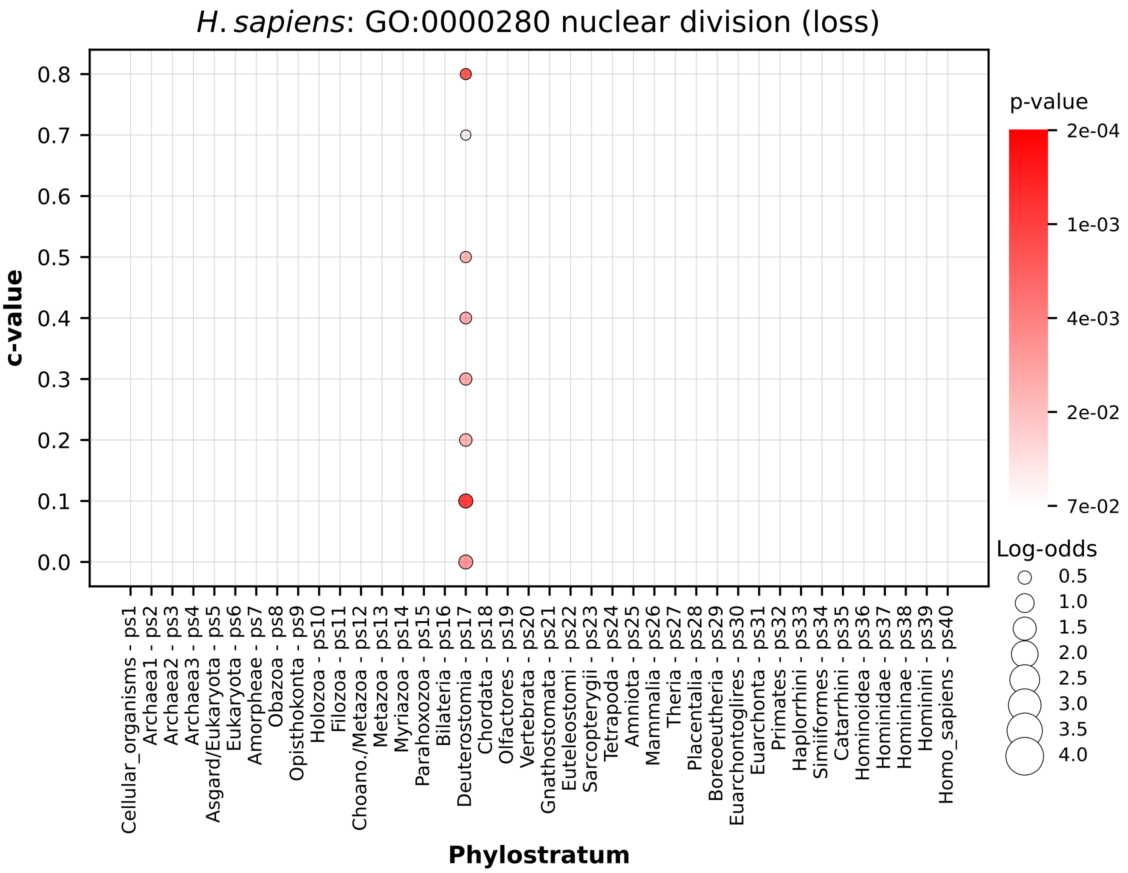

H. sapiens: GO:0000280 nuclear division (loss)
c-value
0.8
0.7
0.6
0.5
0.4
0.3
0.2
0.1
0.0
Phylostratum
Cellular_organisms - ps1
Archaea1 - ps2
Archaea2 - ps3
Archaea3 - ps4
Asgard/Eukaryota - ps5
Eukaryota - ps6
Amorpheae - ps7
Obazoa - ps8
Opisthokonta - ps9
Holozoa - ps10
Filozoa - ps11
Choano./Metazoa - ps12
Metazoa - ps13
Myriazoa - ps14
Parahoxozoa - ps15
Bilateria - ps16
Deuterostomia - ps17
Chordata - ps18
Olfactores - ps19
Vertebrata - ps20
Gnathostomata - ps21
Euteleostomi - ps22
Sarcopterygii - ps23
Tetrapoda - ps24
Amniota - ps25
Mammalia - ps26
Theria - ps27
Placentalia - ps28
Boreoeutheria - ps29
Euarchontoglires - ps30
Euarchonta - ps31
Primates - ps32
Haplorrhini - ps33
Simiiformes - ps34
Catarrhini - ps35
Hominoidea - ps36
Hominidae - ps37
Homininae - ps38
Hominini - ps39
Homo_sapiens - ps40
p-value
2e-04
1e-03
4e-03
2e-02
7e-02
Log-odds
0.5
1.0
1.5
2.0
2.5
3.0
3.5
4.0

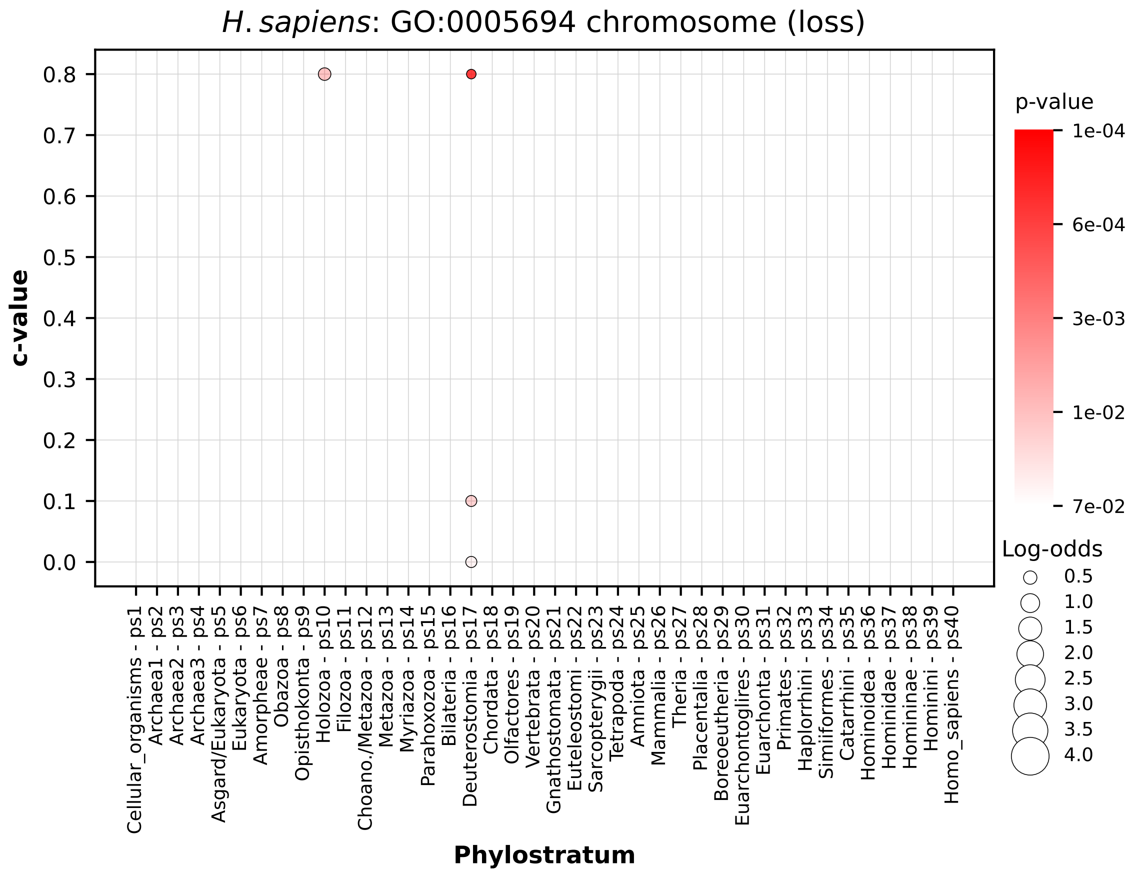

H. sapiens: GO:0005694 chromosome (loss)
c-value
0.8
0.7
0.6
0.5
0.4
0.3
0.2
0.1
0.0
Phylostratum
Cellular_organisms - ps1
Archaea1 - ps2
Archaea2 - ps3
Archaea3 - ps4
Asgard/Eukaryota - ps5
Eukaryota - ps6
Amorpheae - ps7
Obazoa - ps8
Opisthokonta - ps9
Holozoa - ps10
Filozoa - ps11
Choano./Metazoa - ps12
Metazoa - ps13
Myriazoa - ps14
Parahoxozoa - ps15
Bilateria - ps16
Deuterostomia - ps17
Chordata - ps18
Olfactores - ps19
Vertebrata - ps20
Gnathostomata - ps21
Euteleostomi - ps22
Sarcopterygii - ps23
Tetrapoda - ps24
Amniota - ps25
Mammalia - ps26
Theria - ps27
Placentalia - ps28
Boreoeutheria - ps29
Euarchontoglires - ps30
Euarchonta - ps31
Primates - ps32
Haplorrhini - ps33
Simiiformes - ps34
Catarrhini - ps35
Hominoidea - ps36
Hominidae - ps37
Homininae - ps38
Hominini - ps39
Homo_sapiens - ps40
p-value
1e-04
6e-04
3e-03
1e-02
7e-02
Log-odds
0.5
1.0
1.5
2.0
2.5
3.0
3.5
4.0

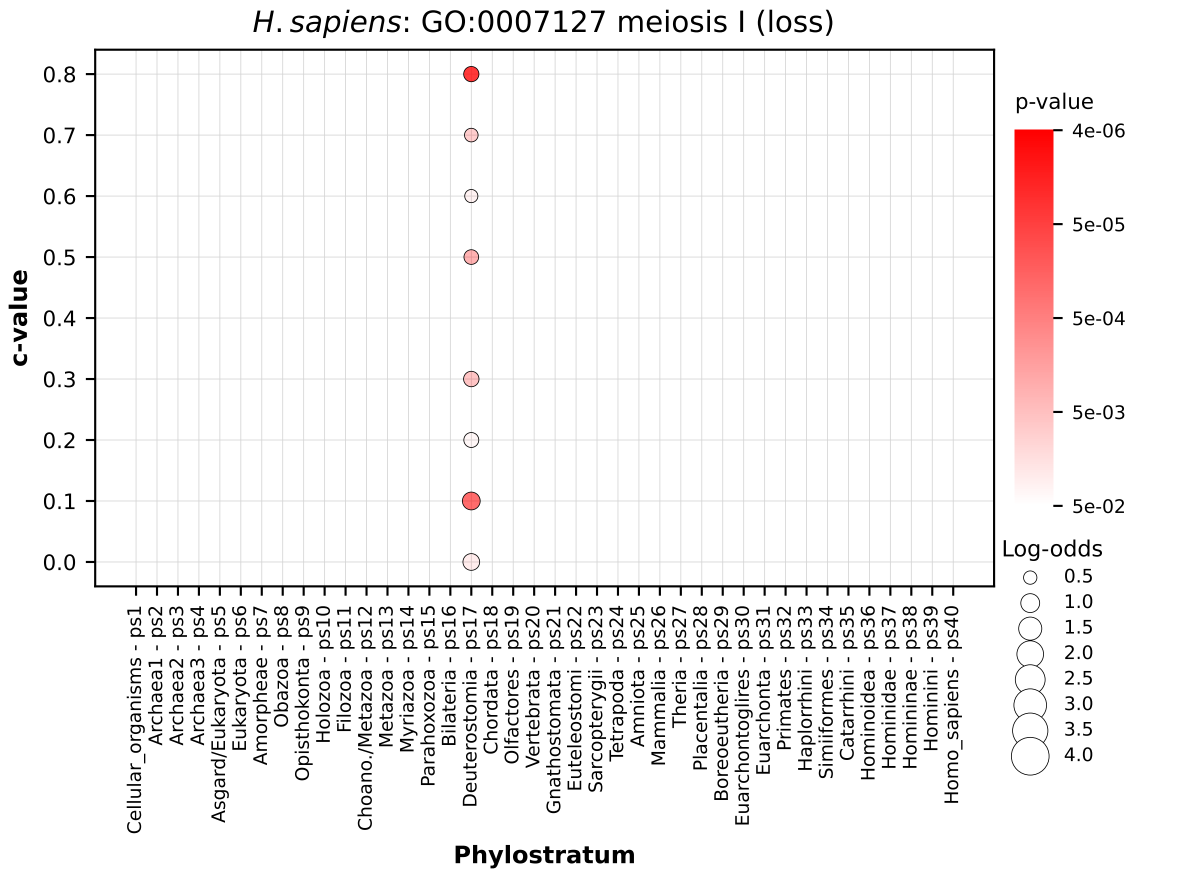

H. sapiens: GO:0007127 meiosis I (loss)
c-value
0.8
0.7
0.6
0.5
0.4
0.3
0.2
0.1
0.0
Phylostratum
Cellular_organisms - ps1
Archaea1 - ps2
Archaea2 - ps3
Archaea3 - ps4
Asgard/Eukaryota - ps5
Eukaryota - ps6
Amorpheae - ps7
Obazoa - ps8
Opisthokonta - ps9
Holozoa - ps10
Filozoa - ps11
Choano./Metazoa - ps12
Metazoa - ps13
Myriazoa - ps14
Parahoxozoa - ps15
Bilateria - ps16
Deuterostomia - ps17
Chordata - ps18
Olfactores - ps19
Vertebrata - ps20
Gnathostomata - ps21
Euteleostomi - ps22
Sarcopterygii - ps23
Tetrapoda - ps24
Amniota - ps25
Mammalia - ps26
Theria - ps27
Placentalia - ps28
Boreoeutheria - ps29
Euarchontoglires - ps30
Euarchonta - ps31
Primates - ps32
Haplorrhini - ps33
Simiiformes - ps34
Catarrhini - ps35
Hominoidea - ps36
Hominidae - ps37
Homininae - ps38
Hominini - ps39
Homo_sapiens - ps40
p-value
4e-06
5e-05
5e-04
5e-03
5e-02
Log-odds
0.5
1.0
1.5
2.0
2.5
3.0
3.5
4.0

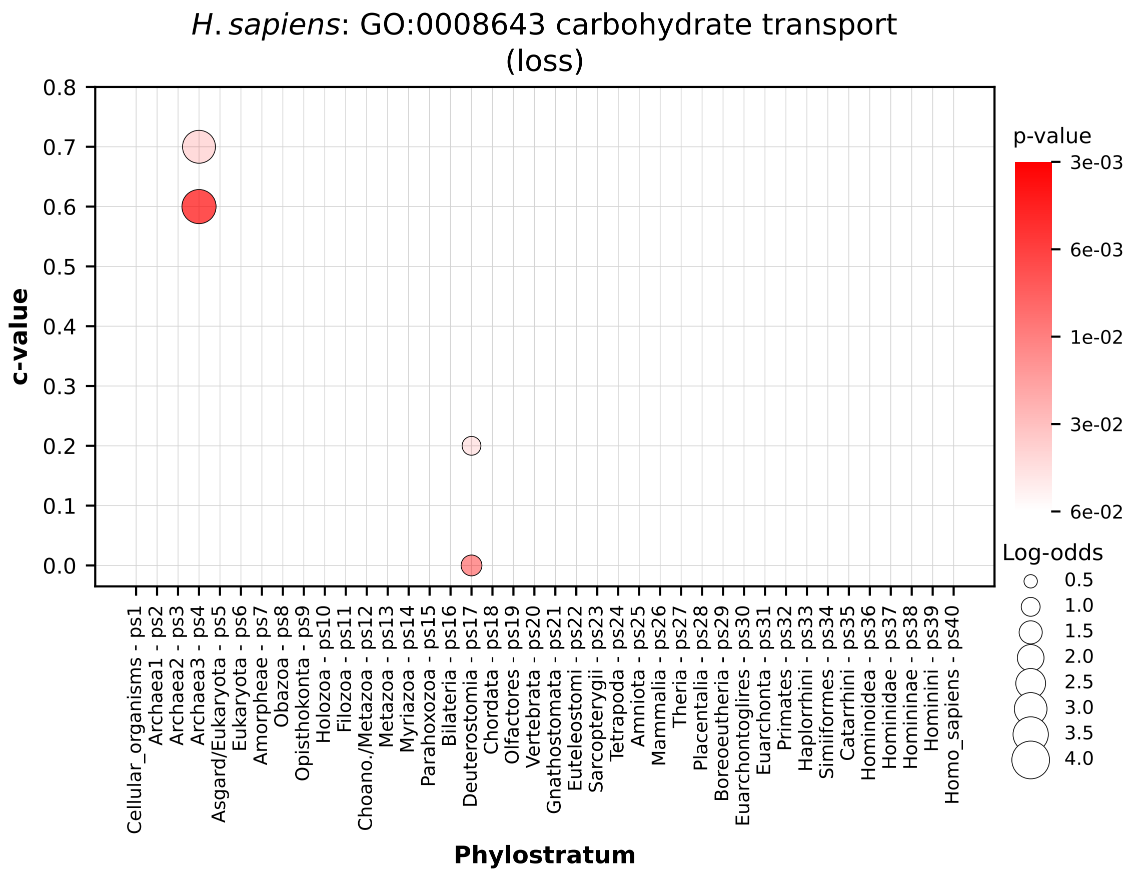

H. sapiens: GO:0008643 carbohydrate transport
(loss)
c-value
0.8
0.7
0.6
0.5
0.4
0.3
0.2
0.1
0.0
Phylostratum
Cellular_organisms - ps1
Archaea1 - ps2
Archaea2 - ps3
Archaea3 - ps4
Asgard/Eukaryota - ps5
Eukaryota - ps6
Amorpheae - ps7
Obazoa - ps8
Opisthokonta - ps9
Holozoa - ps10
Filozoa - ps11
Choano./Metazoa - ps12
Metazoa - ps13
Myriazoa - ps14
Parahoxozoa - ps15
Bilateria - ps16
Deuterostomia - ps17
Chordata - ps18
Olfactores - ps19
Vertebrata - ps20
Gnathostomata - ps21
Euteleostomi - ps22
Sarcopterygii - ps23
Tetrapoda - ps24
Amniota - ps25
Mammalia - ps26
Theria - ps27
Placentalia - ps28
Boreoeutheria - ps29
Euarchontoglires - ps30
Euarchonta - ps31
Primates - ps32
Haplorrhini - ps33
Simiiformes - ps34
Catarrhini - ps35
Hominoidea - ps36
Hominidae - ps37
Homininae - ps38
Hominini - ps39
Homo_sapiens - ps40
p-value
3e-03
6e-03
1e-02
3e-02
6e-02
Log-odds
0.5
1.0
1.5
2.0
2.5
3.0
3.5
4.0

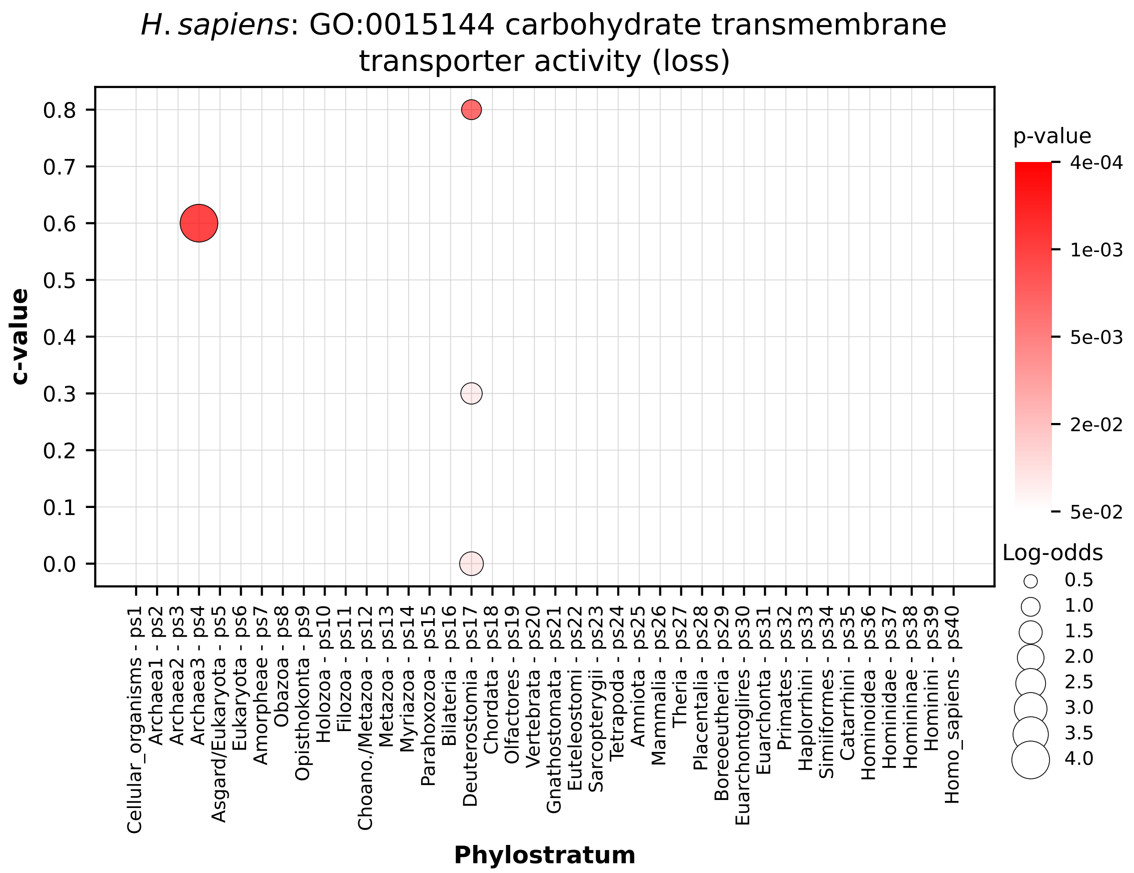

H. sapiens: GO:0015144 carbohydrate transmembrane transporter activity (loss)
c-value
0.8
0.7
0.6
0.5
0.4
0.3
0.2
0.1
0.0
Phylostratum
Cellular_organisms - ps1
Archaea1 - ps2
Archaea2 - ps3
Archaea3 - ps4
Asgard/Eukaryota - ps5
Eukaryota - ps6
Amorpheae - ps7
Obazoa - ps8
Opisthokonta - ps9
Holozoa - ps10
Filozoa - ps11
Choano./Metazoa - ps12
Metazoa - ps13
Myriazoa - ps14
Parahoxozoa - ps15
Bilateria - ps16
Deuterostomia - ps17
Chordata - ps18
Olfactores - ps19
Vertebrata - ps20
Gnathostomata - ps21
Euteleostomi - ps22
Sarcopterygii - ps23
Tetrapoda - ps24
Amniota - ps25
Mammalia - ps26
Theria - ps27
Placentalia - ps28
Boreoeutheria - ps29
Euarchontoglires - ps30
Euarchonta - ps31
Primates - ps32
Haplorrhini - ps33
Simiiformes - ps34
Catarrhini - ps35
Hominoidea - ps36
Hominidae - ps37
Homininae - ps38
Hominini - ps39
Homo_sapiens - ps40
p-value
4e-04
1e-03
5e-03
2e-02
5e-02
Log-odds
0.5
1.0
1.5
2.0
2.5
3.0
3.5
4.0

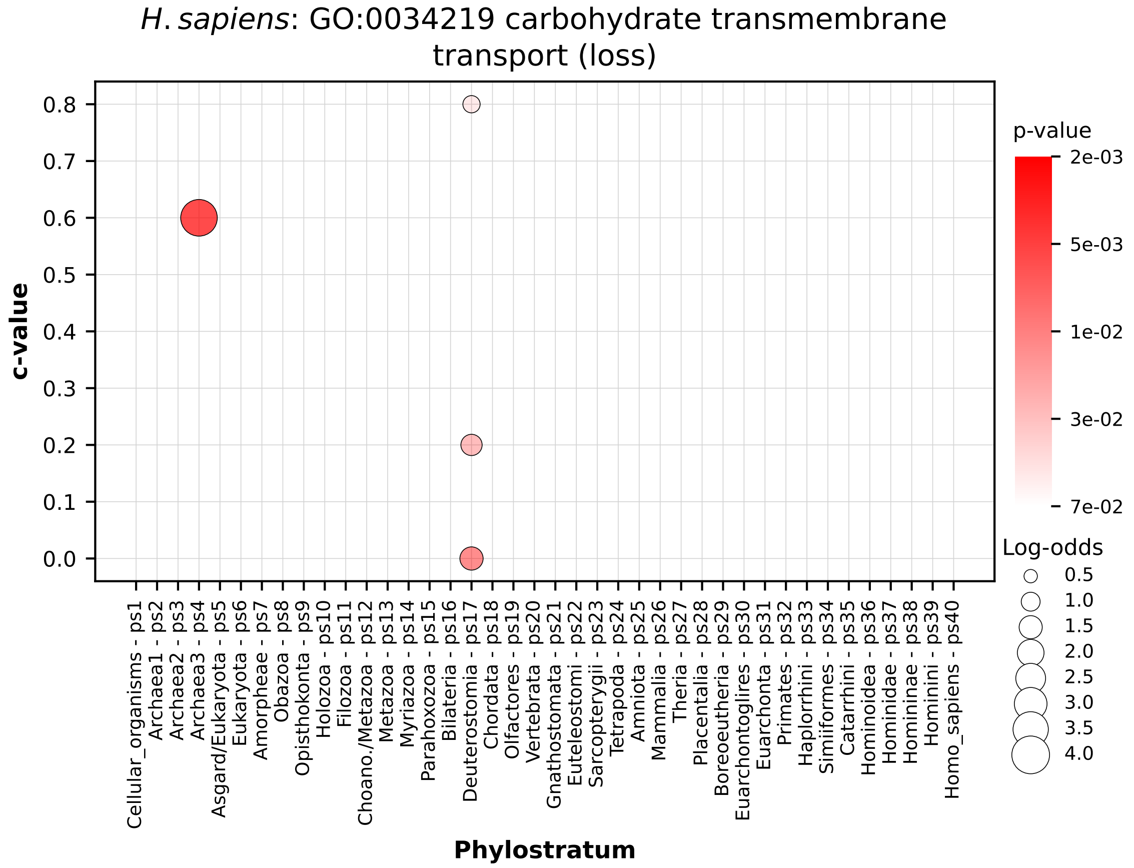

H. sapiens: GO:0034219 carbohydrate transmembrane transport (loss)
c-value
0.0
0.1
0.2
0.3
0.4
0.5
0.6
0.7
0.8
Phylostratum
Cellular_organisms - ps1
Archaea1 - ps2
Archaea2 - ps3
Archaea3 - ps4
Asgard/Eukaryota - ps5
Eukaryota - ps6
Amorpheae - ps7
Obazoa - ps8
Opisthokonta - ps9
Holozoa - ps10
Filozoa - ps11
Choano./Metazoa - ps12
Metazoa - ps13
Myriazoa - ps14
Parahoxozoa - ps15
Bilateria - ps16
Deuterostomia - ps17
Chordata - ps18
Olfactores - ps19
Vertebrata - ps20
Gnathostomata - ps21
Euteleostomi - ps22
Sarcopterygii - ps23
Tetrapoda - ps24
Amniota - ps25
Mammalia - ps26
Theria - ps27
Placentalia - ps28
Boreoeutheria - ps29
Euarchontoglires - ps30
Euarchonta - ps31
Primates - ps32
Haplorrhini - ps33
Simiiformes - ps34
Catarrhini - ps35
Hominoidea - ps36
Hominidae - ps37
Homininae - ps38
Hominini - ps39
Homo_sapiens - ps40
p-value
2e-03
5e-03
1e-02
3e-02
7e-02
Log-odds
0.5
1.0
1.5
2.0
2.5
3.0
3.5
4.0

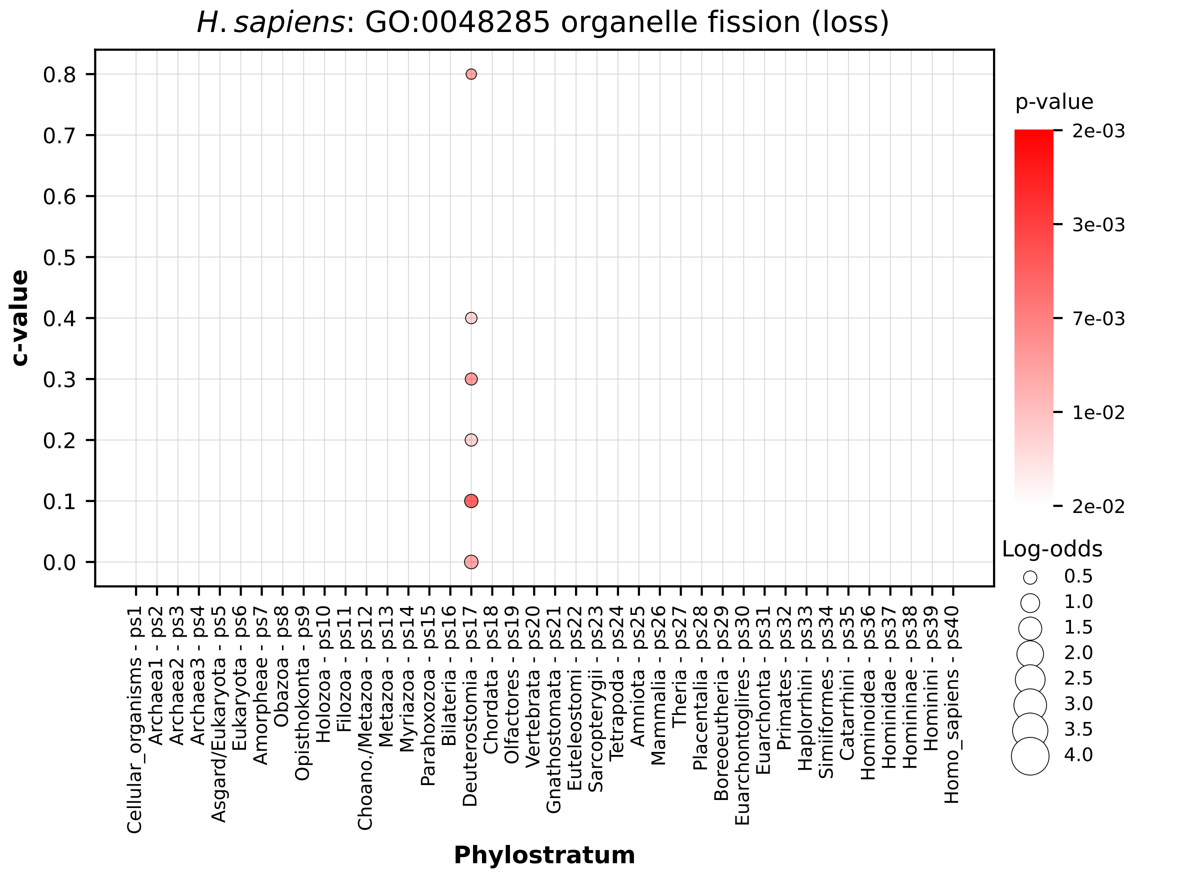

H. sapiens: GO:0048285 organelle fission (loss)
c-value
0.0
0.1
0.2
0.3
0.4
0.5
0.6
0.7
0.8
p-value
2e-03
3e-03
7e-03
1e-02
2e-02
Log-odds
0.5
1.0
1.5
2.0
2.5
3.0
3.5
4.0
Cellular_organisms - ps1
Archaea1 - ps2
Archaea2 - ps3
Archaea3 - ps4
Asgard/Eukaryota - ps5
Eukaryota - ps6
Amorpheae - ps7
Obazoa - ps8
Opisthokonta - ps9
Holozoa - ps10
Filozoa - ps11
Choano./Metazoa - ps12
Metazoa - ps13
Myriazoa - ps14
Parahoxozoa - ps15
Bilateria - ps16
Deuterostomia - ps17
Chordata - ps18
Olfactores - ps19
Vertebrata - ps20
Gnathostomata - ps21
Euteleostomi - ps22
Sarcopterygii - ps23
Tetrapoda - ps24
Amniota - ps25
Mammalia - ps26
Theria - ps27
Placentalia - ps28
Boreoeutheria - ps29
Euarchontoglires - ps30
Euarchonta - ps31
Primates - ps32
Haplorrhini - ps33
Simiiformes - ps34
Catarrhini - ps35
Hominoidea - ps36
Hominidae - ps37
Homininae - ps38
Hominini - ps39
Homo_sapiens - ps40
Phylostratum

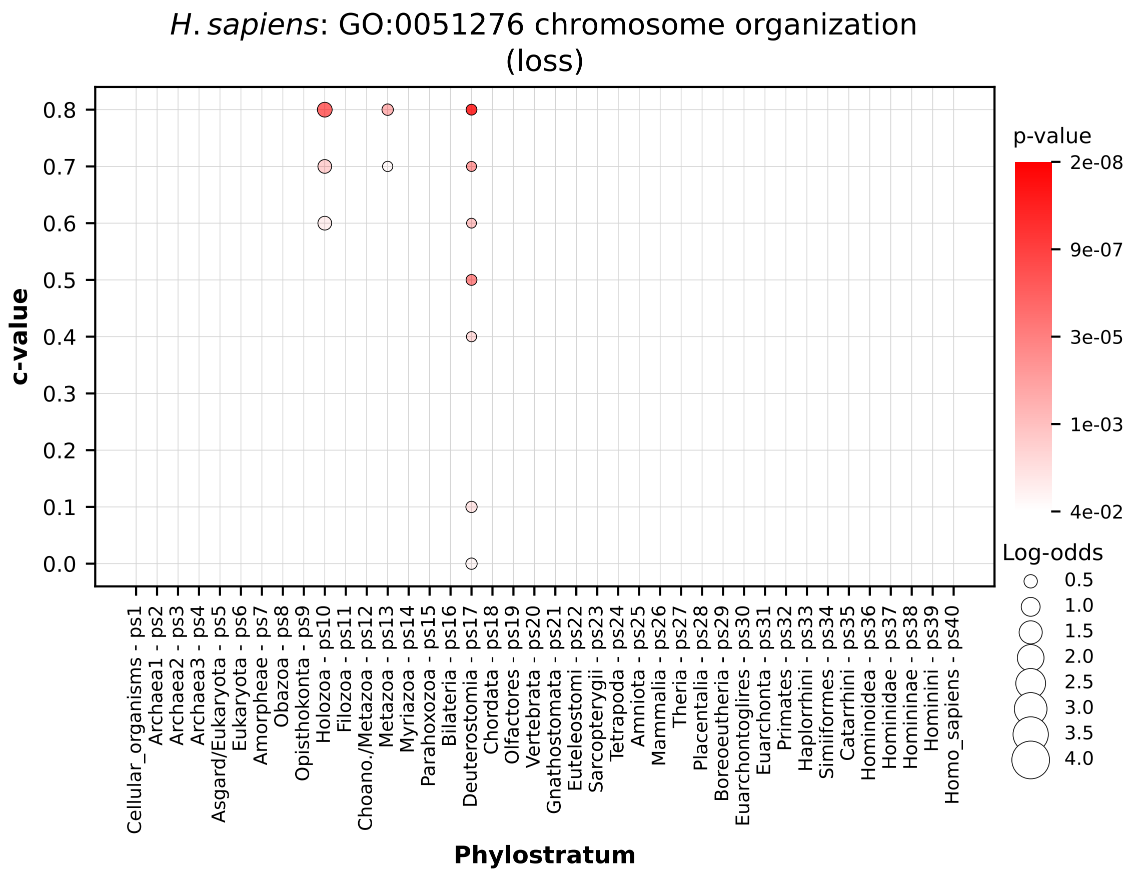

H. sapiens: GO:0051276 chromosome organization
(loss)
c-value
0.8
0.7
0.6
0.5
0.4
0.3
0.2
0.1
0.0
p-value
2e-08
9e-07
3e-05
1e-03
4e-02
Log-odds
0.5
1.0
1.5
2.0
2.5
3.0
3.5
4.0
Cellular_organisms - ps1
Archaea1 - ps2
Archaea2 - ps3
Archaea3 - ps4
Asgard/Eukaryota - ps5
Eukaryota - ps6
Amorpheae - ps7
Obazoa - ps8
Opisthokonta - ps9
Holozoa - ps10
Filozoa - ps11
Choano./Metazoa - ps12
Metazoa - ps13
Myriazoa - ps14
Parahoxozoa - ps15
Bilateria - ps16
Deuterostomia - ps17
Chordata - ps18
Olfactores - ps19
Vertebrata - ps20
Gnathostomata - ps21
Euteleostomi - ps22
Sarcopterygii - ps23
Tetrapoda - ps24
Amniota - ps25
Mammalia - ps26
Theria - ps27
Placentalia - ps28
Boreoeutheria - ps29
Euarchontoglires - ps30
Euarchonta - ps31
Primates - ps32
Haplorrhini - ps33
Simiiformes - ps34
Catarrhini - ps35
Hominoidea - ps36
Hominidae - ps37
Homininae - ps38
Hominini - ps39
Homo_sapiens - ps40
Phylostratum

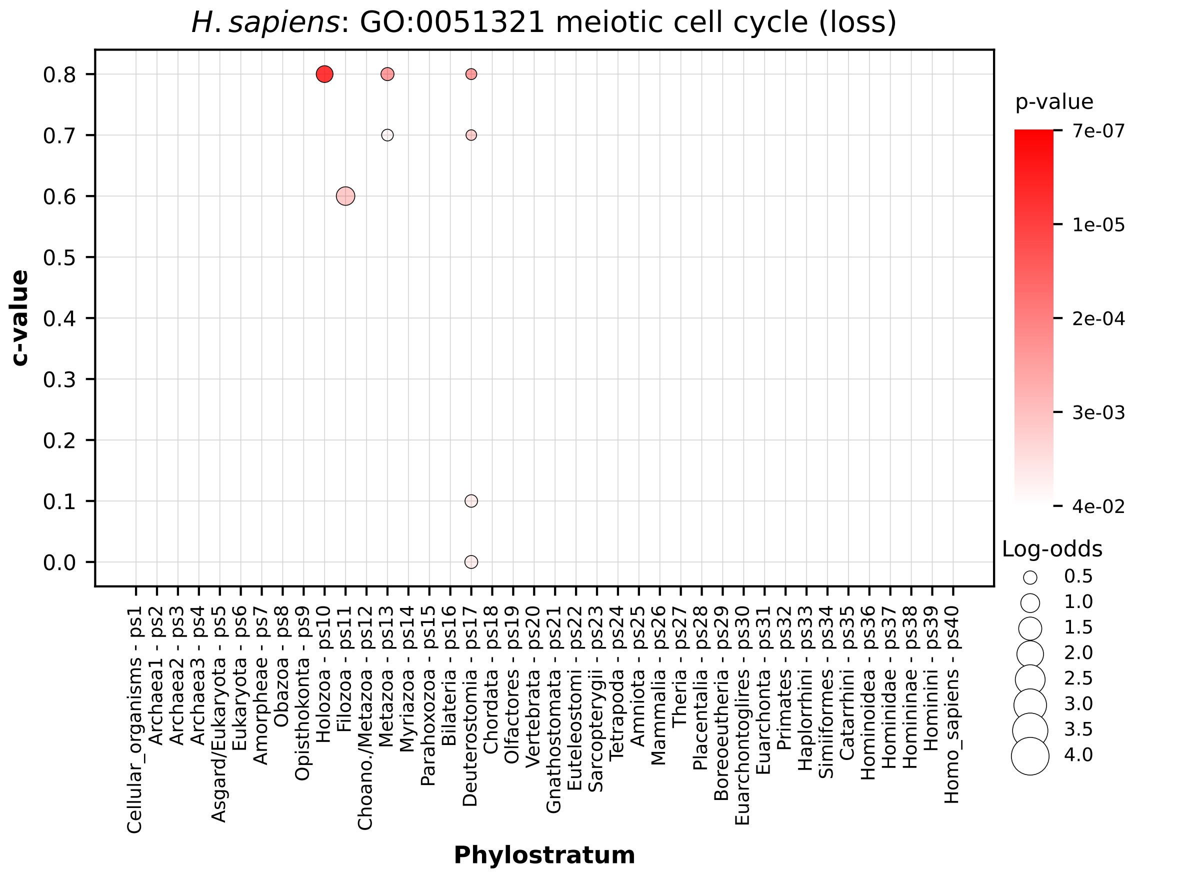
H. sapiens: GO:0051321 meiotic cell cycle (loss)
c-value
0.0
0.1
0.2
0.3
0.4
0.5
0.6
0.7
0.8
Phylostratum
Cellular_organisms - ps1
Archaea1 - ps2
Archaea2 - ps3
Archaea3 - ps4
Asgard/Eukaryota - ps5
Eukaryota - ps6
Amorpheae - ps7
Obazoa - ps8
Opisthokonta - ps9
Holozoa - ps10
Filozoa - ps11
Choano./Metazoa - ps12
Metazoa - ps13
Myriazoa - ps14
Parahoxozoa - ps15
Bilateria - ps16
Deuterostomia - ps17
Chordata - ps18
Olfactores - ps19
Vertebrata - ps20
Gnathostomata - ps21
Euteleostomi - ps22
Sarcopterygii - ps23
Tetrapoda - ps24
Amniota - ps25
Mammalia - ps26
Theria - ps27
Placentalia - ps28
Boreoeutheria - ps29
Euarchontoglires - ps30
Euarchonta - ps31
Primates - ps32
Haplorrhini - ps33
Simiiformes - ps34
Catarrhini - ps35
Hominoidea - ps36
Hominidae - ps37
Homininae - ps38
Hominini - ps39
Homo_sapiens - ps40
p-value
7e-07
1e-05
2e-04
3e-03
4e-02
Log-odds
0.5
1.0
1.5
2.0
2.5
3.0
3.5
4.0

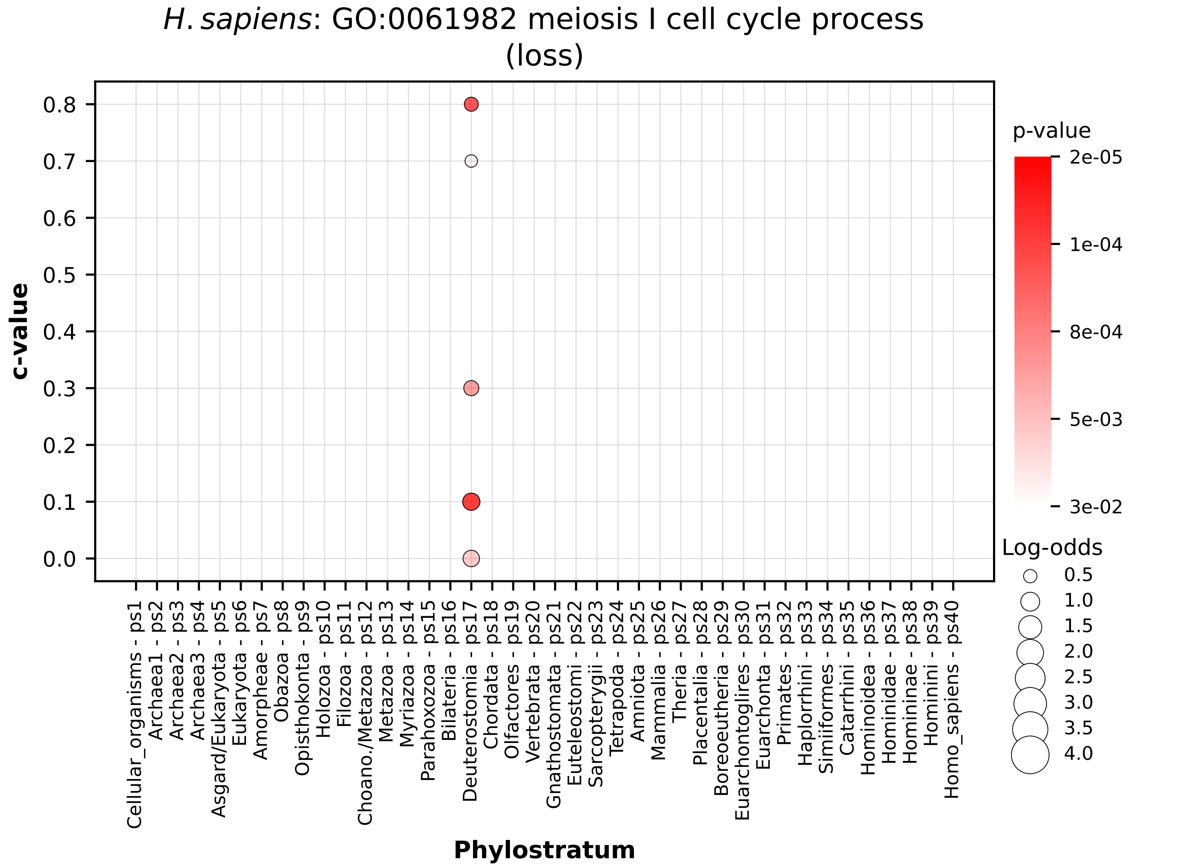
H. sapiens: GO:0061982 meiosis I cell cycle process
(loss)
c-value
0.8
0.7
0.6
0.5
0.4
0.3
0.2
0.1
0.0
Phylostratum
Cellular_organisms - ps1
Archaea1 - ps2
Archaea2 - ps3
Archaea3 - ps4
Asgard/Eukaryota - ps5
Eukaryota - ps6
Amorpheae - ps7
Obazoa - ps8
Opisthokonta - ps9
Holozoa - ps10
Filozoa - ps11
Choano./Metazoa - ps12
Metazoa - ps13
Myriazoa - ps14
Parahoxozoa - ps15
Bilateria - ps16
Deuterostomia - ps17
Chordata - ps18
Olfactores - ps19
Vertebrata - ps20
Gnathostomata - ps21
Euteleostomi - ps22
Sarcopterygii - ps23
Tetrapoda - ps24
Amniota - ps25
Mammalia - ps26
Theria - ps27
Placentalia - ps28
Boreoeutheria - ps29
Euarchontoglires - ps30
Euarchonta - ps31
Primates - ps32
Haplorrhini - ps33
Simiiformes - ps34
Catarrhini - ps35
Hominoidea - ps36
Hominidae - ps37
Homininae - ps38
Hominini - ps39
Homo_sapiens - ps40
p-value
2e-05
1e-04
8e-04
5e-03
3e-02
Log-odds
0.5
1.0
1.5
2.0
2.5
3.0
3.5
4.0

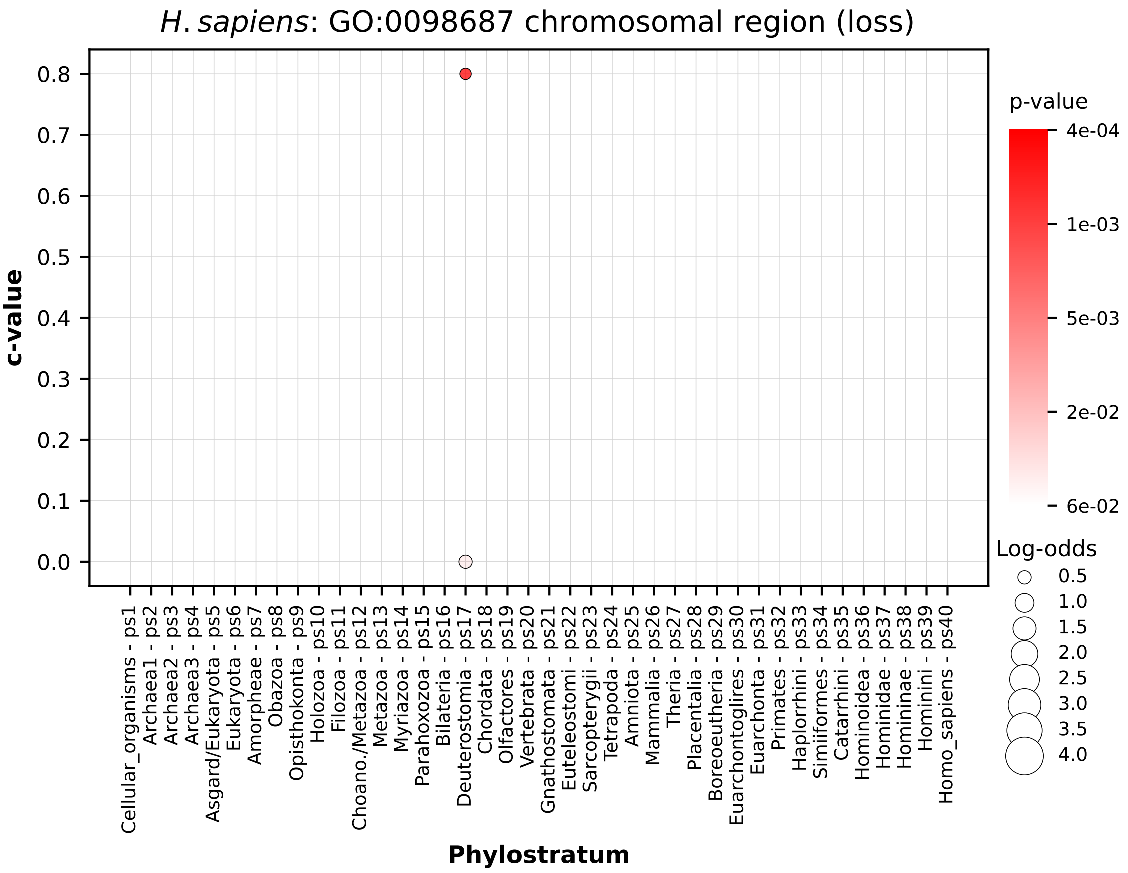

H. sapiens: GO:0098687 chromosomal region (loss)
c-value
0.0
0.1
0.2
0.3
0.4
0.5
0.6
0.7
0.8
p-value
4e-04
1e-03
5e-03
2e-02
6e-02
Log-odds
0.5
1.0
1.5
2.0
2.5
3.0
3.5
4.0
Cellular_organisms - ps1
Archaea1 - ps2
Archaea2 - ps3
Archaea3 - ps4
Asgard/Eukaryota - ps5
Eukaryota - ps6
Amorpheae - ps7
Obazoa - ps8
Opisthokonta - ps9
Holozoa - ps10
Filozoa - ps11
Choano./Metazoa - ps12
Metazoa - ps13
Myriazoa - ps14
Parahoxozoa - ps15
Bilateria - ps16
Deuterostomia - ps17
Chordata - ps18
Olfactores - ps19
Vertebrata - ps20
Gnathostomata - ps21
Euteleostomi - ps22
Sarcopterygii - ps23
Tetrapoda - ps24
Amniota - ps25
Mammalia - ps26
Theria - ps27
Placentalia - ps28
Boreoeutheria - ps29
Euarchontoglires - ps30
Euarchonta - ps31
Primates - ps32
Haplorrhini - ps33
Simiiformes - ps34
Catarrhini - ps35
Hominoidea - ps36
Hominidae - ps37
Homininae - ps38
Hominini - ps39
Homo_sapiens - ps40
Phylostratum

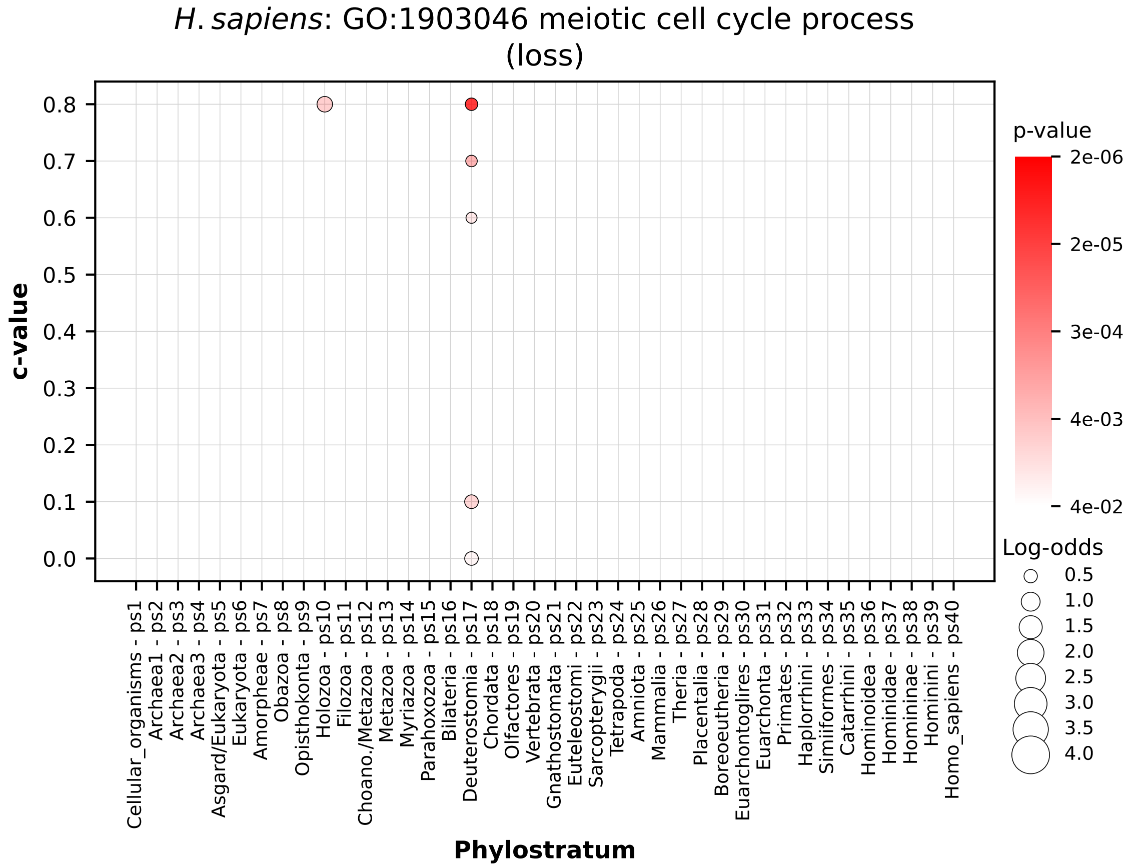
H. sapiens: GO:1903046 meiotic cell cycle process
(loss)
c-value
0.8
0.7
0.6
0.5
0.4
0.3
0.2
0.1
0.0
p-value
2e-06
2e-05
3e-04
4e-03
4e-02
Log-odds
0.5
1.0
1.5
2.0
2.5
3.0
3.5
4.0
Cellular_organisms - ps1
Archaea1 - ps2
Archaea2 - ps3
Archaea3 - ps4
Asgard/Eukaryota - ps5
Eukaryota - ps6
Amorpheae - ps7
Obazoa - ps8
Opisthokonta - ps9
Holozoa - ps10
Filozoa - ps11
Choano./Metazoa - ps12
Metazoa - ps13
Myriazoa - ps14
Parahoxozoa - ps15
Bilateria - ps16
Deuterostomia - ps17
Chordata - ps18
Olfactores - ps19
Vertebrata - ps20
Gnathostomata - ps21
Euteleostomi - ps22
Sarcopterygii - ps23
Tetrapoda - ps24
Amniota - ps25
Mammalia - ps26
Theria - ps27
Placentalia - ps28
Boreoeutheria - ps29
Euarchontoglires - ps30
Euarchonta - ps31
Primates - ps32
Haplorrhini - ps33
Simiiformes - ps34
Catarrhini - ps35
Hominoidea - ps36
Hominidae - ps37
Homininae - ps38
Hominini - ps39
Homo_sapiens - ps40
Phylostratum

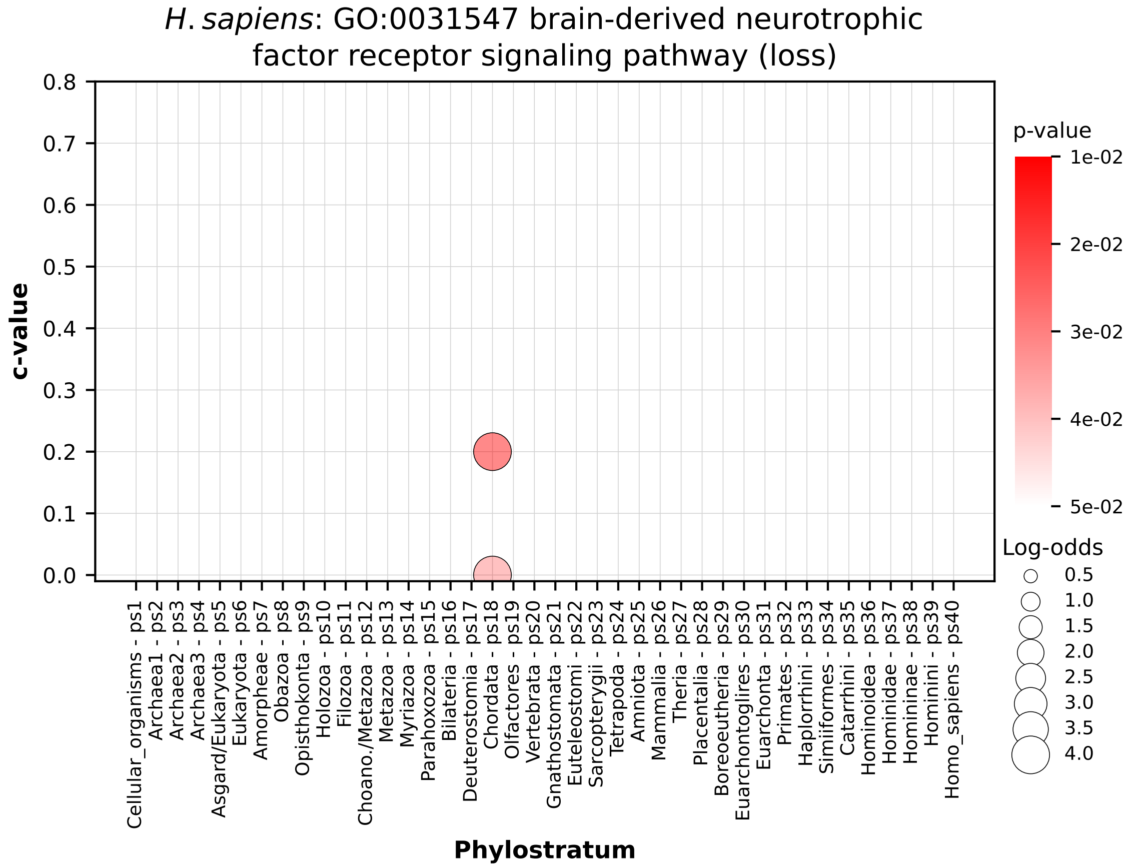

H. sapiens: GO:0031547 brain-derived neurotrophic factor receptor signaling pathway (loss)
c-value
0.8
0.7
0.6
0.5
0.4
0.3
0.2
0.1
0.0
p-value
1e-02
2e-02
3e-02
4e-02
5e-02
Log-odds
0.5
1.0
1.5
2.0
2.5
3.0
3.5
4.0
Cellular_organisms - ps1
Archaea1 - ps2
Archaea2 - ps3
Archaea3 - ps4
Asgard/Eukaryota - ps5
Eukaryota - ps6
Amorpheae - ps7
Obazoa - ps8
Opisthokonta - ps9
Holozoa - ps10
Filozoa - ps11
Choano./Metazoa - ps12
Metazoa - ps13
Myriazoa - ps14
Parahoxozoa - ps15
Bilateria - ps16
Deuterostomia - ps17
Chordata - ps18
Olfactores - ps19
Vertebrata - ps20
Gnathostomata - ps21
Euteleostomi - ps22
Sarcopterygii - ps23
Tetrapoda - ps24
Amniota - ps25
Mammalia - ps26
Theria - ps27
Placentalia - ps28
Boreoeutheria - ps29
Euarchontoglires - ps30
Euarchonta - ps31
Primates - ps32
Haplorrhini - ps33
Simiiformes - ps34
Catarrhini - ps35
Hominoidea - ps36
Hominidae - ps37
Homininae - ps38
Hominini - ps39
Homo_sapiens - ps40
Phylostratum

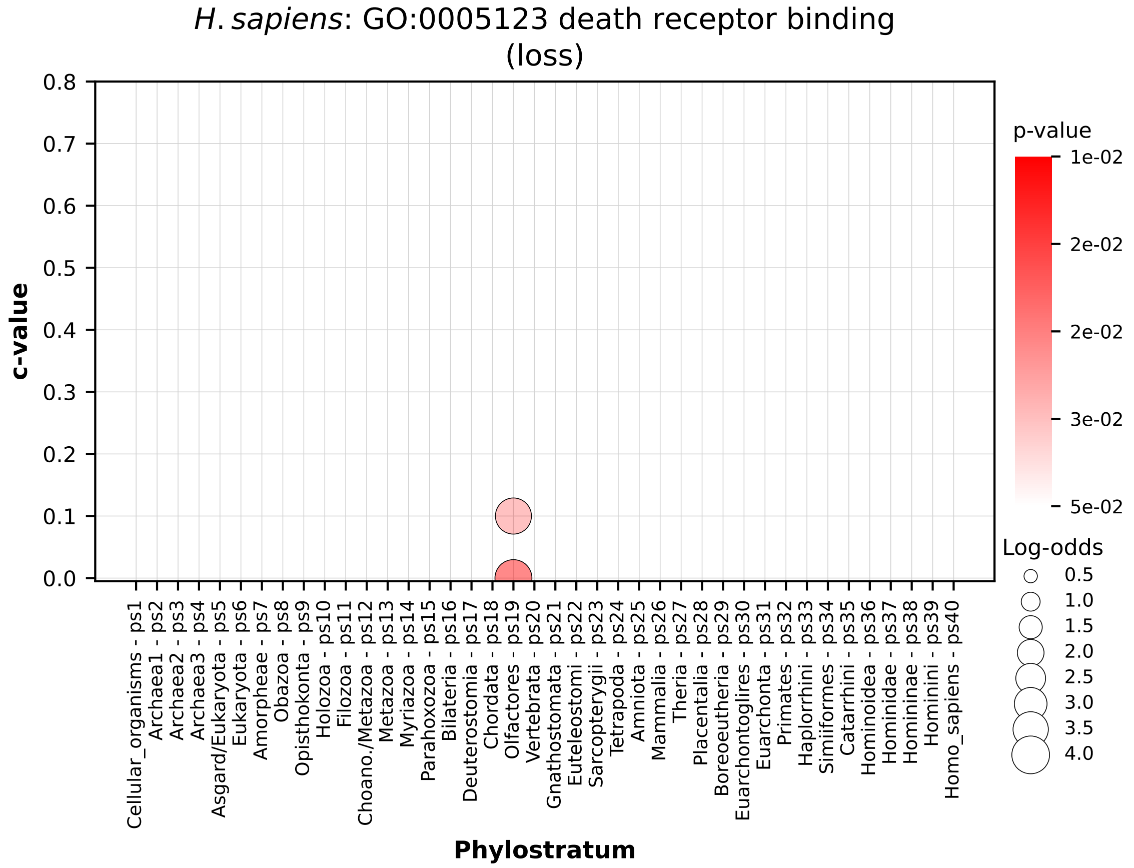

H. sapiens: GO:0005123 death receptor binding
(loss)
c-value
0.0
0.1
0.2
0.3
0.4
0.5
0.6
0.7
0.8
Phylostratum
Cellular_organisms - ps1
Archaea1 - ps2
Archaea2 - ps3
Archaea3 - ps4
Asgard/Eukaryota - ps5
Eukaryota - ps6
Amorpheae - ps7
Obazoa - ps8
Opisthokonta - ps9
Holozoa - ps10
Filozoa - ps11
Choano./Metazoa - ps12
Metazoa - ps13
Myriazoa - ps14
Parahoxozoa - ps15
Bilateria - ps16
Deuterostomia - ps17
Chordata - ps18
Olfactores - ps19
Vertebrata - ps20
Gnathostomata - ps21
Euteleostomi - ps22
Sarcopterygii - ps23
Tetrapoda - ps24
Amniota - ps25
Mammalia - ps26
Theria - ps27
Placentalia - ps28
Boreoeutheria - ps29
Euarchontoglires - ps30
Euarchonta - ps31
Primates - ps32
Haplorrhini - ps33
Simiiformes - ps34
Catarrhini - ps35
Hominoidea - ps36
Hominidae - ps37
Homininae - ps38
Hominini - ps39
Homo_sapiens - ps40
p-value
1e-02
2e-02
2e-02
3e-02
5e-02
Log-odds
0.5
1.0
1.5
2.0
2.5
3.0
3.5
4.0

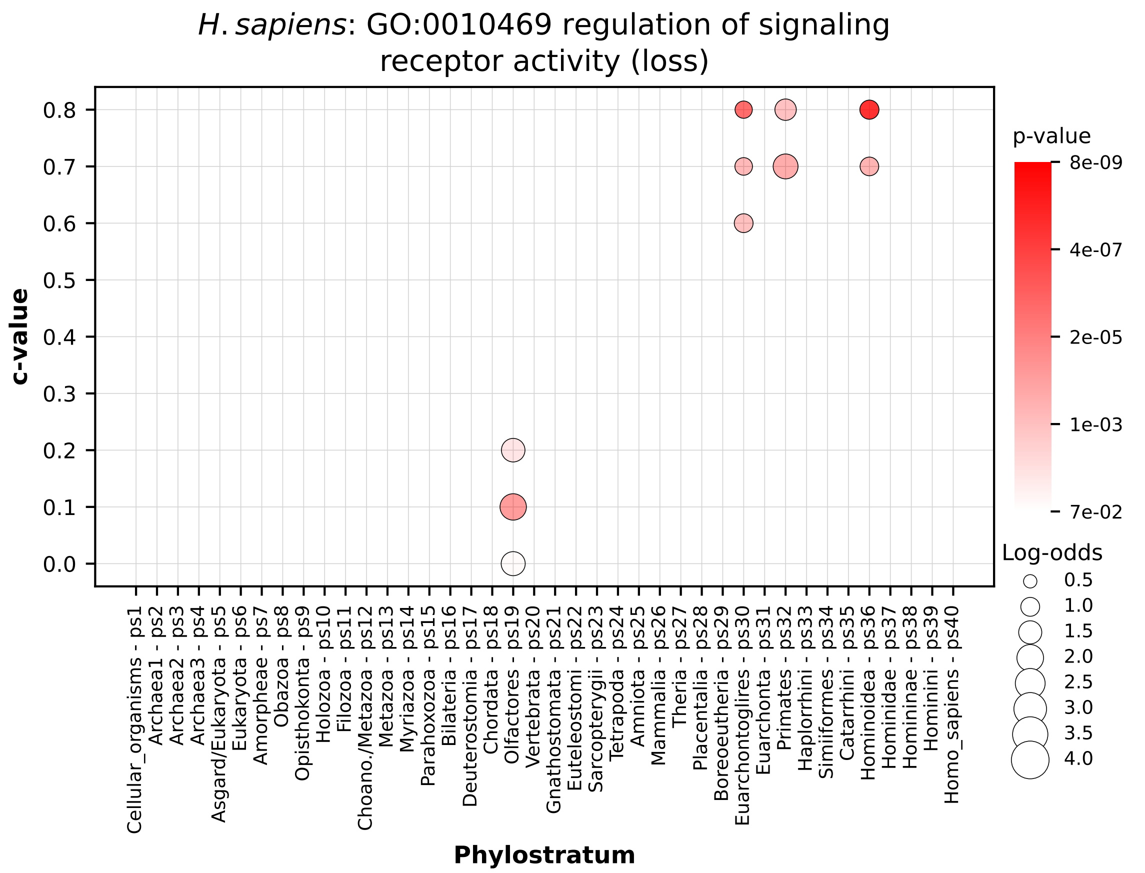

H. sapiens: GO:0010469 regulation of signaling receptor activity (loss)
c-value
0.0
0.1
0.2
0.3
0.4
0.5
0.6
0.7
0.8
Phylostratum
Cellular_organisms - ps1
Archaea1 - ps2
Archaea2 - ps3
Archaea3 - ps4
Asgard/Eukaryota - ps5
Eukaryota - ps6
Amorpheae - ps7
Obazoa - ps8
Opisthokonta - ps9
Holozoa - ps10
Filozoa - ps11
Choano./Metazoa - ps12
Metazoa - ps13
Myriazoa - ps14
Parahoxozoa - ps15
Bilateria - ps16
Deuterostomia - ps17
Chordata - ps18
Olfactores - ps19
Vertebrata - ps20
Gnathostomata - ps21
Euteleostomi - ps22
Sarcopterygii - ps23
Tetrapoda - ps24
Amniota - ps25
Mammalia - ps26
Theria - ps27
Placentalia - ps28
Boreoeutheria - ps29
Euarchontoglires - ps30
Euarchonta - ps31
Primates - ps32
Haplorrhini - ps33
Simiiformes - ps34
Catarrhini - ps35
Hominoidea - ps36
Hominidae - ps37
Homininae - ps38
Hominini - ps39
Homo_sapiens - ps40
p-value
8e-09
4e-07
2e-05
1e-03
7e-02
Log-odds
0.5
1.0
1.5
2.0
2.5
3.0
3.5
4.0

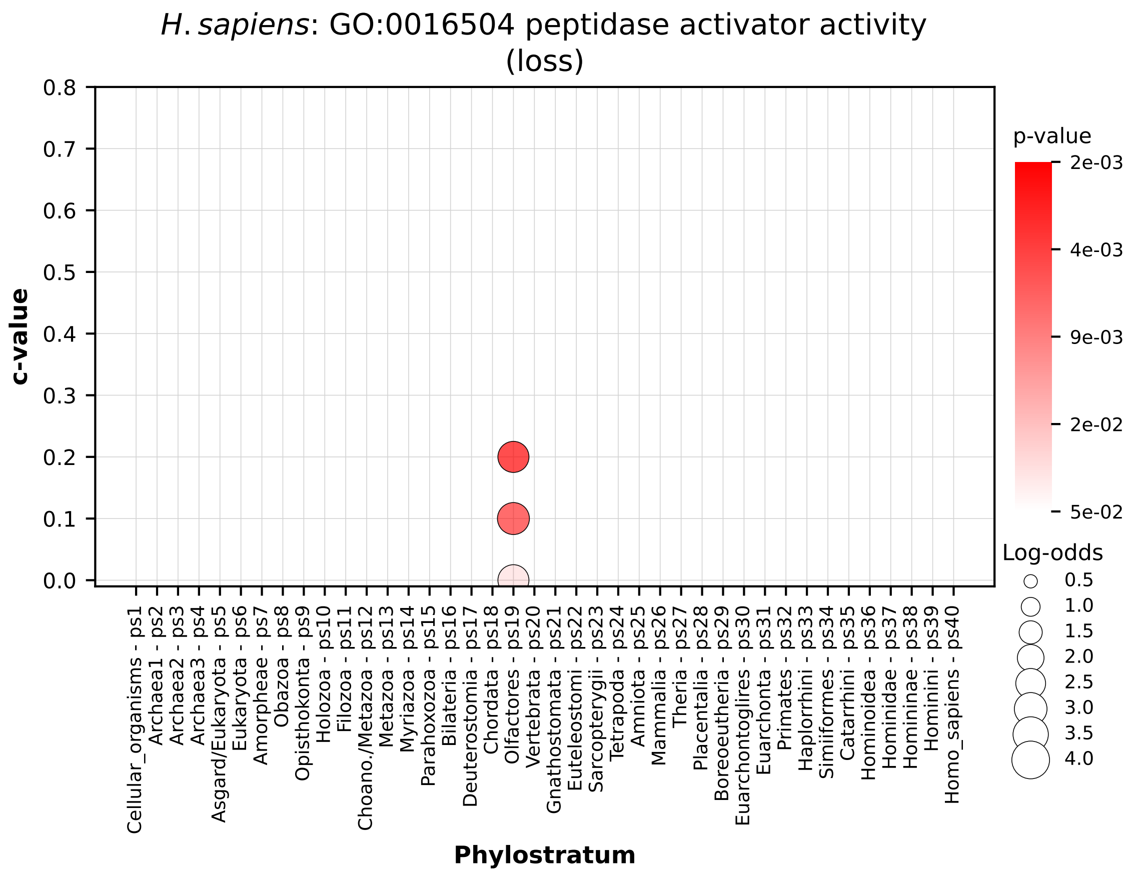

H. sapiens: GO:0016504 peptidase activator activity
(loss)
c-value
0.8
0.7
0.6
0.5
0.4
0.3
0.2
0.1
0.0
Phylostratum
Cellular_organisms - ps1
Archaea1 - ps2
Archaea2 - ps3
Archaea3 - ps4
Asgard/Eukaryota - ps5
Eukaryota - ps6
Amorpheae - ps7
Obazoa - ps8
Opisthokonta - ps9
Holozoa - ps10
Filozoa - ps11
Choano./Metazoa - ps12
Metazoa - ps13
Myriazoa - ps14
Parahoxozoa - ps15
Bilateria - ps16
Deuterostomia - ps17
Chordata - ps18
Olfactores - ps19
Vertebrata - ps20
Gnathostomata - ps21
Euteleostomi - ps22
Sarcopterygii - ps23
Tetrapoda - ps24
Amniota - ps25
Mammalia - ps26
Theria - ps27
Placentalia - ps28
Boreoeutheria - ps29
Euarchontoglires - ps30
Euarchonta - ps31
Primates - ps32
Haplorrhini - ps33
Simiiformes - ps34
Catarrhini - ps35
Hominoidea - ps36
Hominidae - ps37
Homininae - ps38
Hominini - ps39
Homo_sapiens - ps40
p-value
2e-03
4e-03
9e-03
2e-02
5e-02
Log-odds
0.5
1.0
1.5
2.0
2.5
3.0
3.5
4.0

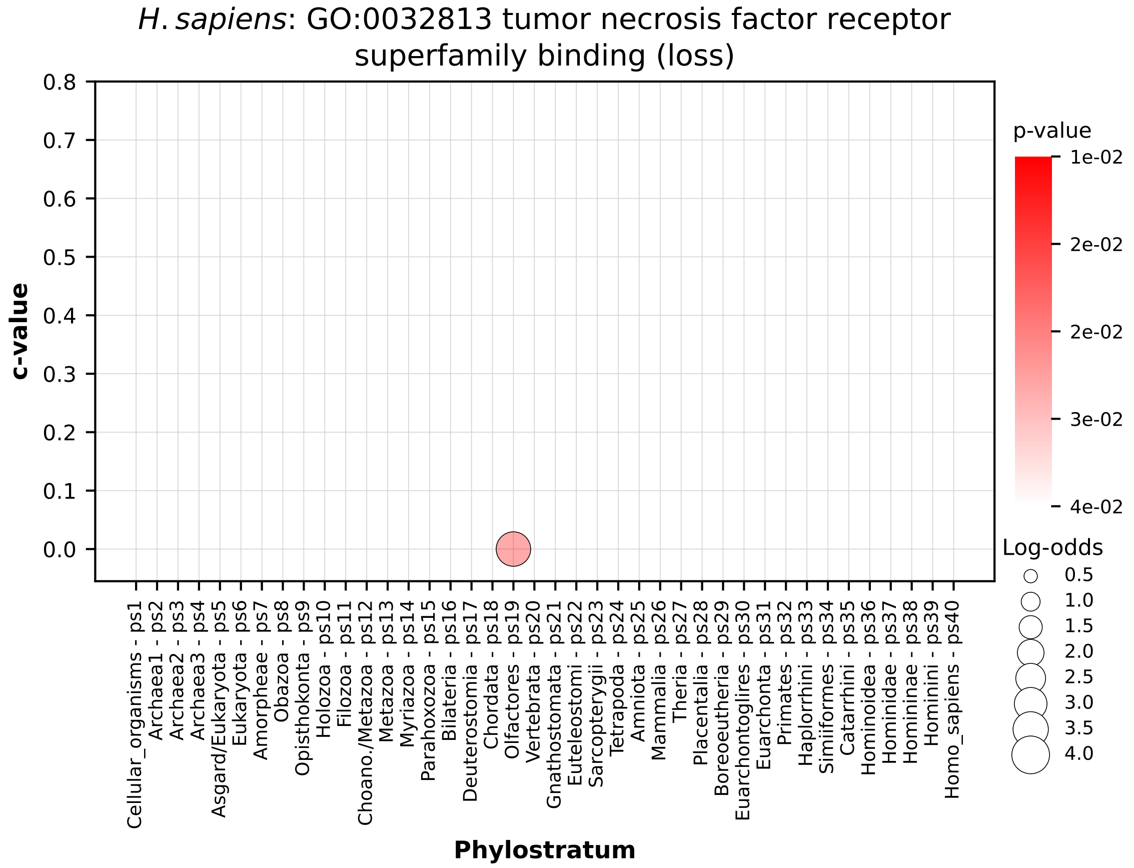

H. sapiens: GO:0032813 tumor necrosis factor receptor superfamily binding (loss)
c-value
0.8
0.7
0.6
0.5
0.4
0.3
0.2
0.1
0.0
p-value
1e-02
2e-02
2e-02
3e-02
4e-02
Log-odds
0.5
1.0
1.5
2.0
2.5
3.0
3.5
4.0
Cellular_organisms - ps1
Archaea1 - ps2
Archaea2 - ps3
Archaea3 - ps4
Asgard/Eukaryota - ps5
Eukaryota - ps6
Amorpheae - ps7
Obazoa - ps8
Opisthokonta - ps9
Holozoa - ps10
Filozoa - ps11
Choano./Metazoa - ps12
Metazoa - ps13
Myriazoa - ps14
Parahoxozoa - ps15
Bilateria - ps16
Deuterostomia - ps17
Chordata - ps18
Olfactores - ps19
Vertebrata - ps20
Gnathostomata - ps21
Euteleostomi - ps22
Sarcopterygii - ps23
Tetrapoda - ps24
Amniota - ps25
Mammalia - ps26
Theria - ps27
Placentalia - ps28
Boreoeutheria - ps29
Euarchontoglires - ps30
Euarchonta - ps31
Primates - ps32
Haplorrhini - ps33
Simiiformes - ps34
Catarrhini - ps35
Hominoidea - ps36
Hominidae - ps37
Homininae - ps38
Hominini - ps39
Homo_sapiens - ps40
Phylostratum

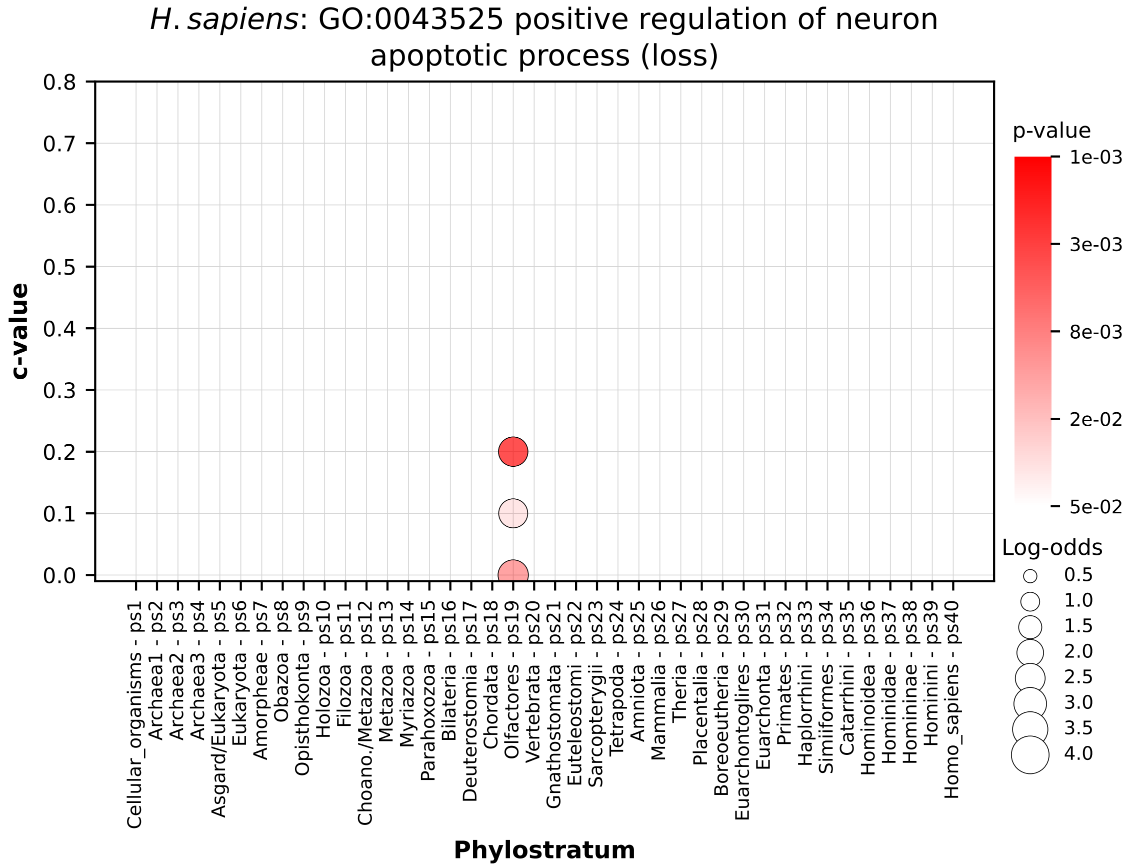

H. sapiens: GO:0043525 positive regulation of neuron apoptotic process (loss)
c-value
0.0
0.1
0.2
0.3
0.4
0.5
0.6
0.7
0.8
Phylostratum
Cellular_organisms - ps1
Archaea1 - ps2
Archaea2 - ps3
Archaea3 - ps4
Asgard/Eukaryota - ps5
Eukaryota - ps6
Amorpheae - ps7
Obazoa - ps8
Opisthokonta - ps9
Holozoa - ps10
Filozoa - ps11
Choano./Metazoa - ps12
Metazoa - ps13
Myriazoa - ps14
Parahoxozoa - ps15
Bilateria - ps16
Deuterostomia - ps17
Chordata - ps18
Olfactores - ps19
Vertebrata - ps20
Gnathostomata - ps21
Euteleostomi - ps22
Sarcopterygii - ps23
Tetrapoda - ps24
Amniota - ps25
Mammalia - ps26
Theria - ps27
Placentalia - ps28
Boreoeutheria - ps29
Euarchontoglires - ps30
Euarchonta - ps31
Primates - ps32
Haplorrhini - ps33
Simiiformes - ps34
Catarrhini - ps35
Hominoidea - ps36
Hominidae - ps37
Homininae - ps38
Hominini - ps39
Homo_sapiens - ps40
p-value
1e-03
3e-03
8e-03
2e-02
5e-02
Log-odds
0.5
1.0
1.5
2.0
2.5
3.0
3.5
4.0

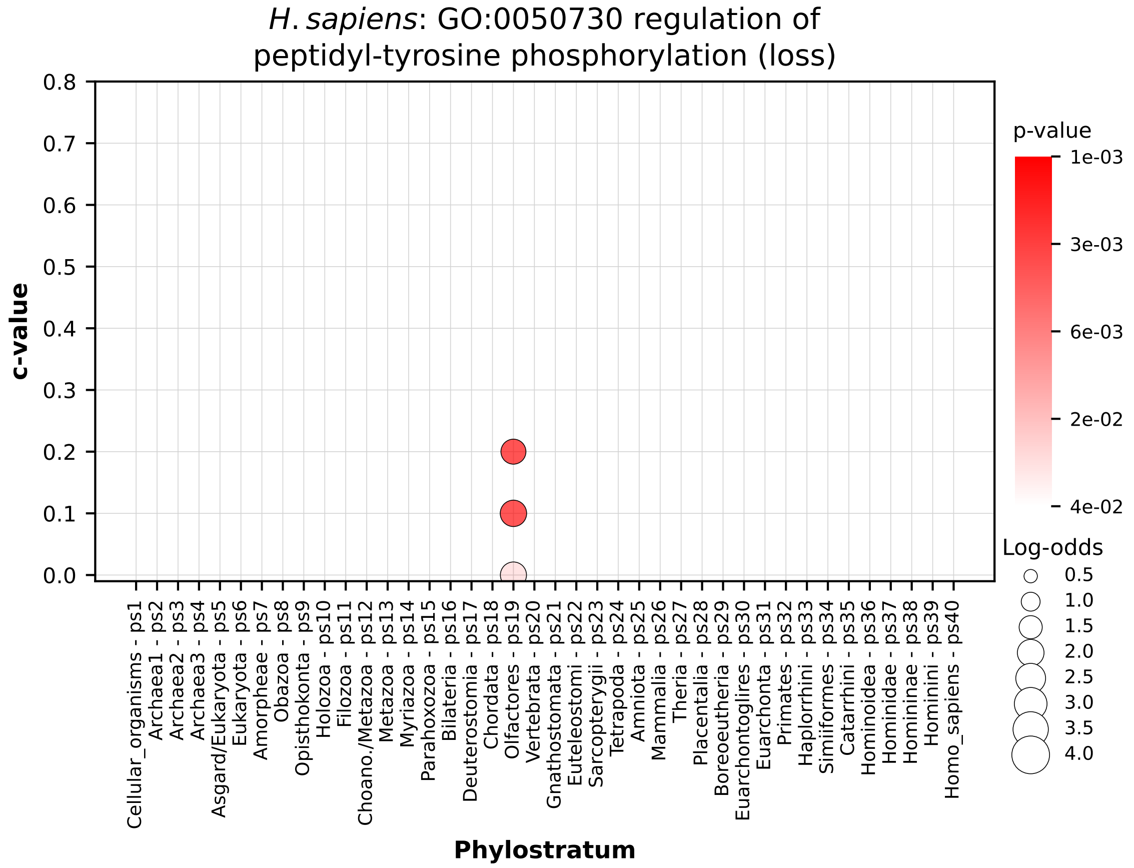

H. sapiens: GO:0050730 regulation of peptidyl-tyrosine phosphorylation (loss)
c-value
0.8
0.7
0.6
0.5
0.4
0.3
0.2
0.1
0.0
p-value
1e-03
3e-03
6e-03
2e-02
4e-02
Log-odds
0.5
1.0
1.5
2.0
2.5
3.0
3.5
4.0
Cellular_organisms - ps1
Archaea1 - ps2
Archaea2 - ps3
Archaea3 - ps4
Asgard/Eukaryota - ps5
Eukaryota - ps6
Amorpheae - ps7
Obazoa - ps8
Opisthokonta - ps9
Holozoa - ps10
Filozoa - ps11
Choano./Metazoa - ps12
Metazoa - ps13
Myriazoa - ps14
Parahoxozoa - ps15
Bilateria - ps16
Deuterostomia - ps17
Chordata - ps18
Olfactores - ps19
Vertebrata - ps20
Gnathostomata - ps21
Euteleostomi - ps22
Sarcopterygii - ps23
Tetrapoda - ps24
Amniota - ps25
Mammalia - ps26
Theria - ps27
Placentalia - ps28
Boreoeutheria - ps29
Euarchontoglires - ps30
Euarchonta - ps31
Primates - ps32
Haplorrhini - ps33
Simiiformes - ps34
Catarrhini - ps35
Hominoidea - ps36
Hominidae - ps37
Homininae - ps38
Hominini - ps39
Homo_sapiens - ps40
Phylostratum

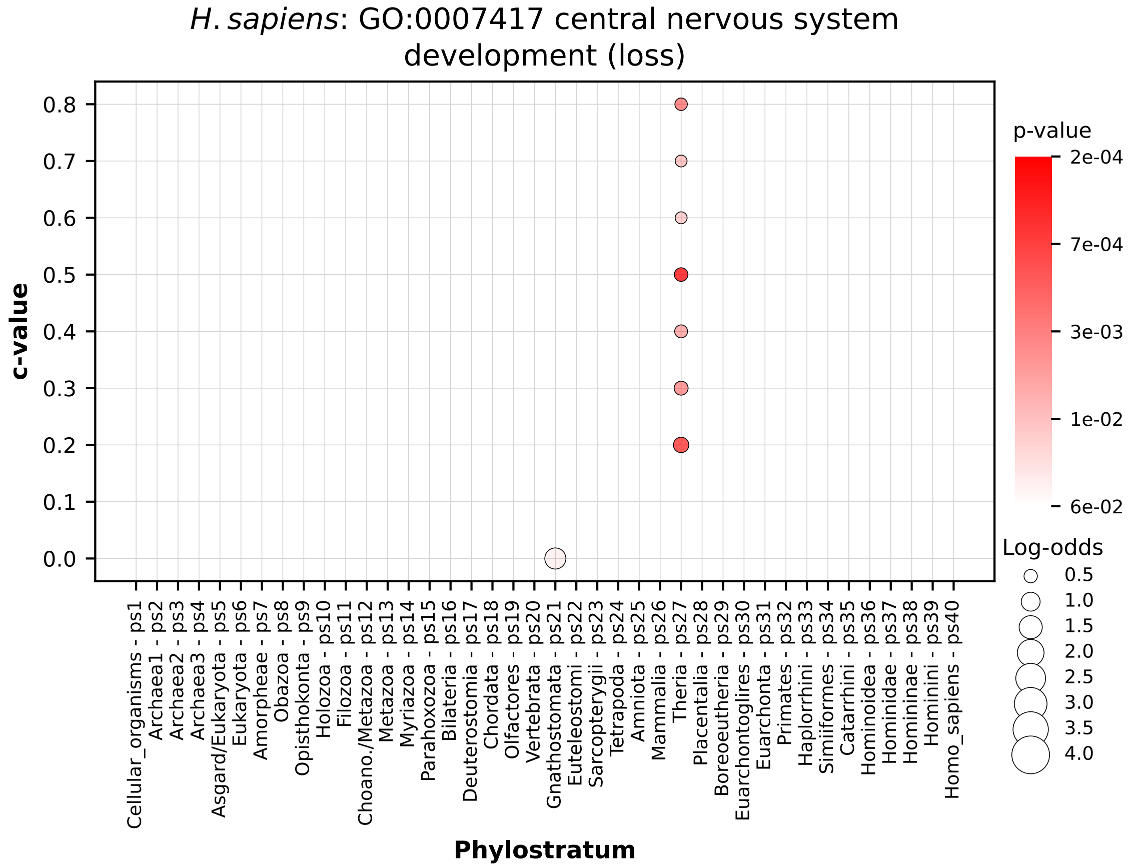

H. sapiens: GO:0007417 central nervous system development (loss)
c-value
0.8
0.7
0.6
0.5
0.4
0.3
0.2
0.1
0.0
p-value
2e-04
7e-04
3e-03
1e-02
6e-02
Log-odds
0.5
1.0
1.5
2.0
2.5
3.0
3.5
4.0
Cellular_organisms - ps1
Archaea1 - ps2
Archaea2 - ps3
Archaea3 - ps4
Asgard/Eukaryota - ps5
Eukaryota - ps6
Amorpheae - ps7
Obazoa - ps8
Opisthokonta - ps9
Holozoa - ps10
Filozoa - ps11
Choano./Metazoa - ps12
Metazoa - ps13
Myriazoa - ps14
Parahoxozoa - ps15
Bilateria - ps16
Deuterostomia - ps17
Chordata - ps18
Olfactores - ps19
Vertebrata - ps20
Gnathostomata - ps21
Euteleostomi - ps22
Sarcopterygii - ps23
Tetrapoda - ps24
Amniota - ps25
Mammalia - ps26
Theria - ps27
Placentalia - ps28
Boreoeutheria - ps29
Euarchontoglires - ps30
Euarchonta - ps31
Primates - ps32
Haplorrhini - ps33
Similformes - ps34
Catarrhini - ps35
Hominoidea - ps36
Hominidae - ps37
Homininae - ps38
Hominini - ps39
Homo_sapiens - ps40
Phylostratum

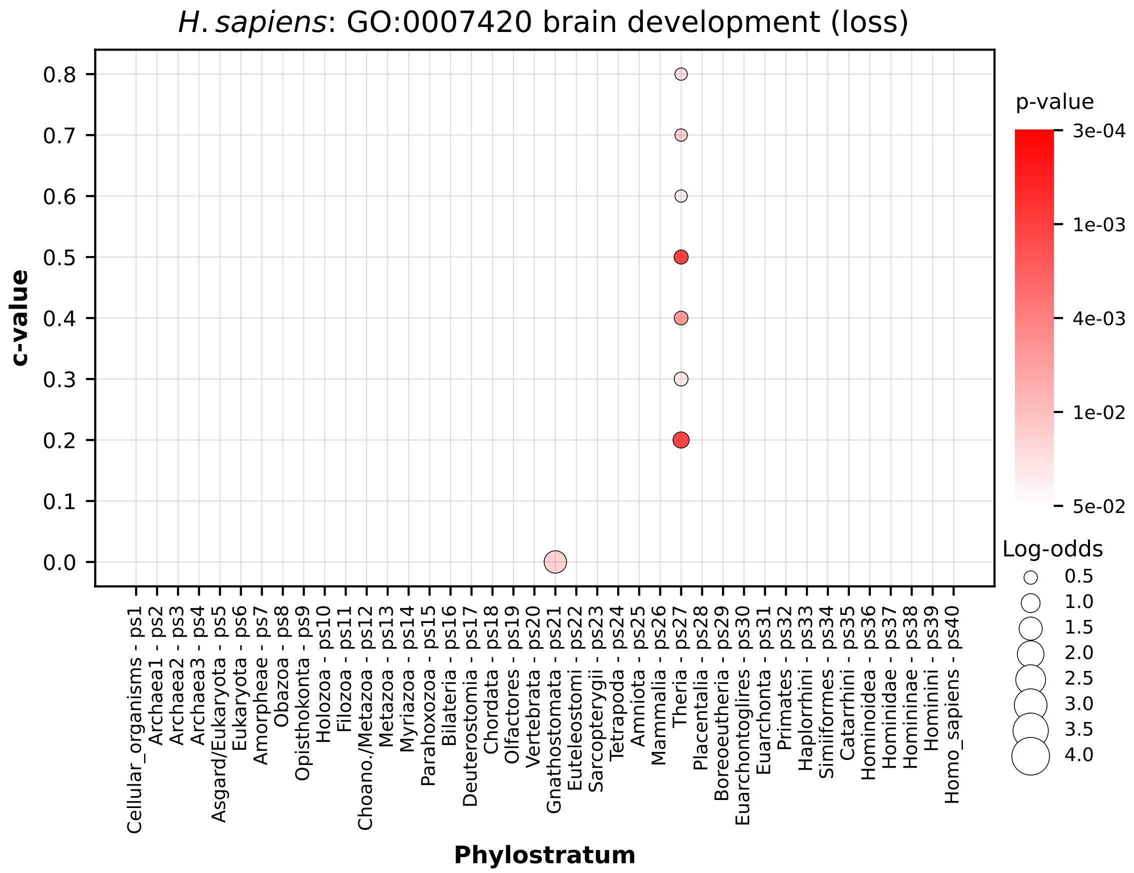

H. sapiens: GO:0007420 brain development (loss)
c-value
0.8
0.7
0.6
0.5
0.4
0.3
0.2
0.1
0.0
Phylostratum
Cellular_organisms - ps1
Archaea1 - ps2
Archaea2 - ps3
Archaea3 - ps4
Asgard/Eukaryota - ps5
Eukaryota - ps6
Amorpheae - ps7
Obazoa - ps8
Opisthokonta - ps9
Holozoa - ps10
Filozoa - ps11
Choano./Metazoa - ps12
Metazoa - ps13
Myriazoa - ps14
Parahoxozoa - ps15
Bilateria - ps16
Deuterostomia - ps17
Chordata - ps18
Olfactores - ps19
Vertebrata - ps20
Gnathostomata - ps21
Euteleostomi - ps22
Sarcopterygii - ps23
Tetrapoda - ps24
Amniota - ps25
Mammalia - ps26
Theria - ps27
Placentalia - ps28
Boreoeutheria - ps29
Euarchontoglires - ps30
Euarchonta - ps31
Primates - ps32
Haplorrhini - ps33
Simiiformes - ps34
Catarrhini - ps35
Hominoidea - ps36
Hominidae - ps37
Homininae - ps38
Hominini - ps39
Homo_sapiens - ps40
p-value
3e-04
1e-03
4e-03
1e-02
5e-02
Log-odds
0.5
1.0
1.5
2.0
2.5
3.0
3.5
4.0

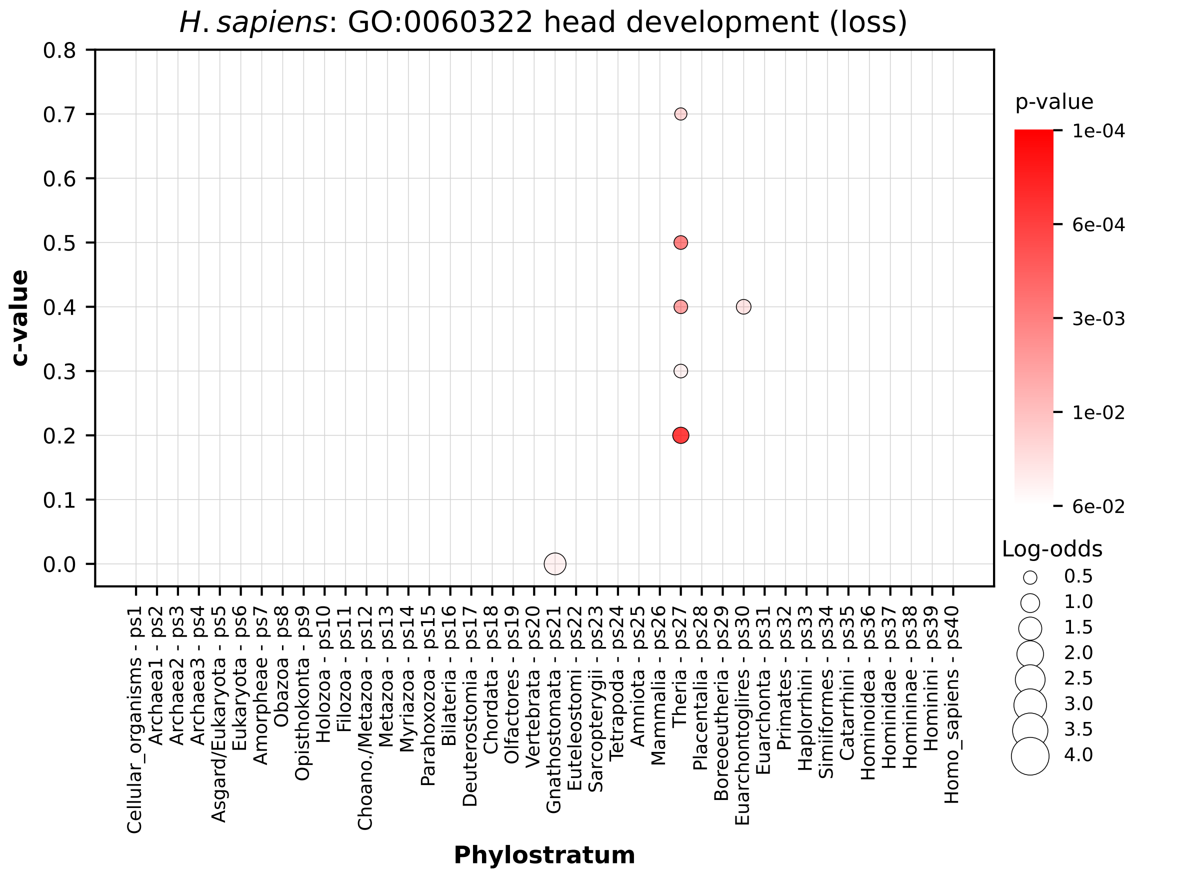

H. sapiens: GO:0060322 head development (loss)
c-value
0.8
0.7
0.6
0.5
0.4
0.3
0.2
0.1
0.0
Phylostratum
Cellular_organisms - ps1
Archaea1 - ps2
Archaea2 - ps3
Archaea3 - ps4
Asgard/Eukaryota - ps5
Eukaryota - ps6
Amorpheae - ps7
Obazoa - ps8
Opisthokonta - ps9
Holozoa - ps10
Filozoa - ps11
Choano./Metazoa - ps12
Metazoa - ps13
Myriazoa - ps14
Parahoxozoa - ps15
Bilateria - ps16
Deuterostomia - ps17
Chordata - ps18
Olfactores - ps19
Vertebrata - ps20
Gnathostomata - ps21
Euteleostomi - ps22
Sarcopterygii - ps23
Tetrapoda - ps24
Amniota - ps25
Mammalia - ps26
Theria - ps27
Placentalia - ps28
Boreoeutheria - ps29
Euarchontoglires - ps30
Euarchonta - ps31
Primates - ps32
Haplorrhini - ps33
Simiiformes - ps34
Catarrhini - ps35
Hominoidea - ps36
Hominidae - ps37
Homininae - ps38
Hominini - ps39
Homo_sapiens - ps40
p-value
1e-04
6e-04
3e-03
1e-02
6e-02
Log-odds
0.5
1.0
1.5
2.0
2.5
3.0
3.5
4.0

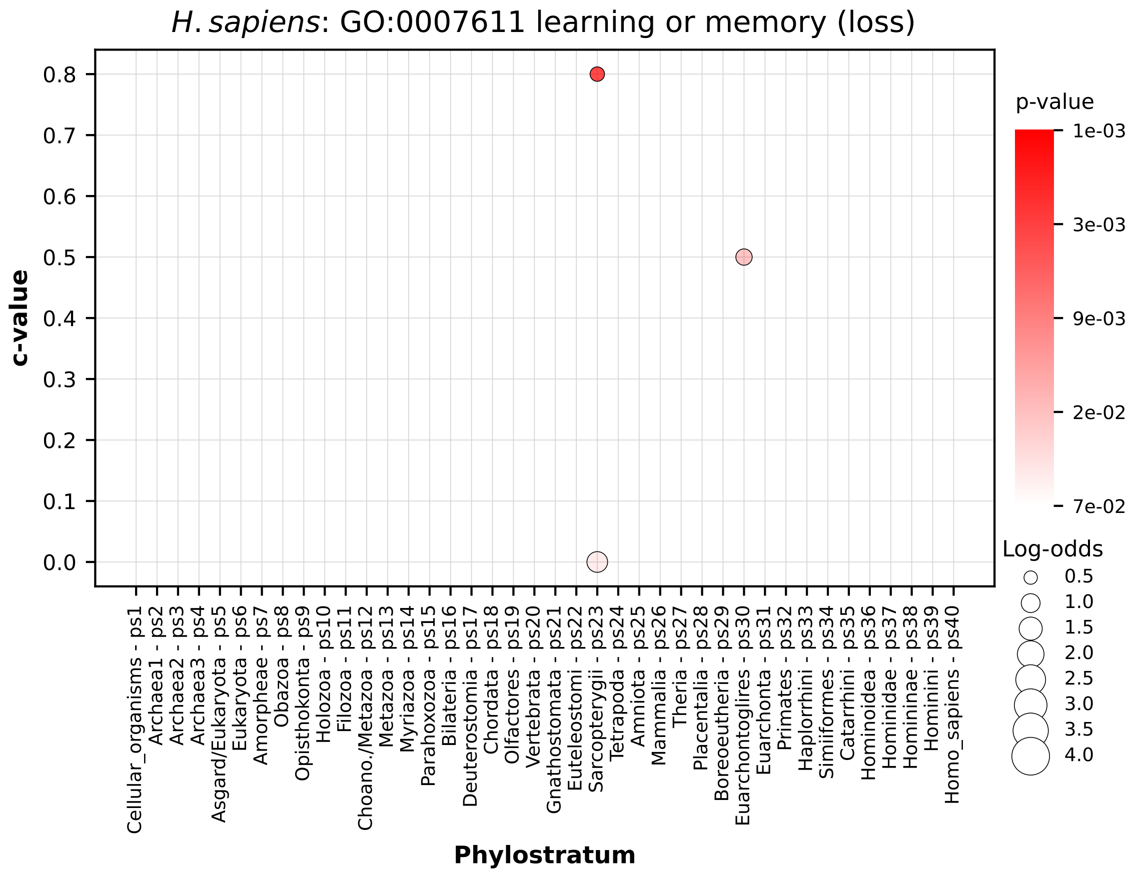

H. sapiens: GO:0007611 learning or memory (loss)
c-value
0.0
0.1
0.2
0.3
0.4
0.5
0.6
0.7
0.8
Phylostratum
Cellular_organisms - ps1
Archaea1 - ps2
Archaea2 - ps3
Archaea3 - ps4
Asgard/Eukaryota - ps5
Eukaryota - ps6
Amorpheae - ps7
Obazoa - ps8
Opisthokonta - ps9
Holozoa - ps10
Filozoa - ps11
Choano./Metazoa - ps12
Metazoa - ps13
Myriazoa - ps14
Parahoxozoa - ps15
Bilateria - ps16
Deuterostomia - ps17
Chordata - ps18
Olfactores - ps19
Vertebrata - ps20
Gnathostomata - ps21
Euteleostomi - ps22
Sarcopterygii - ps23
Tetrapoda - ps24
Amniota - ps25
Mammalia - ps26
Theria - ps27
Placentalia - ps28
Boreoeutheria - ps29
Euarchontoglires - ps30
Euarchonta - ps31
Primates - ps32
Haplorrhini - ps33
Simiiformes - ps34
Catarrhini - ps35
Hominoidea - ps36
Hominidae - ps37
Homininae - ps38
Hominini - ps39
Homo_sapiens - ps40
p-value
1e-03
3e-03
9e-03
2e-02
7e-02
Log-odds
0.5
1.0
1.5
2.0
2.5
3.0
3.5
4.0

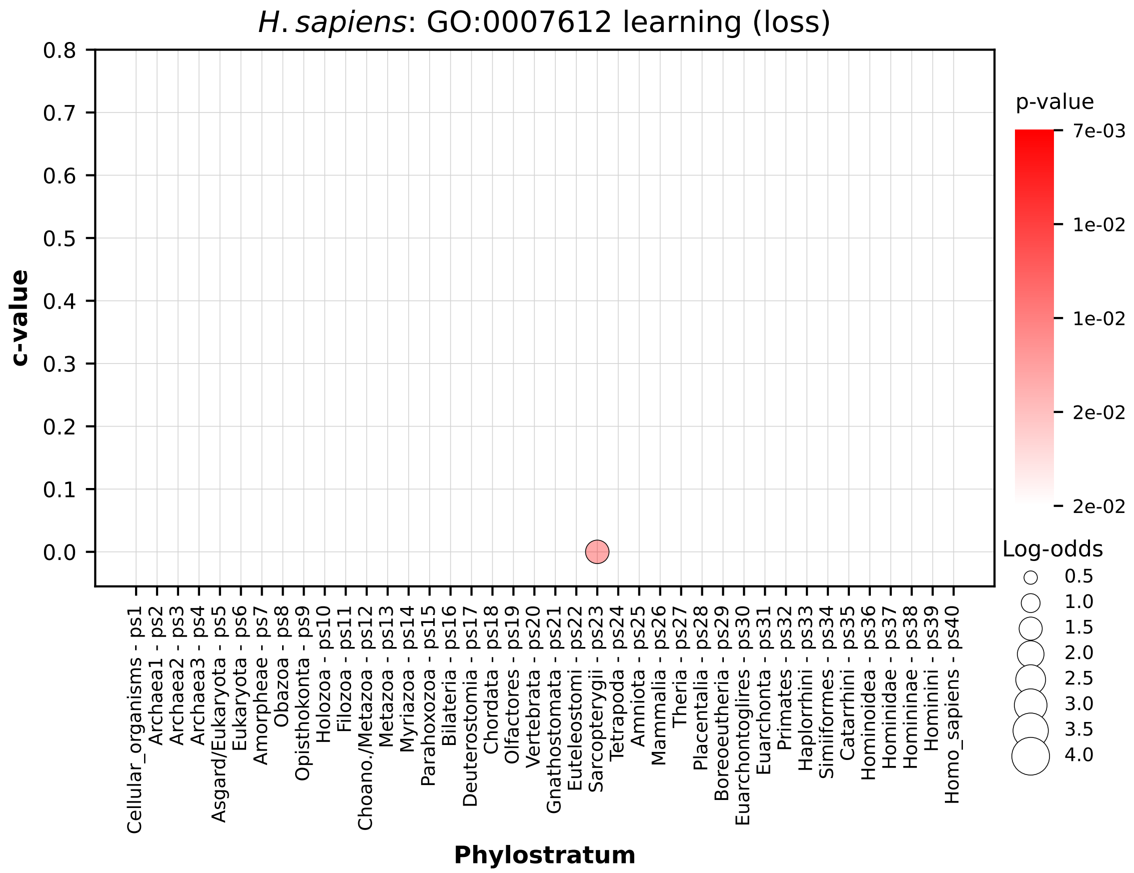
H. sapiens: GO:0007612 learning (loss)
c-value
0.8
0.7
0.6
0.5
0.4
0.3
0.2
0.1
0.0
p-value
7e-03
1e-02
1e-02
2e-02
2e-02
Log-odds
0.5
1.0
1.5
2.0
2.5
3.0
3.5
4.0
Cellular_organisms - ps1
Archaea1 - ps2
Archaea2 - ps3
Archaea3 - ps4
Asgard/Eukaryota - ps5
Eukaryota - ps6
Amorpheae - ps7
Obazoa - ps8
Opisthokonta - ps9
Holozoa - ps10
Filozoa - ps11
Choano./Metazoa - ps12
Metazoa - ps13
Myriazoa - ps14
Parahoxozoa - ps15
Bilateria - ps16
Deuterostomia - ps17
Chordata - ps18
Olfactores - ps19
Vertebrata - ps20
Gnathostomata - ps21
Euteleostomi - ps22
Sarcopterygii - ps23
Tetrapoda - ps24
Amniota - ps25
Mammalia - ps26
Theria - ps27
Placentalia - ps28
Boreoeutheria - ps29
Euarchontoglires - ps30
Euarchonta - ps31
Primates - ps32
Haplorrhini - ps33
Simiiformes - ps34
Catarrhini - ps35
Hominoidea - ps36
Hominidae - ps37
Homininae - ps38
Hominini - ps39
Homo_sapiens - ps40
Phylostratum

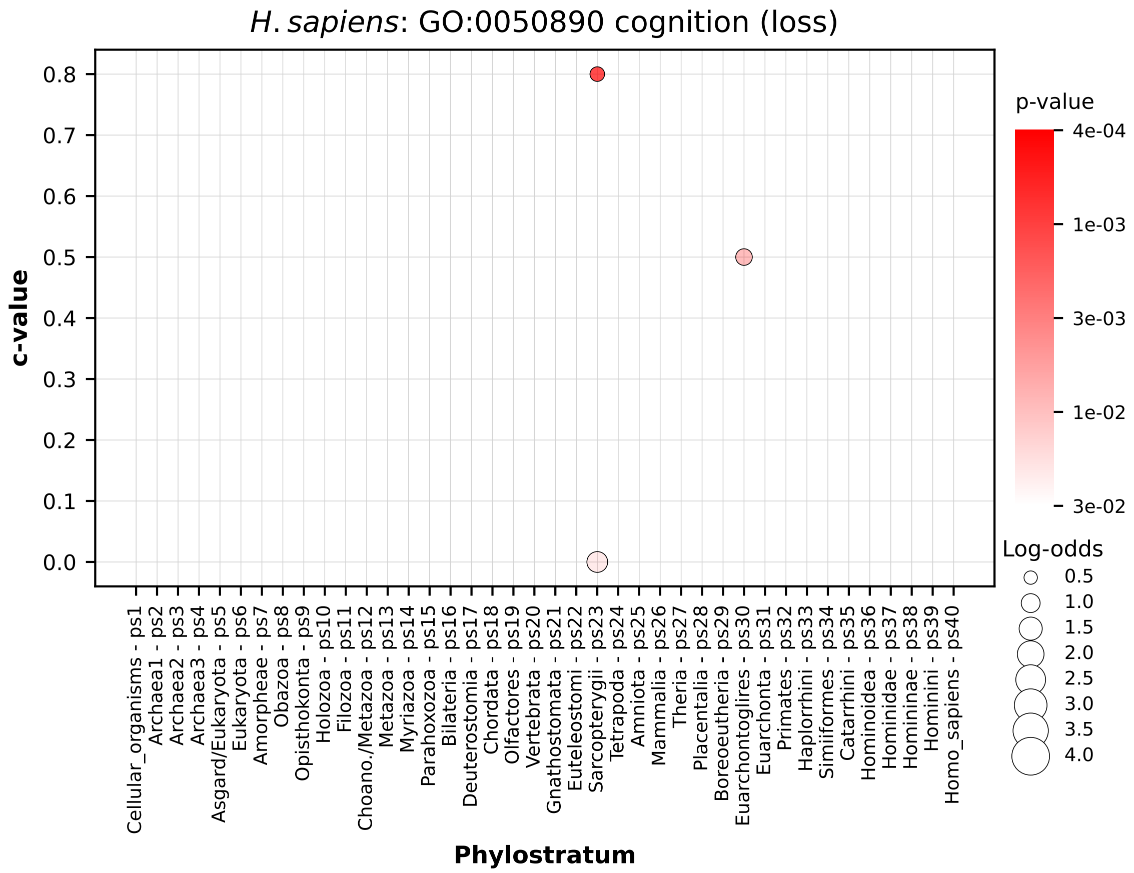

H. sapiens: GO:0050890 cognition (loss)
c-value
0.0
0.1
0.2
0.3
0.4
0.5
0.6
0.7
0.8
Phylostratum
Cellular_organisms - ps1
Archaea1 - ps2
Archaea2 - ps3
Archaea3 - ps4
Asgard/Eukaryota - ps5
Eukaryota - ps6
Amorpheae - ps7
Obazoa - ps8
Opisthokonta - ps9
Holozoa - ps10
Filozoa - ps11
Choano./Metazoa - ps12
Metazoa - ps13
Myriazoa - ps14
Parahoxozoa - ps15
Bilateria - ps16
Deuterostomia - ps17
Chordata - ps18
Olfactores - ps19
Vertebrata - ps20
Gnathostomata - ps21
Euteleostomi - ps22
Sarcopterygii - ps23
Tetrapoda - ps24
Amniota - ps25
Mammalia - ps26
Theria - ps27
Placentalia - ps28
Boreoeutheria - ps29
Euarchontoglires - ps30
Euarchonta - ps31
Primates - ps32
Haplorrhini - ps33
Simiiformes - ps34
Catarrhini - ps35
Hominoidea - ps36
Hominidae - ps37
Homininae - ps38
Hominini - ps39
Homo_sapiens - ps40
p-value
4e-04
1e-03
3e-03
1e-02
3e-02
Log-odds
0.5
1.0
1.5
2.0
2.5
3.0
3.5
4.0

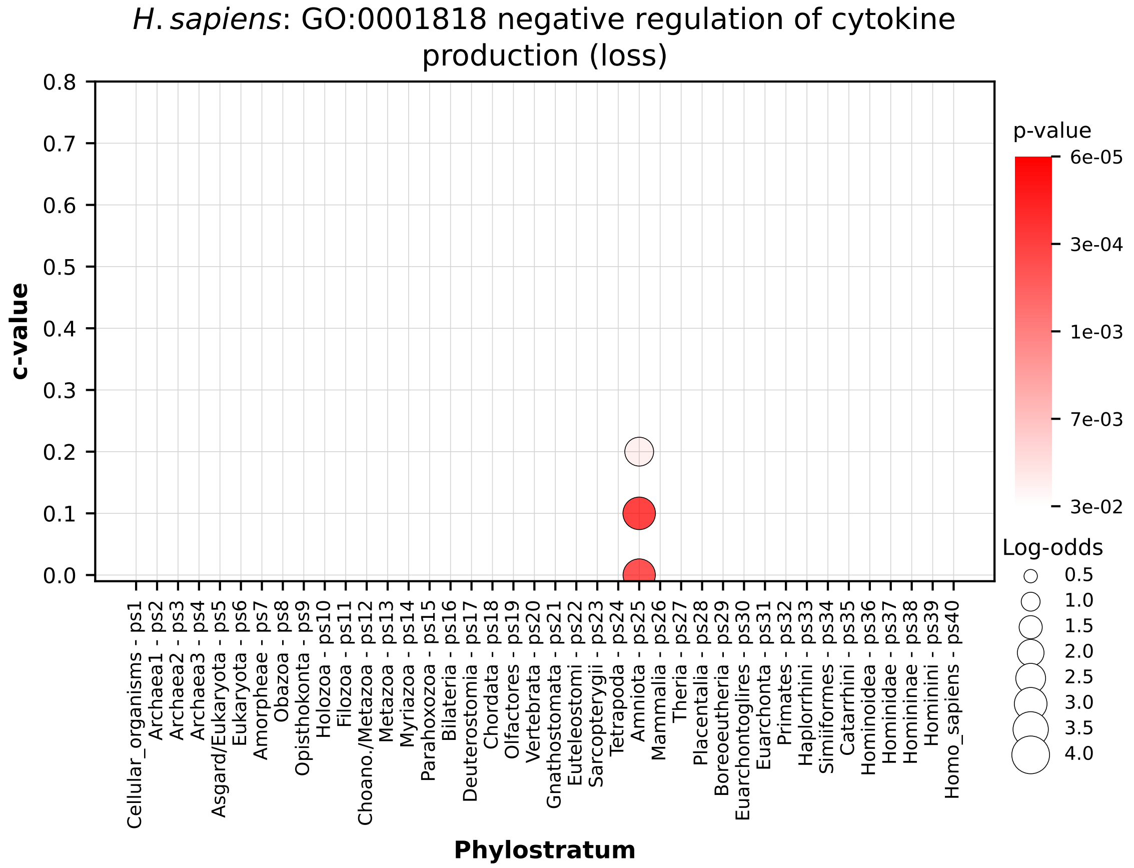

H. sapiens: GO:0001818 negative regulation of cytokine production (loss)
c-value
0.8
0.7
0.6
0.5
0.4
0.3
0.2
0.1
0.0
Phylostratum
Cellular_organisms - ps1
Archaea1 - ps2
Archaea2 - ps3
Archaea3 - ps4
Asgard/Eukaryota - ps5
Eukaryota - ps6
Amorpheae - ps7
Obazoa - ps8
Opisthokonta - ps9
Holozoa - ps10
Filozoa - ps11
Choano./Metazoa - ps12
Metazoa - ps13
Myriazoa - ps14
Parahoxozoa - ps15
Bilateria - ps16
Deuterostomia - ps17
Chordata - ps18
Olfactores - ps19
Vertebrata - ps20
Gnathostomata - ps21
Euteleostomi - ps22
Sarcopterygii - ps23
Tetrapoda - ps24
Amniota - ps25
Mammalia - ps26
Theria - ps27
Placentalia - ps28
Boreoeutheria - ps29
Euarchontoglires - ps30
Euarchonta - ps31
Primates - ps32
Haplorrhini - ps33
Simiiformes - ps34
Catarrhini - ps35
Hominoidea - ps36
Hominidae - ps37
Homininae - ps38
Hominini - ps39
Homo_sapiens - ps40
p-value
6e-05
3e-04
1e-03
7e-03
3e-02
Log-odds
0.5
1.0
1.5
2.0
2.5
3.0
3.5
4.0

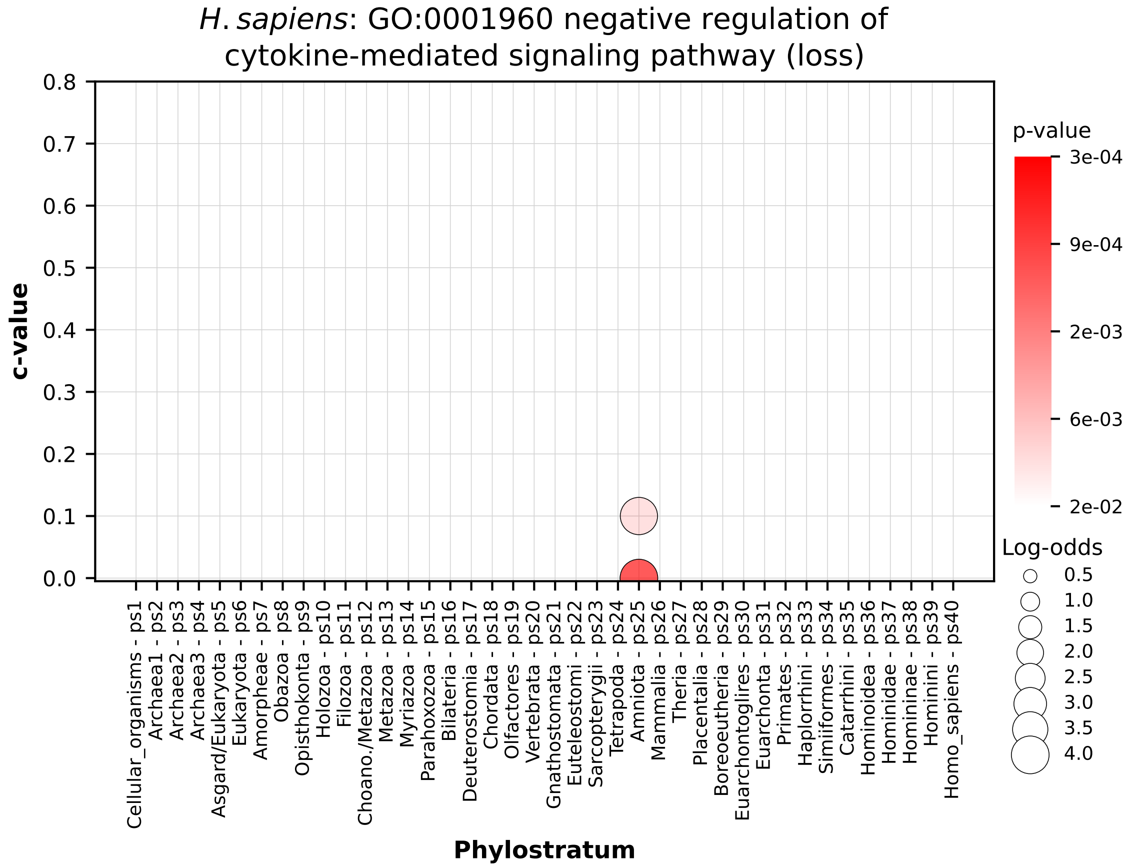

H. sapiens: GO:0001960 negative regulation of cytokine-mediated signaling pathway (loss)
c-value
0.0
0.1
0.2
0.3
0.4
0.5
0.6
0.7
0.8
Phylostratum
Cellular_organisms - ps1
Archaea1 - ps2
Archaea2 - ps3
Archaea3 - ps4
Asgard/Eukaryota - ps5
Eukaryota - ps6
Amorpheae - ps7
Obazoa - ps8
Opisthokonta - ps9
Holozoa - ps10
Filozoa - ps11
Choano./Metazoa - ps12
Metazoa - ps13
Myriazoa - ps14
Parahoxozoa - ps15
Bilateria - ps16
Deuterostomia - ps17
Chordata - ps18
Olfactores - ps19
Vertebrata - ps20
Gnathostomata - ps21
Euteleostomi - ps22
Sarcopterygii - ps23
Tetrapoda - ps24
Amniota - ps25
Mammalia - ps26
Theria - ps27
Placentalia - ps28
Boreoeutheria - ps29
Euarchontoglires - ps30
Euarchonta - ps31
Primates - ps32
Haplorrhini - ps33
Simiiformes - ps34
Catarrhini - ps35
Hominoidea - ps36
Hominidae - ps37
Homininae - ps38
Hominini - ps39
Homo_sapiens - ps40
p-value
3e-04
9e-04
2e-03
6e-03
2e-02
Log-odds
0.5
1.0
1.5
2.0
2.5
3.0
3.5
4.0

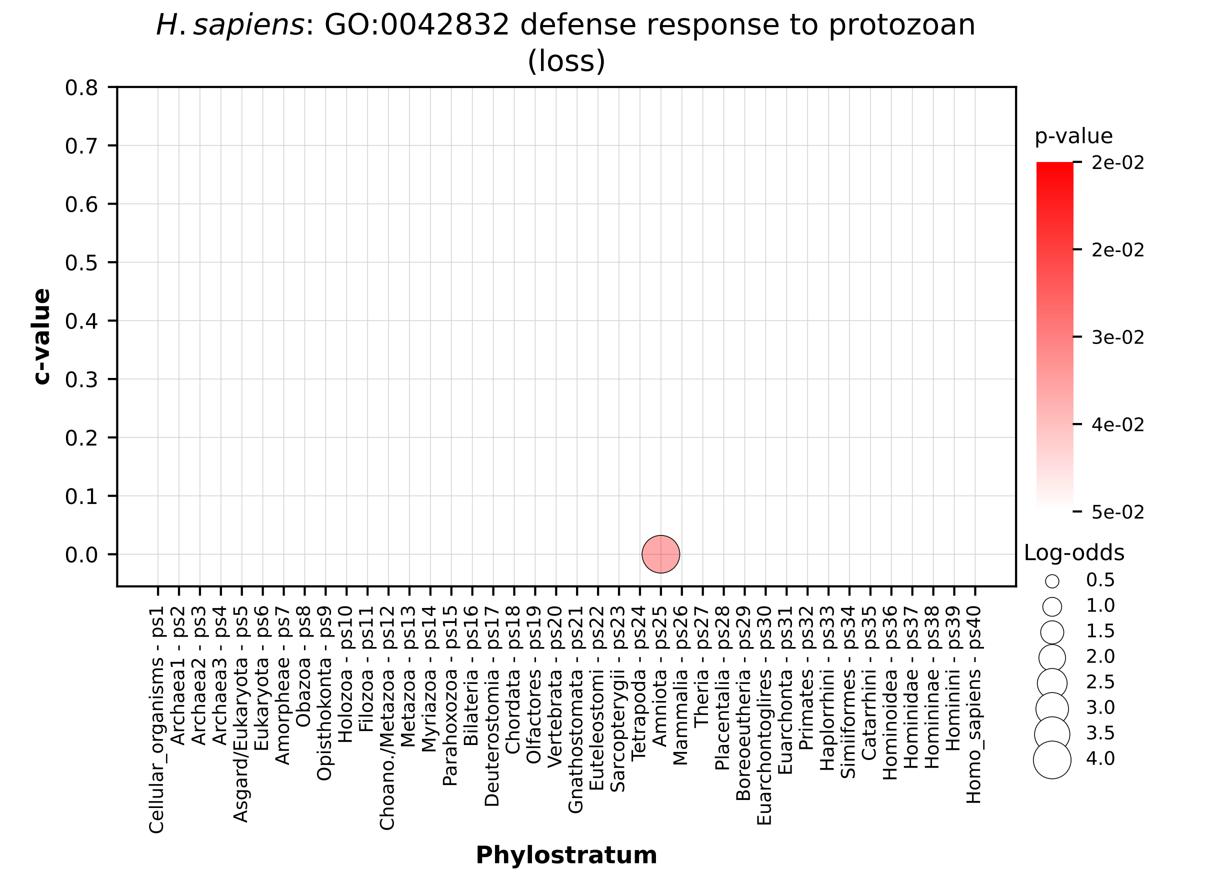

H. sapiens: GO:0042832 defense response to protozoan (loss)
c-value
0.8
0.7
0.6
0.5
0.4
0.3
0.2
0.1
0.0
p-value
2e-02
2e-02
3e-02
4e-02
5e-02
Log-odds
0.5
1.0
1.5
2.0
2.5
3.0
3.5
4.0
Cellular_organisms - ps1
Archaea1 - ps2
Archaea2 - ps3
Archaea3 - ps4
Asgard/Eukaryota - ps5
Eukaryota - ps6
Amorpheae - ps7
Obazoa - ps8
Opisthokonta - ps9
Holozoa - ps10
Filozoa - ps11
Choano./Metazoa - ps12
Metazoa - ps13
Myriazoa - ps14
Parahoxozoa - ps15
Bilateria - ps16
Deuterostomia - ps17
Chordata - ps18
Olfactores - ps19
Vertebrata - ps20
Gnathostomata - ps21
Euteleostomi - ps22
Sarcopterygii - ps23
Tetrapoda - ps24
Amniota - ps25
Mammalia - ps26
Theria - ps27
Placentalia - ps28
Boreoeutheria - ps29
Euarchontoglires - ps30
Euarchonta - ps31
Primates - ps32
Haplorrhini - ps33
Simiiformes - ps34
Catarrhini - ps35
Hominoidea - ps36
Hominidae - ps37
Homininae - ps38
Hominini - ps39
Homo_sapiens - ps40
Phylostratum

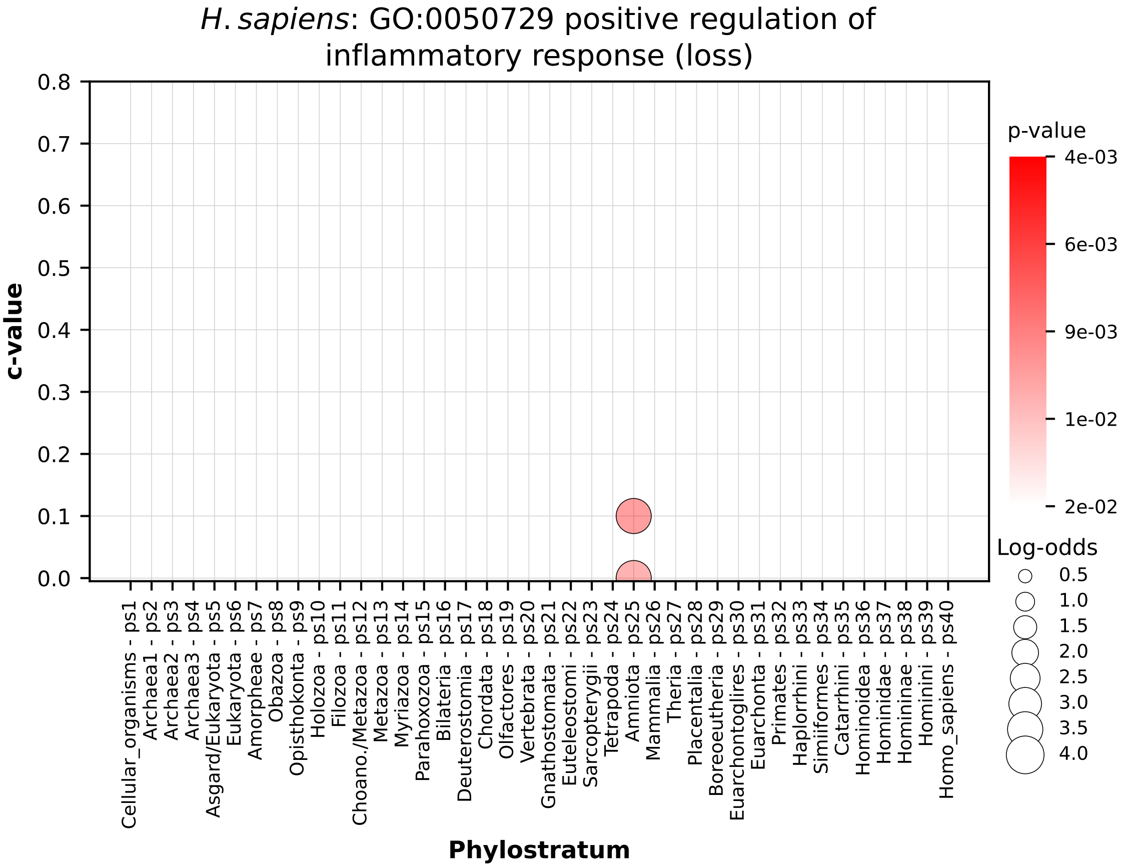

H. sapiens: GO:0050729 positive regulation of inflammatory response (loss)
c-value
0.8
0.7
0.6
0.5
0.4
0.3
0.2
0.1
0.0
p-value
4e-03
6e-03
9e-03
1e-02
2e-02
Log-odds
0.5
1.0
1.5
2.0
2.5
3.0
3.5
4.0
Cellular_organisms - ps1
Archaea1 - ps2
Archaea2 - ps3
Archaea3 - ps4
Asgard/Eukaryota - ps5
Eukaryota - ps6
Amorpheae - ps7
Obazoa - ps8
Opisthokonta - ps9
Holozoa - ps10
Filozoa - ps11
Choano./Metazoa - ps12
Metazoa - ps13
Myriazoa - ps14
Parahoxozoa - ps15
Bilateria - ps16
Deuterostomia - ps17
Chordata - ps18
Olfactores - ps19
Vertebrata - ps20
Gnathostomata - ps21
Euteleostomi - ps22
Sarcopterygii - ps23
Tetrapoda - ps24
Amniota - ps25
Mammalia - ps26
Theria - ps27
Placentalia - ps28
Boreoeutheria - ps29
Euarchontoglires - ps30
Euarchonta - ps31
Primates - ps32
Haplorrhini - ps33
Simiiformes - ps34
Catarrhini - ps35
Hominoidea - ps36
Hominidae - ps37
Homininae - ps38
Hominini - ps39
Homo_sapiens - ps40
Phylostratum

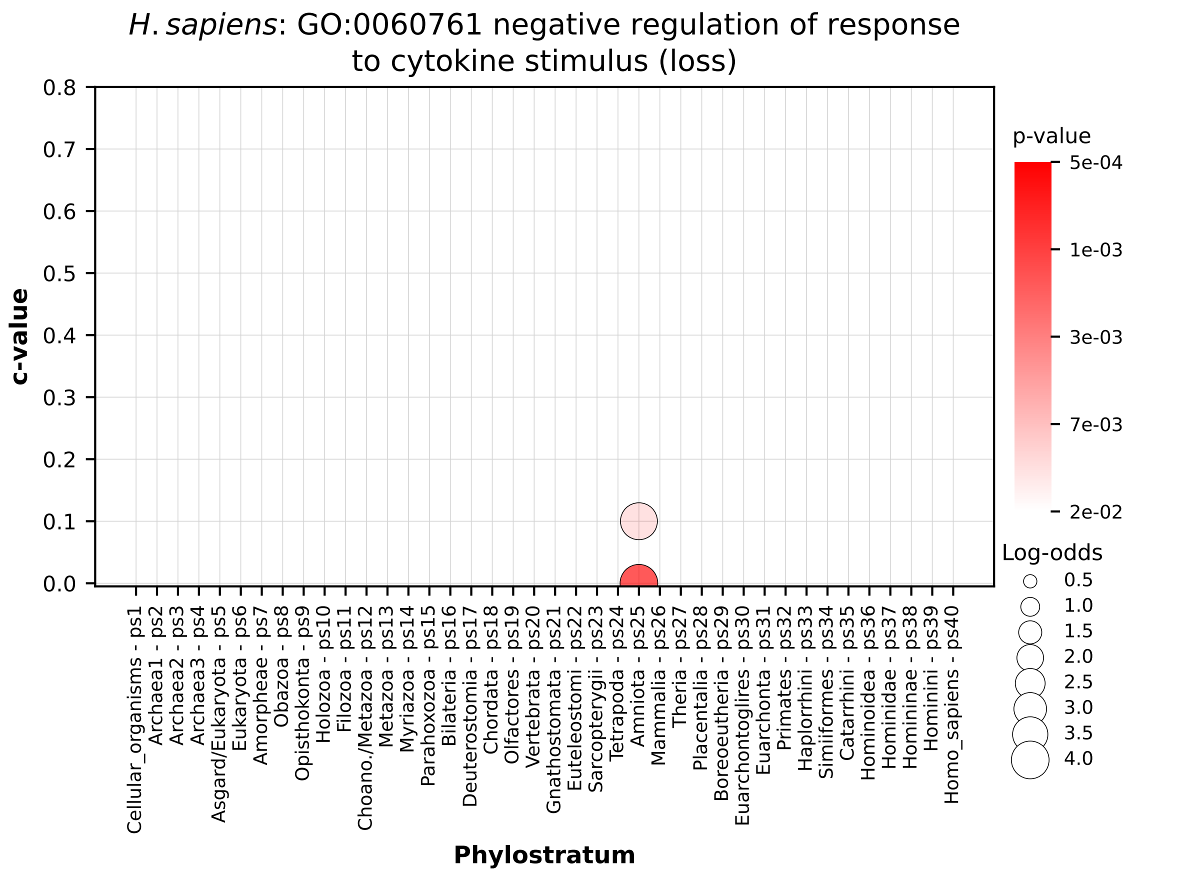

H. sapiens: GO:0060761 negative regulation of response to cytokine stimulus (loss)
c-value
0.0
0.1
0.2
0.3
0.4
0.5
0.6
0.7
0.8
Phylostratum
Cellular_organisms - ps1
Archaea1 - ps2
Archaea2 - ps3
Archaea3 - ps4
Asgard/Eukaryota - ps5
Eukaryota - ps6
Amorpheae - ps7
Obazoa - ps8
Opisthokonta - ps9
Holozoa - ps10
Filozoa - ps11
Choano./Metazoa - ps12
Metazoa - ps13
Myriazoa - ps14
Parahoxozoa - ps15
Bilateria - ps16
Deuterostomia - ps17
Chordata - ps18
Olfactores - ps19
Vertebrata - ps20
Gnathostomata - ps21
Euteleostomi - ps22
Sarcopterygii - ps23
Tetrapoda - ps24
Amniota - ps25
Mammalia - ps26
Theria - ps27
Placentalia - ps28
Boreoeutheria - ps29
Euarchontoglires - ps30
Euarchonta - ps31
Primates - ps32
Haplorrhini - ps33
Simiiformes - ps34
Catarrhini - ps35
Hominoidea - ps36
Hominidae - ps37
Homininae - ps38
Hominini - ps39
Homo_sapiens - ps40
p-value
5e-04
1e-03
3e-03
7e-03
2e-02
Log-odds
0.5
1.0
1.5
2.0
2.5
3.0
3.5
4.0

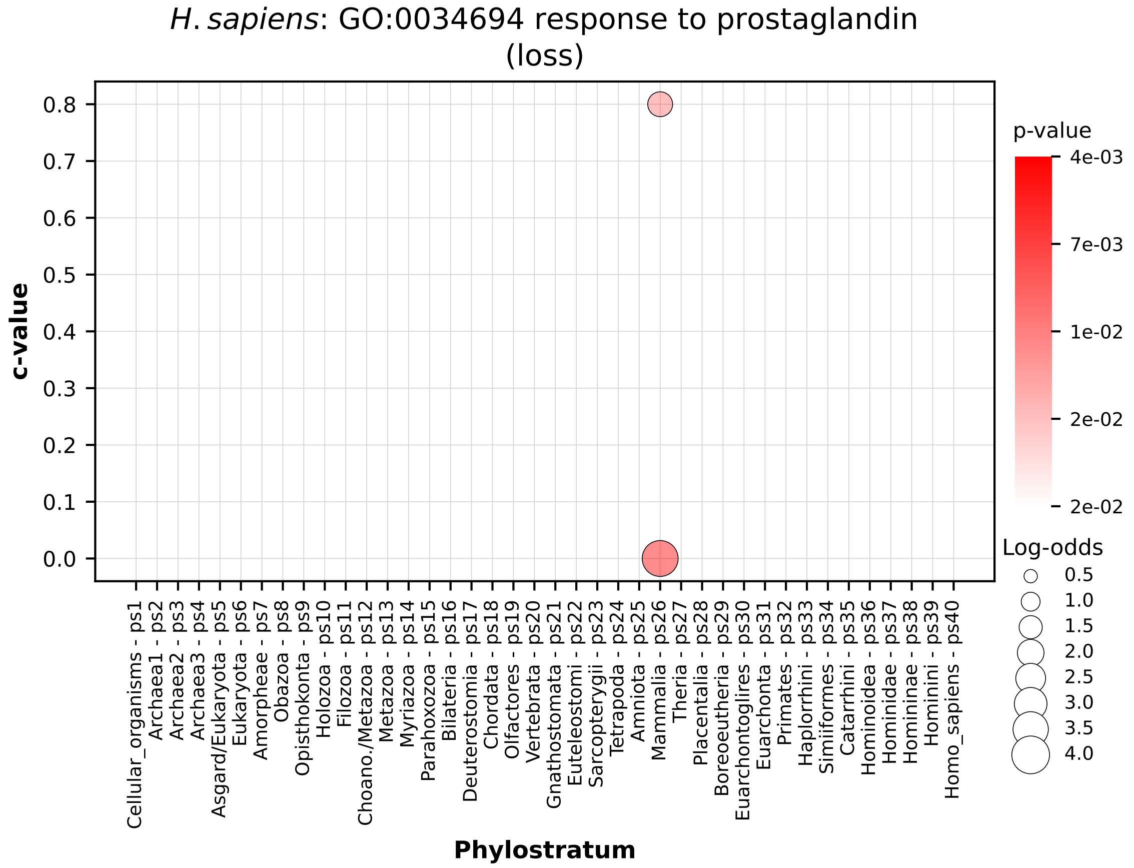

H. sapiens: GO:0034694 response to prostaglandin
(loss)
c-value
0.8
0.7
0.6
0.5
0.4
0.3
0.2
0.1
0.0
p-value
4e-03
7e-03
1e-02
2e-02
2e-02
Log-odds
0.5
1.0
1.5
2.0
2.5
3.0
3.5
4.0
Cellular_organisms - ps1
Archaea1 - ps2
Archaea2 - ps3
Archaea3 - ps4
Asgard/Eukaryota - ps5
Eukaryota - ps6
Amorpheae - ps7
Obazoa - ps8
Opisthokonta - ps9
Holozoa - ps10
Filozoa - ps11
Choano./Metazoa - ps12
Metazoa - ps13
Myriazoa - ps14
Parahoxozoa - ps15
Bilateria - ps16
Deuterostomia - ps17
Chordata - ps18
Olfactores - ps19
Vertebrata - ps20
Gnathostomata - ps21
Euteleostomi - ps22
Sarcopterygii - ps23
Tetrapoda - ps24
Amniota - ps25
Mammalia - ps26
Theria - ps27
Placentalia - ps28
Boreoeutheria - ps29
Euarchontoglires - ps30
Euarchonta - ps31
Primates - ps32
Haplorrhini - ps33
Simiiformes - ps34
Catarrhini - ps35
Hominoidea - ps36
Hominidae - ps37
Homininae - ps38
Hominini - ps39
Homo_sapiens - ps40
Phylostratum

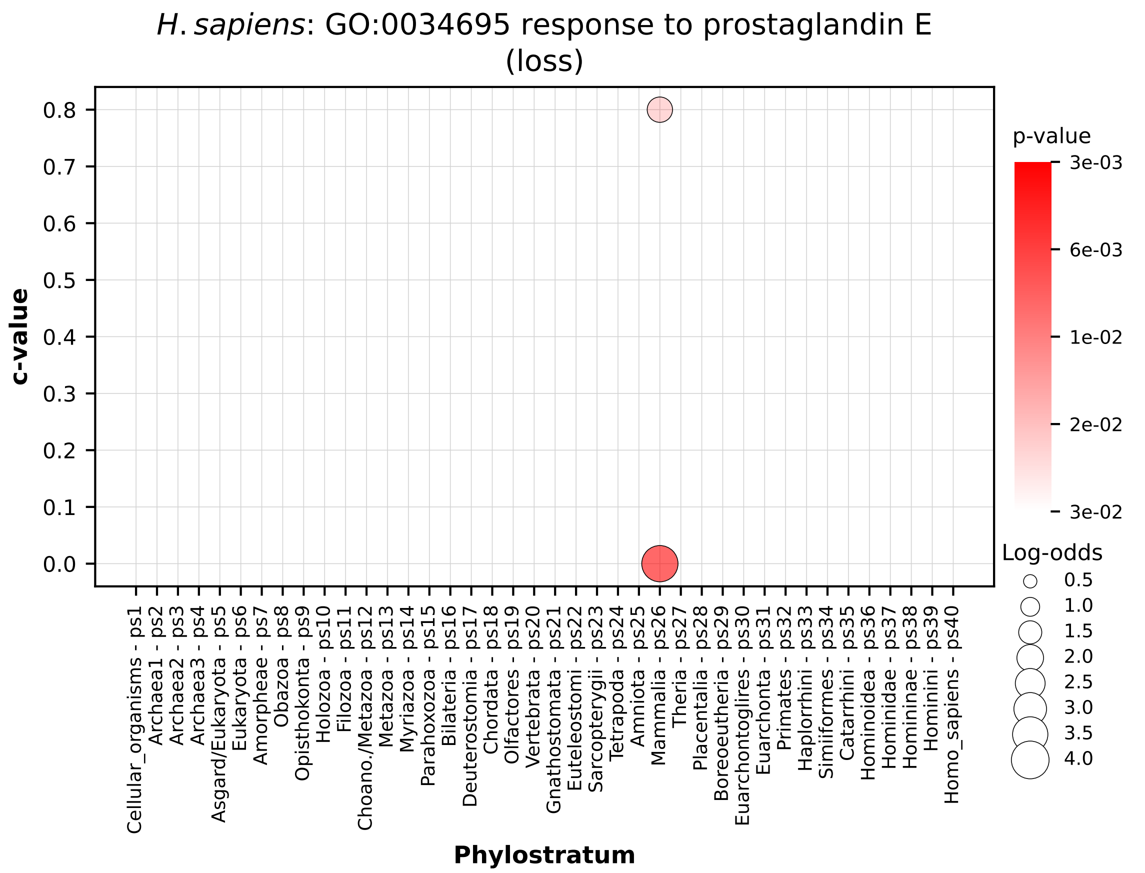

H. sapiens: GO:0034695 response to prostaglandin E
(loss)
c-value
0.8
0.7
0.6
0.5
0.4
0.3
0.2
0.1
0.0
p-value
3e-03
6e-03
1e-02
2e-02
3e-02
Log-odds
0.5
1.0
1.5
2.0
2.5
3.0
3.5
4.0
Cellular_organisms - ps1
Archaea1 - ps2
Archaea2 - ps3
Archaea3 - ps4
Asgard/Eukaryota - ps5
Eukaryota - ps6
Amorpheae - ps7
Obazoa - ps8
Opisthokonta - ps9
Holozoa - ps10
Filozoa - ps11
Choano./Metazoa - ps12
Metazoa - ps13
Myriazoa - ps14
Parahoxozoa - ps15
Bilateria - ps16
Deuterostomia - ps17
Chordata - ps18
Olfactores - ps19
Vertebrata - ps20
Gnathostomata - ps21
Euteleostomi - ps22
Sarcopterygii - ps23
Tetrapoda - ps24
Amniota - ps25
Mammalia - ps26
Theria - ps27
Placentalia - ps28
Boreoeutheria - ps29
Euarchontoglires - ps30
Euarchonta - ps31
Primates - ps32
Haplorrhini - ps33
Simiiformes - ps34
Catarrhini - ps35
Hominoidea - ps36
Hominidae - ps37
Homininae - ps38
Hominini - ps39
Homo_sapiens - ps40
Phylostratum

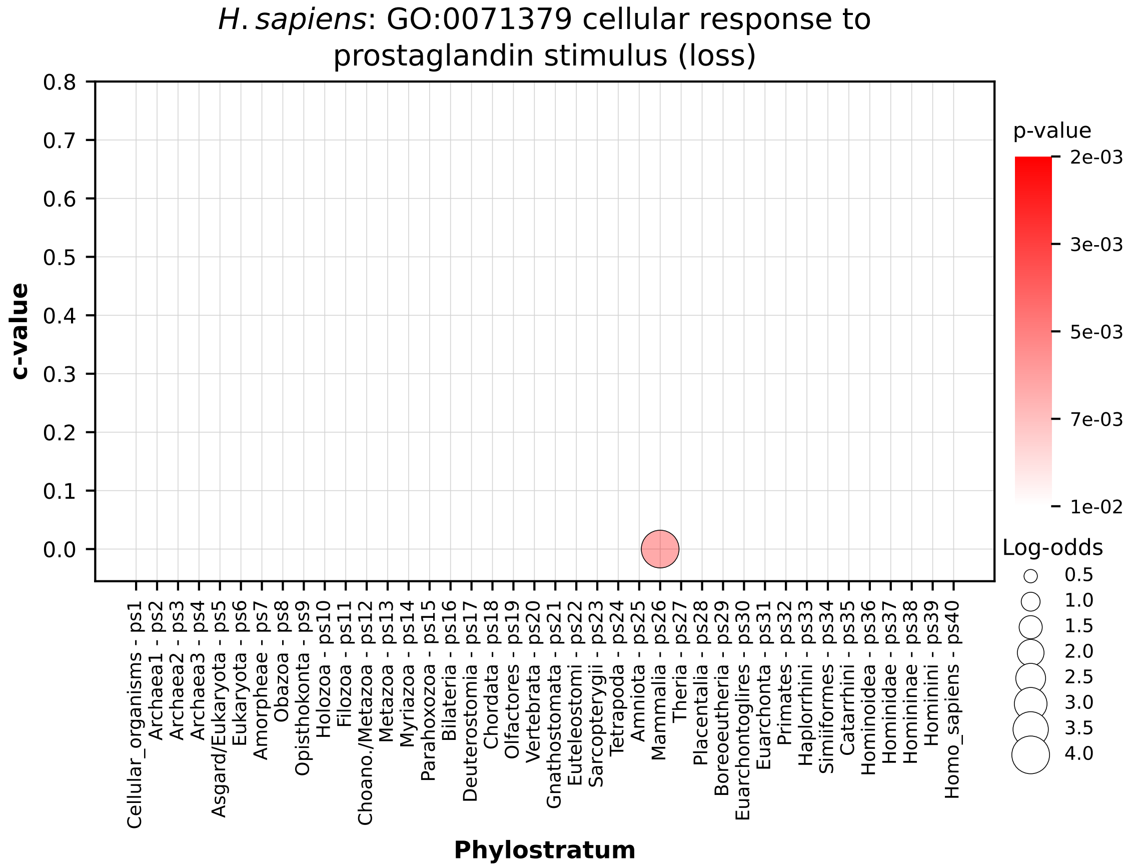

H. sapiens: GO:0071379 cellular response to prostaglandin stimulus (loss)
c-value
0.8
0.7
0.6
0.5
0.4
0.3
0.2
0.1
0.0
Phylostratum
Cellular_organisms - ps1
Archaea1 - ps2
Archaea2 - ps3
Archaea3 - ps4
Asgard/Eukaryota - ps5
Eukaryota - ps6
Amorpheae - ps7
Obazoa - ps8
Opisthokonta - ps9
Holozoa - ps10
Filozoa - ps11
Choano./Metazoa - ps12
Metazoa - ps13
Myriazoa - ps14
Parahoxozoa - ps15
Bilateria - ps16
Deuterostomia - ps17
Chordata - ps18
Olfactores - ps19
Vertebrata - ps20
Gnathostomata - ps21
Euteleostomi - ps22
Sarcopterygii - ps23
Tetrapoda - ps24
Amniota - ps25
Mammalia - ps26
Theria - ps27
Placentalia - ps28
Boreoeutheria - ps29
Euarchontoglires - ps30
Euarchonta - ps31
Primates - ps32
Haplorrhini - ps33
Simiiformes - ps34
Catarrhini - ps35
Hominoidea - ps36
Hominidae - ps37
Homininae - ps38
Hominini - ps39
Homo_sapiens - ps40
p-value
2e-03
3e-03
5e-03
7e-03
1e-02
Log-odds
0.5
1.0
1.5
2.0
2.5
3.0
3.5
4.0

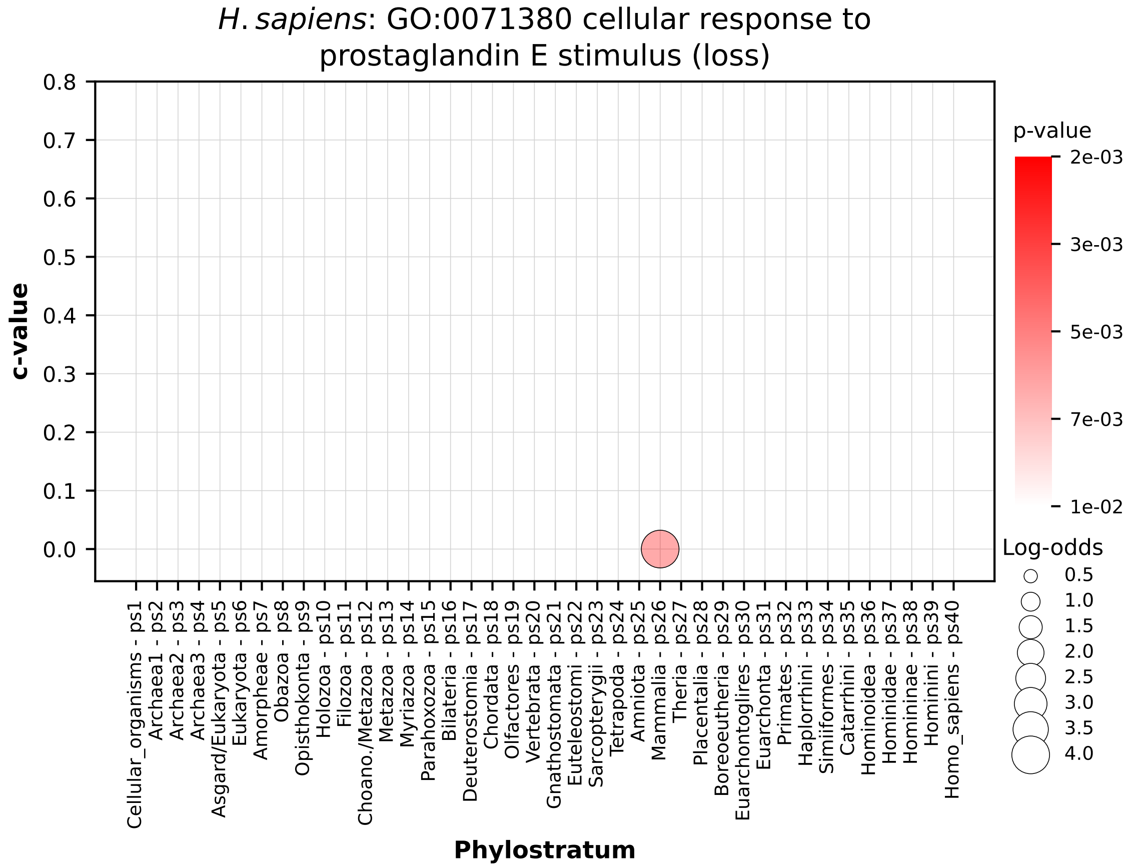

H. sapiens: GO:0071380 cellular response to prostaglandin E stimulus (loss)
c-value
0.8
0.7
0.6
0.5
0.4
0.3
0.2
0.1
0.0
p-value
2e-03
3e-03
5e-03
7e-03
1e-02
Log-odds
0.5
1.0
1.5
2.0
2.5
3.0
3.5
4.0
Cellular_organisms - ps1
Archaea1 - ps2
Archaea2 - ps3
Archaea3 - ps4
Asgard/Eukaryota - ps5
Eukaryota - ps6
Amorpheae - ps7
Obazoa - ps8
Opisthokonta - ps9
Holozoa - ps10
Filozoa - ps11
Choano./Metazoa - ps12
Metazoa - ps13
Myriazoa - ps14
Parahoxozoa - ps15
Bilateria - ps16
Deuterostomia - ps17
Chordata - ps18
Olfactores - ps19
Vertebrata - ps20
Gnathostomata - ps21
Euteleostomi - ps22
Sarcopterygii - ps23
Tetrapoda - ps24
Amniota - ps25
Mammalia - ps26
Theria - ps27
Placentalia - ps28
Boreoeutheria - ps29
Euarchontoglires - ps30
Euarchonta - ps31
Primates - ps32
Haplorrhini - ps33
Simiiformes - ps34
Catarrhini - ps35
Hominoidea - ps36
Hominidae - ps37
Homininae - ps38
Hominini - ps39
Homo_sapiens - ps40
Phylostratum

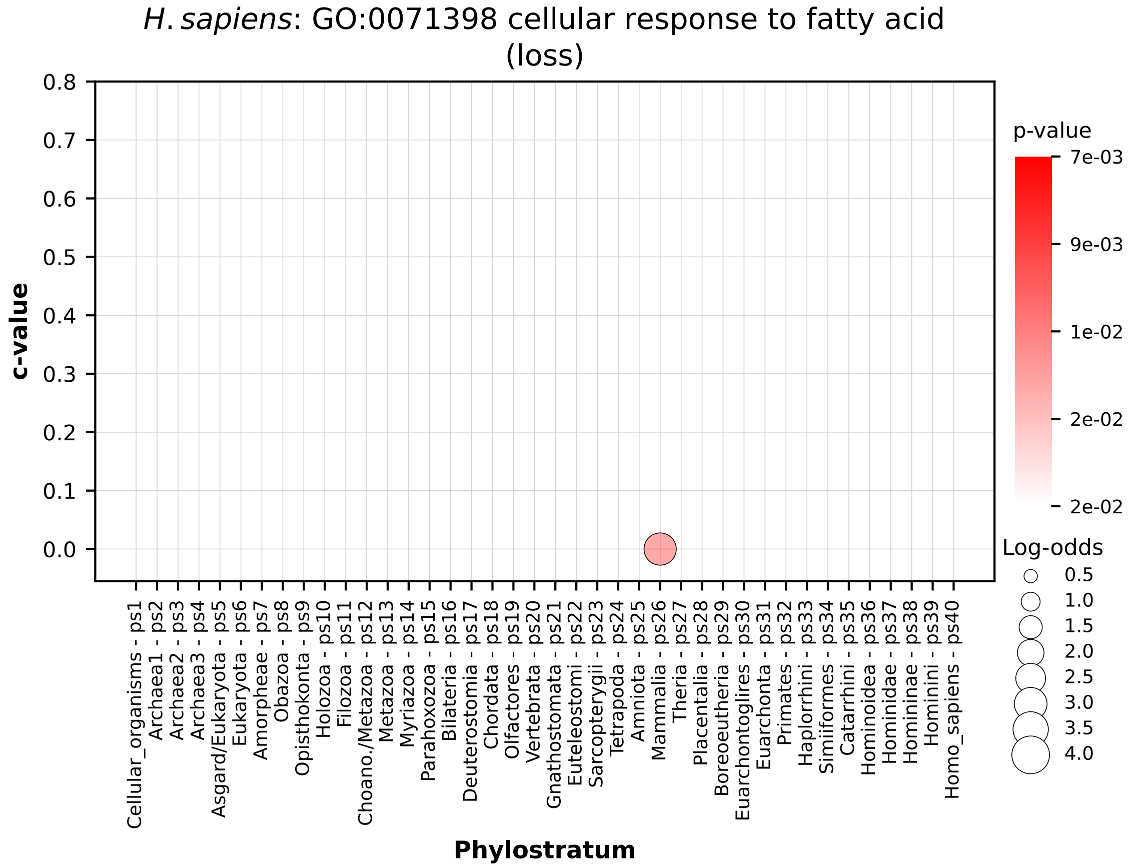
H. sapiens: GO:0071398 cellular response to fatty acid
(loss)
c-value
0.8
0.7
0.6
0.5
0.4
0.3
0.2
0.1
0.0
p-value
7e-03
9e-03
1e-02
2e-02
2e-02
Log-odds
0.5
1.0
1.5
2.0
2.5
3.0
3.5
4.0
Cellular_organisms - ps1
Archaea1 - ps2
Archaea2 - ps3
Archaea3 - ps4
Asgard/Eukaryota - ps5
Eukaryota - ps6
Amorpheae - ps7
Obazoa - ps8
Opisthokonta - ps9
Holozoa - ps10
Filozoa - ps11
Choano./Metazoa - ps12
Metazoa - ps13
Myriazoa - ps14
Parahoxozoa - ps15
Bilateria - ps16
Deuterostomia - ps17
Chordata - ps18
Olfactores - ps19
Vertebrata - ps20
Gnathostomata - ps21
Euteleostomi - ps22
Sarcopterygii - ps23
Tetrapoda - ps24
Amniota - ps25
Mammalia - ps26
Theria - ps27
Placentalia - ps28
Boreoeutheria - ps29
Euarchontoglires - ps30
Euarchonta - ps31
Primates - ps32
Haplorrhini - ps33
Simiiformes - ps34
Catarrhini - ps35
Hominoidea - ps36
Hominidae - ps37
Homininae - ps38
Hominini - ps39
Homo_sapiens - ps40
Phylostratum

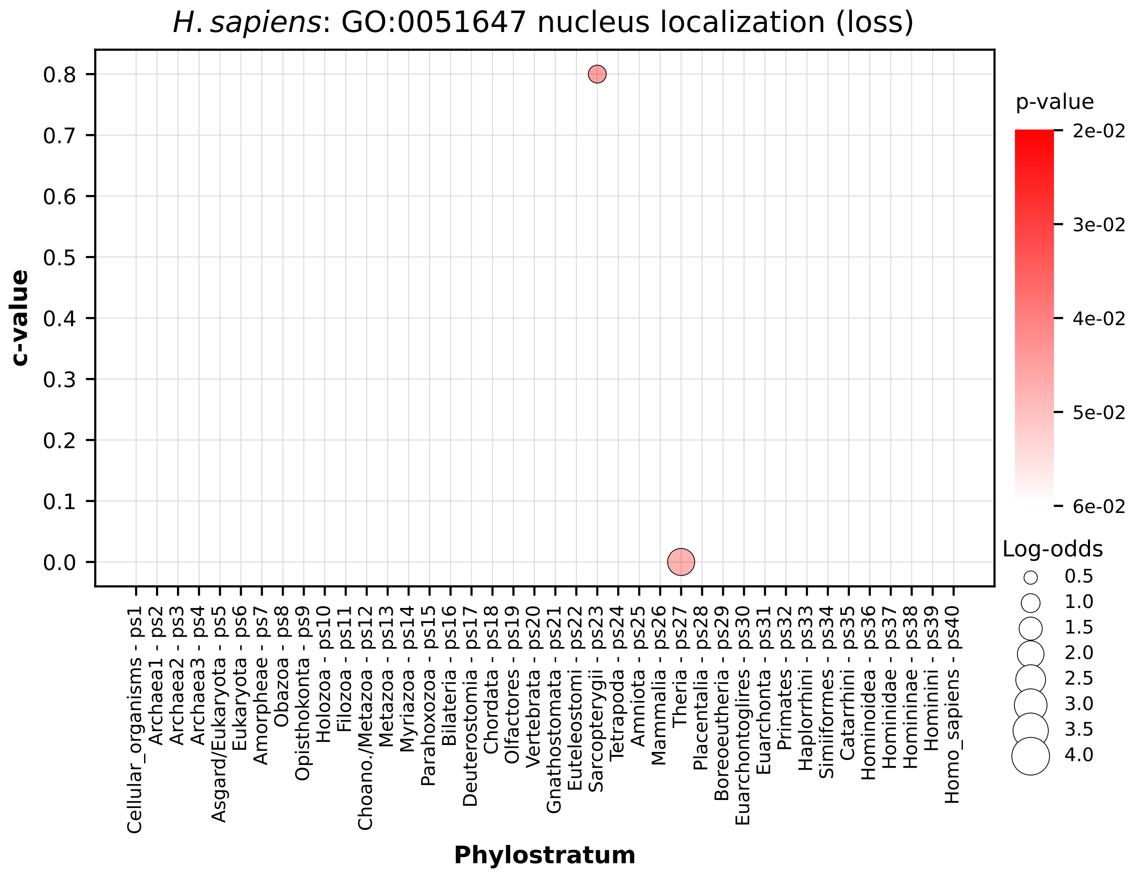

H. sapiens: GO:0051647 nucleus localization (loss)
c-value
0.8
0.7
0.6
0.5
0.4
0.3
0.2
0.1
0.0
Phylostratum
Cellular_organisms - ps1
Archaea1 - ps2
Archaea2 - ps3
Archaea3 - ps4
Asgard/Eukaryota - ps5
Eukaryota - ps6
Amorpheae - ps7
Obazoa - ps8
Opisthokonta - ps9
Holozoa - ps10
Filozoa - ps11
Choano./Metazoa - ps12
Metazoa - ps13
Myriazoa - ps14
Parahoxozoa - ps15
Bilateria - ps16
Deuterostomia - ps17
Chordata - ps18
Olfactores - ps19
Vertebrata - ps20
Gnathostomata - ps21
Euteleostomi - ps22
Sarcopterygii - ps23
Tetrapoda - ps24
Amniota - ps25
Mammalia - ps26
Theria - ps27
Placentalia - ps28
Boreoeutheria - ps29
Euarchontoglires - ps30
Euarchonta - ps31
Primates - ps32
Haplorrhini - ps33
Simiiformes - ps34
Catarrhini - ps35
Hominoidea - ps36
Hominidae - ps37
Homininae - ps38
Hominini - ps39
Homo_sapiens - ps40
p-value
2e-02
3e-02
4e-02
5e-02
6e-02
Log-odds
0.5
1.0
1.5
2.0
2.5
3.0
3.5
4.0

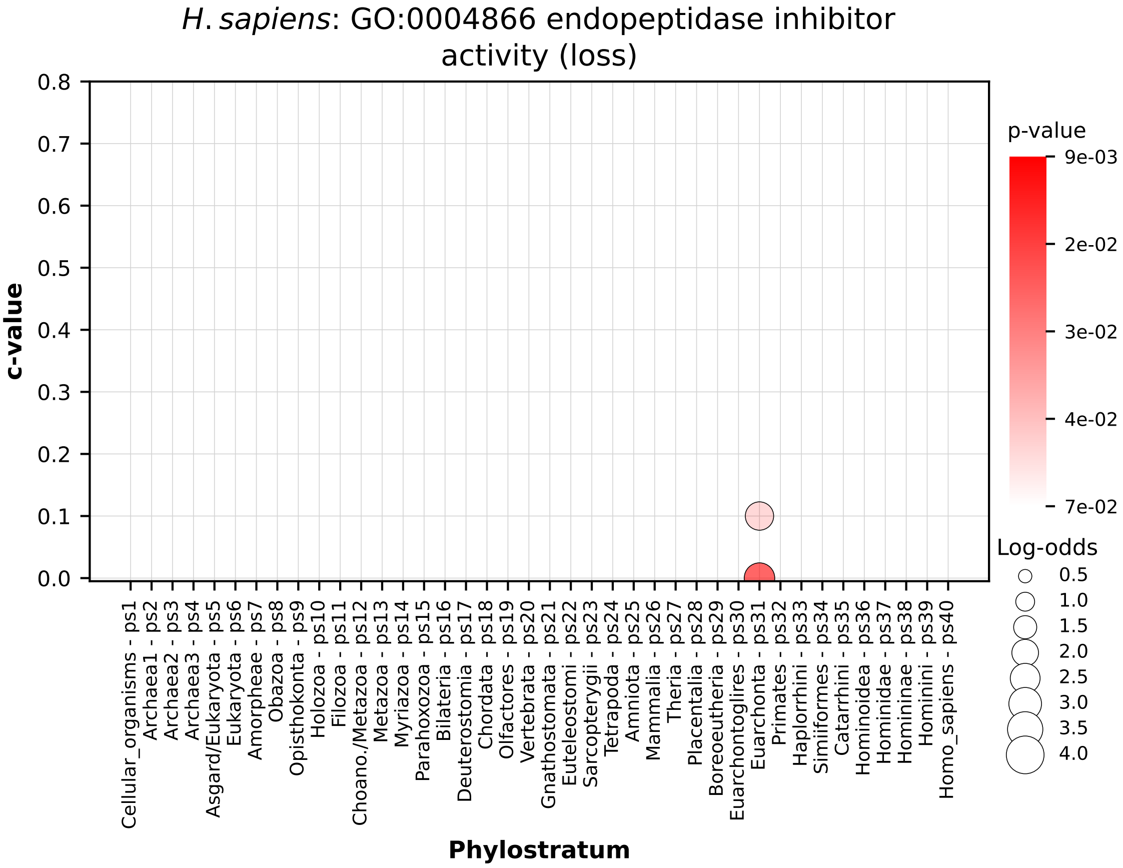

H. sapiens: GO:0004866 endopeptidase inhibitor activity (loss)
c-value
0.8
0.7
0.6
0.5
0.4
0.3
0.2
0.1
0.0
p-value
9e-03
2e-02
3e-02
4e-02
7e-02
Log-odds
0.5
1.0
1.5
2.0
2.5
3.0
3.5
4.0
Cellular_organisms - ps1
Archaea1 - ps2
Archaea2 - ps3
Archaea3 - ps4
Asgard/Eukaryota - ps5
Eukaryota - ps6
Amorpheae - ps7
Obazoa - ps8
Opisthokonta - ps9
Holozoa - ps10
Filozoa - ps11
Choano./Metazoa - ps12
Metazoa - ps13
Myriazoa - ps14
Parahoxozoa - ps15
Bilateria - ps16
Deuterostomia - ps17
Chordata - ps18
Olfactores - ps19
Vertebrata - ps20
Gnathostomata - ps21
Euteleostomi - ps22
Sarcopterygii - ps23
Tetrapoda - ps24
Amniota - ps25
Mammalia - ps26
Theria - ps27
Placentalia - ps28
Boreoeutheria - ps29
Euarchontoglires - ps30
Euarchonta - ps31
Primates - ps32
Haplorrhini - ps33
Simiiformes - ps34
Catarrhini - ps35
Hominoidea - ps36
Hominidae - ps37
Homininae - ps38
Hominini - ps39
Homo_sapiens - ps40
Phylostratum

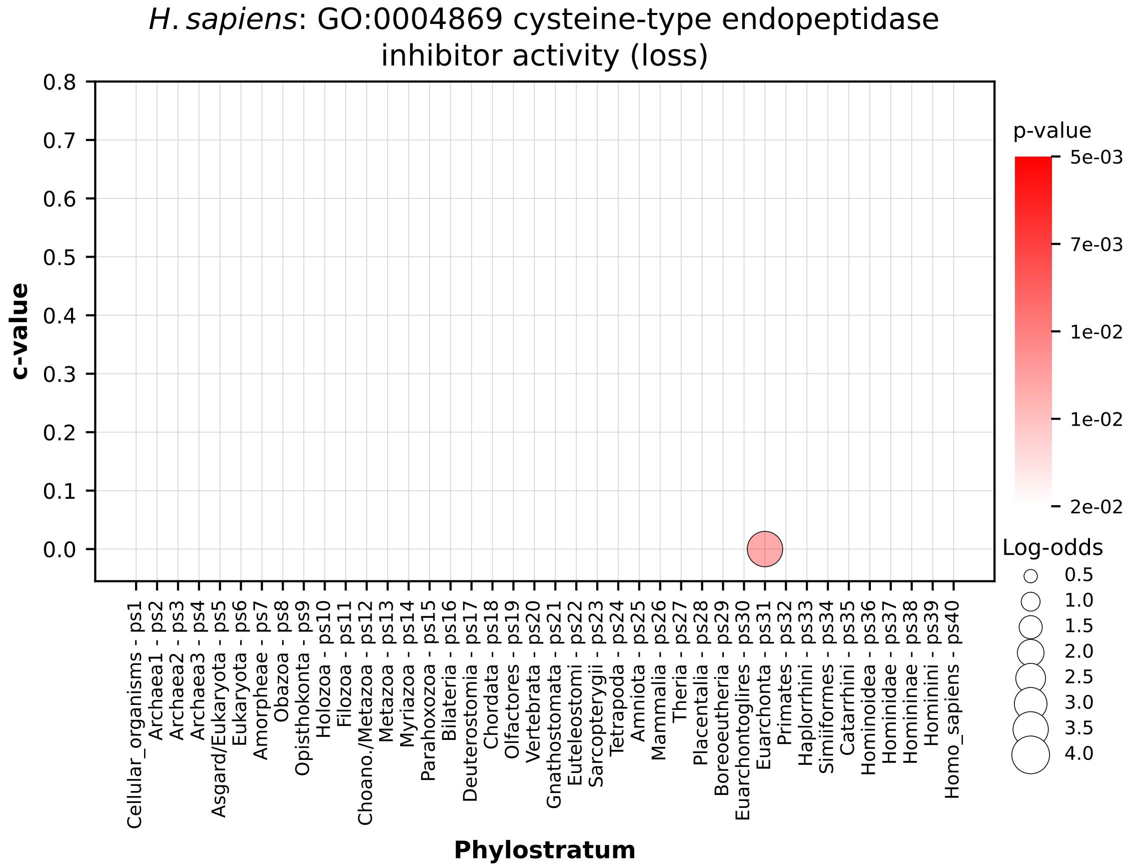

H. sapiens: GO:0004869 cysteine-type endopeptidase inhibitor activity (loss)
c-value
0.8
0.7
0.6
0.5
0.4
0.3
0.2
0.1
0.0
p-value
5e-03
7e-03
1e-02
1e-02
2e-02
Log-odds
0.5
1.0
1.5
2.0
2.5
3.0
3.5
4.0
Cellular_organisms - ps1
Archaea1 - ps2
Archaea2 - ps3
Archaea3 - ps4
Asgard/Eukaryota - ps5
Eukaryota - ps6
Amorpheae - ps7
Obazoa - ps8
Opisthokonta - ps9
Holozoa - ps10
Filozoa - ps11
Choano./Metazoa - ps12
Metazoa - ps13
Myriazoa - ps14
Parahoxozoa - ps15
Bilateria - ps16
Deuterostomia - ps17
Chordata - ps18
Olfactores - ps19
Vertebrata - ps20
Gnathostomata - ps21
Euteleostomi - ps22
Sarcopterygii - ps23
Tetrapoda - ps24
Amniota - ps25
Mammalia - ps26
Theria - ps27
Placentalia - ps28
Boreoeutheria - ps29
Euarchontoglires - ps30
Euarchonta - ps31
Primates - ps32
Haplorrhini - ps33
Simiiformes - ps34
Catarrhini - ps35
Hominoidea - ps36
Hominidae - ps37
Homininae - ps38
Hominini - ps39
Homo_sapiens - ps40
Phylostratum

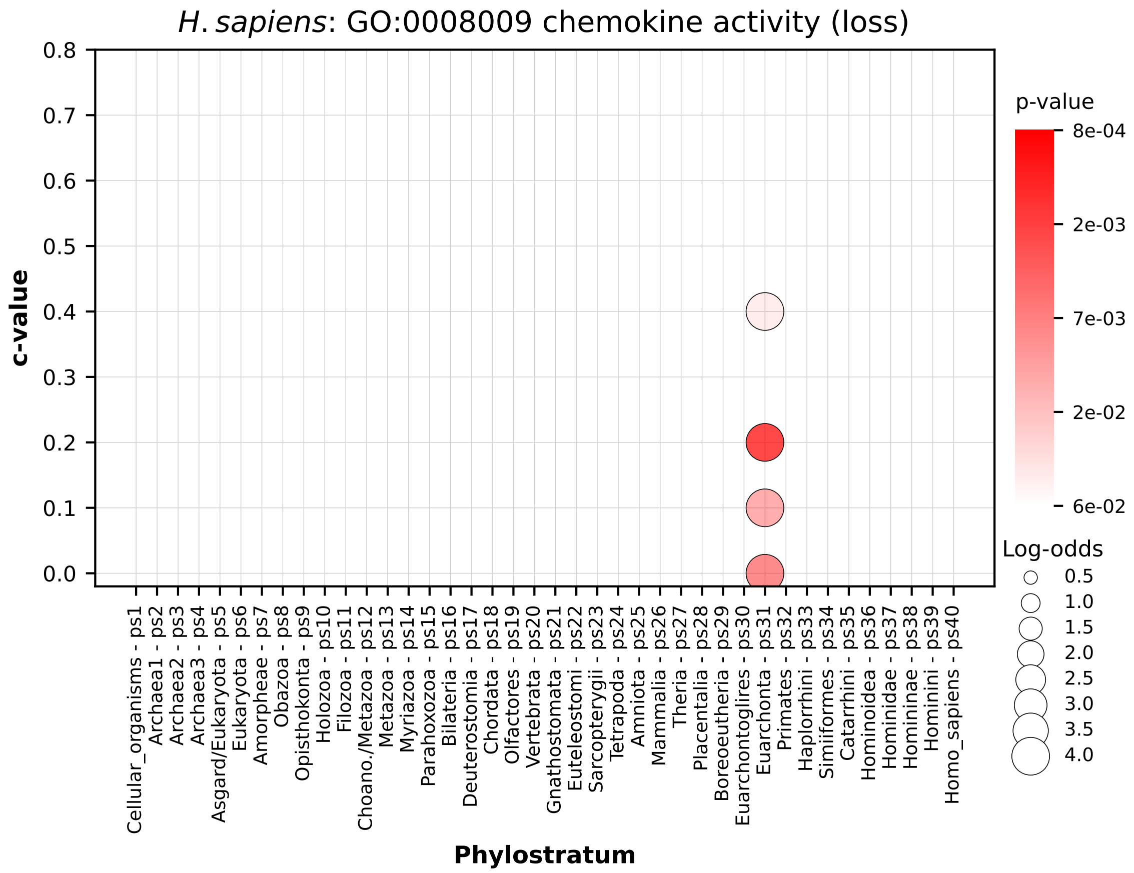

H. sapiens: GO:0008009 chemokine activity (loss)
c-value
0.8
0.7
0.6
0.5
0.4
0.3
0.2
0.1
0.0
Phylostratum
Cellular_organisms - ps1
Archaea1 - ps2
Archaea2 - ps3
Archaea3 - ps4
Asgard/Eukaryota - ps5
Eukaryota - ps6
Amorpheae - ps7
Obazoa - ps8
Opisthokonta - ps9
Holozoa - ps10
Filozoa - ps11
Choano./Metazoa - ps12
Metazoa - ps13
Myriazoa - ps14
Parahoxozoa - ps15
Bilateria - ps16
Deuterostomia - ps17
Chordata - ps18
Olfactores - ps19
Vertebrata - ps20
Gnathostomata - ps21
Euteleostomi - ps22
Sarcopterygii - ps23
Tetrapoda - ps24
Amniota - ps25
Mammalia - ps26
Theria - ps27
Placentalia - ps28
Boreoeutheria - ps29
Euarchontoglires - ps30
Euarchonta - ps31
Primates - ps32
Haplorrhini - ps33
Simiiformes - ps34
Catarrhini - ps35
Hominoidea - ps36
Hominidae - ps37
Homininae - ps38
Hominini - ps39
Homo_sapiens - ps40
p-value
8e-04
2e-03
7e-03
2e-02
6e-02
Log-odds
0.5
1.0
1.5
2.0
2.5
3.0
3.5
4.0

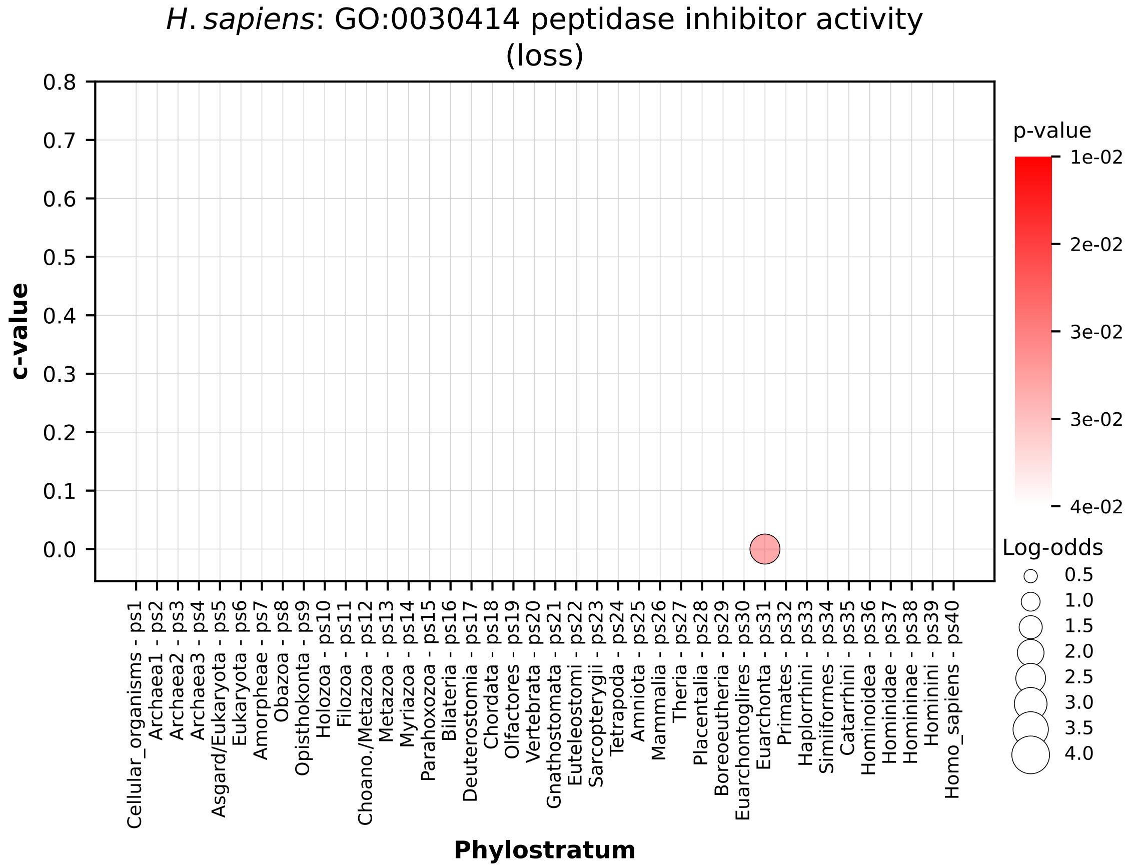

H. sapiens: GO:0030414 peptidase inhibitor activity (loss)
c-value
0.8
0.7
0.6
0.5
0.4
0.3
0.2
0.1
0.0
p-value
1e-02
2e-02
3e-02
3e-02
4e-02
Log-odds
0.5
1.0
1.5
2.0
2.5
3.0
3.5
4.0
Cellular_organisms - ps1
Archaea1 - ps2
Archaea2 - ps3
Archaea3 - ps4
Asgard/Eukaryota - ps5
Eukaryota - ps6
Amorpheae - ps7
Obazoa - ps8
Opisthokonta - ps9
Holozoa - ps10
Filozoa - ps11
Choano./Metazoa - ps12
Metazoa - ps13
Myriazoa - ps14
Parahoxozoa - ps15
Bilateria - ps16
Deuterostomia - ps17
Chordata - ps18
Olfactores - ps19
Vertebrata - ps20
Gnathostomata - ps21
Euteleostomi - ps22
Sarcopterygii - ps23
Tetrapoda - ps24
Amniota - ps25
Mammalia - ps26
Theria - ps27
Placentalia - ps28
Boreoeutheria - ps29
Euarchontoglires - ps30
Euarchonta - ps31
Primates - ps32
Haplorrhini - ps33
Simiiformes - ps34
Catarrhini - ps35
Hominoidea - ps36
Hominidae - ps37
Homininae - ps38
Hominini - ps39
Homo_sapiens - ps40
Phylostratum

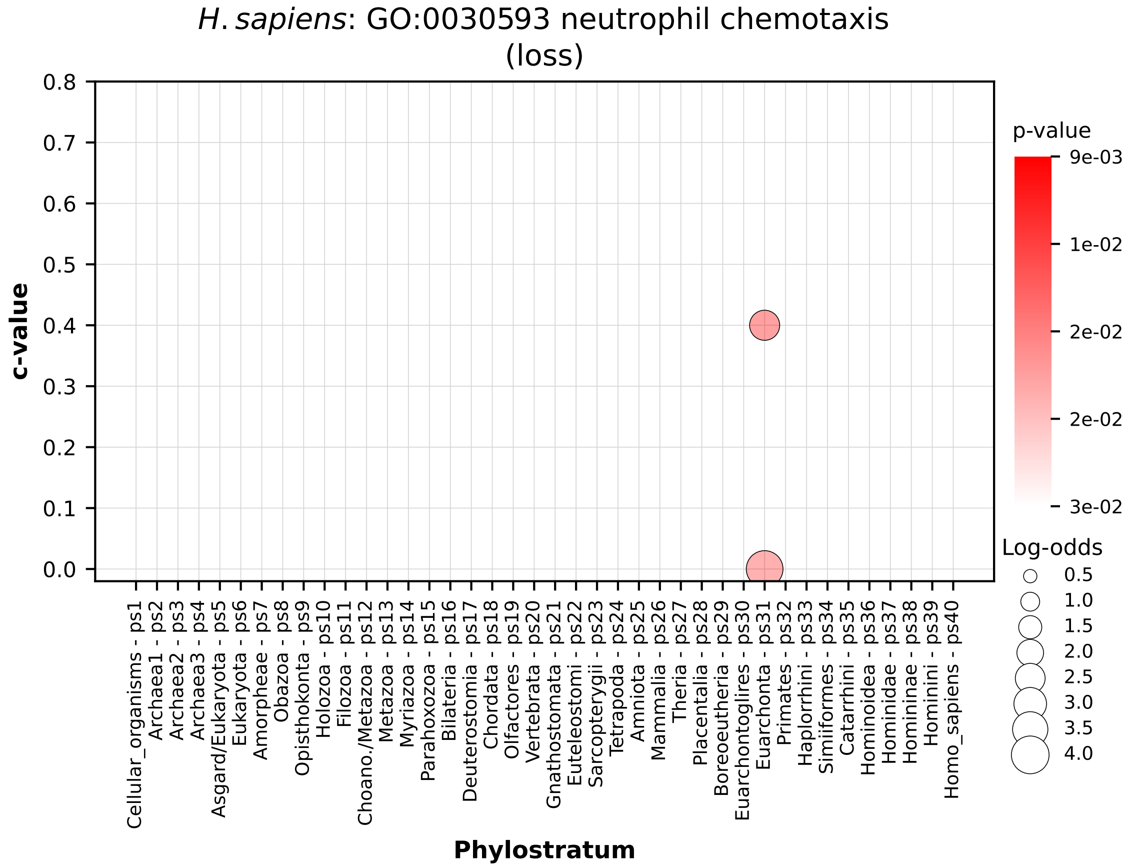

H. sapiens: GO:0030593 neutrophil chemotaxis
(loss)
c-value
0.8
0.7
0.6
0.5
0.4
0.3
0.2
0.1
0.0
p-value
9e-03
1e-02
2e-02
2e-02
3e-02
Log-odds
0.5
1.0
1.5
2.0
2.5
3.0
3.5
4.0
Cellular_organisms - ps1
Archaea1 - ps2
Archaea2 - ps3
Archaea3 - ps4
Asgard/Eukaryota - ps5
Eukaryota - ps6
Amorpheae - ps7
Obazoa - ps8
Opisthokonta - ps9
Holozoa - ps10
Filozoa - ps11
Choano./Metazoa - ps12
Metazoa - ps13
Myriazoa - ps14
Parahoxozoa - ps15
Bilateria - ps16
Deuterostomia - ps17
Chordata - ps18
Olfactores - ps19
Vertebrata - ps20
Gnathostomata - ps21
Euteleostomi - ps22
Sarcopterygii - ps23
Tetrapoda - ps24
Amniota - ps25
Mammalia - ps26
Theria - ps27
Placentalia - ps28
Boreoeutheria - ps29
Euarchontoglires - ps30
Euarchonta - ps31
Primates - ps32
Haplorrhini - ps33
Simiiformes - ps34
Catarrhini - ps35
Hominoidea - ps36
Hominidae - ps37
Homininae - ps38
Hominini - ps39
Homo_sapiens - ps40
Phylostratum

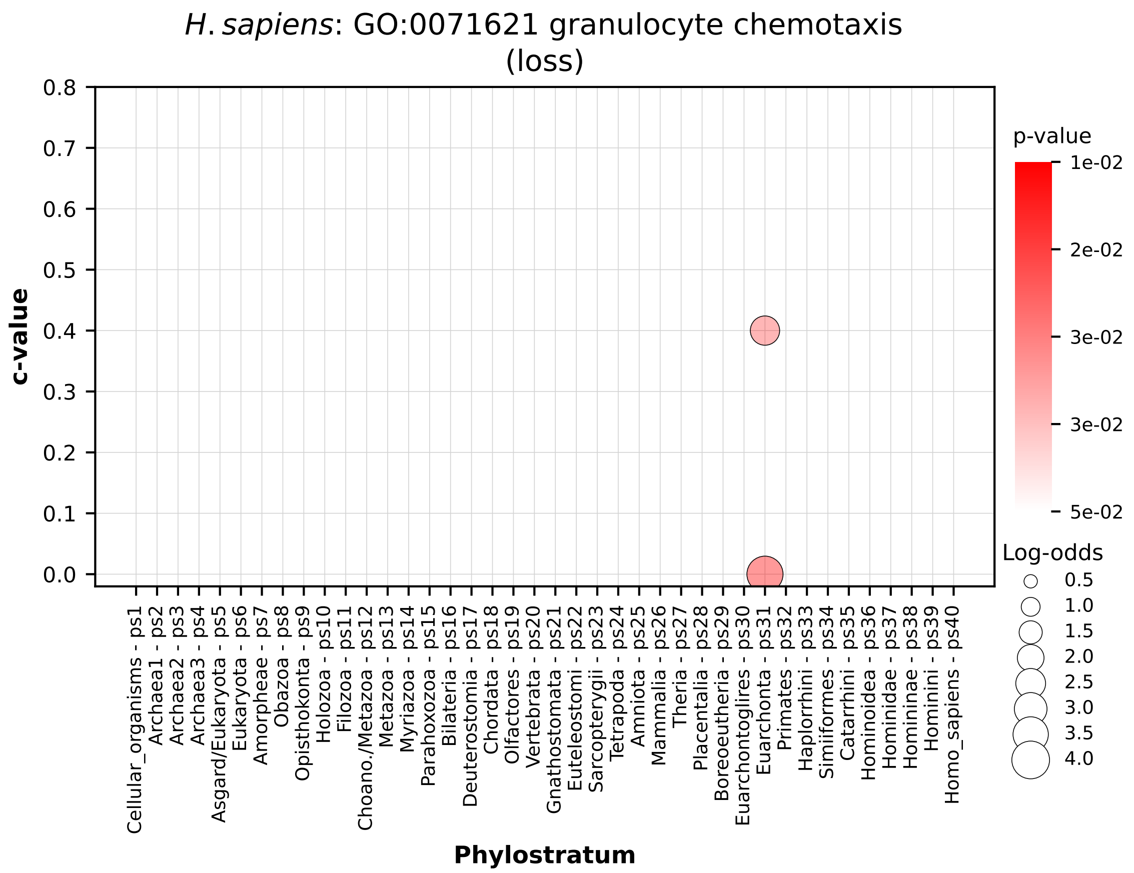

H. sapiens: GO:0071621 granulocyte chemotaxis
(loss)
c-value
0.8
0.7
0.6
0.5
0.4
0.3
0.2
0.1
0.0
p-value
1e-02
2e-02
3e-02
3e-02
5e-02
Log-odds
0.5
1.0
1.5
2.0
2.5
3.0
3.5
4.0
Cellular_organisms - ps1
Archaea1 - ps2
Archaea2 - ps3
Archaea3 - ps4
Asgard/Eukaryota - ps5
Eukaryota - ps6
Amorpheae - ps7
Obazoa - ps8
Opisthokonta - ps9
Holozoa - ps10
Filozoa - ps11
Choano./Metazoa - ps12
Metazoa - ps13
Myriazoa - ps14
Parahoxozoa - ps15
Bilateria - ps16
Deuterostomia - ps17
Chordata - ps18
Olfactores - ps19
Vertebrata - ps20
Gnathostomata - ps21
Euteleostomi - ps22
Sarcopterygii - ps23
Tetrapoda - ps24
Amniota - ps25
Mammalia - ps26
Theria - ps27
Placentalia - ps28
Boreoeutheria - ps29
Euarchontoglires - ps30
Euarchonta - ps31
Primates - ps32
Haplorrhini - ps33
Simiiformes - ps34
Catarrhini - ps35
Hominoidea - ps36
Hominidae - ps37
Homininae - ps38
Hominini - ps39
Homo_sapiens - ps40
Phylostratum

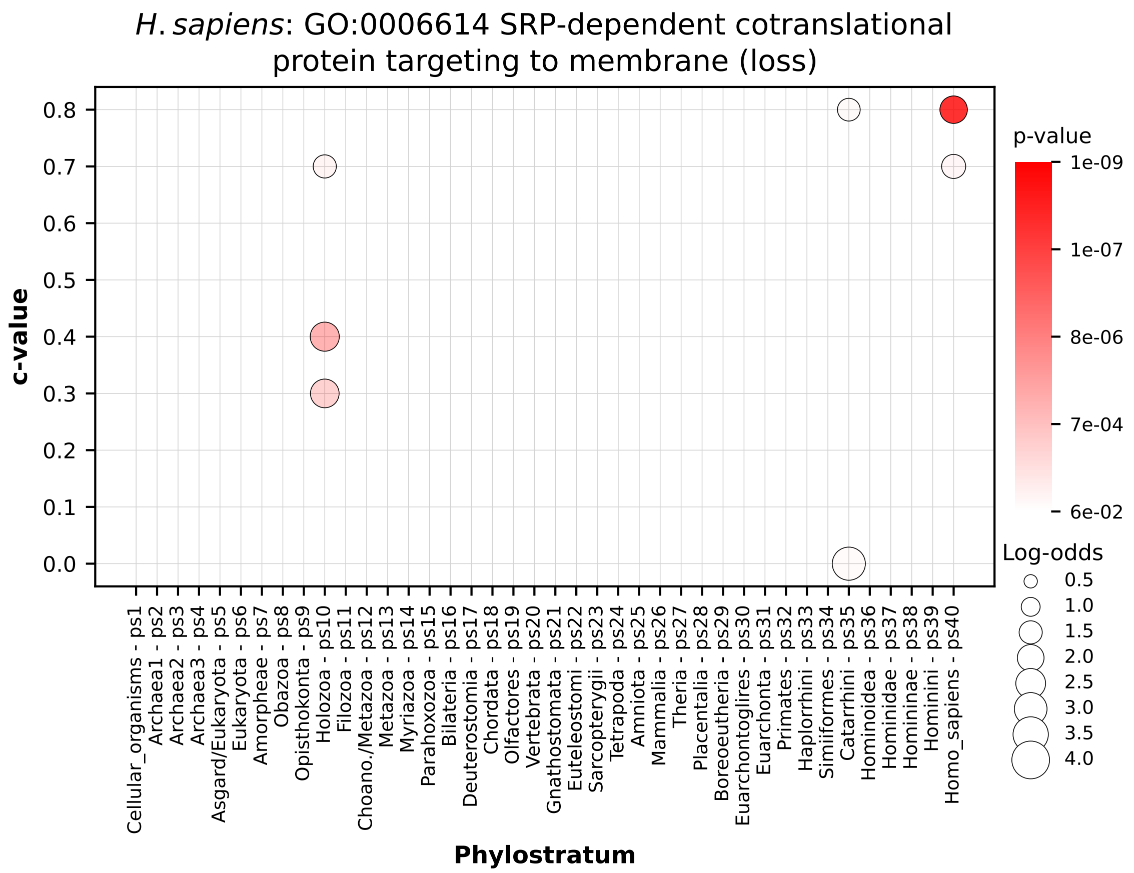
H. sapiens: GO:0006614 SRP-dependent cotranslational
protein targeting to membrane (loss)
c-value
0.8
0.7
0.6
0.5
0.4
0.3
0.2
0.1
0.0
p-value
1e-09
1e-07
8e-06
7e-04
6e-02
Log-odds
0.5
1.0
1.5
2.0
2.5
3.0
3.5
4.0
Cellular_organisms - ps1
Archaea1 - ps2
Archaea2 - ps3
Archaea3 - ps4
Asgard/Eukaryota - ps5
Eukaryota - ps6
Amorpheae - ps7
Obazoa - ps8
Opisthokonta - ps9
Holozoa - ps10
Filozoa - ps11
Choano./Metazoa - ps12
Metazoa - ps13
Myriazoa - ps14
Parahoxozoa - ps15
Bilateria - ps16
Deuterostomia - ps17
Chordata - ps18
Olfactores - ps19
Vertebrata - ps20
Gnathostomata - ps21
Euteleostomi - ps22
Sarcopterygii - ps23
Tetrapoda - ps24
Amniota - ps25
Mammalia - ps26
Theria - ps27
Placentalia - ps28
Boreoeutheria - ps29
Euarchontoglires - ps30
Euarchonta - ps31
Primates - ps32
Haplorrhini - ps33
Simiiformes - ps34
Catarrhini - ps35
Hominoidea - ps36
Hominidae - ps37
Homininae - ps38
Hominini - ps39
Homo_sapiens - ps40
Phylostratum

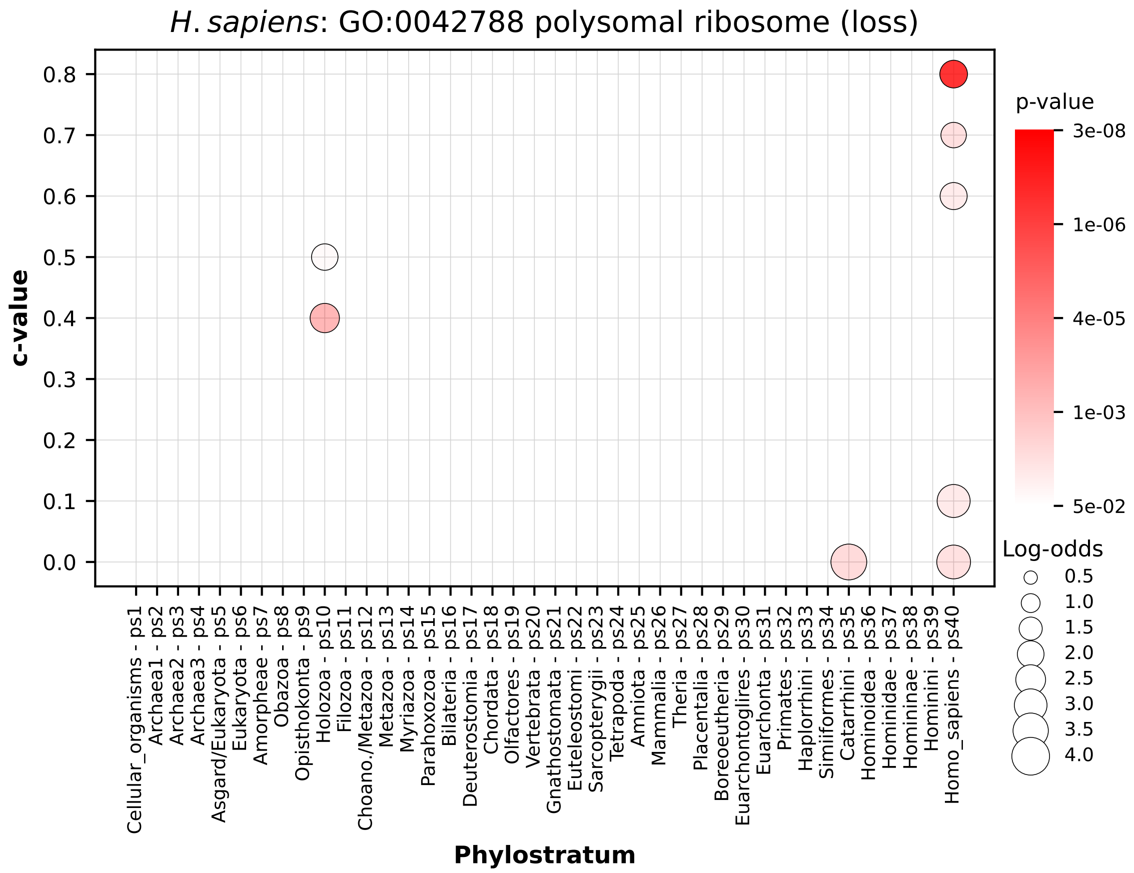

H. sapiens: GO:0042788 polysomal ribosome (loss)
c-value
0.0
0.1
0.2
0.3
0.4
0.5
0.6
0.7
0.8
Phylostratum
Cellular_organisms - ps1
Archaea1 - ps2
Archaea2 - ps3
Archaea3 - ps4
Asgard/Eukaryota - ps5
Eukaryota - ps6
Amorpheae - ps7
Obazoa - ps8
Opisthokonta - ps9
Holozoa - ps10
Filozoa - ps11
Choano./Metazoa - ps12
Metazoa - ps13
Myriazoa - ps14
Parahoxozoa - ps15
Bilateria - ps16
Deuterostomia - ps17
Chordata - ps18
Olfactores - ps19
Vertebrata - ps20
Gnathostomata - ps21
Euteleostomi - ps22
Sarcopterygii - ps23
Tetrapoda - ps24
Amniota - ps25
Mammalia - ps26
Theria - ps27
Placentalia - ps28
Boreoeutheria - ps29
Euarchontoglires - ps30
Euarchonta - ps31
Primates - ps32
Haplorrhini - ps33
Simiiformes - ps34
Catarrhini - ps35
Hominoidea - ps36
Hominidae - ps37
Homininae - ps38
Hominini - ps39
Homo_sapiens - ps40
p-value
3e-08
1e-06
4e-05
1e-03
5e-02
Log-odds
0.5
1.0
1.5
2.0
2.5
3.0
3.5
4.0

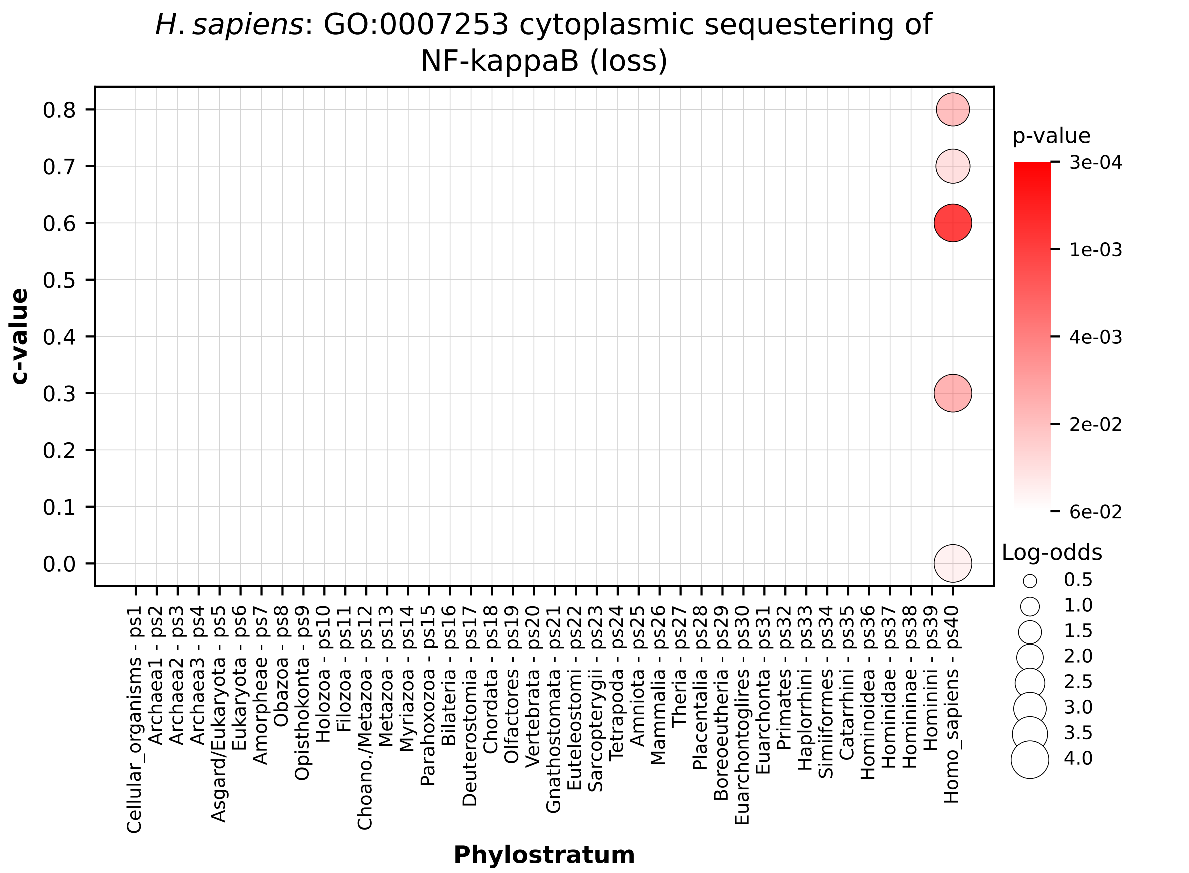

H. sapiens: GO:0007253 cytoplasmic sequestering of NF-kappaB (loss)
c-value
0.8
0.7
0.6
0.5
0.4
0.3
0.2
0.1
0.0
Phylostratum
Cellular_organisms - ps1
Archaea1 - ps2
Archaea2 - ps3
Archaea3 - ps4
Asgard/Eukaryota - ps5
Eukaryota - ps6
Amorpheae - ps7
Obazoa - ps8
Opisthokonta - ps9
Holozoa - ps10
Filozoa - ps11
Choano./Metazoa - ps12
Metazoa - ps13
Myriazoa - ps14
Parahoxozoa - ps15
Bilateria - ps16
Deuterostomia - ps17
Chordata - ps18
Olfactores - ps19
Vertebrata - ps20
Gnathostomata - ps21
Euteleostomi - ps22
Sarcopterygii - ps23
Tetrapoda - ps24
Amniota - ps25
Mammalia - ps26
Theria - ps27
Placentalia - ps28
Boreoeutheria - ps29
Euarchontoglires - ps30
Euarchonta - ps31
Primates - ps32
Haplorrhini - ps33
Simiiformes - ps34
Catarrhini - ps35
Hominoidea - ps36
Hominidae - ps37
Homininae - ps38
Hominini - ps39
Homo_sapiens - ps40
p-value
3e-04
1e-03
4e-03
2e-02
6e-02
Log-odds
0.5
1.0
1.5
2.0
2.5
3.0
3.5
4.0

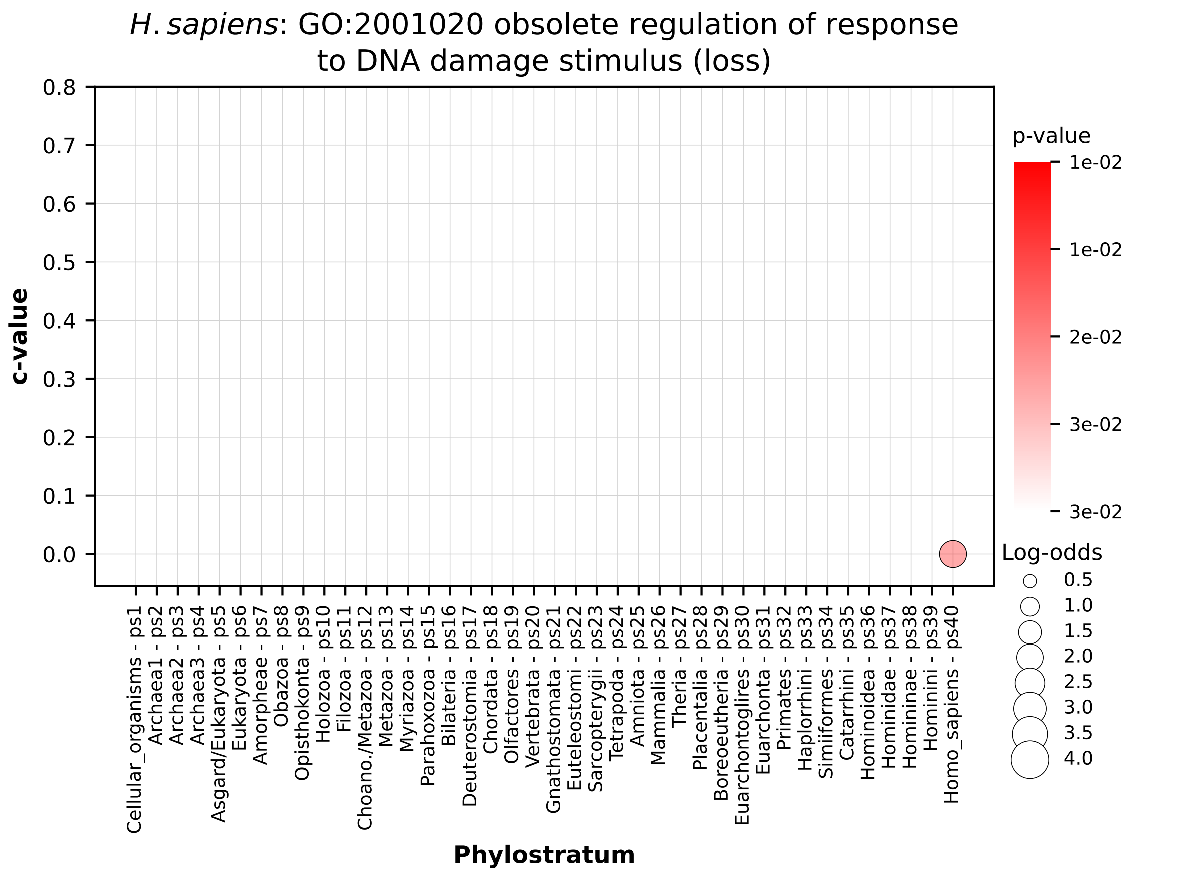
H. sapiens: GO:2001020 obsolete regulation of response to DNA damage stimulus (loss)
c-value
0.8
0.7
0.6
0.5
0.4
0.3
0.2
0.1
0.0
Phylostratum
Cellular_organisms - ps1
Archaea1 - ps2
Archaea2 - ps3
Archaea3 - ps4
Asgard/Eukaryota - ps5
Eukaryota - ps6
Amorpheae - ps7
Obazoa - ps8
Opisthokonta - ps9
Holozoa - ps10
Filozoa - ps11
Choano./Metazoa - ps12
Metazoa - ps13
Myriazoa - ps14
Parahoxozoa - ps15
Bilateria - ps16
Deuterostomia - ps17
Chordata - ps18
Olfactores - ps19
Vertebrata - ps20
Gnathostomata - ps21
Euteleostomi - ps22
Sarcopterygii - ps23
Tetrapoda - ps24
Amniota - ps25
Mammalia - ps26
Theria - ps27
Placentalia - ps28
Boreoeutheria - ps29
Euarchontoglires - ps30
Euarchonta - ps31
Primates - ps32
Haplorrhini - ps33
Simiiformes - ps34
Catarrhini - ps35
Hominoidea - ps36
Hominidae - ps37
Homininae - ps38
Hominini - ps39
Homo_sapiens - ps40
p-value
1e-02
1e-02
2e-02
3e-02
3e-02
Log-odds
0.5
1.0
1.5
2.0
2.5
3.0
3.5
4.0

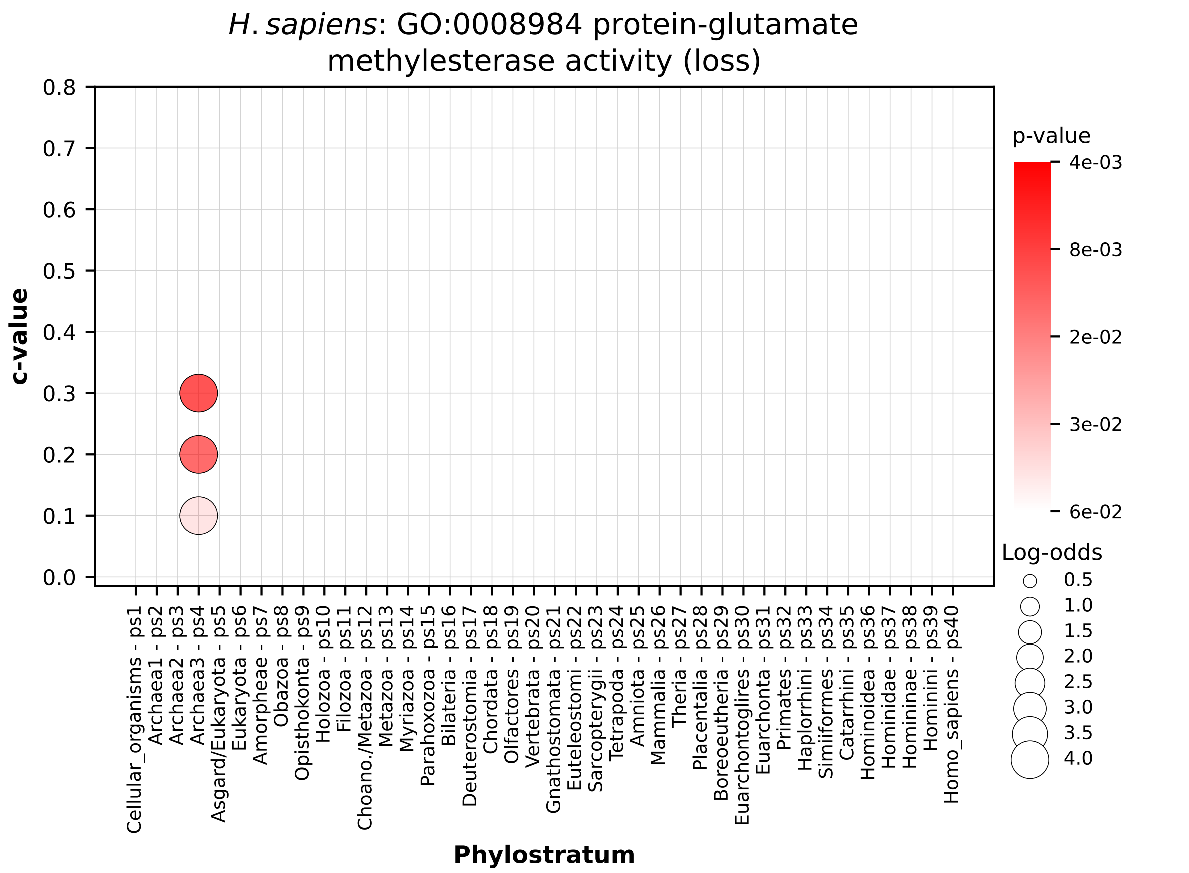

H. sapiens: GO:0008984 protein-glutamate methylesterase activity (loss)
c-value
0.0
0.1
0.2
0.3
0.4
0.5
0.6
0.7
0.8
Phylostratum
Cellular_organisms - ps1
Archaea1 - ps2
Archaea2 - ps3
Archaea3 - ps4
Asgard/Eukaryota - ps5
Eukaryota - ps6
Amorpheae - ps7
Obazoa - ps8
Opisthokonta - ps9
Holozoa - ps10
Filozoa - ps11
Choano./Metazoa - ps12
Metazoa - ps13
Myriazoa - ps14
Parahoxozoa - ps15
Bilateria - ps16
Deuterostomia - ps17
Chordata - ps18
Olfactores - ps19
Vertebrata - ps20
Gnathostomata - ps21
Euteleostomi - ps22
Sarcopterygii - ps23
Tetrapoda - ps24
Amniota - ps25
Mammalia - ps26
Theria - ps27
Placentalia - ps28
Boreoeutheria - ps29
Euarchontoglires - ps30
Euarchonta - ps31
Primates - ps32
Haplorrhini - ps33
Simiiformes - ps34
Catarrhini - ps35
Hominoidea - ps36
Hominidae - ps37
Homininae - ps38
Hominini - ps39
Homo_sapiens - ps40
p-value
4e-03
8e-03
2e-02
3e-02
6e-02
Log-odds
0.5
1.0
1.5
2.0
2.5
3.0
3.5
4.0

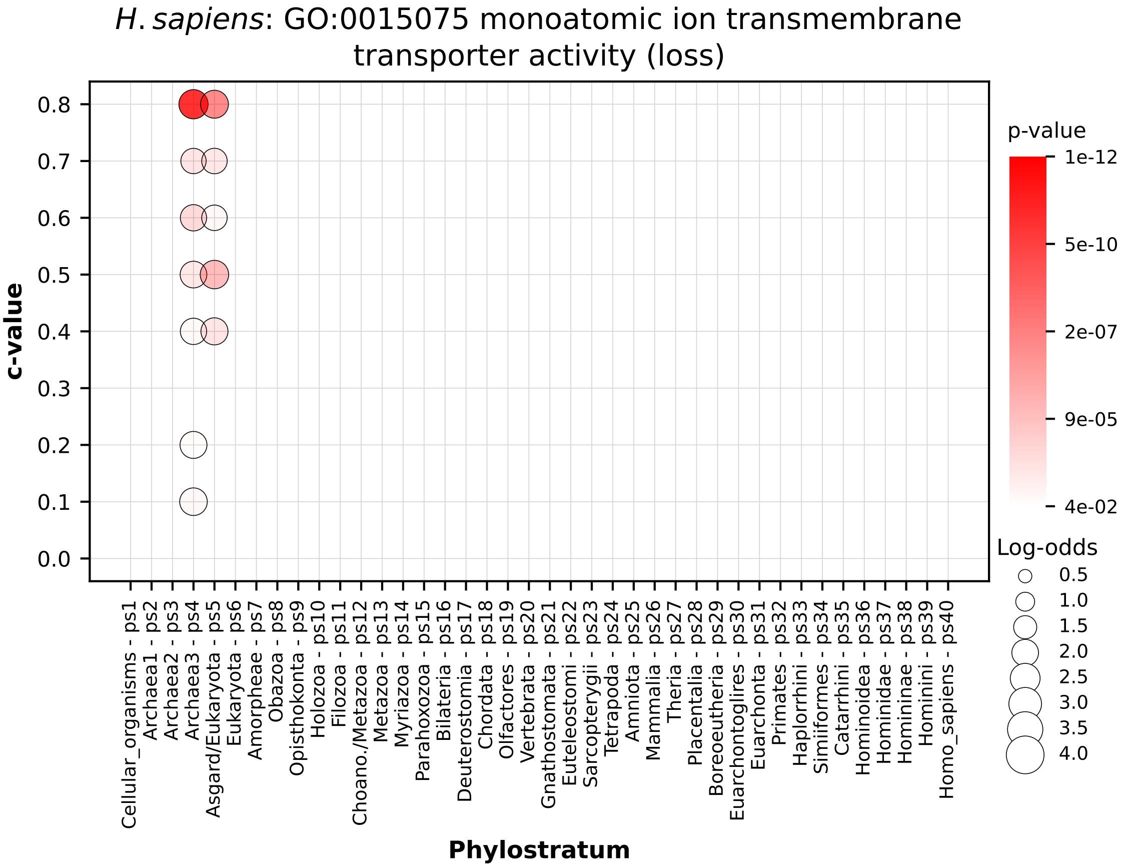

H. sapiens: GO:0015075 monoatomic ion transmembrane transporter activity (loss)
c-value
0.8
0.7
0.6
0.5
0.4
0.3
0.2
0.1
0.0
p-value
1e-12
5e-10
2e-07
9e-05
4e-02
Log-odds
0.5
1.0
1.5
2.0
2.5
3.0
3.5
4.0
Cellular_organisms - ps1
Archaea1 - ps2
Archaea2 - ps3
Archaea3 - ps4
Asgard/Eukaryota - ps5
Eukaryota - ps6
Amorpheae - ps7
Obazoa - ps8
Opisthokonta - ps9
Holozoa - ps10
Filozoa - ps11
Choano./Metazoa - ps12
Metazoa - ps13
Myriazoa - ps14
Parahoxozoa - ps15
Bilateria - ps16
Deuterostomia - ps17
Chordata - ps18
Olfactores - ps19
Vertebrata - ps20
Gnathostomata - ps21
Euteleostomi - ps22
Sarcopterygii - ps23
Tetrapoda - ps24
Amniota - ps25
Mammalia - ps26
Theria - ps27
Placentalia - ps28
Boreoeutheria - ps29
Euarchontoglires - ps30
Euarchonta - ps31
Primates - ps32
Haplorrhini - ps33
Simiiformes - ps34
Catarrhini - ps35
Hominoidea - ps36
Hominidae - ps37
Homininae - ps38
Hominini - ps39
Homo_sapiens - ps40
Phylostratum

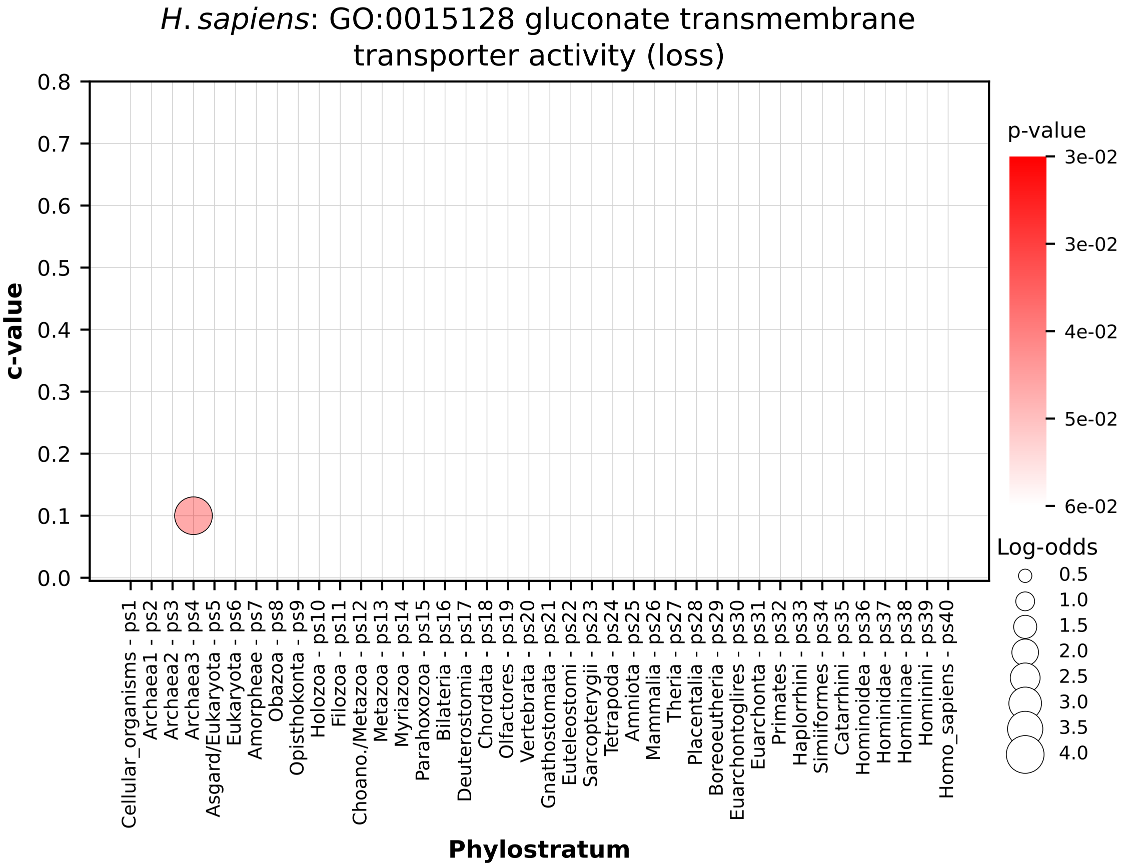

H. sapiens: GO:0015128 gluconate transmembrane transporter activity (loss)
c-value
0.0
0.1
0.2
0.3
0.4
0.5
0.6
0.7
0.8
Phylostratum
Cellular_organisms - ps1
Archaea1 - ps2
Archaea2 - ps3
Archaea3 - ps4
Asgard/Eukaryota - ps5
Eukaryota - ps6
Amorpheae - ps7
Obazoa - ps8
Opisthokonta - ps9
Holozoa - ps10
Filozoa - ps11
Choano./Metazoa - ps12
Metazoa - ps13
Myriazoa - ps14
Parahoxozoa - ps15
Bilateria - ps16
Deuterostomia - ps17
Chordata - ps18
Olfactores - ps19
Vertebrata - ps20
Gnathostomata - ps21
Euteleostomi - ps22
Sarcopterygii - ps23
Tetrapoda - ps24
Amniota - ps25
Mammalia - ps26
Theria - ps27
Placentalia - ps28
Boreoeutheria - ps29
Euarchontoglires - ps30
Euarchonta - ps31
Primates - ps32
Haplorrhini - ps33
Simiiformes - ps34
Catarrhini - ps35
Hominoidea - ps36
Hominidae - ps37
Homininae - ps38
Hominini - ps39
Homo_sapiens - ps40
p-value
3e-02
3e-02
4e-02
5e-02
6e-02
Log-odds
0.5
1.0
1.5
2.0
2.5
3.0
3.5
4.0

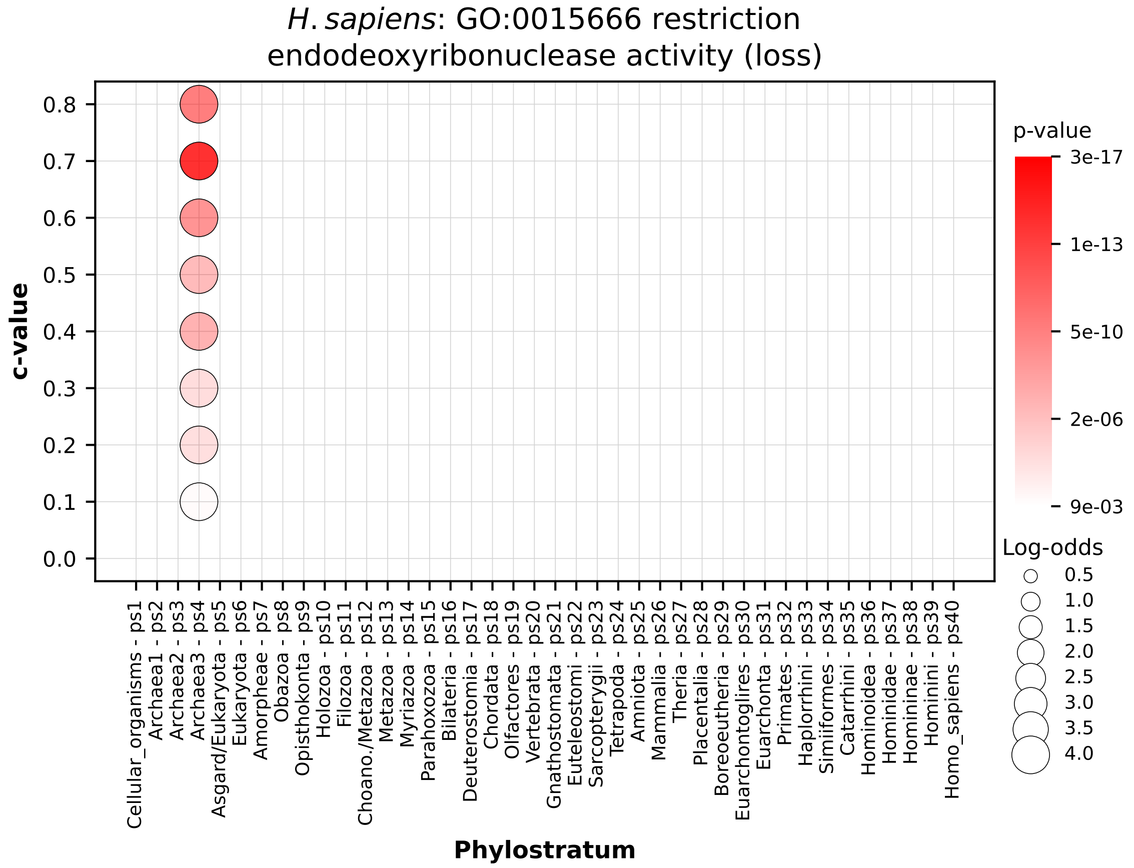

H. sapiens: GO:0015666 restriction endodeoxyribonuclease activity (loss)
c-value
0.0
0.1
0.2
0.3
0.4
0.5
0.6
0.7
0.8
Phylostratum
Cellular_organisms - ps1
Archaea1 - ps2
Archaea2 - ps3
Archaea3 - ps4
Asgard/Eukaryota - ps5
Eukaryota - ps6
Amorpheae - ps7
Obazoa - ps8
Opisthokonta - ps9
Holozoa - ps10
Filozoa - ps11
Choano./Metazoa - ps12
Metazoa - ps13
Myriazoa - ps14
Parahoxozoa - ps15
Bilateria - ps16
Deuterostomia - ps17
Chordata - ps18
Olfactores - ps19
Vertebrata - ps20
Gnathostomata - ps21
Euteleostomi - ps22
Sarcopterygii - ps23
Tetrapoda - ps24
Amniota - ps25
Mammalia - ps26
Theria - ps27
Placentalia - ps28
Boreoeutheria - ps29
Euarchontoglires - ps30
Euarchonta - ps31
Primates - ps32
Haplorrhini - ps33
Simiiformes - ps34
Catarrhini - ps35
Hominoidea - ps36
Hominidae - ps37
Homininae - ps38
Hominini - ps39
Homo_sapiens - ps40
p-value
3e-17
1e-13
5e-10
2e-06
9e-03
Log-odds
0.5
1.0
1.5
2.0
2.5
3.0
3.5
4.0

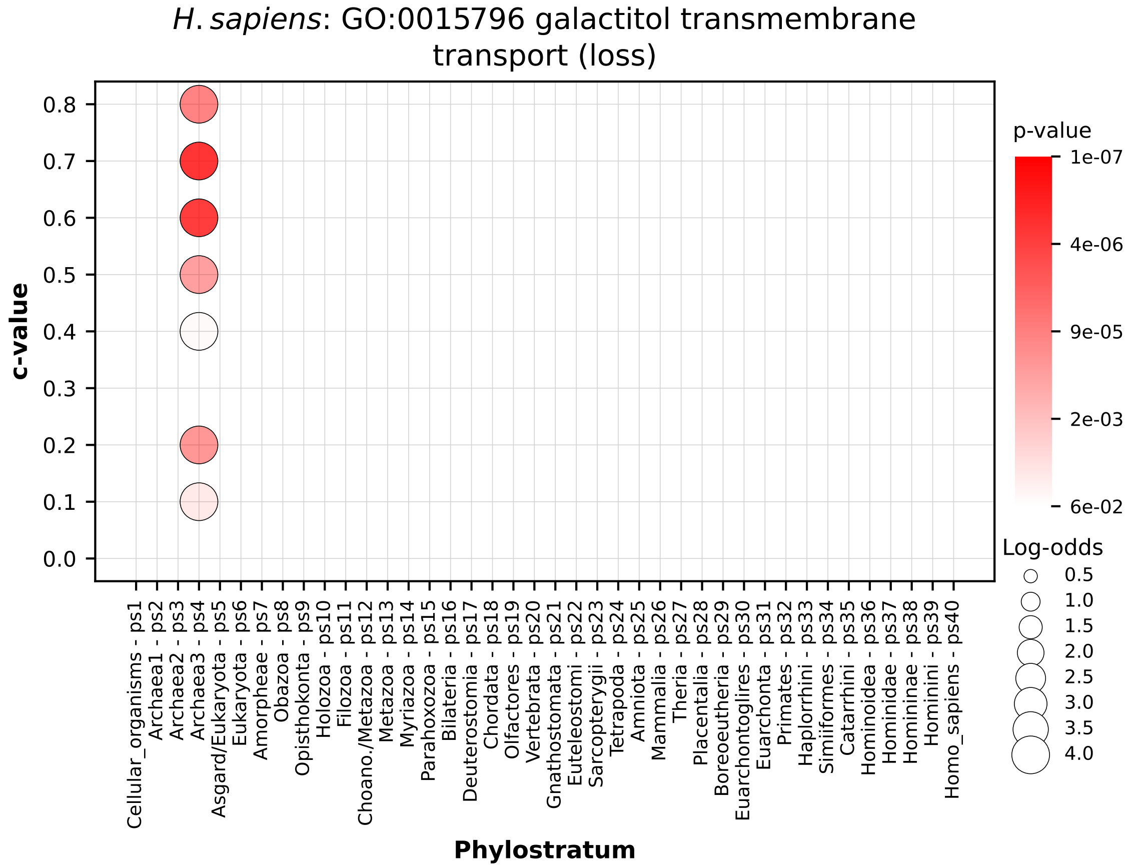

H. sapiens: GO:0015796 galactitol transmembrane transport (loss)
c-value
0.0
0.1
0.2
0.3
0.4
0.5
0.6
0.7
0.8
Phylostratum
Cellular_organisms - ps1
Archaea1 - ps2
Archaea2 - ps3
Archaea3 - ps4
Asgard/Eukaryota - ps5
Eukaryota - ps6
Amorpheae - ps7
Obazoa - ps8
Opisthokonta - ps9
Holozoa - ps10
Filozoa - ps11
Choano./Metazoa - ps12
Metazoa - ps13
Myriazoa - ps14
Parahoxozoa - ps15
Bilateria - ps16
Deuterostomia - ps17
Chordata - ps18
Olfactores - ps19
Vertebrata - ps20
Gnathostomata - ps21
Euteleostomi - ps22
Sarcopterygii - ps23
Tetrapoda - ps24
Amniota - ps25
Mammalia - ps26
Theria - ps27
Placentalia - ps28
Boreoeutheria - ps29
Euarchontoglires - ps30
Euarchonta - ps31
Primates - ps32
Haplorrhini - ps33
Simiiformes - ps34
Catarrhini - ps35
Hominoidea - ps36
Hominidae - ps37
Homininae - ps38
Hominini - ps39
Homo_sapiens - ps40
p-value
1e-07
4e-06
9e-05
2e-03
6e-02
Log-odds
0.5
1.0
1.5
2.0
2.5
3.0
3.5
4.0

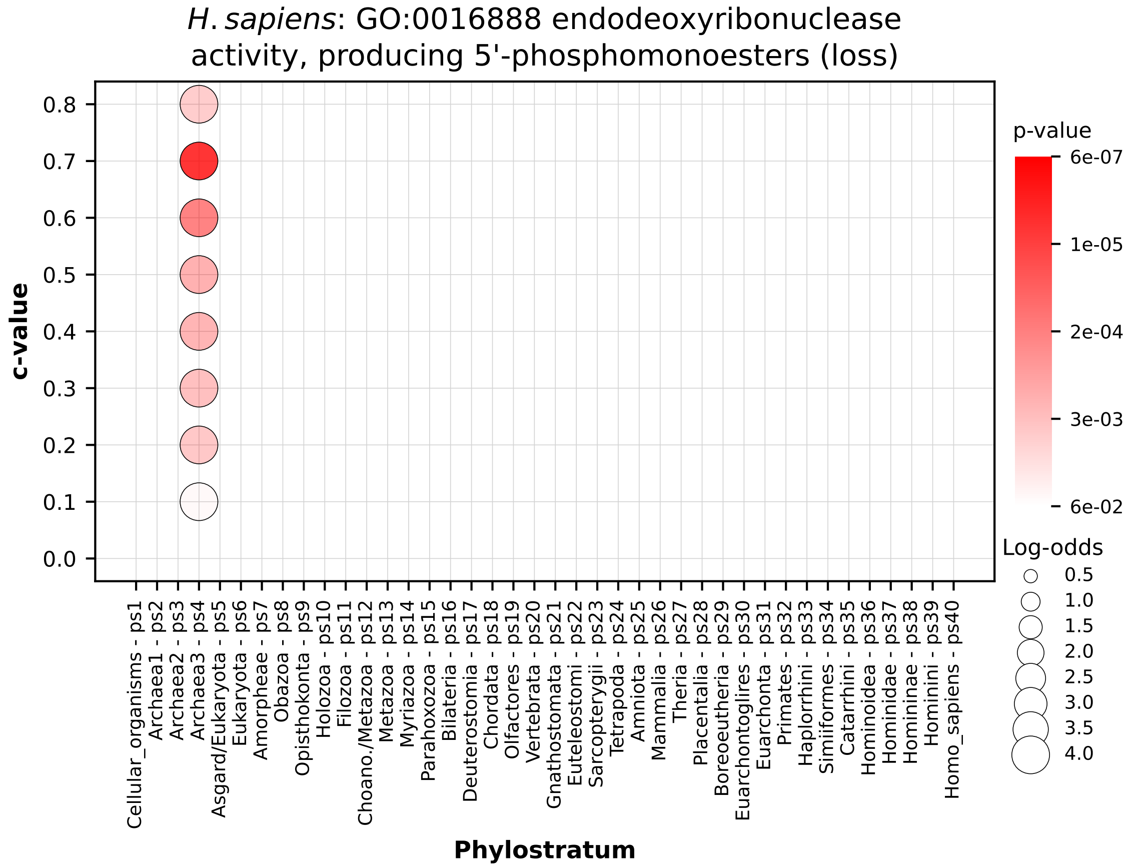

H. sapiens: GO:0016888 endodeoxyribonuclease activity, producing 5'-phosphomonoesters (loss)
c-value
0.0
0.1
0.2
0.3
0.4
0.5
0.6
0.7
0.8
p-value
6e-07
1e-05
2e-04
3e-03
6e-02
Log-odds
0.5
1.0
1.5
2.0
2.5
3.0
3.5
4.0
Cellular_organisms - ps1
Archaea1 - ps2
Archaea2 - ps3
Archaea3 - ps4
Asgard/Eukaryota - ps5
Eukaryota - ps6
Amorpheae - ps7
Obazoa - ps8
Opisthokonta - ps9
Holozoa - ps10
Filozoa - ps11
Choano./Metazoa - ps12
Metazoa - ps13
Myriazoa - ps14
Parahoxozoa - ps15
Bilateria - ps16
Deuterostomia - ps17
Chordata - ps18
Olfactores - ps19
Vertebrata - ps20
Gnathostomata - ps21
Euteleostomi - ps22
Sarcopterygii - ps23
Tetrapoda - ps24
Amniota - ps25
Mammalia - ps26
Theria - ps27
Placentalia - ps28
Boreoeutheria - ps29
Euarchontoglires - ps30
Euarchonta - ps31
Primates - ps32
Haplorrhini - ps33
Simiiformes - ps34
Catarrhini - ps35
Hominoidea - ps36
Hominidae - ps37
Homininae - ps38
Hominini - ps39
Homo_sapiens - ps40
Phylostratum

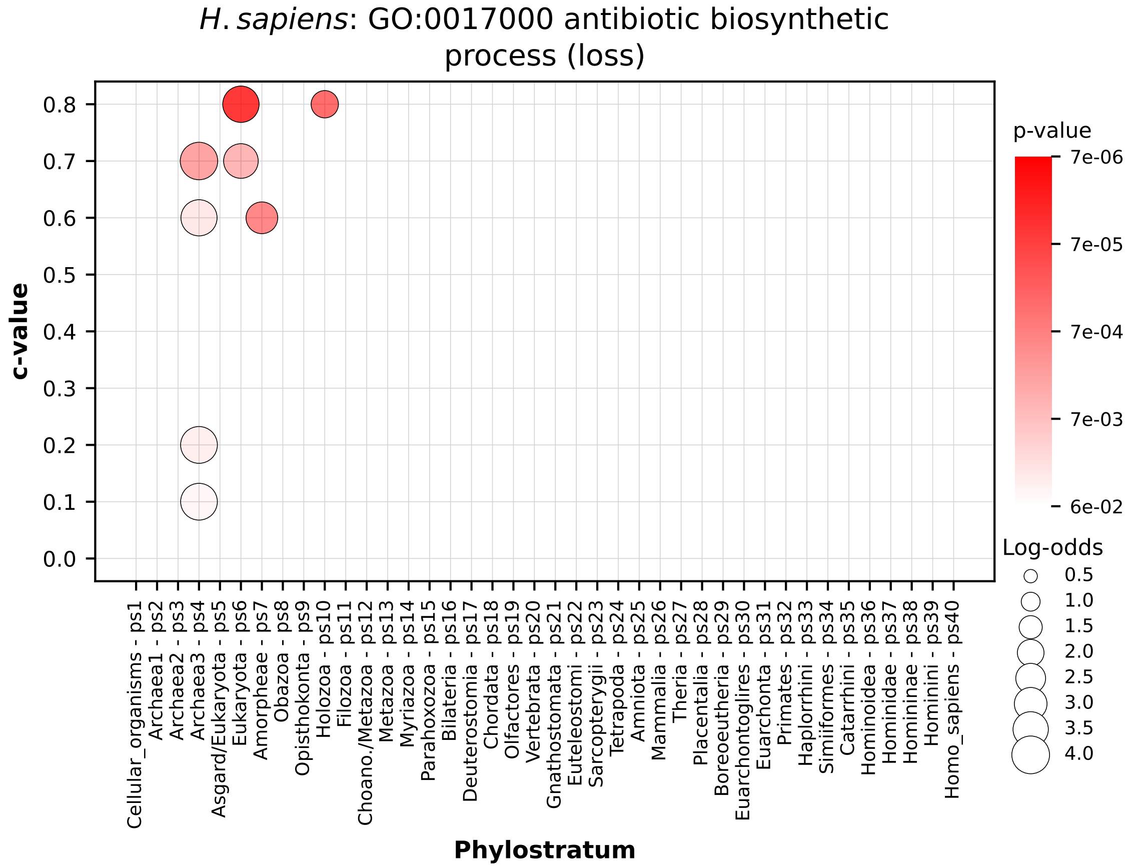

H. sapiens: GO:0017000 antibiotic biosynthetic process (loss)
c-value
0.0
0.1
0.2
0.3
0.4
0.5
0.6
0.7
0.8
Phylostratum
Cellular_organisms - ps1
Archaea1 - ps2
Archaea2 - ps3
Archaea3 - ps4
Asgard/Eukaryota - ps5
Eukaryota - ps6
Amorpheae - ps7
Obazoa - ps8
Opisthokonta - ps9
Holozoa - ps10
Filozoa - ps11
Choano./Metazoa - ps12
Metazoa - ps13
Myriazoa - ps14
Parahoxozoa - ps15
Bilateria - ps16
Deuterostomia - ps17
Chordata - ps18
Olfactores - ps19
Vertebrata - ps20
Gnathostomata - ps21
Euteleostomi - ps22
Sarcopterygii - ps23
Tetrapoda - ps24
Amniota - ps25
Mammalia - ps26
Theria - ps27
Placentalia - ps28
Boreoeutheria - ps29
Euarchontoglires - ps30
Euarchonta - ps31
Primates - ps32
Haplorrhini - ps33
Simiiformes - ps34
Catarrhini - ps35
Hominoidea - ps36
Hominidae - ps37
Homininae - ps38
Hominini - ps39
Homo_sapiens - ps40
p-value
7e-06
7e-05
7e-04
7e-03
6e-02
Log-odds
0.5
1.0
1.5
2.0
2.5
3.0
3.5
4.0

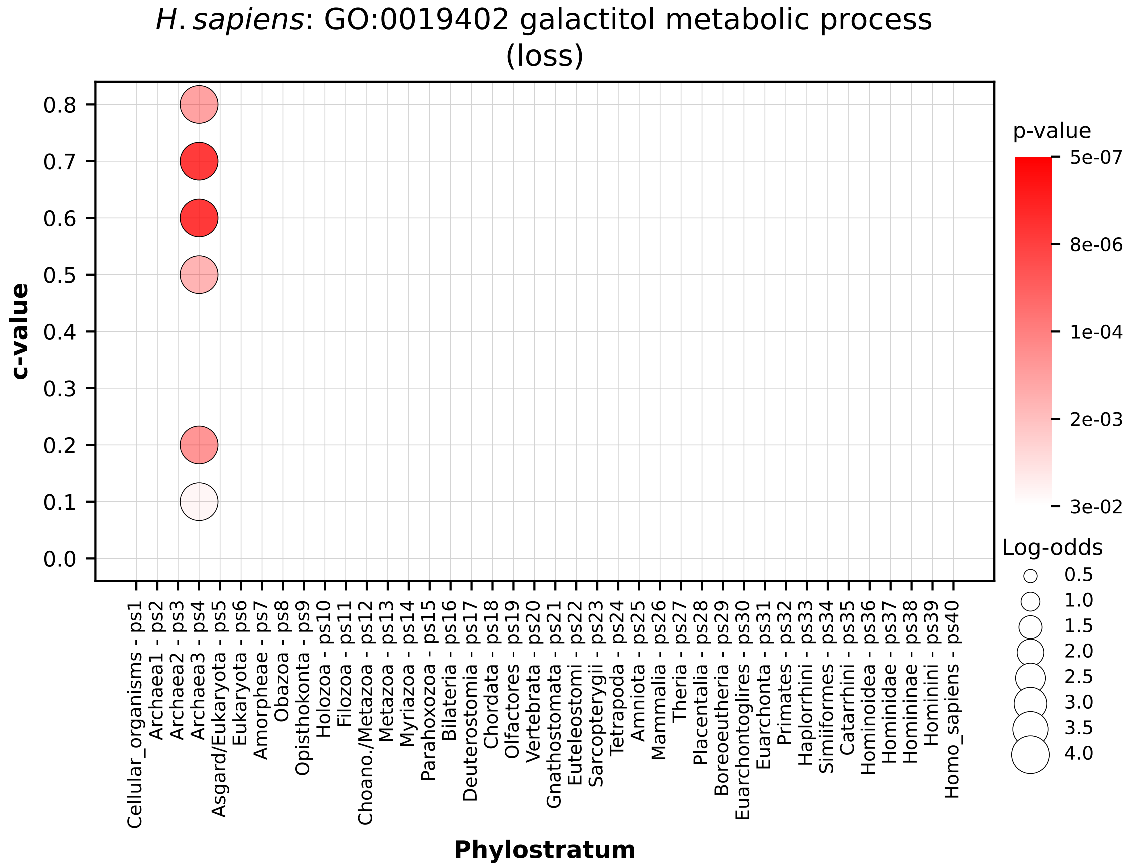

H. sapiens: GO:0019402 galactitol metabolic process
(loss)
c-value
0.0
0.1
0.2
0.3
0.4
0.5
0.6
0.7
0.8
Phylostratum
Cellular_organisms - ps1
Archaea1 - ps2
Archaea2 - ps3
Archaea3 - ps4
Asgard/Eukaryota - ps5
Eukaryota - ps6
Amorpheae - ps7
Obazoa - ps8
Opisthokonta - ps9
Holozoa - ps10
Filozoa - ps11
Choano./Metazoa - ps12
Metazoa - ps13
Myriazoa - ps14
Parahoxozoa - ps15
Bilateria - ps16
Deuterostomia - ps17
Chordata - ps18
Olfactores - ps19
Vertebrata - ps20
Gnathostomata - ps21
Euteleostomi - ps22
Sarcopterygii - ps23
Tetrapoda - ps24
Amniota - ps25
Mammalia - ps26
Theria - ps27
Placentalia - ps28
Boreoeutheria - ps29
Euarchontoglires - ps30
Euarchonta - ps31
Primates - ps32
Haplorrhini - ps33
Simiiformes - ps34
Catarrhini - ps35
Hominoidea - ps36
Hominidae - ps37
Homininae - ps38
Hominini - ps39
Homo_sapiens - ps40
p-value
5e-07
8e-06
1e-04
2e-03
3e-02
Log-odds
0.5
1.0
1.5
2.0
2.5
3.0
3.5
4.0

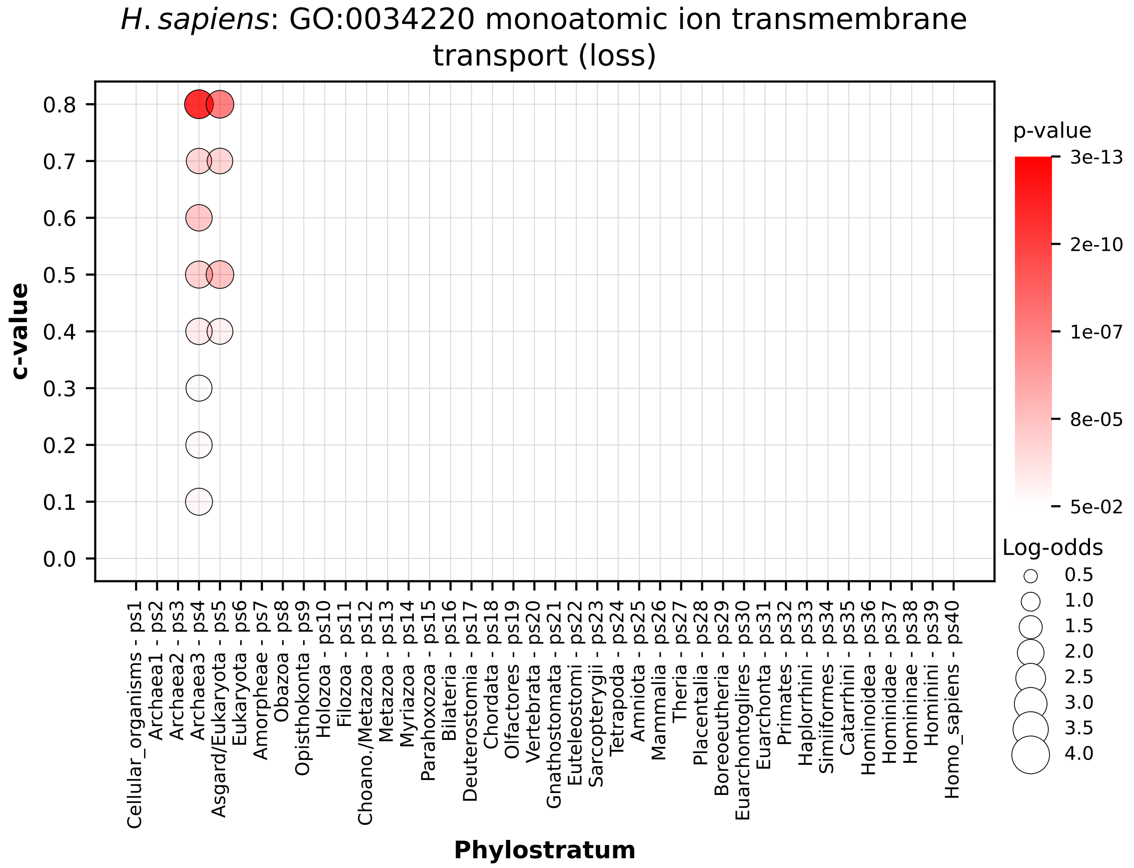

H. sapiens: GO:0034220 monoatomic ion transmembrane transport (loss)
c-value
0.0
0.1
0.2
0.3
0.4
0.5
0.6
0.7
0.8
Phylostratum
Cellular_organisms - ps1
Archaea1 - ps2
Archaea2 - ps3
Archaea3 - ps4
Asgard/Eukaryota - ps5
Eukaryota - ps6
Amorpheae - ps7
Obazoa - ps8
Opisthokonta - ps9
Holozoa - ps10
Filozoa - ps11
Choano./Metazoa - ps12
Metazoa - ps13
Myriazoa - ps14
Parahoxozoa - ps15
Bilateria - ps16
Deuterostomia - ps17
Chordata - ps18
Olfactores - ps19
Vertebrata - ps20
Gnathostomata - ps21
Euteleostomi - ps22
Sarcopterygii - ps23
Tetrapoda - ps24
Amniota - ps25
Mammalia - ps26
Theria - ps27
Placentalia - ps28
Boreoeutheria - ps29
Euarchontoglires - ps30
Euarchonta - ps31
Primates - ps32
Haplorrhini - ps33
Simiiformes - ps34
Catarrhini - ps35
Hominoidea - ps36
Hominidae - ps37
Homininae - ps38
Hominini - ps39
Homo_sapiens - ps40
p-value
3e-13
2e-10
1e-07
8e-05
5e-02
Log-odds
0.5
1.0
1.5
2.0
2.5
3.0
3.5
4.0

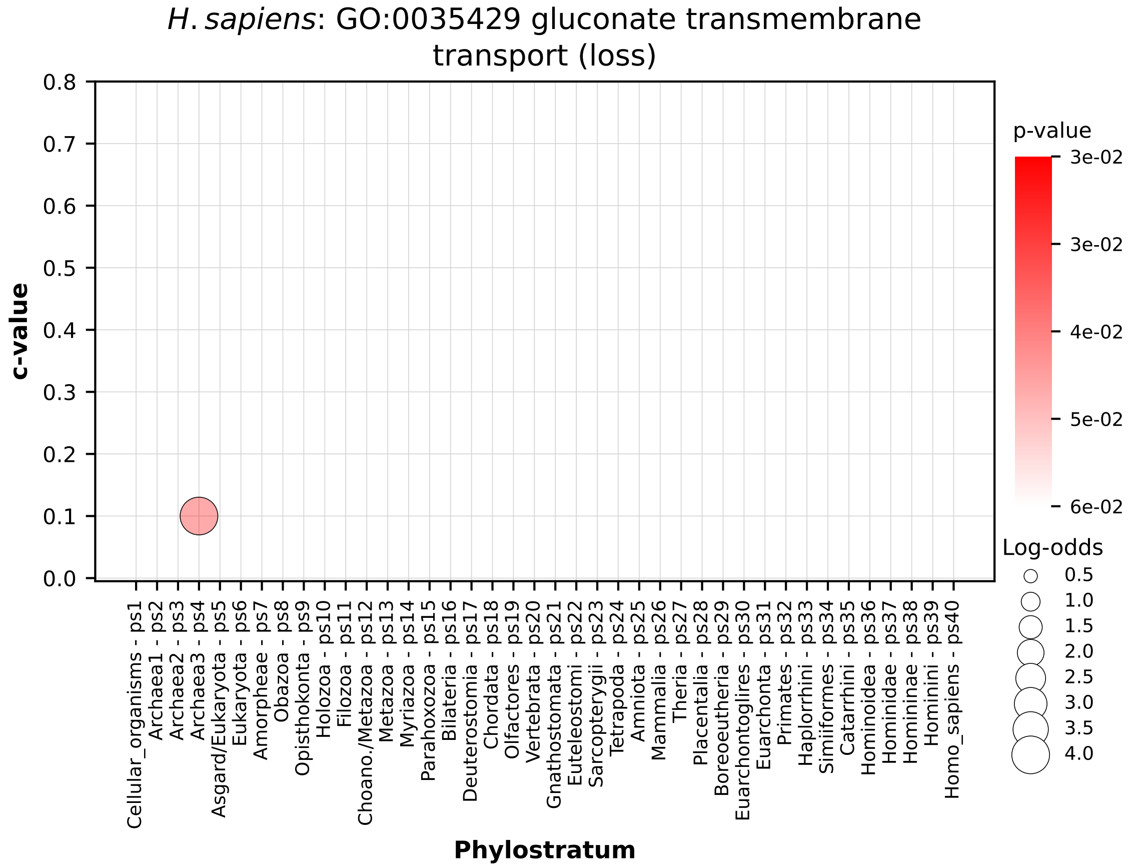

H. sapiens: GO:0035429 gluconate transmembrane transport (loss)
c-value
0.8
0.7
0.6
0.5
0.4
0.3
0.2
0.1
0.0
p-value
3e-02
3e-02
4e-02
5e-02
6e-02
Log-odds
0.5
1.0
1.5
2.0
2.5
3.0
3.5
4.0
Cellular_organisms - ps1
Archaea1 - ps2
Archaea2 - ps3
Archaea3 - ps4
Asgard/Eukaryota - ps5
Eukaryota - ps6
Amorpheae - ps7
Obazoa - ps8
Opisthokonta - ps9
Holozoa - ps10
Filozoa - ps11
Choano./Metazoa - ps12
Metazoa - ps13
Myriazoa - ps14
Parahoxozoa - ps15
Bilateria - ps16
Deuterostomia - ps17
Chordata - ps18
Olfactores - ps19
Vertebrata - ps20
Gnathostomata - ps21
Euteleostomi - ps22
Sarcopterygii - ps23
Tetrapoda - ps24
Amniota - ps25
Mammalia - ps26
Theria - ps27
Placentalia - ps28
Boreoeutheria - ps29
Euarchontoglires - ps30
Euarchonta - ps31
Primates - ps32
Haplorrhini - ps33
Simiiformes - ps34
Catarrhini - ps35
Hominoidea - ps36
Hominidae - ps37
Homininae - ps38
Hominini - ps39
Homo_sapiens - ps40
Phylostratum

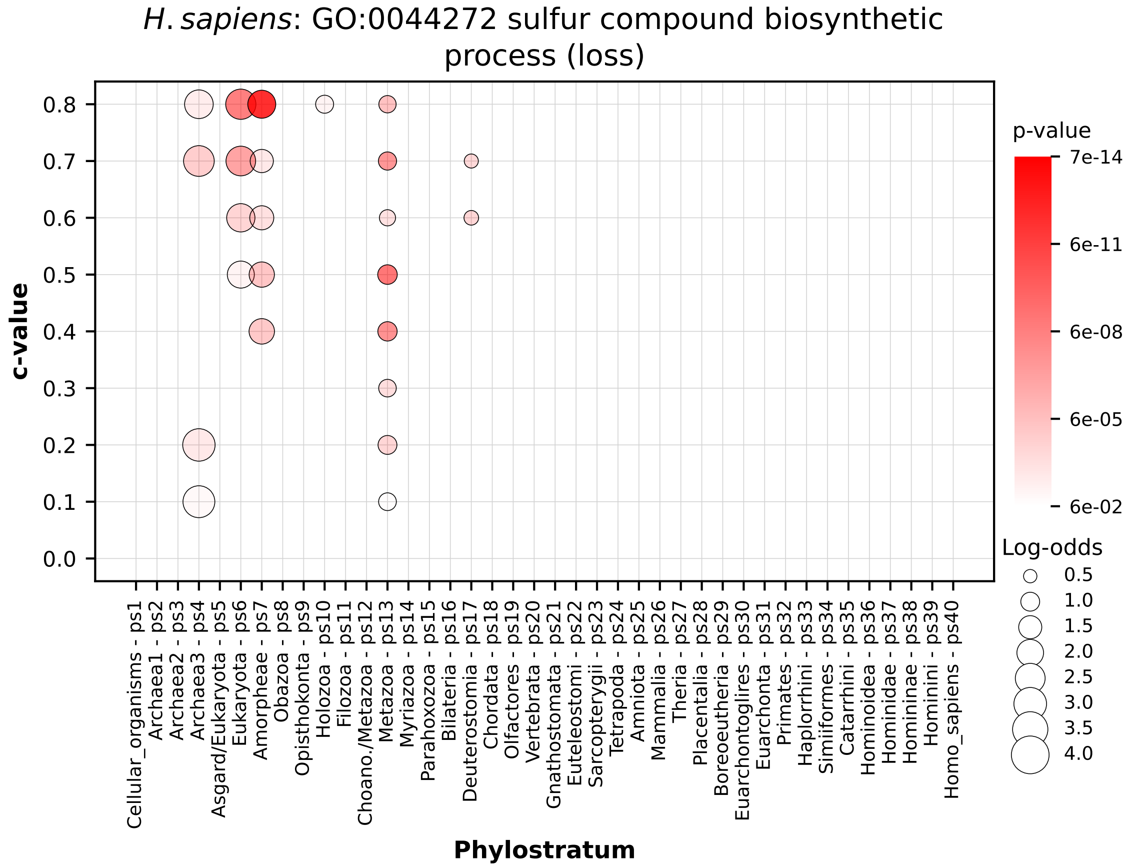
H. sapiens: GO:0044272 sulfur compound biosynthetic process (loss)
c-value
0.8
0.7
0.6
0.5
0.4
0.3
0.2
0.1
0.0
p-value
7e-14
6e-11
6e-08
6e-05
6e-02
Log-odds
0.5
1.0
1.5
2.0
2.5
3.0
3.5
4.0
Cellular_organisms - ps1
Archaea1 - ps2
Archaea2 - ps3
Archaea3 - ps4
Asgard/Eukaryota - ps5
Eukaryota - ps6
Amorpheae - ps7
Obazoa - ps8
Opisthokonta - ps9
Holozoa - ps10
Filozoa - ps11
Choano./Metazoa - ps12
Metazoa - ps13
Myriazoa - ps14
Parahoxozoa - ps15
Bilateria - ps16
Deuterostomia - ps17
Chordata - ps18
Olfactores - ps19
Vertebrata - ps20
Gnathostomata - ps21
Euteleostomi - ps22
Sarcopterygii - ps23
Tetrapoda - ps24
Amniota - ps25
Mammalia - ps26
Theria - ps27
Placentalia - ps28
Boreoeutheria - ps29
Euarchontoglires - ps30
Euarchonta - ps31
Primates - ps32
Haplorrhini - ps33
Simiiformes - ps34
Catarrhini - ps35
Hominoidea - ps36
Hominidae - ps37
Homininae - ps38
Hominini - ps39
Homo_sapiens - ps40
Phylostratum

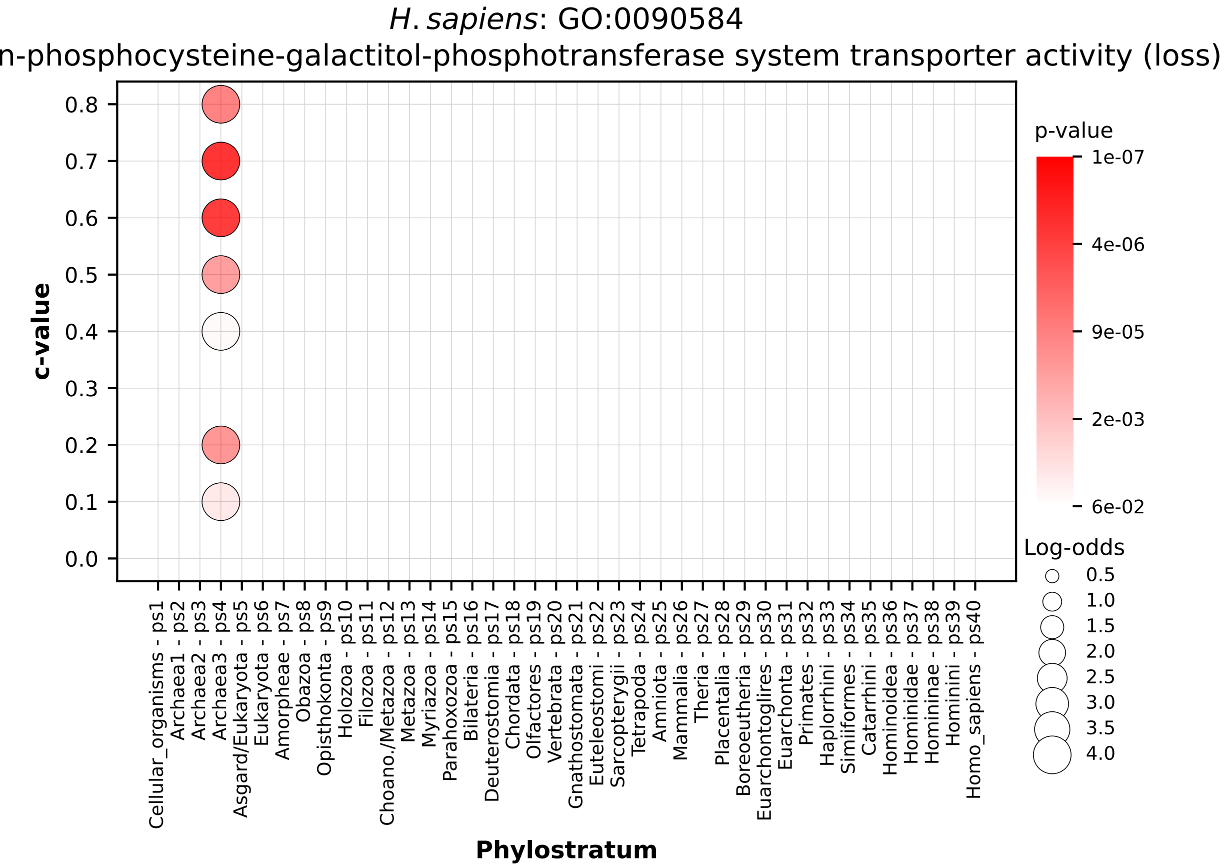

H. sapiens: GO:0090584
n-phosphocysteine-galactitol-phosphotransferase system transporter activity (loss)
c-value
0.8
0.7
0.6
0.5
0.4
0.3
0.2
0.1
0.0
p-value
1e-07
4e-06
9e-05
2e-03
6e-02
Log-odds
0.5
1.0
1.5
2.0
2.5
3.0
3.5
4.0
Cellular_organisms - ps1
Archaea1 - ps2
Archaea2 - ps3
Archaea3 - ps4
Asgard/Eukaryota - ps5
Eukaryota - ps6
Amorpheae - ps7
Obazoa - ps8
Opisthokonta - ps9
Holozoa - ps10
Filozoa - ps11
Choano./Metazoa - ps12
Metazoa - ps13
Myriazoa - ps14
Parahoxozoa - ps15
Bilateria - ps16
Deuterostomia - ps17
Chordata - ps18
Olfactores - ps19
Vertebrata - ps20
Gnathostomata - ps21
Euteleostomi - ps22
Sarcopterygii - ps23
Tetrapoda - ps24
Amniota - ps25
Mammalia - ps26
Theria - ps27
Placentalia - ps28
Boreoeutheria - ps29
Euarchontoglires - ps30
Euarchonta - ps31
Primates - ps32
Haplorrhini - ps33
Simiiformes - ps34
Catarrhini - ps35
Hominoidea - ps36
Hominidae - ps37
Homininae - ps38
Hominini - ps39
Homo_sapiens - ps40
Phylostratum

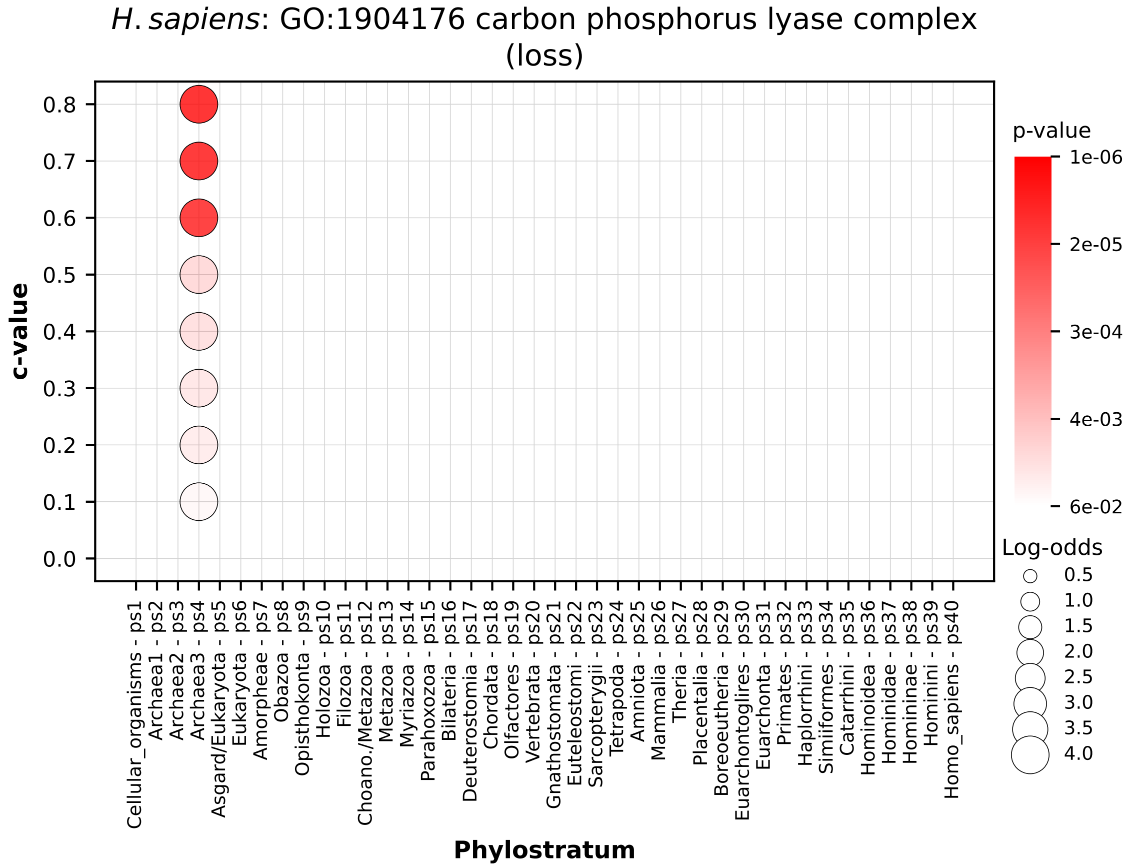
H. sapiens: GO:1904176 carbon phosphorus lyase complex
(loss)
c-value
0.8
0.7
0.6
0.5
0.4
0.3
0.2
0.1
0.0
p-value
1e-06
2e-05
3e-04
4e-03
6e-02
Log-odds
0.5
1.0
1.5
2.0
2.5
3.0
3.5
4.0
Cellular_organisms - ps1
Archaea1 - ps2
Archaea2 - ps3
Archaea3 - ps4
Asgard/Eukaryota - ps5
Eukaryota - ps6
Amorpheae - ps7
Obazoa - ps8
Opisthokonta - ps9
Holozoa - ps10
Filozoa - ps11
Choano./Metazoa - ps12
Metazoa - ps13
Myriazoa - ps14
Parahoxozoa - ps15
Bilateria - ps16
Deuterostomia - ps17
Chordata - ps18
Olfactores - ps19
Vertebrata - ps20
Gnathostomata - ps21
Euteleostomi - ps22
Sarcopterygii - ps23
Tetrapoda - ps24
Amniota - ps25
Mammalia - ps26
Theria - ps27
Placentalia - ps28
Boreoeutheria - ps29
Euarchontoglires - ps30
Euarchonta - ps31
Primates - ps32
Haplorrhini - ps33
Simiiformes - ps34
Catarrhini - ps35
Hominoidea - ps36
Hominidae - ps37
Homininae - ps38
Hominini - ps39
Homo_sapiens - ps40
Phylostratum

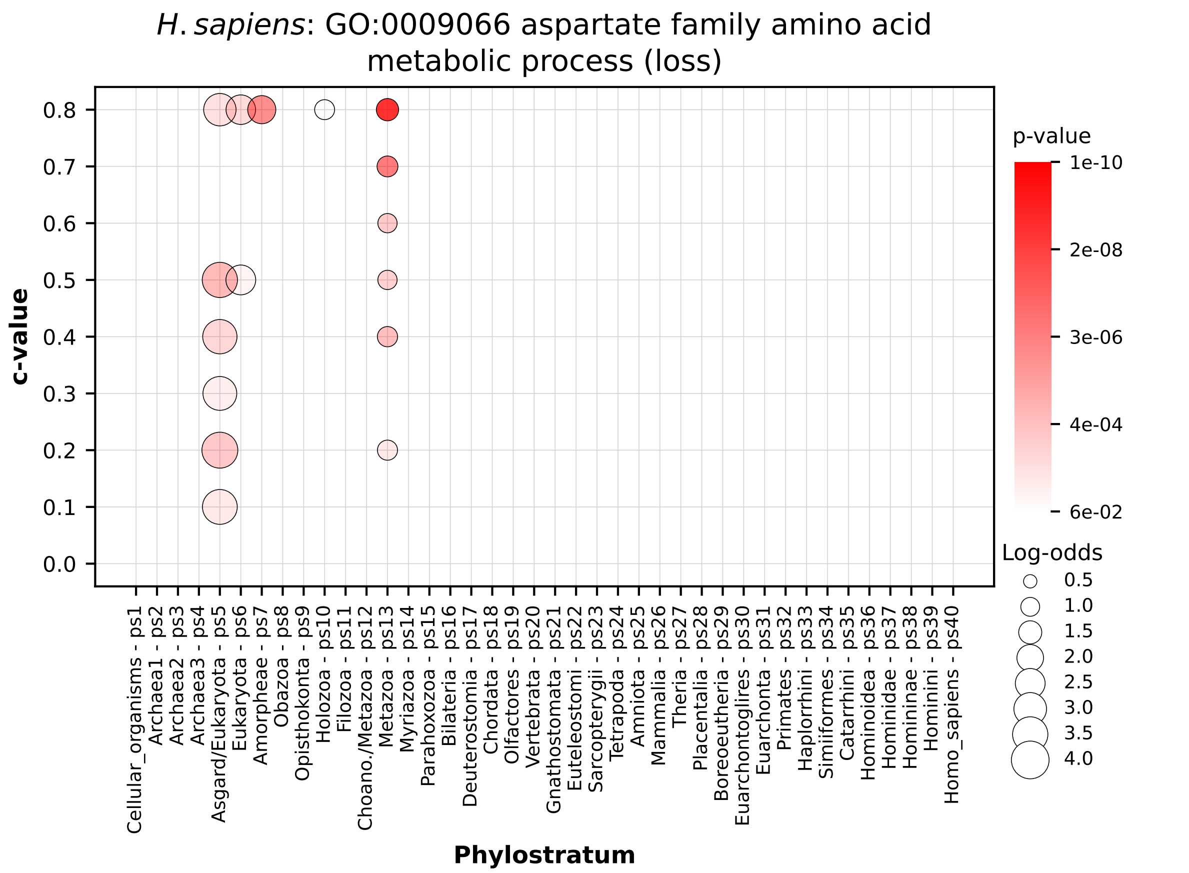

H. sapiens: GO:0009066 aspartate family amino acid metabolic process (loss)
c-value
0.0
0.1
0.2
0.3
0.4
0.5
0.6
0.7
0.8
p-value
1e-10
2e-08
3e-06
4e-04
6e-02
Log-odds
0.5
1.0
1.5
2.0
2.5
3.0
3.5
4.0
Cellular_organisms - ps1
Archaea1 - ps2
Archaea2 - ps3
Archaea3 - ps4
Asgard/Eukaryota - ps5
Eukaryota - ps6
Amorpheae - ps7
Obazoa - ps8
Opisthokonta - ps9
Holozoa - ps10
Filozoa - ps11
Choano./Metazoa - ps12
Metazoa - ps13
Myriazoa - ps14
Parahoxozoa - ps15
Bilateria - ps16
Deuterostomia - ps17
Chordata - ps18
Olfactores - ps19
Vertebrata - ps20
Gnathostomata - ps21
Euteleostomi - ps22
Sarcopterygii - ps23
Tetrapoda - ps24
Amniota - ps25
Mammalia - ps26
Theria - ps27
Placentalia - ps28
Boreoeutheria - ps29
Euarchontoglires - ps30
Euarchonta - ps31
Primates - ps32
Haplorrhini - ps33
Simiiformes - ps34
Catarrhini - ps35
Hominoidea - ps36
Hominidae - ps37
Homininae - ps38
Hominini - ps39
Homo_sapiens - ps40
Phylostratum

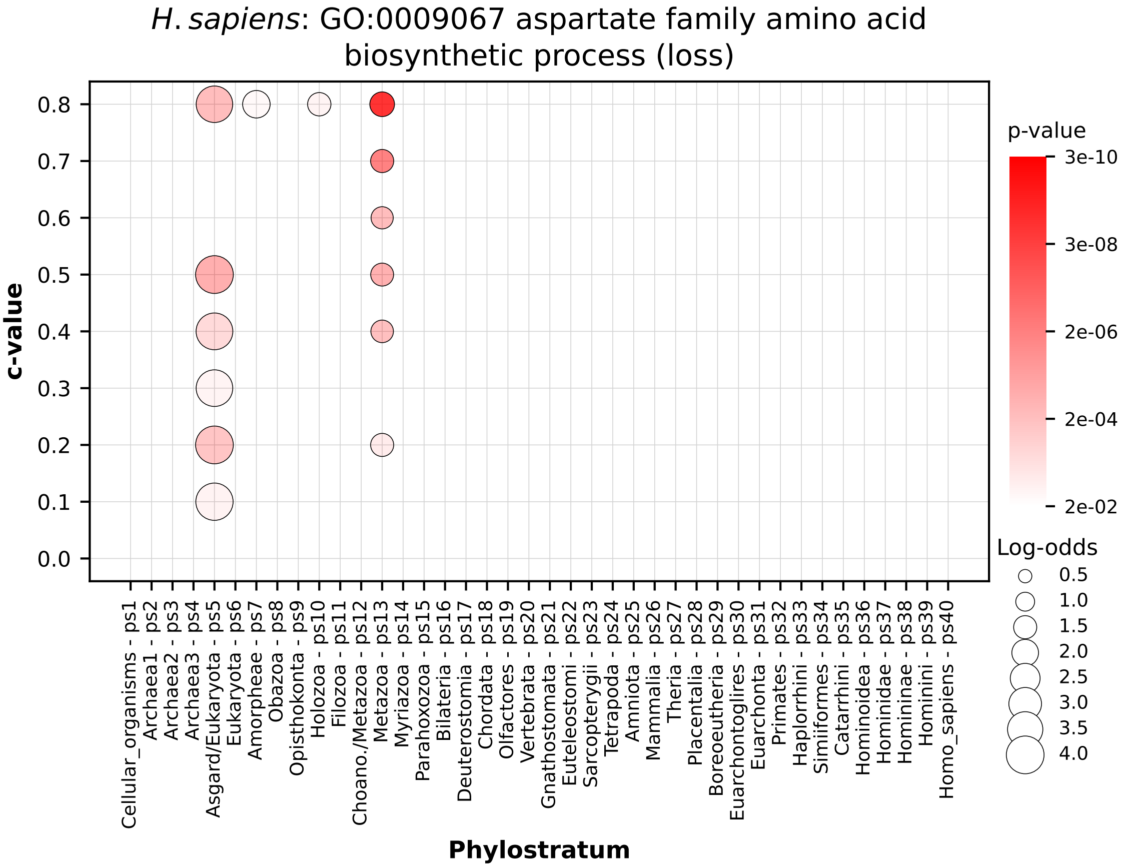

H. sapiens: GO:0009067 aspartate family amino acid biosynthetic process (loss)
c-value
0.0
0.1
0.2
0.3
0.4
0.5
0.6
0.7
0.8
Phylostratum
Cellular_organisms - ps1
Archaea1 - ps2
Archaea2 - ps3
Archaea3 - ps4
Asgard/Eukaryota - ps5
Eukaryota - ps6
Amorpheae - ps7
Obazoa - ps8
Opisthokonta - ps9
Holozoa - ps10
Filozoa - ps11
Choano./Metazoa - ps12
Metazoa - ps13
Myriazoa - ps14
Parahoxozoa - ps15
Bilateria - ps16
Deuterostomia - ps17
Chordata - ps18
Olfactores - ps19
Vertebrata - ps20
Gnathostomata - ps21
Euteleostomi - ps22
Sarcopterygii - ps23
Tetrapoda - ps24
Amniota - ps25
Mammalia - ps26
Theria - ps27
Placentalia - ps28
Boreoeutheria - ps29
Euarchontoglires - ps30
Euarchonta - ps31
Primates - ps32
Haplorrhini - ps33
Simiiformes - ps34
Catarrhini - ps35
Hominoidea - ps36
Hominidae - ps37
Homininae - ps38
Hominini - ps39
Homo_sapiens - ps40
p-value
3e-10
3e-08
2e-06
2e-04
2e-02
Log-odds
0.5
1.0
1.5
2.0
2.5
3.0
3.5
4.0

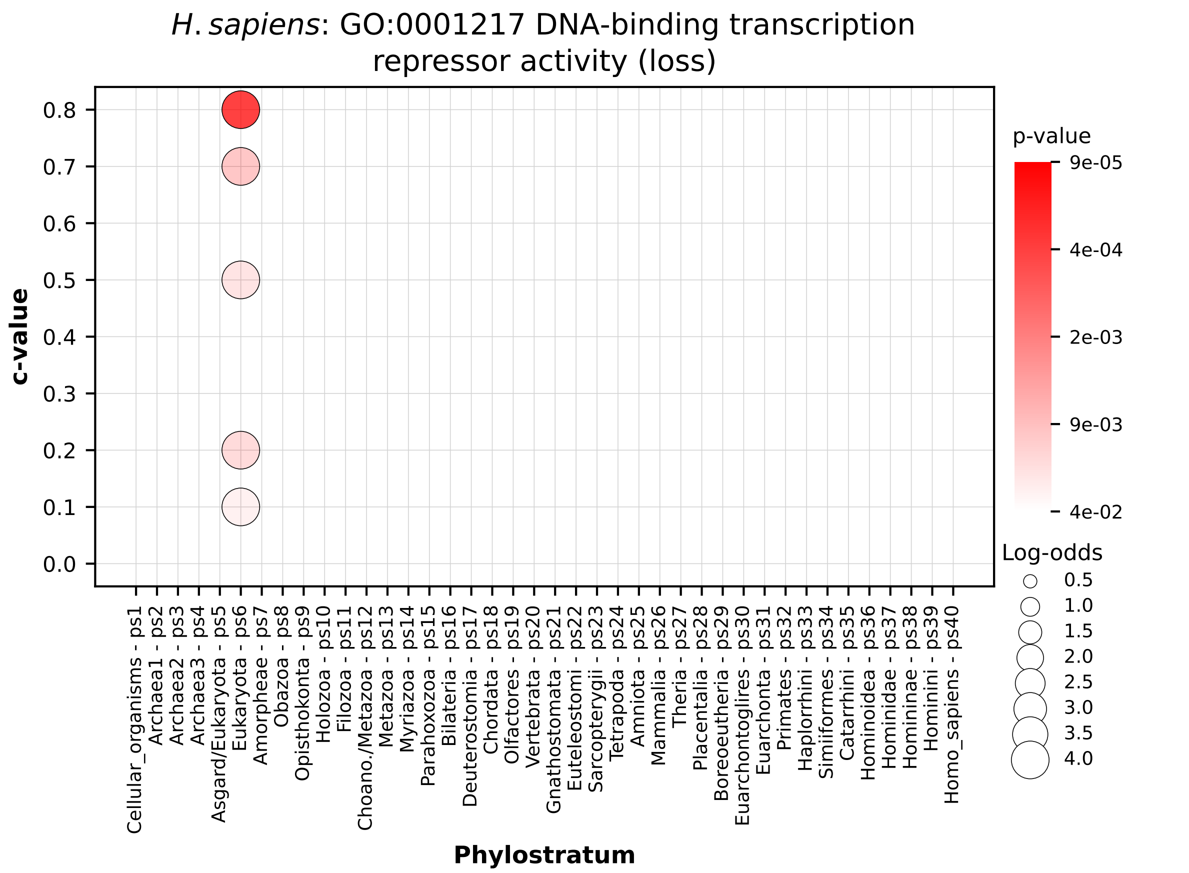

H. sapiens: GO:0001217 DNA-binding transcription repressor activity (loss)
c-value
0.8
0.7
0.6
0.5
0.4
0.3
0.2
0.1
0.0
p-value
9e-05
4e-04
2e-03
9e-03
4e-02
Log-odds
0.5
1.0
1.5
2.0
2.5
3.0
3.5
4.0
Cellular_organisms - ps1
Archaea1 - ps2
Archaea2 - ps3
Archaea3 - ps4
Asgard/Eukaryota - ps5
Eukaryota - ps6
Amorpheae - ps7
Obazoa - ps8
Opisthokonta - ps9
Holozoa - ps10
Filozoa - ps11
Choano./Metazoa - ps12
Metazoa - ps13
Myriazoa - ps14
Parahoxozoa - ps15
Bilateria - ps16
Deuterostomia - ps17
Chordata - ps18
Olfactores - ps19
Vertebrata - ps20
Gnathostomata - ps21
Euteleostomi - ps22
Sarcopterygii - ps23
Tetrapoda - ps24
Amniota - ps25
Mammalia - ps26
Theria - ps27
Placentalia - ps28
Boreoeutheria - ps29
Euarchontoglires - ps30
Euarchonta - ps31
Primates - ps32
Haplorrhini - ps33
Simiiformes - ps34
Catarrhini - ps35
Hominoidea - ps36
Hominidae - ps37
Homininae - ps38
Hominini - ps39
Homo_sapiens - ps40
Phylostratum

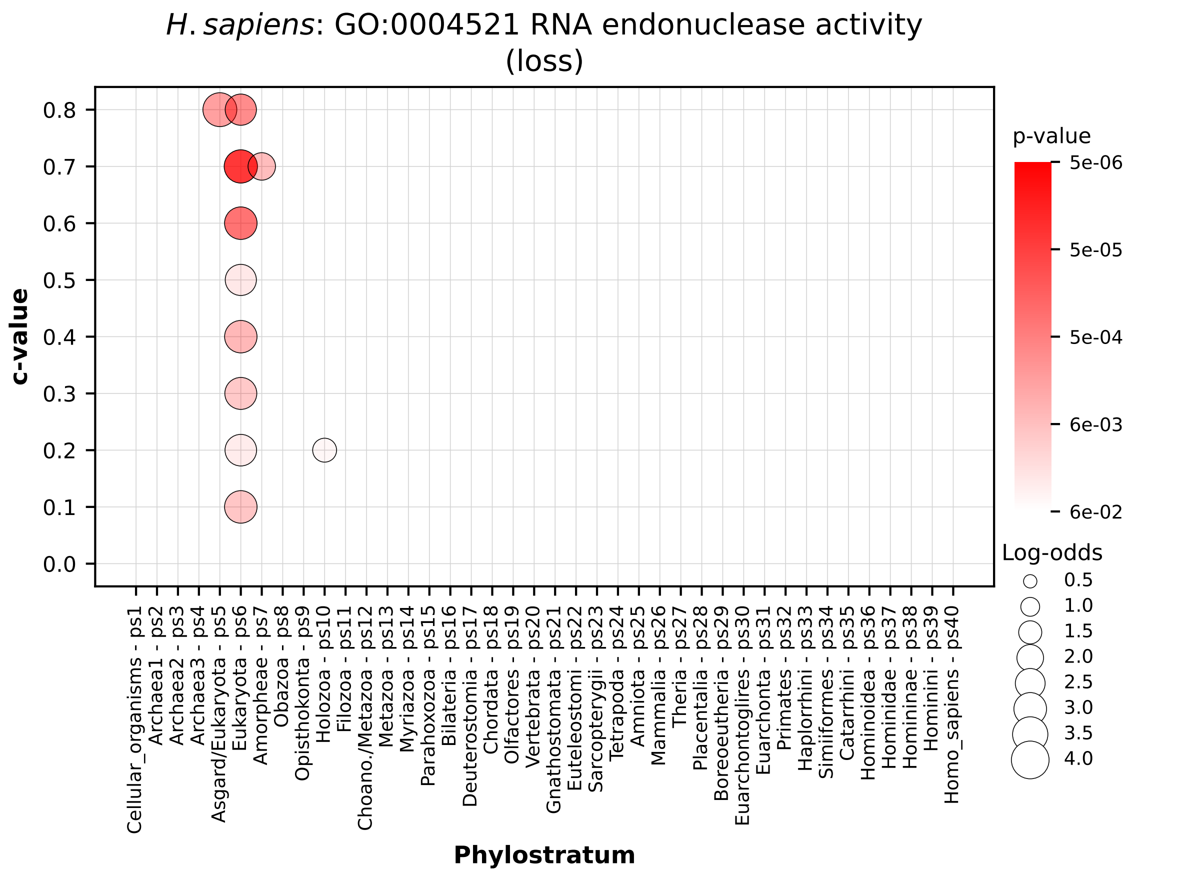

H. sapiens: GO:0004521 RNA endonuclease activity
(loss)
c-value
0.0
0.1
0.2
0.3
0.4
0.5
0.6
0.7
0.8
Phylostratum
Cellular_organisms - ps1
Archaea1 - ps2
Archaea2 - ps3
Archaea3 - ps4
Asgard/Eukaryota - ps5
Eukaryota - ps6
Amorpheae - ps7
Obazoa - ps8
Opisthokonta - ps9
Holozoa - ps10
Filozoa - ps11
Choano./Metazoa - ps12
Metazoa - ps13
Myriazoa - ps14
Parahoxozoa - ps15
Bilateria - ps16
Deuterostomia - ps17
Chordata - ps18
Olfactores - ps19
Vertebrata - ps20
Gnathostomata - ps21
Euteleostomi - ps22
Sarcopterygii - ps23
Tetrapoda - ps24
Amniota - ps25
Mammalia - ps26
Theria - ps27
Placentalia - ps28
Boreoeutheria - ps29
Euarchontoglires - ps30
Euarchonta - ps31
Primates - ps32
Haplorrhini - ps33
Simiiformes - ps34
Catarrhini - ps35
Hominoidea - ps36
Hominidae - ps37
Homininae - ps38
Hominini - ps39
Homo_sapiens - ps40
p-value
5e-06
5e-05
5e-04
6e-03
6e-02
Log-odds
0.5
1.0
1.5
2.0
2.5
3.0
3.5
4.0

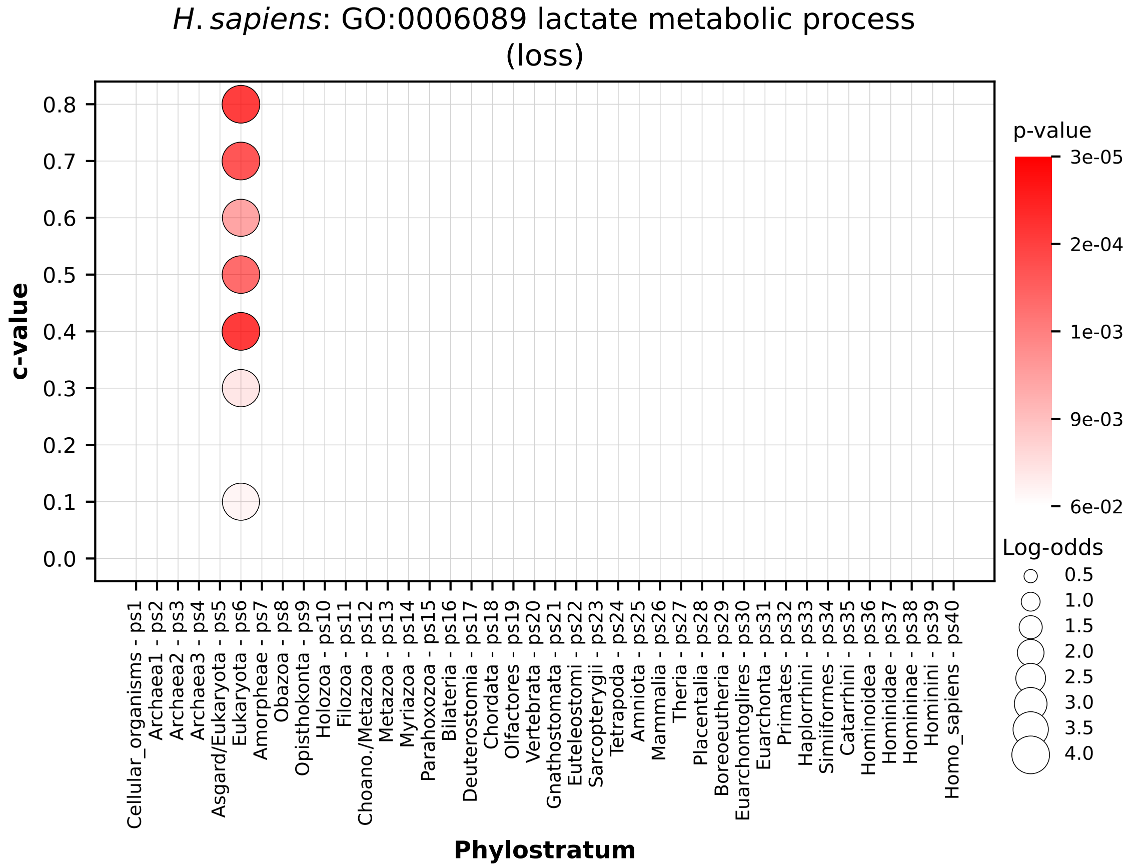

H. sapiens: GO:0006089 lactate metabolic process
(loss)
c-value
0.8
0.7
0.6
0.5
0.4
0.3
0.2
0.1
0.0
p-value
3e-05
2e-04
1e-03
9e-03
6e-02
Log-odds
0.5
1.0
1.5
2.0
2.5
3.0
3.5
4.0
Cellular_organisms - ps1
Archaea1 - ps2
Archaea2 - ps3
Archaea3 - ps4
Asgard/Eukaryota - ps5
Eukaryota - ps6
Amorpheae - ps7
Obazoa - ps8
Opisthokonta - ps9
Holozoa - ps10
Filozoa - ps11
Choano./Metazoa - ps12
Metazoa - ps13
Myriazoa - ps14
Parahoxozoa - ps15
Bilateria - ps16
Deuterostomia - ps17
Chordata - ps18
Olfactores - ps19
Vertebrata - ps20
Gnathostomata - ps21
Euteleostomi - ps22
Sarcopterygii - ps23
Tetrapoda - ps24
Amniota - ps25
Mammalia - ps26
Theria - ps27
Placentalia - ps28
Boreoeutheria - ps29
Euarchontoglires - ps30
Euarchonta - ps31
Primates - ps32
Haplorrhini - ps33
Simiiformes - ps34
Catarrhini - ps35
Hominoidea - ps36
Hominidae - ps37
Homininae - ps38
Hominini - ps39
Homo_sapiens - ps40
Phylostratum

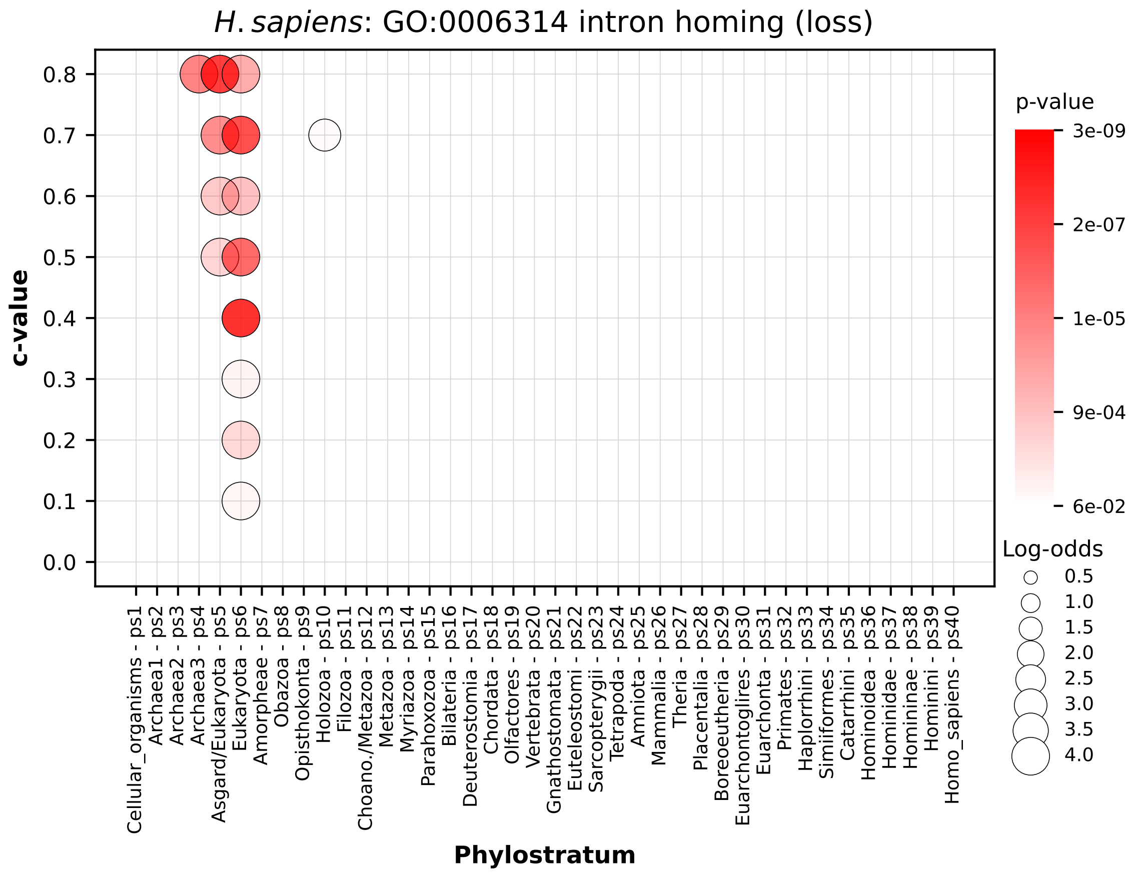
H. sapiens: GO:0006314 intron homing (loss)
c-value
0.8
0.7
0.6
0.5
0.4
0.3
0.2
0.1
0.0
Phylostratum
Cellular_organisms - ps1
Archaea1 - ps2
Archaea2 - ps3
Archaea3 - ps4
Asgard/Eukaryota - ps5
Eukaryota - ps6
Amorpheae - ps7
Obazoa - ps8
Opisthokonta - ps9
Holozoa - ps10
Filozoa - ps11
Choano./Metazoa - ps12
Metazoa - ps13
Myriazoa - ps14
Parahoxozoa - ps15
Bilateria - ps16
Deuterostomia - ps17
Chordata - ps18
Olfactores - ps19
Vertebrata - ps20
Gnathostomata - ps21
Euteleostomi - ps22
Sarcopterygii - ps23
Tetrapoda - ps24
Amniota - ps25
Mammalia - ps26
Theria - ps27
Placentalia - ps28
Boreoeutheria - ps29
Euarchontoglires - ps30
Euarchonta - ps31
Primates - ps32
Haplorrhini - ps33
Simiiformes - ps34
Catarrhini - ps35
Hominoidea - ps36
Hominidae - ps37
Homininae - ps38
Hominini - ps39
Homo_sapiens - ps40
p-value
3e-09
2e-07
1e-05
9e-04
6e-02
Log-odds
0.5
1.0
1.5
2.0
2.5
3.0
3.5
4.0

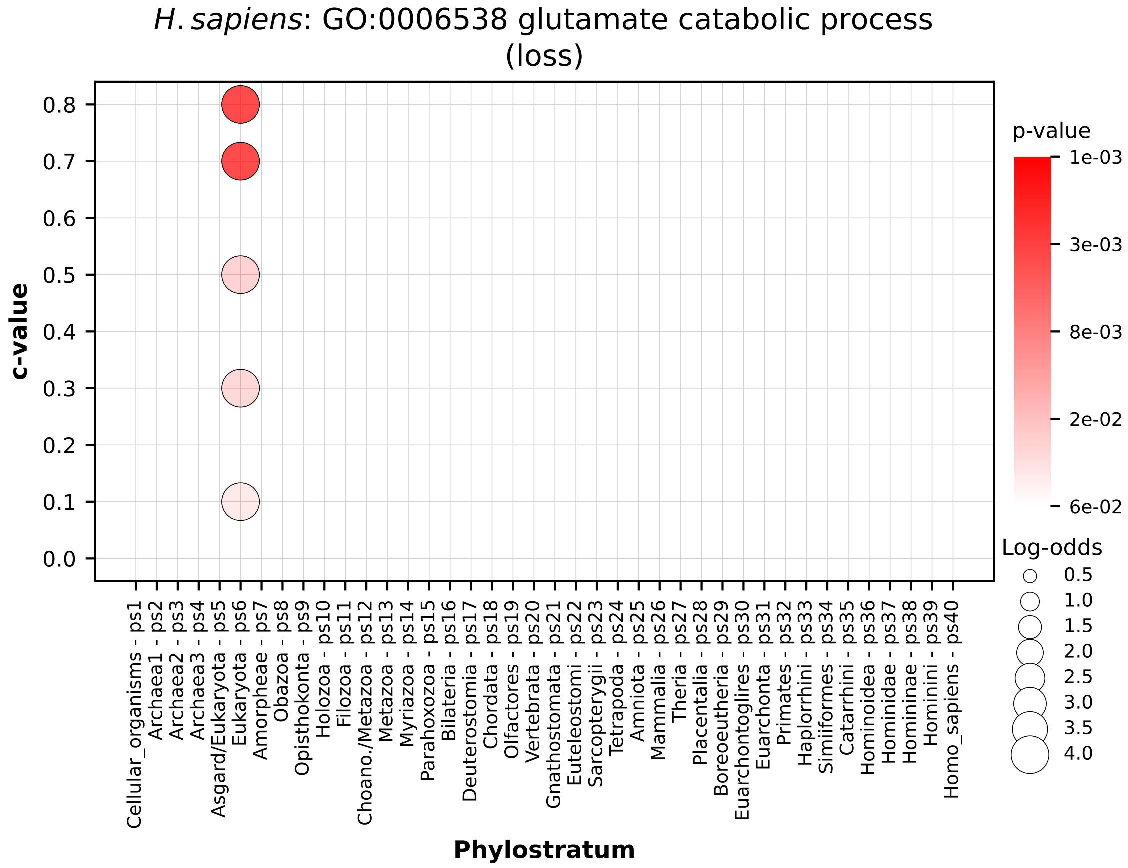

H. sapiens: GO:0006538 glutamate catabolic process
(loss)
c-value
0.8
0.7
0.6
0.5
0.4
0.3
0.2
0.1
0.0
p-value
1e-03
3e-03
8e-03
2e-02
6e-02
Log-odds
0.5
1.0
1.5
2.0
2.5
3.0
3.5
4.0
Cellular_organisms - ps1
Archaea1 - ps2
Archaea2 - ps3
Archaea3 - ps4
Asgard/Eukaryota - ps5
Eukaryota - ps6
Amorpheae - ps7
Obazoa - ps8
Opisthokonta - ps9
Holozoa - ps10
Filozoa - ps11
Choano./Metazoa - ps12
Metazoa - ps13
Myriazoa - ps14
Parahoxozoa - ps15
Bilateria - ps16
Deuterostomia - ps17
Chordata - ps18
Olfactores - ps19
Vertebrata - ps20
Gnathostomata - ps21
Euteleostomi - ps22
Sarcopterygii - ps23
Tetrapoda - ps24
Amniota - ps25
Mammalia - ps26
Theria - ps27
Placentalia - ps28
Boreoeutheria - ps29
Euarchontoglires - ps30
Euarchonta - ps31
Primates - ps32
Haplorrhini - ps33
Simiiformes - ps34
Catarrhini - ps35
Hominoidea - ps36
Hominidae - ps37
Homininae - ps38
Hominini - ps39
Homo_sapiens - ps40
Phylostratum

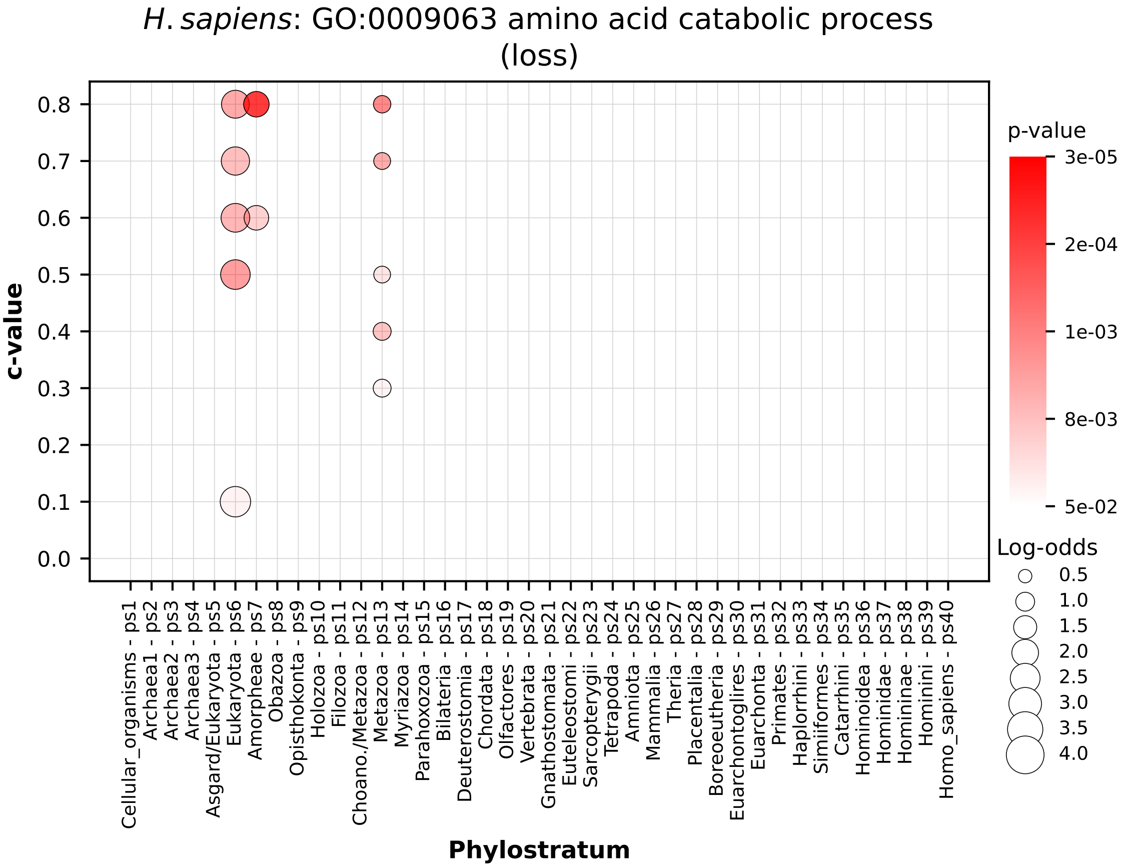

H. sapiens: GO:0009063 amino acid catabolic process
(loss)
c-value
0.0
0.1
0.2
0.3
0.4
0.5
0.6
0.7
0.8
Phylostratum
Cellular_organisms - ps1
Archaea1 - ps2
Archaea2 - ps3
Archaea3 - ps4
Asgard/Eukaryota - ps5
Eukaryota - ps6
Amorpheae - ps7
Obazoa - ps8
Opisthokonta - ps9
Holozoa - ps10
Filozoa - ps11
Choano./Metazoa - ps12
Metazoa - ps13
Myriazoa - ps14
Parahoxozoa - ps15
Bilateria - ps16
Deuterostomia - ps17
Chordata - ps18
Olfactores - ps19
Vertebrata - ps20
Gnathostomata - ps21
Euteleostomi - ps22
Sarcopterygii - ps23
Tetrapoda - ps24
Amniota - ps25
Mammalia - ps26
Theria - ps27
Placentalia - ps28
Boreoeutheria - ps29
Euarchontoglires - ps30
Euarchonta - ps31
Primates - ps32
Haplorrhini - ps33
Simiiformes - ps34
Catarrhini - ps35
Hominoidea - ps36
Hominidae - ps37
Homininae - ps38
Hominini - ps39
Homo_sapiens - ps40
p-value
3e-05
2e-04
1e-03
8e-03
5e-02
Log-odds
0.5
1.0
1.5
2.0
2.5
3.0
3.5
4.0

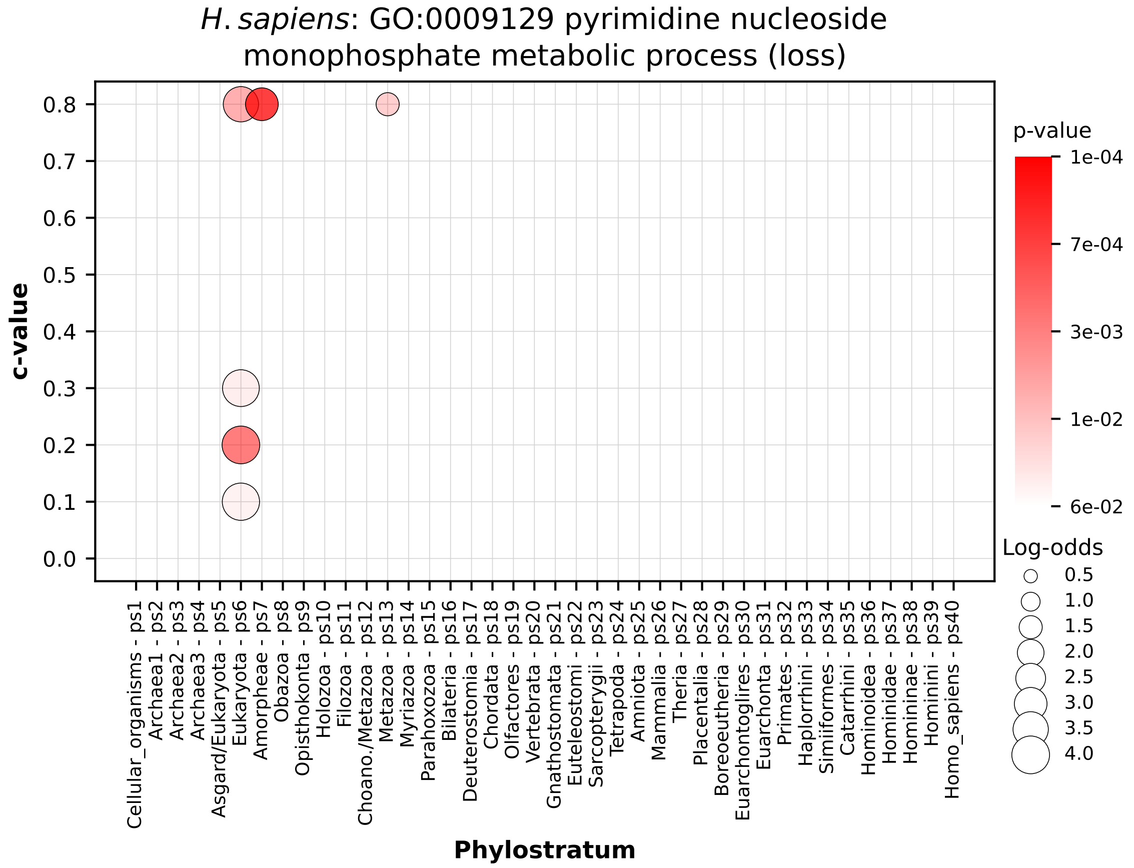

H. sapiens: GO:0009129 pyrimidine nucleoside monophosphate metabolic process (loss)
c-value
0.0
0.1
0.2
0.3
0.4
0.5
0.6
0.7
0.8
Phylostratum
Cellular_organisms - ps1
Archaea1 - ps2
Archaea2 - ps3
Archaea3 - ps4
Asgard/Eukaryota - ps5
Eukaryota - ps6
Amorpheae - ps7
Obazoa - ps8
Opisthokonta - ps9
Holozoa - ps10
Filozoa - ps11
Choano./Metazoa - ps12
Metazoa - ps13
Myriazoa - ps14
Parahoxozoa - ps15
Bilateria - ps16
Deuterostomia - ps17
Chordata - ps18
Olfactores - ps19
Vertebrata - ps20
Gnathostomata - ps21
Euteleostomi - ps22
Sarcopterygii - ps23
Tetrapoda - ps24
Amniota - ps25
Mammalia - ps26
Theria - ps27
Placentalia - ps28
Boreoeutheria - ps29
Euarchontoglires - ps30
Euarchonta - ps31
Primates - ps32
Haplorrhini - ps33
Simiiformes - ps34
Catarrhini - ps35
Hominoidea - ps36
Hominidae - ps37
Homininae - ps38
Hominini - ps39
Homo_sapiens - ps40
p-value
1e-04
7e-04
3e-03
1e-02
6e-02
Log-odds
0.5
1.0
1.5
2.0
2.5
3.0
3.5
4.0

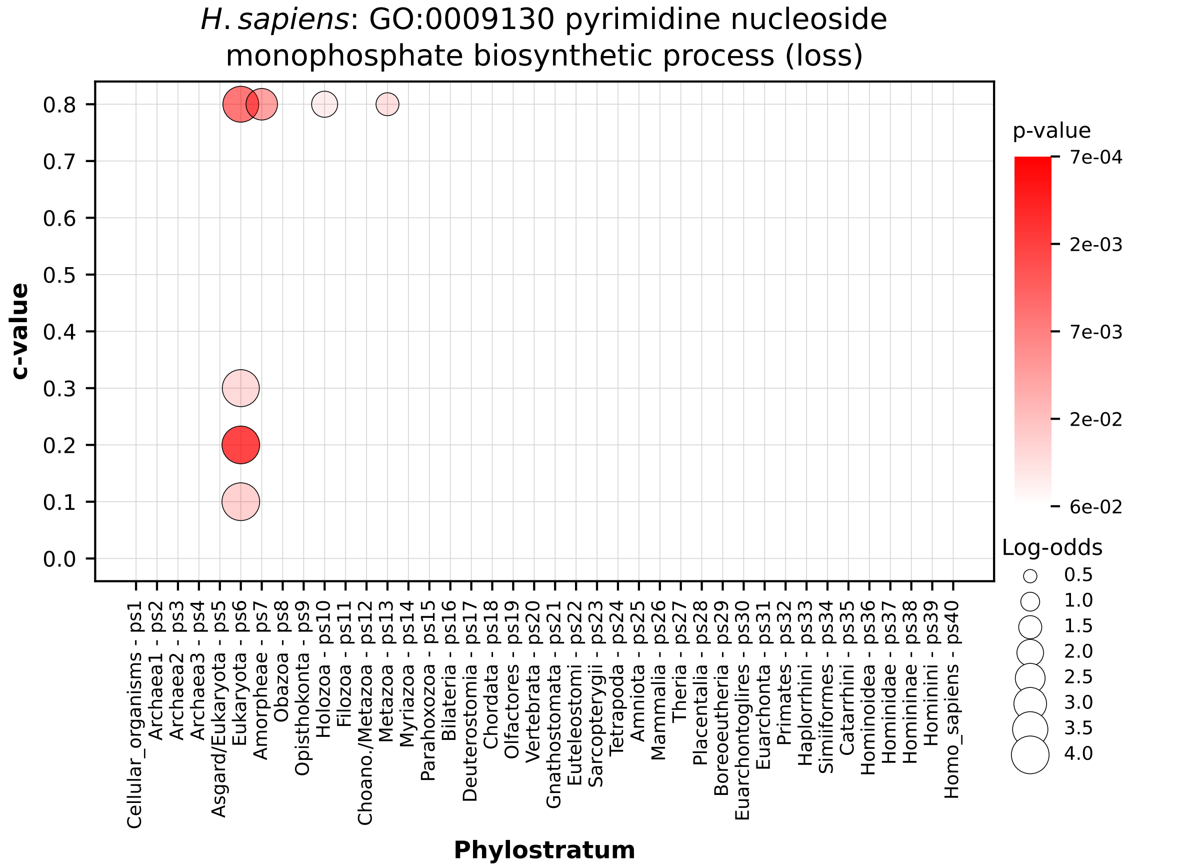

H. sapiens: GO:0009130 pyrimidine nucleoside monophosphate biosynthetic process (loss)
c-value
0.8
0.7
0.6
0.5
0.4
0.3
0.2
0.1
0.0
p-value
7e-04
2e-03
7e-03
2e-02
6e-02
Log-odds
0.5
1.0
1.5
2.0
2.5
3.0
3.5
4.0
Cellular_organisms - ps1
Archaea1 - ps2
Archaea2 - ps3
Archaea3 - ps4
Asgard/Eukaryota - ps5
Eukaryota - ps6
Amorpheae - ps7
Obazoa - ps8
Opisthokonta - ps9
Holozoa - ps10
Filozoa - ps11
Choano./Metazoa - ps12
Metazoa - ps13
Myriazoa - ps14
Parahoxozoa - ps15
Bilateria - ps16
Deuterostomia - ps17
Chordata - ps18
Olfactores - ps19
Vertebrata - ps20
Gnathostomata - ps21
Euteleostomi - ps22
Sarcopterygii - ps23
Tetrapoda - ps24
Amniota - ps25
Mammalia - ps26
Theria - ps27
Placentalia - ps28
Boreoeutheria - ps29
Euarchontoglires - ps30
Euarchonta - ps31
Primates - ps32
Haplorrhini - ps33
Simiiformes - ps34
Catarrhini - ps35
Hominoidea - ps36
Hominidae - ps37
Homininae - ps38
Hominini - ps39
Homo_sapiens - ps40
Phylostratum

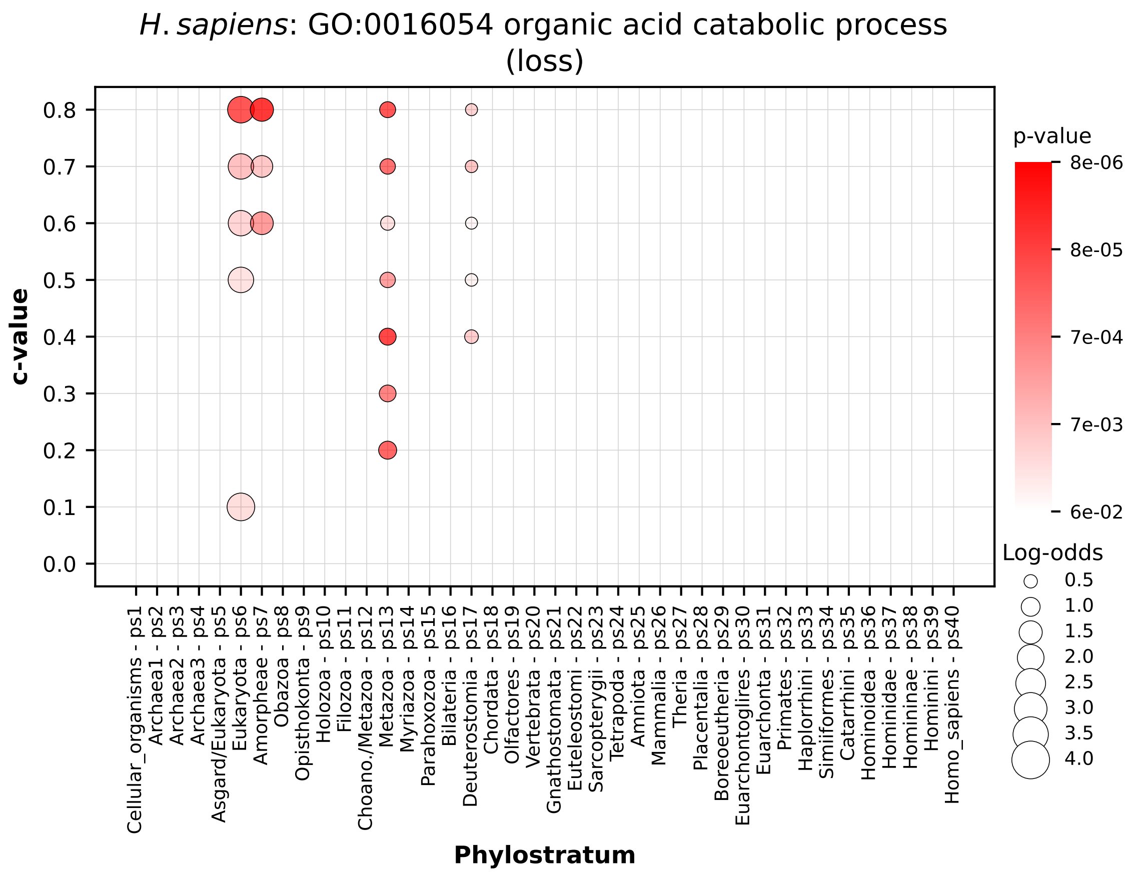
H. sapiens: GO:0016054 organic acid catabolic process
(loss)
c-value
0.8
0.7
0.6
0.5
0.4
0.3
0.2
0.1
0.0
Phylostratum
Cellular_organisms - ps1
Archaea1 - ps2
Archaea2 - ps3
Archaea3 - ps4
Asgard/Eukaryota - ps5
Eukaryota - ps6
Amorpheae - ps7
Obazoa - ps8
Opisthokonta - ps9
Holozoa - ps10
Filozoa - ps11
Choano./Metazoa - ps12
Metazoa - ps13
Myriazoa - ps14
Parahoxozoa - ps15
Bilateria - ps16
Deuterostomia - ps17
Chordata - ps18
Olfactores - ps19
Vertebrata - ps20
Gnathostomata - ps21
Euteleostomi - ps22
Sarcopterygii - ps23
Tetrapoda - ps24
Amniota - ps25
Mammalia - ps26
Theria - ps27
Placentalia - ps28
Boreoeutheria - ps29
Euarchontoglires - ps30
Euarchonta - ps31
Primates - ps32
Haplorrhini - ps33
Simiiformes - ps34
Catarrhini - ps35
Hominoidea - ps36
Hominidae - ps37
Homininae - ps38
Hominini - ps39
Homo_sapiens - ps40
p-value
8e-06
8e-05
7e-04
7e-03
6e-02
Log-odds
0.5
1.0
1.5
2.0
2.5
3.0
3.5
4.0

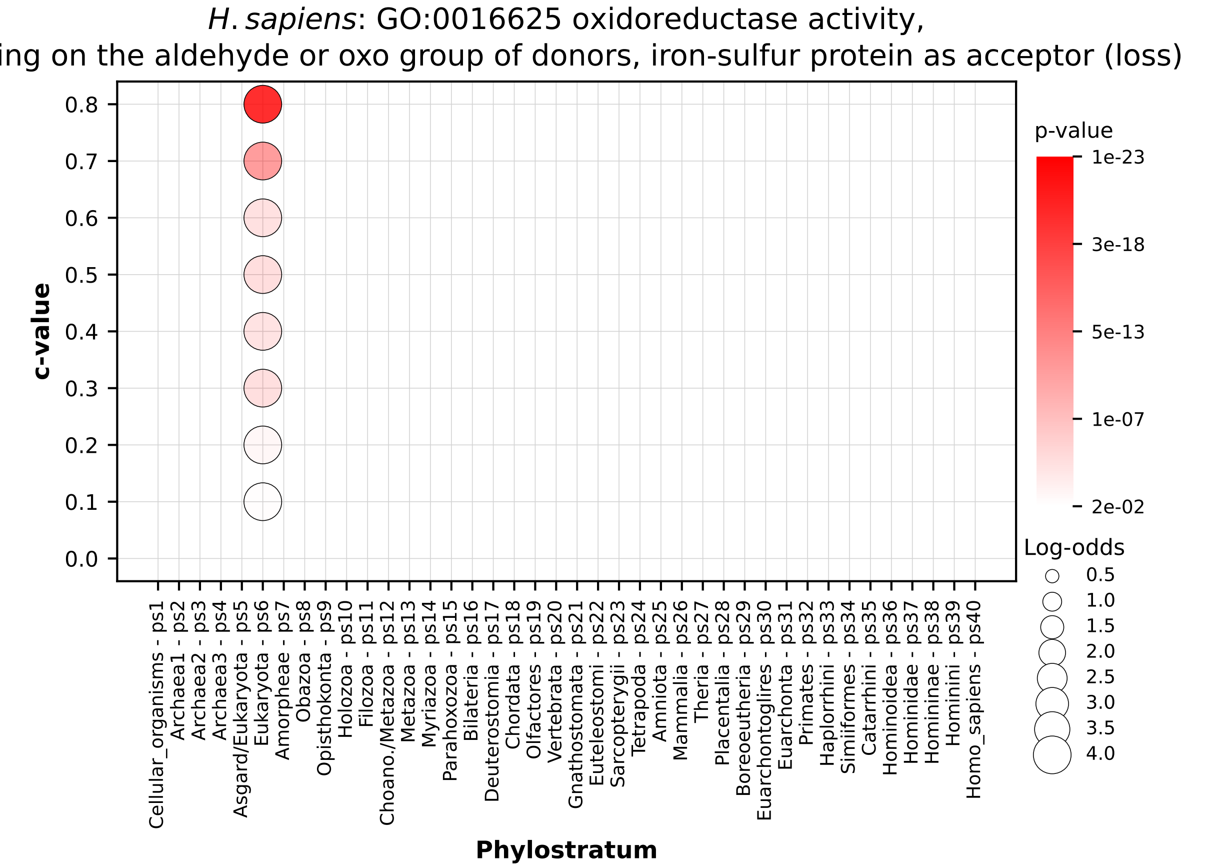

H. sapiens: GO:0016625 oxidoreductase activity,
ing on the aldehyde or oxo group of donors, iron-sulfur protein as acceptor (loss)
c-value
0.8
0.7
0.6
0.5
0.4
0.3
0.2
0.1
0.0
p-value
1e-23
3e-18
5e-13
1e-07
2e-02
Log-odds
0.5
1.0
1.5
2.0
2.5
3.0
3.5
4.0
Cellular_organisms - ps1
Archaea1 - ps2
Archaea2 - ps3
Archaea3 - ps4
Asgard/Eukaryota - ps5
Eukaryota - ps6
Amorpheae - ps7
Obazoa - ps8
Opisthokonta - ps9
Holozoa - ps10
Filozoa - ps11
Choano./Metazoa - ps12
Metazoa - ps13
Myriazoa - ps14
Parahoxozoa - ps15
Bilateria - ps16
Deuterostomia - ps17
Chordata - ps18
Olfactores - ps19
Vertebrata - ps20
Gnathostomata - ps21
Euteleostomi - ps22
Sarcopterygii - ps23
Tetrapoda - ps24
Amniota - ps25
Mammalia - ps26
Theria - ps27
Placentalia - ps28
Boreoeutheria - ps29
Euarchontoglires - ps30
Euarchonta - ps31
Primates - ps32
Haplorrhini - ps33
Simiiformes - ps34
Catarrhini - ps35
Hominoidea - ps36
Hominidae - ps37
Homininae - ps38
Hominini - ps39
Homo_sapiens - ps40
Phylostratum

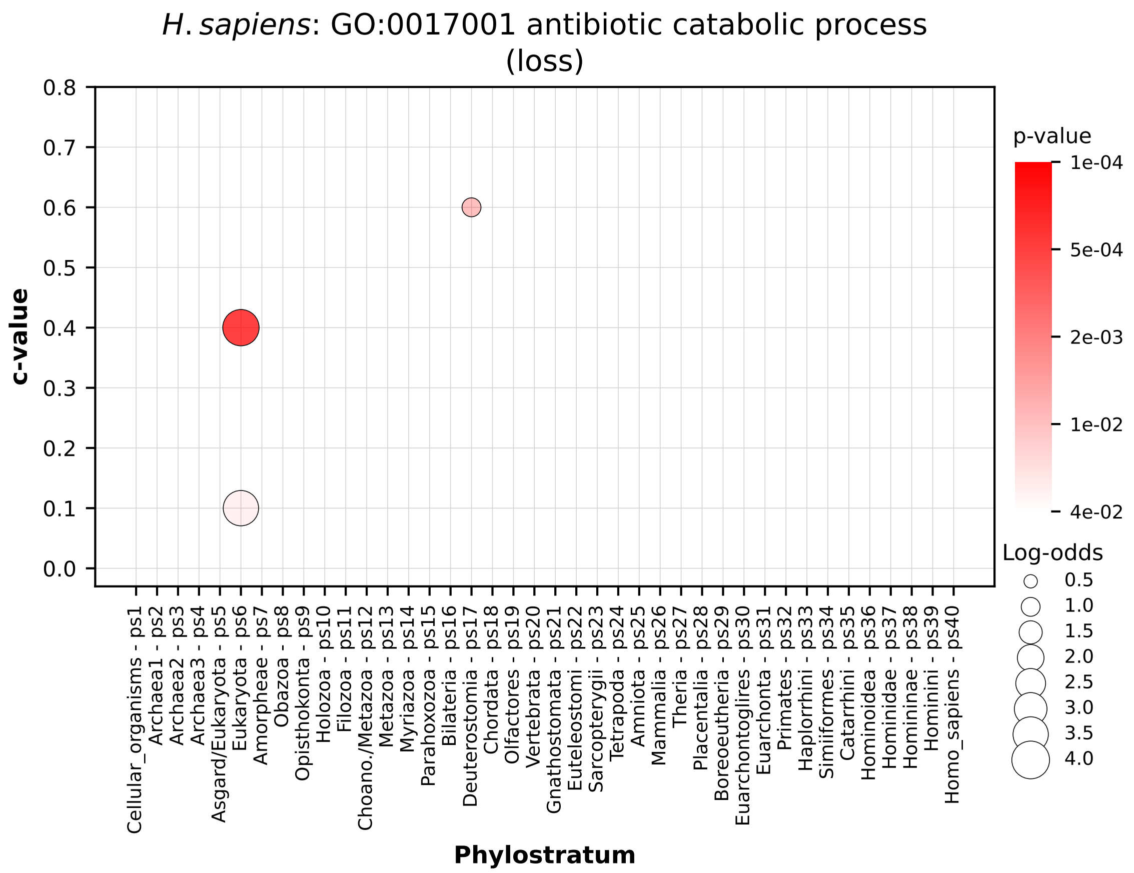

H. sapiens: GO:0017001 antibiotic catabolic process
(loss)
c-value
0.8
0.7
0.6
0.5
0.4
0.3
0.2
0.1
0.0
Phylostratum
Cellular_organisms - ps1
Archaea1 - ps2
Archaea2 - ps3
Archaea3 - ps4
Asgard/Eukaryota - ps5
Eukaryota - ps6
Amorpheae - ps7
Obazoa - ps8
Opisthokonta - ps9
Holozoa - ps10
Filozoa - ps11
Choano./Metazoa - ps12
Metazoa - ps13
Myriazoa - ps14
Parahoxozoa - ps15
Bilateria - ps16
Deuterostomia - ps17
Chordata - ps18
Olfactores - ps19
Vertebrata - ps20
Gnathostomata - ps21
Euteleostomi - ps22
Sarcopterygii - ps23
Tetrapoda - ps24
Amniota - ps25
Mammalia - ps26
Theria - ps27
Placentalia - ps28
Boreoeutheria - ps29
Euarchontoglires - ps30
Euarchonta - ps31
Primates - ps32
Haplorrhini - ps33
Simiiformes - ps34
Catarrhini - ps35
Hominoidea - ps36
Hominidae - ps37
Homininae - ps38
Hominini - ps39
Homo_sapiens - ps40
p-value
1e-04
5e-04
2e-03
1e-02
4e-02
Log-odds
0.5
1.0
1.5
2.0
2.5
3.0
3.5
4.0

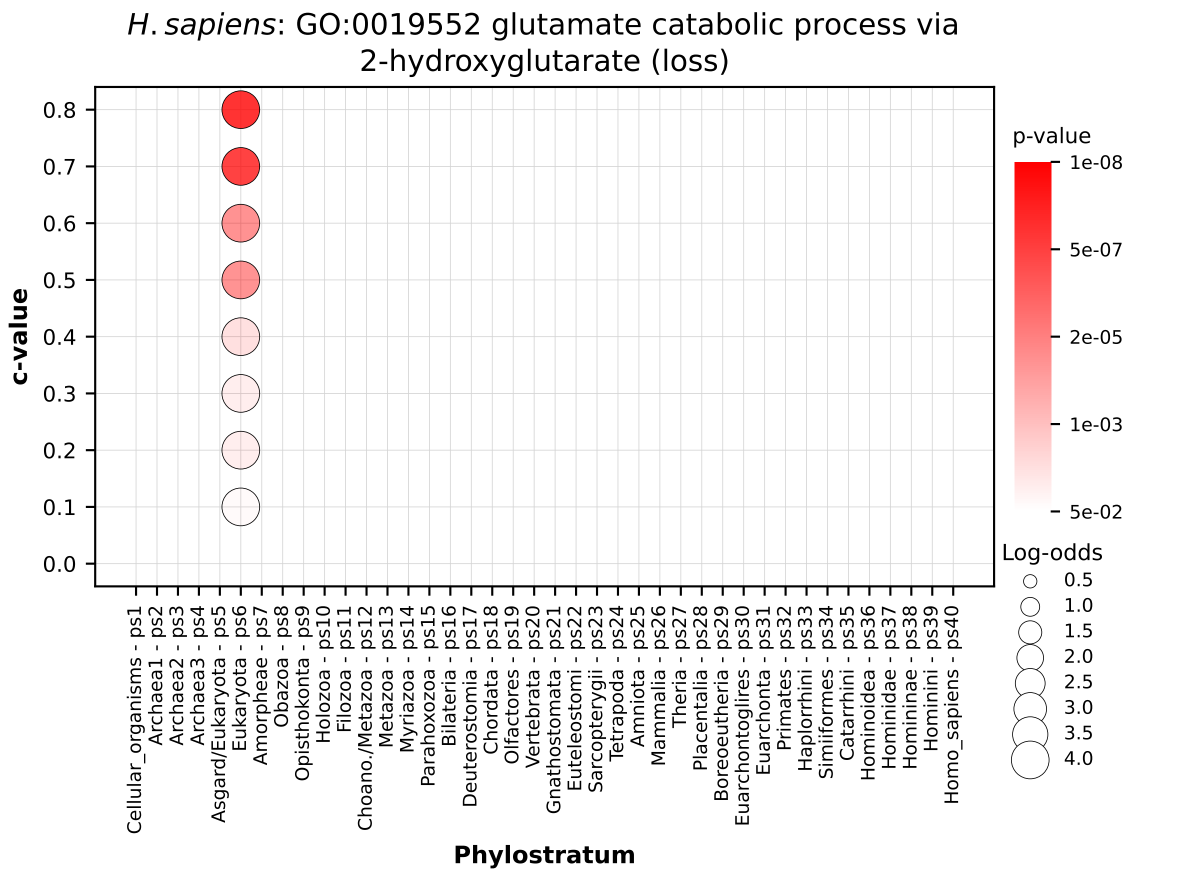

H. sapiens: GO:0019552 glutamate catabolic process via 2-hydroxyglutarate (loss)
c-value
0.0
0.1
0.2
0.3
0.4
0.5
0.6
0.7
0.8
Phylostratum
Cellular_organisms - ps1
Archaea1 - ps2
Archaea2 - ps3
Archaea3 - ps4
Asgard/Eukaryota - ps5
Eukaryota - ps6
Amorpheae - ps7
Obazoa - ps8
Opisthokonta - ps9
Holozoa - ps10
Filozoa - ps11
Choano./Metazoa - ps12
Metazoa - ps13
Myriazoa - ps14
Parahoxozoa - ps15
Bilateria - ps16
Deuterostomia - ps17
Chordata - ps18
Olfactores - ps19
Vertebrata - ps20
Gnathostomata - ps21
Euteleostomi - ps22
Sarcopterygii - ps23
Tetrapoda - ps24
Amniota - ps25
Mammalia - ps26
Theria - ps27
Placentalia - ps28
Boreoeutheria - ps29
Euarchontoglires - ps30
Euarchonta - ps31
Primates - ps32
Haplorrhini - ps33
Simiiformes - ps34
Catarrhini - ps35
Hominoidea - ps36
Hominidae - ps37
Homininae - ps38
Hominini - ps39
Homo_sapiens - ps40
p-value
1e-08
5e-07
2e-05
1e-03
5e-02
Log-odds
0.5
1.0
1.5
2.0
2.5
3.0
3.5
4.0

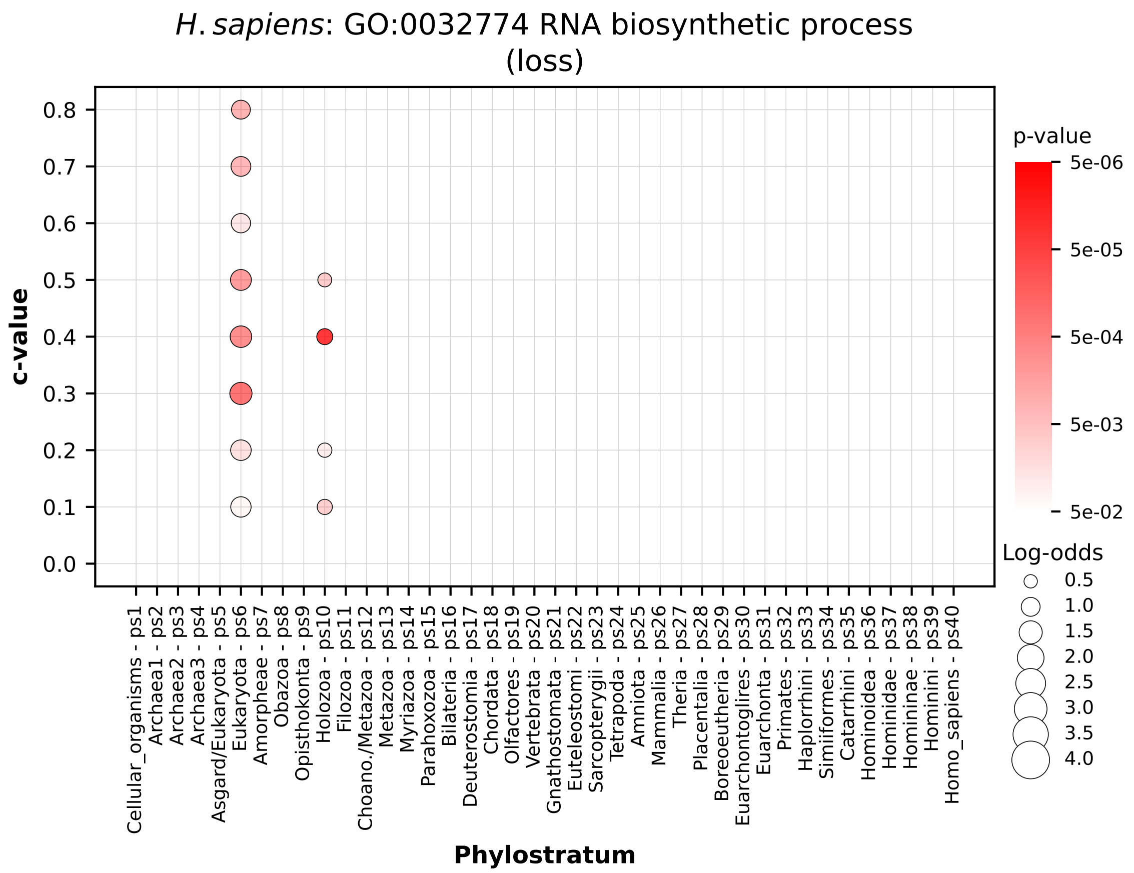

H. sapiens: GO:0032774 RNA biosynthetic process
(loss)
c-value
0.0
0.1
0.2
0.3
0.4
0.5
0.6
0.7
0.8
Phylostratum
Cellular_organisms - ps1
Archaea1 - ps2
Archaea2 - ps3
Archaea3 - ps4
Asgard/Eukaryota - ps5
Eukaryota - ps6
Amorpheae - ps7
Obazoa - ps8
Opisthokonta - ps9
Holozoa - ps10
Filozoa - ps11
Choano./Metazoa - ps12
Metazoa - ps13
Myriazoa - ps14
Parahoxozoa - ps15
Bilateria - ps16
Deuterostomia - ps17
Chordata - ps18
Olfactores - ps19
Vertebrata - ps20
Gnathostomata - ps21
Euteleostomi - ps22
Sarcopterygii - ps23
Tetrapoda - ps24
Amniota - ps25
Mammalia - ps26
Theria - ps27
Placentalia - ps28
Boreoeutheria - ps29
Euarchontoglires - ps30
Euarchonta - ps31
Primates - ps32
Haplorrhini - ps33
Simiiformes - ps34
Catarrhini - ps35
Hominoidea - ps36
Hominidae - ps37
Homininae - ps38
Hominini - ps39
Homo_sapiens - ps40
p-value
5e-06
5e-05
5e-04
5e-03
5e-02
Log-odds
0.5
1.0
1.5
2.0
2.5
3.0
3.5
4.0

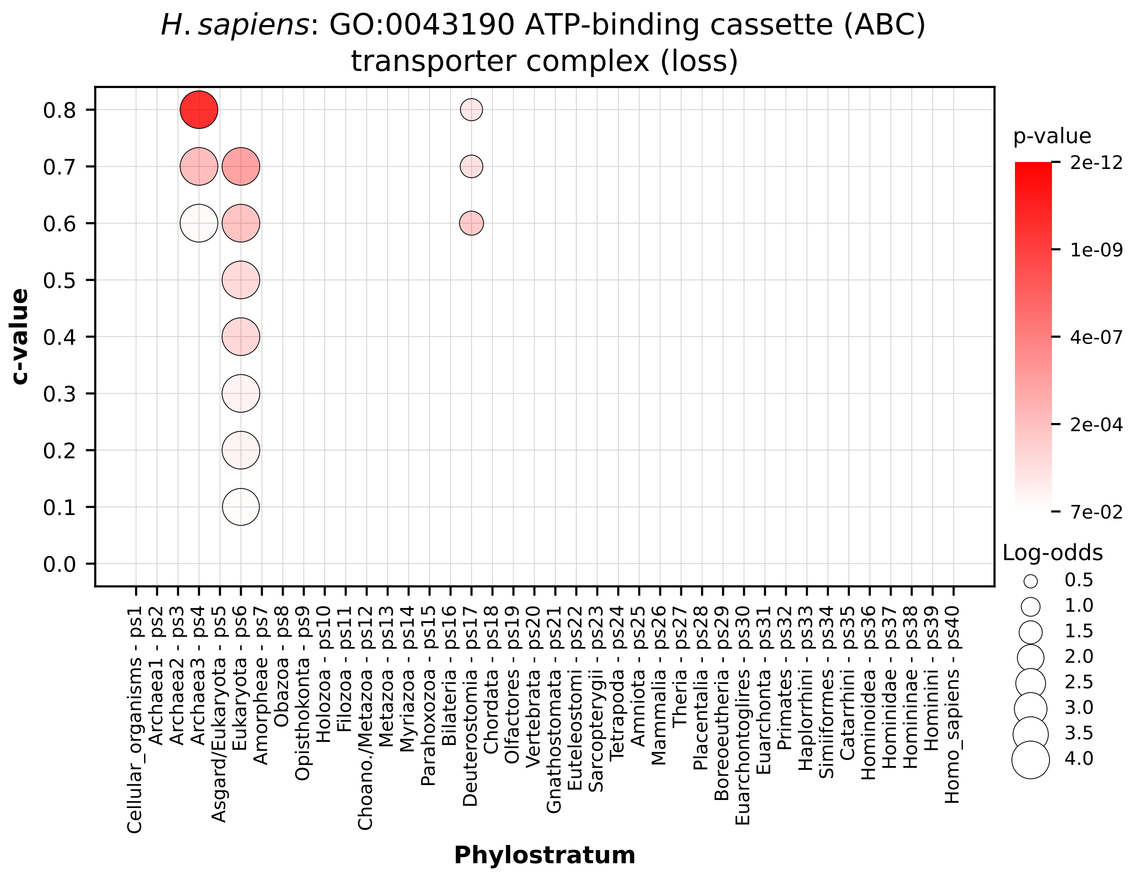

H. sapiens: GO:0043190 ATP-binding cassette (ABC) transporter complex (loss)
c-value
0.8
0.7
0.6
0.5
0.4
0.3
0.2
0.1
0.0
Phylostratum
Cellular_organisms - ps1
Archaea1 - ps2
Archaea2 - ps3
Archaea3 - ps4
Asgard/Eukaryota - ps5
Eukaryota - ps6
Amorpheae - ps7
Obazoa - ps8
Opisthokonta - ps9
Holozoa - ps10
Filozoa - ps11
Choano./Metazoa - ps12
Metazoa - ps13
Myriazoa - ps14
Parahoxozoa - ps15
Bilateria - ps16
Deuterostomia - ps17
Chordata - ps18
Olfactores - ps19
Vertebrata - ps20
Gnathostomata - ps21
Euteleostomi - ps22
Sarcopterygii - ps23
Tetrapoda - ps24
Amniota - ps25
Mammalia - ps26
Theria - ps27
Placentalia - ps28
Boreoeutheria - ps29
Euarchontoglires - ps30
Euarchonta - ps31
Primates - ps32
Haplorrhini - ps33
Simiiformes - ps34
Catarrhini - ps35
Hominoidea - ps36
Hominidae - ps37
Homininae - ps38
Hominini - ps39
Homo_sapiens - ps40
p-value
2e-12
1e-09
4e-07
2e-04
7e-02
Log-odds
0.5
1.0
1.5
2.0
2.5
3.0
3.5
4.0

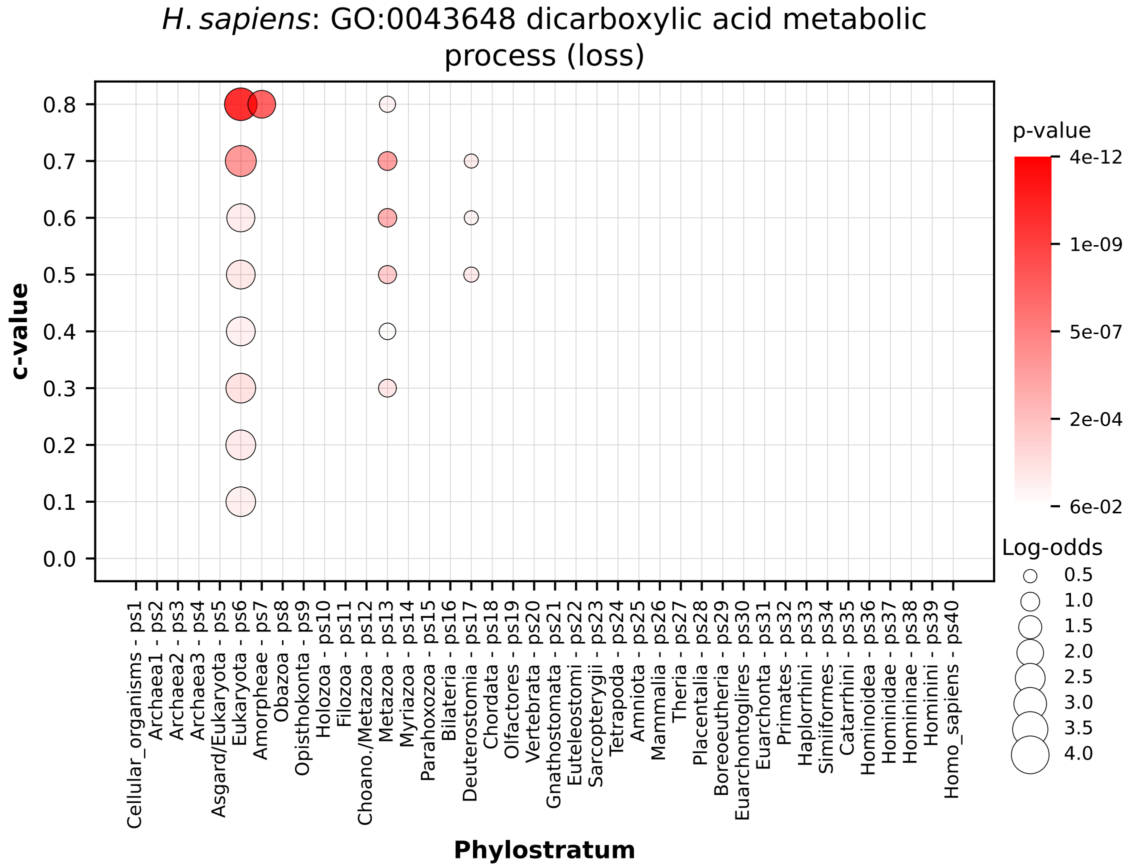

H. sapiens: GO:0043648 dicarboxylic acid metabolic process (loss)
c-value
0.0
0.1
0.2
0.3
0.4
0.5
0.6
0.7
0.8
Phylostratum
Cellular_organisms - ps1
Archaea1 - ps2
Archaea2 - ps3
Archaea3 - ps4
Asgard/Eukaryota - ps5
Eukaryota - ps6
Amorpheae - ps7
Obazoa - ps8
Opisthokonta - ps9
Holozoa - ps10
Filozoa - ps11
Choano./Metazoa - ps12
Metazoa - ps13
Myriazoa - ps14
Parahoxozoa - ps15
Bilateria - ps16
Deuterostomia - ps17
Chordata - ps18
Olfactores - ps19
Vertebrata - ps20
Gnathostomata - ps21
Euteleostomi - ps22
Sarcopterygii - ps23
Tetrapoda - ps24
Amniota - ps25
Mammalia - ps26
Theria - ps27
Placentalia - ps28
Boreoeutheria - ps29
Euarchontoglires - ps30
Euarchonta - ps31
Primates - ps32
Haplorrhini - ps33
Simiiformes - ps34
Catarrhini - ps35
Hominoidea - ps36
Hominidae - ps37
Homininae - ps38
Hominini - ps39
Homo_sapiens - ps40
p-value
4e-12
1e-09
5e-07
2e-04
6e-02
Log-odds
0.5
1.0
1.5
2.0
2.5
3.0
3.5
4.0

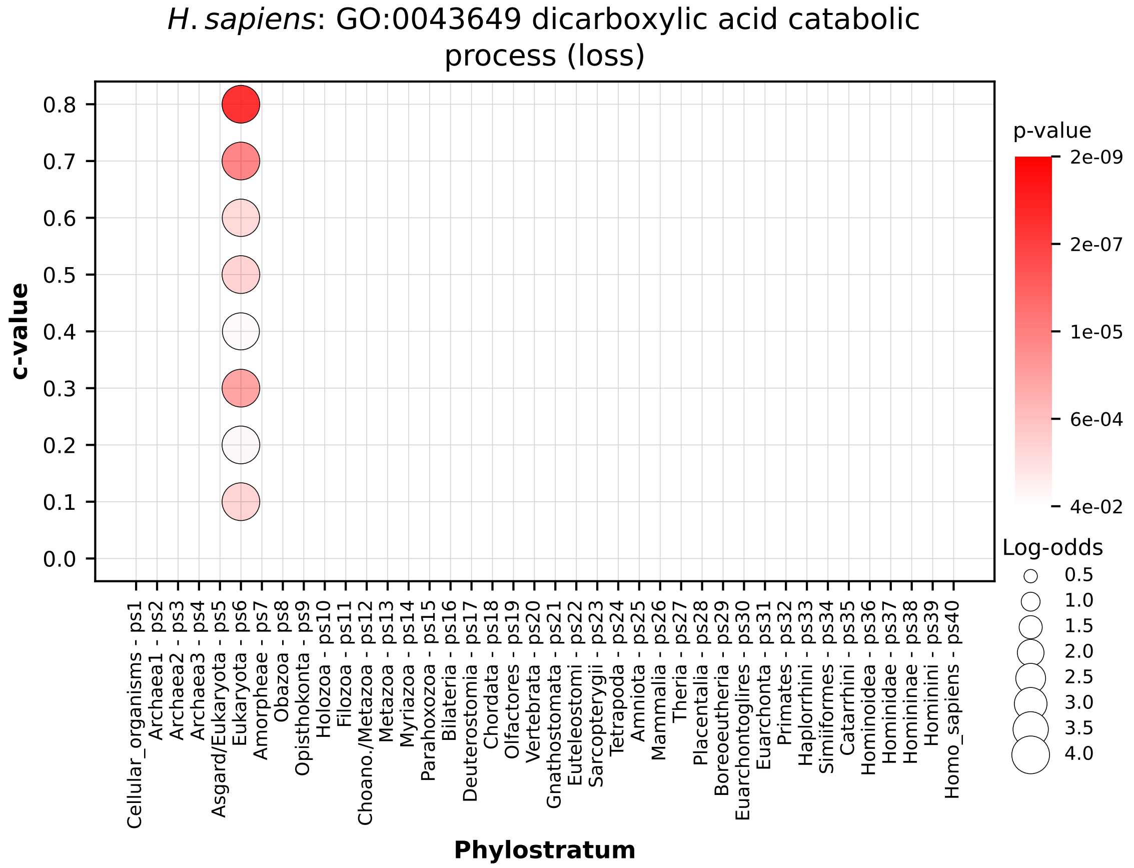

H. sapiens: GO:0043649 dicarboxylic acid catabolic process (loss)
c-value
0.8
0.7
0.6
0.5
0.4
0.3
0.2
0.1
0.0
p-value
2e-09
2e-07
1e-05
6e-04
4e-02
Log-odds
0.5
1.0
1.5
2.0
2.5
3.0
3.5
4.0
Cellular_organisms - ps1
Archaea1 - ps2
Archaea2 - ps3
Archaea3 - ps4
Asgard/Eukaryota - ps5
Eukaryota - ps6
Amorpheae - ps7
Obazoa - ps8
Opisthokonta - ps9
Holozoa - ps10
Filozoa - ps11
Choano./Metazoa - ps12
Metazoa - ps13
Myriazoa - ps14
Parahoxozoa - ps15
Bilateria - ps16
Deuterostomia - ps17
Chordata - ps18
Olfactores - ps19
Vertebrata - ps20
Gnathostomata - ps21
Euteleostomi - ps22
Sarcopterygii - ps23
Tetrapoda - ps24
Amniota - ps25
Mammalia - ps26
Theria - ps27
Placentalia - ps28
Boreoeutheria - ps29
Euarchontoglires - ps30
Euarchonta - ps31
Primates - ps32
Haplorrhini - ps33
Simiiformes - ps34
Catarrhini - ps35
Hominoidea - ps36
Hominidae - ps37
Homininae - ps38
Hominini - ps39
Homo_sapiens - ps40
Phylostratum

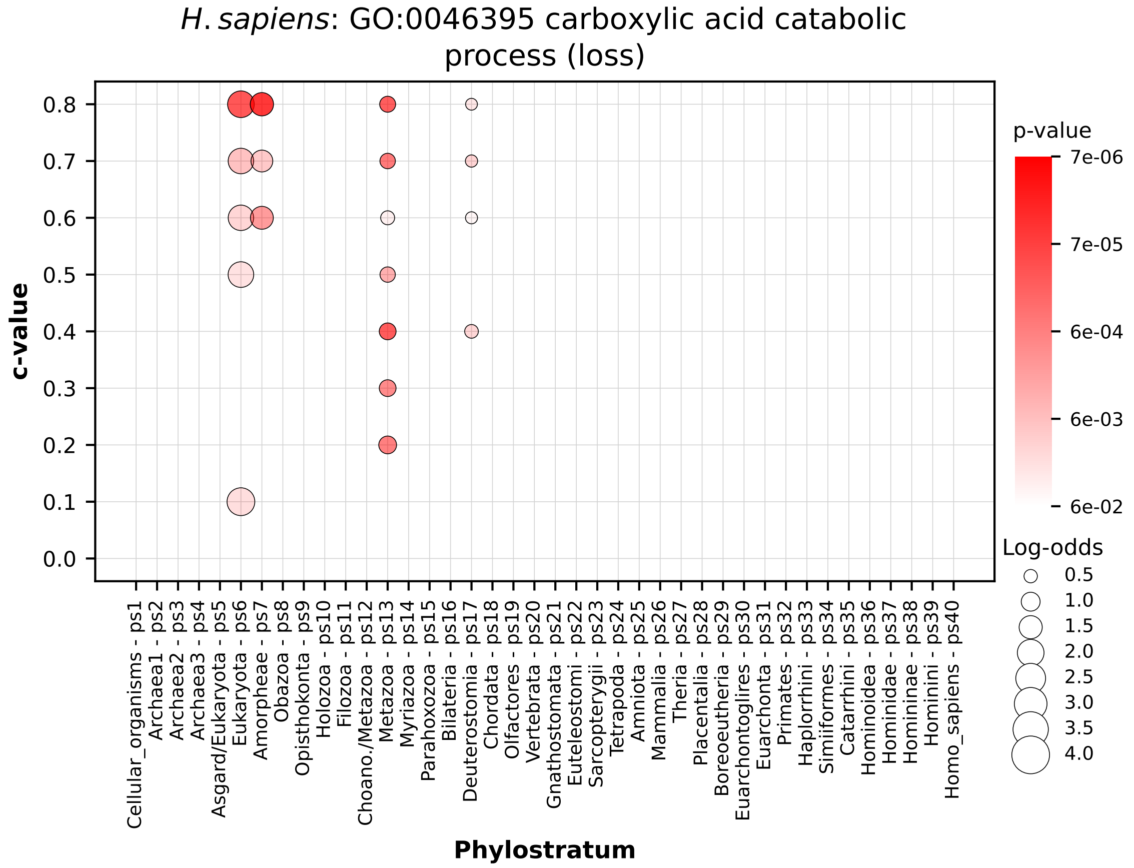

H. sapiens: GO:0046395 carboxylic acid catabolic process (loss)
c-value
0.0
0.1
0.2
0.3
0.4
0.5
0.6
0.7
0.8
Phylostratum
Cellular_organisms - ps1
Archaea1 - ps2
Archaea2 - ps3
Archaea3 - ps4
Asgard/Eukaryota - ps5
Eukaryota - ps6
Amorpheae - ps7
Obazoa - ps8
Opisthokonta - ps9
Holozoa - ps10
Filozoa - ps11
Choano./Metazoa - ps12
Metazoa - ps13
Myriazoa - ps14
Parahoxozoa - ps15
Bilateria - ps16
Deuterostomia - ps17
Chordata - ps18
Olfactores - ps19
Vertebrata - ps20
Gnathostomata - ps21
Euteleostomi - ps22
Sarcopterygii - ps23
Tetrapoda - ps24
Amniota - ps25
Mammalia - ps26
Theria - ps27
Placentalia - ps28
Boreoeutheria - ps29
Euarchontoglires - ps30
Euarchonta - ps31
Primates - ps32
Haplorrhini - ps33
Simiiformes - ps34
Catarrhini - ps35
Hominoidea - ps36
Hominidae - ps37
Homininae - ps38
Hominini - ps39
Homo_sapiens - ps40
p-value
7e-06
7e-05
6e-04
6e-03
6e-02
Log-odds
0.5
1.0
1.5
2.0
2.5
3.0
3.5
4.0

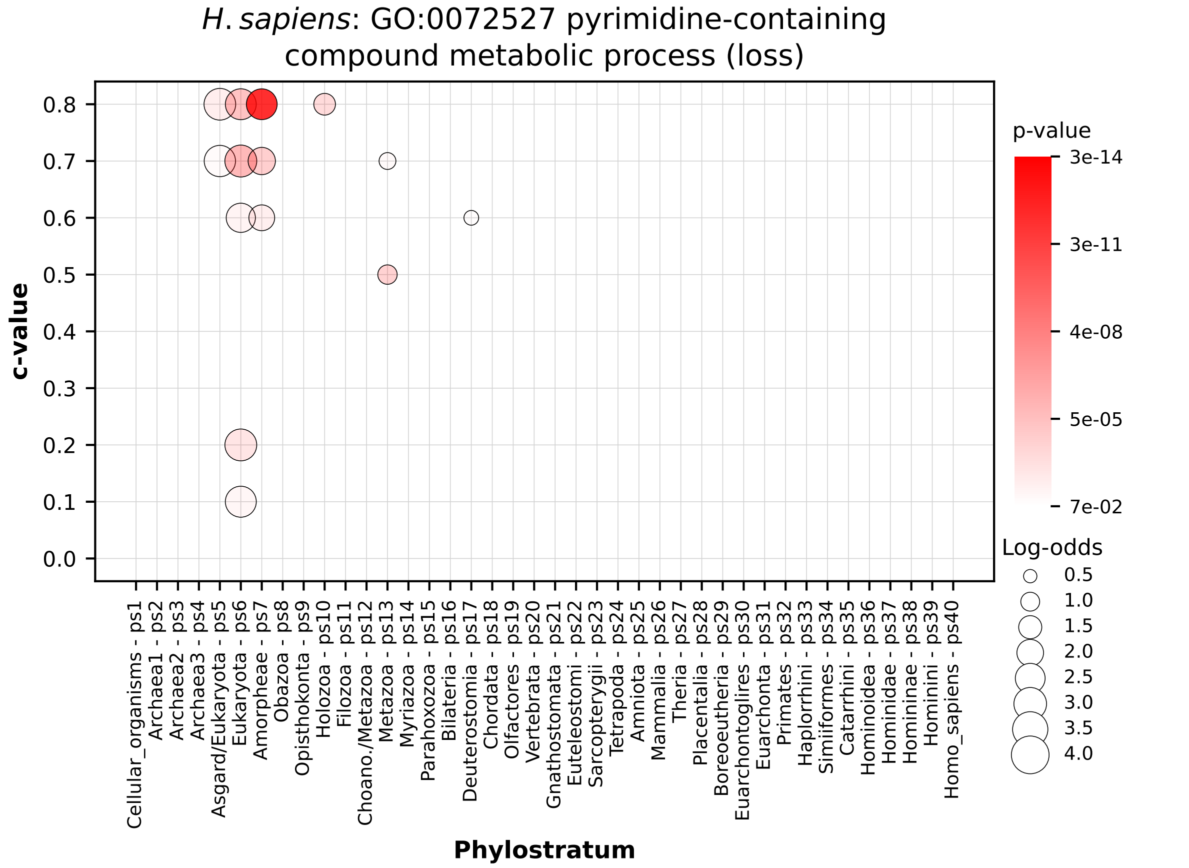

H. sapiens: GO:0072527 pyrimidine-containing compound metabolic process (loss)
c-value
0.0
0.1
0.2
0.3
0.4
0.5
0.6
0.7
0.8
Phylostratum
Cellular_organisms - ps1
Archaea1 - ps2
Archaea2 - ps3
Archaea3 - ps4
Asgard/Eukaryota - ps5
Eukaryota - ps6
Amorpheae - ps7
Obazoa - ps8
Opisthokonta - ps9
Holozoa - ps10
Filozoa - ps11
Choano./Metazoa - ps12
Metazoa - ps13
Myriazoa - ps14
Parahoxozoa - ps15
Bilateria - ps16
Deuterostomia - ps17
Chordata - ps18
Olfactores - ps19
Vertebrata - ps20
Gnathostomata - ps21
Euteleostomi - ps22
Sarcopterygii - ps23
Tetrapoda - ps24
Amniota - ps25
Mammalia - ps26
Theria - ps27
Placentalia - ps28
Boreoeutheria - ps29
Euarchontoglires - ps30
Euarchonta - ps31
Primates - ps32
Haplorrhini - ps33
Simiiformes - ps34
Catarrhini - ps35
Hominoidea - ps36
Hominidae - ps37
Homininae - ps38
Hominini - ps39
Homo_sapiens - ps40
p-value
3e-14
3e-11
4e-08
5e-05
7e-02
Log-odds
0.5
1.0
1.5
2.0
2.5
3.0
3.5
4.0

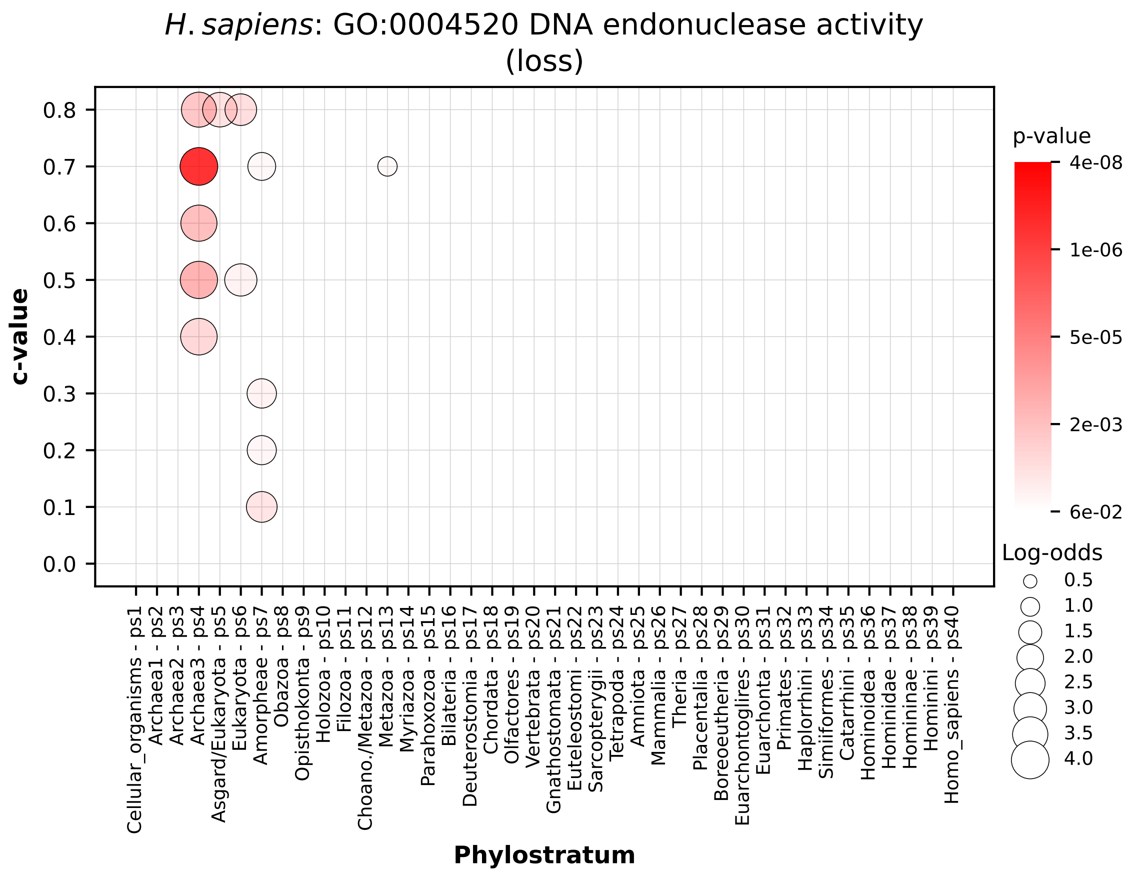

H. sapiens: GO:0004520 DNA endonuclease activity
(loss)
c-value
0.0
0.1
0.2
0.3
0.4
0.5
0.6
0.7
0.8
Phylostratum
Cellular_organisms - ps1
Archaea1 - ps2
Archaea2 - ps3
Archaea3 - ps4
Asgard/Eukaryota - ps5
Eukaryota - ps6
Amorpheae - ps7
Obazoa - ps8
Opisthokonta - ps9
Holozoa - ps10
Filozoa - ps11
Choano./Metazoa - ps12
Metazoa - ps13
Myriazoa - ps14
Parahoxozoa - ps15
Bilateria - ps16
Deuterostomia - ps17
Chordata - ps18
Olfactores - ps19
Vertebrata - ps20
Gnathostomata - ps21
Euteleostomi - ps22
Sarcopterygii - ps23
Tetrapoda - ps24
Amniota - ps25
Mammalia - ps26
Theria - ps27
Placentalia - ps28
Boreoeutheria - ps29
Euarchontoglires - ps30
Euarchonta - ps31
Primates - ps32
Haplorrhini - ps33
Simiiformes - ps34
Catarrhini - ps35
Hominoidea - ps36
Hominidae - ps37
Homininae - ps38
Hominini - ps39
Homo_sapiens - ps40
p-value
4e-08
1e-06
5e-05
2e-03
6e-02
Log-odds
0.5
1.0
1.5
2.0
2.5
3.0
3.5
4.0

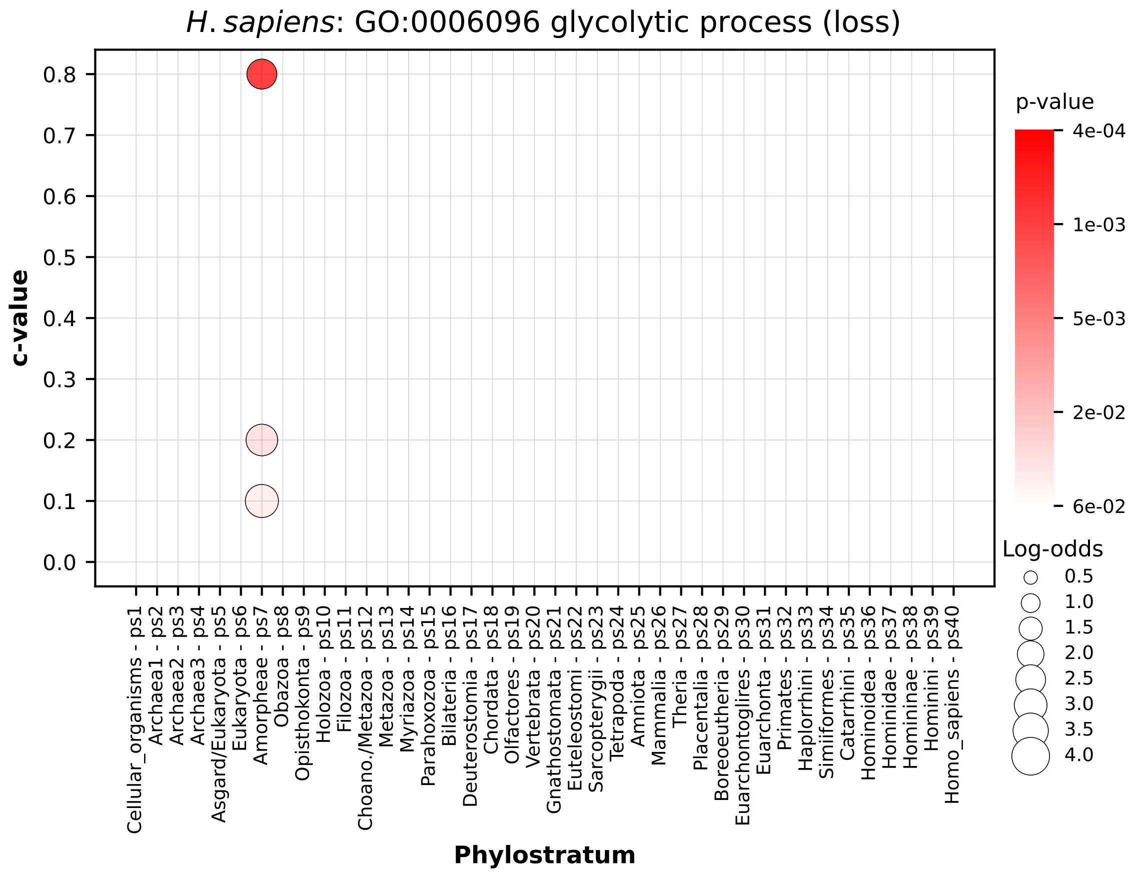

H. sapiens: GO:0006096 glycolytic process (loss)
c-value
0.0
0.1
0.2
0.3
0.4
0.5
0.6
0.7
0.8
p-value
4e-04
1e-03
5e-03
2e-02
6e-02
Log-odds
0.5
1.0
1.5
2.0
2.5
3.0
3.5
4.0
Cellular_organisms - ps1
Archaea1 - ps2
Archaea2 - ps3
Archaea3 - ps4
Asgard/Eukaryota - ps5
Eukaryota - ps6
Amorpheae - ps7
Obazoa - ps8
Opisthokonta - ps9
Holozoa - ps10
Filozoa - ps11
Choano./Metazoa - ps12
Metazoa - ps13
Myriazoa - ps14
Parahoxozoa - ps15
Bilateria - ps16
Deuterostomia - ps17
Chordata - ps18
Olfactores - ps19
Vertebrata - ps20
Gnathostomata - ps21
Euteleostomi - ps22
Sarcopterygii - ps23
Tetrapoda - ps24
Amniota - ps25
Mammalia - ps26
Theria - ps27
Placentalia - ps28
Boreoeutheria - ps29
Euarchontoglires - ps30
Euarchonta - ps31
Primates - ps32
Haplorrhini - ps33
Simiiformes - ps34
Catarrhini - ps35
Hominoidea - ps36
Hominidae - ps37
Homininae - ps38
Hominini - ps39
Homo_sapiens - ps40
Phylostratum

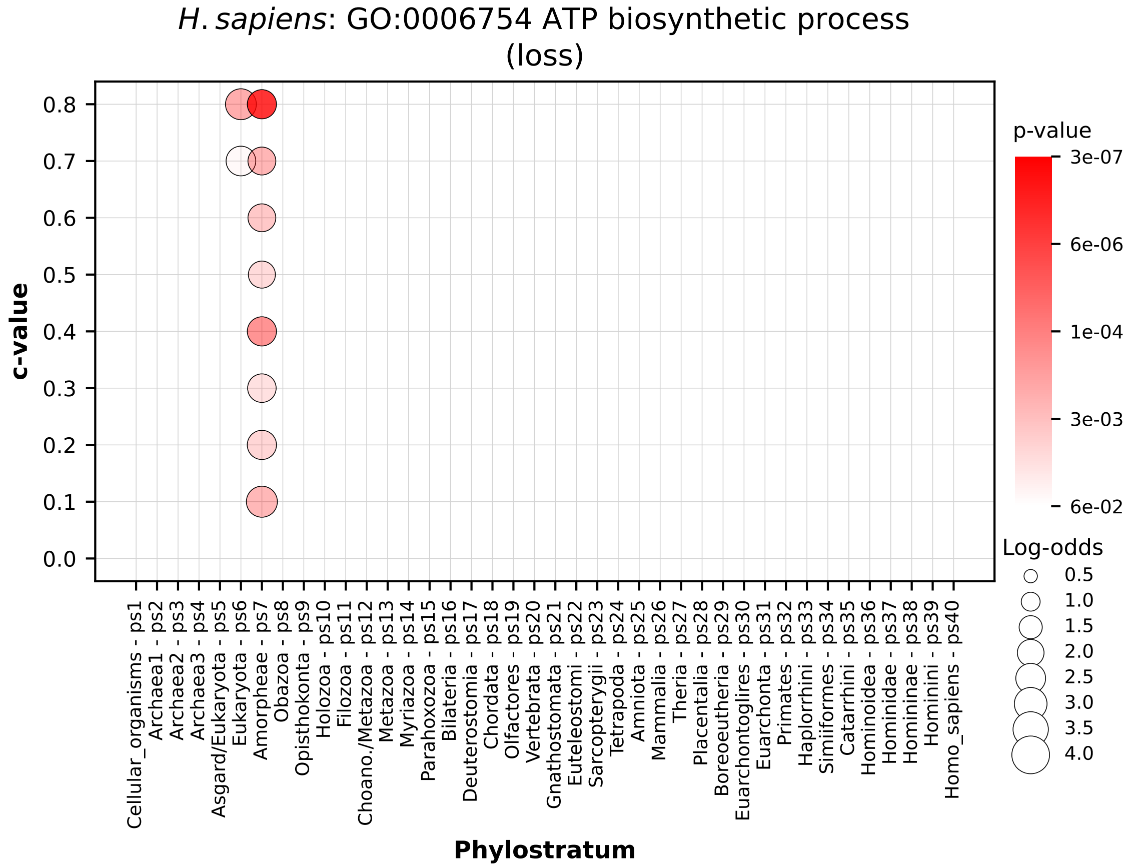

H. sapiens: GO:0006754 ATP biosynthetic process
(loss)
c-value
0.8
0.7
0.6
0.5
0.4
0.3
0.2
0.1
0.0
p-value
3e-07
6e-06
1e-04
3e-03
6e-02
Log-odds
0.5
1.0
1.5
2.0
2.5
3.0
3.5
4.0
Cellular_organisms - ps1
Archaea1 - ps2
Archaea2 - ps3
Archaea3 - ps4
Asgard/Eukaryota - ps5
Eukaryota - ps6
Amorpheae - ps7
Obazoa - ps8
Opisthokonta - ps9
Holozoa - ps10
Filozoa - ps11
Choano./Metazoa - ps12
Metazoa - ps13
Myriazoa - ps14
Parahoxozoa - ps15
Bilateria - ps16
Deuterostomia - ps17
Chordata - ps18
Olfactores - ps19
Vertebrata - ps20
Gnathostomata - ps21
Euteleostomi - ps22
Sarcopterygii - ps23
Tetrapoda - ps24
Amniota - ps25
Mammalia - ps26
Theria - ps27
Placentalia - ps28
Boreoeutheria - ps29
Euarchontoglires - ps30
Euarchonta - ps31
Primates - ps32
Haplorrhini - ps33
Simiiformes - ps34
Catarrhini - ps35
Hominoidea - ps36
Hominidae - ps37
Homininae - ps38
Hominini - ps39
Homo_sapiens - ps40
Phylostratum

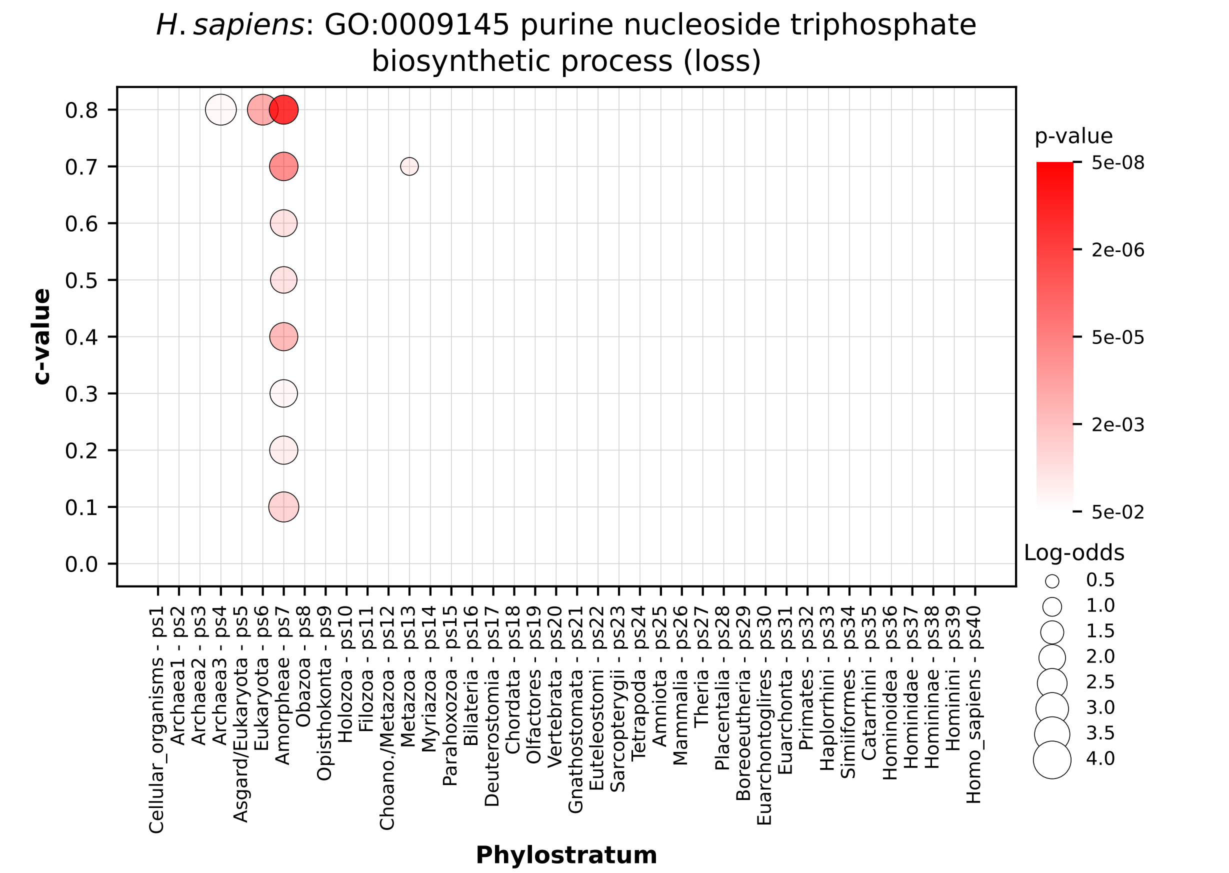

H. sapiens: GO:0009145 purine nucleoside triphosphate biosynthetic process (loss)
c-value
0.0
0.1
0.2
0.3
0.4
0.5
0.6
0.7
0.8
Phylostratum
Cellular_organisms - ps1
Archaea1 - ps2
Archaea2 - ps3
Archaea3 - ps4
Asgard/Eukaryota - ps5
Eukaryota - ps6
Amorpheae - ps7
Obazoa - ps8
Opisthokonta - ps9
Holozoa - ps10
Filozoa - ps11
Choano./Metazoa - ps12
Metazoa - ps13
Myriazoa - ps14
Parahoxozoa - ps15
Bilateria - ps16
Deuterostomia - ps17
Chordata - ps18
Olfactores - ps19
Vertebrata - ps20
Gnathostomata - ps21
Euteleostomi - ps22
Sarcopterygii - ps23
Tetrapoda - ps24
Amniota - ps25
Mammalia - ps26
Theria - ps27
Placentalia - ps28
Boreoeutheria - ps29
Euarchontoglires - ps30
Euarchonta - ps31
Primates - ps32
Haplorrhini - ps33
Simiiformes - ps34
Catarrhini - ps35
Hominoidea - ps36
Hominidae - ps37
Homininae - ps38
Hominini - ps39
Homo_sapiens - ps40
p-value
5e-08
2e-06
5e-05
2e-03
5e-02
Log-odds
0.5
1.0
1.5
2.0
2.5
3.0
3.5
4.0

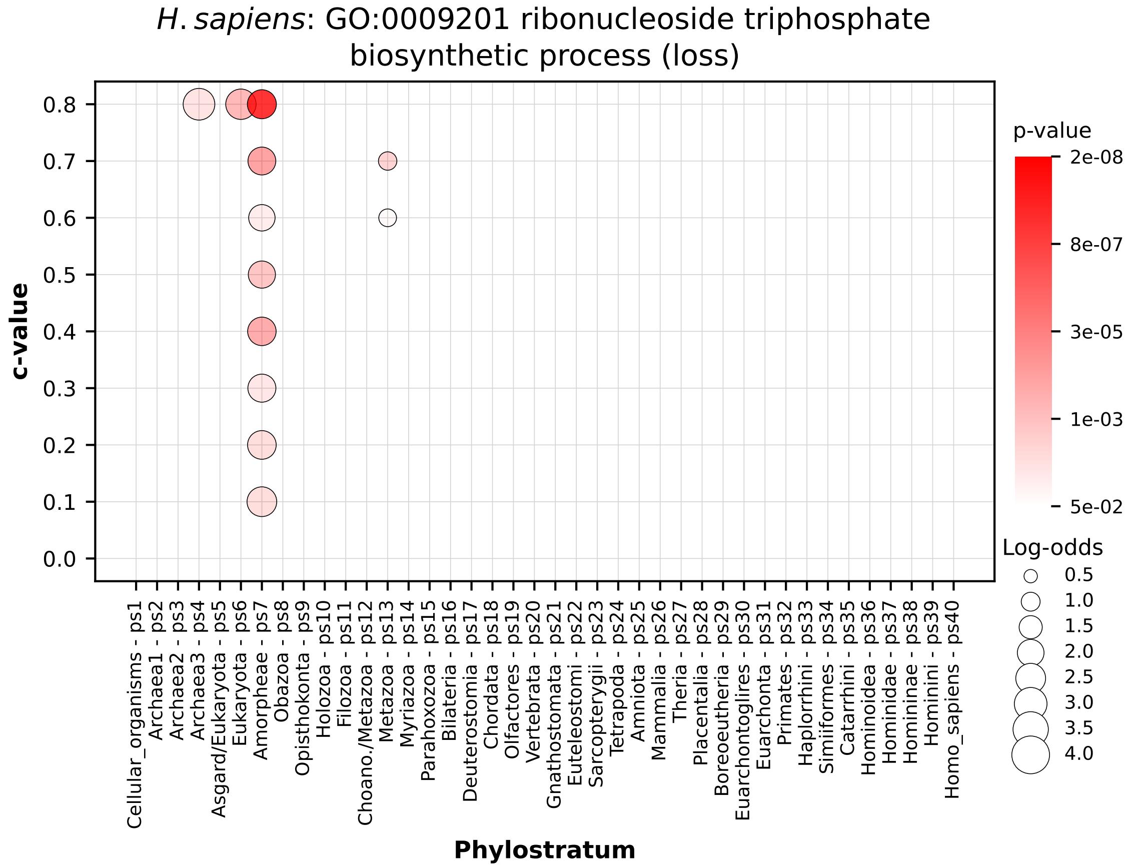

H. sapiens: GO:0009201 ribonucleoside triphosphate biosynthetic process (loss)
c-value
0.8
0.7
0.6
0.5
0.4
0.3
0.2
0.1
0.0
Phylostratum
Cellular_organisms - ps1
Archaea1 - ps2
Archaea2 - ps3
Archaea3 - ps4
Asgard/Eukaryota - ps5
Eukaryota - ps6
Amorpheae - ps7
Obazoa - ps8
Opisthokonta - ps9
Holozoa - ps10
Filozoa - ps11
Choano./Metazoa - ps12
Metazoa - ps13
Myriazoa - ps14
Parahoxozoa - ps15
Bilateria - ps16
Deuterostomia - ps17
Chordata - ps18
Olfactores - ps19
Vertebrata - ps20
Gnathostomata - ps21
Euteleostomi - ps22
Sarcopterygii - ps23
Tetrapoda - ps24
Amniota - ps25
Mammalia - ps26
Theria - ps27
Placentalia - ps28
Boreoeutheria - ps29
Euarchontoglires - ps30
Euarchonta - ps31
Primates - ps32
Haplorrhini - ps33
Simiiformes - ps34
Catarrhini - ps35
Hominoidea - ps36
Hominidae - ps37
Homininae - ps38
Hominini - ps39
Homo_sapiens - ps40
p-value
2e-08
8e-07
3e-05
1e-03
5e-02
Log-odds
0.5
1.0
1.5
2.0
2.5
3.0
3.5
4.0

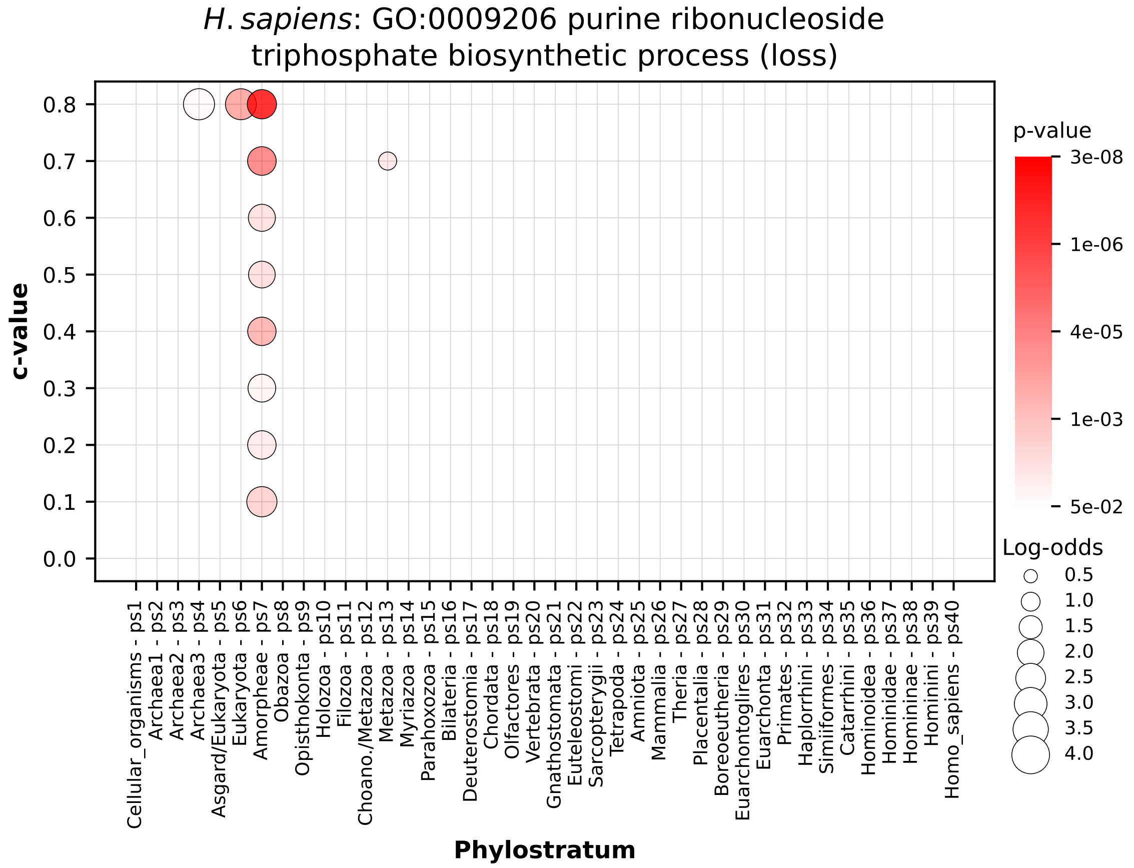

H. sapiens: GO:0009206 purine ribonucleoside triphosphate biosynthetic process (loss)
c-value
0.0
0.1
0.2
0.3
0.4
0.5
0.6
0.7
0.8
Phylostratum
Cellular_organisms - ps1
Archaea1 - ps2
Archaea2 - ps3
Archaea3 - ps4
Asgard/Eukaryota - ps5
Eukaryota - ps6
Amorpheae - ps7
Obazoa - ps8
Opisthokonta - ps9
Holozoa - ps10
Filozoa - ps11
Choano./Metazoa - ps12
Metazoa - ps13
Myriazoa - ps14
Parahoxozoa - ps15
Bilateria - ps16
Deuterostomia - ps17
Chordata - ps18
Olfactores - ps19
Vertebrata - ps20
Gnathostomata - ps21
Euteleostomi - ps22
Sarcopterygii - ps23
Tetrapoda - ps24
Amniota - ps25
Mammalia - ps26
Theria - ps27
Placentalia - ps28
Boreoeutheria - ps29
Euarchontoglires - ps30
Euarchonta - ps31
Primates - ps32
Haplorrhini - ps33
Simiiformes - ps34
Catarrhini - ps35
Hominoidea - ps36
Hominidae - ps37
Homininae - ps38
Hominini - ps39
Homo_sapiens - ps40
p-value
3e-08
1e-06
4e-05
1e-03
5e-02
Log-odds
0.5
1.0
1.5
2.0
2.5
3.0
3.5
4.0

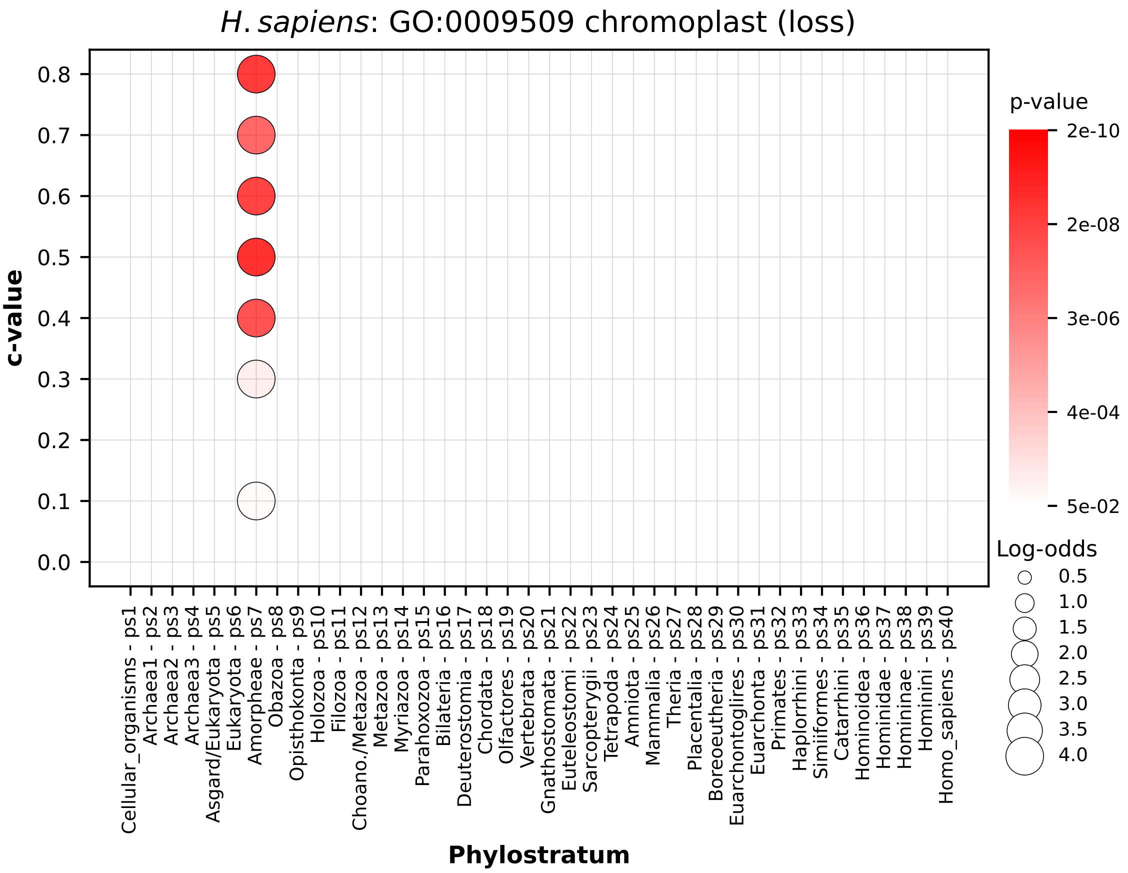

H. sapiens: GO:0009509 chromoplast (loss)
c-value
0.8
0.7
0.6
0.5
0.4
0.3
0.2
0.1
0.0
Cellular_organisms - ps1
Archaea1 - ps2
Archaea2 - ps3
Archaea3 - ps4
Asgard/Eukaryota - ps5
Eukaryota - ps6
Amorpheae - ps7
Obazoa - ps8
Opisthokonta - ps9
Holozoa - ps10
Filozoa - ps11
Choano./Metazoa - ps12
Metazoa - ps13
Myriazoa - ps14
Parahoxozoa - ps15
Bilateria - ps16
Deuterostomia - ps17
Chordata - ps18
Olfactores - ps19
Vertebrata - ps20
Gnathostomata - ps21
Euteleostomi - ps22
Sarcopterygii - ps23
Tetrapoda - ps24
Amniota - ps25
Mammalia - ps26
Theria - ps27
Placentalia - ps28
Boreoeutheria - ps29
Euarchontoglires - ps30
Euarchonta - ps31
Primates - ps32
Haplorrhini - ps33
Simiiformes - ps34
Catarrhini - ps35
Hominoidea - ps36
Hominidae - ps37
Homininae - ps38
Hominini - ps39
Homo_sapiens - ps40
Phylostratum
p-value
2e-10
2e-08
3e-06
4e-04
5e-02
Log-odds
0.5
1.0
1.5
2.0
2.5
3.0
3.5
4.0

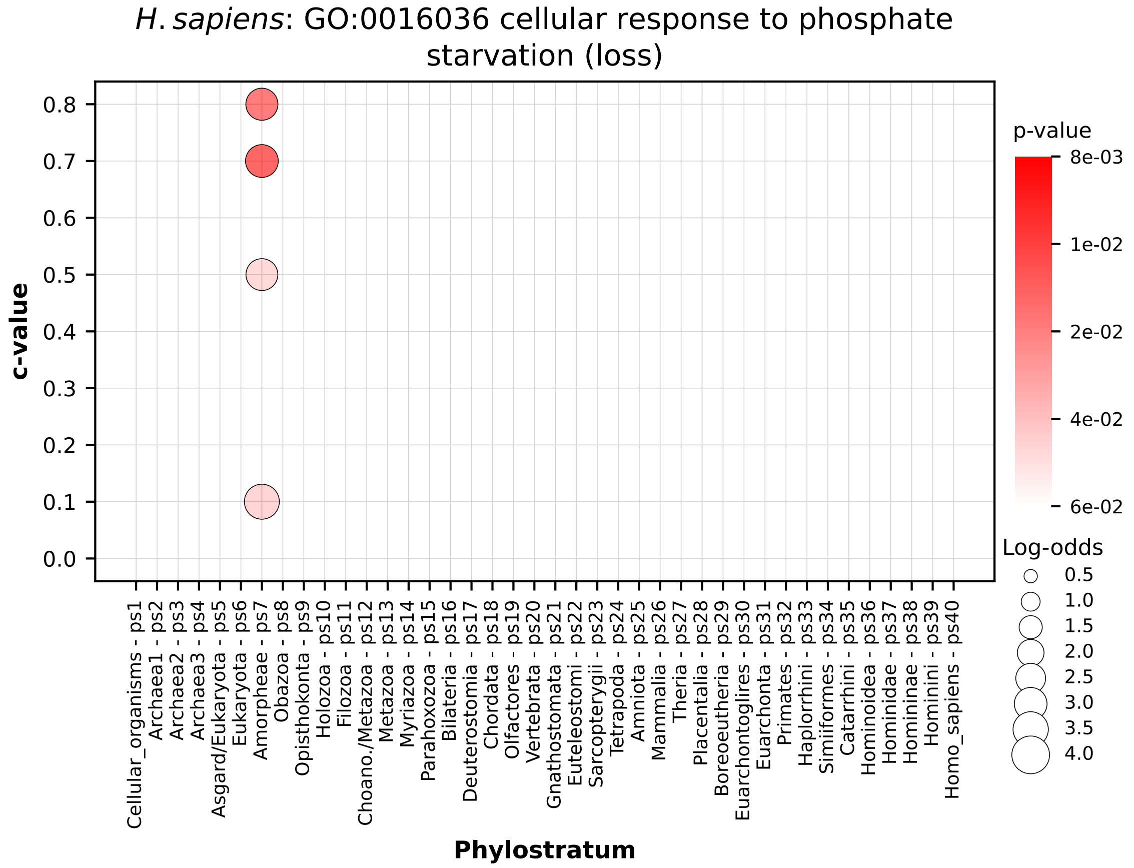

H. sapiens: GO:0016036 cellular response to phosphate starvation (loss)
c-value
0.0
0.1
0.2
0.3
0.4
0.5
0.6
0.7
0.8
p-value
8e-03
1e-02
2e-02
4e-02
6e-02
Log-odds
0.5
1.0
1.5
2.0
2.5
3.0
3.5
4.0
Cellular_organisms - ps1
Archaea1 - ps2
Archaea2 - ps3
Archaea3 - ps4
Asgard/Eukaryota - ps5
Eukaryota - ps6
Amorpheae - ps7
Obazoa - ps8
Opisthokonta - ps9
Holozoa - ps10
Filozoa - ps11
Choano./Metazoa - ps12
Metazoa - ps13
Myriazoa - ps14
Parahoxozoa - ps15
Bilateria - ps16
Deuterostomia - ps17
Chordata - ps18
Olfactores - ps19
Vertebrata - ps20
Gnathostomata - ps21
Euteleostomi - ps22
Sarcopterygii - ps23
Tetrapoda - ps24
Amniota - ps25
Mammalia - ps26
Theria - ps27
Placentalia - ps28
Boreoeutheria - ps29
Euarchontoglires - ps30
Euarchonta - ps31
Primates - ps32
Haplorrhini - ps33
Simiiformes - ps34
Catarrhini - ps35
Hominoidea - ps36
Hominidae - ps37
Homininae - ps38
Hominini - ps39
Homo_sapiens - ps40
Phylostratum

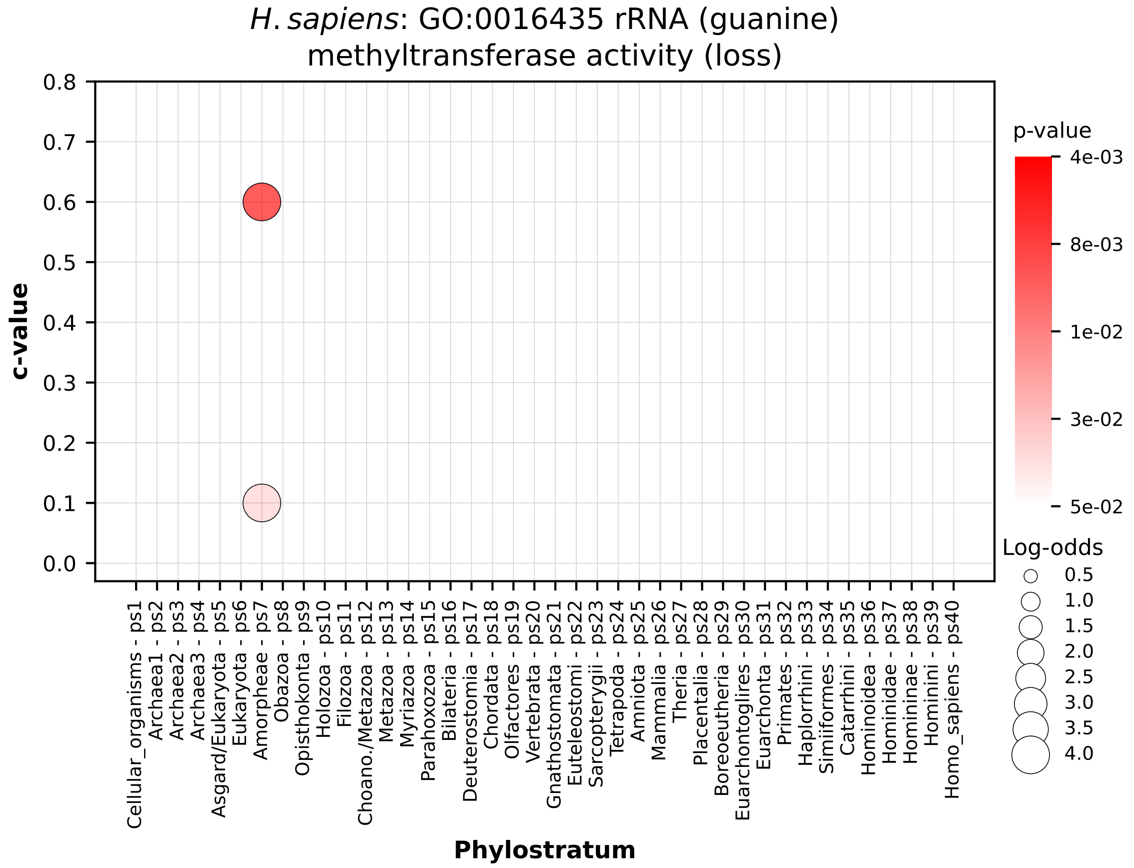

H. sapiens: GO:0016435 rRNA (guanine) methyltransferase activity (loss)
c-value
0.8
0.7
0.6
0.5
0.4
0.3
0.2
0.1
0.0
Phylostratum
Cellular_organisms - ps1
Archaea1 - ps2
Archaea2 - ps3
Archaea3 - ps4
Asgard/Eukaryota - ps5
Eukaryota - ps6
Amorpheae - ps7
Obazoa - ps8
Opisthokonta - ps9
Holozoa - ps10
Filozoa - ps11
Choano./Metazoa - ps12
Metazoa - ps13
Myriazoa - ps14
Parahoxozoa - ps15
Bilateria - ps16
Deuterostomia - ps17
Chordata - ps18
Olfactores - ps19
Vertebrata - ps20
Gnathostomata - ps21
Euteleostomi - ps22
Sarcopterygii - ps23
Tetrapoda - ps24
Amniota - ps25
Mammalia - ps26
Theria - ps27
Placentalia - ps28
Boreoeutheria - ps29
Euarchontoglires - ps30
Euarchonta - ps31
Primates - ps32
Haplorrhini - ps33
Simiiformes - ps34
Catarrhini - ps35
Hominoidea - ps36
Hominidae - ps37
Homininae - ps38
Hominini - ps39
Homo_sapiens - ps40
p-value
4e-03
8e-03
1e-02
3e-02
5e-02
Log-odds
0.5
1.0
1.5
2.0
2.5
3.0
3.5
4.0

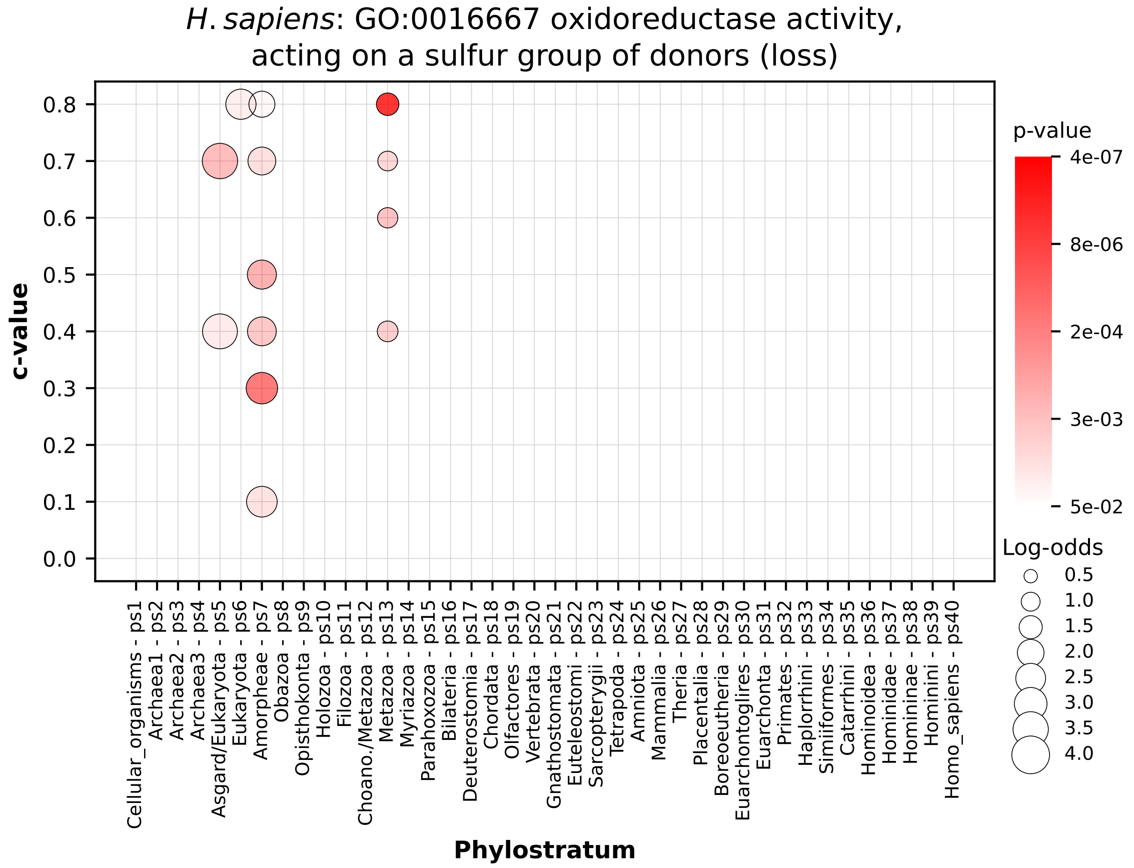
H. sapiens: GO:0016667 oxidoreductase activity,
acting on a sulfur group of donors (loss)
c-value
0.8
0.7
0.6
0.5
0.4
0.3
0.2
0.1
0.0
Phylostratum
Cellular_organisms - ps1
Archaea1 - ps2
Archaea2 - ps3
Archaea3 - ps4
Asgard/Eukaryota - ps5
Eukaryota - ps6
Amorpheae - ps7
Obazoa - ps8
Opisthokonta - ps9
Holozoa - ps10
Filozoa - ps11
Choano./Metazoa - ps12
Metazoa - ps13
Myriazoa - ps14
Parahoxozoa - ps15
Bilateria - ps16
Deuterostomia - ps17
Chordata - ps18
Olfactores - ps19
Vertebrata - ps20
Gnathostomata - ps21
Euteleostomi - ps22
Sarcopterygii - ps23
Tetrapoda - ps24
Amniota - ps25
Mammalia - ps26
Theria - ps27
Placentalia - ps28
Boreoeutheria - ps29
Euarchontoglires - ps30
Euarchonta - ps31
Primates - ps32
Haplorrhini - ps33
Simiiformes - ps34
Catarrhini - ps35
Hominoidea - ps36
Hominidae - ps37
Homininae - ps38
Hominini - ps39
Homo_sapiens - ps40
p-value
4e-07
8e-06
2e-04
3e-03
5e-02
Log-odds
0.5
1.0
1.5
2.0
2.5
3.0
3.5
4.0

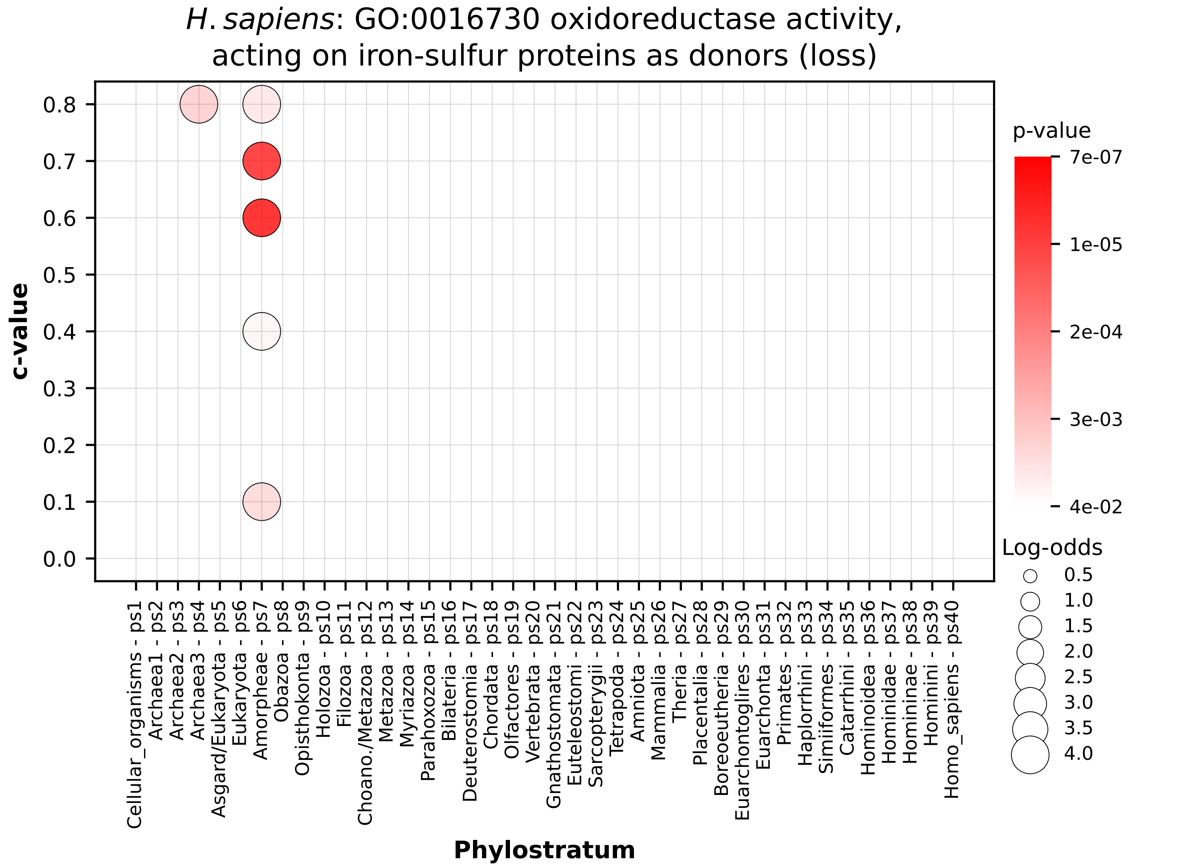

H. sapiens: GO:0016730 oxidoreductase activity,
acting on iron-sulfur proteins as donors (loss)
c-value
0.8
0.7
0.6
0.5
0.4
0.3
0.2
0.1
0.0
p-value
7e-07
1e-05
2e-04
3e-03
4e-02
Log-odds
0.5
1.0
1.5
2.0
2.5
3.0
3.5
4.0
Cellular_organisms - ps1
Archaea1 - ps2
Archaea2 - ps3
Archaea3 - ps4
Asgard/Eukaryota - ps5
Eukaryota - ps6
Amorpheae - ps7
Obazoa - ps8
Opisthokonta - ps9
Holozoa - ps10
Filozoa - ps11
Choano./Metazoa - ps12
Metazoa - ps13
Myriazoa - ps14
Parahoxozoa - ps15
Bilateria - ps16
Deuterostomia - ps17
Chordata - ps18
Olfactores - ps19
Vertebrata - ps20
Gnathostomata - ps21
Euteleostomi - ps22
Sarcopterygii - ps23
Tetrapoda - ps24
Amniota - ps25
Mammalia - ps26
Theria - ps27
Placentalia - ps28
Boreoeutheria - ps29
Euarchontoglires - ps30
Euarchonta - ps31
Primates - ps32
Haplorrhini - ps33
Simiiformes - ps34
Catarrhini - ps35
Hominoidea - ps36
Hominidae - ps37
Homininae - ps38
Hominini - ps39
Homo_sapiens - ps40
Phylostratum

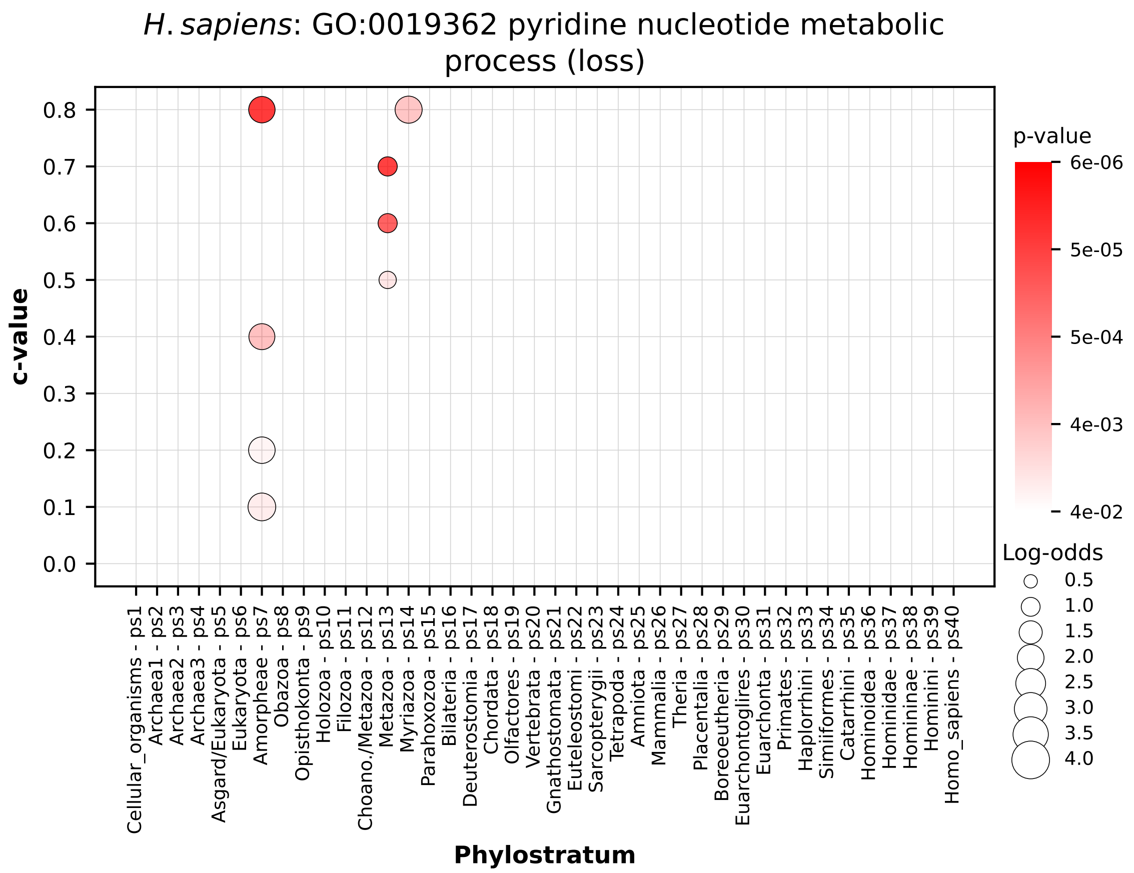

H. sapiens: GO:0019362 pyridine nucleotide metabolic process (loss)
c-value
0.0
0.1
0.2
0.3
0.4
0.5
0.6
0.7
0.8
Phylostratum
Cellular_organisms - ps1
Archaea1 - ps2
Archaea2 - ps3
Archaea3 - ps4
Asgard/Eukaryota - ps5
Eukaryota - ps6
Amorpheae - ps7
Obazoa - ps8
Opisthokonta - ps9
Holozoa - ps10
Filozoa - ps11
Choano./Metazoa - ps12
Metazoa - ps13
Myriazoa - ps14
Parahoxozoa - ps15
Bilateria - ps16
Deuterostomia - ps17
Chordata - ps18
Olfactores - ps19
Vertebrata - ps20
Gnathostomata - ps21
Euteleostomi - ps22
Sarcopterygii - ps23
Tetrapoda - ps24
Amniota - ps25
Mammalia - ps26
Theria - ps27
Placentalia - ps28
Boreoeutheria - ps29
Euarchontoglires - ps30
Euarchonta - ps31
Primates - ps32
Haplorrhini - ps33
Simiiformes - ps34
Catarrhini - ps35
Hominoidea - ps36
Hominidae - ps37
Homininae - ps38
Hominini - ps39
Homo_sapiens - ps40
p-value
6e-06
5e-05
5e-04
4e-03
4e-02
Log-odds
0.5
1.0
1.5
2.0
2.5
3.0
3.5
4.0

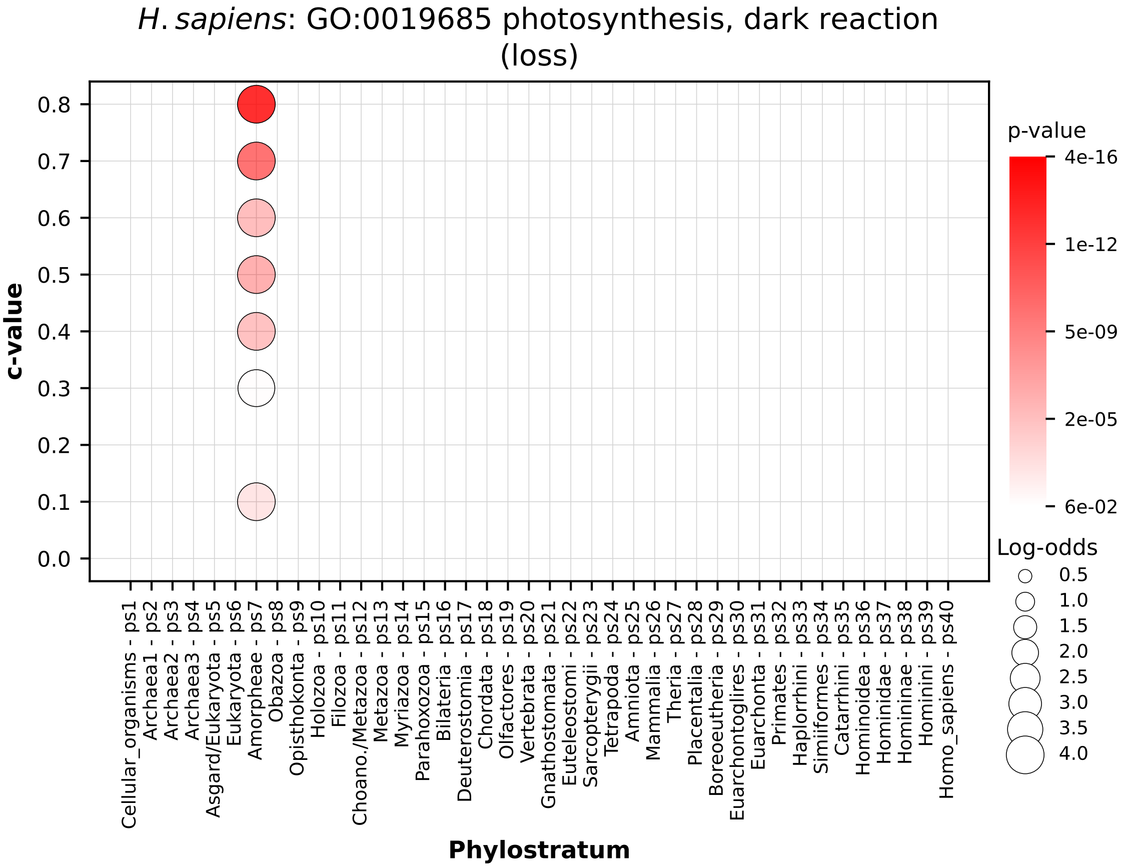

H. sapiens: GO:0019685 photosynthesis, dark reaction (loss)
c-value
0.8
0.7
0.6
0.5
0.4
0.3
0.2
0.1
0.0
p-value
4e-16
1e-12
5e-09
2e-05
6e-02
Log-odds
0.5
1.0
1.5
2.0
2.5
3.0
3.5
4.0
Cellular_organisms - ps1
Archaea1 - ps2
Archaea2 - ps3
Archaea3 - ps4
Asgard/Eukaryota - ps5
Eukaryota - ps6
Amorpheae - ps7
Obazoa - ps8
Opisthokonta - ps9
Holozoa - ps10
Filozoa - ps11
Choano./Metazoa - ps12
Metazoa - ps13
Myriazoa - ps14
Parahoxozoa - ps15
Bilateria - ps16
Deuterostomia - ps17
Chordata - ps18
Olfactores - ps19
Vertebrata - ps20
Gnathostomata - ps21
Euteleostomi - ps22
Sarcopterygii - ps23
Tetrapoda - ps24
Amniota - ps25
Mammalia - ps26
Theria - ps27
Placentalia - ps28
Boreoeutheria - ps29
Euarchontoglires - ps30
Euarchonta - ps31
Primates - ps32
Haplorrhini - ps33
Simiiformes - ps34
Catarrhini - ps35
Hominoidea - ps36
Hominidae - ps37
Homininae - ps38
Hominini - ps39
Homo_sapiens - ps40
Phylostratum

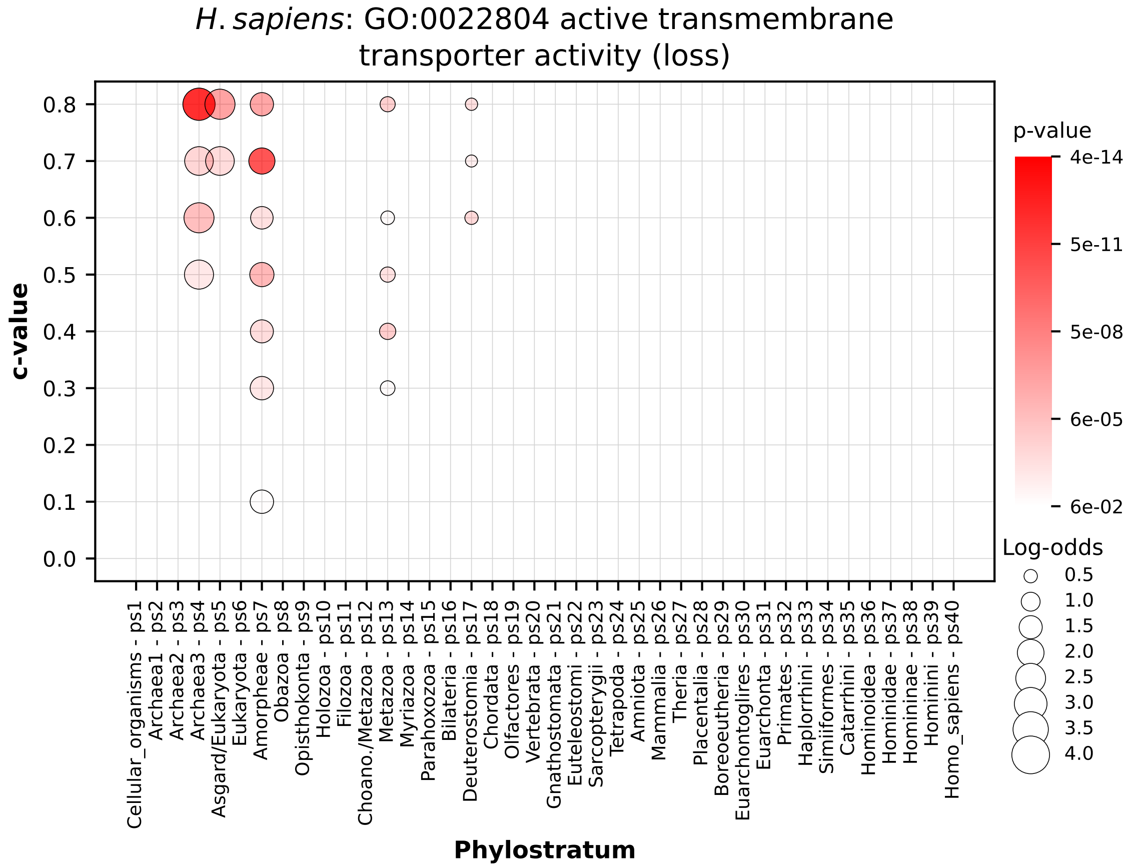

H. sapiens: GO:0022804 active transmembrane transporter activity (loss)
c-value
0.0
0.1
0.2
0.3
0.4
0.5
0.6
0.7
0.8
p-value
4e-14
5e-11
5e-08
6e-05
6e-02
Log-odds
0.5
1.0
1.5
2.0
2.5
3.0
3.5
4.0
Cellular_organisms - ps1
Archaea1 - ps2
Archaea2 - ps3
Archaea3 - ps4
Asgard/Eukaryota - ps5
Eukaryota - ps6
Amorpheae - ps7
Obazoa - ps8
Opisthokonta - ps9
Holozoa - ps10
Filozoa - ps11
Choano./Metazoa - ps12
Metazoa - ps13
Myriazoa - ps14
Parahoxozoa - ps15
Bilateria - ps16
Deuterostomia - ps17
Chordata - ps18
Olfactores - ps19
Vertebrata - ps20
Gnathostomata - ps21
Euteleostomi - ps22
Sarcopterygii - ps23
Tetrapoda - ps24
Amniota - ps25
Mammalia - ps26
Theria - ps27
Placentalia - ps28
Boreoeutheria - ps29
Euarchontoglires - ps30
Euarchonta - ps31
Primates - ps32
Haplorrhini - ps33
Simiiformes - ps34
Catarrhini - ps35
Hominoidea - ps36
Hominidae - ps37
Homininae - ps38
Hominini - ps39
Homo_sapiens - ps40
Phylostratum

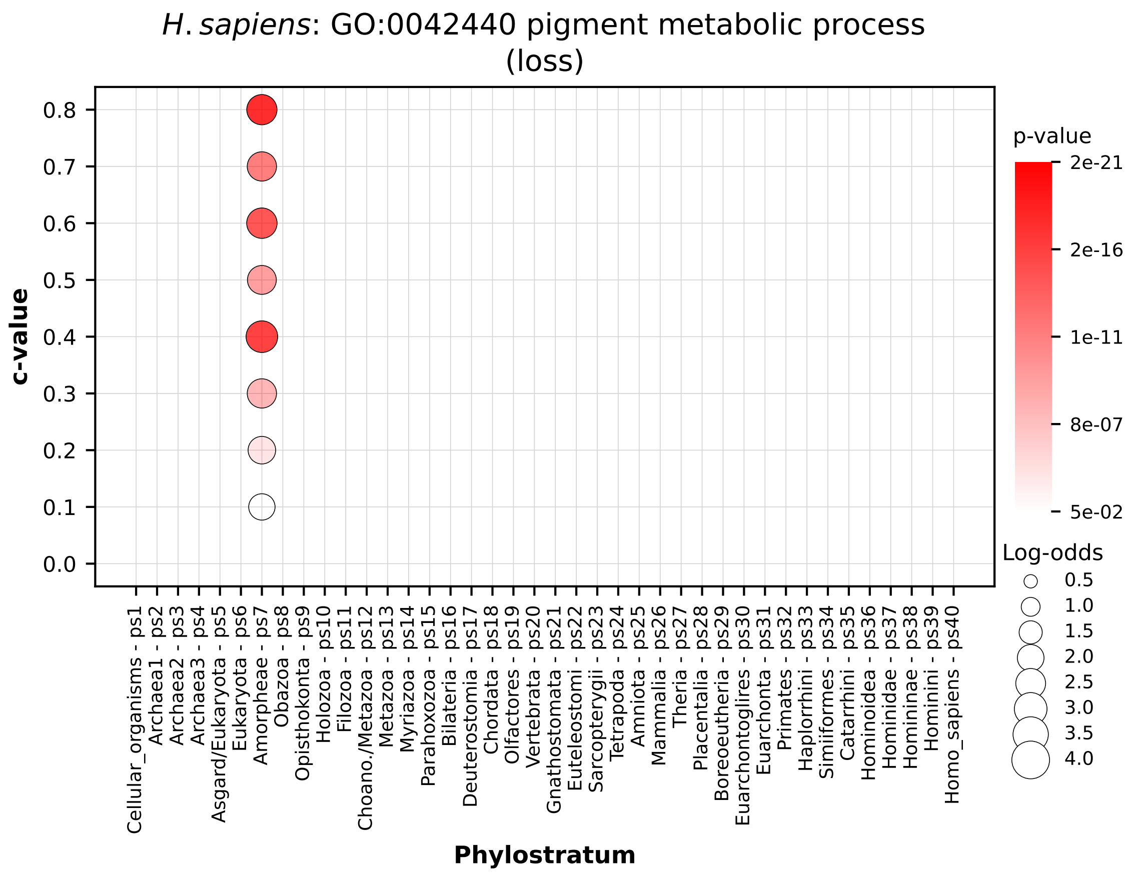

H. sapiens: GO:0042440 pigment metabolic process
(loss)
c-value
0.0
0.1
0.2
0.3
0.4
0.5
0.6
0.7
0.8
p-value
2e-21
2e-16
1e-11
8e-07
5e-02
Log-odds
0.5
1.0
1.5
2.0
2.5
3.0
3.5
4.0
Cellular_organisms - ps1
Archaea1 - ps2
Archaea2 - ps3
Archaea3 - ps4
Asgard/Eukaryota - ps5
Eukaryota - ps6
Amorpheae - ps7
Obazoa - ps8
Opisthokonta - ps9
Holozoa - ps10
Filozoa - ps11
Choano./Metazoa - ps12
Metazoa - ps13
Myriazoa - ps14
Parahoxozoa - ps15
Bilateria - ps16
Deuterostomia - ps17
Chordata - ps18
Olfactores - ps19
Vertebrata - ps20
Gnathostomata - ps21
Euteleostomi - ps22
Sarcopterygii - ps23
Tetrapoda - ps24
Amniota - ps25
Mammalia - ps26
Theria - ps27
Placentalia - ps28
Boreoeutheria - ps29
Euarchontoglires - ps30
Euarchonta - ps31
Primates - ps32
Haplorrhini - ps33
Simiiformes - ps34
Catarrhini - ps35
Hominoidea - ps36
Hominidae - ps37
Homininae - ps38
Hominini - ps39
Homo_sapiens - ps40
Phylostratum

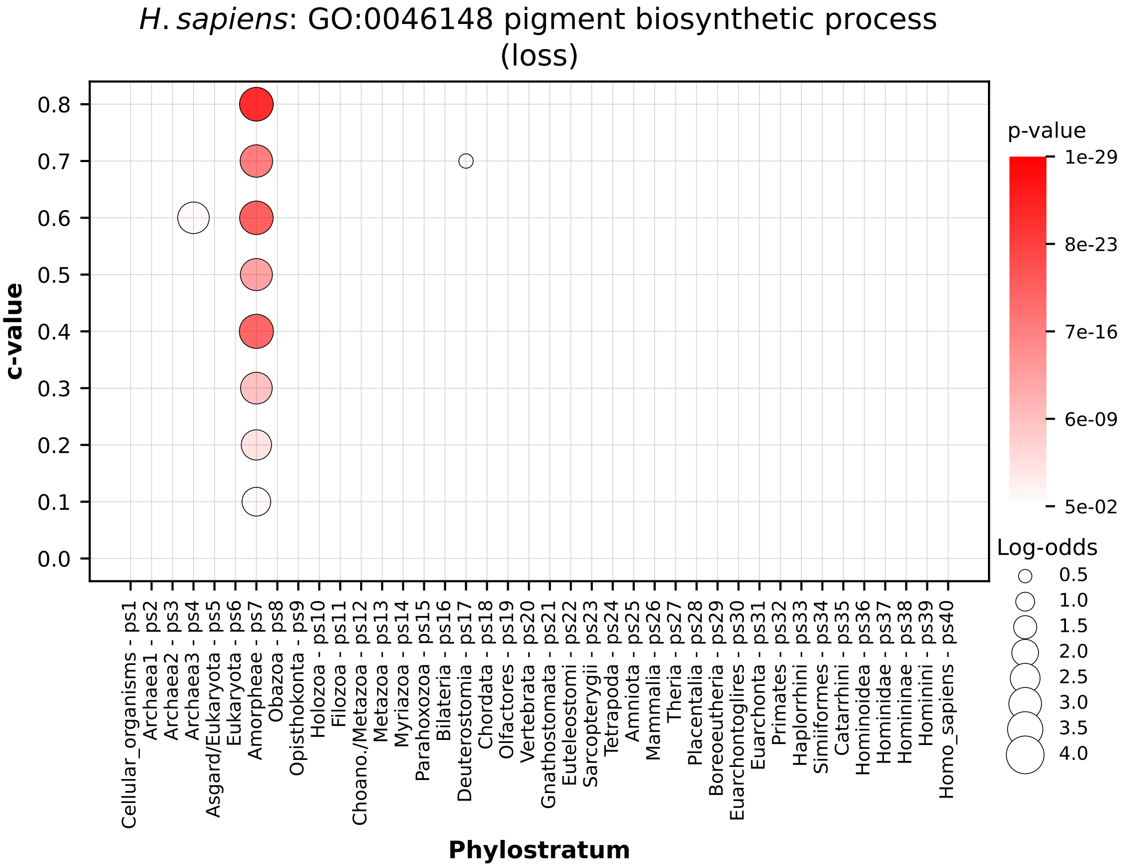

H. sapiens: GO:0046148 pigment biosynthetic process
(loss)
c-value
0.0
0.1
0.2
0.3
0.4
0.5
0.6
0.7
0.8
Phylostratum
Cellular_organisms - ps1
Archaea1 - ps2
Archaea2 - ps3
Archaea3 - ps4
Asgard/Eukaryota - ps5
Eukaryota - ps6
Amorpheae - ps7
Obazoa - ps8
Opisthokonta - ps9
Holozoa - ps10
Filozoa - ps11
Choano./Metazoa - ps12
Metazoa - ps13
Myriazoa - ps14
Parahoxozoa - ps15
Bilateria - ps16
Deuterostomia - ps17
Chordata - ps18
Olfactores - ps19
Vertebrata - ps20
Gnathostomata - ps21
Euteleostomi - ps22
Sarcopterygii - ps23
Tetrapoda - ps24
Amniota - ps25
Mammalia - ps26
Theria - ps27
Placentalia - ps28
Boreoeutheria - ps29
Euarchontoglires - ps30
Euarchonta - ps31
Primates - ps32
Haplorrhini - ps33
Simiiformes - ps34
Catarrhini - ps35
Hominoidea - ps36
Hominidae - ps37
Homininae - ps38
Hominini - ps39
Homo_sapiens - ps40
p-value
1e-29
8e-23
7e-16
6e-09
5e-02
Log-odds
0.5
1.0
1.5
2.0
2.5
3.0
3.5
4.0

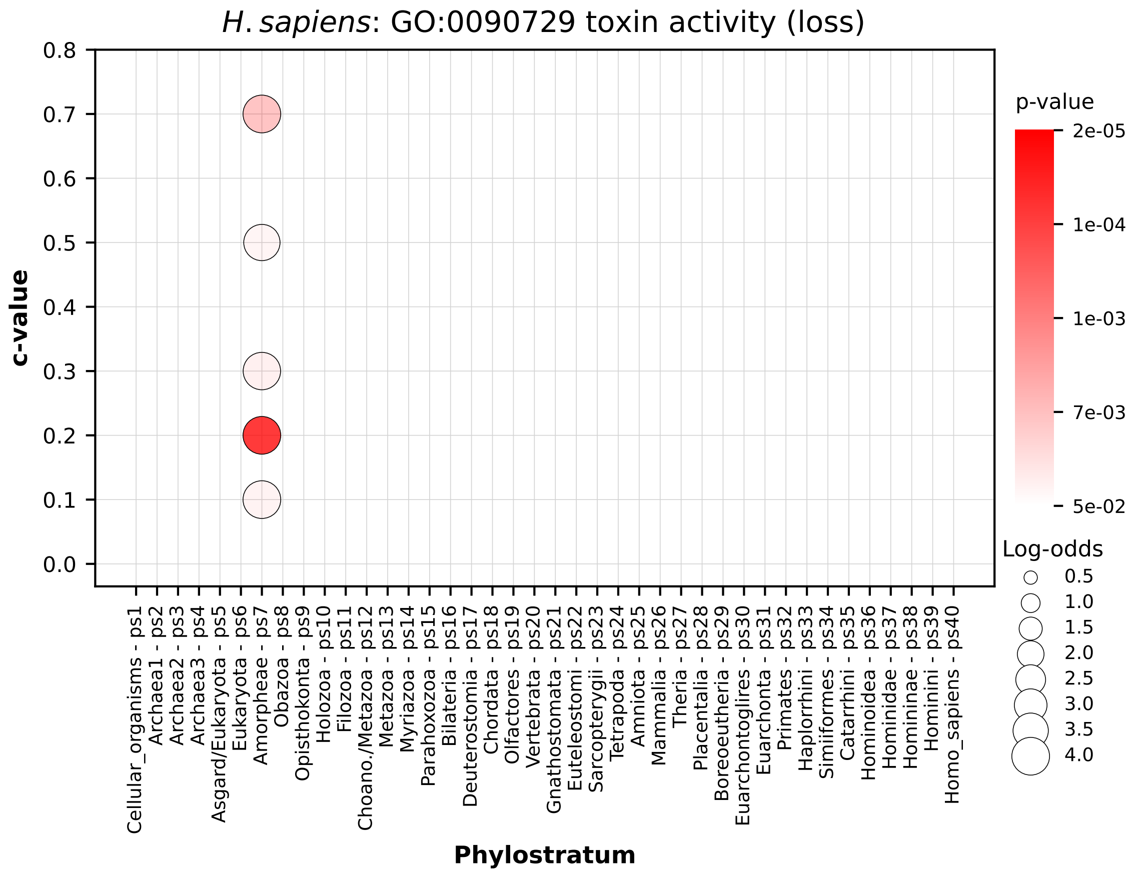

H. sapiens: GO:0090729 toxin activity (loss)
c-value
0.0
0.1
0.2
0.3
0.4
0.5
0.6
0.7
0.8
Phylostratum
Cellular_organisms - ps1
Archaea1 - ps2
Archaea2 - ps3
Archaea3 - ps4
Asgard/Eukaryota - ps5
Eukaryota - ps6
Amorpheae - ps7
Obazoa - ps8
Opisthokonta - ps9
Holozoa - ps10
Filozoa - ps11
Choano./Metazoa - ps12
Metazoa - ps13
Myriazoa - ps14
Parahoxozoa - ps15
Bilateria - ps16
Deuterostomia - ps17
Chordata - ps18
Olfactores - ps19
Vertebrata - ps20
Gnathostomata - ps21
Euteleostomi - ps22
Sarcopterygii - ps23
Tetrapoda - ps24
Amniota - ps25
Mammalia - ps26
Theria - ps27
Placentalia - ps28
Boreoeutheria - ps29
Euarchontoglires - ps30
Euarchonta - ps31
Primates - ps32
Haplorrhini - ps33
Simiiformes - ps34
Catarrhini - ps35
Hominoidea - ps36
Hominidae - ps37
Homininae - ps38
Hominini - ps39
Homo_sapiens - ps40
p-value
2e-05
1e-04
1e-03
7e-03
5e-02
Log-odds
0.5
1.0
1.5
2.0
2.5
3.0
3.5
4.0

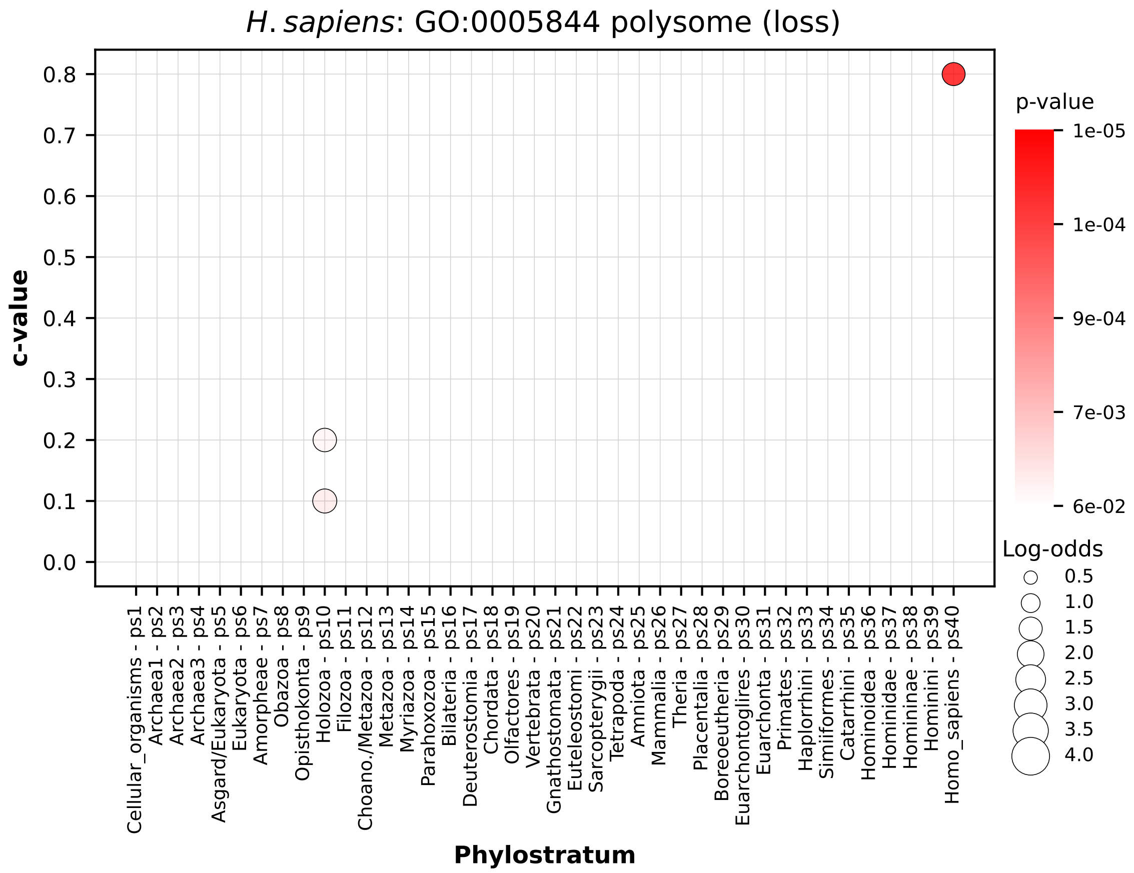

H. sapiens: GO:0005844 polysome (loss)
c-value
0.8
0.7
0.6
0.5
0.4
0.3
0.2
0.1
0.0
p-value
1e-05
1e-04
9e-04
7e-03
6e-02
Log-odds
0.5
1.0
1.5
2.0
2.5
3.0
3.5
4.0
Cellular_organisms - ps1
Archaea1 - ps2
Archaea2 - ps3
Archaea3 - ps4
Asgard/Eukaryota - ps5
Eukaryota - ps6
Amorpheae - ps7
Obazoa - ps8
Opisthokonta - ps9
Holozoa - ps10
Filozoa - ps11
Choano./Metazoa - ps12
Metazoa - ps13
Myriazoa - ps14
Parahoxozoa - ps15
Bilateria - ps16
Deuterostomia - ps17
Chordata - ps18
Olfactores - ps19
Vertebrata - ps20
Gnathostomata - ps21
Euteleostomi - ps22
Sarcopterygii - ps23
Tetrapoda - ps24
Amniota - ps25
Mammalia - ps26
Theria - ps27
Placentalia - ps28
Boreoeutheria - ps29
Euarchontoglires - ps30
Euarchonta - ps31
Primates - ps32
Haplorrhini - ps33
Simiiformes - ps34
Catarrhini - ps35
Hominoidea - ps36
Hominidae - ps37
Homininae - ps38
Hominini - ps39
Homo_sapiens - ps40
Phylostratum

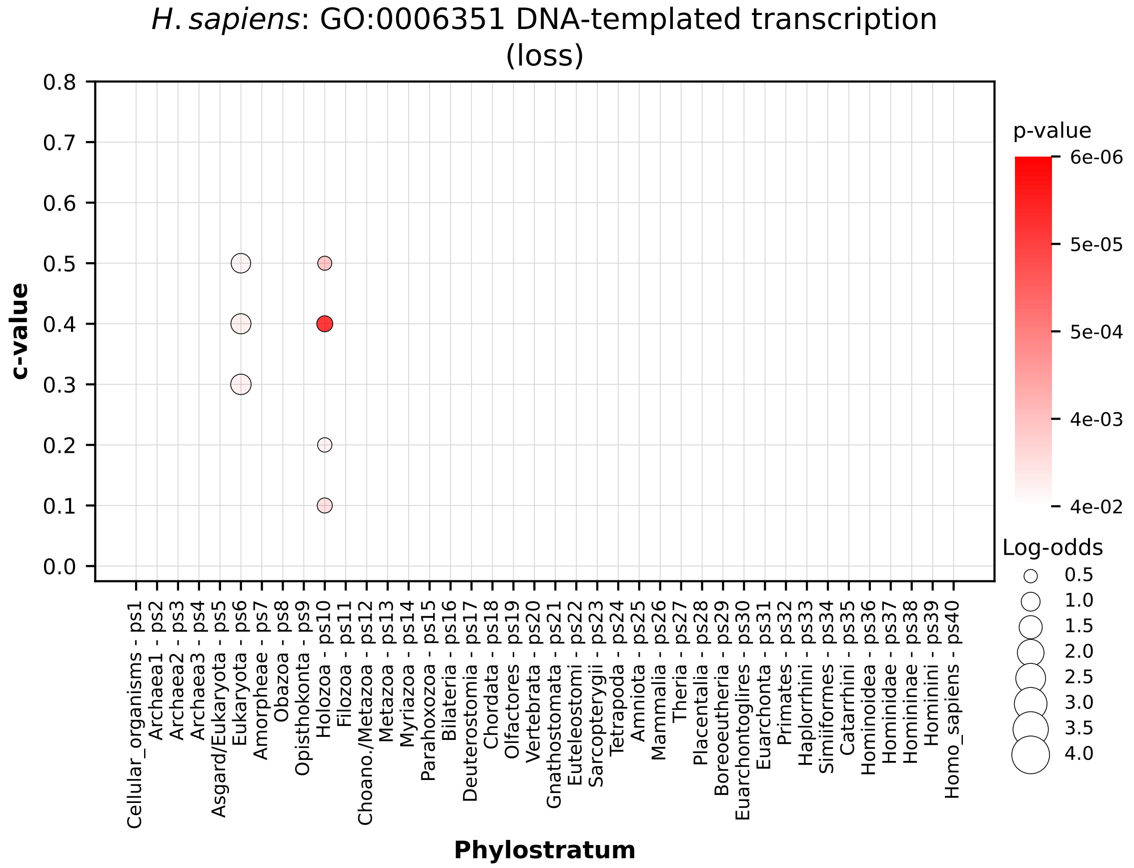

H. sapiens: GO:0006351 DNA-templated transcription
(loss)
c-value
0.0
0.1
0.2
0.3
0.4
0.5
0.6
0.7
0.8
Phylostratum
Cellular_organisms - ps1
Archaea1 - ps2
Archaea2 - ps3
Archaea3 - ps4
Asgard/Eukaryota - ps5
Eukaryota - ps6
Amorpheae - ps7
Obazoa - ps8
Opisthokonta - ps9
Holozoa - ps10
Filozoa - ps11
Choano./Metazoa - ps12
Metazoa - ps13
Myriazoa - ps14
Parahoxozoa - ps15
Bilateria - ps16
Deuterostomia - ps17
Chordata - ps18
Olfactores - ps19
Vertebrata - ps20
Gnathostomata - ps21
Euteleostomi - ps22
Sarcopterygii - ps23
Tetrapoda - ps24
Amniota - ps25
Mammalia - ps26
Theria - ps27
Placentalia - ps28
Boreoeutheria - ps29
Euarchontoglires - ps30
Euarchonta - ps31
Primates - ps32
Haplorrhini - ps33
Simiiformes - ps34
Catarrhini - ps35
Hominoidea - ps36
Hominidae - ps37
Homininae - ps38
Hominini - ps39
Homo_sapiens - ps40
p-value
6e-06
5e-05
5e-04
4e-03
4e-02
Log-odds
0.5
1.0
1.5
2.0
2.5
3.0
3.5
4.0

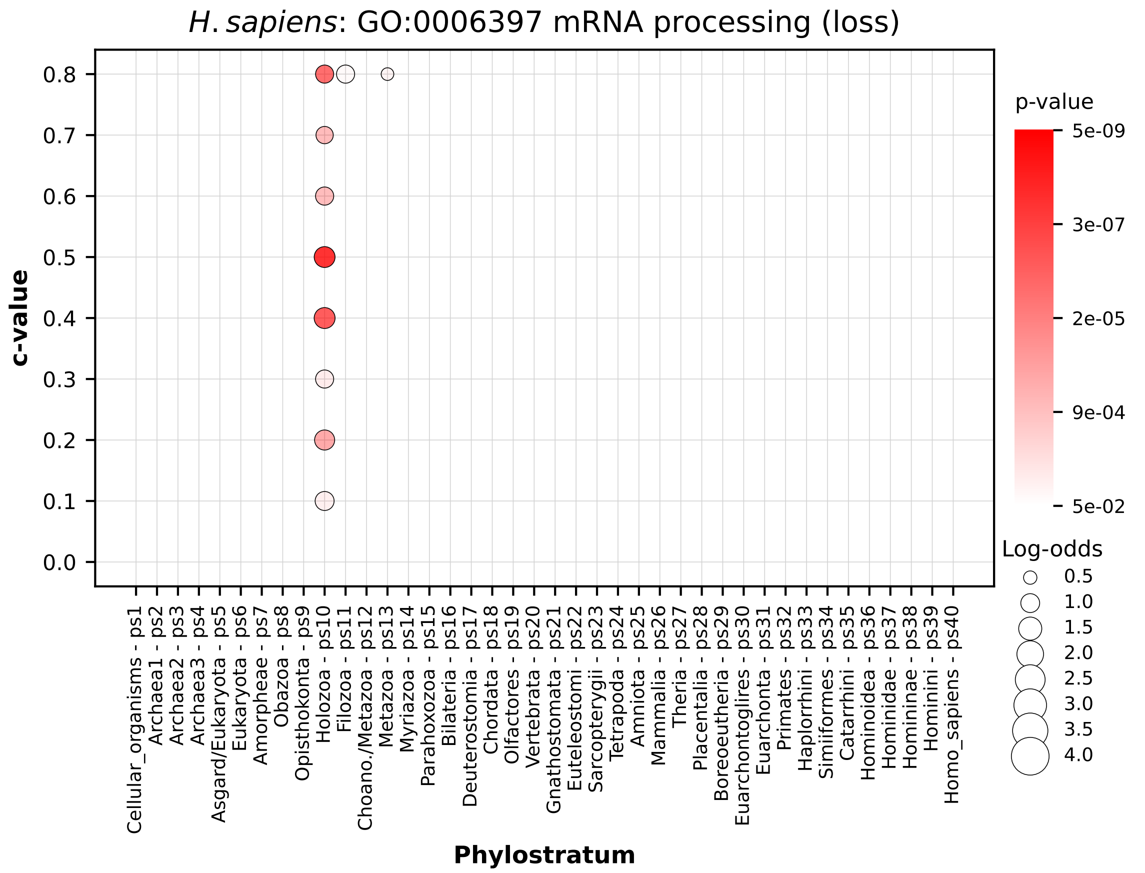

H. sapiens: GO:0006397 mRNA processing (loss)
c-value
0.0
0.1
0.2
0.3
0.4
0.5
0.6
0.7
0.8
Phylostratum
Cellular_organisms - ps1
Archaea1 - ps2
Archaea2 - ps3
Archaea3 - ps4
Asgard/Eukaryota - ps5
Eukaryota - ps6
Amorpheae - ps7
Obazoa - ps8
Opisthokonta - ps9
Holozoa - ps10
Filozoa - ps11
Choano./Metazoa - ps12
Metazoa - ps13
Myriazoa - ps14
Parahoxozoa - ps15
Bilateria - ps16
Deuterostomia - ps17
Chordata - ps18
Olfactores - ps19
Vertebrata - ps20
Gnathostomata - ps21
Euteleostomi - ps22
Sarcopterygii - ps23
Tetrapoda - ps24
Amniota - ps25
Mammalia - ps26
Theria - ps27
Placentalia - ps28
Boreoeutheria - ps29
Euarchontoglires - ps30
Euarchonta - ps31
Primates - ps32
Haplorrhini - ps33
Simiiformes - ps34
Catarrhini - ps35
Hominoidea - ps36
Hominidae - ps37
Homininae - ps38
Hominini - ps39
Homo_sapiens - ps40
p-value
5e-09
3e-07
2e-05
9e-04
5e-02
Log-odds
0.5
1.0
1.5
2.0
2.5
3.0
3.5
4.0

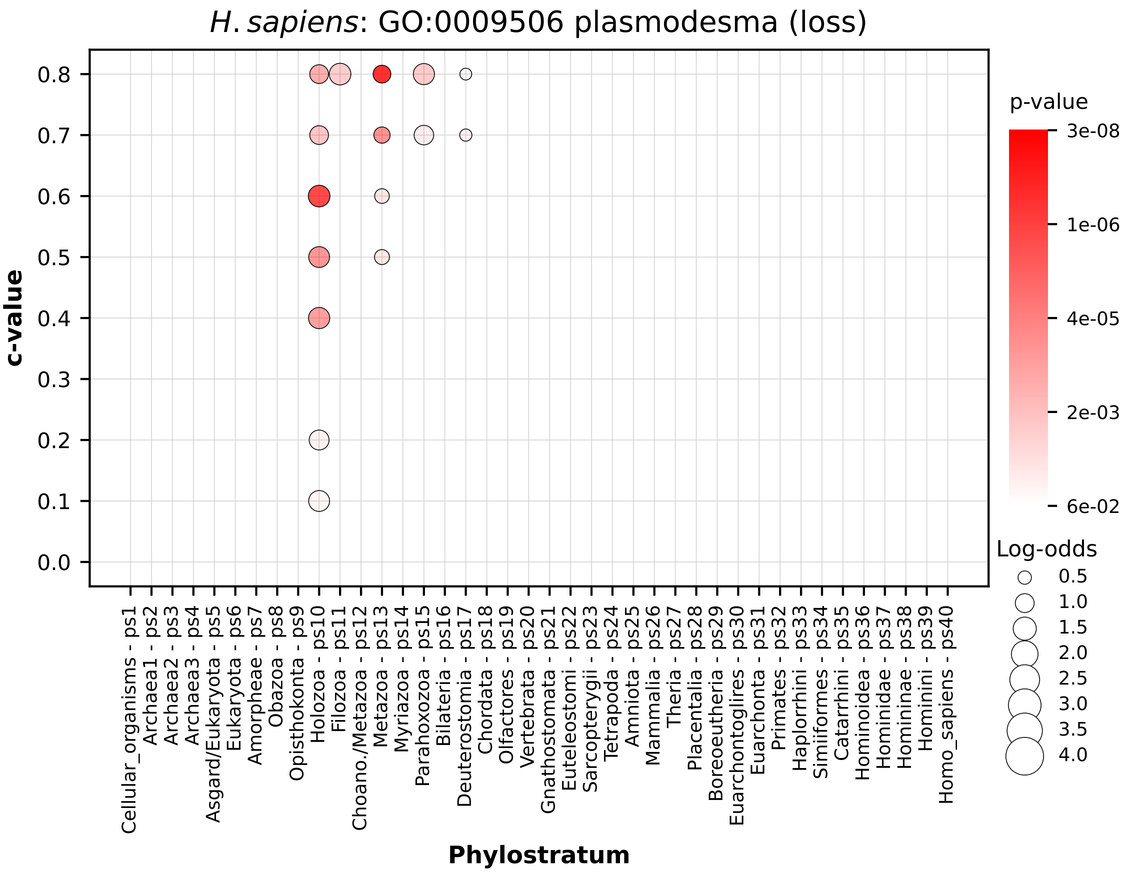
H. sapiens: GO:0009506 plasmodesma (loss)
c-value
0.0
0.1
0.2
0.3
0.4
0.5
0.6
0.7
0.8
Phylostratum
Cellular_organisms - ps1
Archaea1 - ps2
Archaea2 - ps3
Archaea3 - ps4
Asgard/Eukaryota - ps5
Eukaryota - ps6
Amorpheae - ps7
Obazoa - ps8
Opisthokonta - ps9
Holozoa - ps10
Filozoa - ps11
Choano./Metazoa - ps12
Metazoa - ps13
Myriazoa - ps14
Parahoxozoa - ps15
Bilateria - ps16
Deuterostomia - ps17
Chordata - ps18
Olfactores - ps19
Vertebrata - ps20
Gnathostomata - ps21
Euteleostomi - ps22
Sarcopterygii - ps23
Tetrapoda - ps24
Amniota - ps25
Mammalia - ps26
Theria - ps27
Placentalia - ps28
Boreoeutheria - ps29
Euarchontoglires - ps30
Euarchonta - ps31
Primates - ps32
Haplorrhini - ps33
Simiiformes - ps34
Catarrhini - ps35
Hominoidea - ps36
Hominidae - ps37
Homininae - ps38
Hominini - ps39
Homo_sapiens - ps40
p-value
3e-08
1e-06
4e-05
2e-03
6e-02
Log-odds
0.5
1.0
1.5
2.0
2.5
3.0
3.5
4.0

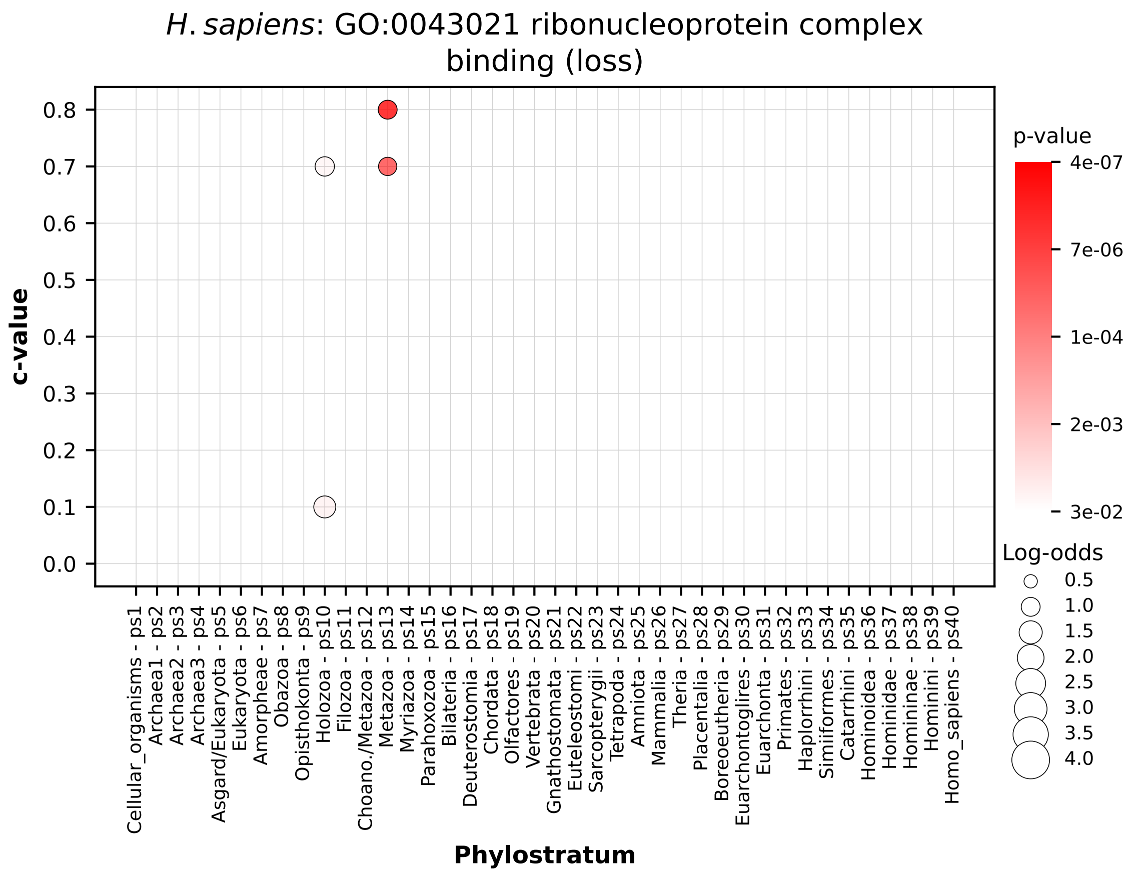

H. sapiens: GO:0043021 ribonucleoprotein complex binding (loss)
c-value
0.0
0.1
0.2
0.3
0.4
0.5
0.6
0.7
0.8
Phylostratum
Cellular_organisms - ps1
Archaea1 - ps2
Archaea2 - ps3
Archaea3 - ps4
Asgard/Eukaryota - ps5
Eukaryota - ps6
Amorpheae - ps7
Obazoa - ps8
Opisthokonta - ps9
Holozoa - ps10
Filozoa - ps11
Choano./Metazoa - ps12
Metazoa - ps13
Myriazoa - ps14
Parahoxozoa - ps15
Bilateria - ps16
Deuterostomia - ps17
Chordata - ps18
Olfactores - ps19
Vertebrata - ps20
Gnathostomata - ps21
Euteleostomi - ps22
Sarcopterygii - ps23
Tetrapoda - ps24
Amniota - ps25
Mammalia - ps26
Theria - ps27
Placentalia - ps28
Boreoeutheria - ps29
Euarchontoglires - ps30
Euarchonta - ps31
Primates - ps32
Haplorrhini - ps33
Simiiformes - ps34
Catarrhini - ps35
Hominoidea - ps36
Hominidae - ps37
Homininae - ps38
Hominini - ps39
Homo_sapiens - ps40
p-value
4e-07
7e-06
1e-04
2e-03
3e-02
Log-odds
0.5
1.0
1.5
2.0
2.5
3.0
3.5
4.0

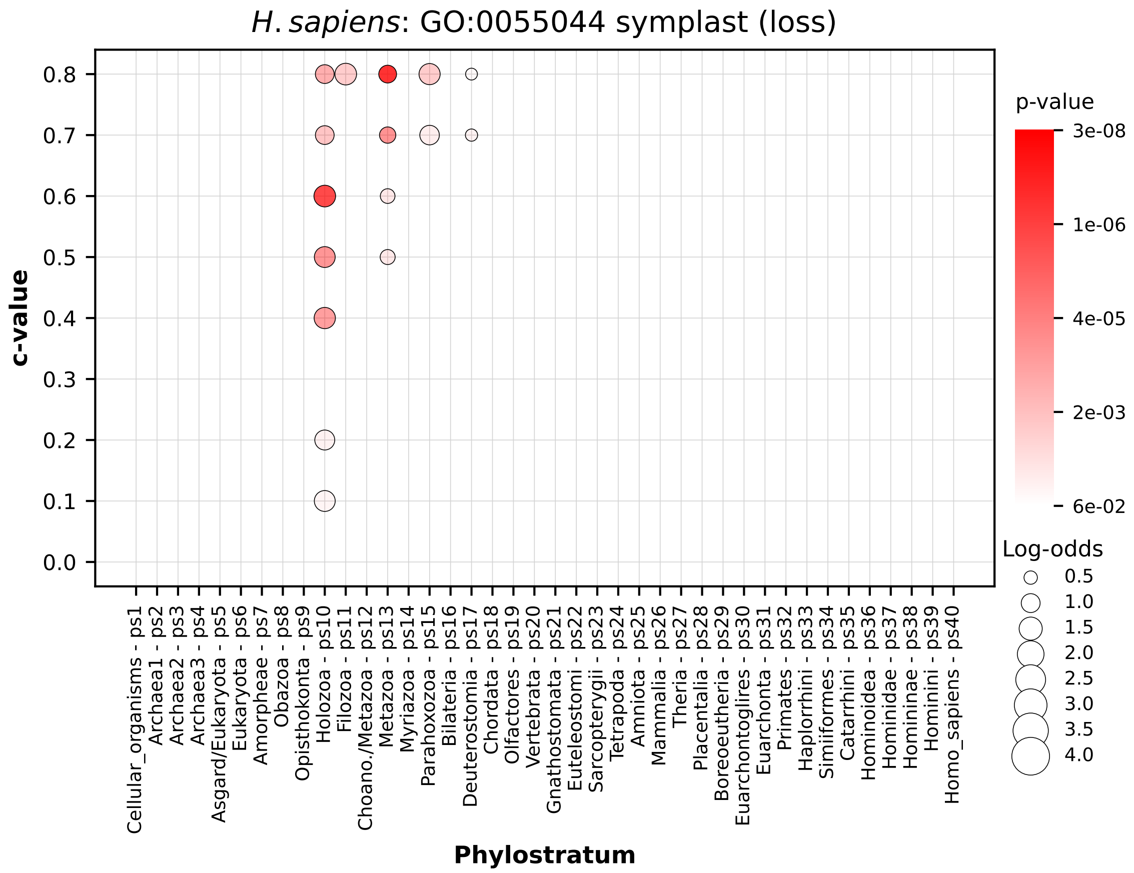

H. sapiens: GO:0055044 symplast (loss)
c-value
0.0
0.1
0.2
0.3
0.4
0.5
0.6
0.7
0.8
Phylostratum
Cellular_organisms - ps1
Archaea1 - ps2
Archaea2 - ps3
Archaea3 - ps4
Asgard/Eukaryota - ps5
Eukaryota - ps6
Amorpheae - ps7
Obazoa - ps8
Opisthokonta - ps9
Holozoa - ps10
Filozoa - ps11
Choano./Metazoa - ps12
Metazoa - ps13
Myriazoa - ps14
Parahoxozoa - ps15
Bilateria - ps16
Deuterostomia - ps17
Chordata - ps18
Olfactores - ps19
Vertebrata - ps20
Gnathostomata - ps21
Euteleostomi - ps22
Sarcopterygii - ps23
Tetrapoda - ps24
Amniota - ps25
Mammalia - ps26
Theria - ps27
Placentalia - ps28
Boreoeutheria - ps29
Euarchontoglires - ps30
Euarchonta - ps31
Primates - ps32
Haplorrhini - ps33
Simiiformes - ps34
Catarrhini - ps35
Hominoidea - ps36
Hominidae - ps37
Homininae - ps38
Hominini - ps39
Homo_sapiens - ps40
p-value
3e-08
1e-06
4e-05
2e-03
6e-02
Log-odds
0.5
1.0
1.5
2.0
2.5
3.0
3.5
4.0

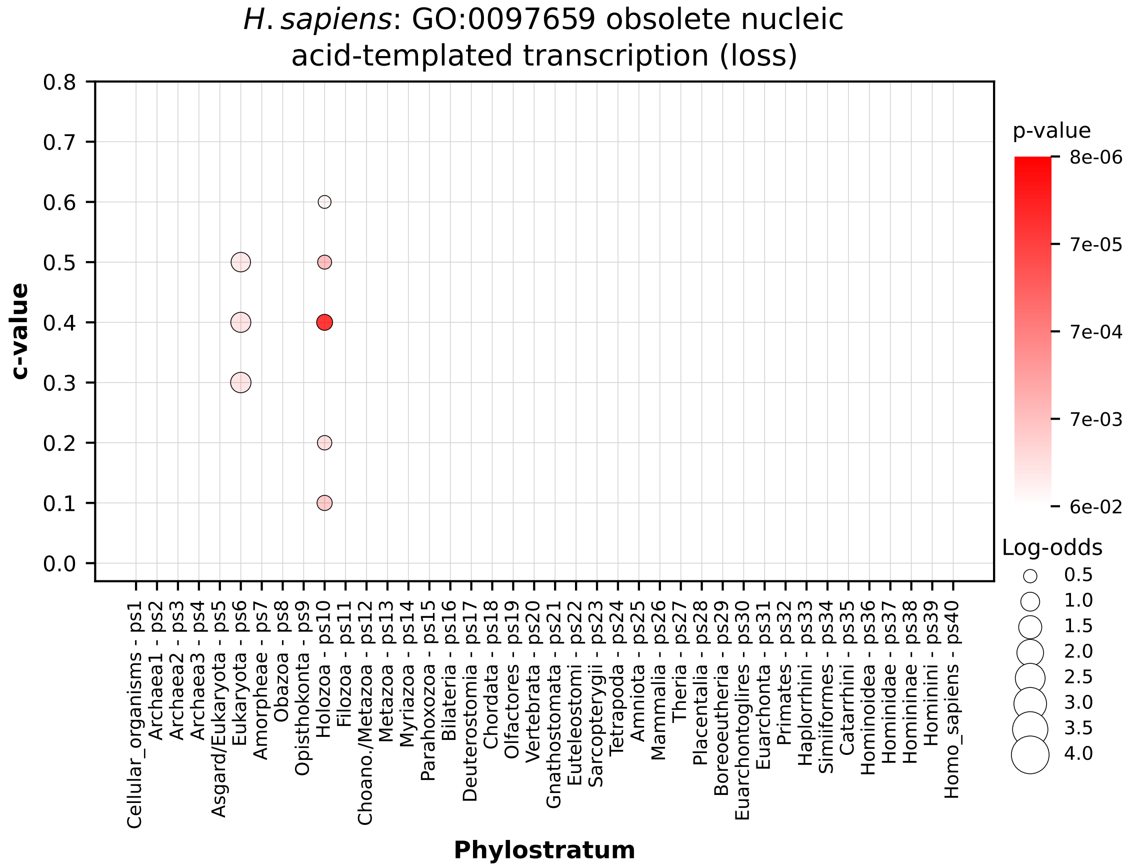

H. sapiens: GO:0097659 obsolete nucleic acid-templated transcription (loss)
c-value
0.0
0.1
0.2
0.3
0.4
0.5
0.6
0.7
0.8
Phylostratum
Cellular_organisms - ps1
Archaea1 - ps2
Archaea2 - ps3
Archaea3 - ps4
Asgard/Eukaryota - ps5
Eukaryota - ps6
Amorpheae - ps7
Obazoa - ps8
Opisthokonta - ps9
Holozoa - ps10
Filozoa - ps11
Choano./Metazoa - ps12
Metazoa - ps13
Myriazoa - ps14
Parahoxozoa - ps15
Bilateria - ps16
Deuterostomia - ps17
Chordata - ps18
Olfactores - ps19
Vertebrata - ps20
Gnathostomata - ps21
Euteleostomi - ps22
Sarcopterygii - ps23
Tetrapoda - ps24
Amniota - ps25
Mammalia - ps26
Theria - ps27
Placentalia - ps28
Boreoeutheria - ps29
Euarchontoglires - ps30
Euarchonta - ps31
Primates - ps32
Haplorrhini - ps33
Simiiformes - ps34
Catarrhini - ps35
Hominoidea - ps36
Hominidae - ps37
Homininae - ps38
Hominini - ps39
Homo_sapiens - ps40
p-value
8e-06
7e-05
7e-04
7e-03
6e-02
Log-odds
0.5
1.0
1.5
2.0
2.5
3.0
3.5
4.0

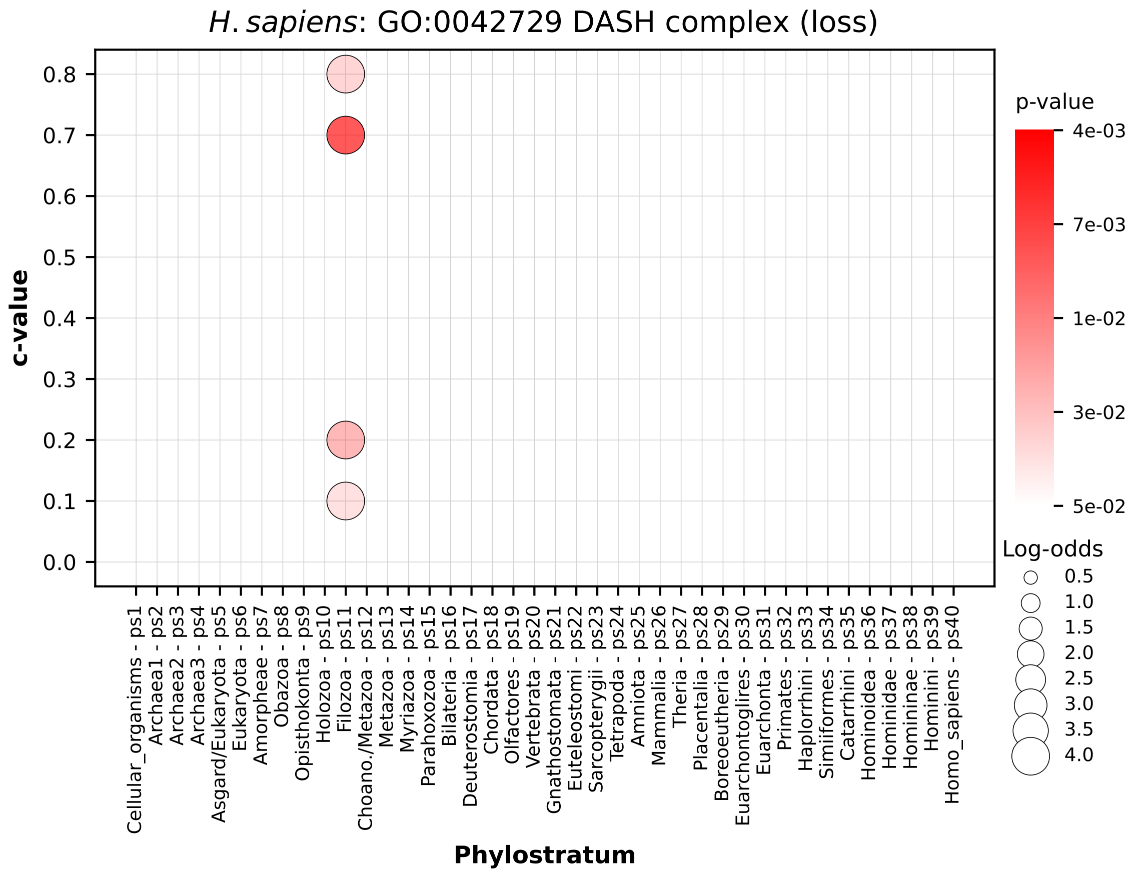

H. sapiens: GO:0042729 DASH complex (loss)
c-value
0.0
0.1
0.2
0.3
0.4
0.5
0.6
0.7
0.8
Phylostratum
Cellular_organisms - ps1
Archaea1 - ps2
Archaea2 - ps3
Archaea3 - ps4
Asgard/Eukaryota - ps5
Eukaryota - ps6
Amorpheae - ps7
Obazoa - ps8
Opisthokonta - ps9
Holozoa - ps10
Filozoa - ps11
Choano./Metazoa - ps12
Metazoa - ps13
Myriazoa - ps14
Parahoxozoa - ps15
Bilateria - ps16
Deuterostomia - ps17
Chordata - ps18
Olfactores - ps19
Vertebrata - ps20
Gnathostomata - ps21
Euteleostomi - ps22
Sarcopterygii - ps23
Tetrapoda - ps24
Amniota - ps25
Mammalia - ps26
Theria - ps27
Placentalia - ps28
Boreoeutheria - ps29
Euarchontoglires - ps30
Euarchonta - ps31
Primates - ps32
Haplorrhini - ps33
Simiiformes - ps34
Catarrhini - ps35
Hominoidea - ps36
Hominidae - ps37
Homininae - ps38
Hominini - ps39
Homo_sapiens - ps40
p-value
4e-03
7e-03
1e-02
3e-02
5e-02
Log-odds
0.5
1.0
1.5
2.0
2.5
3.0
3.5
4.0

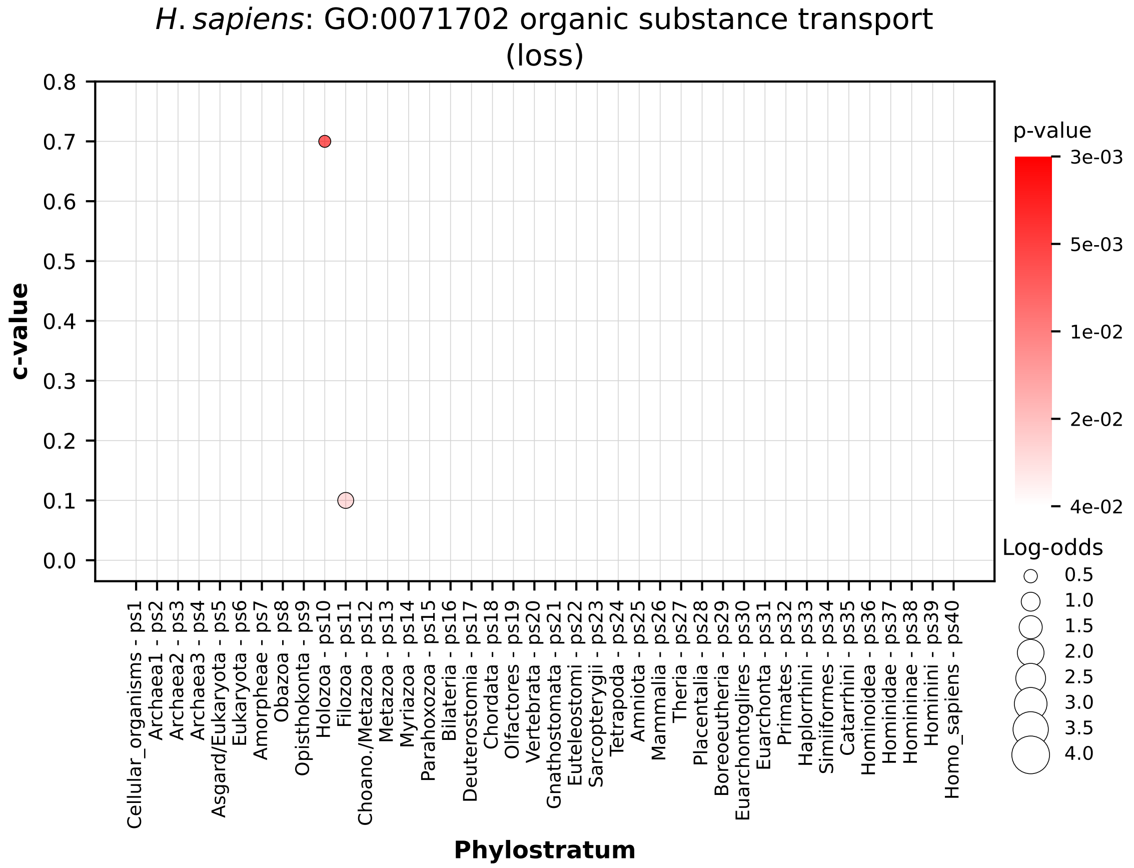

H. sapiens: GO:0071702 organic substance transport
(loss)
c-value
0.8
0.7
0.6
0.5
0.4
0.3
0.2
0.1
0.0
p-value
3e-03
5e-03
1e-02
2e-02
4e-02
Log-odds
0.5
1.0
1.5
2.0
2.5
3.0
3.5
4.0
Cellular_organisms - ps1
Archaea1 - ps2
Archaea2 - ps3
Archaea3 - ps4
Asgard/Eukaryota - ps5
Eukaryota - ps6
Amorpheae - ps7
Obazoa - ps8
Opisthokonta - ps9
Holozoa - ps10
Filozoa - ps11
Choano./Metazoa - ps12
Metazoa - ps13
Myriazoa - ps14
Parahoxozoa - ps15
Bilateria - ps16
Deuterostomia - ps17
Chordata - ps18
Olfactores - ps19
Vertebrata - ps20
Gnathostomata - ps21
Euteleostomi - ps22
Sarcopterygii - ps23
Tetrapoda - ps24
Amniota - ps25
Mammalia - ps26
Theria - ps27
Placentalia - ps28
Boreoeutheria - ps29
Euarchontoglires - ps30
Euarchonta - ps31
Primates - ps32
Haplorrhini - ps33
Simiiformes - ps34
Catarrhini - ps35
Hominoidea - ps36
Hominidae - ps37
Homininae - ps38
Hominini - ps39
Homo_sapiens - ps40
Phylostratum

H. sapiens: GO:0000160 phosphorelay signal transduction system (loss)

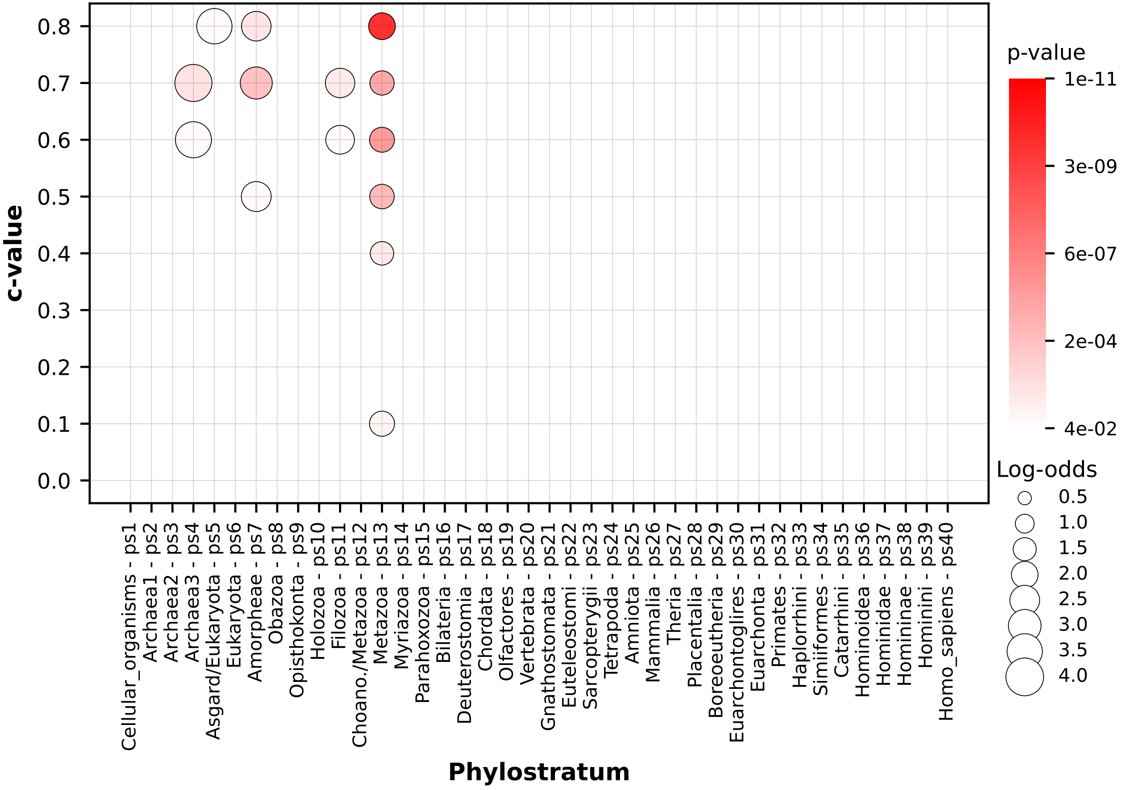

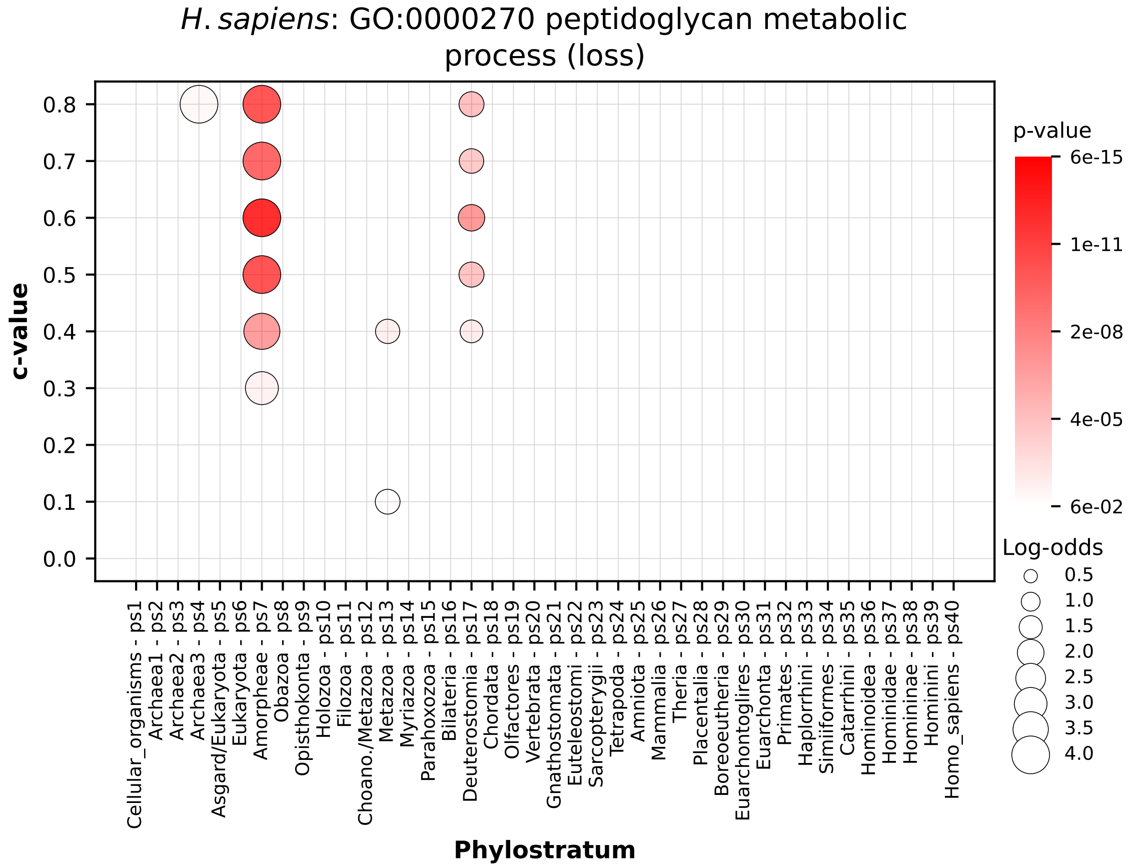

H. sapiens: GO:0000270 peptidoglycan metabolic process (loss)
c-value
0.8
0.7
0.6
0.5
0.4
0.3
0.2
0.1
0.0
Phylostratum
Cellular_organisms - ps1
Archaea1 - ps2
Archaea2 - ps3
Archaea3 - ps4
Asgard/Eukaryota - ps5
Eukaryota - ps6
Amorpheae - ps7
Obazoa - ps8
Opisthokonta - ps9
Holozoa - ps10
Filozoa - ps11
Choano./Metazoa - ps12
Metazoa - ps13
Myriazoa - ps14
Parahoxozoa - ps15
Bilateria - ps16
Deuterostomia - ps17
Chordata - ps18
Olfactores - ps19
Vertebrata - ps20
Gnathostomata - ps21
Euteleostomi - ps22
Sarcopterygii - ps23
Tetrapoda - ps24
Amniota - ps25
Mammalia - ps26
Theria - ps27
Placentalia - ps28
Boreoeutheria - ps29
Euarchontoglires - ps30
Euarchonta - ps31
Primates - ps32
Haplorrhini - ps33
Simiiformes - ps34
Catarrhini - ps35
Hominoidea - ps36
Hominidae - ps37
Homininae - ps38
Hominini - ps39
Homo_sapiens - ps40
p-value
6e-15
1e-11
2e-08
4e-05
6e-02
Log-odds
0.5
1.0
1.5
2.0
2.5
3.0
3.5
4.0

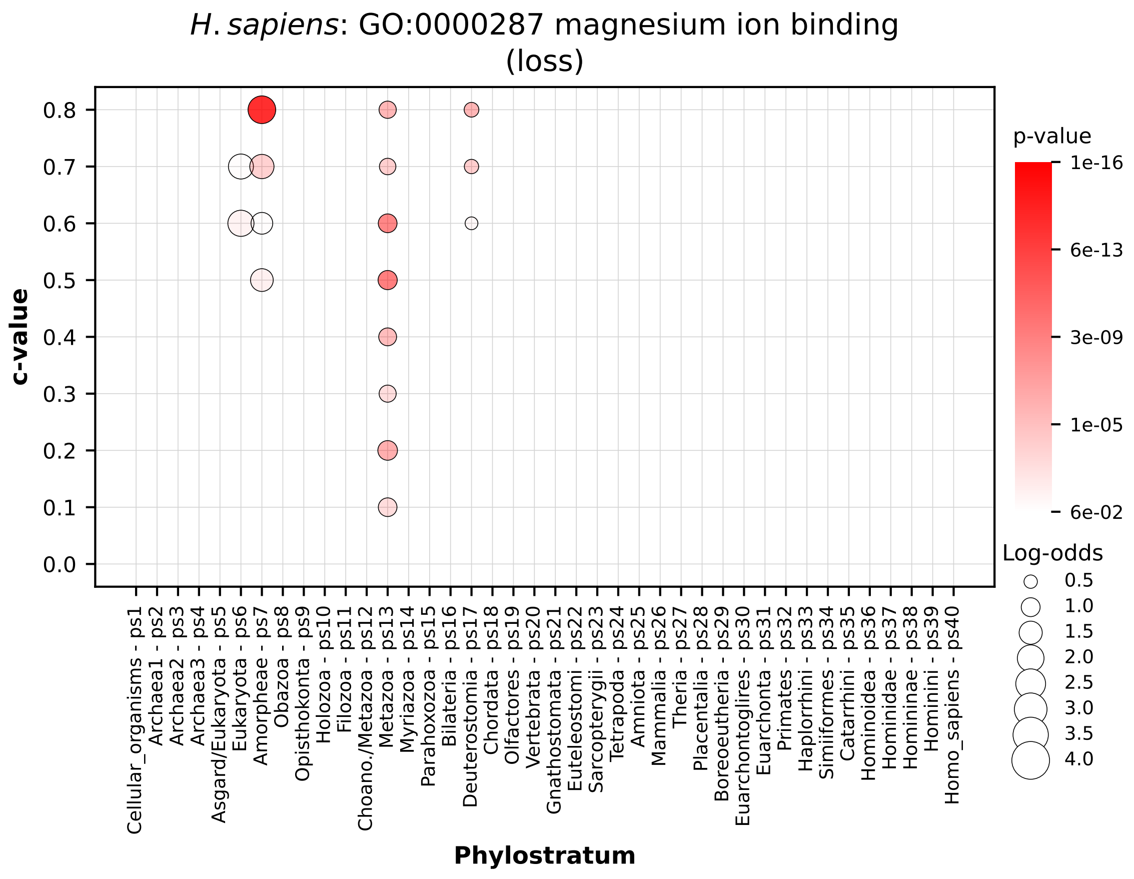

H. sapiens: GO:0000287 magnesium ion binding
(loss)
c-value
0.8
0.7
0.6
0.5
0.4
0.3
0.2
0.1
0.0
p-value
1e-16
6e-13
3e-09
1e-05
6e-02
Log-odds
0.5
1.0
1.5
2.0
2.5
3.0
3.5
4.0
Phylostratum
Cellular_organisms - ps1
Archaea1 - ps2
Archaea2 - ps3
Archaea3 - ps4
Asgard/Eukaryota - ps5
Eukaryota - ps6
Amorpheae - ps7
Obazoa - ps8
Opisthokonta - ps9
Holozoa - ps10
Filozoa - ps11
Choano./Metazoa - ps12
Metazoa - ps13
Myriazoa - ps14
Parahoxozoa - ps15
Bilateria - ps16
Deuterostomia - ps17
Chordata - ps18
Olfactores - ps19
Vertebrata - ps20
Gnathostomata - ps21
Euteleostomi - ps22
Sarcopterygii - ps23
Tetrapoda - ps24
Amniota - ps25
Mammalia - ps26
Theria - ps27
Placentalia - ps28
Boreoeutheria - ps29
Euarchontoglires - ps30
Euarchonta - ps31
Primates - ps32
Haplorrhini - ps33
Simiiformes - ps34
Catarrhini - ps35
Hominoidea - ps36
Hominidae - ps37
Homininae - ps38
Hominini - ps39
Homo_sapiens - ps40

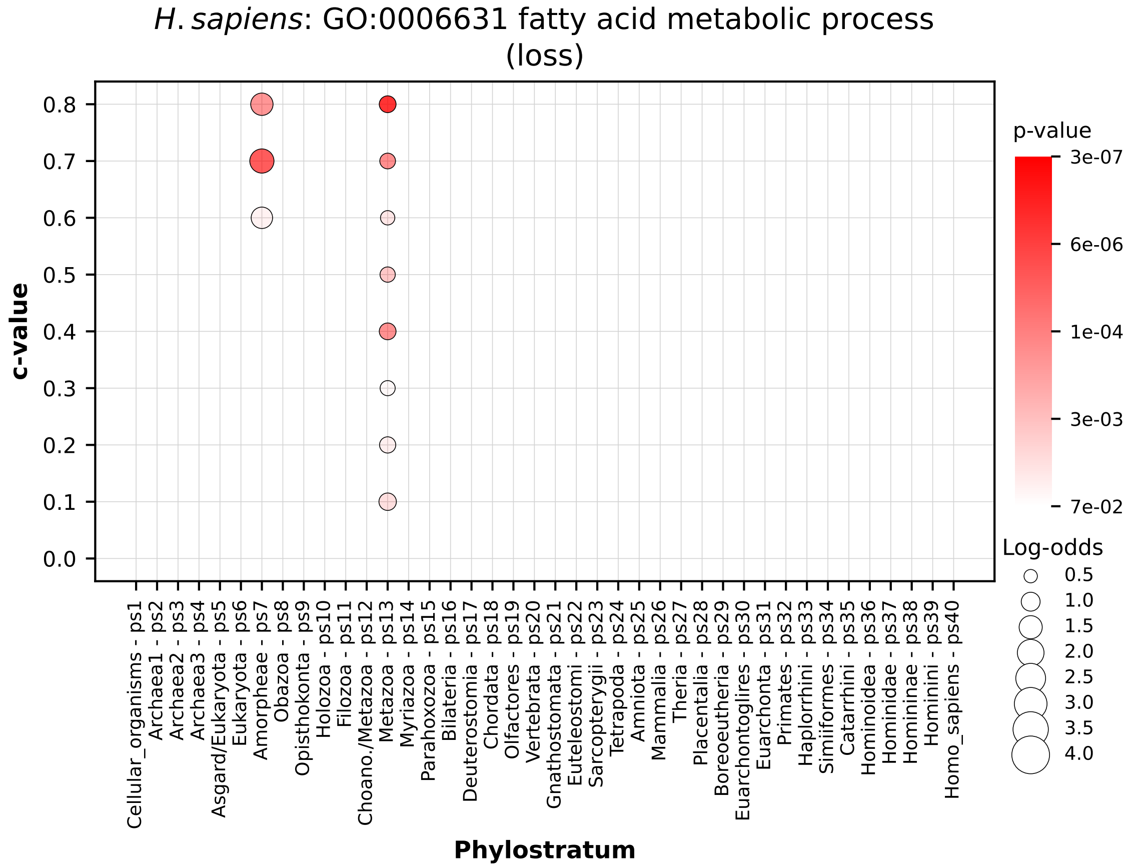

H. sapiens: GO:0006631 fatty acid metabolic process
(loss)
c-value
0.8
0.7
0.6
0.5
0.4
0.3
0.2
0.1
0.0
p-value
3e-07
6e-06
1e-04
3e-03
7e-02
Log-odds
0.5
1.0
1.5
2.0
2.5
3.0
3.5
4.0
Cellular_organisms - ps1
Archaea1 - ps2
Archaea2 - ps3
Archaea3 - ps4
Asgard/Eukaryota - ps5
Eukaryota - ps6
Amorpheae - ps7
Obazoa - ps8
Opisthokonta - ps9
Holozoa - ps10
Filozoa - ps11
Choano./Metazoa - ps12
Metazoa - ps13
Myriazoa - ps14
Parahoxozoa - ps15
Bilateria - ps16
Deuterostomia - ps17
Chordata - ps18
Olfactores - ps19
Vertebrata - ps20
Gnathostomata - ps21
Euteleostomi - ps22
Sarcopterygii - ps23
Tetrapoda - ps24
Amniota - ps25
Mammalia - ps26
Theria - ps27
Placentalia - ps28
Boreoeutheria - ps29
Euarchontoglires - ps30
Euarchonta - ps31
Primates - ps32
Haplorrhini - ps33
Simiiformes - ps34
Catarrhini - ps35
Hominoidea - ps36
Hominidae - ps37
Homininae - ps38
Hominini - ps39
Homo_sapiens - ps40
Phylostratum

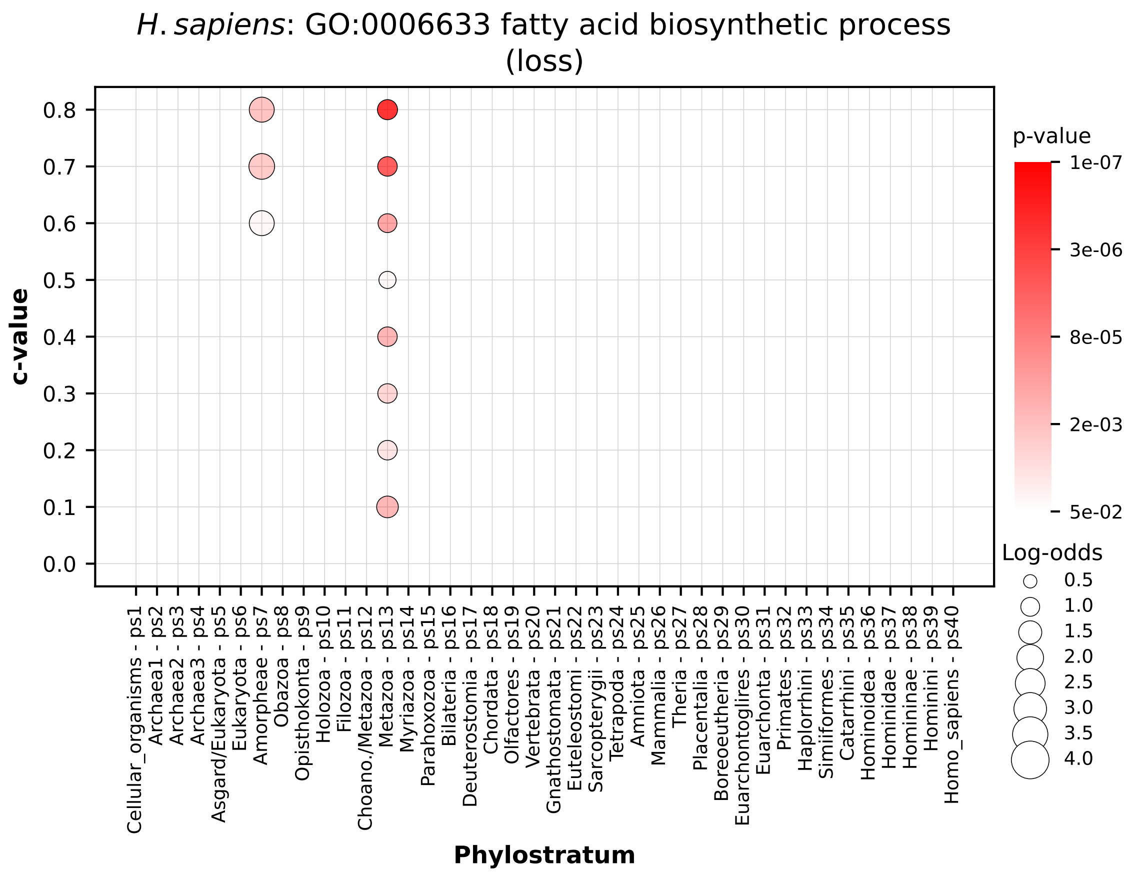

H. sapiens: GO:0006633 fatty acid biosynthetic process
(loss)
c-value
0.0
0.1
0.2
0.3
0.4
0.5
0.6
0.7
0.8
Phylostratum
Cellular_organisms - ps1
Archaea1 - ps2
Archaea2 - ps3
Archaea3 - ps4
Asgard/Eukaryota - ps5
Eukaryota - ps6
Amorpheae - ps7
Obazoa - ps8
Opisthokonta - ps9
Holozoa - ps10
Filozoa - ps11
Choano./Metazoa - ps12
Metazoa - ps13
Myriazoa - ps14
Parahoxozoa - ps15
Bilateria - ps16
Deuterostomia - ps17
Chordata - ps18
Olfactores - ps19
Vertebrata - ps20
Gnathostomata - ps21
Euteleostomi - ps22
Sarcopterygii - ps23
Tetrapoda - ps24
Amniota - ps25
Mammalia - ps26
Theria - ps27
Placentalia - ps28
Boreoeutheria - ps29
Euarchontoglires - ps30
Euarchonta - ps31
Primates - ps32
Haplorrhini - ps33
Simiiformes - ps34
Catarrhini - ps35
Hominoidea - ps36
Hominidae - ps37
Homininae - ps38
Hominini - ps39
Homo_sapiens - ps40
p-value
1e-07
3e-06
8e-05
2e-03
5e-02
Log-odds
0.5
1.0
1.5
2.0
2.5
3.0
3.5
4.0

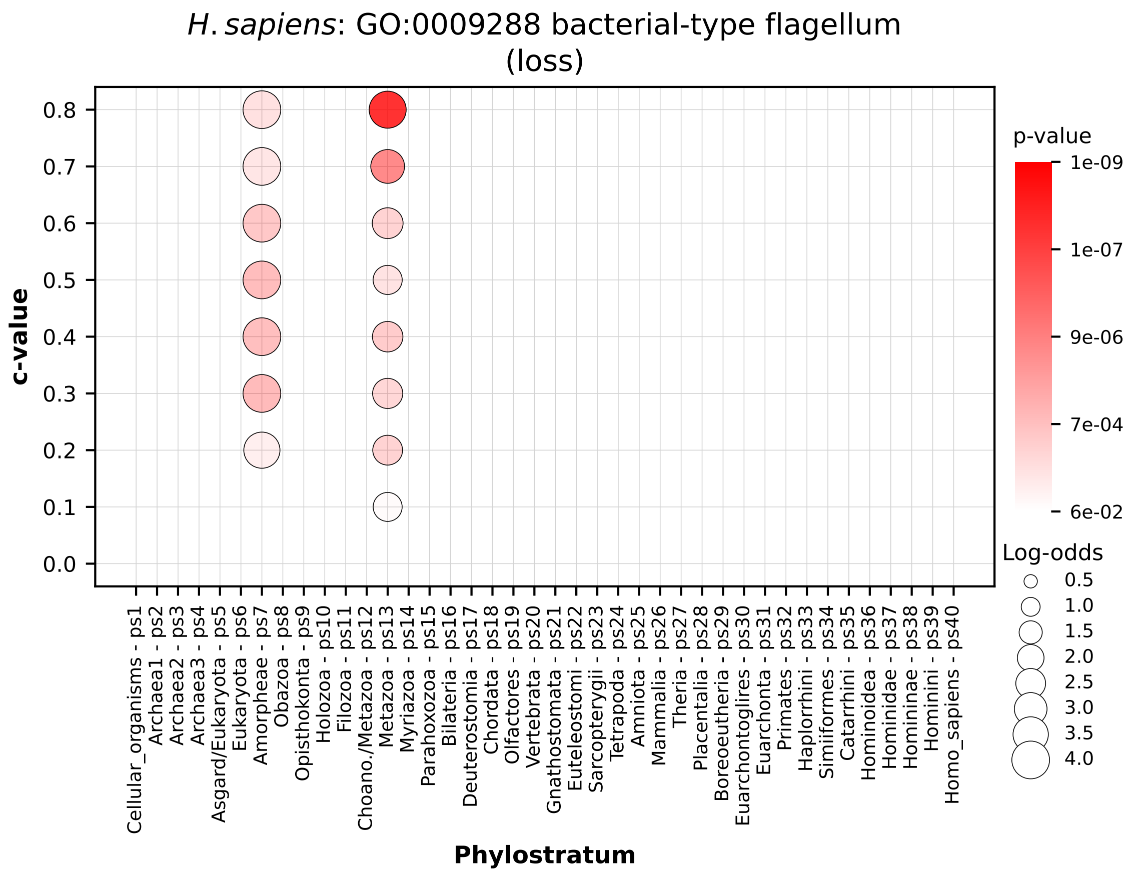

H. sapiens: GO:0009288 bacterial-type flagellum
(loss)
c-value
0.0
0.1
0.2
0.3
0.4
0.5
0.6
0.7
0.8
p-value
1e-09
1e-07
9e-06
7e-04
6e-02
Log-odds
0.5
1.0
1.5
2.0
2.5
3.0
3.5
4.0
Cellular_organisms - ps1
Archaea1 - ps2
Archaea2 - ps3
Archaea3 - ps4
Asgard/Eukaryota - ps5
Eukaryota - ps6
Amorpheae - ps7
Obazoa - ps8
Opisthokonta - ps9
Holozoa - ps10
Filozoa - ps11
Choano./Metazoa - ps12
Metazoa - ps13
Myriazoa - ps14
Parahoxozoa - ps15
Bilateria - ps16
Deuterostomia - ps17
Chordata - ps18
Olfactores - ps19
Vertebrata - ps20
Gnathostomata - ps21
Euteleostomi - ps22
Sarcopterygii - ps23
Tetrapoda - ps24
Amniota - ps25
Mammalia - ps26
Theria - ps27
Placentalia - ps28
Boreoeutheria - ps29
Euarchontoglires - ps30
Euarchonta - ps31
Primates - ps32
Haplorrhini - ps33
Simiiformes - ps34
Catarrhini - ps35
Hominoidea - ps36
Hominidae - ps37
Homininae - ps38
Hominini - ps39
Homo_sapiens - ps40
Phylostratum

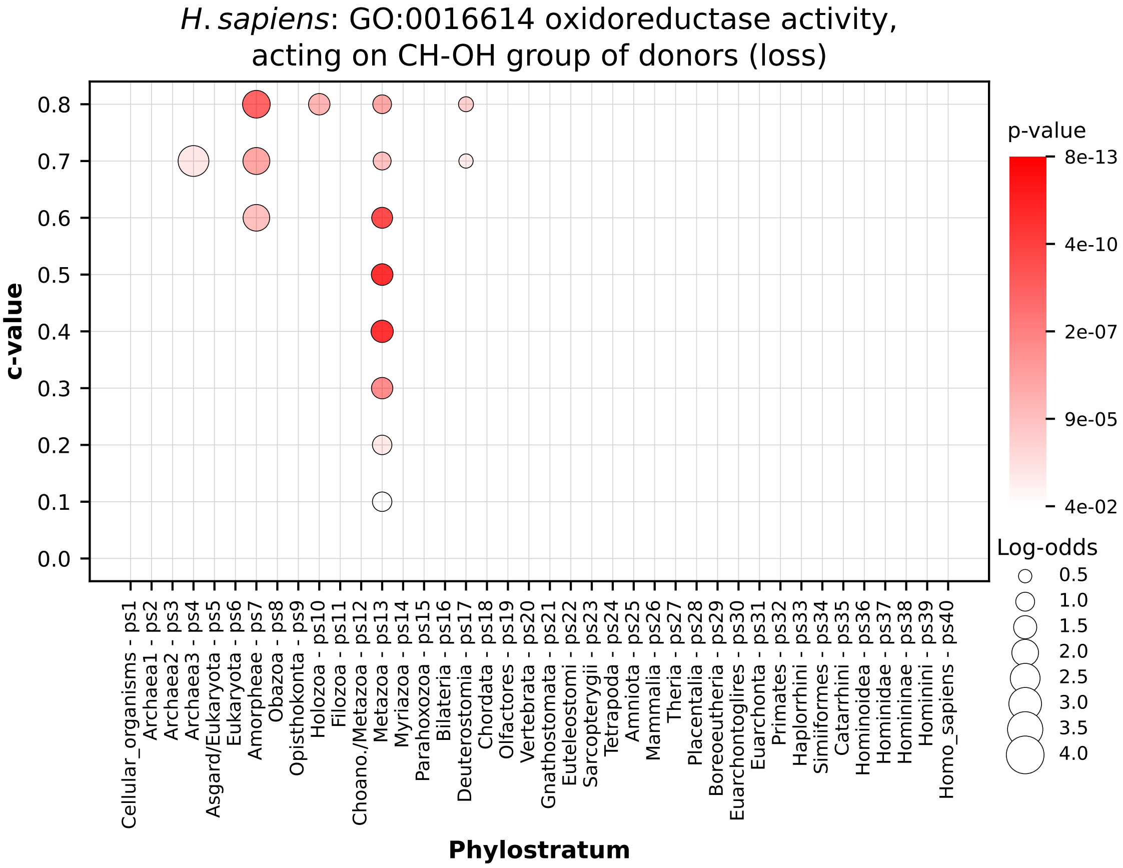

H. sapiens: GO:0016614 oxidoreductase activity, acting on CH-OH group of donors (loss)
c-value
0.8
0.7
0.6
0.5
0.4
0.3
0.2
0.1
0.0
Phylostratum
Cellular_organisms - ps1
Archaea1 - ps2
Archaea2 - ps3
Archaea3 - ps4
Asgard/Eukaryota - ps5
Eukaryota - ps6
Amorpheae - ps7
Obazoa - ps8
Opisthokonta - ps9
Holozoa - ps10
Filozoa - ps11
Choano./Metazoa - ps12
Metazoa - ps13
Myriazoa - ps14
Parahoxozoa - ps15
Bilateria - ps16
Deuterostomia - ps17
Chordata - ps18
Olfactores - ps19
Vertebrata - ps20
Gnathostomata - ps21
Euteleostomi - ps22
Sarcopterygii - ps23
Tetrapoda - ps24
Amniota - ps25
Mammalia - ps26
Theria - ps27
Placentalia - ps28
Boreoeutheria - ps29
Euarchontoglires - ps30
Euarchonta - ps31
Primates - ps32
Haplorrhini - ps33
Simiiformes - ps34
Catarrhini - ps35
Hominoidea - ps36
Hominidae - ps37
Homininae - ps38
Hominini - ps39
Homo_sapiens - ps40
p-value
8e-13
4e-10
2e-07
9e-05
4e-02
Log-odds
0.5
1.0
1.5
2.0
2.5
3.0
3.5
4.0

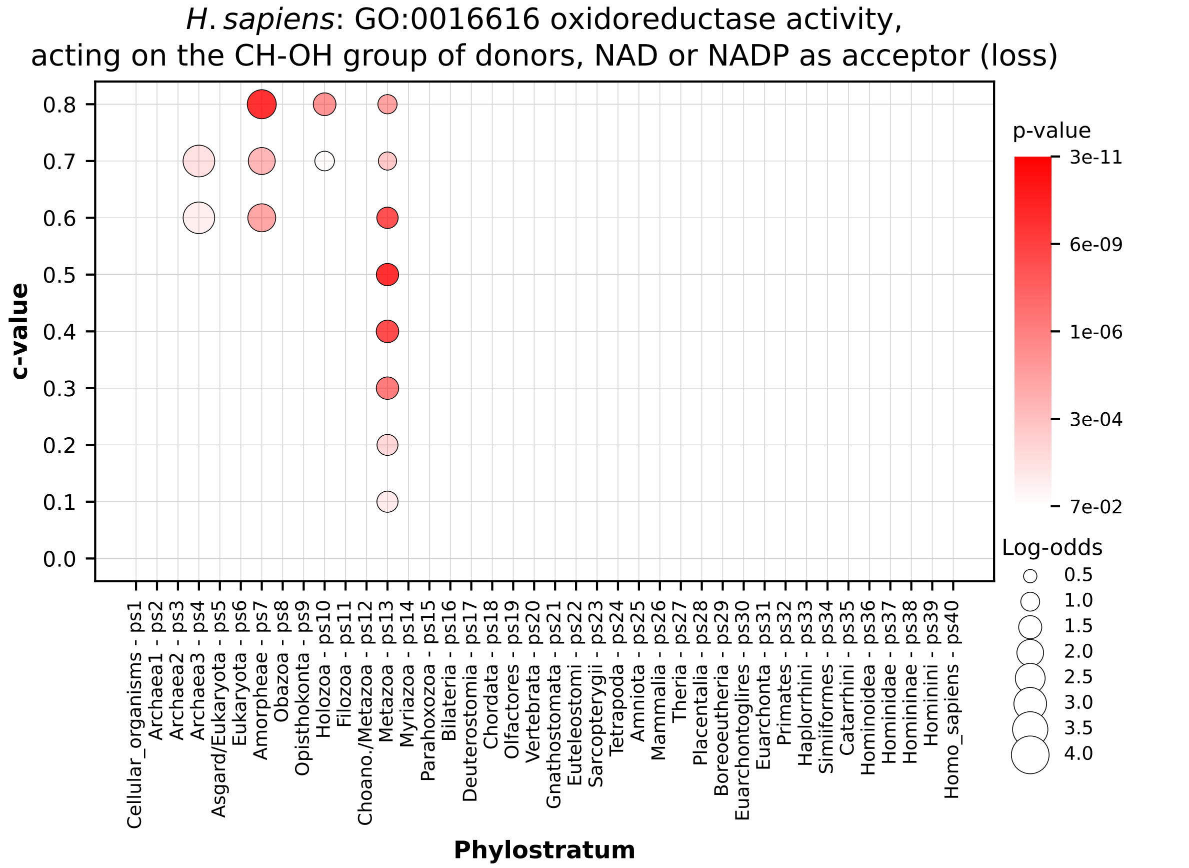

H. sapiens: GO:0016616 oxidoreductase activity, acting on the CH-OH group of donors, NAD or NADP as acceptor (loss)
c-value
0.0
0.1
0.2
0.3
0.4
0.5
0.6
0.7
0.8
Phylostratum
Cellular_organisms - ps1
Archaea1 - ps2
Archaea2 - ps3
Archaea3 - ps4
Asgard/Eukaryota - ps5
Eukaryota - ps6
Amorpheae - ps7
Obazoa - ps8
Opisthokonta - ps9
Holozoa - ps10
Filozoa - ps11
Choano./Metazoa - ps12
Metazoa - ps13
Myriazoa - ps14
Parahoxozoa - ps15
Bilateria - ps16
Deuterostomia - ps17
Chordata - ps18
Olfactores - ps19
Vertebrata - ps20
Gnathostomata - ps21
Euteleostomi - ps22
Sarcopterygii - ps23
Tetrapoda - ps24
Amniota - ps25
Mammalia - ps26
Theria - ps27
Placentalia - ps28
Boreoeutheria - ps29
Euarchontoglires - ps30
Euarchonta - ps31
Primates - ps32
Haplorrhini - ps33
Simiiformes - ps34
Catarrhini - ps35
Hominoidea - ps36
Hominidae - ps37
Homininae - ps38
Hominini - ps39
Homo_sapiens - ps40
p-value
3e-11
6e-09
1e-06
3e-04
7e-02
Log-odds
0.5
1.0
1.5
2.0
2.5
3.0
3.5
4.0

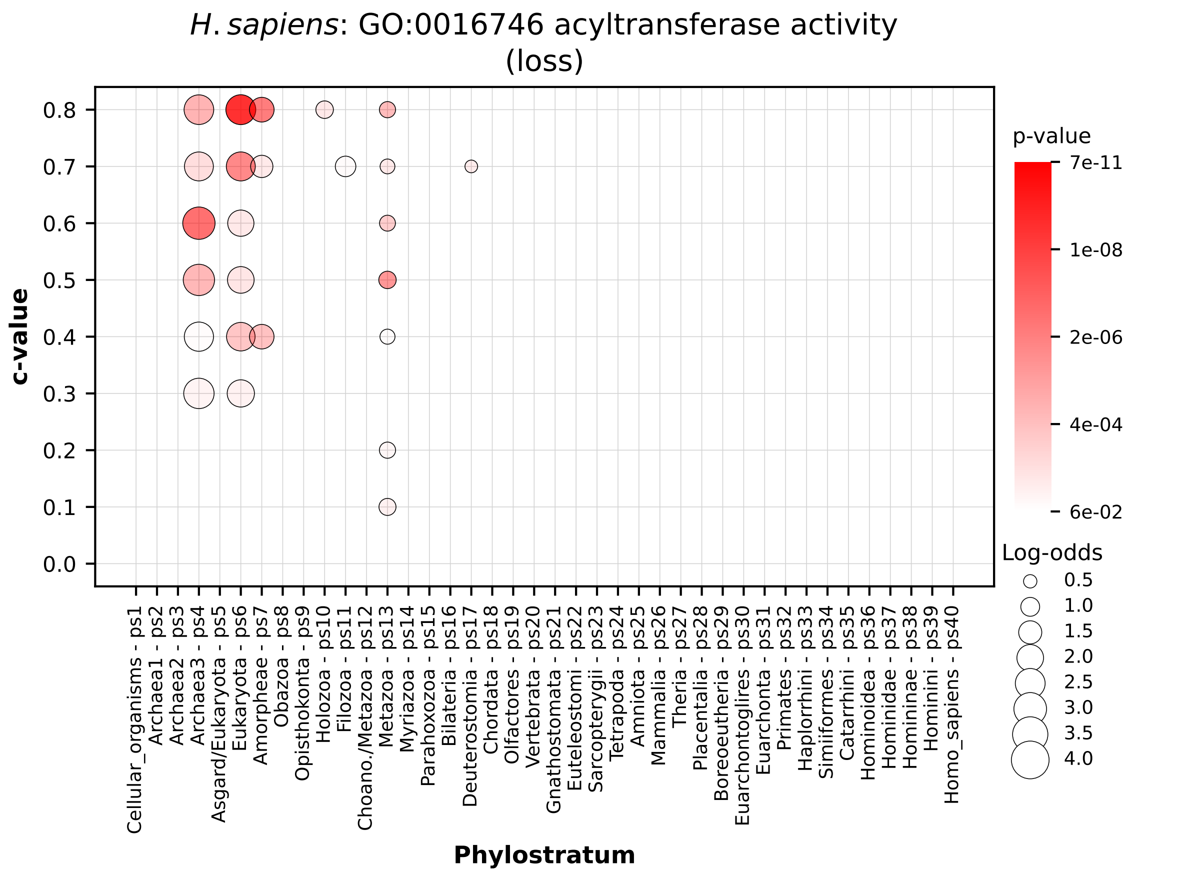
H. sapiens: GO:0016746 acyltransferase activity
(loss)
c-value
0.8
0.7
0.6
0.5
0.4
0.3
0.2
0.1
0.0
Phylostratum
Cellular_organisms - ps1
Archaea1 - ps2
Archaea2 - ps3
Archaea3 - ps4
Asgard/Eukaryota - ps5
Eukaryota - ps6
Amorpheae - ps7
Obazoa - ps8
Opisthokonta - ps9
Holozoa - ps10
Filozoa - ps11
Choano./Metazoa - ps12
Metazoa - ps13
Myriazoa - ps14
Parahoxozoa - ps15
Bilateria - ps16
Deuterostomia - ps17
Chordata - ps18
Olfactores - ps19
Vertebrata - ps20
Gnathostomata - ps21
Euteleostomi - ps22
Sarcopterygii - ps23
Tetrapoda - ps24
Amniota - ps25
Mammalia - ps26
Theria - ps27
Placentalia - ps28
Boreoeutheria - ps29
Euarchontoglires - ps30
Euarchonta - ps31
Primates - ps32
Haplorrhini - ps33
Simiiformes - ps34
Catarrhini - ps35
Hominoidea - ps36
Hominidae - ps37
Homininae - ps38
Hominini - ps39
Homo_sapiens - ps40
p-value
7e-11
1e-08
2e-06
4e-04
6e-02
Log-odds
0.5
1.0
1.5
2.0
2.5
3.0
3.5
4.0

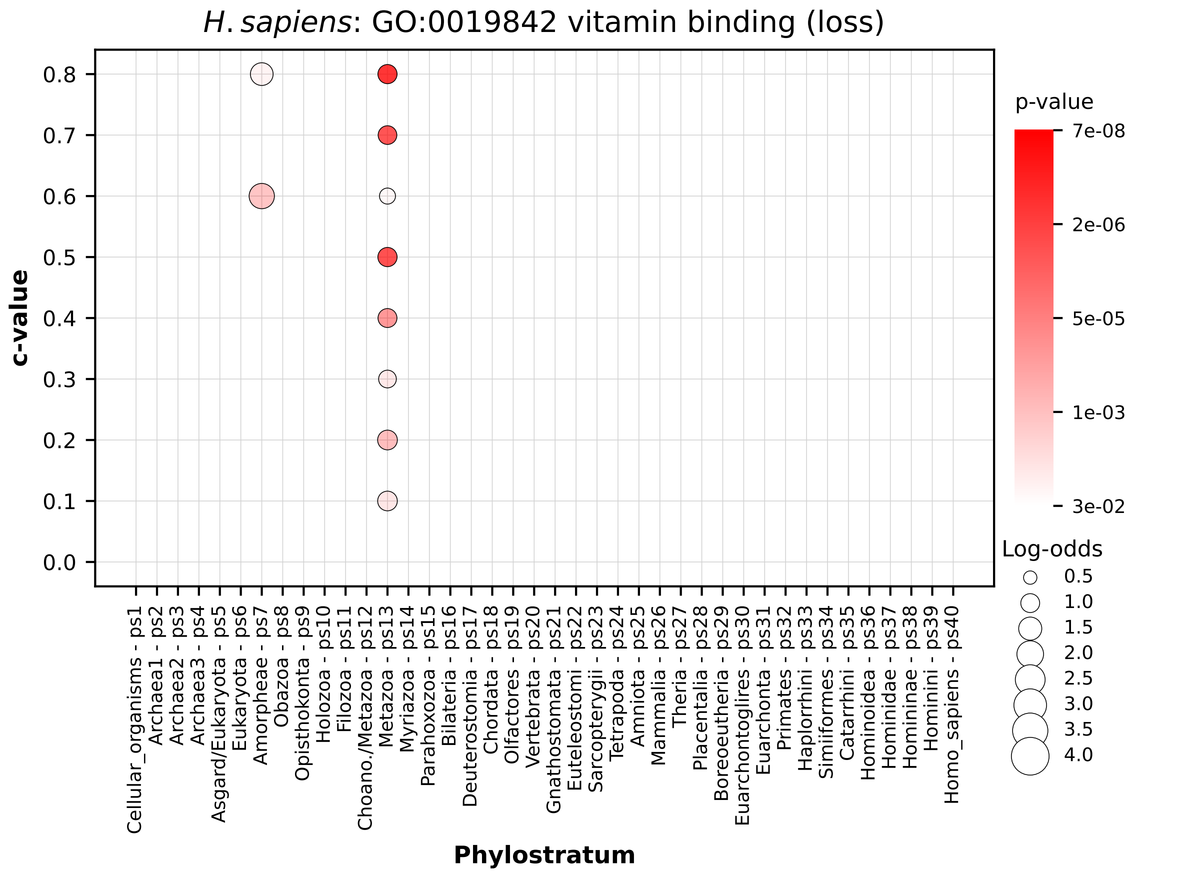
H. sapiens: GO:0019842 vitamin binding (loss)
c-value
0.0
0.1
0.2
0.3
0.4
0.5
0.6
0.7
0.8
Phylostratum
Cellular_organisms - ps1
Archaea1 - ps2
Archaea2 - ps3
Archaea3 - ps4
Asgard/Eukaryota - ps5
Eukaryota - ps6
Amorpheae - ps7
Obazoa - ps8
Opisthokonta - ps9
Holozoa - ps10
Filozoa - ps11
Choano./Metazoa - ps12
Metazoa - ps13
Myriazoa - ps14
Parahoxozoa - ps15
Bilateria - ps16
Deuterostomia - ps17
Chordata - ps18
Olfactores - ps19
Vertebrata - ps20
Gnathostomata - ps21
Euteleostomi - ps22
Sarcopterygii - ps23
Tetrapoda - ps24
Amniota - ps25
Mammalia - ps26
Theria - ps27
Placentalia - ps28
Boreoeutheria - ps29
Euarchontoglires - ps30
Euarchonta - ps31
Primates - ps32
Haplorrhini - ps33
Simiiformes - ps34
Catarrhini - ps35
Hominoidea - ps36
Hominidae - ps37
Homininae - ps38
Hominini - ps39
Homo_sapiens - ps40
p-value
7e-08
2e-06
5e-05
1e-03
3e-02
Log-odds
0.5
1.0
1.5
2.0
2.5
3.0
3.5
4.0

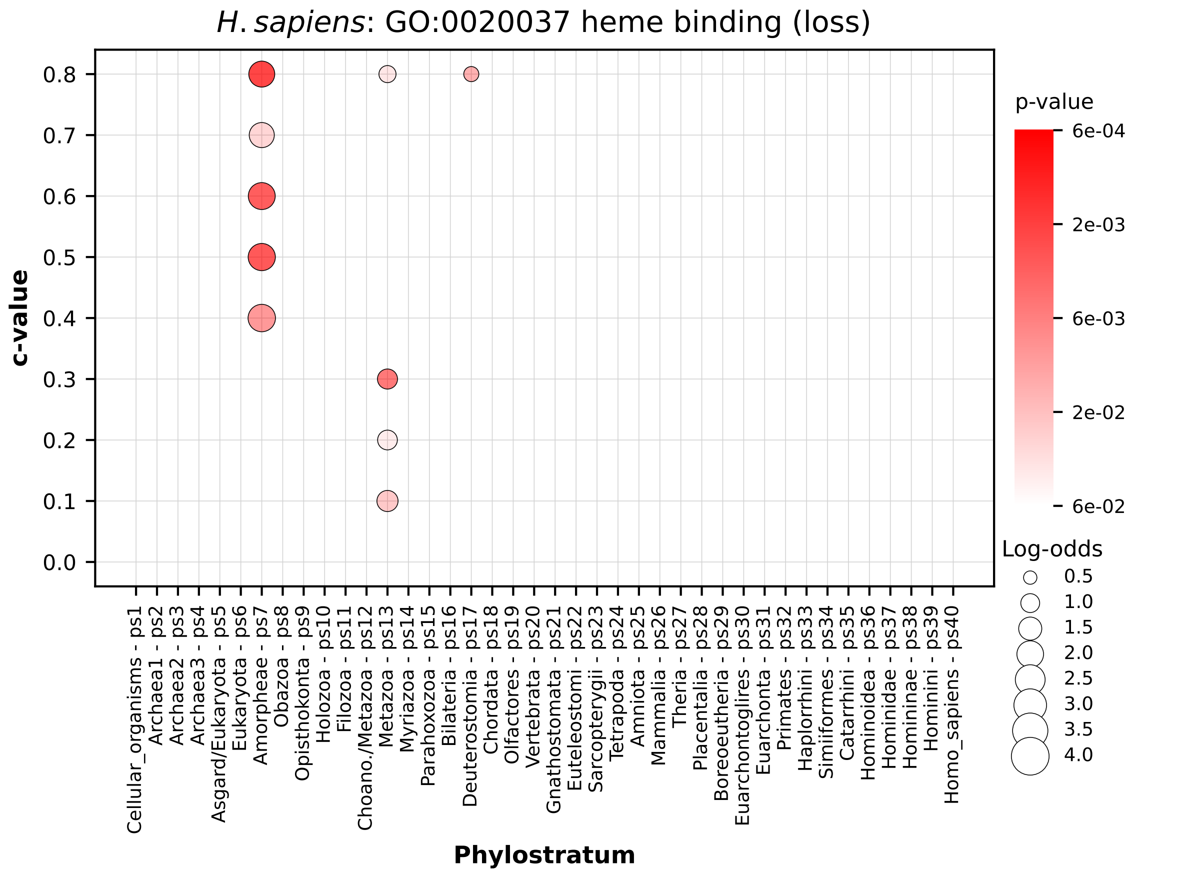

H. sapiens: GO:0020037 heme binding (loss)
c-value
0.0
0.1
0.2
0.3
0.4
0.5
0.6
0.7
0.8
Phylostratum
Cellular_organisms - ps1
Archaea1 - ps2
Archaea2 - ps3
Archaea3 - ps4
Asgard/Eukaryota - ps5
Eukaryota - ps6
Amorpheae - ps7
Obazoa - ps8
Opisthokonta - ps9
Holozoa - ps10
Filozoa - ps11
Choano./Metazoa - ps12
Metazoa - ps13
Myriazoa - ps14
Parahoxozoa - ps15
Bilateria - ps16
Deuterostomia - ps17
Chordata - ps18
Olfactores - ps19
Vertebrata - ps20
Gnathostomata - ps21
Euteleostomi - ps22
Sarcopterygii - ps23
Tetrapoda - ps24
Amniota - ps25
Mammalia - ps26
Theria - ps27
Placentalia - ps28
Boreoeutheria - ps29
Euarchontoglires - ps30
Euarchonta - ps31
Primates - ps32
Haplorrhini - ps33
Simiiformes - ps34
Catarrhini - ps35
Hominoidea - ps36
Hominidae - ps37
Homininae - ps38
Hominini - ps39
Homo_sapiens - ps40
p-value
6e-04
2e-03
6e-03
2e-02
6e-02
Log-odds
0.5
1.0
1.5
2.0
2.5
3.0
3.5
4.0

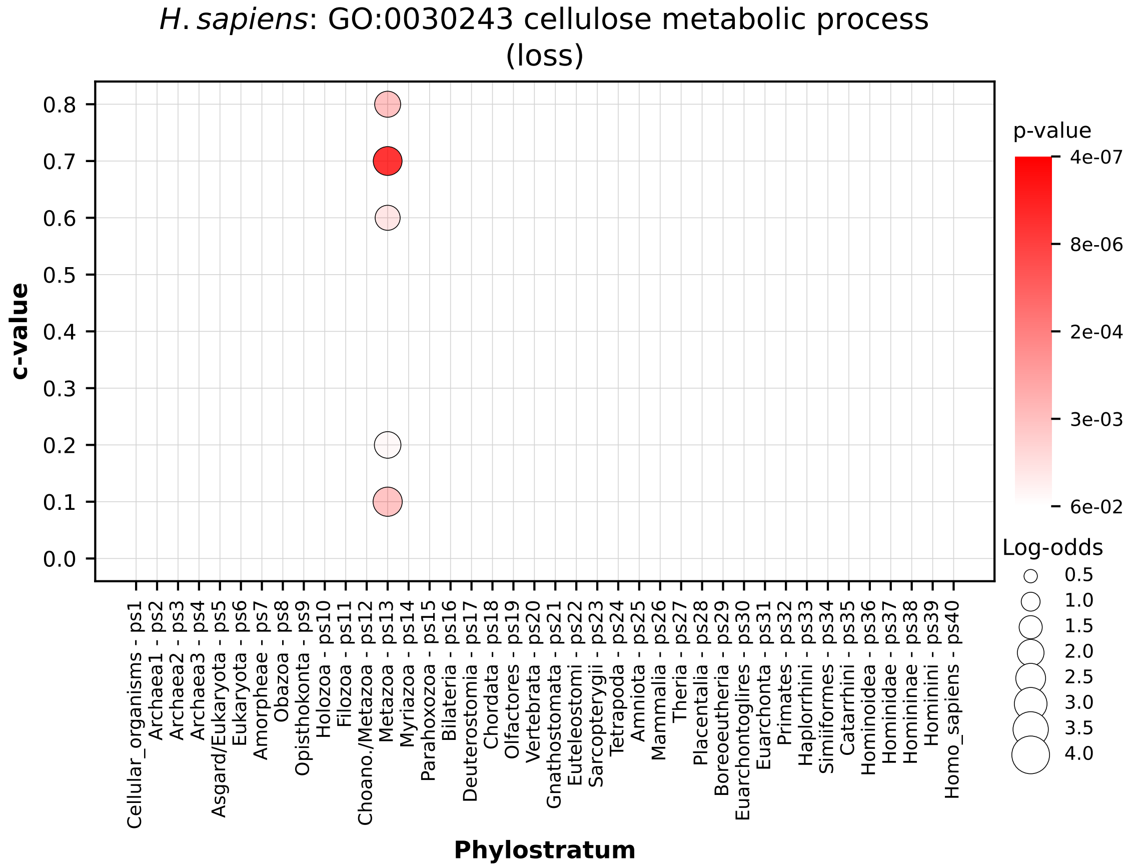

H. sapiens: GO:0030243 cellulose metabolic process
(loss)
c-value
0.8
0.7
0.6
0.5
0.4
0.3
0.2
0.1
0.0
p-value
4e-07
8e-06
2e-04
3e-03
6e-02
Log-odds
0.5
1.0
1.5
2.0
2.5
3.0
3.5
4.0
Cellular_organisms - ps1
Archaea1 - ps2
Archaea2 - ps3
Archaea3 - ps4
Asgard/Eukaryota - ps5
Eukaryota - ps6
Amorpheae - ps7
Obazoa - ps8
Opisthokonta - ps9
Holozoa - ps10
Filozoa - ps11
Choano./Metazoa - ps12
Metazoa - ps13
Myriazoa - ps14
Parahoxozoa - ps15
Bilateria - ps16
Deuterostomia - ps17
Chordata - ps18
Olfactores - ps19
Vertebrata - ps20
Gnathostomata - ps21
Euteleostomi - ps22
Sarcopterygii - ps23
Tetrapoda - ps24
Amniota - ps25
Mammalia - ps26
Theria - ps27
Placentalia - ps28
Boreoeutheria - ps29
Euarchontoglires - ps30
Euarchonta - ps31
Primates - ps32
Haplorrhini - ps33
Simiiformes - ps34
Catarrhini - ps35
Hominoidea - ps36
Hominidae - ps37
Homininae - ps38
Hominini - ps39
Homo_sapiens - ps40
Phylostratum

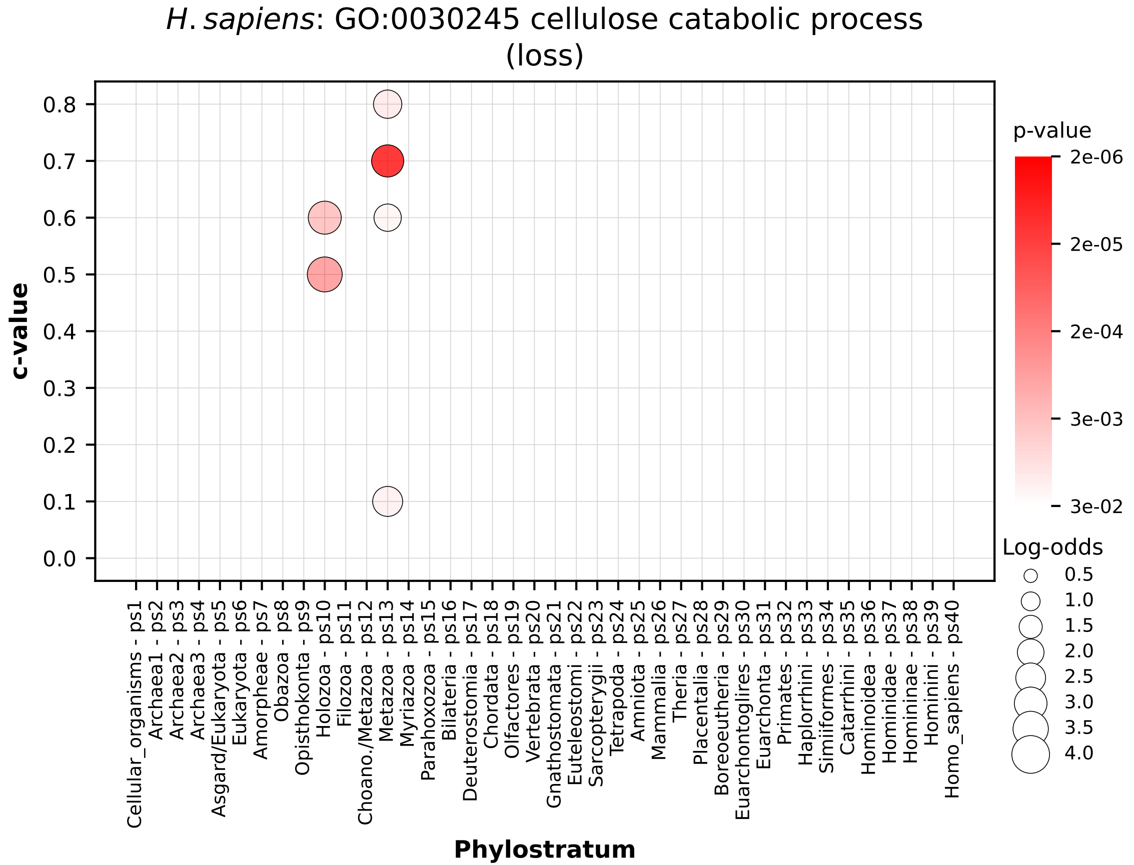

H. sapiens: GO:0030245 cellulose catabolic process
(loss)
c-value
0.0
0.1
0.2
0.3
0.4
0.5
0.6
0.7
0.8
Phylostratum
Cellular_organisms - ps1
Archaea1 - ps2
Archaea2 - ps3
Archaea3 - ps4
Asgard/Eukaryota - ps5
Eukaryota - ps6
Amorpheae - ps7
Obazoa - ps8
Opisthokonta - ps9
Holozoa - ps10
Filozoa - ps11
Choano./Metazoa - ps12
Metazoa - ps13
Myriazoa - ps14
Parahoxozoa - ps15
Bilateria - ps16
Deuterostomia - ps17
Chordata - ps18
Olfactores - ps19
Vertebrata - ps20
Gnathostomata - ps21
Euteleostomi - ps22
Sarcopterygii - ps23
Tetrapoda - ps24
Amniota - ps25
Mammalia - ps26
Theria - ps27
Placentalia - ps28
Boreoeutheria - ps29
Euarchontoglires - ps30
Euarchonta - ps31
Primates - ps32
Haplorrhini - ps33
Simiiformes - ps34
Catarrhini - ps35
Hominoidea - ps36
Hominidae - ps37
Homininae - ps38
Hominini - ps39
Homo_sapiens - ps40
p-value
2e-06
2e-05
2e-04
3e-03
3e-02
Log-odds
0.5
1.0
1.5
2.0
2.5
3.0
3.5
4.0

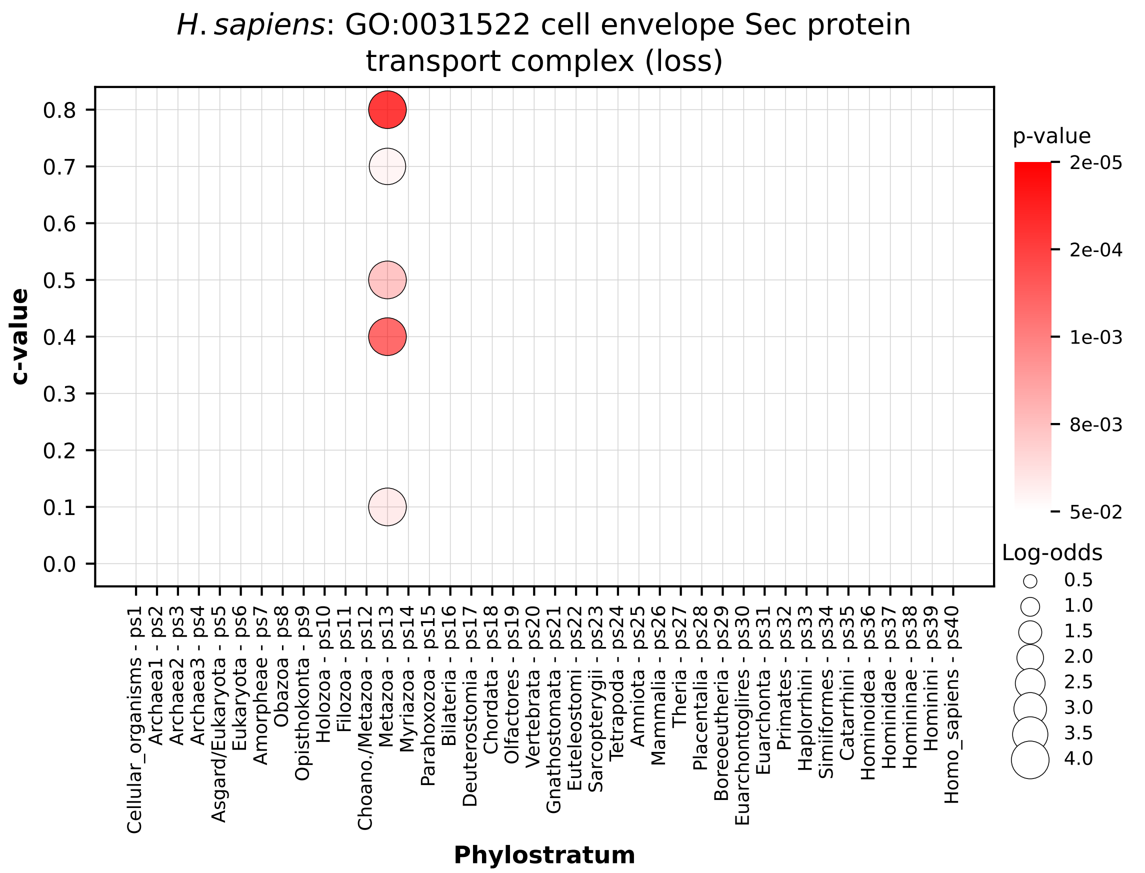

H. sapiens: GO:0031522 cell envelope Sec protein transport complex (loss)
c-value
0.8
0.7
0.6
0.5
0.4
0.3
0.2
0.1
0.0
Phylostratum
Cellular_organisms - ps1
Archaea1 - ps2
Archaea2 - ps3
Archaea3 - ps4
Asgard/Eukaryota - ps5
Eukaryota - ps6
Amorpheae - ps7
Obazoa - ps8
Opisthokonta - ps9
Holozoa - ps10
Filozoa - ps11
Choano./Metazoa - ps12
Metazoa - ps13
Myriazoa - ps14
Parahoxozoa - ps15
Bilateria - ps16
Deuterostomia - ps17
Chordata - ps18
Olfactores - ps19
Vertebrata - ps20
Gnathostomata - ps21
Euteleostomi - ps22
Sarcopterygii - ps23
Tetrapoda - ps24
Amniota - ps25
Mammalia - ps26
Theria - ps27
Placentalia - ps28
Boreoeutheria - ps29
Euarchontoglires - ps30
Euarchonta - ps31
Primates - ps32
Haplorrhini - ps33
Simiiformes - ps34
Catarrhini - ps35
Hominoidea - ps36
Hominidae - ps37
Homininae - ps38
Hominini - ps39
Homo_sapiens - ps40
p-value
2e-05
2e-04
1e-03
8e-03
5e-02
Log-odds
0.5
1.0
1.5
2.0
2.5
3.0
3.5
4.0

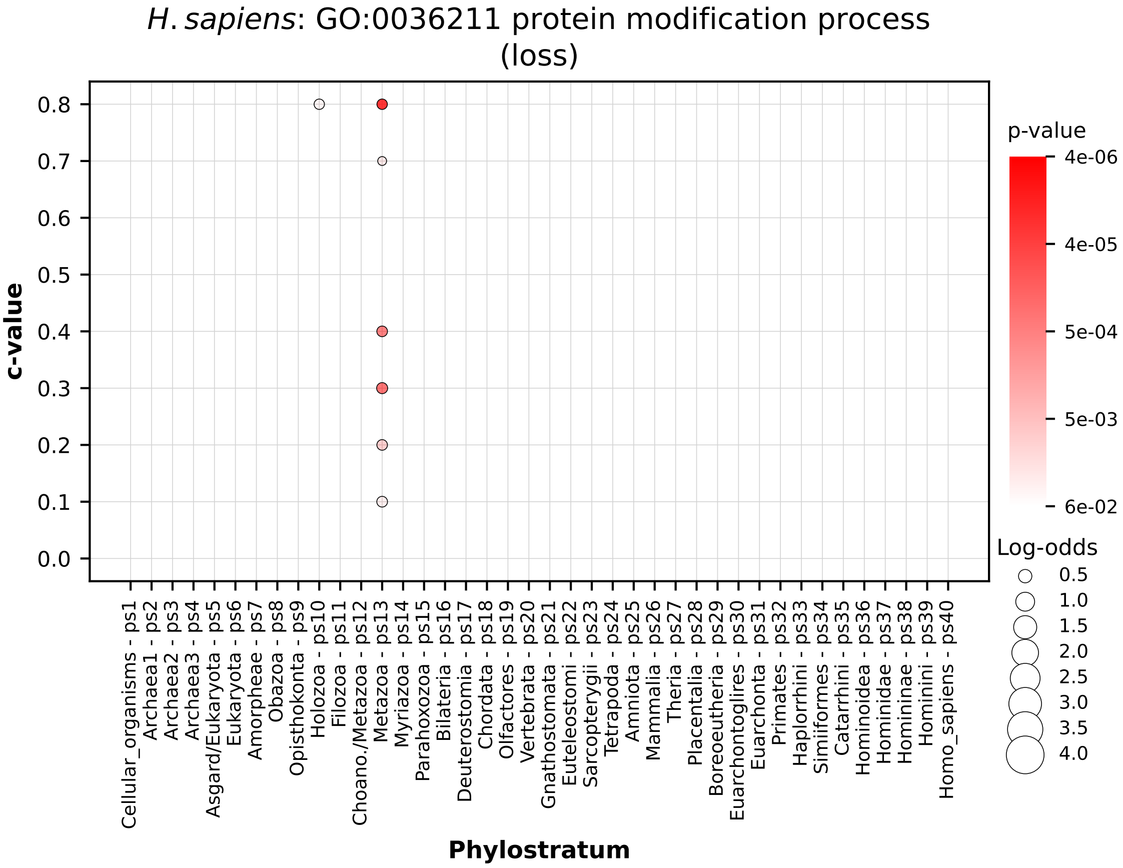

H. sapiens: GO:0036211 protein modification process
(loss)
c-value
0.0
0.1
0.2
0.3
0.4
0.5
0.6
0.7
0.8
Phylostratum
Cellular_organisms - ps1
Archaea1 - ps2
Archaea2 - ps3
Archaea3 - ps4
Asgard/Eukaryota - ps5
Eukaryota - ps6
Amorpheae - ps7
Obazoa - ps8
Opisthokonta - ps9
Holozoa - ps10
Filozoa - ps11
Choano./Metazoa - ps12
Metazoa - ps13
Myriazoa - ps14
Parahoxozoa - ps15
Bilateria - ps16
Deuterostomia - ps17
Chordata - ps18
Olfactores - ps19
Vertebrata - ps20
Gnathostomata - ps21
Euteleostomi - ps22
Sarcopterygii - ps23
Tetrapoda - ps24
Amniota - ps25
Mammalia - ps26
Theria - ps27
Placentalia - ps28
Boreoeutheria - ps29
Euarchontoglires - ps30
Euarchonta - ps31
Primates - ps32
Haplorrhini - ps33
Simiiformes - ps34
Catarrhini - ps35
Hominoidea - ps36
Hominidae - ps37
Homininae - ps38
Hominini - ps39
Homo_sapiens - ps40
p-value
4e-06
4e-05
5e-04
5e-03
6e-02
Log-odds
0.5
1.0
1.5
2.0
2.5
3.0
3.5
4.0

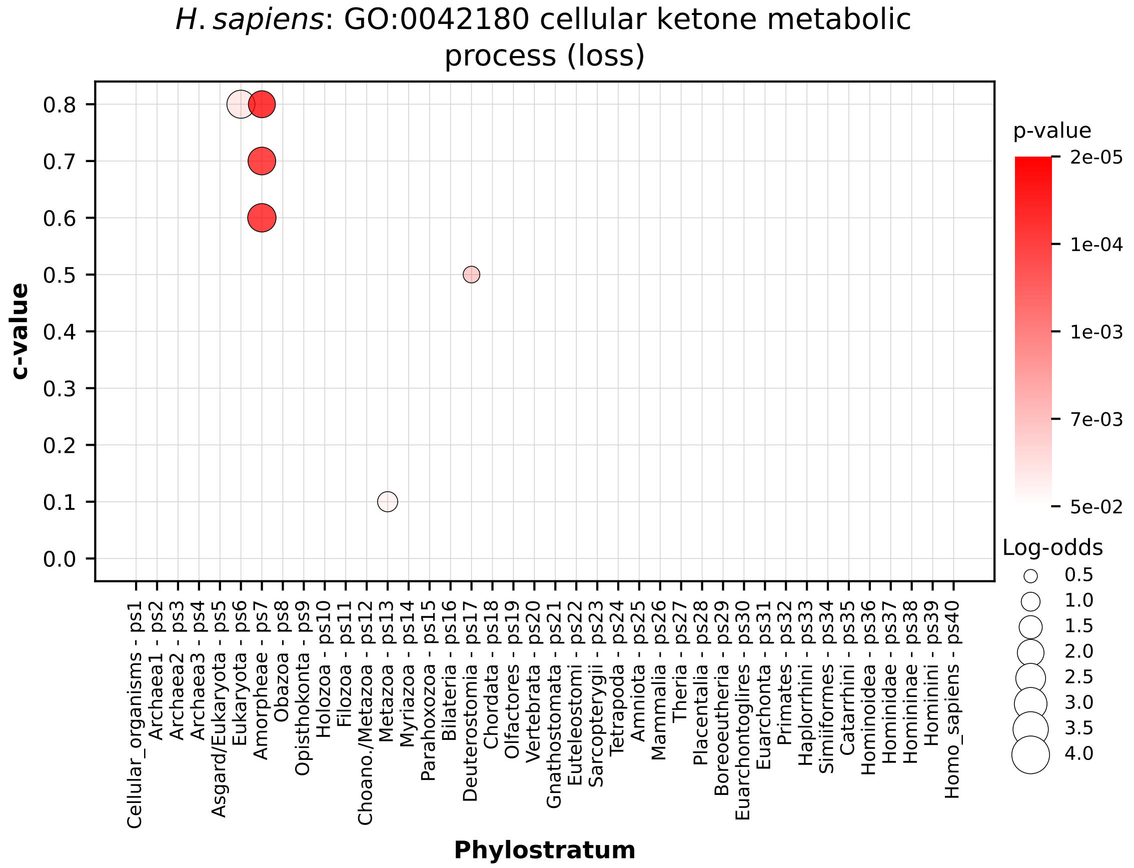

H. sapiens: GO:0042180 cellular ketone metabolic process (loss)
c-value
0.8
0.7
0.6
0.5
0.4
0.3
0.2
0.1
0.0
p-value
2e-05
1e-04
1e-03
7e-03
5e-02
Log-odds
0.5
1.0
1.5
2.0
2.5
3.0
3.5
4.0
Cellular_organisms - ps1
Archaea1 - ps2
Archaea2 - ps3
Archaea3 - ps4
Asgard/Eukaryota - ps5
Eukaryota - ps6
Amorpheae - ps7
Obazoa - ps8
Opisthokonta - ps9
Holozoa - ps10
Filozoa - ps11
Choano./Metazoa - ps12
Metazoa - ps13
Myriazoa - ps14
Parahoxozoa - ps15
Bilateria - ps16
Deuterostomia - ps17
Chordata - ps18
Olfactores - ps19
Vertebrata - ps20
Gnathostomata - ps21
Euteleostomi - ps22
Sarcopterygii - ps23
Tetrapoda - ps24
Amniota - ps25
Mammalia - ps26
Theria - ps27
Placentalia - ps28
Boreoeutheria - ps29
Euarchontoglires - ps30
Euarchonta - ps31
Primates - ps32
Haplorrhini - ps33
Simiiformes - ps34
Catarrhini - ps35
Hominoidea - ps36
Hominidae - ps37
Homininae - ps38
Hominini - ps39
Homo_sapiens - ps40
Phylostratum

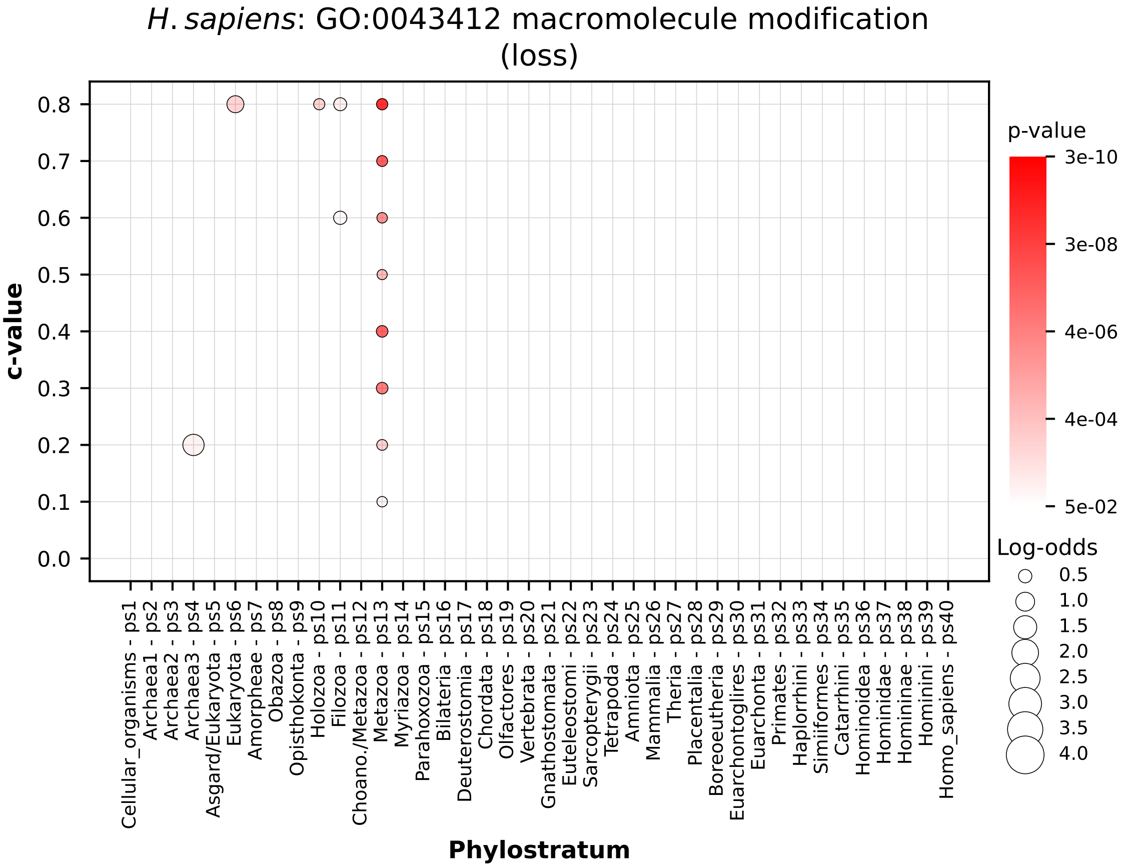

H. sapiens: GO:0043412 macromolecule modification (loss)
c-value
0.0
0.1
0.2
0.3
0.4
0.5
0.6
0.7
0.8
Phylostratum
Cellular_organisms - ps1
Archaea1 - ps2
Archaea2 - ps3
Archaea3 - ps4
Asgard/Eukaryota - ps5
Eukaryota - ps6
Amorpheae - ps7
Obazoa - ps8
Opisthokonta - ps9
Holozoa - ps10
Filozoa - ps11
Choano./Metazoa - ps12
Metazoa - ps13
Myriazoa - ps14
Parahoxozoa - ps15
Bilateria - ps16
Deuterostomia - ps17
Chordata - ps18
Olfactores - ps19
Vertebrata - ps20
Gnathostomata - ps21
Euteleostomi - ps22
Sarcopterygii - ps23
Tetrapoda - ps24
Amniota - ps25
Mammalia - ps26
Theria - ps27
Placentalia - ps28
Boreoeutheria - ps29
Euarchontoglires - ps30
Euarchonta - ps31
Primates - ps32
Haplorrhini - ps33
Simiiformes - ps34
Catarrhini - ps35
Hominoidea - ps36
Hominidae - ps37
Homininae - ps38
Hominini - ps39
Homo_sapiens - ps40
p-value
3e-10
3e-08
4e-06
4e-04
5e-02
Log-odds
0.5
1.0
1.5
2.0
2.5
3.0
3.5
4.0

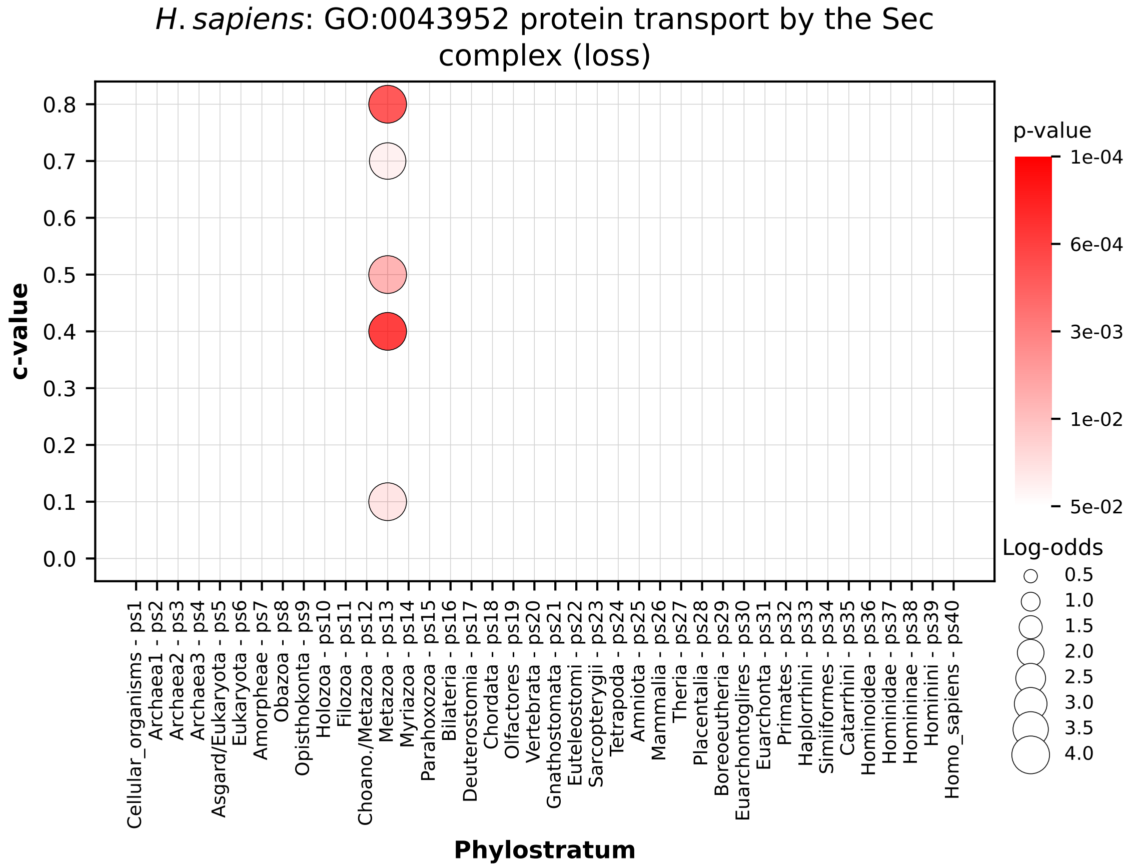

H. sapiens: GO:0043952 protein transport by the Sec complex (loss)
c-value
0.8
0.7
0.6
0.5
0.4
0.3
0.2
0.1
0.0
p-value
1e-04
6e-04
3e-03
1e-02
5e-02
Log-odds
0.5
1.0
1.5
2.0
2.5
3.0
3.5
4.0
Cellular_organisms - ps1
Archaea1 - ps2
Archaea2 - ps3
Archaea3 - ps4
Asgard/Eukaryota - ps5
Eukaryota - ps6
Amorpheae - ps7
Obazoa - ps8
Opisthokonta - ps9
Holozoa - ps10
Filozoa - ps11
Choano./Metazoa - ps12
Metazoa - ps13
Myriazoa - ps14
Parahoxozoa - ps15
Bilateria - ps16
Deuterostomia - ps17
Chordata - ps18
Olfactores - ps19
Vertebrata - ps20
Gnathostomata - ps21
Euteleostomi - ps22
Sarcopterygii - ps23
Tetrapoda - ps24
Amniota - ps25
Mammalia - ps26
Theria - ps27
Placentalia - ps28
Boreoeutheria - ps29
Euarchontoglires - ps30
Euarchonta - ps31
Primates - ps32
Haplorrhini - ps33
Simiiformes - ps34
Catarrhini - ps35
Hominoidea - ps36
Hominidae - ps37
Homininae - ps38
Hominini - ps39
Homo_sapiens - ps40
Phylostratum

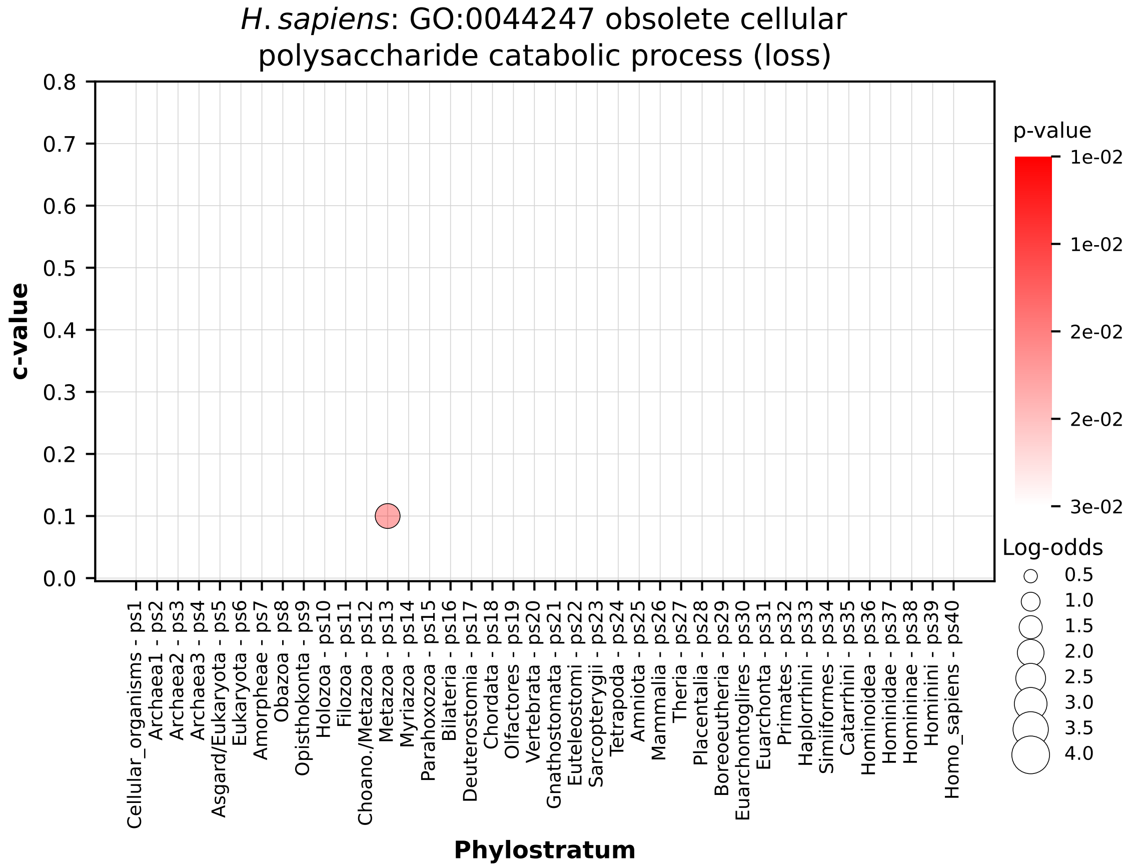
H. sapiens: GO:0044247 obsolete cellular polysaccharide catabolic process (loss)
c-value
0.0
0.1
0.2
0.3
0.4
0.5
0.6
0.7
0.8
p-value
1e-02
1e-02
2e-02
2e-02
3e-02
Log-odds
0.5
1.0
1.5
2.0
2.5
3.0
3.5
4.0
Cellular_organisms - ps1
Archaea1 - ps2
Archaea2 - ps3
Archaea3 - ps4
Asgard/Eukaryota - ps5
Eukaryota - ps6
Amorpheae - ps7
Obazoa - ps8
Opisthokonta - ps9
Holozoa - ps10
Filozoa - ps11
Choano./Metazoa - ps12
Metazoa - ps13
Myriazoa - ps14
Parahoxozoa - ps15
Bilateria - ps16
Deuterostomia - ps17
Chordata - ps18
Olfactores - ps19
Vertebrata - ps20
Gnathostomata - ps21
Euteleostomi - ps22
Sarcopterygii - ps23
Tetrapoda - ps24
Amniota - ps25
Mammalia - ps26
Theria - ps27
Placentalia - ps28
Boreoeutheria - ps29
Euarchontoglires - ps30
Euarchonta - ps31
Primates - ps32
Haplorrhini - ps33
Simiiformes - ps34
Catarrhini - ps35
Hominoidea - ps36
Hominidae - ps37
Homininae - ps38
Hominini - ps39
Homo_sapiens - ps40
Phylostratum

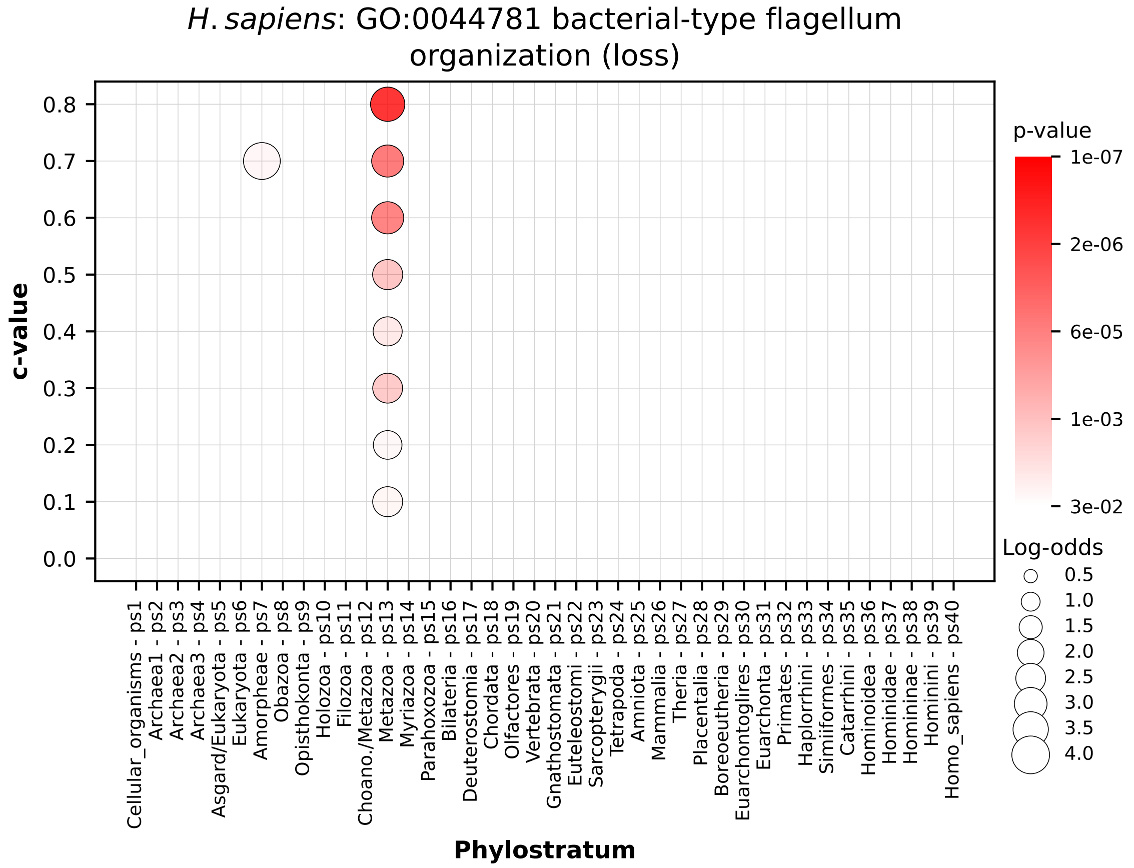

H. sapiens: GO:0044781 bacterial-type flagellum organization (loss)
c-value
0.8
0.7
0.6
0.5
0.4
0.3
0.2
0.1
0.0
p-value
1e-07
2e-06
6e-05
1e-03
3e-02
Log-odds
0.5
1.0
1.5
2.0
2.5
3.0
3.5
4.0
Cellular_organisms - ps1
Archaea1 - ps2
Archaea2 - ps3
Archaea3 - ps4
Asgard/Eukaryota - ps5
Eukaryota - ps6
Amorpheae - ps7
Obazoa - ps8
Opisthokonta - ps9
Holozoa - ps10
Filozoa - ps11
Choano./Metazoa - ps12
Metazoa - ps13
Myriazoa - ps14
Parahoxozoa - ps15
Bilateria - ps16
Deuterostomia - ps17
Chordata - ps18
Olfactores - ps19
Vertebrata - ps20
Gnathostomata - ps21
Euteleostomi - ps22
Sarcopterygii - ps23
Tetrapoda - ps24
Amniota - ps25
Mammalia - ps26
Theria - ps27
Placentalia - ps28
Boreoeutheria - ps29
Euarchontoglires - ps30
Euarchonta - ps31
Primates - ps32
Haplorrhini - ps33
Simiiformes - ps34
Catarrhini - ps35
Hominoidea - ps36
Hominidae - ps37
Homininae - ps38
Hominini - ps39
Homo_sapiens - ps40
Phylostratum

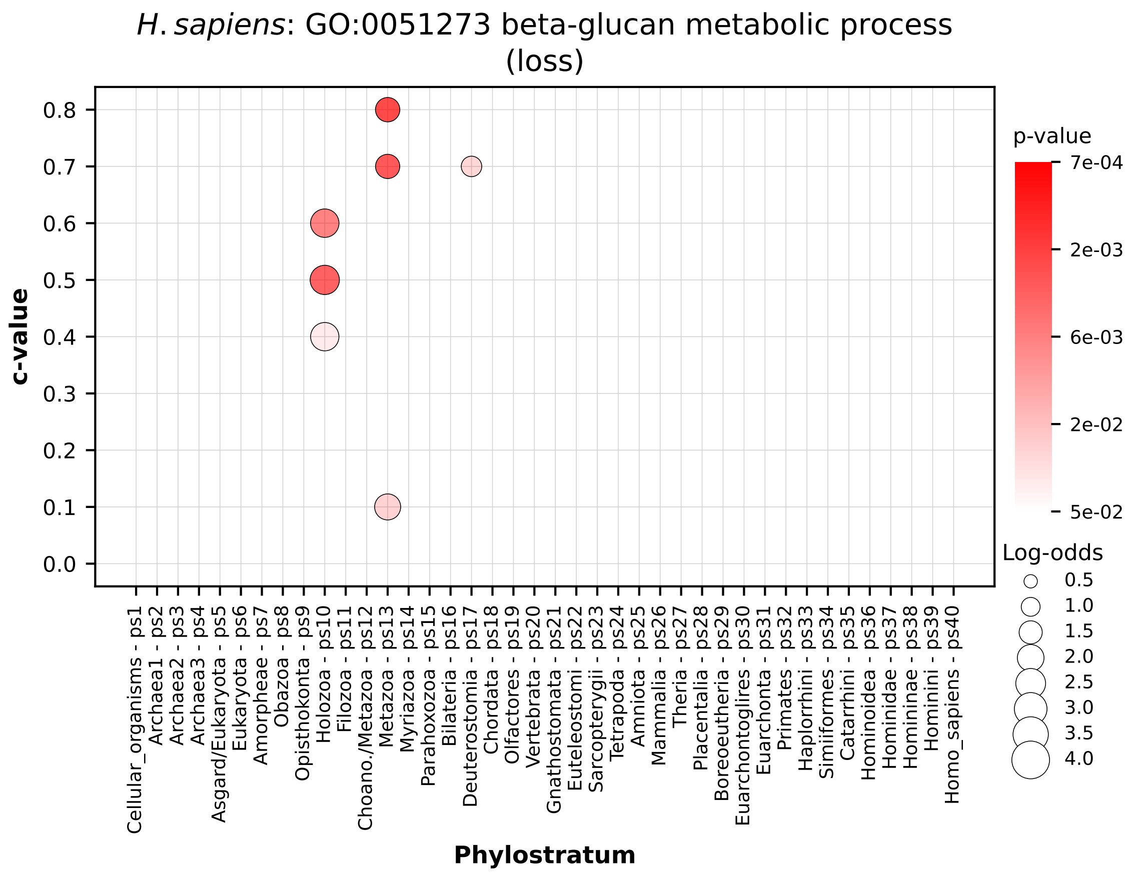

H. sapiens: GO:0051273 beta-glucan metabolic process
(loss)
c-value
0.8
0.7
0.6
0.5
0.4
0.3
0.2
0.1
0.0
p-value
7e-04
2e-03
6e-03
2e-02
5e-02
Log-odds
0.5
1.0
1.5
2.0
2.5
3.0
3.5
4.0
Cellular_organisms - ps1
Archaea1 - ps2
Archaea2 - ps3
Archaea3 - ps4
Asgard/Eukaryota - ps5
Eukaryota - ps6
Amorpheae - ps7
Obazoa - ps8
Opisthokonta - ps9
Holozoa - ps10
Filozoa - ps11
Choano./Metazoa - ps12
Metazoa - ps13
Myriazoa - ps14
Parahoxozoa - ps15
Bilateria - ps16
Deuterostomia - ps17
Chordata - ps18
Olfactores - ps19
Vertebrata - ps20
Gnathostomata - ps21
Euteleostomi - ps22
Sarcopterygii - ps23
Tetrapoda - ps24
Amniota - ps25
Mammalia - ps26
Theria - ps27
Placentalia - ps28
Boreoeutheria - ps29
Euarchontoglires - ps30
Euarchonta - ps31
Primates - ps32
Haplorrhini - ps33
Simiiformes - ps34
Catarrhini - ps35
Hominoidea - ps36
Hominidae - ps37
Homininae - ps38
Hominini - ps39
Homo_sapiens - ps40
Phylostratum

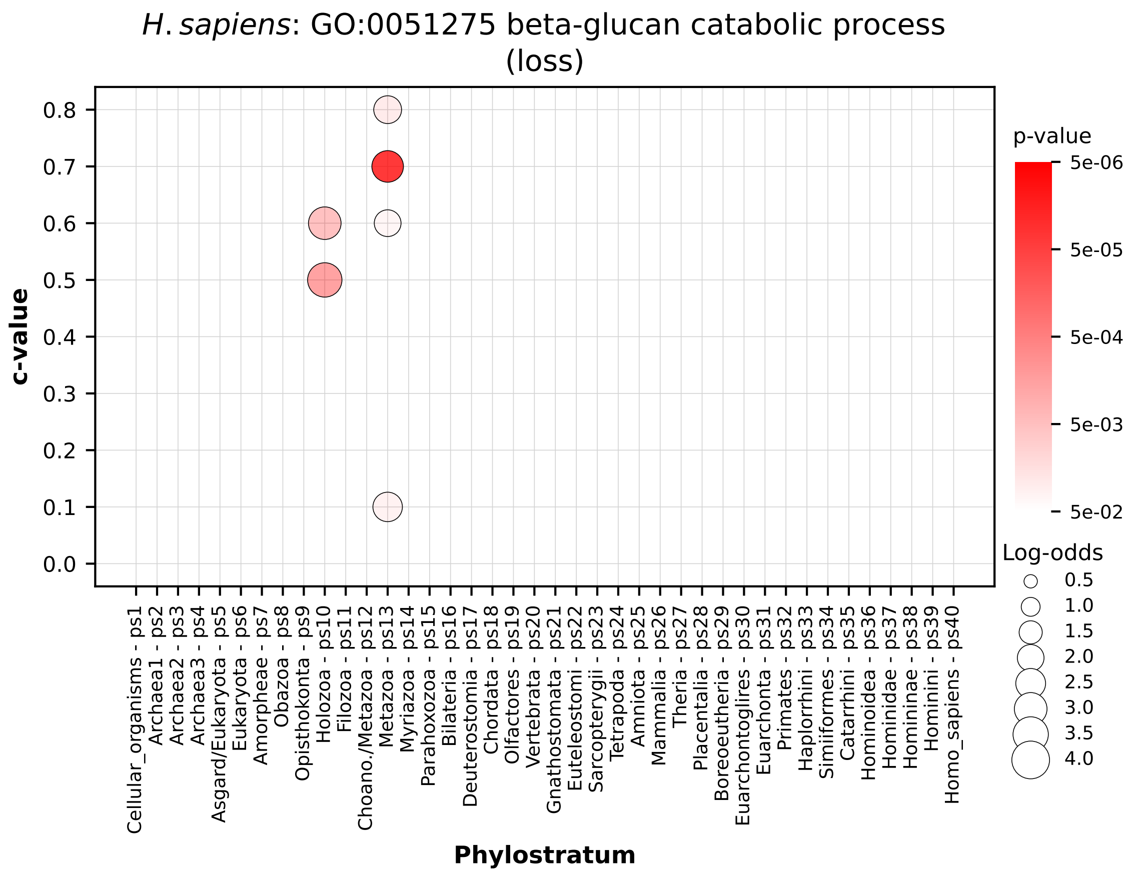

H. sapiens: GO:0051275 beta-glucan catabolic process
(loss)
c-value
0.0
0.1
0.2
0.3
0.4
0.5
0.6
0.7
0.8
Phylostratum
Cellular_organisms - ps1
Archaea1 - ps2
Archaea2 - ps3
Archaea3 - ps4
Asgard/Eukaryota - ps5
Eukaryota - ps6
Amorpheae - ps7
Obazoa - ps8
Opisthokonta - ps9
Holozoa - ps10
Filozoa - ps11
Choano./Metazoa - ps12
Metazoa - ps13
Myriazoa - ps14
Parahoxozoa - ps15
Bilateria - ps16
Deuterostomia - ps17
Chordata - ps18
Olfactores - ps19
Vertebrata - ps20
Gnathostomata - ps21
Euteleostomi - ps22
Sarcopterygii - ps23
Tetrapoda - ps24
Amniota - ps25
Mammalia - ps26
Theria - ps27
Placentalia - ps28
Boreoeutheria - ps29
Euarchontoglires - ps30
Euarchonta - ps31
Primates - ps32
Haplorrhini - ps33
Simiiformes - ps34
Catarrhini - ps35
Hominoidea - ps36
Hominidae - ps37
Homininae - ps38
Hominini - ps39
Homo_sapiens - ps40
p-value
5e-06
5e-05
5e-04
5e-03
5e-02
Log-odds
0.5
1.0
1.5
2.0
2.5
3.0
3.5
4.0

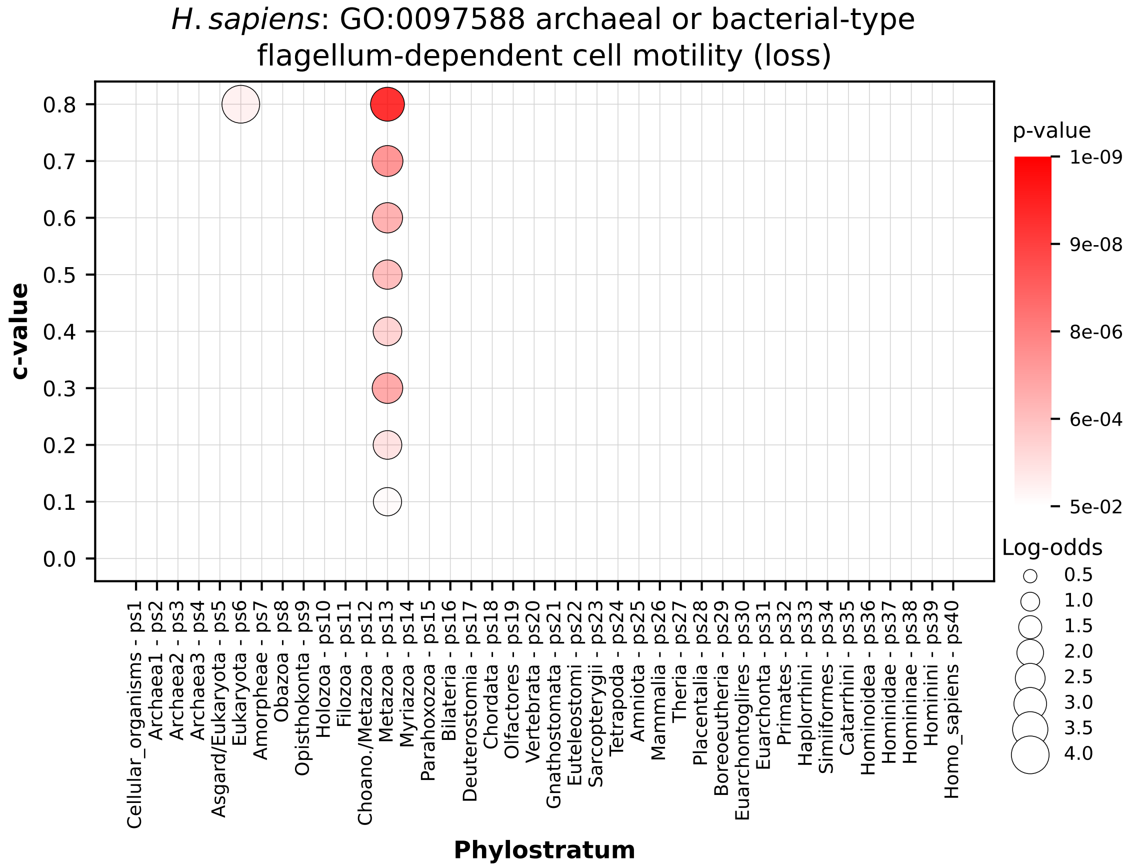

H. sapiens: GO:0097588 archaeal or bacterial-type flagellum-dependent cell motility (loss)
c-value
0.0
0.1
0.2
0.3
0.4
0.5
0.6
0.7
0.8
p-value
1e-09
9e-08
8e-06
6e-04
5e-02
Log-odds
0.5
1.0
1.5
2.0
2.5
3.0
3.5
4.0
Cellular_organisms - ps1
Archaea1 - ps2
Archaea2 - ps3
Archaea3 - ps4
Asgard/Eukaryota - ps5
Eukaryota - ps6
Amorpheae - ps7
Obazoa - ps8
Opisthokonta - ps9
Holozoa - ps10
Filozoa - ps11
Choano./Metazoa - ps12
Metazoa - ps13
Myriazoa - ps14
Parahoxozoa - ps15
Bilateria - ps16
Deuterostomia - ps17
Chordata - ps18
Olfactores - ps19
Vertebrata - ps20
Gnathostomata - ps21
Euteleostomi - ps22
Sarcopterygii - ps23
Tetrapoda - ps24
Amniota - ps25
Mammalia - ps26
Theria - ps27
Placentalia - ps28
Boreoeutheria - ps29
Euarchontoglires - ps30
Euarchonta - ps31
Primates - ps32
Haplorrhini - ps33
Simiiformes - ps34
Catarrhini - ps35
Hominoidea - ps36
Hominidae - ps37
Homininae - ps38
Hominini - ps39
Homo_sapiens - ps40
Phylostratum

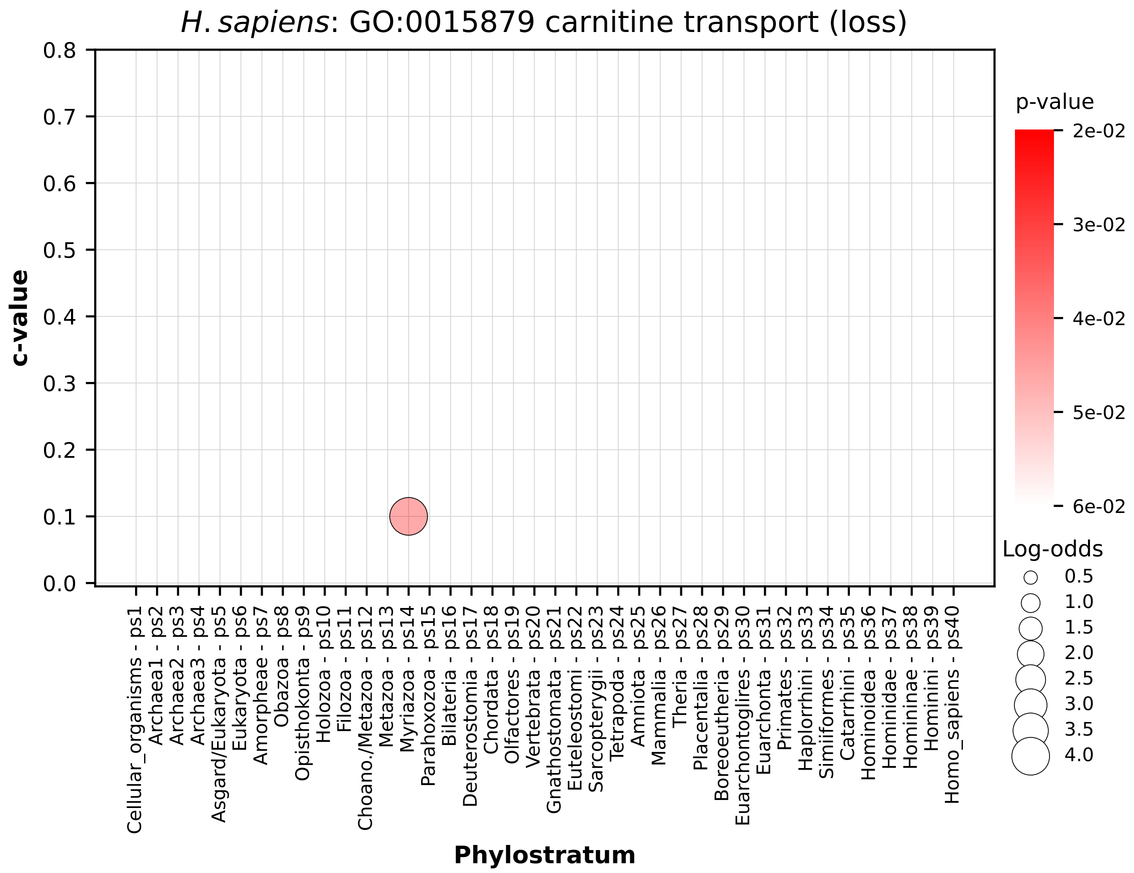

H. sapiens: GO:0015879 carnitine transport (loss)
c-value
0.0
0.1
0.2
0.3
0.4
0.5
0.6
0.7
0.8
Phylostratum
Cellular_organisms - ps1
Archaea1 - ps2
Archaea2 - ps3
Archaea3 - ps4
Asgard/Eukaryota - ps5
Eukaryota - ps6
Amorpheae - ps7
Obazoa - ps8
Opisthokonta - ps9
Holozoa - ps10
Filozoa - ps11
Choano./Metazoa - ps12
Metazoa - ps13
Myriazoa - ps14
Parahoxozoa - ps15
Bilateria - ps16
Deuterostomia - ps17
Chordata - ps18
Olfactores - ps19
Vertebrata - ps20
Gnathostomata - ps21
Euteleostomi - ps22
Sarcopterygii - ps23
Tetrapoda - ps24
Amniota - ps25
Mammalia - ps26
Theria - ps27
Placentalia - ps28
Boreoeutheria - ps29
Euarchontoglires - ps30
Euarchonta - ps31
Primates - ps32
Haplorrhini - ps33
Simiiformes - ps34
Catarrhini - ps35
Hominoidea - ps36
Hominidae - ps37
Homininae - ps38
Hominini - ps39
Homo_sapiens - ps40
p-value
2e-02
3e-02
4e-02
5e-02
6e-02
Log-odds
0.5
1.0
1.5
2.0
2.5
3.0
3.5
4.0

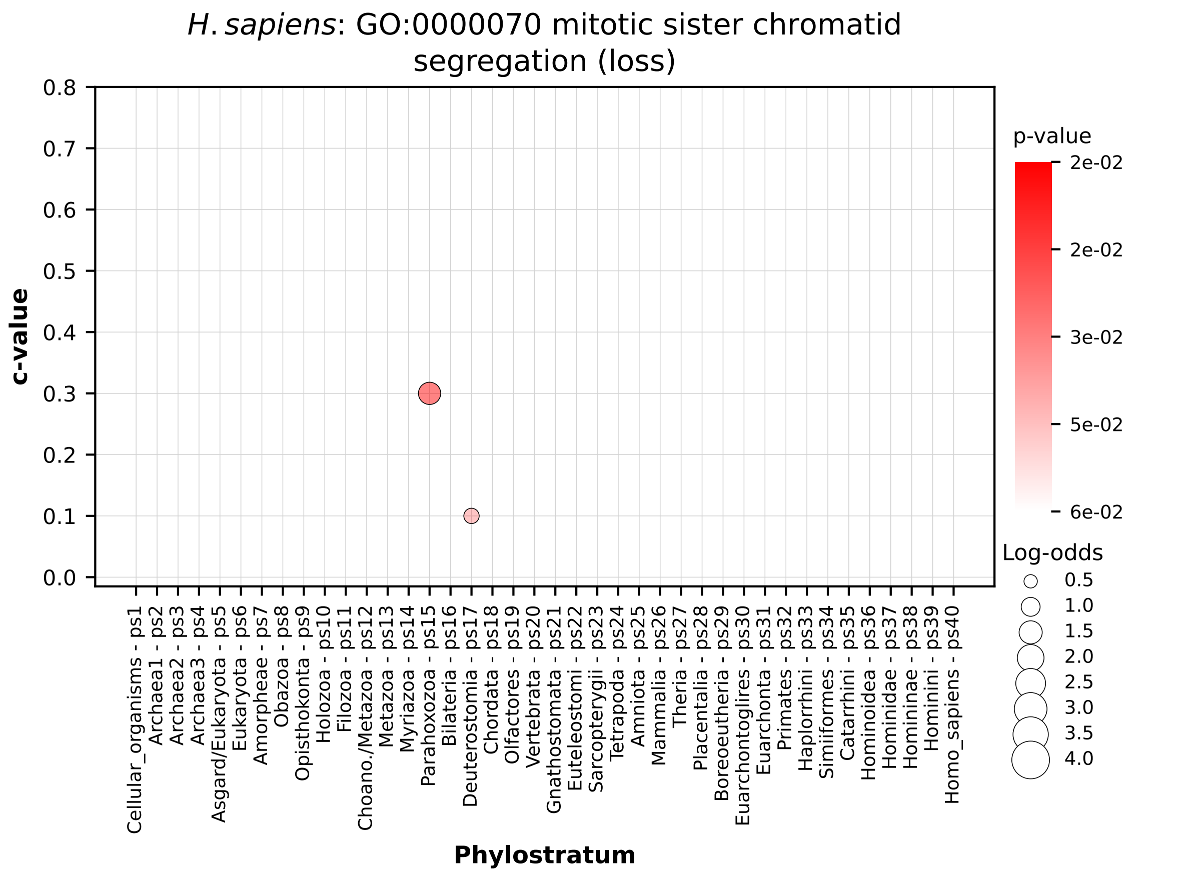

H. sapiens: GO:0000070 mitotic sister chromatid segregation (loss)
c-value
0.8
0.7
0.6
0.5
0.4
0.3
0.2
0.1
0.0
Phylostratum
Cellular_organisms - ps1
Archaea1 - ps2
Archaea2 - ps3
Archaea3 - ps4
Asgard/Eukaryota - ps5
Eukaryota - ps6
Amorpheae - ps7
Obazoa - ps8
Opisthokonta - ps9
Holozoa - ps10
Filozoa - ps11
Choano./Metazoa - ps12
Metazoa - ps13
Myriazoa - ps14
Parahoxozoa - ps15
Bilateria - ps16
Deuterostomia - ps17
Chordata - ps18
Olfactores - ps19
Vertebrata - ps20
Gnathostomata - ps21
Euteleostomi - ps22
Sarcopterygii - ps23
Tetrapoda - ps24
Amniota - ps25
Mammalia - ps26
Theria - ps27
Placentalia - ps28
Boreoeutheria - ps29
Euarchontoglires - ps30
Euarchonta - ps31
Primates - ps32
Haplorrhini - ps33
Simiiformes - ps34
Catarrhini - ps35
Hominoidea - ps36
Hominidae - ps37
Homininae - ps38
Hominini - ps39
Homo_sapiens - ps40
p-value
2e-02
2e-02
3e-02
5e-02
6e-02
Log-odds
0.5
1.0
1.5
2.0
2.5
3.0
3.5
4.0

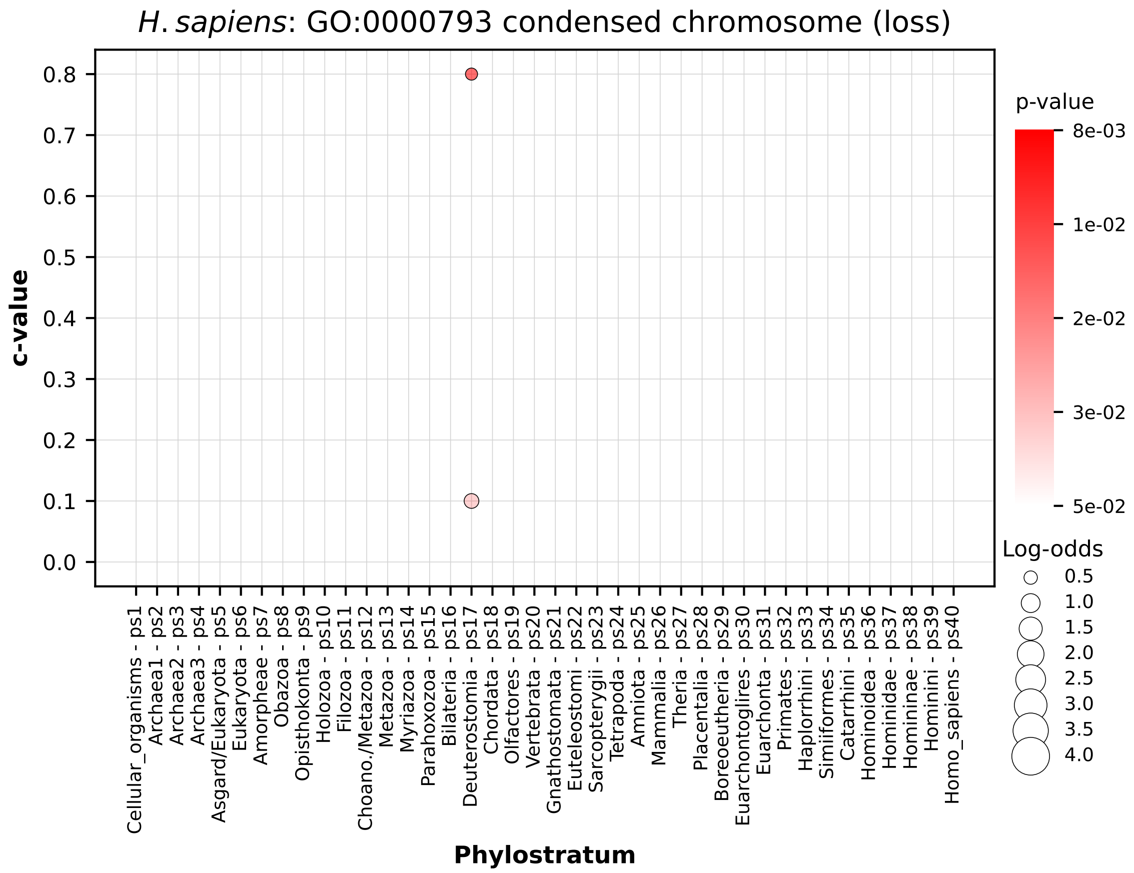

H. sapiens: GO:0000793 condensed chromosome (loss)
c-value
0.0
0.1
0.2
0.3
0.4
0.5
0.6
0.7
0.8
Phylostratum
Cellular_organisms - ps1
Archaea1 - ps2
Archaea2 - ps3
Archaea3 - ps4
Asgard/Eukaryota - ps5
Eukaryota - ps6
Amorpheae - ps7
Obazoa - ps8
Opisthokonta - ps9
Holozoa - ps10
Filozoa - ps11
Choano./Metazoa - ps12
Metazoa - ps13
Myriazoa - ps14
Parahoxozoa - ps15
Bilateria - ps16
Deuterostomia - ps17
Chordata - ps18
Olfactores - ps19
Vertebrata - ps20
Gnathostomata - ps21
Euteleostomi - ps22
Sarcopterygii - ps23
Tetrapoda - ps24
Amniota - ps25
Mammalia - ps26
Theria - ps27
Placentalia - ps28
Boreoeutheria - ps29
Euarchontoglires - ps30
Euarchonta - ps31
Primates - ps32
Haplorrhini - ps33
Simiiformes - ps34
Catarrhini - ps35
Hominoidea - ps36
Hominidae - ps37
Homininae - ps38
Hominini - ps39
Homo_sapiens - ps40
p-value
8e-03
1e-02
2e-02
3e-02
5e-02
Log-odds
0.5
1.0
1.5
2.0
2.5
3.0
3.5
4.0

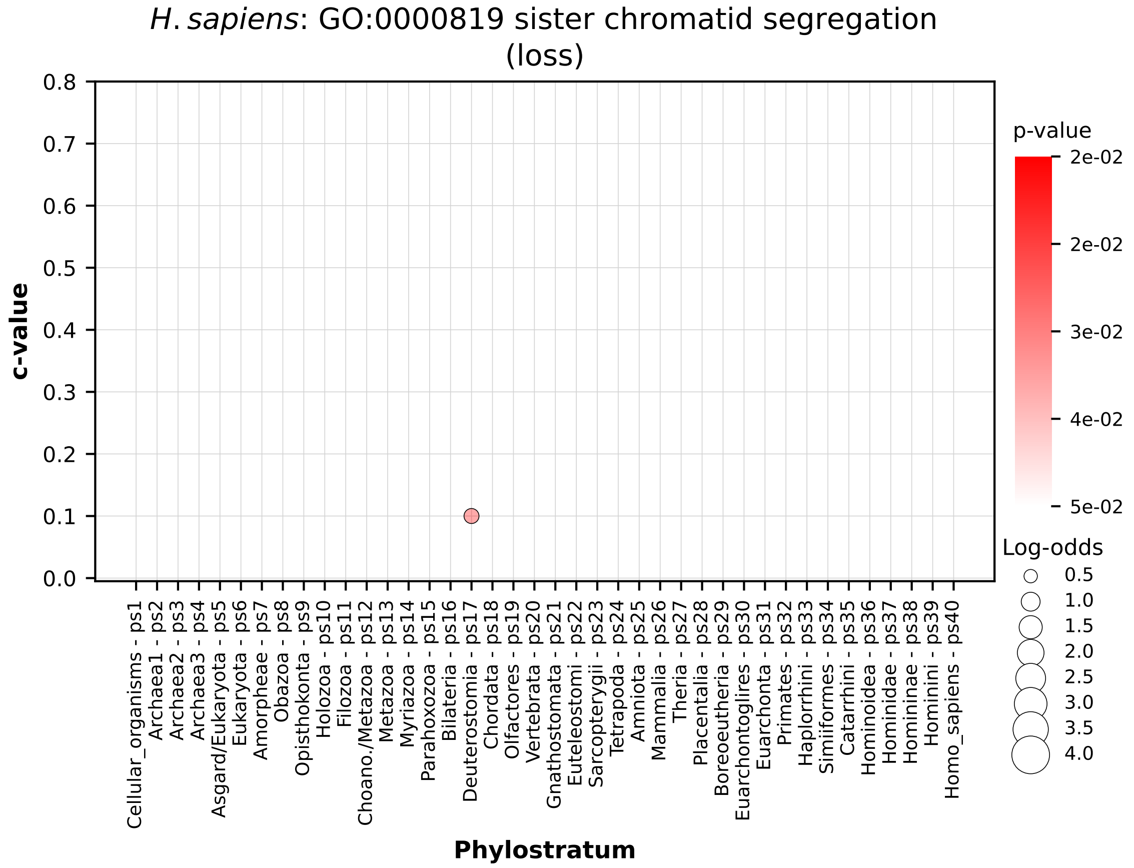
H. sapiens: GO:0000819 sister chromatid segregation (loss)
c-value
0.8
0.7
0.6
0.5
0.4
0.3
0.2
0.1
0.0
Phylostratum
Cellular_organisms - ps1
Archaea1 - ps2
Archaea2 - ps3
Archaea3 - ps4
Asgard/Eukaryota - ps5
Eukaryota - ps6
Amorpheae - ps7
Obazoa - ps8
Opisthokonta - ps9
Holozoa - ps10
Filozoa - ps11
Choano./Metazoa - ps12
Metazoa - ps13
Myriazoa - ps14
Parahoxozoa - ps15
Bilateria - ps16
Deuterostomia - ps17
Chordata - ps18
Olfactores - ps19
Vertebrata - ps20
Gnathostomata - ps21
Euteleostomi - ps22
Sarcopterygii - ps23
Tetrapoda - ps24
Amniota - ps25
Mammalia - ps26
Theria - ps27
Placentalia - ps28
Boreoeutheria - ps29
Euarchontoglires - ps30
Euarchonta - ps31
Primates - ps32
Haplorrhini - ps33
Simiiformes - ps34
Catarrhini - ps35
Hominoidea - ps36
Hominidae - ps37
Homininae - ps38
Hominini - ps39
Homo_sapiens - ps40
p-value
2e-02
2e-02
3e-02
4e-02
5e-02
Log-odds
0.5
1.0
1.5
2.0
2.5
3.0
3.5
4.0

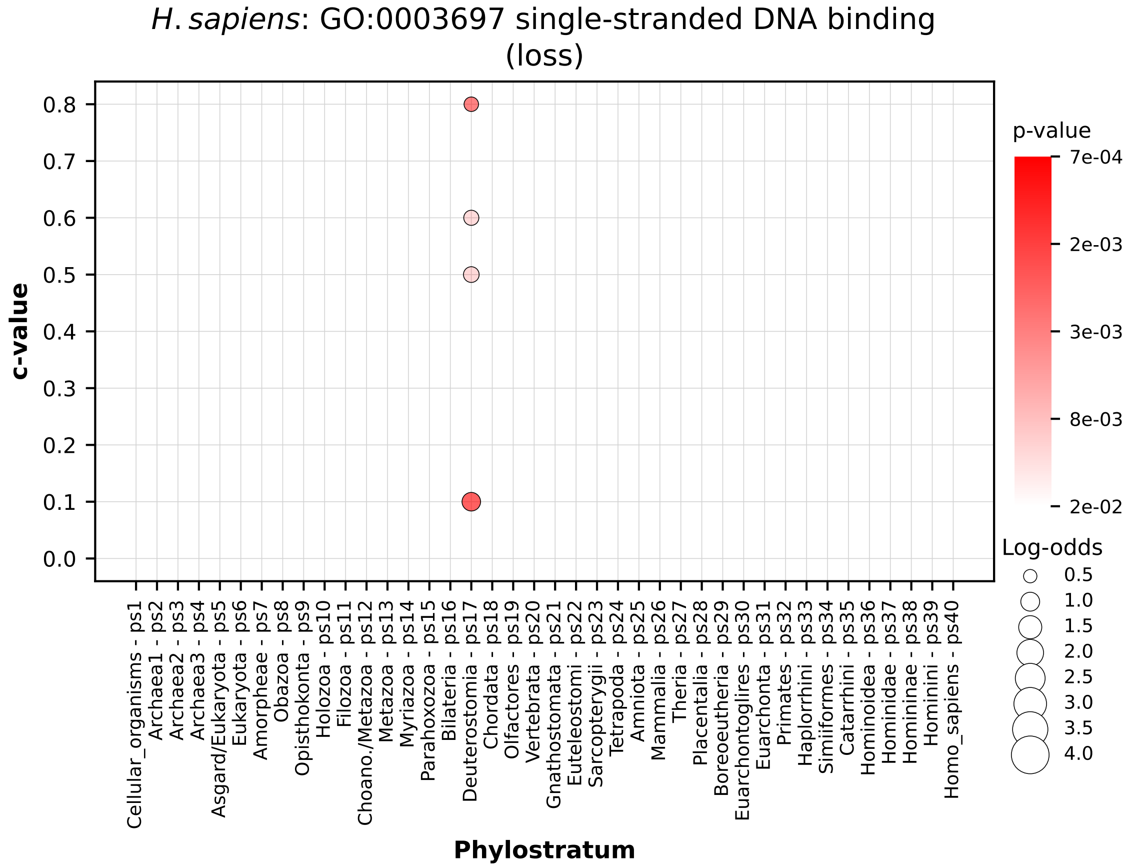

H. sapiens: GO:0003697 single-stranded DNA binding
(loss)
c-value
0.8
0.7
0.6
0.5
0.4
0.3
0.2
0.1
0.0
Phylostratum
Cellular_organisms - ps1
Archaea1 - ps2
Archaea2 - ps3
Archaea3 - ps4
Asgard/Eukaryota - ps5
Eukaryota - ps6
Amorpheae - ps7
Obazoa - ps8
Opisthokonta - ps9
Holozoa - ps10
Filozoa - ps11
Choano./Metazoa - ps12
Metazoa - ps13
Myriazoa - ps14
Parahoxozoa - ps15
Bilateria - ps16
Deuterostomia - ps17
Chordata - ps18
Olfactores - ps19
Vertebrata - ps20
Gnathostomata - ps21
Euteleostomi - ps22
Sarcopterygii - ps23
Tetrapoda - ps24
Amniota - ps25
Mammalia - ps26
Theria - ps27
Placentalia - ps28
Boreoeutheria - ps29
Euarchontoglires - ps30
Euarchonta - ps31
Primates - ps32
Haplorrhini - ps33
Simiiformes - ps34
Catarrhini - ps35
Hominoidea - ps36
Hominidae - ps37
Homininae - ps38
Hominini - ps39
Homo_sapiens - ps40
p-value
7e-04
2e-03
3e-03
8e-03
2e-02
Log-odds
0.5
1.0
1.5
2.0
2.5
3.0
3.5
4.0

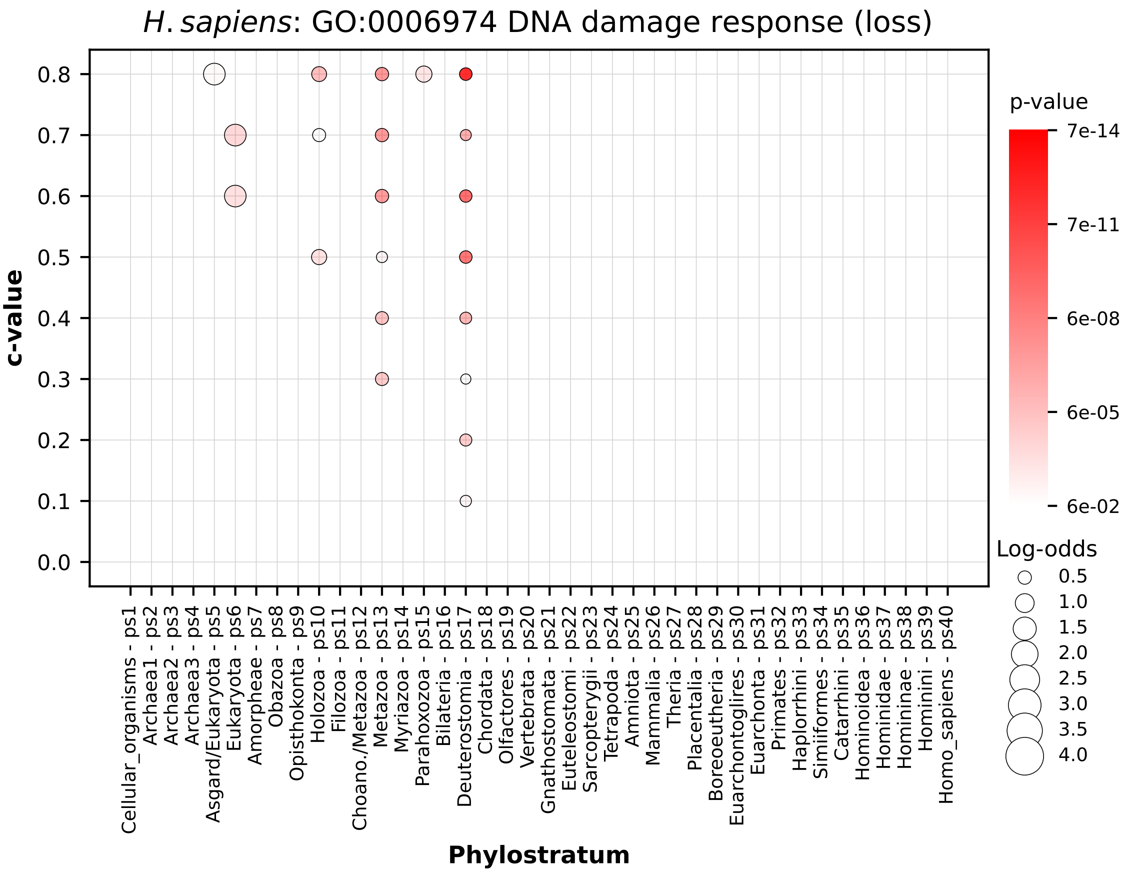

H. sapiens: GO:0006974 DNA damage response (loss)
c-value
0.8
0.7
0.6
0.5
0.4
0.3
0.2
0.1
0.0
p-value
7e-14
7e-11
6e-08
6e-05
6e-02
Log-odds
0.5
1.0
1.5
2.0
2.5
3.0
3.5
4.0
Cellular_organisms - ps1
Archaea1 - ps2
Archaea2 - ps3
Archaea3 - ps4
Asgard/Eukaryota - ps5
Eukaryota - ps6
Amorpheae - ps7
Obazoa - ps8
Opisthokonta - ps9
Holozoa - ps10
Filozoa - ps11
Choano./Metazoa - ps12
Metazoa - ps13
Myriazoa - ps14
Parahoxozoa - ps15
Bilateria - ps16
Deuterostomia - ps17
Chordata - ps18
Olfactores - ps19
Vertebrata - ps20
Gnathostomata - ps21
Euteleostomi - ps22
Sarcopterygii - ps23
Tetrapoda - ps24
Amniota - ps25
Mammalia - ps26
Theria - ps27
Placentalia - ps28
Boreoeutheria - ps29
Euarchontoglires - ps30
Euarchonta - ps31
Primates - ps32
Haplorrhini - ps33
Simiiformes - ps34
Catarrhini - ps35
Hominoidea - ps36
Hominidae - ps37
Homininae - ps38
Hominini - ps39
Homo_sapiens - ps40
Phylostratum

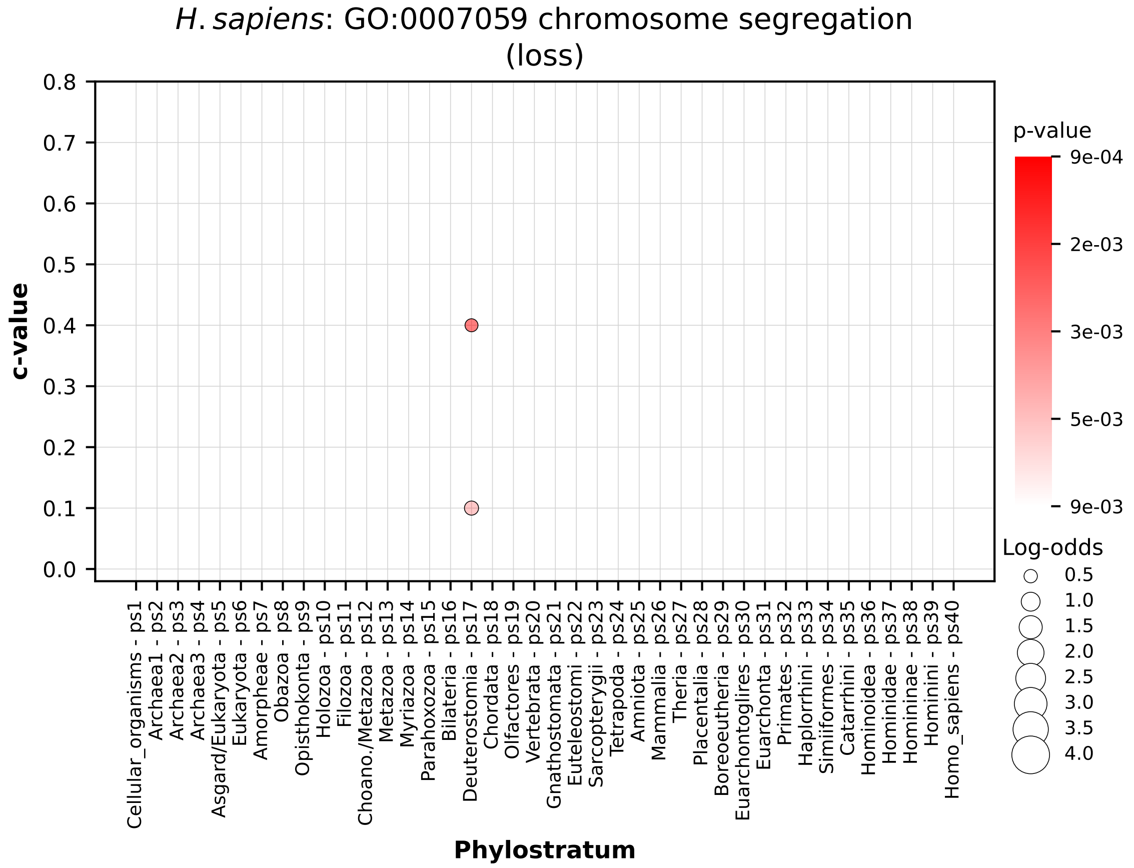

H. sapiens: GO:0007059 chromosome segregation
(loss)
c-value
0.0
0.1
0.2
0.3
0.4
0.5
0.6
0.7
0.8
Phylostratum
Cellular_organisms - ps1
Archaea1 - ps2
Archaea2 - ps3
Archaea3 - ps4
Asgard/Eukaryota - ps5
Eukaryota - ps6
Amorpheae - ps7
Obazoa - ps8
Opisthokonta - ps9
Holozoa - ps10
Filozoa - ps11
Choano./Metazoa - ps12
Metazoa - ps13
Myriazoa - ps14
Parahoxozoa - ps15
Bilateria - ps16
Deuterostomia - ps17
Chordata - ps18
Olfactores - ps19
Vertebrata - ps20
Gnathostomata - ps21
Euteleostomi - ps22
Sarcopterygii - ps23
Tetrapoda - ps24
Amniota - ps25
Mammalia - ps26
Theria - ps27
Placentalia - ps28
Boreoeutheria - ps29
Euarchontoglires - ps30
Euarchonta - ps31
Primates - ps32
Haplorrhini - ps33
Simiiformes - ps34
Catarrhini - ps35
Hominoidea - ps36
Hominidae - ps37
Homininae - ps38
Hominini - ps39
Homo_sapiens - ps40
p-value
9e-04
2e-03
3e-03
5e-03
9e-03
Log-odds
0.5
1.0
1.5
2.0
2.5
3.0
3.5
4.0

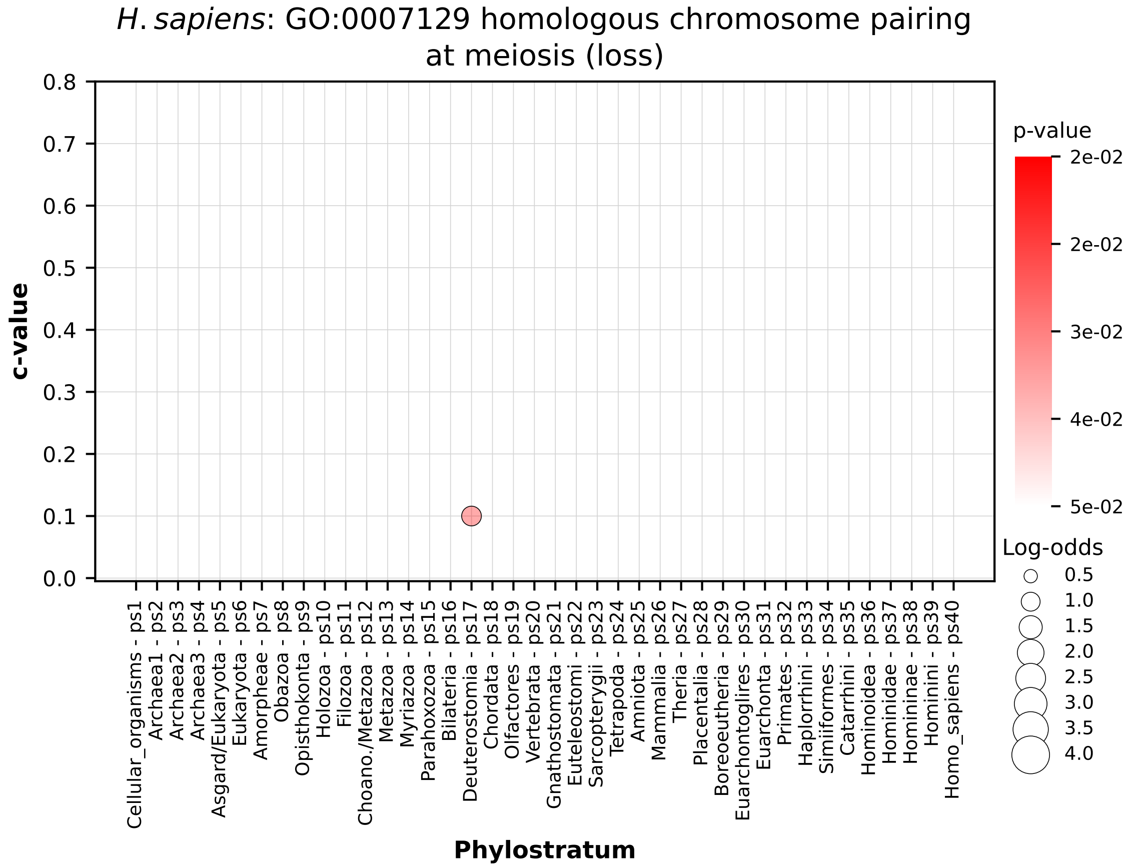

H. sapiens: GO:0007129 homologous chromosome pairing at meiosis (loss)
c-value
0.8
0.7
0.6
0.5
0.4
0.3
0.2
0.1
0.0
Phylostratum
Cellular_organisms - ps1
Archaea1 - ps2
Archaea2 - ps3
Archaea3 - ps4
Asgard/Eukaryota - ps5
Eukaryota - ps6
Amorpheae - ps7
Obazoa - ps8
Opisthokonta - ps9
Holozoa - ps10
Filozoa - ps11
Choano./Metazoa - ps12
Metazoa - ps13
Myriazoa - ps14
Parahoxozoa - ps15
Bilateria - ps16
Deuterostomia - ps17
Chordata - ps18
Olfactores - ps19
Vertebrata - ps20
Gnathostomata - ps21
Euteleostomi - ps22
Sarcopterygii - ps23
Tetrapoda - ps24
Amniota - ps25
Mammalia - ps26
Theria - ps27
Placentalia - ps28
Boreoeutheria - ps29
Euarchontoglires - ps30
Euarchonta - ps31
Primates - ps32
Haplorrhini - ps33
Simiiformes - ps34
Catarrhini - ps35
Hominoidea - ps36
Hominidae - ps37
Homininae - ps38
Hominini - ps39
Homo_sapiens - ps40
p-value
2e-02
2e-02
3e-02
4e-02
5e-02
Log-odds
0.5
1.0
1.5
2.0
2.5
3.0
3.5
4.0

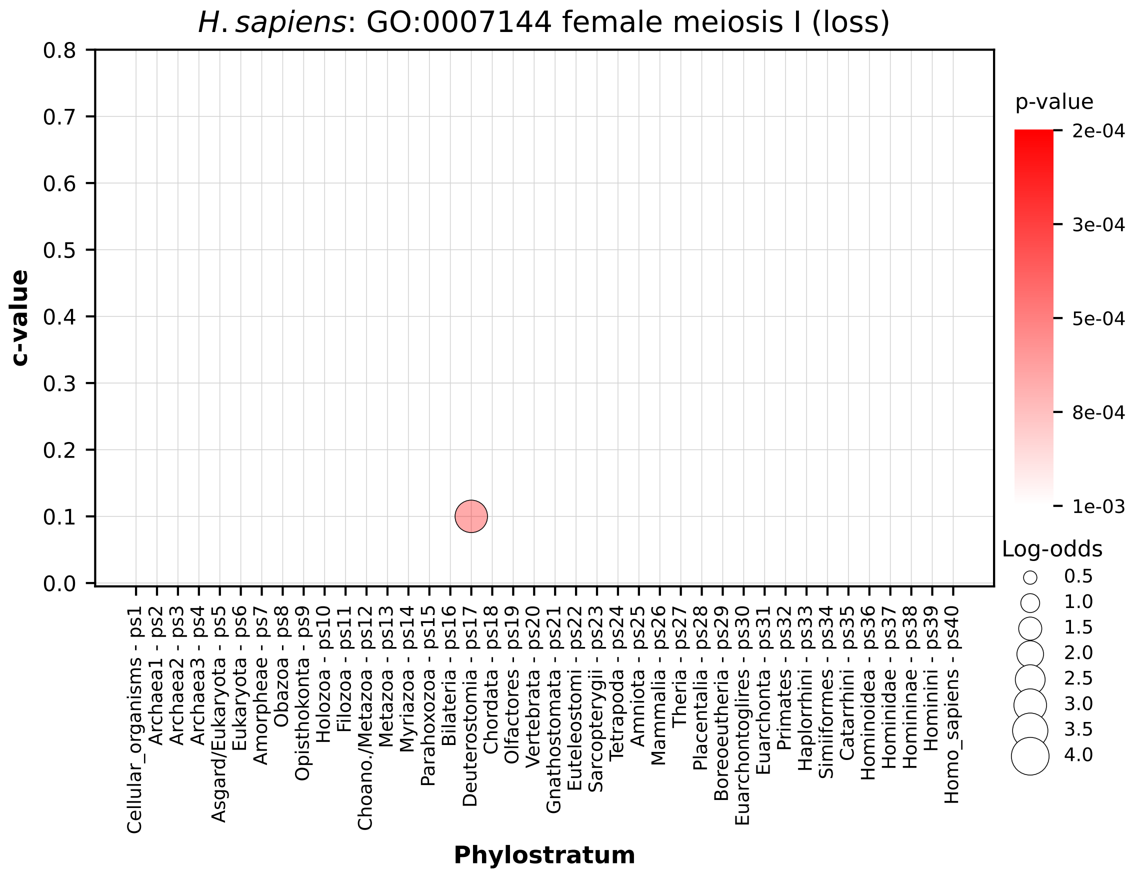

H. sapiens: GO:0007144 female meiosis I (loss)
c-value
0.0
0.1
0.2
0.3
0.4
0.5
0.6
0.7
0.8
Phylostratum
Cellular_organisms - ps1
Archaea1 - ps2
Archaea2 - ps3
Archaea3 - ps4
Asgard/Eukaryota - ps5
Eukaryota - ps6
Amorpheae - ps7
Obazoa - ps8
Opisthokonta - ps9
Holozoa - ps10
Filozoa - ps11
Choano./Metazoa - ps12
Metazoa - ps13
Myriazoa - ps14
Parahoxozoa - ps15
Bilateria - ps16
Deuterostomia - ps17
Chordata - ps18
Olfactores - ps19
Vertebrata - ps20
Gnathostomata - ps21
Euteleostomi - ps22
Sarcopterygii - ps23
Tetrapoda - ps24
Amniota - ps25
Mammalia - ps26
Theria - ps27
Placentalia - ps28
Boreoeutheria - ps29
Euarchontoglires - ps30
Euarchonta - ps31
Primates - ps32
Haplorrhini - ps33
Simiiformes - ps34
Catarrhini - ps35
Hominoidea - ps36
Hominidae - ps37
Homininae - ps38
Hominini - ps39
Homo_sapiens - ps40
p-value
2e-04
3e-04
5e-04
8e-04
1e-03
Log-odds
0.5
1.0
1.5
2.0
2.5
3.0
3.5
4.0

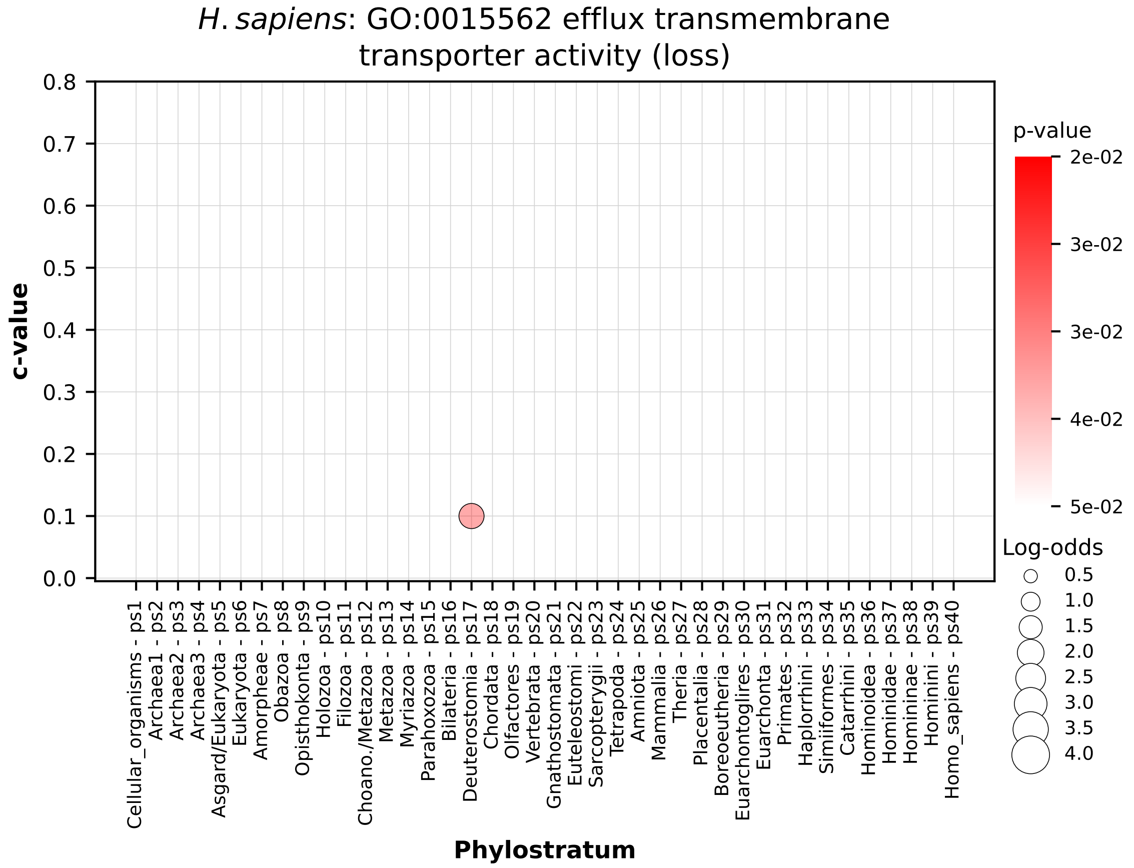

H. sapiens: GO:0015562 efflux transmembrane transporter activity (loss)
c-value
0.0
0.1
0.2
0.3
0.4
0.5
0.6
0.7
0.8
Phylostratum
Cellular_organisms - ps1
Archaea1 - ps2
Archaea2 - ps3
Archaea3 - ps4
Asgard/Eukaryota - ps5
Eukaryota - ps6
Amorpheae - ps7
Obazoa - ps8
Opisthokonta - ps9
Holozoa - ps10
Filozoa - ps11
Choano./Metazoa - ps12
Metazoa - ps13
Myriazoa - ps14
Parahoxozoa - ps15
Bilateria - ps16
Deuterostomia - ps17
Chordata - ps18
Olfactores - ps19
Vertebrata - ps20
Gnathostomata - ps21
Euteleostomi - ps22
Sarcopterygii - ps23
Tetrapoda - ps24
Amniota - ps25
Mammalia - ps26
Theria - ps27
Placentalia - ps28
Boreoeutheria - ps29
Euarchontoglires - ps30
Euarchonta - ps31
Primates - ps32
Haplorrhini - ps33
Simiiformes - ps34
Catarrhini - ps35
Hominoidea - ps36
Hominidae - ps37
Homininae - ps38
Hominini - ps39
Homo_sapiens - ps40
p-value
2e-02
3e-02
3e-02
4e-02
5e-02
Log-odds
0.5
1.0
1.5
2.0
2.5
3.0
3.5
4.0

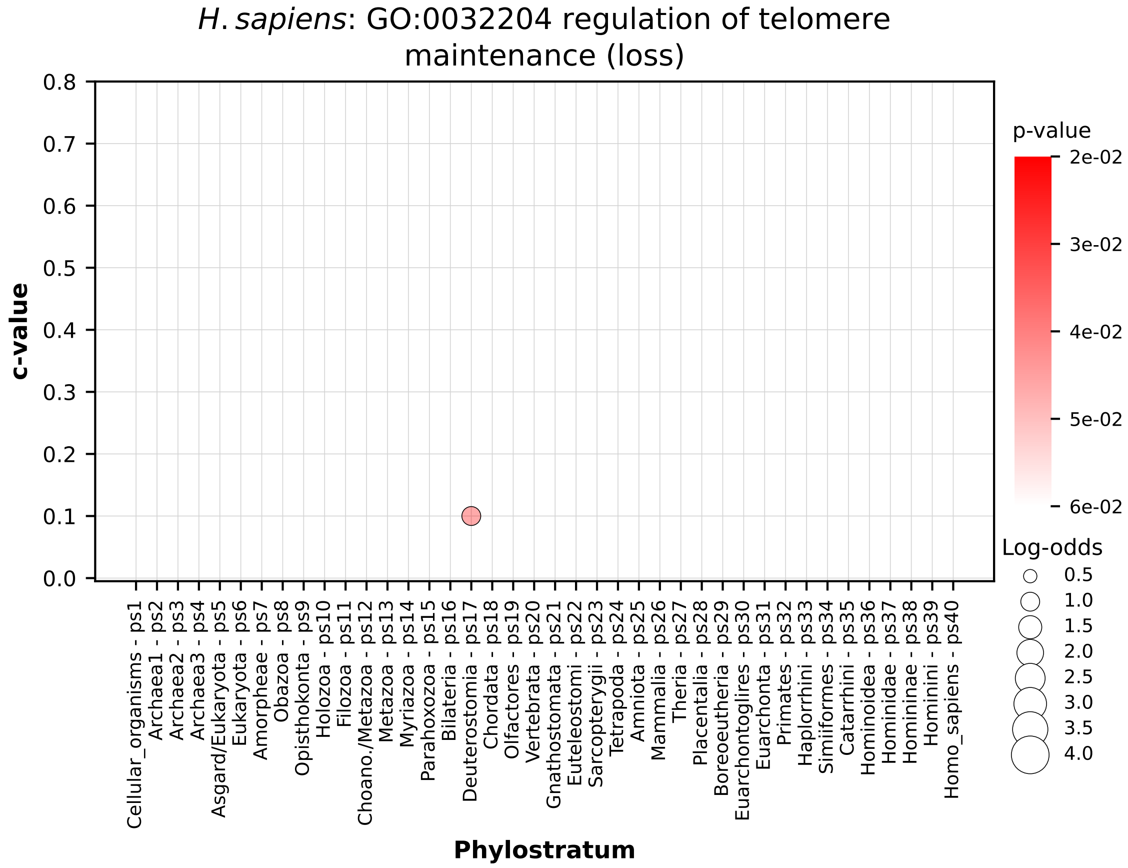
H. sapiens: GO:0032204 regulation of telomere maintenance (loss)
c-value
0.0
0.1
0.2
0.3
0.4
0.5
0.6
0.7
0.8
Phylostratum
Cellular_organisms - ps1
Archaea1 - ps2
Archaea2 - ps3
Archaea3 - ps4
Asgard/Eukaryota - ps5
Eukaryota - ps6
Amorpheae - ps7
Obazoa - ps8
Opisthokonta - ps9
Holozoa - ps10
Filozoa - ps11
Choano./Metazoa - ps12
Metazoa - ps13
Myriazoa - ps14
Parahoxozoa - ps15
Bilateria - ps16
Deuterostomia - ps17
Chordata - ps18
Olfactores - ps19
Vertebrata - ps20
Gnathostomata - ps21
Euteleostomi - ps22
Sarcopterygii - ps23
Tetrapoda - ps24
Amniota - ps25
Mammalia - ps26
Theria - ps27
Placentalia - ps28
Boreoeutheria - ps29
Euarchontoglires - ps30
Euarchonta - ps31
Primates - ps32
Haplorrhini - ps33
Simiiformes - ps34
Catarrhini - ps35
Hominoidea - ps36
Hominidae - ps37
Homininae - ps38
Hominini - ps39
Homo_sapiens - ps40
p-value
2e-02
3e-02
4e-02
5e-02
6e-02
Log-odds
0.5
1.0
1.5
2.0
2.5
3.0
3.5
4.0

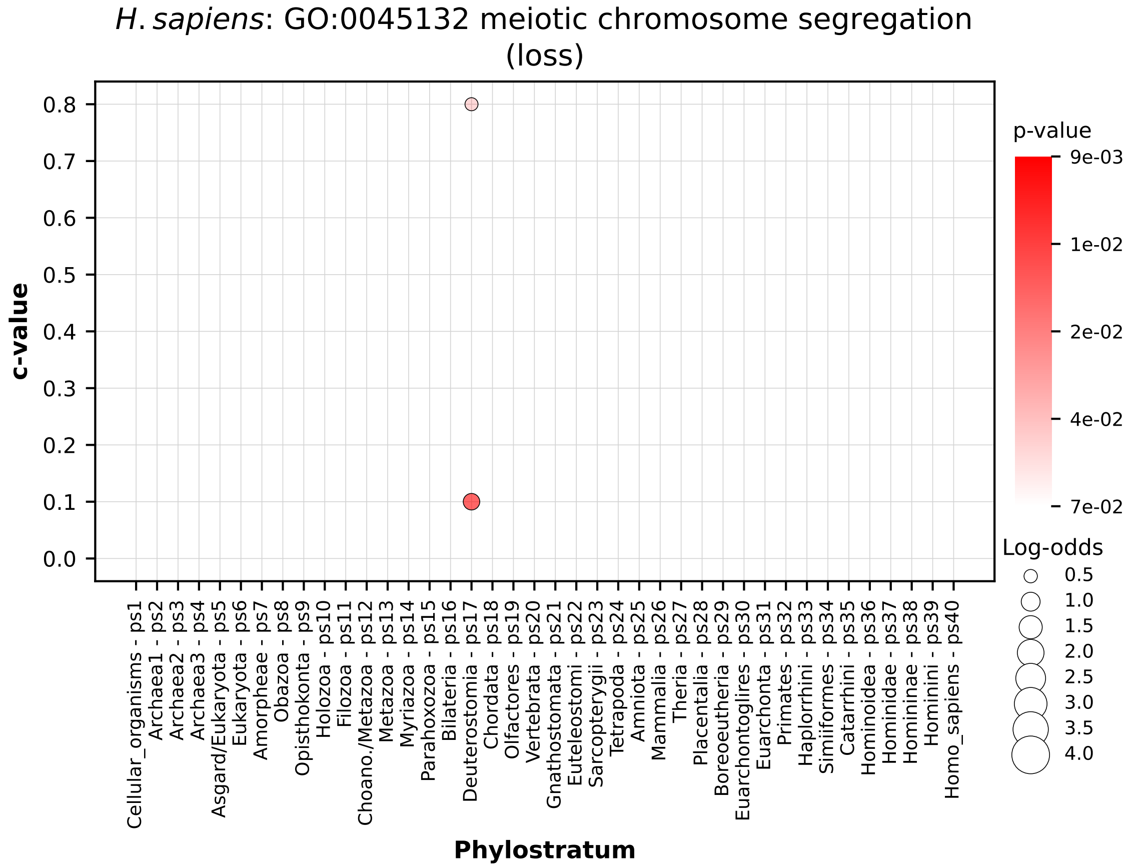

H. sapiens: GO:0045132 meiotic chromosome segregation (loss)
c-value
0.0
0.1
0.2
0.3
0.4
0.5
0.6
0.7
0.8
Phylostratum
Cellular_organisms - ps1
Archaea1 - ps2
Archaea2 - ps3
Archaea3 - ps4
Asgard/Eukaryota - ps5
Eukaryota - ps6
Amorpheae - ps7
Obazoa - ps8
Opisthokonta - ps9
Holozoa - ps10
Filozoa - ps11
Choano./Metazoa - ps12
Metazoa - ps13
Myriazoa - ps14
Parahoxozoa - ps15
Bilateria - ps16
Deuterostomia - ps17
Chordata - ps18
Olfactores - ps19
Vertebrata - ps20
Gnathostomata - ps21
Euteleostomi - ps22
Sarcopterygii - ps23
Tetrapoda - ps24
Amniota - ps25
Mammalia - ps26
Theria - ps27
Placentalia - ps28
Boreoeutheria - ps29
Euarchontoglires - ps30
Euarchonta - ps31
Primates - ps32
Haplorrhini - ps33
Simiiformes - ps34
Catarrhini - ps35
Hominoidea - ps36
Hominidae - ps37
Homininae - ps38
Hominini - ps39
Homo_sapiens - ps40
p-value
9e-03
1e-02
2e-02
4e-02
7e-02
Log-odds
0.5
1.0
1.5
2.0
2.5
3.0
3.5
4.0

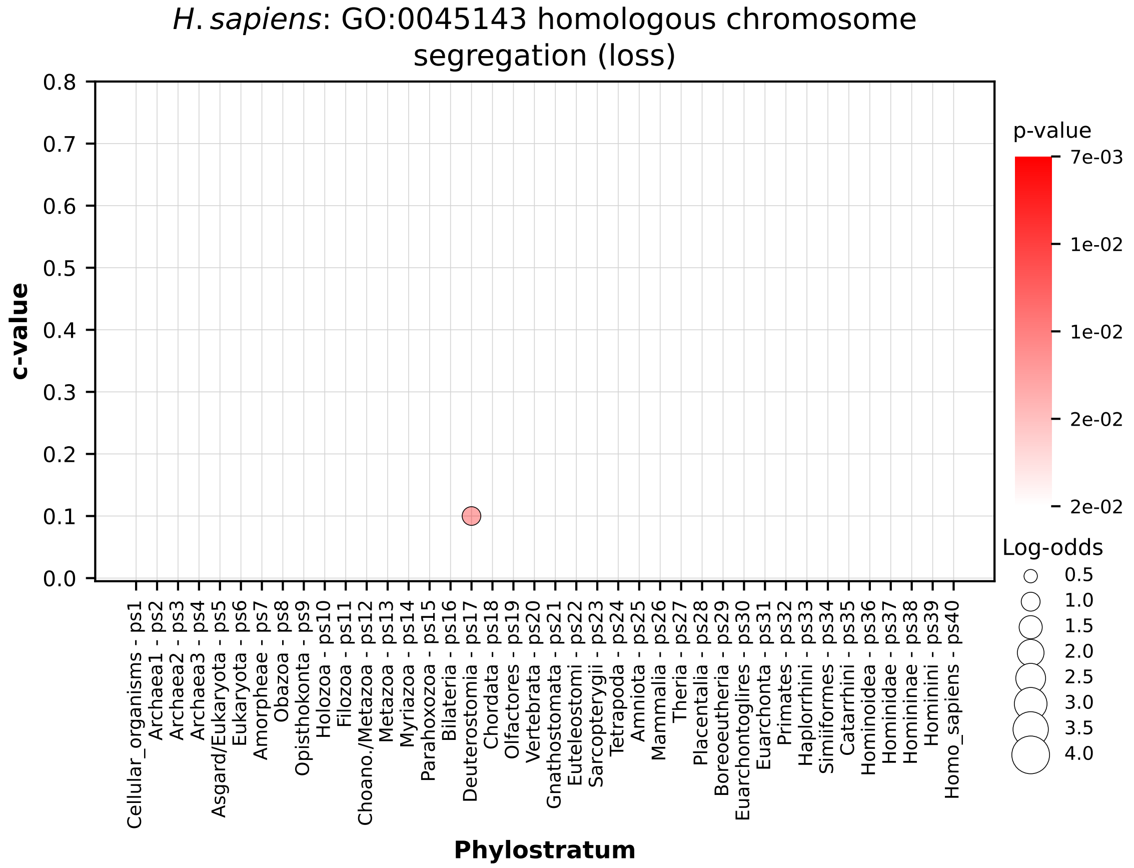

H. sapiens: GO:0045143 homologous chromosome segregation (loss)
c-value
0.0
0.1
0.2
0.3
0.4
0.5
0.6
0.7
0.8
Phylostratum
Cellular_organisms - ps1
Archaea1 - ps2
Archaea2 - ps3
Archaea3 - ps4
Asgard/Eukaryota - ps5
Eukaryota - ps6
Amorpheae - ps7
Obazoa - ps8
Opisthokonta - ps9
Holozoa - ps10
Filozoa - ps11
Choano./Metazoa - ps12
Metazoa - ps13
Myriazoa - ps14
Parahoxozoa - ps15
Bilateria - ps16
Deuterostomia - ps17
Chordata - ps18
Olfactores - ps19
Vertebrata - ps20
Gnathostomata - ps21
Euteleostomi - ps22
Sarcopterygii - ps23
Tetrapoda - ps24
Amniota - ps25
Mammalia - ps26
Theria - ps27
Placentalia - ps28
Boreoeutheria - ps29
Euarchontoglires - ps30
Euarchonta - ps31
Primates - ps32
Haplorrhini - ps33
Simiiformes - ps34
Catarrhini - ps35
Hominoidea - ps36
Hominidae - ps37
Homininae - ps38
Hominini - ps39
Homo_sapiens - ps40
p-value
7e-03
1e-02
1e-02
2e-02
2e-02
Log-odds
0.5
1.0
1.5
2.0
2.5
3.0
3.5
4.0

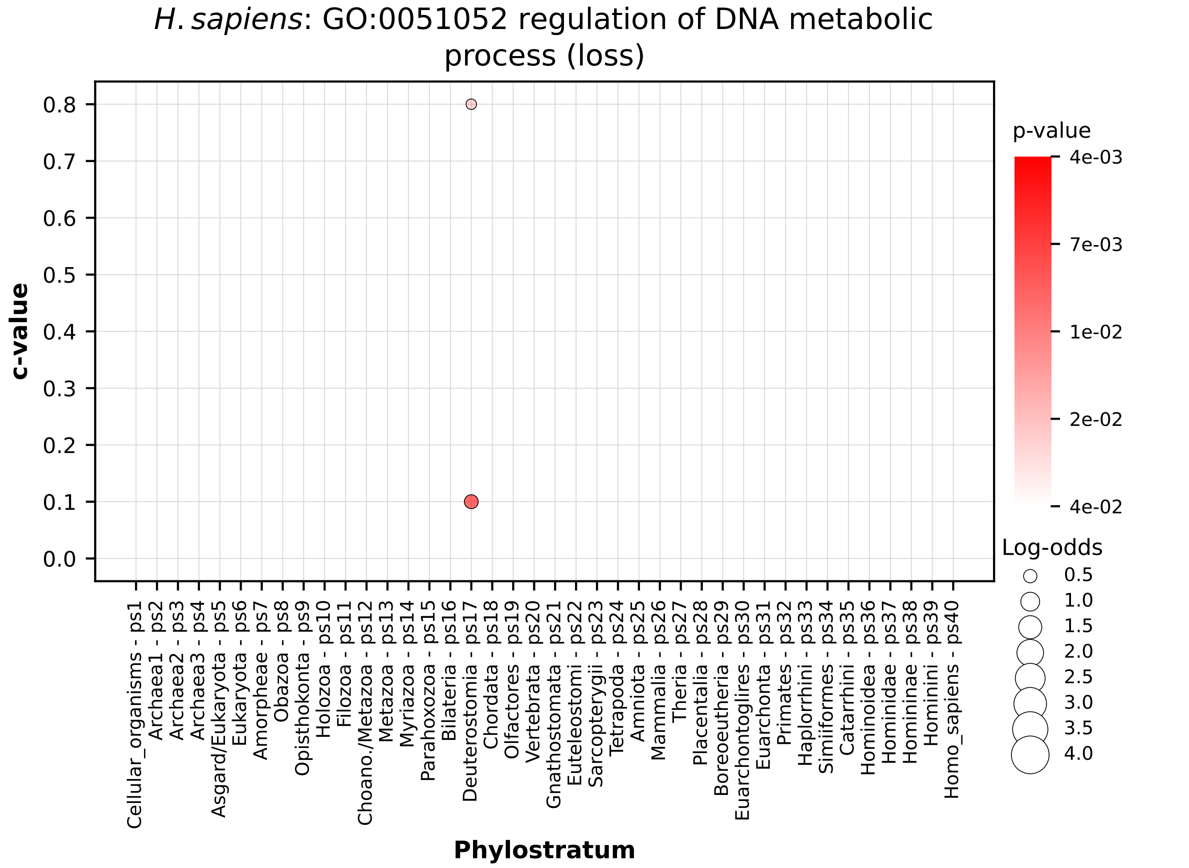

H. sapiens: GO:0051052 regulation of DNA metabolic process (loss)
c-value
0.0
0.1
0.2
0.3
0.4
0.5
0.6
0.7
0.8
Phylostratum
Cellular_organisms - ps1
Archaea1 - ps2
Archaea2 - ps3
Archaea3 - ps4
Asgard/Eukaryota - ps5
Eukaryota - ps6
Amorpheae - ps7
Obazoa - ps8
Opisthokonta - ps9
Holozoa - ps10
Filozoa - ps11
Choano./Metazoa - ps12
Metazoa - ps13
Myriazoa - ps14
Parahoxozoa - ps15
Bilateria - ps16
Deuterostomia - ps17
Chordata - ps18
Olfactores - ps19
Vertebrata - ps20
Gnathostomata - ps21
Euteleostomi - ps22
Sarcopterygii - ps23
Tetrapoda - ps24
Amniota - ps25
Mammalia - ps26
Theria - ps27
Placentalia - ps28
Boreoeutheria - ps29
Euarchontoglires - ps30
Euarchonta - ps31
Primates - ps32
Haplorrhini - ps33
Simiiformes - ps34
Catarrhini - ps35
Hominoidea - ps36
Hominidae - ps37
Homininae - ps38
Hominini - ps39
Homo_sapiens - ps40
p-value
4e-03
7e-03
1e-02
2e-02
4e-02
Log-odds
0.5
1.0
1.5
2.0
2.5
3.0
3.5
4.0

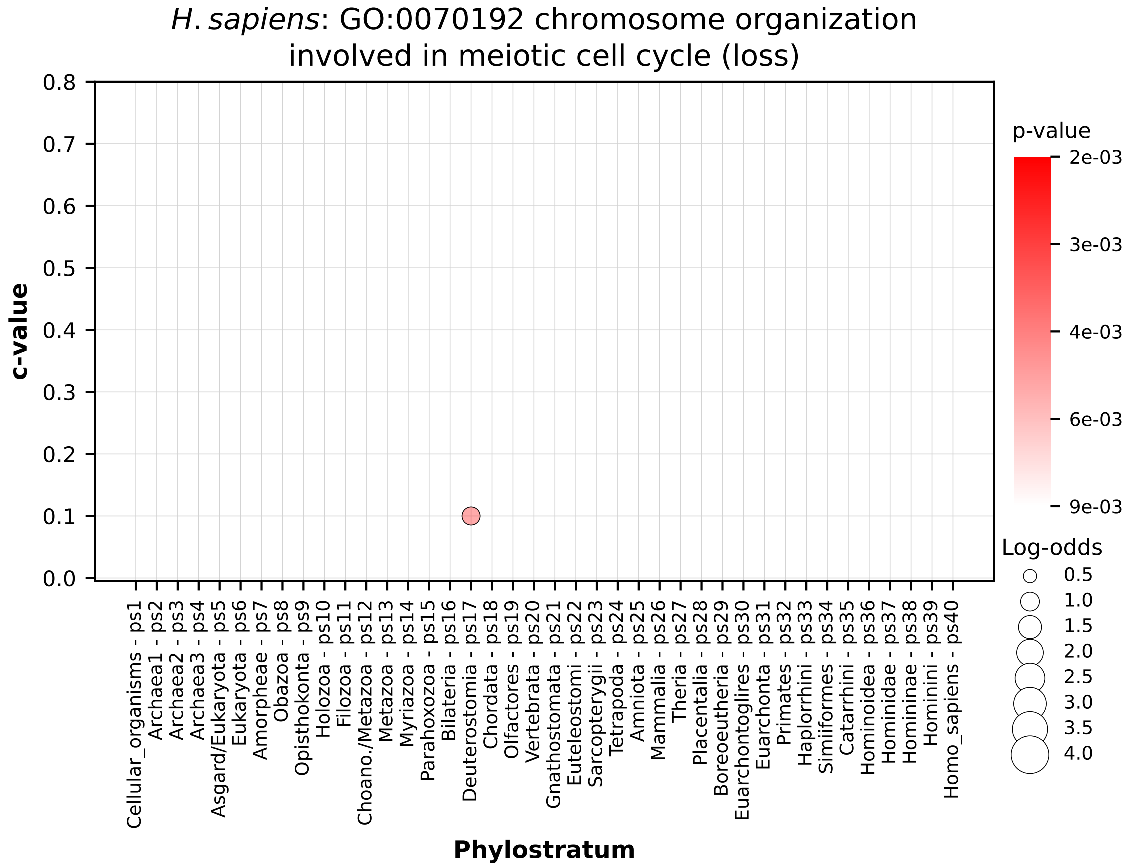

H. sapiens: GO:0070192 chromosome organization involved in meiotic cell cycle (loss)
c-value
0.0
0.1
0.2
0.3
0.4
0.5
0.6
0.7
0.8
Phylostratum
Cellular_organisms - ps1
Archaea1 - ps2
Archaea2 - ps3
Archaea3 - ps4
Asgard/Eukaryota - ps5
Eukaryota - ps6
Amorpheae - ps7
Obazoa - ps8
Opisthokonta - ps9
Holozoa - ps10
Filozoa - ps11
Choano./Metazoa - ps12
Metazoa - ps13
Myriazoa - ps14
Parahoxozoa - ps15
Bilateria - ps16
Deuterostomia - ps17
Chordata - ps18
Olfactores - ps19
Vertebrata - ps20
Gnathostomata - ps21
Euteleostomi - ps22
Sarcopterygii - ps23
Tetrapoda - ps24
Amniota - ps25
Mammalia - ps26
Theria - ps27
Placentalia - ps28
Boreoeutheria - ps29
Euarchontoglires - ps30
Euarchonta - ps31
Primates - ps32
Haplorrhini - ps33
Simiiformes - ps34
Catarrhini - ps35
Hominoidea - ps36
Hominidae - ps37
Homininae - ps38
Hominini - ps39
Homo_sapiens - ps40
p-value
2e-03
3e-03
4e-03
6e-03
9e-03
Log-odds
0.5
1.0
1.5
2.0
2.5
3.0
3.5
4.0

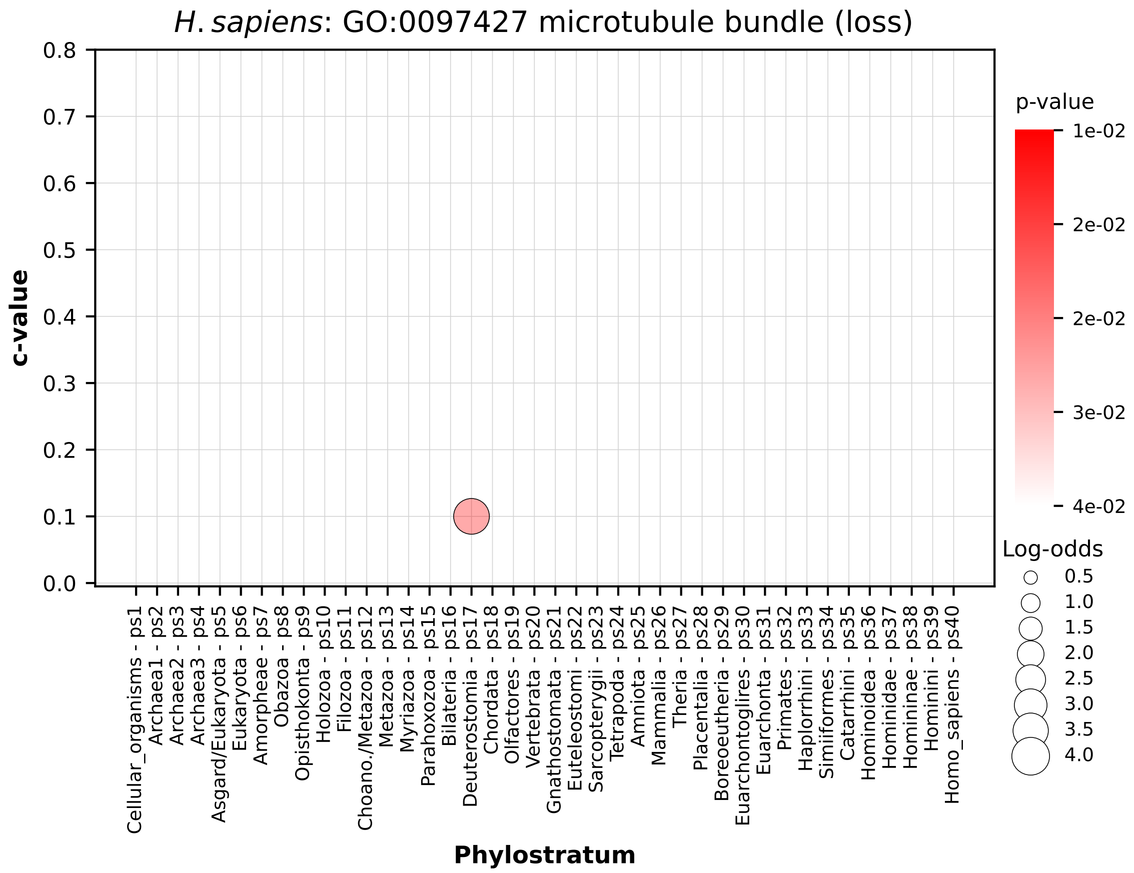
H. sapiens: GO:0097427 microtubule bundle (loss)
c-value
0.8
0.7
0.6
0.5
0.4
0.3
0.2
0.1
0.0
p-value
1e-02
2e-02
2e-02
3e-02
4e-02
Log-odds
0.5
1.0
1.5
2.0
2.5
3.0
3.5
4.0
Cellular_organisms - ps1
Archaea1 - ps2
Archaea2 - ps3
Archaea3 - ps4
Asgard/Eukaryota - ps5
Eukaryota - ps6
Amorpheae - ps7
Obazoa - ps8
Opisthokonta - ps9
Holozoa - ps10
Filozoa - ps11
Choano./Metazoa - ps12
Metazoa - ps13
Myriazoa - ps14
Parahoxozoa - ps15
Bilateria - ps16
Deuterostomia - ps17
Chordata - ps18
Olfactores - ps19
Vertebrata - ps20
Gnathostomata - ps21
Euteleostomi - ps22
Sarcopterygii - ps23
Tetrapoda - ps24
Amniota - ps25
Mammalia - ps26
Theria - ps27
Placentalia - ps28
Boreoeutheria - ps29
Euarchontoglires - ps30
Euarchonta - ps31
Primates - ps32
Haplorrhini - ps33
Simiiformes - ps34
Catarrhini - ps35
Hominoidea - ps36
Hominidae - ps37
Homininae - ps38
Hominini - ps39
Homo_sapiens - ps40
Phylostratum

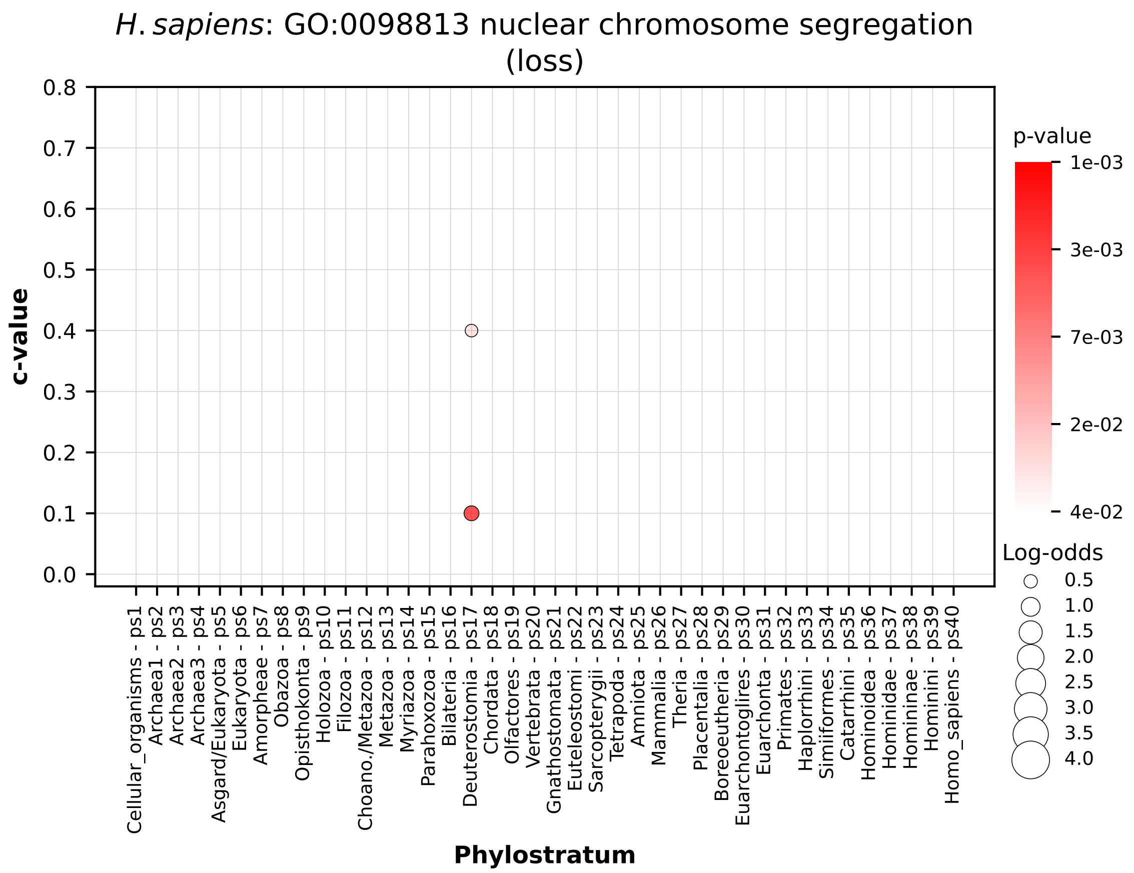

H. sapiens: GO:0098813 nuclear chromosome segregation (loss)
c-value
0.0
0.1
0.2
0.3
0.4
0.5
0.6
0.7
0.8
Phylostratum
Cellular_organisms - ps1
Archaea1 - ps2
Archaea2 - ps3
Archaea3 - ps4
Asgard/Eukaryota - ps5
Eukaryota - ps6
Amorpheae - ps7
Obazoa - ps8
Opisthokonta - ps9
Holozoa - ps10
Filozoa - ps11
Choano./Metazoa - ps12
Metazoa - ps13
Myriazoa - ps14
Parahoxozoa - ps15
Bilateria - ps16
Deuterostomia - ps17
Chordata - ps18
Olfactores - ps19
Vertebrata - ps20
Gnathostomata - ps21
Euteleostomi - ps22
Sarcopterygii - ps23
Tetrapoda - ps24
Amniota - ps25
Mammalia - ps26
Theria - ps27
Placentalia - ps28
Boreoeutheria - ps29
Euarchontoglires - ps30
Euarchonta - ps31
Primates - ps32
Haplorrhini - ps33
Simiiformes - ps34
Catarrhini - ps35
Hominoidea - ps36
Hominidae - ps37
Homininae - ps38
Hominini - ps39
Homo_sapiens - ps40
p-value
1e-03
3e-03
7e-03
2e-02
4e-02
Log-odds
0.5
1.0
1.5
2.0
2.5
3.0
3.5
4.0

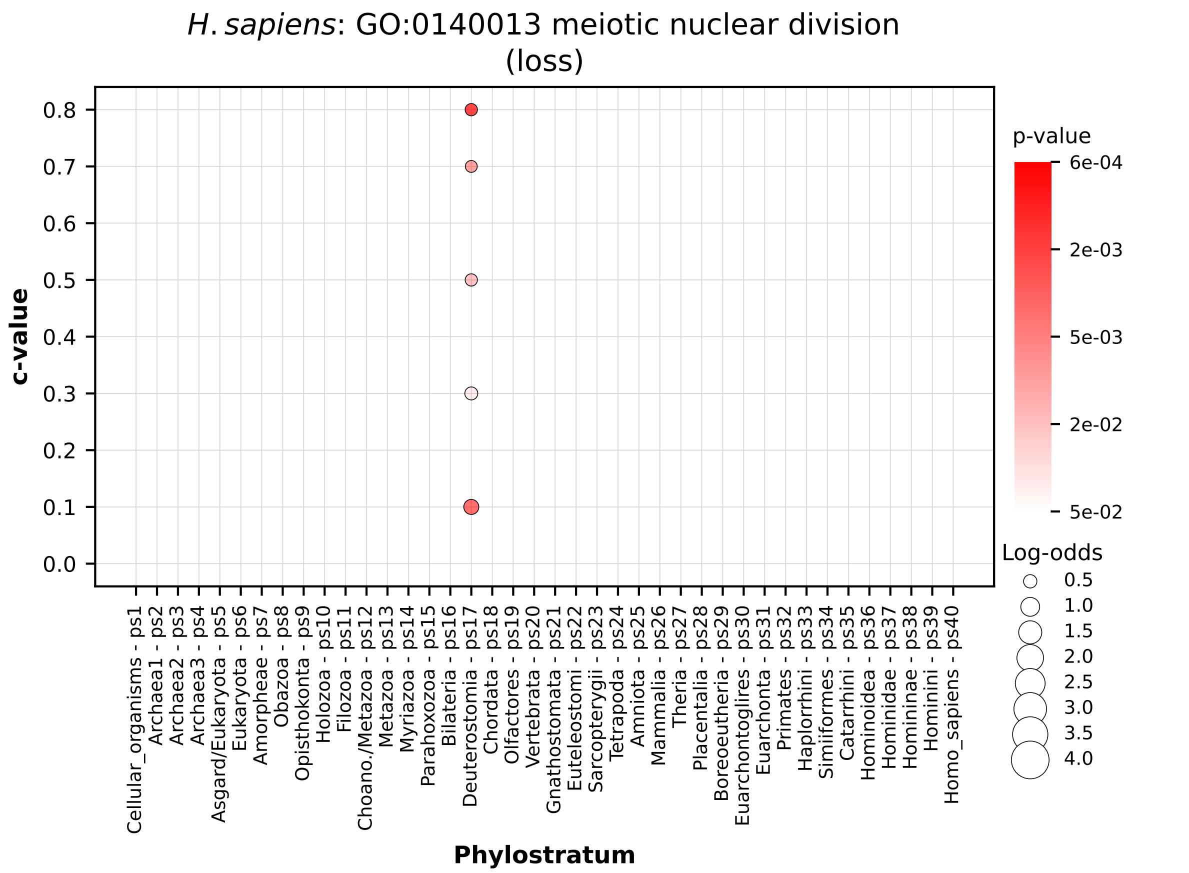

H. sapiens: GO:0140013 meiotic nuclear division
(loss)
c-value
0.0
0.1
0.2
0.3
0.4
0.5
0.6
0.7
0.8
Phylostratum
Cellular_organisms - ps1
Archaea1 - ps2
Archaea2 - ps3
Archaea3 - ps4
Asgard/Eukaryota - ps5
Eukaryota - ps6
Amorpheae - ps7
Obazoa - ps8
Opisthokonta - ps9
Holozoa - ps10
Filozoa - ps11
Choano./Metazoa - ps12
Metazoa - ps13
Myriazoa - ps14
Parahoxozoa - ps15
Bilateria - ps16
Deuterostomia - ps17
Chordata - ps18
Olfactores - ps19
Vertebrata - ps20
Gnathostomata - ps21
Euteleostomi - ps22
Sarcopterygii - ps23
Tetrapoda - ps24
Amniota - ps25
Mammalia - ps26
Theria - ps27
Placentalia - ps28
Boreoeutheria - ps29
Euarchontoglires - ps30
Euarchonta - ps31
Primates - ps32
Haplorrhini - ps33
Simiiformes - ps34
Catarrhini - ps35
Hominoidea - ps36
Hominidae - ps37
Homininae - ps38
Hominini - ps39
Homo_sapiens - ps40
p-value
6e-04
2e-03
5e-03
2e-02
5e-02
Log-odds
0.5
1.0
1.5
2.0
2.5
3.0
3.5
4.0

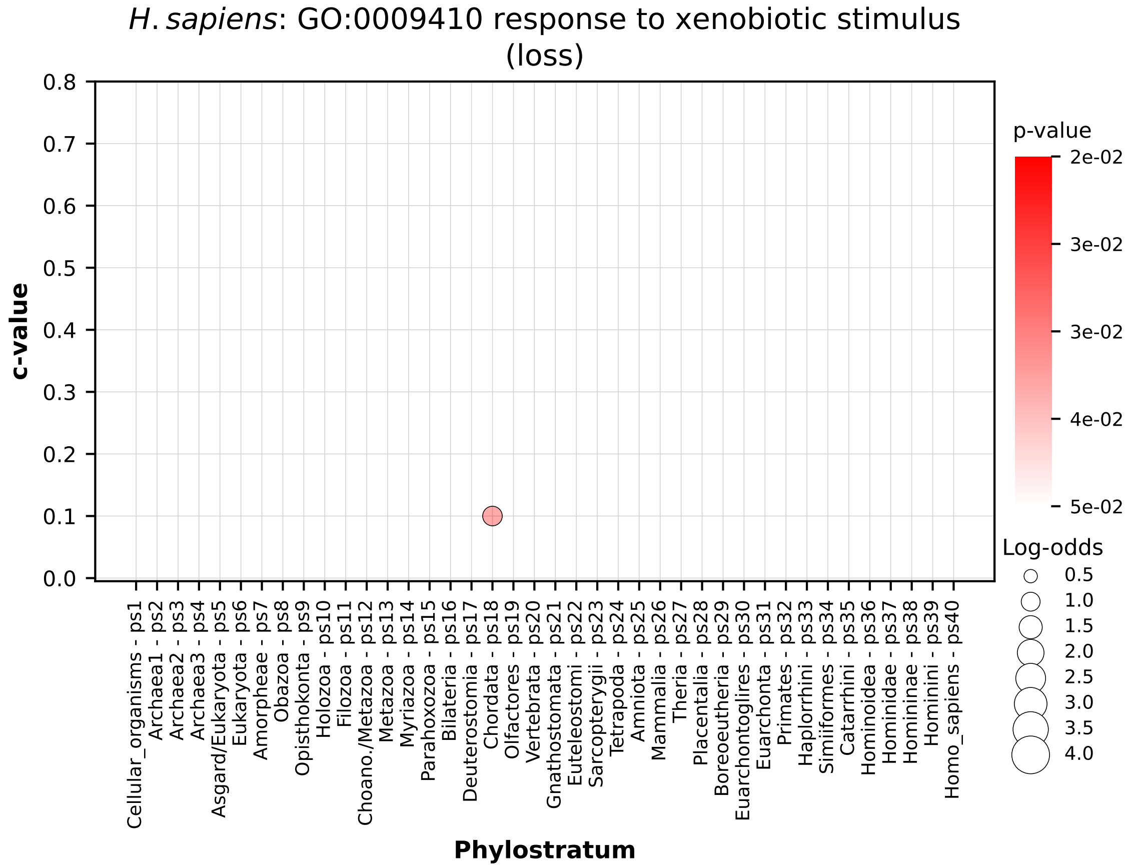

H. sapiens: GO:0009410 response to xenobiotic stimulus (loss)
c-value
0.8
0.7
0.6
0.5
0.4
0.3
0.2
0.1
0.0
Cellular_organisms - ps1
Archaea1 - ps2
Archaea2 - ps3
Archaea3 - ps4
Asgard/Eukaryota - ps5
Eukaryota - ps6
Amorpheae - ps7
Obazoa - ps8
Opisthokonta - ps9
Holozoa - ps10
Filozoa - ps11
Choano./Metazoa - ps12
Metazoa - ps13
Myriazoa - ps14
Parahoxozoa - ps15
Bilateria - ps16
Deuterostomia - ps17
Chordata - ps18
Olfactores - ps19
Vertebrata - ps20
Gnathostomata - ps21
Euteleostomi - ps22
Sarcopterygii - ps23
Tetrapoda - ps24
Amniota - ps25
Mammalia - ps26
Theria - ps27
Placentalia - ps28
Boreoeutheria - ps29
Euarchontoglires - ps30
Euarchonta - ps31
Primates - ps32
Haplorrhini - ps33
Simiiformes - ps34
Catarrhini - ps35
Hominoidea - ps36
Hominidae - ps37
Homininae - ps38
Hominini - ps39
Homo_sapiens - ps40
Phylostratum
p-value
2e-02
3e-02
3e-02
4e-02
5e-02
Log-odds
0.5
1.0
1.5
2.0
2.5
3.0
3.5
4.0

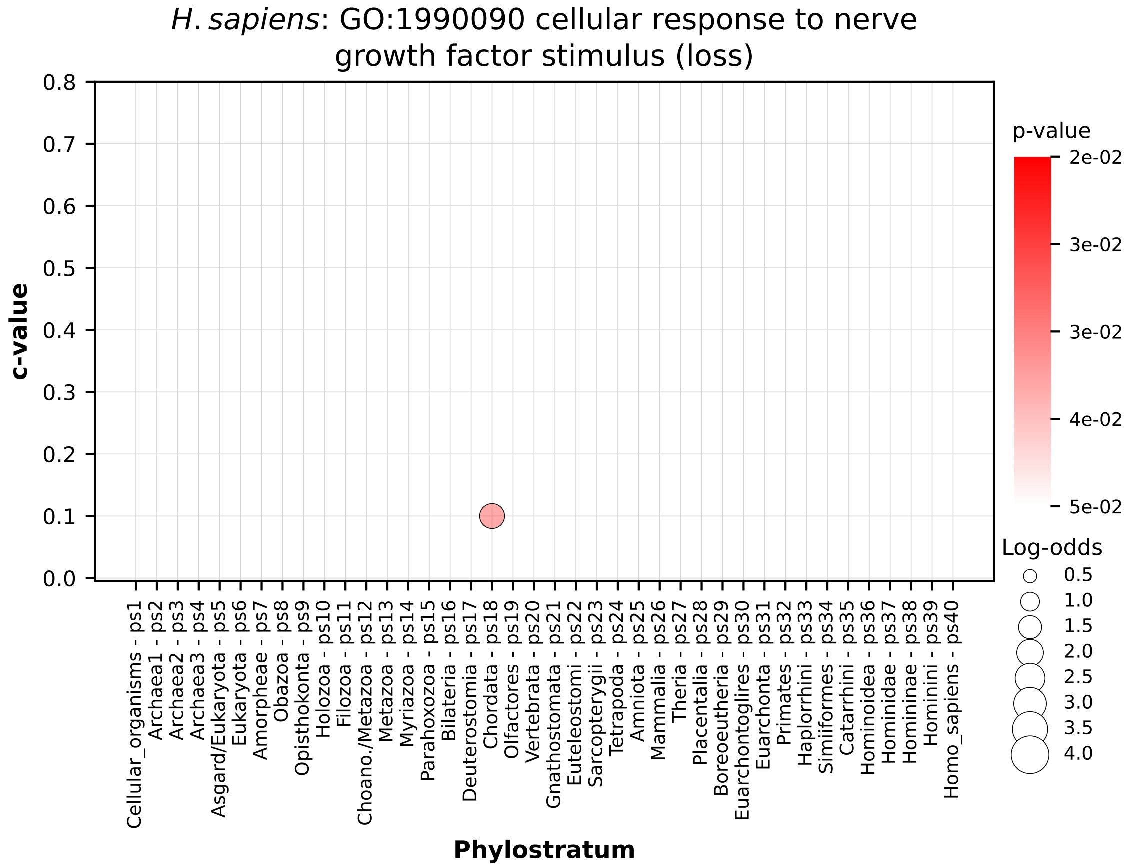

H. sapiens: GO:1990090 cellular response to nerve growth factor stimulus (loss)
c-value
0.0
0.1
0.2
0.3
0.4
0.5
0.6
0.7
0.8
Phylostratum
Cellular_organisms - ps1
Archaea1 - ps2
Archaea2 - ps3
Archaea3 - ps4
Asgard/Eukaryota - ps5
Eukaryota - ps6
Amorpheae - ps7
Obazoa - ps8
Opisthokonta - ps9
Holozoa - ps10
Filozoa - ps11
Choano./Metazoa - ps12
Metazoa - ps13
Myriazoa - ps14
Parahoxozoa - ps15
Bilateria - ps16
Deuterostomia - ps17
Chordata - ps18
Olfactores - ps19
Vertebrata - ps20
Gnathostomata - ps21
Euteleostomi - ps22
Sarcopterygii - ps23
Tetrapoda - ps24
Amniota - ps25
Mammalia - ps26
Theria - ps27
Placentalia - ps28
Boreoeutheria - ps29
Euarchontoglires - ps30
Euarchonta - ps31
Primates - ps32
Haplorrhini - ps33
Simiiformes - ps34
Catarrhini - ps35
Hominoidea - ps36
Hominidae - ps37
Homininae - ps38
Hominini - ps39
Homo_sapiens - ps40
p-value
2e-02
3e-02
3e-02
4e-02
5e-02
Log-odds
0.5
1.0
1.5
2.0
2.5
3.0
3.5
4.0

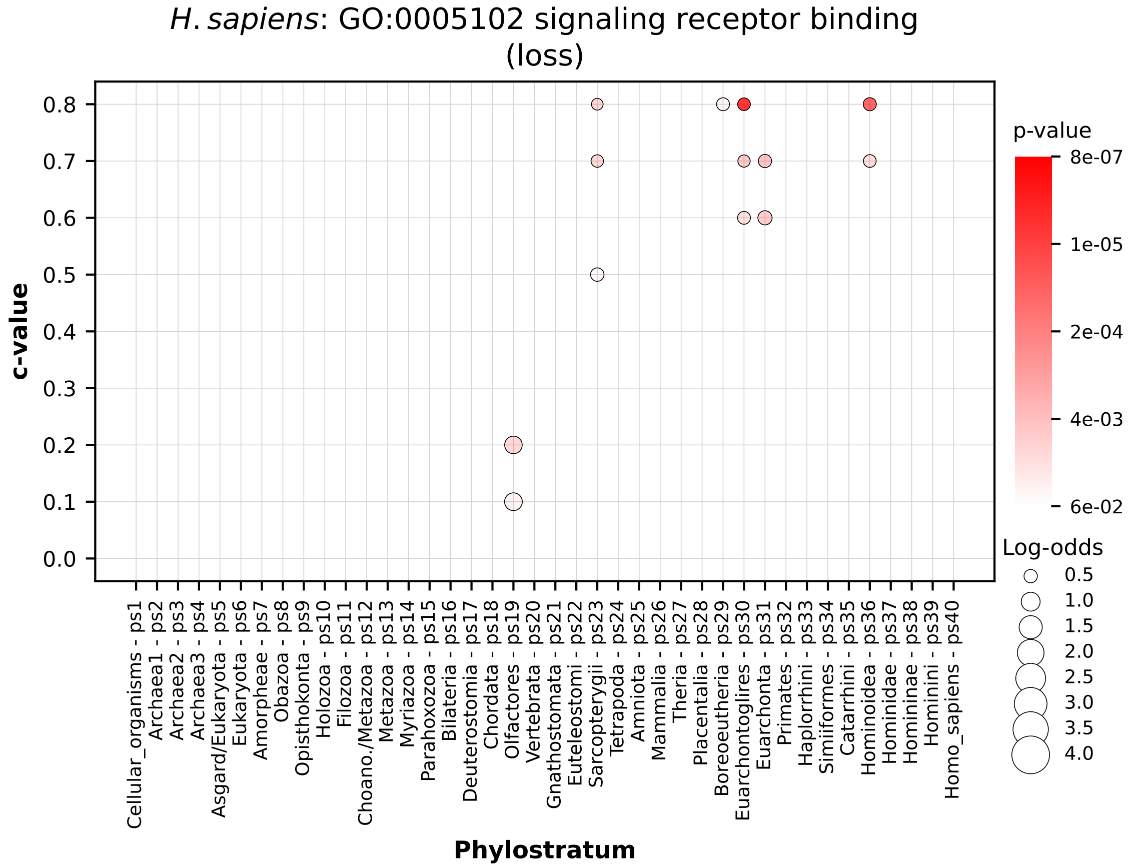

H. sapiens: GO:0005102 signaling receptor binding
(loss)
c-value
0.8
0.7
0.6
0.5
0.4
0.3
0.2
0.1
0.0
Phylostratum
Cellular_organisms - ps1
Archaea1 - ps2
Archaea2 - ps3
Archaea3 - ps4
Asgard/Eukaryota - ps5
Eukaryota - ps6
Amorpheae - ps7
Obazoa - ps8
Opisthokonta - ps9
Holozoa - ps10
Filozoa - ps11
Choano./Metazoa - ps12
Metazoa - ps13
Myriazoa - ps14
Parahoxozoa - ps15
Bilateria - ps16
Deuterostomia - ps17
Chordata - ps18
Olfactores - ps19
Vertebrata - ps20
Gnathostomata - ps21
Euteleostomi - ps22
Sarcopterygii - ps23
Tetrapoda - ps24
Amniota - ps25
Mammalia - ps26
Theria - ps27
Placentalia - ps28
Boreoeutheria - ps29
Euarchontoglires - ps30
Euarchonta - ps31
Primates - ps32
Haplorrhini - ps33
Simiiformes - ps34
Catarrhini - ps35
Hominoidea - ps36
Hominidae - ps37
Homininae - ps38
Hominini - ps39
Homo_sapiens - ps40
p-value
8e-07
1e-05
2e-04
4e-03
6e-02
Log-odds
0.5
1.0
1.5
2.0
2.5
3.0
3.5
4.0

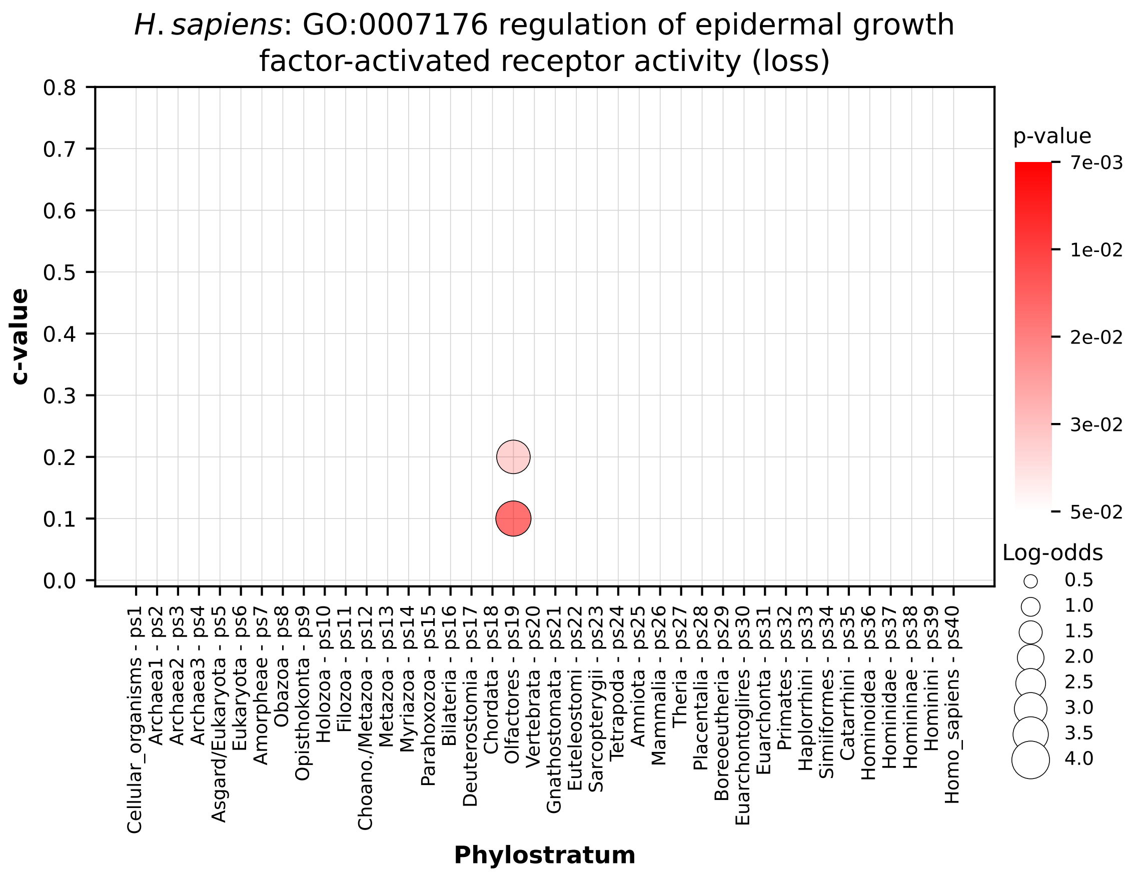

H. sapiens: GO:0007176 regulation of epidermal growth factor-activated receptor activity (loss)
c-value
0.0
0.1
0.2
0.3
0.4
0.5
0.6
0.7
0.8
Phylostratum
Cellular_organisms - ps1
Archaea1 - ps2
Archaea2 - ps3
Archaea3 - ps4
Asgard/Eukaryota - ps5
Eukaryota - ps6
Amorpheae - ps7
Obazoa - ps8
Opisthokonta - ps9
Holozoa - ps10
Filozoa - ps11
Choano./Metazoa - ps12
Metazoa - ps13
Myriazoa - ps14
Parahoxozoa - ps15
Bilateria - ps16
Deuterostomia - ps17
Chordata - ps18
Olfactores - ps19
Vertebrata - ps20
Gnathostomata - ps21
Euteleostomi - ps22
Sarcopterygii - ps23
Tetrapoda - ps24
Amniota - ps25
Mammalia - ps26
Theria - ps27
Placentalia - ps28
Boreoeutheria - ps29
Euarchontoglires - ps30
Euarchonta - ps31
Primates - ps32
Haplorrhini - ps33
Simiiformes - ps34
Catarrhini - ps35
Hominoidea - ps36
Hominidae - ps37
Homininae - ps38
Hominini - ps39
Homo_sapiens - ps40
p-value
7e-03
1e-02
2e-02
3e-02
5e-02
Log-odds
0.5
1.0
1.5
2.0
2.5
3.0
3.5
4.0

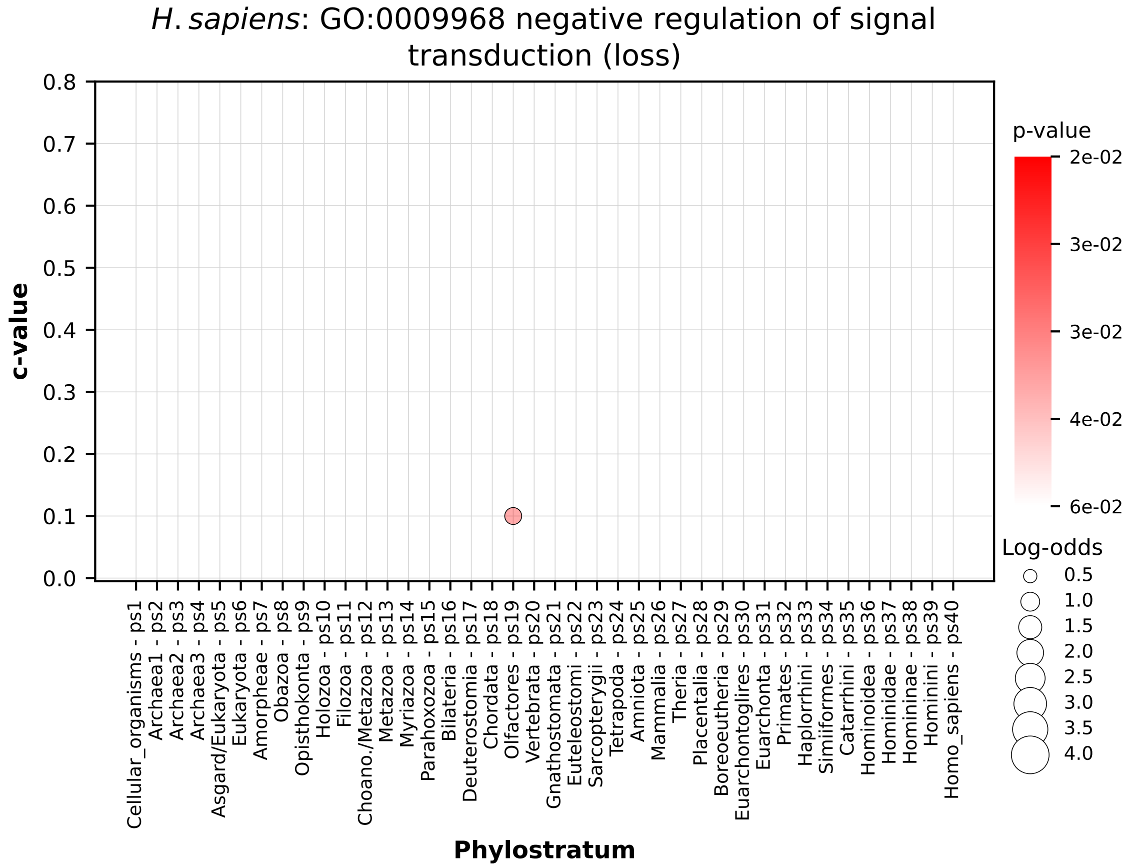

H. sapiens: GO:0009968 negative regulation of signal transduction (loss)
c-value
0.8
0.7
0.6
0.5
0.4
0.3
0.2
0.1
0.0
Phylostratum
Cellular_organisms - ps1
Archaea1 - ps2
Archaea2 - ps3
Archaea3 - ps4
Asgard/Eukaryota - ps5
Eukaryota - ps6
Amorpheae - ps7
Obazoa - ps8
Opisthokonta - ps9
Holozoa - ps10
Filozoa - ps11
Choano./Metazoa - ps12
Metazoa - ps13
Myriazoa - ps14
Parahoxozoa - ps15
Bilateria - ps16
Deuterostomia - ps17
Chordata - ps18
Olfactores - ps19
Vertebrata - ps20
Gnathostomata - ps21
Euteleostomi - ps22
Sarcopterygii - ps23
Tetrapoda - ps24
Amniota - ps25
Mammalia - ps26
Theria - ps27
Placentalia - ps28
Boreoeutheria - ps29
Euarchontoglires - ps30
Euarchonta - ps31
Primates - ps32
Haplorrhini - ps33
Simiiformes - ps34
Catarrhini - ps35
Hominoidea - ps36
Hominidae - ps37
Homininae - ps38
Hominini - ps39
Homo_sapiens - ps40
p-value
2e-02
3e-02
3e-02
4e-02
6e-02
Log-odds
0.5
1.0
1.5
2.0
2.5
3.0
3.5
4.0

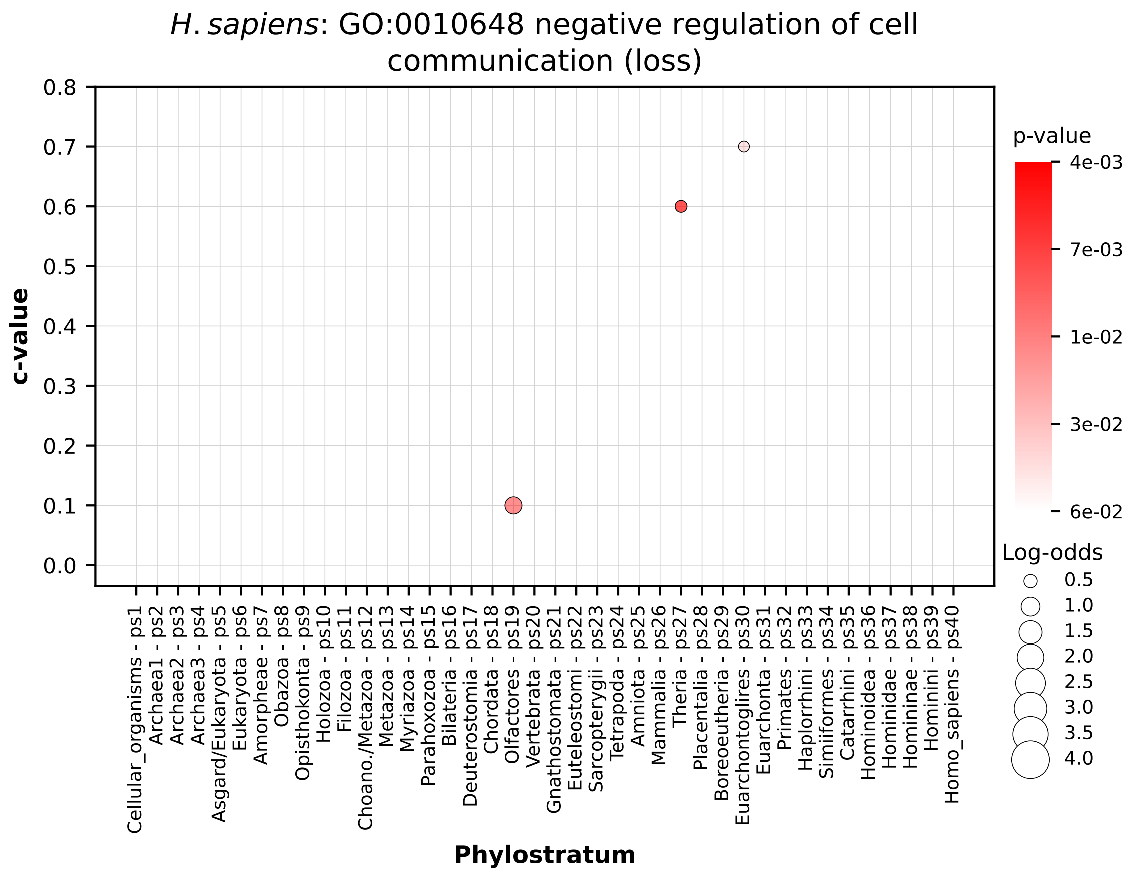

H. sapiens: GO:0010648 negative regulation of cell communication (loss)
c-value
0.8
0.7
0.6
0.5
0.4
0.3
0.2
0.1
0.0
Phylostratum
Cellular_organisms - ps1
Archaea1 - ps2
Archaea2 - ps3
Archaea3 - ps4
Asgard/Eukaryota - ps5
Eukaryota - ps6
Amorpheae - ps7
Obazoa - ps8
Opisthokonta - ps9
Holozoa - ps10
Filozoa - ps11
Choano./Metazoa - ps12
Metazoa - ps13
Myriazoa - ps14
Parahoxozoa - ps15
Bilateria - ps16
Deuterostomia - ps17
Chordata - ps18
Olfactores - ps19
Vertebrata - ps20
Gnathostomata - ps21
Euteleostomi - ps22
Sarcopterygii - ps23
Tetrapoda - ps24
Amniota - ps25
Mammalia - ps26
Theria - ps27
Placentalia - ps28
Boreoeutheria - ps29
Euarchontoglires - ps30
Euarchonta - ps31
Primates - ps32
Haplorrhini - ps33
Simiiformes - ps34
Catarrhini - ps35
Hominoidea - ps36
Hominidae - ps37
Homininae - ps38
Hominini - ps39
Homo_sapiens - ps40
p-value
4e-03
7e-03
1e-02
3e-02
6e-02
Log-odds
0.5
1.0
1.5
2.0
2.5
3.0
3.5
4.0

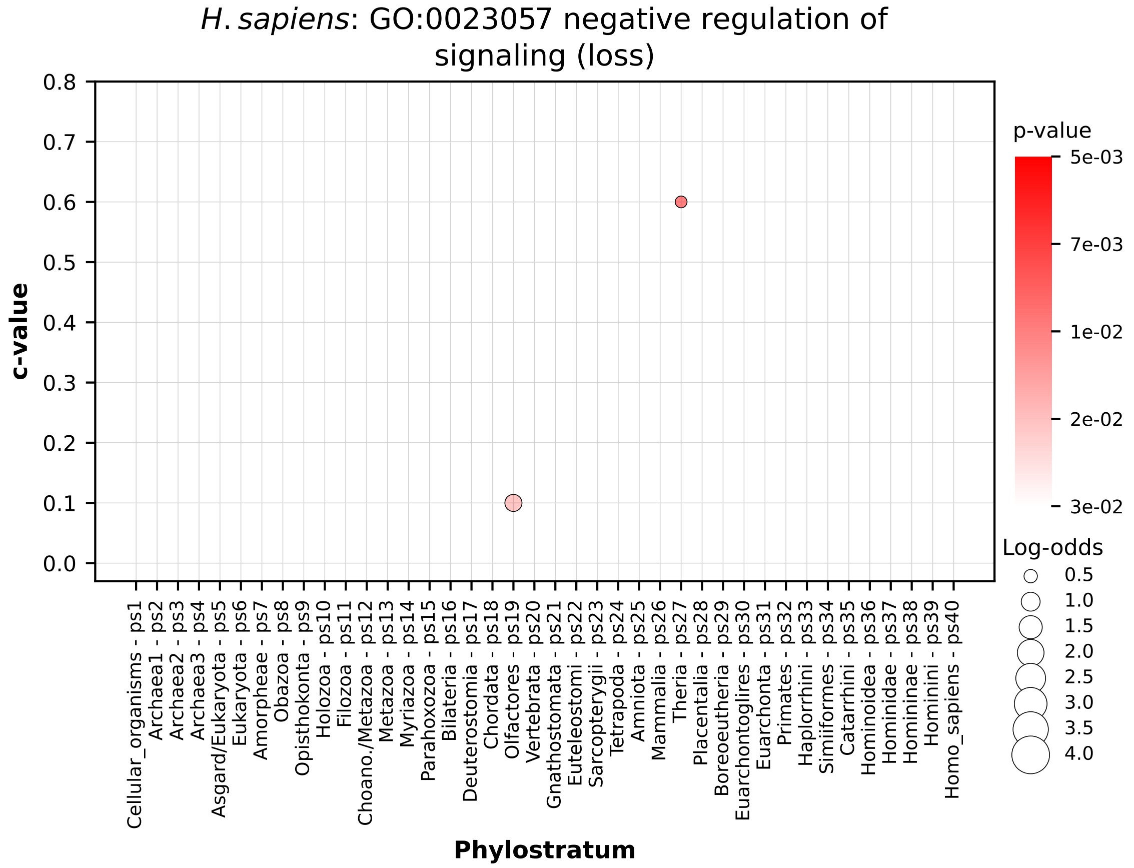

H. sapiens: GO:0023057 negative regulation of signaling (loss)
c-value
0.8
0.7
0.6
0.5
0.4
0.3
0.2
0.1
0.0
Phylostratum
Cellular_organisms - ps1
Archaea1 - ps2
Archaea2 - ps3
Archaea3 - ps4
Asgard/Eukaryota - ps5
Eukaryota - ps6
Amorpheae - ps7
Obazoa - ps8
Opisthokonta - ps9
Holozoa - ps10
Filozoa - ps11
Choano./Metazoa - ps12
Metazoa - ps13
Myriazoa - ps14
Parahoxozoa - ps15
Bilateria - ps16
Deuterostomia - ps17
Chordata - ps18
Olfactores - ps19
Vertebrata - ps20
Gnathostomata - ps21
Euteleostomi - ps22
Sarcopterygii - ps23
Tetrapoda - ps24
Amniota - ps25
Mammalia - ps26
Theria - ps27
Placentalia - ps28
Boreoeutheria - ps29
Euarchontoglires - ps30
Euarchonta - ps31
Primates - ps32
Haplorrhini - ps33
Simiiformes - ps34
Catarrhini - ps35
Hominoidea - ps36
Hominidae - ps37
Homininae - ps38
Hominini - ps39
Homo_sapiens - ps40
p-value
5e-03
7e-03
1e-02
2e-02
3e-02
Log-odds
0.5
1.0
1.5
2.0
2.5
3.0
3.5
4.0

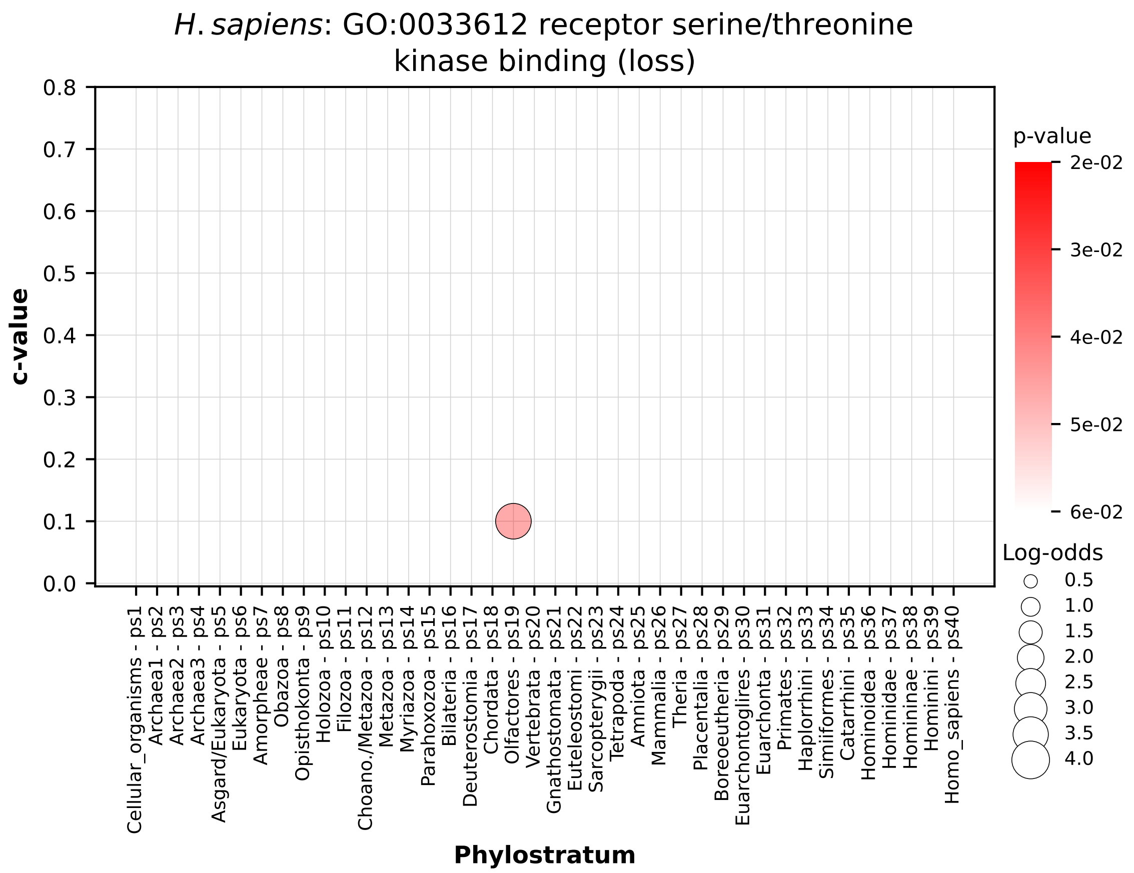

H. sapiens: GO:0033612 receptor serine/threonine kinase binding (loss)
c-value
0.8
0.7
0.6
0.5
0.4
0.3
0.2
0.1
0.0
Phylostratum
Cellular_organisms - ps1
Archaea1 - ps2
Archaea2 - ps3
Archaea3 - ps4
Asgard/Eukaryota - ps5
Eukaryota - ps6
Amorpheae - ps7
Obazoa - ps8
Opisthokonta - ps9
Holozoa - ps10
Filozoa - ps11
Choano./Metazoa - ps12
Metazoa - ps13
Myriazoa - ps14
Parahoxozoa - ps15
Bilateria - ps16
Deuterostomia - ps17
Chordata - ps18
Olfactores - ps19
Vertebrata - ps20
Gnathostomata - ps21
Euteleostomi - ps22
Sarcopterygii - ps23
Tetrapoda - ps24
Amniota - ps25
Mammalia - ps26
Theria - ps27
Placentalia - ps28
Boreoeutheria - ps29
Euarchontoglires - ps30
Euarchonta - ps31
Primates - ps32
Haplorrhini - ps33
Simiiformes - ps34
Catarrhini - ps35
Hominoidea - ps36
Hominidae - ps37
Homininae - ps38
Hominini - ps39
Homo_sapiens - ps40
p-value
2e-02
3e-02
4e-02
5e-02
6e-02
Log-odds
0.5
1.0
1.5
2.0
2.5
3.0
3.5
4.0

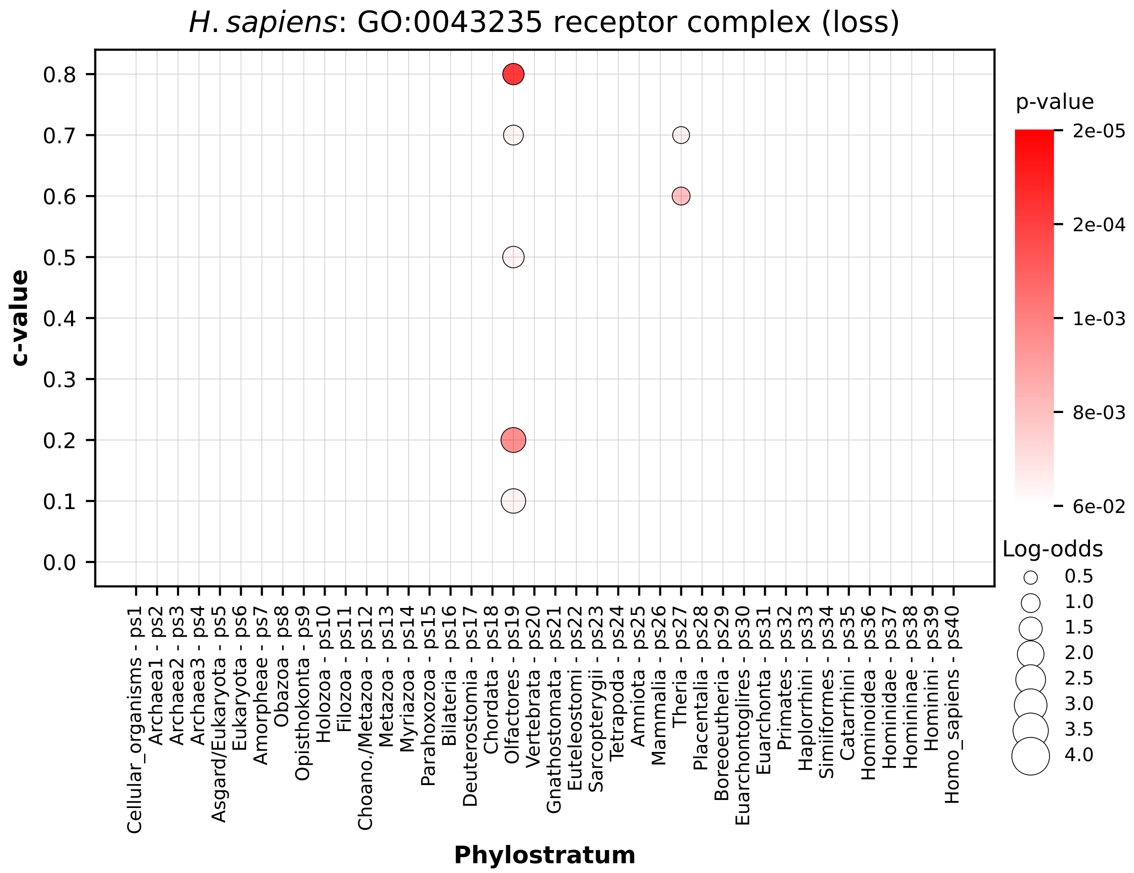

H. sapiens: GO:0043235 receptor complex (loss)
c-value
0.0
0.1
0.2
0.3
0.4
0.5
0.6
0.7
0.8
Phylostratum
Cellular_organisms - ps1
Archaea1 - ps2
Archaea2 - ps3
Archaea3 - ps4
Asgard/Eukaryota - ps5
Eukaryota - ps6
Amorpheae - ps7
Obazoa - ps8
Opisthokonta - ps9
Holozoa - ps10
Filozoa - ps11
Choano./Metazoa - ps12
Metazoa - ps13
Myriazoa - ps14
Parahoxozoa - ps15
Bilateria - ps16
Deuterostomia - ps17
Chordata - ps18
Olfactores - ps19
Vertebrata - ps20
Gnathostomata - ps21
Euteleostomi - ps22
Sarcopterygii - ps23
Tetrapoda - ps24
Amniota - ps25
Mammalia - ps26
Theria - ps27
Placentalia - ps28
Boreoeutheria - ps29
Euarchontoglires - ps30
Euarchonta - ps31
Primates - ps32
Haplorrhini - ps33
Simiiformes - ps34
Catarrhini - ps35
Hominoidea - ps36
Hominidae - ps37
Homininae - ps38
Hominini - ps39
Homo_sapiens - ps40
p-value
2e-05
2e-04
1e-03
8e-03
6e-02
Log-odds
0.5
1.0
1.5
2.0
2.5
3.0
3.5
4.0

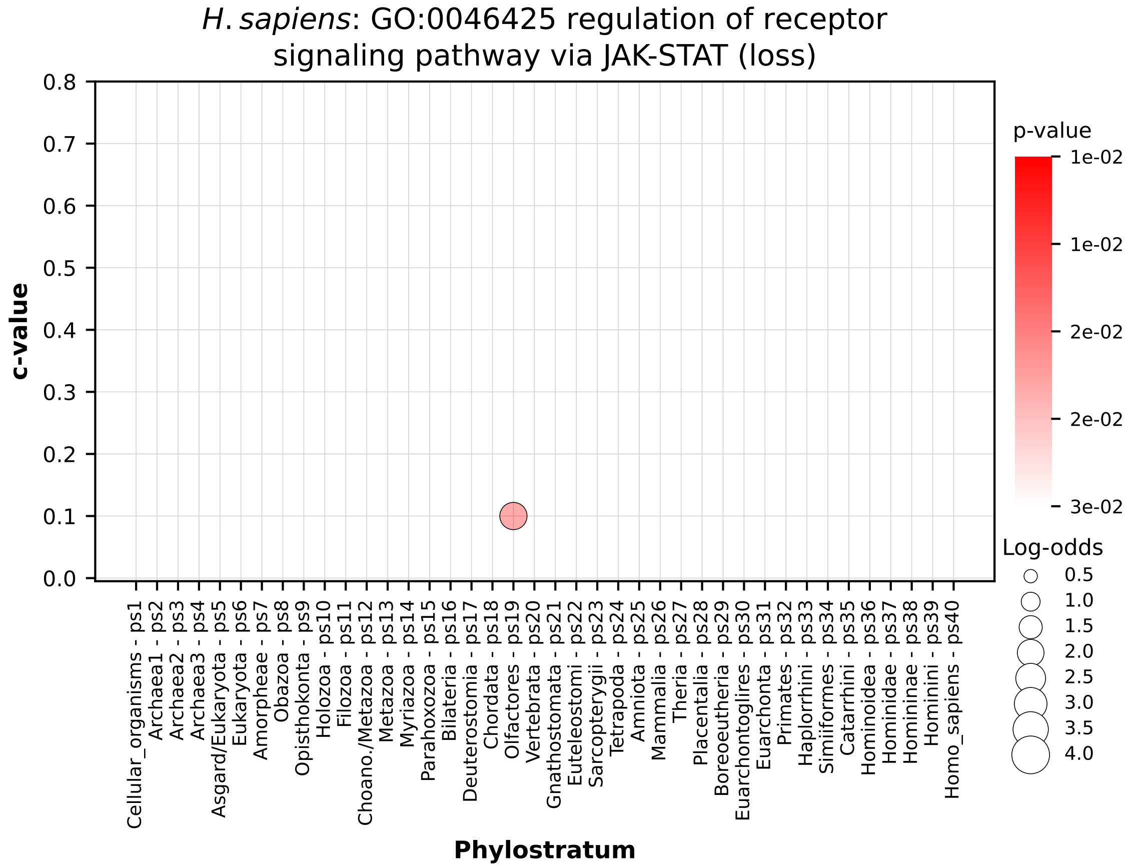

H. sapiens: GO:0046425 regulation of receptor signaling pathway via JAK-STAT (loss)
c-value
0.0
0.1
0.2
0.3
0.4
0.5
0.6
0.7
0.8
Phylostratum
Cellular_organisms - ps1
Archaea1 - ps2
Archaea2 - ps3
Archaea3 - ps4
Asgard/Eukaryota - ps5
Eukaryota - ps6
Amorpheae - ps7
Obazoa - ps8
Opisthokonta - ps9
Holozoa - ps10
Filozoa - ps11
Choano./Metazoa - ps12
Metazoa - ps13
Myriazoa - ps14
Parahoxozoa - ps15
Bilateria - ps16
Deuterostomia - ps17
Chordata - ps18
Olfactores - ps19
Vertebrata - ps20
Gnathostomata - ps21
Euteleostomi - ps22
Sarcopterygii - ps23
Tetrapoda - ps24
Amniota - ps25
Mammalia - ps26
Theria - ps27
Placentalia - ps28
Boreoeutheria - ps29
Euarchontoglires - ps30
Euarchonta - ps31
Primates - ps32
Haplorrhini - ps33
Simiiformes - ps34
Catarrhini - ps35
Hominoidea - ps36
Hominidae - ps37
Homininae - ps38
Hominini - ps39
Homo_sapiens - ps40
p-value
1e-02
1e-02
2e-02
2e-02
3e-02
Log-odds
0.5
1.0
1.5
2.0
2.5
3.0
3.5
4.0

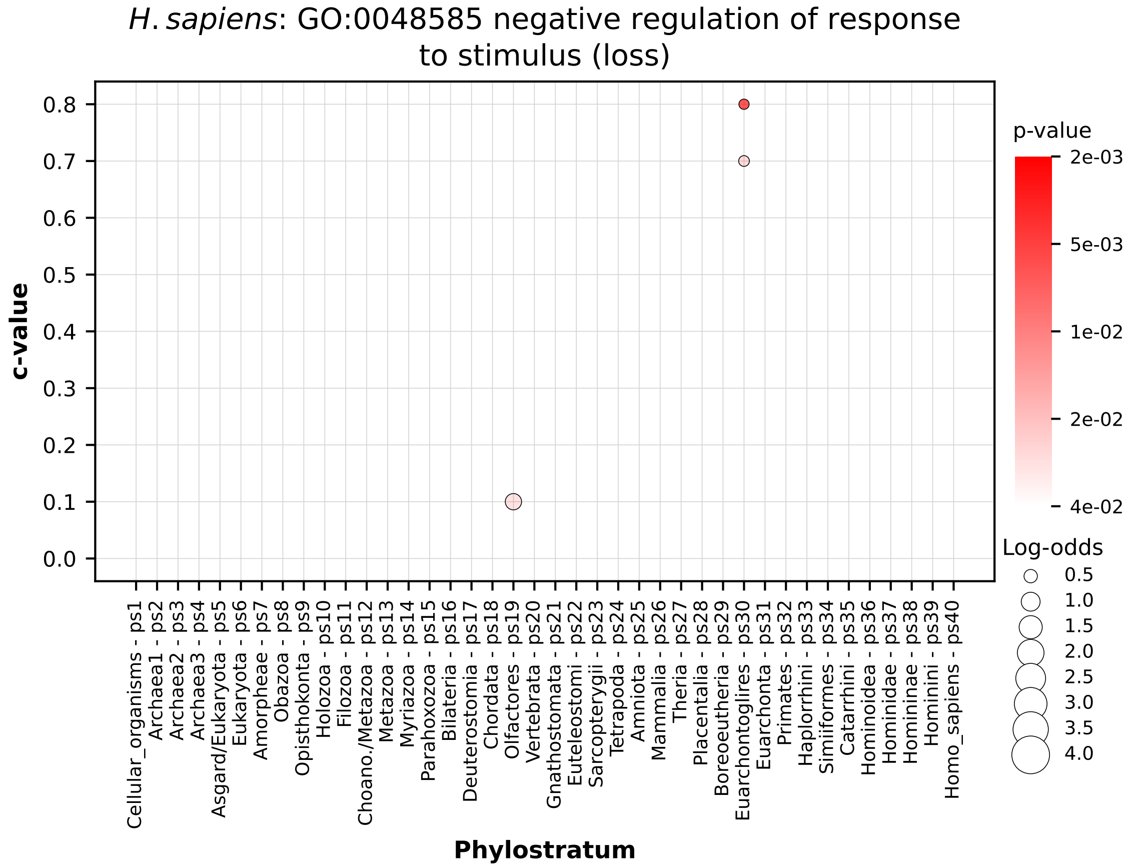

H. sapiens: GO:0048585 negative regulation of response to stimulus (loss)
c-value
0.0
0.1
0.2
0.3
0.4
0.5
0.6
0.7
0.8
p-value
2e-03
5e-03
1e-02
2e-02
4e-02
Log-odds
0.5
1.0
1.5
2.0
2.5
3.0
3.5
4.0
Cellular_organisms - ps1
Archaea1 - ps2
Archaea2 - ps3
Archaea3 - ps4
Asgard/Eukaryota - ps5
Eukaryota - ps6
Amorpheae - ps7
Obazoa - ps8
Opisthokonta - ps9
Holozoa - ps10
Filozoa - ps11
Choano./Metazoa - ps12
Metazoa - ps13
Myriazoa - ps14
Parahoxozoa - ps15
Bilateria - ps16
Deuterostomia - ps17
Chordata - ps18
Olfactores - ps19
Vertebrata - ps20
Gnathostomata - ps21
Euteleostomi - ps22
Sarcopterygii - ps23
Tetrapoda - ps24
Amniota - ps25
Mammalia - ps26
Theria - ps27
Placentalia - ps28
Boreoeutheria - ps29
Euarchontoglires - ps30
Euarchonta - ps31
Primates - ps32
Haplorrhini - ps33
Simiiformes - ps34
Catarrhini - ps35
Hominoidea - ps36
Hominidae - ps37
Homininae - ps38
Hominini - ps39
Homo_sapiens - ps40
Phylostratum

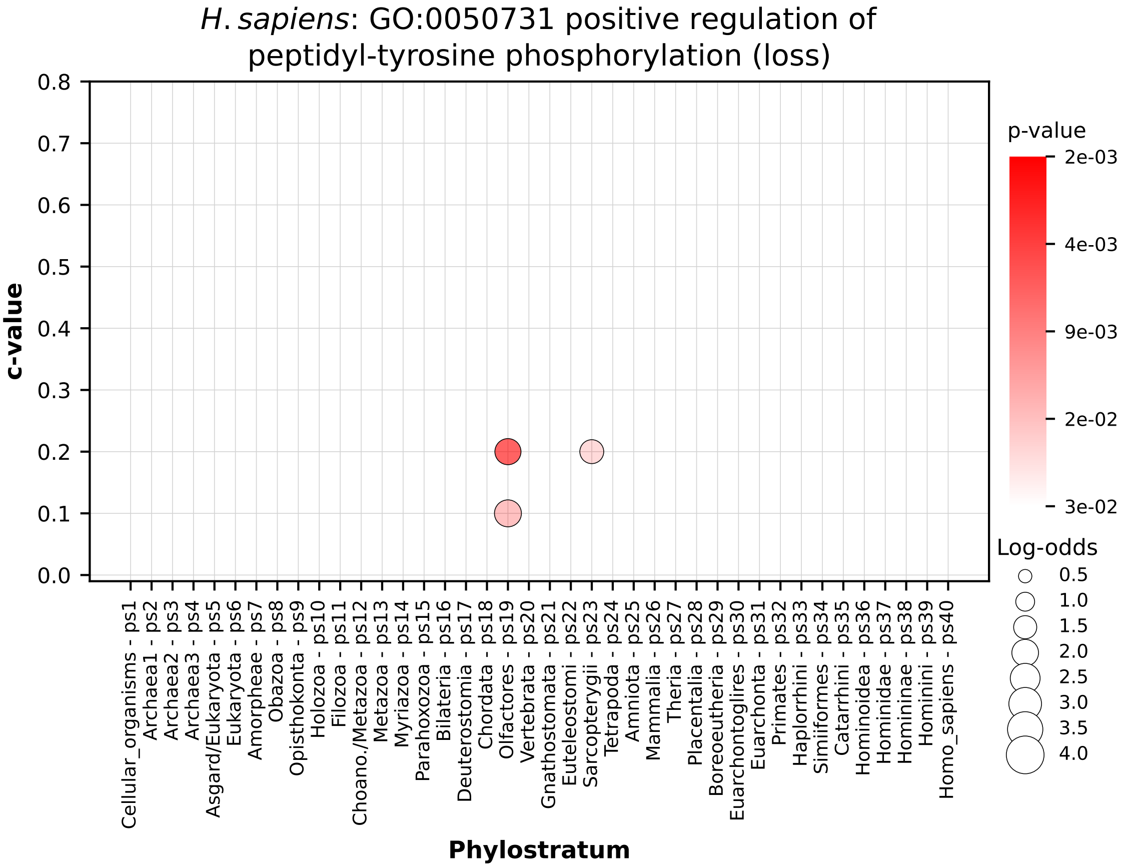

H. sapiens: GO:0050731 positive regulation of peptidyl-tyrosine phosphorylation (loss)
c-value
0.8
0.7
0.6
0.5
0.4
0.3
0.2
0.1
0.0
Phylostratum
Cellular_organisms - ps1
Archaea1 - ps2
Archaea2 - ps3
Archaea3 - ps4
Asgard/Eukaryota - ps5
Eukaryota - ps6
Amorpheae - ps7
Obazoa - ps8
Opisthokonta - ps9
Holozoa - ps10
Filozoa - ps11
Choano./Metazoa - ps12
Metazoa - ps13
Myriazoa - ps14
Parahoxozoa - ps15
Bilateria - ps16
Deuterostomia - ps17
Chordata - ps18
Olfactores - ps19
Vertebrata - ps20
Gnathostomata - ps21
Euteleostomi - ps22
Sarcopterygii - ps23
Tetrapoda - ps24
Amniota - ps25
Mammalia - ps26
Theria - ps27
Placentalia - ps28
Boreoeutheria - ps29
Euarchontoglires - ps30
Euarchonta - ps31
Primates - ps32
Haplorrhini - ps33
Simiiformes - ps34
Catarrhini - ps35
Hominoidea - ps36
Hominidae - ps37
Homininae - ps38
Hominini - ps39
Homo_sapiens - ps40
p-value
2e-03
4e-03
9e-03
2e-02
3e-02
Log-odds
0.5
1.0
1.5
2.0
2.5
3.0
3.5
4.0

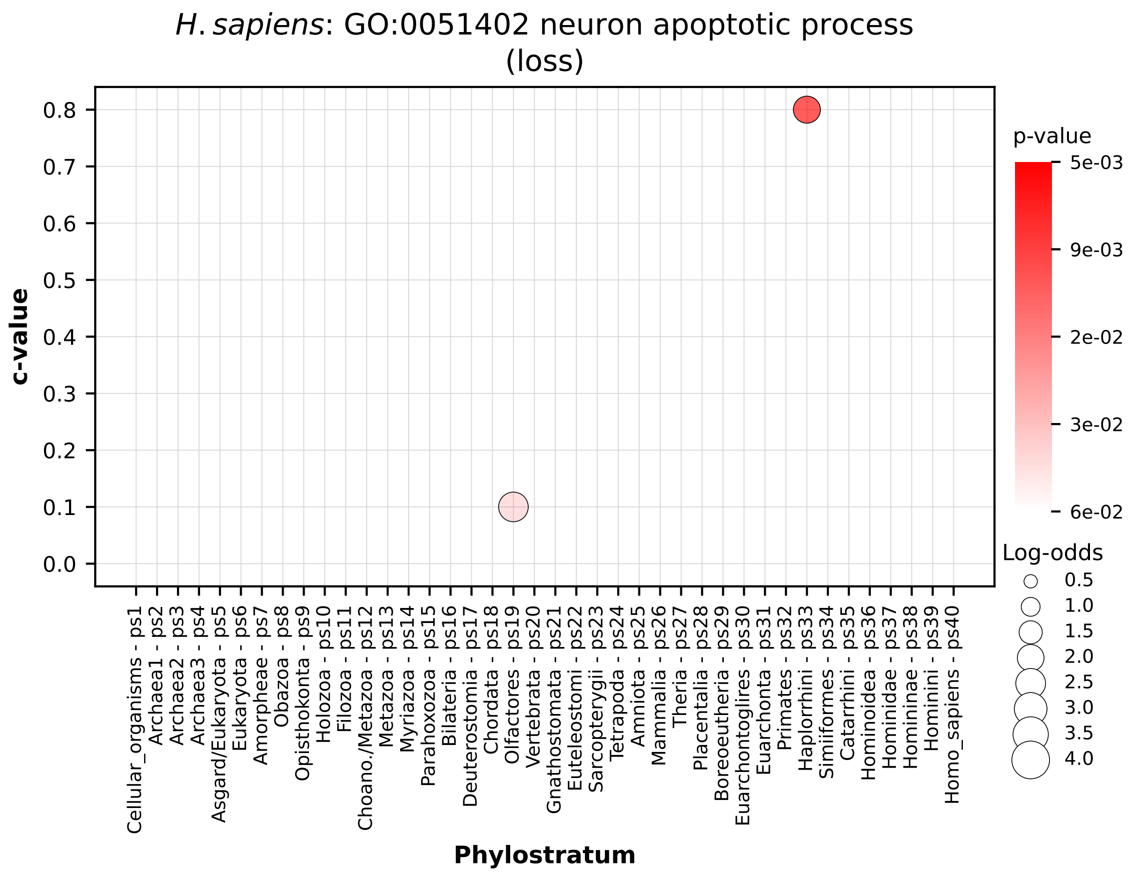

H. sapiens: GO:0051402 neuron apoptotic process
(loss)
c-value
0.0
0.1
0.2
0.3
0.4
0.5
0.6
0.7
0.8
p-value
5e-03
9e-03
2e-02
3e-02
6e-02
Log-odds
0.5
1.0
1.5
2.0
2.5
3.0
3.5
4.0
Cellular_organisms - ps1
Archaea1 - ps2
Archaea2 - ps3
Archaea3 - ps4
Asgard/Eukaryota - ps5
Eukaryota - ps6
Amorpheae - ps7
Obazoa - ps8
Opisthokonta - ps9
Holozoa - ps10
Filozoa - ps11
Choano./Metazoa - ps12
Metazoa - ps13
Myriazoa - ps14
Parahoxozoa - ps15
Bilateria - ps16
Deuterostomia - ps17
Chordata - ps18
Olfactores - ps19
Vertebrata - ps20
Gnathostomata - ps21
Euteleostomi - ps22
Sarcopterygii - ps23
Tetrapoda - ps24
Amniota - ps25
Mammalia - ps26
Theria - ps27
Placentalia - ps28
Boreoeutheria - ps29
Euarchontoglires - ps30
Euarchonta - ps31
Primates - ps32
Haplorrhini - ps33
Simiiformes - ps34
Catarrhini - ps35
Hominoidea - ps36
Hominidae - ps37
Homininae - ps38
Hominini - ps39
Homo_sapiens - ps40
Phylostratum

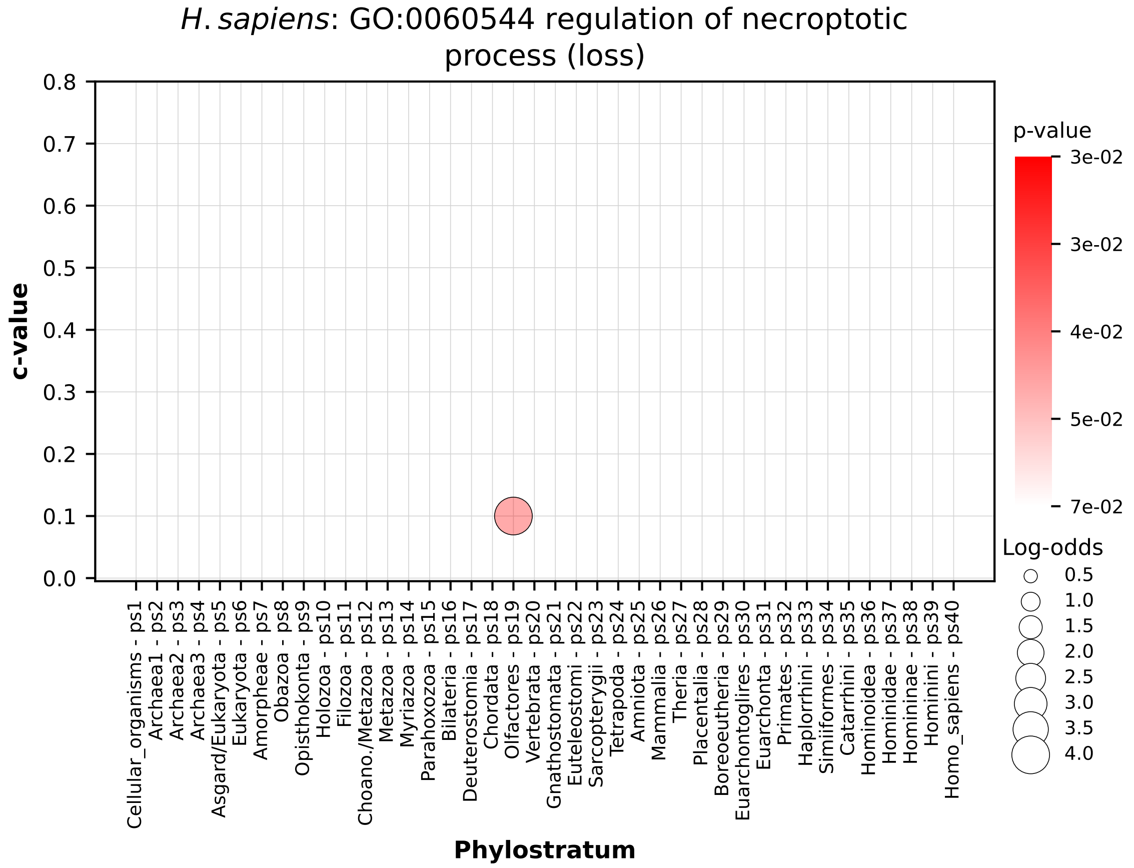

H. sapiens: GO:0060544 regulation of necroptotic process (loss)
c-value
0.0
0.1
0.2
0.3
0.4
0.5
0.6
0.7
0.8
Phylostratum
Cellular_organisms - ps1
Archaea1 - ps2
Archaea2 - ps3
Archaea3 - ps4
Asgard/Eukaryota - ps5
Eukaryota - ps6
Amorpheae - ps7
Obazoa - ps8
Opisthokonta - ps9
Holozoa - ps10
Filozoa - ps11
Choano./Metazoa - ps12
Metazoa - ps13
Myriazoa - ps14
Parahoxozoa - ps15
Bilateria - ps16
Deuterostomia - ps17
Chordata - ps18
Olfactores - ps19
Vertebrata - ps20
Gnathostomata - ps21
Euteleostomi - ps22
Sarcopterygii - ps23
Tetrapoda - ps24
Amniota - ps25
Mammalia - ps26
Theria - ps27
Placentalia - ps28
Boreoeutheria - ps29
Euarchontoglires - ps30
Euarchonta - ps31
Primates - ps32
Haplorrhini - ps33
Simiiformes - ps34
Catarrhini - ps35
Hominoidea - ps36
Hominidae - ps37
Homininae - ps38
Hominini - ps39
Homo_sapiens - ps40
p-value
3e-02
3e-02
4e-02
5e-02
7e-02
Log-odds
0.5
1.0
1.5
2.0
2.5
3.0
3.5
4.0

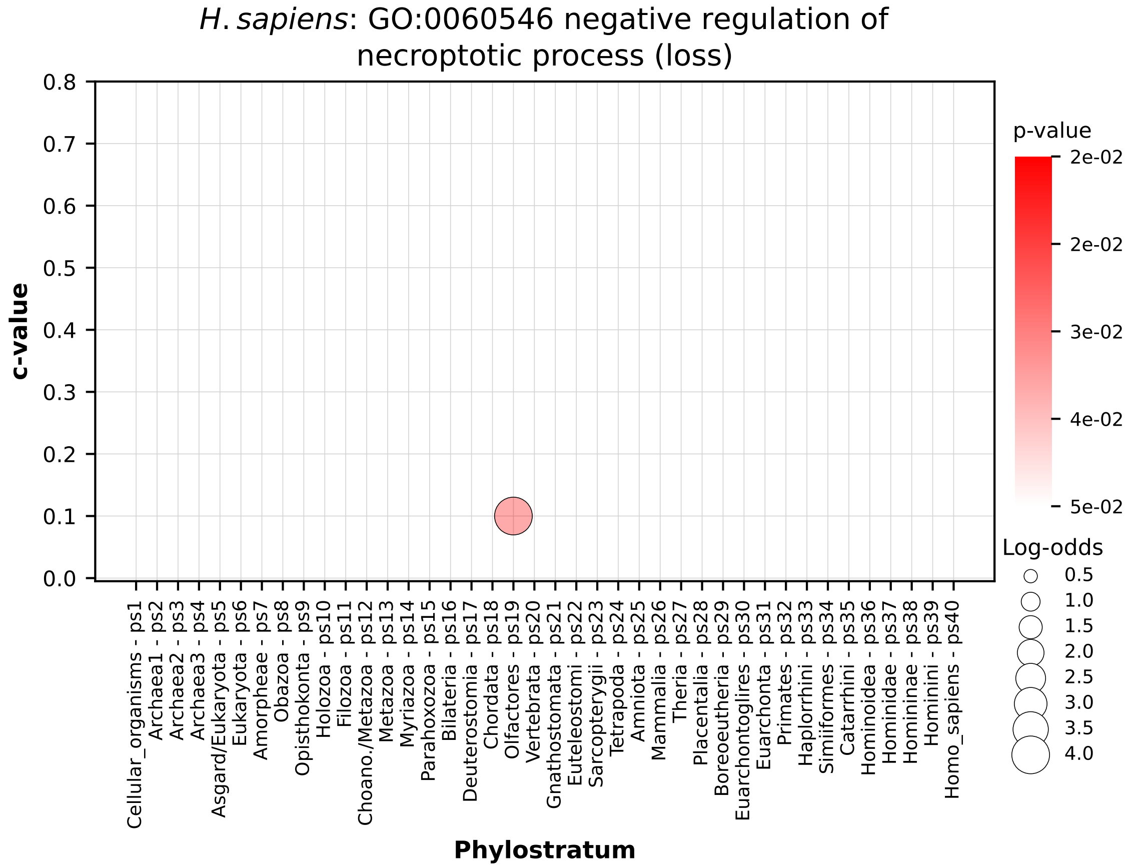

H. sapiens: GO:0060546 negative regulation of necroptotic process (loss)
c-value
0.8
0.7
0.6
0.5
0.4
0.3
0.2
0.1
0.0
Cellular_organisms - ps1
Archaea1 - ps2
Archaea2 - ps3
Archaea3 - ps4
Asgard/Eukaryota - ps5
Eukaryota - ps6
Amorpheae - ps7
Obazoa - ps8
Opisthokonta - ps9
Holozoa - ps10
Filozoa - ps11
Choano./Metazoa - ps12
Metazoa - ps13
Myriazoa - ps14
Parahoxozoa - ps15
Bilateria - ps16
Deuterostomia - ps17
Chordata - ps18
Olfactores - ps19
Vertebrata - ps20
Gnathostomata - ps21
Euteleostomi - ps22
Sarcopterygii - ps23
Tetrapoda - ps24
Amniota - ps25
Mammalia - ps26
Theria - ps27
Placentalia - ps28
Boreoeutheria - ps29
Euarchontoglires - ps30
Euarchonta - ps31
Primates - ps32
Haplorrhini - ps33
Simiiformes - ps34
Catarrhini - ps35
Hominoidea - ps36
Hominidae - ps37
Homininae - ps38
Hominini - ps39
Homo_sapiens - ps40
Phylostratum
p-value
2e-02
2e-02
3e-02
4e-02
5e-02
Log-odds
0.5
1.0
1.5
2.0
2.5
3.0
3.5
4.0

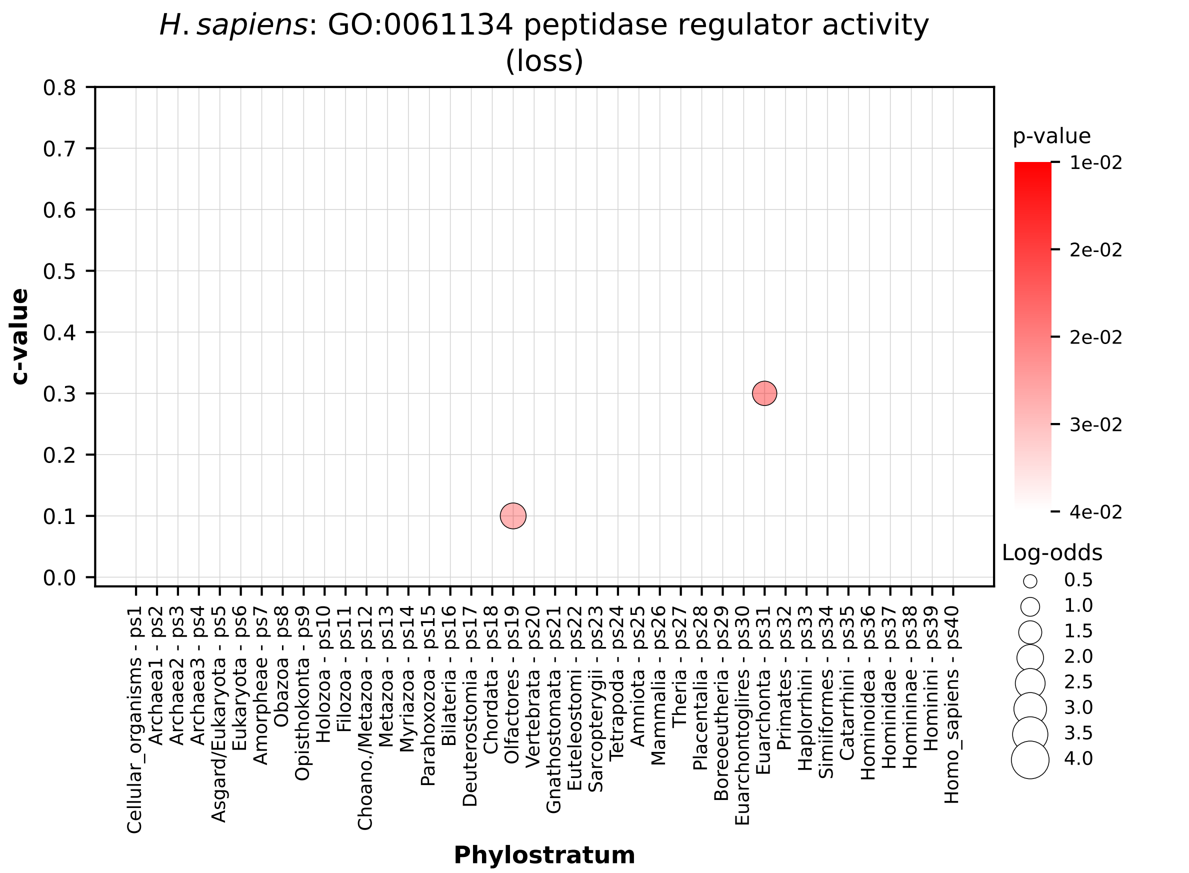

H. sapiens: GO:0061134 peptidase regulator activity
(loss)
c-value
0.0
0.1
0.2
0.3
0.4
0.5
0.6
0.7
0.8
Phylostratum
Cellular_organisms - ps1
Archaea1 - ps2
Archaea2 - ps3
Archaea3 - ps4
Asgard/Eukaryota - ps5
Eukaryota - ps6
Amorpheae - ps7
Obazoa - ps8
Opisthokonta - ps9
Holozoa - ps10
Filozoa - ps11
Choano./Metazoa - ps12
Metazoa - ps13
Myriazoa - ps14
Parahoxozoa - ps15
Bilateria - ps16
Deuterostomia - ps17
Chordata - ps18
Olfactores - ps19
Vertebrata - ps20
Gnathostomata - ps21
Euteleostomi - ps22
Sarcopterygii - ps23
Tetrapoda - ps24
Amniota - ps25
Mammalia - ps26
Theria - ps27
Placentalia - ps28
Boreoeutheria - ps29
Euarchontoglires - ps30
Euarchonta - ps31
Primates - ps32
Haplorrhini - ps33
Simiiformes - ps34
Catarrhini - ps35
Hominoidea - ps36
Hominidae - ps37
Homininae - ps38
Hominini - ps39
Homo_sapiens - ps40
p-value
1e-02
2e-02
2e-02
3e-02
4e-02
Log-odds
0.5
1.0
1.5
2.0
2.5
3.0
3.5
4.0

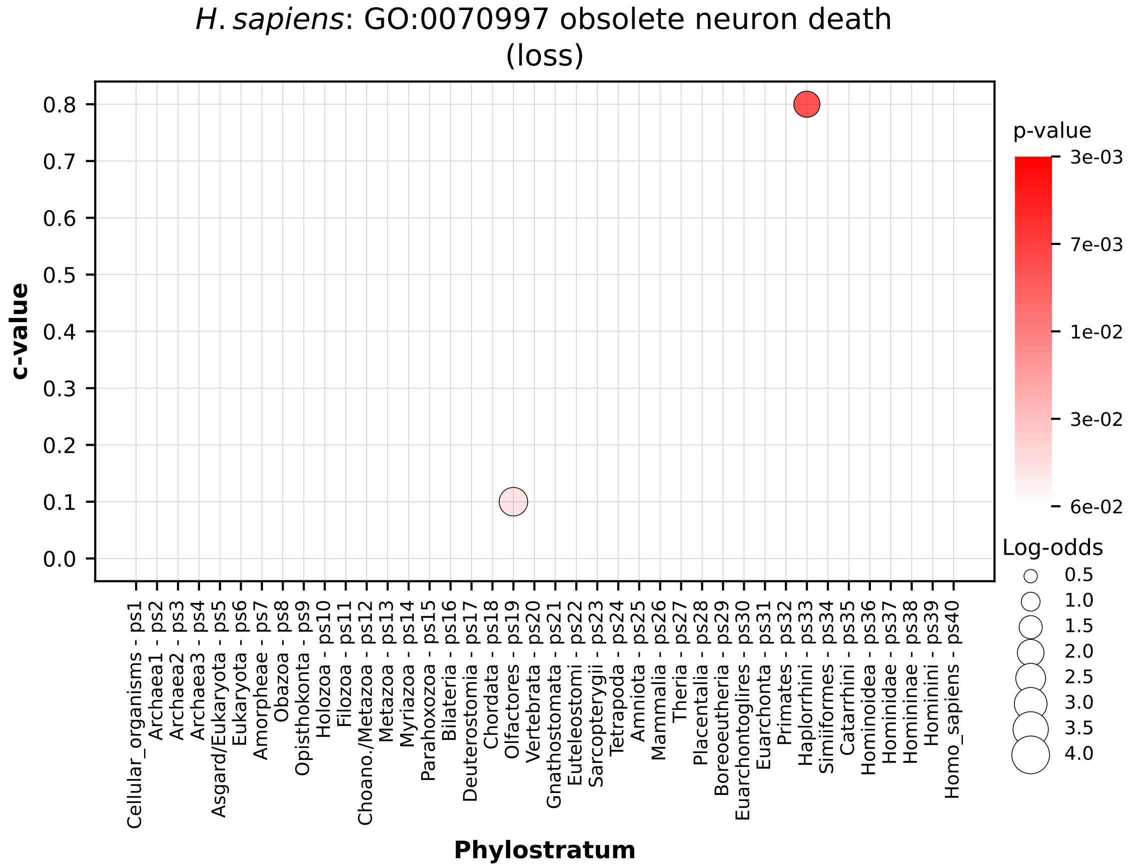

H. sapiens: GO:0070997 obsolete neuron death
(loss)
c-value
0.0
0.1
0.2
0.3
0.4
0.5
0.6
0.7
0.8
p-value
3e-03
7e-03
1e-02
3e-02
6e-02
Log-odds
0.5
1.0
1.5
2.0
2.5
3.0
3.5
4.0
Cellular_organisms - ps1
Archaea1 - ps2
Archaea2 - ps3
Archaea3 - ps4
Asgard/Eukaryota - ps5
Eukaryota - ps6
Amorpheae - ps7
Obazoa - ps8
Opisthokonta - ps9
Holozoa - ps10
Filozoa - ps11
Choano./Metazoa - ps12
Metazoa - ps13
Myriazoa - ps14
Parahoxozoa - ps15
Bilateria - ps16
Deuterostomia - ps17
Chordata - ps18
Olfactores - ps19
Vertebrata - ps20
Gnathostomata - ps21
Euteleostomi - ps22
Sarcopterygii - ps23
Tetrapoda - ps24
Amniota - ps25
Mammalia - ps26
Theria - ps27
Placentalia - ps28
Boreoeutheria - ps29
Euarchontoglires - ps30
Euarchonta - ps31
Primates - ps32
Haplorrhini - ps33
Simiiformes - ps34
Catarrhini - ps35
Hominoidea - ps36
Hominidae - ps37
Homininae - ps38
Hominini - ps39
Homo_sapiens - ps40
Phylostratum

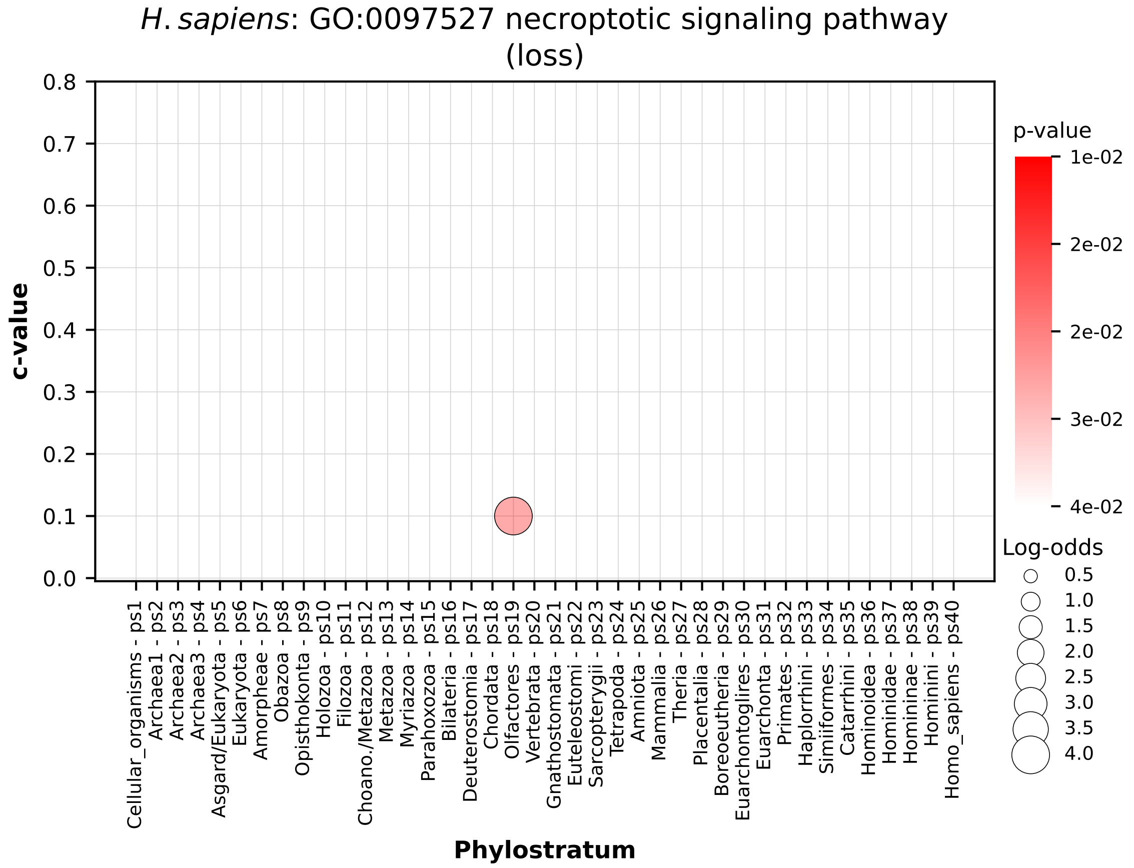

H. sapiens: GO:0097527 necroptotic signaling pathway
(loss)
c-value
0.8
0.7
0.6
0.5
0.4
0.3
0.2
0.1
0.0
Phylostratum
Cellular_organisms - ps1
Archaea1 - ps2
Archaea2 - ps3
Archaea3 - ps4
Asgard/Eukaryota - ps5
Eukaryota - ps6
Amorpheae - ps7
Obazoa - ps8
Opisthokonta - ps9
Holozoa - ps10
Filozoa - ps11
Choano./Metazoa - ps12
Metazoa - ps13
Myriazoa - ps14
Parahoxozoa - ps15
Bilateria - ps16
Deuterostomia - ps17
Chordata - ps18
Olfactores - ps19
Vertebrata - ps20
Gnathostomata - ps21
Euteleostomi - ps22
Sarcopterygii - ps23
Tetrapoda - ps24
Amniota - ps25
Mammalia - ps26
Theria - ps27
Placentalia - ps28
Boreoeutheria - ps29
Euarchontoglires - ps30
Euarchonta - ps31
Primates - ps32
Haplorrhini - ps33
Simiiformes - ps34
Catarrhini - ps35
Hominoidea - ps36
Hominidae - ps37
Homininae - ps38
Hominini - ps39
Homo_sapiens - ps40
p-value
1e-02
2e-02
2e-02
3e-02
4e-02
Log-odds
0.5
1.0
1.5
2.0
2.5
3.0
3.5
4.0

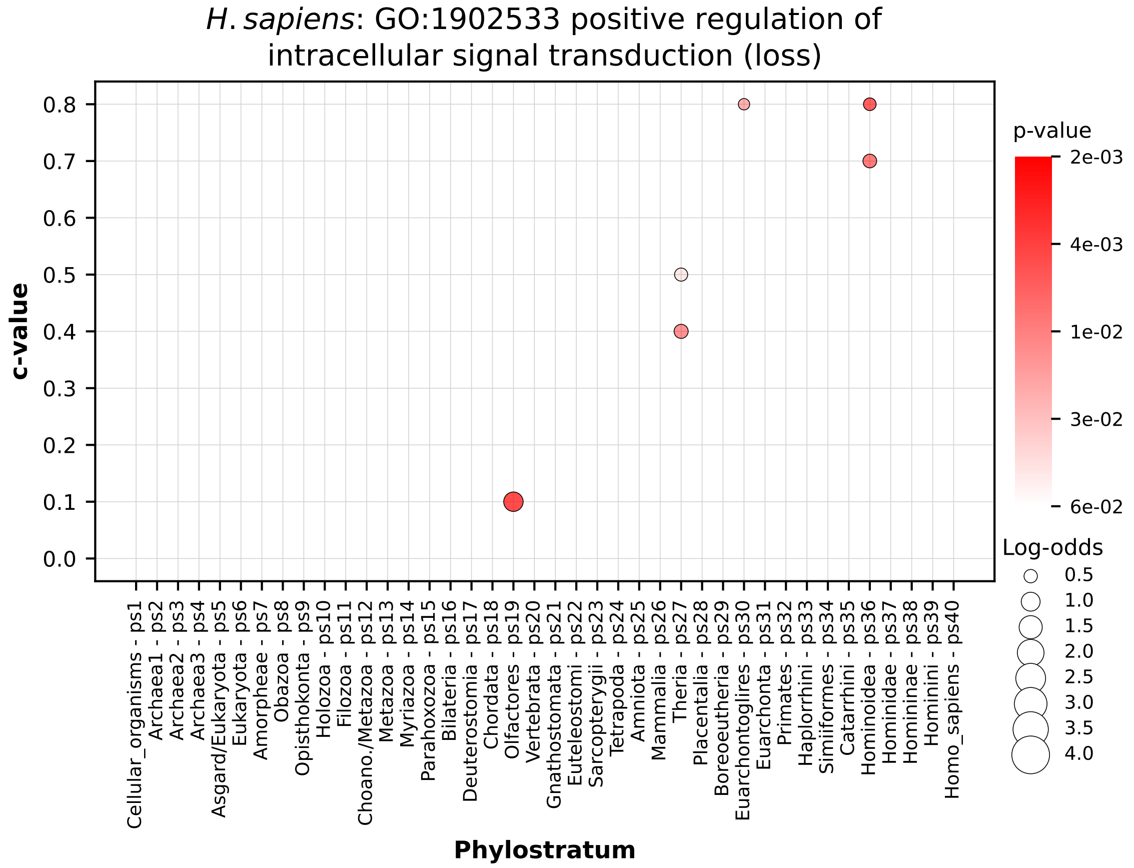

H. sapiens: GO:1902533 positive regulation of intracellular signal transduction (loss)
c-value
0.8
0.7
0.6
0.5
0.4
0.3
0.2
0.1
0.0
Phylostratum
Cellular_organisms - ps1
Archaea1 - ps2
Archaea2 - ps3
Archaea3 - ps4
Asgard/Eukaryota - ps5
Eukaryota - ps6
Amorpheae - ps7
Obazoa - ps8
Opisthokonta - ps9
Holozoa - ps10
Filozoa - ps11
Choano./Metazoa - ps12
Metazoa - ps13
Myriazoa - ps14
Parahoxozoa - ps15
Bilateria - ps16
Deuterostomia - ps17
Chordata - ps18
Olfactores - ps19
Vertebrata - ps20
Gnathostomata - ps21
Euteleostomi - ps22
Sarcopterygii - ps23
Tetrapoda - ps24
Amniota - ps25
Mammalia - ps26
Theria - ps27
Placentalia - ps28
Boreoeutheria - ps29
Euarchontoglires - ps30
Euarchonta - ps31
Primates - ps32
Haplorrhini - ps33
Simiiformes - ps34
Catarrhini - ps35
Hominoidea - ps36
Hominidae - ps37
Homininae - ps38
Hominini - ps39
Homo_sapiens - ps40
p-value
2e-03
4e-03
1e-02
3e-02
6e-02
Log-odds
0.5
1.0
1.5
2.0
2.5
3.0
3.5
4.0

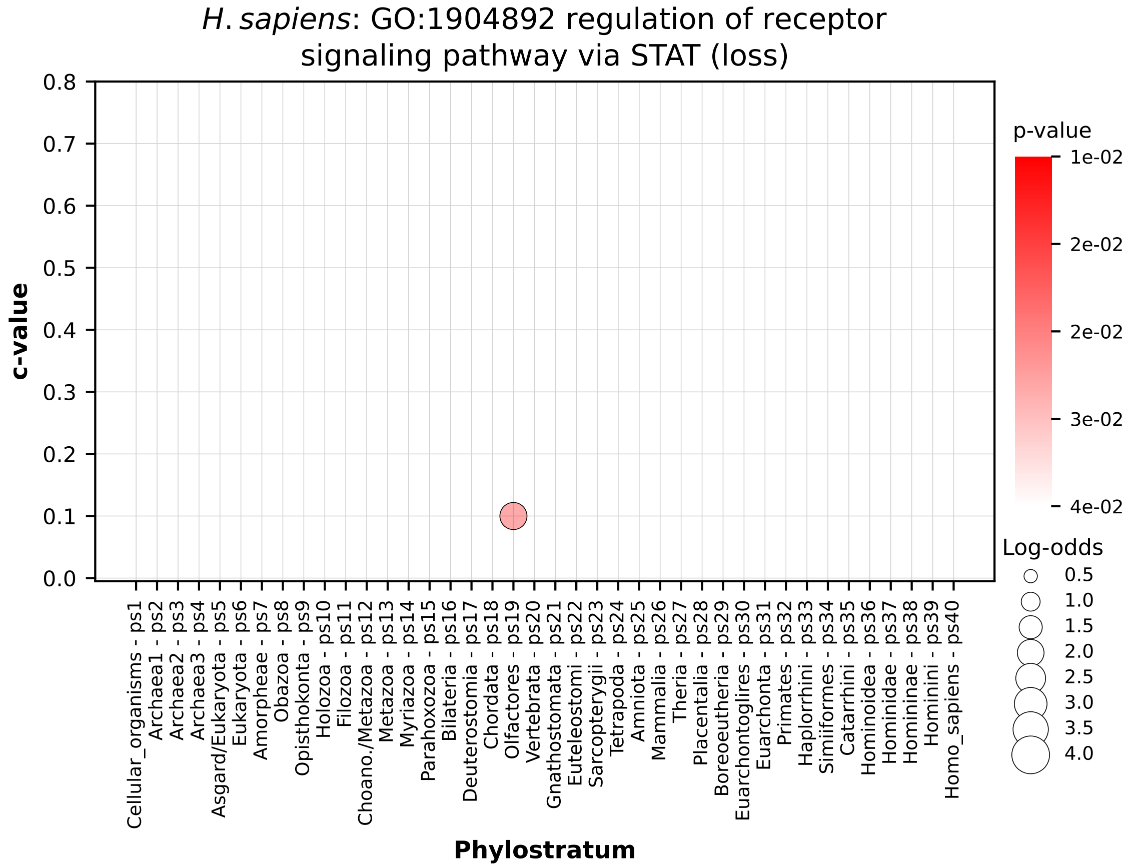

H. sapiens: GO:1904892 regulation of receptor signaling pathway via STAT (loss)
c-value
0.0
0.1
0.2
0.3
0.4
0.5
0.6
0.7
0.8
Phylostratum
Cellular_organisms - ps1
Archaea1 - ps2
Archaea2 - ps3
Archaea3 - ps4
Asgard/Eukaryota - ps5
Eukaryota - ps6
Amorpheae - ps7
Obazoa - ps8
Opisthokonta - ps9
Holozoa - ps10
Filozoa - ps11
Choano./Metazoa - ps12
Metazoa - ps13
Myriazoa - ps14
Parahoxozoa - ps15
Bilateria - ps16
Deuterostomia - ps17
Chordata - ps18
Olfactores - ps19
Vertebrata - ps20
Gnathostomata - ps21
Euteleostomi - ps22
Sarcopterygii - ps23
Tetrapoda - ps24
Amniota - ps25
Mammalia - ps26
Theria - ps27
Placentalia - ps28
Boreoeutheria - ps29
Euarchontoglires - ps30
Euarchonta - ps31
Primates - ps32
Haplorrhini - ps33
Simiiformes - ps34
Catarrhini - ps35
Hominoidea - ps36
Hominidae - ps37
Homininae - ps38
Hominini - ps39
Homo_sapiens - ps40
p-value
1e-02
2e-02
2e-02
3e-02
4e-02
Log-odds
0.5
1.0
1.5
2.0
2.5
3.0
3.5
4.0

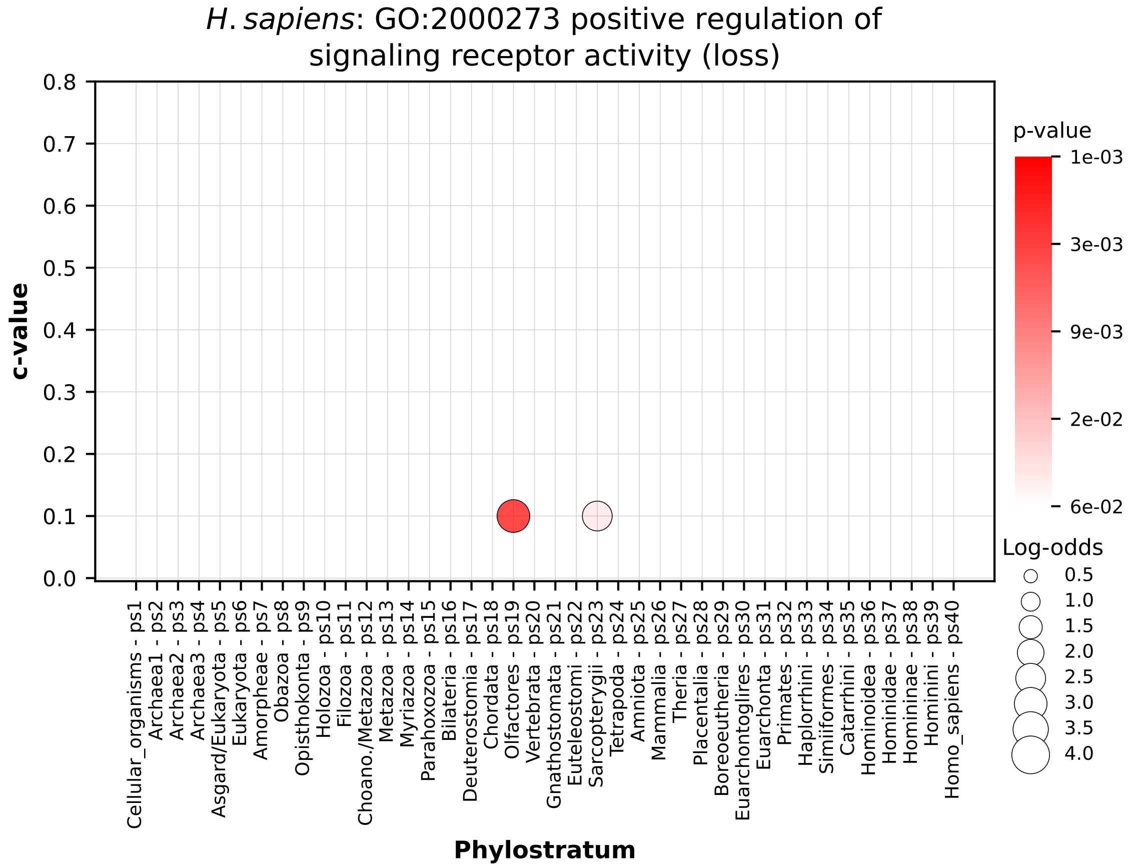

H. sapiens: GO:2000273 positive regulation of signaling receptor activity (loss)
c-value
0.0
0.1
0.2
0.3
0.4
0.5
0.6
0.7
0.8
Phylostratum
Cellular_organisms - ps1
Archaea1 - ps2
Archaea2 - ps3
Archaea3 - ps4
Asgard/Eukaryota - ps5
Eukaryota - ps6
Amorpheae - ps7
Obazoa - ps8
Opisthokonta - ps9
Holozoa - ps10
Filozoa - ps11
Choano./Metazoa - ps12
Metazoa - ps13
Myriazoa - ps14
Parahoxozoa - ps15
Bilateria - ps16
Deuterostomia - ps17
Chordata - ps18
Olfactores - ps19
Vertebrata - ps20
Gnathostomata - ps21
Euteleostomi - ps22
Sarcopterygii - ps23
Tetrapoda - ps24
Amniota - ps25
Mammalia - ps26
Theria - ps27
Placentalia - ps28
Boreoeutheria - ps29
Euarchontoglires - ps30
Euarchonta - ps31
Primates - ps32
Haplorrhini - ps33
Simiiformes - ps34
Catarrhini - ps35
Hominoidea - ps36
Hominidae - ps37
Homininae - ps38
Hominini - ps39
Homo_sapiens - ps40
p-value
1e-03
3e-03
9e-03
2e-02
6e-02
Log-odds
0.5
1.0
1.5
2.0
2.5
3.0
3.5
4.0

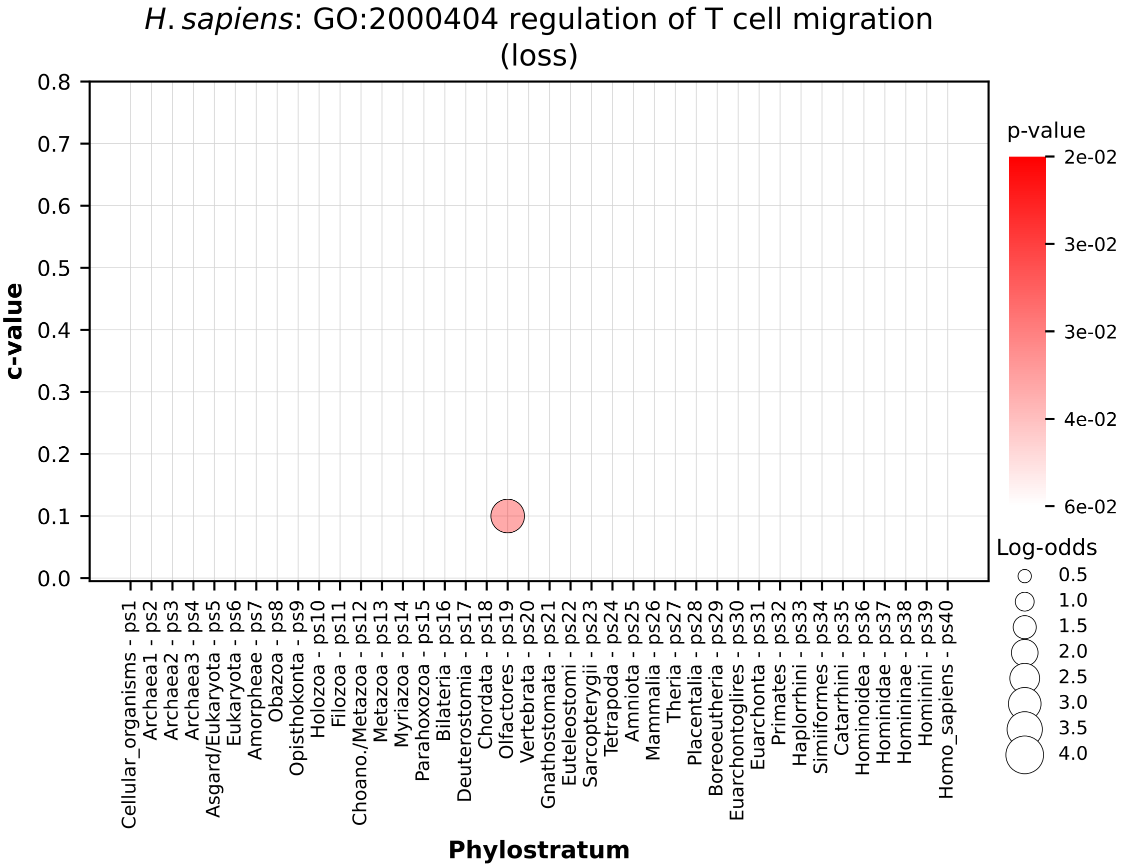

H. sapiens: GO:2000404 regulation of T cell migration
(loss)
c-value
0.0
0.1
0.2
0.3
0.4
0.5
0.6
0.7
0.8
Phylostratum
Cellular_organisms - ps1
Archaea1 - ps2
Archaea2 - ps3
Archaea3 - ps4
Asgard/Eukaryota - ps5
Eukaryota - ps6
Amorpheae - ps7
Obazoa - ps8
Opisthokonta - ps9
Holozoa - ps10
Filozoa - ps11
Choano./Metazoa - ps12
Metazoa - ps13
Myriazoa - ps14
Parahoxozoa - ps15
Bilateria - ps16
Deuterostomia - ps17
Chordata - ps18
Olfactores - ps19
Vertebrata - ps20
Gnathostomata - ps21
Euteleostomi - ps22
Sarcopterygii - ps23
Tetrapoda - ps24
Amniota - ps25
Mammalia - ps26
Theria - ps27
Placentalia - ps28
Boreoeutheria - ps29
Euarchontoglires - ps30
Euarchonta - ps31
Primates - ps32
Haplorrhini - ps33
Simiiformes - ps34
Catarrhini - ps35
Hominoidea - ps36
Hominidae - ps37
Homininae - ps38
Hominini - ps39
Homo_sapiens - ps40
p-value
2e-02
3e-02
3e-02
4e-02
6e-02
Log-odds
0.5
1.0
1.5
2.0
2.5
3.0
3.5
4.0

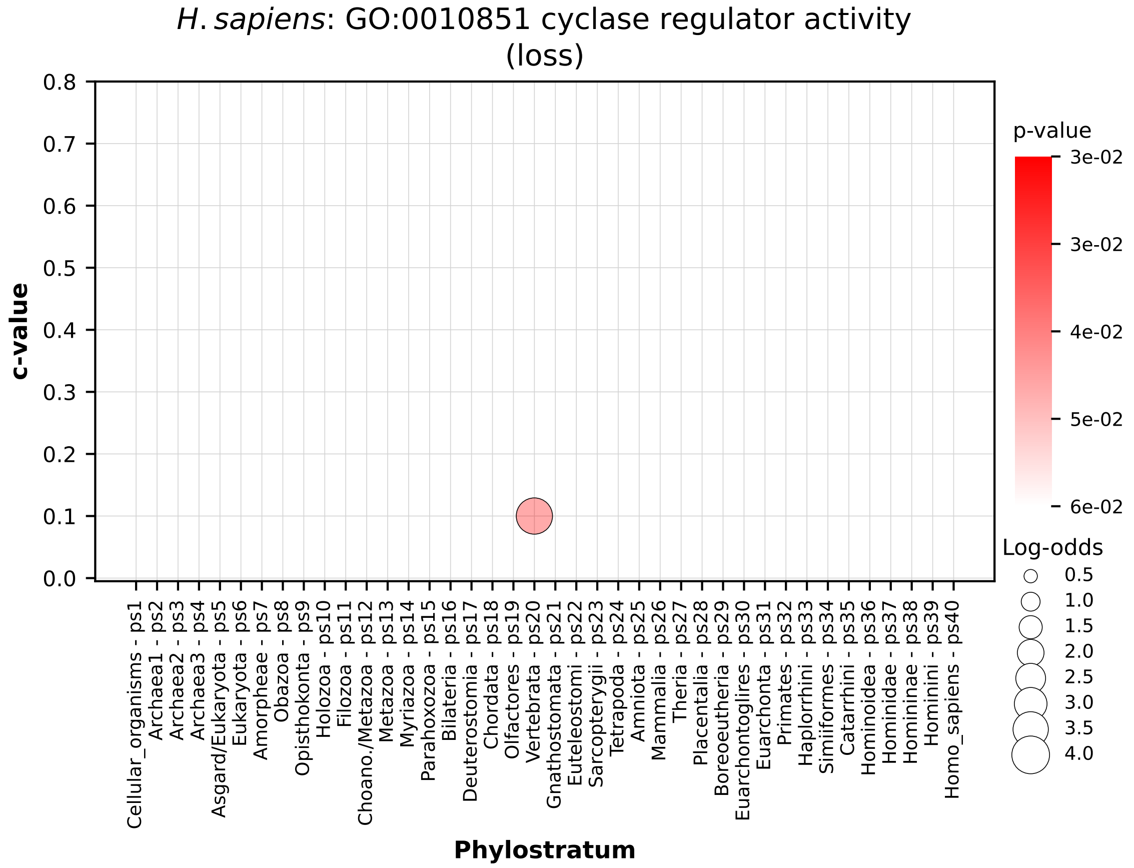

H. sapiens: GO:0010851 cyclase regulator activity
(loss)
c-value
0.8
0.7
0.6
0.5
0.4
0.3
0.2
0.1
0.0
Phylostratum
Cellular_organisms - ps1
Archaea1 - ps2
Archaea2 - ps3
Archaea3 - ps4
Asgard/Eukaryota - ps5
Eukaryota - ps6
Amorpheae - ps7
Obazoa - ps8
Opisthokonta - ps9
Holozoa - ps10
Filozoa - ps11
Choano./Metazoa - ps12
Metazoa - ps13
Myriazoa - ps14
Parahoxozoa - ps15
Bilateria - ps16
Deuterostomia - ps17
Chordata - ps18
Olfactores - ps19
Vertebrata - ps20
Gnathostomata - ps21
Euteleostomi - ps22
Sarcopterygii - ps23
Tetrapoda - ps24
Amniota - ps25
Mammalia - ps26
Theria - ps27
Placentalia - ps28
Boreoeutheria - ps29
Euarchontoglires - ps30
Euarchonta - ps31
Primates - ps32
Haplorrhini - ps33
Simiiformes - ps34
Catarrhini - ps35
Hominoidea - ps36
Hominidae - ps37
Homininae - ps38
Hominini - ps39
Homo_sapiens - ps40
p-value
3e-02
3e-02
4e-02
5e-02
6e-02
Log-odds
0.5
1.0
1.5
2.0
2.5
3.0
3.5
4.0

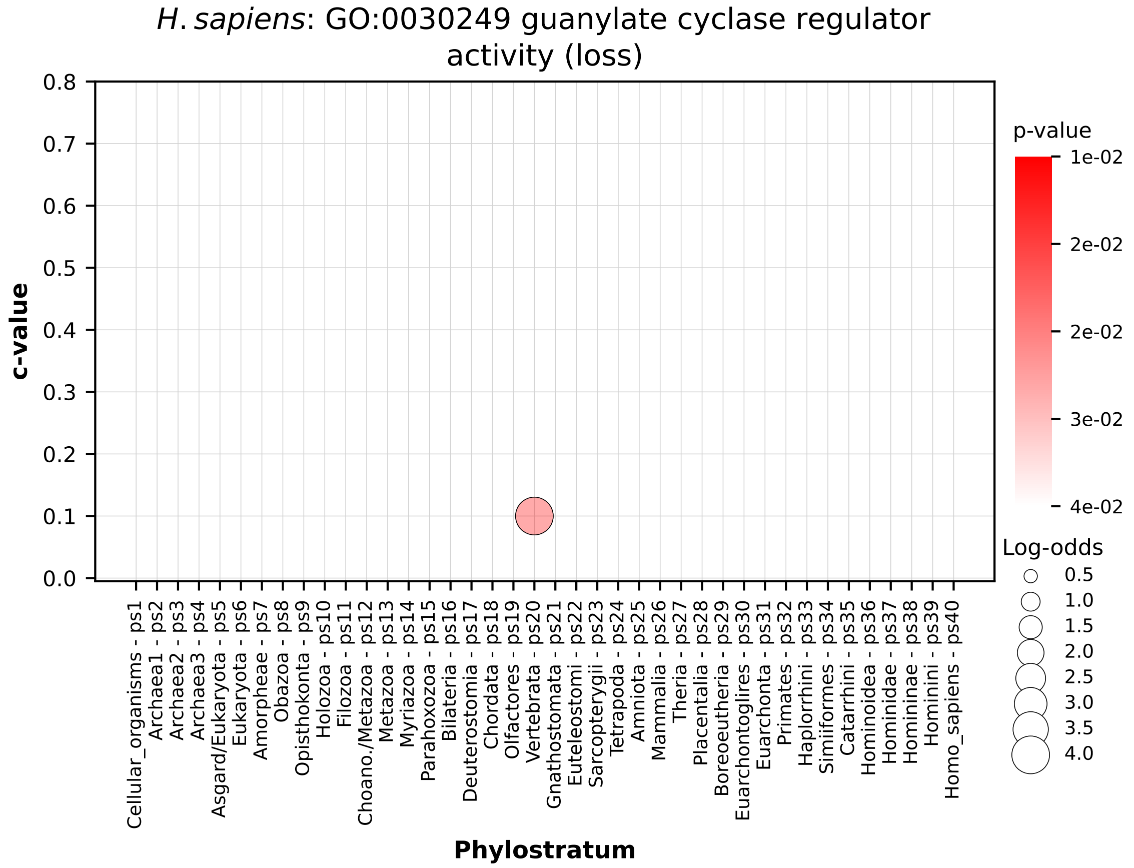

H. sapiens: GO:0030249 guanylate cyclase regulator activity (loss)
c-value
0.0
0.1
0.2
0.3
0.4
0.5
0.6
0.7
0.8
Phylostratum
Cellular_organisms - ps1
Archaea1 - ps2
Archaea2 - ps3
Archaea3 - ps4
Asgard/Eukaryota - ps5
Eukaryota - ps6
Amorpheae - ps7
Obazoa - ps8
Opisthokonta - ps9
Holozoa - ps10
Filozoa - ps11
Choano./Metazoa - ps12
Metazoa - ps13
Myriazoa - ps14
Parahoxozoa - ps15
Bilateria - ps16
Deuterostomia - ps17
Chordata - ps18
Olfactores - ps19
Vertebrata - ps20
Gnathostomata - ps21
Euteleostomi - ps22
Sarcopterygii - ps23
Tetrapoda - ps24
Amniota - ps25
Mammalia - ps26
Theria - ps27
Placentalia - ps28
Boreoeutheria - ps29
Euarchontoglires - ps30
Euarchonta - ps31
Primates - ps32
Haplorrhini - ps33
Simiiformes - ps34
Catarrhini - ps35
Hominoidea - ps36
Hominidae - ps37
Homininae - ps38
Hominini - ps39
Homo_sapiens - ps40
p-value
1e-02
2e-02
2e-02
3e-02
4e-02
Log-odds
0.5
1.0
1.5
2.0
2.5
3.0
3.5
4.0

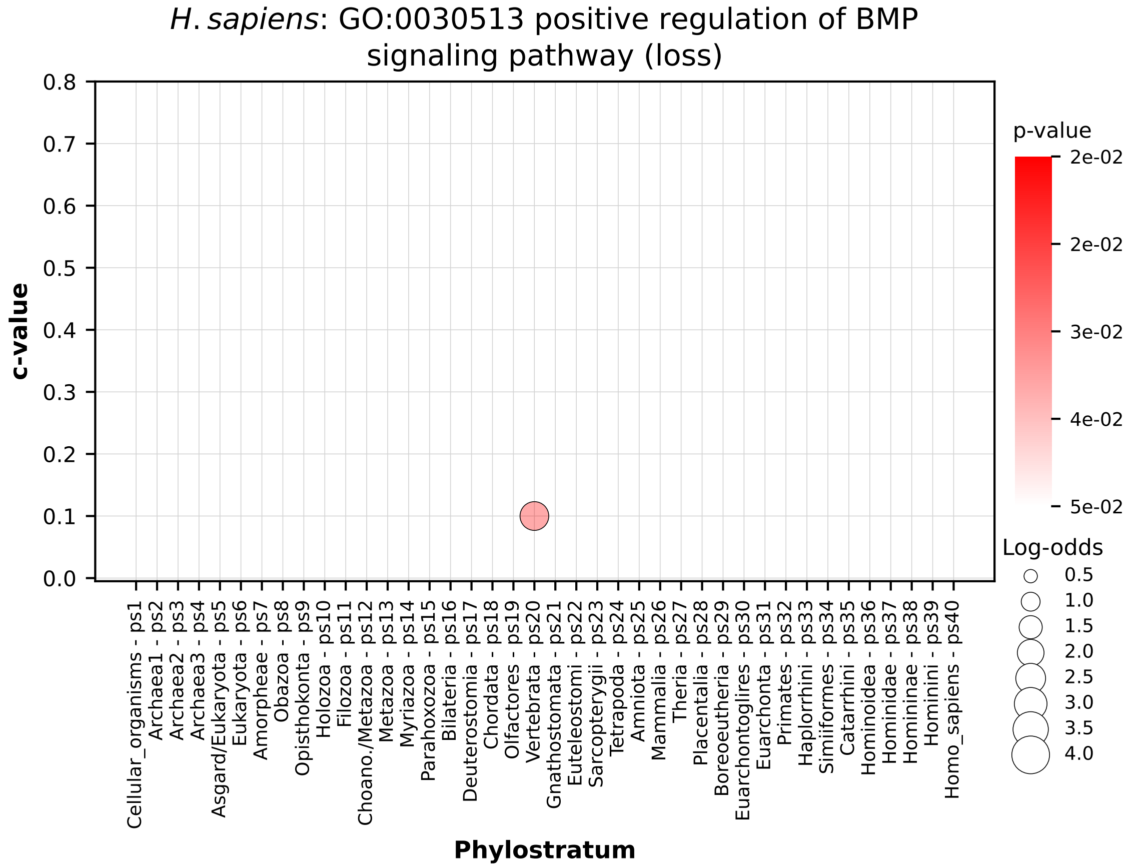

H. sapiens: GO:0030513 positive regulation of BMP signaling pathway (loss)
c-value
0.0
0.1
0.2
0.3
0.4
0.5
0.6
0.7
0.8
Phylostratum
Cellular_organisms - ps1
Archaea1 - ps2
Archaea2 - ps3
Archaea3 - ps4
Asgard/Eukaryota - ps5
Eukaryota - ps6
Amorpheae - ps7
Obazoa - ps8
Opisthokonta - ps9
Holozoa - ps10
Filozoa - ps11
Choano./Metazoa - ps12
Metazoa - ps13
Myriazoa - ps14
Parahoxozoa - ps15
Bilateria - ps16
Deuterostomia - ps17
Chordata - ps18
Olfactores - ps19
Vertebrata - ps20
Gnathostomata - ps21
Euteleostomi - ps22
Sarcopterygii - ps23
Tetrapoda - ps24
Amniota - ps25
Mammalia - ps26
Theria - ps27
Placentalia - ps28
Boreoeutheria - ps29
Euarchontoglires - ps30
Euarchonta - ps31
Primates - ps32
Haplorrhini - ps33
Simiiformes - ps34
Catarrhini - ps35
Hominoidea - ps36
Hominidae - ps37
Homininae - ps38
Hominini - ps39
Homo_sapiens - ps40
p-value
2e-02
2e-02
3e-02
4e-02
5e-02
Log-odds
0.5
1.0
1.5
2.0
2.5
3.0
3.5
4.0

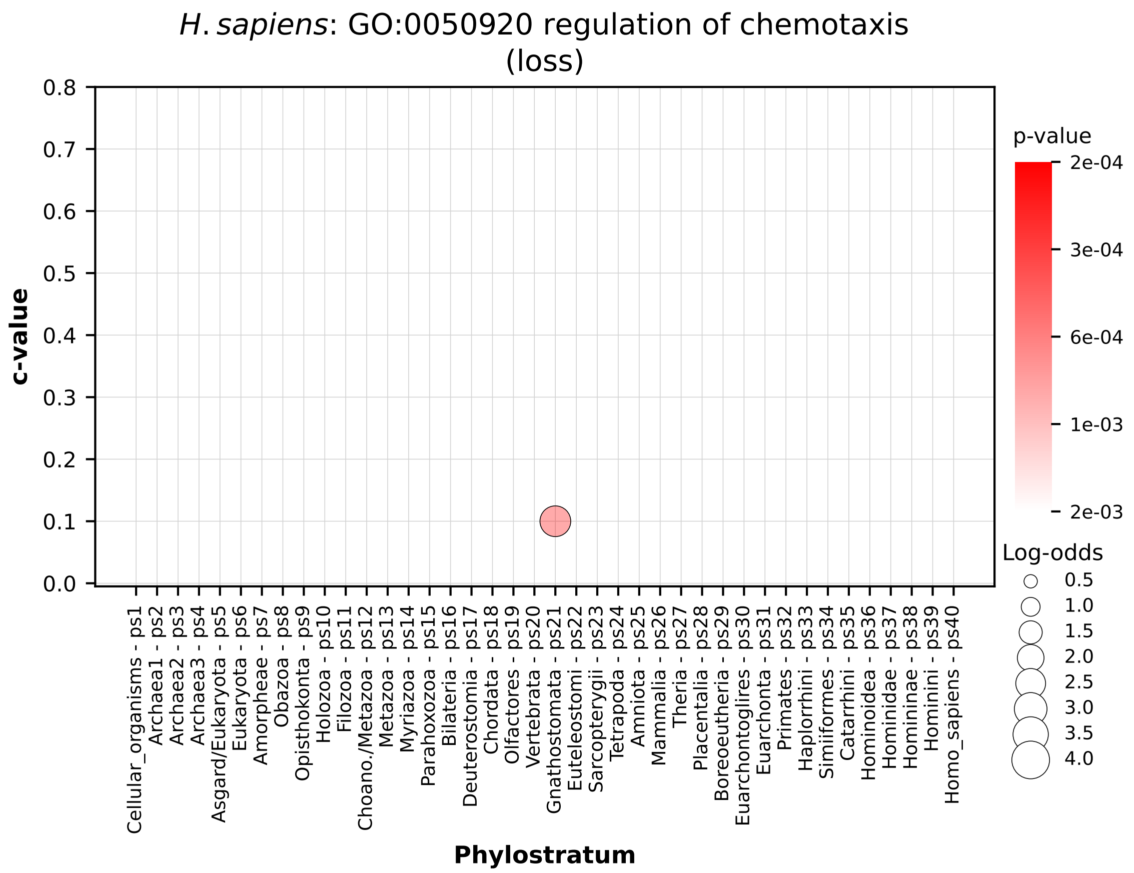

H. sapiens: GO:0050920 regulation of chemotaxis
(loss)
c-value
0.8
0.7
0.6
0.5
0.4
0.3
0.2
0.1
0.0
Phylostratum
Cellular_organisms - ps1
Archaea1 - ps2
Archaea2 - ps3
Archaea3 - ps4
Asgard/Eukaryota - ps5
Eukaryota - ps6
Amorpheae - ps7
Obazoa - ps8
Opisthokonta - ps9
Holozoa - ps10
Filozoa - ps11
Choano./Metazoa - ps12
Metazoa - ps13
Myriazoa - ps14
Parahoxozoa - ps15
Bilateria - ps16
Deuterostomia - ps17
Chordata - ps18
Olfactores - ps19
Vertebrata - ps20
Gnathostomata - ps21
Euteleostomi - ps22
Sarcopterygii - ps23
Tetrapoda - ps24
Amniota - ps25
Mammalia - ps26
Theria - ps27
Placentalia - ps28
Boreoeutheria - ps29
Euarchontoglires - ps30
Euarchonta - ps31
Primates - ps32
Haplorrhini - ps33
Simiiformes - ps34
Catarrhini - ps35
Hominoidea - ps36
Hominidae - ps37
Homininae - ps38
Hominini - ps39
Homo_sapiens - ps40
p-value
2e-04
3e-04
6e-04
1e-03
2e-03
Log-odds
0.5
1.0
1.5
2.0
2.5
3.0
3.5
4.0

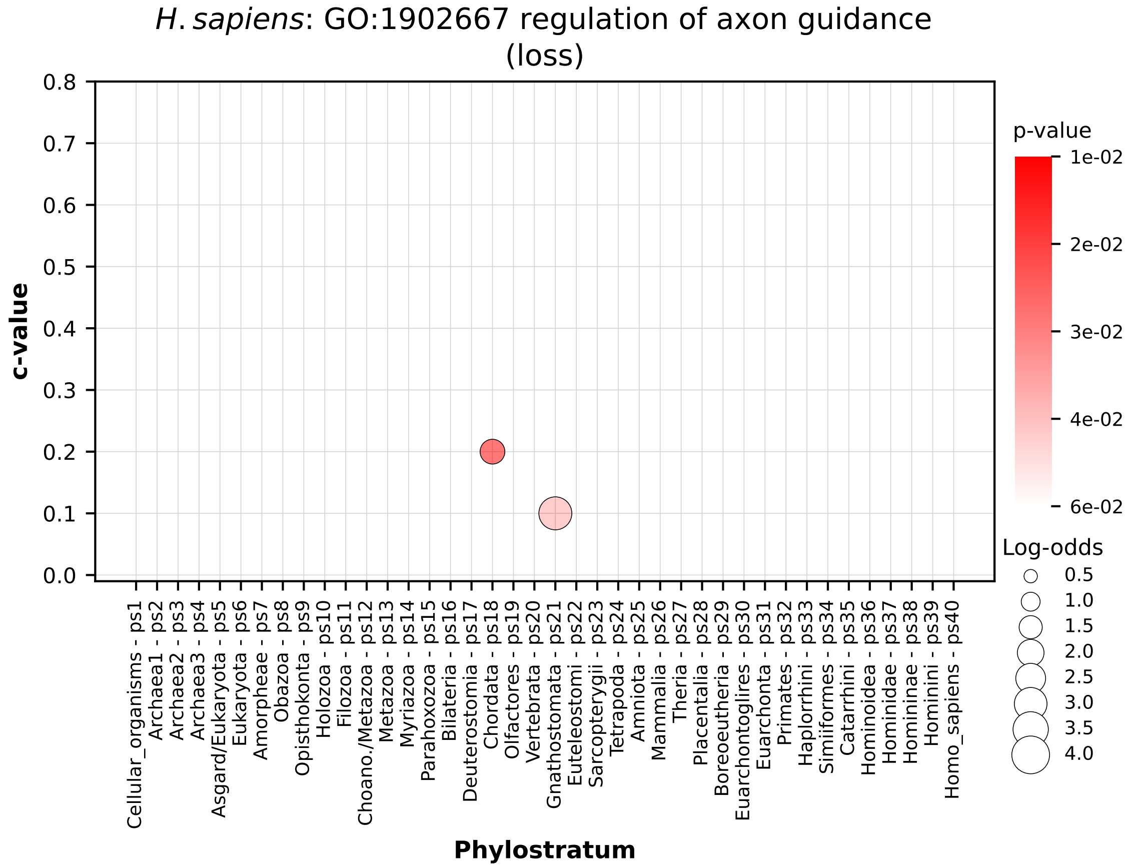

H. sapiens: GO:1902667 regulation of axon guidance
(loss)
c-value
0.0
0.1
0.2
0.3
0.4
0.5
0.6
0.7
0.8
p-value
1e-02
2e-02
3e-02
4e-02
6e-02
Log-odds
0.5
1.0
1.5
2.0
2.5
3.0
3.5
4.0
Cellular_organisms - ps1
Archaea1 - ps2
Archaea2 - ps3
Archaea3 - ps4
Asgard/Eukaryota - ps5
Eukaryota - ps6
Amorpheae - ps7
Obazoa - ps8
Opisthokonta - ps9
Holozoa - ps10
Filozoa - ps11
Choano./Metazoa - ps12
Metazoa - ps13
Myriazoa - ps14
Parahoxozoa - ps15
Bilateria - ps16
Deuterostomia - ps17
Chordata - ps18
Olfactores - ps19
Vertebrata - ps20
Gnathostomata - ps21
Euteleostomi - ps22
Sarcopterygii - ps23
Tetrapoda - ps24
Amniota - ps25
Mammalia - ps26
Theria - ps27
Placentalia - ps28
Boreoeutheria - ps29
Euarchontoglires - ps30
Euarchonta - ps31
Primates - ps32
Haplorrhini - ps33
Simiiformes - ps34
Catarrhini - ps35
Hominoidea - ps36
Hominidae - ps37
Homininae - ps38
Hominini - ps39
Homo_sapiens - ps40
Phylostratum

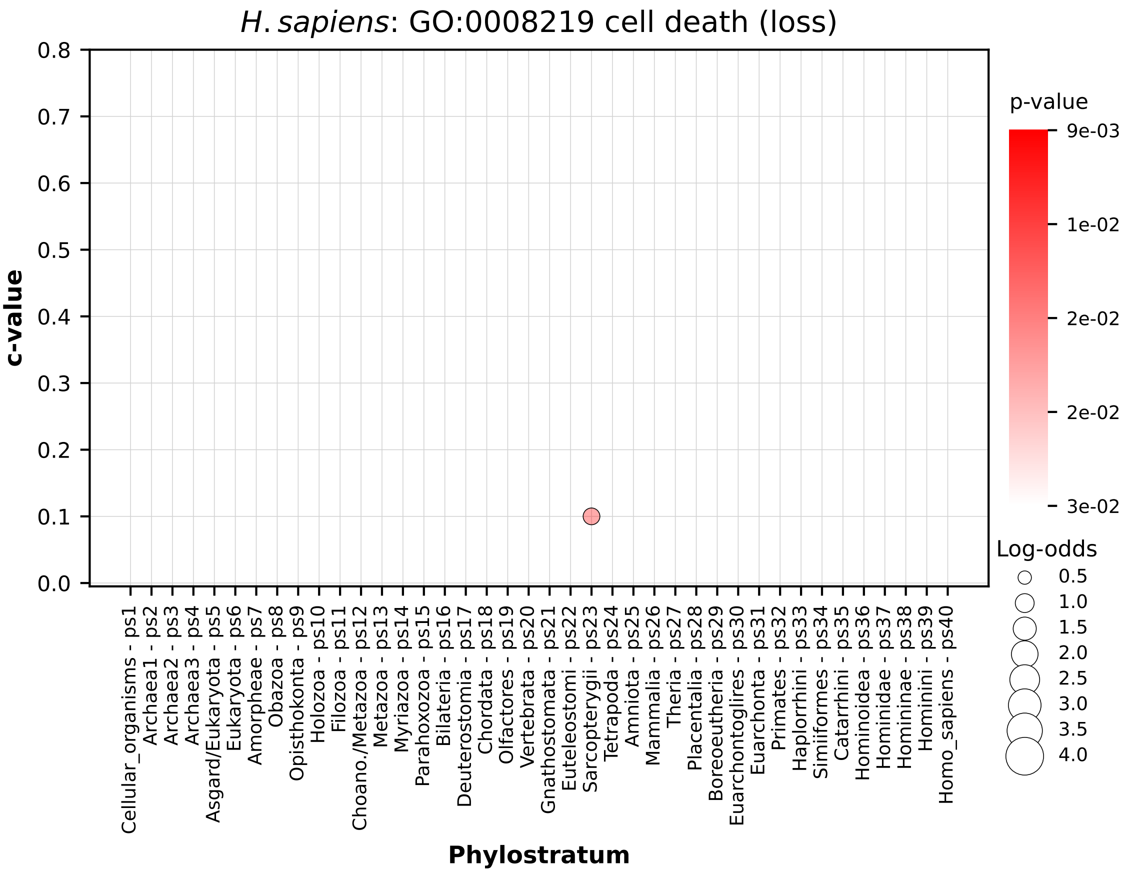

H. sapiens: GO:0008219 cell death (loss)
c-value
0.8
0.7
0.6
0.5
0.4
0.3
0.2
0.1
0.0
p-value
9e-03
1e-02
2e-02
2e-02
3e-02
Log-odds
0.5
1.0
1.5
2.0
2.5
3.0
3.5
4.0
Cellular_organisms - ps1
Archaea1 - ps2
Archaea2 - ps3
Archaea3 - ps4
Asgard/Eukaryota - ps5
Eukaryota - ps6
Amorpheae - ps7
Obazoa - ps8
Opisthokonta - ps9
Holozoa - ps10
Filozoa - ps11
Choano./Metazoa - ps12
Metazoa - ps13
Myriazoa - ps14
Parahoxozoa - ps15
Bilateria - ps16
Deuterostomia - ps17
Chordata - ps18
Olfactores - ps19
Vertebrata - ps20
Gnathostomata - ps21
Euteleostomi - ps22
Sarcopterygii - ps23
Tetrapoda - ps24
Amniota - ps25
Mammalia - ps26
Theria - ps27
Placentalia - ps28
Boreoeutheria - ps29
Euarchontoglires - ps30
Euarchonta - ps31
Primates - ps32
Haplorrhini - ps33
Simiiformes - ps34
Catarrhini - ps35
Hominoidea - ps36
Hominidae - ps37
Homininae - ps38
Hominini - ps39
Homo_sapiens - ps40
Phylostratum

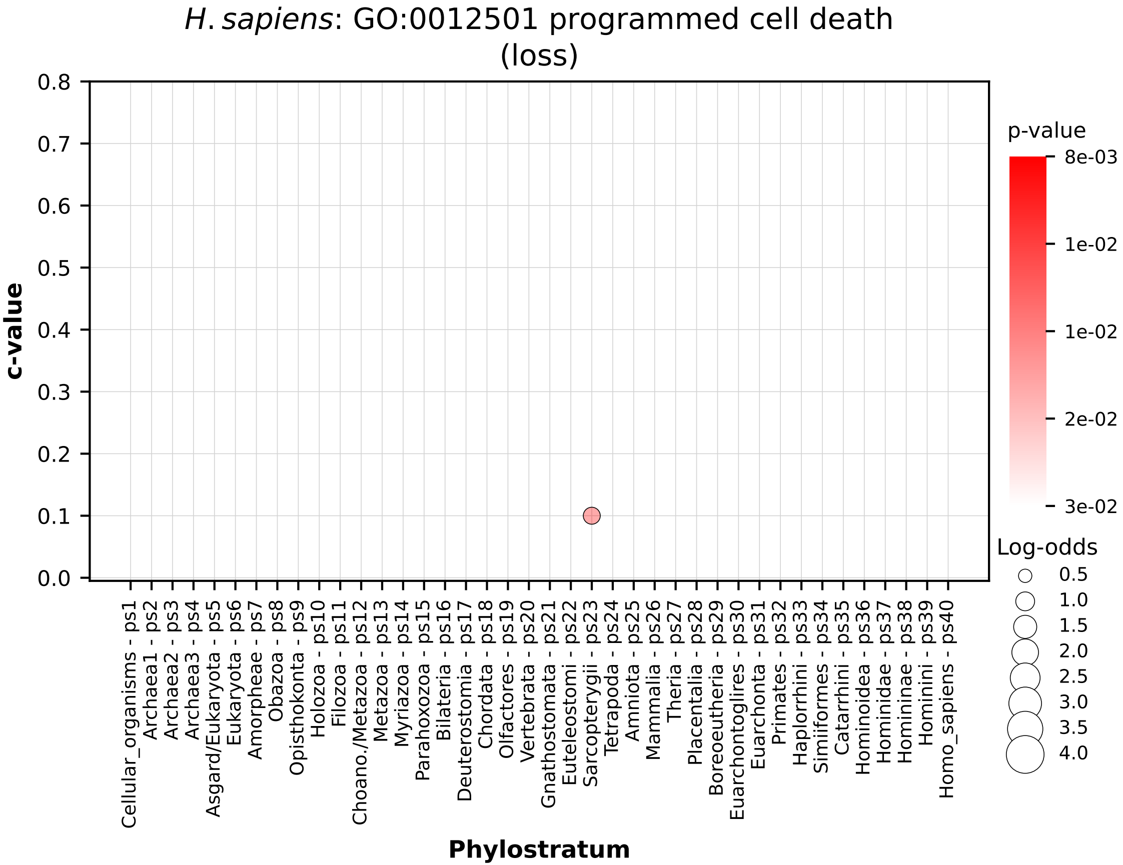

H. sapiens: GO:0012501 programmed cell death
(loss)
c-value
0.0
0.1
0.2
0.3
0.4
0.5
0.6
0.7
0.8
p-value
8e-03
1e-02
1e-02
2e-02
3e-02
Log-odds
0.5
1.0
1.5
2.0
2.5
3.0
3.5
4.0
Cellular_organisms - ps1
Archaea1 - ps2
Archaea2 - ps3
Archaea3 - ps4
Asgard/Eukaryota - ps5
Eukaryota - ps6
Amorpheae - ps7
Obazoa - ps8
Opisthokonta - ps9
Holozoa - ps10
Filozoa - ps11
Choano./Metazoa - ps12
Metazoa - ps13
Myriazoa - ps14
Parahoxozoa - ps15
Bilateria - ps16
Deuterostomia - ps17
Chordata - ps18
Olfactores - ps19
Vertebrata - ps20
Gnathostomata - ps21
Euteleostomi - ps22
Sarcopterygii - ps23
Tetrapoda - ps24
Amniota - ps25
Mammalia - ps26
Theria - ps27
Placentalia - ps28
Boreoeutheria - ps29
Euarchontoglires - ps30
Euarchonta - ps31
Primates - ps32
Haplorrhini - ps33
Simiiformes - ps34
Catarrhini - ps35
Hominoidea - ps36
Hominidae - ps37
Homininae - ps38
Hominini - ps39
Homo_sapiens - ps40
Phylostratum

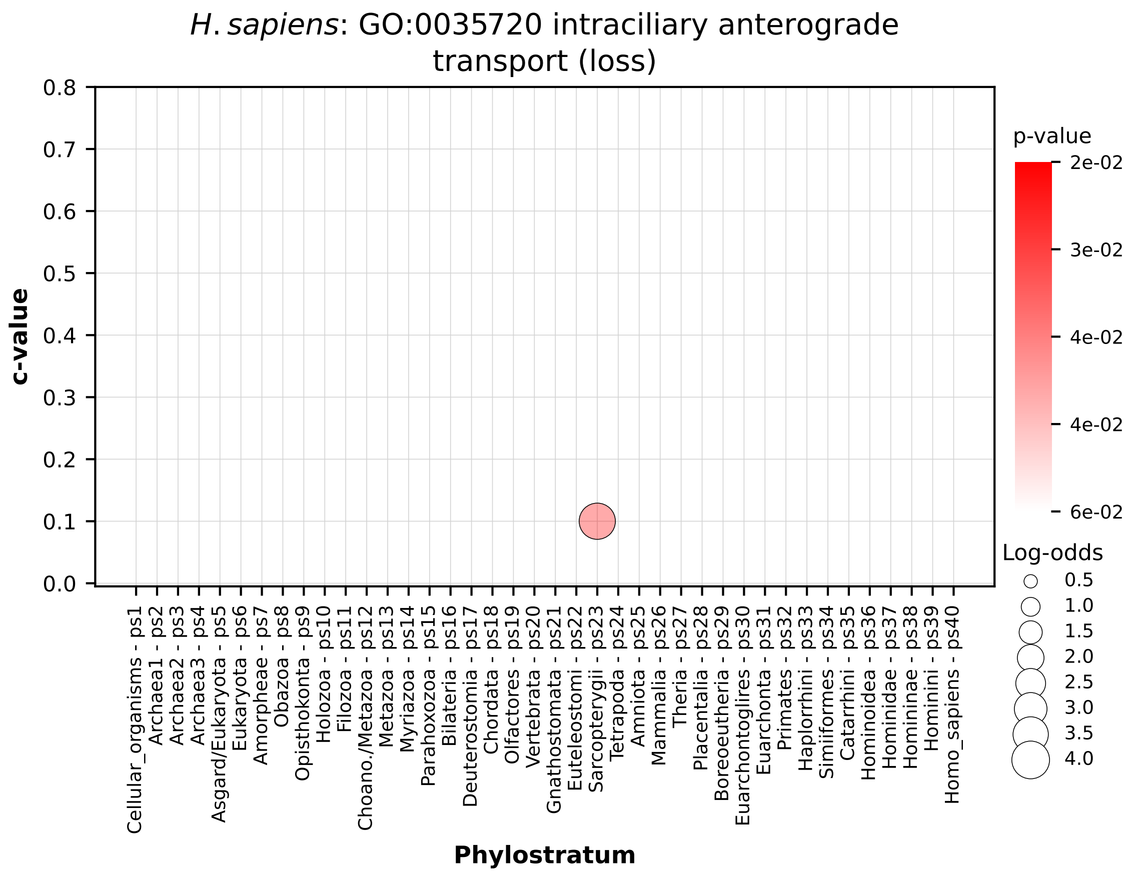

H. sapiens: GO:0035720 intraciliary anterograde transport (loss)
c-value
0.8
0.7
0.6
0.5
0.4
0.3
0.2
0.1
0.0
Phylostratum
Cellular_organisms - ps1
Archaea1 - ps2
Archaea2 - ps3
Archaea3 - ps4
Asgard/Eukaryota - ps5
Eukaryota - ps6
Amorpheae - ps7
Obazoa - ps8
Opisthokonta - ps9
Holozoa - ps10
Filozoa - ps11
Choano./Metazoa - ps12
Metazoa - ps13
Myriazoa - ps14
Parahoxozoa - ps15
Bilateria - ps16
Deuterostomia - ps17
Chordata - ps18
Olfactores - ps19
Vertebrata - ps20
Gnathostomata - ps21
Euteleostomi - ps22
Sarcopterygii - ps23
Tetrapoda - ps24
Amniota - ps25
Mammalia - ps26
Theria - ps27
Placentalia - ps28
Boreoeutheria - ps29
Euarchontoglires - ps30
Euarchonta - ps31
Primates - ps32
Haplorrhini - ps33
Simiiformes - ps34
Catarrhini - ps35
Hominoidea - ps36
Hominidae - ps37
Homininae - ps38
Hominini - ps39
Homo_sapiens - ps40
p-value
2e-02
3e-02
4e-02
4e-02
6e-02
Log-odds
0.5
1.0
1.5
2.0
2.5
3.0
3.5
4.0

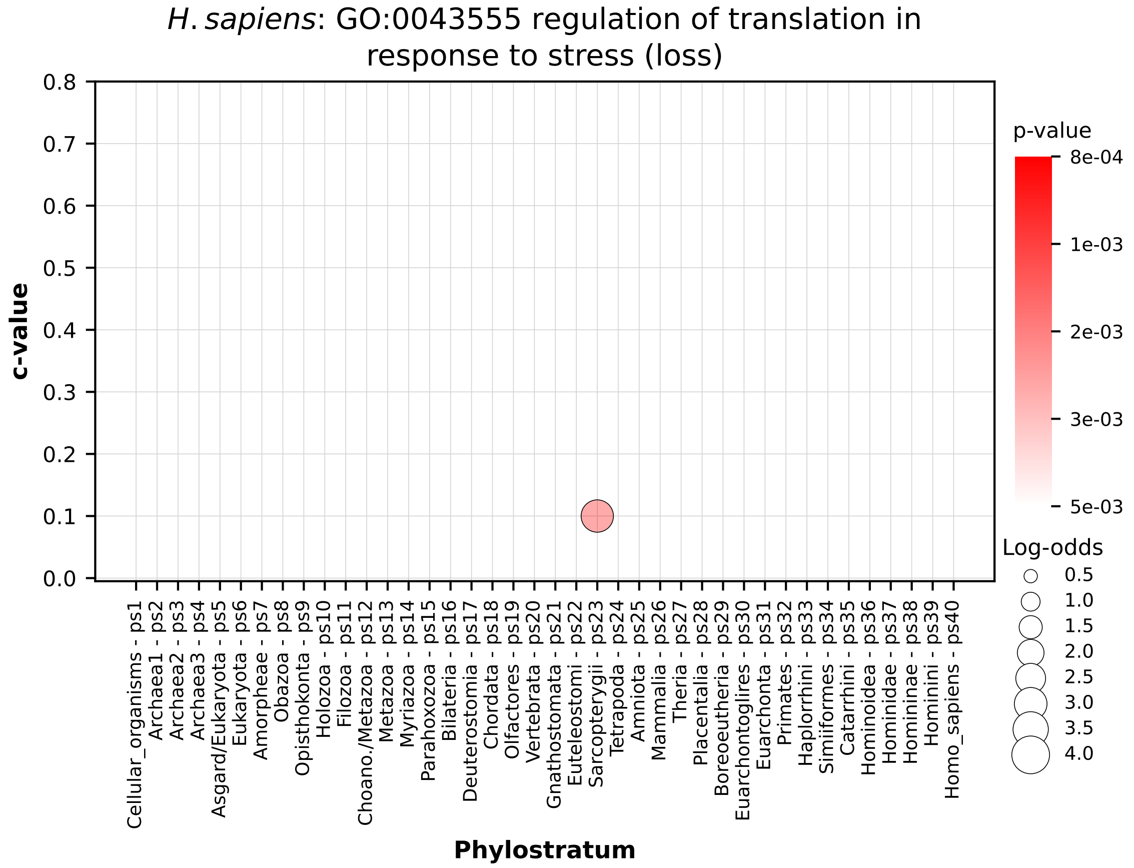

H. sapiens: GO:0043555 regulation of translation in response to stress (loss)
c-value
0.0
0.1
0.2
0.3
0.4
0.5
0.6
0.7
0.8
Phylostratum
Cellular_organisms - ps1
Archaea1 - ps2
Archaea2 - ps3
Archaea3 - ps4
Asgard/Eukaryota - ps5
Eukaryota - ps6
Amorpheae - ps7
Obazoa - ps8
Opisthokonta - ps9
Holozoa - ps10
Filozoa - ps11
Choano./Metazoa - ps12
Metazoa - ps13
Myriazoa - ps14
Parahoxozoa - ps15
Bilateria - ps16
Deuterostomia - ps17
Chordata - ps18
Olfactores - ps19
Vertebrata - ps20
Gnathostomata - ps21
Euteleostomi - ps22
Sarcopterygii - ps23
Tetrapoda - ps24
Amniota - ps25
Mammalia - ps26
Theria - ps27
Placentalia - ps28
Boreoeutheria - ps29
Euarchontoglires - ps30
Euarchonta - ps31
Primates - ps32
Haplorrhini - ps33
Simiiformes - ps34
Catarrhini - ps35
Hominoidea - ps36
Hominidae - ps37
Homininae - ps38
Hominini - ps39
Homo_sapiens - ps40
p-value
8e-04
1e-03
2e-03
3e-03
5e-03
Log-odds
0.5
1.0
1.5
2.0
2.5
3.0
3.5
4.0

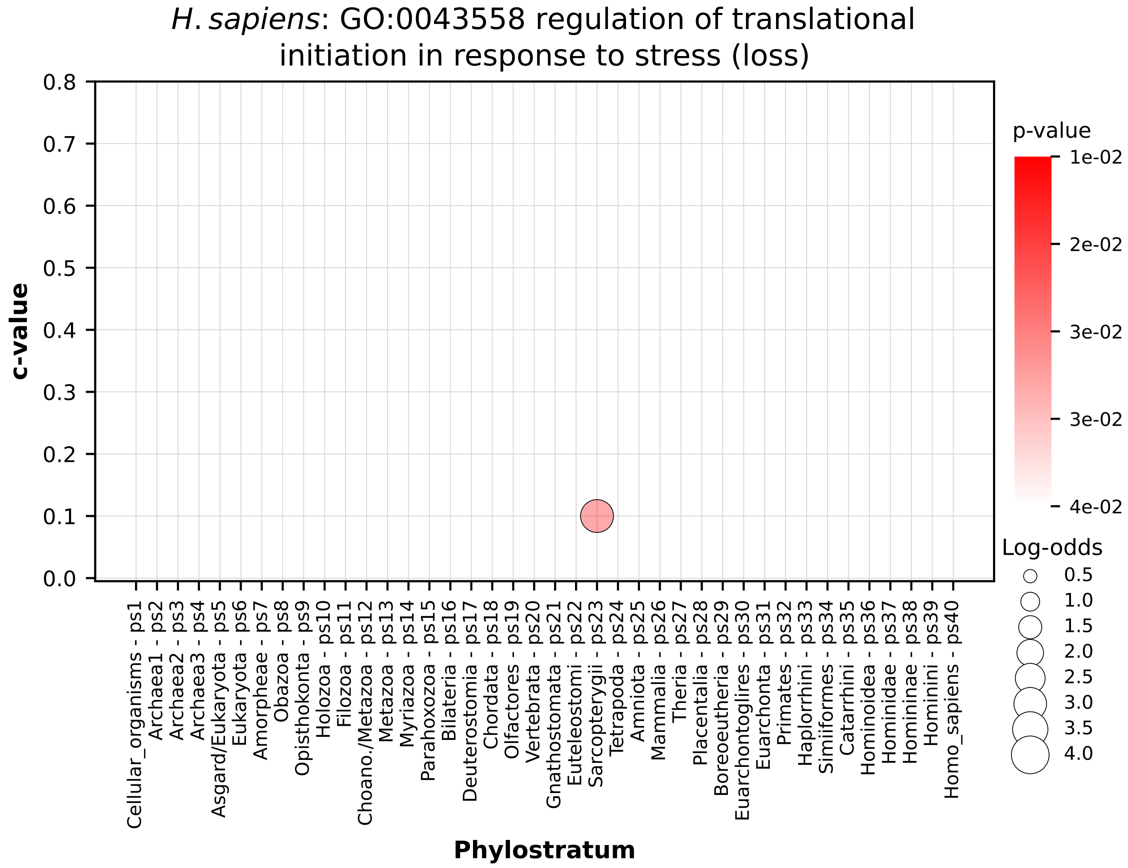

H. sapiens: GO:0043558 regulation of translational initiation in response to stress (loss)
c-value
0.0
0.1
0.2
0.3
0.4
0.5
0.6
0.7
0.8
Phylostratum
Cellular_organisms - ps1
Archaea1 - ps2
Archaea2 - ps3
Archaea3 - ps4
Asgard/Eukaryota - ps5
Eukaryota - ps6
Amorpheae - ps7
Obazoa - ps8
Opisthokonta - ps9
Holozoa - ps10
Filozoa - ps11
Choano./Metazoa - ps12
Metazoa - ps13
Myriazoa - ps14
Parahoxozoa - ps15
Bilateria - ps16
Deuterostomia - ps17
Chordata - ps18
Olfactores - ps19
Vertebrata - ps20
Gnathostomata - ps21
Euteleostomi - ps22
Sarcopterygii - ps23
Tetrapoda - ps24
Amniota - ps25
Mammalia - ps26
Theria - ps27
Placentalia - ps28
Boreoeutheria - ps29
Euarchontoglires - ps30
Euarchonta - ps31
Primates - ps32
Haplorrhini - ps33
Simiiformes - ps34
Catarrhini - ps35
Hominoidea - ps36
Hominidae - ps37
Homininae - ps38
Hominini - ps39
Homo_sapiens - ps40
p-value
1e-02
2e-02
3e-02
3e-02
4e-02
Log-odds
0.5
1.0
1.5
2.0
2.5
3.0
3.5
4.0

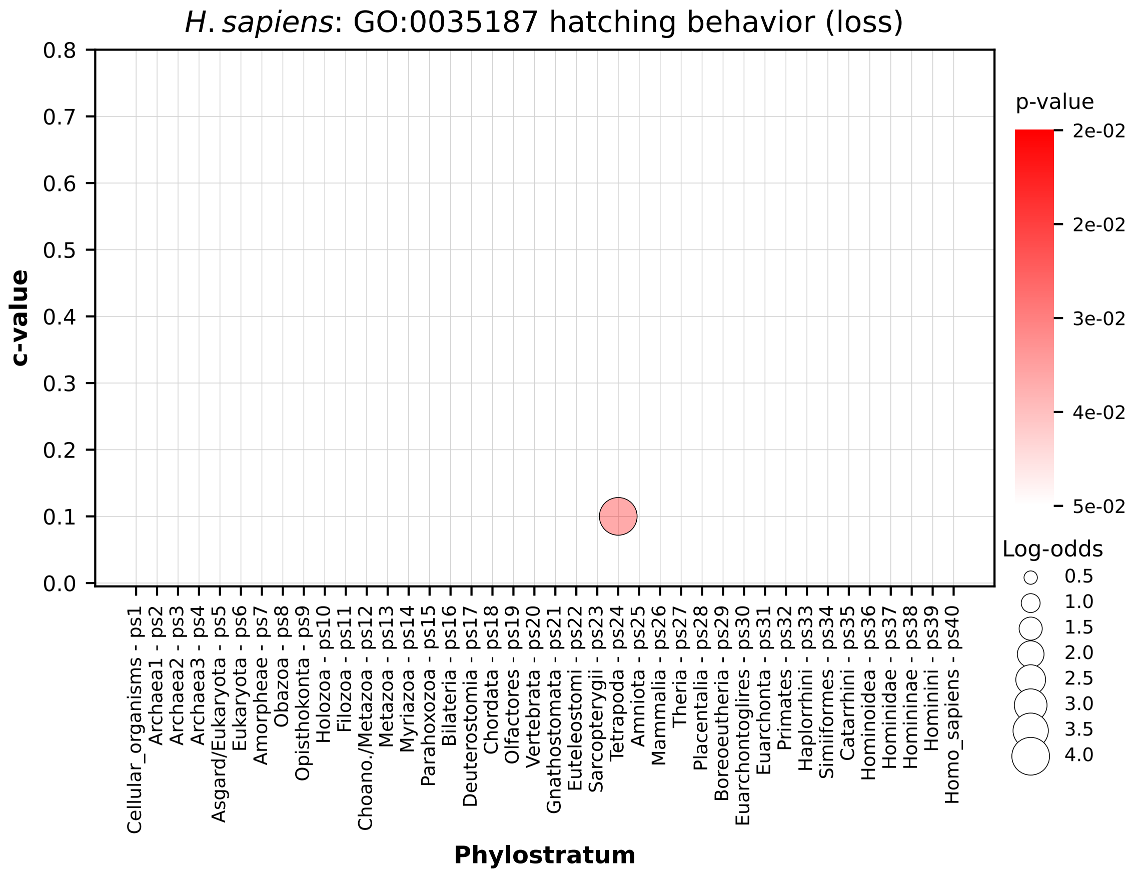

H. sapiens: GO:0035187 hatching behavior (loss)
c-value
0.8
0.7
0.6
0.5
0.4
0.3
0.2
0.1
0.0
p-value
2e-02
2e-02
3e-02
4e-02
5e-02
Log-odds
0.5
1.0
1.5
2.0
2.5
3.0
3.5
4.0
Cellular_organisms - ps1
Archaea1 - ps2
Archaea2 - ps3
Archaea3 - ps4
Asgard/Eukaryota - ps5
Eukaryota - ps6
Amorpheae - ps7
Obazoa - ps8
Opisthokonta - ps9
Holozoa - ps10
Filozoa - ps11
Choano./Metazoa - ps12
Metazoa - ps13
Myriazoa - ps14
Parahoxozoa - ps15
Bilateria - ps16
Deuterostomia - ps17
Chordata - ps18
Olfactores - ps19
Vertebrata - ps20
Gnathostomata - ps21
Euteleostomi - ps22
Sarcopterygii - ps23
Tetrapoda - ps24
Amniota - ps25
Mammalia - ps26
Theria - ps27
Placentalia - ps28
Boreoeutheria - ps29
Euarchontoglires - ps30
Euarchonta - ps31
Primates - ps32
Haplorrhini - ps33
Simiiformes - ps34
Catarrhini - ps35
Hominoidea - ps36
Hominidae - ps37
Homininae - ps38
Hominini - ps39
Homo_sapiens - ps40
Phylostratum

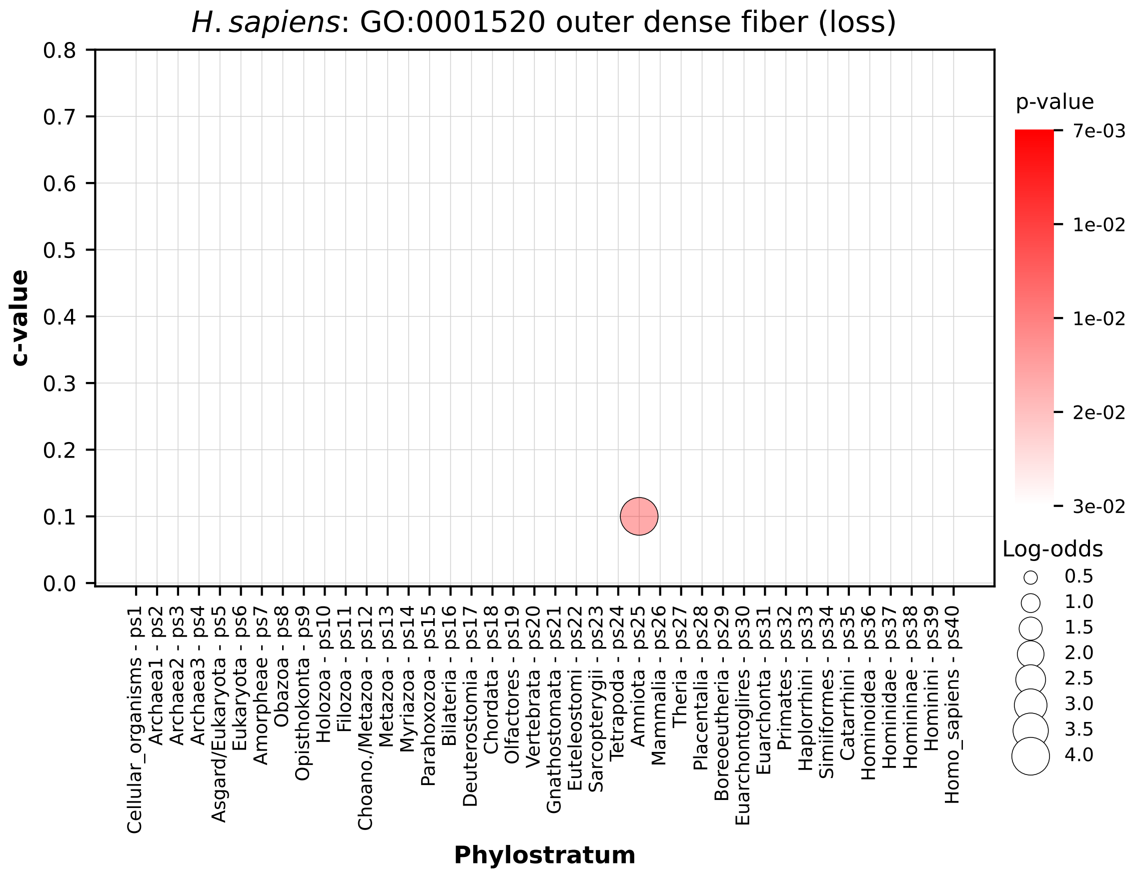

H. sapiens: GO:0001520 outer dense fiber (loss)
c-value
0.0
0.1
0.2
0.3
0.4
0.5
0.6
0.7
0.8
Phylostratum
Cellular_organisms - ps1
Archaea1 - ps2
Archaea2 - ps3
Archaea3 - ps4
Asgard/Eukaryota - ps5
Eukaryota - ps6
Amorpheae - ps7
Obazoa - ps8
Opisthokonta - ps9
Holozoa - ps10
Filozoa - ps11
Choano./Metazoa - ps12
Metazoa - ps13
Myriazoa - ps14
Parahoxozoa - ps15
Bilateria - ps16
Deuterostomia - ps17
Chordata - ps18
Olfactores - ps19
Vertebrata - ps20
Gnathostomata - ps21
Euteleostomi - ps22
Sarcopterygii - ps23
Tetrapoda - ps24
Amniota - ps25
Mammalia - ps26
Theria - ps27
Placentalia - ps28
Boreoeutheria - ps29
Euarchontoglires - ps30
Euarchonta - ps31
Primates - ps32
Haplorrhini - ps33
Simiiformes - ps34
Catarrhini - ps35
Hominoidea - ps36
Hominidae - ps37
Homininae - ps38
Hominini - ps39
Homo_sapiens - ps40
p-value
7e-03
1e-02
1e-02
2e-02
3e-02
Log-odds
0.5
1.0
1.5
2.0
2.5
3.0
3.5
4.0

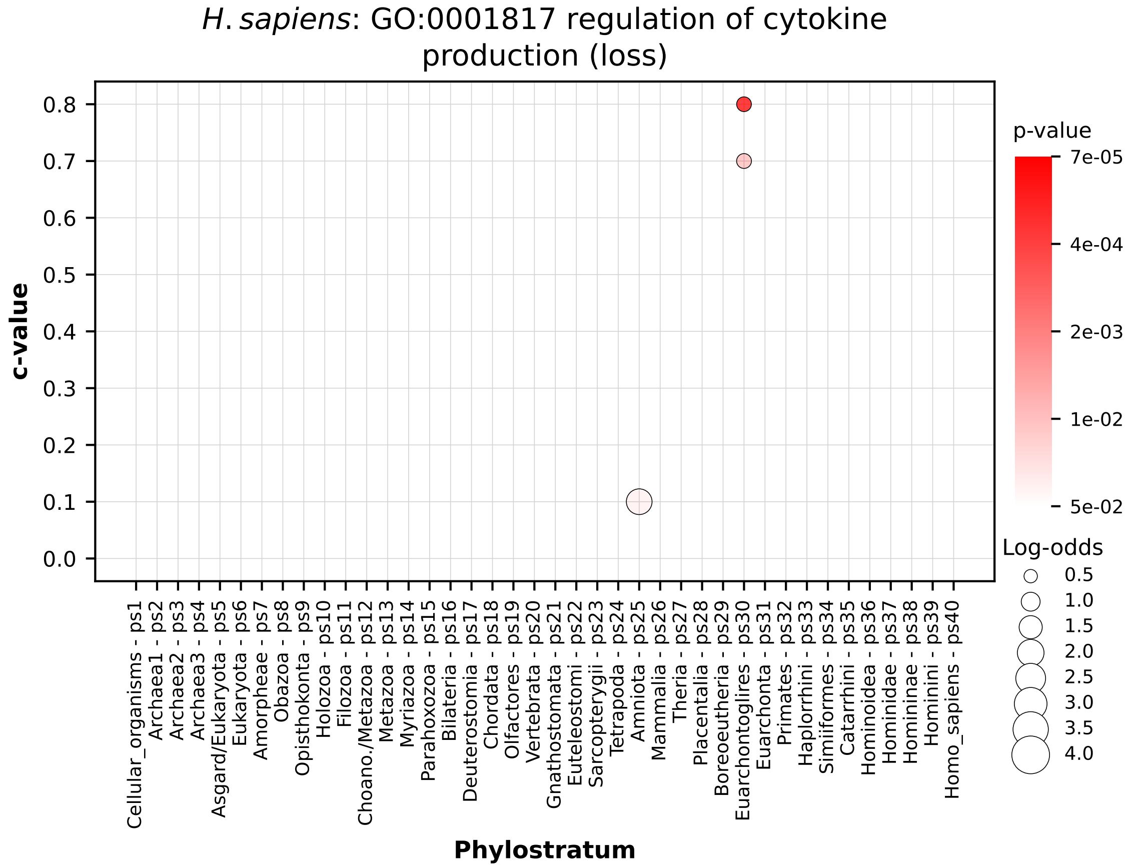
H. sapiens: GO:0001817 regulation of cytokine production (loss)
c-value
0.8
0.7
0.6
0.5
0.4
0.3
0.2
0.1
0.0
Phylostratum
Cellular_organisms - ps1
Archaea1 - ps2
Archaea2 - ps3
Archaea3 - ps4
Asgard/Eukaryota - ps5
Eukaryota - ps6
Amorpheae - ps7
Obazoa - ps8
Opisthokonta - ps9
Holozoa - ps10
Filozoa - ps11
Choano./Metazoa - ps12
Metazoa - ps13
Myriazoa - ps14
Parahoxozoa - ps15
Bilateria - ps16
Deuterostomia - ps17
Chordata - ps18
Olfactores - ps19
Vertebrata - ps20
Gnathostomata - ps21
Euteleostomi - ps22
Sarcopterygii - ps23
Tetrapoda - ps24
Amniota - ps25
Mammalia - ps26
Theria - ps27
Placentalia - ps28
Boreoeutheria - ps29
Euarchontoglires - ps30
Euarchonta - ps31
Primates - ps32
Haplorrhini - ps33
Simiiformes - ps34
Catarrhini - ps35
Hominoidea - ps36
Hominidae - ps37
Homininae - ps38
Hominini - ps39
Homo_sapiens - ps40
p-value
7e-05
4e-04
2e-03
1e-02
5e-02
Log-odds
0.5
1.0
1.5
2.0
2.5
3.0
3.5
4.0

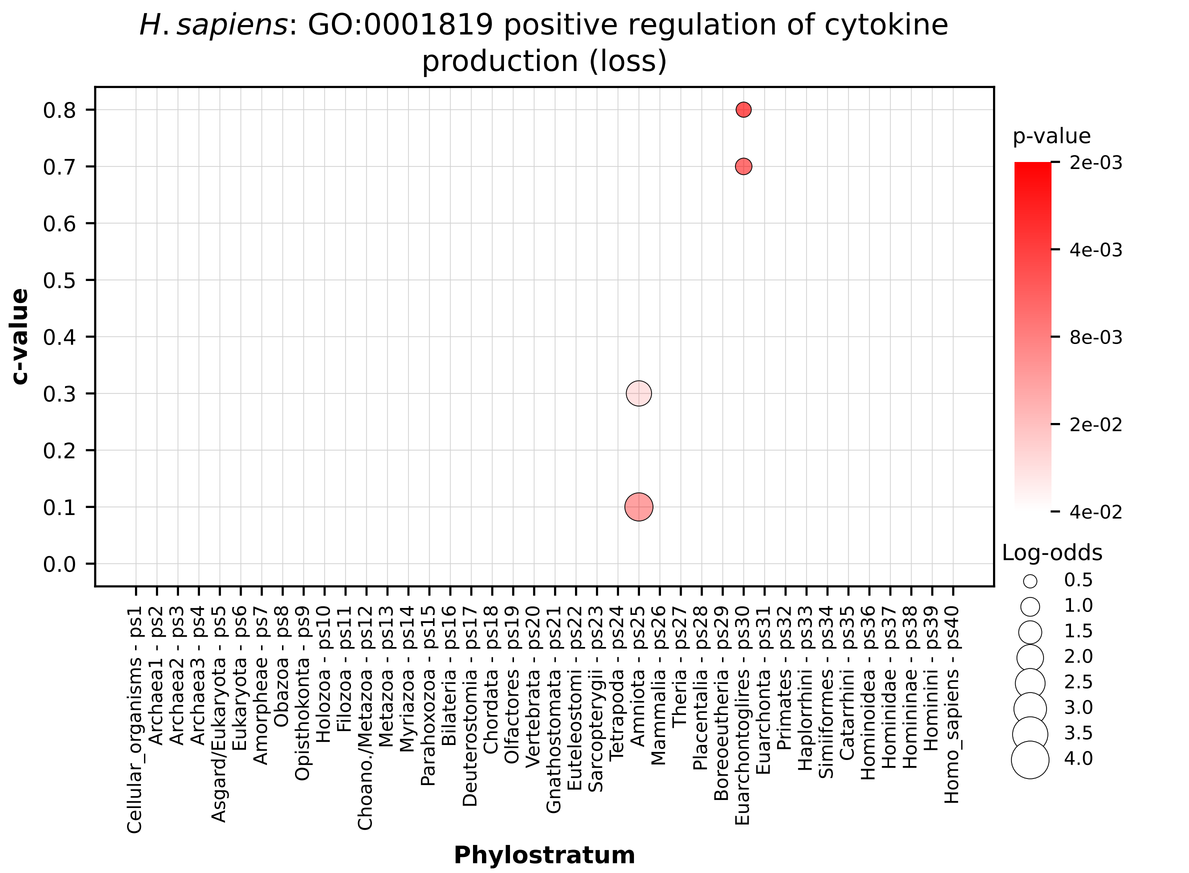

H. sapiens: GO:0001819 positive regulation of cytokine production (loss)
c-value
0.0
0.1
0.2
0.3
0.4
0.5
0.6
0.7
0.8
Phylostratum
Cellular_organisms - ps1
Archaea1 - ps2
Archaea2 - ps3
Archaea3 - ps4
Asgard/Eukaryota - ps5
Eukaryota - ps6
Amorpheae - ps7
Obazoa - ps8
Opisthokonta - ps9
Holozoa - ps10
Filozoa - ps11
Choano./Metazoa - ps12
Metazoa - ps13
Myriazoa - ps14
Parahoxozoa - ps15
Bilateria - ps16
Deuterostomia - ps17
Chordata - ps18
Olfactores - ps19
Vertebrata - ps20
Gnathostomata - ps21
Euteleostomi - ps22
Sarcopterygii - ps23
Tetrapoda - ps24
Amniota - ps25
Mammalia - ps26
Theria - ps27
Placentalia - ps28
Boreoeutheria - ps29
Euarchontoglires - ps30
Euarchonta - ps31
Primates - ps32
Haplorrhini - ps33
Simiiformes - ps34
Catarrhini - ps35
Hominoidea - ps36
Hominidae - ps37
Homininae - ps38
Hominini - ps39
Homo_sapiens - ps40
p-value
2e-03
4e-03
8e-03
2e-02
4e-02
Log-odds
0.5
1.0
1.5
2.0
2.5
3.0
3.5
4.0

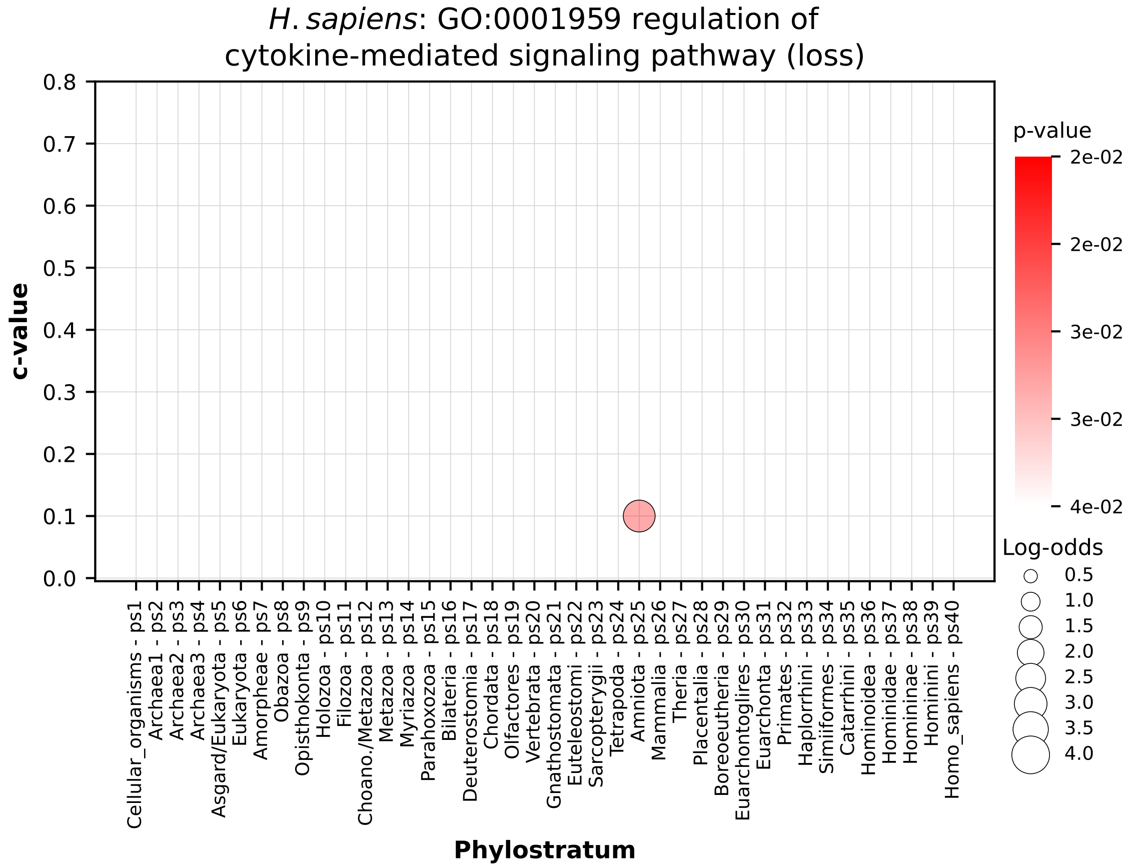

H. sapiens: GO:0001959 regulation of cytokine-mediated signaling pathway (loss)
c-value
0.0
0.1
0.2
0.3
0.4
0.5
0.6
0.7
0.8
Phylostratum
Cellular_organisms - ps1
Archaea1 - ps2
Archaea2 - ps3
Archaea3 - ps4
Asgard/Eukaryota - ps5
Eukaryota - ps6
Amorpheae - ps7
Obazoa - ps8
Opisthokonta - ps9
Holozoa - ps10
Filozoa - ps11
Choano./Metazoa - ps12
Metazoa - ps13
Myriazoa - ps14
Parahoxozoa - ps15
Bilateria - ps16
Deuterostomia - ps17
Chordata - ps18
Olfactores - ps19
Vertebrata - ps20
Gnathostomata - ps21
Euteleostomi - ps22
Sarcopterygii - ps23
Tetrapoda - ps24
Amniota - ps25
Mammalia - ps26
Theria - ps27
Placentalia - ps28
Boreoeutheria - ps29
Euarchontoglires - ps30
Euarchonta - ps31
Primates - ps32
Haplorrhini - ps33
Simiiformes - ps34
Catarrhini - ps35
Hominoidea - ps36
Hominidae - ps37
Homininae - ps38
Hominini - ps39
Homo_sapiens - ps40
p-value
2e-02
2e-02
3e-02
3e-02
4e-02
Log-odds
0.5
1.0
1.5
2.0
2.5
3.0
3.5
4.0

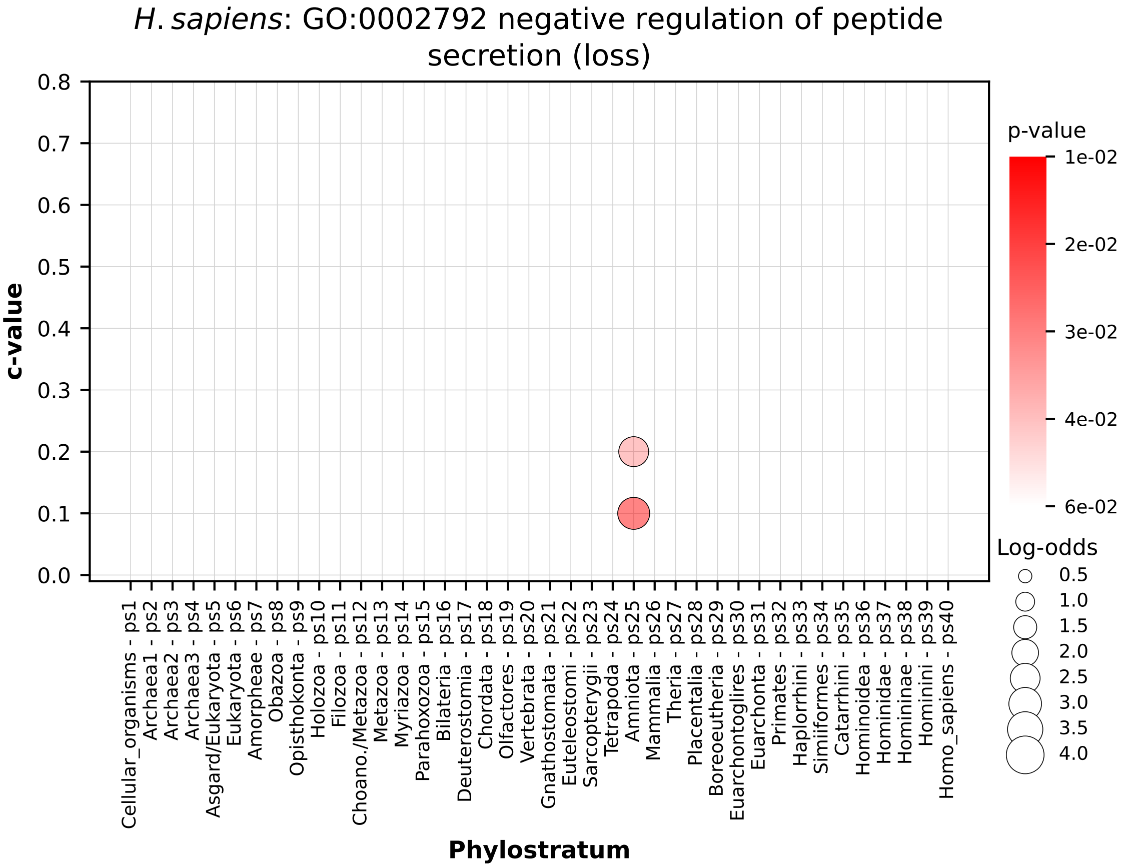

H. sapiens: GO:0002792 negative regulation of peptide secretion (loss)
c-value
0.0
0.1
0.2
0.3
0.4
0.5
0.6
0.7
0.8
Phylostratum
Cellular_organisms - ps1
Archaea1 - ps2
Archaea2 - ps3
Archaea3 - ps4
Asgard/Eukaryota - ps5
Eukaryota - ps6
Amorpheae - ps7
Obazoa - ps8
Opisthokonta - ps9
Holozoa - ps10
Filozoa - ps11
Choano./Metazoa - ps12
Metazoa - ps13
Myriazoa - ps14
Parahoxozoa - ps15
Bilateria - ps16
Deuterostomia - ps17
Chordata - ps18
Olfactores - ps19
Vertebrata - ps20
Gnathostomata - ps21
Euteleostomi - ps22
Sarcopterygii - ps23
Tetrapoda - ps24
Amniota - ps25
Mammalia - ps26
Theria - ps27
Placentalia - ps28
Boreoeutheria - ps29
Euarchontoglires - ps30
Euarchonta - ps31
Primates - ps32
Haplorrhini - ps33
Simiiformes - ps34
Catarrhini - ps35
Hominoidea - ps36
Hominidae - ps37
Homininae - ps38
Hominini - ps39
Homo_sapiens - ps40
p-value
1e-02
2e-02
3e-02
4e-02
6e-02
Log-odds
0.5
1.0
1.5
2.0
2.5
3.0
3.5
4.0

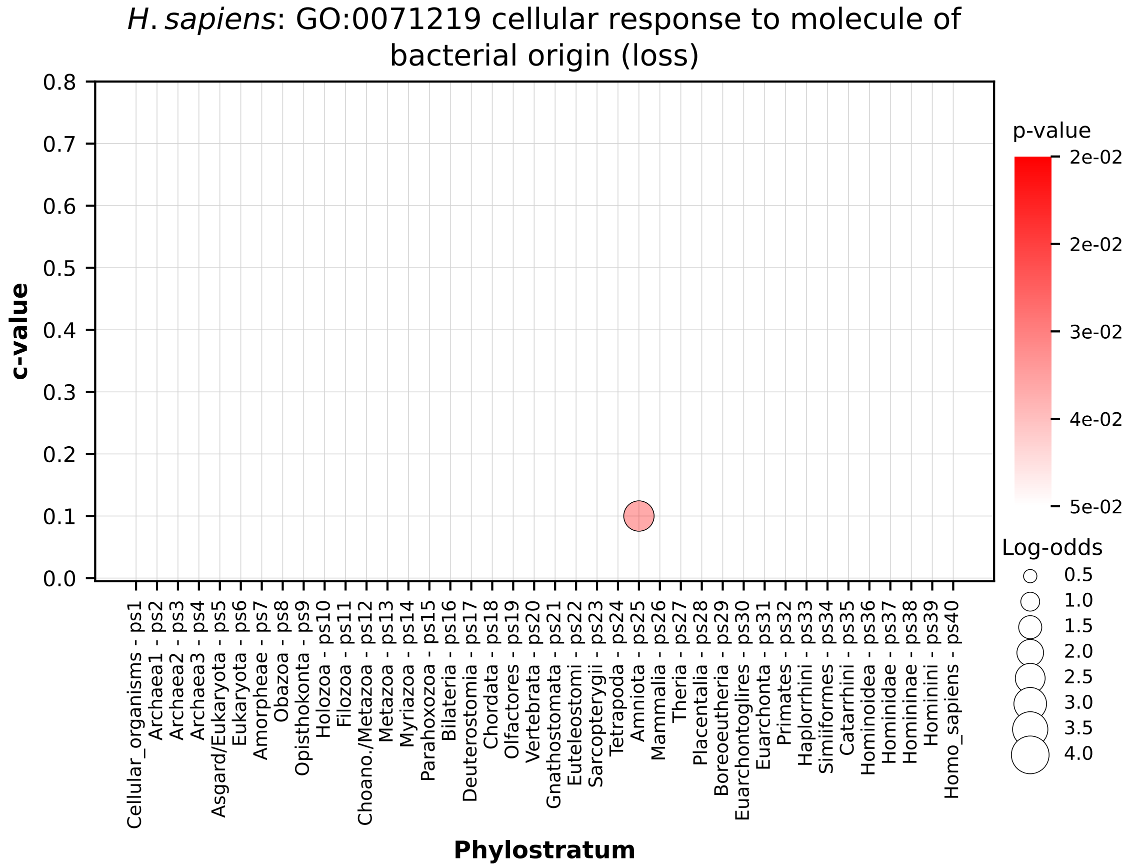
H. sapiens: GO:0071219 cellular response to molecule of bacterial origin (loss)
c-value
0.0
0.1
0.2
0.3
0.4
0.5
0.6
0.7
0.8
Phylostratum
Cellular_organisms - ps1
Archaea1 - ps2
Archaea2 - ps3
Archaea3 - ps4
Asgard/Eukaryota - ps5
Eukaryota - ps6
Amorpheae - ps7
Obazoa - ps8
Opisthokonta - ps9
Holozoa - ps10
Filozoa - ps11
Choano./Metazoa - ps12
Metazoa - ps13
Myriazoa - ps14
Parahoxozoa - ps15
Bilateria - ps16
Deuterostomia - ps17
Chordata - ps18
Olfactores - ps19
Vertebrata - ps20
Gnathostomata - ps21
Euteleostomi - ps22
Sarcopterygii - ps23
Tetrapoda - ps24
Amniota - ps25
Mammalia - ps26
Theria - ps27
Placentalia - ps28
Boreoeutheria - ps29
Euarchontoglires - ps30
Euarchonta - ps31
Primates - ps32
Haplorrhini - ps33
Simiiformes - ps34
Catarrhini - ps35
Hominoidea - ps36
Hominidae - ps37
Homininae - ps38
Hominini - ps39
Homo_sapiens - ps40
p-value
2e-02
2e-02
3e-02
4e-02
5e-02
Log-odds
0.5
1.0
1.5
2.0
2.5
3.0
3.5
4.0

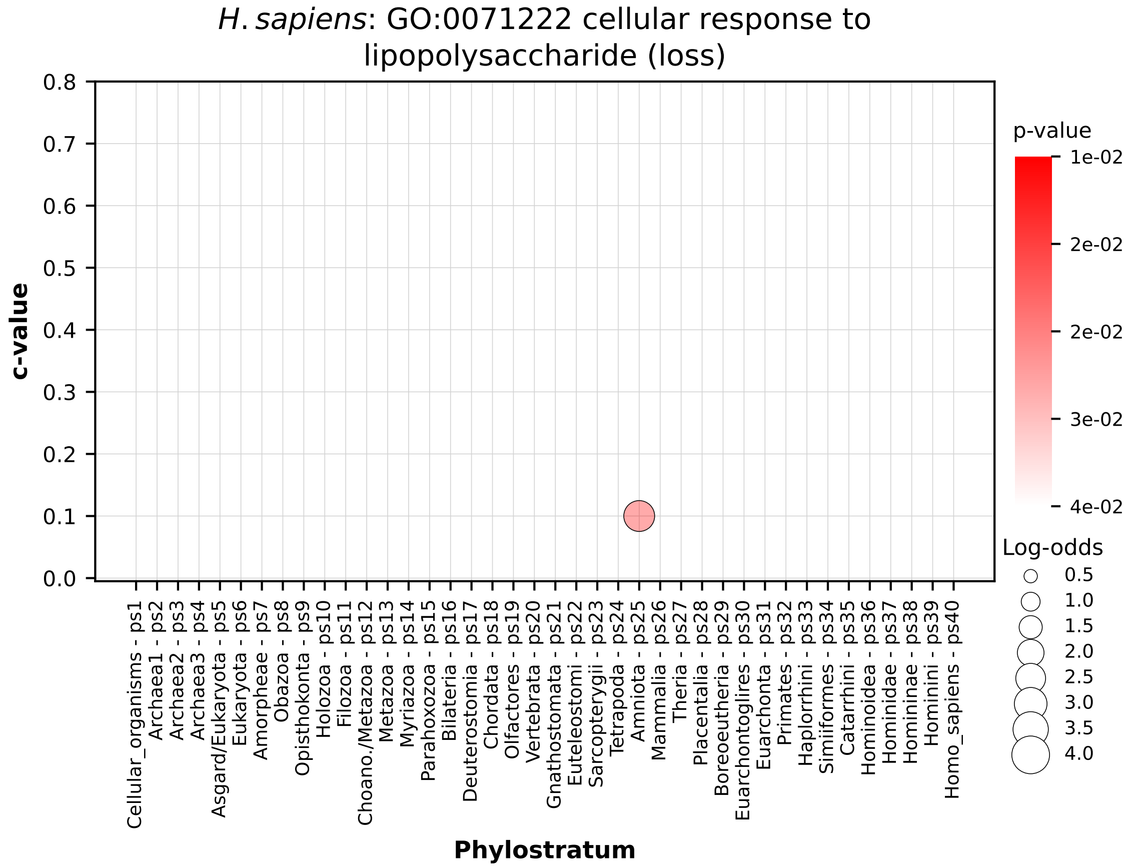

H. sapiens: GO:0071222 cellular response to lipopolysaccharide (loss)
c-value
0.0
0.1
0.2
0.3
0.4
0.5
0.6
0.7
0.8
Phylostratum
Cellular_organisms - ps1
Archaea1 - ps2
Archaea2 - ps3
Archaea3 - ps4
Asgard/Eukaryota - ps5
Eukaryota - ps6
Amorpheae - ps7
Obazoa - ps8
Opisthokonta - ps9
Holozoa - ps10
Filozoa - ps11
Choano./Metazoa - ps12
Metazoa - ps13
Myriazoa - ps14
Parahoxozoa - ps15
Bilateria - ps16
Deuterostomia - ps17
Chordata - ps18
Olfactores - ps19
Vertebrata - ps20
Gnathostomata - ps21
Euteleostomi - ps22
Sarcopterygii - ps23
Tetrapoda - ps24
Amniota - ps25
Mammalia - ps26
Theria - ps27
Placentalia - ps28
Boreoeutheria - ps29
Euarchontoglires - ps30
Euarchonta - ps31
Primates - ps32
Haplorrhini - ps33
Simiiformes - ps34
Catarrhini - ps35
Hominoidea - ps36
Hominidae - ps37
Homininae - ps38
Hominini - ps39
Homo_sapiens - ps40
p-value
1e-02
2e-02
2e-02
3e-02
4e-02
Log-odds
0.5
1.0
1.5
2.0
2.5
3.0
3.5
4.0

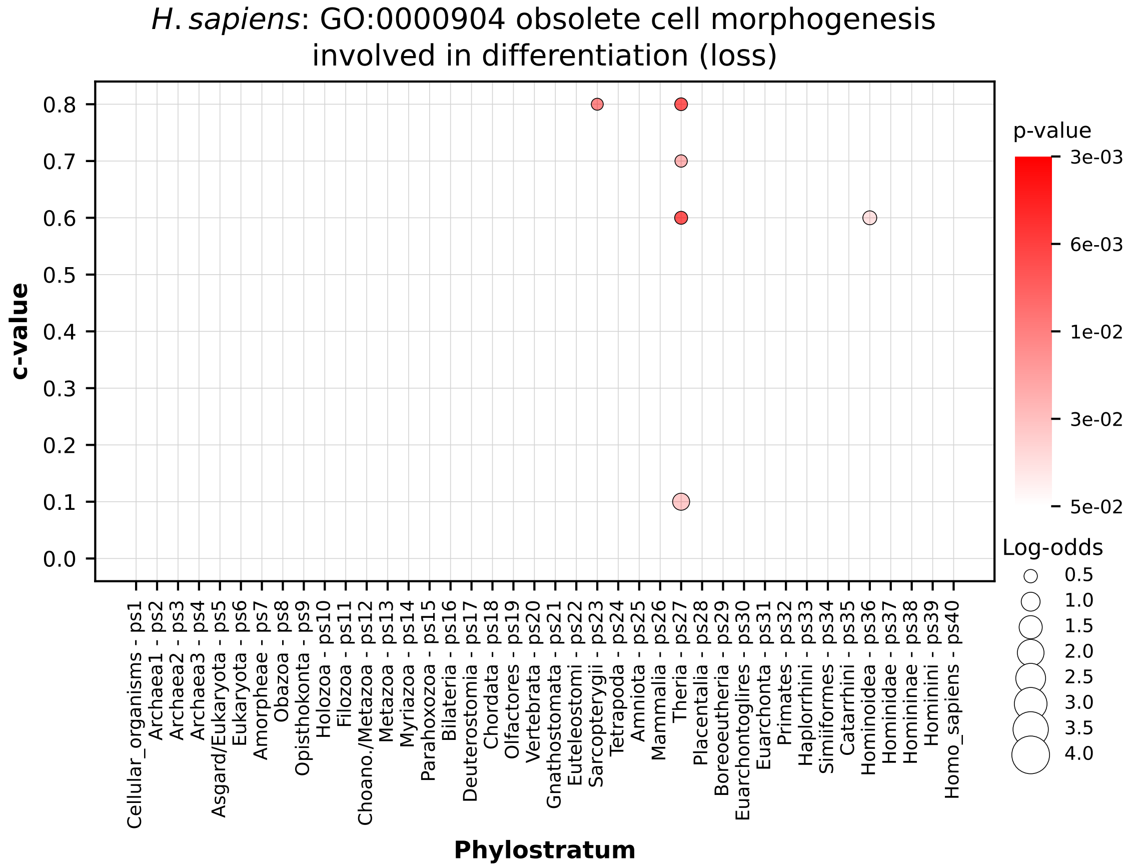

H. sapiens: GO:0000904 obsolete cell morphogenesis involved in differentiation (loss)
c-value
0.0
0.1
0.2
0.3
0.4
0.5
0.6
0.7
0.8
Phylostratum
Cellular_organisms - ps1
Archaea1 - ps2
Archaea2 - ps3
Archaea3 - ps4
Asgard/Eukaryota - ps5
Eukaryota - ps6
Amorpheae - ps7
Obazoa - ps8
Opisthokonta - ps9
Holozoa - ps10
Filozoa - ps11
Choano./Metazoa - ps12
Metazoa - ps13
Myriazoa - ps14
Parahoxozoa - ps15
Bilateria - ps16
Deuterostomia - ps17
Chordata - ps18
Olfactores - ps19
Vertebrata - ps20
Gnathostomata - ps21
Euteleostomi - ps22
Sarcopterygii - ps23
Tetrapoda - ps24
Amniota - ps25
Mammalia - ps26
Theria - ps27
Placentalia - ps28
Boreoeutheria - ps29
Euarchontoglires - ps30
Euarchonta - ps31
Primates - ps32
Haplorrhini - ps33
Simiiformes - ps34
Catarrhini - ps35
Hominoidea - ps36
Hominidae - ps37
Homininae - ps38
Hominini - ps39
Homo_sapiens - ps40
p-value
3e-03
6e-03
1e-02
3e-02
5e-02
Log-odds
0.5
1.0
1.5
2.0
2.5
3.0
3.5
4.0

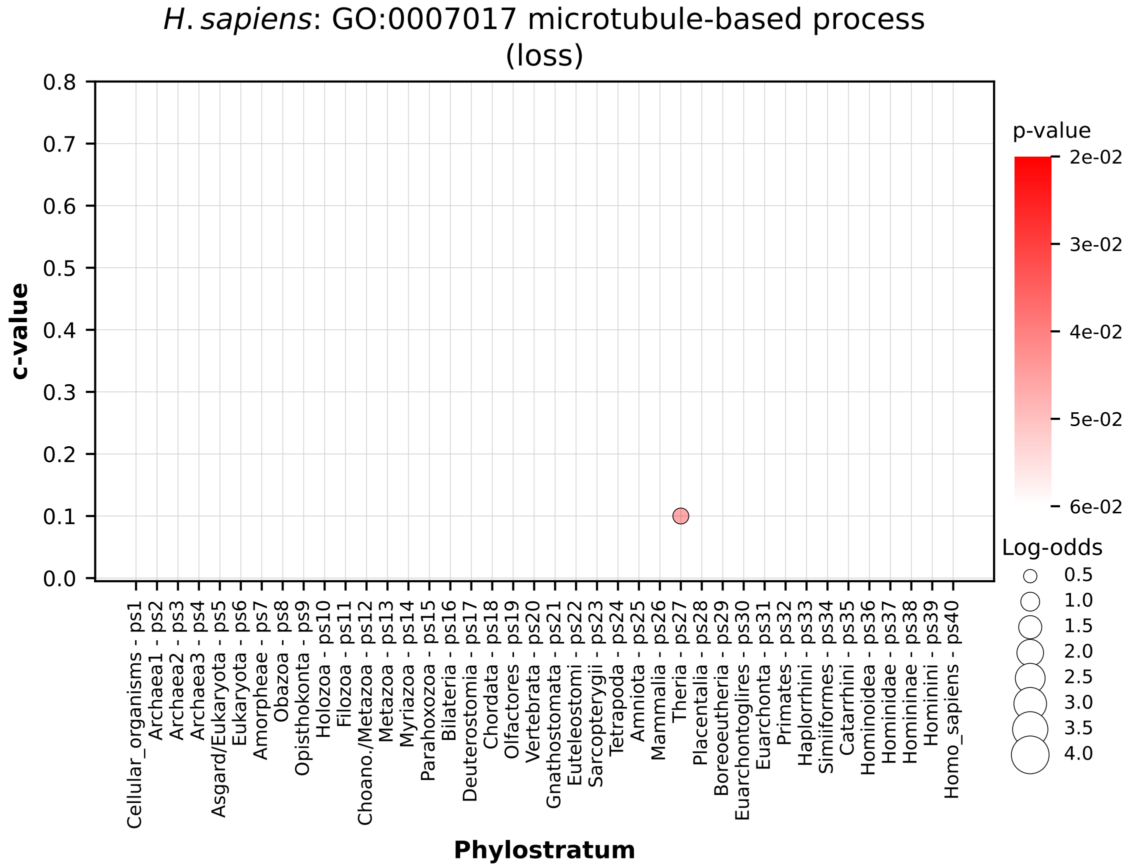

H. sapiens: GO:0007017 microtubule-based process
(loss)
c-value
0.0
0.1
0.2
0.3
0.4
0.5
0.6
0.7
0.8
Phylostratum
Cellular_organisms - ps1
Archaea1 - ps2
Archaea2 - ps3
Archaea3 - ps4
Asgard/Eukaryota - ps5
Eukaryota - ps6
Amorpheae - ps7
Obazoa - ps8
Opisthokonta - ps9
Holozoa - ps10
Filozoa - ps11
Choano./Metazoa - ps12
Metazoa - ps13
Myriazoa - ps14
Parahoxozoa - ps15
Bilateria - ps16
Deuterostomia - ps17
Chordata - ps18
Olfactores - ps19
Vertebrata - ps20
Gnathostomata - ps21
Euteleostomi - ps22
Sarcopterygii - ps23
Tetrapoda - ps24
Amniota - ps25
Mammalia - ps26
Theria - ps27
Placentalia - ps28
Boreoeutheria - ps29
Euarchontoglires - ps30
Euarchonta - ps31
Primates - ps32
Haplorrhini - ps33
Simiiformes - ps34
Catarrhini - ps35
Hominoidea - ps36
Hominidae - ps37
Homininae - ps38
Hominini - ps39
Homo_sapiens - ps40
p-value
2e-02
3e-02
4e-02
5e-02
6e-02
Log-odds
0.5
1.0
1.5
2.0
2.5
3.0
3.5
4.0

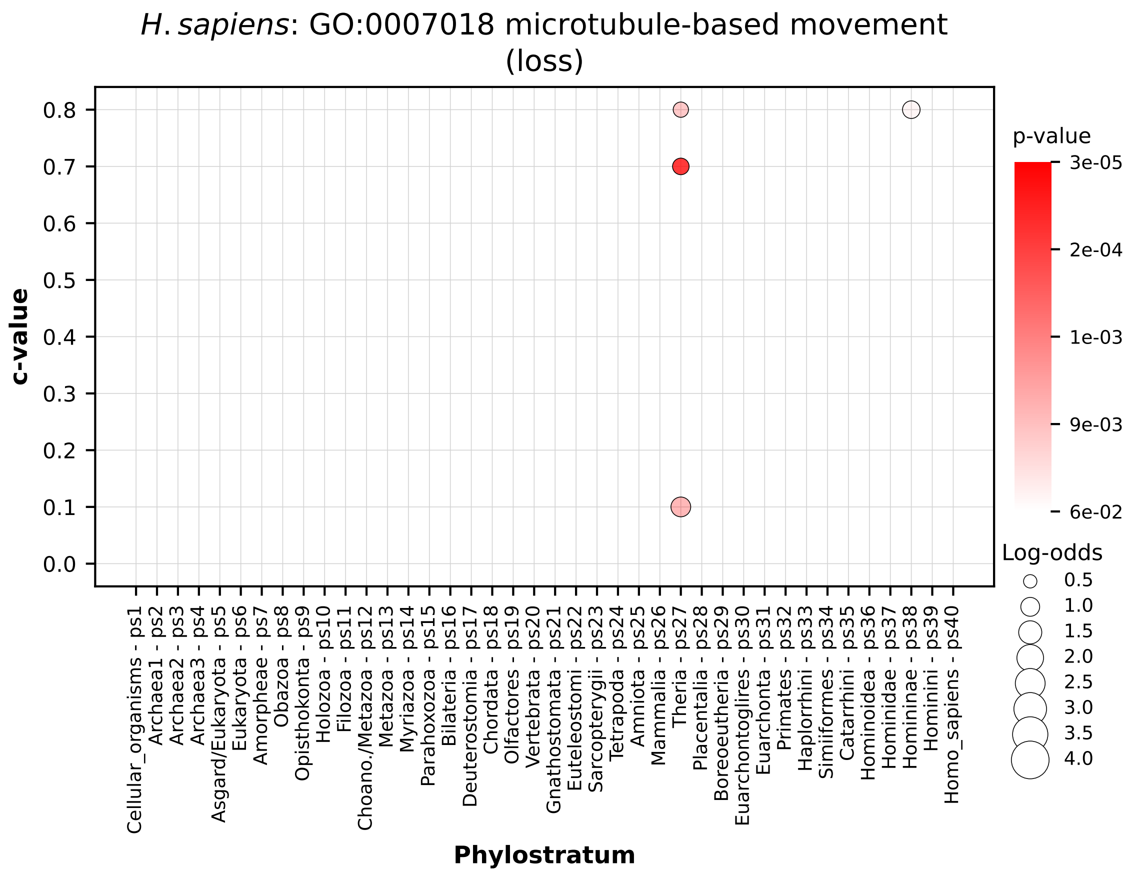

H. sapiens: GO:0007018 microtubule-based movement
(loss)
c-value
0.0
0.1
0.2
0.3
0.4
0.5
0.6
0.7
0.8
Phylostratum
Cellular_organisms - ps1
Archaea1 - ps2
Archaea2 - ps3
Archaea3 - ps4
Asgard/Eukaryota - ps5
Eukaryota - ps6
Amorpheae - ps7
Obazoa - ps8
Opisthokonta - ps9
Holozoa - ps10
Filozoa - ps11
Choano./Metazoa - ps12
Metazoa - ps13
Myriazoa - ps14
Parahoxozoa - ps15
Bilateria - ps16
Deuterostomia - ps17
Chordata - ps18
Olfactores - ps19
Vertebrata - ps20
Gnathostomata - ps21
Euteleostomi - ps22
Sarcopterygii - ps23
Tetrapoda - ps24
Amniota - ps25
Mammalia - ps26
Theria - ps27
Placentalia - ps28
Boreoeutheria - ps29
Euarchontoglires - ps30
Euarchonta - ps31
Primates - ps32
Haplorrhini - ps33
Simiiformes - ps34
Catarrhini - ps35
Hominoidea - ps36
Hominidae - ps37
Homininae - ps38
Hominini - ps39
Homo_sapiens - ps40
p-value
3e-05
2e-04
1e-03
9e-03
6e-02
Log-odds
0.5
1.0
1.5
2.0
2.5
3.0
3.5
4.0

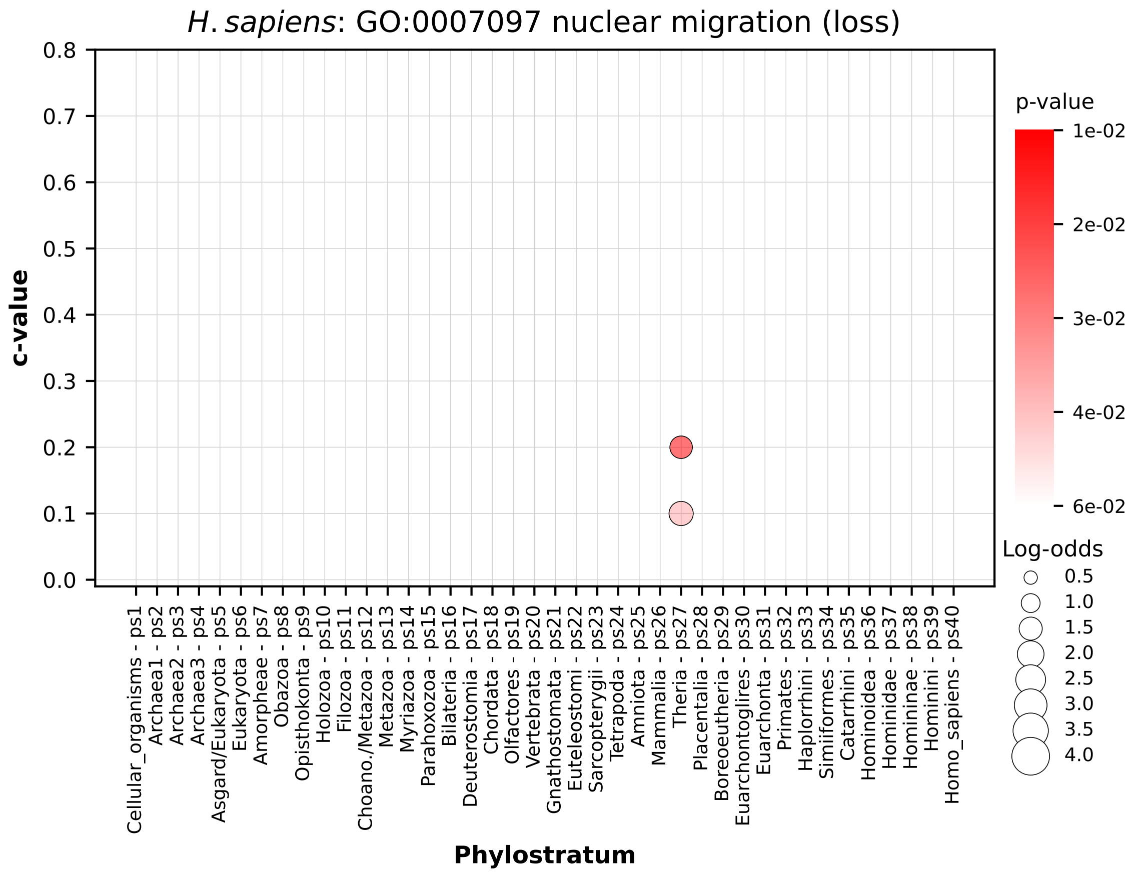

H. sapiens: GO:0007097 nuclear migration (loss)
c-value
0.0
0.1
0.2
0.3
0.4
0.5
0.6
0.7
0.8
Phylostratum
Cellular_organisms - ps1
Archaea1 - ps2
Archaea2 - ps3
Archaea3 - ps4
Asgard/Eukaryota - ps5
Eukaryota - ps6
Amorpheae - ps7
Obazoa - ps8
Opisthokonta - ps9
Holozoa - ps10
Filozoa - ps11
Choano./Metazoa - ps12
Metazoa - ps13
Myriazoa - ps14
Parahoxozoa - ps15
Bilateria - ps16
Deuterostomia - ps17
Chordata - ps18
Olfactores - ps19
Vertebrata - ps20
Gnathostomata - ps21
Euteleostomi - ps22
Sarcopterygii - ps23
Tetrapoda - ps24
Amniota - ps25
Mammalia - ps26
Theria - ps27
Placentalia - ps28
Boreoeutheria - ps29
Euarchontoglires - ps30
Euarchonta - ps31
Primates - ps32
Haplorrhini - ps33
Simiiformes - ps34
Catarrhini - ps35
Hominoidea - ps36
Hominidae - ps37
Homininae - ps38
Hominini - ps39
Homo_sapiens - ps40
p-value
1e-02
2e-02
3e-02
4e-02
6e-02
Log-odds
0.5
1.0
1.5
2.0
2.5
3.0
3.5
4.0

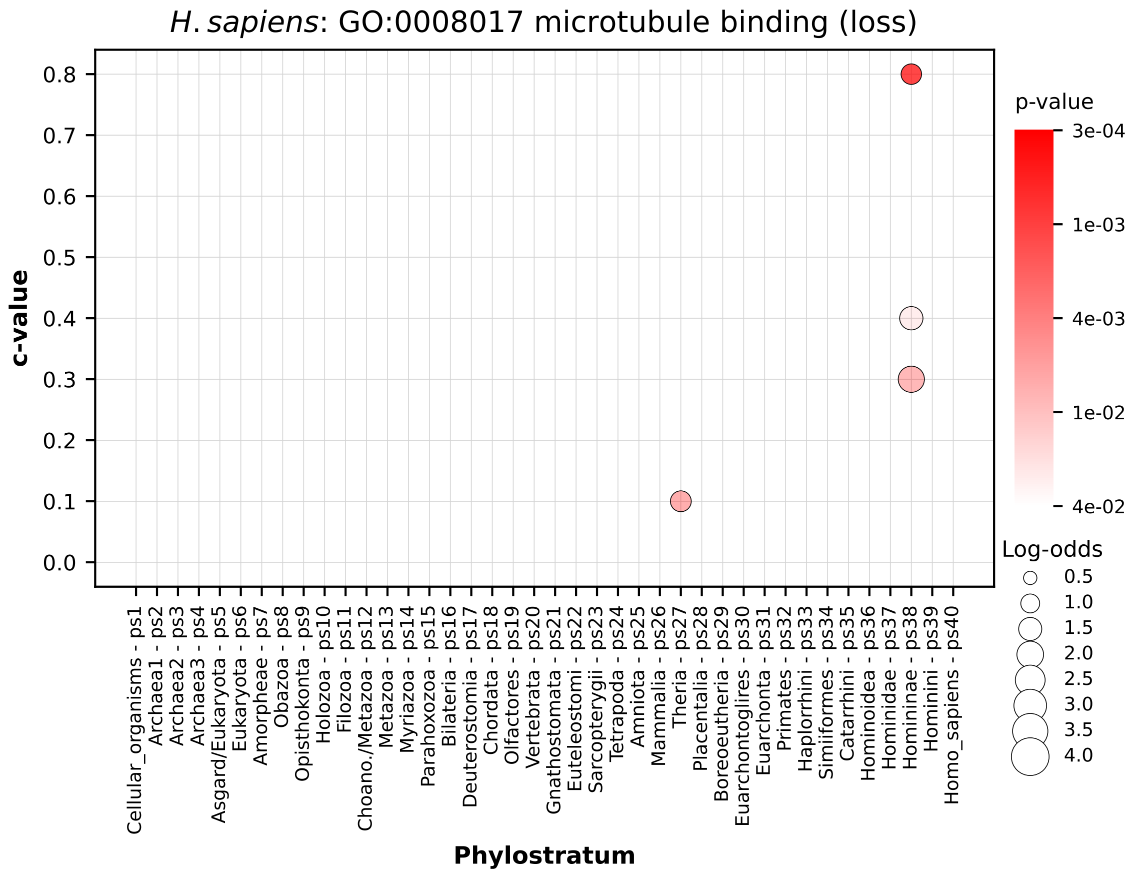

H. sapiens: GO:0008017 microtubule binding (loss)
c-value
0.8
0.7
0.6
0.5
0.4
0.3
0.2
0.1
0.0
p-value
3e-04
1e-03
4e-03
1e-02
4e-02
Log-odds
0.5
1.0
1.5
2.0
2.5
3.0
3.5
4.0
Cellular_organisms - ps1
Archaea1 - ps2
Archaea2 - ps3
Archaea3 - ps4
Asgard/Eukaryota - ps5
Eukaryota - ps6
Amorpheae - ps7
Obazoa - ps8
Opisthokonta - ps9
Holozoa - ps10
Filozoa - ps11
Choano./Metazoa - ps12
Metazoa - ps13
Myriazoa - ps14
Parahoxozoa - ps15
Bilateria - ps16
Deuterostomia - ps17
Chordata - ps18
Olfactores - ps19
Vertebrata - ps20
Gnathostomata - ps21
Euteleostomi - ps22
Sarcopterygii - ps23
Tetrapoda - ps24
Amniota - ps25
Mammalia - ps26
Theria - ps27
Placentalia - ps28
Boreoeutheria - ps29
Euarchontoglires - ps30
Euarchonta - ps31
Primates - ps32
Haplorrhini - ps33
Simiiformes - ps34
Catarrhini - ps35
Hominoidea - ps36
Hominidae - ps37
Homininae - ps38
Hominini - ps39
Homo_sapiens - ps40
Phylostratum

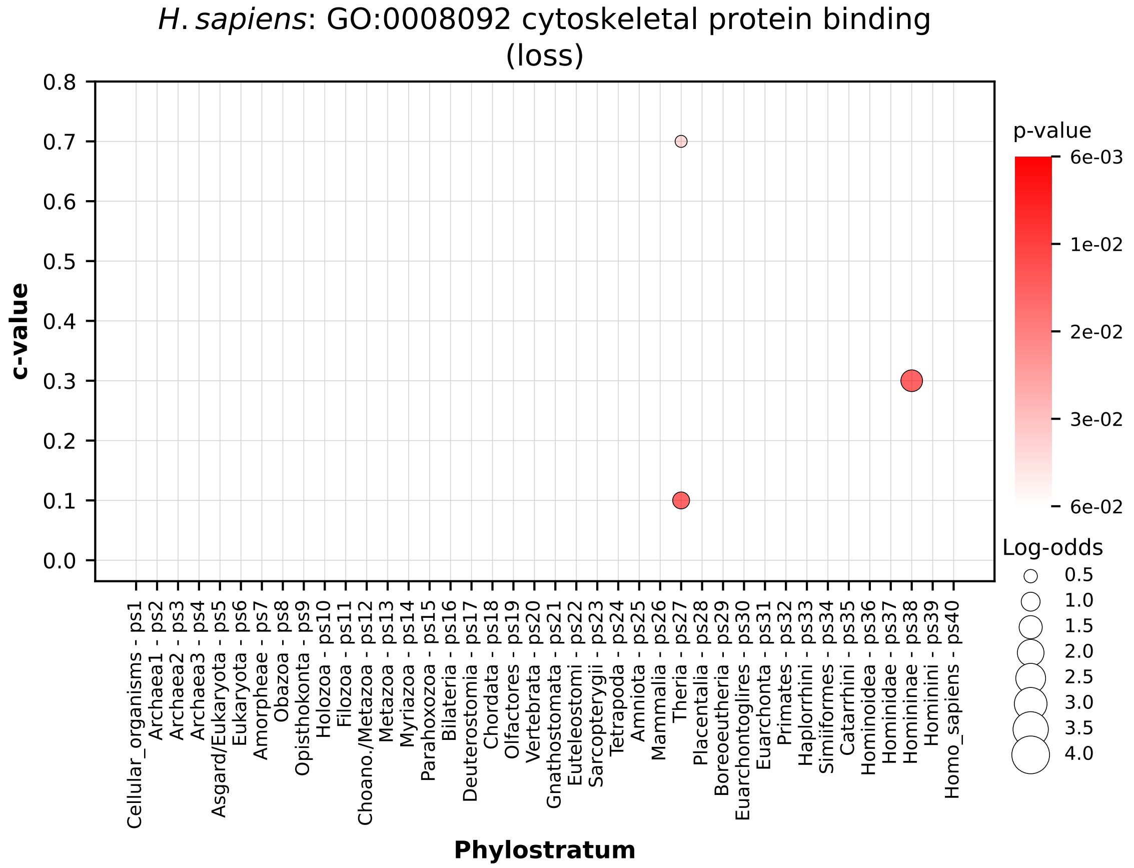

H. sapiens: GO:0008092 cytoskeletal protein binding
(loss)
c-value
0.8
0.7
0.6
0.5
0.4
0.3
0.2
0.1
0.0
p-value
6e-03
1e-02
2e-02
3e-02
6e-02
Log-odds
0.5
1.0
1.5
2.0
2.5
3.0
3.5
4.0
Cellular_organisms - ps1
Archaea1 - ps2
Archaea2 - ps3
Archaea3 - ps4
Asgard/Eukaryota - ps5
Eukaryota - ps6
Amorpheae - ps7
Obazoa - ps8
Opisthokonta - ps9
Holozoa - ps10
Filozoa - ps11
Choano./Metazoa - ps12
Metazoa - ps13
Myriazoa - ps14
Parahoxozoa - ps15
Bilateria - ps16
Deuterostomia - ps17
Chordata - ps18
Olfactores - ps19
Vertebrata - ps20
Gnathostomata - ps21
Euteleostomi - ps22
Sarcopterygii - ps23
Tetrapoda - ps24
Amniota - ps25
Mammalia - ps26
Theria - ps27
Placentalia - ps28
Boreoeutheria - ps29
Euarchontoglires - ps30
Euarchonta - ps31
Primates - ps32
Haplorrhini - ps33
Simiiformes - ps34
Catarrhini - ps35
Hominoidea - ps36
Hominidae - ps37
Homininae - ps38
Hominini - ps39
Homo_sapiens - ps40
Phylostratum

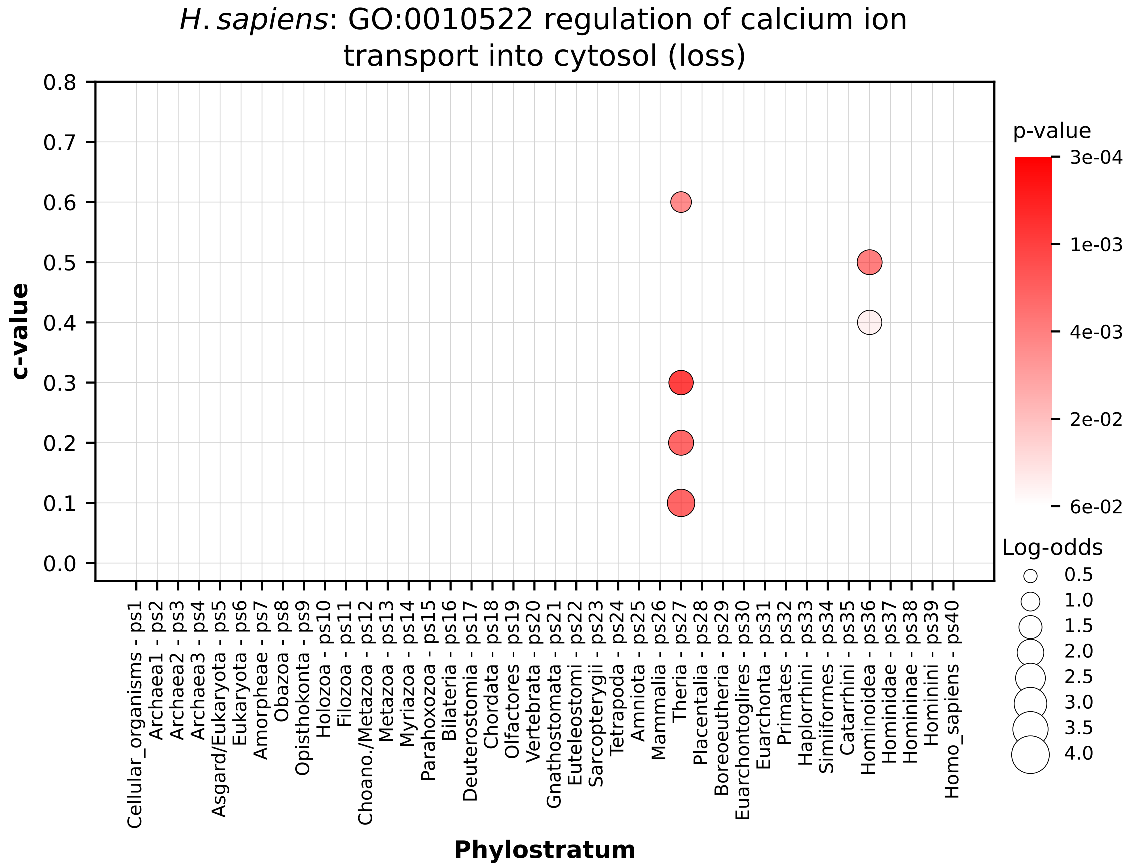

H. sapiens: GO:0010522 regulation of calcium ion transport into cytosol (loss)
c-value
0.8
0.7
0.6
0.5
0.4
0.3
0.2
0.1
0.0
Phylostratum
Cellular_organisms - ps1
Archaea1 - ps2
Archaea2 - ps3
Archaea3 - ps4
Asgard/Eukaryota - ps5
Eukaryota - ps6
Amorpheae - ps7
Obazoa - ps8
Opisthokonta - ps9
Holozoa - ps10
Filozoa - ps11
Choano./Metazoa - ps12
Metazoa - ps13
Myriazoa - ps14
Parahoxozoa - ps15
Bilateria - ps16
Deuterostomia - ps17
Chordata - ps18
Olfactores - ps19
Vertebrata - ps20
Gnathostomata - ps21
Euteleostomi - ps22
Sarcopterygii - ps23
Tetrapoda - ps24
Amniota - ps25
Mammalia - ps26
Theria - ps27
Placentalia - ps28
Boreoeutheria - ps29
Euarchontoglires - ps30
Euarchonta - ps31
Primates - ps32
Haplorrhini - ps33
Simiiformes - ps34
Catarrhini - ps35
Hominoidea - ps36
Hominidae - ps37
Homininae - ps38
Hominini - ps39
Homo_sapiens - ps40
p-value
3e-04
1e-03
4e-03
2e-02
6e-02
Log-odds
0.5
1.0
1.5
2.0
2.5
3.0
3.5
4.0

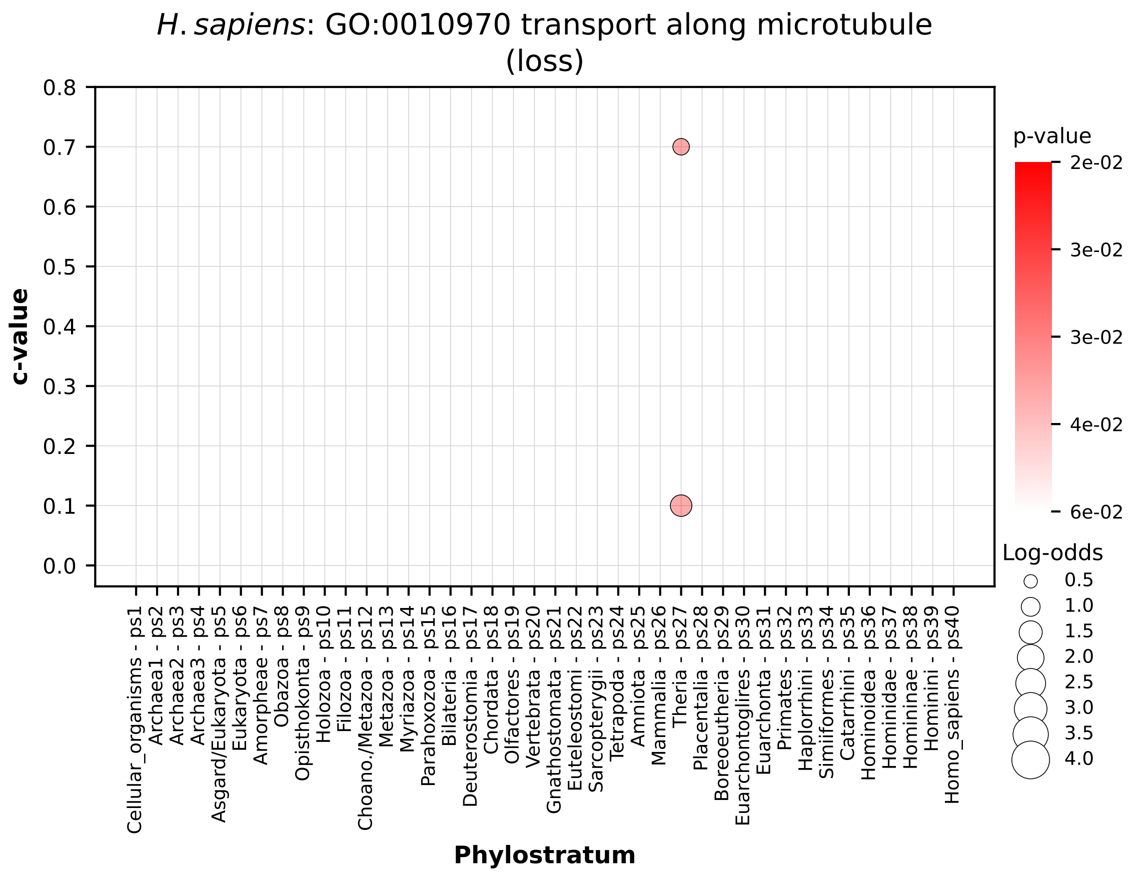

H. sapiens: GO:0010970 transport along microtubule (loss)
c-value
0.8
0.7
0.6
0.5
0.4
0.3
0.2
0.1
0.0
Phylostratum
Cellular_organisms - ps1
Archaea1 - ps2
Archaea2 - ps3
Archaea3 - ps4
Asgard/Eukaryota - ps5
Eukaryota - ps6
Amorpheae - ps7
Obazoa - ps8
Opisthokonta - ps9
Holozoa - ps10
Filozoa - ps11
Choano./Metazoa - ps12
Metazoa - ps13
Myriazoa - ps14
Parahoxozoa - ps15
Bilateria - ps16
Deuterostomia - ps17
Chordata - ps18
Olfactores - ps19
Vertebrata - ps20
Gnathostomata - ps21
Euteleostomi - ps22
Sarcopterygii - ps23
Tetrapoda - ps24
Amniota - ps25
Mammalia - ps26
Theria - ps27
Placentalia - ps28
Boreoeutheria - ps29
Euarchontoglires - ps30
Euarchonta - ps31
Primates - ps32
Haplorrhini - ps33
Simiiformes - ps34
Catarrhini - ps35
Hominoidea - ps36
Hominidae - ps37
Homininae - ps38
Hominini - ps39
Homo_sapiens - ps40
p-value
2e-02
3e-02
3e-02
4e-02
6e-02
Log-odds
0.5
1.0
1.5
2.0
2.5
3.0
3.5
4.0

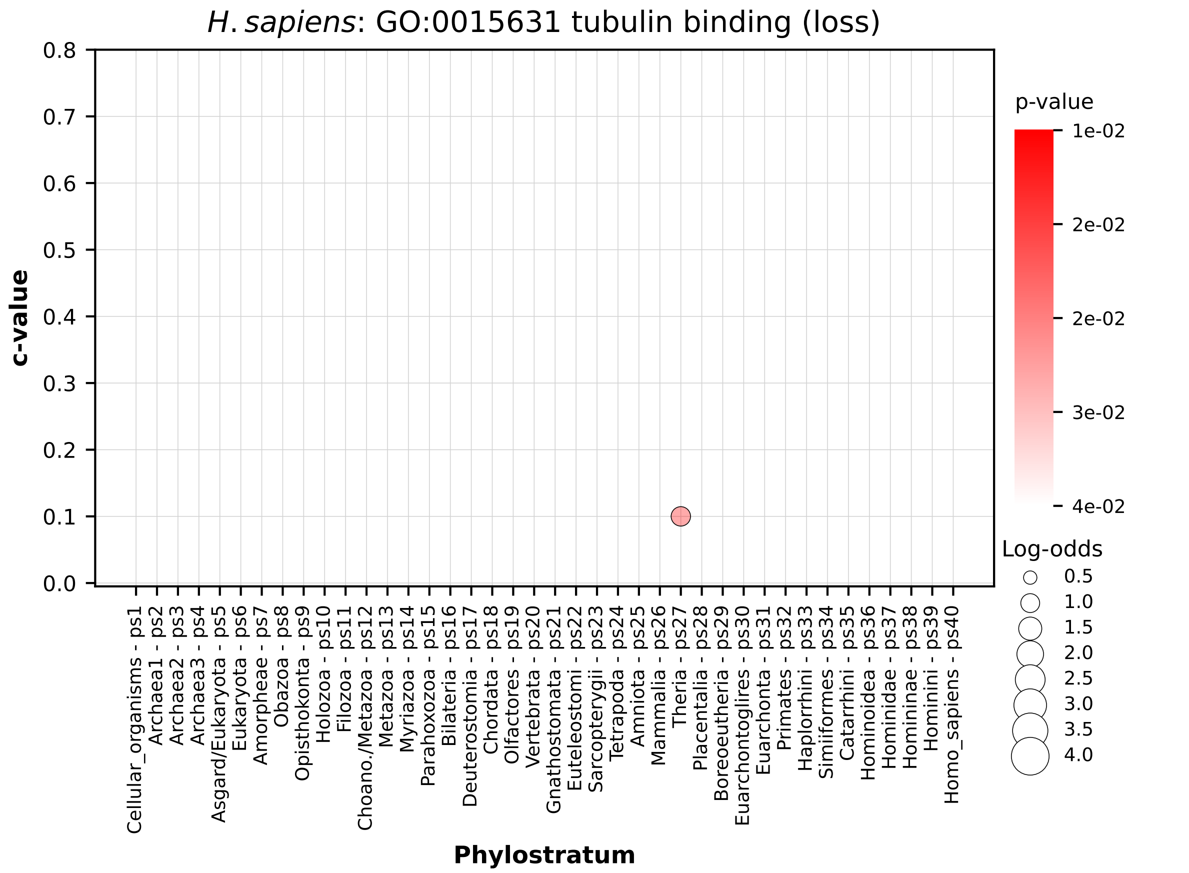
H. sapiens: GO:0015631 tubulin binding (loss)
c-value
0.8
0.7
0.6
0.5
0.4
0.3
0.2
0.1
0.0
Phylostratum
Cellular_organisms - ps1
Archaea1 - ps2
Archaea2 - ps3
Archaea3 - ps4
Asgard/Eukaryota - ps5
Eukaryota - ps6
Amorpheae - ps7
Obazoa - ps8
Opisthokonta - ps9
Holozoa - ps10
Filozoa - ps11
Choano./Metazoa - ps12
Metazoa - ps13
Myriazoa - ps14
Parahoxozoa - ps15
Bilateria - ps16
Deuterostomia - ps17
Chordata - ps18
Olfactores - ps19
Vertebrata - ps20
Gnathostomata - ps21
Euteleostomi - ps22
Sarcopterygii - ps23
Tetrapoda - ps24
Amniota - ps25
Mammalia - ps26
Theria - ps27
Placentalia - ps28
Boreoeutheria - ps29
Euarchontoglires - ps30
Euarchonta - ps31
Primates - ps32
Haplorrhini - ps33
Simiiformes - ps34
Catarrhini - ps35
Hominoidea - ps36
Hominidae - ps37
Homininae - ps38
Hominini - ps39
Homo_sapiens - ps40
p-value
1e-02
2e-02
2e-02
3e-02
4e-02
Log-odds
0.5
1.0
1.5
2.0
2.5
3.0
3.5
4.0

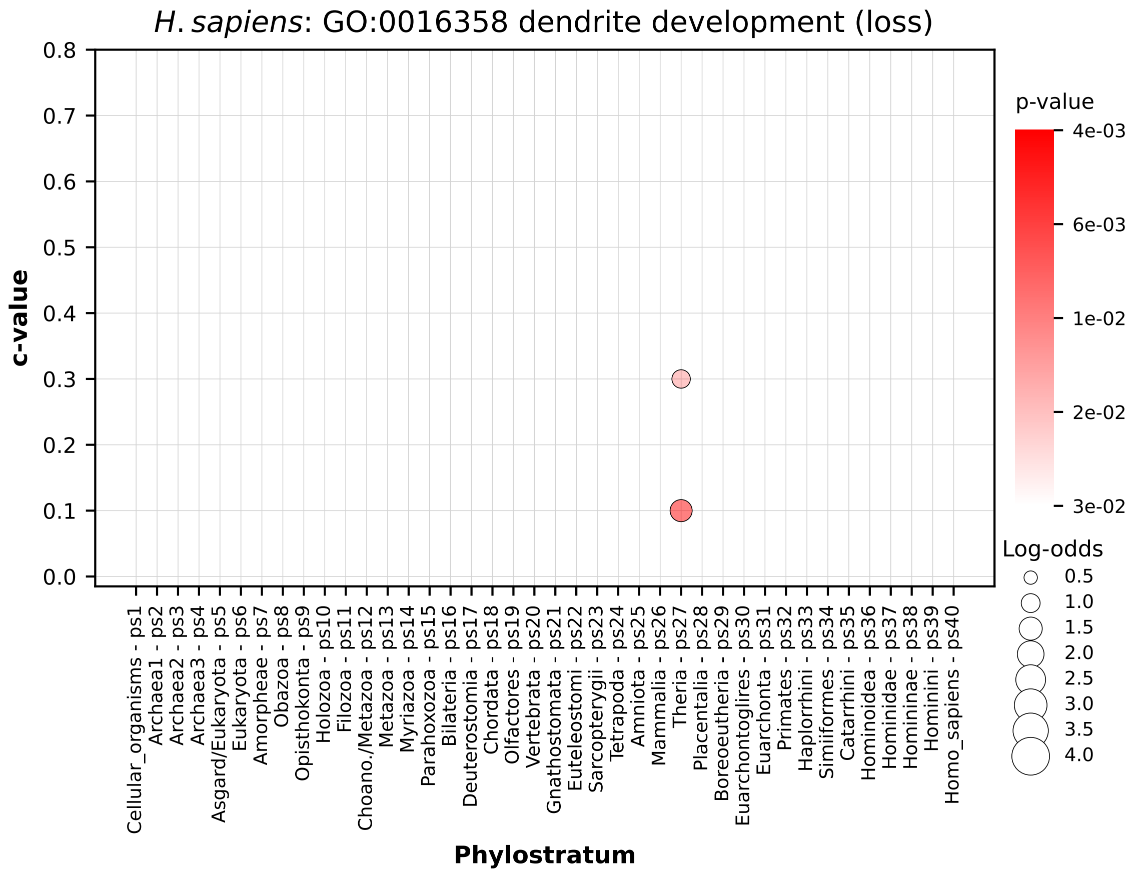

H. sapiens: GO:0016358 dendrite development (loss)
c-value
0.0
0.1
0.2
0.3
0.4
0.5
0.6
0.7
0.8
Phylostratum
Cellular_organisms - ps1
Archaea1 - ps2
Archaea2 - ps3
Archaea3 - ps4
Asgard/Eukaryota - ps5
Eukaryota - ps6
Amorpheae - ps7
Obazoa - ps8
Opisthokonta - ps9
Holozoa - ps10
Filozoa - ps11
Choano./Metazoa - ps12
Metazoa - ps13
Myriazoa - ps14
Parahoxozoa - ps15
Bilateria - ps16
Deuterostomia - ps17
Chordata - ps18
Olfactores - ps19
Vertebrata - ps20
Gnathostomata - ps21
Euteleostomi - ps22
Sarcopterygii - ps23
Tetrapoda - ps24
Amniota - ps25
Mammalia - ps26
Theria - ps27
Placentalia - ps28
Boreoeutheria - ps29
Euarchontoglires - ps30
Euarchonta - ps31
Primates - ps32
Haplorrhini - ps33
Simiiformes - ps34
Catarrhini - ps35
Hominoidea - ps36
Hominidae - ps37
Homininae - ps38
Hominini - ps39
Homo_sapiens - ps40
p-value
4e-03
6e-03
1e-02
2e-02
3e-02
Log-odds
0.5
1.0
1.5
2.0
2.5
3.0
3.5
4.0

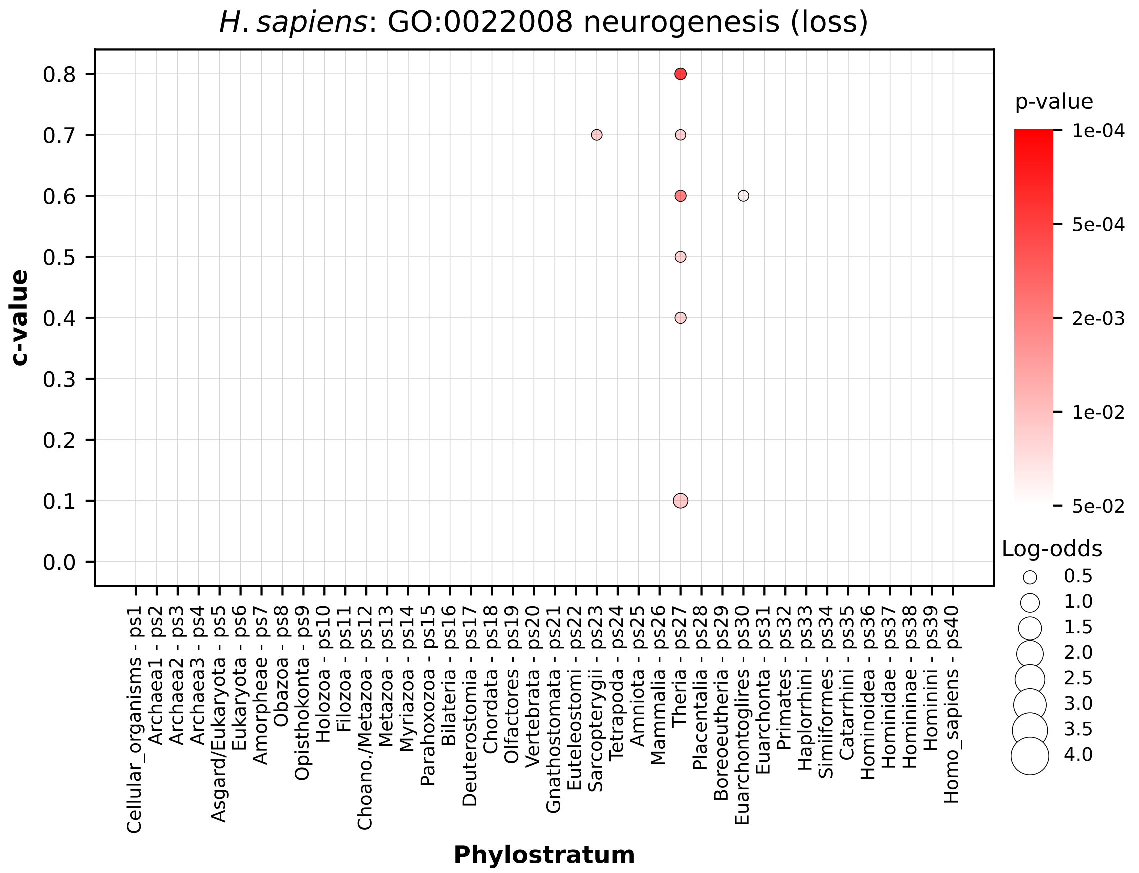

H. sapiens: GO:0022008 neurogenesis (loss)
c-value
0.0
0.1
0.2
0.3
0.4
0.5
0.6
0.7
0.8
Phylostratum
Cellular_organisms - ps1
Archaea1 - ps2
Archaea2 - ps3
Archaea3 - ps4
Asgard/Eukaryota - ps5
Eukaryota - ps6
Amorpheae - ps7
Obazoa - ps8
Opisthokonta - ps9
Holozoa - ps10
Filozoa - ps11
Choano./Metazoa - ps12
Metazoa - ps13
Myriazoa - ps14
Parahoxozoa - ps15
Bilateria - ps16
Deuterostomia - ps17
Chordata - ps18
Olfactores - ps19
Vertebrata - ps20
Gnathostomata - ps21
Euteleostomi - ps22
Sarcopterygii - ps23
Tetrapoda - ps24
Amniota - ps25
Mammalia - ps26
Theria - ps27
Placentalia - ps28
Boreoeutheria - ps29
Euarchontoglires - ps30
Euarchonta - ps31
Primates - ps32
Haplorrhini - ps33
Simiiformes - ps34
Catarrhini - ps35
Hominoidea - ps36
Hominidae - ps37
Homininae - ps38
Hominini - ps39
Homo_sapiens - ps40
p-value
1e-04
5e-04
2e-03
1e-02
5e-02
Log-odds
0.5
1.0
1.5
2.0
2.5
3.0
3.5
4.0

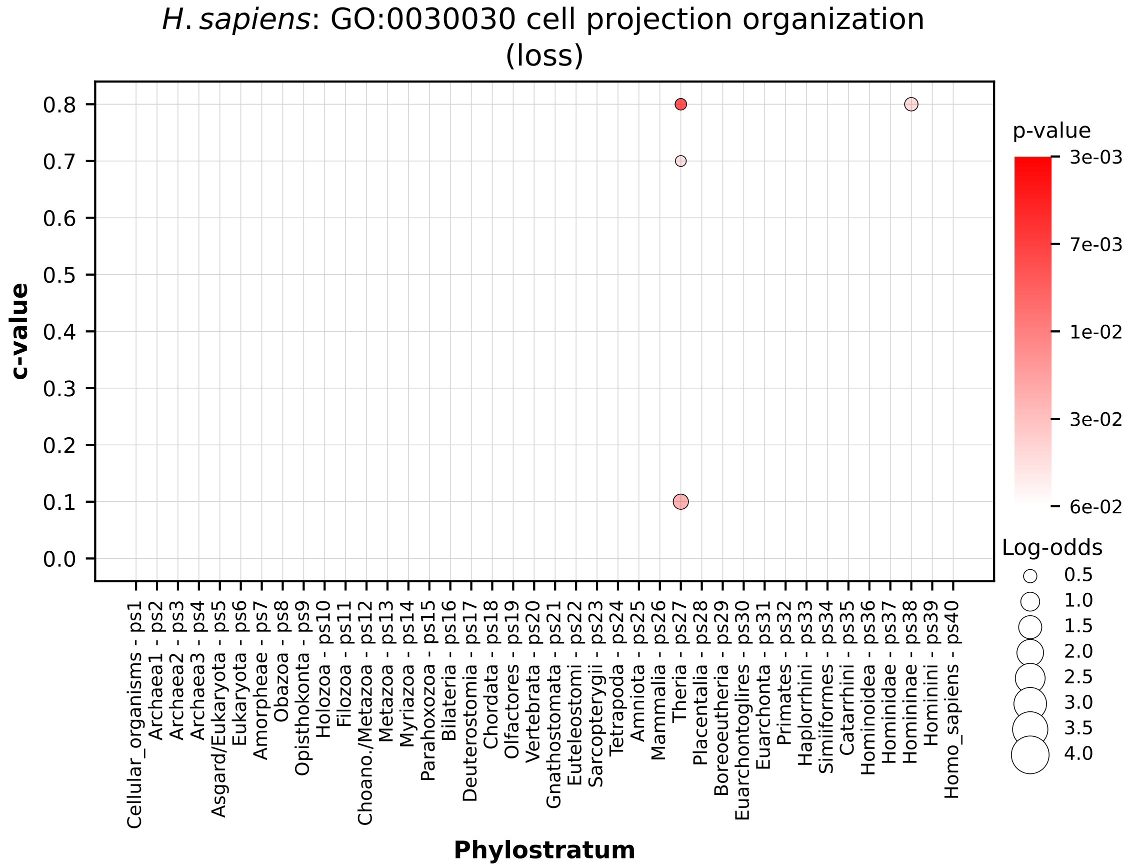

H. sapiens: GO:0030030 cell projection organization
(loss)
c-value
0.0
0.1
0.2
0.3
0.4
0.5
0.6
0.7
0.8
p-value
3e-03
7e-03
1e-02
3e-02
6e-02
Log-odds
0.5
1.0
1.5
2.0
2.5
3.0
3.5
4.0
Cellular_organisms - ps1
Archaea1 - ps2
Archaea2 - ps3
Archaea3 - ps4
Asgard/Eukaryota - ps5
Eukaryota - ps6
Amorpheae - ps7
Obazoa - ps8
Opisthokonta - ps9
Holozoa - ps10
Filozoa - ps11
Choano./Metazoa - ps12
Metazoa - ps13
Myriazoa - ps14
Parahoxozoa - ps15
Bilateria - ps16
Deuterostomia - ps17
Chordata - ps18
Olfactores - ps19
Vertebrata - ps20
Gnathostomata - ps21
Euteleostomi - ps22
Sarcopterygii - ps23
Tetrapoda - ps24
Amniota - ps25
Mammalia - ps26
Theria - ps27
Placentalia - ps28
Boreoeutheria - ps29
Euarchontoglires - ps30
Euarchonta - ps31
Primates - ps32
Haplorrhini - ps33
Simiiformes - ps34
Catarrhini - ps35
Hominoidea - ps36
Hominidae - ps37
Homininae - ps38
Hominini - ps39
Homo_sapiens - ps40
Phylostratum

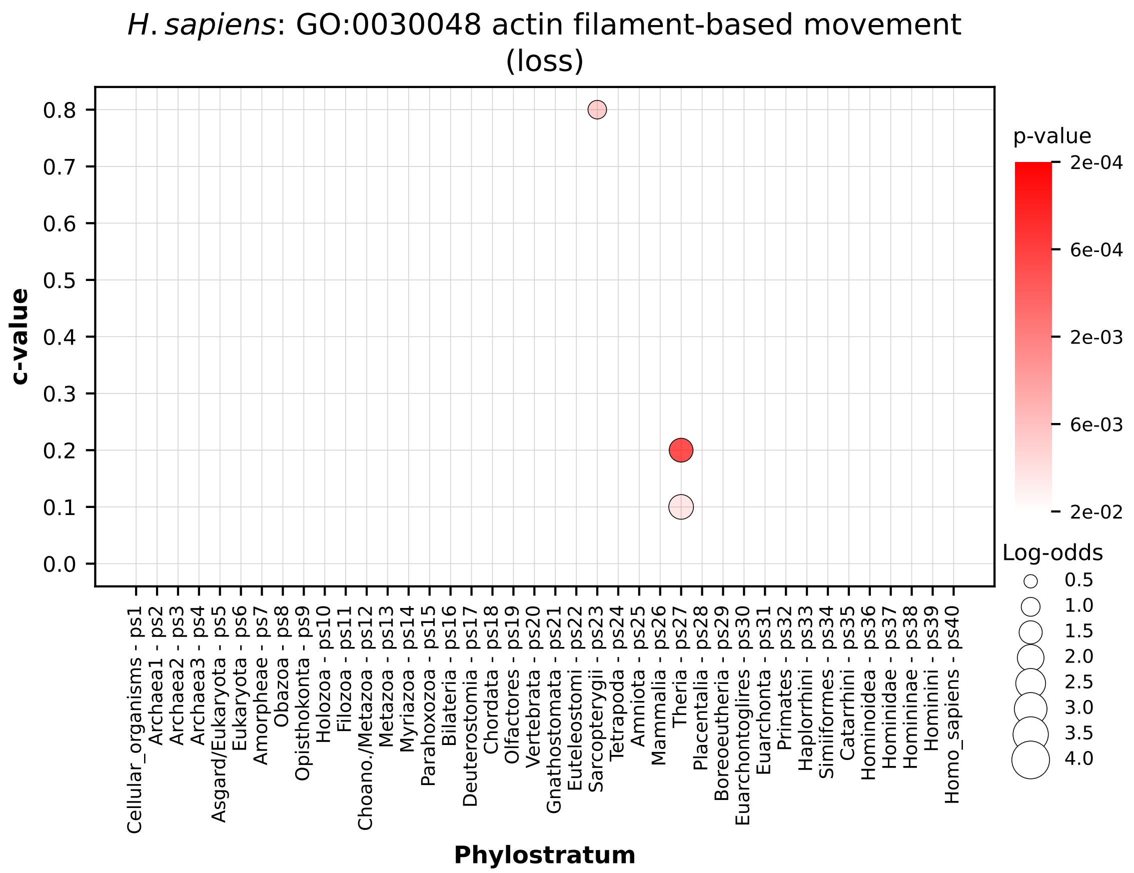

H. sapiens: GO:0030048 actin filament-based movement
(loss)
c-value
0.0
0.1
0.2
0.3
0.4
0.5
0.6
0.7
0.8
Phylostratum
Cellular_organisms - ps1
Archaea1 - ps2
Archaea2 - ps3
Archaea3 - ps4
Asgard/Eukaryota - ps5
Eukaryota - ps6
Amorpheae - ps7
Obazoa - ps8
Opisthokonta - ps9
Holozoa - ps10
Filozoa - ps11
Choano./Metazoa - ps12
Metazoa - ps13
Myriazoa - ps14
Parahoxozoa - ps15
Bilateria - ps16
Deuterostomia - ps17
Chordata - ps18
Olfactores - ps19
Vertebrata - ps20
Gnathostomata - ps21
Euteleostomi - ps22
Sarcopterygii - ps23
Tetrapoda - ps24
Amniota - ps25
Mammalia - ps26
Theria - ps27
Placentalia - ps28
Boreoeutheria - ps29
Euarchontoglires - ps30
Euarchonta - ps31
Primates - ps32
Haplorrhini - ps33
Simiiformes - ps34
Catarrhini - ps35
Hominoidea - ps36
Hominidae - ps37
Homininae - ps38
Hominini - ps39
Homo_sapiens - ps40
p-value
2e-04
6e-04
2e-03
6e-03
2e-02
Log-odds
0.5
1.0
1.5
2.0
2.5
3.0
3.5
4.0

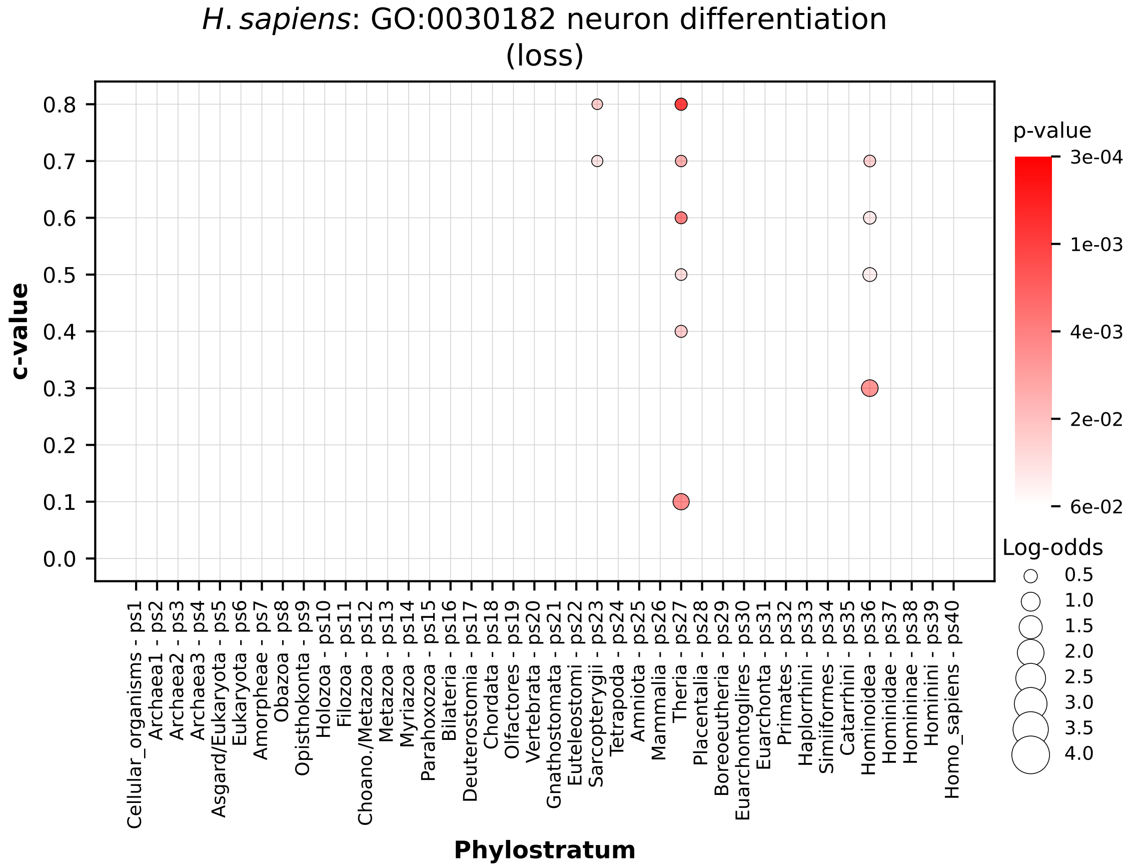

H. sapiens: GO:0030182 neuron differentiation
(loss)
c-value
0.8
0.7
0.6
0.5
0.4
0.3
0.2
0.1
0.0
p-value
3e-04
1e-03
4e-03
2e-02
6e-02
Log-odds
0.5
1.0
1.5
2.0
2.5
3.0
3.5
4.0
Cellular_organisms - ps1
Archaea1 - ps2
Archaea2 - ps3
Archaea3 - ps4
Asgard/Eukaryota - ps5
Eukaryota - ps6
Amorpheae - ps7
Obazoa - ps8
Opisthokonta - ps9
Holozoa - ps10
Filozoa - ps11
Choano./Metazoa - ps12
Metazoa - ps13
Myriazoa - ps14
Parahoxozoa - ps15
Bilateria - ps16
Deuterostomia - ps17
Chordata - ps18
Olfactores - ps19
Vertebrata - ps20
Gnathostomata - ps21
Euteleostomi - ps22
Sarcopterygii - ps23
Tetrapoda - ps24
Amniota - ps25
Mammalia - ps26
Theria - ps27
Placentalia - ps28
Boreoeutheria - ps29
Euarchontoglires - ps30
Euarchonta - ps31
Primates - ps32
Haplorrhini - ps33
Simiiformes - ps34
Catarrhini - ps35
Hominoidea - ps36
Hominidae - ps37
Homininae - ps38
Hominini - ps39
Homo_sapiens - ps40
Phylostratum

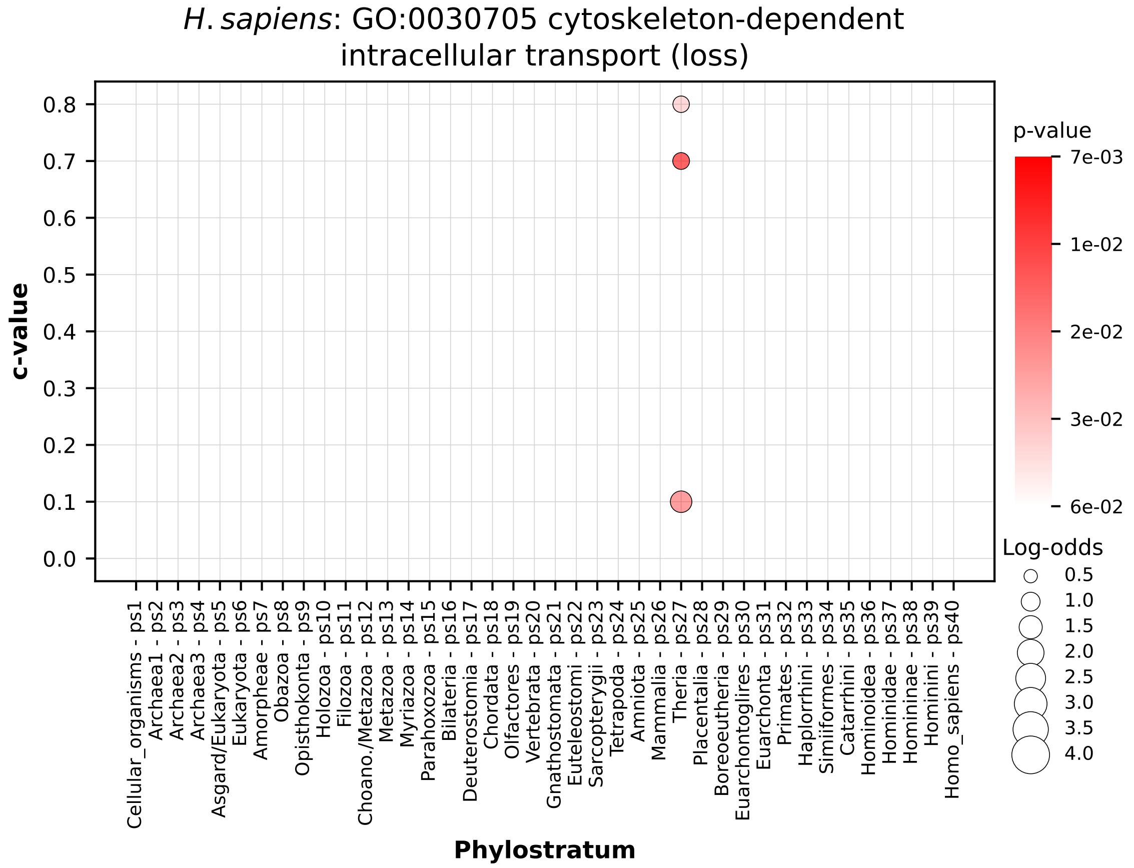

H. sapiens: GO:0030705 cytoskeleton-dependent intracellular transport (loss)
c-value
0.0
0.1
0.2
0.3
0.4
0.5
0.6
0.7
0.8
p-value
7e-03
1e-02
2e-02
3e-02
6e-02
Log-odds
0.5
1.0
1.5
2.0
2.5
3.0
3.5
4.0
Cellular_organisms - ps1
Archaea1 - ps2
Archaea2 - ps3
Archaea3 - ps4
Asgard/Eukaryota - ps5
Eukaryota - ps6
Amorpheae - ps7
Obazoa - ps8
Opisthokonta - ps9
Holozoa - ps10
Filozoa - ps11
Choano./Metazoa - ps12
Metazoa - ps13
Myriazoa - ps14
Parahoxozoa - ps15
Bilateria - ps16
Deuterostomia - ps17
Chordata - ps18
Olfactores - ps19
Vertebrata - ps20
Gnathostomata - ps21
Euteleostomi - ps22
Sarcopterygii - ps23
Tetrapoda - ps24
Amniota - ps25
Mammalia - ps26
Theria - ps27
Placentalia - ps28
Boreoeutheria - ps29
Euarchontoglires - ps30
Euarchonta - ps31
Primates - ps32
Haplorrhini - ps33
Simiiformes - ps34
Catarrhini - ps35
Hominoidea - ps36
Hominidae - ps37
Homininae - ps38
Hominini - ps39
Homo_sapiens - ps40
Phylostratum

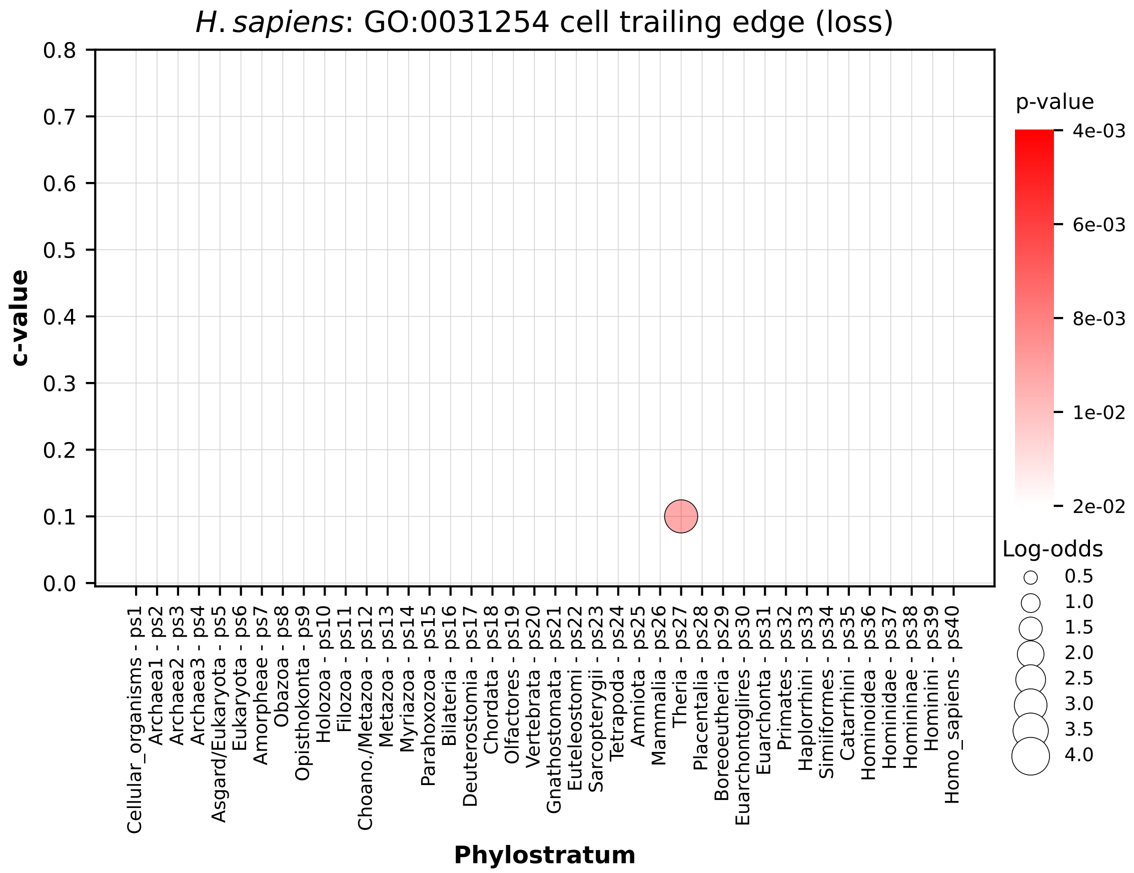

H. sapiens: GO:0031254 cell trailing edge (loss)
c-value
0.0
0.1
0.2
0.3
0.4
0.5
0.6
0.7
0.8
p-value
4e-03
6e-03
8e-03
1e-02
2e-02
Log-odds
0.5
1.0
1.5
2.0
2.5
3.0
3.5
4.0
Cellular_organisms - ps1
Archaea1 - ps2
Archaea2 - ps3
Archaea3 - ps4
Asgard/Eukaryota - ps5
Eukaryota - ps6
Amorpheae - ps7
Obazoa - ps8
Opisthokonta - ps9
Holozoa - ps10
Filozoa - ps11
Choano./Metazoa - ps12
Metazoa - ps13
Myriazoa - ps14
Parahoxozoa - ps15
Bilateria - ps16
Deuterostomia - ps17
Chordata - ps18
Olfactores - ps19
Vertebrata - ps20
Gnathostomata - ps21
Euteleostomi - ps22
Sarcopterygii - ps23
Tetrapoda - ps24
Amniota - ps25
Mammalia - ps26
Theria - ps27
Placentalia - ps28
Boreoeutheria - ps29
Euarchontoglires - ps30
Euarchonta - ps31
Primates - ps32
Haplorrhini - ps33
Simiiformes - ps34
Catarrhini - ps35
Hominoidea - ps36
Hominidae - ps37
Homininae - ps38
Hominini - ps39
Homo_sapiens - ps40
Phylostratum

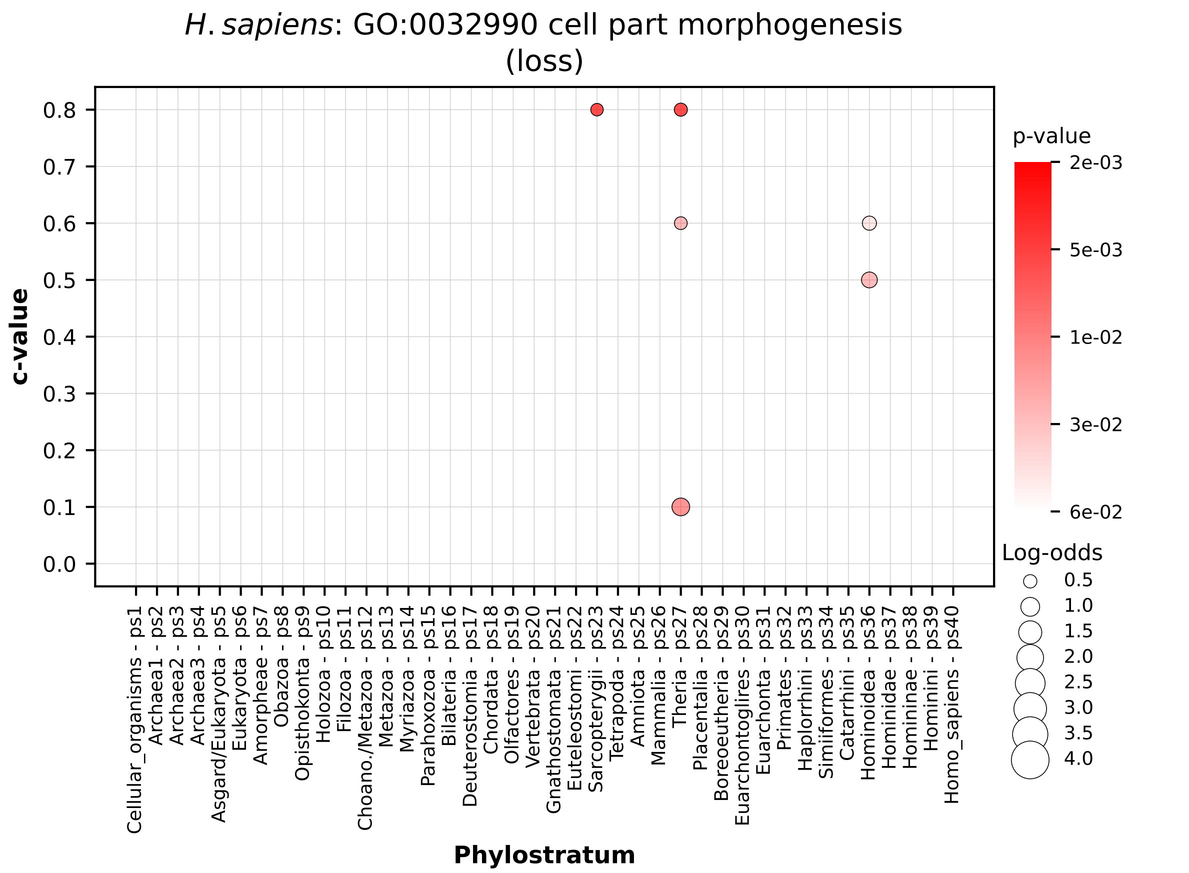

H. sapiens: GO:0032990 cell part morphogenesis
(loss)
c-value
0.8
0.7
0.6
0.5
0.4
0.3
0.2
0.1
0.0
p-value
2e-03
5e-03
1e-02
3e-02
6e-02
Log-odds
0.5
1.0
1.5
2.0
2.5
3.0
3.5
4.0
Cellular_organisms - ps1
Archaea1 - ps2
Archaea2 - ps3
Archaea3 - ps4
Asgard/Eukaryota - ps5
Eukaryota - ps6
Amorpheae - ps7
Obazoa - ps8
Opisthokonta - ps9
Holozoa - ps10
Filozoa - ps11
Choano./Metazoa - ps12
Metazoa - ps13
Myriazoa - ps14
Parahoxozoa - ps15
Bilateria - ps16
Deuterostomia - ps17
Chordata - ps18
Olfactores - ps19
Vertebrata - ps20
Gnathostomata - ps21
Euteleostomi - ps22
Sarcopterygii - ps23
Tetrapoda - ps24
Amniota - ps25
Mammalia - ps26
Theria - ps27
Placentalia - ps28
Boreoeutheria - ps29
Euarchontoglires - ps30
Euarchonta - ps31
Primates - ps32
Haplorrhini - ps33
Simiiformes - ps34
Catarrhini - ps35
Hominoidea - ps36
Hominidae - ps37
Homininae - ps38
Hominini - ps39
Homo_sapiens - ps40
Phylostratum

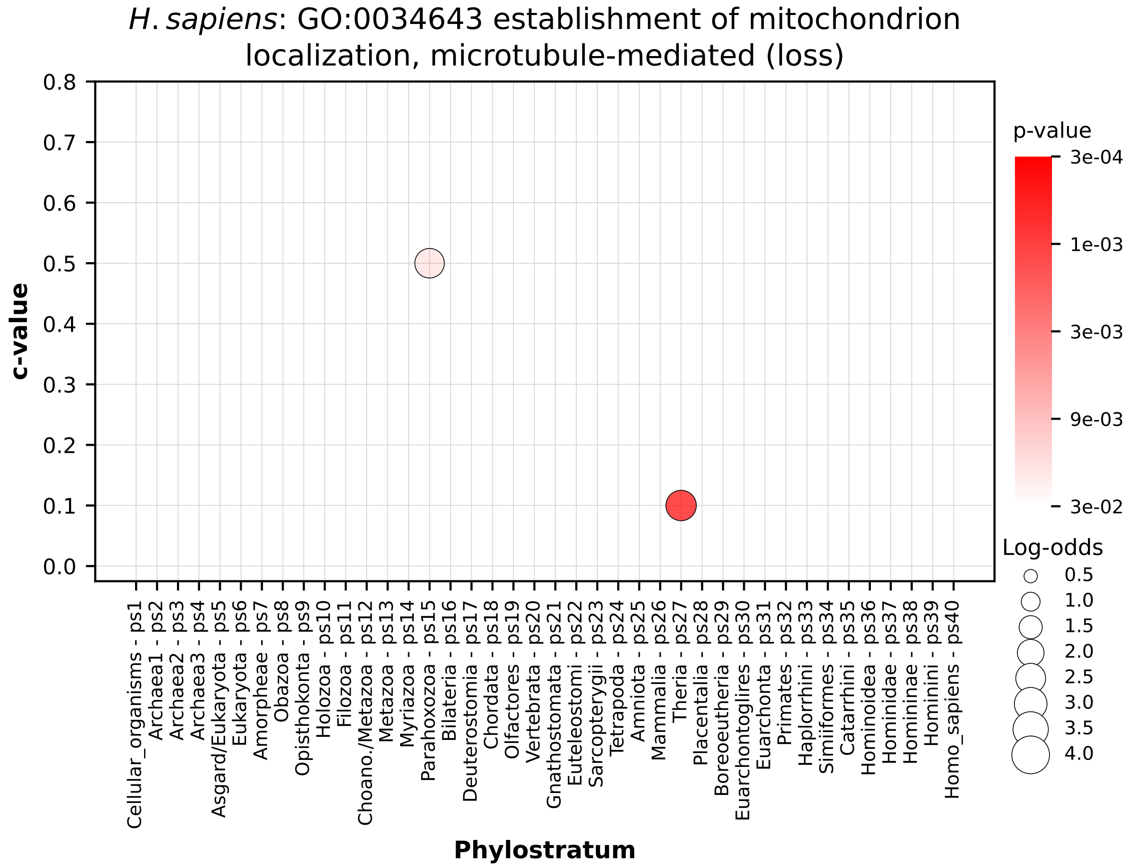
H. sapiens: GO:0034643 establishment of mitochondrion localization, microtubule-mediated (loss)
c-value
0.8
0.7
0.6
0.5
0.4
0.3
0.2
0.1
0.0
Phylostratum
Cellular_organisms - ps1
Archaea1 - ps2
Archaea2 - ps3
Archaea3 - ps4
Asgard/Eukaryota - ps5
Eukaryota - ps6
Amorpheae - ps7
Obazoa - ps8
Opisthokonta - ps9
Holozoa - ps10
Filozoa - ps11
Choano./Metazoa - ps12
Metazoa - ps13
Myriazoa - ps14
Parahoxozoa - ps15
Bilateria - ps16
Deuterostomia - ps17
Chordata - ps18
Olfactores - ps19
Vertebrata - ps20
Gnathostomata - ps21
Euteleostomi - ps22
Sarcopterygii - ps23
Tetrapoda - ps24
Amniota - ps25
Mammalia - ps26
Theria - ps27
Placentalia - ps28
Boreoeutheria - ps29
Euarchontoglires - ps30
Euarchonta - ps31
Primates - ps32
Haplorrhini - ps33
Simiiformes - ps34
Catarrhini - ps35
Hominoidea - ps36
Hominidae - ps37
Homininae - ps38
Hominini - ps39
Homo_sapiens - ps40
p-value
3e-04
1e-03
3e-03
9e-03
3e-02
Log-odds
0.5
1.0
1.5
2.0
2.5
3.0
3.5
4.0

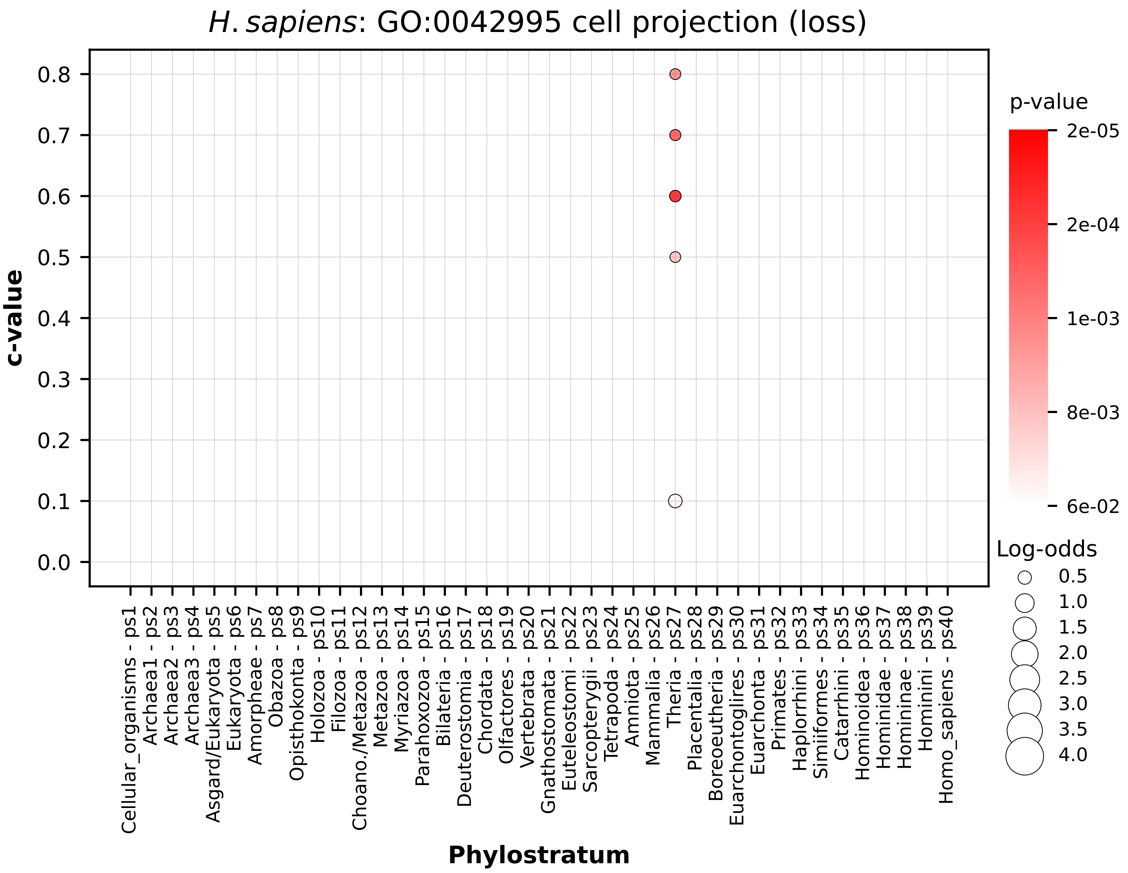

H. sapiens: GO:0042995 cell projection (loss)
c-value
0.0
0.1
0.2
0.3
0.4
0.5
0.6
0.7
0.8
Phylostratum
Cellular_organisms - ps1
Archaea1 - ps2
Archaea2 - ps3
Archaea3 - ps4
Asgard/Eukaryota - ps5
Eukaryota - ps6
Amorpheae - ps7
Obazoa - ps8
Opisthokonta - ps9
Holozoa - ps10
Filozoa - ps11
Choano./Metazoa - ps12
Metazoa - ps13
Myriazoa - ps14
Parahoxozoa - ps15
Bilateria - ps16
Deuterostomia - ps17
Chordata - ps18
Olfactores - ps19
Vertebrata - ps20
Gnathostomata - ps21
Euteleostomi - ps22
Sarcopterygii - ps23
Tetrapoda - ps24
Amniota - ps25
Mammalia - ps26
Theria - ps27
Placentalia - ps28
Boreoeutheria - ps29
Euarchontoglires - ps30
Euarchonta - ps31
Primates - ps32
Haplorrhini - ps33
Simiiformes - ps34
Catarrhini - ps35
Hominoidea - ps36
Hominidae - ps37
Homininae - ps38
Hominini - ps39
Homo_sapiens - ps40
p-value
2e-05
2e-04
1e-03
8e-03
6e-02
Log-odds
0.5
1.0
1.5
2.0
2.5
3.0
3.5
4.0

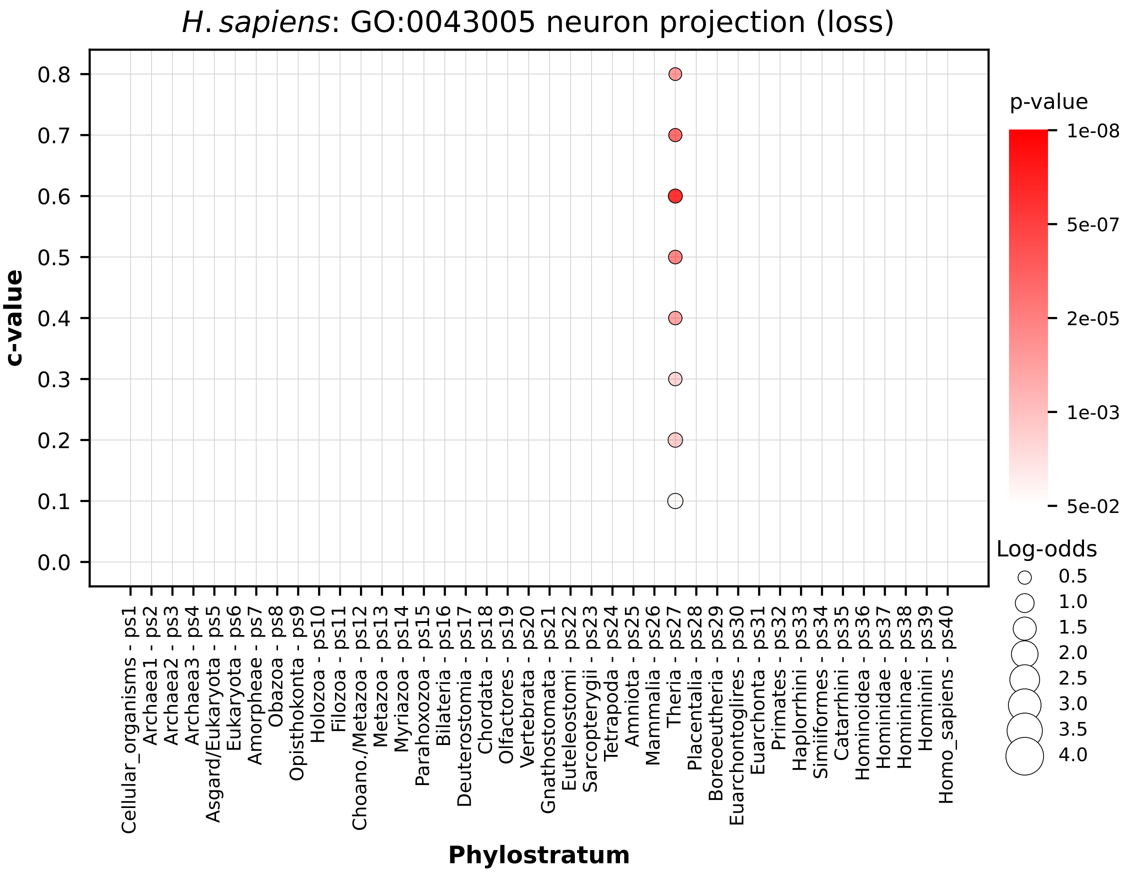

H. sapiens: GO:0043005 neuron projection (loss)
c-value
0.8
0.7
0.6
0.5
0.4
0.3
0.2
0.1
0.0
p-value
1e-08
5e-07
2e-05
1e-03
5e-02
Log-odds
0.5
1.0
1.5
2.0
2.5
3.0
3.5
4.0
Cellular_organisms - ps1
Archaea1 - ps2
Archaea2 - ps3
Archaea3 - ps4
Asgard/Eukaryota - ps5
Eukaryota - ps6
Amorpheae - ps7
Obazoa - ps8
Opisthokonta - ps9
Holozoa - ps10
Filozoa - ps11
Choano./Metazoa - ps12
Metazoa - ps13
Myriazoa - ps14
Parahoxozoa - ps15
Bilateria - ps16
Deuterostomia - ps17
Chordata - ps18
Olfactores - ps19
Vertebrata - ps20
Gnathostomata - ps21
Euteleostomi - ps22
Sarcopterygii - ps23
Tetrapoda - ps24
Amniota - ps25
Mammalia - ps26
Theria - ps27
Placentalia - ps28
Boreoeutheria - ps29
Euarchontoglires - ps30
Euarchonta - ps31
Primates - ps32
Haplorrhini - ps33
Simiiformes - ps34
Catarrhini - ps35
Hominoidea - ps36
Hominidae - ps37
Homininae - ps38
Hominini - ps39
Homo_sapiens - ps40
Phylostratum

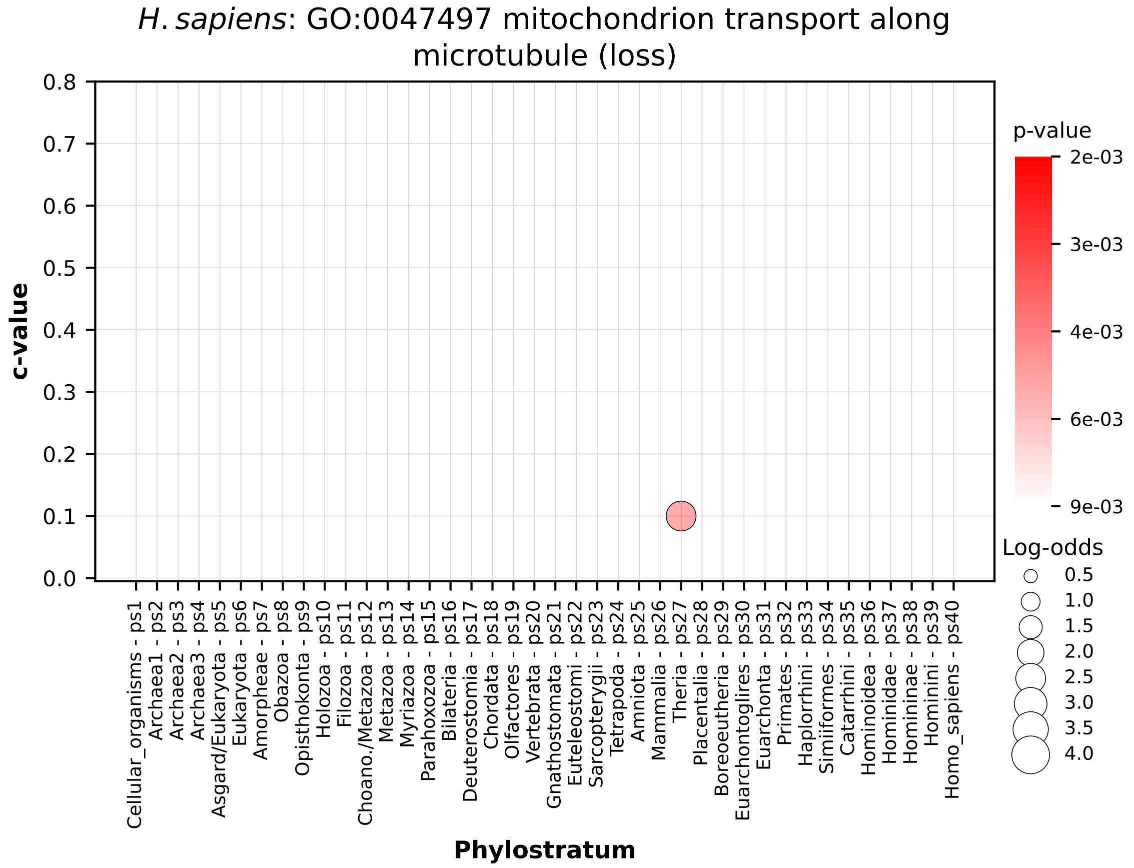

H. sapiens: GO:0047497 mitochondrion transport along microtubule (loss)
c-value
0.8
0.7
0.6
0.5
0.4
0.3
0.2
0.1
0.0
p-value
2e-03
3e-03
4e-03
6e-03
9e-03
Log-odds
0.5
1.0
1.5
2.0
2.5
3.0
3.5
4.0
Cellular_organisms - ps1
Archaea1 - ps2
Archaea2 - ps3
Archaea3 - ps4
Asgard/Eukaryota - ps5
Eukaryota - ps6
Amorpheae - ps7
Obazoa - ps8
Opisthokonta - ps9
Holozoa - ps10
Filozoa - ps11
Choano./Metazoa - ps12
Metazoa - ps13
Myriazoa - ps14
Parahoxozoa - ps15
Bilateria - ps16
Deuterostomia - ps17
Chordata - ps18
Olfactores - ps19
Vertebrata - ps20
Gnathostomata - ps21
Euteleostomi - ps22
Sarcopterygii - ps23
Tetrapoda - ps24
Amniota - ps25
Mammalia - ps26
Theria - ps27
Placentalia - ps28
Boreoeutheria - ps29
Euarchontoglires - ps30
Euarchonta - ps31
Primates - ps32
Haplorrhini - ps33
Simiiformes - ps34
Catarrhini - ps35
Hominoidea - ps36
Hominidae - ps37
Homininae - ps38
Hominini - ps39
Homo_sapiens - ps40
Phylostratum

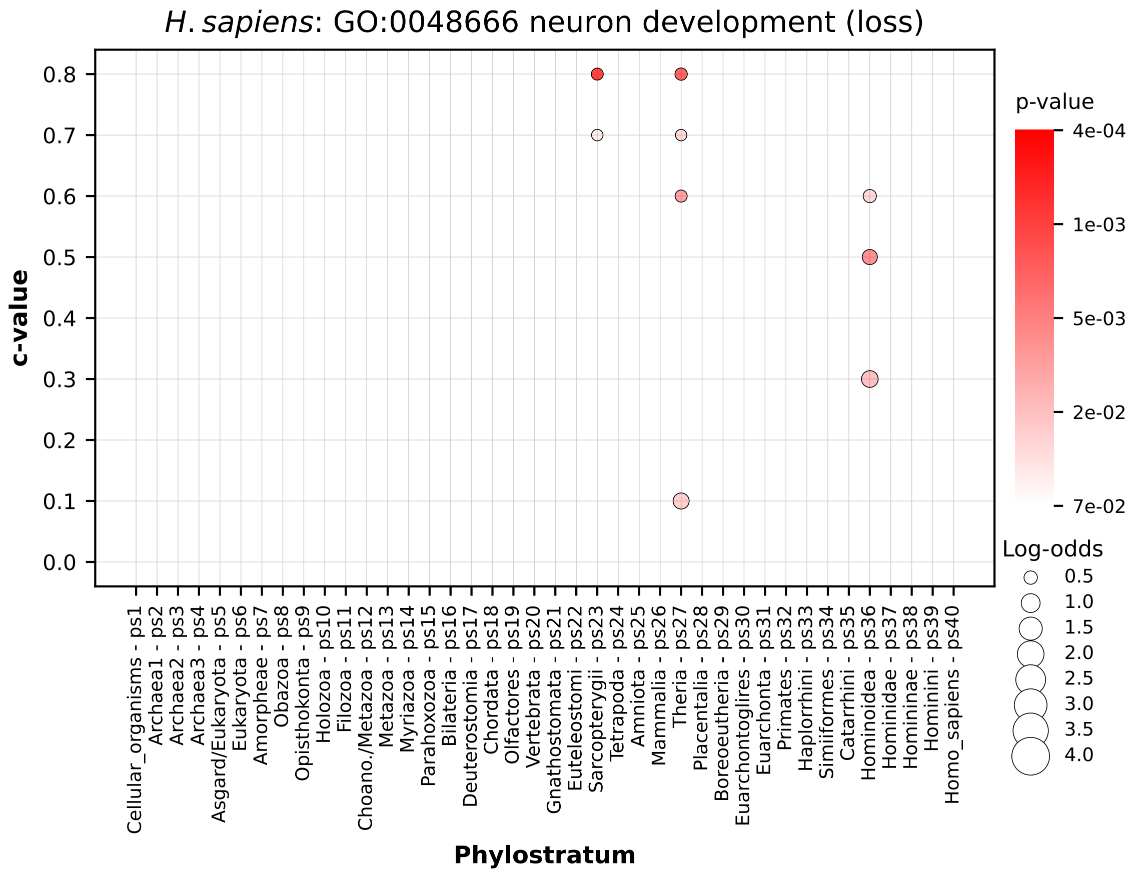

H. sapiens: GO:0048666 neuron development (loss)
c-value
0.0
0.1
0.2
0.3
0.4
0.5
0.6
0.7
0.8
Phylostratum
Cellular_organisms - ps1
Archaea1 - ps2
Archaea2 - ps3
Archaea3 - ps4
Asgard/Eukaryota - ps5
Eukaryota - ps6
Amorpheae - ps7
Obazoa - ps8
Opisthokonta - ps9
Holozoa - ps10
Filozoa - ps11
Choano./Metazoa - ps12
Metazoa - ps13
Myriazoa - ps14
Parahoxozoa - ps15
Bilateria - ps16
Deuterostomia - ps17
Chordata - ps18
Olfactores - ps19
Vertebrata - ps20
Gnathostomata - ps21
Euteleostomi - ps22
Sarcopterygii - ps23
Tetrapoda - ps24
Amniota - ps25
Mammalia - ps26
Theria - ps27
Placentalia - ps28
Boreoeutheria - ps29
Euarchontoglires - ps30
Euarchonta - ps31
Primates - ps32
Haplorrhini - ps33
Simiiformes - ps34
Catarrhini - ps35
Hominoidea - ps36
Hominidae - ps37
Homininae - ps38
Hominini - ps39
Homo_sapiens - ps40
p-value
4e-04
1e-03
5e-03
2e-02
7e-02
Log-odds
0.5
1.0
1.5
2.0
2.5
3.0
3.5
4.0

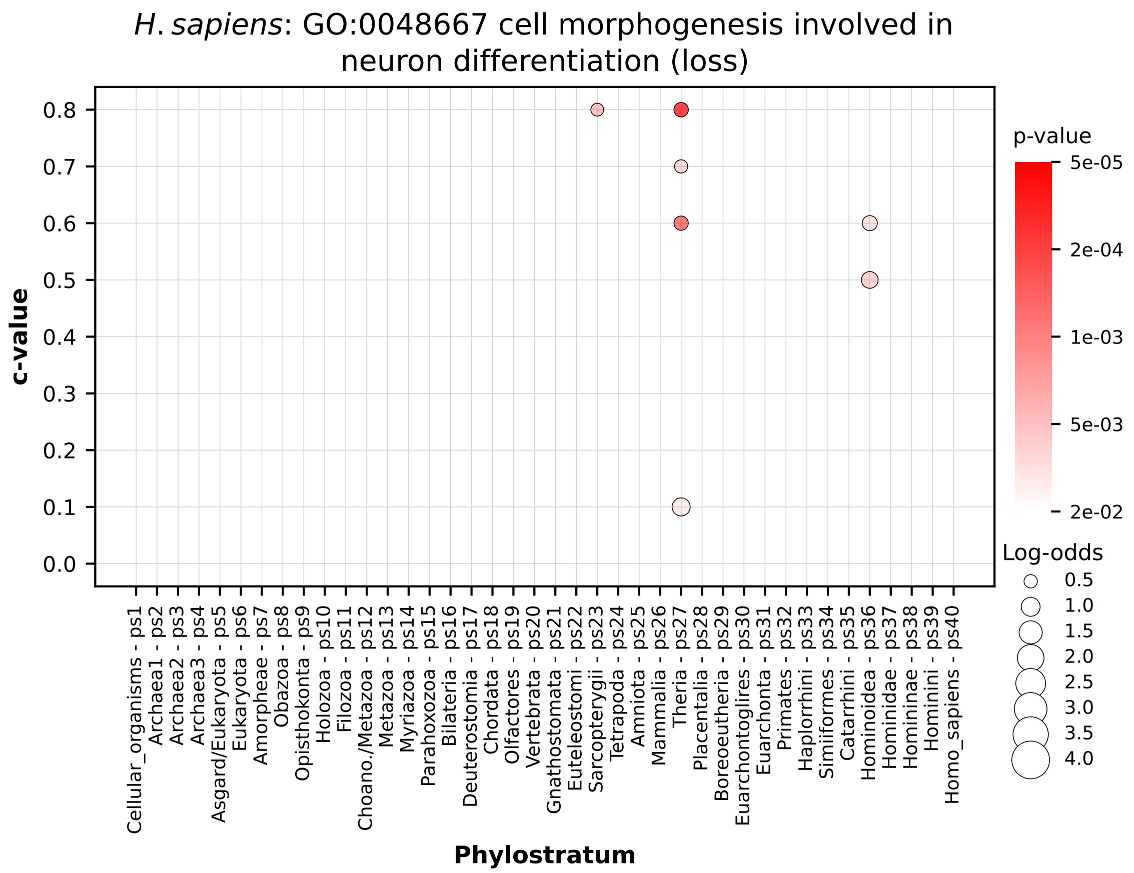

H. sapiens: GO:0048667 cell morphogenesis involved in neuron differentiation (loss)
c-value
0.0
0.1
0.2
0.3
0.4
0.5
0.6
0.7
0.8
Phylostratum
Cellular_organisms - ps1
Archaea1 - ps2
Archaea2 - ps3
Archaea3 - ps4
Asgard/Eukaryota - ps5
Eukaryota - ps6
Amorpheae - ps7
Obazoa - ps8
Opisthokonta - ps9
Holozoa - ps10
Filozoa - ps11
Choano./Metazoa - ps12
Metazoa - ps13
Myriazoa - ps14
Parahoxozoa - ps15
Bilateria - ps16
Deuterostomia - ps17
Chordata - ps18
Olfactores - ps19
Vertebrata - ps20
Gnathostomata - ps21
Euteleostomi - ps22
Sarcopterygii - ps23
Tetrapoda - ps24
Amniota - ps25
Mammalia - ps26
Theria - ps27
Placentalia - ps28
Boreoeutheria - ps29
Euarchontoglires - ps30
Euarchonta - ps31
Primates - ps32
Haplorrhini - ps33
Simiiformes - ps34
Catarrhini - ps35
Hominoidea - ps36
Hominidae - ps37
Homininae - ps38
Hominini - ps39
Homo_sapiens - ps40
p-value
5e-05
2e-04
1e-03
5e-03
2e-02
Log-odds
0.5
1.0
1.5
2.0
2.5
3.0
3.5
4.0

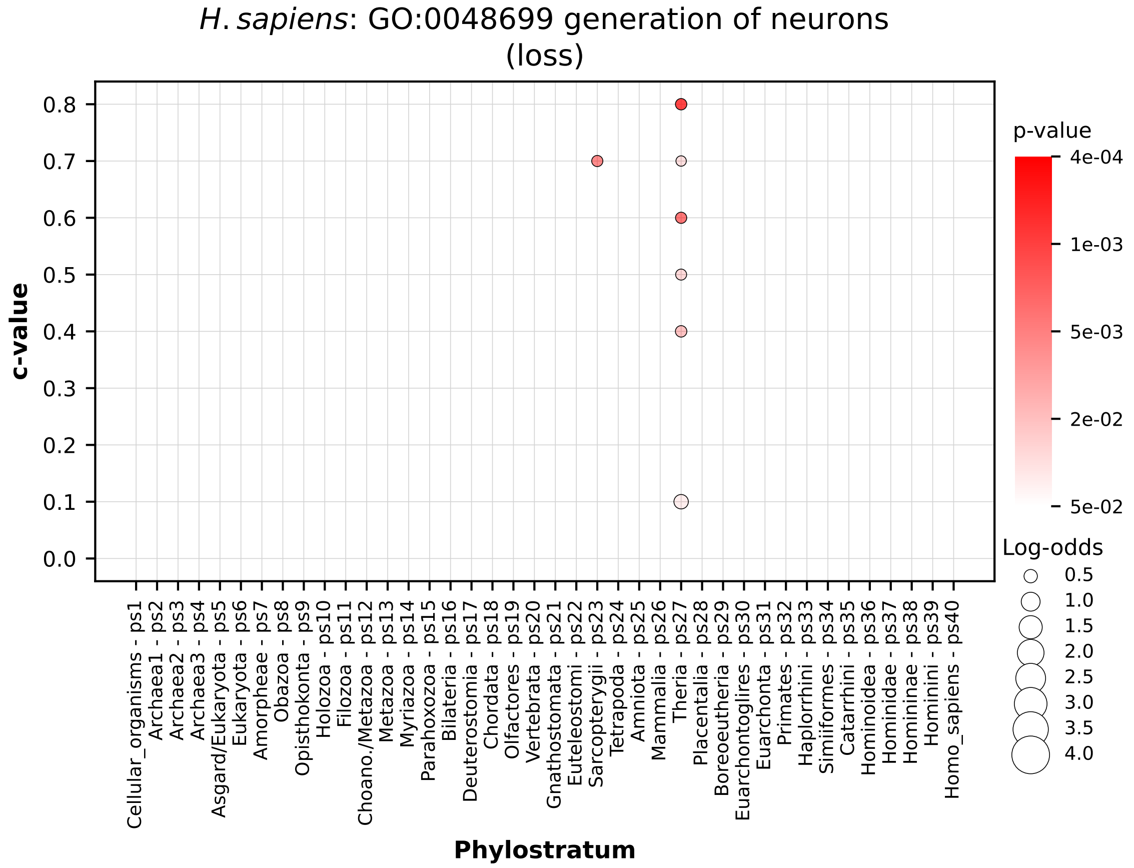

H. sapiens: GO:0048699 generation of neurons (loss)
c-value
0.0
0.1
0.2
0.3
0.4
0.5
0.6
0.7
0.8
Phylostratum
Cellular_organisms - ps1
Archaea1 - ps2
Archaea2 - ps3
Archaea3 - ps4
Asgard/Eukaryota - ps5
Eukaryota - ps6
Amorpheae - ps7
Obazoa - ps8
Opisthokonta - ps9
Holozoa - ps10
Filozoa - ps11
Choano./Metazoa - ps12
Metazoa - ps13
Myriazoa - ps14
Parahoxozoa - ps15
Bilateria - ps16
Deuterostomia - ps17
Chordata - ps18
Olfactores - ps19
Vertebrata - ps20
Gnathostomata - ps21
Euteleostomi - ps22
Sarcopterygii - ps23
Tetrapoda - ps24
Amniota - ps25
Mammalia - ps26
Theria - ps27
Placentalia - ps28
Boreoeutheria - ps29
Euarchontoglires - ps30
Euarchonta - ps31
Primates - ps32
Haplorrhini - ps33
Simiiformes - ps34
Catarrhini - ps35
Hominoidea - ps36
Hominidae - ps37
Homininae - ps38
Hominini - ps39
Homo_sapiens - ps40
p-value
4e-04
1e-03
5e-03
2e-02
5e-02
Log-odds
0.5
1.0
1.5
2.0
2.5
3.0
3.5
4.0

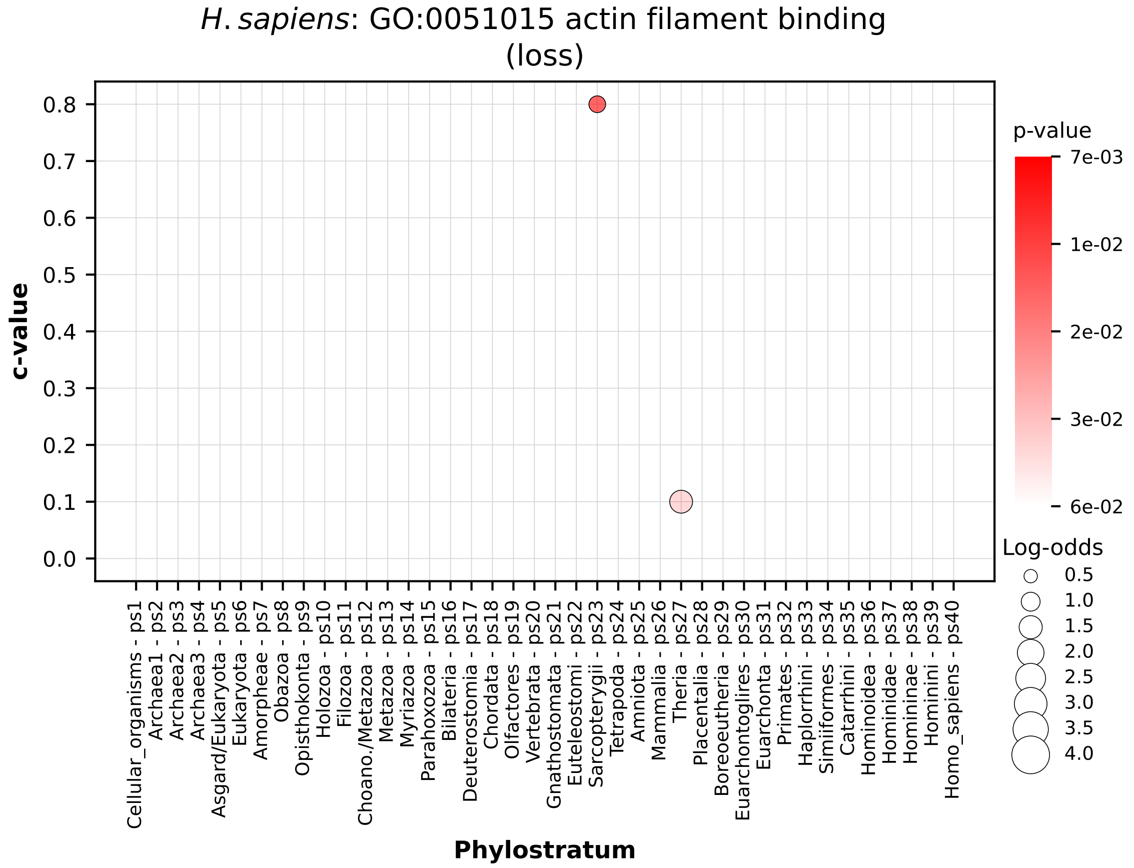

H. sapiens: GO:0051015 actin filament binding
(loss)
c-value
0.0
0.1
0.2
0.3
0.4
0.5
0.6
0.7
0.8
p-value
7e-03
1e-02
2e-02
3e-02
6e-02
Log-odds
0.5
1.0
1.5
2.0
2.5
3.0
3.5
4.0
Cellular_organisms - ps1
Archaea1 - ps2
Archaea2 - ps3
Archaea3 - ps4
Asgard/Eukaryota - ps5
Eukaryota - ps6
Amorpheae - ps7
Obazoa - ps8
Opisthokonta - ps9
Holozoa - ps10
Filozoa - ps11
Choano./Metazoa - ps12
Metazoa - ps13
Myriazoa - ps14
Parahoxozoa - ps15
Bilateria - ps16
Deuterostomia - ps17
Chordata - ps18
Olfactores - ps19
Vertebrata - ps20
Gnathostomata - ps21
Euteleostomi - ps22
Sarcopterygii - ps23
Tetrapoda - ps24
Amniota - ps25
Mammalia - ps26
Theria - ps27
Placentalia - ps28
Boreoeutheria - ps29
Euarchontoglires - ps30
Euarchonta - ps31
Primates - ps32
Haplorrhini - ps33
Simiiformes - ps34
Catarrhini - ps35
Hominoidea - ps36
Hominidae - ps37
Homininae - ps38
Hominini - ps39
Homo_sapiens - ps40
Phylostratum

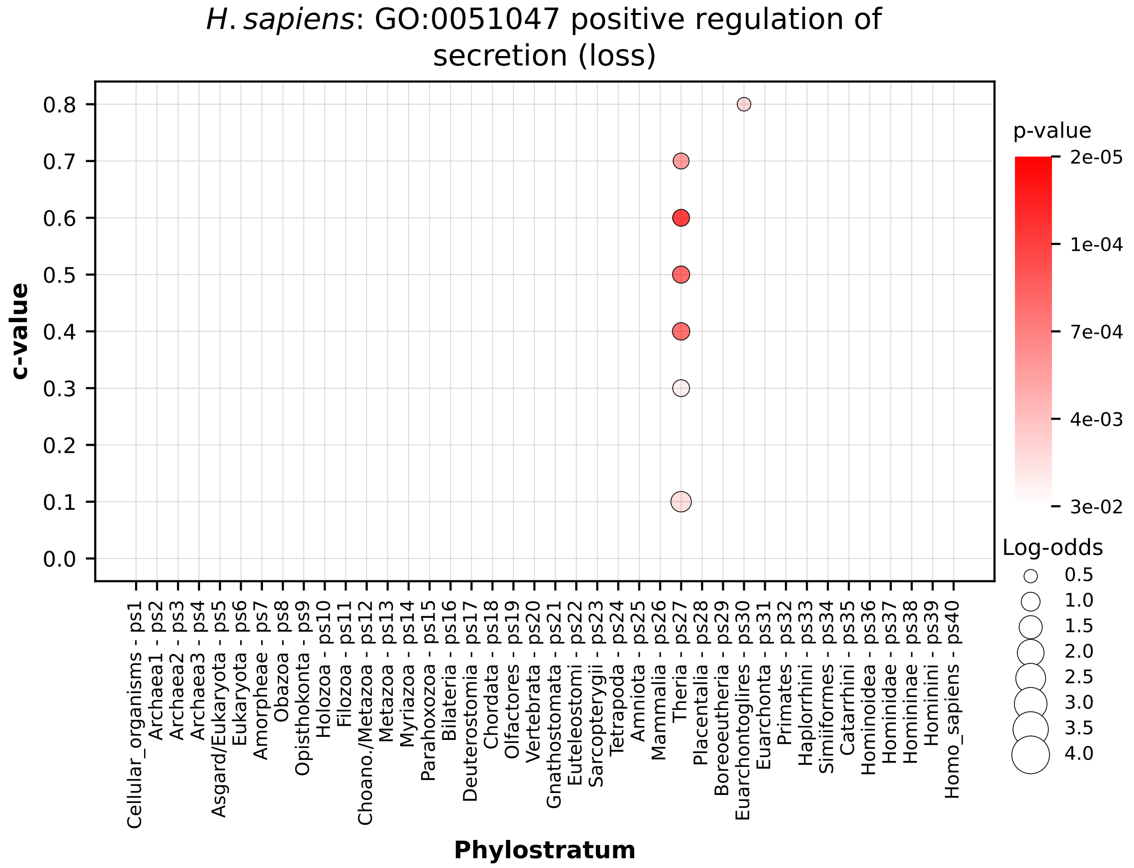

H. sapiens: GO:0051047 positive regulation of secretion (loss)
c-value
0.8
0.7
0.6
0.5
0.4
0.3
0.2
0.1
0.0
p-value
2e-05
1e-04
7e-04
4e-03
3e-02
Log-odds
0.5
1.0
1.5
2.0
2.5
3.0
3.5
4.0
Cellular_organisms - ps1
Archaea1 - ps2
Archaea2 - ps3
Archaea3 - ps4
Asgard/Eukaryota - ps5
Eukaryota - ps6
Amorpheae - ps7
Obazoa - ps8
Opisthokonta - ps9
Holozoa - ps10
Filozoa - ps11
Choano./Metazoa - ps12
Metazoa - ps13
Myriazoa - ps14
Parahoxozoa - ps15
Bilateria - ps16
Deuterostomia - ps17
Chordata - ps18
Olfactores - ps19
Vertebrata - ps20
Gnathostomata - ps21
Euteleostomi - ps22
Sarcopterygii - ps23
Tetrapoda - ps24
Amniota - ps25
Mammalia - ps26
Theria - ps27
Placentalia - ps28
Boreoeutheria - ps29
Euarchontoglires - ps30
Euarchonta - ps31
Primates - ps32
Haplorrhini - ps33
Simiiformes - ps34
Catarrhini - ps35
Hominoidea - ps36
Hominidae - ps37
Homininae - ps38
Hominini - ps39
Homo_sapiens - ps40
Phylostratum

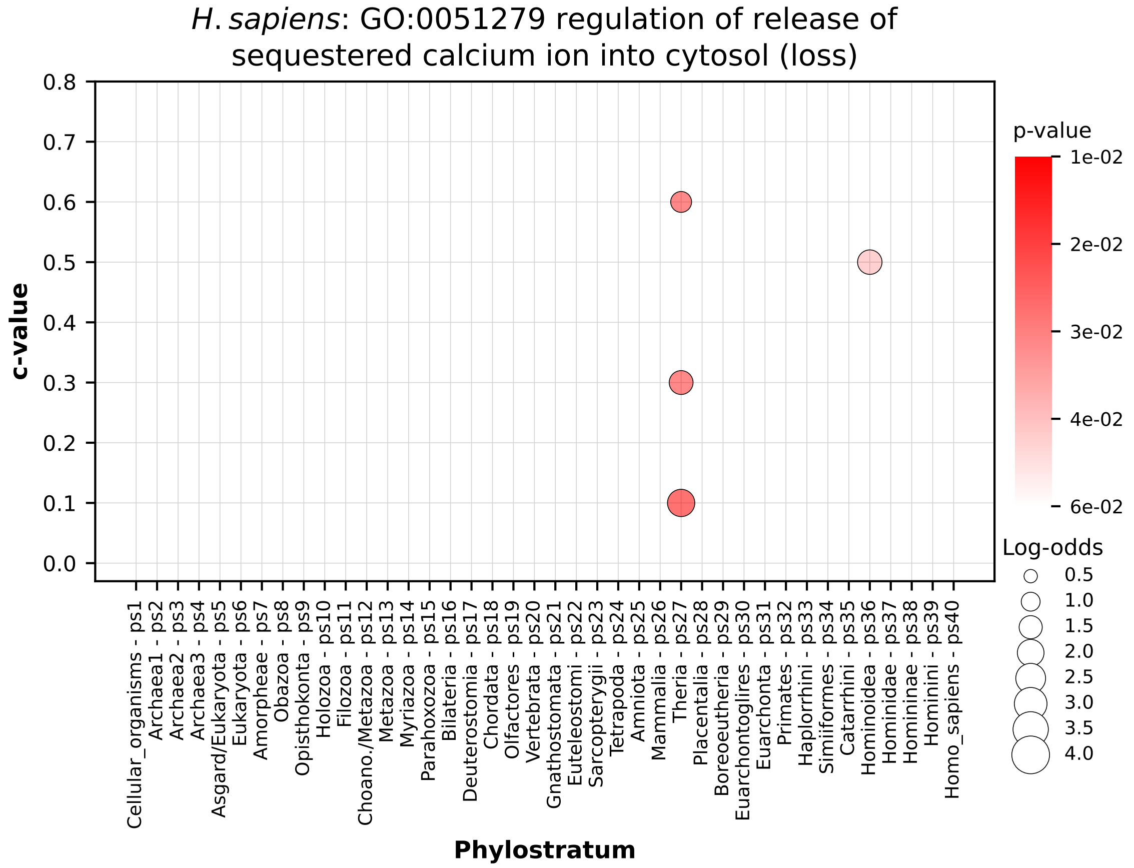

H. sapiens: GO:0051279 regulation of release of sequestered calcium ion into cytosol (loss)
c-value
0.8
0.7
0.6
0.5
0.4
0.3
0.2
0.1
0.0
p-value
1e-02
2e-02
3e-02
4e-02
6e-02
Log-odds
0.5
1.0
1.5
2.0
2.5
3.0
3.5
4.0
Cellular_organisms - ps1
Archaea1 - ps2
Archaea2 - ps3
Archaea3 - ps4
Asgard/Eukaryota - ps5
Eukaryota - ps6
Amorpheae - ps7
Obazoa - ps8
Opisthokonta - ps9
Holozoa - ps10
Filozoa - ps11
Choano./Metazoa - ps12
Metazoa - ps13
Myriazoa - ps14
Parahoxozoa - ps15
Bilateria - ps16
Deuterostomia - ps17
Chordata - ps18
Olfactores - ps19
Vertebrata - ps20
Gnathostomata - ps21
Euteleostomi - ps22
Sarcopterygii - ps23
Tetrapoda - ps24
Amniota - ps25
Mammalia - ps26
Theria - ps27
Placentalia - ps28
Boreoeutheria - ps29
Euarchontoglires - ps30
Euarchonta - ps31
Primates - ps32
Haplorrhini - ps33
Simiiformes - ps34
Catarrhini - ps35
Hominoidea - ps36
Hominidae - ps37
Homininae - ps38
Hominini - ps39
Homo_sapiens - ps40
Phylostratum

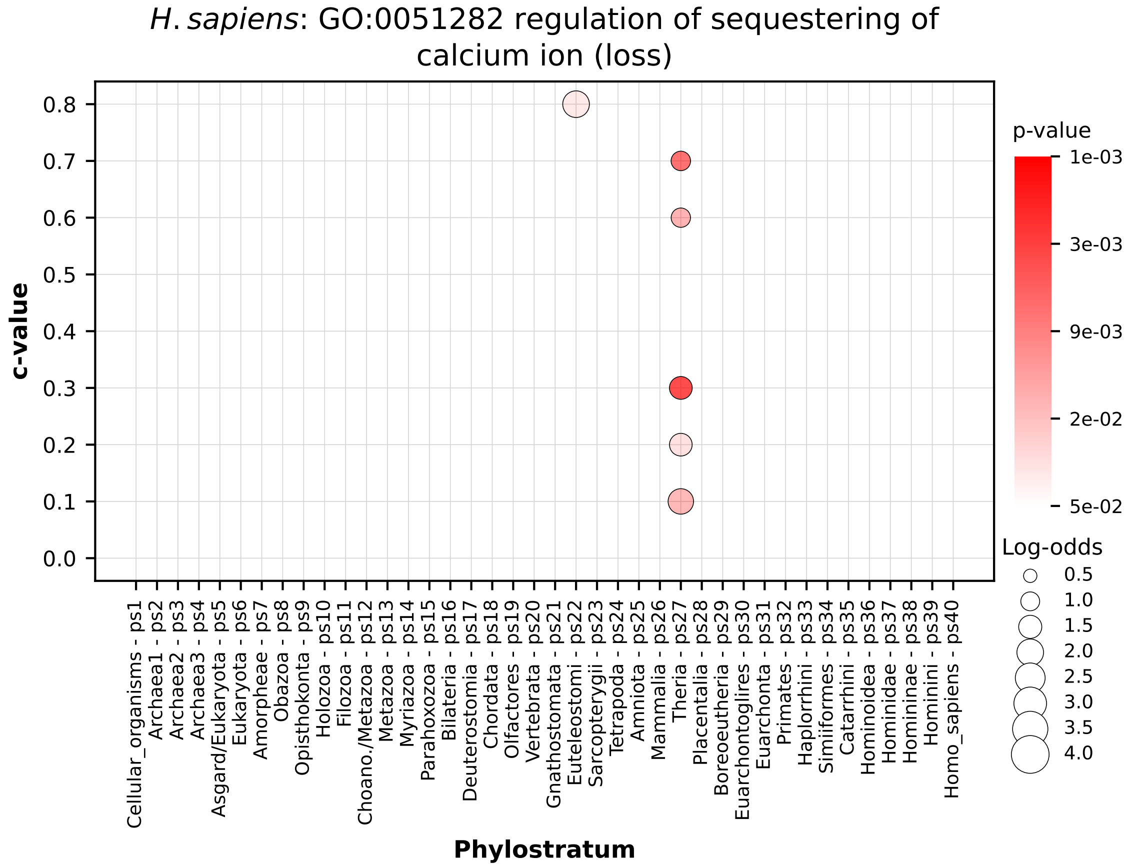

H. sapiens: GO:0051282 regulation of sequestering of calcium ion (loss)
c-value
0.8
0.7
0.6
0.5
0.4
0.3
0.2
0.1
0.0
p-value
1e-03
3e-03
9e-03
2e-02
5e-02
Log-odds
0.5
1.0
1.5
2.0
2.5
3.0
3.5
4.0
Cellular_organisms - ps1
Archaea1 - ps2
Archaea2 - ps3
Archaea3 - ps4
Asgard/Eukaryota - ps5
Eukaryota - ps6
Amorpheae - ps7
Obazoa - ps8
Opisthokonta - ps9
Holozoa - ps10
Filozoa - ps11
Choano./Metazoa - ps12
Metazoa - ps13
Myriazoa - ps14
Parahoxozoa - ps15
Bilateria - ps16
Deuterostomia - ps17
Chordata - ps18
Olfactores - ps19
Vertebrata - ps20
Gnathostomata - ps21
Euteleostomi - ps22
Sarcopterygii - ps23
Tetrapoda - ps24
Amniota - ps25
Mammalia - ps26
Theria - ps27
Placentalia - ps28
Boreoeutheria - ps29
Euarchontoglires - ps30
Euarchonta - ps31
Primates - ps32
Haplorrhini - ps33
Simiiformes - ps34
Catarrhini - ps35
Hominoidea - ps36
Hominidae - ps37
Homininae - ps38
Hominini - ps39
Homo_sapiens - ps40
Phylostratum

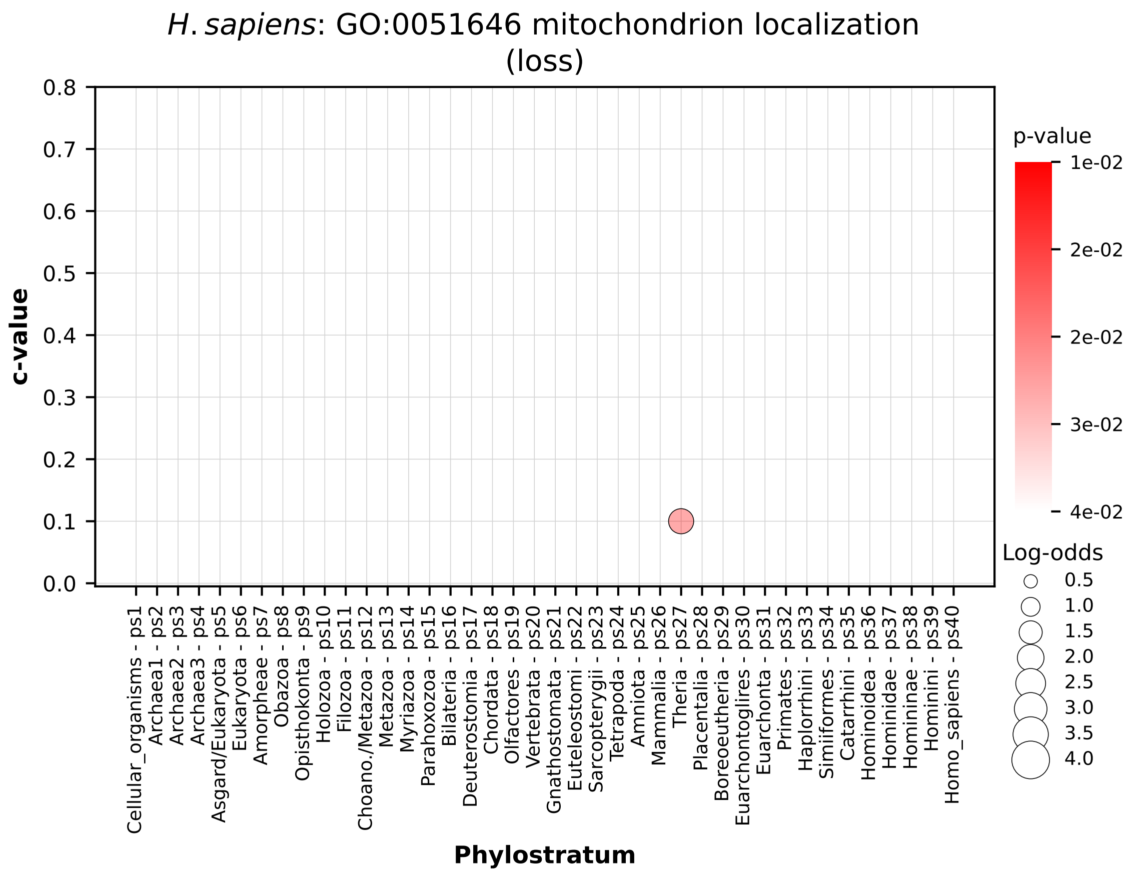

H. sapiens: GO:0051646 mitochondrion localization
(loss)
c-value
0.8
0.7
0.6
0.5
0.4
0.3
0.2
0.1
0.0
Phylostratum
Cellular_organisms - ps1
Archaea1 - ps2
Archaea2 - ps3
Archaea3 - ps4
Asgard/Eukaryota - ps5
Eukaryota - ps6
Amorpheae - ps7
Obazoa - ps8
Opisthokonta - ps9
Holozoa - ps10
Filozoa - ps11
Choano./Metazoa - ps12
Metazoa - ps13
Myriazoa - ps14
Parahoxozoa - ps15
Bilateria - ps16
Deuterostomia - ps17
Chordata - ps18
Olfactores - ps19
Vertebrata - ps20
Gnathostomata - ps21
Euteleostomi - ps22
Sarcopterygii - ps23
Tetrapoda - ps24
Amniota - ps25
Mammalia - ps26
Theria - ps27
Placentalia - ps28
Boreoeutheria - ps29
Euarchontoglires - ps30
Euarchonta - ps31
Primates - ps32
Haplorrhini - ps33
Simiiformes - ps34
Catarrhini - ps35
Hominoidea - ps36
Hominidae - ps37
Homininae - ps38
Hominini - ps39
Homo_sapiens - ps40
p-value
1e-02
2e-02
2e-02
3e-02
4e-02
Log-odds
0.5
1.0
1.5
2.0
2.5
3.0
3.5
4.0

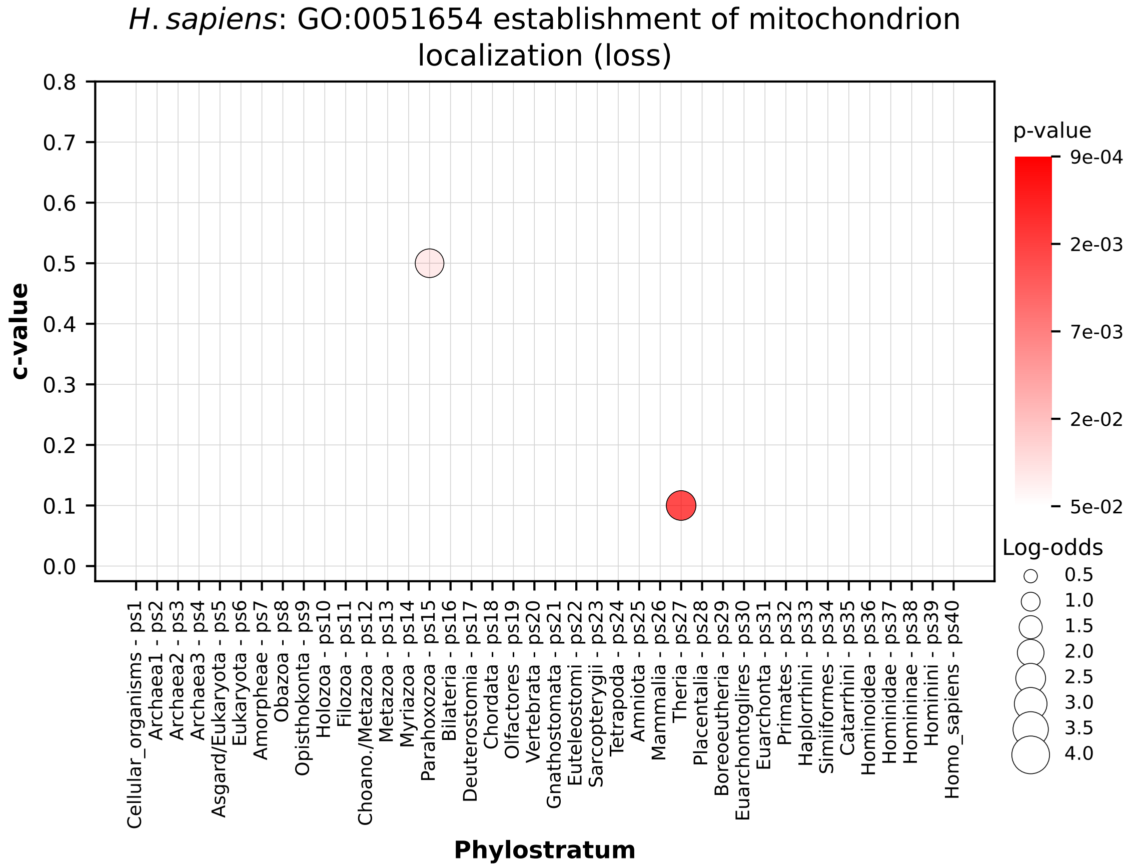

H. sapiens: GO:0051654 establishment of mitochondrion localization (loss)
c-value
0.8
0.7
0.6
0.5
0.4
0.3
0.2
0.1
0.0
Phylostratum
Cellular_organisms - ps1
Archaea1 - ps2
Archaea2 - ps3
Archaea3 - ps4
Asgard/Eukaryota - ps5
Eukaryota - ps6
Amorpheae - ps7
Obazoa - ps8
Opisthokonta - ps9
Holozoa - ps10
Filozoa - ps11
Choano./Metazoa - ps12
Metazoa - ps13
Myriazoa - ps14
Parahoxozoa - ps15
Bilateria - ps16
Deuterostomia - ps17
Chordata - ps18
Olfactores - ps19
Vertebrata - ps20
Gnathostomata - ps21
Euteleostomi - ps22
Sarcopterygii - ps23
Tetrapoda - ps24
Amniota - ps25
Mammalia - ps26
Theria - ps27
Placentalia - ps28
Boreoeutheria - ps29
Euarchontoglires - ps30
Euarchonta - ps31
Primates - ps32
Haplorrhini - ps33
Simiiformes - ps34
Catarrhini - ps35
Hominoidea - ps36
Hominidae - ps37
Homininae - ps38
Hominini - ps39
Homo_sapiens - ps40
p-value
9e-04
2e-03
7e-03
2e-02
5e-02
Log-odds
0.5
1.0
1.5
2.0
2.5
3.0
3.5
4.0

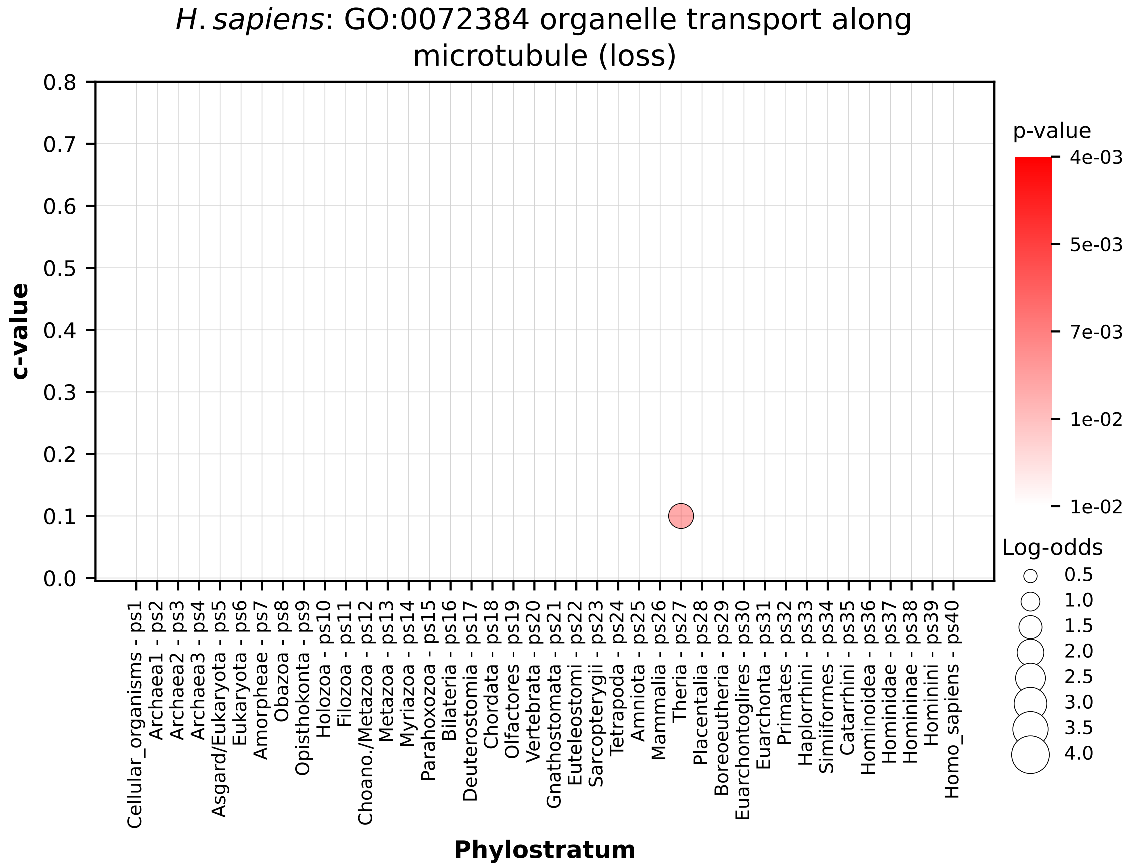

H. sapiens: GO:0072384 organelle transport along microtubule (loss)
c-value
0.0
0.1
0.2
0.3
0.4
0.5
0.6
0.7
0.8
Phylostratum
Cellular_organisms - ps1
Archaea1 - ps2
Archaea2 - ps3
Archaea3 - ps4
Asgard/Eukaryota - ps5
Eukaryota - ps6
Amorpheae - ps7
Obazoa - ps8
Opisthokonta - ps9
Holozoa - ps10
Filozoa - ps11
Choano./Metazoa - ps12
Metazoa - ps13
Myriazoa - ps14
Parahoxozoa - ps15
Bilateria - ps16
Deuterostomia - ps17
Chordata - ps18
Olfactores - ps19
Vertebrata - ps20
Gnathostomata - ps21
Euteleostomi - ps22
Sarcopterygii - ps23
Tetrapoda - ps24
Amniota - ps25
Mammalia - ps26
Theria - ps27
Placentalia - ps28
Boreoeutheria - ps29
Euarchontoglires - ps30
Euarchonta - ps31
Primates - ps32
Haplorrhini - ps33
Simiiformes - ps34
Catarrhini - ps35
Hominoidea - ps36
Hominidae - ps37
Homininae - ps38
Hominini - ps39
Homo_sapiens - ps40
p-value
4e-03
5e-03
7e-03
1e-02
1e-02
Log-odds
0.5
1.0
1.5
2.0
2.5
3.0
3.5
4.0

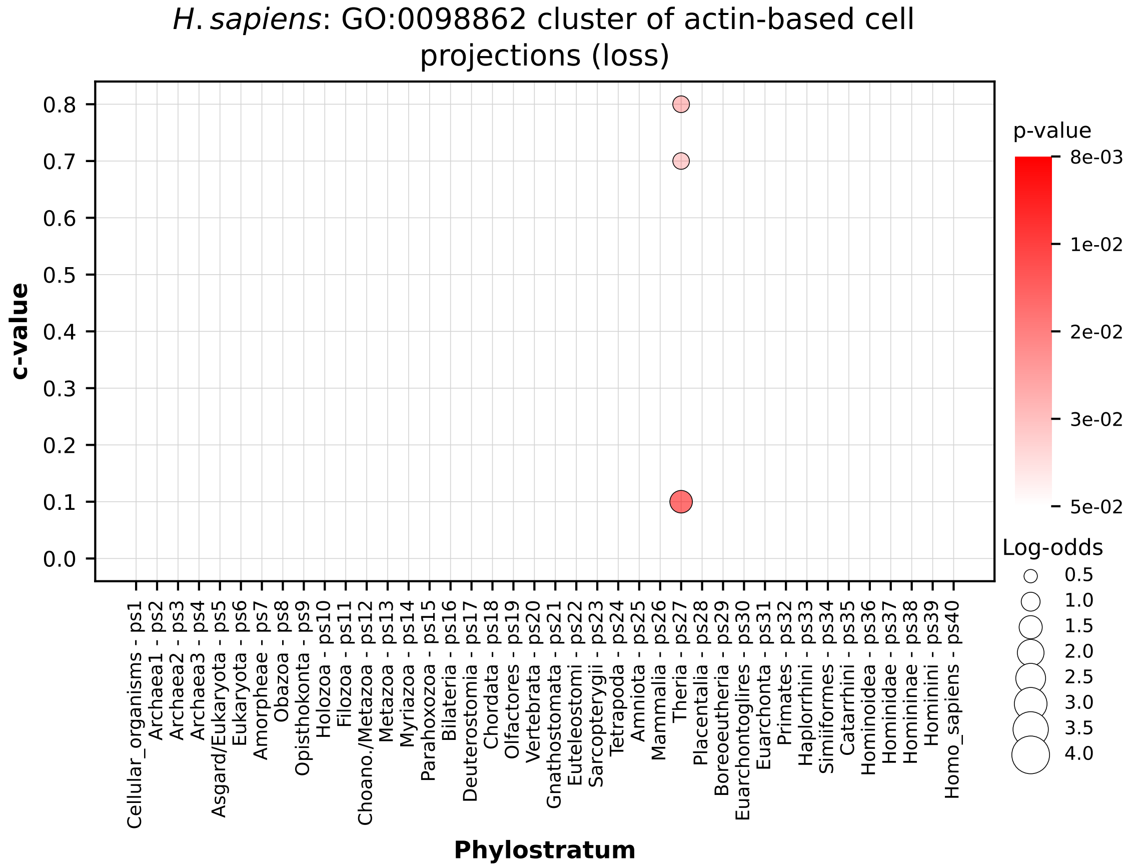

H. sapiens: GO:0098862 cluster of actin-based cell projections (loss)
c-value
0.0
0.1
0.2
0.3
0.4
0.5
0.6
0.7
0.8
Phylostratum
Cellular_organisms - ps1
Archaea1 - ps2
Archaea2 - ps3
Archaea3 - ps4
Asgard/Eukaryota - ps5
Eukaryota - ps6
Amorpheae - ps7
Obazoa - ps8
Opisthokonta - ps9
Holozoa - ps10
Filozoa - ps11
Choano./Metazoa - ps12
Metazoa - ps13
Myriazoa - ps14
Parahoxozoa - ps15
Bilateria - ps16
Deuterostomia - ps17
Chordata - ps18
Olfactores - ps19
Vertebrata - ps20
Gnathostomata - ps21
Euteleostomi - ps22
Sarcopterygii - ps23
Tetrapoda - ps24
Amniota - ps25
Mammalia - ps26
Theria - ps27
Placentalia - ps28
Boreoeutheria - ps29
Euarchontoglires - ps30
Euarchonta - ps31
Primates - ps32
Haplorrhini - ps33
Simiiformes - ps34
Catarrhini - ps35
Hominoidea - ps36
Hominidae - ps37
Homininae - ps38
Hominini - ps39
Homo_sapiens - ps40
p-value
8e-03
1e-02
2e-02
3e-02
5e-02
Log-odds
0.5
1.0
1.5
2.0
2.5
3.0
3.5
4.0

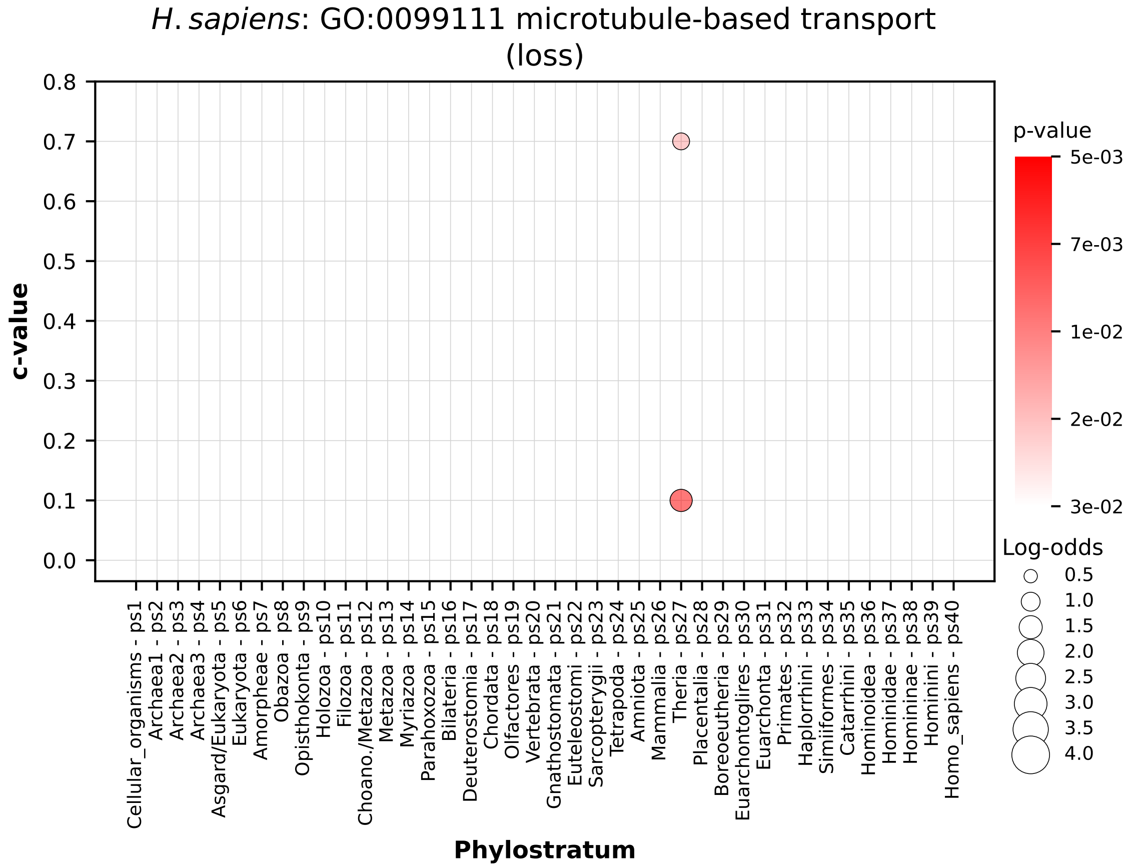

H. sapiens: GO:0099111 microtubule-based transport
(loss)
c-value
0.8
0.7
0.6
0.5
0.4
0.3
0.2
0.1
0.0
p-value
5e-03
7e-03
1e-02
2e-02
3e-02
Log-odds
0.5
1.0
1.5
2.0
2.5
3.0
3.5
4.0
Cellular_organisms - ps1
Archaea1 - ps2
Archaea2 - ps3
Archaea3 - ps4
Asgard/Eukaryota - ps5
Eukaryota - ps6
Amorpheae - ps7
Obazoa - ps8
Opisthokonta - ps9
Holozoa - ps10
Filozoa - ps11
Choano./Metazoa - ps12
Metazoa - ps13
Myriazoa - ps14
Parahoxozoa - ps15
Bilateria - ps16
Deuterostomia - ps17
Chordata - ps18
Olfactores - ps19
Vertebrata - ps20
Gnathostomata - ps21
Euteleostomi - ps22
Sarcopterygii - ps23
Tetrapoda - ps24
Amniota - ps25
Mammalia - ps26
Theria - ps27
Placentalia - ps28
Boreoeutheria - ps29
Euarchontoglires - ps30
Euarchonta - ps31
Primates - ps32
Haplorrhini - ps33
Simiiformes - ps34
Catarrhini - ps35
Hominoidea - ps36
Hominidae - ps37
Homininae - ps38
Hominini - ps39
Homo_sapiens - ps40
Phylostratum

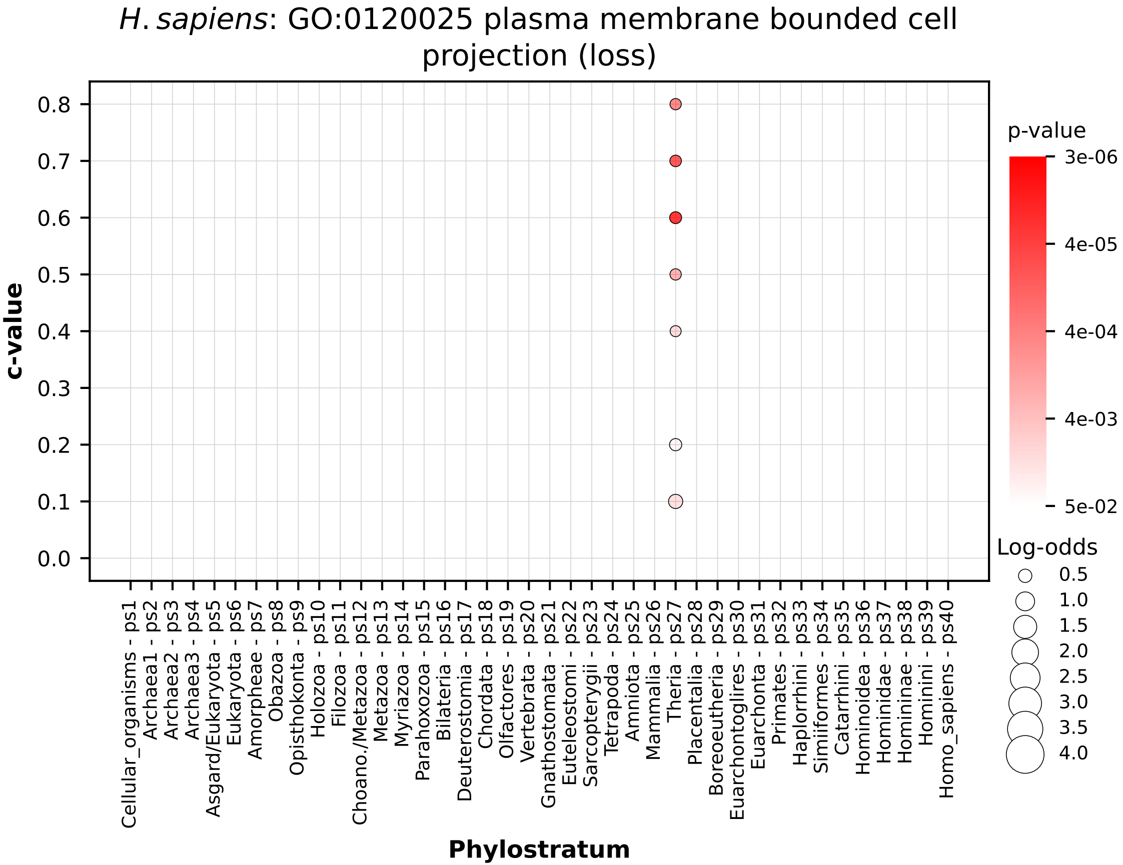

H. sapiens: GO:0120025 plasma membrane bounded cell projection (loss)
c-value
0.0
0.1
0.2
0.3
0.4
0.5
0.6
0.7
0.8
p-value
3e-06
4e-05
4e-04
4e-03
5e-02
Log-odds
0.5
1.0
1.5
2.0
2.5
3.0
3.5
4.0
Cellular_organisms - ps1
Archaea1 - ps2
Archaea2 - ps3
Archaea3 - ps4
Asgard/Eukaryota - ps5
Eukaryota - ps6
Amorpheae - ps7
Obazoa - ps8
Opisthokonta - ps9
Holozoa - ps10
Filozoa - ps11
Choano./Metazoa - ps12
Metazoa - ps13
Myriazoa - ps14
Parahoxozoa - ps15
Bilateria - ps16
Deuterostomia - ps17
Chordata - ps18
Olfactores - ps19
Vertebrata - ps20
Gnathostomata - ps21
Euteleostomi - ps22
Sarcopterygii - ps23
Tetrapoda - ps24
Amniota - ps25
Mammalia - ps26
Theria - ps27
Placentalia - ps28
Boreoeutheria - ps29
Euarchontoglires - ps30
Euarchonta - ps31
Primates - ps32
Haplorrhini - ps33
Simiiformes - ps34
Catarrhini - ps35
Hominoidea - ps36
Hominidae - ps37
Homininae - ps38
Hominini - ps39
Homo_sapiens - ps40
Phylostratum

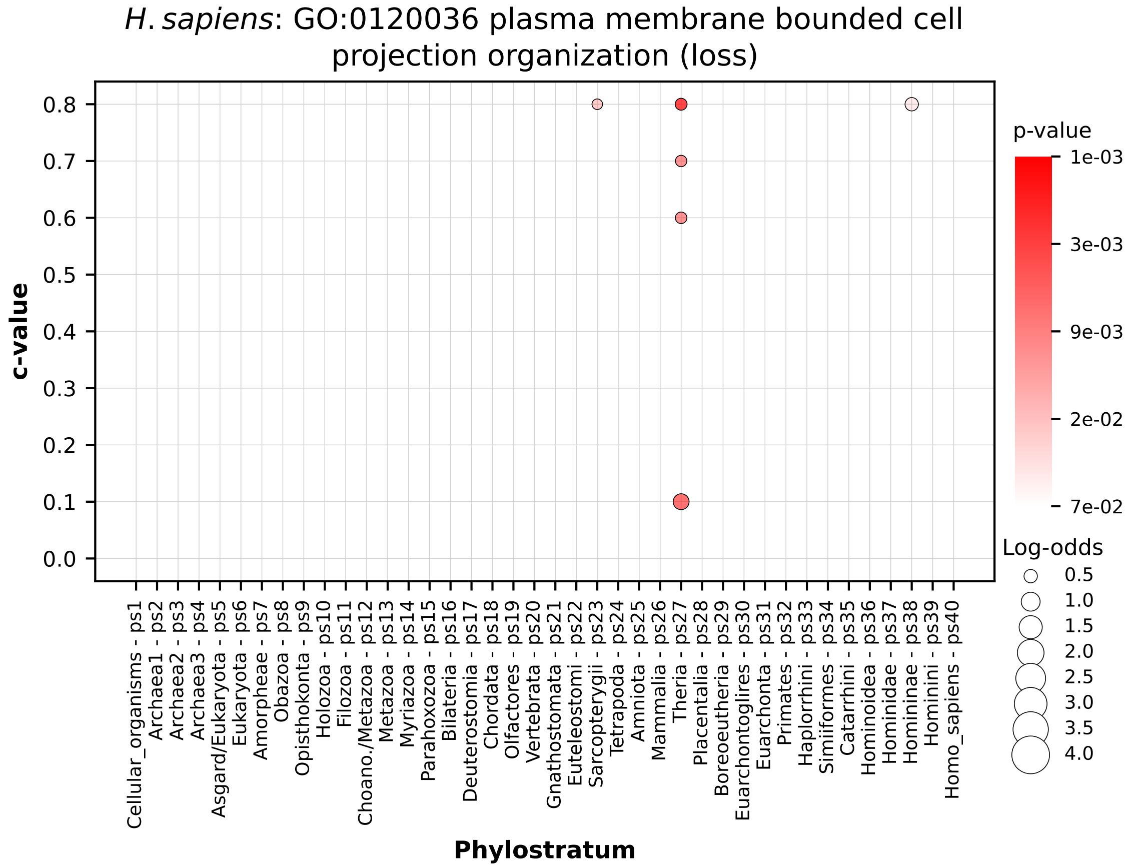

H. sapiens: GO:0120036 plasma membrane bounded cell projection organization (loss)
c-value
0.0
0.1
0.2
0.3
0.4
0.5
0.6
0.7
0.8
Phylostratum
Cellular_organisms - ps1
Archaea1 - ps2
Archaea2 - ps3
Archaea3 - ps4
Asgard/Eukaryota - ps5
Eukaryota - ps6
Amorpheae - ps7
Obazoa - ps8
Opisthokonta - ps9
Holozoa - ps10
Filozoa - ps11
Choano./Metazoa - ps12
Metazoa - ps13
Myriazoa - ps14
Parahoxozoa - ps15
Bilateria - ps16
Deuterostomia - ps17
Chordata - ps18
Olfactores - ps19
Vertebrata - ps20
Gnathostomata - ps21
Euteleostomi - ps22
Sarcopterygii - ps23
Tetrapoda - ps24
Amniota - ps25
Mammalia - ps26
Theria - ps27
Placentalia - ps28
Boreoeutheria - ps29
Euarchontoglires - ps30
Euarchonta - ps31
Primates - ps32
Haplorrhini - ps33
Simiiformes - ps34
Catarrhini - ps35
Hominoidea - ps36
Hominidae - ps37
Homininae - ps38
Hominini - ps39
Homo_sapiens - ps40
p-value
1e-03
3e-03
9e-03
2e-02
7e-02
Log-odds
0.5
1.0
1.5
2.0
2.5
3.0
3.5
4.0

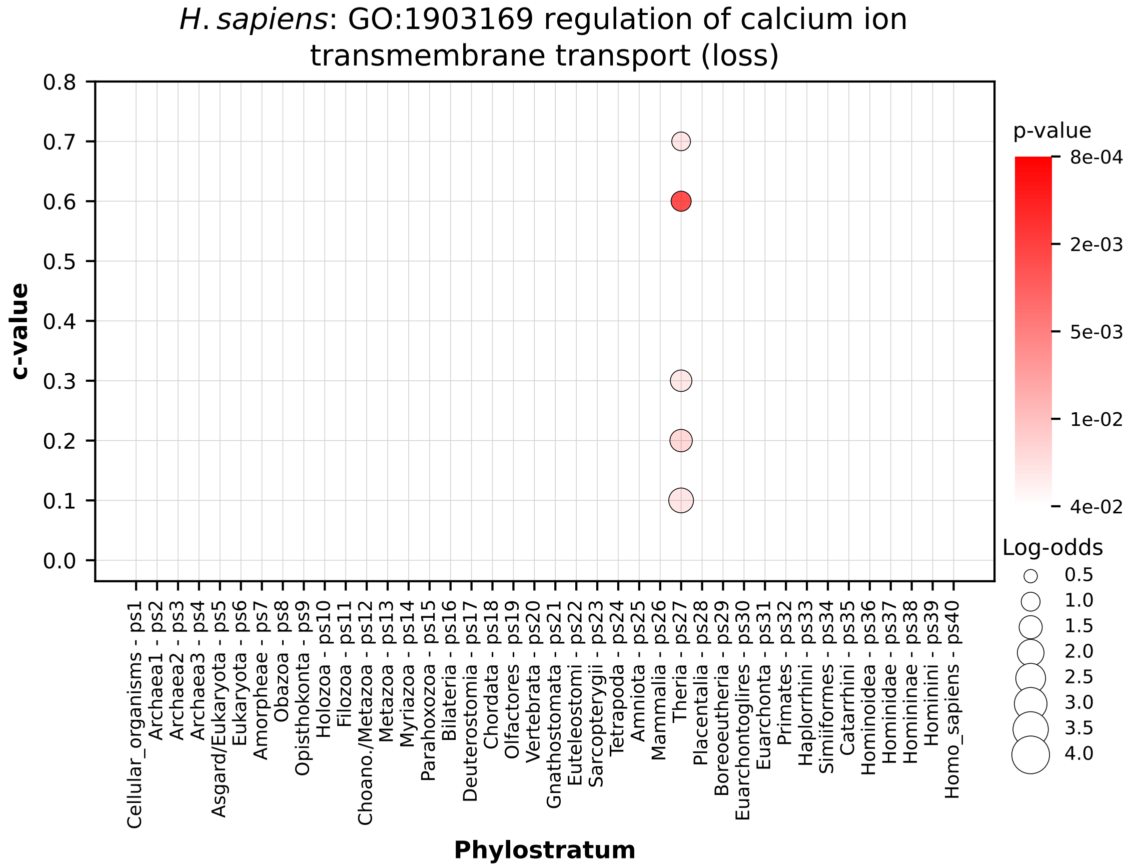

H. sapiens: GO:1903169 regulation of calcium ion transmembrane transport (loss)
c-value
0.8
0.7
0.6
0.5
0.4
0.3
0.2
0.1
0.0
Phylostratum
Cellular_organisms - ps1
Archaea1 - ps2
Archaea2 - ps3
Archaea3 - ps4
Asgard/Eukaryota - ps5
Eukaryota - ps6
Amorpheae - ps7
Obazoa - ps8
Opisthokonta - ps9
Holozoa - ps10
Filozoa - ps11
Choano./Metazoa - ps12
Metazoa - ps13
Myriazoa - ps14
Parahoxozoa - ps15
Bilateria - ps16
Deuterostomia - ps17
Chordata - ps18
Olfactores - ps19
Vertebrata - ps20
Gnathostomata - ps21
Euteleostomi - ps22
Sarcopterygii - ps23
Tetrapoda - ps24
Amniota - ps25
Mammalia - ps26
Theria - ps27
Placentalia - ps28
Boreoeutheria - ps29
Euarchontoglires - ps30
Euarchonta - ps31
Primates - ps32
Haplorrhini - ps33
Simiiformes - ps34
Catarrhini - ps35
Hominoidea - ps36
Hominidae - ps37
Homininae - ps38
Hominini - ps39
Homo_sapiens - ps40
p-value
8e-04
2e-03
5e-03
1e-02
4e-02
Log-odds
0.5
1.0
1.5
2.0
2.5
3.0
3.5
4.0

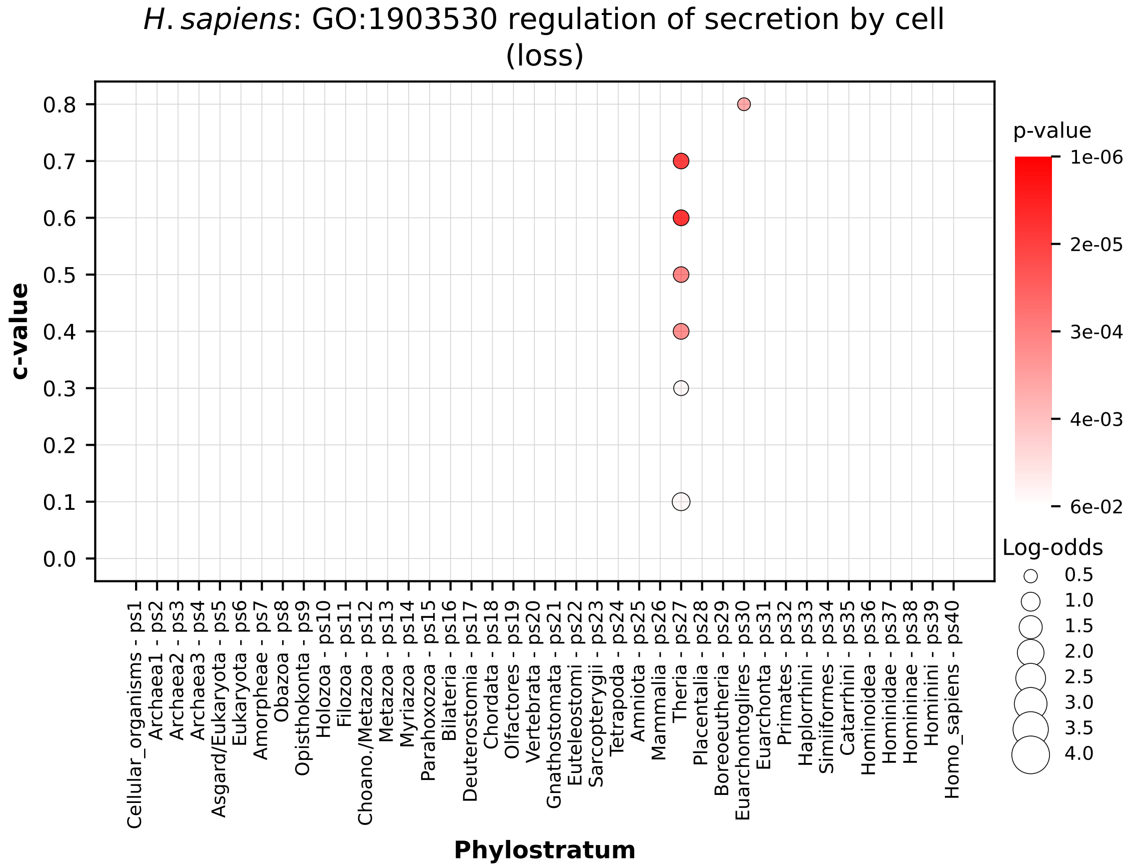

H. sapiens: GO:1903530 regulation of secretion by cell
(loss)
c-value
0.0
0.1
0.2
0.3
0.4
0.5
0.6
0.7
0.8
Phylostratum
Cellular_organisms - ps1
Archaea1 - ps2
Archaea2 - ps3
Archaea3 - ps4
Asgard/Eukaryota - ps5
Eukaryota - ps6
Amorpheae - ps7
Obazoa - ps8
Opisthokonta - ps9
Holozoa - ps10
Filozoa - ps11
Choano./Metazoa - ps12
Metazoa - ps13
Myriazoa - ps14
Parahoxozoa - ps15
Bilateria - ps16
Deuterostomia - ps17
Chordata - ps18
Olfactores - ps19
Vertebrata - ps20
Gnathostomata - ps21
Euteleostomi - ps22
Sarcopterygii - ps23
Tetrapoda - ps24
Amniota - ps25
Mammalia - ps26
Theria - ps27
Placentalia - ps28
Boreoeutheria - ps29
Euarchontoglires - ps30
Euarchonta - ps31
Primates - ps32
Haplorrhini - ps33
Simiiformes - ps34
Catarrhini - ps35
Hominoidea - ps36
Hominidae - ps37
Homininae - ps38
Hominini - ps39
Homo_sapiens - ps40
p-value
1e-06
2e-05
3e-04
4e-03
6e-02
Log-odds
0.5
1.0
1.5
2.0
2.5
3.0
3.5
4.0

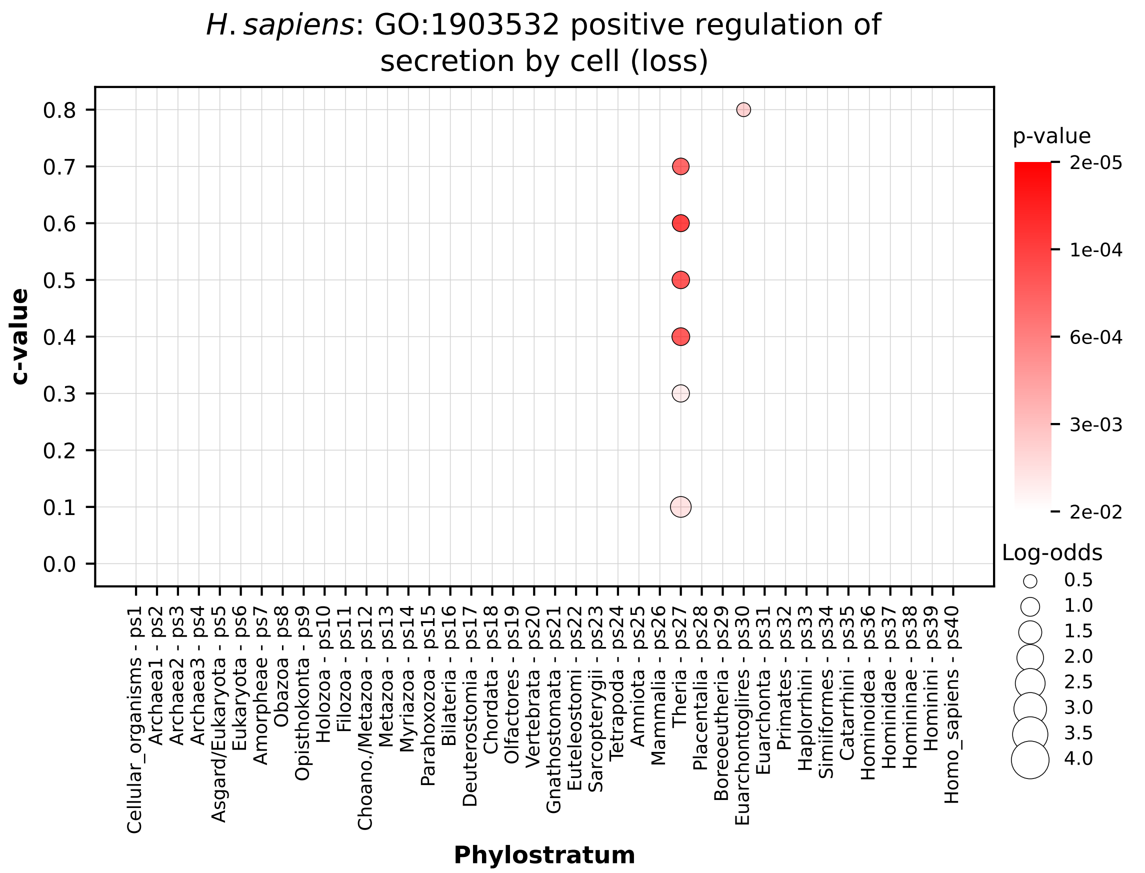

H. sapiens: GO:1903532 positive regulation of secretion by cell (loss)
c-value
0.8
0.7
0.6
0.5
0.4
0.3
0.2
0.1
0.0
p-value
2e-05
1e-04
6e-04
3e-03
2e-02
Log-odds
0.5
1.0
1.5
2.0
2.5
3.0
3.5
4.0
Cellular_organisms - ps1
Archaea1 - ps2
Archaea2 - ps3
Archaea3 - ps4
Asgard/Eukaryota - ps5
Eukaryota - ps6
Amorpheae - ps7
Obazoa - ps8
Opisthokonta - ps9
Holozoa - ps10
Filozoa - ps11
Choano./Metazoa - ps12
Metazoa - ps13
Myriazoa - ps14
Parahoxozoa - ps15
Bilateria - ps16
Deuterostomia - ps17
Chordata - ps18
Olfactores - ps19
Vertebrata - ps20
Gnathostomata - ps21
Euteleostomi - ps22
Sarcopterygii - ps23
Tetrapoda - ps24
Amniota - ps25
Mammalia - ps26
Theria - ps27
Placentalia - ps28
Boreoeutheria - ps29
Euarchontoglires - ps30
Euarchonta - ps31
Primates - ps32
Haplorrhini - ps33
Simiiformes - ps34
Catarrhini - ps35
Hominoidea - ps36
Hominidae - ps37
Homininae - ps38
Hominini - ps39
Homo_sapiens - ps40
Phylostratum

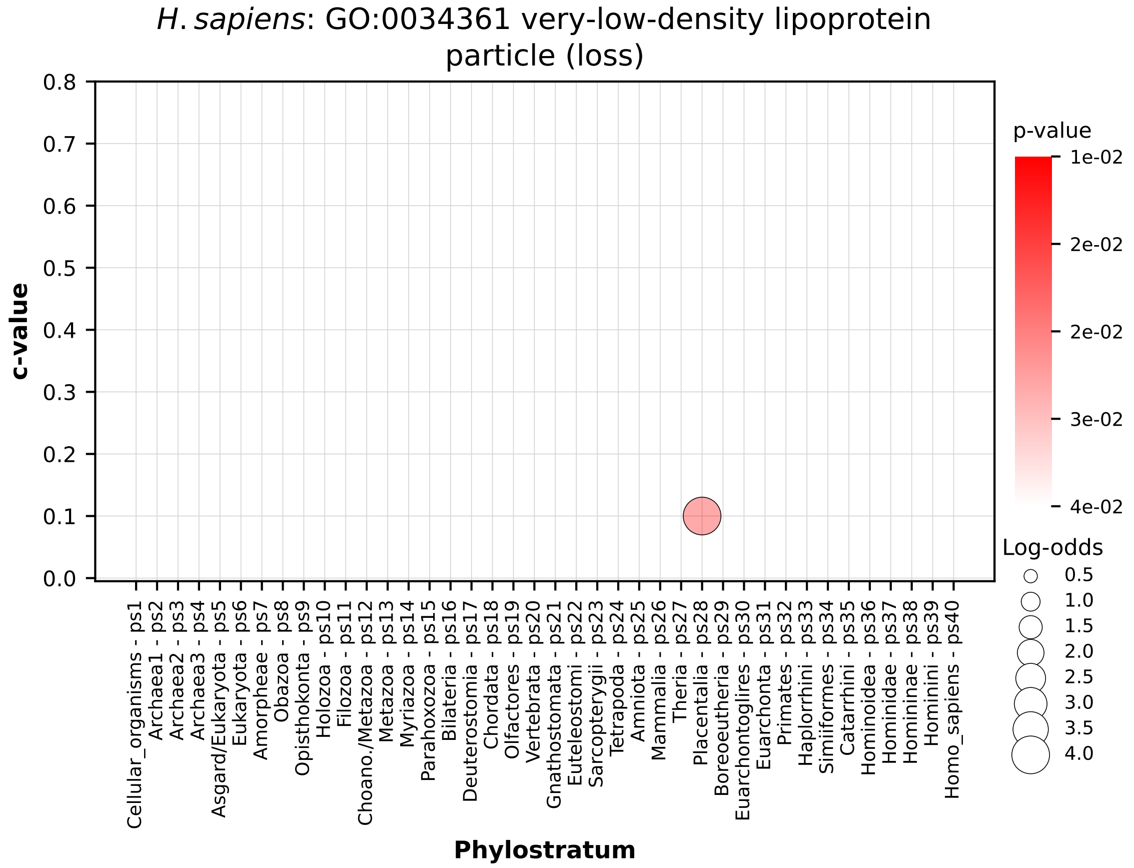
H. sapiens: GO:0034361 very-low-density lipoprotein particle (loss)
c-value
0.8
0.7
0.6
0.5
0.4
0.3
0.2
0.1
0.0
p-value
1e-02
2e-02
2e-02
3e-02
4e-02
Log-odds
0.5
1.0
1.5
2.0
2.5
3.0
3.5
4.0
Cellular_organisms - ps1
Archaea1 - ps2
Archaea2 - ps3
Archaea3 - ps4
Asgard/Eukaryota - ps5
Eukaryota - ps6
Amorpheae - ps7
Obazoa - ps8
Opisthokonta - ps9
Holozoa - ps10
Filozoa - ps11
Choano./Metazoa - ps12
Metazoa - ps13
Myriazoa - ps14
Parahoxozoa - ps15
Bilateria - ps16
Deuterostomia - ps17
Chordata - ps18
Olfactores - ps19
Vertebrata - ps20
Gnathostomata - ps21
Euteleostomi - ps22
Sarcopterygii - ps23
Tetrapoda - ps24
Amniota - ps25
Mammalia - ps26
Theria - ps27
Placentalia - ps28
Boreoeutheria - ps29
Euarchontoglires - ps30
Euarchonta - ps31
Primates - ps32
Haplorrhini - ps33
Simiiformes - ps34
Catarrhini - ps35
Hominoidea - ps36
Hominidae - ps37
Homininae - ps38
Hominini - ps39
Homo_sapiens - ps40
Phylostratum

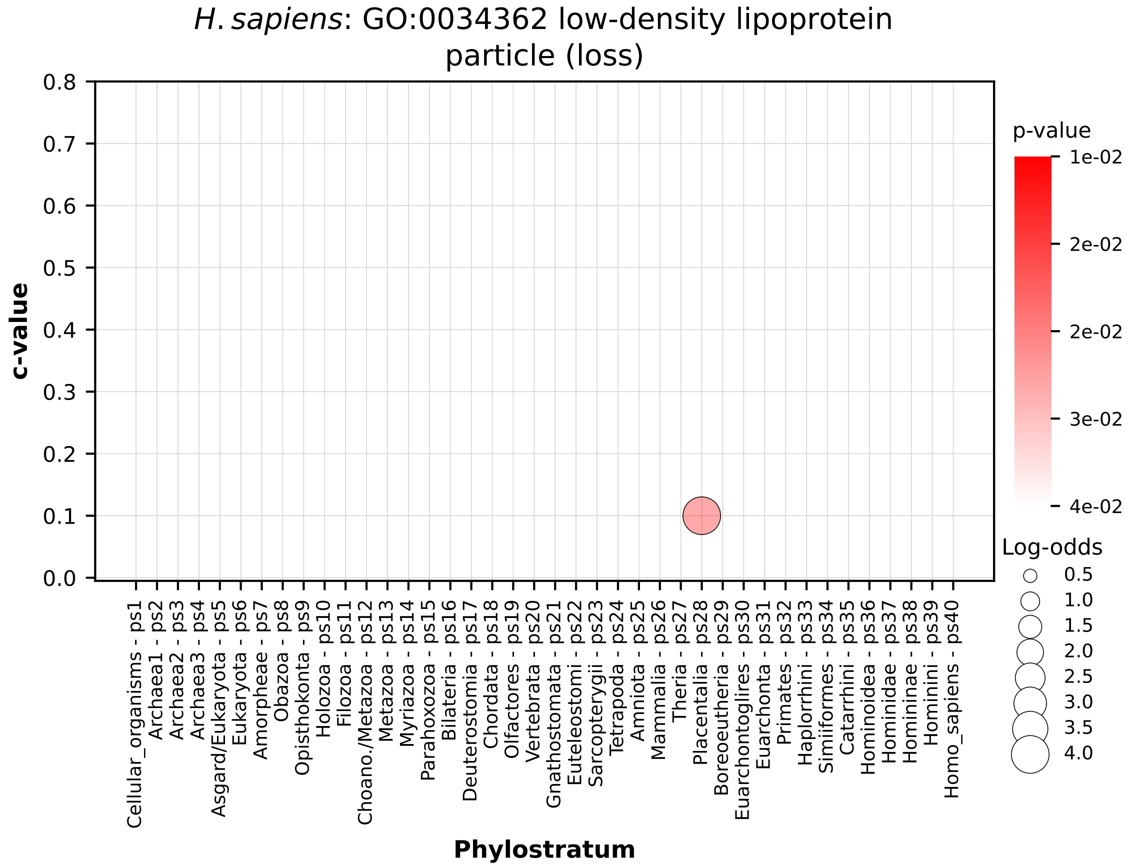

H. sapiens: GO:0034362 low-density lipoprotein particle (loss)
c-value
0.0
0.1
0.2
0.3
0.4
0.5
0.6
0.7
0.8
Phylostratum
Cellular_organisms - ps1
Archaea1 - ps2
Archaea2 - ps3
Archaea3 - ps4
Asgard/Eukaryota - ps5
Eukaryota - ps6
Amorpheae - ps7
Obazoa - ps8
Opisthokonta - ps9
Holozoa - ps10
Filozoa - ps11
Choano./Metazoa - ps12
Metazoa - ps13
Myriazoa - ps14
Parahoxozoa - ps15
Bilateria - ps16
Deuterostomia - ps17
Chordata - ps18
Olfactores - ps19
Vertebrata - ps20
Gnathostomata - ps21
Euteleostomi - ps22
Sarcopterygii - ps23
Tetrapoda - ps24
Amniota - ps25
Mammalia - ps26
Theria - ps27
Placentalia - ps28
Boreoeutheria - ps29
Euarchontoglires - ps30
Euarchonta - ps31
Primates - ps32
Haplorrhini - ps33
Simiiformes - ps34
Catarrhini - ps35
Hominoidea - ps36
Hominidae - ps37
Homininae - ps38
Hominini - ps39
Homo_sapiens - ps40
p-value
1e-02
2e-02
2e-02
3e-02
4e-02
Log-odds
0.5
1.0
1.5
2.0
2.5
3.0
3.5
4.0

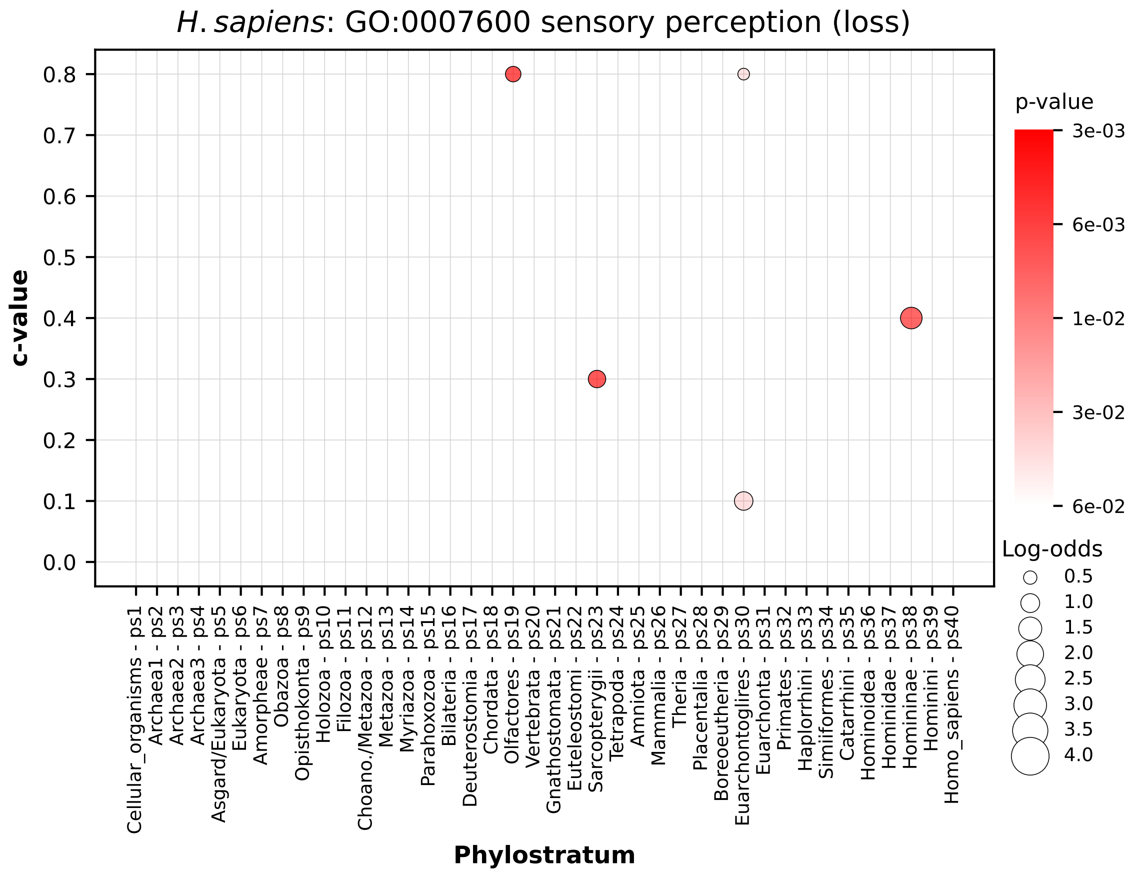

H. sapiens: GO:0007600 sensory perception (loss)
c-value
0.0
0.1
0.2
0.3
0.4
0.5
0.6
0.7
0.8
Phylostratum
Cellular_organisms - ps1
Archaea1 - ps2
Archaea2 - ps3
Archaea3 - ps4
Asgard/Eukaryota - ps5
Eukaryota - ps6
Amorpheae - ps7
Obazoa - ps8
Opisthokonta - ps9
Holozoa - ps10
Filozoa - ps11
Choano./Metazoa - ps12
Metazoa - ps13
Myriazoa - ps14
Parahoxozoa - ps15
Bilateria - ps16
Deuterostomia - ps17
Chordata - ps18
Olfactores - ps19
Vertebrata - ps20
Gnathostomata - ps21
Euteleostomi - ps22
Sarcopterygii - ps23
Tetrapoda - ps24
Amniota - ps25
Mammalia - ps26
Theria - ps27
Placentalia - ps28
Boreoeutheria - ps29
Euarchontoglires - ps30
Euarchonta - ps31
Primates - ps32
Haplorrhini - ps33
Simiiformes - ps34
Catarrhini - ps35
Hominoidea - ps36
Hominidae - ps37
Homininae - ps38
Hominini - ps39
Homo_sapiens - ps40
p-value
3e-03
6e-03
1e-02
3e-02
6e-02
Log-odds
0.5
1.0
1.5
2.0
2.5
3.0
3.5
4.0

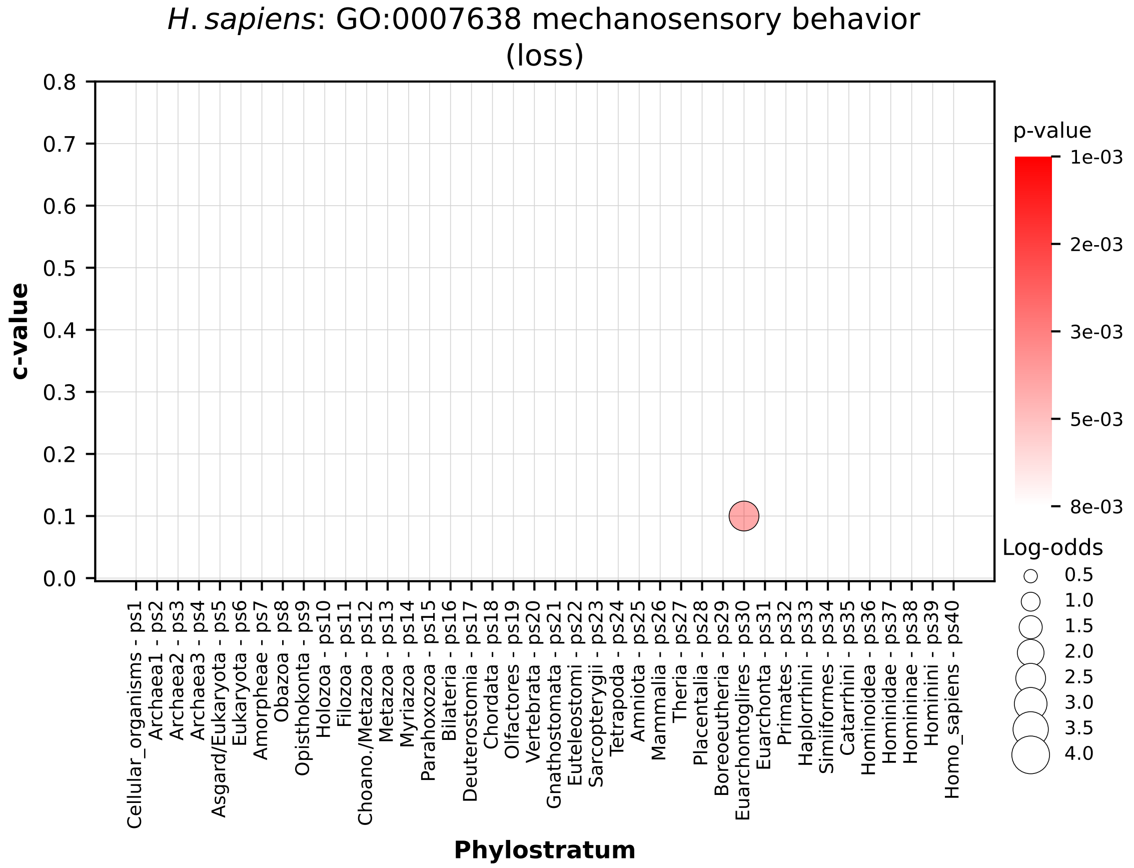
H. sapiens: GO:0007638 mechanosensory behavior (loss)
c-value
0.0
0.1
0.2
0.3
0.4
0.5
0.6
0.7
0.8
Phylostratum
Cellular_organisms - ps1
Archaea1 - ps2
Archaea2 - ps3
Archaea3 - ps4
Asgard/Eukaryota - ps5
Eukaryota - ps6
Amorpheae - ps7
Obazoa - ps8
Opisthokonta - ps9
Holozoa - ps10
Filozoa - ps11
Choano./Metazoa - ps12
Metazoa - ps13
Myriazoa - ps14
Parahoxozoa - ps15
Bilateria - ps16
Deuterostomia - ps17
Chordata - ps18
Olfactores - ps19
Vertebrata - ps20
Gnathostomata - ps21
Euteleostomi - ps22
Sarcopterygii - ps23
Tetrapoda - ps24
Amniota - ps25
Mammalia - ps26
Theria - ps27
Placentalia - ps28
Boreoeutheria - ps29
Euarchontoglires - ps30
Euarchonta - ps31
Primates - ps32
Haplorrhini - ps33
Simiiformes - ps34
Catarrhini - ps35
Hominoidea - ps36
Hominidae - ps37
Homininae - ps38
Hominini - ps39
Homo_sapiens - ps40
p-value
1e-03
2e-03
3e-03
5e-03
8e-03
Log-odds
0.5
1.0
1.5
2.0
2.5
3.0
3.5
4.0

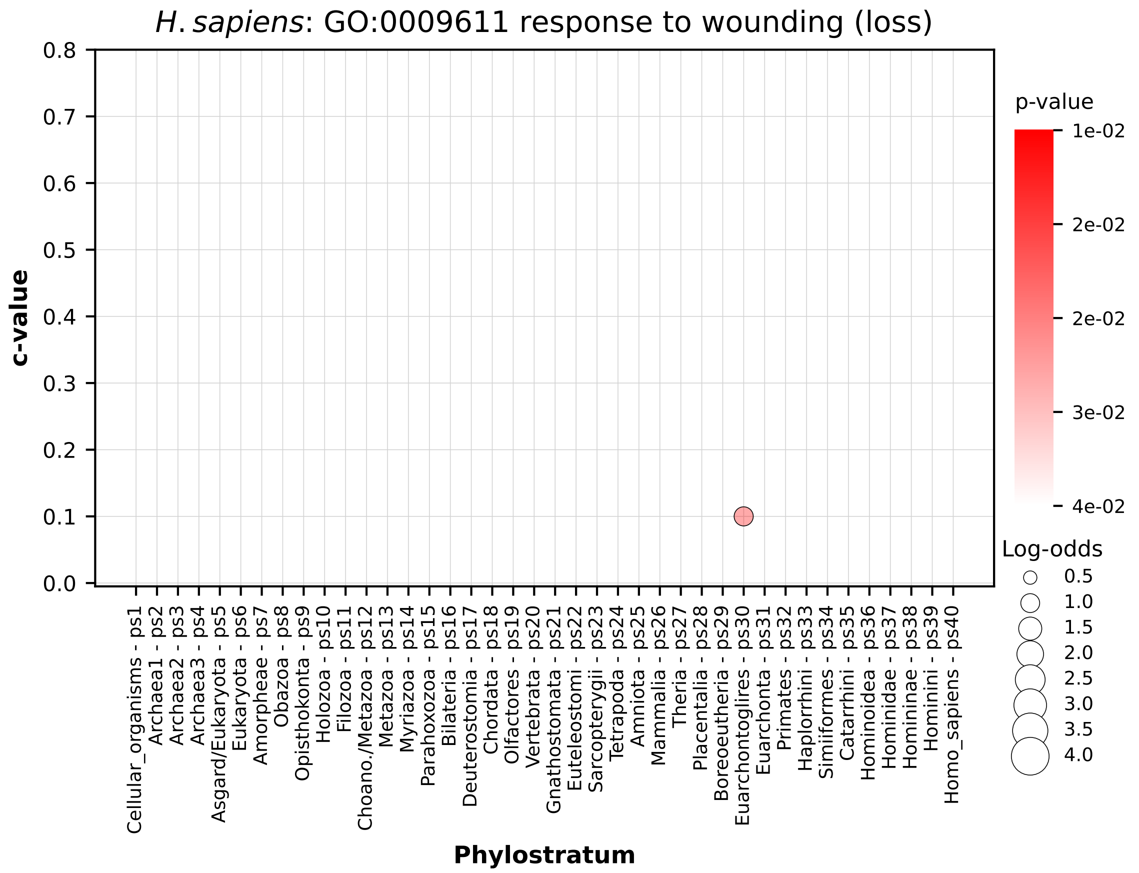

H. sapiens: GO:0009611 response to wounding (loss)
c-value
0.8
0.7
0.6
0.5
0.4
0.3
0.2
0.1
0.0
Phylostratum
Cellular_organisms - ps1
Archaea1 - ps2
Archaea2 - ps3
Archaea3 - ps4
Asgard/Eukaryota - ps5
Eukaryota - ps6
Amorpheae - ps7
Obazoa - ps8
Opisthokonta - ps9
Holozoa - ps10
Filozoa - ps11
Choano./Metazoa - ps12
Metazoa - ps13
Myriazoa - ps14
Parahoxozoa - ps15
Bilateria - ps16
Deuterostomia - ps17
Chordata - ps18
Olfactores - ps19
Vertebrata - ps20
Gnathostomata - ps21
Euteleostomi - ps22
Sarcopterygii - ps23
Tetrapoda - ps24
Amniota - ps25
Mammalia - ps26
Theria - ps27
Placentalia - ps28
Boreoeutheria - ps29
Euarchontoglires - ps30
Euarchonta - ps31
Primates - ps32
Haplorrhini - ps33
Simiiformes - ps34
Catarrhini - ps35
Hominoidea - ps36
Hominidae - ps37
Homininae - ps38
Hominini - ps39
Homo_sapiens - ps40
p-value
1e-02
2e-02
2e-02
3e-02
4e-02
Log-odds
0.5
1.0
1.5
2.0
2.5
3.0
3.5
4.0

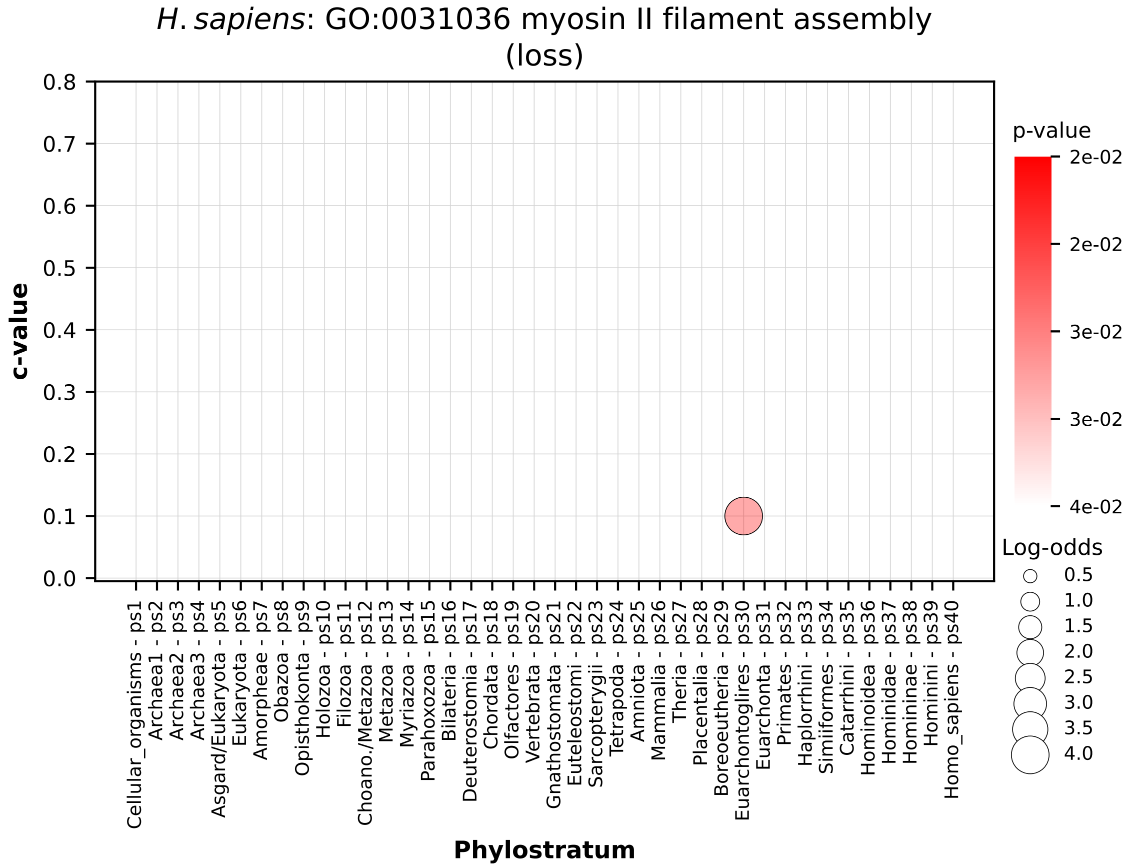
H. sapiens: GO:0031036 myosin II filament assembly
(loss)
c-value
0.8
0.7
0.6
0.5
0.4
0.3
0.2
0.1
0.0
Phylostratum
Cellular_organisms - ps1
Archaea1 - ps2
Archaea2 - ps3
Archaea3 - ps4
Asgard/Eukaryota - ps5
Eukaryota - ps6
Amorpheae - ps7
Obazoa - ps8
Opisthokonta - ps9
Holozoa - ps10
Filozoa - ps11
Choano./Metazoa - ps12
Metazoa - ps13
Myriazoa - ps14
Parahoxozoa - ps15
Bilateria - ps16
Deuterostomia - ps17
Chordata - ps18
Olfactores - ps19
Vertebrata - ps20
Gnathostomata - ps21
Euteleostomi - ps22
Sarcopterygii - ps23
Tetrapoda - ps24
Amniota - ps25
Mammalia - ps26
Theria - ps27
Placentalia - ps28
Boreoeutheria - ps29
Euarchontoglires - ps30
Euarchonta - ps31
Primates - ps32
Haplorrhini - ps33
Simiiformes - ps34
Catarrhini - ps35
Hominoidea - ps36
Hominidae - ps37
Homininae - ps38
Hominini - ps39
Homo_sapiens - ps40
p-value
2e-02
2e-02
3e-02
3e-02
4e-02
Log-odds
0.5
1.0
1.5
2.0
2.5
3.0
3.5
4.0

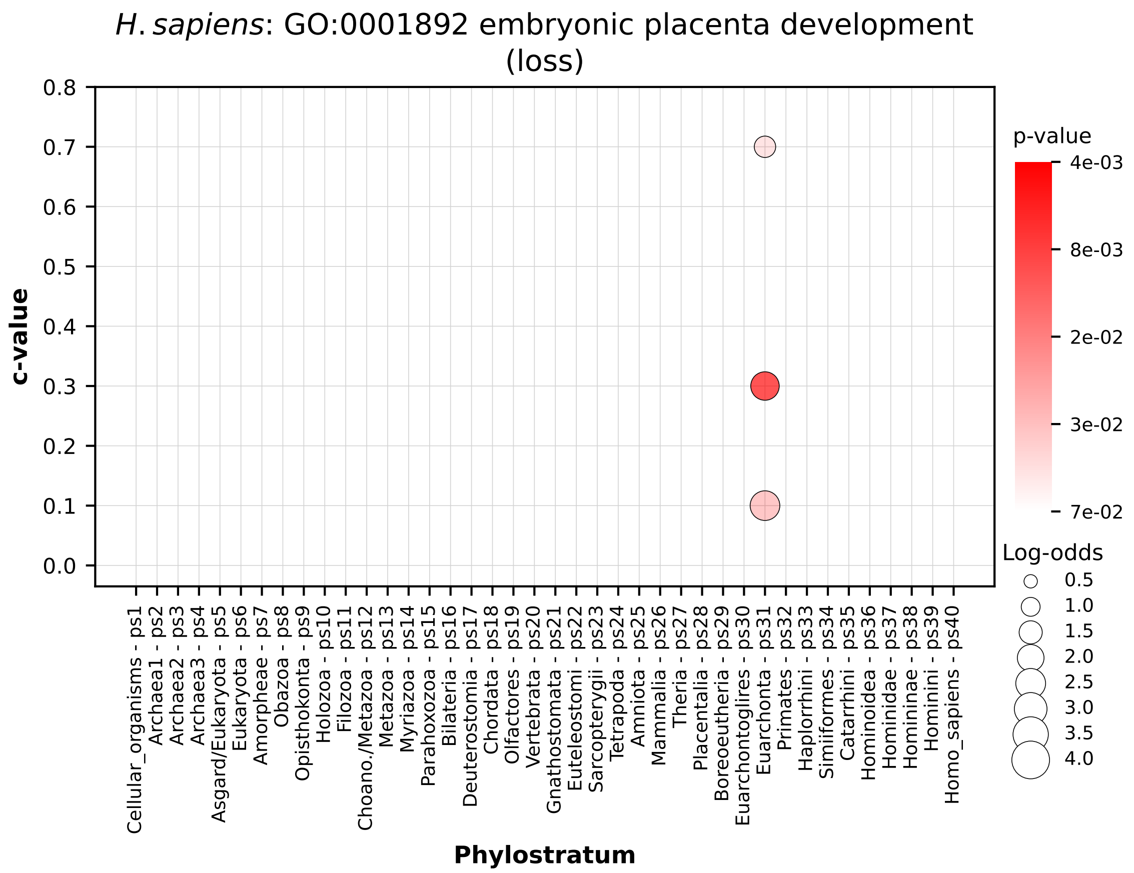

H. sapiens: GO:0001892 embryonic placenta development
(loss)
c-value
0.0
0.1
0.2
0.3
0.4
0.5
0.6
0.7
0.8
p-value
4e-03
8e-03
2e-02
3e-02
7e-02
Log-odds
0.5
1.0
1.5
2.0
2.5
3.0
3.5
4.0
Cellular_organisms - ps1
Archaea1 - ps2
Archaea2 - ps3
Archaea3 - ps4
Asgard/Eukaryota - ps5
Eukaryota - ps6
Amorpheae - ps7
Obazoa - ps8
Opisthokonta - ps9
Holozoa - ps10
Filozoa - ps11
Choano./Metazoa - ps12
Metazoa - ps13
Myriazoa - ps14
Parahoxozoa - ps15
Bilateria - ps16
Deuterostomia - ps17
Chordata - ps18
Olfactores - ps19
Vertebrata - ps20
Gnathostomata - ps21
Euteleostomi - ps22
Sarcopterygii - ps23
Tetrapoda - ps24
Amniota - ps25
Mammalia - ps26
Theria - ps27
Placentalia - ps28
Boreoeutheria - ps29
Euarchontoglires - ps30
Euarchonta - ps31
Primates - ps32
Haplorrhini - ps33
Simiiformes - ps34
Catarrhini - ps35
Hominoidea - ps36
Hominidae - ps37
Homininae - ps38
Hominini - ps39
Homo_sapiens - ps40
Phylostratum

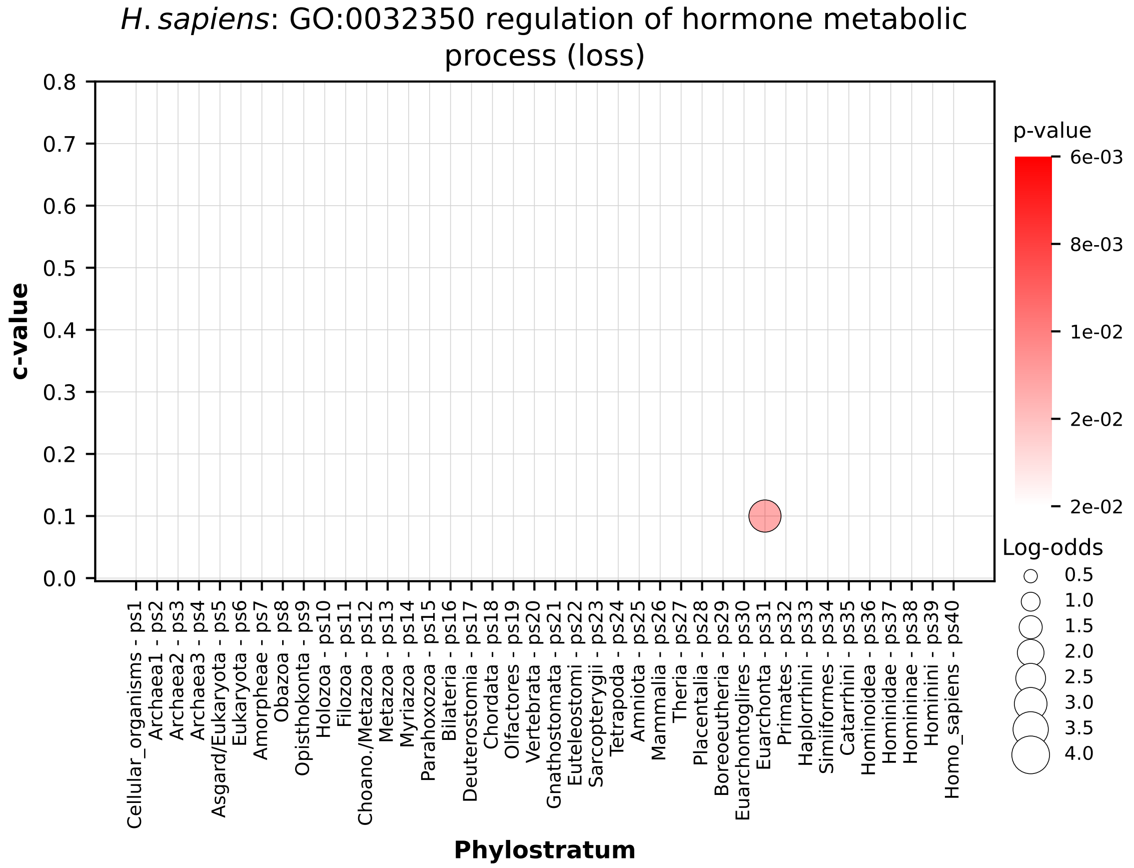
H. sapiens: GO:0032350 regulation of hormone metabolic process (loss)
c-value
0.8
0.7
0.6
0.5
0.4
0.3
0.2
0.1
0.0
Phylostratum
Cellular_organisms - ps1
Archaea1 - ps2
Archaea2 - ps3
Archaea3 - ps4
Asgard/Eukaryota - ps5
Eukaryota - ps6
Amorpheae - ps7
Obazoa - ps8
Opisthokonta - ps9
Holozoa - ps10
Filozoa - ps11
Choano./Metazoa - ps12
Metazoa - ps13
Myriazoa - ps14
Parahoxozoa - ps15
Bilateria - ps16
Deuterostomia - ps17
Chordata - ps18
Olfactores - ps19
Vertebrata - ps20
Gnathostomata - ps21
Euteleostomi - ps22
Sarcopterygii - ps23
Tetrapoda - ps24
Amniota - ps25
Mammalia - ps26
Theria - ps27
Placentalia - ps28
Boreoeutheria - ps29
Euarchontoglires - ps30
Euarchonta - ps31
Primates - ps32
Haplorrhini - ps33
Simiiformes - ps34
Catarrhini - ps35
Hominoidea - ps36
Hominidae - ps37
Homininae - ps38
Hominini - ps39
Homo_sapiens - ps40
p-value
6e-03
8e-03
1e-02
2e-02
2e-02
Log-odds
0.5
1.0
1.5
2.0
2.5
3.0
3.5
4.0

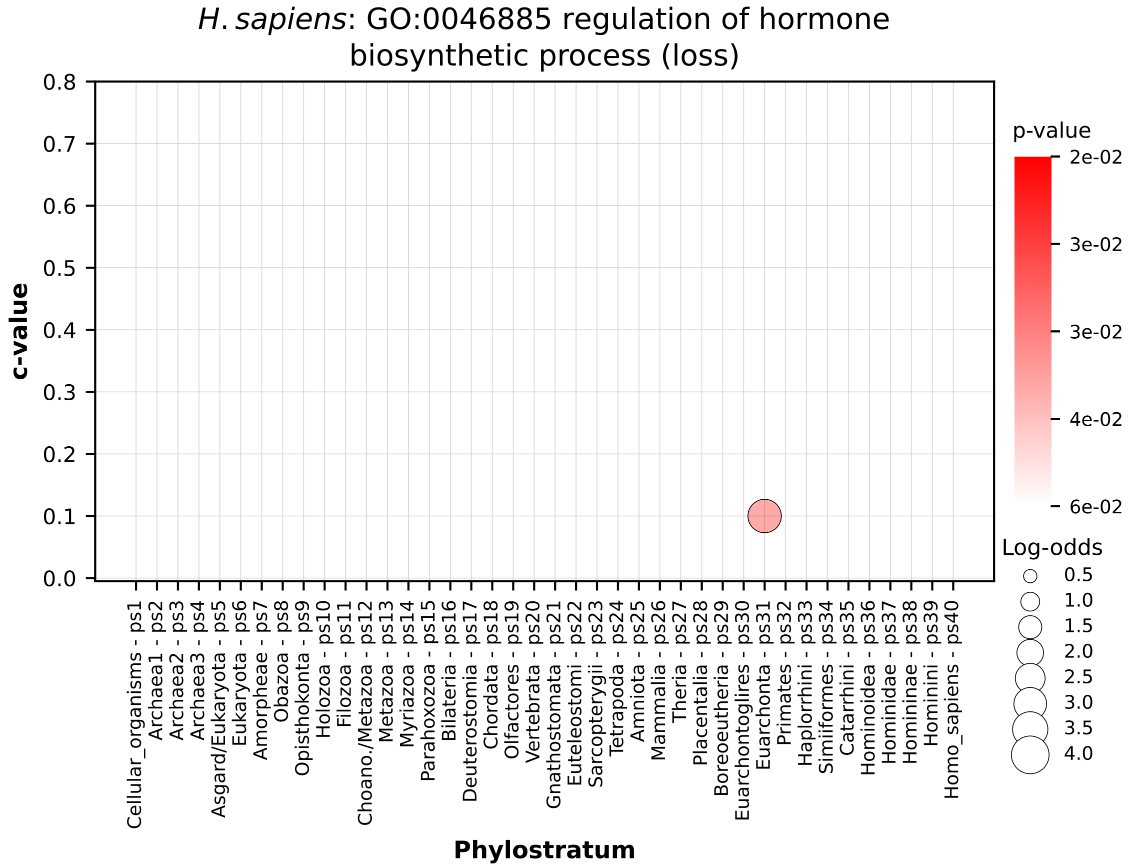

H. sapiens: GO:0046885 regulation of hormone biosynthetic process (loss)
c-value
0.8
0.7
0.6
0.5
0.4
0.3
0.2
0.1
0.0
Phylostratum
Cellular_organisms - ps1
Archaea1 - ps2
Archaea2 - ps3
Archaea3 - ps4
Asgard/Eukaryota - ps5
Eukaryota - ps6
Amorpheae - ps7
Obazoa - ps8
Opisthokonta - ps9
Holozoa - ps10
Filozoa - ps11
Choano./Metazoa - ps12
Metazoa - ps13
Myriazoa - ps14
Parahoxozoa - ps15
Bilateria - ps16
Deuterostomia - ps17
Chordata - ps18
Olfactores - ps19
Vertebrata - ps20
Gnathostomata - ps21
Euteleostomi - ps22
Sarcopterygii - ps23
Tetrapoda - ps24
Amniota - ps25
Mammalia - ps26
Theria - ps27
Placentalia - ps28
Boreoeutheria - ps29
Euarchontoglires - ps30
Euarchonta - ps31
Primates - ps32
Haplorrhini - ps33
Simiiformes - ps34
Catarrhini - ps35
Hominoidea - ps36
Hominidae - ps37
Homininae - ps38
Hominini - ps39
Homo_sapiens - ps40
p-value
2e-02
3e-02
3e-02
4e-02
6e-02
Log-odds
0.5
1.0
1.5
2.0
2.5
3.0
3.5
4.0

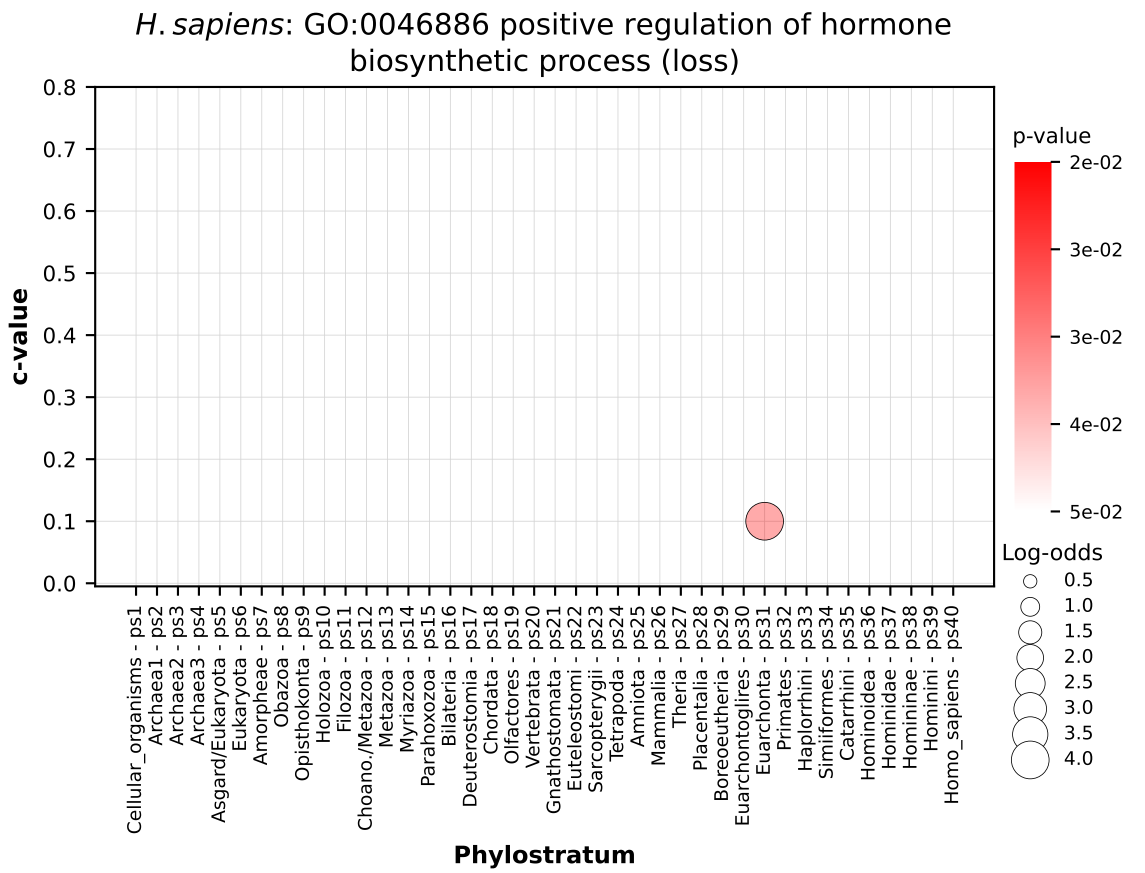

H. sapiens: GO:0046886 positive regulation of hormone biosynthetic process (loss)
c-value
0.0
0.1
0.2
0.3
0.4
0.5
0.6
0.7
0.8
Phylostratum
Cellular_organisms - ps1
Archaea1 - ps2
Archaea2 - ps3
Archaea3 - ps4
Asgard/Eukaryota - ps5
Eukaryota - ps6
Amorpheae - ps7
Obazoa - ps8
Opisthokonta - ps9
Holozoa - ps10
Filozoa - ps11
Choano./Metazoa - ps12
Metazoa - ps13
Myriazoa - ps14
Parahoxozoa - ps15
Bilateria - ps16
Deuterostomia - ps17
Chordata - ps18
Olfactores - ps19
Vertebrata - ps20
Gnathostomata - ps21
Euteleostomi - ps22
Sarcopterygii - ps23
Tetrapoda - ps24
Amniota - ps25
Mammalia - ps26
Theria - ps27
Placentalia - ps28
Boreoeutheria - ps29
Euarchontoglires - ps30
Euarchonta - ps31
Primates - ps32
Haplorrhini - ps33
Simiiformes - ps34
Catarrhini - ps35
Hominoidea - ps36
Hominidae - ps37
Homininae - ps38
Hominini - ps39
Homo_sapiens - ps40
p-value
2e-02
3e-02
3e-02
4e-02
5e-02
Log-odds
0.5
1.0
1.5
2.0
2.5
3.0
3.5
4.0

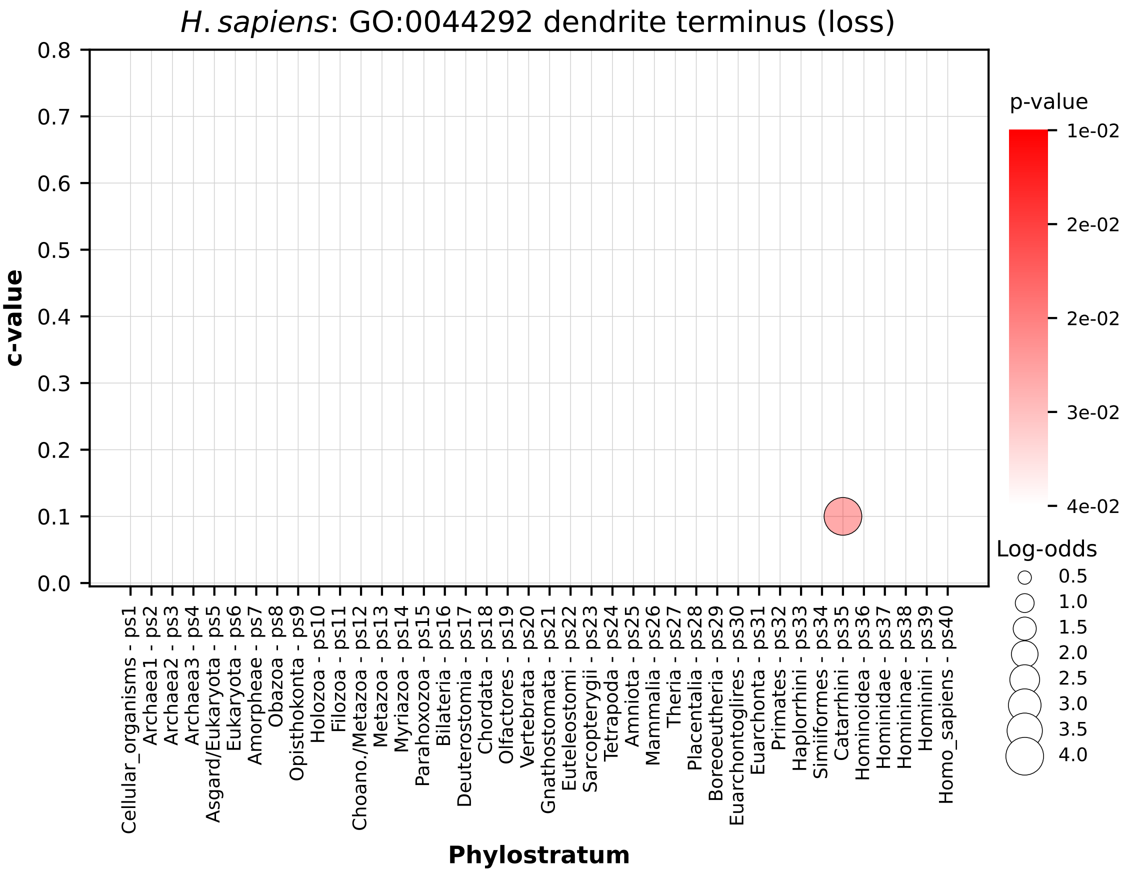

H. sapiens: GO:0044292 dendrite terminus (loss)
c-value
0.0
0.1
0.2
0.3
0.4
0.5
0.6
0.7
0.8
p-value
1e-02
2e-02
2e-02
3e-02
4e-02
Log-odds
0.5
1.0
1.5
2.0
2.5
3.0
3.5
4.0
Cellular_organisms - ps1
Archaea1 - ps2
Archaea2 - ps3
Archaea3 - ps4
Asgard/Eukaryota - ps5
Eukaryota - ps6
Amorpheae - ps7
Obazoa - ps8
Opisthokonta - ps9
Holozoa - ps10
Filozoa - ps11
Choano./Metazoa - ps12
Metazoa - ps13
Myriazoa - ps14
Parahoxozoa - ps15
Bilateria - ps16
Deuterostomia - ps17
Chordata - ps18
Olfactores - ps19
Vertebrata - ps20
Gnathostomata - ps21
Euteleostomi - ps22
Sarcopterygii - ps23
Tetrapoda - ps24
Amniota - ps25
Mammalia - ps26
Theria - ps27
Placentalia - ps28
Boreoeutheria - ps29
Euarchontoglires - ps30
Euarchonta - ps31
Primates - ps32
Haplorrhini - ps33
Simiiformes - ps34
Catarrhini - ps35
Hominoidea - ps36
Hominidae - ps37
Homininae - ps38
Hominini - ps39
Homo_sapiens - ps40
Phylostratum

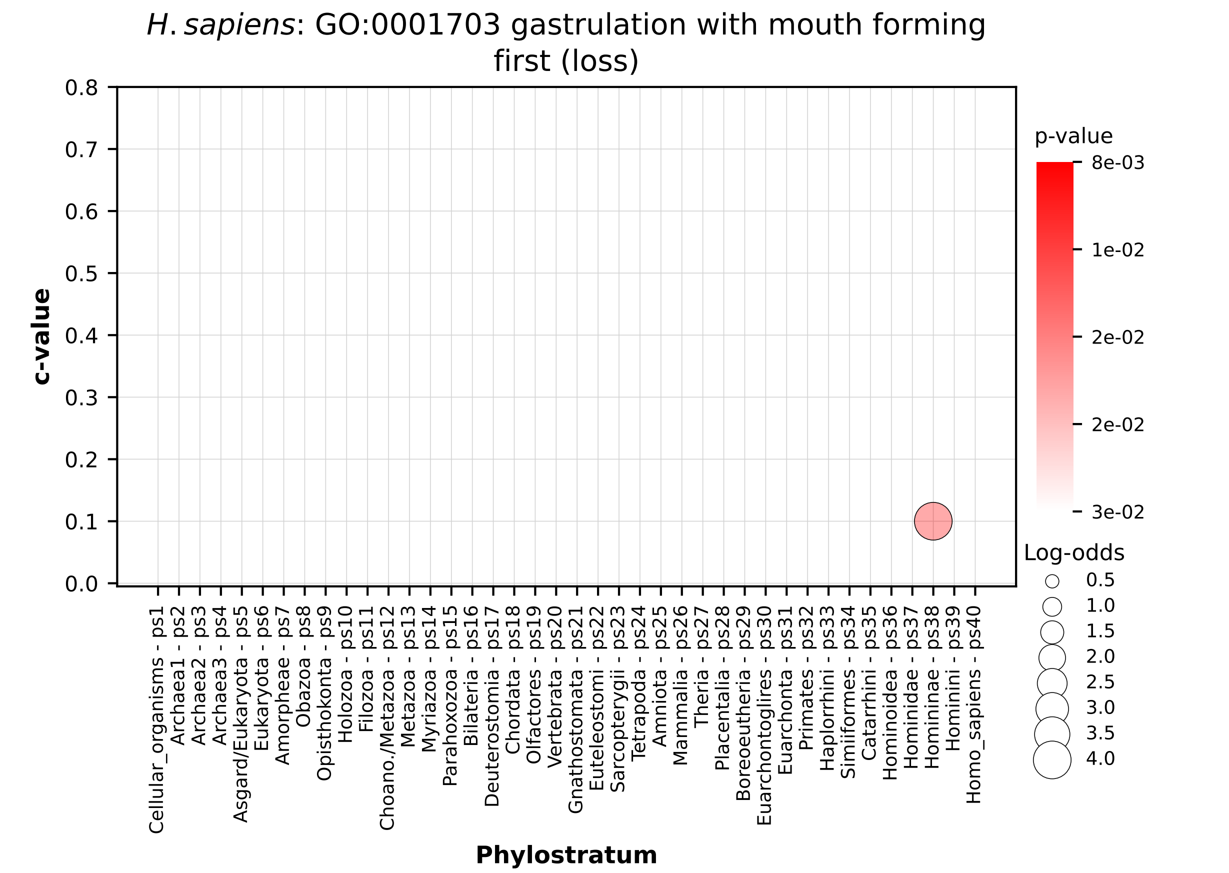

H. sapiens: GO:0001703 gastrulation with mouth forming first (loss)
c-value
0.0
0.1
0.2
0.3
0.4
0.5
0.6
0.7
0.8
Phylostratum
Cellular_organisms - ps1
Archaea1 - ps2
Archaea2 - ps3
Archaea3 - ps4
Asgard/Eukaryota - ps5
Eukaryota - ps6
Amorpheae - ps7
Obazoa - ps8
Opisthokonta - ps9
Holozoa - ps10
Filozoa - ps11
Choano./Metazoa - ps12
Metazoa - ps13
Myriazoa - ps14
Parahoxozoa - ps15
Bilateria - ps16
Deuterostomia - ps17
Chordata - ps18
Olfactores - ps19
Vertebrata - ps20
Gnathostomata - ps21
Euteleostomi - ps22
Sarcopterygii - ps23
Tetrapoda - ps24
Amniota - ps25
Mammalia - ps26
Theria - ps27
Placentalia - ps28
Boreoeutheria - ps29
Euarchontoglires - ps30
Euarchonta - ps31
Primates - ps32
Haplorrhini - ps33
Simiiformes - ps34
Catarrhini - ps35
Hominoidea - ps36
Hominidae - ps37
Homininae - ps38
Hominini - ps39
Homo_sapiens - ps40
p-value
8e-03
1e-02
2e-02
2e-02
3e-02
Log-odds
0.5
1.0
1.5
2.0
2.5
3.0
3.5
4.0

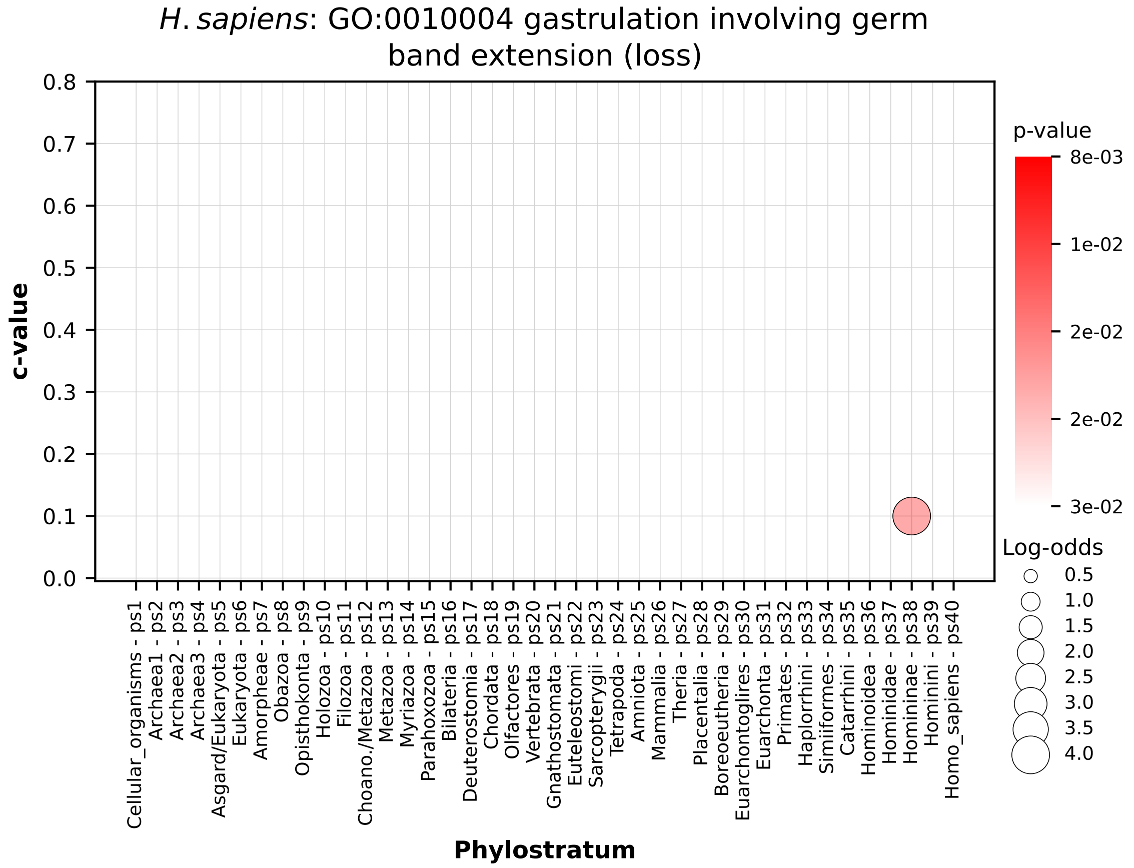

H. sapiens: GO:0010004 gastrulation involving germ band extension (loss)
c-value
0.8
0.7
0.6
0.5
0.4
0.3
0.2
0.1
0.0
Phylostratum
Cellular_organisms - ps1
Archaea1 - ps2
Archaea2 - ps3
Archaea3 - ps4
Asgard/Eukaryota - ps5
Eukaryota - ps6
Amorpheae - ps7
Obazoa - ps8
Opisthokonta - ps9
Holozoa - ps10
Filozoa - ps11
Choano./Metazoa - ps12
Metazoa - ps13
Myriazoa - ps14
Parahoxozoa - ps15
Bilateria - ps16
Deuterostomia - ps17
Chordata - ps18
Olfactores - ps19
Vertebrata - ps20
Gnathostomata - ps21
Euteleostomi - ps22
Sarcopterygii - ps23
Tetrapoda - ps24
Amniota - ps25
Mammalia - ps26
Theria - ps27
Placentalia - ps28
Boreoeutheria - ps29
Euarchontoglires - ps30
Euarchonta - ps31
Primates - ps32
Haplorrhini - ps33
Simiiformes - ps34
Catarrhini - ps35
Hominoidea - ps36
Hominidae - ps37
Homininae - ps38
Hominini - ps39
Homo_sapiens - ps40
p-value
8e-03
1e-02
2e-02
2e-02
3e-02
Log-odds
0.5
1.0
1.5
2.0
2.5
3.0
3.5
4.0

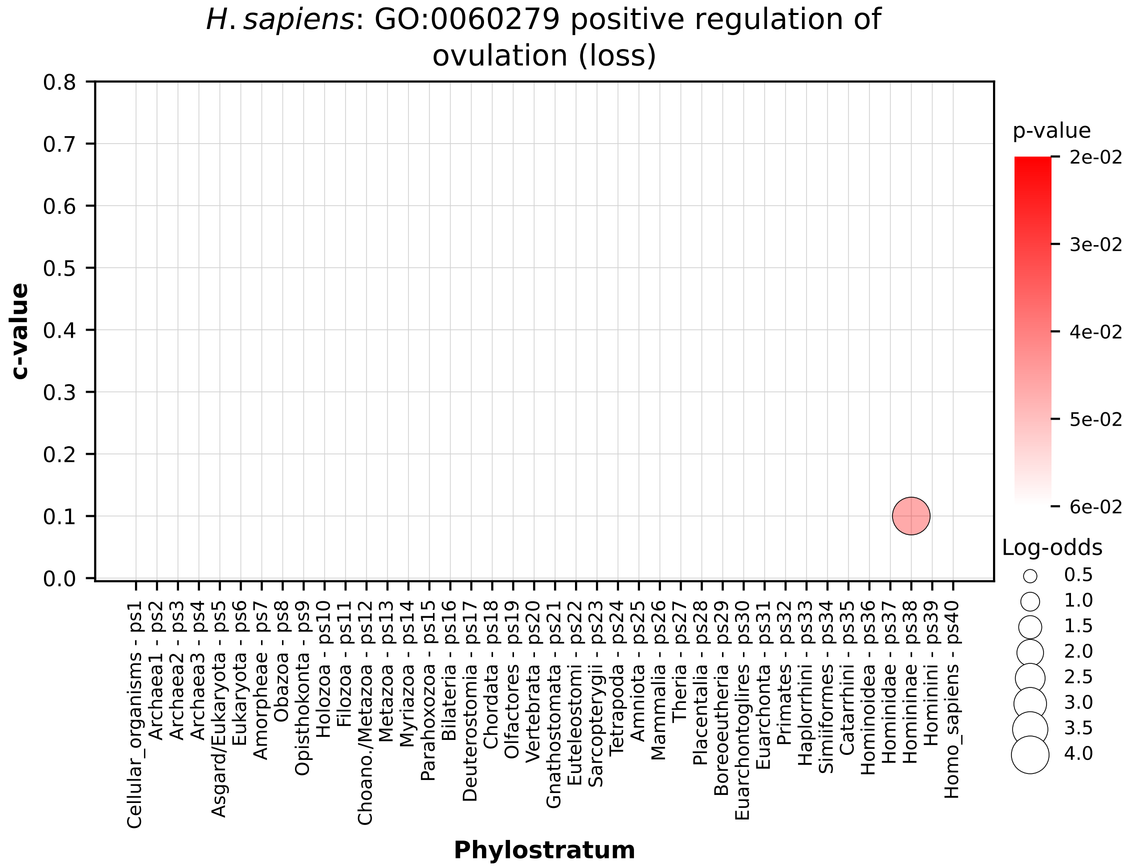

H. sapiens: GO:0060279 positive regulation of ovulation (loss)
c-value
0.8
0.7
0.6
0.5
0.4
0.3
0.2
0.1
0.0
Cellular_organisms - ps1
Archaea1 - ps2
Archaea2 - ps3
Archaea3 - ps4
Asgard/Eukaryota - ps5
Eukaryota - ps6
Amorpheae - ps7
Obazoa - ps8
Opisthokonta - ps9
Holozoa - ps10
Filozoa - ps11
Choano./Metazoa - ps12
Metazoa - ps13
Myriazoa - ps14
Parahoxozoa - ps15
Bilateria - ps16
Deuterostomia - ps17
Chordata - ps18
Olfactores - ps19
Vertebrata - ps20
Gnathostomata - ps21
Euteleostomi - ps22
Sarcopterygii - ps23
Tetrapoda - ps24
Amniota - ps25
Mammalia - ps26
Theria - ps27
Placentalia - ps28
Boreoeutheria - ps29
Euarchontoglires - ps30
Euarchonta - ps31
Primates - ps32
Haplorrhini - ps33
Simiiformes - ps34
Catarrhini - ps35
Hominoidea - ps36
Hominidae - ps37
Homininae - ps38
Hominini - ps39
Homo_sapiens - ps40
Phylostratum
p-value
2e-02
3e-02
4e-02
5e-02
6e-02
Log-odds
0.5
1.0
1.5
2.0
2.5
3.0
3.5
4.0

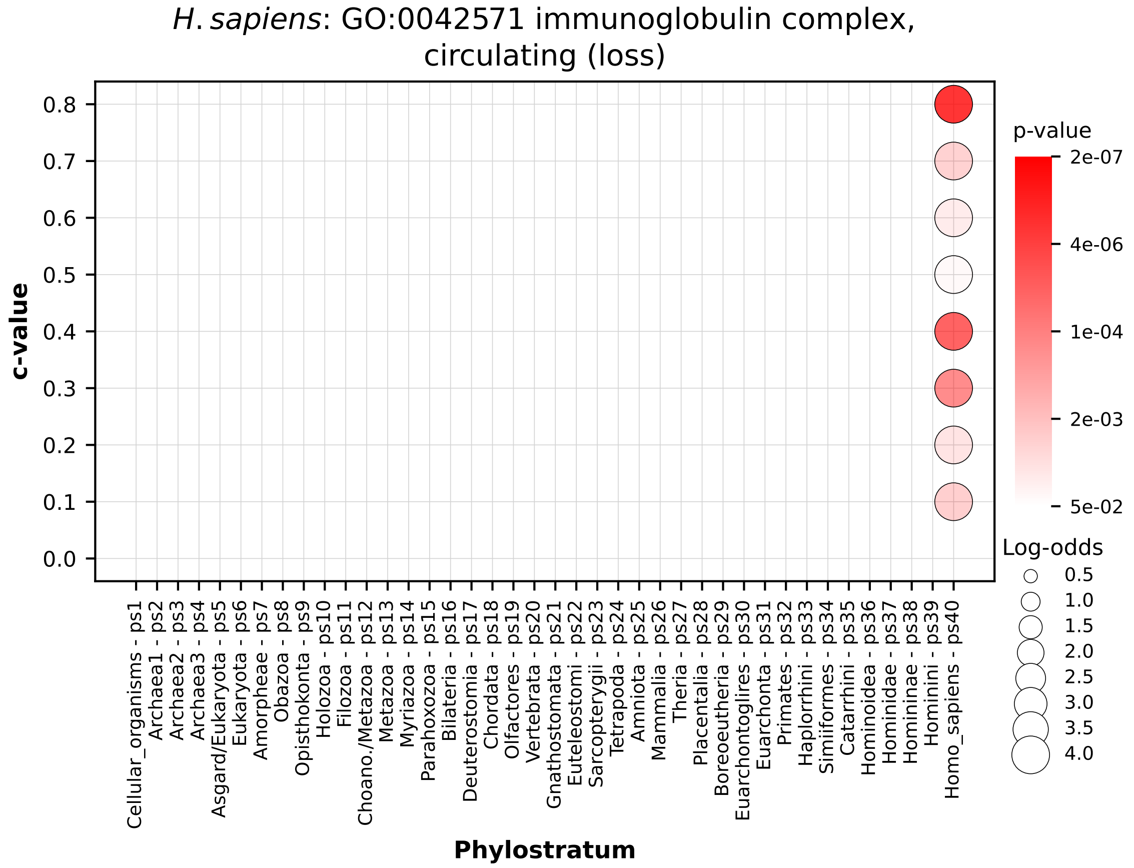
H. sapiens: GO:0042571 immunoglobulin complex, circulating (loss)
c-value
0.0
0.1
0.2
0.3
0.4
0.5
0.6
0.7
0.8
p-value
2e-07
4e-06
1e-04
2e-03
5e-02
Log-odds
0.5
1.0
1.5
2.0
2.5
3.0
3.5
4.0
Cellular_organisms - ps1
Archaea1 - ps2
Archaea2 - ps3
Archaea3 - ps4
Asgard/Eukaryota - ps5
Eukaryota - ps6
Amorpheae - ps7
Obazoa - ps8
Opisthokonta - ps9
Holozoa - ps10
Filozoa - ps11
Choano./Metazoa - ps12
Metazoa - ps13
Myriazoa - ps14
Parahoxozoa - ps15
Bilateria - ps16
Deuterostomia - ps17
Chordata - ps18
Olfactores - ps19
Vertebrata - ps20
Gnathostomata - ps21
Euteleostomi - ps22
Sarcopterygii - ps23
Tetrapoda - ps24
Amniota - ps25
Mammalia - ps26
Theria - ps27
Placentalia - ps28
Boreoeutheria - ps29
Euarchontoglires - ps30
Euarchonta - ps31
Primates - ps32
Haplorrhini - ps33
Simiiformes - ps34
Catarrhini - ps35
Hominoidea - ps36
Hominidae - ps37
Homininae - ps38
Hominini - ps39
Homo_sapiens - ps40
Phylostratum

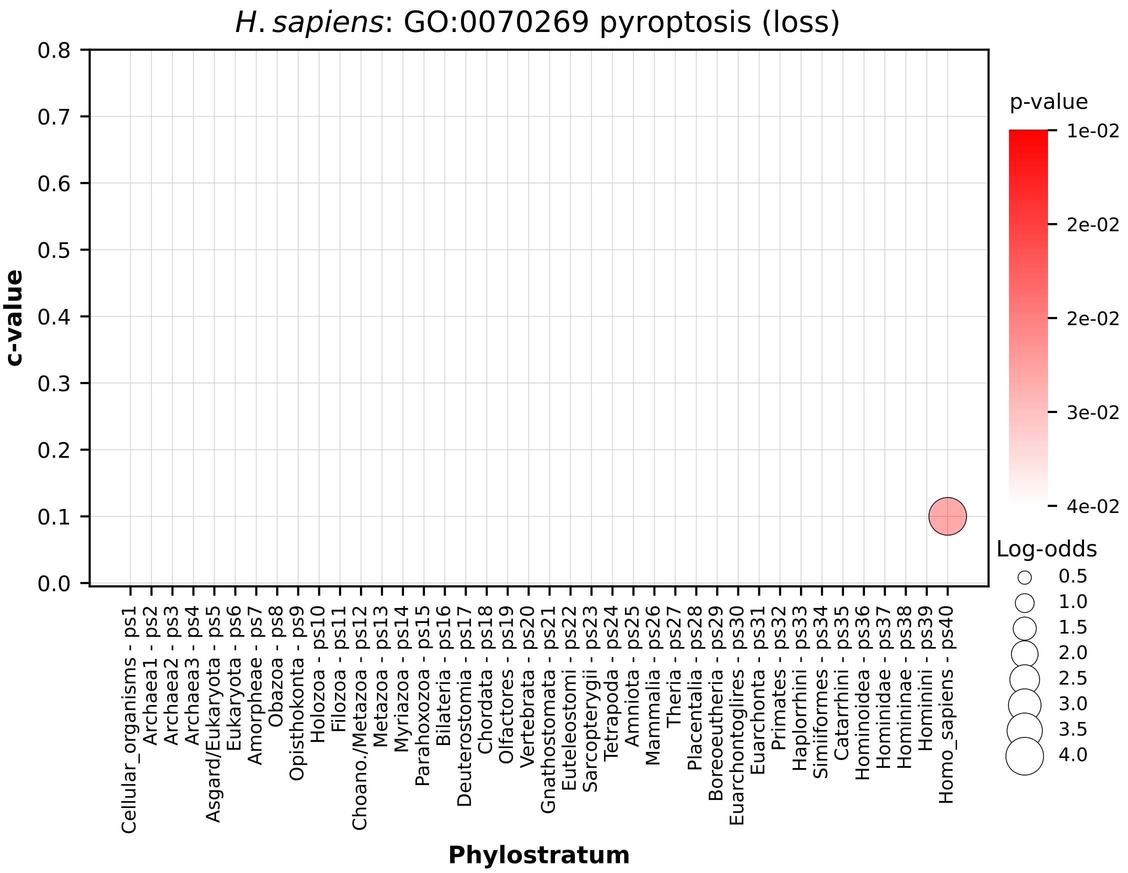

H. sapiens: GO:0070269 pyroptosis (loss)
c-value
0.0
0.1
0.2
0.3
0.4
0.5
0.6
0.7
0.8
Phylostratum
Cellular_organisms - ps1
Archaea1 - ps2
Archaea2 - ps3
Archaea3 - ps4
Asgard/Eukaryota - ps5
Eukaryota - ps6
Amorpheae - ps7
Obazoa - ps8
Opisthokonta - ps9
Holozoa - ps10
Filozoa - ps11
Choano./Metazoa - ps12
Metazoa - ps13
Myriazoa - ps14
Parahoxozoa - ps15
Bilateria - ps16
Deuterostomia - ps17
Chordata - ps18
Olfactores - ps19
Vertebrata - ps20
Gnathostomata - ps21
Euteleostomi - ps22
Sarcopterygii - ps23
Tetrapoda - ps24
Amniota - ps25
Mammalia - ps26
Theria - ps27
Placentalia - ps28
Boreoeutheria - ps29
Euarchontoglires - ps30
Euarchonta - ps31
Primates - ps32
Haplorrhini - ps33
Simiiformes - ps34
Catarrhini - ps35
Hominoidea - ps36
Hominidae - ps37
Homininae - ps38
Hominini - ps39
Homo_sapiens - ps40
p-value
1e-02
2e-02
2e-02
3e-02
4e-02
Log-odds
0.5
1.0
1.5
2.0
2.5
3.0
3.5
4.0

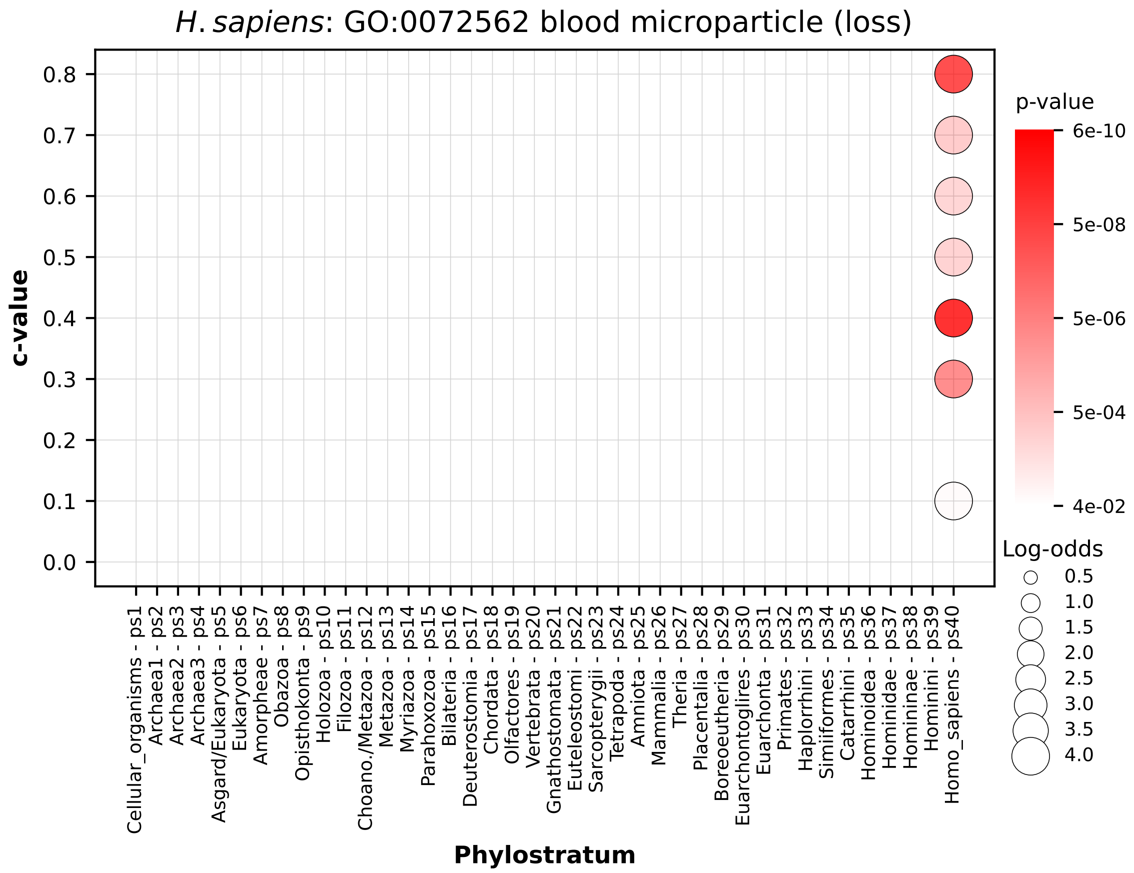

H. sapiens: GO:0072562 blood microparticle (loss)
c-value
0.0
0.1
0.2
0.3
0.4
0.5
0.6
0.7
0.8
p-value
6e-10
5e-08
5e-06
5e-04
4e-02
Log-odds
0.5
1.0
1.5
2.0
2.5
3.0
3.5
4.0
Cellular_organisms - ps1
Archaea1 - ps2
Archaea2 - ps3
Archaea3 - ps4
Asgard/Eukaryota - ps5
Eukaryota - ps6
Amorpheae - ps7
Obazoa - ps8
Opisthokonta - ps9
Holozoa - ps10
Filozoa - ps11
Choano./Metazoa - ps12
Metazoa - ps13
Myriazoa - ps14
Parahoxozoa - ps15
Bilateria - ps16
Deuterostomia - ps17
Chordata - ps18
Olfactores - ps19
Vertebrata - ps20
Gnathostomata - ps21
Euteleostomi - ps22
Sarcopterygii - ps23
Tetrapoda - ps24
Amniota - ps25
Mammalia - ps26
Theria - ps27
Placentalia - ps28
Boreoeutheria - ps29
Euarchontoglires - ps30
Euarchonta - ps31
Primates - ps32
Haplorrhini - ps33
Simiiformes - ps34
Catarrhini - ps35
Hominoidea - ps36
Hominidae - ps37
Homininae - ps38
Hominini - ps39
Homo_sapiens - ps40
Phylostratum

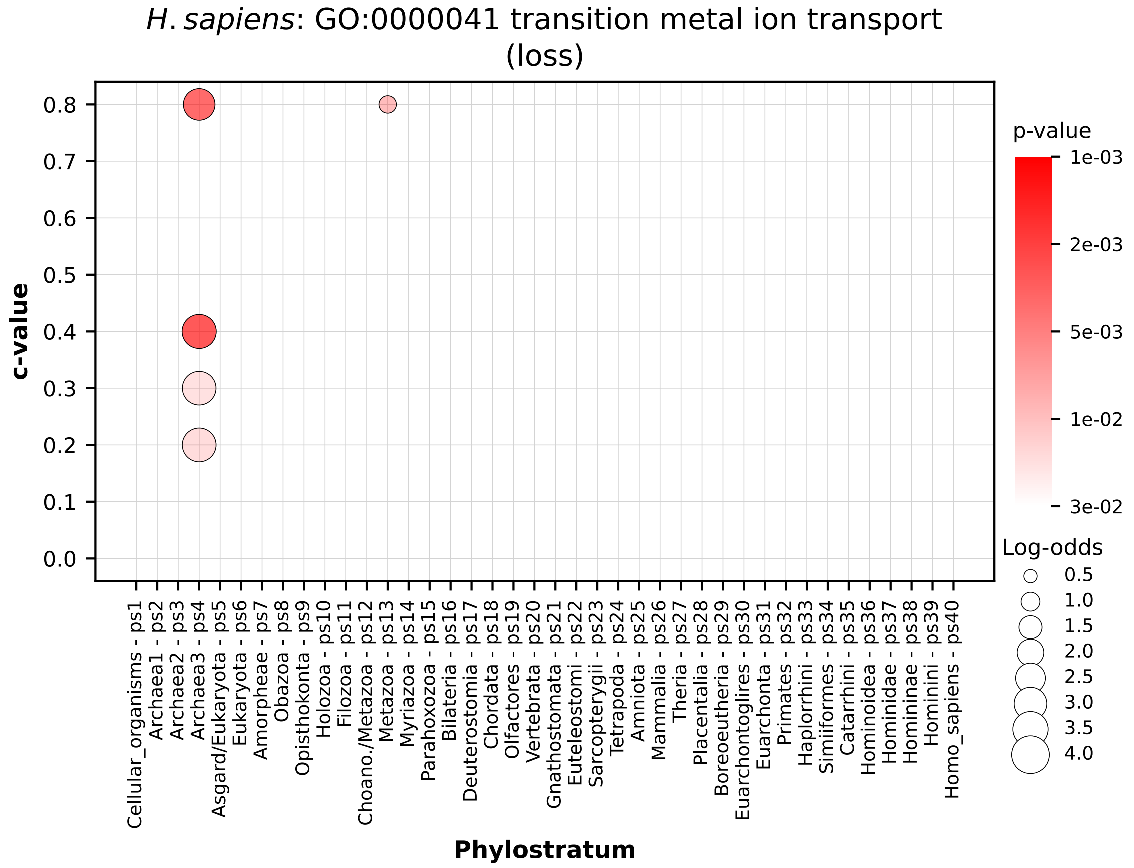

H. sapiens: GO:0000041 transition metal ion transport (loss)
c-value
0.0
0.1
0.2
0.3
0.4
0.5
0.6
0.7
0.8
Phylostratum
Cellular_organisms - ps1
Archaea1 - ps2
Archaea2 - ps3
Archaea3 - ps4
Asgard/Eukaryota - ps5
Eukaryota - ps6
Amorpheae - ps7
Obazoa - ps8
Opisthokonta - ps9
Holozoa - ps10
Filozoa - ps11
Choano./Metazoa - ps12
Metazoa - ps13
Myriazoa - ps14
Parahoxozoa - ps15
Bilateria - ps16
Deuterostomia - ps17
Chordata - ps18
Olfactores - ps19
Vertebrata - ps20
Gnathostomata - ps21
Euteleostomi - ps22
Sarcopterygii - ps23
Tetrapoda - ps24
Amniota - ps25
Mammalia - ps26
Theria - ps27
Placentalia - ps28
Boreoeutheria - ps29
Euarchontoglires - ps30
Euarchonta - ps31
Primates - ps32
Haplorrhini - ps33
Simiiformes - ps34
Catarrhini - ps35
Hominoidea - ps36
Hominidae - ps37
Homininae - ps38
Hominini - ps39
Homo_sapiens - ps40
p-value
1e-03
2e-03
5e-03
1e-02
3e-02
Log-odds
0.5
1.0
1.5
2.0
2.5
3.0
3.5
4.0

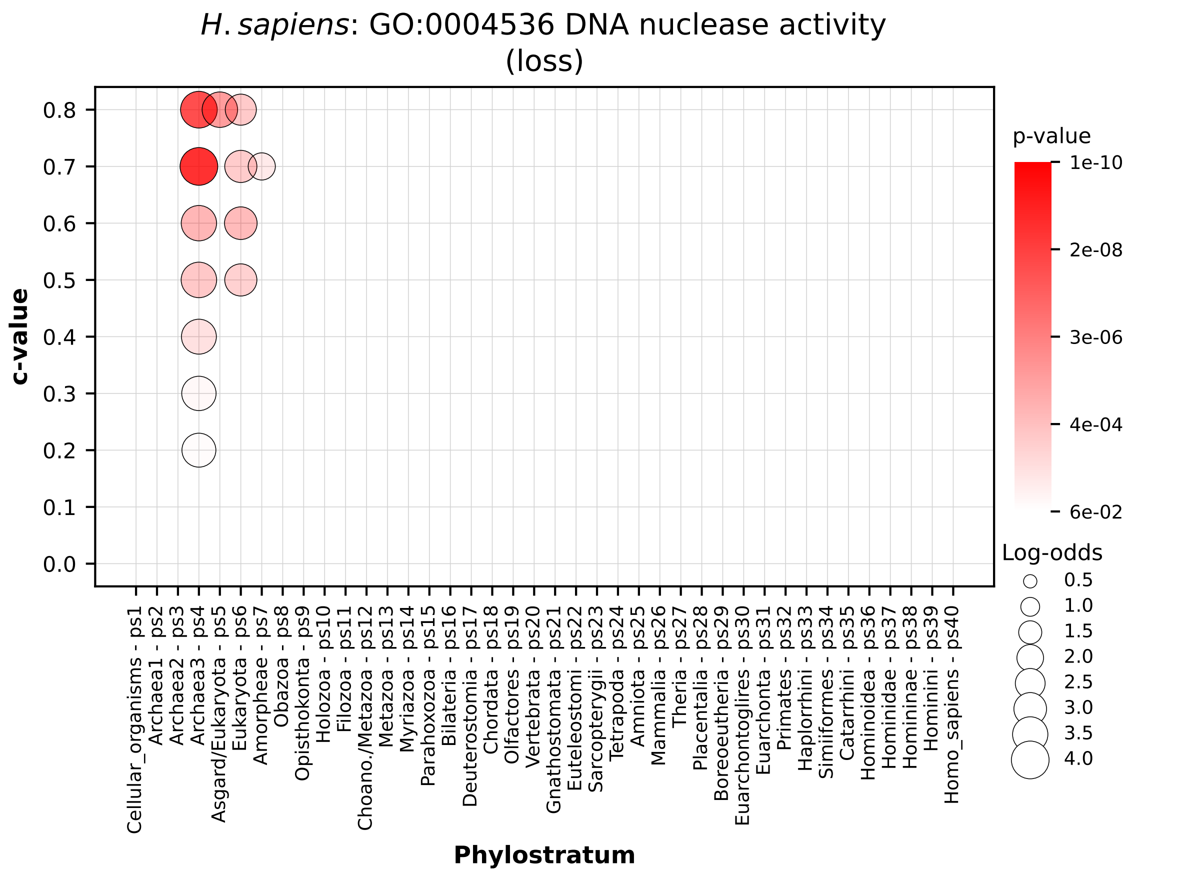

H. sapiens: GO:0004536 DNA nuclease activity
(loss)
c-value
0.8
0.7
0.6
0.5
0.4
0.3
0.2
0.1
0.0
p-value
1e-10
2e-08
3e-06
4e-04
6e-02
Log-odds
0.5
1.0
1.5
2.0
2.5
3.0
3.5
4.0
Cellular_organisms - ps1
Archaea1 - ps2
Archaea2 - ps3
Archaea3 - ps4
Asgard/Eukaryota - ps5
Eukaryota - ps6
Amorpheae - ps7
Obazoa - ps8
Opisthokonta - ps9
Holozoa - ps10
Filozoa - ps11
Choano./Metazoa - ps12
Metazoa - ps13
Myriazoa - ps14
Parahoxozoa - ps15
Bilateria - ps16
Deuterostomia - ps17
Chordata - ps18
Olfactores - ps19
Vertebrata - ps20
Gnathostomata - ps21
Euteleostomi - ps22
Sarcopterygii - ps23
Tetrapoda - ps24
Amniota - ps25
Mammalia - ps26
Theria - ps27
Placentalia - ps28
Boreoeutheria - ps29
Euarchontoglires - ps30
Euarchonta - ps31
Primates - ps32
Haplorrhini - ps33
Simiiformes - ps34
Catarrhini - ps35
Hominoidea - ps36
Hominidae - ps37
Homininae - ps38
Hominini - ps39
Homo_sapiens - ps40
Phylostratum

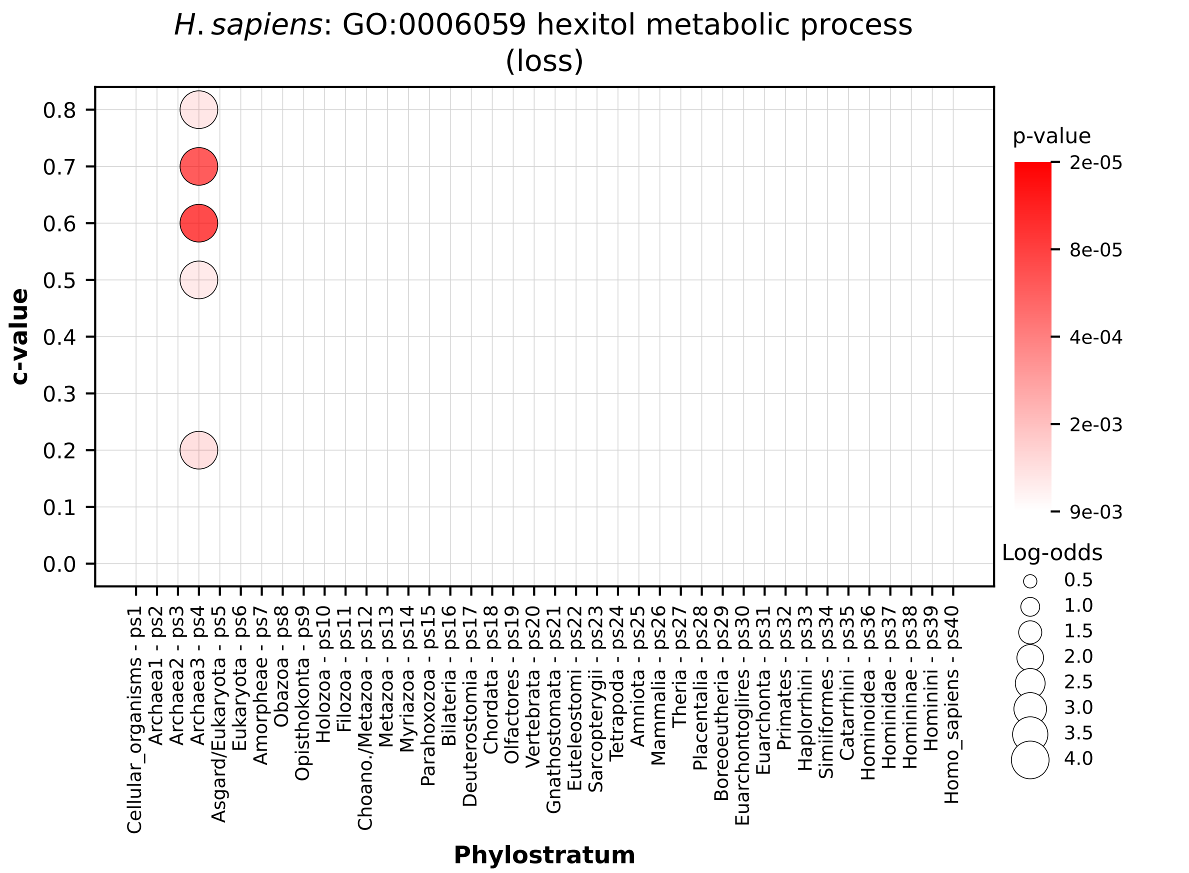

H. sapiens: GO:0006059 hexitol metabolic process
(loss)
c-value
0.8
0.7
0.6
0.5
0.4
0.3
0.2
0.1
0.0
p-value
2e-05
8e-05
4e-04
2e-03
9e-03
Log-odds
0.5
1.0
1.5
2.0
2.5
3.0
3.5
4.0
Cellular_organisms - ps1
Archaea1 - ps2
Archaea2 - ps3
Archaea3 - ps4
Asgard/Eukaryota - ps5
Eukaryota - ps6
Amorpheae - ps7
Obazoa - ps8
Opisthokonta - ps9
Holozoa - ps10
Filozoa - ps11
Choano./Metazoa - ps12
Metazoa - ps13
Myriazoa - ps14
Parahoxozoa - ps15
Bilateria - ps16
Deuterostomia - ps17
Chordata - ps18
Olfactores - ps19
Vertebrata - ps20
Gnathostomata - ps21
Euteleostomi - ps22
Sarcopterygii - ps23
Tetrapoda - ps24
Amniota - ps25
Mammalia - ps26
Theria - ps27
Placentalia - ps28
Boreoeutheria - ps29
Euarchontoglires - ps30
Euarchonta - ps31
Primates - ps32
Haplorrhini - ps33
Simiiformes - ps34
Catarrhini - ps35
Hominoidea - ps36
Hominidae - ps37
Homininae - ps38
Hominini - ps39
Homo_sapiens - ps40
Phylostratum

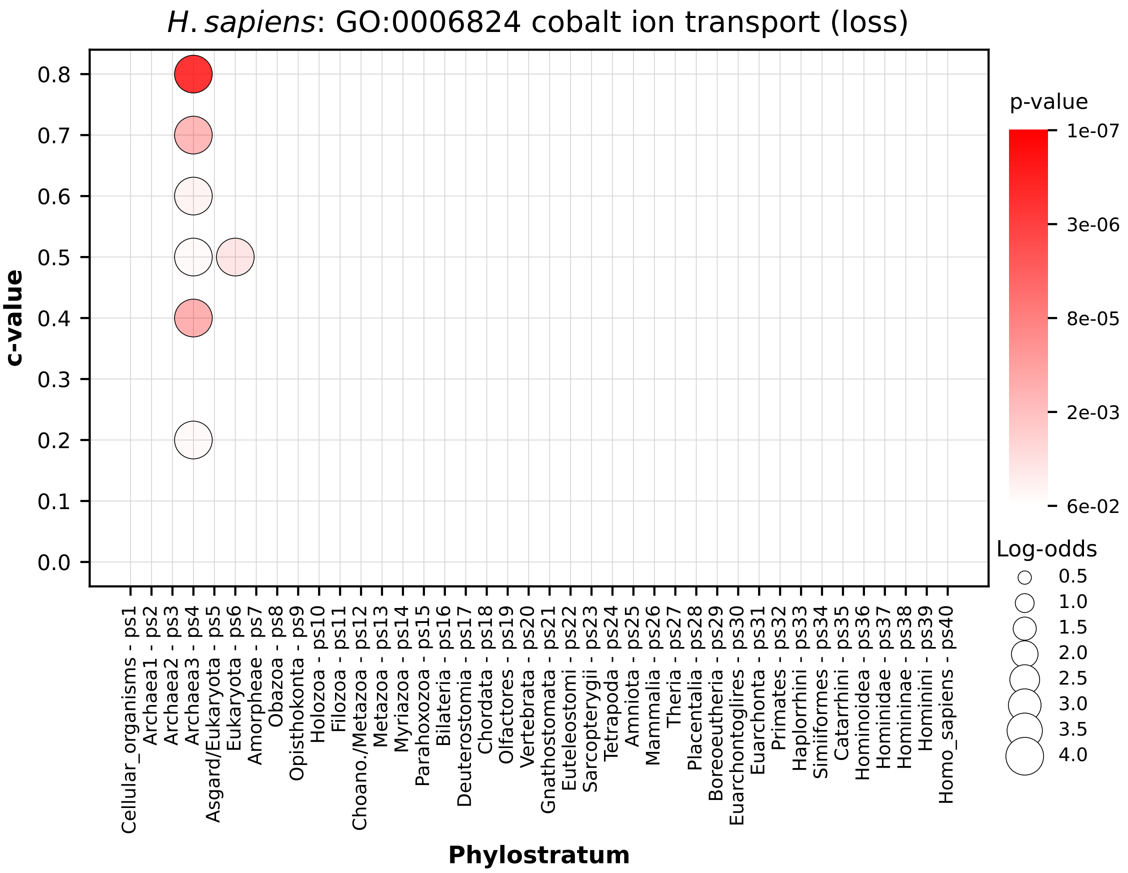

H. sapiens: GO:0006824 cobalt ion transport (loss)
c-value
0.8
0.7
0.6
0.5
0.4
0.3
0.2
0.1
0.0
Phylostratum
Cellular_organisms - ps1
Archaea1 - ps2
Archaea2 - ps3
Archaea3 - ps4
Asgard/Eukaryota - ps5
Eukaryota - ps6
Amorpheae - ps7
Obazoa - ps8
Opisthokonta - ps9
Holozoa - ps10
Filozoa - ps11
Choano./Metazoa - ps12
Metazoa - ps13
Myriazoa - ps14
Parahoxozoa - ps15
Bilateria - ps16
Deuterostomia - ps17
Chordata - ps18
Olfactores - ps19
Vertebrata - ps20
Gnathostomata - ps21
Euteleostomi - ps22
Sarcopterygii - ps23
Tetrapoda - ps24
Amniota - ps25
Mammalia - ps26
Theria - ps27
Placentalia - ps28
Boreoeutheria - ps29
Euarchontoglires - ps30
Euarchonta - ps31
Primates - ps32
Haplorrhini - ps33
Simiiformes - ps34
Catarrhini - ps35
Hominoidea - ps36
Hominidae - ps37
Homininae - ps38
Hominini - ps39
Homo_sapiens - ps40
p-value
1e-07
3e-06
8e-05
2e-03
6e-02
Log-odds
0.5
1.0
1.5
2.0
2.5
3.0
3.5
4.0

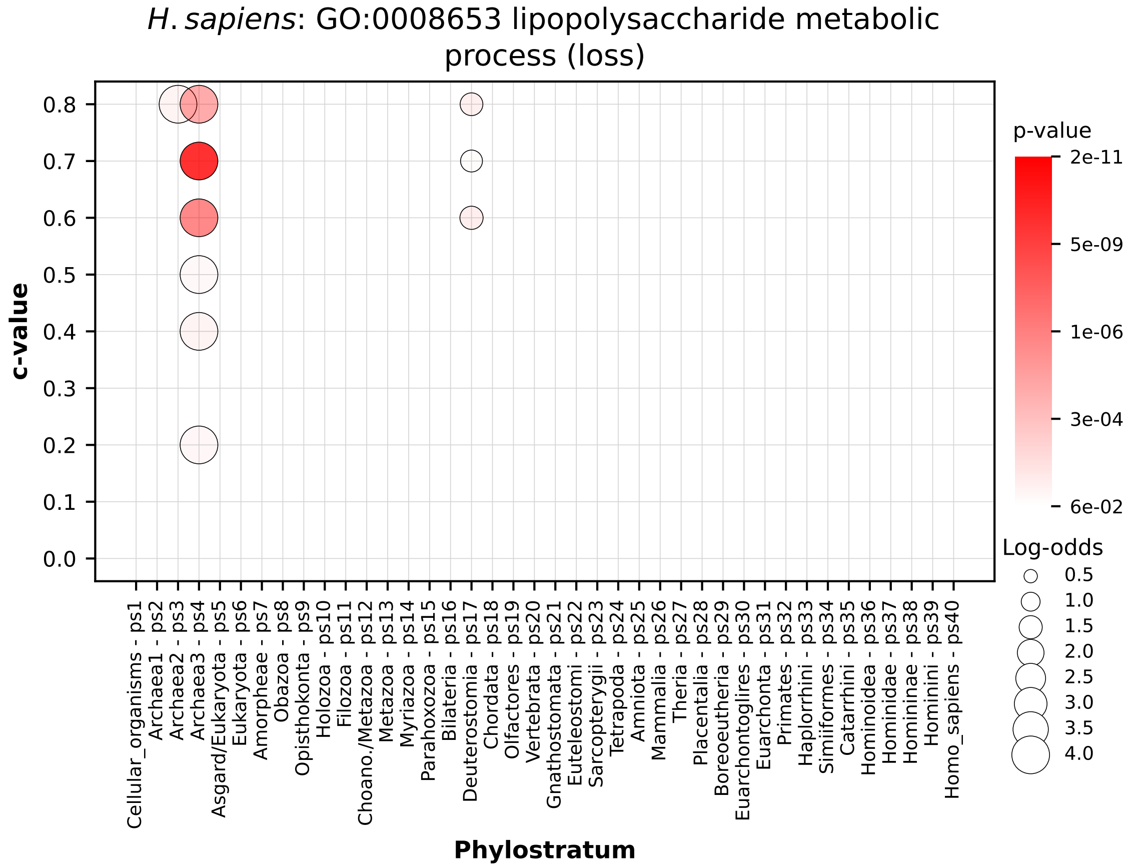
H. sapiens: GO:0008653 lipopolysaccharide metabolic process (loss)
c-value
0.0
0.1
0.2
0.3
0.4
0.5
0.6
0.7
0.8
Phylostratum
Cellular_organisms - ps1
Archaea1 - ps2
Archaea2 - ps3
Archaea3 - ps4
Asgard/Eukaryota - ps5
Eukaryota - ps6
Amorpheae - ps7
Obazoa - ps8
Opisthokonta - ps9
Holozoa - ps10
Filozoa - ps11
Choano./Metazoa - ps12
Metazoa - ps13
Myriazoa - ps14
Parahoxozoa - ps15
Bilateria - ps16
Deuterostomia - ps17
Chordata - ps18
Olfactores - ps19
Vertebrata - ps20
Gnathostomata - ps21
Euteleostomi - ps22
Sarcopterygii - ps23
Tetrapoda - ps24
Amniota - ps25
Mammalia - ps26
Theria - ps27
Placentalia - ps28
Boreoeutheria - ps29
Euarchontoglires - ps30
Euarchonta - ps31
Primates - ps32
Haplorrhini - ps33
Simiiformes - ps34
Catarrhini - ps35
Hominoidea - ps36
Hominidae - ps37
Homininae - ps38
Hominini - ps39
Homo_sapiens - ps40
p-value
2e-11
5e-09
1e-06
3e-04
6e-02
Log-odds
0.5
1.0
1.5
2.0
2.5
3.0
3.5
4.0

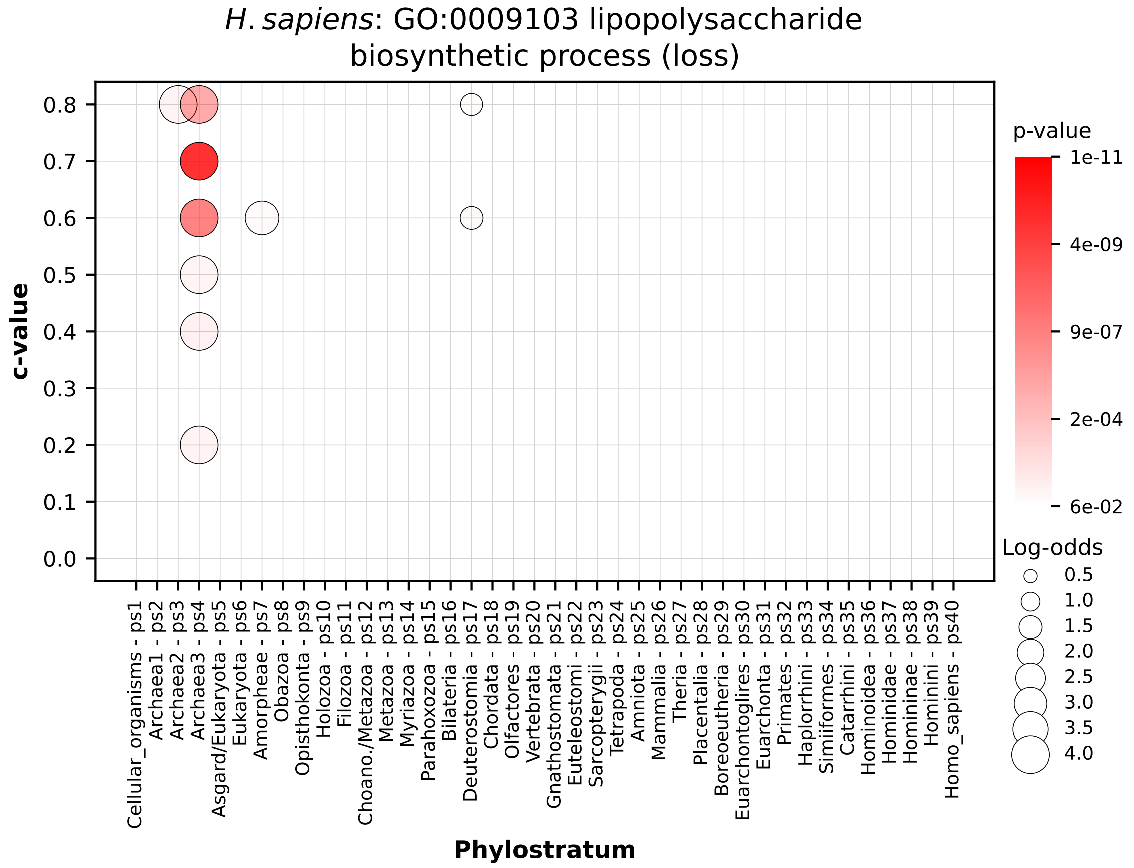

H. sapiens: GO:0009103 lipopolysaccharide biosynthetic process (loss)
c-value
0.0
0.1
0.2
0.3
0.4
0.5
0.6
0.7
0.8
Phylostratum
Cellular_organisms - ps1
Archaea1 - ps2
Archaea2 - ps3
Archaea3 - ps4
Asgard/Eukaryota - ps5
Eukaryota - ps6
Amorpheae - ps7
Obazoa - ps8
Opisthokonta - ps9
Holozoa - ps10
Filozoa - ps11
Choano./Metazoa - ps12
Metazoa - ps13
Myriazoa - ps14
Parahoxozoa - ps15
Bilateria - ps16
Deuterostomia - ps17
Chordata - ps18
Olfactores - ps19
Vertebrata - ps20
Gnathostomata - ps21
Euteleostomi - ps22
Sarcopterygii - ps23
Tetrapoda - ps24
Amniota - ps25
Mammalia - ps26
Theria - ps27
Placentalia - ps28
Boreoeutheria - ps29
Euarchontoglires - ps30
Euarchonta - ps31
Primates - ps32
Haplorrhini - ps33
Simiiformes - ps34
Catarrhini - ps35
Hominoidea - ps36
Hominidae - ps37
Homininae - ps38
Hominini - ps39
Homo_sapiens - ps40
p-value
1e-11
4e-09
9e-07
2e-04
6e-02
Log-odds
0.5
1.0
1.5
2.0
2.5
3.0
3.5
4.0

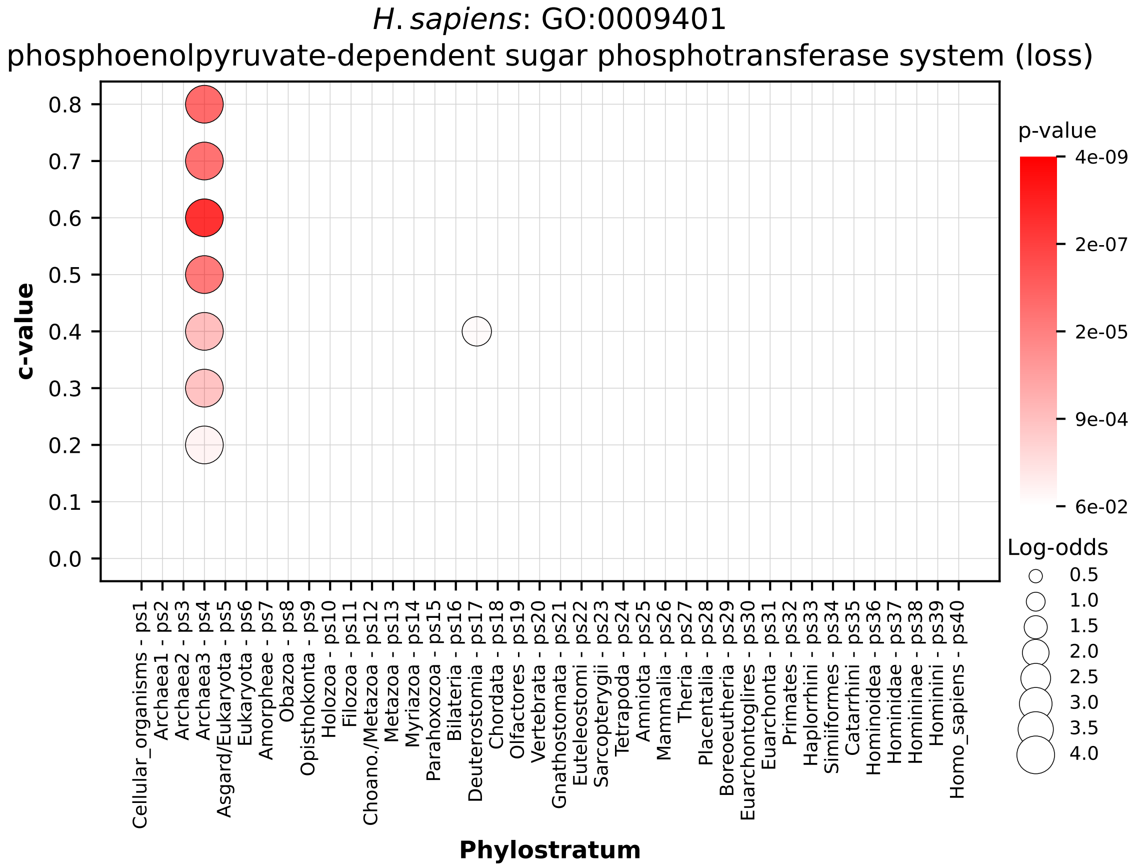

H. sapiens: GO:0009401
phosphoenolpyruvate-dependent sugar phosphotransferase system (loss)
c-value
0.0
0.1
0.2
0.3
0.4
0.5
0.6
0.7
0.8
Phylostratum
Cellular_organisms - ps1
Archaea1 - ps2
Archaea2 - ps3
Archaea3 - ps4
Asgard/Eukaryota - ps5
Eukaryota - ps6
Amorpheae - ps7
Obazoa - ps8
Opisthokonta - ps9
Holozoa - ps10
Filozoa - ps11
Choano./Metazoa - ps12
Metazoa - ps13
Myriazoa - ps14
Parahoxozoa - ps15
Bilateria - ps16
Deuterostomia - ps17
Chordata - ps18
Olfactores - ps19
Vertebrata - ps20
Gnathostomata - ps21
Euteleostomi - ps22
Sarcopterygii - ps23
Tetrapoda - ps24
Amniota - ps25
Mammalia - ps26
Theria - ps27
Placentalia - ps28
Boreoeutheria - ps29
Euarchontoglires - ps30
Euarchonta - ps31
Primates - ps32
Haplorrhini - ps33
Simiiformes - ps34
Catarrhini - ps35
Hominoidea - ps36
Hominidae - ps37
Homininae - ps38
Hominini - ps39
Homo_sapiens - ps40
p-value
4e-09
2e-07
2e-05
9e-04
6e-02
Log-odds
0.5
1.0
1.5
2.0
2.5
3.0
3.5
4.0

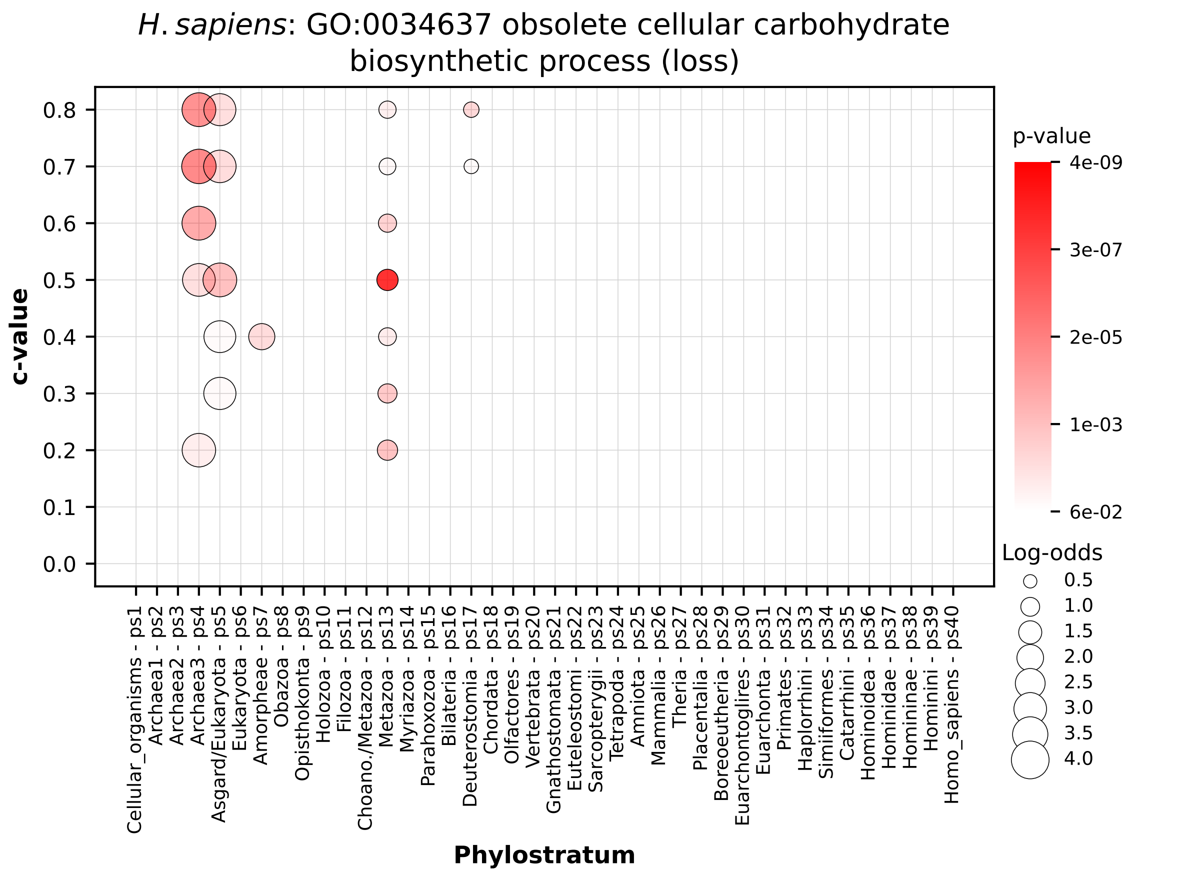

H. sapiens: GO:0034637 obsolete cellular carbohydrate biosynthetic process (loss)
c-value
0.0
0.1
0.2
0.3
0.4
0.5
0.6
0.7
0.8
Phylostratum
Cellular_organisms - ps1
Archaea1 - ps2
Archaea2 - ps3
Archaea3 - ps4
Asgard/Eukaryota - ps5
Eukaryota - ps6
Amorpheae - ps7
Obazoa - ps8
Opisthokonta - ps9
Holozoa - ps10
Filozoa - ps11
Choano./Metazoa - ps12
Metazoa - ps13
Myriazoa - ps14
Parahoxozoa - ps15
Bilateria - ps16
Deuterostomia - ps17
Chordata - ps18
Olfactores - ps19
Vertebrata - ps20
Gnathostomata - ps21
Euteleostomi - ps22
Sarcopterygii - ps23
Tetrapoda - ps24
Amniota - ps25
Mammalia - ps26
Theria - ps27
Placentalia - ps28
Boreoeutheria - ps29
Euarchontoglires - ps30
Euarchonta - ps31
Primates - ps32
Haplorrhini - ps33
Simiiformes - ps34
Catarrhini - ps35
Hominoidea - ps36
Hominidae - ps37
Homininae - ps38
Hominini - ps39
Homo_sapiens - ps40
p-value
4e-09
3e-07
2e-05
1e-03
6e-02
Log-odds
0.5
1.0
1.5
2.0
2.5
3.0
3.5
4.0

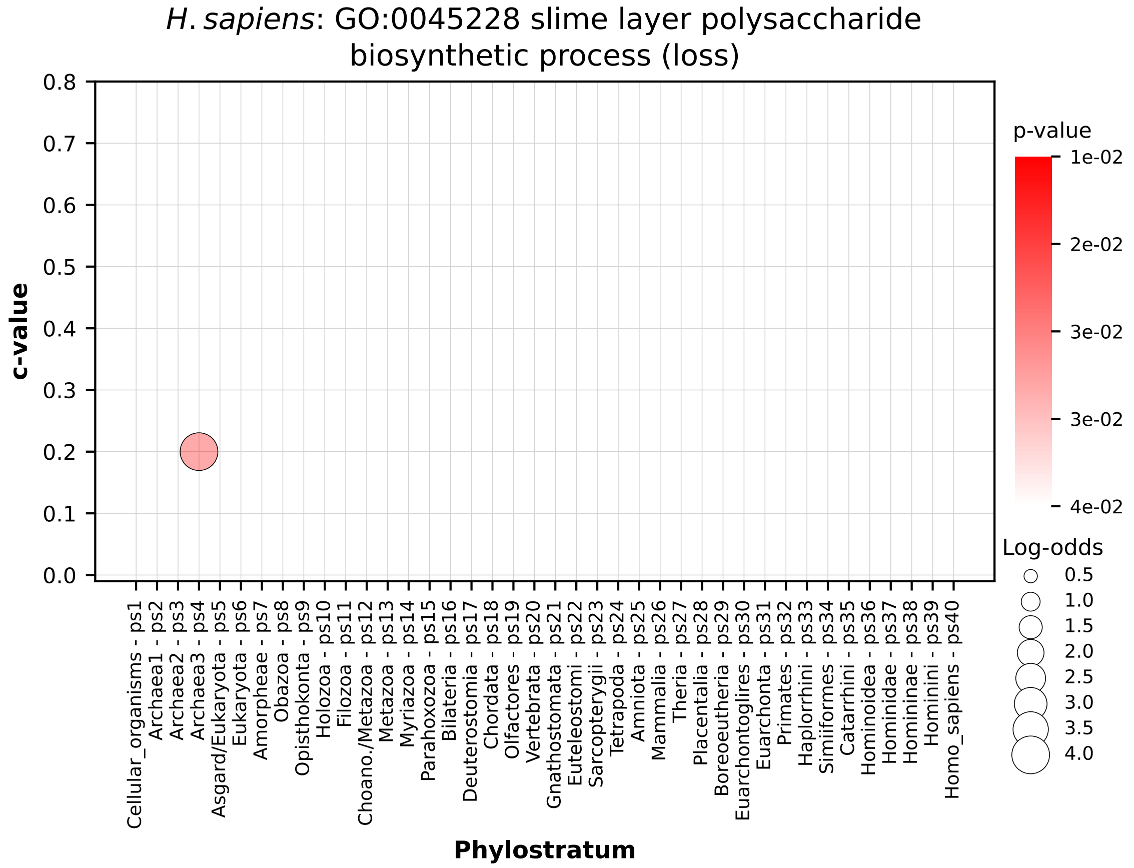
H. sapiens: GO:0045228 slime layer polysaccharide biosynthetic process (loss)
c-value
0.0
0.1
0.2
0.3
0.4
0.5
0.6
0.7
0.8
Phylostratum
Cellular_organisms - ps1
Archaea1 - ps2
Archaea2 - ps3
Archaea3 - ps4
Asgard/Eukaryota - ps5
Eukaryota - ps6
Amorpheae - ps7
Obazoa - ps8
Opisthokonta - ps9
Holozoa - ps10
Filozoa - ps11
Choano./Metazoa - ps12
Metazoa - ps13
Myriazoa - ps14
Parahoxozoa - ps15
Bilateria - ps16
Deuterostomia - ps17
Chordata - ps18
Olfactores - ps19
Vertebrata - ps20
Gnathostomata - ps21
Euteleostomi - ps22
Sarcopterygii - ps23
Tetrapoda - ps24
Amniota - ps25
Mammalia - ps26
Theria - ps27
Placentalia - ps28
Boreoeutheria - ps29
Euarchontoglires - ps30
Euarchonta - ps31
Primates - ps32
Haplorrhini - ps33
Simiiformes - ps34
Catarrhini - ps35
Hominoidea - ps36
Hominidae - ps37
Homininae - ps38
Hominini - ps39
Homo_sapiens - ps40
p-value
1e-02
2e-02
3e-02
3e-02
4e-02
Log-odds
0.5
1.0
1.5
2.0
2.5
3.0
3.5
4.0

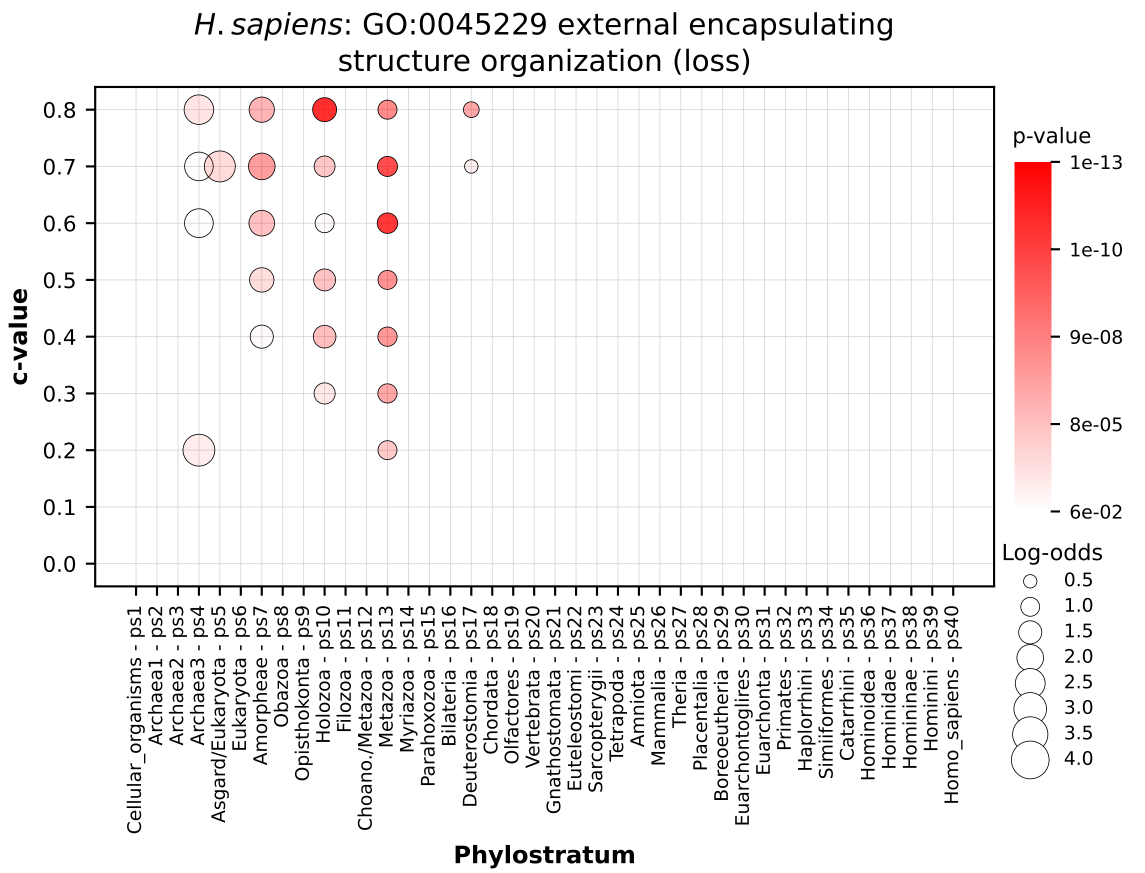

H. sapiens: GO:0045229 external encapsulating structure organization (loss)
c-value
0.8
0.7
0.6
0.5
0.4
0.3
0.2
0.1
0.0
Phylostratum
Cellular_organisms - ps1
Archaea1 - ps2
Archaea2 - ps3
Archaea3 - ps4
Asgard/Eukaryota - ps5
Eukaryota - ps6
Amorpheae - ps7
Obazoa - ps8
Opisthokonta - ps9
Holozoa - ps10
Filozoa - ps11
Choano./Metazoa - ps12
Metazoa - ps13
Myriazoa - ps14
Parahoxozoa - ps15
Bilateria - ps16
Deuterostomia - ps17
Chordata - ps18
Olfactores - ps19
Vertebrata - ps20
Gnathostomata - ps21
Euteleostomi - ps22
Sarcopterygii - ps23
Tetrapoda - ps24
Amniota - ps25
Mammalia - ps26
Theria - ps27
Placentalia - ps28
Boreoeutheria - ps29
Euarchontoglires - ps30
Euarchonta - ps31
Primates - ps32
Haplorrhini - ps33
Simiiformes - ps34
Catarrhini - ps35
Hominoidea - ps36
Hominidae - ps37
Homininae - ps38
Hominini - ps39
Homo_sapiens - ps40
p-value
1e-13
1e-10
9e-08
8e-05
6e-02
Log-odds
0.5
1.0
1.5
2.0
2.5
3.0
3.5
4.0

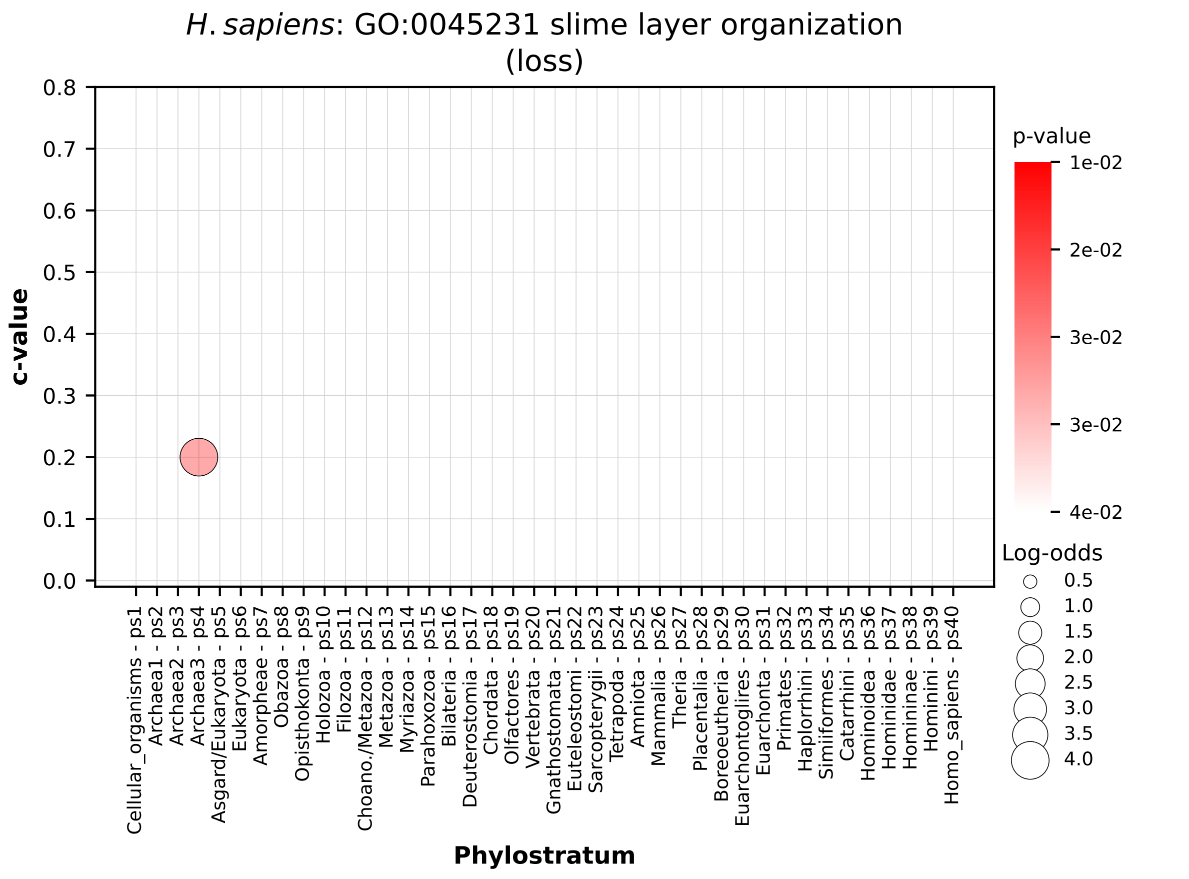
H. sapiens: GO:0045231 slime layer organization
(loss)
c-value
0.0
0.1
0.2
0.3
0.4
0.5
0.6
0.7
0.8
Phylostratum
Cellular_organisms - ps1
Archaea1 - ps2
Archaea2 - ps3
Archaea3 - ps4
Asgard/Eukaryota - ps5
Eukaryota - ps6
Amorpheae - ps7
Obazoa - ps8
Opisthokonta - ps9
Holozoa - ps10
Filozoa - ps11
Choano./Metazoa - ps12
Metazoa - ps13
Myriazoa - ps14
Parahoxozoa - ps15
Bilateria - ps16
Deuterostomia - ps17
Chordata - ps18
Olfactores - ps19
Vertebrata - ps20
Gnathostomata - ps21
Euteleostomi - ps22
Sarcopterygii - ps23
Tetrapoda - ps24
Amniota - ps25
Mammalia - ps26
Theria - ps27
Placentalia - ps28
Boreoeutheria - ps29
Euarchontoglires - ps30
Euarchonta - ps31
Primates - ps32
Haplorrhini - ps33
Simiiformes - ps34
Catarrhini - ps35
Hominoidea - ps36
Hominidae - ps37
Homininae - ps38
Hominini - ps39
Homo_sapiens - ps40
p-value
1e-02
2e-02
3e-02
3e-02
4e-02
Log-odds
0.5
1.0
1.5
2.0
2.5
3.0
3.5
4.0

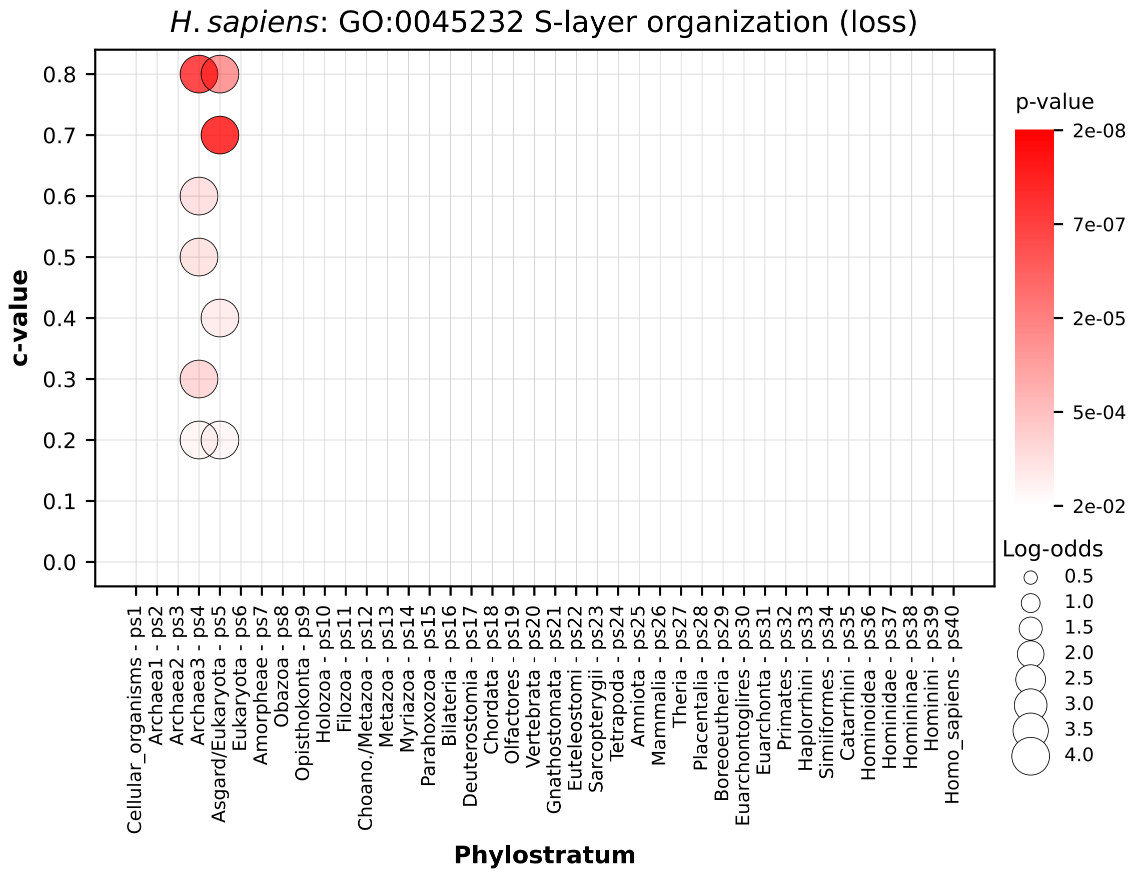
H. sapiens: GO:0045232 S-layer organization (loss)
c-value
0.0
0.1
0.2
0.3
0.4
0.5
0.6
0.7
0.8
Phylostratum
Cellular_organisms - ps1
Archaea1 - ps2
Archaea2 - ps3
Archaea3 - ps4
Asgard/Eukaryota - ps5
Eukaryota - ps6
Amorpheae - ps7
Obazoa - ps8
Opisthokonta - ps9
Holozoa - ps10
Filozoa - ps11
Choano./Metazoa - ps12
Metazoa - ps13
Myriazoa - ps14
Parahoxozoa - ps15
Bilateria - ps16
Deuterostomia - ps17
Chordata - ps18
Olfactores - ps19
Vertebrata - ps20
Gnathostomata - ps21
Euteleostomi - ps22
Sarcopterygii - ps23
Tetrapoda - ps24
Amniota - ps25
Mammalia - ps26
Theria - ps27
Placentalia - ps28
Boreoeutheria - ps29
Euarchontoglires - ps30
Euarchonta - ps31
Primates - ps32
Haplorrhini - ps33
Simiiformes - ps34
Catarrhini - ps35
Hominoidea - ps36
Hominidae - ps37
Homininae - ps38
Hominini - ps39
Homo_sapiens - ps40
p-value
2e-08
7e-07
2e-05
5e-04
2e-02
Log-odds
0.5
1.0
1.5
2.0
2.5
3.0
3.5
4.0

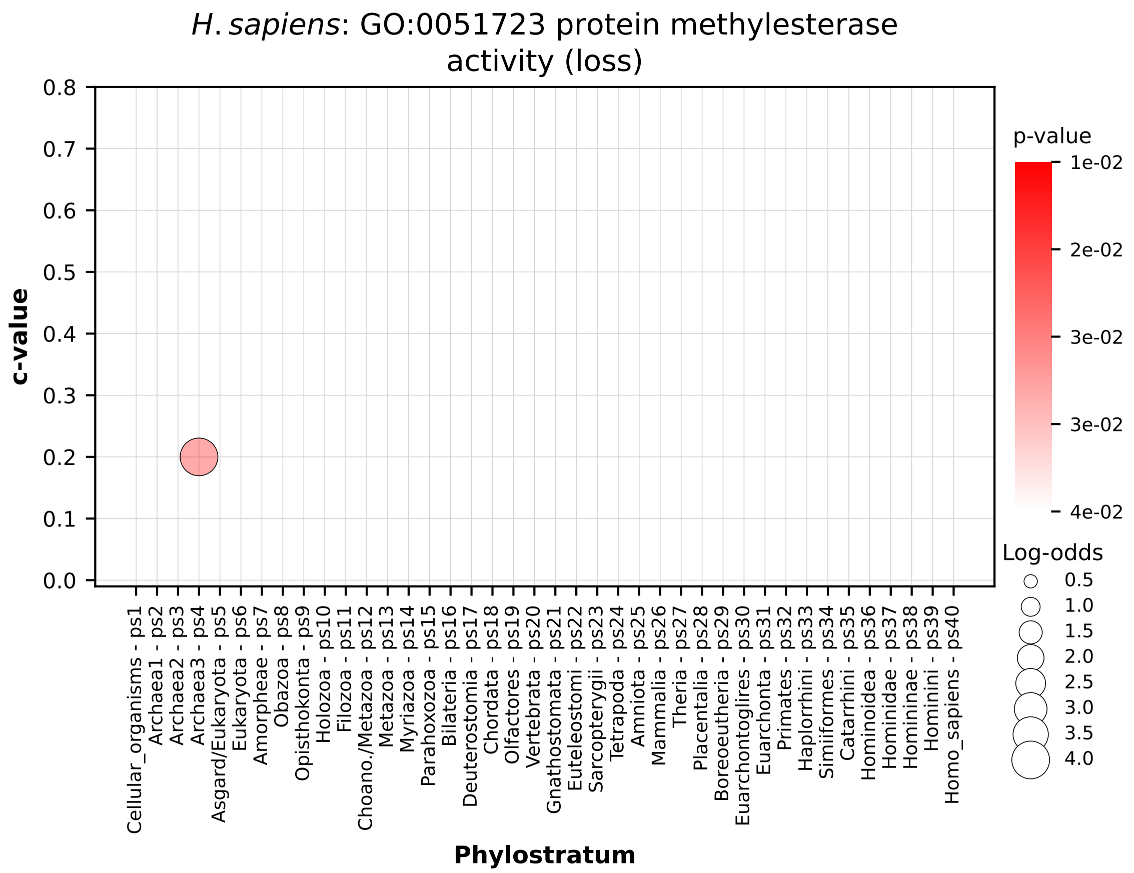

H. sapiens: GO:0051723 protein methylesterase activity (loss)
c-value
0.0
0.1
0.2
0.3
0.4
0.5
0.6
0.7
0.8
Phylostratum
Cellular_organisms - ps1
Archaea1 - ps2
Archaea2 - ps3
Archaea3 - ps4
Asgard/Eukaryota - ps5
Eukaryota - ps6
Amorpheae - ps7
Obazoa - ps8
Opisthokonta - ps9
Holozoa - ps10
Filozoa - ps11
Choano./Metazoa - ps12
Metazoa - ps13
Myriazoa - ps14
Parahoxozoa - ps15
Bilateria - ps16
Deuterostomia - ps17
Chordata - ps18
Olfactores - ps19
Vertebrata - ps20
Gnathostomata - ps21
Euteleostomi - ps22
Sarcopterygii - ps23
Tetrapoda - ps24
Amniota - ps25
Mammalia - ps26
Theria - ps27
Placentalia - ps28
Boreoeutheria - ps29
Euarchontoglires - ps30
Euarchonta - ps31
Primates - ps32
Haplorrhini - ps33
Simiiformes - ps34
Catarrhini - ps35
Hominoidea - ps36
Hominidae - ps37
Homininae - ps38
Hominini - ps39
Homo_sapiens - ps40
p-value
1e-02
2e-02
3e-02
3e-02
4e-02
Log-odds
0.5
1.0
1.5
2.0
2.5
3.0
3.5
4.0

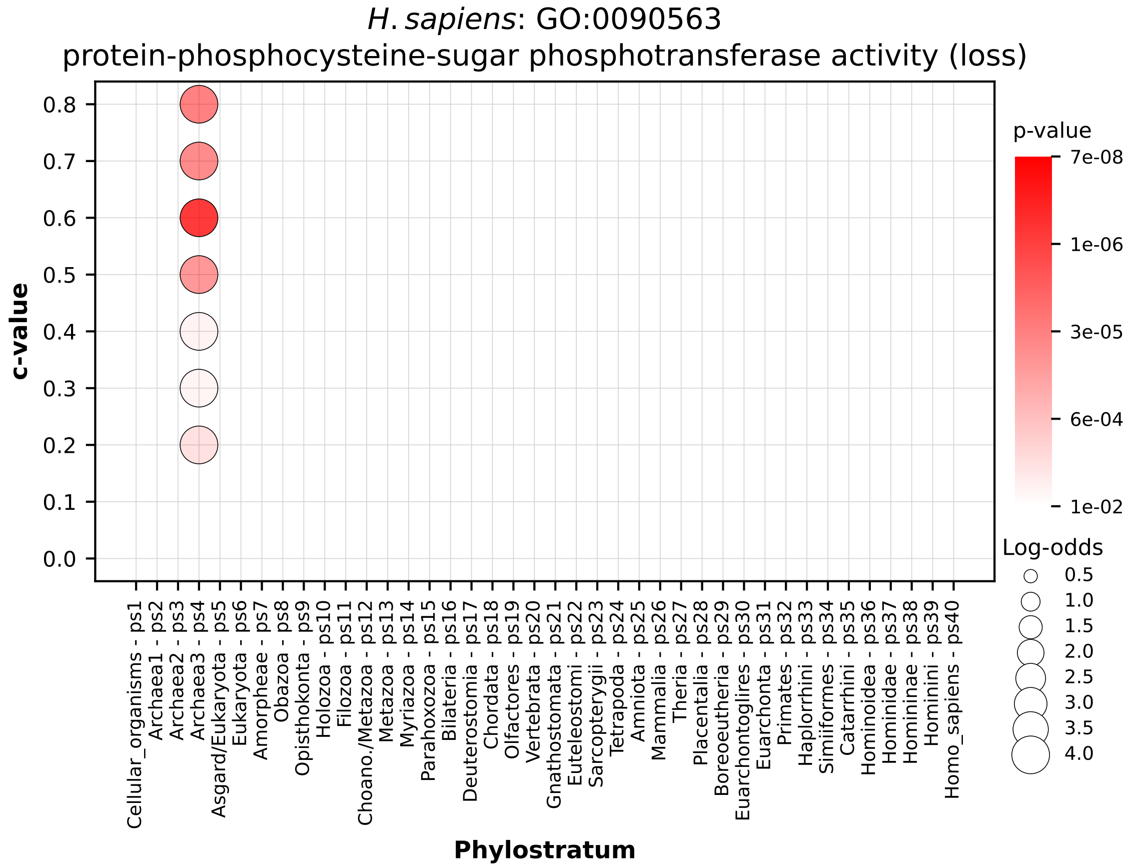

H. sapiens: GO:0090563
protein-phosphocysteine-sugar phosphotransferase activity (loss)
c-value
0.8
0.7
0.6
0.5
0.4
0.3
0.2
0.1
0.0
p-value
7e-08
1e-06
3e-05
6e-04
1e-02
Log-odds
0.5
1.0
1.5
2.0
2.5
3.0
3.5
4.0
Cellular_organisms - ps1
Archaea1 - ps2
Archaea2 - ps3
Archaea3 - ps4
Asgard/Eukaryota - ps5
Eukaryota - ps6
Amorpheae - ps7
Obazoa - ps8
Opisthokonta - ps9
Holozoa - ps10
Filozoa - ps11
Choano./Metazoa - ps12
Metazoa - ps13
Myriazoa - ps14
Parahoxozoa - ps15
Bilateria - ps16
Deuterostomia - ps17
Chordata - ps18
Olfactores - ps19
Vertebrata - ps20
Gnathostomata - ps21
Euteleostomi - ps22
Sarcopterygii - ps23
Tetrapoda - ps24
Amniota - ps25
Mammalia - ps26
Theria - ps27
Placentalia - ps28
Boreoeutheria - ps29
Euarchontoglires - ps30
Euarchonta - ps31
Primates - ps32
Haplorrhini - ps33
Simiiformes - ps34
Catarrhini - ps35
Hominoidea - ps36
Hominidae - ps37
Homininae - ps38
Hominini - ps39
Homo_sapiens - ps40
Phylostratum

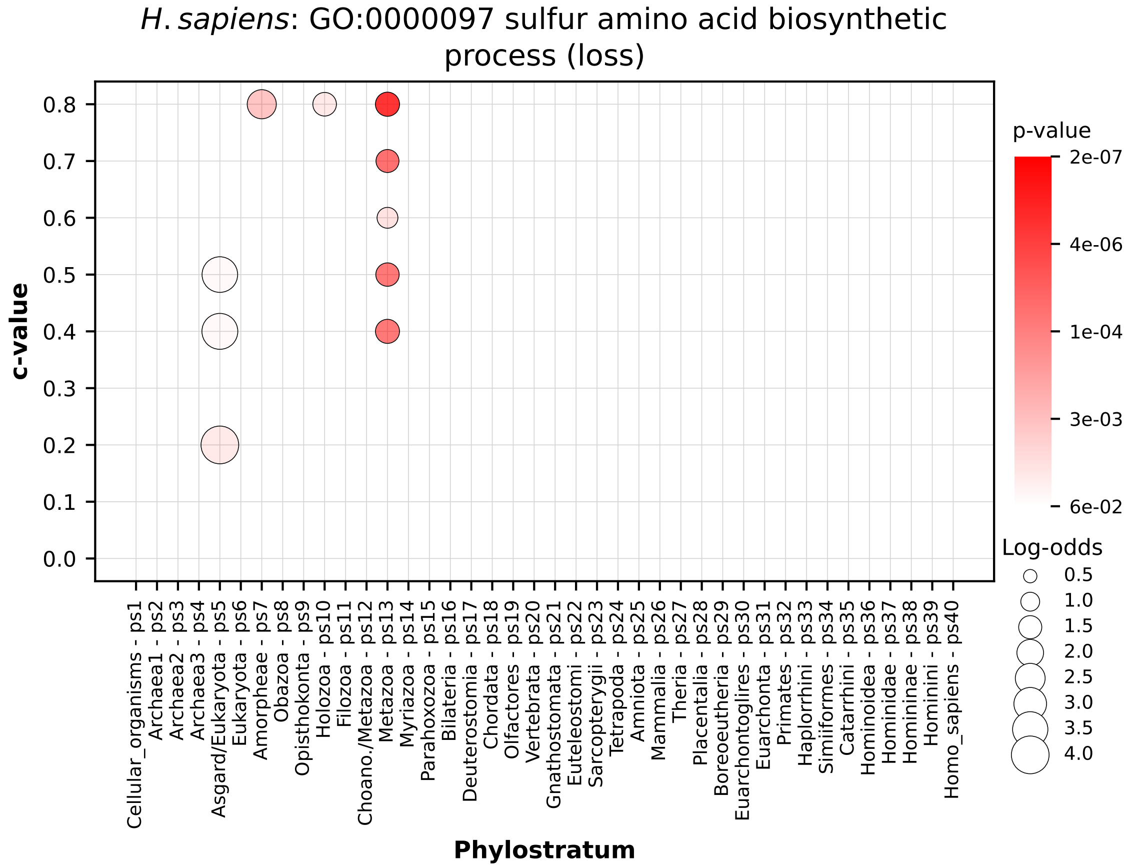

H. sapiens: GO:0000097 sulfur amino acid biosynthetic process (loss)
c-value
0.8
0.7
0.6
0.5
0.4
0.3
0.2
0.1
0.0
Phylostratum
Cellular_organisms - ps1
Archaea1 - ps2
Archaea2 - ps3
Archaea3 - ps4
Asgard/Eukaryota - ps5
Eukaryota - ps6
Amorpheae - ps7
Obazoa - ps8
Opisthokonta - ps9
Holozoa - ps10
Filozoa - ps11
Choano./Metazoa - ps12
Metazoa - ps13
Myriazoa - ps14
Parahoxozoa - ps15
Bilateria - ps16
Deuterostomia - ps17
Chordata - ps18
Olfactores - ps19
Vertebrata - ps20
Gnathostomata - ps21
Euteleostomi - ps22
Sarcopterygii - ps23
Tetrapoda - ps24
Amniota - ps25
Mammalia - ps26
Theria - ps27
Placentalia - ps28
Boreoeutheria - ps29
Euarchontoglires - ps30
Euarchonta - ps31
Primates - ps32
Haplorrhini - ps33
Simiiformes - ps34
Catarrhini - ps35
Hominoidea - ps36
Hominidae - ps37
Homininae - ps38
Hominini - ps39
Homo_sapiens - ps40
p-value
2e-07
4e-06
1e-04
3e-03
6e-02
Log-odds
0.5
1.0
1.5
2.0
2.5
3.0
3.5
4.0

H. sapiens: GO:0004452 isopentenyl-diphosphate delta-isomerase activity (loss)

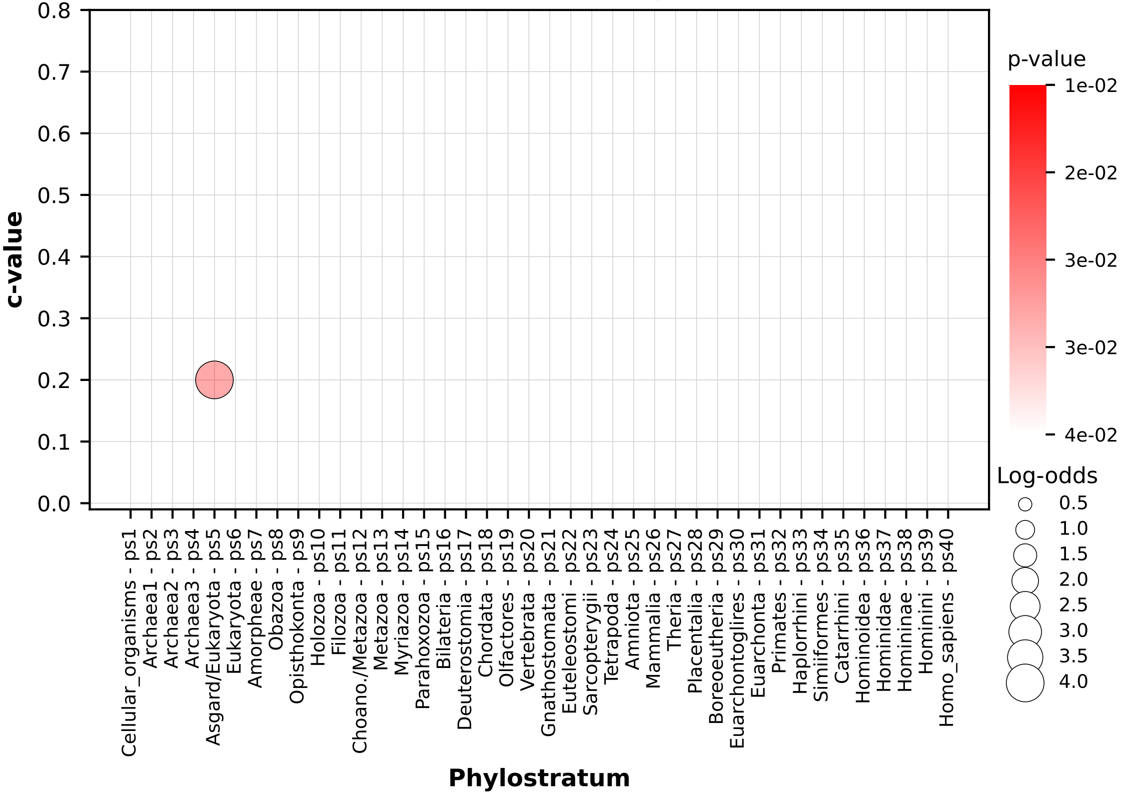

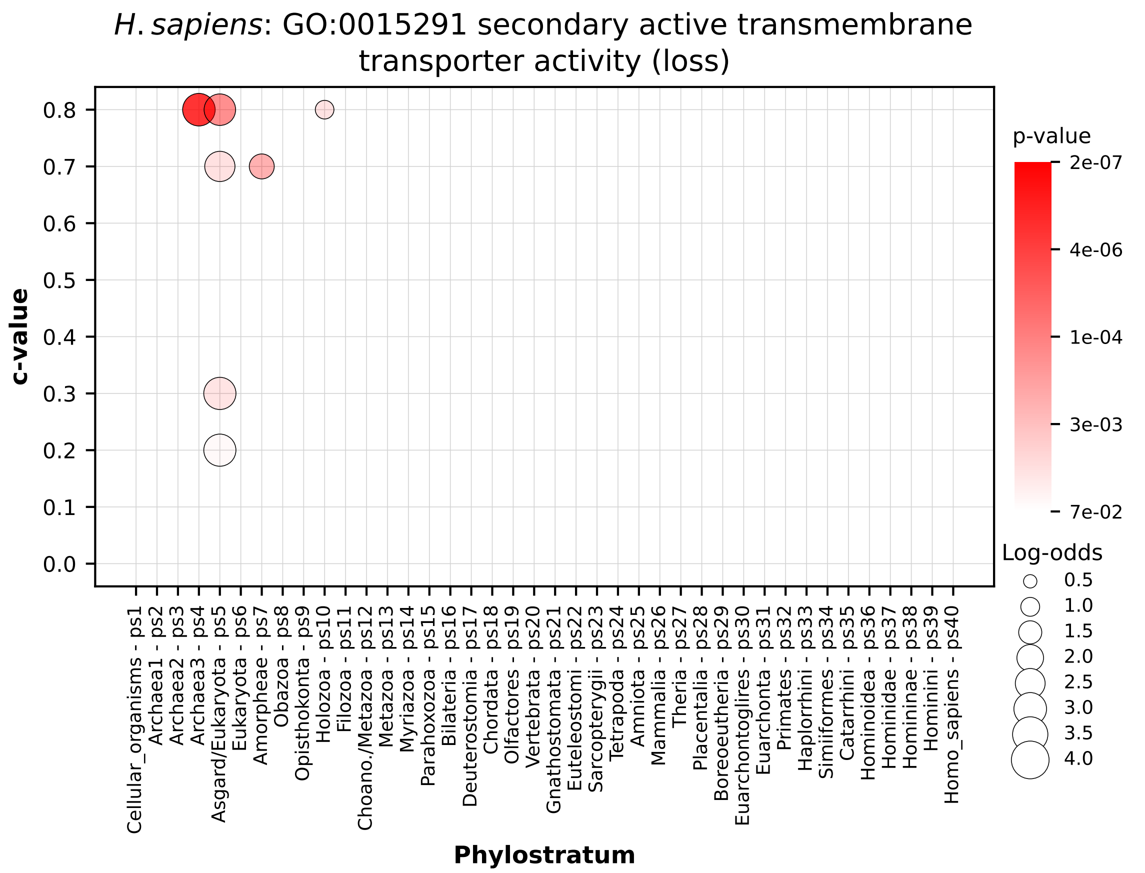

H. sapiens: GO:0015291 secondary active transmembrane transporter activity (loss)
c-value
0.0
0.1
0.2
0.3
0.4
0.5
0.6
0.7
0.8
Phylostratum
Cellular_organisms - ps1
Archaea1 - ps2
Archaea2 - ps3
Archaea3 - ps4
Asgard/Eukaryota - ps5
Eukaryota - ps6
Amorpheae - ps7
Obazoa - ps8
Opisthokonta - ps9
Holozoa - ps10
Filozoa - ps11
Choano./Metazoa - ps12
Metazoa - ps13
Myriazoa - ps14
Parahoxozoa - ps15
Bilateria - ps16
Deuterostomia - ps17
Chordata - ps18
Olfactores - ps19
Vertebrata - ps20
Gnathostomata - ps21
Euteleostomi - ps22
Sarcopterygii - ps23
Tetrapoda - ps24
Amniota - ps25
Mammalia - ps26
Theria - ps27
Placentalia - ps28
Boreoeutheria - ps29
Euarchontoglires - ps30
Euarchonta - ps31
Primates - ps32
Haplorrhini - ps33
Simiiformes - ps34
Catarrhini - ps35
Hominoidea - ps36
Hominidae - ps37
Homininae - ps38
Hominini - ps39
Homo_sapiens - ps40
p-value
2e-07
4e-06
1e-04
3e-03
7e-02
Log-odds
0.5
1.0
1.5
2.0
2.5
3.0
3.5
4.0

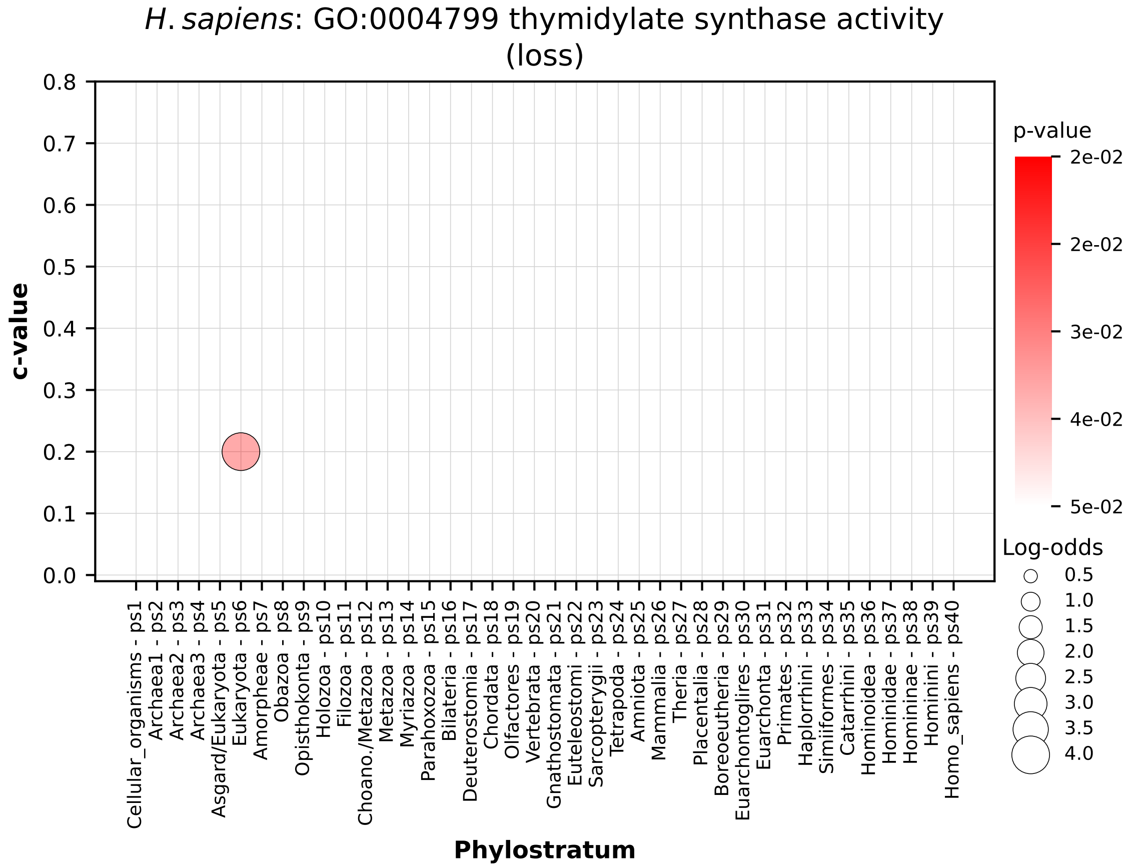

H. sapiens: GO:0004799 thymidylate synthase activity (loss)
c-value
0.0
0.1
0.2
0.3
0.4
0.5
0.6
0.7
0.8
p-value
2e-02
2e-02
3e-02
4e-02
5e-02
Log-odds
0.5
1.0
1.5
2.0
2.5
3.0
3.5
4.0
Cellular_organisms - ps1
Archaea1 - ps2
Archaea2 - ps3
Archaea3 - ps4
Asgard/Eukaryota - ps5
Eukaryota - ps6
Amorpheae - ps7
Obazoa - ps8
Opisthokonta - ps9
Holozoa - ps10
Filozoa - ps11
Choano./Metazoa - ps12
Metazoa - ps13
Myriazoa - ps14
Parahoxozoa - ps15
Bilateria - ps16
Deuterostomia - ps17
Chordata - ps18
Olfactores - ps19
Vertebrata - ps20
Gnathostomata - ps21
Euteleostomi - ps22
Sarcopterygii - ps23
Tetrapoda - ps24
Amniota - ps25
Mammalia - ps26
Theria - ps27
Placentalia - ps28
Boreoeutheria - ps29
Euarchontoglires - ps30
Euarchonta - ps31
Primates - ps32
Haplorrhini - ps33
Simiiformes - ps34
Catarrhini - ps35
Hominoidea - ps36
Hominidae - ps37
Homininae - ps38
Hominini - ps39
Homo_sapiens - ps40
Phylostratum

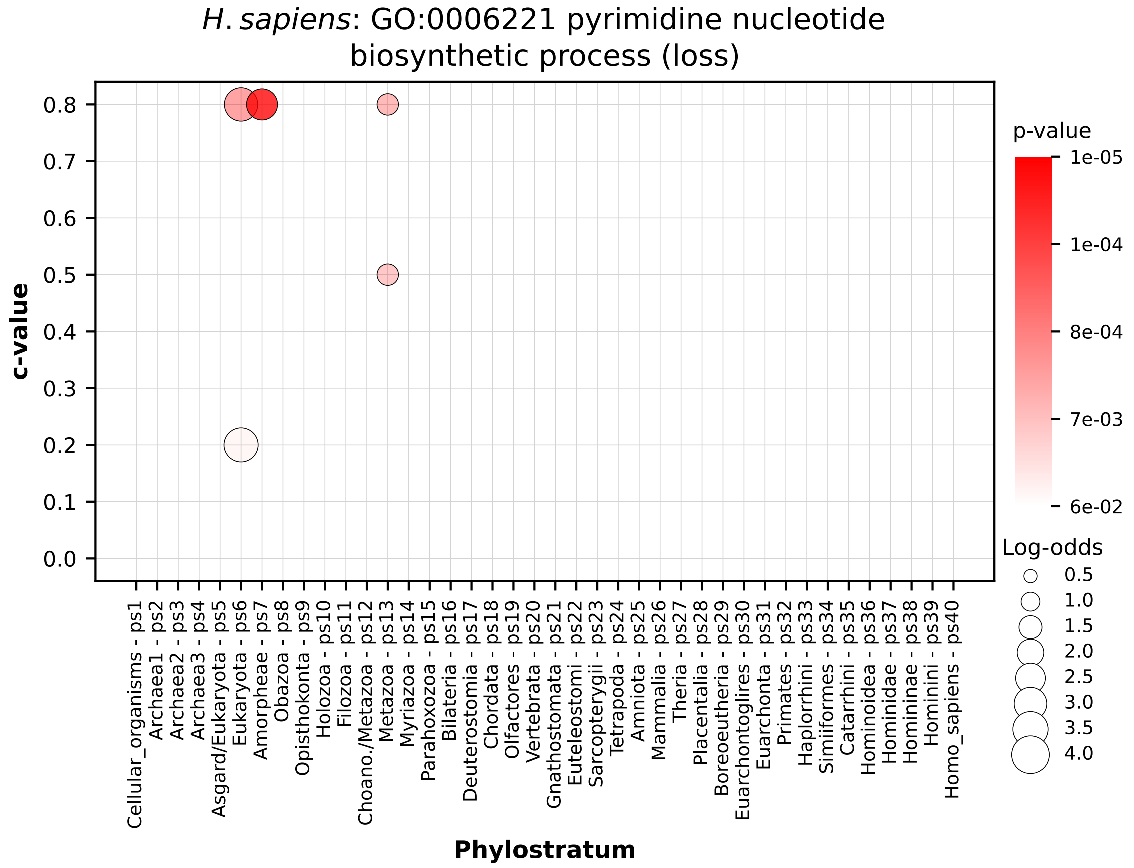

H. sapiens: GO:0006221 pyrimidine nucleotide biosynthetic process (loss)
c-value
0.0
0.1
0.2
0.3
0.4
0.5
0.6
0.7
0.8
Phylostratum
Cellular_organisms - ps1
Archaea1 - ps2
Archaea2 - ps3
Archaea3 - ps4
Asgard/Eukaryota - ps5
Eukaryota - ps6
Amorpheae - ps7
Obazoa - ps8
Opisthokonta - ps9
Holozoa - ps10
Filozoa - ps11
Choano./Metazoa - ps12
Metazoa - ps13
Myriazoa - ps14
Parahoxozoa - ps15
Bilateria - ps16
Deuterostomia - ps17
Chordata - ps18
Olfactores - ps19
Vertebrata - ps20
Gnathostomata - ps21
Euteleostomi - ps22
Sarcopterygii - ps23
Tetrapoda - ps24
Amniota - ps25
Mammalia - ps26
Theria - ps27
Placentalia - ps28
Boreoeutheria - ps29
Euarchontoglires - ps30
Euarchonta - ps31
Primates - ps32
Haplorrhini - ps33
Simiiformes - ps34
Catarrhini - ps35
Hominoidea - ps36
Hominidae - ps37
Homininae - ps38
Hominini - ps39
Homo_sapiens - ps40
p-value
1e-05
1e-04
8e-04
7e-03
6e-02
Log-odds
0.5
1.0
1.5
2.0
2.5
3.0
3.5
4.0

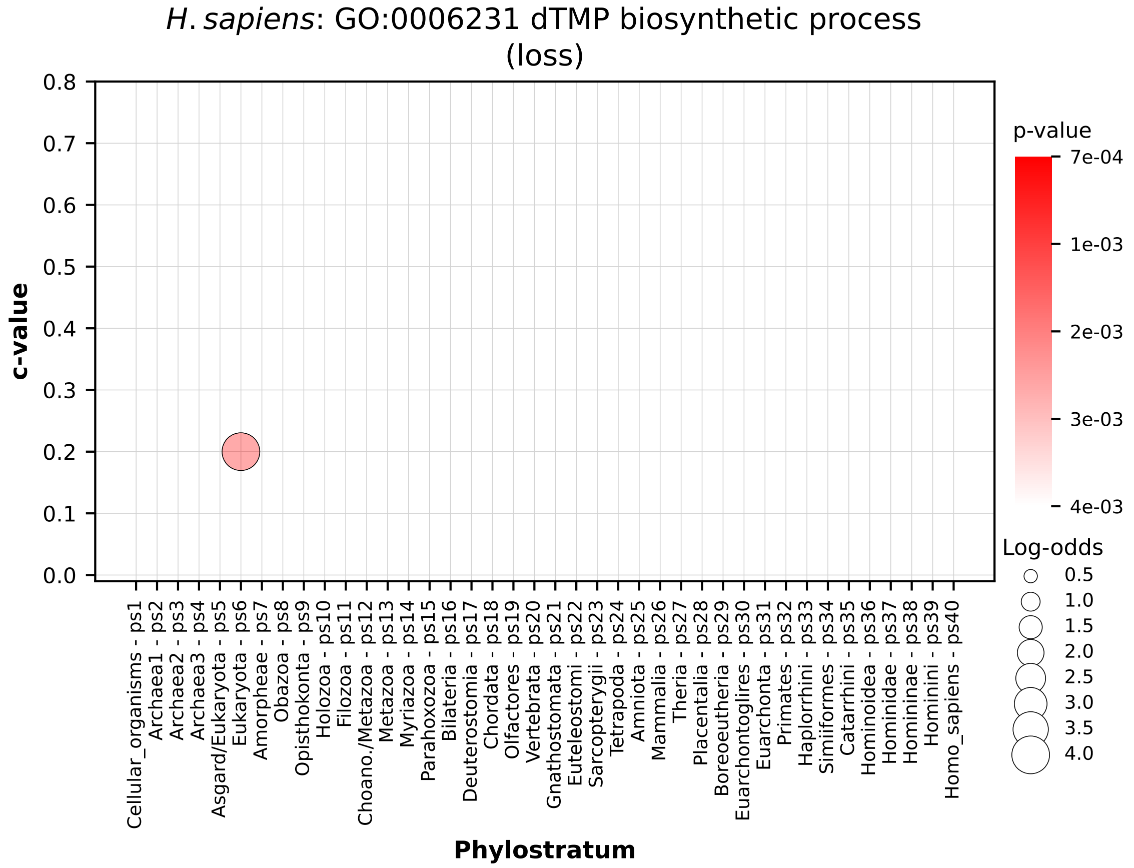

H. sapiens: GO:0006231 dTMP biosynthetic process
(loss)
c-value
0.8
0.7
0.6
0.5
0.4
0.3
0.2
0.1
0.0
p-value
7e-04
1e-03
2e-03
3e-03
4e-03
Log-odds
0.5
1.0
1.5
2.0
2.5
3.0
3.5
4.0
Cellular_organisms - ps1
Archaea1 - ps2
Archaea2 - ps3
Archaea3 - ps4
Asgard/Eukaryota - ps5
Eukaryota - ps6
Amorpheae - ps7
Obazoa - ps8
Opisthokonta - ps9
Holozoa - ps10
Filozoa - ps11
Choano./Metazoa - ps12
Metazoa - ps13
Myriazoa - ps14
Parahoxozoa - ps15
Bilateria - ps16
Deuterostomia - ps17
Chordata - ps18
Olfactores - ps19
Vertebrata - ps20
Gnathostomata - ps21
Euteleostomi - ps22
Sarcopterygii - ps23
Tetrapoda - ps24
Amniota - ps25
Mammalia - ps26
Theria - ps27
Placentalia - ps28
Boreoeutheria - ps29
Euarchontoglires - ps30
Euarchonta - ps31
Primates - ps32
Haplorrhini - ps33
Simiiformes - ps34
Catarrhini - ps35
Hominoidea - ps36
Hominidae - ps37
Homininae - ps38
Hominini - ps39
Homo_sapiens - ps40
Phylostratum

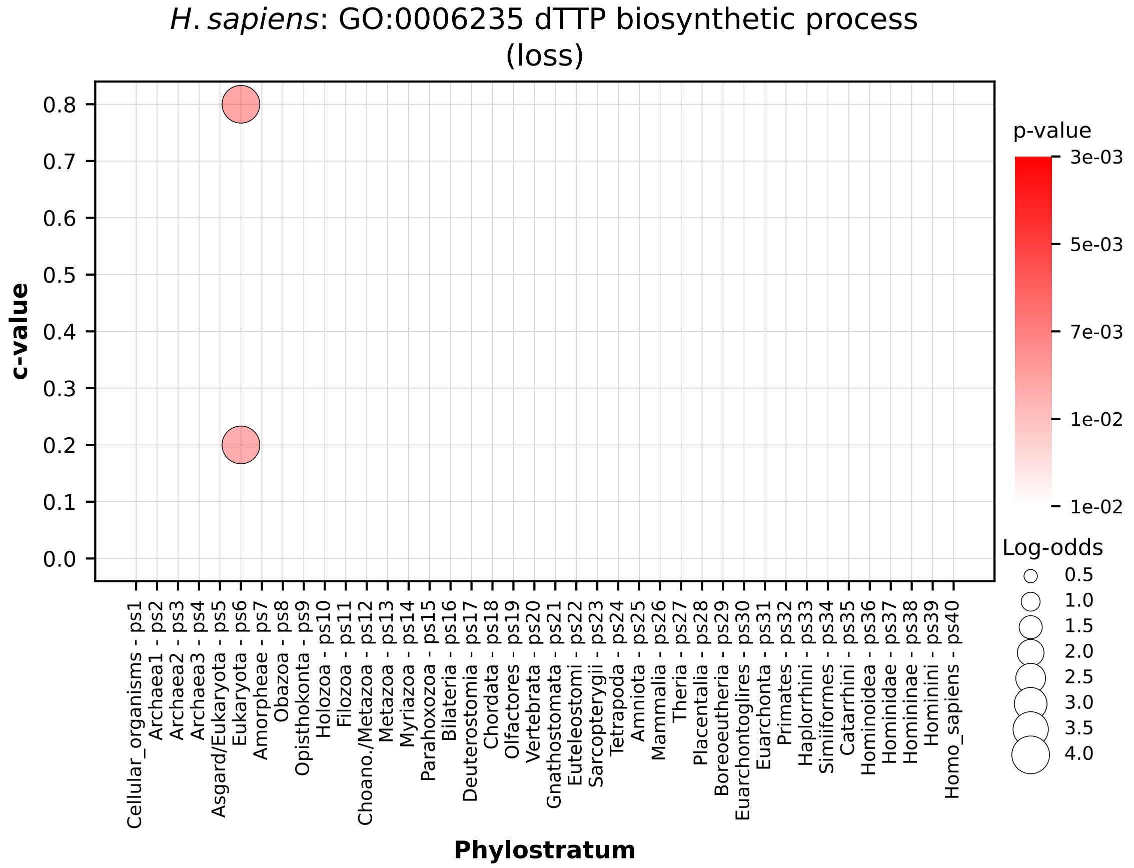

H. sapiens: GO:0006235 dTTP biosynthetic process
(loss)
c-value
0.0
0.1
0.2
0.3
0.4
0.5
0.6
0.7
0.8
Phylostratum
Cellular_organisms - ps1
Archaea1 - ps2
Archaea2 - ps3
Archaea3 - ps4
Asgard/Eukaryota - ps5
Eukaryota - ps6
Amorpheae - ps7
Obazoa - ps8
Opisthokonta - ps9
Holozoa - ps10
Filozoa - ps11
Choano./Metazoa - ps12
Metazoa - ps13
Myriazoa - ps14
Parahoxozoa - ps15
Bilateria - ps16
Deuterostomia - ps17
Chordata - ps18
Olfactores - ps19
Vertebrata - ps20
Gnathostomata - ps21
Euteleostomi - ps22
Sarcopterygii - ps23
Tetrapoda - ps24
Amniota - ps25
Mammalia - ps26
Theria - ps27
Placentalia - ps28
Boreoeutheria - ps29
Euarchontoglires - ps30
Euarchonta - ps31
Primates - ps32
Haplorrhini - ps33
Simiiformes - ps34
Catarrhini - ps35
Hominoidea - ps36
Hominidae - ps37
Homininae - ps38
Hominini - ps39
Homo_sapiens - ps40
p-value
3e-03
5e-03
7e-03
1e-02
1e-02
Log-odds
0.5
1.0
1.5
2.0
2.5
3.0
3.5
4.0

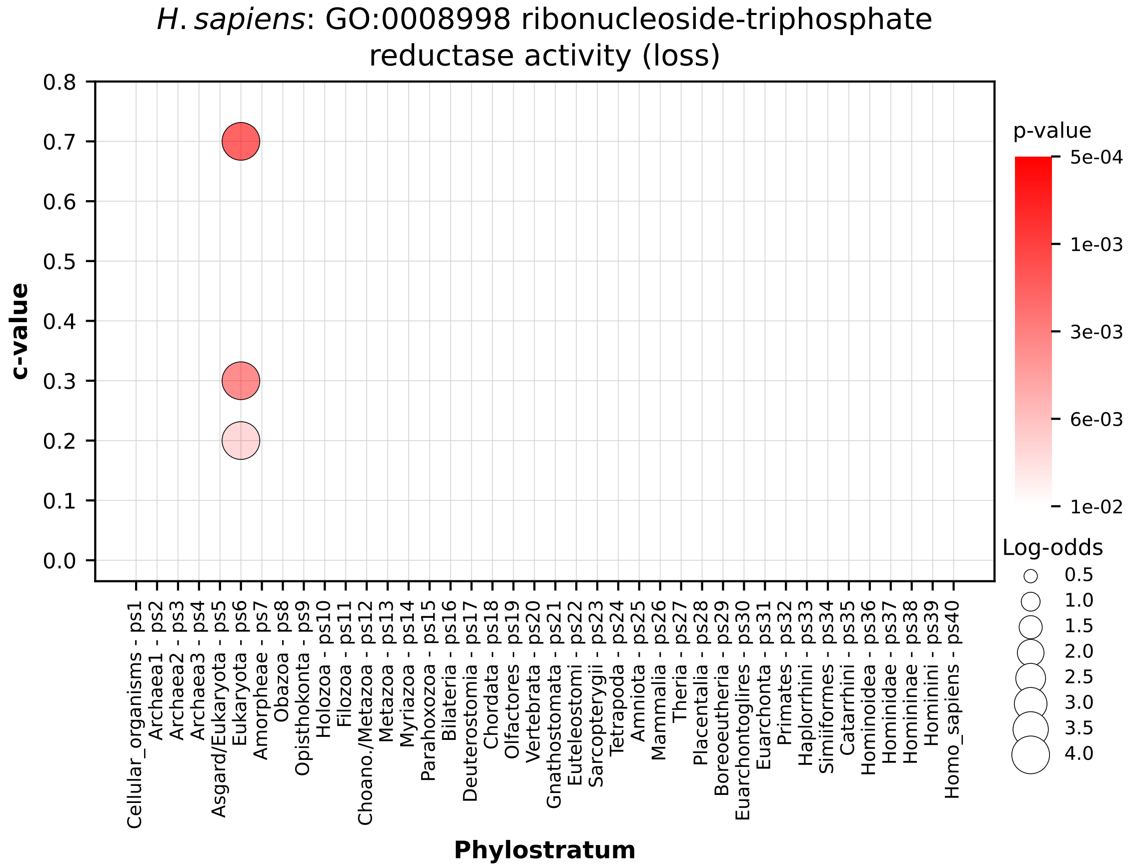
H. sapiens: GO:0008998 ribonucleoside-triphosphate reductase activity (loss)
c-value
0.8
0.7
0.6
0.5
0.4
0.3
0.2
0.1
0.0
Phylostratum
Cellular_organisms - ps1
Archaea1 - ps2
Archaea2 - ps3
Archaea3 - ps4
Asgard/Eukaryota - ps5
Eukaryota - ps6
Amorpheae - ps7
Obazoa - ps8
Opisthokonta - ps9
Holozoa - ps10
Filozoa - ps11
Choano./Metazoa - ps12
Metazoa - ps13
Myriazoa - ps14
Parahoxozoa - ps15
Bilateria - ps16
Deuterostomia - ps17
Chordata - ps18
Olfactores - ps19
Vertebrata - ps20
Gnathostomata - ps21
Euteleostomi - ps22
Sarcopterygii - ps23
Tetrapoda - ps24
Amniota - ps25
Mammalia - ps26
Theria - ps27
Placentalia - ps28
Boreoeutheria - ps29
Euarchontoglires - ps30
Euarchonta - ps31
Primates - ps32
Haplorrhini - ps33
Simiiformes - ps34
Catarrhini - ps35
Hominoidea - ps36
Hominidae - ps37
Homininae - ps38
Hominini - ps39
Homo_sapiens - ps40
p-value
5e-04
1e-03
3e-03
6e-03
1e-02
Log-odds
0.5
1.0
1.5
2.0
2.5
3.0
3.5
4.0

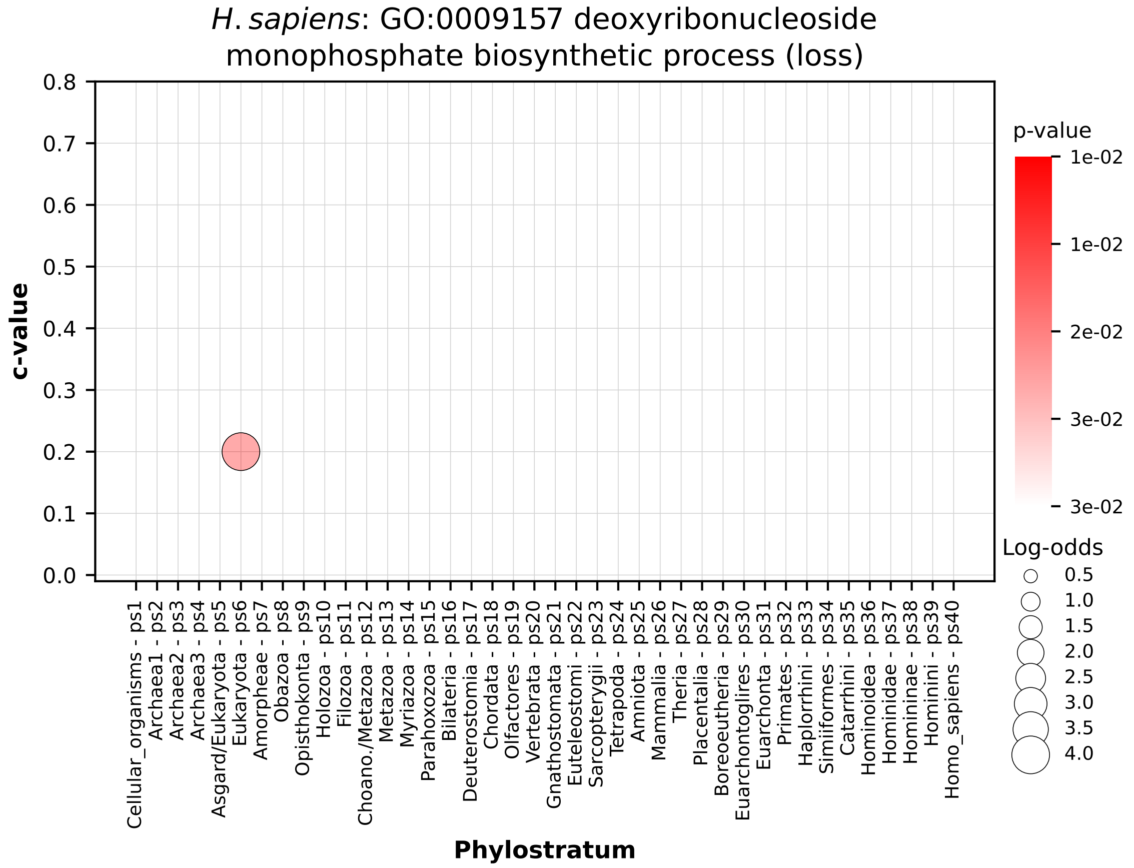

H. sapiens: GO:0009157 deoxyribonucleoside monophosphate biosynthetic process (loss)
c-value
0.0
0.1
0.2
0.3
0.4
0.5
0.6
0.7
0.8
Phylostratum
Cellular_organisms - ps1
Archaea1 - ps2
Archaea2 - ps3
Archaea3 - ps4
Asgard/Eukaryota - ps5
Eukaryota - ps6
Amorpheae - ps7
Obazoa - ps8
Opisthokonta - ps9
Holozoa - ps10
Filozoa - ps11
Choano./Metazoa - ps12
Metazoa - ps13
Myriazoa - ps14
Parahoxozoa - ps15
Bilateria - ps16
Deuterostomia - ps17
Chordata - ps18
Olfactores - ps19
Vertebrata - ps20
Gnathostomata - ps21
Euteleostomi - ps22
Sarcopterygii - ps23
Tetrapoda - ps24
Amniota - ps25
Mammalia - ps26
Theria - ps27
Placentalia - ps28
Boreoeutheria - ps29
Euarchontoglires - ps30
Euarchonta - ps31
Primates - ps32
Haplorrhini - ps33
Simiiformes - ps34
Catarrhini - ps35
Hominoidea - ps36
Hominidae - ps37
Homininae - ps38
Hominini - ps39
Homo_sapiens - ps40
p-value
1e-02
1e-02
2e-02
3e-02
3e-02
Log-odds
0.5
1.0
1.5
2.0
2.5
3.0
3.5
4.0

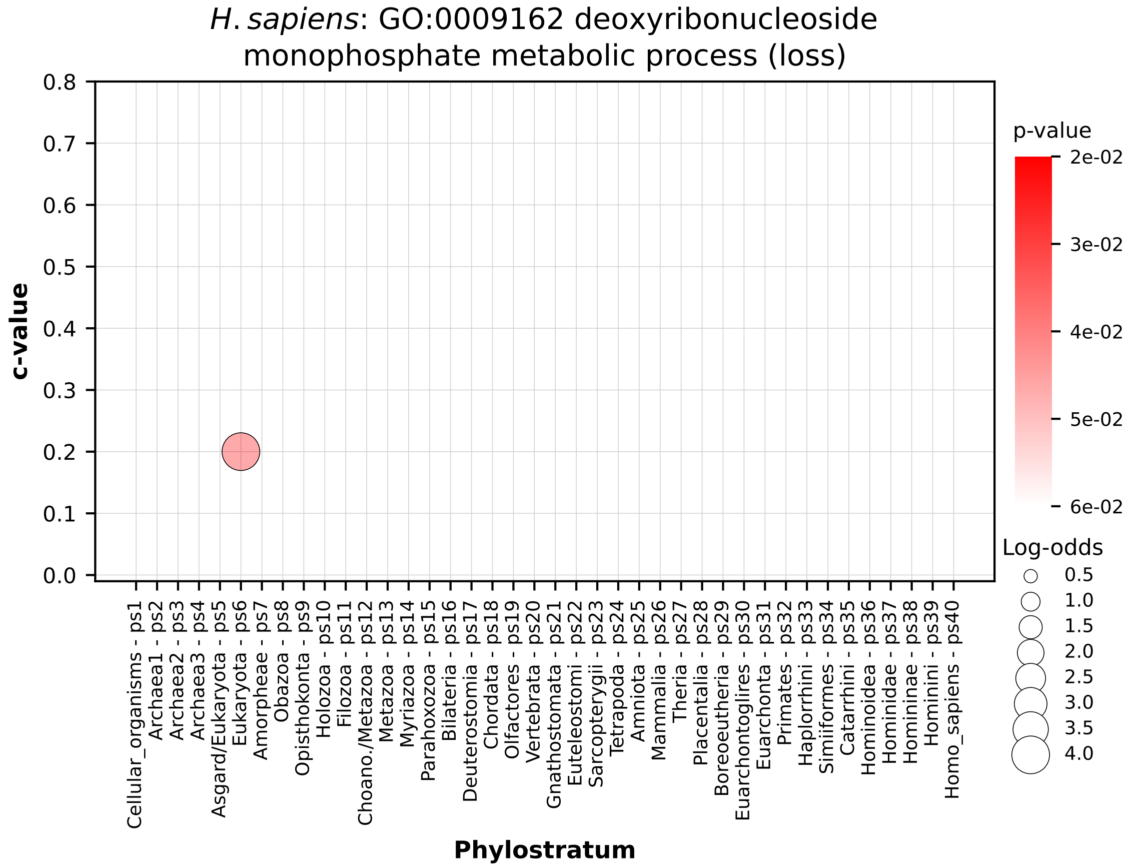

H. sapiens: GO:0009162 deoxyribonucleoside monophosphate metabolic process (loss)
c-value
0.0
0.1
0.2
0.3
0.4
0.5
0.6
0.7
0.8
Phylostratum
Cellular_organisms - ps1
Archaea1 - ps2
Archaea2 - ps3
Archaea3 - ps4
Asgard/Eukaryota - ps5
Eukaryota - ps6
Amorpheae - ps7
Obazoa - ps8
Opisthokonta - ps9
Holozoa - ps10
Filozoa - ps11
Choano./Metazoa - ps12
Metazoa - ps13
Myriazoa - ps14
Parahoxozoa - ps15
Bilateria - ps16
Deuterostomia - ps17
Chordata - ps18
Olfactores - ps19
Vertebrata - ps20
Gnathostomata - ps21
Euteleostomi - ps22
Sarcopterygii - ps23
Tetrapoda - ps24
Amniota - ps25
Mammalia - ps26
Theria - ps27
Placentalia - ps28
Boreoeutheria - ps29
Euarchontoglires - ps30
Euarchonta - ps31
Primates - ps32
Haplorrhini - ps33
Simiiformes - ps34
Catarrhini - ps35
Hominoidea - ps36
Hominidae - ps37
Homininae - ps38
Hominini - ps39
Homo_sapiens - ps40
p-value
2e-02
3e-02
4e-02
5e-02
6e-02
Log-odds
0.5
1.0
1.5
2.0
2.5
3.0
3.5
4.0

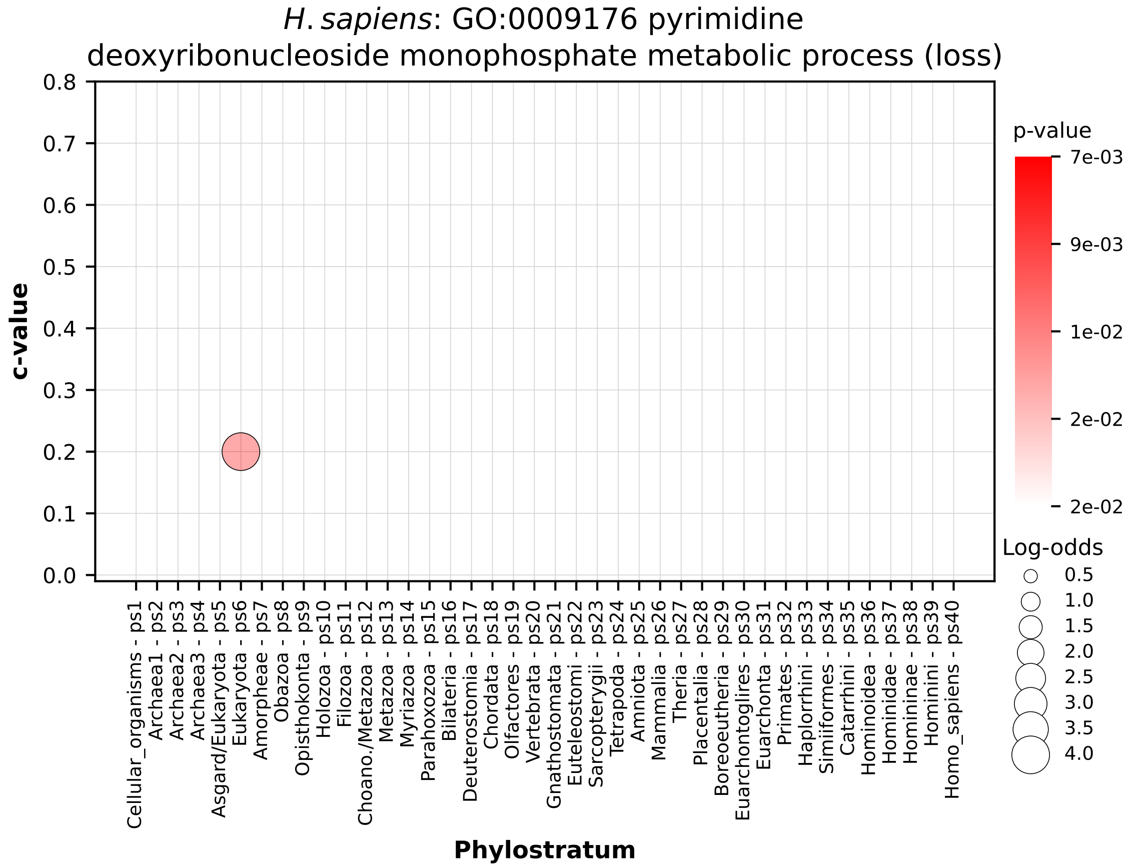

H. sapiens: GO:0009176 pyrimidine deoxyribonucleoside monophosphate metabolic process (loss)
c-value
0.0
0.1
0.2
0.3
0.4
0.5
0.6
0.7
0.8
Phylostratum
Cellular_organisms - ps1
Archaea1 - ps2
Archaea2 - ps3
Archaea3 - ps4
Asgard/Eukaryota - ps5
Eukaryota - ps6
Amorpheae - ps7
Obazoa - ps8
Opisthokonta - ps9
Holozoa - ps10
Filozoa - ps11
Choano./Metazoa - ps12
Metazoa - ps13
Myriazoa - ps14
Parahoxozoa - ps15
Bilateria - ps16
Deuterostomia - ps17
Chordata - ps18
Olfactores - ps19
Vertebrata - ps20
Gnathostomata - ps21
Euteleostomi - ps22
Sarcopterygii - ps23
Tetrapoda - ps24
Amniota - ps25
Mammalia - ps26
Theria - ps27
Placentalia - ps28
Boreoeutheria - ps29
Euarchontoglires - ps30
Euarchonta - ps31
Primates - ps32
Haplorrhini - ps33
Simiiformes - ps34
Catarrhini - ps35
Hominoidea - ps36
Hominidae - ps37
Homininae - ps38
Hominini - ps39
Homo_sapiens - ps40
p-value
7e-03
9e-03
1e-02
2e-02
2e-02
Log-odds
0.5
1.0
1.5
2.0
2.5
3.0
3.5
4.0

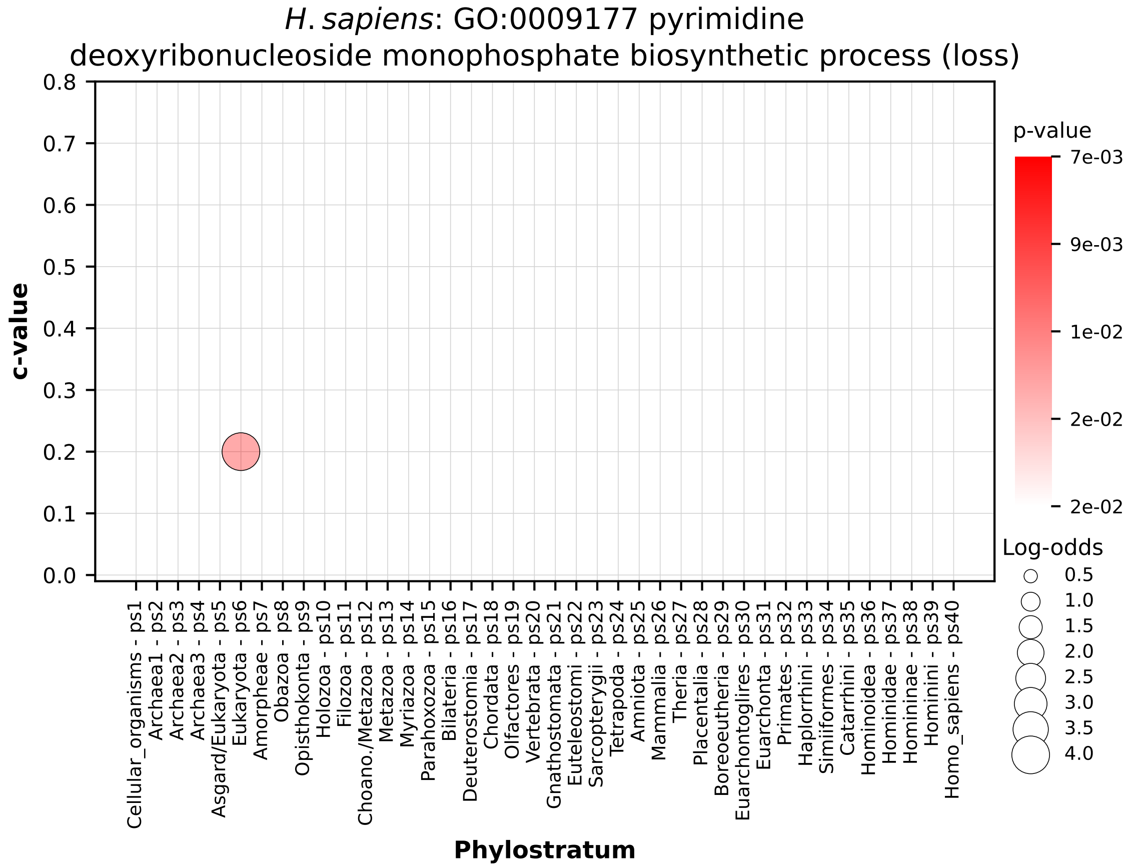

H. sapiens: GO:0009177 pyrimidine deoxyribonucleoside monophosphate biosynthetic process (loss)
c-value
0.8
0.7
0.6
0.5
0.4
0.3
0.2
0.1
0.0
Phylostratum
Cellular_organisms - ps1
Archaea1 - ps2
Archaea2 - ps3
Archaea3 - ps4
Asgard/Eukaryota - ps5
Eukaryota - ps6
Amorpheae - ps7
Obazoa - ps8
Opisthokonta - ps9
Holozoa - ps10
Filozoa - ps11
Choano./Metazoa - ps12
Metazoa - ps13
Myriazoa - ps14
Parahoxozoa - ps15
Bilateria - ps16
Deuterostomia - ps17
Chordata - ps18
Olfactores - ps19
Vertebrata - ps20
Gnathostomata - ps21
Euteleostomi - ps22
Sarcopterygii - ps23
Tetrapoda - ps24
Amniota - ps25
Mammalia - ps26
Theria - ps27
Placentalia - ps28
Boreoeutheria - ps29
Euarchontoglires - ps30
Euarchonta - ps31
Primates - ps32
Haplorrhini - ps33
Simiiformes - ps34
Catarrhini - ps35
Hominoidea - ps36
Hominidae - ps37
Homininae - ps38
Hominini - ps39
Homo_sapiens - ps40
p-value
7e-03
9e-03
1e-02
2e-02
2e-02
Log-odds
0.5
1.0
1.5
2.0
2.5
3.0
3.5
4.0

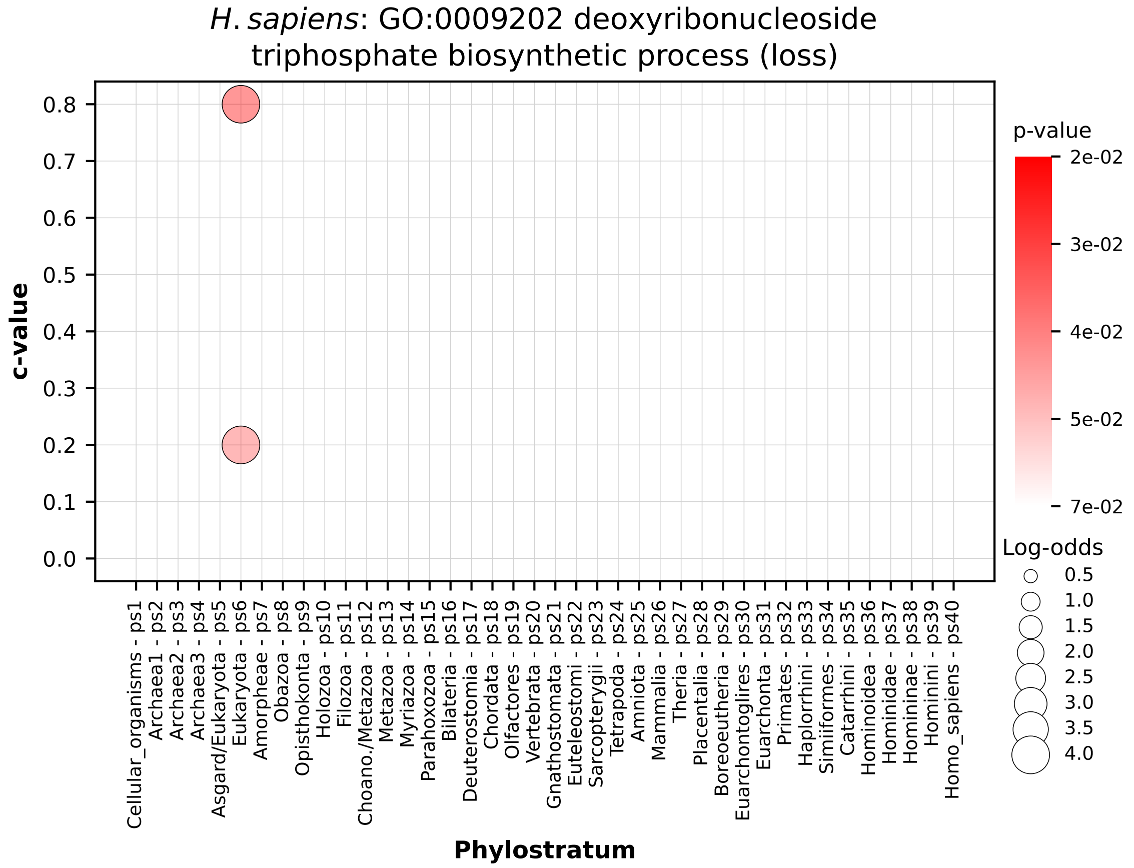

H. sapiens: GO:0009202 deoxyribonucleoside triphosphate biosynthetic process (loss)
c-value
0.0
0.1
0.2
0.3
0.4
0.5
0.6
0.7
0.8
Phylostratum
Cellular_organisms - ps1
Archaea1 - ps2
Archaea2 - ps3
Archaea3 - ps4
Asgard/Eukaryota - ps5
Eukaryota - ps6
Amorpheae - ps7
Obazoa - ps8
Opisthokonta - ps9
Holozoa - ps10
Filozoa - ps11
Choano./Metazoa - ps12
Metazoa - ps13
Myriazoa - ps14
Parahoxozoa - ps15
Bilateria - ps16
Deuterostomia - ps17
Chordata - ps18
Olfactores - ps19
Vertebrata - ps20
Gnathostomata - ps21
Euteleostomi - ps22
Sarcopterygii - ps23
Tetrapoda - ps24
Amniota - ps25
Mammalia - ps26
Theria - ps27
Placentalia - ps28
Boreoeutheria - ps29
Euarchontoglires - ps30
Euarchonta - ps31
Primates - ps32
Haplorrhini - ps33
Simiiformes - ps34
Catarrhini - ps35
Hominoidea - ps36
Hominidae - ps37
Homininae - ps38
Hominini - ps39
Homo_sapiens - ps40
p-value
2e-02
3e-02
4e-02
5e-02
7e-02
Log-odds
0.5
1.0
1.5
2.0
2.5
3.0
3.5
4.0

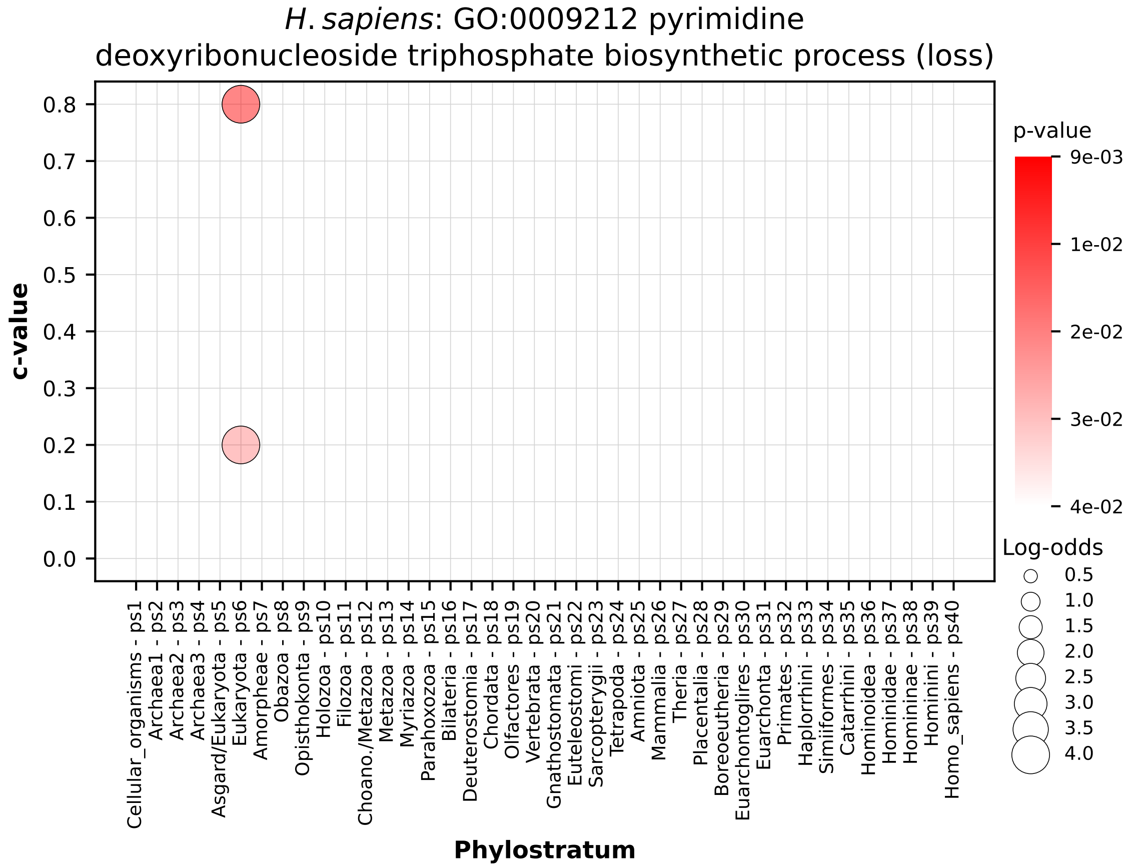

H. sapiens: GO:0009212 pyrimidine deoxyribonucleoside triphosphate biosynthetic process (loss)
c-value
0.8
0.7
0.6
0.5
0.4
0.3
0.2
0.1
0.0
Phylostratum
Cellular_organisms - ps1
Archaea1 - ps2
Archaea2 - ps3
Archaea3 - ps4
Asgard/Eukaryota - ps5
Eukaryota - ps6
Amorpheae - ps7
Obazoa - ps8
Opisthokonta - ps9
Holozoa - ps10
Filozoa - ps11
Choano./Metazoa - ps12
Metazoa - ps13
Myriazoa - ps14
Parahoxozoa - ps15
Bilateria - ps16
Deuterostomia - ps17
Chordata - ps18
Olfactores - ps19
Vertebrata - ps20
Gnathostomata - ps21
Euteleostomi - ps22
Sarcopterygii - ps23
Tetrapoda - ps24
Amniota - ps25
Mammalia - ps26
Theria - ps27
Placentalia - ps28
Boreoeutheria - ps29
Euarchontoglires - ps30
Euarchonta - ps31
Primates - ps32
Haplorrhini - ps33
Simiiformes - ps34
Catarrhini - ps35
Hominoidea - ps36
Hominidae - ps37
Homininae - ps38
Hominini - ps39
Homo_sapiens - ps40
p-value
9e-03
1e-02
2e-02
3e-02
4e-02
Log-odds
0.5
1.0
1.5
2.0
2.5
3.0
3.5
4.0

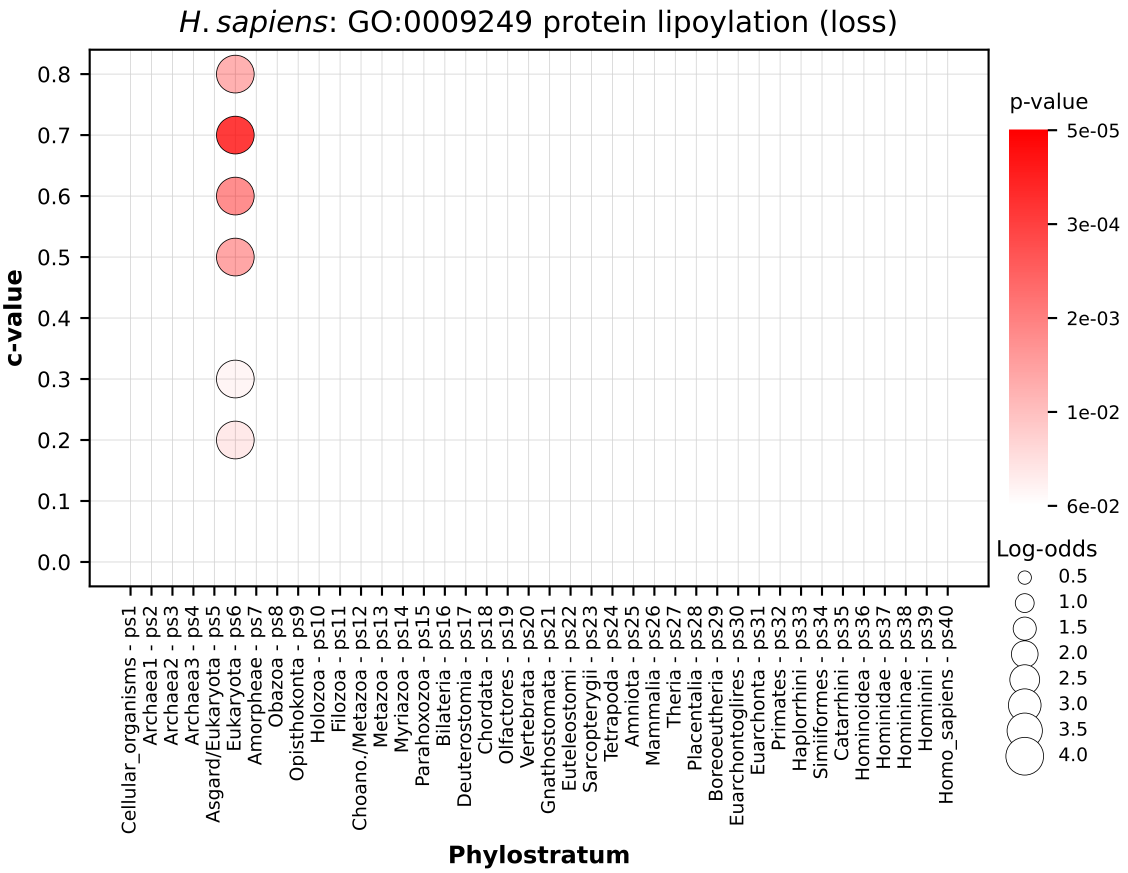
H. sapiens: GO:0009249 protein lipoylation (loss)
c-value
0.0
0.1
0.2
0.3
0.4
0.5
0.6
0.7
0.8
p-value
5e-05
3e-04
2e-03
1e-02
6e-02
Log-odds
0.5
1.0
1.5
2.0
2.5
3.0
3.5
4.0
Cellular_organisms - ps1
Archaea1 - ps2
Archaea2 - ps3
Archaea3 - ps4
Asgard/Eukaryota - ps5
Eukaryota - ps6
Amorpheae - ps7
Obazoa - ps8
Opisthokonta - ps9
Holozoa - ps10
Filozoa - ps11
Choano./Metazoa - ps12
Metazoa - ps13
Myriazoa - ps14
Parahoxozoa - ps15
Bilateria - ps16
Deuterostomia - ps17
Chordata - ps18
Olfactores - ps19
Vertebrata - ps20
Gnathostomata - ps21
Euteleostomi - ps22
Sarcopterygii - ps23
Tetrapoda - ps24
Amniota - ps25
Mammalia - ps26
Theria - ps27
Placentalia - ps28
Boreoeutheria - ps29
Euarchontoglires - ps30
Euarchonta - ps31
Primates - ps32
Haplorrhini - ps33
Simiiformes - ps34
Catarrhini - ps35
Hominoidea - ps36
Hominidae - ps37
Homininae - ps38
Hominini - ps39
Homo_sapiens - ps40
Phylostratum

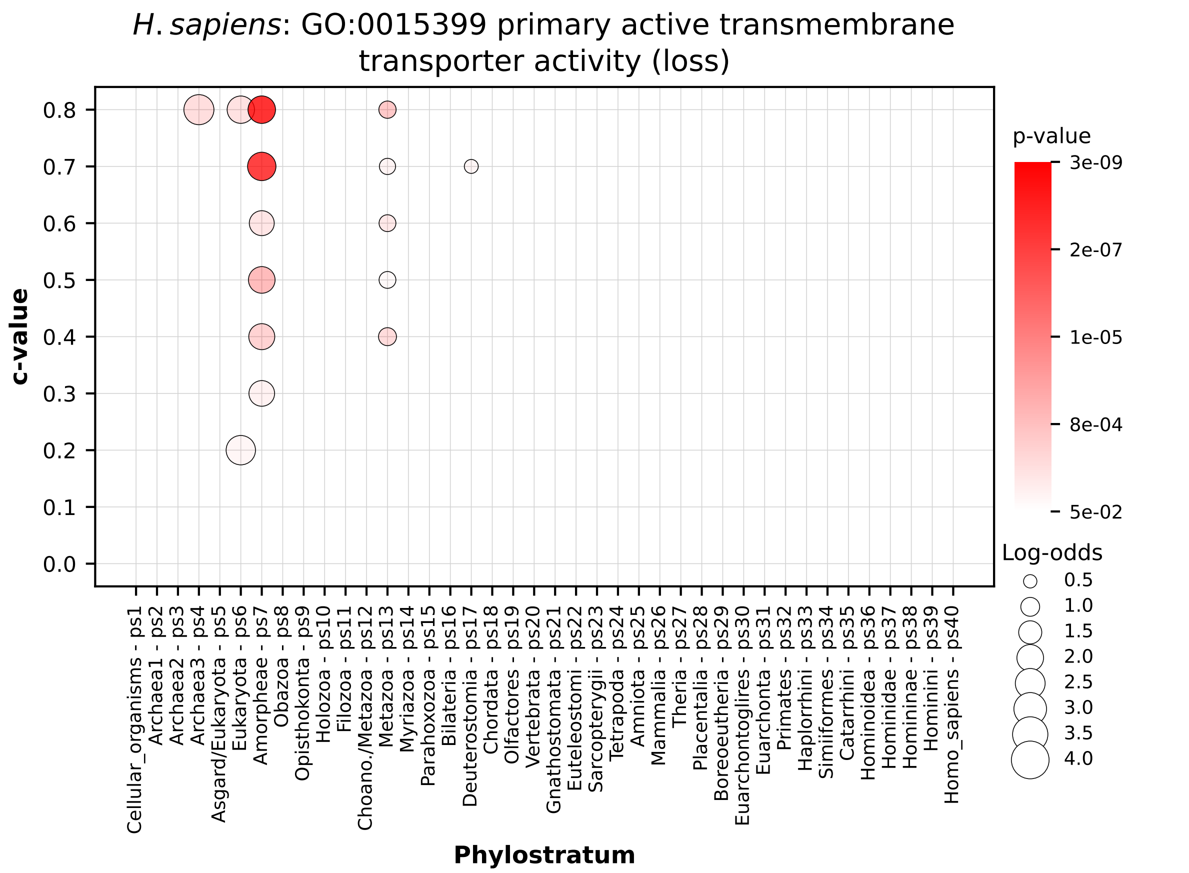

H. sapiens: GO:0015399 primary active transmembrane transporter activity (loss)
c-value
0.0
0.1
0.2
0.3
0.4
0.5
0.6
0.7
0.8
Phylostratum
Cellular_organisms - ps1
Archaea1 - ps2
Archaea2 - ps3
Archaea3 - ps4
Asgard/Eukaryota - ps5
Eukaryota - ps6
Amorpheae - ps7
Obazoa - ps8
Opisthokonta - ps9
Holozoa - ps10
Filozoa - ps11
Choano./Metazoa - ps12
Metazoa - ps13
Myriazoa - ps14
Parahoxozoa - ps15
Bilateria - ps16
Deuterostomia - ps17
Chordata - ps18
Olfactores - ps19
Vertebrata - ps20
Gnathostomata - ps21
Euteleostomi - ps22
Sarcopterygii - ps23
Tetrapoda - ps24
Amniota - ps25
Mammalia - ps26
Theria - ps27
Placentalia - ps28
Boreoeutheria - ps29
Euarchontoglires - ps30
Euarchonta - ps31
Primates - ps32
Haplorrhini - ps33
Simiiformes - ps34
Catarrhini - ps35
Hominoidea - ps36
Hominidae - ps37
Homininae - ps38
Hominini - ps39
Homo_sapiens - ps40
p-value
3e-09
2e-07
1e-05
8e-04
5e-02
Log-odds
0.5
1.0
1.5
2.0
2.5
3.0
3.5
4.0

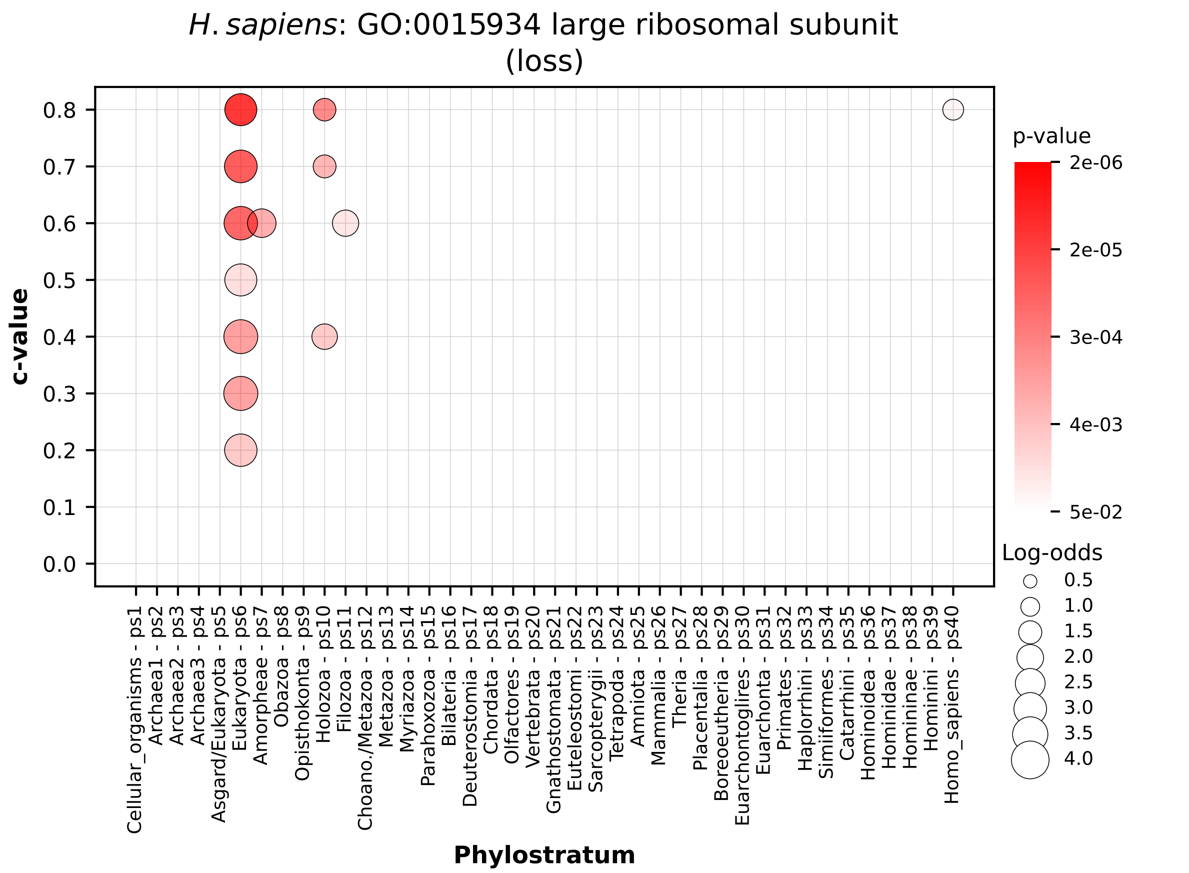

H. sapiens: GO:0015934 large ribosomal subunit
(loss)
c-value
0.8
0.7
0.6
0.5
0.4
0.3
0.2
0.1
0.0
Phylostratum
Cellular_organisms - ps1
Archaea1 - ps2
Archaea2 - ps3
Archaea3 - ps4
Asgard/Eukaryota - ps5
Eukaryota - ps6
Amorpheae - ps7
Obazoa - ps8
Opisthokonta - ps9
Holozoa - ps10
Filozoa - ps11
Choano./Metazoa - ps12
Metazoa - ps13
Myriazoa - ps14
Parahoxozoa - ps15
Bilateria - ps16
Deuterostomia - ps17
Chordata - ps18
Olfactores - ps19
Vertebrata - ps20
Gnathostomata - ps21
Euteleostomi - ps22
Sarcopterygii - ps23
Tetrapoda - ps24
Amniota - ps25
Mammalia - ps26
Theria - ps27
Placentalia - ps28
Boreoeutheria - ps29
Euarchontoglires - ps30
Euarchonta - ps31
Primates - ps32
Haplorrhini - ps33
Simiiformes - ps34
Catarrhini - ps35
Hominoidea - ps36
Hominidae - ps37
Homininae - ps38
Hominini - ps39
Homo_sapiens - ps40
p-value
2e-06
2e-05
3e-04
4e-03
5e-02
Log-odds
0.5
1.0
1.5
2.0
2.5
3.0
3.5
4.0

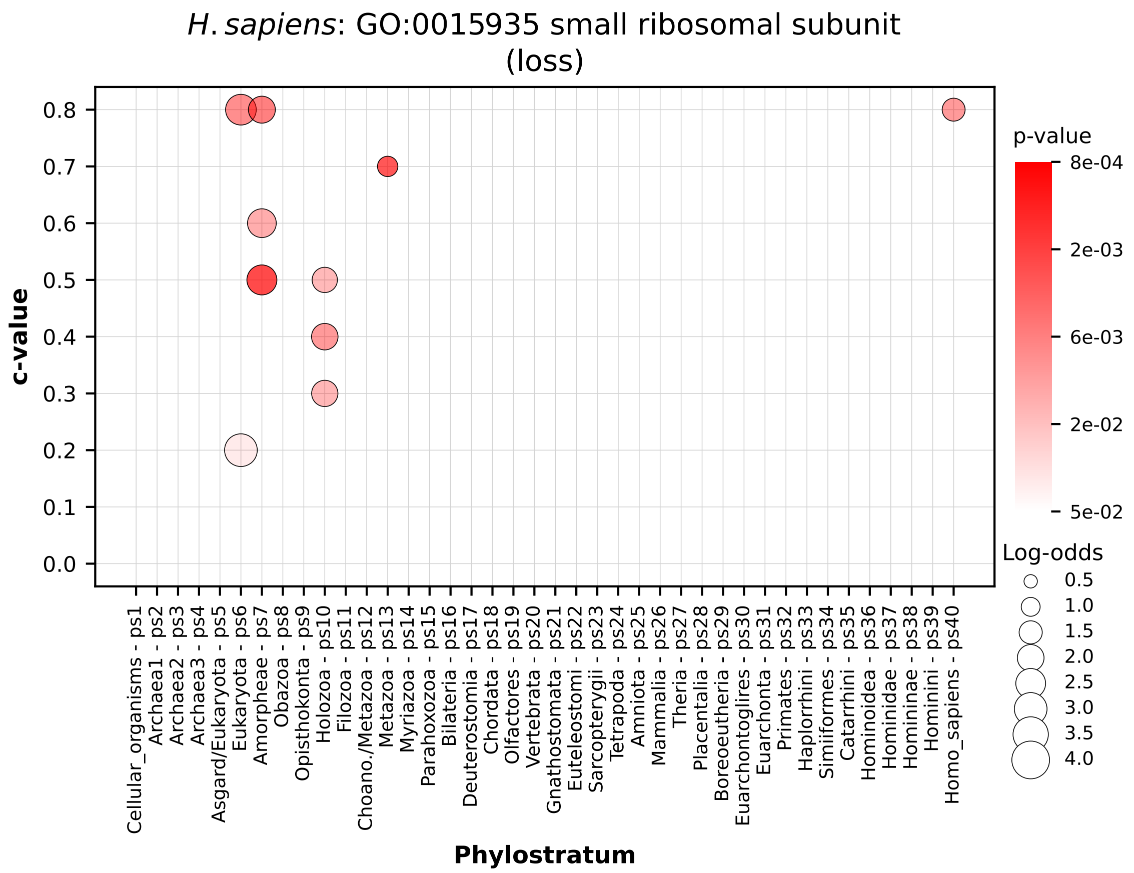

H. sapiens: GO:0015935 small ribosomal subunit (loss)
c-value
0.0
0.1
0.2
0.3
0.4
0.5
0.6
0.7
0.8
Phylostratum
Cellular_organisms - ps1
Archaea1 - ps2
Archaea2 - ps3
Archaea3 - ps4
Asgard/Eukaryota - ps5
Eukaryota - ps6
Amorpheae - ps7
Obazoa - ps8
Opisthokonta - ps9
Holozoa - ps10
Filozoa - ps11
Choano./Metazoa - ps12
Metazoa - ps13
Myriazoa - ps14
Parahoxozoa - ps15
Bilateria - ps16
Deuterostomia - ps17
Chordata - ps18
Olfactores - ps19
Vertebrata - ps20
Gnathostomata - ps21
Euteleostomi - ps22
Sarcopterygii - ps23
Tetrapoda - ps24
Amniota - ps25
Mammalia - ps26
Theria - ps27
Placentalia - ps28
Boreoeutheria - ps29
Euarchontoglires - ps30
Euarchonta - ps31
Primates - ps32
Haplorrhini - ps33
Simiiformes - ps34
Catarrhini - ps35
Hominoidea - ps36
Hominidae - ps37
Homininae - ps38
Hominini - ps39
Homo_sapiens - ps40
p-value
8e-04
2e-03
6e-03
2e-02
5e-02
Log-odds
0.5
1.0
1.5
2.0
2.5
3.0
3.5
4.0

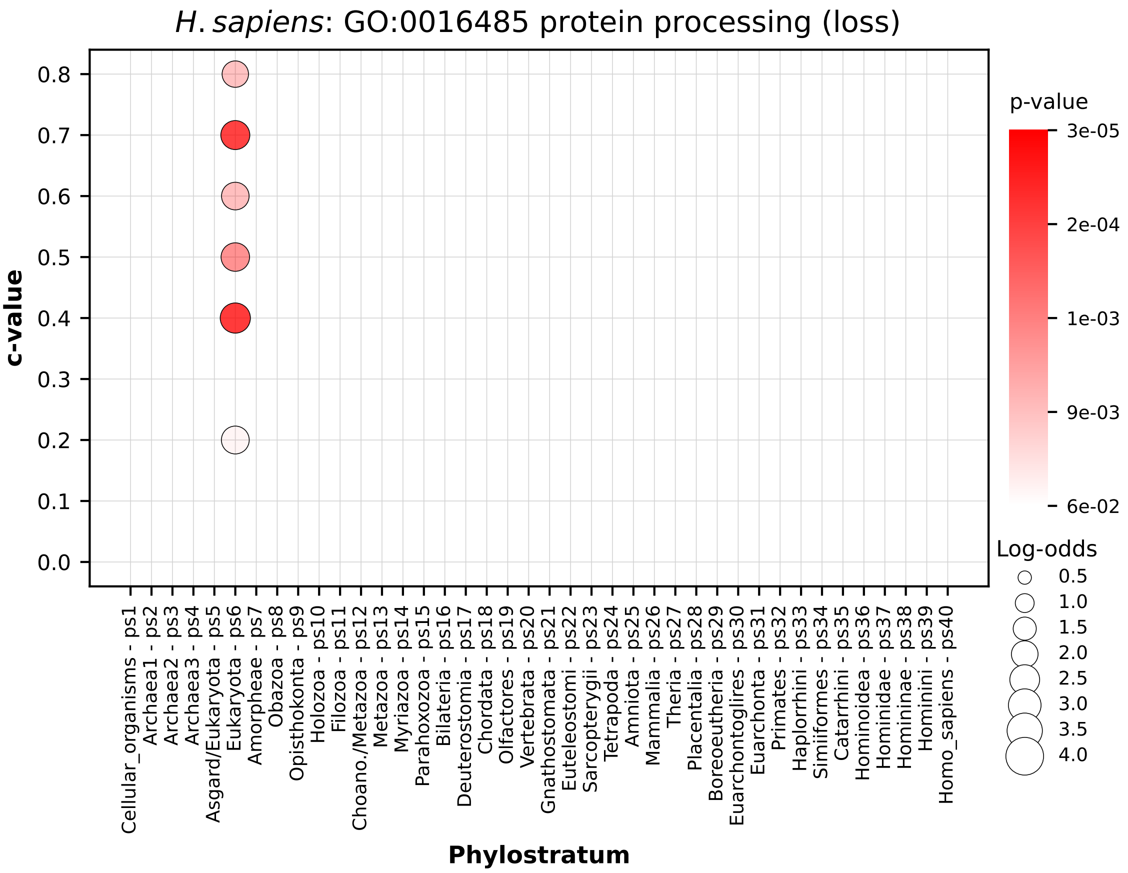

H. sapiens: GO:0016485 protein processing (loss)
c-value
0.0
0.1
0.2
0.3
0.4
0.5
0.6
0.7
0.8
Phylostratum
Cellular_organisms - ps1
Archaea1 - ps2
Archaea2 - ps3
Archaea3 - ps4
Asgard/Eukaryota - ps5
Eukaryota - ps6
Amorpheae - ps7
Obazoa - ps8
Opisthokonta - ps9
Holozoa - ps10
Filozoa - ps11
Choano./Metazoa - ps12
Metazoa - ps13
Myriazoa - ps14
Parahoxozoa - ps15
Bilateria - ps16
Deuterostomia - ps17
Chordata - ps18
Olfactores - ps19
Vertebrata - ps20
Gnathostomata - ps21
Euteleostomi - ps22
Sarcopterygii - ps23
Tetrapoda - ps24
Amniota - ps25
Mammalia - ps26
Theria - ps27
Placentalia - ps28
Boreoeutheria - ps29
Euarchontoglires - ps30
Euarchonta - ps31
Primates - ps32
Haplorrhini - ps33
Simiiformes - ps34
Catarrhini - ps35
Hominoidea - ps36
Hominidae - ps37
Homininae - ps38
Hominini - ps39
Homo_sapiens - ps40
p-value
3e-05
2e-04
1e-03
9e-03
6e-02
Log-odds
0.5
1.0
1.5
2.0
2.5
3.0
3.5
4.0

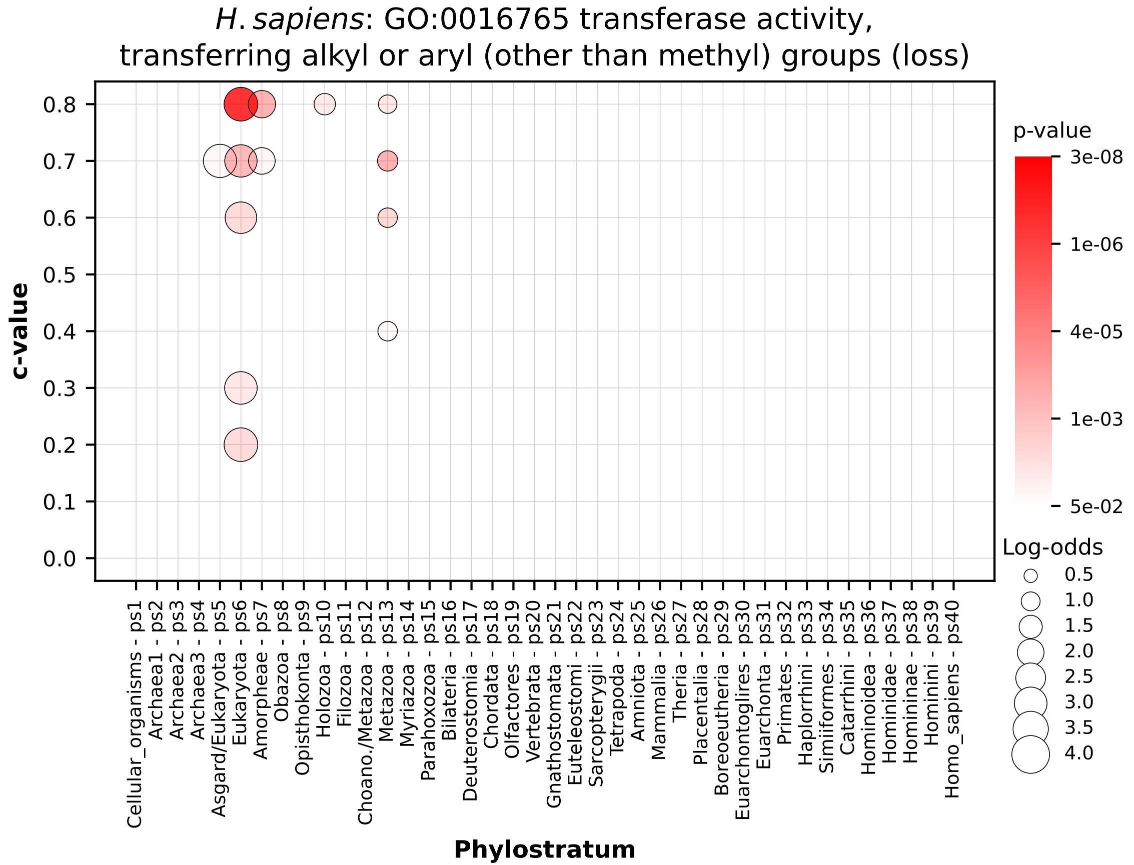

H. sapiens: GO:0016765 transferase activity,
transferring alkyl or aryl (other than methyl) groups (loss)
c-value
0.0
0.1
0.2
0.3
0.4
0.5
0.6
0.7
0.8
Phylostratum
Cellular_organisms - ps1
Archaea1 - ps2
Archaea2 - ps3
Archaea3 - ps4
Asgard/Eukaryota - ps5
Eukaryota - ps6
Amorpheae - ps7
Obazoa - ps8
Opisthokonta - ps9
Holozoa - ps10
Filozoa - ps11
Choano./Metazoa - ps12
Metazoa - ps13
Myriazoa - ps14
Parahoxozoa - ps15
Bilateria - ps16
Deuterostomia - ps17
Chordata - ps18
Olfactores - ps19
Vertebrata - ps20
Gnathostomata - ps21
Euteleostomi - ps22
Sarcopterygii - ps23
Tetrapoda - ps24
Amniota - ps25
Mammalia - ps26
Theria - ps27
Placentalia - ps28
Boreoeutheria - ps29
Euarchontoglires - ps30
Euarchonta - ps31
Primates - ps32
Haplorrhini - ps33
Simiiformes - ps34
Catarrhini - ps35
Hominoidea - ps36
Hominidae - ps37
Homininae - ps38
Hominini - ps39
Homo_sapiens - ps40
p-value
3e-08
1e-06
4e-05
1e-03
5e-02
Log-odds
0.5
1.0
1.5
2.0
2.5
3.0
3.5
4.0

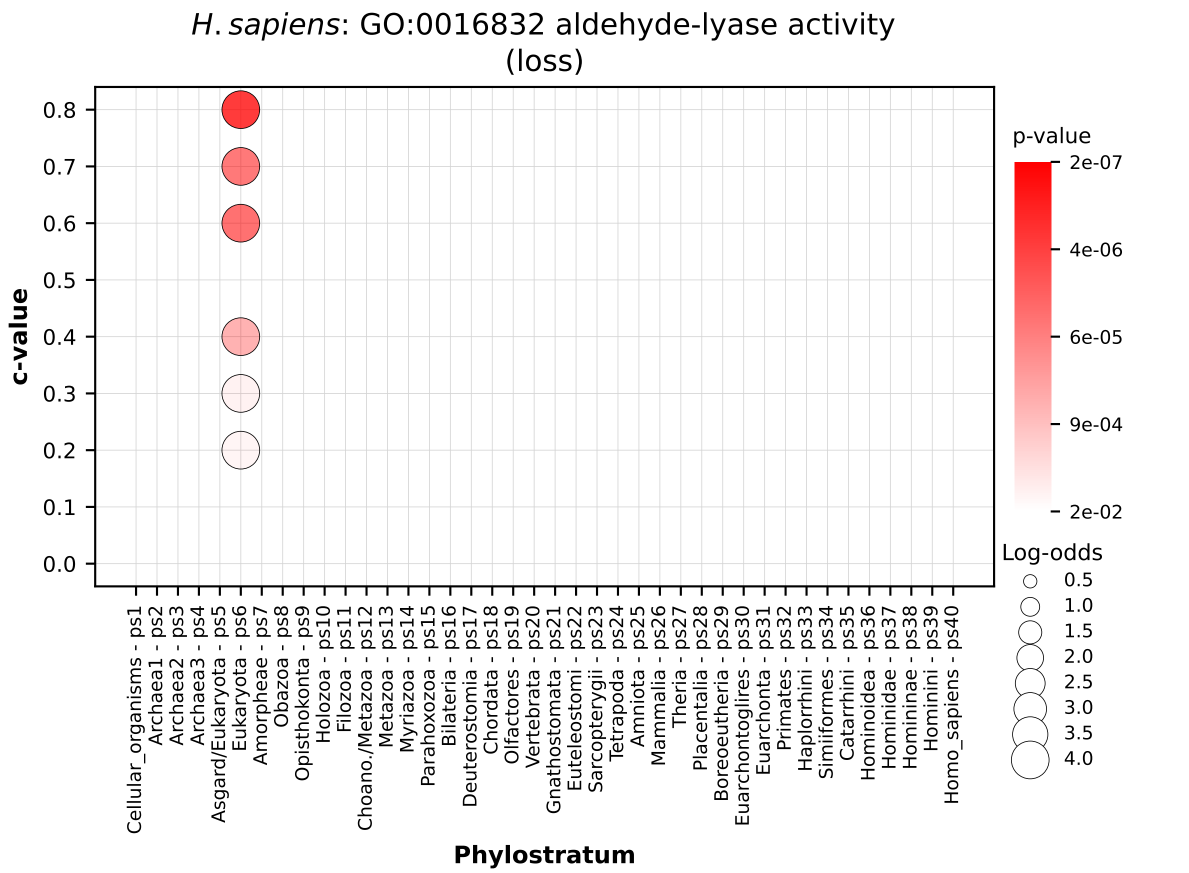
H. sapiens: GO:0016832 aldehyde-lyase activity
(loss)
c-value
0.8
0.7
0.6
0.5
0.4
0.3
0.2
0.1
0.0
p-value
2e-07
4e-06
6e-05
9e-04
2e-02
Log-odds
0.5
1.0
1.5
2.0
2.5
3.0
3.5
4.0
Cellular_organisms - ps1
Archaea1 - ps2
Archaea2 - ps3
Archaea3 - ps4
Asgard/Eukaryota - ps5
Eukaryota - ps6
Amorpheae - ps7
Obazoa - ps8
Opisthokonta - ps9
Holozoa - ps10
Filozoa - ps11
Choano./Metazoa - ps12
Metazoa - ps13
Myriazoa - ps14
Parahoxozoa - ps15
Bilateria - ps16
Deuterostomia - ps17
Chordata - ps18
Olfactores - ps19
Vertebrata - ps20
Gnathostomata - ps21
Euteleostomi - ps22
Sarcopterygii - ps23
Tetrapoda - ps24
Amniota - ps25
Mammalia - ps26
Theria - ps27
Placentalia - ps28
Boreoeutheria - ps29
Euarchontoglires - ps30
Euarchonta - ps31
Primates - ps32
Haplorrhini - ps33
Simiiformes - ps34
Catarrhini - ps35
Hominoidea - ps36
Hominidae - ps37
Homininae - ps38
Hominini - ps39
Homo_sapiens - ps40
Phylostratum

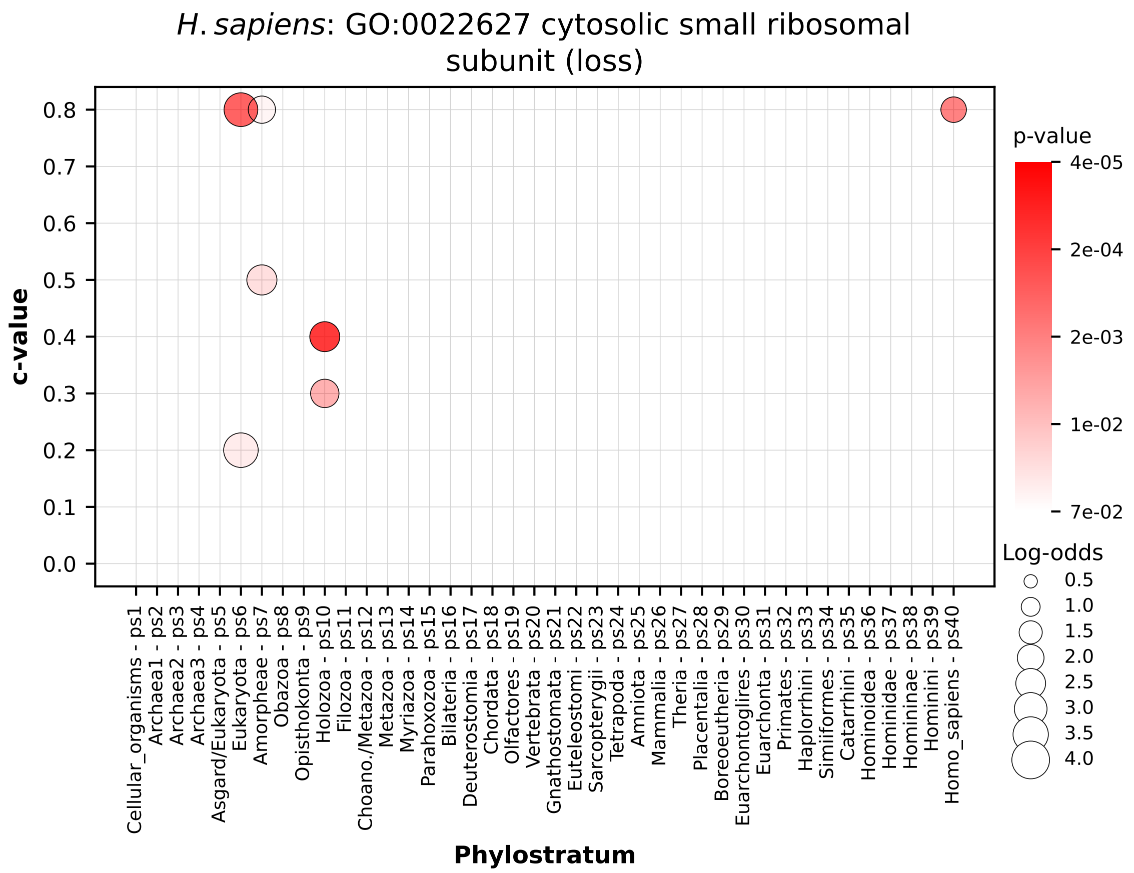

H. sapiens: GO:0022627 cytosolic small ribosomal subunit (loss)
c-value
0.0
0.1
0.2
0.3
0.4
0.5
0.6
0.7
0.8
Phylostratum
Cellular_organisms - ps1
Archaea1 - ps2
Archaea2 - ps3
Archaea3 - ps4
Asgard/Eukaryota - ps5
Eukaryota - ps6
Amorpheae - ps7
Obazoa - ps8
Opisthokonta - ps9
Holozoa - ps10
Filozoa - ps11
Choano./Metazoa - ps12
Metazoa - ps13
Myriazoa - ps14
Parahoxozoa - ps15
Bilateria - ps16
Deuterostomia - ps17
Chordata - ps18
Olfactores - ps19
Vertebrata - ps20
Gnathostomata - ps21
Euteleostomi - ps22
Sarcopterygii - ps23
Tetrapoda - ps24
Amniota - ps25
Mammalia - ps26
Theria - ps27
Placentalia - ps28
Boreoeutheria - ps29
Euarchontoglires - ps30
Euarchonta - ps31
Primates - ps32
Haplorrhini - ps33
Simiiformes - ps34
Catarrhini - ps35
Hominoidea - ps36
Hominidae - ps37
Homininae - ps38
Hominini - ps39
Homo_sapiens - ps40
p-value
4e-05
2e-04
2e-03
1e-02
7e-02
Log-odds
0.5
1.0
1.5
2.0
2.5
3.0
3.5
4.0

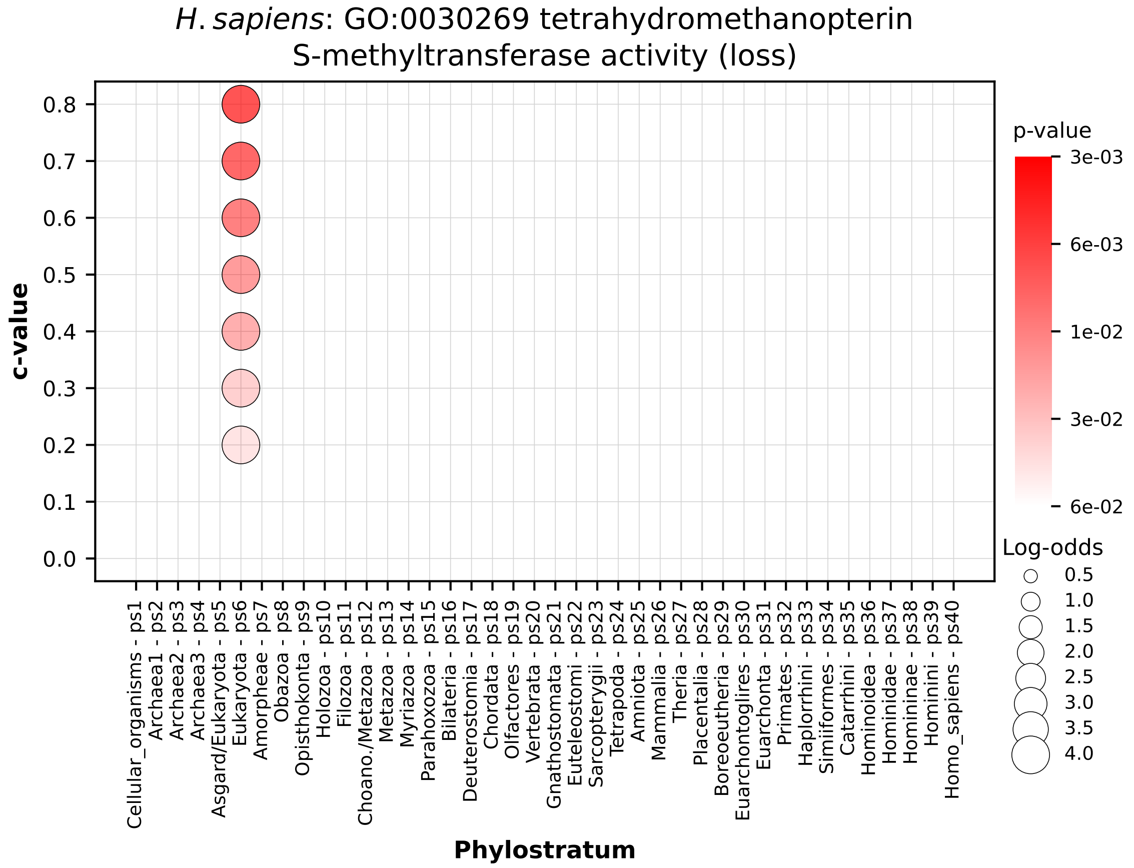

H. sapiens: GO:0030269 tetrahydromethanopterin S-methyltransferase activity (loss)
c-value
0.0
0.1
0.2
0.3
0.4
0.5
0.6
0.7
0.8
Phylostratum
Cellular_organisms - ps1
Archaea1 - ps2
Archaea2 - ps3
Archaea3 - ps4
Asgard/Eukaryota - ps5
Eukaryota - ps6
Amorpheae - ps7
Obazoa - ps8
Opisthokonta - ps9
Holozoa - ps10
Filozoa - ps11
Choano./Metazoa - ps12
Metazoa - ps13
Myriazoa - ps14
Parahoxozoa - ps15
Bilateria - ps16
Deuterostomia - ps17
Chordata - ps18
Olfactores - ps19
Vertebrata - ps20
Gnathostomata - ps21
Euteleostomi - ps22
Sarcopterygii - ps23
Tetrapoda - ps24
Amniota - ps25
Mammalia - ps26
Theria - ps27
Placentalia - ps28
Boreoeutheria - ps29
Euarchontoglires - ps30
Euarchonta - ps31
Primates - ps32
Haplorrhini - ps33
Simiiformes - ps34
Catarrhini - ps35
Hominoidea - ps36
Hominidae - ps37
Homininae - ps38
Hominini - ps39
Homo_sapiens - ps40
p-value
3e-03
6e-03
1e-02
3e-02
6e-02
Log-odds
0.5
1.0
1.5
2.0
2.5
3.0
3.5
4.0

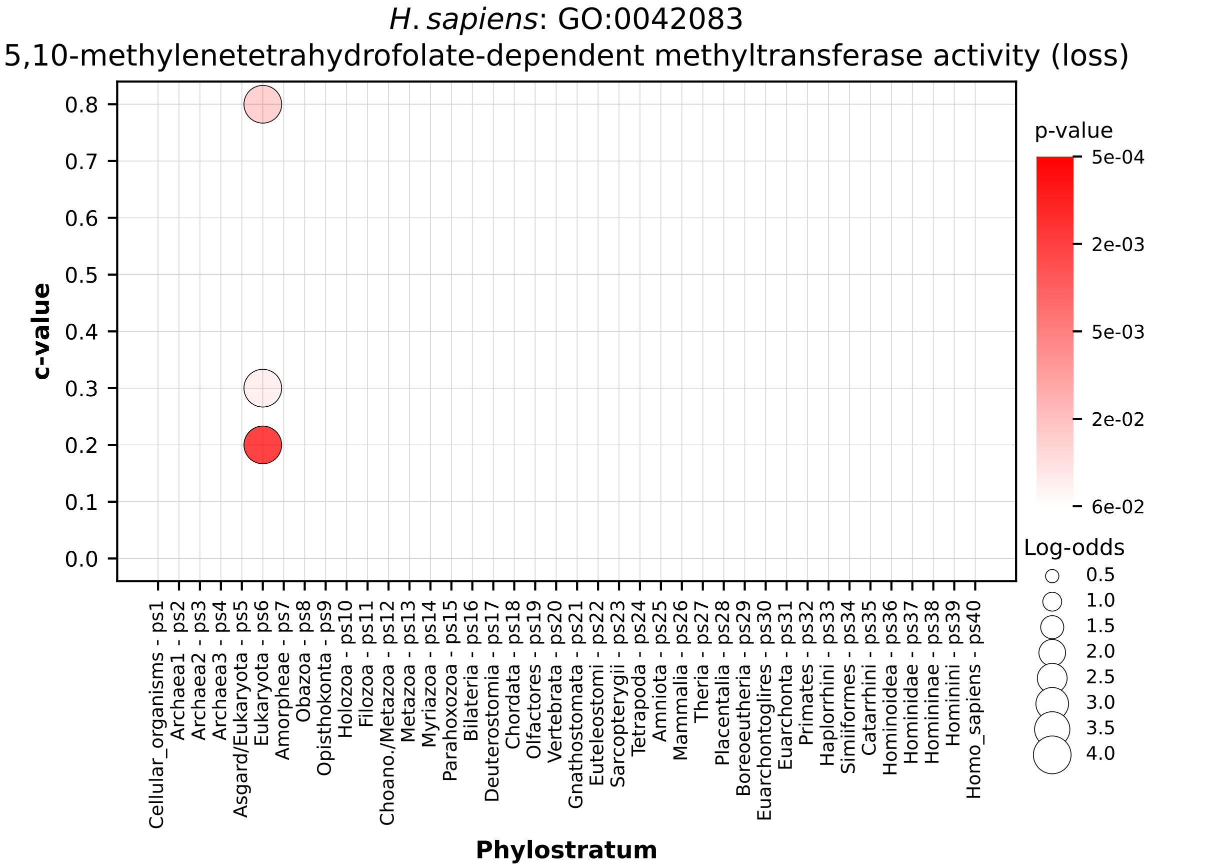

H. sapiens: GO:0042083
5,10-methylenetetrahydrofolate-dependent methyltransferase activity (loss)
c-value
0.0
0.1
0.2
0.3
0.4
0.5
0.6
0.7
0.8
p-value
5e-04
2e-03
5e-03
2e-02
6e-02
Log-odds
0.5
1.0
1.5
2.0
2.5
3.0
3.5
4.0
Cellular_organisms - ps1
Archaea1 - ps2
Archaea2 - ps3
Archaea3 - ps4
Asgard/Eukaryota - ps5
Eukaryota - ps6
Amorpheae - ps7
Obazoa - ps8
Opisthokonta - ps9
Holozoa - ps10
Filozoa - ps11
Choano./Metazoa - ps12
Metazoa - ps13
Myriazoa - ps14
Parahoxozoa - ps15
Bilateria - ps16
Deuterostomia - ps17
Chordata - ps18
Olfactores - ps19
Vertebrata - ps20
Gnathostomata - ps21
Euteleostomi - ps22
Sarcopterygii - ps23
Tetrapoda - ps24
Amniota - ps25
Mammalia - ps26
Theria - ps27
Placentalia - ps28
Boreoeutheria - ps29
Euarchontoglires - ps30
Euarchonta - ps31
Primates - ps32
Haplorrhini - ps33
Simiiformes - ps34
Catarrhini - ps35
Hominoidea - ps36
Hominidae - ps37
Homininae - ps38
Hominini - ps39
Homo_sapiens - ps40
Phylostratum

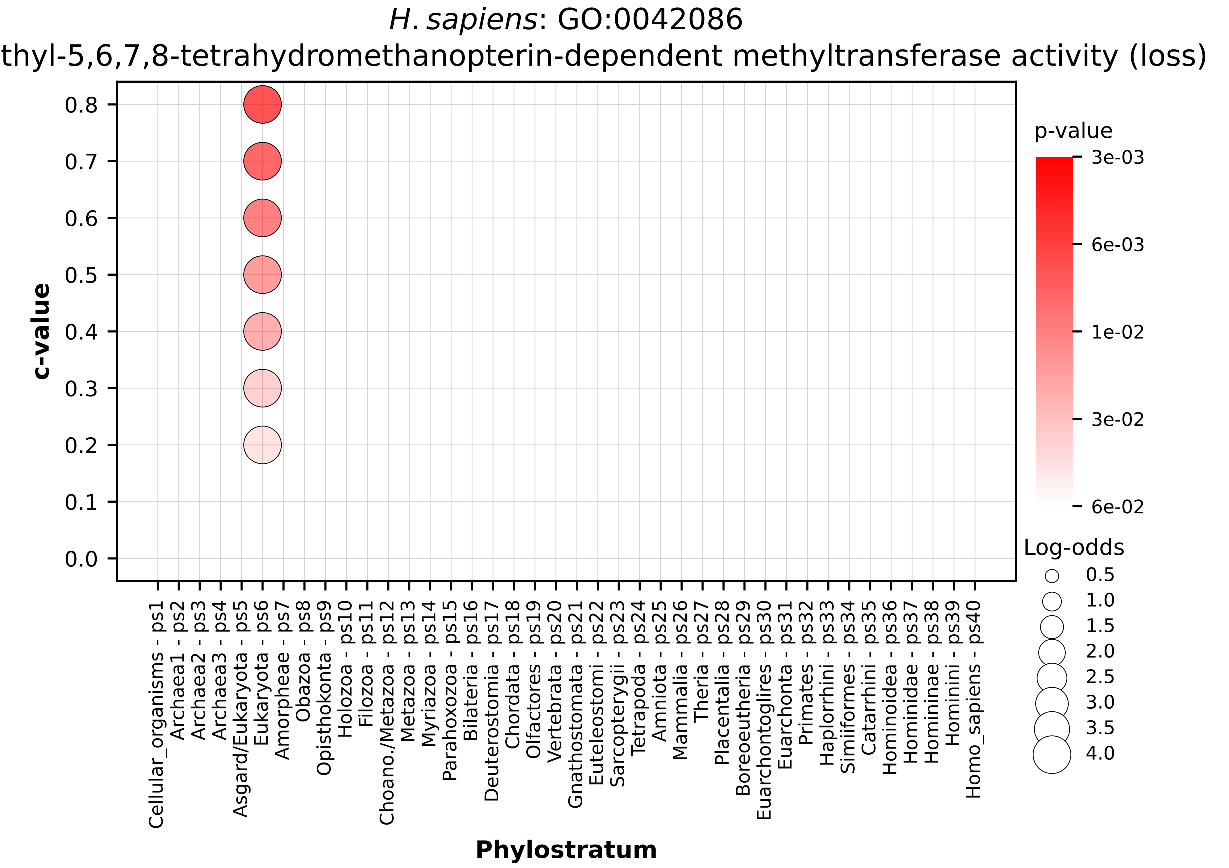

H. sapiens: GO:0042086
thyl-5,6,7,8-tetrahydromethanopterin-dependent methyltransferase activity (loss)
c-value
0.8
0.7
0.6
0.5
0.4
0.3
0.2
0.1
0.0
p-value
3e-03
6e-03
1e-02
3e-02
6e-02
Log-odds
0.5
1.0
1.5
2.0
2.5
3.0
3.5
4.0
Cellular_organisms - ps1
Archaea1 - ps2
Archaea2 - ps3
Archaea3 - ps4
Asgard/Eukaryota - ps5
Eukaryota - ps6
Amorpheae - ps7
Obazoa - ps8
Opisthokonta - ps9
Holozoa - ps10
Filozoa - ps11
Choano./Metazoa - ps12
Metazoa - ps13
Myriazoa - ps14
Parahoxozoa - ps15
Bilateria - ps16
Deuterostomia - ps17
Chordata - ps18
Olfactores - ps19
Vertebrata - ps20
Gnathostomata - ps21
Euteleostomi - ps22
Sarcopterygii - ps23
Tetrapoda - ps24
Amniota - ps25
Mammalia - ps26
Theria - ps27
Placentalia - ps28
Boreoeutheria - ps29
Euarchontoglires - ps30
Euarchonta - ps31
Primates - ps32
Haplorrhini - ps33
Simiiformes - ps34
Catarrhini - ps35
Hominoidea - ps36
Hominidae - ps37
Homininae - ps38
Hominini - ps39
Homo_sapiens - ps40
Phylostratum

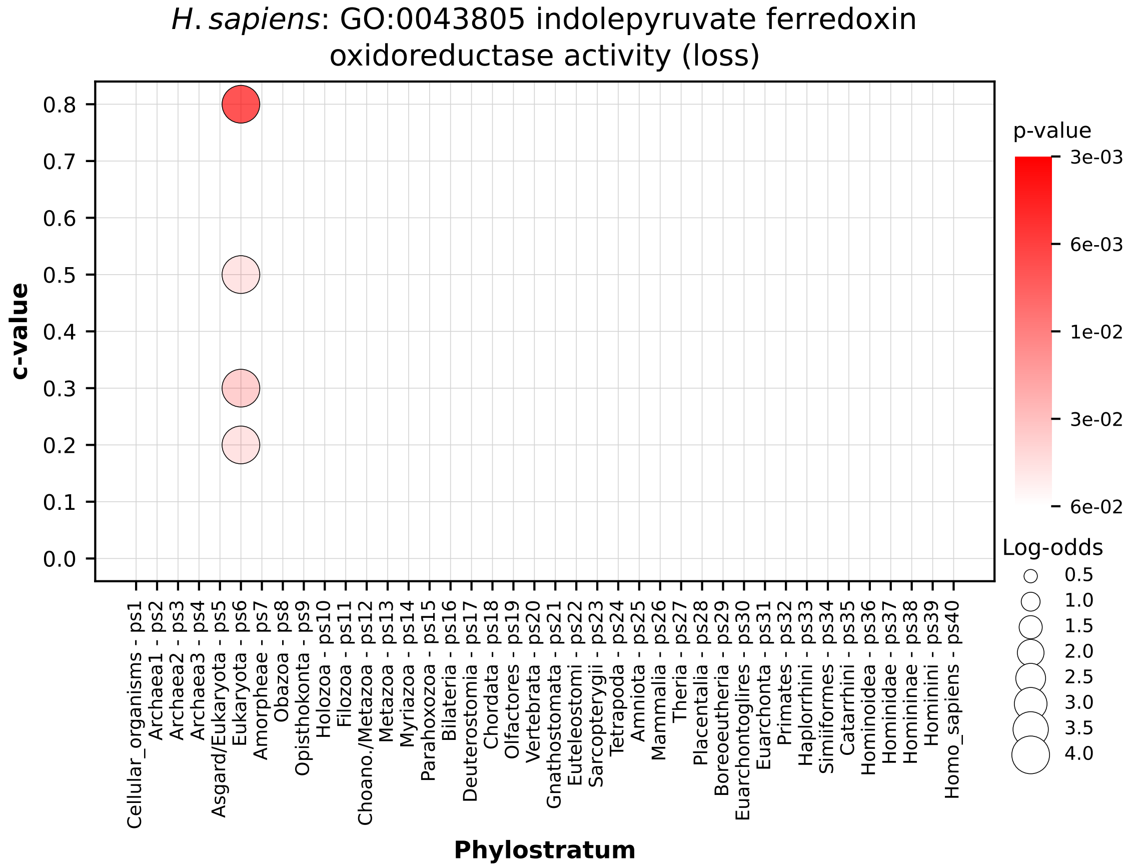

H. sapiens: GO:0043805 indolepyruvate ferredoxin oxidoreductase activity (loss)
c-value
0.0
0.1
0.2
0.3
0.4
0.5
0.6
0.7
0.8
p-value
3e-03
6e-03
1e-02
3e-02
6e-02
Log-odds
0.5
1.0
1.5
2.0
2.5
3.0
3.5
4.0
Cellular_organisms - ps1
Archaea1 - ps2
Archaea2 - ps3
Archaea3 - ps4
Asgard/Eukaryota - ps5
Eukaryota - ps6
Amorpheae - ps7
Obazoa - ps8
Opisthokonta - ps9
Holozoa - ps10
Filozoa - ps11
Choano./Metazoa - ps12
Metazoa - ps13
Myriazoa - ps14
Parahoxozoa - ps15
Bilateria - ps16
Deuterostomia - ps17
Chordata - ps18
Olfactores - ps19
Vertebrata - ps20
Gnathostomata - ps21
Euteleostomi - ps22
Sarcopterygii - ps23
Tetrapoda - ps24
Amniota - ps25
Mammalia - ps26
Theria - ps27
Placentalia - ps28
Boreoeutheria - ps29
Euarchontoglires - ps30
Euarchonta - ps31
Primates - ps32
Haplorrhini - ps33
Simiiformes - ps34
Catarrhini - ps35
Hominoidea - ps36
Hominidae - ps37
Homininae - ps38
Hominini - ps39
Homo_sapiens - ps40
Phylostratum

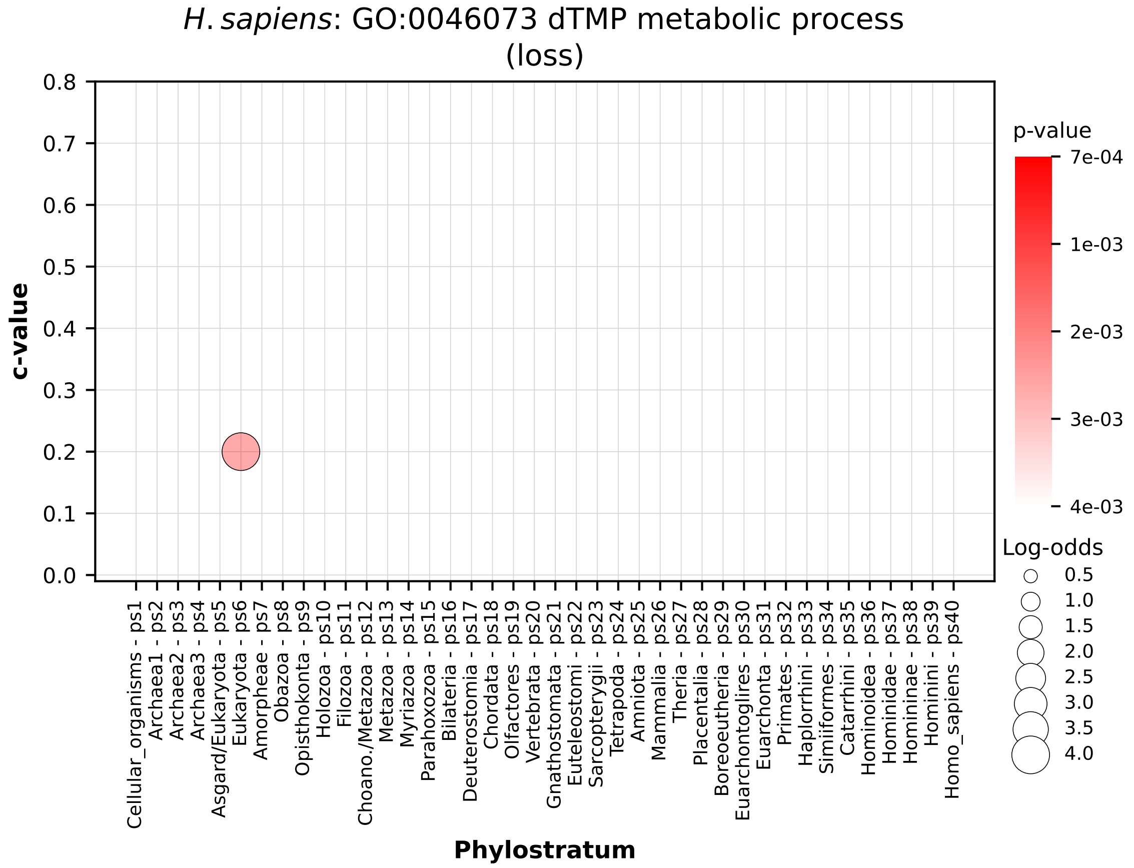

H. sapiens: GO:0046073 dTMP metabolic process
(loss)
c-value
0.0
0.1
0.2
0.3
0.4
0.5
0.6
0.7
0.8
Phylostratum
Cellular_organisms - ps1
Archaea1 - ps2
Archaea2 - ps3
Archaea3 - ps4
Asgard/Eukaryota - ps5
Eukaryota - ps6
Amorpheae - ps7
Obazoa - ps8
Opisthokonta - ps9
Holozoa - ps10
Filozoa - ps11
Choano./Metazoa - ps12
Metazoa - ps13
Myriazoa - ps14
Parahoxozoa - ps15
Bilateria - ps16
Deuterostomia - ps17
Chordata - ps18
Olfactores - ps19
Vertebrata - ps20
Gnathostomata - ps21
Euteleostomi - ps22
Sarcopterygii - ps23
Tetrapoda - ps24
Amniota - ps25
Mammalia - ps26
Theria - ps27
Placentalia - ps28
Boreoeutheria - ps29
Euarchontoglires - ps30
Euarchonta - ps31
Primates - ps32
Haplorrhini - ps33
Simiiformes - ps34
Catarrhini - ps35
Hominoidea - ps36
Hominidae - ps37
Homininae - ps38
Hominini - ps39
Homo_sapiens - ps40
p-value
7e-04
1e-03
2e-03
3e-03
4e-03
Log-odds
0.5
1.0
1.5
2.0
2.5
3.0
3.5
4.0

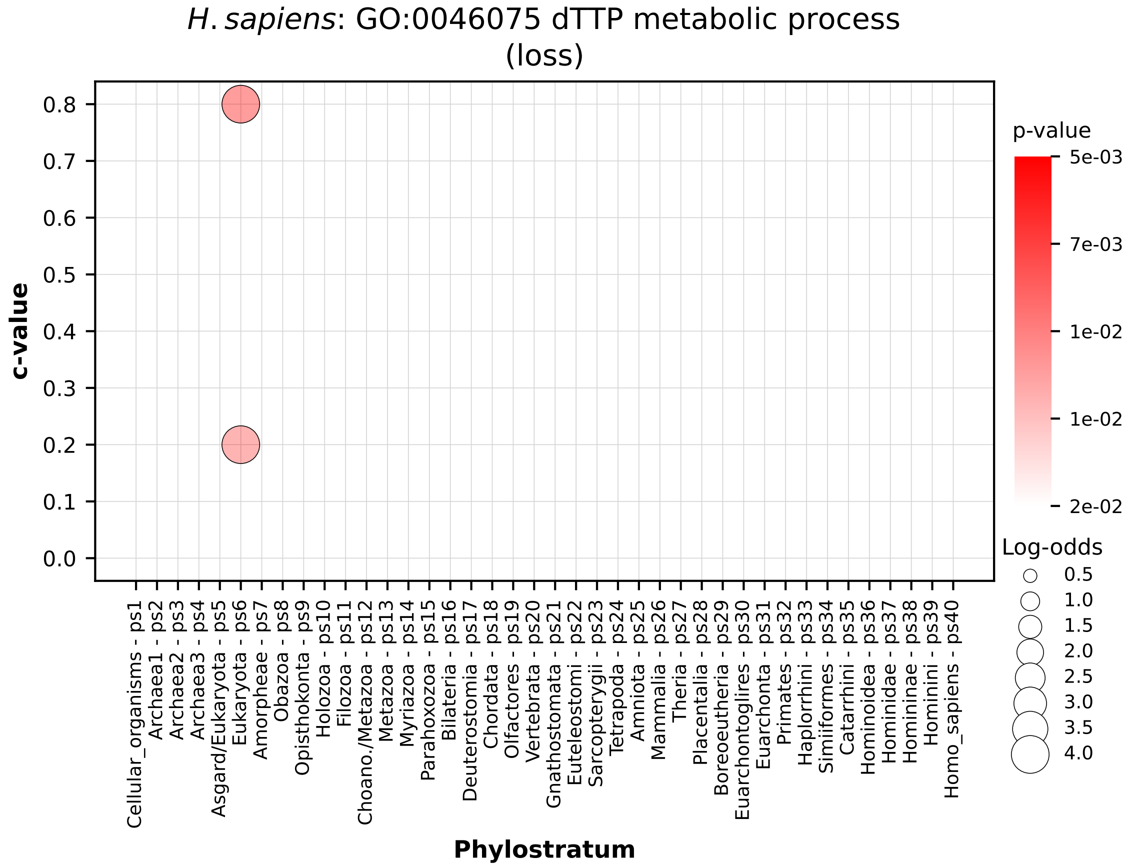
H. sapiens: GO:0046075 dTTP metabolic process
(loss)
c-value
0.0
0.1
0.2
0.3
0.4
0.5
0.6
0.7
0.8
Phylostratum
Cellular_organisms - ps1
Archaea1 - ps2
Archaea2 - ps3
Archaea3 - ps4
Asgard/Eukaryota - ps5
Eukaryota - ps6
Amorpheae - ps7
Obazoa - ps8
Opisthokonta - ps9
Holozoa - ps10
Filozoa - ps11
Choano./Metazoa - ps12
Metazoa - ps13
Myriazoa - ps14
Parahoxozoa - ps15
Bilateria - ps16
Deuterostomia - ps17
Chordata - ps18
Olfactores - ps19
Vertebrata - ps20
Gnathostomata - ps21
Euteleostomi - ps22
Sarcopterygii - ps23
Tetrapoda - ps24
Amniota - ps25
Mammalia - ps26
Theria - ps27
Placentalia - ps28
Boreoeutheria - ps29
Euarchontoglires - ps30
Euarchonta - ps31
Primates - ps32
Haplorrhini - ps33
Simiiformes - ps34
Catarrhini - ps35
Hominoidea - ps36
Hominidae - ps37
Homininae - ps38
Hominini - ps39
Homo_sapiens - ps40
p-value
5e-03
7e-03
1e-02
1e-02
2e-02
Log-odds
0.5
1.0
1.5
2.0
2.5
3.0
3.5
4.0

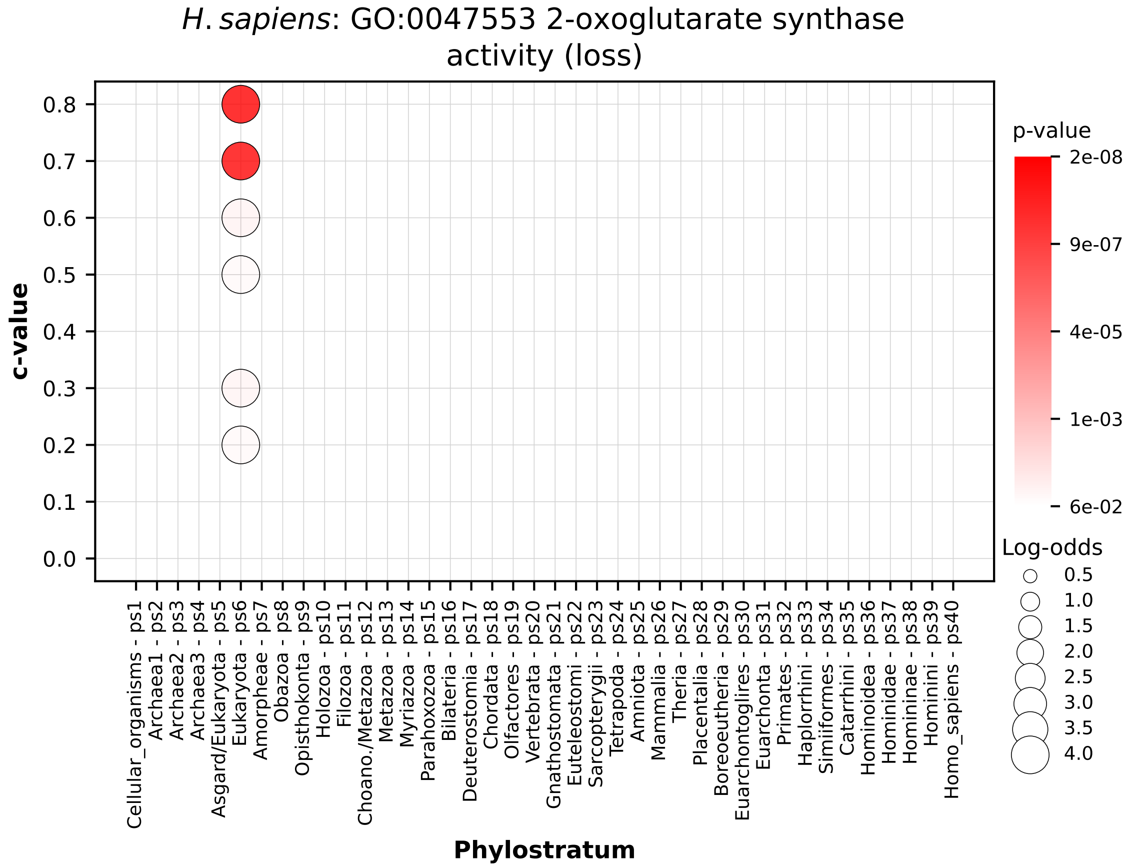

H. sapiens: GO:0047553 2-oxoglutarate synthase activity (loss)
c-value
0.8
0.7
0.6
0.5
0.4
0.3
0.2
0.1
0.0
p-value
2e-08
9e-07
4e-05
1e-03
6e-02
Log-odds
0.5
1.0
1.5
2.0
2.5
3.0
3.5
4.0
Cellular_organisms - ps1
Archaea1 - ps2
Archaea2 - ps3
Archaea3 - ps4
Asgard/Eukaryota - ps5
Eukaryota - ps6
Amorpheae - ps7
Obazoa - ps8
Opisthokonta - ps9
Holozoa - ps10
Filozoa - ps11
Choano./Metazoa - ps12
Metazoa - ps13
Myriazoa - ps14
Parahoxozoa - ps15
Bilateria - ps16
Deuterostomia - ps17
Chordata - ps18
Olfactores - ps19
Vertebrata - ps20
Gnathostomata - ps21
Euteleostomi - ps22
Sarcopterygii - ps23
Tetrapoda - ps24
Amniota - ps25
Mammalia - ps26
Theria - ps27
Placentalia - ps28
Boreoeutheria - ps29
Euarchontoglires - ps30
Euarchonta - ps31
Primates - ps32
Haplorrhini - ps33
Simiiformes - ps34
Catarrhini - ps35
Hominoidea - ps36
Hominidae - ps37
Homininae - ps38
Hominini - ps39
Homo_sapiens - ps40
Phylostratum

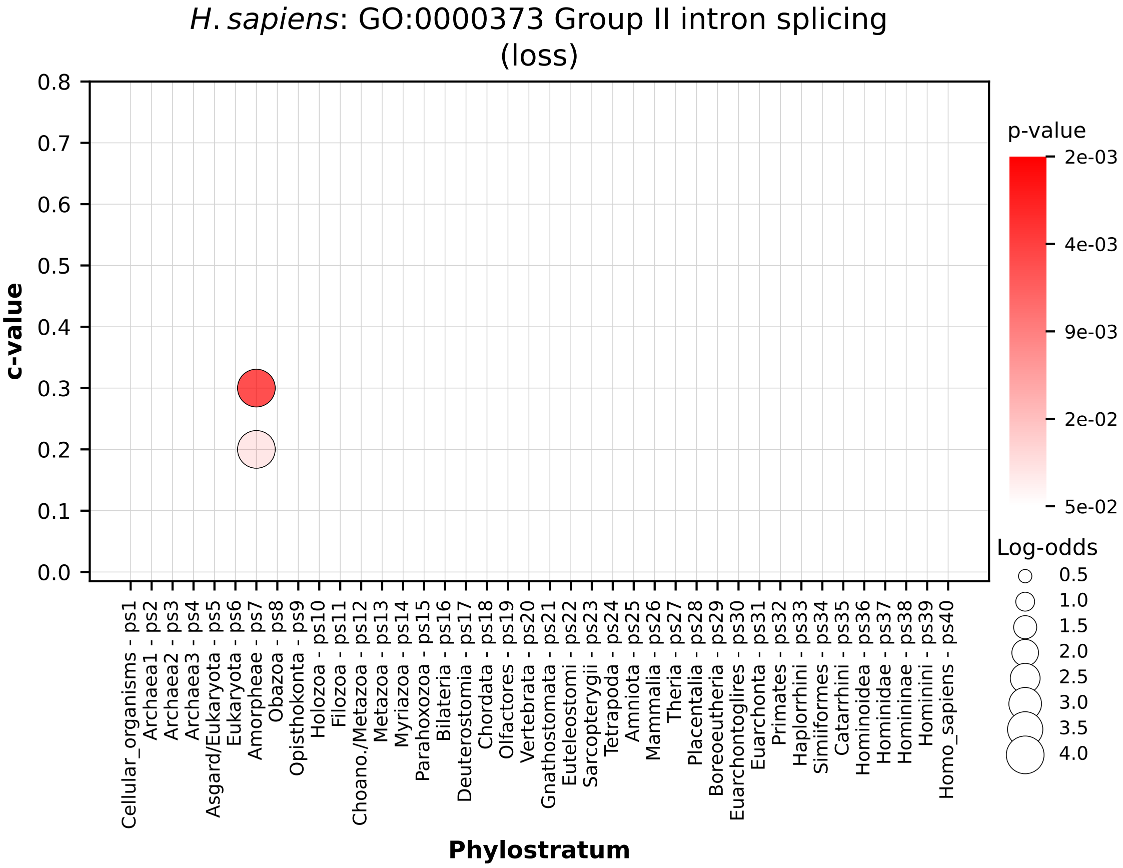

H. sapiens: GO:0000373 Group II intron splicing
(loss)
c-value
0.8
0.7
0.6
0.5
0.4
0.3
0.2
0.1
0.0
p-value
2e-03
4e-03
9e-03
2e-02
5e-02
Log-odds
0.5
1.0
1.5
2.0
2.5
3.0
3.5
4.0
Cellular_organisms - ps1
Archaea1 - ps2
Archaea2 - ps3
Archaea3 - ps4
Asgard/Eukaryota - ps5
Eukaryota - ps6
Amorpheae - ps7
Obazoa - ps8
Opisthokonta - ps9
Holozoa - ps10
Filozoa - ps11
Choano./Metazoa - ps12
Metazoa - ps13
Myriazoa - ps14
Parahoxozoa - ps15
Bilateria - ps16
Deuterostomia - ps17
Chordata - ps18
Olfactores - ps19
Vertebrata - ps20
Gnathostomata - ps21
Euteleostomi - ps22
Sarcopterygii - ps23
Tetrapoda - ps24
Amniota - ps25
Mammalia - ps26
Theria - ps27
Placentalia - ps28
Boreoeutheria - ps29
Euarchontoglires - ps30
Euarchonta - ps31
Primates - ps32
Haplorrhini - ps33
Simiiformes - ps34
Catarrhini - ps35
Hominoidea - ps36
Hominidae - ps37
Homininae - ps38
Hominini - ps39
Homo_sapiens - ps40
Phylostratum

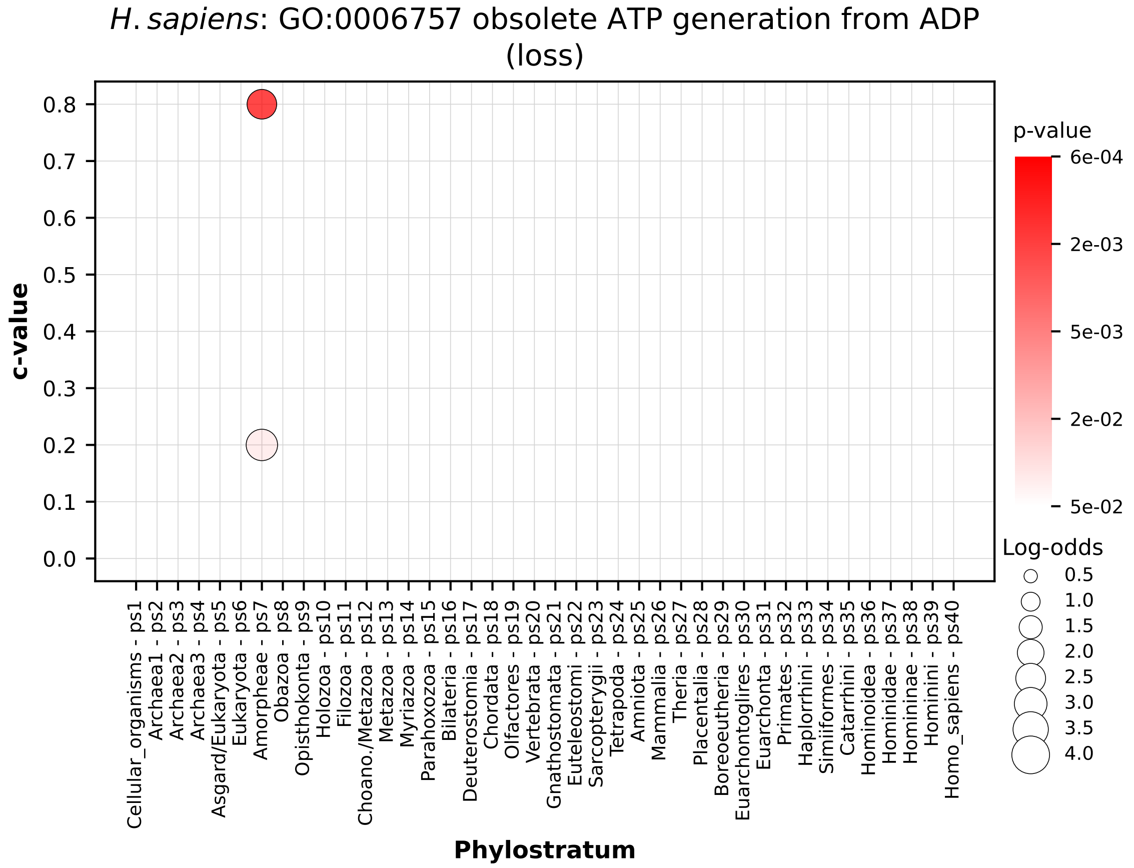

H. sapiens: GO:0006757 obsolete ATP generation from ADP
(loss)
c-value
0.0
0.1
0.2
0.3
0.4
0.5
0.6
0.7
0.8
p-value
6e-04
2e-03
5e-03
2e-02
5e-02
Log-odds
0.5
1.0
1.5
2.0
2.5
3.0
3.5
4.0
Cellular_organisms - ps1
Archaea1 - ps2
Archaea2 - ps3
Archaea3 - ps4
Asgard/Eukaryota - ps5
Eukaryota - ps6
Amorpheae - ps7
Obazoa - ps8
Opisthokonta - ps9
Holozoa - ps10
Filozoa - ps11
Choano./Metazoa - ps12
Metazoa - ps13
Myriazoa - ps14
Parahoxozoa - ps15
Bilateria - ps16
Deuterostomia - ps17
Chordata - ps18
Olfactores - ps19
Vertebrata - ps20
Gnathostomata - ps21
Euteleostomi - ps22
Sarcopterygii - ps23
Tetrapoda - ps24
Amniota - ps25
Mammalia - ps26
Theria - ps27
Placentalia - ps28
Boreoeutheria - ps29
Euarchontoglires - ps30
Euarchonta - ps31
Primates - ps32
Haplorrhini - ps33
Simiiformes - ps34
Catarrhini - ps35
Hominoidea - ps36
Hominidae - ps37
Homininae - ps38
Hominini - ps39
Homo_sapiens - ps40
Phylostratum

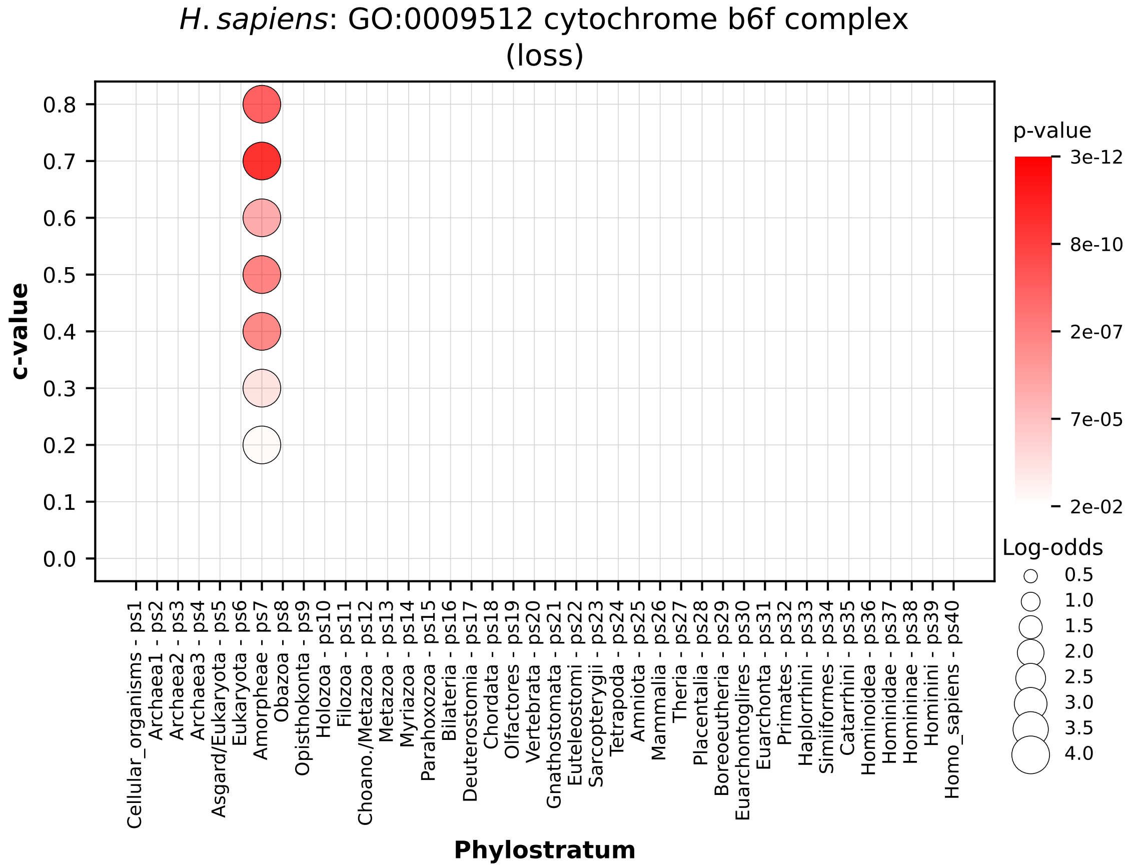

H. sapiens: GO:0009512 cytochrome b6f complex
(loss)
c-value
0.8
0.7
0.6
0.5
0.4
0.3
0.2
0.1
0.0
p-value
3e-12
8e-10
2e-07
7e-05
2e-02
Log-odds
0.5
1.0
1.5
2.0
2.5
3.0
3.5
4.0
Cellular_organisms - ps1
Archaea1 - ps2
Archaea2 - ps3
Archaea3 - ps4
Asgard/Eukaryota - ps5
Eukaryota - ps6
Amorpheae - ps7
Obazoa - ps8
Opisthokonta - ps9
Holozoa - ps10
Filozoa - ps11
Choano./Metazoa - ps12
Metazoa - ps13
Myriazoa - ps14
Parahoxozoa - ps15
Bilateria - ps16
Deuterostomia - ps17
Chordata - ps18
Olfactores - ps19
Vertebrata - ps20
Gnathostomata - ps21
Euteleostomi - ps22
Sarcopterygii - ps23
Tetrapoda - ps24
Amniota - ps25
Mammalia - ps26
Theria - ps27
Placentalia - ps28
Boreoeutheria - ps29
Euarchontoglires - ps30
Euarchonta - ps31
Primates - ps32
Haplorrhini - ps33
Simiiformes - ps34
Catarrhini - ps35
Hominoidea - ps36
Hominidae - ps37
Homininae - ps38
Hominini - ps39
Homo_sapiens - ps40
Phylostratum

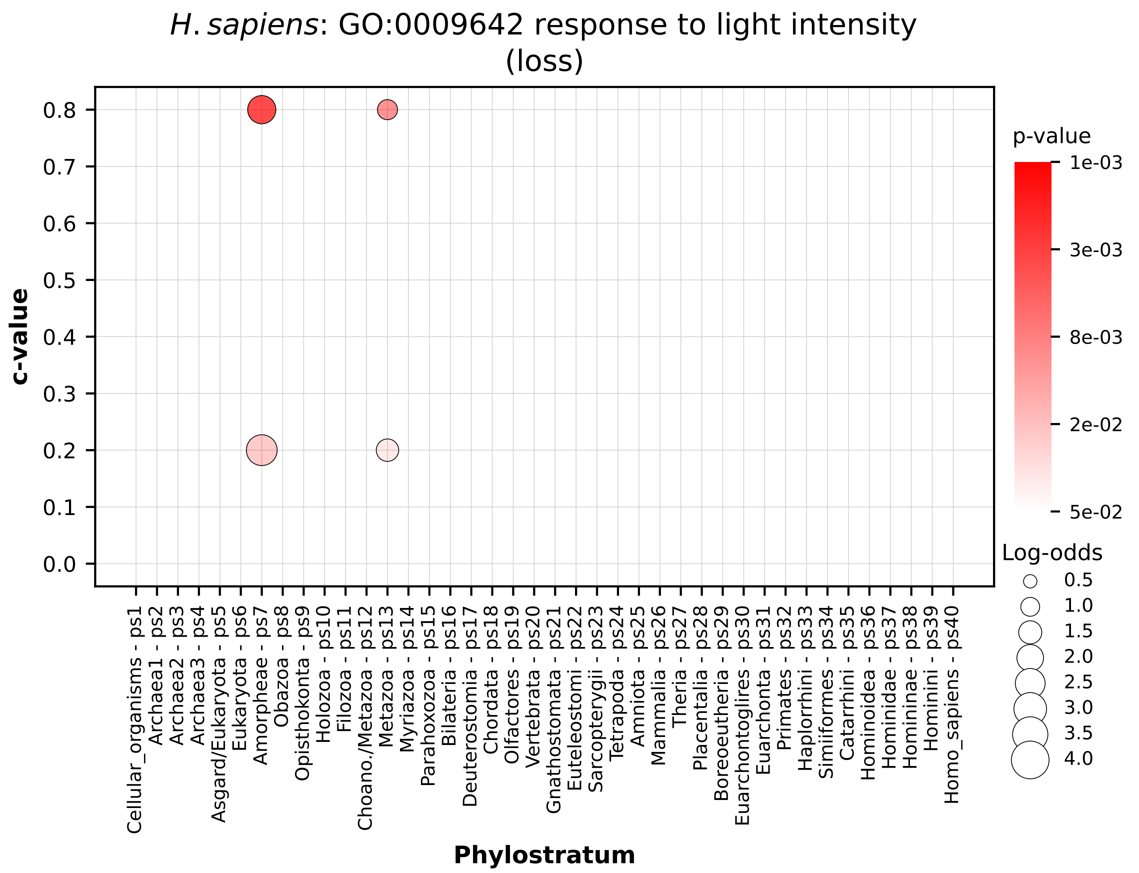

H. sapiens: GO:0009642 response to light intensity
(loss)
c-value
0.0
0.1
0.2
0.3
0.4
0.5
0.6
0.7
0.8
Phylostratum
Cellular_organisms - ps1
Archaea1 - ps2
Archaea2 - ps3
Archaea3 - ps4
Asgard/Eukaryota - ps5
Eukaryota - ps6
Amorpheae - ps7
Obazoa - ps8
Opisthokonta - ps9
Holozoa - ps10
Filozoa - ps11
Choano./Metazoa - ps12
Metazoa - ps13
Myriazoa - ps14
Parahoxozoa - ps15
Bilateria - ps16
Deuterostomia - ps17
Chordata - ps18
Olfactores - ps19
Vertebrata - ps20
Gnathostomata - ps21
Euteleostomi - ps22
Sarcopterygii - ps23
Tetrapoda - ps24
Amniota - ps25
Mammalia - ps26
Theria - ps27
Placentalia - ps28
Boreoeutheria - ps29
Euarchontoglires - ps30
Euarchonta - ps31
Primates - ps32
Haplorrhini - ps33
Simiiformes - ps34
Catarrhini - ps35
Hominoidea - ps36
Hominidae - ps37
Homininae - ps38
Hominini - ps39
Homo_sapiens - ps40
p-value
1e-03
3e-03
8e-03
2e-02
5e-02
Log-odds
0.5
1.0
1.5
2.0
2.5
3.0
3.5
4.0

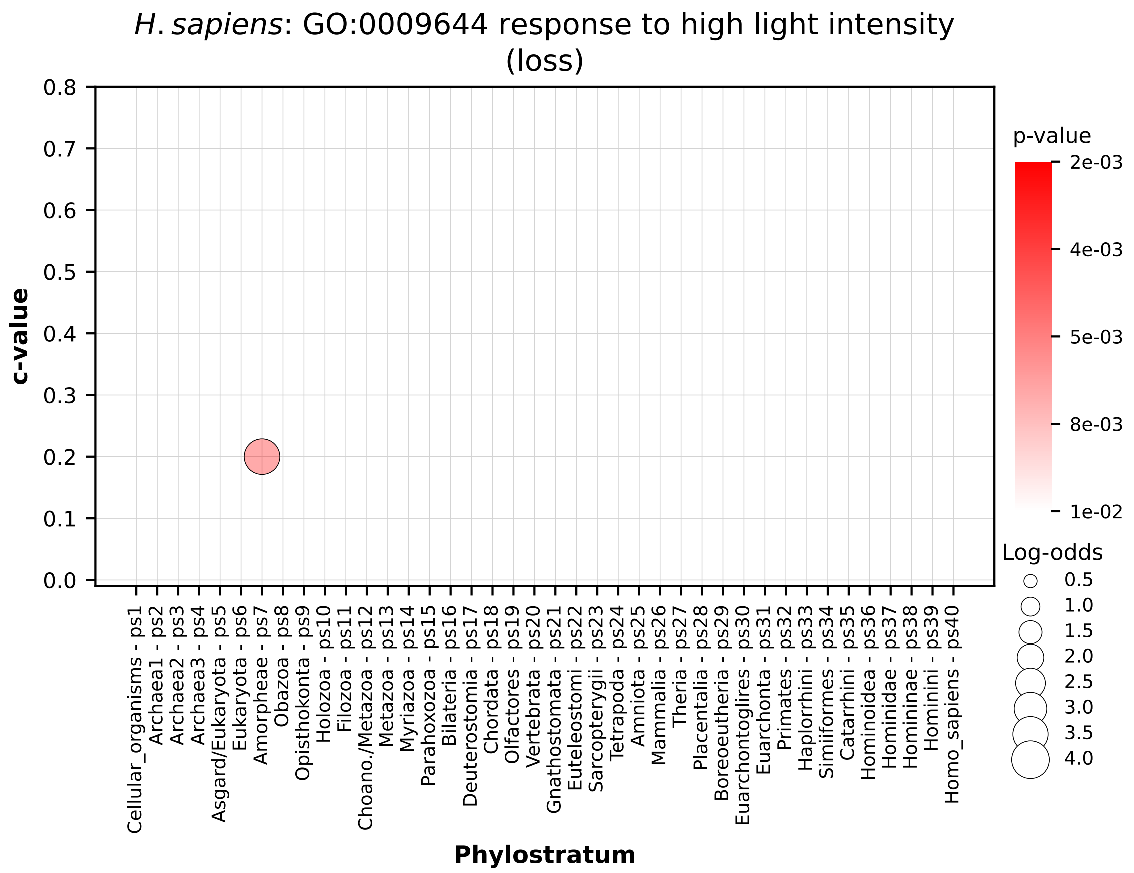

H. sapiens: GO:0009644 response to high light intensity
(loss)
c-value
0.0
0.1
0.2
0.3
0.4
0.5
0.6
0.7
0.8
Phylostratum
Cellular_organisms - ps1
Archaea1 - ps2
Archaea2 - ps3
Archaea3 - ps4
Asgard/Eukaryota - ps5
Eukaryota - ps6
Amorpheae - ps7
Obazoa - ps8
Opisthokonta - ps9
Holozoa - ps10
Filozoa - ps11
Choano./Metazoa - ps12
Metazoa - ps13
Myriazoa - ps14
Parahoxozoa - ps15
Bilateria - ps16
Deuterostomia - ps17
Chordata - ps18
Olfactores - ps19
Vertebrata - ps20
Gnathostomata - ps21
Euteleostomi - ps22
Sarcopterygii - ps23
Tetrapoda - ps24
Amniota - ps25
Mammalia - ps26
Theria - ps27
Placentalia - ps28
Boreoeutheria - ps29
Euarchontoglires - ps30
Euarchonta - ps31
Primates - ps32
Haplorrhini - ps33
Simiiformes - ps34
Catarrhini - ps35
Hominoidea - ps36
Hominidae - ps37
Homininae - ps38
Hominini - ps39
Homo_sapiens - ps40
p-value
2e-03
4e-03
5e-03
8e-03
1e-02
Log-odds
0.5
1.0
1.5
2.0
2.5
3.0
3.5
4.0

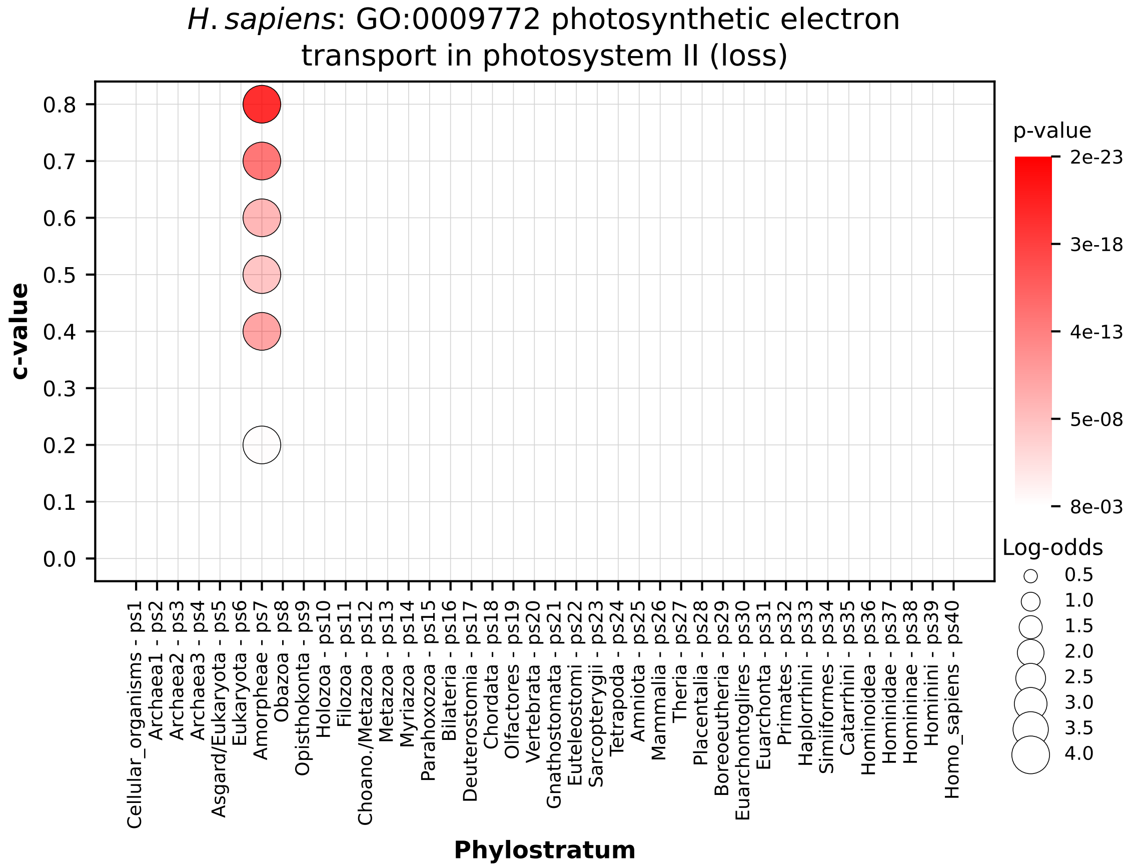

H. sapiens: GO:0009772 photosynthetic electron transport in photosystem II (loss)
c-value
0.0
0.1
0.2
0.3
0.4
0.5
0.6
0.7
0.8
Phylostratum
Cellular_organisms - ps1
Archaea1 - ps2
Archaea2 - ps3
Archaea3 - ps4
Asgard/Eukaryota - ps5
Eukaryota - ps6
Amorpheae - ps7
Obazoa - ps8
Opisthokonta - ps9
Holozoa - ps10
Filozoa - ps11
Choano./Metazoa - ps12
Metazoa - ps13
Myriazoa - ps14
Parahoxozoa - ps15
Bilateria - ps16
Deuterostomia - ps17
Chordata - ps18
Olfactores - ps19
Vertebrata - ps20
Gnathostomata - ps21
Euteleostomi - ps22
Sarcopterygii - ps23
Tetrapoda - ps24
Amniota - ps25
Mammalia - ps26
Theria - ps27
Placentalia - ps28
Boreoeutheria - ps29
Euarchontoglires - ps30
Euarchonta - ps31
Primates - ps32
Haplorrhini - ps33
Simiiformes - ps34
Catarrhini - ps35
Hominoidea - ps36
Hominidae - ps37
Homininae - ps38
Hominini - ps39
Homo_sapiens - ps40
p-value
2e-23
3e-18
4e-13
5e-08
8e-03
Log-odds
0.5
1.0
1.5
2.0
2.5
3.0
3.5
4.0

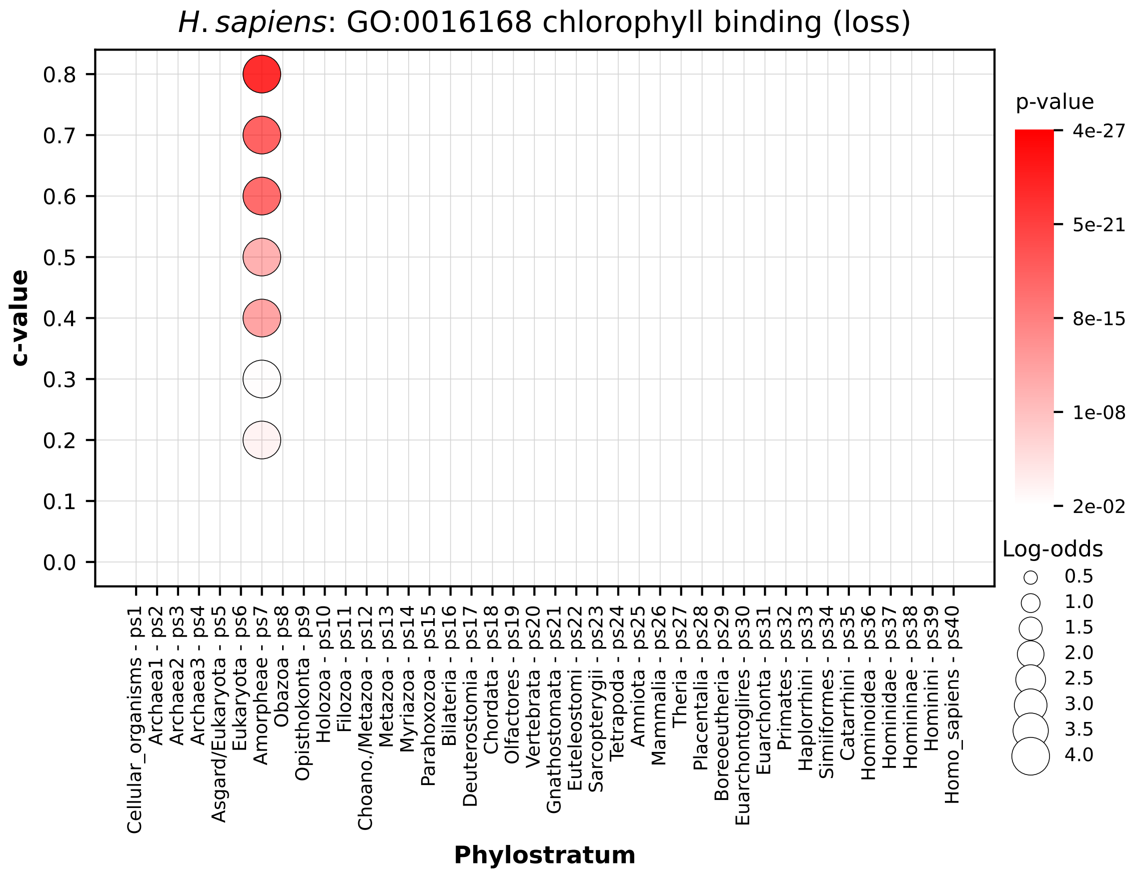

H. sapiens: GO:0016168 chlorophyll binding (loss)
c-value
0.0
0.1
0.2
0.3
0.4
0.5
0.6
0.7
0.8
Phylostratum
Cellular_organisms - ps1
Archaea1 - ps2
Archaea2 - ps3
Archaea3 - ps4
Asgard/Eukaryota - ps5
Eukaryota - ps6
Amorpheae - ps7
Obazoa - ps8
Opisthokonta - ps9
Holozoa - ps10
Filozoa - ps11
Choano./Metazoa - ps12
Metazoa - ps13
Myriazoa - ps14
Parahoxozoa - ps15
Bilateria - ps16
Deuterostomia - ps17
Chordata - ps18
Olfactores - ps19
Vertebrata - ps20
Gnathostomata - ps21
Euteleostomi - ps22
Sarcopterygii - ps23
Tetrapoda - ps24
Amniota - ps25
Mammalia - ps26
Theria - ps27
Placentalia - ps28
Boreoeutheria - ps29
Euarchontoglires - ps30
Euarchonta - ps31
Primates - ps32
Haplorrhini - ps33
Simiiformes - ps34
Catarrhini - ps35
Hominoidea - ps36
Hominidae - ps37
Homininae - ps38
Hominini - ps39
Homo_sapiens - ps40
p-value
4e-27
5e-21
8e-15
1e-08
2e-02
Log-odds
0.5
1.0
1.5
2.0
2.5
3.0
3.5
4.0

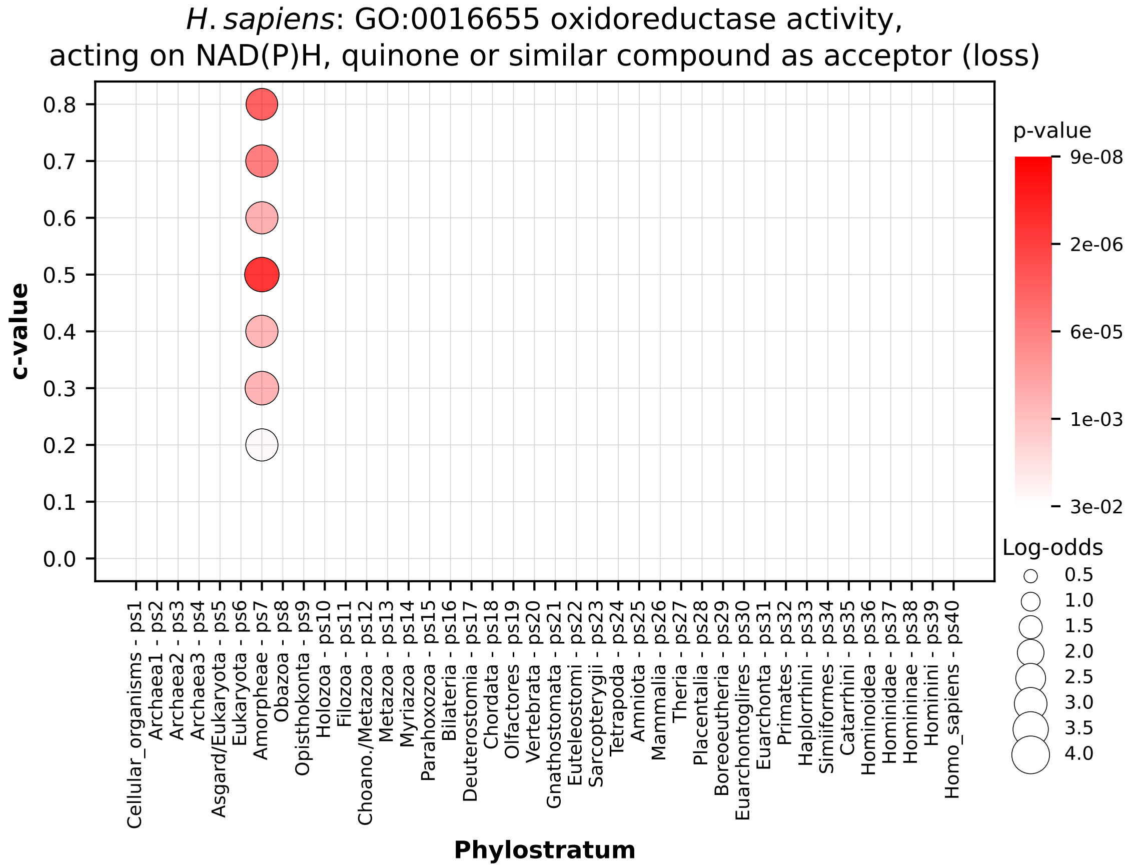

H. sapiens: GO:0016655 oxidoreductase activity,
acting on NAD(P)H, quinone or similar compound as acceptor (loss)
c-value
0.0
0.1
0.2
0.3
0.4
0.5
0.6
0.7
0.8
Phylostratum
Cellular_organisms - ps1
Archaea1 - ps2
Archaea2 - ps3
Archaea3 - ps4
Asgard/Eukaryota - ps5
Eukaryota - ps6
Amorpheae - ps7
Obazoa - ps8
Opisthokonta - ps9
Holozoa - ps10
Filozoa - ps11
Choano./Metazoa - ps12
Metazoa - ps13
Myriazoa - ps14
Parahoxozoa - ps15
Bilateria - ps16
Deuterostomia - ps17
Chordata - ps18
Olfactores - ps19
Vertebrata - ps20
Gnathostomata - ps21
Euteleostomi - ps22
Sarcopterygii - ps23
Tetrapoda - ps24
Amniota - ps25
Mammalia - ps26
Theria - ps27
Placentalia - ps28
Boreoeutheria - ps29
Euarchontoglires - ps30
Euarchonta - ps31
Primates - ps32
Haplorrhini - ps33
Simiiformes - ps34
Catarrhini - ps35
Hominoidea - ps36
Hominidae - ps37
Homininae - ps38
Hominini - ps39
Homo_sapiens - ps40
p-value
9e-08
2e-06
6e-05
1e-03
3e-02
Log-odds
0.5
1.0
1.5
2.0
2.5
3.0
3.5
4.0

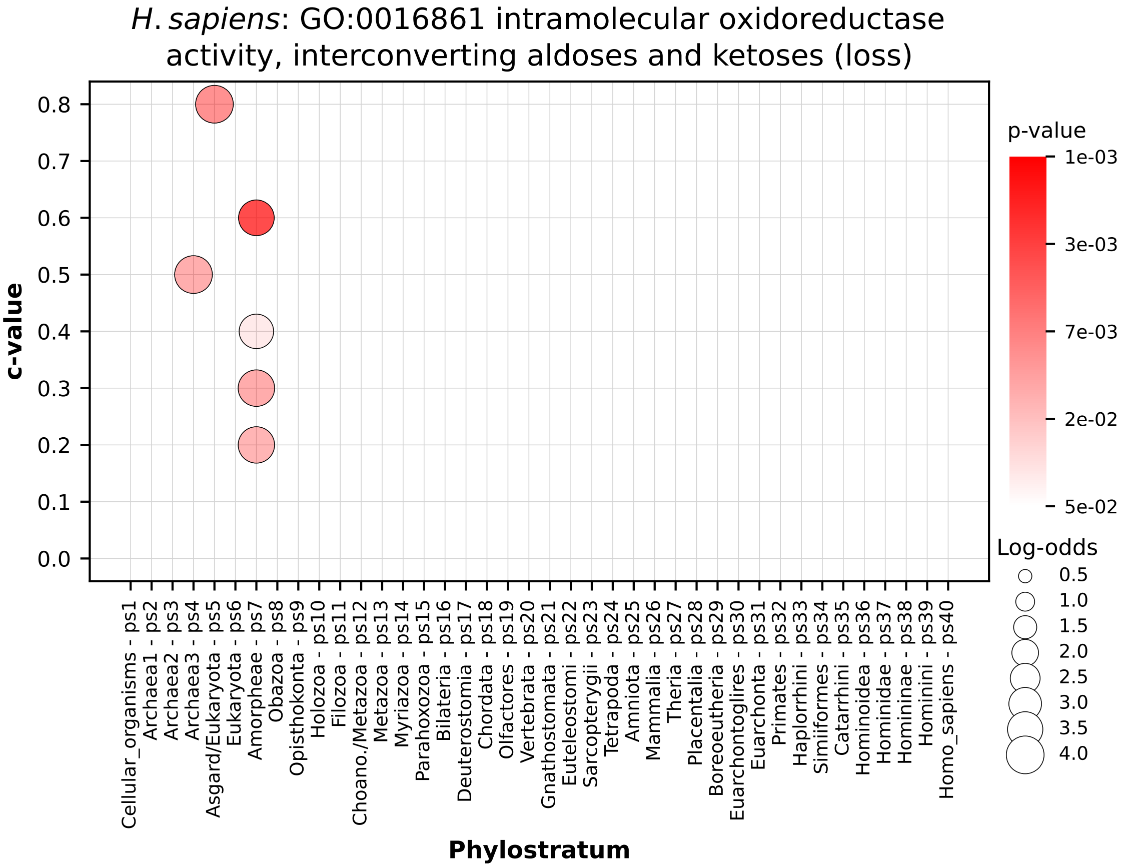

H. sapiens: GO:0016861 intramolecular oxidoreductase activity, interconverting aldoses and ketoses (loss)
c-value
0.0
0.1
0.2
0.3
0.4
0.5
0.6
0.7
0.8
Phylostratum
Cellular_organisms - ps1
Archaea1 - ps2
Archaea2 - ps3
Archaea3 - ps4
Asgard/Eukaryota - ps5
Eukaryota - ps6
Amorpheae - ps7
Obazoa - ps8
Opisthokonta - ps9
Holozoa - ps10
Filozoa - ps11
Choano./Metazoa - ps12
Metazoa - ps13
Myriazoa - ps14
Parahoxozoa - ps15
Bilateria - ps16
Deuterostomia - ps17
Chordata - ps18
Olfactores - ps19
Vertebrata - ps20
Gnathostomata - ps21
Euteleostomi - ps22
Sarcopterygii - ps23
Tetrapoda - ps24
Amniota - ps25
Mammalia - ps26
Theria - ps27
Placentalia - ps28
Boreoeutheria - ps29
Euarchontoglires - ps30
Euarchonta - ps31
Primates - ps32
Haplorrhini - ps33
Simiiformes - ps34
Catarrhini - ps35
Hominoidea - ps36
Hominidae - ps37
Homininae - ps38
Hominini - ps39
Homo_sapiens - ps40
p-value
1e-03
3e-03
7e-03
2e-02
5e-02
Log-odds
0.5
1.0
1.5
2.0
2.5
3.0
3.5
4.0

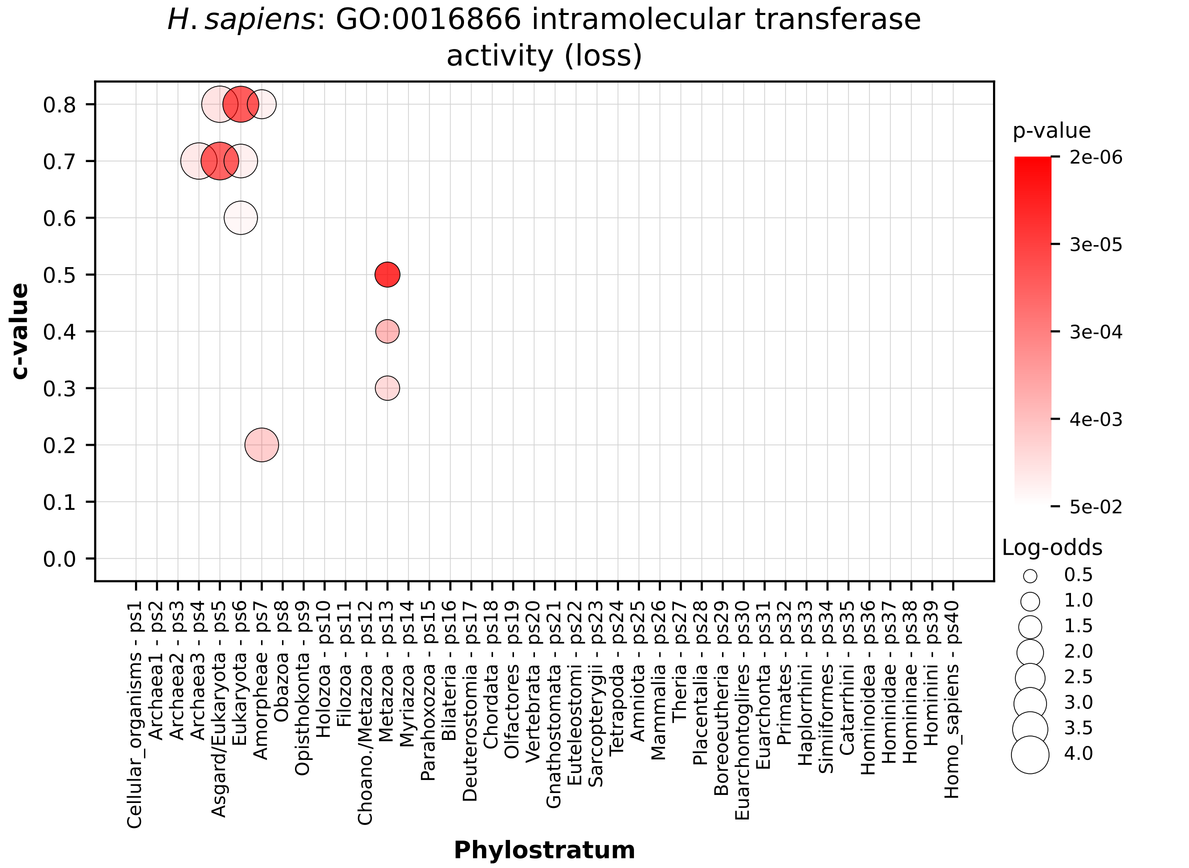

H. sapiens: GO:0016866 intramolecular transferase activity (loss)
c-value
0.8
0.7
0.6
0.5
0.4
0.3
0.2
0.1
0.0
p-value
2e-06
3e-05
3e-04
4e-03
5e-02
Log-odds
0.5
1.0
1.5
2.0
2.5
3.0
3.5
4.0
Cellular_organisms - ps1
Archaea1 - ps2
Archaea2 - ps3
Archaea3 - ps4
Asgard/Eukaryota - ps5
Eukaryota - ps6
Amorpheae - ps7
Obazoa - ps8
Opisthokonta - ps9
Holozoa - ps10
Filozoa - ps11
Choano./Metazoa - ps12
Metazoa - ps13
Myriazoa - ps14
Parahoxozoa - ps15
Bilateria - ps16
Deuterostomia - ps17
Chordata - ps18
Olfactores - ps19
Vertebrata - ps20
Gnathostomata - ps21
Euteleostomi - ps22
Sarcopterygii - ps23
Tetrapoda - ps24
Amniota - ps25
Mammalia - ps26
Theria - ps27
Placentalia - ps28
Boreoeutheria - ps29
Euarchontoglires - ps30
Euarchonta - ps31
Primates - ps32
Haplorrhini - ps33
Simiiformes - ps34
Catarrhini - ps35
Hominoidea - ps36
Hominidae - ps37
Homininae - ps38
Hominini - ps39
Homo_sapiens - ps40
Phylostratum

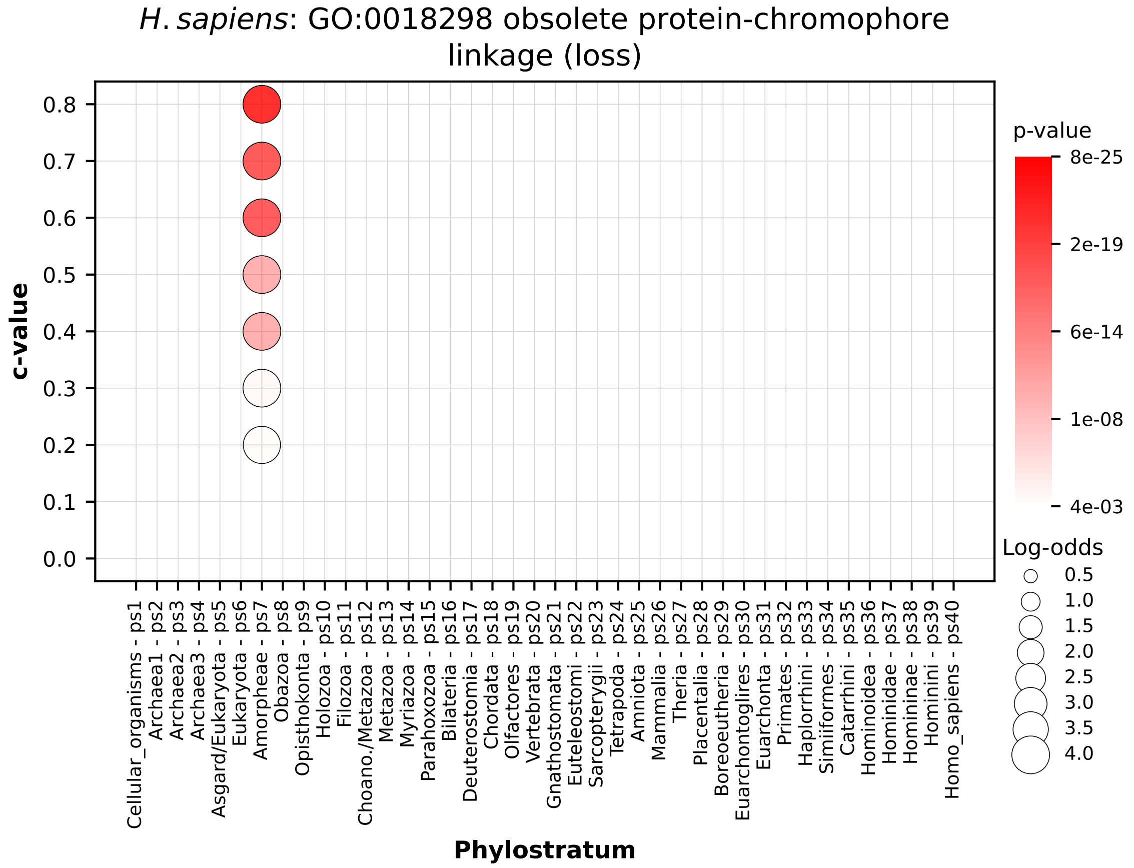

H. sapiens: GO:0018298 obsolete protein-chromophore linkage (loss)
c-value
0.8
0.7
0.6
0.5
0.4
0.3
0.2
0.1
0.0
p-value
8e-25
2e-19
6e-14
1e-08
4e-03
Log-odds
0.5
1.0
1.5
2.0
2.5
3.0
3.5
4.0
Cellular_organisms - ps1
Archaea1 - ps2
Archaea2 - ps3
Archaea3 - ps4
Asgard/Eukaryota - ps5
Eukaryota - ps6
Amorpheae - ps7
Obazoa - ps8
Opisthokonta - ps9
Holozoa - ps10
Filozoa - ps11
Choano./Metazoa - ps12
Metazoa - ps13
Myriazoa - ps14
Parahoxozoa - ps15
Bilateria - ps16
Deuterostomia - ps17
Chordata - ps18
Olfactores - ps19
Vertebrata - ps20
Gnathostomata - ps21
Euteleostomi - ps22
Sarcopterygii - ps23
Tetrapoda - ps24
Amniota - ps25
Mammalia - ps26
Theria - ps27
Placentalia - ps28
Boreoeutheria - ps29
Euarchontoglires - ps30
Euarchonta - ps31
Primates - ps32
Haplorrhini - ps33
Simiiformes - ps34
Catarrhini - ps35
Hominoidea - ps36
Hominidae - ps37
Homininae - ps38
Hominini - ps39
Homo_sapiens - ps40
Phylostratum

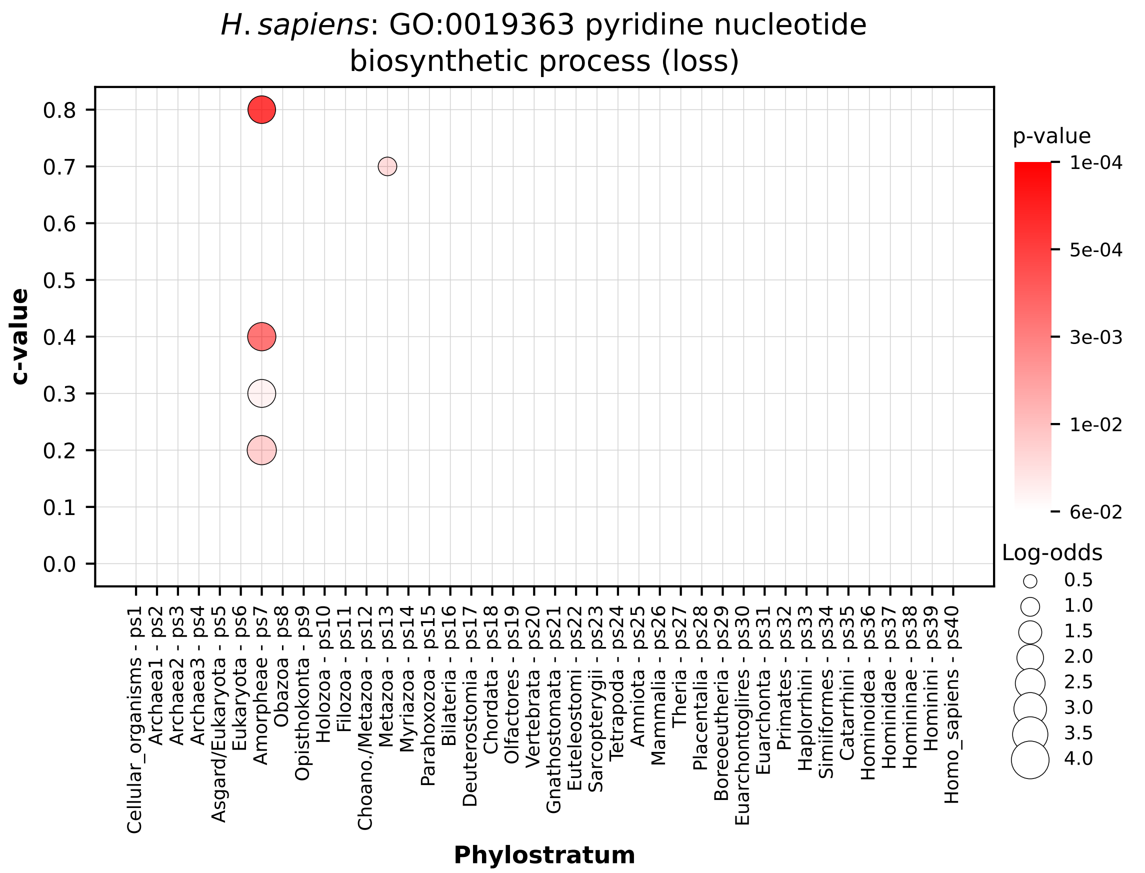

H. sapiens: GO:0019363 pyridine nucleotide biosynthetic process (loss)
c-value
0.0
0.1
0.2
0.3
0.4
0.5
0.6
0.7
0.8
Phylostratum
Cellular_organisms - ps1
Archaea1 - ps2
Archaea2 - ps3
Archaea3 - ps4
Asgard/Eukaryota - ps5
Eukaryota - ps6
Amorpheae - ps7
Obazoa - ps8
Opisthokonta - ps9
Holozoa - ps10
Filozoa - ps11
Choano./Metazoa - ps12
Metazoa - ps13
Myriazoa - ps14
Parahoxozoa - ps15
Bilateria - ps16
Deuterostomia - ps17
Chordata - ps18
Olfactores - ps19
Vertebrata - ps20
Gnathostomata - ps21
Euteleostomi - ps22
Sarcopterygii - ps23
Tetrapoda - ps24
Amniota - ps25
Mammalia - ps26
Theria - ps27
Placentalia - ps28
Boreoeutheria - ps29
Euarchontoglires - ps30
Euarchonta - ps31
Primates - ps32
Haplorrhini - ps33
Simiiformes - ps34
Catarrhini - ps35
Hominoidea - ps36
Hominidae - ps37
Homininae - ps38
Hominini - ps39
Homo_sapiens - ps40
p-value
1e-04
5e-04
3e-03
1e-02
6e-02
Log-odds
0.5
1.0
1.5
2.0
2.5
3.0
3.5
4.0

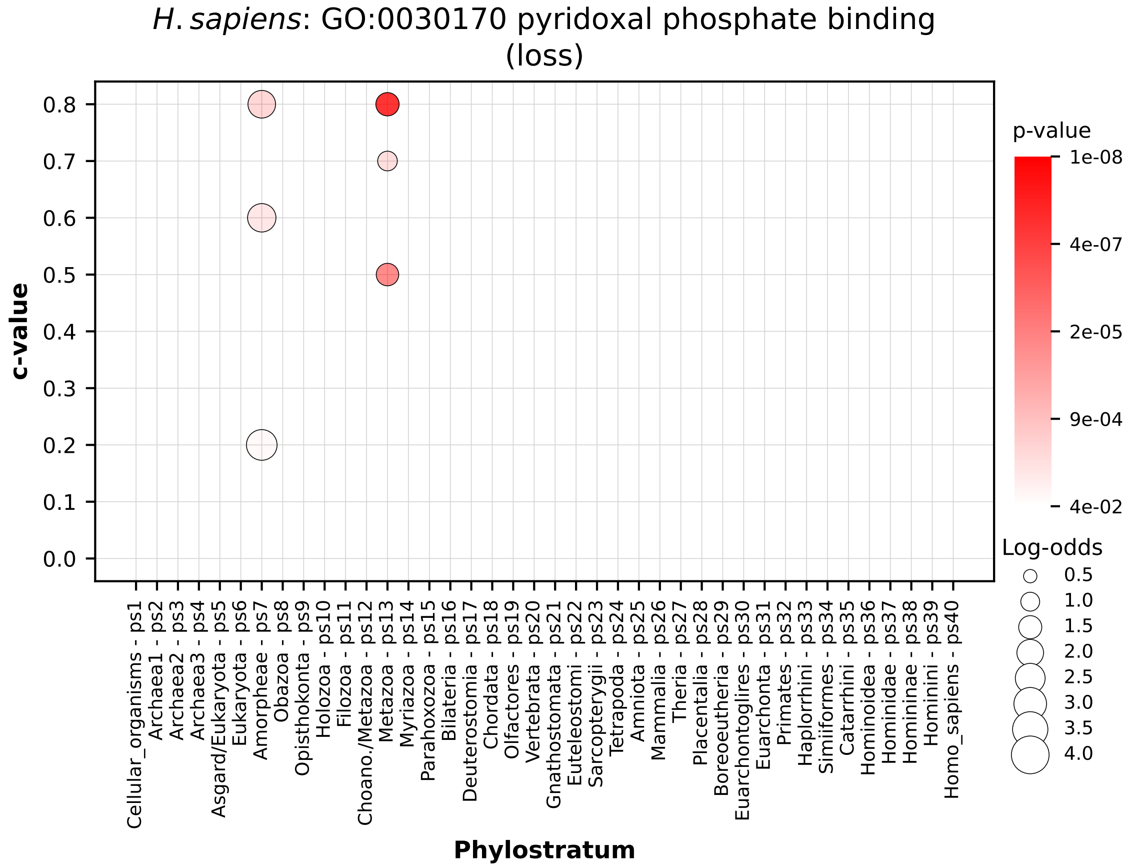

H. sapiens: GO:0030170 pyridoxal phosphate binding (loss)
c-value
0.0
0.1
0.2
0.3
0.4
0.5
0.6
0.7
0.8
Phylostratum
Cellular_organisms - ps1
Archaea1 - ps2
Archaea2 - ps3
Archaea3 - ps4
Asgard/Eukaryota - ps5
Eukaryota - ps6
Amorpheae - ps7
Obazoa - ps8
Opisthokonta - ps9
Holozoa - ps10
Filozoa - ps11
Choano./Metazoa - ps12
Metazoa - ps13
Myriazoa - ps14
Parahoxozoa - ps15
Bilateria - ps16
Deuterostomia - ps17
Chordata - ps18
Olfactores - ps19
Vertebrata - ps20
Gnathostomata - ps21
Euteleostomi - ps22
Sarcopterygii - ps23
Tetrapoda - ps24
Amniota - ps25
Mammalia - ps26
Theria - ps27
Placentalia - ps28
Boreoeutheria - ps29
Euarchontoglires - ps30
Euarchonta - ps31
Primates - ps32
Haplorrhini - ps33
Simiiformes - ps34
Catarrhini - ps35
Hominoidea - ps36
Hominidae - ps37
Homininae - ps38
Hominini - ps39
Homo_sapiens - ps40
p-value
1e-08
4e-07
2e-05
9e-04
4e-02
Log-odds
0.5
1.0
1.5
2.0
2.5
3.0
3.5
4.0

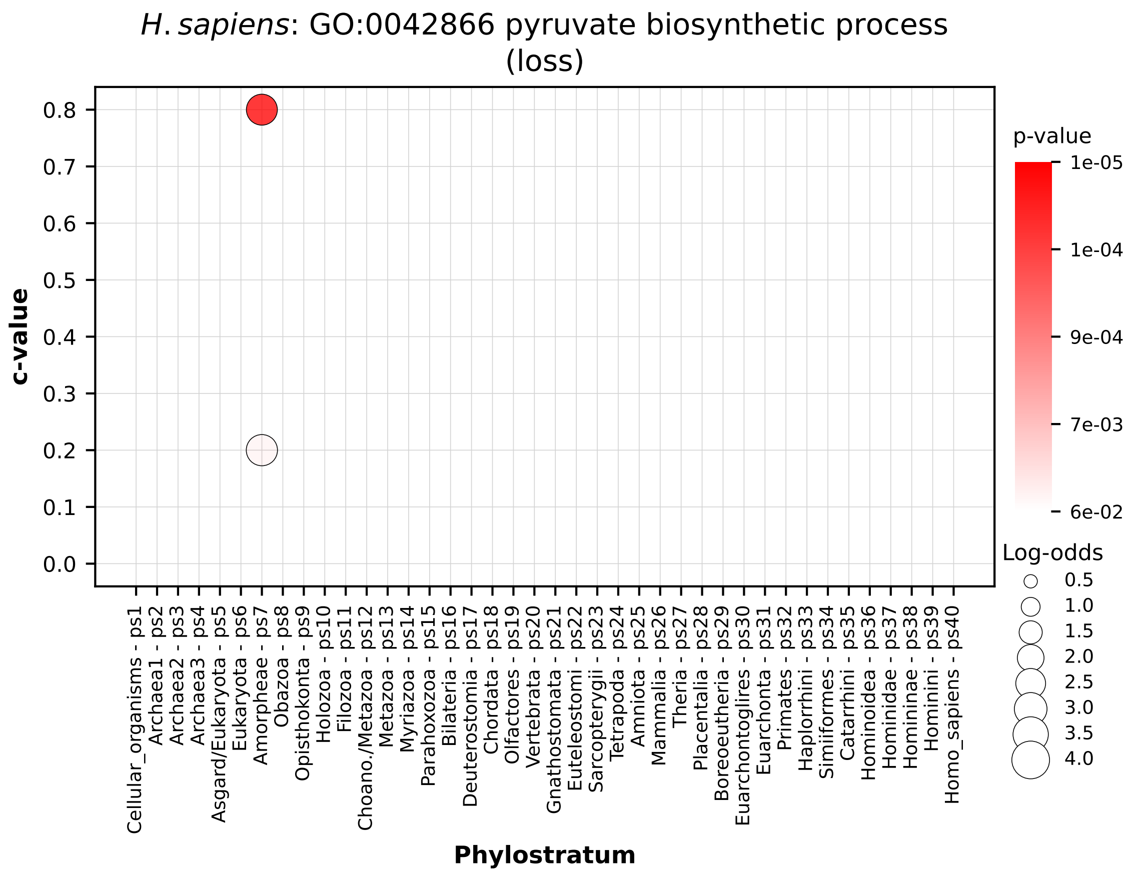

H. sapiens: GO:0042866 pyruvate biosynthetic process (loss)
c-value
0.0
0.1
0.2
0.3
0.4
0.5
0.6
0.7
0.8
Phylostratum
Cellular_organisms - ps1
Archaea1 - ps2
Archaea2 - ps3
Archaea3 - ps4
Asgard/Eukaryota - ps5
Eukaryota - ps6
Amorpheae - ps7
Obazoa - ps8
Opisthokonta - ps9
Holozoa - ps10
Filozoa - ps11
Choano./Metazoa - ps12
Metazoa - ps13
Myriazoa - ps14
Parahoxozoa - ps15
Bilateria - ps16
Deuterostomia - ps17
Chordata - ps18
Olfactores - ps19
Vertebrata - ps20
Gnathostomata - ps21
Euteleostomi - ps22
Sarcopterygii - ps23
Tetrapoda - ps24
Amniota - ps25
Mammalia - ps26
Theria - ps27
Placentalia - ps28
Boreoeutheria - ps29
Euarchontoglires - ps30
Euarchonta - ps31
Primates - ps32
Haplorrhini - ps33
Simiiformes - ps34
Catarrhini - ps35
Hominoidea - ps36
Hominidae - ps37
Homininae - ps38
Hominini - ps39
Homo_sapiens - ps40
p-value
1e-05
1e-04
9e-04
7e-03
6e-02
Log-odds
0.5
1.0
1.5
2.0
2.5
3.0
3.5
4.0

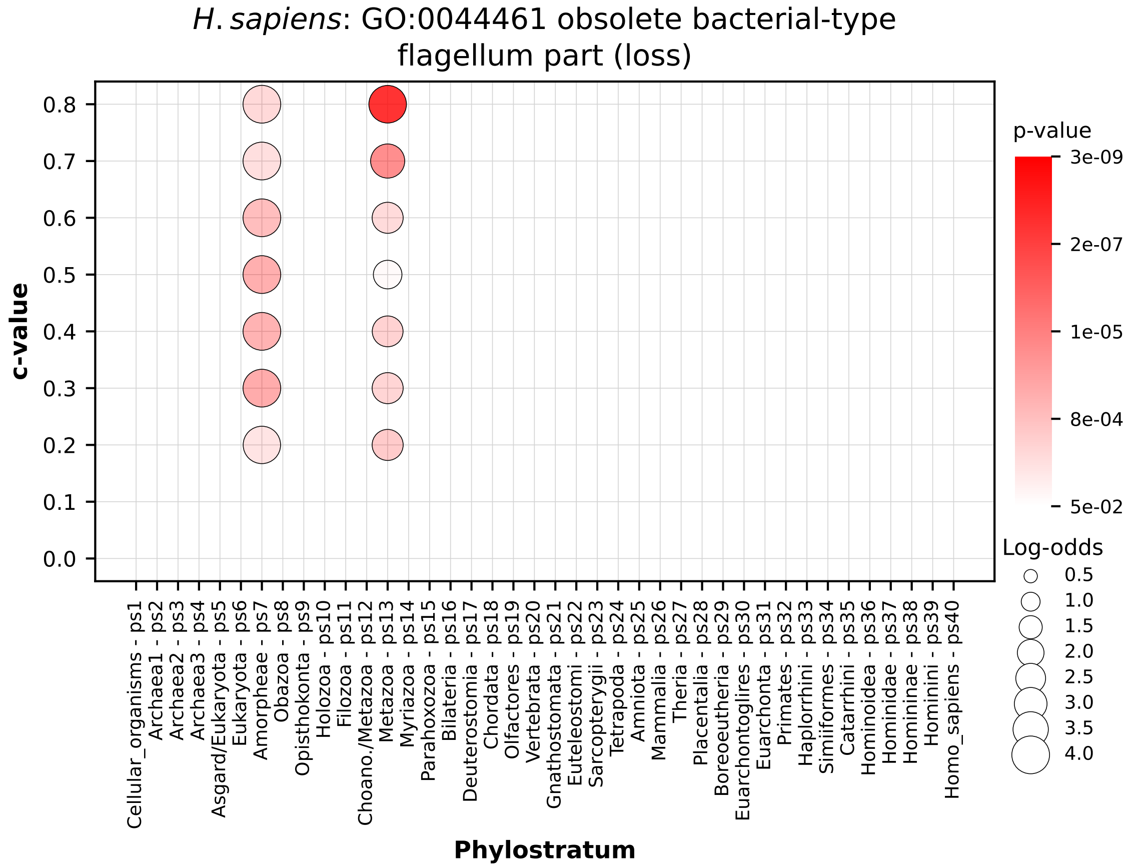

H. sapiens: GO:0044461 obsolete bacterial-type flagellum part (loss)
c-value
0.8
0.7
0.6
0.5
0.4
0.3
0.2
0.1
0.0
Phylostratum
Cellular_organisms - ps1
Archaea1 - ps2
Archaea2 - ps3
Archaea3 - ps4
Asgard/Eukaryota - ps5
Eukaryota - ps6
Amorpheae - ps7
Obazoa - ps8
Opisthokonta - ps9
Holozoa - ps10
Filozoa - ps11
Choano./Metazoa - ps12
Metazoa - ps13
Myriazoa - ps14
Parahoxozoa - ps15
Bilateria - ps16
Deuterostomia - ps17
Chordata - ps18
Olfactores - ps19
Vertebrata - ps20
Gnathostomata - ps21
Euteleostomi - ps22
Sarcopterygii - ps23
Tetrapoda - ps24
Amniota - ps25
Mammalia - ps26
Theria - ps27
Placentalia - ps28
Boreoeutheria - ps29
Euarchontoglires - ps30
Euarchonta - ps31
Primates - ps32
Haplorrhini - ps33
Simiiformes - ps34
Catarrhini - ps35
Hominoidea - ps36
Hominidae - ps37
Homininae - ps38
Hominini - ps39
Homo_sapiens - ps40
p-value
3e-09
2e-07
1e-05
8e-04
5e-02
Log-odds
0.5
1.0
1.5
2.0
2.5
3.0
3.5
4.0

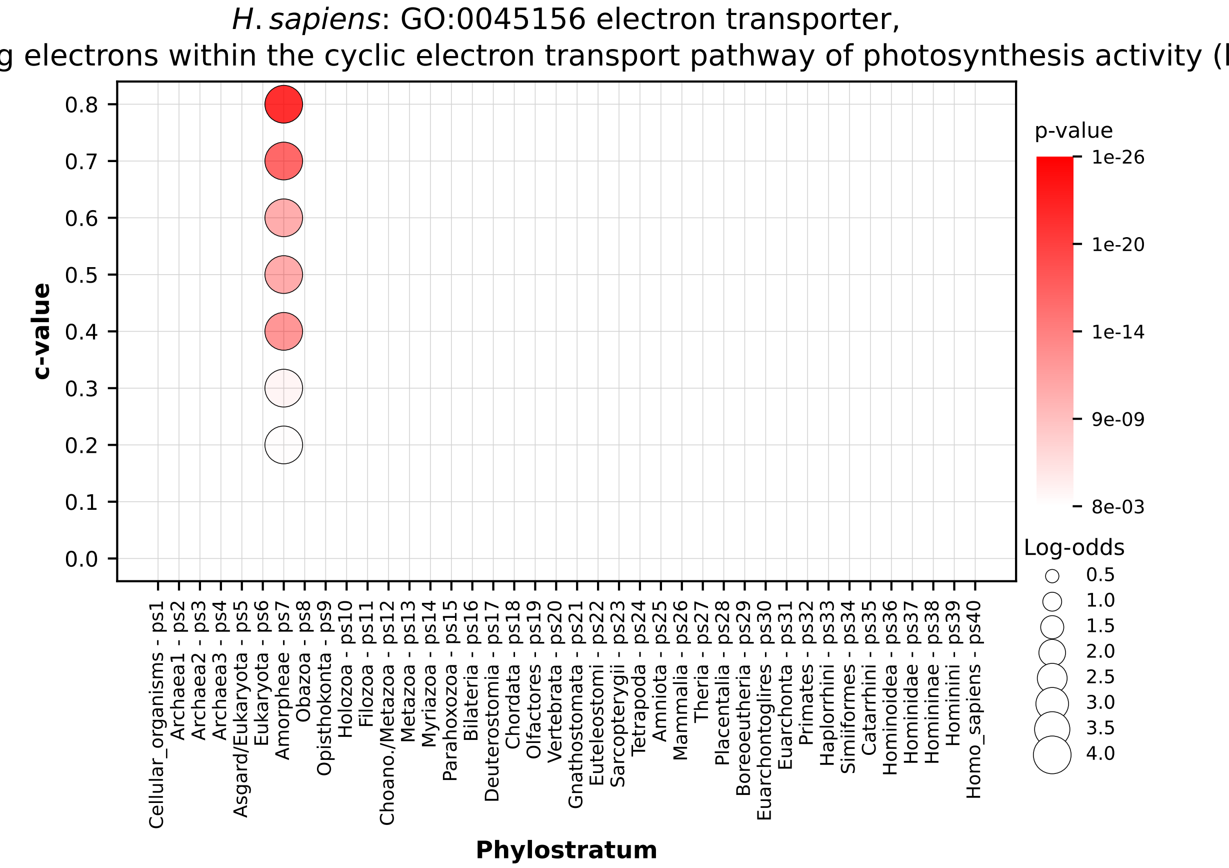

H. sapiens: GO:0045156 electron transporter,
g electrons within the cyclic electron transport pathway of photosynthesis activity (I
c-value
0.8
0.7
0.6
0.5
0.4
0.3
0.2
0.1
0.0
p-value
1e-26
1e-20
1e-14
9e-09
8e-03
Log-odds
0.5
1.0
1.5
2.0
2.5
3.0
3.5
4.0
Cellular_organisms - ps1
Archaea1 - ps2
Archaea2 - ps3
Archaea3 - ps4
Asgard/Eukaryota - ps5
Eukaryota - ps6
Amorpheae - ps7
Obazoa - ps8
Opisthokonta - ps9
Holozoa - ps10
Filozoa - ps11
Choano./Metazoa - ps12
Metazoa - ps13
Myriazoa - ps14
Parahoxozoa - ps15
Bilateria - ps16
Deuterostomia - ps17
Chordata - ps18
Olfactores - ps19
Vertebrata - ps20
Gnathostomata - ps21
Euteleostomi - ps22
Sarcopterygii - ps23
Tetrapoda - ps24
Amniota - ps25
Mammalia - ps26
Theria - ps27
Placentalia - ps28
Boreoeutheria - ps29
Euarchontoglires - ps30
Euarchonta - ps31
Primates - ps32
Haplorrhini - ps33
Simiiformes - ps34
Catarrhini - ps35
Hominoidea - ps36
Hominidae - ps37
Homininae - ps38
Hominini - ps39
Homo_sapiens - ps40
Phylostratum

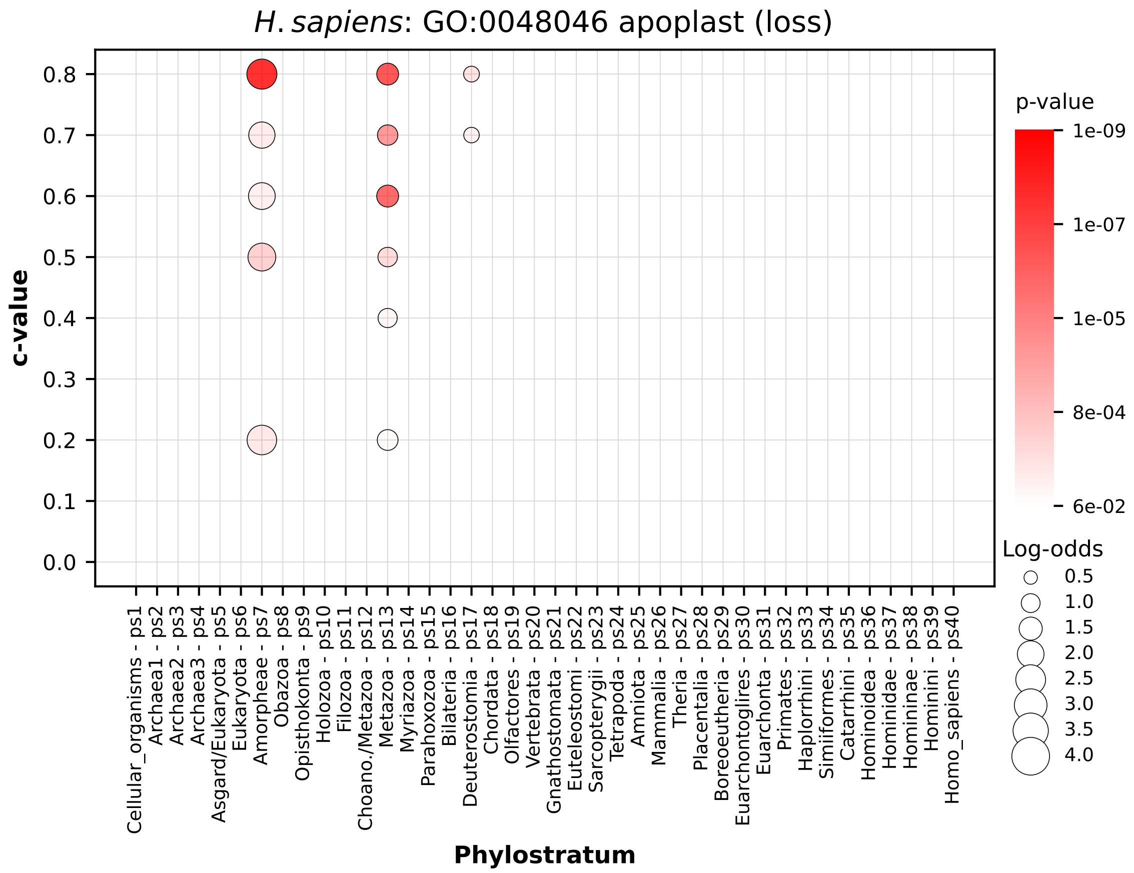

H. sapiens: GO:0048046 apoplast (loss)
c-value
Phylostratum
p-value
1e-09
1e-07
1e-05
8e-04
6e-02
Log-odds
0.5
1.0
1.5
2.0
2.5
3.0
3.5
4.0
Cellular_organisms - ps1
Archaea1 - ps2
Archaea2 - ps3
Archaea3 - ps4
Asgard/Eukaryota - ps5
Eukaryota - ps6
Amorpheae - ps7
Obazoa - ps8
Opisthokonta - ps9
Holozoa - ps10
Filozoa - ps11
Choano./Metazoa - ps12
Metazoa - ps13
Myriazoa - ps14
Parahoxozoa - ps15
Bilateria - ps16
Deuterostomia - ps17
Chordata - ps18
Olfactores - ps19
Vertebrata - ps20
Gnathostomata - ps21
Euteleostomi - ps22
Sarcopterygii - ps23
Tetrapoda - ps24
Amniota - ps25
Mammalia - ps26
Theria - ps27
Placentalia - ps28
Boreoeutheria - ps29
Euarchontoglires - ps30
Euarchonta - ps31
Primates - ps32
Haplorrhini - ps33
Simiiformes - ps34
Catarrhini - ps35
Hominoidea - ps36
Hominidae - ps37
Homininae - ps38
Hominini - ps39
Homo_sapiens - ps40

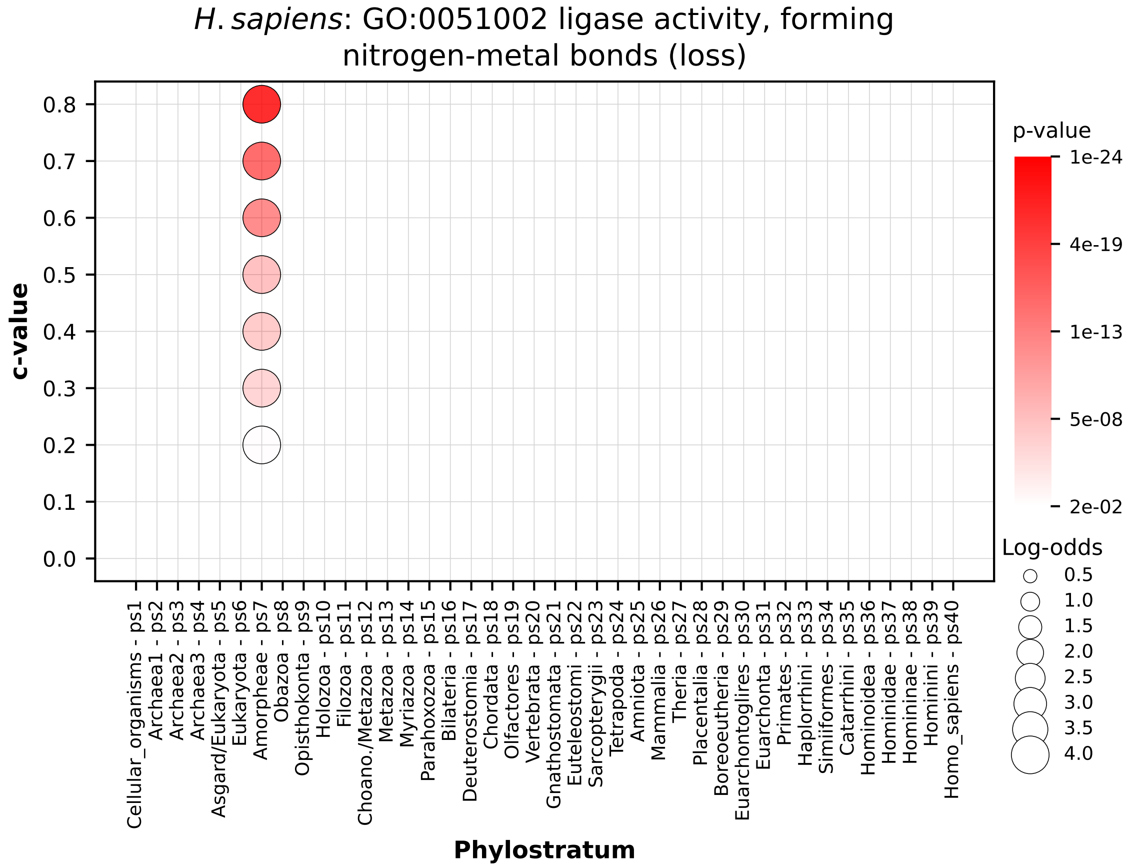

H. sapiens: GO:0051002 ligase activity, forming nitrogen-metal bonds (loss)
c-value
0.8
0.7
0.6
0.5
0.4
0.3
0.2
0.1
0.0
p-value
1e-24
4e-19
1e-13
5e-08
2e-02
Log-odds
0.5
1.0
1.5
2.0
2.5
3.0
3.5
4.0
Cellular_organisms - ps1
Archaea1 - ps2
Archaea2 - ps3
Archaea3 - ps4
Asgard/Eukaryota - ps5
Eukaryota - ps6
Amorpheae - ps7
Obazoa - ps8
Opisthokonta - ps9
Holozoa - ps10
Filozoa - ps11
Choano./Metazoa - ps12
Metazoa - ps13
Myriazoa - ps14
Parahoxozoa - ps15
Bilateria - ps16
Deuterostomia - ps17
Chordata - ps18
Olfactores - ps19
Vertebrata - ps20
Gnathostomata - ps21
Euteleostomi - ps22
Sarcopterygii - ps23
Tetrapoda - ps24
Amniota - ps25
Mammalia - ps26
Theria - ps27
Placentalia - ps28
Boreoeutheria - ps29
Euarchontoglires - ps30
Euarchonta - ps31
Primates - ps32
Haplorrhini - ps33
Simiiformes - ps34
Catarrhini - ps35
Hominoidea - ps36
Hominidae - ps37
Homininae - ps38
Hominini - ps39
Homo_sapiens - ps40
Phylostratum

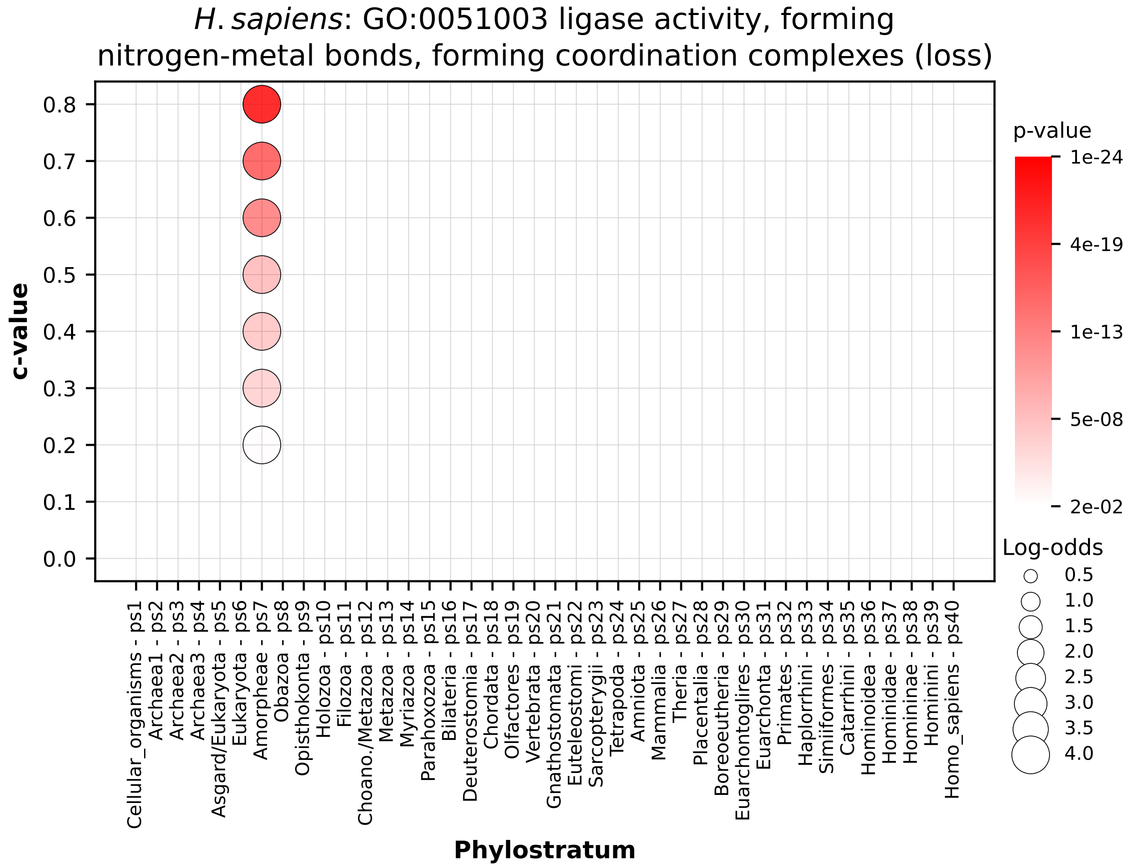

H. sapiens: GO:0051003 ligase activity, forming nitrogen-metal bonds, forming coordination complexes (loss)
c-value
0.8
0.7
0.6
0.5
0.4
0.3
0.2
0.1
0.0
p-value
1e-24
4e-19
1e-13
5e-08
2e-02
Log-odds
0.5
1.0
1.5
2.0
2.5
3.0
3.5
4.0
Cellular_organisms - ps1
Archaea1 - ps2
Archaea2 - ps3
Archaea3 - ps4
Asgard/Eukaryota - ps5
Eukaryota - ps6
Amorpheae - ps7
Obazoa - ps8
Opisthokonta - ps9
Holozoa - ps10
Filozoa - ps11
Choano./Metazoa - ps12
Metazoa - ps13
Myriazoa - ps14
Parahoxozoa - ps15
Bilateria - ps16
Deuterostomia - ps17
Chordata - ps18
Olfactores - ps19
Vertebrata - ps20
Gnathostomata - ps21
Euteleostomi - ps22
Sarcopterygii - ps23
Tetrapoda - ps24
Amniota - ps25
Mammalia - ps26
Theria - ps27
Placentalia - ps28
Boreoeutheria - ps29
Euarchontoglires - ps30
Euarchonta - ps31
Primates - ps32
Haplorrhini - ps33
Simiiformes - ps34
Catarrhini - ps35
Hominoidea - ps36
Hominidae - ps37
Homininae - ps38
Hominini - ps39
Homo_sapiens - ps40
Phylostratum

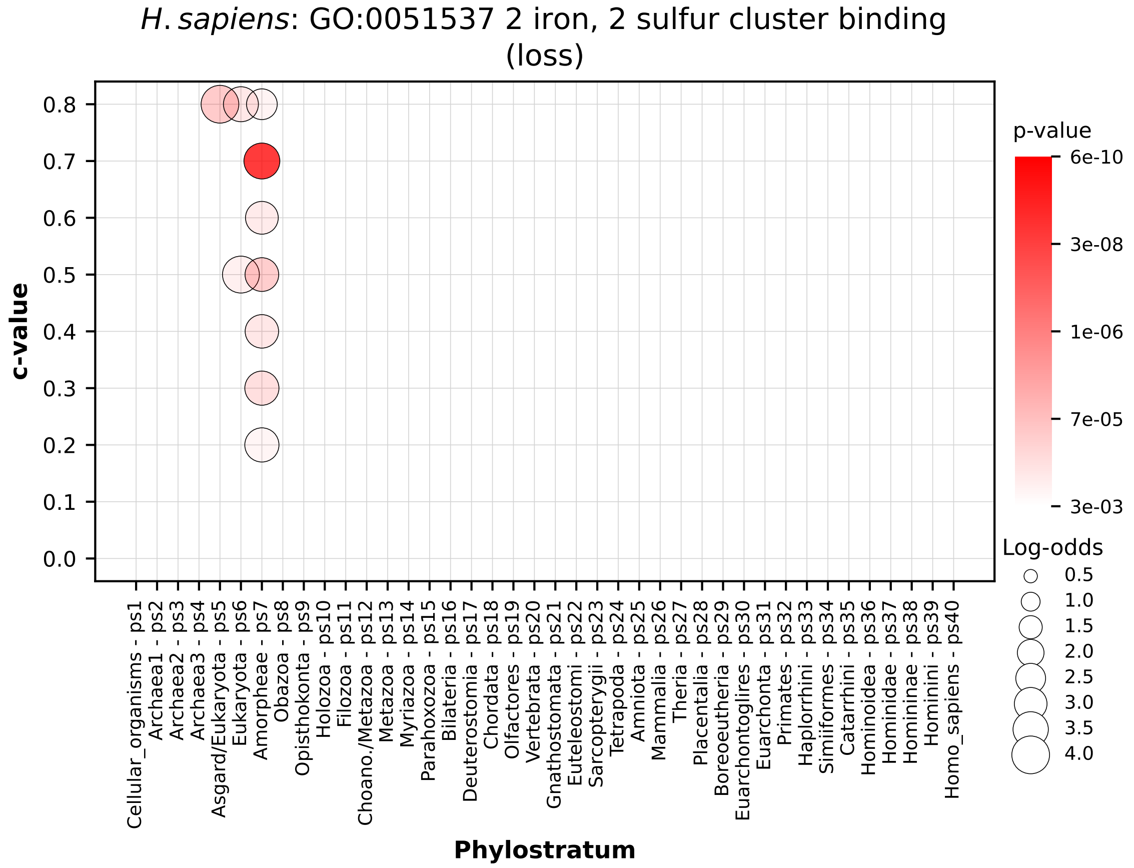

H. sapiens: GO:0051537 2 iron, 2 sulfur cluster binding
(loss)
c-value
0.0
0.1
0.2
0.3
0.4
0.5
0.6
0.7
0.8
p-value
6e-10
3e-08
1e-06
7e-05
3e-03
Log-odds
0.5
1.0
1.5
2.0
2.5
3.0
3.5
4.0
Cellular_organisms - ps1
Archaea1 - ps2
Archaea2 - ps3
Archaea3 - ps4
Asgard/Eukaryota - ps5
Eukaryota - ps6
Amorpheae - ps7
Obazoa - ps8
Opisthokonta - ps9
Holozoa - ps10
Filozoa - ps11
Choano./Metazoa - ps12
Metazoa - ps13
Myriazoa - ps14
Parahoxozoa - ps15
Bilateria - ps16
Deuterostomia - ps17
Chordata - ps18
Olfactores - ps19
Vertebrata - ps20
Gnathostomata - ps21
Euteleostomi - ps22
Sarcopterygii - ps23
Tetrapoda - ps24
Amniota - ps25
Mammalia - ps26
Theria - ps27
Placentalia - ps28
Boreoeutheria - ps29
Euarchontoglires - ps30
Euarchonta - ps31
Primates - ps32
Haplorrhini - ps33
Simiiformes - ps34
Catarrhini - ps35
Hominoidea - ps36
Hominidae - ps37
Homininae - ps38
Hominini - ps39
Homo_sapiens - ps40
Phylostratum

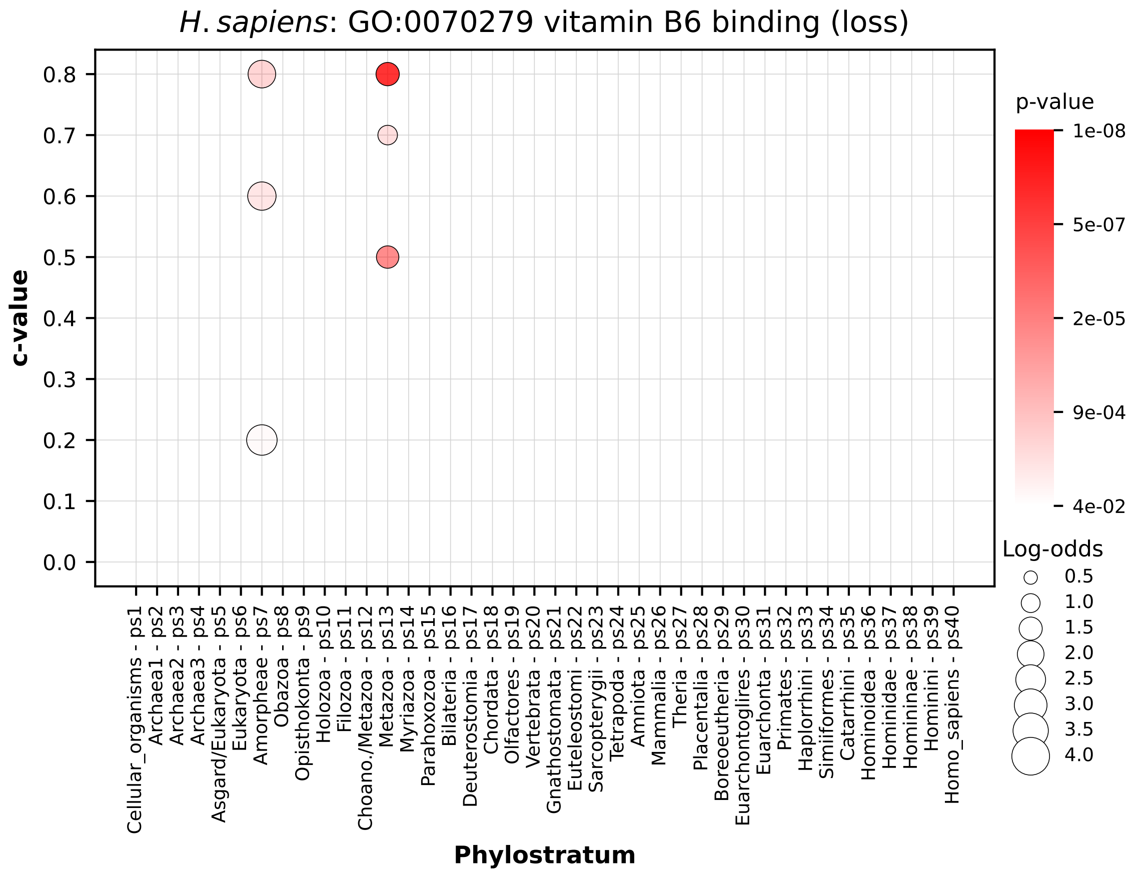

H. sapiens: GO:0070279 vitamin B6 binding (loss)
c-value
0.0
0.1
0.2
0.3
0.4
0.5
0.6
0.7
0.8
Phylostratum
Cellular_organisms - ps1
Archaea1 - ps2
Archaea2 - ps3
Archaea3 - ps4
Asgard/Eukaryota - ps5
Eukaryota - ps6
Amorpheae - ps7
Obazoa - ps8
Opisthokonta - ps9
Holozoa - ps10
Filozoa - ps11
Choano./Metazoa - ps12
Metazoa - ps13
Myriazoa - ps14
Parahoxozoa - ps15
Bilateria - ps16
Deuterostomia - ps17
Chordata - ps18
Olfactores - ps19
Vertebrata - ps20
Gnathostomata - ps21
Euteleostomi - ps22
Sarcopterygii - ps23
Tetrapoda - ps24
Amniota - ps25
Mammalia - ps26
Theria - ps27
Placentalia - ps28
Boreoeutheria - ps29
Euarchontoglires - ps30
Euarchonta - ps31
Primates - ps32
Haplorrhini - ps33
Simiiformes - ps34
Catarrhini - ps35
Hominoidea - ps36
Hominidae - ps37
Homininae - ps38
Hominini - ps39
Homo_sapiens - ps40
p-value
1e-08
5e-07
2e-05
9e-04
4e-02
Log-odds
0.5
1.0
1.5
2.0
2.5
3.0
3.5
4.0

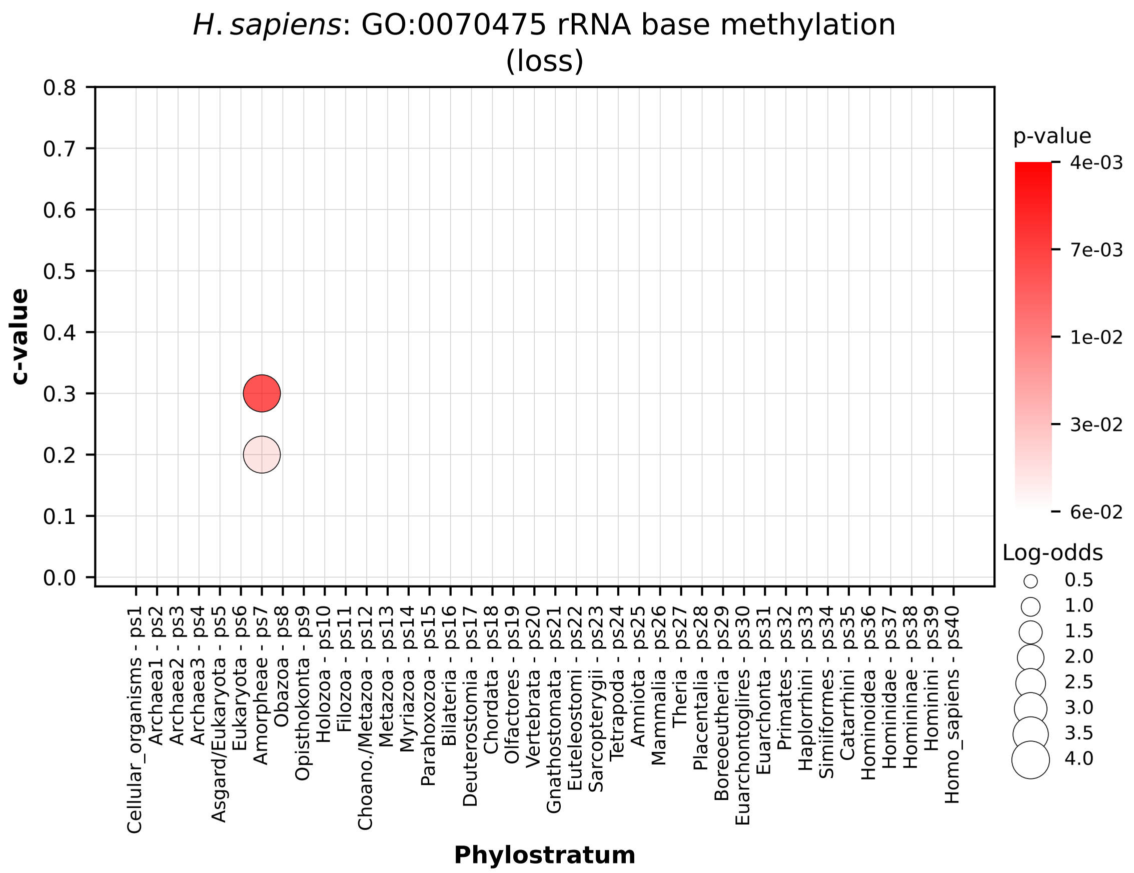

H. sapiens: GO:0070475 rRNA base methylation
(loss)
c-value
0.0
0.1
0.2
0.3
0.4
0.5
0.6
0.7
0.8
Phylostratum
Cellular_organisms - ps1
Archaea1 - ps2
Archaea2 - ps3
Archaea3 - ps4
Asgard/Eukaryota - ps5
Eukaryota - ps6
Amorpheae - ps7
Obazoa - ps8
Opisthokonta - ps9
Holozoa - ps10
Filozoa - ps11
Choano./Metazoa - ps12
Metazoa - ps13
Myriazoa - ps14
Parahoxozoa - ps15
Bilateria - ps16
Deuterostomia - ps17
Chordata - ps18
Olfactores - ps19
Vertebrata - ps20
Gnathostomata - ps21
Euteleostomi - ps22
Sarcopterygii - ps23
Tetrapoda - ps24
Amniota - ps25
Mammalia - ps26
Theria - ps27
Placentalia - ps28
Boreoeutheria - ps29
Euarchontoglires - ps30
Euarchonta - ps31
Primates - ps32
Haplorrhini - ps33
Simiiformes - ps34
Catarrhini - ps35
Hominoidea - ps36
Hominidae - ps37
Homininae - ps38
Hominini - ps39
Homo_sapiens - ps40
p-value
4e-03
7e-03
1e-02
3e-02
6e-02
Log-odds
0.5
1.0
1.5
2.0
2.5
3.0
3.5
4.0

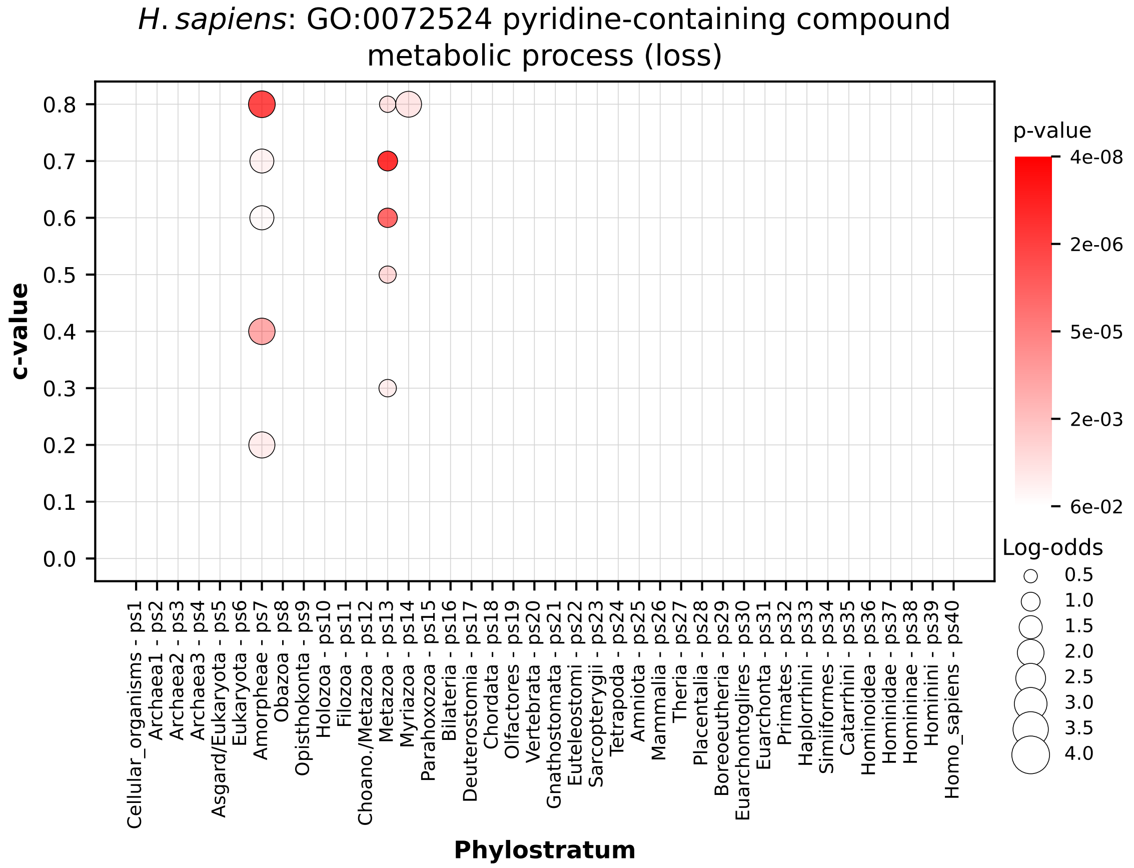
H. sapiens: GO:0072524 pyridine-containing compound metabolic process (loss)
c-value
0.8
0.7
0.6
0.5
0.4
0.3
0.2
0.1
0.0
p-value
4e-08
2e-06
5e-05
2e-03
6e-02
Log-odds
0.5
1.0
1.5
2.0
2.5
3.0
3.5
4.0
Cellular_organisms - ps1
Archaea1 - ps2
Archaea2 - ps3
Archaea3 - ps4
Asgard/Eukaryota - ps5
Eukaryota - ps6
Amorpheae - ps7
Obazoa - ps8
Opisthokonta - ps9
Holozoa - ps10
Filozoa - ps11
Choano./Metazoa - ps12
Metazoa - ps13
Myriazoa - ps14
Parahoxozoa - ps15
Bilateria - ps16
Deuterostomia - ps17
Chordata - ps18
Olfactores - ps19
Vertebrata - ps20
Gnathostomata - ps21
Euteleostomi - ps22
Sarcopterygii - ps23
Tetrapoda - ps24
Amniota - ps25
Mammalia - ps26
Theria - ps27
Placentalia - ps28
Boreoeutheria - ps29
Euarchontoglires - ps30
Euarchonta - ps31
Primates - ps32
Haplorrhini - ps33
Simiiformes - ps34
Catarrhini - ps35
Hominoidea - ps36
Hominidae - ps37
Homininae - ps38
Hominini - ps39
Homo_sapiens - ps40
Phylostratum

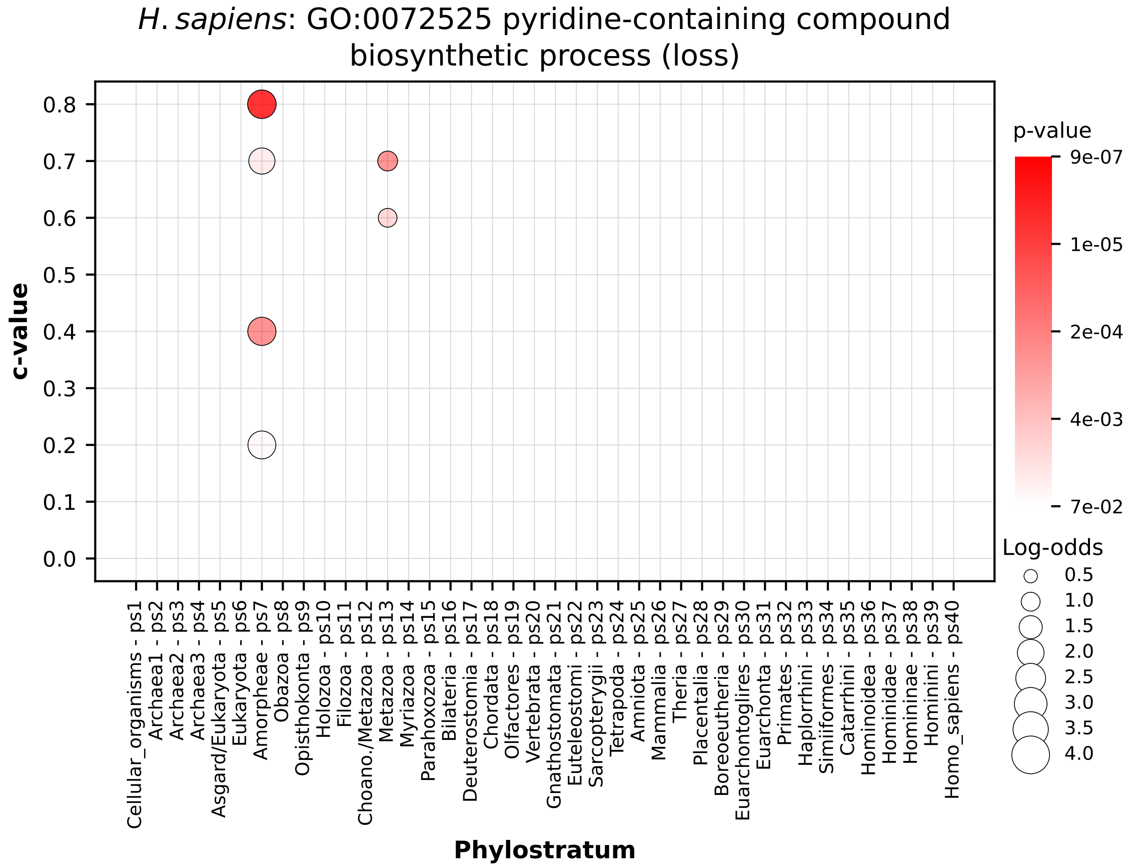

H. sapiens: GO:0072525 pyridine-containing compound biosynthetic process (loss)
c-value
0.0
0.1
0.2
0.3
0.4
0.5
0.6
0.7
0.8
Phylostratum
Cellular_organisms - ps1
Archaea1 - ps2
Archaea2 - ps3
Archaea3 - ps4
Asgard/Eukaryota - ps5
Eukaryota - ps6
Amorpheae - ps7
Obazoa - ps8
Opisthokonta - ps9
Holozoa - ps10
Filozoa - ps11
Choano./Metazoa - ps12
Metazoa - ps13
Myriazoa - ps14
Parahoxozoa - ps15
Bilateria - ps16
Deuterostomia - ps17
Chordata - ps18
Olfactores - ps19
Vertebrata - ps20
Gnathostomata - ps21
Euteleostomi - ps22
Sarcopterygii - ps23
Tetrapoda - ps24
Amniota - ps25
Mammalia - ps26
Theria - ps27
Placentalia - ps28
Boreoeutheria - ps29
Euarchontoglires - ps30
Euarchonta - ps31
Primates - ps32
Haplorrhini - ps33
Simiiformes - ps34
Catarrhini - ps35
Hominoidea - ps36
Hominidae - ps37
Homininae - ps38
Hominini - ps39
Homo_sapiens - ps40
p-value
9e-07
1e-05
2e-04
4e-03
7e-02
Log-odds
0.5
1.0
1.5
2.0
2.5
3.0
3.5
4.0

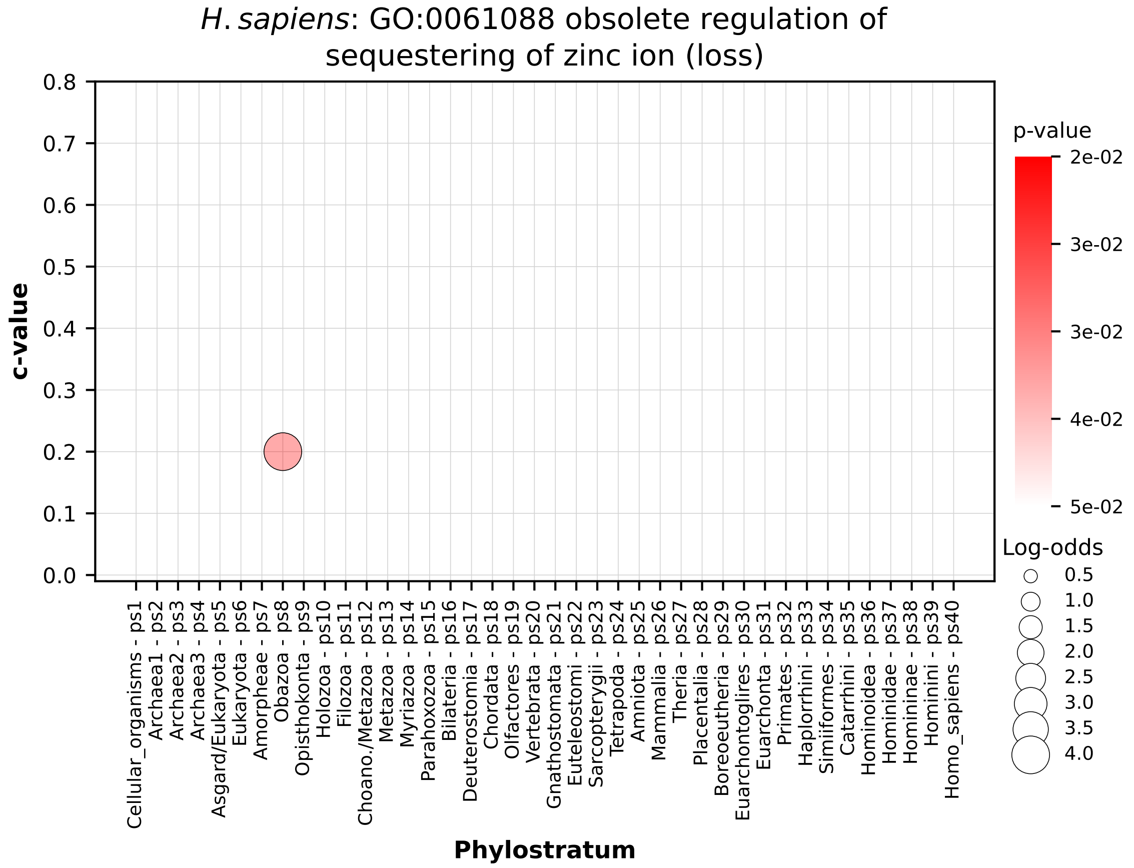

H. sapiens: GO:0061088 obsolete regulation of sequestering of zinc ion (loss)
c-value
0.0
0.1
0.2
0.3
0.4
0.5
0.6
0.7
0.8
Phylostratum
Cellular_organisms - ps1
Archaea1 - ps2
Archaea2 - ps3
Archaea3 - ps4
Asgard/Eukaryota - ps5
Eukaryota - ps6
Amorpheae - ps7
Obazoa - ps8
Opisthokonta - ps9
Holozoa - ps10
Filozoa - ps11
Choano./Metazoa - ps12
Metazoa - ps13
Myriazoa - ps14
Parahoxozoa - ps15
Bilateria - ps16
Deuterostomia - ps17
Chordata - ps18
Olfactores - ps19
Vertebrata - ps20
Gnathostomata - ps21
Euteleostomi - ps22
Sarcopterygii - ps23
Tetrapoda - ps24
Amniota - ps25
Mammalia - ps26
Theria - ps27
Placentalia - ps28
Boreoeutheria - ps29
Euarchontoglires - ps30
Euarchonta - ps31
Primates - ps32
Haplorrhini - ps33
Simiiformes - ps34
Catarrhini - ps35
Hominoidea - ps36
Hominidae - ps37
Homininae - ps38
Hominini - ps39
Homo_sapiens - ps40
p-value
2e-02
3e-02
3e-02
4e-02
5e-02
Log-odds
0.5
1.0
1.5
2.0
2.5
3.0
3.5
4.0

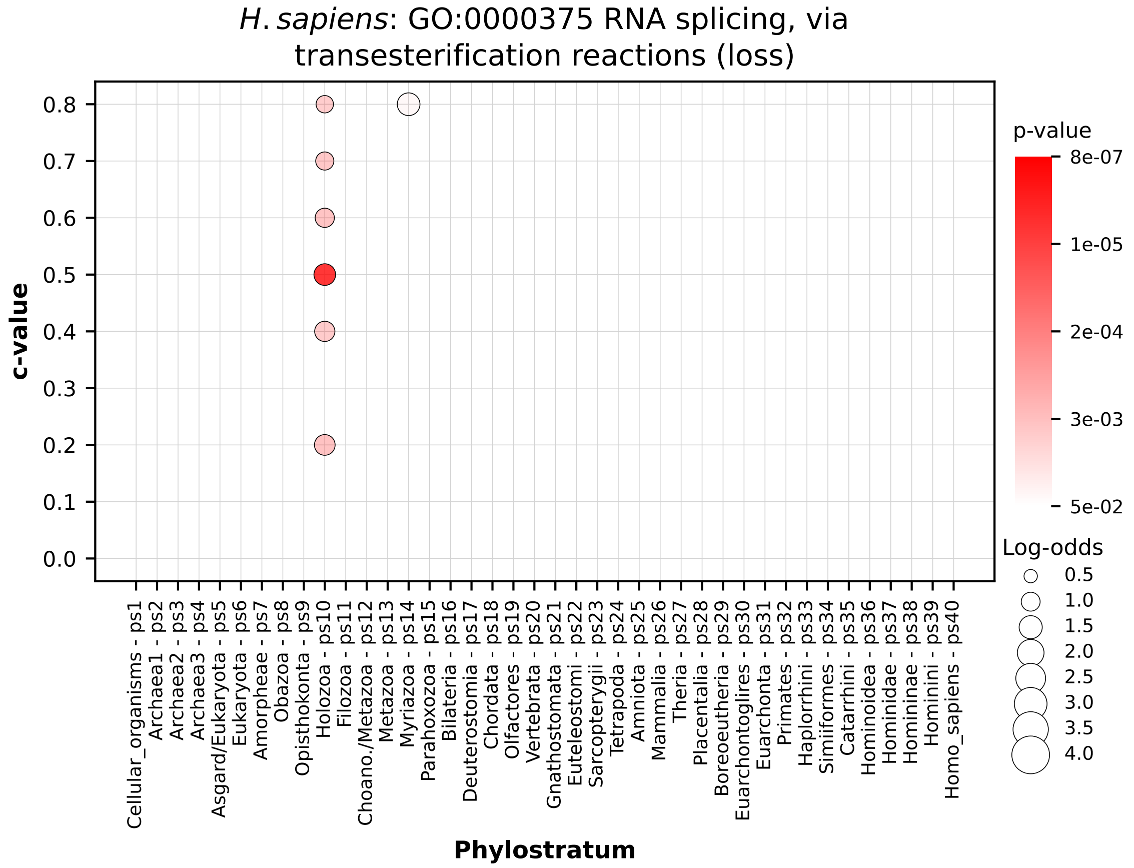

H. sapiens: GO:0000375 RNA splicing, via transesterification reactions (loss)
c-value
0.0
0.1
0.2
0.3
0.4
0.5
0.6
0.7
0.8
Phylostratum
Cellular_organisms - ps1
Archaea1 - ps2
Archaea2 - ps3
Archaea3 - ps4
Asgard/Eukaryota - ps5
Eukaryota - ps6
Amorpheae - ps7
Obazoa - ps8
Opisthokonta - ps9
Holozoa - ps10
Filozoa - ps11
Choano./Metazoa - ps12
Metazoa - ps13
Myriazoa - ps14
Parahoxozoa - ps15
Bilateria - ps16
Deuterostomia - ps17
Chordata - ps18
Olfactores - ps19
Vertebrata - ps20
Gnathostomata - ps21
Euteleostomi - ps22
Sarcopterygii - ps23
Tetrapoda - ps24
Amniota - ps25
Mammalia - ps26
Theria - ps27
Placentalia - ps28
Boreoeutheria - ps29
Euarchontoglires - ps30
Euarchonta - ps31
Primates - ps32
Haplorrhini - ps33
Simiiformes - ps34
Catarrhini - ps35
Hominoidea - ps36
Hominidae - ps37
Homininae - ps38
Hominini - ps39
Homo_sapiens - ps40
p-value
8e-07
1e-05
2e-04
3e-03
5e-02
Log-odds
0.5
1.0
1.5
2.0
2.5
3.0
3.5
4.0

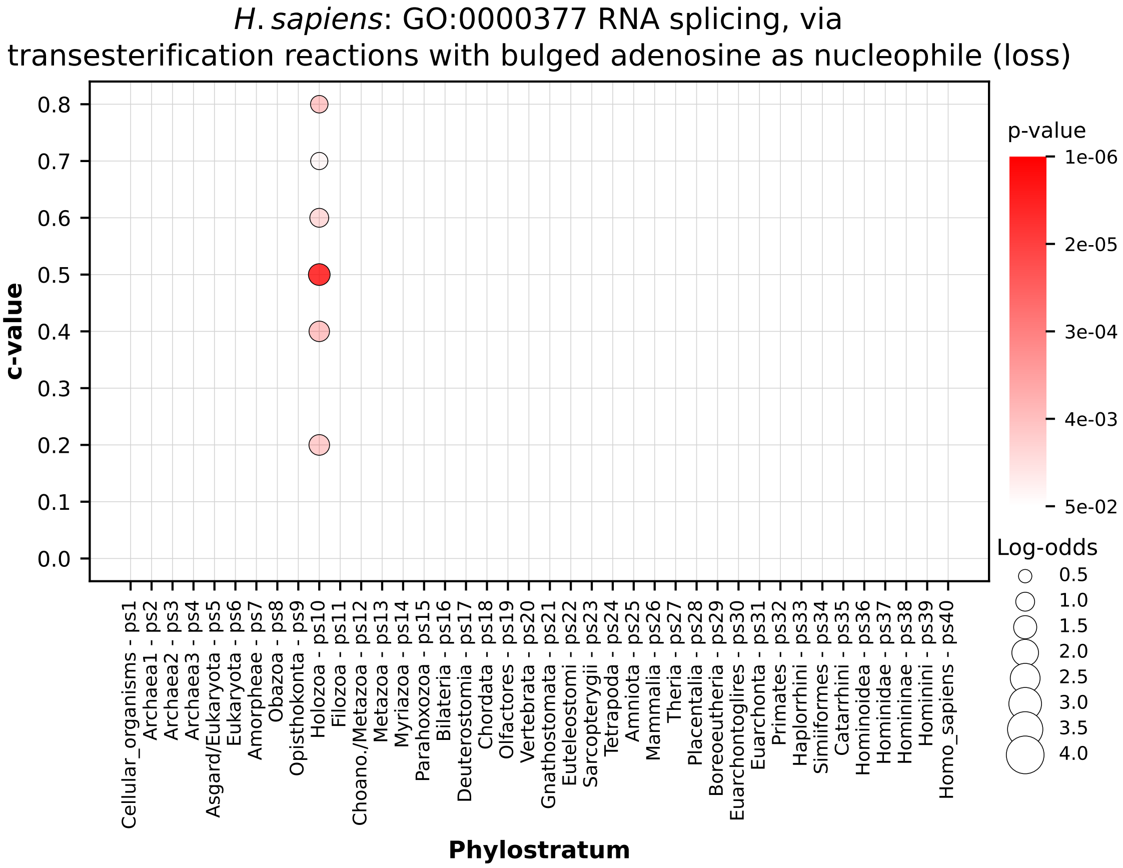

H. sapiens: GO:0000377 RNA splicing, via transesterification reactions with bulged adenosine as nucleophile (loss)
c-value
0.8
0.7
0.6
0.5
0.4
0.3
0.2
0.1
0.0
p-value
1e-06
2e-05
3e-04
4e-03
5e-02
Log-odds
0.5
1.0
1.5
2.0
2.5
3.0
3.5
4.0
Cellular_organisms - ps1
Archaea1 - ps2
Archaea2 - ps3
Archaea3 - ps4
Asgard/Eukaryota - ps5
Eukaryota - ps6
Amorpheae - ps7
Obazoa - ps8
Opisthokonta - ps9
Holozoa - ps10
Filozoa - ps11
Choano./Metazoa - ps12
Metazoa - ps13
Myriazoa - ps14
Parahoxozoa - ps15
Bilateria - ps16
Deuterostomia - ps17
Chordata - ps18
Olfactores - ps19
Vertebrata - ps20
Gnathostomata - ps21
Euteleostomi - ps22
Sarcopterygii - ps23
Tetrapoda - ps24
Amniota - ps25
Mammalia - ps26
Theria - ps27
Placentalia - ps28
Boreoeutheria - ps29
Euarchontoglires - ps30
Euarchonta - ps31
Primates - ps32
Haplorrhini - ps33
Simiiformes - ps34
Catarrhini - ps35
Hominoidea - ps36
Hominidae - ps37
Homininae - ps38
Hominini - ps39
Homo_sapiens - ps40
Phylostratum

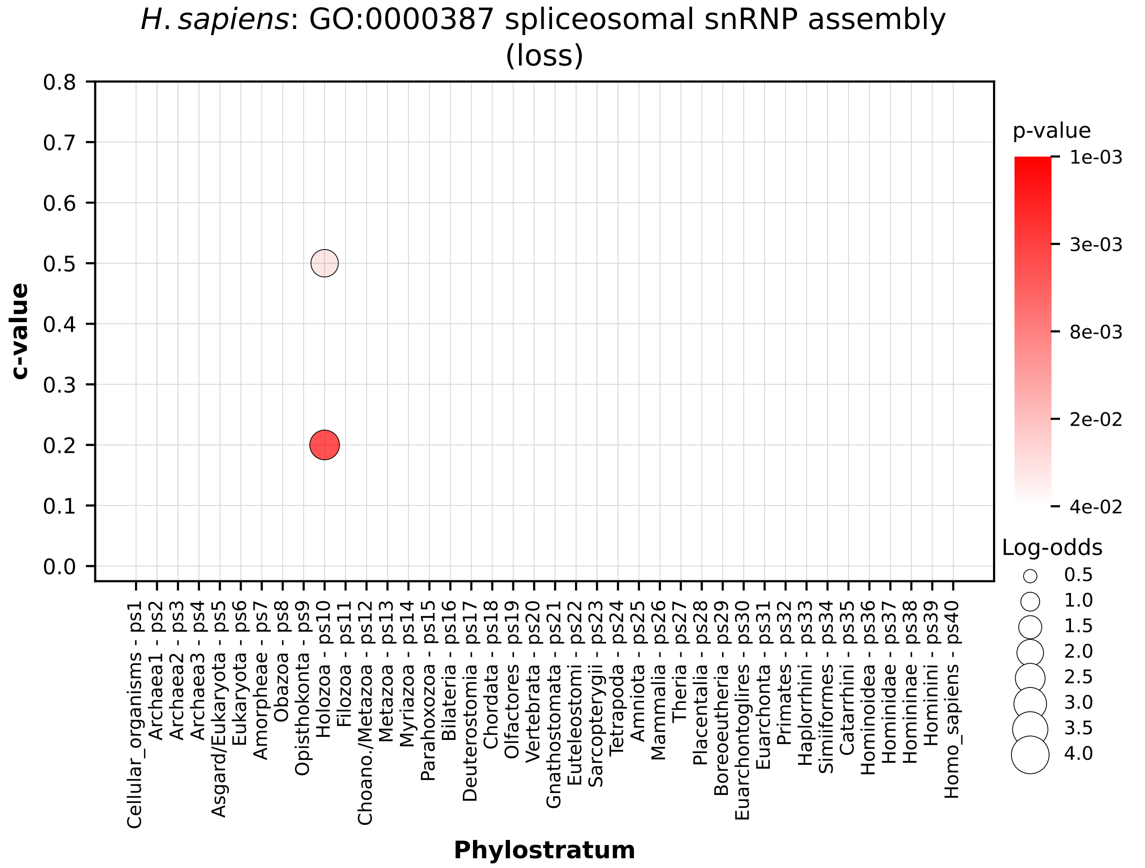

H. sapiens: GO:0000387 spliceosomal snRNP assembly (loss)
c-value
0.8
0.7
0.6
0.5
0.4
0.3
0.2
0.1
0.0
p-value
1e-03
3e-03
8e-03
2e-02
4e-02
Log-odds
0.5
1.0
1.5
2.0
2.5
3.0
3.5
4.0
Cellular_organisms - ps1
Archaea1 - ps2
Archaea2 - ps3
Archaea3 - ps4
Asgard/Eukaryota - ps5
Eukaryota - ps6
Amorpheae - ps7
Obazoa - ps8
Opisthokonta - ps9
Holozoa - ps10
Filozoa - ps11
Choano./Metazoa - ps12
Metazoa - ps13
Myriazoa - ps14
Parahoxozoa - ps15
Bilateria - ps16
Deuterostomia - ps17
Chordata - ps18
Olfactores - ps19
Vertebrata - ps20
Gnathostomata - ps21
Euteleostomi - ps22
Sarcopterygii - ps23
Tetrapoda - ps24
Amniota - ps25
Mammalia - ps26
Theria - ps27
Placentalia - ps28
Boreoeutheria - ps29
Euarchontoglires - ps30
Euarchonta - ps31
Primates - ps32
Haplorrhini - ps33
Simiiformes - ps34
Catarrhini - ps35
Hominoidea - ps36
Hominidae - ps37
Homininae - ps38
Hominini - ps39
Homo_sapiens - ps40
Phylostratum

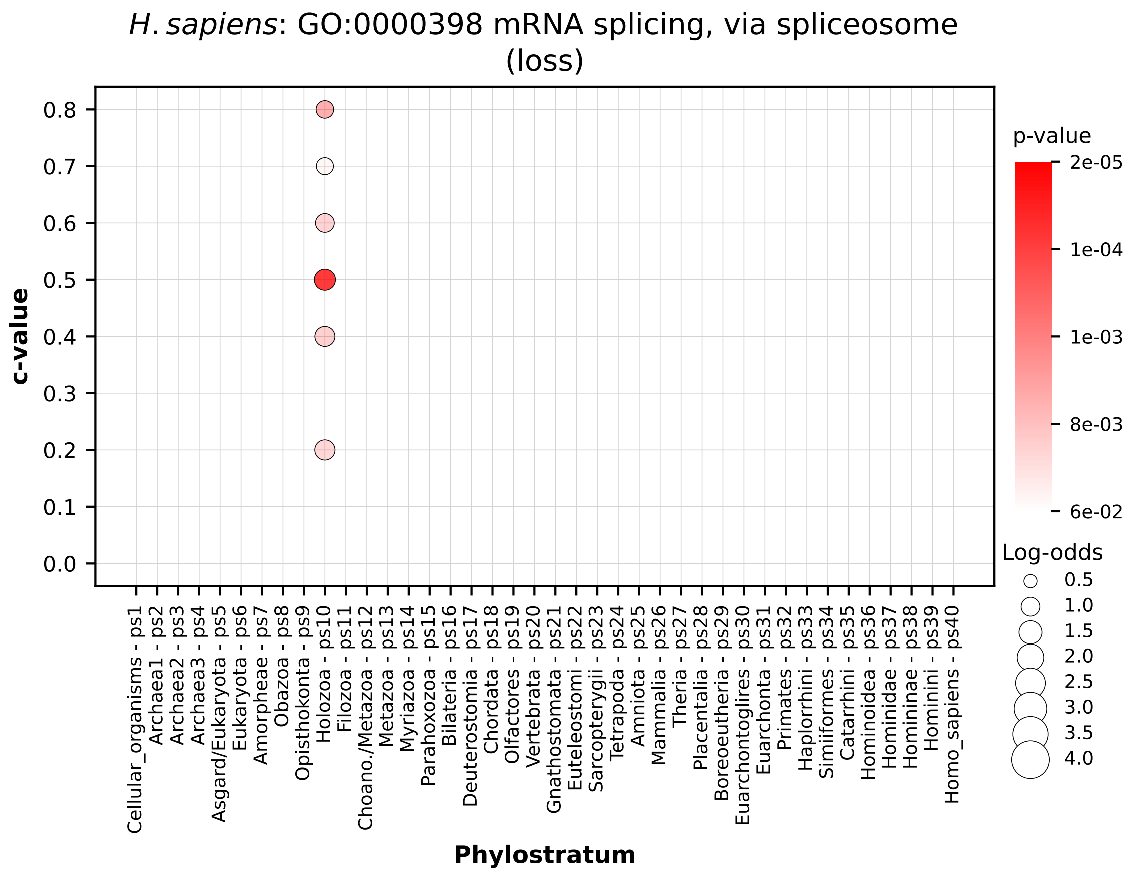

H. sapiens: GO:0000398 mRNA splicing, via spliceosome
(loss)
c-value
0.0
0.1
0.2
0.3
0.4
0.5
0.6
0.7
0.8
Phylostratum
Cellular_organisms - ps1
Archaea1 - ps2
Archaea2 - ps3
Archaea3 - ps4
Asgard/Eukaryota - ps5
Eukaryota - ps6
Amorpheae - ps7
Obazoa - ps8
Opisthokonta - ps9
Holozoa - ps10
Filozoa - ps11
Choano./Metazoa - ps12
Metazoa - ps13
Myriazoa - ps14
Parahoxozoa - ps15
Bilateria - ps16
Deuterostomia - ps17
Chordata - ps18
Olfactores - ps19
Vertebrata - ps20
Gnathostomata - ps21
Euteleostomi - ps22
Sarcopterygii - ps23
Tetrapoda - ps24
Amniota - ps25
Mammalia - ps26
Theria - ps27
Placentalia - ps28
Boreoeutheria - ps29
Euarchontoglires - ps30
Euarchonta - ps31
Primates - ps32
Haplorrhini - ps33
Simiiformes - ps34
Catarrhini - ps35
Hominoidea - ps36
Hominidae - ps37
Homininae - ps38
Hominini - ps39
Homo_sapiens - ps40
p-value
2e-05
1e-04
1e-03
8e-03
6e-02
Log-odds
0.5
1.0
1.5
2.0
2.5
3.0
3.5
4.0

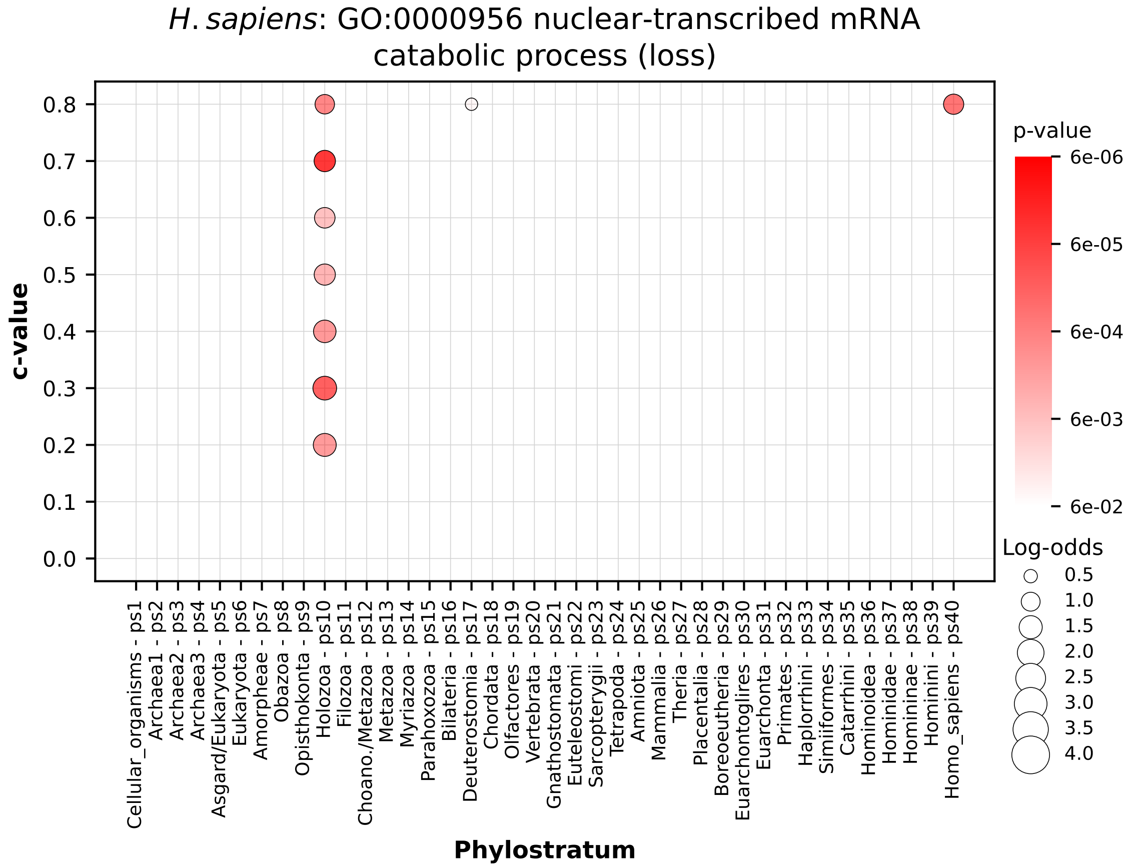

H. sapiens: GO:0000956 nuclear-transcribed mRNA catabolic process (loss)
c-value
0.0
0.1
0.2
0.3
0.4
0.5
0.6
0.7
0.8
p-value
6e-06
6e-05
6e-04
6e-03
6e-02
Log-odds
0.5
1.0
1.5
2.0
2.5
3.0
3.5
4.0
Cellular_organisms - ps1
Archaea1 - ps2
Archaea2 - ps3
Archaea3 - ps4
Asgard/Eukaryota - ps5
Eukaryota - ps6
Amorpheae - ps7
Obazoa - ps8
Opisthokonta - ps9
Holozoa - ps10
Filozoa - ps11
Choano./Metazoa - ps12
Metazoa - ps13
Myriazoa - ps14
Parahoxozoa - ps15
Bilateria - ps16
Deuterostomia - ps17
Chordata - ps18
Olfactores - ps19
Vertebrata - ps20
Gnathostomata - ps21
Euteleostomi - ps22
Sarcopterygii - ps23
Tetrapoda - ps24
Amniota - ps25
Mammalia - ps26
Theria - ps27
Placentalia - ps28
Boreoeutheria - ps29
Euarchontoglires - ps30
Euarchonta - ps31
Primates - ps32
Haplorrhini - ps33
Simiiformes - ps34
Catarrhini - ps35
Hominoidea - ps36
Hominidae - ps37
Homininae - ps38
Hominini - ps39
Homo_sapiens - ps40
Phylostratum

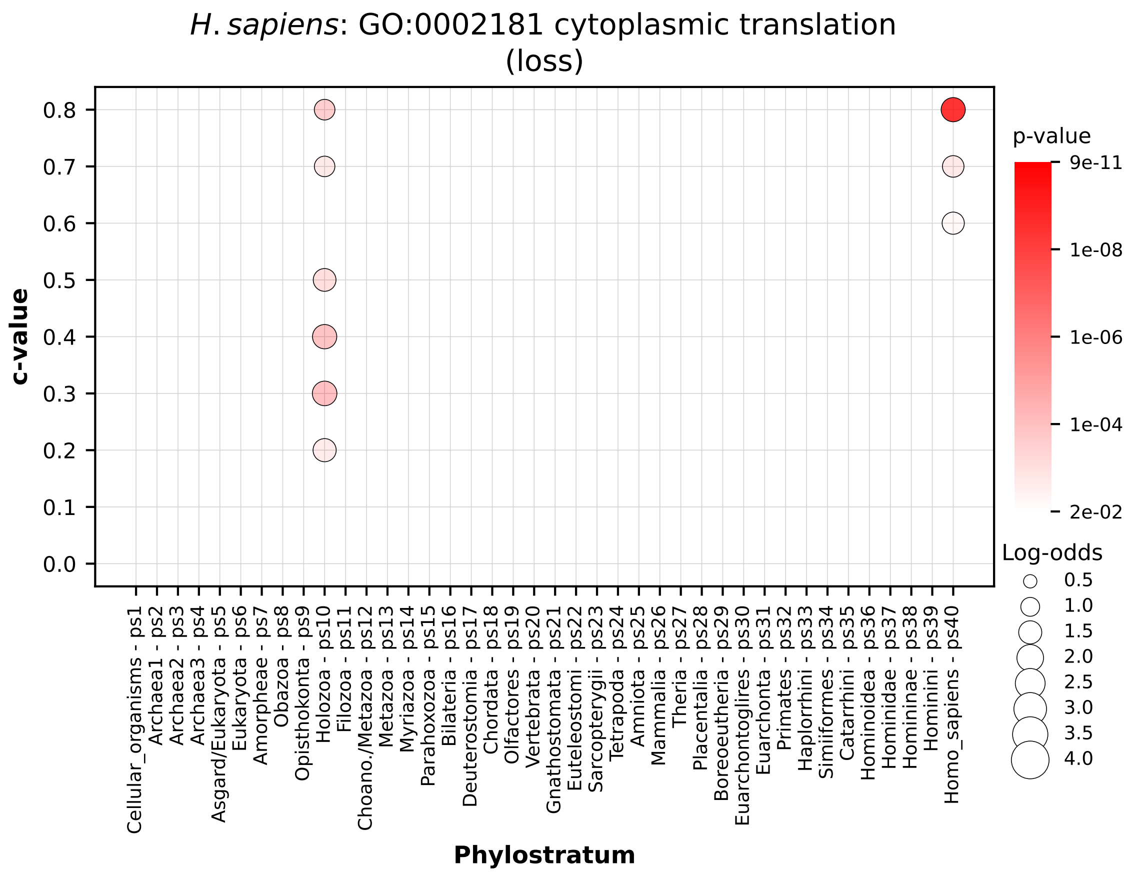
H. sapiens: GO:0002181 cytoplasmic translation
(loss)
c-value
0.8
0.7
0.6
0.5
0.4
0.3
0.2
0.1
0.0
Phylostratum
Cellular_organisms - ps1
Archaea1 - ps2
Archaea2 - ps3
Archaea3 - ps4
Asgard/Eukaryota - ps5
Eukaryota - ps6
Amorpheae - ps7
Obazoa - ps8
Opisthokonta - ps9
Holozoa - ps10
Filozoa - ps11
Choano./Metazoa - ps12
Metazoa - ps13
Myriazoa - ps14
Parahoxozoa - ps15
Bilateria - ps16
Deuterostomia - ps17
Chordata - ps18
Olfactores - ps19
Vertebrata - ps20
Gnathostomata - ps21
Euteleostomi - ps22
Sarcopterygii - ps23
Tetrapoda - ps24
Amniota - ps25
Mammalia - ps26
Theria - ps27
Placentalia - ps28
Boreoeutheria - ps29
Euarchontoglires - ps30
Euarchonta - ps31
Primates - ps32
Haplorrhini - ps33
Simiiformes - ps34
Catarrhini - ps35
Hominoidea - ps36
Hominidae - ps37
Homininae - ps38
Hominini - ps39
Homo_sapiens - ps40
p-value
9e-11
1e-08
1e-06
1e-04
2e-02
Log-odds
0.5
1.0
1.5
2.0
2.5
3.0
3.5
4.0

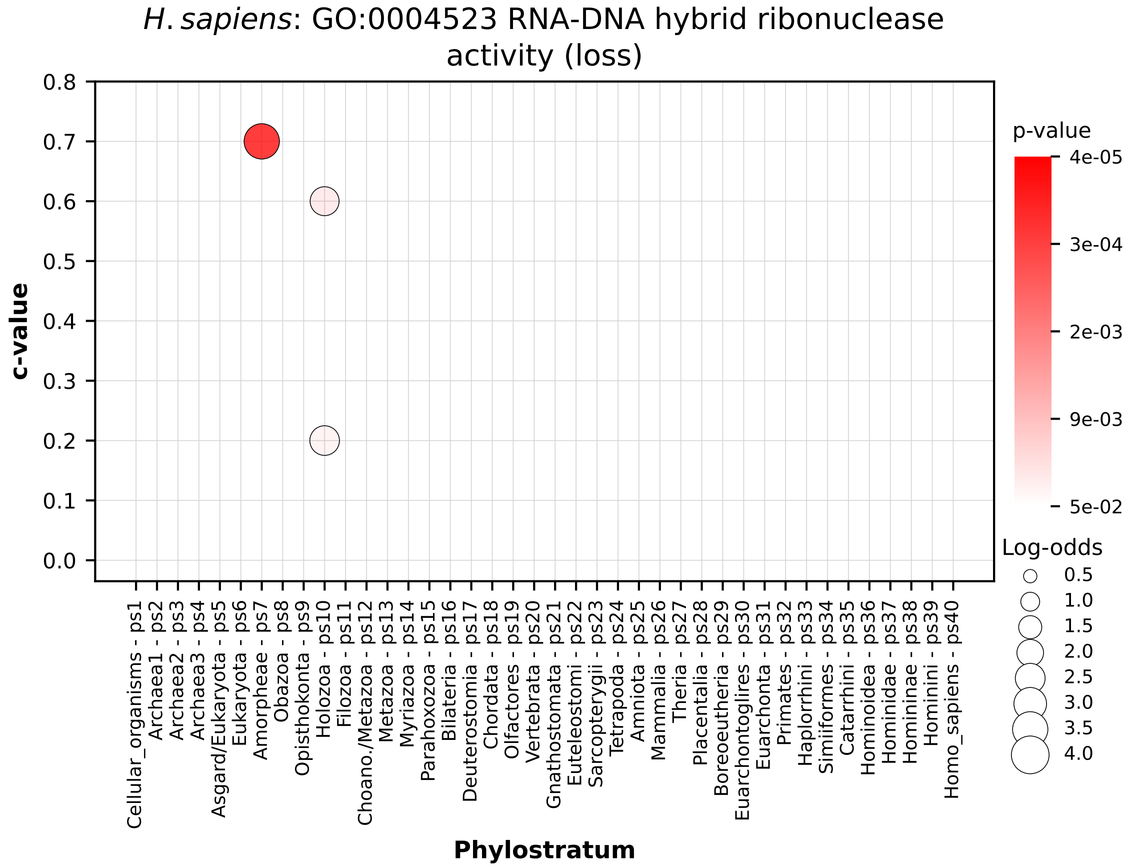

H. sapiens: GO:0004523 RNA-DNA hybrid ribonuclease activity (loss)
c-value
0.8
0.7
0.6
0.5
0.4
0.3
0.2
0.1
0.0
Phylostratum
Cellular_organisms - ps1
Archaea1 - ps2
Archaea2 - ps3
Archaea3 - ps4
Asgard/Eukaryota - ps5
Eukaryota - ps6
Amorpheae - ps7
Obazoa - ps8
Opisthokonta - ps9
Holozoa - ps10
Filozoa - ps11
Choano./Metazoa - ps12
Metazoa - ps13
Myriazoa - ps14
Parahoxozoa - ps15
Bilateria - ps16
Deuterostomia - ps17
Chordata - ps18
Olfactores - ps19
Vertebrata - ps20
Gnathostomata - ps21
Euteleostomi - ps22
Sarcopterygii - ps23
Tetrapoda - ps24
Amniota - ps25
Mammalia - ps26
Theria - ps27
Placentalia - ps28
Boreoeutheria - ps29
Euarchontoglires - ps30
Euarchonta - ps31
Primates - ps32
Haplorrhini - ps33
Simiiformes - ps34
Catarrhini - ps35
Hominoidea - ps36
Hominidae - ps37
Homininae - ps38
Hominini - ps39
Homo_sapiens - ps40
p-value
4e-05
3e-04
2e-03
9e-03
5e-02
Log-odds
0.5
1.0
1.5
2.0
2.5
3.0
3.5
4.0

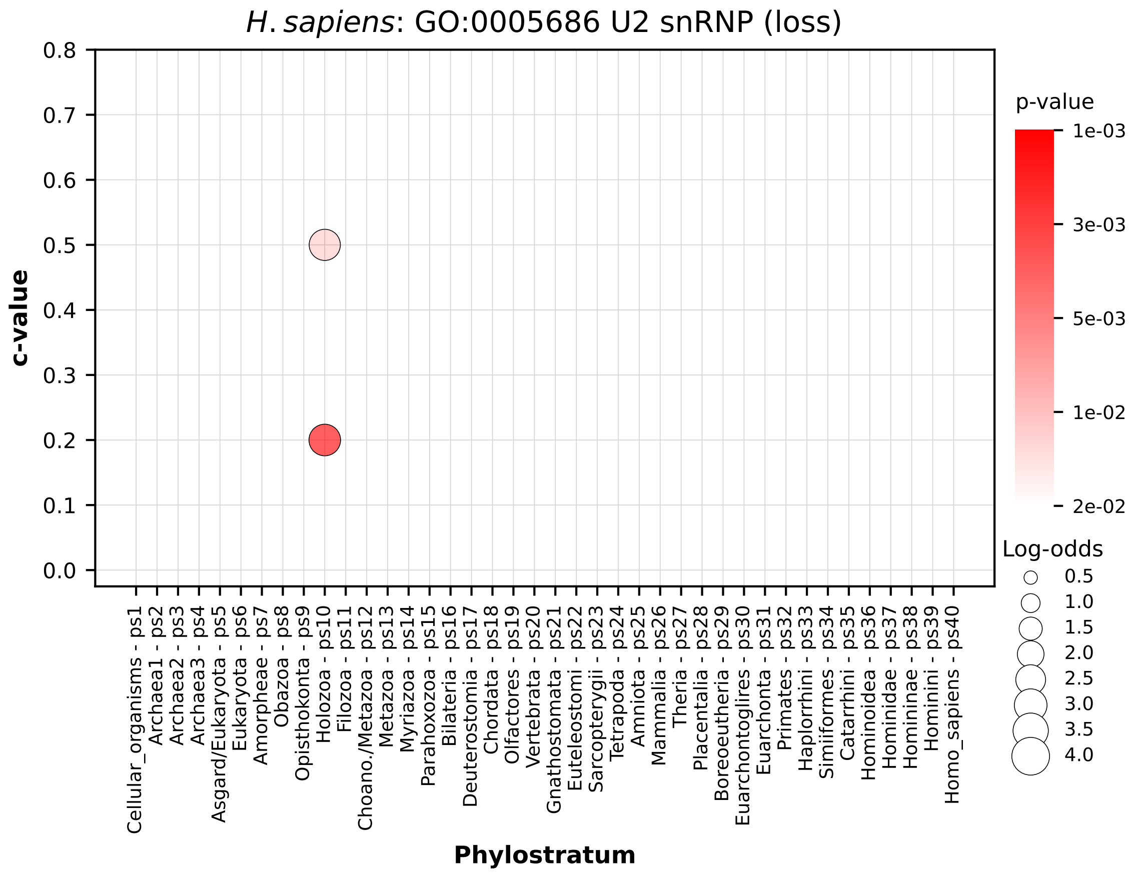
H. sapiens: GO:0005686 U2 snRNP (loss)
c-value
0.0
0.1
0.2
0.3
0.4
0.5
0.6
0.7
0.8
Phylostratum
Cellular_organisms - ps1
Archaea1 - ps2
Archaea2 - ps3
Archaea3 - ps4
Asgard/Eukaryota - ps5
Eukaryota - ps6
Amorpheae - ps7
Obazoa - ps8
Opisthokonta - ps9
Holozoa - ps10
Filozoa - ps11
Choano./Metazoa - ps12
Metazoa - ps13
Myriazoa - ps14
Parahoxozoa - ps15
Bilateria - ps16
Deuterostomia - ps17
Chordata - ps18
Olfactores - ps19
Vertebrata - ps20
Gnathostomata - ps21
Euteleostomi - ps22
Sarcopterygii - ps23
Tetrapoda - ps24
Amniota - ps25
Mammalia - ps26
Theria - ps27
Placentalia - ps28
Boreoeutheria - ps29
Euarchontoglires - ps30
Euarchonta - ps31
Primates - ps32
Haplorrhini - ps33
Simiiformes - ps34
Catarrhini - ps35
Hominoidea - ps36
Hominidae - ps37
Homininae - ps38
Hominini - ps39
Homo_sapiens - ps40
p-value
1e-03
3e-03
5e-03
1e-02
2e-02
Log-odds
0.5
1.0
1.5
2.0
2.5
3.0
3.5
4.0

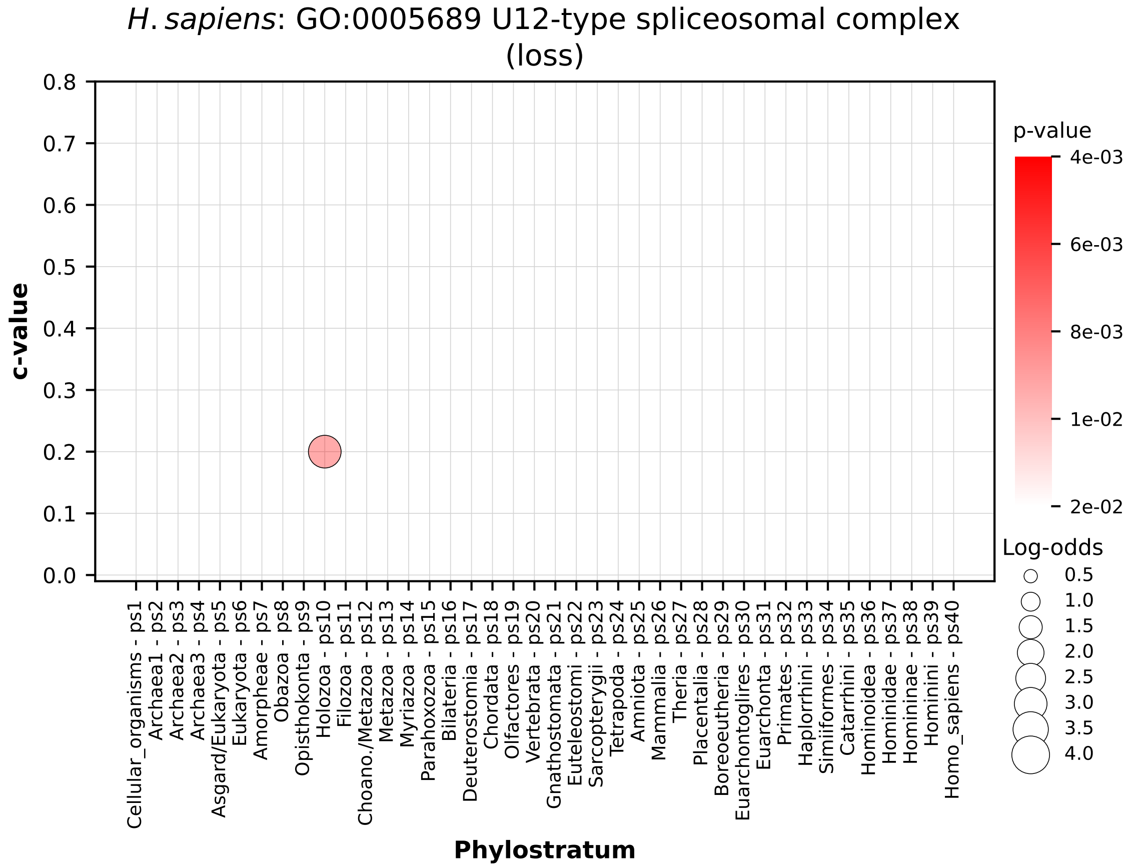

H. sapiens: GO:0005689 U12-type spliceosomal complex
(loss)
c-value
0.8
0.7
0.6
0.5
0.4
0.3
0.2
0.1
0.0
Phylostratum
Cellular_organisms - ps1
Archaea1 - ps2
Archaea2 - ps3
Archaea3 - ps4
Asgard/Eukaryota - ps5
Eukaryota - ps6
Amorpheae - ps7
Obazoa - ps8
Opisthokonta - ps9
Holozoa - ps10
Filozoa - ps11
Choano./Metazoa - ps12
Metazoa - ps13
Myriazoa - ps14
Parahoxozoa - ps15
Bilateria - ps16
Deuterostomia - ps17
Chordata - ps18
Olfactores - ps19
Vertebrata - ps20
Gnathostomata - ps21
Euteleostomi - ps22
Sarcopterygii - ps23
Tetrapoda - ps24
Amniota - ps25
Mammalia - ps26
Theria - ps27
Placentalia - ps28
Boreoeutheria - ps29
Euarchontoglires - ps30
Euarchonta - ps31
Primates - ps32
Haplorrhini - ps33
Simiiformes - ps34
Catarrhini - ps35
Hominoidea - ps36
Hominidae - ps37
Homininae - ps38
Hominini - ps39
Homo_sapiens - ps40
p-value
4e-03
6e-03
8e-03
1e-02
2e-02
Log-odds
0.5
1.0
1.5
2.0
2.5
3.0
3.5
4.0

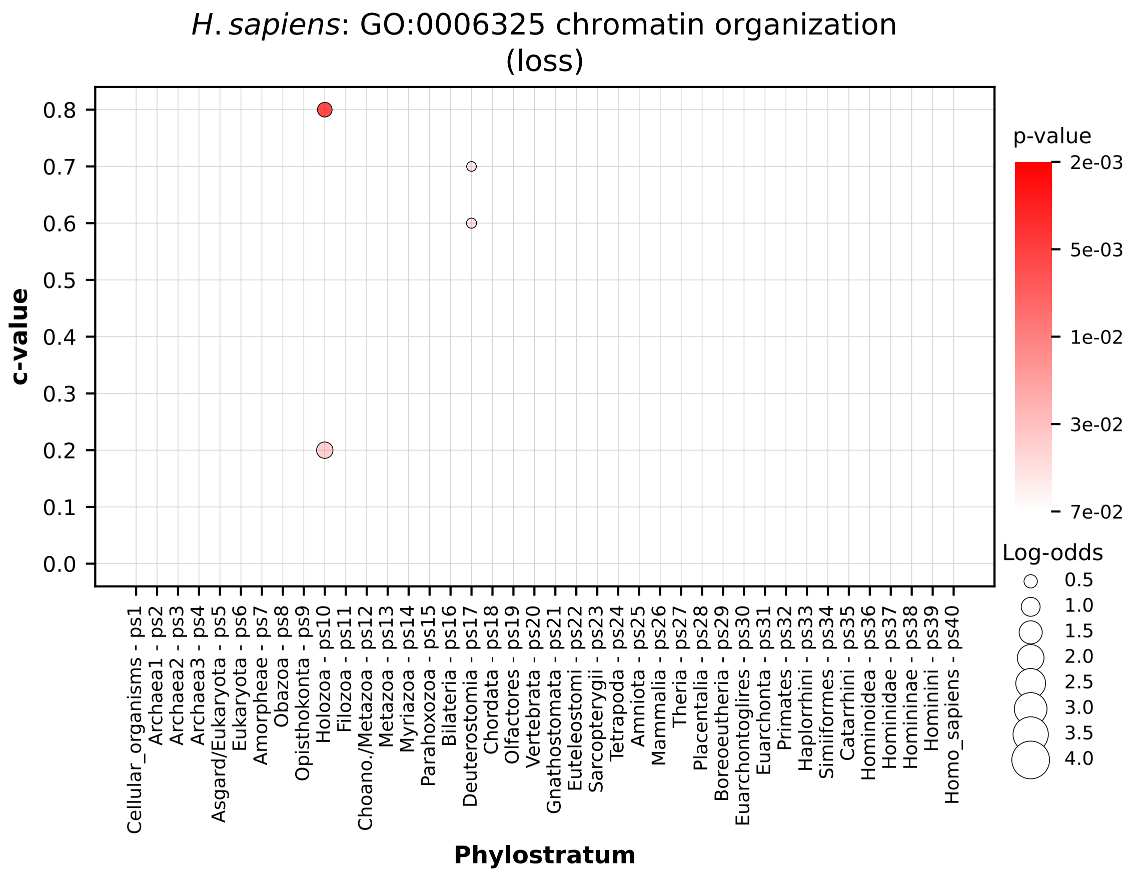

H. sapiens: GO:0006325 chromatin organization
(loss)
c-value
0.0
0.1
0.2
0.3
0.4
0.5
0.6
0.7
0.8
Phylostratum
Cellular_organisms - ps1
Archaea1 - ps2
Archaea2 - ps3
Archaea3 - ps4
Asgard/Eukaryota - ps5
Eukaryota - ps6
Amorpheae - ps7
Obazoa - ps8
Opisthokonta - ps9
Holozoa - ps10
Filozoa - ps11
Choano./Metazoa - ps12
Metazoa - ps13
Myriazoa - ps14
Parahoxozoa - ps15
Bilateria - ps16
Deuterostomia - ps17
Chordata - ps18
Olfactores - ps19
Vertebrata - ps20
Gnathostomata - ps21
Euteleostomi - ps22
Sarcopterygii - ps23
Tetrapoda - ps24
Amniota - ps25
Mammalia - ps26
Theria - ps27
Placentalia - ps28
Boreoeutheria - ps29
Euarchontoglires - ps30
Euarchonta - ps31
Primates - ps32
Haplorrhini - ps33
Simiiformes - ps34
Catarrhini - ps35
Hominoidea - ps36
Hominidae - ps37
Homininae - ps38
Hominini - ps39
Homo_sapiens - ps40
p-value
2e-03
5e-03
1e-02
3e-02
7e-02
Log-odds
0.5
1.0
1.5
2.0
2.5
3.0
3.5
4.0

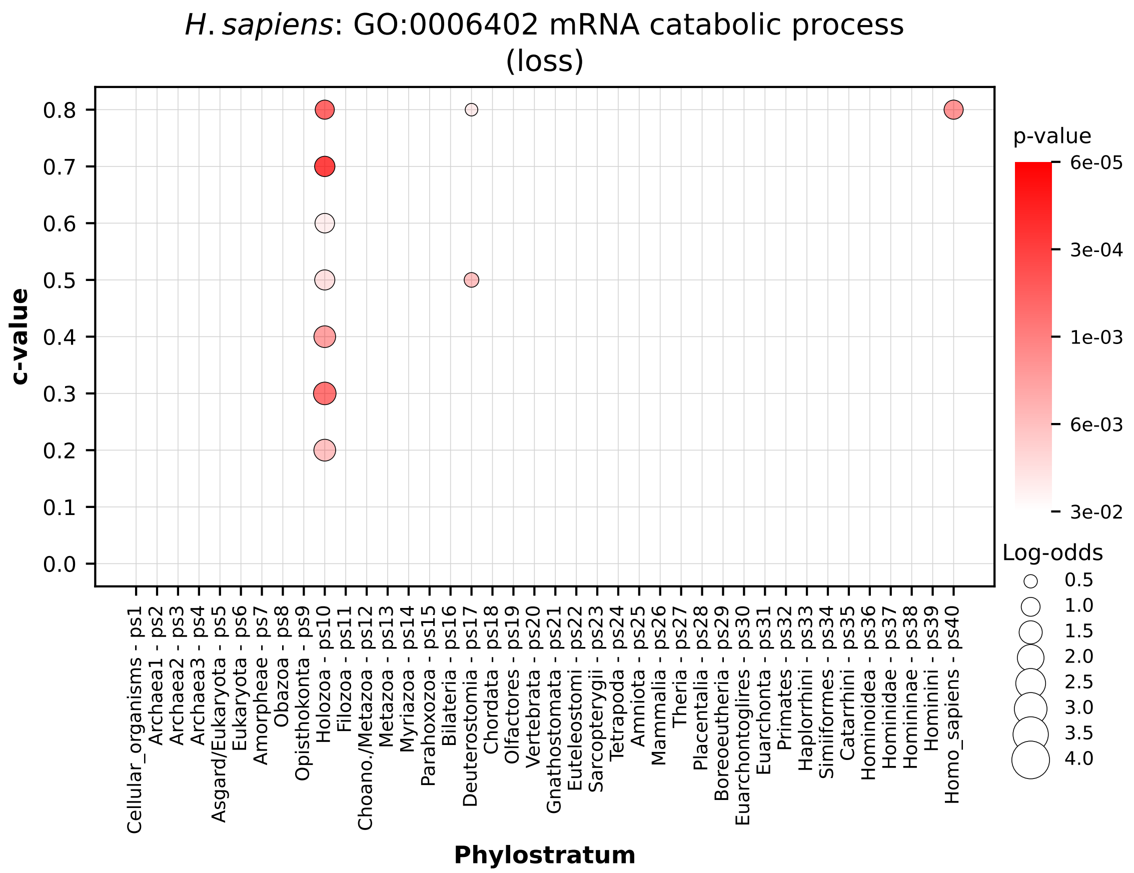

H. sapiens: GO:0006402 mRNA catabolic process
(loss)
c-value
0.8
0.7
0.6
0.5
0.4
0.3
0.2
0.1
0.0
p-value
6e-05
3e-04
1e-03
6e-03
3e-02
Log-odds
0.5
1.0
1.5
2.0
2.5
3.0
3.5
4.0
Cellular_organisms - ps1
Archaea1 - ps2
Archaea2 - ps3
Archaea3 - ps4
Asgard/Eukaryota - ps5
Eukaryota - ps6
Amorpheae - ps7
Obazoa - ps8
Opisthokonta - ps9
Holozoa - ps10
Filozoa - ps11
Choano./Metazoa - ps12
Metazoa - ps13
Myriazoa - ps14
Parahoxozoa - ps15
Bilateria - ps16
Deuterostomia - ps17
Chordata - ps18
Olfactores - ps19
Vertebrata - ps20
Gnathostomata - ps21
Euteleostomi - ps22
Sarcopterygii - ps23
Tetrapoda - ps24
Amniota - ps25
Mammalia - ps26
Theria - ps27
Placentalia - ps28
Boreoeutheria - ps29
Euarchontoglires - ps30
Euarchonta - ps31
Primates - ps32
Haplorrhini - ps33
Simiiformes - ps34
Catarrhini - ps35
Hominoidea - ps36
Hominidae - ps37
Homininae - ps38
Hominini - ps39
Homo_sapiens - ps40
Phylostratum

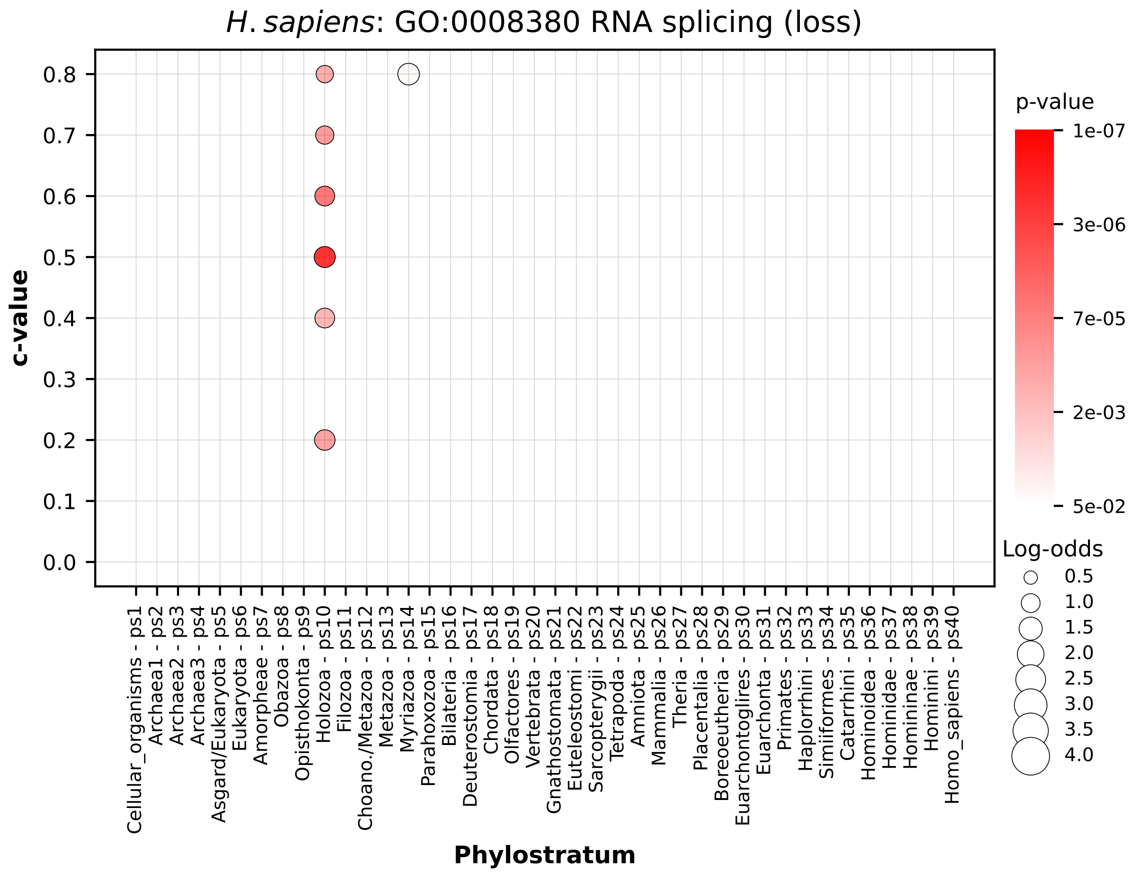

H. sapiens: GO:0008380 RNA splicing (loss)
c-value
0.0
0.1
0.2
0.3
0.4
0.5
0.6
0.7
0.8
Phylostratum
Cellular_organisms - ps1
Archaea1 - ps2
Archaea2 - ps3
Archaea3 - ps4
Asgard/Eukaryota - ps5
Eukaryota - ps6
Amorpheae - ps7
Obazoa - ps8
Opisthokonta - ps9
Holozoa - ps10
Filozoa - ps11
Choano./Metazoa - ps12
Metazoa - ps13
Myriazoa - ps14
Parahoxozoa - ps15
Bilateria - ps16
Deuterostomia - ps17
Chordata - ps18
Olfactores - ps19
Vertebrata - ps20
Gnathostomata - ps21
Euteleostomi - ps22
Sarcopterygii - ps23
Tetrapoda - ps24
Amniota - ps25
Mammalia - ps26
Theria - ps27
Placentalia - ps28
Boreoeutheria - ps29
Euarchontoglires - ps30
Euarchonta - ps31
Primates - ps32
Haplorrhini - ps33
Simiiformes - ps34
Catarrhini - ps35
Hominoidea - ps36
Hominidae - ps37
Homininae - ps38
Hominini - ps39
Homo_sapiens - ps40
p-value
1e-07
3e-06
7e-05
2e-03
5e-02
Log-odds
0.5
1.0
1.5
2.0
2.5
3.0
3.5
4.0

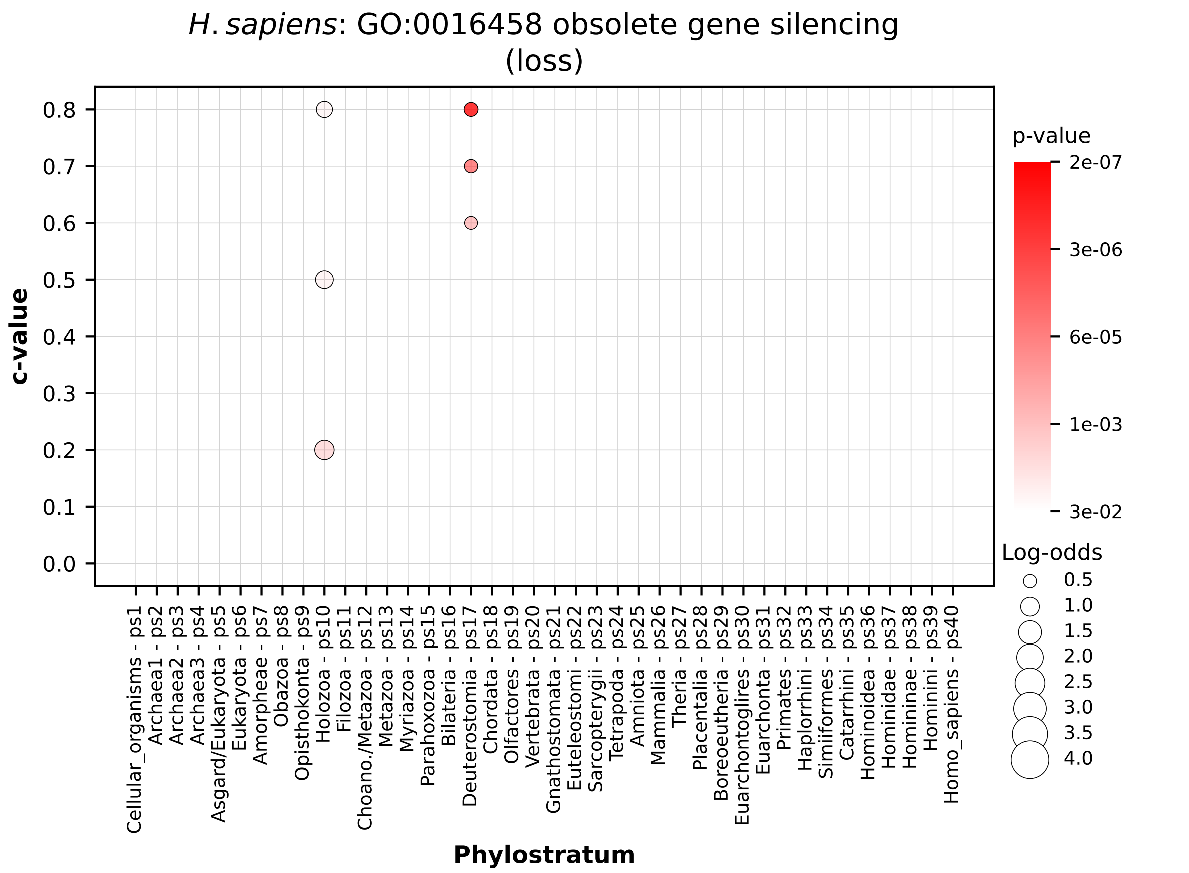

H. sapiens: GO:0016458 obsolete gene silencing (loss)
c-value
0.8
0.7
0.6
0.5
0.4
0.3
0.2
0.1
0.0
p-value
2e-07
3e-06
6e-05
1e-03
3e-02
Log-odds
0.5
1.0
1.5
2.0
2.5
3.0
3.5
4.0
Cellular_organisms - ps1
Archaea1 - ps2
Archaea2 - ps3
Archaea3 - ps4
Asgard/Eukaryota - ps5
Eukaryota - ps6
Amorpheae - ps7
Obazoa - ps8
Opisthokonta - ps9
Holozoa - ps10
Filozoa - ps11
Choano./Metazoa - ps12
Metazoa - ps13
Myriazoa - ps14
Parahoxozoa - ps15
Bilateria - ps16
Deuterostomia - ps17
Chordata - ps18
Olfactores - ps19
Vertebrata - ps20
Gnathostomata - ps21
Euteleostomi - ps22
Sarcopterygii - ps23
Tetrapoda - ps24
Amniota - ps25
Mammalia - ps26
Theria - ps27
Placentalia - ps28
Boreoeutheria - ps29
Euarchontoglires - ps30
Euarchonta - ps31
Primates - ps32
Haplorrhini - ps33
Simiiformes - ps34
Catarrhini - ps35
Hominoidea - ps36
Hominidae - ps37
Homininae - ps38
Hominini - ps39
Homo_sapiens - ps40
Phylostratum

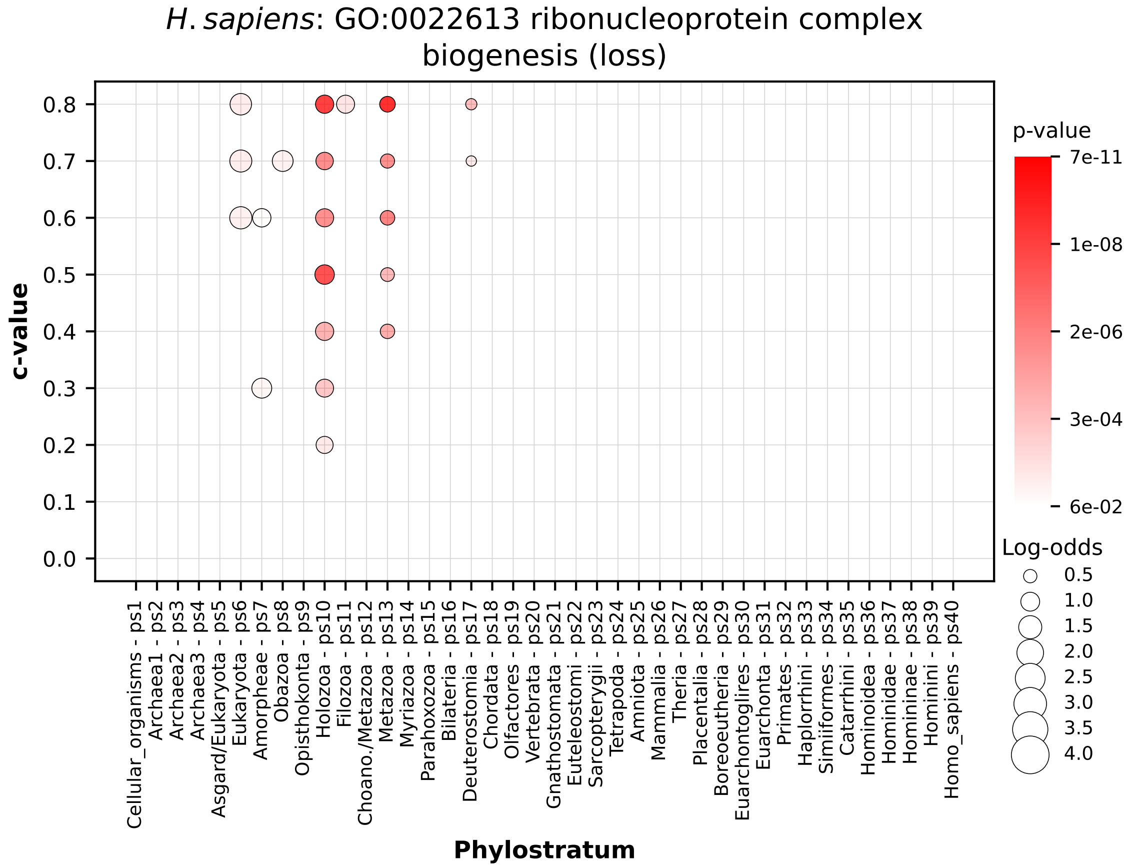

H. sapiens: GO:0022613 ribonucleoprotein complex biogenesis (loss)
c-value
0.8
0.7
0.6
0.5
0.4
0.3
0.2
0.1
0.0
p-value
7e-11
1e-08
2e-06
3e-04
6e-02
Log-odds
0.5
1.0
1.5
2.0
2.5
3.0
3.5
4.0
Cellular_organisms - ps1
Archaea1 - ps2
Archaea2 - ps3
Archaea3 - ps4
Asgard/Eukaryota - ps5
Eukaryota - ps6
Amorpheae - ps7
Obazoa - ps8
Opisthokonta - ps9
Holozoa - ps10
Filozoa - ps11
Choano./Metazoa - ps12
Metazoa - ps13
Myriazoa - ps14
Parahoxozoa - ps15
Bilateria - ps16
Deuterostomia - ps17
Chordata - ps18
Olfactores - ps19
Vertebrata - ps20
Gnathostomata - ps21
Euteleostomi - ps22
Sarcopterygii - ps23
Tetrapoda - ps24
Amniota - ps25
Mammalia - ps26
Theria - ps27
Placentalia - ps28
Boreoeutheria - ps29
Euarchontoglires - ps30
Euarchonta - ps31
Primates - ps32
Haplorrhini - ps33
Simiiformes - ps34
Catarrhini - ps35
Hominoidea - ps36
Hominidae - ps37
Homininae - ps38
Hominini - ps39
Homo_sapiens - ps40
Phylostratum

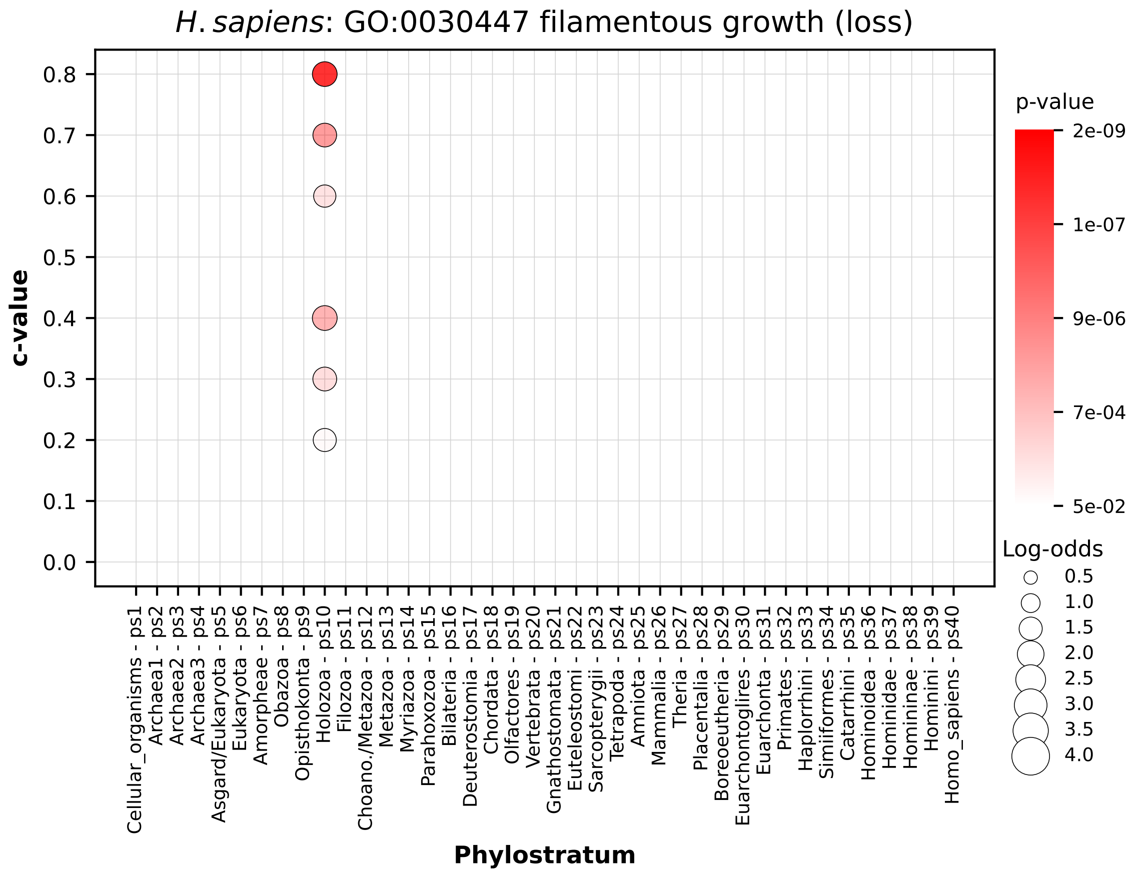

H. sapiens: GO:0030447 filamentous growth (loss)
c-value
0.8
0.7
0.6
0.5
0.4
0.3
0.2
0.1
0.0
Phylostratum
Cellular_organisms - ps1
Archaea1 - ps2
Archaea2 - ps3
Archaea3 - ps4
Asgard/Eukaryota - ps5
Eukaryota - ps6
Amorpheae - ps7
Obazoa - ps8
Opisthokonta - ps9
Holozoa - ps10
Filozoa - ps11
Choano./Metazoa - ps12
Metazoa - ps13
Myriazoa - ps14
Parahoxozoa - ps15
Bilateria - ps16
Deuterostomia - ps17
Chordata - ps18
Olfactores - ps19
Vertebrata - ps20
Gnathostomata - ps21
Euteleostomi - ps22
Sarcopterygii - ps23
Tetrapoda - ps24
Amniota - ps25
Mammalia - ps26
Theria - ps27
Placentalia - ps28
Boreoeutheria - ps29
Euarchontoglires - ps30
Euarchonta - ps31
Primates - ps32
Haplorrhini - ps33
Simiiformes - ps34
Catarrhini - ps35
Hominoidea - ps36
Hominidae - ps37
Homininae - ps38
Hominini - ps39
Homo_sapiens - ps40
p-value
2e-09
1e-07
9e-06
7e-04
5e-02
Log-odds
0.5
1.0
1.5
2.0
2.5
3.0
3.5
4.0

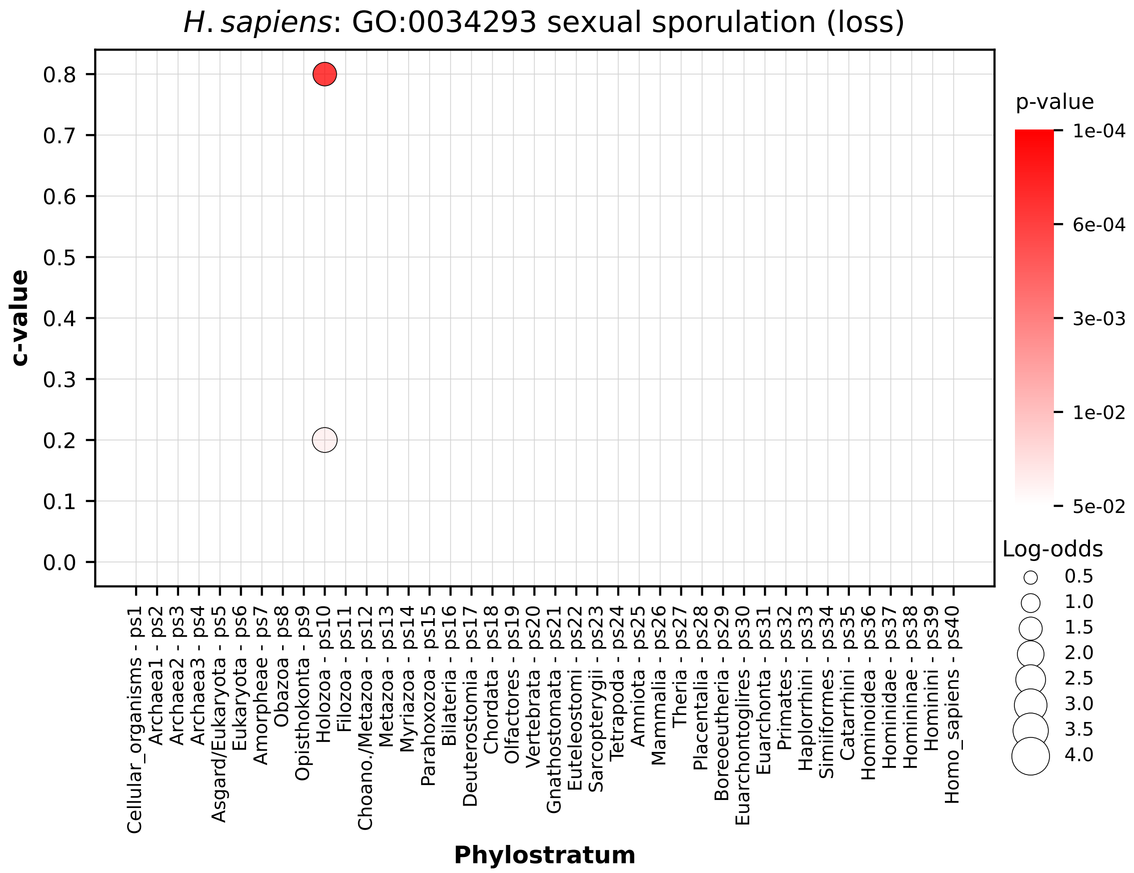

H. sapiens: GO:0034293 sexual sporulation (loss)
c-value
0.8
0.7
0.6
0.5
0.4
0.3
0.2
0.1
0.0
p-value
1e-04
6e-04
3e-03
1e-02
5e-02
Log-odds
0.5
1.0
1.5
2.0
2.5
3.0
3.5
4.0
Cellular_organisms - ps1
Archaea1 - ps2
Archaea2 - ps3
Archaea3 - ps4
Asgard/Eukaryota - ps5
Eukaryota - ps6
Amorpheae - ps7
Obazoa - ps8
Opisthokonta - ps9
Holozoa - ps10
Filozoa - ps11
Choano./Metazoa - ps12
Metazoa - ps13
Myriazoa - ps14
Parahoxozoa - ps15
Bilateria - ps16
Deuterostomia - ps17
Chordata - ps18
Olfactores - ps19
Vertebrata - ps20
Gnathostomata - ps21
Euteleostomi - ps22
Sarcopterygii - ps23
Tetrapoda - ps24
Amniota - ps25
Mammalia - ps26
Theria - ps27
Placentalia - ps28
Boreoeutheria - ps29
Euarchontoglires - ps30
Euarchonta - ps31
Primates - ps32
Haplorrhini - ps33
Simiiformes - ps34
Catarrhini - ps35
Hominoidea - ps36
Hominidae - ps37
Homininae - ps38
Hominini - ps39
Homo_sapiens - ps40
Phylostratum

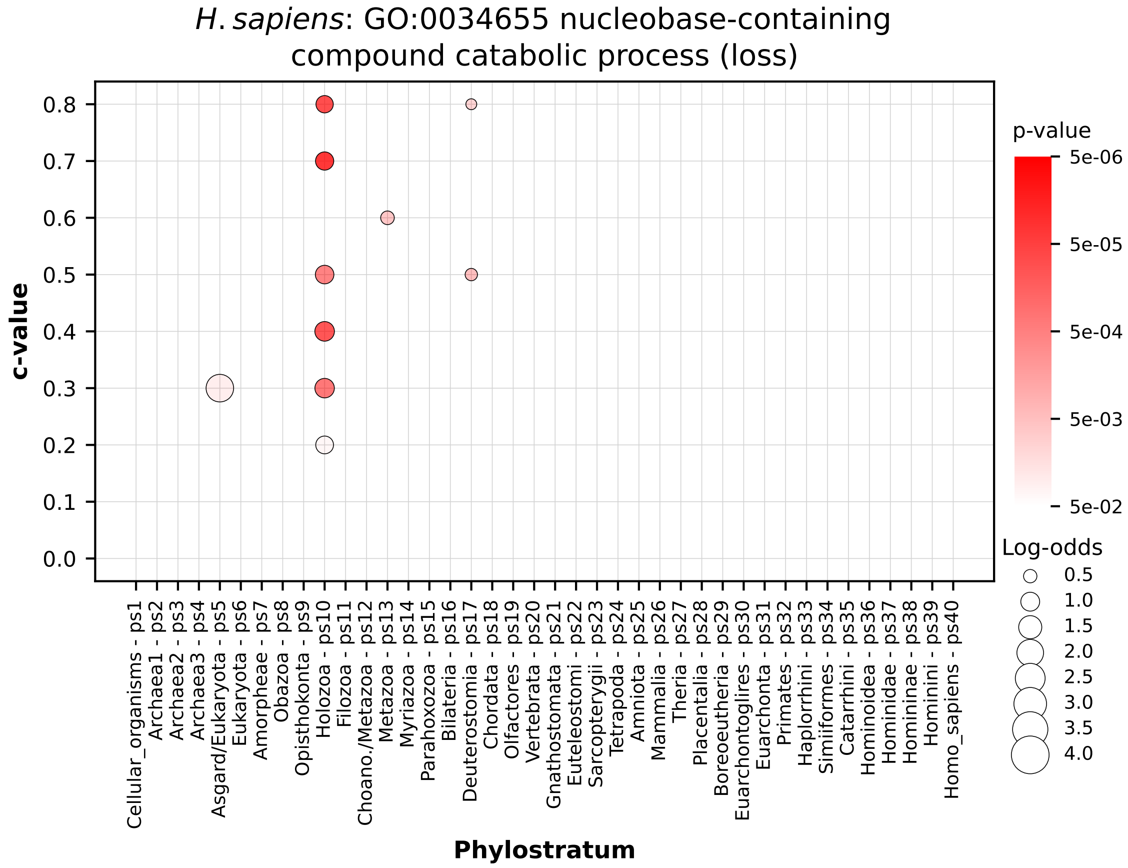

H. sapiens: GO:0034655 nucleobase-containing compound catabolic process (loss)
c-value
0.0
0.1
0.2
0.3
0.4
0.5
0.6
0.7
0.8
Phylostratum
Cellular_organisms - ps1
Archaea1 - ps2
Archaea2 - ps3
Archaea3 - ps4
Asgard/Eukaryota - ps5
Eukaryota - ps6
Amorpheae - ps7
Obazoa - ps8
Opisthokonta - ps9
Holozoa - ps10
Filozoa - ps11
Choano./Metazoa - ps12
Metazoa - ps13
Myriazoa - ps14
Parahoxozoa - ps15
Bilateria - ps16
Deuterostomia - ps17
Chordata - ps18
Olfactores - ps19
Vertebrata - ps20
Gnathostomata - ps21
Euteleostomi - ps22
Sarcopterygii - ps23
Tetrapoda - ps24
Amniota - ps25
Mammalia - ps26
Theria - ps27
Placentalia - ps28
Boreoeutheria - ps29
Euarchontoglires - ps30
Euarchonta - ps31
Primates - ps32
Haplorrhini - ps33
Simiiformes - ps34
Catarrhini - ps35
Hominoidea - ps36
Hominidae - ps37
Homininae - ps38
Hominini - ps39
Homo_sapiens - ps40
p-value
5e-06
5e-05
5e-04
5e-03
5e-02
Log-odds
0.5
1.0
1.5
2.0
2.5
3.0
3.5
4.0

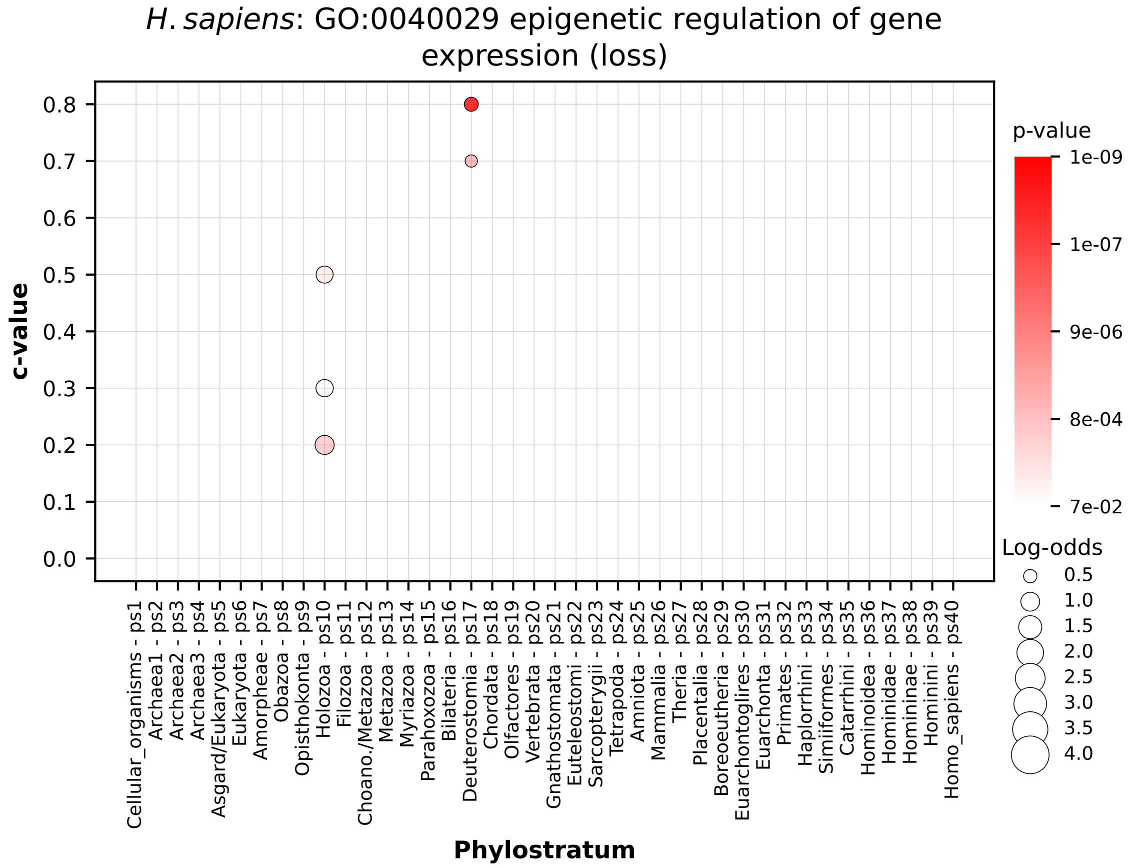

H. sapiens: GO:0040029 epigenetic regulation of gene expression (loss)
c-value
0.0
0.1
0.2
0.3
0.4
0.5
0.6
0.7
0.8
Phylostratum
Cellular_organisms - ps1
Archaea1 - ps2
Archaea2 - ps3
Archaea3 - ps4
Asgard/Eukaryota - ps5
Eukaryota - ps6
Amorpheae - ps7
Obazoa - ps8
Opisthokonta - ps9
Holozoa - ps10
Filozoa - ps11
Choano./Metazoa - ps12
Metazoa - ps13
Myriazoa - ps14
Parahoxozoa - ps15
Bilateria - ps16
Deuterostomia - ps17
Chordata - ps18
Olfactores - ps19
Vertebrata - ps20
Gnathostomata - ps21
Euteleostomi - ps22
Sarcopterygii - ps23
Tetrapoda - ps24
Amniota - ps25
Mammalia - ps26
Theria - ps27
Placentalia - ps28
Boreoeutheria - ps29
Euarchontoglires - ps30
Euarchonta - ps31
Primates - ps32
Haplorrhini - ps33
Simiiformes - ps34
Catarrhini - ps35
Hominoidea - ps36
Hominidae - ps37
Homininae - ps38
Hominini - ps39
Homo_sapiens - ps40
p-value
1e-09
1e-07
9e-06
8e-04
7e-02
Log-odds
0.5
1.0
1.5
2.0
2.5
3.0
3.5
4.0

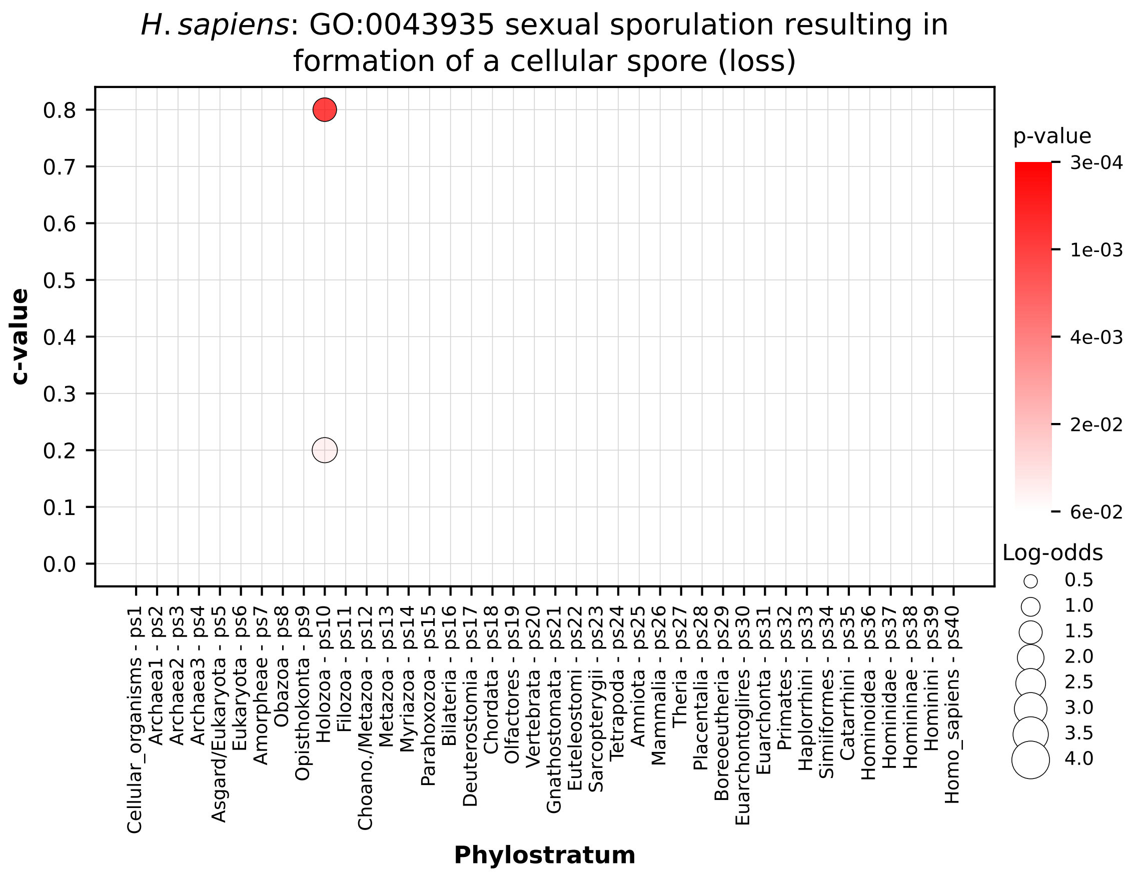

H. sapiens: GO:0043935 sexual sporulation resulting in formation of a cellular spore (loss)
c-value
0.0
0.1
0.2
0.3
0.4
0.5
0.6
0.7
0.8
Phylostratum
Cellular_organisms - ps1
Archaea1 - ps2
Archaea2 - ps3
Archaea3 - ps4
Asgard/Eukaryota - ps5
Eukaryota - ps6
Amorpheae - ps7
Obazoa - ps8
Opisthokonta - ps9
Holozoa - ps10
Filozoa - ps11
Choano./Metazoa - ps12
Metazoa - ps13
Myriazoa - ps14
Parahoxozoa - ps15
Bilateria - ps16
Deuterostomia - ps17
Chordata - ps18
Olfactores - ps19
Vertebrata - ps20
Gnathostomata - ps21
Euteleostomi - ps22
Sarcopterygii - ps23
Tetrapoda - ps24
Amniota - ps25
Mammalia - ps26
Theria - ps27
Placentalia - ps28
Boreoeutheria - ps29
Euarchontoglires - ps30
Euarchonta - ps31
Primates - ps32
Haplorrhini - ps33
Simiiformes - ps34
Catarrhini - ps35
Hominoidea - ps36
Hominidae - ps37
Homininae - ps38
Hominini - ps39
Homo_sapiens - ps40
p-value
3e-04
1e-03
4e-03
2e-02
6e-02
Log-odds
0.5
1.0
1.5
2.0
2.5
3.0
3.5
4.0

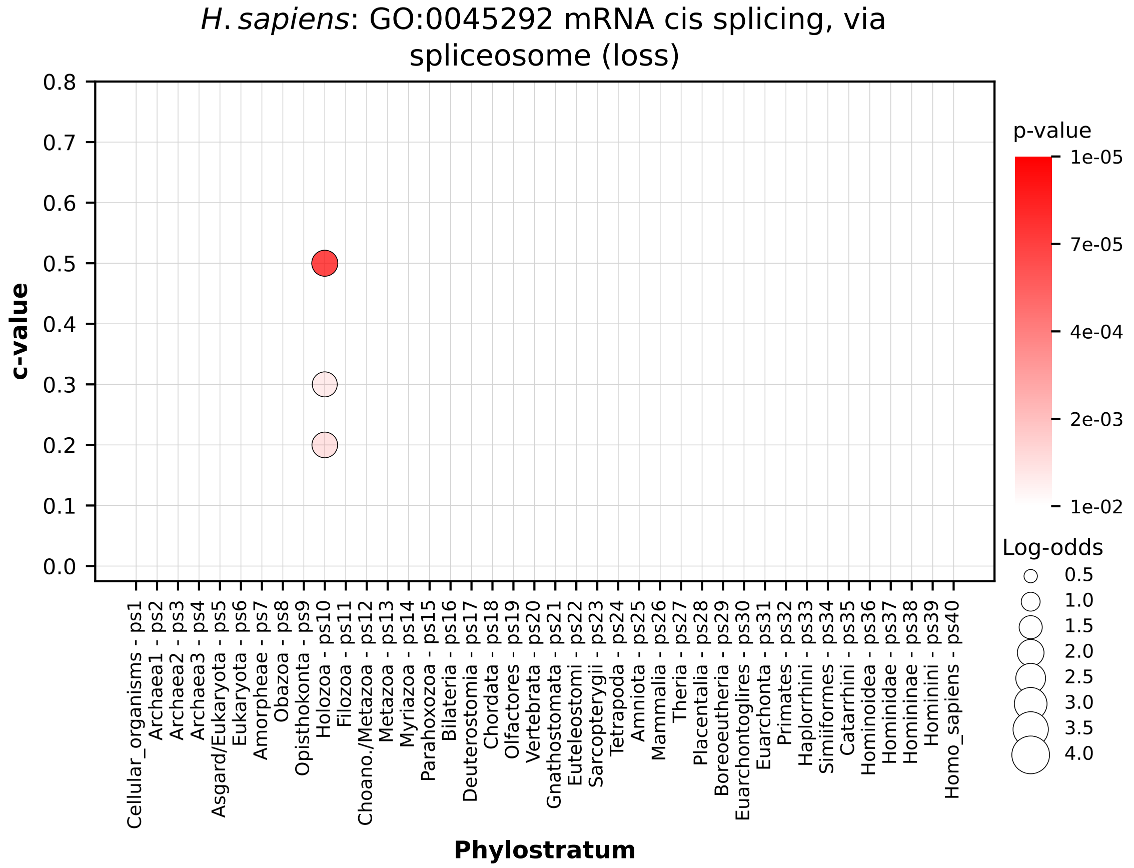

H. sapiens: GO:0045292 mRNA cis splicing, via spliceosome (loss)
c-value
0.8
0.7
0.6
0.5
0.4
0.3
0.2
0.1
0.0
Phylostratum
Cellular_organisms - ps1
Archaea1 - ps2
Archaea2 - ps3
Archaea3 - ps4
Asgard/Eukaryota - ps5
Eukaryota - ps6
Amorpheae - ps7
Obazoa - ps8
Opisthokonta - ps9
Holozoa - ps10
Filozoa - ps11
Choano./Metazoa - ps12
Metazoa - ps13
Myriazoa - ps14
Parahoxozoa - ps15
Bilateria - ps16
Deuterostomia - ps17
Chordata - ps18
Olfactores - ps19
Vertebrata - ps20
Gnathostomata - ps21
Euteleostomi - ps22
Sarcopterygii - ps23
Tetrapoda - ps24
Amniota - ps25
Mammalia - ps26
Theria - ps27
Placentalia - ps28
Boreoeutheria - ps29
Euarchontoglires - ps30
Euarchonta - ps31
Primates - ps32
Haplorrhini - ps33
Simiiformes - ps34
Catarrhini - ps35
Hominoidea - ps36
Hominidae - ps37
Homininae - ps38
Hominini - ps39
Homo_sapiens - ps40
p-value
1e-05
7e-05
4e-04
2e-03
1e-02
Log-odds
0.5
1.0
1.5
2.0
2.5
3.0
3.5
4.0

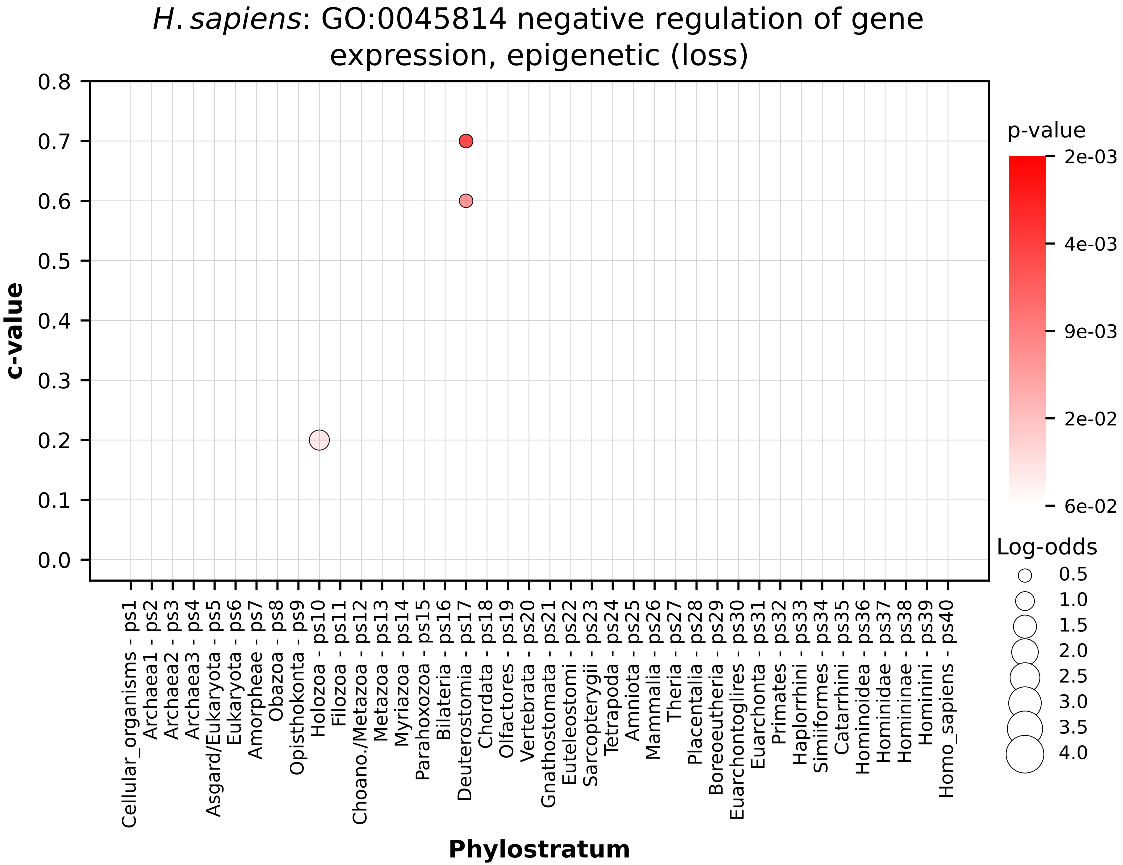

H. sapiens: GO:0045814 negative regulation of gene expression, epigenetic (loss)
c-value
0.8
0.7
0.6
0.5
0.4
0.3
0.2
0.1
0.0
Phylostratum
Cellular_organisms - ps1
Archaea1 - ps2
Archaea2 - ps3
Archaea3 - ps4
Asgard/Eukaryota - ps5
Eukaryota - ps6
Amorpheae - ps7
Obazoa - ps8
Opisthokonta - ps9
Holozoa - ps10
Filozoa - ps11
Choano./Metazoa - ps12
Metazoa - ps13
Myriazoa - ps14
Parahoxozoa - ps15
Bilateria - ps16
Deuterostomia - ps17
Chordata - ps18
Olfactores - ps19
Vertebrata - ps20
Gnathostomata - ps21
Euteleostomi - ps22
Sarcopterygii - ps23
Tetrapoda - ps24
Amniota - ps25
Mammalia - ps26
Theria - ps27
Placentalia - ps28
Boreoeutheria - ps29
Euarchontoglires - ps30
Euarchonta - ps31
Primates - ps32
Haplorrhini - ps33
Simiiformes - ps34
Catarrhini - ps35
Hominoidea - ps36
Hominidae - ps37
Homininae - ps38
Hominini - ps39
Homo_sapiens - ps40
p-value
2e-03
4e-03
9e-03
2e-02
6e-02
Log-odds
0.5
1.0
1.5
2.0
2.5
3.0
3.5
4.0

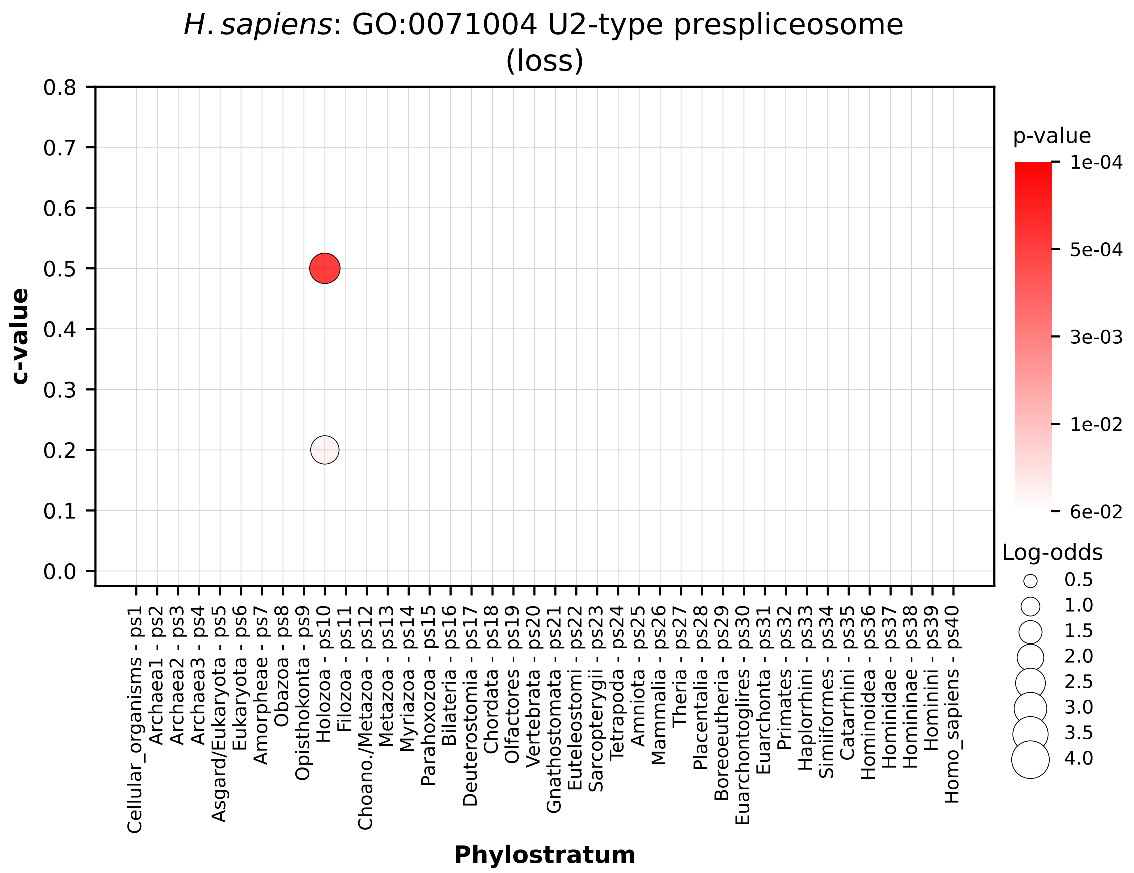
H. sapiens: GO:0071004 U2-type prespliceosome
(loss)
c-value
0.8
0.7
0.6
0.5
0.4
0.3
0.2
0.1
0.0
Phylostratum
Cellular_organisms - ps1
Archaea1 - ps2
Archaea2 - ps3
Archaea3 - ps4
Asgard/Eukaryota - ps5
Eukaryota - ps6
Amorpheae - ps7
Obazoa - ps8
Opisthokonta - ps9
Holozoa - ps10
Filozoa - ps11
Choano./Metazoa - ps12
Metazoa - ps13
Myriazoa - ps14
Parahoxozoa - ps15
Bilateria - ps16
Deuterostomia - ps17
Chordata - ps18
Olfactores - ps19
Vertebrata - ps20
Gnathostomata - ps21
Euteleostomi - ps22
Sarcopterygii - ps23
Tetrapoda - ps24
Amniota - ps25
Mammalia - ps26
Theria - ps27
Placentalia - ps28
Boreoeutheria - ps29
Euarchontoglires - ps30
Euarchonta - ps31
Primates - ps32
Haplorrhini - ps33
Simiiformes - ps34
Catarrhini - ps35
Hominoidea - ps36
Hominidae - ps37
Homininae - ps38
Hominini - ps39
Homo_sapiens - ps40
p-value
1e-04
5e-04
3e-03
1e-02
6e-02
Log-odds
0.5
1.0
1.5
2.0
2.5
3.0
3.5
4.0

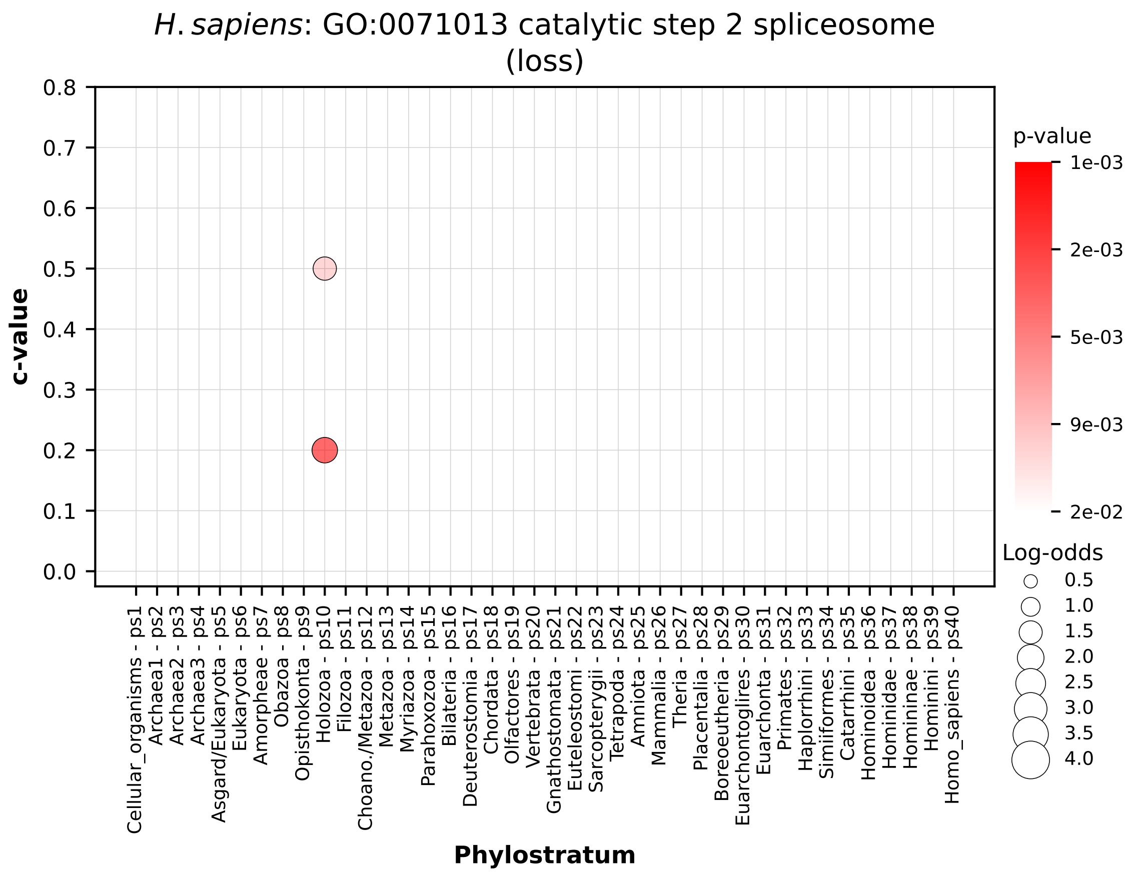

H. sapiens: GO:0071013 catalytic step 2 spliceosome (loss)
c-value
0.8
0.7
0.6
0.5
0.4
0.3
0.2
0.1
0.0
Phylostratum
Cellular_organisms - ps1
Archaea1 - ps2
Archaea2 - ps3
Archaea3 - ps4
Asgard/Eukaryota - ps5
Eukaryota - ps6
Amorpheae - ps7
Obazoa - ps8
Opisthokonta - ps9
Holozoa - ps10
Filozoa - ps11
Choano./Metazoa - ps12
Metazoa - ps13
Myriazoa - ps14
Parahoxozoa - ps15
Bilateria - ps16
Deuterostomia - ps17
Chordata - ps18
Olfactores - ps19
Vertebrata - ps20
Gnathostomata - ps21
Euteleostomi - ps22
Sarcopterygii - ps23
Tetrapoda - ps24
Amniota - ps25
Mammalia - ps26
Theria - ps27
Placentalia - ps28
Boreoeutheria - ps29
Euarchontoglires - ps30
Euarchonta - ps31
Primates - ps32
Haplorrhini - ps33
Simiiformes - ps34
Catarrhini - ps35
Hominoidea - ps36
Hominidae - ps37
Homininae - ps38
Hominini - ps39
Homo_sapiens - ps40
p-value
1e-03
2e-03
5e-03
9e-03
2e-02
Log-odds
0.5
1.0
1.5
2.0
2.5
3.0
3.5
4.0

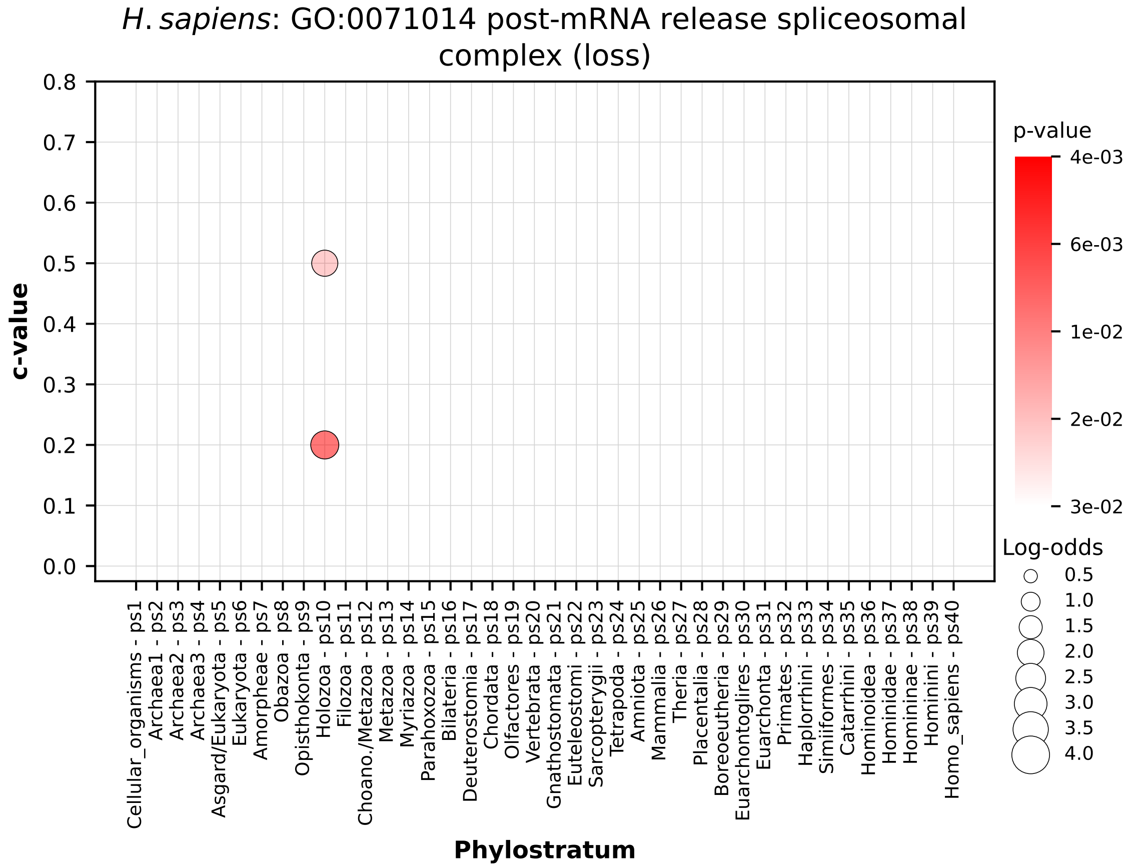

H. sapiens: GO:0071014 post-mRNA release spliceosomal complex (loss)
c-value
0.0
0.1
0.2
0.3
0.4
0.5
0.6
0.7
0.8
Phylostratum
Cellular_organisms - ps1
Archaea1 - ps2
Archaea2 - ps3
Archaea3 - ps4
Asgard/Eukaryota - ps5
Eukaryota - ps6
Amorpheae - ps7
Obazoa - ps8
Opisthokonta - ps9
Holozoa - ps10
Filozoa - ps11
Choano./Metazoa - ps12
Metazoa - ps13
Myriazoa - ps14
Parahoxozoa - ps15
Bilateria - ps16
Deuterostomia - ps17
Chordata - ps18
Olfactores - ps19
Vertebrata - ps20
Gnathostomata - ps21
Euteleostomi - ps22
Sarcopterygii - ps23
Tetrapoda - ps24
Amniota - ps25
Mammalia - ps26
Theria - ps27
Placentalia - ps28
Boreoeutheria - ps29
Euarchontoglires - ps30
Euarchonta - ps31
Primates - ps32
Haplorrhini - ps33
Simiiformes - ps34
Catarrhini - ps35
Hominoidea - ps36
Hominidae - ps37
Homininae - ps38
Hominini - ps39
Homo_sapiens - ps40
p-value
4e-03
6e-03
1e-02
2e-02
3e-02
Log-odds
0.5
1.0
1.5
2.0
2.5
3.0
3.5
4.0

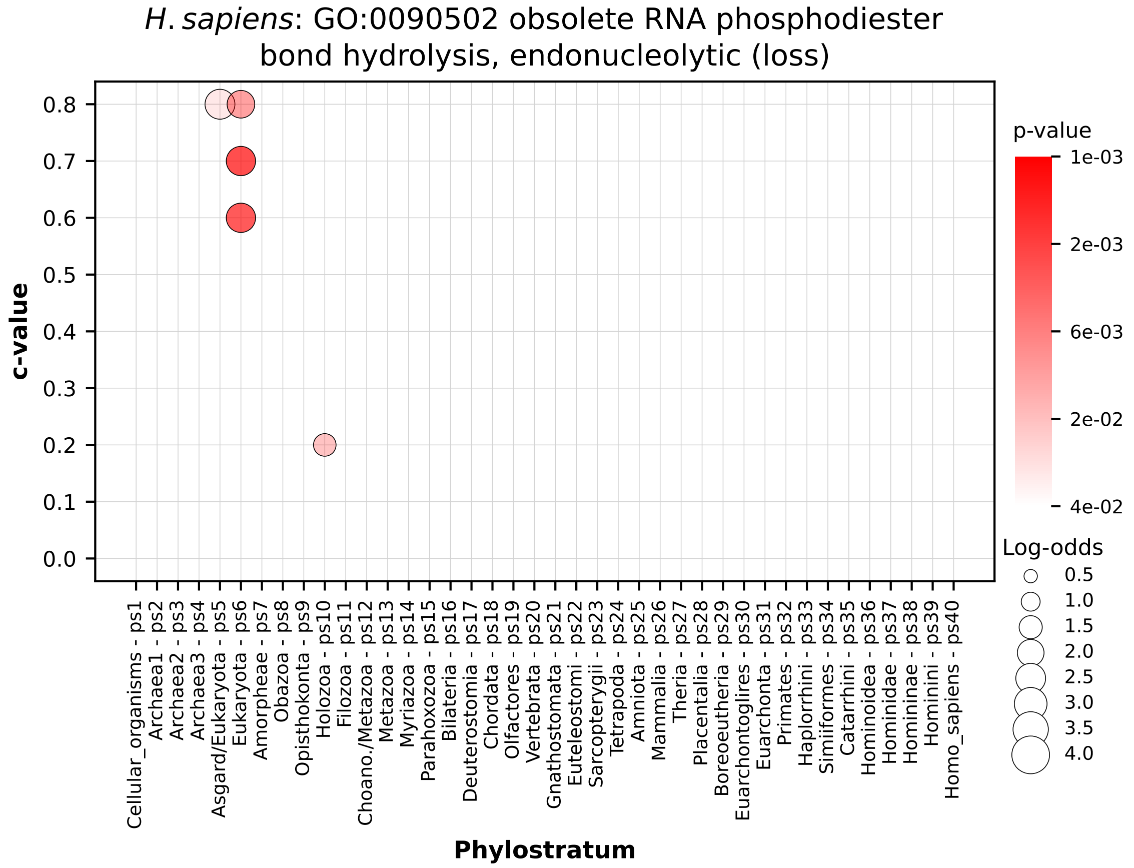

H. sapiens: GO:0090502 obsolete RNA phosphodiester bond hydrolysis, endonucleolytic (loss)
c-value
0.8
0.7
0.6
0.5
0.4
0.3
0.2
0.1
0.0
Phylostratum
Cellular_organisms - ps1
Archaea1 - ps2
Archaea2 - ps3
Archaea3 - ps4
Asgard/Eukaryota - ps5
Eukaryota - ps6
Amorpheae - ps7
Obazoa - ps8
Opisthokonta - ps9
Holozoa - ps10
Filozoa - ps11
Choano./Metazoa - ps12
Metazoa - ps13
Myriazoa - ps14
Parahoxozoa - ps15
Bilateria - ps16
Deuterostomia - ps17
Chordata - ps18
Olfactores - ps19
Vertebrata - ps20
Gnathostomata - ps21
Euteleostomi - ps22
Sarcopterygii - ps23
Tetrapoda - ps24
Amniota - ps25
Mammalia - ps26
Theria - ps27
Placentalia - ps28
Boreoeutheria - ps29
Euarchontoglires - ps30
Euarchonta - ps31
Primates - ps32
Haplorrhini - ps33
Simiiformes - ps34
Catarrhini - ps35
Hominoidea - ps36
Hominidae - ps37
Homininae - ps38
Hominini - ps39
Homo_sapiens - ps40
p-value
1e-03
2e-03
6e-03
2e-02
4e-02
Log-odds
0.5
1.0
1.5
2.0
2.5
3.0
3.5
4.0

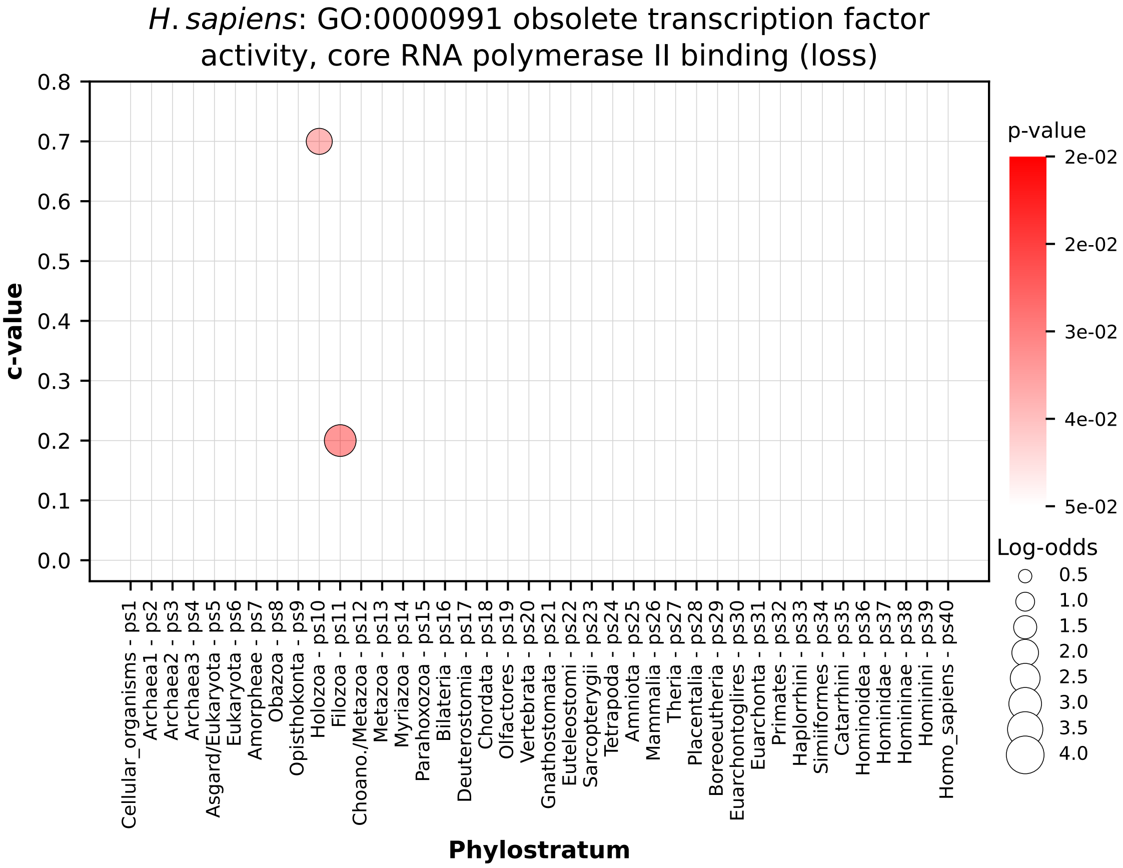

H. sapiens: GO:0000991 obsolete transcription factor activity, core RNA polymerase II binding (loss)
c-value
0.8
0.7
0.6
0.5
0.4
0.3
0.2
0.1
0.0
Phylostratum
Cellular_organisms - ps1
Archaea1 - ps2
Archaea2 - ps3
Archaea3 - ps4
Asgard/Eukaryota - ps5
Eukaryota - ps6
Amorpheae - ps7
Obazoa - ps8
Opisthokonta - ps9
Holozoa - ps10
Filozoa - ps11
Choano./Metazoa - ps12
Metazoa - ps13
Myriazoa - ps14
Parahoxozoa - ps15
Bilateria - ps16
Deuterostomia - ps17
Chordata - ps18
Olfactores - ps19
Vertebrata - ps20
Gnathostomata - ps21
Euteleostomi - ps22
Sarcopterygii - ps23
Tetrapoda - ps24
Amniota - ps25
Mammalia - ps26
Theria - ps27
Placentalia - ps28
Boreoeutheria - ps29
Euarchontoglires - ps30
Euarchonta - ps31
Primates - ps32
Haplorrhini - ps33
Simiiformes - ps34
Catarrhini - ps35
Hominoidea - ps36
Hominidae - ps37
Homininae - ps38
Hominini - ps39
Homo_sapiens - ps40
p-value
2e-02
2e-02
3e-02
4e-02
5e-02
Log-odds
0.5
1.0
1.5
2.0
2.5
3.0
3.5
4.0

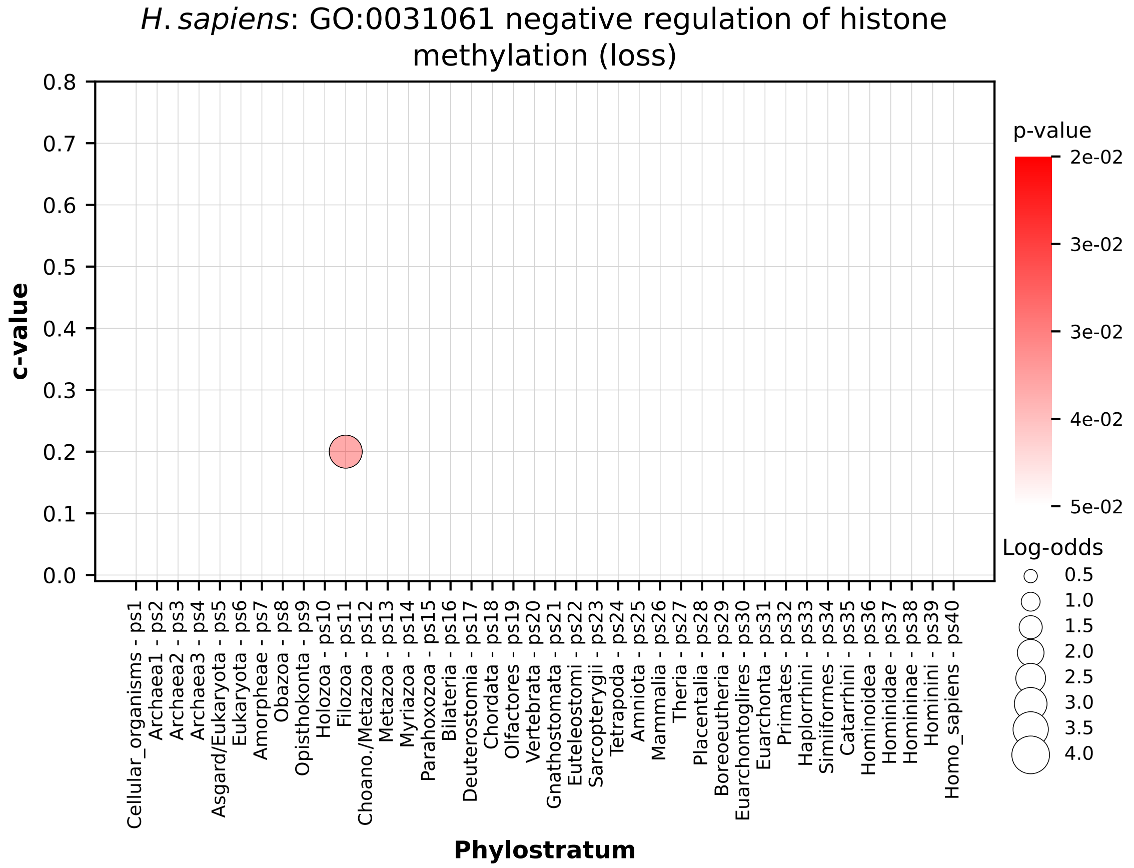

H. sapiens: GO:0031061 negative regulation of histone methylation (loss)
c-value
0.0
0.1
0.2
0.3
0.4
0.5
0.6
0.7
0.8
Phylostratum
Cellular_organisms - ps1
Archaea1 - ps2
Archaea2 - ps3
Archaea3 - ps4
Asgard/Eukaryota - ps5
Eukaryota - ps6
Amorpheae - ps7
Obazoa - ps8
Opisthokonta - ps9
Holozoa - ps10
Filozoa - ps11
Choano./Metazoa - ps12
Metazoa - ps13
Myriazoa - ps14
Parahoxozoa - ps15
Bilateria - ps16
Deuterostomia - ps17
Chordata - ps18
Olfactores - ps19
Vertebrata - ps20
Gnathostomata - ps21
Euteleostomi - ps22
Sarcopterygii - ps23
Tetrapoda - ps24
Amniota - ps25
Mammalia - ps26
Theria - ps27
Placentalia - ps28
Boreoeutheria - ps29
Euarchontoglires - ps30
Euarchonta - ps31
Primates - ps32
Haplorrhini - ps33
Simiiformes - ps34
Catarrhini - ps35
Hominoidea - ps36
Hominidae - ps37
Homininae - ps38
Hominini - ps39
Homo_sapiens - ps40
p-value
2e-02
3e-02
3e-02
4e-02
5e-02
Log-odds
0.5
1.0
1.5
2.0
2.5
3.0
3.5
4.0

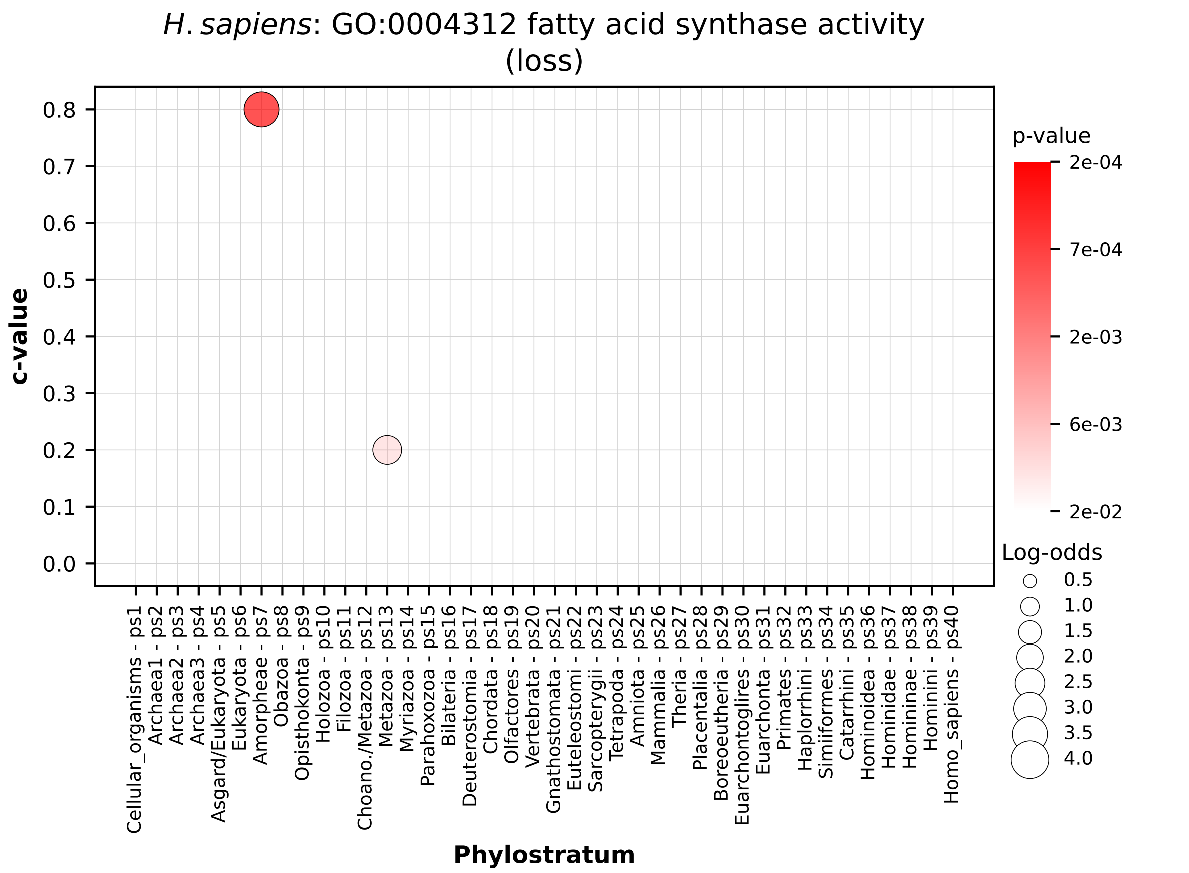
H. sapiens: GO:0004312 fatty acid synthase activity
(loss)
c-value
0.0
0.1
0.2
0.3
0.4
0.5
0.6
0.7
0.8
Phylostratum
Cellular_organisms - ps1
Archaea1 - ps2
Archaea2 - ps3
Archaea3 - ps4
Asgard/Eukaryota - ps5
Eukaryota - ps6
Amorpheae - ps7
Obazoa - ps8
Opisthokonta - ps9
Holozoa - ps10
Filozoa - ps11
Choano./Metazoa - ps12
Metazoa - ps13
Myriazoa - ps14
Parahoxozoa - ps15
Bilateria - ps16
Deuterostomia - ps17
Chordata - ps18
Olfactores - ps19
Vertebrata - ps20
Gnathostomata - ps21
Euteleostomi - ps22
Sarcopterygii - ps23
Tetrapoda - ps24
Amniota - ps25
Mammalia - ps26
Theria - ps27
Placentalia - ps28
Boreoeutheria - ps29
Euarchontoglires - ps30
Euarchonta - ps31
Primates - ps32
Haplorrhini - ps33
Simiiformes - ps34
Catarrhini - ps35
Hominoidea - ps36
Hominidae - ps37
Homininae - ps38
Hominini - ps39
Homo_sapiens - ps40
p-value
2e-04
7e-04
2e-03
6e-03
2e-02
Log-odds
0.5
1.0
1.5
2.0
2.5
3.0
3.5
4.0

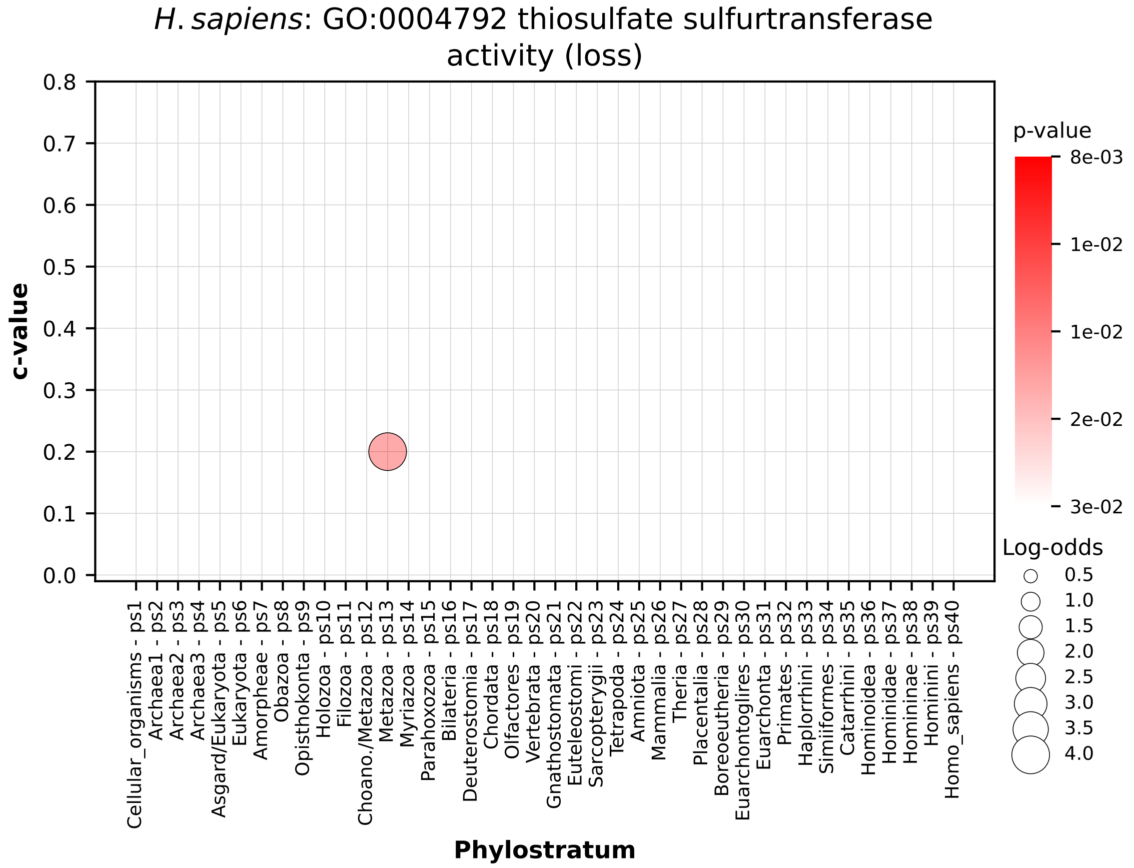

H. sapiens: GO:0004792 thiosulfate sulfurtransferase activity (loss)
c-value
0.8
0.7
0.6
0.5
0.4
0.3
0.2
0.1
0.0
Phylostratum
Cellular_organisms - ps1
Archaea1 - ps2
Archaea2 - ps3
Archaea3 - ps4
Asgard/Eukaryota - ps5
Eukaryota - ps6
Amorpheae - ps7
Obazoa - ps8
Opisthokonta - ps9
Holozoa - ps10
Filozoa - ps11
Choano./Metazoa - ps12
Metazoa - ps13
Myriazoa - ps14
Parahoxozoa - ps15
Bilateria - ps16
Deuterostomia - ps17
Chordata - ps18
Olfactores - ps19
Vertebrata - ps20
Gnathostomata - ps21
Euteleostomi - ps22
Sarcopterygii - ps23
Tetrapoda - ps24
Amniota - ps25
Mammalia - ps26
Theria - ps27
Placentalia - ps28
Boreoeutheria - ps29
Euarchontoglires - ps30
Euarchonta - ps31
Primates - ps32
Haplorrhini - ps33
Simiiformes - ps34
Catarrhini - ps35
Hominoidea - ps36
Hominidae - ps37
Homininae - ps38
Hominini - ps39
Homo_sapiens - ps40
p-value
8e-03
1e-02
1e-02
2e-02
3e-02
Log-odds
0.5
1.0
1.5
2.0
2.5
3.0
3.5
4.0

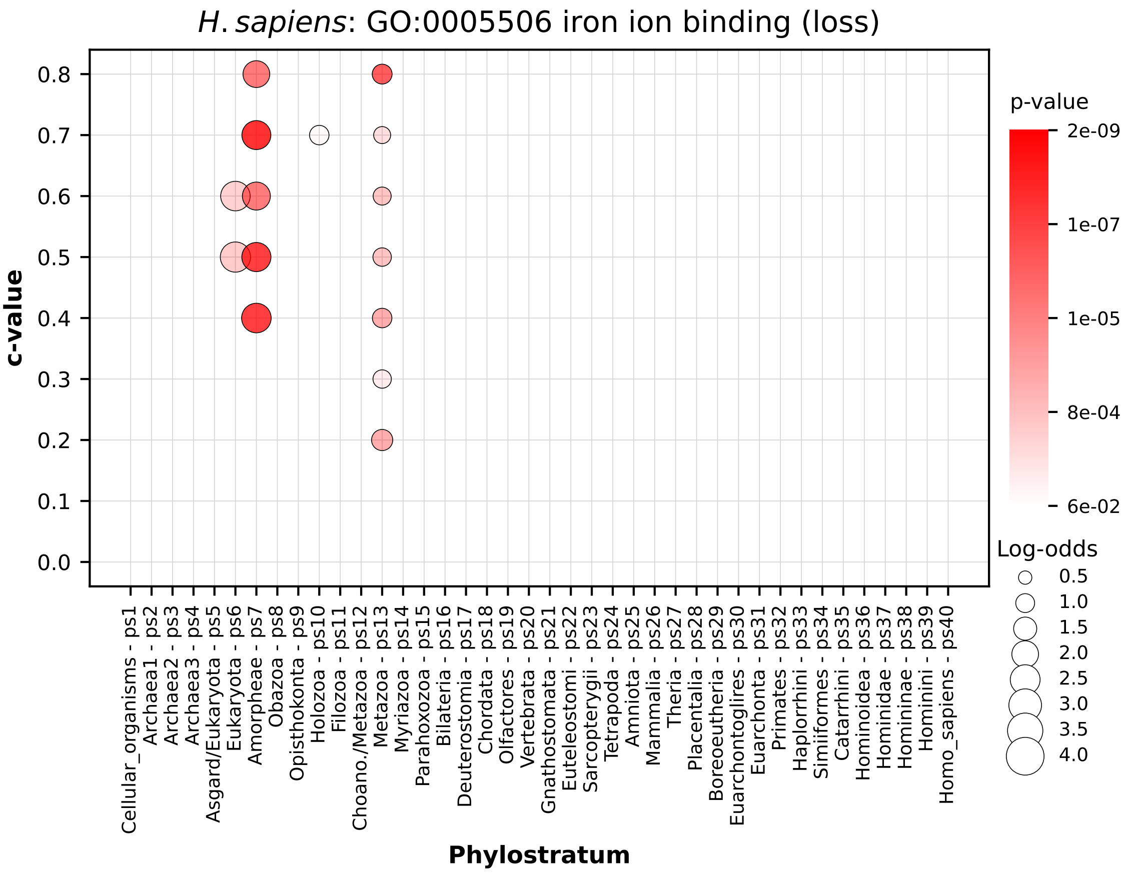
H. sapiens: GO:0005506 iron ion binding (loss)
c-value
0.8
0.7
0.6
0.5
0.4
0.3
0.2
0.1
0.0
p-value
2e-09
1e-07
1e-05
8e-04
6e-02
Log-odds
0.5
1.0
1.5
2.0
2.5
3.0
3.5
4.0
Cellular_organisms - ps1
Archaea1 - ps2
Archaea2 - ps3
Archaea3 - ps4
Asgard/Eukaryota - ps5
Eukaryota - ps6
Amorpheae - ps7
Obazoa - ps8
Opisthokonta - ps9
Holozoa - ps10
Filozoa - ps11
Choano./Metazoa - ps12
Metazoa - ps13
Myriazoa - ps14
Parahoxozoa - ps15
Bilateria - ps16
Deuterostomia - ps17
Chordata - ps18
Olfactores - ps19
Vertebrata - ps20
Gnathostomata - ps21
Euteleostomi - ps22
Sarcopterygii - ps23
Tetrapoda - ps24
Amniota - ps25
Mammalia - ps26
Theria - ps27
Placentalia - ps28
Boreoeutheria - ps29
Euarchontoglires - ps30
Euarchonta - ps31
Primates - ps32
Haplorrhini - ps33
Simiiformes - ps34
Catarrhini - ps35
Hominoidea - ps36
Hominidae - ps37
Homininae - ps38
Hominini - ps39
Homo_sapiens - ps40
Phylostratum

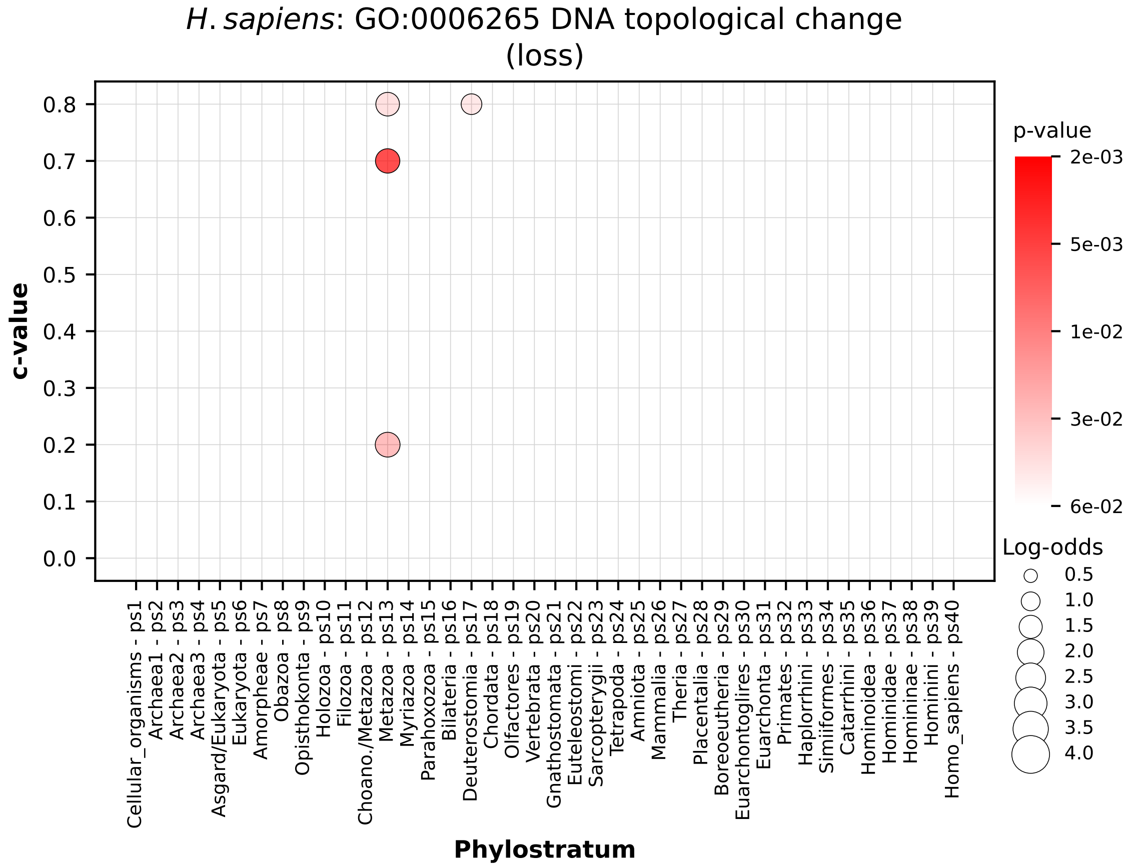

H. sapiens: GO:0006265 DNA topological change
(loss)
c-value
0.8
0.7
0.6
0.5
0.4
0.3
0.2
0.1
0.0
p-value
2e-03
5e-03
1e-02
3e-02
6e-02
Log-odds
0.5
1.0
1.5
2.0
2.5
3.0
3.5
4.0
Cellular_organisms - ps1
Archaea1 - ps2
Archaea2 - ps3
Archaea3 - ps4
Asgard/Eukaryota - ps5
Eukaryota - ps6
Amorpheae - ps7
Obazoa - ps8
Opisthokonta - ps9
Holozoa - ps10
Filozoa - ps11
Choano./Metazoa - ps12
Metazoa - ps13
Myriazoa - ps14
Parahoxozoa - ps15
Bilateria - ps16
Deuterostomia - ps17
Chordata - ps18
Olfactores - ps19
Vertebrata - ps20
Gnathostomata - ps21
Euteleostomi - ps22
Sarcopterygii - ps23
Tetrapoda - ps24
Amniota - ps25
Mammalia - ps26
Theria - ps27
Placentalia - ps28
Boreoeutheria - ps29
Euarchontoglires - ps30
Euarchonta - ps31
Primates - ps32
Haplorrhini - ps33
Simiiformes - ps34
Catarrhini - ps35
Hominoidea - ps36
Hominidae - ps37
Homininae - ps38
Hominini - ps39
Homo_sapiens - ps40
Phylostratum

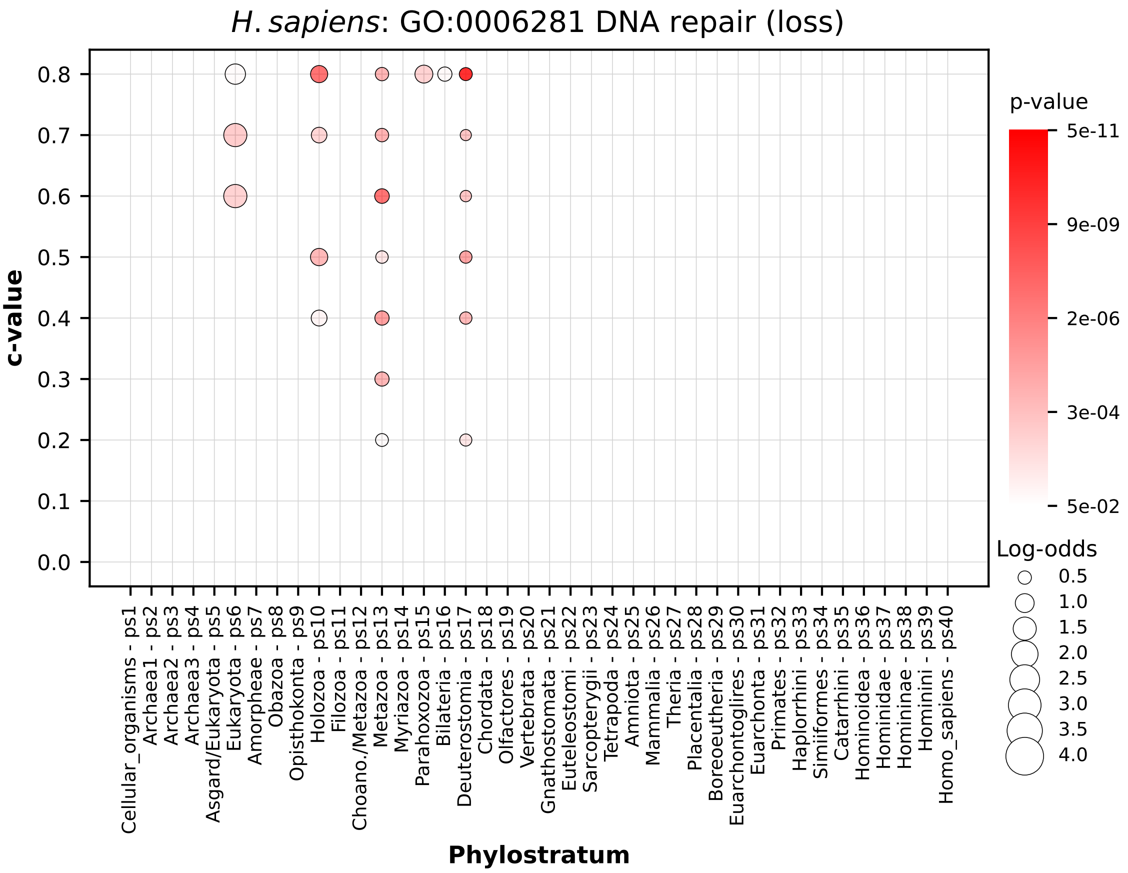

H. sapiens: GO:0006281 DNA repair (loss)
c-value
0.8
0.7
0.6
0.5
0.4
0.3
0.2
0.1
0.0
p-value
5e-11
9e-09
2e-06
3e-04
5e-02
Log-odds
0.5
1.0
1.5
2.0
2.5
3.0
3.5
4.0
Cellular_organisms - ps1
Archaea1 - ps2
Archaea2 - ps3
Archaea3 - ps4
Asgard/Eukaryota - ps5
Eukaryota - ps6
Amorpheae - ps7
Obazoa - ps8
Opisthokonta - ps9
Holozoa - ps10
Filozoa - ps11
Choano./Metazoa - ps12
Metazoa - ps13
Myriazoa - ps14
Parahoxozoa - ps15
Bilateria - ps16
Deuterostomia - ps17
Chordata - ps18
Olfactores - ps19
Vertebrata - ps20
Gnathostomata - ps21
Euteleostomi - ps22
Sarcopterygii - ps23
Tetrapoda - ps24
Amniota - ps25
Mammalia - ps26
Theria - ps27
Placentalia - ps28
Boreoeutheria - ps29
Euarchontoglires - ps30
Euarchonta - ps31
Primates - ps32
Haplorrhini - ps33
Simiiformes - ps34
Catarrhini - ps35
Hominoidea - ps36
Hominidae - ps37
Homininae - ps38
Hominini - ps39
Homo_sapiens - ps40
Phylostratum

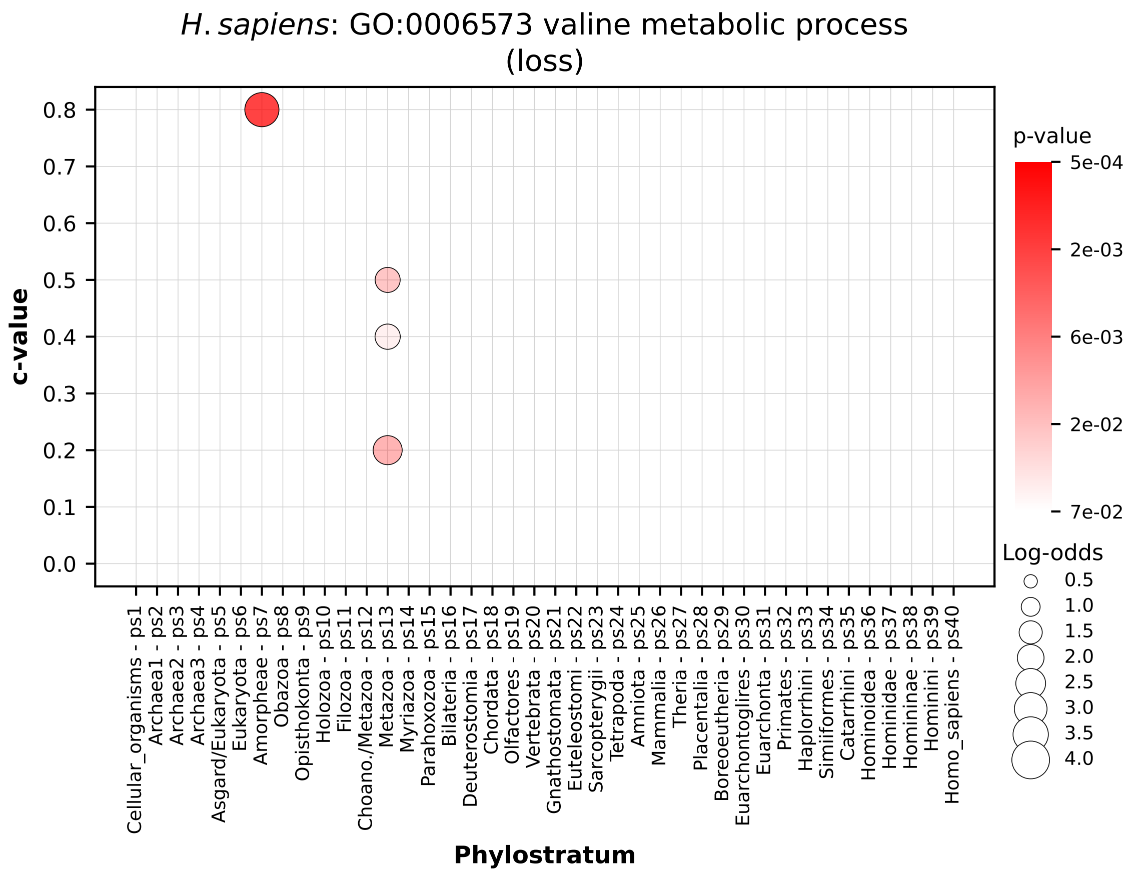

H. sapiens: GO:0006573 valine metabolic process
(loss)
c-value
0.8
0.7
0.6
0.5
0.4
0.3
0.2
0.1
0.0
p-value
5e-04
2e-03
6e-03
2e-02
7e-02
Log-odds
0.5
1.0
1.5
2.0
2.5
3.0
3.5
4.0
Cellular_organisms - ps1
Archaea1 - ps2
Archaea2 - ps3
Archaea3 - ps4
Asgard/Eukaryota - ps5
Eukaryota - ps6
Amorpheae - ps7
Obazoa - ps8
Opisthokonta - ps9
Holozoa - ps10
Filozoa - ps11
Choano./Metazoa - ps12
Metazoa - ps13
Myriazoa - ps14
Parahoxozoa - ps15
Bilateria - ps16
Deuterostomia - ps17
Chordata - ps18
Olfactores - ps19
Vertebrata - ps20
Gnathostomata - ps21
Euteleostomi - ps22
Sarcopterygii - ps23
Tetrapoda - ps24
Amniota - ps25
Mammalia - ps26
Theria - ps27
Placentalia - ps28
Boreoeutheria - ps29
Euarchontoglires - ps30
Euarchonta - ps31
Primates - ps32
Haplorrhini - ps33
Simiiformes - ps34
Catarrhini - ps35
Hominoidea - ps36
Hominidae - ps37
Homininae - ps38
Hominini - ps39
Homo_sapiens - ps40
Phylostratum

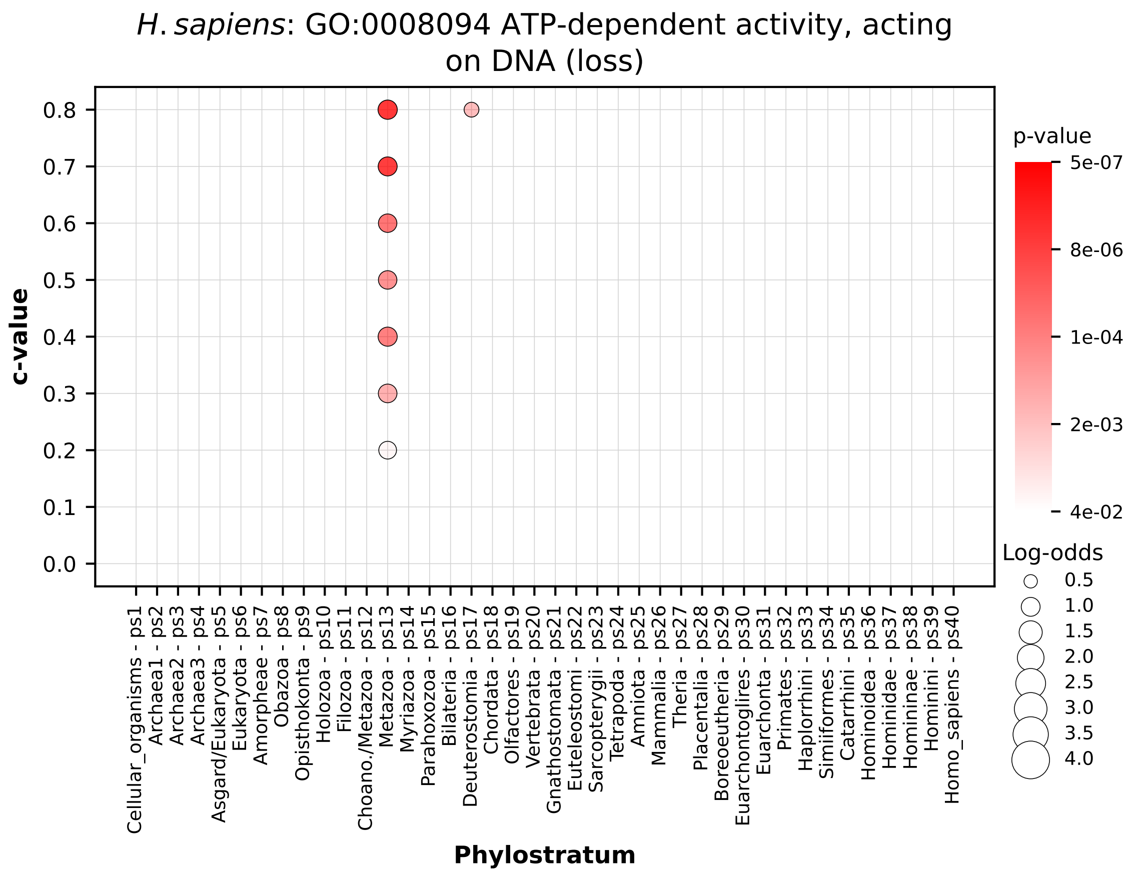

H. sapiens: GO:0008094 ATP-dependent activity, acting on DNA (loss)
c-value
0.0
0.1
0.2
0.3
0.4
0.5
0.6
0.7
0.8
Phylostratum
Cellular_organisms - ps1
Archaea1 - ps2
Archaea2 - ps3
Archaea3 - ps4
Asgard/Eukaryota - ps5
Eukaryota - ps6
Amorpheae - ps7
Obazoa - ps8
Opisthokonta - ps9
Holozoa - ps10
Filozoa - ps11
Choano./Metazoa - ps12
Metazoa - ps13
Myriazoa - ps14
Parahoxozoa - ps15
Bilateria - ps16
Deuterostomia - ps17
Chordata - ps18
Olfactores - ps19
Vertebrata - ps20
Gnathostomata - ps21
Euteleostomi - ps22
Sarcopterygii - ps23
Tetrapoda - ps24
Amniota - ps25
Mammalia - ps26
Theria - ps27
Placentalia - ps28
Boreoeutheria - ps29
Euarchontoglires - ps30
Euarchonta - ps31
Primates - ps32
Haplorrhini - ps33
Simiiformes - ps34
Catarrhini - ps35
Hominoidea - ps36
Hominidae - ps37
Homininae - ps38
Hominini - ps39
Homo_sapiens - ps40
p-value
5e-07
8e-06
1e-04
2e-03
4e-02
Log-odds
0.5
1.0
1.5
2.0
2.5
3.0
3.5
4.0

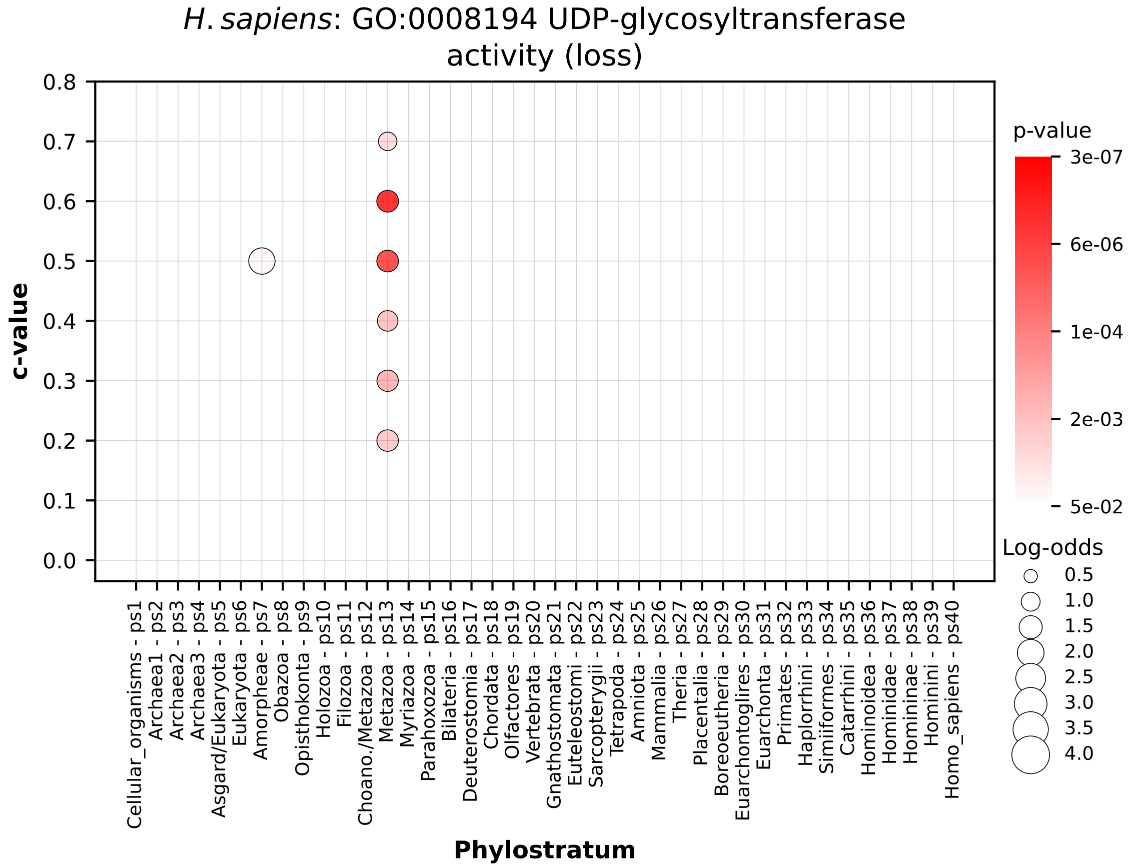

H. sapiens: GO:0008194 UDP-glycosyltransferase activity (loss)
c-value
0.8
0.7
0.6
0.5
0.4
0.3
0.2
0.1
0.0
Phylostratum
Cellular_organisms - ps1
Archaea1 - ps2
Archaea2 - ps3
Archaea3 - ps4
Asgard/Eukaryota - ps5
Eukaryota - ps6
Amorpheae - ps7
Obazoa - ps8
Opisthokonta - ps9
Holozoa - ps10
Filozoa - ps11
Choano./Metazoa - ps12
Metazoa - ps13
Myriazoa - ps14
Parahoxozoa - ps15
Bilateria - ps16
Deuterostomia - ps17
Chordata - ps18
Olfactores - ps19
Vertebrata - ps20
Gnathostomata - ps21
Euteleostomi - ps22
Sarcopterygii - ps23
Tetrapoda - ps24
Amniota - ps25
Mammalia - ps26
Theria - ps27
Placentalia - ps28
Boreoeutheria - ps29
Euarchontoglires - ps30
Euarchonta - ps31
Primates - ps32
Haplorrhini - ps33
Simiiformes - ps34
Catarrhini - ps35
Hominoidea - ps36
Hominidae - ps37
Homininae - ps38
Hominini - ps39
Homo_sapiens - ps40
p-value
3e-07
6e-06
1e-04
2e-03
5e-02
Log-odds
0.5
1.0
1.5
2.0
2.5
3.0
3.5
4.0

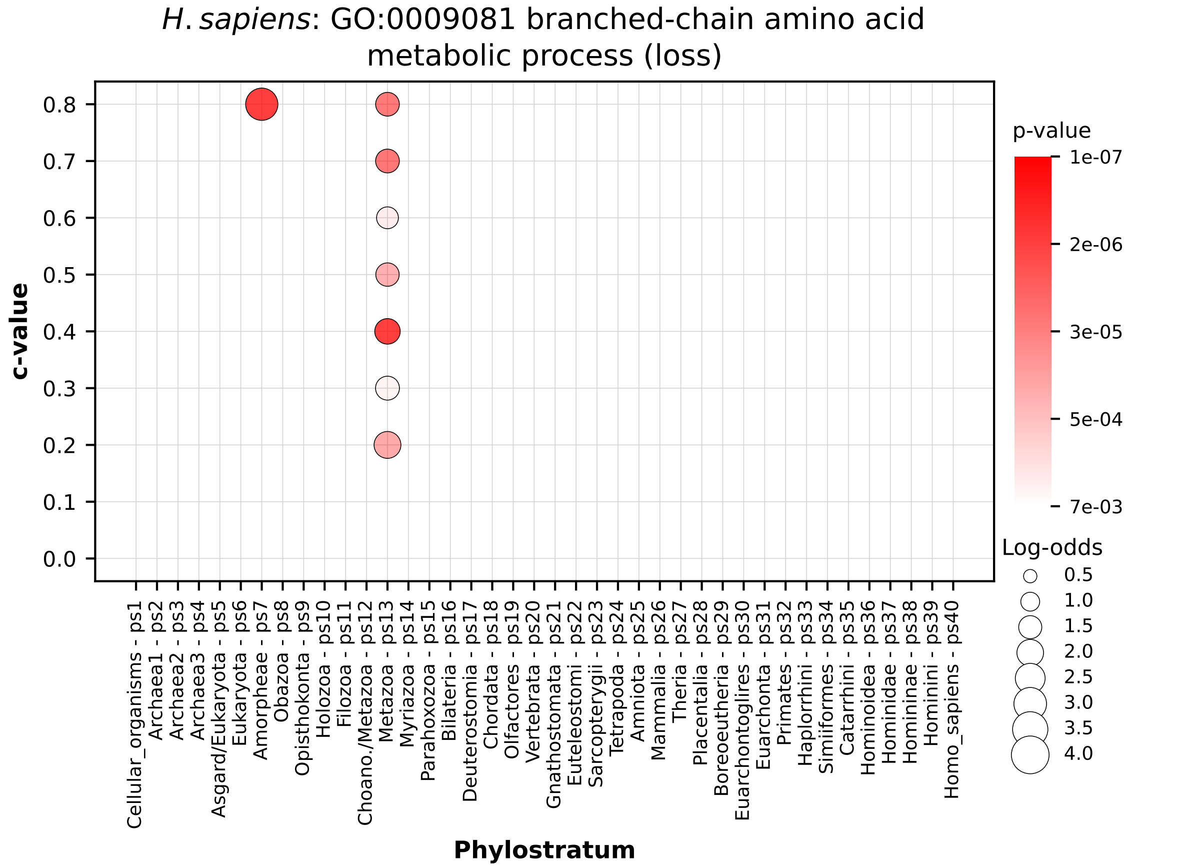

H. sapiens: GO:0009081 branched-chain amino acid metabolic process (loss)
c-value
0.8
0.7
0.6
0.5
0.4
0.3
0.2
0.1
0.0
p-value
1e-07
2e-06
3e-05
5e-04
7e-03
Log-odds
0.5
1.0
1.5
2.0
2.5
3.0
3.5
4.0
Cellular_organisms - ps1
Archaea1 - ps2
Archaea2 - ps3
Archaea3 - ps4
Asgard/Eukaryota - ps5
Eukaryota - ps6
Amorpheae - ps7
Obazoa - ps8
Opisthokonta - ps9
Holozoa - ps10
Filozoa - ps11
Choano./Metazoa - ps12
Metazoa - ps13
Myriazoa - ps14
Parahoxozoa - ps15
Bilateria - ps16
Deuterostomia - ps17
Chordata - ps18
Olfactores - ps19
Vertebrata - ps20
Gnathostomata - ps21
Euteleostomi - ps22
Sarcopterygii - ps23
Tetrapoda - ps24
Amniota - ps25
Mammalia - ps26
Theria - ps27
Placentalia - ps28
Boreoeutheria - ps29
Euarchontoglires - ps30
Euarchonta - ps31
Primates - ps32
Haplorrhini - ps33
Simiiformes - ps34
Catarrhini - ps35
Hominoidea - ps36
Hominidae - ps37
Homininae - ps38
Hominini - ps39
Homo_sapiens - ps40
Phylostratum

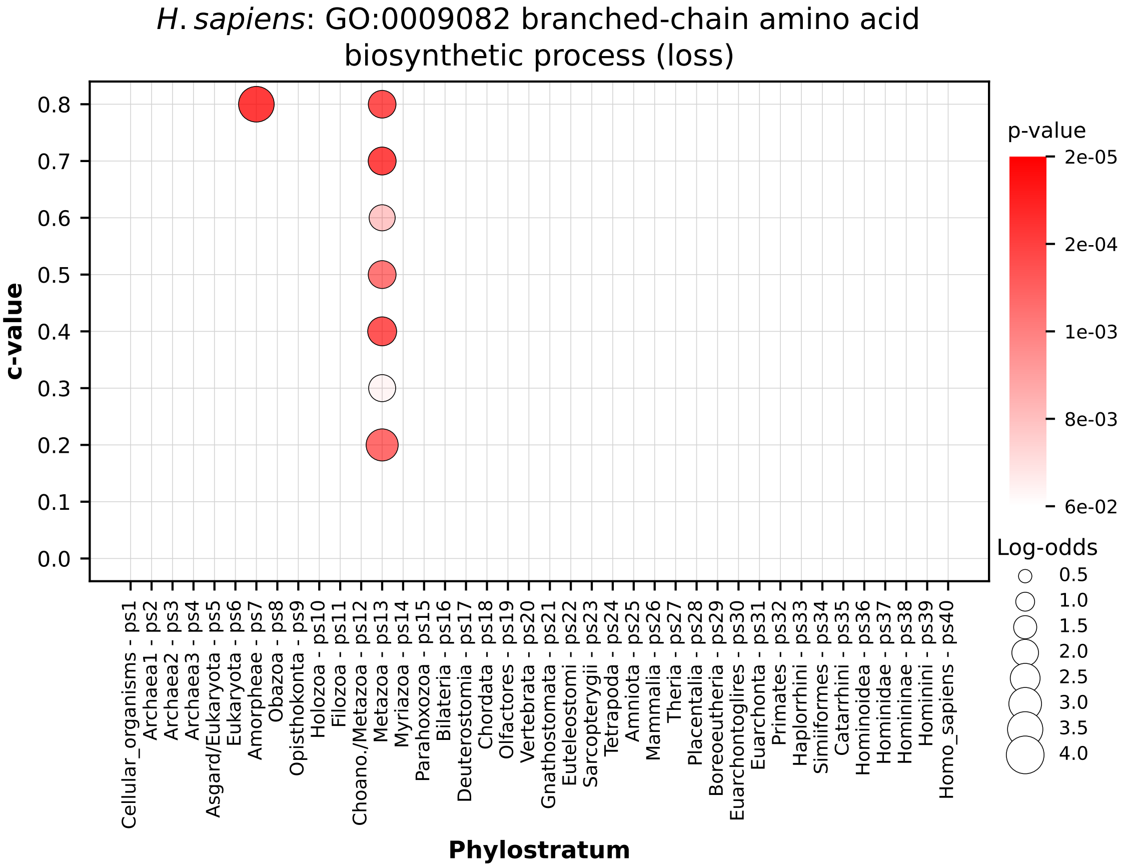

H. sapiens: GO:0009082 branched-chain amino acid biosynthetic process (loss)
c-value
0.0
0.1
0.2
0.3
0.4
0.5
0.6
0.7
0.8
p-value
2e-05
2e-04
1e-03
8e-03
6e-02
Log-odds
0.5
1.0
1.5
2.0
2.5
3.0
3.5
4.0
Cellular_organisms - ps1
Archaea1 - ps2
Archaea2 - ps3
Archaea3 - ps4
Asgard/Eukaryota - ps5
Eukaryota - ps6
Amorpheae - ps7
Obazoa - ps8
Opisthokonta - ps9
Holozoa - ps10
Filozoa - ps11
Choano./Metazoa - ps12
Metazoa - ps13
Myriazoa - ps14
Parahoxozoa - ps15
Bilateria - ps16
Deuterostomia - ps17
Chordata - ps18
Olfactores - ps19
Vertebrata - ps20
Gnathostomata - ps21
Euteleostomi - ps22
Sarcopterygii - ps23
Tetrapoda - ps24
Amniota - ps25
Mammalia - ps26
Theria - ps27
Placentalia - ps28
Boreoeutheria - ps29
Euarchontoglires - ps30
Euarchonta - ps31
Primates - ps32
Haplorrhini - ps33
Simiiformes - ps34
Catarrhini - ps35
Hominoidea - ps36
Hominidae - ps37
Homininae - ps38
Hominini - ps39
Homo_sapiens - ps40
Phylostratum

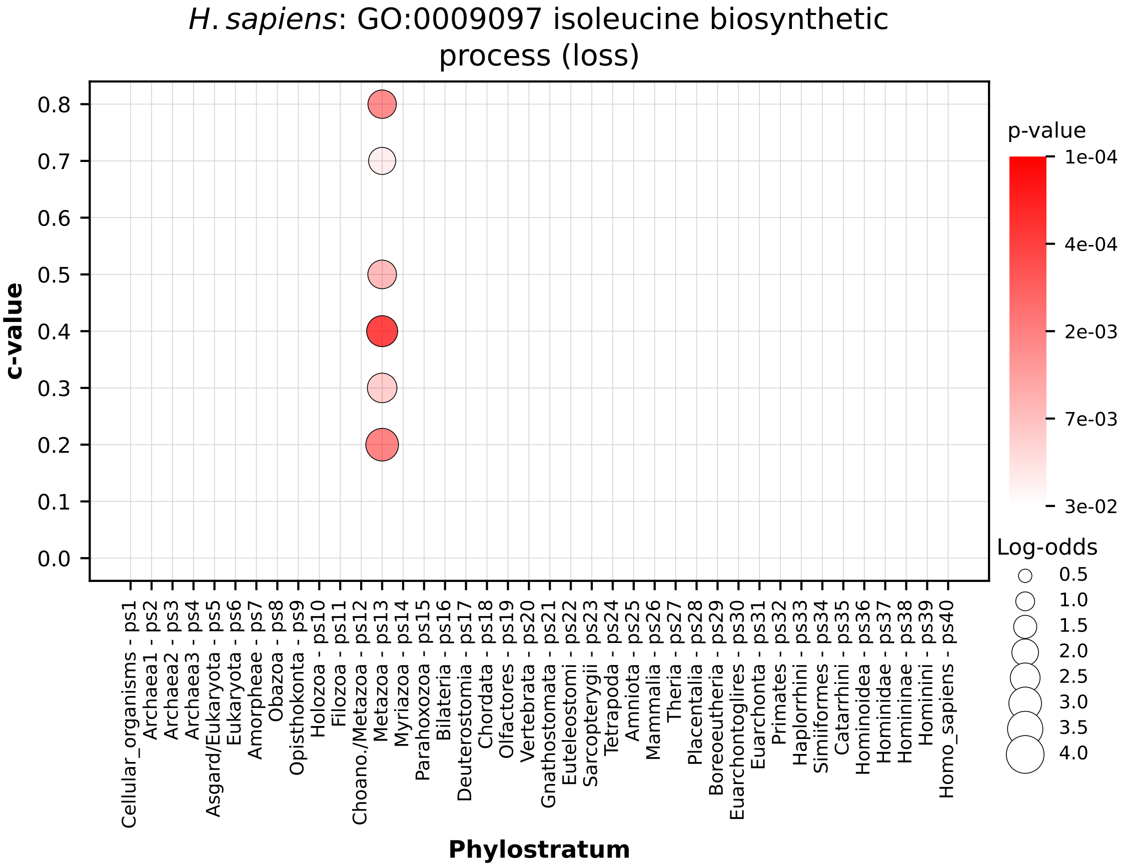

H. sapiens: GO:0009097 isoleucine biosynthetic process (loss)
c-value
0.8
0.7
0.6
0.5
0.4
0.3
0.2
0.1
0.0
p-value
1e-04
4e-04
2e-03
7e-03
3e-02
Log-odds
0.5
1.0
1.5
2.0
2.5
3.0
3.5
4.0
Cellular_organisms - ps1
Archaea1 - ps2
Archaea2 - ps3
Archaea3 - ps4
Asgard/Eukaryota - ps5
Eukaryota - ps6
Amorpheae - ps7
Obazoa - ps8
Opisthokonta - ps9
Holozoa - ps10
Filozoa - ps11
Choano./Metazoa - ps12
Metazoa - ps13
Myriazoa - ps14
Parahoxozoa - ps15
Bilateria - ps16
Deuterostomia - ps17
Chordata - ps18
Olfactores - ps19
Vertebrata - ps20
Gnathostomata - ps21
Euteleostomi - ps22
Sarcopterygii - ps23
Tetrapoda - ps24
Amniota - ps25
Mammalia - ps26
Theria - ps27
Placentalia - ps28
Boreoeutheria - ps29
Euarchontoglires - ps30
Euarchonta - ps31
Primates - ps32
Haplorrhini - ps33
Simiiformes - ps34
Catarrhini - ps35
Hominoidea - ps36
Hominidae - ps37
Homininae - ps38
Hominini - ps39
Homo_sapiens - ps40
Phylostratum

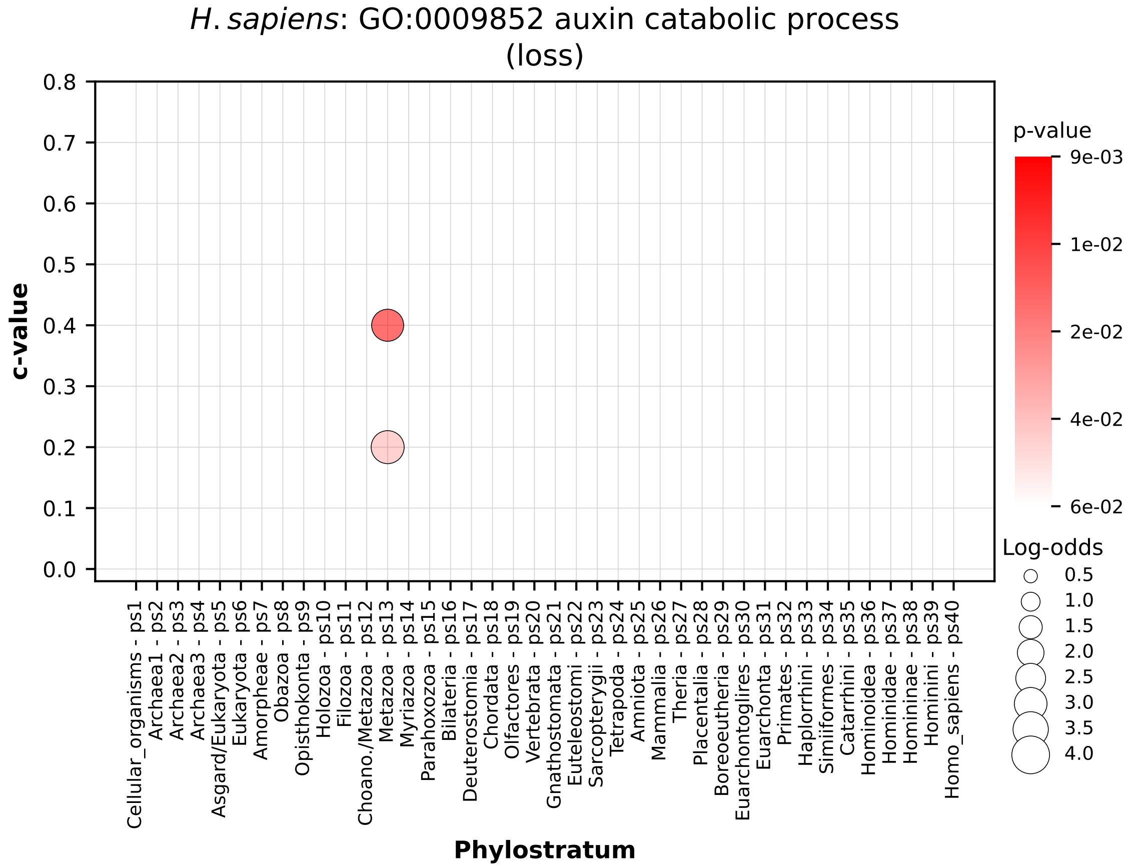

H. sapiens: GO:0009852 auxin catabolic process
(loss)
c-value
0.0
0.1
0.2
0.3
0.4
0.5
0.6
0.7
0.8
Phylostratum
Cellular_organisms - ps1
Archaea1 - ps2
Archaea2 - ps3
Archaea3 - ps4
Asgard/Eukaryota - ps5
Eukaryota - ps6
Amorpheae - ps7
Obazoa - ps8
Opisthokonta - ps9
Holozoa - ps10
Filozoa - ps11
Choano./Metazoa - ps12
Metazoa - ps13
Myriazoa - ps14
Parahoxozoa - ps15
Bilateria - ps16
Deuterostomia - ps17
Chordata - ps18
Olfactores - ps19
Vertebrata - ps20
Gnathostomata - ps21
Euteleostomi - ps22
Sarcopterygii - ps23
Tetrapoda - ps24
Amniota - ps25
Mammalia - ps26
Theria - ps27
Placentalia - ps28
Boreoeutheria - ps29
Euarchontoglires - ps30
Euarchonta - ps31
Primates - ps32
Haplorrhini - ps33
Simiiformes - ps34
Catarrhini - ps35
Hominoidea - ps36
Hominidae - ps37
Homininae - ps38
Hominini - ps39
Homo_sapiens - ps40
p-value
9e-03
1e-02
2e-02
4e-02
6e-02
Log-odds
0.5
1.0
1.5
2.0
2.5
3.0
3.5
4.0

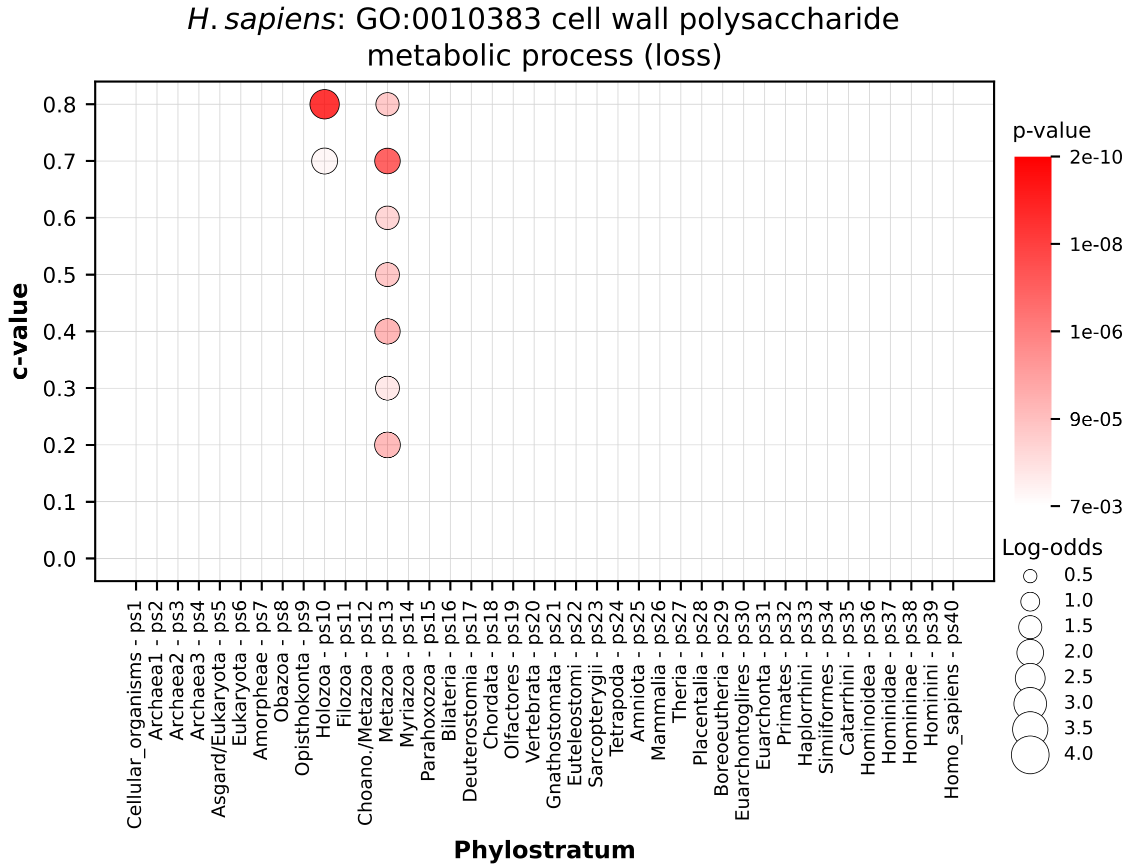
H. sapiens: GO:0010383 cell wall polysaccharide metabolic process (loss)
c-value
0.8
0.7
0.6
0.5
0.4
0.3
0.2
0.1
0.0
Phylostratum
Cellular_organisms - ps1
Archaea1 - ps2
Archaea2 - ps3
Archaea3 - ps4
Asgard/Eukaryota - ps5
Eukaryota - ps6
Amorpheae - ps7
Obazoa - ps8
Opisthokonta - ps9
Holozoa - ps10
Filozoa - ps11
Choano./Metazoa - ps12
Metazoa - ps13
Myriazoa - ps14
Parahoxozoa - ps15
Bilateria - ps16
Deuterostomia - ps17
Chordata - ps18
Olfactores - ps19
Vertebrata - ps20
Gnathostomata - ps21
Euteleostomi - ps22
Sarcopterygii - ps23
Tetrapoda - ps24
Amniota - ps25
Mammalia - ps26
Theria - ps27
Placentalia - ps28
Boreoeutheria - ps29
Euarchontoglires - ps30
Euarchonta - ps31
Primates - ps32
Haplorrhini - ps33
Simiiformes - ps34
Catarrhini - ps35
Hominoidea - ps36
Hominidae - ps37
Homininae - ps38
Hominini - ps39
Homo_sapiens - ps40
p-value
2e-10
1e-08
1e-06
9e-05
7e-03
Log-odds
0.5
1.0
1.5
2.0
2.5
3.0
3.5
4.0

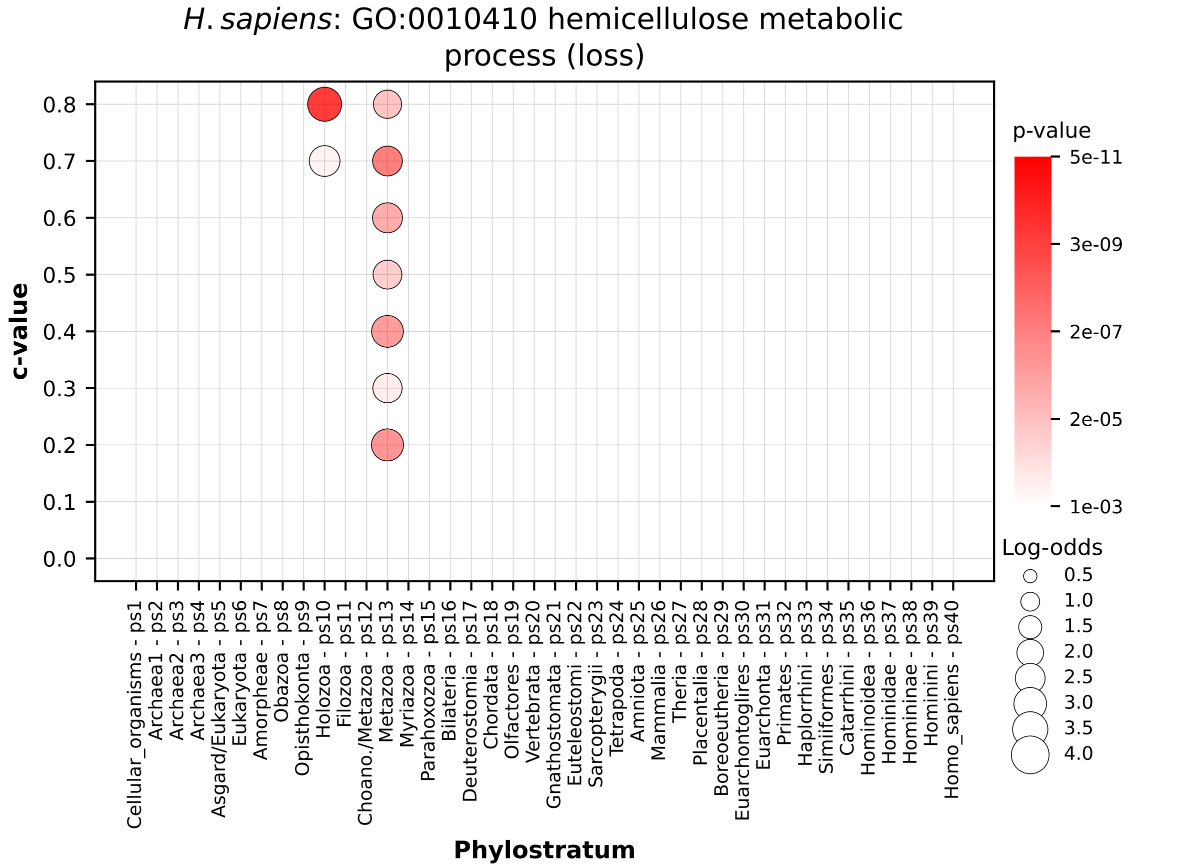
H. sapiens: GO:0010410 hemicellulose metabolic process (loss)
c-value
0.0
0.1
0.2
0.3
0.4
0.5
0.6
0.7
0.8
Phylostratum
Cellular_organisms - ps1
Archaea1 - ps2
Archaea2 - ps3
Archaea3 - ps4
Asgard/Eukaryota - ps5
Eukaryota - ps6
Amorpheae - ps7
Obazoa - ps8
Opisthokonta - ps9
Holozoa - ps10
Filozoa - ps11
Choano./Metazoa - ps12
Metazoa - ps13
Myriazoa - ps14
Parahoxozoa - ps15
Bilateria - ps16
Deuterostomia - ps17
Chordata - ps18
Olfactores - ps19
Vertebrata - ps20
Gnathostomata - ps21
Euteleostomi - ps22
Sarcopterygii - ps23
Tetrapoda - ps24
Amniota - ps25
Mammalia - ps26
Theria - ps27
Placentalia - ps28
Boreoeutheria - ps29
Euarchontoglires - ps30
Euarchonta - ps31
Primates - ps32
Haplorrhini - ps33
Simiiformes - ps34
Catarrhini - ps35
Hominoidea - ps36
Hominidae - ps37
Homininae - ps38
Hominini - ps39
Homo_sapiens - ps40
p-value
5e-11
3e-09
2e-07
2e-05
1e-03
Log-odds
0.5
1.0
1.5
2.0
2.5
3.0
3.5
4.0

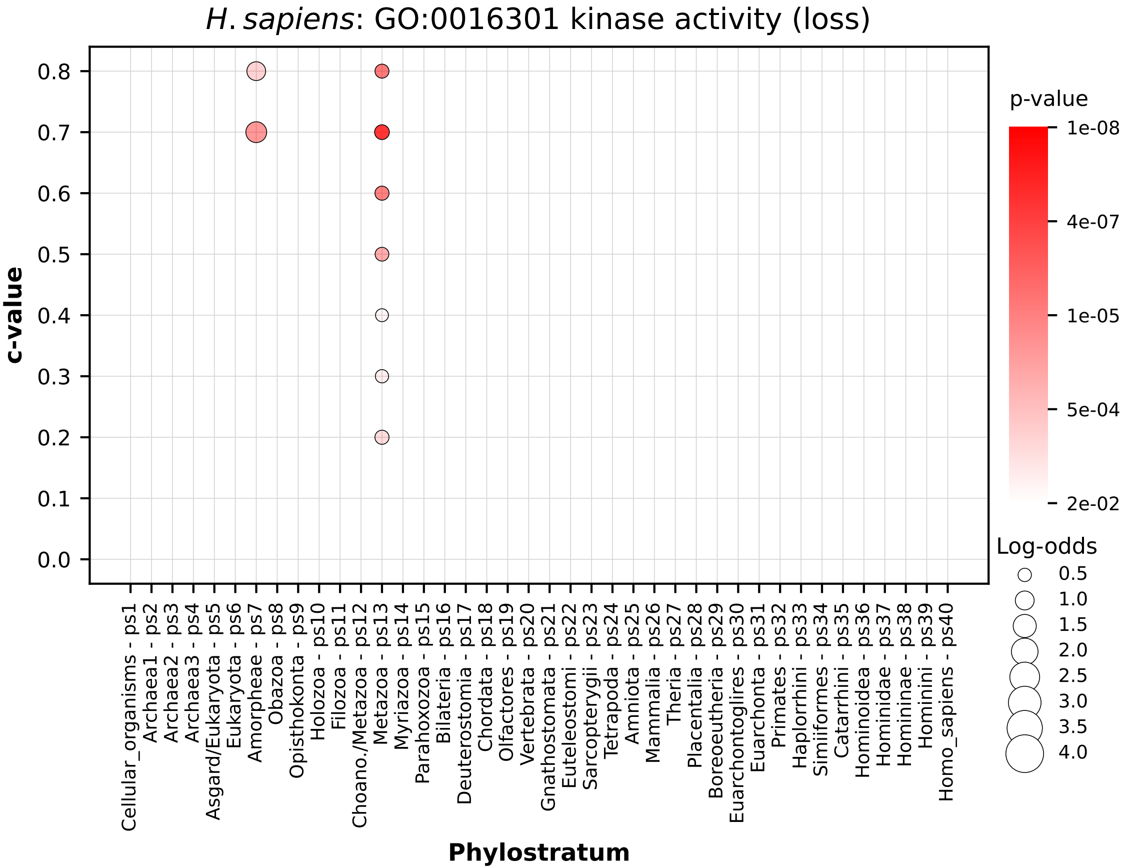

H. sapiens: GO:0016301 kinase activity (loss)
c-value
0.0
0.1
0.2
0.3
0.4
0.5
0.6
0.7
0.8
Phylostratum
Cellular_organisms - ps1
Archaea1 - ps2
Archaea2 - ps3
Archaea3 - ps4
Asgard/Eukaryota - ps5
Eukaryota - ps6
Amorpheae - ps7
Obazoa - ps8
Opisthokonta - ps9
Holozoa - ps10
Filozoa - ps11
Choano./Metazoa - ps12
Metazoa - ps13
Myriazoa - ps14
Parahoxozoa - ps15
Bilateria - ps16
Deuterostomia - ps17
Chordata - ps18
Olfactores - ps19
Vertebrata - ps20
Gnathostomata - ps21
Euteleostomi - ps22
Sarcopterygii - ps23
Tetrapoda - ps24
Amniota - ps25
Mammalia - ps26
Theria - ps27
Placentalia - ps28
Boreoeutheria - ps29
Euarchontoglires - ps30
Euarchonta - ps31
Primates - ps32
Haplorrhini - ps33
Simiiformes - ps34
Catarrhini - ps35
Hominoidea - ps36
Hominidae - ps37
Homininae - ps38
Hominini - ps39
Homo_sapiens - ps40
p-value
1e-08
4e-07
1e-05
5e-04
2e-02
Log-odds
0.5
1.0
1.5
2.0
2.5
3.0
3.5
4.0

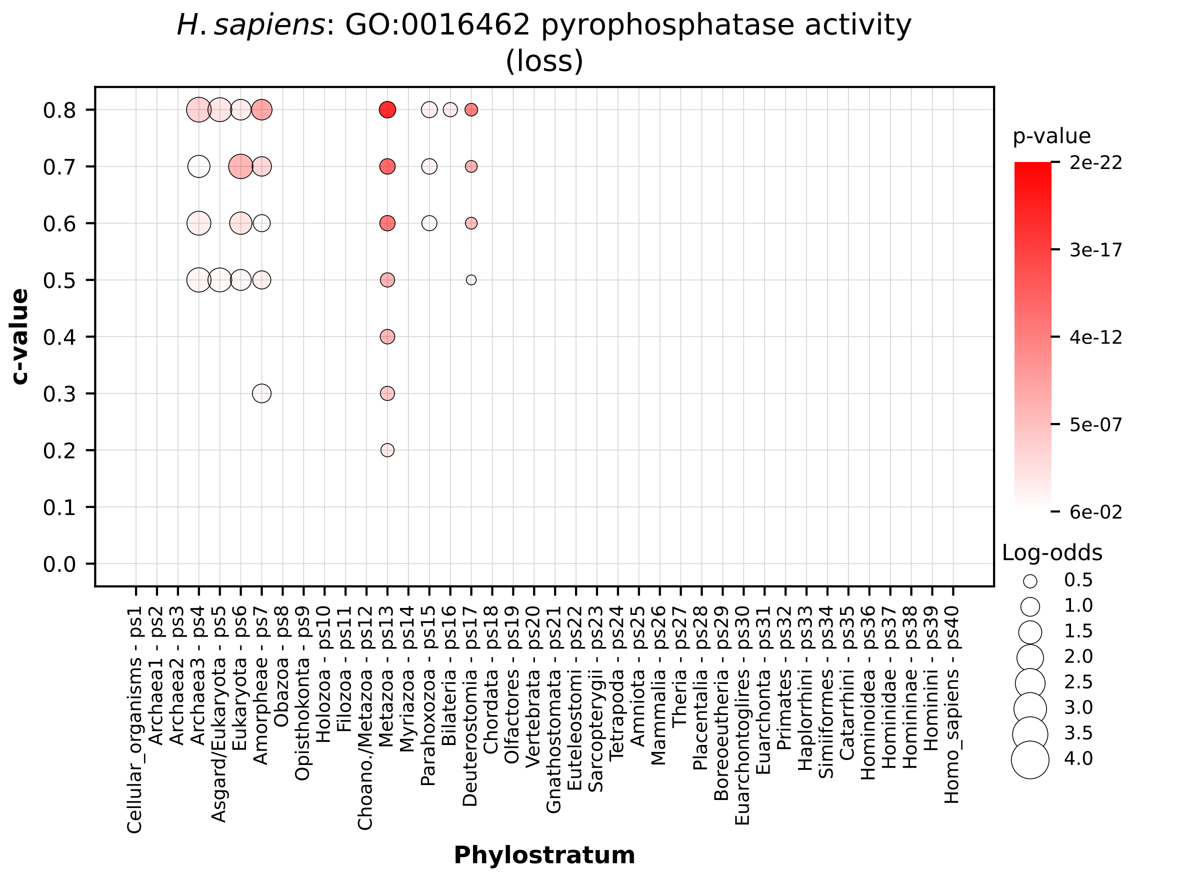

H. sapiens: GO:0016462 pyrophosphatase activity
(loss)
c-value
0.0
0.1
0.2
0.3
0.4
0.5
0.6
0.7
0.8
Phylostratum
Cellular_organisms - ps1
Archaea1 - ps2
Archaea2 - ps3
Archaea3 - ps4
Asgard/Eukaryota - ps5
Eukaryota - ps6
Amorpheae - ps7
Obazoa - ps8
Opisthokonta - ps9
Holozoa - ps10
Filozoa - ps11
Choano./Metazoa - ps12
Metazoa - ps13
Myriazoa - ps14
Parahoxozoa - ps15
Bilateria - ps16
Deuterostomia - ps17
Chordata - ps18
Olfactores - ps19
Vertebrata - ps20
Gnathostomata - ps21
Euteleostomi - ps22
Sarcopterygii - ps23
Tetrapoda - ps24
Amniota - ps25
Mammalia - ps26
Theria - ps27
Placentalia - ps28
Boreoeutheria - ps29
Euarchontoglires - ps30
Euarchonta - ps31
Primates - ps32
Haplorrhini - ps33
Simiiformes - ps34
Catarrhini - ps35
Hominoidea - ps36
Hominidae - ps37
Homininae - ps38
Hominini - ps39
Homo_sapiens - ps40
p-value
2e-22
3e-17
4e-12
5e-07
6e-02
Log-odds
0.5
1.0
1.5
2.0
2.5
3.0
3.5
4.0

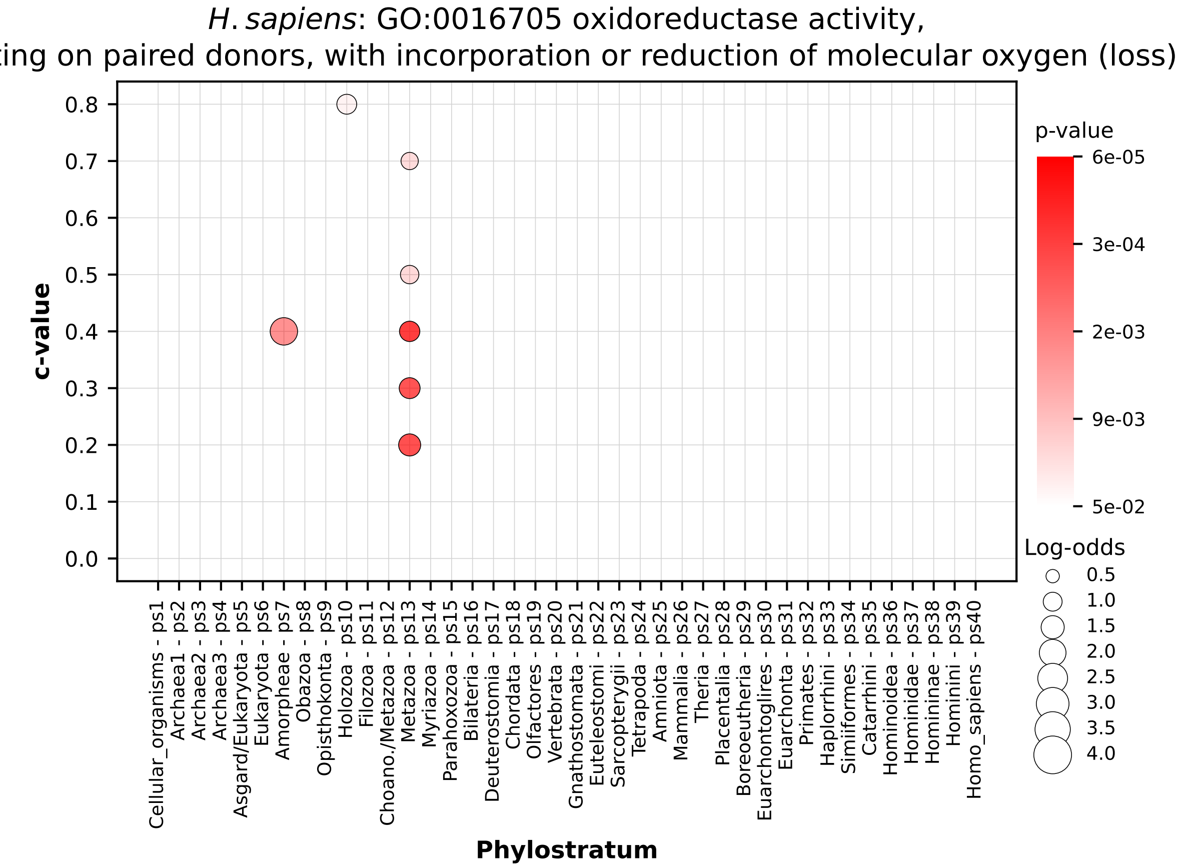
H. sapiens: GO:0016705 oxidoreductase activity,
ting on paired donors, with incorporation or reduction of molecular oxygen (loss)
c-value
0.8
0.7
0.6
0.5
0.4
0.3
0.2
0.1
0.0
p-value
6e-05
3e-04
2e-03
9e-03
5e-02
Log-odds
0.5
1.0
1.5
2.0
2.5
3.0
3.5
4.0
Cellular_organisms - ps1
Archaea1 - ps2
Archaea2 - ps3
Archaea3 - ps4
Asgard/Eukaryota - ps5
Eukaryota - ps6
Amorpheae - ps7
Obazoa - ps8
Opisthokonta - ps9
Holozoa - ps10
Filozoa - ps11
Choano./Metazoa - ps12
Metazoa - ps13
Myriazoa - ps14
Parahoxozoa - ps15
Bilateria - ps16
Deuterostomia - ps17
Chordata - ps18
Olfactores - ps19
Vertebrata - ps20
Gnathostomata - ps21
Euteleostomi - ps22
Sarcopterygii - ps23
Tetrapoda - ps24
Amniota - ps25
Mammalia - ps26
Theria - ps27
Placentalia - ps28
Boreoeutheria - ps29
Euarchontoglires - ps30
Euarchonta - ps31
Primates - ps32
Haplorrhini - ps33
Simiiformes - ps34
Catarrhini - ps35
Hominoidea - ps36
Hominidae - ps37
Homininae - ps38
Hominini - ps39
Homo_sapiens - ps40
Phylostratum

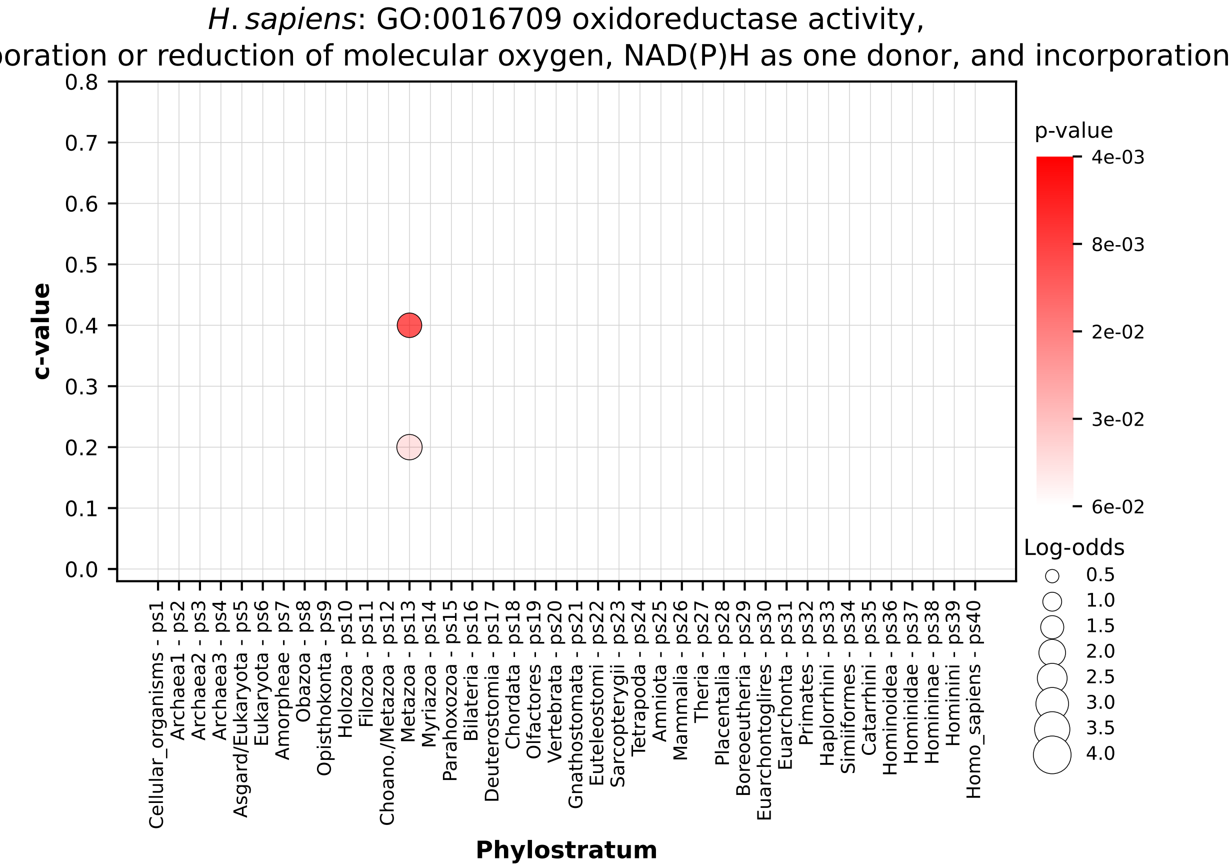

H. sapiens: GO:0016709 oxidoreductase activity,
poration or reduction of molecular oxygen, NAD(P)H as one donor, and incorporation
c-value
0.8
0.7
0.6
0.5
0.4
0.3
0.2
0.1
0.0
p-value
4e-03
8e-03
2e-02
3e-02
6e-02
Log-odds
0.5
1.0
1.5
2.0
2.5
3.0
3.5
4.0
Cellular_organisms - ps1
Archaea1 - ps2
Archaea2 - ps3
Archaea3 - ps4
Asgard/Eukaryota - ps5
Eukaryota - ps6
Amorpheae - ps7
Obazoa - ps8
Opisthokonta - ps9
Holozoa - ps10
Filozoa - ps11
Choano./Metazoa - ps12
Metazoa - ps13
Myriazoa - ps14
Parahoxozoa - ps15
Bilateria - ps16
Deuterostomia - ps17
Chordata - ps18
Olfactores - ps19
Vertebrata - ps20
Gnathostomata - ps21
Euteleostomi - ps22
Sarcopterygii - ps23
Tetrapoda - ps24
Amniota - ps25
Mammalia - ps26
Theria - ps27
Placentalia - ps28
Boreoeutheria - ps29
Euarchontoglires - ps30
Euarchonta - ps31
Primates - ps32
Haplorrhini - ps33
Simiiformes - ps34
Catarrhini - ps35
Hominoidea - ps36
Hominidae - ps37
Homininae - ps38
Hominini - ps39
Homo_sapiens - ps40
Phylostratum

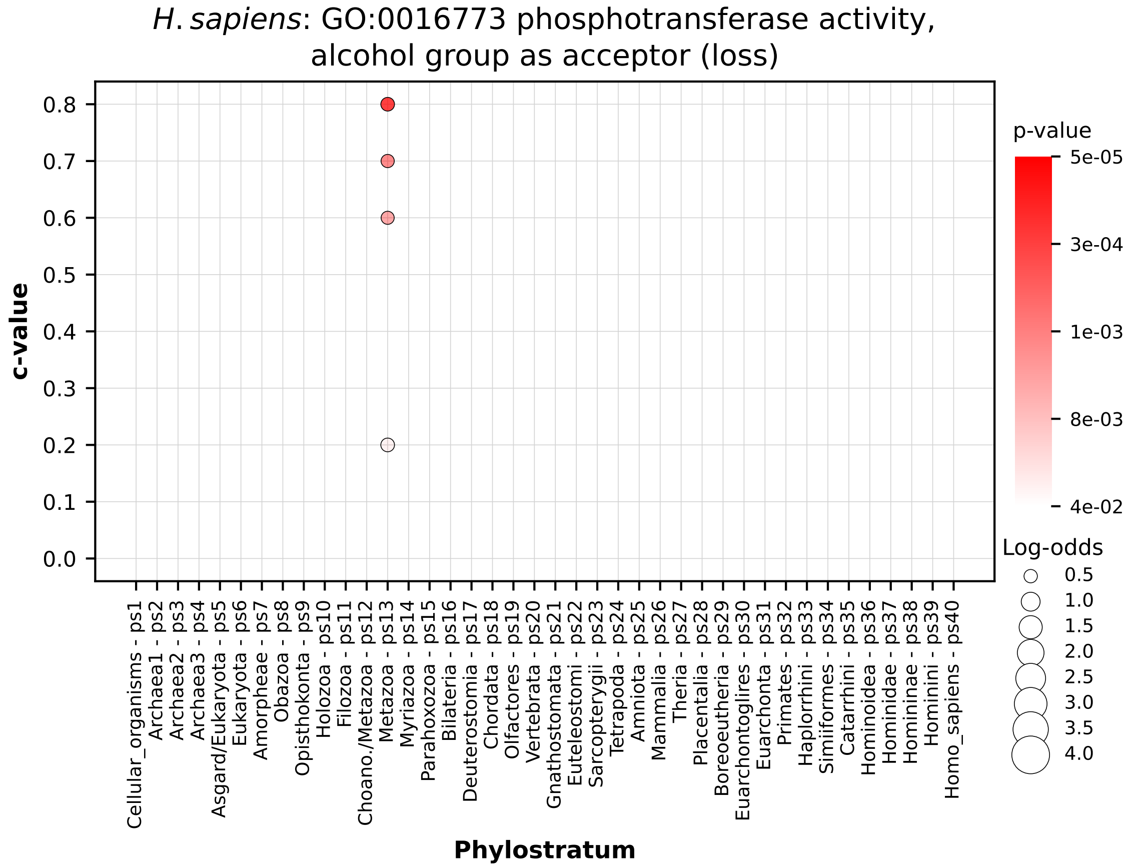

H. sapiens: GO:0016773 phosphotransferase activity, alcohol group as acceptor (loss)
c-value
0.0
0.1
0.2
0.3
0.4
0.5
0.6
0.7
0.8
Phylostratum
Cellular_organisms - ps1
Archaea1 - ps2
Archaea2 - ps3
Archaea3 - ps4
Asgard/Eukaryota - ps5
Eukaryota - ps6
Amorpheae - ps7
Obazoa - ps8
Opisthokonta - ps9
Holozoa - ps10
Filozoa - ps11
Choano./Metazoa - ps12
Metazoa - ps13
Myriazoa - ps14
Parahoxozoa - ps15
Bilateria - ps16
Deuterostomia - ps17
Chordata - ps18
Olfactores - ps19
Vertebrata - ps20
Gnathostomata - ps21
Euteleostomi - ps22
Sarcopterygii - ps23
Tetrapoda - ps24
Amniota - ps25
Mammalia - ps26
Theria - ps27
Placentalia - ps28
Boreoeutheria - ps29
Euarchontoglires - ps30
Euarchonta - ps31
Primates - ps32
Haplorrhini - ps33
Simiiformes - ps34
Catarrhini - ps35
Hominoidea - ps36
Hominidae - ps37
Homininae - ps38
Hominini - ps39
Homo_sapiens - ps40
p-value
5e-05
3e-04
1e-03
8e-03
4e-02
Log-odds
0.5
1.0
1.5
2.0
2.5
3.0
3.5
4.0

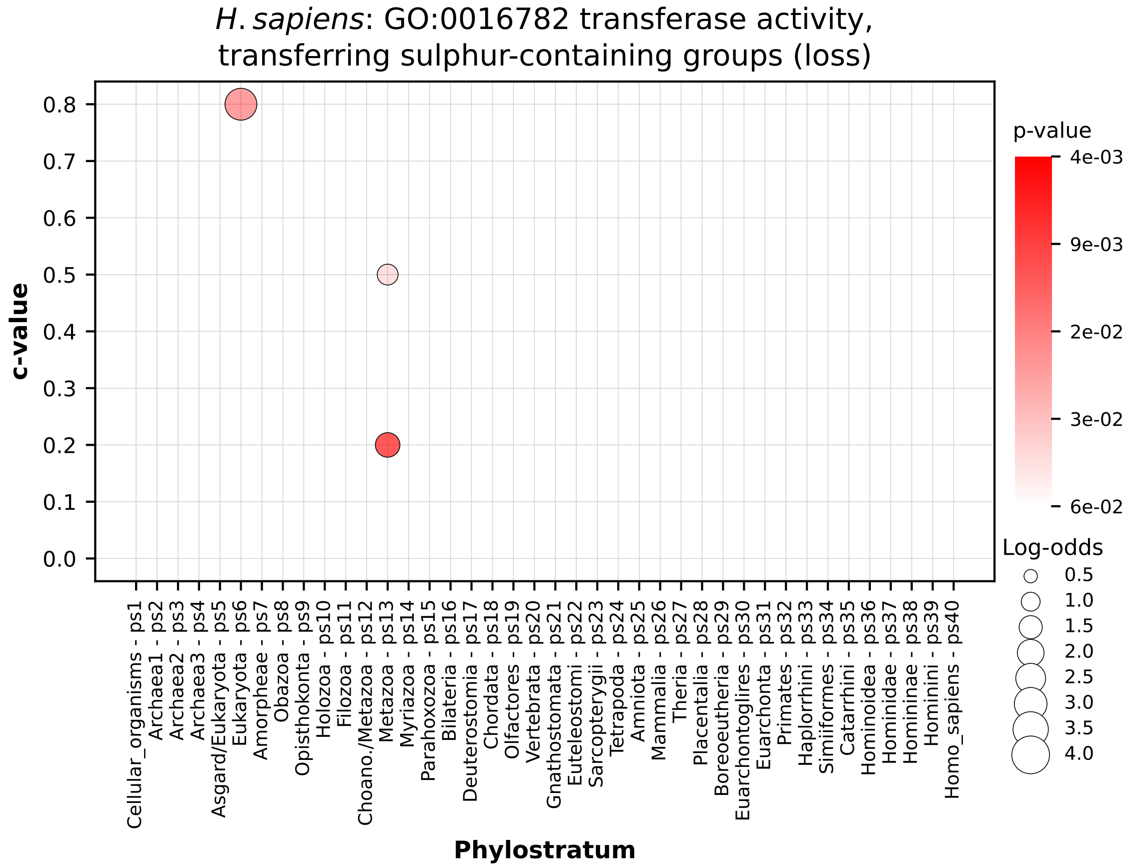

H. sapiens: GO:0016782 transferase activity, transferring sulphur-containing groups (loss)
c-value
0.0
0.1
0.2
0.3
0.4
0.5
0.6
0.7
0.8
Phylostratum
Cellular_organisms - ps1
Archaea1 - ps2
Archaea2 - ps3
Archaea3 - ps4
Asgard/Eukaryota - ps5
Eukaryota - ps6
Amorpheae - ps7
Obazoa - ps8
Opisthokonta - ps9
Holozoa - ps10
Filozoa - ps11
Choano./Metazoa - ps12
Metazoa - ps13
Myriazoa - ps14
Parahoxozoa - ps15
Bilateria - ps16
Deuterostomia - ps17
Chordata - ps18
Olfactores - ps19
Vertebrata - ps20
Gnathostomata - ps21
Euteleostomi - ps22
Sarcopterygii - ps23
Tetrapoda - ps24
Amniota - ps25
Mammalia - ps26
Theria - ps27
Placentalia - ps28
Boreoeutheria - ps29
Euarchontoglires - ps30
Euarchonta - ps31
Primates - ps32
Haplorrhini - ps33
Simiiformes - ps34
Catarrhini - ps35
Hominoidea - ps36
Hominidae - ps37
Homininae - ps38
Hominini - ps39
Homo_sapiens - ps40
p-value
4e-03
9e-03
2e-02
3e-02
6e-02
Log-odds
0.5
1.0
1.5
2.0
2.5
3.0
3.5
4.0

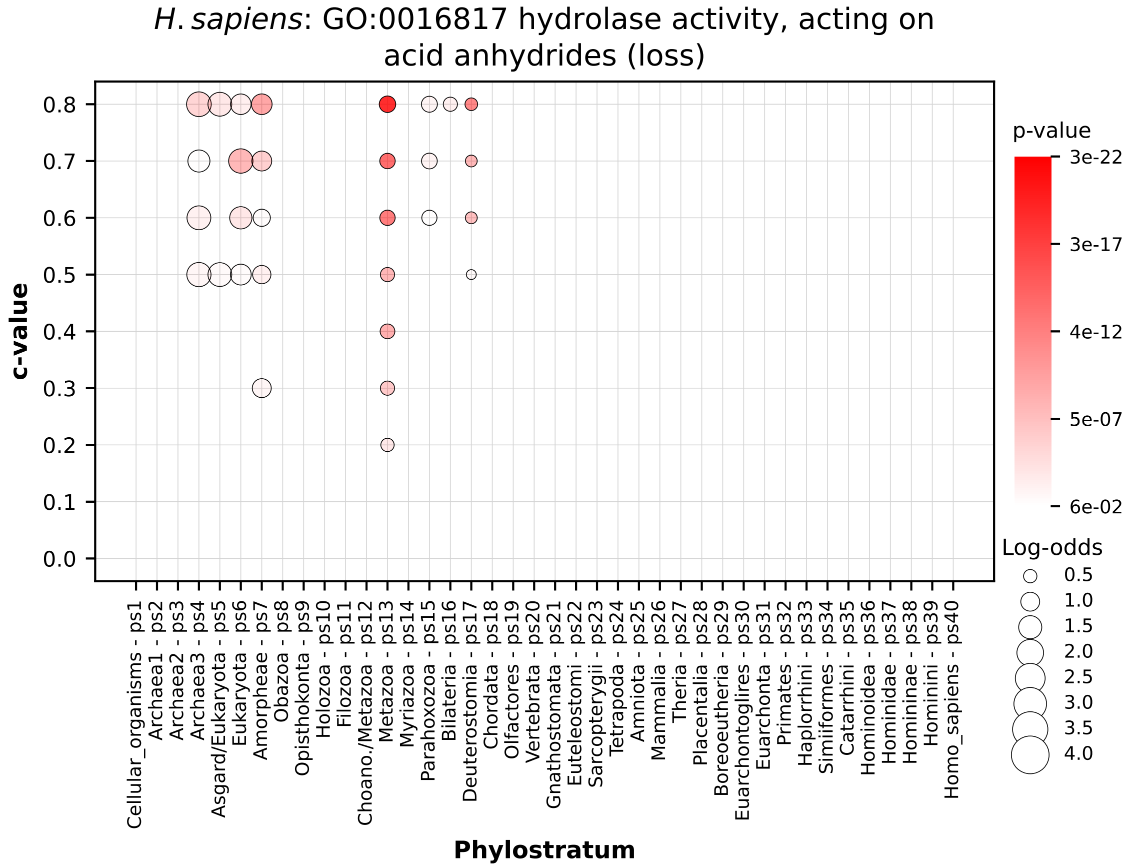

H. sapiens: GO:0016817 hydrolase activity, acting on acid anhydrides (loss)
c-value
0.0
0.1
0.2
0.3
0.4
0.5
0.6
0.7
0.8
Phylostratum
Cellular_organisms - ps1
Archaea1 - ps2
Archaea2 - ps3
Archaea3 - ps4
Asgard/Eukaryota - ps5
Eukaryota - ps6
Amorpheae - ps7
Obazoa - ps8
Opisthokonta - ps9
Holozoa - ps10
Filozoa - ps11
Choano./Metazoa - ps12
Metazoa - ps13
Myriazoa - ps14
Parahoxozoa - ps15
Bilateria - ps16
Deuterostomia - ps17
Chordata - ps18
Olfactores - ps19
Vertebrata - ps20
Gnathostomata - ps21
Euteleostomi - ps22
Sarcopterygii - ps23
Tetrapoda - ps24
Amniota - ps25
Mammalia - ps26
Theria - ps27
Placentalia - ps28
Boreoeutheria - ps29
Euarchontoglires - ps30
Euarchonta - ps31
Primates - ps32
Haplorrhini - ps33
Simiiformes - ps34
Catarrhini - ps35
Hominoidea - ps36
Hominidae - ps37
Homininae - ps38
Hominini - ps39
Homo_sapiens - ps40
p-value
3e-22
3e-17
4e-12
5e-07
6e-02
Log-odds
0.5
1.0
1.5
2.0
2.5
3.0
3.5
4.0

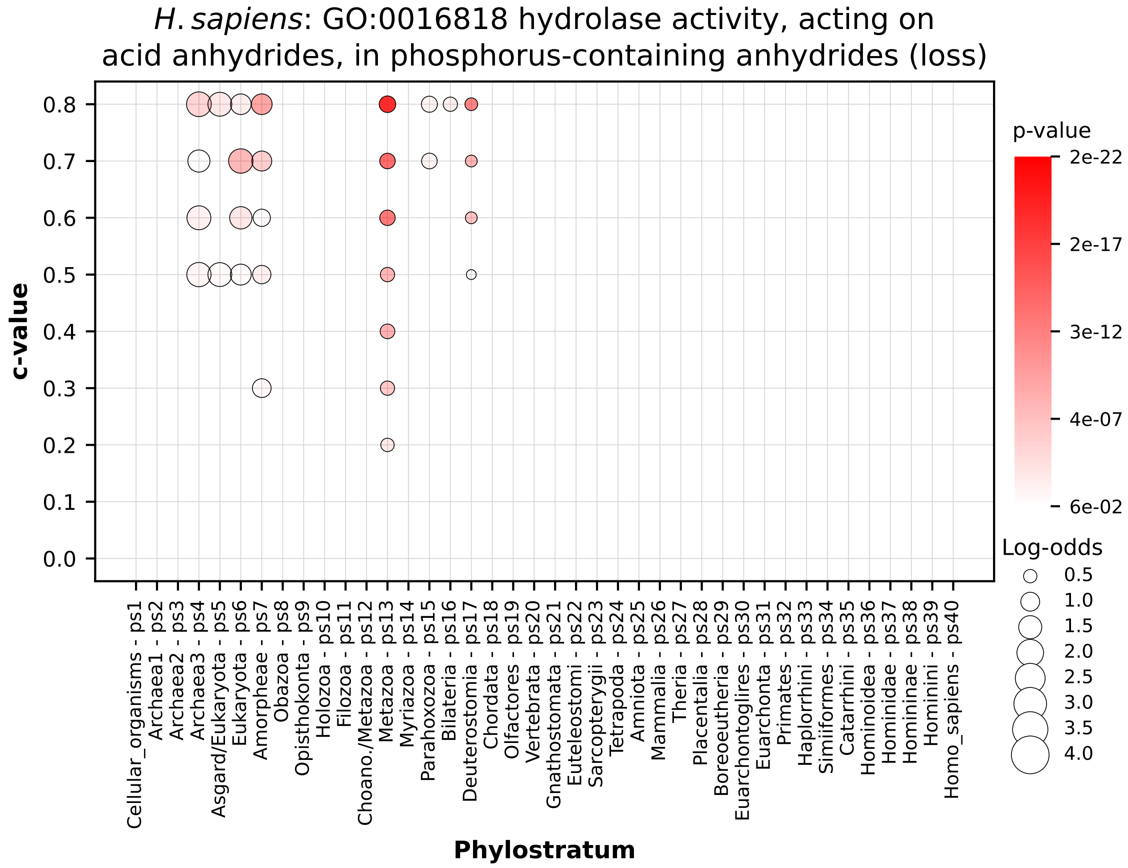
H. sapiens: GO:0016818 hydrolase activity, acting on acid anhydrides, in phosphorus-containing anhydrides (loss)
c-value
0.0
0.1
0.2
0.3
0.4
0.5
0.6
0.7
0.8
Phylostratum
Cellular_organisms - ps1
Archaea1 - ps2
Archaea2 - ps3
Archaea3 - ps4
Asgard/Eukaryota - ps5
Eukaryota - ps6
Amorpheae - ps7
Obazoa - ps8
Opisthokonta - ps9
Holozoa - ps10
Filozoa - ps11
Choano./Metazoa - ps12
Metazoa - ps13
Myriazoa - ps14
Parahoxozoa - ps15
Bilateria - ps16
Deuterostomia - ps17
Chordata - ps18
Olfactores - ps19
Vertebrata - ps20
Gnathostomata - ps21
Euteleostomi - ps22
Sarcopterygii - ps23
Tetrapoda - ps24
Amniota - ps25
Mammalia - ps26
Theria - ps27
Placentalia - ps28
Boreoeutheria - ps29
Euarchontoglires - ps30
Euarchonta - ps31
Primates - ps32
Haplorrhini - ps33
Simiiformes - ps34
Catarrhini - ps35
Hominoidea - ps36
Hominidae - ps37
Homininae - ps38
Hominini - ps39
Homo_sapiens - ps40
p-value
2e-22
2e-17
3e-12
4e-07
6e-02
Log-odds
0.5
1.0
1.5
2.0
2.5
3.0
3.5
4.0

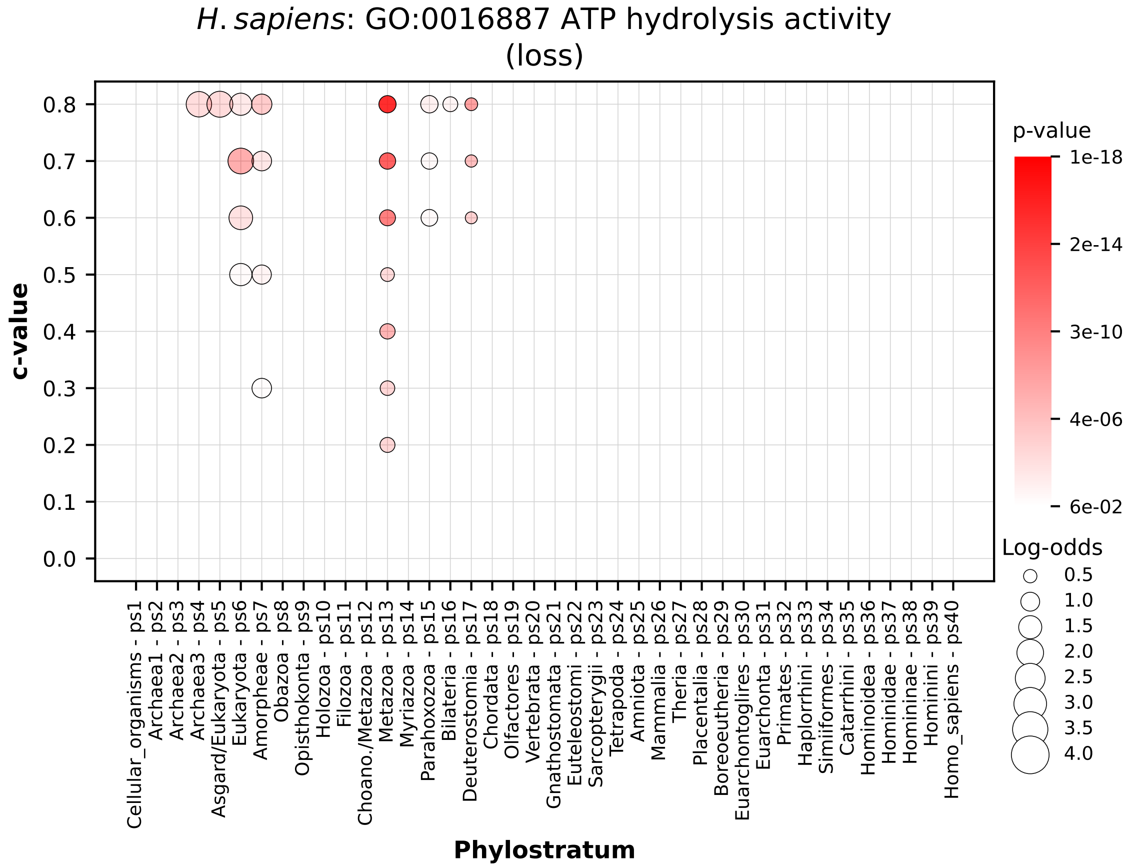

H. sapiens: GO:0016887 ATP hydrolysis activity
(loss)
c-value
0.8
0.7
0.6
0.5
0.4
0.3
0.2
0.1
0.0
Phylostratum
Cellular_organisms - ps1
Archaea1 - ps2
Archaea2 - ps3
Archaea3 - ps4
Asgard/Eukaryota - ps5
Eukaryota - ps6
Amorpheae - ps7
Obazoa - ps8
Opisthokonta - ps9
Holozoa - ps10
Filozoa - ps11
Choano./Metazoa - ps12
Metazoa - ps13
Myriazoa - ps14
Parahoxozoa - ps15
Bilateria - ps16
Deuterostomia - ps17
Chordata - ps18
Olfactores - ps19
Vertebrata - ps20
Gnathostomata - ps21
Euteleostomi - ps22
Sarcopterygii - ps23
Tetrapoda - ps24
Amniota - ps25
Mammalia - ps26
Theria - ps27
Placentalia - ps28
Boreoeutheria - ps29
Euarchontoglires - ps30
Euarchonta - ps31
Primates - ps32
Haplorrhini - ps33
Simiiformes - ps34
Catarrhini - ps35
Hominoidea - ps36
Hominidae - ps37
Homininae - ps38
Hominini - ps39
Homo_sapiens - ps40
p-value
1e-18
2e-14
3e-10
4e-06
6e-02
Log-odds
0.5
1.0
1.5
2.0
2.5
3.0
3.5
4.0

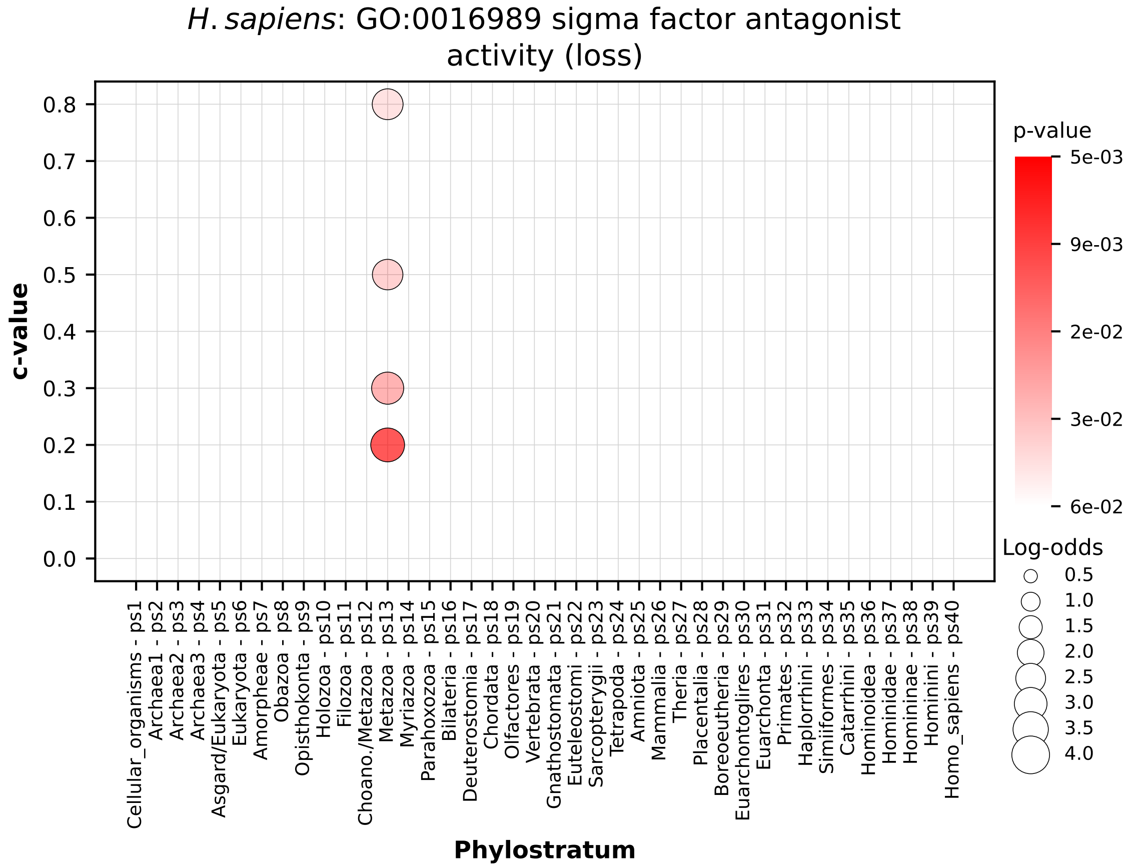

H. sapiens: GO:0016989 sigma factor antagonist activity (loss)
c-value
0.8
0.7
0.6
0.5
0.4
0.3
0.2
0.1
0.0
p-value
5e-03
9e-03
2e-02
3e-02
6e-02
Log-odds
0.5
1.0
1.5
2.0
2.5
3.0
3.5
4.0
Cellular_organisms - ps1
Archaea1 - ps2
Archaea2 - ps3
Archaea3 - ps4
Asgard/Eukaryota - ps5
Eukaryota - ps6
Amorpheae - ps7
Obazoa - ps8
Opisthokonta - ps9
Holozoa - ps10
Filozoa - ps11
Choano./Metazoa - ps12
Metazoa - ps13
Myriazoa - ps14
Parahoxozoa - ps15
Bilateria - ps16
Deuterostomia - ps17
Chordata - ps18
Olfactores - ps19
Vertebrata - ps20
Gnathostomata - ps21
Euteleostomi - ps22
Sarcopterygii - ps23
Tetrapoda - ps24
Amniota - ps25
Mammalia - ps26
Theria - ps27
Placentalia - ps28
Boreoeutheria - ps29
Euarchontoglires - ps30
Euarchonta - ps31
Primates - ps32
Haplorrhini - ps33
Simiiformes - ps34
Catarrhini - ps35
Hominoidea - ps36
Hominidae - ps37
Homininae - ps38
Hominini - ps39
Homo_sapiens - ps40
Phylostratum

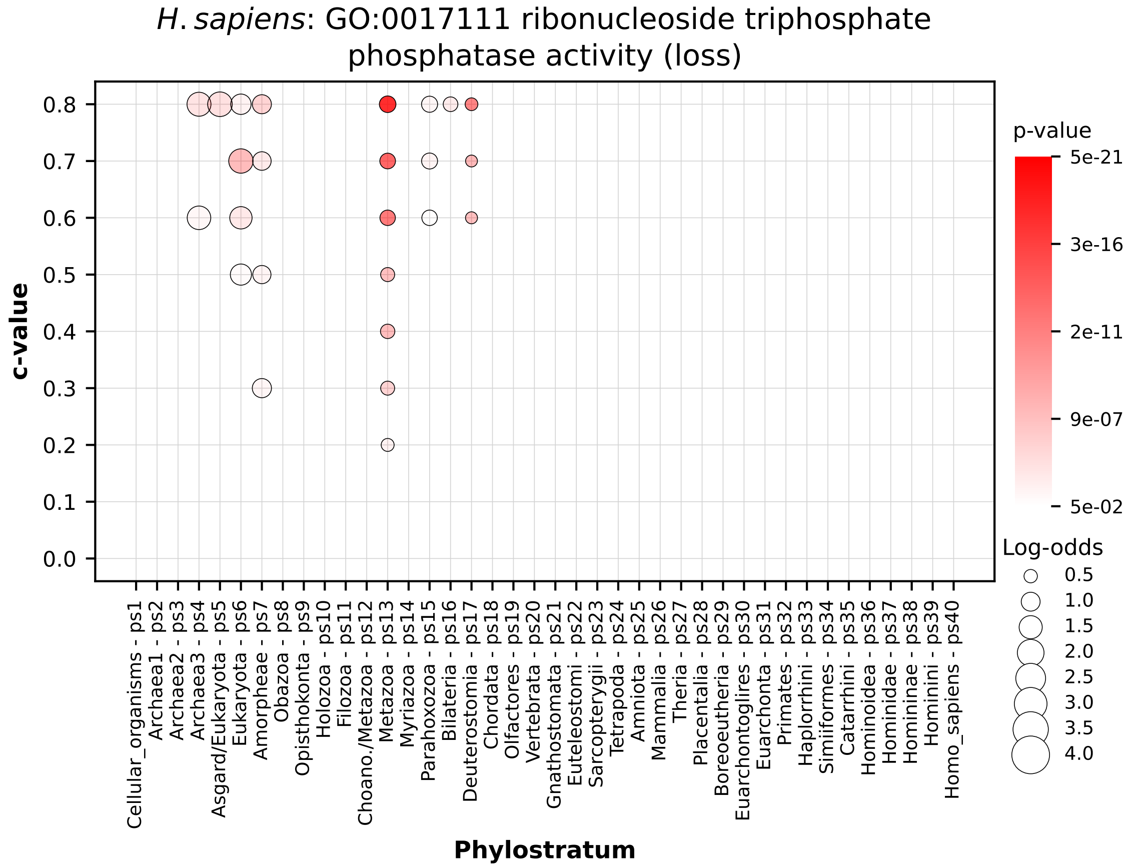

H. sapiens: GO:0017111 ribonucleoside triphosphate phosphatase activity (loss)
c-value
0.8
0.7
0.6
0.5
0.4
0.3
0.2
0.1
0.0
Phylostratum
Cellular_organisms - ps1
Archaea1 - ps2
Archaea2 - ps3
Archaea3 - ps4
Asgard/Eukaryota - ps5
Eukaryota - ps6
Amorpheae - ps7
Obazoa - ps8
Opisthokonta - ps9
Holozoa - ps10
Filozoa - ps11
Choano./Metazoa - ps12
Metazoa - ps13
Myriazoa - ps14
Parahoxozoa - ps15
Bilateria - ps16
Deuterostomia - ps17
Chordata - ps18
Olfactores - ps19
Vertebrata - ps20
Gnathostomata - ps21
Euteleostomi - ps22
Sarcopterygii - ps23
Tetrapoda - ps24
Amniota - ps25
Mammalia - ps26
Theria - ps27
Placentalia - ps28
Boreoeutheria - ps29
Euarchontoglires - ps30
Euarchonta - ps31
Primates - ps32
Haplorrhini - ps33
Simiiformes - ps34
Catarrhini - ps35
Hominoidea - ps36
Hominidae - ps37
Homininae - ps38
Hominini - ps39
Homo_sapiens - ps40
p-value
5e-21
3e-16
2e-11
9e-07
5e-02
Log-odds
0.5
1.0
1.5
2.0
2.5
3.0
3.5
4.0

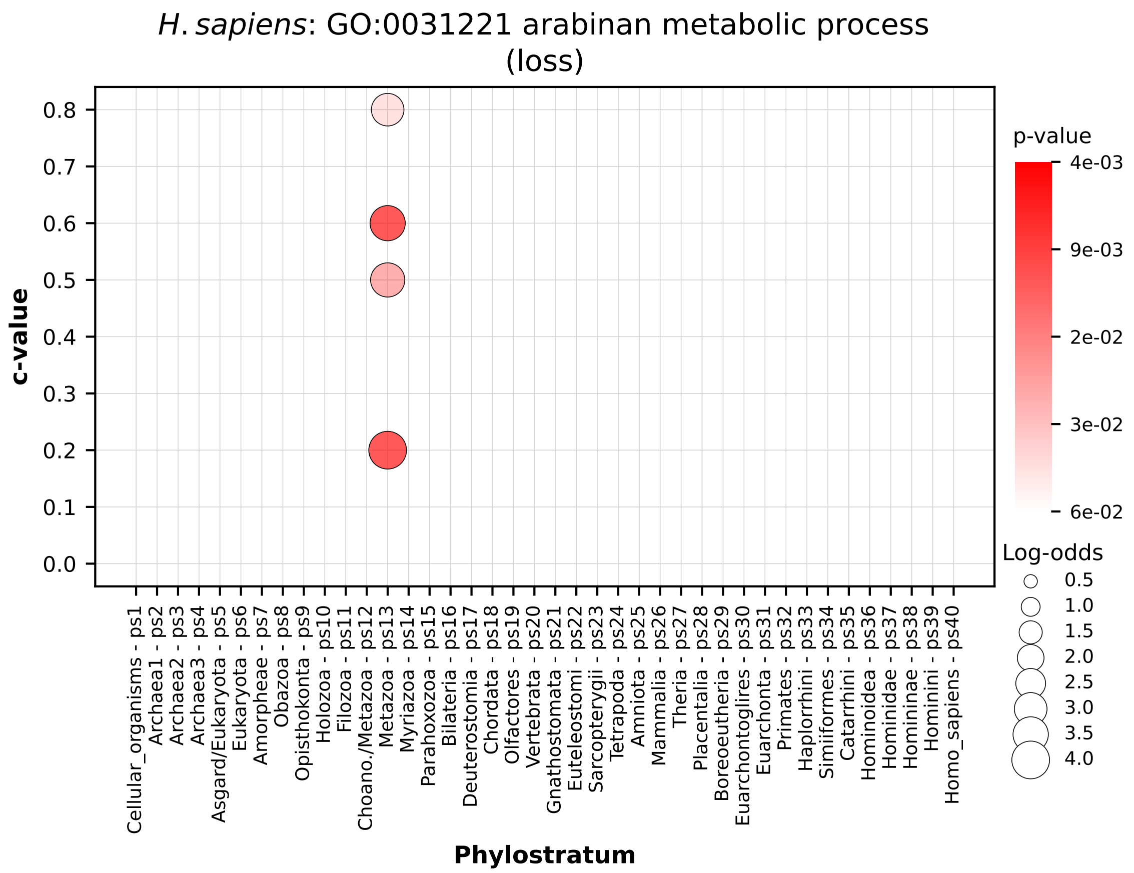

H. sapiens: GO:0031221 arabinan metabolic process
(loss)
c-value
0.0
0.1
0.2
0.3
0.4
0.5
0.6
0.7
0.8
p-value
4e-03
9e-03
2e-02
3e-02
6e-02
Log-odds
0.5
1.0
1.5
2.0
2.5
3.0
3.5
4.0
Cellular_organisms - ps1
Archaea1 - ps2
Archaea2 - ps3
Archaea3 - ps4
Asgard/Eukaryota - ps5
Eukaryota - ps6
Amorpheae - ps7
Obazoa - ps8
Opisthokonta - ps9
Holozoa - ps10
Filozoa - ps11
Choano./Metazoa - ps12
Metazoa - ps13
Myriazoa - ps14
Parahoxozoa - ps15
Bilateria - ps16
Deuterostomia - ps17
Chordata - ps18
Olfactores - ps19
Vertebrata - ps20
Gnathostomata - ps21
Euteleostomi - ps22
Sarcopterygii - ps23
Tetrapoda - ps24
Amniota - ps25
Mammalia - ps26
Theria - ps27
Placentalia - ps28
Boreoeutheria - ps29
Euarchontoglires - ps30
Euarchonta - ps31
Primates - ps32
Haplorrhini - ps33
Simiiformes - ps34
Catarrhini - ps35
Hominoidea - ps36
Hominidae - ps37
Homininae - ps38
Hominini - ps39
Homo_sapiens - ps40
Phylostratum

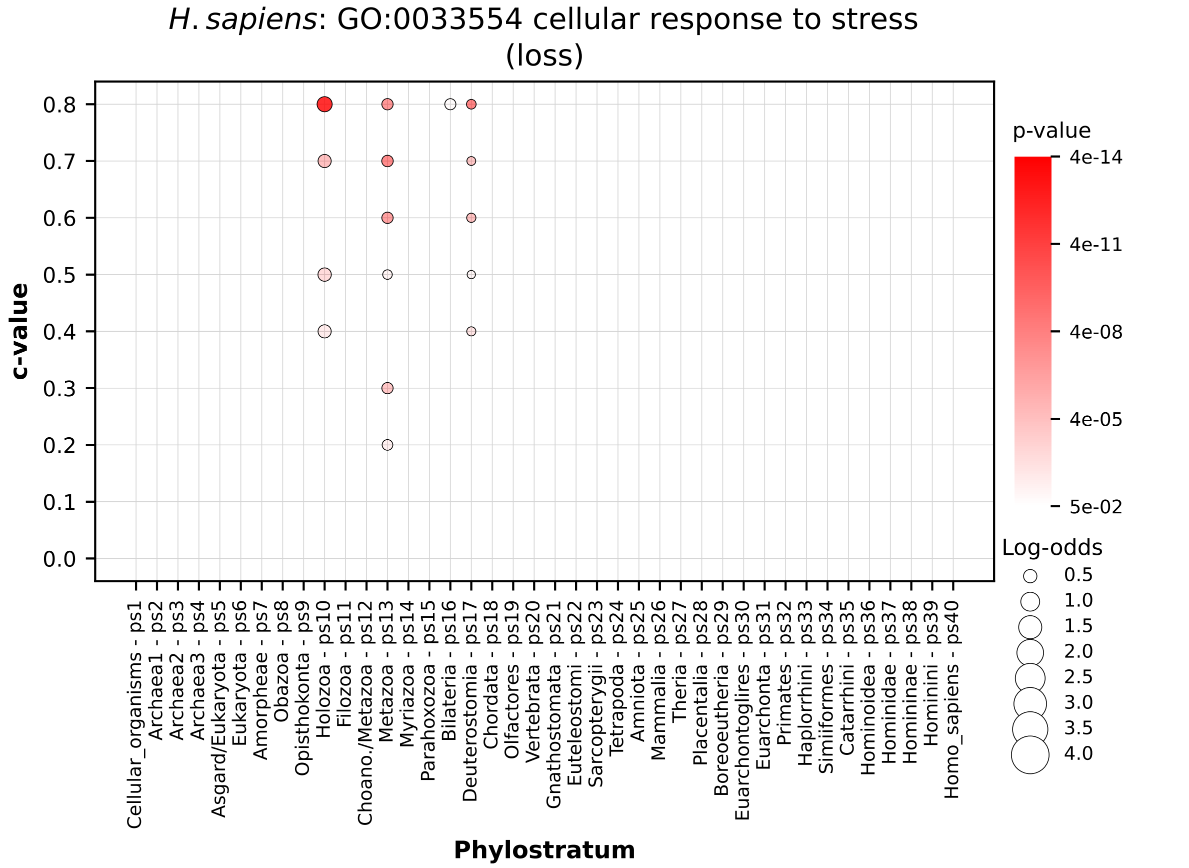

H. sapiens: GO:0033554 cellular response to stress (loss)
c-value
0.8
0.7
0.6
0.5
0.4
0.3
0.2
0.1
0.0
Phylostratum
Cellular_organisms - ps1
Archaea1 - ps2
Archaea2 - ps3
Archaea3 - ps4
Asgard/Eukaryota - ps5
Eukaryota - ps6
Amorpheae - ps7
Obazoa - ps8
Opisthokonta - ps9
Holozoa - ps10
Filozoa - ps11
Choano./Metazoa - ps12
Metazoa - ps13
Myriazoa - ps14
Parahoxozoa - ps15
Bilateria - ps16
Deuterostomia - ps17
Chordata - ps18
Olfactores - ps19
Vertebrata - ps20
Gnathostomata - ps21
Euteleostomi - ps22
Sarcopterygii - ps23
Tetrapoda - ps24
Amniota - ps25
Mammalia - ps26
Theria - ps27
Placentalia - ps28
Boreoeutheria - ps29
Euarchontoglires - ps30
Euarchonta - ps31
Primates - ps32
Haplorrhini - ps33
Simiiformes - ps34
Catarrhini - ps35
Hominoidea - ps36
Hominidae - ps37
Homininae - ps38
Hominini - ps39
Homo_sapiens - ps40
p-value
4e-14
4e-11
4e-08
4e-05
5e-02
Log-odds
0.5
1.0
1.5
2.0
2.5
3.0
3.5
4.0

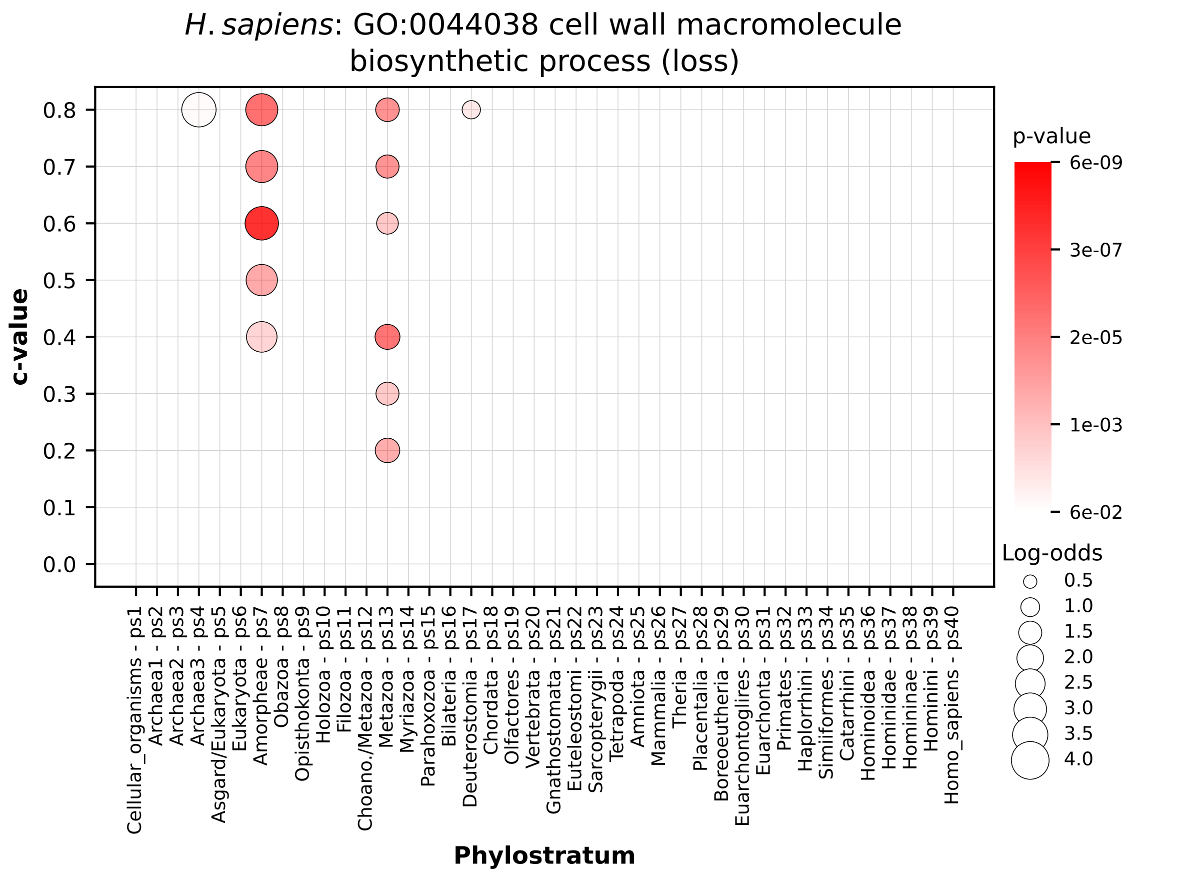

H. sapiens: GO:0044038 cell wall macromolecule biosynthetic process (loss)
c-value
0.8
0.7
0.6
0.5
0.4
0.3
0.2
0.1
0.0
p-value
6e-09
3e-07
2e-05
1e-03
6e-02
Log-odds
0.5
1.0
1.5
2.0
2.5
3.0
3.5
4.0
Cellular_organisms - ps1
Archaea1 - ps2
Archaea2 - ps3
Archaea3 - ps4
Asgard/Eukaryota - ps5
Eukaryota - ps6
Amorpheae - ps7
Obazoa - ps8
Opisthokonta - ps9
Holozoa - ps10
Filozoa - ps11
Choano./Metazoa - ps12
Metazoa - ps13
Myriazoa - ps14
Parahoxozoa - ps15
Bilateria - ps16
Deuterostomia - ps17
Chordata - ps18
Olfactores - ps19
Vertebrata - ps20
Gnathostomata - ps21
Euteleostomi - ps22
Sarcopterygii - ps23
Tetrapoda - ps24
Amniota - ps25
Mammalia - ps26
Theria - ps27
Placentalia - ps28
Boreoeutheria - ps29
Euarchontoglires - ps30
Euarchonta - ps31
Primates - ps32
Haplorrhini - ps33
Simiiformes - ps34
Catarrhini - ps35
Hominoidea - ps36
Hominidae - ps37
Homininae - ps38
Hominini - ps39
Homo_sapiens - ps40
Phylostratum

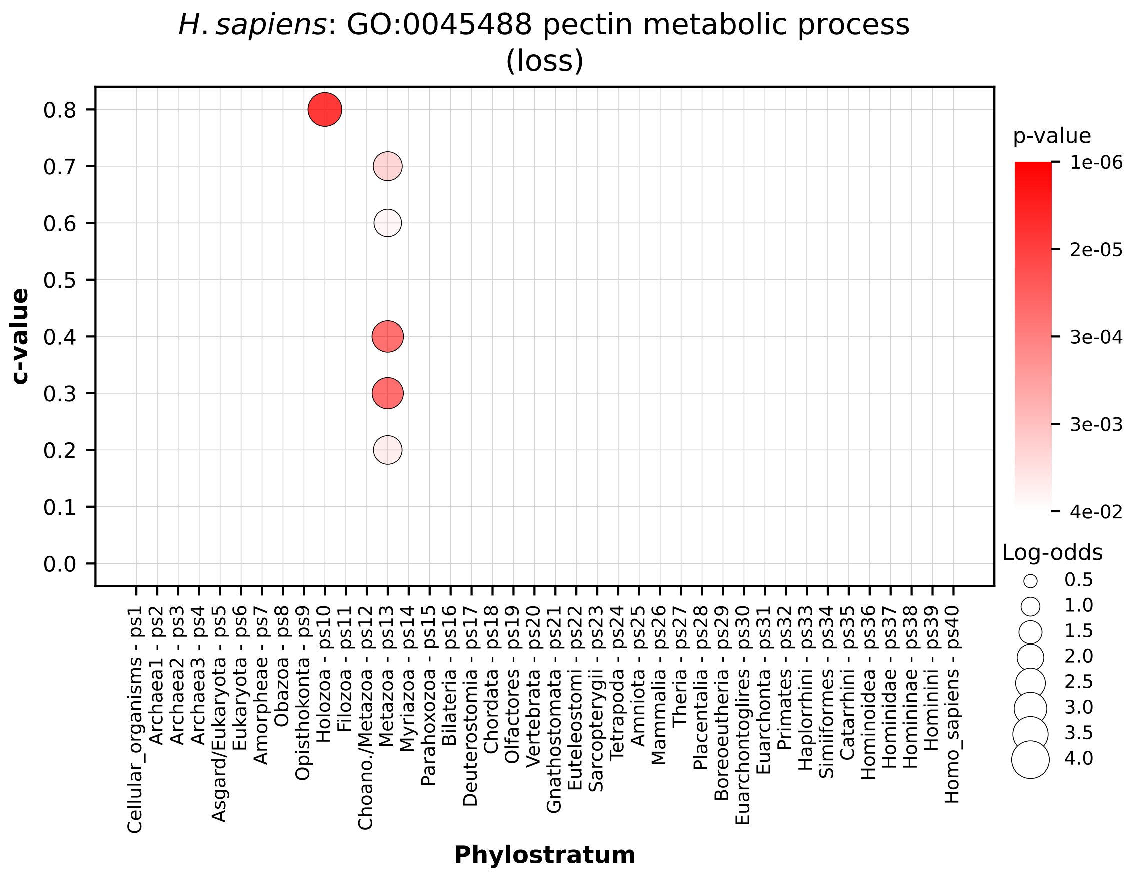

H. sapiens: GO:0045488 pectin metabolic process
(loss)
c-value
0.0
0.1
0.2
0.3
0.4
0.5
0.6
0.7
0.8
Phylostratum
Cellular_organisms - ps1
Archaea1 - ps2
Archaea2 - ps3
Archaea3 - ps4
Asgard/Eukaryota - ps5
Eukaryota - ps6
Amorpheae - ps7
Obazoa - ps8
Opisthokonta - ps9
Holozoa - ps10
Filozoa - ps11
Choano./Metazoa - ps12
Metazoa - ps13
Myriazoa - ps14
Parahoxozoa - ps15
Bilateria - ps16
Deuterostomia - ps17
Chordata - ps18
Olfactores - ps19
Vertebrata - ps20
Gnathostomata - ps21
Euteleostomi - ps22
Sarcopterygii - ps23
Tetrapoda - ps24
Amniota - ps25
Mammalia - ps26
Theria - ps27
Placentalia - ps28
Boreoeutheria - ps29
Euarchontoglires - ps30
Euarchonta - ps31
Primates - ps32
Haplorrhini - ps33
Simiiformes - ps34
Catarrhini - ps35
Hominoidea - ps36
Hominidae - ps37
Homininae - ps38
Hominini - ps39
Homo_sapiens - ps40
p-value
1e-06
2e-05
3e-04
3e-03
4e-02
Log-odds
0.5
1.0
1.5
2.0
2.5
3.0
3.5
4.0

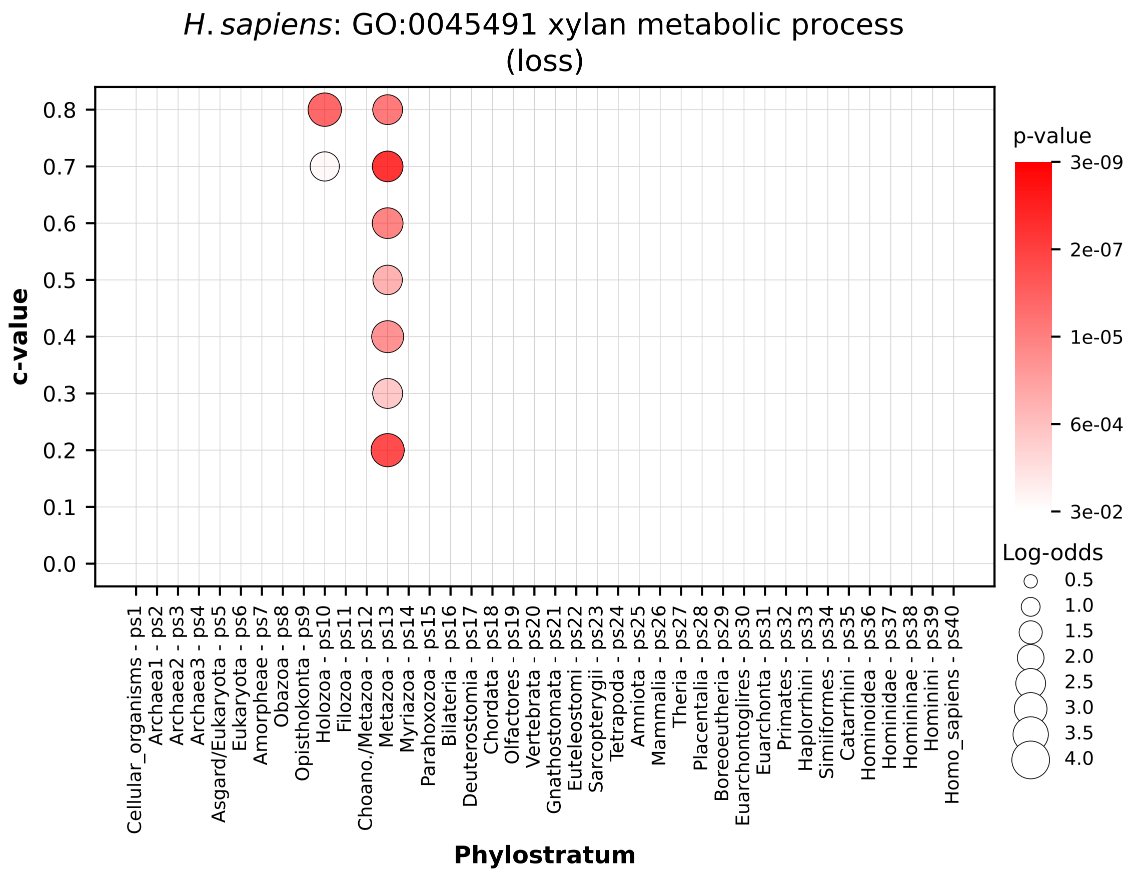

H. sapiens: GO:0045491 xylan metabolic process
(loss)
c-value
0.8
0.7
0.6
0.5
0.4
0.3
0.2
0.1
0.0
p-value
3e-09
2e-07
1e-05
6e-04
3e-02
Log-odds
0.5
1.0
1.5
2.0
2.5
3.0
3.5
4.0
Cellular_organisms - ps1
Archaea1 - ps2
Archaea2 - ps3
Archaea3 - ps4
Asgard/Eukaryota - ps5
Eukaryota - ps6
Amorpheae - ps7
Obazoa - ps8
Opisthokonta - ps9
Holozoa - ps10
Filozoa - ps11
Choano./Metazoa - ps12
Metazoa - ps13
Myriazoa - ps14
Parahoxozoa - ps15
Bilateria - ps16
Deuterostomia - ps17
Chordata - ps18
Olfactores - ps19
Vertebrata - ps20
Gnathostomata - ps21
Euteleostomi - ps22
Sarcopterygii - ps23
Tetrapoda - ps24
Amniota - ps25
Mammalia - ps26
Theria - ps27
Placentalia - ps28
Boreoeutheria - ps29
Euarchontoglires - ps30
Euarchonta - ps31
Primates - ps32
Haplorrhini - ps33
Simiiformes - ps34
Catarrhini - ps35
Hominoidea - ps36
Hominidae - ps37
Homininae - ps38
Hominini - ps39
Homo_sapiens - ps40
Phylostratum

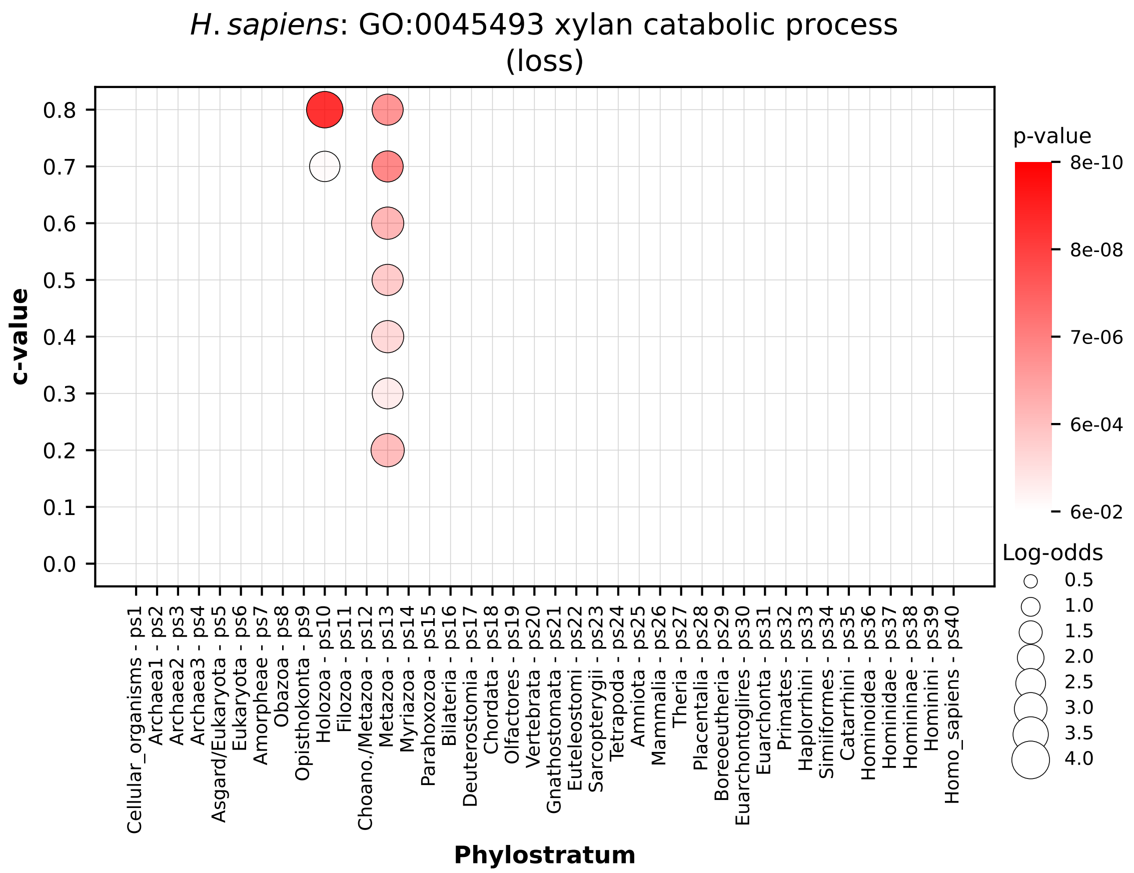

$H. sapiens$: GO:0045493 xylan catabolic process
(loss)
c-value
0.8
0.7
0.6
0.5
0.4
0.3
0.2
0.1
0.0
p-value
8e-10
8e-08
7e-06
6e-04
6e-02
Log-odds
0.5
1.0
1.5
2.0
2.5
3.0
3.5
4.0
Cellular_organisms - ps1
Archaea1 - ps2
Archaea2 - ps3
Archaea3 - ps4
Asgard/Eukaryota - ps5
Eukaryota - ps6
Amorpheae - ps7
Obazoa - ps8
Opisthokonta - ps9
Holozoa - ps10
Filozoa - ps11
Choano./Metazoa - ps12
Metazoa - ps13
Myriazoa - ps14
Parahoxozoa - ps15
Bilateria - ps16
Deuterostomia - ps17
Chordata - ps18
Olfactores - ps19
Vertebrata - ps20
Gnathostomata - ps21
Euteleostomi - ps22
Sarcopterygii - ps23
Tetrapoda - ps24
Amniota - ps25
Mammalia - ps26
Theria - ps27
Placentalia - ps28
Boreoeutheria - ps29
Euarchontoglires - ps30
Euarchonta - ps31
Primates - ps32
Haplorrhini - ps33
Simiiformes - ps34
Catarrhini - ps35
Hominoidea - ps36
Hominidae - ps37
Homininae - ps38
Hominini - ps39
Homo_sapiens - ps40
Phylostratum

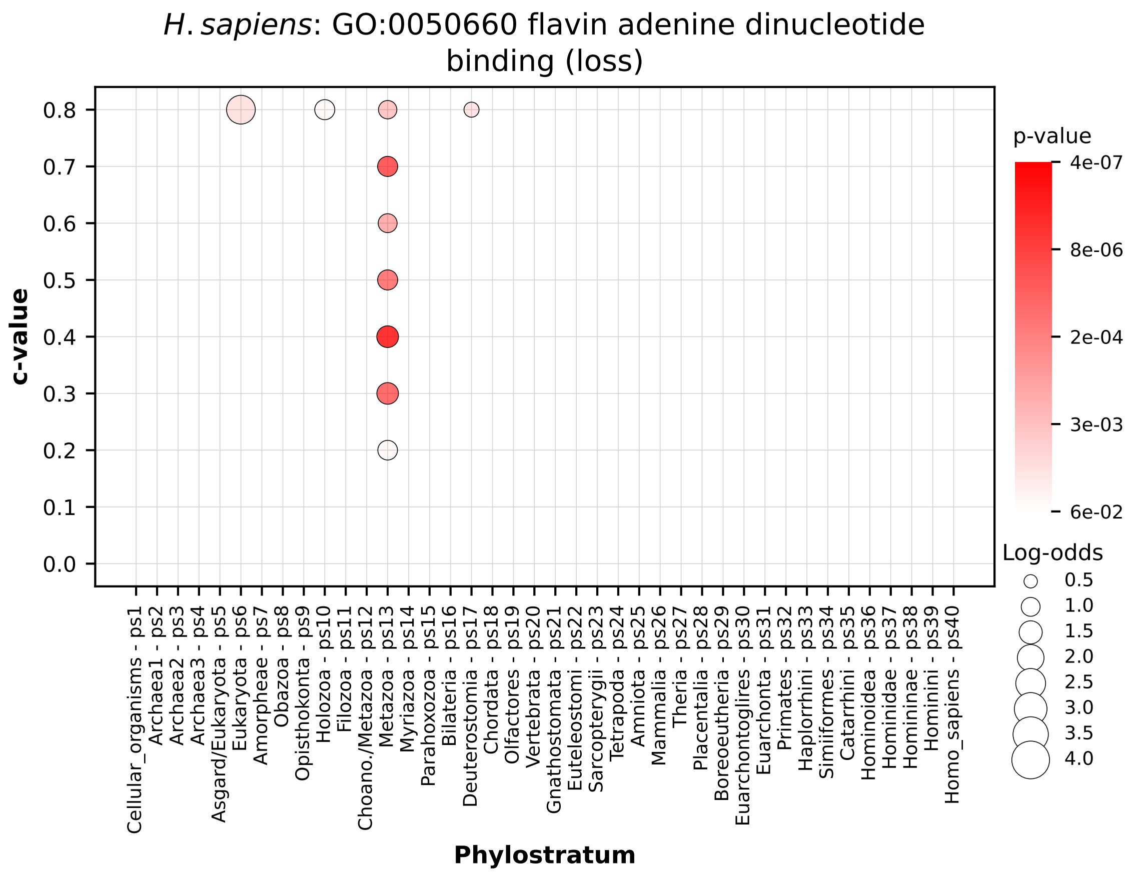

H. sapiens: GO:0050660 flavin adenine dinucleotide binding (loss)
c-value
0.0
0.1
0.2
0.3
0.4
0.5
0.6
0.7
0.8
Phylostratum
Cellular_organisms - ps1
Archaea1 - ps2
Archaea2 - ps3
Archaea3 - ps4
Asgard/Eukaryota - ps5
Eukaryota - ps6
Amorpheae - ps7
Obazoa - ps8
Opisthokonta - ps9
Holozoa - ps10
Filozoa - ps11
Choano./Metazoa - ps12
Metazoa - ps13
Myriazoa - ps14
Parahoxozoa - ps15
Bilateria - ps16
Deuterostomia - ps17
Chordata - ps18
Olfactores - ps19
Vertebrata - ps20
Gnathostomata - ps21
Euteleostomi - ps22
Sarcopterygii - ps23
Tetrapoda - ps24
Amniota - ps25
Mammalia - ps26
Theria - ps27
Placentalia - ps28
Boreoeutheria - ps29
Euarchontoglires - ps30
Euarchonta - ps31
Primates - ps32
Haplorrhini - ps33
Simiiformes - ps34
Catarrhini - ps35
Hominoidea - ps36
Hominidae - ps37
Homininae - ps38
Hominini - ps39
Homo_sapiens - ps40
p-value
4e-07
8e-06
2e-04
3e-03
6e-02
Log-odds
0.5
1.0
1.5
2.0
2.5
3.0
3.5
4.0

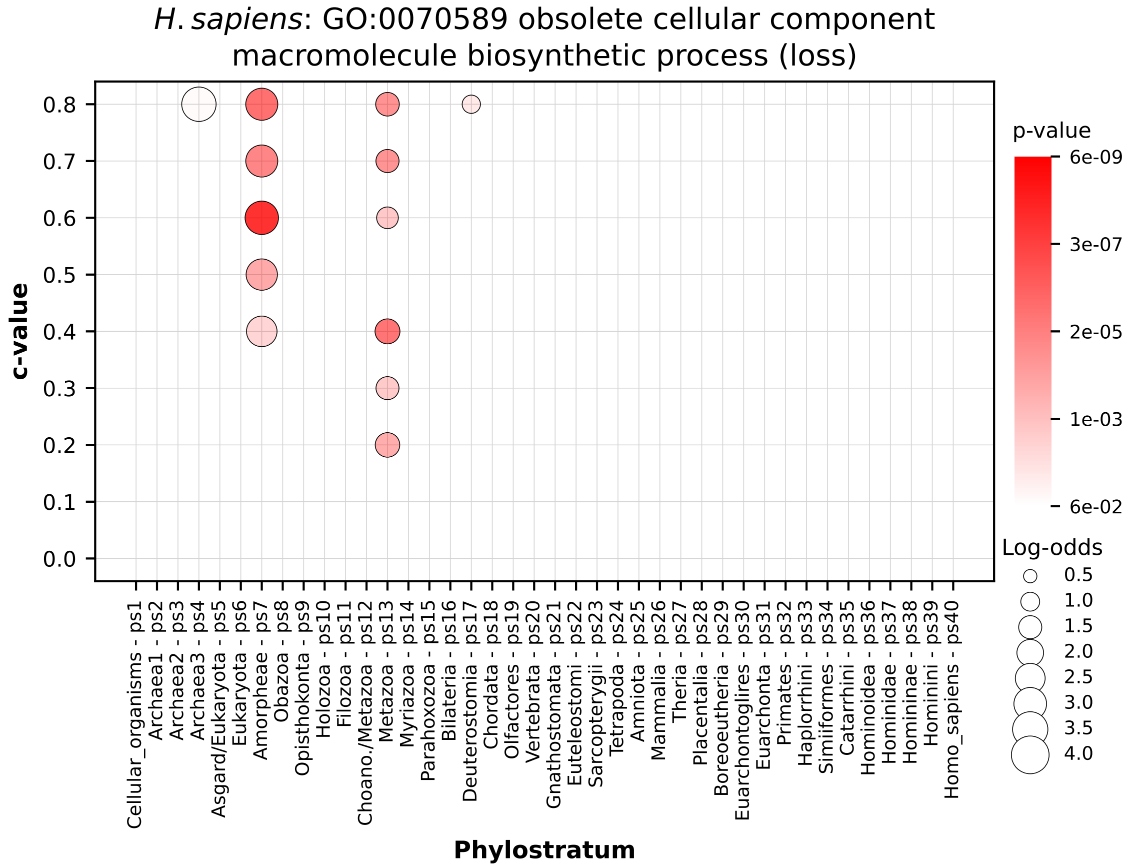

H. sapiens: GO:0070589 obsolete cellular component macromolecule biosynthetic process (loss)
c-value
0.0
0.1
0.2
0.3
0.4
0.5
0.6
0.7
0.8
Phylostratum
Cellular_organisms - ps1
Archaea1 - ps2
Archaea2 - ps3
Archaea3 - ps4
Asgard/Eukaryota - ps5
Eukaryota - ps6
Amorpheae - ps7
Obazoa - ps8
Opisthokonta - ps9
Holozoa - ps10
Filozoa - ps11
Choano./Metazoa - ps12
Metazoa - ps13
Myriazoa - ps14
Parahoxozoa - ps15
Bilateria - ps16
Deuterostomia - ps17
Chordata - ps18
Olfactores - ps19
Vertebrata - ps20
Gnathostomata - ps21
Euteleostomi - ps22
Sarcopterygii - ps23
Tetrapoda - ps24
Amniota - ps25
Mammalia - ps26
Theria - ps27
Placentalia - ps28
Boreoeutheria - ps29
Euarchontoglires - ps30
Euarchonta - ps31
Primates - ps32
Haplorrhini - ps33
Simiiformes - ps34
Catarrhini - ps35
Hominoidea - ps36
Hominidae - ps37
Homininae - ps38
Hominini - ps39
Homo_sapiens - ps40
p-value
6e-09
3e-07
2e-05
1e-03
6e-02
Log-odds
0.5
1.0
1.5
2.0
2.5
3.0
3.5
4.0

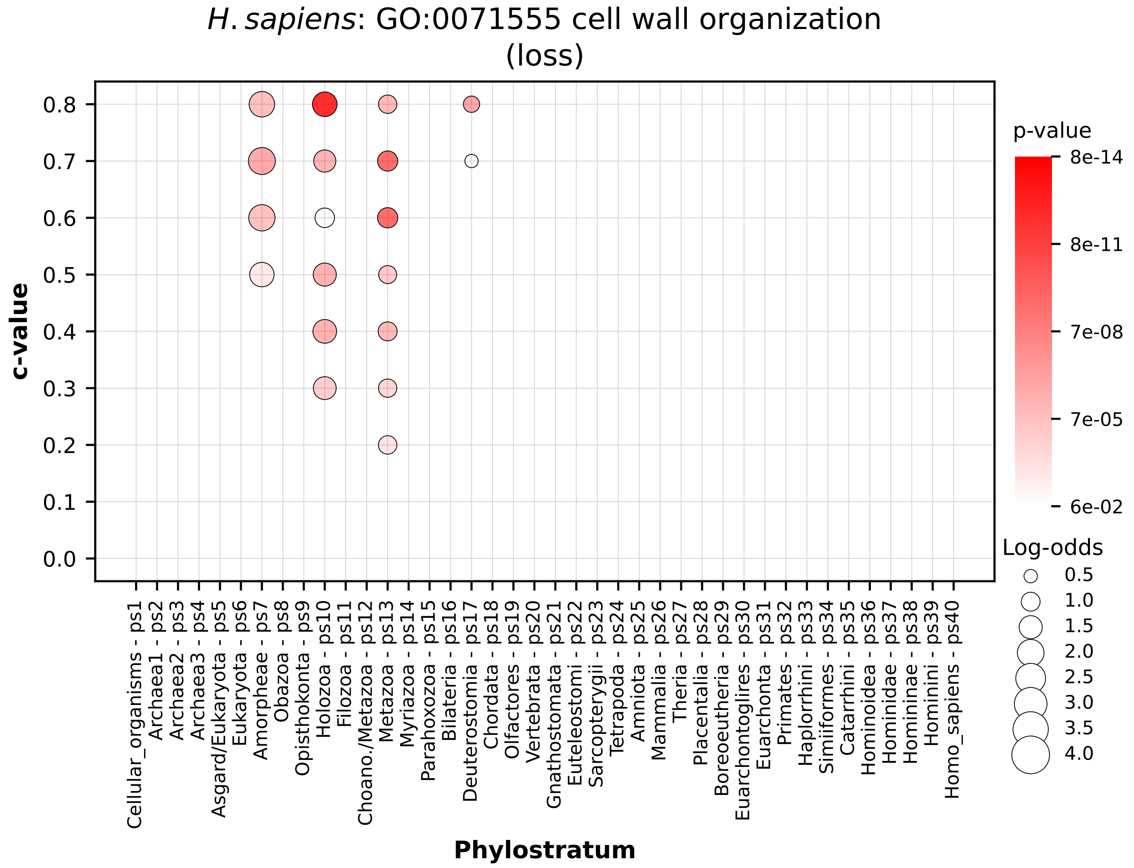
H. sapiens: GO:0071555 cell wall organization
(loss)
c-value
0.8
0.7
0.6
0.5
0.4
0.3
0.2
0.1
0.0
p-value
8e-14
8e-11
7e-08
7e-05
6e-02
Log-odds
0.5
1.0
1.5
2.0
2.5
3.0
3.5
4.0
Cellular_organisms - ps1
Archaea1 - ps2
Archaea2 - ps3
Archaea3 - ps4
Asgard/Eukaryota - ps5
Eukaryota - ps6
Amorpheae - ps7
Obazoa - ps8
Opisthokonta - ps9
Holozoa - ps10
Filozoa - ps11
Choano./Metazoa - ps12
Metazoa - ps13
Myriazoa - ps14
Parahoxozoa - ps15
Bilateria - ps16
Deuterostomia - ps17
Chordata - ps18
Olfactores - ps19
Vertebrata - ps20
Gnathostomata - ps21
Euteleostomi - ps22
Sarcopterygii - ps23
Tetrapoda - ps24
Amniota - ps25
Mammalia - ps26
Theria - ps27
Placentalia - ps28
Boreoeutheria - ps29
Euarchontoglires - ps30
Euarchonta - ps31
Primates - ps32
Haplorrhini - ps33
Simiiformes - ps34
Catarrhini - ps35
Hominoidea - ps36
Hominidae - ps37
Homininae - ps38
Hominini - ps39
Homo_sapiens - ps40
Phylostratum

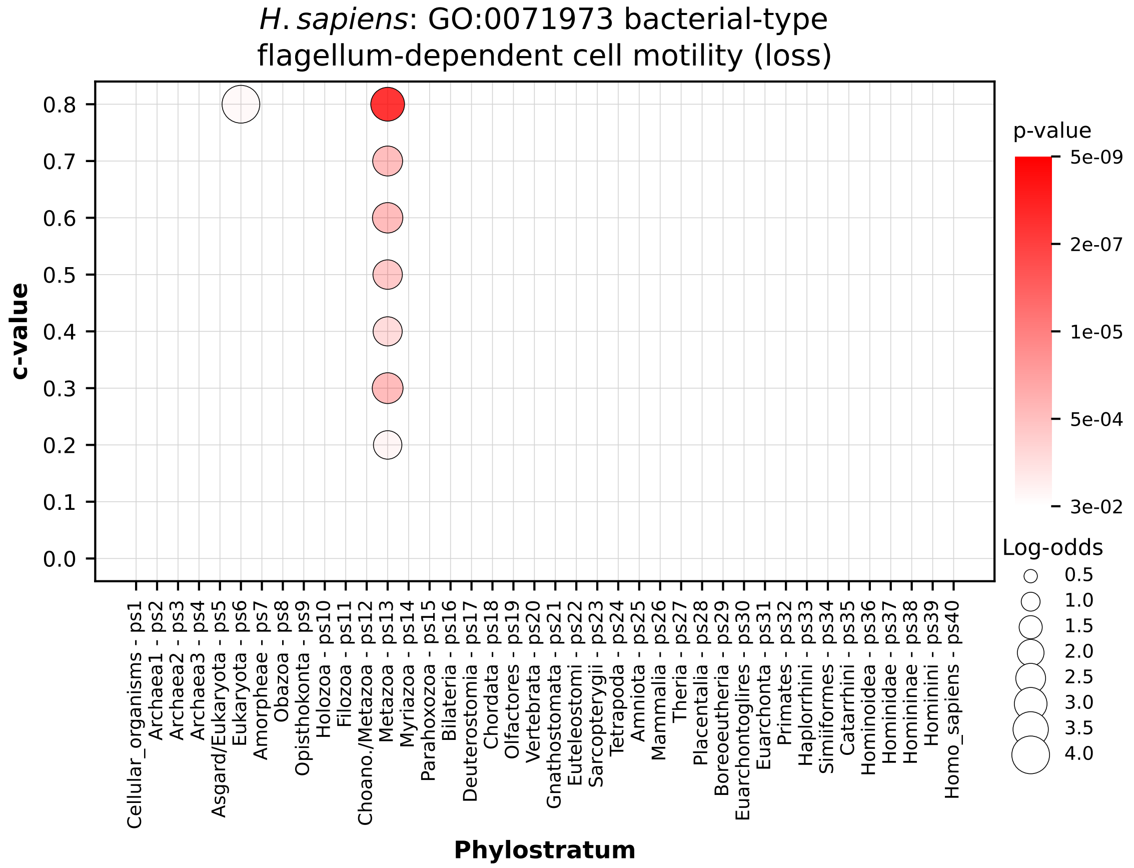

H. sapiens: GO:0071973 bacterial-type flagellum-dependent cell motility (loss)
c-value
0.8
0.7
0.6
0.5
0.4
0.3
0.2
0.1
0.0
p-value
5e-09
2e-07
1e-05
5e-04
3e-02
Log-odds
0.5
1.0
1.5
2.0
2.5
3.0
3.5
4.0
Cellular_organisms - ps1
Archaea1 - ps2
Archaea2 - ps3
Archaea3 - ps4
Asgard/Eukaryota - ps5
Eukaryota - ps6
Amorpheae - ps7
Obazoa - ps8
Opisthokonta - ps9
Holozoa - ps10
Filozoa - ps11
Choano./Metazoa - ps12
Metazoa - ps13
Myriazoa - ps14
Parahoxozoa - ps15
Bilateria - ps16
Deuterostomia - ps17
Chordata - ps18
Olfactores - ps19
Vertebrata - ps20
Gnathostomata - ps21
Euteleostomi - ps22
Sarcopterygii - ps23
Tetrapoda - ps24
Amniota - ps25
Mammalia - ps26
Theria - ps27
Placentalia - ps28
Boreoeutheria - ps29
Euarchontoglires - ps30
Euarchonta - ps31
Primates - ps32
Haplorrhini - ps33
Simiiformes - ps34
Catarrhini - ps35
Hominoidea - ps36
Hominidae - ps37
Homininae - ps38
Hominini - ps39
Homo_sapiens - ps40
Phylostratum

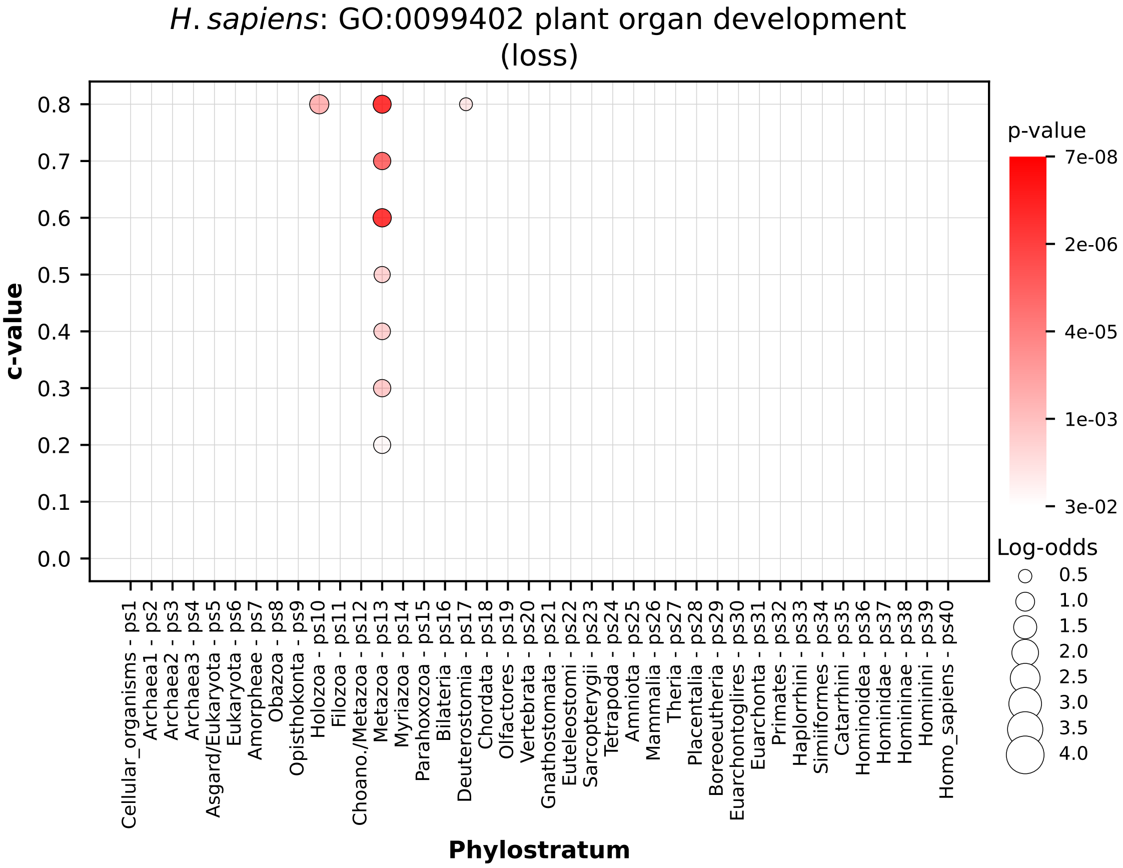

H. sapiens: GO:0099402 plant organ development (loss)
c-value
0.0
0.1
0.2
0.3
0.4
0.5
0.6
0.7
0.8
Phylostratum
Cellular_organisms - ps1
Archaea1 - ps2
Archaea2 - ps3
Archaea3 - ps4
Asgard/Eukaryota - ps5
Eukaryota - ps6
Amorpheae - ps7
Obazoa - ps8
Opisthokonta - ps9
Holozoa - ps10
Filozoa - ps11
Choano./Metazoa - ps12
Metazoa - ps13
Myriazoa - ps14
Parahoxozoa - ps15
Bilateria - ps16
Deuterostomia - ps17
Chordata - ps18
Olfactores - ps19
Vertebrata - ps20
Gnathostomata - ps21
Euteleostomi - ps22
Sarcopterygii - ps23
Tetrapoda - ps24
Amniota - ps25
Mammalia - ps26
Theria - ps27
Placentalia - ps28
Boreoeutheria - ps29
Euarchontoglires - ps30
Euarchonta - ps31
Primates - ps32
Haplorrhini - ps33
Simiiformes - ps34
Catarrhini - ps35
Hominoidea - ps36
Hominidae - ps37
Homininae - ps38
Hominini - ps39
Homo_sapiens - ps40
p-value
7e-08
2e-06
4e-05
1e-03
3e-02
Log-odds
0.5
1.0
1.5
2.0
2.5
3.0
3.5
4.0

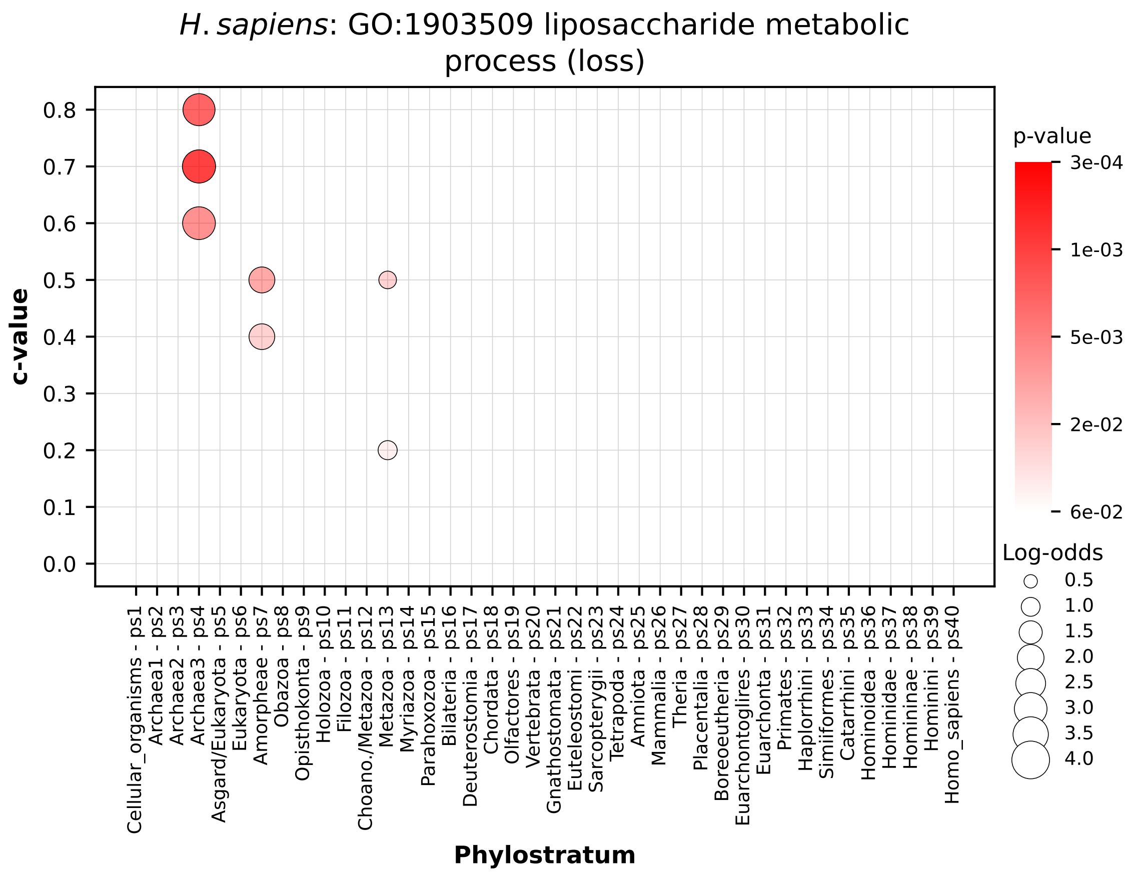

H. sapiens: GO:1903509 liposaccharide metabolic process (loss)
c-value
0.0
0.1
0.2
0.3
0.4
0.5
0.6
0.7
0.8
Phylostratum
Cellular_organisms - ps1
Archaea1 - ps2
Archaea2 - ps3
Archaea3 - ps4
Asgard/Eukaryota - ps5
Eukaryota - ps6
Amorpheae - ps7
Obazoa - ps8
Opisthokonta - ps9
Holozoa - ps10
Filozoa - ps11
Choano./Metazoa - ps12
Metazoa - ps13
Myriazoa - ps14
Parahoxozoa - ps15
Bilateria - ps16
Deuterostomia - ps17
Chordata - ps18
Olfactores - ps19
Vertebrata - ps20
Gnathostomata - ps21
Euteleostomi - ps22
Sarcopterygii - ps23
Tetrapoda - ps24
Amniota - ps25
Mammalia - ps26
Theria - ps27
Placentalia - ps28
Boreoeutheria - ps29
Euarchontoglires - ps30
Euarchonta - ps31
Primates - ps32
Haplorrhini - ps33
Simiiformes - ps34
Catarrhini - ps35
Hominoidea - ps36
Hominidae - ps37
Homininae - ps38
Hominini - ps39
Homo_sapiens - ps40
p-value
3e-04
1e-03
5e-03
2e-02
6e-02
Log-odds
0.5
1.0
1.5
2.0
2.5
3.0
3.5
4.0

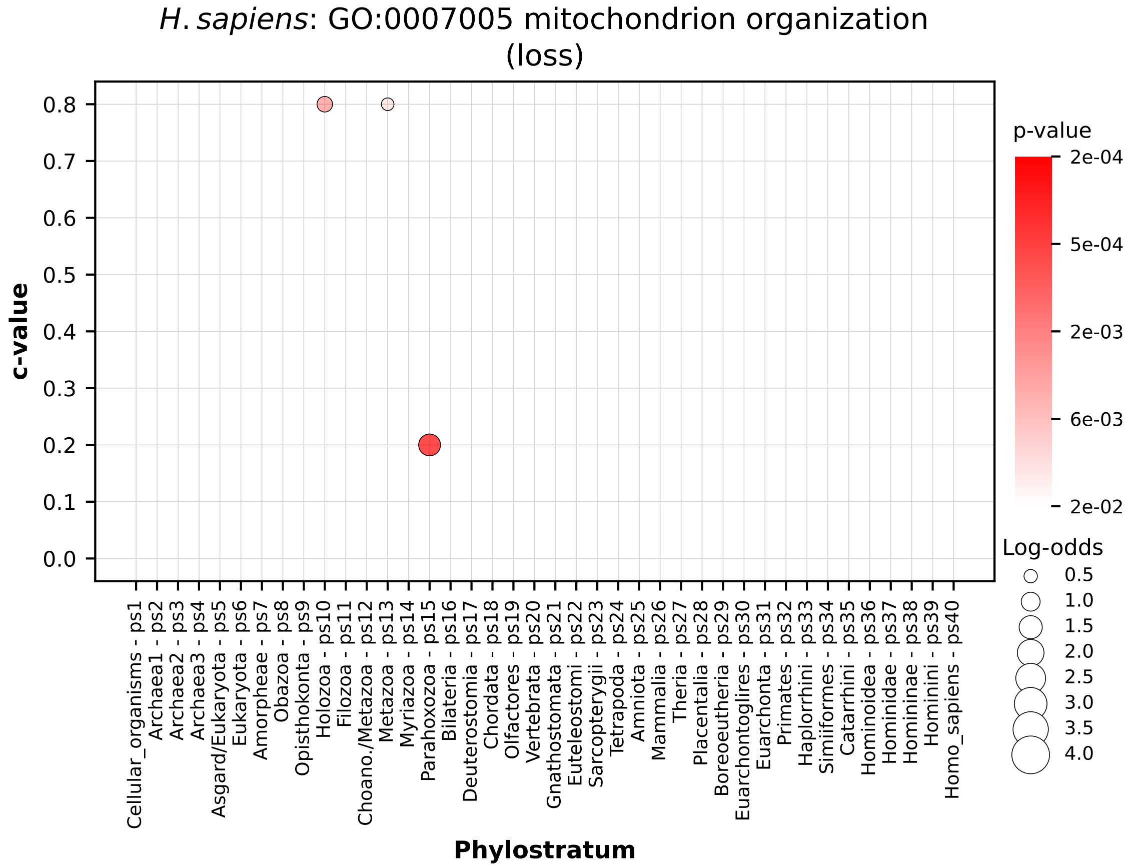

H. sapiens: GO:0007005 mitochondrion organization
(loss)
c-value
0.8
0.7
0.6
0.5
0.4
0.3
0.2
0.1
0.0
p-value
2e-04
5e-04
2e-03
6e-03
2e-02
Log-odds
0.5
1.0
1.5
2.0
2.5
3.0
3.5
4.0
Cellular_organisms - ps1
Archaea1 - ps2
Archaea2 - ps3
Archaea3 - ps4
Asgard/Eukaryota - ps5
Eukaryota - ps6
Amorpheae - ps7
Obazoa - ps8
Opisthokonta - ps9
Holozoa - ps10
Filozoa - ps11
Choano./Metazoa - ps12
Metazoa - ps13
Myriazoa - ps14
Parahoxozoa - ps15
Bilateria - ps16
Deuterostomia - ps17
Chordata - ps18
Olfactores - ps19
Vertebrata - ps20
Gnathostomata - ps21
Euteleostomi - ps22
Sarcopterygii - ps23
Tetrapoda - ps24
Amniota - ps25
Mammalia - ps26
Theria - ps27
Placentalia - ps28
Boreoeutheria - ps29
Euarchontoglires - ps30
Euarchonta - ps31
Primates - ps32
Haplorrhini - ps33
Simiiformes - ps34
Catarrhini - ps35
Hominoidea - ps36
Hominidae - ps37
Homininae - ps38
Hominini - ps39
Homo_sapiens - ps40
Phylostratum

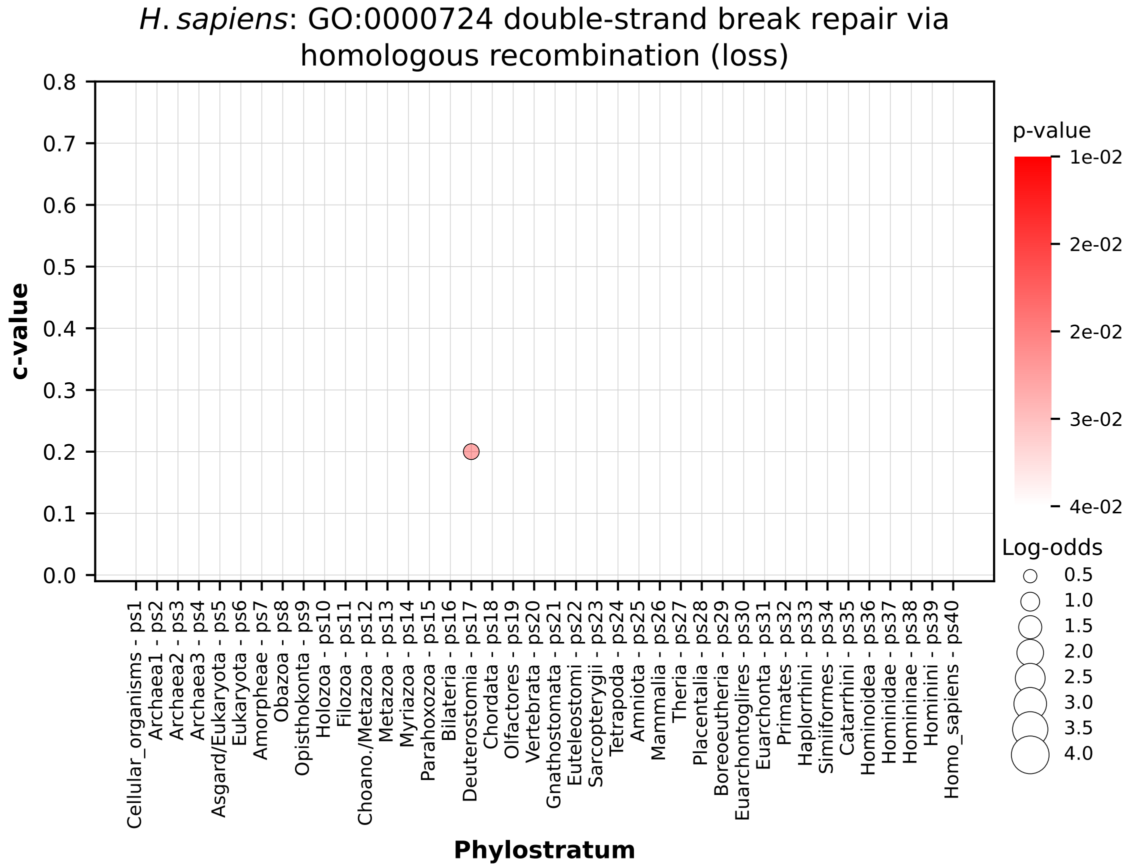

H. sapiens: GO:0000724 double-strand break repair via homologous recombination (loss)
c-value
0.0
0.1
0.2
0.3
0.4
0.5
0.6
0.7
0.8
Phylostratum
Cellular_organisms - ps1
Archaea1 - ps2
Archaea2 - ps3
Archaea3 - ps4
Asgard/Eukaryota - ps5
Eukaryota - ps6
Amorpheae - ps7
Obazoa - ps8
Opisthokonta - ps9
Holozoa - ps10
Filozoa - ps11
Choano./Metazoa - ps12
Metazoa - ps13
Myriazoa - ps14
Parahoxozoa - ps15
Bilateria - ps16
Deuterostomia - ps17
Chordata - ps18
Olfactores - ps19
Vertebrata - ps20
Gnathostomata - ps21
Euteleostomi - ps22
Sarcopterygii - ps23
Tetrapoda - ps24
Amniota - ps25
Mammalia - ps26
Theria - ps27
Placentalia - ps28
Boreoeutheria - ps29
Euarchontoglires - ps30
Euarchonta - ps31
Primates - ps32
Haplorrhini - ps33
Simiiformes - ps34
Catarrhini - ps35
Hominoidea - ps36
Hominidae - ps37
Homininae - ps38
Hominini - ps39
Homo_sapiens - ps40
p-value
1e-02
2e-02
2e-02
3e-02
4e-02
Log-odds
0.5
1.0
1.5
2.0
2.5
3.0
3.5
4.0

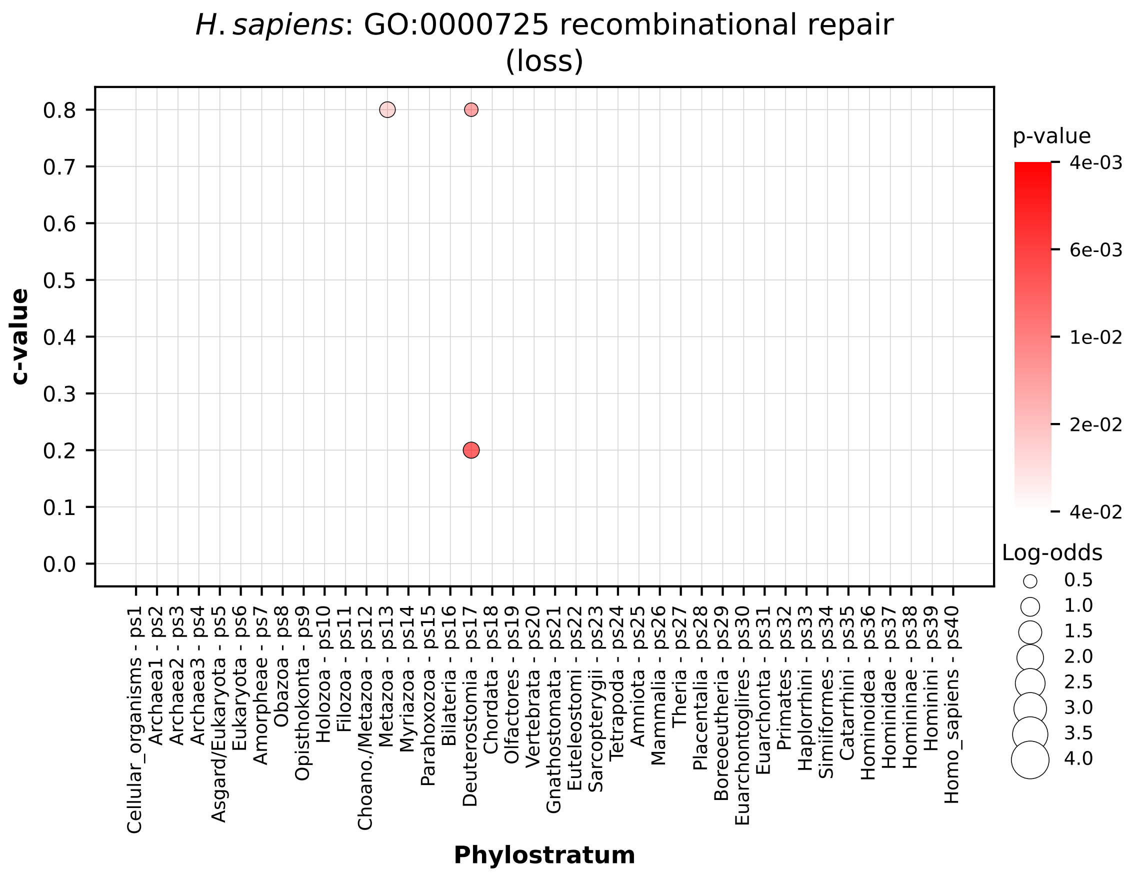

H. sapiens: GO:0000725 recombinational repair
(loss)
c-value
0.0
0.1
0.2
0.3
0.4
0.5
0.6
0.7
0.8
Phylostratum
Cellular_organisms - ps1
Archaea1 - ps2
Archaea2 - ps3
Archaea3 - ps4
Asgard/Eukaryota - ps5
Eukaryota - ps6
Amorpheae - ps7
Obazoa - ps8
Opisthokonta - ps9
Holozoa - ps10
Filozoa - ps11
Choano./Metazoa - ps12
Metazoa - ps13
Myriazoa - ps14
Parahoxozoa - ps15
Bilateria - ps16
Deuterostomia - ps17
Chordata - ps18
Olfactores - ps19
Vertebrata - ps20
Gnathostomata - ps21
Euteleostomi - ps22
Sarcopterygii - ps23
Tetrapoda - ps24
Amniota - ps25
Mammalia - ps26
Theria - ps27
Placentalia - ps28
Boreoeutheria - ps29
Euarchontoglires - ps30
Euarchonta - ps31
Primates - ps32
Haplorrhini - ps33
Simiiformes - ps34
Catarrhini - ps35
Hominoidea - ps36
Hominidae - ps37
Homininae - ps38
Hominini - ps39
Homo_sapiens - ps40
p-value
4e-03
6e-03
1e-02
2e-02
4e-02
Log-odds
0.5
1.0
1.5
2.0
2.5
3.0
3.5
4.0

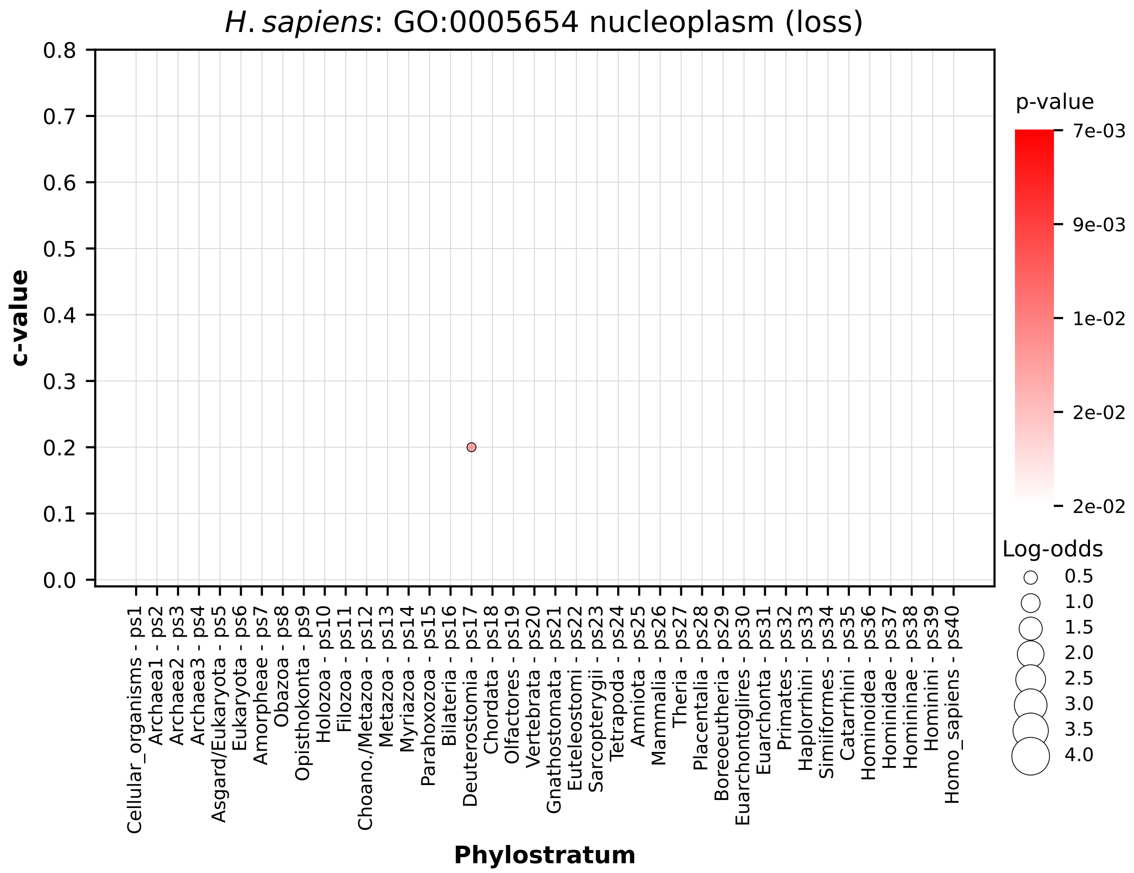
H. sapiens: GO:0005654 nucleoplasm (loss)
c-value
0.0
0.1
0.2
0.3
0.4
0.5
0.6
0.7
0.8
Phylostratum
Cellular_organisms - ps1
Archaea1 - ps2
Archaea2 - ps3
Archaea3 - ps4
Asgard/Eukaryota - ps5
Eukaryota - ps6
Amorpheae - ps7
Obazoa - ps8
Opisthokonta - ps9
Holozoa - ps10
Filozoa - ps11
Choano./Metazoa - ps12
Metazoa - ps13
Myriazoa - ps14
Parahoxozoa - ps15
Bilateria - ps16
Deuterostomia - ps17
Chordata - ps18
Olfactores - ps19
Vertebrata - ps20
Gnathostomata - ps21
Euteleostomi - ps22
Sarcopterygii - ps23
Tetrapoda - ps24
Amniota - ps25
Mammalia - ps26
Theria - ps27
Placentalia - ps28
Boreoeutheria - ps29
Euarchontoglires - ps30
Euarchonta - ps31
Primates - ps32
Haplorrhini - ps33
Simiiformes - ps34
Catarrhini - ps35
Hominoidea - ps36
Hominidae - ps37
Homininae - ps38
Hominini - ps39
Homo_sapiens - ps40
p-value
7e-03
9e-03
1e-02
2e-02
2e-02
Log-odds
0.5
1.0
1.5
2.0
2.5
3.0
3.5
4.0

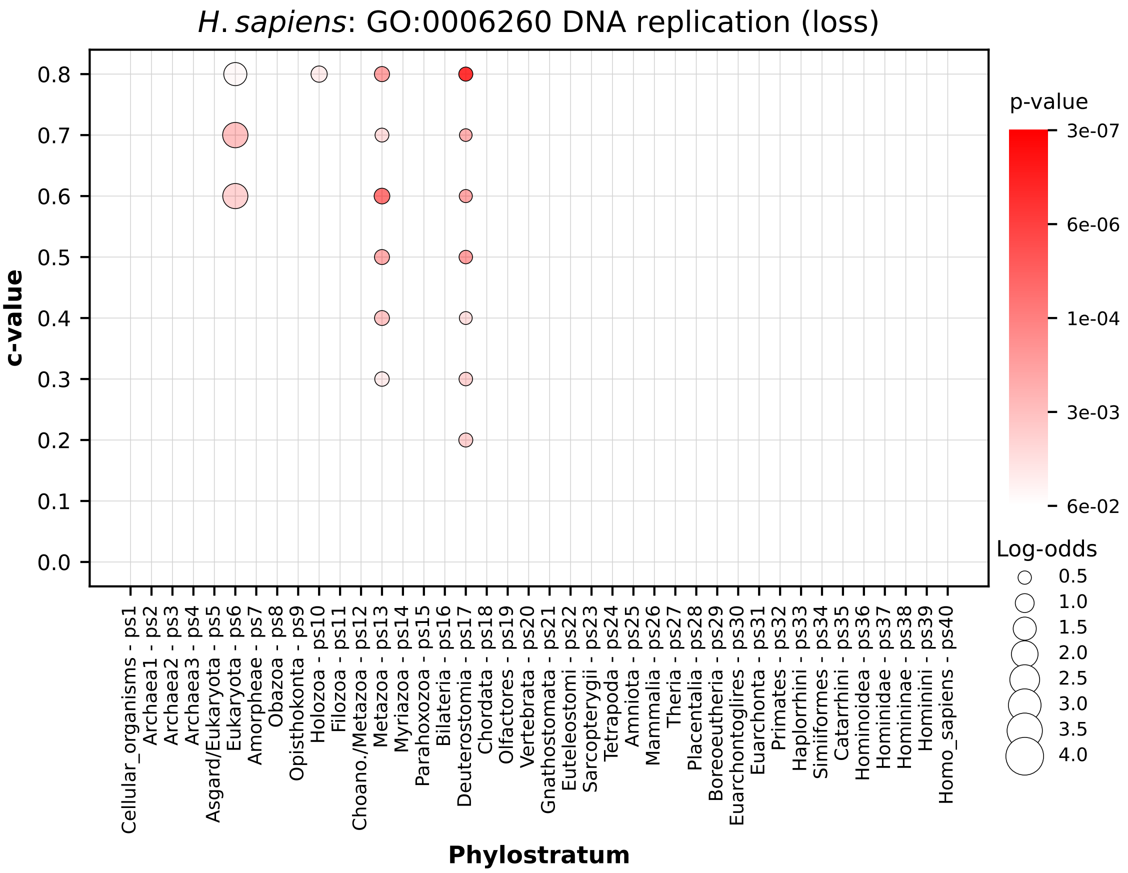

H. sapiens: GO:0006260 DNA replication (loss)
c-value
0.0
0.1
0.2
0.3
0.4
0.5
0.6
0.7
0.8
p-value
3e-07
6e-06
1e-04
3e-03
6e-02
Log-odds
0.5
1.0
1.5
2.0
2.5
3.0
3.5
4.0
Cellular_organisms - ps1
Archaea1 - ps2
Archaea2 - ps3
Archaea3 - ps4
Asgard/Eukaryota - ps5
Eukaryota - ps6
Amorpheae - ps7
Obazoa - ps8
Opisthokonta - ps9
Holozoa - ps10
Filozoa - ps11
Choano./Metazoa - ps12
Metazoa - ps13
Myriazoa - ps14
Parahoxozoa - ps15
Bilateria - ps16
Deuterostomia - ps17
Chordata - ps18
Olfactores - ps19
Vertebrata - ps20
Gnathostomata - ps21
Euteleostomi - ps22
Sarcopterygii - ps23
Tetrapoda - ps24
Amniota - ps25
Mammalia - ps26
Theria - ps27
Placentalia - ps28
Boreoeutheria - ps29
Euarchontoglires - ps30
Euarchonta - ps31
Primates - ps32
Haplorrhini - ps33
Simiiformes - ps34
Catarrhini - ps35
Hominoidea - ps36
Hominidae - ps37
Homininae - ps38
Hominini - ps39
Homo_sapiens - ps40
Phylostratum

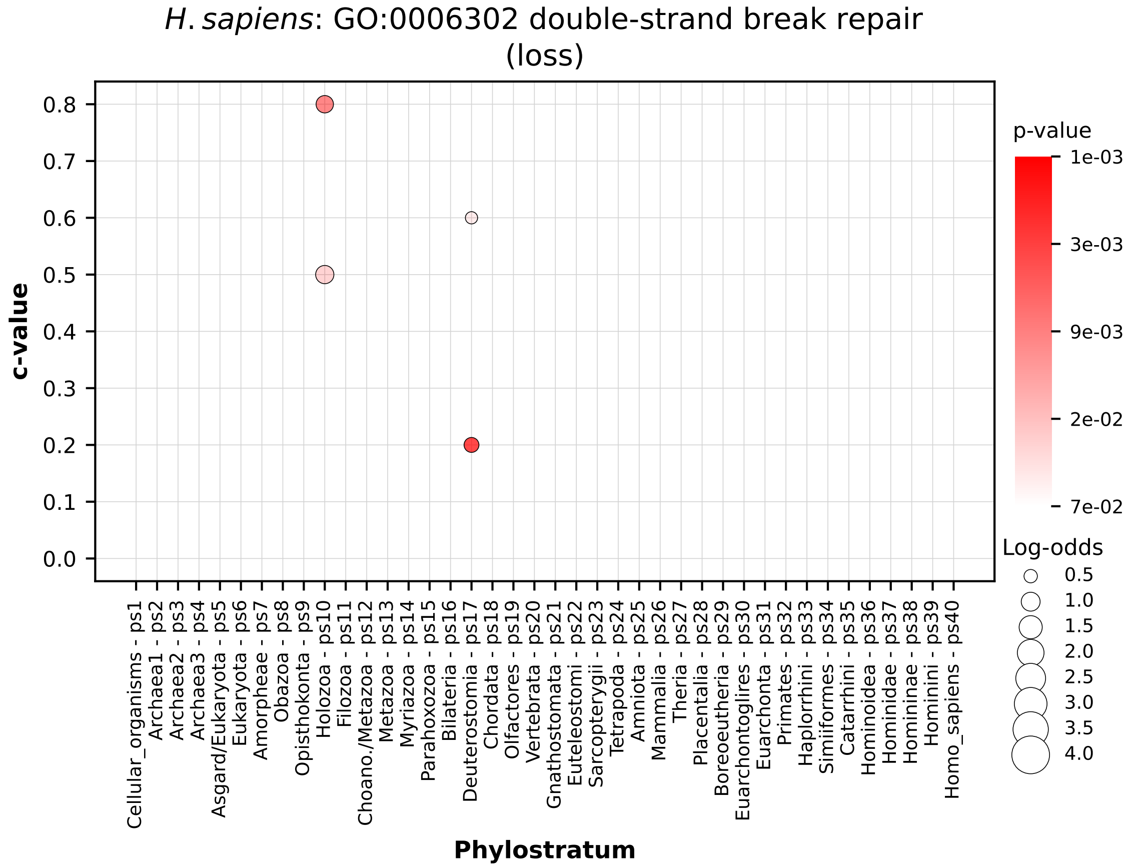

H. sapiens: GO:0006302 double-strand break repair
(loss)
c-value
0.8
0.7
0.6
0.5
0.4
0.3
0.2
0.1
0.0
Phylostratum
Cellular_organisms - ps1
Archaea1 - ps2
Archaea2 - ps3
Archaea3 - ps4
Asgard/Eukaryota - ps5
Eukaryota - ps6
Amorpheae - ps7
Obazoa - ps8
Opisthokonta - ps9
Holozoa - ps10
Filozoa - ps11
Choano./Metazoa - ps12
Metazoa - ps13
Myriazoa - ps14
Parahoxozoa - ps15
Bilateria - ps16
Deuterostomia - ps17
Chordata - ps18
Olfactores - ps19
Vertebrata - ps20
Gnathostomata - ps21
Euteleostomi - ps22
Sarcopterygii - ps23
Tetrapoda - ps24
Amniota - ps25
Mammalia - ps26
Theria - ps27
Placentalia - ps28
Boreoeutheria - ps29
Euarchontoglires - ps30
Euarchonta - ps31
Primates - ps32
Haplorrhini - ps33
Simiiformes - ps34
Catarrhini - ps35
Hominoidea - ps36
Hominidae - ps37
Homininae - ps38
Hominini - ps39
Homo_sapiens - ps40
p-value
1e-03
3e-03
9e-03
2e-02
7e-02
Log-odds
0.5
1.0
1.5
2.0
2.5
3.0
3.5
4.0

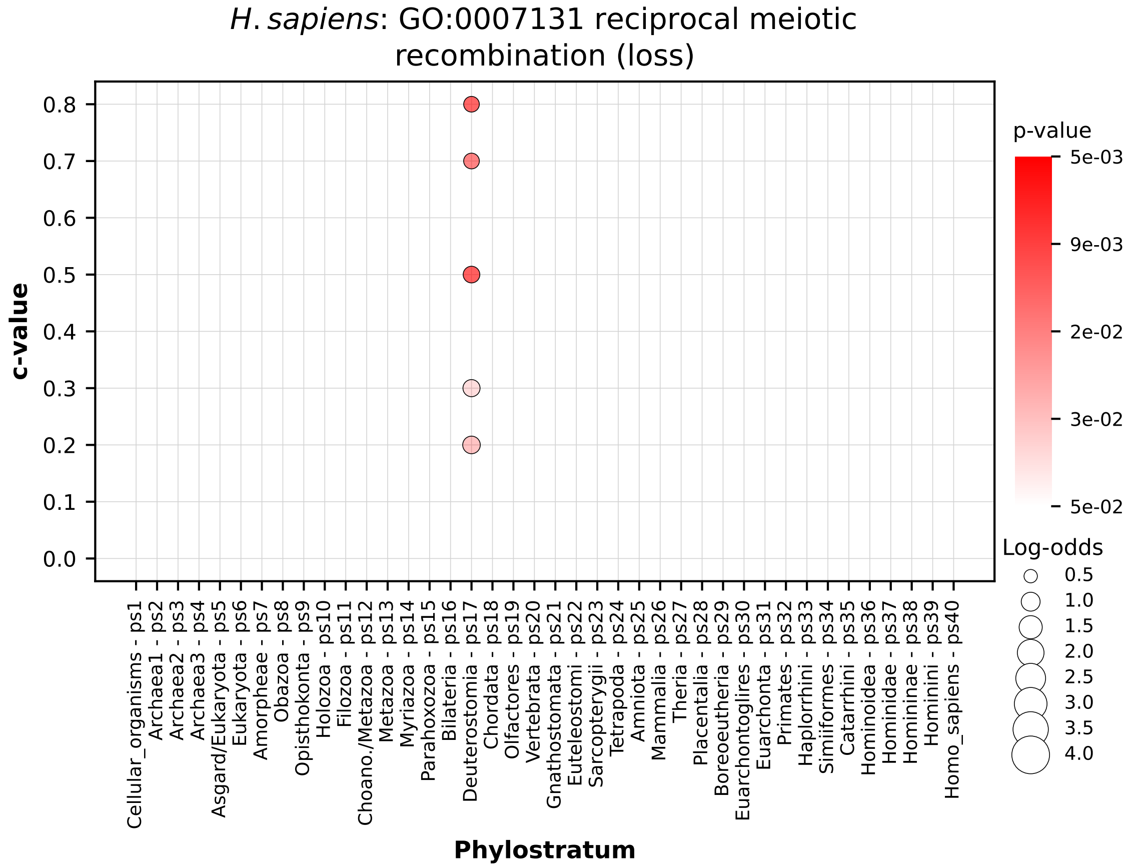

H. sapiens: GO:0007131 reciprocal meiotic recombination (loss)
c-value
0.8
0.7
0.6
0.5
0.4
0.3
0.2
0.1
0.0
Phylostratum
Cellular_organisms - ps1
Archaea1 - ps2
Archaea2 - ps3
Archaea3 - ps4
Asgard/Eukaryota - ps5
Eukaryota - ps6
Amorpheae - ps7
Obazoa - ps8
Opisthokonta - ps9
Holozoa - ps10
Filozoa - ps11
Choano./Metazoa - ps12
Metazoa - ps13
Myriazoa - ps14
Parahoxozoa - ps15
Bilateria - ps16
Deuterostomia - ps17
Chordata - ps18
Olfactores - ps19
Vertebrata - ps20
Gnathostomata - ps21
Euteleostomi - ps22
Sarcopterygii - ps23
Tetrapoda - ps24
Amniota - ps25
Mammalia - ps26
Theria - ps27
Placentalia - ps28
Boreoeutheria - ps29
Euarchontoglires - ps30
Euarchonta - ps31
Primates - ps32
Haplorrhini - ps33
Simiiformes - ps34
Catarrhini - ps35
Hominoidea - ps36
Hominidae - ps37
Homininae - ps38
Hominini - ps39
Homo_sapiens - ps40
p-value
5e-03
9e-03
2e-02
3e-02
5e-02
Log-odds
0.5
1.0
1.5
2.0
2.5
3.0
3.5
4.0

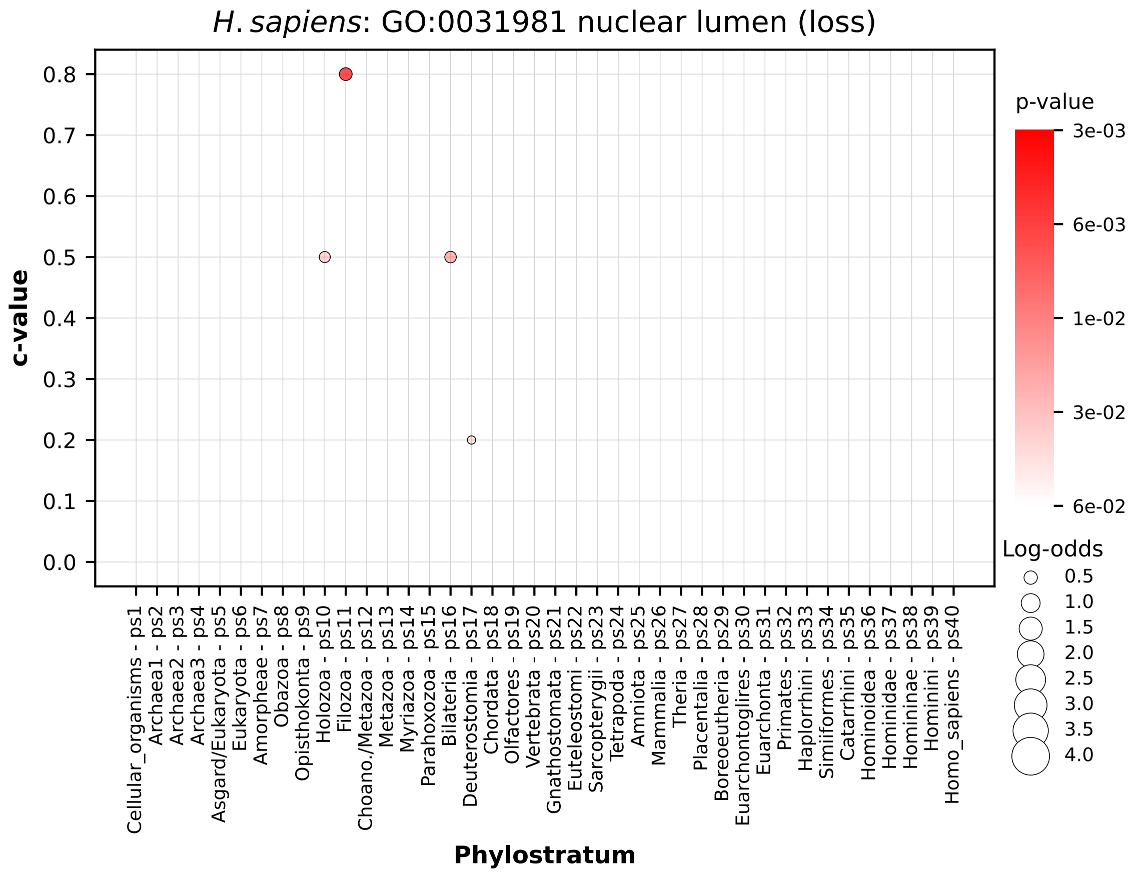

H. sapiens: GO:0031981 nuclear lumen (loss)
c-value
0.0
0.1
0.2
0.3
0.4
0.5
0.6
0.7
0.8
Phylostratum
Cellular_organisms - ps1
Archaea1 - ps2
Archaea2 - ps3
Archaea3 - ps4
Asgard/Eukaryota - ps5
Eukaryota - ps6
Amorpheae - ps7
Obazoa - ps8
Opisthokonta - ps9
Holozoa - ps10
Filozoa - ps11
Choano./Metazoa - ps12
Metazoa - ps13
Myriazoa - ps14
Parahoxozoa - ps15
Bilateria - ps16
Deuterostomia - ps17
Chordata - ps18
Olfactores - ps19
Vertebrata - ps20
Gnathostomata - ps21
Euteleostomi - ps22
Sarcopterygii - ps23
Tetrapoda - ps24
Amniota - ps25
Mammalia - ps26
Theria - ps27
Placentalia - ps28
Boreoeutheria - ps29
Euarchontoglires - ps30
Euarchonta - ps31
Primates - ps32
Haplorrhini - ps33
Simiiformes - ps34
Catarrhini - ps35
Hominoidea - ps36
Hominidae - ps37
Homininae - ps38
Hominini - ps39
Homo_sapiens - ps40
p-value
3e-03
6e-03
1e-02
3e-02
6e-02
Log-odds
0.5
1.0
1.5
2.0
2.5
3.0
3.5
4.0

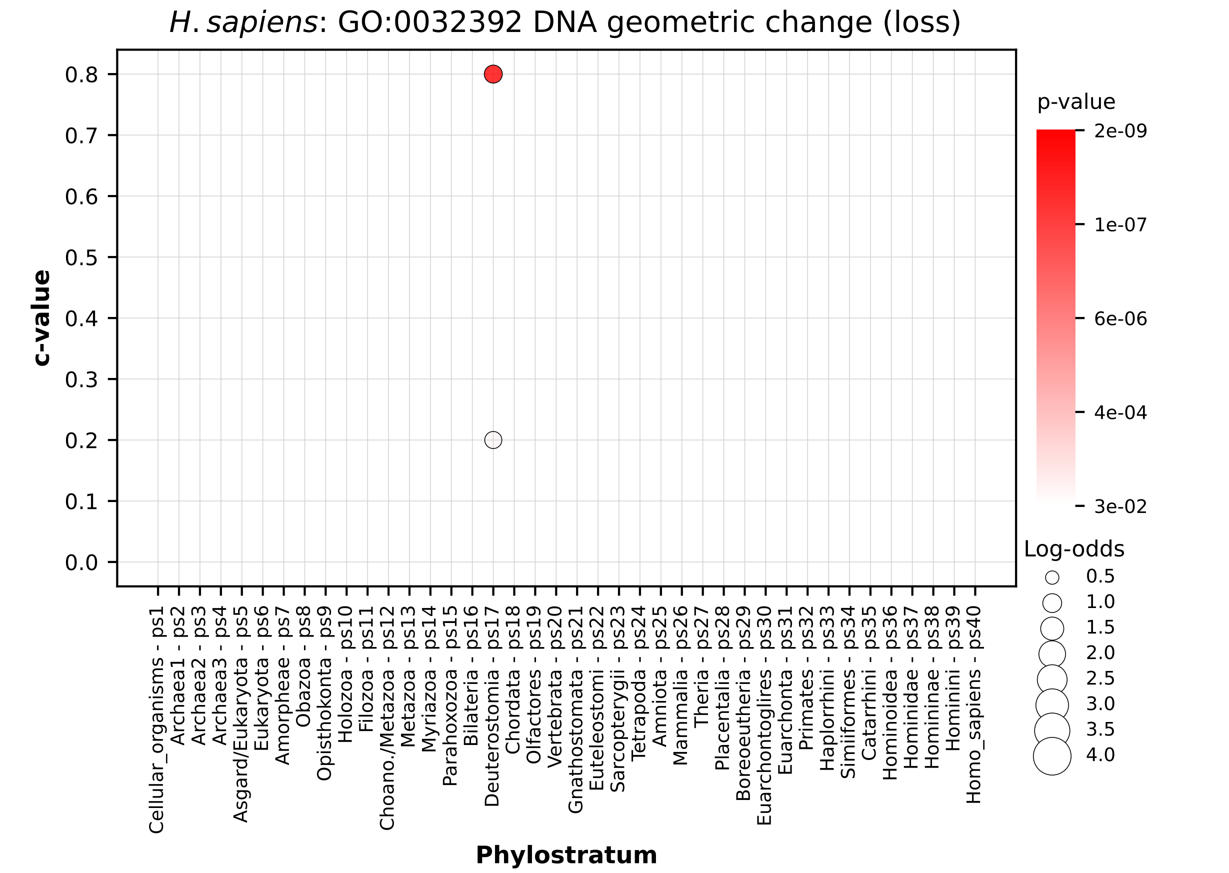

H. sapiens: GO:0032392 DNA geometric change (loss)
c-value
0.8
0.7
0.6
0.5
0.4
0.3
0.2
0.1
0.0
p-value
2e-09
1e-07
6e-06
4e-04
3e-02
Log-odds
0.5
1.0
1.5
2.0
2.5
3.0
3.5
4.0
Cellular_organisms - ps1
Archaea1 - ps2
Archaea2 - ps3
Archaea3 - ps4
Asgard/Eukaryota - ps5
Eukaryota - ps6
Amorpheae - ps7
Obazoa - ps8
Opisthokonta - ps9
Holozoa - ps10
Filozoa - ps11
Choano./Metazoa - ps12
Metazoa - ps13
Myriazoa - ps14
Parahoxozoa - ps15
Bilateria - ps16
Deuterostomia - ps17
Chordata - ps18
Olfactores - ps19
Vertebrata - ps20
Gnathostomata - ps21
Euteleostomi - ps22
Sarcopterygii - ps23
Tetrapoda - ps24
Amniota - ps25
Mammalia - ps26
Theria - ps27
Placentalia - ps28
Boreoeutheria - ps29
Euarchontoglires - ps30
Euarchonta - ps31
Primates - ps32
Haplorrhini - ps33
Simiiformes - ps34
Catarrhini - ps35
Hominoidea - ps36
Hominidae - ps37
Homininae - ps38
Hominini - ps39
Homo_sapiens - ps40
Phylostratum

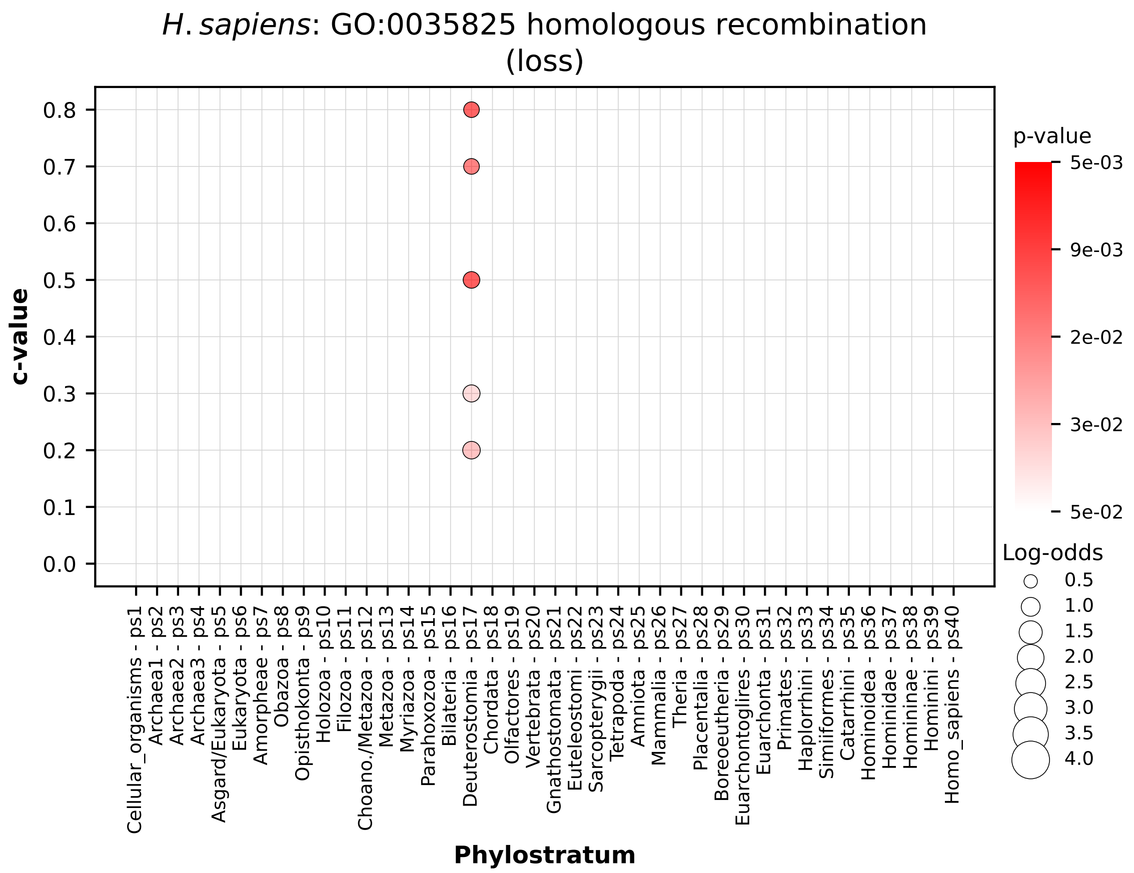

H. sapiens: GO:0035825 homologous recombination
(loss)
c-value
0.8
0.7
0.6
0.5
0.4
0.3
0.2
0.1
0.0
p-value
5e-03
9e-03
2e-02
3e-02
5e-02
Log-odds
0.5
1.0
1.5
2.0
2.5
3.0
3.5
4.0
Cellular_organisms - ps1
Archaea1 - ps2
Archaea2 - ps3
Archaea3 - ps4
Asgard/Eukaryota - ps5
Eukaryota - ps6
Amorpheae - ps7
Obazoa - ps8
Opisthokonta - ps9
Holozoa - ps10
Filozoa - ps11
Choano./Metazoa - ps12
Metazoa - ps13
Myriazoa - ps14
Parahoxozoa - ps15
Bilateria - ps16
Deuterostomia - ps17
Chordata - ps18
Olfactores - ps19
Vertebrata - ps20
Gnathostomata - ps21
Euteleostomi - ps22
Sarcopterygii - ps23
Tetrapoda - ps24
Amniota - ps25
Mammalia - ps26
Theria - ps27
Placentalia - ps28
Boreoeutheria - ps29
Euarchontoglires - ps30
Euarchonta - ps31
Primates - ps32
Haplorrhini - ps33
Simiiformes - ps34
Catarrhini - ps35
Hominoidea - ps36
Hominidae - ps37
Homininae - ps38
Hominini - ps39
Homo_sapiens - ps40
Phylostratum

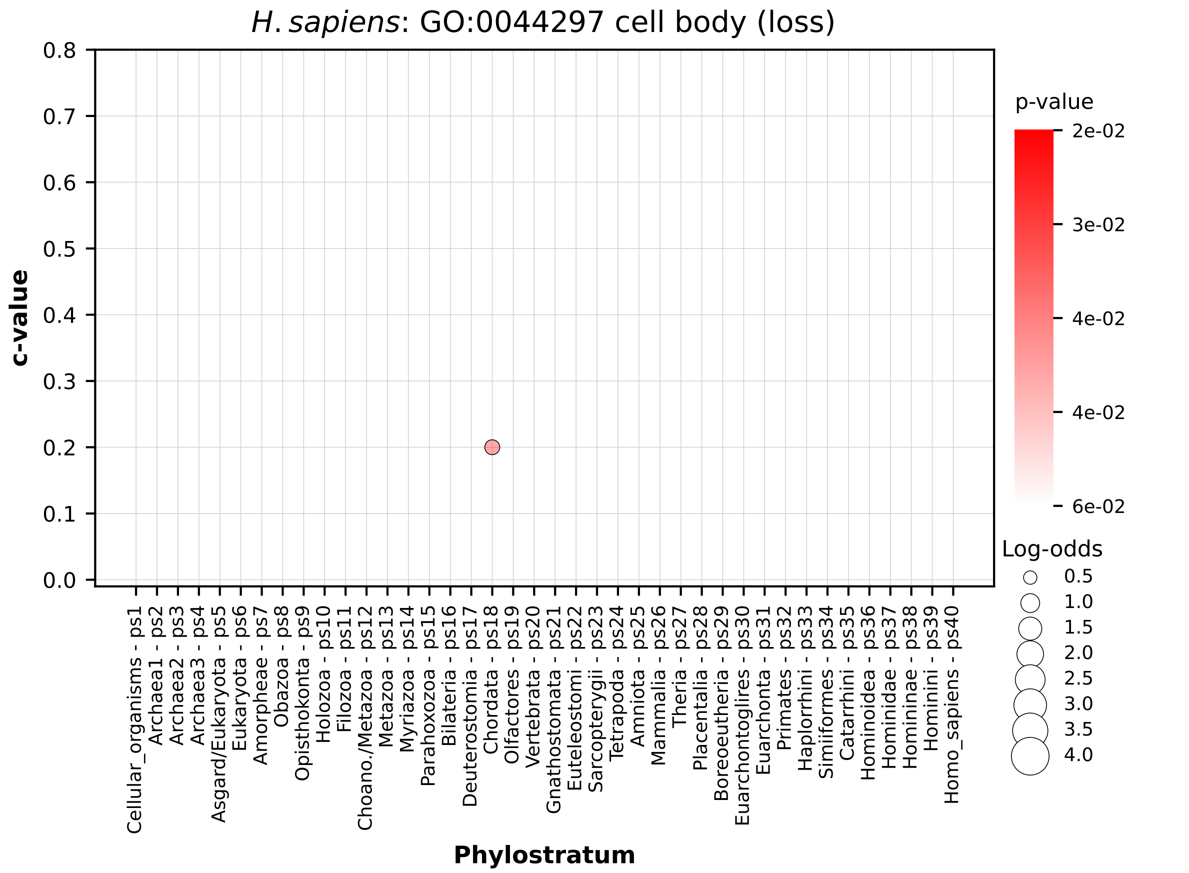

H. sapiens: GO:0044297 cell body (loss)
c-value
0.0
0.1
0.2
0.3
0.4
0.5
0.6
0.7
0.8
Phylostratum
Cellular_organisms - ps1
Archaea1 - ps2
Archaea2 - ps3
Archaea3 - ps4
Asgard/Eukaryota - ps5
Eukaryota - ps6
Amorpheae - ps7
Obazoa - ps8
Opisthokonta - ps9
Holozoa - ps10
Filozoa - ps11
Choano./Metazoa - ps12
Metazoa - ps13
Myriazoa - ps14
Parahoxozoa - ps15
Bilateria - ps16
Deuterostomia - ps17
Chordata - ps18
Olfactores - ps19
Vertebrata - ps20
Gnathostomata - ps21
Euteleostomi - ps22
Sarcopterygii - ps23
Tetrapoda - ps24
Amniota - ps25
Mammalia - ps26
Theria - ps27
Placentalia - ps28
Boreoeutheria - ps29
Euarchontoglires - ps30
Euarchonta - ps31
Primates - ps32
Haplorrhini - ps33
Simiiformes - ps34
Catarrhini - ps35
Hominoidea - ps36
Hominidae - ps37
Homininae - ps38
Hominini - ps39
Homo_sapiens - ps40
p-value
2e-02
3e-02
4e-02
4e-02
6e-02
Log-odds
0.5
1.0
1.5
2.0
2.5
3.0
3.5
4.0

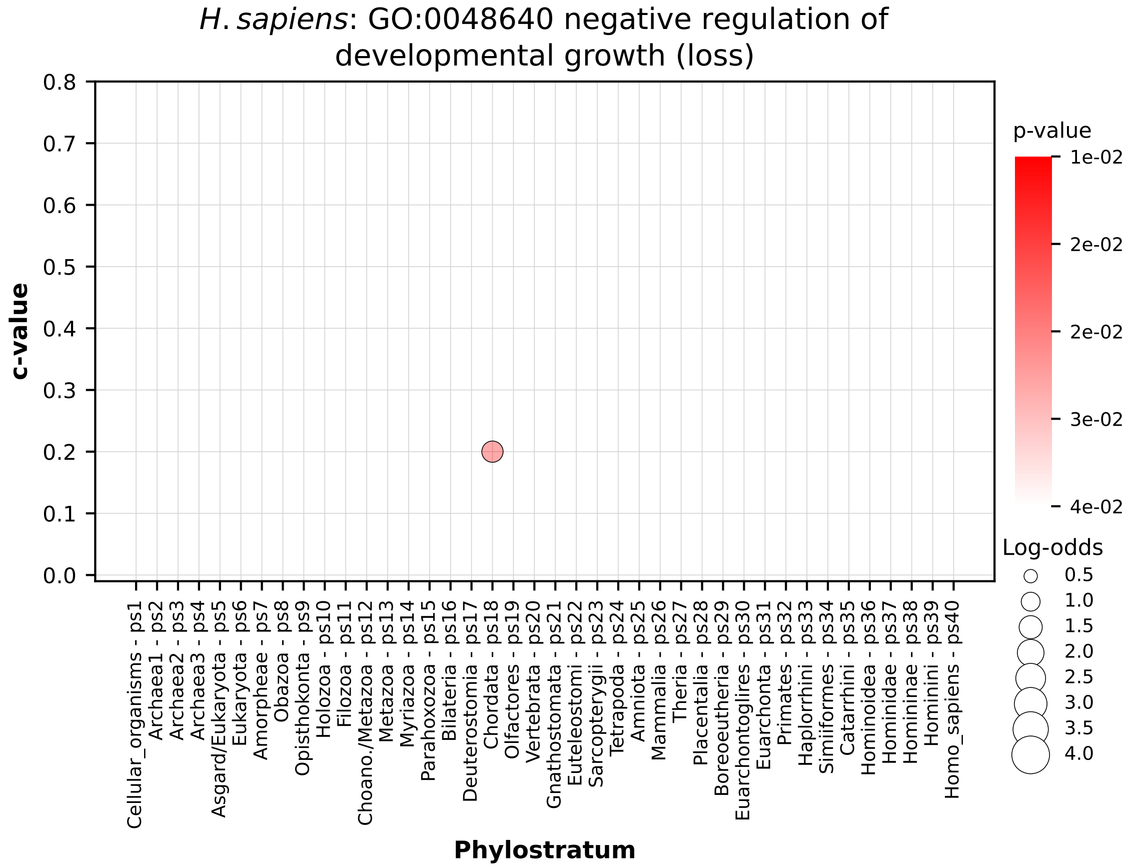
H. sapiens: GO:0048640 negative regulation of developmental growth (loss)
c-value
0.8
0.7
0.6
0.5
0.4
0.3
0.2
0.1
0.0
Phylostratum
Cellular_organisms - ps1
Archaea1 - ps2
Archaea2 - ps3
Archaea3 - ps4
Asgard/Eukaryota - ps5
Eukaryota - ps6
Amorpheae - ps7
Obazoa - ps8
Opisthokonta - ps9
Holozoa - ps10
Filozoa - ps11
Choano./Metazoa - ps12
Metazoa - ps13
Myriazoa - ps14
Parahoxozoa - ps15
Bilateria - ps16
Deuterostomia - ps17
Chordata - ps18
Olfactores - ps19
Vertebrata - ps20
Gnathostomata - ps21
Euteleostomi - ps22
Sarcopterygii - ps23
Tetrapoda - ps24
Amniota - ps25
Mammalia - ps26
Theria - ps27
Placentalia - ps28
Boreoeutheria - ps29
Euarchontoglires - ps30
Euarchonta - ps31
Primates - ps32
Haplorrhini - ps33
Simiiformes - ps34
Catarrhini - ps35
Hominoidea - ps36
Hominidae - ps37
Homininae - ps38
Hominini - ps39
Homo_sapiens - ps40
p-value
1e-02
2e-02
2e-02
3e-02
4e-02
Log-odds
0.5
1.0
1.5
2.0
2.5
3.0
3.5
4.0

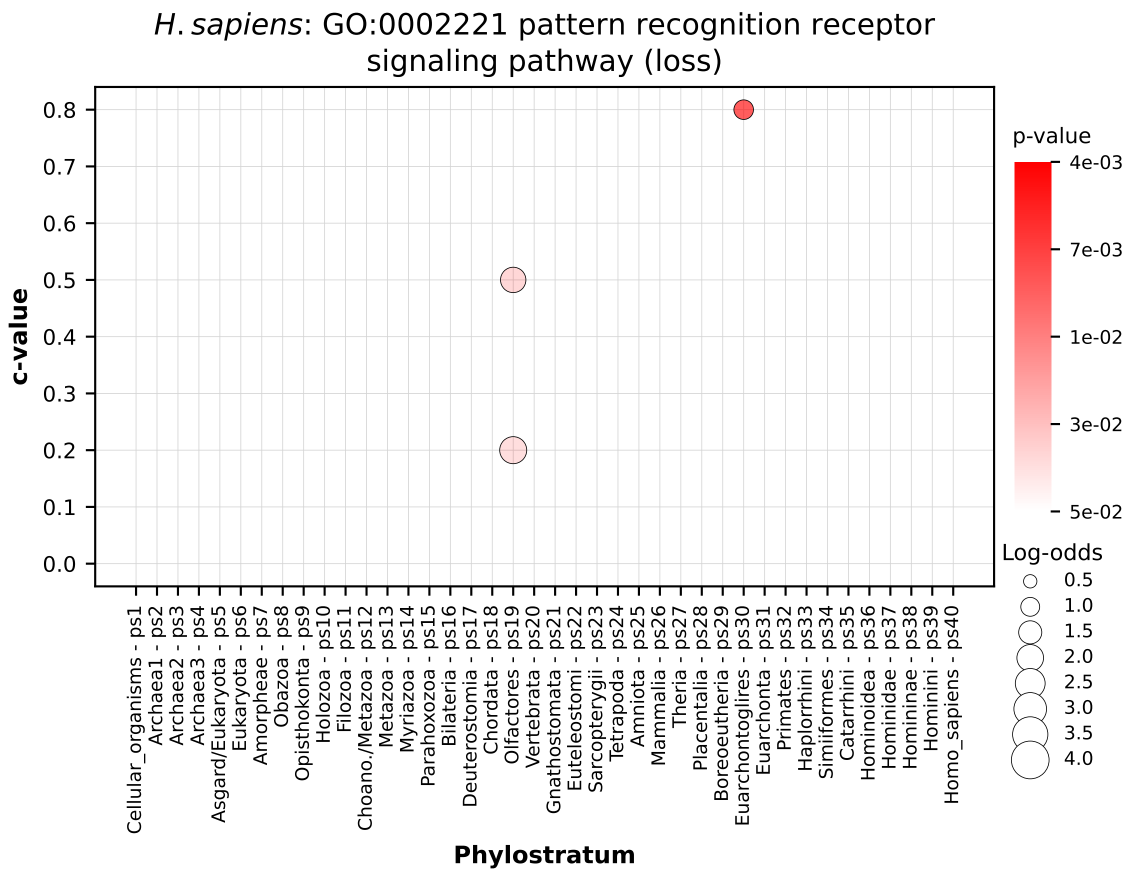

H. sapiens: GO:0002221 pattern recognition receptor signaling pathway (loss)
c-value
0.0
0.1
0.2
0.3
0.4
0.5
0.6
0.7
0.8
Phylostratum
Cellular_organisms - ps1
Archaea1 - ps2
Archaea2 - ps3
Archaea3 - ps4
Asgard/Eukaryota - ps5
Eukaryota - ps6
Amorpheae - ps7
Obazoa - ps8
Opisthokonta - ps9
Holozoa - ps10
Filozoa - ps11
Choano./Metazoa - ps12
Metazoa - ps13
Myriazoa - ps14
Parahoxozoa - ps15
Bilateria - ps16
Deuterostomia - ps17
Chordata - ps18
Olfactores - ps19
Vertebrata - ps20
Gnathostomata - ps21
Euteleostomi - ps22
Sarcopterygii - ps23
Tetrapoda - ps24
Amniota - ps25
Mammalia - ps26
Theria - ps27
Placentalia - ps28
Boreoeutheria - ps29
Euarchontoglires - ps30
Euarchonta - ps31
Primates - ps32
Haplorrhini - ps33
Simiiformes - ps34
Catarrhini - ps35
Hominoidea - ps36
Hominidae - ps37
Homininae - ps38
Hominini - ps39
Homo_sapiens - ps40
p-value
4e-03
7e-03
1e-02
3e-02
5e-02
Log-odds
0.5
1.0
1.5
2.0
2.5
3.0
3.5
4.0

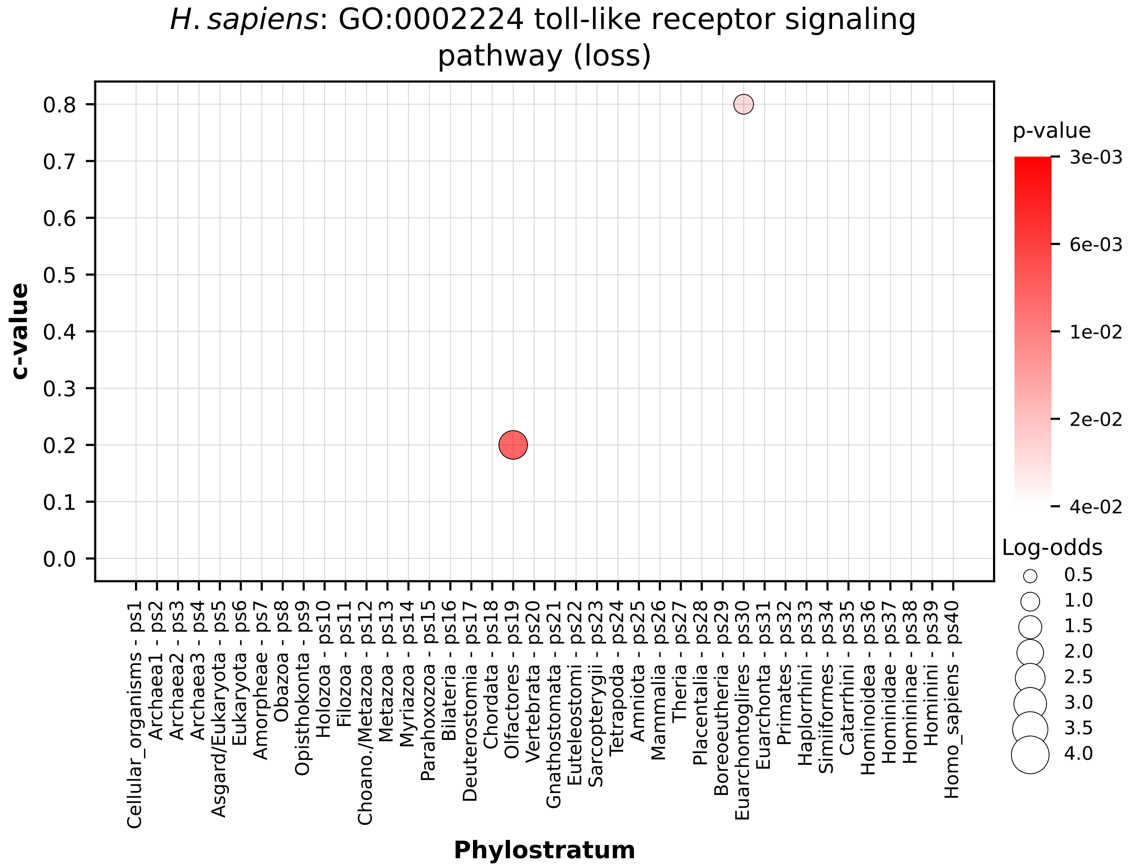

H. sapiens: GO:0002224 toll-like receptor signaling pathway (loss)
c-value
0.8
0.7
0.6
0.5
0.4
0.3
0.2
0.1
0.0
p-value
3e-03
6e-03
1e-02
2e-02
4e-02
Log-odds
0.5
1.0
1.5
2.0
2.5
3.0
3.5
4.0
Cellular_organisms - ps1
Archaea1 - ps2
Archaea2 - ps3
Archaea3 - ps4
Asgard/Eukaryota - ps5
Eukaryota - ps6
Amorpheae - ps7
Obazoa - ps8
Opisthokonta - ps9
Holozoa - ps10
Filozoa - ps11
Choano./Metazoa - ps12
Metazoa - ps13
Myriazoa - ps14
Parahoxozoa - ps15
Bilateria - ps16
Deuterostomia - ps17
Chordata - ps18
Olfactores - ps19
Vertebrata - ps20
Gnathostomata - ps21
Euteleostomi - ps22
Sarcopterygii - ps23
Tetrapoda - ps24
Amniota - ps25
Mammalia - ps26
Theria - ps27
Placentalia - ps28
Boreoeutheria - ps29
Euarchontoglires - ps30
Euarchonta - ps31
Primates - ps32
Haplorrhini - ps33
Simiiformes - ps34
Catarrhini - ps35
Hominoidea - ps36
Hominidae - ps37
Homininae - ps38
Hominini - ps39
Homo_sapiens - ps40
Phylostratum

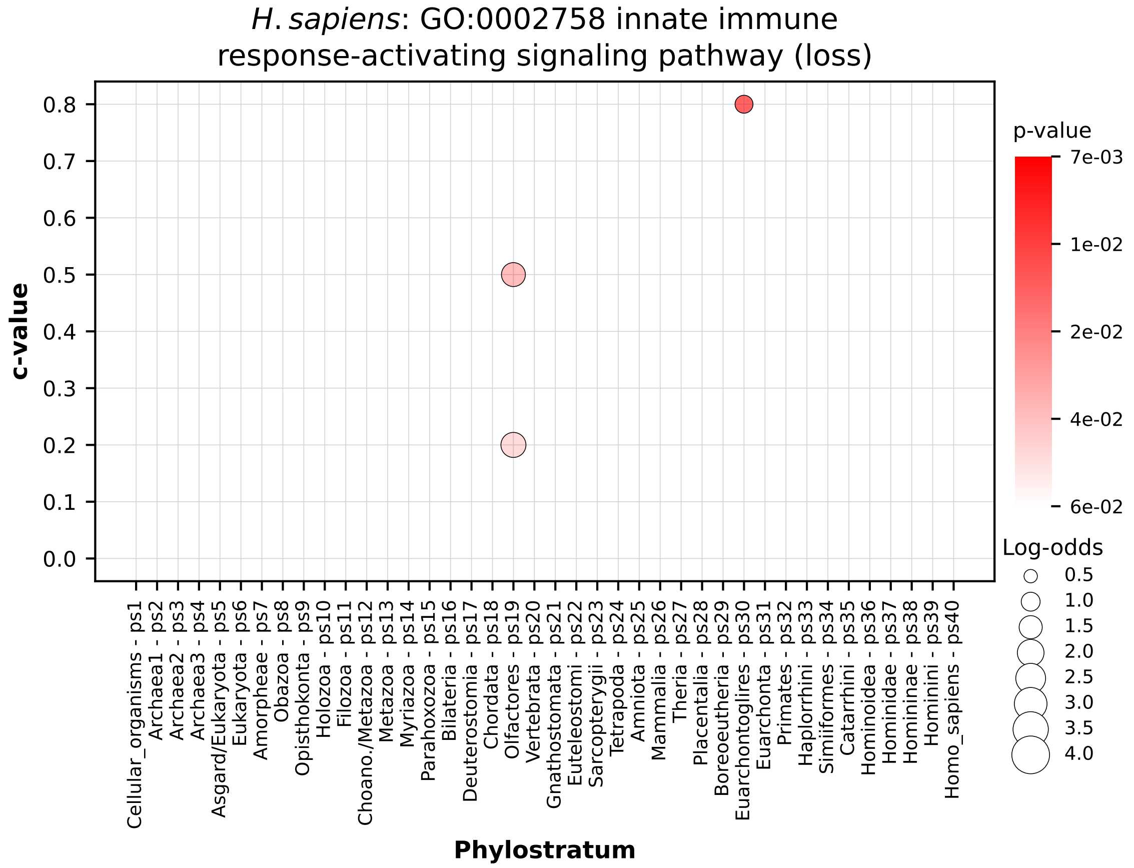

H. sapiens: GO:0002758 innate immune response-activating signaling pathway (loss)
c-value
0.0
0.1
0.2
0.3
0.4
0.5
0.6
0.7
0.8
p-value
7e-03
1e-02
2e-02
4e-02
6e-02
Log-odds
0.5
1.0
1.5
2.0
2.5
3.0
3.5
4.0
Cellular_organisms - ps1
Archaea1 - ps2
Archaea2 - ps3
Archaea3 - ps4
Asgard/Eukaryota - ps5
Eukaryota - ps6
Amorpheae - ps7
Obazoa - ps8
Opisthokonta - ps9
Holozoa - ps10
Filozoa - ps11
Choano./Metazoa - ps12
Metazoa - ps13
Myriazoa - ps14
Parahoxozoa - ps15
Bilateria - ps16
Deuterostomia - ps17
Chordata - ps18
Olfactores - ps19
Vertebrata - ps20
Gnathostomata - ps21
Euteleostomi - ps22
Sarcopterygii - ps23
Tetrapoda - ps24
Amniota - ps25
Mammalia - ps26
Theria - ps27
Placentalia - ps28
Boreoeutheria - ps29
Euarchontoglires - ps30
Euarchonta - ps31
Primates - ps32
Haplorrhini - ps33
Simiiformes - ps34
Catarrhini - ps35
Hominoidea - ps36
Hominidae - ps37
Homininae - ps38
Hominini - ps39
Homo_sapiens - ps40
Phylostratum

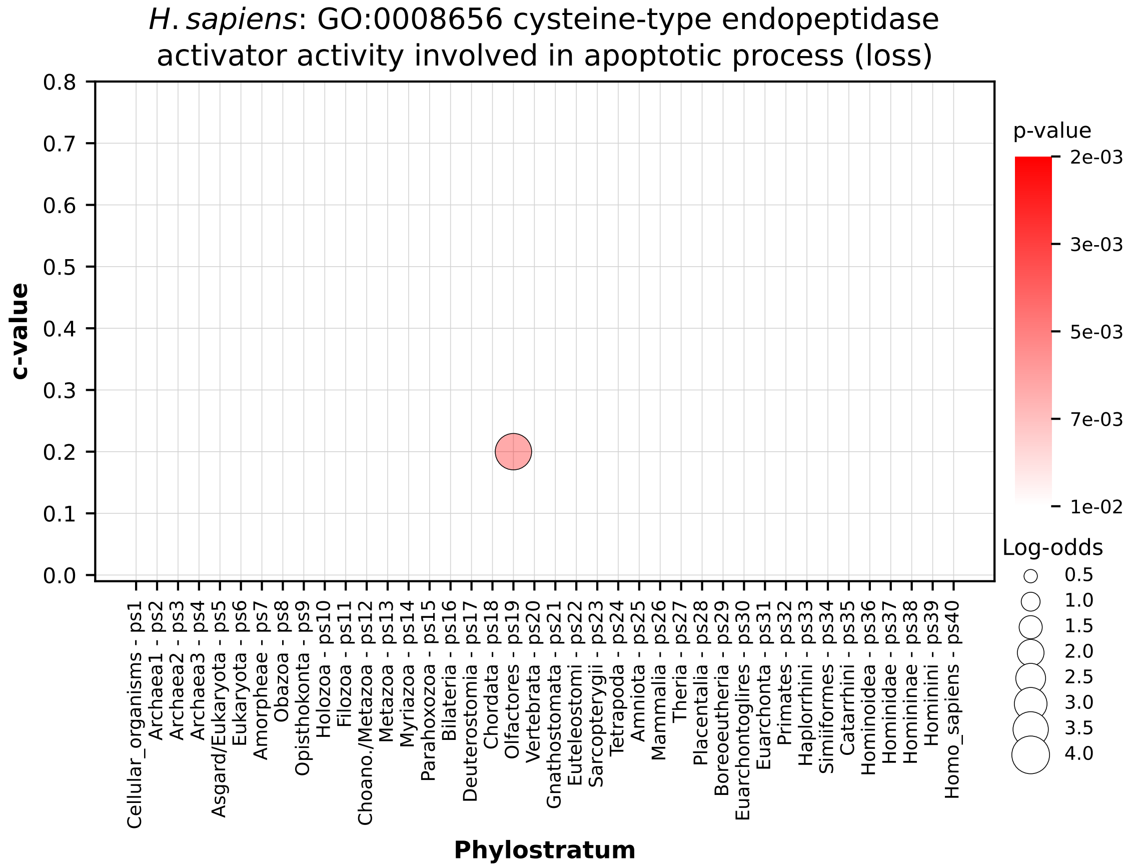
H. sapiens: GO:0008656 cysteine-type endopeptidase activator activity involved in apoptotic process (loss)
c-value
0.8
0.7
0.6
0.5
0.4
0.3
0.2
0.1
0.0
Phylostratum
Cellular_organisms - ps1
Archaea1 - ps2
Archaea2 - ps3
Archaea3 - ps4
Asgard/Eukaryota - ps5
Eukaryota - ps6
Amorpheae - ps7
Obazoa - ps8
Opisthokonta - ps9
Holozoa - ps10
Filozoa - ps11
Choano./Metazoa - ps12
Metazoa - ps13
Myriazoa - ps14
Parahoxozoa - ps15
Bilateria - ps16
Deuterostomia - ps17
Chordata - ps18
Olfactores - ps19
Vertebrata - ps20
Gnathostomata - ps21
Euteleostomi - ps22
Sarcopterygii - ps23
Tetrapoda - ps24
Amniota - ps25
Mammalia - ps26
Theria - ps27
Placentalia - ps28
Boreoeutheria - ps29
Euarchontoglires - ps30
Euarchonta - ps31
Primates - ps32
Haplorrhini - ps33
Simiiformes - ps34
Catarrhini - ps35
Hominoidea - ps36
Hominidae - ps37
Homininae - ps38
Hominini - ps39
Homo_sapiens - ps40
p-value
2e-03
3e-03
5e-03
7e-03
1e-02
Log-odds
0.5
1.0
1.5
2.0
2.5
3.0
3.5
4.0

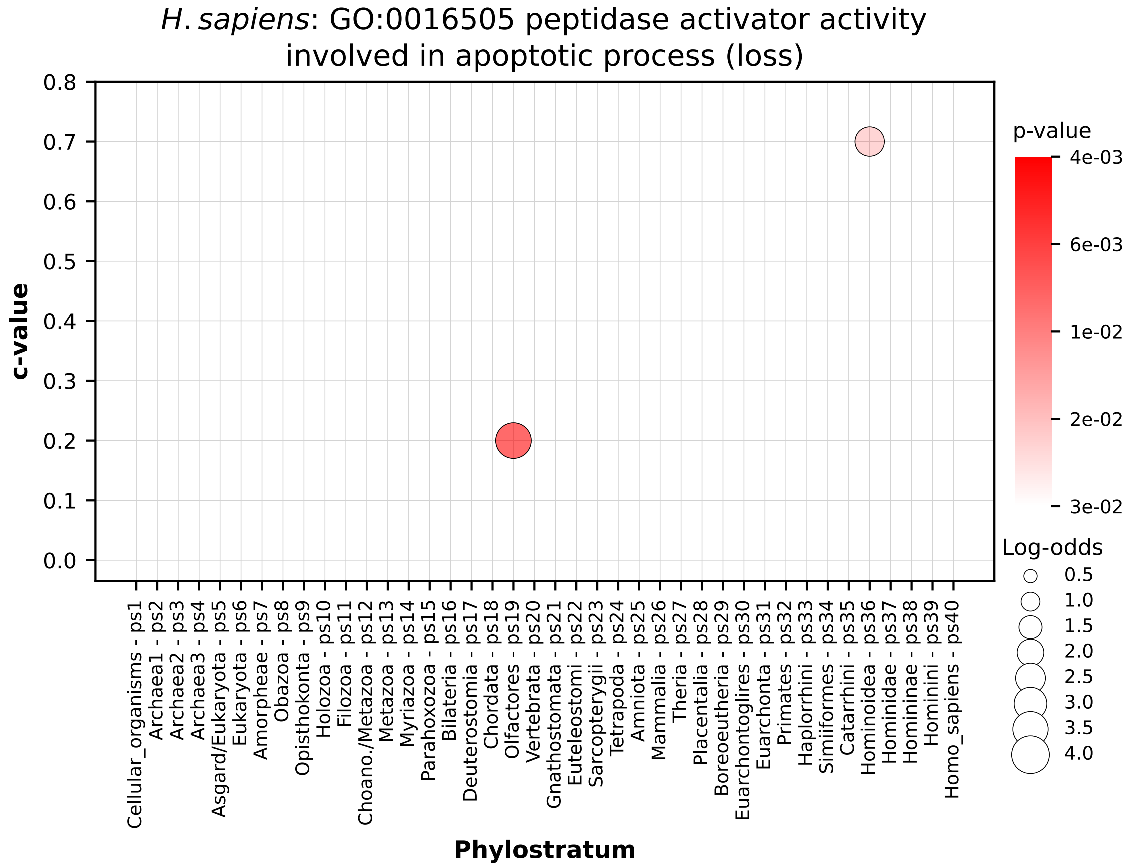

H. sapiens: GO:0016505 peptidase activator activity involved in apoptotic process (loss)
c-value
0.0
0.1
0.2
0.3
0.4
0.5
0.6
0.7
0.8
Phylostratum
Cellular_organisms - ps1
Archaea1 - ps2
Archaea2 - ps3
Archaea3 - ps4
Asgard/Eukaryota - ps5
Eukaryota - ps6
Amorpheae - ps7
Obazoa - ps8
Opisthokonta - ps9
Holozoa - ps10
Filozoa - ps11
Choano./Metazoa - ps12
Metazoa - ps13
Myriazoa - ps14
Parahoxozoa - ps15
Bilateria - ps16
Deuterostomia - ps17
Chordata - ps18
Olfactores - ps19
Vertebrata - ps20
Gnathostomata - ps21
Euteleostomi - ps22
Sarcopterygii - ps23
Tetrapoda - ps24
Amniota - ps25
Mammalia - ps26
Theria - ps27
Placentalia - ps28
Boreoeutheria - ps29
Euarchontoglires - ps30
Euarchonta - ps31
Primates - ps32
Haplorrhini - ps33
Simiiformes - ps34
Catarrhini - ps35
Hominoidea - ps36
Hominidae - ps37
Homininae - ps38
Hominini - ps39
Homo_sapiens - ps40
p-value
4e-03
6e-03
1e-02
2e-02
3e-02
Log-odds
0.5
1.0
1.5
2.0
2.5
3.0
3.5
4.0

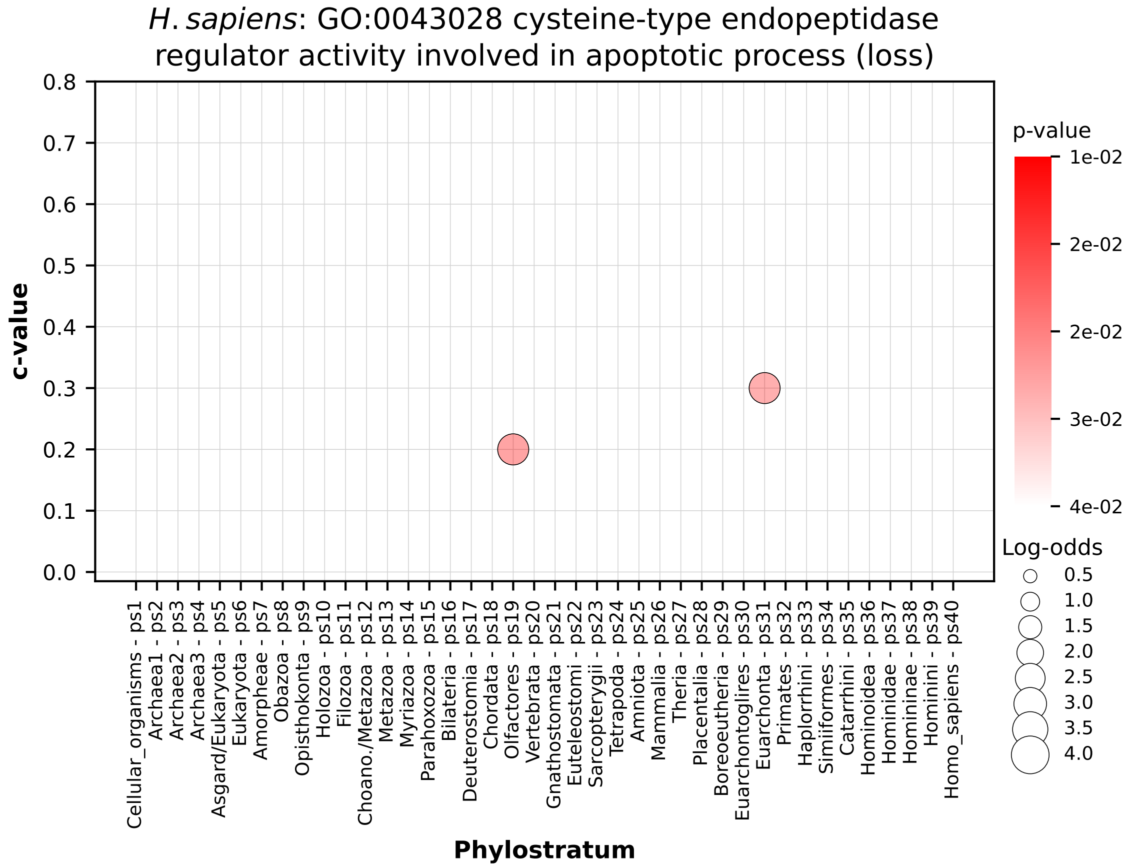
H. sapiens: GO:0043028 cysteine-type endopeptidase regulator activity involved in apoptotic process (loss)
c-value
0.8
0.7
0.6
0.5
0.4
0.3
0.2
0.1
0.0
p-value
1e-02
2e-02
2e-02
3e-02
4e-02
Log-odds
0.5
1.0
1.5
2.0
2.5
3.0
3.5
4.0
Cellular_organisms - ps1
Archaea1 - ps2
Archaea2 - ps3
Archaea3 - ps4
Asgard/Eukaryota - ps5
Eukaryota - ps6
Amorpheae - ps7
Obazoa - ps8
Opisthokonta - ps9
Holozoa - ps10
Filozoa - ps11
Choano./Metazoa - ps12
Metazoa - ps13
Myriazoa - ps14
Parahoxozoa - ps15
Bilateria - ps16
Deuterostomia - ps17
Chordata - ps18
Olfactores - ps19
Vertebrata - ps20
Gnathostomata - ps21
Euteleostomi - ps22
Sarcopterygii - ps23
Tetrapoda - ps24
Amniota - ps25
Mammalia - ps26
Theria - ps27
Placentalia - ps28
Boreoeutheria - ps29
Euarchontoglires - ps30
Euarchonta - ps31
Primates - ps32
Haplorrhini - ps33
Simiiformes - ps34
Catarrhini - ps35
Hominoidea - ps36
Hominidae - ps37
Homininae - ps38
Hominini - ps39
Homo_sapiens - ps40
Phylostratum

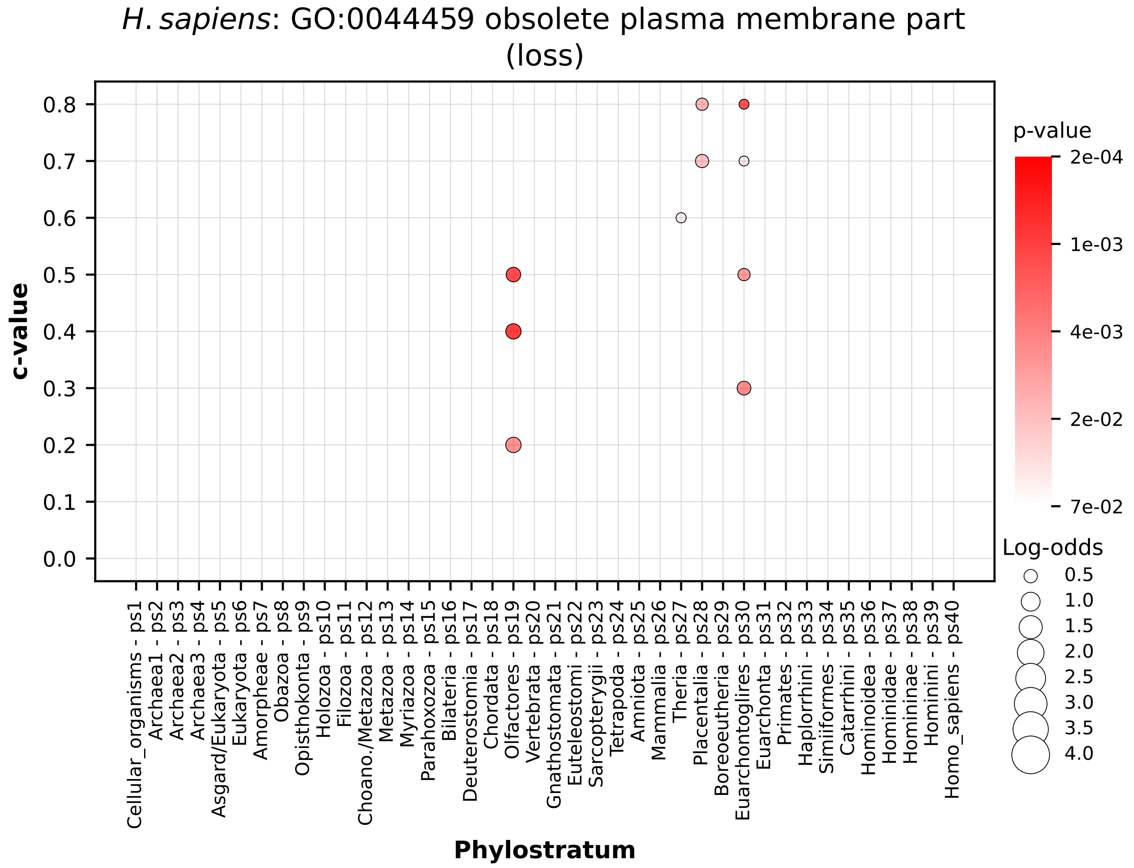

H. sapiens: GO:0044459 obsolete plasma membrane part
(loss)
c-value
0.0
0.1
0.2
0.3
0.4
0.5
0.6
0.7
0.8
p-value
2e-04
1e-03
4e-03
2e-02
7e-02
Log-odds
0.5
1.0
1.5
2.0
2.5
3.0
3.5
4.0
Cellular_organisms - ps1
Archaea1 - ps2
Archaea2 - ps3
Archaea3 - ps4
Asgard/Eukaryota - ps5
Eukaryota - ps6
Amorpheae - ps7
Obazoa - ps8
Opisthokonta - ps9
Holozoa - ps10
Filozoa - ps11
Choano./Metazoa - ps12
Metazoa - ps13
Myriazoa - ps14
Parahoxozoa - ps15
Bilateria - ps16
Deuterostomia - ps17
Chordata - ps18
Olfactores - ps19
Vertebrata - ps20
Gnathostomata - ps21
Euteleostomi - ps22
Sarcopterygii - ps23
Tetrapoda - ps24
Amniota - ps25
Mammalia - ps26
Theria - ps27
Placentalia - ps28
Boreoeutheria - ps29
Euarchontoglires - ps30
Euarchonta - ps31
Primates - ps32
Haplorrhini - ps33
Simiiformes - ps34
Catarrhini - ps35
Hominoidea - ps36
Hominidae - ps37
Homininae - ps38
Hominini - ps39
Homo_sapiens - ps40
Phylostratum

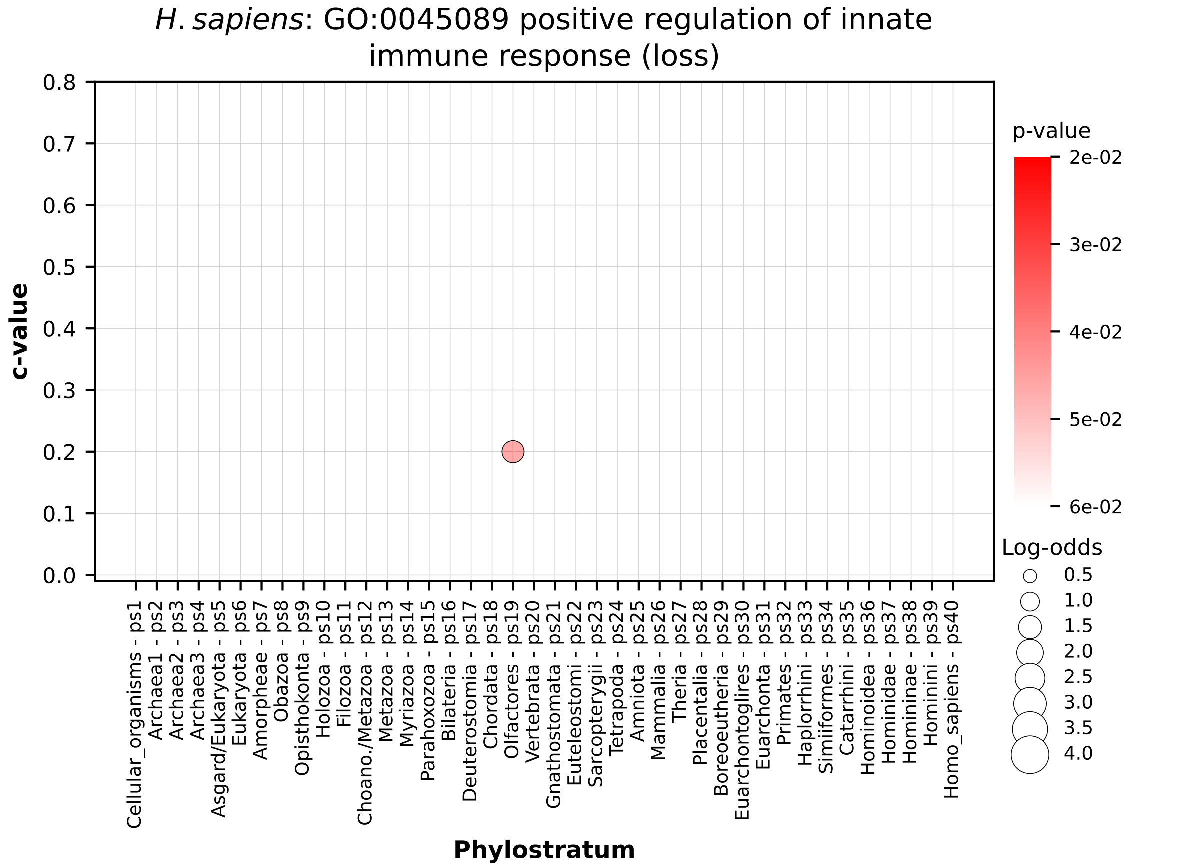
H. sapiens: GO:0045089 positive regulation of innate immune response (loss)
c-value
0.8
0.7
0.6
0.5
0.4
0.3
0.2
0.1
0.0
Phylostratum
Cellular_organisms - ps1
Archaea1 - ps2
Archaea2 - ps3
Archaea3 - ps4
Asgard/Eukaryota - ps5
Eukaryota - ps6
Amorpheae - ps7
Obazoa - ps8
Opisthokonta - ps9
Holozoa - ps10
Filozoa - ps11
Choano./Metazoa - ps12
Metazoa - ps13
Myriazoa - ps14
Parahoxozoa - ps15
Bilateria - ps16
Deuterostomia - ps17
Chordata - ps18
Olfactores - ps19
Vertebrata - ps20
Gnathostomata - ps21
Euteleostomi - ps22
Sarcopterygii - ps23
Tetrapoda - ps24
Amniota - ps25
Mammalia - ps26
Theria - ps27
Placentalia - ps28
Boreoeutheria - ps29
Euarchontoglires - ps30
Euarchonta - ps31
Primates - ps32
Haplorrhini - ps33
Simiiformes - ps34
Catarrhini - ps35
Hominoidea - ps36
Hominidae - ps37
Homininae - ps38
Hominini - ps39
Homo_sapiens - ps40
p-value
2e-02
3e-02
4e-02
5e-02
6e-02
Log-odds
0.5
1.0
1.5
2.0
2.5
3.0
3.5
4.0

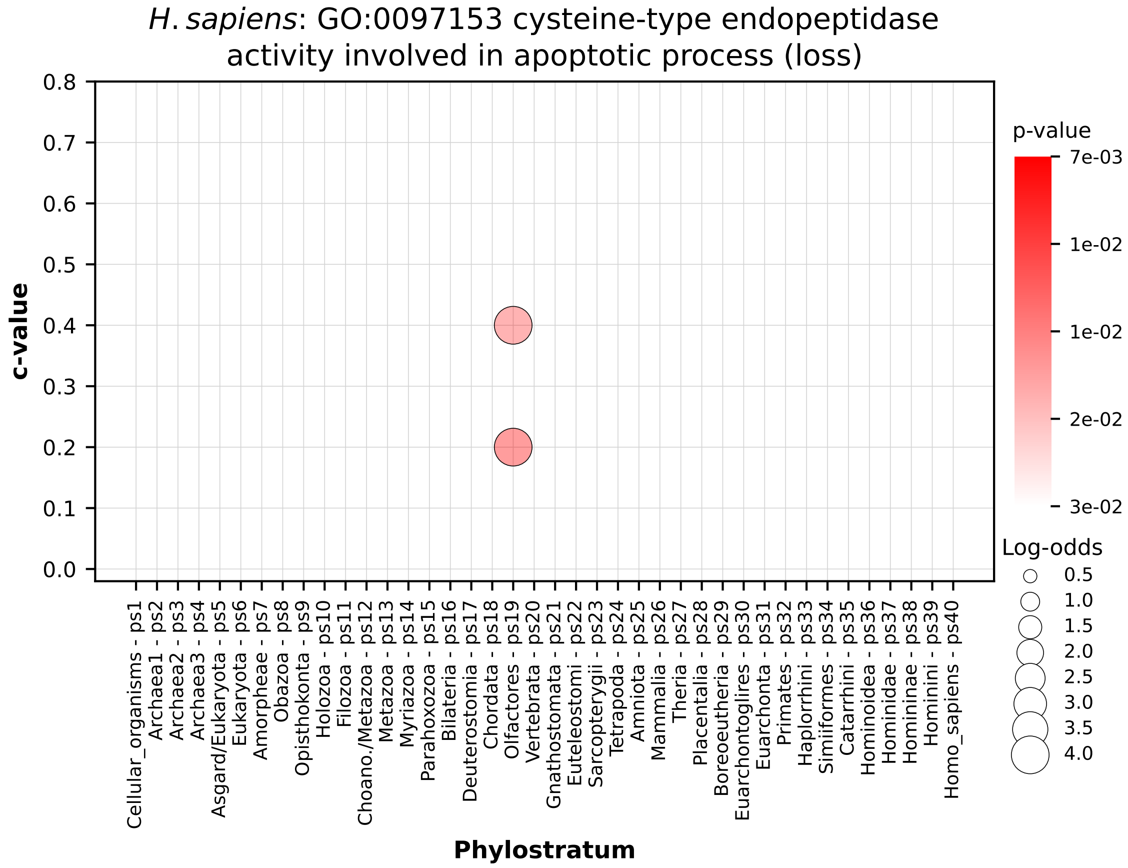

H. sapiens: GO:0097153 cysteine-type endopeptidase activity involved in apoptotic process (loss)
c-value
0.0
0.1
0.2
0.3
0.4
0.5
0.6
0.7
0.8
Phylostratum
Cellular_organisms - ps1
Archaea1 - ps2
Archaea2 - ps3
Archaea3 - ps4
Asgard/Eukaryota - ps5
Eukaryota - ps6
Amorpheae - ps7
Obazoa - ps8
Opisthokonta - ps9
Holozoa - ps10
Filozoa - ps11
Choano./Metazoa - ps12
Metazoa - ps13
Myriazoa - ps14
Parahoxozoa - ps15
Bilateria - ps16
Deuterostomia - ps17
Chordata - ps18
Olfactores - ps19
Vertebrata - ps20
Gnathostomata - ps21
Euteleostomi - ps22
Sarcopterygii - ps23
Tetrapoda - ps24
Amniota - ps25
Mammalia - ps26
Theria - ps27
Placentalia - ps28
Boreoeutheria - ps29
Euarchontoglires - ps30
Euarchonta - ps31
Primates - ps32
Haplorrhini - ps33
Simiiformes - ps34
Catarrhini - ps35
Hominoidea - ps36
Hominidae - ps37
Homininae - ps38
Hominini - ps39
Homo_sapiens - ps40
p-value
7e-03
1e-02
1e-02
2e-02
3e-02
Log-odds
0.5
1.0
1.5
2.0
2.5
3.0
3.5
4.0

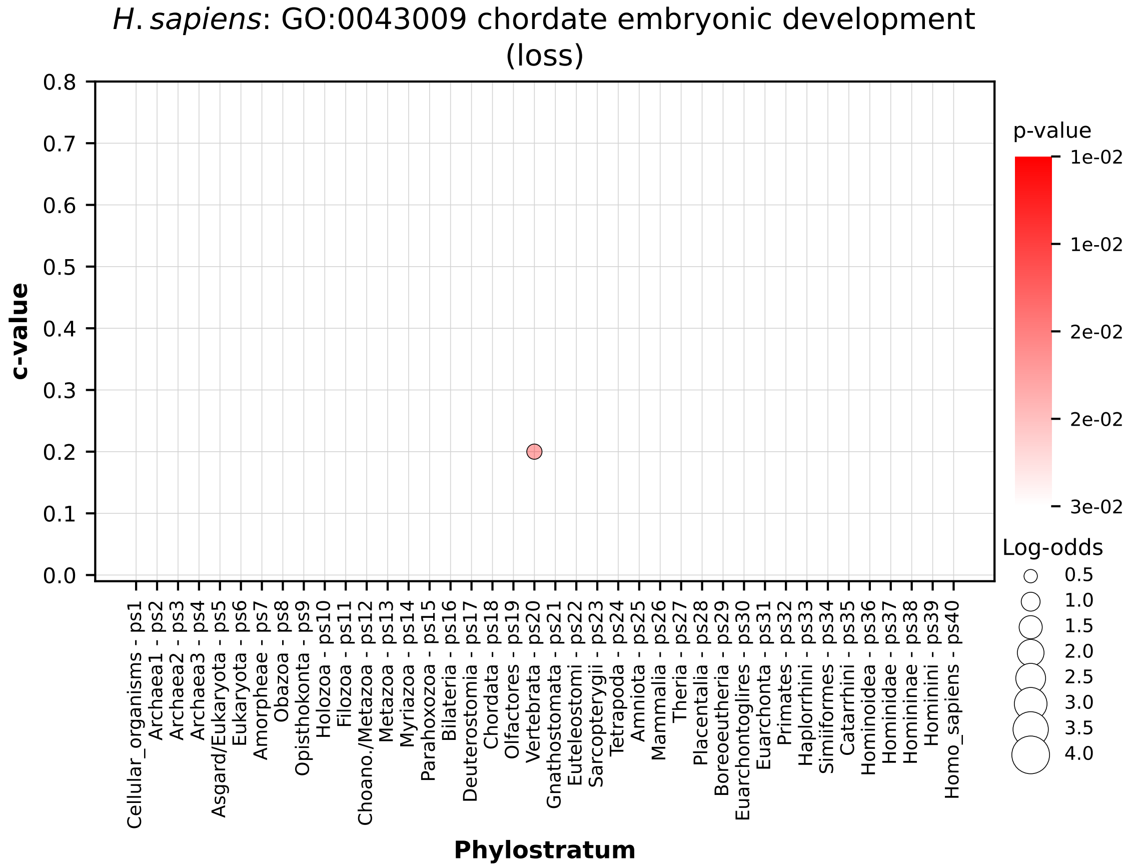

H. sapiens: GO:0043009 chordate embryonic development (loss)
c-value
0.8
0.7
0.6
0.5
0.4
0.3
0.2
0.1
0.0
Phylostratum
Cellular_organisms - ps1
Archaea1 - ps2
Archaea2 - ps3
Archaea3 - ps4
Asgard/Eukaryota - ps5
Eukaryota - ps6
Amorpheae - ps7
Obazoa - ps8
Opisthokonta - ps9
Holozoa - ps10
Filozoa - ps11
Choano./Metazoa - ps12
Metazoa - ps13
Myriazoa - ps14
Parahoxozoa - ps15
Bilateria - ps16
Deuterostomia - ps17
Chordata - ps18
Olfactores - ps19
Vertebrata - ps20
Gnathostomata - ps21
Euteleostomi - ps22
Sarcopterygii - ps23
Tetrapoda - ps24
Amniota - ps25
Mammalia - ps26
Theria - ps27
Placentalia - ps28
Boreoeutheria - ps29
Euarchontoglires - ps30
Euarchonta - ps31
Primates - ps32
Haplorrhini - ps33
Simiiformes - ps34
Catarrhini - ps35
Hominoidea - ps36
Hominidae - ps37
Homininae - ps38
Hominini - ps39
Homo_sapiens - ps40
p-value
1e-02
1e-02
2e-02
2e-02
3e-02
Log-odds
0.5
1.0
1.5
2.0
2.5
3.0
3.5
4.0

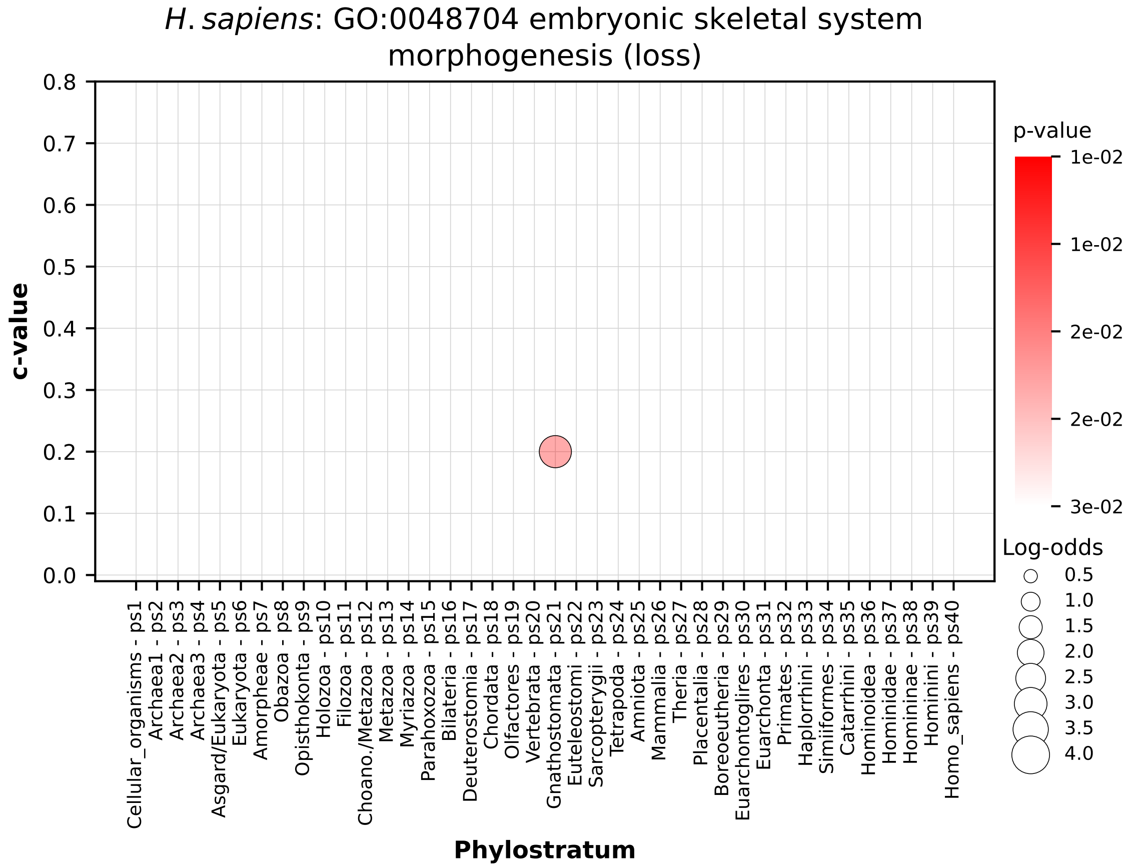

H. sapiens: GO:0048704 embryonic skeletal system morphogenesis (loss)
c-value
0.0
0.1
0.2
0.3
0.4
0.5
0.6
0.7
0.8
Phylostratum
Cellular_organisms - ps1
Archaea1 - ps2
Archaea2 - ps3
Archaea3 - ps4
Asgard/Eukaryota - ps5
Eukaryota - ps6
Amorpheae - ps7
Obazoa - ps8
Opisthokonta - ps9
Holozoa - ps10
Filozoa - ps11
Choano./Metazoa - ps12
Metazoa - ps13
Myriazoa - ps14
Parahoxozoa - ps15
Bilateria - ps16
Deuterostomia - ps17
Chordata - ps18
Olfactores - ps19
Vertebrata - ps20
Gnathostomata - ps21
Euteleostomi - ps22
Sarcopterygii - ps23
Tetrapoda - ps24
Amniota - ps25
Mammalia - ps26
Theria - ps27
Placentalia - ps28
Boreoeutheria - ps29
Euarchontoglires - ps30
Euarchonta - ps31
Primates - ps32
Haplorrhini - ps33
Simiiformes - ps34
Catarrhini - ps35
Hominoidea - ps36
Hominidae - ps37
Homininae - ps38
Hominini - ps39
Homo_sapiens - ps40
p-value
1e-02
1e-02
2e-02
2e-02
3e-02
Log-odds
0.5
1.0
1.5
2.0
2.5
3.0
3.5
4.0

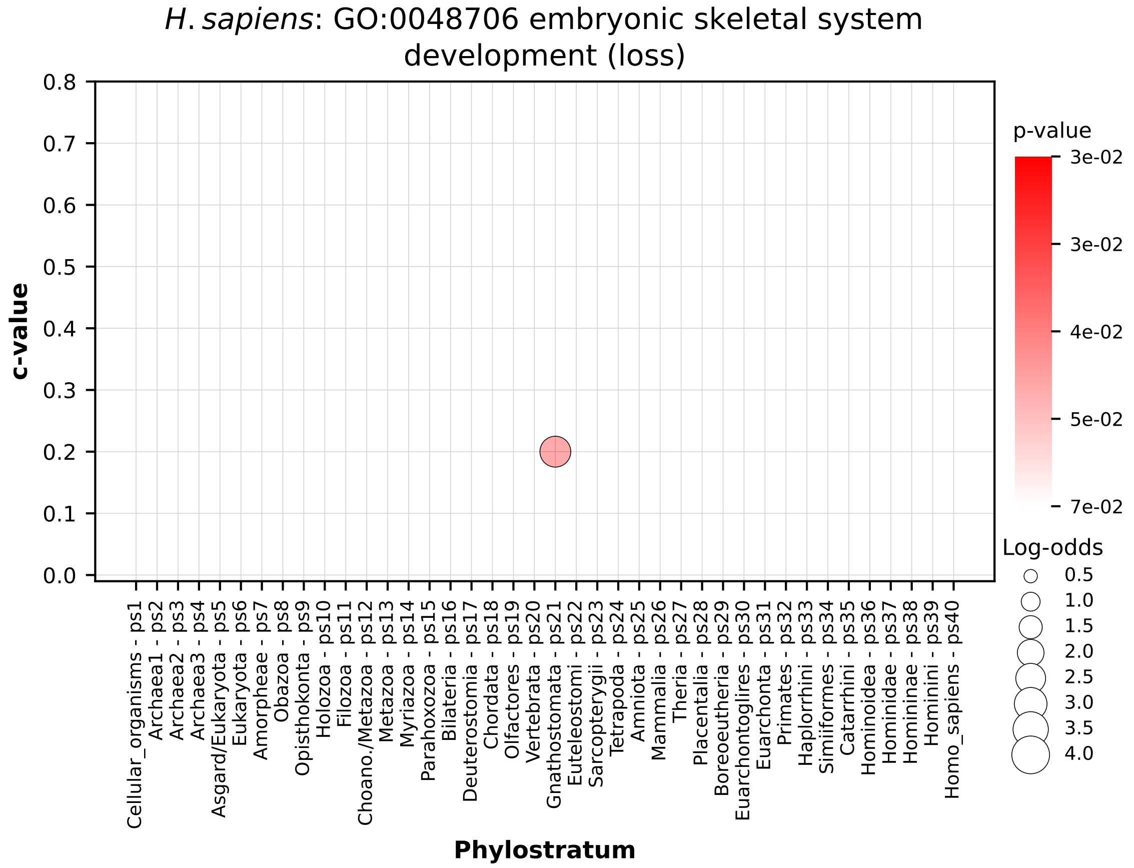

H. sapiens: GO:0048706 embryonic skeletal system development (loss)
c-value
0.0
0.1
0.2
0.3
0.4
0.5
0.6
0.7
0.8
Phylostratum
Cellular_organisms - ps1
Archaea1 - ps2
Archaea2 - ps3
Archaea3 - ps4
Asgard/Eukaryota - ps5
Eukaryota - ps6
Amorpheae - ps7
Obazoa - ps8
Opisthokonta - ps9
Holozoa - ps10
Filozoa - ps11
Choano./Metazoa - ps12
Metazoa - ps13
Myriazoa - ps14
Parahoxozoa - ps15
Bilateria - ps16
Deuterostomia - ps17
Chordata - ps18
Olfactores - ps19
Vertebrata - ps20
Gnathostomata - ps21
Euteleostomi - ps22
Sarcopterygii - ps23
Tetrapoda - ps24
Amniota - ps25
Mammalia - ps26
Theria - ps27
Placentalia - ps28
Boreoeutheria - ps29
Euarchontoglires - ps30
Euarchonta - ps31
Primates - ps32
Haplorrhini - ps33
Simiiformes - ps34
Catarrhini - ps35
Hominoidea - ps36
Hominidae - ps37
Homininae - ps38
Hominini - ps39
Homo_sapiens - ps40
p-value
3e-02
3e-02
4e-02
5e-02
7e-02
Log-odds
0.5
1.0
1.5
2.0
2.5
3.0
3.5
4.0

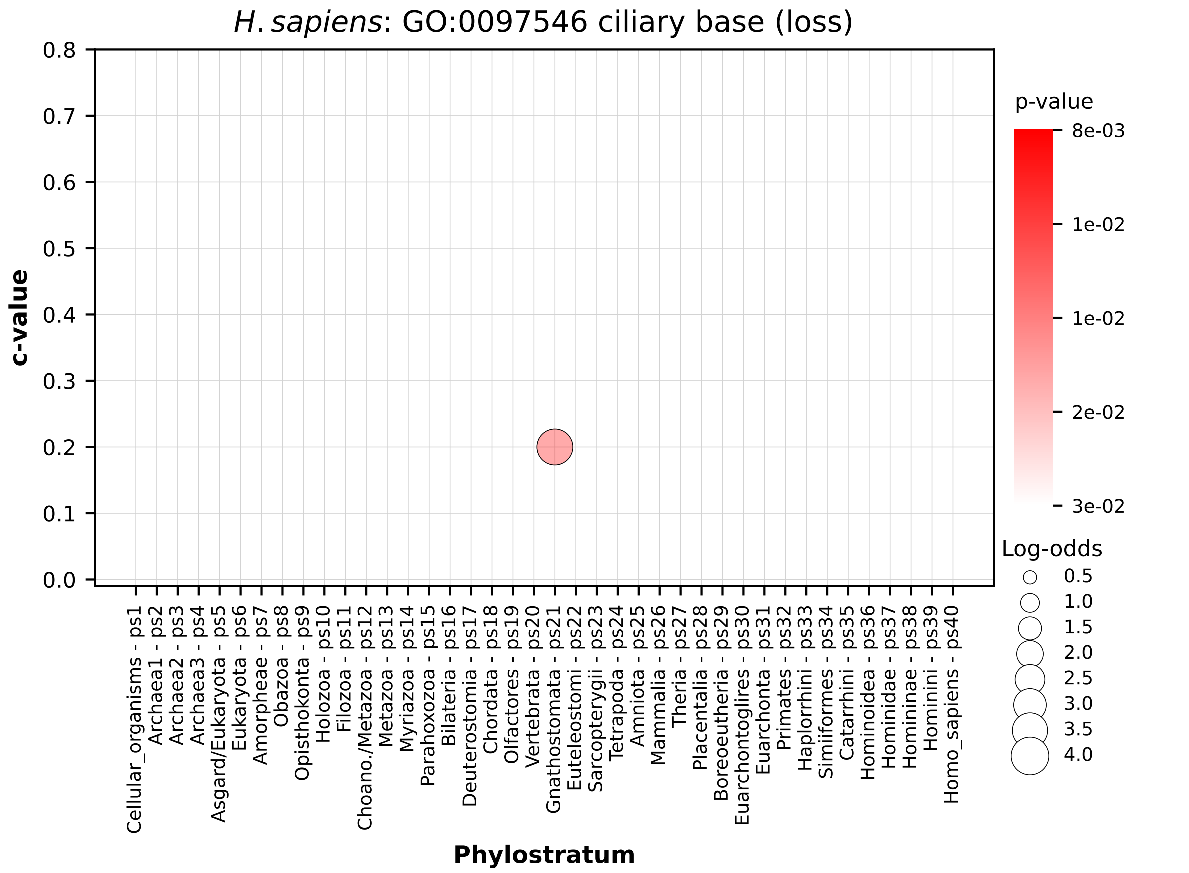

H. sapiens: GO:0097546 ciliary base (loss)
c-value
0.8
0.7
0.6
0.5
0.4
0.3
0.2
0.1
0.0
p-value
8e-03
1e-02
1e-02
2e-02
3e-02
Log-odds
0.5
1.0
1.5
2.0
2.5
3.0
3.5
4.0
Cellular_organisms - ps1
Archaea1 - ps2
Archaea2 - ps3
Archaea3 - ps4
Asgard/Eukaryota - ps5
Eukaryota - ps6
Amorpheae - ps7
Obazoa - ps8
Opisthokonta - ps9
Holozoa - ps10
Filozoa - ps11
Choano./Metazoa - ps12
Metazoa - ps13
Myriazoa - ps14
Parahoxozoa - ps15
Bilateria - ps16
Deuterostomia - ps17
Chordata - ps18
Olfactores - ps19
Vertebrata - ps20
Gnathostomata - ps21
Euteleostomi - ps22
Sarcopterygii - ps23
Tetrapoda - ps24
Amniota - ps25
Mammalia - ps26
Theria - ps27
Placentalia - ps28
Boreoeutheria - ps29
Euarchontoglires - ps30
Euarchonta - ps31
Primates - ps32
Haplorrhini - ps33
Simiiformes - ps34
Catarrhini - ps35
Hominoidea - ps36
Hominidae - ps37
Homininae - ps38
Hominini - ps39
Homo_sapiens - ps40
Phylostratum

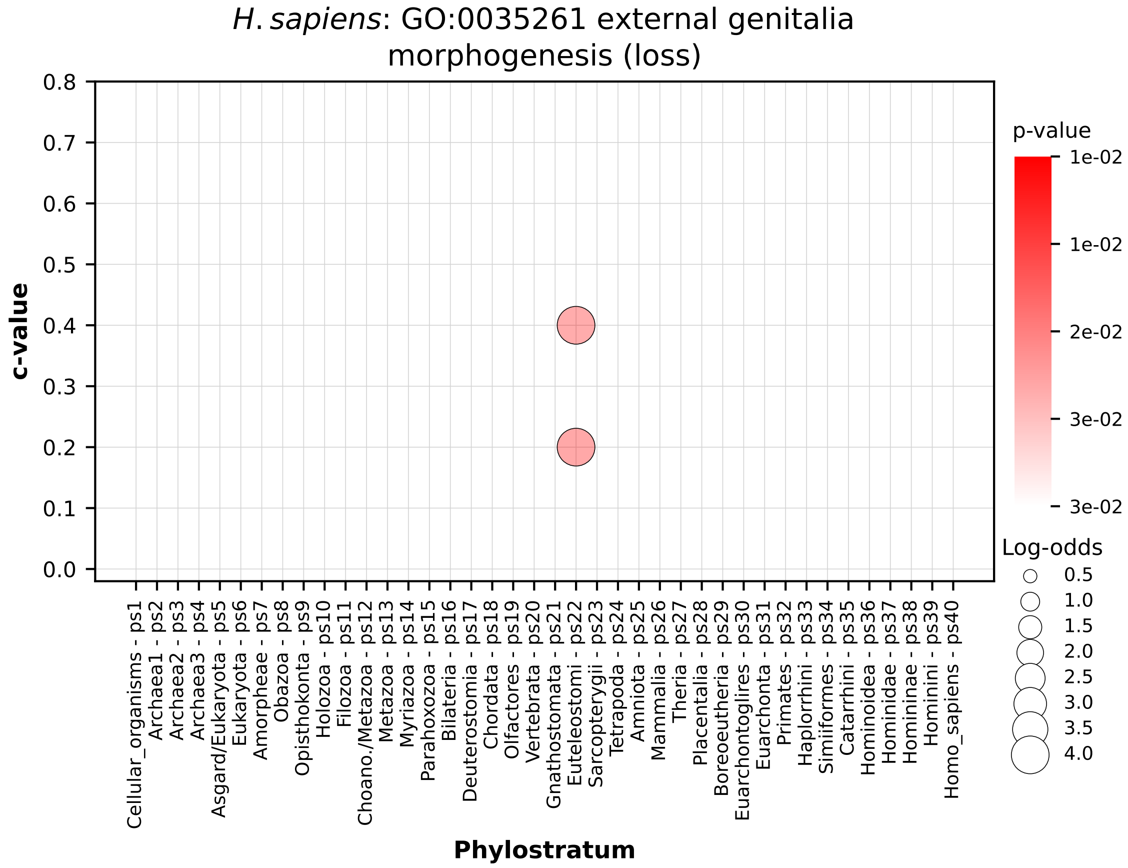

H. sapiens: GO:0035261 external genitalia morphogenesis (loss)
c-value
0.8
0.7
0.6
0.5
0.4
0.3
0.2
0.1
0.0
Cellular_organisms - ps1
Archaea1 - ps2
Archaea2 - ps3
Archaea3 - ps4
Asgard/Eukaryota - ps5
Eukaryota - ps6
Amorpheae - ps7
Obazoa - ps8
Opisthokonta - ps9
Holozoa - ps10
Filozoa - ps11
Choano./Metazoa - ps12
Metazoa - ps13
Myriazoa - ps14
Parahoxozoa - ps15
Bilateria - ps16
Deuterostomia - ps17
Chordata - ps18
Olfactores - ps19
Vertebrata - ps20
Gnathostomata - ps21
Euteleostomi - ps22
Sarcopterygii - ps23
Tetrapoda - ps24
Amniota - ps25
Mammalia - ps26
Theria - ps27
Placentalia - ps28
Boreoeutheria - ps29
Euarchontoglires - ps30
Euarchonta - ps31
Primates - ps32
Haplorrhini - ps33
Simiiformes - ps34
Catarrhini - ps35
Hominoidea - ps36
Hominidae - ps37
Homininae - ps38
Hominini - ps39
Homo_sapiens - ps40
Phylostratum
p-value
1e-02
1e-02
2e-02
3e-02
3e-02
Log-odds
0.5
1.0
1.5
2.0
2.5
3.0
3.5
4.0

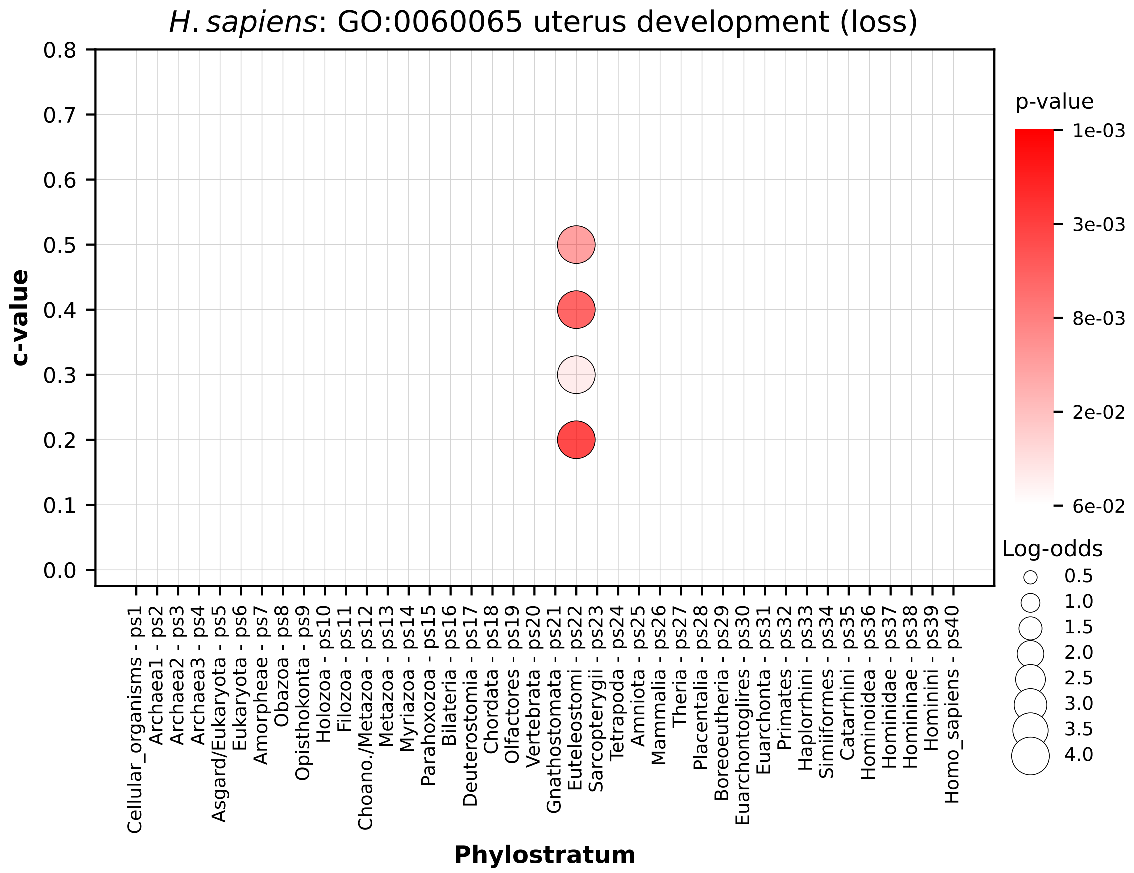

H. sapiens: GO:0060065 uterus development (loss)
c-value
0.8
0.7
0.6
0.5
0.4
0.3
0.2
0.1
0.0
p-value
1e-03
3e-03
8e-03
2e-02
6e-02
Log-odds
0.5
1.0
1.5
2.0
2.5
3.0
3.5
4.0
Cellular_organisms - ps1
Archaea1 - ps2
Archaea2 - ps3
Archaea3 - ps4
Asgard/Eukaryota - ps5
Eukaryota - ps6
Amorpheae - ps7
Obazoa - ps8
Opisthokonta - ps9
Holozoa - ps10
Filozoa - ps11
Choano./Metazoa - ps12
Metazoa - ps13
Myriazoa - ps14
Parahoxozoa - ps15
Bilateria - ps16
Deuterostomia - ps17
Chordata - ps18
Olfactores - ps19
Vertebrata - ps20
Gnathostomata - ps21
Euteleostomi - ps22
Sarcopterygii - ps23
Tetrapoda - ps24
Amniota - ps25
Mammalia - ps26
Theria - ps27
Placentalia - ps28
Boreoeutheria - ps29
Euarchontoglires - ps30
Euarchonta - ps31
Primates - ps32
Haplorrhini - ps33
Simiiformes - ps34
Catarrhini - ps35
Hominoidea - ps36
Hominidae - ps37
Homininae - ps38
Hominini - ps39
Homo_sapiens - ps40
Phylostratum

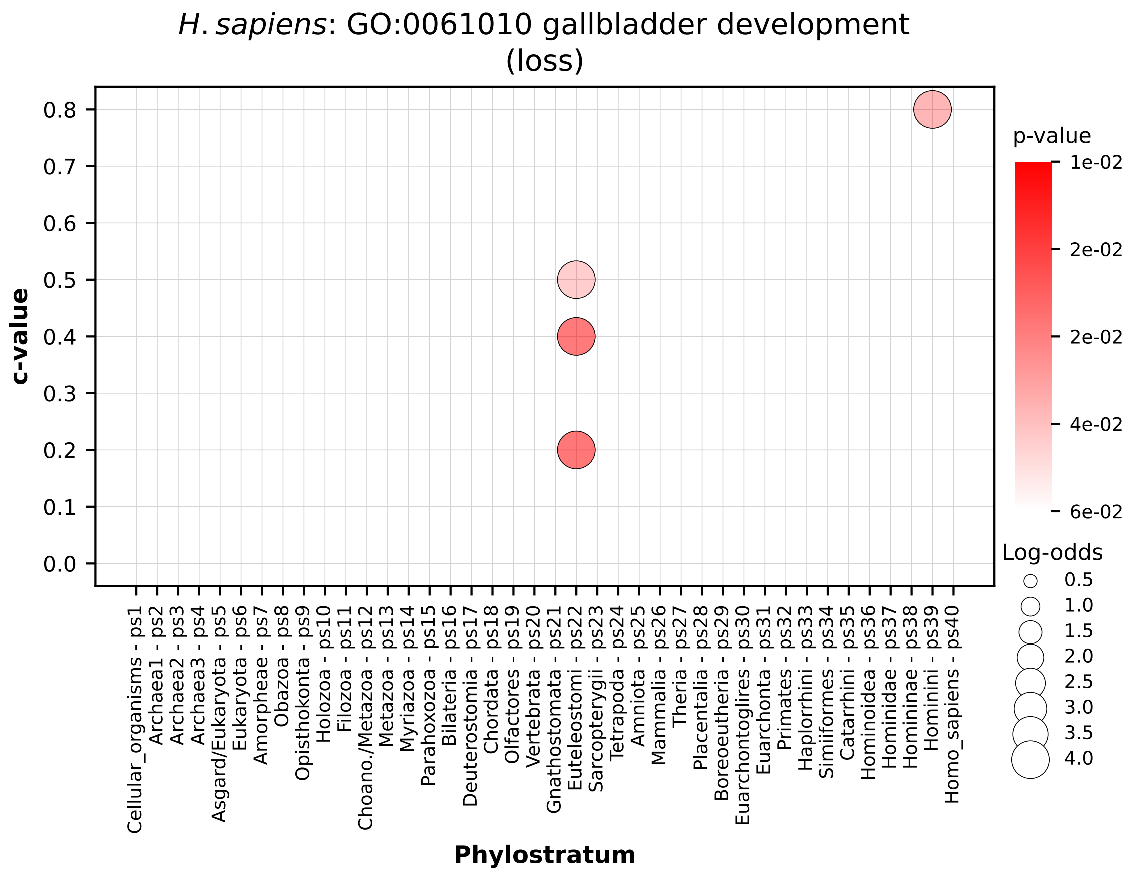

H. sapiens: GO:0061010 gallbladder development
(loss)
c-value
0.0
0.1
0.2
0.3
0.4
0.5
0.6
0.7
0.8
p-value
1e-02
2e-02
2e-02
4e-02
6e-02
Log-odds
0.5
1.0
1.5
2.0
2.5
3.0
3.5
4.0
Cellular_organisms - ps1
Archaea1 - ps2
Archaea2 - ps3
Archaea3 - ps4
Asgard/Eukaryota - ps5
Eukaryota - ps6
Amorpheae - ps7
Obazoa - ps8
Opisthokonta - ps9
Holozoa - ps10
Filozoa - ps11
Choano./Metazoa - ps12
Metazoa - ps13
Myriazoa - ps14
Parahoxozoa - ps15
Bilateria - ps16
Deuterostomia - ps17
Chordata - ps18
Olfactores - ps19
Vertebrata - ps20
Gnathostomata - ps21
Euteleostomi - ps22
Sarcopterygii - ps23
Tetrapoda - ps24
Amniota - ps25
Mammalia - ps26
Theria - ps27
Placentalia - ps28
Boreoeutheria - ps29
Euarchontoglires - ps30
Euarchonta - ps31
Primates - ps32
Haplorrhini - ps33
Simiiformes - ps34
Catarrhini - ps35
Hominoidea - ps36
Hominidae - ps37
Homininae - ps38
Hominini - ps39
Homo_sapiens - ps40
Phylostratum

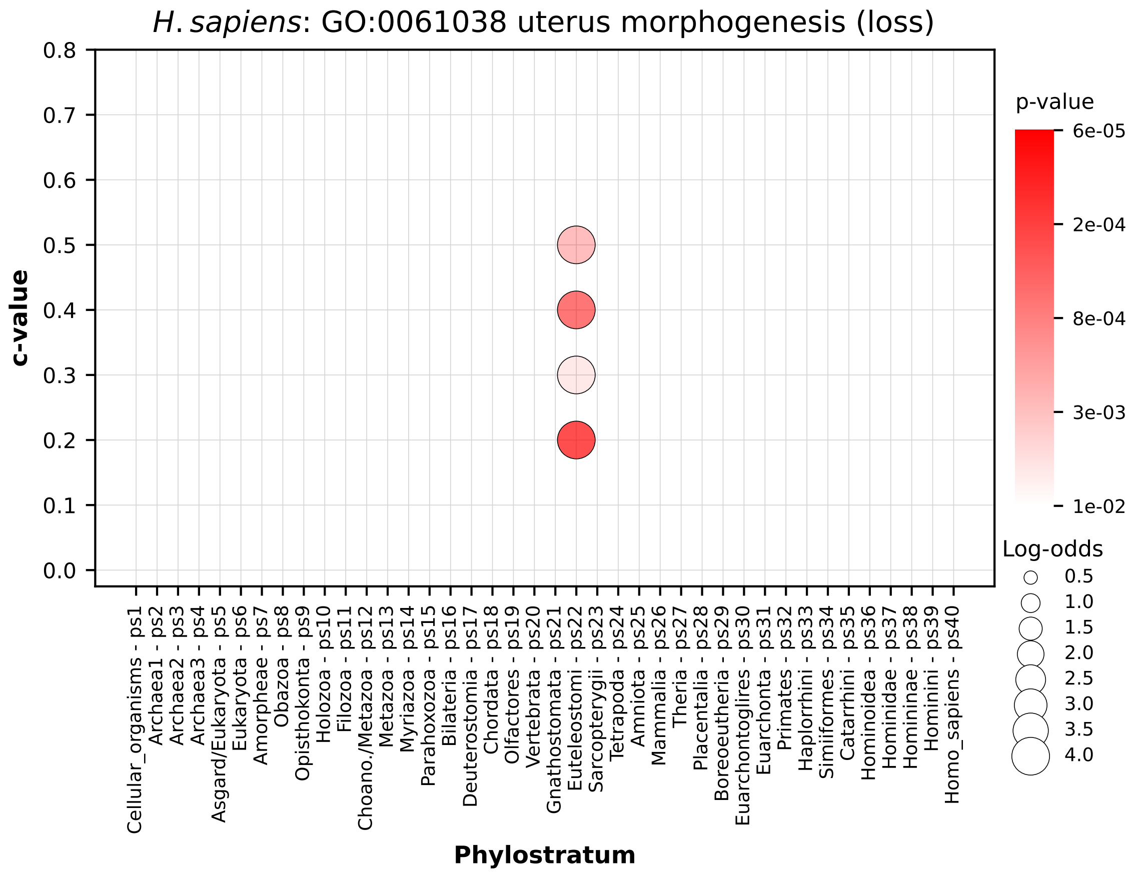

H. sapiens: GO:0061038 uterus morphogenesis (loss)
c-value
0.8
0.7
0.6
0.5
0.4
0.3
0.2
0.1
0.0
p-value
6e-05
2e-04
8e-04
3e-03
1e-02
Log-odds
0.5
1.0
1.5
2.0
2.5
3.0
3.5
4.0
Cellular_organisms - ps1
Archaea1 - ps2
Archaea2 - ps3
Archaea3 - ps4
Asgard/Eukaryota - ps5
Eukaryota - ps6
Amorpheae - ps7
Obazoa - ps8
Opisthokonta - ps9
Holozoa - ps10
Filozoa - ps11
Choano./Metazoa - ps12
Metazoa - ps13
Myriazoa - ps14
Parahoxozoa - ps15
Bilateria - ps16
Deuterostomia - ps17
Chordata - ps18
Olfactores - ps19
Vertebrata - ps20
Gnathostomata - ps21
Euteleostomi - ps22
Sarcopterygii - ps23
Tetrapoda - ps24
Amniota - ps25
Mammalia - ps26
Theria - ps27
Placentalia - ps28
Boreoeutheria - ps29
Euarchontoglires - ps30
Euarchonta - ps31
Primates - ps32
Haplorrhini - ps33
Simiiformes - ps34
Catarrhini - ps35
Hominoidea - ps36
Hominidae - ps37
Homininae - ps38
Hominini - ps39
Homo_sapiens - ps40
Phylostratum

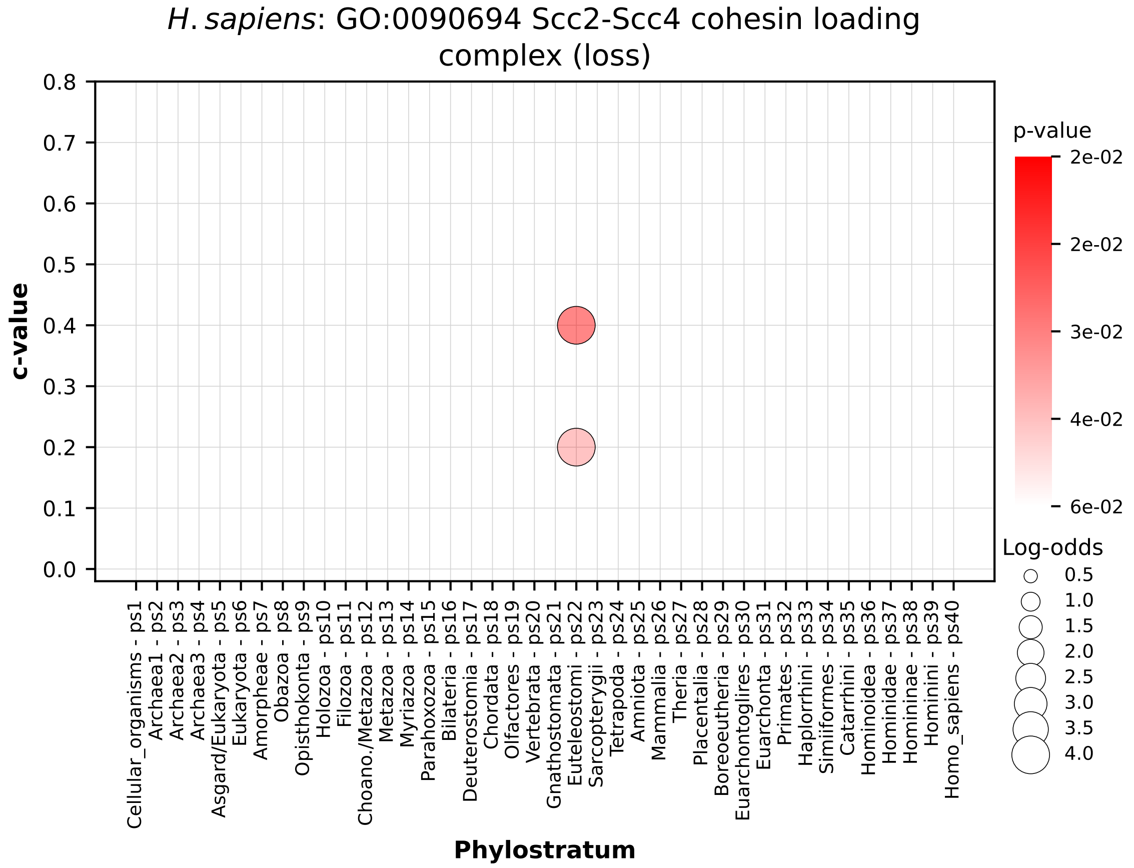

H. sapiens: GO:0090694 Scc2-Scc4 cohesin loading complex (loss)
c-value
0.8
0.7
0.6
0.5
0.4
0.3
0.2
0.1
0.0
Cellular_organisms - ps1
Archaea1 - ps2
Archaea2 - ps3
Archaea3 - ps4
Asgard/Eukaryota - ps5
Eukaryota - ps6
Amorpheae - ps7
Obazoa - ps8
Opisthokonta - ps9
Holozoa - ps10
Filozoa - ps11
Choano./Metazoa - ps12
Metazoa - ps13
Myriazoa - ps14
Parahoxozoa - ps15
Bilateria - ps16
Deuterostomia - ps17
Chordata - ps18
Olfactores - ps19
Vertebrata - ps20
Gnathostomata - ps21
Euteleostomi - ps22
Sarcopterygii - ps23
Tetrapoda - ps24
Amniota - ps25
Mammalia - ps26
Theria - ps27
Placentalia - ps28
Boreoeutheria - ps29
Euarchontoglires - ps30
Euarchonta - ps31
Primates - ps32
Haplorrhini - ps33
Simiiformes - ps34
Catarrhini - ps35
Hominoidea - ps36
Hominidae - ps37
Homininae - ps38
Hominini - ps39
Homo_sapiens - ps40
Phylostratum
p-value
2e-02
2e-02
3e-02
4e-02
6e-02
Log-odds
0.5
1.0
1.5
2.0
2.5
3.0
3.5
4.0

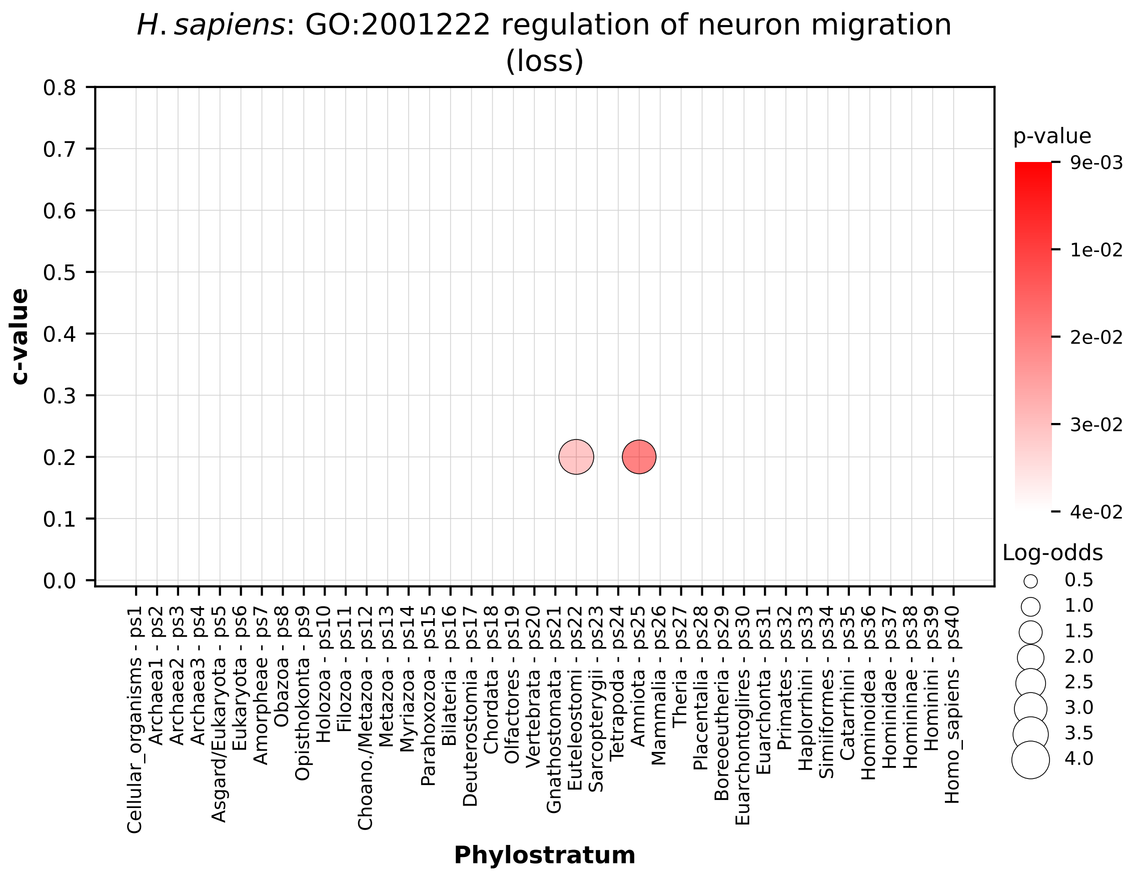

H. sapiens: GO:2001222 regulation of neuron migration
(loss)
c-value
0.0
0.1
0.2
0.3
0.4
0.5
0.6
0.7
0.8
Phylostratum
Cellular_organisms - ps1
Archaea1 - ps2
Archaea2 - ps3
Archaea3 - ps4
Asgard/Eukaryota - ps5
Eukaryota - ps6
Amorpheae - ps7
Obazoa - ps8
Opisthokonta - ps9
Holozoa - ps10
Filozoa - ps11
Choano./Metazoa - ps12
Metazoa - ps13
Myriazoa - ps14
Parahoxozoa - ps15
Bilateria - ps16
Deuterostomia - ps17
Chordata - ps18
Olfactores - ps19
Vertebrata - ps20
Gnathostomata - ps21
Euteleostomi - ps22
Sarcopterygii - ps23
Tetrapoda - ps24
Amniota - ps25
Mammalia - ps26
Theria - ps27
Placentalia - ps28
Boreoeutheria - ps29
Euarchontoglires - ps30
Euarchonta - ps31
Primates - ps32
Haplorrhini - ps33
Simiiformes - ps34
Catarrhini - ps35
Hominoidea - ps36
Hominidae - ps37
Homininae - ps38
Hominini - ps39
Homo_sapiens - ps40
p-value
9e-03
1e-02
2e-02
3e-02
4e-02
Log-odds
0.5
1.0
1.5
2.0
2.5
3.0
3.5
4.0

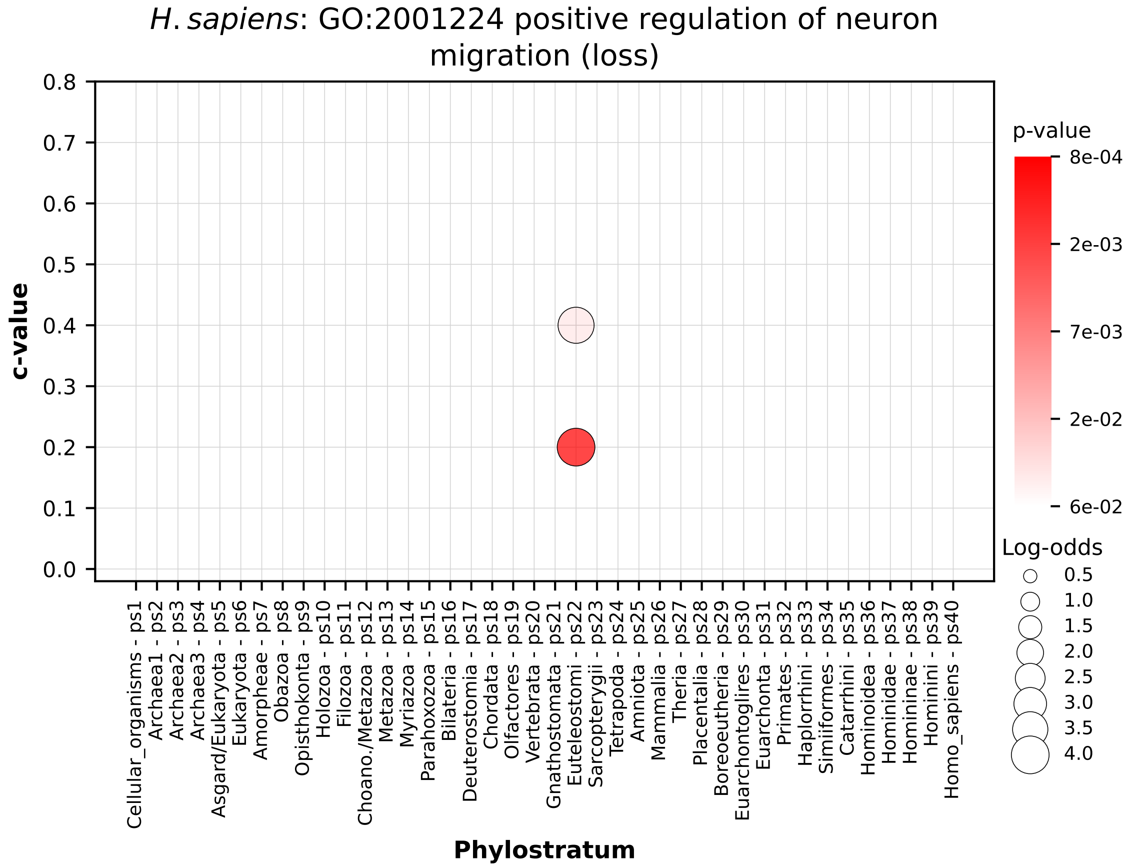

H. sapiens: GO:2001224 positive regulation of neuron migration (loss)
c-value
0.8
0.7
0.6
0.5
0.4
0.3
0.2
0.1
0.0
Phylostratum
Cellular_organisms - ps1
Archaea1 - ps2
Archaea2 - ps3
Archaea3 - ps4
Asgard/Eukaryota - ps5
Eukaryota - ps6
Amorpheae - ps7
Obazoa - ps8
Opisthokonta - ps9
Holozoa - ps10
Filozoa - ps11
Choano./Metazoa - ps12
Metazoa - ps13
Myriazoa - ps14
Parahoxozoa - ps15
Bilateria - ps16
Deuterostomia - ps17
Chordata - ps18
Olfactores - ps19
Vertebrata - ps20
Gnathostomata - ps21
Euteleostomi - ps22
Sarcopterygii - ps23
Tetrapoda - ps24
Amniota - ps25
Mammalia - ps26
Theria - ps27
Placentalia - ps28
Boreoeutheria - ps29
Euarchontoglires - ps30
Euarchonta - ps31
Primates - ps32
Haplorrhini - ps33
Simiiformes - ps34
Catarrhini - ps35
Hominoidea - ps36
Hominidae - ps37
Homininae - ps38
Hominini - ps39
Homo_sapiens - ps40
p-value
8e-04
2e-03
7e-03
2e-02
6e-02
Log-odds
0.5
1.0
1.5
2.0
2.5
3.0
3.5
4.0

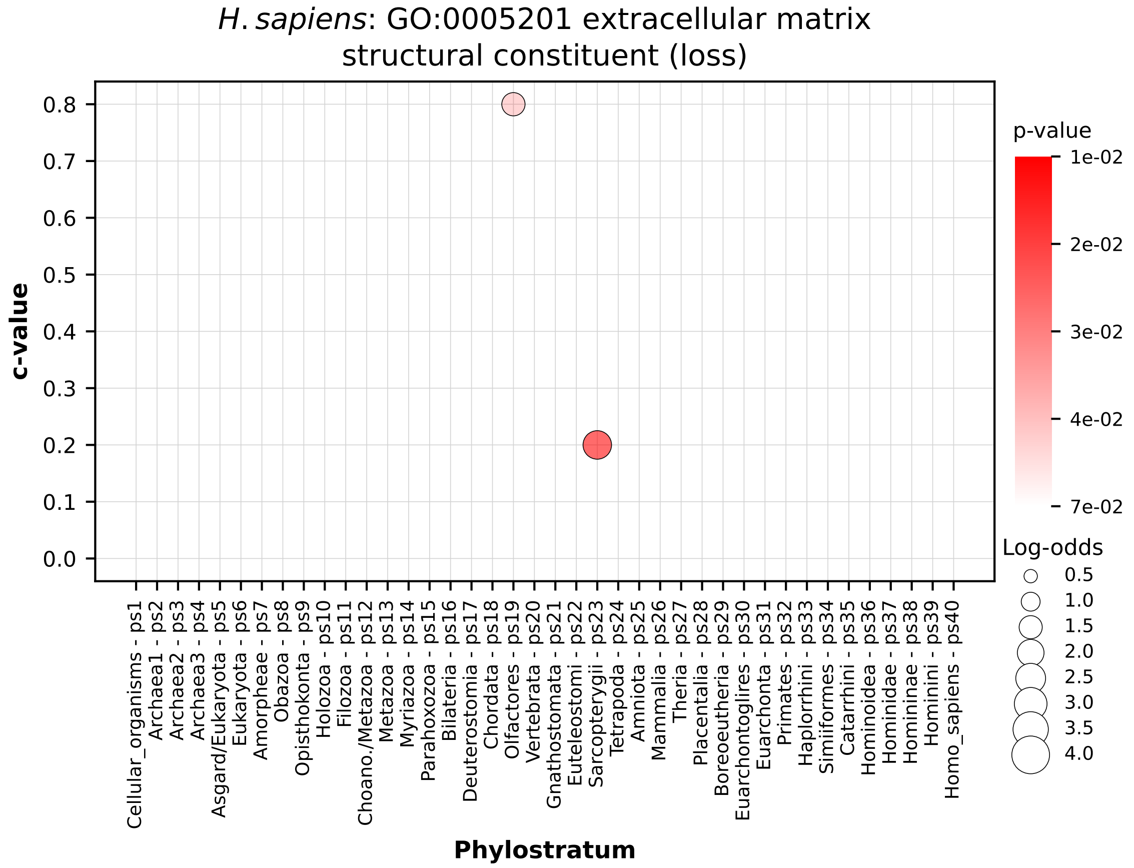

H. sapiens: GO:0005201 extracellular matrix structural constituent (loss)
c-value
0.0
0.1
0.2
0.3
0.4
0.5
0.6
0.7
0.8
Phylostratum
Cellular_organisms - ps1
Archaea1 - ps2
Archaea2 - ps3
Archaea3 - ps4
Asgard/Eukaryota - ps5
Eukaryota - ps6
Amorpheae - ps7
Obazoa - ps8
Opisthokonta - ps9
Holozoa - ps10
Filozoa - ps11
Choano./Metazoa - ps12
Metazoa - ps13
Myriazoa - ps14
Parahoxozoa - ps15
Bilateria - ps16
Deuterostomia - ps17
Chordata - ps18
Olfactores - ps19
Vertebrata - ps20
Gnathostomata - ps21
Euteleostomi - ps22
Sarcopterygii - ps23
Tetrapoda - ps24
Amniota - ps25
Mammalia - ps26
Theria - ps27
Placentalia - ps28
Boreoeutheria - ps29
Euarchontoglires - ps30
Euarchonta - ps31
Primates - ps32
Haplorrhini - ps33
Simiiformes - ps34
Catarrhini - ps35
Hominoidea - ps36
Hominidae - ps37
Homininae - ps38
Hominini - ps39
Homo_sapiens - ps40
p-value
1e-02
2e-02
3e-02
4e-02
7e-02
Log-odds
0.5
1.0
1.5
2.0
2.5
3.0
3.5
4.0

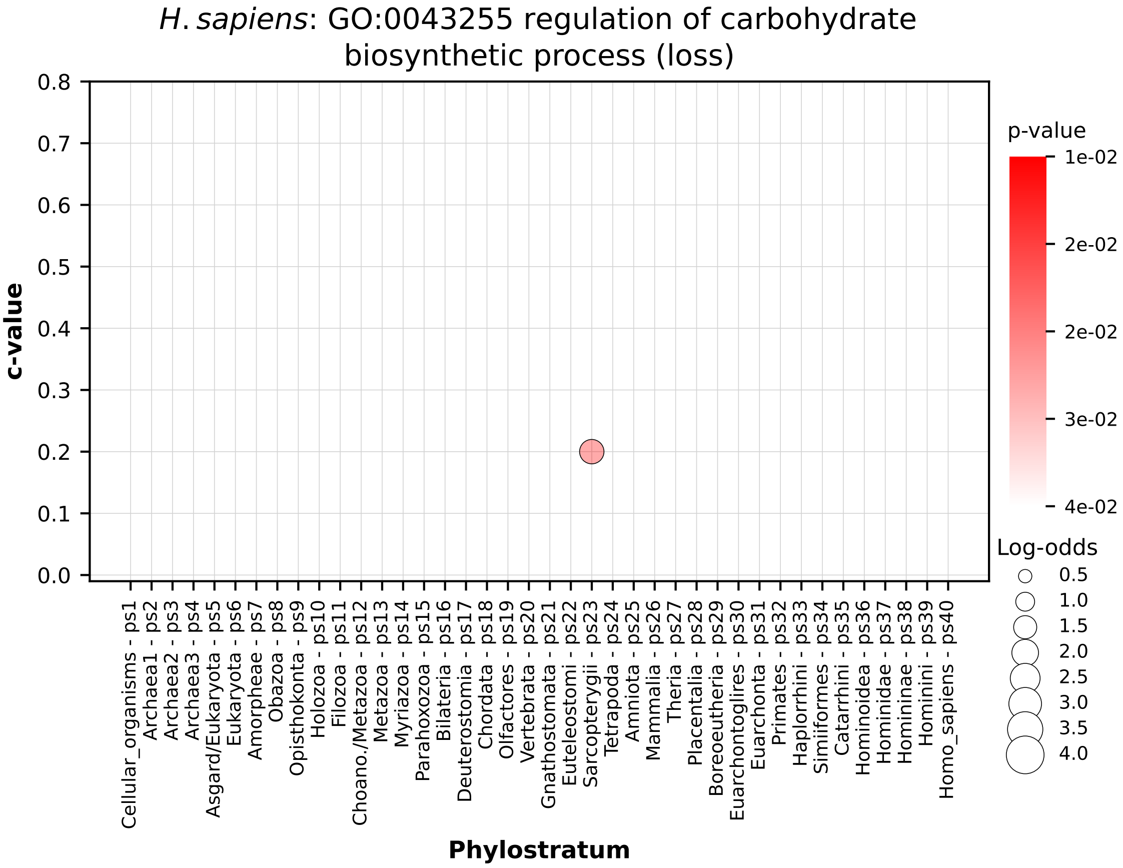

H. sapiens: GO:0043255 regulation of carbohydrate biosynthetic process (loss)
c-value
0.8
0.7
0.6
0.5
0.4
0.3
0.2
0.1
0.0
p-value
1e-02
2e-02
2e-02
3e-02
4e-02
Log-odds
0.5
1.0
1.5
2.0
2.5
3.0
3.5
4.0
Cellular_organisms - ps1
Archaea1 - ps2
Archaea2 - ps3
Archaea3 - ps4
Asgard/Eukaryota - ps5
Eukaryota - ps6
Amorpheae - ps7
Obazoa - ps8
Opisthokonta - ps9
Holozoa - ps10
Filozoa - ps11
Choano./Metazoa - ps12
Metazoa - ps13
Myriazoa - ps14
Parahoxozoa - ps15
Bilateria - ps16
Deuterostomia - ps17
Chordata - ps18
Olfactores - ps19
Vertebrata - ps20
Gnathostomata - ps21
Euteleostomi - ps22
Sarcopterygii - ps23
Tetrapoda - ps24
Amniota - ps25
Mammalia - ps26
Theria - ps27
Placentalia - ps28
Boreoeutheria - ps29
Euarchontoglires - ps30
Euarchonta - ps31
Primates - ps32
Haplorrhini - ps33
Simiiformes - ps34
Catarrhini - ps35
Hominoidea - ps36
Hominidae - ps37
Homininae - ps38
Hominini - ps39
Homo_sapiens - ps40
Phylostratum

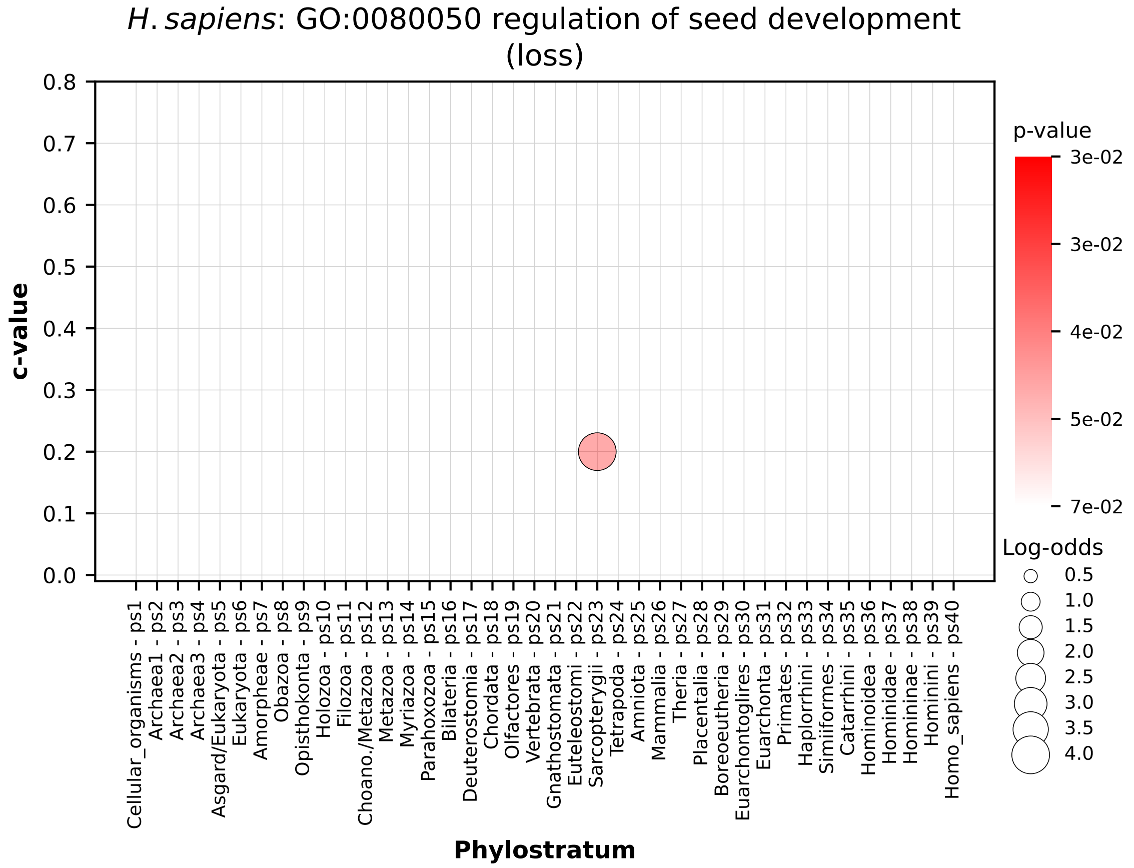

H. sapiens: GO:0080050 regulation of seed development (loss)
c-value
0.8
0.7
0.6
0.5
0.4
0.3
0.2
0.1
0.0
Phylostratum
Cellular_organisms - ps1
Archaea1 - ps2
Archaea2 - ps3
Archaea3 - ps4
Asgard/Eukaryota - ps5
Eukaryota - ps6
Amorpheae - ps7
Obazoa - ps8
Opisthokonta - ps9
Holozoa - ps10
Filozoa - ps11
Choano./Metazoa - ps12
Metazoa - ps13
Myriazoa - ps14
Parahoxozoa - ps15
Bilateria - ps16
Deuterostomia - ps17
Chordata - ps18
Olfactores - ps19
Vertebrata - ps20
Gnathostomata - ps21
Euteleostomi - ps22
Sarcopterygii - ps23
Tetrapoda - ps24
Amniota - ps25
Mammalia - ps26
Theria - ps27
Placentalia - ps28
Boreoeutheria - ps29
Euarchontoglires - ps30
Euarchonta - ps31
Primates - ps32
Haplorrhini - ps33
Simiiformes - ps34
Catarrhini - ps35
Hominoidea - ps36
Hominidae - ps37
Homininae - ps38
Hominini - ps39
Homo_sapiens - ps40
p-value
3e-02
3e-02
4e-02
5e-02
7e-02
Log-odds
0.5
1.0
1.5
2.0
2.5
3.0
3.5
4.0

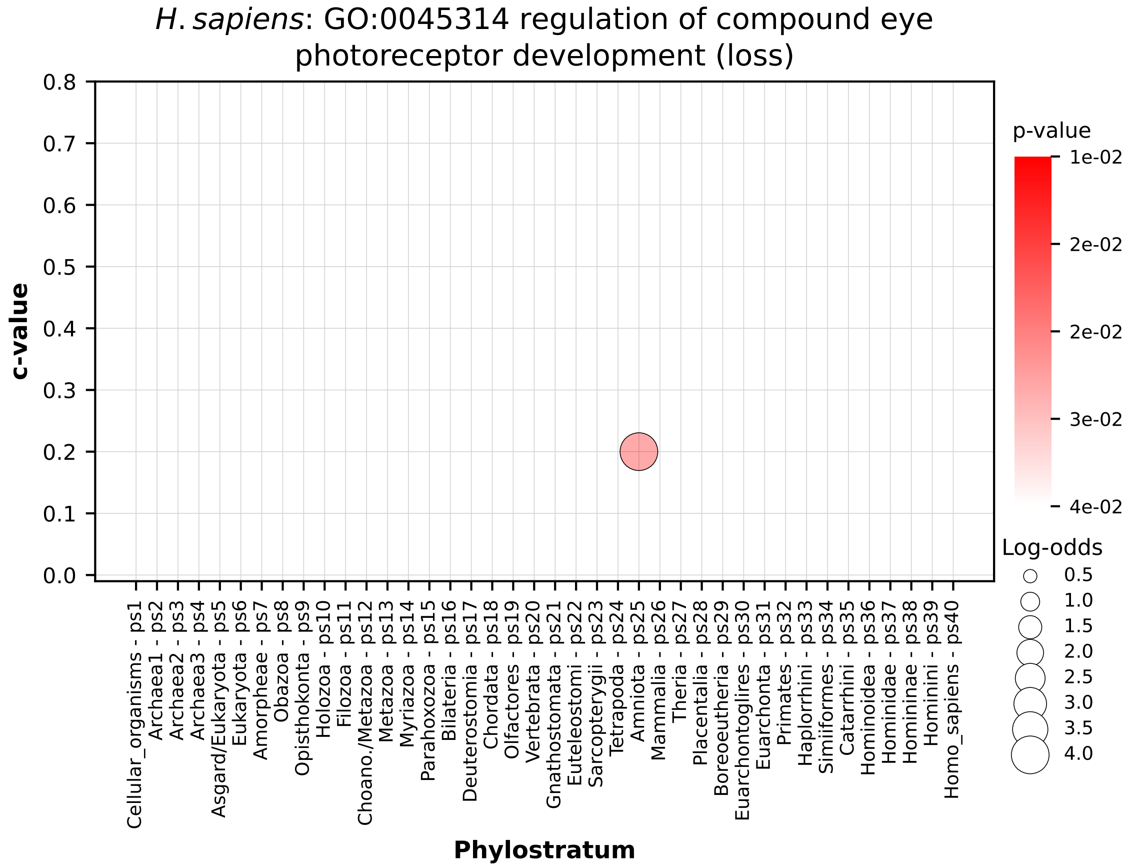

H. sapiens: GO:0045314 regulation of compound eye photoreceptor development (loss)
c-value
0.0
0.1
0.2
0.3
0.4
0.5
0.6
0.7
0.8
Phylostratum
Cellular_organisms - ps1
Archaea1 - ps2
Archaea2 - ps3
Archaea3 - ps4
Asgard/Eukaryota - ps5
Eukaryota - ps6
Amorpheae - ps7
Obazoa - ps8
Opisthokonta - ps9
Holozoa - ps10
Filozoa - ps11
Choano./Metazoa - ps12
Metazoa - ps13
Myriazoa - ps14
Parahoxozoa - ps15
Bilateria - ps16
Deuterostomia - ps17
Chordata - ps18
Olfactores - ps19
Vertebrata - ps20
Gnathostomata - ps21
Euteleostomi - ps22
Sarcopterygii - ps23
Tetrapoda - ps24
Amniota - ps25
Mammalia - ps26
Theria - ps27
Placentalia - ps28
Boreoeutheria - ps29
Euarchontoglires - ps30
Euarchonta - ps31
Primates - ps32
Haplorrhini - ps33
Simiiformes - ps34
Catarrhini - ps35
Hominoidea - ps36
Hominidae - ps37
Homininae - ps38
Hominini - ps39
Homo_sapiens - ps40
p-value
1e-02
2e-02
2e-02
3e-02
4e-02
Log-odds
0.5
1.0
1.5
2.0
2.5
3.0
3.5
4.0

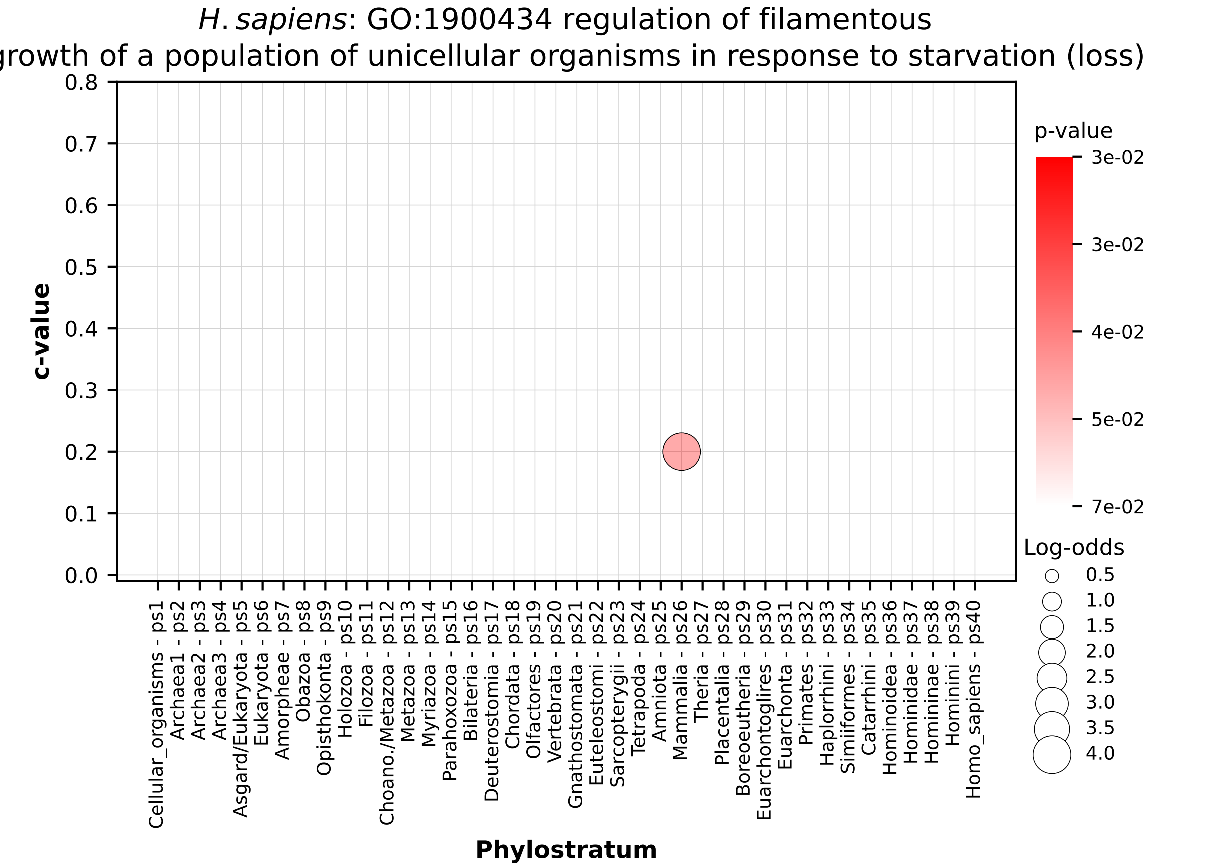

H. sapiens: GO:1900434 regulation of filamentous growth of a population of unicellular organisms in response to starvation (loss)
c-value
0.0
0.1
0.2
0.3
0.4
0.5
0.6
0.7
0.8
Phylostratum
Cellular_organisms - ps1
Archaea1 - ps2
Archaea2 - ps3
Archaea3 - ps4
Asgard/Eukaryota - ps5
Eukaryota - ps6
Amorpheae - ps7
Obazoa - ps8
Opisthokonta - ps9
Holozoa - ps10
Filozoa - ps11
Choano./Metazoa - ps12
Metazoa - ps13
Myriazoa - ps14
Parahoxozoa - ps15
Bilateria - ps16
Deuterostomia - ps17
Chordata - ps18
Olfactores - ps19
Vertebrata - ps20
Gnathostomata - ps21
Euteleostomi - ps22
Sarcopterygii - ps23
Tetrapoda - ps24
Amniota - ps25
Mammalia - ps26
Theria - ps27
Placentalia - ps28
Boreoeutheria - ps29
Euarchontoglires - ps30
Euarchonta - ps31
Primates - ps32
Haplorrhini - ps33
Simiiformes - ps34
Catarrhini - ps35
Hominoidea - ps36
Hominidae - ps37
Homininae - ps38
Hominini - ps39
Homo_sapiens - ps40
p-value
3e-02
3e-02
4e-02
5e-02
7e-02
Log-odds
0.5
1.0
1.5
2.0
2.5
3.0
3.5
4.0

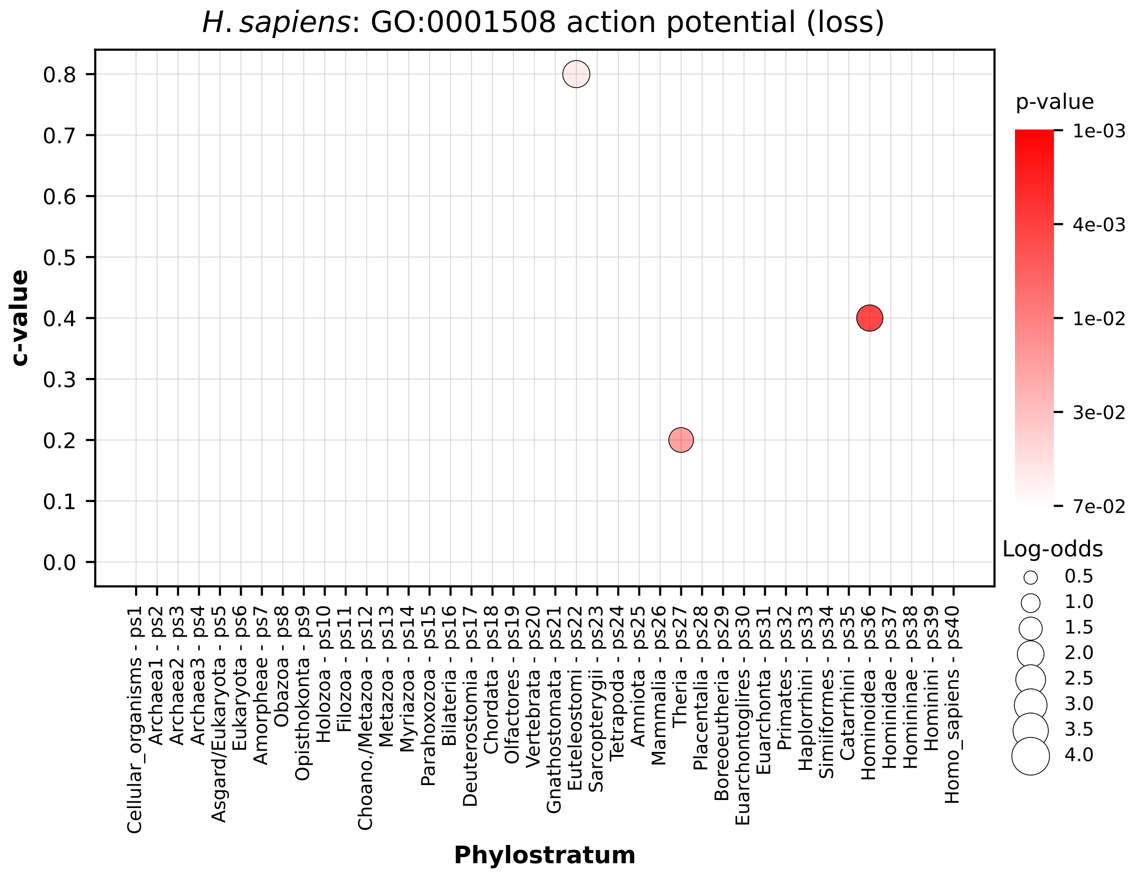
H. sapiens: GO:0001508 action potential (loss)
c-value
0.0
0.1
0.2
0.3
0.4
0.5
0.6
0.7
0.8
Phylostratum
Cellular_organisms - ps1
Archaea1 - ps2
Archaea2 - ps3
Archaea3 - ps4
Asgard/Eukaryota - ps5
Eukaryota - ps6
Amorpheae - ps7
Obazoa - ps8
Opisthokonta - ps9
Holozoa - ps10
Filozoa - ps11
Choano./Metazoa - ps12
Metazoa - ps13
Myriazoa - ps14
Parahoxozoa - ps15
Bilateria - ps16
Deuterostomia - ps17
Chordata - ps18
Olfactores - ps19
Vertebrata - ps20
Gnathostomata - ps21
Euteleostomi - ps22
Sarcopterygii - ps23
Tetrapoda - ps24
Amniota - ps25
Mammalia - ps26
Theria - ps27
Placentalia - ps28
Boreoeutheria - ps29
Euarchontoglires - ps30
Euarchonta - ps31
Primates - ps32
Haplorrhini - ps33
Simiiformes - ps34
Catarrhini - ps35
Hominoidea - ps36
Hominidae - ps37
Homininae - ps38
Hominini - ps39
Homo_sapiens - ps40
p-value
1e-03
4e-03
1e-02
3e-02
7e-02
Log-odds
0.5
1.0
1.5
2.0
2.5
3.0
3.5
4.0

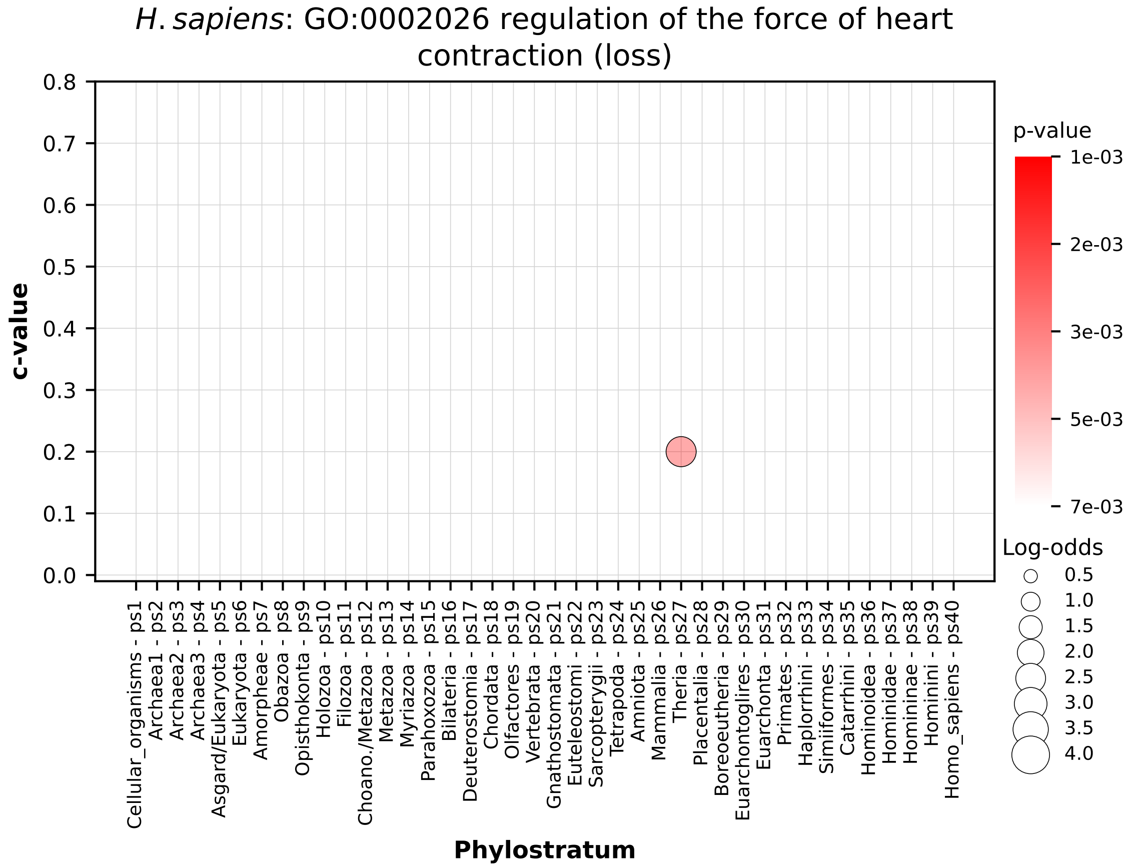
H. sapiens: GO:0002026 regulation of the force of heart contraction (loss)
c-value
0.8
0.7
0.6
0.5
0.4
0.3
0.2
0.1
0.0
Phylostratum
Cellular_organisms - ps1
Archaea1 - ps2
Archaea2 - ps3
Archaea3 - ps4
Asgard/Eukaryota - ps5
Eukaryota - ps6
Amorpheae - ps7
Obazoa - ps8
Opisthokonta - ps9
Holozoa - ps10
Filozoa - ps11
Choano./Metazoa - ps12
Metazoa - ps13
Myriazoa - ps14
Parahoxozoa - ps15
Bilateria - ps16
Deuterostomia - ps17
Chordata - ps18
Olfactores - ps19
Vertebrata - ps20
Gnathostomata - ps21
Euteleostomi - ps22
Sarcopterygii - ps23
Tetrapoda - ps24
Amniota - ps25
Mammalia - ps26
Theria - ps27
Placentalia - ps28
Boreoeutheria - ps29
Euarchontoglires - ps30
Euarchonta - ps31
Primates - ps32
Haplorrhini - ps33
Simiiformes - ps34
Catarrhini - ps35
Hominoidea - ps36
Hominidae - ps37
Homininae - ps38
Hominini - ps39
Homo_sapiens - ps40
p-value
1e-03
2e-03
3e-03
5e-03
7e-03
Log-odds
0.5
1.0
1.5
2.0
2.5
3.0
3.5
4.0

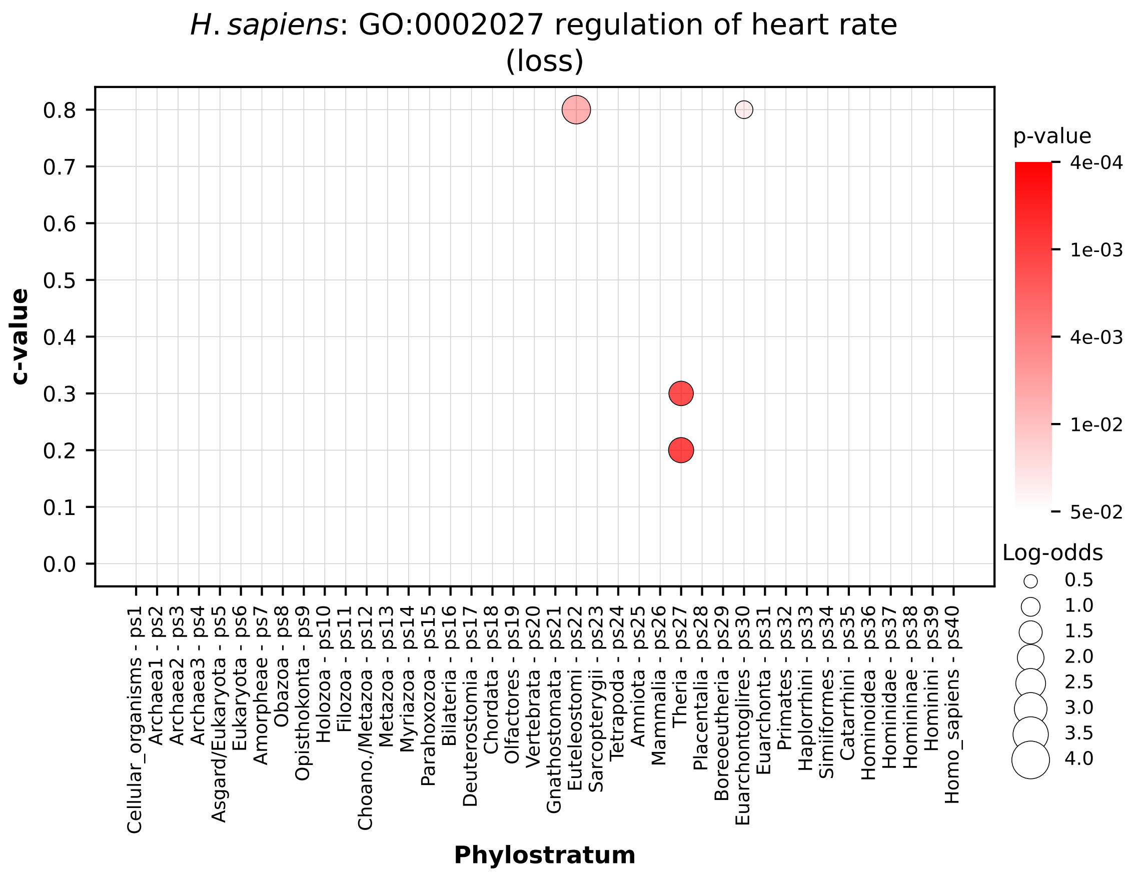

H. sapiens: GO:0002027 regulation of heart rate
(loss)
c-value
0.0
0.1
0.2
0.3
0.4
0.5
0.6
0.7
0.8
Phylostratum
Cellular_organisms - ps1
Archaea1 - ps2
Archaea2 - ps3
Archaea3 - ps4
Asgard/Eukaryota - ps5
Eukaryota - ps6
Amorpheae - ps7
Obazoa - ps8
Opisthokonta - ps9
Holozoa - ps10
Filozoa - ps11
Choano./Metazoa - ps12
Metazoa - ps13
Myriazoa - ps14
Parahoxozoa - ps15
Bilateria - ps16
Deuterostomia - ps17
Chordata - ps18
Olfactores - ps19
Vertebrata - ps20
Gnathostomata - ps21
Euteleostomi - ps22
Sarcopterygii - ps23
Tetrapoda - ps24
Amniota - ps25
Mammalia - ps26
Theria - ps27
Placentalia - ps28
Boreoeutheria - ps29
Euarchontoglires - ps30
Euarchonta - ps31
Primates - ps32
Haplorrhini - ps33
Simiiformes - ps34
Catarrhini - ps35
Hominoidea - ps36
Hominidae - ps37
Homininae - ps38
Hominini - ps39
Homo_sapiens - ps40
p-value
4e-04
1e-03
4e-03
1e-02
5e-02
Log-odds
0.5
1.0
1.5
2.0
2.5
3.0
3.5
4.0

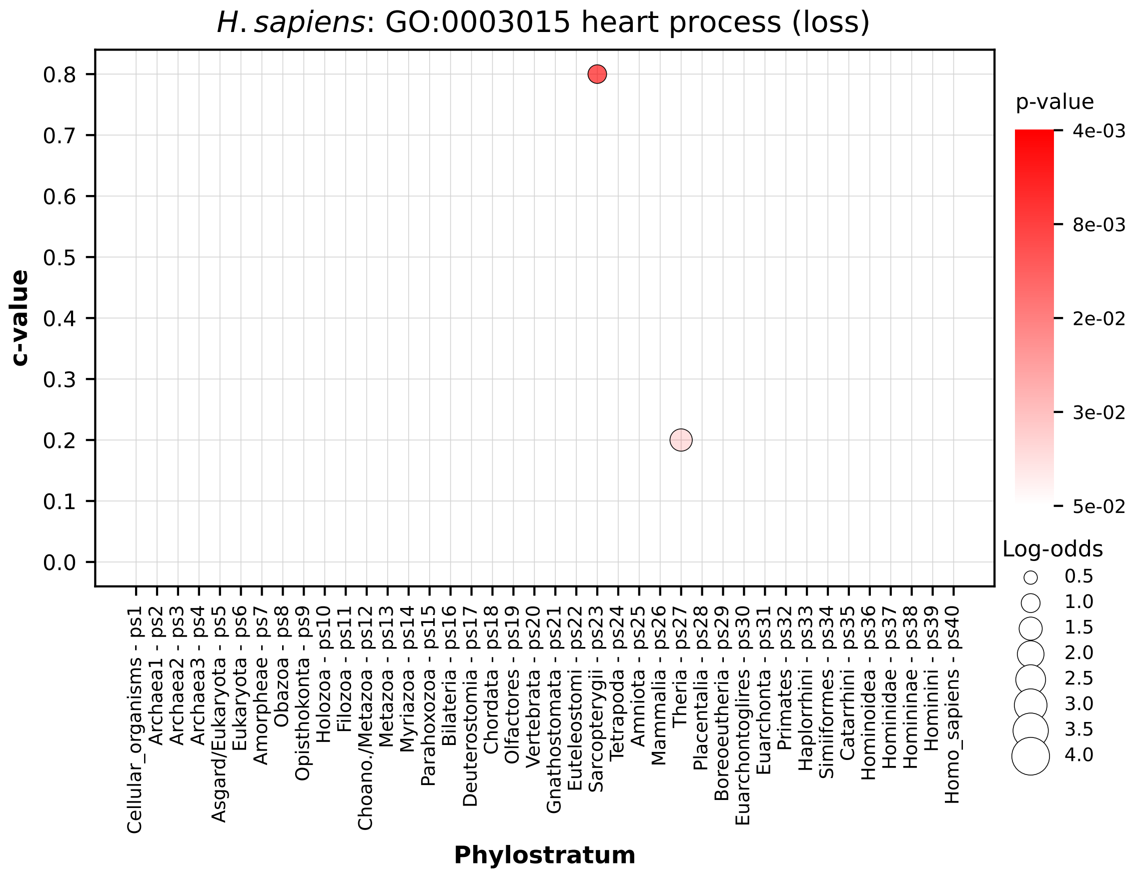

H. sapiens: GO:0003015 heart process (loss)
c-value
0.8
0.7
0.6
0.5
0.4
0.3
0.2
0.1
0.0
Phylostratum
Cellular_organisms - ps1
Archaea1 - ps2
Archaea2 - ps3
Archaea3 - ps4
Asgard/Eukaryota - ps5
Eukaryota - ps6
Amorpheae - ps7
Obazoa - ps8
Opisthokonta - ps9
Holozoa - ps10
Filozoa - ps11
Choano./Metazoa - ps12
Metazoa - ps13
Myriazoa - ps14
Parahoxozoa - ps15
Bilateria - ps16
Deuterostomia - ps17
Chordata - ps18
Olfactores - ps19
Vertebrata - ps20
Gnathostomata - ps21
Euteleostomi - ps22
Sarcopterygii - ps23
Tetrapoda - ps24
Amniota - ps25
Mammalia - ps26
Theria - ps27
Placentalia - ps28
Boreoeutheria - ps29
Euarchontoglires - ps30
Euarchonta - ps31
Primates - ps32
Haplorrhini - ps33
Simiiformes - ps34
Catarrhini - ps35
Hominoidea - ps36
Hominidae - ps37
Homininae - ps38
Hominini - ps39
Homo_sapiens - ps40
p-value
4e-03
8e-03
2e-02
3e-02
5e-02
Log-odds
0.5
1.0
1.5
2.0
2.5
3.0
3.5
4.0

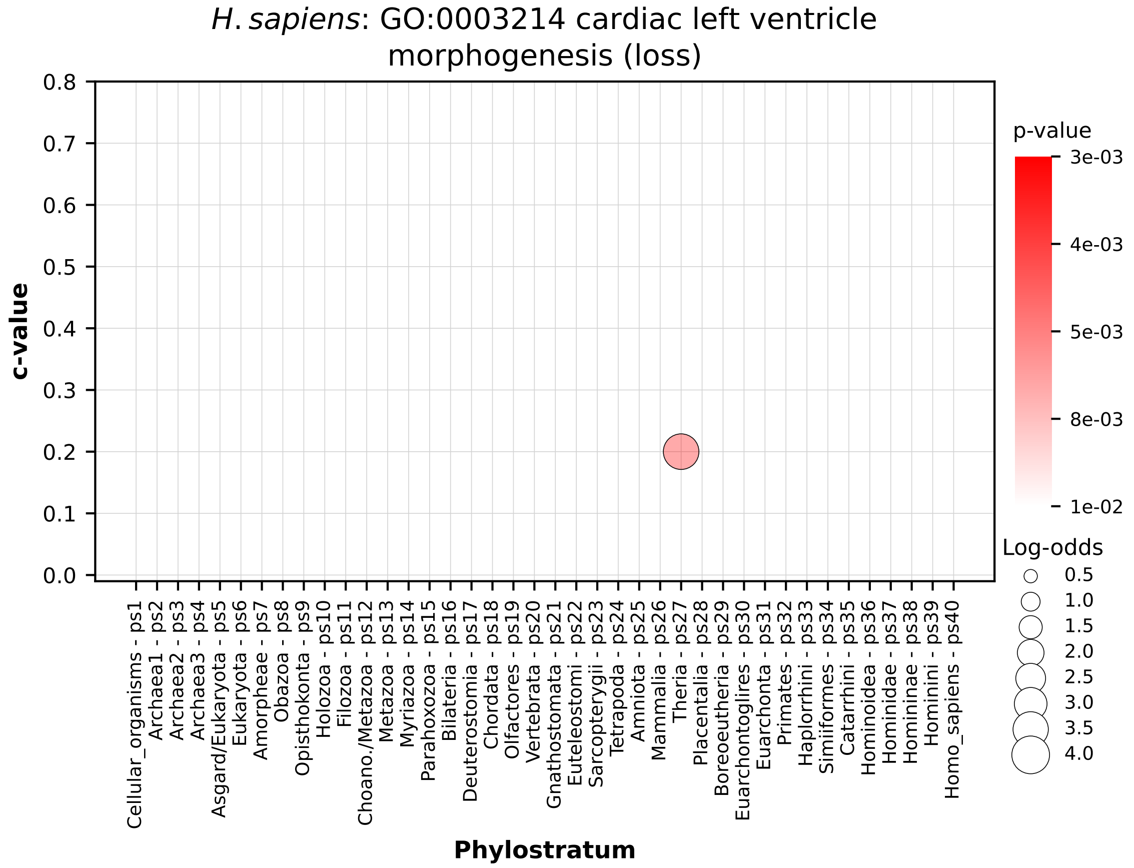
H. sapiens: GO:0003214 cardiac left ventricle morphogenesis (loss)
c-value
0.8
0.7
0.6
0.5
0.4
0.3
0.2
0.1
0.0
Cellular_organisms - ps1
Archaea1 - ps2
Archaea2 - ps3
Archaea3 - ps4
Asgard/Eukaryota - ps5
Eukaryota - ps6
Amorpheae - ps7
Obazoa - ps8
Opisthokonta - ps9
Holozoa - ps10
Filozoa - ps11
Choano./Metazoa - ps12
Metazoa - ps13
Myriazoa - ps14
Parahoxozoa - ps15
Bilateria - ps16
Deuterostomia - ps17
Chordata - ps18
Olfactores - ps19
Vertebrata - ps20
Gnathostomata - ps21
Euteleostomi - ps22
Sarcopterygii - ps23
Tetrapoda - ps24
Amniota - ps25
Mammalia - ps26
Theria - ps27
Placentalia - ps28
Boreoeutheria - ps29
Euarchontoglires - ps30
Euarchonta - ps31
Primates - ps32
Haplorrhini - ps33
Simiiformes - ps34
Catarrhini - ps35
Hominoidea - ps36
Hominidae - ps37
Homininae - ps38
Hominini - ps39
Homo_sapiens - ps40
Phylostratum
p-value
3e-03
4e-03
5e-03
8e-03
1e-02
Log-odds
0.5
1.0
1.5
2.0
2.5
3.0
3.5
4.0

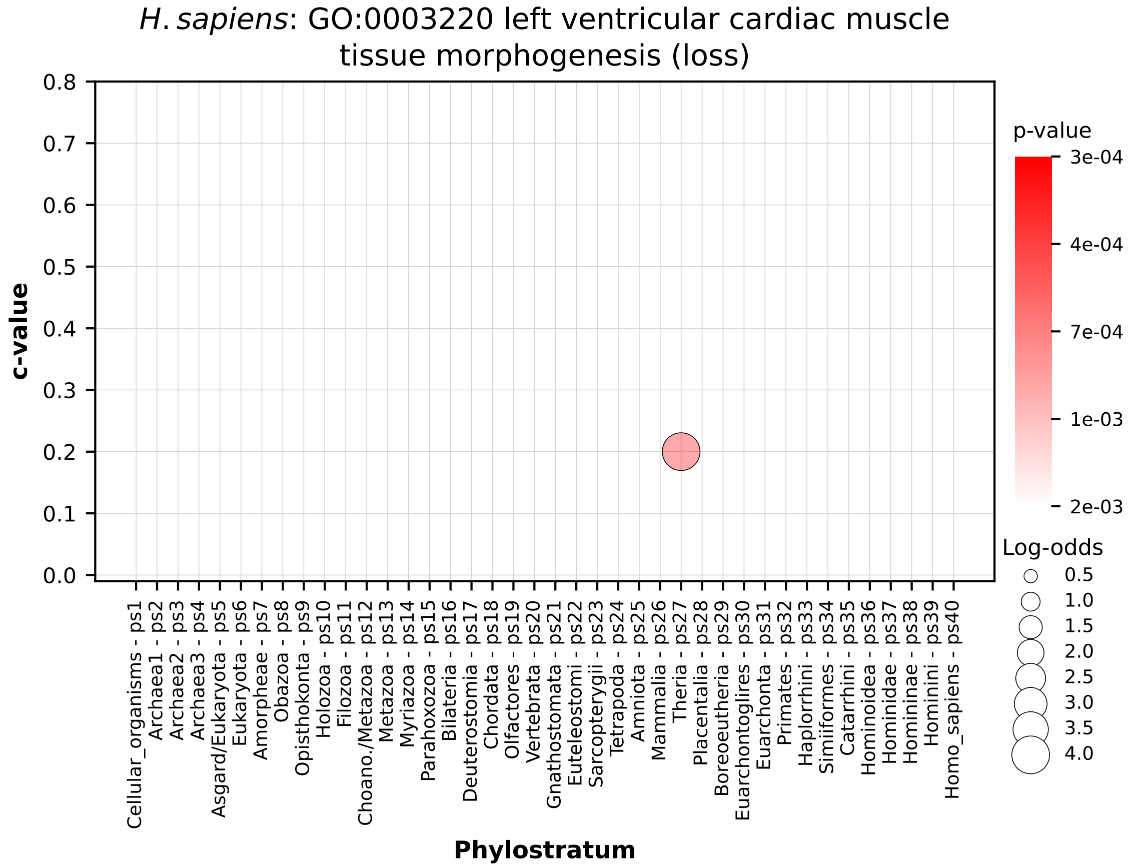

H. sapiens: GO:0003220 left ventricular cardiac muscle tissue morphogenesis (loss)
c-value
0.0
0.1
0.2
0.3
0.4
0.5
0.6
0.7
0.8
Phylostratum
Cellular_organisms - ps1
Archaea1 - ps2
Archaea2 - ps3
Archaea3 - ps4
Asgard/Eukaryota - ps5
Eukaryota - ps6
Amorpheae - ps7
Obazoa - ps8
Opisthokonta - ps9
Holozoa - ps10
Filozoa - ps11
Choano./Metazoa - ps12
Metazoa - ps13
Myriazoa - ps14
Parahoxozoa - ps15
Bilateria - ps16
Deuterostomia - ps17
Chordata - ps18
Olfactores - ps19
Vertebrata - ps20
Gnathostomata - ps21
Euteleostomi - ps22
Sarcopterygii - ps23
Tetrapoda - ps24
Amniota - ps25
Mammalia - ps26
Theria - ps27
Placentalia - ps28
Boreoeutheria - ps29
Euarchontoglires - ps30
Euarchonta - ps31
Primates - ps32
Haplorrhini - ps33
Simiiformes - ps34
Catarrhini - ps35
Hominoidea - ps36
Hominidae - ps37
Homininae - ps38
Hominini - ps39
Homo_sapiens - ps40
p-value
3e-04
4e-04
7e-04
1e-03
2e-03
Log-odds
0.5
1.0
1.5
2.0
2.5
3.0
3.5
4.0

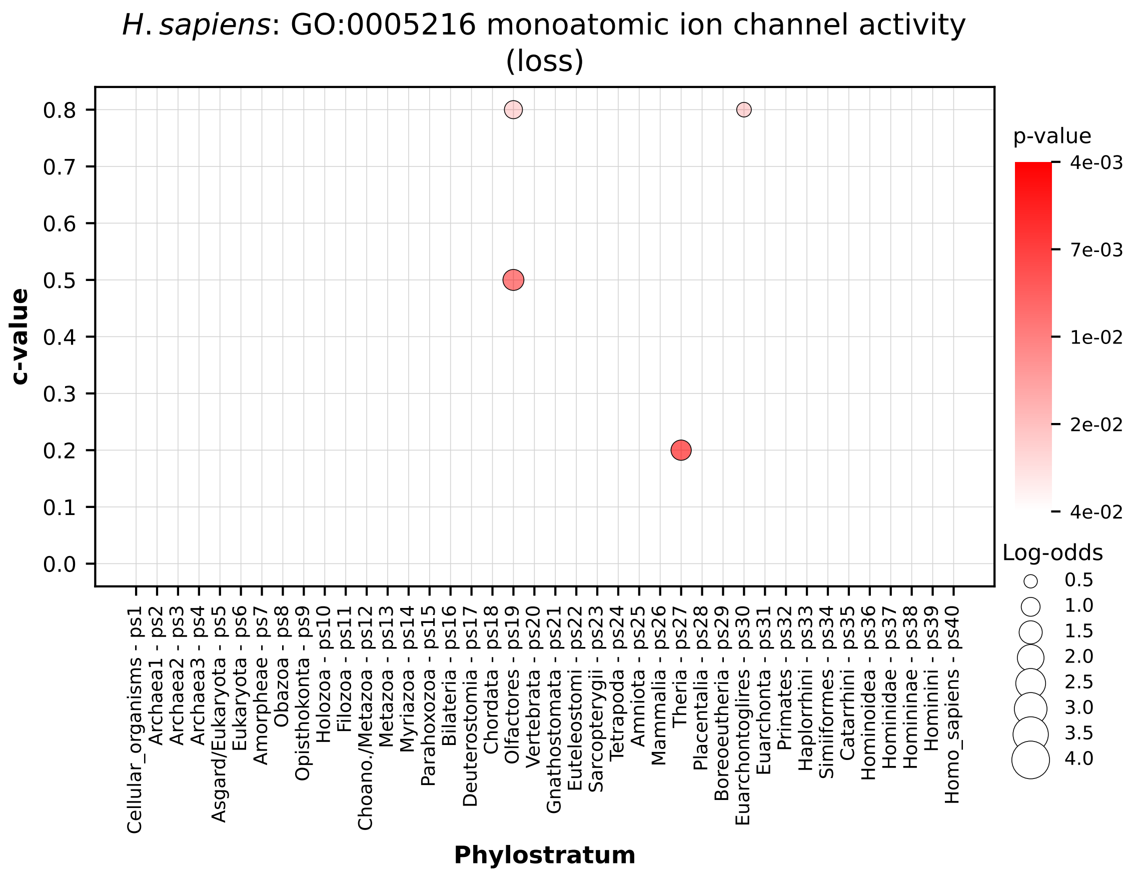

H. sapiens: GO:0005216 monoatomic ion channel activity
(loss)
c-value
0.0
0.1
0.2
0.3
0.4
0.5
0.6
0.7
0.8
Phylostratum
Cellular_organisms - ps1
Archaea1 - ps2
Archaea2 - ps3
Archaea3 - ps4
Asgard/Eukaryota - ps5
Eukaryota - ps6
Amorpheae - ps7
Obazoa - ps8
Opisthokonta - ps9
Holozoa - ps10
Filozoa - ps11
Choano./Metazoa - ps12
Metazoa - ps13
Myriazoa - ps14
Parahoxozoa - ps15
Bilateria - ps16
Deuterostomia - ps17
Chordata - ps18
Olfactores - ps19
Vertebrata - ps20
Gnathostomata - ps21
Euteleostomi - ps22
Sarcopterygii - ps23
Tetrapoda - ps24
Amniota - ps25
Mammalia - ps26
Theria - ps27
Placentalia - ps28
Boreoeutheria - ps29
Euarchontoglires - ps30
Euarchonta - ps31
Primates - ps32
Haplorrhini - ps33
Simiiformes - ps34
Catarrhini - ps35
Hominoidea - ps36
Hominidae - ps37
Homininae - ps38
Hominini - ps39
Homo_sapiens - ps40
p-value
4e-03
7e-03
1e-02
2e-02
4e-02
Log-odds
0.5
1.0
1.5
2.0
2.5
3.0
3.5
4.0

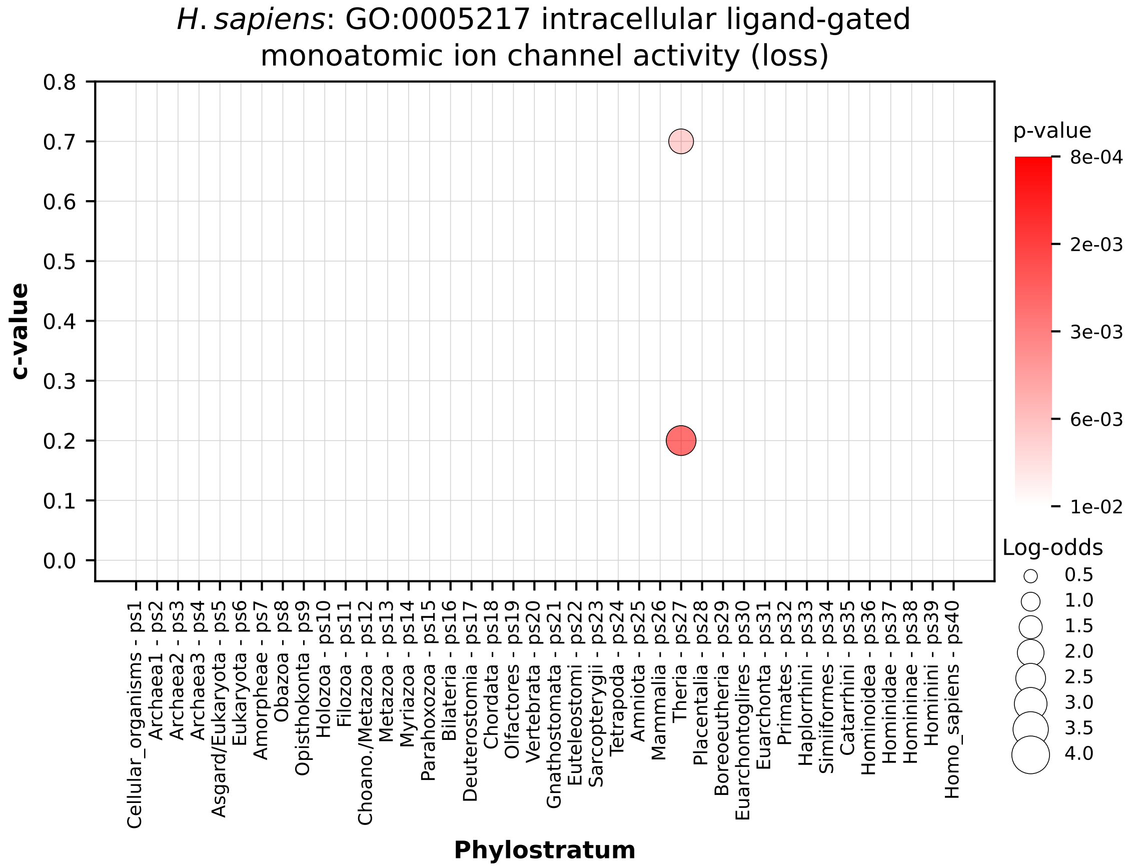

H. sapiens: GO:0005217 intracellular ligand-gated monoatomic ion channel activity (loss)
c-value
0.8
0.7
0.6
0.5
0.4
0.3
0.2
0.1
0.0
p-value
8e-04
2e-03
3e-03
6e-03
1e-02
Log-odds
0.5
1.0
1.5
2.0
2.5
3.0
3.5
4.0
Cellular_organisms - ps1
Archaea1 - ps2
Archaea2 - ps3
Archaea3 - ps4
Asgard/Eukaryota - ps5
Eukaryota - ps6
Amorpheae - ps7
Obazoa - ps8
Opisthokonta - ps9
Holozoa - ps10
Filozoa - ps11
Choano./Metazoa - ps12
Metazoa - ps13
Myriazoa - ps14
Parahoxozoa - ps15
Bilateria - ps16
Deuterostomia - ps17
Chordata - ps18
Olfactores - ps19
Vertebrata - ps20
Gnathostomata - ps21
Euteleostomi - ps22
Sarcopterygii - ps23
Tetrapoda - ps24
Amniota - ps25
Mammalia - ps26
Theria - ps27
Placentalia - ps28
Boreoeutheria - ps29
Euarchontoglires - ps30
Euarchonta - ps31
Primates - ps32
Haplorrhini - ps33
Simiiformes - ps34
Catarrhini - ps35
Hominoidea - ps36
Hominidae - ps37
Homininae - ps38
Hominini - ps39
Homo_sapiens - ps40
Phylostratum

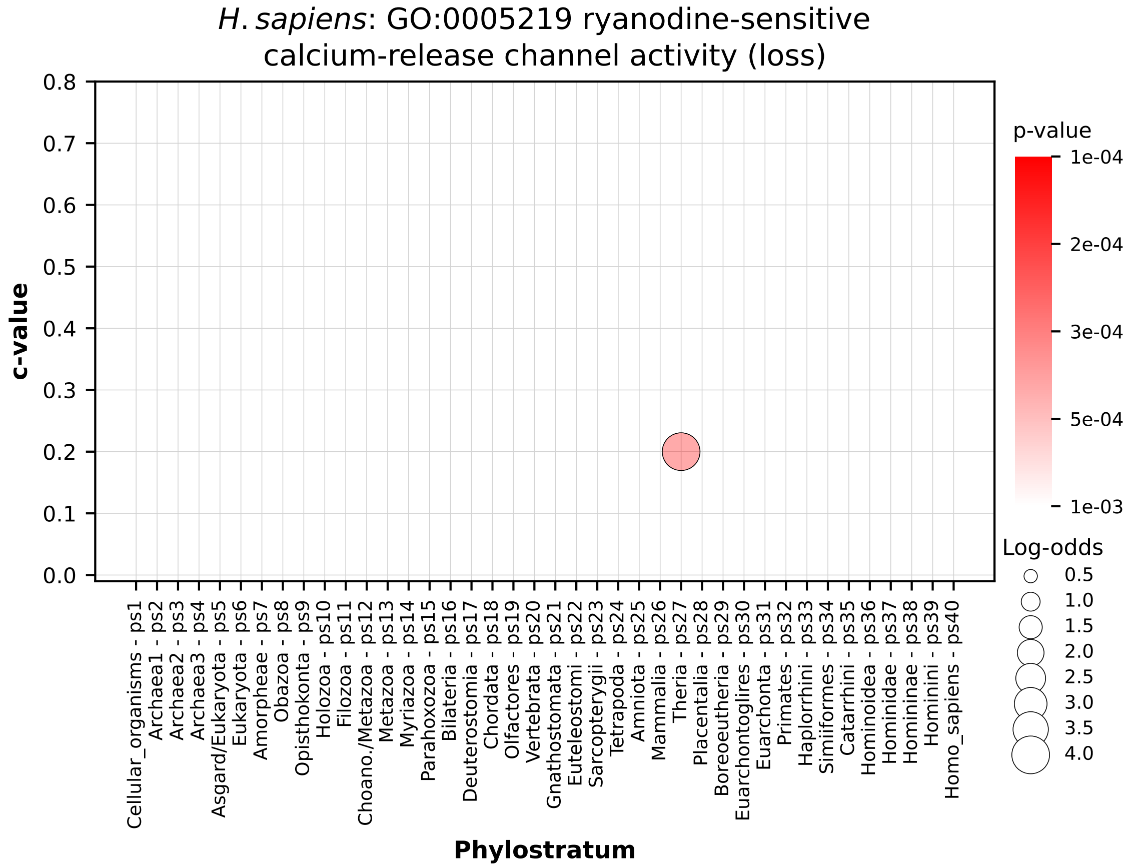

H. sapiens: GO:0005219 ryanodine-sensitive calcium-release channel activity (loss)
c-value
0.0
0.1
0.2
0.3
0.4
0.5
0.6
0.7
0.8
Phylostratum
Cellular_organisms - ps1
Archaea1 - ps2
Archaea2 - ps3
Archaea3 - ps4
Asgard/Eukaryota - ps5
Eukaryota - ps6
Amorpheae - ps7
Obazoa - ps8
Opisthokonta - ps9
Holozoa - ps10
Filozoa - ps11
Choano./Metazoa - ps12
Metazoa - ps13
Myriazoa - ps14
Parahoxozoa - ps15
Bilateria - ps16
Deuterostomia - ps17
Chordata - ps18
Olfactores - ps19
Vertebrata - ps20
Gnathostomata - ps21
Euteleostomi - ps22
Sarcopterygii - ps23
Tetrapoda - ps24
Amniota - ps25
Mammalia - ps26
Theria - ps27
Placentalia - ps28
Boreoeutheria - ps29
Euarchontoglires - ps30
Euarchonta - ps31
Primates - ps32
Haplorrhini - ps33
Simiiformes - ps34
Catarrhini - ps35
Hominoidea - ps36
Hominidae - ps37
Homininae - ps38
Hominini - ps39
Homo_sapiens - ps40
p-value
1e-04
2e-04
3e-04
5e-04
1e-03
Log-odds
0.5
1.0
1.5
2.0
2.5
3.0
3.5
4.0

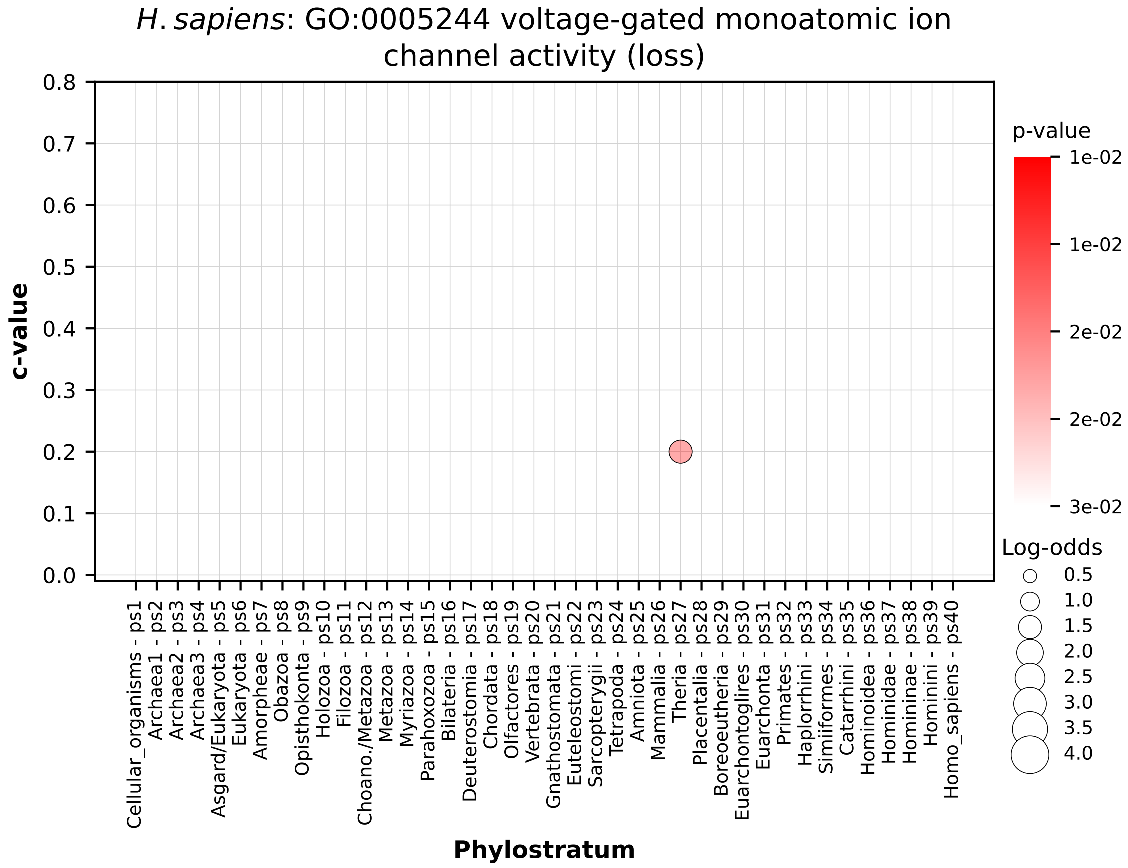

H. sapiens: GO:0005244 voltage-gated monoatomic ion channel activity (loss)
c-value
0.0
0.1
0.2
0.3
0.4
0.5
0.6
0.7
0.8
Phylostratum
Cellular_organisms - ps1
Archaea1 - ps2
Archaea2 - ps3
Archaea3 - ps4
Asgard/Eukaryota - ps5
Eukaryota - ps6
Amorpheae - ps7
Obazoa - ps8
Opisthokonta - ps9
Holozoa - ps10
Filozoa - ps11
Choano./Metazoa - ps12
Metazoa - ps13
Myriazoa - ps14
Parahoxozoa - ps15
Bilateria - ps16
Deuterostomia - ps17
Chordata - ps18
Olfactores - ps19
Vertebrata - ps20
Gnathostomata - ps21
Euteleostomi - ps22
Sarcopterygii - ps23
Tetrapoda - ps24
Amniota - ps25
Mammalia - ps26
Theria - ps27
Placentalia - ps28
Boreoeutheria - ps29
Euarchontoglires - ps30
Euarchonta - ps31
Primates - ps32
Haplorrhini - ps33
Simiiformes - ps34
Catarrhini - ps35
Hominoidea - ps36
Hominidae - ps37
Homininae - ps38
Hominini - ps39
Homo_sapiens - ps40
p-value
1e-02
1e-02
2e-02
2e-02
3e-02
Log-odds
0.5
1.0
1.5
2.0
2.5
3.0
3.5
4.0

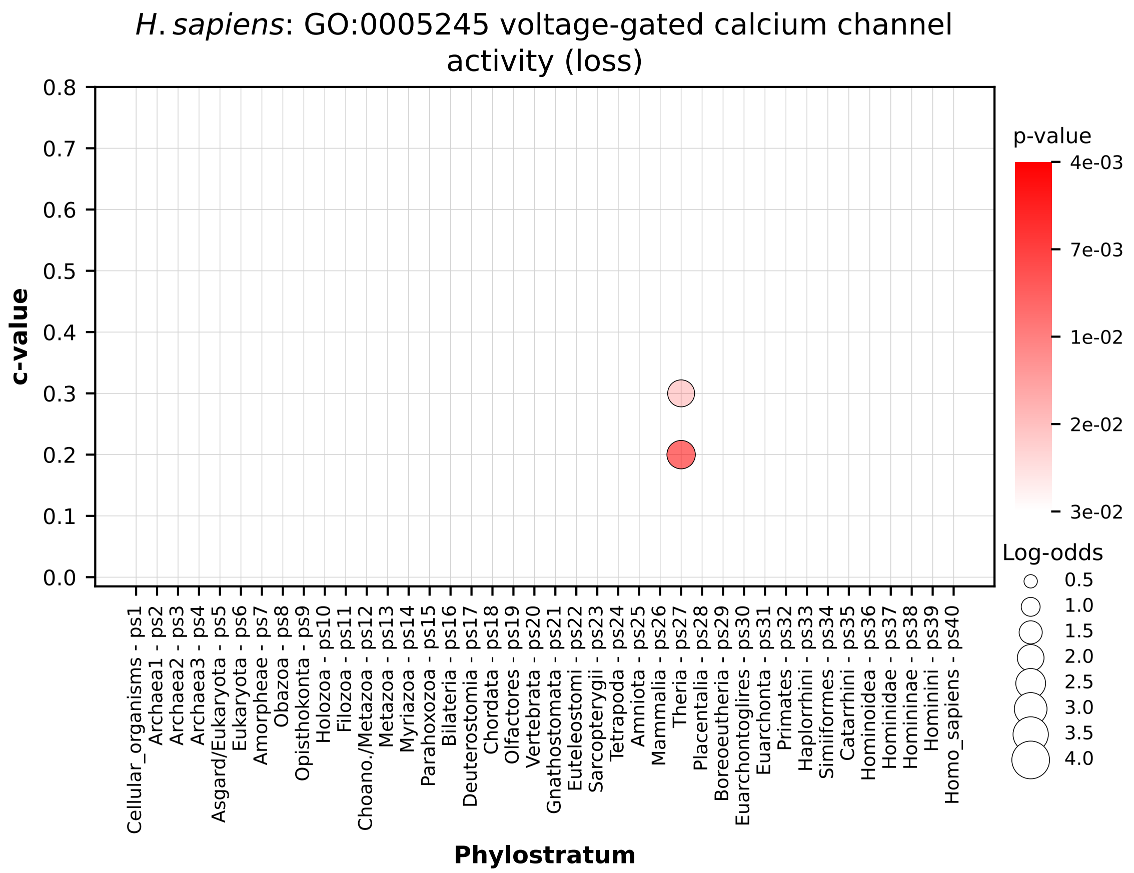

H. sapiens: GO:0005245 voltage-gated calcium channel activity (loss)
c-value
0.8
0.7
0.6
0.5
0.4
0.3
0.2
0.1
0.0
Cellular_organisms - ps1
Archaea1 - ps2
Archaea2 - ps3
Archaea3 - ps4
Asgard/Eukaryota - ps5
Eukaryota - ps6
Amorpheae - ps7
Obazoa - ps8
Opisthokonta - ps9
Holozoa - ps10
Filozoa - ps11
Choano./Metazoa - ps12
Metazoa - ps13
Myriazoa - ps14
Parahoxozoa - ps15
Bilateria - ps16
Deuterostomia - ps17
Chordata - ps18
Olfactores - ps19
Vertebrata - ps20
Gnathostomata - ps21
Euteleostomi - ps22
Sarcopterygii - ps23
Tetrapoda - ps24
Amniota - ps25
Mammalia - ps26
Theria - ps27
Placentalia - ps28
Boreoeutheria - ps29
Euarchontoglires - ps30
Euarchonta - ps31
Primates - ps32
Haplorrhini - ps33
Simiiformes - ps34
Catarrhini - ps35
Hominoidea - ps36
Hominidae - ps37
Homininae - ps38
Hominini - ps39
Homo_sapiens - ps40
Phylostratum
p-value
4e-03
7e-03
1e-02
2e-02
3e-02
Log-odds
0.5
1.0
1.5
2.0
2.5
3.0
3.5
4.0

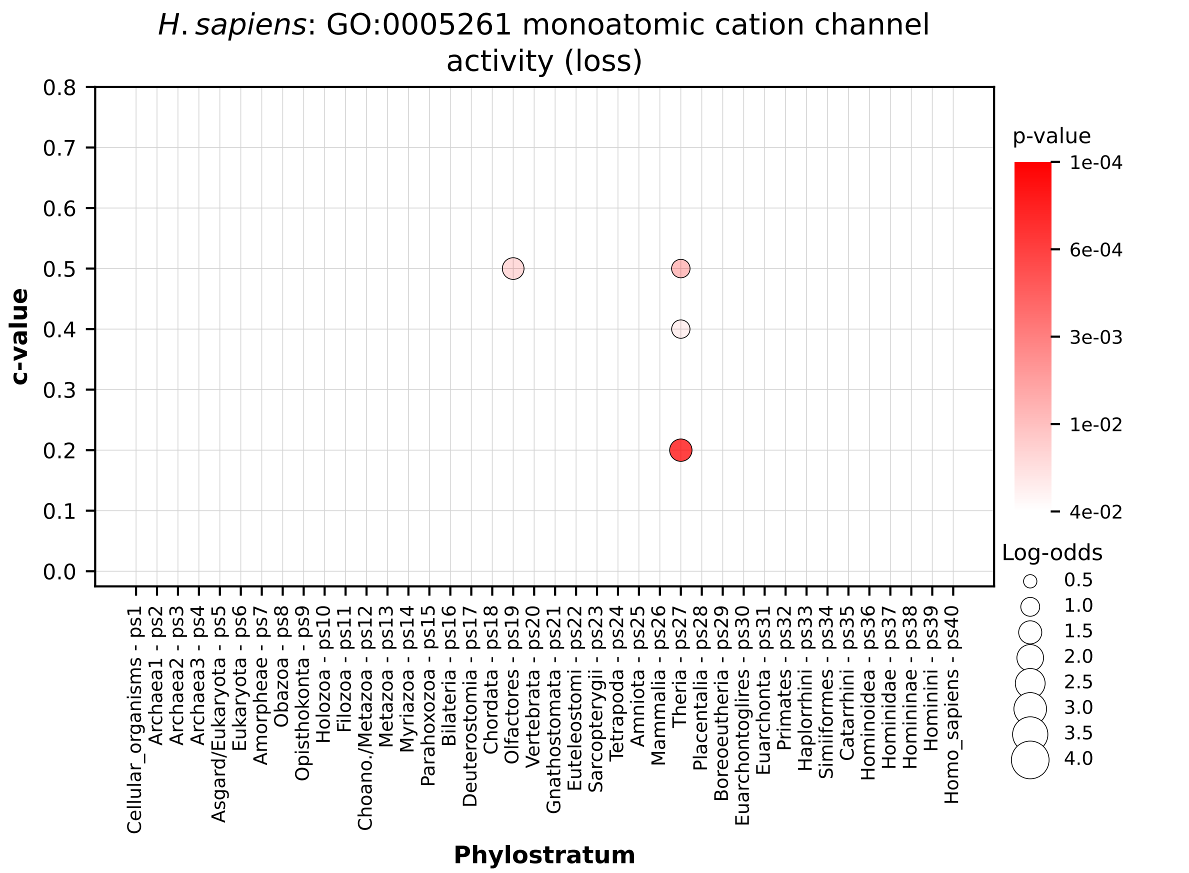

H. sapiens: GO:0005261 monoatomic cation channel activity (loss)
c-value
0.8
0.7
0.6
0.5
0.4
0.3
0.2
0.1
0.0
Phylostratum
Cellular_organisms - ps1
Archaea1 - ps2
Archaea2 - ps3
Archaea3 - ps4
Asgard/Eukaryota - ps5
Eukaryota - ps6
Amorpheae - ps7
Obazoa - ps8
Opisthokonta - ps9
Holozoa - ps10
Filozoa - ps11
Choano./Metazoa - ps12
Metazoa - ps13
Myriazoa - ps14
Parahoxozoa - ps15
Bilateria - ps16
Deuterostomia - ps17
Chordata - ps18
Olfactores - ps19
Vertebrata - ps20
Gnathostomata - ps21
Euteleostomi - ps22
Sarcopterygii - ps23
Tetrapoda - ps24
Amniota - ps25
Mammalia - ps26
Theria - ps27
Placentalia - ps28
Boreoeutheria - ps29
Euarchontoglires - ps30
Euarchonta - ps31
Primates - ps32
Haplorrhini - ps33
Simiiformes - ps34
Catarrhini - ps35
Hominoidea - ps36
Hominidae - ps37
Homininae - ps38
Hominini - ps39
Homo_sapiens - ps40
p-value
1e-04
6e-04
3e-03
1e-02
4e-02
Log-odds
0.5
1.0
1.5
2.0
2.5
3.0
3.5
4.0

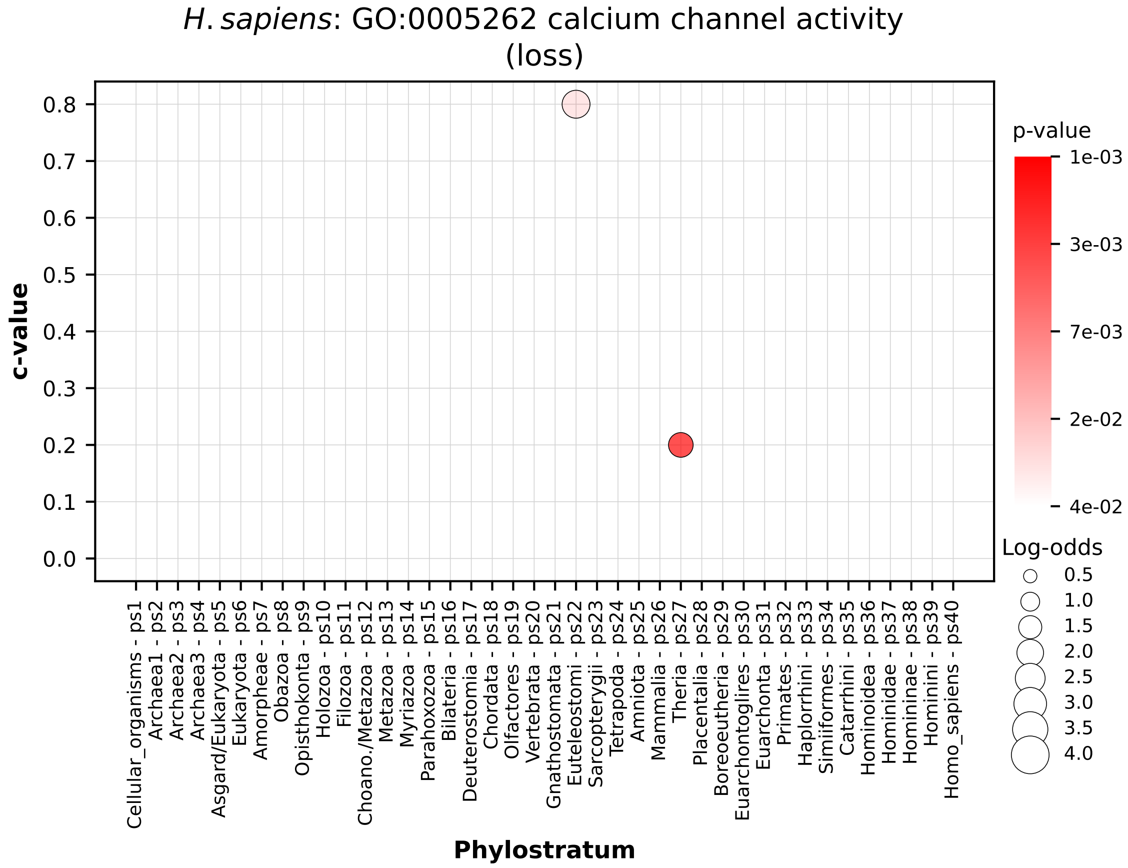

H. sapiens: GO:0005262 calcium channel activity
(loss)
c-value
0.8
0.7
0.6
0.5
0.4
0.3
0.2
0.1
0.0
p-value
1e-03
3e-03
7e-03
2e-02
4e-02
Log-odds
0.5
1.0
1.5
2.0
2.5
3.0
3.5
4.0
Cellular_organisms - ps1
Archaea1 - ps2
Archaea2 - ps3
Archaea3 - ps4
Asgard/Eukaryota - ps5
Eukaryota - ps6
Amorpheae - ps7
Obazoa - ps8
Opisthokonta - ps9
Holozoa - ps10
Filozoa - ps11
Choano./Metazoa - ps12
Metazoa - ps13
Myriazoa - ps14
Parahoxozoa - ps15
Bilateria - ps16
Deuterostomia - ps17
Chordata - ps18
Olfactores - ps19
Vertebrata - ps20
Gnathostomata - ps21
Euteleostomi - ps22
Sarcopterygii - ps23
Tetrapoda - ps24
Amniota - ps25
Mammalia - ps26
Theria - ps27
Placentalia - ps28
Boreoeutheria - ps29
Euarchontoglires - ps30
Euarchonta - ps31
Primates - ps32
Haplorrhini - ps33
Simiiformes - ps34
Catarrhini - ps35
Hominoidea - ps36
Hominidae - ps37
Homininae - ps38
Hominini - ps39
Homo_sapiens - ps40
Phylostratum

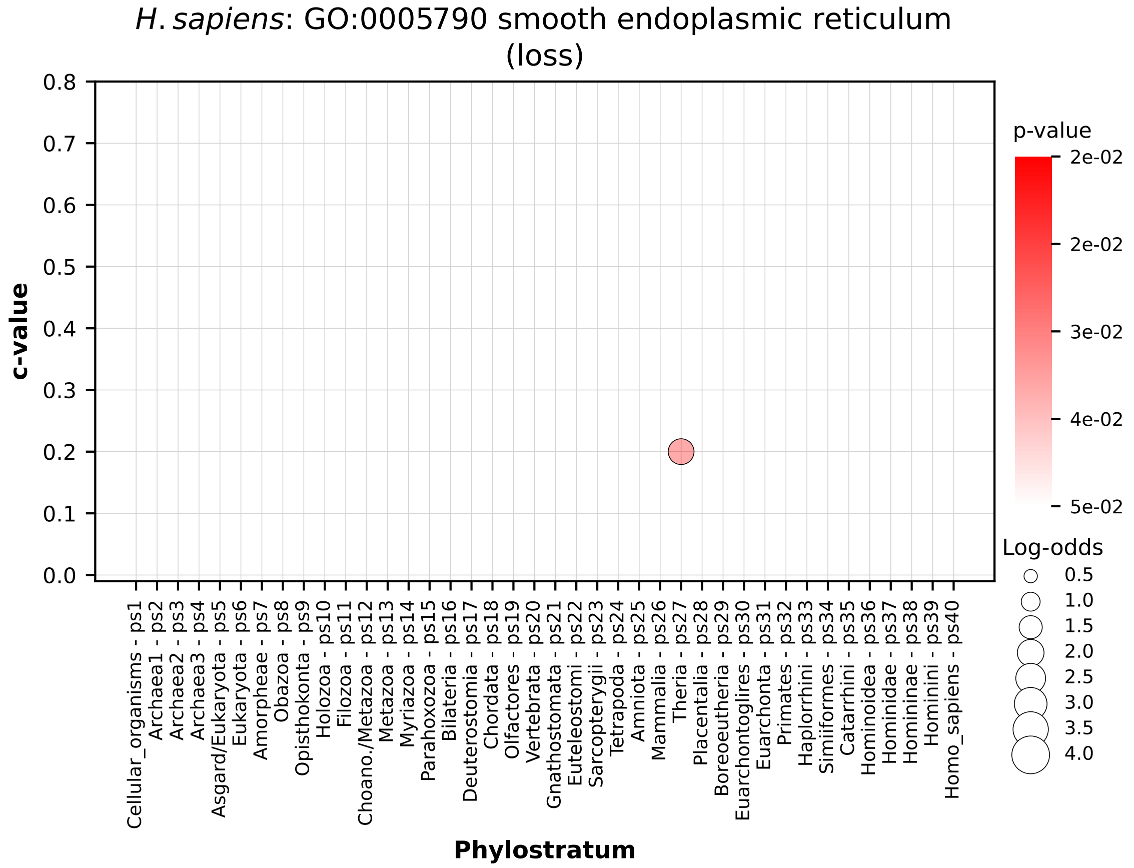

H. sapiens: GO:0005790 smooth endoplasmic reticulum
(loss)
c-value
0.0
0.1
0.2
0.3
0.4
0.5
0.6
0.7
0.8
Phylostratum
Cellular_organisms - ps1
Archaea1 - ps2
Archaea2 - ps3
Archaea3 - ps4
Asgard/Eukaryota - ps5
Eukaryota - ps6
Amorpheae - ps7
Obazoa - ps8
Opisthokonta - ps9
Holozoa - ps10
Filozoa - ps11
Choano./Metazoa - ps12
Metazoa - ps13
Myriazoa - ps14
Parahoxozoa - ps15
Bilateria - ps16
Deuterostomia - ps17
Chordata - ps18
Olfactores - ps19
Vertebrata - ps20
Gnathostomata - ps21
Euteleostomi - ps22
Sarcopterygii - ps23
Tetrapoda - ps24
Amniota - ps25
Mammalia - ps26
Theria - ps27
Placentalia - ps28
Boreoeutheria - ps29
Euarchontoglires - ps30
Euarchonta - ps31
Primates - ps32
Haplorrhini - ps33
Simiiformes - ps34
Catarrhini - ps35
Hominoidea - ps36
Hominidae - ps37
Homininae - ps38
Hominini - ps39
Homo_sapiens - ps40
p-value
2e-02
2e-02
3e-02
4e-02
5e-02
Log-odds
0.5
1.0
1.5
2.0
2.5
3.0
3.5
4.0

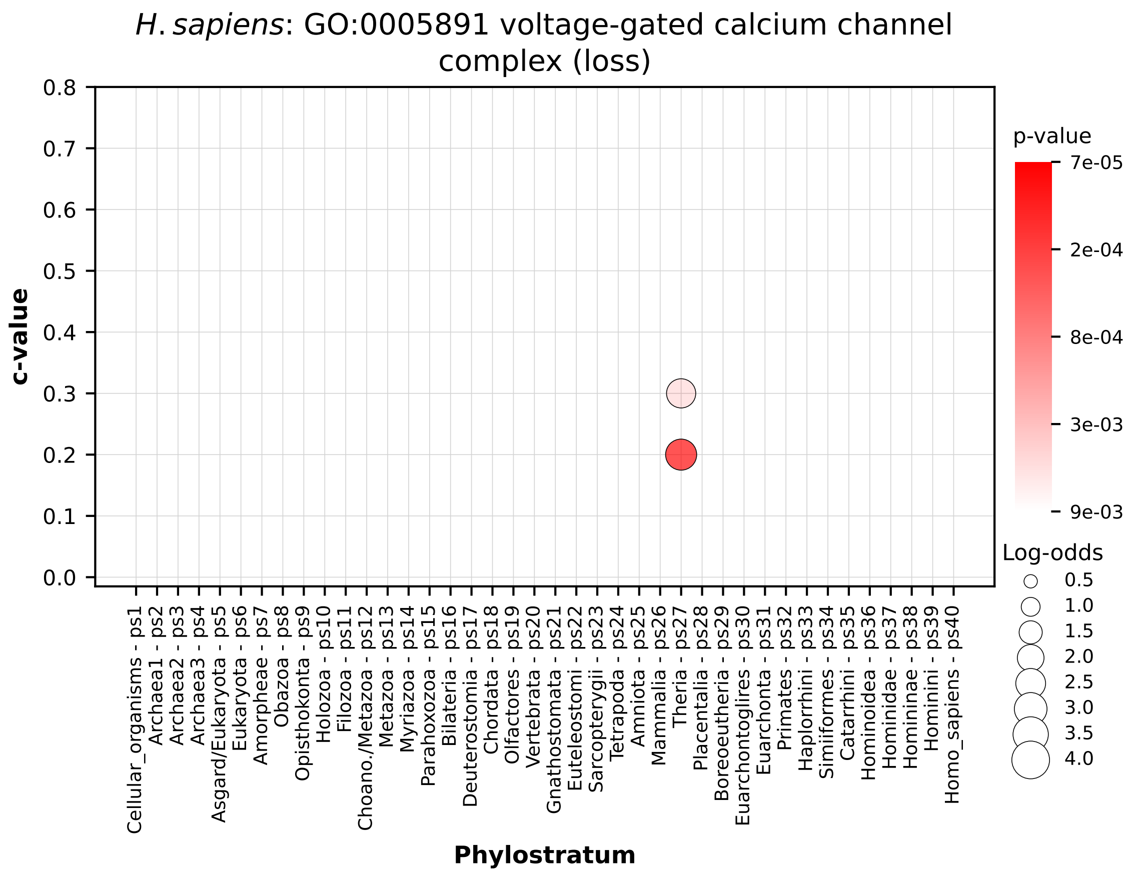

H. sapiens: GO:0005891 voltage-gated calcium channel complex (loss)
c-value
0.0
0.1
0.2
0.3
0.4
0.5
0.6
0.7
0.8
Phylostratum
Cellular_organisms - ps1
Archaea1 - ps2
Archaea2 - ps3
Archaea3 - ps4
Asgard/Eukaryota - ps5
Eukaryota - ps6
Amorpheae - ps7
Obazoa - ps8
Opisthokonta - ps9
Holozoa - ps10
Filozoa - ps11
Choano./Metazoa - ps12
Metazoa - ps13
Myriazoa - ps14
Parahoxozoa - ps15
Bilateria - ps16
Deuterostomia - ps17
Chordata - ps18
Olfactores - ps19
Vertebrata - ps20
Gnathostomata - ps21
Euteleostomi - ps22
Sarcopterygii - ps23
Tetrapoda - ps24
Amniota - ps25
Mammalia - ps26
Theria - ps27
Placentalia - ps28
Boreoeutheria - ps29
Euarchontoglires - ps30
Euarchonta - ps31
Primates - ps32
Haplorrhini - ps33
Simiiformes - ps34
Catarrhini - ps35
Hominoidea - ps36
Hominidae - ps37
Homininae - ps38
Hominini - ps39
Homo_sapiens - ps40
p-value
7e-05
2e-04
8e-04
3e-03
9e-03
Log-odds
0.5
1.0
1.5
2.0
2.5
3.0
3.5
4.0

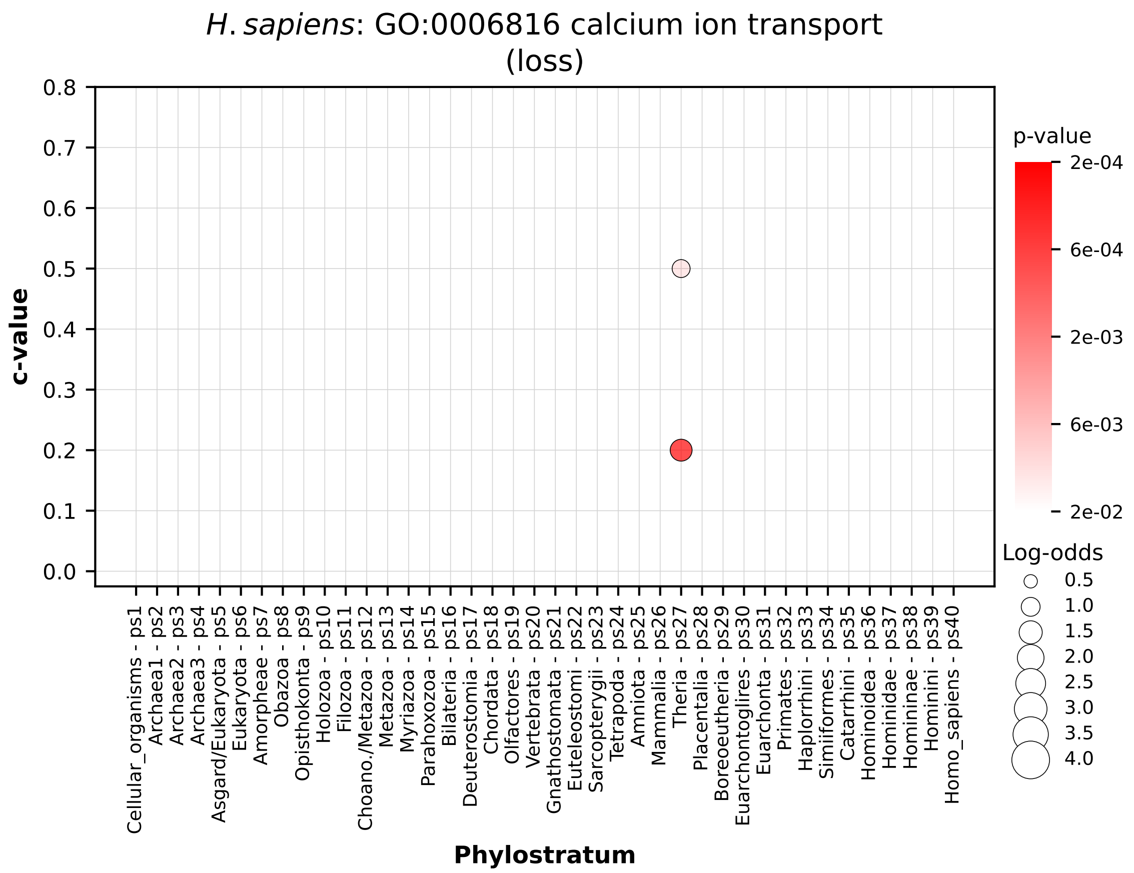

H. sapiens: GO:0006816 calcium ion transport
(loss)
c-value
0.8
0.7
0.6
0.5
0.4
0.3
0.2
0.1
0.0
p-value
2e-04
6e-04
2e-03
6e-03
2e-02
Log-odds
0.5
1.0
1.5
2.0
2.5
3.0
3.5
4.0
Cellular_organisms - ps1
Archaea1 - ps2
Archaea2 - ps3
Archaea3 - ps4
Asgard/Eukaryota - ps5
Eukaryota - ps6
Amorpheae - ps7
Obazoa - ps8
Opisthokonta - ps9
Holozoa - ps10
Filozoa - ps11
Choano./Metazoa - ps12
Metazoa - ps13
Myriazoa - ps14
Parahoxozoa - ps15
Bilateria - ps16
Deuterostomia - ps17
Chordata - ps18
Olfactores - ps19
Vertebrata - ps20
Gnathostomata - ps21
Euteleostomi - ps22
Sarcopterygii - ps23
Tetrapoda - ps24
Amniota - ps25
Mammalia - ps26
Theria - ps27
Placentalia - ps28
Boreoeutheria - ps29
Euarchontoglires - ps30
Euarchonta - ps31
Primates - ps32
Haplorrhini - ps33
Simiiformes - ps34
Catarrhini - ps35
Hominoidea - ps36
Hominidae - ps37
Homininae - ps38
Hominini - ps39
Homo_sapiens - ps40
Phylostratum

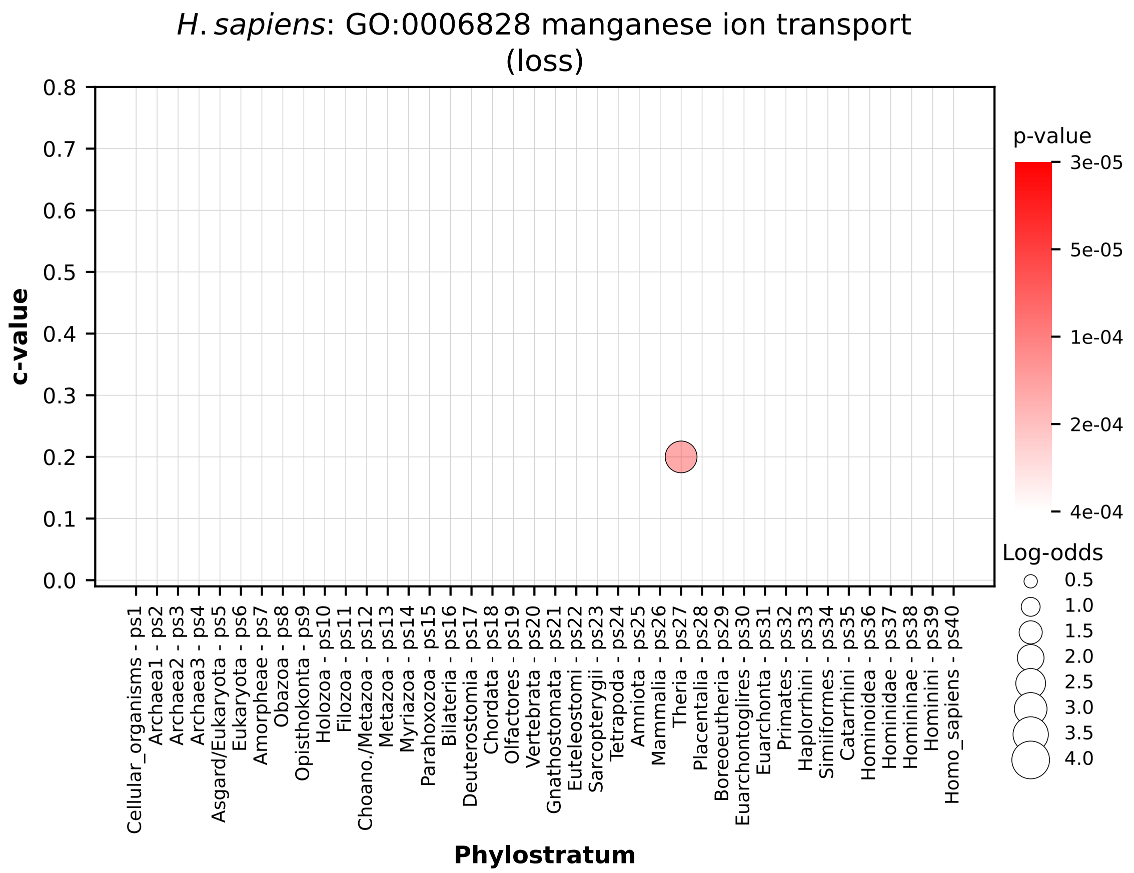

H. sapiens: GO:0006828 manganese ion transport
(loss)
c-value
0.0
0.1
0.2
0.3
0.4
0.5
0.6
0.7
0.8
Phylostratum
Cellular_organisms - ps1
Archaea1 - ps2
Archaea2 - ps3
Archaea3 - ps4
Asgard/Eukaryota - ps5
Eukaryota - ps6
Amorpheae - ps7
Obazoa - ps8
Opisthokonta - ps9
Holozoa - ps10
Filozoa - ps11
Choano./Metazoa - ps12
Metazoa - ps13
Myriazoa - ps14
Parahoxozoa - ps15
Bilateria - ps16
Deuterostomia - ps17
Chordata - ps18
Olfactores - ps19
Vertebrata - ps20
Gnathostomata - ps21
Euteleostomi - ps22
Sarcopterygii - ps23
Tetrapoda - ps24
Amniota - ps25
Mammalia - ps26
Theria - ps27
Placentalia - ps28
Boreoeutheria - ps29
Euarchontoglires - ps30
Euarchonta - ps31
Primates - ps32
Haplorrhini - ps33
Simiiformes - ps34
Catarrhini - ps35
Hominoidea - ps36
Hominidae - ps37
Homininae - ps38
Hominini - ps39
Homo_sapiens - ps40
p-value
3e-05
5e-05
1e-04
2e-04
4e-04
Log-odds
0.5
1.0
1.5
2.0
2.5
3.0
3.5
4.0

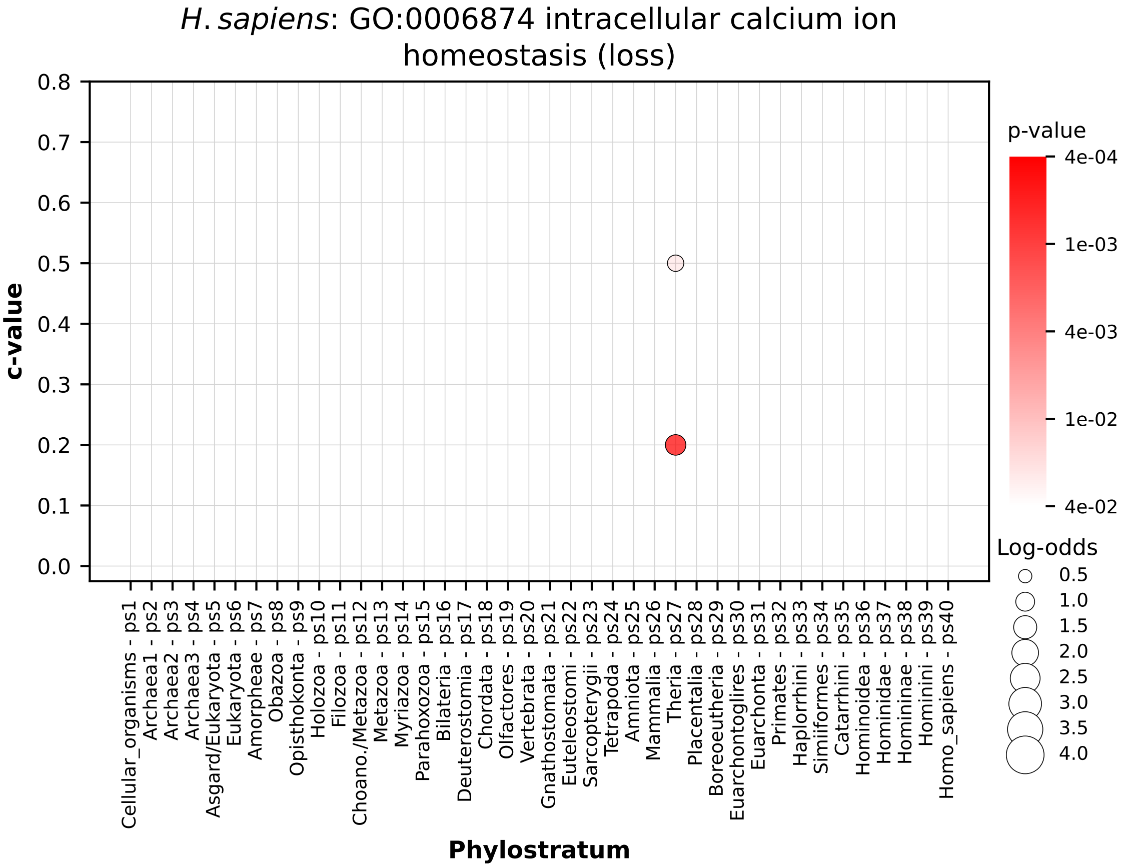

H. sapiens: GO:0006874 intracellular calcium ion homeostasis (loss)
c-value
0.8
0.7
0.6
0.5
0.4
0.3
0.2
0.1
0.0
p-value
4e-04
1e-03
4e-03
1e-02
4e-02
Log-odds
0.5
1.0
1.5
2.0
2.5
3.0
3.5
4.0
Cellular_organisms - ps1
Archaea1 - ps2
Archaea2 - ps3
Archaea3 - ps4
Asgard/Eukaryota - ps5
Eukaryota - ps6
Amorpheae - ps7
Obazoa - ps8
Opisthokonta - ps9
Holozoa - ps10
Filozoa - ps11
Choano./Metazoa - ps12
Metazoa - ps13
Myriazoa - ps14
Parahoxozoa - ps15
Bilateria - ps16
Deuterostomia - ps17
Chordata - ps18
Olfactores - ps19
Vertebrata - ps20
Gnathostomata - ps21
Euteleostomi - ps22
Sarcopterygii - ps23
Tetrapoda - ps24
Amniota - ps25
Mammalia - ps26
Theria - ps27
Placentalia - ps28
Boreoeutheria - ps29
Euarchontoglires - ps30
Euarchonta - ps31
Primates - ps32
Haplorrhini - ps33
Simiiformes - ps34
Catarrhini - ps35
Hominoidea - ps36
Hominidae - ps37
Homininae - ps38
Hominini - ps39
Homo_sapiens - ps40
Phylostratum

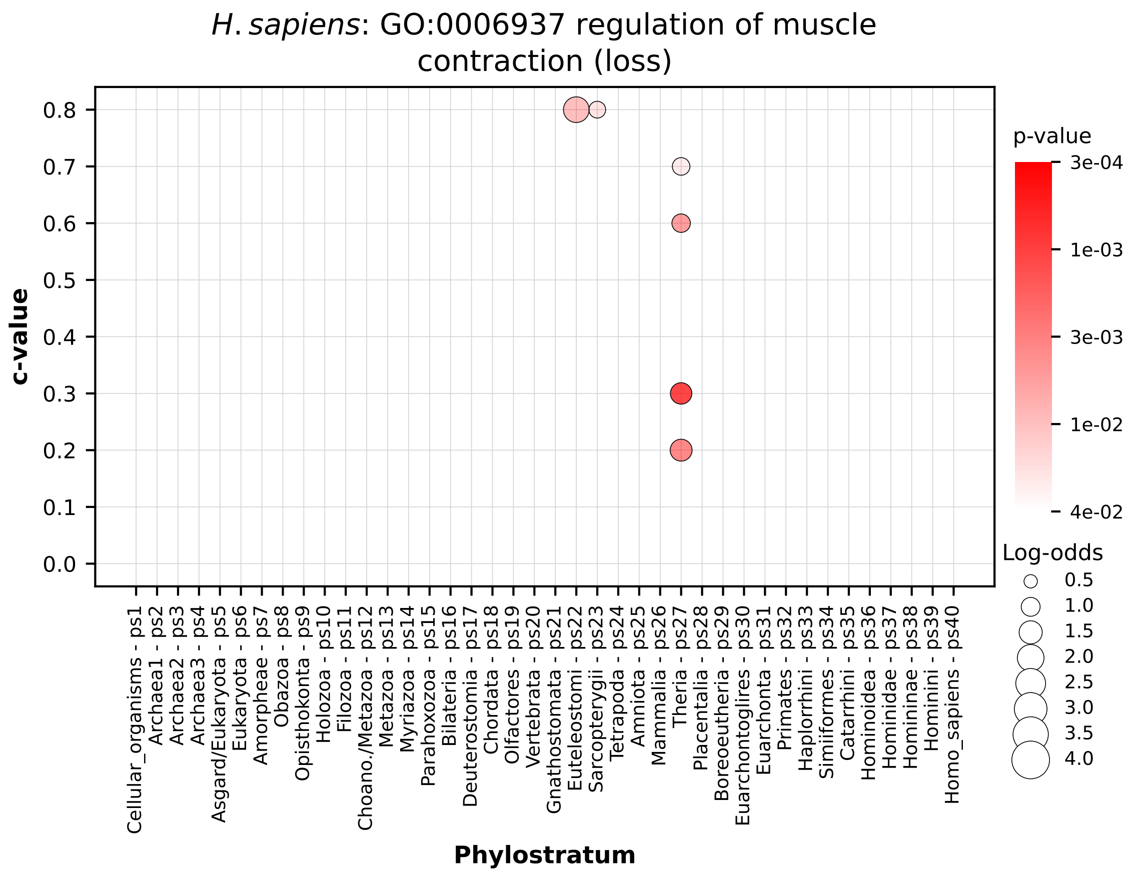

H. sapiens: GO:0006937 regulation of muscle contraction (loss)
c-value
0.0
0.1
0.2
0.3
0.4
0.5
0.6
0.7
0.8
Phylostratum
Cellular_organisms - ps1
Archaea1 - ps2
Archaea2 - ps3
Archaea3 - ps4
Asgard/Eukaryota - ps5
Eukaryota - ps6
Amorpheae - ps7
Obazoa - ps8
Opisthokonta - ps9
Holozoa - ps10
Filozoa - ps11
Choano./Metazoa - ps12
Metazoa - ps13
Myriazoa - ps14
Parahoxozoa - ps15
Bilateria - ps16
Deuterostomia - ps17
Chordata - ps18
Olfactores - ps19
Vertebrata - ps20
Gnathostomata - ps21
Euteleostomi - ps22
Sarcopterygii - ps23
Tetrapoda - ps24
Amniota - ps25
Mammalia - ps26
Theria - ps27
Placentalia - ps28
Boreoeutheria - ps29
Euarchontoglires - ps30
Euarchonta - ps31
Primates - ps32
Haplorrhini - ps33
Simiiformes - ps34
Catarrhini - ps35
Hominoidea - ps36
Hominidae - ps37
Homininae - ps38
Hominini - ps39
Homo_sapiens - ps40
p-value
3e-04
1e-03
3e-03
1e-02
4e-02
Log-odds
0.5
1.0
1.5
2.0
2.5
3.0
3.5
4.0

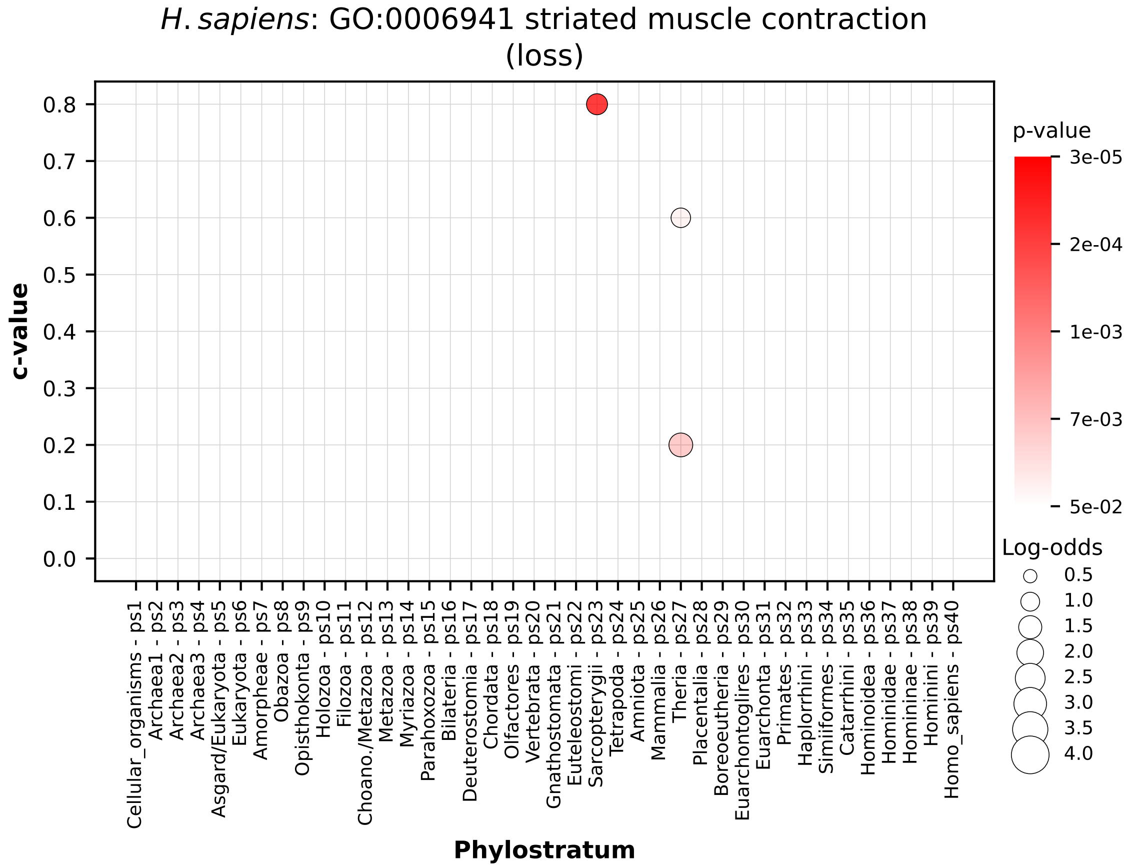

H. sapiens: GO:0006941 striated muscle contraction
(loss)
c-value
0.0
0.1
0.2
0.3
0.4
0.5
0.6
0.7
0.8
p-value
3e-05
2e-04
1e-03
7e-03
5e-02
Log-odds
0.5
1.0
1.5
2.0
2.5
3.0
3.5
4.0
Cellular_organisms - ps1
Archaea1 - ps2
Archaea2 - ps3
Archaea3 - ps4
Asgard/Eukaryota - ps5
Eukaryota - ps6
Amorpheae - ps7
Obazoa - ps8
Opisthokonta - ps9
Holozoa - ps10
Filozoa - ps11
Choano./Metazoa - ps12
Metazoa - ps13
Myriazoa - ps14
Parahoxozoa - ps15
Bilateria - ps16
Deuterostomia - ps17
Chordata - ps18
Olfactores - ps19
Vertebrata - ps20
Gnathostomata - ps21
Euteleostomi - ps22
Sarcopterygii - ps23
Tetrapoda - ps24
Amniota - ps25
Mammalia - ps26
Theria - ps27
Placentalia - ps28
Boreoeutheria - ps29
Euarchontoglires - ps30
Euarchonta - ps31
Primates - ps32
Haplorrhini - ps33
Simiiformes - ps34
Catarrhini - ps35
Hominoidea - ps36
Hominidae - ps37
Homininae - ps38
Hominini - ps39
Homo_sapiens - ps40
Phylostratum

H. sapiens: GO:0006942 regulation of striated muscle contraction (loss)

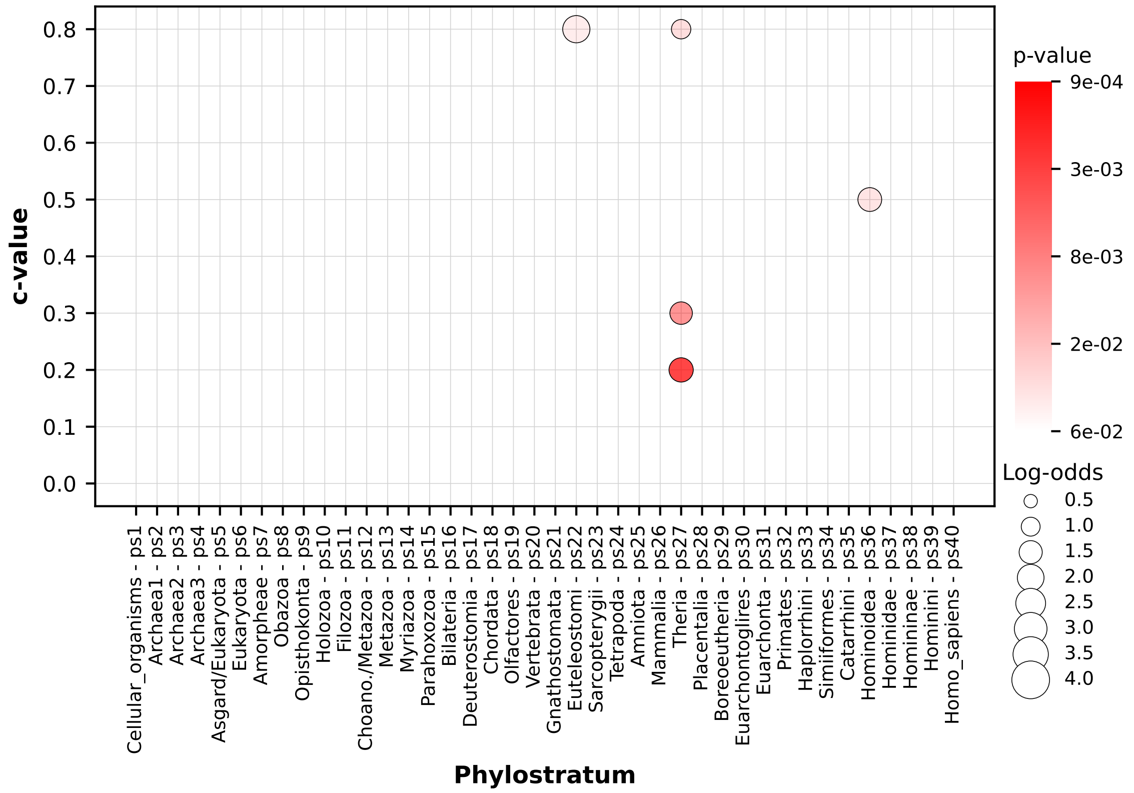

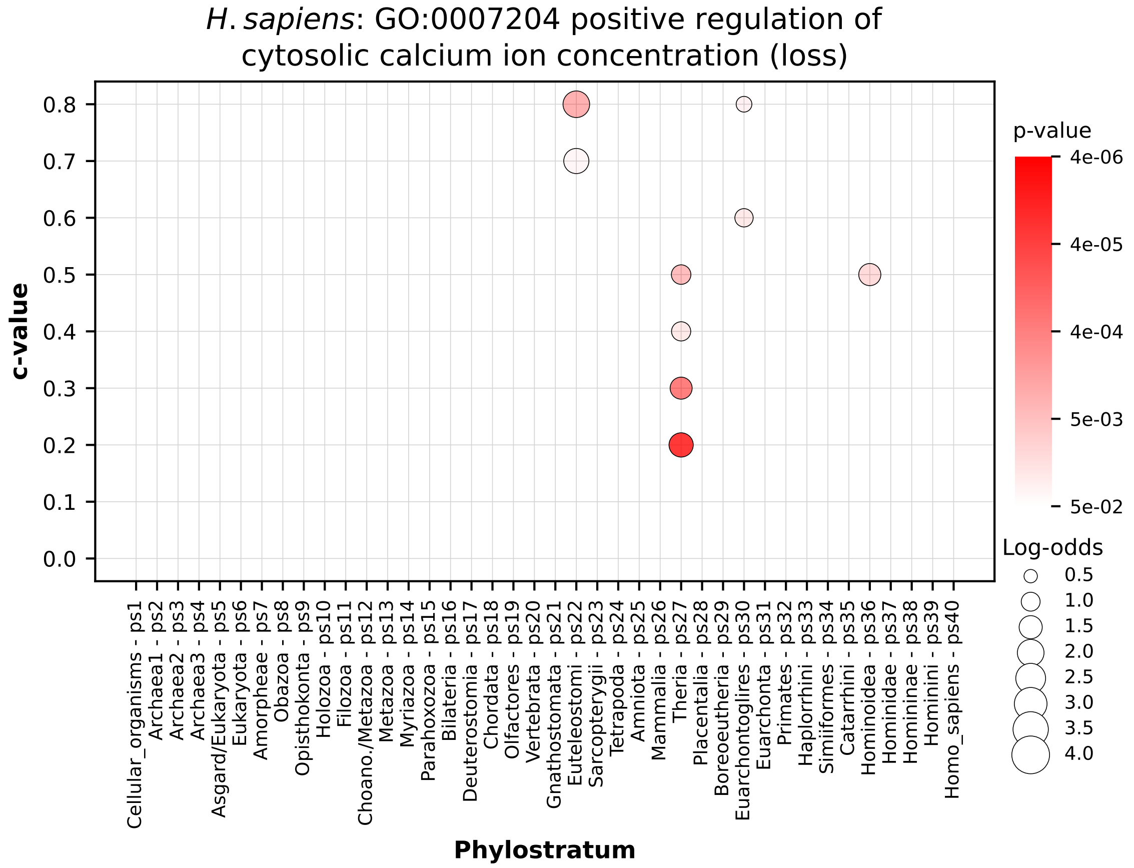

H. sapiens: GO:0007204 positive regulation of cytosolic calcium ion concentration (loss)
c-value
0.0
0.1
0.2
0.3
0.4
0.5
0.6
0.7
0.8
p-value
4e-06
4e-05
4e-04
5e-03
5e-02
Log-odds
0.5
1.0
1.5
2.0
2.5
3.0
3.5
4.0
Cellular_organisms - ps1
Archaea1 - ps2
Archaea2 - ps3
Archaea3 - ps4
Asgard/Eukaryota - ps5
Eukaryota - ps6
Amorpheae - ps7
Obazoa - ps8
Opisthokonta - ps9
Holozoa - ps10
Filozoa - ps11
Choano./Metazoa - ps12
Metazoa - ps13
Myriazoa - ps14
Parahoxozoa - ps15
Bilateria - ps16
Deuterostomia - ps17
Chordata - ps18
Olfactores - ps19
Vertebrata - ps20
Gnathostomata - ps21
Euteleostomi - ps22
Sarcopterygii - ps23
Tetrapoda - ps24
Amniota - ps25
Mammalia - ps26
Theria - ps27
Placentalia - ps28
Boreoeutheria - ps29
Euarchontoglires - ps30
Euarchonta - ps31
Primates - ps32
Haplorrhini - ps33
Simiiformes - ps34
Catarrhini - ps35
Hominoidea - ps36
Hominidae - ps37
Homininae - ps38
Hominini - ps39
Homo_sapiens - ps40
Phylostratum

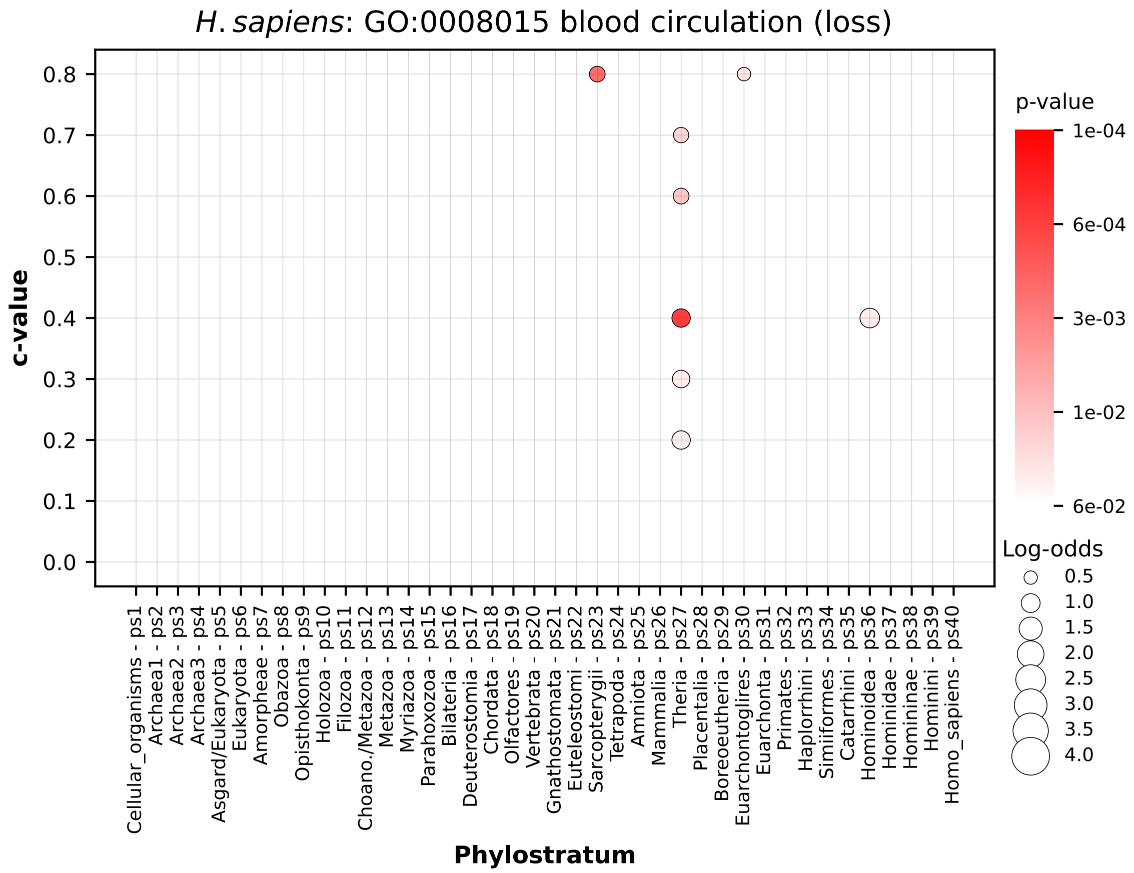

H. sapiens: GO:0008015 blood circulation (loss)
c-value
0.0
0.1
0.2
0.3
0.4
0.5
0.6
0.7
0.8
Phylostratum
Cellular_organisms - ps1
Archaea1 - ps2
Archaea2 - ps3
Archaea3 - ps4
Asgard/Eukaryota - ps5
Eukaryota - ps6
Amorpheae - ps7
Obazoa - ps8
Opisthokonta - ps9
Holozoa - ps10
Filozoa - ps11
Choano./Metazoa - ps12
Metazoa - ps13
Myriazoa - ps14
Parahoxozoa - ps15
Bilateria - ps16
Deuterostomia - ps17
Chordata - ps18
Olfactores - ps19
Vertebrata - ps20
Gnathostomata - ps21
Euteleostomi - ps22
Sarcopterygii - ps23
Tetrapoda - ps24
Amniota - ps25
Mammalia - ps26
Theria - ps27
Placentalia - ps28
Boreoeutheria - ps29
Euarchontoglires - ps30
Euarchonta - ps31
Primates - ps32
Haplorrhini - ps33
Simiiformes - ps34
Catarrhini - ps35
Hominoidea - ps36
Hominidae - ps37
Homininae - ps38
Hominini - ps39
Homo_sapiens - ps40
p-value
1e-04
6e-04
3e-03
1e-02
6e-02
Log-odds
0.5
1.0
1.5
2.0
2.5
3.0
3.5
4.0

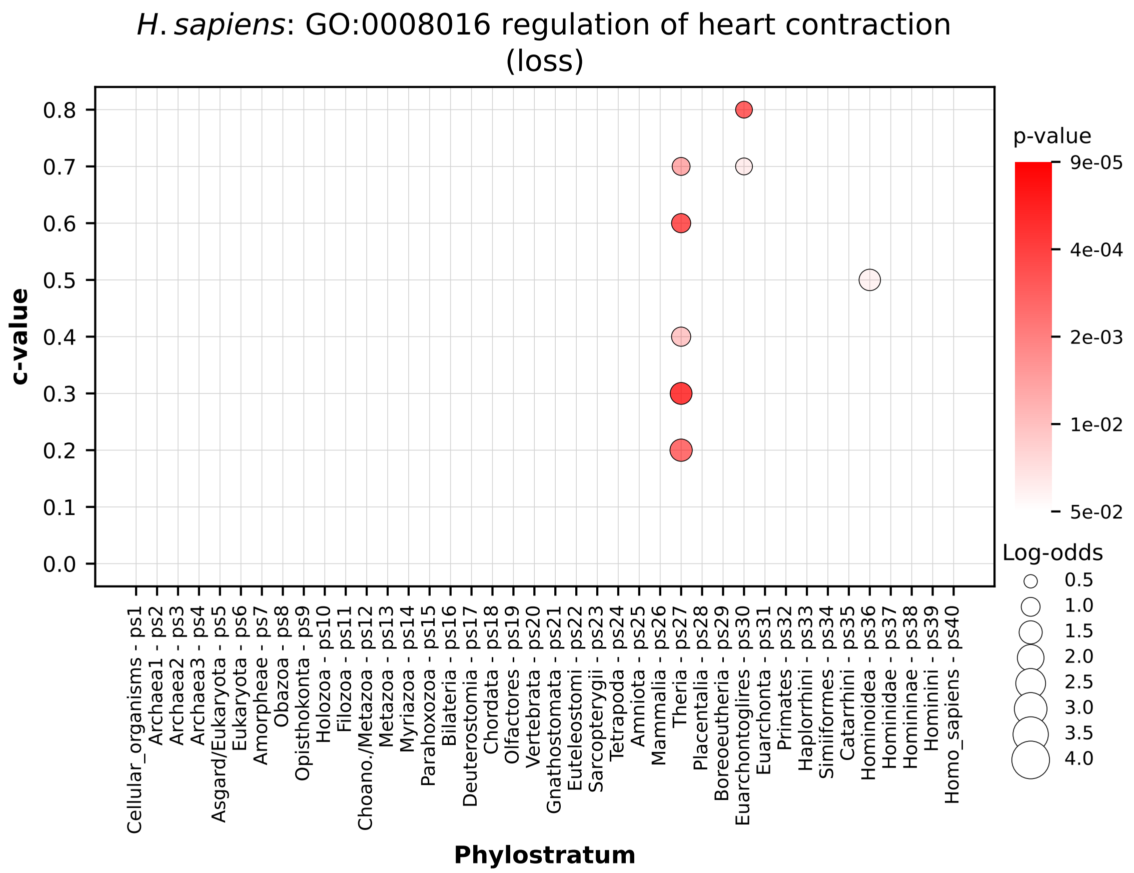

H. sapiens: GO:0008016 regulation of heart contraction
(loss)
c-value
0.0
0.1
0.2
0.3
0.4
0.5
0.6
0.7
0.8
p-value
9e-05
4e-04
2e-03
1e-02
5e-02
Log-odds
0.5
1.0
1.5
2.0
2.5
3.0
3.5
4.0
Cellular_organisms - ps1
Archaea1 - ps2
Archaea2 - ps3
Archaea3 - ps4
Asgard/Eukaryota - ps5
Eukaryota - ps6
Amorpheae - ps7
Obazoa - ps8
Opisthokonta - ps9
Holozoa - ps10
Filozoa - ps11
Choano./Metazoa - ps12
Metazoa - ps13
Myriazoa - ps14
Parahoxozoa - ps15
Bilateria - ps16
Deuterostomia - ps17
Chordata - ps18
Olfactores - ps19
Vertebrata - ps20
Gnathostomata - ps21
Euteleostomi - ps22
Sarcopterygii - ps23
Tetrapoda - ps24
Amniota - ps25
Mammalia - ps26
Theria - ps27
Placentalia - ps28
Boreoeutheria - ps29
Euarchontoglires - ps30
Euarchonta - ps31
Primates - ps32
Haplorrhini - ps33
Simiiformes - ps34
Catarrhini - ps35
Hominoidea - ps36
Hominidae - ps37
Homininae - ps38
Hominini - ps39
Homo_sapiens - ps40
Phylostratum

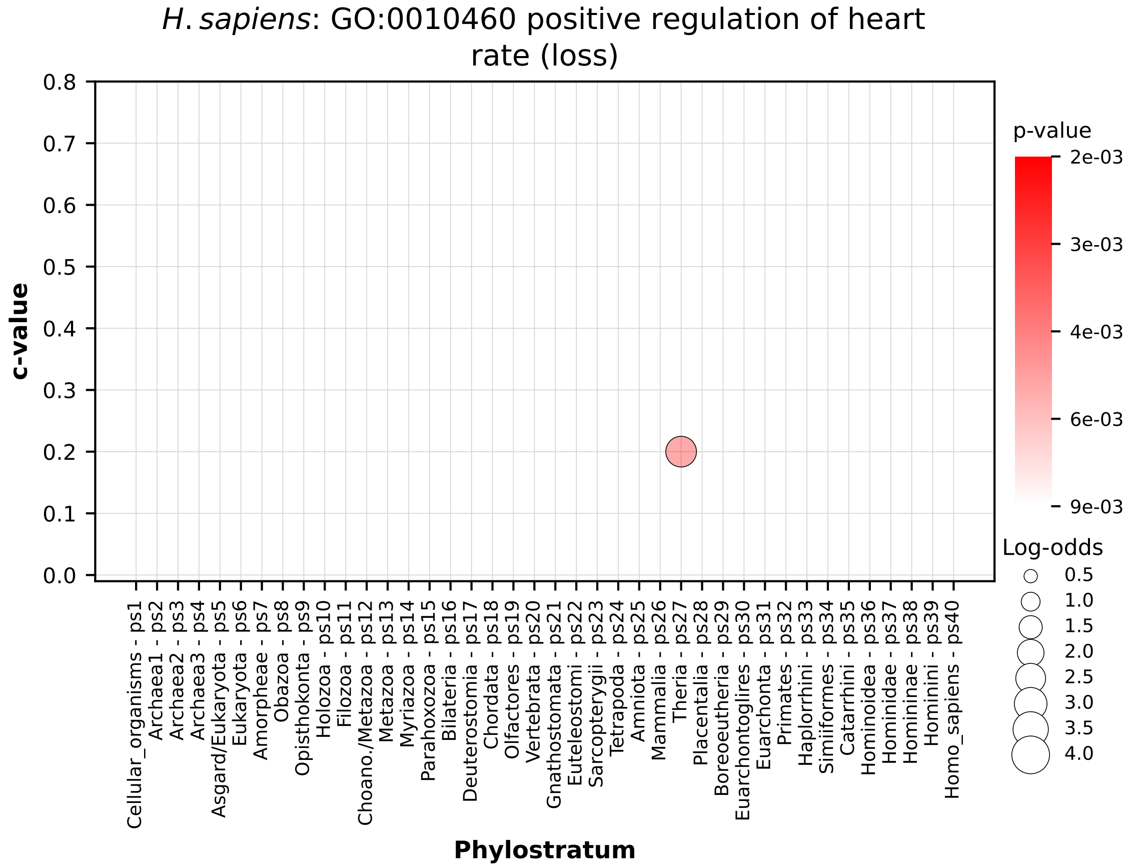

H. sapiens: GO:0010460 positive regulation of heart rate (loss)
c-value
0.0
0.1
0.2
0.3
0.4
0.5
0.6
0.7
0.8
p-value
2e-03
3e-03
4e-03
6e-03
9e-03
Log-odds
0.5
1.0
1.5
2.0
2.5
3.0
3.5
4.0
Cellular_organisms - ps1
Archaea1 - ps2
Archaea2 - ps3
Archaea3 - ps4
Asgard/Eukaryota - ps5
Eukaryota - ps6
Amorpheae - ps7
Obazoa - ps8
Opisthokonta - ps9
Holozoa - ps10
Filozoa - ps11
Choano./Metazoa - ps12
Metazoa - ps13
Myriazoa - ps14
Parahoxozoa - ps15
Bilateria - ps16
Deuterostomia - ps17
Chordata - ps18
Olfactores - ps19
Vertebrata - ps20
Gnathostomata - ps21
Euteleostomi - ps22
Sarcopterygii - ps23
Tetrapoda - ps24
Amniota - ps25
Mammalia - ps26
Theria - ps27
Placentalia - ps28
Boreoeutheria - ps29
Euarchontoglires - ps30
Euarchonta - ps31
Primates - ps32
Haplorrhini - ps33
Simiiformes - ps34
Catarrhini - ps35
Hominoidea - ps36
Hominidae - ps37
Homininae - ps38
Hominini - ps39
Homo_sapiens - ps40
Phylostratum

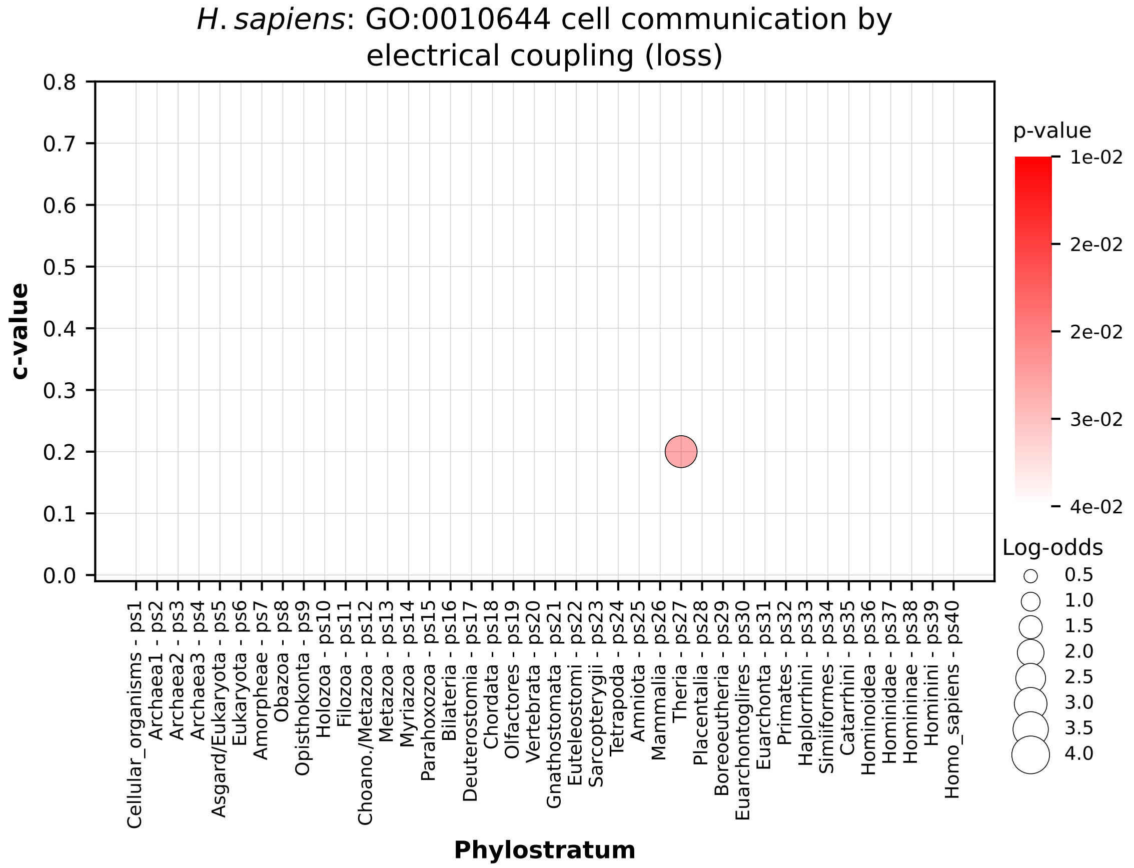
H. sapiens: GO:0010644 cell communication by electrical coupling (loss)
c-value
0.0
0.1
0.2
0.3
0.4
0.5
0.6
0.7
0.8
p-value
1e-02
2e-02
2e-02
3e-02
4e-02
Log-odds
0.5
1.0
1.5
2.0
2.5
3.0
3.5
4.0
Cellular_organisms - ps1
Archaea1 - ps2
Archaea2 - ps3
Archaea3 - ps4
Asgard/Eukaryota - ps5
Eukaryota - ps6
Amorpheae - ps7
Obazoa - ps8
Opisthokonta - ps9
Holozoa - ps10
Filozoa - ps11
Choano./Metazoa - ps12
Metazoa - ps13
Myriazoa - ps14
Parahoxozoa - ps15
Bilateria - ps16
Deuterostomia - ps17
Chordata - ps18
Olfactores - ps19
Vertebrata - ps20
Gnathostomata - ps21
Euteleostomi - ps22
Sarcopterygii - ps23
Tetrapoda - ps24
Amniota - ps25
Mammalia - ps26
Theria - ps27
Placentalia - ps28
Boreoeutheria - ps29
Euarchontoglires - ps30
Euarchonta - ps31
Primates - ps32
Haplorrhini - ps33
Simiiformes - ps34
Catarrhini - ps35
Hominoidea - ps36
Hominidae - ps37
Homininae - ps38
Hominini - ps39
Homo_sapiens - ps40
Phylostratum

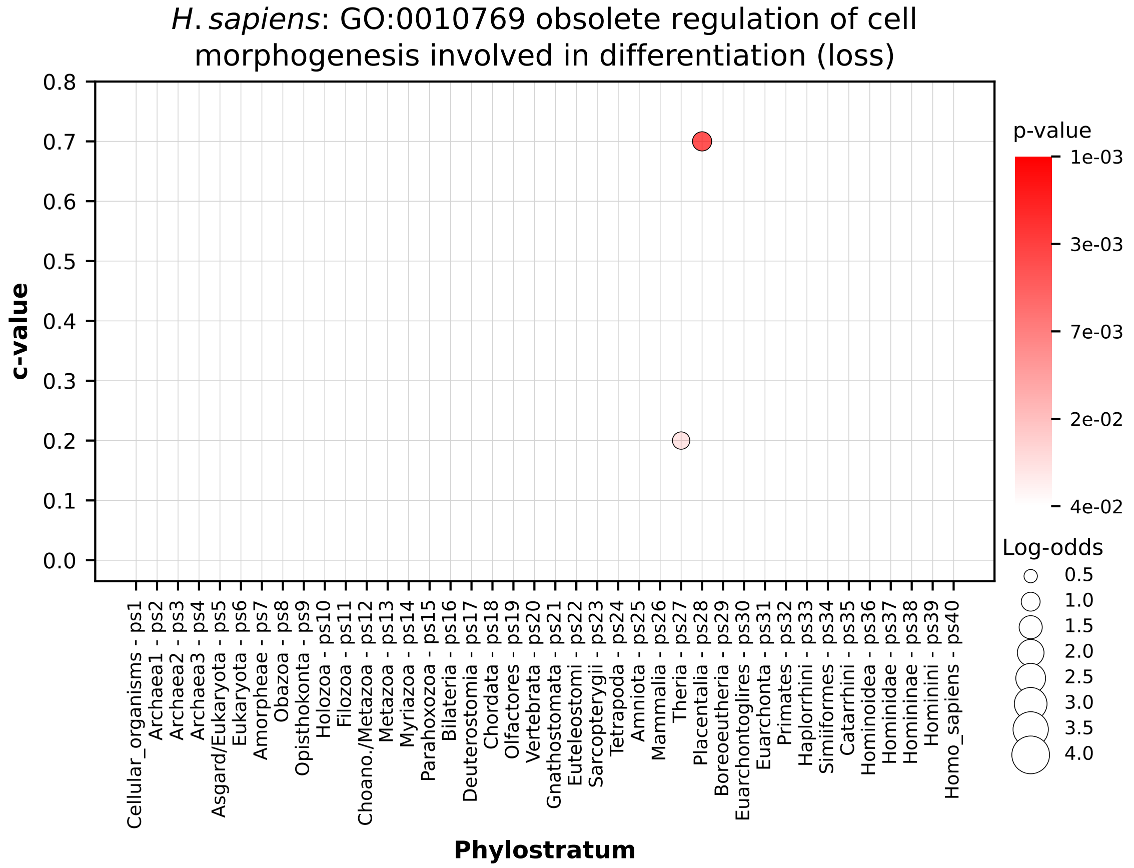
H. sapiens: GO:0010769 obsolete regulation of cell morphogenesis involved in differentiation (loss)
c-value
0.8
0.7
0.6
0.5
0.4
0.3
0.2
0.1
0.0
p-value
1e-03
3e-03
7e-03
2e-02
4e-02
Log-odds
0.5
1.0
1.5
2.0
2.5
3.0
3.5
4.0
Cellular_organisms - ps1
Archaea1 - ps2
Archaea2 - ps3
Archaea3 - ps4
Asgard/Eukaryota - ps5
Eukaryota - ps6
Amorpheae - ps7
Obazoa - ps8
Opisthokonta - ps9
Holozoa - ps10
Filozoa - ps11
Choano./Metazoa - ps12
Metazoa - ps13
Myriazoa - ps14
Parahoxozoa - ps15
Bilateria - ps16
Deuterostomia - ps17
Chordata - ps18
Olfactores - ps19
Vertebrata - ps20
Gnathostomata - ps21
Euteleostomi - ps22
Sarcopterygii - ps23
Tetrapoda - ps24
Amniota - ps25
Mammalia - ps26
Theria - ps27
Placentalia - ps28
Boreoeutheria - ps29
Euarchontoglires - ps30
Euarchonta - ps31
Primates - ps32
Haplorrhini - ps33
Simiiformes - ps34
Catarrhini - ps35
Hominoidea - ps36
Hominidae - ps37
Homininae - ps38
Hominini - ps39
Homo_sapiens - ps40
Phylostratum

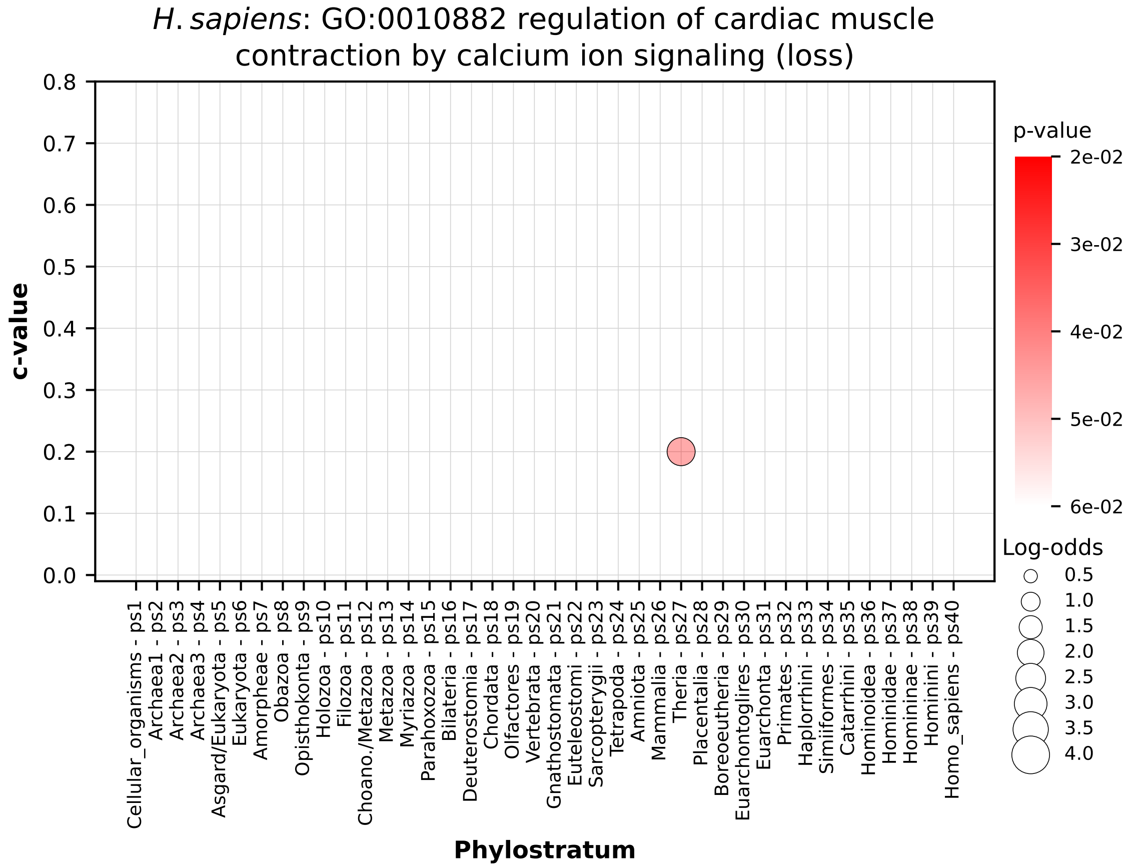

H. sapiens: GO:0010882 regulation of cardiac muscle contraction by calcium ion signaling (loss)
c-value
0.0
0.1
0.2
0.3
0.4
0.5
0.6
0.7
0.8
Phylostratum
Cellular_organisms - ps1
Archaea1 - ps2
Archaea2 - ps3
Archaea3 - ps4
Asgard/Eukaryota - ps5
Eukaryota - ps6
Amorpheae - ps7
Obazoa - ps8
Opisthokonta - ps9
Holozoa - ps10
Filozoa - ps11
Choano./Metazoa - ps12
Metazoa - ps13
Myriazoa - ps14
Parahoxozoa - ps15
Bilateria - ps16
Deuterostomia - ps17
Chordata - ps18
Olfactores - ps19
Vertebrata - ps20
Gnathostomata - ps21
Euteleostomi - ps22
Sarcopterygii - ps23
Tetrapoda - ps24
Amniota - ps25
Mammalia - ps26
Theria - ps27
Placentalia - ps28
Boreoeutheria - ps29
Euarchontoglires - ps30
Euarchonta - ps31
Primates - ps32
Haplorrhini - ps33
Simiiformes - ps34
Catarrhini - ps35
Hominoidea - ps36
Hominidae - ps37
Homininae - ps38
Hominini - ps39
Homo_sapiens - ps40
p-value
2e-02
3e-02
4e-02
5e-02
6e-02
Log-odds
0.5
1.0
1.5
2.0
2.5
3.0
3.5
4.0

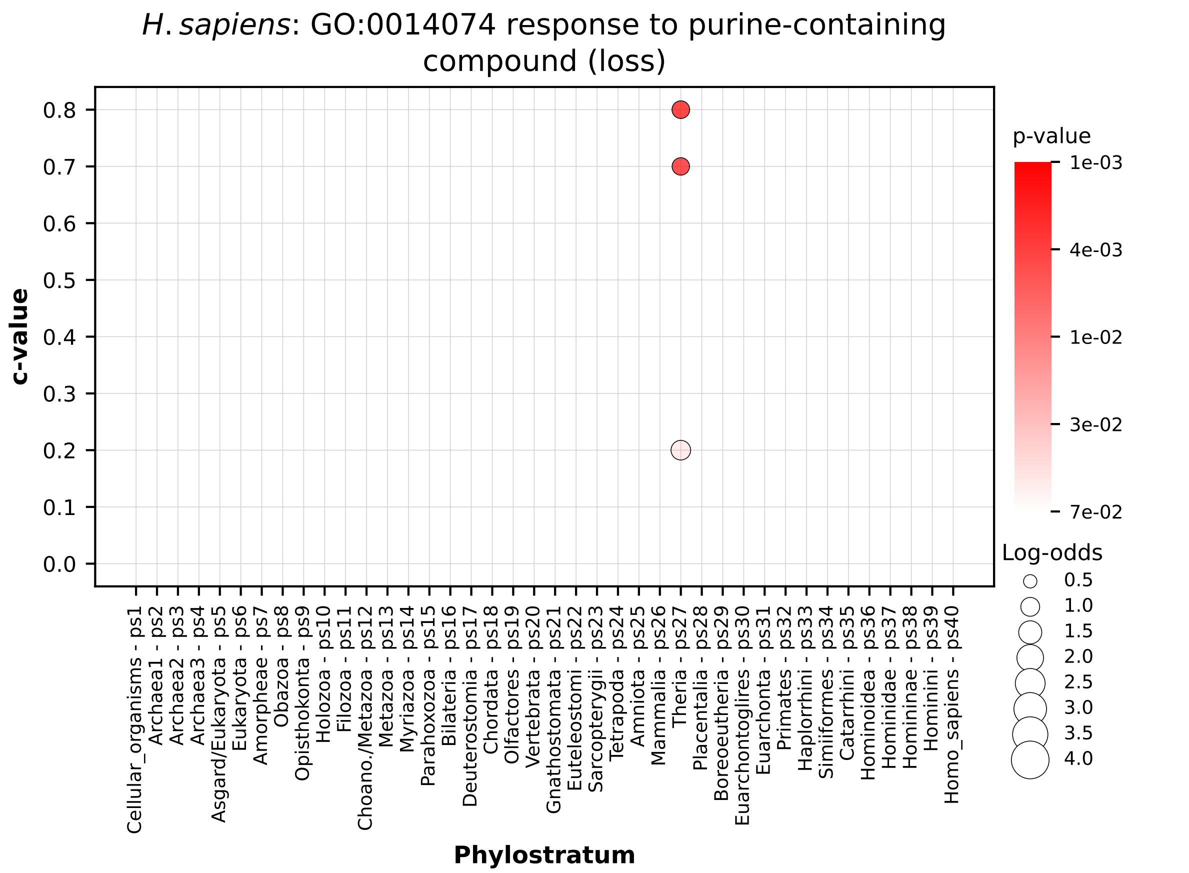

H. sapiens: GO:0014074 response to purine-containing compound (loss)
c-value
0.8
0.7
0.6
0.5
0.4
0.3
0.2
0.1
0.0
p-value
1e-03
4e-03
1e-02
3e-02
7e-02
Log-odds
0.5
1.0
1.5
2.0
2.5
3.0
3.5
4.0
Cellular_organisms - ps1
Archaea1 - ps2
Archaea2 - ps3
Archaea3 - ps4
Asgard/Eukaryota - ps5
Eukaryota - ps6
Amorpheae - ps7
Obazoa - ps8
Opisthokonta - ps9
Holozoa - ps10
Filozoa - ps11
Choano./Metazoa - ps12
Metazoa - ps13
Myriazoa - ps14
Parahoxozoa - ps15
Bilateria - ps16
Deuterostomia - ps17
Chordata - ps18
Olfactores - ps19
Vertebrata - ps20
Gnathostomata - ps21
Euteleostomi - ps22
Sarcopterygii - ps23
Tetrapoda - ps24
Amniota - ps25
Mammalia - ps26
Theria - ps27
Placentalia - ps28
Boreoeutheria - ps29
Euarchontoglires - ps30
Euarchonta - ps31
Primates - ps32
Haplorrhini - ps33
Simiiformes - ps34
Catarrhini - ps35
Hominoidea - ps36
Hominidae - ps37
Homininae - ps38
Hominini - ps39
Homo_sapiens - ps40
Phylostratum

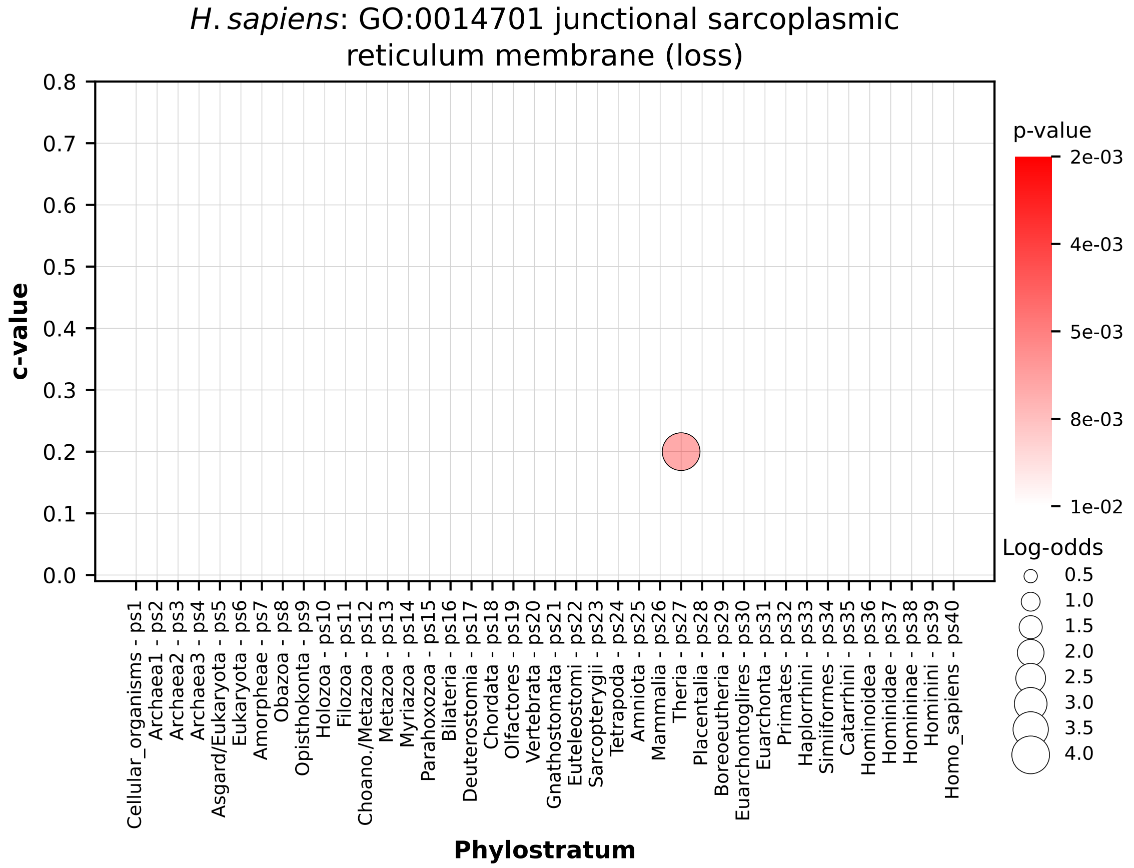
H. sapiens: GO:0014701 junctional sarcoplasmic reticulum membrane (loss)
c-value
0.0
0.1
0.2
0.3
0.4
0.5
0.6
0.7
0.8
Phylostratum
Cellular_organisms - ps1
Archaea1 - ps2
Archaea2 - ps3
Archaea3 - ps4
Asgard/Eukaryota - ps5
Eukaryota - ps6
Amorpheae - ps7
Obazoa - ps8
Opisthokonta - ps9
Holozoa - ps10
Filozoa - ps11
Choano./Metazoa - ps12
Metazoa - ps13
Myriazoa - ps14
Parahoxozoa - ps15
Bilateria - ps16
Deuterostomia - ps17
Chordata - ps18
Olfactores - ps19
Vertebrata - ps20
Gnathostomata - ps21
Euteleostomi - ps22
Sarcopterygii - ps23
Tetrapoda - ps24
Amniota - ps25
Mammalia - ps26
Theria - ps27
Placentalia - ps28
Boreoeutheria - ps29
Euarchontoglires - ps30
Euarchonta - ps31
Primates - ps32
Haplorrhini - ps33
Simiiformes - ps34
Catarrhini - ps35
Hominoidea - ps36
Hominidae - ps37
Homininae - ps38
Hominini - ps39
Homo_sapiens - ps40
p-value
2e-03
4e-03
5e-03
8e-03
1e-02
Log-odds
0.5
1.0
1.5
2.0
2.5
3.0
3.5
4.0

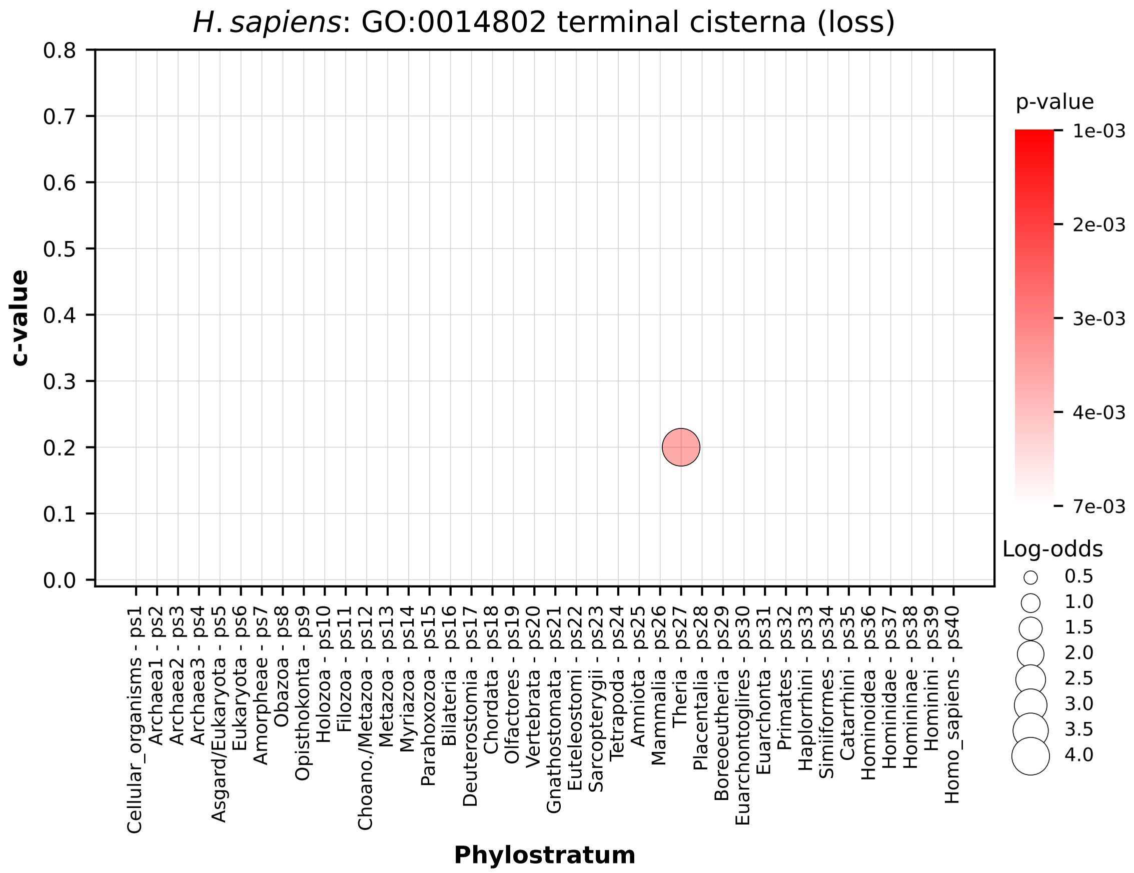

H. sapiens: GO:0014802 terminal cisterna (loss)
c-value
0.8
0.7
0.6
0.5
0.4
0.3
0.2
0.1
0.0
Phylostratum
Cellular_organisms - ps1
Archaea1 - ps2
Archaea2 - ps3
Archaea3 - ps4
Asgard/Eukaryota - ps5
Eukaryota - ps6
Amorpheae - ps7
Obazoa - ps8
Opisthokonta - ps9
Holozoa - ps10
Filozoa - ps11
Choano./Metazoa - ps12
Metazoa - ps13
Myriazoa - ps14
Parahoxozoa - ps15
Bilateria - ps16
Deuterostomia - ps17
Chordata - ps18
Olfactores - ps19
Vertebrata - ps20
Gnathostomata - ps21
Euteleostomi - ps22
Sarcopterygii - ps23
Tetrapoda - ps24
Amniota - ps25
Mammalia - ps26
Theria - ps27
Placentalia - ps28
Boreoeutheria - ps29
Euarchontoglires - ps30
Euarchonta - ps31
Primates - ps32
Haplorrhini - ps33
Simiiformes - ps34
Catarrhini - ps35
Hominoidea - ps36
Hominidae - ps37
Homininae - ps38
Hominini - ps39
Homo_sapiens - ps40
p-value
1e-03
2e-03
3e-03
4e-03
7e-03
Log-odds
0.5
1.0
1.5
2.0
2.5
3.0
3.5
4.0

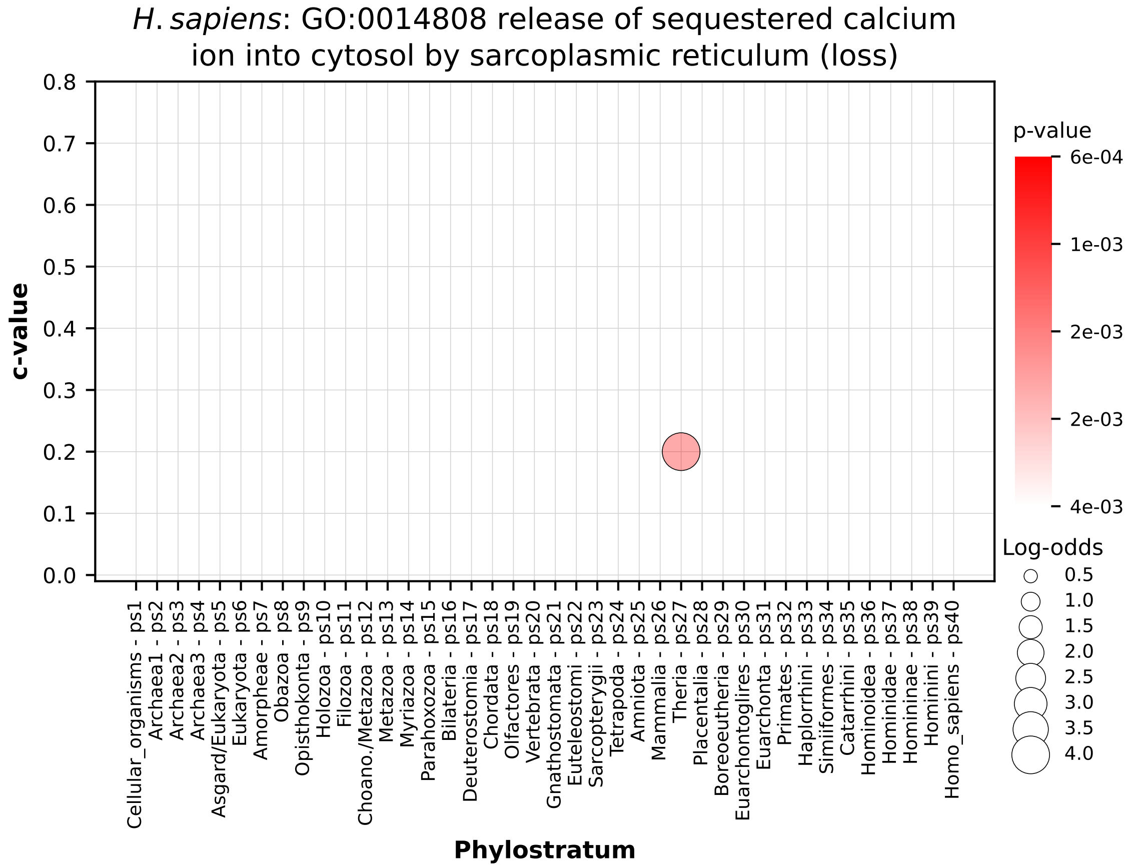

H. sapiens: GO:0014808 release of sequestered calcium ion into cytosol by sarcoplasmic reticulum (loss)
c-value
0.8
0.7
0.6
0.5
0.4
0.3
0.2
0.1
0.0
p-value
6e-04
1e-03
2e-03
2e-03
4e-03
Log-odds
0.5
1.0
1.5
2.0
2.5
3.0
3.5
4.0
Cellular_organisms - ps1
Archaea1 - ps2
Archaea2 - ps3
Archaea3 - ps4
Asgard/Eukaryota - ps5
Eukaryota - ps6
Amorpheae - ps7
Obazoa - ps8
Opisthokonta - ps9
Holozoa - ps10
Filozoa - ps11
Choano./Metazoa - ps12
Metazoa - ps13
Myriazoa - ps14
Parahoxozoa - ps15
Bilateria - ps16
Deuterostomia - ps17
Chordata - ps18
Olfactores - ps19
Vertebrata - ps20
Gnathostomata - ps21
Euteleostomi - ps22
Sarcopterygii - ps23
Tetrapoda - ps24
Amniota - ps25
Mammalia - ps26
Theria - ps27
Placentalia - ps28
Boreoeutheria - ps29
Euarchontoglires - ps30
Euarchonta - ps31
Primates - ps32
Haplorrhini - ps33
Simiiformes - ps34
Catarrhini - ps35
Hominoidea - ps36
Hominidae - ps37
Homininae - ps38
Hominini - ps39
Homo_sapiens - ps40
Phylostratum

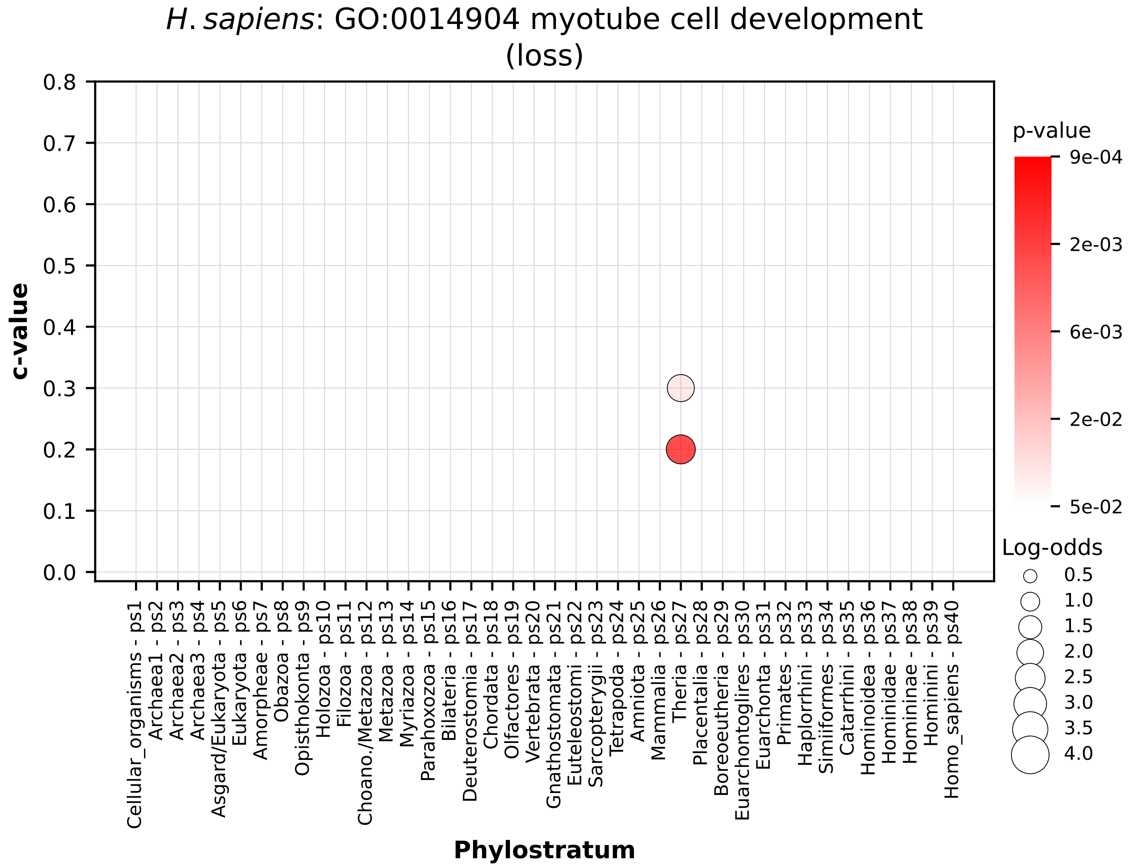

H. sapiens: GO:0014904 myotube cell development
(loss)
c-value
0.8
0.7
0.6
0.5
0.4
0.3
0.2
0.1
0.0
p-value
9e-04
2e-03
6e-03
2e-02
5e-02
Log-odds
0.5
1.0
1.5
2.0
2.5
3.0
3.5
4.0
Cellular_organisms - ps1
Archaea1 - ps2
Archaea2 - ps3
Archaea3 - ps4
Asgard/Eukaryota - ps5
Eukaryota - ps6
Amorpheae - ps7
Obazoa - ps8
Opisthokonta - ps9
Holozoa - ps10
Filozoa - ps11
Choano./Metazoa - ps12
Metazoa - ps13
Myriazoa - ps14
Parahoxozoa - ps15
Bilateria - ps16
Deuterostomia - ps17
Chordata - ps18
Olfactores - ps19
Vertebrata - ps20
Gnathostomata - ps21
Euteleostomi - ps22
Sarcopterygii - ps23
Tetrapoda - ps24
Amniota - ps25
Mammalia - ps26
Theria - ps27
Placentalia - ps28
Boreoeutheria - ps29
Euarchontoglires - ps30
Euarchonta - ps31
Primates - ps32
Haplorrhini - ps33
Simiiformes - ps34
Catarrhini - ps35
Hominoidea - ps36
Hominidae - ps37
Homininae - ps38
Hominini - ps39
Homo_sapiens - ps40
Phylostratum

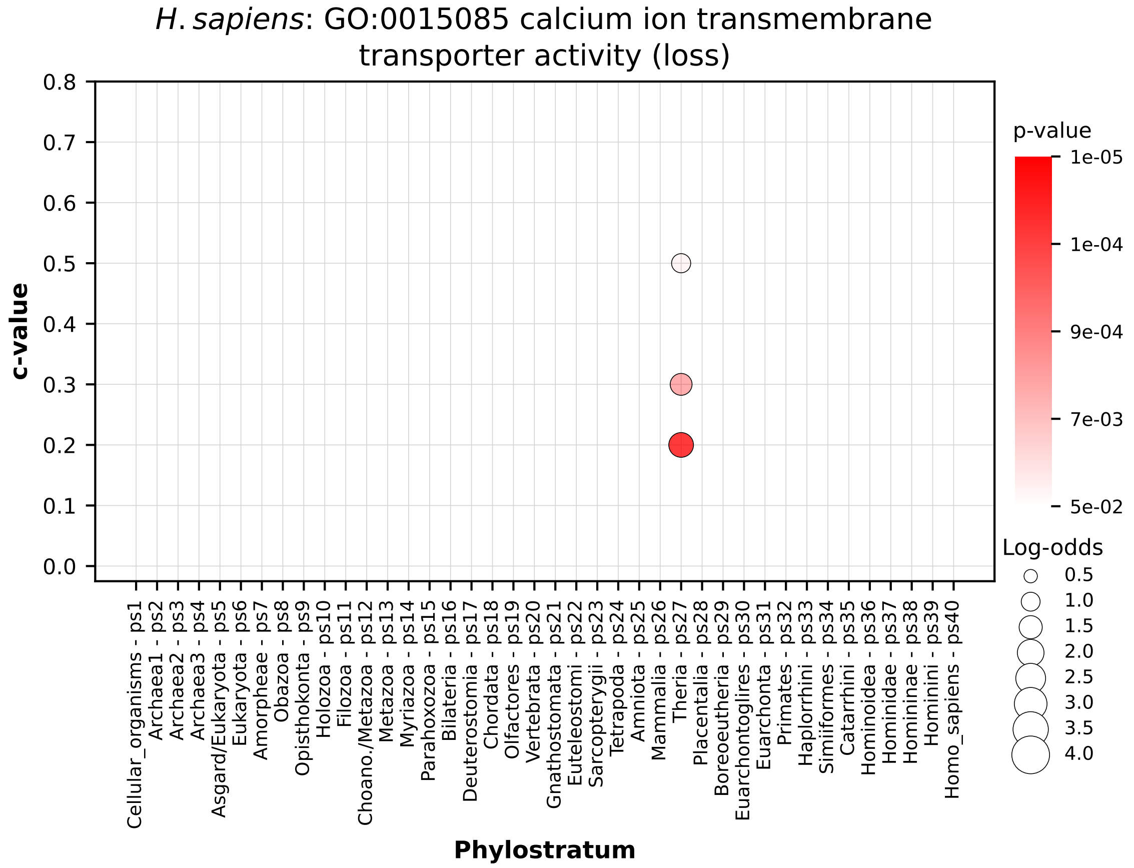

H. sapiens: GO:0015085 calcium ion transmembrane transporter activity (loss)
c-value
0.0
0.1
0.2
0.3
0.4
0.5
0.6
0.7
0.8
Phylostratum
Cellular_organisms - ps1
Archaea1 - ps2
Archaea2 - ps3
Archaea3 - ps4
Asgard/Eukaryota - ps5
Eukaryota - ps6
Amorpheae - ps7
Obazoa - ps8
Opisthokonta - ps9
Holozoa - ps10
Filozoa - ps11
Choano./Metazoa - ps12
Metazoa - ps13
Myriazoa - ps14
Parahoxozoa - ps15
Bilateria - ps16
Deuterostomia - ps17
Chordata - ps18
Olfactores - ps19
Vertebrata - ps20
Gnathostomata - ps21
Euteleostomi - ps22
Sarcopterygii - ps23
Tetrapoda - ps24
Amniota - ps25
Mammalia - ps26
Theria - ps27
Placentalia - ps28
Boreoeutheria - ps29
Euarchontoglires - ps30
Euarchonta - ps31
Primates - ps32
Haplorrhini - ps33
Simiiformes - ps34
Catarrhini - ps35
Hominoidea - ps36
Hominidae - ps37
Homininae - ps38
Hominini - ps39
Homo_sapiens - ps40
p-value
1e-05
1e-04
9e-04
7e-03
5e-02
Log-odds
0.5
1.0
1.5
2.0
2.5
3.0
3.5
4.0

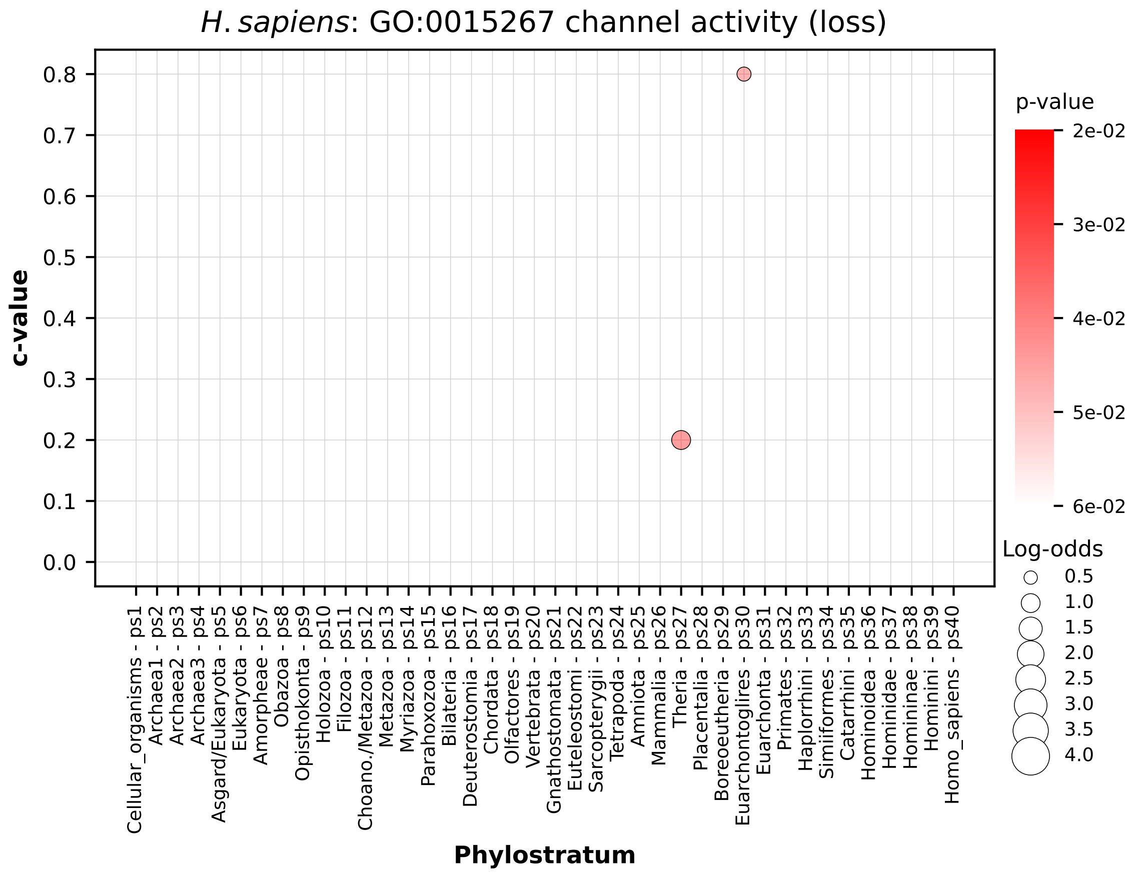

H. sapiens: GO:0015267 channel activity (loss)
c-value
0.0
0.1
0.2
0.3
0.4
0.5
0.6
0.7
0.8
Phylostratum
Cellular_organisms - ps1
Archaea1 - ps2
Archaea2 - ps3
Archaea3 - ps4
Asgard/Eukaryota - ps5
Eukaryota - ps6
Amorpheae - ps7
Obazoa - ps8
Opisthokonta - ps9
Holozoa - ps10
Filozoa - ps11
Choano./Metazoa - ps12
Metazoa - ps13
Myriazoa - ps14
Parahoxozoa - ps15
Bilateria - ps16
Deuterostomia - ps17
Chordata - ps18
Olfactores - ps19
Vertebrata - ps20
Gnathostomata - ps21
Euteleostomi - ps22
Sarcopterygii - ps23
Tetrapoda - ps24
Amniota - ps25
Mammalia - ps26
Theria - ps27
Placentalia - ps28
Boreoeutheria - ps29
Euarchontoglires - ps30
Euarchonta - ps31
Primates - ps32
Haplorrhini - ps33
Simiiformes - ps34
Catarrhini - ps35
Hominoidea - ps36
Hominidae - ps37
Homininae - ps38
Hominini - ps39
Homo_sapiens - ps40
p-value
2e-02
3e-02
4e-02
5e-02
6e-02
Log-odds
0.5
1.0
1.5
2.0
2.5
3.0
3.5
4.0

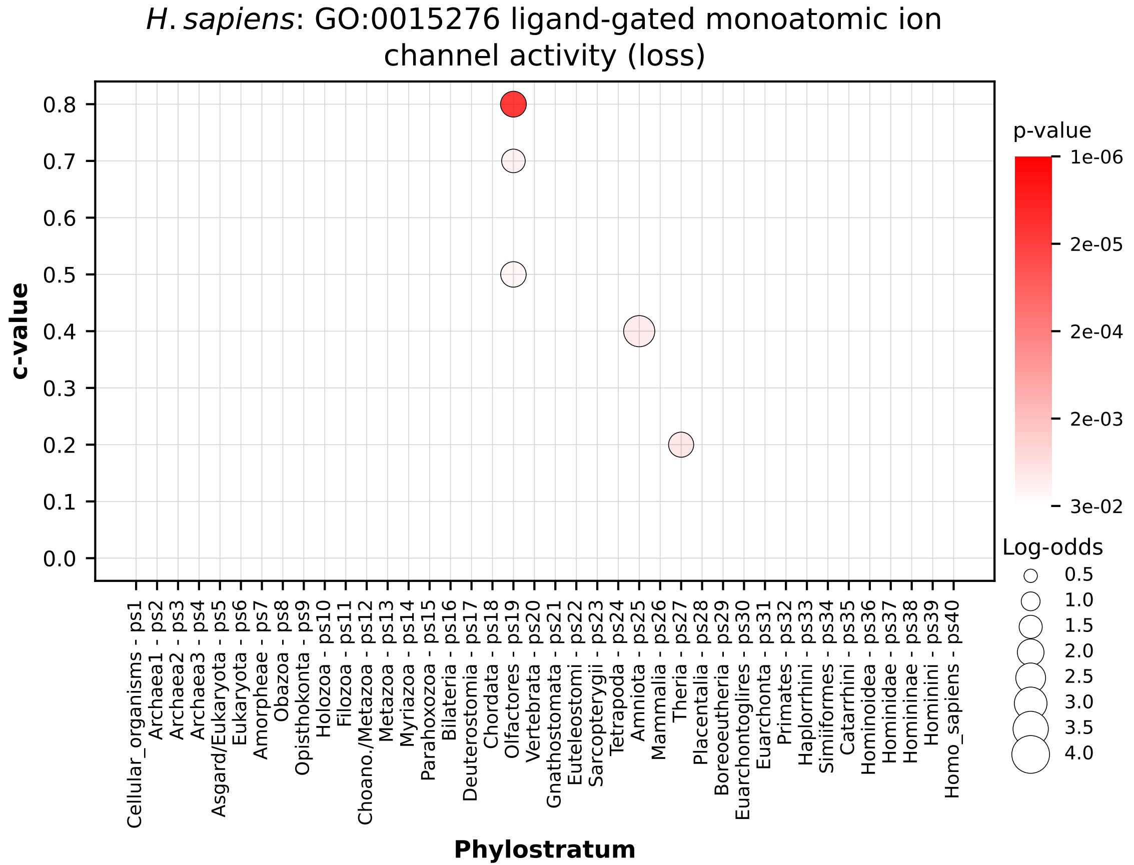

H. sapiens: GO:0015276 ligand-gated monoatomic ion channel activity (loss)
c-value
0.8
0.7
0.6
0.5
0.4
0.3
0.2
0.1
0.0
p-value
1e-06
2e-05
2e-04
2e-03
3e-02
Log-odds
0.5
1.0
1.5
2.0
2.5
3.0
3.5
4.0
Cellular_organisms - ps1
Archaea1 - ps2
Archaea2 - ps3
Archaea3 - ps4
Asgard/Eukaryota - ps5
Eukaryota - ps6
Amorpheae - ps7
Obazoa - ps8
Opisthokonta - ps9
Holozoa - ps10
Filozoa - ps11
Choano./Metazoa - ps12
Metazoa - ps13
Myriazoa - ps14
Parahoxozoa - ps15
Bilateria - ps16
Deuterostomia - ps17
Chordata - ps18
Olfactores - ps19
Vertebrata - ps20
Gnathostomata - ps21
Euteleostomi - ps22
Sarcopterygii - ps23
Tetrapoda - ps24
Amniota - ps25
Mammalia - ps26
Theria - ps27
Placentalia - ps28
Boreoeutheria - ps29
Euarchontoglires - ps30
Euarchonta - ps31
Primates - ps32
Haplorrhini - ps33
Simiiformes - ps34
Catarrhini - ps35
Hominoidea - ps36
Hominidae - ps37
Homininae - ps38
Hominini - ps39
Homo_sapiens - ps40
Phylostratum

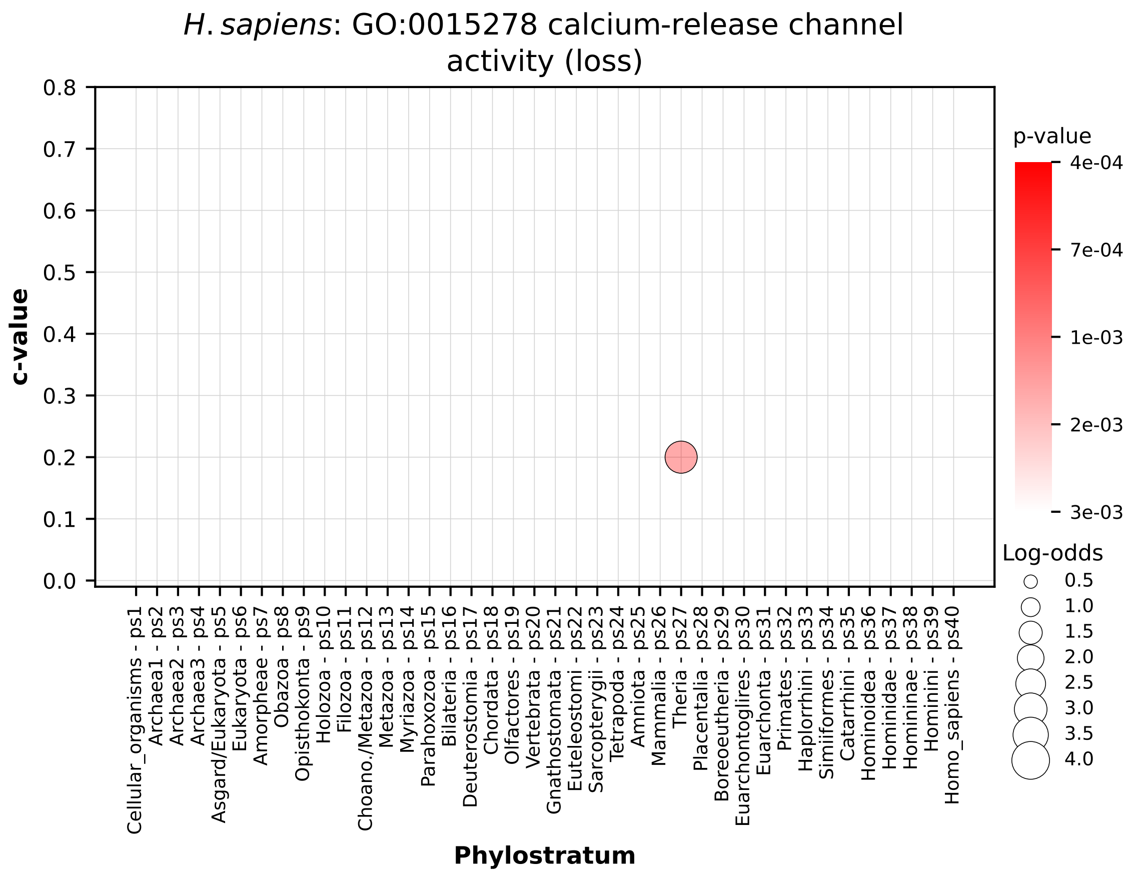

$H. sapiens$: GO:0015278 calcium-release channel activity (loss)
c-value
0.0
0.1
0.2
0.3
0.4
0.5
0.6
0.7
0.8
Phylostratum
Cellular_organisms - ps1
Archaea1 - ps2
Archaea2 - ps3
Archaea3 - ps4
Asgard/Eukaryota - ps5
Eukaryota - ps6
Amorpheae - ps7
Obazoa - ps8
Opisthokonta - ps9
Holozoa - ps10
Filozoa - ps11
Choano./Metazoa - ps12
Metazoa - ps13
Myriazoa - ps14
Parahoxozoa - ps15
Bilateria - ps16
Deuterostomia - ps17
Chordata - ps18
Olfactores - ps19
Vertebrata - ps20
Gnathostomata - ps21
Euteleostomi - ps22
Sarcopterygii - ps23
Tetrapoda - ps24
Amniota - ps25
Mammalia - ps26
Theria - ps27
Placentalia - ps28
Boreoeutheria - ps29
Euarchontoglires - ps30
Euarchonta - ps31
Primates - ps32
Haplorrhini - ps33
Simiiformes - ps34
Catarrhini - ps35
Hominoidea - ps36
Hominidae - ps37
Homininae - ps38
Hominini - ps39
Homo_sapiens - ps40
p-value
4e-04
7e-04
1e-03
2e-03
3e-03
Log-odds
0.5
1.0
1.5
2.0
2.5
3.0
3.5
4.0

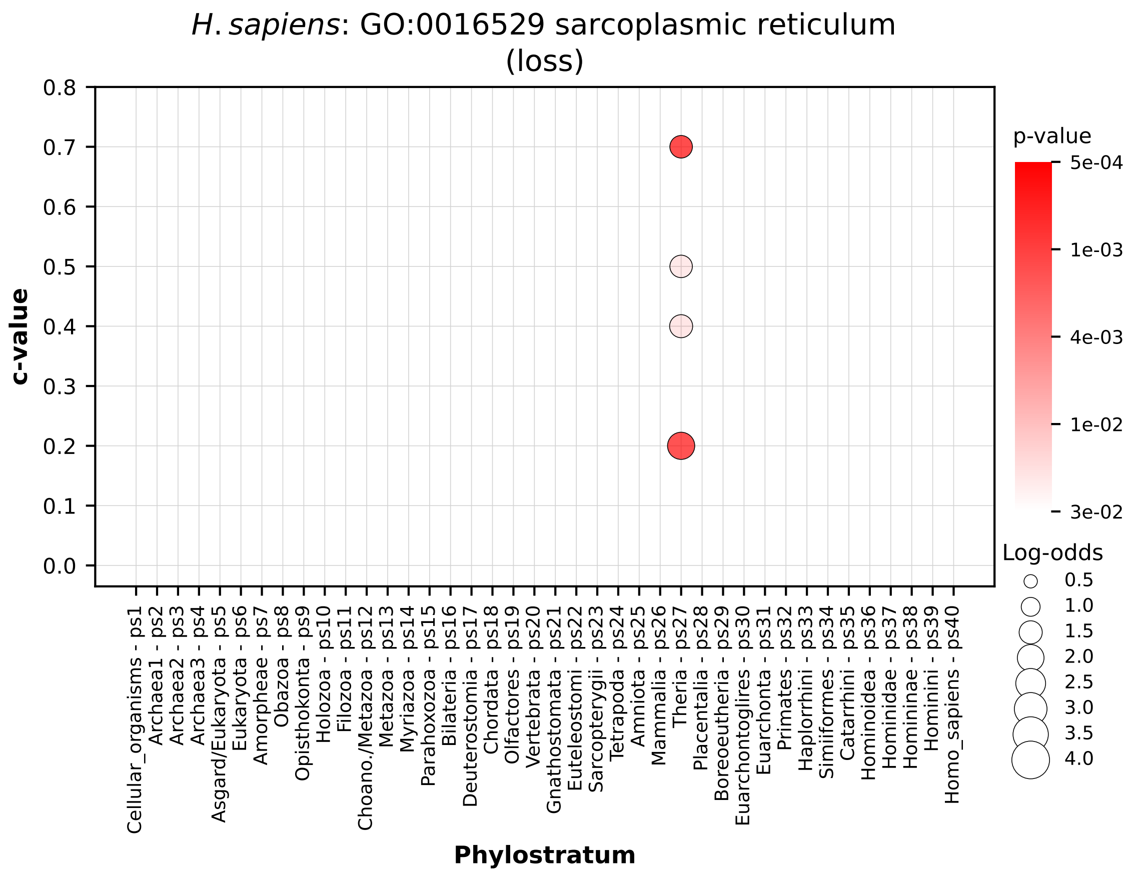

H. sapiens: GO:0016529 sarcoplasmic reticulum
(loss)
c-value
0.8
0.7
0.6
0.5
0.4
0.3
0.2
0.1
0.0
p-value
5e-04
1e-03
4e-03
1e-02
3e-02
Log-odds
0.5
1.0
1.5
2.0
2.5
3.0
3.5
4.0
Cellular_organisms - ps1
Archaea1 - ps2
Archaea2 - ps3
Archaea3 - ps4
Asgard/Eukaryota - ps5
Eukaryota - ps6
Amorpheae - ps7
Obazoa - ps8
Opisthokonta - ps9
Holozoa - ps10
Filozoa - ps11
Choano./Metazoa - ps12
Metazoa - ps13
Myriazoa - ps14
Parahoxozoa - ps15
Bilateria - ps16
Deuterostomia - ps17
Chordata - ps18
Olfactores - ps19
Vertebrata - ps20
Gnathostomata - ps21
Euteleostomi - ps22
Sarcopterygii - ps23
Tetrapoda - ps24
Amniota - ps25
Mammalia - ps26
Theria - ps27
Placentalia - ps28
Boreoeutheria - ps29
Euarchontoglires - ps30
Euarchonta - ps31
Primates - ps32
Haplorrhini - ps33
Simiiformes - ps34
Catarrhini - ps35
Hominoidea - ps36
Hominidae - ps37
Homininae - ps38
Hominini - ps39
Homo_sapiens - ps40
Phylostratum

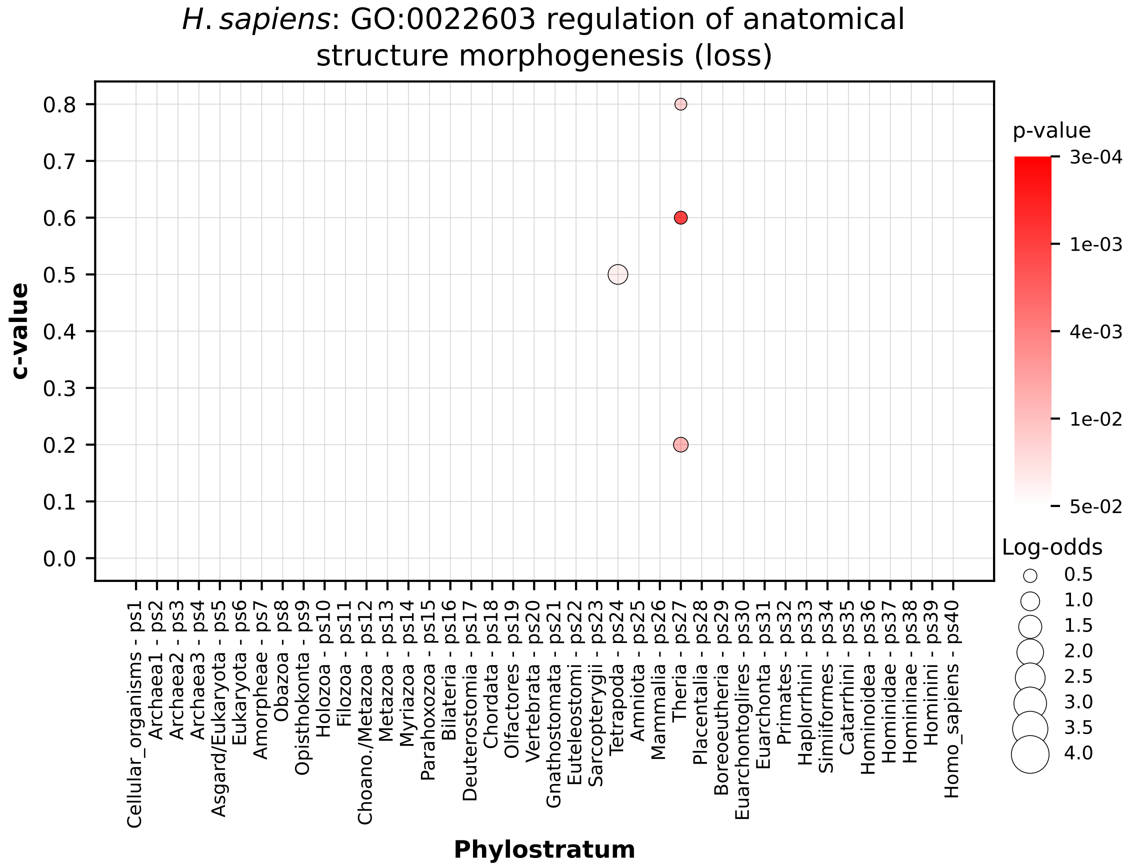

H. sapiens: GO:0022603 regulation of anatomical structure morphogenesis (loss)
c-value
0.8
0.7
0.6
0.5
0.4
0.3
0.2
0.1
0.0
Phylostratum
Cellular_organisms - ps1
Archaea1 - ps2
Archaea2 - ps3
Archaea3 - ps4
Asgard/Eukaryota - ps5
Eukaryota - ps6
Amorpheae - ps7
Obazoa - ps8
Opisthokonta - ps9
Holozoa - ps10
Filozoa - ps11
Choano./Metazoa - ps12
Metazoa - ps13
Myriazoa - ps14
Parahoxozoa - ps15
Bilateria - ps16
Deuterostomia - ps17
Chordata - ps18
Olfactores - ps19
Vertebrata - ps20
Gnathostomata - ps21
Euteleostomi - ps22
Sarcopterygii - ps23
Tetrapoda - ps24
Amniota - ps25
Mammalia - ps26
Theria - ps27
Placentalia - ps28
Boreoeutheria - ps29
Euarchontoglires - ps30
Euarchonta - ps31
Primates - ps32
Haplorrhini - ps33
Simiiformes - ps34
Catarrhini - ps35
Hominoidea - ps36
Hominidae - ps37
Homininae - ps38
Hominini - ps39
Homo_sapiens - ps40
p-value
3e-04
1e-03
4e-03
1e-02
5e-02
Log-odds
0.5
1.0
1.5
2.0
2.5
3.0
3.5
4.0

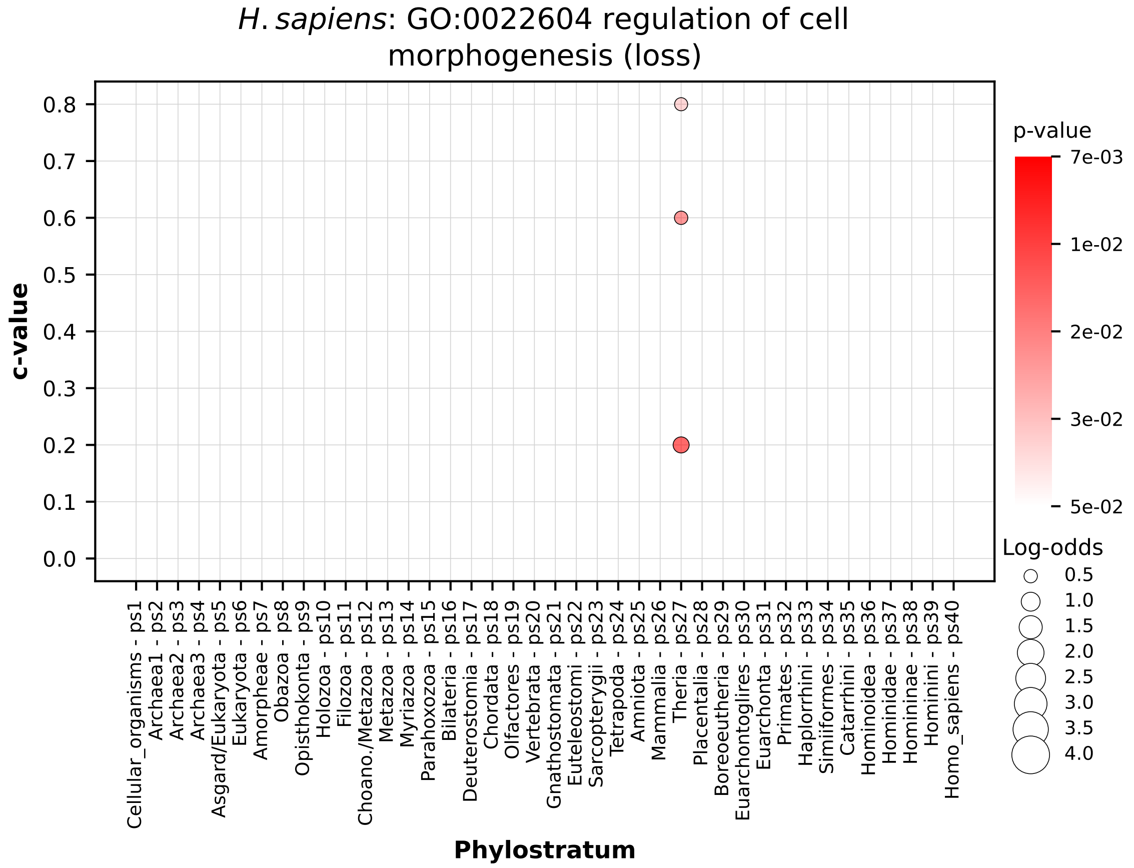

H. sapiens: GO:0022604 regulation of cell morphogenesis (loss)
c-value
0.0
0.1
0.2
0.3
0.4
0.5
0.6
0.7
0.8
Phylostratum
Cellular_organisms - ps1
Archaea1 - ps2
Archaea2 - ps3
Archaea3 - ps4
Asgard/Eukaryota - ps5
Eukaryota - ps6
Amorpheae - ps7
Obazoa - ps8
Opisthokonta - ps9
Holozoa - ps10
Filozoa - ps11
Choano./Metazoa - ps12
Metazoa - ps13
Myriazoa - ps14
Parahoxozoa - ps15
Bilateria - ps16
Deuterostomia - ps17
Chordata - ps18
Olfactores - ps19
Vertebrata - ps20
Gnathostomata - ps21
Euteleostomi - ps22
Sarcopterygii - ps23
Tetrapoda - ps24
Amniota - ps25
Mammalia - ps26
Theria - ps27
Placentalia - ps28
Boreoeutheria - ps29
Euarchontoglires - ps30
Euarchonta - ps31
Primates - ps32
Haplorrhini - ps33
Simiiformes - ps34
Catarrhini - ps35
Hominoidea - ps36
Hominidae - ps37
Homininae - ps38
Hominini - ps39
Homo_sapiens - ps40
p-value
7e-03
1e-02
2e-02
3e-02
5e-02
Log-odds
0.5
1.0
1.5
2.0
2.5
3.0
3.5
4.0

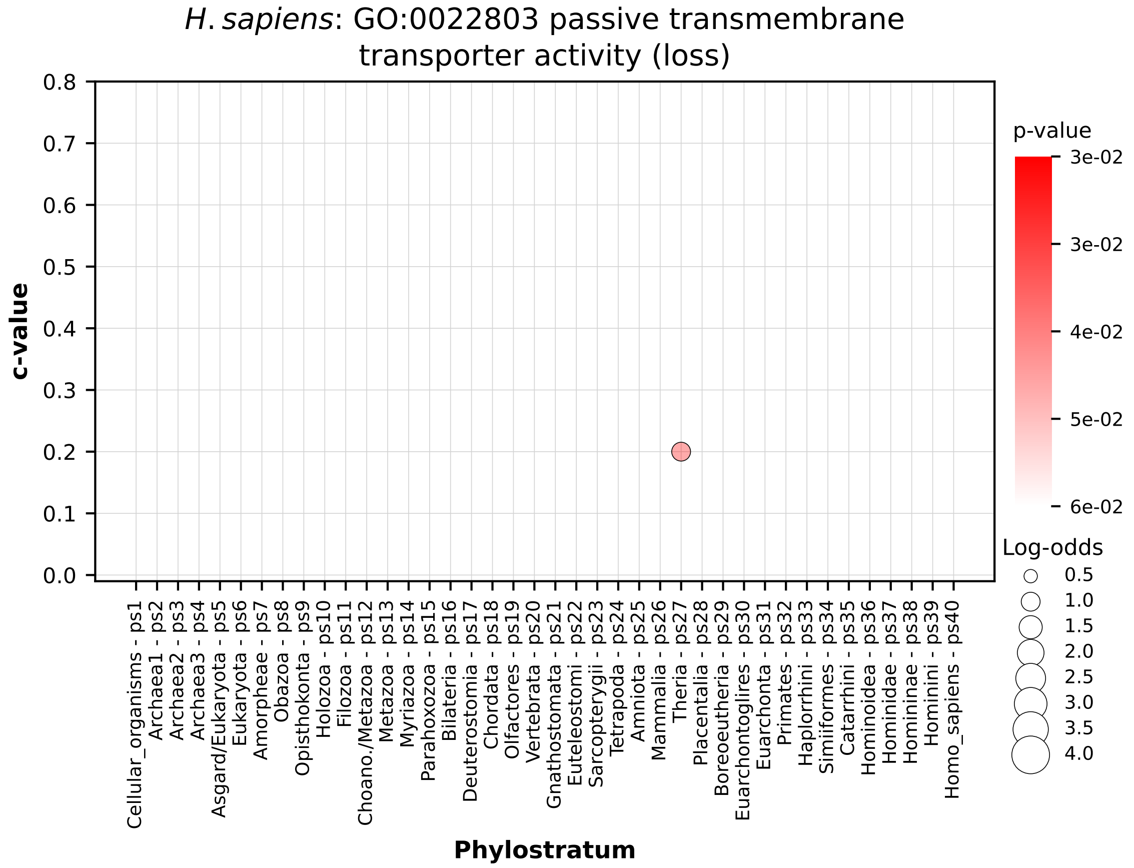

H. sapiens: GO:0022803 passive transmembrane transporter activity (loss)
c-value
0.8
0.7
0.6
0.5
0.4
0.3
0.2
0.1
0.0
p-value
3e-02
3e-02
4e-02
5e-02
6e-02
Log-odds
0.5
1.0
1.5
2.0
2.5
3.0
3.5
4.0
Cellular_organisms - ps1
Archaea1 - ps2
Archaea2 - ps3
Archaea3 - ps4
Asgard/Eukaryota - ps5
Eukaryota - ps6
Amorpheae - ps7
Obazoa - ps8
Opisthokonta - ps9
Holozoa - ps10
Filozoa - ps11
Choano./Metazoa - ps12
Metazoa - ps13
Myriazoa - ps14
Parahoxozoa - ps15
Bilateria - ps16
Deuterostomia - ps17
Chordata - ps18
Olfactores - ps19
Vertebrata - ps20
Gnathostomata - ps21
Euteleostomi - ps22
Sarcopterygii - ps23
Tetrapoda - ps24
Amniota - ps25
Mammalia - ps26
Theria - ps27
Placentalia - ps28
Boreoeutheria - ps29
Euarchontoglires - ps30
Euarchonta - ps31
Primates - ps32
Haplorrhini - ps33
Simiiformes - ps34
Catarrhini - ps35
Hominoidea - ps36
Hominidae - ps37
Homininae - ps38
Hominini - ps39
Homo_sapiens - ps40
Phylostratum

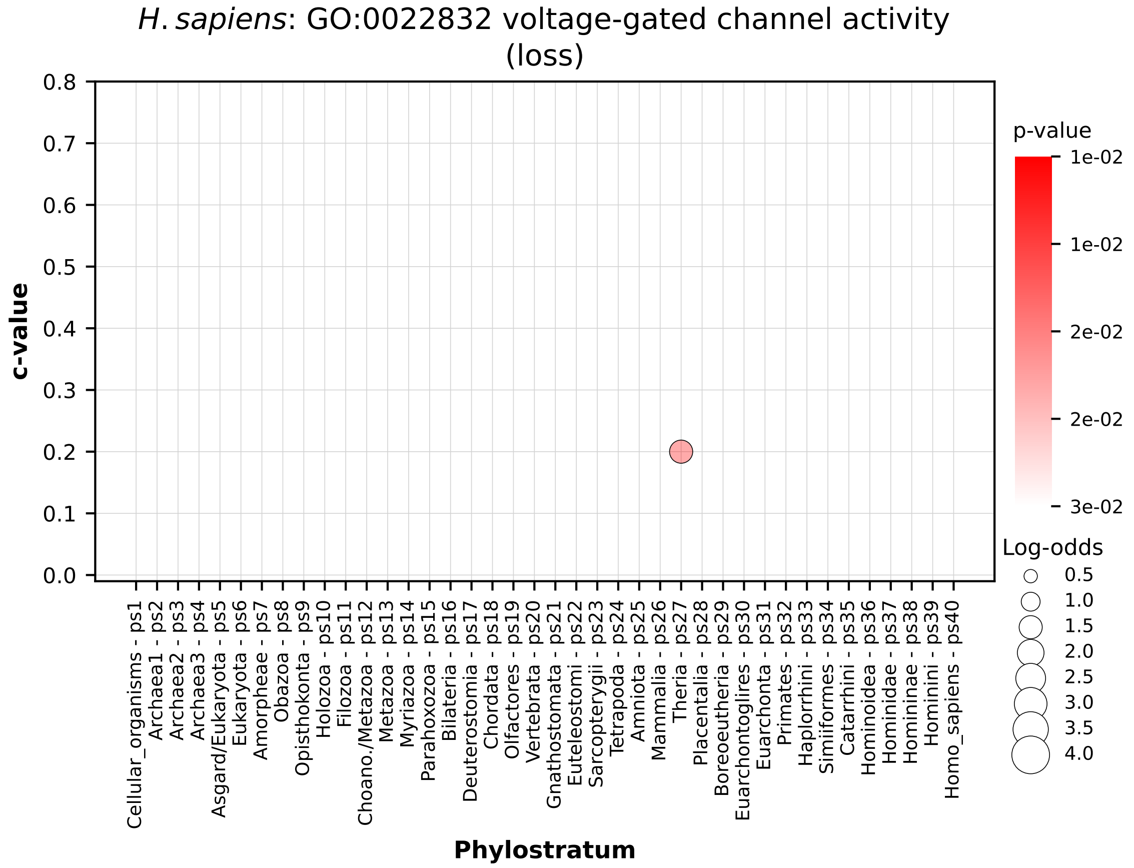

H. sapiens: GO:0022832 voltage-gated channel activity
(loss)
c-value
0.0
0.1
0.2
0.3
0.4
0.5
0.6
0.7
0.8
Phylostratum
Cellular_organisms - ps1
Archaea1 - ps2
Archaea2 - ps3
Archaea3 - ps4
Asgard/Eukaryota - ps5
Eukaryota - ps6
Amorpheae - ps7
Obazoa - ps8
Opisthokonta - ps9
Holozoa - ps10
Filozoa - ps11
Choano./Metazoa - ps12
Metazoa - ps13
Myriazoa - ps14
Parahoxozoa - ps15
Bilateria - ps16
Deuterostomia - ps17
Chordata - ps18
Olfactores - ps19
Vertebrata - ps20
Gnathostomata - ps21
Euteleostomi - ps22
Sarcopterygii - ps23
Tetrapoda - ps24
Amniota - ps25
Mammalia - ps26
Theria - ps27
Placentalia - ps28
Boreoeutheria - ps29
Euarchontoglires - ps30
Euarchonta - ps31
Primates - ps32
Haplorrhini - ps33
Simiiformes - ps34
Catarrhini - ps35
Hominoidea - ps36
Hominidae - ps37
Homininae - ps38
Hominini - ps39
Homo_sapiens - ps40
p-value
1e-02
1e-02
2e-02
2e-02
3e-02
Log-odds
0.5
1.0
1.5
2.0
2.5
3.0
3.5
4.0

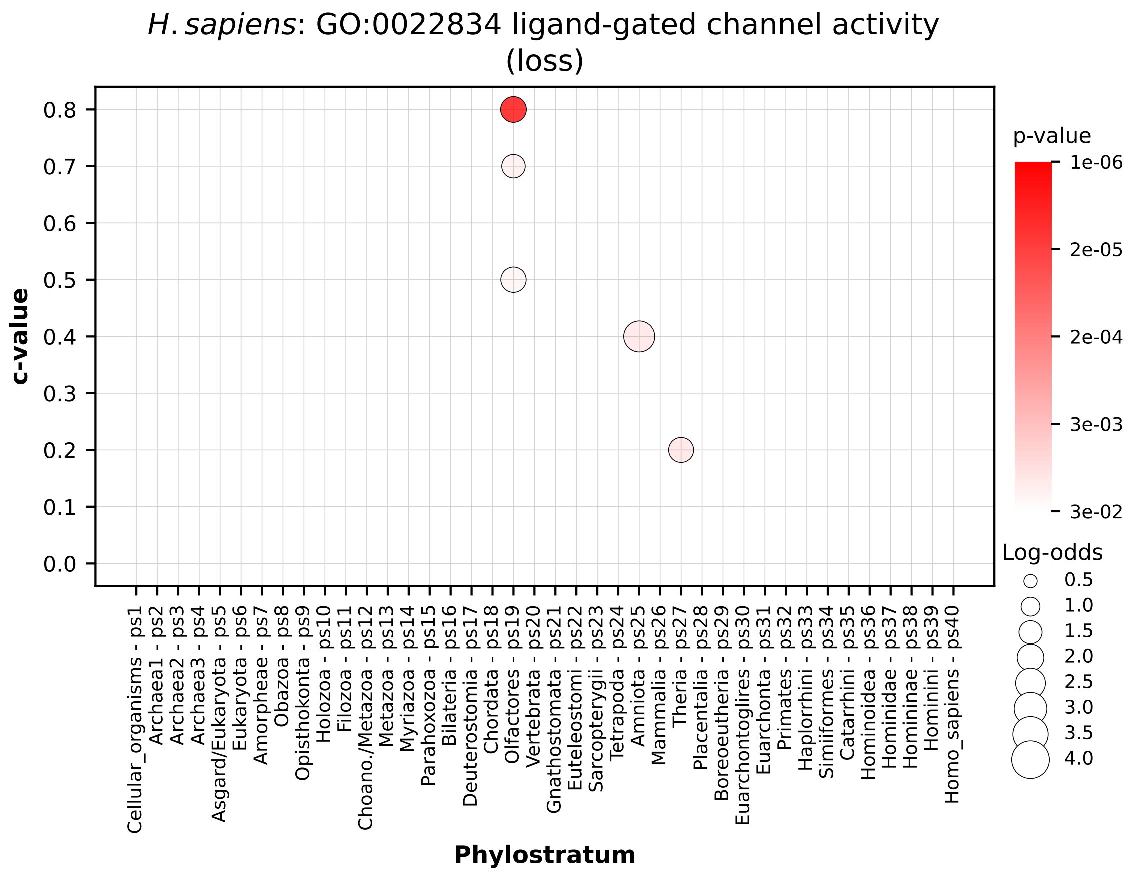

H. sapiens: GO:0022834 ligand-gated channel activity (loss)
c-value
0.0
0.1
0.2
0.3
0.4
0.5
0.6
0.7
0.8
p-value
1e-06
2e-05
2e-04
3e-03
3e-02
Log-odds
0.5
1.0
1.5
2.0
2.5
3.0
3.5
4.0
Cellular_organisms - ps1
Archaea1 - ps2
Archaea2 - ps3
Archaea3 - ps4
Asgard/Eukaryota - ps5
Eukaryota - ps6
Amorpheae - ps7
Obazoa - ps8
Opisthokonta - ps9
Holozoa - ps10
Filozoa - ps11
Choano./Metazoa - ps12
Metazoa - ps13
Myriazoa - ps14
Parahoxozoa - ps15
Bilateria - ps16
Deuterostomia - ps17
Chordata - ps18
Olfactores - ps19
Vertebrata - ps20
Gnathostomata - ps21
Euteleostomi - ps22
Sarcopterygii - ps23
Tetrapoda - ps24
Amniota - ps25
Mammalia - ps26
Theria - ps27
Placentalia - ps28
Boreoeutheria - ps29
Euarchontoglires - ps30
Euarchonta - ps31
Primates - ps32
Haplorrhini - ps33
Simiiformes - ps34
Catarrhini - ps35
Hominoidea - ps36
Hominidae - ps37
Homininae - ps38
Hominini - ps39
Homo_sapiens - ps40
Phylostratum

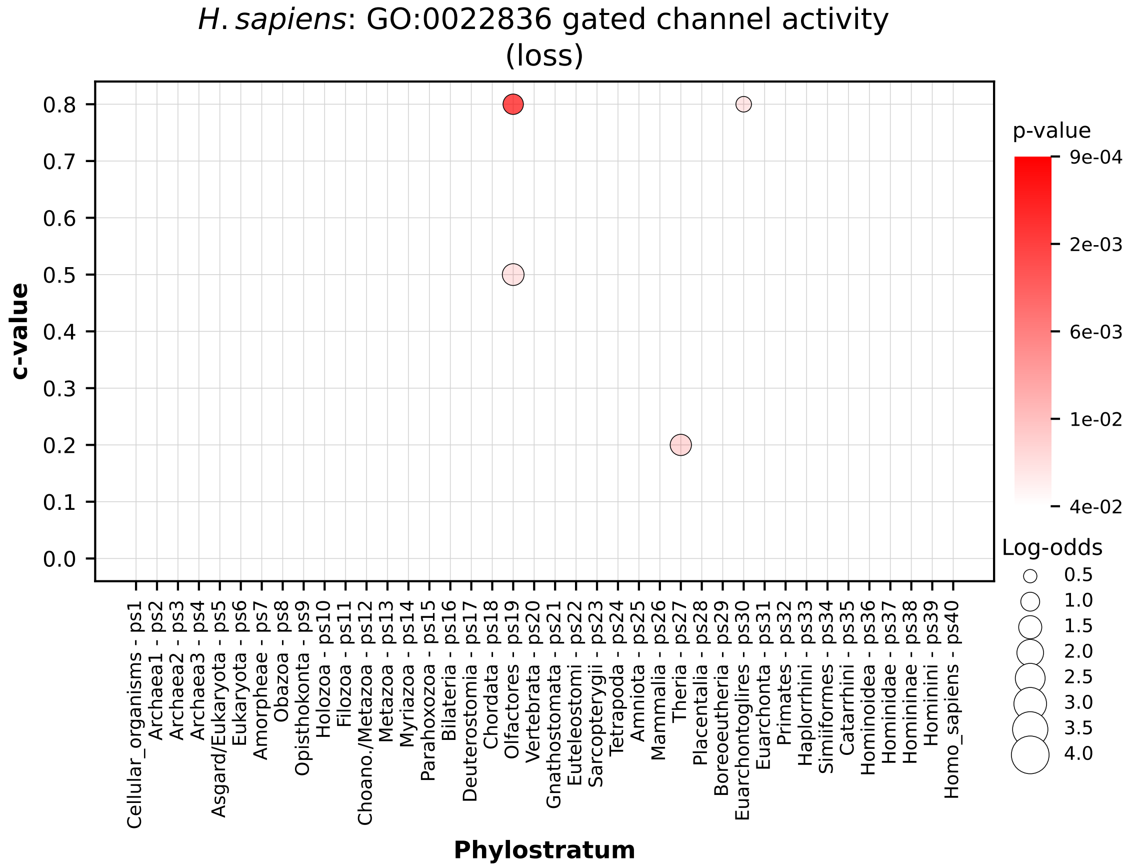

H. sapiens: GO:0022836 gated channel activity
(loss)
c-value
0.0
0.1
0.2
0.3
0.4
0.5
0.6
0.7
0.8
p-value
9e-04
2e-03
6e-03
1e-02
4e-02
Log-odds
0.5
1.0
1.5
2.0
2.5
3.0
3.5
4.0
Cellular_organisms - ps1
Archaea1 - ps2
Archaea2 - ps3
Archaea3 - ps4
Asgard/Eukaryota - ps5
Eukaryota - ps6
Amorpheae - ps7
Obazoa - ps8
Opisthokonta - ps9
Holozoa - ps10
Filozoa - ps11
Choano./Metazoa - ps12
Metazoa - ps13
Myriazoa - ps14
Parahoxozoa - ps15
Bilateria - ps16
Deuterostomia - ps17
Chordata - ps18
Olfactores - ps19
Vertebrata - ps20
Gnathostomata - ps21
Euteleostomi - ps22
Sarcopterygii - ps23
Tetrapoda - ps24
Amniota - ps25
Mammalia - ps26
Theria - ps27
Placentalia - ps28
Boreoeutheria - ps29
Euarchontoglires - ps30
Euarchonta - ps31
Primates - ps32
Haplorrhini - ps33
Simiiformes - ps34
Catarrhini - ps35
Hominoidea - ps36
Hominidae - ps37
Homininae - ps38
Hominini - ps39
Homo_sapiens - ps40
Phylostratum

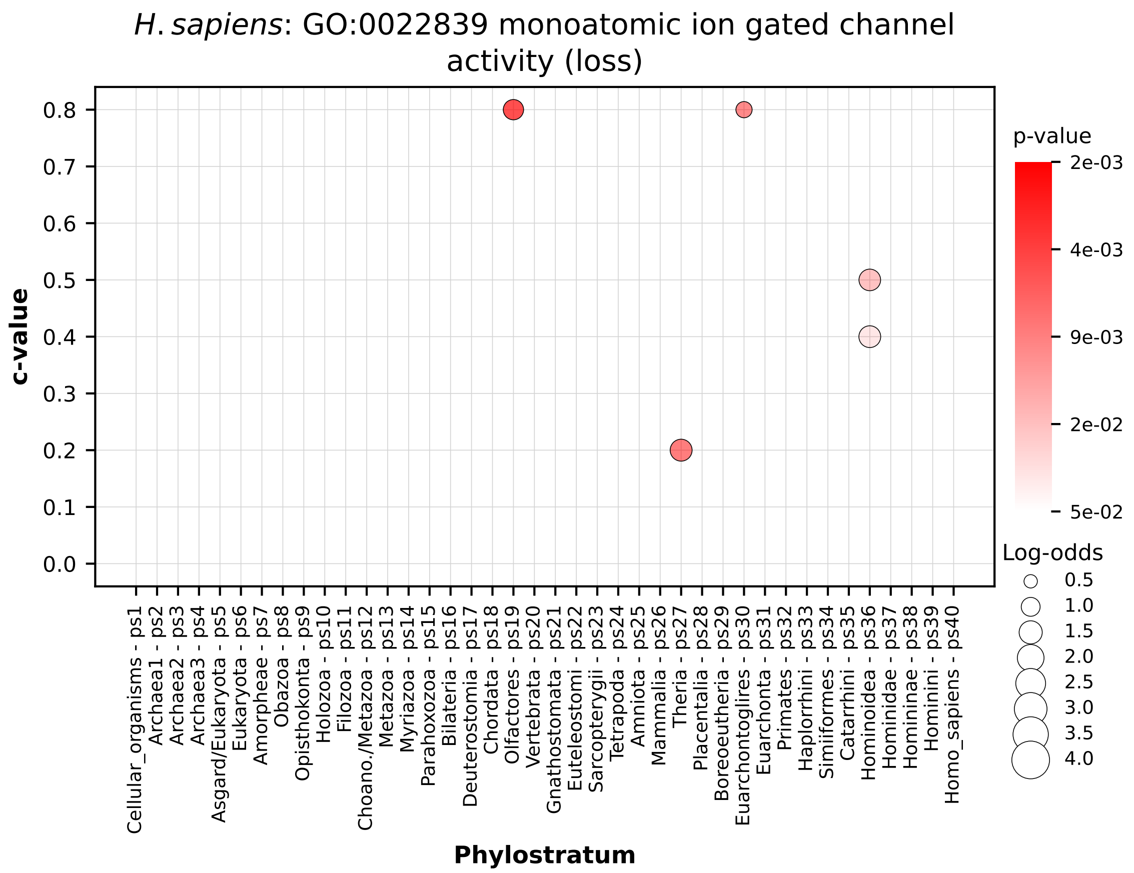

H. sapiens: GO:0022839 monoatomic ion gated channel activity (loss)
c-value
0.8
0.7
0.6
0.5
0.4
0.3
0.2
0.1
0.0
p-value
2e-03
4e-03
9e-03
2e-02
5e-02
Log-odds
0.5
1.0
1.5
2.0
2.5
3.0
3.5
4.0
Cellular_organisms - ps1
Archaea1 - ps2
Archaea2 - ps3
Archaea3 - ps4
Asgard/Eukaryota - ps5
Eukaryota - ps6
Amorpheae - ps7
Obazoa - ps8
Opisthokonta - ps9
Holozoa - ps10
Filozoa - ps11
Choano./Metazoa - ps12
Metazoa - ps13
Myriazoa - ps14
Parahoxozoa - ps15
Bilateria - ps16
Deuterostomia - ps17
Chordata - ps18
Olfactores - ps19
Vertebrata - ps20
Gnathostomata - ps21
Euteleostomi - ps22
Sarcopterygii - ps23
Tetrapoda - ps24
Amniota - ps25
Mammalia - ps26
Theria - ps27
Placentalia - ps28
Boreoeutheria - ps29
Euarchontoglires - ps30
Euarchonta - ps31
Primates - ps32
Haplorrhini - ps33
Simiiformes - ps34
Catarrhini - ps35
Hominoidea - ps36
Hominidae - ps37
Homininae - ps38
Hominini - ps39
Homo_sapiens - ps40
Phylostratum

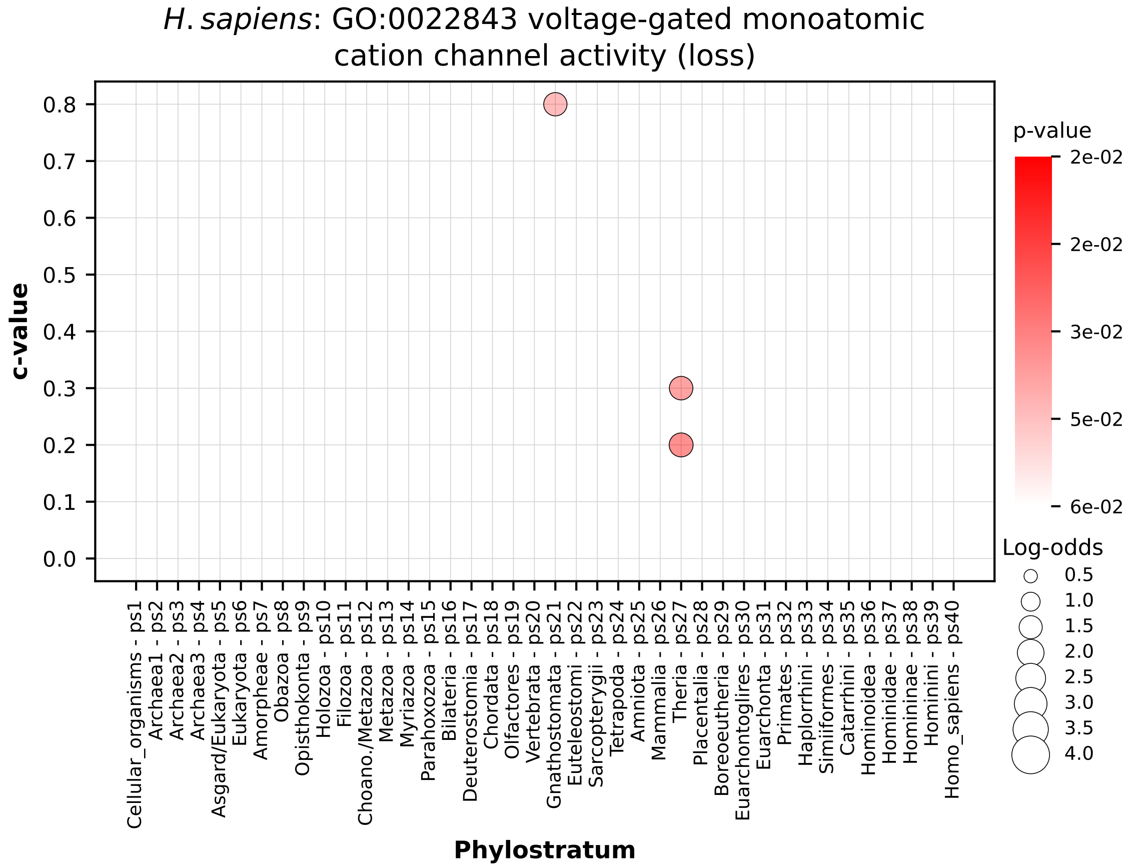

H. sapiens: GO:0022843 voltage-gated monoatomic cation channel activity (loss)
c-value
0.0
0.1
0.2
0.3
0.4
0.5
0.6
0.7
0.8
p-value
2e-02
2e-02
3e-02
5e-02
6e-02
Log-odds
0.5
1.0
1.5
2.0
2.5
3.0
3.5
4.0
Cellular_organisms - ps1
Archaea1 - ps2
Archaea2 - ps3
Archaea3 - ps4
Asgard/Eukaryota - ps5
Eukaryota - ps6
Amorpheae - ps7
Obazoa - ps8
Opisthokonta - ps9
Holozoa - ps10
Filozoa - ps11
Choano./Metazoa - ps12
Metazoa - ps13
Myriazoa - ps14
Parahoxozoa - ps15
Bilateria - ps16
Deuterostomia - ps17
Chordata - ps18
Olfactores - ps19
Vertebrata - ps20
Gnathostomata - ps21
Euteleostomi - ps22
Sarcopterygii - ps23
Tetrapoda - ps24
Amniota - ps25
Mammalia - ps26
Theria - ps27
Placentalia - ps28
Boreoeutheria - ps29
Euarchontoglires - ps30
Euarchonta - ps31
Primates - ps32
Haplorrhini - ps33
Simiiformes - ps34
Catarrhini - ps35
Hominoidea - ps36
Hominidae - ps37
Homininae - ps38
Hominini - ps39
Homo_sapiens - ps40
Phylostratum

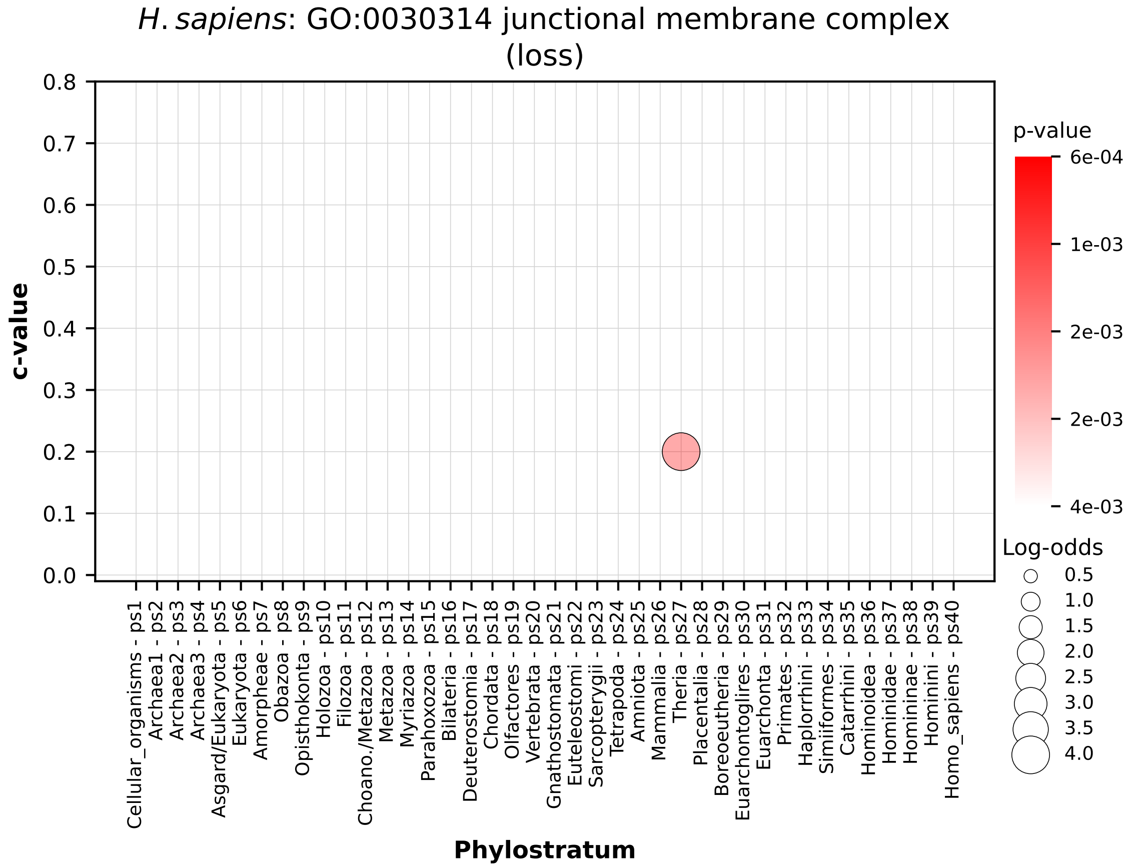

H. sapiens: GO:0030314 junctional membrane complex
(loss)
c-value
0.0
0.1
0.2
0.3
0.4
0.5
0.6
0.7
0.8
Phylostratum
Cellular_organisms - ps1
Archaea1 - ps2
Archaea2 - ps3
Archaea3 - ps4
Asgard/Eukaryota - ps5
Eukaryota - ps6
Amorpheae - ps7
Obazoa - ps8
Opisthokonta - ps9
Holozoa - ps10
Filozoa - ps11
Choano./Metazoa - ps12
Metazoa - ps13
Myriazoa - ps14
Parahoxozoa - ps15
Bilateria - ps16
Deuterostomia - ps17
Chordata - ps18
Olfactores - ps19
Vertebrata - ps20
Gnathostomata - ps21
Euteleostomi - ps22
Sarcopterygii - ps23
Tetrapoda - ps24
Amniota - ps25
Mammalia - ps26
Theria - ps27
Placentalia - ps28
Boreoeutheria - ps29
Euarchontoglires - ps30
Euarchonta - ps31
Primates - ps32
Haplorrhini - ps33
Simiiformes - ps34
Catarrhini - ps35
Hominoidea - ps36
Hominidae - ps37
Homininae - ps38
Hominini - ps39
Homo_sapiens - ps40
p-value
6e-04
1e-03
2e-03
2e-03
4e-03
Log-odds
0.5
1.0
1.5
2.0
2.5
3.0
3.5
4.0

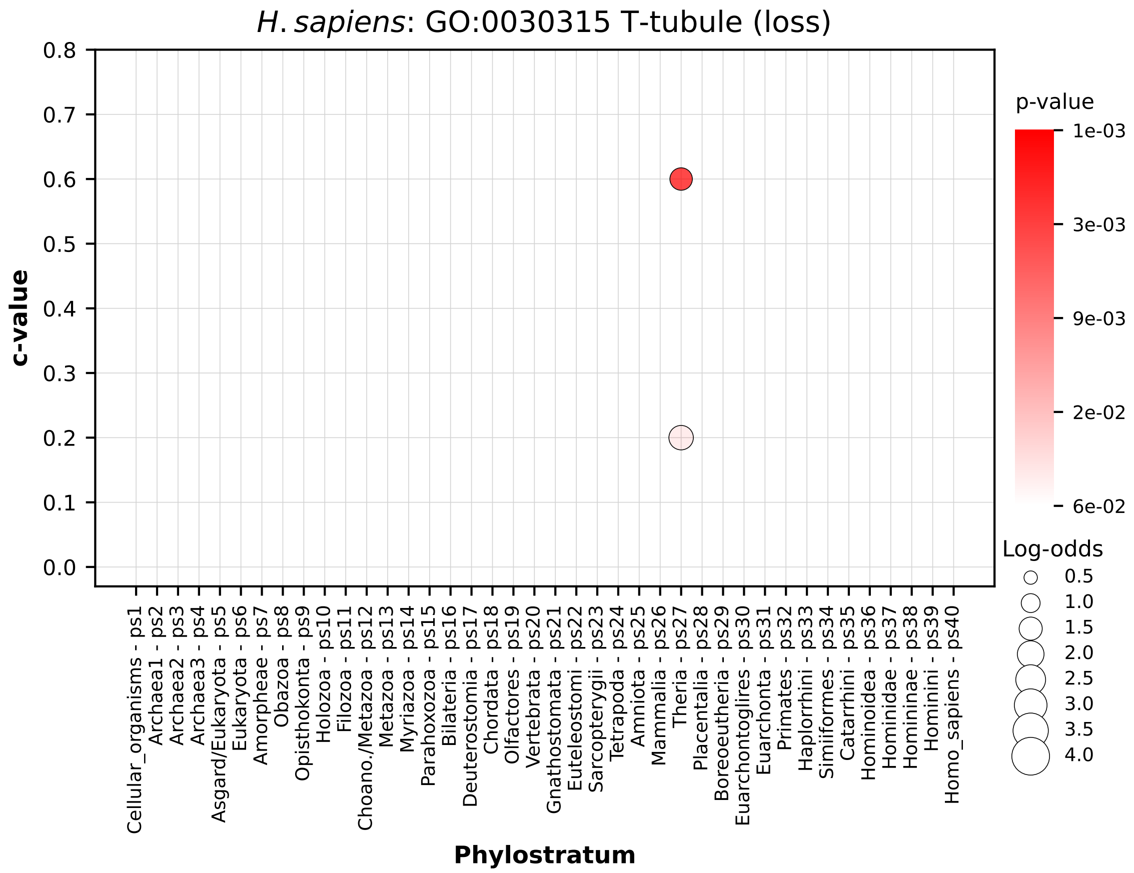

H. sapiens: GO:0030315 T-tubule (loss)
c-value
0.8
0.7
0.6
0.5
0.4
0.3
0.2
0.1
0.0
Phylostratum
Cellular_organisms - ps1
Archaea1 - ps2
Archaea2 - ps3
Archaea3 - ps4
Asgard/Eukaryota - ps5
Eukaryota - ps6
Amorpheae - ps7
Obazoa - ps8
Opisthokonta - ps9
Holozoa - ps10
Filozoa - ps11
Choano./Metazoa - ps12
Metazoa - ps13
Myriazoa - ps14
Parahoxozoa - ps15
Bilateria - ps16
Deuterostomia - ps17
Chordata - ps18
Olfactores - ps19
Vertebrata - ps20
Gnathostomata - ps21
Euteleostomi - ps22
Sarcopterygii - ps23
Tetrapoda - ps24
Amniota - ps25
Mammalia - ps26
Theria - ps27
Placentalia - ps28
Boreoeutheria - ps29
Euarchontoglires - ps30
Euarchonta - ps31
Primates - ps32
Haplorrhini - ps33
Simiiformes - ps34
Catarrhini - ps35
Hominoidea - ps36
Hominidae - ps37
Homininae - ps38
Hominini - ps39
Homo_sapiens - ps40
p-value
1e-03
3e-03
9e-03
2e-02
6e-02
Log-odds
0.5
1.0
1.5
2.0
2.5
3.0
3.5
4.0

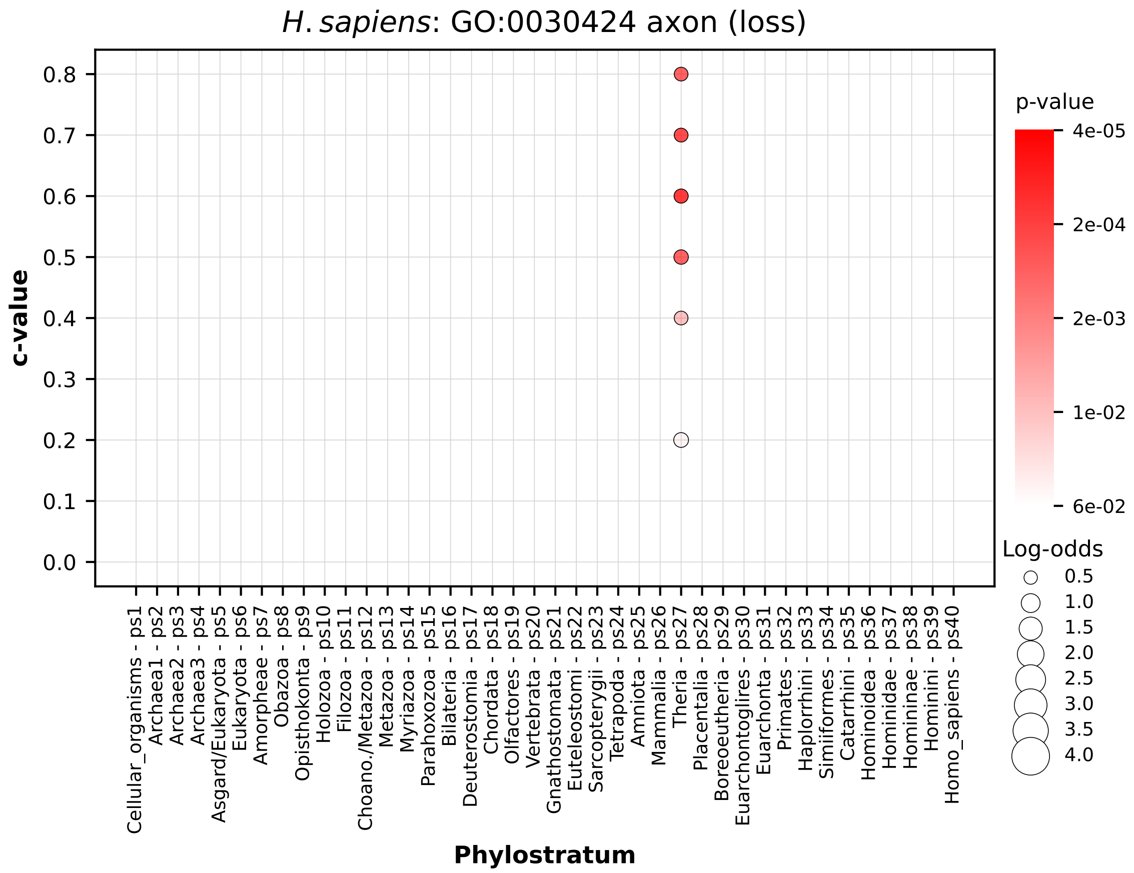

H. sapiens: GO:0030424 axon (loss)
c-value
0.8
0.7
0.6
0.5
0.4
0.3
0.2
0.1
0.0
p-value
4e-05
2e-04
2e-03
1e-02
6e-02
Log-odds
0.5
1.0
1.5
2.0
2.5
3.0
3.5
4.0
Cellular_organisms - ps1
Archaea1 - ps2
Archaea2 - ps3
Archaea3 - ps4
Asgard/Eukaryota - ps5
Eukaryota - ps6
Amorpheae - ps7
Obazoa - ps8
Opisthokonta - ps9
Holozoa - ps10
Filozoa - ps11
Choano./Metazoa - ps12
Metazoa - ps13
Myriazoa - ps14
Parahoxozoa - ps15
Bilateria - ps16
Deuterostomia - ps17
Chordata - ps18
Olfactores - ps19
Vertebrata - ps20
Gnathostomata - ps21
Euteleostomi - ps22
Sarcopterygii - ps23
Tetrapoda - ps24
Amniota - ps25
Mammalia - ps26
Theria - ps27
Placentalia - ps28
Boreoeutheria - ps29
Euarchontoglires - ps30
Euarchonta - ps31
Primates - ps32
Haplorrhini - ps33
Simiiformes - ps34
Catarrhini - ps35
Hominoidea - ps36
Hominidae - ps37
Homininae - ps38
Hominini - ps39
Homo_sapiens - ps40
Phylostratum

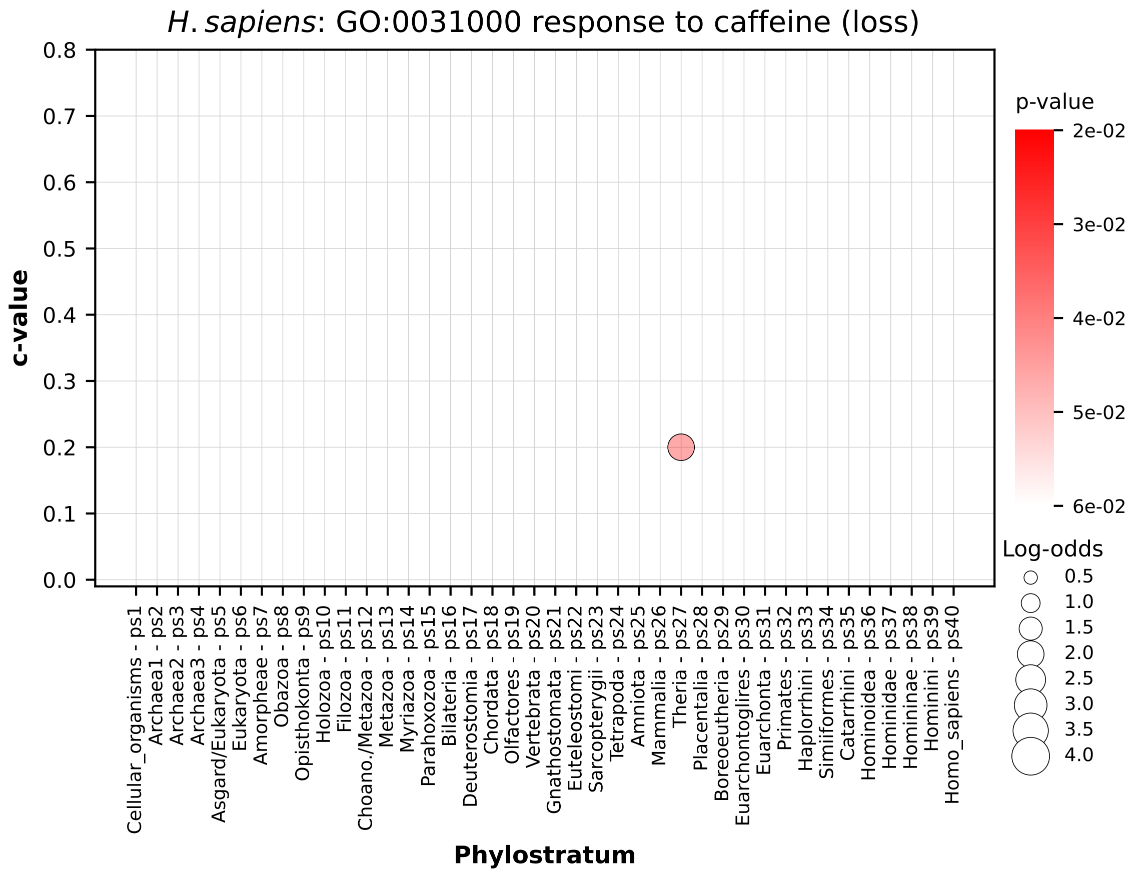
H. sapiens: GO:0031000 response to caffeine (loss)
c-value
0.8
0.7
0.6
0.5
0.4
0.3
0.2
0.1
0.0
p-value
2e-02
3e-02
4e-02
5e-02
6e-02
Log-odds
0.5
1.0
1.5
2.0
2.5
3.0
3.5
4.0
Cellular_organisms - ps1
Archaea1 - ps2
Archaea2 - ps3
Archaea3 - ps4
Asgard/Eukaryota - ps5
Eukaryota - ps6
Amorpheae - ps7
Obazoa - ps8
Opisthokonta - ps9
Holozoa - ps10
Filozoa - ps11
Choano./Metazoa - ps12
Metazoa - ps13
Myriazoa - ps14
Parahoxozoa - ps15
Bilateria - ps16
Deuterostomia - ps17
Chordata - ps18
Olfactores - ps19
Vertebrata - ps20
Gnathostomata - ps21
Euteleostomi - ps22
Sarcopterygii - ps23
Tetrapoda - ps24
Amniota - ps25
Mammalia - ps26
Theria - ps27
Placentalia - ps28
Boreoeutheria - ps29
Euarchontoglires - ps30
Euarchonta - ps31
Primates - ps32
Haplorrhini - ps33
Simiiformes - ps34
Catarrhini - ps35
Hominoidea - ps36
Hominidae - ps37
Homininae - ps38
Hominini - ps39
Homo_sapiens - ps40
Phylostratum

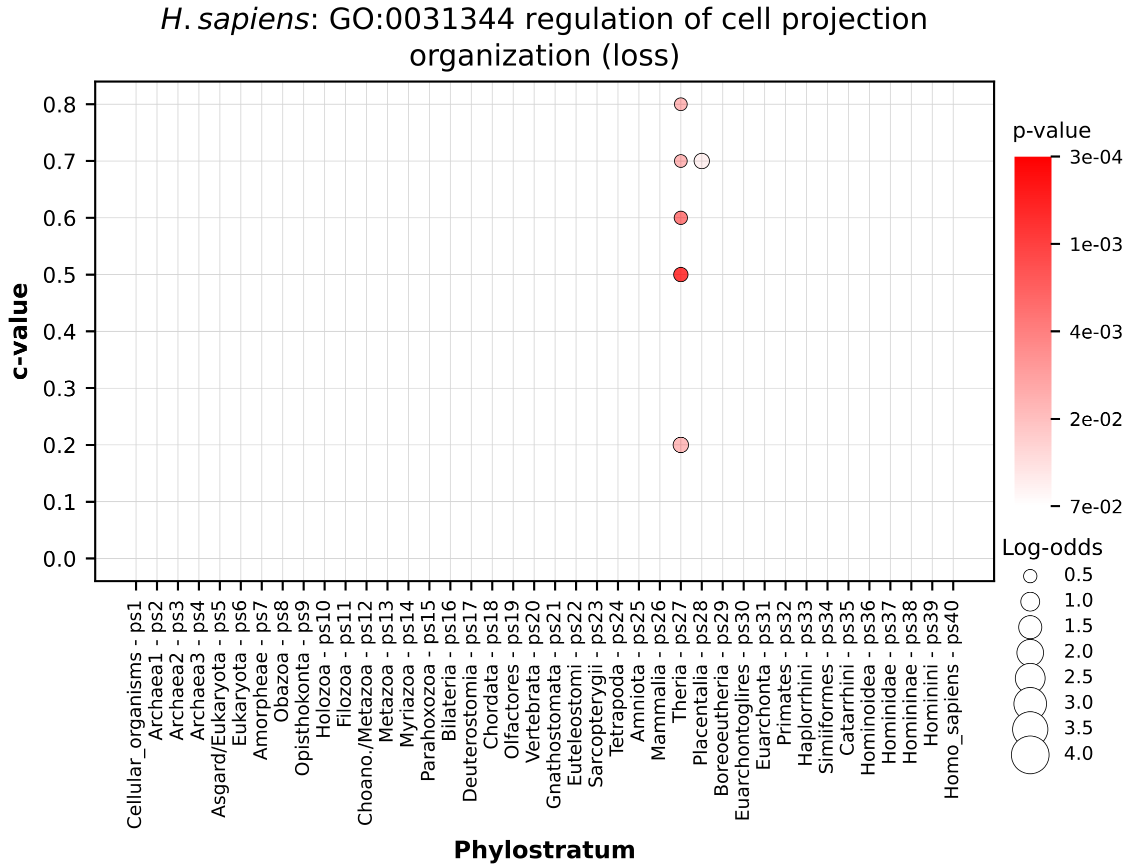

H. sapiens: GO:0031344 regulation of cell projection organization (loss)
c-value
0.0
0.1
0.2
0.3
0.4
0.5
0.6
0.7
0.8
Phylostratum
Cellular_organisms - ps1
Archaea1 - ps2
Archaea2 - ps3
Archaea3 - ps4
Asgard/Eukaryota - ps5
Eukaryota - ps6
Amorpheae - ps7
Obazoa - ps8
Opisthokonta - ps9
Holozoa - ps10
Filozoa - ps11
Choano./Metazoa - ps12
Metazoa - ps13
Myriazoa - ps14
Parahoxozoa - ps15
Bilateria - ps16
Deuterostomia - ps17
Chordata - ps18
Olfactores - ps19
Vertebrata - ps20
Gnathostomata - ps21
Euteleostomi - ps22
Sarcopterygii - ps23
Tetrapoda - ps24
Amniota - ps25
Mammalia - ps26
Theria - ps27
Placentalia - ps28
Boreoeutheria - ps29
Euarchontoglires - ps30
Euarchonta - ps31
Primates - ps32
Haplorrhini - ps33
Simiiformes - ps34
Catarrhini - ps35
Hominoidea - ps36
Hominidae - ps37
Homininae - ps38
Hominini - ps39
Homo_sapiens - ps40
p-value
3e-04
1e-03
4e-03
2e-02
7e-02
Log-odds
0.5
1.0
1.5
2.0
2.5
3.0
3.5
4.0

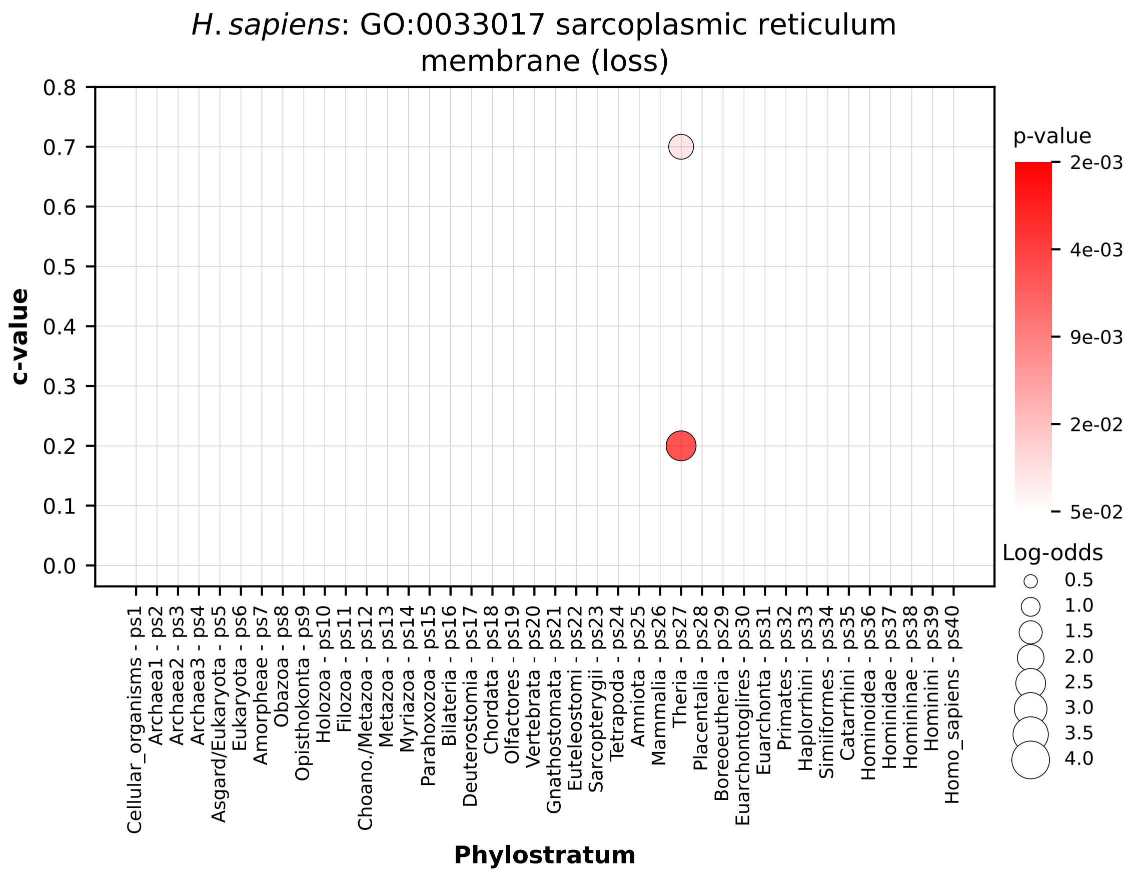

H. sapiens: GO:0033017 sarcoplasmic reticulum membrane (loss)
c-value
0.8
0.7
0.6
0.5
0.4
0.3
0.2
0.1
0.0
p-value
2e-03
4e-03
9e-03
2e-02
5e-02
Log-odds
0.5
1.0
1.5
2.0
2.5
3.0
3.5
4.0
Cellular_organisms - ps1
Archaea1 - ps2
Archaea2 - ps3
Archaea3 - ps4
Asgard/Eukaryota - ps5
Eukaryota - ps6
Amorpheae - ps7
Obazoa - ps8
Opisthokonta - ps9
Holozoa - ps10
Filozoa - ps11
Choano./Metazoa - ps12
Metazoa - ps13
Myriazoa - ps14
Parahoxozoa - ps15
Bilateria - ps16
Deuterostomia - ps17
Chordata - ps18
Olfactores - ps19
Vertebrata - ps20
Gnathostomata - ps21
Euteleostomi - ps22
Sarcopterygii - ps23
Tetrapoda - ps24
Amniota - ps25
Mammalia - ps26
Theria - ps27
Placentalia - ps28
Boreoeutheria - ps29
Euarchontoglires - ps30
Euarchonta - ps31
Primates - ps32
Haplorrhini - ps33
Simiiformes - ps34
Catarrhini - ps35
Hominoidea - ps36
Hominidae - ps37
Homininae - ps38
Hominini - ps39
Homo_sapiens - ps40
Phylostratum

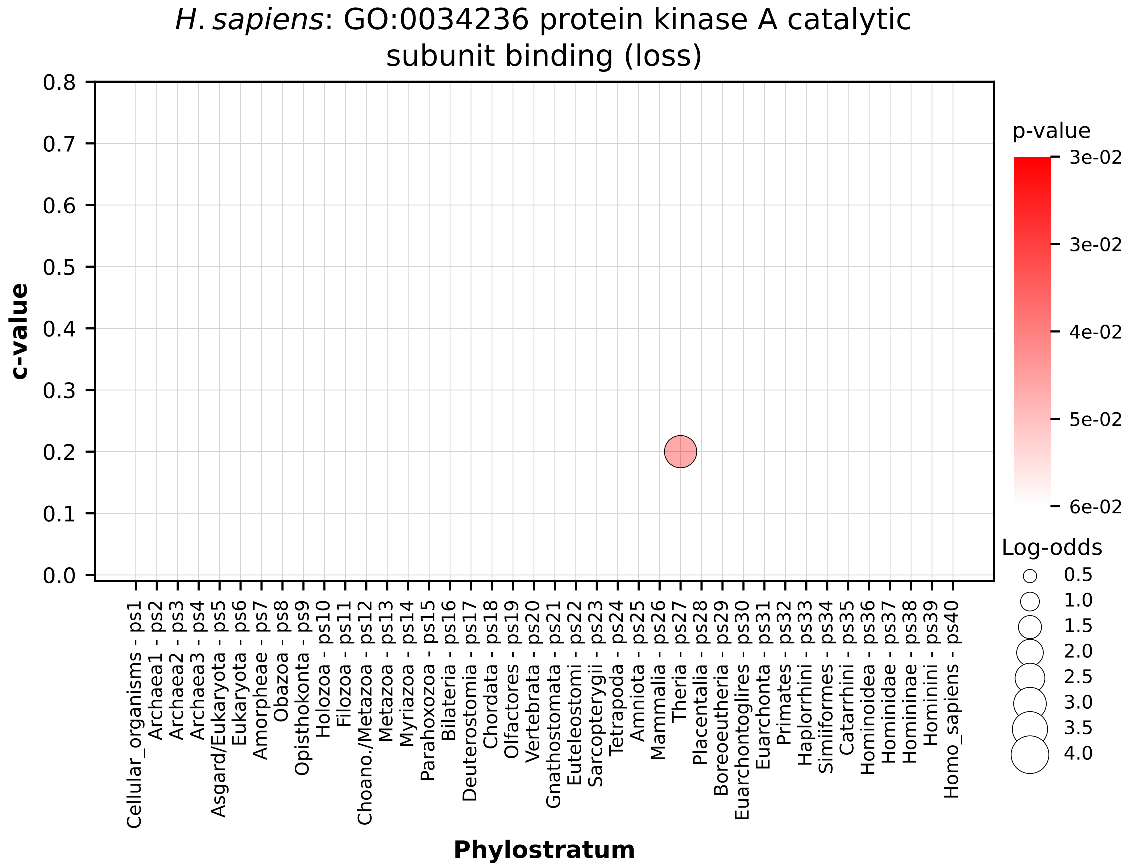

H. sapiens: GO:0034236 protein kinase A catalytic subunit binding (loss)
c-value
0.8
0.7
0.6
0.5
0.4
0.3
0.2
0.1
0.0
p-value
3e-02
3e-02
4e-02
5e-02
6e-02
Log-odds
0.5
1.0
1.5
2.0
2.5
3.0
3.5
4.0
Cellular_organisms - ps1
Archaea1 - ps2
Archaea2 - ps3
Archaea3 - ps4
Asgard/Eukaryota - ps5
Eukaryota - ps6
Amorpheae - ps7
Obazoa - ps8
Opisthokonta - ps9
Holozoa - ps10
Filozoa - ps11
Choano./Metazoa - ps12
Metazoa - ps13
Myriazoa - ps14
Parahoxozoa - ps15
Bilateria - ps16
Deuterostomia - ps17
Chordata - ps18
Olfactores - ps19
Vertebrata - ps20
Gnathostomata - ps21
Euteleostomi - ps22
Sarcopterygii - ps23
Tetrapoda - ps24
Amniota - ps25
Mammalia - ps26
Theria - ps27
Placentalia - ps28
Boreoeutheria - ps29
Euarchontoglires - ps30
Euarchonta - ps31
Primates - ps32
Haplorrhini - ps33
Simiiformes - ps34
Catarrhini - ps35
Hominoidea - ps36
Hominidae - ps37
Homininae - ps38
Hominini - ps39
Homo_sapiens - ps40
Phylostratum

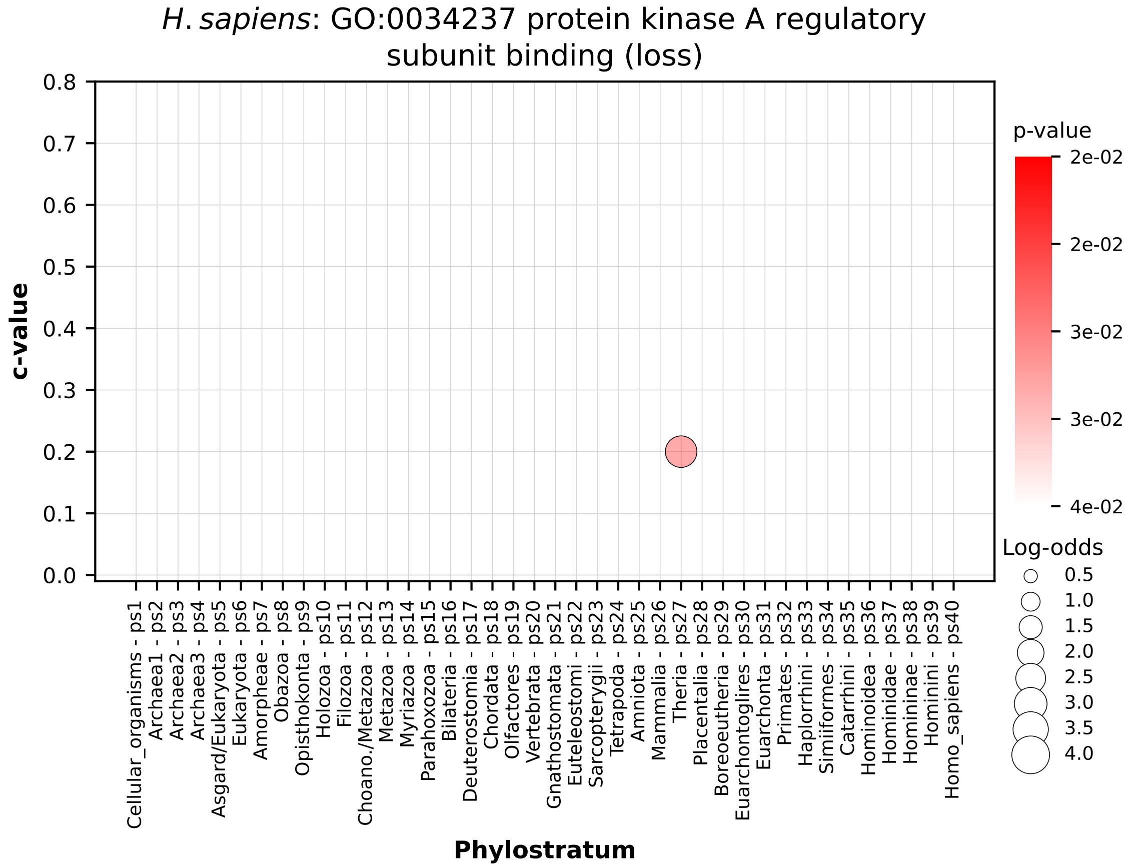

H. sapiens: GO:0034237 protein kinase A regulatory subunit binding (loss)
c-value
0.0
0.1
0.2
0.3
0.4
0.5
0.6
0.7
0.8
Phylostratum
Cellular_organisms - ps1
Archaea1 - ps2
Archaea2 - ps3
Archaea3 - ps4
Asgard/Eukaryota - ps5
Eukaryota - ps6
Amorpheae - ps7
Obazoa - ps8
Opisthokonta - ps9
Holozoa - ps10
Filozoa - ps11
Choano./Metazoa - ps12
Metazoa - ps13
Myriazoa - ps14
Parahoxozoa - ps15
Bilateria - ps16
Deuterostomia - ps17
Chordata - ps18
Olfactores - ps19
Vertebrata - ps20
Gnathostomata - ps21
Euteleostomi - ps22
Sarcopterygii - ps23
Tetrapoda - ps24
Amniota - ps25
Mammalia - ps26
Theria - ps27
Placentalia - ps28
Boreoeutheria - ps29
Euarchontoglires - ps30
Euarchonta - ps31
Primates - ps32
Haplorrhini - ps33
Simiiformes - ps34
Catarrhini - ps35
Hominoidea - ps36
Hominidae - ps37
Homininae - ps38
Hominini - ps39
Homo_sapiens - ps40
p-value
2e-02
2e-02
3e-02
3e-02
4e-02
Log-odds
0.5
1.0
1.5
2.0
2.5
3.0
3.5
4.0

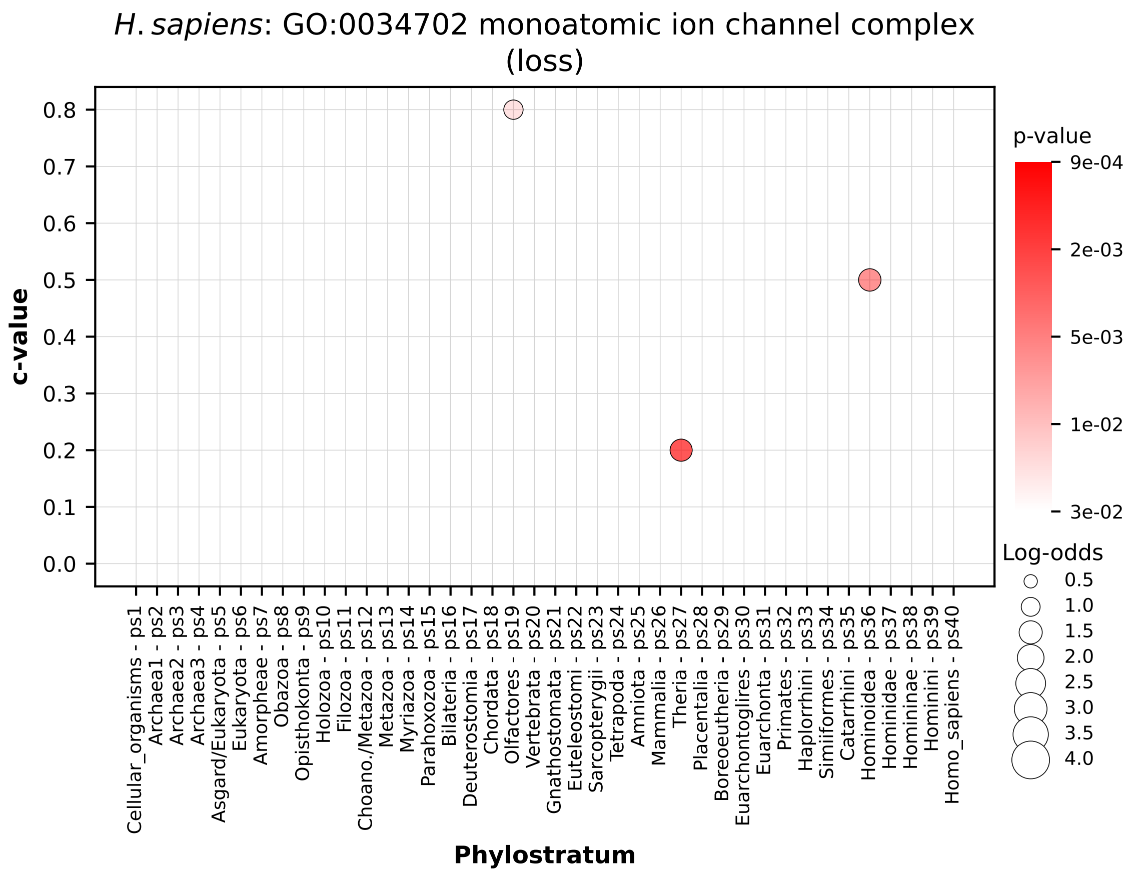

H. sapiens: GO:0034702 monoatomic ion channel complex (loss)
c-value
0.0
0.1
0.2
0.3
0.4
0.5
0.6
0.7
0.8
p-value
9e-04
2e-03
5e-03
1e-02
3e-02
Log-odds
0.5
1.0
1.5
2.0
2.5
3.0
3.5
4.0
Cellular_organisms - ps1
Archaea1 - ps2
Archaea2 - ps3
Archaea3 - ps4
Asgard/Eukaryota - ps5
Eukaryota - ps6
Amorpheae - ps7
Obazoa - ps8
Opisthokonta - ps9
Holozoa - ps10
Filozoa - ps11
Choano./Metazoa - ps12
Metazoa - ps13
Myriazoa - ps14
Parahoxozoa - ps15
Bilateria - ps16
Deuterostomia - ps17
Chordata - ps18
Olfactores - ps19
Vertebrata - ps20
Gnathostomata - ps21
Euteleostomi - ps22
Sarcopterygii - ps23
Tetrapoda - ps24
Amniota - ps25
Mammalia - ps26
Theria - ps27
Placentalia - ps28
Boreoeutheria - ps29
Euarchontoglires - ps30
Euarchonta - ps31
Primates - ps32
Haplorrhini - ps33
Simiiformes - ps34
Catarrhini - ps35
Hominoidea - ps36
Hominidae - ps37
Homininae - ps38
Hominini - ps39
Homo_sapiens - ps40
Phylostratum

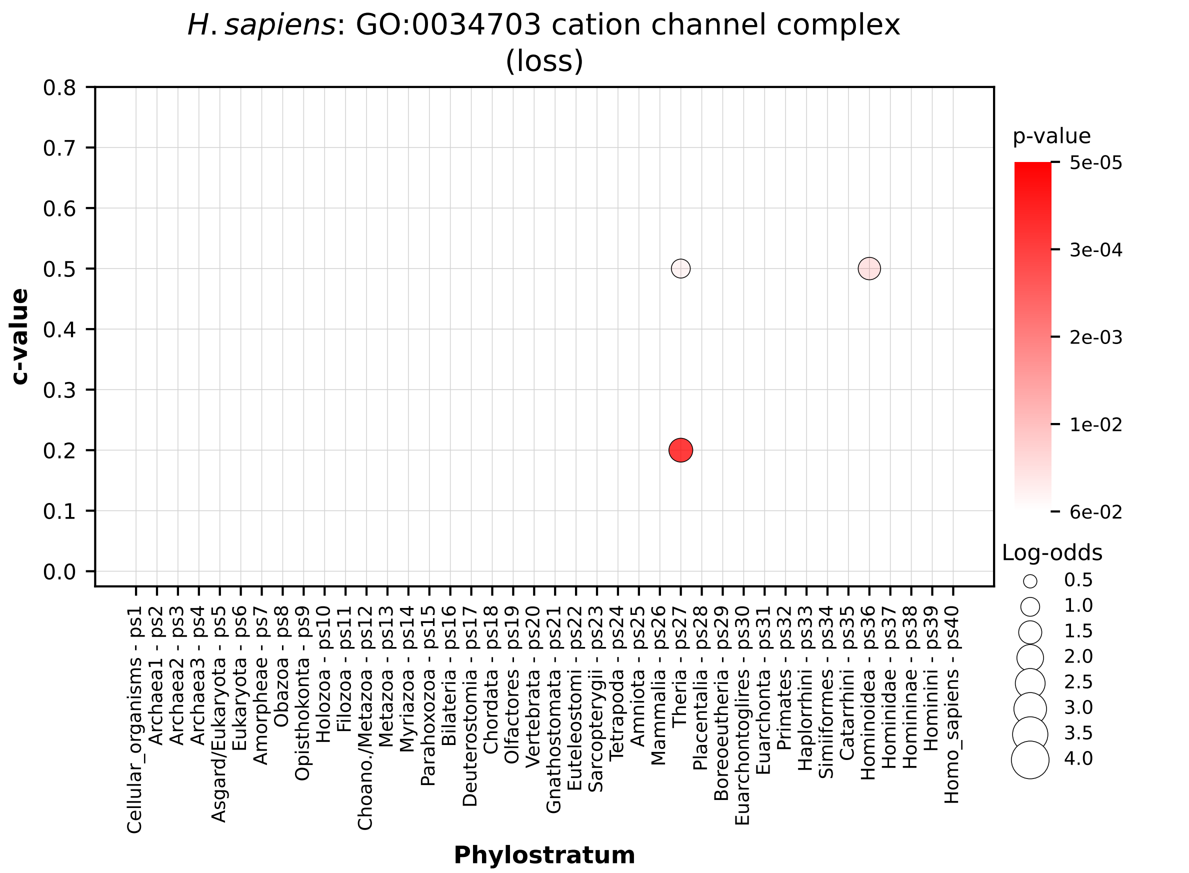

H. sapiens: GO:0034703 cation channel complex
(loss)
c-value
0.8
0.7
0.6
0.5
0.4
0.3
0.2
0.1
0.0
p-value
5e-05
3e-04
2e-03
1e-02
6e-02
Log-odds
0.5
1.0
1.5
2.0
2.5
3.0
3.5
4.0
Cellular_organisms - ps1
Archaea1 - ps2
Archaea2 - ps3
Archaea3 - ps4
Asgard/Eukaryota - ps5
Eukaryota - ps6
Amorpheae - ps7
Obazoa - ps8
Opisthokonta - ps9
Holozoa - ps10
Filozoa - ps11
Choano./Metazoa - ps12
Metazoa - ps13
Myriazoa - ps14
Parahoxozoa - ps15
Bilateria - ps16
Deuterostomia - ps17
Chordata - ps18
Olfactores - ps19
Vertebrata - ps20
Gnathostomata - ps21
Euteleostomi - ps22
Sarcopterygii - ps23
Tetrapoda - ps24
Amniota - ps25
Mammalia - ps26
Theria - ps27
Placentalia - ps28
Boreoeutheria - ps29
Euarchontoglires - ps30
Euarchonta - ps31
Primates - ps32
Haplorrhini - ps33
Simiiformes - ps34
Catarrhini - ps35
Hominoidea - ps36
Hominidae - ps37
Homininae - ps38
Hominini - ps39
Homo_sapiens - ps40
Phylostratum

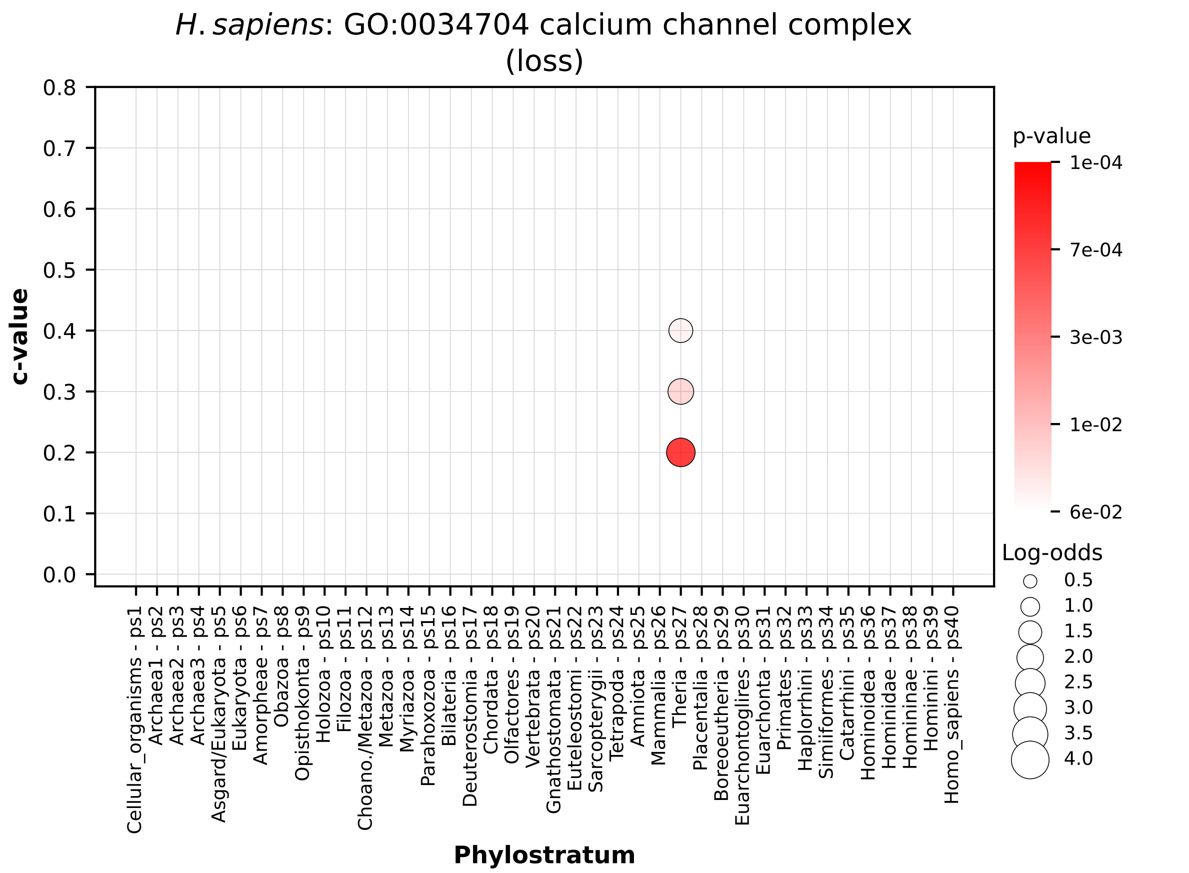

H. sapiens: GO:0034704 calcium channel complex
(loss)
c-value
0.8
0.7
0.6
0.5
0.4
0.3
0.2
0.1
0.0
p-value
1e-04
7e-04
3e-03
1e-02
6e-02
Log-odds
0.5
1.0
1.5
2.0
2.5
3.0
3.5
4.0
Cellular_organisms - ps1
Archaea1 - ps2
Archaea2 - ps3
Archaea3 - ps4
Asgard/Eukaryota - ps5
Eukaryota - ps6
Amorpheae - ps7
Obazoa - ps8
Opisthokonta - ps9
Holozoa - ps10
Filozoa - ps11
Choano./Metazoa - ps12
Metazoa - ps13
Myriazoa - ps14
Parahoxozoa - ps15
Bilateria - ps16
Deuterostomia - ps17
Chordata - ps18
Olfactores - ps19
Vertebrata - ps20
Gnathostomata - ps21
Euteleostomi - ps22
Sarcopterygii - ps23
Tetrapoda - ps24
Amniota - ps25
Mammalia - ps26
Theria - ps27
Placentalia - ps28
Boreoeutheria - ps29
Euarchontoglires - ps30
Euarchonta - ps31
Primates - ps32
Haplorrhini - ps33
Simiiformes - ps34
Catarrhini - ps35
Hominoidea - ps36
Hominidae - ps37
Homininae - ps38
Hominini - ps39
Homo_sapiens - ps40
Phylostratum

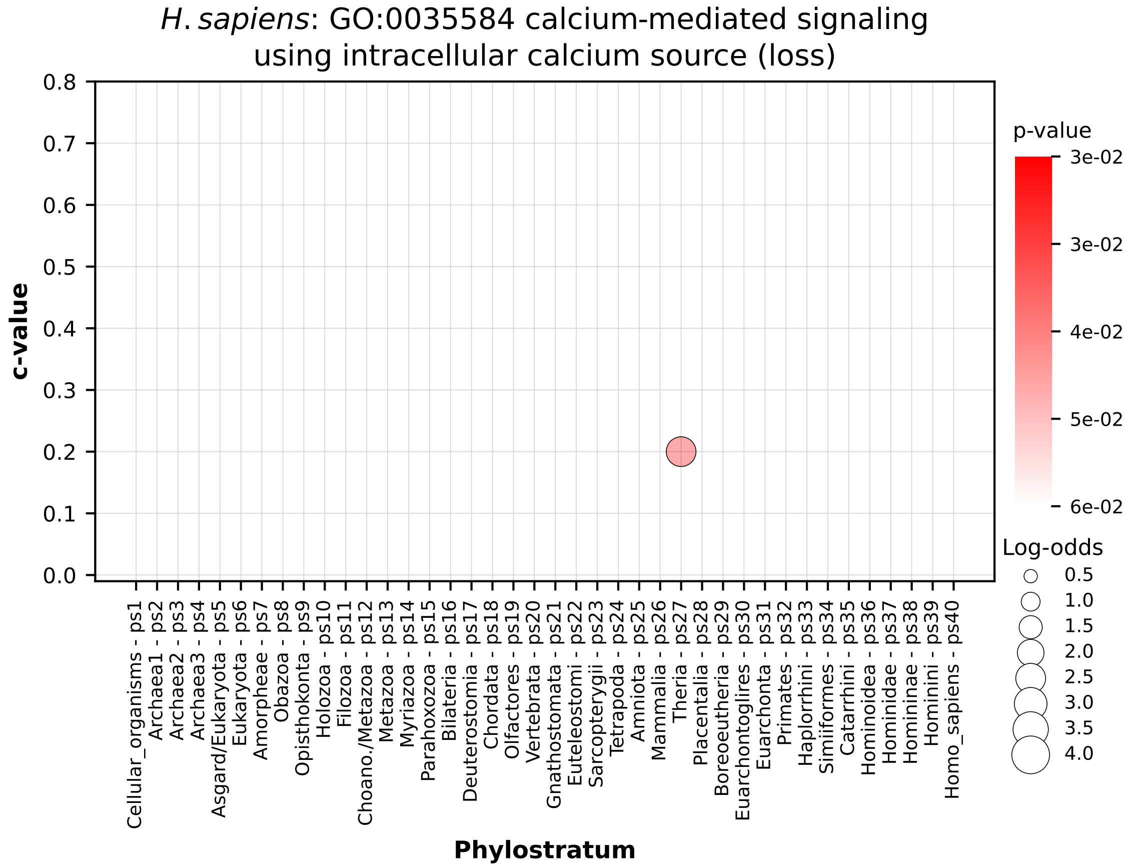

H. sapiens: GO:0035584 calcium-mediated signaling using intracellular calcium source (loss)
c-value
0.0
0.1
0.2
0.3
0.4
0.5
0.6
0.7
0.8
Phylostratum
Cellular_organisms - ps1
Archaea1 - ps2
Archaea2 - ps3
Archaea3 - ps4
Asgard/Eukaryota - ps5
Eukaryota - ps6
Amorpheae - ps7
Obazoa - ps8
Opisthokonta - ps9
Holozoa - ps10
Filozoa - ps11
Choano./Metazoa - ps12
Metazoa - ps13
Myriazoa - ps14
Parahoxozoa - ps15
Bilateria - ps16
Deuterostomia - ps17
Chordata - ps18
Olfactores - ps19
Vertebrata - ps20
Gnathostomata - ps21
Euteleostomi - ps22
Sarcopterygii - ps23
Tetrapoda - ps24
Amniota - ps25
Mammalia - ps26
Theria - ps27
Placentalia - ps28
Boreoeutheria - ps29
Euarchontoglires - ps30
Euarchonta - ps31
Primates - ps32
Haplorrhini - ps33
Simiiformes - ps34
Catarrhini - ps35
Hominoidea - ps36
Hominidae - ps37
Homininae - ps38
Hominini - ps39
Homo_sapiens - ps40
p-value
3e-02
3e-02
4e-02
5e-02
6e-02
Log-odds
0.5
1.0
1.5
2.0
2.5
3.0
3.5
4.0

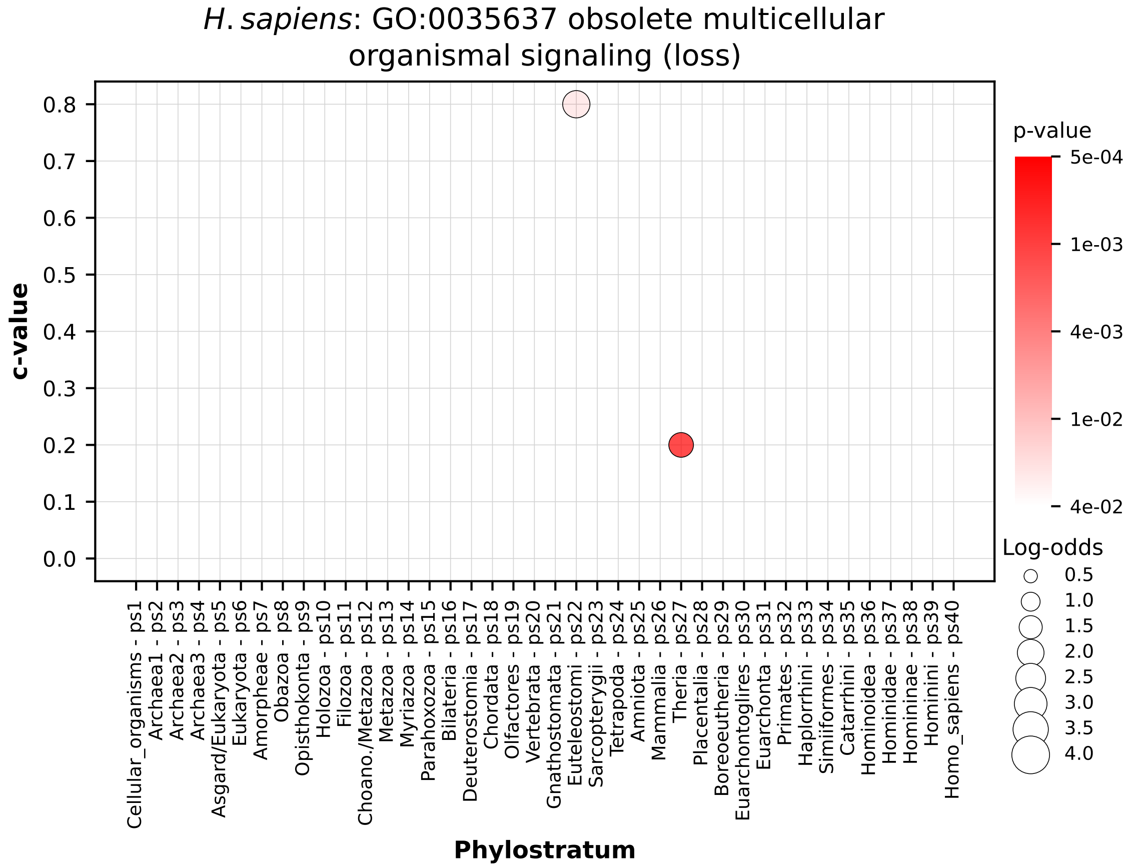

H. sapiens: GO:0035637 obsolete multicellular organismal signaling (loss)
c-value
0.0
0.1
0.2
0.3
0.4
0.5
0.6
0.7
0.8
Phylostratum
Cellular_organisms - ps1
Archaea1 - ps2
Archaea2 - ps3
Archaea3 - ps4
Asgard/Eukaryota - ps5
Eukaryota - ps6
Amorpheae - ps7
Obazoa - ps8
Opisthokonta - ps9
Holozoa - ps10
Filozoa - ps11
Choano./Metazoa - ps12
Metazoa - ps13
Myriazoa - ps14
Parahoxozoa - ps15
Bilateria - ps16
Deuterostomia - ps17
Chordata - ps18
Olfactores - ps19
Vertebrata - ps20
Gnathostomata - ps21
Euteleostomi - ps22
Sarcopterygii - ps23
Tetrapoda - ps24
Amniota - ps25
Mammalia - ps26
Theria - ps27
Placentalia - ps28
Boreoeutheria - ps29
Euarchontoglires - ps30
Euarchonta - ps31
Primates - ps32
Haplorrhini - ps33
Simiiformes - ps34
Catarrhini - ps35
Hominoidea - ps36
Hominidae - ps37
Homininae - ps38
Hominini - ps39
Homo_sapiens - ps40
p-value
5e-04
1e-03
4e-03
1e-02
4e-02
Log-odds
0.5
1.0
1.5
2.0
2.5
3.0
3.5
4.0

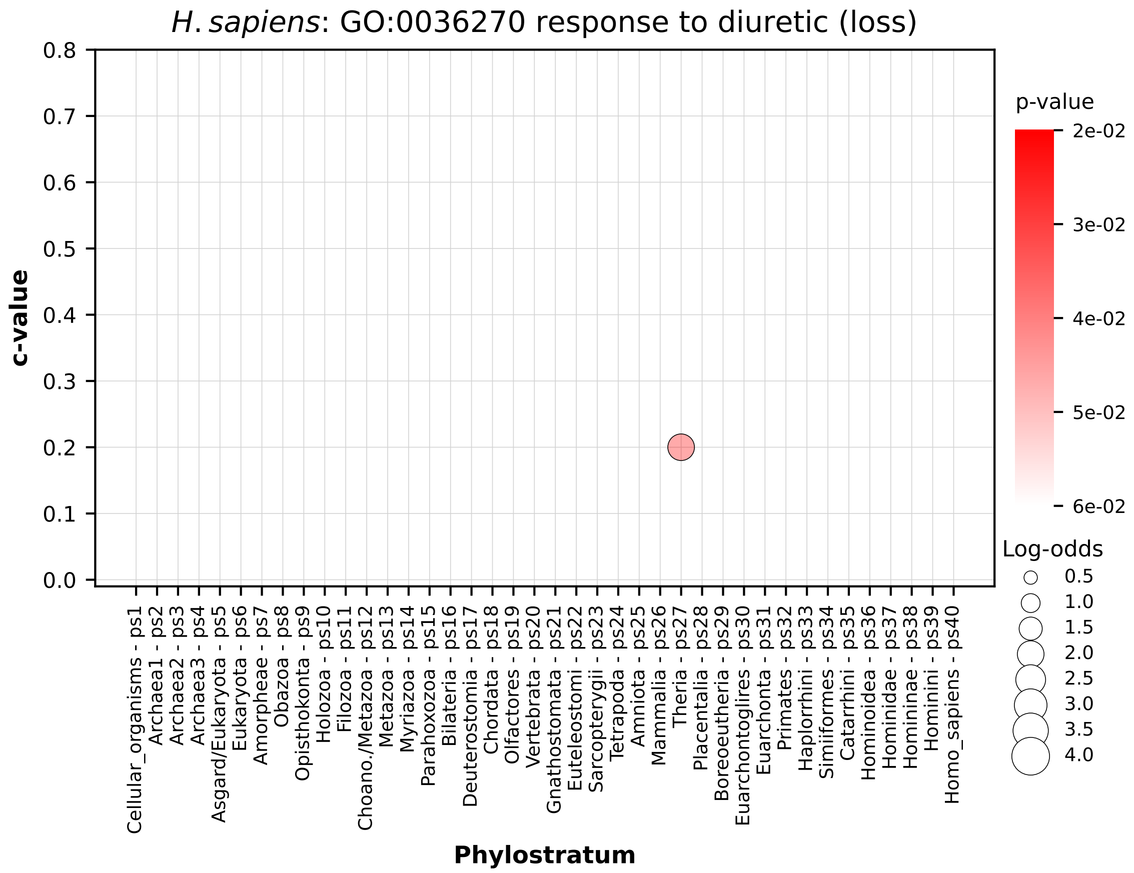

H. sapiens: GO:0036270 response to diuretic (loss)
c-value
0.8
0.7
0.6
0.5
0.4
0.3
0.2
0.1
0.0
Phylostratum
Cellular_organisms - ps1
Archaea1 - ps2
Archaea2 - ps3
Archaea3 - ps4
Asgard/Eukaryota - ps5
Eukaryota - ps6
Amorpheae - ps7
Obazoa - ps8
Opisthokonta - ps9
Holozoa - ps10
Filozoa - ps11
Choano./Metazoa - ps12
Metazoa - ps13
Myriazoa - ps14
Parahoxozoa - ps15
Bilateria - ps16
Deuterostomia - ps17
Chordata - ps18
Olfactores - ps19
Vertebrata - ps20
Gnathostomata - ps21
Euteleostomi - ps22
Sarcopterygii - ps23
Tetrapoda - ps24
Amniota - ps25
Mammalia - ps26
Theria - ps27
Placentalia - ps28
Boreoeutheria - ps29
Euarchontoglires - ps30
Euarchonta - ps31
Primates - ps32
Haplorrhini - ps33
Simiiformes - ps34
Catarrhini - ps35
Hominoidea - ps36
Hominidae - ps37
Homininae - ps38
Hominini - ps39
Homo_sapiens - ps40
p-value
2e-02
3e-02
4e-02
5e-02
6e-02
Log-odds
0.5
1.0
1.5
2.0
2.5
3.0
3.5
4.0

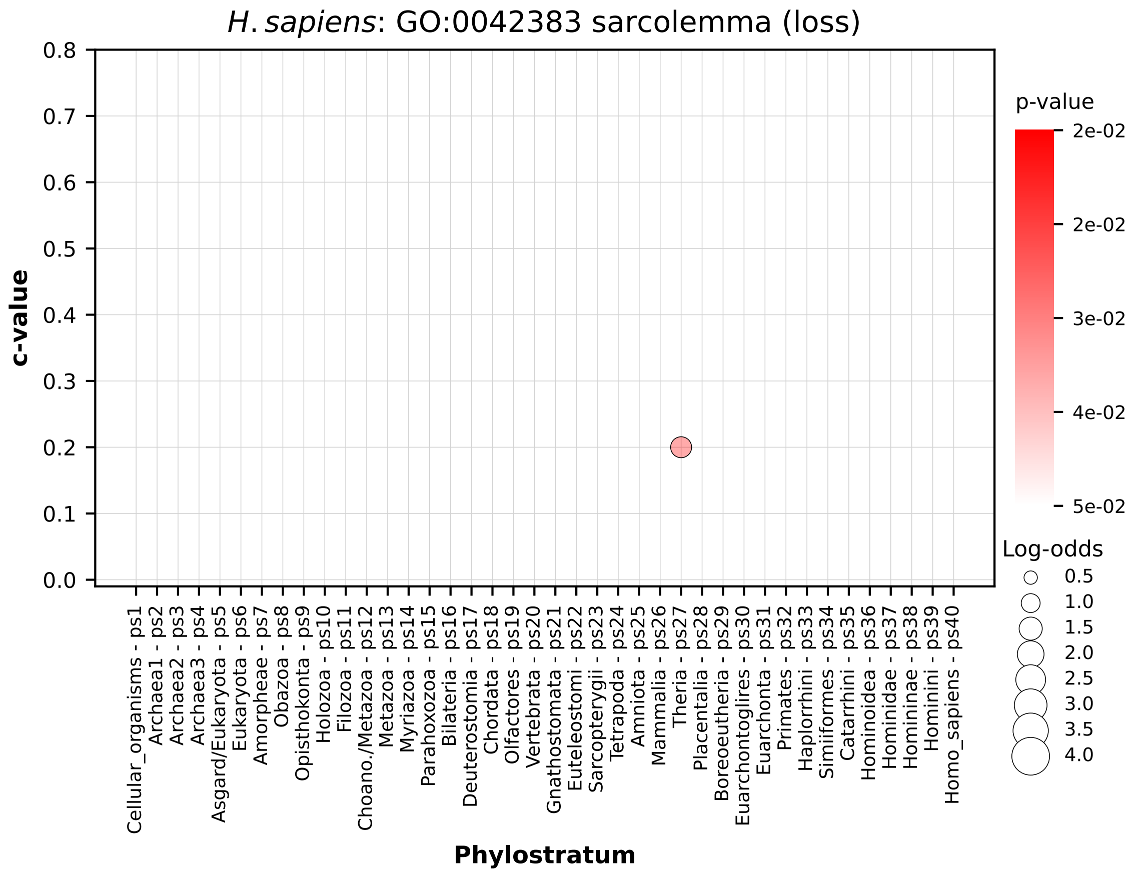
H. sapiens: GO:0042383 sarcolemma (loss)
c-value
0.0
0.1
0.2
0.3
0.4
0.5
0.6
0.7
0.8
p-value
2e-02
2e-02
3e-02
4e-02
5e-02
Log-odds
0.5
1.0
1.5
2.0
2.5
3.0
3.5
4.0
Cellular_organisms - ps1
Archaea1 - ps2
Archaea2 - ps3
Archaea3 - ps4
Asgard/Eukaryota - ps5
Eukaryota - ps6
Amorpheae - ps7
Obazoa - ps8
Opisthokonta - ps9
Holozoa - ps10
Filozoa - ps11
Choano./Metazoa - ps12
Metazoa - ps13
Myriazoa - ps14
Parahoxozoa - ps15
Bilateria - ps16
Deuterostomia - ps17
Chordata - ps18
Olfactores - ps19
Vertebrata - ps20
Gnathostomata - ps21
Euteleostomi - ps22
Sarcopterygii - ps23
Tetrapoda - ps24
Amniota - ps25
Mammalia - ps26
Theria - ps27
Placentalia - ps28
Boreoeutheria - ps29
Euarchontoglires - ps30
Euarchonta - ps31
Primates - ps32
Haplorrhini - ps33
Simiiformes - ps34
Catarrhini - ps35
Hominoidea - ps36
Hominidae - ps37
Homininae - ps38
Hominini - ps39
Homo_sapiens - ps40
Phylostratum

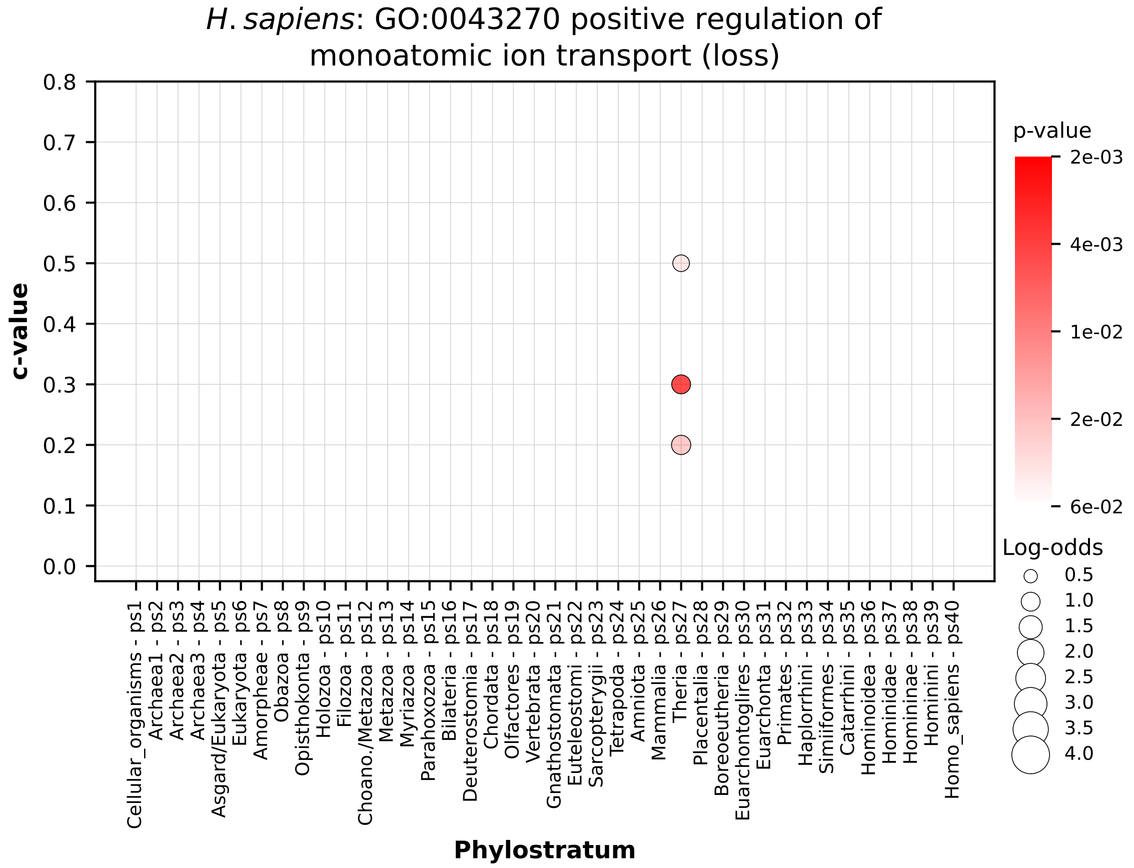

H. sapiens: GO:0043270 positive regulation of monoatomic ion transport (loss)
c-value
0.0
0.1
0.2
0.3
0.4
0.5
0.6
0.7
0.8
Phylostratum
Cellular_organisms - ps1
Archaea1 - ps2
Archaea2 - ps3
Archaea3 - ps4
Asgard/Eukaryota - ps5
Eukaryota - ps6
Amorpheae - ps7
Obazoa - ps8
Opisthokonta - ps9
Holozoa - ps10
Filozoa - ps11
Choano./Metazoa - ps12
Metazoa - ps13
Myriazoa - ps14
Parahoxozoa - ps15
Bilateria - ps16
Deuterostomia - ps17
Chordata - ps18
Olfactores - ps19
Vertebrata - ps20
Gnathostomata - ps21
Euteleostomi - ps22
Sarcopterygii - ps23
Tetrapoda - ps24
Amniota - ps25
Mammalia - ps26
Theria - ps27
Placentalia - ps28
Boreoeutheria - ps29
Euarchontoglires - ps30
Euarchonta - ps31
Primates - ps32
Haplorrhini - ps33
Simiiformes - ps34
Catarrhini - ps35
Hominoidea - ps36
Hominidae - ps37
Homininae - ps38
Hominini - ps39
Homo_sapiens - ps40
p-value
2e-03
4e-03
1e-02
2e-02
6e-02
Log-odds
0.5
1.0
1.5
2.0
2.5
3.0
3.5
4.0

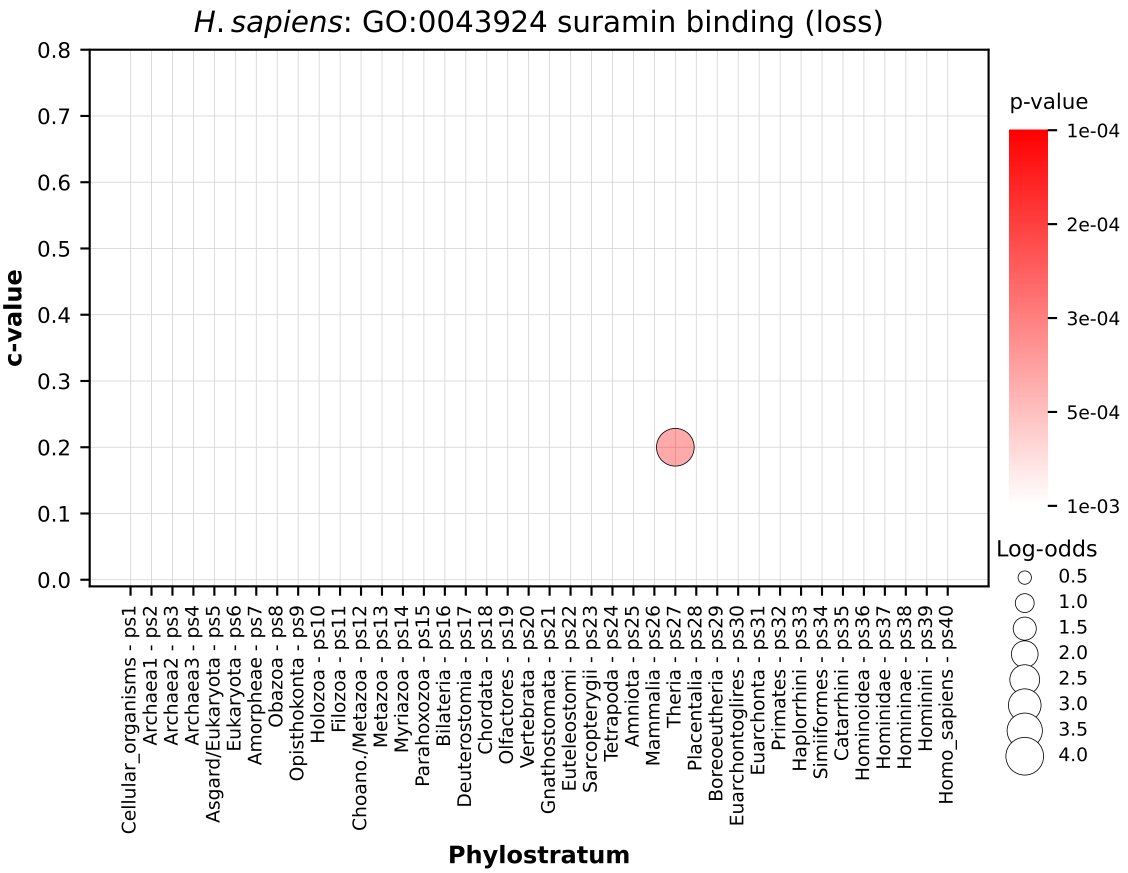

H. sapiens: GO:0043924 suramin binding (loss)
c-value
0.0
0.1
0.2
0.3
0.4
0.5
0.6
0.7
0.8
Phylostratum
Cellular_organisms - ps1
Archaea1 - ps2
Archaea2 - ps3
Archaea3 - ps4
Asgard/Eukaryota - ps5
Eukaryota - ps6
Amorpheae - ps7
Obazoa - ps8
Opisthokonta - ps9
Holozoa - ps10
Filozoa - ps11
Choano./Metazoa - ps12
Metazoa - ps13
Myriazoa - ps14
Parahoxozoa - ps15
Bilateria - ps16
Deuterostomia - ps17
Chordata - ps18
Olfactores - ps19
Vertebrata - ps20
Gnathostomata - ps21
Euteleostomi - ps22
Sarcopterygii - ps23
Tetrapoda - ps24
Amniota - ps25
Mammalia - ps26
Theria - ps27
Placentalia - ps28
Boreoeutheria - ps29
Euarchontoglires - ps30
Euarchonta - ps31
Primates - ps32
Haplorrhini - ps33
Simiiformes - ps34
Catarrhini - ps35
Hominoidea - ps36
Hominidae - ps37
Homininae - ps38
Hominini - ps39
Homo_sapiens - ps40
p-value
1e-04
2e-04
3e-04
5e-04
1e-03
Log-odds
0.5
1.0
1.5
2.0
2.5
3.0
3.5
4.0

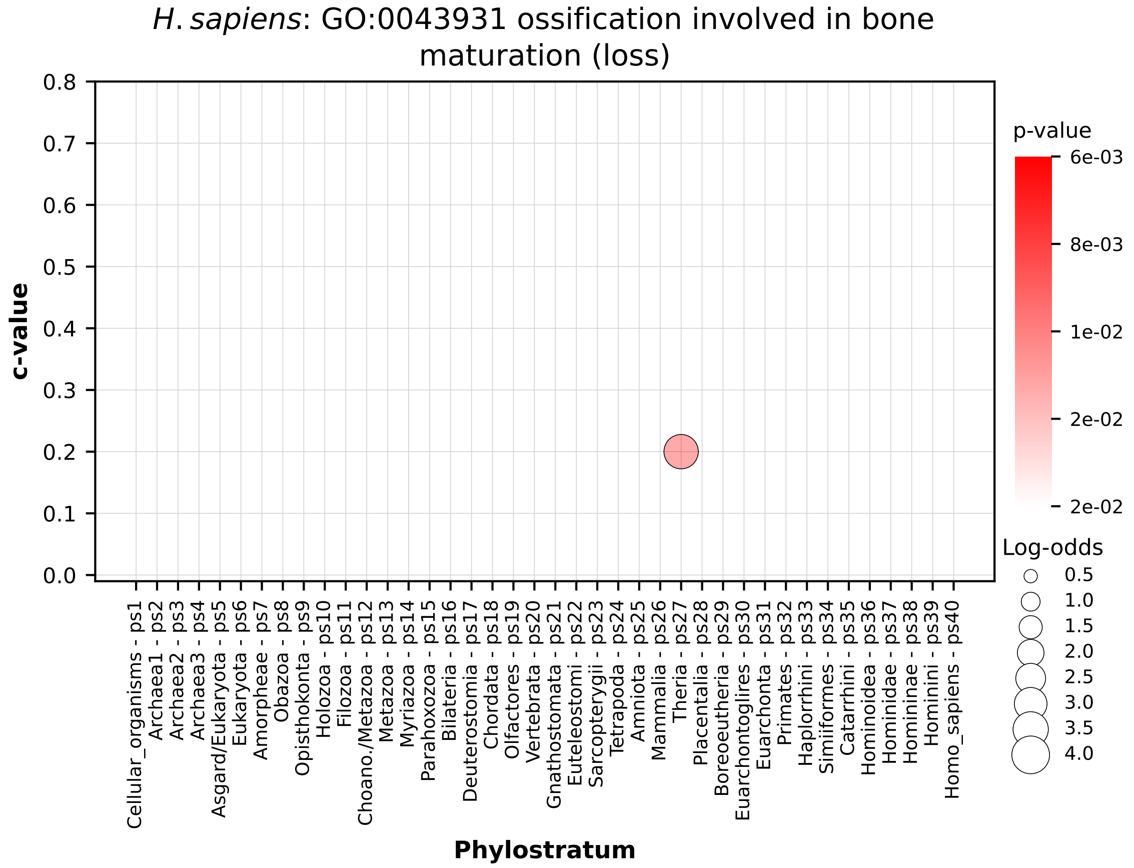
H. sapiens: GO:0043931 ossification involved in bone maturation (loss)
c-value
0.0
0.1
0.2
0.3
0.4
0.5
0.6
0.7
0.8
Phylostratum
Cellular_organisms - ps1
Archaea1 - ps2
Archaea2 - ps3
Archaea3 - ps4
Asgard/Eukaryota - ps5
Eukaryota - ps6
Amorpheae - ps7
Obazoa - ps8
Opisthokonta - ps9
Holozoa - ps10
Filozoa - ps11
Choano./Metazoa - ps12
Metazoa - ps13
Myriazoa - ps14
Parahoxozoa - ps15
Bilateria - ps16
Deuterostomia - ps17
Chordata - ps18
Olfactores - ps19
Vertebrata - ps20
Gnathostomata - ps21
Euteleostomi - ps22
Sarcopterygii - ps23
Tetrapoda - ps24
Amniota - ps25
Mammalia - ps26
Theria - ps27
Placentalia - ps28
Boreoeutheria - ps29
Euarchontoglires - ps30
Euarchonta - ps31
Primates - ps32
Haplorrhini - ps33
Simiiformes - ps34
Catarrhini - ps35
Hominoidea - ps36
Hominidae - ps37
Homininae - ps38
Hominini - ps39
Homo_sapiens - ps40
p-value
6e-03
8e-03
1e-02
2e-02
2e-02
Log-odds
0.5
1.0
1.5
2.0
2.5
3.0
3.5
4.0

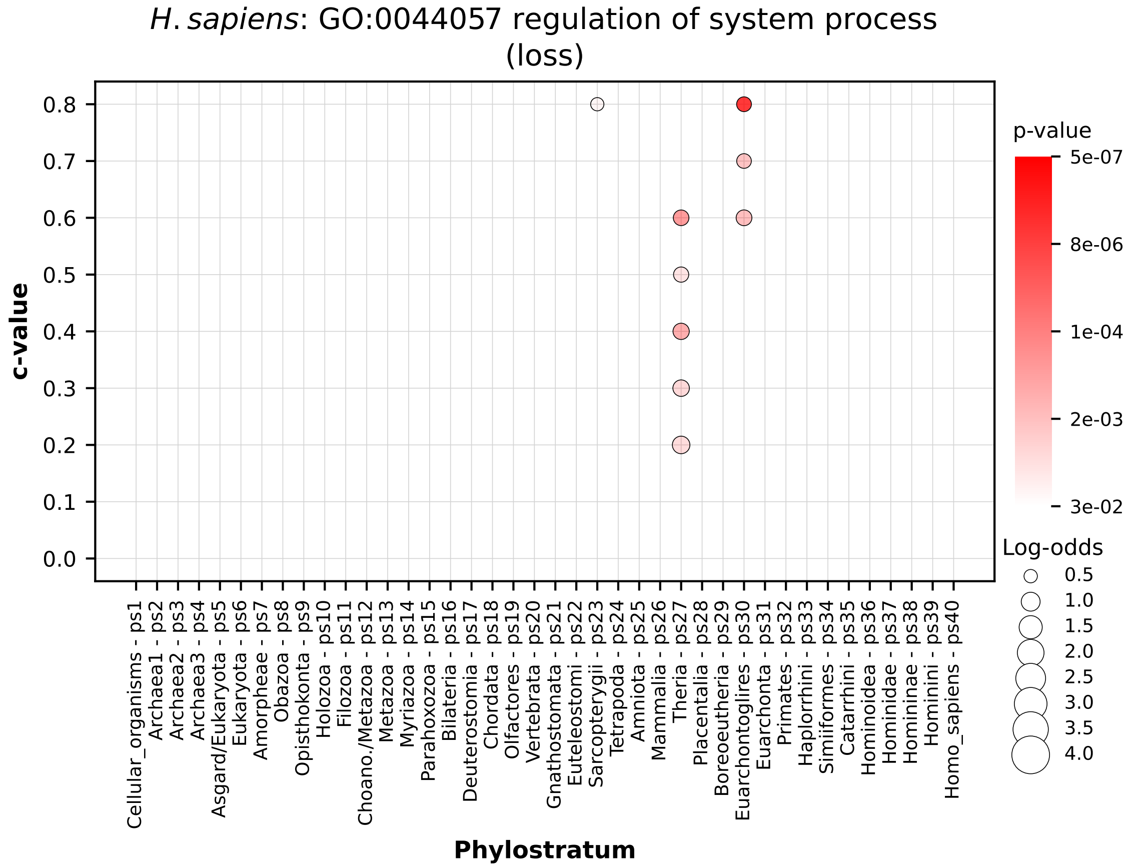

H. sapiens: GO:0044057 regulation of system process (loss)
c-value
0.0
0.1
0.2
0.3
0.4
0.5
0.6
0.7
0.8
Phylostratum
Cellular_organisms - ps1
Archaea1 - ps2
Archaea2 - ps3
Archaea3 - ps4
Asgard/Eukaryota - ps5
Eukaryota - ps6
Amorpheae - ps7
Obazoa - ps8
Opisthokonta - ps9
Holozoa - ps10
Filozoa - ps11
Choano./Metazoa - ps12
Metazoa - ps13
Myriazoa - ps14
Parahoxozoa - ps15
Bilateria - ps16
Deuterostomia - ps17
Chordata - ps18
Olfactores - ps19
Vertebrata - ps20
Gnathostomata - ps21
Euteleostomi - ps22
Sarcopterygii - ps23
Tetrapoda - ps24
Amniota - ps25
Mammalia - ps26
Theria - ps27
Placentalia - ps28
Boreoeutheria - ps29
Euarchontoglires - ps30
Euarchonta - ps31
Primates - ps32
Haplorrhini - ps33
Simiiformes - ps34
Catarrhini - ps35
Hominoidea - ps36
Hominidae - ps37
Homininae - ps38
Hominini - ps39
Homo_sapiens - ps40
p-value
5e-07
8e-06
1e-04
2e-03
3e-02
Log-odds
0.5
1.0
1.5
2.0
2.5
3.0
3.5
4.0

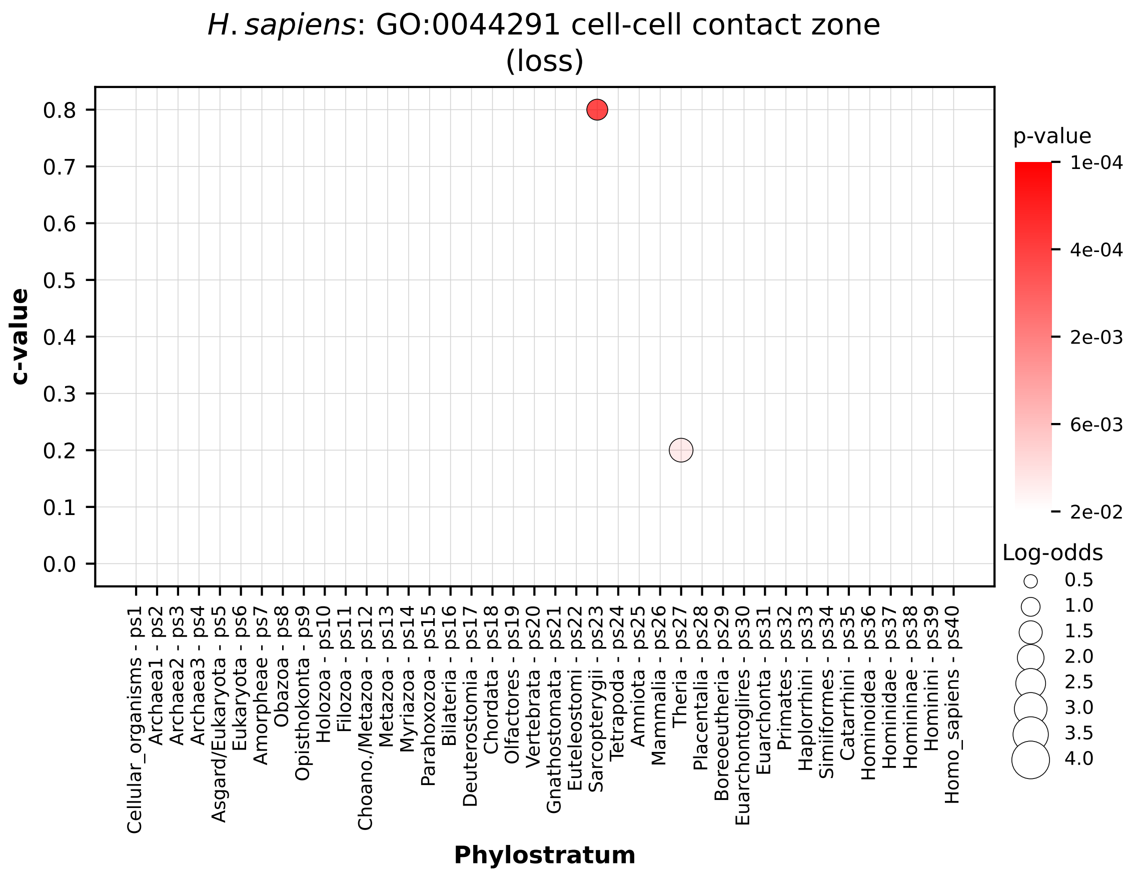

H. sapiens: GO:0044291 cell-cell contact zone
(loss)
c-value
0.0
0.1
0.2
0.3
0.4
0.5
0.6
0.7
0.8
p-value
1e-04
4e-04
2e-03
6e-03
2e-02
Log-odds
0.5
1.0
1.5
2.0
2.5
3.0
3.5
4.0
Cellular_organisms - ps1
Archaea1 - ps2
Archaea2 - ps3
Archaea3 - ps4
Asgard/Eukaryota - ps5
Eukaryota - ps6
Amorpheae - ps7
Obazoa - ps8
Opisthokonta - ps9
Holozoa - ps10
Filozoa - ps11
Choano./Metazoa - ps12
Metazoa - ps13
Myriazoa - ps14
Parahoxozoa - ps15
Bilateria - ps16
Deuterostomia - ps17
Chordata - ps18
Olfactores - ps19
Vertebrata - ps20
Gnathostomata - ps21
Euteleostomi - ps22
Sarcopterygii - ps23
Tetrapoda - ps24
Amniota - ps25
Mammalia - ps26
Theria - ps27
Placentalia - ps28
Boreoeutheria - ps29
Euarchontoglires - ps30
Euarchonta - ps31
Primates - ps32
Haplorrhini - ps33
Simiiformes - ps34
Catarrhini - ps35
Hominoidea - ps36
Hominidae - ps37
Homininae - ps38
Hominini - ps39
Homo_sapiens - ps40
Phylostratum

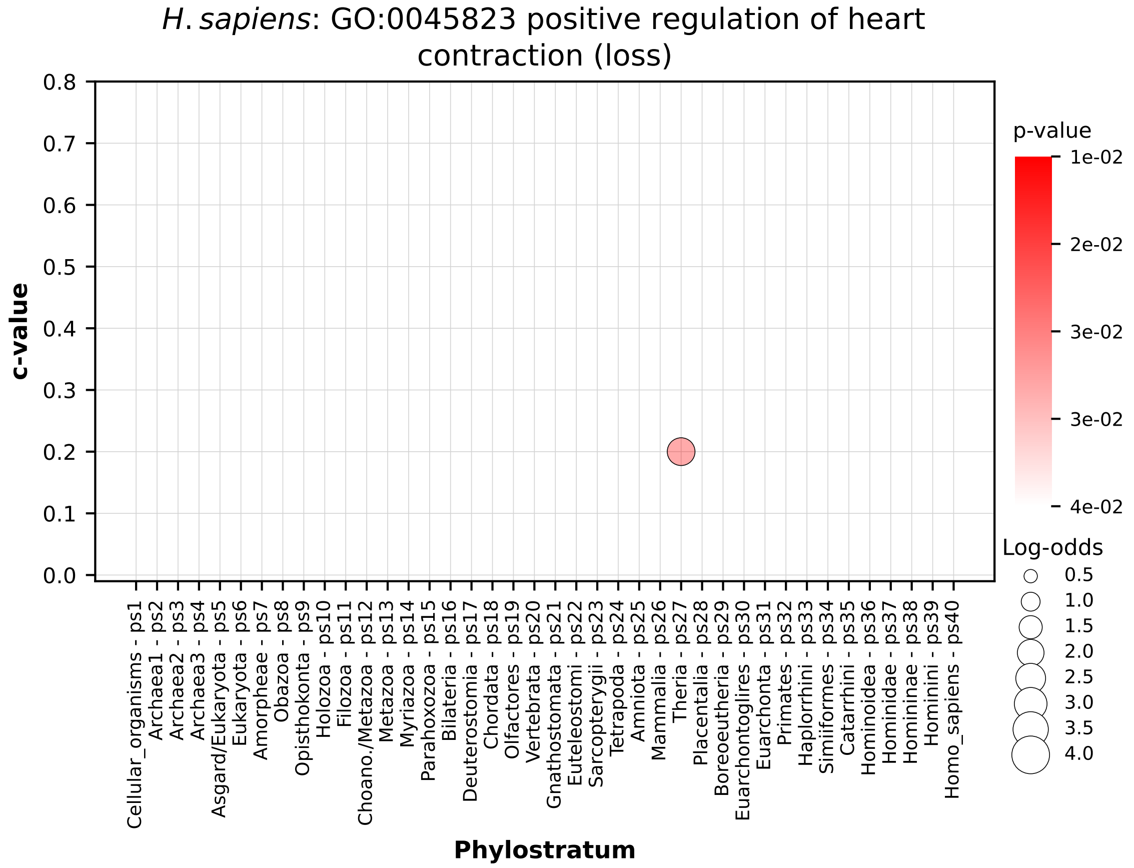

H. sapiens: GO:0045823 positive regulation of heart contraction (loss)
c-value
0.0
0.1
0.2
0.3
0.4
0.5
0.6
0.7
0.8
p-value
1e-02
2e-02
3e-02
3e-02
4e-02
Log-odds
0.5
1.0
1.5
2.0
2.5
3.0
3.5
4.0
Cellular_organisms - ps1
Archaea1 - ps2
Archaea2 - ps3
Archaea3 - ps4
Asgard/Eukaryota - ps5
Eukaryota - ps6
Amorpheae - ps7
Obazoa - ps8
Opisthokonta - ps9
Holozoa - ps10
Filozoa - ps11
Choano./Metazoa - ps12
Metazoa - ps13
Myriazoa - ps14
Parahoxozoa - ps15
Bilateria - ps16
Deuterostomia - ps17
Chordata - ps18
Olfactores - ps19
Vertebrata - ps20
Gnathostomata - ps21
Euteleostomi - ps22
Sarcopterygii - ps23
Tetrapoda - ps24
Amniota - ps25
Mammalia - ps26
Theria - ps27
Placentalia - ps28
Boreoeutheria - ps29
Euarchontoglires - ps30
Euarchonta - ps31
Primates - ps32
Haplorrhini - ps33
Simiiformes - ps34
Catarrhini - ps35
Hominoidea - ps36
Hominidae - ps37
Homininae - ps38
Hominini - ps39
Homo_sapiens - ps40
Phylostratum

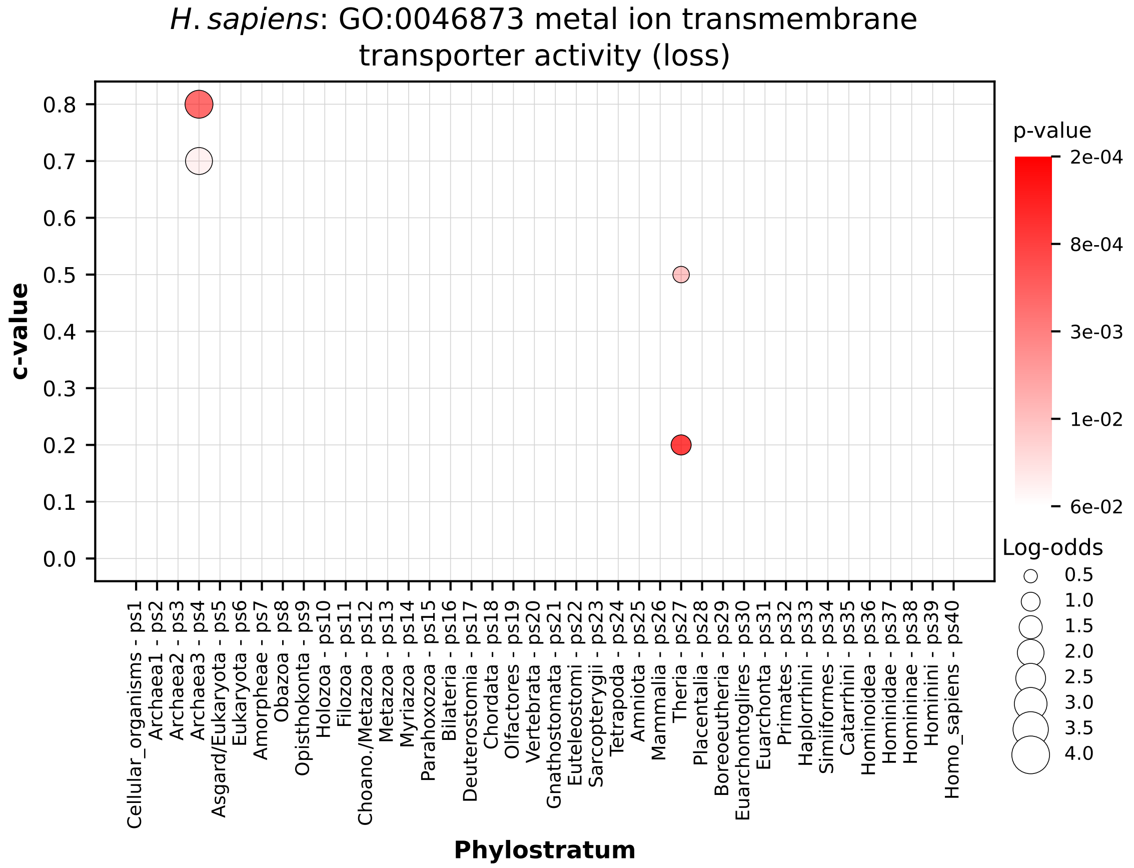

H. sapiens: GO:0046873 metal ion transmembrane transporter activity (loss)
c-value
0.8
0.7
0.6
0.5
0.4
0.3
0.2
0.1
0.0
Phylostratum
Cellular_organisms - ps1
Archaea1 - ps2
Archaea2 - ps3
Archaea3 - ps4
Asgard/Eukaryota - ps5
Eukaryota - ps6
Amorpheae - ps7
Obazoa - ps8
Opisthokonta - ps9
Holozoa - ps10
Filozoa - ps11
Choano./Metazoa - ps12
Metazoa - ps13
Myriazoa - ps14
Parahoxozoa - ps15
Bilateria - ps16
Deuterostomia - ps17
Chordata - ps18
Olfactores - ps19
Vertebrata - ps20
Gnathostomata - ps21
Euteleostomi - ps22
Sarcopterygii - ps23
Tetrapoda - ps24
Amniota - ps25
Mammalia - ps26
Theria - ps27
Placentalia - ps28
Boreoeutheria - ps29
Euarchontoglires - ps30
Euarchonta - ps31
Primates - ps32
Haplorrhini - ps33
Simiiformes - ps34
Catarrhini - ps35
Hominoidea - ps36
Hominidae - ps37
Homininae - ps38
Hominini - ps39
Homo_sapiens - ps40
p-value
2e-04
8e-04
3e-03
1e-02
6e-02
Log-odds
0.5
1.0
1.5
2.0
2.5
3.0
3.5
4.0

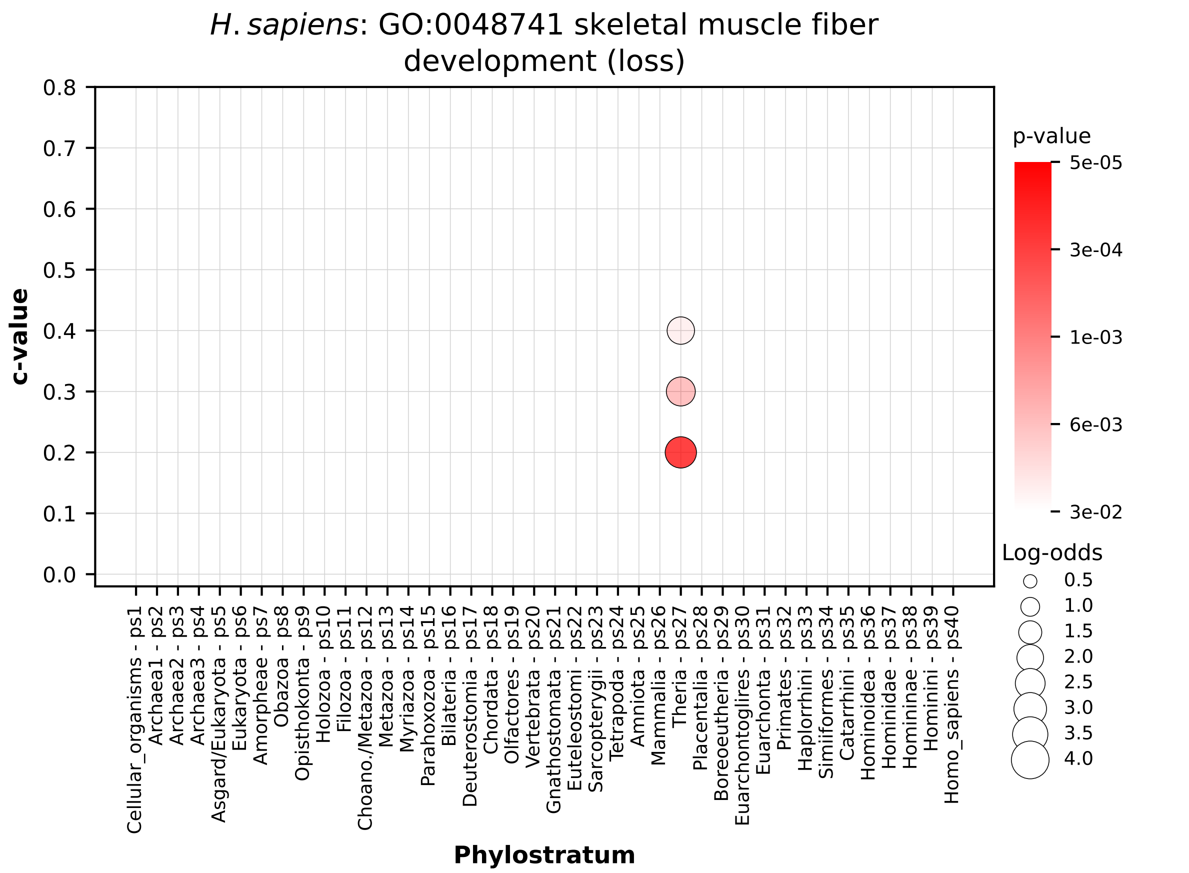

H. sapiens: GO:0048741 skeletal muscle fiber development (loss)
c-value
0.0
0.1
0.2
0.3
0.4
0.5
0.6
0.7
0.8
Phylostratum
Cellular_organisms - ps1
Archaea1 - ps2
Archaea2 - ps3
Archaea3 - ps4
Asgard/Eukaryota - ps5
Eukaryota - ps6
Amorpheae - ps7
Obazoa - ps8
Opisthokonta - ps9
Holozoa - ps10
Filozoa - ps11
Choano./Metazoa - ps12
Metazoa - ps13
Myriazoa - ps14
Parahoxozoa - ps15
Bilateria - ps16
Deuterostomia - ps17
Chordata - ps18
Olfactores - ps19
Vertebrata - ps20
Gnathostomata - ps21
Euteleostomi - ps22
Sarcopterygii - ps23
Tetrapoda - ps24
Amniota - ps25
Mammalia - ps26
Theria - ps27
Placentalia - ps28
Boreoeutheria - ps29
Euarchontoglires - ps30
Euarchonta - ps31
Primates - ps32
Haplorrhini - ps33
Simiiformes - ps34
Catarrhini - ps35
Hominoidea - ps36
Hominidae - ps37
Homininae - ps38
Hominini - ps39
Homo_sapiens - ps40
p-value
5e-05
3e-04
1e-03
6e-03
3e-02
Log-odds
0.5
1.0
1.5
2.0
2.5
3.0
3.5
4.0

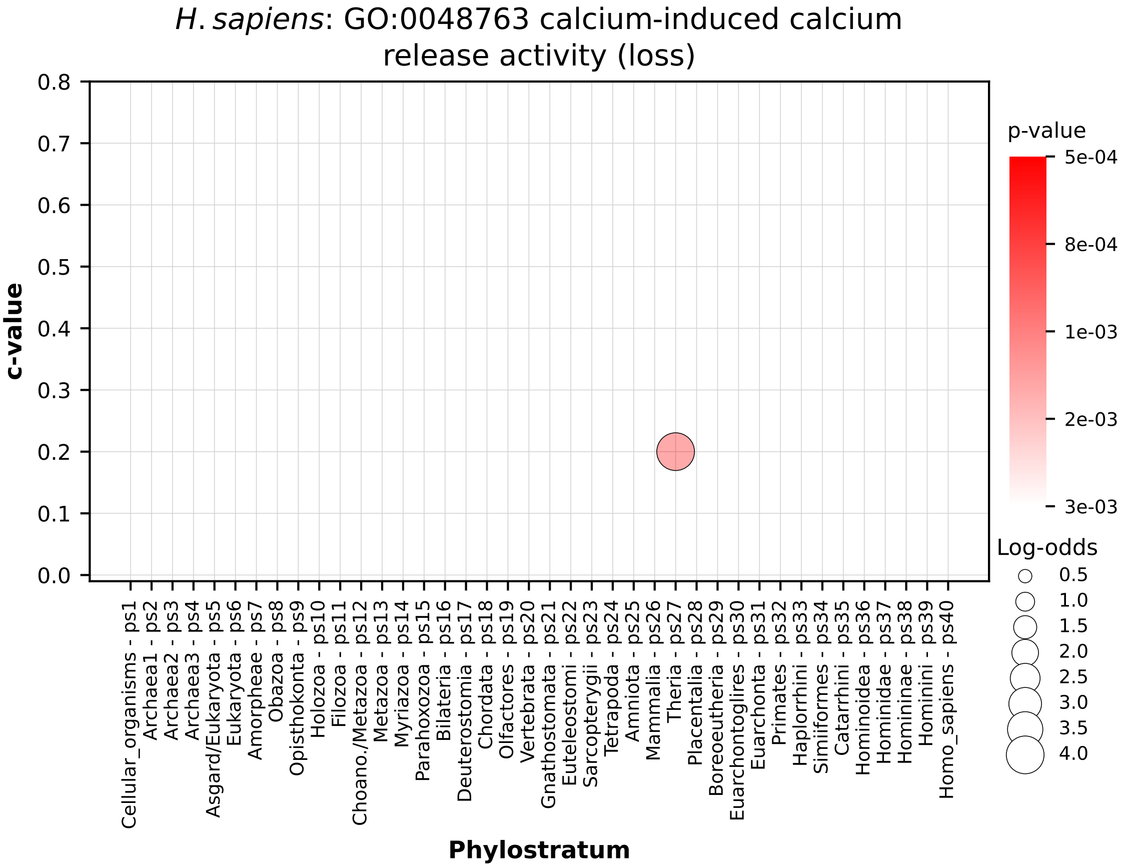

H. sapiens: GO:0048763 calcium-induced calcium release activity (loss)
c-value
0.0
0.1
0.2
0.3
0.4
0.5
0.6
0.7
0.8
Phylostratum
Cellular_organisms - ps1
Archaea1 - ps2
Archaea2 - ps3
Archaea3 - ps4
Asgard/Eukaryota - ps5
Eukaryota - ps6
Amorpheae - ps7
Obazoa - ps8
Opisthokonta - ps9
Holozoa - ps10
Filozoa - ps11
Choano./Metazoa - ps12
Metazoa - ps13
Myriazoa - ps14
Parahoxozoa - ps15
Bilateria - ps16
Deuterostomia - ps17
Chordata - ps18
Olfactores - ps19
Vertebrata - ps20
Gnathostomata - ps21
Euteleostomi - ps22
Sarcopterygii - ps23
Tetrapoda - ps24
Amniota - ps25
Mammalia - ps26
Theria - ps27
Placentalia - ps28
Boreoeutheria - ps29
Euarchontoglires - ps30
Euarchonta - ps31
Primates - ps32
Haplorrhini - ps33
Simiiformes - ps34
Catarrhini - ps35
Hominoidea - ps36
Hominidae - ps37
Homininae - ps38
Hominini - ps39
Homo_sapiens - ps40
p-value
5e-04
8e-04
1e-03
2e-03
3e-03
Log-odds
0.5
1.0
1.5
2.0
2.5
3.0
3.5
4.0

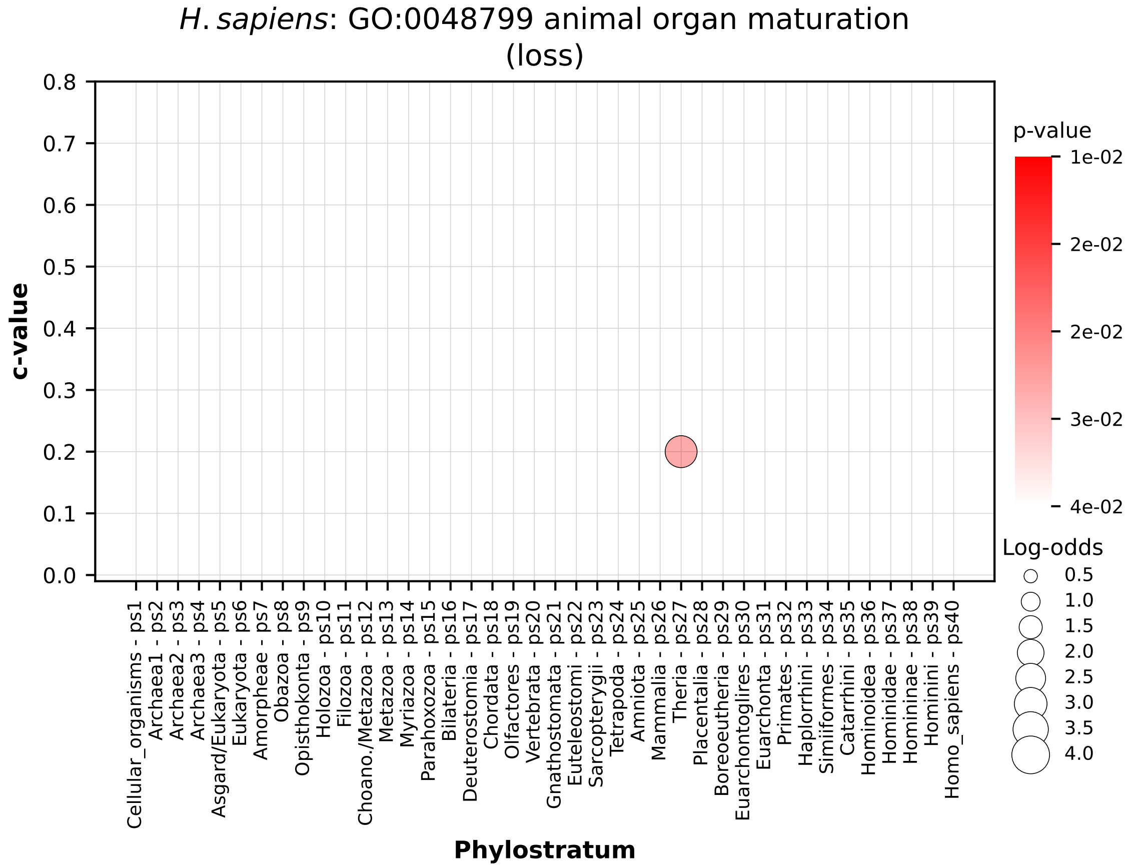

H. sapiens: GO:0048799 animal organ maturation
(loss)
c-value
0.0
0.1
0.2
0.3
0.4
0.5
0.6
0.7
0.8
Phylostratum
Cellular_organisms - ps1
Archaea1 - ps2
Archaea2 - ps3
Archaea3 - ps4
Asgard/Eukaryota - ps5
Eukaryota - ps6
Amorpheae - ps7
Obazoa - ps8
Opisthokonta - ps9
Holozoa - ps10
Filozoa - ps11
Choano./Metazoa - ps12
Metazoa - ps13
Myriazoa - ps14
Parahoxozoa - ps15
Bilateria - ps16
Deuterostomia - ps17
Chordata - ps18
Olfactores - ps19
Vertebrata - ps20
Gnathostomata - ps21
Euteleostomi - ps22
Sarcopterygii - ps23
Tetrapoda - ps24
Amniota - ps25
Mammalia - ps26
Theria - ps27
Placentalia - ps28
Boreoeutheria - ps29
Euarchontoglires - ps30
Euarchonta - ps31
Primates - ps32
Haplorrhini - ps33
Simiiformes - ps34
Catarrhini - ps35
Hominoidea - ps36
Hominidae - ps37
Homininae - ps38
Hominini - ps39
Homo_sapiens - ps40
p-value
1e-02
2e-02
2e-02
3e-02
4e-02
Log-odds
0.5
1.0
1.5
2.0
2.5
3.0
3.5
4.0

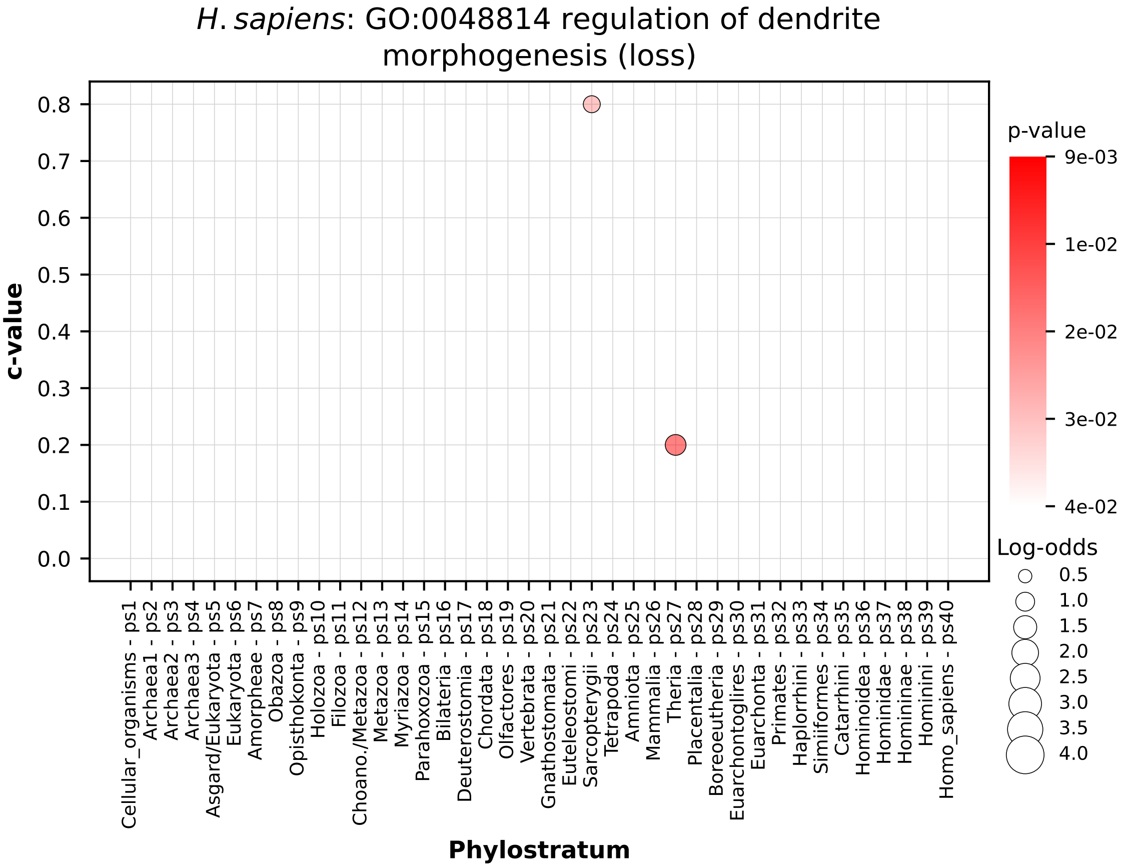

H. sapiens: GO:0048814 regulation of dendrite morphogenesis (loss)
c-value
0.8
0.7
0.6
0.5
0.4
0.3
0.2
0.1
0.0
p-value
9e-03
1e-02
2e-02
3e-02
4e-02
Log-odds
0.5
1.0
1.5
2.0
2.5
3.0
3.5
4.0
Cellular_organisms - ps1
Archaea1 - ps2
Archaea2 - ps3
Archaea3 - ps4
Asgard/Eukaryota - ps5
Eukaryota - ps6
Amorpheae - ps7
Obazoa - ps8
Opisthokonta - ps9
Holozoa - ps10
Filozoa - ps11
Choano./Metazoa - ps12
Metazoa - ps13
Myriazoa - ps14
Parahoxozoa - ps15
Bilateria - ps16
Deuterostomia - ps17
Chordata - ps18
Olfactores - ps19
Vertebrata - ps20
Gnathostomata - ps21
Euteleostomi - ps22
Sarcopterygii - ps23
Tetrapoda - ps24
Amniota - ps25
Mammalia - ps26
Theria - ps27
Placentalia - ps28
Boreoeutheria - ps29
Euarchontoglires - ps30
Euarchonta - ps31
Primates - ps32
Haplorrhini - ps33
Simiiformes - ps34
Catarrhini - ps35
Hominoidea - ps36
Hominidae - ps37
Homininae - ps38
Hominini - ps39
Homo_sapiens - ps40
Phylostratum

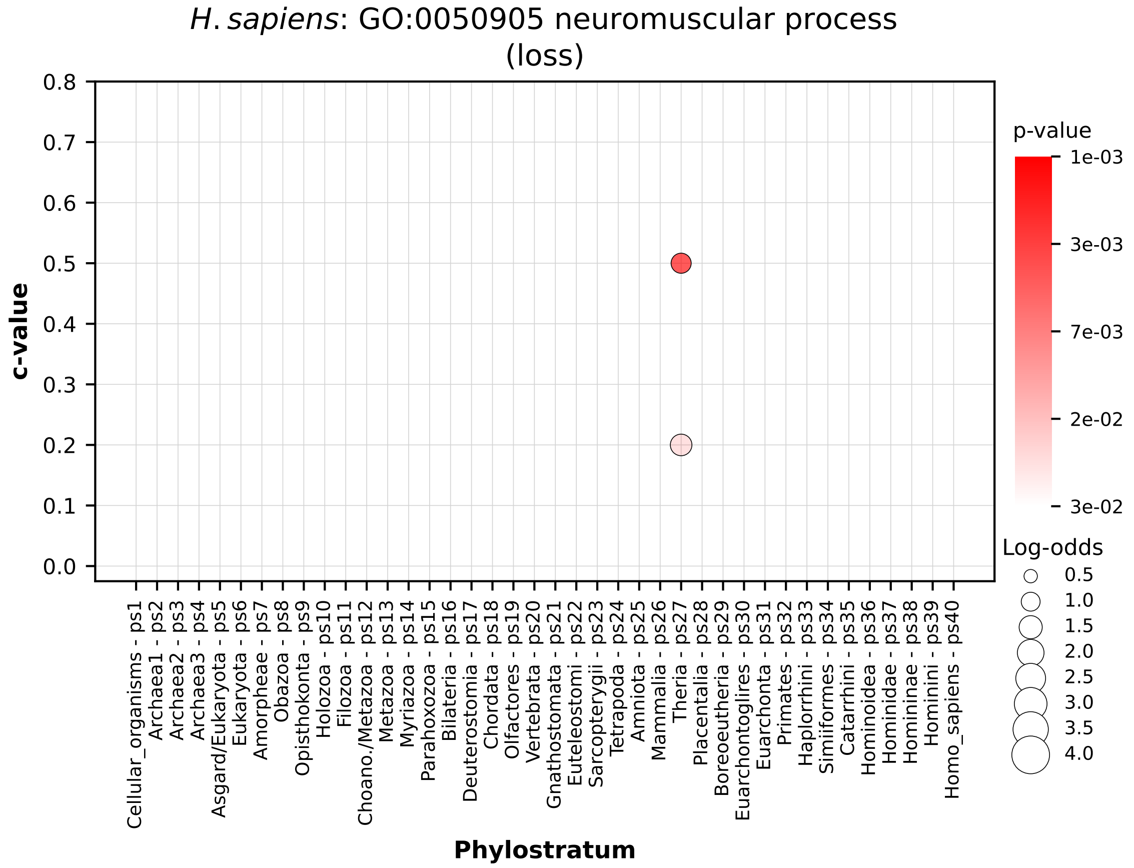

H. sapiens: GO:0050905 neuromuscular process
(loss)
c-value
0.8
0.7
0.6
0.5
0.4
0.3
0.2
0.1
0.0
p-value
1e-03
3e-03
7e-03
2e-02
3e-02
Log-odds
0.5
1.0
1.5
2.0
2.5
3.0
3.5
4.0
Cellular_organisms - ps1
Archaea1 - ps2
Archaea2 - ps3
Archaea3 - ps4
Asgard/Eukaryota - ps5
Eukaryota - ps6
Amorpheae - ps7
Obazoa - ps8
Opisthokonta - ps9
Holozoa - ps10
Filozoa - ps11
Choano./Metazoa - ps12
Metazoa - ps13
Myriazoa - ps14
Parahoxozoa - ps15
Bilateria - ps16
Deuterostomia - ps17
Chordata - ps18
Olfactores - ps19
Vertebrata - ps20
Gnathostomata - ps21
Euteleostomi - ps22
Sarcopterygii - ps23
Tetrapoda - ps24
Amniota - ps25
Mammalia - ps26
Theria - ps27
Placentalia - ps28
Boreoeutheria - ps29
Euarchontoglires - ps30
Euarchonta - ps31
Primates - ps32
Haplorrhini - ps33
Simiiformes - ps34
Catarrhini - ps35
Hominoidea - ps36
Hominidae - ps37
Homininae - ps38
Hominini - ps39
Homo_sapiens - ps40
Phylostratum

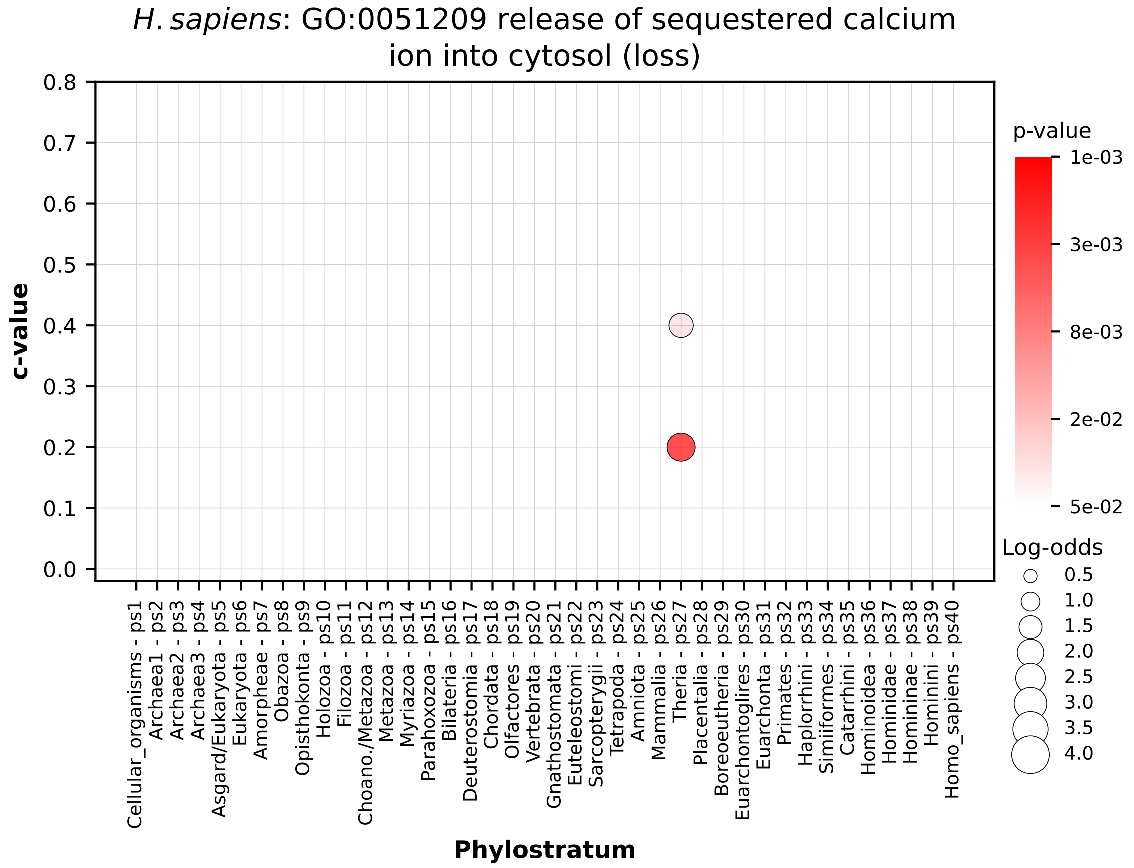

H. sapiens: GO:0051209 release of sequestered calcium ion into cytosol (loss)
c-value
0.8
0.7
0.6
0.5
0.4
0.3
0.2
0.1
0.0
Phylostratum
Cellular_organisms - ps1
Archaea1 - ps2
Archaea2 - ps3
Archaea3 - ps4
Asgard/Eukaryota - ps5
Eukaryota - ps6
Amorpheae - ps7
Obazoa - ps8
Opisthokonta - ps9
Holozoa - ps10
Filozoa - ps11
Choano./Metazoa - ps12
Metazoa - ps13
Myriazoa - ps14
Parahoxozoa - ps15
Bilateria - ps16
Deuterostomia - ps17
Chordata - ps18
Olfactores - ps19
Vertebrata - ps20
Gnathostomata - ps21
Euteleostomi - ps22
Sarcopterygii - ps23
Tetrapoda - ps24
Amniota - ps25
Mammalia - ps26
Theria - ps27
Placentalia - ps28
Boreoeutheria - ps29
Euarchontoglires - ps30
Euarchonta - ps31
Primates - ps32
Haplorrhini - ps33
Simiiformes - ps34
Catarrhini - ps35
Hominoidea - ps36
Hominidae - ps37
Homininae - ps38
Hominini - ps39
Homo_sapiens - ps40
p-value
1e-03
3e-03
8e-03
2e-02
5e-02
Log-odds
0.5
1.0
1.5
2.0
2.5
3.0
3.5
4.0

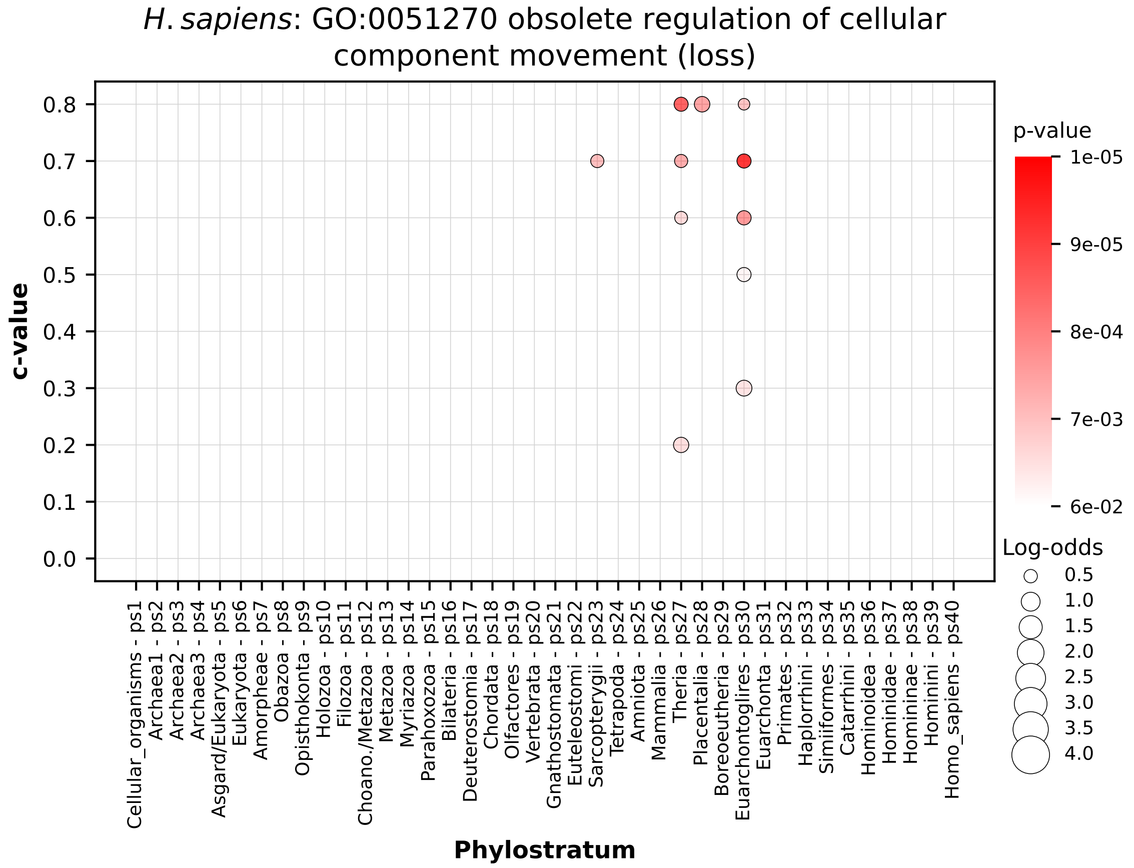

H. sapiens: GO:0051270 obsolete regulation of cellular component movement (loss)
c-value
0.0
0.1
0.2
0.3
0.4
0.5
0.6
0.7
0.8
Phylostratum
Cellular_organisms - ps1
Archaea1 - ps2
Archaea2 - ps3
Archaea3 - ps4
Asgard/Eukaryota - ps5
Eukaryota - ps6
Amorpheae - ps7
Obazoa - ps8
Opisthokonta - ps9
Holozoa - ps10
Filozoa - ps11
Choano./Metazoa - ps12
Metazoa - ps13
Myriazoa - ps14
Parahoxozoa - ps15
Bilateria - ps16
Deuterostomia - ps17
Chordata - ps18
Olfactores - ps19
Vertebrata - ps20
Gnathostomata - ps21
Euteleostomi - ps22
Sarcopterygii - ps23
Tetrapoda - ps24
Amniota - ps25
Mammalia - ps26
Theria - ps27
Placentalia - ps28
Boreoeutheria - ps29
Euarchontoglires - ps30
Euarchonta - ps31
Primates - ps32
Haplorrhini - ps33
Simiiformes - ps34
Catarrhini - ps35
Hominoidea - ps36
Hominidae - ps37
Homininae - ps38
Hominini - ps39
Homo_sapiens - ps40
p-value
1e-05
9e-05
8e-04
7e-03
6e-02
Log-odds
0.5
1.0
1.5
2.0
2.5
3.0
3.5
4.0

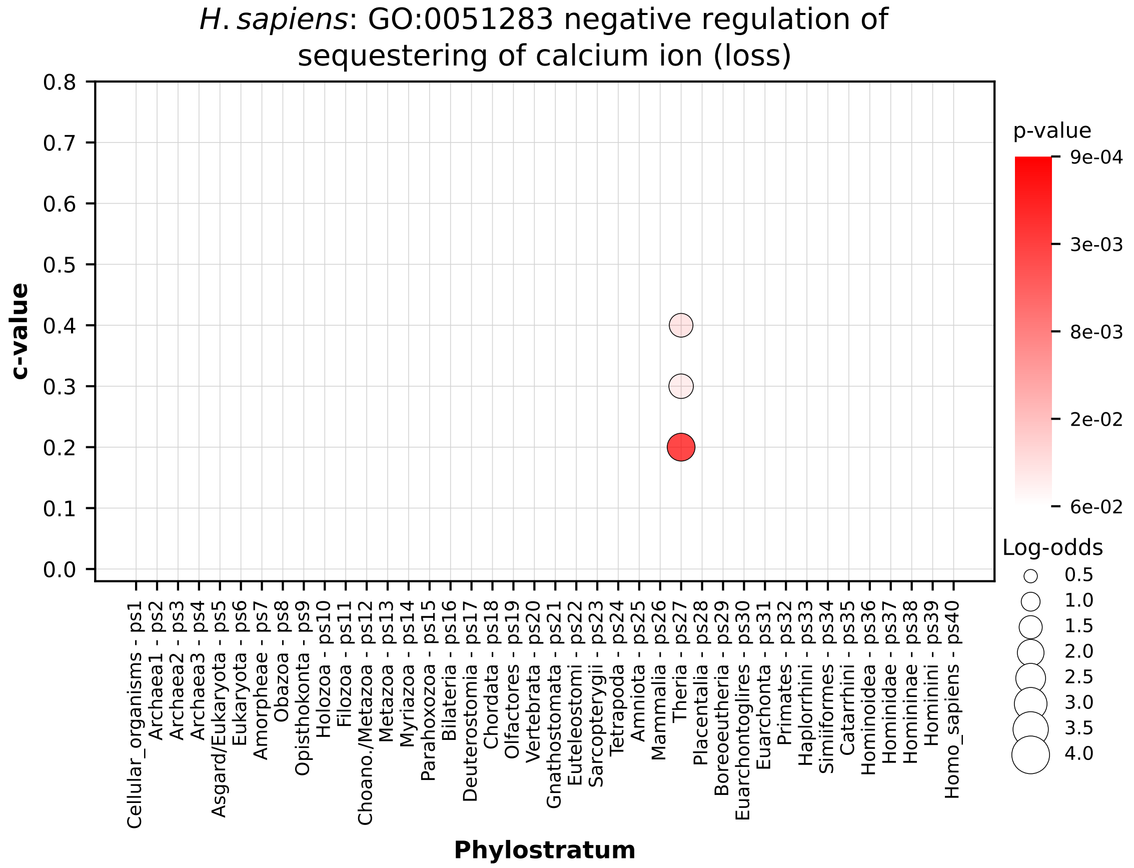

H. sapiens: GO:0051283 negative regulation of sequestering of calcium ion (loss)
c-value
0.8
0.7
0.6
0.5
0.4
0.3
0.2
0.1
0.0
p-value
9e-04
3e-03
8e-03
2e-02
6e-02
Log-odds
0.5
1.0
1.5
2.0
2.5
3.0
3.5
4.0
Cellular_organisms - ps1
Archaea1 - ps2
Archaea2 - ps3
Archaea3 - ps4
Asgard/Eukaryota - ps5
Eukaryota - ps6
Amorpheae - ps7
Obazoa - ps8
Opisthokonta - ps9
Holozoa - ps10
Filozoa - ps11
Choano./Metazoa - ps12
Metazoa - ps13
Myriazoa - ps14
Parahoxozoa - ps15
Bilateria - ps16
Deuterostomia - ps17
Chordata - ps18
Olfactores - ps19
Vertebrata - ps20
Gnathostomata - ps21
Euteleostomi - ps22
Sarcopterygii - ps23
Tetrapoda - ps24
Amniota - ps25
Mammalia - ps26
Theria - ps27
Placentalia - ps28
Boreoeutheria - ps29
Euarchontoglires - ps30
Euarchonta - ps31
Primates - ps32
Haplorrhini - ps33
Simiiformes - ps34
Catarrhini - ps35
Hominoidea - ps36
Hominidae - ps37
Homininae - ps38
Hominini - ps39
Homo_sapiens - ps40
Phylostratum

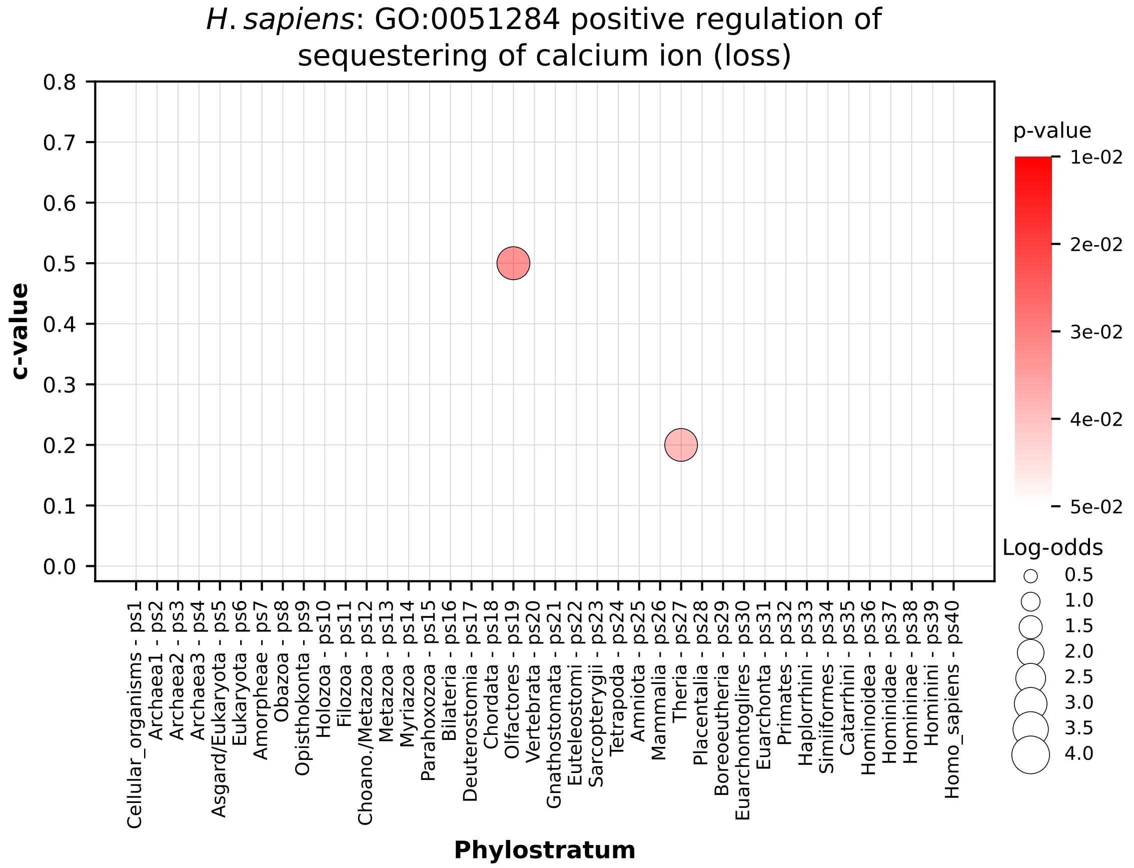
H. sapiens: GO:0051284 positive regulation of sequestering of calcium ion (loss)
c-value
0.0
0.1
0.2
0.3
0.4
0.5
0.6
0.7
0.8
Phylostratum
Cellular_organisms - ps1
Archaea1 - ps2
Archaea2 - ps3
Archaea3 - ps4
Asgard/Eukaryota - ps5
Eukaryota - ps6
Amorpheae - ps7
Obazoa - ps8
Opisthokonta - ps9
Holozoa - ps10
Filozoa - ps11
Choano./Metazoa - ps12
Metazoa - ps13
Myriazoa - ps14
Parahoxozoa - ps15
Bilateria - ps16
Deuterostomia - ps17
Chordata - ps18
Olfactores - ps19
Vertebrata - ps20
Gnathostomata - ps21
Euteleostomi - ps22
Sarcopterygii - ps23
Tetrapoda - ps24
Amniota - ps25
Mammalia - ps26
Theria - ps27
Placentalia - ps28
Boreoeutheria - ps29
Euarchontoglires - ps30
Euarchonta - ps31
Primates - ps32
Haplorrhini - ps33
Simiiformes - ps34
Catarrhini - ps35
Hominoidea - ps36
Hominidae - ps37
Homininae - ps38
Hominini - ps39
Homo_sapiens - ps40
p-value
1e-02
2e-02
3e-02
4e-02
5e-02
Log-odds
0.5
1.0
1.5
2.0
2.5
3.0
3.5
4.0

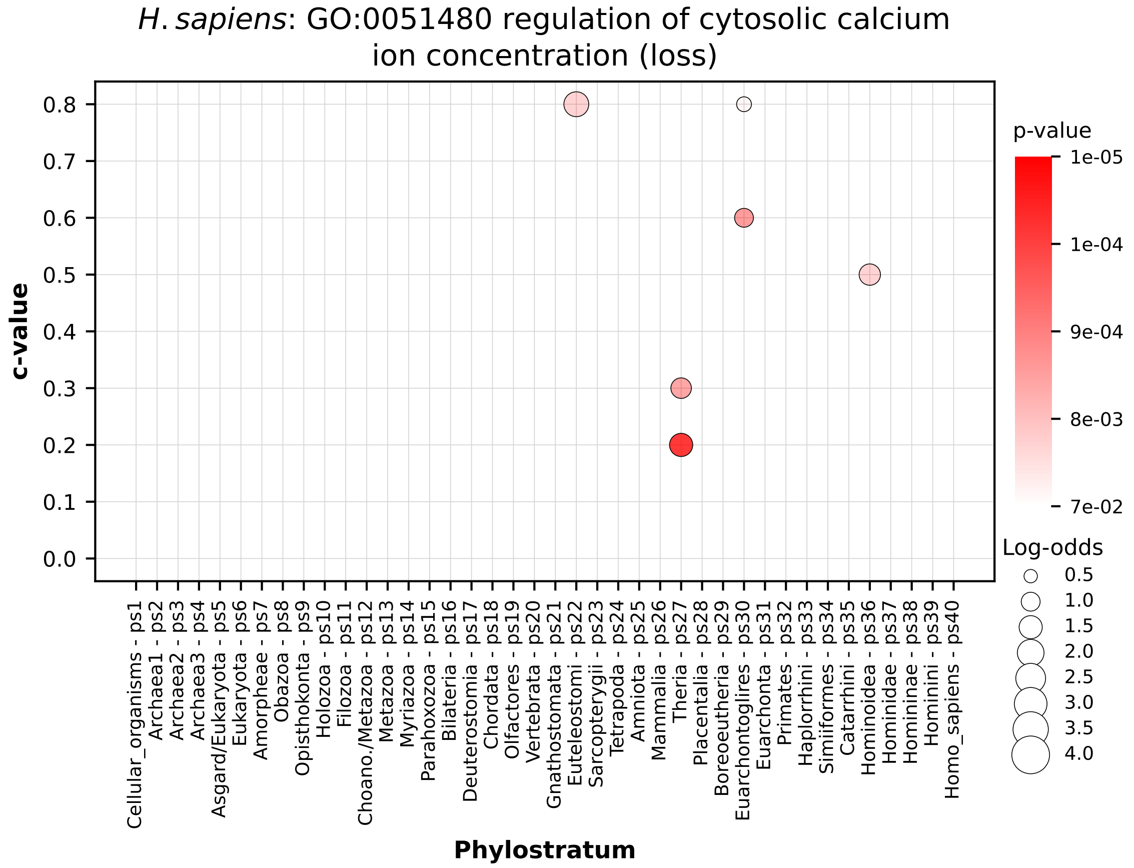

H. sapiens: GO:0051480 regulation of cytosolic calcium ion concentration (loss)
c-value
0.8
0.7
0.6
0.5
0.4
0.3
0.2
0.1
0.0
p-value
1e-05
1e-04
9e-04
8e-03
7e-02
Log-odds
0.5
1.0
1.5
2.0
2.5
3.0
3.5
4.0
Cellular_organisms - ps1
Archaea1 - ps2
Archaea2 - ps3
Archaea3 - ps4
Asgard/Eukaryota - ps5
Eukaryota - ps6
Amorpheae - ps7
Obazoa - ps8
Opisthokonta - ps9
Holozoa - ps10
Filozoa - ps11
Choano./Metazoa - ps12
Metazoa - ps13
Myriazoa - ps14
Parahoxozoa - ps15
Bilateria - ps16
Deuterostomia - ps17
Chordata - ps18
Olfactores - ps19
Vertebrata - ps20
Gnathostomata - ps21
Euteleostomi - ps22
Sarcopterygii - ps23
Tetrapoda - ps24
Amniota - ps25
Mammalia - ps26
Theria - ps27
Placentalia - ps28
Boreoeutheria - ps29
Euarchontoglires - ps30
Euarchonta - ps31
Primates - ps32
Haplorrhini - ps33
Simiiformes - ps34
Catarrhini - ps35
Hominoidea - ps36
Hominidae - ps37
Homininae - ps38
Hominini - ps39
Homo_sapiens - ps40
Phylostratum

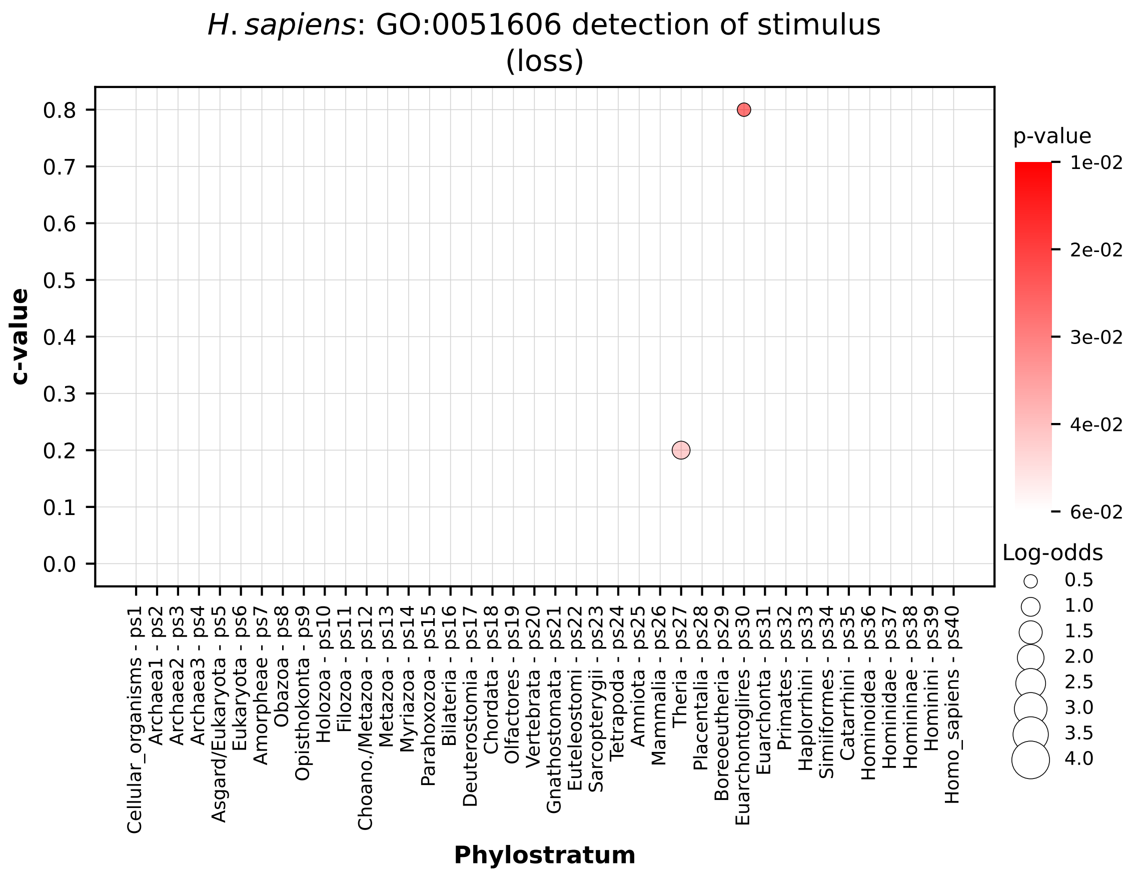

H. sapiens: GO:0051606 detection of stimulus
(loss)
c-value
0.0
0.1
0.2
0.3
0.4
0.5
0.6
0.7
0.8
p-value
1e-02
2e-02
3e-02
4e-02
6e-02
Log-odds
0.5
1.0
1.5
2.0
2.5
3.0
3.5
4.0
Cellular_organisms - ps1
Archaea1 - ps2
Archaea2 - ps3
Archaea3 - ps4
Asgard/Eukaryota - ps5
Eukaryota - ps6
Amorpheae - ps7
Obazoa - ps8
Opisthokonta - ps9
Holozoa - ps10
Filozoa - ps11
Choano./Metazoa - ps12
Metazoa - ps13
Myriazoa - ps14
Parahoxozoa - ps15
Bilateria - ps16
Deuterostomia - ps17
Chordata - ps18
Olfactores - ps19
Vertebrata - ps20
Gnathostomata - ps21
Euteleostomi - ps22
Sarcopterygii - ps23
Tetrapoda - ps24
Amniota - ps25
Mammalia - ps26
Theria - ps27
Placentalia - ps28
Boreoeutheria - ps29
Euarchontoglires - ps30
Euarchonta - ps31
Primates - ps32
Haplorrhini - ps33
Simiiformes - ps34
Catarrhini - ps35
Hominoidea - ps36
Hominidae - ps37
Homininae - ps38
Hominini - ps39
Homo_sapiens - ps40
Phylostratum

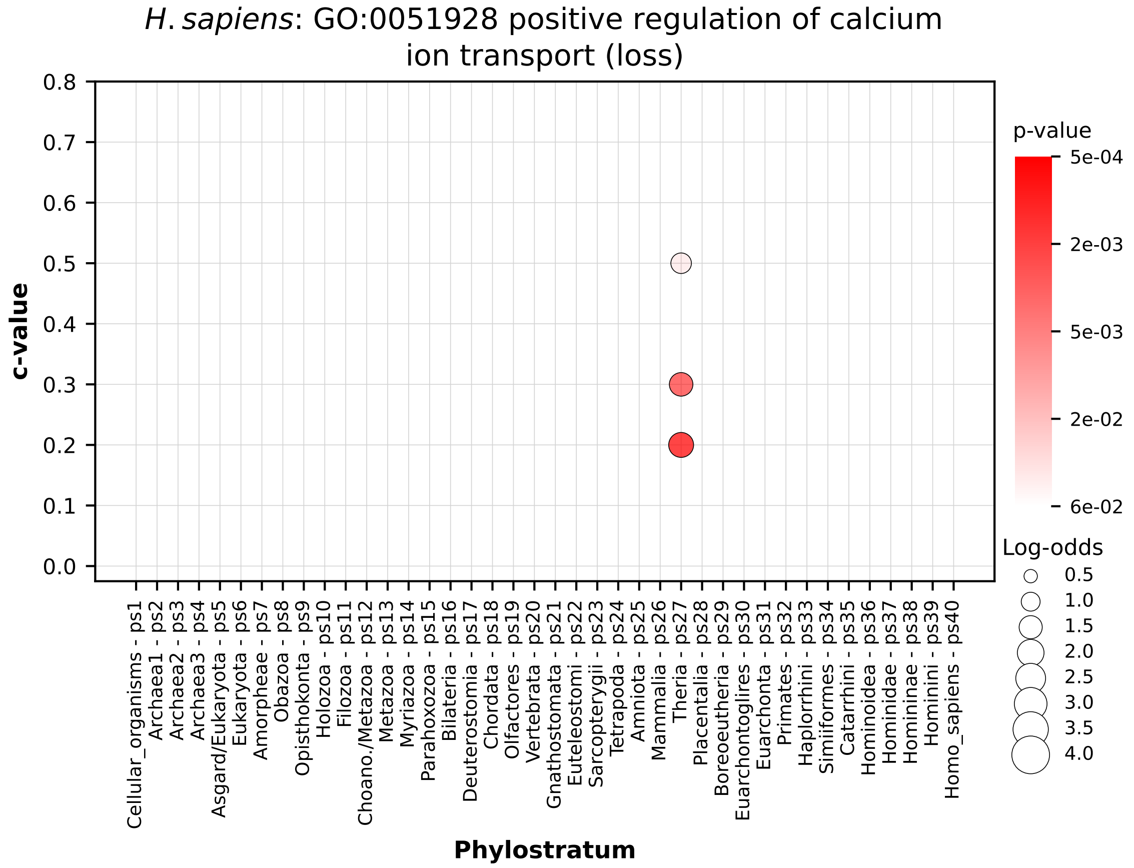

H. sapiens: GO:0051928 positive regulation of calcium ion transport (loss)
c-value
0.8
0.7
0.6
0.5
0.4
0.3
0.2
0.1
0.0
Phylostratum
Cellular_organisms - ps1
Archaea1 - ps2
Archaea2 - ps3
Archaea3 - ps4
Asgard/Eukaryota - ps5
Eukaryota - ps6
Amorpheae - ps7
Obazoa - ps8
Opisthokonta - ps9
Holozoa - ps10
Filozoa - ps11
Choano./Metazoa - ps12
Metazoa - ps13
Myriazoa - ps14
Parahoxozoa - ps15
Bilateria - ps16
Deuterostomia - ps17
Chordata - ps18
Olfactores - ps19
Vertebrata - ps20
Gnathostomata - ps21
Euteleostomi - ps22
Sarcopterygii - ps23
Tetrapoda - ps24
Amniota - ps25
Mammalia - ps26
Theria - ps27
Placentalia - ps28
Boreoeutheria - ps29
Euarchontoglires - ps30
Euarchonta - ps31
Primates - ps32
Haplorrhini - ps33
Simiiformes - ps34
Catarrhini - ps35
Hominoidea - ps36
Hominidae - ps37
Homininae - ps38
Hominini - ps39
Homo_sapiens - ps40
p-value
5e-04
2e-03
5e-03
2e-02
6e-02
Log-odds
0.5
1.0
1.5
2.0
2.5
3.0
3.5
4.0

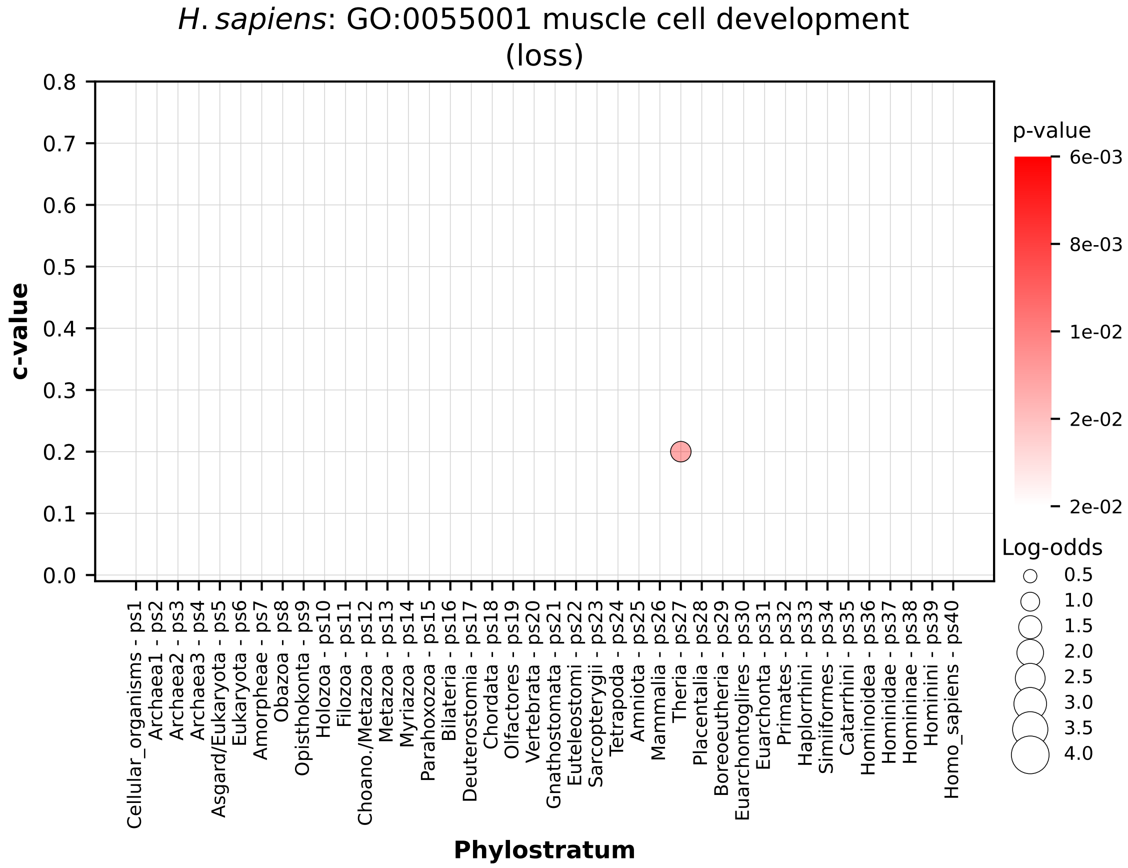

H. sapiens: GO:0055001 muscle cell development (loss)
c-value
0.8
0.7
0.6
0.5
0.4
0.3
0.2
0.1
0.0
Phylostratum
Cellular_organisms - ps1
Archaea1 - ps2
Archaea2 - ps3
Archaea3 - ps4
Asgard/Eukaryota - ps5
Eukaryota - ps6
Amorpheae - ps7
Obazoa - ps8
Opisthokonta - ps9
Holozoa - ps10
Filozoa - ps11
Choano./Metazoa - ps12
Metazoa - ps13
Myriazoa - ps14
Parahoxozoa - ps15
Bilateria - ps16
Deuterostomia - ps17
Chordata - ps18
Olfactores - ps19
Vertebrata - ps20
Gnathostomata - ps21
Euteleostomi - ps22
Sarcopterygii - ps23
Tetrapoda - ps24
Amniota - ps25
Mammalia - ps26
Theria - ps27
Placentalia - ps28
Boreoeutheria - ps29
Euarchontoglires - ps30
Euarchonta - ps31
Primates - ps32
Haplorrhini - ps33
Simiiformes - ps34
Catarrhini - ps35
Hominoidea - ps36
Hominidae - ps37
Homininae - ps38
Hominini - ps39
Homo_sapiens - ps40
p-value
6e-03
8e-03
1e-02
2e-02
2e-02
Log-odds
0.5
1.0
1.5
2.0
2.5
3.0
3.5
4.0

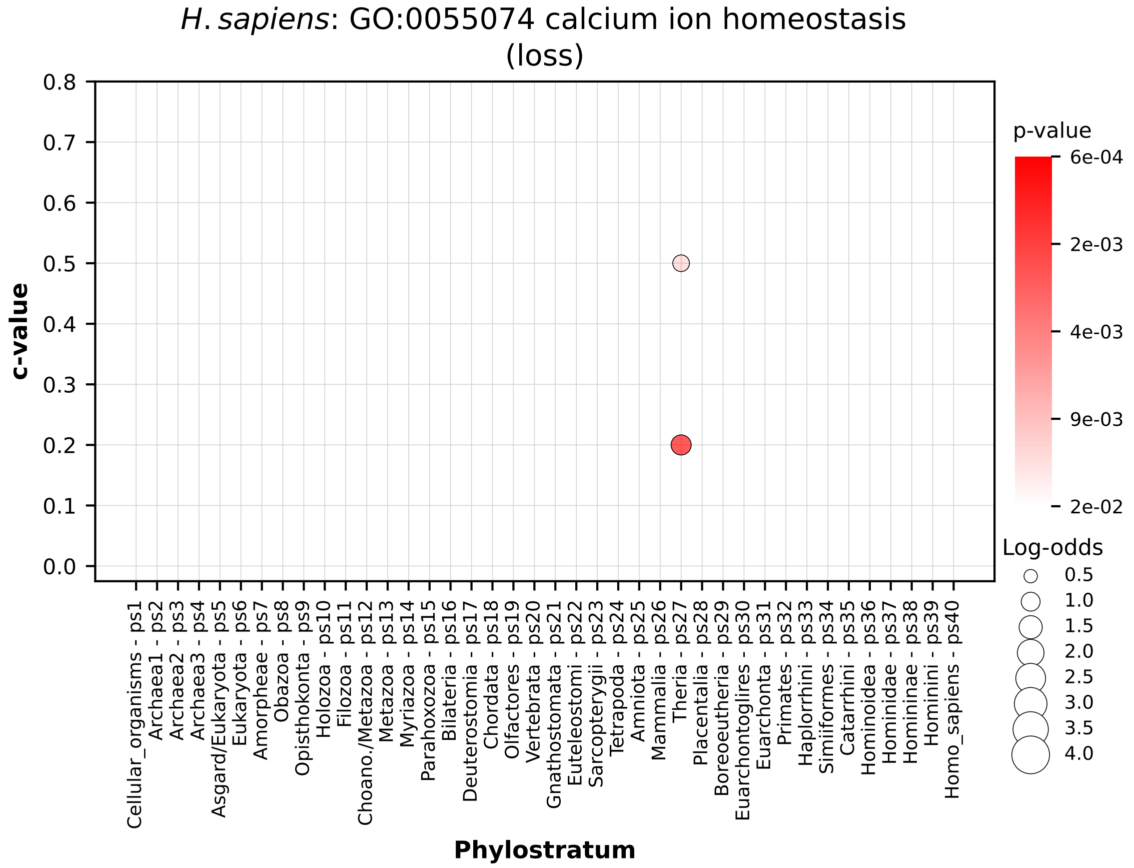
H. sapiens: GO:0055074 calcium ion homeostasis
(loss)
c-value
0.8
0.7
0.6
0.5
0.4
0.3
0.2
0.1
0.0
Phylostratum
Cellular_organisms - ps1
Archaea1 - ps2
Archaea2 - ps3
Archaea3 - ps4
Asgard/Eukaryota - ps5
Eukaryota - ps6
Amorpheae - ps7
Obazoa - ps8
Opisthokonta - ps9
Holozoa - ps10
Filozoa - ps11
Choano./Metazoa - ps12
Metazoa - ps13
Myriazoa - ps14
Parahoxozoa - ps15
Bilateria - ps16
Deuterostomia - ps17
Chordata - ps18
Olfactores - ps19
Vertebrata - ps20
Gnathostomata - ps21
Euteleostomi - ps22
Sarcopterygii - ps23
Tetrapoda - ps24
Amniota - ps25
Mammalia - ps26
Theria - ps27
Placentalia - ps28
Boreoeutheria - ps29
Euarchontoglires - ps30
Euarchonta - ps31
Primates - ps32
Haplorrhini - ps33
Simiiformes - ps34
Catarrhini - ps35
Hominoidea - ps36
Hominidae - ps37
Homininae - ps38
Hominini - ps39
Homo_sapiens - ps40
p-value
6e-04
2e-03
4e-03
9e-03
2e-02
Log-odds
0.5
1.0
1.5
2.0
2.5
3.0
3.5
4.0

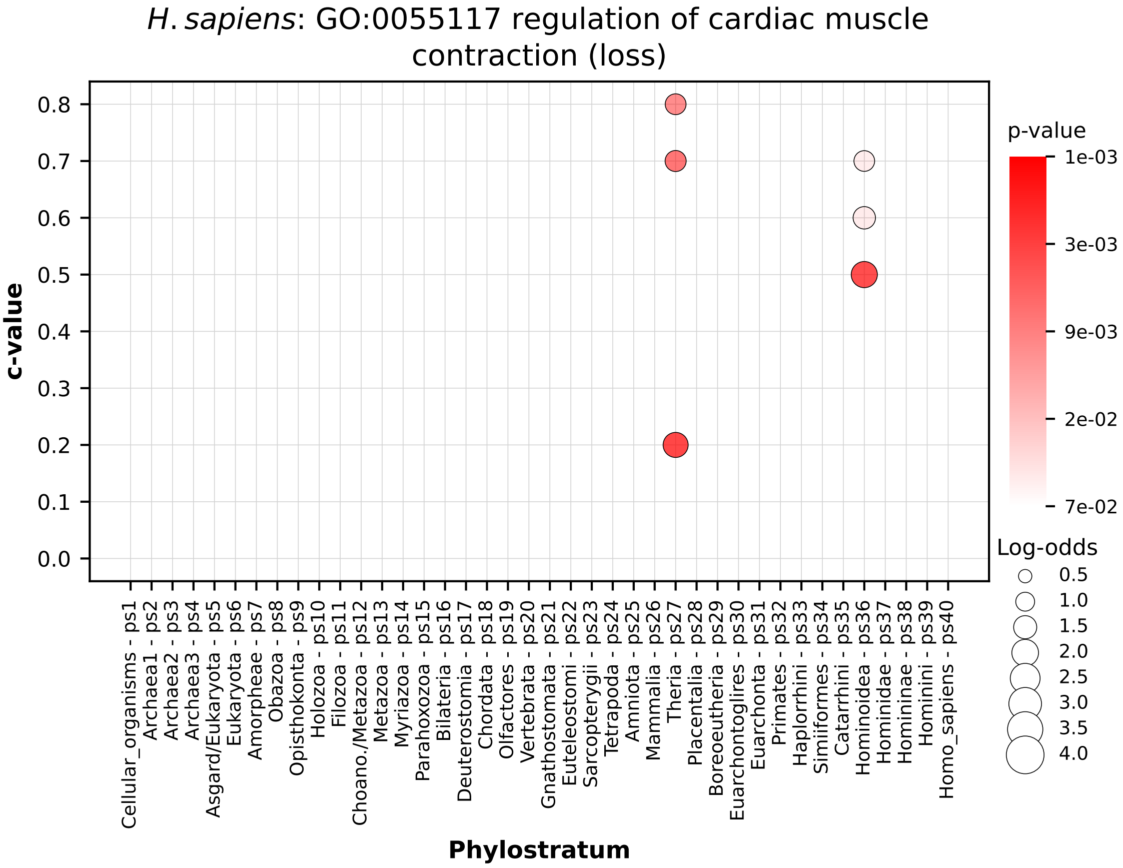

H. sapiens: GO:0055117 regulation of cardiac muscle contraction (loss)
c-value
0.0
0.1
0.2
0.3
0.4
0.5
0.6
0.7
0.8
Phylostratum
Cellular_organisms - ps1
Archaea1 - ps2
Archaea2 - ps3
Archaea3 - ps4
Asgard/Eukaryota - ps5
Eukaryota - ps6
Amorpheae - ps7
Obazoa - ps8
Opisthokonta - ps9
Holozoa - ps10
Filozoa - ps11
Choano./Metazoa - ps12
Metazoa - ps13
Myriazoa - ps14
Parahoxozoa - ps15
Bilateria - ps16
Deuterostomia - ps17
Chordata - ps18
Olfactores - ps19
Vertebrata - ps20
Gnathostomata - ps21
Euteleostomi - ps22
Sarcopterygii - ps23
Tetrapoda - ps24
Amniota - ps25
Mammalia - ps26
Theria - ps27
Placentalia - ps28
Boreoeutheria - ps29
Euarchontoglires - ps30
Euarchonta - ps31
Primates - ps32
Haplorrhini - ps33
Simiiformes - ps34
Catarrhini - ps35
Hominoidea - ps36
Hominidae - ps37
Homininae - ps38
Hominini - ps39
Homo_sapiens - ps40
p-value
1e-03
3e-03
9e-03
2e-02
7e-02
Log-odds
0.5
1.0
1.5
2.0
2.5
3.0
3.5
4.0

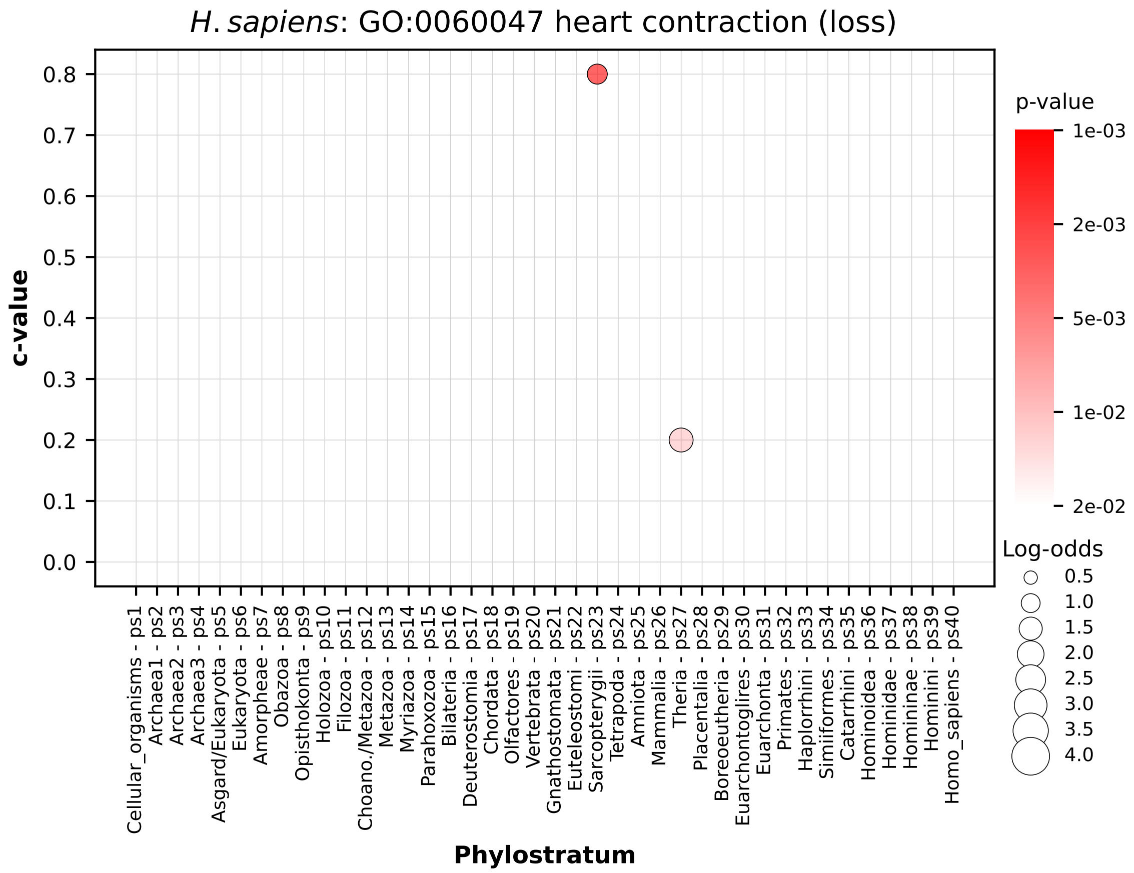

H. sapiens: GO:0060047 heart contraction (loss)
c-value
0.8
0.7
0.6
0.5
0.4
0.3
0.2
0.1
0.0
p-value
1e-03
2e-03
5e-03
1e-02
2e-02
Log-odds
0.5
1.0
1.5
2.0
2.5
3.0
3.5
4.0
Cellular_organisms - ps1
Archaea1 - ps2
Archaea2 - ps3
Archaea3 - ps4
Asgard/Eukaryota - ps5
Eukaryota - ps6
Amorpheae - ps7
Obazoa - ps8
Opisthokonta - ps9
Holozoa - ps10
Filozoa - ps11
Choano./Metazoa - ps12
Metazoa - ps13
Myriazoa - ps14
Parahoxozoa - ps15
Bilateria - ps16
Deuterostomia - ps17
Chordata - ps18
Olfactores - ps19
Vertebrata - ps20
Gnathostomata - ps21
Euteleostomi - ps22
Sarcopterygii - ps23
Tetrapoda - ps24
Amniota - ps25
Mammalia - ps26
Theria - ps27
Placentalia - ps28
Boreoeutheria - ps29
Euarchontoglires - ps30
Euarchonta - ps31
Primates - ps32
Haplorrhini - ps33
Simiiformes - ps34
Catarrhini - ps35
Hominoidea - ps36
Hominidae - ps37
Homininae - ps38
Hominini - ps39
Homo_sapiens - ps40
Phylostratum

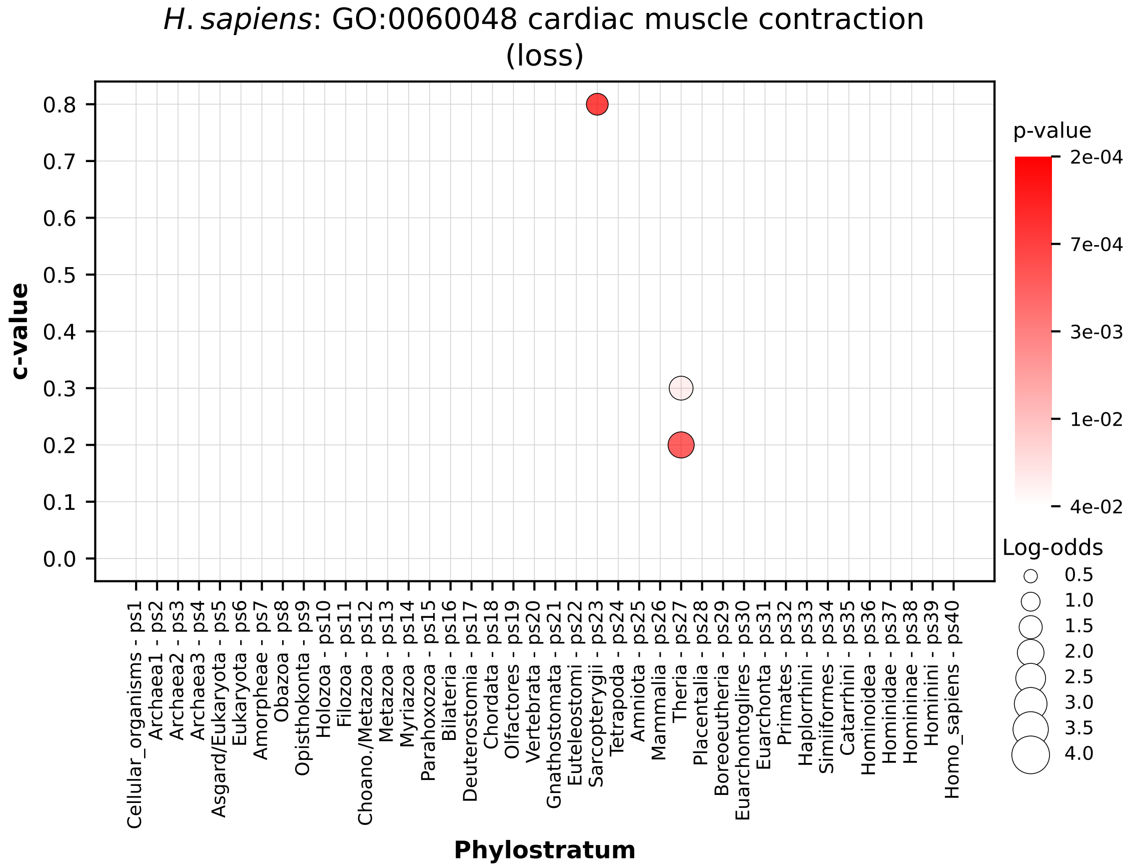

H. sapiens: GO:0060048 cardiac muscle contraction
(loss)
c-value
0.8
0.7
0.6
0.5
0.4
0.3
0.2
0.1
0.0
p-value
2e-04
7e-04
3e-03
1e-02
4e-02
Log-odds
0.5
1.0
1.5
2.0
2.5
3.0
3.5
4.0
Cellular_organisms - ps1
Archaea1 - ps2
Archaea2 - ps3
Archaea3 - ps4
Asgard/Eukaryota - ps5
Eukaryota - ps6
Amorpheae - ps7
Obazoa - ps8
Opisthokonta - ps9
Holozoa - ps10
Filozoa - ps11
Choano./Metazoa - ps12
Metazoa - ps13
Myriazoa - ps14
Parahoxozoa - ps15
Bilateria - ps16
Deuterostomia - ps17
Chordata - ps18
Olfactores - ps19
Vertebrata - ps20
Gnathostomata - ps21
Euteleostomi - ps22
Sarcopterygii - ps23
Tetrapoda - ps24
Amniota - ps25
Mammalia - ps26
Theria - ps27
Placentalia - ps28
Boreoeutheria - ps29
Euarchontoglires - ps30
Euarchonta - ps31
Primates - ps32
Haplorrhini - ps33
Simiiformes - ps34
Catarrhini - ps35
Hominoidea - ps36
Hominidae - ps37
Homininae - ps38
Hominini - ps39
Homo_sapiens - ps40
Phylostratum

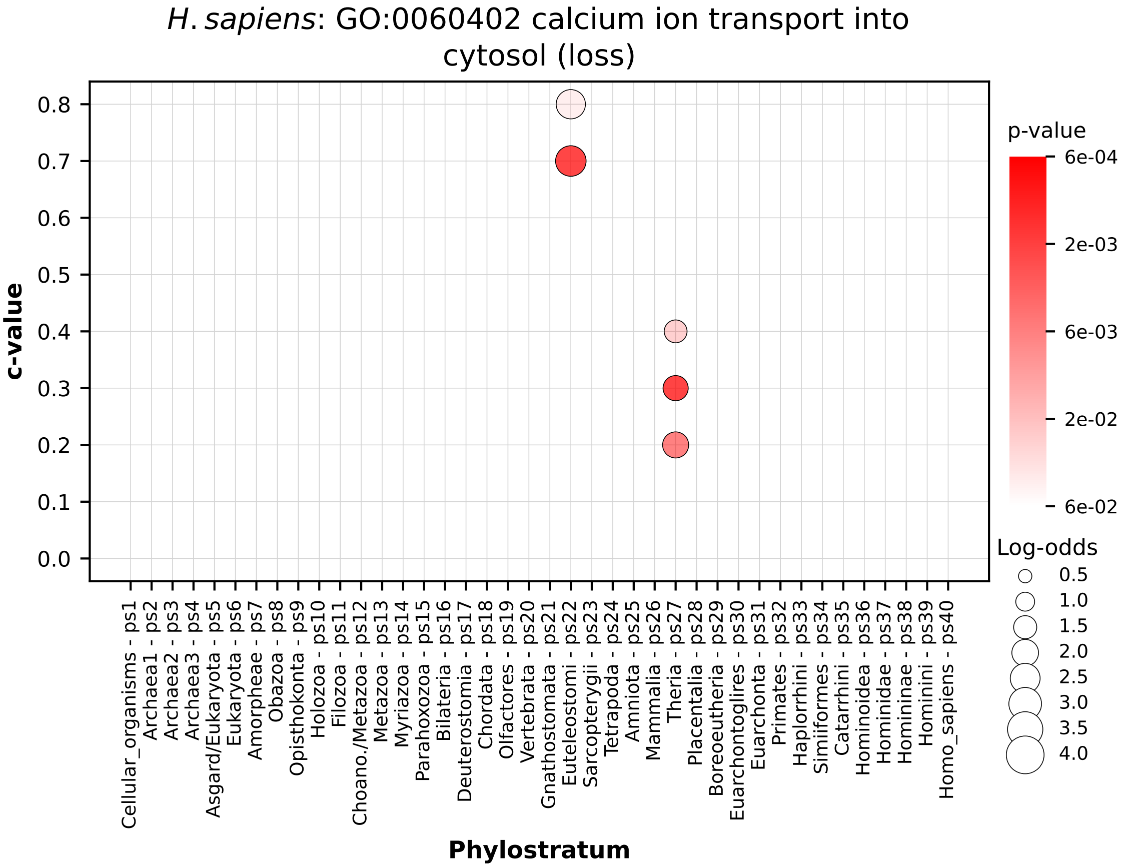

H. sapiens: GO:0060402 calcium ion transport into cytosol (loss)
c-value
0.0
0.1
0.2
0.3
0.4
0.5
0.6
0.7
0.8
Phylostratum
Cellular_organisms - ps1
Archaea1 - ps2
Archaea2 - ps3
Archaea3 - ps4
Asgard/Eukaryota - ps5
Eukaryota - ps6
Amorpheae - ps7
Obazoa - ps8
Opisthokonta - ps9
Holozoa - ps10
Filozoa - ps11
Choano./Metazoa - ps12
Metazoa - ps13
Myriazoa - ps14
Parahoxozoa - ps15
Bilateria - ps16
Deuterostomia - ps17
Chordata - ps18
Olfactores - ps19
Vertebrata - ps20
Gnathostomata - ps21
Euteleostomi - ps22
Sarcopterygii - ps23
Tetrapoda - ps24
Amniota - ps25
Mammalia - ps26
Theria - ps27
Placentalia - ps28
Boreoeutheria - ps29
Euarchontoglires - ps30
Euarchonta - ps31
Primates - ps32
Haplorrhini - ps33
Simiiformes - ps34
Catarrhini - ps35
Hominoidea - ps36
Hominidae - ps37
Homininae - ps38
Hominini - ps39
Homo_sapiens - ps40
p-value
6e-04
2e-03
6e-03
2e-02
6e-02
Log-odds
0.5
1.0
1.5
2.0
2.5
3.0
3.5
4.0

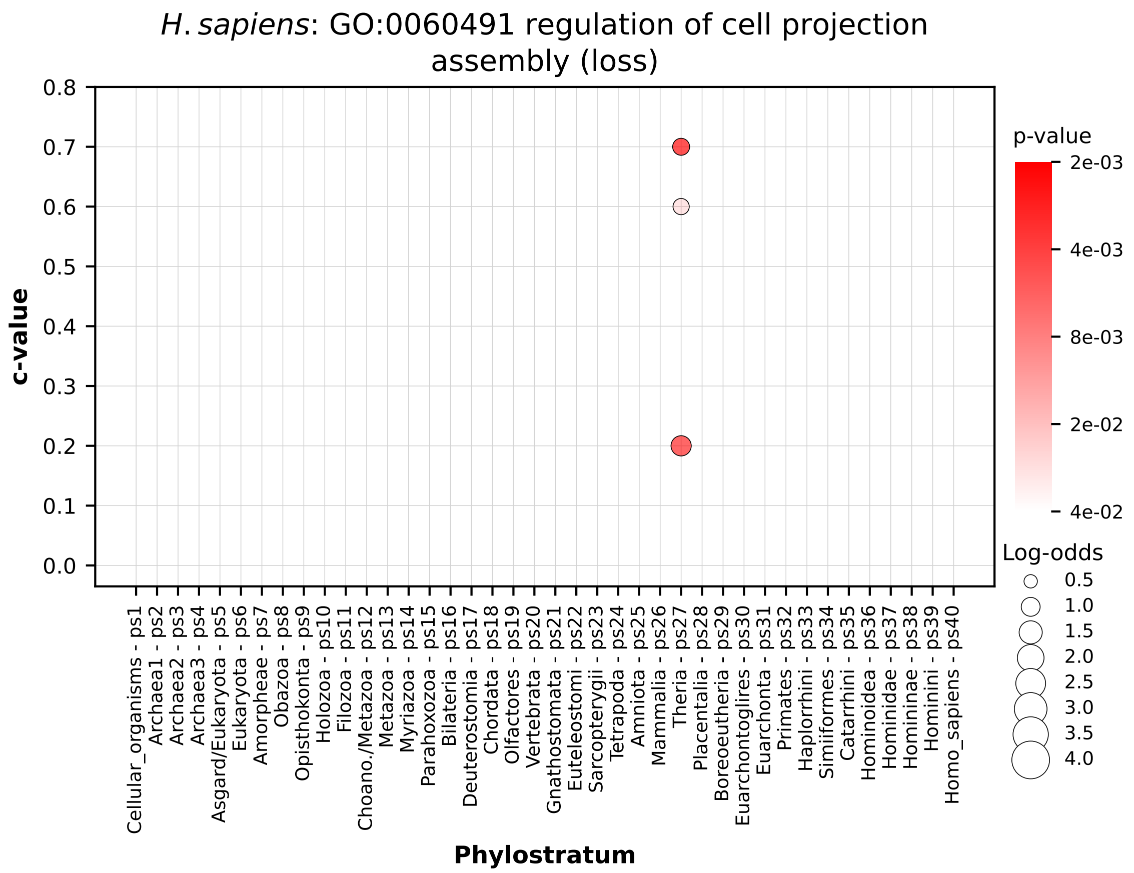

H. sapiens: GO:0060491 regulation of cell projection assembly (loss)
c-value
0.8
0.7
0.6
0.5
0.4
0.3
0.2
0.1
0.0
p-value
2e-03
4e-03
8e-03
2e-02
4e-02
Log-odds
0.5
1.0
1.5
2.0
2.5
3.0
3.5
4.0
Cellular_organisms - ps1
Archaea1 - ps2
Archaea2 - ps3
Archaea3 - ps4
Asgard/Eukaryota - ps5
Eukaryota - ps6
Amorpheae - ps7
Obazoa - ps8
Opisthokonta - ps9
Holozoa - ps10
Filozoa - ps11
Choano./Metazoa - ps12
Metazoa - ps13
Myriazoa - ps14
Parahoxozoa - ps15
Bilateria - ps16
Deuterostomia - ps17
Chordata - ps18
Olfactores - ps19
Vertebrata - ps20
Gnathostomata - ps21
Euteleostomi - ps22
Sarcopterygii - ps23
Tetrapoda - ps24
Amniota - ps25
Mammalia - ps26
Theria - ps27
Placentalia - ps28
Boreoeutheria - ps29
Euarchontoglires - ps30
Euarchonta - ps31
Primates - ps32
Haplorrhini - ps33
Simiiformes - ps34
Catarrhini - ps35
Hominoidea - ps36
Hominidae - ps37
Homininae - ps38
Hominini - ps39
Homo_sapiens - ps40
Phylostratum

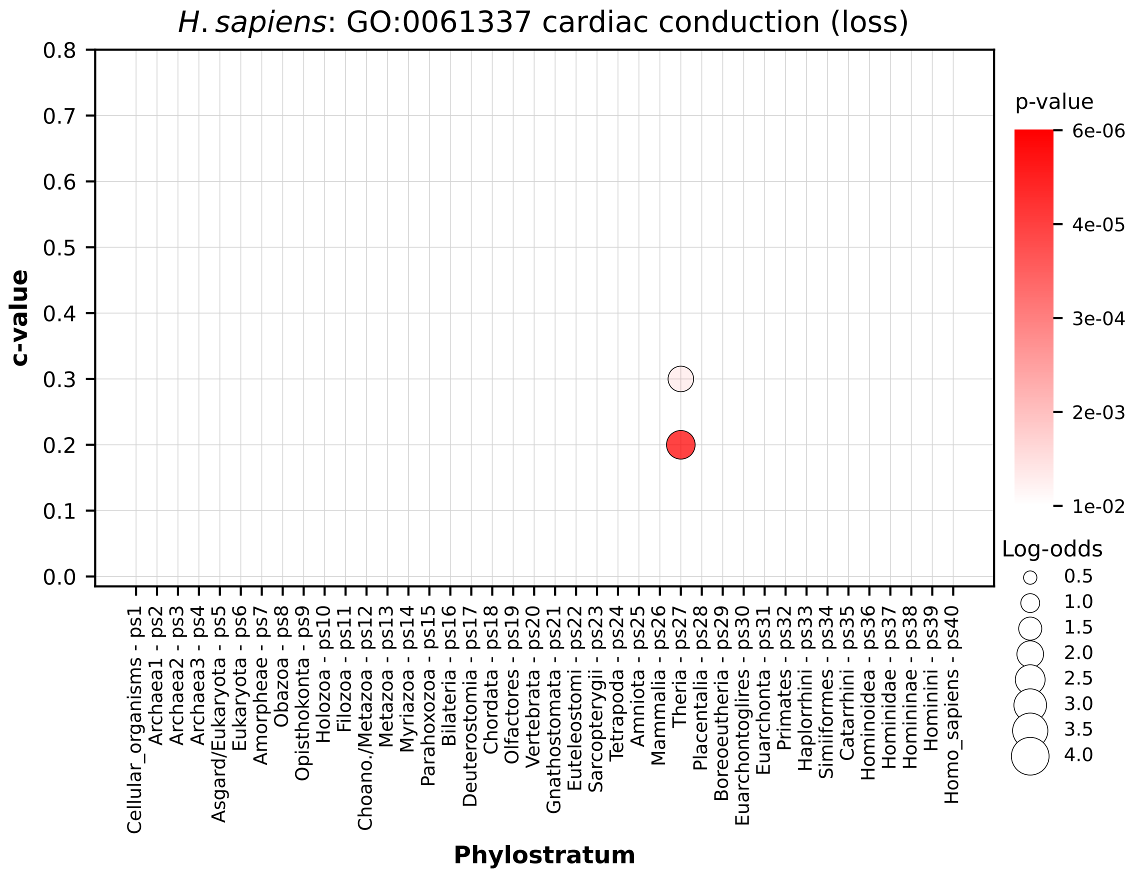

H. sapiens: GO:0061337 cardiac conduction (loss)
c-value
0.8
0.7
0.6
0.5
0.4
0.3
0.2
0.1
0.0
p-value
6e-06
4e-05
3e-04
2e-03
1e-02
Log-odds
0.5
1.0
1.5
2.0
2.5
3.0
3.5
4.0
Cellular_organisms - ps1
Archaea1 - ps2
Archaea2 - ps3
Archaea3 - ps4
Asgard/Eukaryota - ps5
Eukaryota - ps6
Amorpheae - ps7
Obazoa - ps8
Opisthokonta - ps9
Holozoa - ps10
Filozoa - ps11
Choano./Metazoa - ps12
Metazoa - ps13
Myriazoa - ps14
Parahoxozoa - ps15
Bilateria - ps16
Deuterostomia - ps17
Chordata - ps18
Olfactores - ps19
Vertebrata - ps20
Gnathostomata - ps21
Euteleostomi - ps22
Sarcopterygii - ps23
Tetrapoda - ps24
Amniota - ps25
Mammalia - ps26
Theria - ps27
Placentalia - ps28
Boreoeutheria - ps29
Euarchontoglires - ps30
Euarchonta - ps31
Primates - ps32
Haplorrhini - ps33
Simiiformes - ps34
Catarrhini - ps35
Hominoidea - ps36
Hominidae - ps37
Homininae - ps38
Hominini - ps39
Homo_sapiens - ps40
Phylostratum

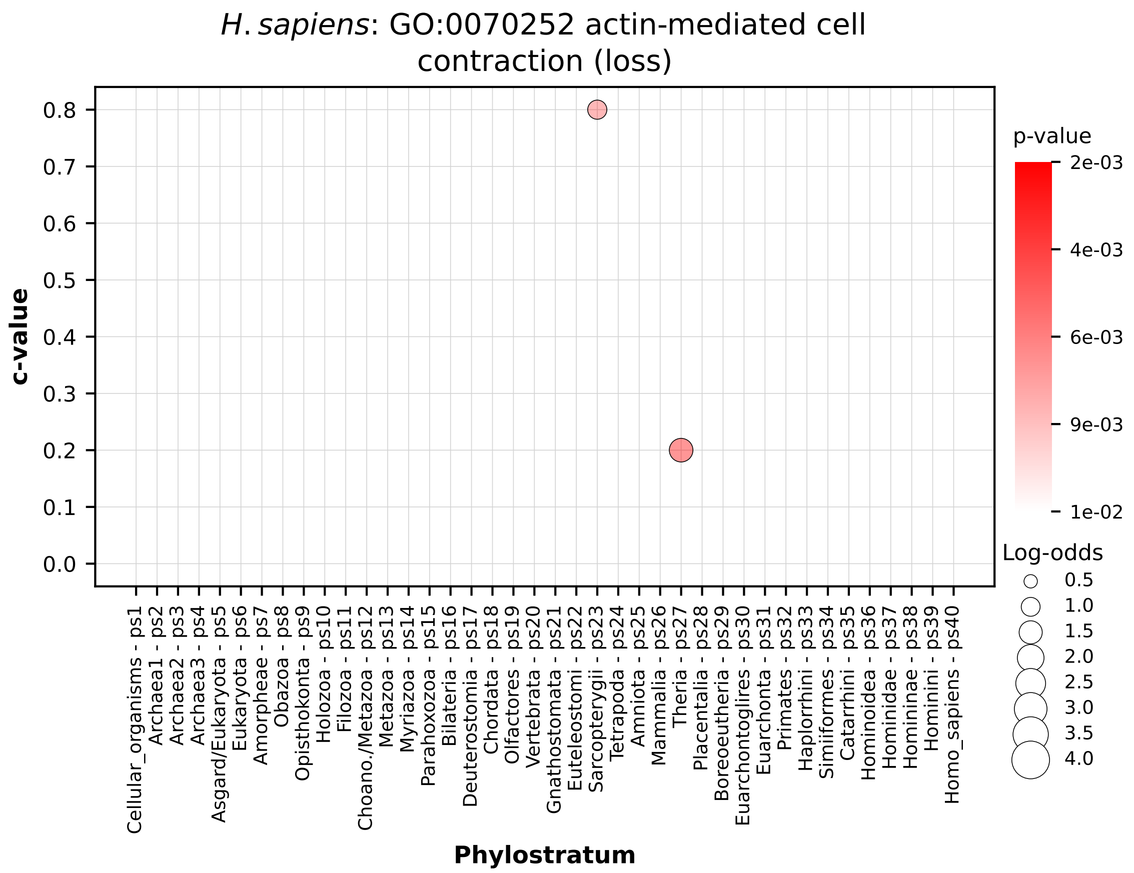
H. sapiens: GO:0070252 actin-mediated cell contraction (loss)
c-value
0.0
0.1
0.2
0.3
0.4
0.5
0.6
0.7
0.8
p-value
2e-03
4e-03
6e-03
9e-03
1e-02
Log-odds
0.5
1.0
1.5
2.0
2.5
3.0
3.5
4.0
Cellular_organisms - ps1
Archaea1 - ps2
Archaea2 - ps3
Archaea3 - ps4
Asgard/Eukaryota - ps5
Eukaryota - ps6
Amorpheae - ps7
Obazoa - ps8
Opisthokonta - ps9
Holozoa - ps10
Filozoa - ps11
Choano./Metazoa - ps12
Metazoa - ps13
Myriazoa - ps14
Parahoxozoa - ps15
Bilateria - ps16
Deuterostomia - ps17
Chordata - ps18
Olfactores - ps19
Vertebrata - ps20
Gnathostomata - ps21
Euteleostomi - ps22
Sarcopterygii - ps23
Tetrapoda - ps24
Amniota - ps25
Mammalia - ps26
Theria - ps27
Placentalia - ps28
Boreoeutheria - ps29
Euarchontoglires - ps30
Euarchonta - ps31
Primates - ps32
Haplorrhini - ps33
Simiiformes - ps34
Catarrhini - ps35
Hominoidea - ps36
Hominidae - ps37
Homininae - ps38
Hominini - ps39
Homo_sapiens - ps40
Phylostratum

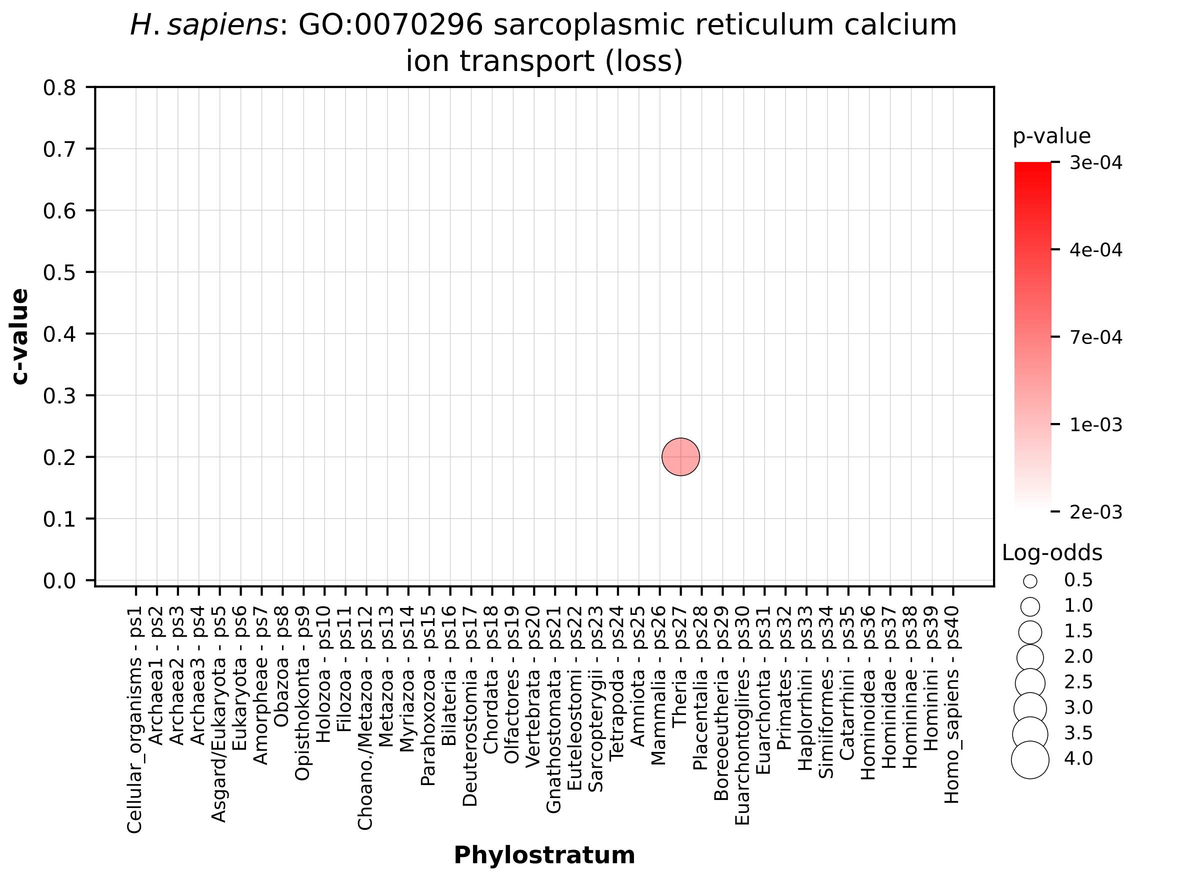

H. sapiens: GO:0070296 sarcoplasmic reticulum calcium ion transport (loss)
c-value
0.0
0.1
0.2
0.3
0.4
0.5
0.6
0.7
0.8
Phylostratum
Cellular_organisms - ps1
Archaea1 - ps2
Archaea2 - ps3
Archaea3 - ps4
Asgard/Eukaryota - ps5
Eukaryota - ps6
Amorpheae - ps7
Obazoa - ps8
Opisthokonta - ps9
Holozoa - ps10
Filozoa - ps11
Choano./Metazoa - ps12
Metazoa - ps13
Myriazoa - ps14
Parahoxozoa - ps15
Bilateria - ps16
Deuterostomia - ps17
Chordata - ps18
Olfactores - ps19
Vertebrata - ps20
Gnathostomata - ps21
Euteleostomi - ps22
Sarcopterygii - ps23
Tetrapoda - ps24
Amniota - ps25
Mammalia - ps26
Theria - ps27
Placentalia - ps28
Boreoeutheria - ps29
Euarchontoglires - ps30
Euarchonta - ps31
Primates - ps32
Haplorrhini - ps33
Simiiformes - ps34
Catarrhini - ps35
Hominoidea - ps36
Hominidae - ps37
Homininae - ps38
Hominini - ps39
Homo_sapiens - ps40
p-value
3e-04
4e-04
7e-04
1e-03
2e-03
Log-odds
0.5
1.0
1.5
2.0
2.5
3.0
3.5
4.0

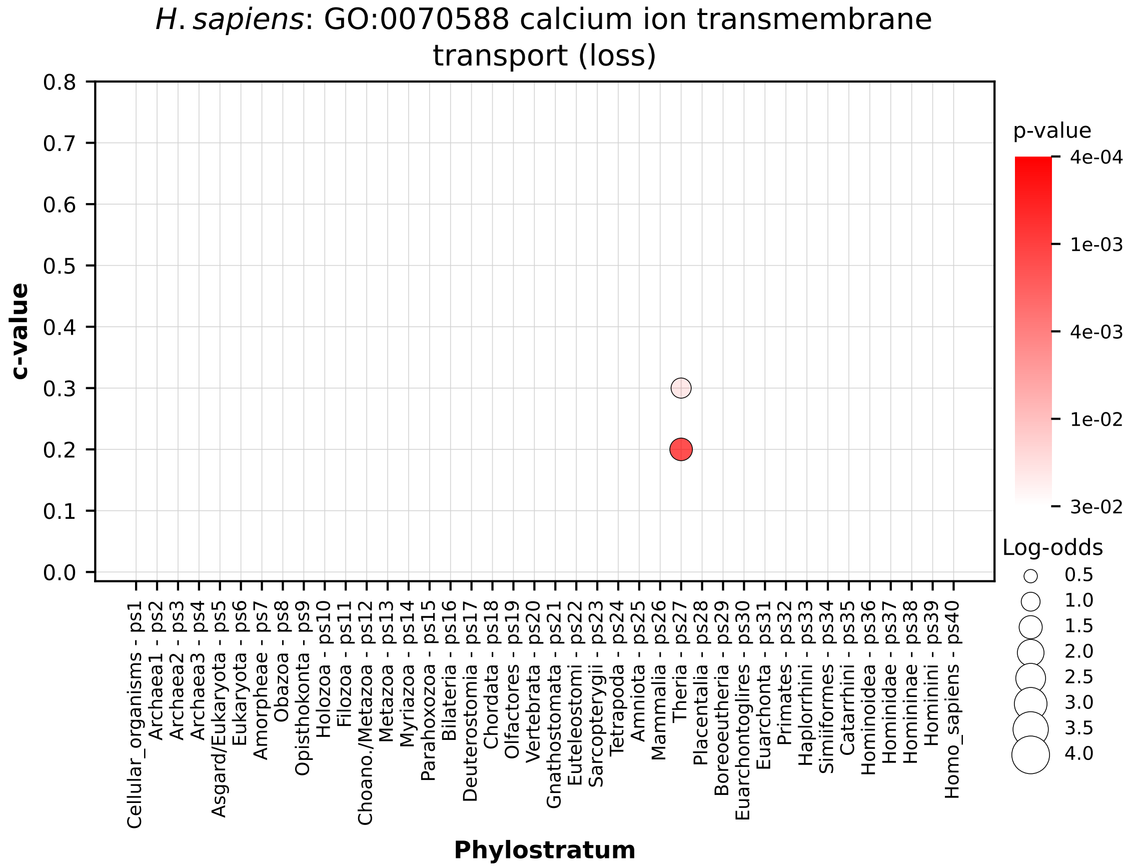

H. sapiens: GO:0070588 calcium ion transmembrane transport (loss)
c-value
0.0
0.1
0.2
0.3
0.4
0.5
0.6
0.7
0.8
Phylostratum
Cellular_organisms - ps1
Archaea1 - ps2
Archaea2 - ps3
Archaea3 - ps4
Asgard/Eukaryota - ps5
Eukaryota - ps6
Amorpheae - ps7
Obazoa - ps8
Opisthokonta - ps9
Holozoa - ps10
Filozoa - ps11
Choano./Metazoa - ps12
Metazoa - ps13
Myriazoa - ps14
Parahoxozoa - ps15
Bilateria - ps16
Deuterostomia - ps17
Chordata - ps18
Olfactores - ps19
Vertebrata - ps20
Gnathostomata - ps21
Euteleostomi - ps22
Sarcopterygii - ps23
Tetrapoda - ps24
Amniota - ps25
Mammalia - ps26
Theria - ps27
Placentalia - ps28
Boreoeutheria - ps29
Euarchontoglires - ps30
Euarchonta - ps31
Primates - ps32
Haplorrhini - ps33
Simiiformes - ps34
Catarrhini - ps35
Hominoidea - ps36
Hominidae - ps37
Homininae - ps38
Hominini - ps39
Homo_sapiens - ps40
p-value
4e-04
1e-03
4e-03
1e-02
3e-02
Log-odds
0.5
1.0
1.5
2.0
2.5
3.0
3.5
4.0

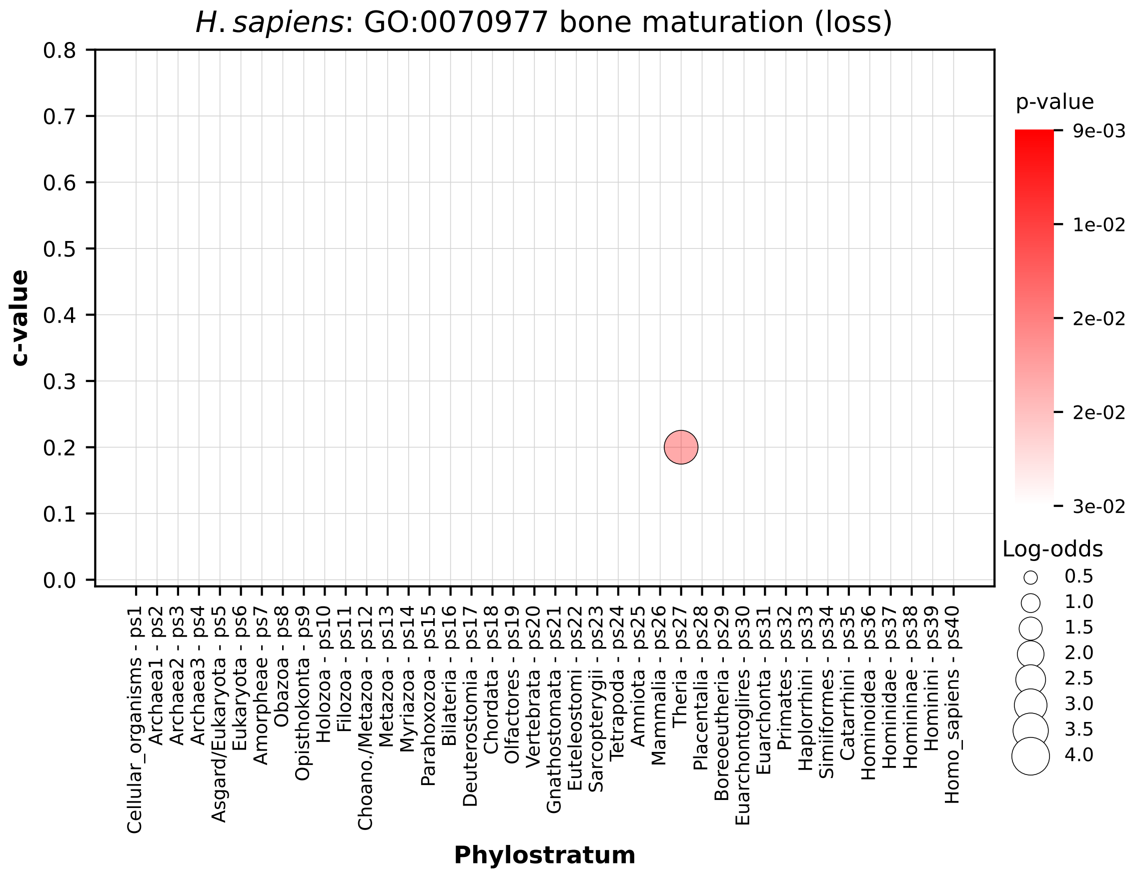

H. sapiens: GO:0070977 bone maturation (loss)
c-value
0.8
0.7
0.6
0.5
0.4
0.3
0.2
0.1
0.0
Phylostratum
Cellular_organisms - ps1
Archaea1 - ps2
Archaea2 - ps3
Archaea3 - ps4
Asgard/Eukaryota - ps5
Eukaryota - ps6
Amorpheae - ps7
Obazoa - ps8
Opisthokonta - ps9
Holozoa - ps10
Filozoa - ps11
Choano./Metazoa - ps12
Metazoa - ps13
Myriazoa - ps14
Parahoxozoa - ps15
Bilateria - ps16
Deuterostomia - ps17
Chordata - ps18
Olfactores - ps19
Vertebrata - ps20
Gnathostomata - ps21
Euteleostomi - ps22
Sarcopterygii - ps23
Tetrapoda - ps24
Amniota - ps25
Mammalia - ps26
Theria - ps27
Placentalia - ps28
Boreoeutheria - ps29
Euarchontoglires - ps30
Euarchonta - ps31
Primates - ps32
Haplorrhini - ps33
Simiiformes - ps34
Catarrhini - ps35
Hominoidea - ps36
Hominidae - ps37
Homininae - ps38
Hominini - ps39
Homo_sapiens - ps40
p-value
9e-03
1e-02
2e-02
2e-02
3e-02
Log-odds
0.5
1.0
1.5
2.0
2.5
3.0
3.5
4.0

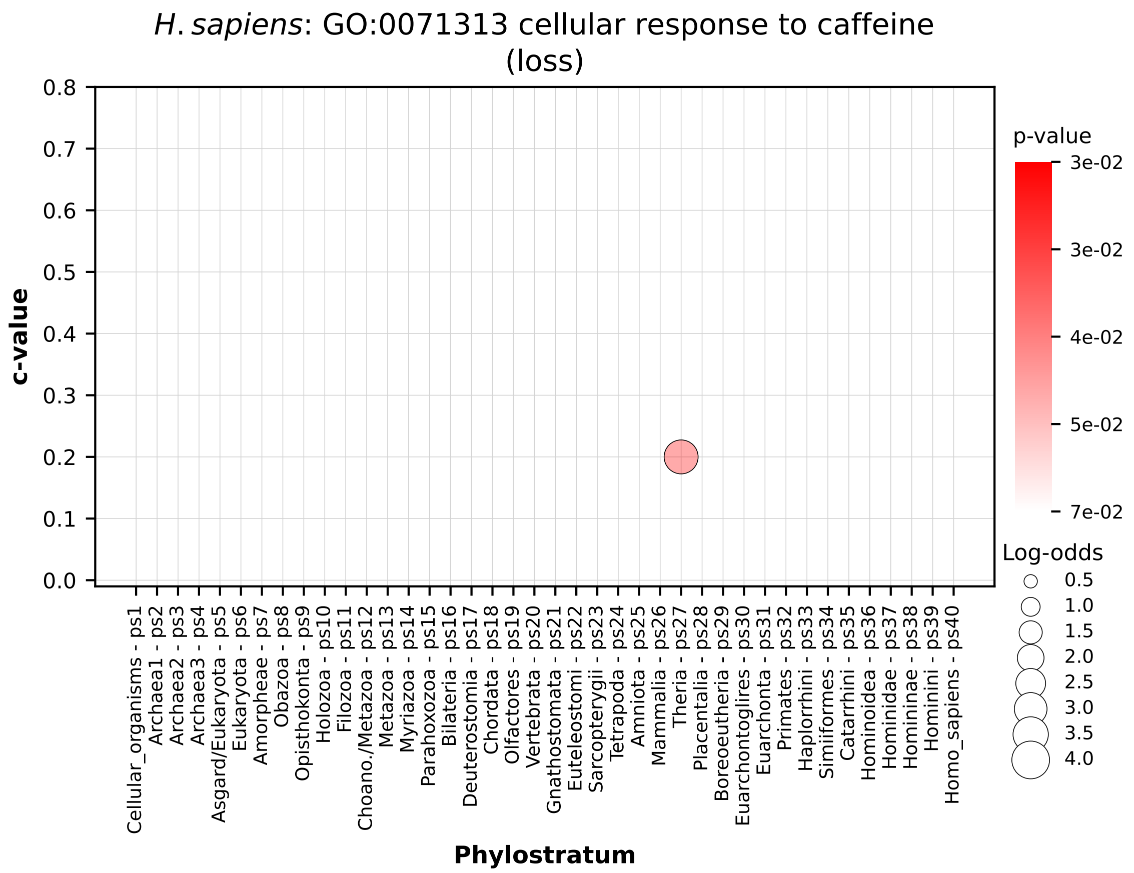

H. sapiens: GO:0071313 cellular response to caffeine
(loss)
c-value
0.0
0.1
0.2
0.3
0.4
0.5
0.6
0.7
0.8
Phylostratum
Cellular_organisms - ps1
Archaea1 - ps2
Archaea2 - ps3
Archaea3 - ps4
Asgard/Eukaryota - ps5
Eukaryota - ps6
Amorpheae - ps7
Obazoa - ps8
Opisthokonta - ps9
Holozoa - ps10
Filozoa - ps11
Choano./Metazoa - ps12
Metazoa - ps13
Myriazoa - ps14
Parahoxozoa - ps15
Bilateria - ps16
Deuterostomia - ps17
Chordata - ps18
Olfactores - ps19
Vertebrata - ps20
Gnathostomata - ps21
Euteleostomi - ps22
Sarcopterygii - ps23
Tetrapoda - ps24
Amniota - ps25
Mammalia - ps26
Theria - ps27
Placentalia - ps28
Boreoeutheria - ps29
Euarchontoglires - ps30
Euarchonta - ps31
Primates - ps32
Haplorrhini - ps33
Simiiformes - ps34
Catarrhini - ps35
Hominoidea - ps36
Hominidae - ps37
Homininae - ps38
Hominini - ps39
Homo_sapiens - ps40
p-value
3e-02
3e-02
4e-02
5e-02
7e-02
Log-odds
0.5
1.0
1.5
2.0
2.5
3.0
3.5
4.0

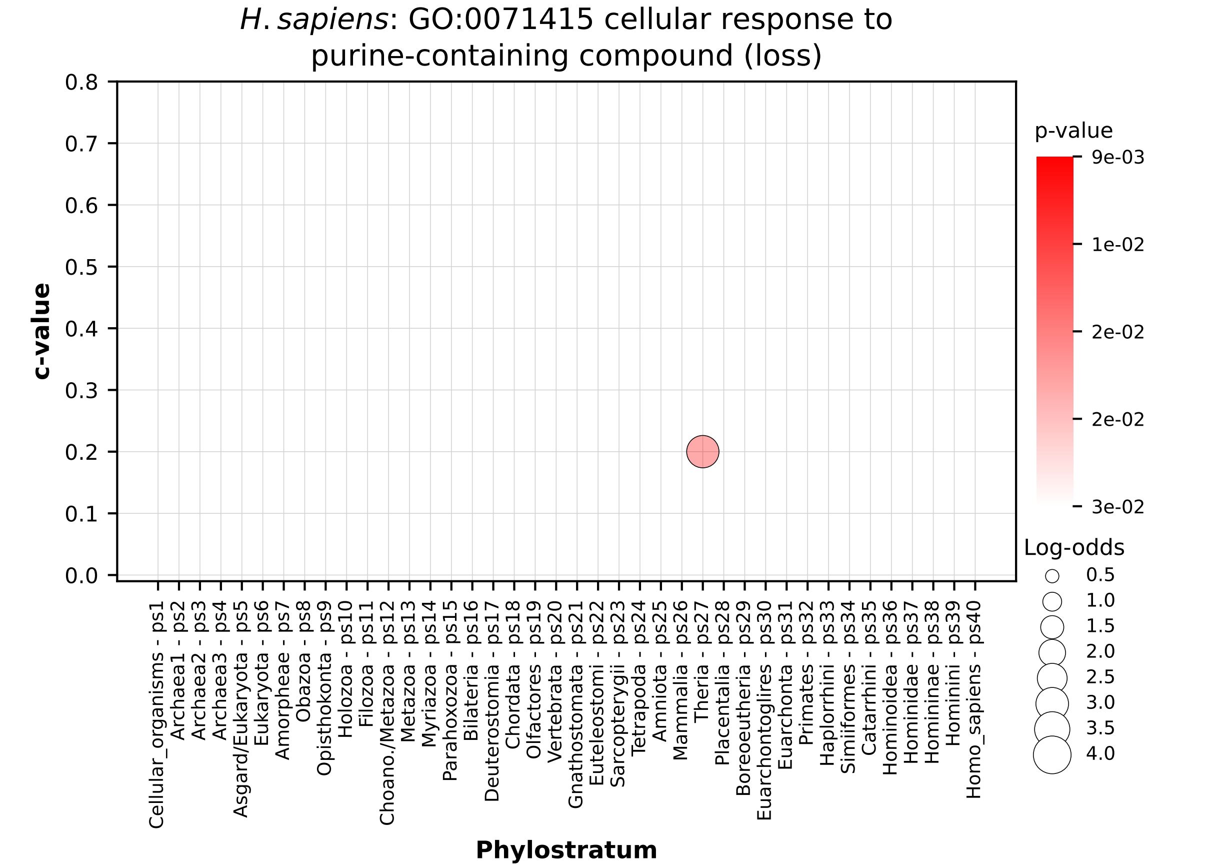

H. sapiens: GO:0071415 cellular response to purine-containing compound (loss)
c-value
0.8
0.7
0.6
0.5
0.4
0.3
0.2
0.1
0.0
p-value
9e-03
1e-02
2e-02
2e-02
3e-02
Log-odds
0.5
1.0
1.5
2.0
2.5
3.0
3.5
4.0
Cellular_organisms - ps1
Archaea1 - ps2
Archaea2 - ps3
Archaea3 - ps4
Asgard/Eukaryota - ps5
Eukaryota - ps6
Amorpheae - ps7
Obazoa - ps8
Opisthokonta - ps9
Holozoa - ps10
Filozoa - ps11
Choano./Metazoa - ps12
Metazoa - ps13
Myriazoa - ps14
Parahoxozoa - ps15
Bilateria - ps16
Deuterostomia - ps17
Chordata - ps18
Olfactores - ps19
Vertebrata - ps20
Gnathostomata - ps21
Euteleostomi - ps22
Sarcopterygii - ps23
Tetrapoda - ps24
Amniota - ps25
Mammalia - ps26
Theria - ps27
Placentalia - ps28
Boreoeutheria - ps29
Euarchontoglires - ps30
Euarchonta - ps31
Primates - ps32
Haplorrhini - ps33
Simiiformes - ps34
Catarrhini - ps35
Hominoidea - ps36
Hominidae - ps37
Homininae - ps38
Hominini - ps39
Homo_sapiens - ps40
Phylostratum

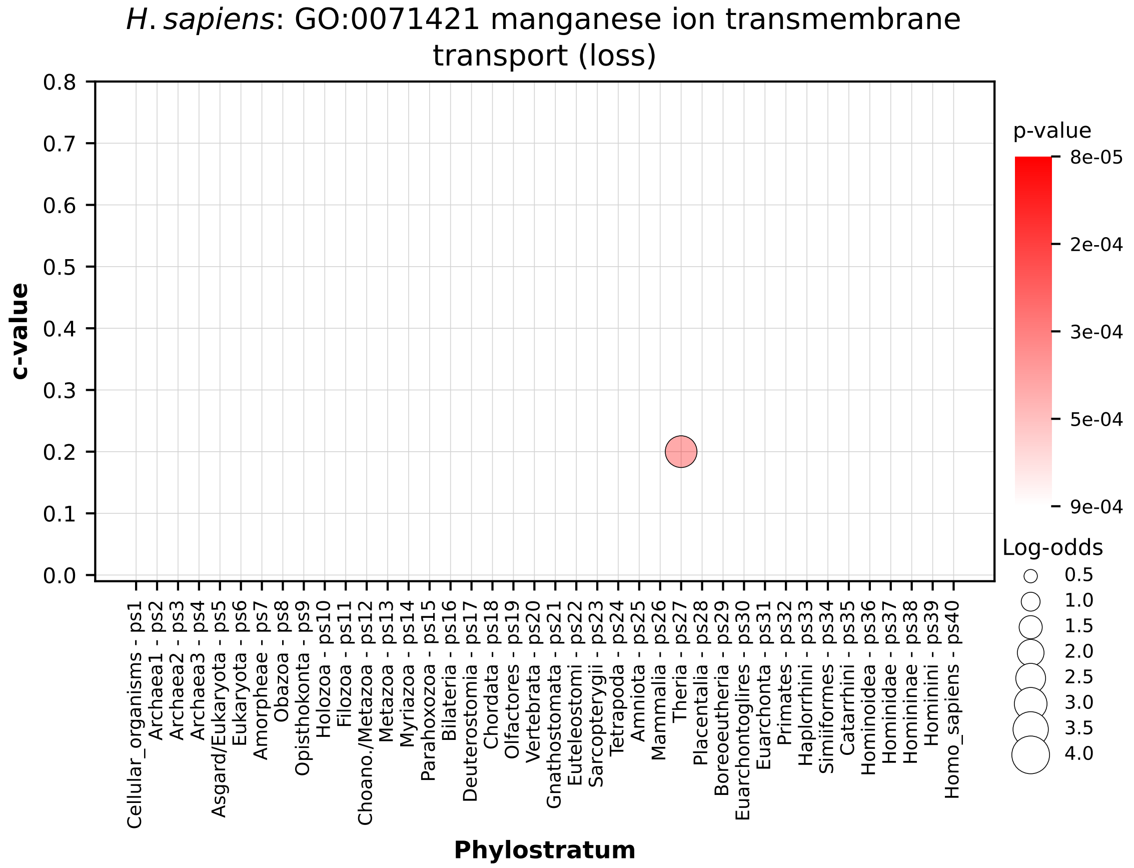

H. sapiens: GO:0071421 manganese ion transmembrane transport (loss)
c-value
0.8
0.7
0.6
0.5
0.4
0.3
0.2
0.1
0.0
Phylostratum
Cellular_organisms - ps1
Archaea1 - ps2
Archaea2 - ps3
Archaea3 - ps4
Asgard/Eukaryota - ps5
Eukaryota - ps6
Amorpheae - ps7
Obazoa - ps8
Opisthokonta - ps9
Holozoa - ps10
Filozoa - ps11
Choano./Metazoa - ps12
Metazoa - ps13
Myriazoa - ps14
Parahoxozoa - ps15
Bilateria - ps16
Deuterostomia - ps17
Chordata - ps18
Olfactores - ps19
Vertebrata - ps20
Gnathostomata - ps21
Euteleostomi - ps22
Sarcopterygii - ps23
Tetrapoda - ps24
Amniota - ps25
Mammalia - ps26
Theria - ps27
Placentalia - ps28
Boreoeutheria - ps29
Euarchontoglires - ps30
Euarchonta - ps31
Primates - ps32
Haplorrhini - ps33
Simiiformes - ps34
Catarrhini - ps35
Hominoidea - ps36
Hominidae - ps37
Homininae - ps38
Hominini - ps39
Homo_sapiens - ps40
p-value
8e-05
2e-04
3e-04
5e-04
9e-04
Log-odds
0.5
1.0
1.5
2.0
2.5
3.0
3.5
4.0

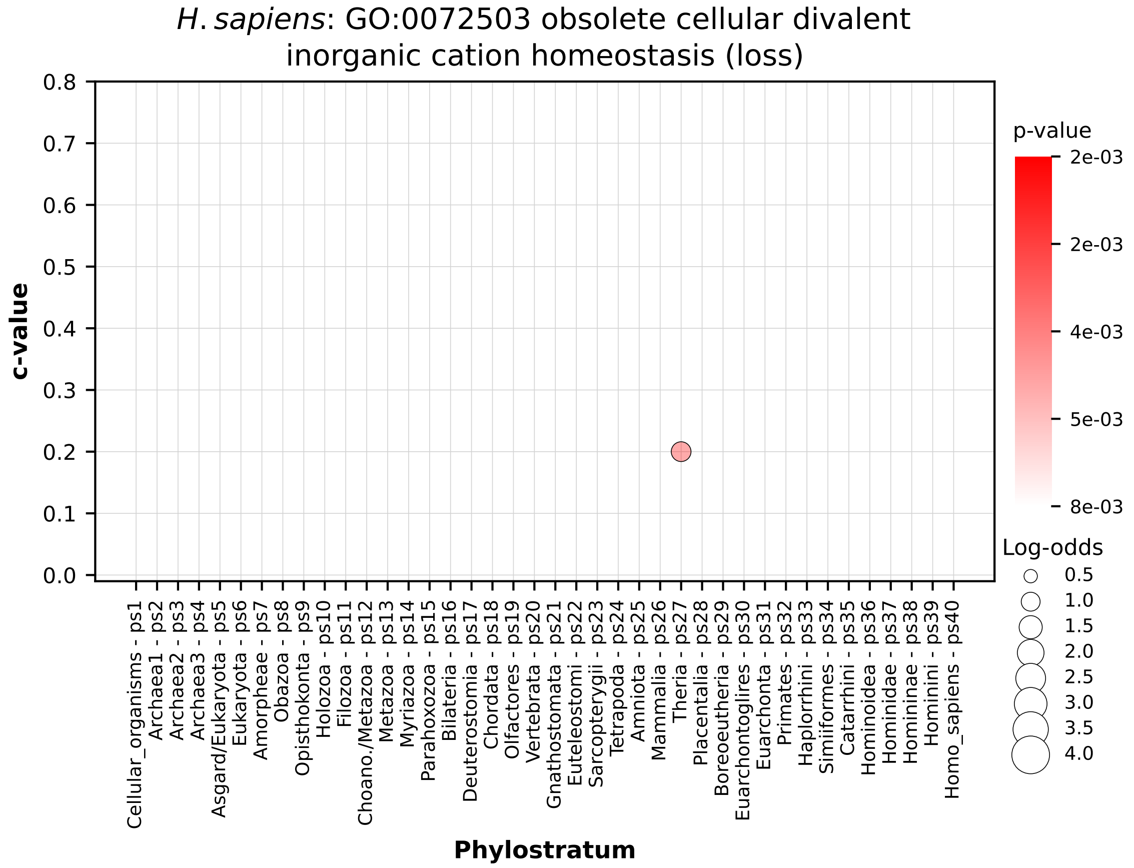

H. sapiens: GO:0072503 obsolete cellular divalent inorganic cation homeostasis (loss)
c-value
0.0
0.1
0.2
0.3
0.4
0.5
0.6
0.7
0.8
Phylostratum
Cellular_organisms - ps1
Archaea1 - ps2
Archaea2 - ps3
Archaea3 - ps4
Asgard/Eukaryota - ps5
Eukaryota - ps6
Amorpheae - ps7
Obazoa - ps8
Opisthokonta - ps9
Holozoa - ps10
Filozoa - ps11
Choano./Metazoa - ps12
Metazoa - ps13
Myriazoa - ps14
Parahoxozoa - ps15
Bilateria - ps16
Deuterostomia - ps17
Chordata - ps18
Olfactores - ps19
Vertebrata - ps20
Gnathostomata - ps21
Euteleostomi - ps22
Sarcopterygii - ps23
Tetrapoda - ps24
Amniota - ps25
Mammalia - ps26
Theria - ps27
Placentalia - ps28
Boreoeutheria - ps29
Euarchontoglires - ps30
Euarchonta - ps31
Primates - ps32
Haplorrhini - ps33
Simiiformes - ps34
Catarrhini - ps35
Hominoidea - ps36
Hominidae - ps37
Homininae - ps38
Hominini - ps39
Homo_sapiens - ps40
p-value
2e-03
2e-03
4e-03
5e-03
8e-03
Log-odds
0.5
1.0
1.5
2.0
2.5
3.0
3.5
4.0

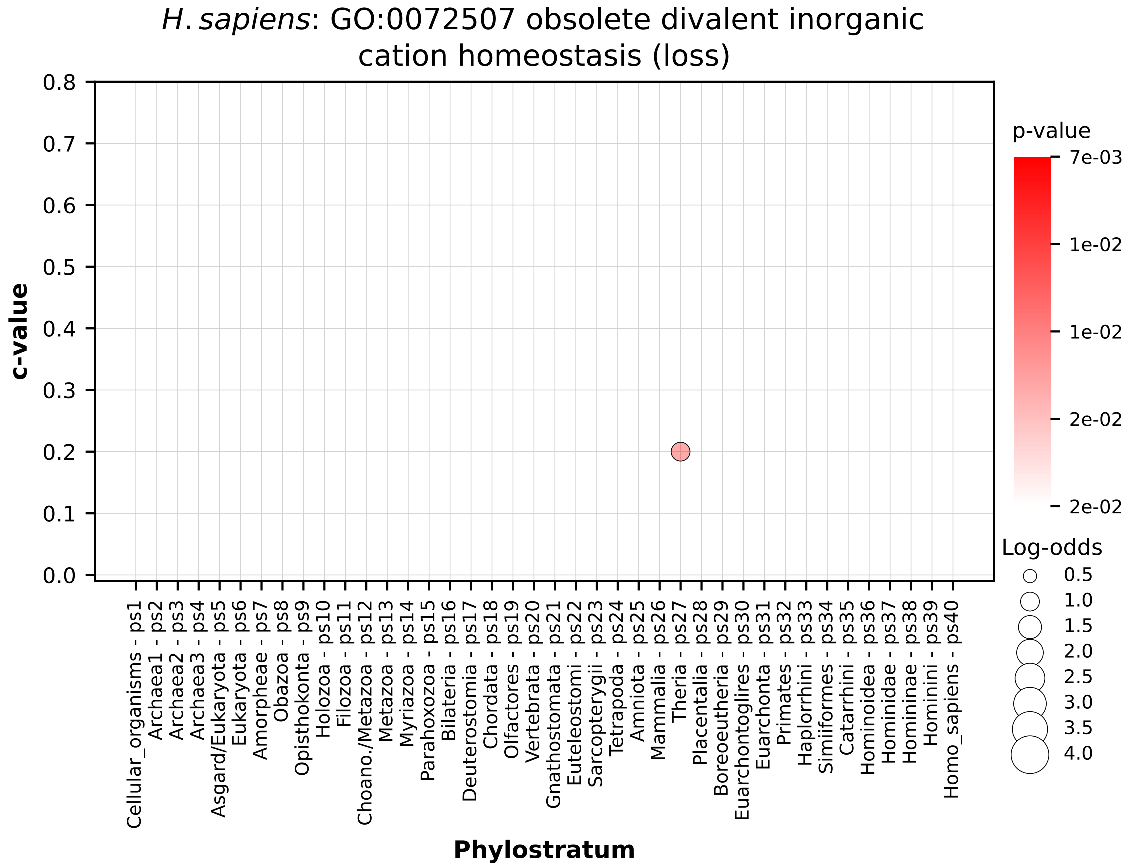

H. sapiens: GO:0072507 obsolete divalent inorganic cation homeostasis (loss)
c-value
0.0
0.1
0.2
0.3
0.4
0.5
0.6
0.7
0.8
Phylostratum
Cellular_organisms - ps1
Archaea1 - ps2
Archaea2 - ps3
Archaea3 - ps4
Asgard/Eukaryota - ps5
Eukaryota - ps6
Amorpheae - ps7
Obazoa - ps8
Opisthokonta - ps9
Holozoa - ps10
Filozoa - ps11
Choano./Metazoa - ps12
Metazoa - ps13
Myriazoa - ps14
Parahoxozoa - ps15
Bilateria - ps16
Deuterostomia - ps17
Chordata - ps18
Olfactores - ps19
Vertebrata - ps20
Gnathostomata - ps21
Euteleostomi - ps22
Sarcopterygii - ps23
Tetrapoda - ps24
Amniota - ps25
Mammalia - ps26
Theria - ps27
Placentalia - ps28
Boreoeutheria - ps29
Euarchontoglires - ps30
Euarchonta - ps31
Primates - ps32
Haplorrhini - ps33
Simiiformes - ps34
Catarrhini - ps35
Hominoidea - ps36
Hominidae - ps37
Homininae - ps38
Hominini - ps39
Homo_sapiens - ps40
p-value
7e-03
1e-02
1e-02
2e-02
2e-02
Log-odds
0.5
1.0
1.5
2.0
2.5
3.0
3.5
4.0

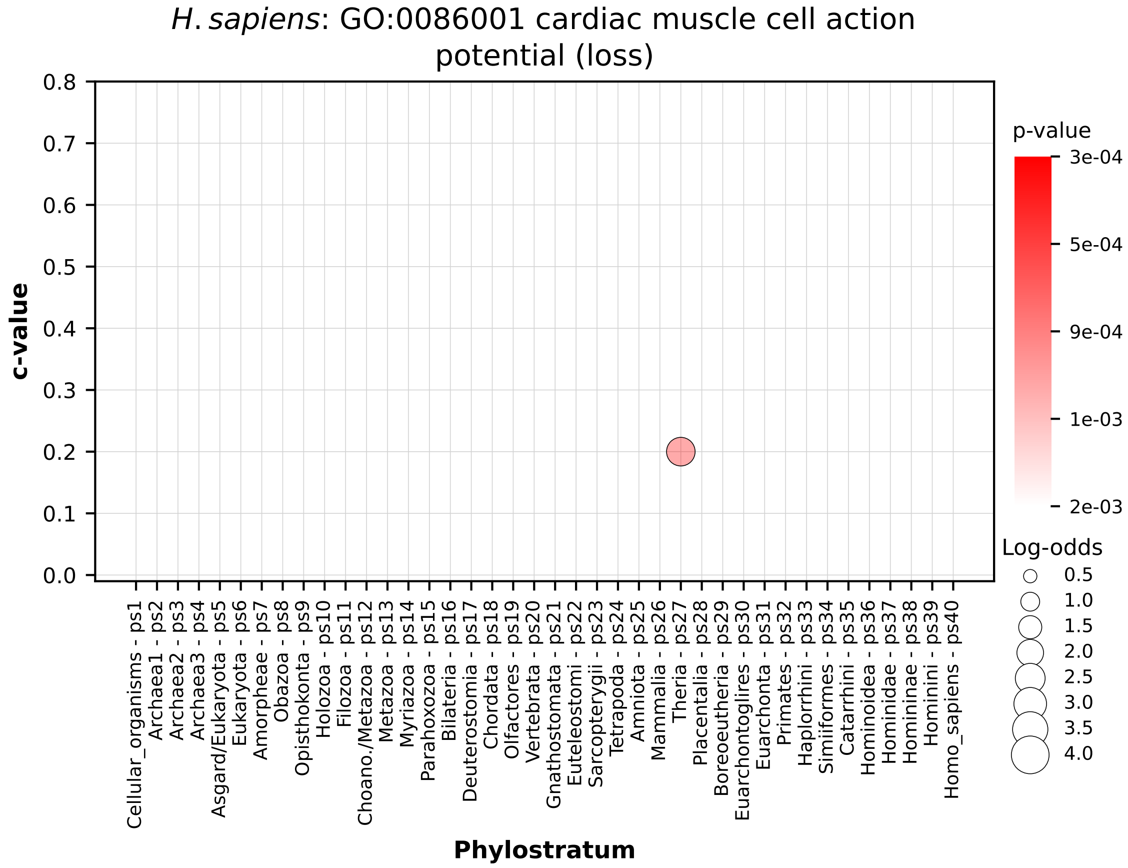

H. sapiens: GO:0086001 cardiac muscle cell action potential (loss)
c-value
0.8
0.7
0.6
0.5
0.4
0.3
0.2
0.1
0.0
p-value
3e-04
5e-04
9e-04
1e-03
2e-03
Log-odds
0.5
1.0
1.5
2.0
2.5
3.0
3.5
4.0
Cellular_organisms - ps1
Archaea1 - ps2
Archaea2 - ps3
Archaea3 - ps4
Asgard/Eukaryota - ps5
Eukaryota - ps6
Amorpheae - ps7
Obazoa - ps8
Opisthokonta - ps9
Holozoa - ps10
Filozoa - ps11
Choano./Metazoa - ps12
Metazoa - ps13
Myriazoa - ps14
Parahoxozoa - ps15
Bilateria - ps16
Deuterostomia - ps17
Chordata - ps18
Olfactores - ps19
Vertebrata - ps20
Gnathostomata - ps21
Euteleostomi - ps22
Sarcopterygii - ps23
Tetrapoda - ps24
Amniota - ps25
Mammalia - ps26
Theria - ps27
Placentalia - ps28
Boreoeutheria - ps29
Euarchontoglires - ps30
Euarchonta - ps31
Primates - ps32
Haplorrhini - ps33
Simiiformes - ps34
Catarrhini - ps35
Hominoidea - ps36
Hominidae - ps37
Homininae - ps38
Hominini - ps39
Homo_sapiens - ps40
Phylostratum

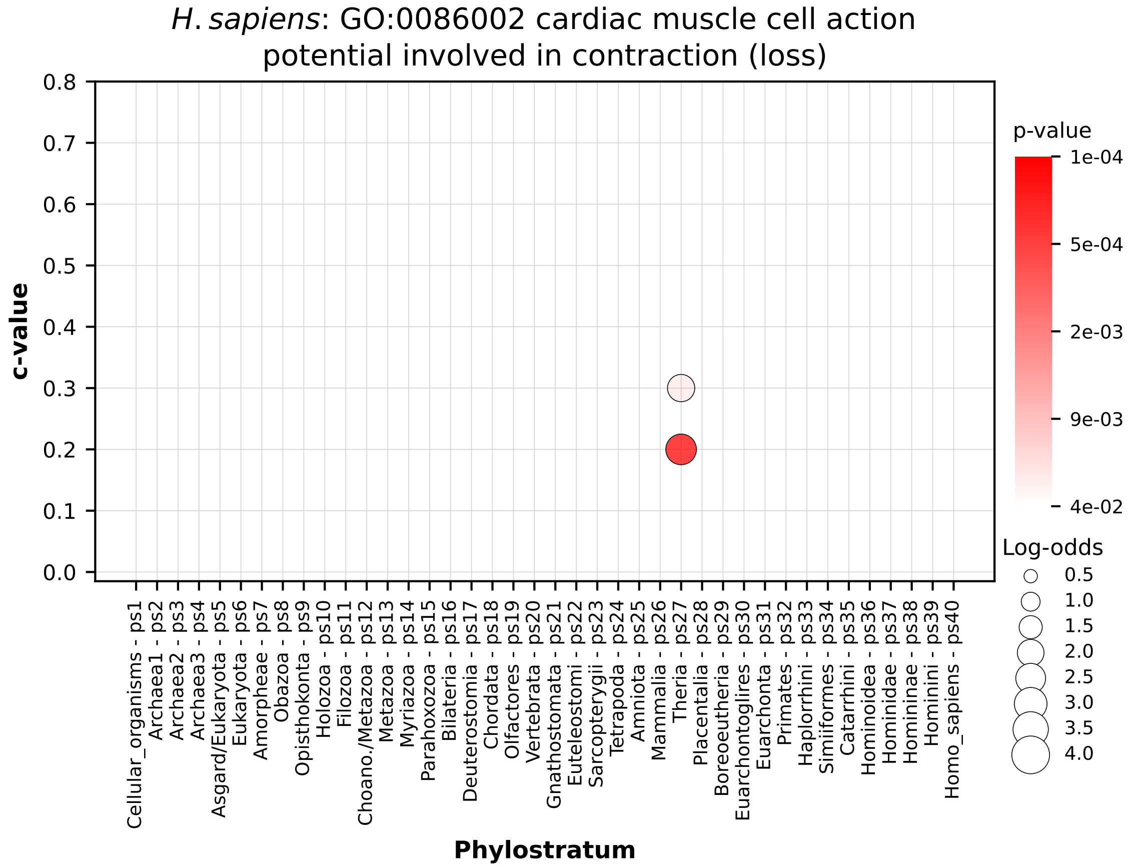

H. sapiens: GO:0086002 cardiac muscle cell action potential involved in contraction (loss)
c-value
0.0
0.1
0.2
0.3
0.4
0.5
0.6
0.7
0.8
Phylostratum
Cellular_organisms - ps1
Archaea1 - ps2
Archaea2 - ps3
Archaea3 - ps4
Asgard/Eukaryota - ps5
Eukaryota - ps6
Amorpheae - ps7
Obazoa - ps8
Opisthokonta - ps9
Holozoa - ps10
Filozoa - ps11
Choano./Metazoa - ps12
Metazoa - ps13
Myriazoa - ps14
Parahoxozoa - ps15
Bilateria - ps16
Deuterostomia - ps17
Chordata - ps18
Olfactores - ps19
Vertebrata - ps20
Gnathostomata - ps21
Euteleostomi - ps22
Sarcopterygii - ps23
Tetrapoda - ps24
Amniota - ps25
Mammalia - ps26
Theria - ps27
Placentalia - ps28
Boreoeutheria - ps29
Euarchontoglires - ps30
Euarchonta - ps31
Primates - ps32
Haplorrhini - ps33
Simiiformes - ps34
Catarrhini - ps35
Hominoidea - ps36
Hominidae - ps37
Homininae - ps38
Hominini - ps39
Homo_sapiens - ps40
p-value
1e-04
5e-04
2e-03
9e-03
4e-02
Log-odds
0.5
1.0
1.5
2.0
2.5
3.0
3.5
4.0

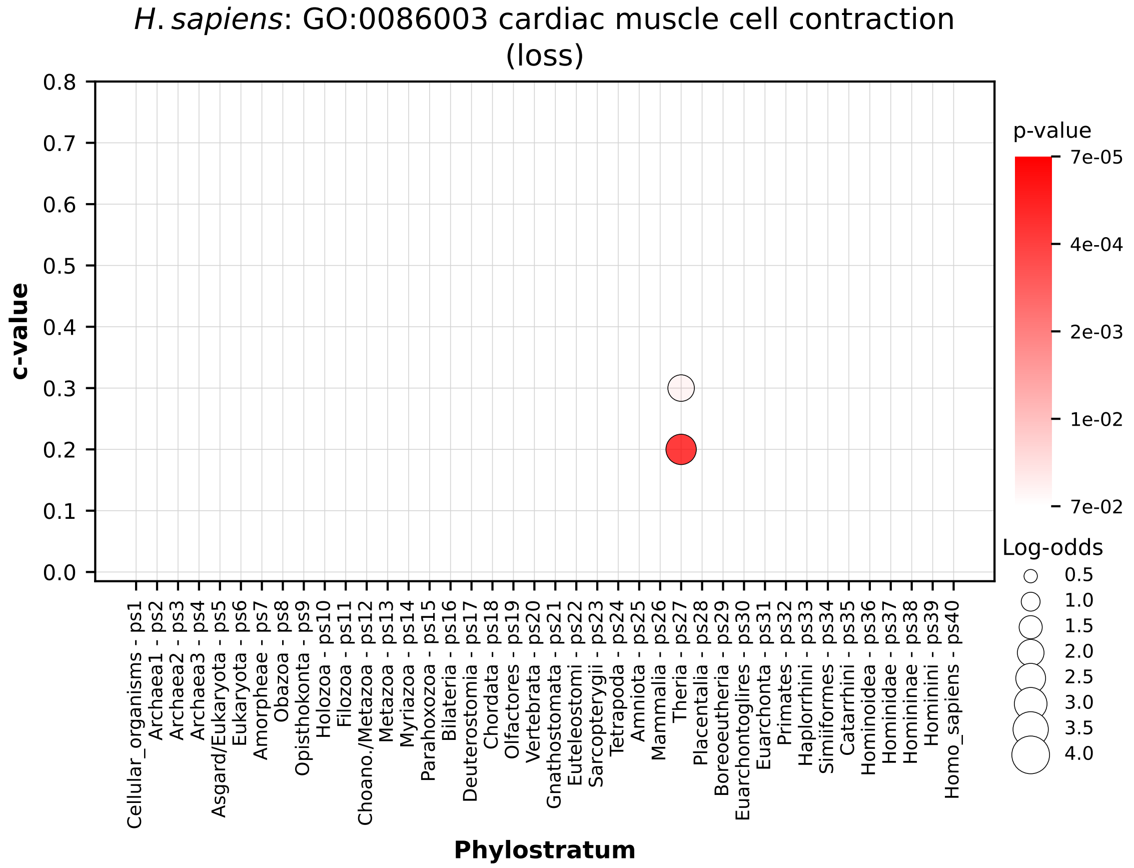

H. sapiens: GO:0086003 cardiac muscle cell contraction (loss)
c-value
0.0
0.1
0.2
0.3
0.4
0.5
0.6
0.7
0.8
Phylostratum
Cellular_organisms - ps1
Archaea1 - ps2
Archaea2 - ps3
Archaea3 - ps4
Asgard/Eukaryota - ps5
Eukaryota - ps6
Amorpheae - ps7
Obazoa - ps8
Opisthokonta - ps9
Holozoa - ps10
Filozoa - ps11
Choano./Metazoa - ps12
Metazoa - ps13
Myriazoa - ps14
Parahoxozoa - ps15
Bilateria - ps16
Deuterostomia - ps17
Chordata - ps18
Olfactores - ps19
Vertebrata - ps20
Gnathostomata - ps21
Euteleostomi - ps22
Sarcopterygii - ps23
Tetrapoda - ps24
Amniota - ps25
Mammalia - ps26
Theria - ps27
Placentalia - ps28
Boreoeutheria - ps29
Euarchontoglires - ps30
Euarchonta - ps31
Primates - ps32
Haplorrhini - ps33
Simiiformes - ps34
Catarrhini - ps35
Hominoidea - ps36
Hominidae - ps37
Homininae - ps38
Hominini - ps39
Homo_sapiens - ps40
p-value
7e-05
4e-04
2e-03
1e-02
7e-02
Log-odds
0.5
1.0
1.5
2.0
2.5
3.0
3.5
4.0

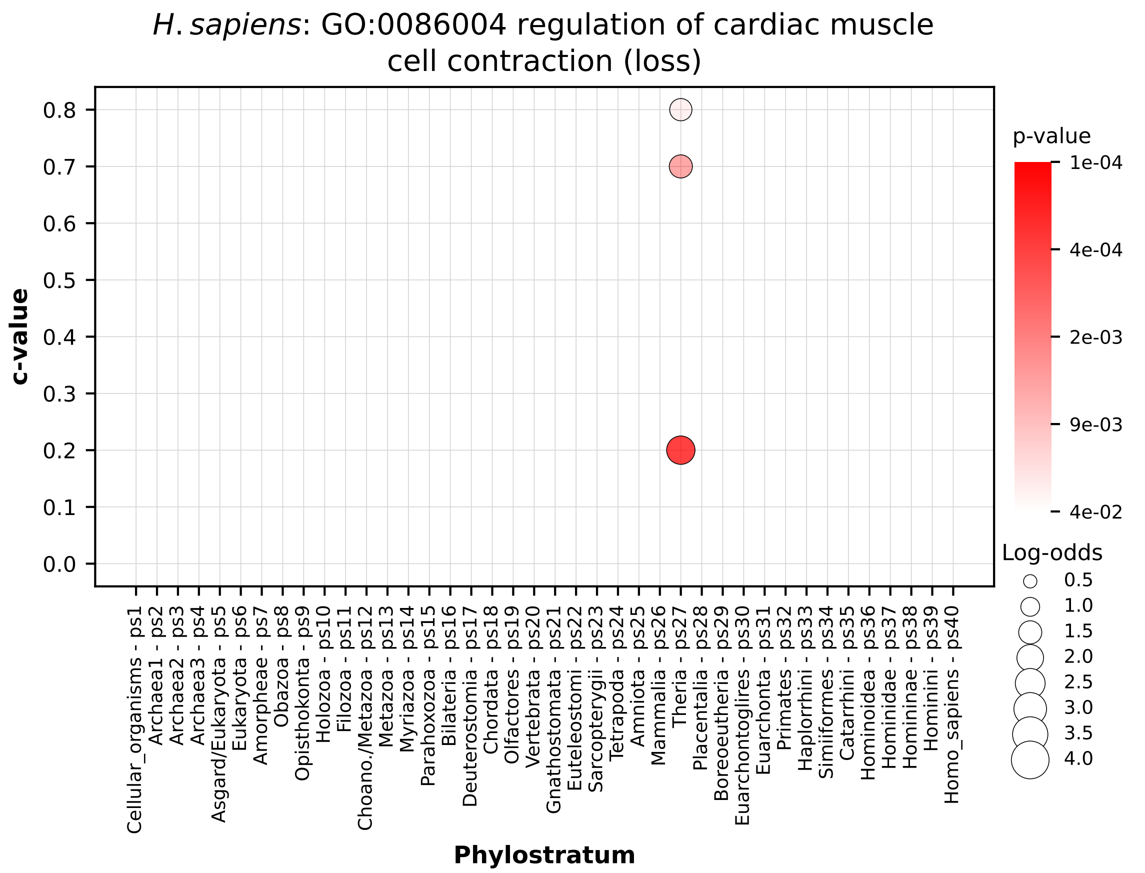

H. sapiens: GO:0086004 regulation of cardiac muscle cell contraction (loss)
c-value
0.0
0.1
0.2
0.3
0.4
0.5
0.6
0.7
0.8
p-value
1e-04
4e-04
2e-03
9e-03
4e-02
Log-odds
0.5
1.0
1.5
2.0
2.5
3.0
3.5
4.0
Cellular_organisms - ps1
Archaea1 - ps2
Archaea2 - ps3
Archaea3 - ps4
Asgard/Eukaryota - ps5
Eukaryota - ps6
Amorpheae - ps7
Obazoa - ps8
Opisthokonta - ps9
Holozoa - ps10
Filozoa - ps11
Choano./Metazoa - ps12
Metazoa - ps13
Myriazoa - ps14
Parahoxozoa - ps15
Bilateria - ps16
Deuterostomia - ps17
Chordata - ps18
Olfactores - ps19
Vertebrata - ps20
Gnathostomata - ps21
Euteleostomi - ps22
Sarcopterygii - ps23
Tetrapoda - ps24
Amniota - ps25
Mammalia - ps26
Theria - ps27
Placentalia - ps28
Boreoeutheria - ps29
Euarchontoglires - ps30
Euarchonta - ps31
Primates - ps32
Haplorrhini - ps33
Simiiformes - ps34
Catarrhini - ps35
Hominoidea - ps36
Hominidae - ps37
Homininae - ps38
Hominini - ps39
Homo_sapiens - ps40
Phylostratum

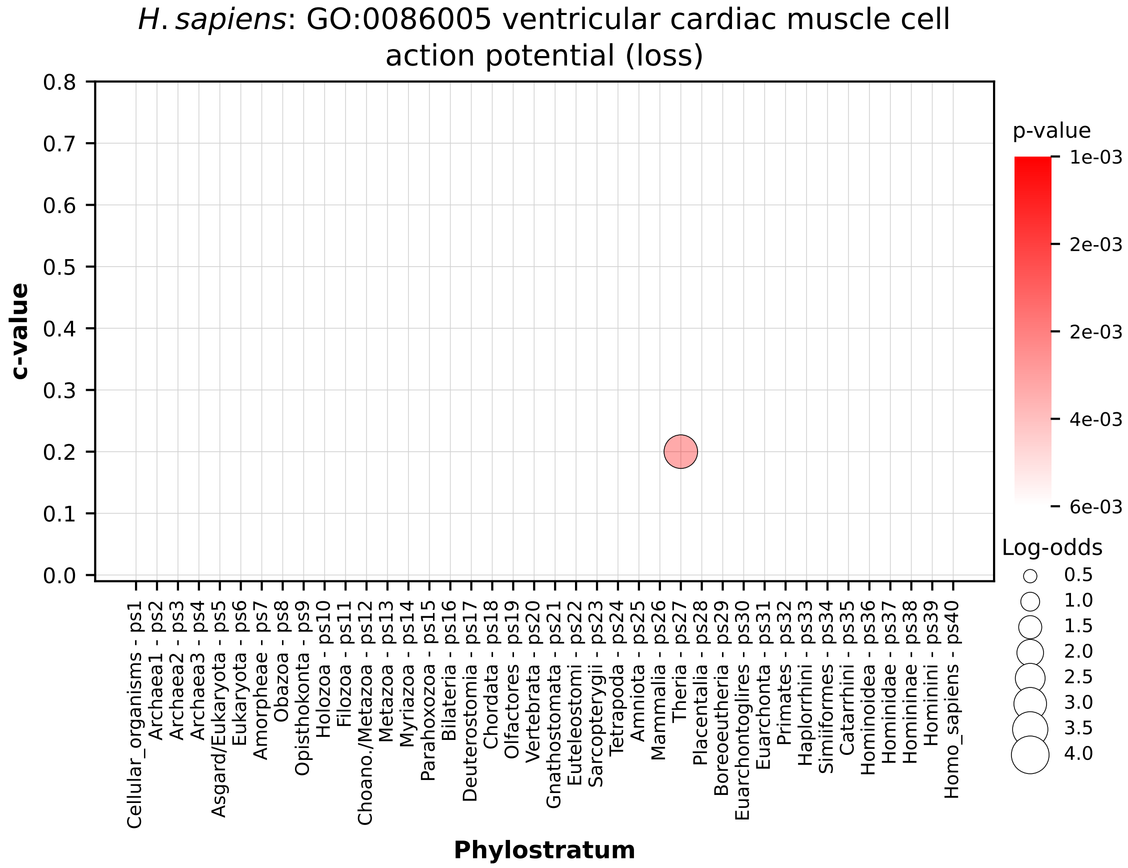
H. sapiens: GO:0086005 ventricular cardiac muscle cell action potential (loss)
c-value
0.0
0.1
0.2
0.3
0.4
0.5
0.6
0.7
0.8
Phylostratum
Cellular_organisms - ps1
Archaea1 - ps2
Archaea2 - ps3
Archaea3 - ps4
Asgard/Eukaryota - ps5
Eukaryota - ps6
Amorpheae - ps7
Obazoa - ps8
Opisthokonta - ps9
Holozoa - ps10
Filozoa - ps11
Choano./Metazoa - ps12
Metazoa - ps13
Myriazoa - ps14
Parahoxozoa - ps15
Bilateria - ps16
Deuterostomia - ps17
Chordata - ps18
Olfactores - ps19
Vertebrata - ps20
Gnathostomata - ps21
Euteleostomi - ps22
Sarcopterygii - ps23
Tetrapoda - ps24
Amniota - ps25
Mammalia - ps26
Theria - ps27
Placentalia - ps28
Boreoeutheria - ps29
Euarchontoglires - ps30
Euarchonta - ps31
Primates - ps32
Haplorrhini - ps33
Simiiformes - ps34
Catarrhini - ps35
Hominoidea - ps36
Hominidae - ps37
Homininae - ps38
Hominini - ps39
Homo_sapiens - ps40
p-value
1e-03
2e-03
2e-03
4e-03
6e-03
Log-odds
0.5
1.0
1.5
2.0
2.5
3.0
3.5
4.0

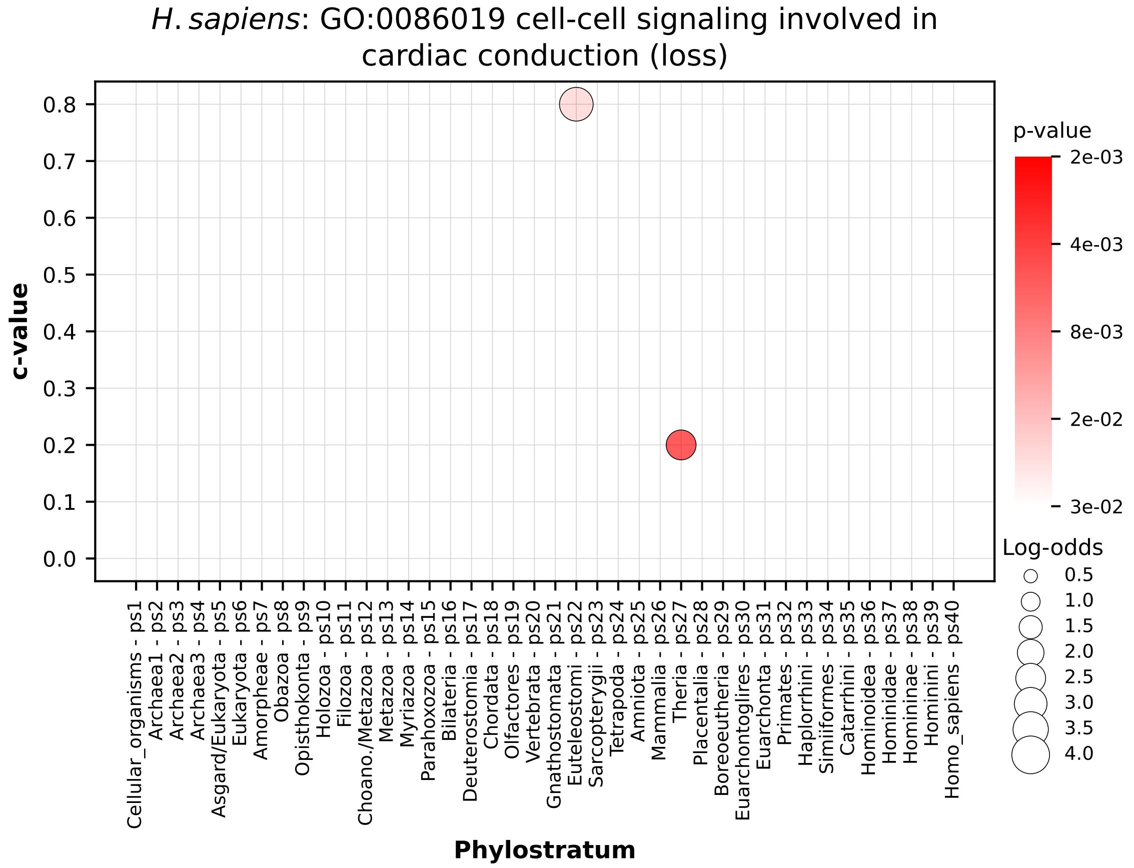

H. sapiens: GO:0086019 cell-cell signaling involved in cardiac conduction (loss)
c-value
0.0
0.1
0.2
0.3
0.4
0.5
0.6
0.7
0.8
Phylostratum
Cellular_organisms - ps1
Archaea1 - ps2
Archaea2 - ps3
Archaea3 - ps4
Asgard/Eukaryota - ps5
Eukaryota - ps6
Amorpheae - ps7
Obazoa - ps8
Opisthokonta - ps9
Holozoa - ps10
Filozoa - ps11
Choano./Metazoa - ps12
Metazoa - ps13
Myriazoa - ps14
Parahoxozoa - ps15
Bilateria - ps16
Deuterostomia - ps17
Chordata - ps18
Olfactores - ps19
Vertebrata - ps20
Gnathostomata - ps21
Euteleostomi - ps22
Sarcopterygii - ps23
Tetrapoda - ps24
Amniota - ps25
Mammalia - ps26
Theria - ps27
Placentalia - ps28
Boreoeutheria - ps29
Euarchontoglires - ps30
Euarchonta - ps31
Primates - ps32
Haplorrhini - ps33
Simiiformes - ps34
Catarrhini - ps35
Hominoidea - ps36
Hominidae - ps37
Homininae - ps38
Hominini - ps39
Homo_sapiens - ps40
p-value
2e-03
4e-03
8e-03
2e-02
3e-02
Log-odds
0.5
1.0
1.5
2.0
2.5
3.0
3.5
4.0

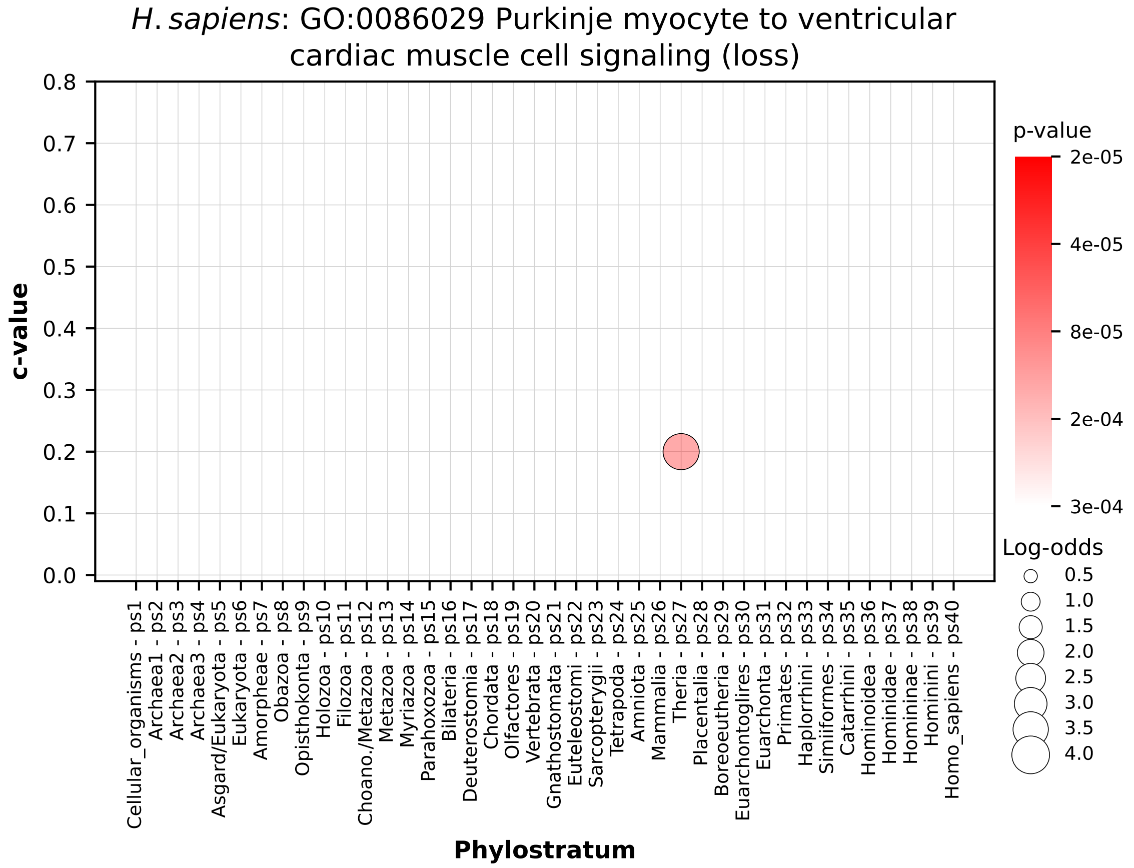

H. sapiens: GO:0086029 Purkinje myocyte to ventricular cardiac muscle cell signaling (loss)
c-value
0.0
0.1
0.2
0.3
0.4
0.5
0.6
0.7
0.8
Phylostratum
Cellular_organisms - ps1
Archaea1 - ps2
Archaea2 - ps3
Archaea3 - ps4
Asgard/Eukaryota - ps5
Eukaryota - ps6
Amorpheae - ps7
Obazoa - ps8
Opisthokonta - ps9
Holozoa - ps10
Filozoa - ps11
Choano./Metazoa - ps12
Metazoa - ps13
Myriazoa - ps14
Parahoxozoa - ps15
Bilateria - ps16
Deuterostomia - ps17
Chordata - ps18
Olfactores - ps19
Vertebrata - ps20
Gnathostomata - ps21
Euteleostomi - ps22
Sarcopterygii - ps23
Tetrapoda - ps24
Amniota - ps25
Mammalia - ps26
Theria - ps27
Placentalia - ps28
Boreoeutheria - ps29
Euarchontoglires - ps30
Euarchonta - ps31
Primates - ps32
Haplorrhini - ps33
Simiiformes - ps34
Catarrhini - ps35
Hominoidea - ps36
Hominidae - ps37
Homininae - ps38
Hominini - ps39
Homo_sapiens - ps40
p-value
2e-05
4e-05
8e-05
2e-04
3e-04
Log-odds
0.5
1.0
1.5
2.0
2.5
3.0
3.5
4.0

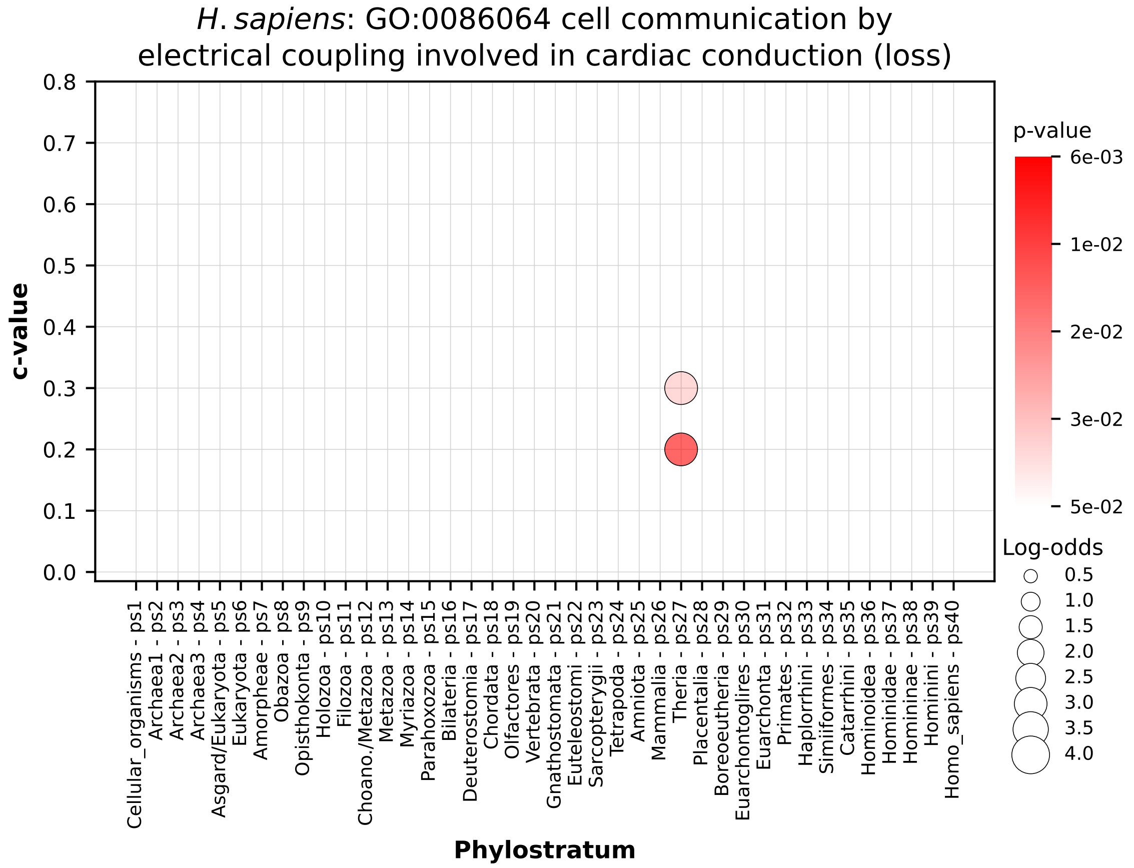

H. sapiens: GO:0086064 cell communication by electrical coupling involved in cardiac conduction (loss)
c-value
0.8
0.7
0.6
0.5
0.4
0.3
0.2
0.1
0.0
p-value
6e-03
1e-02
2e-02
3e-02
5e-02
Log-odds
0.5
1.0
1.5
2.0
2.5
3.0
3.5
4.0
Cellular_organisms - ps1
Archaea1 - ps2
Archaea2 - ps3
Archaea3 - ps4
Asgard/Eukaryota - ps5
Eukaryota - ps6
Amorpheae - ps7
Obazoa - ps8
Opisthokonta - ps9
Holozoa - ps10
Filozoa - ps11
Choano./Metazoa - ps12
Metazoa - ps13
Myriazoa - ps14
Parahoxozoa - ps15
Bilateria - ps16
Deuterostomia - ps17
Chordata - ps18
Olfactores - ps19
Vertebrata - ps20
Gnathostomata - ps21
Euteleostomi - ps22
Sarcopterygii - ps23
Tetrapoda - ps24
Amniota - ps25
Mammalia - ps26
Theria - ps27
Placentalia - ps28
Boreoeutheria - ps29
Euarchontoglires - ps30
Euarchonta - ps31
Primates - ps32
Haplorrhini - ps33
Simiiformes - ps34
Catarrhini - ps35
Hominoidea - ps36
Hominidae - ps37
Homininae - ps38
Hominini - ps39
Homo_sapiens - ps40
Phylostratum

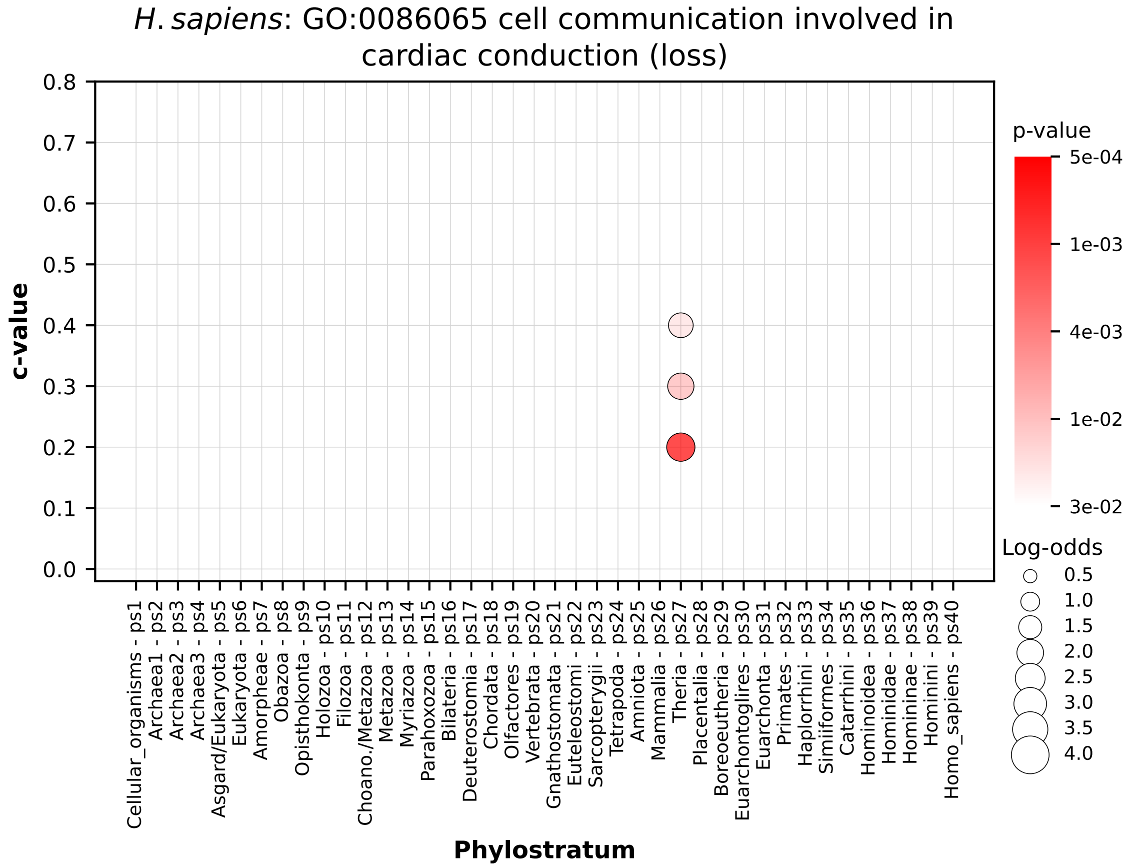

H. sapiens: GO:0086065 cell communication involved in cardiac conduction (loss)
c-value
0.0
0.1
0.2
0.3
0.4
0.5
0.6
0.7
0.8
Phylostratum
Cellular_organisms - ps1
Archaea1 - ps2
Archaea2 - ps3
Archaea3 - ps4
Asgard/Eukaryota - ps5
Eukaryota - ps6
Amorpheae - ps7
Obazoa - ps8
Opisthokonta - ps9
Holozoa - ps10
Filozoa - ps11
Choano./Metazoa - ps12
Metazoa - ps13
Myriazoa - ps14
Parahoxozoa - ps15
Bilateria - ps16
Deuterostomia - ps17
Chordata - ps18
Olfactores - ps19
Vertebrata - ps20
Gnathostomata - ps21
Euteleostomi - ps22
Sarcopterygii - ps23
Tetrapoda - ps24
Amniota - ps25
Mammalia - ps26
Theria - ps27
Placentalia - ps28
Boreoeutheria - ps29
Euarchontoglires - ps30
Euarchonta - ps31
Primates - ps32
Haplorrhini - ps33
Simiiformes - ps34
Catarrhini - ps35
Hominoidea - ps36
Hominidae - ps37
Homininae - ps38
Hominini - ps39
Homo_sapiens - ps40
p-value
5e-04
1e-03
4e-03
1e-02
3e-02
Log-odds
0.5
1.0
1.5
2.0
2.5
3.0
3.5
4.0

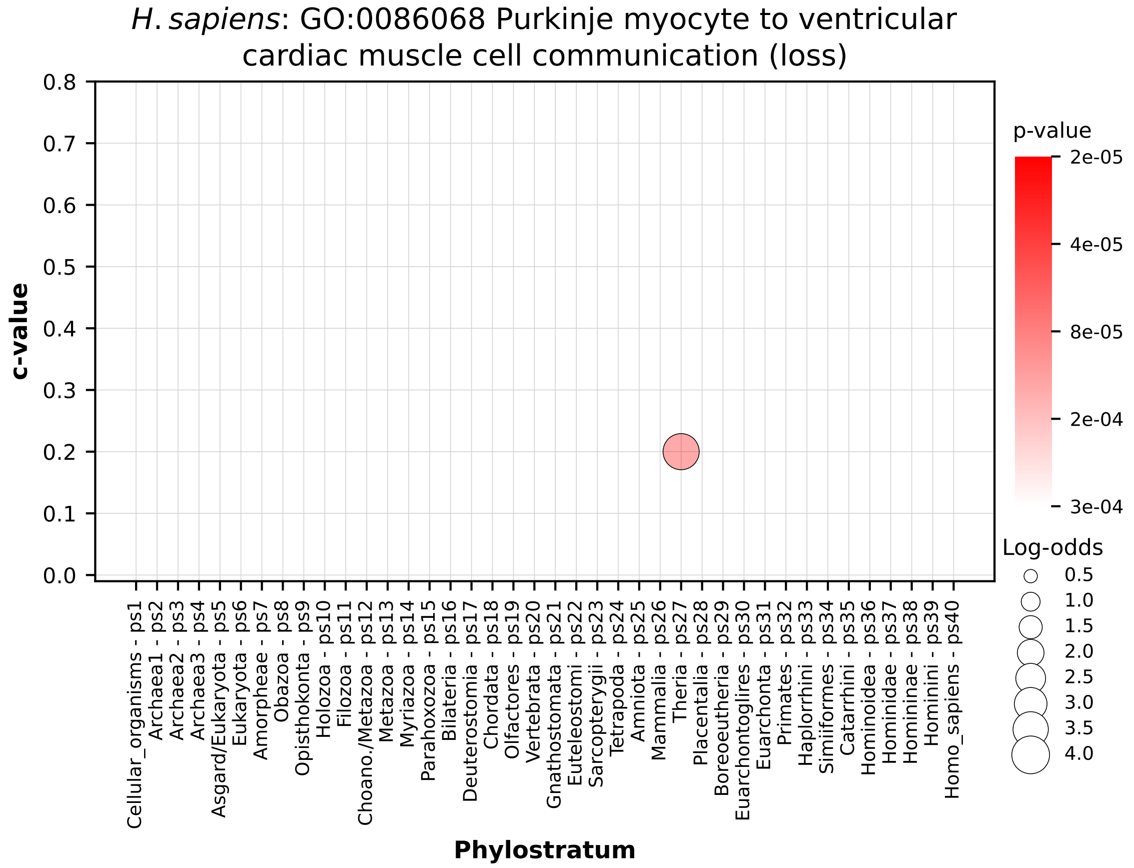

H. sapiens: GO:0086068 Purkinje myocyte to ventricular cardiac muscle cell communication (loss)
c-value
0.8
0.7
0.6
0.5
0.4
0.3
0.2
0.1
0.0
Phylostratum
Cellular_organisms - ps1
Archaea1 - ps2
Archaea2 - ps3
Archaea3 - ps4
Asgard/Eukaryota - ps5
Eukaryota - ps6
Amorpheae - ps7
Obazoa - ps8
Opisthokonta - ps9
Holozoa - ps10
Filozoa - ps11
Choano./Metazoa - ps12
Metazoa - ps13
Myriazoa - ps14
Parahoxozoa - ps15
Bilateria - ps16
Deuterostomia - ps17
Chordata - ps18
Olfactores - ps19
Vertebrata - ps20
Gnathostomata - ps21
Euteleostomi - ps22
Sarcopterygii - ps23
Tetrapoda - ps24
Amniota - ps25
Mammalia - ps26
Theria - ps27
Placentalia - ps28
Boreoeutheria - ps29
Euarchontoglires - ps30
Euarchonta - ps31
Primates - ps32
Haplorrhini - ps33
Simiiformes - ps34
Catarrhini - ps35
Hominoidea - ps36
Hominidae - ps37
Homininae - ps38
Hominini - ps39
Homo_sapiens - ps40
p-value
2e-05
4e-05
8e-05
2e-04
3e-04
Log-odds
0.5
1.0
1.5
2.0
2.5
3.0
3.5
4.0

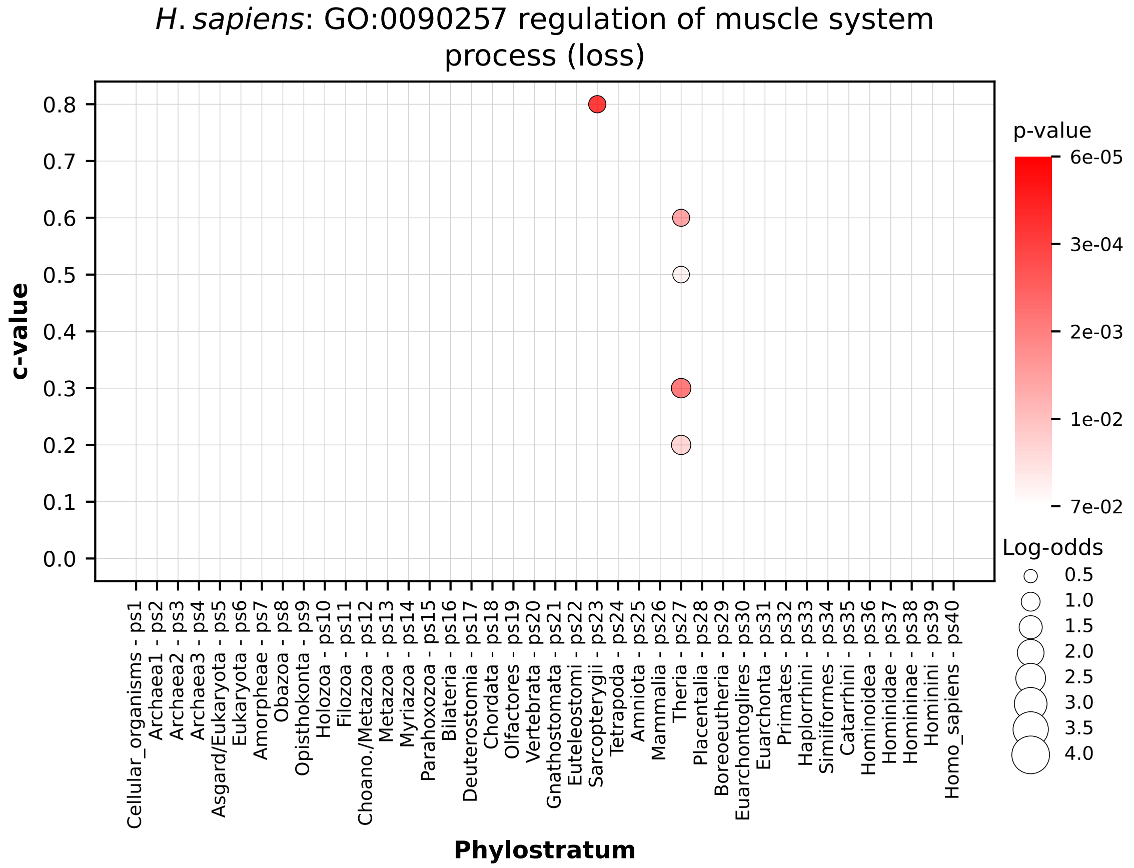

H. sapiens: GO:0090257 regulation of muscle system process (loss)
c-value
0.0
0.1
0.2
0.3
0.4
0.5
0.6
0.7
0.8
Phylostratum
Cellular_organisms - ps1
Archaea1 - ps2
Archaea2 - ps3
Archaea3 - ps4
Asgard/Eukaryota - ps5
Eukaryota - ps6
Amorpheae - ps7
Obazoa - ps8
Opisthokonta - ps9
Holozoa - ps10
Filozoa - ps11
Choano./Metazoa - ps12
Metazoa - ps13
Myriazoa - ps14
Parahoxozoa - ps15
Bilateria - ps16
Deuterostomia - ps17
Chordata - ps18
Olfactores - ps19
Vertebrata - ps20
Gnathostomata - ps21
Euteleostomi - ps22
Sarcopterygii - ps23
Tetrapoda - ps24
Amniota - ps25
Mammalia - ps26
Theria - ps27
Placentalia - ps28
Boreoeutheria - ps29
Euarchontoglires - ps30
Euarchonta - ps31
Primates - ps32
Haplorrhini - ps33
Simiiformes - ps34
Catarrhini - ps35
Hominoidea - ps36
Hominidae - ps37
Homininae - ps38
Hominini - ps39
Homo_sapiens - ps40
p-value
6e-05
3e-04
2e-03
1e-02
7e-02
Log-odds
0.5
1.0
1.5
2.0
2.5
3.0
3.5
4.0

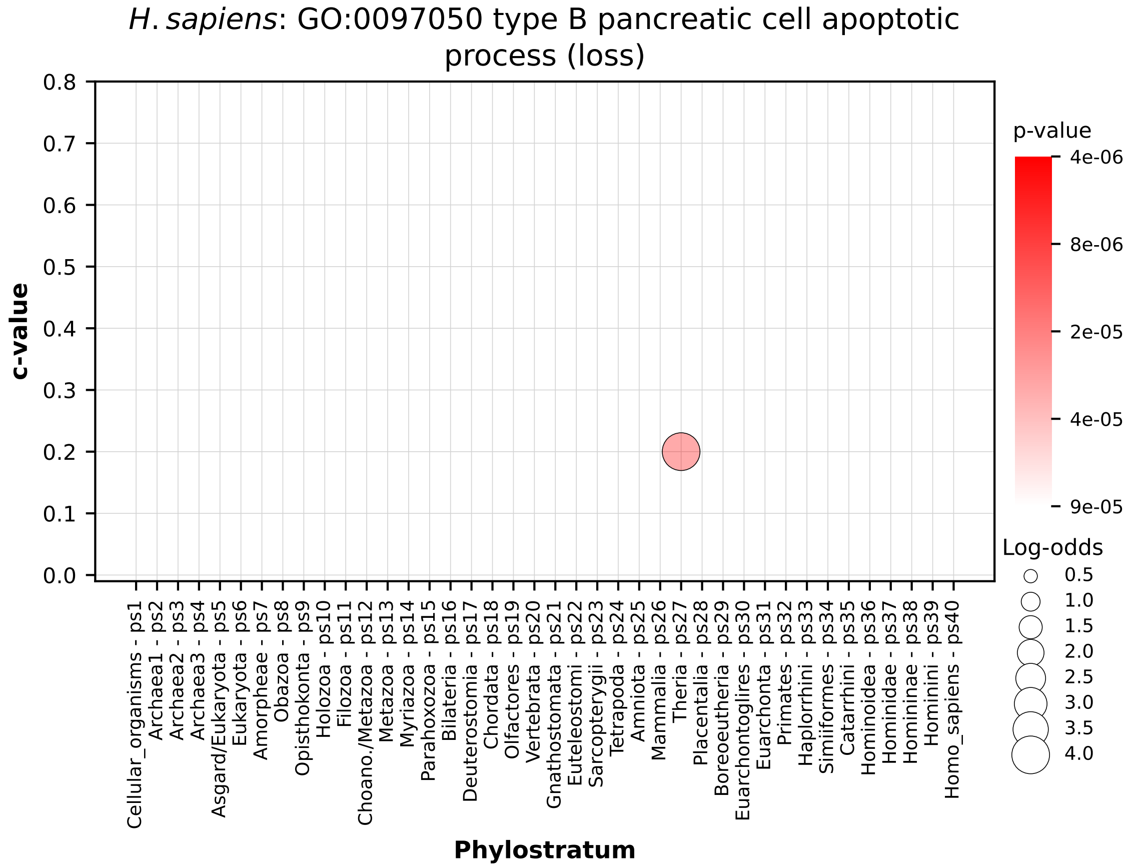

H. sapiens: GO:0097050 type B pancreatic cell apoptotic process (loss)
c-value
0.0
0.1
0.2
0.3
0.4
0.5
0.6
0.7
0.8
Phylostratum
Cellular_organisms - ps1
Archaea1 - ps2
Archaea2 - ps3
Archaea3 - ps4
Asgard/Eukaryota - ps5
Eukaryota - ps6
Amorpheae - ps7
Obazoa - ps8
Opisthokonta - ps9
Holozoa - ps10
Filozoa - ps11
Choano./Metazoa - ps12
Metazoa - ps13
Myriazoa - ps14
Parahoxozoa - ps15
Bilateria - ps16
Deuterostomia - ps17
Chordata - ps18
Olfactores - ps19
Vertebrata - ps20
Gnathostomata - ps21
Euteleostomi - ps22
Sarcopterygii - ps23
Tetrapoda - ps24
Amniota - ps25
Mammalia - ps26
Theria - ps27
Placentalia - ps28
Boreoeutheria - ps29
Euarchontoglires - ps30
Euarchonta - ps31
Primates - ps32
Haplorrhini - ps33
Simiiformes - ps34
Catarrhini - ps35
Hominoidea - ps36
Hominidae - ps37
Homininae - ps38
Hominini - ps39
Homo_sapiens - ps40
p-value
4e-06
8e-06
2e-05
4e-05
9e-05
Log-odds
0.5
1.0
1.5
2.0
2.5
3.0
3.5
4.0

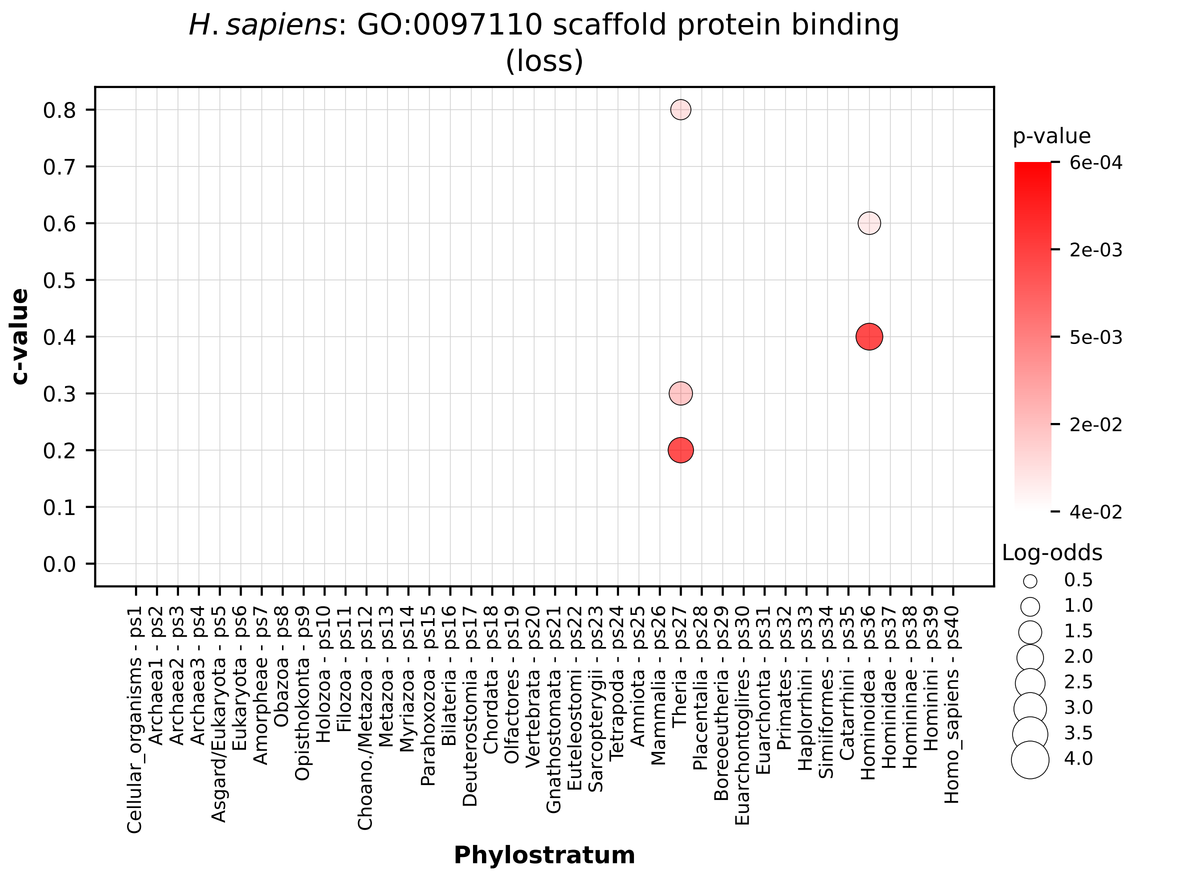

H. sapiens: GO:0097110 scaffold protein binding
(loss)
c-value
0.0
0.1
0.2
0.3
0.4
0.5
0.6
0.7
0.8
Phylostratum
Cellular_organisms - ps1
Archaea1 - ps2
Archaea2 - ps3
Archaea3 - ps4
Asgard/Eukaryota - ps5
Eukaryota - ps6
Amorpheae - ps7
Obazoa - ps8
Opisthokonta - ps9
Holozoa - ps10
Filozoa - ps11
Choano./Metazoa - ps12
Metazoa - ps13
Myriazoa - ps14
Parahoxozoa - ps15
Bilateria - ps16
Deuterostomia - ps17
Chordata - ps18
Olfactores - ps19
Vertebrata - ps20
Gnathostomata - ps21
Euteleostomi - ps22
Sarcopterygii - ps23
Tetrapoda - ps24
Amniota - ps25
Mammalia - ps26
Theria - ps27
Placentalia - ps28
Boreoeutheria - ps29
Euarchontoglires - ps30
Euarchonta - ps31
Primates - ps32
Haplorrhini - ps33
Simiiformes - ps34
Catarrhini - ps35
Hominoidea - ps36
Hominidae - ps37
Homininae - ps38
Hominini - ps39
Homo_sapiens - ps40
p-value
6e-04
2e-03
5e-03
2e-02
4e-02
Log-odds
0.5
1.0
1.5
2.0
2.5
3.0
3.5
4.0

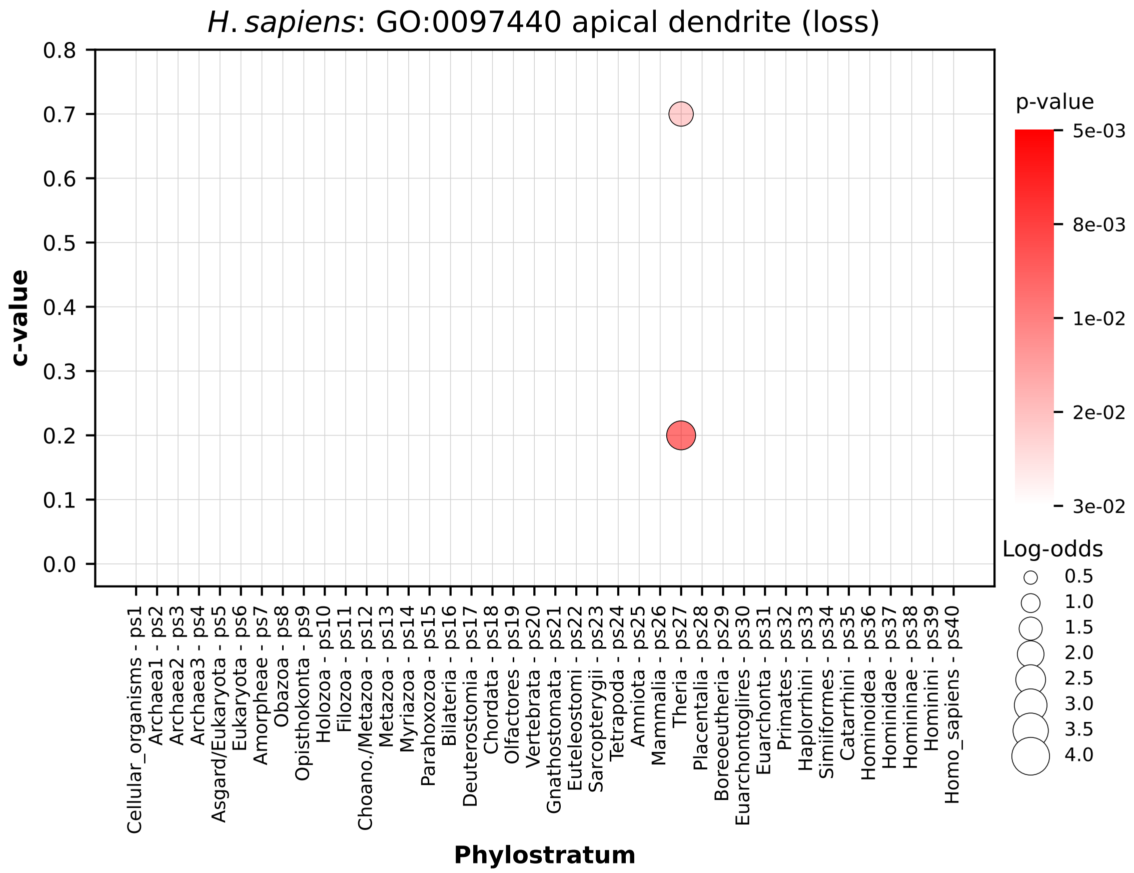

H. sapiens: GO:0097440 apical dendrite (loss)
c-value
0.8
0.7
0.6
0.5
0.4
0.3
0.2
0.1
0.0
Phylostratum
Cellular_organisms - ps1
Archaea1 - ps2
Archaea2 - ps3
Archaea3 - ps4
Asgard/Eukaryota - ps5
Eukaryota - ps6
Amorpheae - ps7
Obazoa - ps8
Opisthokonta - ps9
Holozoa - ps10
Filozoa - ps11
Choano./Metazoa - ps12
Metazoa - ps13
Myriazoa - ps14
Parahoxozoa - ps15
Bilateria - ps16
Deuterostomia - ps17
Chordata - ps18
Olfactores - ps19
Vertebrata - ps20
Gnathostomata - ps21
Euteleostomi - ps22
Sarcopterygii - ps23
Tetrapoda - ps24
Amniota - ps25
Mammalia - ps26
Theria - ps27
Placentalia - ps28
Boreoeutheria - ps29
Euarchontoglires - ps30
Euarchonta - ps31
Primates - ps32
Haplorrhini - ps33
Simiiformes - ps34
Catarrhini - ps35
Hominoidea - ps36
Hominidae - ps37
Homininae - ps38
Hominini - ps39
Homo_sapiens - ps40
p-value
5e-03
8e-03
1e-02
2e-02
3e-02
Log-odds
0.5
1.0
1.5
2.0
2.5
3.0
3.5
4.0

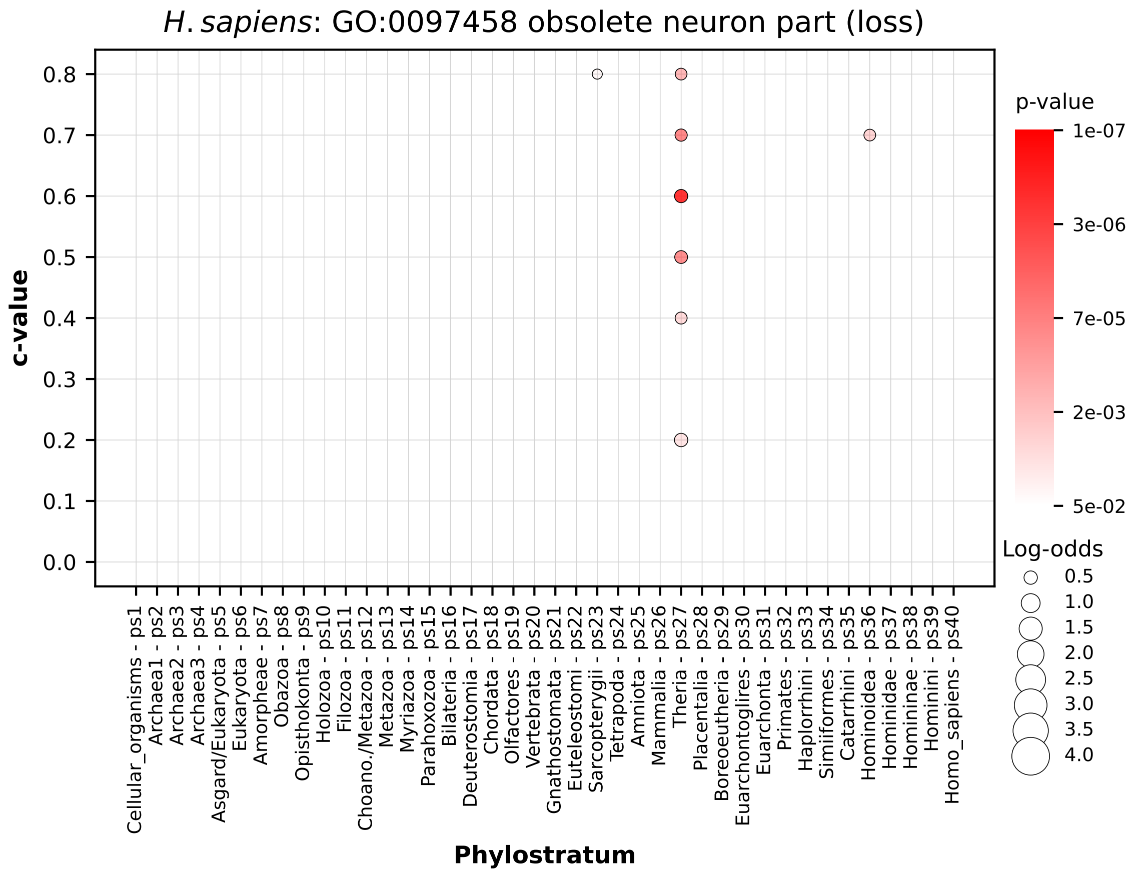

H. sapiens: GO:0097458 obsolete neuron part (loss)
c-value
Phylostratum
p-value
1e-07
3e-06
7e-05
2e-03
5e-02
Log-odds
0.5
1.0
1.5
2.0
2.5
3.0
3.5
4.0
0.0
0.1
0.2
0.3
0.4
0.5
0.6
0.7
0.8
Cellular_organisms - ps1
Archaea1 - ps2
Archaea2 - ps3
Archaea3 - ps4
Asgard/Eukaryota - ps5
Eukaryota - ps6
Amorpheae - ps7
Obazoa - ps8
Opisthokonta - ps9
Holozoa - ps10
Filozoa - ps11
Choano./Metazoa - ps12
Metazoa - ps13
Myriazoa - ps14
Parahoxozoa - ps15
Bilateria - ps16
Deuterostomia - ps17
Chordata - ps18
Olfactores - ps19
Vertebrata - ps20
Gnathostomata - ps21
Euteleostomi - ps22
Sarcopterygii - ps23
Tetrapoda - ps24
Amniota - ps25
Mammalia - ps26
Theria - ps27
Placentalia - ps28
Boreoeutheria - ps29
Euarchontoglires - ps30
Euarchonta - ps31
Primates - ps32
Haplorrhini - ps33
Simiiformes - ps34
Catarrhini - ps35
Hominoidea - ps36
Hominidae - ps37
Homininae - ps38
Hominini - ps39
Homo_sapiens - ps40

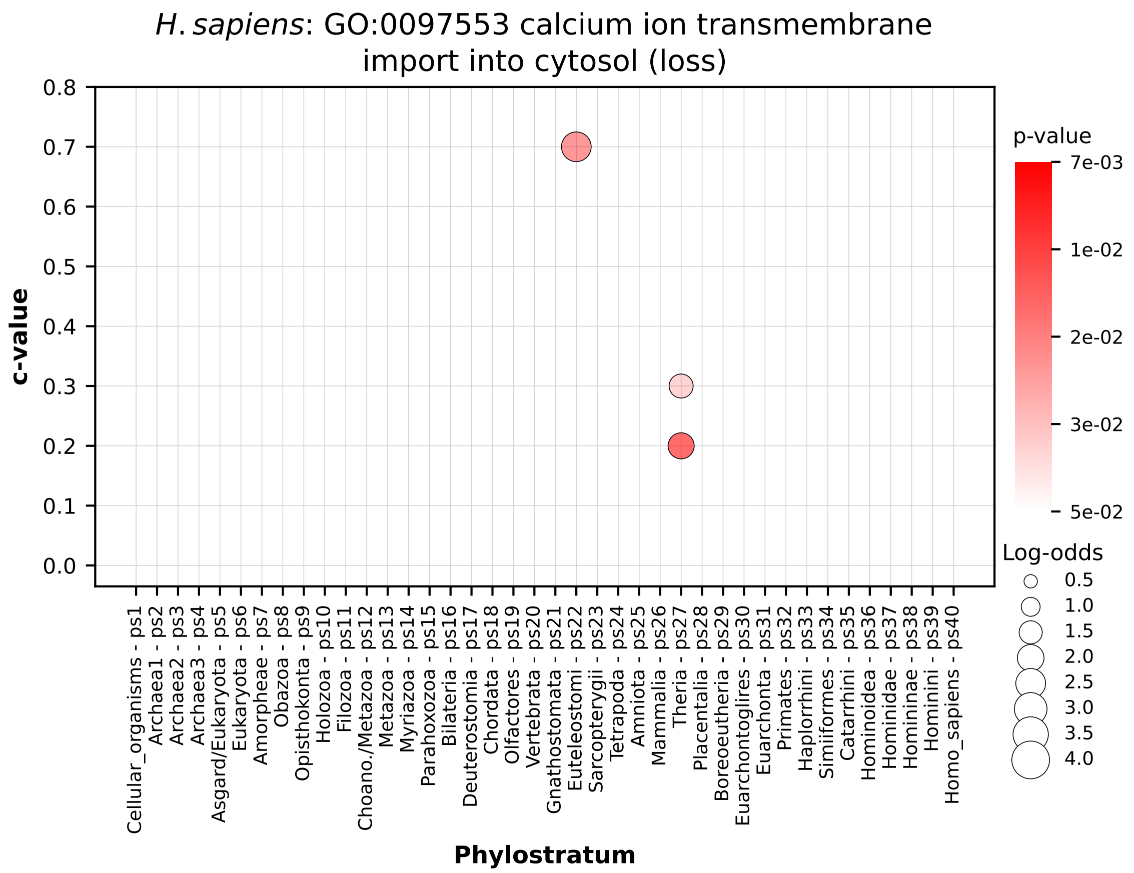

H. sapiens: GO:0097553 calcium ion transmembrane import into cytosol (loss)
c-value
0.8
0.7
0.6
0.5
0.4
0.3
0.2
0.1
0.0
Phylostratum
Cellular_organisms - ps1
Archaea1 - ps2
Archaea2 - ps3
Archaea3 - ps4
Asgard/Eukaryota - ps5
Eukaryota - ps6
Amorpheae - ps7
Obazoa - ps8
Opisthokonta - ps9
Holozoa - ps10
Filozoa - ps11
Choano./Metazoa - ps12
Metazoa - ps13
Myriazoa - ps14
Parahoxozoa - ps15
Bilateria - ps16
Deuterostomia - ps17
Chordata - ps18
Olfactores - ps19
Vertebrata - ps20
Gnathostomata - ps21
Euteleostomi - ps22
Sarcopterygii - ps23
Tetrapoda - ps24
Amniota - ps25
Mammalia - ps26
Theria - ps27
Placentalia - ps28
Boreoeutheria - ps29
Euarchontoglires - ps30
Euarchonta - ps31
Primates - ps32
Haplorrhini - ps33
Simiiformes - ps34
Catarrhini - ps35
Hominoidea - ps36
Hominidae - ps37
Homininae - ps38
Hominini - ps39
Homo_sapiens - ps40
p-value
7e-03
1e-02
2e-02
3e-02
5e-02
Log-odds
0.5
1.0
1.5
2.0
2.5
3.0
3.5
4.0

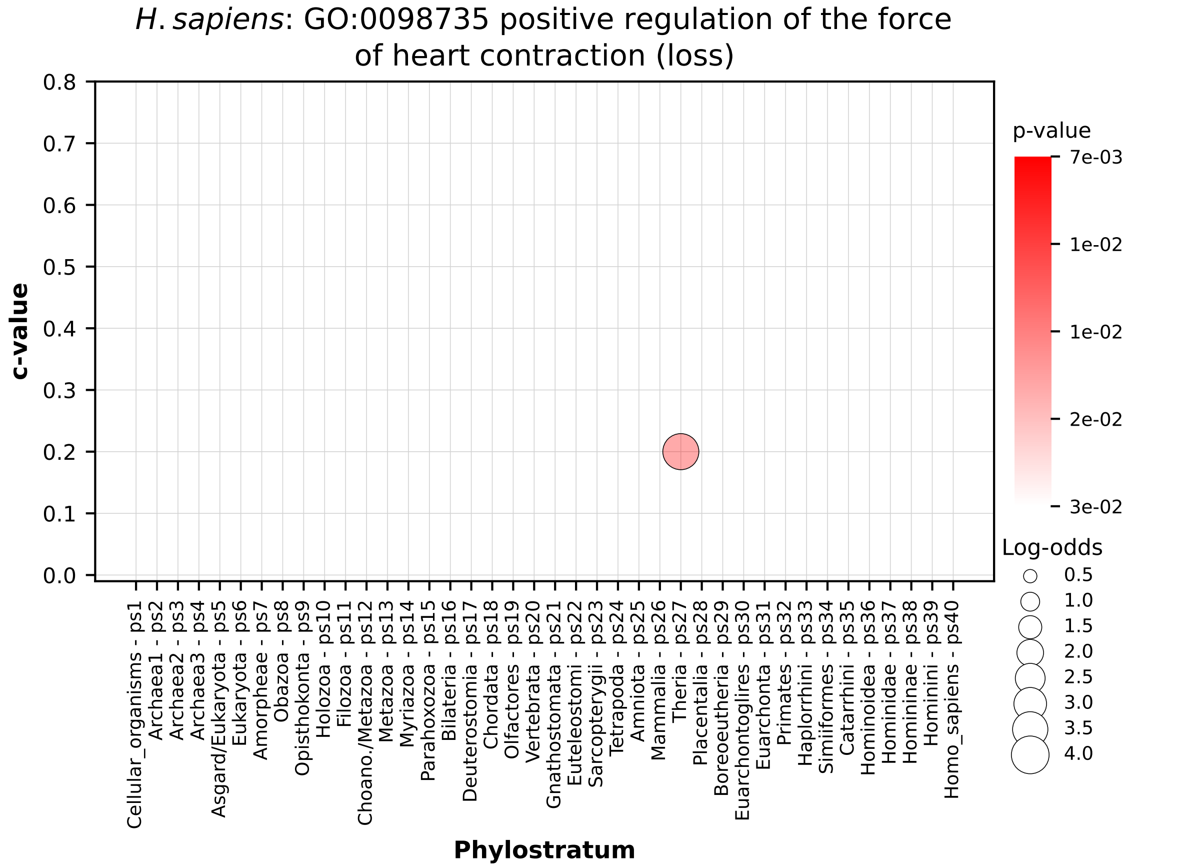

H. sapiens: GO:0098735 positive regulation of the force of heart contraction (loss)
c-value
0.8
0.7
0.6
0.5
0.4
0.3
0.2
0.1
0.0
Phylostratum
Cellular_organisms - ps1
Archaea1 - ps2
Archaea2 - ps3
Archaea3 - ps4
Asgard/Eukaryota - ps5
Eukaryota - ps6
Amorpheae - ps7
Obazoa - ps8
Opisthokonta - ps9
Holozoa - ps10
Filozoa - ps11
Choano./Metazoa - ps12
Metazoa - ps13
Myriazoa - ps14
Parahoxozoa - ps15
Bilateria - ps16
Deuterostomia - ps17
Chordata - ps18
Olfactores - ps19
Vertebrata - ps20
Gnathostomata - ps21
Euteleostomi - ps22
Sarcopterygii - ps23
Tetrapoda - ps24
Amniota - ps25
Mammalia - ps26
Theria - ps27
Placentalia - ps28
Boreoeutheria - ps29
Euarchontoglires - ps30
Euarchonta - ps31
Primates - ps32
Haplorrhini - ps33
Simiiformes - ps34
Catarrhini - ps35
Hominoidea - ps36
Hominidae - ps37
Homininae - ps38
Hominini - ps39
Homo_sapiens - ps40
p-value
7e-03
1e-02
1e-02
2e-02
3e-02
Log-odds
0.5
1.0
1.5
2.0
2.5
3.0
3.5
4.0

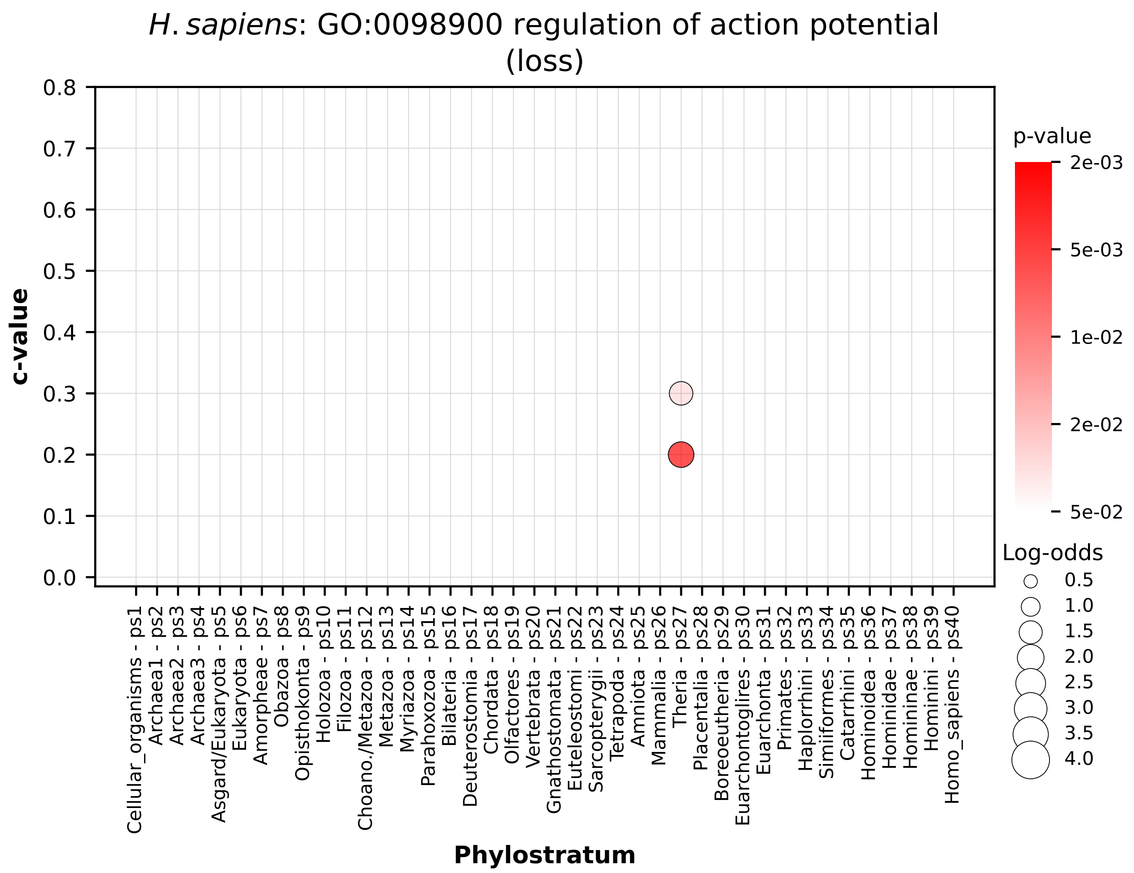
H. sapiens: GO:0098900 regulation of action potential
(loss)
c-value
0.0
0.1
0.2
0.3
0.4
0.5
0.6
0.7
0.8
Phylostratum
Cellular_organisms - ps1
Archaea1 - ps2
Archaea2 - ps3
Archaea3 - ps4
Asgard/Eukaryota - ps5
Eukaryota - ps6
Amorpheae - ps7
Obazoa - ps8
Opisthokonta - ps9
Holozoa - ps10
Filozoa - ps11
Choano./Metazoa - ps12
Metazoa - ps13
Myriazoa - ps14
Parahoxozoa - ps15
Bilateria - ps16
Deuterostomia - ps17
Chordata - ps18
Olfactores - ps19
Vertebrata - ps20
Gnathostomata - ps21
Euteleostomi - ps22
Sarcopterygii - ps23
Tetrapoda - ps24
Amniota - ps25
Mammalia - ps26
Theria - ps27
Placentalia - ps28
Boreoeutheria - ps29
Euarchontoglires - ps30
Euarchonta - ps31
Primates - ps32
Haplorrhini - ps33
Simiiformes - ps34
Catarrhini - ps35
Hominoidea - ps36
Hominidae - ps37
Homininae - ps38
Hominini - ps39
Homo_sapiens - ps40
p-value
2e-03
5e-03
1e-02
2e-02
5e-02
Log-odds
0.5
1.0
1.5
2.0
2.5
3.0
3.5
4.0

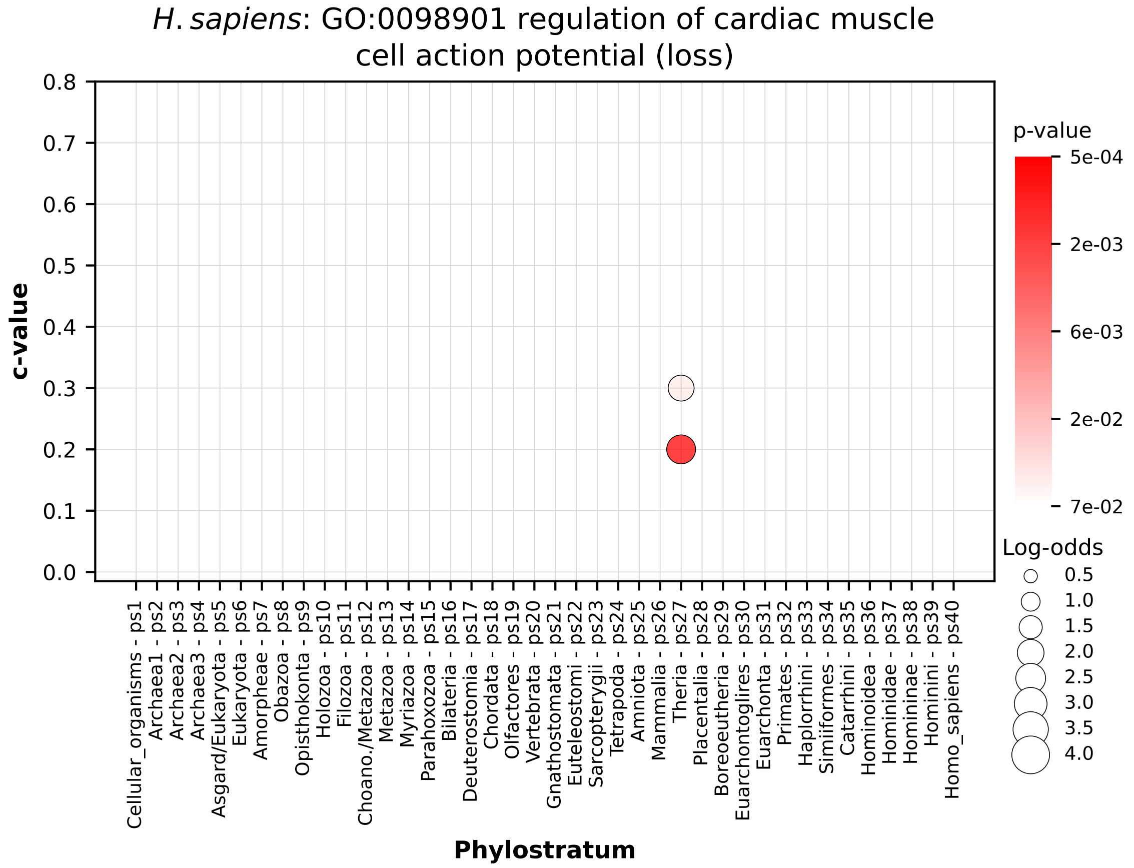

H. sapiens: GO:0098901 regulation of cardiac muscle cell action potential (loss)
c-value
0.8
0.7
0.6
0.5
0.4
0.3
0.2
0.1
0.0
Phylostratum
Cellular_organisms - ps1
Archaea1 - ps2
Archaea2 - ps3
Archaea3 - ps4
Asgard/Eukaryota - ps5
Eukaryota - ps6
Amorpheae - ps7
Obazoa - ps8
Opisthokonta - ps9
Holozoa - ps10
Filozoa - ps11
Choano./Metazoa - ps12
Metazoa - ps13
Myriazoa - ps14
Parahoxozoa - ps15
Bilateria - ps16
Deuterostomia - ps17
Chordata - ps18
Olfactores - ps19
Vertebrata - ps20
Gnathostomata - ps21
Euteleostomi - ps22
Sarcopterygii - ps23
Tetrapoda - ps24
Amniota - ps25
Mammalia - ps26
Theria - ps27
Placentalia - ps28
Boreoeutheria - ps29
Euarchontoglires - ps30
Euarchonta - ps31
Primates - ps32
Haplorrhini - ps33
Simiiformes - ps34
Catarrhini - ps35
Hominoidea - ps36
Hominidae - ps37
Homininae - ps38
Hominini - ps39
Homo_sapiens - ps40
p-value
5e-04
2e-03
6e-03
2e-02
7e-02
Log-odds
0.5
1.0
1.5
2.0
2.5
3.0
3.5
4.0

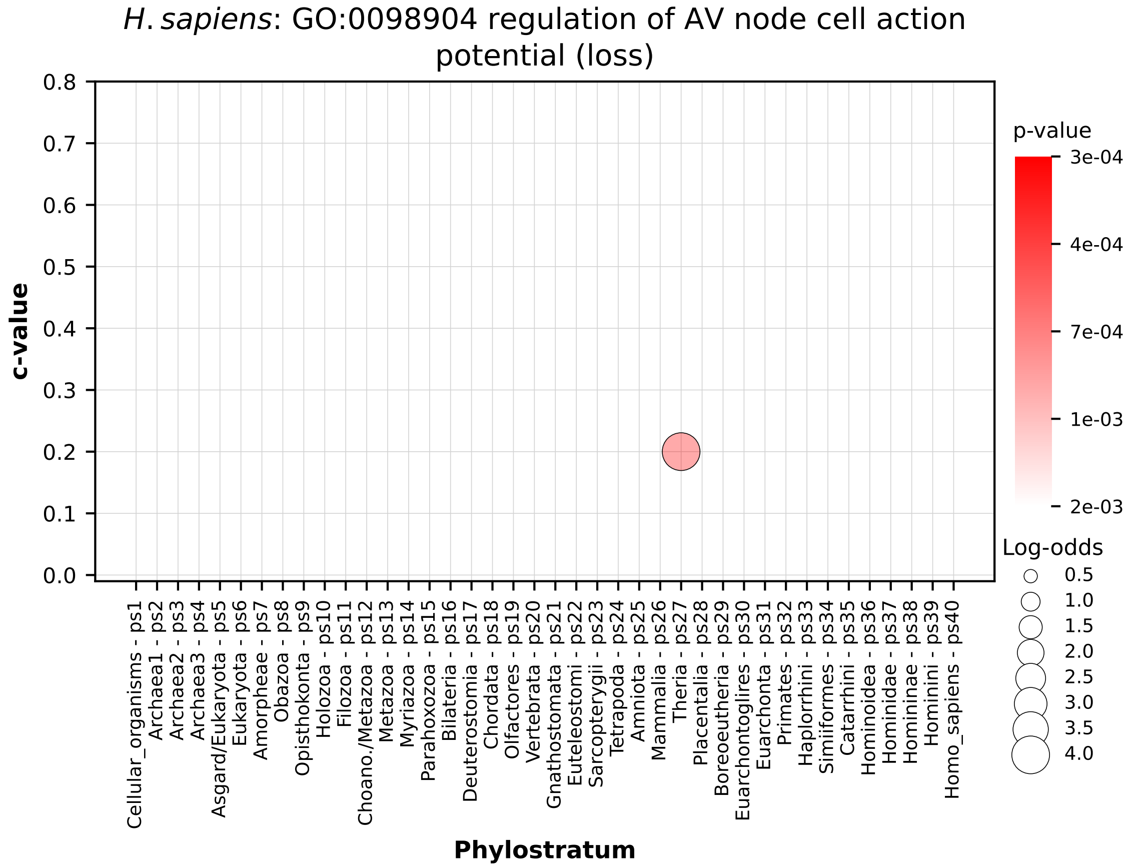

H. sapiens: GO:0098904 regulation of AV node cell action potential (loss)
c-value
0.0
0.1
0.2
0.3
0.4
0.5
0.6
0.7
0.8
Phylostratum
Cellular_organisms - ps1
Archaea1 - ps2
Archaea2 - ps3
Archaea3 - ps4
Asgard/Eukaryota - ps5
Eukaryota - ps6
Amorpheae - ps7
Obazoa - ps8
Opisthokonta - ps9
Holozoa - ps10
Filozoa - ps11
Choano./Metazoa - ps12
Metazoa - ps13
Myriazoa - ps14
Parahoxozoa - ps15
Bilateria - ps16
Deuterostomia - ps17
Chordata - ps18
Olfactores - ps19
Vertebrata - ps20
Gnathostomata - ps21
Euteleostomi - ps22
Sarcopterygii - ps23
Tetrapoda - ps24
Amniota - ps25
Mammalia - ps26
Theria - ps27
Placentalia - ps28
Boreoeutheria - ps29
Euarchontoglires - ps30
Euarchonta - ps31
Primates - ps32
Haplorrhini - ps33
Simiiformes - ps34
Catarrhini - ps35
Hominoidea - ps36
Hominidae - ps37
Homininae - ps38
Hominini - ps39
Homo_sapiens - ps40
p-value
3e-04
4e-04
7e-04
1e-03
2e-03
Log-odds
0.5
1.0
1.5
2.0
2.5
3.0
3.5
4.0

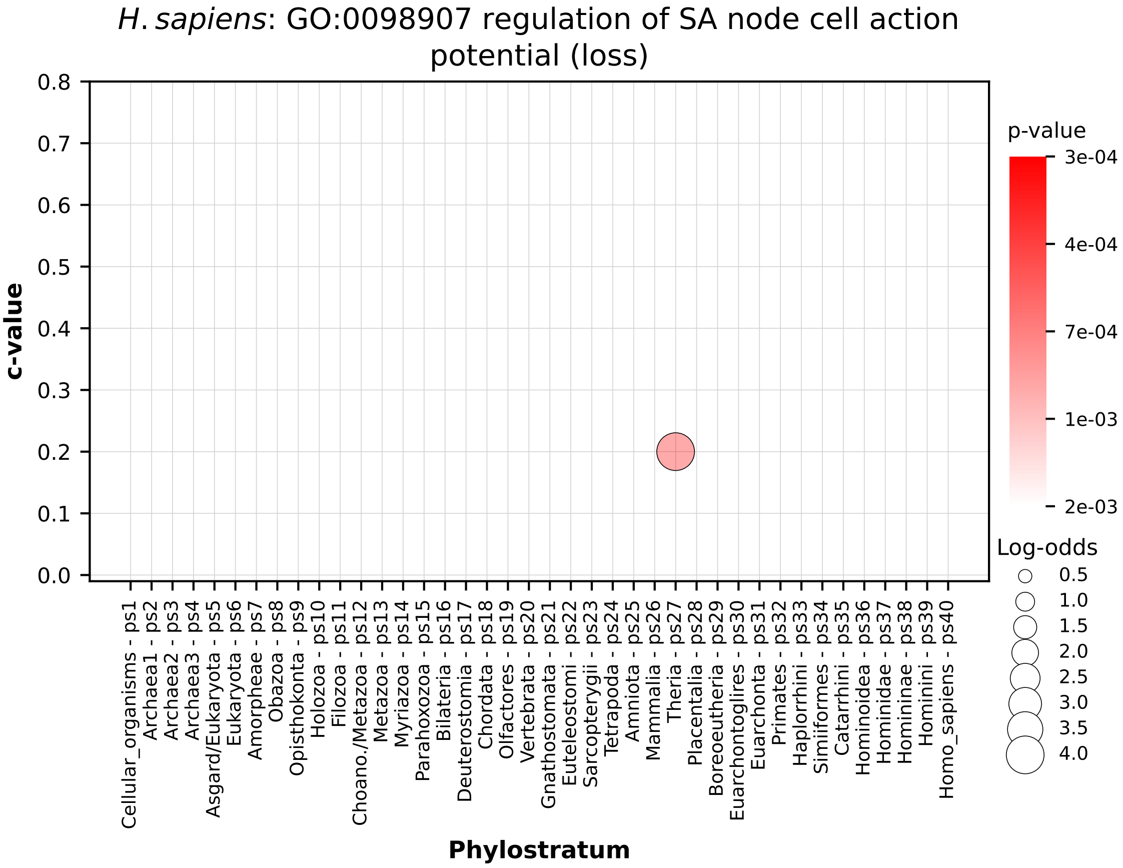
H. sapiens: GO:0098907 regulation of SA node cell action potential (loss)
c-value
0.8
0.7
0.6
0.5
0.4
0.3
0.2
0.1
0.0
Cellular_organisms - ps1
Archaea1 - ps2
Archaea2 - ps3
Archaea3 - ps4
Asgard/Eukaryota - ps5
Eukaryota - ps6
Amorpheae - ps7
Obazoa - ps8
Opisthokonta - ps9
Holozoa - ps10
Filozoa - ps11
Choano./Metazoa - ps12
Metazoa - ps13
Myriazoa - ps14
Parahoxozoa - ps15
Bilateria - ps16
Deuterostomia - ps17
Chordata - ps18
Olfactores - ps19
Vertebrata - ps20
Gnathostomata - ps21
Euteleostomi - ps22
Sarcopterygii - ps23
Tetrapoda - ps24
Amniota - ps25
Mammalia - ps26
Theria - ps27
Placentalia - ps28
Boreoeutheria - ps29
Euarchontoglires - ps30
Euarchonta - ps31
Primates - ps32
Haplorrhini - ps33
Simiiformes - ps34
Catarrhini - ps35
Hominoidea - ps36
Hominidae - ps37
Homininae - ps38
Hominini - ps39
Homo_sapiens - ps40
Phylostratum
p-value
3e-04
4e-04
7e-04
1e-03
2e-03
Log-odds
0.5
1.0
1.5
2.0
2.5
3.0
3.5
4.0

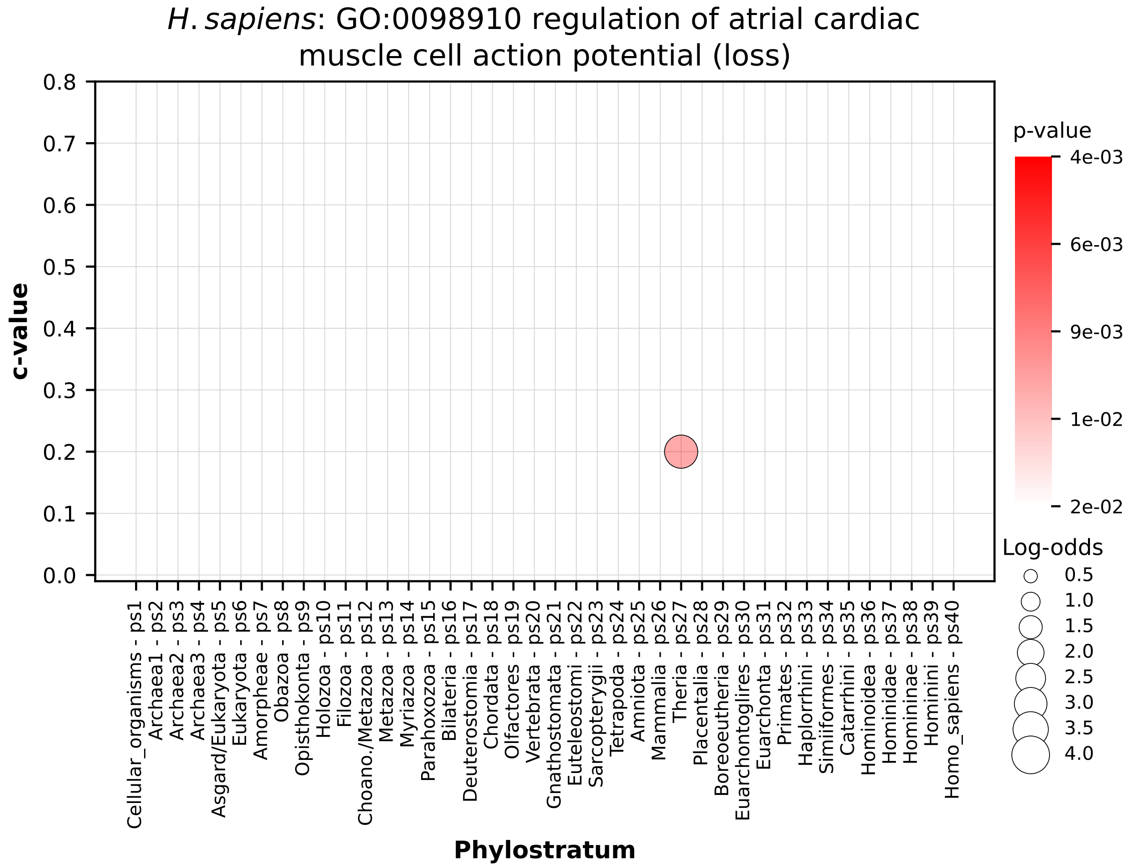

H. sapiens: GO:0098910 regulation of atrial cardiac muscle cell action potential (loss)
c-value
0.8
0.7
0.6
0.5
0.4
0.3
0.2
0.1
0.0
Phylostratum
Cellular_organisms - ps1
Archaea1 - ps2
Archaea2 - ps3
Archaea3 - ps4
Asgard/Eukaryota - ps5
Eukaryota - ps6
Amorpheae - ps7
Obazoa - ps8
Opisthokonta - ps9
Holozoa - ps10
Filozoa - ps11
Choano./Metazoa - ps12
Metazoa - ps13
Myriazoa - ps14
Parahoxozoa - ps15
Bilateria - ps16
Deuterostomia - ps17
Chordata - ps18
Olfactores - ps19
Vertebrata - ps20
Gnathostomata - ps21
Euteleostomi - ps22
Sarcopterygii - ps23
Tetrapoda - ps24
Amniota - ps25
Mammalia - ps26
Theria - ps27
Placentalia - ps28
Boreoeutheria - ps29
Euarchontoglires - ps30
Euarchonta - ps31
Primates - ps32
Haplorrhini - ps33
Simiiformes - ps34
Catarrhini - ps35
Hominoidea - ps36
Hominidae - ps37
Homininae - ps38
Hominini - ps39
Homo_sapiens - ps40
p-value
4e-03
6e-03
9e-03
1e-02
2e-02
Log-odds
0.5
1.0
1.5
2.0
2.5
3.0
3.5
4.0

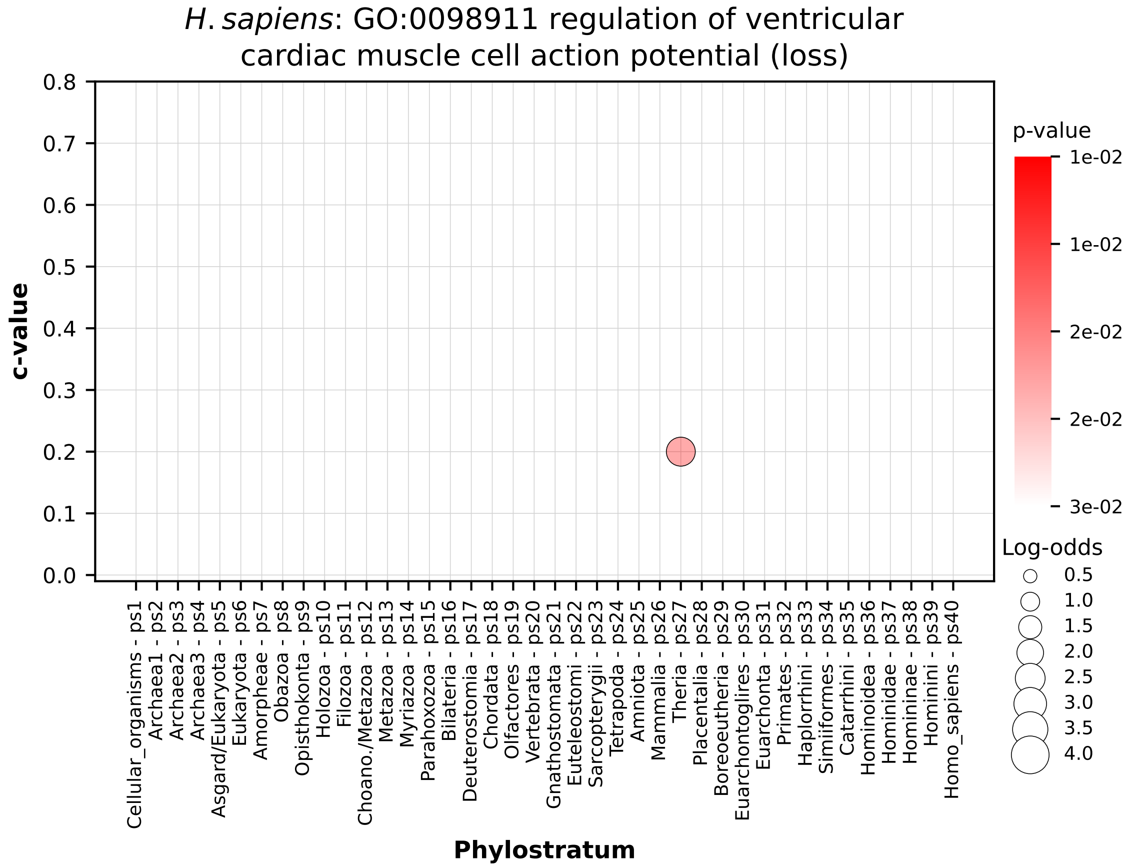
H. sapiens: GO:0098911 regulation of ventricular cardiac muscle cell action potential (loss)
c-value
0.0
0.1
0.2
0.3
0.4
0.5
0.6
0.7
0.8
Phylostratum
Cellular_organisms - ps1
Archaea1 - ps2
Archaea2 - ps3
Archaea3 - ps4
Asgard/Eukaryota - ps5
Eukaryota - ps6
Amorpheae - ps7
Obazoa - ps8
Opisthokonta - ps9
Holozoa - ps10
Filozoa - ps11
Choano./Metazoa - ps12
Metazoa - ps13
Myriazoa - ps14
Parahoxozoa - ps15
Bilateria - ps16
Deuterostomia - ps17
Chordata - ps18
Olfactores - ps19
Vertebrata - ps20
Gnathostomata - ps21
Euteleostomi - ps22
Sarcopterygii - ps23
Tetrapoda - ps24
Amniota - ps25
Mammalia - ps26
Theria - ps27
Placentalia - ps28
Boreoeutheria - ps29
Euarchontoglires - ps30
Euarchonta - ps31
Primates - ps32
Haplorrhini - ps33
Simiiformes - ps34
Catarrhini - ps35
Hominoidea - ps36
Hominidae - ps37
Homininae - ps38
Hominini - ps39
Homo_sapiens - ps40
p-value
1e-02
1e-02
2e-02
2e-02
3e-02
Log-odds
0.5
1.0
1.5
2.0
2.5
3.0
3.5
4.0

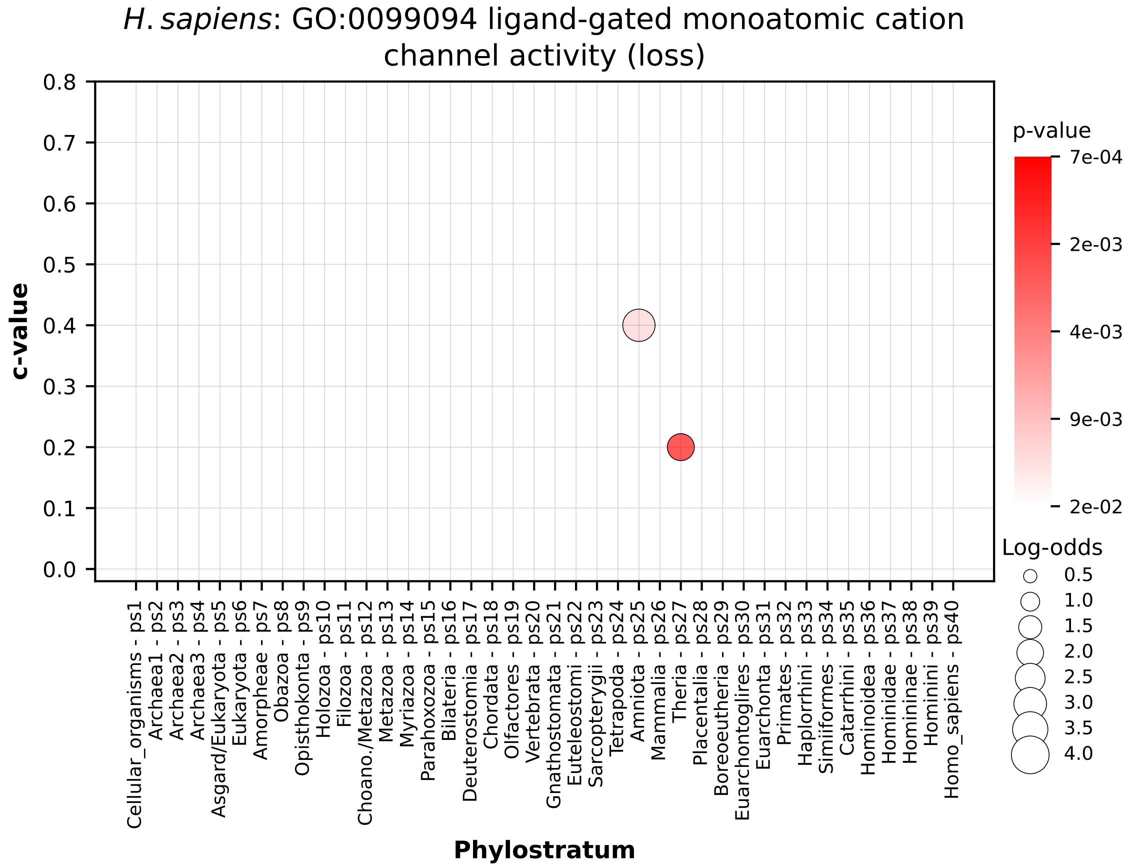

H. sapiens: GO:0099094 ligand-gated monoatomic cation channel activity (loss)
c-value
0.8
0.7
0.6
0.5
0.4
0.3
0.2
0.1
0.0
Phylostratum
Cellular_organisms - ps1
Archaea1 - ps2
Archaea2 - ps3
Archaea3 - ps4
Asgard/Eukaryota - ps5
Eukaryota - ps6
Amorpheae - ps7
Obazoa - ps8
Opisthokonta - ps9
Holozoa - ps10
Filozoa - ps11
Choano./Metazoa - ps12
Metazoa - ps13
Myriazoa - ps14
Parahoxozoa - ps15
Bilateria - ps16
Deuterostomia - ps17
Chordata - ps18
Olfactores - ps19
Vertebrata - ps20
Gnathostomata - ps21
Euteleostomi - ps22
Sarcopterygii - ps23
Tetrapoda - ps24
Amniota - ps25
Mammalia - ps26
Theria - ps27
Placentalia - ps28
Boreoeutheria - ps29
Euarchontoglires - ps30
Euarchonta - ps31
Primates - ps32
Haplorrhini - ps33
Simiiformes - ps34
Catarrhini - ps35
Hominoidea - ps36
Hominidae - ps37
Homininae - ps38
Hominini - ps39
Homo_sapiens - ps40
p-value
7e-04
2e-03
4e-03
9e-03
2e-02
Log-odds
0.5
1.0
1.5
2.0
2.5
3.0
3.5
4.0

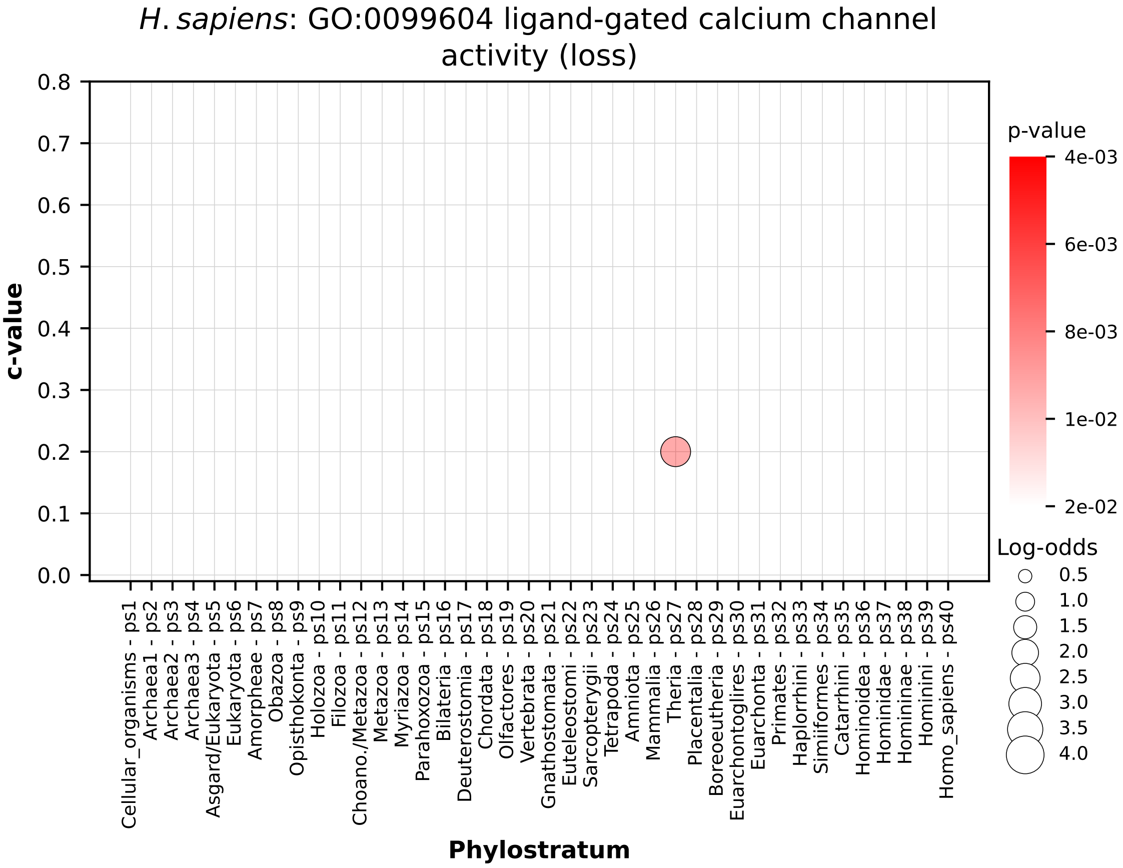

H. sapiens: GO:0099604 ligand-gated calcium channel activity (loss)
c-value
0.0
0.1
0.2
0.3
0.4
0.5
0.6
0.7
0.8
Phylostratum
Cellular_organisms - ps1
Archaea1 - ps2
Archaea2 - ps3
Archaea3 - ps4
Asgard/Eukaryota - ps5
Eukaryota - ps6
Amorpheae - ps7
Obazoa - ps8
Opisthokonta - ps9
Holozoa - ps10
Filozoa - ps11
Choano./Metazoa - ps12
Metazoa - ps13
Myriazoa - ps14
Parahoxozoa - ps15
Bilateria - ps16
Deuterostomia - ps17
Chordata - ps18
Olfactores - ps19
Vertebrata - ps20
Gnathostomata - ps21
Euteleostomi - ps22
Sarcopterygii - ps23
Tetrapoda - ps24
Amniota - ps25
Mammalia - ps26
Theria - ps27
Placentalia - ps28
Boreoeutheria - ps29
Euarchontoglires - ps30
Euarchonta - ps31
Primates - ps32
Haplorrhini - ps33
Simiiformes - ps34
Catarrhini - ps35
Hominoidea - ps36
Hominidae - ps37
Homininae - ps38
Hominini - ps39
Homo_sapiens - ps40
p-value
4e-03
6e-03
8e-03
1e-02
2e-02
Log-odds
0.5
1.0
1.5
2.0
2.5
3.0
3.5
4.0

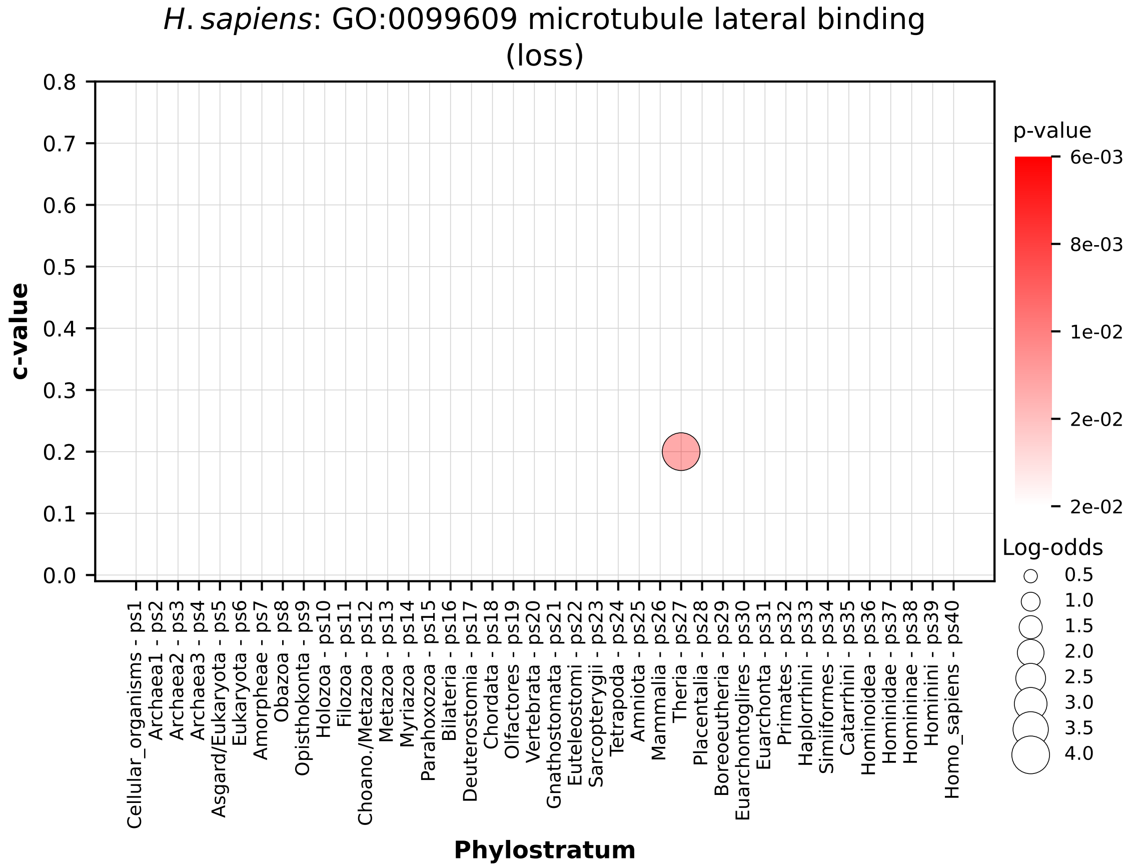

H. sapiens: GO:0099609 microtubule lateral binding
(loss)
c-value
0.0
0.1
0.2
0.3
0.4
0.5
0.6
0.7
0.8
Phylostratum
Cellular_organisms - ps1
Archaea1 - ps2
Archaea2 - ps3
Archaea3 - ps4
Asgard/Eukaryota - ps5
Eukaryota - ps6
Amorpheae - ps7
Obazoa - ps8
Opisthokonta - ps9
Holozoa - ps10
Filozoa - ps11
Choano./Metazoa - ps12
Metazoa - ps13
Myriazoa - ps14
Parahoxozoa - ps15
Bilateria - ps16
Deuterostomia - ps17
Chordata - ps18
Olfactores - ps19
Vertebrata - ps20
Gnathostomata - ps21
Euteleostomi - ps22
Sarcopterygii - ps23
Tetrapoda - ps24
Amniota - ps25
Mammalia - ps26
Theria - ps27
Placentalia - ps28
Boreoeutheria - ps29
Euarchontoglires - ps30
Euarchonta - ps31
Primates - ps32
Haplorrhini - ps33
Simiiformes - ps34
Catarrhini - ps35
Hominoidea - ps36
Hominidae - ps37
Homininae - ps38
Hominini - ps39
Homo_sapiens - ps40
p-value
6e-03
8e-03
1e-02
2e-02
2e-02
Log-odds
0.5
1.0
1.5
2.0
2.5
3.0
3.5
4.0

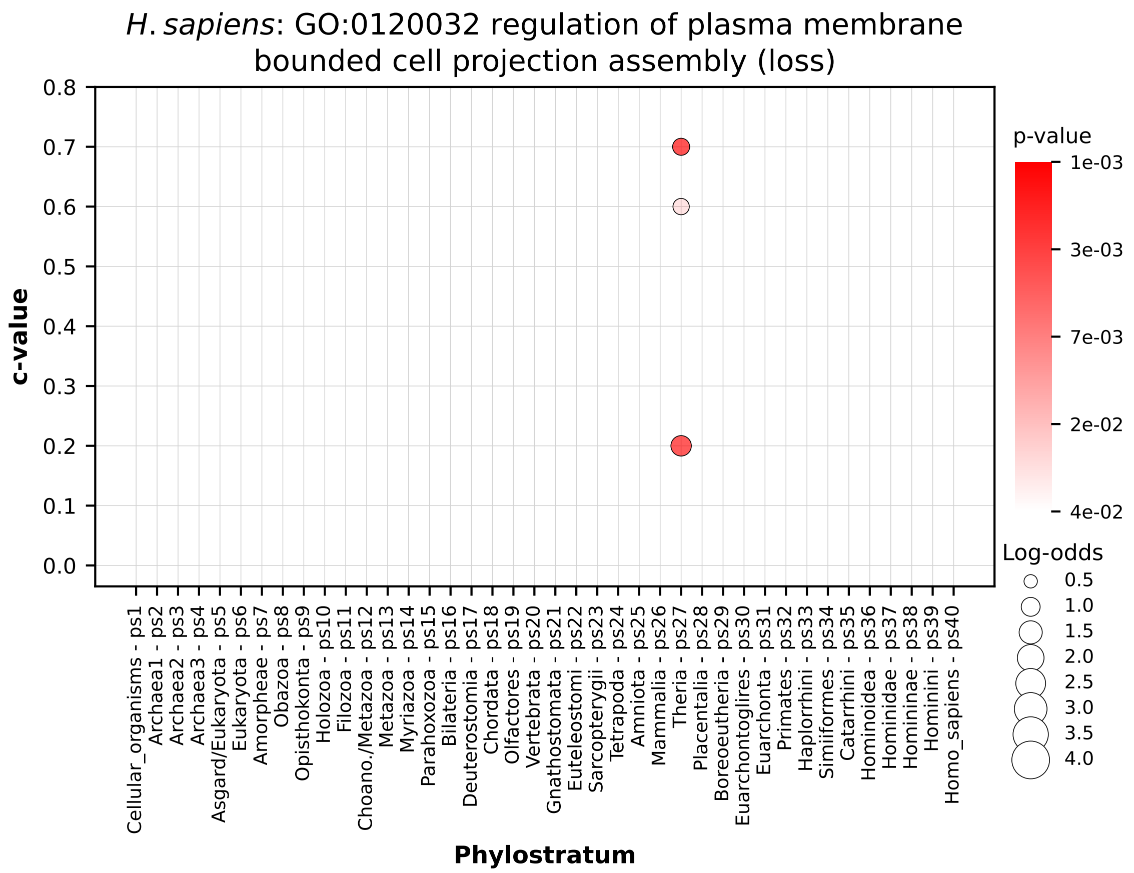
H. sapiens: GO:0120032 regulation of plasma membrane bounded cell projection assembly (loss)
c-value
0.0
0.1
0.2
0.3
0.4
0.5
0.6
0.7
0.8
Phylostratum
Cellular_organisms - ps1
Archaea1 - ps2
Archaea2 - ps3
Archaea3 - ps4
Asgard/Eukaryota - ps5
Eukaryota - ps6
Amorpheae - ps7
Obazoa - ps8
Opisthokonta - ps9
Holozoa - ps10
Filozoa - ps11
Choano./Metazoa - ps12
Metazoa - ps13
Myriazoa - ps14
Parahoxozoa - ps15
Bilateria - ps16
Deuterostomia - ps17
Chordata - ps18
Olfactores - ps19
Vertebrata - ps20
Gnathostomata - ps21
Euteleostomi - ps22
Sarcopterygii - ps23
Tetrapoda - ps24
Amniota - ps25
Mammalia - ps26
Theria - ps27
Placentalia - ps28
Boreoeutheria - ps29
Euarchontoglires - ps30
Euarchonta - ps31
Primates - ps32
Haplorrhini - ps33
Simiiformes - ps34
Catarrhini - ps35
Hominoidea - ps36
Hominidae - ps37
Homininae - ps38
Hominini - ps39
Homo_sapiens - ps40
p-value
1e-03
3e-03
7e-03
2e-02
4e-02
Log-odds
0.5
1.0
1.5
2.0
2.5
3.0
3.5
4.0

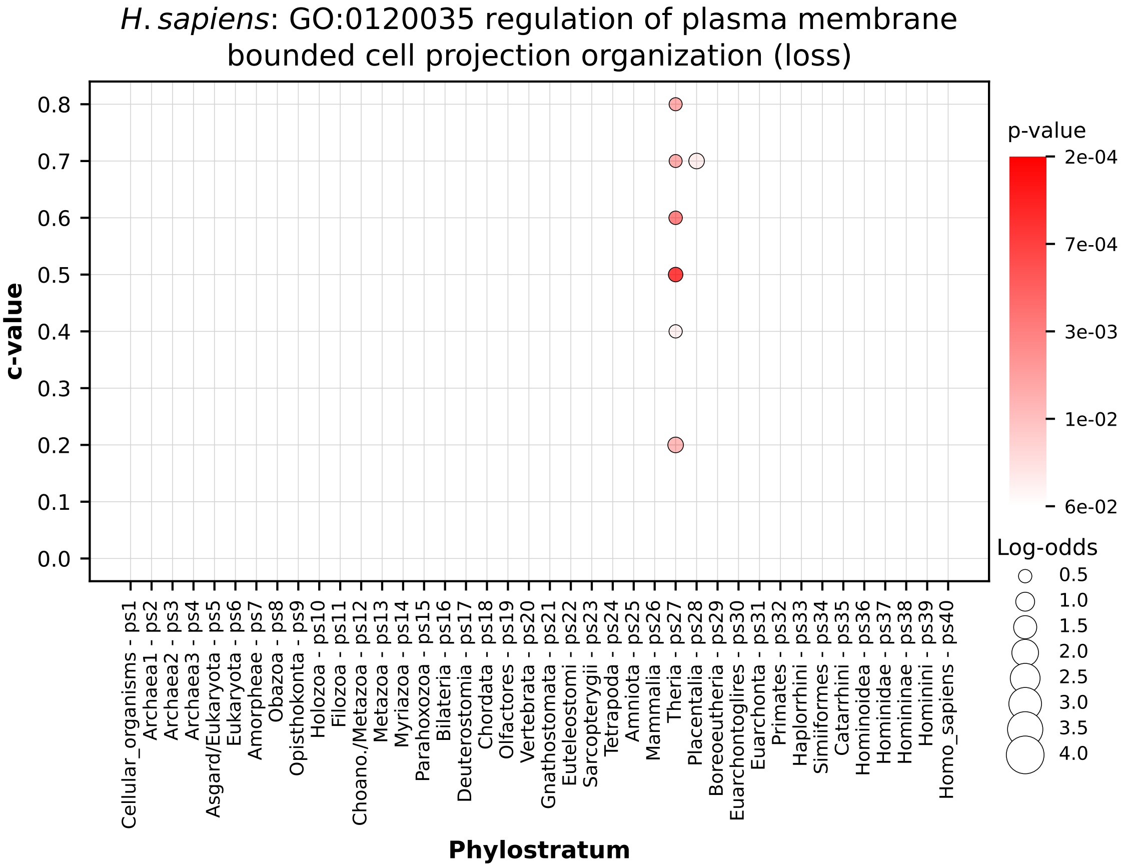

H. sapiens: GO:0120035 regulation of plasma membrane bounded cell projection organization (loss)
c-value
0.0
0.1
0.2
0.3
0.4
0.5
0.6
0.7
0.8
Phylostratum
Cellular_organisms - ps1
Archaea1 - ps2
Archaea2 - ps3
Archaea3 - ps4
Asgard/Eukaryota - ps5
Eukaryota - ps6
Amorpheae - ps7
Obazoa - ps8
Opisthokonta - ps9
Holozoa - ps10
Filozoa - ps11
Choano./Metazoa - ps12
Metazoa - ps13
Myriazoa - ps14
Parahoxozoa - ps15
Bilateria - ps16
Deuterostomia - ps17
Chordata - ps18
Olfactores - ps19
Vertebrata - ps20
Gnathostomata - ps21
Euteleostomi - ps22
Sarcopterygii - ps23
Tetrapoda - ps24
Amniota - ps25
Mammalia - ps26
Theria - ps27
Placentalia - ps28
Boreoeutheria - ps29
Euarchontoglires - ps30
Euarchonta - ps31
Primates - ps32
Haplorrhini - ps33
Simiiformes - ps34
Catarrhini - ps35
Hominoidea - ps36
Hominidae - ps37
Homininae - ps38
Hominini - ps39
Homo_sapiens - ps40
p-value
2e-04
7e-04
3e-03
1e-02
6e-02
Log-odds
0.5
1.0
1.5
2.0
2.5
3.0
3.5
4.0

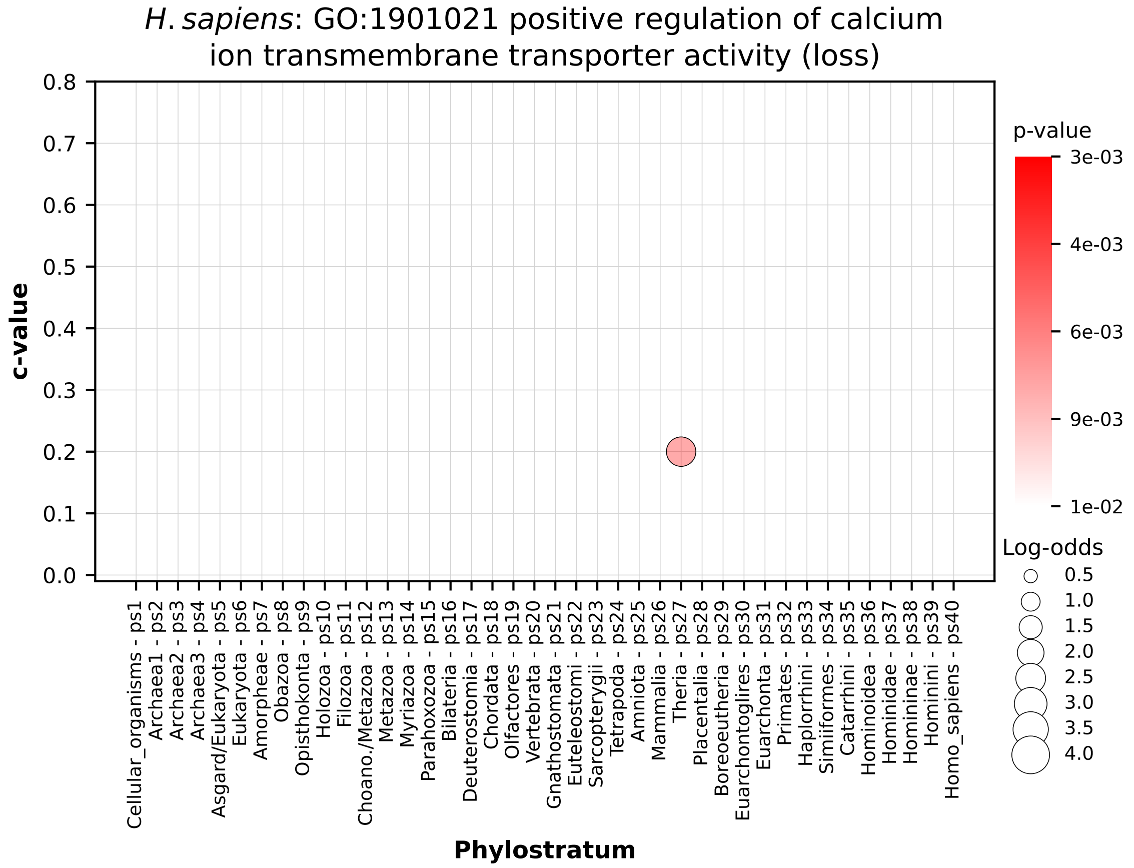

H. sapiens: GO:1901021 positive regulation of calcium ion transmembrane transporter activity (loss)
c-value
0.0
0.1
0.2
0.3
0.4
0.5
0.6
0.7
0.8
Phylostratum
Cellular_organisms - ps1
Archaea1 - ps2
Archaea2 - ps3
Archaea3 - ps4
Asgard/Eukaryota - ps5
Eukaryota - ps6
Amorpheae - ps7
Obazoa - ps8
Opisthokonta - ps9
Holozoa - ps10
Filozoa - ps11
Choano./Metazoa - ps12
Metazoa - ps13
Myriazoa - ps14
Parahoxozoa - ps15
Bilateria - ps16
Deuterostomia - ps17
Chordata - ps18
Olfactores - ps19
Vertebrata - ps20
Gnathostomata - ps21
Euteleostomi - ps22
Sarcopterygii - ps23
Tetrapoda - ps24
Amniota - ps25
Mammalia - ps26
Theria - ps27
Placentalia - ps28
Boreoeutheria - ps29
Euarchontoglires - ps30
Euarchonta - ps31
Primates - ps32
Haplorrhini - ps33
Simiiformes - ps34
Catarrhini - ps35
Hominoidea - ps36
Hominidae - ps37
Homininae - ps38
Hominini - ps39
Homo_sapiens - ps40
p-value
3e-03
4e-03
6e-03
9e-03
1e-02
Log-odds
0.5
1.0
1.5
2.0
2.5
3.0
3.5
4.0

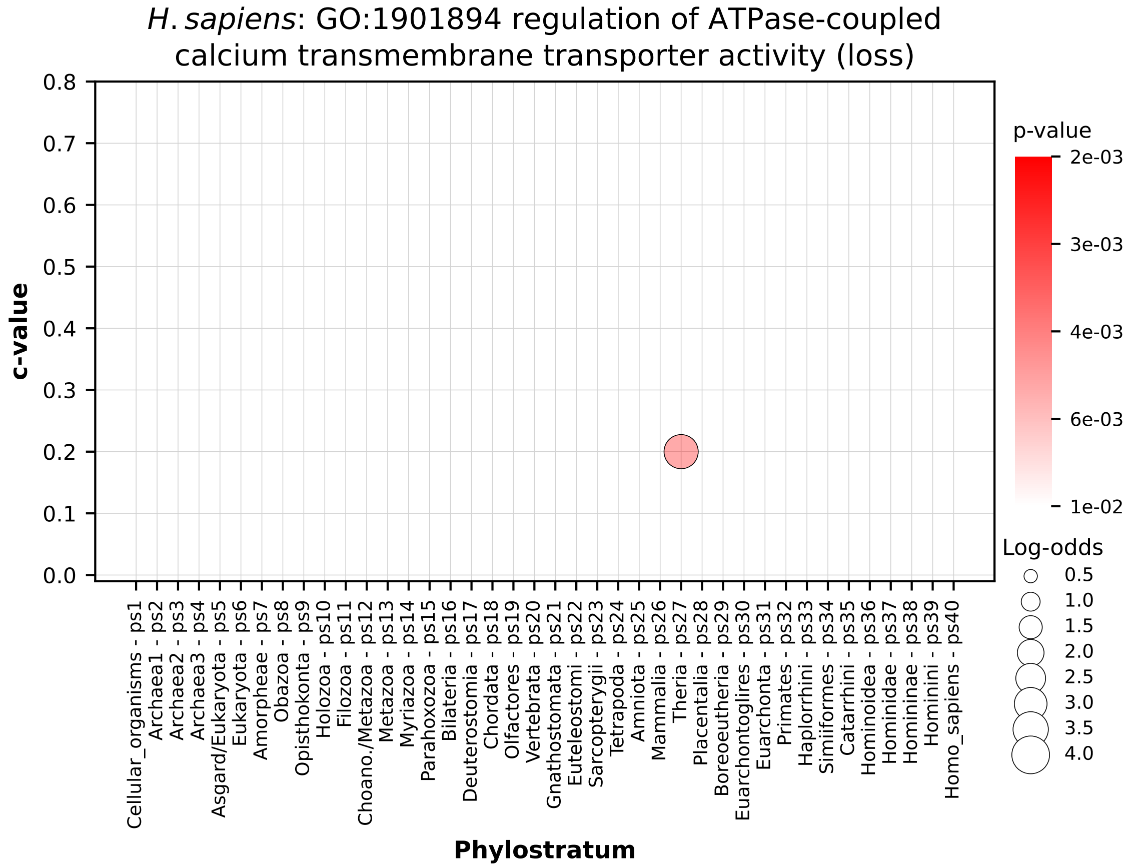

H. sapiens: GO:1901894 regulation of ATPase-coupled calcium transmembrane transporter activity (loss)
c-value
0.0
0.1
0.2
0.3
0.4
0.5
0.6
0.7
0.8
Phylostratum
Cellular_organisms - ps1
Archaea1 - ps2
Archaea2 - ps3
Archaea3 - ps4
Asgard/Eukaryota - ps5
Eukaryota - ps6
Amorpheae - ps7
Obazoa - ps8
Opisthokonta - ps9
Holozoa - ps10
Filozoa - ps11
Choano./Metazoa - ps12
Metazoa - ps13
Myriazoa - ps14
Parahoxozoa - ps15
Bilateria - ps16
Deuterostomia - ps17
Chordata - ps18
Olfactores - ps19
Vertebrata - ps20
Gnathostomata - ps21
Euteleostomi - ps22
Sarcopterygii - ps23
Tetrapoda - ps24
Amniota - ps25
Mammalia - ps26
Theria - ps27
Placentalia - ps28
Boreoeutheria - ps29
Euarchontoglires - ps30
Euarchonta - ps31
Primates - ps32
Haplorrhini - ps33
Simiiformes - ps34
Catarrhini - ps35
Hominoidea - ps36
Hominidae - ps37
Homininae - ps38
Hominini - ps39
Homo_sapiens - ps40
p-value
2e-03
3e-03
4e-03
6e-03
1e-02
Log-odds
0.5
1.0
1.5
2.0
2.5
3.0
3.5
4.0

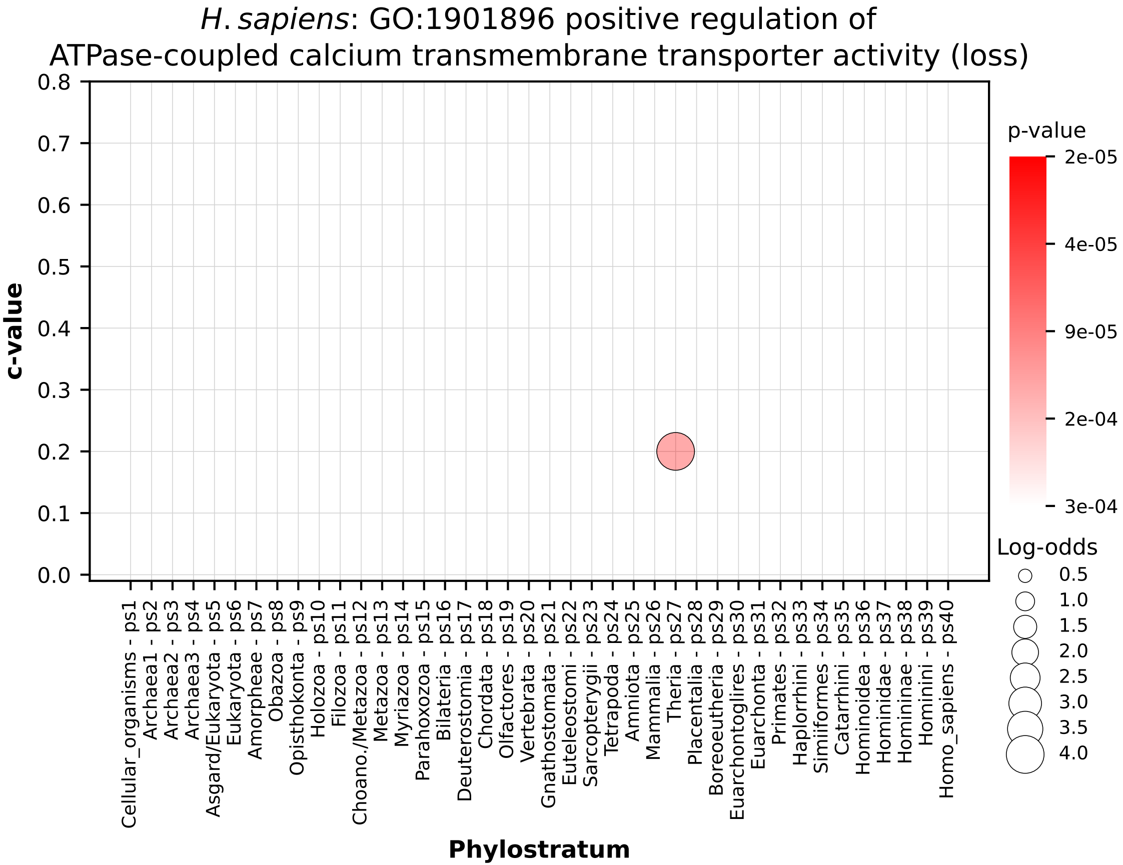

H. sapiens: GO:1901896 positive regulation of
ATPase-coupled calcium transmembrane transporter activity (loss)
c-value
0.8
0.7
0.6
0.5
0.4
0.3
0.2
0.1
0.0
p-value
2e-05
4e-05
9e-05
2e-04
3e-04
Log-odds
0.5
1.0
1.5
2.0
2.5
3.0
3.5
4.0
Cellular_organisms - ps1
Archaea1 - ps2
Archaea2 - ps3
Archaea3 - ps4
Asgard/Eukaryota - ps5
Eukaryota - ps6
Amorpheae - ps7
Obazoa - ps8
Opisthokonta - ps9
Holozoa - ps10
Filozoa - ps11
Choano./Metazoa - ps12
Metazoa - ps13
Myriazoa - ps14
Parahoxozoa - ps15
Bilateria - ps16
Deuterostomia - ps17
Chordata - ps18
Olfactores - ps19
Vertebrata - ps20
Gnathostomata - ps21
Euteleostomi - ps22
Sarcopterygii - ps23
Tetrapoda - ps24
Amniota - ps25
Mammalia - ps26
Theria - ps27
Placentalia - ps28
Boreoeutheria - ps29
Euarchontoglires - ps30
Euarchonta - ps31
Primates - ps32
Haplorrhini - ps33
Simiiformes - ps34
Catarrhini - ps35
Hominoidea - ps36
Hominidae - ps37
Homininae - ps38
Hominini - ps39
Homo_sapiens - ps40
Phylostratum

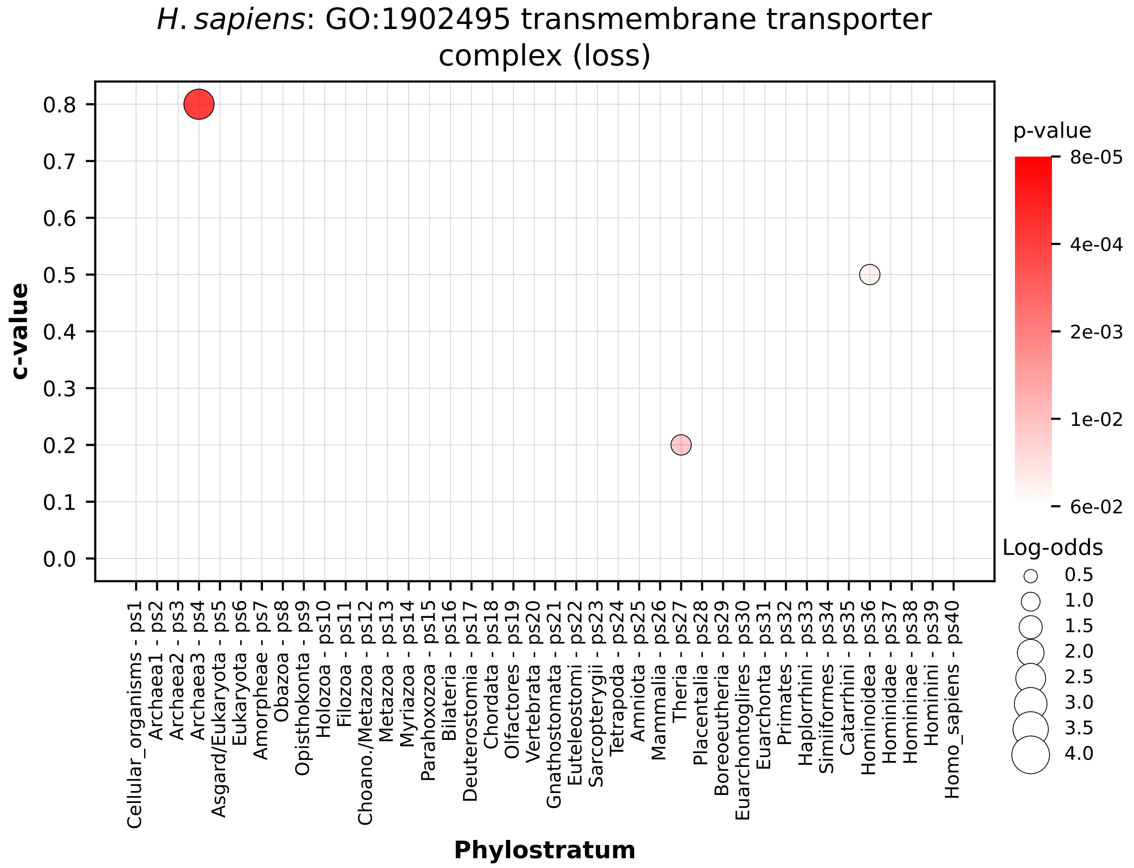

H. sapiens: GO:1902495 transmembrane transporter complex (loss)
c-value
0.0
0.1
0.2
0.3
0.4
0.5
0.6
0.7
0.8
Phylostratum
Cellular_organisms - ps1
Archaea1 - ps2
Archaea2 - ps3
Archaea3 - ps4
Asgard/Eukaryota - ps5
Eukaryota - ps6
Amorpheae - ps7
Obazoa - ps8
Opisthokonta - ps9
Holozoa - ps10
Filozoa - ps11
Choano./Metazoa - ps12
Metazoa - ps13
Myriazoa - ps14
Parahoxozoa - ps15
Bilateria - ps16
Deuterostomia - ps17
Chordata - ps18
Olfactores - ps19
Vertebrata - ps20
Gnathostomata - ps21
Euteleostomi - ps22
Sarcopterygii - ps23
Tetrapoda - ps24
Amniota - ps25
Mammalia - ps26
Theria - ps27
Placentalia - ps28
Boreoeutheria - ps29
Euarchontoglires - ps30
Euarchonta - ps31
Primates - ps32
Haplorrhini - ps33
Simiiformes - ps34
Catarrhini - ps35
Hominoidea - ps36
Hominidae - ps37
Homininae - ps38
Hominini - ps39
Homo_sapiens - ps40
p-value
8e-05
4e-04
2e-03
1e-02
6e-02
Log-odds
0.5
1.0
1.5
2.0
2.5
3.0
3.5
4.0

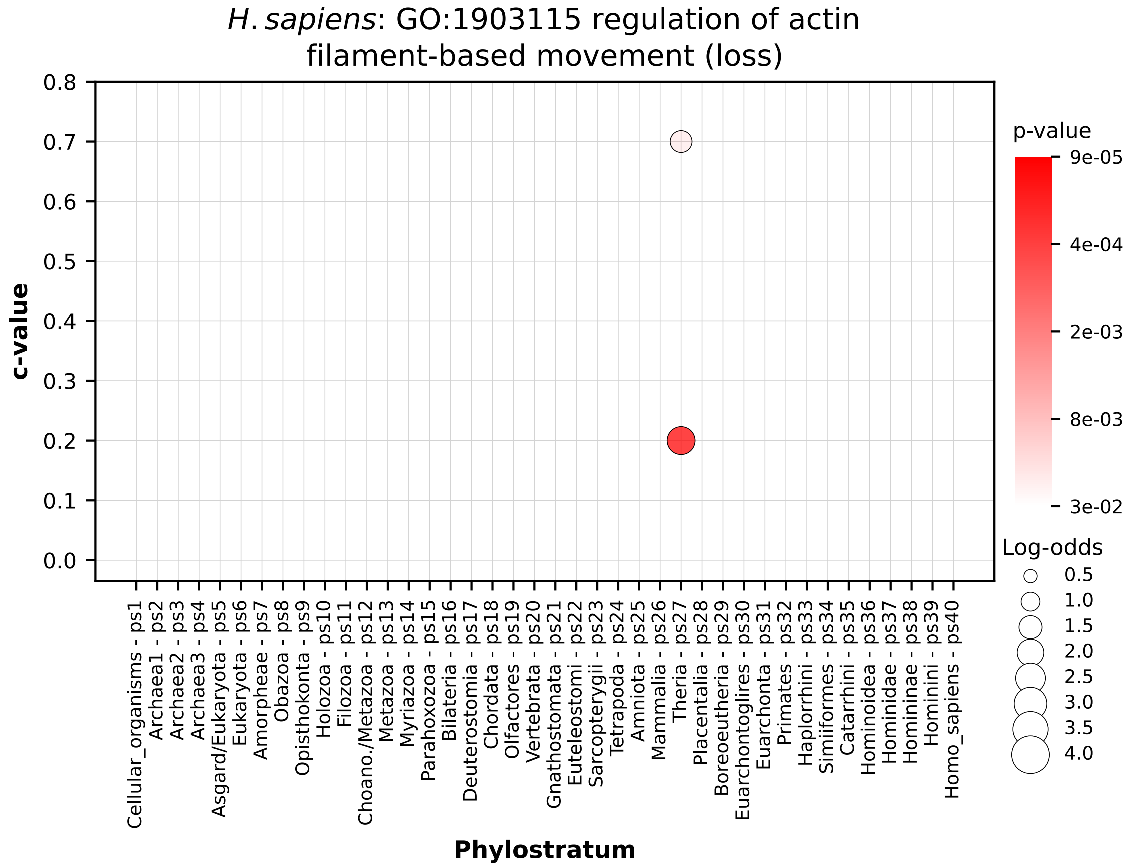

H. sapiens: GO:1903115 regulation of actin filament-based movement (loss)
c-value
0.8
0.7
0.6
0.5
0.4
0.3
0.2
0.1
0.0
Phylostratum
Cellular_organisms - ps1
Archaea1 - ps2
Archaea2 - ps3
Archaea3 - ps4
Asgard/Eukaryota - ps5
Eukaryota - ps6
Amorpheae - ps7
Obazoa - ps8
Opisthokonta - ps9
Holozoa - ps10
Filozoa - ps11
Choano./Metazoa - ps12
Metazoa - ps13
Myriazoa - ps14
Parahoxozoa - ps15
Bilateria - ps16
Deuterostomia - ps17
Chordata - ps18
Olfactores - ps19
Vertebrata - ps20
Gnathostomata - ps21
Euteleostomi - ps22
Sarcopterygii - ps23
Tetrapoda - ps24
Amniota - ps25
Mammalia - ps26
Theria - ps27
Placentalia - ps28
Boreoeutheria - ps29
Euarchontoglires - ps30
Euarchonta - ps31
Primates - ps32
Haplorrhini - ps33
Simiiformes - ps34
Catarrhini - ps35
Hominoidea - ps36
Hominidae - ps37
Homininae - ps38
Hominini - ps39
Homo_sapiens - ps40
p-value
9e-05
4e-04
2e-03
8e-03
3e-02
Log-odds
0.5
1.0
1.5
2.0
2.5
3.0
3.5
4.0

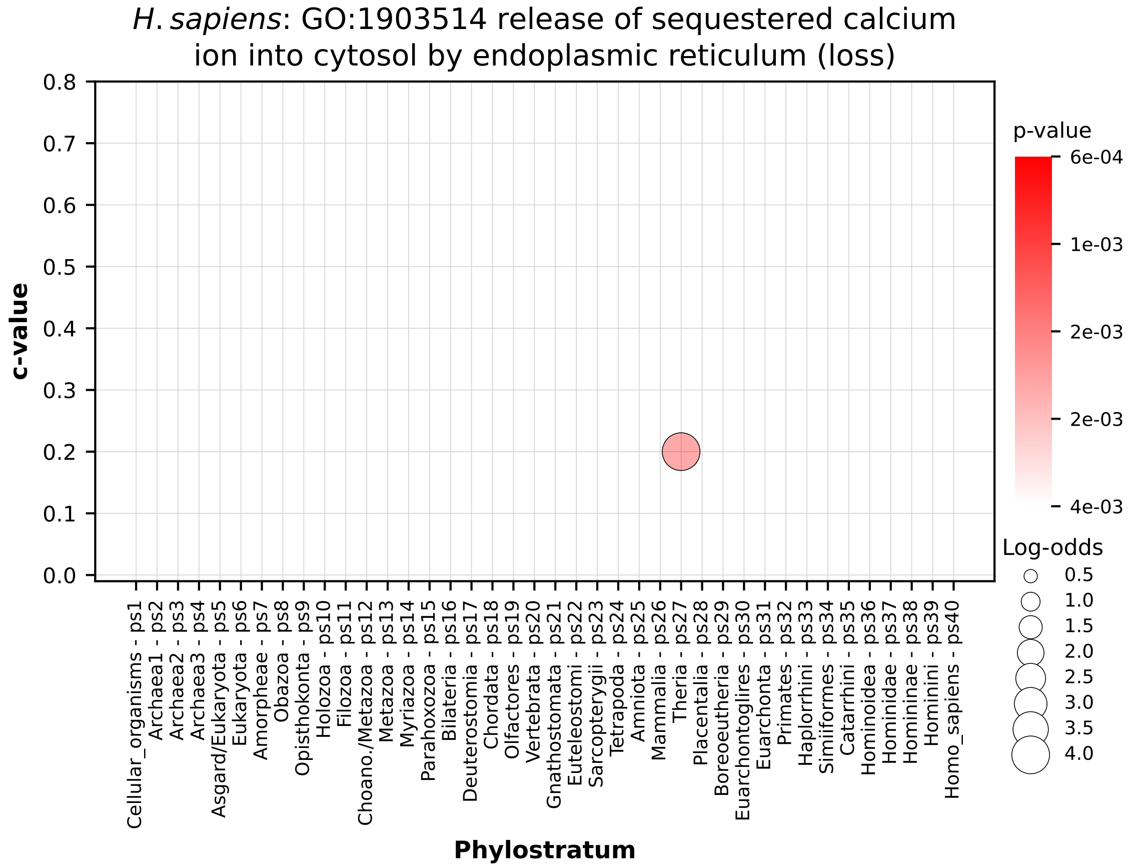

H. sapiens: GO:1903514 release of sequestered calcium ion into cytosol by endoplasmic reticulum (loss)
c-value
0.0
0.1
0.2
0.3
0.4
0.5
0.6
0.7
0.8
Phylostratum
Cellular_organisms - ps1
Archaea1 - ps2
Archaea2 - ps3
Archaea3 - ps4
Asgard/Eukaryota - ps5
Eukaryota - ps6
Amorpheae - ps7
Obazoa - ps8
Opisthokonta - ps9
Holozoa - ps10
Filozoa - ps11
Choano./Metazoa - ps12
Metazoa - ps13
Myriazoa - ps14
Parahoxozoa - ps15
Bilateria - ps16
Deuterostomia - ps17
Chordata - ps18
Olfactores - ps19
Vertebrata - ps20
Gnathostomata - ps21
Euteleostomi - ps22
Sarcopterygii - ps23
Tetrapoda - ps24
Amniota - ps25
Mammalia - ps26
Theria - ps27
Placentalia - ps28
Boreoeutheria - ps29
Euarchontoglires - ps30
Euarchonta - ps31
Primates - ps32
Haplorrhini - ps33
Simiiformes - ps34
Catarrhini - ps35
Hominoidea - ps36
Hominidae - ps37
Homininae - ps38
Hominini - ps39
Homo_sapiens - ps40
p-value
6e-04
1e-03
2e-03
2e-03
4e-03
Log-odds
0.5
1.0
1.5
2.0
2.5
3.0
3.5
4.0

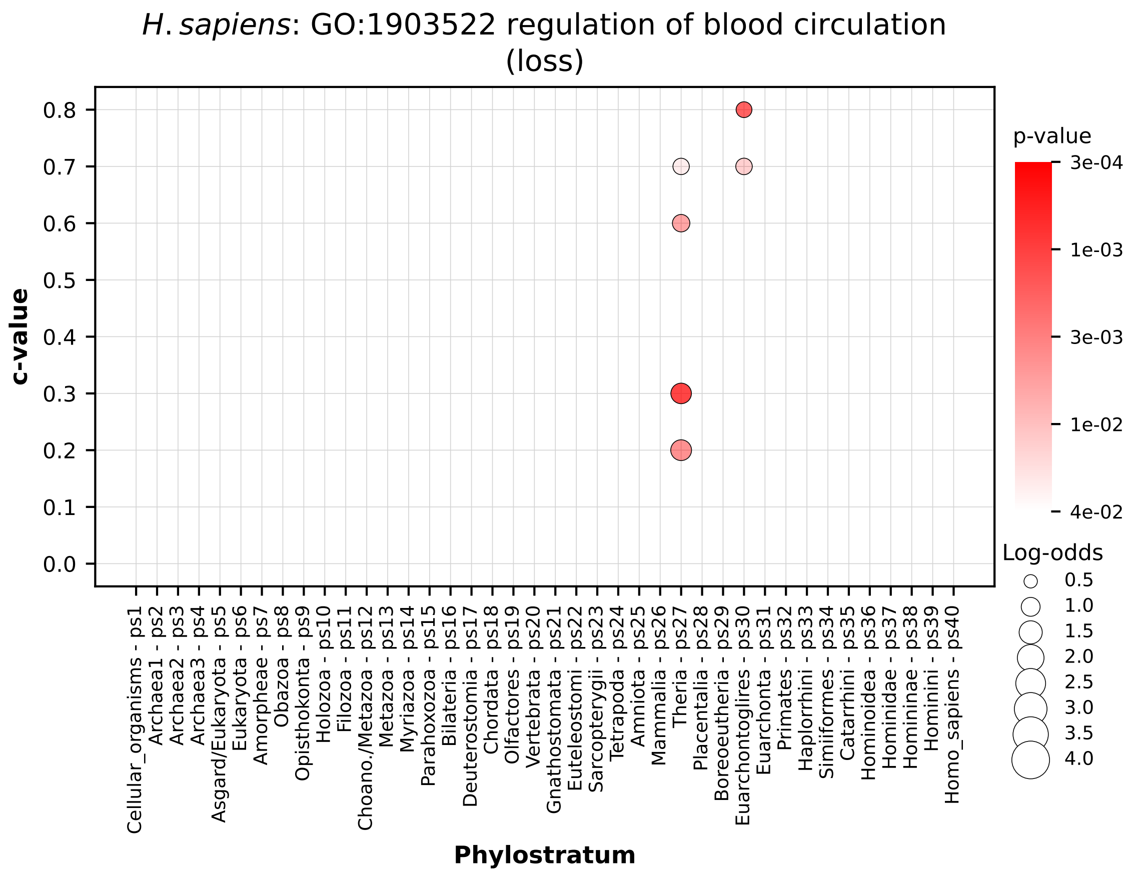

H. sapiens: GO:1903522 regulation of blood circulation (loss)
c-value
0.0
0.1
0.2
0.3
0.4
0.5
0.6
0.7
0.8
Phylostratum
Cellular_organisms - ps1
Archaea1 - ps2
Archaea2 - ps3
Archaea3 - ps4
Asgard/Eukaryota - ps5
Eukaryota - ps6
Amorpheae - ps7
Obazoa - ps8
Opisthokonta - ps9
Holozoa - ps10
Filozoa - ps11
Choano./Metazoa - ps12
Metazoa - ps13
Myriazoa - ps14
Parahoxozoa - ps15
Bilateria - ps16
Deuterostomia - ps17
Chordata - ps18
Olfactores - ps19
Vertebrata - ps20
Gnathostomata - ps21
Euteleostomi - ps22
Sarcopterygii - ps23
Tetrapoda - ps24
Amniota - ps25
Mammalia - ps26
Theria - ps27
Placentalia - ps28
Boreoeutheria - ps29
Euarchontoglires - ps30
Euarchonta - ps31
Primates - ps32
Haplorrhini - ps33
Simiiformes - ps34
Catarrhini - ps35
Hominoidea - ps36
Hominidae - ps37
Homininae - ps38
Hominini - ps39
Homo_sapiens - ps40
p-value
3e-04
1e-03
3e-03
1e-02
4e-02
Log-odds
0.5
1.0
1.5
2.0
2.5
3.0
3.5
4.0

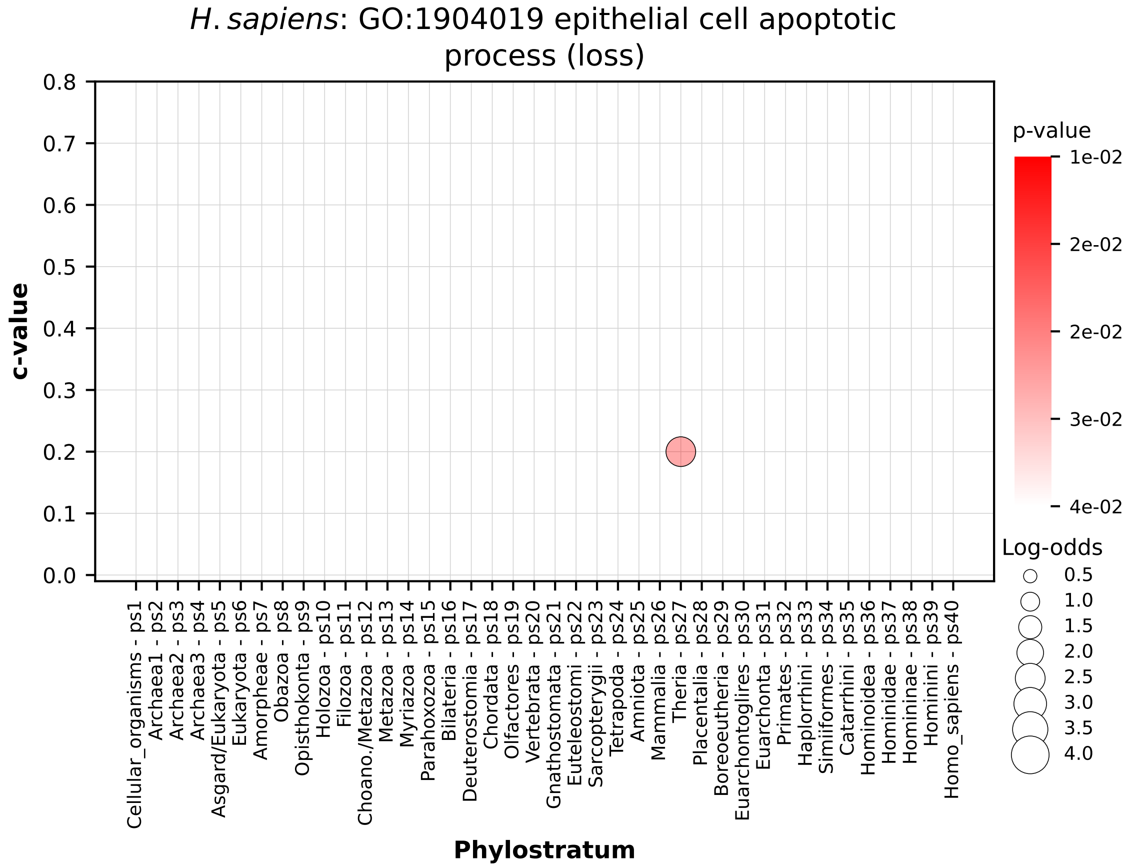

H. sapiens: GO:1904019 epithelial cell apoptotic process (loss)
c-value
0.8
0.7
0.6
0.5
0.4
0.3
0.2
0.1
0.0
p-value
1e-02
2e-02
2e-02
3e-02
4e-02
Log-odds
0.5
1.0
1.5
2.0
2.5
3.0
3.5
4.0
Cellular_organisms - ps1
Archaea1 - ps2
Archaea2 - ps3
Archaea3 - ps4
Asgard/Eukaryota - ps5
Eukaryota - ps6
Amorpheae - ps7
Obazoa - ps8
Opisthokonta - ps9
Holozoa - ps10
Filozoa - ps11
Choano./Metazoa - ps12
Metazoa - ps13
Myriazoa - ps14
Parahoxozoa - ps15
Bilateria - ps16
Deuterostomia - ps17
Chordata - ps18
Olfactores - ps19
Vertebrata - ps20
Gnathostomata - ps21
Euteleostomi - ps22
Sarcopterygii - ps23
Tetrapoda - ps24
Amniota - ps25
Mammalia - ps26
Theria - ps27
Placentalia - ps28
Boreoeutheria - ps29
Euarchontoglires - ps30
Euarchonta - ps31
Primates - ps32
Haplorrhini - ps33
Simiiformes - ps34
Catarrhini - ps35
Hominoidea - ps36
Hominidae - ps37
Homininae - ps38
Hominini - ps39
Homo_sapiens - ps40
Phylostratum

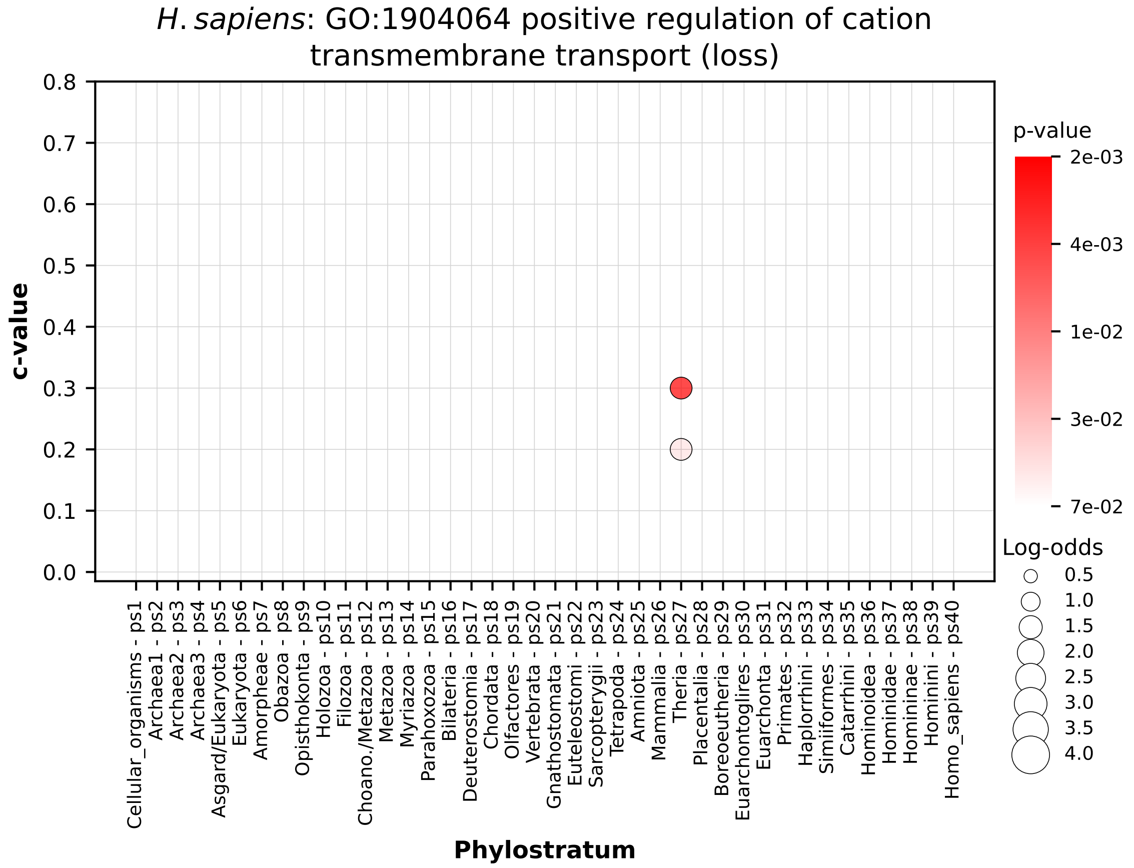

H. sapiens: GO:1904064 positive regulation of cation transmembrane transport (loss)
c-value
0.0
0.1
0.2
0.3
0.4
0.5
0.6
0.7
0.8
Phylostratum
Cellular_organisms - ps1
Archaea1 - ps2
Archaea2 - ps3
Archaea3 - ps4
Asgard/Eukaryota - ps5
Eukaryota - ps6
Amorpheae - ps7
Obazoa - ps8
Opisthokonta - ps9
Holozoa - ps10
Filozoa - ps11
Choano./Metazoa - ps12
Metazoa - ps13
Myriazoa - ps14
Parahoxozoa - ps15
Bilateria - ps16
Deuterostomia - ps17
Chordata - ps18
Olfactores - ps19
Vertebrata - ps20
Gnathostomata - ps21
Euteleostomi - ps22
Sarcopterygii - ps23
Tetrapoda - ps24
Amniota - ps25
Mammalia - ps26
Theria - ps27
Placentalia - ps28
Boreoeutheria - ps29
Euarchontoglires - ps30
Euarchonta - ps31
Primates - ps32
Haplorrhini - ps33
Simiiformes - ps34
Catarrhini - ps35
Hominoidea - ps36
Hominidae - ps37
Homininae - ps38
Hominini - ps39
Homo_sapiens - ps40
p-value
2e-03
4e-03
1e-02
3e-02
7e-02
Log-odds
0.5
1.0
1.5
2.0
2.5
3.0
3.5
4.0

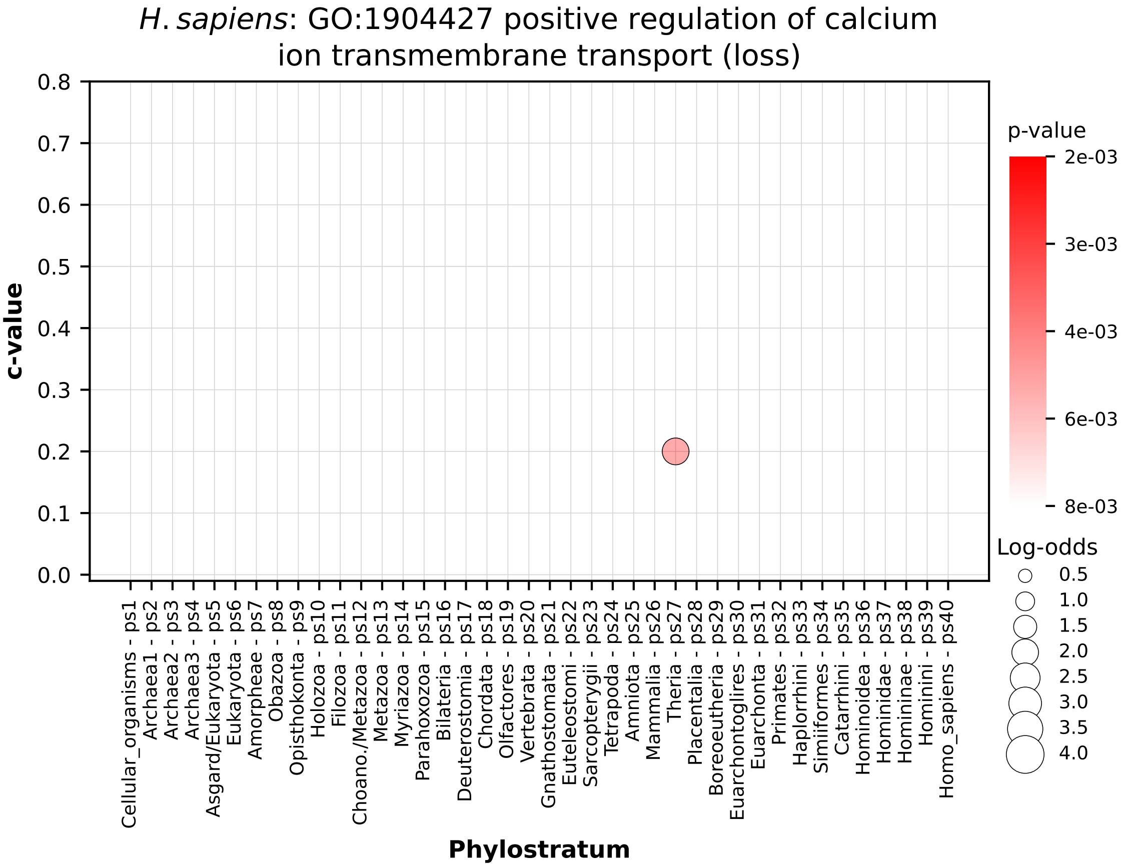

H. sapiens: GO:1904427 positive regulation of calcium ion transmembrane transport (loss)
c-value
0.8
0.7
0.6
0.5
0.4
0.3
0.2
0.1
0.0
Phylostratum
Cellular_organisms - ps1
Archaea1 - ps2
Archaea2 - ps3
Archaea3 - ps4
Asgard/Eukaryota - ps5
Eukaryota - ps6
Amorpheae - ps7
Obazoa - ps8
Opisthokonta - ps9
Holozoa - ps10
Filozoa - ps11
Choano./Metazoa - ps12
Metazoa - ps13
Myriazoa - ps14
Parahoxozoa - ps15
Bilateria - ps16
Deuterostomia - ps17
Chordata - ps18
Olfactores - ps19
Vertebrata - ps20
Gnathostomata - ps21
Euteleostomi - ps22
Sarcopterygii - ps23
Tetrapoda - ps24
Amniota - ps25
Mammalia - ps26
Theria - ps27
Placentalia - ps28
Boreoeutheria - ps29
Euarchontoglires - ps30
Euarchonta - ps31
Primates - ps32
Haplorrhini - ps33
Simiiformes - ps34
Catarrhini - ps35
Hominoidea - ps36
Hominidae - ps37
Homininae - ps38
Hominini - ps39
Homo_sapiens - ps40
p-value
2e-03
3e-03
4e-03
6e-03
8e-03
Log-odds
0.5
1.0
1.5
2.0
2.5
3.0
3.5
4.0

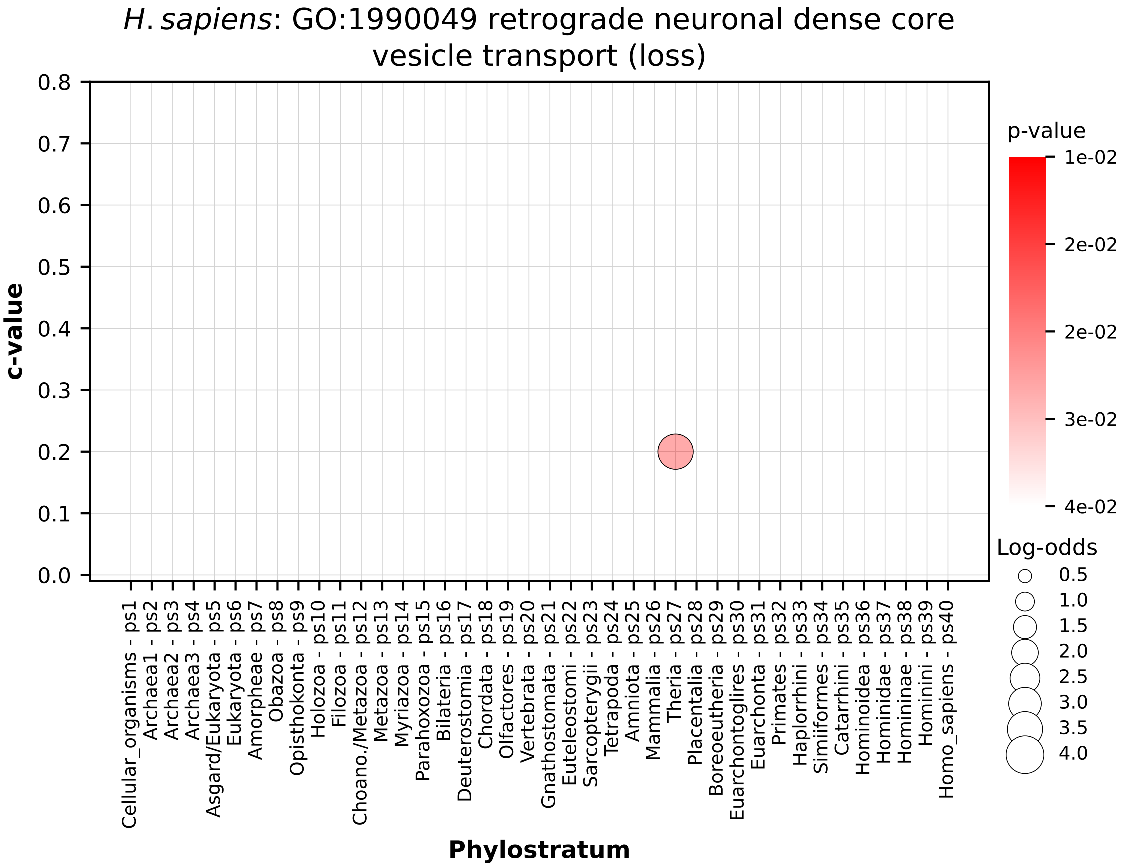
H. sapiens: GO:1990049 retrograde neuronal dense core vesicle transport (loss)
c-value
0.0
0.1
0.2
0.3
0.4
0.5
0.6
0.7
0.8
Phylostratum
Cellular_organisms - ps1
Archaea1 - ps2
Archaea2 - ps3
Archaea3 - ps4
Asgard/Eukaryota - ps5
Eukaryota - ps6
Amorpheae - ps7
Obazoa - ps8
Opisthokonta - ps9
Holozoa - ps10
Filozoa - ps11
Choano./Metazoa - ps12
Metazoa - ps13
Myriazoa - ps14
Parahoxozoa - ps15
Bilateria - ps16
Deuterostomia - ps17
Chordata - ps18
Olfactores - ps19
Vertebrata - ps20
Gnathostomata - ps21
Euteleostomi - ps22
Sarcopterygii - ps23
Tetrapoda - ps24
Amniota - ps25
Mammalia - ps26
Theria - ps27
Placentalia - ps28
Boreoeutheria - ps29
Euarchontoglires - ps30
Euarchonta - ps31
Primates - ps32
Haplorrhini - ps33
Simiiformes - ps34
Catarrhini - ps35
Hominoidea - ps36
Hominidae - ps37
Homininae - ps38
Hominini - ps39
Homo_sapiens - ps40
p-value
1e-02
2e-02
2e-02
3e-02
4e-02
Log-odds
0.5
1.0
1.5
2.0
2.5
3.0
3.5
4.0

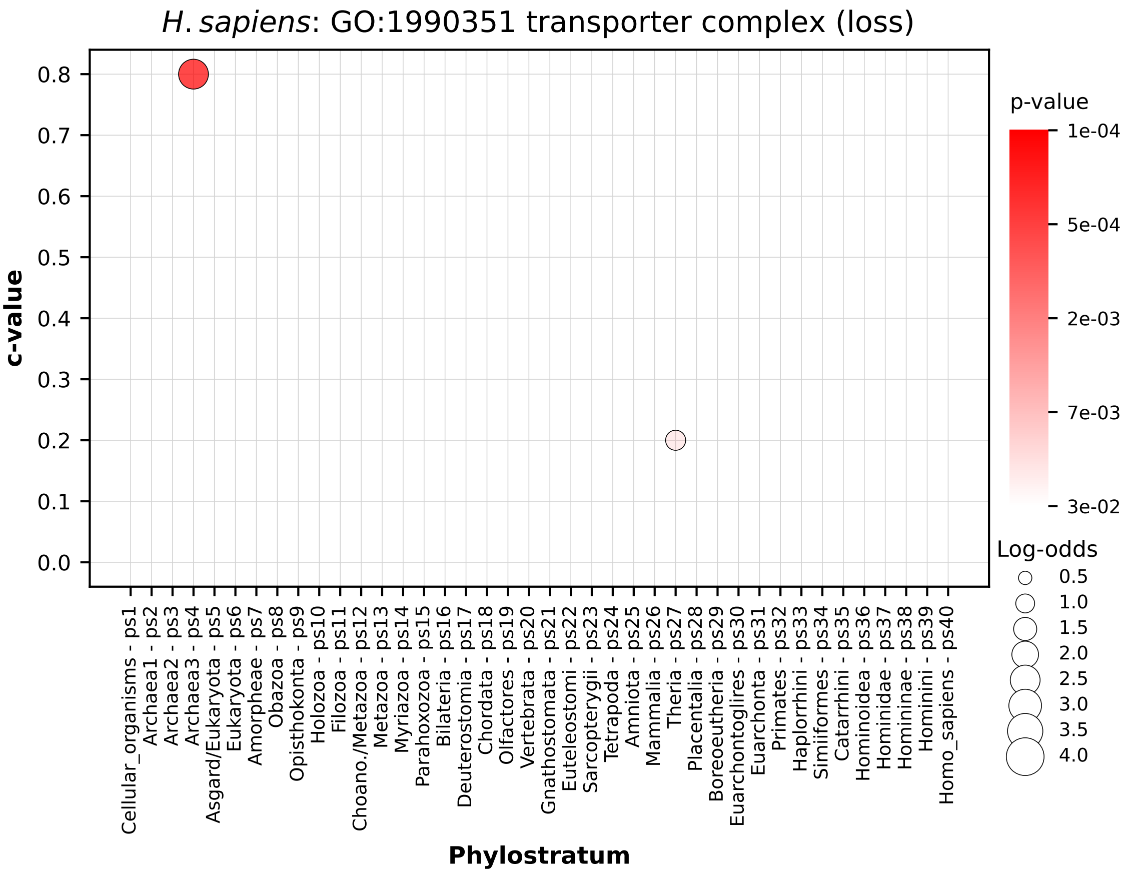

H. sapiens: GO:1990351 transporter complex (loss)
c-value
0.0
0.1
0.2
0.3
0.4
0.5
0.6
0.7
0.8
Phylostratum
Cellular_organisms - ps1
Archaea1 - ps2
Archaea2 - ps3
Archaea3 - ps4
Asgard/Eukaryota - ps5
Eukaryota - ps6
Amorpheae - ps7
Obazoa - ps8
Opisthokonta - ps9
Holozoa - ps10
Filozoa - ps11
Choano./Metazoa - ps12
Metazoa - ps13
Myriazoa - ps14
Parahoxozoa - ps15
Bilateria - ps16
Deuterostomia - ps17
Chordata - ps18
Olfactores - ps19
Vertebrata - ps20
Gnathostomata - ps21
Euteleostomi - ps22
Sarcopterygii - ps23
Tetrapoda - ps24
Amniota - ps25
Mammalia - ps26
Theria - ps27
Placentalia - ps28
Boreoeutheria - ps29
Euarchontoglires - ps30
Euarchonta - ps31
Primates - ps32
Haplorrhini - ps33
Simiiformes - ps34
Catarrhini - ps35
Hominoidea - ps36
Hominidae - ps37
Homininae - ps38
Hominini - ps39
Homo_sapiens - ps40
p-value
1e-04
5e-04
2e-03
7e-03
3e-02
Log-odds
0.5
1.0
1.5
2.0
2.5
3.0
3.5
4.0

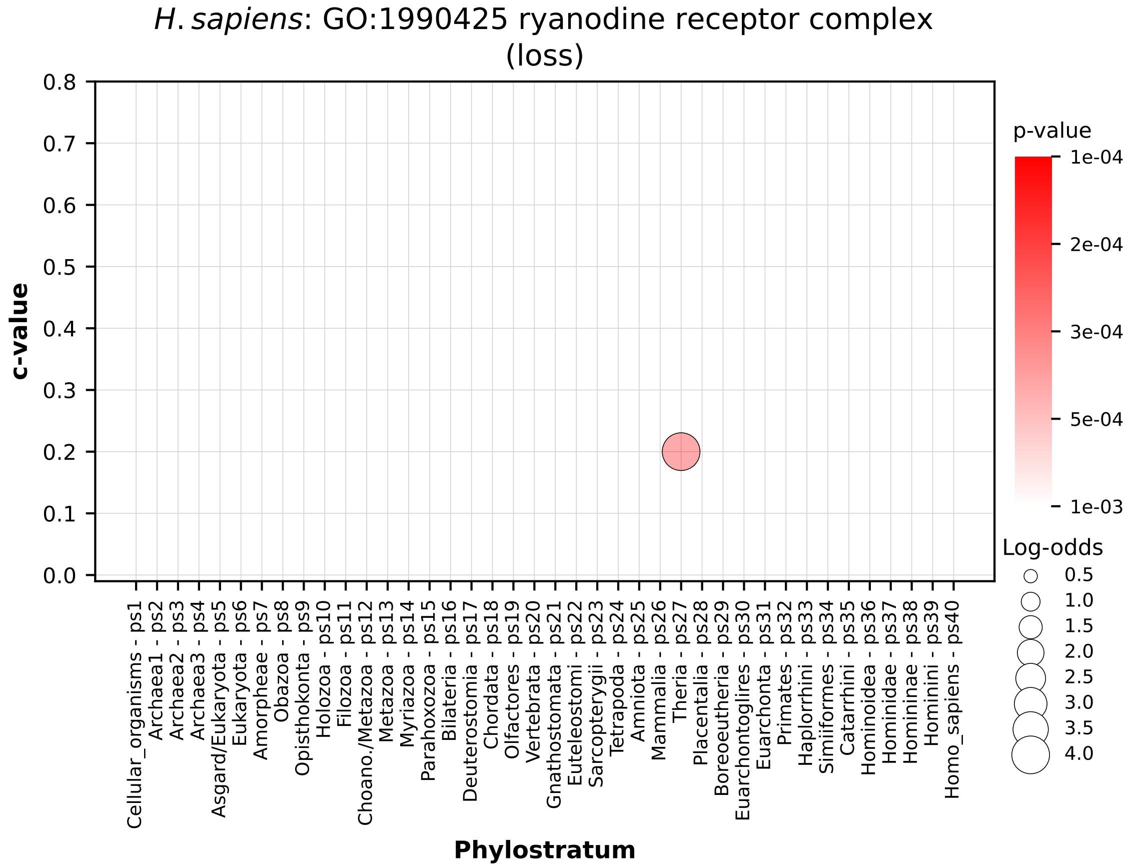

H. sapiens: GO:1990425 ryanodine receptor complex
(loss)
c-value
0.0
0.1
0.2
0.3
0.4
0.5
0.6
0.7
0.8
Phylostratum
Cellular_organisms - ps1
Archaea1 - ps2
Archaea2 - ps3
Archaea3 - ps4
Asgard/Eukaryota - ps5
Eukaryota - ps6
Amorpheae - ps7
Obazoa - ps8
Opisthokonta - ps9
Holozoa - ps10
Filozoa - ps11
Choano./Metazoa - ps12
Metazoa - ps13
Myriazoa - ps14
Parahoxozoa - ps15
Bilateria - ps16
Deuterostomia - ps17
Chordata - ps18
Olfactores - ps19
Vertebrata - ps20
Gnathostomata - ps21
Euteleostomi - ps22
Sarcopterygii - ps23
Tetrapoda - ps24
Amniota - ps25
Mammalia - ps26
Theria - ps27
Placentalia - ps28
Boreoeutheria - ps29
Euarchontoglires - ps30
Euarchonta - ps31
Primates - ps32
Haplorrhini - ps33
Simiiformes - ps34
Catarrhini - ps35
Hominoidea - ps36
Hominidae - ps37
Homininae - ps38
Hominini - ps39
Homo_sapiens - ps40
p-value
1e-04
2e-04
3e-04
5e-04
1e-03
Log-odds
0.5
1.0
1.5
2.0
2.5
3.0
3.5
4.0

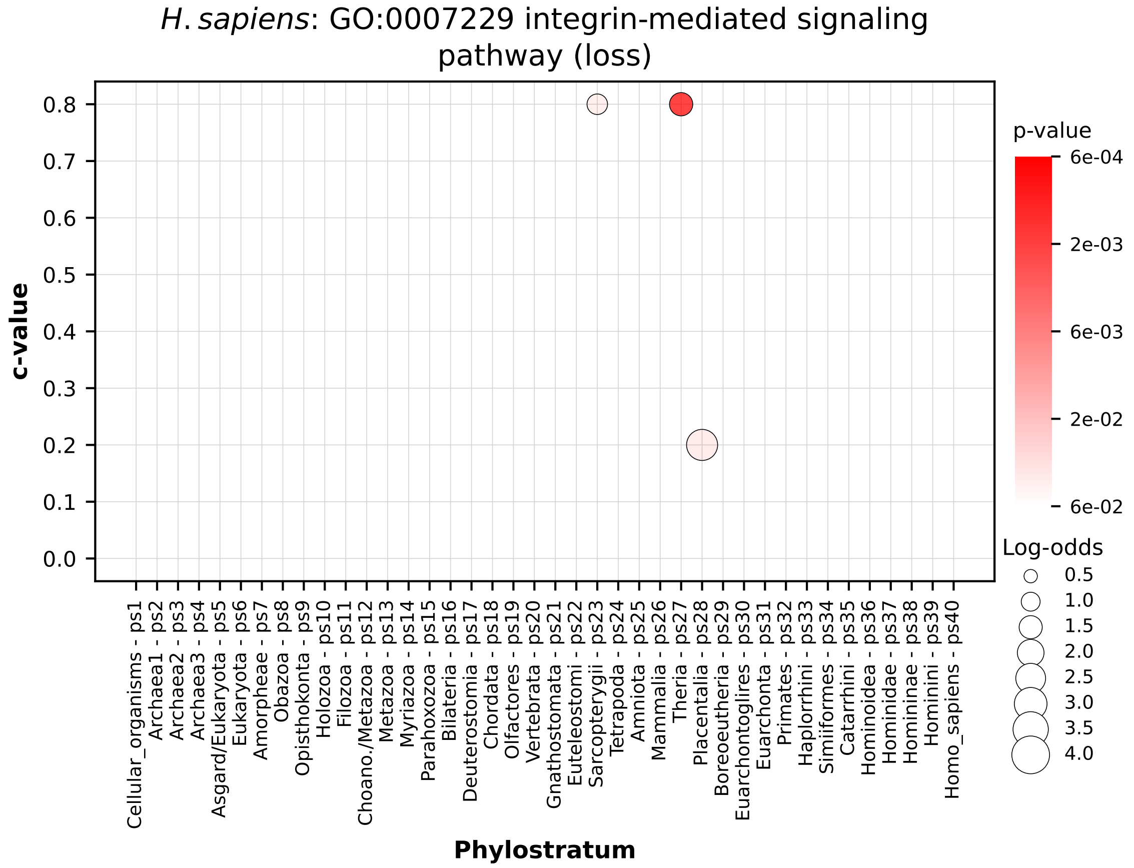
H. sapiens: GO:0007229 integrin-mediated signaling pathway (loss)
c-value
0.0
0.1
0.2
0.3
0.4
0.5
0.6
0.7
0.8
Phylostratum
Cellular_organisms - ps1
Archaea1 - ps2
Archaea2 - ps3
Archaea3 - ps4
Asgard/Eukaryota - ps5
Eukaryota - ps6
Amorpheae - ps7
Obazoa - ps8
Opisthokonta - ps9
Holozoa - ps10
Filozoa - ps11
Choano./Metazoa - ps12
Metazoa - ps13
Myriazoa - ps14
Parahoxozoa - ps15
Bilateria - ps16
Deuterostomia - ps17
Chordata - ps18
Olfactores - ps19
Vertebrata - ps20
Gnathostomata - ps21
Euteleostomi - ps22
Sarcopterygii - ps23
Tetrapoda - ps24
Amniota - ps25
Mammalia - ps26
Theria - ps27
Placentalia - ps28
Boreoeutheria - ps29
Euarchontoglires - ps30
Euarchonta - ps31
Primates - ps32
Haplorrhini - ps33
Simiiformes - ps34
Catarrhini - ps35
Hominoidea - ps36
Hominidae - ps37
Homininae - ps38
Hominini - ps39
Homo_sapiens - ps40
p-value
6e-04
2e-03
6e-03
2e-02
6e-02
Log-odds
0.5
1.0
1.5
2.0
2.5
3.0
3.5
4.0

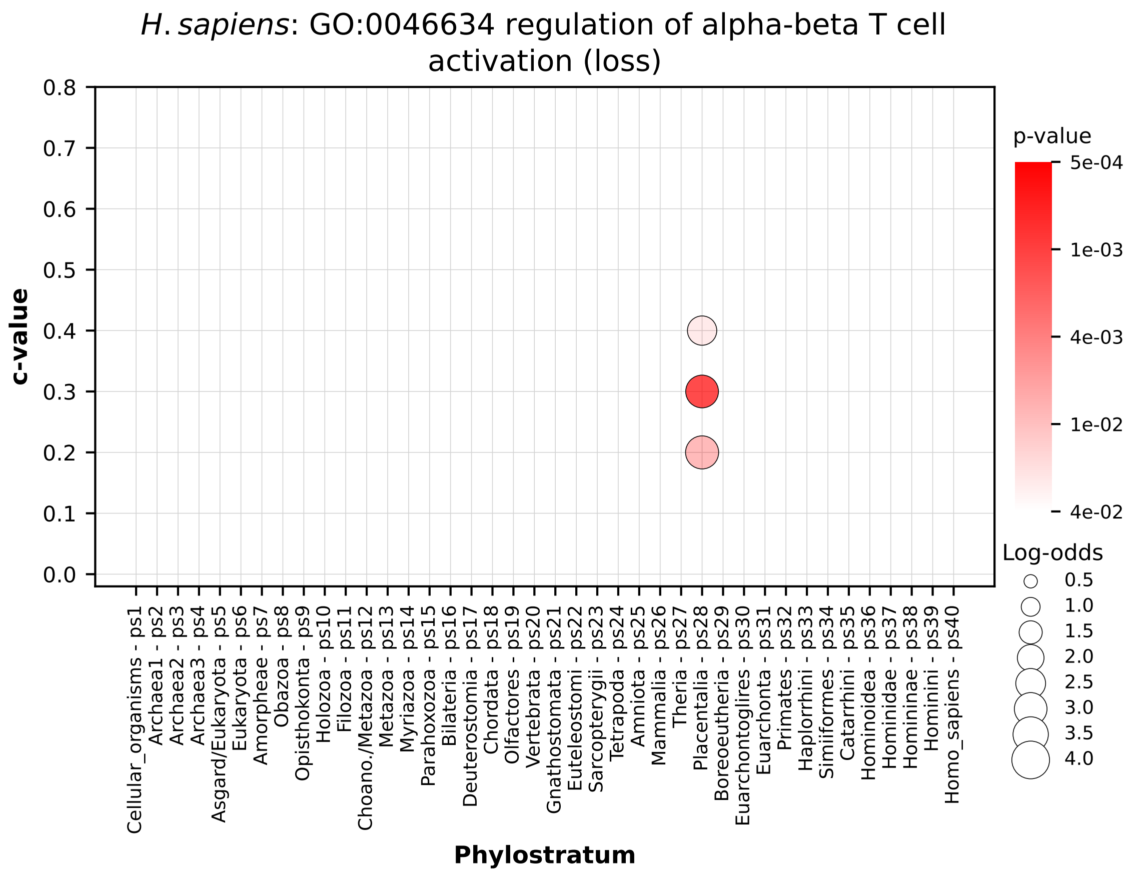

H. sapiens: GO:0046634 regulation of alpha-beta T cell activation (loss)
c-value
0.8
0.7
0.6
0.5
0.4
0.3
0.2
0.1
0.0
p-value
5e-04
1e-03
4e-03
1e-02
4e-02
Log-odds
0.5
1.0
1.5
2.0
2.5
3.0
3.5
4.0
Cellular_organisms - ps1
Archaea1 - ps2
Archaea2 - ps3
Archaea3 - ps4
Asgard/Eukaryota - ps5
Eukaryota - ps6
Amorpheae - ps7
Obazoa - ps8
Opisthokonta - ps9
Holozoa - ps10
Filozoa - ps11
Choano./Metazoa - ps12
Metazoa - ps13
Myriazoa - ps14
Parahoxozoa - ps15
Bilateria - ps16
Deuterostomia - ps17
Chordata - ps18
Olfactores - ps19
Vertebrata - ps20
Gnathostomata - ps21
Euteleostomi - ps22
Sarcopterygii - ps23
Tetrapoda - ps24
Amniota - ps25
Mammalia - ps26
Theria - ps27
Placentalia - ps28
Boreoeutheria - ps29
Euarchontoglires - ps30
Euarchonta - ps31
Primates - ps32
Haplorrhini - ps33
Simiiformes - ps34
Catarrhini - ps35
Hominoidea - ps36
Hominidae - ps37
Homininae - ps38
Hominini - ps39
Homo_sapiens - ps40
Phylostratum

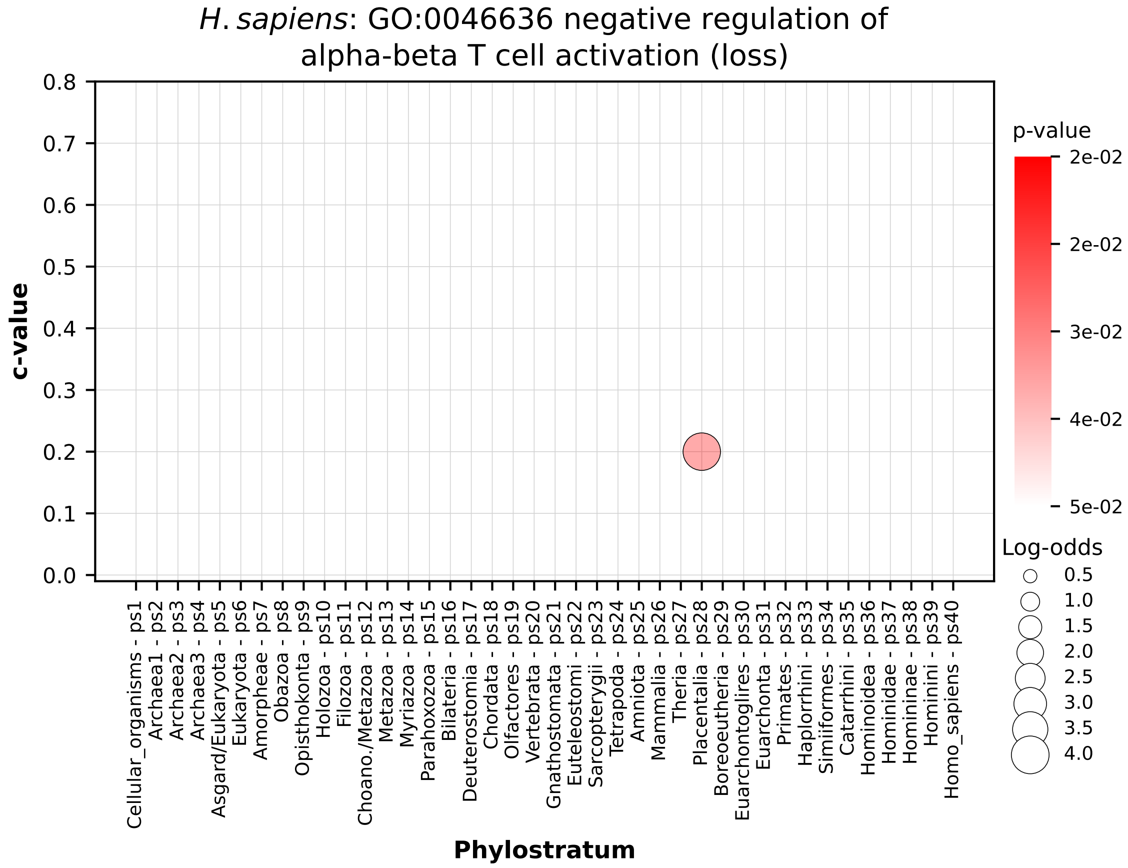

H. sapiens: GO:0046636 negative regulation of alpha-beta T cell activation (loss)
c-value
0.8
0.7
0.6
0.5
0.4
0.3
0.2
0.1
0.0
Phylostratum
Cellular_organisms - ps1
Archaea1 - ps2
Archaea2 - ps3
Archaea3 - ps4
Asgard/Eukaryota - ps5
Eukaryota - ps6
Amorpheae - ps7
Obazoa - ps8
Opisthokonta - ps9
Holozoa - ps10
Filozoa - ps11
Choano./Metazoa - ps12
Metazoa - ps13
Myriazoa - ps14
Parahoxozoa - ps15
Bilateria - ps16
Deuterostomia - ps17
Chordata - ps18
Olfactores - ps19
Vertebrata - ps20
Gnathostomata - ps21
Euteleostomi - ps22
Sarcopterygii - ps23
Tetrapoda - ps24
Amniota - ps25
Mammalia - ps26
Theria - ps27
Placentalia - ps28
Boreoeutheria - ps29
Euarchontoglires - ps30
Euarchonta - ps31
Primates - ps32
Haplorrhini - ps33
Simiiformes - ps34
Catarrhini - ps35
Hominoidea - ps36
Hominidae - ps37
Homininae - ps38
Hominini - ps39
Homo_sapiens - ps40
p-value
2e-02
2e-02
3e-02
4e-02
5e-02
Log-odds
0.5
1.0
1.5
2.0
2.5
3.0
3.5
4.0

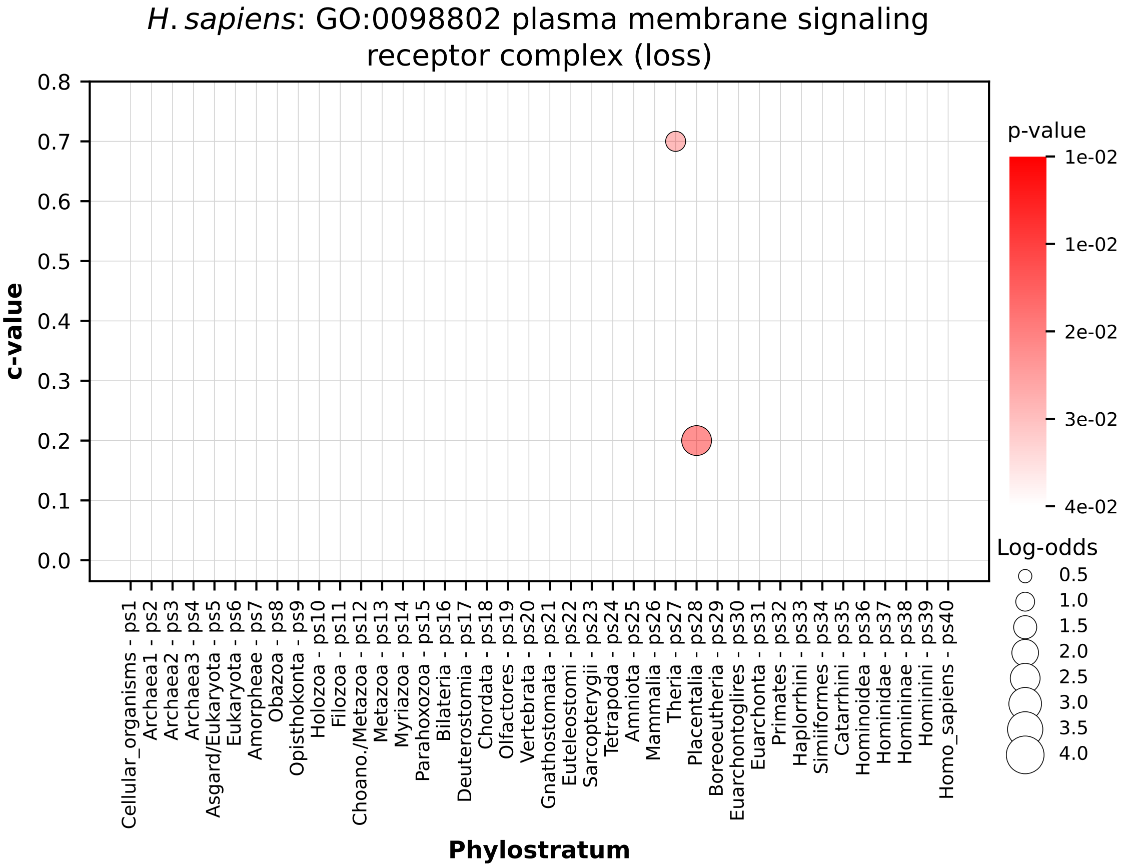

H. sapiens: GO:0098802 plasma membrane signaling receptor complex (loss)
c-value
0.8
0.7
0.6
0.5
0.4
0.3
0.2
0.1
0.0
p-value
1e-02
1e-02
2e-02
3e-02
4e-02
Log-odds
0.5
1.0
1.5
2.0
2.5
3.0
3.5
4.0
Cellular_organisms - ps1
Archaea1 - ps2
Archaea2 - ps3
Archaea3 - ps4
Asgard/Eukaryota - ps5
Eukaryota - ps6
Amorpheae - ps7
Obazoa - ps8
Opisthokonta - ps9
Holozoa - ps10
Filozoa - ps11
Choano./Metazoa - ps12
Metazoa - ps13
Myriazoa - ps14
Parahoxozoa - ps15
Bilateria - ps16
Deuterostomia - ps17
Chordata - ps18
Olfactores - ps19
Vertebrata - ps20
Gnathostomata - ps21
Euteleostomi - ps22
Sarcopterygii - ps23
Tetrapoda - ps24
Amniota - ps25
Mammalia - ps26
Theria - ps27
Placentalia - ps28
Boreoeutheria - ps29
Euarchontoglires - ps30
Euarchonta - ps31
Primates - ps32
Haplorrhini - ps33
Simiiformes - ps34
Catarrhini - ps35
Hominoidea - ps36
Hominidae - ps37
Homininae - ps38
Hominini - ps39
Homo_sapiens - ps40
Phylostratum

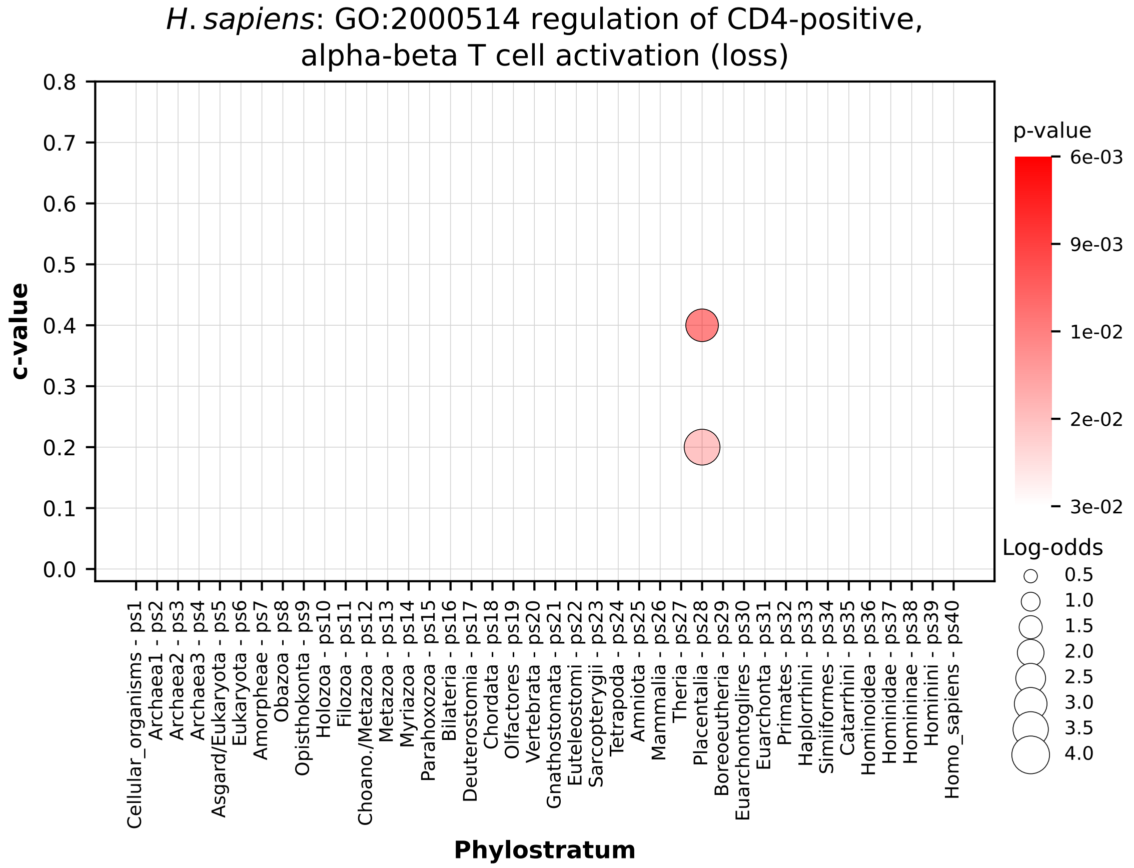

H. sapiens: GO:2000514 regulation of CD4-positive, alpha-beta T cell activation (loss)
c-value
0.0
0.1
0.2
0.3
0.4
0.5
0.6
0.7
0.8
p-value
6e-03
9e-03
1e-02
2e-02
3e-02
Log-odds
0.5
1.0
1.5
2.0
2.5
3.0
3.5
4.0
Cellular_organisms - ps1
Archaea1 - ps2
Archaea2 - ps3
Archaea3 - ps4
Asgard/Eukaryota - ps5
Eukaryota - ps6
Amorpheae - ps7
Obazoa - ps8
Opisthokonta - ps9
Holozoa - ps10
Filozoa - ps11
Choano./Metazoa - ps12
Metazoa - ps13
Myriazoa - ps14
Parahoxozoa - ps15
Bilateria - ps16
Deuterostomia - ps17
Chordata - ps18
Olfactores - ps19
Vertebrata - ps20
Gnathostomata - ps21
Euteleostomi - ps22
Sarcopterygii - ps23
Tetrapoda - ps24
Amniota - ps25
Mammalia - ps26
Theria - ps27
Placentalia - ps28
Boreoeutheria - ps29
Euarchontoglires - ps30
Euarchonta - ps31
Primates - ps32
Haplorrhini - ps33
Simiiformes - ps34
Catarrhini - ps35
Hominoidea - ps36
Hominidae - ps37
Homininae - ps38
Hominini - ps39
Homo_sapiens - ps40
Phylostratum

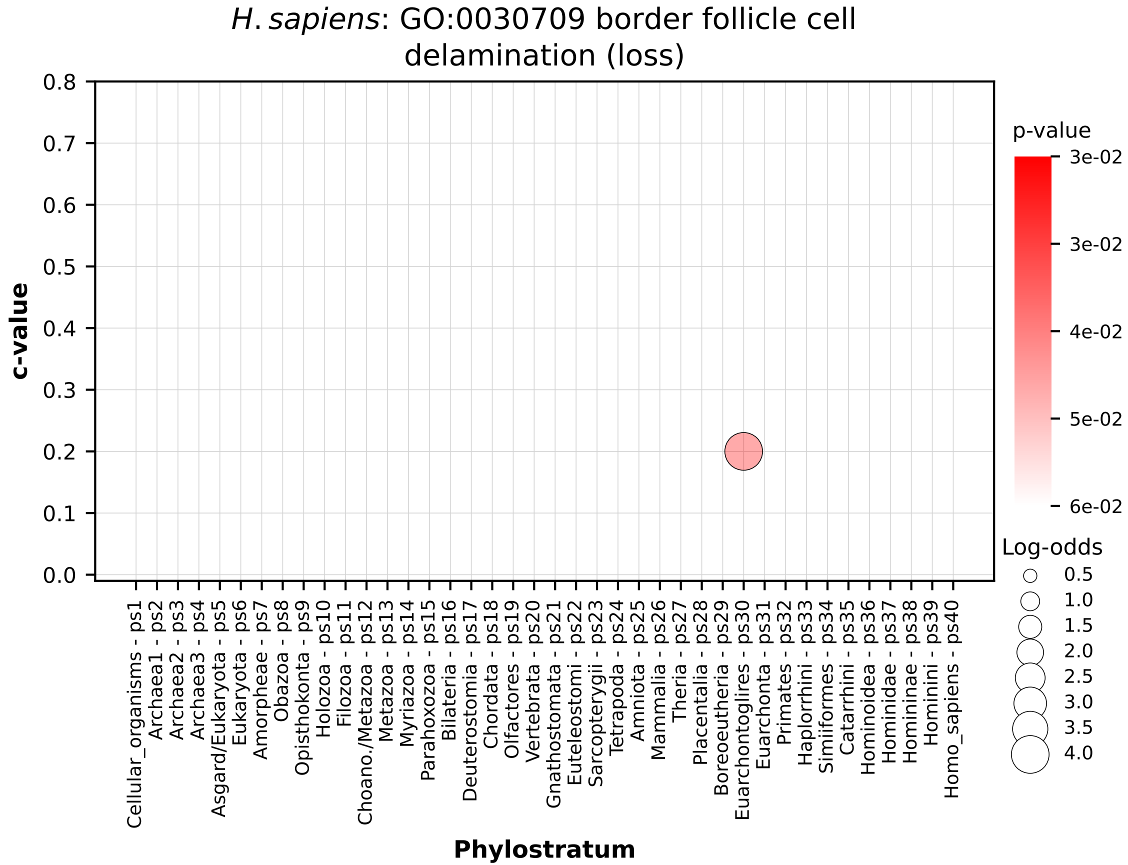

H. sapiens: GO:0030709 border follicle cell delamination (loss)
c-value
0.8
0.7
0.6
0.5
0.4
0.3
0.2
0.1
0.0
p-value
3e-02
3e-02
4e-02
5e-02
6e-02
Log-odds
0.5
1.0
1.5
2.0
2.5
3.0
3.5
4.0
Cellular_organisms - ps1
Archaea1 - ps2
Archaea2 - ps3
Archaea3 - ps4
Asgard/Eukaryota - ps5
Eukaryota - ps6
Amorpheae - ps7
Obazoa - ps8
Opisthokonta - ps9
Holozoa - ps10
Filozoa - ps11
Choano./Metazoa - ps12
Metazoa - ps13
Myriazoa - ps14
Parahoxozoa - ps15
Bilateria - ps16
Deuterostomia - ps17
Chordata - ps18
Olfactores - ps19
Vertebrata - ps20
Gnathostomata - ps21
Euteleostomi - ps22
Sarcopterygii - ps23
Tetrapoda - ps24
Amniota - ps25
Mammalia - ps26
Theria - ps27
Placentalia - ps28
Boreoeutheria - ps29
Euarchontoglires - ps30
Euarchonta - ps31
Primates - ps32
Haplorrhini - ps33
Simiiformes - ps34
Catarrhini - ps35
Hominoidea - ps36
Hominidae - ps37
Homininae - ps38
Hominini - ps39
Homo_sapiens - ps40
Phylostratum

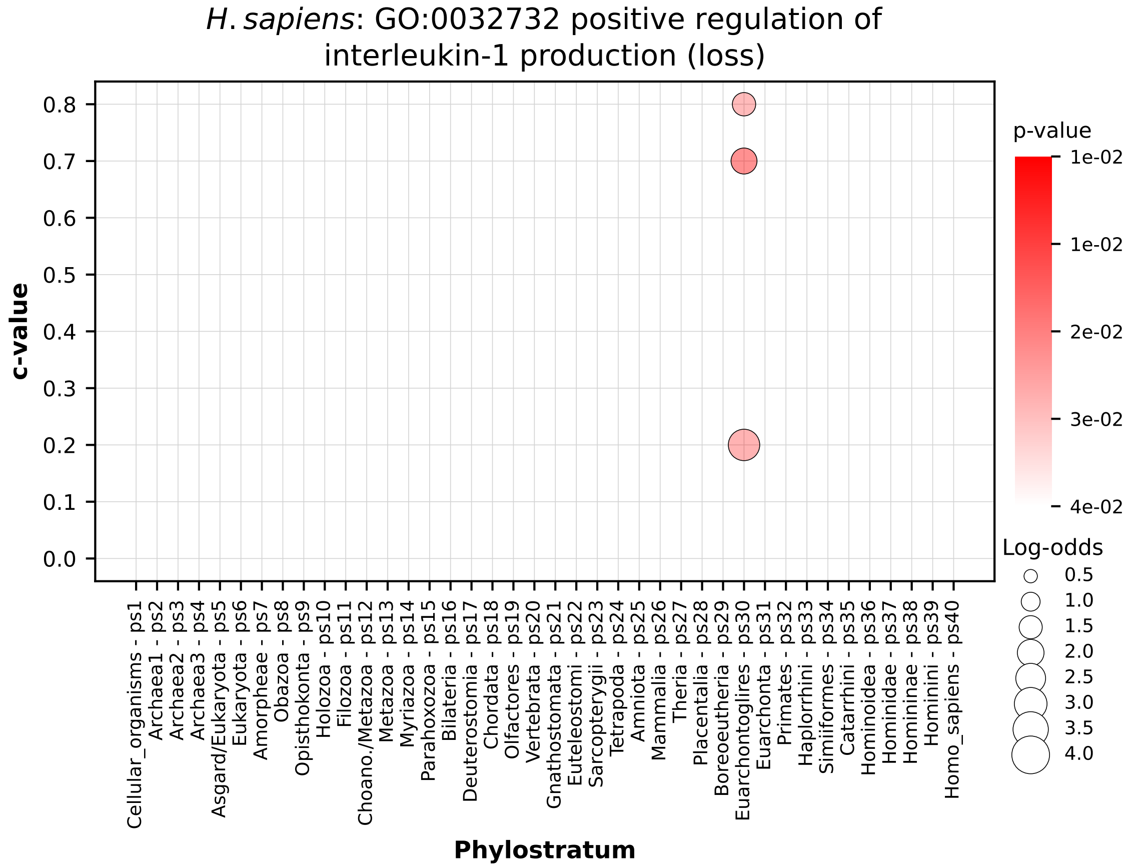

H. sapiens: GO:0032732 positive regulation of interleukin-1 production (loss)
c-value
0.8
0.7
0.6
0.5
0.4
0.3
0.2
0.1
0.0
p-value
1e-02
1e-02
2e-02
3e-02
4e-02
Log-odds
0.5
1.0
1.5
2.0
2.5
3.0
3.5
4.0
Cellular_organisms - ps1
Archaea1 - ps2
Archaea2 - ps3
Archaea3 - ps4
Asgard/Eukaryota - ps5
Eukaryota - ps6
Amorpheae - ps7
Obazoa - ps8
Opisthokonta - ps9
Holozoa - ps10
Filozoa - ps11
Choano./Metazoa - ps12
Metazoa - ps13
Myriazoa - ps14
Parahoxozoa - ps15
Bilateria - ps16
Deuterostomia - ps17
Chordata - ps18
Olfactores - ps19
Vertebrata - ps20
Gnathostomata - ps21
Euteleostomi - ps22
Sarcopterygii - ps23
Tetrapoda - ps24
Amniota - ps25
Mammalia - ps26
Theria - ps27
Placentalia - ps28
Boreoeutheria - ps29
Euarchontoglires - ps30
Euarchonta - ps31
Primates - ps32
Haplorrhini - ps33
Simiiformes - ps34
Catarrhini - ps35
Hominoidea - ps36
Hominidae - ps37
Homininae - ps38
Hominini - ps39
Homo_sapiens - ps40
Phylostratum

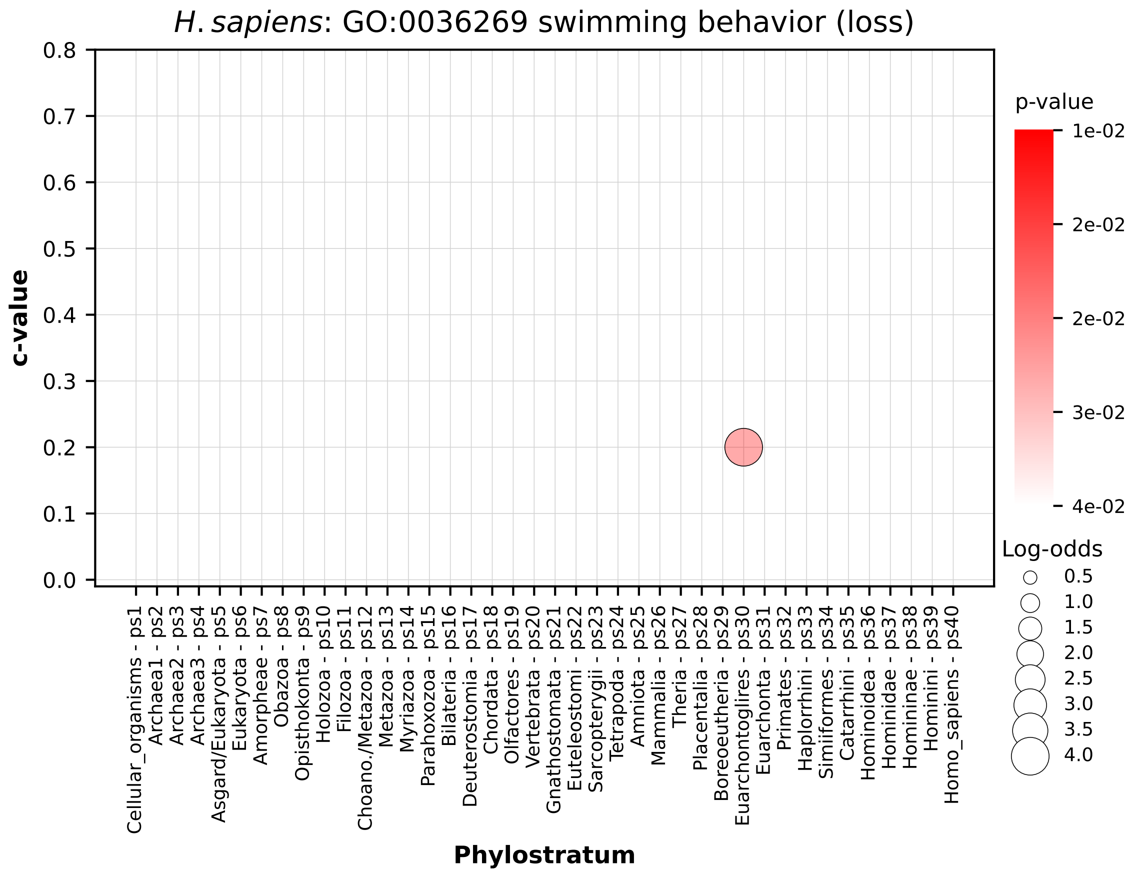

H. sapiens: GO:0036269 swimming behavior (loss)
c-value
0.0
0.1
0.2
0.3
0.4
0.5
0.6
0.7
0.8
Phylostratum
Cellular_organisms - ps1
Archaea1 - ps2
Archaea2 - ps3
Archaea3 - ps4
Asgard/Eukaryota - ps5
Eukaryota - ps6
Amorpheae - ps7
Obazoa - ps8
Opisthokonta - ps9
Holozoa - ps10
Filozoa - ps11
Choano./Metazoa - ps12
Metazoa - ps13
Myriazoa - ps14
Parahoxozoa - ps15
Bilateria - ps16
Deuterostomia - ps17
Chordata - ps18
Olfactores - ps19
Vertebrata - ps20
Gnathostomata - ps21
Euteleostomi - ps22
Sarcopterygii - ps23
Tetrapoda - ps24
Amniota - ps25
Mammalia - ps26
Theria - ps27
Placentalia - ps28
Boreoeutheria - ps29
Euarchontoglires - ps30
Euarchonta - ps31
Primates - ps32
Haplorrhini - ps33
Simiiformes - ps34
Catarrhini - ps35
Hominoidea - ps36
Hominidae - ps37
Homininae - ps38
Hominini - ps39
Homo_sapiens - ps40
p-value
1e-02
2e-02
2e-02
3e-02
4e-02
Log-odds
0.5
1.0
1.5
2.0
2.5
3.0
3.5
4.0

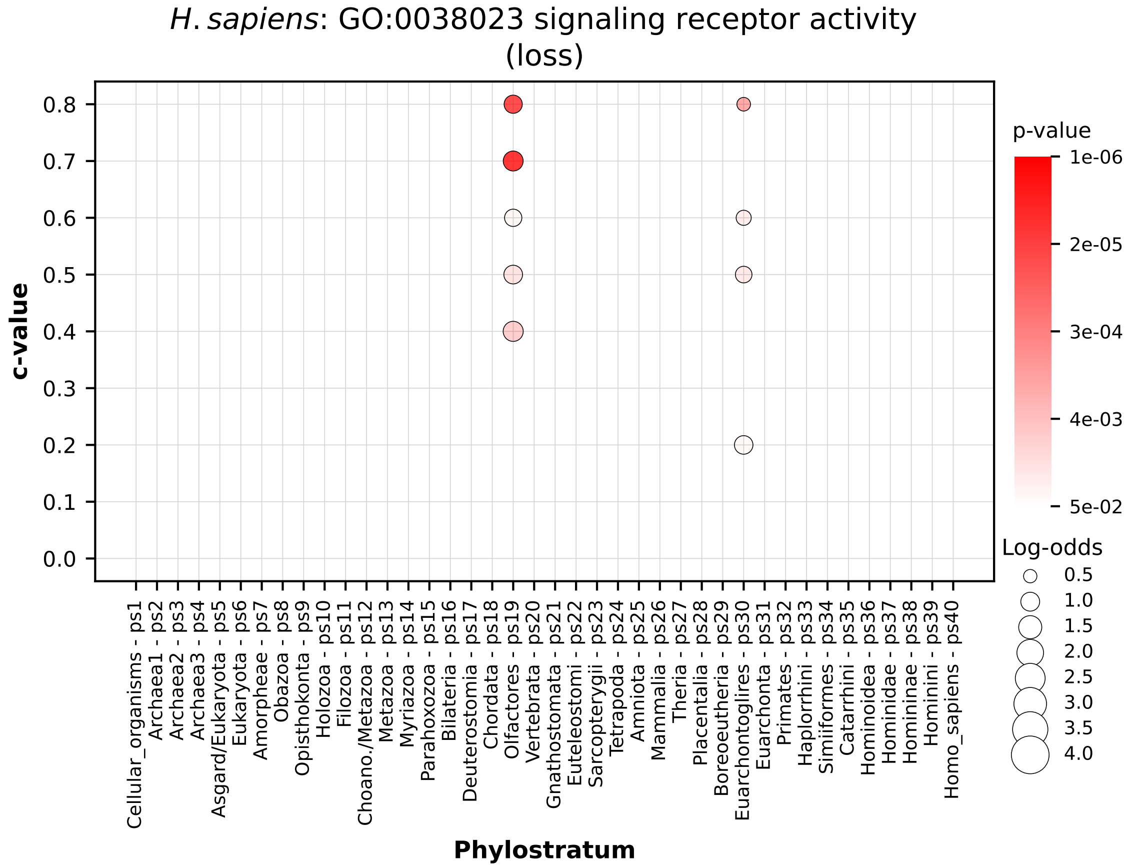

H. sapiens: GO:0038023 signaling receptor activity
(loss)
c-value
0.8
0.7
0.6
0.5
0.4
0.3
0.2
0.1
0.0
p-value
1e-06
2e-05
3e-04
4e-03
5e-02
Log-odds
0.5
1.0
1.5
2.0
2.5
3.0
3.5
4.0
Cellular_organisms - ps1
Archaea1 - ps2
Archaea2 - ps3
Archaea3 - ps4
Asgard/Eukaryota - ps5
Eukaryota - ps6
Amorpheae - ps7
Obazoa - ps8
Opisthokonta - ps9
Holozoa - ps10
Filozoa - ps11
Choano./Metazoa - ps12
Metazoa - ps13
Myriazoa - ps14
Parahoxozoa - ps15
Bilateria - ps16
Deuterostomia - ps17
Chordata - ps18
Olfactores - ps19
Vertebrata - ps20
Gnathostomata - ps21
Euteleostomi - ps22
Sarcopterygii - ps23
Tetrapoda - ps24
Amniota - ps25
Mammalia - ps26
Theria - ps27
Placentalia - ps28
Boreoeutheria - ps29
Euarchontoglires - ps30
Euarchonta - ps31
Primates - ps32
Haplorrhini - ps33
Simiiformes - ps34
Catarrhini - ps35
Hominoidea - ps36
Hominidae - ps37
Homininae - ps38
Hominini - ps39
Homo_sapiens - ps40
Phylostratum

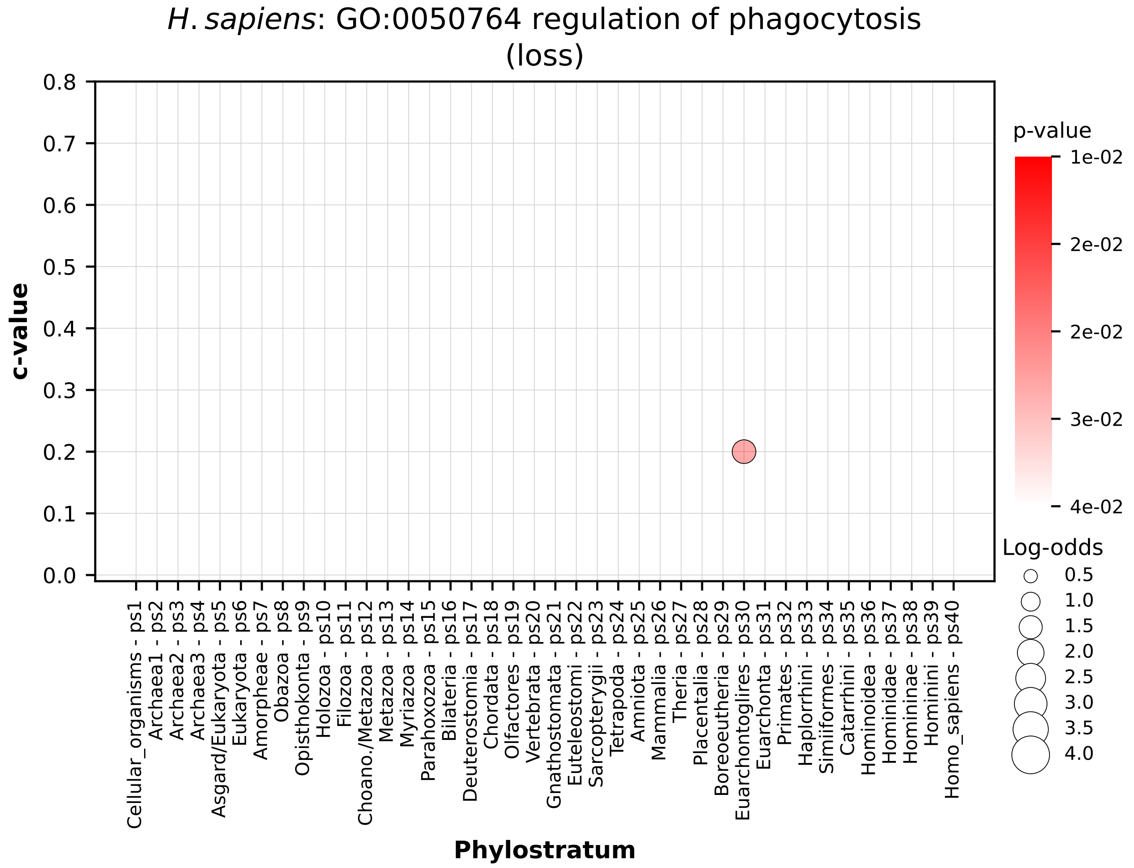

H. sapiens: GO:0050764 regulation of phagocytosis
(loss)
c-value
0.8
0.7
0.6
0.5
0.4
0.3
0.2
0.1
0.0
p-value
1e-02
2e-02
2e-02
3e-02
4e-02
Log-odds
0.5
1.0
1.5
2.0
2.5
3.0
3.5
4.0
Cellular_organisms - ps1
Archaea1 - ps2
Archaea2 - ps3
Archaea3 - ps4
Asgard/Eukaryota - ps5
Eukaryota - ps6
Amorpheae - ps7
Obazoa - ps8
Opisthokonta - ps9
Holozoa - ps10
Filozoa - ps11
Choano./Metazoa - ps12
Metazoa - ps13
Myriazoa - ps14
Parahoxozoa - ps15
Bilateria - ps16
Deuterostomia - ps17
Chordata - ps18
Olfactores - ps19
Vertebrata - ps20
Gnathostomata - ps21
Euteleostomi - ps22
Sarcopterygii - ps23
Tetrapoda - ps24
Amniota - ps25
Mammalia - ps26
Theria - ps27
Placentalia - ps28
Boreoeutheria - ps29
Euarchontoglires - ps30
Euarchonta - ps31
Primates - ps32
Haplorrhini - ps33
Simiiformes - ps34
Catarrhini - ps35
Hominoidea - ps36
Hominidae - ps37
Homininae - ps38
Hominini - ps39
Homo_sapiens - ps40
Phylostratum

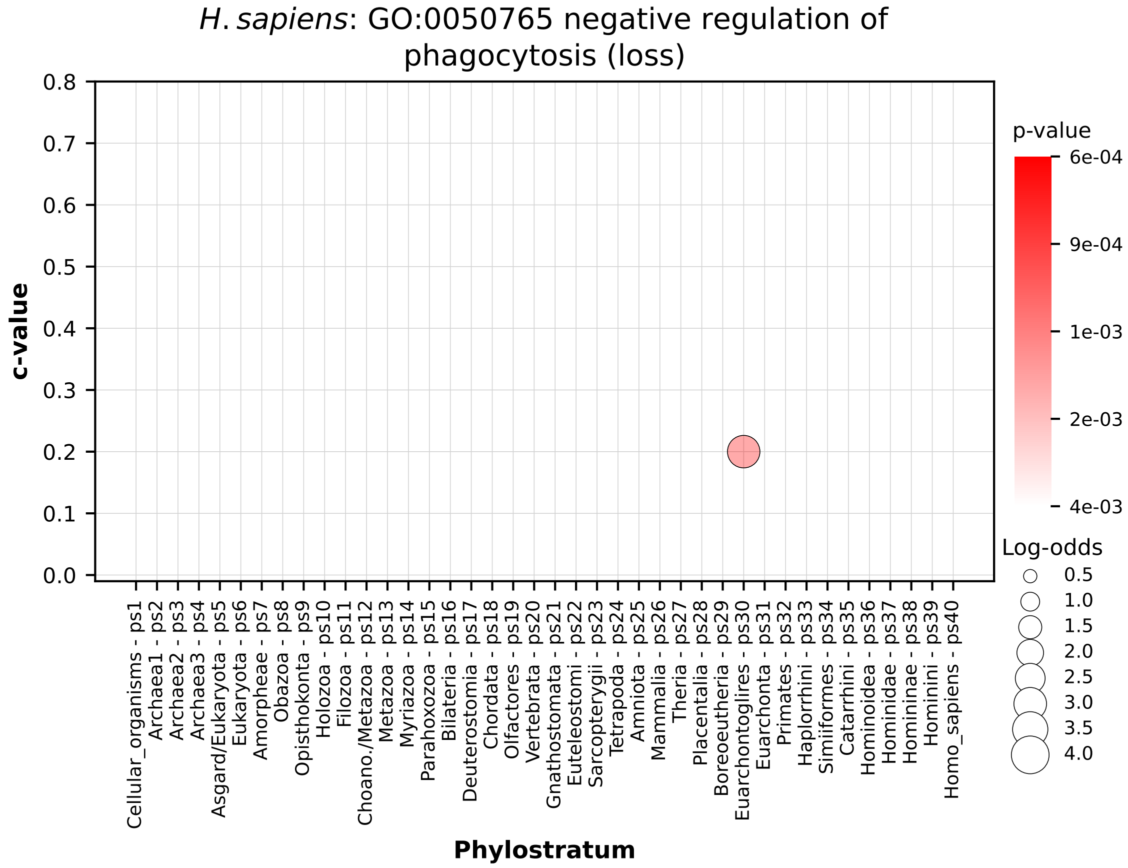
H. sapiens: GO:0050765 negative regulation of phagocytosis (loss)
c-value
0.0
0.1
0.2
0.3
0.4
0.5
0.6
0.7
0.8
Phylostratum
Cellular_organisms - ps1
Archaea1 - ps2
Archaea2 - ps3
Archaea3 - ps4
Asgard/Eukaryota - ps5
Eukaryota - ps6
Amorpheae - ps7
Obazoa - ps8
Opisthokonta - ps9
Holozoa - ps10
Filozoa - ps11
Choano./Metazoa - ps12
Metazoa - ps13
Myriazoa - ps14
Parahoxozoa - ps15
Bilateria - ps16
Deuterostomia - ps17
Chordata - ps18
Olfactores - ps19
Vertebrata - ps20
Gnathostomata - ps21
Euteleostomi - ps22
Sarcopterygii - ps23
Tetrapoda - ps24
Amniota - ps25
Mammalia - ps26
Theria - ps27
Placentalia - ps28
Boreoeutheria - ps29
Euarchontoglires - ps30
Euarchonta - ps31
Primates - ps32
Haplorrhini - ps33
Simiiformes - ps34
Catarrhini - ps35
Hominoidea - ps36
Hominidae - ps37
Homininae - ps38
Hominini - ps39
Homo_sapiens - ps40
p-value
6e-04
9e-04
1e-03
2e-03
4e-03
Log-odds
0.5
1.0
1.5
2.0
2.5
3.0
3.5
4.0

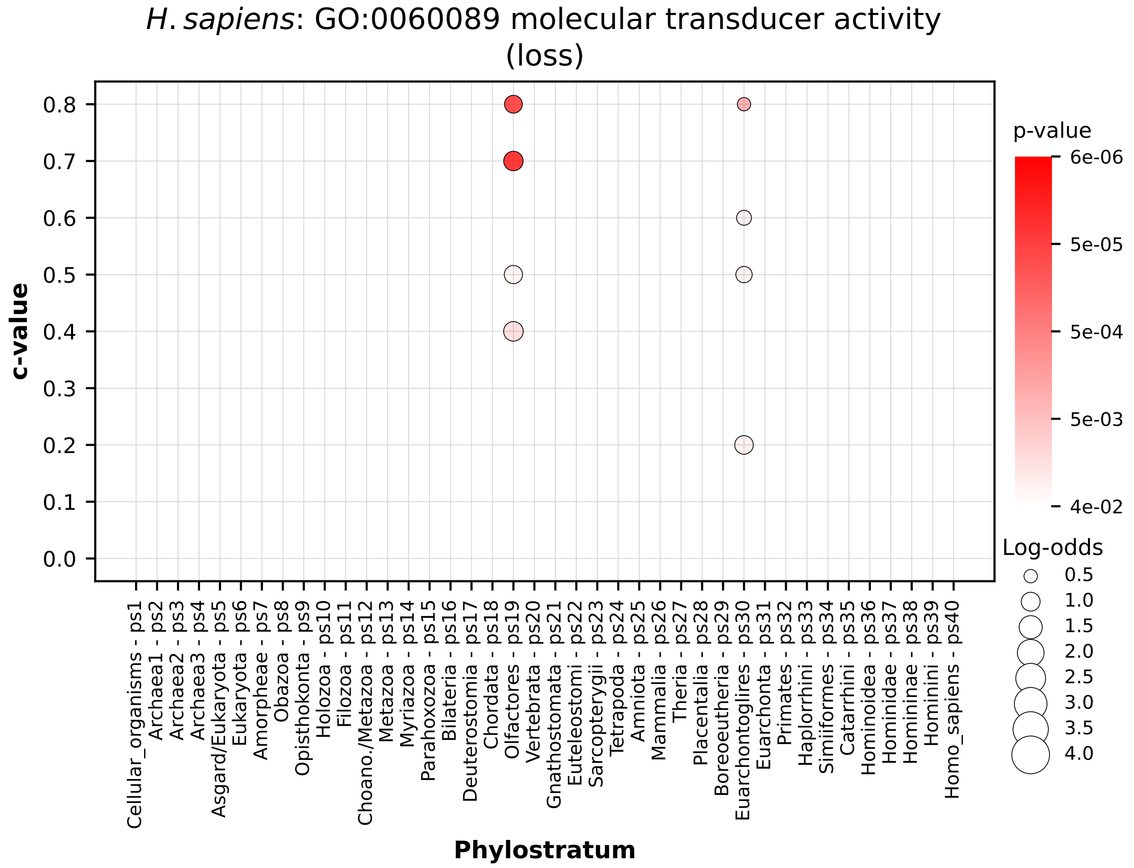

H. sapiens: GO:0060089 molecular transducer activity
(loss)
c-value
0.8
0.7
0.6
0.5
0.4
0.3
0.2
0.1
0.0
Phylostratum
Cellular_organisms - ps1
Archaea1 - ps2
Archaea2 - ps3
Archaea3 - ps4
Asgard/Eukaryota - ps5
Eukaryota - ps6
Amorpheae - ps7
Obazoa - ps8
Opisthokonta - ps9
Holozoa - ps10
Filozoa - ps11
Choano./Metazoa - ps12
Metazoa - ps13
Myriazoa - ps14
Parahoxozoa - ps15
Bilateria - ps16
Deuterostomia - ps17
Chordata - ps18
Olfactores - ps19
Vertebrata - ps20
Gnathostomata - ps21
Euteleostomi - ps22
Sarcopterygii - ps23
Tetrapoda - ps24
Amniota - ps25
Mammalia - ps26
Theria - ps27
Placentalia - ps28
Boreoeutheria - ps29
Euarchontoglires - ps30
Euarchonta - ps31
Primates - ps32
Haplorrhini - ps33
Simiiformes - ps34
Catarrhini - ps35
Hominoidea - ps36
Hominidae - ps37
Homininae - ps38
Hominini - ps39
Homo_sapiens - ps40
p-value
6e-06
5e-05
5e-04
5e-03
4e-02
Log-odds
0.5
1.0
1.5
2.0
2.5
3.0
3.5
4.0

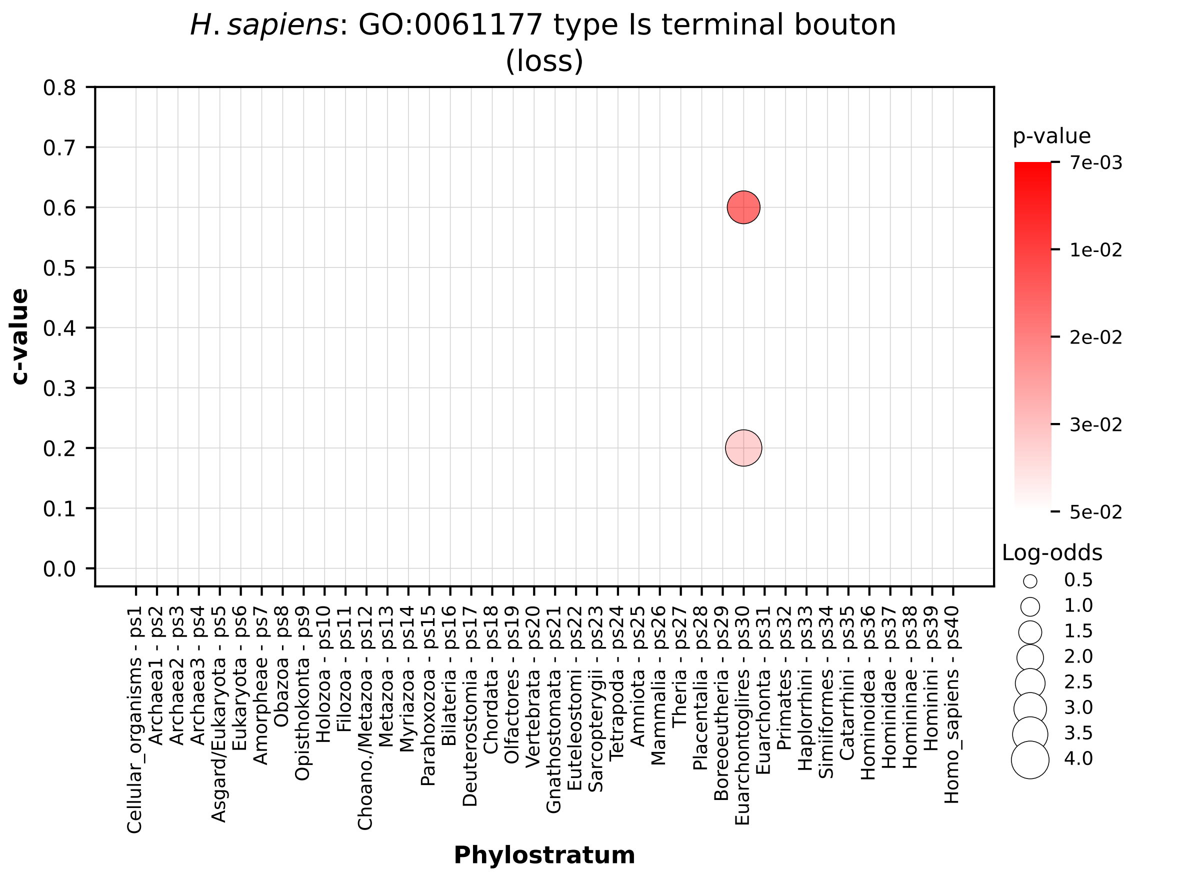

H. sapiens: GO:0061177 type Is terminal bouton
(loss)
c-value
0.0
0.1
0.2
0.3
0.4
0.5
0.6
0.7
0.8
Phylostratum
Cellular_organisms - ps1
Archaea1 - ps2
Archaea2 - ps3
Archaea3 - ps4
Asgard/Eukaryota - ps5
Eukaryota - ps6
Amorpheae - ps7
Obazoa - ps8
Opisthokonta - ps9
Holozoa - ps10
Filozoa - ps11
Choano./Metazoa - ps12
Metazoa - ps13
Myriazoa - ps14
Parahoxozoa - ps15
Bilateria - ps16
Deuterostomia - ps17
Chordata - ps18
Olfactores - ps19
Vertebrata - ps20
Gnathostomata - ps21
Euteleostomi - ps22
Sarcopterygii - ps23
Tetrapoda - ps24
Amniota - ps25
Mammalia - ps26
Theria - ps27
Placentalia - ps28
Boreoeutheria - ps29
Euarchontoglires - ps30
Euarchonta - ps31
Primates - ps32
Haplorrhini - ps33
Simiiformes - ps34
Catarrhini - ps35
Hominoidea - ps36
Hominidae - ps37
Homininae - ps38
Hominini - ps39
Homo_sapiens - ps40
p-value
7e-03
1e-02
2e-02
3e-02
5e-02
Log-odds
0.5
1.0
1.5
2.0
2.5
3.0
3.5
4.0

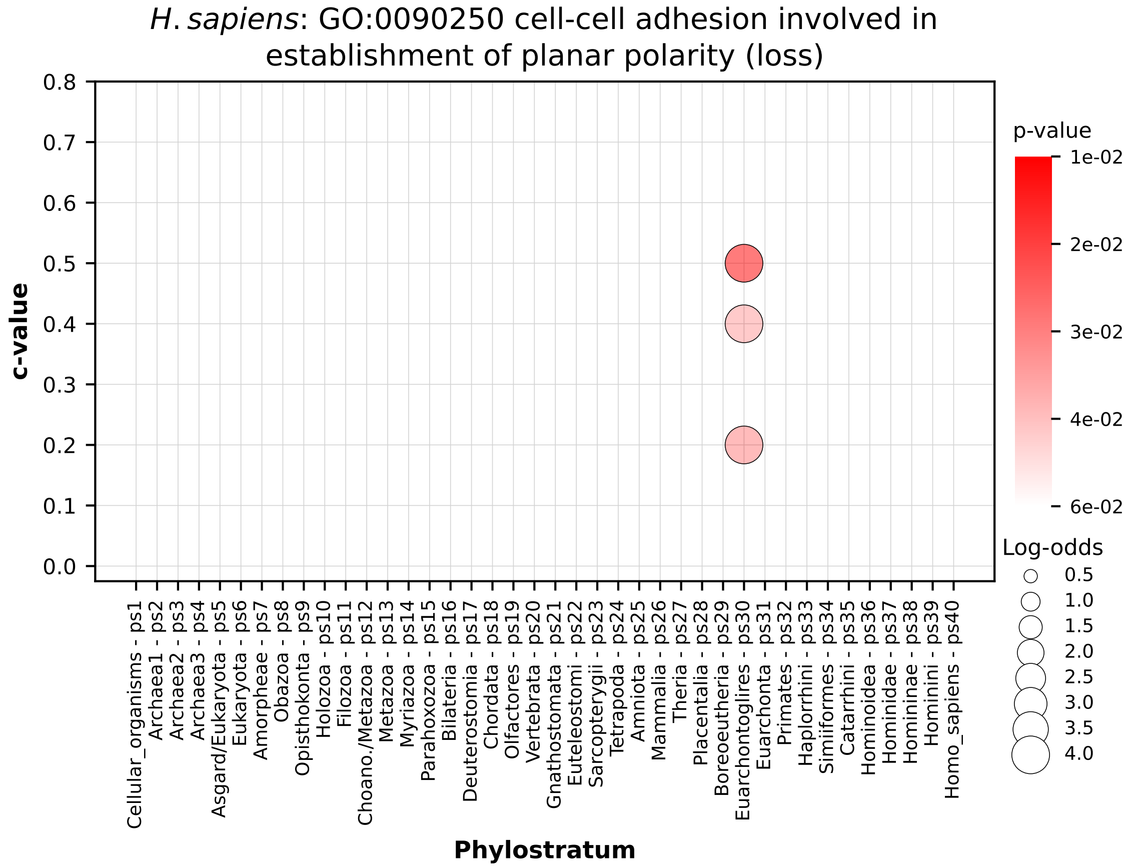

H. sapiens: GO:0090250 cell-cell adhesion involved in establishment of planar polarity (loss)
c-value
0.8
0.7
0.6
0.5
0.4
0.3
0.2
0.1
0.0
Phylostratum
Cellular_organisms - ps1
Archaea1 - ps2
Archaea2 - ps3
Archaea3 - ps4
Asgard/Eukaryota - ps5
Eukaryota - ps6
Amorpheae - ps7
Obazoa - ps8
Opisthokonta - ps9
Holozoa - ps10
Filozoa - ps11
Choano./Metazoa - ps12
Metazoa - ps13
Myriazoa - ps14
Parahoxozoa - ps15
Bilateria - ps16
Deuterostomia - ps17
Chordata - ps18
Olfactores - ps19
Vertebrata - ps20
Gnathostomata - ps21
Euteleostomi - ps22
Sarcopterygii - ps23
Tetrapoda - ps24
Amniota - ps25
Mammalia - ps26
Theria - ps27
Placentalia - ps28
Boreoeutheria - ps29
Euarchontoglires - ps30
Euarchonta - ps31
Primates - ps32
Haplorrhini - ps33
Simiiformes - ps34
Catarrhini - ps35
Hominoidea - ps36
Hominidae - ps37
Homininae - ps38
Hominini - ps39
Homo_sapiens - ps40
p-value
1e-02
2e-02
3e-02
4e-02
6e-02
Log-odds
0.5
1.0
1.5
2.0
2.5
3.0
3.5
4.0

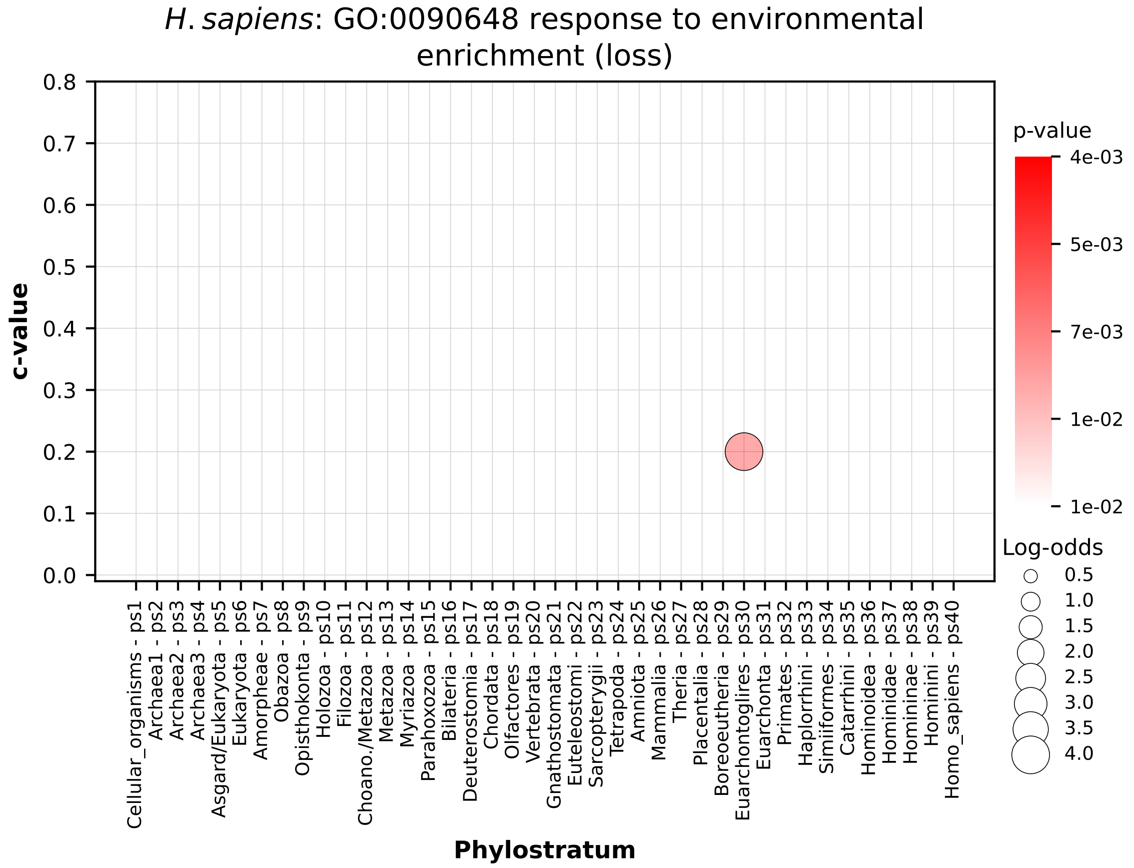

H. sapiens: GO:0090648 response to environmental enrichment (loss)
c-value
0.8
0.7
0.6
0.5
0.4
0.3
0.2
0.1
0.0
p-value
4e-03
5e-03
7e-03
1e-02
1e-02
Log-odds
0.5
1.0
1.5
2.0
2.5
3.0
3.5
4.0
Cellular_organisms - ps1
Archaea1 - ps2
Archaea2 - ps3
Archaea3 - ps4
Asgard/Eukaryota - ps5
Eukaryota - ps6
Amorpheae - ps7
Obazoa - ps8
Opisthokonta - ps9
Holozoa - ps10
Filozoa - ps11
Choano./Metazoa - ps12
Metazoa - ps13
Myriazoa - ps14
Parahoxozoa - ps15
Bilateria - ps16
Deuterostomia - ps17
Chordata - ps18
Olfactores - ps19
Vertebrata - ps20
Gnathostomata - ps21
Euteleostomi - ps22
Sarcopterygii - ps23
Tetrapoda - ps24
Amniota - ps25
Mammalia - ps26
Theria - ps27
Placentalia - ps28
Boreoeutheria - ps29
Euarchontoglires - ps30
Euarchonta - ps31
Primates - ps32
Haplorrhini - ps33
Simiiformes - ps34
Catarrhini - ps35
Hominoidea - ps36
Hominidae - ps37
Homininae - ps38
Hominini - ps39
Homo_sapiens - ps40
Phylostratum

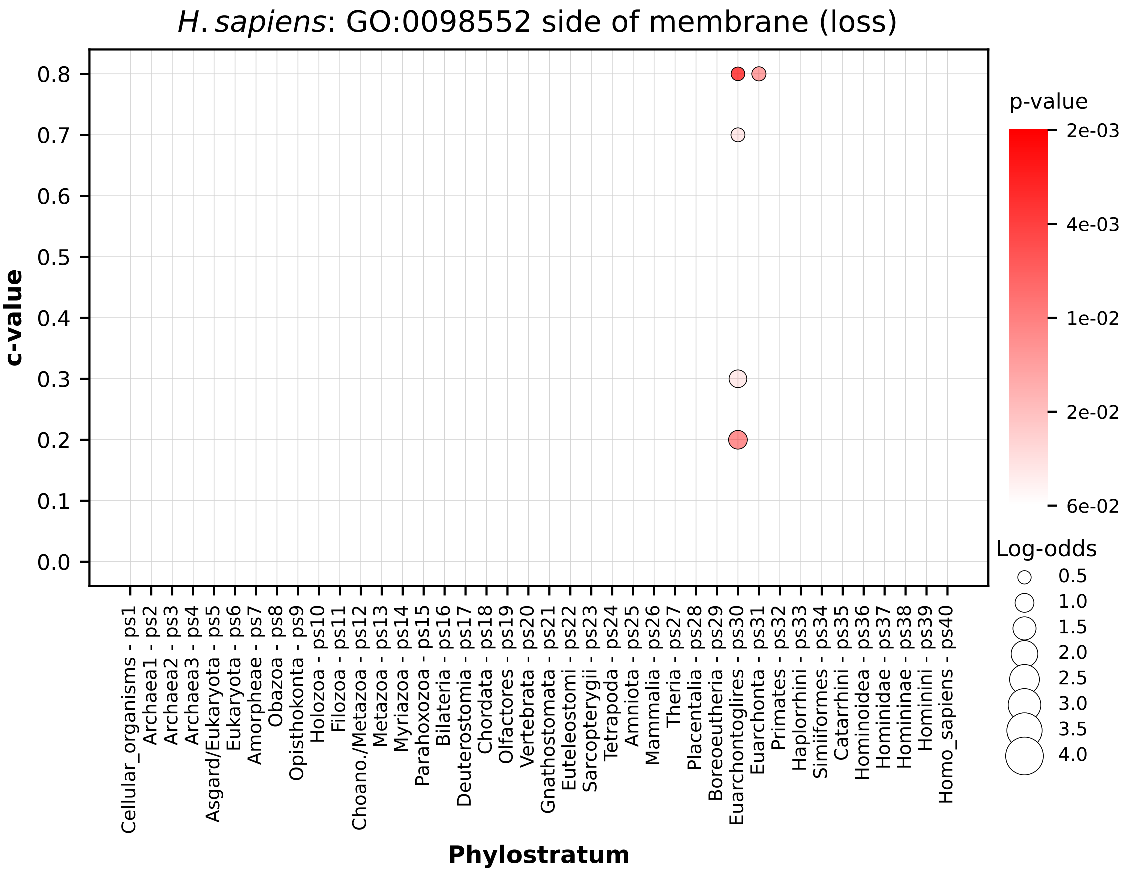

H. sapiens: GO:0098552 side of membrane (loss)
c-value
0.8
0.7
0.6
0.5
0.4
0.3
0.2
0.1
0.0
Phylostratum
Cellular_organisms - ps1
Archaea1 - ps2
Archaea2 - ps3
Archaea3 - ps4
Asgard/Eukaryota - ps5
Eukaryota - ps6
Amorpheae - ps7
Obazoa - ps8
Opisthokonta - ps9
Holozoa - ps10
Filozoa - ps11
Choano./Metazoa - ps12
Metazoa - ps13
Myriazoa - ps14
Parahoxozoa - ps15
Bilateria - ps16
Deuterostomia - ps17
Chordata - ps18
Olfactores - ps19
Vertebrata - ps20
Gnathostomata - ps21
Euteleostomi - ps22
Sarcopterygii - ps23
Tetrapoda - ps24
Amniota - ps25
Mammalia - ps26
Theria - ps27
Placentalia - ps28
Boreoeutheria - ps29
Euarchontoglires - ps30
Euarchonta - ps31
Primates - ps32
Haplorrhini - ps33
Simiiformes - ps34
Catarrhini - ps35
Hominoidea - ps36
Hominidae - ps37
Homininae - ps38
Hominini - ps39
Homo_sapiens - ps40
p-value
2e-03
4e-03
1e-02
2e-02
6e-02
Log-odds
0.5
1.0
1.5
2.0
2.5
3.0
3.5
4.0

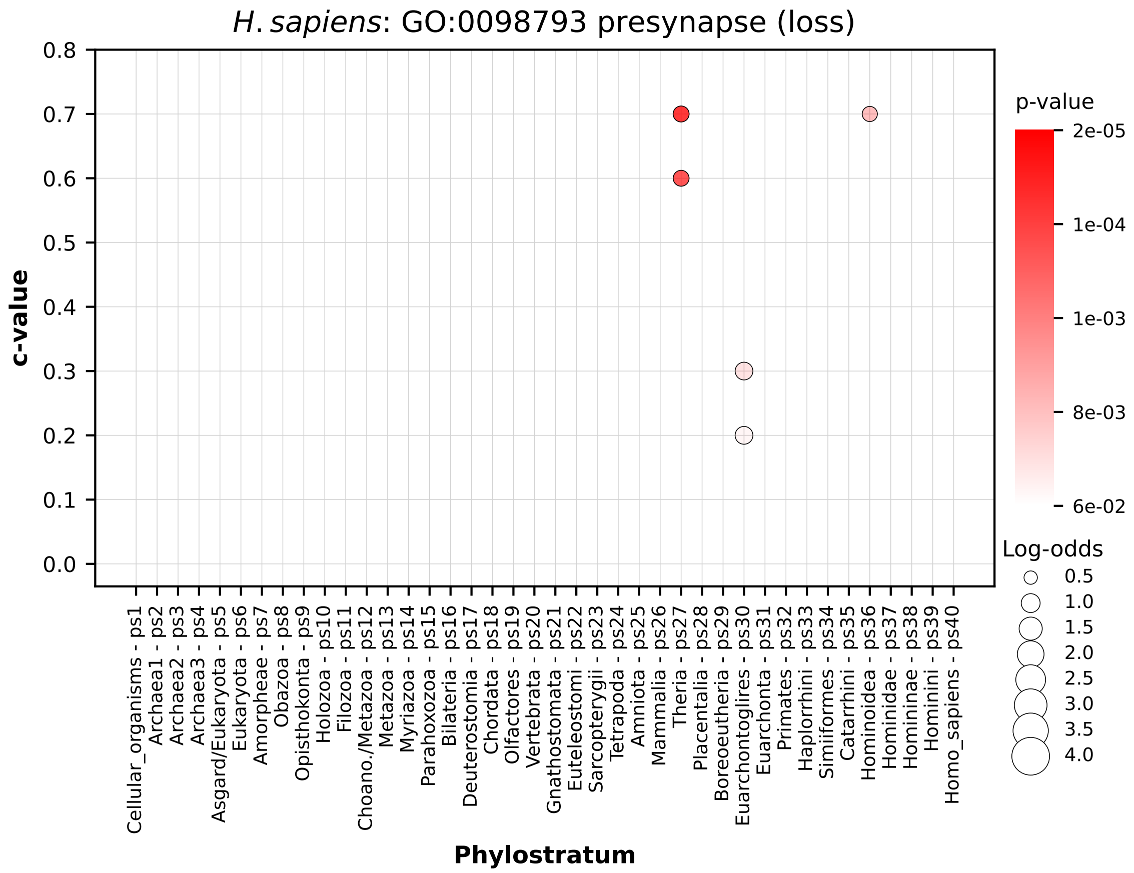

H. sapiens: GO:0098793 presynapse (loss)
c-value
0.8
0.7
0.6
0.5
0.4
0.3
0.2
0.1
0.0
p-value
2e-05
1e-04
1e-03
8e-03
6e-02
Log-odds
0.5
1.0
1.5
2.0
2.5
3.0
3.5
4.0
Cellular_organisms - ps1
Archaea1 - ps2
Archaea2 - ps3
Archaea3 - ps4
Asgard/Eukaryota - ps5
Eukaryota - ps6
Amorpheae - ps7
Obazoa - ps8
Opisthokonta - ps9
Holozoa - ps10
Filozoa - ps11
Choano./Metazoa - ps12
Metazoa - ps13
Myriazoa - ps14
Parahoxozoa - ps15
Bilateria - ps16
Deuterostomia - ps17
Chordata - ps18
Olfactores - ps19
Vertebrata - ps20
Gnathostomata - ps21
Euteleostomi - ps22
Sarcopterygii - ps23
Tetrapoda - ps24
Amniota - ps25
Mammalia - ps26
Theria - ps27
Placentalia - ps28
Boreoeutheria - ps29
Euarchontoglires - ps30
Euarchonta - ps31
Primates - ps32
Haplorrhini - ps33
Simiiformes - ps34
Catarrhini - ps35
Hominoidea - ps36
Hominidae - ps37
Homininae - ps38
Hominini - ps39
Homo_sapiens - ps40
Phylostratum

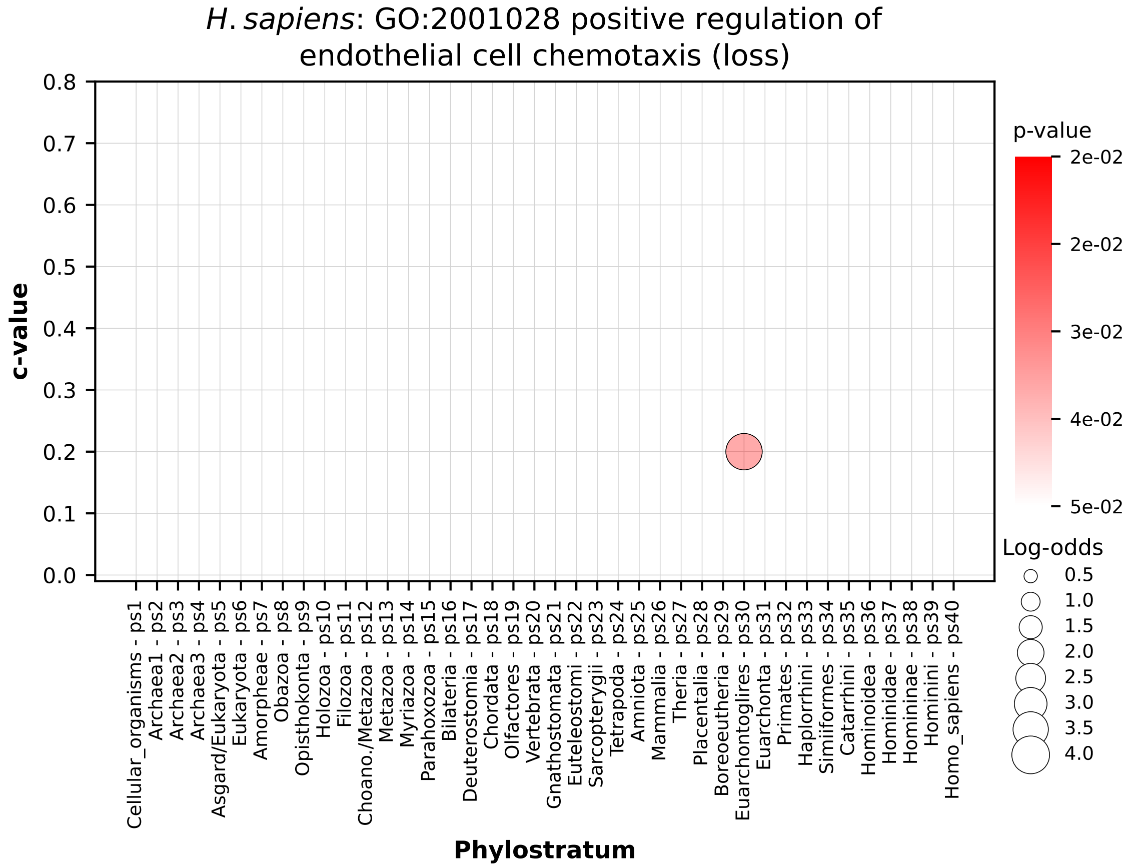

H. sapiens: GO:2001028 positive regulation of endothelial cell chemotaxis (loss)
c-value
0.0
0.1
0.2
0.3
0.4
0.5
0.6
0.7
0.8
Phylostratum
Cellular_organisms - ps1
Archaea1 - ps2
Archaea2 - ps3
Archaea3 - ps4
Asgard/Eukaryota - ps5
Eukaryota - ps6
Amorpheae - ps7
Obazoa - ps8
Opisthokonta - ps9
Holozoa - ps10
Filozoa - ps11
Choano./Metazoa - ps12
Metazoa - ps13
Myriazoa - ps14
Parahoxozoa - ps15
Bilateria - ps16
Deuterostomia - ps17
Chordata - ps18
Olfactores - ps19
Vertebrata - ps20
Gnathostomata - ps21
Euteleostomi - ps22
Sarcopterygii - ps23
Tetrapoda - ps24
Amniota - ps25
Mammalia - ps26
Theria - ps27
Placentalia - ps28
Boreoeutheria - ps29
Euarchontoglires - ps30
Euarchonta - ps31
Primates - ps32
Haplorrhini - ps33
Simiiformes - ps34
Catarrhini - ps35
Hominoidea - ps36
Hominidae - ps37
Homininae - ps38
Hominini - ps39
Homo_sapiens - ps40
p-value
2e-02
2e-02
3e-02
4e-02
5e-02
Log-odds
0.5
1.0
1.5
2.0
2.5
3.0
3.5
4.0

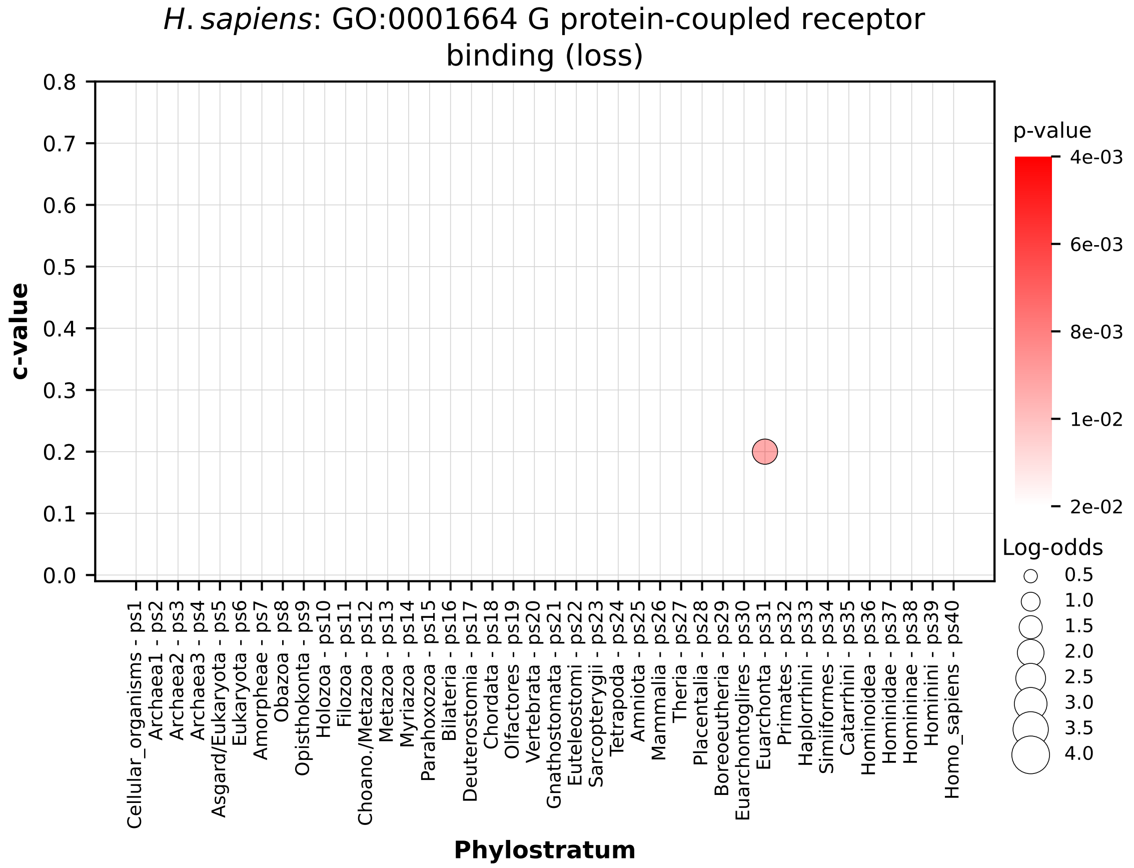

H. sapiens: GO:0001664 G protein-coupled receptor binding (loss)
c-value
0.8
0.7
0.6
0.5
0.4
0.3
0.2
0.1
0.0
Phylostratum
Cellular_organisms - ps1
Archaea1 - ps2
Archaea2 - ps3
Archaea3 - ps4
Asgard/Eukaryota - ps5
Eukaryota - ps6
Amorpheae - ps7
Obazoa - ps8
Opisthokonta - ps9
Holozoa - ps10
Filozoa - ps11
Choano./Metazoa - ps12
Metazoa - ps13
Myriazoa - ps14
Parahoxozoa - ps15
Bilateria - ps16
Deuterostomia - ps17
Chordata - ps18
Olfactores - ps19
Vertebrata - ps20
Gnathostomata - ps21
Euteleostomi - ps22
Sarcopterygii - ps23
Tetrapoda - ps24
Amniota - ps25
Mammalia - ps26
Theria - ps27
Placentalia - ps28
Boreoeutheria - ps29
Euarchontoglires - ps30
Euarchonta - ps31
Primates - ps32
Haplorrhini - ps33
Simiiformes - ps34
Catarrhini - ps35
Hominoidea - ps36
Hominidae - ps37
Homininae - ps38
Hominini - ps39
Homo_sapiens - ps40
p-value
4e-03
6e-03
8e-03
1e-02
2e-02
Log-odds
0.5
1.0
1.5
2.0
2.5
3.0
3.5
4.0

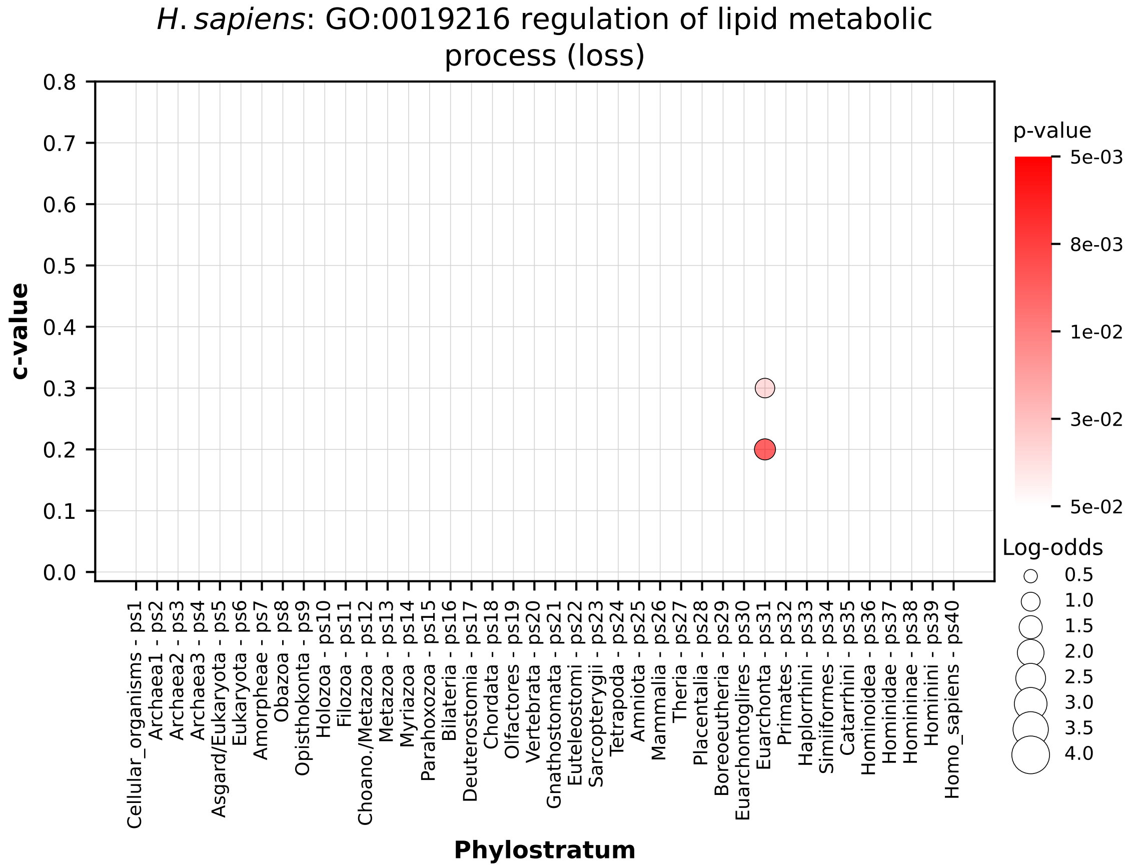

H. sapiens: GO:0019216 regulation of lipid metabolic process (loss)
c-value
0.0
0.1
0.2
0.3
0.4
0.5
0.6
0.7
0.8
p-value
5e-03
8e-03
1e-02
3e-02
5e-02
Log-odds
0.5
1.0
1.5
2.0
2.5
3.0
3.5
4.0
Cellular_organisms - ps1
Archaea1 - ps2
Archaea2 - ps3
Archaea3 - ps4
Asgard/Eukaryota - ps5
Eukaryota - ps6
Amorpheae - ps7
Obazoa - ps8
Opisthokonta - ps9
Holozoa - ps10
Filozoa - ps11
Choano./Metazoa - ps12
Metazoa - ps13
Myriazoa - ps14
Parahoxozoa - ps15
Bilateria - ps16
Deuterostomia - ps17
Chordata - ps18
Olfactores - ps19
Vertebrata - ps20
Gnathostomata - ps21
Euteleostomi - ps22
Sarcopterygii - ps23
Tetrapoda - ps24
Amniota - ps25
Mammalia - ps26
Theria - ps27
Placentalia - ps28
Boreoeutheria - ps29
Euarchontoglires - ps30
Euarchonta - ps31
Primates - ps32
Haplorrhini - ps33
Simiiformes - ps34
Catarrhini - ps35
Hominoidea - ps36
Hominidae - ps37
Homininae - ps38
Hominini - ps39
Homo_sapiens - ps40
Phylostratum

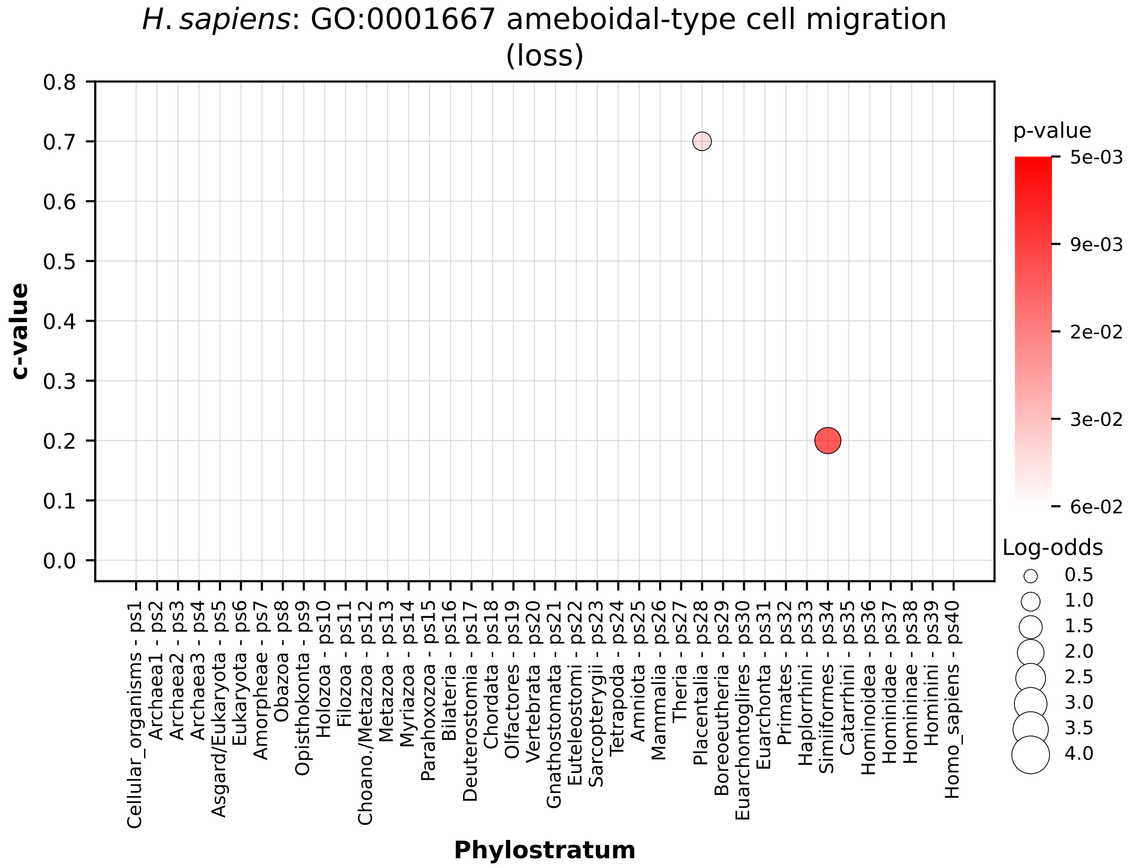

H. sapiens: GO:0001667 ameboidal-type cell migration (loss)
c-value
0.8
0.7
0.6
0.5
0.4
0.3
0.2
0.1
0.0
Phylostratum
Cellular_organisms - ps1
Archaea1 - ps2
Archaea2 - ps3
Archaea3 - ps4
Asgard/Eukaryota - ps5
Eukaryota - ps6
Amorpheae - ps7
Obazoa - ps8
Opisthokonta - ps9
Holozoa - ps10
Filozoa - ps11
Choano./Metazoa - ps12
Metazoa - ps13
Myriazoa - ps14
Parahoxozoa - ps15
Bilateria - ps16
Deuterostomia - ps17
Chordata - ps18
Olfactores - ps19
Vertebrata - ps20
Gnathostomata - ps21
Euteleostomi - ps22
Sarcopterygii - ps23
Tetrapoda - ps24
Amniota - ps25
Mammalia - ps26
Theria - ps27
Placentalia - ps28
Boreoeutheria - ps29
Euarchontoglires - ps30
Euarchonta - ps31
Primates - ps32
Haplorrhini - ps33
Simiiformes - ps34
Catarrhini - ps35
Hominoidea - ps36
Hominidae - ps37
Homininae - ps38
Hominini - ps39
Homo_sapiens - ps40
p-value
5e-03
9e-03
2e-02
3e-02
6e-02
Log-odds
0.5
1.0
1.5
2.0
2.5
3.0
3.5
4.0

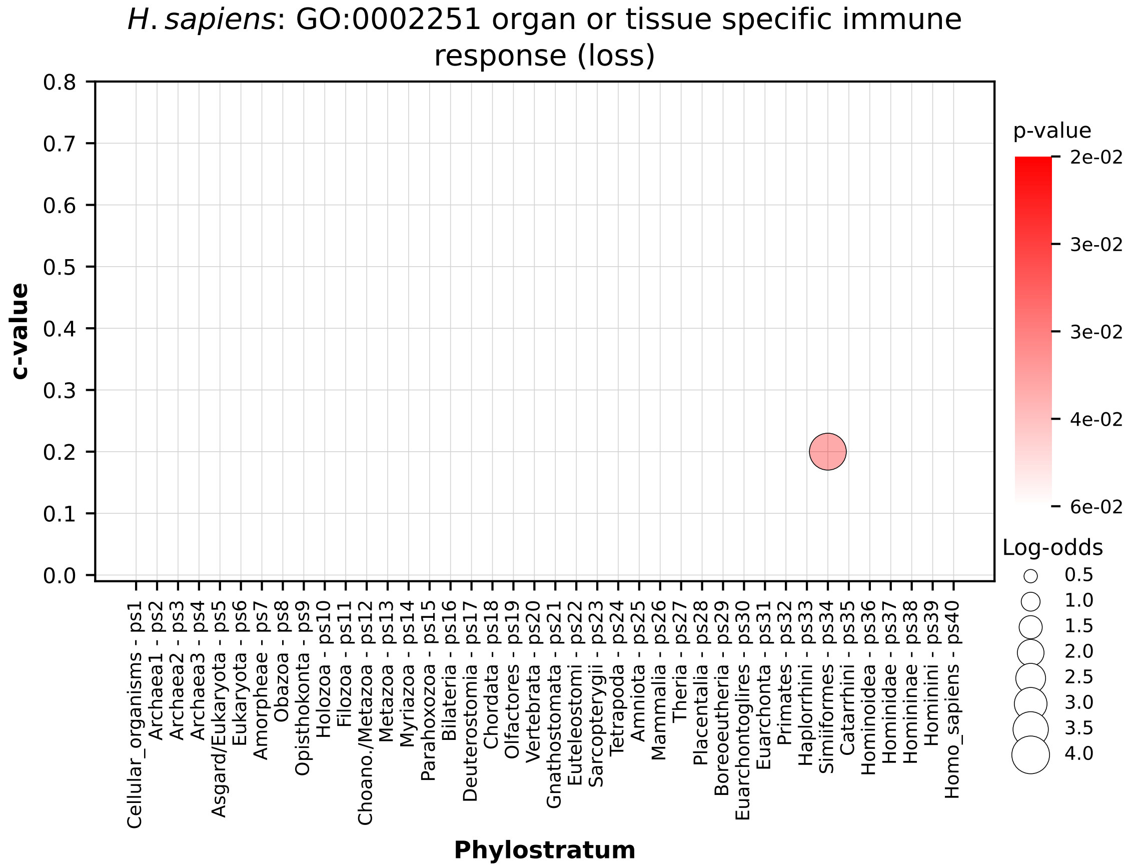

H. sapiens: GO:0002251 organ or tissue specific immune response (loss)
c-value
0.0
0.1
0.2
0.3
0.4
0.5
0.6
0.7
0.8
Phylostratum
Cellular_organisms - ps1
Archaea1 - ps2
Archaea2 - ps3
Archaea3 - ps4
Asgard/Eukaryota - ps5
Eukaryota - ps6
Amorpheae - ps7
Obazoa - ps8
Opisthokonta - ps9
Holozoa - ps10
Filozoa - ps11
Choano./Metazoa - ps12
Metazoa - ps13
Myriazoa - ps14
Parahoxozoa - ps15
Bilateria - ps16
Deuterostomia - ps17
Chordata - ps18
Olfactores - ps19
Vertebrata - ps20
Gnathostomata - ps21
Euteleostomi - ps22
Sarcopterygii - ps23
Tetrapoda - ps24
Amniota - ps25
Mammalia - ps26
Theria - ps27
Placentalia - ps28
Boreoeutheria - ps29
Euarchontoglires - ps30
Euarchonta - ps31
Primates - ps32
Haplorrhini - ps33
Simiiformes - ps34
Catarrhini - ps35
Hominoidea - ps36
Hominidae - ps37
Homininae - ps38
Hominini - ps39
Homo_sapiens - ps40
p-value
2e-02
3e-02
3e-02
4e-02
6e-02
Log-odds
0.5
1.0
1.5
2.0
2.5
3.0
3.5
4.0

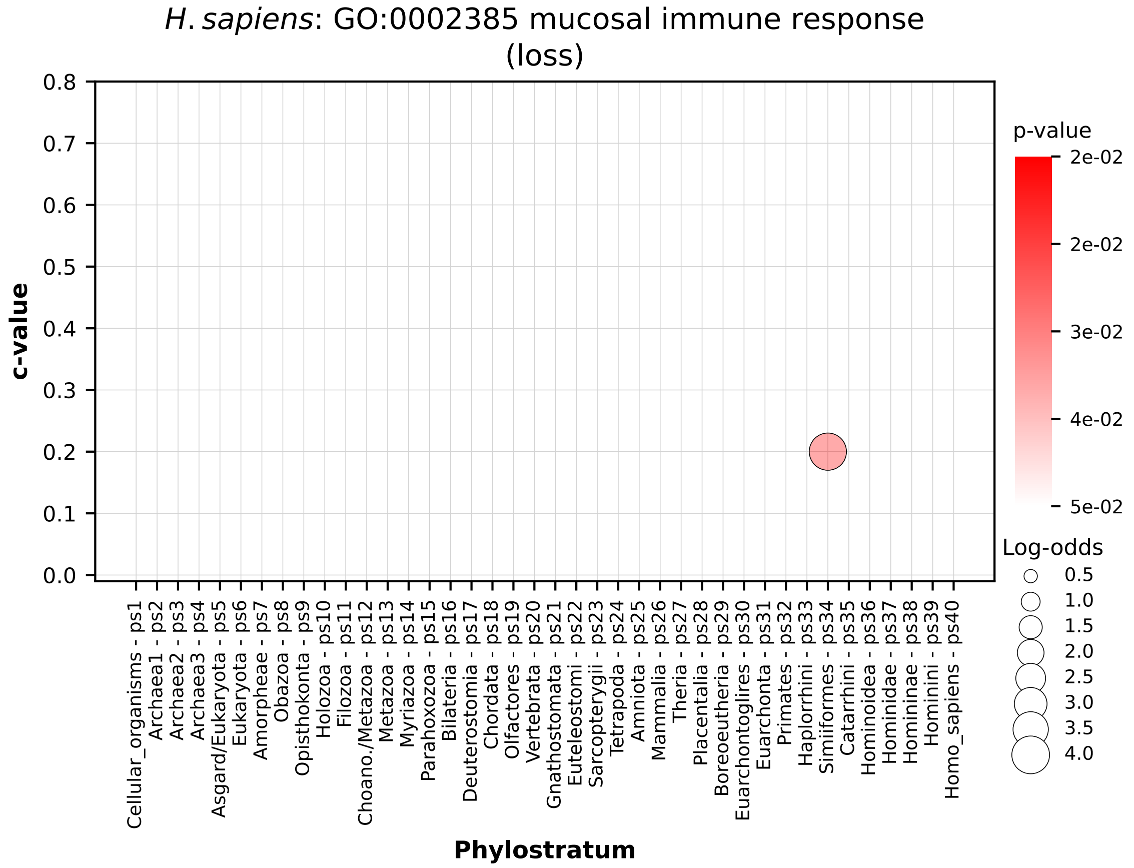

H. sapiens: GO:0002385 mucosal immune response
(loss)
c-value
0.0
0.1
0.2
0.3
0.4
0.5
0.6
0.7
0.8
Phylostratum
Cellular_organisms - ps1
Archaea1 - ps2
Archaea2 - ps3
Archaea3 - ps4
Asgard/Eukaryota - ps5
Eukaryota - ps6
Amorpheae - ps7
Obazoa - ps8
Opisthokonta - ps9
Holozoa - ps10
Filozoa - ps11
Choano./Metazoa - ps12
Metazoa - ps13
Myriazoa - ps14
Parahoxozoa - ps15
Bilateria - ps16
Deuterostomia - ps17
Chordata - ps18
Olfactores - ps19
Vertebrata - ps20
Gnathostomata - ps21
Euteleostomi - ps22
Sarcopterygii - ps23
Tetrapoda - ps24
Amniota - ps25
Mammalia - ps26
Theria - ps27
Placentalia - ps28
Boreoeutheria - ps29
Euarchontoglires - ps30
Euarchonta - ps31
Primates - ps32
Haplorrhini - ps33
Simiiformes - ps34
Catarrhini - ps35
Hominoidea - ps36
Hominidae - ps37
Homininae - ps38
Hominini - ps39
Homo_sapiens - ps40
p-value
2e-02
2e-02
3e-02
4e-02
5e-02
Log-odds
0.5
1.0
1.5
2.0
2.5
3.0
3.5
4.0

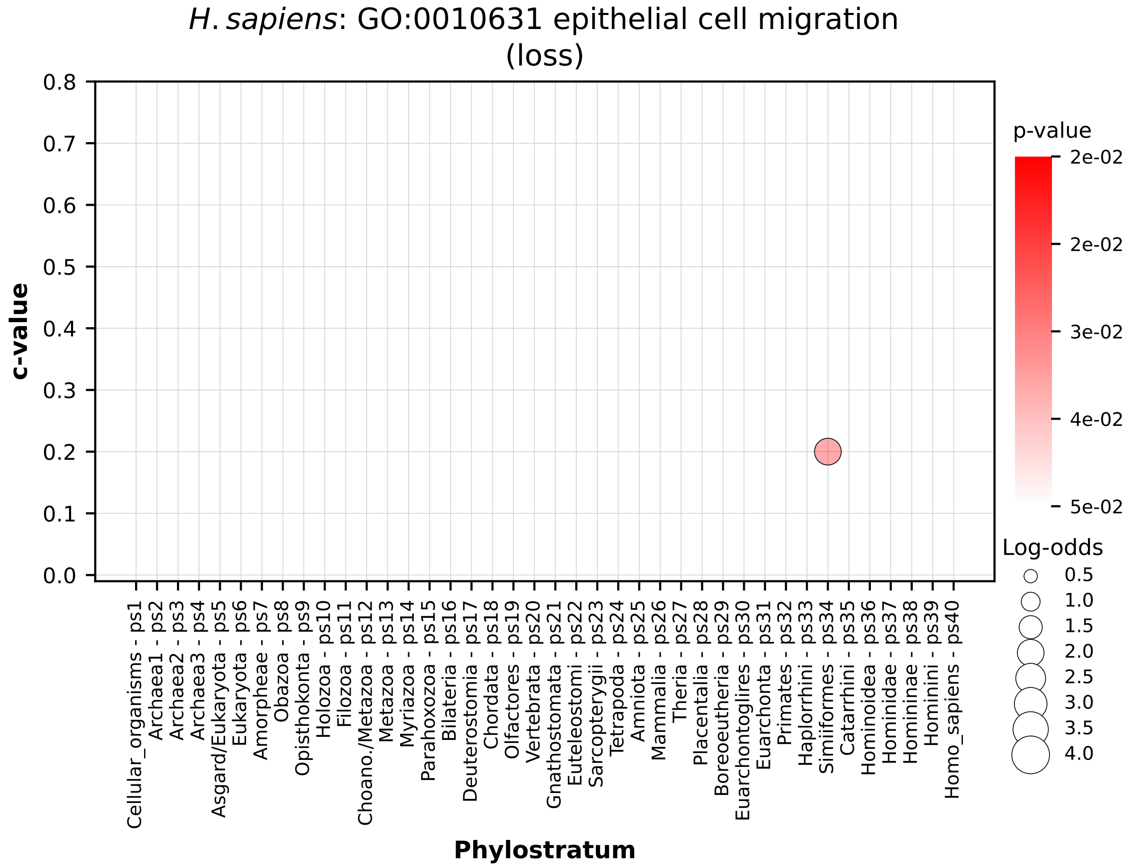

H. sapiens: GO:0010631 epithelial cell migration
(loss)
c-value
0.0
0.1
0.2
0.3
0.4
0.5
0.6
0.7
0.8
Phylostratum
Cellular_organisms - ps1
Archaea1 - ps2
Archaea2 - ps3
Archaea3 - ps4
Asgard/Eukaryota - ps5
Eukaryota - ps6
Amorpheae - ps7
Obazoa - ps8
Opisthokonta - ps9
Holozoa - ps10
Filozoa - ps11
Choano./Metazoa - ps12
Metazoa - ps13
Myriazoa - ps14
Parahoxozoa - ps15
Bilateria - ps16
Deuterostomia - ps17
Chordata - ps18
Olfactores - ps19
Vertebrata - ps20
Gnathostomata - ps21
Euteleostomi - ps22
Sarcopterygii - ps23
Tetrapoda - ps24
Amniota - ps25
Mammalia - ps26
Theria - ps27
Placentalia - ps28
Boreoeutheria - ps29
Euarchontoglires - ps30
Euarchonta - ps31
Primates - ps32
Haplorrhini - ps33
Simiiformes - ps34
Catarrhini - ps35
Hominoidea - ps36
Hominidae - ps37
Homininae - ps38
Hominini - ps39
Homo_sapiens - ps40
p-value
2e-02
2e-02
3e-02
4e-02
5e-02
Log-odds
0.5
1.0
1.5
2.0
2.5
3.0
3.5
4.0

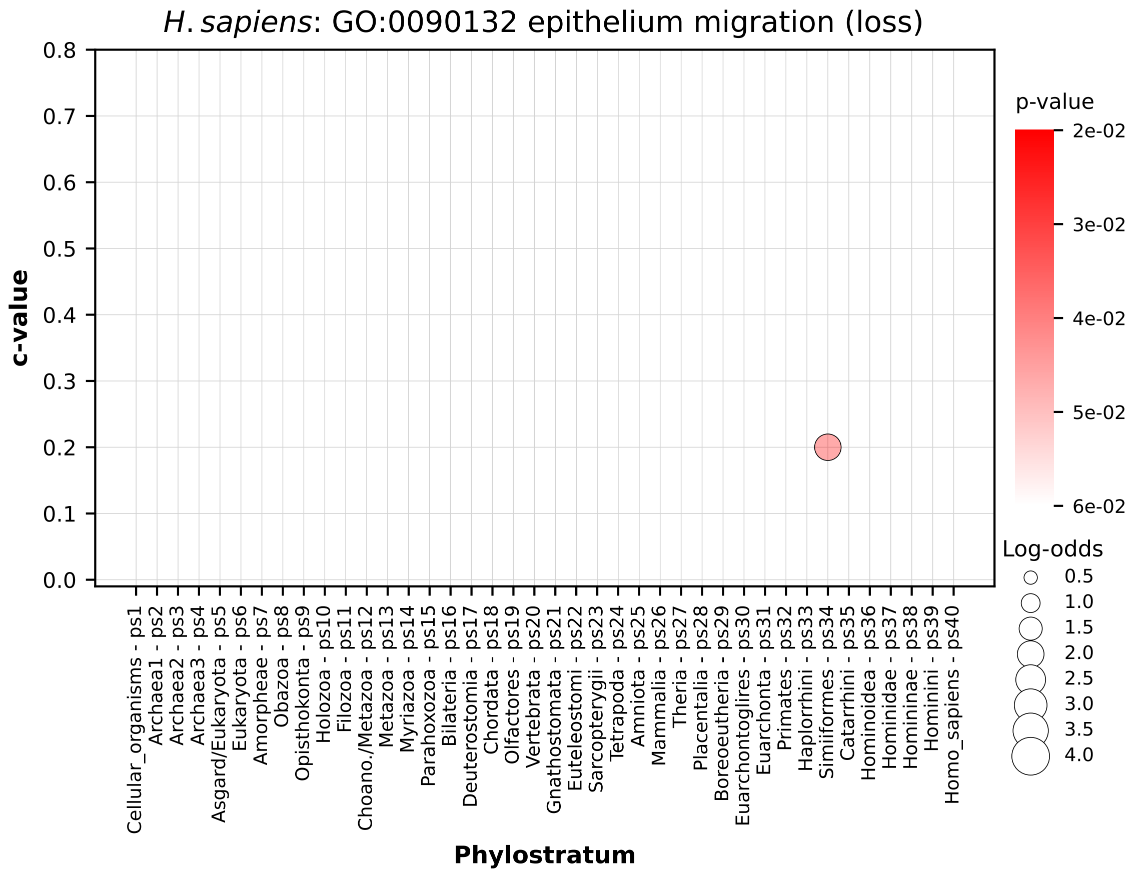

H. sapiens: GO:0090132 epithelium migration (loss)
c-value
0.8
0.7
0.6
0.5
0.4
0.3
0.2
0.1
0.0
p-value
2e-02
3e-02
4e-02
5e-02
6e-02
Log-odds
0.5
1.0
1.5
2.0
2.5
3.0
3.5
4.0
Cellular_organisms - ps1
Archaea1 - ps2
Archaea2 - ps3
Archaea3 - ps4
Asgard/Eukaryota - ps5
Eukaryota - ps6
Amorpheae - ps7
Obazoa - ps8
Opisthokonta - ps9
Holozoa - ps10
Filozoa - ps11
Choano./Metazoa - ps12
Metazoa - ps13
Myriazoa - ps14
Parahoxozoa - ps15
Bilateria - ps16
Deuterostomia - ps17
Chordata - ps18
Olfactores - ps19
Vertebrata - ps20
Gnathostomata - ps21
Euteleostomi - ps22
Sarcopterygii - ps23
Tetrapoda - ps24
Amniota - ps25
Mammalia - ps26
Theria - ps27
Placentalia - ps28
Boreoeutheria - ps29
Euarchontoglires - ps30
Euarchonta - ps31
Primates - ps32
Haplorrhini - ps33
Simiiformes - ps34
Catarrhini - ps35
Hominoidea - ps36
Hominidae - ps37
Homininae - ps38
Hominini - ps39
Homo_sapiens - ps40
Phylostratum

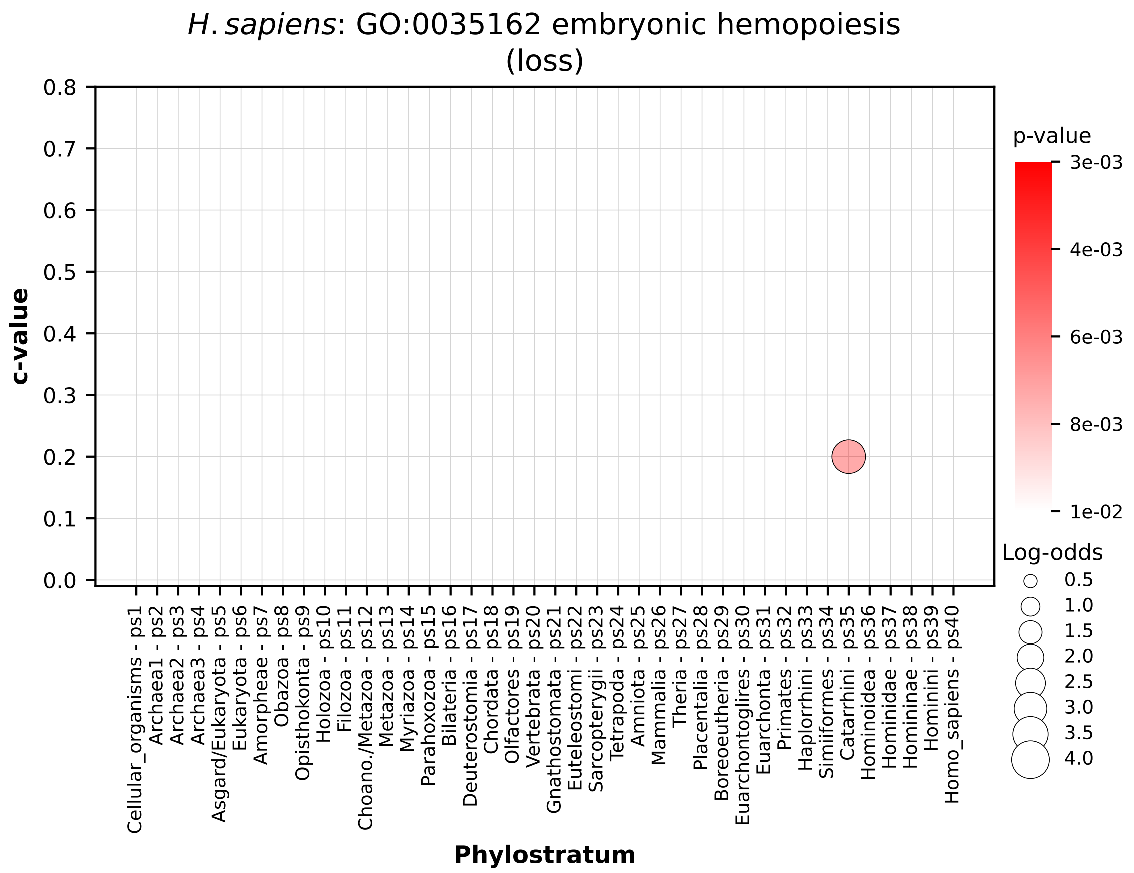

H. sapiens: GO:0035162 embryonic hemopoiesis
(loss)
c-value
0.0
0.1
0.2
0.3
0.4
0.5
0.6
0.7
0.8
Phylostratum
Cellular_organisms - ps1
Archaea1 - ps2
Archaea2 - ps3
Archaea3 - ps4
Asgard/Eukaryota - ps5
Eukaryota - ps6
Amorpheae - ps7
Obazoa - ps8
Opisthokonta - ps9
Holozoa - ps10
Filozoa - ps11
Choano./Metazoa - ps12
Metazoa - ps13
Myriazoa - ps14
Parahoxozoa - ps15
Bilateria - ps16
Deuterostomia - ps17
Chordata - ps18
Olfactores - ps19
Vertebrata - ps20
Gnathostomata - ps21
Euteleostomi - ps22
Sarcopterygii - ps23
Tetrapoda - ps24
Amniota - ps25
Mammalia - ps26
Theria - ps27
Placentalia - ps28
Boreoeutheria - ps29
Euarchontoglires - ps30
Euarchonta - ps31
Primates - ps32
Haplorrhini - ps33
Simiiformes - ps34
Catarrhini - ps35
Hominoidea - ps36
Hominidae - ps37
Homininae - ps38
Hominini - ps39
Homo_sapiens - ps40
p-value
3e-03
4e-03
6e-03
8e-03
1e-02
Log-odds
0.5
1.0
1.5
2.0
2.5
3.0
3.5
4.0

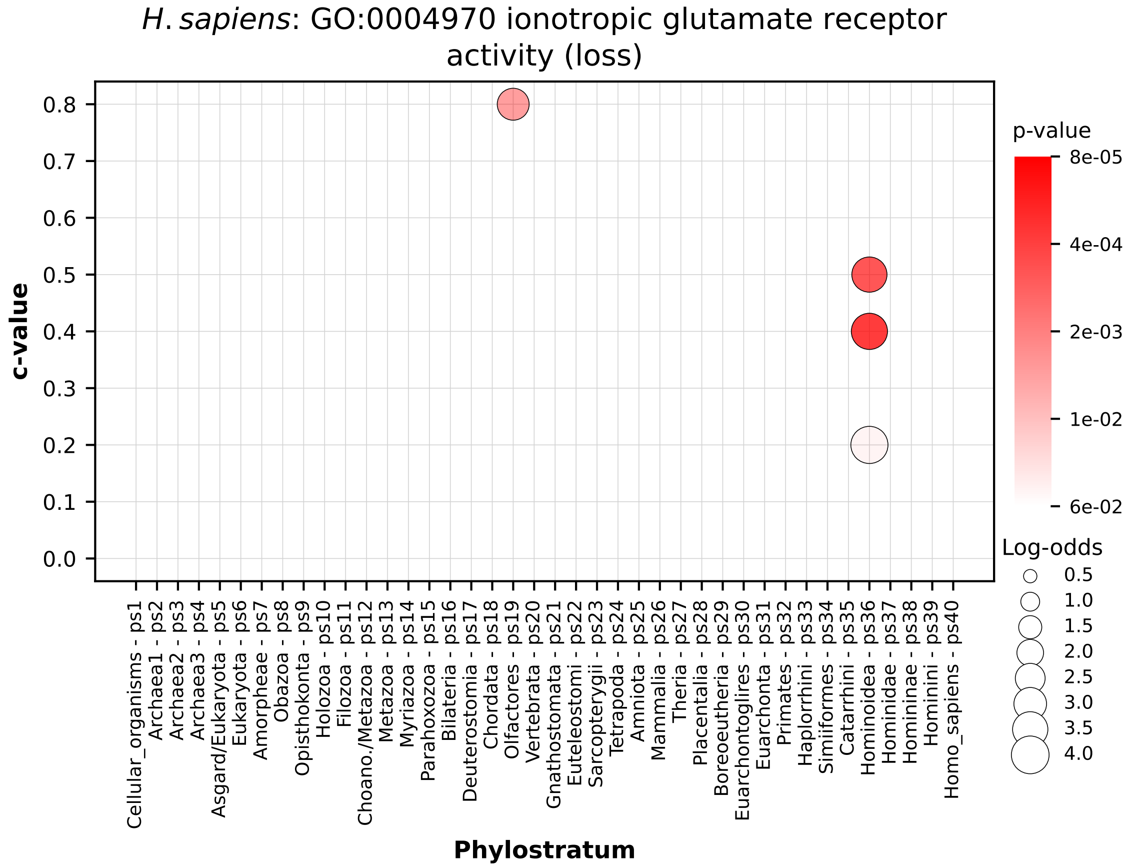

H. sapiens: GO:0004970 ionotropic glutamate receptor activity (loss)
c-value
0.8
0.7
0.6
0.5
0.4
0.3
0.2
0.1
0.0
Phylostratum
Cellular_organisms - ps1
Archaea1 - ps2
Archaea2 - ps3
Archaea3 - ps4
Asgard/Eukaryota - ps5
Eukaryota - ps6
Amorpheae - ps7
Obazoa - ps8
Opisthokonta - ps9
Holozoa - ps10
Filozoa - ps11
Choano./Metazoa - ps12
Metazoa - ps13
Myriazoa - ps14
Parahoxozoa - ps15
Bilateria - ps16
Deuterostomia - ps17
Chordata - ps18
Olfactores - ps19
Vertebrata - ps20
Gnathostomata - ps21
Euteleostomi - ps22
Sarcopterygii - ps23
Tetrapoda - ps24
Amniota - ps25
Mammalia - ps26
Theria - ps27
Placentalia - ps28
Boreoeutheria - ps29
Euarchontoglires - ps30
Euarchonta - ps31
Primates - ps32
Haplorrhini - ps33
Simiiformes - ps34
Catarrhini - ps35
Hominoidea - ps36
Hominidae - ps37
Homininae - ps38
Hominini - ps39
Homo_sapiens - ps40
p-value
8e-05
4e-04
2e-03
1e-02
6e-02
Log-odds
0.5
1.0
1.5
2.0
2.5
3.0
3.5
4.0

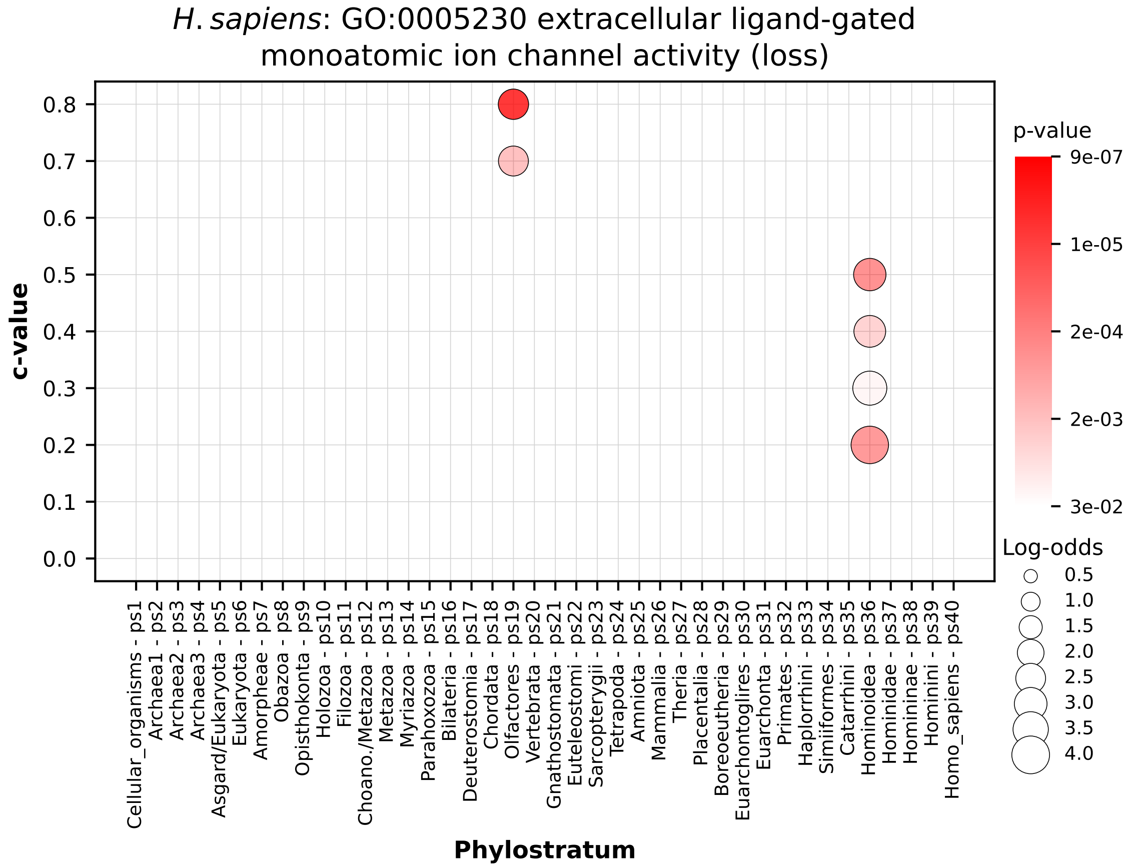

H. sapiens: GO:0005230 extracellular ligand-gated monoatomic ion channel activity (loss)
c-value
0.0
0.1
0.2
0.3
0.4
0.5
0.6
0.7
0.8
Phylostratum
Cellular_organisms - ps1
Archaea1 - ps2
Archaea2 - ps3
Archaea3 - ps4
Asgard/Eukaryota - ps5
Eukaryota - ps6
Amorpheae - ps7
Obazoa - ps8
Opisthokonta - ps9
Holozoa - ps10
Filozoa - ps11
Choano./Metazoa - ps12
Metazoa - ps13
Myriazoa - ps14
Parahoxozoa - ps15
Bilateria - ps16
Deuterostomia - ps17
Chordata - ps18
Olfactores - ps19
Vertebrata - ps20
Gnathostomata - ps21
Euteleostomi - ps22
Sarcopterygii - ps23
Tetrapoda - ps24
Amniota - ps25
Mammalia - ps26
Theria - ps27
Placentalia - ps28
Boreoeutheria - ps29
Euarchontoglires - ps30
Euarchonta - ps31
Primates - ps32
Haplorrhini - ps33
Simiiformes - ps34
Catarrhini - ps35
Hominoidea - ps36
Hominidae - ps37
Homininae - ps38
Hominini - ps39
Homo_sapiens - ps40
p-value
9e-07
1e-05
2e-04
2e-03
3e-02
Log-odds
0.5
1.0
1.5
2.0
2.5
3.0
3.5
4.0

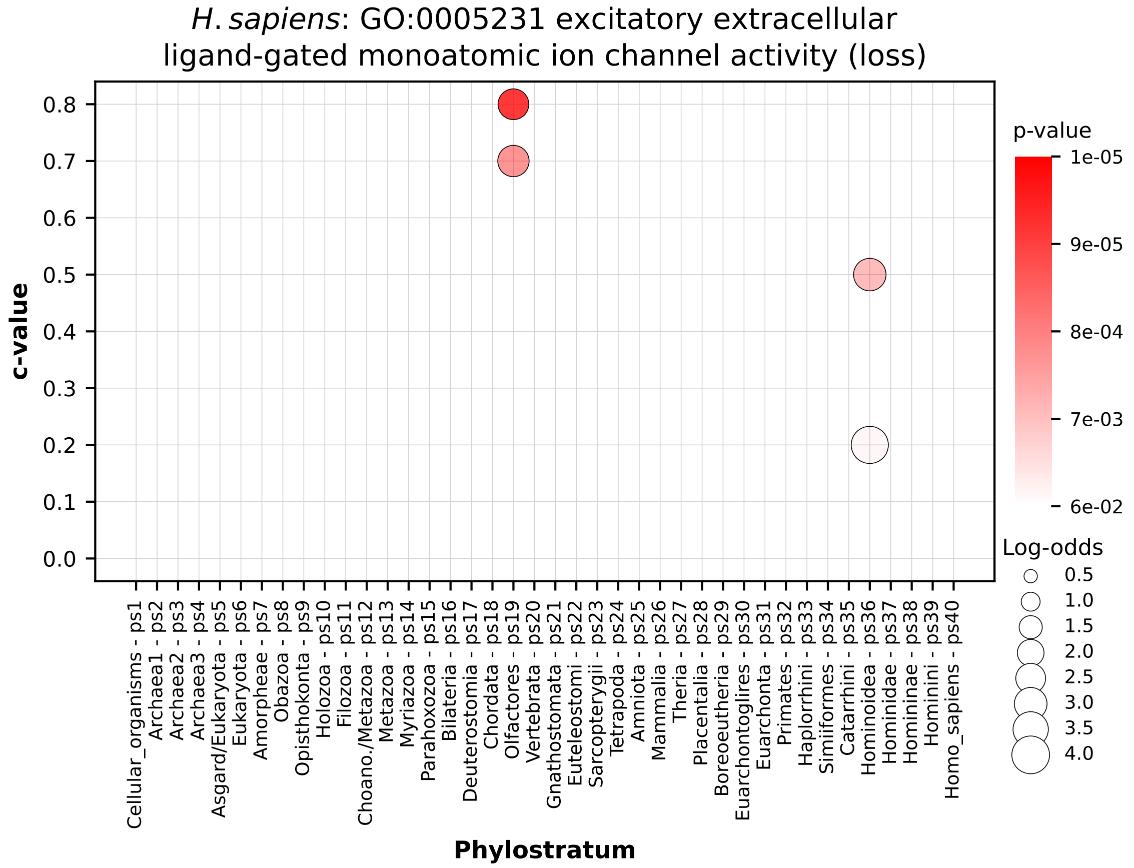

H. sapiens: GO:0005231 excitatory extracellular ligand-gated monoatomic ion channel activity (loss)
c-value
0.0
0.1
0.2
0.3
0.4
0.5
0.6
0.7
0.8
Phylostratum
Cellular_organisms - ps1
Archaea1 - ps2
Archaea2 - ps3
Archaea3 - ps4
Asgard/Eukaryota - ps5
Eukaryota - ps6
Amorpheae - ps7
Obazoa - ps8
Opisthokonta - ps9
Holozoa - ps10
Filozoa - ps11
Choano./Metazoa - ps12
Metazoa - ps13
Myriazoa - ps14
Parahoxozoa - ps15
Bilateria - ps16
Deuterostomia - ps17
Chordata - ps18
Olfactores - ps19
Vertebrata - ps20
Gnathostomata - ps21
Euteleostomi - ps22
Sarcopterygii - ps23
Tetrapoda - ps24
Amniota - ps25
Mammalia - ps26
Theria - ps27
Placentalia - ps28
Boreoeutheria - ps29
Euarchontoglires - ps30
Euarchonta - ps31
Primates - ps32
Haplorrhini - ps33
Simiiformes - ps34
Catarrhini - ps35
Hominoidea - ps36
Hominidae - ps37
Homininae - ps38
Hominini - ps39
Homo_sapiens - ps40
p-value
1e-05
9e-05
8e-04
7e-03
6e-02
Log-odds
0.5
1.0
1.5
2.0
2.5
3.0
3.5
4.0

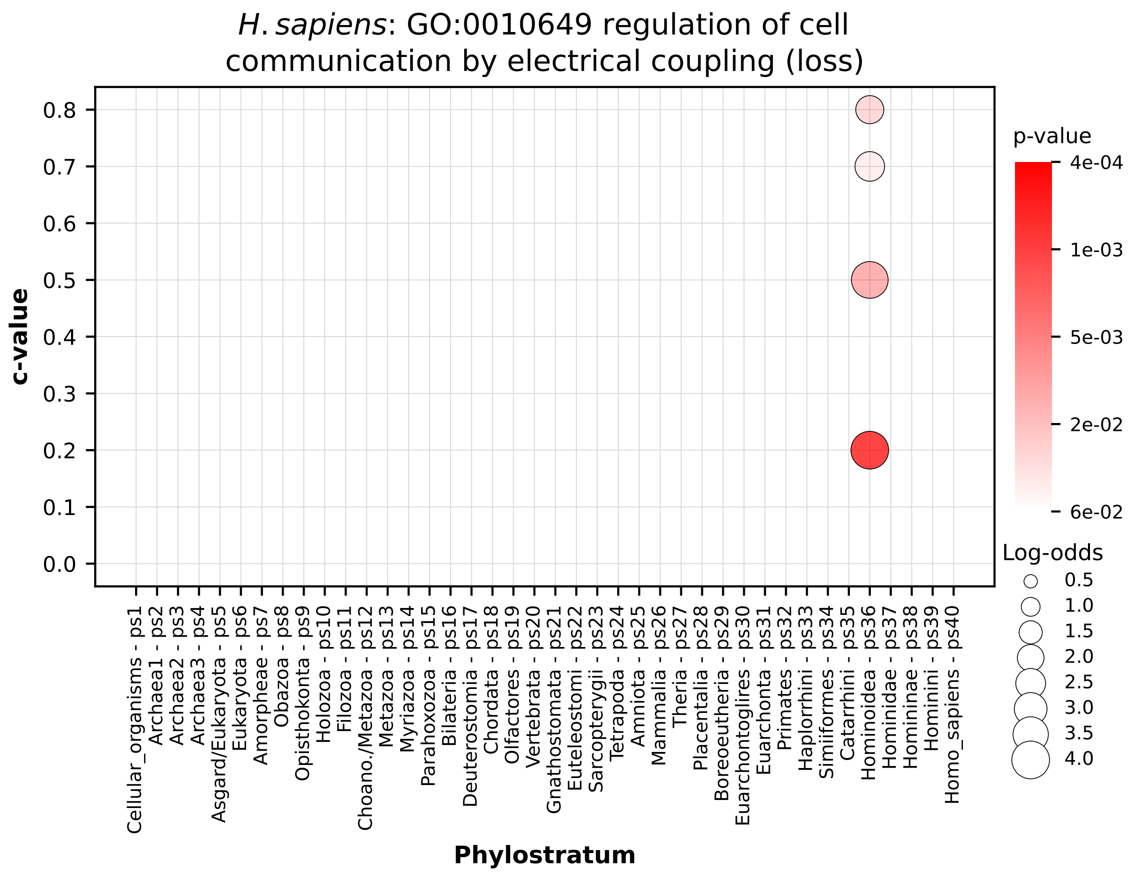

H. sapiens: GO:0010649 regulation of cell communication by electrical coupling (loss)
c-value
0.0
0.1
0.2
0.3
0.4
0.5
0.6
0.7
0.8
p-value
4e-04
1e-03
5e-03
2e-02
6e-02
Log-odds
0.5
1.0
1.5
2.0
2.5
3.0
3.5
4.0
Cellular_organisms - ps1
Archaea1 - ps2
Archaea2 - ps3
Archaea3 - ps4
Asgard/Eukaryota - ps5
Eukaryota - ps6
Amorpheae - ps7
Obazoa - ps8
Opisthokonta - ps9
Holozoa - ps10
Filozoa - ps11
Choano./Metazoa - ps12
Metazoa - ps13
Myriazoa - ps14
Parahoxozoa - ps15
Bilateria - ps16
Deuterostomia - ps17
Chordata - ps18
Olfactores - ps19
Vertebrata - ps20
Gnathostomata - ps21
Euteleostomi - ps22
Sarcopterygii - ps23
Tetrapoda - ps24
Amniota - ps25
Mammalia - ps26
Theria - ps27
Placentalia - ps28
Boreoeutheria - ps29
Euarchontoglires - ps30
Euarchonta - ps31
Primates - ps32
Haplorrhini - ps33
Simiiformes - ps34
Catarrhini - ps35
Hominoidea - ps36
Hominidae - ps37
Homininae - ps38
Hominini - ps39
Homo_sapiens - ps40
Phylostratum

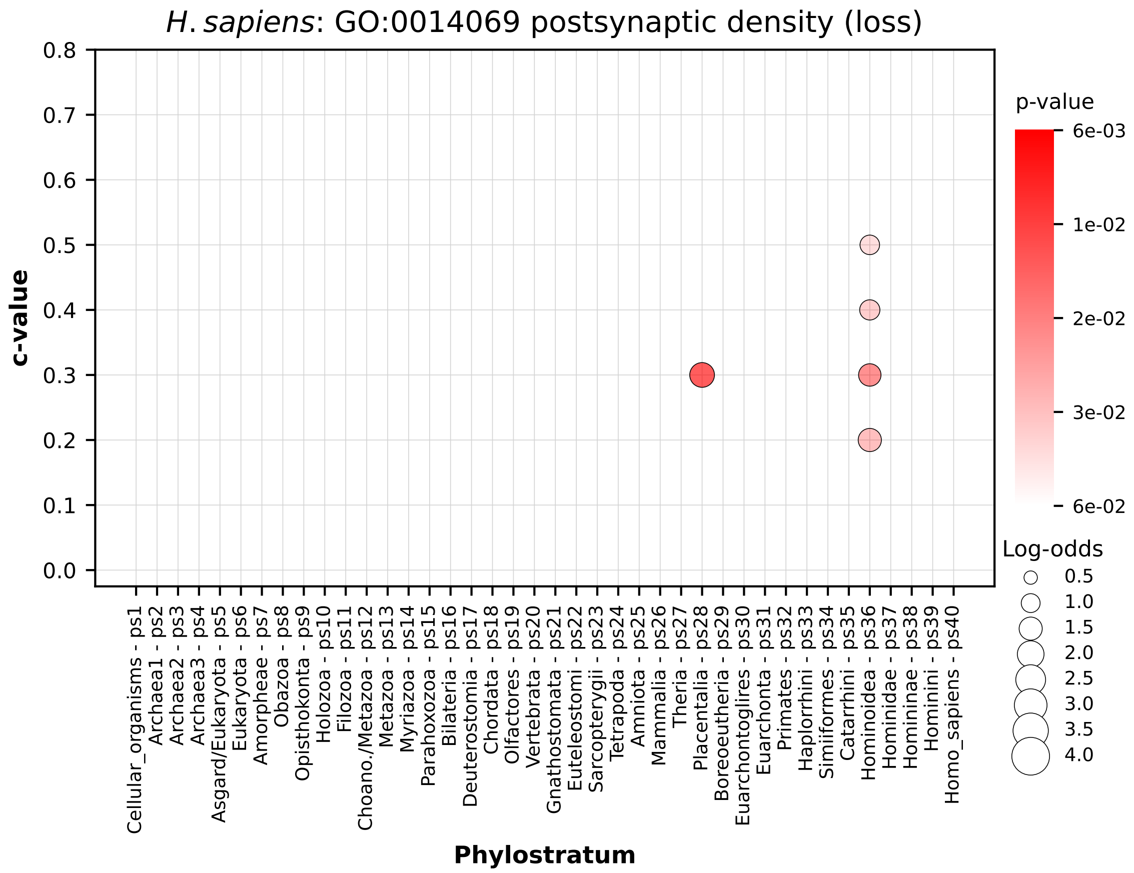

H. sapiens: GO:0014069 postsynaptic density (loss)
c-value
0.0
0.1
0.2
0.3
0.4
0.5
0.6
0.7
0.8
Phylostratum
Cellular_organisms - ps1
Archaea1 - ps2
Archaea2 - ps3
Archaea3 - ps4
Asgard/Eukaryota - ps5
Eukaryota - ps6
Amorpheae - ps7
Obazoa - ps8
Opisthokonta - ps9
Holozoa - ps10
Filozoa - ps11
Choano./Metazoa - ps12
Metazoa - ps13
Myriazoa - ps14
Parahoxozoa - ps15
Bilateria - ps16
Deuterostomia - ps17
Chordata - ps18
Olfactores - ps19
Vertebrata - ps20
Gnathostomata - ps21
Euteleostomi - ps22
Sarcopterygii - ps23
Tetrapoda - ps24
Amniota - ps25
Mammalia - ps26
Theria - ps27
Placentalia - ps28
Boreoeutheria - ps29
Euarchontoglires - ps30
Euarchonta - ps31
Primates - ps32
Haplorrhini - ps33
Simiiformes - ps34
Catarrhini - ps35
Hominoidea - ps36
Hominidae - ps37
Homininae - ps38
Hominini - ps39
Homo_sapiens - ps40
p-value
6e-03
1e-02
2e-02
3e-02
6e-02
Log-odds
0.5
1.0
1.5
2.0
2.5
3.0
3.5
4.0

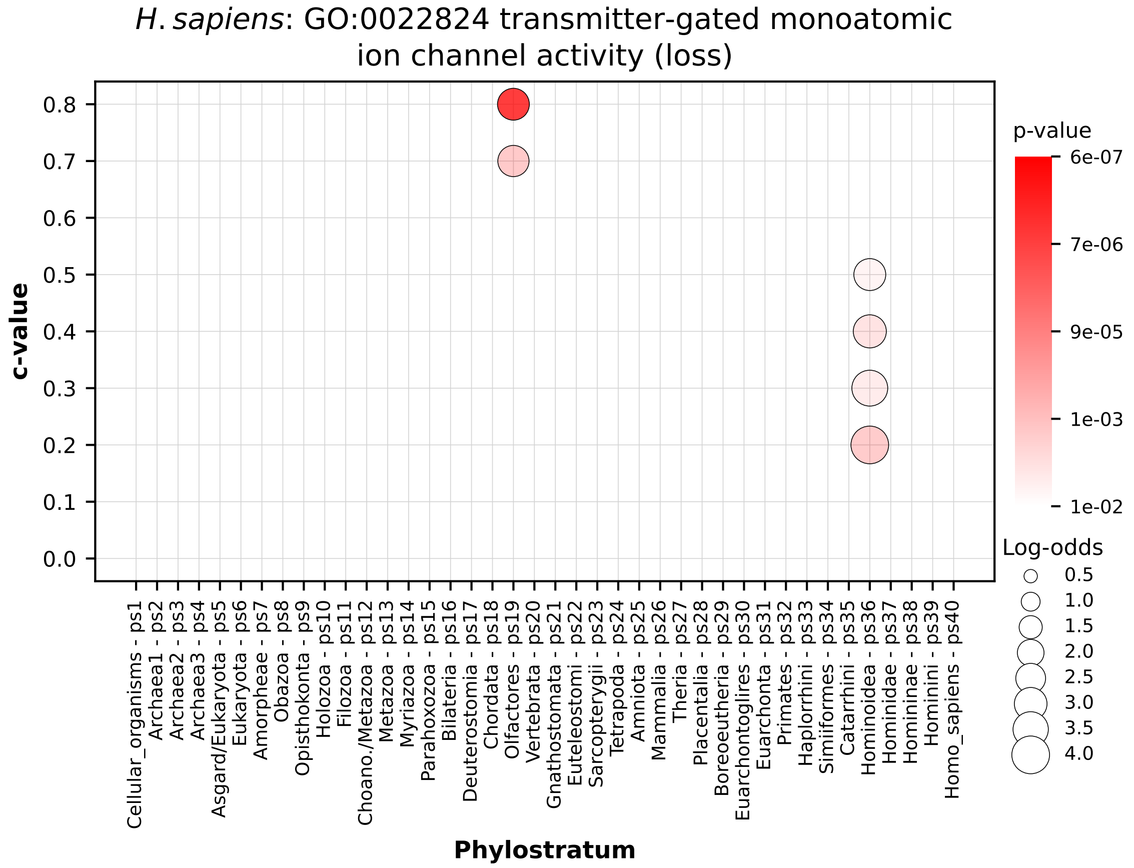
H. sapiens: GO:0022824 transmitter-gated monoatomic ion channel activity (loss)
c-value
0.8
0.7
0.6
0.5
0.4
0.3
0.2
0.1
0.0
Phylostratum
Cellular_organisms - ps1
Archaea1 - ps2
Archaea2 - ps3
Archaea3 - ps4
Asgard/Eukaryota - ps5
Eukaryota - ps6
Amorpheae - ps7
Obazoa - ps8
Opisthokonta - ps9
Holozoa - ps10
Filozoa - ps11
Choano./Metazoa - ps12
Metazoa - ps13
Myriazoa - ps14
Parahoxozoa - ps15
Bilateria - ps16
Deuterostomia - ps17
Chordata - ps18
Olfactores - ps19
Vertebrata - ps20
Gnathostomata - ps21
Euteleostomi - ps22
Sarcopterygii - ps23
Tetrapoda - ps24
Amniota - ps25
Mammalia - ps26
Theria - ps27
Placentalia - ps28
Boreoeutheria - ps29
Euarchontoglires - ps30
Euarchonta - ps31
Primates - ps32
Haplorrhini - ps33
Simiiformes - ps34
Catarrhini - ps35
Hominoidea - ps36
Hominidae - ps37
Homininae - ps38
Hominini - ps39
Homo_sapiens - ps40
p-value
6e-07
7e-06
9e-05
1e-03
1e-02
Log-odds
0.5
1.0
1.5
2.0
2.5
3.0
3.5
4.0

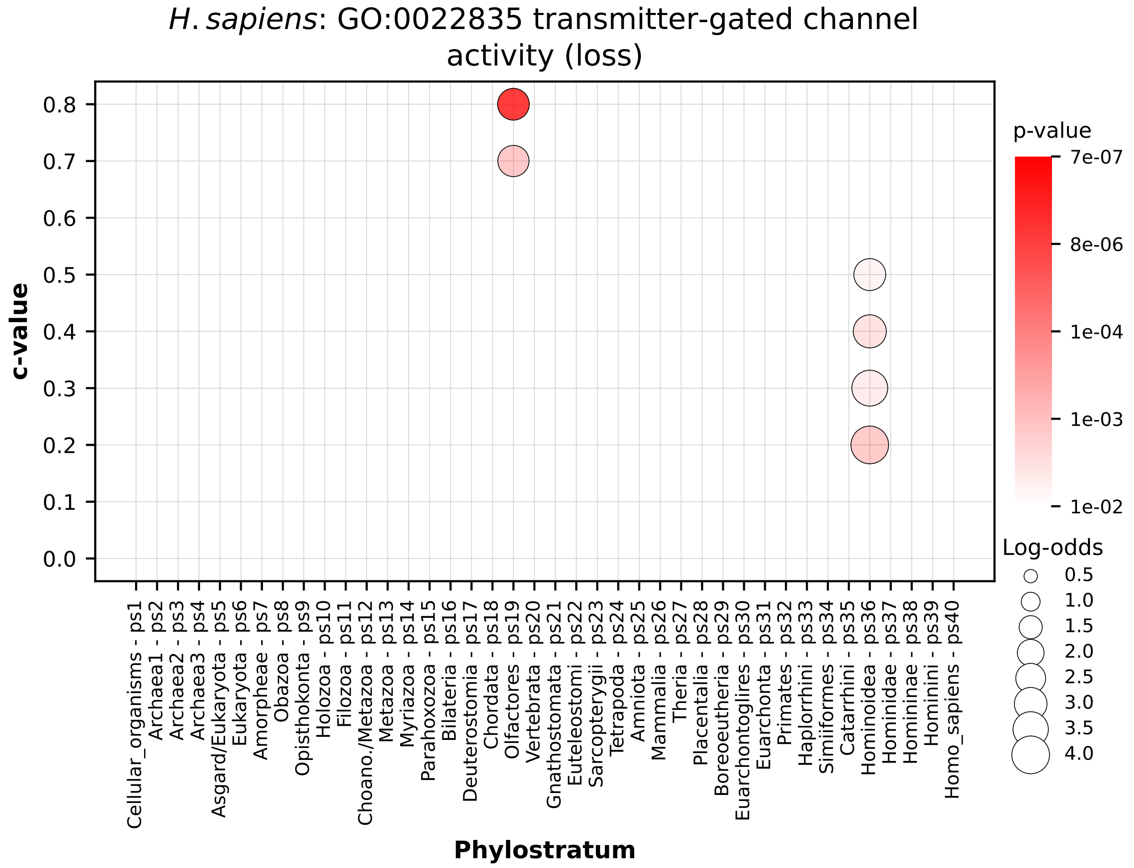

H. sapiens: GO:0022835 transmitter-gated channel activity (loss)
c-value
0.8
0.7
0.6
0.5
0.4
0.3
0.2
0.1
0.0
p-value
7e-07
8e-06
1e-04
1e-03
1e-02
Log-odds
0.5
1.0
1.5
2.0
2.5
3.0
3.5
4.0
Cellular_organisms - ps1
Archaea1 - ps2
Archaea2 - ps3
Archaea3 - ps4
Asgard/Eukaryota - ps5
Eukaryota - ps6
Amorpheae - ps7
Obazoa - ps8
Opisthokonta - ps9
Holozoa - ps10
Filozoa - ps11
Choano./Metazoa - ps12
Metazoa - ps13
Myriazoa - ps14
Parahoxozoa - ps15
Bilateria - ps16
Deuterostomia - ps17
Chordata - ps18
Olfactores - ps19
Vertebrata - ps20
Gnathostomata - ps21
Euteleostomi - ps22
Sarcopterygii - ps23
Tetrapoda - ps24
Amniota - ps25
Mammalia - ps26
Theria - ps27
Placentalia - ps28
Boreoeutheria - ps29
Euarchontoglires - ps30
Euarchonta - ps31
Primates - ps32
Haplorrhini - ps33
Simiiformes - ps34
Catarrhini - ps35
Hominoidea - ps36
Hominidae - ps37
Homininae - ps38
Hominini - ps39
Homo_sapiens - ps40
Phylostratum

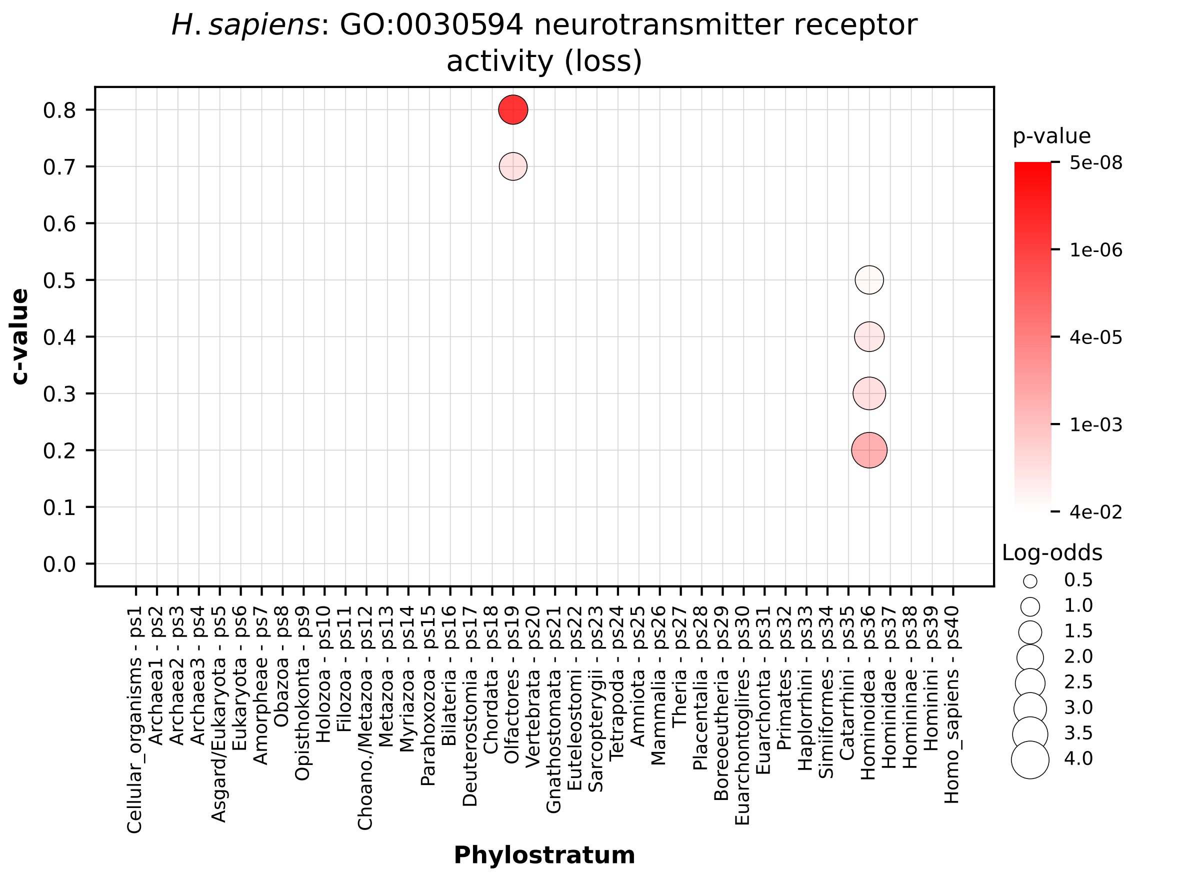

H. sapiens: GO:0030594 neurotransmitter receptor activity (loss)
c-value
0.0
0.1
0.2
0.3
0.4
0.5
0.6
0.7
0.8
p-value
5e-08
1e-06
4e-05
1e-03
4e-02
Log-odds
0.5
1.0
1.5
2.0
2.5
3.0
3.5
4.0
Cellular_organisms - ps1
Archaea1 - ps2
Archaea2 - ps3
Archaea3 - ps4
Asgard/Eukaryota - ps5
Eukaryota - ps6
Amorpheae - ps7
Obazoa - ps8
Opisthokonta - ps9
Holozoa - ps10
Filozoa - ps11
Choano./Metazoa - ps12
Metazoa - ps13
Myriazoa - ps14
Parahoxozoa - ps15
Bilateria - ps16
Deuterostomia - ps17
Chordata - ps18
Olfactores - ps19
Vertebrata - ps20
Gnathostomata - ps21
Euteleostomi - ps22
Sarcopterygii - ps23
Tetrapoda - ps24
Amniota - ps25
Mammalia - ps26
Theria - ps27
Placentalia - ps28
Boreoeutheria - ps29
Euarchontoglires - ps30
Euarchonta - ps31
Primates - ps32
Haplorrhini - ps33
Simiiformes - ps34
Catarrhini - ps35
Hominoidea - ps36
Hominidae - ps37
Homininae - ps38
Hominini - ps39
Homo_sapiens - ps40
Phylostratum

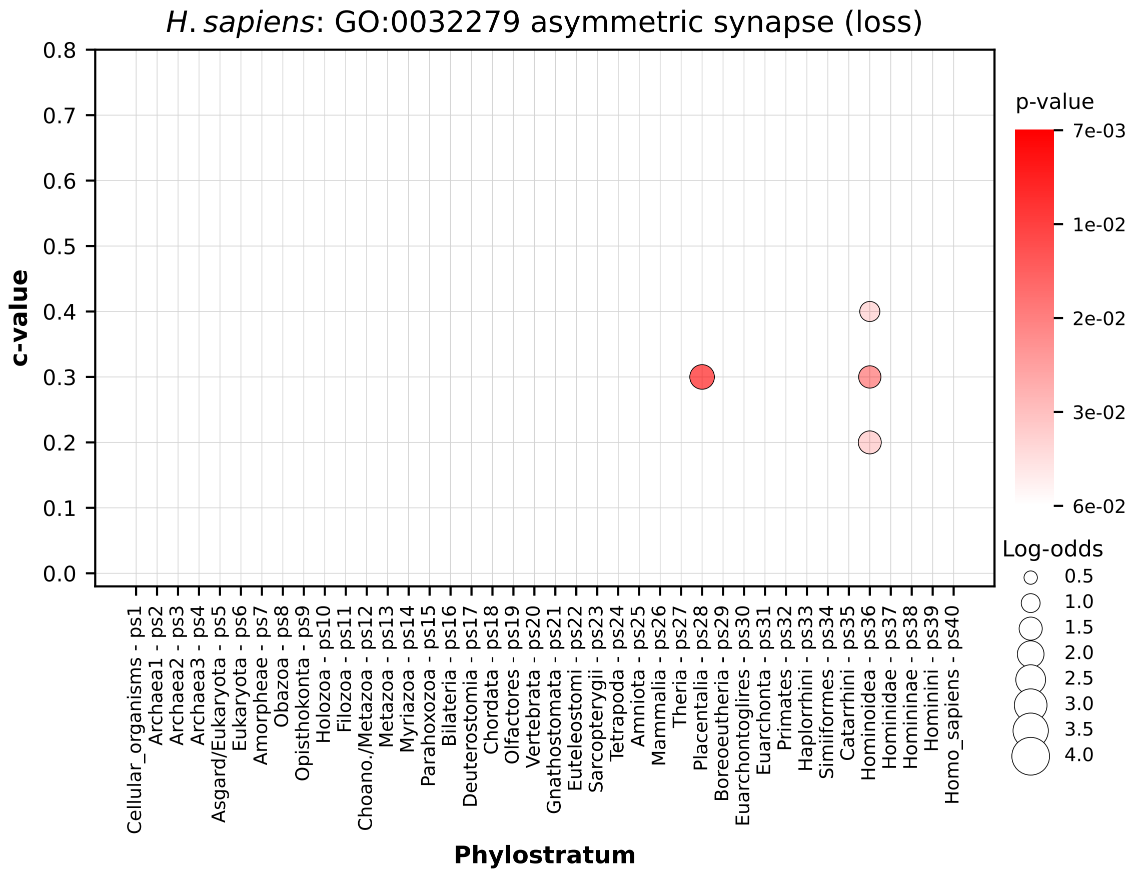

H. sapiens: GO:0032279 asymmetric synapse (loss)
c-value
0.8
0.7
0.6
0.5
0.4
0.3
0.2
0.1
0.0
p-value
7e-03
1e-02
2e-02
3e-02
6e-02
Log-odds
0.5
1.0
1.5
2.0
2.5
3.0
3.5
4.0
Cellular_organisms - ps1
Archaea1 - ps2
Archaea2 - ps3
Archaea3 - ps4
Asgard/Eukaryota - ps5
Eukaryota - ps6
Amorpheae - ps7
Obazoa - ps8
Opisthokonta - ps9
Holozoa - ps10
Filozoa - ps11
Choano./Metazoa - ps12
Metazoa - ps13
Myriazoa - ps14
Parahoxozoa - ps15
Bilateria - ps16
Deuterostomia - ps17
Chordata - ps18
Olfactores - ps19
Vertebrata - ps20
Gnathostomata - ps21
Euteleostomi - ps22
Sarcopterygii - ps23
Tetrapoda - ps24
Amniota - ps25
Mammalia - ps26
Theria - ps27
Placentalia - ps28
Boreoeutheria - ps29
Euarchontoglires - ps30
Euarchonta - ps31
Primates - ps32
Haplorrhini - ps33
Simiiformes - ps34
Catarrhini - ps35
Hominoidea - ps36
Hominidae - ps37
Homininae - ps38
Hominini - ps39
Homo_sapiens - ps40
Phylostratum

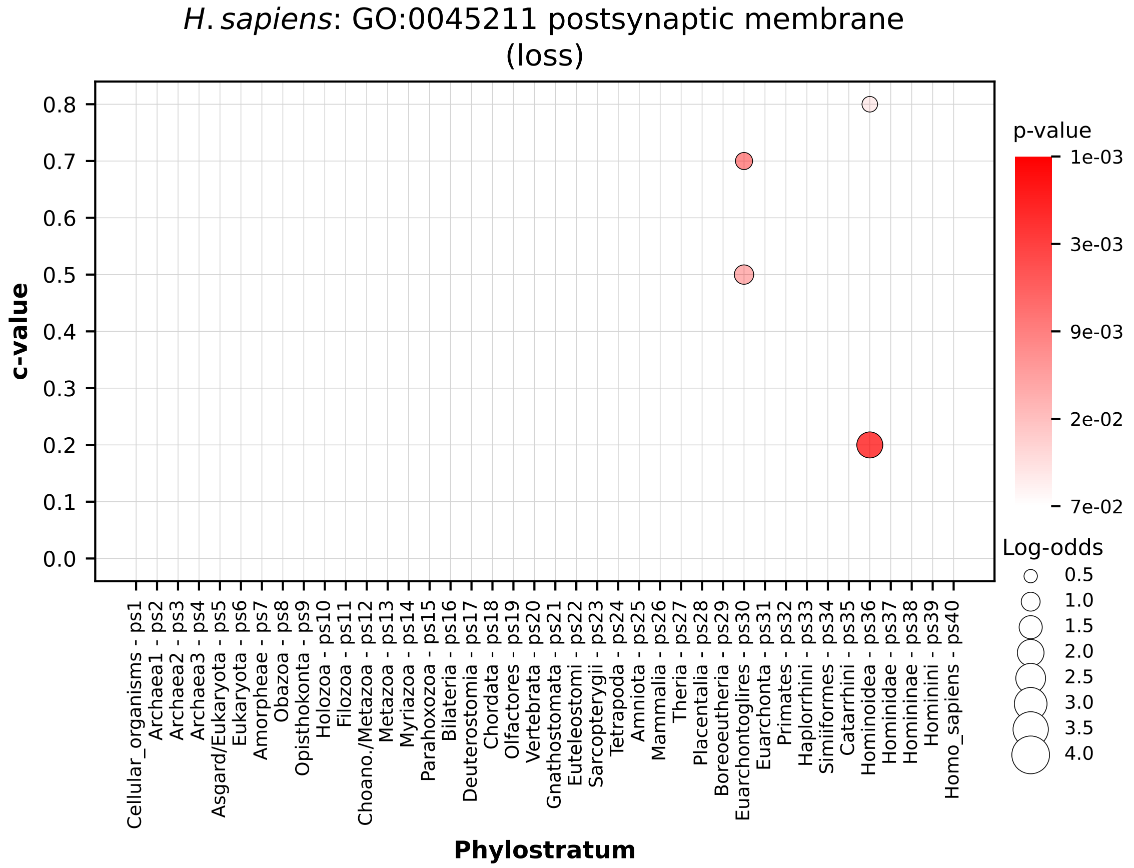
H. sapiens: GO:0045211 postsynaptic membrane
(loss)
c-value
0.0
0.1
0.2
0.3
0.4
0.5
0.6
0.7
0.8
Phylostratum
Cellular_organisms - ps1
Archaea1 - ps2
Archaea2 - ps3
Archaea3 - ps4
Asgard/Eukaryota - ps5
Eukaryota - ps6
Amorpheae - ps7
Obazoa - ps8
Opisthokonta - ps9
Holozoa - ps10
Filozoa - ps11
Choano./Metazoa - ps12
Metazoa - ps13
Myriazoa - ps14
Parahoxozoa - ps15
Bilateria - ps16
Deuterostomia - ps17
Chordata - ps18
Olfactores - ps19
Vertebrata - ps20
Gnathostomata - ps21
Euteleostomi - ps22
Sarcopterygii - ps23
Tetrapoda - ps24
Amniota - ps25
Mammalia - ps26
Theria - ps27
Placentalia - ps28
Boreoeutheria - ps29
Euarchontoglires - ps30
Euarchonta - ps31
Primates - ps32
Haplorrhini - ps33
Simiiformes - ps34
Catarrhini - ps35
Hominoidea - ps36
Hominidae - ps37
Homininae - ps38
Hominini - ps39
Homo_sapiens - ps40
p-value
1e-03
3e-03
9e-03
2e-02
7e-02
Log-odds
0.5
1.0
1.5
2.0
2.5
3.0
3.5
4.0

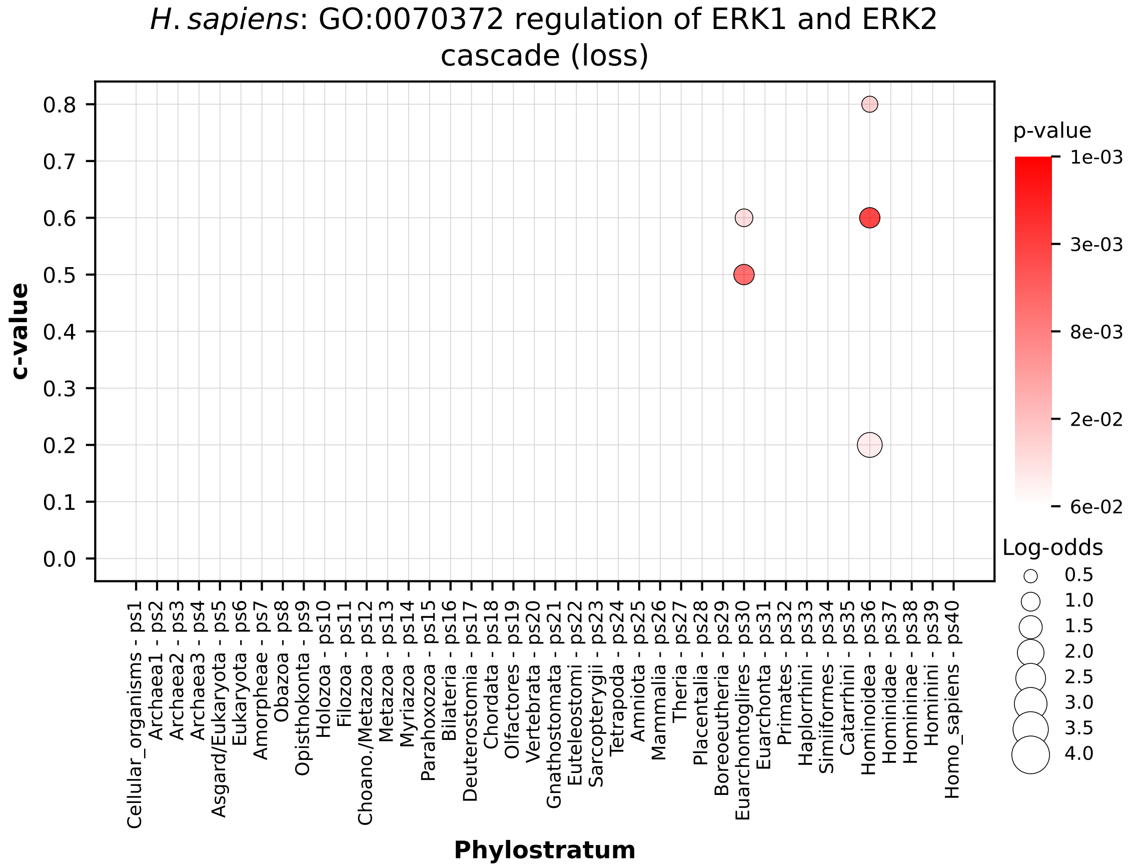

H. sapiens: GO:0070372 regulation of ERK1 and ERK2 cascade (loss)
c-value
0.8
0.7
0.6
0.5
0.4
0.3
0.2
0.1
0.0
Phylostratum
Cellular_organisms - ps1
Archaea1 - ps2
Archaea2 - ps3
Archaea3 - ps4
Asgard/Eukaryota - ps5
Eukaryota - ps6
Amorpheae - ps7
Obazoa - ps8
Opisthokonta - ps9
Holozoa - ps10
Filozoa - ps11
Choano./Metazoa - ps12
Metazoa - ps13
Myriazoa - ps14
Parahoxozoa - ps15
Bilateria - ps16
Deuterostomia - ps17
Chordata - ps18
Olfactores - ps19
Vertebrata - ps20
Gnathostomata - ps21
Euteleostomi - ps22
Sarcopterygii - ps23
Tetrapoda - ps24
Amniota - ps25
Mammalia - ps26
Theria - ps27
Placentalia - ps28
Boreoeutheria - ps29
Euarchontoglires - ps30
Euarchonta - ps31
Primates - ps32
Haplorrhini - ps33
Simiiformes - ps34
Catarrhini - ps35
Hominoidea - ps36
Hominidae - ps37
Homininae - ps38
Hominini - ps39
Homo_sapiens - ps40
p-value
1e-03
3e-03
8e-03
2e-02
6e-02
Log-odds
0.5
1.0
1.5
2.0
2.5
3.0
3.5
4.0

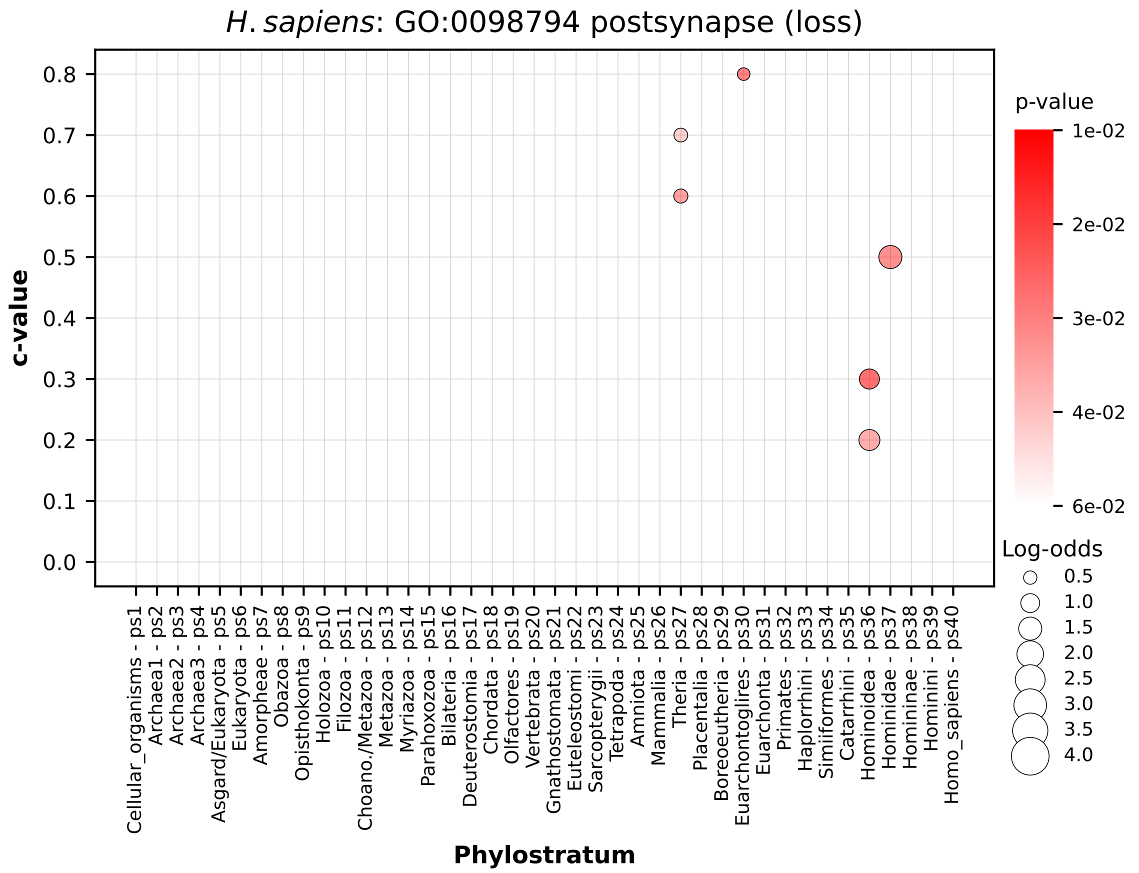

H. sapiens: GO:0098794 postsynapse (loss)
c-value
0.0
0.1
0.2
0.3
0.4
0.5
0.6
0.7
0.8
Phylostratum
Cellular_organisms - ps1
Archaea1 - ps2
Archaea2 - ps3
Archaea3 - ps4
Asgard/Eukaryota - ps5
Eukaryota - ps6
Amorpheae - ps7
Obazoa - ps8
Opisthokonta - ps9
Holozoa - ps10
Filozoa - ps11
Choano./Metazoa - ps12
Metazoa - ps13
Myriazoa - ps14
Parahoxozoa - ps15
Bilateria - ps16
Deuterostomia - ps17
Chordata - ps18
Olfactores - ps19
Vertebrata - ps20
Gnathostomata - ps21
Euteleostomi - ps22
Sarcopterygii - ps23
Tetrapoda - ps24
Amniota - ps25
Mammalia - ps26
Theria - ps27
Placentalia - ps28
Boreoeutheria - ps29
Euarchontoglires - ps30
Euarchonta - ps31
Primates - ps32
Haplorrhini - ps33
Simiiformes - ps34
Catarrhini - ps35
Hominoidea - ps36
Hominidae - ps37
Homininae - ps38
Hominini - ps39
Homo_sapiens - ps40
p-value
1e-02
2e-02
3e-02
4e-02
6e-02
Log-odds
0.5
1.0
1.5
2.0
2.5
3.0
3.5
4.0

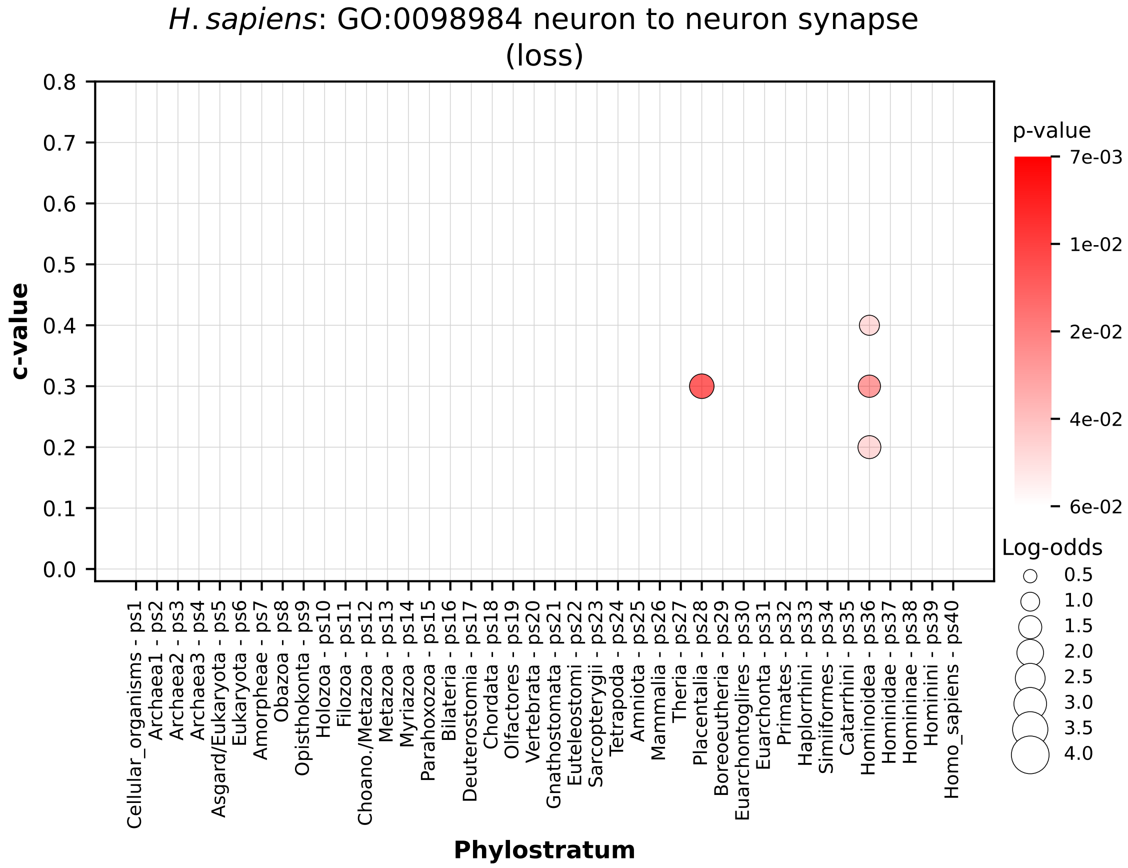

H. sapiens: GO:0098984 neuron to neuron synapse
(loss)
c-value
0.0
0.1
0.2
0.3
0.4
0.5
0.6
0.7
0.8
Phylostratum
Cellular_organisms - ps1
Archaea1 - ps2
Archaea2 - ps3
Archaea3 - ps4
Asgard/Eukaryota - ps5
Eukaryota - ps6
Amorpheae - ps7
Obazoa - ps8
Opisthokonta - ps9
Holozoa - ps10
Filozoa - ps11
Choano./Metazoa - ps12
Metazoa - ps13
Myriazoa - ps14
Parahoxozoa - ps15
Bilateria - ps16
Deuterostomia - ps17
Chordata - ps18
Olfactores - ps19
Vertebrata - ps20
Gnathostomata - ps21
Euteleostomi - ps22
Sarcopterygii - ps23
Tetrapoda - ps24
Amniota - ps25
Mammalia - ps26
Theria - ps27
Placentalia - ps28
Boreoeutheria - ps29
Euarchontoglires - ps30
Euarchonta - ps31
Primates - ps32
Haplorrhini - ps33
Simiiformes - ps34
Catarrhini - ps35
Hominoidea - ps36
Hominidae - ps37
Homininae - ps38
Hominini - ps39
Homo_sapiens - ps40
p-value
7e-03
1e-02
2e-02
4e-02
6e-02
Log-odds
0.5
1.0
1.5
2.0
2.5
3.0
3.5
4.0

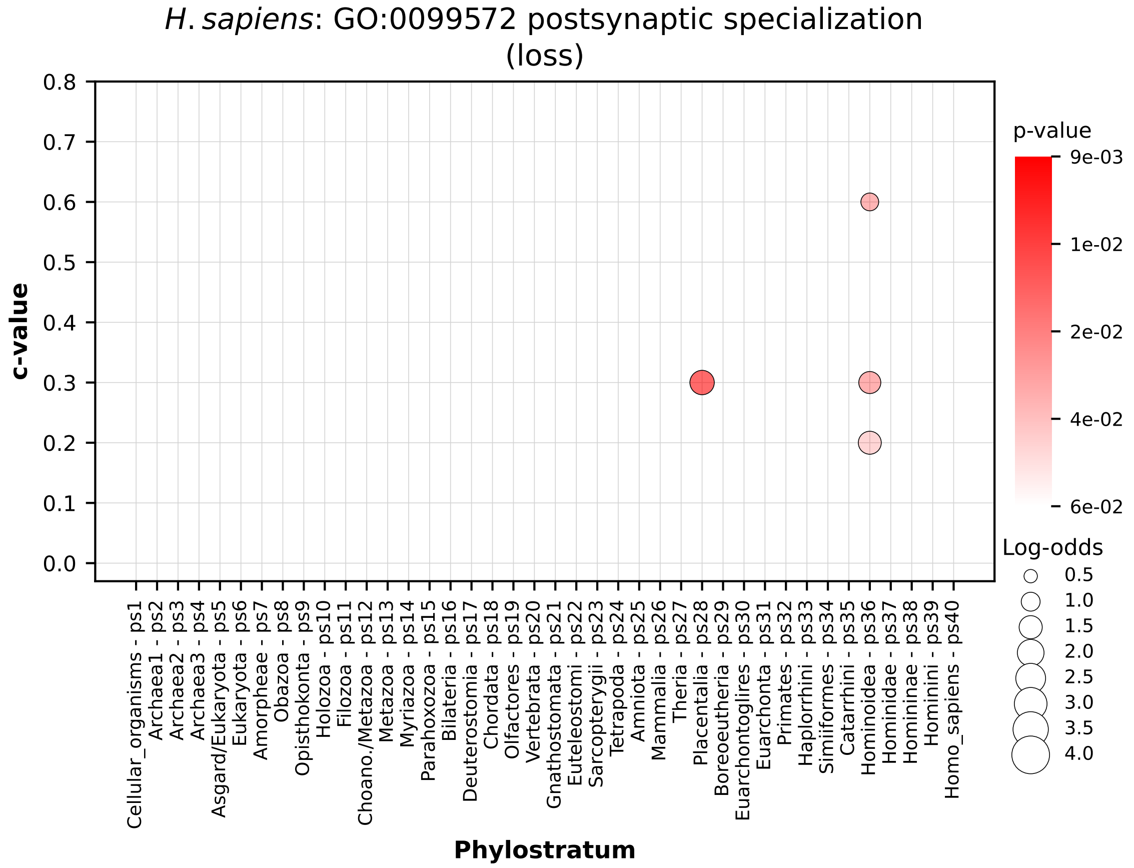
H. sapiens: GO:0099572 postsynaptic specialization
(loss)
c-value
0.8
0.7
0.6
0.5
0.4
0.3
0.2
0.1
0.0
Phylostratum
Cellular_organisms - ps1
Archaea1 - ps2
Archaea2 - ps3
Archaea3 - ps4
Asgard/Eukaryota - ps5
Eukaryota - ps6
Amorpheae - ps7
Obazoa - ps8
Opisthokonta - ps9
Holozoa - ps10
Filozoa - ps11
Choano./Metazoa - ps12
Metazoa - ps13
Myriazoa - ps14
Parahoxozoa - ps15
Bilateria - ps16
Deuterostomia - ps17
Chordata - ps18
Olfactores - ps19
Vertebrata - ps20
Gnathostomata - ps21
Euteleostomi - ps22
Sarcopterygii - ps23
Tetrapoda - ps24
Amniota - ps25
Mammalia - ps26
Theria - ps27
Placentalia - ps28
Boreoeutheria - ps29
Euarchontoglires - ps30
Euarchonta - ps31
Primates - ps32
Haplorrhini - ps33
Simiiformes - ps34
Catarrhini - ps35
Hominoidea - ps36
Hominidae - ps37
Homininae - ps38
Hominini - ps39
Homo_sapiens - ps40
p-value
9e-03
1e-02
2e-02
4e-02
6e-02
Log-odds
0.5
1.0
1.5
2.0
2.5
3.0
3.5
4.0

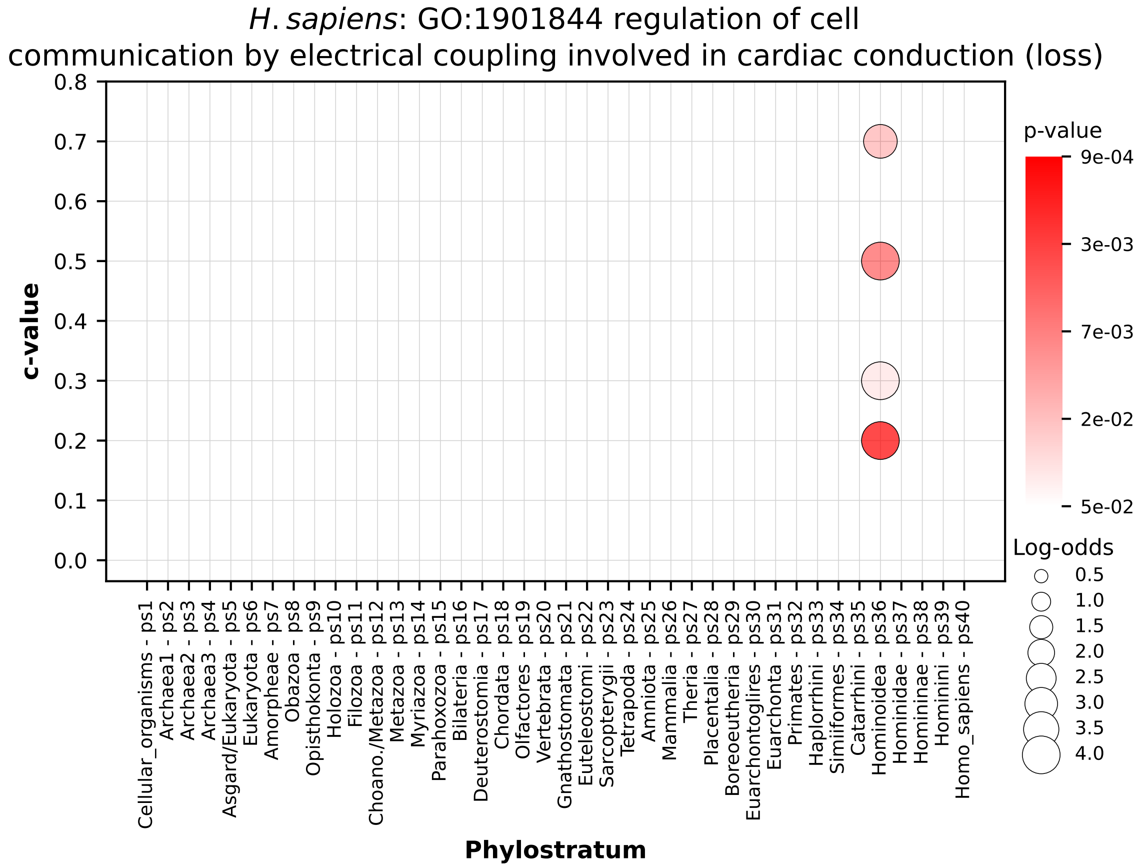

H. sapiens: GO:1901844 regulation of cell communication by electrical coupling involved in cardiac conduction (loss)
c-value
0.8
0.7
0.6
0.5
0.4
0.3
0.2
0.1
0.0
Phylostratum
Cellular_organisms - ps1
Archaea1 - ps2
Archaea2 - ps3
Archaea3 - ps4
Asgard/Eukaryota - ps5
Eukaryota - ps6
Amorpheae - ps7
Obazoa - ps8
Opisthokonta - ps9
Holozoa - ps10
Filozoa - ps11
Choano./Metazoa - ps12
Metazoa - ps13
Myriazoa - ps14
Parahoxozoa - ps15
Bilateria - ps16
Deuterostomia - ps17
Chordata - ps18
Olfactores - ps19
Vertebrata - ps20
Gnathostomata - ps21
Euteleostomi - ps22
Sarcopterygii - ps23
Tetrapoda - ps24
Amniota - ps25
Mammalia - ps26
Theria - ps27
Placentalia - ps28
Boreoeutheria - ps29
Euarchontoglires - ps30
Euarchonta - ps31
Primates - ps32
Haplorrhini - ps33
Simiiformes - ps34
Catarrhini - ps35
Hominoidea - ps36
Hominidae - ps37
Homininae - ps38
Hominini - ps39
Homo_sapiens - ps40
p-value
9e-04
3e-03
7e-03
2e-02
5e-02
Log-odds
0.5
1.0
1.5
2.0
2.5
3.0
3.5
4.0

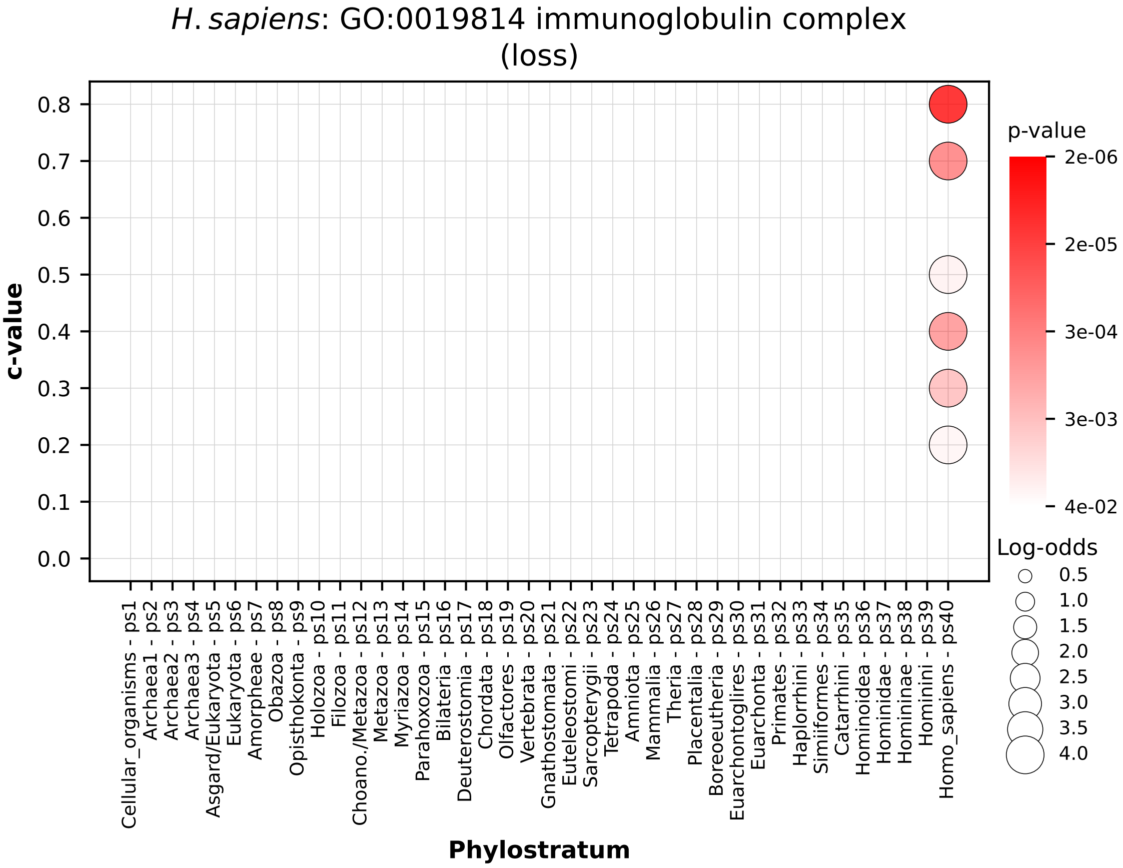

H. sapiens: GO:0019814 immunoglobulin complex
(loss)
c-value
0.0
0.1
0.2
0.3
0.4
0.5
0.6
0.7
0.8
p-value
2e-06
2e-05
3e-04
3e-03
4e-02
Log-odds
0.5
1.0
1.5
2.0
2.5
3.0
3.5
4.0
Cellular_organisms - ps1
Archaea1 - ps2
Archaea2 - ps3
Archaea3 - ps4
Asgard/Eukaryota - ps5
Eukaryota - ps6
Amorpheae - ps7
Obazoa - ps8
Opisthokonta - ps9
Holozoa - ps10
Filozoa - ps11
Choano./Metazoa - ps12
Metazoa - ps13
Myriazoa - ps14
Parahoxozoa - ps15
Bilateria - ps16
Deuterostomia - ps17
Chordata - ps18
Olfactores - ps19
Vertebrata - ps20
Gnathostomata - ps21
Euteleostomi - ps22
Sarcopterygii - ps23
Tetrapoda - ps24
Amniota - ps25
Mammalia - ps26
Theria - ps27
Placentalia - ps28
Boreoeutheria - ps29
Euarchontoglires - ps30
Euarchonta - ps31
Primates - ps32
Haplorrhini - ps33
Simiiformes - ps34
Catarrhini - ps35
Hominoidea - ps36
Hominidae - ps37
Homininae - ps38
Hominini - ps39
Homo_sapiens - ps40
Phylostratum

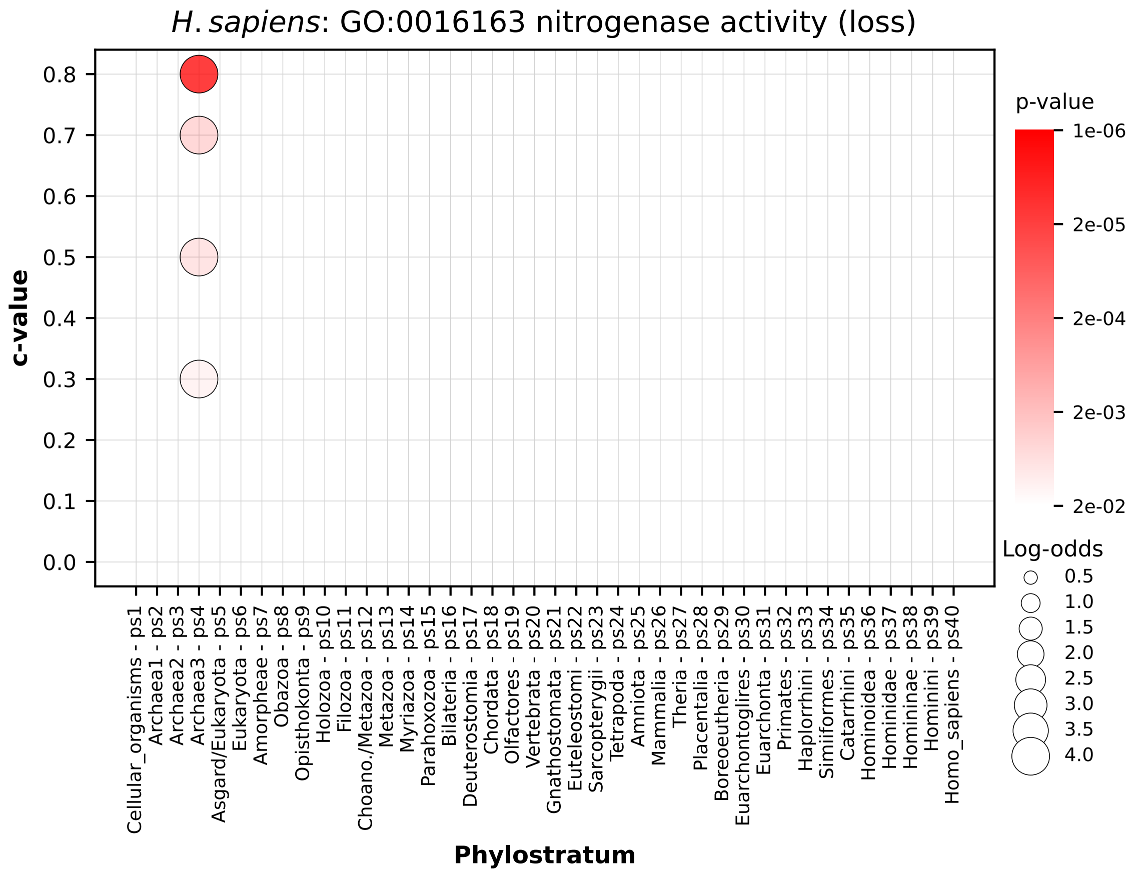

H. sapiens: GO:0016163 nitrogenase activity (loss)
c-value
0.0
0.1
0.2
0.3
0.4
0.5
0.6
0.7
0.8
Phylostratum
Cellular_organisms - ps1
Archaea1 - ps2
Archaea2 - ps3
Archaea3 - ps4
Asgard/Eukaryota - ps5
Eukaryota - ps6
Amorpheae - ps7
Obazoa - ps8
Opisthokonta - ps9
Holozoa - ps10
Filozoa - ps11
Choano./Metazoa - ps12
Metazoa - ps13
Myriazoa - ps14
Parahoxozoa - ps15
Bilateria - ps16
Deuterostomia - ps17
Chordata - ps18
Olfactores - ps19
Vertebrata - ps20
Gnathostomata - ps21
Euteleostomi - ps22
Sarcopterygii - ps23
Tetrapoda - ps24
Amniota - ps25
Mammalia - ps26
Theria - ps27
Placentalia - ps28
Boreoeutheria - ps29
Euarchontoglires - ps30
Euarchonta - ps31
Primates - ps32
Haplorrhini - ps33
Simiiformes - ps34
Catarrhini - ps35
Hominoidea - ps36
Hominidae - ps37
Homininae - ps38
Hominini - ps39
Homo_sapiens - ps40
p-value
1e-06
2e-05
2e-04
2e-03
2e-02
Log-odds
0.5
1.0
1.5
2.0
2.5
3.0
3.5
4.0

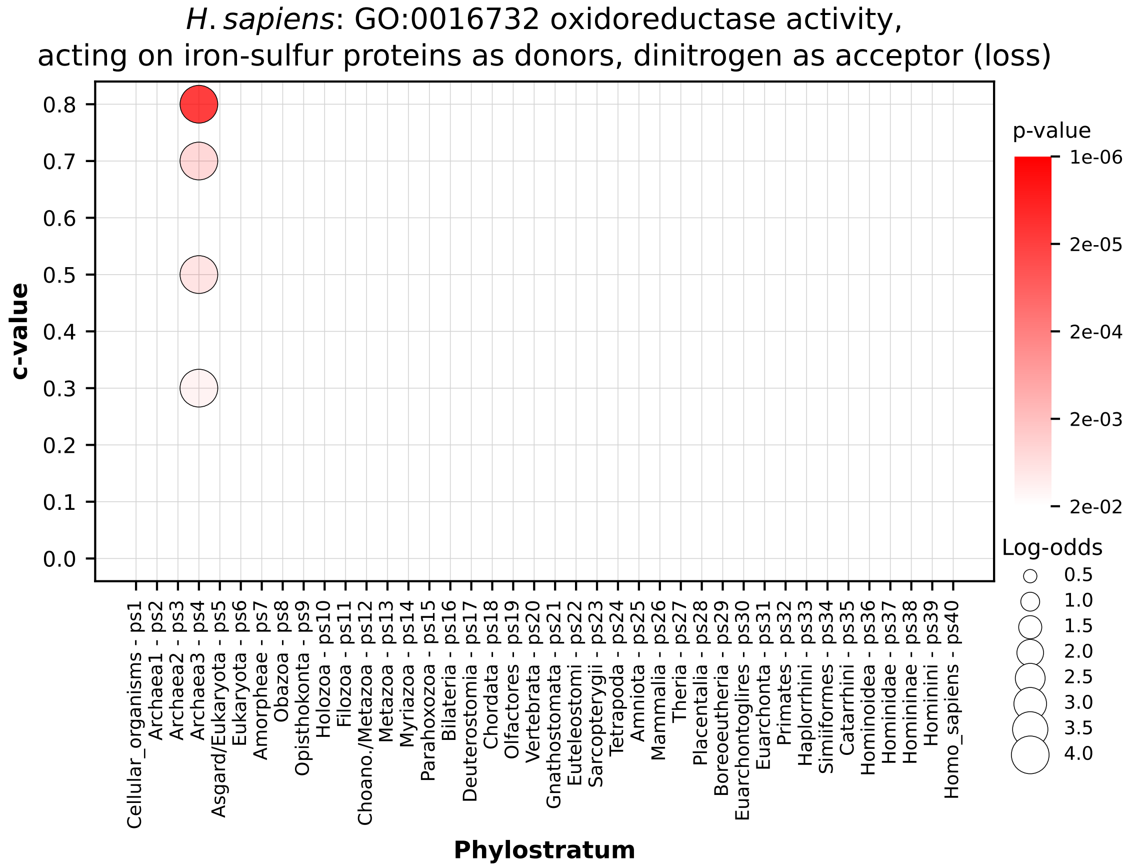

H. sapiens: GO:0016732 oxidoreductase activity,
acting on iron-sulfur proteins as donors, dinitrogen as acceptor (loss)
c-value
0.8
0.7
0.6
0.5
0.4
0.3
0.2
0.1
0.0
Phylostratum
Cellular_organisms - ps1
Archaea1 - ps2
Archaea2 - ps3
Archaea3 - ps4
Asgard/Eukaryota - ps5
Eukaryota - ps6
Amorpheae - ps7
Obazoa - ps8
Opisthokonta - ps9
Holozoa - ps10
Filozoa - ps11
Choano./Metazoa - ps12
Metazoa - ps13
Myriazoa - ps14
Parahoxozoa - ps15
Bilateria - ps16
Deuterostomia - ps17
Chordata - ps18
Olfactores - ps19
Vertebrata - ps20
Gnathostomata - ps21
Euteleostomi - ps22
Sarcopterygii - ps23
Tetrapoda - ps24
Amniota - ps25
Mammalia - ps26
Theria - ps27
Placentalia - ps28
Boreoeutheria - ps29
Euarchontoglires - ps30
Euarchonta - ps31
Primates - ps32
Haplorrhini - ps33
Simiiformes - ps34
Catarrhini - ps35
Hominoidea - ps36
Hominidae - ps37
Homininae - ps38
Hominini - ps39
Homo_sapiens - ps40
p-value
1e-06
2e-05
2e-04
2e-03
2e-02
Log-odds
0.5
1.0
1.5
2.0
2.5
3.0
3.5
4.0

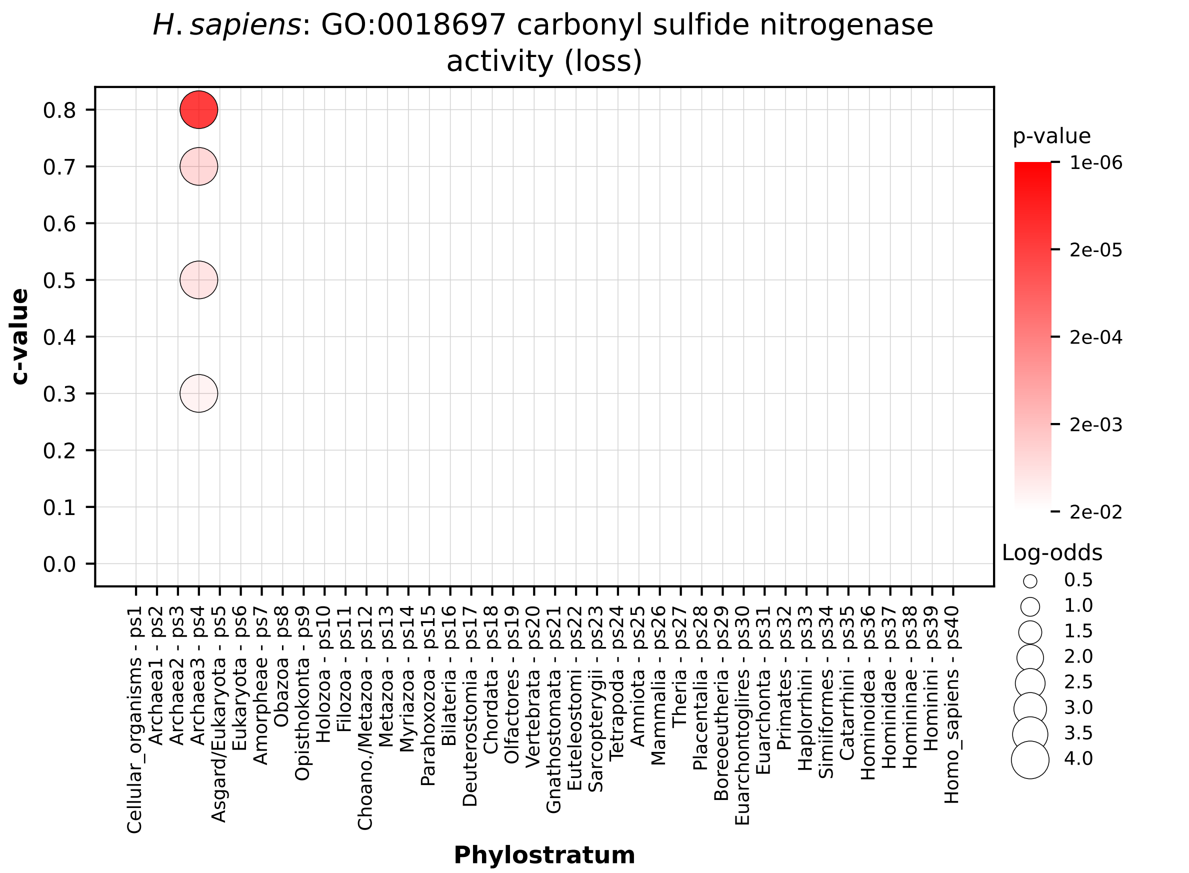

H. sapiens: GO:0018697 carbonyl sulfide nitrogenase activity (loss)
c-value
0.8
0.7
0.6
0.5
0.4
0.3
0.2
0.1
0.0
p-value
1e-06
2e-05
2e-04
2e-03
2e-02
Log-odds
0.5
1.0
1.5
2.0
2.5
3.0
3.5
4.0
Cellular_organisms - ps1
Archaea1 - ps2
Archaea2 - ps3
Archaea3 - ps4
Asgard/Eukaryota - ps5
Eukaryota - ps6
Amorpheae - ps7
Obazoa - ps8
Opisthokonta - ps9
Holozoa - ps10
Filozoa - ps11
Choano./Metazoa - ps12
Metazoa - ps13
Myriazoa - ps14
Parahoxozoa - ps15
Bilateria - ps16
Deuterostomia - ps17
Chordata - ps18
Olfactores - ps19
Vertebrata - ps20
Gnathostomata - ps21
Euteleostomi - ps22
Sarcopterygii - ps23
Tetrapoda - ps24
Amniota - ps25
Mammalia - ps26
Theria - ps27
Placentalia - ps28
Boreoeutheria - ps29
Euarchontoglires - ps30
Euarchonta - ps31
Primates - ps32
Haplorrhini - ps33
Simiiformes - ps34
Catarrhini - ps35
Hominoidea - ps36
Hominidae - ps37
Homininae - ps38
Hominini - ps39
Homo_sapiens - ps40
Phylostratum

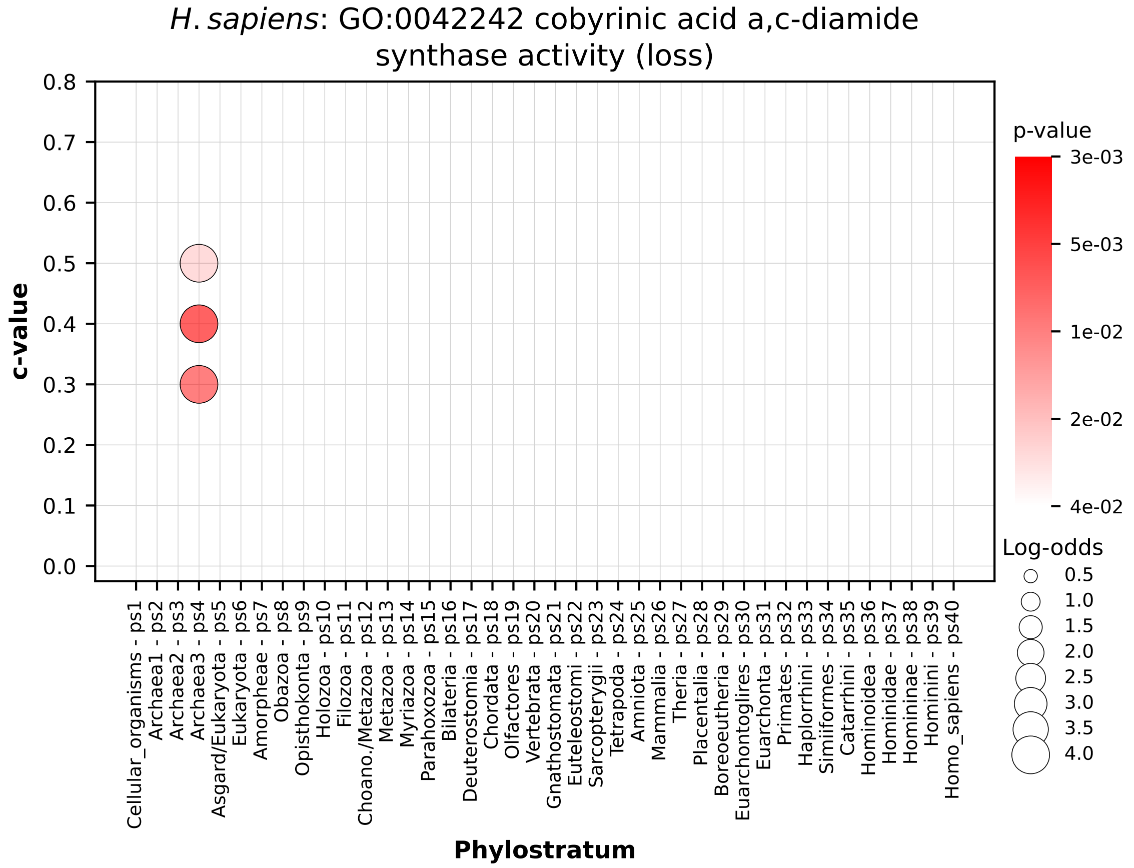

H. sapiens: GO:0042242 cobyrinic acid a,c-diamide synthase activity (loss)
c-value
0.8
0.7
0.6
0.5
0.4
0.3
0.2
0.1
0.0
Phylostratum
Cellular_organisms - ps1
Archaea1 - ps2
Archaea2 - ps3
Archaea3 - ps4
Asgard/Eukaryota - ps5
Eukaryota - ps6
Amorpheae - ps7
Obazoa - ps8
Opisthokonta - ps9
Holozoa - ps10
Filozoa - ps11
Choano./Metazoa - ps12
Metazoa - ps13
Myriazoa - ps14
Parahoxozoa - ps15
Bilateria - ps16
Deuterostomia - ps17
Chordata - ps18
Olfactores - ps19
Vertebrata - ps20
Gnathostomata - ps21
Euteleostomi - ps22
Sarcopterygii - ps23
Tetrapoda - ps24
Amniota - ps25
Mammalia - ps26
Theria - ps27
Placentalia - ps28
Boreoeutheria - ps29
Euarchontoglires - ps30
Euarchonta - ps31
Primates - ps32
Haplorrhini - ps33
Simiiformes - ps34
Catarrhini - ps35
Hominoidea - ps36
Hominidae - ps37
Homininae - ps38
Hominini - ps39
Homo_sapiens - ps40
p-value
3e-03
5e-03
1e-02
2e-02
4e-02
Log-odds
0.5
1.0
1.5
2.0
2.5
3.0
3.5
4.0

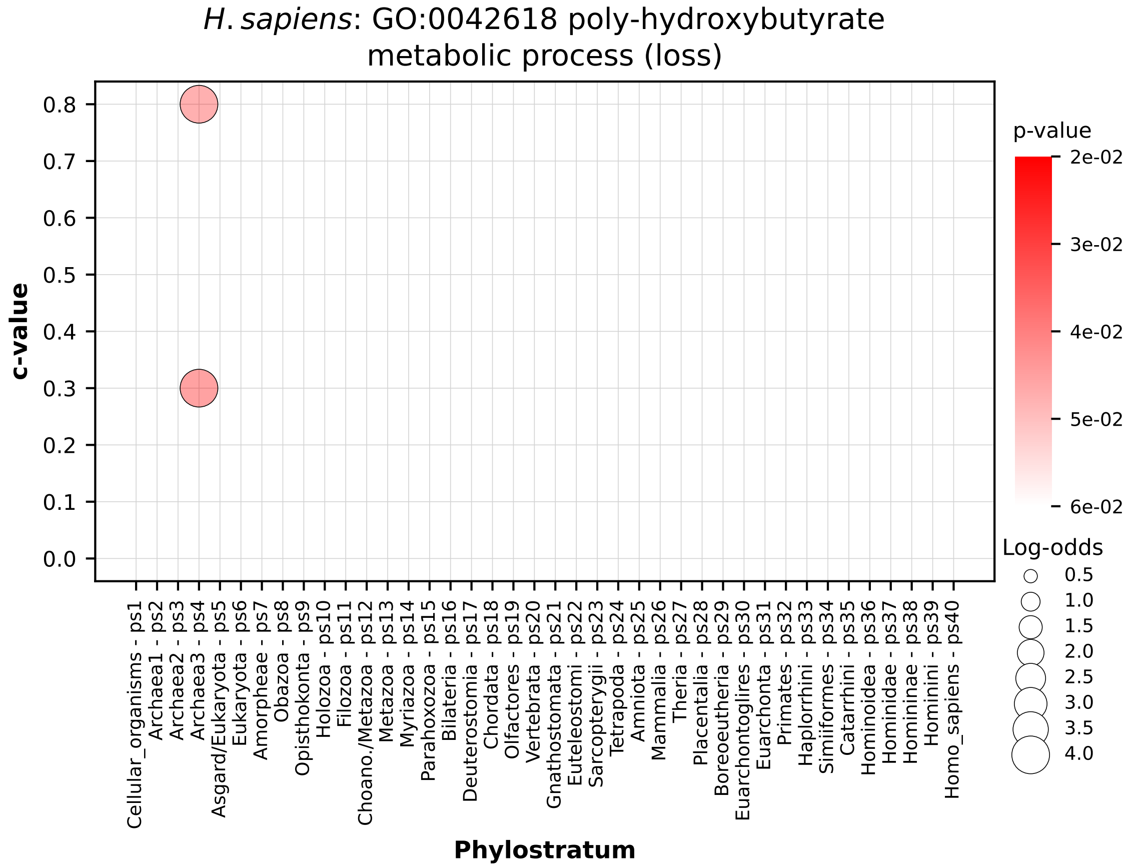

H. sapiens: GO:0042618 poly-hydroxybutyrate metabolic process (loss)
c-value
0.0
0.1
0.2
0.3
0.4
0.5
0.6
0.7
0.8
Phylostratum
Cellular_organisms - ps1
Archaea1 - ps2
Archaea2 - ps3
Archaea3 - ps4
Asgard/Eukaryota - ps5
Eukaryota - ps6
Amorpheae - ps7
Obazoa - ps8
Opisthokonta - ps9
Holozoa - ps10
Filozoa - ps11
Choano./Metazoa - ps12
Metazoa - ps13
Myriazoa - ps14
Parahoxozoa - ps15
Bilateria - ps16
Deuterostomia - ps17
Chordata - ps18
Olfactores - ps19
Vertebrata - ps20
Gnathostomata - ps21
Euteleostomi - ps22
Sarcopterygii - ps23
Tetrapoda - ps24
Amniota - ps25
Mammalia - ps26
Theria - ps27
Placentalia - ps28
Boreoeutheria - ps29
Euarchontoglires - ps30
Euarchonta - ps31
Primates - ps32
Haplorrhini - ps33
Simiiformes - ps34
Catarrhini - ps35
Hominoidea - ps36
Hominidae - ps37
Homininae - ps38
Hominini - ps39
Homo_sapiens - ps40
p-value
2e-02
3e-02
4e-02
5e-02
6e-02
Log-odds
0.5
1.0
1.5
2.0
2.5
3.0
3.5
4.0

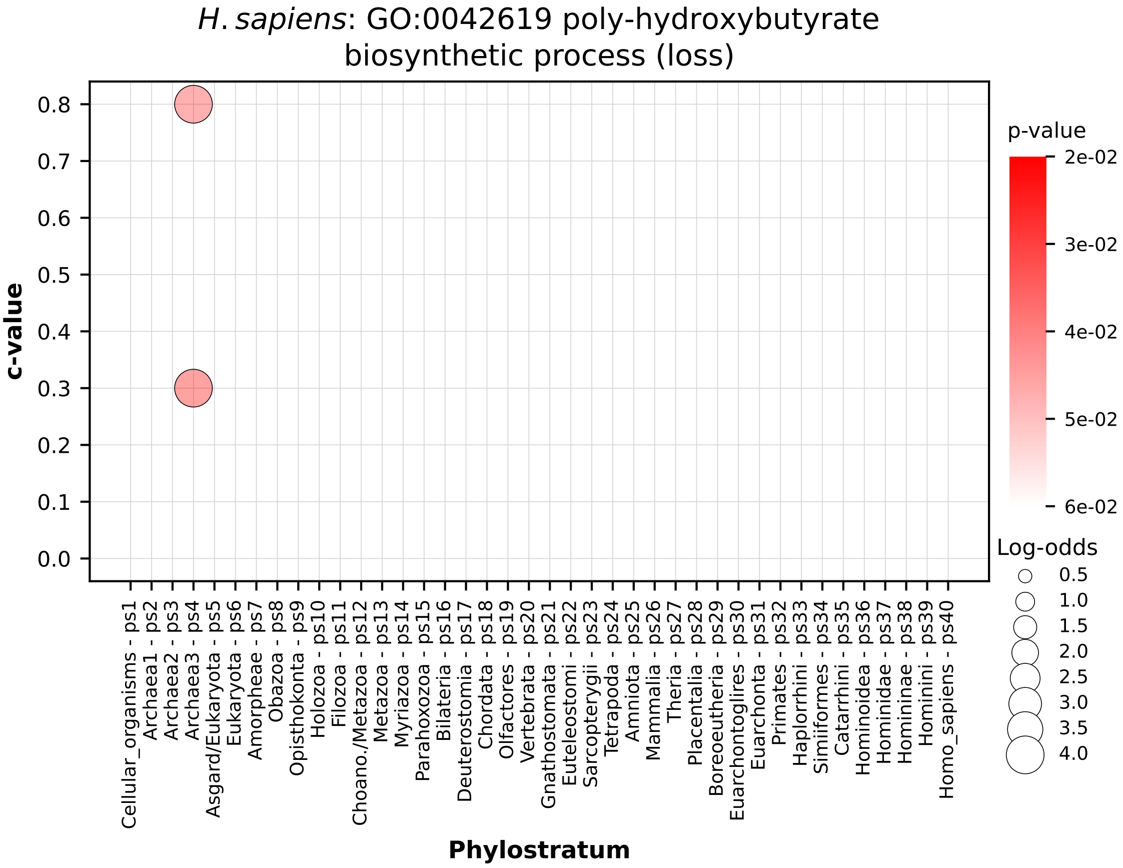

H. sapiens: GO:0042619 poly-hydroxybutyrate biosynthetic process (loss)
c-value
0.0
0.1
0.2
0.3
0.4
0.5
0.6
0.7
0.8
Phylostratum
Cellular_organisms - ps1
Archaea1 - ps2
Archaea2 - ps3
Archaea3 - ps4
Asgard/Eukaryota - ps5
Eukaryota - ps6
Amorpheae - ps7
Obazoa - ps8
Opisthokonta - ps9
Holozoa - ps10
Filozoa - ps11
Choano./Metazoa - ps12
Metazoa - ps13
Myriazoa - ps14
Parahoxozoa - ps15
Bilateria - ps16
Deuterostomia - ps17
Chordata - ps18
Olfactores - ps19
Vertebrata - ps20
Gnathostomata - ps21
Euteleostomi - ps22
Sarcopterygii - ps23
Tetrapoda - ps24
Amniota - ps25
Mammalia - ps26
Theria - ps27
Placentalia - ps28
Boreoeutheria - ps29
Euarchontoglires - ps30
Euarchonta - ps31
Primates - ps32
Haplorrhini - ps33
Simiiformes - ps34
Catarrhini - ps35
Hominoidea - ps36
Hominidae - ps37
Homininae - ps38
Hominini - ps39
Homo_sapiens - ps40
p-value
2e-02
3e-02
4e-02
5e-02
6e-02
Log-odds
0.5
1.0
1.5
2.0
2.5
3.0
3.5
4.0

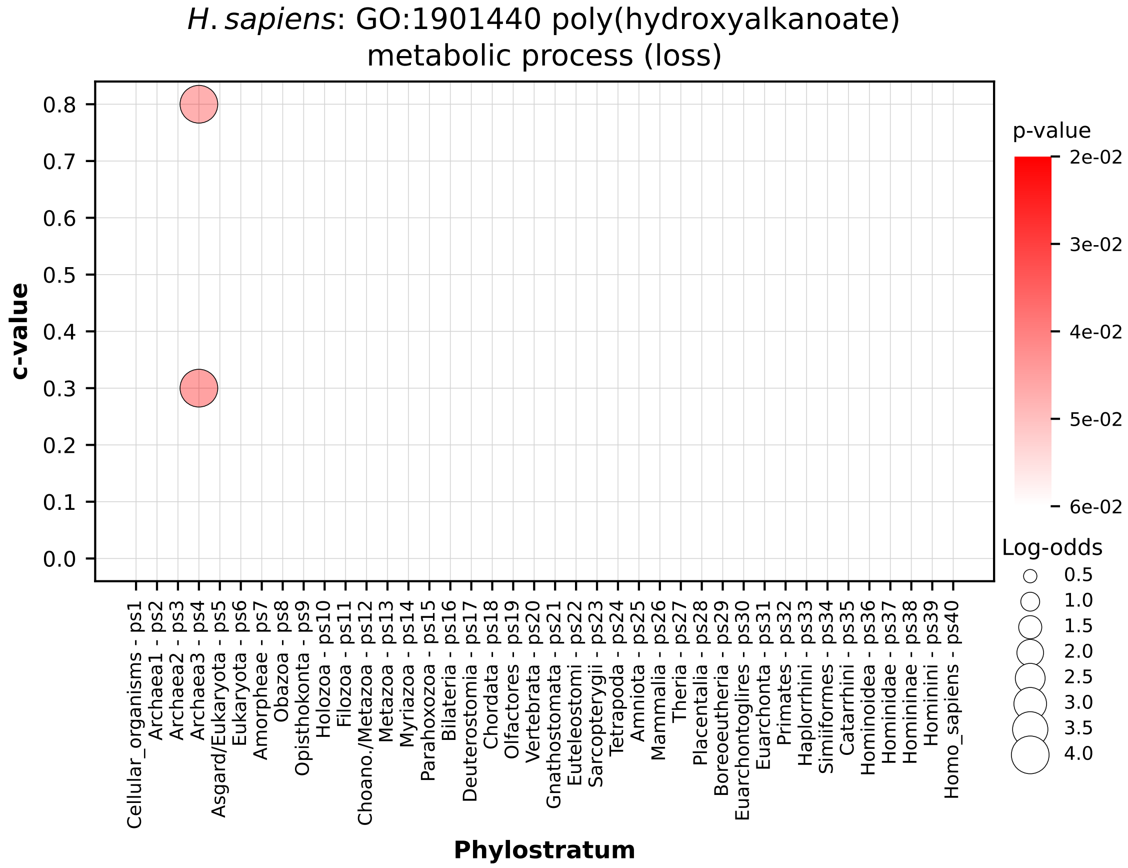

H. sapiens: GO:1901440 poly(hydroxyalkanoate) metabolic process (loss)
c-value
0.8
0.7
0.6
0.5
0.4
0.3
0.2
0.1
0.0
p-value
2e-02
3e-02
4e-02
5e-02
6e-02
Log-odds
0.5
1.0
1.5
2.0
2.5
3.0
3.5
4.0
Cellular_organisms - ps1
Archaea1 - ps2
Archaea2 - ps3
Archaea3 - ps4
Asgard/Eukaryota - ps5
Eukaryota - ps6
Amorpheae - ps7
Obazoa - ps8
Opisthokonta - ps9
Holozoa - ps10
Filozoa - ps11
Choano./Metazoa - ps12
Metazoa - ps13
Myriazoa - ps14
Parahoxozoa - ps15
Bilateria - ps16
Deuterostomia - ps17
Chordata - ps18
Olfactores - ps19
Vertebrata - ps20
Gnathostomata - ps21
Euteleostomi - ps22
Sarcopterygii - ps23
Tetrapoda - ps24
Amniota - ps25
Mammalia - ps26
Theria - ps27
Placentalia - ps28
Boreoeutheria - ps29
Euarchontoglires - ps30
Euarchonta - ps31
Primates - ps32
Haplorrhini - ps33
Simiiformes - ps34
Catarrhini - ps35
Hominoidea - ps36
Hominidae - ps37
Homininae - ps38
Hominini - ps39
Homo_sapiens - ps40
Phylostratum

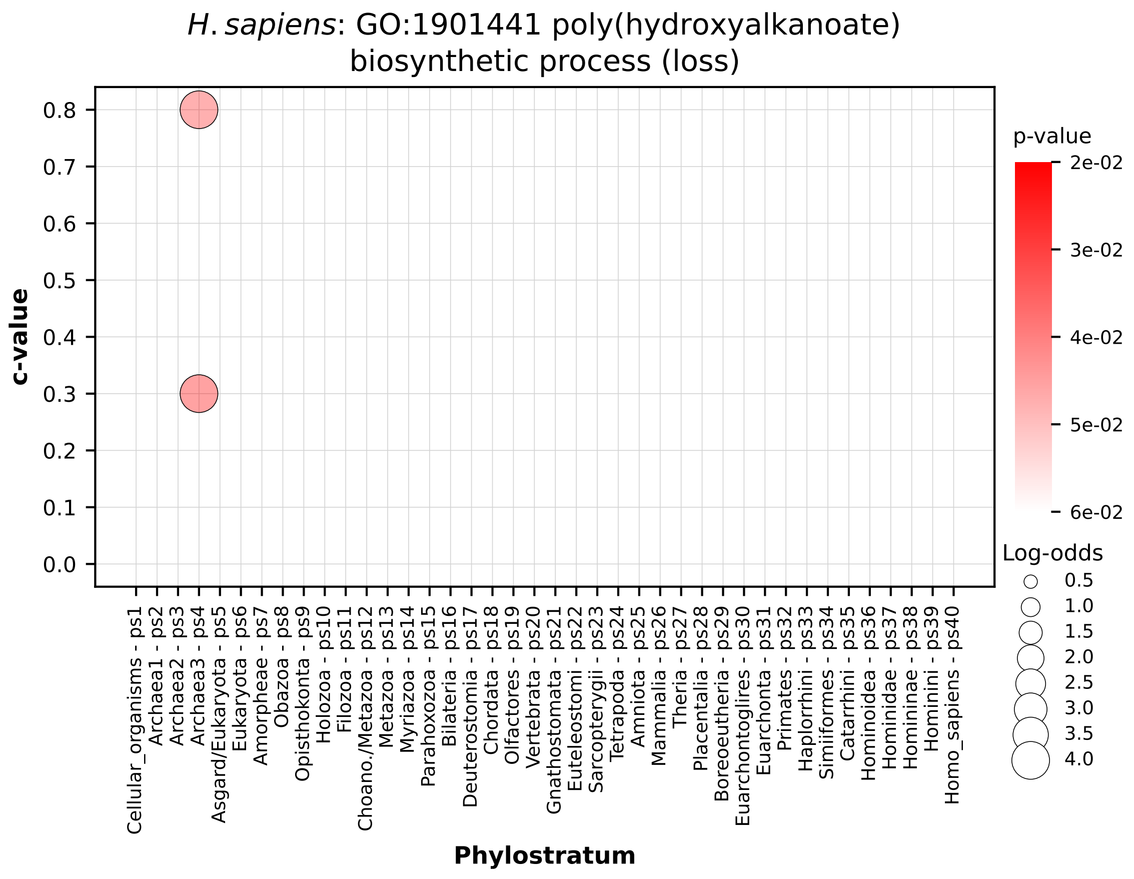

H. sapiens: GO:1901441 poly(hydroxyalkanoate) biosynthetic process (loss)
c-value
0.0
0.1
0.2
0.3
0.4
0.5
0.6
0.7
0.8
Phylostratum
Cellular_organisms - ps1
Archaea1 - ps2
Archaea2 - ps3
Archaea3 - ps4
Asgard/Eukaryota - ps5
Eukaryota - ps6
Amorpheae - ps7
Obazoa - ps8
Opisthokonta - ps9
Holozoa - ps10
Filozoa - ps11
Choano./Metazoa - ps12
Metazoa - ps13
Myriazoa - ps14
Parahoxozoa - ps15
Bilateria - ps16
Deuterostomia - ps17
Chordata - ps18
Olfactores - ps19
Vertebrata - ps20
Gnathostomata - ps21
Euteleostomi - ps22
Sarcopterygii - ps23
Tetrapoda - ps24
Amniota - ps25
Mammalia - ps26
Theria - ps27
Placentalia - ps28
Boreoeutheria - ps29
Euarchontoglires - ps30
Euarchonta - ps31
Primates - ps32
Haplorrhini - ps33
Similformes - ps34
Catarrhini - ps35
Hominoidea - ps36
Hominidae - ps37
Homininae - ps38
Hominini - ps39
Homo_sapiens - ps40
p-value
2e-02
3e-02
4e-02
5e-02
6e-02
Log-odds
0.5
1.0
1.5
2.0
2.5
3.0
3.5
4.0

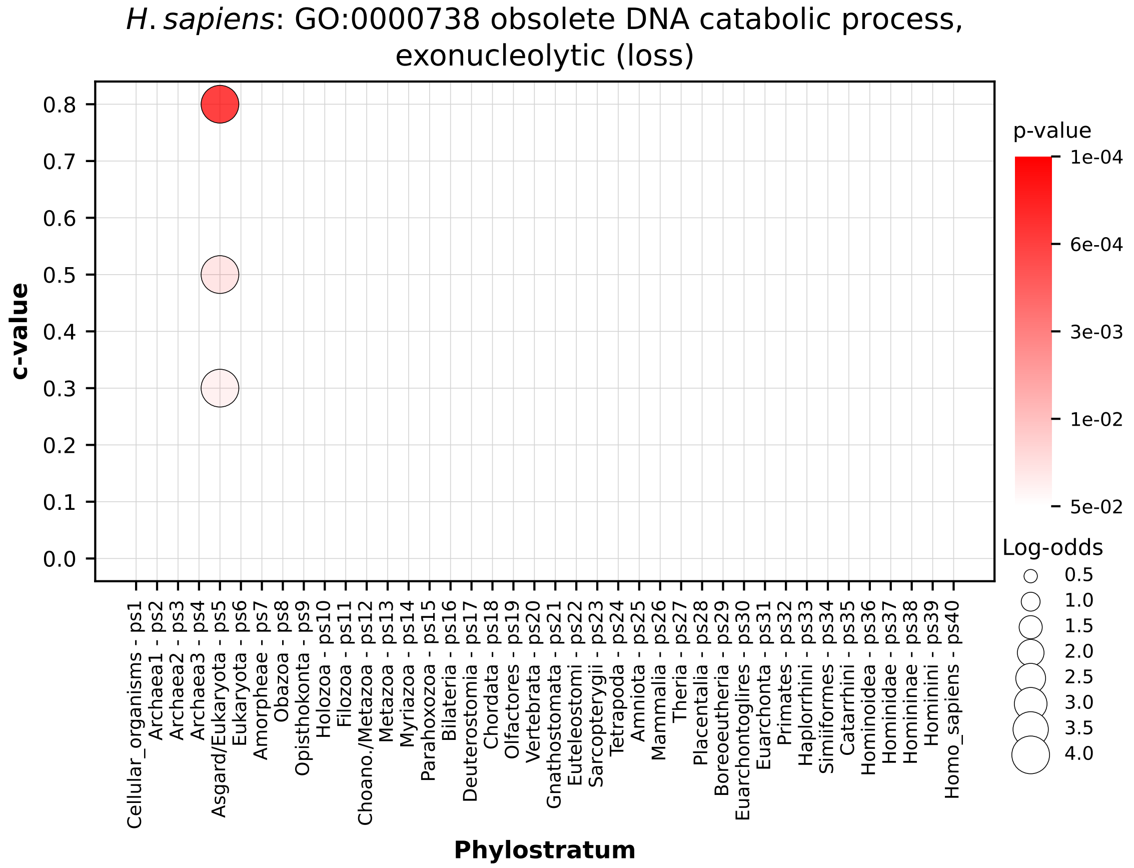

H. sapiens: GO:0000738 obsolete DNA catabolic process, exonucleolytic (loss)
c-value
0.8
0.7
0.6
0.5
0.4
0.3
0.2
0.1
0.0
p-value
1e-04
6e-04
3e-03
1e-02
5e-02
Log-odds
0.5
1.0
1.5
2.0
2.5
3.0
3.5
4.0
Cellular_organisms - ps1
Archaea1 - ps2
Archaea2 - ps3
Archaea3 - ps4
Asgard/Eukaryota - ps5
Eukaryota - ps6
Amorpheae - ps7
Obazoa - ps8
Opisthokonta - ps9
Holozoa - ps10
Filozoa - ps11
Choano./Metazoa - ps12
Metazoa - ps13
Myriazoa - ps14
Parahoxozoa - ps15
Bilateria - ps16
Deuterostomia - ps17
Chordata - ps18
Olfactores - ps19
Vertebrata - ps20
Gnathostomata - ps21
Euteleostomi - ps22
Sarcopterygii - ps23
Tetrapoda - ps24
Amniota - ps25
Mammalia - ps26
Theria - ps27
Placentalia - ps28
Boreoeutheria - ps29
Euarchontoglires - ps30
Euarchonta - ps31
Primates - ps32
Haplorrhini - ps33
Simiiformes - ps34
Catarrhini - ps35
Hominoidea - ps36
Hominidae - ps37
Homininae - ps38
Hominini - ps39
Homo_sapiens - ps40
Phylostratum

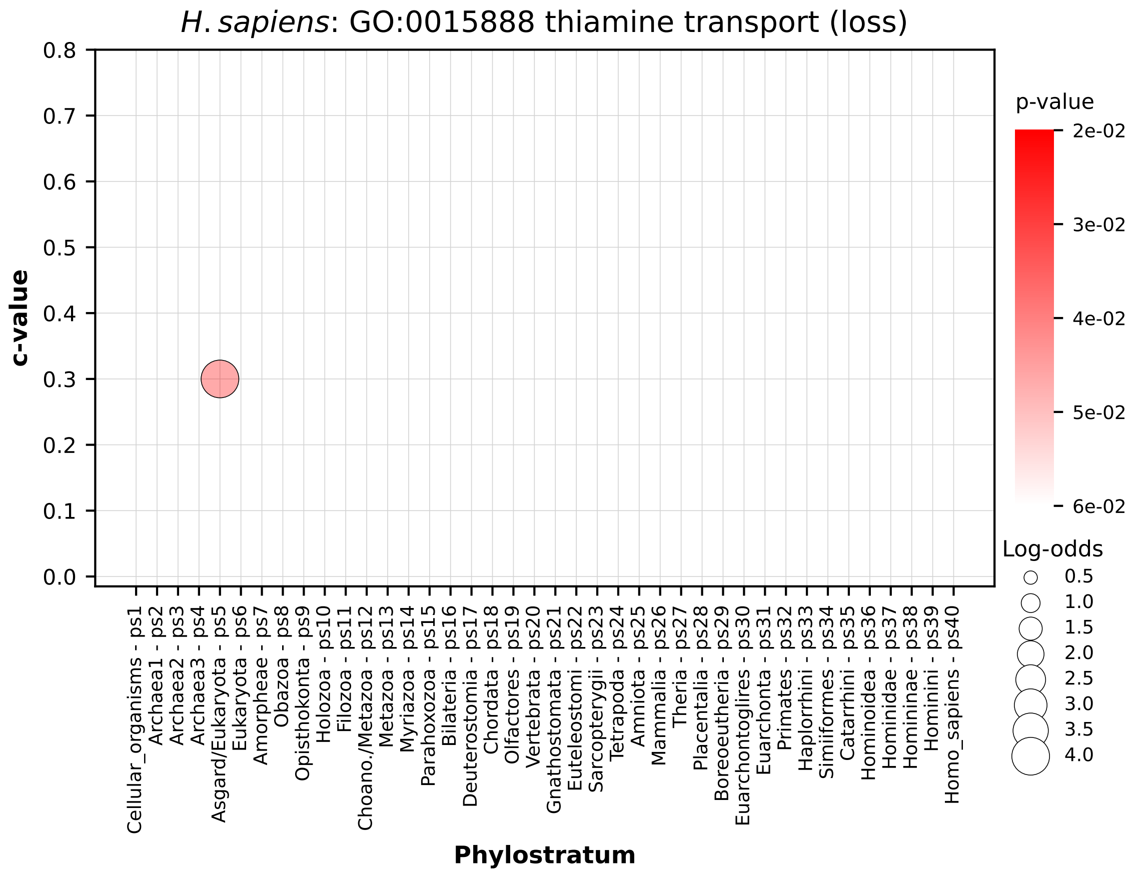
H. sapiens: GO:0015888 thiamine transport (loss)
c-value
Phylostratum
p-value
2e-02
3e-02
4e-02
5e-02
6e-02
Log-odds
0.5
1.0
1.5
2.0
2.5
3.0
3.5
4.0
Cellular_organisms - ps1
Archaea1 - ps2
Archaea2 - ps3
Archaea3 - ps4
Asgard/Eukaryota - ps5
Eukaryota - ps6
Amorpheae - ps7
Obazoa - ps8
Opisthokonta - ps9
Holozoa - ps10
Filozoa - ps11
Choano./Metazoa - ps12
Metazoa - ps13
Myriazoa - ps14
Parahoxozoa - ps15
Bilateria - ps16
Deuterostomia - ps17
Chordata - ps18
Olfactores - ps19
Vertebrata - ps20
Gnathostomata - ps21
Euteleostomi - ps22
Sarcopterygii - ps23
Tetrapoda - ps24
Amniota - ps25
Mammalia - ps26
Theria - ps27
Placentalia - ps28
Boreoeutheria - ps29
Euarchontoglires - ps30
Euarchonta - ps31
Primates - ps32
Haplorrhini - ps33
Simiiformes - ps34
Catarrhini - ps35
Hominoidea - ps36
Hominidae - ps37
Homininae - ps38
Hominini - ps39
Homo_sapiens - ps40

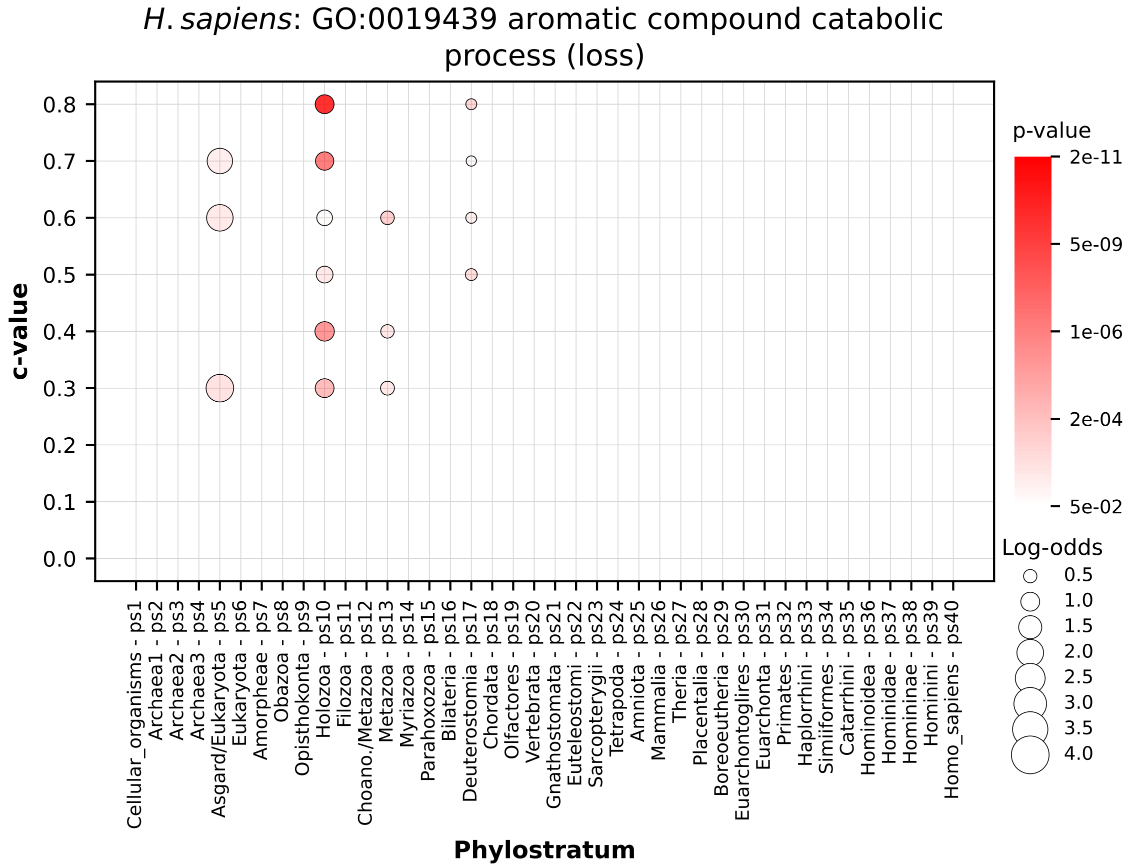

H. sapiens: GO:0019439 aromatic compound catabolic process (loss)
c-value
0.0
0.1
0.2
0.3
0.4
0.5
0.6
0.7
0.8
p-value
2e-11
5e-09
1e-06
2e-04
5e-02
Log-odds
0.5
1.0
1.5
2.0
2.5
3.0
3.5
4.0
Cellular_organisms - ps1
Archaea1 - ps2
Archaea2 - ps3
Archaea3 - ps4
Asgard/Eukaryota - ps5
Eukaryota - ps6
Amorpheae - ps7
Obazoa - ps8
Opisthokonta - ps9
Holozoa - ps10
Filozoa - ps11
Choano./Metazoa - ps12
Metazoa - ps13
Myriazoa - ps14
Parahoxozoa - ps15
Bilateria - ps16
Deuterostomia - ps17
Chordata - ps18
Olfactores - ps19
Vertebrata - ps20
Gnathostomata - ps21
Euteleostomi - ps22
Sarcopterygii - ps23
Tetrapoda - ps24
Amniota - ps25
Mammalia - ps26
Theria - ps27
Placentalia - ps28
Boreoeutheria - ps29
Euarchontoglires - ps30
Euarchonta - ps31
Primates - ps32
Haplorrhini - ps33
Simiiformes - ps34
Catarrhini - ps35
Hominoidea - ps36
Hominidae - ps37
Homininae - ps38
Hominini - ps39
Homo_sapiens - ps40
Phylostratum

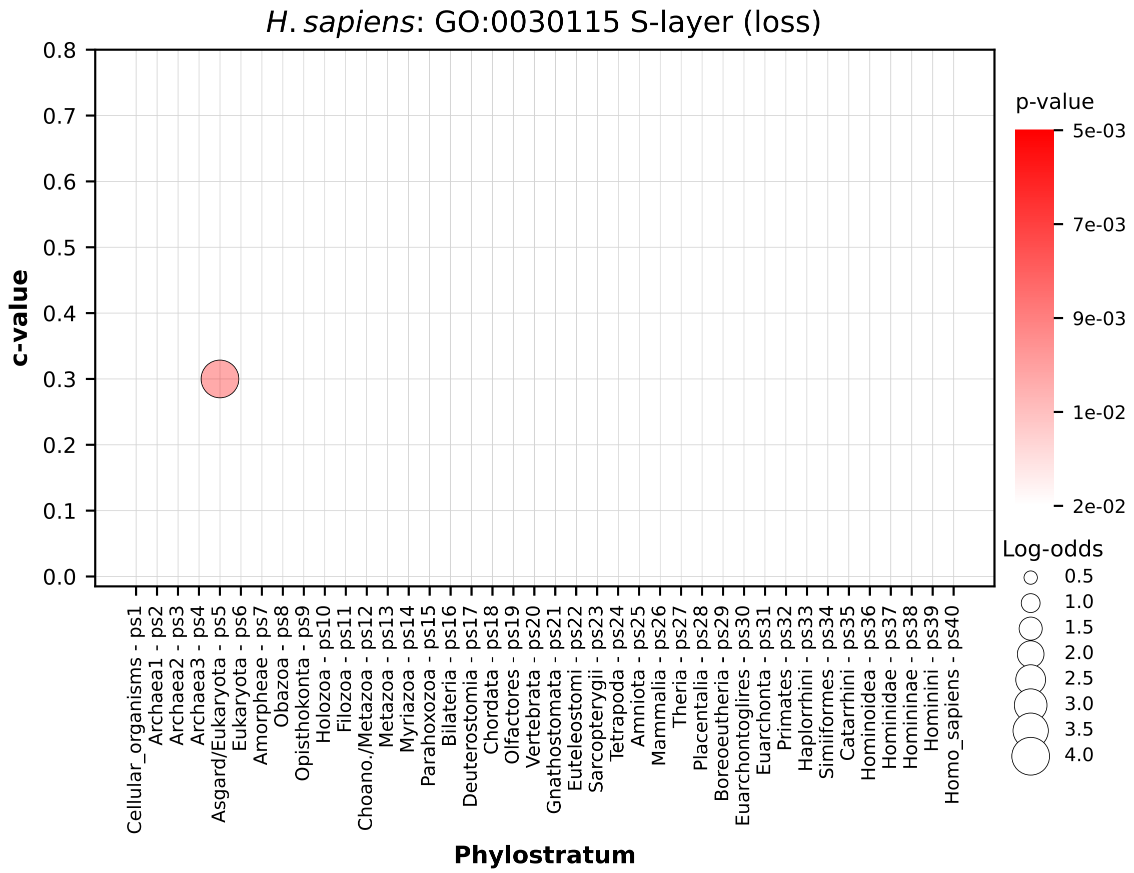
H. sapiens: GO:0030115 S-layer (loss)
c-value
0.8
0.7
0.6
0.5
0.4
0.3
0.2
0.1
0.0
p-value
5e-03
7e-03
9e-03
1e-02
2e-02
Log-odds
0.5
1.0
1.5
2.0
2.5
3.0
3.5
4.0
Cellular_organisms - ps1
Archaea1 - ps2
Archaea2 - ps3
Archaea3 - ps4
Asgard/Eukaryota - ps5
Eukaryota - ps6
Amorpheae - ps7
Obazoa - ps8
Opisthokonta - ps9
Holozoa - ps10
Filozoa - ps11
Choano./Metazoa - ps12
Metazoa - ps13
Myriazoa - ps14
Parahoxozoa - ps15
Bilateria - ps16
Deuterostomia - ps17
Chordata - ps18
Olfactores - ps19
Vertebrata - ps20
Gnathostomata - ps21
Euteleostomi - ps22
Sarcopterygii - ps23
Tetrapoda - ps24
Amniota - ps25
Mammalia - ps26
Theria - ps27
Placentalia - ps28
Boreoeutheria - ps29
Euarchontoglires - ps30
Euarchonta - ps31
Primates - ps32
Haplorrhini - ps33
Simiiformes - ps34
Catarrhini - ps35
Hominoidea - ps36
Hominidae - ps37
Homininae - ps38
Hominini - ps39
Homo_sapiens - ps40
Phylostratum

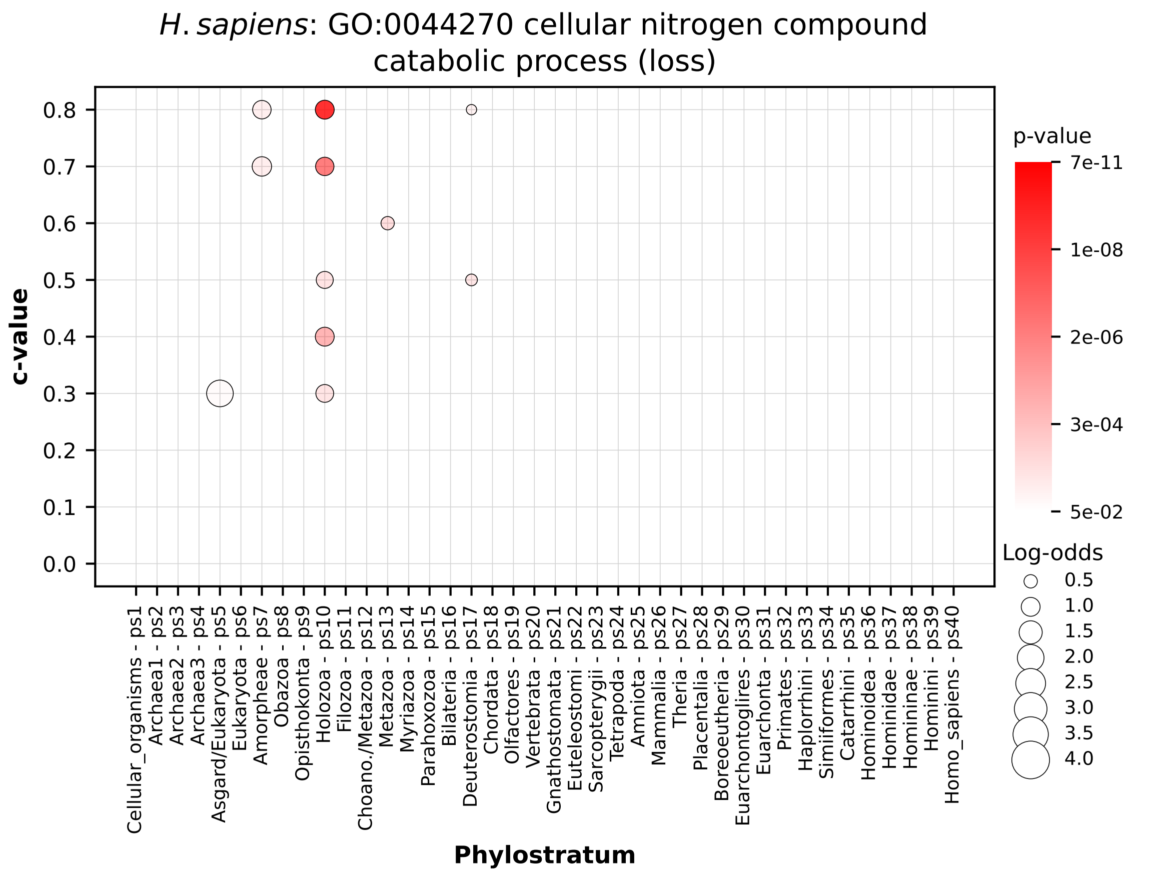

H. sapiens: GO:0044270 cellular nitrogen compound catabolic process (loss)
c-value
0.8
0.7
0.6
0.5
0.4
0.3
0.2
0.1
0.0
p-value
7e-11
1e-08
2e-06
3e-04
5e-02
Log-odds
0.5
1.0
1.5
2.0
2.5
3.0
3.5
4.0
Cellular_organisms - ps1
Archaea1 - ps2
Archaea2 - ps3
Archaea3 - ps4
Asgard/Eukaryota - ps5
Eukaryota - ps6
Amorpheae - ps7
Obazoa - ps8
Opisthokonta - ps9
Holozoa - ps10
Filozoa - ps11
Choano./Metazoa - ps12
Metazoa - ps13
Myriazoa - ps14
Parahoxozoa - ps15
Bilateria - ps16
Deuterostomia - ps17
Chordata - ps18
Olfactores - ps19
Vertebrata - ps20
Gnathostomata - ps21
Euteleostomi - ps22
Sarcopterygii - ps23
Tetrapoda - ps24
Amniota - ps25
Mammalia - ps26
Theria - ps27
Placentalia - ps28
Boreoeutheria - ps29
Euarchontoglires - ps30
Euarchonta - ps31
Primates - ps32
Haplorrhini - ps33
Simiiformes - ps34
Catarrhini - ps35
Hominoidea - ps36
Hominidae - ps37
Homininae - ps38
Hominini - ps39
Homo_sapiens - ps40
Phylostratum

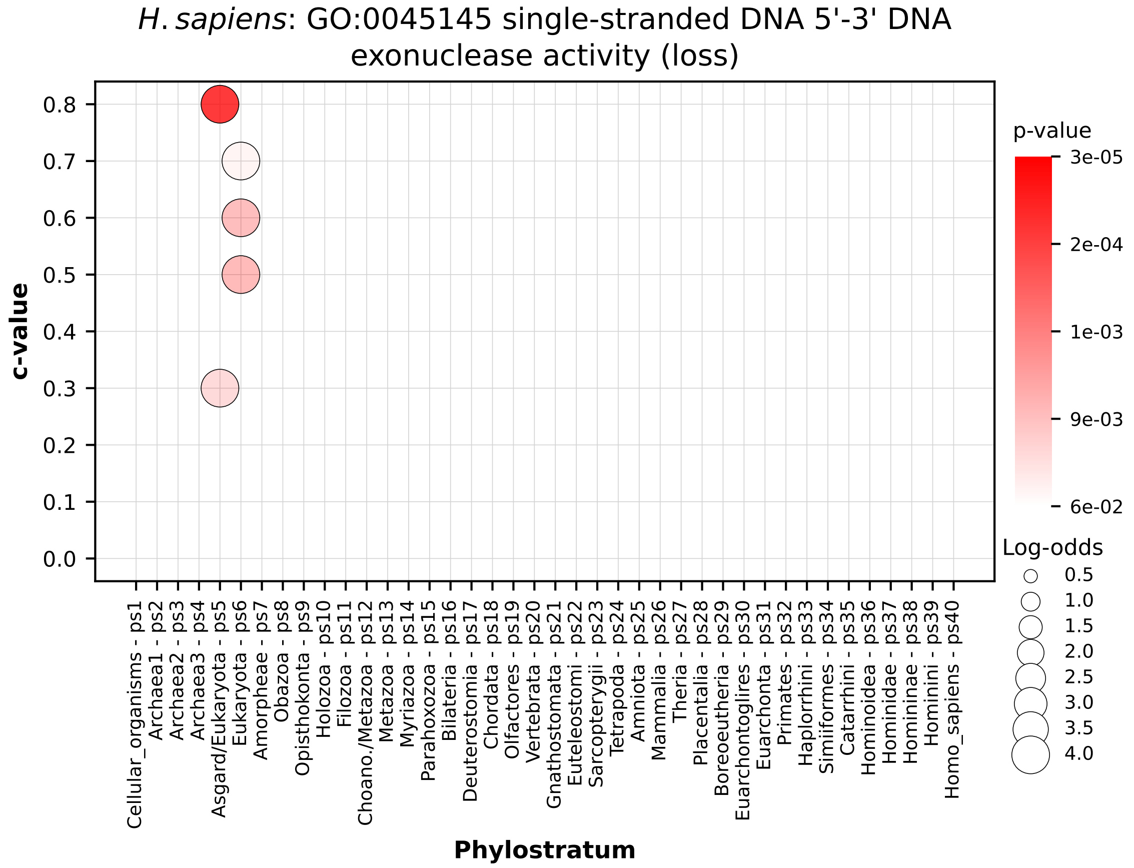

H. sapiens: GO:0045145 single-stranded DNA 5'-3' DNA exonuclease activity (loss)
c-value
0.8
0.7
0.6
0.5
0.4
0.3
0.2
0.1
0.0
p-value
3e-05
2e-04
1e-03
9e-03
6e-02
Log-odds
0.5
1.0
1.5
2.0
2.5
3.0
3.5
4.0
Cellular_organisms - ps1
Archaea1 - ps2
Archaea2 - ps3
Archaea3 - ps4
Asgard/Eukaryota - ps5
Eukaryota - ps6
Amorpheae - ps7
Obazoa - ps8
Opisthokonta - ps9
Holozoa - ps10
Filozoa - ps11
Choano./Metazoa - ps12
Metazoa - ps13
Myriazoa - ps14
Parahoxozoa - ps15
Bilateria - ps16
Deuterostomia - ps17
Chordata - ps18
Olfactores - ps19
Vertebrata - ps20
Gnathostomata - ps21
Euteleostomi - ps22
Sarcopterygii - ps23
Tetrapoda - ps24
Amniota - ps25
Mammalia - ps26
Theria - ps27
Placentalia - ps28
Boreoeutheria - ps29
Euarchontoglires - ps30
Euarchonta - ps31
Primates - ps32
Haplorrhini - ps33
Simiiformes - ps34
Catarrhini - ps35
Hominoidea - ps36
Hominidae - ps37
Homininae - ps38
Hominini - ps39
Homo_sapiens - ps40
Phylostratum

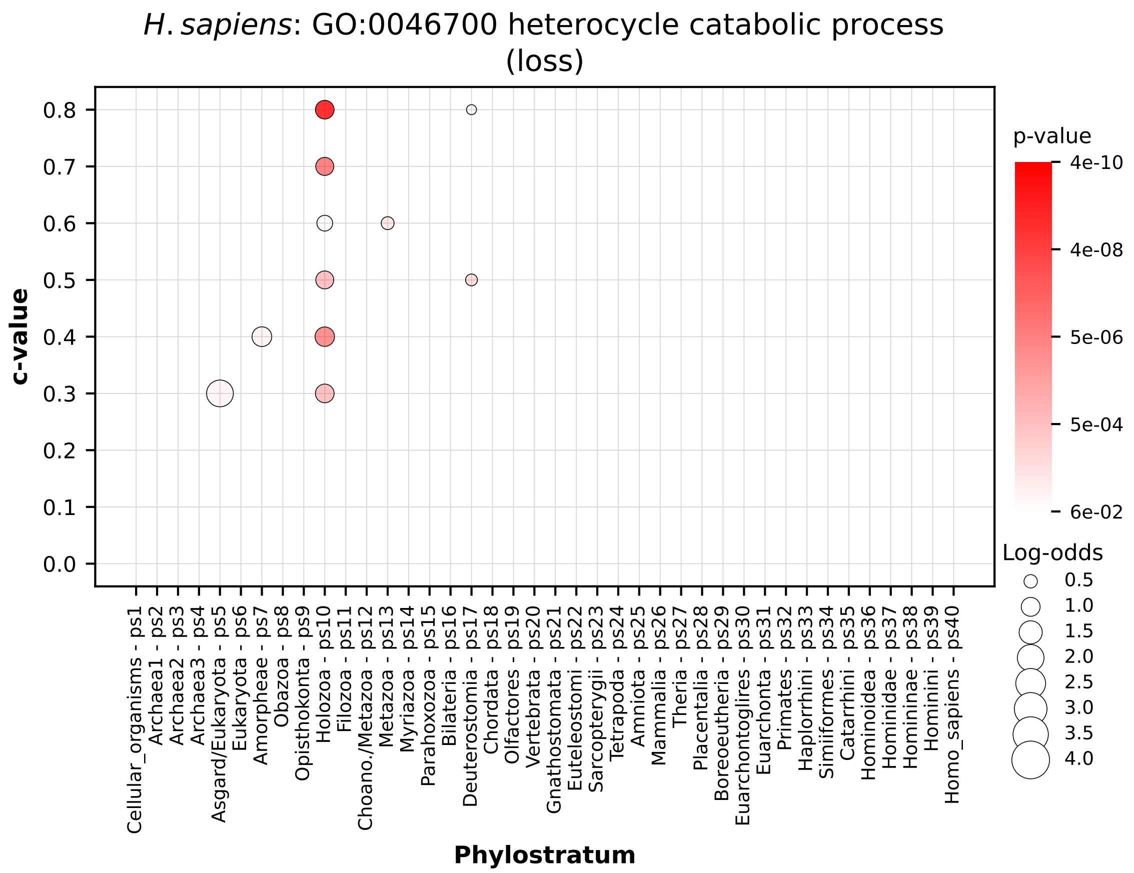

H. sapiens: GO:0046700 heterocycle catabolic process
(loss)
c-value
0.0
0.1
0.2
0.3
0.4
0.5
0.6
0.7
0.8
p-value
4e-10
4e-08
5e-06
5e-04
6e-02
Log-odds
0.5
1.0
1.5
2.0
2.5
3.0
3.5
4.0
Cellular_organisms - ps1
Archaea1 - ps2
Archaea2 - ps3
Archaea3 - ps4
Asgard/Eukaryota - ps5
Eukaryota - ps6
Amorpheae - ps7
Obazoa - ps8
Opisthokonta - ps9
Holozoa - ps10
Filozoa - ps11
Choano./Metazoa - ps12
Metazoa - ps13
Myriazoa - ps14
Parahoxozoa - ps15
Bilateria - ps16
Deuterostomia - ps17
Chordata - ps18
Olfactores - ps19
Vertebrata - ps20
Gnathostomata - ps21
Euteleostomi - ps22
Sarcopterygii - ps23
Tetrapoda - ps24
Amniota - ps25
Mammalia - ps26
Theria - ps27
Placentalia - ps28
Boreoeutheria - ps29
Euarchontoglires - ps30
Euarchonta - ps31
Primates - ps32
Haplorrhini - ps33
Simiiformes - ps34
Catarrhini - ps35
Hominoidea - ps36
Hominidae - ps37
Homininae - ps38
Hominini - ps39
Homo_sapiens - ps40
Phylostratum

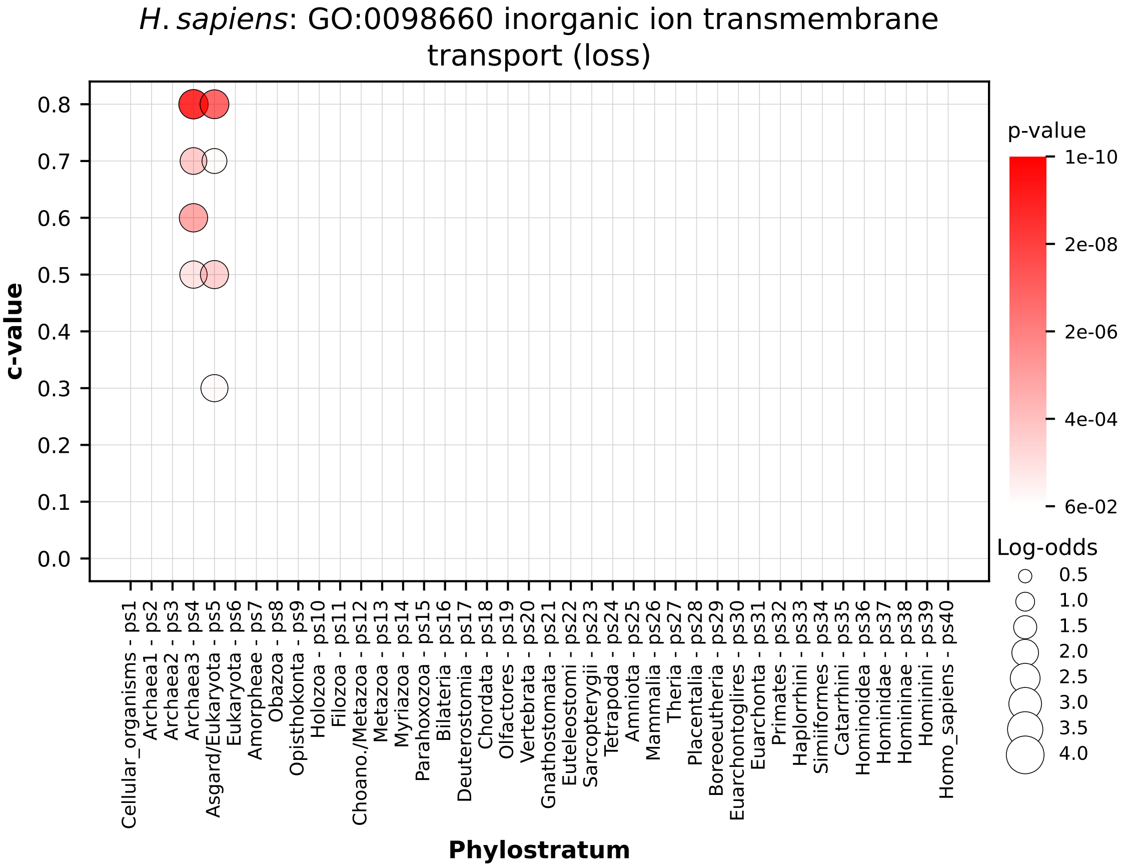

H. sapiens: GO:0098660 inorganic ion transmembrane transport (loss)
c-value
0.0
0.1
0.2
0.3
0.4
0.5
0.6
0.7
0.8
Phylostratum
Cellular_organisms - ps1
Archaea1 - ps2
Archaea2 - ps3
Archaea3 - ps4
Asgard/Eukaryota - ps5
Eukaryota - ps6
Amorpheae - ps7
Obazoa - ps8
Opisthokonta - ps9
Holozoa - ps10
Filozoa - ps11
Choano./Metazoa - ps12
Metazoa - ps13
Myriazoa - ps14
Parahoxozoa - ps15
Bilateria - ps16
Deuterostomia - ps17
Chordata - ps18
Olfactores - ps19
Vertebrata - ps20
Gnathostomata - ps21
Euteleostomi - ps22
Sarcopterygii - ps23
Tetrapoda - ps24
Amniota - ps25
Mammalia - ps26
Theria - ps27
Placentalia - ps28
Boreoeutheria - ps29
Euarchontoglires - ps30
Euarchonta - ps31
Primates - ps32
Haplorrhini - ps33
Simiiformes - ps34
Catarrhini - ps35
Hominoidea - ps36
Hominidae - ps37
Homininae - ps38
Hominini - ps39
Homo_sapiens - ps40
p-value
1e-10
2e-08
2e-06
4e-04
6e-02
Log-odds
0.5
1.0
1.5
2.0
2.5
3.0
3.5
4.0

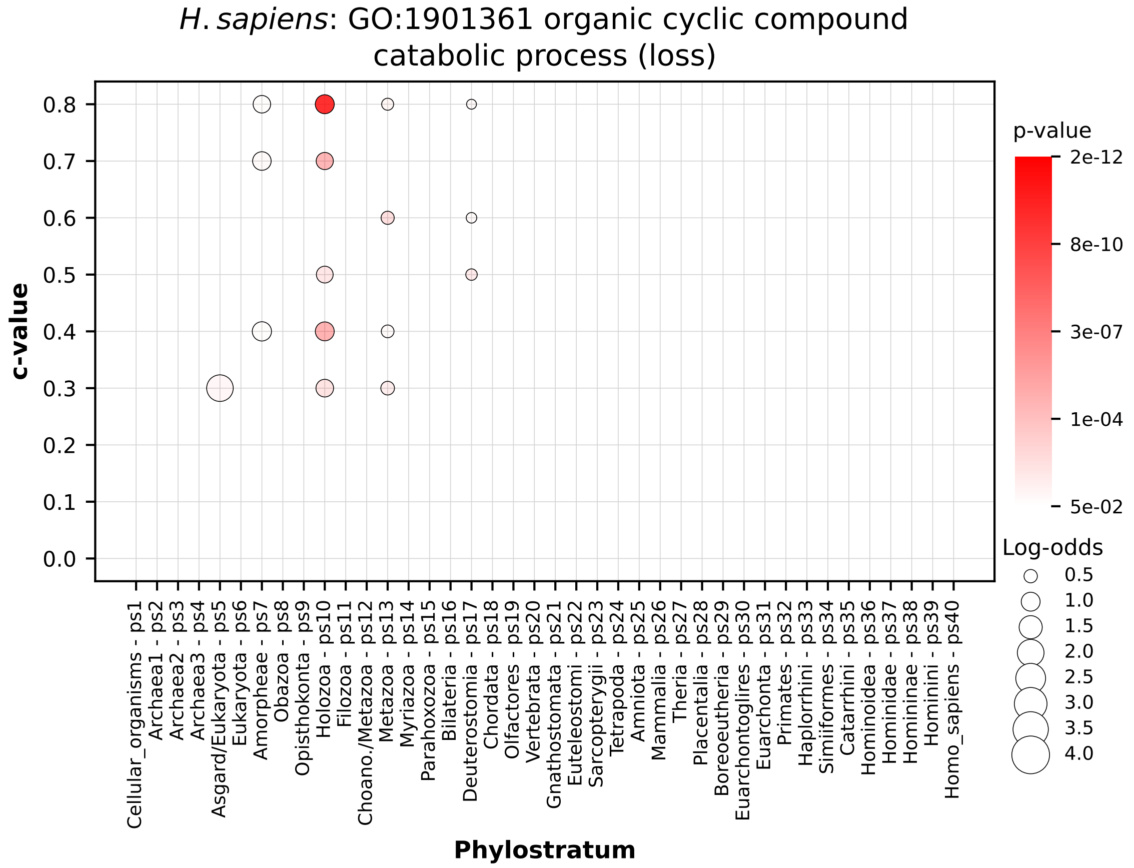

H. sapiens: GO:1901361 organic cyclic compound catabolic process (loss)
c-value
0.0
0.1
0.2
0.3
0.4
0.5
0.6
0.7
0.8
Phylostratum
Cellular_organisms - ps1
Archaea1 - ps2
Archaea2 - ps3
Archaea3 - ps4
Asgard/Eukaryota - ps5
Eukaryota - ps6
Amorpheae - ps7
Obazoa - ps8
Opisthokonta - ps9
Holozoa - ps10
Filozoa - ps11
Choano./Metazoa - ps12
Metazoa - ps13
Myriazoa - ps14
Parahoxozoa - ps15
Bilateria - ps16
Deuterostomia - ps17
Chordata - ps18
Olfactores - ps19
Vertebrata - ps20
Gnathostomata - ps21
Euteleostomi - ps22
Sarcopterygii - ps23
Tetrapoda - ps24
Amniota - ps25
Mammalia - ps26
Theria - ps27
Placentalia - ps28
Boreoeutheria - ps29
Euarchontoglires - ps30
Euarchonta - ps31
Primates - ps32
Haplorrhini - ps33
Simiiformes - ps34
Catarrhini - ps35
Hominoidea - ps36
Hominidae - ps37
Homininae - ps38
Hominini - ps39
Homo_sapiens - ps40
p-value
2e-12
8e-10
3e-07
1e-04
5e-02
Log-odds
0.5
1.0
1.5
2.0
2.5
3.0
3.5
4.0

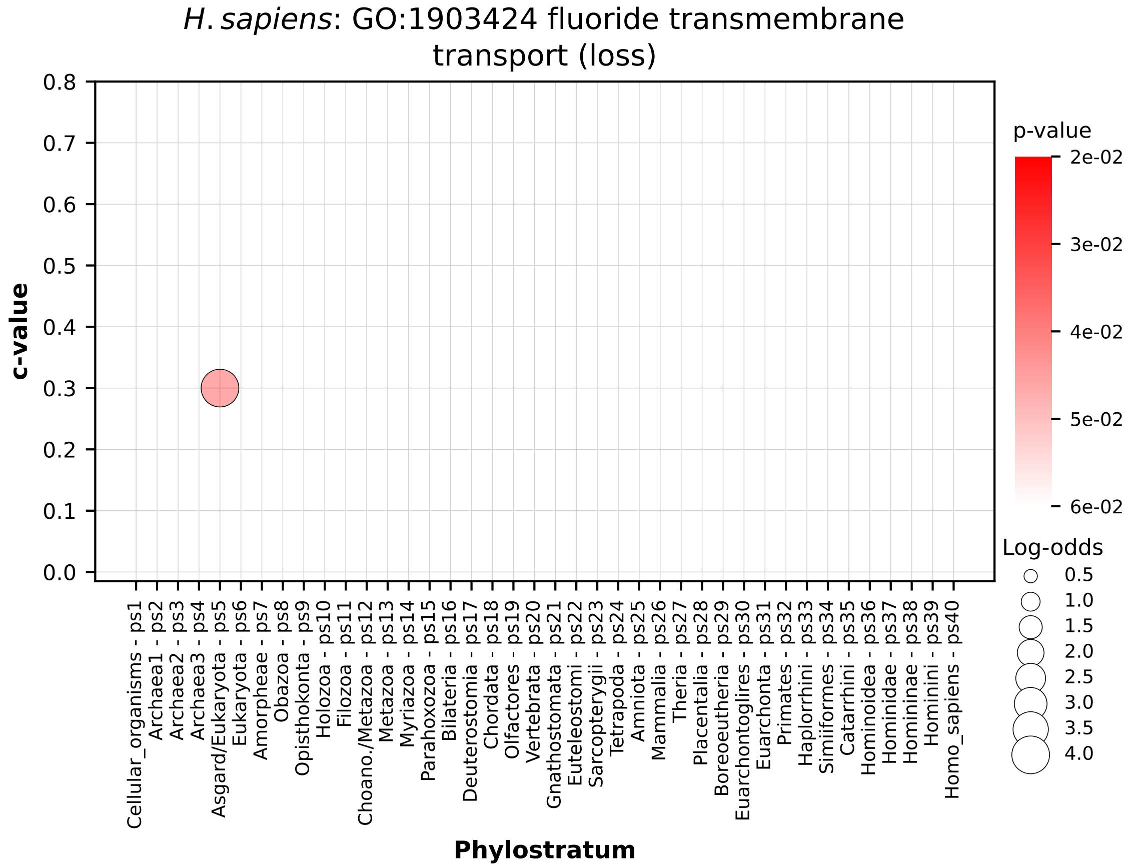
H. sapiens: GO:1903424 fluoride transmembrane transport (loss)
c-value
0.0
0.1
0.2
0.3
0.4
0.5
0.6
0.7
0.8
Phylostratum
Cellular_organisms - ps1
Archaea1 - ps2
Archaea2 - ps3
Archaea3 - ps4
Asgard/Eukaryota - ps5
Eukaryota - ps6
Amorpheae - ps7
Obazoa - ps8
Opisthokonta - ps9
Holozoa - ps10
Filozoa - ps11
Choano./Metazoa - ps12
Metazoa - ps13
Myriazoa - ps14
Parahoxozoa - ps15
Bilateria - ps16
Deuterostomia - ps17
Chordata - ps18
Olfactores - ps19
Vertebrata - ps20
Gnathostomata - ps21
Euteleostomi - ps22
Sarcopterygii - ps23
Tetrapoda - ps24
Amniota - ps25
Mammalia - ps26
Theria - ps27
Placentalia - ps28
Boreoeutheria - ps29
Euarchontoglires - ps30
Euarchonta - ps31
Primates - ps32
Haplorrhini - ps33
Simiiformes - ps34
Catarrhini - ps35
Hominoidea - ps36
Hominidae - ps37
Homininae - ps38
Hominini - ps39
Homo_sapiens - ps40
p-value
2e-02
3e-02
4e-02
5e-02
6e-02
Log-odds
0.5
1.0
1.5
2.0
2.5
3.0
3.5
4.0

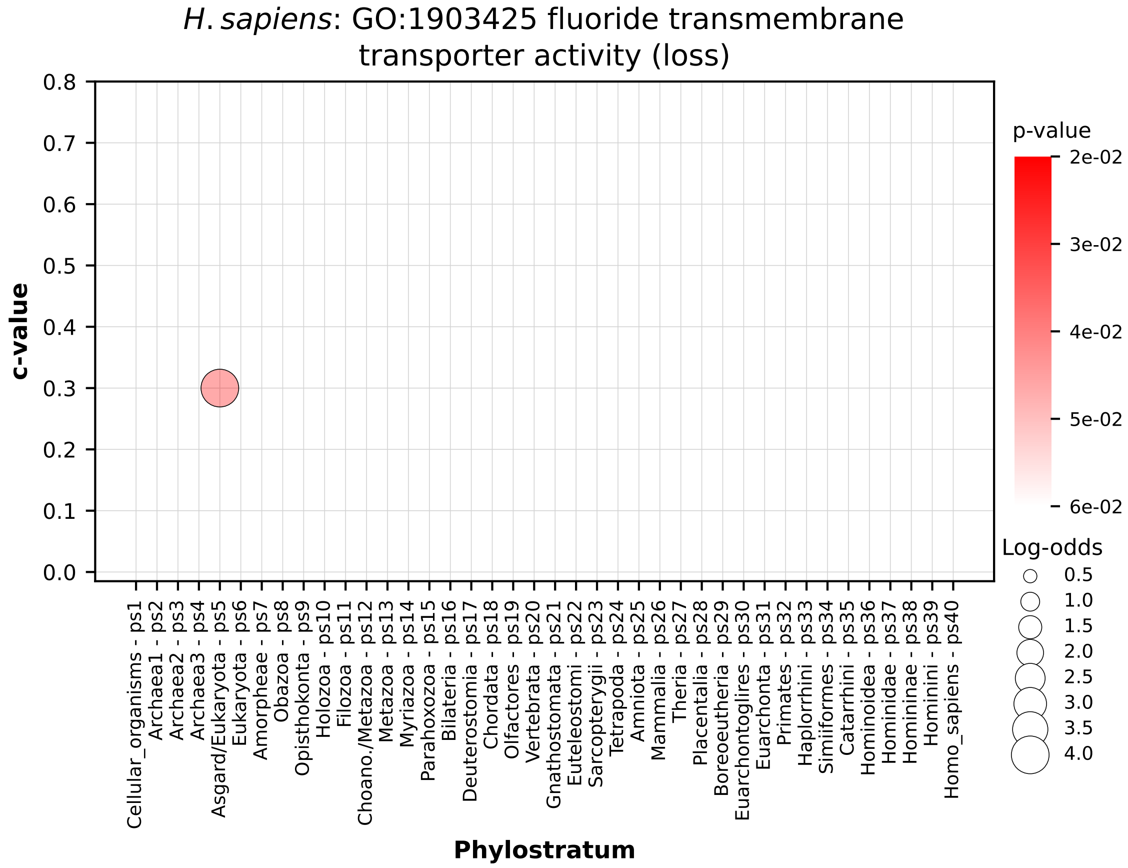

H. sapiens: GO:1903425 fluoride transmembrane transporter activity (loss)
c-value
0.0
0.1
0.2
0.3
0.4
0.5
0.6
0.7
0.8
Phylostratum
Cellular_organisms - ps1
Archaea1 - ps2
Archaea2 - ps3
Archaea3 - ps4
Asgard/Eukaryota - ps5
Eukaryota - ps6
Amorpheae - ps7
Obazoa - ps8
Opisthokonta - ps9
Holozoa - ps10
Filozoa - ps11
Choano./Metazoa - ps12
Metazoa - ps13
Myriazoa - ps14
Parahoxozoa - ps15
Bilateria - ps16
Deuterostomia - ps17
Chordata - ps18
Olfactores - ps19
Vertebrata - ps20
Gnathostomata - ps21
Euteleostomi - ps22
Sarcopterygii - ps23
Tetrapoda - ps24
Amniota - ps25
Mammalia - ps26
Theria - ps27
Placentalia - ps28
Boreoeutheria - ps29
Euarchontoglires - ps30
Euarchonta - ps31
Primates - ps32
Haplorrhini - ps33
Simiiformes - ps34
Catarrhini - ps35
Hominoidea - ps36
Hominidae - ps37
Homininae - ps38
Hominini - ps39
Homo_sapiens - ps40
p-value
2e-02
3e-02
4e-02
5e-02
6e-02
Log-odds
0.5
1.0
1.5
2.0
2.5
3.0
3.5
4.0

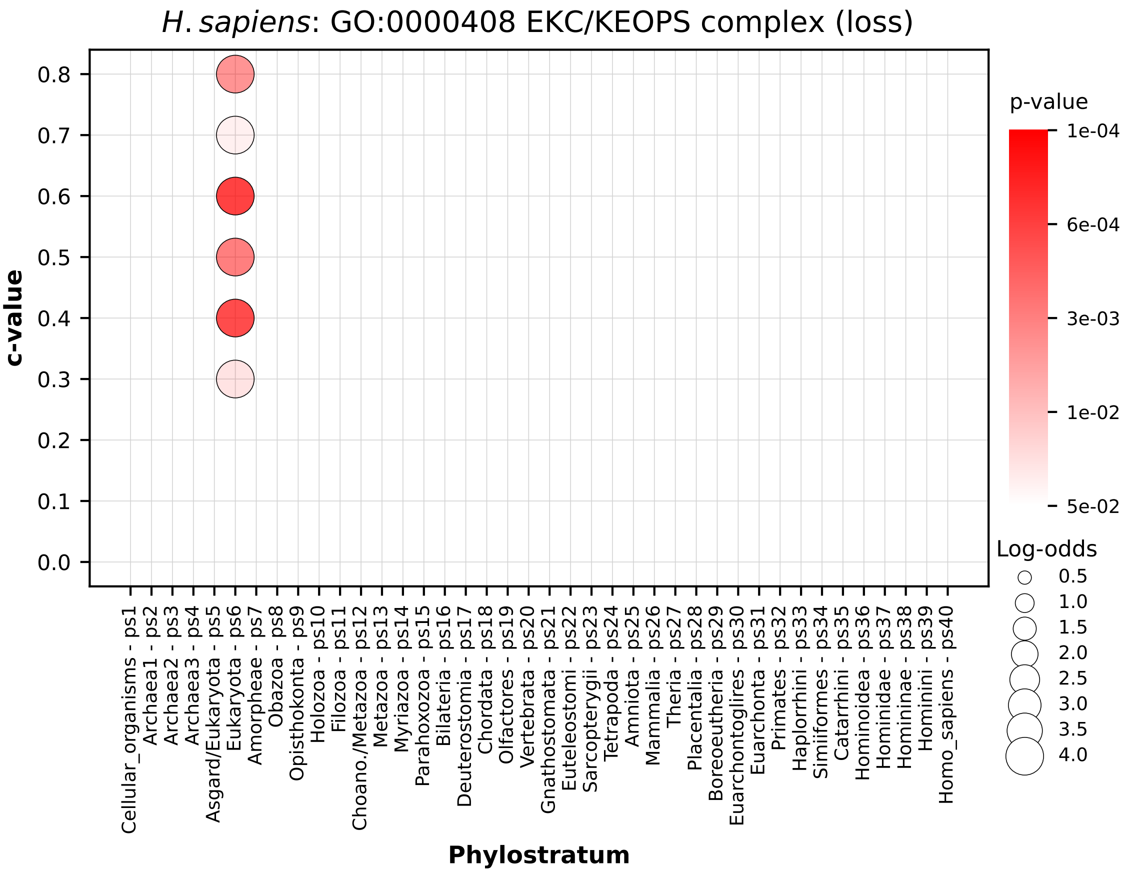
H. sapiens: GO:0000408 EKC/KEOPS complex (loss)
c-value
0.8
0.7
0.6
0.5
0.4
0.3
0.2
0.1
0.0
Phylostratum
Cellular_organisms - ps1
Archaea1 - ps2
Archaea2 - ps3
Archaea3 - ps4
Asgard/Eukaryota - ps5
Eukaryota - ps6
Amorpheae - ps7
Obazoa - ps8
Opisthokonta - ps9
Holozoa - ps10
Filozoa - ps11
Choano./Metazoa - ps12
Metazoa - ps13
Myriazoa - ps14
Parahoxozoa - ps15
Bilateria - ps16
Deuterostomia - ps17
Chordata - ps18
Olfactores - ps19
Vertebrata - ps20
Gnathostomata - ps21
Euteleostomi - ps22
Sarcopterygii - ps23
Tetrapoda - ps24
Amniota - ps25
Mammalia - ps26
Theria - ps27
Placentalia - ps28
Boreoeutheria - ps29
Euarchontoglires - ps30
Euarchonta - ps31
Primates - ps32
Haplorrhini - ps33
Simiiformes - ps34
Catarrhini - ps35
Hominoidea - ps36
Hominidae - ps37
Homininae - ps38
Hominini - ps39
Homo_sapiens - ps40
p-value
1e-04
6e-04
3e-03
1e-02
5e-02
Log-odds
0.5
1.0
1.5
2.0
2.5
3.0
3.5
4.0

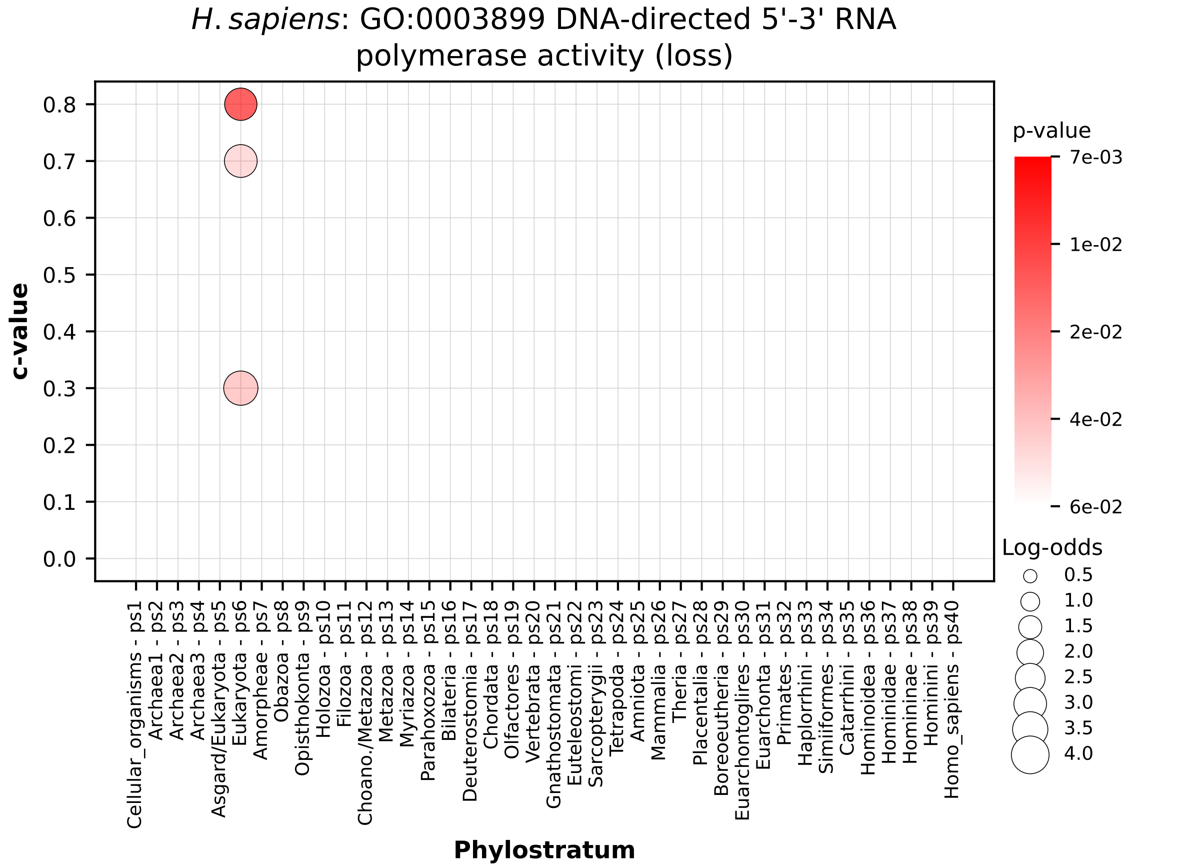

H. sapiens: GO:0003899 DNA-directed 5'-3' RNA polymerase activity (loss)
c-value
0.8
0.7
0.6
0.5
0.4
0.3
0.2
0.1
0.0
Phylostratum
Cellular_organisms - ps1
Archaea1 - ps2
Archaea2 - ps3
Archaea3 - ps4
Asgard/Eukaryota - ps5
Eukaryota - ps6
Amorpheae - ps7
Obazoa - ps8
Opisthokonta - ps9
Holozoa - ps10
Filozoa - ps11
Choano./Metazoa - ps12
Metazoa - ps13
Myriazoa - ps14
Parahoxozoa - ps15
Bilateria - ps16
Deuterostomia - ps17
Chordata - ps18
Olfactores - ps19
Vertebrata - ps20
Gnathostomata - ps21
Euteleostomi - ps22
Sarcopterygii - ps23
Tetrapoda - ps24
Amniota - ps25
Mammalia - ps26
Theria - ps27
Placentalia - ps28
Boreoeutheria - ps29
Euarchontoglires - ps30
Euarchonta - ps31
Primates - ps32
Haplorrhini - ps33
Simiiformes - ps34
Catarrhini - ps35
Hominoidea - ps36
Hominidae - ps37
Homininae - ps38
Hominini - ps39
Homo_sapiens - ps40
p-value
7e-03
1e-02
2e-02
4e-02
6e-02
Log-odds
0.5
1.0
1.5
2.0
2.5
3.0
3.5
4.0

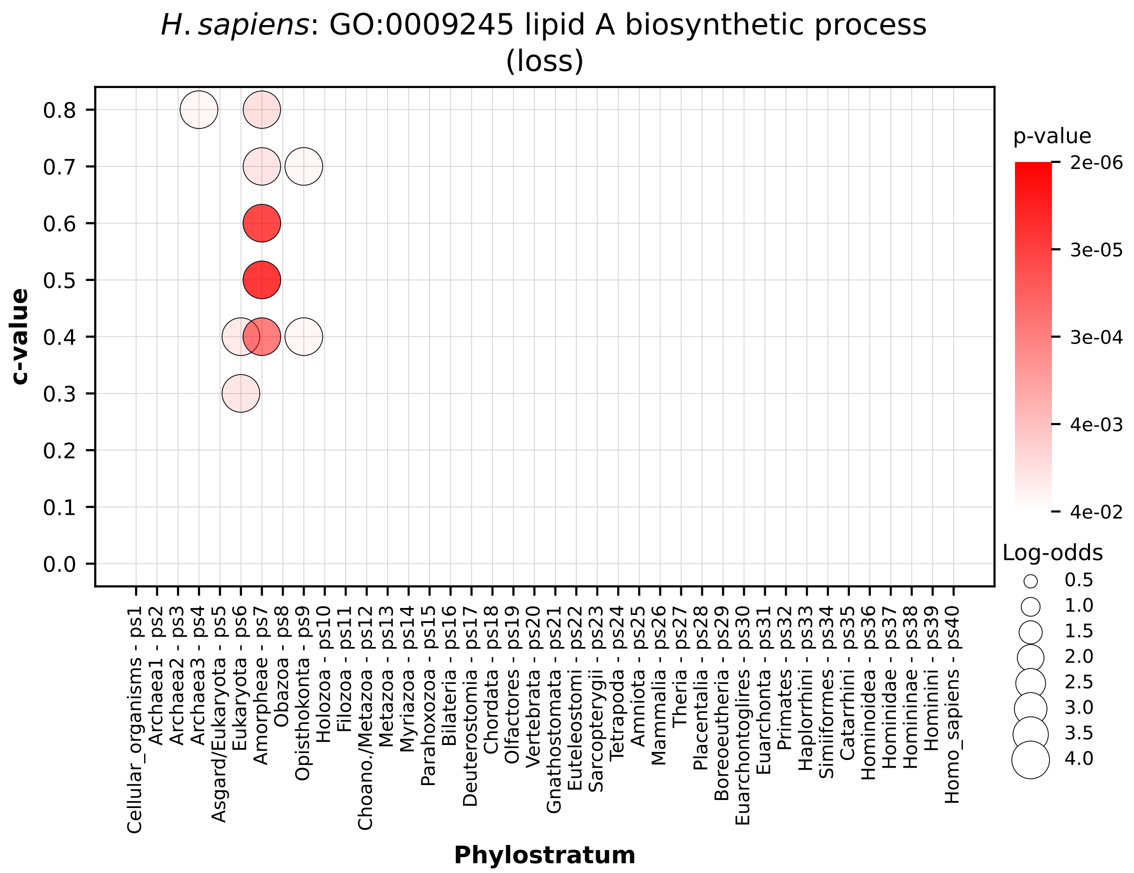

H. sapiens: GO:0009245 lipid A biosynthetic process
(loss)
c-value
0.0
0.1
0.2
0.3
0.4
0.5
0.6
0.7
0.8
Phylostratum
Cellular_organisms - ps1
Archaea1 - ps2
Archaea2 - ps3
Archaea3 - ps4
Asgard/Eukaryota - ps5
Eukaryota - ps6
Amorpheae - ps7
Obazoa - ps8
Opisthokonta - ps9
Holozoa - ps10
Filozoa - ps11
Choano./Metazoa - ps12
Metazoa - ps13
Myriazoa - ps14
Parahoxozoa - ps15
Bilateria - ps16
Deuterostomia - ps17
Chordata - ps18
Olfactores - ps19
Vertebrata - ps20
Gnathostomata - ps21
Euteleostomi - ps22
Sarcopterygii - ps23
Tetrapoda - ps24
Amniota - ps25
Mammalia - ps26
Theria - ps27
Placentalia - ps28
Boreoeutheria - ps29
Euarchontoglires - ps30
Euarchonta - ps31
Primates - ps32
Haplorrhini - ps33
Simiiformes - ps34
Catarrhini - ps35
Hominoidea - ps36
Hominidae - ps37
Homininae - ps38
Hominini - ps39
Homo_sapiens - ps40
p-value
2e-06
3e-05
3e-04
4e-03
4e-02
Log-odds
0.5
1.0
1.5
2.0
2.5
3.0
3.5
4.0

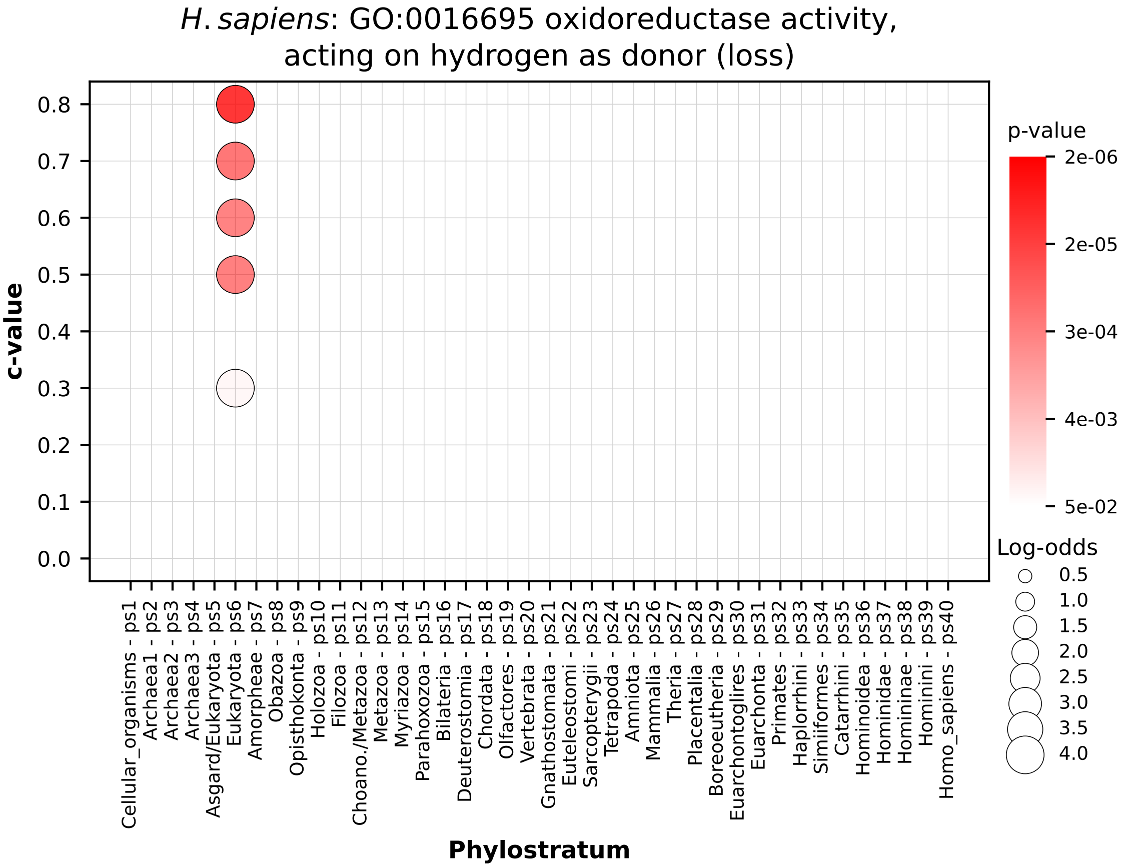

H. sapiens: GO:0016695 oxidoreductase activity, acting on hydrogen as donor (loss)
c-value
0.8
0.7
0.6
0.5
0.4
0.3
0.2
0.1
0.0
p-value
2e-06
2e-05
3e-04
4e-03
5e-02
Log-odds
0.5
1.0
1.5
2.0
2.5
3.0
3.5
4.0
Cellular_organisms - ps1
Archaea1 - ps2
Archaea2 - ps3
Archaea3 - ps4
Asgard/Eukaryota - ps5
Eukaryota - ps6
Amorpheae - ps7
Obazoa - ps8
Opisthokonta - ps9
Holozoa - ps10
Filozoa - ps11
Choano./Metazoa - ps12
Metazoa - ps13
Myriazoa - ps14
Parahoxozoa - ps15
Bilateria - ps16
Deuterostomia - ps17
Chordata - ps18
Olfactores - ps19
Vertebrata - ps20
Gnathostomata - ps21
Euteleostomi - ps22
Sarcopterygii - ps23
Tetrapoda - ps24
Amniota - ps25
Mammalia - ps26
Theria - ps27
Placentalia - ps28
Boreoeutheria - ps29
Euarchontoglires - ps30
Euarchonta - ps31
Primates - ps32
Haplorrhini - ps33
Simiiformes - ps34
Catarrhini - ps35
Hominoidea - ps36
Hominidae - ps37
Homininae - ps38
Hominini - ps39
Homo_sapiens - ps40
Phylostratum

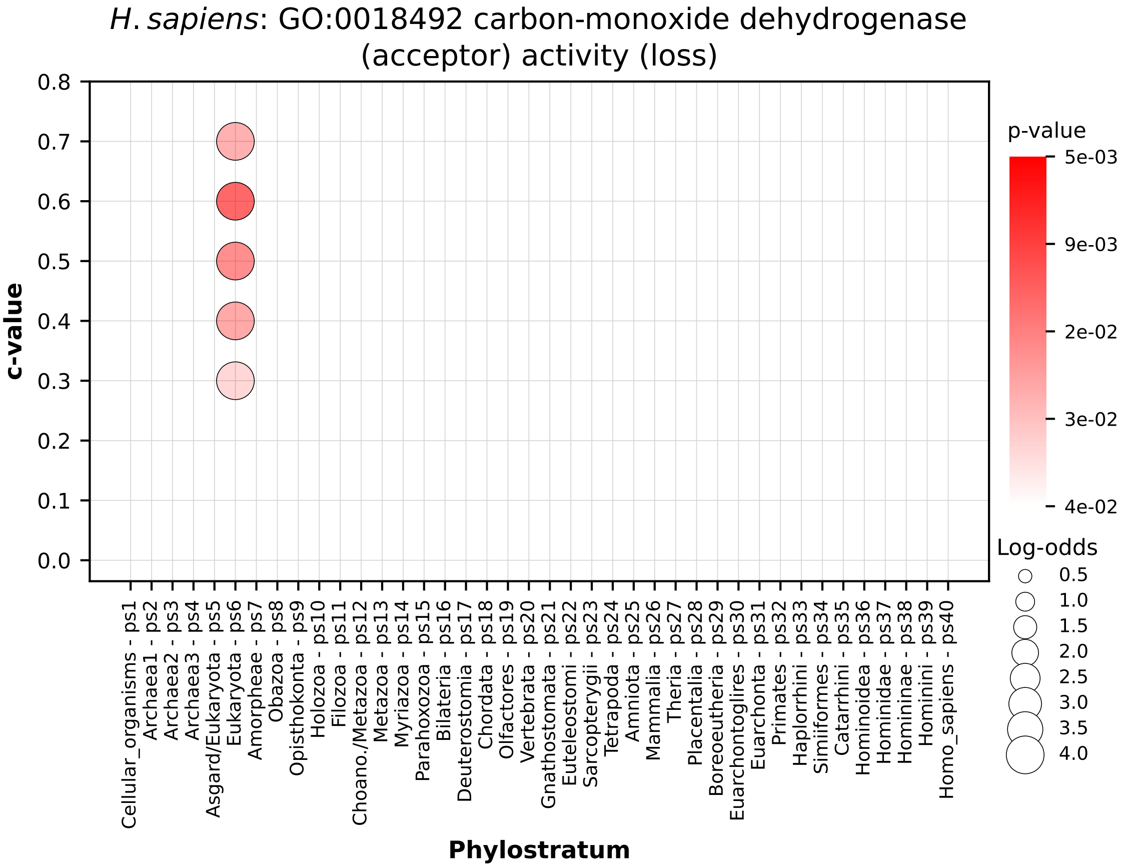

H. sapiens: GO:0018492 carbon-monoxide dehydrogenase (acceptor) activity (loss)
c-value
0.8
0.7
0.6
0.5
0.4
0.3
0.2
0.1
0.0
p-value
5e-03
9e-03
2e-02
3e-02
4e-02
Log-odds
0.5
1.0
1.5
2.0
2.5
3.0
3.5
4.0
Cellular_organisms - ps1
Archaea1 - ps2
Archaea2 - ps3
Archaea3 - ps4
Asgard/Eukaryota - ps5
Eukaryota - ps6
Amorpheae - ps7
Obazoa - ps8
Opisthokonta - ps9
Holozoa - ps10
Filozoa - ps11
Choano./Metazoa - ps12
Metazoa - ps13
Myriazoa - ps14
Parahoxozoa - ps15
Bilateria - ps16
Deuterostomia - ps17
Chordata - ps18
Olfactores - ps19
Vertebrata - ps20
Gnathostomata - ps21
Euteleostomi - ps22
Sarcopterygii - ps23
Tetrapoda - ps24
Amniota - ps25
Mammalia - ps26
Theria - ps27
Placentalia - ps28
Boreoeutheria - ps29
Euarchontoglires - ps30
Euarchonta - ps31
Primates - ps32
Haplorrhini - ps33
Simiiformes - ps34
Catarrhini - ps35
Hominoidea - ps36
Hominidae - ps37
Homininae - ps38
Hominini - ps39
Homo_sapiens - ps40
Phylostratum

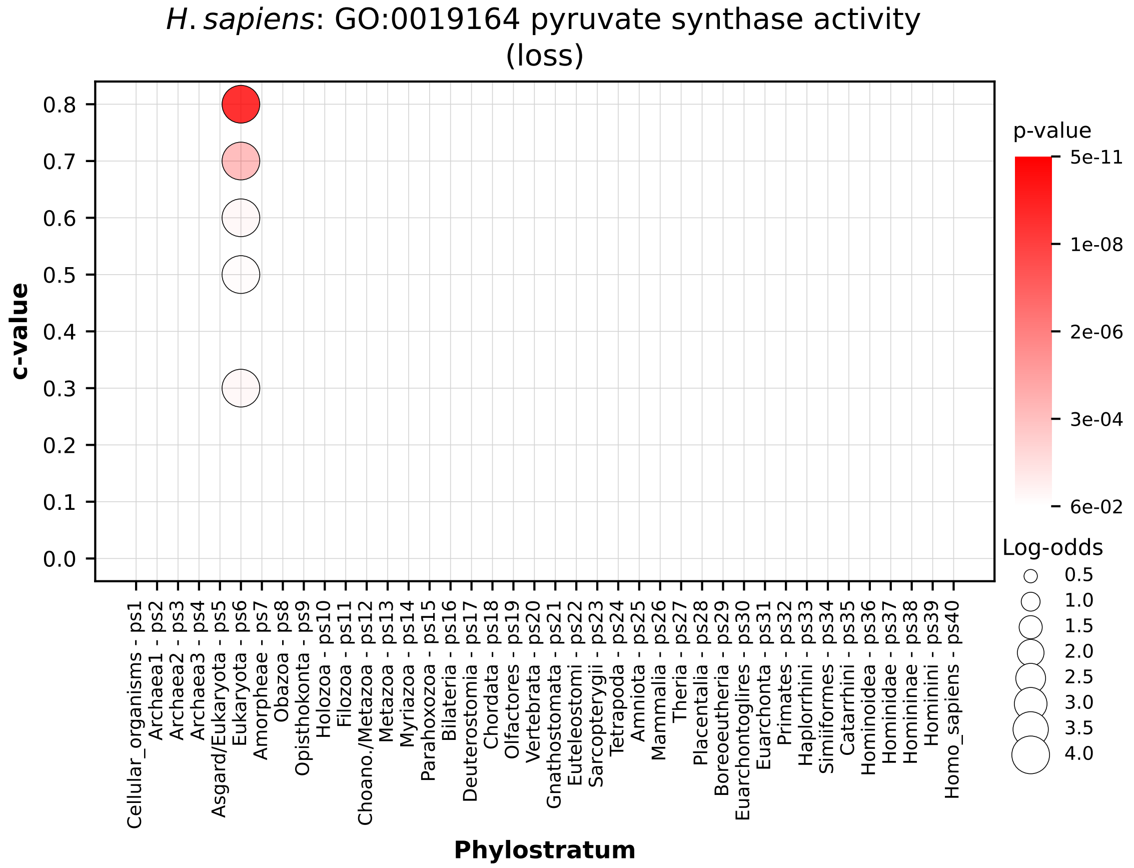
H. sapiens: GO:0019164 pyruvate synthase activity
(loss)
c-value
0.8
0.7
0.6
0.5
0.4
0.3
0.2
0.1
0.0
p-value
5e-11
1e-08
2e-06
3e-04
6e-02
Log-odds
0.5
1.0
1.5
2.0
2.5
3.0
3.5
4.0
Cellular_organisms - ps1
Archaea1 - ps2
Archaea2 - ps3
Archaea3 - ps4
Asgard/Eukaryota - ps5
Eukaryota - ps6
Amorpheae - ps7
Obazoa - ps8
Opisthokonta - ps9
Holozoa - ps10
Filozoa - ps11
Choano./Metazoa - ps12
Metazoa - ps13
Myriazoa - ps14
Parahoxozoa - ps15
Bilateria - ps16
Deuterostomia - ps17
Chordata - ps18
Olfactores - ps19
Vertebrata - ps20
Gnathostomata - ps21
Euteleostomi - ps22
Sarcopterygii - ps23
Tetrapoda - ps24
Amniota - ps25
Mammalia - ps26
Theria - ps27
Placentalia - ps28
Boreoeutheria - ps29
Euarchontoglires - ps30
Euarchonta - ps31
Primates - ps32
Haplorrhini - ps33
Simiiformes - ps34
Catarrhini - ps35
Hominoidea - ps36
Hominidae - ps37
Homininae - ps38
Hominini - ps39
Homo_sapiens - ps40
Phylostratum

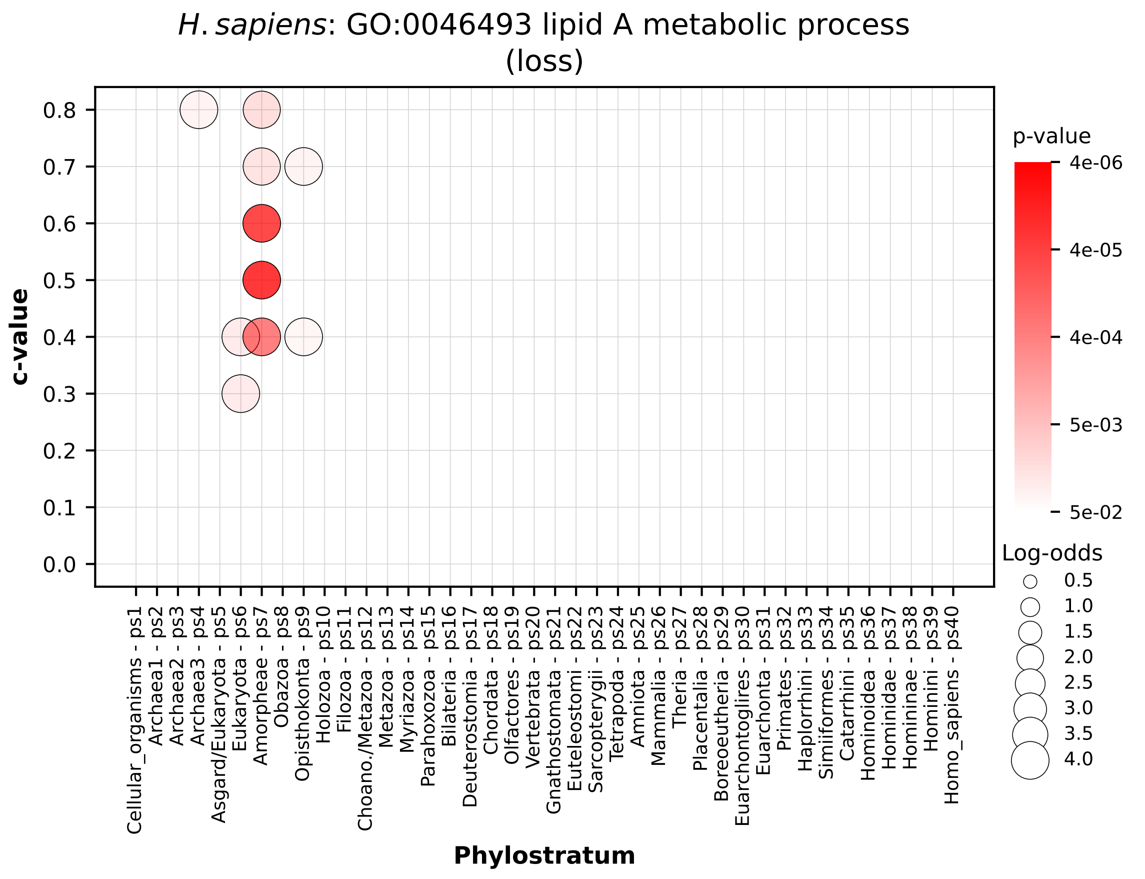

H. sapiens: GO:0046493 lipid A metabolic process
(loss)
c-value
0.0
0.1
0.2
0.3
0.4
0.5
0.6
0.7
0.8
p-value
4e-06
4e-05
4e-04
5e-03
5e-02
Log-odds
0.5
1.0
1.5
2.0
2.5
3.0
3.5
4.0
Cellular_organisms - ps1
Archaea1 - ps2
Archaea2 - ps3
Archaea3 - ps4
Asgard/Eukaryota - ps5
Eukaryota - ps6
Amorpheae - ps7
Obazoa - ps8
Opisthokonta - ps9
Holozoa - ps10
Filozoa - ps11
Choano./Metazoa - ps12
Metazoa - ps13
Myriazoa - ps14
Parahoxozoa - ps15
Bilateria - ps16
Deuterostomia - ps17
Chordata - ps18
Olfactores - ps19
Vertebrata - ps20
Gnathostomata - ps21
Euteleostomi - ps22
Sarcopterygii - ps23
Tetrapoda - ps24
Amniota - ps25
Mammalia - ps26
Theria - ps27
Placentalia - ps28
Boreoeutheria - ps29
Euarchontoglires - ps30
Euarchonta - ps31
Primates - ps32
Haplorrhini - ps33
Simiiformes - ps34
Catarrhini - ps35
Hominoidea - ps36
Hominidae - ps37
Homininae - ps38
Hominini - ps39
Homo_sapiens - ps40
Phylostratum

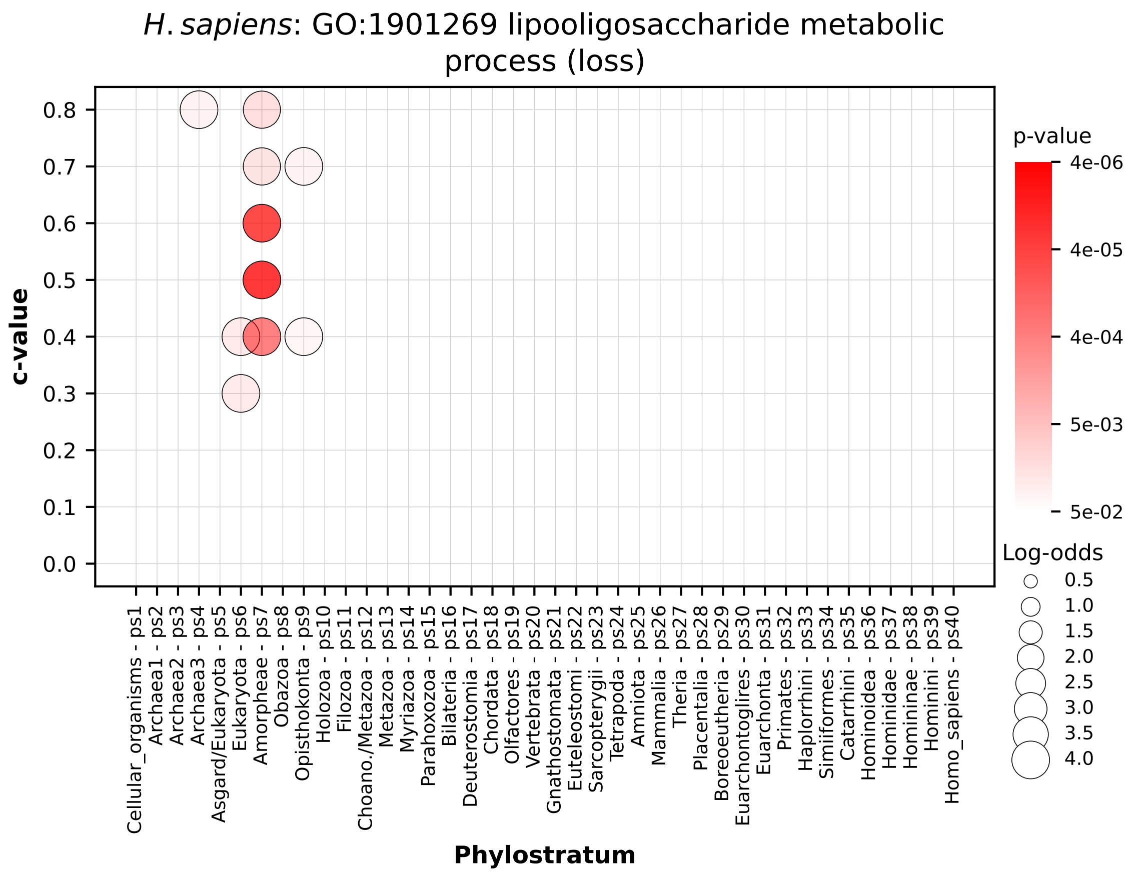

H. sapiens: GO:1901269 lipooligosaccharide metabolic process (loss)
c-value
0.8
0.7
0.6
0.5
0.4
0.3
0.2
0.1
0.0
p-value
4e-06
4e-05
4e-04
5e-03
5e-02
Log-odds
0.5
1.0
1.5
2.0
2.5
3.0
3.5
4.0
Cellular_organisms - ps1
Archaea1 - ps2
Archaea2 - ps3
Archaea3 - ps4
Asgard/Eukaryota - ps5
Eukaryota - ps6
Amorpheae - ps7
Obazoa - ps8
Opisthokonta - ps9
Holozoa - ps10
Filozoa - ps11
Choano./Metazoa - ps12
Metazoa - ps13
Myriazoa - ps14
Parahoxozoa - ps15
Bilateria - ps16
Deuterostomia - ps17
Chordata - ps18
Olfactores - ps19
Vertebrata - ps20
Gnathostomata - ps21
Euteleostomi - ps22
Sarcopterygii - ps23
Tetrapoda - ps24
Amniota - ps25
Mammalia - ps26
Theria - ps27
Placentalia - ps28
Boreoeutheria - ps29
Euarchontoglires - ps30
Euarchonta - ps31
Primates - ps32
Haplorrhini - ps33
Simiiformes - ps34
Catarrhini - ps35
Hominoidea - ps36
Hominidae - ps37
Homininae - ps38
Hominini - ps39
Homo_sapiens - ps40
Phylostratum

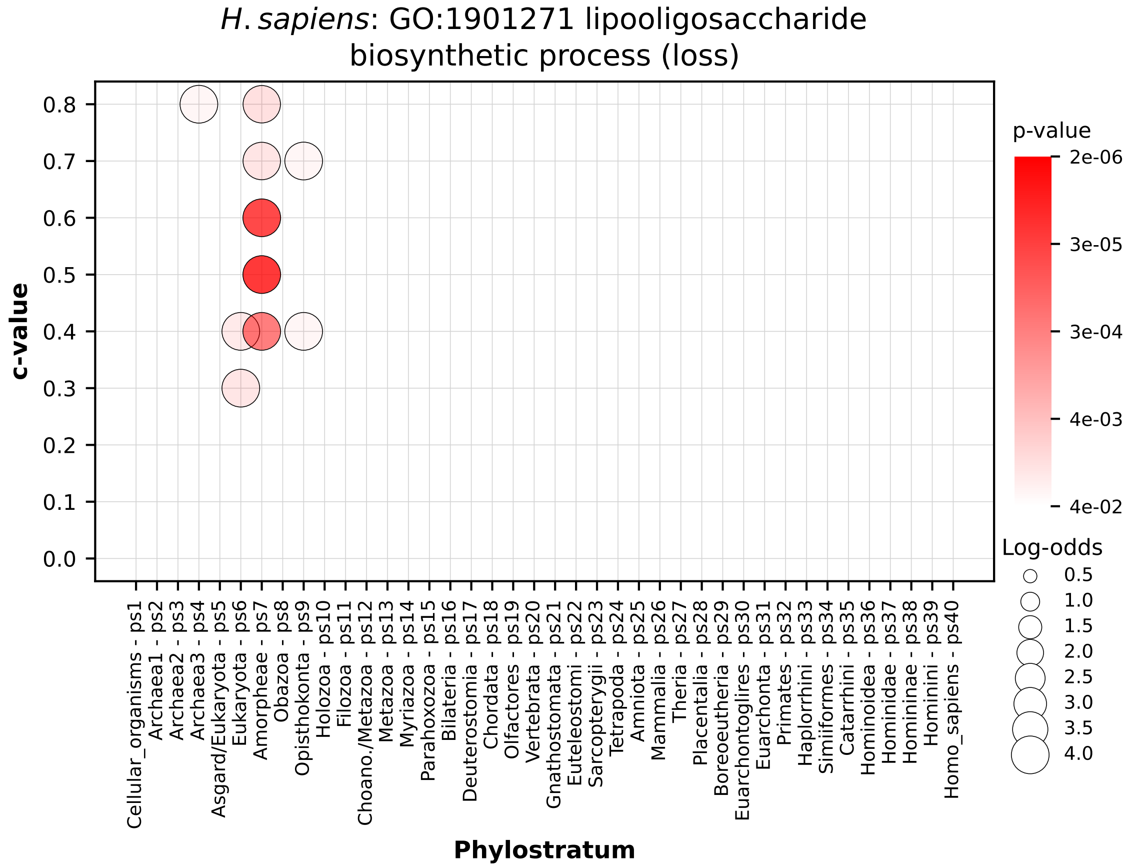
H. sapiens: GO:1901271 lipooligosaccharide biosynthetic process (loss)
c-value
0.0
0.1
0.2
0.3
0.4
0.5
0.6
0.7
0.8
p-value
2e-06
3e-05
3e-04
4e-03
4e-02
Log-odds
0.5
1.0
1.5
2.0
2.5
3.0
3.5
4.0
Cellular_organisms - ps1
Archaea1 - ps2
Archaea2 - ps3
Archaea3 - ps4
Asgard/Eukaryota - ps5
Eukaryota - ps6
Amorpheae - ps7
Obazoa - ps8
Opisthokonta - ps9
Holozoa - ps10
Filozoa - ps11
Choano./Metazoa - ps12
Metazoa - ps13
Myriazoa - ps14
Parahoxozoa - ps15
Bilateria - ps16
Deuterostomia - ps17
Chordata - ps18
Olfactores - ps19
Vertebrata - ps20
Gnathostomata - ps21
Euteleostomi - ps22
Sarcopterygii - ps23
Tetrapoda - ps24
Amniota - ps25
Mammalia - ps26
Theria - ps27
Placentalia - ps28
Boreoeutheria - ps29
Euarchontoglires - ps30
Euarchonta - ps31
Primates - ps32
Haplorrhini - ps33
Simiiformes - ps34
Catarrhini - ps35
Hominoidea - ps36
Hominidae - ps37
Homininae - ps38
Hominini - ps39
Homo_sapiens - ps40
Phylostratum

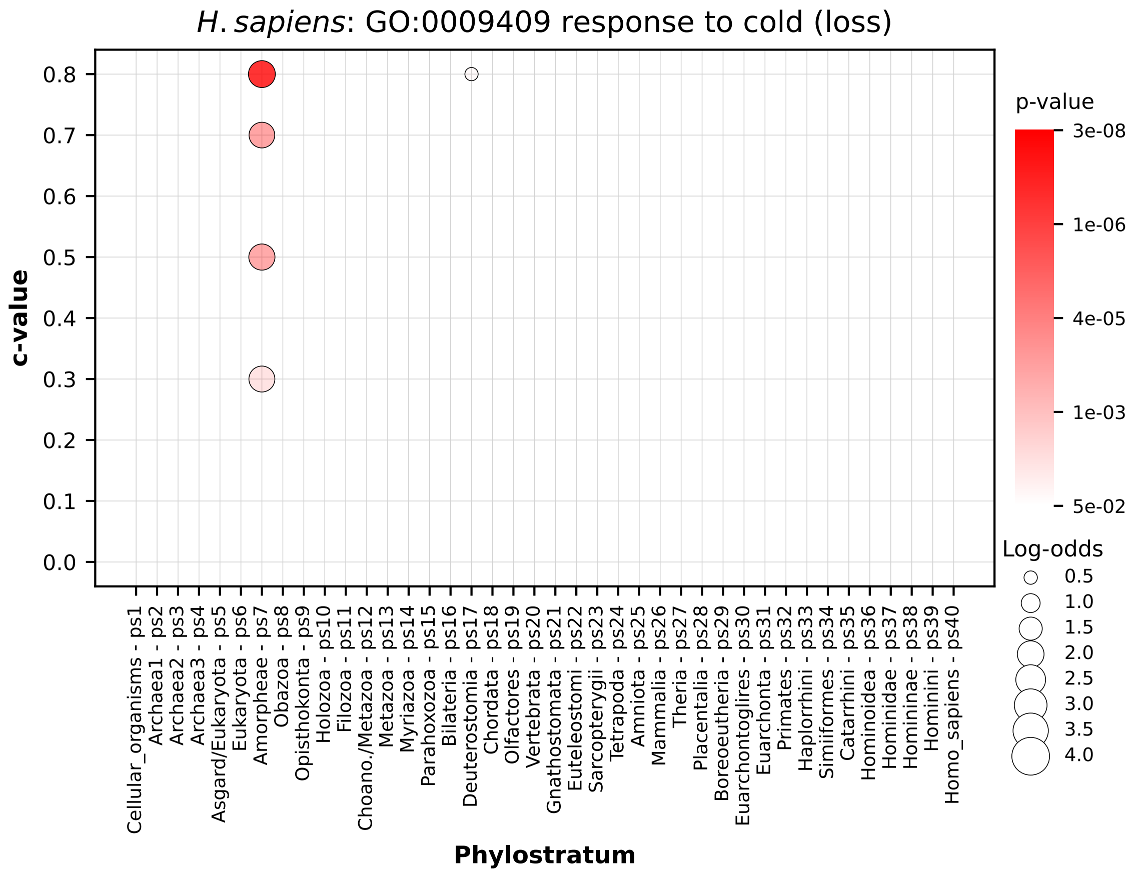

H. sapiens: GO:0009409 response to cold (loss)
c-value
0.0
0.1
0.2
0.3
0.4
0.5
0.6
0.7
0.8
Phylostratum
Cellular_organisms - ps1
Archaea1 - ps2
Archaea2 - ps3
Archaea3 - ps4
Asgard/Eukaryota - ps5
Eukaryota - ps6
Amorpheae - ps7
Obazoa - ps8
Opisthokonta - ps9
Holozoa - ps10
Filozoa - ps11
Choano./Metazoa - ps12
Metazoa - ps13
Myriazoa - ps14
Parahoxozoa - ps15
Bilateria - ps16
Deuterostomia - ps17
Chordata - ps18
Olfactores - ps19
Vertebrata - ps20
Gnathostomata - ps21
Euteleostomi - ps22
Sarcopterygii - ps23
Tetrapoda - ps24
Amniota - ps25
Mammalia - ps26
Theria - ps27
Placentalia - ps28
Boreoeutheria - ps29
Euarchontoglires - ps30
Euarchonta - ps31
Primates - ps32
Haplorrhini - ps33
Simiiformes - ps34
Catarrhini - ps35
Hominoidea - ps36
Hominidae - ps37
Homininae - ps38
Hominini - ps39
Homo_sapiens - ps40
p-value
3e-08
1e-06
4e-05
1e-03
5e-02
Log-odds
0.5
1.0
1.5
2.0
2.5
3.0
3.5
4.0

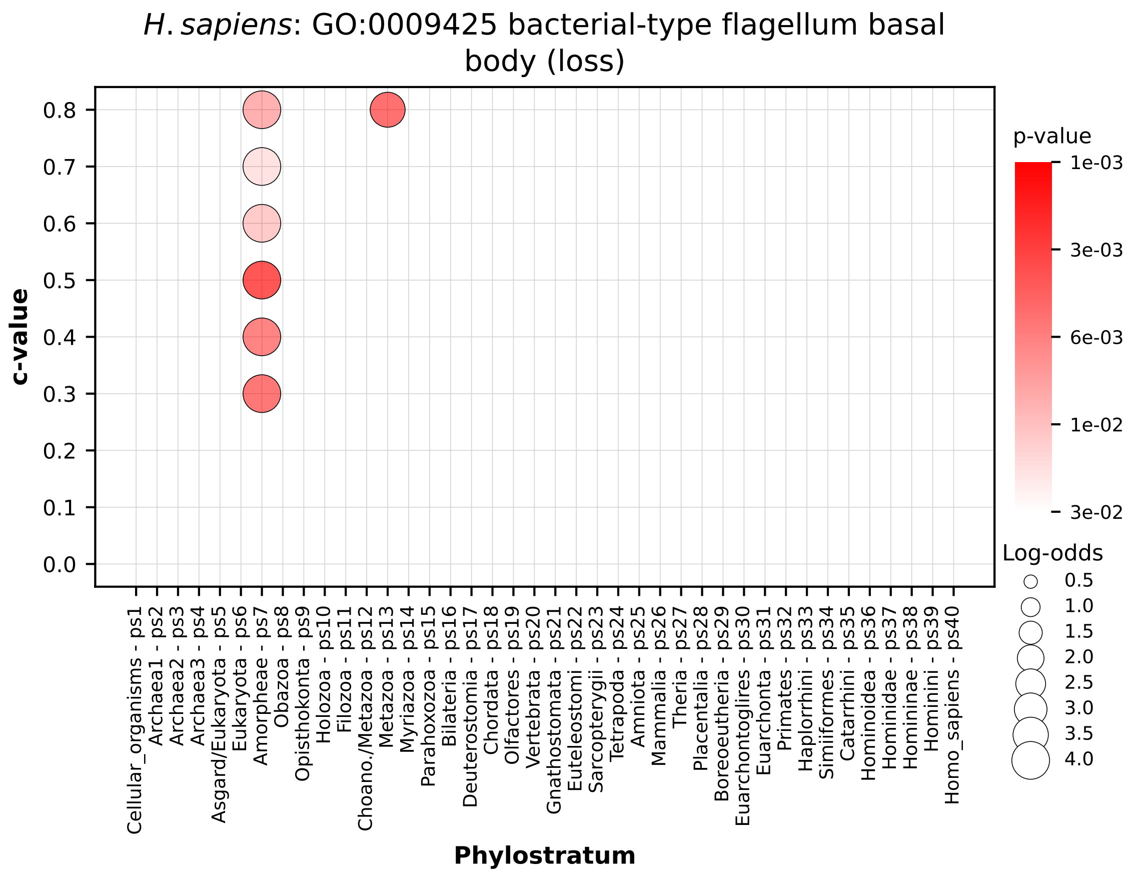

H. sapiens: GO:0009425 bacterial-type flagellum basal body (loss)
c-value
0.8
0.7
0.6
0.5
0.4
0.3
0.2
0.1
0.0
p-value
1e-03
3e-03
6e-03
1e-02
3e-02
Log-odds
0.5
1.0
1.5
2.0
2.5
3.0
3.5
4.0
Cellular_organisms - ps1
Archaea1 - ps2
Archaea2 - ps3
Archaea3 - ps4
Asgard/Eukaryota - ps5
Eukaryota - ps6
Amorpheae - ps7
Obazoa - ps8
Opisthokonta - ps9
Holozoa - ps10
Filozoa - ps11
Choano./Metazoa - ps12
Metazoa - ps13
Myriazoa - ps14
Parahoxozoa - ps15
Bilateria - ps16
Deuterostomia - ps17
Chordata - ps18
Olfactores - ps19
Vertebrata - ps20
Gnathostomata - ps21
Euteleostomi - ps22
Sarcopterygii - ps23
Tetrapoda - ps24
Amniota - ps25
Mammalia - ps26
Theria - ps27
Placentalia - ps28
Boreoeutheria - ps29
Euarchontoglires - ps30
Euarchonta - ps31
Primates - ps32
Haplorrhini - ps33
Simiiformes - ps34
Catarrhini - ps35
Hominoidea - ps36
Hominidae - ps37
Homininae - ps38
Hominini - ps39
Homo_sapiens - ps40
Phylostratum

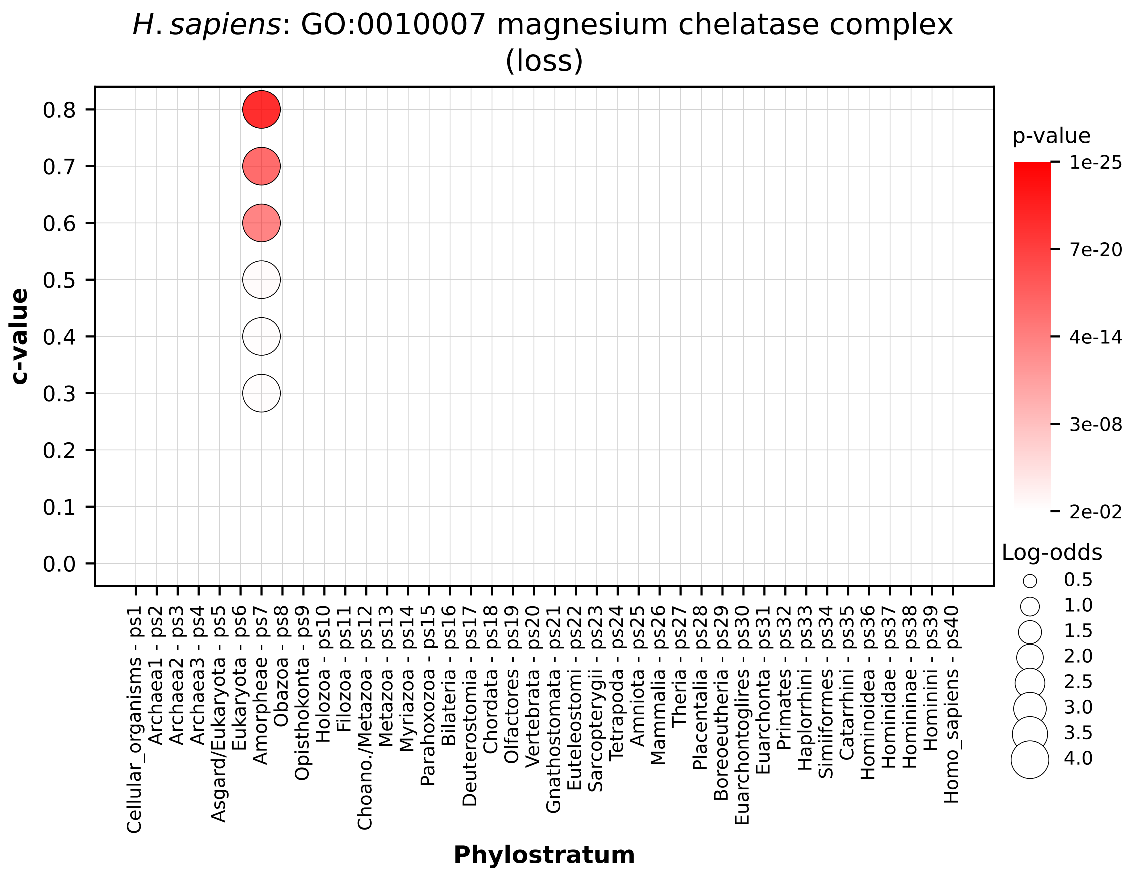

H. sapiens: GO:0010007 magnesium chelatase complex
(loss)
c-value
0.8
0.7
0.6
0.5
0.4
0.3
0.2
0.1
0.0
p-value
1e-25
7e-20
4e-14
3e-08
2e-02
Log-odds
0.5
1.0
1.5
2.0
2.5
3.0
3.5
4.0
Phylostratum
Cellular_organisms - ps1
Archaea1 - ps2
Archaea2 - ps3
Archaea3 - ps4
Asgard/Eukaryota - ps5
Eukaryota - ps6
Amorpheae - ps7
Obazoa - ps8
Opisthokonta - ps9
Holozoa - ps10
Filozoa - ps11
Choano./Metazoa - ps12
Metazoa - ps13
Myriazoa - ps14
Parahoxozoa - ps15
Bilateria - ps16
Deuterostomia - ps17
Chordata - ps18
Olfactores - ps19
Vertebrata - ps20
Gnathostomata - ps21
Euteleostomi - ps22
Sarcopterygii - ps23
Tetrapoda - ps24
Amniota - ps25
Mammalia - ps26
Theria - ps27
Placentalia - ps28
Boreoeutheria - ps29
Euarchontoglires - ps30
Euarchonta - ps31
Primates - ps32
Haplorrhini - ps33
Simiiformes - ps34
Catarrhini - ps35
Hominoidea - ps36
Hominidae - ps37
Homininae - ps38
Hominini - ps39
Homo_sapiens - ps40

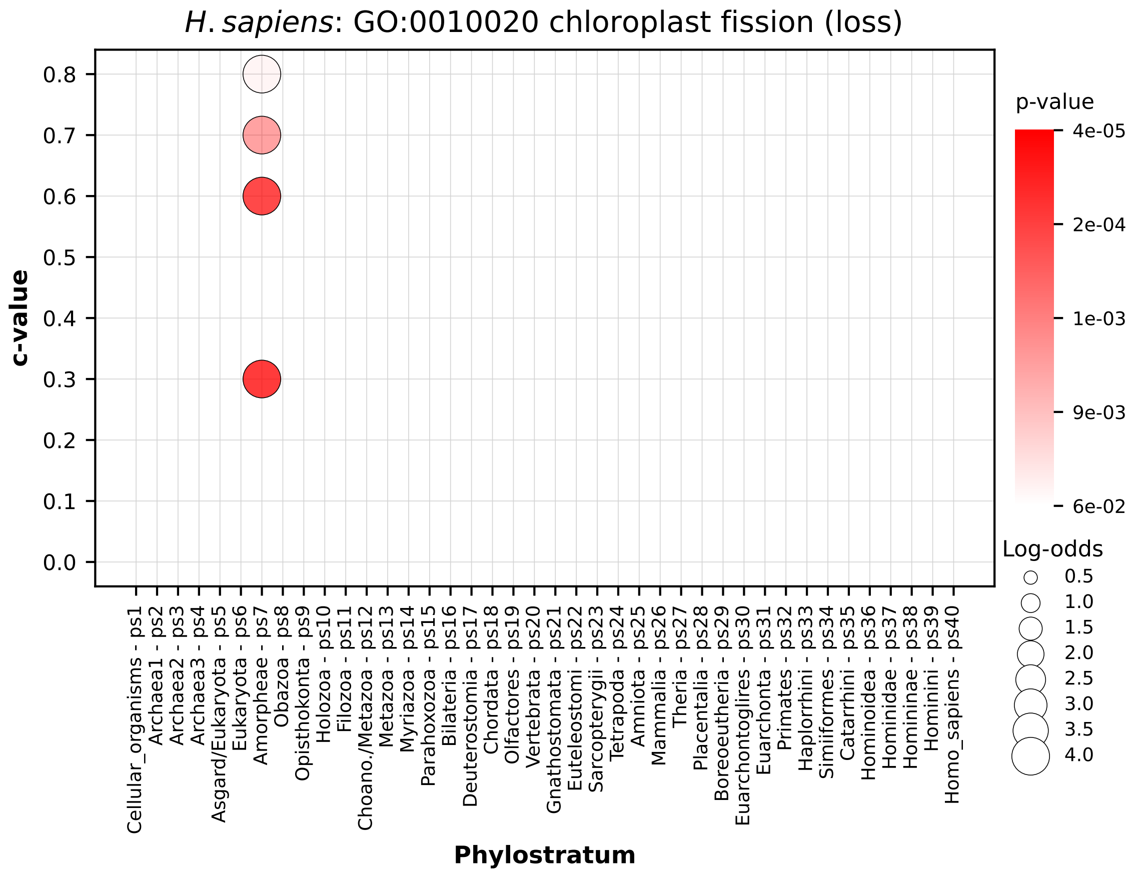

H. sapiens: GO:0010020 chloroplast fission (loss)
c-value
0.8
0.7
0.6
0.5
0.4
0.3
0.2
0.1
0.0
Phylostratum
Cellular_organisms - ps1
Archaea1 - ps2
Archaea2 - ps3
Archaea3 - ps4
Asgard/Eukaryota - ps5
Eukaryota - ps6
Amorpheae - ps7
Obazoa - ps8
Opisthokonta - ps9
Holozoa - ps10
Filozoa - ps11
Choano./Metazoa - ps12
Metazoa - ps13
Myriazoa - ps14
Parahoxozoa - ps15
Bilateria - ps16
Deuterostomia - ps17
Chordata - ps18
Olfactores - ps19
Vertebrata - ps20
Gnathostomata - ps21
Euteleostomi - ps22
Sarcopterygii - ps23
Tetrapoda - ps24
Amniota - ps25
Mammalia - ps26
Theria - ps27
Placentalia - ps28
Boreoeutheria - ps29
Euarchontoglires - ps30
Euarchonta - ps31
Primates - ps32
Haplorrhini - ps33
Simiiformes - ps34
Catarrhini - ps35
Hominoidea - ps36
Hominidae - ps37
Homininae - ps38
Hominini - ps39
Homo_sapiens - ps40
p-value
4e-05
2e-04
1e-03
9e-03
6e-02
Log-odds
0.5
1.0
1.5
2.0
2.5
3.0
3.5
4.0

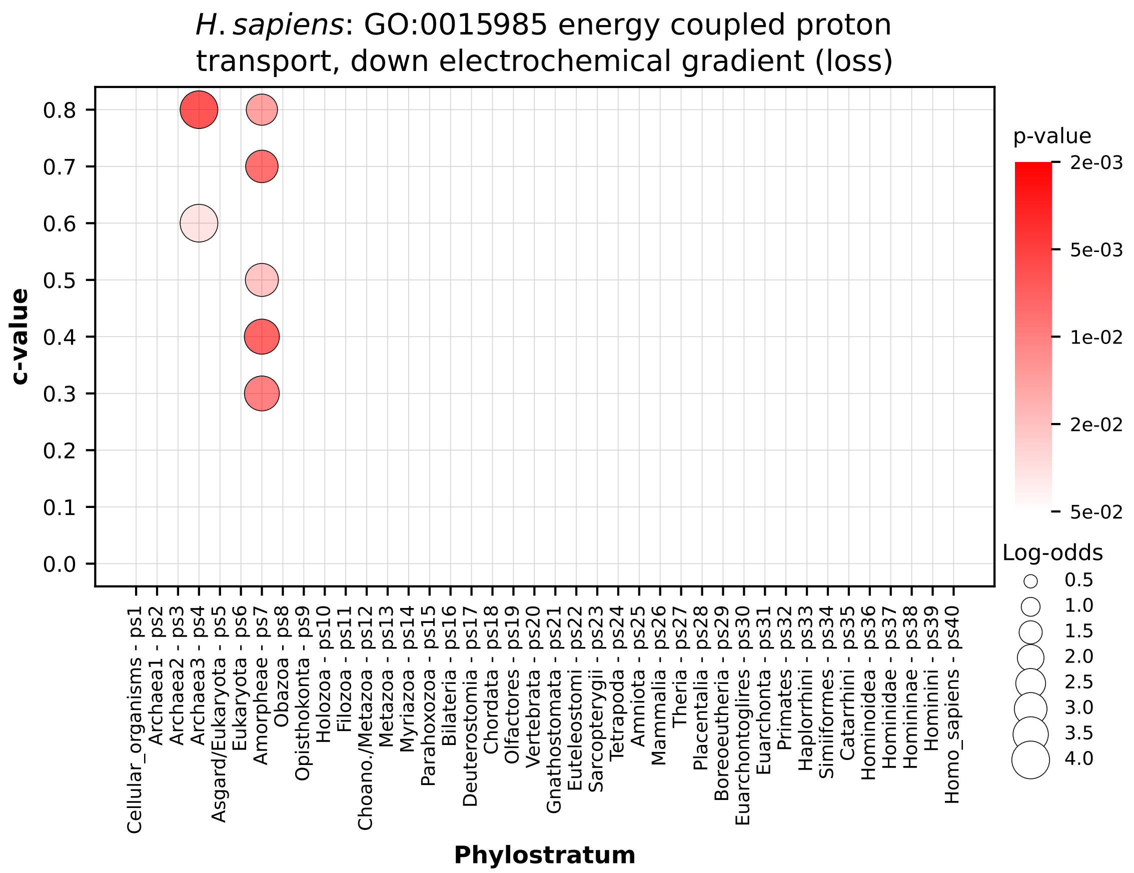

H. sapiens: GO:0015985 energy coupled proton transport, down electrochemical gradient (loss)
c-value
0.0
0.1
0.2
0.3
0.4
0.5
0.6
0.7
0.8
p-value
2e-03
5e-03
1e-02
2e-02
5e-02
Log-odds
0.5
1.0
1.5
2.0
2.5
3.0
3.5
4.0
Cellular_organisms - ps1
Archaea1 - ps2
Archaea2 - ps3
Archaea3 - ps4
Asgard/Eukaryota - ps5
Eukaryota - ps6
Amorpheae - ps7
Obazoa - ps8
Opisthokonta - ps9
Holozoa - ps10
Filozoa - ps11
Choano./Metazoa - ps12
Metazoa - ps13
Myriazoa - ps14
Parahoxozoa - ps15
Bilateria - ps16
Deuterostomia - ps17
Chordata - ps18
Olfactores - ps19
Vertebrata - ps20
Gnathostomata - ps21
Euteleostomi - ps22
Sarcopterygii - ps23
Tetrapoda - ps24
Amniota - ps25
Mammalia - ps26
Theria - ps27
Placentalia - ps28
Boreoeutheria - ps29
Euarchontoglires - ps30
Euarchonta - ps31
Primates - ps32
Haplorrhini - ps33
Simiiformes - ps34
Catarrhini - ps35
Hominoidea - ps36
Hominidae - ps37
Homininae - ps38
Hominini - ps39
Homo_sapiens - ps40
Phylostratum

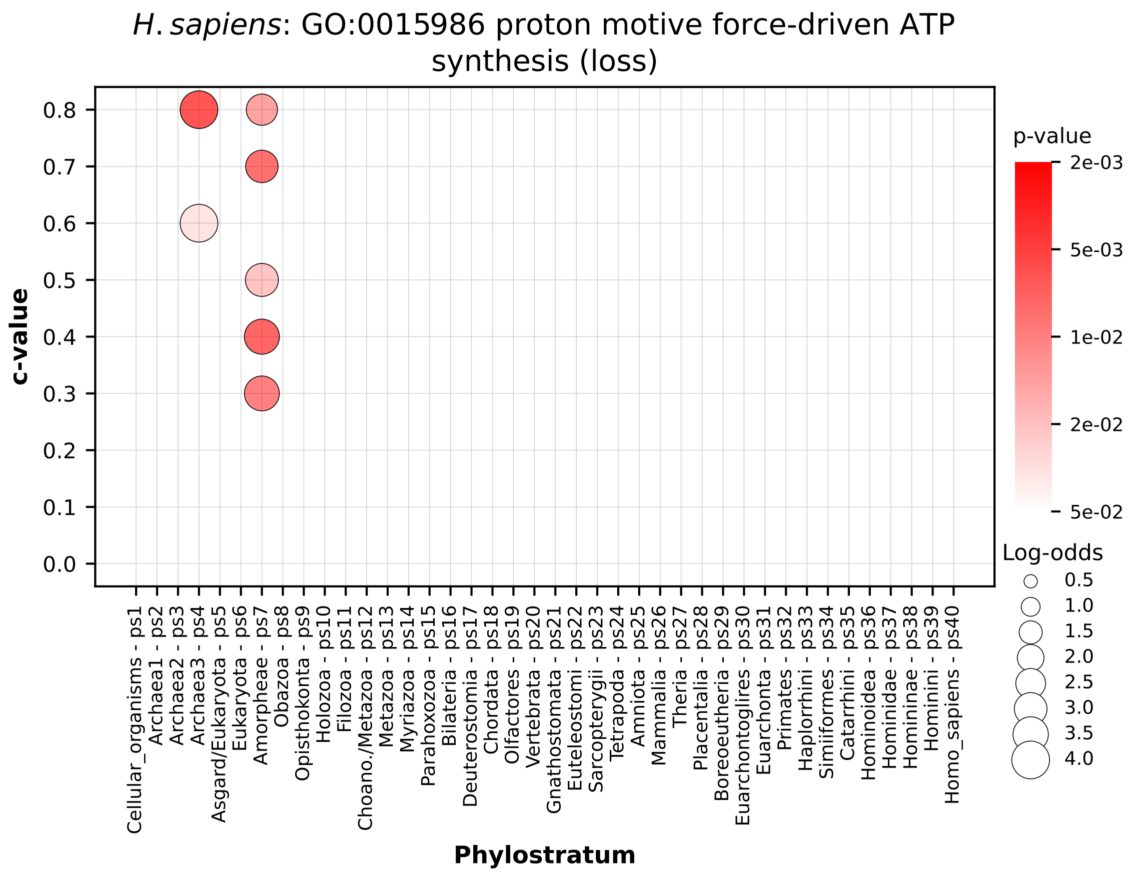

H. sapiens: GO:0015986 proton motive force-driven ATP synthesis (loss)
c-value
0.0
0.1
0.2
0.3
0.4
0.5
0.6
0.7
0.8
Phylostratum
Cellular_organisms - ps1
Archaea1 - ps2
Archaea2 - ps3
Archaea3 - ps4
Asgard/Eukaryota - ps5
Eukaryota - ps6
Amorpheae - ps7
Obazoa - ps8
Opisthokonta - ps9
Holozoa - ps10
Filozoa - ps11
Choano./Metazoa - ps12
Metazoa - ps13
Myriazoa - ps14
Parahoxozoa - ps15
Bilateria - ps16
Deuterostomia - ps17
Chordata - ps18
Olfactores - ps19
Vertebrata - ps20
Gnathostomata - ps21
Euteleostomi - ps22
Sarcopterygii - ps23
Tetrapoda - ps24
Amniota - ps25
Mammalia - ps26
Theria - ps27
Placentalia - ps28
Boreoeutheria - ps29
Euarchontoglires - ps30
Euarchonta - ps31
Primates - ps32
Haplorrhini - ps33
Simiiformes - ps34
Catarrhini - ps35
Hominoidea - ps36
Hominidae - ps37
Homininae - ps38
Hominini - ps39
Homo_sapiens - ps40
p-value
2e-03
5e-03
1e-02
2e-02
5e-02
Log-odds
0.5
1.0
1.5
2.0
2.5
3.0
3.5
4.0

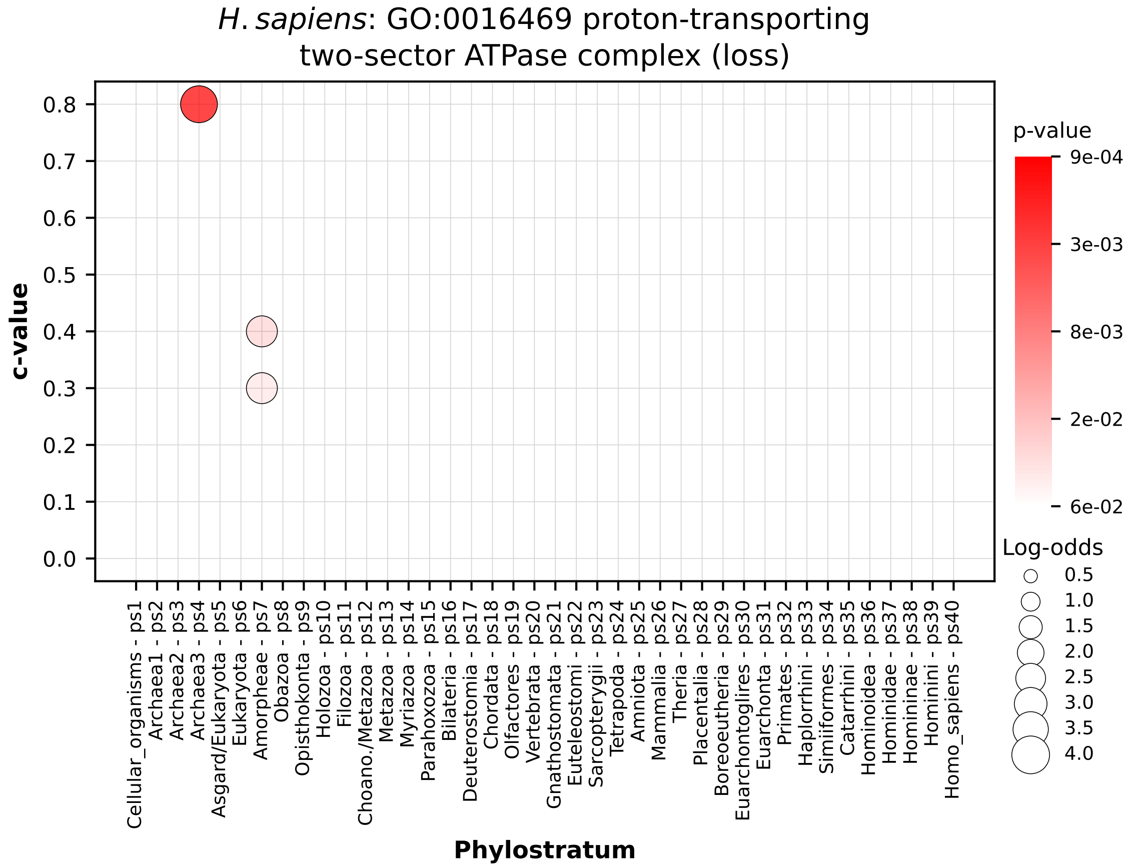

H. sapiens: GO:0016469 proton-transporting two-sector ATPase complex (loss)
c-value
0.8
0.7
0.6
0.5
0.4
0.3
0.2
0.1
0.0
p-value
9e-04
3e-03
8e-03
2e-02
6e-02
Log-odds
0.5
1.0
1.5
2.0
2.5
3.0
3.5
4.0
Cellular_organisms - ps1
Archaea1 - ps2
Archaea2 - ps3
Archaea3 - ps4
Asgard/Eukaryota - ps5
Eukaryota - ps6
Amorpheae - ps7
Obazoa - ps8
Opisthokonta - ps9
Holozoa - ps10
Filozoa - ps11
Choano./Metazoa - ps12
Metazoa - ps13
Myriazoa - ps14
Parahoxozoa - ps15
Bilateria - ps16
Deuterostomia - ps17
Chordata - ps18
Olfactores - ps19
Vertebrata - ps20
Gnathostomata - ps21
Euteleostomi - ps22
Sarcopterygii - ps23
Tetrapoda - ps24
Amniota - ps25
Mammalia - ps26
Theria - ps27
Placentalia - ps28
Boreoeutheria - ps29
Euarchontoglires - ps30
Euarchonta - ps31
Primates - ps32
Haplorrhini - ps33
Simiiformes - ps34
Catarrhini - ps35
Hominoidea - ps36
Hominidae - ps37
Homininae - ps38
Hominini - ps39
Homo_sapiens - ps40
Phylostratum

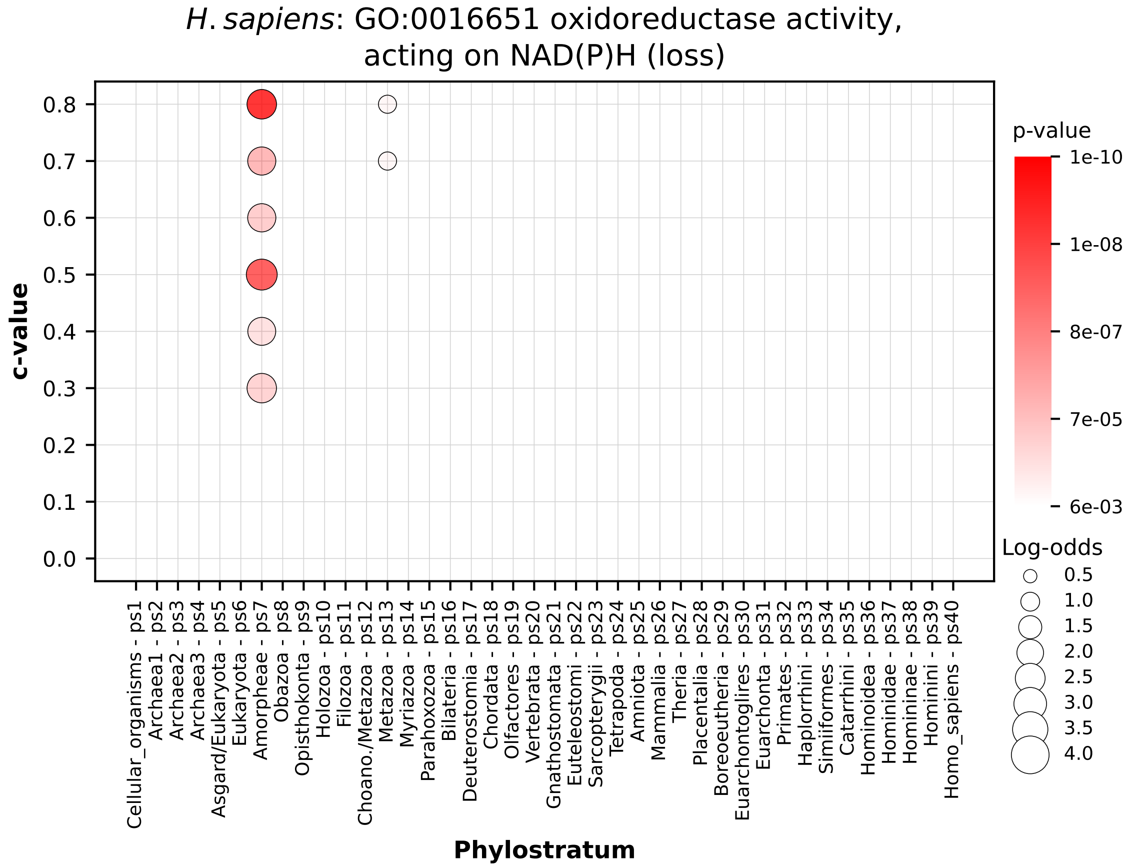

H. sapiens: GO:0016651 oxidoreductase activity, acting on NAD(P)H (loss)
c-value
0.8
0.7
0.6
0.5
0.4
0.3
0.2
0.1
0.0
p-value
1e-10
1e-08
8e-07
7e-05
6e-03
Log-odds
0.5
1.0
1.5
2.0
2.5
3.0
3.5
4.0
Cellular_organisms - ps1
Archaea1 - ps2
Archaea2 - ps3
Archaea3 - ps4
Asgard/Eukaryota - ps5
Eukaryota - ps6
Amorpheae - ps7
Obazoa - ps8
Opisthokonta - ps9
Holozoa - ps10
Filozoa - ps11
Choano./Metazoa - ps12
Metazoa - ps13
Myriazoa - ps14
Parahoxozoa - ps15
Bilateria - ps16
Deuterostomia - ps17
Chordata - ps18
Olfactores - ps19
Vertebrata - ps20
Gnathostomata - ps21
Euteleostomi - ps22
Sarcopterygii - ps23
Tetrapoda - ps24
Amniota - ps25
Mammalia - ps26
Theria - ps27
Placentalia - ps28
Boreoeutheria - ps29
Euarchontoglires - ps30
Euarchonta - ps31
Primates - ps32
Haplorrhini - ps33
Simiiformes - ps34
Catarrhini - ps35
Hominoidea - ps36
Hominidae - ps37
Homininae - ps38
Hominini - ps39
Homo_sapiens - ps40
Phylostratum

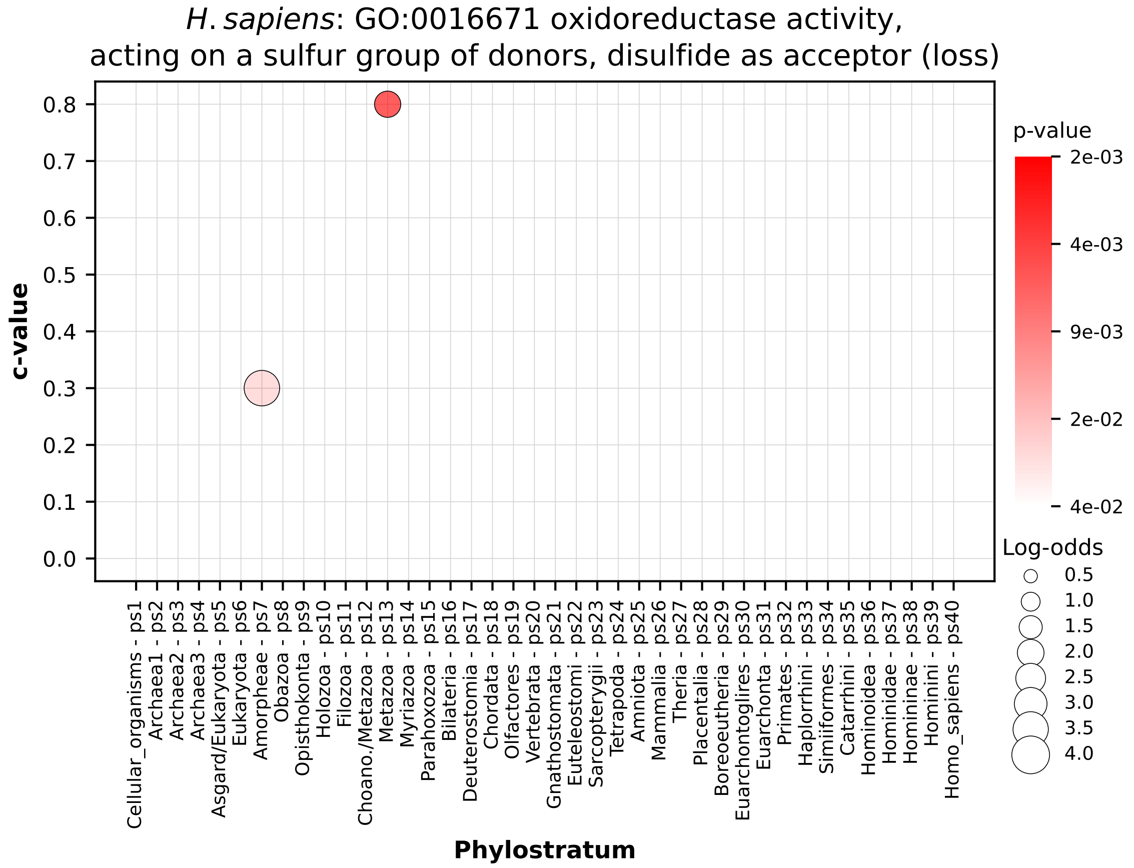

H. sapiens: GO:0016671 oxidoreductase activity,
acting on a sulfur group of donors, disulfide as acceptor (loss)
c-value
0.8
0.7
0.6
0.5
0.4
0.3
0.2
0.1
0.0
p-value
2e-03
4e-03
9e-03
2e-02
4e-02
Log-odds
0.5
1.0
1.5
2.0
2.5
3.0
3.5
4.0
Cellular_organisms - ps1
Archaea1 - ps2
Archaea2 - ps3
Archaea3 - ps4
Asgard/Eukaryota - ps5
Eukaryota - ps6
Amorpheae - ps7
Obazoa - ps8
Opisthokonta - ps9
Holozoa - ps10
Filozoa - ps11
Choano./Metazoa - ps12
Metazoa - ps13
Myriazoa - ps14
Parahoxozoa - ps15
Bilateria - ps16
Deuterostomia - ps17
Chordata - ps18
Olfactores - ps19
Vertebrata - ps20
Gnathostomata - ps21
Euteleostomi - ps22
Sarcopterygii - ps23
Tetrapoda - ps24
Amniota - ps25
Mammalia - ps26
Theria - ps27
Placentalia - ps28
Boreoeutheria - ps29
Euarchontoglires - ps30
Euarchonta - ps31
Primates - ps32
Haplorrhini - ps33
Simiiformes - ps34
Catarrhini - ps35
Hominoidea - ps36
Hominidae - ps37
Homininae - ps38
Hominini - ps39
Homo_sapiens - ps40
Phylostratum

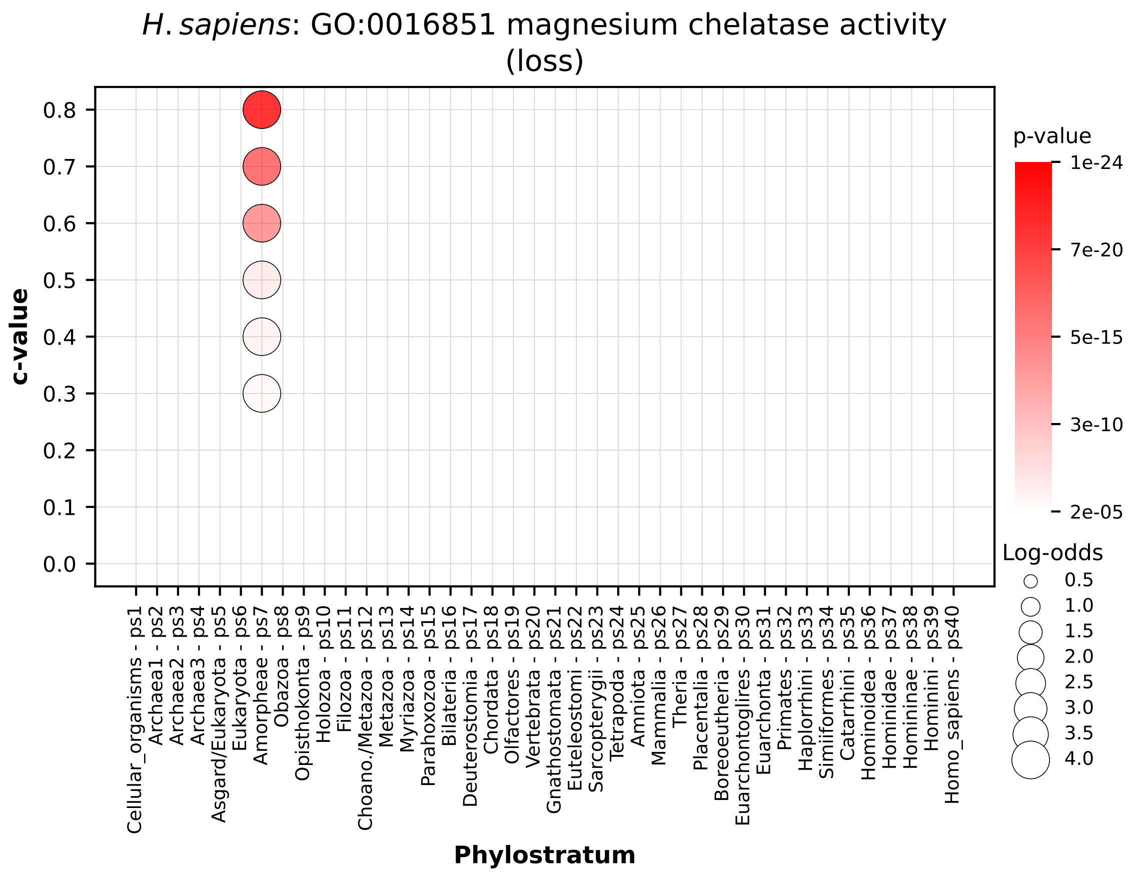

H. sapiens: GO:0016851 magnesium chelatase activity
(loss)
c-value
0.8
0.7
0.6
0.5
0.4
0.3
0.2
0.1
0.0
p-value
1e-24
7e-20
5e-15
3e-10
2e-05
Log-odds
0.5
1.0
1.5
2.0
2.5
3.0
3.5
4.0
Cellular_organisms - ps1
Archaea1 - ps2
Archaea2 - ps3
Archaea3 - ps4
Asgard/Eukaryota - ps5
Eukaryota - ps6
Amorpheae - ps7
Obazoa - ps8
Opisthokonta - ps9
Holozoa - ps10
Filozoa - ps11
Choano./Metazoa - ps12
Metazoa - ps13
Myriazoa - ps14
Parahoxozoa - ps15
Bilateria - ps16
Deuterostomia - ps17
Chordata - ps18
Olfactores - ps19
Vertebrata - ps20
Gnathostomata - ps21
Euteleostomi - ps22
Sarcopterygii - ps23
Tetrapoda - ps24
Amniota - ps25
Mammalia - ps26
Theria - ps27
Placentalia - ps28
Boreoeutheria - ps29
Euarchontoglires - ps30
Euarchonta - ps31
Primates - ps32
Haplorrhini - ps33
Simiiformes - ps34
Catarrhini - ps35
Hominoidea - ps36
Hominidae - ps37
Homininae - ps38
Hominini - ps39
Homo_sapiens - ps40
Phylostratum

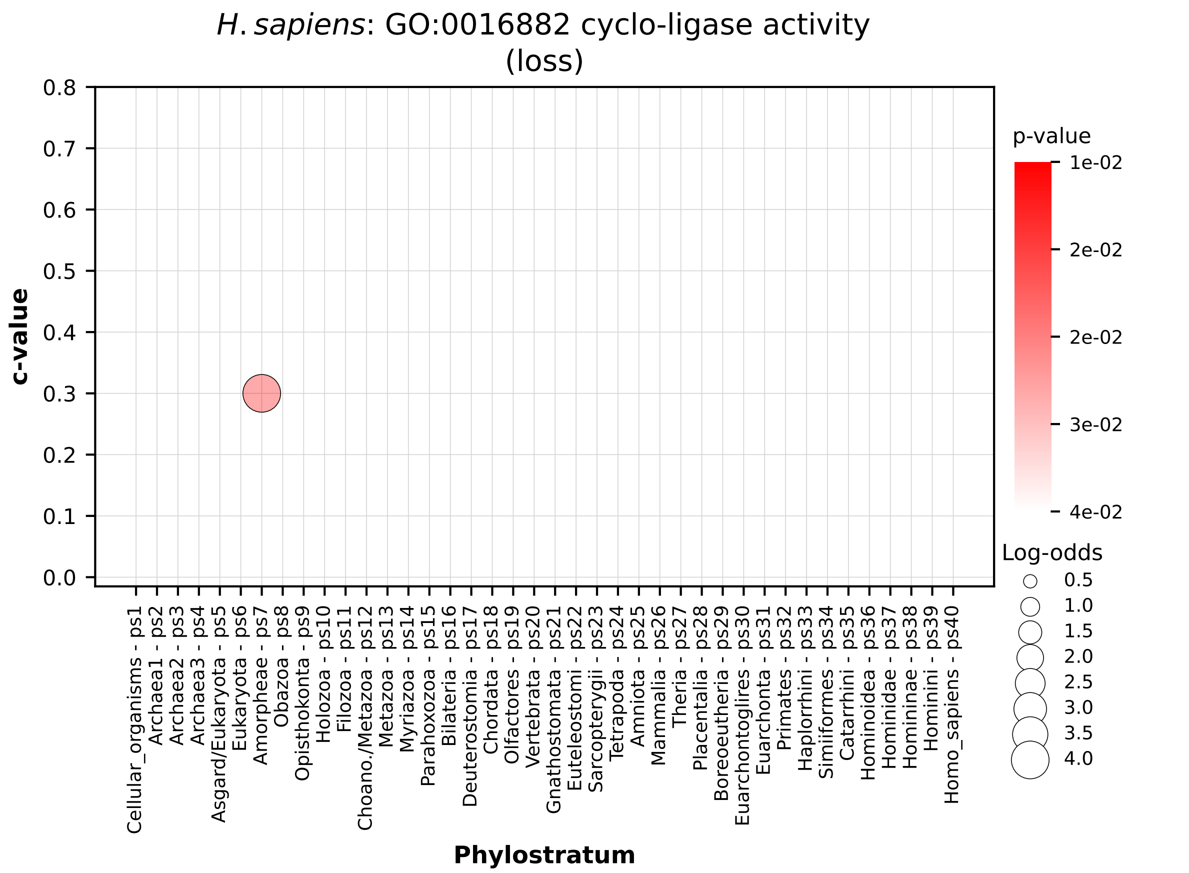

H. sapiens: GO:0016882 cyclo-ligase activity
(loss)
c-value
0.0
0.1
0.2
0.3
0.4
0.5
0.6
0.7
0.8
p-value
1e-02
2e-02
2e-02
3e-02
4e-02
Log-odds
0.5
1.0
1.5
2.0
2.5
3.0
3.5
4.0
Cellular_organisms - ps1
Archaea1 - ps2
Archaea2 - ps3
Archaea3 - ps4
Asgard/Eukaryota - ps5
Eukaryota - ps6
Amorpheae - ps7
Obazoa - ps8
Opisthokonta - ps9
Holozoa - ps10
Filozoa - ps11
Choano./Metazoa - ps12
Metazoa - ps13
Myriazoa - ps14
Parahoxozoa - ps15
Bilateria - ps16
Deuterostomia - ps17
Chordata - ps18
Olfactores - ps19
Vertebrata - ps20
Gnathostomata - ps21
Euteleostomi - ps22
Sarcopterygii - ps23
Tetrapoda - ps24
Amniota - ps25
Mammalia - ps26
Theria - ps27
Placentalia - ps28
Boreoeutheria - ps29
Euarchontoglires - ps30
Euarchonta - ps31
Primates - ps32
Haplorrhini - ps33
Simiiformes - ps34
Catarrhini - ps35
Hominoidea - ps36
Hominidae - ps37
Homininae - ps38
Hominini - ps39
Homo_sapiens - ps40
Phylostratum

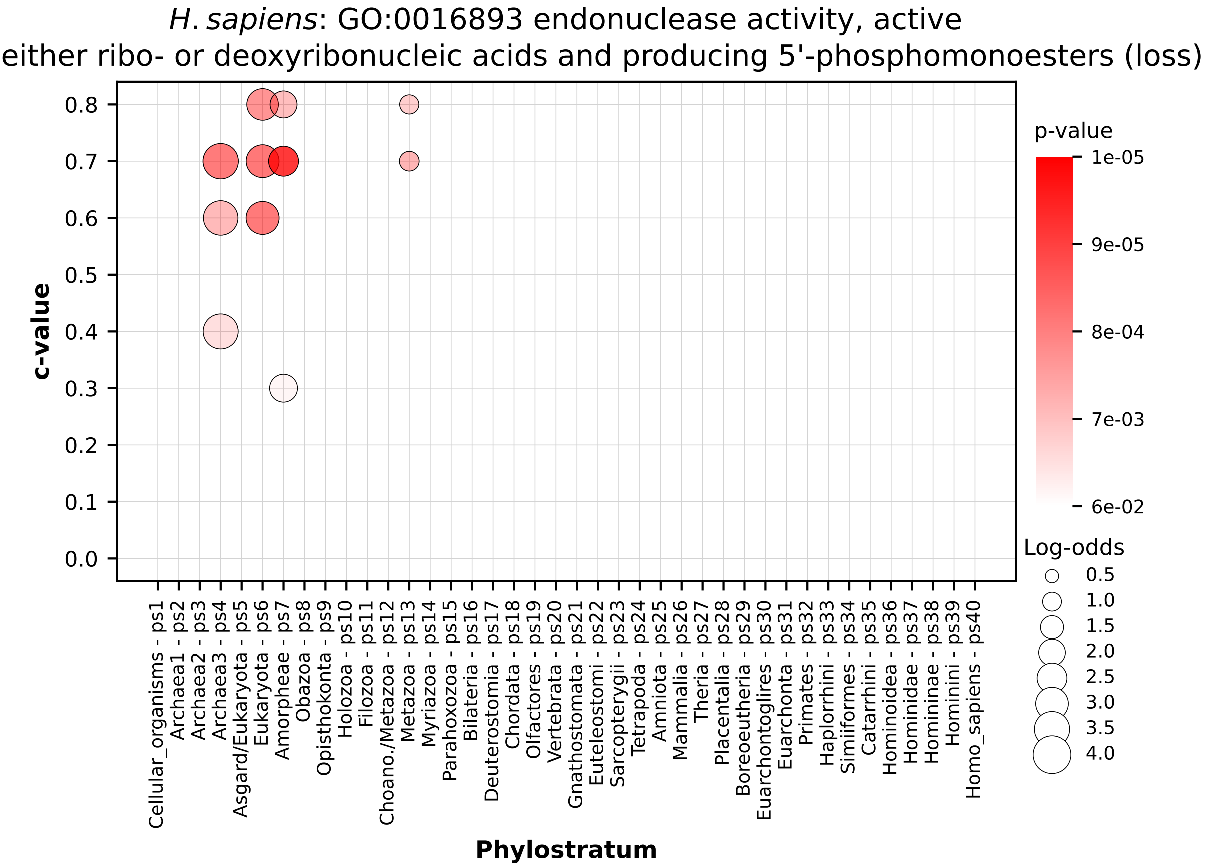

H. sapiens: GO:0016893 endonuclease activity, active
either ribo- or deoxyribonucleic acids and producing 5'-phosphomonoesters (loss)
c-value
0.8
0.7
0.6
0.5
0.4
0.3
0.2
0.1
0.0
p-value
1e-05
9e-05
8e-04
7e-03
6e-02
Log-odds
0.5
1.0
1.5
2.0
2.5
3.0
3.5
4.0
Cellular_organisms - ps1
Archaea1 - ps2
Archaea2 - ps3
Archaea3 - ps4
Asgard/Eukaryota - ps5
Eukaryota - ps6
Amorpheae - ps7
Obazoa - ps8
Opisthokonta - ps9
Holozoa - ps10
Filozoa - ps11
Choano./Metazoa - ps12
Metazoa - ps13
Myriazoa - ps14
Parahoxozoa - ps15
Bilateria - ps16
Deuterostomia - ps17
Chordata - ps18
Olfactores - ps19
Vertebrata - ps20
Gnathostomata - ps21
Euteleostomi - ps22
Sarcopterygii - ps23
Tetrapoda - ps24
Amniota - ps25
Mammalia - ps26
Theria - ps27
Placentalia - ps28
Boreoeutheria - ps29
Euarchontoglires - ps30
Euarchonta - ps31
Primates - ps32
Haplorrhini - ps33
Simiiformes - ps34
Catarrhini - ps35
Hominoidea - ps36
Hominidae - ps37
Homininae - ps38
Hominini - ps39
Homo_sapiens - ps40
Phylostratum

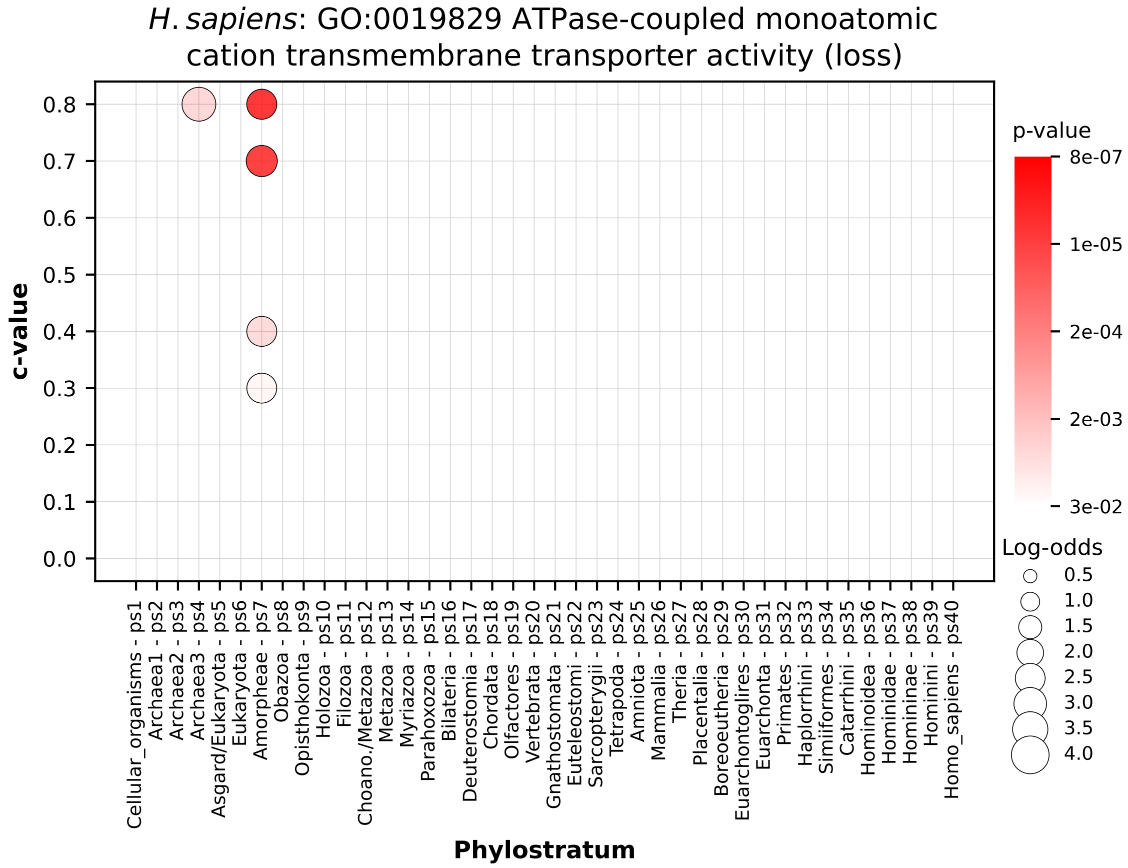

H. sapiens: GO:0019829 ATPase-coupled monoatomic cation transmembrane transporter activity (loss)
c-value
0.8
0.7
0.6
0.5
0.4
0.3
0.2
0.1
0.0
Phylostratum
Cellular_organisms - ps1
Archaea1 - ps2
Archaea2 - ps3
Archaea3 - ps4
Asgard/Eukaryota - ps5
Eukaryota - ps6
Amorpheae - ps7
Obazoa - ps8
Opisthokonta - ps9
Holozoa - ps10
Filozoa - ps11
Choano./Metazoa - ps12
Metazoa - ps13
Myriazoa - ps14
Parahoxozoa - ps15
Bilateria - ps16
Deuterostomia - ps17
Chordata - ps18
Olfactores - ps19
Vertebrata - ps20
Gnathostomata - ps21
Euteleostomi - ps22
Sarcopterygii - ps23
Tetrapoda - ps24
Amniota - ps25
Mammalia - ps26
Theria - ps27
Placentalia - ps28
Boreoeutheria - ps29
Euarchontoglires - ps30
Euarchonta - ps31
Primates - ps32
Haplorrhini - ps33
Simiiformes - ps34
Catarrhini - ps35
Hominoidea - ps36
Hominidae - ps37
Homininae - ps38
Hominini - ps39
Homo_sapiens - ps40
p-value
8e-07
1e-05
2e-04
2e-03
3e-02
Log-odds
0.5
1.0
1.5
2.0
2.5
3.0
3.5
4.0

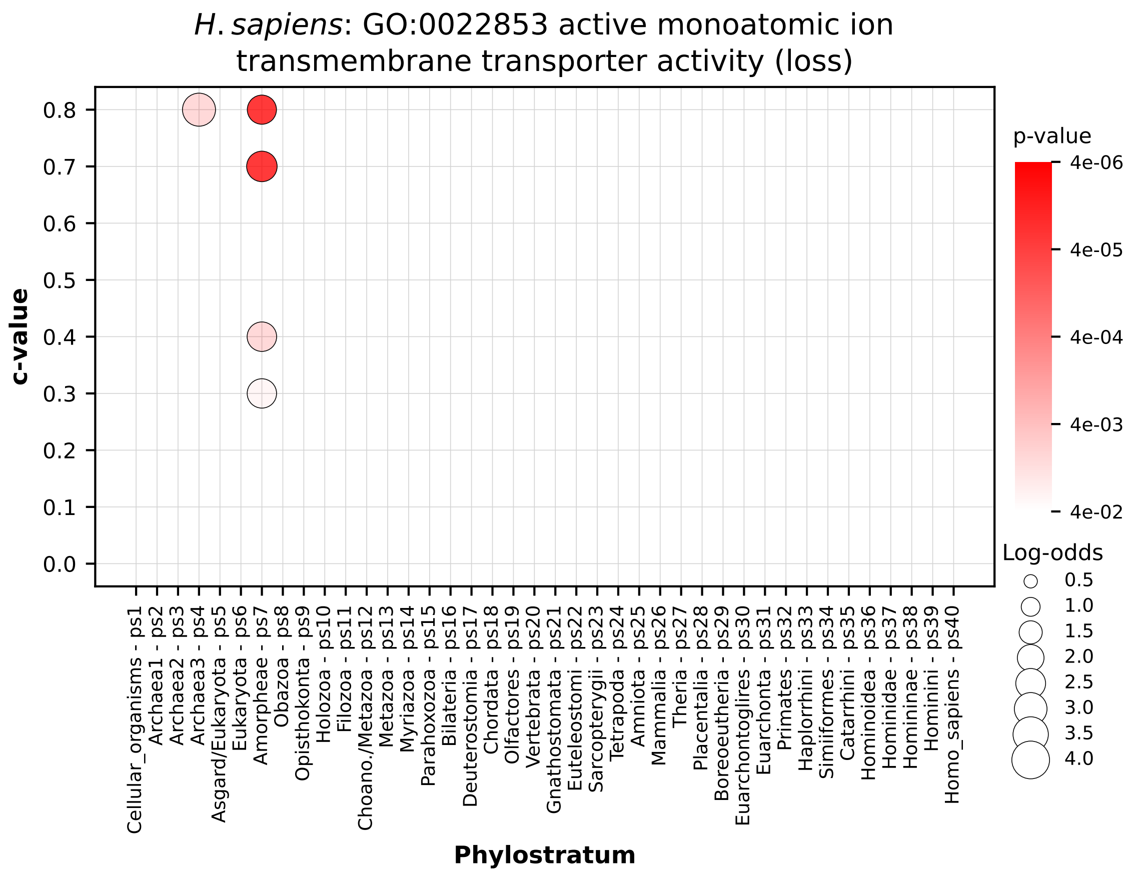

H. sapiens: GO:0022853 active monoatomic ion transmembrane transporter activity (loss)
c-value
0.8
0.7
0.6
0.5
0.4
0.3
0.2
0.1
0.0
p-value
4e-06
4e-05
4e-04
4e-03
4e-02
Log-odds
0.5
1.0
1.5
2.0
2.5
3.0
3.5
4.0
Cellular_organisms - ps1
Archaea1 - ps2
Archaea2 - ps3
Archaea3 - ps4
Asgard/Eukaryota - ps5
Eukaryota - ps6
Amorpheae - ps7
Obazoa - ps8
Opisthokonta - ps9
Holozoa - ps10
Filozoa - ps11
Choano./Metazoa - ps12
Metazoa - ps13
Myriazoa - ps14
Parahoxozoa - ps15
Bilateria - ps16
Deuterostomia - ps17
Chordata - ps18
Olfactores - ps19
Vertebrata - ps20
Gnathostomata - ps21
Euteleostomi - ps22
Sarcopterygii - ps23
Tetrapoda - ps24
Amniota - ps25
Mammalia - ps26
Theria - ps27
Placentalia - ps28
Boreoeutheria - ps29
Euarchontoglires - ps30
Euarchonta - ps31
Primates - ps32
Haplorrhini - ps33
Simiiformes - ps34
Catarrhini - ps35
Hominoidea - ps36
Hominidae - ps37
Homininae - ps38
Hominini - ps39
Homo_sapiens - ps40
Phylostratum

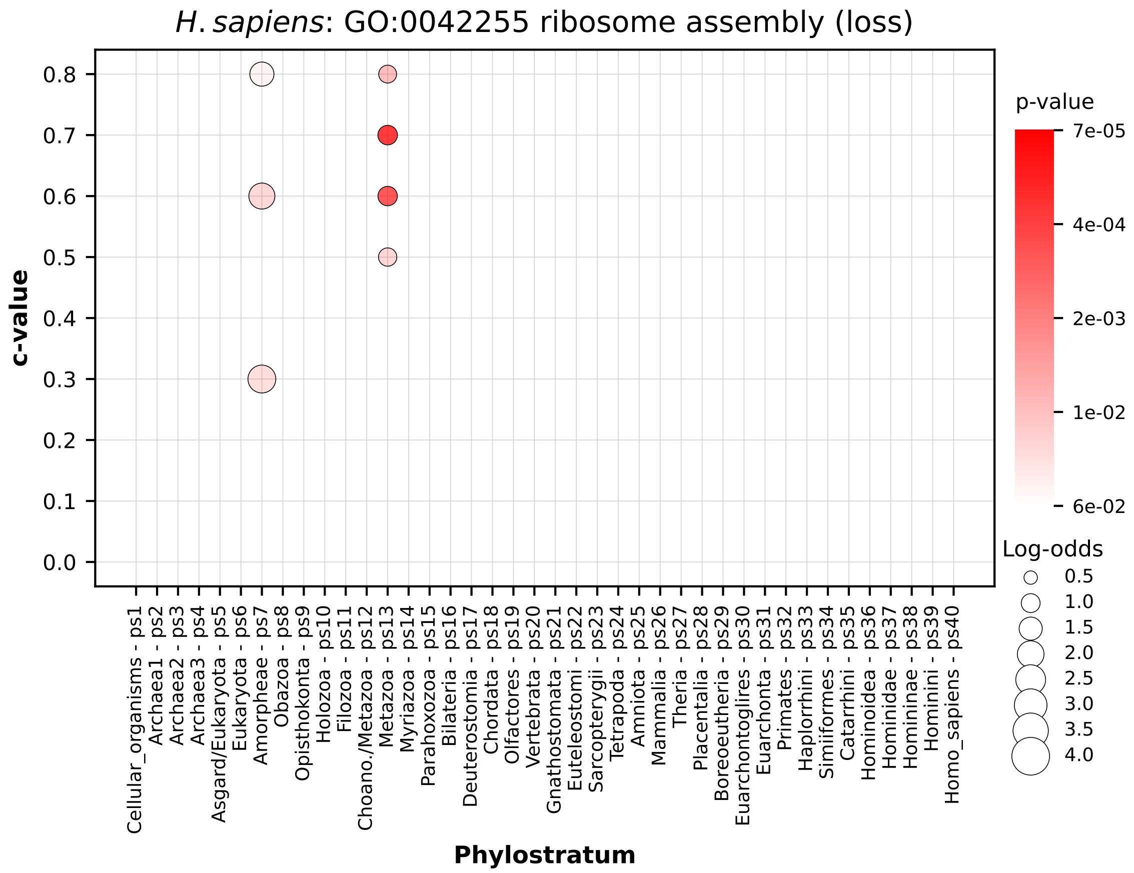

H. sapiens: GO:0042255 ribosome assembly (loss)
c-value
0.0
0.1
0.2
0.3
0.4
0.5
0.6
0.7
0.8
Phylostratum
Cellular_organisms - ps1
Archaea1 - ps2
Archaea2 - ps3
Archaea3 - ps4
Asgard/Eukaryota - ps5
Eukaryota - ps6
Amorpheae - ps7
Obazoa - ps8
Opisthokonta - ps9
Holozoa - ps10
Filozoa - ps11
Choano./Metazoa - ps12
Metazoa - ps13
Myriazoa - ps14
Parahoxozoa - ps15
Bilateria - ps16
Deuterostomia - ps17
Chordata - ps18
Olfactores - ps19
Vertebrata - ps20
Gnathostomata - ps21
Euteleostomi - ps22
Sarcopterygii - ps23
Tetrapoda - ps24
Amniota - ps25
Mammalia - ps26
Theria - ps27
Placentalia - ps28
Boreoeutheria - ps29
Euarchontoglires - ps30
Euarchonta - ps31
Primates - ps32
Haplorrhini - ps33
Simiiformes - ps34
Catarrhini - ps35
Hominoidea - ps36
Hominidae - ps37
Homininae - ps38
Hominini - ps39
Homo_sapiens - ps40
p-value
7e-05
4e-04
2e-03
1e-02
6e-02
Log-odds
0.5
1.0
1.5
2.0
2.5
3.0
3.5
4.0

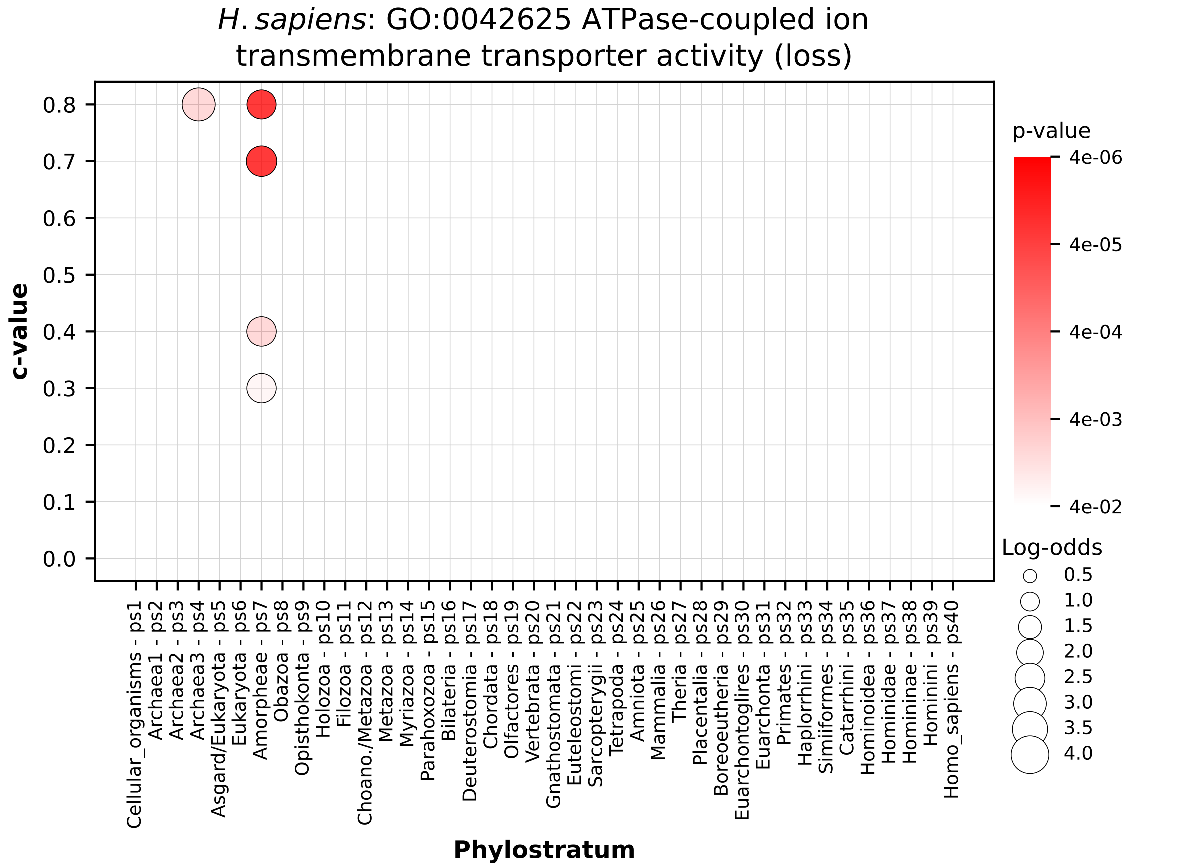

H. sapiens: GO:0042625 ATPase-coupled ion transmembrane transporter activity (loss)
c-value
0.8
0.7
0.6
0.5
0.4
0.3
0.2
0.1
0.0
p-value
4e-06
4e-05
4e-04
4e-03
4e-02
Log-odds
0.5
1.0
1.5
2.0
2.5
3.0
3.5
4.0
Cellular_organisms - ps1
Archaea1 - ps2
Archaea2 - ps3
Archaea3 - ps4
Asgard/Eukaryota - ps5
Eukaryota - ps6
Amorpheae - ps7
Obazoa - ps8
Opisthokonta - ps9
Holozoa - ps10
Filozoa - ps11
Choano./Metazoa - ps12
Metazoa - ps13
Myriazoa - ps14
Parahoxozoa - ps15
Bilateria - ps16
Deuterostomia - ps17
Chordata - ps18
Olfactores - ps19
Vertebrata - ps20
Gnathostomata - ps21
Euteleostomi - ps22
Sarcopterygii - ps23
Tetrapoda - ps24
Amniota - ps25
Mammalia - ps26
Theria - ps27
Placentalia - ps28
Boreoeutheria - ps29
Euarchontoglires - ps30
Euarchonta - ps31
Primates - ps32
Haplorrhini - ps33
Simiiformes - ps34
Catarrhini - ps35
Hominoidea - ps36
Hominidae - ps37
Homininae - ps38
Hominini - ps39
Homo_sapiens - ps40
Phylostratum

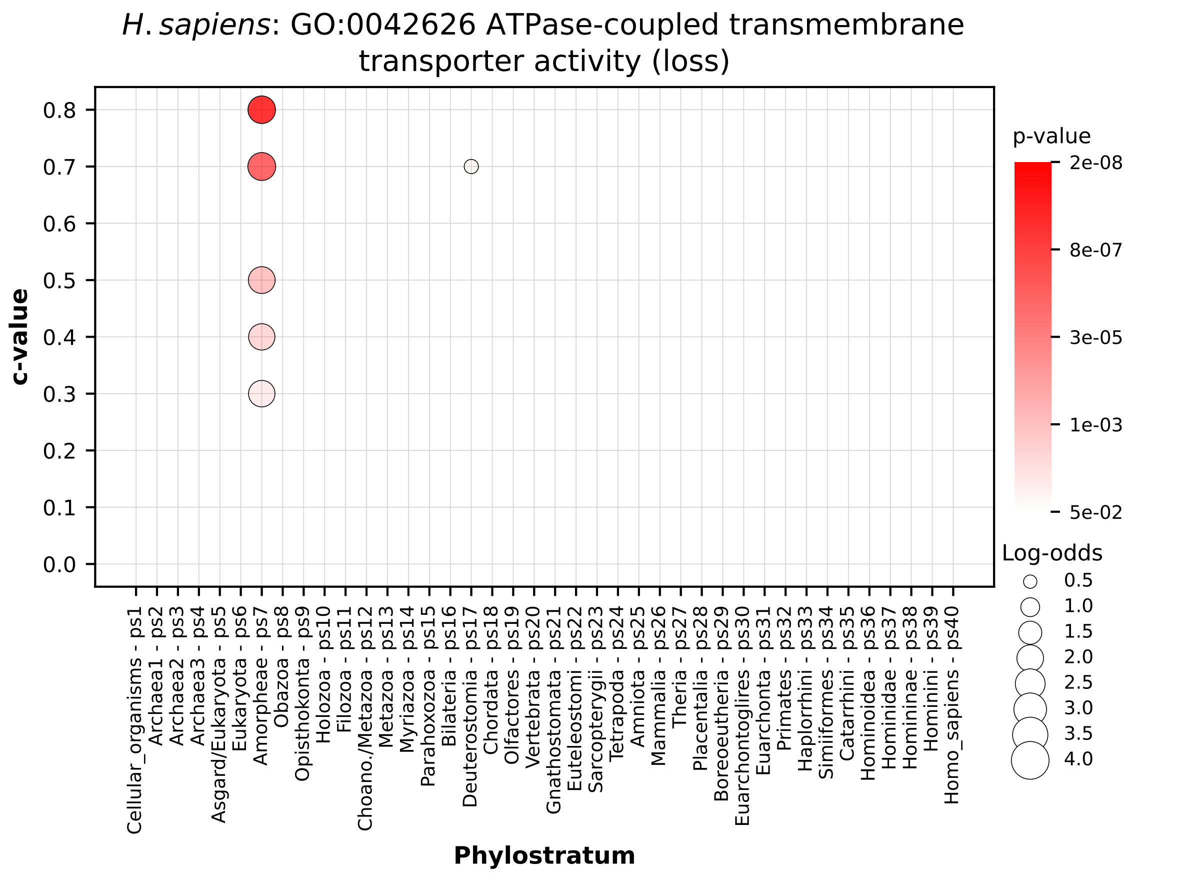

H. sapiens: GO:0042626 ATPase-coupled transmembrane transporter activity (loss)
c-value
0.8
0.7
0.6
0.5
0.4
0.3
0.2
0.1
0.0
p-value
2e-08
8e-07
3e-05
1e-03
5e-02
Log-odds
0.5
1.0
1.5
2.0
2.5
3.0
3.5
4.0
Cellular_organisms - ps1
Archaea1 - ps2
Archaea2 - ps3
Archaea3 - ps4
Asgard/Eukaryota - ps5
Eukaryota - ps6
Amorpheae - ps7
Obazoa - ps8
Opisthokonta - ps9
Holozoa - ps10
Filozoa - ps11
Choano./Metazoa - ps12
Metazoa - ps13
Myriazoa - ps14
Parahoxozoa - ps15
Bilateria - ps16
Deuterostomia - ps17
Chordata - ps18
Olfactores - ps19
Vertebrata - ps20
Gnathostomata - ps21
Euteleostomi - ps22
Sarcopterygii - ps23
Tetrapoda - ps24
Amniota - ps25
Mammalia - ps26
Theria - ps27
Placentalia - ps28
Boreoeutheria - ps29
Euarchontoglires - ps30
Euarchonta - ps31
Primates - ps32
Haplorrhini - ps33
Simiiformes - ps34
Catarrhini - ps35
Hominoidea - ps36
Hominidae - ps37
Homininae - ps38
Hominini - ps39
Homo_sapiens - ps40
Phylostratum

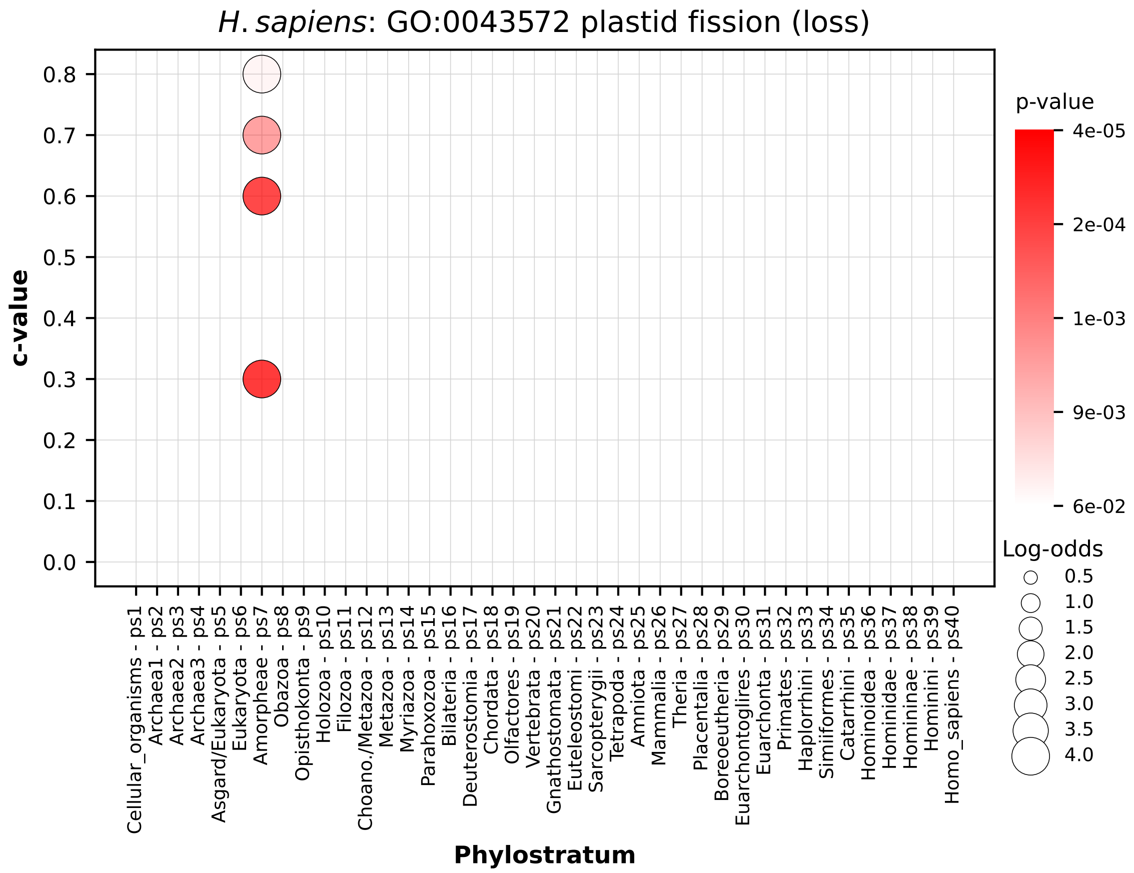

H. sapiens: GO:0043572 plastid fission (loss)
c-value
0.8
0.7
0.6
0.5
0.4
0.3
0.2
0.1
0.0
p-value
4e-05
2e-04
1e-03
9e-03
6e-02
Log-odds
0.5
1.0
1.5
2.0
2.5
3.0
3.5
4.0
Cellular_organisms - ps1
Archaea1 - ps2
Archaea2 - ps3
Archaea3 - ps4
Asgard/Eukaryota - ps5
Eukaryota - ps6
Amorpheae - ps7
Obazoa - ps8
Opisthokonta - ps9
Holozoa - ps10
Filozoa - ps11
Choano./Metazoa - ps12
Metazoa - ps13
Myriazoa - ps14
Parahoxozoa - ps15
Bilateria - ps16
Deuterostomia - ps17
Chordata - ps18
Olfactores - ps19
Vertebrata - ps20
Gnathostomata - ps21
Euteleostomi - ps22
Sarcopterygii - ps23
Tetrapoda - ps24
Amniota - ps25
Mammalia - ps26
Theria - ps27
Placentalia - ps28
Boreoeutheria - ps29
Euarchontoglires - ps30
Euarchonta - ps31
Primates - ps32
Haplorrhini - ps33
Simiiformes - ps34
Catarrhini - ps35
Hominoidea - ps36
Hominidae - ps37
Homininae - ps38
Hominini - ps39
Homo_sapiens - ps40
Phylostratum

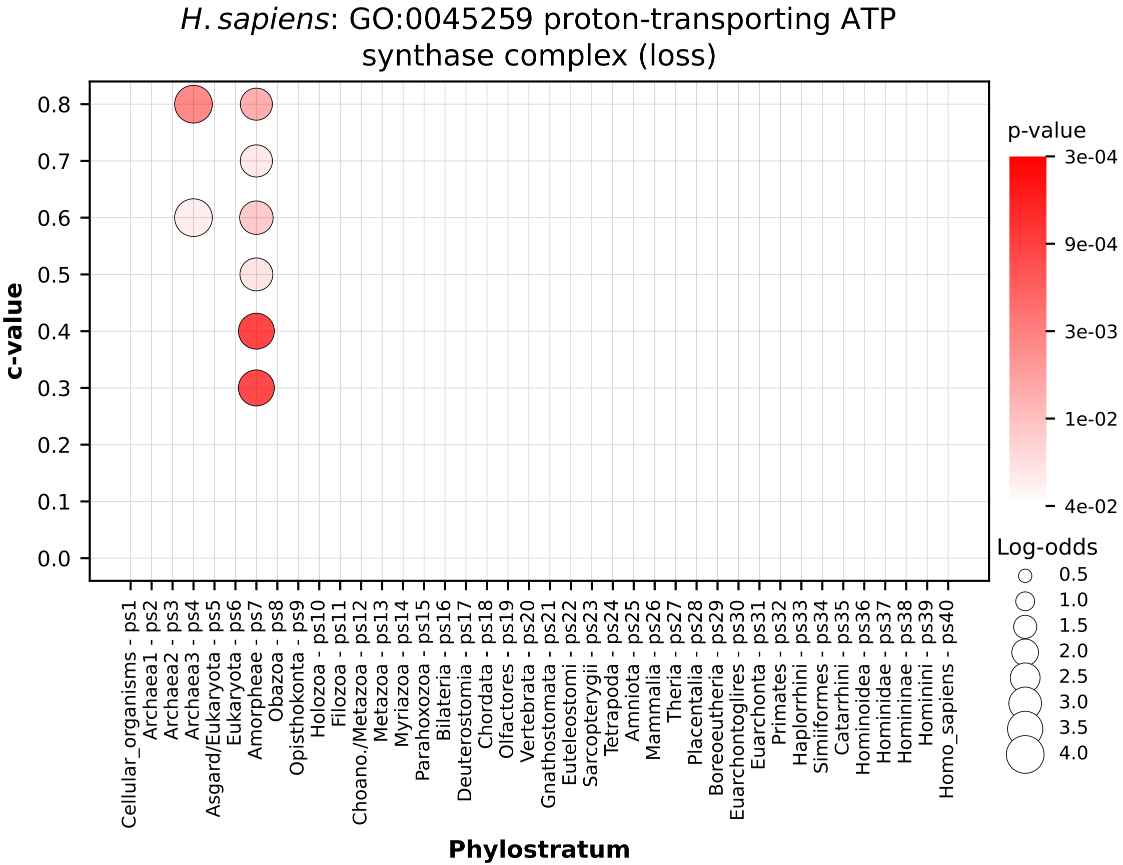

H. sapiens: GO:0045259 proton-transporting ATP synthase complex (loss)
c-value
0.8
0.7
0.6
0.5
0.4
0.3
0.2
0.1
0.0
Phylostratum
Cellular_organisms - ps1
Archaea1 - ps2
Archaea2 - ps3
Archaea3 - ps4
Asgard/Eukaryota - ps5
Eukaryota - ps6
Amorpheae - ps7
Obazoa - ps8
Opisthokonta - ps9
Holozoa - ps10
Filozoa - ps11
Choano./Metazoa - ps12
Metazoa - ps13
Myriazoa - ps14
Parahoxozoa - ps15
Bilateria - ps16
Deuterostomia - ps17
Chordata - ps18
Olfactores - ps19
Vertebrata - ps20
Gnathostomata - ps21
Euteleostomi - ps22
Sarcopterygii - ps23
Tetrapoda - ps24
Amniota - ps25
Mammalia - ps26
Theria - ps27
Placentalia - ps28
Boreoeutheria - ps29
Euarchontoglires - ps30
Euarchonta - ps31
Primates - ps32
Haplorrhini - ps33
Simiiformes - ps34
Catarrhini - ps35
Hominoidea - ps36
Hominidae - ps37
Homininae - ps38
Hominini - ps39
Homo_sapiens - ps40
p-value
3e-04
9e-04
3e-03
1e-02
4e-02
Log-odds
0.5
1.0
1.5
2.0
2.5
3.0
3.5
4.0

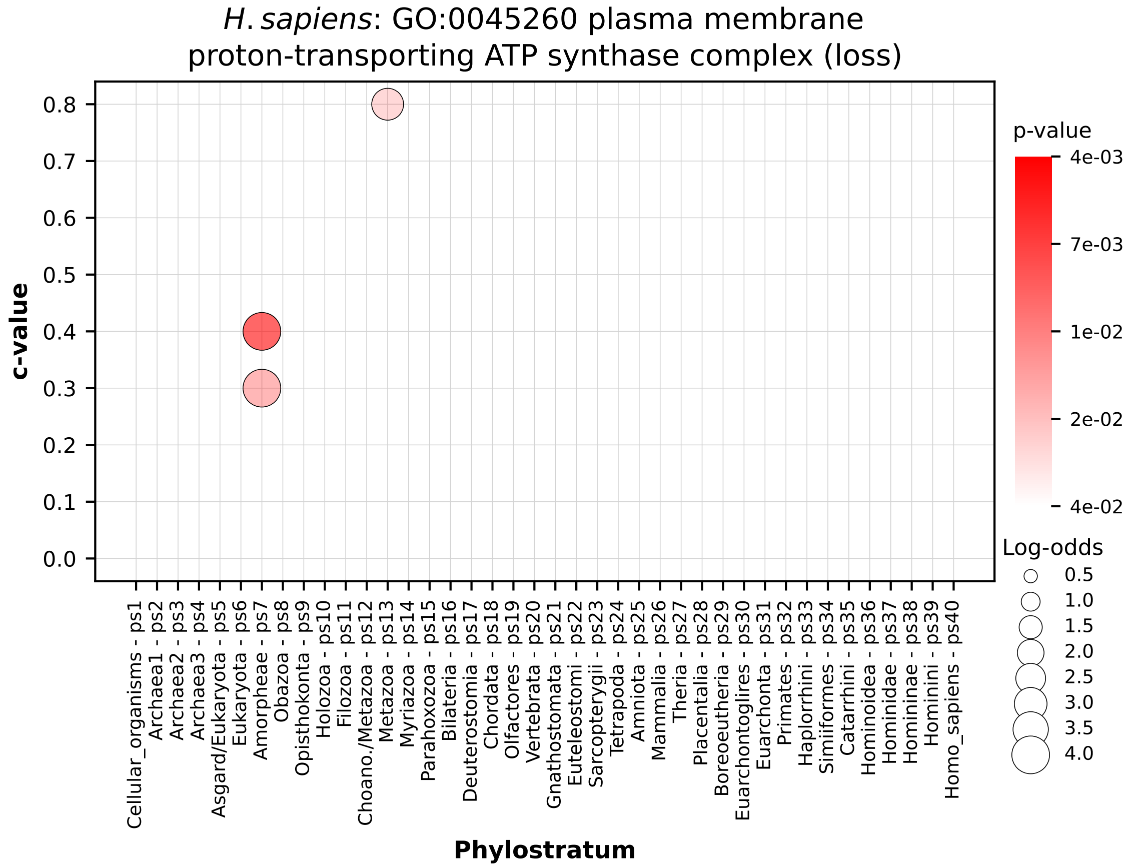

H. sapiens: GO:0045260 plasma membrane proton-transporting ATP synthase complex (loss)
c-value
0.8
0.7
0.6
0.5
0.4
0.3
0.2
0.1
0.0
Phylostratum
Cellular_organisms - ps1
Archaea1 - ps2
Archaea2 - ps3
Archaea3 - ps4
Asgard/Eukaryota - ps5
Eukaryota - ps6
Amorpheae - ps7
Obazoa - ps8
Opisthokonta - ps9
Holozoa - ps10
Filozoa - ps11
Choano./Metazoa - ps12
Metazoa - ps13
Myriazoa - ps14
Parahoxozoa - ps15
Bilateria - ps16
Deuterostomia - ps17
Chordata - ps18
Olfactores - ps19
Vertebrata - ps20
Gnathostomata - ps21
Euteleostomi - ps22
Sarcopterygii - ps23
Tetrapoda - ps24
Amniota - ps25
Mammalia - ps26
Theria - ps27
Placentalia - ps28
Boreoeutheria - ps29
Euarchontoglires - ps30
Euarchonta - ps31
Primates - ps32
Haplorrhini - ps33
Simiiformes - ps34
Catarrhini - ps35
Hominoidea - ps36
Hominidae - ps37
Homininae - ps38
Hominini - ps39
Homo_sapiens - ps40
p-value
4e-03
7e-03
1e-02
2e-02
4e-02
Log-odds
0.5
1.0
1.5
2.0
2.5
3.0
3.5
4.0

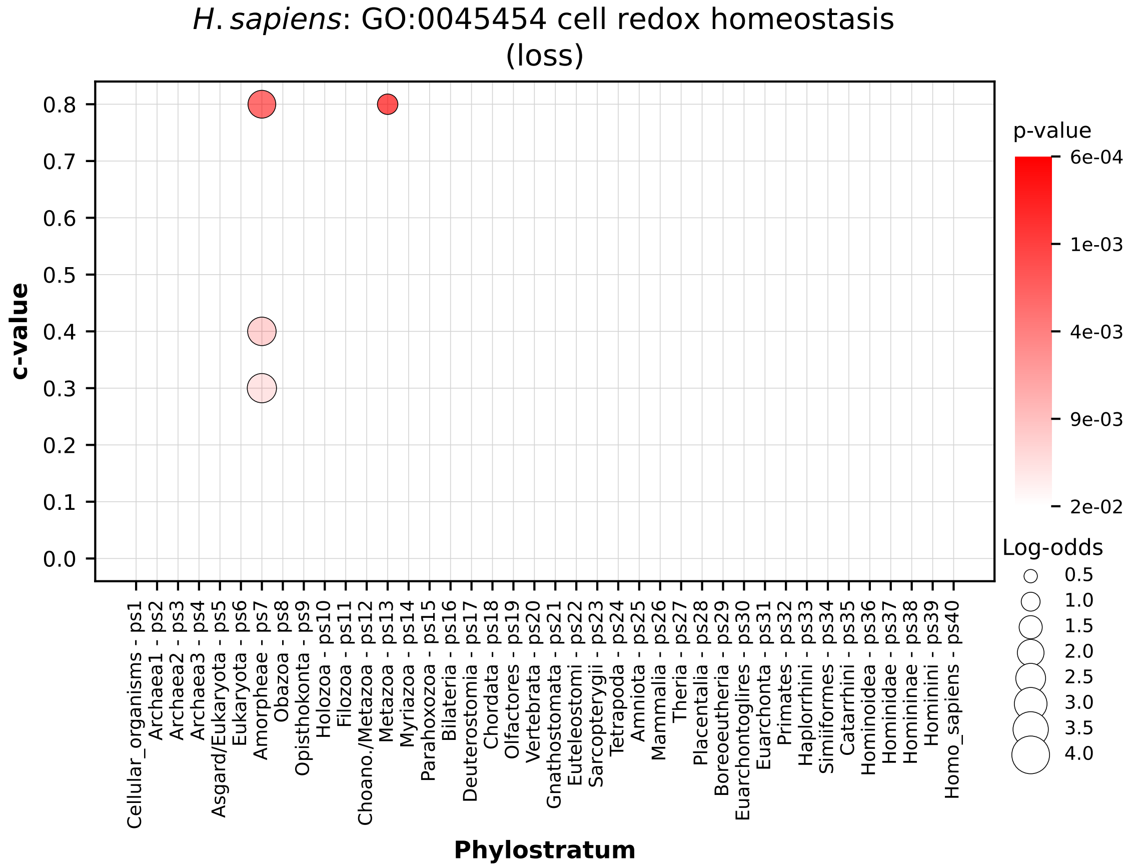

H. sapiens: GO:0045454 cell redox homeostasis
(loss)
c-value
0.0
0.1
0.2
0.3
0.4
0.5
0.6
0.7
0.8
Phylostratum
Cellular_organisms - ps1
Archaea1 - ps2
Archaea2 - ps3
Archaea3 - ps4
Asgard/Eukaryota - ps5
Eukaryota - ps6
Amorpheae - ps7
Obazoa - ps8
Opisthokonta - ps9
Holozoa - ps10
Filozoa - ps11
Choano./Metazoa - ps12
Metazoa - ps13
Myriazoa - ps14
Parahoxozoa - ps15
Bilateria - ps16
Deuterostomia - ps17
Chordata - ps18
Olfactores - ps19
Vertebrata - ps20
Gnathostomata - ps21
Euteleostomi - ps22
Sarcopterygii - ps23
Tetrapoda - ps24
Amniota - ps25
Mammalia - ps26
Theria - ps27
Placentalia - ps28
Boreoeutheria - ps29
Euarchontoglires - ps30
Euarchonta - ps31
Primates - ps32
Haplorrhini - ps33
Simiiformes - ps34
Catarrhini - ps35
Hominoidea - ps36
Hominidae - ps37
Homininae - ps38
Hominini - ps39
Homo_sapiens - ps40
p-value
6e-04
1e-03
4e-03
9e-03
2e-02
Log-odds
0.5
1.0
1.5
2.0
2.5
3.0
3.5
4.0

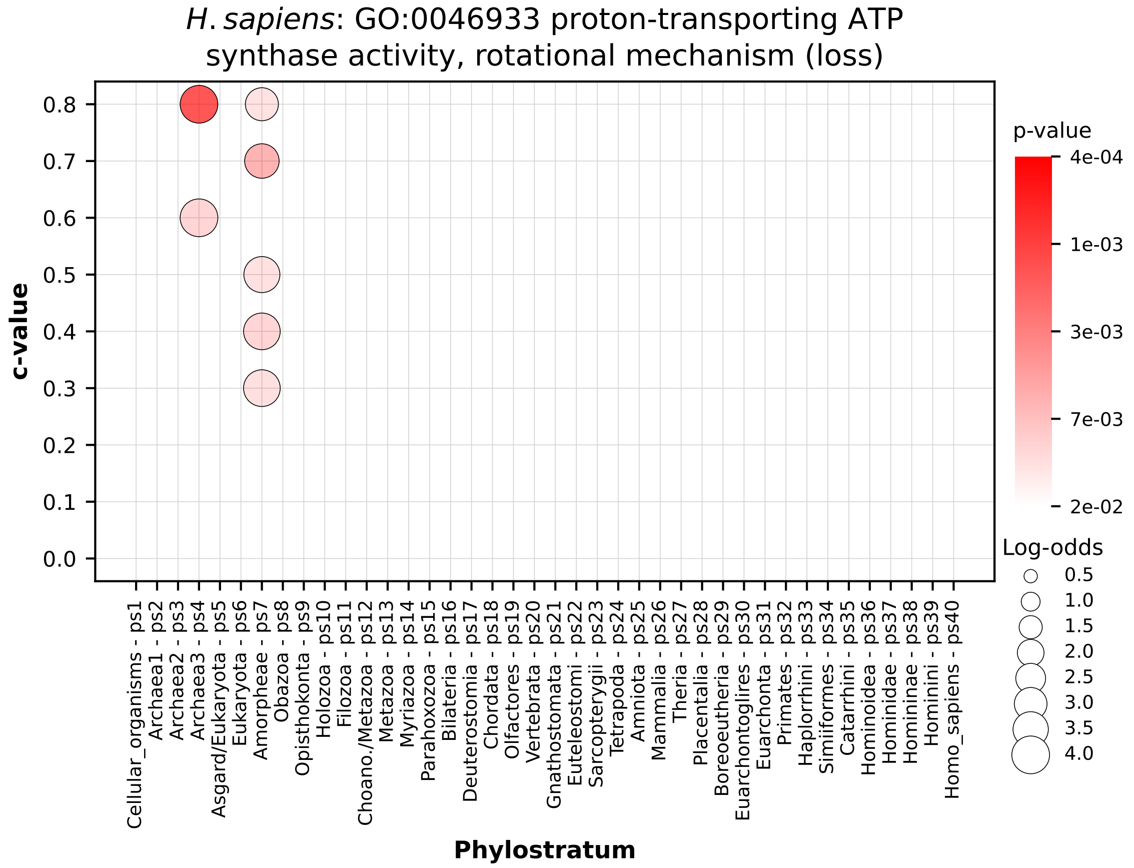
H. sapiens: GO:0046933 proton-transporting ATP synthase activity, rotational mechanism (loss)
c-value
0.8
0.7
0.6
0.5
0.4
0.3
0.2
0.1
0.0
p-value
4e-04
1e-03
3e-03
7e-03
2e-02
Log-odds
0.5
1.0
1.5
2.0
2.5
3.0
3.5
4.0
Cellular_organisms - ps1
Archaea1 - ps2
Archaea2 - ps3
Archaea3 - ps4
Asgard/Eukaryota - ps5
Eukaryota - ps6
Amorpheae - ps7
Obazoa - ps8
Opisthokonta - ps9
Holozoa - ps10
Filozoa - ps11
Choano./Metazoa - ps12
Metazoa - ps13
Myriazoa - ps14
Parahoxozoa - ps15
Bilateria - ps16
Deuterostomia - ps17
Chordata - ps18
Olfactores - ps19
Vertebrata - ps20
Gnathostomata - ps21
Euteleostomi - ps22
Sarcopterygii - ps23
Tetrapoda - ps24
Amniota - ps25
Mammalia - ps26
Theria - ps27
Placentalia - ps28
Boreoeutheria - ps29
Euarchontoglires - ps30
Euarchonta - ps31
Primates - ps32
Haplorrhini - ps33
Simiiformes - ps34
Catarrhini - ps35
Hominoidea - ps36
Hominidae - ps37
Homininae - ps38
Hominini - ps39
Homo_sapiens - ps40
Phylostratum

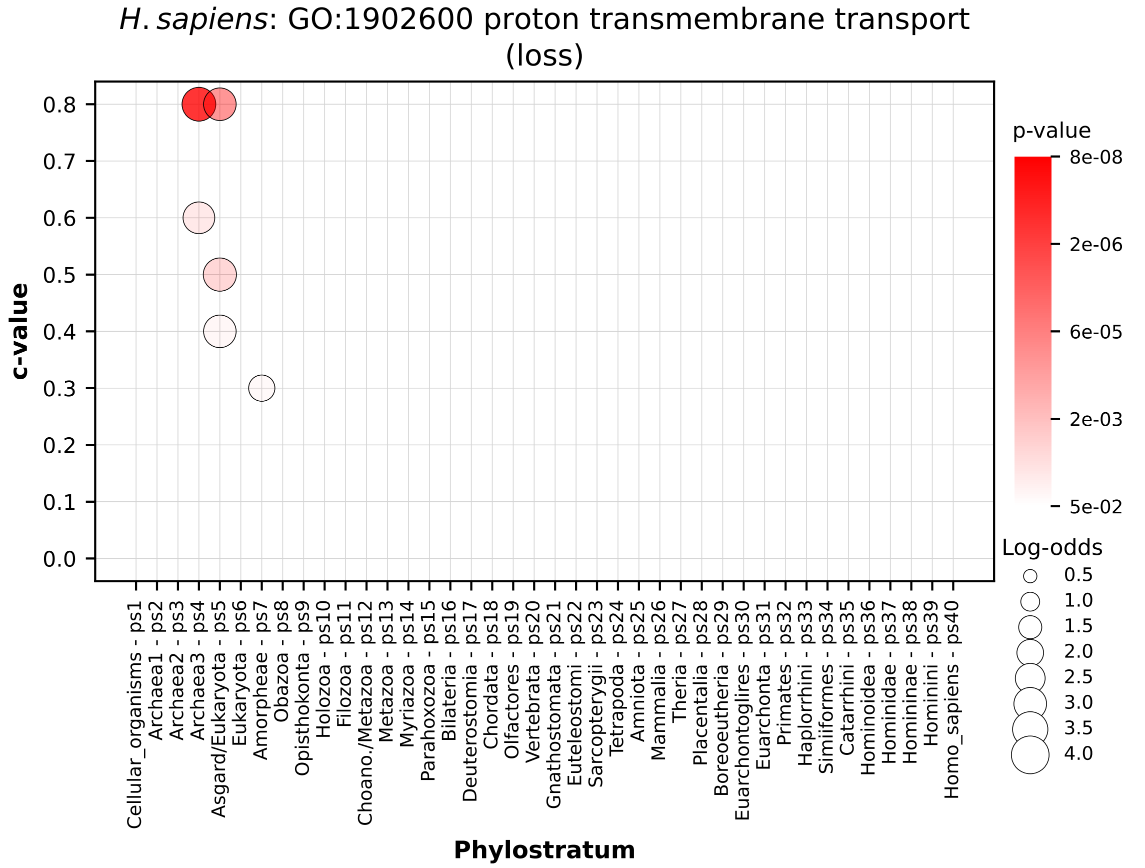

H. sapiens: GO:1902600 proton transmembrane transport (loss)
c-value
0.8
0.7
0.6
0.5
0.4
0.3
0.2
0.1
0.0
Phylostratum
Cellular_organisms - ps1
Archaea1 - ps2
Archaea2 - ps3
Archaea3 - ps4
Asgard/Eukaryota - ps5
Eukaryota - ps6
Amorpheae - ps7
Obazoa - ps8
Opisthokonta - ps9
Holozoa - ps10
Filozoa - ps11
Choano./Metazoa - ps12
Metazoa - ps13
Myriazoa - ps14
Parahoxozoa - ps15
Bilateria - ps16
Deuterostomia - ps17
Chordata - ps18
Olfactores - ps19
Vertebrata - ps20
Gnathostomata - ps21
Euteleostomi - ps22
Sarcopterygii - ps23
Tetrapoda - ps24
Amniota - ps25
Mammalia - ps26
Theria - ps27
Placentalia - ps28
Boreoeutheria - ps29
Euarchontoglires - ps30
Euarchonta - ps31
Primates - ps32
Haplorrhini - ps33
Simiiformes - ps34
Catarrhini - ps35
Hominoidea - ps36
Hominidae - ps37
Homininae - ps38
Hominini - ps39
Homo_sapiens - ps40
p-value
8e-08
2e-06
6e-05
2e-03
5e-02
Log-odds
0.5
1.0
1.5
2.0
2.5
3.0
3.5
4.0

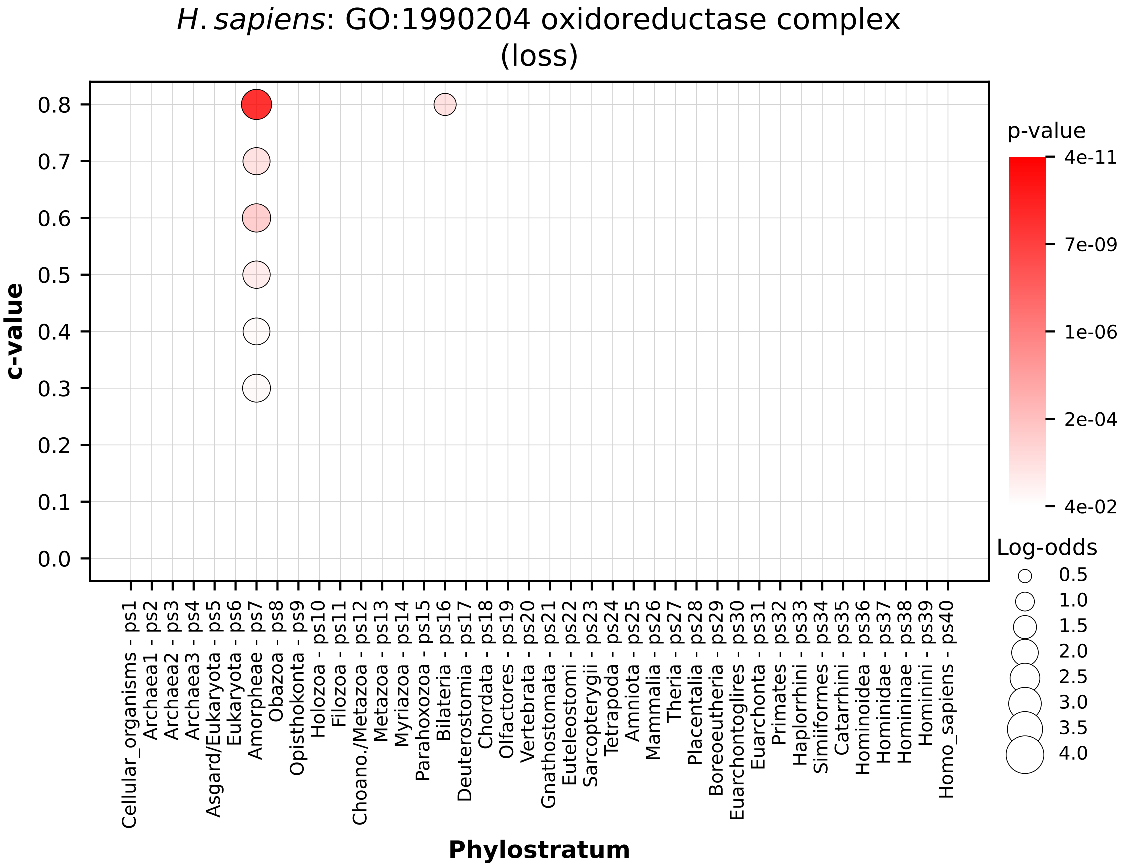
H. sapiens: GO:1990204 oxidoreductase complex (loss)
c-value
0.0
0.1
0.2
0.3
0.4
0.5
0.6
0.7
0.8
Phylostratum
Cellular_organisms - ps1
Archaea1 - ps2
Archaea2 - ps3
Archaea3 - ps4
Asgard/Eukaryota - ps5
Eukaryota - ps6
Amorpheae - ps7
Obazoa - ps8
Opisthokonta - ps9
Holozoa - ps10
Filozoa - ps11
Choano./Metazoa - ps12
Metazoa - ps13
Myriazoa - ps14
Parahoxozoa - ps15
Bilateria - ps16
Deuterostomia - ps17
Chordata - ps18
Olfactores - ps19
Vertebrata - ps20
Gnathostomata - ps21
Euteleostomi - ps22
Sarcopterygii - ps23
Tetrapoda - ps24
Amniota - ps25
Mammalia - ps26
Theria - ps27
Placentalia - ps28
Boreoeutheria - ps29
Euarchontoglires - ps30
Euarchonta - ps31
Primates - ps32
Haplorrhini - ps33
Simiiformes - ps34
Catarrhini - ps35
Hominoidea - ps36
Hominidae - ps37
Homininae - ps38
Hominini - ps39
Homo_sapiens - ps40
p-value
4e-11
7e-09
1e-06
2e-04
4e-02
Log-odds
0.5
1.0
1.5
2.0
2.5
3.0
3.5
4.0

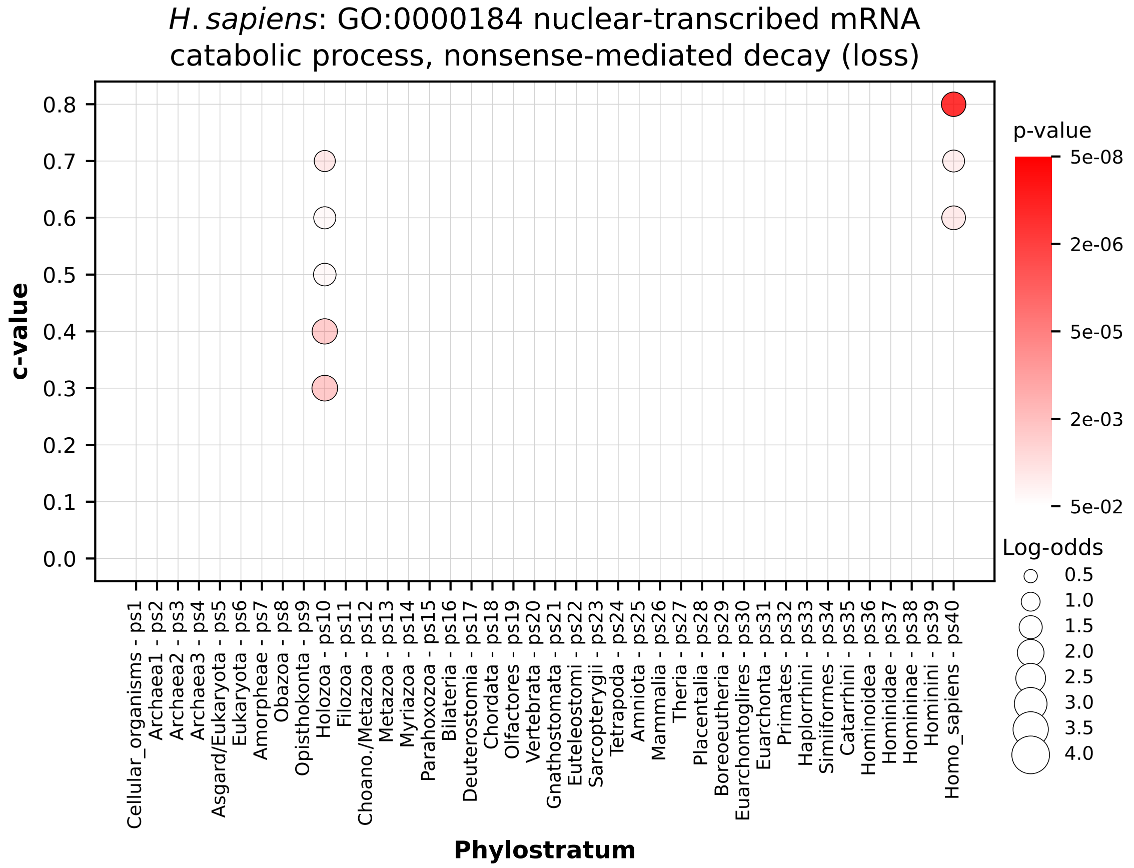

H. sapiens: GO:0000184 nuclear-transcribed mRNA catabolic process, nonsense-mediated decay (loss)
c-value
Phylostratum
p-value
5e-08
2e-06
5e-05
2e-03
5e-02
Log-odds
0.5
1.0
1.5
2.0
2.5
3.0
3.5
4.0
0.0
0.1
0.2
0.3
0.4
0.5
0.6
0.7
0.8
Cellular_organisms - ps1
Archaea1 - ps2
Archaea2 - ps3
Archaea3 - ps4
Asgard/Eukaryota - ps5
Eukaryota - ps6
Amorpheae - ps7
Obazoa - ps8
Opisthokonta - ps9
Holozoa - ps10
Filozoa - ps11
Choano./Metazoa - ps12
Metazoa - ps13
Myriazoa - ps14
Parahoxozoa - ps15
Bilateria - ps16
Deuterostomia - ps17
Chordata - ps18
Olfactores - ps19
Vertebrata - ps20
Gnathostomata - ps21
Euteleostomi - ps22
Sarcopterygii - ps23
Tetrapoda - ps24
Amniota - ps25
Mammalia - ps26
Theria - ps27
Placentalia - ps28
Boreoeutheria - ps29
Euarchontoglires - ps30
Euarchonta - ps31
Primates - ps32
Haplorrhini - ps33
Simiiformes - ps34
Catarrhini - ps35
Hominoidea - ps36
Hominidae - ps37
Homininae - ps38
Hominini - ps39
Homo_sapiens - ps40

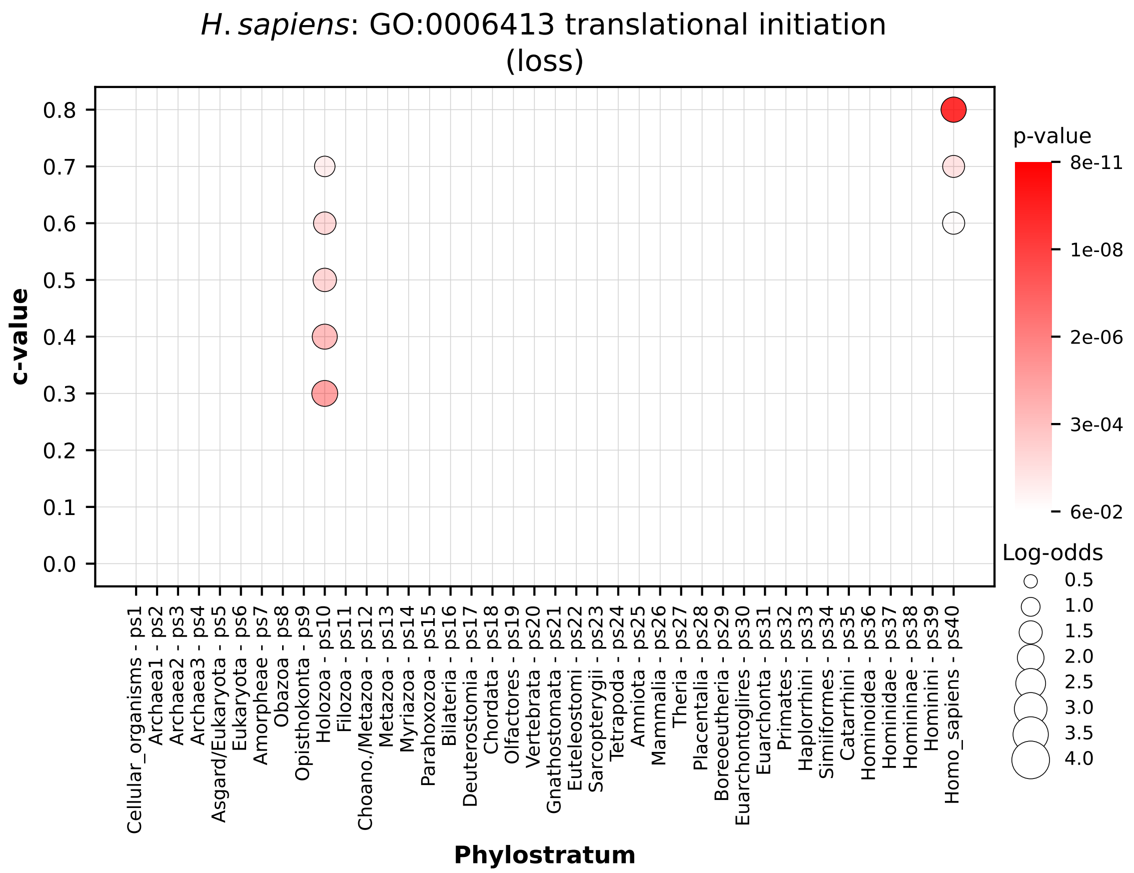

H. sapiens: GO:0006413 translational initiation
(loss)
c-value
0.8
0.7
0.6
0.5
0.4
0.3
0.2
0.1
0.0
p-value
8e-11
1e-08
2e-06
3e-04
6e-02
Log-odds
0.5
1.0
1.5
2.0
2.5
3.0
3.5
4.0
Cellular_organisms - ps1
Archaea1 - ps2
Archaea2 - ps3
Archaea3 - ps4
Asgard/Eukaryota - ps5
Eukaryota - ps6
Amorpheae - ps7
Obazoa - ps8
Opisthokonta - ps9
Holozoa - ps10
Filozoa - ps11
Choano./Metazoa - ps12
Metazoa - ps13
Myriazoa - ps14
Parahoxozoa - ps15
Bilateria - ps16
Deuterostomia - ps17
Chordata - ps18
Olfactores - ps19
Vertebrata - ps20
Gnathostomata - ps21
Euteleostomi - ps22
Sarcopterygii - ps23
Tetrapoda - ps24
Amniota - ps25
Mammalia - ps26
Theria - ps27
Placentalia - ps28
Boreoeutheria - ps29
Euarchontoglires - ps30
Euarchonta - ps31
Primates - ps32
Haplorrhini - ps33
Simiiformes - ps34
Catarrhini - ps35
Hominoidea - ps36
Hominidae - ps37
Homininae - ps38
Hominini - ps39
Homo_sapiens - ps40
Phylostratum

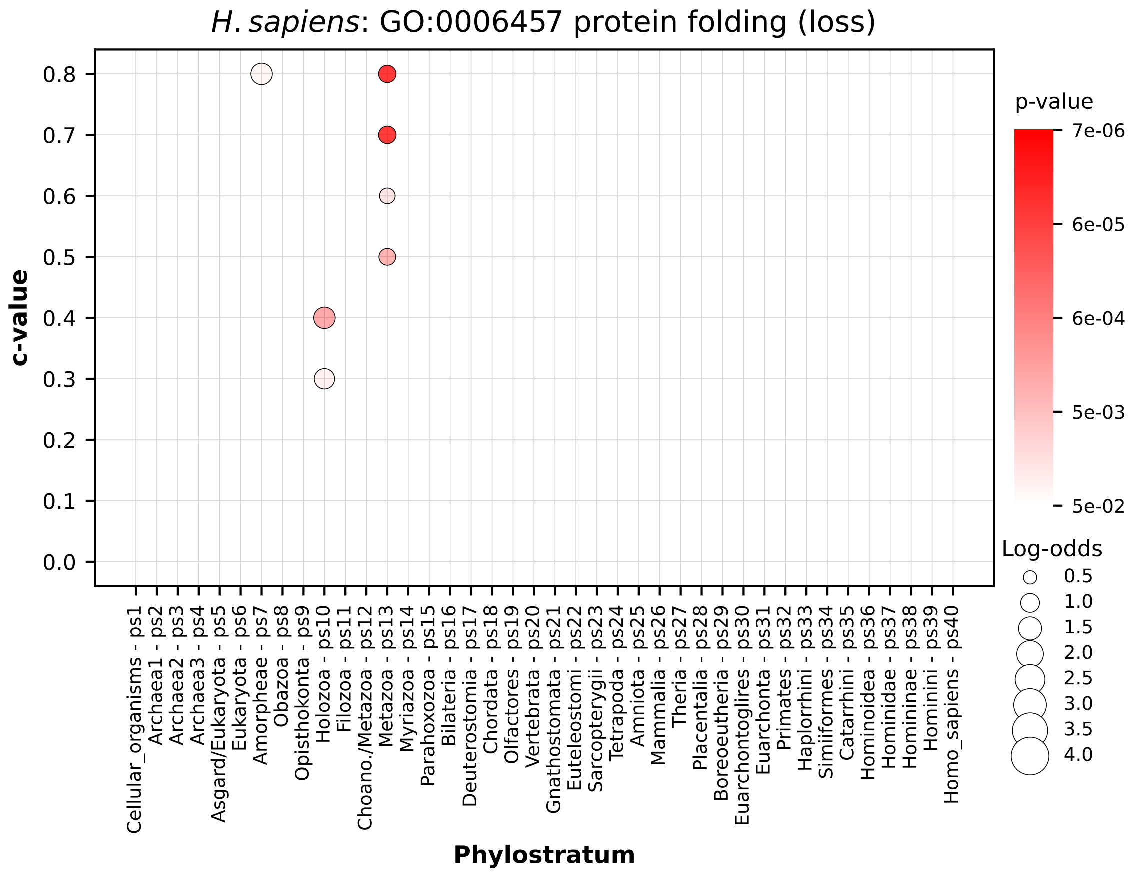
H. sapiens: GO:0006457 protein folding (loss)
c-value
0.0
0.1
0.2
0.3
0.4
0.5
0.6
0.7
0.8
Phylostratum
Cellular_organisms - ps1
Archaea1 - ps2
Archaea2 - ps3
Archaea3 - ps4
Asgard/Eukaryota - ps5
Eukaryota - ps6
Amorpheae - ps7
Obazoa - ps8
Opisthokonta - ps9
Holozoa - ps10
Filozoa - ps11
Choano./Metazoa - ps12
Metazoa - ps13
Myriazoa - ps14
Parahoxozoa - ps15
Bilateria - ps16
Deuterostomia - ps17
Chordata - ps18
Olfactores - ps19
Vertebrata - ps20
Gnathostomata - ps21
Euteleostomi - ps22
Sarcopterygii - ps23
Tetrapoda - ps24
Amniota - ps25
Mammalia - ps26
Theria - ps27
Placentalia - ps28
Boreoeutheria - ps29
Euarchontoglires - ps30
Euarchonta - ps31
Primates - ps32
Haplorrhini - ps33
Simiiformes - ps34
Catarrhini - ps35
Hominoidea - ps36
Hominidae - ps37
Homininae - ps38
Hominini - ps39
Homo_sapiens - ps40
p-value
7e-06
6e-05
6e-04
5e-03
5e-02
Log-odds
0.5
1.0
1.5
2.0
2.5
3.0
3.5
4.0

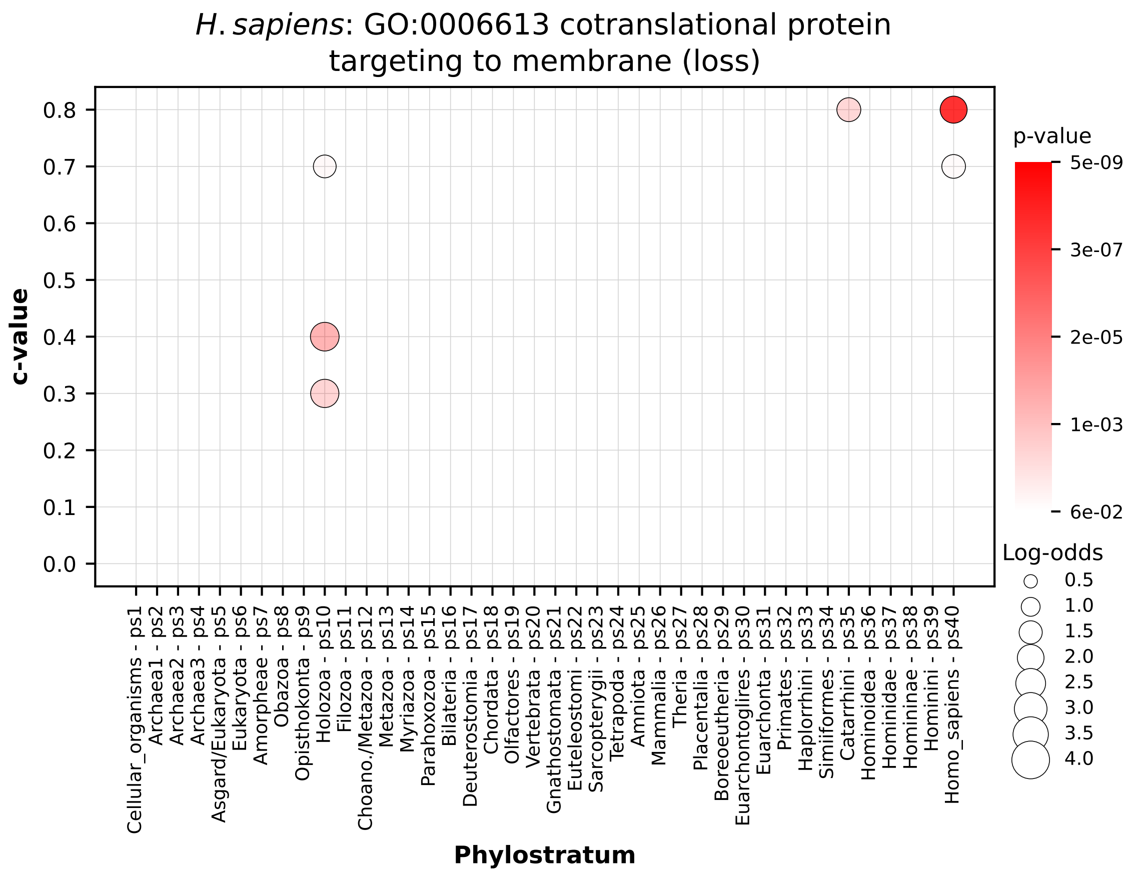

H. sapiens: GO:0006613 cotranslational protein targeting to membrane (loss)
c-value
0.0
0.1
0.2
0.3
0.4
0.5
0.6
0.7
0.8
p-value
5e-09
3e-07
2e-05
1e-03
6e-02
Log-odds
0.5
1.0
1.5
2.0
2.5
3.0
3.5
4.0
Cellular_organisms - ps1
Archaea1 - ps2
Archaea2 - ps3
Archaea3 - ps4
Asgard/Eukaryota - ps5
Eukaryota - ps6
Amorpheae - ps7
Obazoa - ps8
Opisthokonta - ps9
Holozoa - ps10
Filozoa - ps11
Choano./Metazoa - ps12
Metazoa - ps13
Myriazoa - ps14
Parahoxozoa - ps15
Bilateria - ps16
Deuterostomia - ps17
Chordata - ps18
Olfactores - ps19
Vertebrata - ps20
Gnathostomata - ps21
Euteleostomi - ps22
Sarcopterygii - ps23
Tetrapoda - ps24
Amniota - ps25
Mammalia - ps26
Theria - ps27
Placentalia - ps28
Boreoeutheria - ps29
Euarchontoglires - ps30
Euarchonta - ps31
Primates - ps32
Haplorrhini - ps33
Simiiformes - ps34
Catarrhini - ps35
Hominoidea - ps36
Hominidae - ps37
Homininae - ps38
Hominini - ps39
Homo_sapiens - ps40
Phylostratum

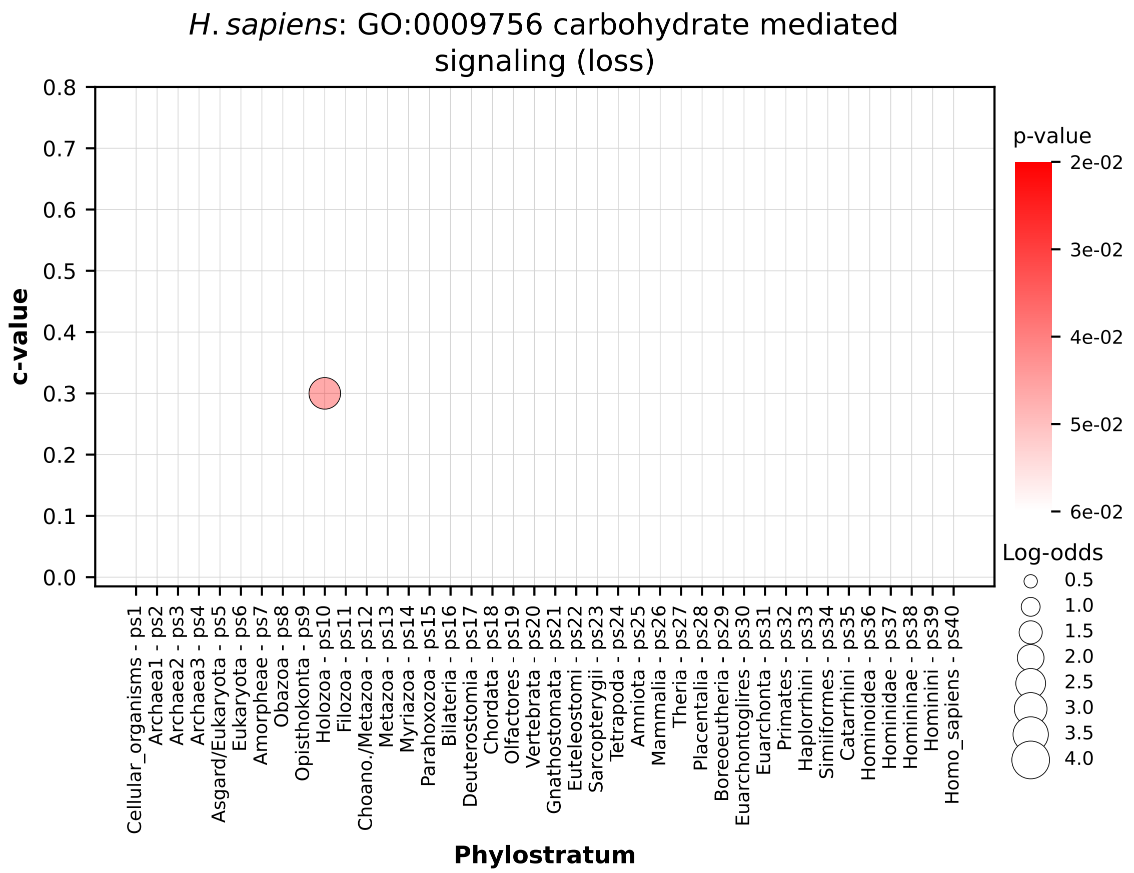

H. sapiens: GO:0009756 carbohydrate mediated signaling (loss)
c-value
0.0
0.1
0.2
0.3
0.4
0.5
0.6
0.7
0.8
Phylostratum
Cellular_organisms - ps1
Archaea1 - ps2
Archaea2 - ps3
Archaea3 - ps4
Asgard/Eukaryota - ps5
Eukaryota - ps6
Amorpheae - ps7
Obazoa - ps8
Opisthokonta - ps9
Holozoa - ps10
Filozoa - ps11
Choano./Metazoa - ps12
Metazoa - ps13
Myriazoa - ps14
Parahoxozoa - ps15
Bilateria - ps16
Deuterostomia - ps17
Chordata - ps18
Olfactores - ps19
Vertebrata - ps20
Gnathostomata - ps21
Euteleostomi - ps22
Sarcopterygii - ps23
Tetrapoda - ps24
Amniota - ps25
Mammalia - ps26
Theria - ps27
Placentalia - ps28
Boreoeutheria - ps29
Euarchontoglires - ps30
Euarchonta - ps31
Primates - ps32
Haplorrhini - ps33
Simiiformes - ps34
Catarrhini - ps35
Hominoidea - ps36
Hominidae - ps37
Homininae - ps38
Hominini - ps39
Homo_sapiens - ps40
p-value
2e-02
3e-02
4e-02
5e-02
6e-02
Log-odds
0.5
1.0
1.5
2.0
2.5
3.0
3.5
4.0

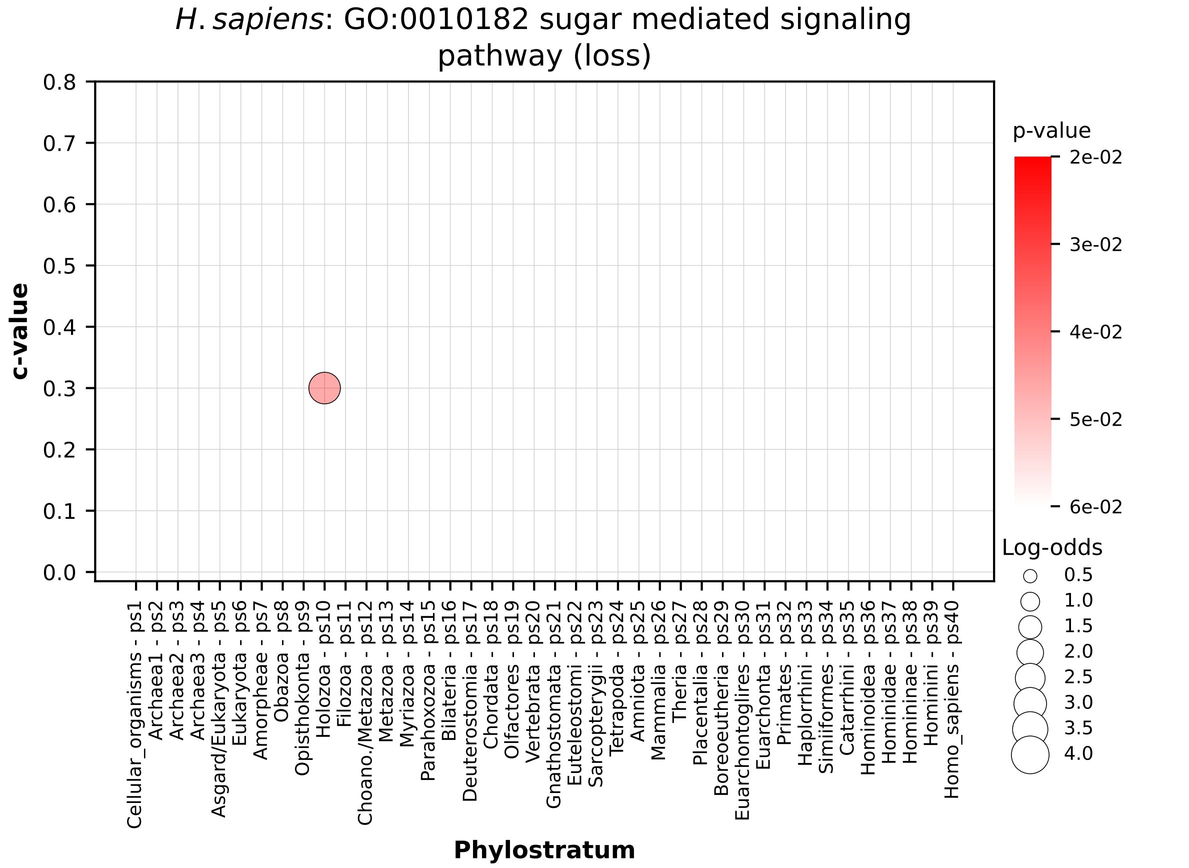

H. sapiens: GO:0010182 sugar mediated signaling pathway (loss)
c-value
0.0
0.1
0.2
0.3
0.4
0.5
0.6
0.7
0.8
Phylostratum
Cellular_organisms - ps1
Archaea1 - ps2
Archaea2 - ps3
Archaea3 - ps4
Asgard/Eukaryota - ps5
Eukaryota - ps6
Amorpheae - ps7
Obazoa - ps8
Opisthokonta - ps9
Holozoa - ps10
Filozoa - ps11
Choano./Metazoa - ps12
Metazoa - ps13
Myriazoa - ps14
Parahoxozoa - ps15
Bilateria - ps16
Deuterostomia - ps17
Chordata - ps18
Olfactores - ps19
Vertebrata - ps20
Gnathostomata - ps21
Euteleostomi - ps22
Sarcopterygii - ps23
Tetrapoda - ps24
Amniota - ps25
Mammalia - ps26
Theria - ps27
Placentalia - ps28
Boreoeutheria - ps29
Euarchontoglires - ps30
Euarchonta - ps31
Primates - ps32
Haplorrhini - ps33
Simiiformes - ps34
Catarrhini - ps35
Hominoidea - ps36
Hominidae - ps37
Homininae - ps38
Hominini - ps39
Homo_sapiens - ps40
p-value
2e-02
3e-02
4e-02
5e-02
6e-02
Log-odds
0.5
1.0
1.5
2.0
2.5
3.0
3.5
4.0

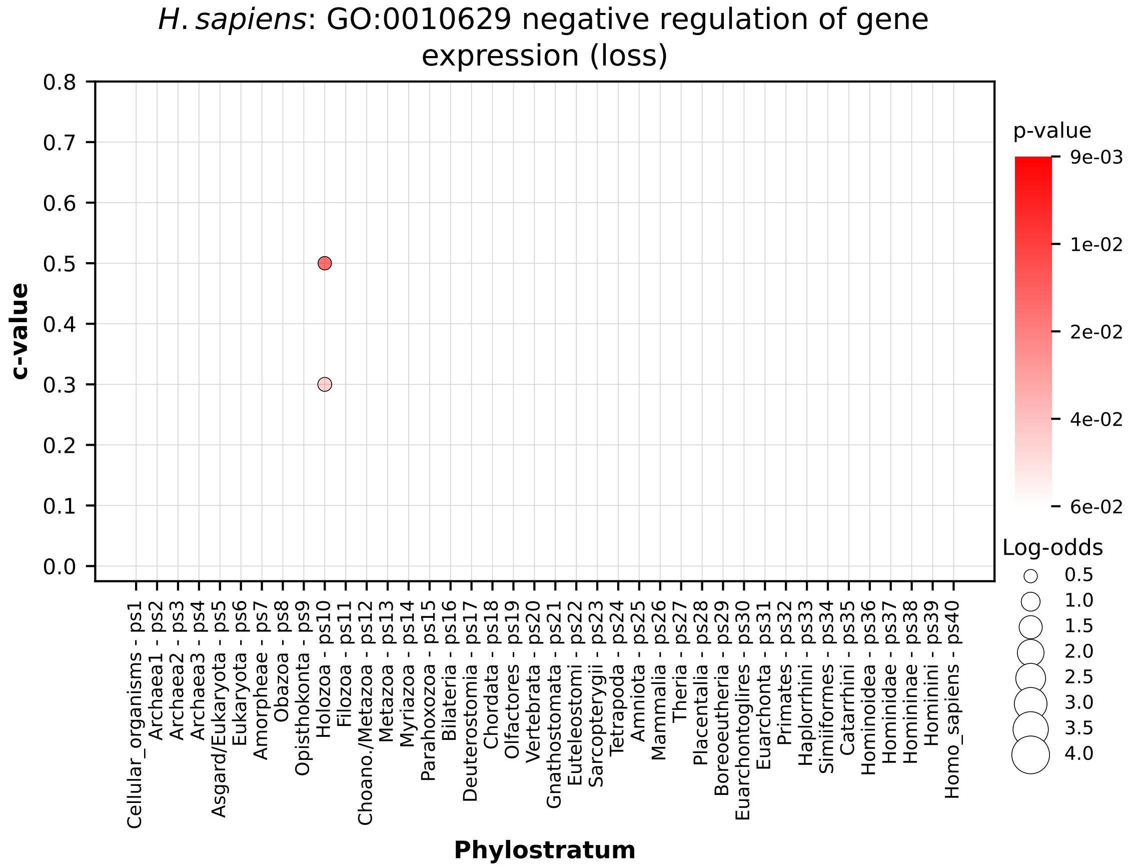

H. sapiens: GO:0010629 negative regulation of gene expression (loss)
c-value
0.8
0.7
0.6
0.5
0.4
0.3
0.2
0.1
0.0
Phylostratum
Cellular_organisms - ps1
Archaea1 - ps2
Archaea2 - ps3
Archaea3 - ps4
Asgard/Eukaryota - ps5
Eukaryota - ps6
Amorpheae - ps7
Obazoa - ps8
Opisthokonta - ps9
Holozoa - ps10
Filozoa - ps11
Choano./Metazoa - ps12
Metazoa - ps13
Myriazoa - ps14
Parahoxozoa - ps15
Bilateria - ps16
Deuterostomia - ps17
Chordata - ps18
Olfactores - ps19
Vertebrata - ps20
Gnathostomata - ps21
Euteleostomi - ps22
Sarcopterygii - ps23
Tetrapoda - ps24
Amniota - ps25
Mammalia - ps26
Theria - ps27
Placentalia - ps28
Boreoeutheria - ps29
Euarchontoglires - ps30
Euarchonta - ps31
Primates - ps32
Haplorrhini - ps33
Simiiformes - ps34
Catarrhini - ps35
Hominoidea - ps36
Hominidae - ps37
Homininae - ps38
Hominini - ps39
Homo_sapiens - ps40
p-value
9e-03
1e-02
2e-02
4e-02
6e-02
Log-odds
0.5
1.0
1.5
2.0
2.5
3.0
3.5
4.0

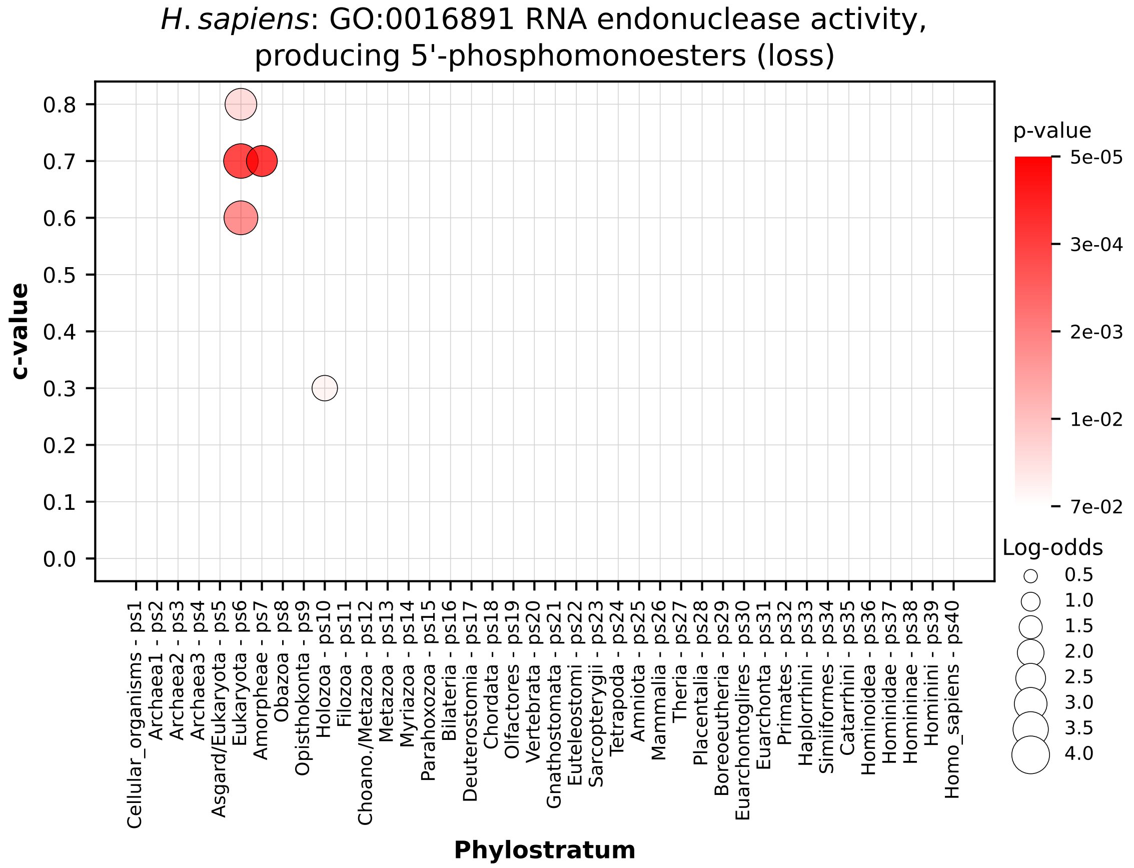

H. sapiens: GO:0016891 RNA endonuclease activity, producing 5'-phosphomonoesters (loss)
c-value
0.8
0.7
0.6
0.5
0.4
0.3
0.2
0.1
0.0
p-value
5e-05
3e-04
2e-03
1e-02
7e-02
Log-odds
0.5
1.0
1.5
2.0
2.5
3.0
3.5
4.0
Cellular_organisms - ps1
Archaea1 - ps2
Archaea2 - ps3
Archaea3 - ps4
Asgard/Eukaryota - ps5
Eukaryota - ps6
Amorpheae - ps7
Obazoa - ps8
Opisthokonta - ps9
Holozoa - ps10
Filozoa - ps11
Choano./Metazoa - ps12
Metazoa - ps13
Myriazoa - ps14
Parahoxozoa - ps15
Bilateria - ps16
Deuterostomia - ps17
Chordata - ps18
Olfactores - ps19
Vertebrata - ps20
Gnathostomata - ps21
Euteleostomi - ps22
Sarcopterygii - ps23
Tetrapoda - ps24
Amniota - ps25
Mammalia - ps26
Theria - ps27
Placentalia - ps28
Boreoeutheria - ps29
Euarchontoglires - ps30
Euarchonta - ps31
Primates - ps32
Haplorrhini - ps33
Simiiformes - ps34
Catarrhini - ps35
Hominoidea - ps36
Hominidae - ps37
Homininae - ps38
Hominini - ps39
Homo_sapiens - ps40
Phylostratum

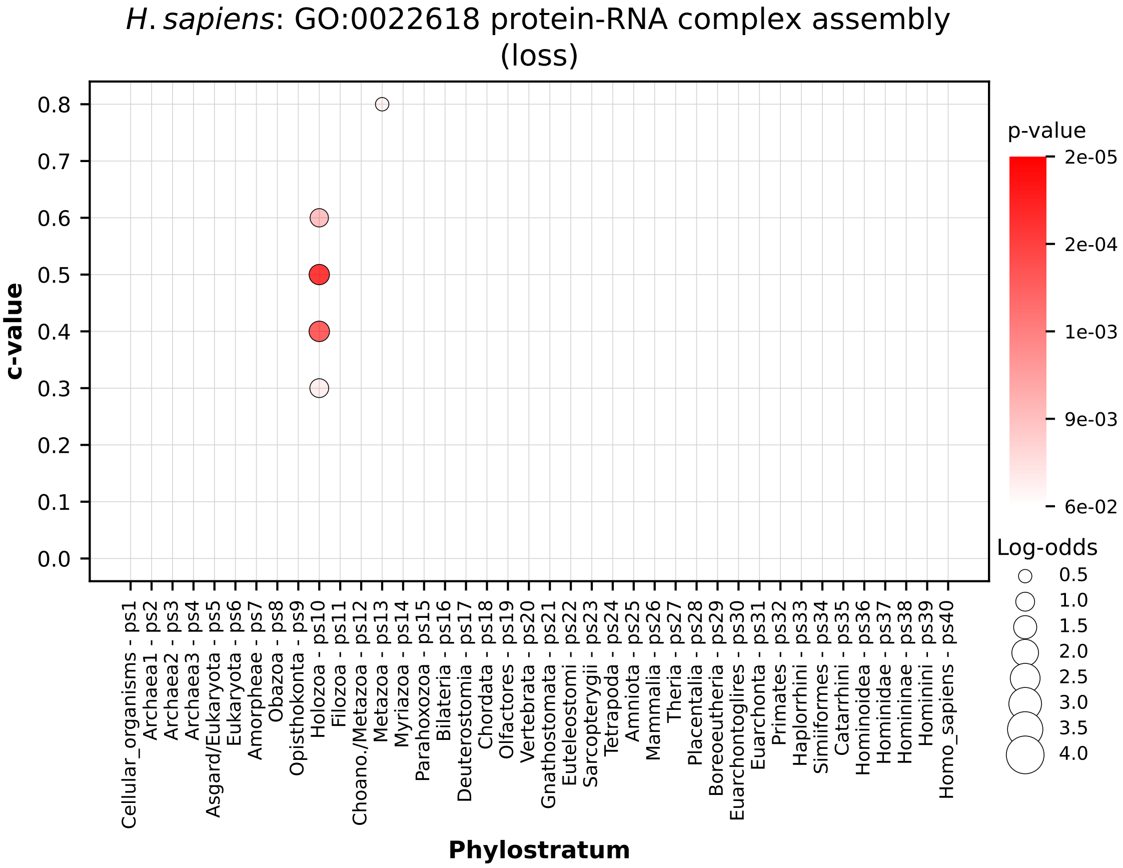

H. sapiens: GO:0022618 protein-RNA complex assembly (loss)
c-value
0.0
0.1
0.2
0.3
0.4
0.5
0.6
0.7
0.8
Phylostratum
Cellular_organisms - ps1
Archaea1 - ps2
Archaea2 - ps3
Archaea3 - ps4
Asgard/Eukaryota - ps5
Eukaryota - ps6
Amorpheae - ps7
Obazoa - ps8
Opisthokonta - ps9
Holozoa - ps10
Filozoa - ps11
Choano./Metazoa - ps12
Metazoa - ps13
Myriazoa - ps14
Parahoxozoa - ps15
Bilateria - ps16
Deuterostomia - ps17
Chordata - ps18
Olfactores - ps19
Vertebrata - ps20
Gnathostomata - ps21
Euteleostomi - ps22
Sarcopterygii - ps23
Tetrapoda - ps24
Amniota - ps25
Mammalia - ps26
Theria - ps27
Placentalia - ps28
Boreoeutheria - ps29
Euarchontoglires - ps30
Euarchonta - ps31
Primates - ps32
Haplorrhini - ps33
Simiiformes - ps34
Catarrhini - ps35
Hominoidea - ps36
Hominidae - ps37
Homininae - ps38
Hominini - ps39
Homo_sapiens - ps40
p-value
2e-05
2e-04
1e-03
9e-03
6e-02
Log-odds
0.5
1.0
1.5
2.0
2.5
3.0
3.5
4.0

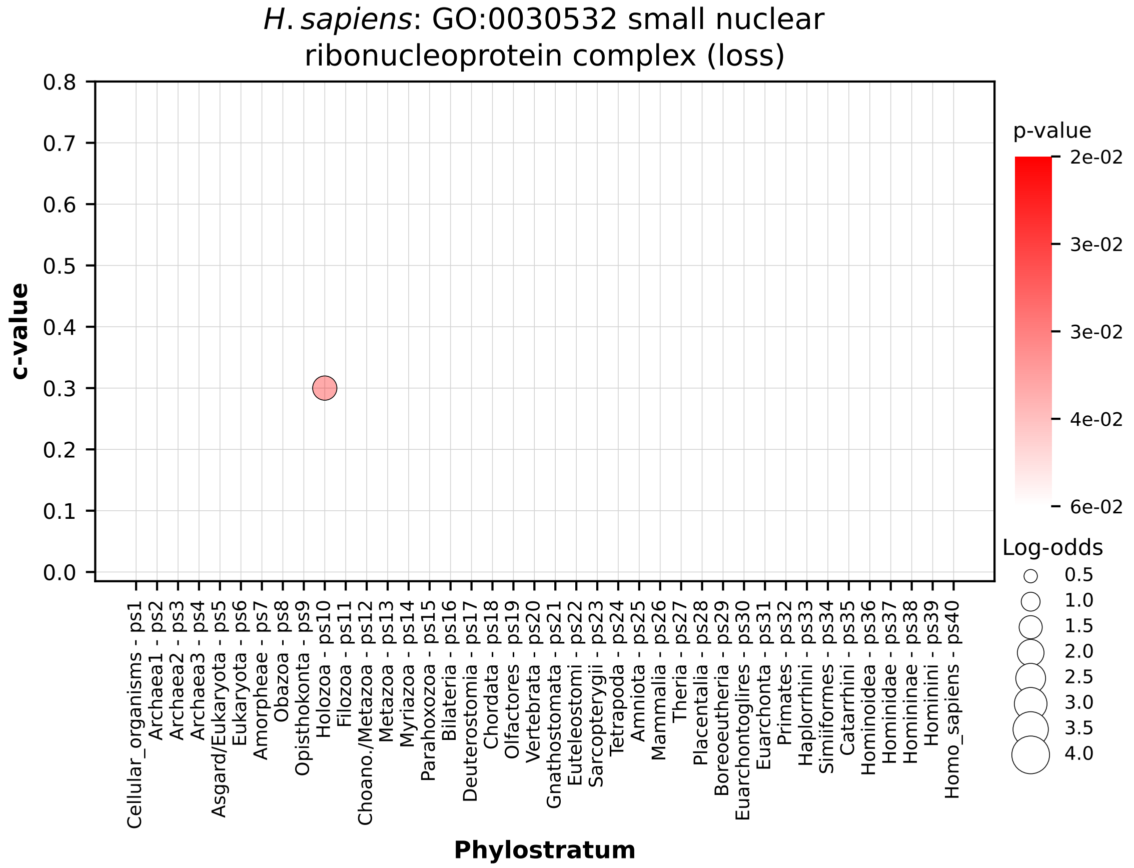

H. sapiens: GO:0030532 small nuclear ribonucleoprotein complex (loss)
c-value
0.8
0.7
0.6
0.5
0.4
0.3
0.2
0.1
0.0
p-value
2e-02
3e-02
3e-02
4e-02
6e-02
Log-odds
0.5
1.0
1.5
2.0
2.5
3.0
3.5
4.0
Cellular_organisms - ps1
Archaea1 - ps2
Archaea2 - ps3
Archaea3 - ps4
Asgard/Eukaryota - ps5
Eukaryota - ps6
Amorpheae - ps7
Obazoa - ps8
Opisthokonta - ps9
Holozoa - ps10
Filozoa - ps11
Choano./Metazoa - ps12
Metazoa - ps13
Myriazoa - ps14
Parahoxozoa - ps15
Bilateria - ps16
Deuterostomia - ps17
Chordata - ps18
Olfactores - ps19
Vertebrata - ps20
Gnathostomata - ps21
Euteleostomi - ps22
Sarcopterygii - ps23
Tetrapoda - ps24
Amniota - ps25
Mammalia - ps26
Theria - ps27
Placentalia - ps28
Boreoeutheria - ps29
Euarchontoglires - ps30
Euarchonta - ps31
Primates - ps32
Haplorrhini - ps33
Simiiformes - ps34
Catarrhini - ps35
Hominoidea - ps36
Hominidae - ps37
Homininae - ps38
Hominini - ps39
Homo_sapiens - ps40
Phylostratum

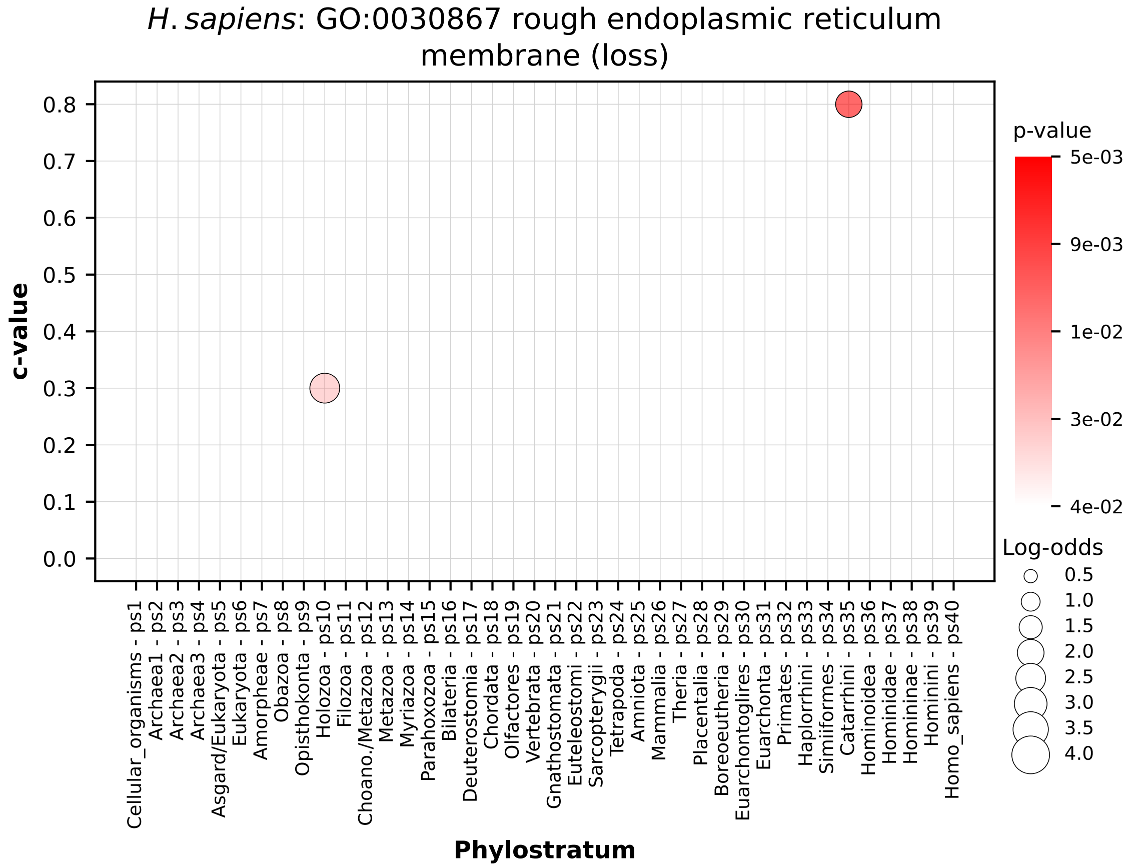

H. sapiens: GO:0030867 rough endoplasmic reticulum membrane (loss)
c-value
0.0
0.1
0.2
0.3
0.4
0.5
0.6
0.7
0.8
Phylostratum
Cellular_organisms - ps1
Archaea1 - ps2
Archaea2 - ps3
Archaea3 - ps4
Asgard/Eukaryota - ps5
Eukaryota - ps6
Amorpheae - ps7
Obazoa - ps8
Opisthokonta - ps9
Holozoa - ps10
Filozoa - ps11
Choano./Metazoa - ps12
Metazoa - ps13
Myriazoa - ps14
Parahoxozoa - ps15
Bilateria - ps16
Deuterostomia - ps17
Chordata - ps18
Olfactores - ps19
Vertebrata - ps20
Gnathostomata - ps21
Euteleostomi - ps22
Sarcopterygii - ps23
Tetrapoda - ps24
Amniota - ps25
Mammalia - ps26
Theria - ps27
Placentalia - ps28
Boreoeutheria - ps29
Euarchontoglires - ps30
Euarchonta - ps31
Primates - ps32
Haplorrhini - ps33
Simiiformes - ps34
Catarrhini - ps35
Hominoidea - ps36
Hominidae - ps37
Homininae - ps38
Hominini - ps39
Homo_sapiens - ps40
p-value
5e-03
9e-03
1e-02
3e-02
4e-02
Log-odds
0.5
1.0
1.5
2.0
2.5
3.0
3.5
4.0

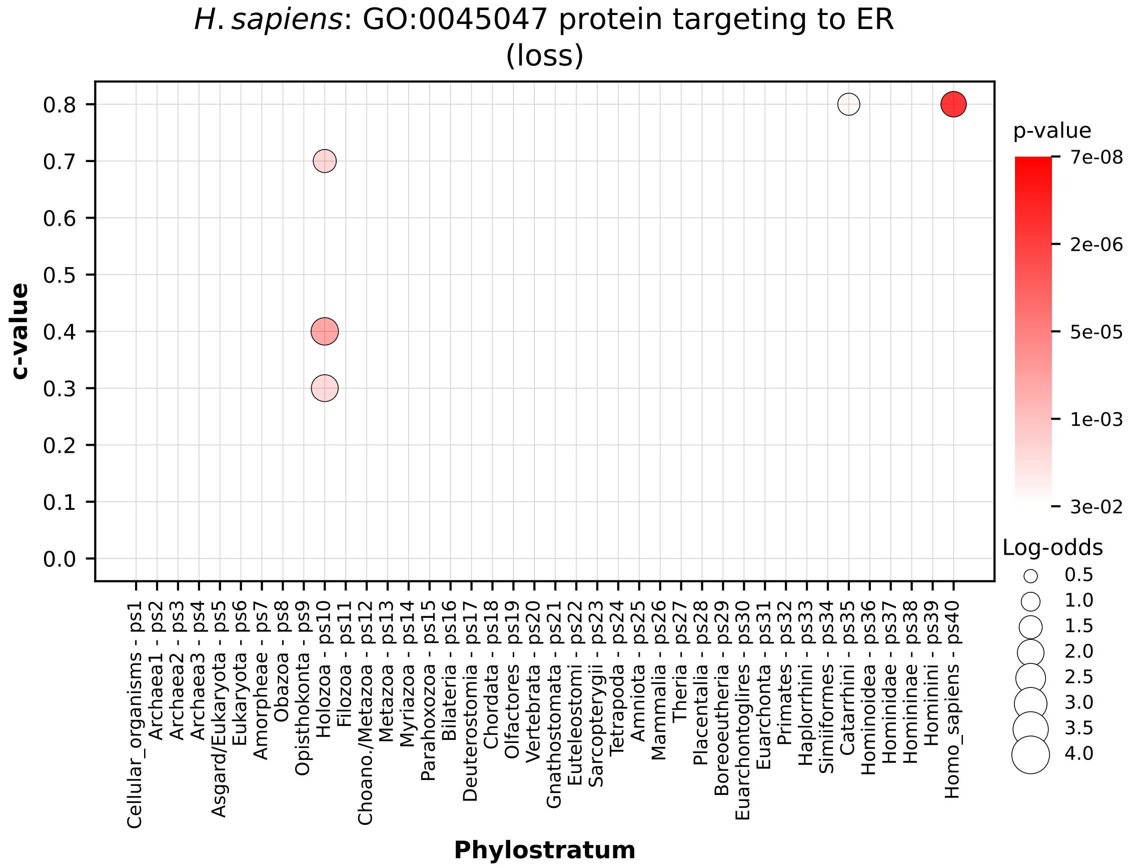

H. sapiens: GO:0045047 protein targeting to ER
(loss)
c-value
0.0
0.1
0.2
0.3
0.4
0.5
0.6
0.7
0.8
p-value
7e-08
2e-06
5e-05
1e-03
3e-02
Log-odds
0.5
1.0
1.5
2.0
2.5
3.0
3.5
4.0
Cellular_organisms - ps1
Archaea1 - ps2
Archaea2 - ps3
Archaea3 - ps4
Asgard/Eukaryota - ps5
Eukaryota - ps6
Amorpheae - ps7
Obazoa - ps8
Opisthokonta - ps9
Holozoa - ps10
Filozoa - ps11
Choano./Metazoa - ps12
Metazoa - ps13
Myriazoa - ps14
Parahoxozoa - ps15
Bilateria - ps16
Deuterostomia - ps17
Chordata - ps18
Olfactores - ps19
Vertebrata - ps20
Gnathostomata - ps21
Euteleostomi - ps22
Sarcopterygii - ps23
Tetrapoda - ps24
Amniota - ps25
Mammalia - ps26
Theria - ps27
Placentalia - ps28
Boreoeutheria - ps29
Euarchontoglires - ps30
Euarchonta - ps31
Primates - ps32
Haplorrhini - ps33
Simiiformes - ps34
Catarrhini - ps35
Hominoidea - ps36
Hominidae - ps37
Homininae - ps38
Hominini - ps39
Homo_sapiens - ps40
Phylostratum

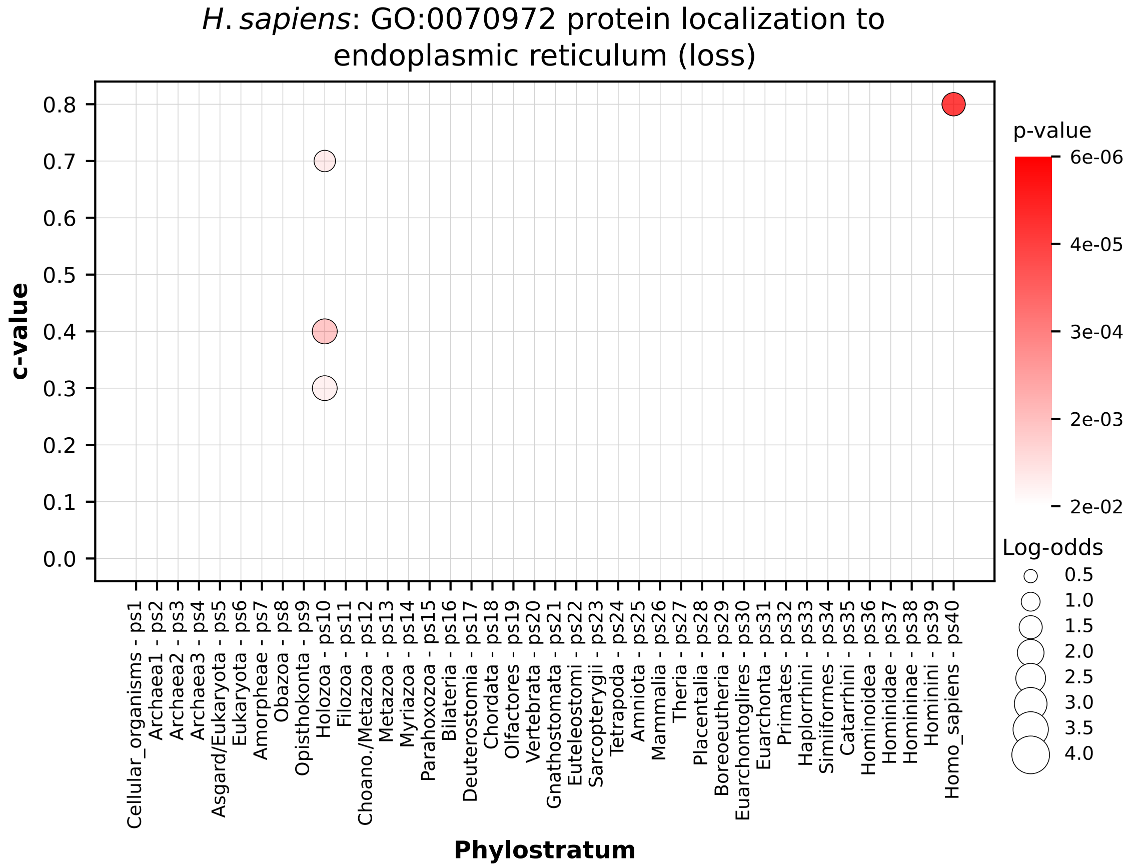

H. sapiens: GO:0070972 protein localization to endoplasmic reticulum (loss)
c-value
0.0
0.1
0.2
0.3
0.4
0.5
0.6
0.7
0.8
p-value
6e-06
4e-05
3e-04
2e-03
2e-02
Log-odds
0.5
1.0
1.5
2.0
2.5
3.0
3.5
4.0
Cellular_organisms - ps1
Archaea1 - ps2
Archaea2 - ps3
Archaea3 - ps4
Asgard/Eukaryota - ps5
Eukaryota - ps6
Amorpheae - ps7
Obazoa - ps8
Opisthokonta - ps9
Holozoa - ps10
Filozoa - ps11
Choano./Metazoa - ps12
Metazoa - ps13
Myriazoa - ps14
Parahoxozoa - ps15
Bilateria - ps16
Deuterostomia - ps17
Chordata - ps18
Olfactores - ps19
Vertebrata - ps20
Gnathostomata - ps21
Euteleostomi - ps22
Sarcopterygii - ps23
Tetrapoda - ps24
Amniota - ps25
Mammalia - ps26
Theria - ps27
Placentalia - ps28
Boreoeutheria - ps29
Euarchontoglires - ps30
Euarchonta - ps31
Primates - ps32
Haplorrhini - ps33
Simiiformes - ps34
Catarrhini - ps35
Hominoidea - ps36
Hominidae - ps37
Homininae - ps38
Hominini - ps39
Homo_sapiens - ps40
Phylostratum

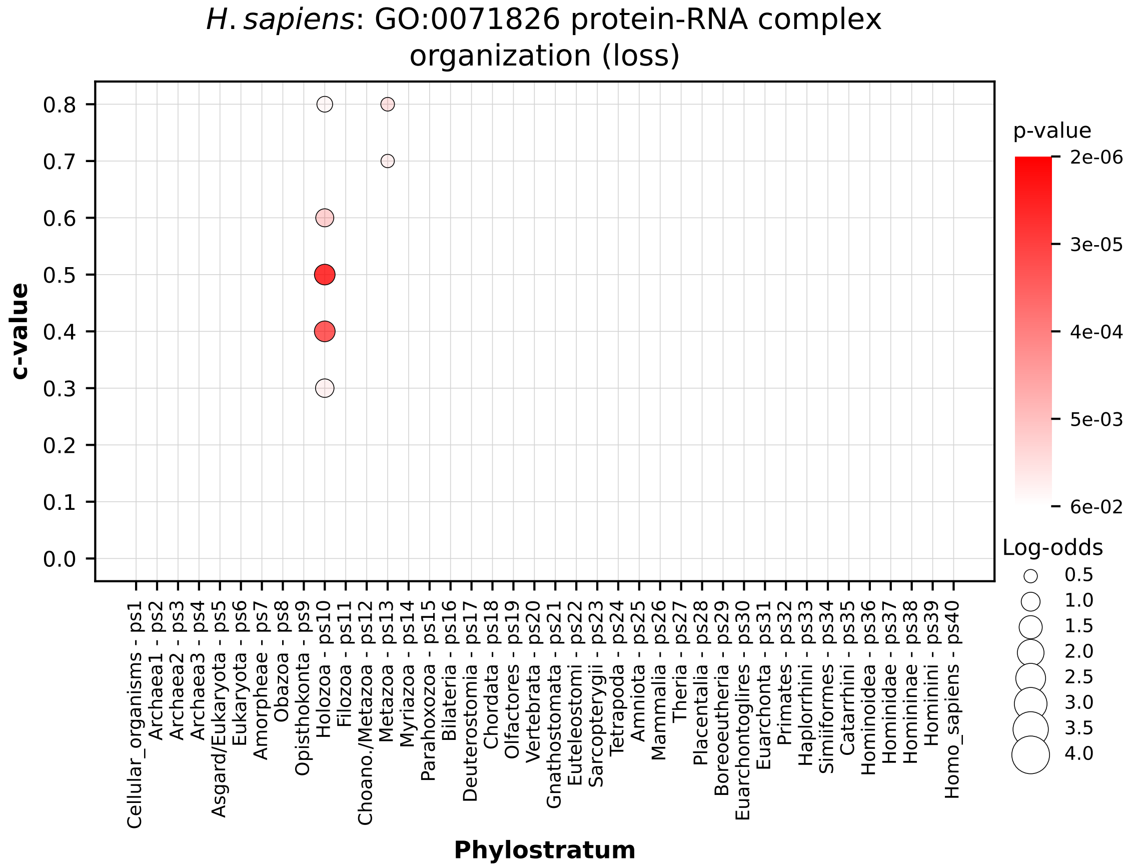

H. sapiens: GO:0071826 protein-RNA complex organization (loss)
c-value
0.0
0.1
0.2
0.3
0.4
0.5
0.6
0.7
0.8
Phylostratum
Cellular_organisms - ps1
Archaea1 - ps2
Archaea2 - ps3
Archaea3 - ps4
Asgard/Eukaryota - ps5
Eukaryota - ps6
Amorpheae - ps7
Obazoa - ps8
Opisthokonta - ps9
Holozoa - ps10
Filozoa - ps11
Choano./Metazoa - ps12
Metazoa - ps13
Myriazoa - ps14
Parahoxozoa - ps15
Bilateria - ps16
Deuterostomia - ps17
Chordata - ps18
Olfactores - ps19
Vertebrata - ps20
Gnathostomata - ps21
Euteleostomi - ps22
Sarcopterygii - ps23
Tetrapoda - ps24
Amniota - ps25
Mammalia - ps26
Theria - ps27
Placentalia - ps28
Boreoeutheria - ps29
Euarchontoglires - ps30
Euarchonta - ps31
Primates - ps32
Haplorrhini - ps33
Simiiformes - ps34
Catarrhini - ps35
Hominoidea - ps36
Hominidae - ps37
Homininae - ps38
Hominini - ps39
Homo_sapiens - ps40
p-value
2e-06
3e-05
4e-04
5e-03
6e-02
Log-odds
0.5
1.0
1.5
2.0
2.5
3.0
3.5
4.0

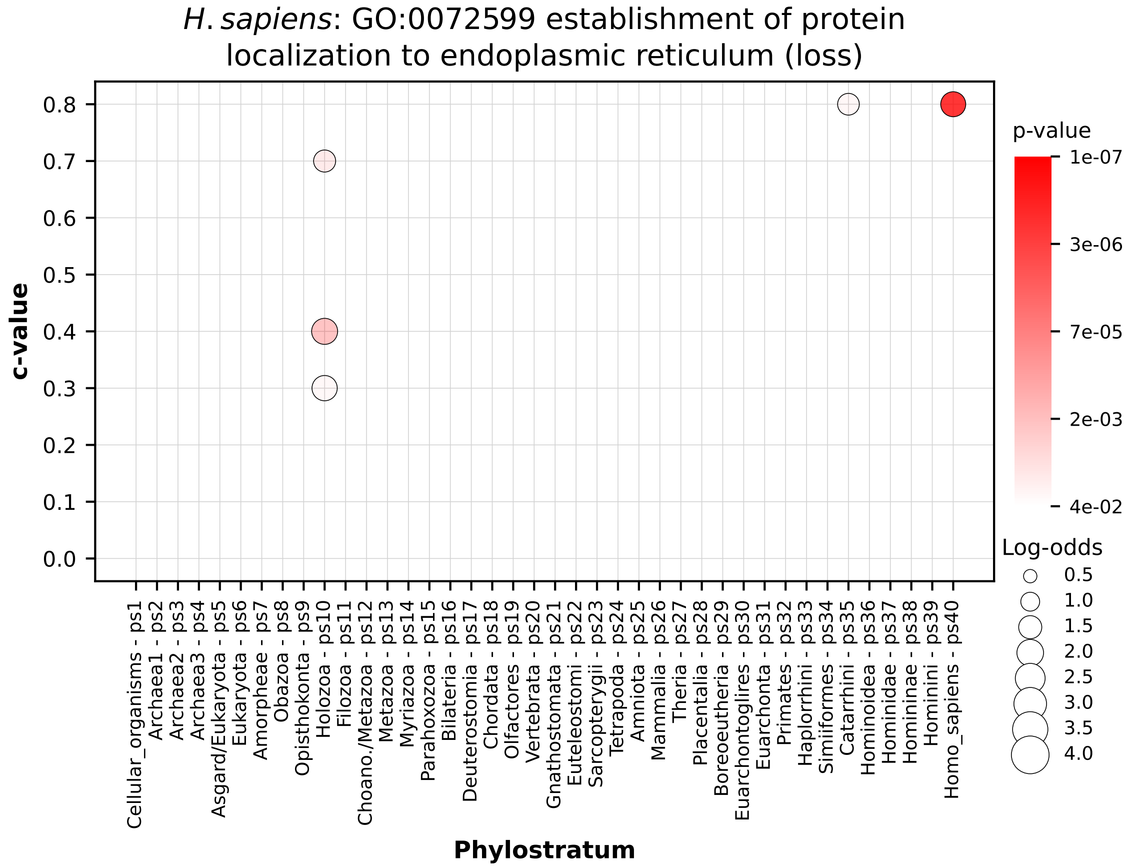

H. sapiens: GO:0072599 establishment of protein localization to endoplasmic reticulum (loss)
c-value
0.0
0.1
0.2
0.3
0.4
0.5
0.6
0.7
0.8
Phylostratum
Cellular_organisms - ps1
Archaea1 - ps2
Archaea2 - ps3
Archaea3 - ps4
Asgard/Eukaryota - ps5
Eukaryota - ps6
Amorpheae - ps7
Obazoa - ps8
Opisthokonta - ps9
Holozoa - ps10
Filozoa - ps11
Choano./Metazoa - ps12
Metazoa - ps13
Myriazoa - ps14
Parahoxozoa - ps15
Bilateria - ps16
Deuterostomia - ps17
Chordata - ps18
Olfactores - ps19
Vertebrata - ps20
Gnathostomata - ps21
Euteleostomi - ps22
Sarcopterygii - ps23
Tetrapoda - ps24
Amniota - ps25
Mammalia - ps26
Theria - ps27
Placentalia - ps28
Boreoeutheria - ps29
Euarchontoglires - ps30
Euarchonta - ps31
Primates - ps32
Haplorrhini - ps33
Simiiformes - ps34
Catarrhini - ps35
Hominoidea - ps36
Hominidae - ps37
Homininae - ps38
Hominini - ps39
Homo_sapiens - ps40
p-value
1e-07
3e-06
7e-05
2e-03
4e-02
Log-odds
0.5
1.0
1.5
2.0
2.5
3.0
3.5
4.0

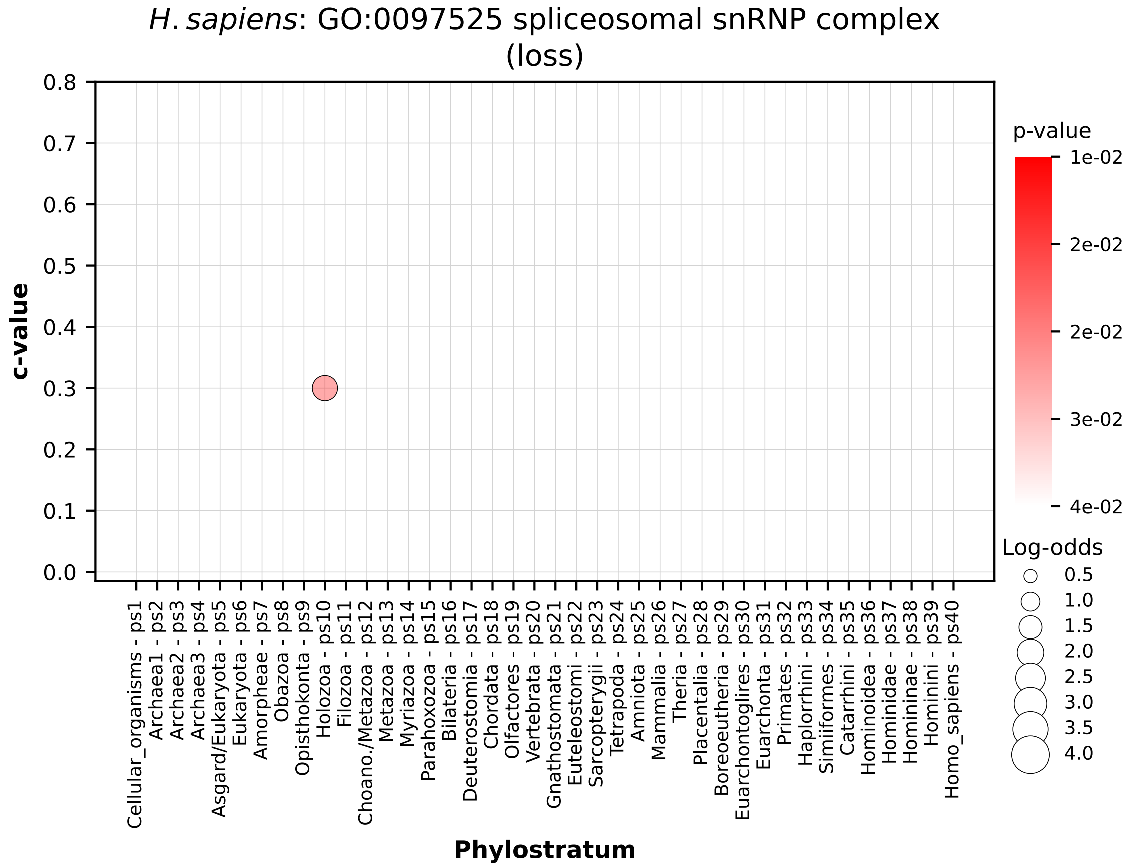
H. sapiens: GO:0097525 spliceosomal snRNP complex
(loss)
c-value
0.0
0.1
0.2
0.3
0.4
0.5
0.6
0.7
0.8
Phylostratum
Cellular_organisms - ps1
Archaea1 - ps2
Archaea2 - ps3
Archaea3 - ps4
Asgard/Eukaryota - ps5
Eukaryota - ps6
Amorpheae - ps7
Obazoa - ps8
Opisthokonta - ps9
Holozoa - ps10
Filozoa - ps11
Choano./Metazoa - ps12
Metazoa - ps13
Myriazoa - ps14
Parahoxozoa - ps15
Bilateria - ps16
Deuterostomia - ps17
Chordata - ps18
Olfactores - ps19
Vertebrata - ps20
Gnathostomata - ps21
Euteleostomi - ps22
Sarcopterygii - ps23
Tetrapoda - ps24
Amniota - ps25
Mammalia - ps26
Theria - ps27
Placentalia - ps28
Boreoeutheria - ps29
Euarchontoglires - ps30
Euarchonta - ps31
Primates - ps32
Haplorrhini - ps33
Simiiformes - ps34
Catarrhini - ps35
Hominoidea - ps36
Hominidae - ps37
Homininae - ps38
Hominini - ps39
Homo_sapiens - ps40
p-value
1e-02
2e-02
2e-02
3e-02
4e-02
Log-odds
0.5
1.0
1.5
2.0
2.5
3.0
3.5
4.0

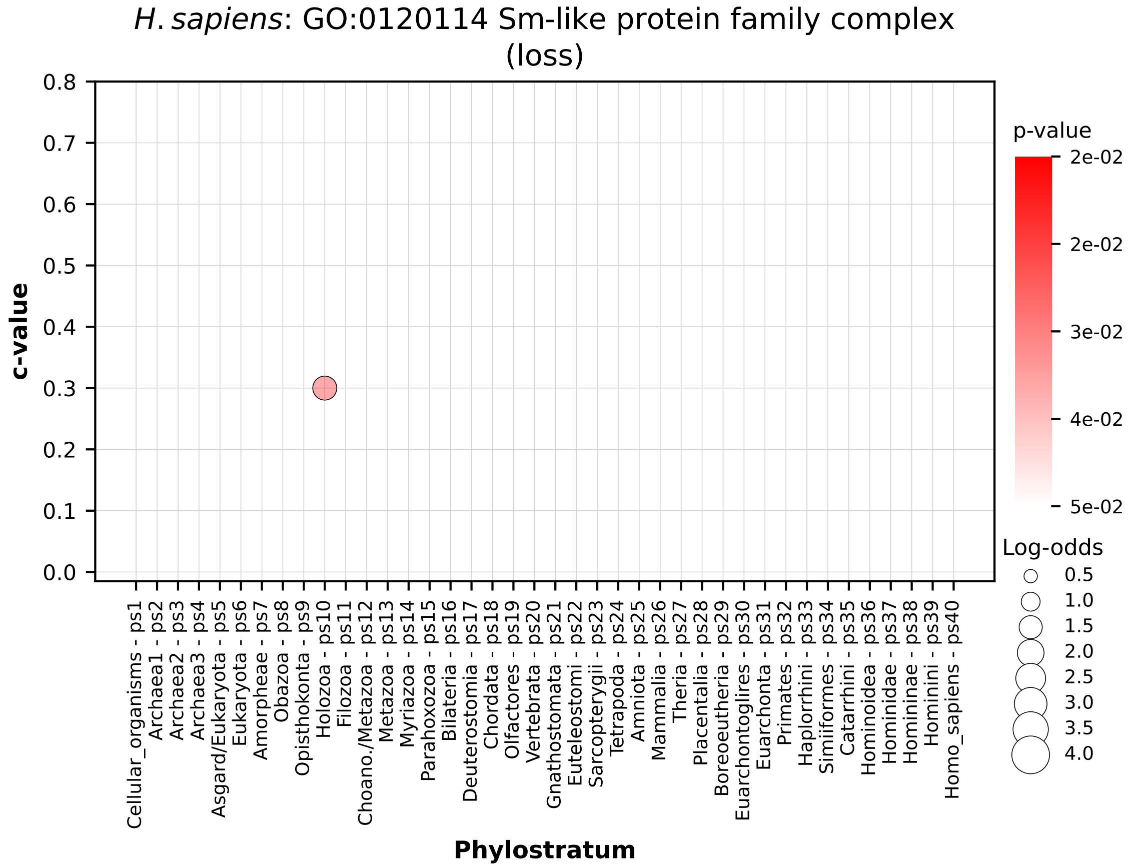
H. sapiens: GO:0120114 Sm-like protein family complex
(loss)
c-value
0.0
0.1
0.2
0.3
0.4
0.5
0.6
0.7
0.8
p-value
2e-02
2e-02
3e-02
4e-02
5e-02
Log-odds
0.5
1.0
1.5
2.0
2.5
3.0
3.5
4.0
Cellular_organisms - ps1
Archaea1 - ps2
Archaea2 - ps3
Archaea3 - ps4
Asgard/Eukaryota - ps5
Eukaryota - ps6
Amorpheae - ps7
Obazoa - ps8
Opisthokonta - ps9
Holozoa - ps10
Filozoa - ps11
Choano./Metazoa - ps12
Metazoa - ps13
Myriazoa - ps14
Parahoxozoa - ps15
Bilateria - ps16
Deuterostomia - ps17
Chordata - ps18
Olfactores - ps19
Vertebrata - ps20
Gnathostomata - ps21
Euteleostomi - ps22
Sarcopterygii - ps23
Tetrapoda - ps24
Amniota - ps25
Mammalia - ps26
Theria - ps27
Placentalia - ps28
Boreoeutheria - ps29
Euarchontoglires - ps30
Euarchonta - ps31
Primates - ps32
Haplorrhini - ps33
Simiiformes - ps34
Catarrhini - ps35
Hominoidea - ps36
Hominidae - ps37
Homininae - ps38
Hominini - ps39
Homo_sapiens - ps40
Phylostratum

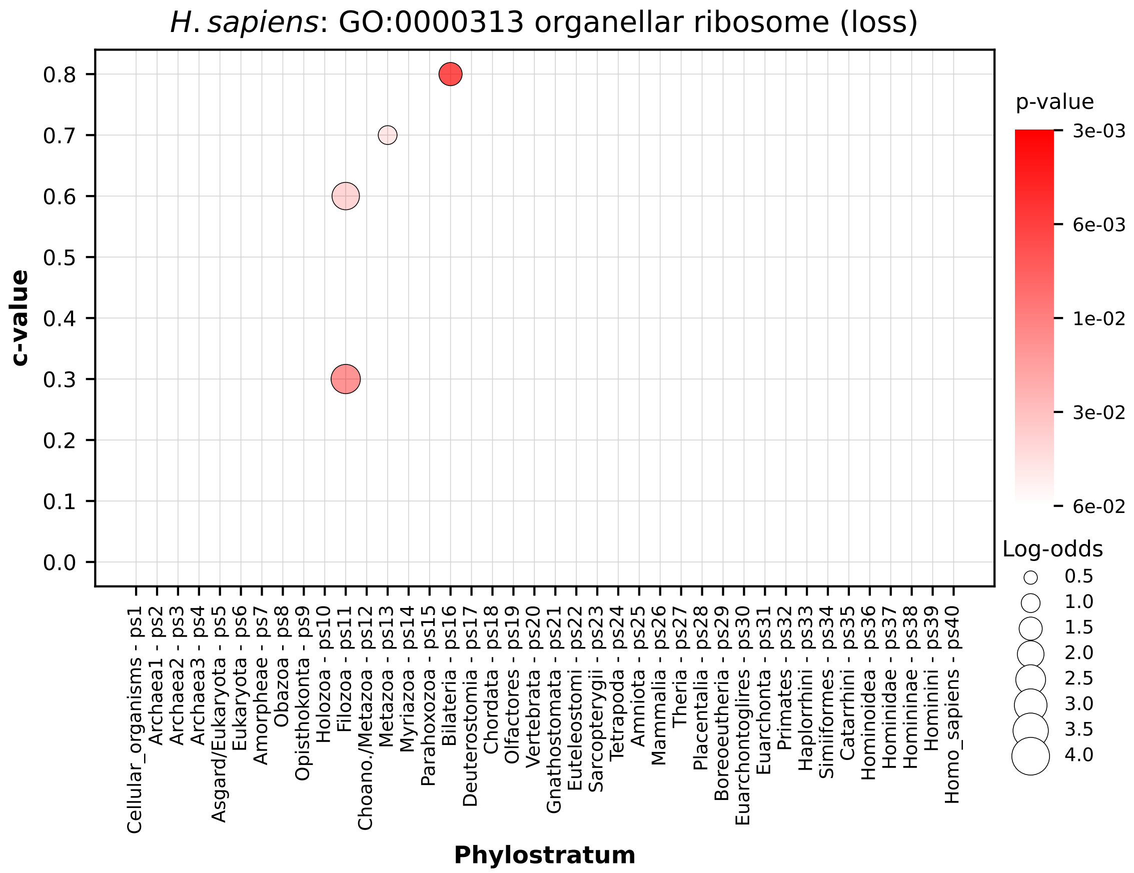

H. sapiens: GO:0000313 organellar ribosome (loss)
c-value
Phylostratum
p-value
3e-03
6e-03
1e-02
3e-02
6e-02
Log-odds
0.5
1.0
1.5
2.0
2.5
3.0
3.5
4.0
Cellular_organisms - ps1
Archaea1 - ps2
Archaea2 - ps3
Archaea3 - ps4
Asgard/Eukaryota - ps5
Eukaryota - ps6
Amorpheae - ps7
Obazoa - ps8
Opisthokonta - ps9
Holozoa - ps10
Filozoa - ps11
Choano./Metazoa - ps12
Metazoa - ps13
Myriazoa - ps14
Parahoxozoa - ps15
Bilateria - ps16
Deuterostomia - ps17
Chordata - ps18
Olfactores - ps19
Vertebrata - ps20
Gnathostomata - ps21
Euteleostomi - ps22
Sarcopterygii - ps23
Tetrapoda - ps24
Amniota - ps25
Mammalia - ps26
Theria - ps27
Placentalia - ps28
Boreoeutheria - ps29
Euarchontoglires - ps30
Euarchonta - ps31
Primates - ps32
Haplorrhini - ps33
Simiiformes - ps34
Catarrhini - ps35
Hominoidea - ps36
Hominidae - ps37
Homininae - ps38
Hominini - ps39
Homo_sapiens - ps40

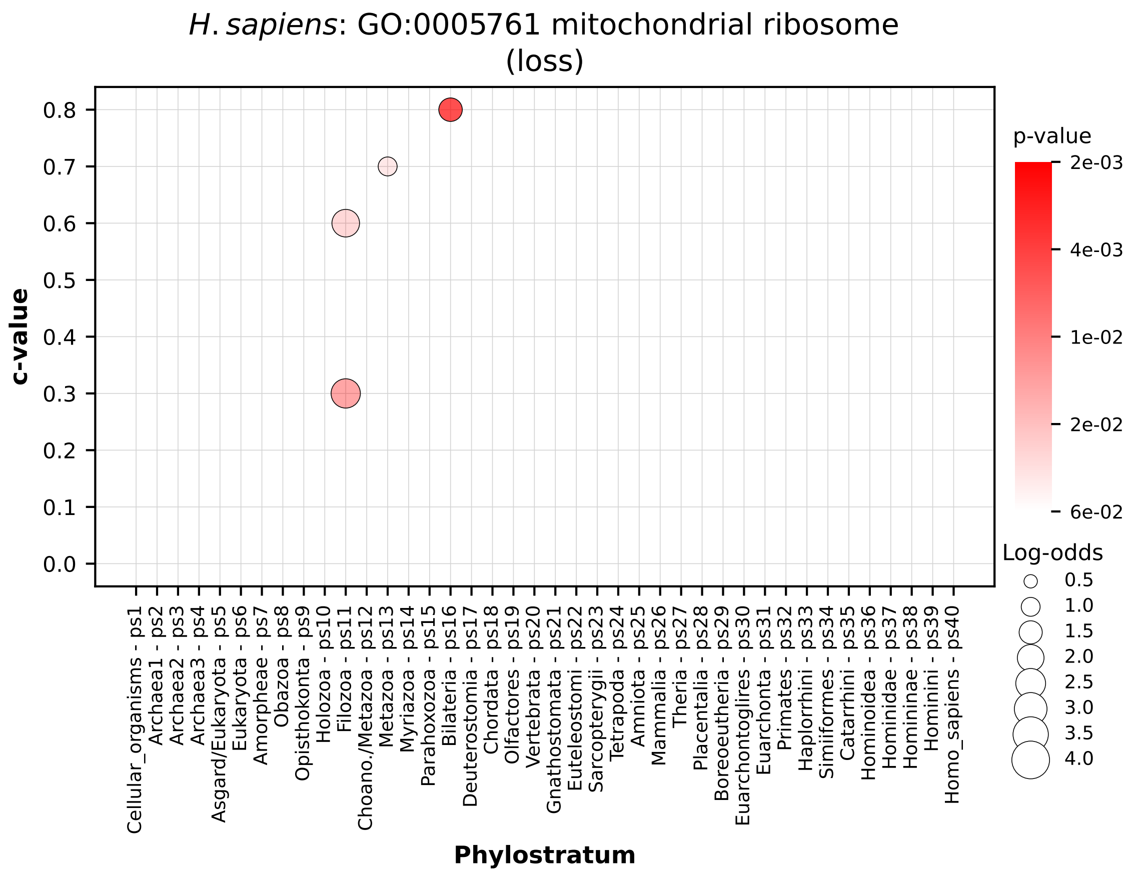

H. sapiens: GO:0005761 mitochondrial ribosome
(loss)
c-value
0.8
0.7
0.6
0.5
0.4
0.3
0.2
0.1
0.0
p-value
2e-03
4e-03
1e-02
2e-02
6e-02
Log-odds
0.5
1.0
1.5
2.0
2.5
3.0
3.5
4.0
Cellular_organisms - ps1
Archaea1 - ps2
Archaea2 - ps3
Archaea3 - ps4
Asgard/Eukaryota - ps5
Eukaryota - ps6
Amorpheae - ps7
Obazoa - ps8
Opisthokonta - ps9
Holozoa - ps10
Filozoa - ps11
Choano./Metazoa - ps12
Metazoa - ps13
Myriazoa - ps14
Parahoxozoa - ps15
Bilateria - ps16
Deuterostomia - ps17
Chordata - ps18
Olfactores - ps19
Vertebrata - ps20
Gnathostomata - ps21
Euteleostomi - ps22
Sarcopterygii - ps23
Tetrapoda - ps24
Amniota - ps25
Mammalia - ps26
Theria - ps27
Placentalia - ps28
Boreoeutheria - ps29
Euarchontoglires - ps30
Euarchonta - ps31
Primates - ps32
Haplorrhini - ps33
Simiiformes - ps34
Catarrhini - ps35
Hominoidea - ps36
Hominidae - ps37
Homininae - ps38
Hominini - ps39
Homo_sapiens - ps40
Phylostratum

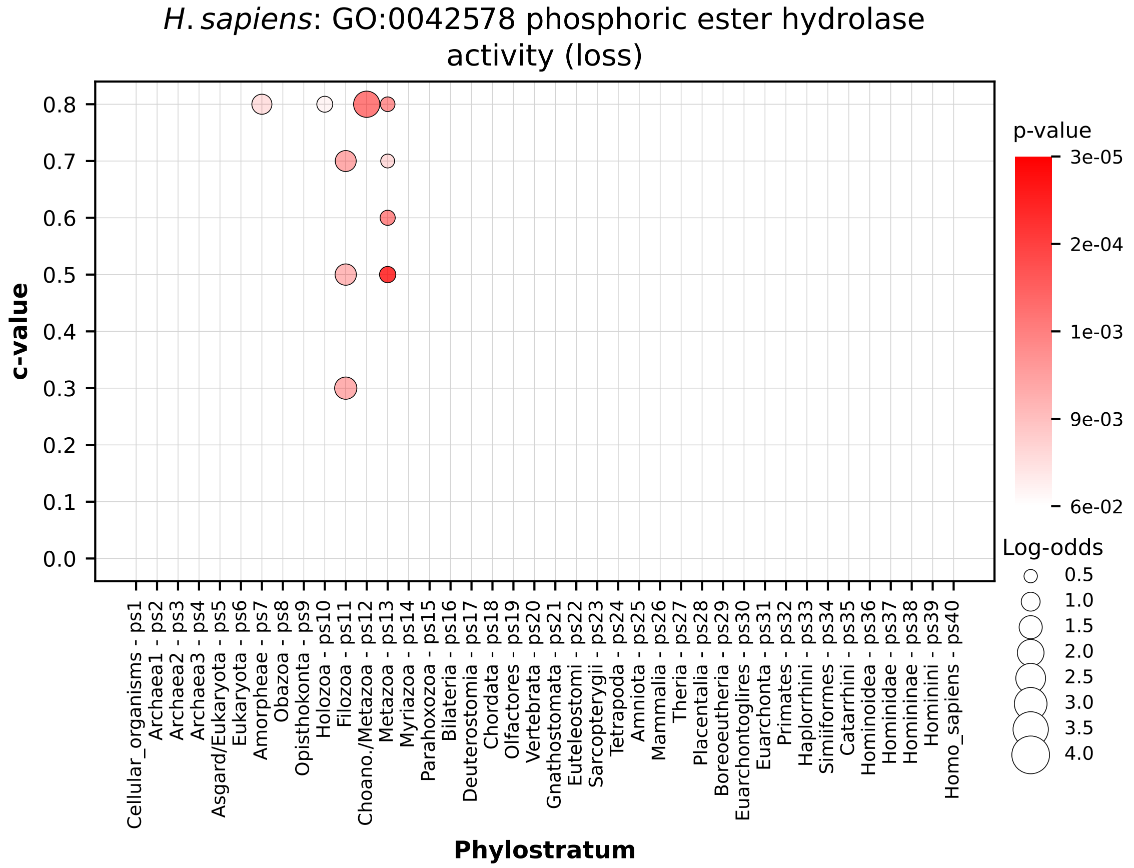

H. sapiens: GO:0042578 phosphoric ester hydrolase activity (loss)
c-value
0.0
0.1
0.2
0.3
0.4
0.5
0.6
0.7
0.8
Phylostratum
Cellular_organisms - ps1
Archaea1 - ps2
Archaea2 - ps3
Archaea3 - ps4
Asgard/Eukaryota - ps5
Eukaryota - ps6
Amorpheae - ps7
Obazoa - ps8
Opisthokonta - ps9
Holozoa - ps10
Filozoa - ps11
Choano./Metazoa - ps12
Metazoa - ps13
Myriazoa - ps14
Parahoxozoa - ps15
Bilateria - ps16
Deuterostomia - ps17
Chordata - ps18
Olfactores - ps19
Vertebrata - ps20
Gnathostomata - ps21
Euteleostomi - ps22
Sarcopterygii - ps23
Tetrapoda - ps24
Amniota - ps25
Mammalia - ps26
Theria - ps27
Placentalia - ps28
Boreoeutheria - ps29
Euarchontoglires - ps30
Euarchonta - ps31
Primates - ps32
Haplorrhini - ps33
Simiiformes - ps34
Catarrhini - ps35
Hominoidea - ps36
Hominidae - ps37
Homininae - ps38
Hominini - ps39
Homo_sapiens - ps40
p-value
3e-05
2e-04
1e-03
9e-03
6e-02
Log-odds
0.5
1.0
1.5
2.0
2.5
3.0
3.5
4.0

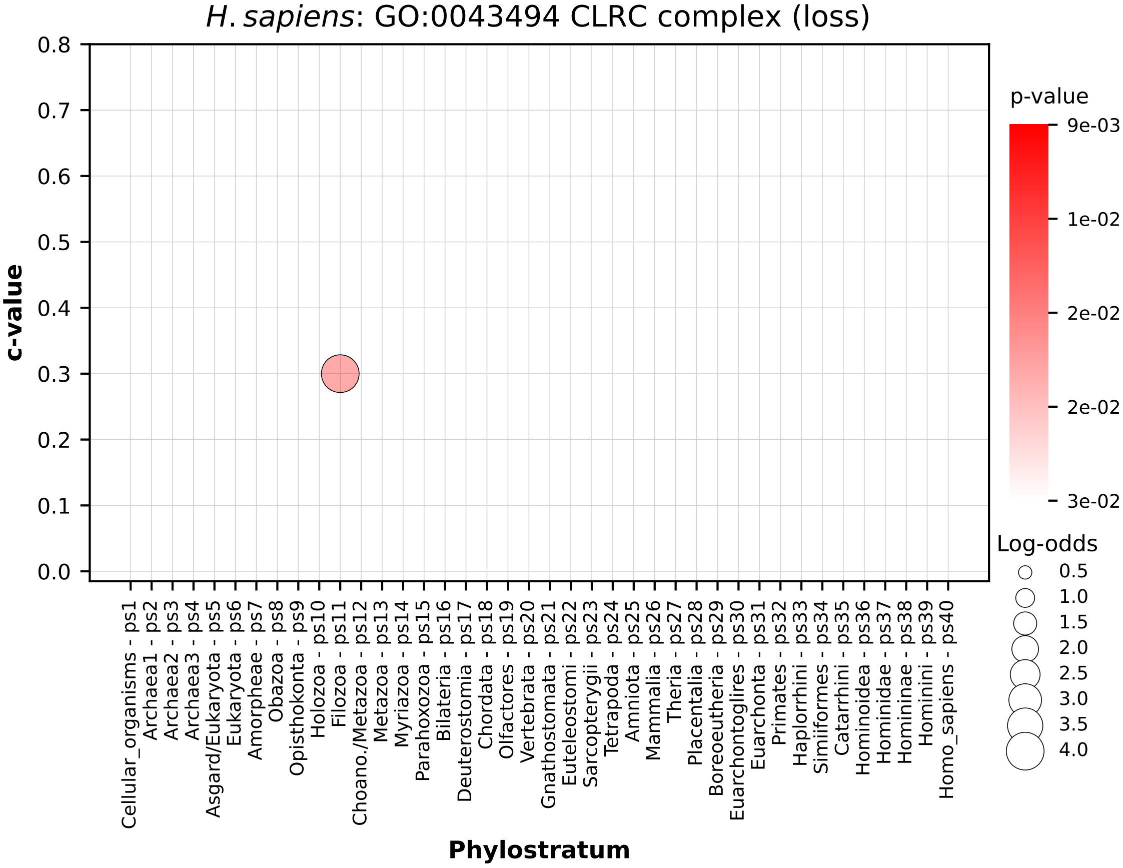
H. sapiens: GO:0043494 CLRC complex (loss)
c-value
0.0
0.1
0.2
0.3
0.4
0.5
0.6
0.7
0.8
Phylostratum
Cellular_organisms - ps1
Archaea1 - ps2
Archaea2 - ps3
Archaea3 - ps4
Asgard/Eukaryota - ps5
Eukaryota - ps6
Amorpheae - ps7
Obazoa - ps8
Opisthokonta - ps9
Holozoa - ps10
Filozoa - ps11
Choano./Metazoa - ps12
Metazoa - ps13
Myriazoa - ps14
Parahoxozoa - ps15
Bilateria - ps16
Deuterostomia - ps17
Chordata - ps18
Olfactores - ps19
Vertebrata - ps20
Gnathostomata - ps21
Euteleostomi - ps22
Sarcopterygii - ps23
Tetrapoda - ps24
Amniota - ps25
Mammalia - ps26
Theria - ps27
Placentalia - ps28
Boreoeutheria - ps29
Euarchontoglires - ps30
Euarchonta - ps31
Primates - ps32
Haplorrhini - ps33
Simiiformes - ps34
Catarrhini - ps35
Hominoidea - ps36
Hominidae - ps37
Homininae - ps38
Hominini - ps39
Homo_sapiens - ps40
p-value
9e-03
1e-02
2e-02
2e-02
3e-02
Log-odds
0.5
1.0
1.5
2.0
2.5
3.0
3.5
4.0

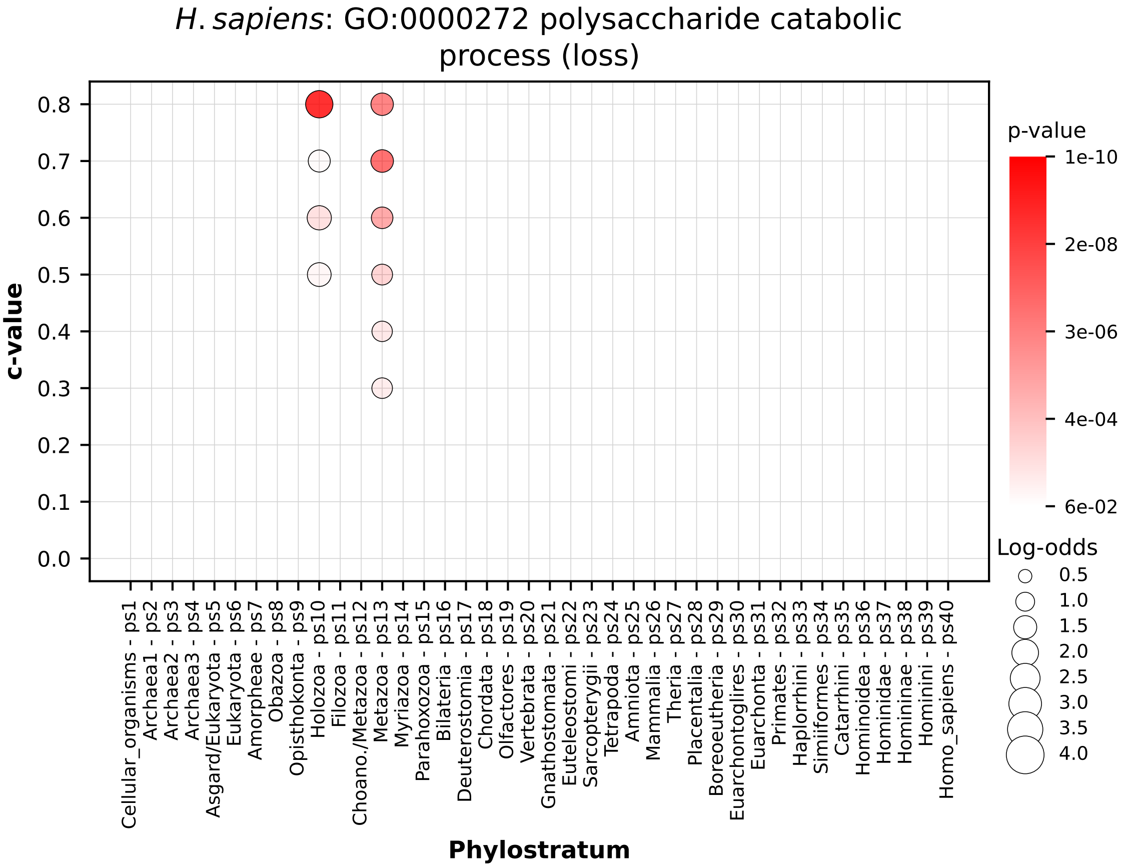

H. sapiens: GO:0000272 polysaccharide catabolic process (loss)
c-value
0.8
0.7
0.6
0.5
0.4
0.3
0.2
0.1
0.0
p-value
1e-10
2e-08
3e-06
4e-04
6e-02
Log-odds
0.5
1.0
1.5
2.0
2.5
3.0
3.5
4.0
Cellular_organisms - ps1
Archaea1 - ps2
Archaea2 - ps3
Archaea3 - ps4
Asgard/Eukaryota - ps5
Eukaryota - ps6
Amorpheae - ps7
Obazoa - ps8
Opisthokonta - ps9
Holozoa - ps10
Filozoa - ps11
Choano./Metazoa - ps12
Metazoa - ps13
Myriazoa - ps14
Parahoxozoa - ps15
Bilateria - ps16
Deuterostomia - ps17
Chordata - ps18
Olfactores - ps19
Vertebrata - ps20
Gnathostomata - ps21
Euteleostomi - ps22
Sarcopterygii - ps23
Tetrapoda - ps24
Amniota - ps25
Mammalia - ps26
Theria - ps27
Placentalia - ps28
Boreoeutheria - ps29
Euarchontoglires - ps30
Euarchonta - ps31
Primates - ps32
Haplorrhini - ps33
Simiiformes - ps34
Catarrhini - ps35
Hominoidea - ps36
Hominidae - ps37
Homininae - ps38
Hominini - ps39
Homo_sapiens - ps40
Phylostratum

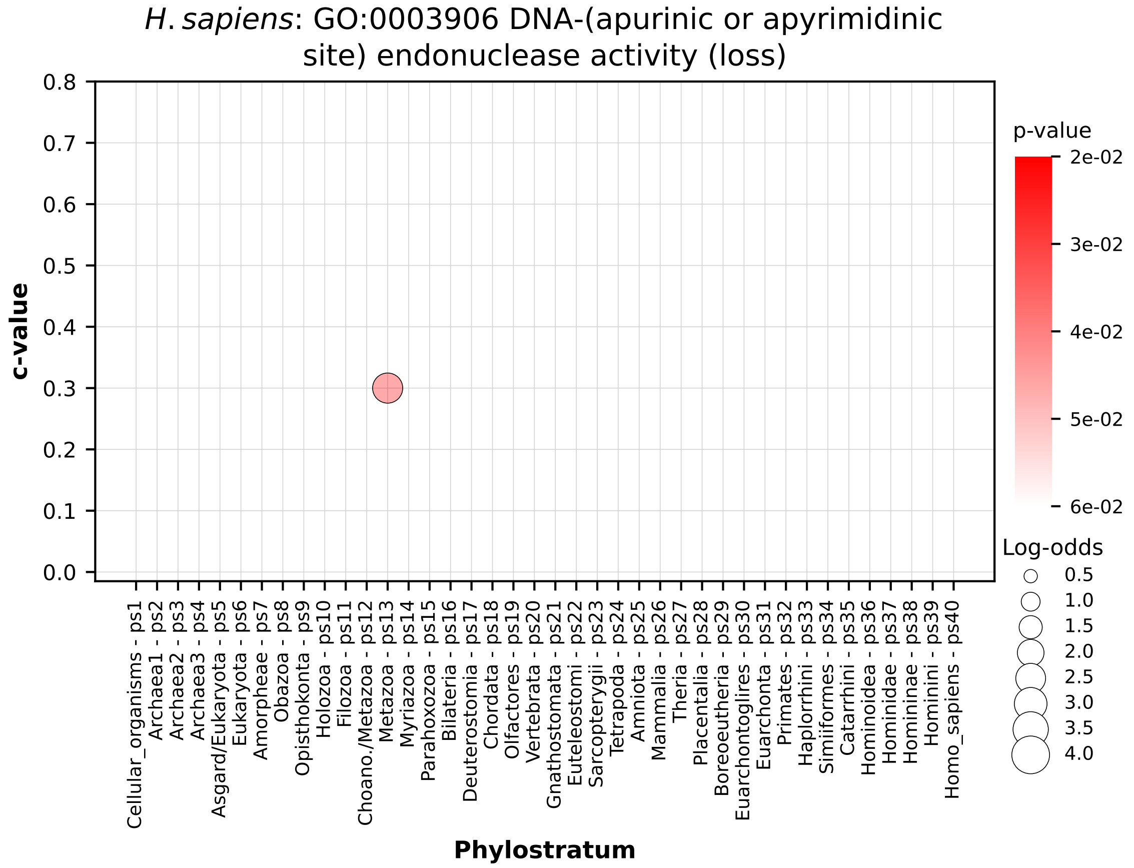
H. sapiens: GO:0003906 DNA-(apurinic or apyrimidinic site) endonuclease activity (loss)
c-value
0.0
0.1
0.2
0.3
0.4
0.5
0.6
0.7
0.8
Phylostratum
Cellular_organisms - ps1
Archaea1 - ps2
Archaea2 - ps3
Archaea3 - ps4
Asgard/Eukaryota - ps5
Eukaryota - ps6
Amorpheae - ps7
Obazoa - ps8
Opisthokonta - ps9
Holozoa - ps10
Filozoa - ps11
Choano./Metazoa - ps12
Metazoa - ps13
Myriazoa - ps14
Parahoxozoa - ps15
Bilateria - ps16
Deuterostomia - ps17
Chordata - ps18
Olfactores - ps19
Vertebrata - ps20
Gnathostomata - ps21
Euteleostomi - ps22
Sarcopterygii - ps23
Tetrapoda - ps24
Amniota - ps25
Mammalia - ps26
Theria - ps27
Placentalia - ps28
Boreoeutheria - ps29
Euarchontoglires - ps30
Euarchonta - ps31
Primates - ps32
Haplorrhini - ps33
Simiiformes - ps34
Catarrhini - ps35
Hominoidea - ps36
Hominidae - ps37
Homininae - ps38
Hominini - ps39
Homo_sapiens - ps40
p-value
2e-02
3e-02
4e-02
5e-02
6e-02
Log-odds
0.5
1.0
1.5
2.0
2.5
3.0
3.5
4.0

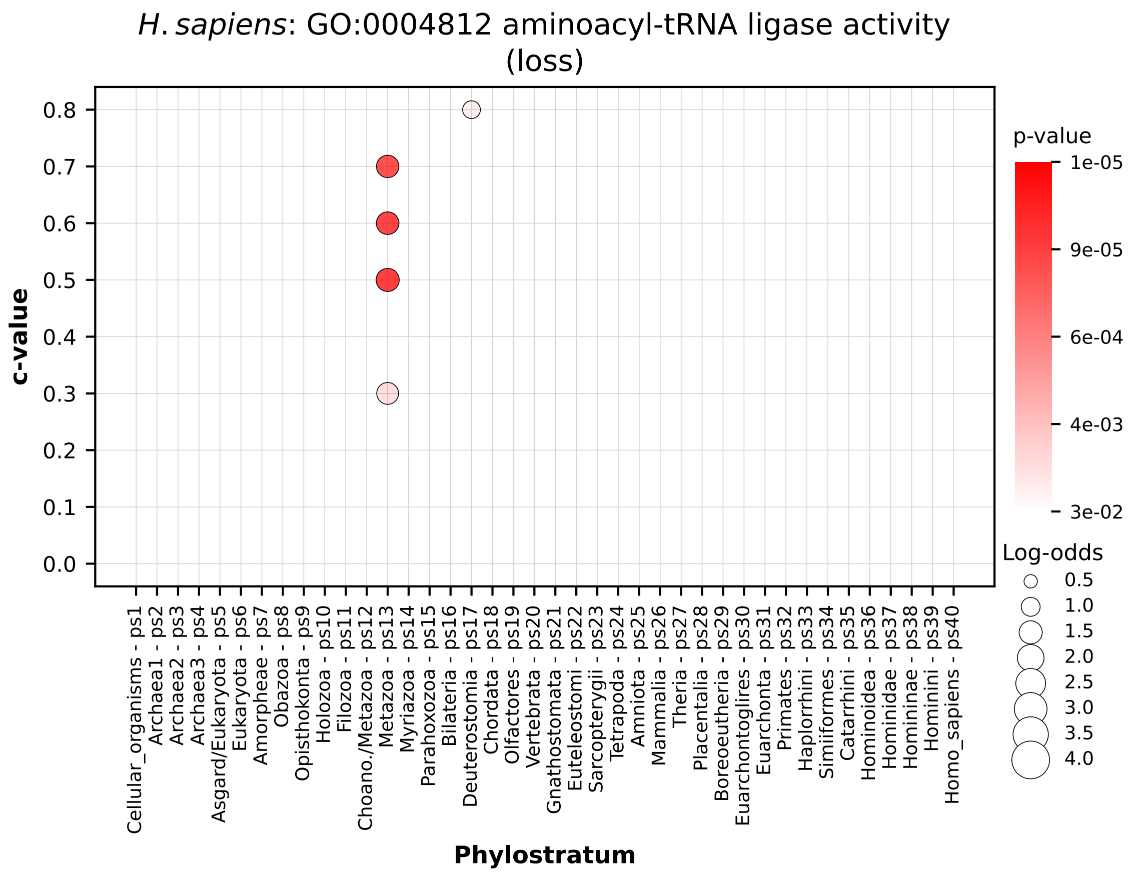

H. sapiens: GO:0004812 aminoacyl-tRNA ligase activity
(loss)
c-value
0.0
0.1
0.2
0.3
0.4
0.5
0.6
0.7
0.8
Phylostratum
Cellular_organisms - ps1
Archaea1 - ps2
Archaea2 - ps3
Archaea3 - ps4
Asgard/Eukaryota - ps5
Eukaryota - ps6
Amorpheae - ps7
Obazoa - ps8
Opisthokonta - ps9
Holozoa - ps10
Filozoa - ps11
Choano./Metazoa - ps12
Metazoa - ps13
Myriazoa - ps14
Parahoxozoa - ps15
Bilateria - ps16
Deuterostomia - ps17
Chordata - ps18
Olfactores - ps19
Vertebrata - ps20
Gnathostomata - ps21
Euteleostomi - ps22
Sarcopterygii - ps23
Tetrapoda - ps24
Amniota - ps25
Mammalia - ps26
Theria - ps27
Placentalia - ps28
Boreoeutheria - ps29
Euarchontoglires - ps30
Euarchonta - ps31
Primates - ps32
Haplorrhini - ps33
Simiiformes - ps34
Catarrhini - ps35
Hominoidea - ps36
Hominidae - ps37
Homininae - ps38
Hominini - ps39
Homo_sapiens - ps40
p-value
1e-05
9e-05
6e-04
4e-03
3e-02
Log-odds
0.5
1.0
1.5
2.0
2.5
3.0
3.5
4.0

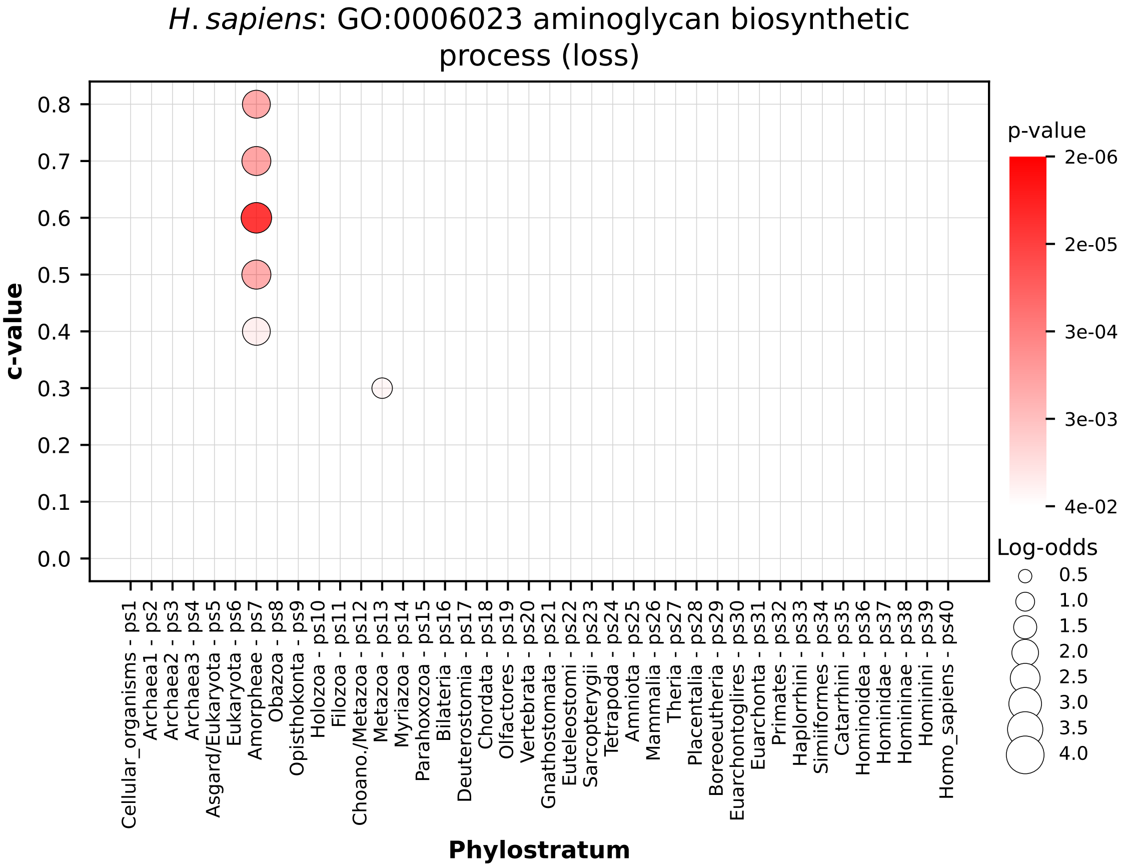

H. sapiens: GO:0006023 aminoglycan biosynthetic process (loss)
c-value
0.8
0.7
0.6
0.5
0.4
0.3
0.2
0.1
0.0
p-value
2e-06
2e-05
3e-04
3e-03
4e-02
Log-odds
0.5
1.0
1.5
2.0
2.5
3.0
3.5
4.0
Cellular_organisms - ps1
Archaea1 - ps2
Archaea2 - ps3
Archaea3 - ps4
Asgard/Eukaryota - ps5
Eukaryota - ps6
Amorpheae - ps7
Obazoa - ps8
Opisthokonta - ps9
Holozoa - ps10
Filozoa - ps11
Choano./Metazoa - ps12
Metazoa - ps13
Myriazoa - ps14
Parahoxozoa - ps15
Bilateria - ps16
Deuterostomia - ps17
Chordata - ps18
Olfactores - ps19
Vertebrata - ps20
Gnathostomata - ps21
Euteleostomi - ps22
Sarcopterygii - ps23
Tetrapoda - ps24
Amniota - ps25
Mammalia - ps26
Theria - ps27
Placentalia - ps28
Boreoeutheria - ps29
Euarchontoglires - ps30
Euarchonta - ps31
Primates - ps32
Haplorrhini - ps33
Simiiformes - ps34
Catarrhini - ps35
Hominoidea - ps36
Hominidae - ps37
Homininae - ps38
Hominini - ps39
Homo_sapiens - ps40
Phylostratum

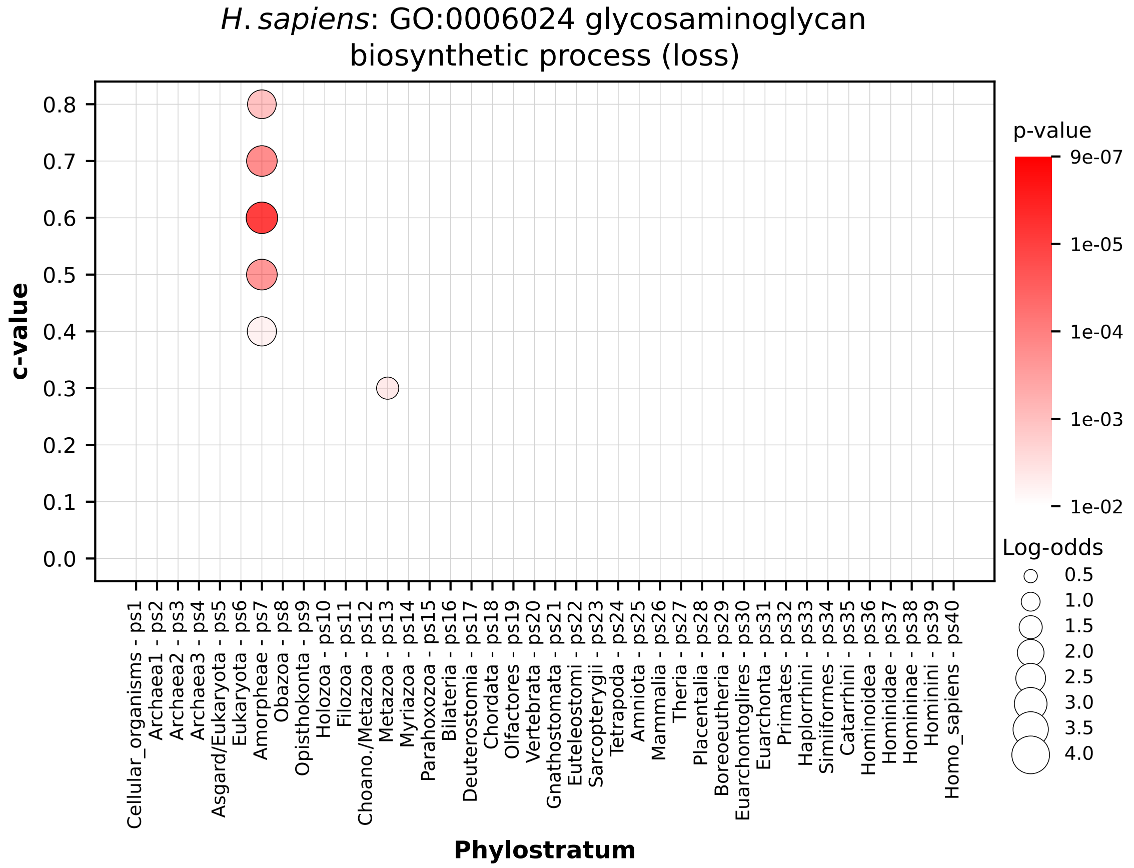

H. sapiens: GO:0006024 glycosaminoglycan biosynthetic process (loss)
c-value
0.8
0.7
0.6
0.5
0.4
0.3
0.2
0.1
0.0
p-value
9e-07
1e-05
1e-04
1e-03
1e-02
Log-odds
0.5
1.0
1.5
2.0
2.5
3.0
3.5
4.0
Cellular_organisms - ps1
Archaea1 - ps2
Archaea2 - ps3
Archaea3 - ps4
Asgard/Eukaryota - ps5
Eukaryota - ps6
Amorpheae - ps7
Obazoa - ps8
Opisthokonta - ps9
Holozoa - ps10
Filozoa - ps11
Choano./Metazoa - ps12
Metazoa - ps13
Myriazoa - ps14
Parahoxozoa - ps15
Bilateria - ps16
Deuterostomia - ps17
Chordata - ps18
Olfactores - ps19
Vertebrata - ps20
Gnathostomata - ps21
Euteleostomi - ps22
Sarcopterygii - ps23
Tetrapoda - ps24
Amniota - ps25
Mammalia - ps26
Theria - ps27
Placentalia - ps28
Boreoeutheria - ps29
Euarchontoglires - ps30
Euarchonta - ps31
Primates - ps32
Haplorrhini - ps33
Simiiformes - ps34
Catarrhini - ps35
Hominoidea - ps36
Hominidae - ps37
Homininae - ps38
Hominini - ps39
Homo_sapiens - ps40
Phylostratum

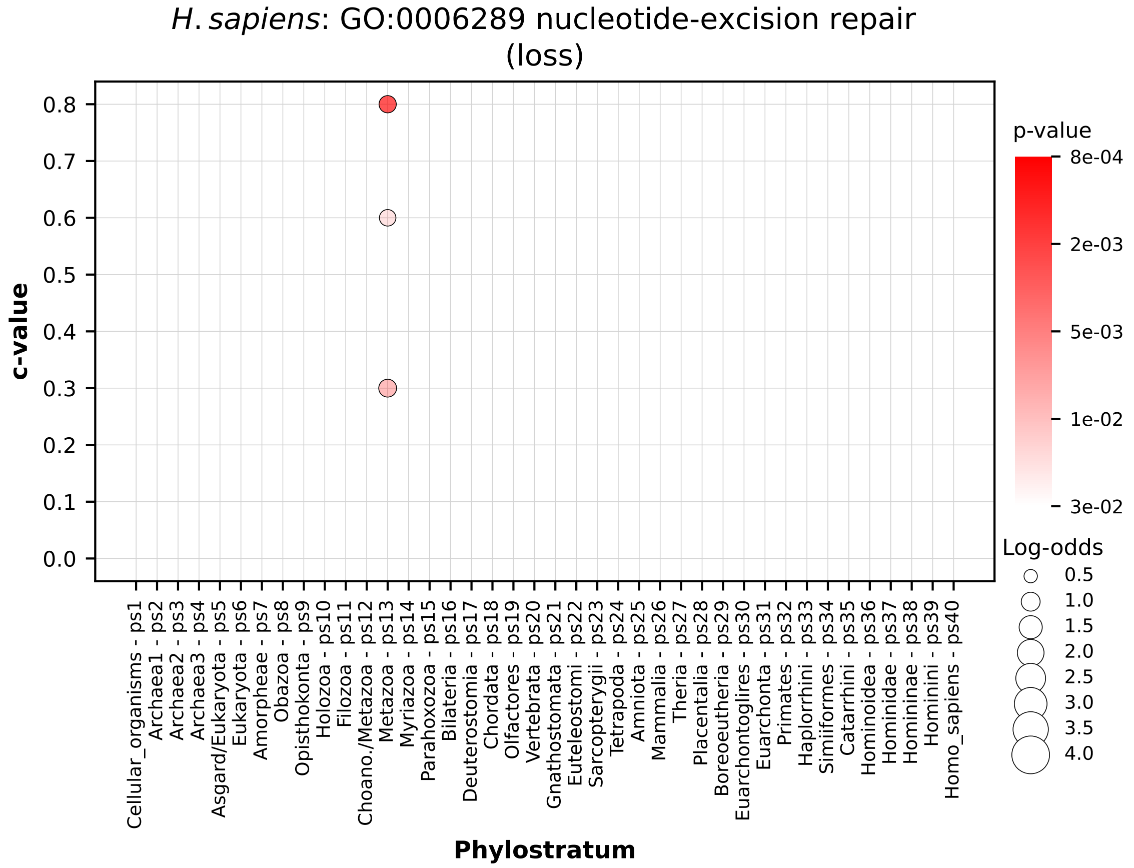

H. sapiens: GO:0006289 nucleotide-excision repair
(loss)
c-value
0.0
0.1
0.2
0.3
0.4
0.5
0.6
0.7
0.8
p-value
8e-04
2e-03
5e-03
1e-02
3e-02
Log-odds
0.5
1.0
1.5
2.0
2.5
3.0
3.5
4.0
Cellular_organisms - ps1
Archaea1 - ps2
Archaea2 - ps3
Archaea3 - ps4
Asgard/Eukaryota - ps5
Eukaryota - ps6
Amorpheae - ps7
Obazoa - ps8
Opisthokonta - ps9
Holozoa - ps10
Filozoa - ps11
Choano./Metazoa - ps12
Metazoa - ps13
Myriazoa - ps14
Parahoxozoa - ps15
Bilateria - ps16
Deuterostomia - ps17
Chordata - ps18
Olfactores - ps19
Vertebrata - ps20
Gnathostomata - ps21
Euteleostomi - ps22
Sarcopterygii - ps23
Tetrapoda - ps24
Amniota - ps25
Mammalia - ps26
Theria - ps27
Placentalia - ps28
Boreoeutheria - ps29
Euarchontoglires - ps30
Euarchonta - ps31
Primates - ps32
Haplorrhini - ps33
Simiiformes - ps34
Catarrhini - ps35
Hominoidea - ps36
Hominidae - ps37
Homininae - ps38
Hominini - ps39
Homo_sapiens - ps40
Phylostratum

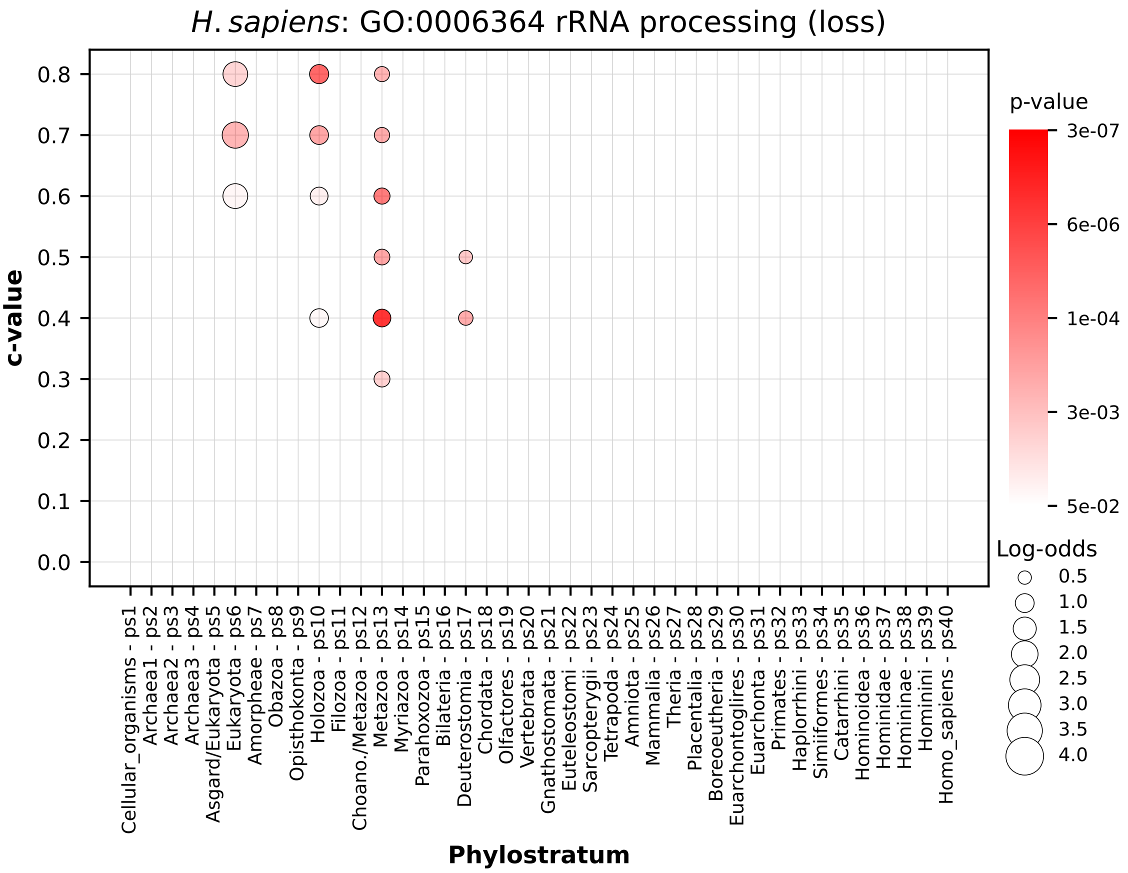

H. sapiens: GO:0006364 rRNA processing (loss)
c-value
0.8
0.7
0.6
0.5
0.4
0.3
0.2
0.1
0.0
Phylostratum
Cellular_organisms - ps1
Archaea1 - ps2
Archaea2 - ps3
Archaea3 - ps4
Asgard/Eukaryota - ps5
Eukaryota - ps6
Amorpheae - ps7
Obazoa - ps8
Opisthokonta - ps9
Holozoa - ps10
Filozoa - ps11
Choano./Metazoa - ps12
Metazoa - ps13
Myriazoa - ps14
Parahoxozoa - ps15
Bilateria - ps16
Deuterostomia - ps17
Chordata - ps18
Olfactores - ps19
Vertebrata - ps20
Gnathostomata - ps21
Euteleostomi - ps22
Sarcopterygii - ps23
Tetrapoda - ps24
Amniota - ps25
Mammalia - ps26
Theria - ps27
Placentalia - ps28
Boreoeutheria - ps29
Euarchontoglires - ps30
Euarchonta - ps31
Primates - ps32
Haplorrhini - ps33
Simiiformes - ps34
Catarrhini - ps35
Hominoidea - ps36
Hominidae - ps37
Homininae - ps38
Hominini - ps39
Homo_sapiens - ps40
p-value
3e-07
6e-06
1e-04
3e-03
5e-02
Log-odds
0.5
1.0
1.5
2.0
2.5
3.0
3.5
4.0

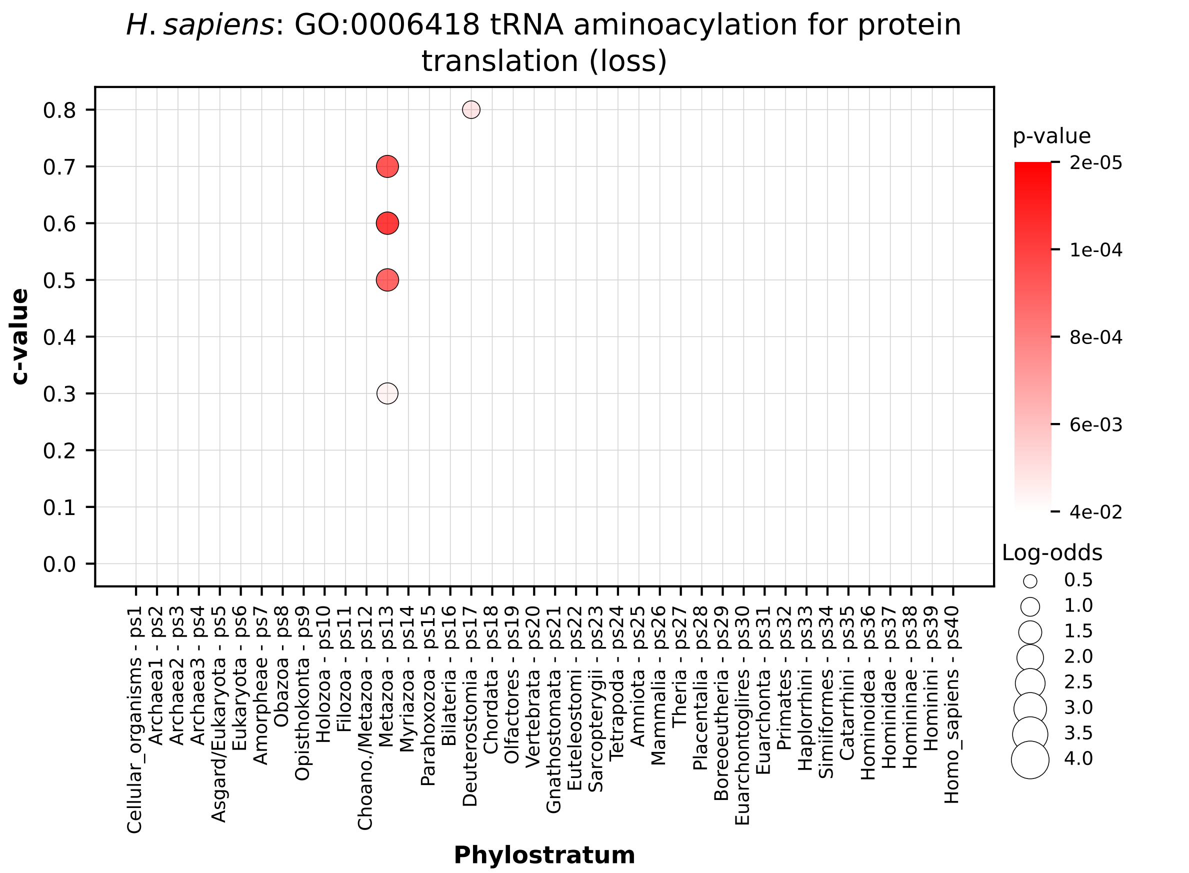

H. sapiens: GO:0006418 tRNA aminoacylation for protein translation (loss)
c-value
0.8
0.7
0.6
0.5
0.4
0.3
0.2
0.1
0.0
Phylostratum
Cellular_organisms - ps1
Archaea1 - ps2
Archaea2 - ps3
Archaea3 - ps4
Asgard/Eukaryota - ps5
Eukaryota - ps6
Amorpheae - ps7
Obazoa - ps8
Opisthokonta - ps9
Holozoa - ps10
Filozoa - ps11
Choano./Metazoa - ps12
Metazoa - ps13
Myriazoa - ps14
Parahoxozoa - ps15
Bilateria - ps16
Deuterostomia - ps17
Chordata - ps18
Olfactores - ps19
Vertebrata - ps20
Gnathostomata - ps21
Euteleostomi - ps22
Sarcopterygii - ps23
Tetrapoda - ps24
Amniota - ps25
Mammalia - ps26
Theria - ps27
Placentalia - ps28
Boreoeutheria - ps29
Euarchontoglires - ps30
Euarchonta - ps31
Primates - ps32
Haplorrhini - ps33
Simiiformes - ps34
Catarrhini - ps35
Hominoidea - ps36
Hominidae - ps37
Homininae - ps38
Hominini - ps39
Homo_sapiens - ps40
p-value
2e-05
1e-04
8e-04
6e-03
4e-02
Log-odds
0.5
1.0
1.5
2.0
2.5
3.0
3.5
4.0

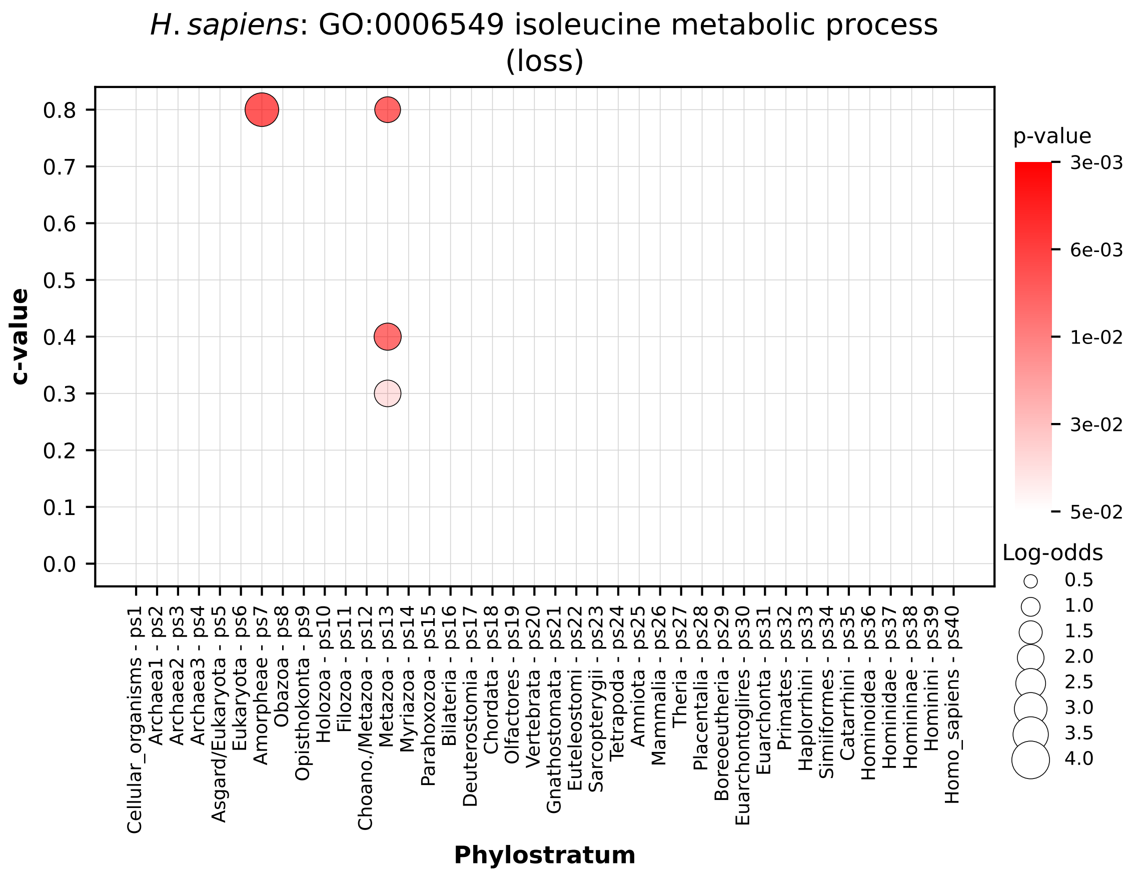

$H. sapiens$: GO:0006549 isoleucine metabolic process
(loss)
c-value
0.0
0.1
0.2
0.3
0.4
0.5
0.6
0.7
0.8
Phylostratum
Cellular_organisms - ps1
Archaea1 - ps2
Archaea2 - ps3
Archaea3 - ps4
Asgard/Eukaryota - ps5
Eukaryota - ps6
Amorpheae - ps7
Obazoa - ps8
Opisthokonta - ps9
Holozoa - ps10
Filozoa - ps11
Choano./Metazoa - ps12
Metazoa - ps13
Myriazoa - ps14
Parahoxozoa - ps15
Bilateria - ps16
Deuterostomia - ps17
Chordata - ps18
Olfactores - ps19
Vertebrata - ps20
Gnathostomata - ps21
Euteleostomi - ps22
Sarcopterygii - ps23
Tetrapoda - ps24
Amniota - ps25
Mammalia - ps26
Theria - ps27
Placentalia - ps28
Boreoeutheria - ps29
Euarchontoglires - ps30
Euarchonta - ps31
Primates - ps32
Haplorrhini - ps33
Simiiformes - ps34
Catarrhini - ps35
Hominoidea - ps36
Hominidae - ps37
Homininae - ps38
Hominini - ps39
Homo_sapiens - ps40
p-value
3e-03
6e-03
1e-02
3e-02
5e-02
Log-odds
0.5
1.0
1.5
2.0
2.5
3.0
3.5
4.0

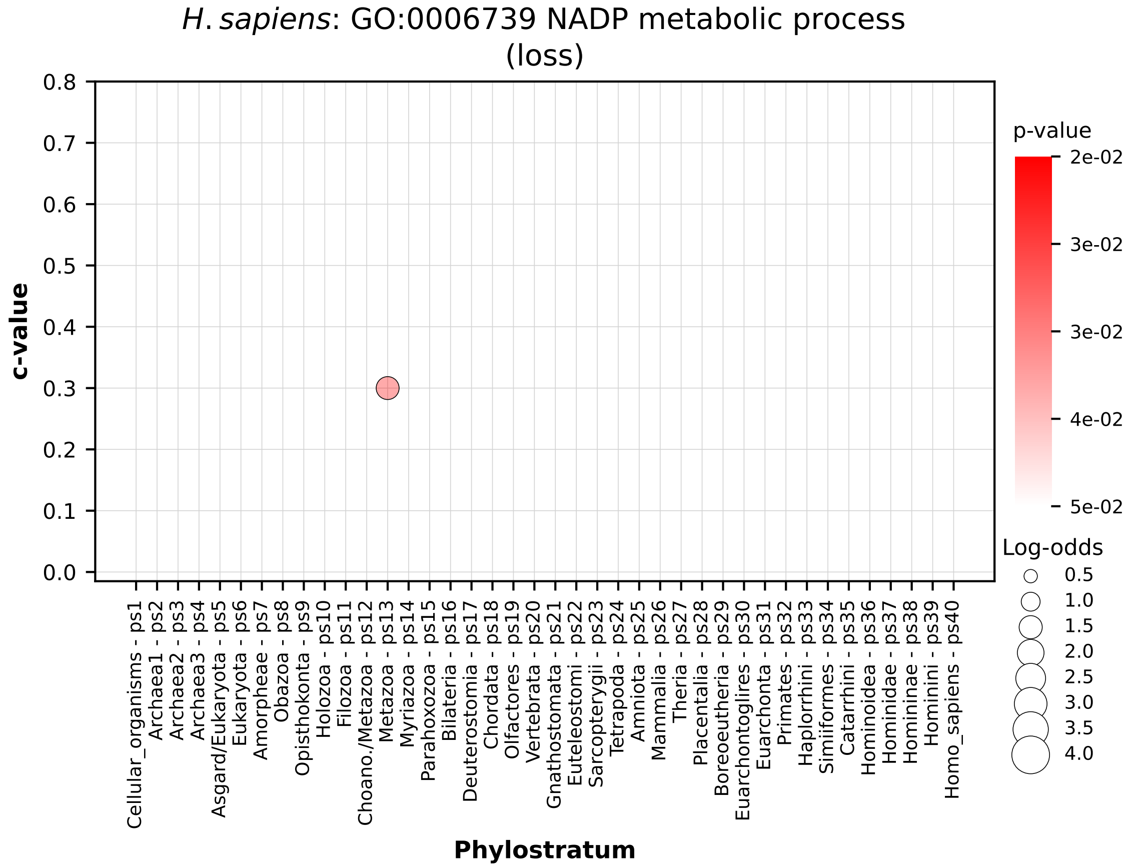
H. sapiens: GO:0006739 NADP metabolic process
(loss)
c-value
0.8
0.7
0.6
0.5
0.4
0.3
0.2
0.1
0.0
Phylostratum
Cellular_organisms - ps1
Archaea1 - ps2
Archaea2 - ps3
Archaea3 - ps4
Asgard/Eukaryota - ps5
Eukaryota - ps6
Amorpheae - ps7
Obazoa - ps8
Opisthokonta - ps9
Holozoa - ps10
Filozoa - ps11
Choano./Metazoa - ps12
Metazoa - ps13
Myriazoa - ps14
Parahoxozoa - ps15
Bilateria - ps16
Deuterostomia - ps17
Chordata - ps18
Olfactores - ps19
Vertebrata - ps20
Gnathostomata - ps21
Euteleostomi - ps22
Sarcopterygii - ps23
Tetrapoda - ps24
Amniota - ps25
Mammalia - ps26
Theria - ps27
Placentalia - ps28
Boreoeutheria - ps29
Euarchontoglires - ps30
Euarchonta - ps31
Primates - ps32
Haplorrhini - ps33
Simiiformes - ps34
Catarrhini - ps35
Hominoidea - ps36
Hominidae - ps37
Homininae - ps38
Hominini - ps39
Homo_sapiens - ps40
p-value
2e-02
3e-02
3e-02
4e-02
5e-02
Log-odds
0.5
1.0
1.5
2.0
2.5
3.0
3.5
4.0

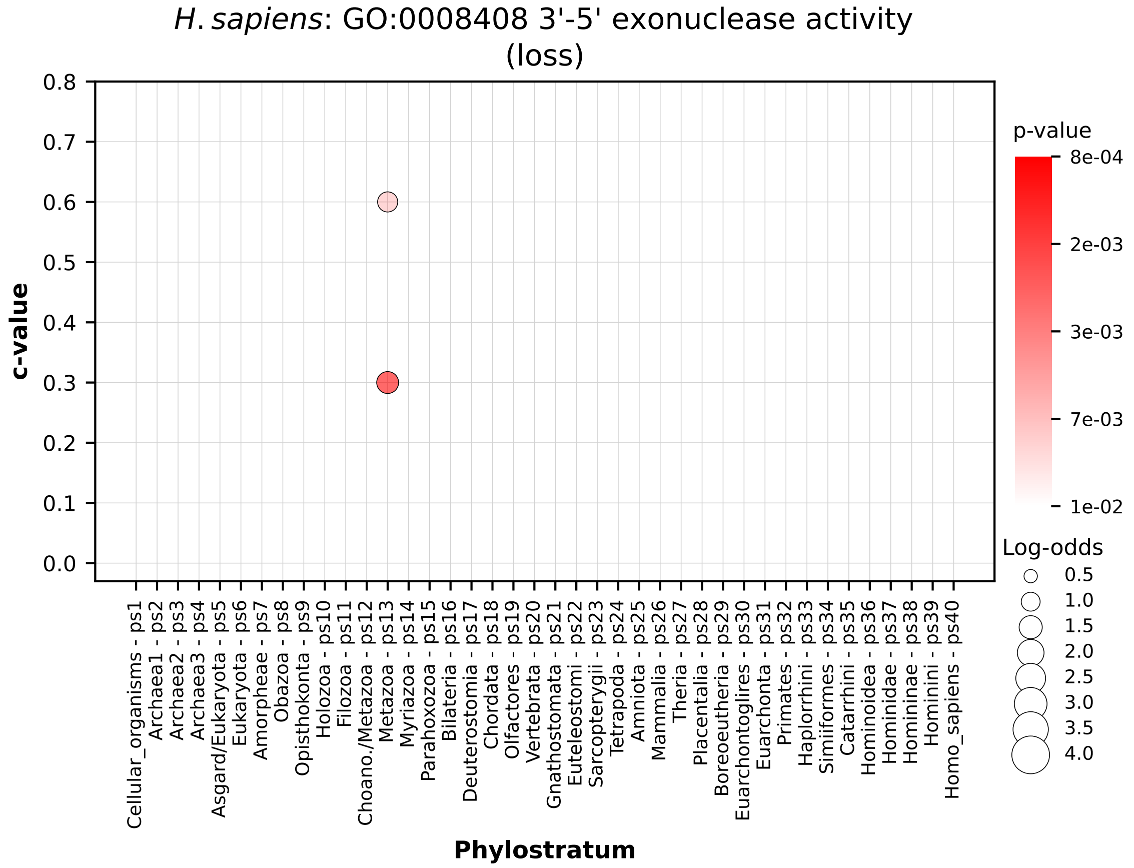

H. sapiens: GO:0008408 3'-5' exonuclease activity (loss)
c-value
0.8
0.7
0.6
0.5
0.4
0.3
0.2
0.1
0.0
p-value
8e-04
2e-03
3e-03
7e-03
1e-02
Log-odds
0.5
1.0
1.5
2.0
2.5
3.0
3.5
4.0
Cellular_organisms - ps1
Archaea1 - ps2
Archaea2 - ps3
Archaea3 - ps4
Asgard/Eukaryota - ps5
Eukaryota - ps6
Amorpheae - ps7
Obazoa - ps8
Opisthokonta - ps9
Holozoa - ps10
Filozoa - ps11
Choano./Metazoa - ps12
Metazoa - ps13
Myriazoa - ps14
Parahoxozoa - ps15
Bilateria - ps16
Deuterostomia - ps17
Chordata - ps18
Olfactores - ps19
Vertebrata - ps20
Gnathostomata - ps21
Euteleostomi - ps22
Sarcopterygii - ps23
Tetrapoda - ps24
Amniota - ps25
Mammalia - ps26
Theria - ps27
Placentalia - ps28
Boreoeutheria - ps29
Euarchontoglires - ps30
Euarchonta - ps31
Primates - ps32
Haplorrhini - ps33
Simiiformes - ps34
Catarrhini - ps35
Hominoidea - ps36
Hominidae - ps37
Homininae - ps38
Hominini - ps39
Homo_sapiens - ps40
Phylostratum

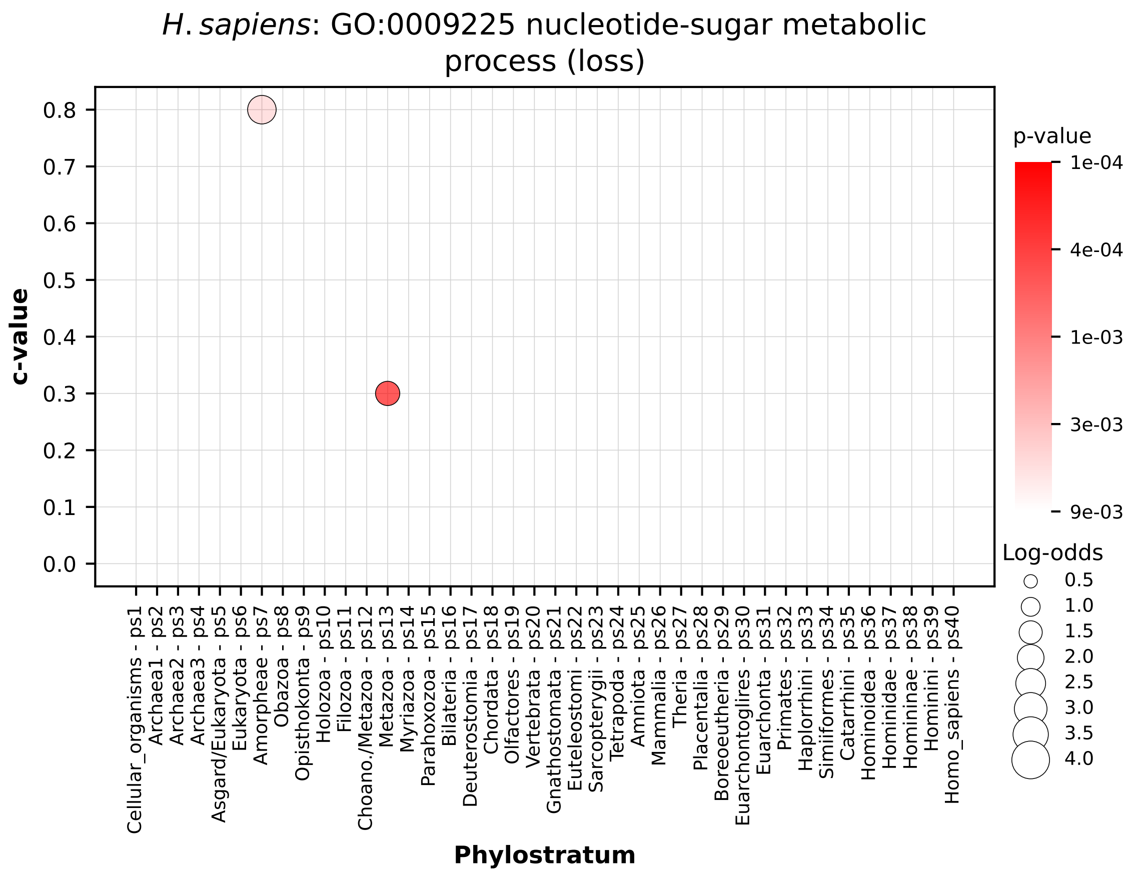

H. sapiens: GO:0009225 nucleotide-sugar metabolic process (loss)
c-value
0.8
0.7
0.6
0.5
0.4
0.3
0.2
0.1
0.0
p-value
1e-04
4e-04
1e-03
3e-03
9e-03
Log-odds
0.5
1.0
1.5
2.0
2.5
3.0
3.5
4.0
Cellular_organisms - ps1
Archaea1 - ps2
Archaea2 - ps3
Archaea3 - ps4
Asgard/Eukaryota - ps5
Eukaryota - ps6
Amorpheae - ps7
Obazoa - ps8
Opisthokonta - ps9
Holozoa - ps10
Filozoa - ps11
Choano./Metazoa - ps12
Metazoa - ps13
Myriazoa - ps14
Parahoxozoa - ps15
Bilateria - ps16
Deuterostomia - ps17
Chordata - ps18
Olfactores - ps19
Vertebrata - ps20
Gnathostomata - ps21
Euteleostomi - ps22
Sarcopterygii - ps23
Tetrapoda - ps24
Amniota - ps25
Mammalia - ps26
Theria - ps27
Placentalia - ps28
Boreoeutheria - ps29
Euarchontoglires - ps30
Euarchonta - ps31
Primates - ps32
Haplorrhini - ps33
Simiiformes - ps34
Catarrhini - ps35
Hominoidea - ps36
Hominidae - ps37
Homininae - ps38
Hominini - ps39
Homo_sapiens - ps40
Phylostratum

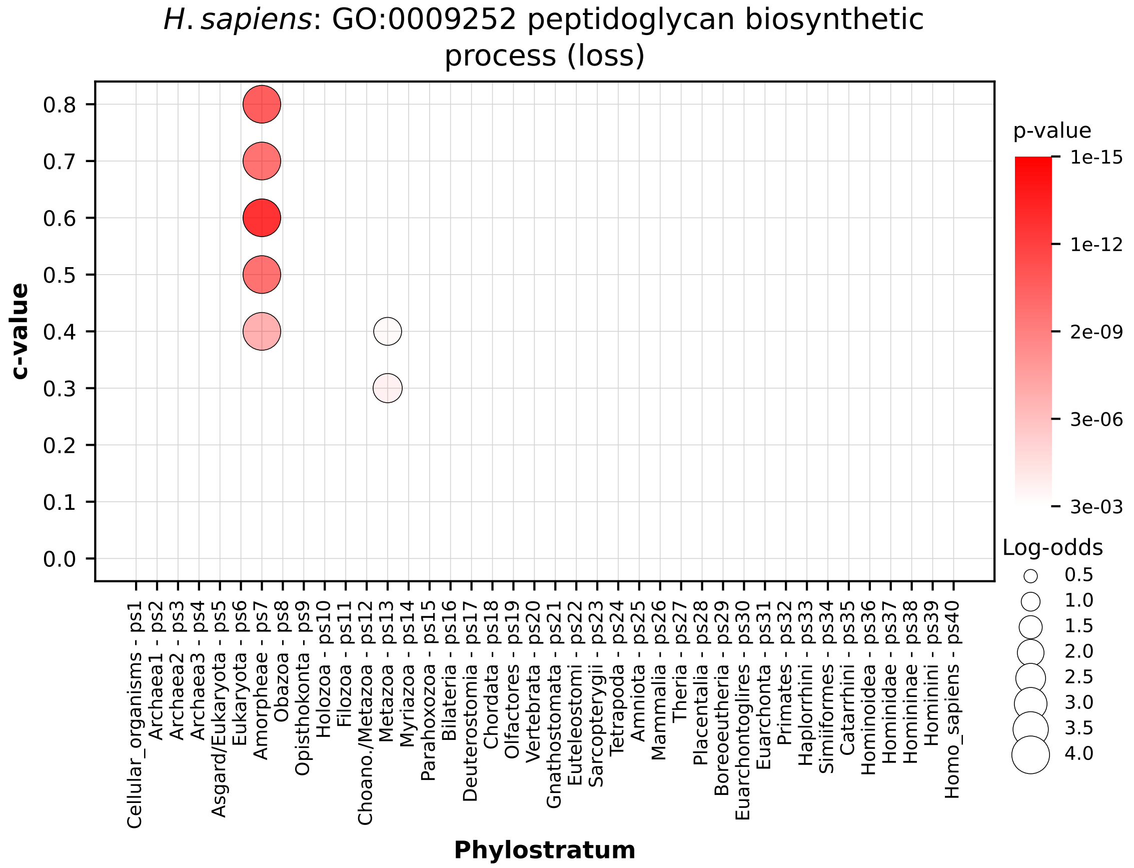

H. sapiens: GO:0009252 peptidoglycan biosynthetic process (loss)
c-value
0.8
0.7
0.6
0.5
0.4
0.3
0.2
0.1
0.0
p-value
1e-15
1e-12
2e-09
3e-06
3e-03
Log-odds
0.5
1.0
1.5
2.0
2.5
3.0
3.5
4.0
Cellular_organisms - ps1
Archaea1 - ps2
Archaea2 - ps3
Archaea3 - ps4
Asgard/Eukaryota - ps5
Eukaryota - ps6
Amorpheae - ps7
Obazoa - ps8
Opisthokonta - ps9
Holozoa - ps10
Filozoa - ps11
Choano./Metazoa - ps12
Metazoa - ps13
Myriazoa - ps14
Parahoxozoa - ps15
Bilateria - ps16
Deuterostomia - ps17
Chordata - ps18
Olfactores - ps19
Vertebrata - ps20
Gnathostomata - ps21
Euteleostomi - ps22
Sarcopterygii - ps23
Tetrapoda - ps24
Amniota - ps25
Mammalia - ps26
Theria - ps27
Placentalia - ps28
Boreoeutheria - ps29
Euarchontoglires - ps30
Euarchonta - ps31
Primates - ps32
Haplorrhini - ps33
Simiiformes - ps34
Catarrhini - ps35
Hominoidea - ps36
Hominidae - ps37
Homininae - ps38
Hominini - ps39
Homo_sapiens - ps40
Phylostratum

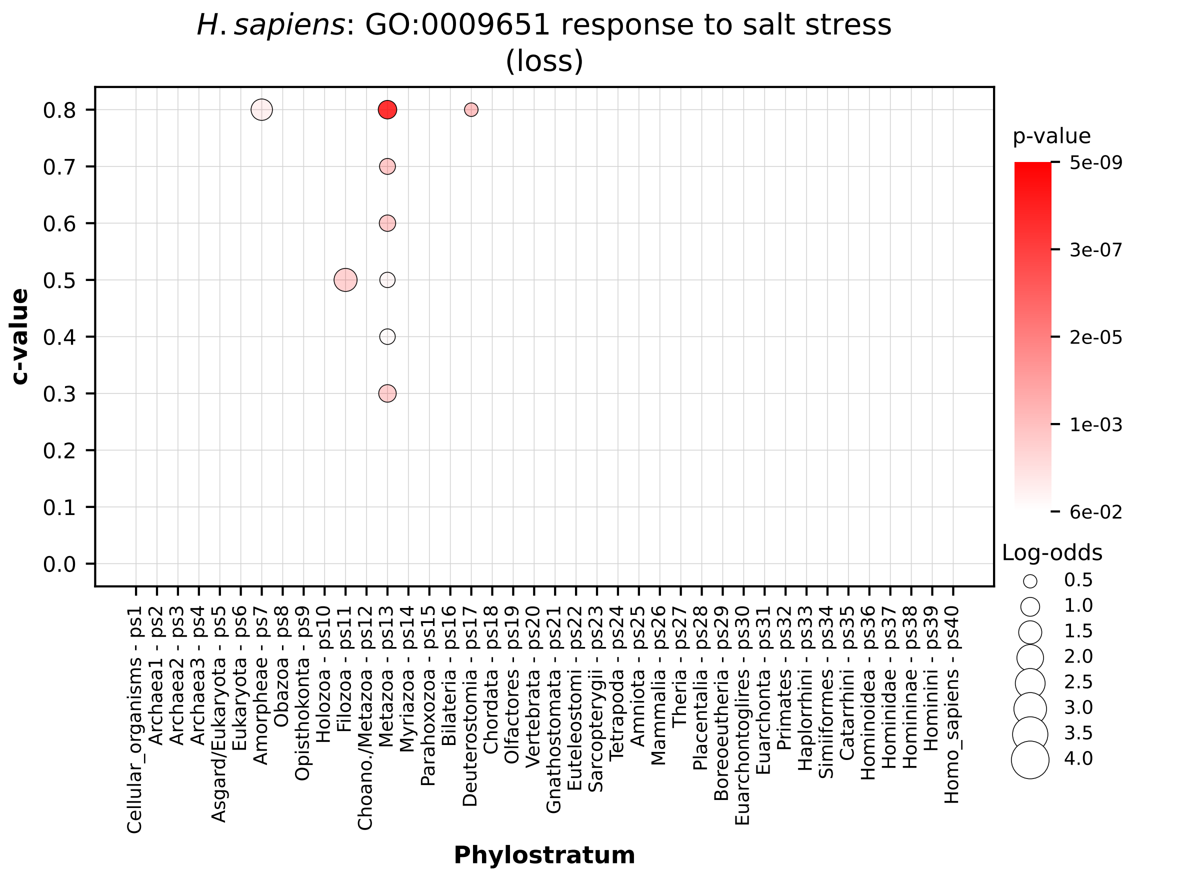

H. sapiens: GO:0009651 response to salt stress
(loss)
c-value
0.0
0.1
0.2
0.3
0.4
0.5
0.6
0.7
0.8
Phylostratum
Cellular_organisms - ps1
Archaea1 - ps2
Archaea2 - ps3
Archaea3 - ps4
Asgard/Eukaryota - ps5
Eukaryota - ps6
Amorpheae - ps7
Obazoa - ps8
Opisthokonta - ps9
Holozoa - ps10
Filozoa - ps11
Choano./Metazoa - ps12
Metazoa - ps13
Myriazoa - ps14
Parahoxozoa - ps15
Bilateria - ps16
Deuterostomia - ps17
Chordata - ps18
Olfactores - ps19
Vertebrata - ps20
Gnathostomata - ps21
Euteleostomi - ps22
Sarcopterygii - ps23
Tetrapoda - ps24
Amniota - ps25
Mammalia - ps26
Theria - ps27
Placentalia - ps28
Boreoeutheria - ps29
Euarchontoglires - ps30
Euarchonta - ps31
Primates - ps32
Haplorrhini - ps33
Simiiformes - ps34
Catarrhini - ps35
Hominoidea - ps36
Hominidae - ps37
Homininae - ps38
Hominini - ps39
Homo_sapiens - ps40
p-value
5e-09
3e-07
2e-05
1e-03
6e-02
Log-odds
0.5
1.0
1.5
2.0
2.5
3.0
3.5
4.0

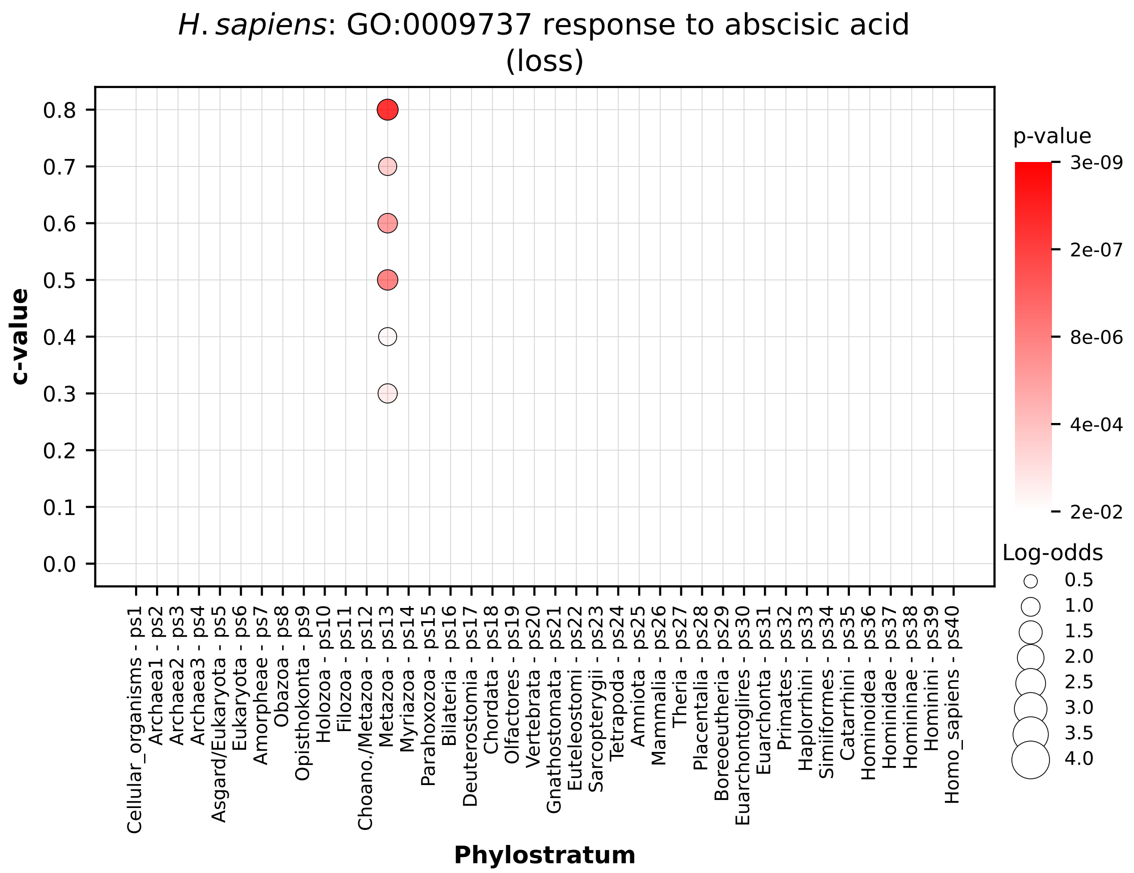
H. sapiens: GO:0009737 response to abscisic acid
(loss)
c-value
0.8
0.7
0.6
0.5
0.4
0.3
0.2
0.1
0.0
p-value
3e-09
2e-07
8e-06
4e-04
2e-02
Log-odds
0.5
1.0
1.5
2.0
2.5
3.0
3.5
4.0
Cellular_organisms - ps1
Archaea1 - ps2
Archaea2 - ps3
Archaea3 - ps4
Asgard/Eukaryota - ps5
Eukaryota - ps6
Amorpheae - ps7
Obazoa - ps8
Opisthokonta - ps9
Holozoa - ps10
Filozoa - ps11
Choano./Metazoa - ps12
Metazoa - ps13
Myriazoa - ps14
Parahoxozoa - ps15
Bilateria - ps16
Deuterostomia - ps17
Chordata - ps18
Olfactores - ps19
Vertebrata - ps20
Gnathostomata - ps21
Euteleostomi - ps22
Sarcopterygii - ps23
Tetrapoda - ps24
Amniota - ps25
Mammalia - ps26
Theria - ps27
Placentalia - ps28
Boreoeutheria - ps29
Euarchontoglires - ps30
Euarchonta - ps31
Primates - ps32
Haplorrhini - ps33
Simiiformes - ps34
Catarrhini - ps35
Hominoidea - ps36
Hominidae - ps37
Homininae - ps38
Hominini - ps39
Homo_sapiens - ps40
Phylostratum

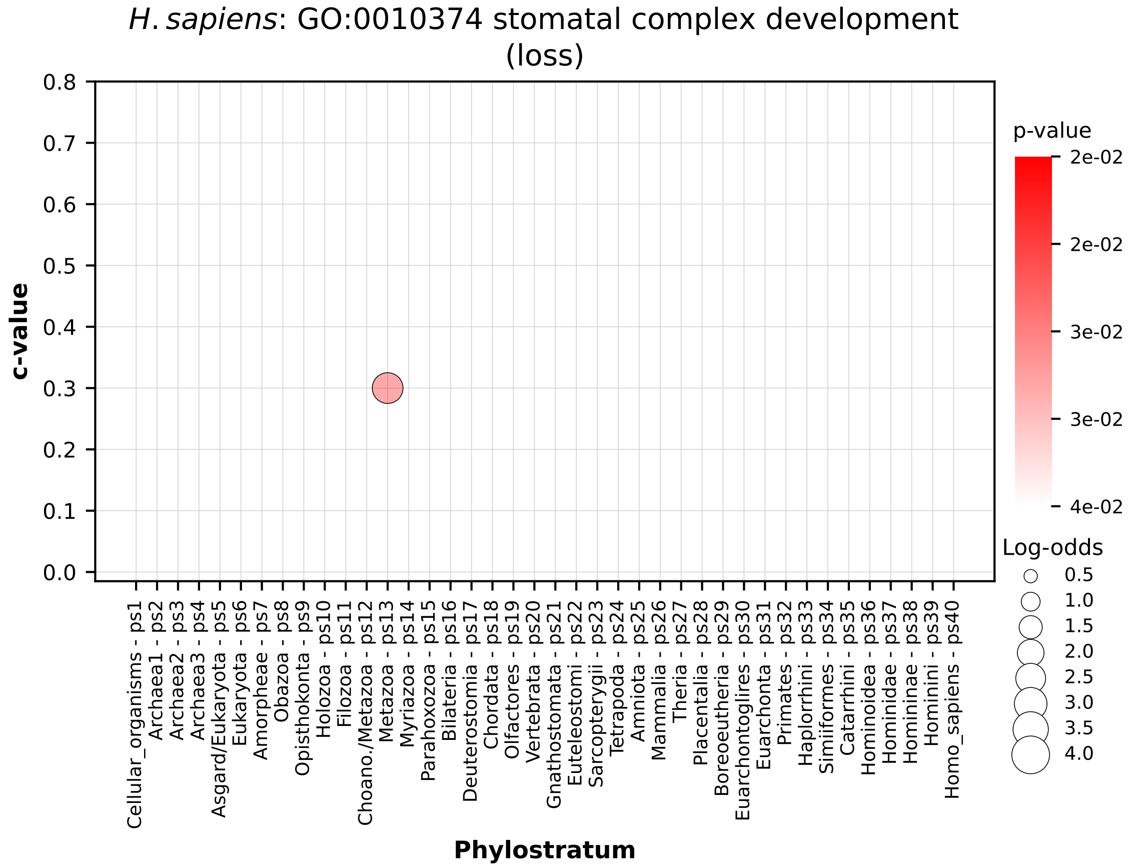

H. sapiens: GO:0010374 stomatal complex development (loss)
c-value
0.8
0.7
0.6
0.5
0.4
0.3
0.2
0.1
0.0
p-value
2e-02
2e-02
3e-02
3e-02
4e-02
Log-odds
0.5
1.0
1.5
2.0
2.5
3.0
3.5
4.0
Cellular_organisms - ps1
Archaea1 - ps2
Archaea2 - ps3
Archaea3 - ps4
Asgard/Eukaryota - ps5
Eukaryota - ps6
Amorpheae - ps7
Obazoa - ps8
Opisthokonta - ps9
Holozoa - ps10
Filozoa - ps11
Choano./Metazoa - ps12
Metazoa - ps13
Myriazoa - ps14
Parahoxozoa - ps15
Bilateria - ps16
Deuterostomia - ps17
Chordata - ps18
Olfactores - ps19
Vertebrata - ps20
Gnathostomata - ps21
Euteleostomi - ps22
Sarcopterygii - ps23
Tetrapoda - ps24
Amniota - ps25
Mammalia - ps26
Theria - ps27
Placentalia - ps28
Boreoeutheria - ps29
Euarchontoglires - ps30
Euarchonta - ps31
Primates - ps32
Haplorrhini - ps33
Simiiformes - ps34
Catarrhini - ps35
Hominoidea - ps36
Hominidae - ps37
Homininae - ps38
Hominini - ps39
Homo_sapiens - ps40
Phylostratum

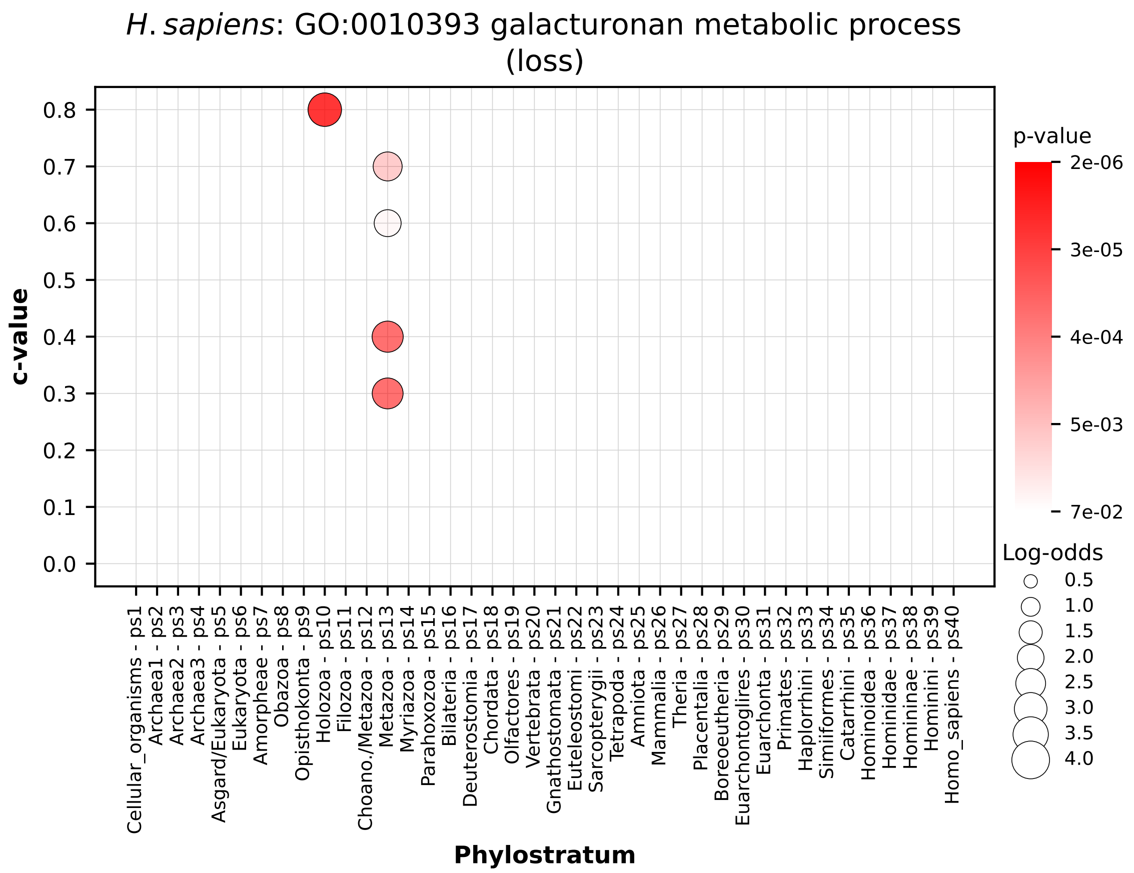

H. sapiens: GO:0010393 galacturonan metabolic process
(loss)
c-value
0.0
0.1
0.2
0.3
0.4
0.5
0.6
0.7
0.8
Phylostratum
Cellular_organisms - ps1
Archaea1 - ps2
Archaea2 - ps3
Archaea3 - ps4
Asgard/Eukaryota - ps5
Eukaryota - ps6
Amorpheae - ps7
Obazoa - ps8
Opisthokonta - ps9
Holozoa - ps10
Filozoa - ps11
Choano./Metazoa - ps12
Metazoa - ps13
Myriazoa - ps14
Parahoxozoa - ps15
Bilateria - ps16
Deuterostomia - ps17
Chordata - ps18
Olfactores - ps19
Vertebrata - ps20
Gnathostomata - ps21
Euteleostomi - ps22
Sarcopterygii - ps23
Tetrapoda - ps24
Amniota - ps25
Mammalia - ps26
Theria - ps27
Placentalia - ps28
Boreoeutheria - ps29
Euarchontoglires - ps30
Euarchonta - ps31
Primates - ps32
Haplorrhini - ps33
Simiiformes - ps34
Catarrhini - ps35
Hominoidea - ps36
Hominidae - ps37
Homininae - ps38
Hominini - ps39
Homo_sapiens - ps40
p-value
2e-06
3e-05
4e-04
5e-03
7e-02
Log-odds
0.5
1.0
1.5
2.0
2.5
3.0
3.5
4.0

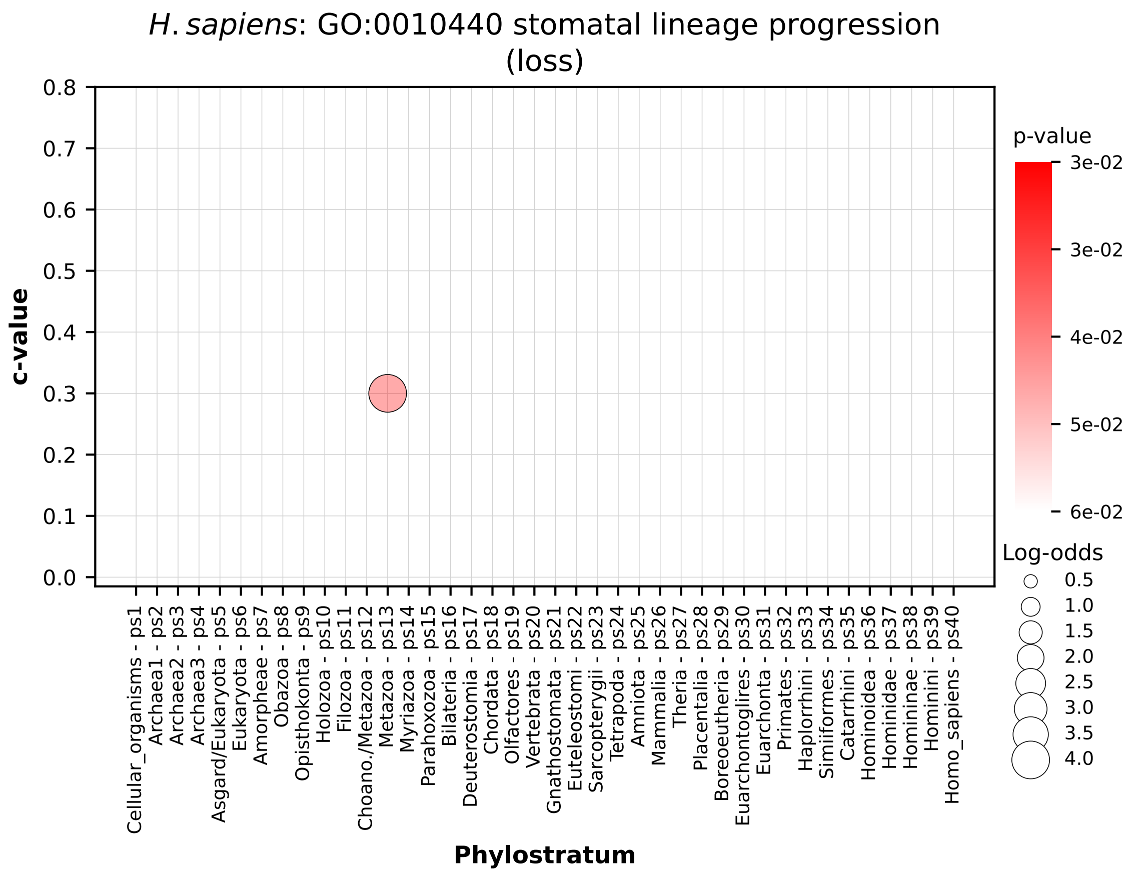

H. sapiens: GO:0010440 stomatal lineage progression
(loss)
c-value
0.0
0.1
0.2
0.3
0.4
0.5
0.6
0.7
0.8
Phylostratum
Cellular_organisms - ps1
Archaea1 - ps2
Archaea2 - ps3
Archaea3 - ps4
Asgard/Eukaryota - ps5
Eukaryota - ps6
Amorpheae - ps7
Obazoa - ps8
Opisthokonta - ps9
Holozoa - ps10
Filozoa - ps11
Choano./Metazoa - ps12
Metazoa - ps13
Myriazoa - ps14
Parahoxozoa - ps15
Bilateria - ps16
Deuterostomia - ps17
Chordata - ps18
Olfactores - ps19
Vertebrata - ps20
Gnathostomata - ps21
Euteleostomi - ps22
Sarcopterygii - ps23
Tetrapoda - ps24
Amniota - ps25
Mammalia - ps26
Theria - ps27
Placentalia - ps28
Boreoeutheria - ps29
Euarchontoglires - ps30
Euarchonta - ps31
Primates - ps32
Haplorrhini - ps33
Simiiformes - ps34
Catarrhini - ps35
Hominoidea - ps36
Hominidae - ps37
Homininae - ps38
Hominini - ps39
Homo_sapiens - ps40
p-value
3e-02
3e-02
4e-02
5e-02
6e-02
Log-odds
0.5
1.0
1.5
2.0
2.5
3.0
3.5
4.0

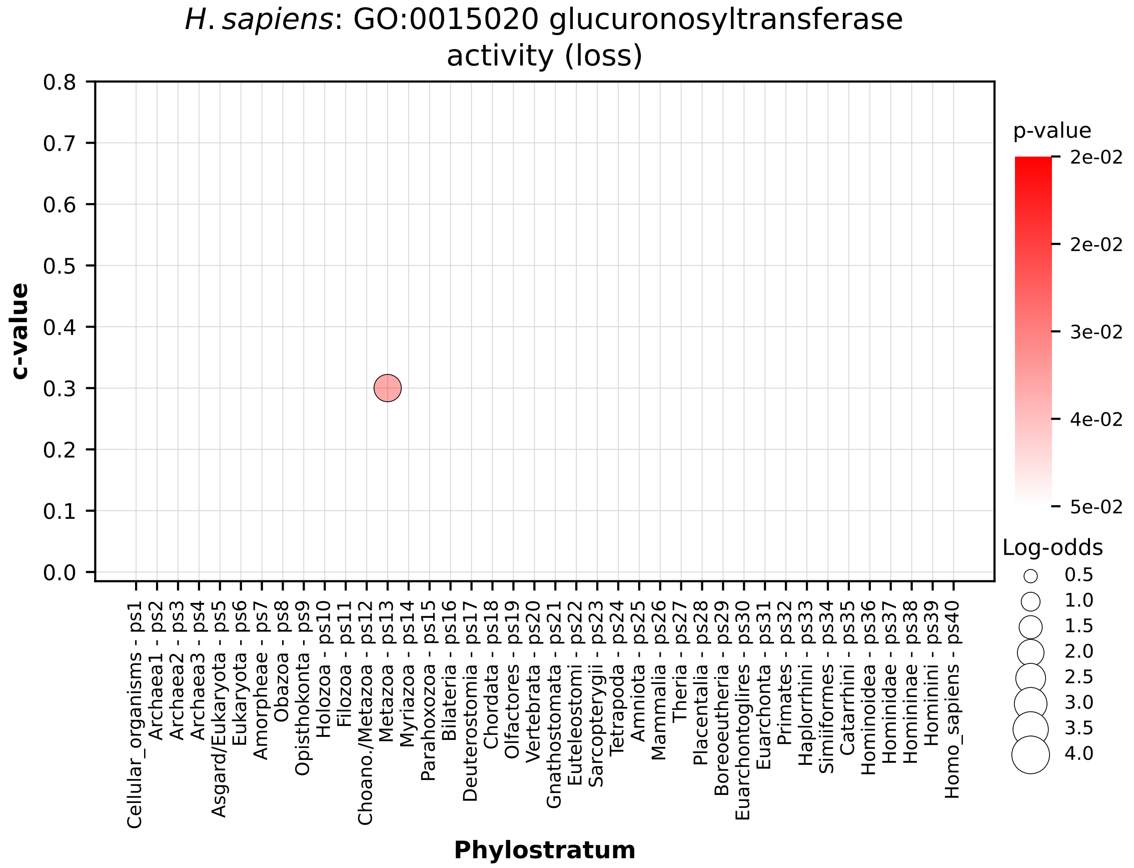

H. sapiens: GO:0015020 glucuronosyltransferase activity (loss)
c-value
0.8
0.7
0.6
0.5
0.4
0.3
0.2
0.1
0.0
p-value
2e-02
2e-02
3e-02
4e-02
5e-02
Log-odds
0.5
1.0
1.5
2.0
2.5
3.0
3.5
4.0
Cellular_organisms - ps1
Archaea1 - ps2
Archaea2 - ps3
Archaea3 - ps4
Asgard/Eukaryota - ps5
Eukaryota - ps6
Amorpheae - ps7
Obazoa - ps8
Opisthokonta - ps9
Holozoa - ps10
Filozoa - ps11
Choano./Metazoa - ps12
Metazoa - ps13
Myriazoa - ps14
Parahoxozoa - ps15
Bilateria - ps16
Deuterostomia - ps17
Chordata - ps18
Olfactores - ps19
Vertebrata - ps20
Gnathostomata - ps21
Euteleostomi - ps22
Sarcopterygii - ps23
Tetrapoda - ps24
Amniota - ps25
Mammalia - ps26
Theria - ps27
Placentalia - ps28
Boreoeutheria - ps29
Euarchontoglires - ps30
Euarchonta - ps31
Primates - ps32
Haplorrhini - ps33
Simiiformes - ps34
Catarrhini - ps35
Hominoidea - ps36
Hominidae - ps37
Homininae - ps38
Hominini - ps39
Homo_sapiens - ps40
Phylostratum

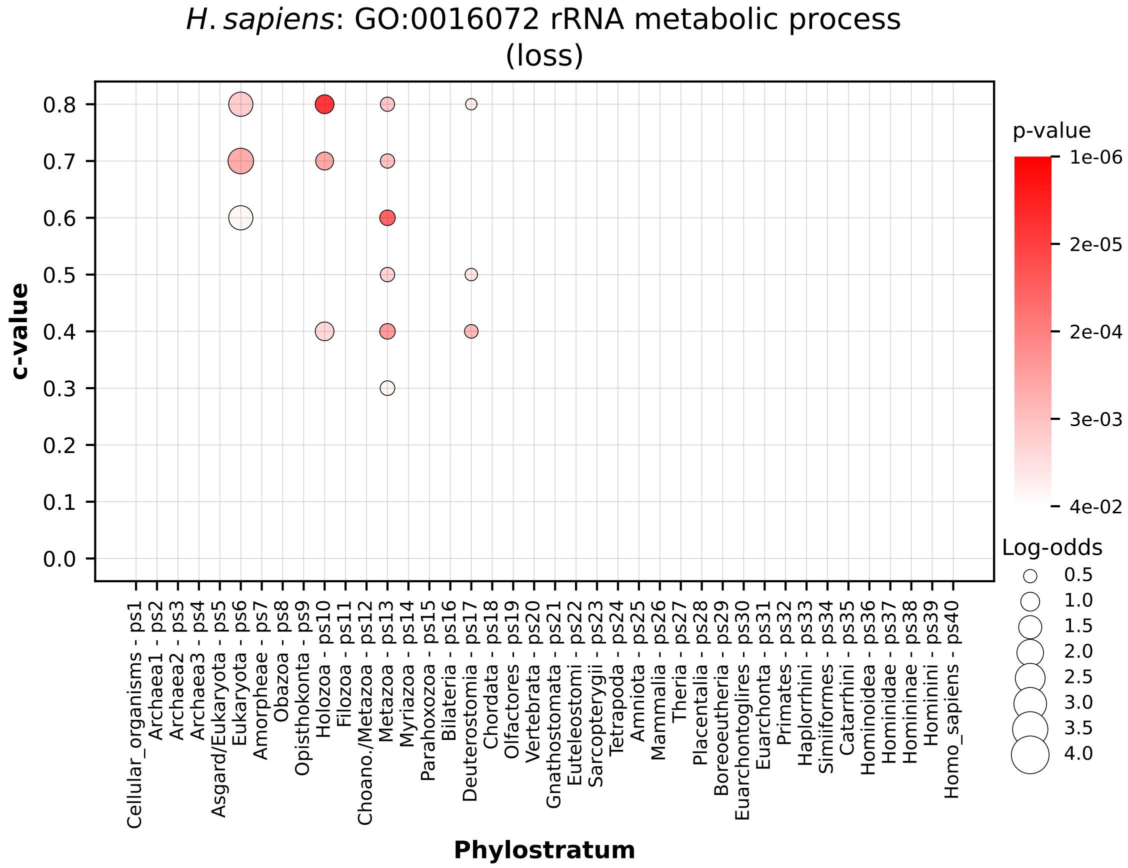

H. sapiens: GO:0016072 rRNA metabolic process (loss)
c-value
0.0
0.1
0.2
0.3
0.4
0.5
0.6
0.7
0.8
Phylostratum
Cellular_organisms - ps1
Archaea1 - ps2
Archaea2 - ps3
Archaea3 - ps4
Asgard/Eukaryota - ps5
Eukaryota - ps6
Amorpheae - ps7
Obazoa - ps8
Opisthokonta - ps9
Holozoa - ps10
Filozoa - ps11
Choano./Metazoa - ps12
Metazoa - ps13
Myriazoa - ps14
Parahoxozoa - ps15
Bilateria - ps16
Deuterostomia - ps17
Chordata - ps18
Olfactores - ps19
Vertebrata - ps20
Gnathostomata - ps21
Euteleostomi - ps22
Sarcopterygii - ps23
Tetrapoda - ps24
Amniota - ps25
Mammalia - ps26
Theria - ps27
Placentalia - ps28
Boreoeutheria - ps29
Euarchontoglires - ps30
Euarchonta - ps31
Primates - ps32
Haplorrhini - ps33
Simiiformes - ps34
Catarrhini - ps35
Hominoidea - ps36
Hominidae - ps37
Homininae - ps38
Hominini - ps39
Homo_sapiens - ps40
p-value
1e-06
2e-05
2e-04
3e-03
4e-02
Log-odds
0.5
1.0
1.5
2.0
2.5
3.0
3.5
4.0

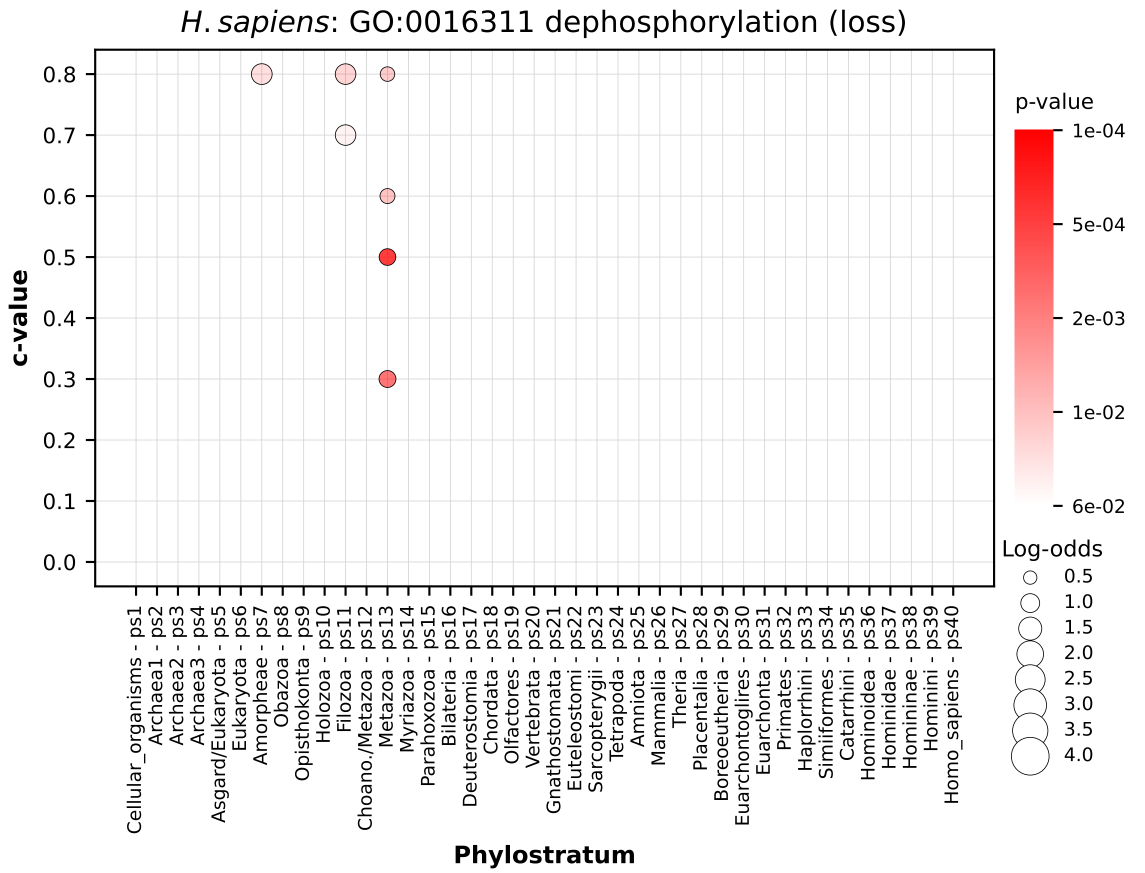

H. sapiens: GO:0016311 dephosphorylation (loss)
c-value
0.8
0.7
0.6
0.5
0.4
0.3
0.2
0.1
0.0
p-value
1e-04
5e-04
2e-03
1e-02
6e-02
Log-odds
0.5
1.0
1.5
2.0
2.5
3.0
3.5
4.0
Cellular_organisms - ps1
Archaea1 - ps2
Archaea2 - ps3
Archaea3 - ps4
Asgard/Eukaryota - ps5
Eukaryota - ps6
Amorpheae - ps7
Obazoa - ps8
Opisthokonta - ps9
Holozoa - ps10
Filozoa - ps11
Choano./Metazoa - ps12
Metazoa - ps13
Myriazoa - ps14
Parahoxozoa - ps15
Bilateria - ps16
Deuterostomia - ps17
Chordata - ps18
Olfactores - ps19
Vertebrata - ps20
Gnathostomata - ps21
Euteleostomi - ps22
Sarcopterygii - ps23
Tetrapoda - ps24
Amniota - ps25
Mammalia - ps26
Theria - ps27
Placentalia - ps28
Boreoeutheria - ps29
Euarchontoglires - ps30
Euarchonta - ps31
Primates - ps32
Haplorrhini - ps33
Simiiformes - ps34
Catarrhini - ps35
Hominoidea - ps36
Hominidae - ps37
Homininae - ps38
Hominini - ps39
Homo_sapiens - ps40
Phylostratum

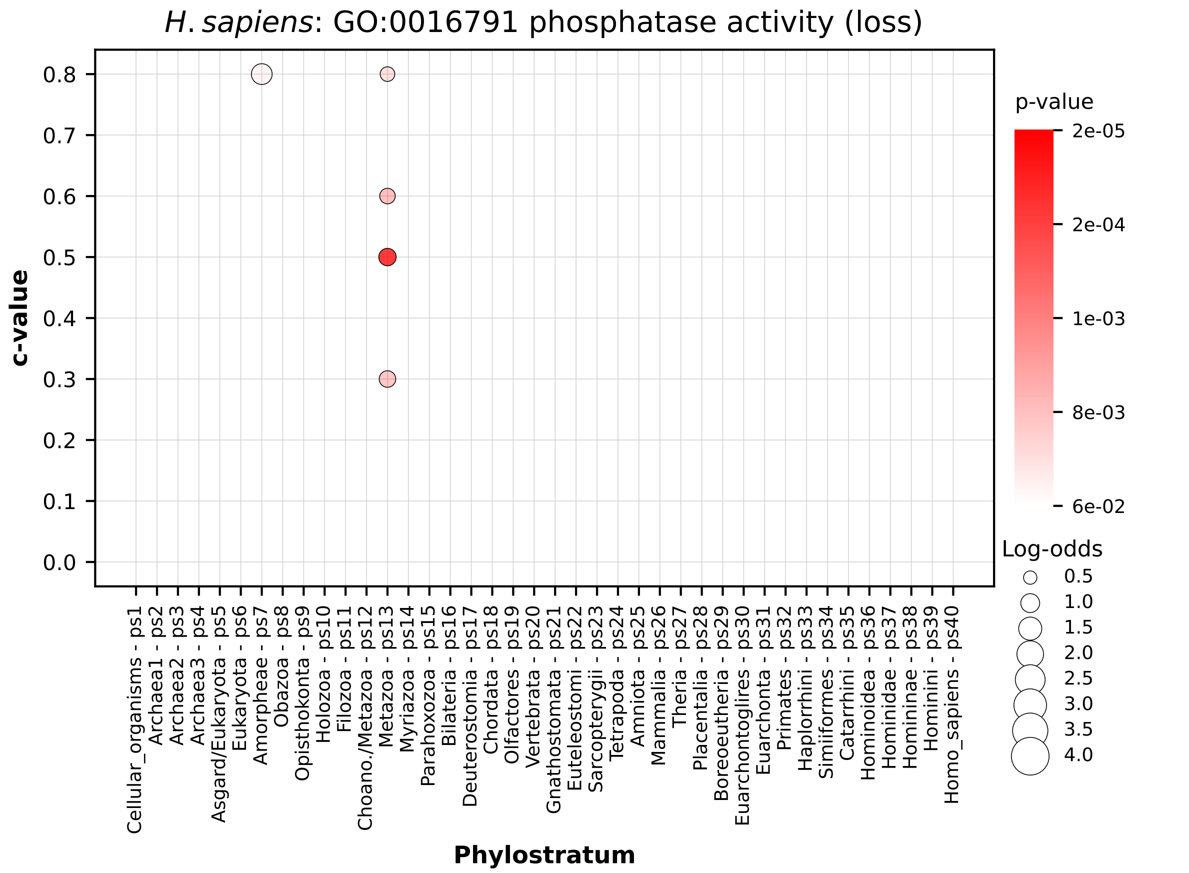

H. sapiens: GO:0016791 phosphatase activity (loss)
c-value
0.8
0.7
0.6
0.5
0.4
0.3
0.2
0.1
0.0
Phylostratum
Cellular_organisms - ps1
Archaea1 - ps2
Archaea2 - ps3
Archaea3 - ps4
Asgard/Eukaryota - ps5
Eukaryota - ps6
Amorpheae - ps7
Obazoa - ps8
Opisthokonta - ps9
Holozoa - ps10
Filozoa - ps11
Choano./Metazoa - ps12
Metazoa - ps13
Myriazoa - ps14
Parahoxozoa - ps15
Bilateria - ps16
Deuterostomia - ps17
Chordata - ps18
Olfactores - ps19
Vertebrata - ps20
Gnathostomata - ps21
Euteleostomi - ps22
Sarcopterygii - ps23
Tetrapoda - ps24
Amniota - ps25
Mammalia - ps26
Theria - ps27
Placentalia - ps28
Boreoeutheria - ps29
Euarchontoglires - ps30
Euarchonta - ps31
Primates - ps32
Haplorrhini - ps33
Simiiformes - ps34
Catarrhini - ps35
Hominoidea - ps36
Hominidae - ps37
Homininae - ps38
Hominini - ps39
Homo_sapiens - ps40
p-value
2e-05
2e-04
1e-03
8e-03
6e-02
Log-odds
0.5
1.0
1.5
2.0
2.5
3.0
3.5
4.0

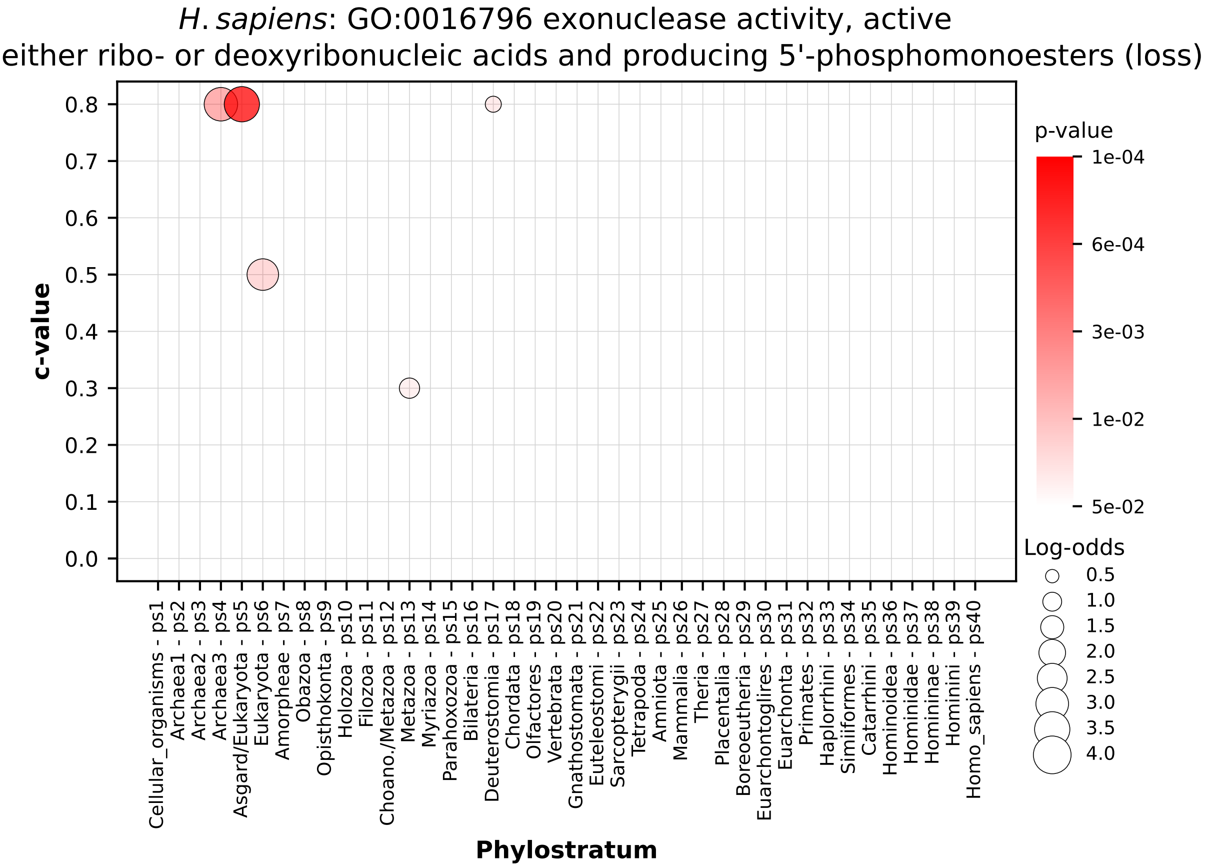
H. sapiens: GO:0016796 exonuclease activity, active
either ribo- or deoxyribonucleic acids and producing 5'-phosphomonoesters (loss)
c-value
0.8
0.7
0.6
0.5
0.4
0.3
0.2
0.1
0.0
p-value
1e-04
6e-04
3e-03
1e-02
5e-02
Log-odds
0.5
1.0
1.5
2.0
2.5
3.0
3.5
4.0
Cellular_organisms - ps1
Archaea1 - ps2
Archaea2 - ps3
Archaea3 - ps4
Asgard/Eukaryota - ps5
Eukaryota - ps6
Amorpheae - ps7
Obazoa - ps8
Opisthokonta - ps9
Holozoa - ps10
Filozoa - ps11
Choano./Metazoa - ps12
Metazoa - ps13
Myriazoa - ps14
Parahoxozoa - ps15
Bilateria - ps16
Deuterostomia - ps17
Chordata - ps18
Olfactores - ps19
Vertebrata - ps20
Gnathostomata - ps21
Euteleostomi - ps22
Sarcopterygii - ps23
Tetrapoda - ps24
Amniota - ps25
Mammalia - ps26
Theria - ps27
Placentalia - ps28
Boreoeutheria - ps29
Euarchontoglires - ps30
Euarchonta - ps31
Primates - ps32
Haplorrhini - ps33
Simiiformes - ps34
Catarrhini - ps35
Hominoidea - ps36
Hominidae - ps37
Homininae - ps38
Hominini - ps39
Homo_sapiens - ps40
Phylostratum

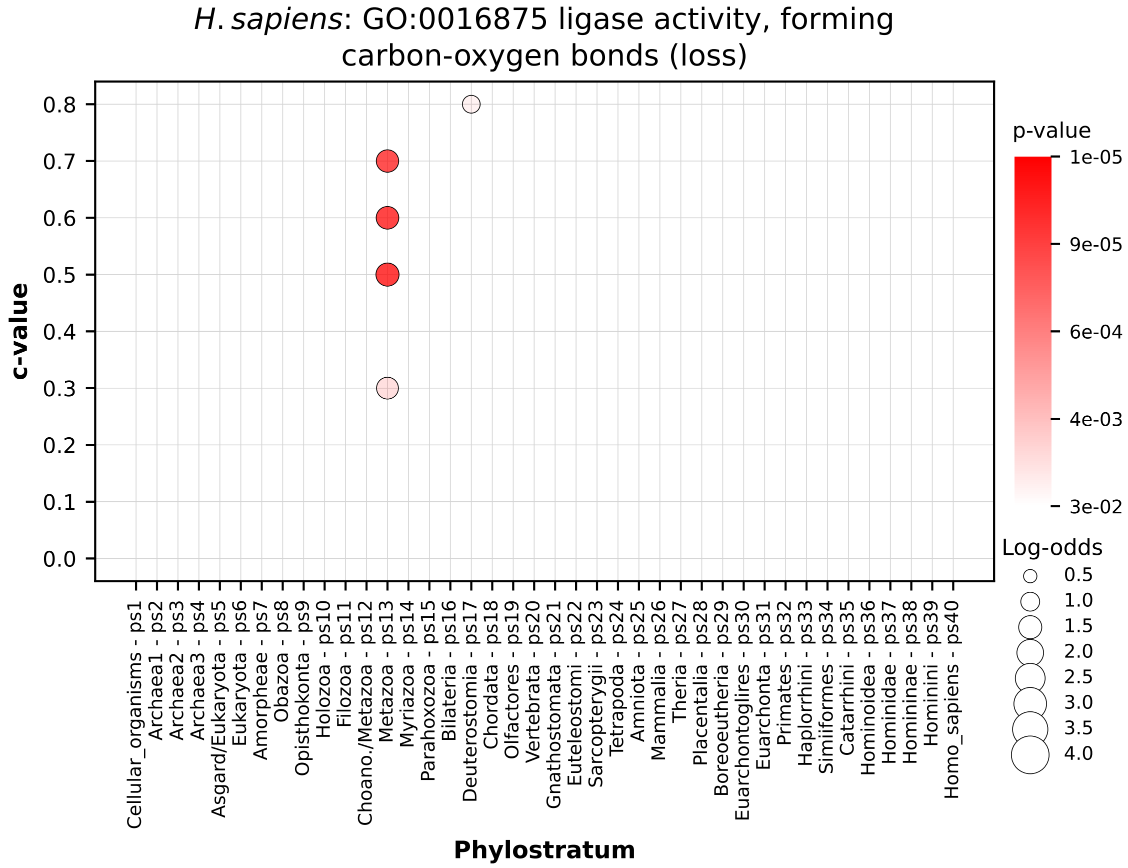
H. sapiens: GO:0016875 ligase activity, forming carbon-oxygen bonds (loss)
c-value
0.8
0.7
0.6
0.5
0.4
0.3
0.2
0.1
0.0
Phylostratum
Cellular_organisms - ps1
Archaea1 - ps2
Archaea2 - ps3
Archaea3 - ps4
Asgard/Eukaryota - ps5
Eukaryota - ps6
Amorpheae - ps7
Obazoa - ps8
Opisthokonta - ps9
Holozoa - ps10
Filozoa - ps11
Choano./Metazoa - ps12
Metazoa - ps13
Myriazoa - ps14
Parahoxozoa - ps15
Bilateria - ps16
Deuterostomia - ps17
Chordata - ps18
Olfactores - ps19
Vertebrata - ps20
Gnathostomata - ps21
Euteleostomi - ps22
Sarcopterygii - ps23
Tetrapoda - ps24
Amniota - ps25
Mammalia - ps26
Theria - ps27
Placentalia - ps28
Boreoeutheria - ps29
Euarchontoglires - ps30
Euarchonta - ps31
Primates - ps32
Haplorrhini - ps33
Simiiformes - ps34
Catarrhini - ps35
Hominoidea - ps36
Hominidae - ps37
Homininae - ps38
Hominini - ps39
Homo_sapiens - ps40
p-value
1e-05
9e-05
6e-04
4e-03
3e-02
Log-odds
0.5
1.0
1.5
2.0
2.5
3.0
3.5
4.0

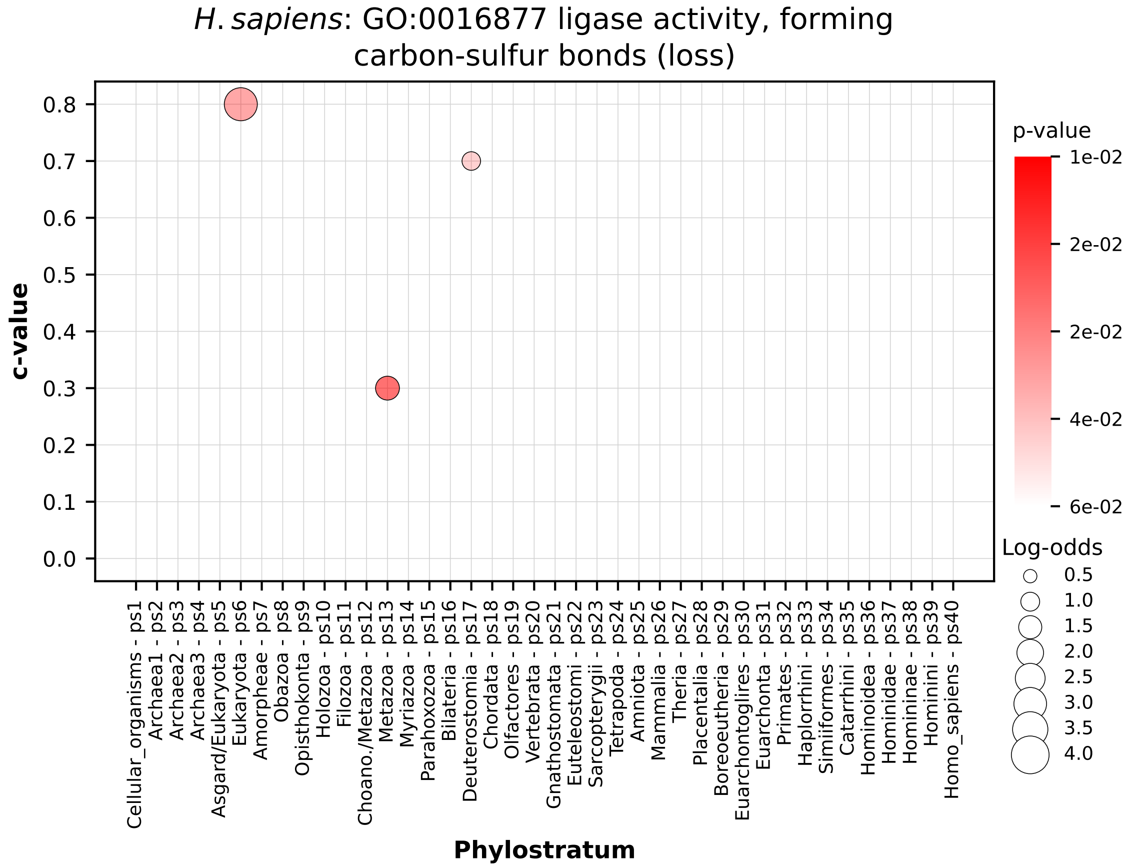

H. sapiens: GO:0016877 ligase activity, forming carbon-sulfur bonds (loss)
c-value
0.0
0.1
0.2
0.3
0.4
0.5
0.6
0.7
0.8
Phylostratum
Cellular_organisms - ps1
Archaea1 - ps2
Archaea2 - ps3
Archaea3 - ps4
Asgard/Eukaryota - ps5
Eukaryota - ps6
Amorpheae - ps7
Obazoa - ps8
Opisthokonta - ps9
Holozoa - ps10
Filozoa - ps11
Choano./Metazoa - ps12
Metazoa - ps13
Myriazoa - ps14
Parahoxozoa - ps15
Bilateria - ps16
Deuterostomia - ps17
Chordata - ps18
Olfactores - ps19
Vertebrata - ps20
Gnathostomata - ps21
Euteleostomi - ps22
Sarcopterygii - ps23
Tetrapoda - ps24
Amniota - ps25
Mammalia - ps26
Theria - ps27
Placentalia - ps28
Boreoeutheria - ps29
Euarchontoglires - ps30
Euarchonta - ps31
Primates - ps32
Haplorrhini - ps33
Simiiformes - ps34
Catarrhini - ps35
Hominoidea - ps36
Hominidae - ps37
Homininae - ps38
Hominini - ps39
Homo_sapiens - ps40
p-value
1e-02
2e-02
2e-02
4e-02
6e-02
Log-odds
0.5
1.0
1.5
2.0
2.5
3.0
3.5
4.0

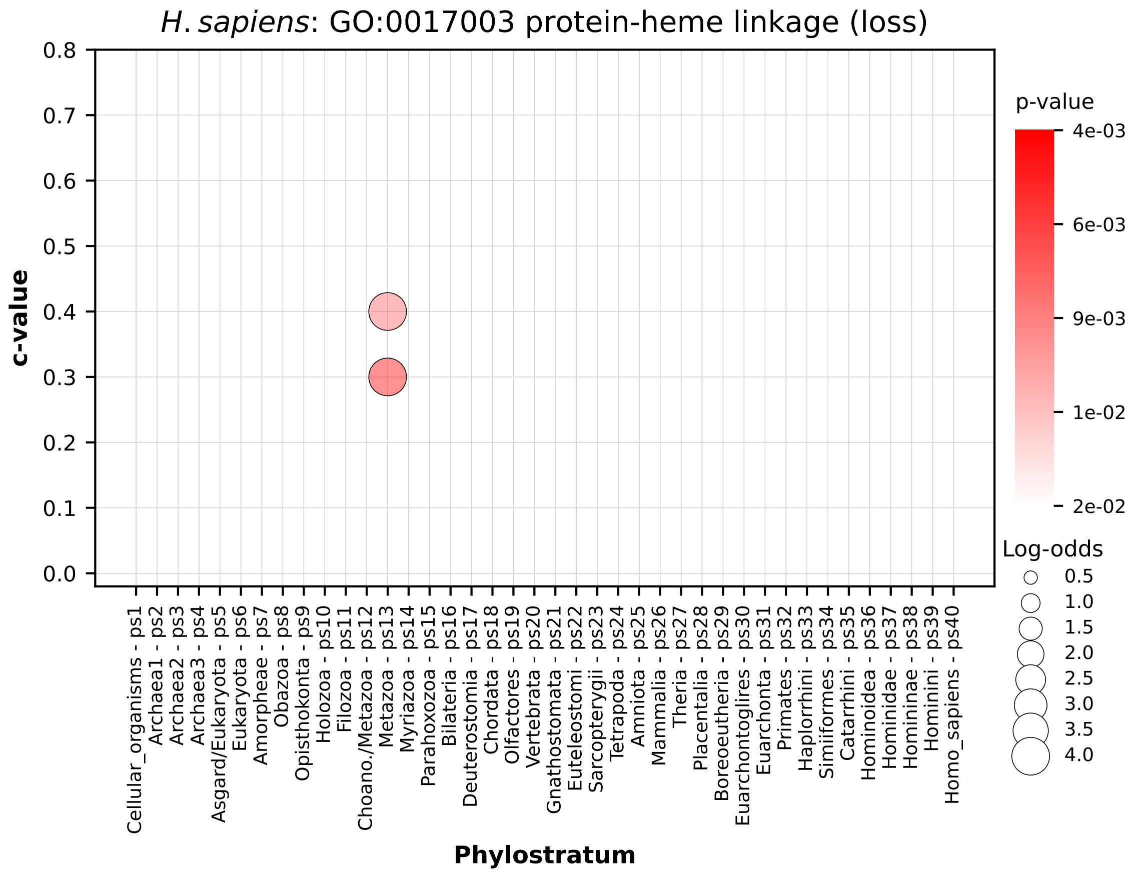

H. sapiens: GO:0017003 protein-heme linkage (loss)
c-value
0.8
0.7
0.6
0.5
0.4
0.3
0.2
0.1
0.0
Phylostratum
Cellular_organisms - ps1
Archaea1 - ps2
Archaea2 - ps3
Archaea3 - ps4
Asgard/Eukaryota - ps5
Eukaryota - ps6
Amorpheae - ps7
Obazoa - ps8
Opisthokonta - ps9
Holozoa - ps10
Filozoa - ps11
Choano./Metazoa - ps12
Metazoa - ps13
Myriazoa - ps14
Parahoxozoa - ps15
Bilateria - ps16
Deuterostomia - ps17
Chordata - ps18
Olfactores - ps19
Vertebrata - ps20
Gnathostomata - ps21
Euteleostomi - ps22
Sarcopterygii - ps23
Tetrapoda - ps24
Amniota - ps25
Mammalia - ps26
Theria - ps27
Placentalia - ps28
Boreoeutheria - ps29
Euarchontoglires - ps30
Euarchonta - ps31
Primates - ps32
Haplorrhini - ps33
Simiiformes - ps34
Catarrhini - ps35
Hominoidea - ps36
Hominidae - ps37
Homininae - ps38
Hominini - ps39
Homo_sapiens - ps40
p-value
4e-03
6e-03
9e-03
1e-02
2e-02
Log-odds
0.5
1.0
1.5
2.0
2.5
3.0
3.5
4.0

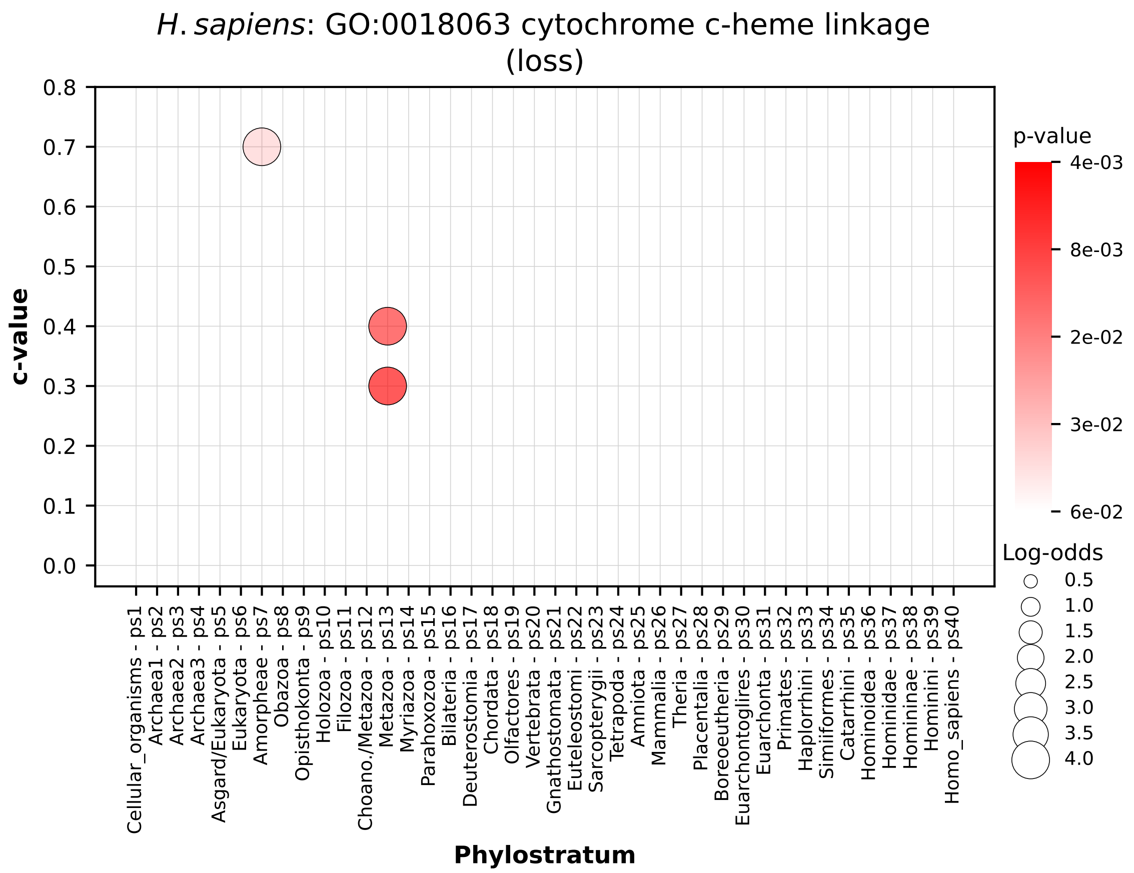

H. sapiens: GO:0018063 cytochrome c-heme linkage
(loss)
c-value
0.8
0.7
0.6
0.5
0.4
0.3
0.2
0.1
0.0
Phylostratum
Cellular_organisms - ps1
Archaea1 - ps2
Archaea2 - ps3
Archaea3 - ps4
Asgard/Eukaryota - ps5
Eukaryota - ps6
Amorpheae - ps7
Obazoa - ps8
Opisthokonta - ps9
Holozoa - ps10
Filozoa - ps11
Choano./Metazoa - ps12
Metazoa - ps13
Myriazoa - ps14
Parahoxozoa - ps15
Bilateria - ps16
Deuterostomia - ps17
Chordata - ps18
Olfactores - ps19
Vertebrata - ps20
Gnathostomata - ps21
Euteleostomi - ps22
Sarcopterygii - ps23
Tetrapoda - ps24
Amniota - ps25
Mammalia - ps26
Theria - ps27
Placentalia - ps28
Boreoeutheria - ps29
Euarchontoglires - ps30
Euarchonta - ps31
Primates - ps32
Haplorrhini - ps33
Simiiformes - ps34
Catarrhini - ps35
Hominoidea - ps36
Hominidae - ps37
Homininae - ps38
Hominini - ps39
Homo_sapiens - ps40
p-value
4e-03
8e-03
2e-02
3e-02
6e-02
Log-odds
0.5
1.0
1.5
2.0
2.5
3.0
3.5
4.0

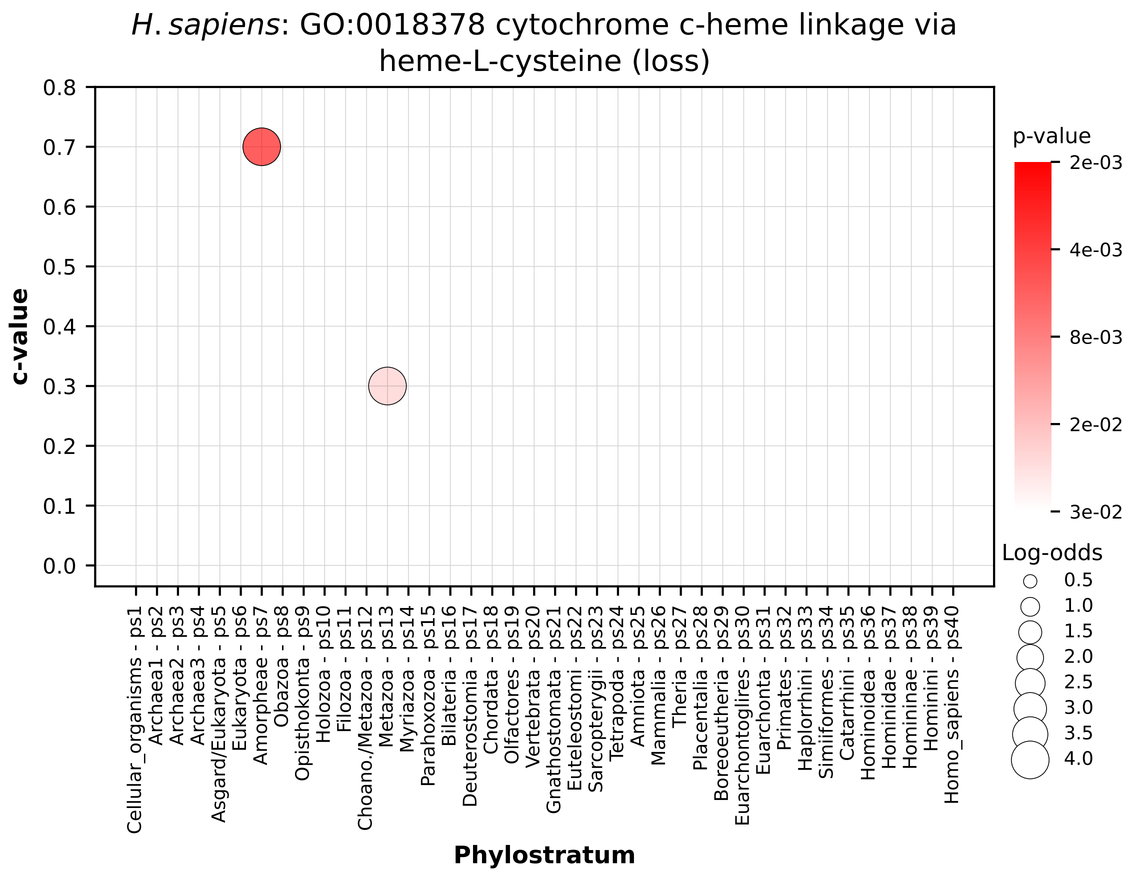

H. sapiens: GO:0018378 cytochrome c-heme linkage via heme-L-cysteine (loss)
c-value
0.8
0.7
0.6
0.5
0.4
0.3
0.2
0.1
0.0
Cellular_organisms - ps1
Archaea1 - ps2
Archaea2 - ps3
Archaea3 - ps4
Asgard/Eukaryota - ps5
Eukaryota - ps6
Amorpheae - ps7
Obazoa - ps8
Opisthokonta - ps9
Holozoa - ps10
Filozoa - ps11
Choano./Metazoa - ps12
Metazoa - ps13
Myriazoa - ps14
Parahoxozoa - ps15
Bilateria - ps16
Deuterostomia - ps17
Chordata - ps18
Olfactores - ps19
Vertebrata - ps20
Gnathostomata - ps21
Euteleostomi - ps22
Sarcopterygii - ps23
Tetrapoda - ps24
Amniota - ps25
Mammalia - ps26
Theria - ps27
Placentalia - ps28
Boreoeutheria - ps29
Euarchontoglires - ps30
Euarchonta - ps31
Primates - ps32
Haplorrhini - ps33
Simiiformes - ps34
Catarrhini - ps35
Hominoidea - ps36
Hominidae - ps37
Homininae - ps38
Hominini - ps39
Homo_sapiens - ps40
Phylostratum
p-value
2e-03
4e-03
8e-03
2e-02
3e-02
Log-odds
0.5
1.0
1.5
2.0
2.5
3.0
3.5
4.0

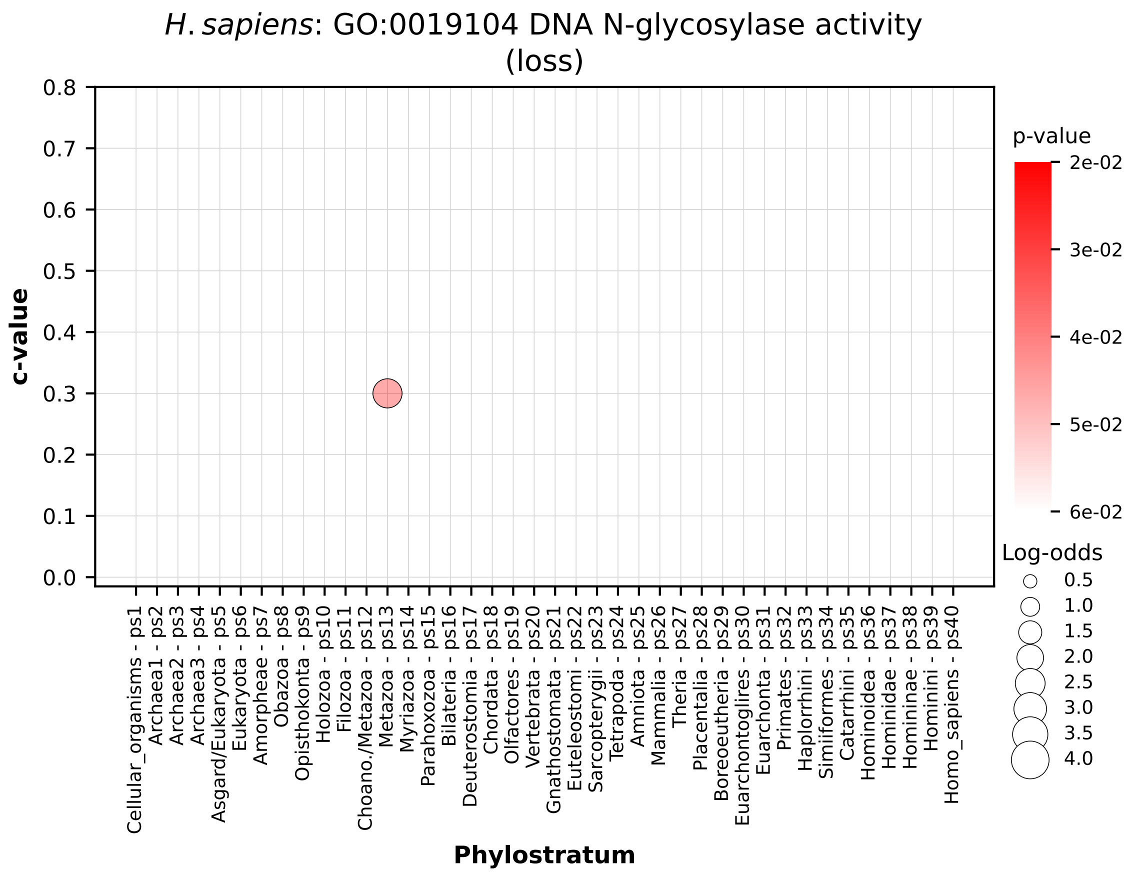
H. sapiens: GO:0019104 DNA N-glycosylase activity (loss)
c-value
0.0
0.1
0.2
0.3
0.4
0.5
0.6
0.7
0.8
Phylostratum
Cellular_organisms - ps1
Archaea1 - ps2
Archaea2 - ps3
Archaea3 - ps4
Asgard/Eukaryota - ps5
Eukaryota - ps6
Amorpheae - ps7
Obazoa - ps8
Opisthokonta - ps9
Holozoa - ps10
Filozoa - ps11
Choano./Metazoa - ps12
Metazoa - ps13
Myriazoa - ps14
Parahoxozoa - ps15
Bilateria - ps16
Deuterostomia - ps17
Chordata - ps18
Olfactores - ps19
Vertebrata - ps20
Gnathostomata - ps21
Euteleostomi - ps22
Sarcopterygii - ps23
Tetrapoda - ps24
Amniota - ps25
Mammalia - ps26
Theria - ps27
Placentalia - ps28
Boreoeutheria - ps29
Euarchontoglires - ps30
Euarchonta - ps31
Primates - ps32
Haplorrhini - ps33
Simiiformes - ps34
Catarrhini - ps35
Hominoidea - ps36
Hominidae - ps37
Homininae - ps38
Hominini - ps39
Homo_sapiens - ps40
p-value
2e-02
3e-02
4e-02
5e-02
6e-02
Log-odds
0.5
1.0
1.5
2.0
2.5
3.0
3.5
4.0

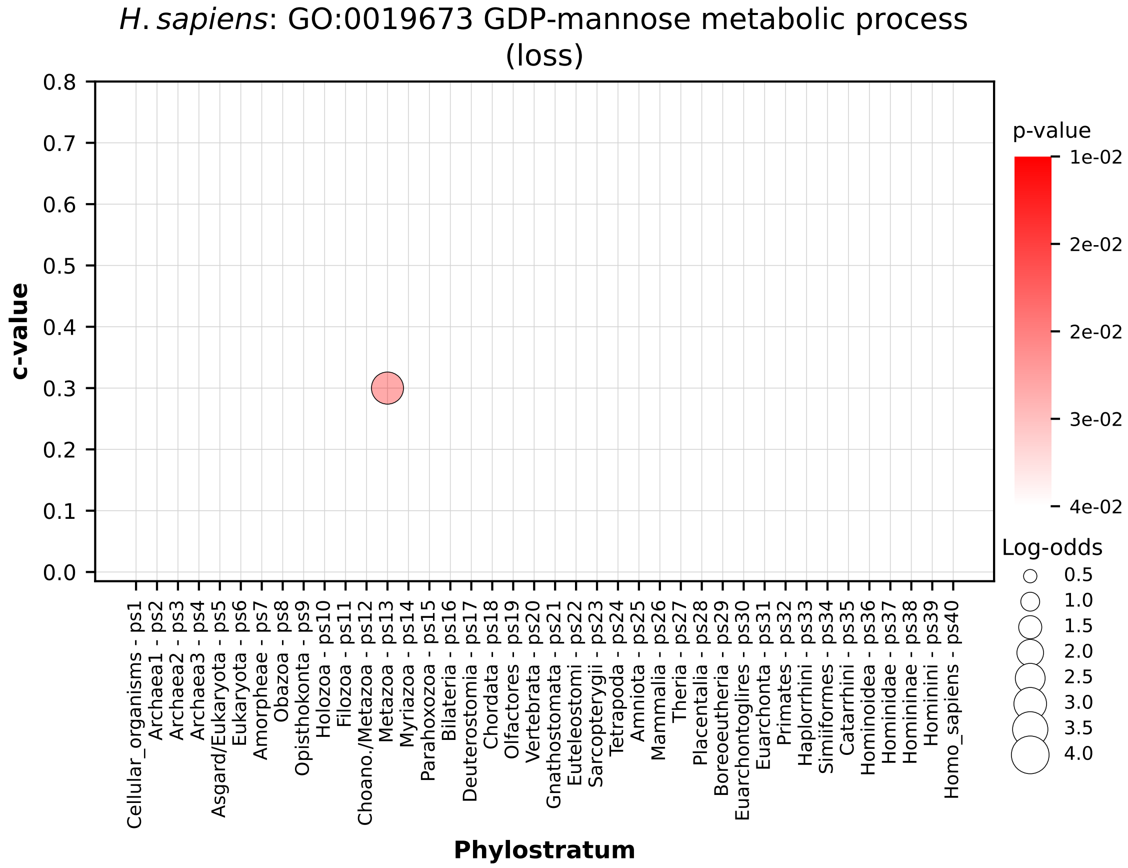
H. sapiens: GO:0019673 GDP-mannose metabolic process
(loss)
c-value
0.8
0.7
0.6
0.5
0.4
0.3
0.2
0.1
0.0
p-value
1e-02
2e-02
2e-02
3e-02
4e-02
Log-odds
0.5
1.0
1.5
2.0
2.5
3.0
3.5
4.0
Cellular_organisms - ps1
Archaea1 - ps2
Archaea2 - ps3
Archaea3 - ps4
Asgard/Eukaryota - ps5
Eukaryota - ps6
Amorpheae - ps7
Obazoa - ps8
Opisthokonta - ps9
Holozoa - ps10
Filozoa - ps11
Choano./Metazoa - ps12
Metazoa - ps13
Myriazoa - ps14
Parahoxozoa - ps15
Bilateria - ps16
Deuterostomia - ps17
Chordata - ps18
Olfactores - ps19
Vertebrata - ps20
Gnathostomata - ps21
Euteleostomi - ps22
Sarcopterygii - ps23
Tetrapoda - ps24
Amniota - ps25
Mammalia - ps26
Theria - ps27
Placentalia - ps28
Boreoeutheria - ps29
Euarchontoglires - ps30
Euarchonta - ps31
Primates - ps32
Haplorrhini - ps33
Simiiformes - ps34
Catarrhini - ps35
Hominoidea - ps36
Hominidae - ps37
Homininae - ps38
Hominini - ps39
Homo_sapiens - ps40
Phylostratum

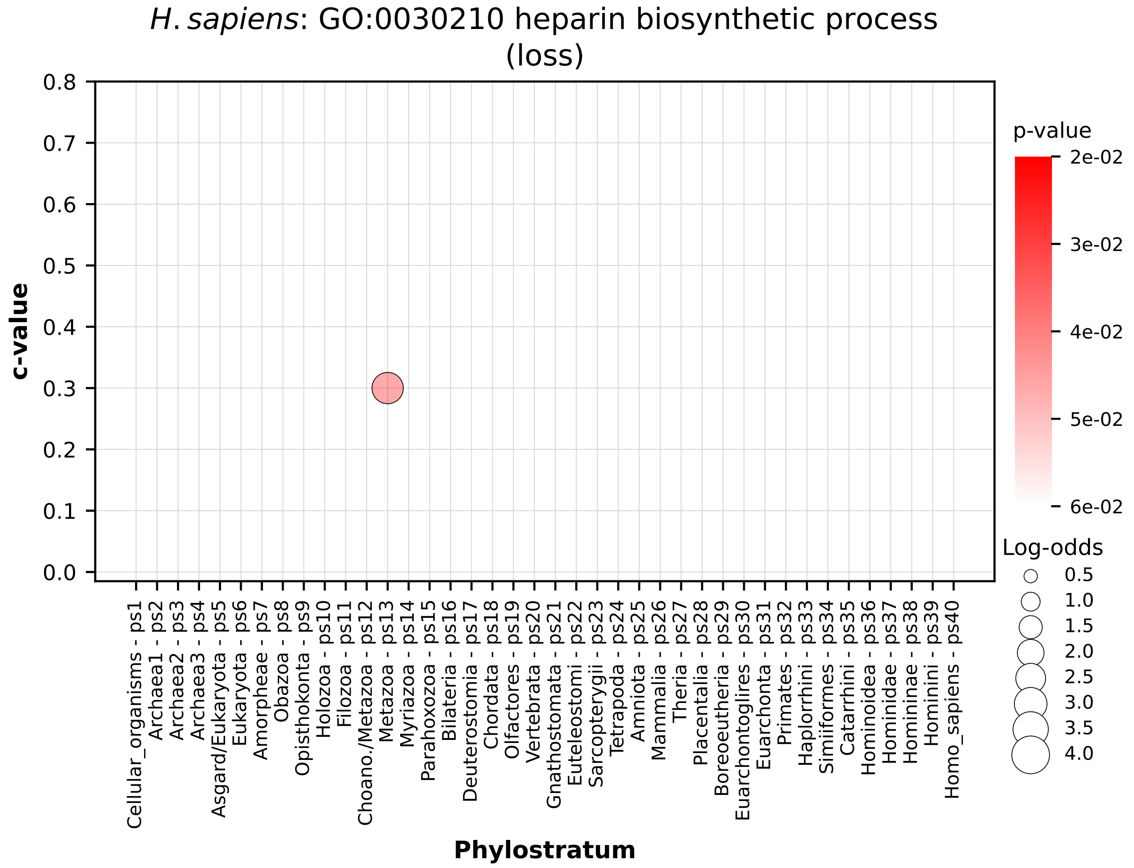

H. sapiens: GO:0030210 heparin biosynthetic process
(loss)
c-value
0.8
0.7
0.6
0.5
0.4
0.3
0.2
0.1
0.0
p-value
2e-02
3e-02
4e-02
5e-02
6e-02
Log-odds
0.5
1.0
1.5
2.0
2.5
3.0
3.5
4.0
Cellular_organisms - ps1
Archaea1 - ps2
Archaea2 - ps3
Archaea3 - ps4
Asgard/Eukaryota - ps5
Eukaryota - ps6
Amorpheae - ps7
Obazoa - ps8
Opisthokonta - ps9
Holozoa - ps10
Filozoa - ps11
Choano./Metazoa - ps12
Metazoa - ps13
Myriazoa - ps14
Parahoxozoa - ps15
Bilateria - ps16
Deuterostomia - ps17
Chordata - ps18
Olfactores - ps19
Vertebrata - ps20
Gnathostomata - ps21
Euteleostomi - ps22
Sarcopterygii - ps23
Tetrapoda - ps24
Amniota - ps25
Mammalia - ps26
Theria - ps27
Placentalia - ps28
Boreoeutheria - ps29
Euarchontoglires - ps30
Euarchonta - ps31
Primates - ps32
Haplorrhini - ps33
Simiiformes - ps34
Catarrhini - ps35
Hominoidea - ps36
Hominidae - ps37
Homininae - ps38
Hominini - ps39
Homo_sapiens - ps40
Phylostratum

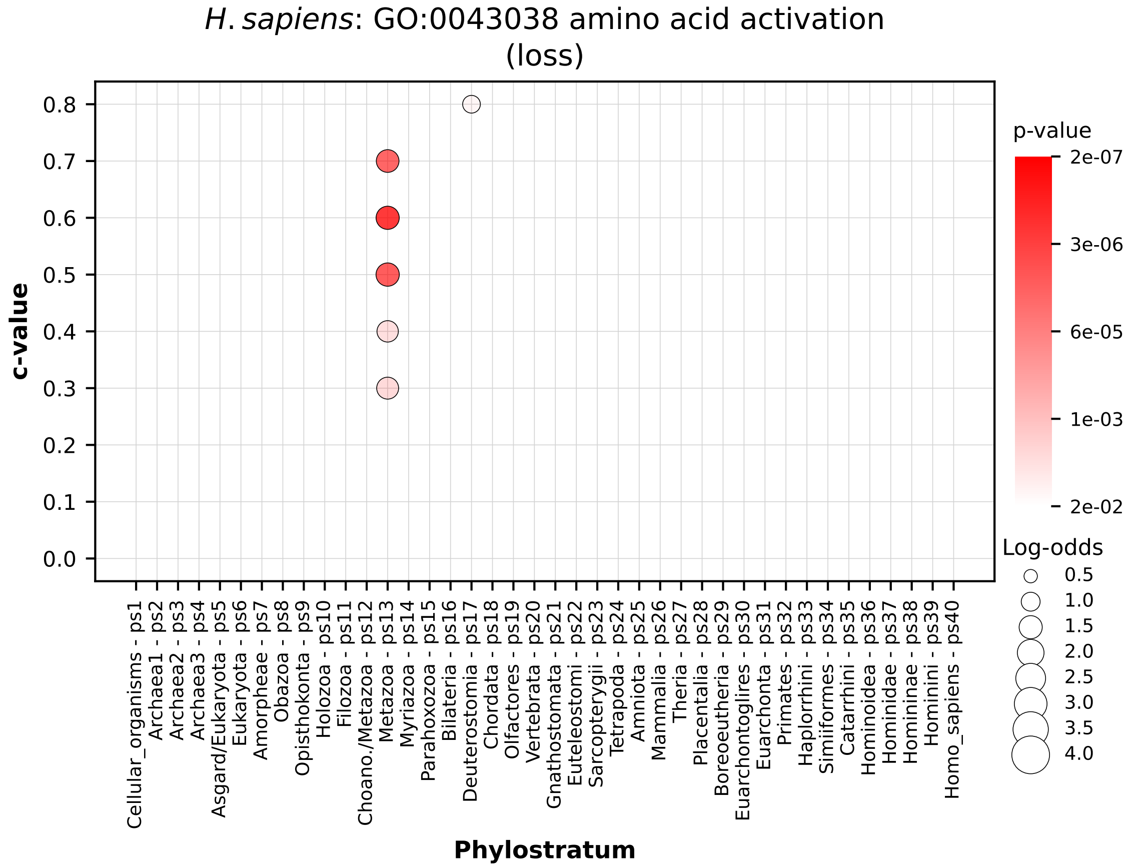

H. sapiens: GO:0043038 amino acid activation
(loss)
c-value
0.8
0.7
0.6
0.5
0.4
0.3
0.2
0.1
0.0
Phylostratum
Cellular_organisms - ps1
Archaea1 - ps2
Archaea2 - ps3
Archaea3 - ps4
Asgard/Eukaryota - ps5
Eukaryota - ps6
Amorpheae - ps7
Obazoa - ps8
Opisthokonta - ps9
Holozoa - ps10
Filozoa - ps11
Choano./Metazoa - ps12
Metazoa - ps13
Myriazoa - ps14
Parahoxozoa - ps15
Bilateria - ps16
Deuterostomia - ps17
Chordata - ps18
Olfactores - ps19
Vertebrata - ps20
Gnathostomata - ps21
Euteleostomi - ps22
Sarcopterygii - ps23
Tetrapoda - ps24
Amniota - ps25
Mammalia - ps26
Theria - ps27
Placentalia - ps28
Boreoeutheria - ps29
Euarchontoglires - ps30
Euarchonta - ps31
Primates - ps32
Haplorrhini - ps33
Simiiformes - ps34
Catarrhini - ps35
Hominoidea - ps36
Hominidae - ps37
Homininae - ps38
Hominini - ps39
Homo_sapiens - ps40
p-value
2e-07
3e-06
6e-05
1e-03
2e-02
Log-odds
0.5
1.0
1.5
2.0
2.5
3.0
3.5
4.0

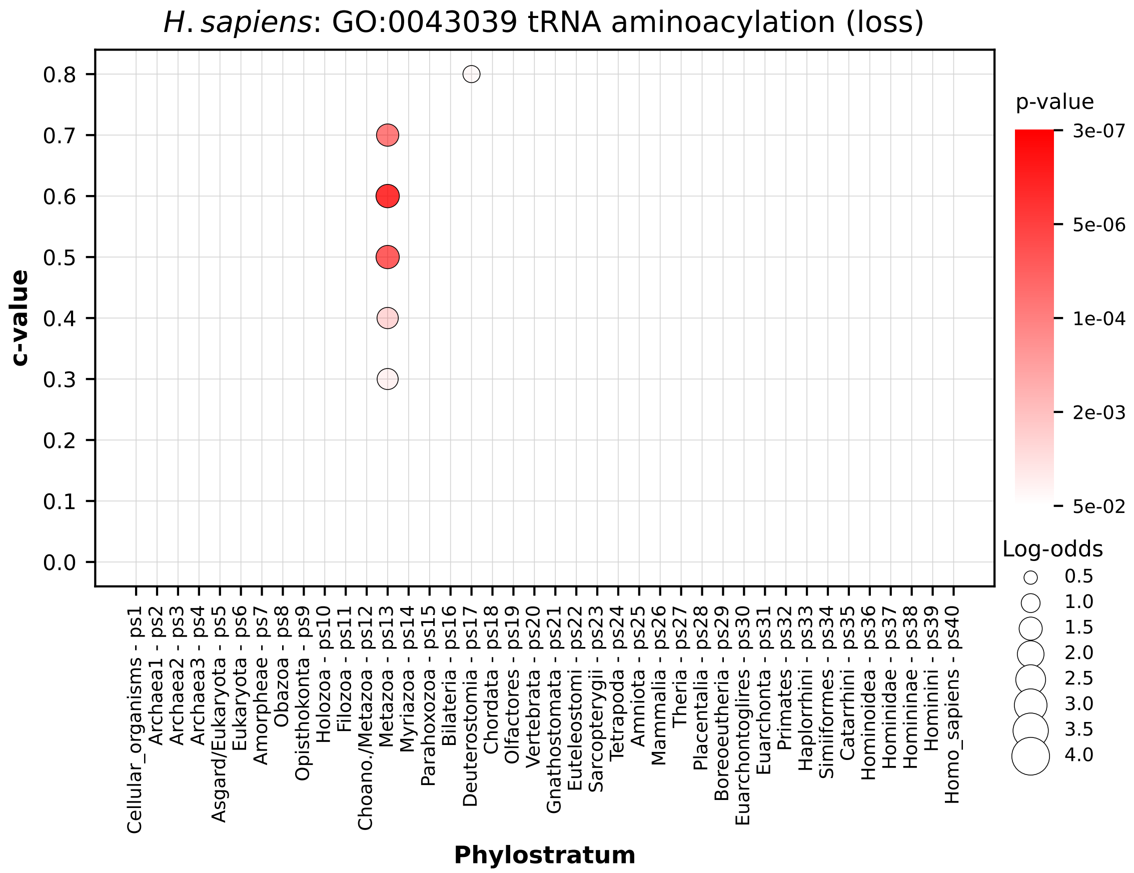

H. sapiens: GO:0043039 tRNA aminoacylation (loss)
c-value
0.8
0.7
0.6
0.5
0.4
0.3
0.2
0.1
0.0
Phylostratum
Cellular_organisms - ps1
Archaea1 - ps2
Archaea2 - ps3
Archaea3 - ps4
Asgard/Eukaryota - ps5
Eukaryota - ps6
Amorpheae - ps7
Obazoa - ps8
Opisthokonta - ps9
Holozoa - ps10
Filozoa - ps11
Choano./Metazoa - ps12
Metazoa - ps13
Myriazoa - ps14
Parahoxozoa - ps15
Bilateria - ps16
Deuterostomia - ps17
Chordata - ps18
Olfactores - ps19
Vertebrata - ps20
Gnathostomata - ps21
Euteleostomi - ps22
Sarcopterygii - ps23
Tetrapoda - ps24
Amniota - ps25
Mammalia - ps26
Theria - ps27
Placentalia - ps28
Boreoeutheria - ps29
Euarchontoglires - ps30
Euarchonta - ps31
Primates - ps32
Haplorrhini - ps33
Simiiformes - ps34
Catarrhini - ps35
Hominoidea - ps36
Hominidae - ps37
Homininae - ps38
Hominini - ps39
Homo_sapiens - ps40
p-value
3e-07
5e-06
1e-04
2e-03
5e-02
Log-odds
0.5
1.0
1.5
2.0
2.5
3.0
3.5
4.0

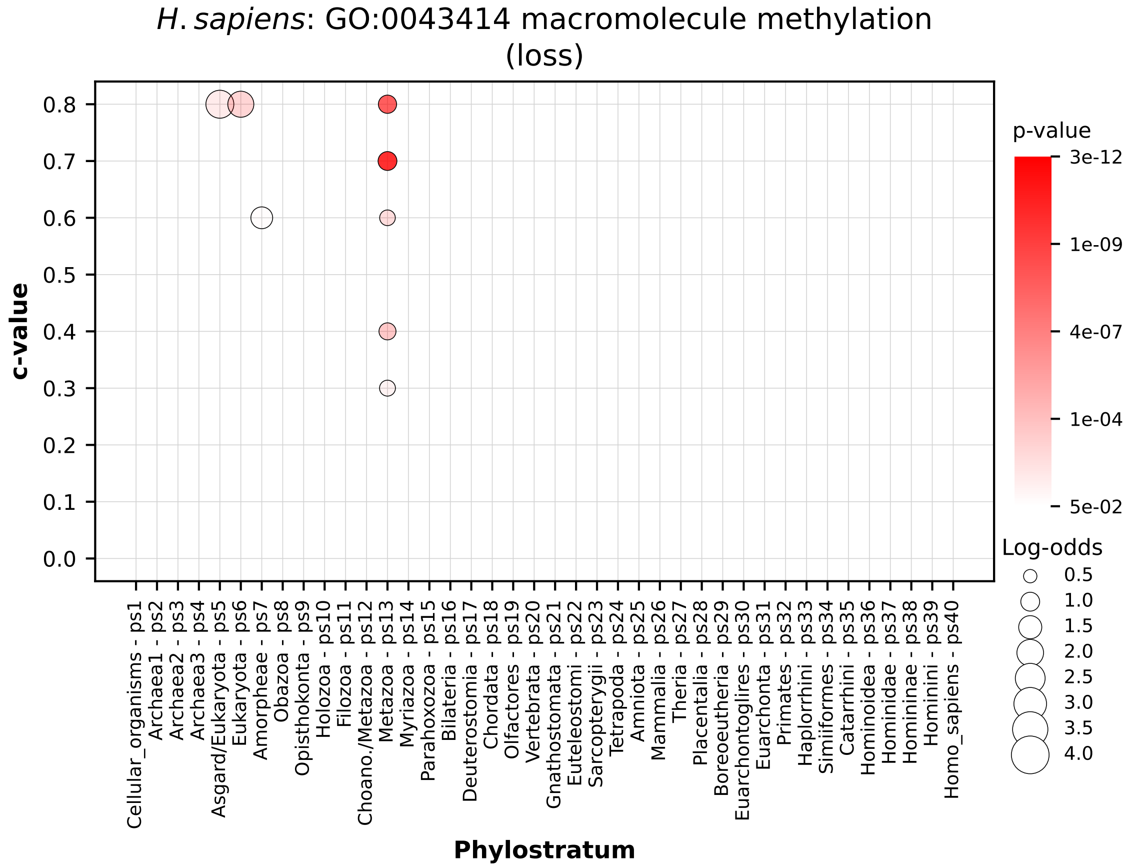

H. sapiens: GO:0043414 macromolecule methylation (loss)
c-value
0.8
0.7
0.6
0.5
0.4
0.3
0.2
0.1
0.0
p-value
3e-12
1e-09
4e-07
1e-04
5e-02
Log-odds
0.5
1.0
1.5
2.0
2.5
3.0
3.5
4.0
Cellular_organisms - ps1
Archaea1 - ps2
Archaea2 - ps3
Archaea3 - ps4
Asgard/Eukaryota - ps5
Eukaryota - ps6
Amorpheae - ps7
Obazoa - ps8
Opisthokonta - ps9
Holozoa - ps10
Filozoa - ps11
Choano./Metazoa - ps12
Metazoa - ps13
Myriazoa - ps14
Parahoxozoa - ps15
Bilateria - ps16
Deuterostomia - ps17
Chordata - ps18
Olfactores - ps19
Vertebrata - ps20
Gnathostomata - ps21
Euteleostomi - ps22
Sarcopterygii - ps23
Tetrapoda - ps24
Amniota - ps25
Mammalia - ps26
Theria - ps27
Placentalia - ps28
Boreoeutheria - ps29
Euarchontoglires - ps30
Euarchonta - ps31
Primates - ps32
Haplorrhini - ps33
Simiiformes - ps34
Catarrhini - ps35
Hominoidea - ps36
Hominidae - ps37
Homininae - ps38
Hominini - ps39
Homo_sapiens - ps40
Phylostratum

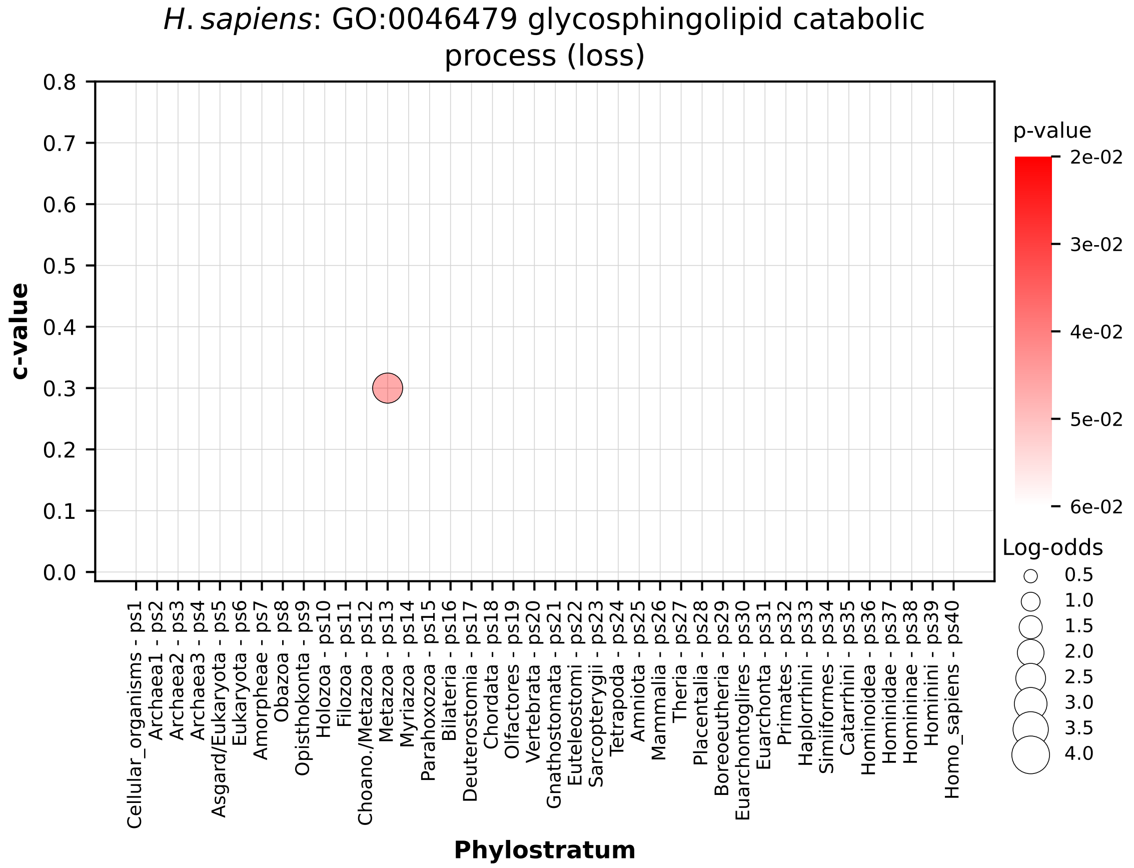

H. sapiens: GO:0046479 glycosphingolipid catabolic process (loss)
c-value
0.8
0.7
0.6
0.5
0.4
0.3
0.2
0.1
0.0
Phylostratum
Cellular_organisms - ps1
Archaea1 - ps2
Archaea2 - ps3
Archaea3 - ps4
Asgard/Eukaryota - ps5
Eukaryota - ps6
Amorpheae - ps7
Obazoa - ps8
Opisthokonta - ps9
Holozoa - ps10
Filozoa - ps11
Choano./Metazoa - ps12
Metazoa - ps13
Myriazoa - ps14
Parahoxozoa - ps15
Bilateria - ps16
Deuterostomia - ps17
Chordata - ps18
Olfactores - ps19
Vertebrata - ps20
Gnathostomata - ps21
Euteleostomi - ps22
Sarcopterygii - ps23
Tetrapoda - ps24
Amniota - ps25
Mammalia - ps26
Theria - ps27
Placentalia - ps28
Boreoeutheria - ps29
Euarchontoglires - ps30
Euarchonta - ps31
Primates - ps32
Haplorrhini - ps33
Simiiformes - ps34
Catarrhini - ps35
Hominoidea - ps36
Hominidae - ps37
Homininae - ps38
Hominini - ps39
Homo_sapiens - ps40
p-value
2e-02
3e-02
4e-02
5e-02
6e-02
Log-odds
0.5
1.0
1.5
2.0
2.5
3.0
3.5
4.0

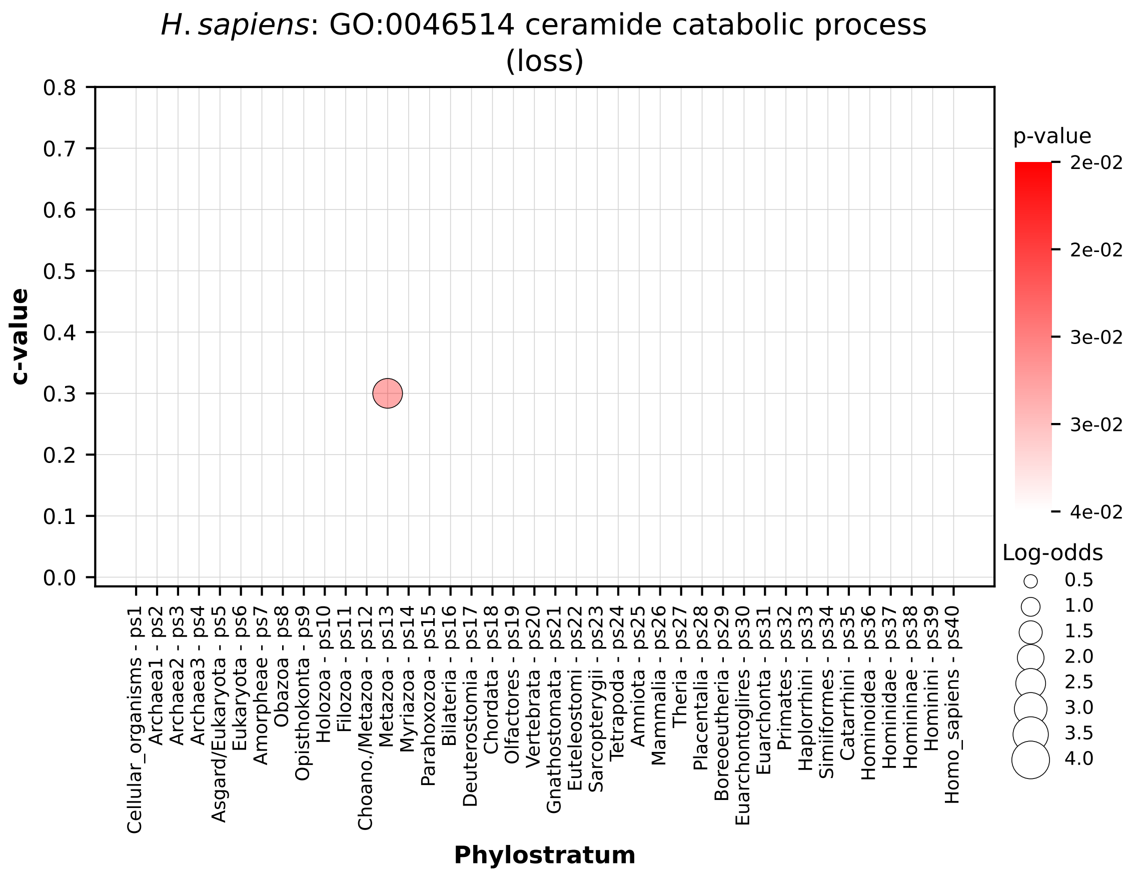

H. sapiens: GO:0046514 ceramide catabolic process
(loss)
c-value
0.8
0.7
0.6
0.5
0.4
0.3
0.2
0.1
0.0
p-value
2e-02
2e-02
3e-02
3e-02
4e-02
Log-odds
0.5
1.0
1.5
2.0
2.5
3.0
3.5
4.0
Cellular_organisms - ps1
Archaea1 - ps2
Archaea2 - ps3
Archaea3 - ps4
Asgard/Eukaryota - ps5
Eukaryota - ps6
Amorpheae - ps7
Obazoa - ps8
Opisthokonta - ps9
Holozoa - ps10
Filozoa - ps11
Choano./Metazoa - ps12
Metazoa - ps13
Myriazoa - ps14
Parahoxozoa - ps15
Bilateria - ps16
Deuterostomia - ps17
Chordata - ps18
Olfactores - ps19
Vertebrata - ps20
Gnathostomata - ps21
Euteleostomi - ps22
Sarcopterygii - ps23
Tetrapoda - ps24
Amniota - ps25
Mammalia - ps26
Theria - ps27
Placentalia - ps28
Boreoeutheria - ps29
Euarchontoglires - ps30
Euarchonta - ps31
Primates - ps32
Haplorrhini - ps33
Simiiformes - ps34
Catarrhini - ps35
Hominoidea - ps36
Hominidae - ps37
Homininae - ps38
Hominini - ps39
Homo_sapiens - ps40
Phylostratum

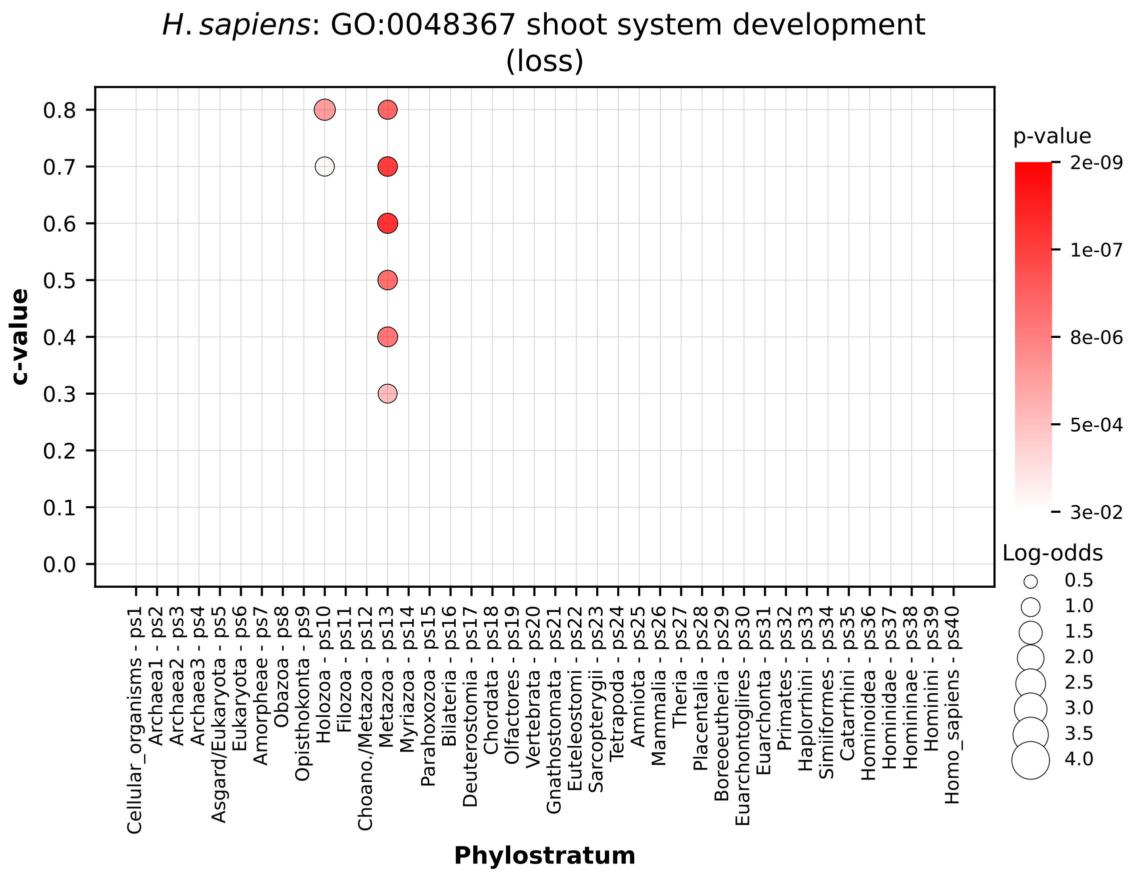

H. sapiens: GO:0048367 shoot system development
(loss)
c-value
0.8
0.7
0.6
0.5
0.4
0.3
0.2
0.1
0.0
Phylostratum
Cellular_organisms - ps1
Archaea1 - ps2
Archaea2 - ps3
Archaea3 - ps4
Asgard/Eukaryota - ps5
Eukaryota - ps6
Amorpheae - ps7
Obazoa - ps8
Opisthokonta - ps9
Holozoa - ps10
Filozoa - ps11
Choano./Metazoa - ps12
Metazoa - ps13
Myriazoa - ps14
Parahoxozoa - ps15
Bilateria - ps16
Deuterostomia - ps17
Chordata - ps18
Olfactores - ps19
Vertebrata - ps20
Gnathostomata - ps21
Euteleostomi - ps22
Sarcopterygii - ps23
Tetrapoda - ps24
Amniota - ps25
Mammalia - ps26
Theria - ps27
Placentalia - ps28
Boreoeutheria - ps29
Euarchontoglires - ps30
Euarchonta - ps31
Primates - ps32
Haplorrhini - ps33
Simiiformes - ps34
Catarrhini - ps35
Hominoidea - ps36
Hominidae - ps37
Homininae - ps38
Hominini - ps39
Homo_sapiens - ps40
p-value
2e-09
1e-07
8e-06
5e-04
3e-02
Log-odds
0.5
1.0
1.5
2.0
2.5
3.0
3.5
4.0

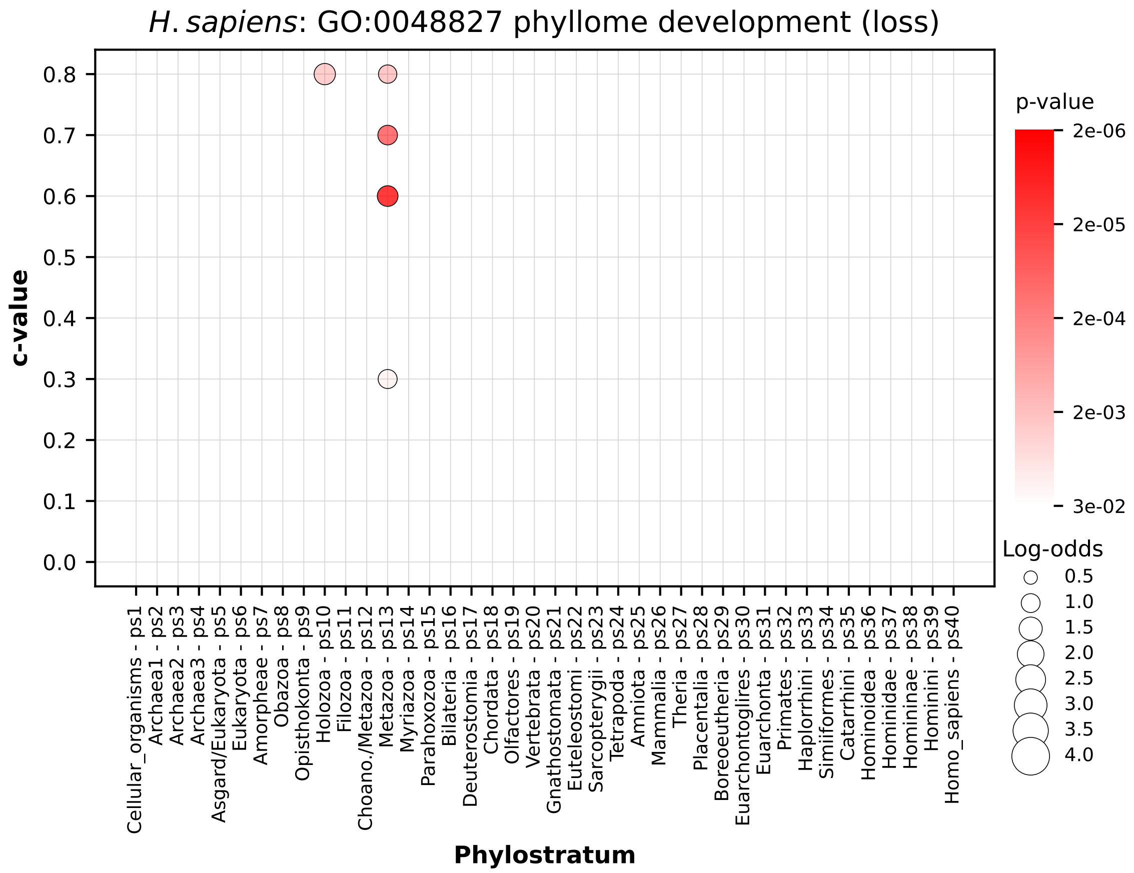
H. sapiens: GO:0048827 phyllome development (loss)
c-value
0.0
0.1
0.2
0.3
0.4
0.5
0.6
0.7
0.8
p-value
2e-06
2e-05
2e-04
2e-03
3e-02
Log-odds
0.5
1.0
1.5
2.0
2.5
3.0
3.5
4.0
Cellular_organisms - ps1
Archaea1 - ps2
Archaea2 - ps3
Archaea3 - ps4
Asgard/Eukaryota - ps5
Eukaryota - ps6
Amorpheae - ps7
Obazoa - ps8
Opisthokonta - ps9
Holozoa - ps10
Filozoa - ps11
Choano./Metazoa - ps12
Metazoa - ps13
Myriazoa - ps14
Parahoxozoa - ps15
Bilateria - ps16
Deuterostomia - ps17
Chordata - ps18
Olfactores - ps19
Vertebrata - ps20
Gnathostomata - ps21
Euteleostomi - ps22
Sarcopterygii - ps23
Tetrapoda - ps24
Amniota - ps25
Mammalia - ps26
Theria - ps27
Placentalia - ps28
Boreoeutheria - ps29
Euarchontoglires - ps30
Euarchonta - ps31
Primates - ps32
Haplorrhini - ps33
Simiiformes - ps34
Catarrhini - ps35
Hominoidea - ps36
Hominidae - ps37
Homininae - ps38
Hominini - ps39
Homo_sapiens - ps40
Phylostratum

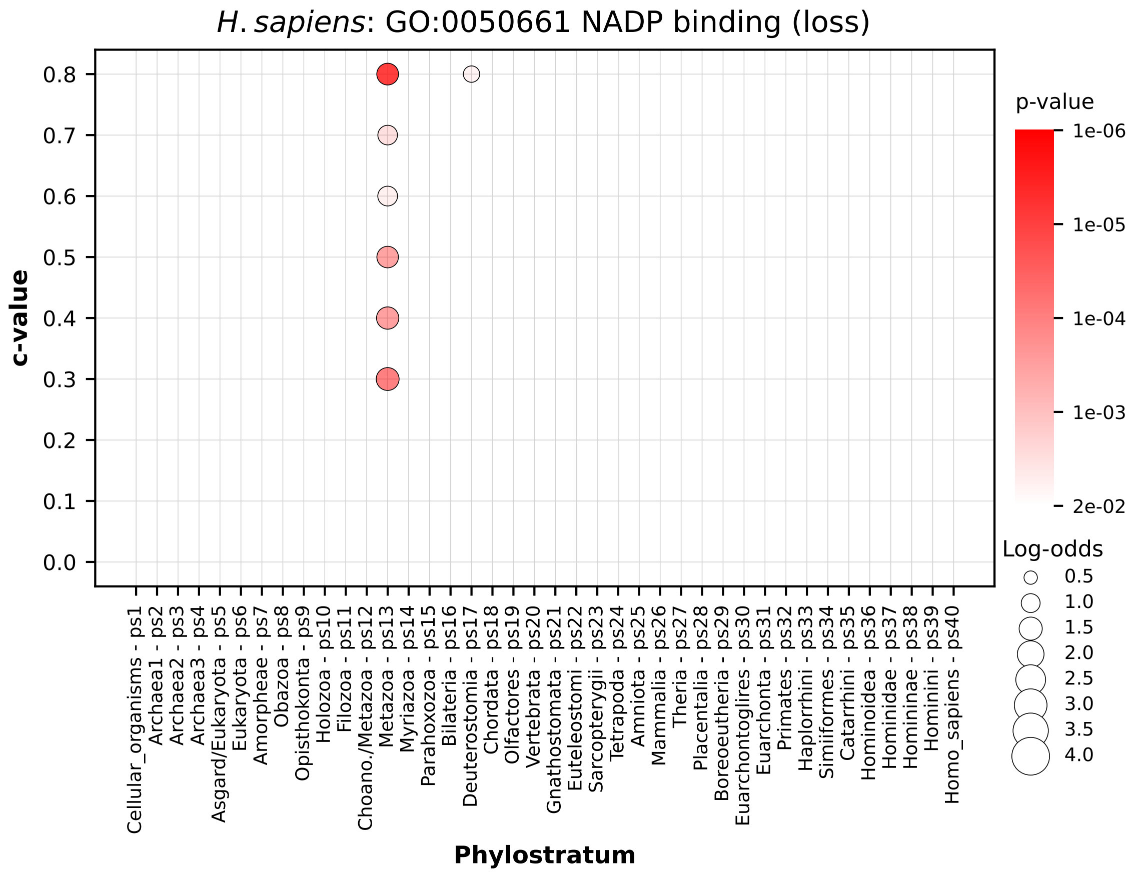

H. sapiens: GO:0050661 NADP binding (loss)
c-value
0.8
0.7
0.6
0.5
0.4
0.3
0.2
0.1
0.0
p-value
1e-06
1e-05
1e-04
1e-03
2e-02
Log-odds
0.5
1.0
1.5
2.0
2.5
3.0
3.5
4.0
Cellular_organisms - ps1
Archaea1 - ps2
Archaea2 - ps3
Archaea3 - ps4
Asgard/Eukaryota - ps5
Eukaryota - ps6
Amorpheae - ps7
Obazoa - ps8
Opisthokonta - ps9
Holozoa - ps10
Filozoa - ps11
Choano./Metazoa - ps12
Metazoa - ps13
Myriazoa - ps14
Parahoxozoa - ps15
Bilateria - ps16
Deuterostomia - ps17
Chordata - ps18
Olfactores - ps19
Vertebrata - ps20
Gnathostomata - ps21
Euteleostomi - ps22
Sarcopterygii - ps23
Tetrapoda - ps24
Amniota - ps25
Mammalia - ps26
Theria - ps27
Placentalia - ps28
Boreoeutheria - ps29
Euarchontoglires - ps30
Euarchonta - ps31
Primates - ps32
Haplorrhini - ps33
Simiiformes - ps34
Catarrhini - ps35
Hominoidea - ps36
Hominidae - ps37
Homininae - ps38
Hominini - ps39
Homo_sapiens - ps40
Phylostratum

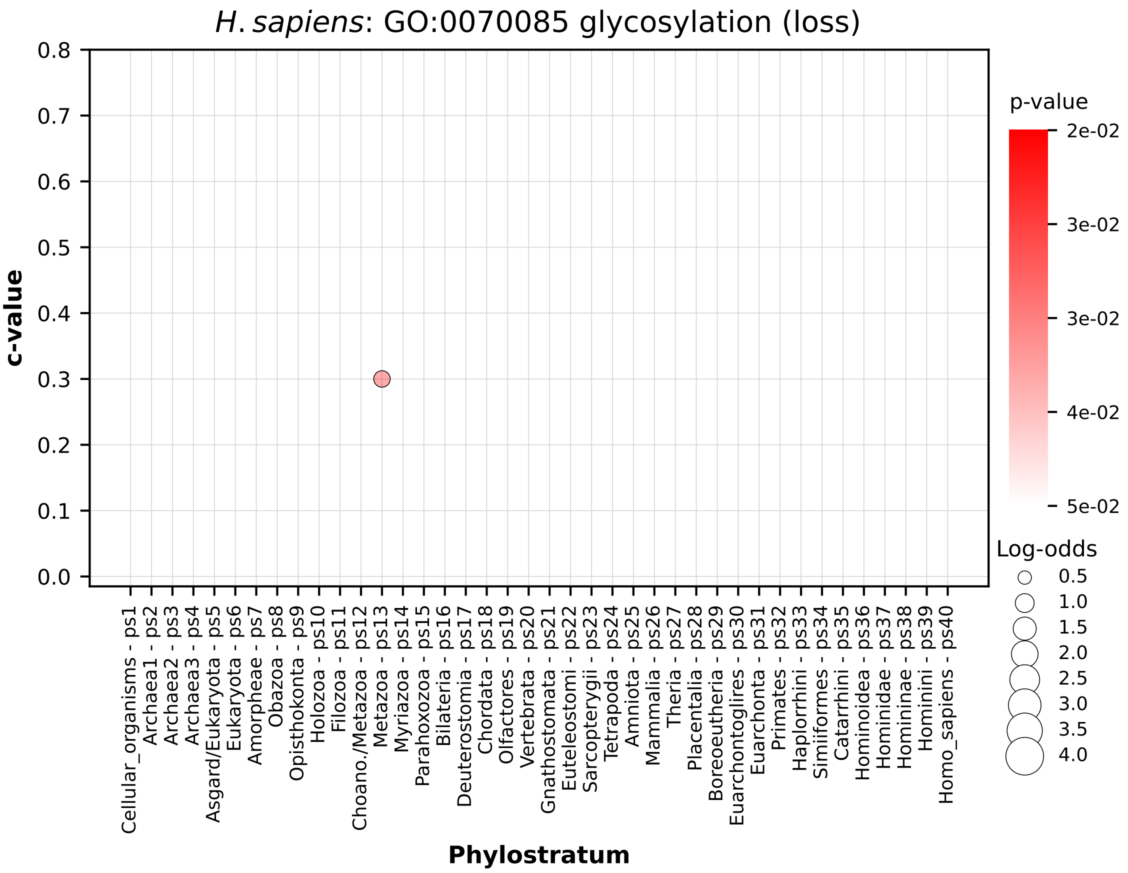

H. sapiens: GO:0070085 glycosylation (loss)
c-value
0.0
0.1
0.2
0.3
0.4
0.5
0.6
0.7
0.8
Phylostratum
Cellular_organisms - ps1
Archaea1 - ps2
Archaea2 - ps3
Archaea3 - ps4
Asgard/Eukaryota - ps5
Eukaryota - ps6
Amorpheae - ps7
Obazoa - ps8
Opisthokonta - ps9
Holozoa - ps10
Filozoa - ps11
Choano./Metazoa - ps12
Metazoa - ps13
Myriazoa - ps14
Parahoxozoa - ps15
Bilateria - ps16
Deuterostomia - ps17
Chordata - ps18
Olfactores - ps19
Vertebrata - ps20
Gnathostomata - ps21
Euteleostomi - ps22
Sarcopterygii - ps23
Tetrapoda - ps24
Amniota - ps25
Mammalia - ps26
Theria - ps27
Placentalia - ps28
Boreoeutheria - ps29
Euarchontoglires - ps30
Euarchonta - ps31
Primates - ps32
Haplorrhini - ps33
Simiiformes - ps34
Catarrhini - ps35
Hominoidea - ps36
Hominidae - ps37
Homininae - ps38
Hominini - ps39
Homo_sapiens - ps40
p-value
2e-02
3e-02
3e-02
4e-02
5e-02
Log-odds
0.5
1.0
1.5
2.0
2.5
3.0
3.5
4.0

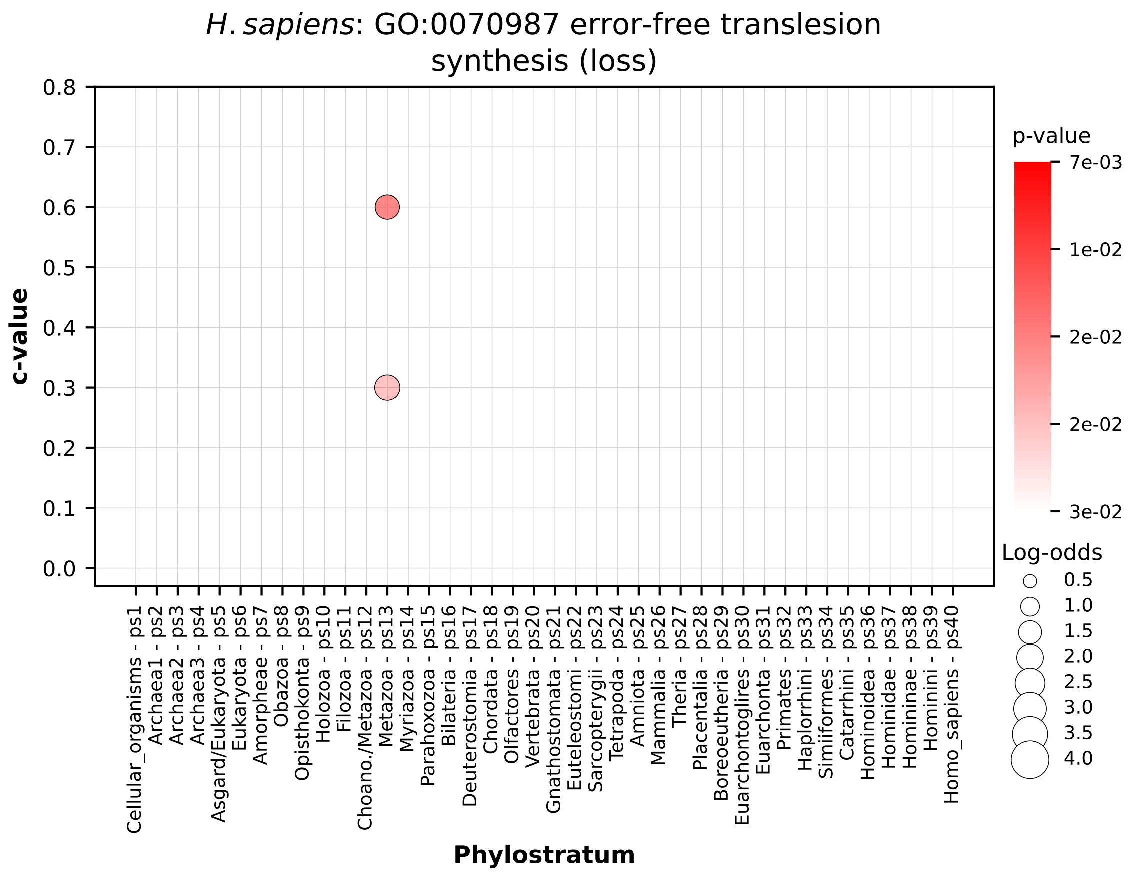

H. sapiens: GO:0070987 error-free translesion synthesis (loss)
c-value
0.8
0.7
0.6
0.5
0.4
0.3
0.2
0.1
0.0
p-value
7e-03
1e-02
2e-02
2e-02
3e-02
Log-odds
0.5
1.0
1.5
2.0
2.5
3.0
3.5
4.0
Cellular_organisms - ps1
Archaea1 - ps2
Archaea2 - ps3
Archaea3 - ps4
Asgard/Eukaryota - ps5
Eukaryota - ps6
Amorpheae - ps7
Obazoa - ps8
Opisthokonta - ps9
Holozoa - ps10
Filozoa - ps11
Choano./Metazoa - ps12
Metazoa - ps13
Myriazoa - ps14
Parahoxozoa - ps15
Bilateria - ps16
Deuterostomia - ps17
Chordata - ps18
Olfactores - ps19
Vertebrata - ps20
Gnathostomata - ps21
Euteleostomi - ps22
Sarcopterygii - ps23
Tetrapoda - ps24
Amniota - ps25
Mammalia - ps26
Theria - ps27
Placentalia - ps28
Boreoeutheria - ps29
Euarchontoglires - ps30
Euarchonta - ps31
Primates - ps32
Haplorrhini - ps33
Simiiformes - ps34
Catarrhini - ps35
Hominoidea - ps36
Hominidae - ps37
Homininae - ps38
Hominini - ps39
Homo_sapiens - ps40
Phylostratum

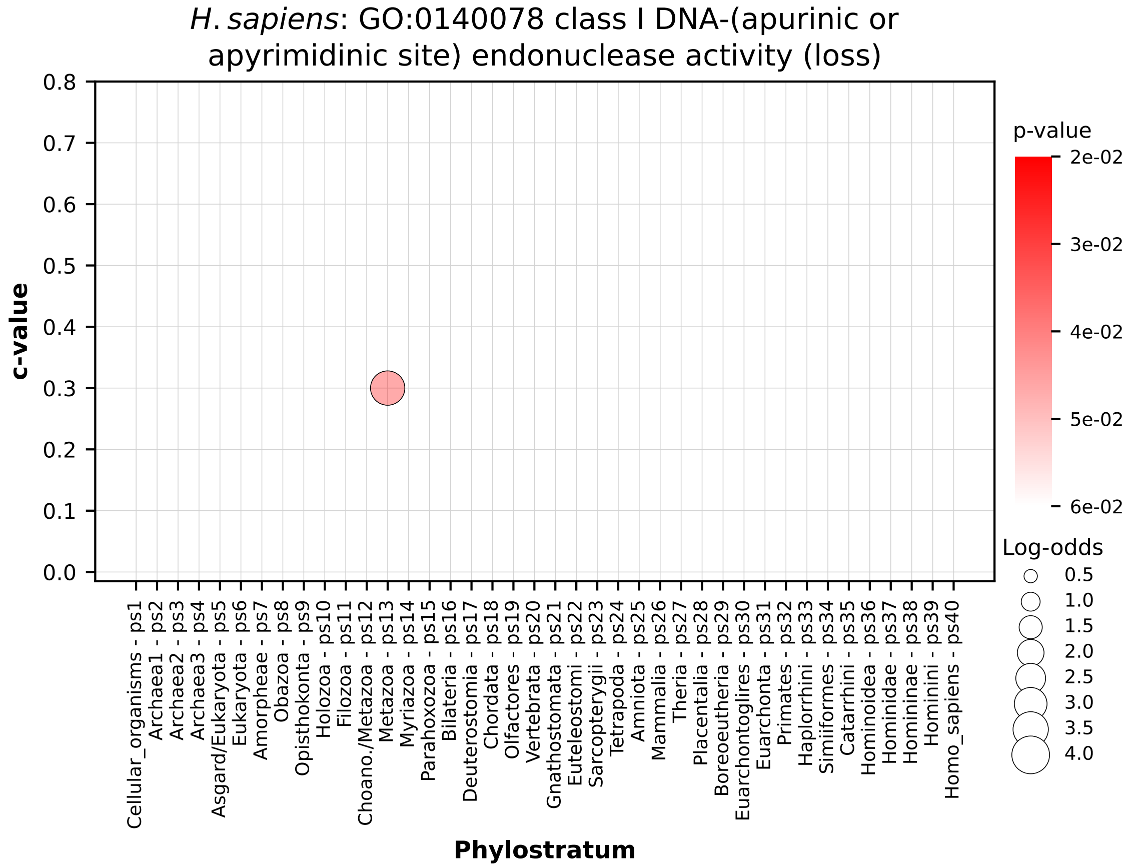

H. sapiens: GO:0140078 class I DNA-(apurinic or apyrimidinic site) endonuclease activity (loss)
c-value
0.8
0.7
0.6
0.5
0.4
0.3
0.2
0.1
0.0
p-value
2e-02
3e-02
4e-02
5e-02
6e-02
Log-odds
0.5
1.0
1.5
2.0
2.5
3.0
3.5
4.0
Cellular_organisms - ps1
Archaea1 - ps2
Archaea2 - ps3
Archaea3 - ps4
Asgard/Eukaryota - ps5
Eukaryota - ps6
Amorpheae - ps7
Obazoa - ps8
Opisthokonta - ps9
Holozoa - ps10
Filozoa - ps11
Choano./Metazoa - ps12
Metazoa - ps13
Myriazoa - ps14
Parahoxozoa - ps15
Bilateria - ps16
Deuterostomia - ps17
Chordata - ps18
Olfactores - ps19
Vertebrata - ps20
Gnathostomata - ps21
Euteleostomi - ps22
Sarcopterygii - ps23
Tetrapoda - ps24
Amniota - ps25
Mammalia - ps26
Theria - ps27
Placentalia - ps28
Boreoeutheria - ps29
Euarchontoglires - ps30
Euarchonta - ps31
Primates - ps32
Haplorrhini - ps33
Simiiformes - ps34
Catarrhini - ps35
Hominoidea - ps36
Hominidae - ps37
Homininae - ps38
Hominini - ps39
Homo_sapiens - ps40
Phylostratum

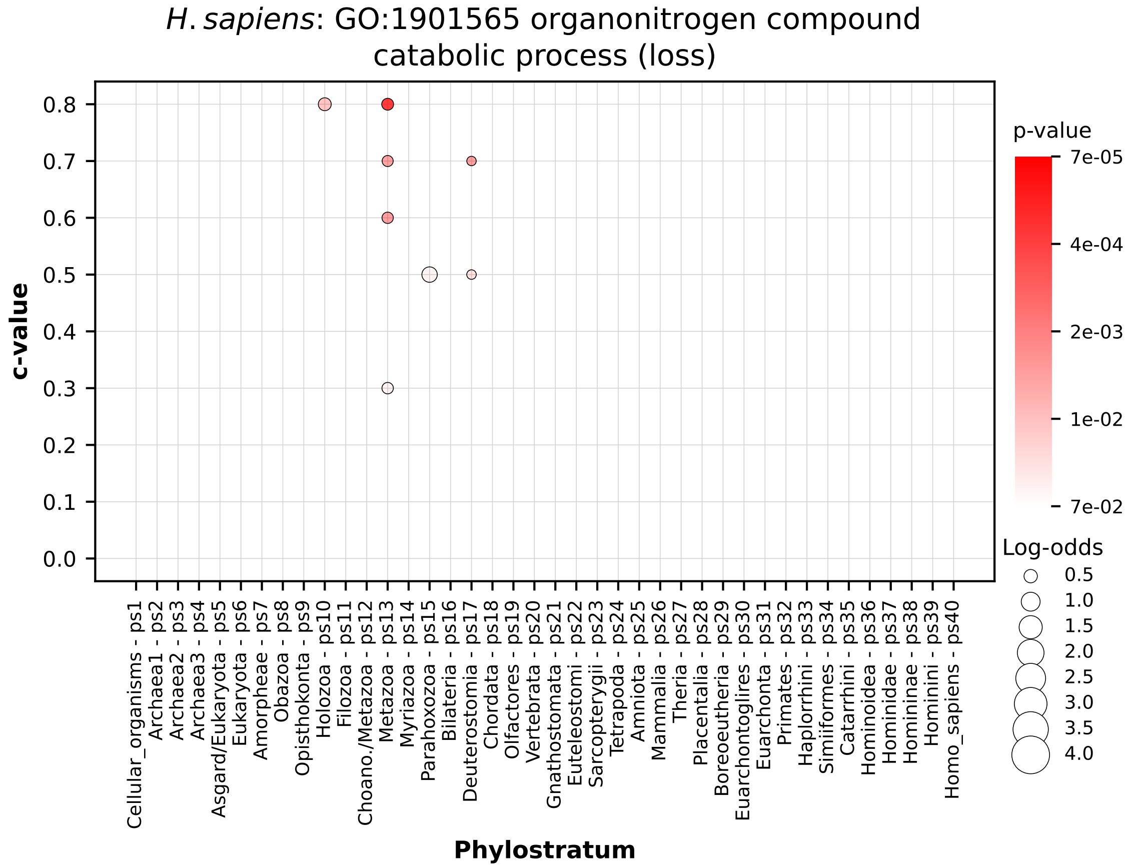
H. sapiens: GO:1901565 organonitrogen compound catabolic process (loss)
c-value
0.8
0.7
0.6
0.5
0.4
0.3
0.2
0.1
0.0
Phylostratum
Cellular_organisms - ps1
Archaea1 - ps2
Archaea2 - ps3
Archaea3 - ps4
Asgard/Eukaryota - ps5
Eukaryota - ps6
Amorpheae - ps7
Obazoa - ps8
Opisthokonta - ps9
Holozoa - ps10
Filozoa - ps11
Choano./Metazoa - ps12
Metazoa - ps13
Myriazoa - ps14
Parahoxozoa - ps15
Bilateria - ps16
Deuterostomia - ps17
Chordata - ps18
Olfactores - ps19
Vertebrata - ps20
Gnathostomata - ps21
Euteleostomi - ps22
Sarcopterygii - ps23
Tetrapoda - ps24
Amniota - ps25
Mammalia - ps26
Theria - ps27
Placentalia - ps28
Boreoeutheria - ps29
Euarchontoglires - ps30
Euarchonta - ps31
Primates - ps32
Haplorrhini - ps33
Simiiformes - ps34
Catarrhini - ps35
Hominoidea - ps36
Hominidae - ps37
Homininae - ps38
Hominini - ps39
Homo_sapiens - ps40
p-value
7e-05
4e-04
2e-03
1e-02
7e-02
Log-odds
0.5
1.0
1.5
2.0
2.5
3.0
3.5
4.0

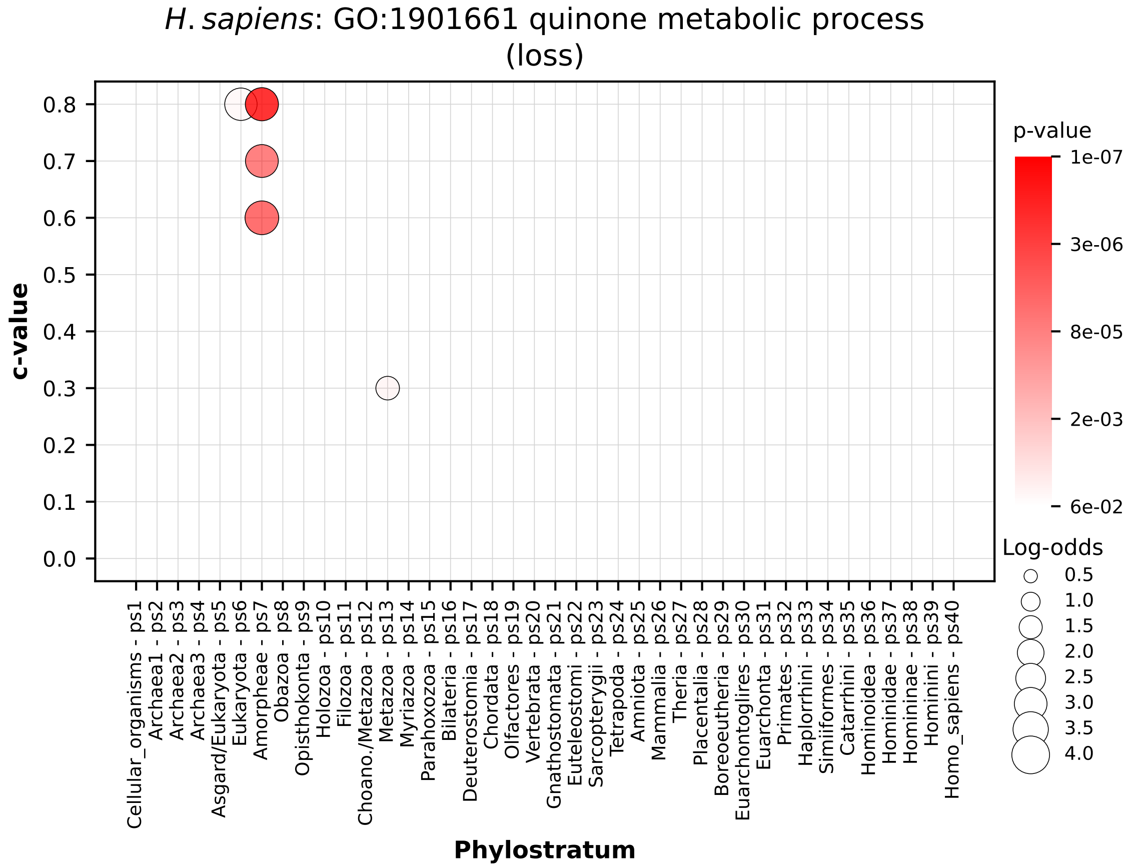

H. sapiens: GO:1901661 quinone metabolic process
(loss)
c-value
0.8
0.7
0.6
0.5
0.4
0.3
0.2
0.1
0.0
Phylostratum
Cellular_organisms - ps1
Archaea1 - ps2
Archaea2 - ps3
Archaea3 - ps4
Asgard/Eukaryota - ps5
Eukaryota - ps6
Amorpheae - ps7
Obazoa - ps8
Opisthokonta - ps9
Holozoa - ps10
Filozoa - ps11
Choano./Metazoa - ps12
Metazoa - ps13
Myriazoa - ps14
Parahoxozoa - ps15
Bilateria - ps16
Deuterostomia - ps17
Chordata - ps18
Olfactores - ps19
Vertebrata - ps20
Gnathostomata - ps21
Euteleostomi - ps22
Sarcopterygii - ps23
Tetrapoda - ps24
Amniota - ps25
Mammalia - ps26
Theria - ps27
Placentalia - ps28
Boreoeutheria - ps29
Euarchontoglires - ps30
Euarchonta - ps31
Primates - ps32
Haplorrhini - ps33
Simiiformes - ps34
Catarrhini - ps35
Hominoidea - ps36
Hominidae - ps37
Homininae - ps38
Hominini - ps39
Homo_sapiens - ps40
p-value
1e-07
3e-06
8e-05
2e-03
6e-02
Log-odds
0.5
1.0
1.5
2.0
2.5
3.0
3.5
4.0

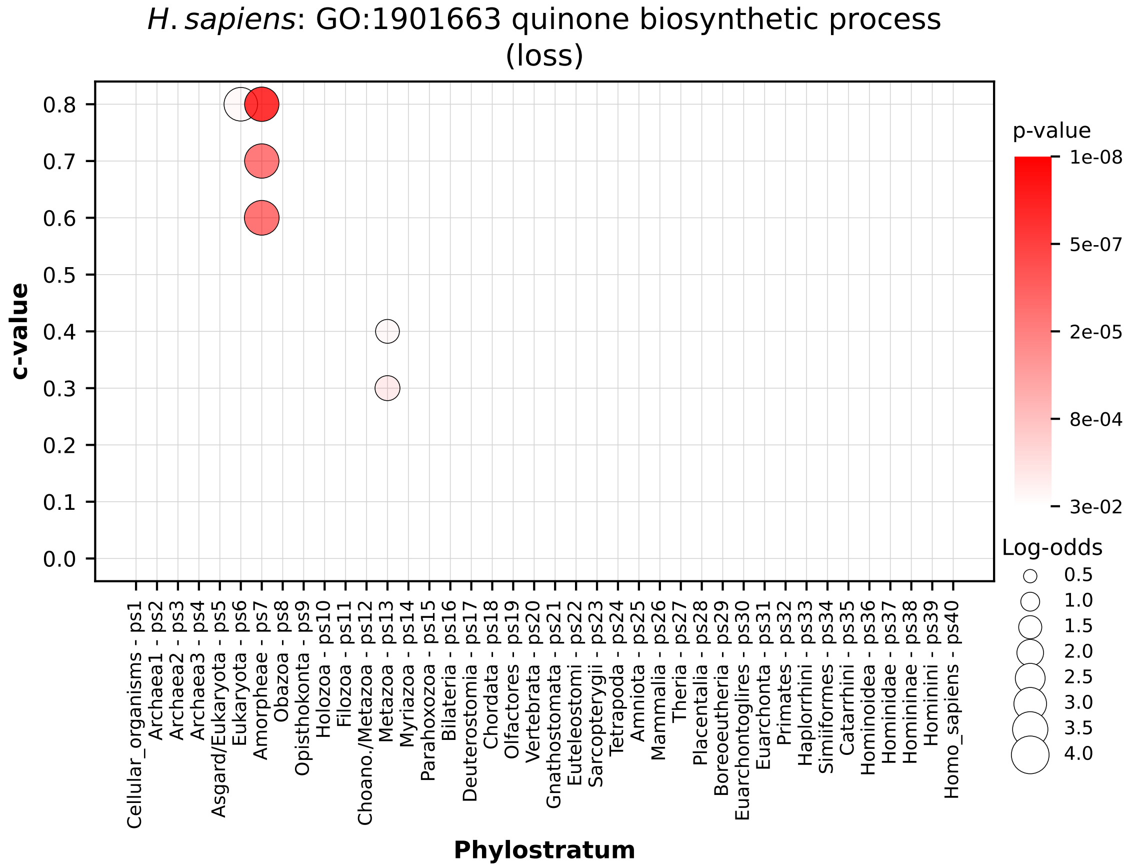
H. sapiens: GO:1901663 quinone biosynthetic process
(loss)
c-value
0.8
0.7
0.6
0.5
0.4
0.3
0.2
0.1
0.0
Phylostratum
Cellular_organisms - ps1
Archaea1 - ps2
Archaea2 - ps3
Archaea3 - ps4
Asgard/Eukaryota - ps5
Eukaryota - ps6
Amorpheae - ps7
Obazoa - ps8
Opisthokonta - ps9
Holozoa - ps10
Filozoa - ps11
Choano./Metazoa - ps12
Metazoa - ps13
Myriazoa - ps14
Parahoxozoa - ps15
Bilateria - ps16
Deuterostomia - ps17
Chordata - ps18
Olfactores - ps19
Vertebrata - ps20
Gnathostomata - ps21
Euteleostomi - ps22
Sarcopterygii - ps23
Tetrapoda - ps24
Amniota - ps25
Mammalia - ps26
Theria - ps27
Placentalia - ps28
Boreoeutheria - ps29
Euarchontoglires - ps30
Euarchonta - ps31
Primates - ps32
Haplorrhini - ps33
Simiiformes - ps34
Catarrhini - ps35
Hominoidea - ps36
Hominidae - ps37
Homininae - ps38
Hominini - ps39
Homo_sapiens - ps40
p-value
1e-08
5e-07
2e-05
8e-04
3e-02
Log-odds
0.5
1.0
1.5
2.0
2.5
3.0
3.5
4.0

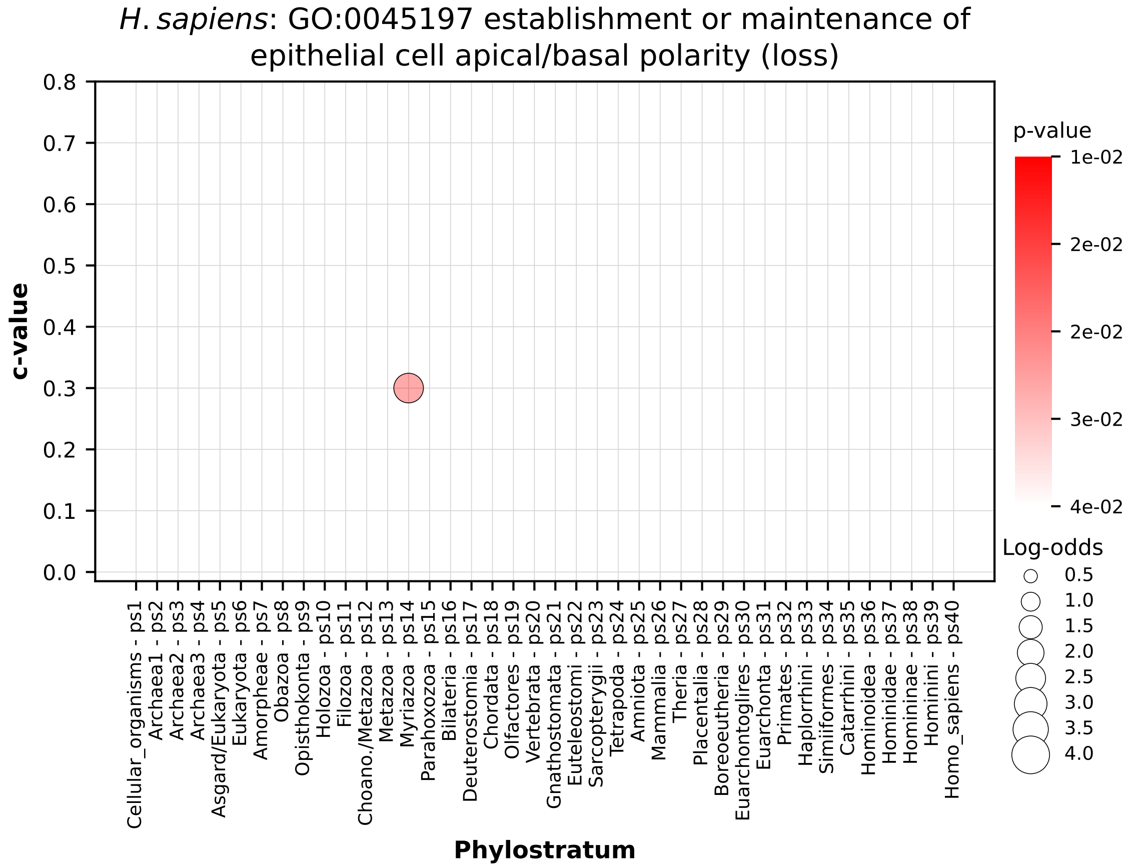

H. sapiens: GO:0045197 establishment or maintenance of epithelial cell apical/basal polarity (loss)
c-value
0.8
0.7
0.6
0.5
0.4
0.3
0.2
0.1
0.0
Phylostratum
Cellular_organisms - ps1
Archaea1 - ps2
Archaea2 - ps3
Archaea3 - ps4
Asgard/Eukaryota - ps5
Eukaryota - ps6
Amorpheae - ps7
Obazoa - ps8
Opisthokonta - ps9
Holozoa - ps10
Filozoa - ps11
Choano./Metazoa - ps12
Metazoa - ps13
Myriazoa - ps14
Parahoxozoa - ps15
Bilateria - ps16
Deuterostomia - ps17
Chordata - ps18
Olfactores - ps19
Vertebrata - ps20
Gnathostomata - ps21
Euteleostomi - ps22
Sarcopterygii - ps23
Tetrapoda - ps24
Amniota - ps25
Mammalia - ps26
Theria - ps27
Placentalia - ps28
Boreoeutheria - ps29
Euarchontoglires - ps30
Euarchonta - ps31
Primates - ps32
Haplorrhini - ps33
Simiiformes - ps34
Catarrhini - ps35
Hominoidea - ps36
Hominidae - ps37
Homininae - ps38
Hominini - ps39
Homo_sapiens - ps40
p-value
1e-02
2e-02
2e-02
3e-02
4e-02
Log-odds
0.5
1.0
1.5
2.0
2.5
3.0
3.5
4.0

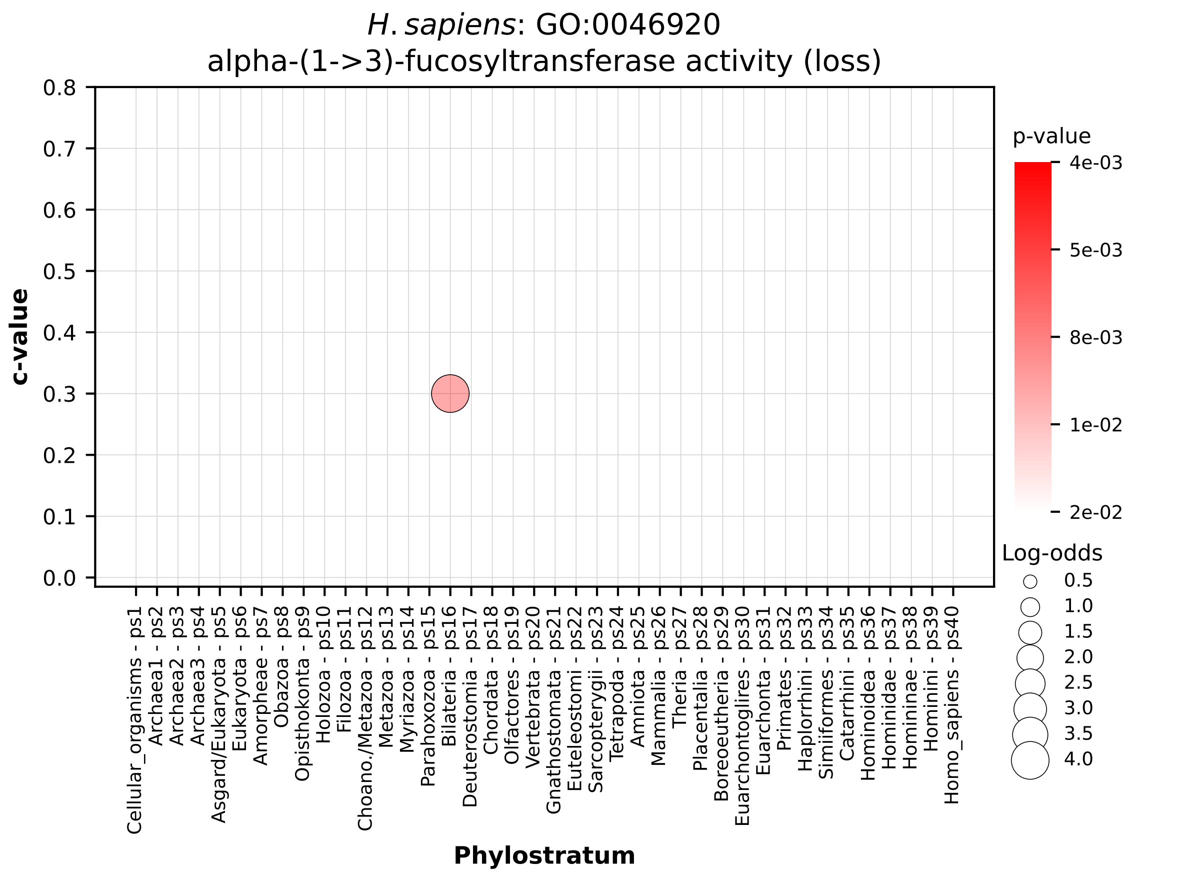

H. sapiens: GO:0046920
alpha-(1->3)-fucosyltransferase activity (loss)
c-value
0.8
0.7
0.6
0.5
0.4
0.3
0.2
0.1
0.0
p-value
4e-03
5e-03
8e-03
1e-02
2e-02
Log-odds
0.5
1.0
1.5
2.0
2.5
3.0
3.5
4.0
Cellular_organisms - ps1
Archaea1 - ps2
Archaea2 - ps3
Archaea3 - ps4
Asgard/Eukaryota - ps5
Eukaryota - ps6
Amorpheae - ps7
Obazoa - ps8
Opisthokonta - ps9
Holozoa - ps10
Filozoa - ps11
Choano./Metazoa - ps12
Metazoa - ps13
Myriazoa - ps14
Parahoxozoa - ps15
Bilateria - ps16
Deuterostomia - ps17
Chordata - ps18
Olfactores - ps19
Vertebrata - ps20
Gnathostomata - ps21
Euteleostomi - ps22
Sarcopterygii - ps23
Tetrapoda - ps24
Amniota - ps25
Mammalia - ps26
Theria - ps27
Placentalia - ps28
Boreoeutheria - ps29
Euarchontoglires - ps30
Euarchonta - ps31
Primates - ps32
Haplorrhini - ps33
Simiiformes - ps34
Catarrhini - ps35
Hominoidea - ps36
Hominidae - ps37
Homininae - ps38
Hominini - ps39
Homo_sapiens - ps40
Phylostratum

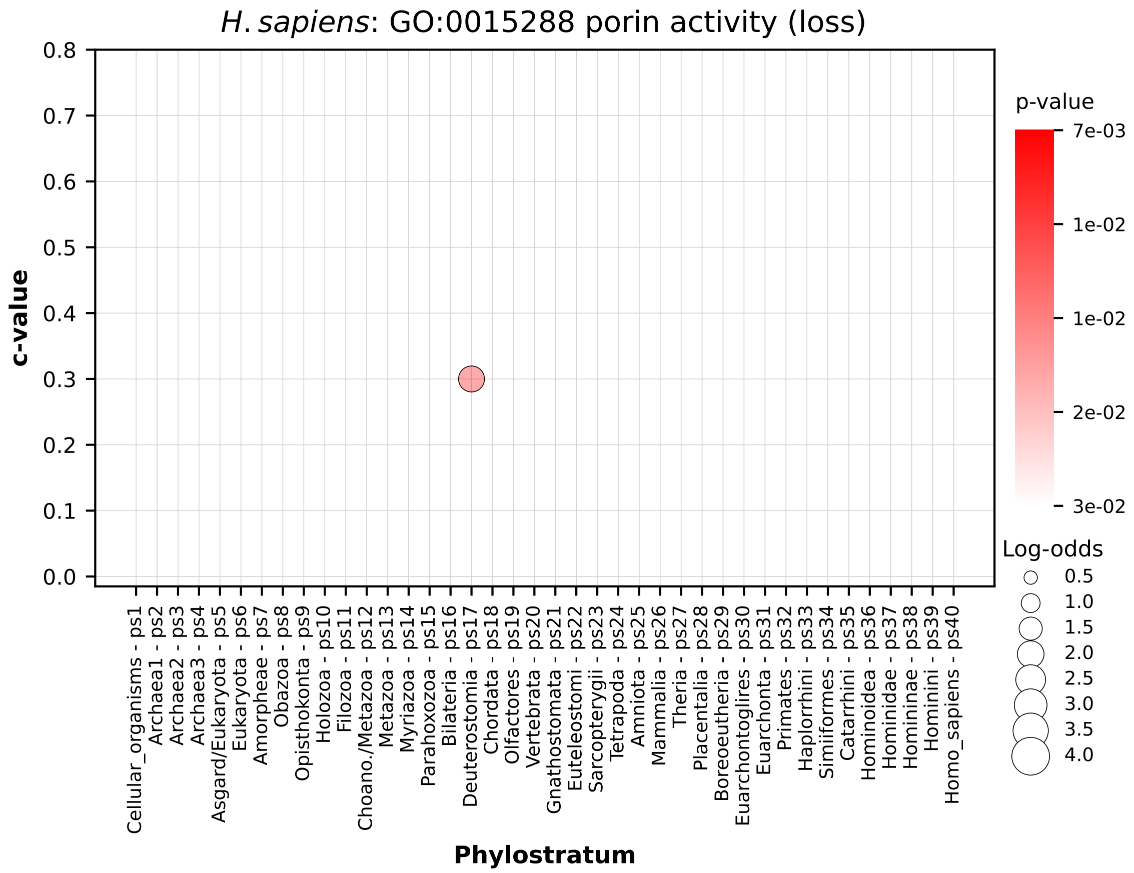

H. sapiens: GO:0015288 porin activity (loss)
c-value
0.0
0.1
0.2
0.3
0.4
0.5
0.6
0.7
0.8
Phylostratum
Cellular_organisms - ps1
Archaea1 - ps2
Archaea2 - ps3
Archaea3 - ps4
Asgard/Eukaryota - ps5
Eukaryota - ps6
Amorpheae - ps7
Obazoa - ps8
Opisthokonta - ps9
Holozoa - ps10
Filozoa - ps11
Choano./Metazoa - ps12
Metazoa - ps13
Myriazoa - ps14
Parahoxozoa - ps15
Bilateria - ps16
Deuterostomia - ps17
Chordata - ps18
Olfactores - ps19
Vertebrata - ps20
Gnathostomata - ps21
Euteleostomi - ps22
Sarcopterygii - ps23
Tetrapoda - ps24
Amniota - ps25
Mammalia - ps26
Theria - ps27
Placentalia - ps28
Boreoeutheria - ps29
Euarchontoglires - ps30
Euarchonta - ps31
Primates - ps32
Haplorrhini - ps33
Simiiformes - ps34
Catarrhini - ps35
Hominoidea - ps36
Hominidae - ps37
Homininae - ps38
Hominini - ps39
Homo_sapiens - ps40
p-value
7e-03
1e-02
1e-02
2e-02
3e-02
Log-odds
0.5
1.0
1.5
2.0
2.5
3.0
3.5
4.0

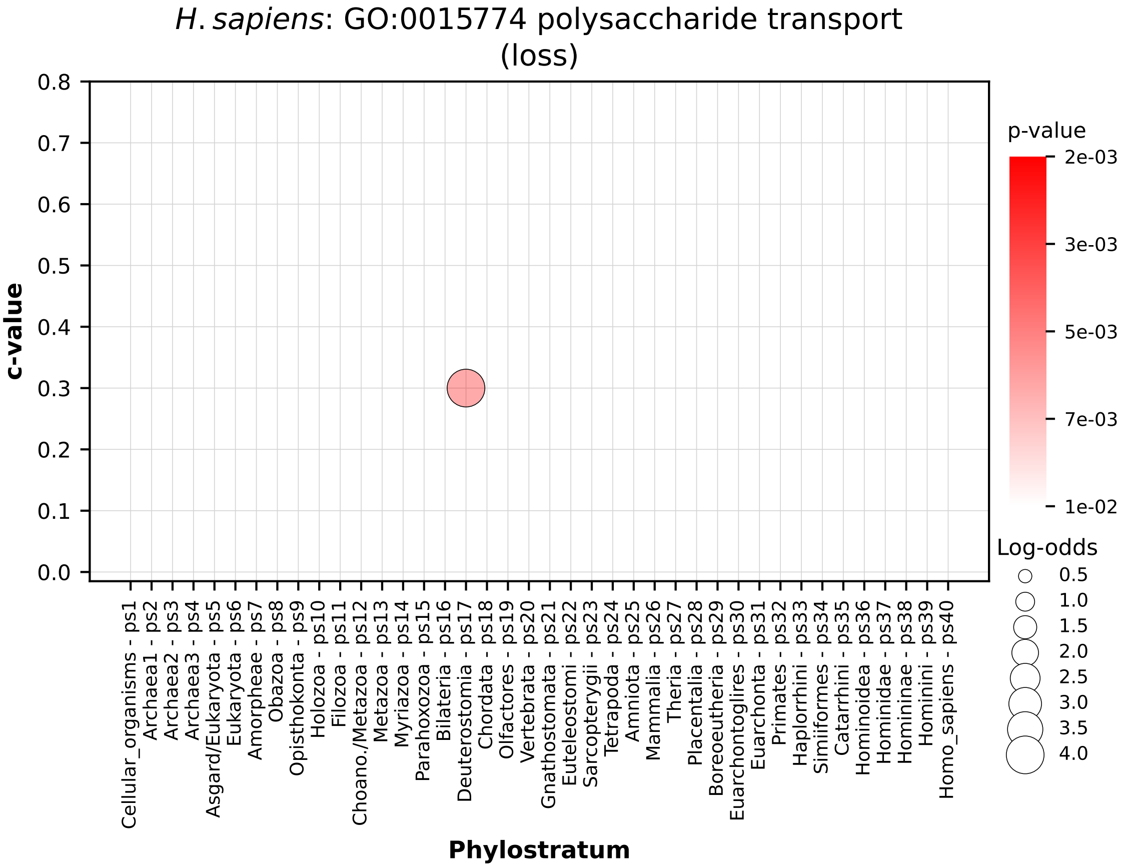
H. sapiens: GO:0015774 polysaccharide transport
(loss)
c-value
0.8
0.7
0.6
0.5
0.4
0.3
0.2
0.1
0.0
p-value
2e-03
3e-03
5e-03
7e-03
1e-02
Log-odds
0.5
1.0
1.5
2.0
2.5
3.0
3.5
4.0
Cellular_organisms - ps1
Archaea1 - ps2
Archaea2 - ps3
Archaea3 - ps4
Asgard/Eukaryota - ps5
Eukaryota - ps6
Amorpheae - ps7
Obazoa - ps8
Opisthokonta - ps9
Holozoa - ps10
Filozoa - ps11
Choano./Metazoa - ps12
Metazoa - ps13
Myriazoa - ps14
Parahoxozoa - ps15
Bilateria - ps16
Deuterostomia - ps17
Chordata - ps18
Olfactores - ps19
Vertebrata - ps20
Gnathostomata - ps21
Euteleostomi - ps22
Sarcopterygii - ps23
Tetrapoda - ps24
Amniota - ps25
Mammalia - ps26
Theria - ps27
Placentalia - ps28
Boreoeutheria - ps29
Euarchontoglires - ps30
Euarchonta - ps31
Primates - ps32
Haplorrhini - ps33
Simiiformes - ps34
Catarrhini - ps35
Hominoidea - ps36
Hominidae - ps37
Homininae - ps38
Hominini - ps39
Homo_sapiens - ps40
Phylostratum

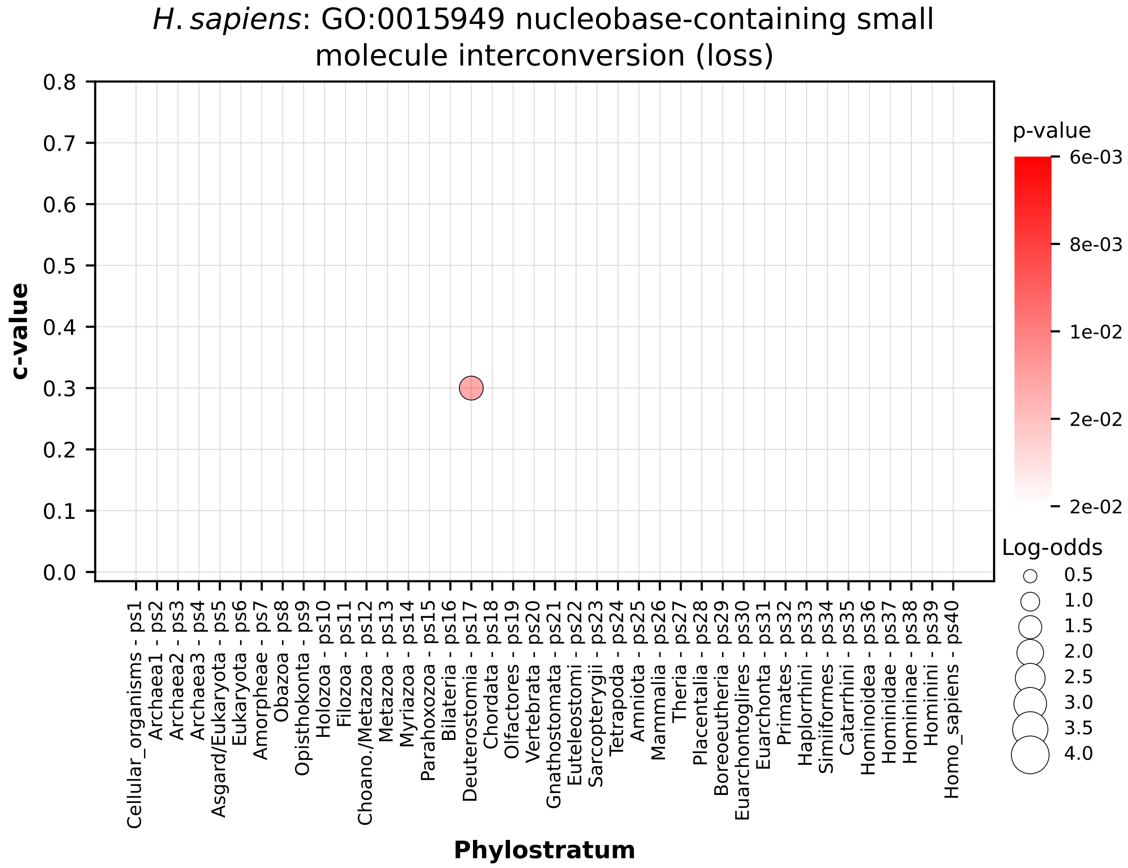

H. sapiens: GO:0015949 nucleobase-containing small molecule interconversion (loss)
c-value
0.0
0.1
0.2
0.3
0.4
0.5
0.6
0.7
0.8
Phylostratum
Cellular_organisms - ps1
Archaea1 - ps2
Archaea2 - ps3
Archaea3 - ps4
Asgard/Eukaryota - ps5
Eukaryota - ps6
Amorpheae - ps7
Obazoa - ps8
Opisthokonta - ps9
Holozoa - ps10
Filozoa - ps11
Choano./Metazoa - ps12
Metazoa - ps13
Myriazoa - ps14
Parahoxozoa - ps15
Bilateria - ps16
Deuterostomia - ps17
Chordata - ps18
Olfactores - ps19
Vertebrata - ps20
Gnathostomata - ps21
Euteleostomi - ps22
Sarcopterygii - ps23
Tetrapoda - ps24
Amniota - ps25
Mammalia - ps26
Theria - ps27
Placentalia - ps28
Boreoeutheria - ps29
Euarchontoglires - ps30
Euarchonta - ps31
Primates - ps32
Haplorrhini - ps33
Simiiformes - ps34
Catarrhini - ps35
Hominoidea - ps36
Hominidae - ps37
Homininae - ps38
Hominini - ps39
Homo_sapiens - ps40
p-value
6e-03
8e-03
1e-02
2e-02
2e-02
Log-odds
0.5
1.0
1.5
2.0
2.5
3.0
3.5
4.0

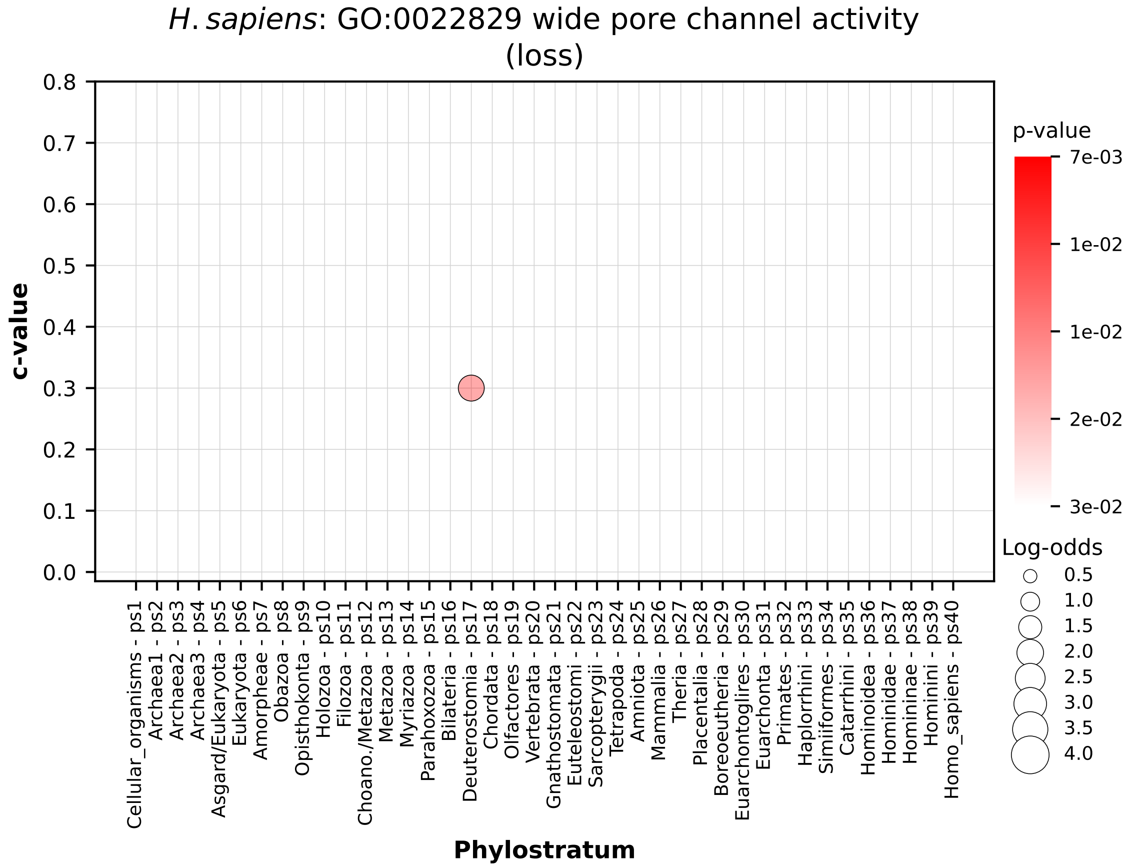

$H. sapiens$: GO:0022829 wide pore channel activity
(loss)
c-value
0.0
0.1
0.2
0.3
0.4
0.5
0.6
0.7
0.8
Phylostratum
Cellular_organisms - ps1
Archaea1 - ps2
Archaea2 - ps3
Archaea3 - ps4
Asgard/Eukaryota - ps5
Eukaryota - ps6
Amorpheae - ps7
Obazoa - ps8
Opisthokonta - ps9
Holozoa - ps10
Filozoa - ps11
Choano./Metazoa - ps12
Metazoa - ps13
Myriazoa - ps14
Parahoxozoa - ps15
Bilateria - ps16
Deuterostomia - ps17
Chordata - ps18
Olfactores - ps19
Vertebrata - ps20
Gnathostomata - ps21
Euteleostomi - ps22
Sarcopterygii - ps23
Tetrapoda - ps24
Amniota - ps25
Mammalia - ps26
Theria - ps27
Placentalia - ps28
Boreoeutheria - ps29
Euarchontoglires - ps30
Euarchonta - ps31
Primates - ps32
Haplorrhini - ps33
Simiiformes - ps34
Catarrhini - ps35
Hominoidea - ps36
Hominidae - ps37
Homininae - ps38
Hominini - ps39
Homo_sapiens - ps40
p-value
7e-03
1e-02
1e-02
2e-02
3e-02
Log-odds
0.5
1.0
1.5
2.0
2.5
3.0
3.5
4.0

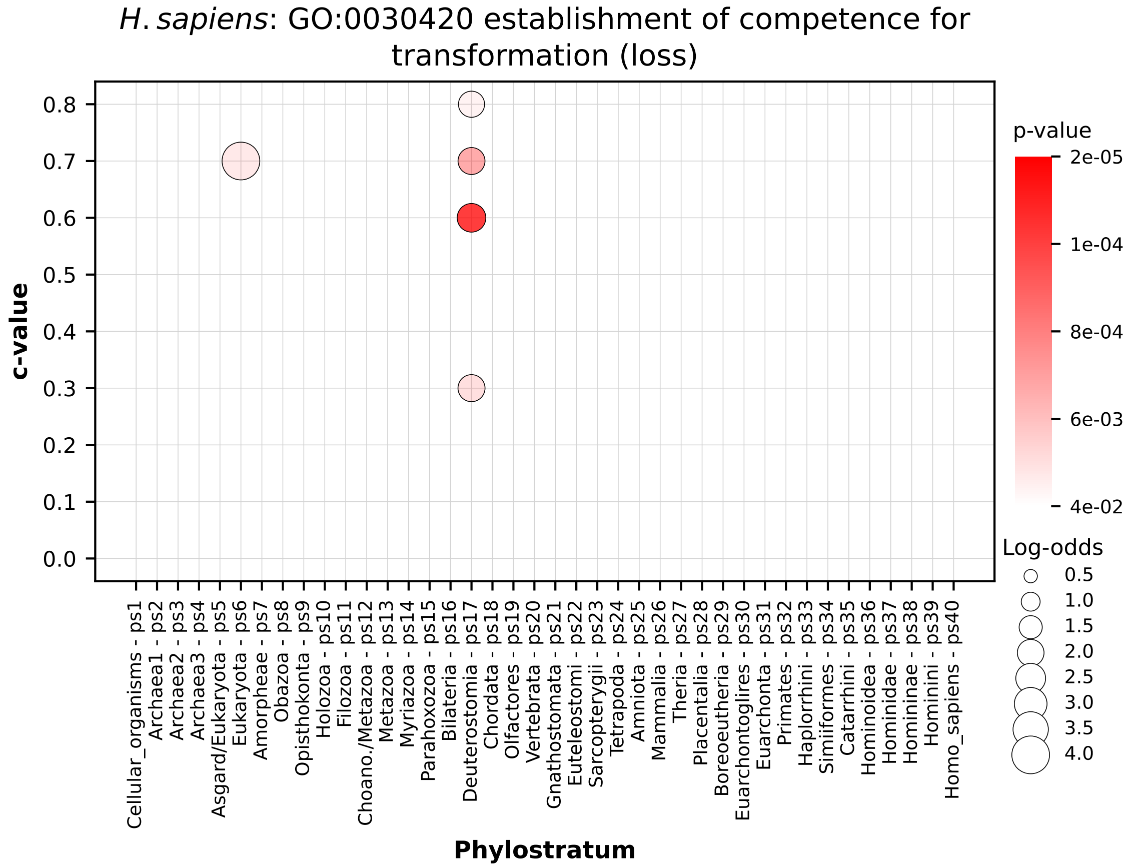

H. sapiens: GO:0030420 establishment of competence for transformation (loss)
c-value
0.8
0.7
0.6
0.5
0.4
0.3
0.2
0.1
0.0
p-value
2e-05
1e-04
8e-04
6e-03
4e-02
Log-odds
0.5
1.0
1.5
2.0
2.5
3.0
3.5
4.0
Cellular_organisms - ps1
Archaea1 - ps2
Archaea2 - ps3
Archaea3 - ps4
Asgard/Eukaryota - ps5
Eukaryota - ps6
Amorpheae - ps7
Obazoa - ps8
Opisthokonta - ps9
Holozoa - ps10
Filozoa - ps11
Choano./Metazoa - ps12
Metazoa - ps13
Myriazoa - ps14
Parahoxozoa - ps15
Bilateria - ps16
Deuterostomia - ps17
Chordata - ps18
Olfactores - ps19
Vertebrata - ps20
Gnathostomata - ps21
Euteleostomi - ps22
Sarcopterygii - ps23
Tetrapoda - ps24
Amniota - ps25
Mammalia - ps26
Theria - ps27
Placentalia - ps28
Boreoeutheria - ps29
Euarchontoglires - ps30
Euarchonta - ps31
Primates - ps32
Haplorrhini - ps33
Simiiformes - ps34
Catarrhini - ps35
Hominoidea - ps36
Hominidae - ps37
Homininae - ps38
Hominini - ps39
Homo_sapiens - ps40
Phylostratum

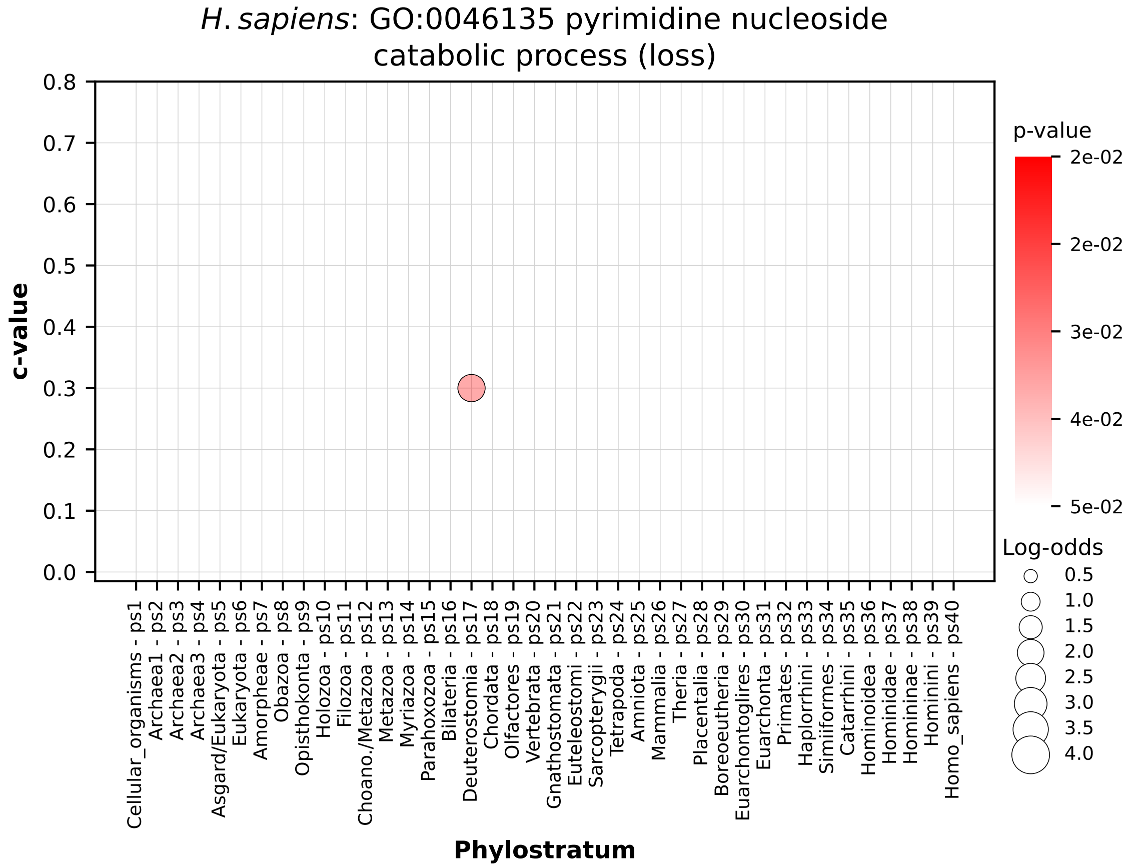

H. sapiens: GO:0046135 pyrimidine nucleoside catabolic process (loss)
c-value
0.0
0.1
0.2
0.3
0.4
0.5
0.6
0.7
0.8
Phylostratum
Cellular_organisms - ps1
Archaea1 - ps2
Archaea2 - ps3
Archaea3 - ps4
Asgard/Eukaryota - ps5
Eukaryota - ps6
Amorpheae - ps7
Obazoa - ps8
Opisthokonta - ps9
Holozoa - ps10
Filozoa - ps11
Choano./Metazoa - ps12
Metazoa - ps13
Myriazoa - ps14
Parahoxozoa - ps15
Bilateria - ps16
Deuterostomia - ps17
Chordata - ps18
Olfactores - ps19
Vertebrata - ps20
Gnathostomata - ps21
Euteleostomi - ps22
Sarcopterygii - ps23
Tetrapoda - ps24
Amniota - ps25
Mammalia - ps26
Theria - ps27
Placentalia - ps28
Boreoeutheria - ps29
Euarchontoglires - ps30
Euarchonta - ps31
Primates - ps32
Haplorrhini - ps33
Simiiformes - ps34
Catarrhini - ps35
Hominoidea - ps36
Hominidae - ps37
Homininae - ps38
Hominini - ps39
Homo_sapiens - ps40
p-value
2e-02
2e-02
3e-02
4e-02
5e-02
Log-odds
0.5
1.0
1.5
2.0
2.5
3.0
3.5
4.0

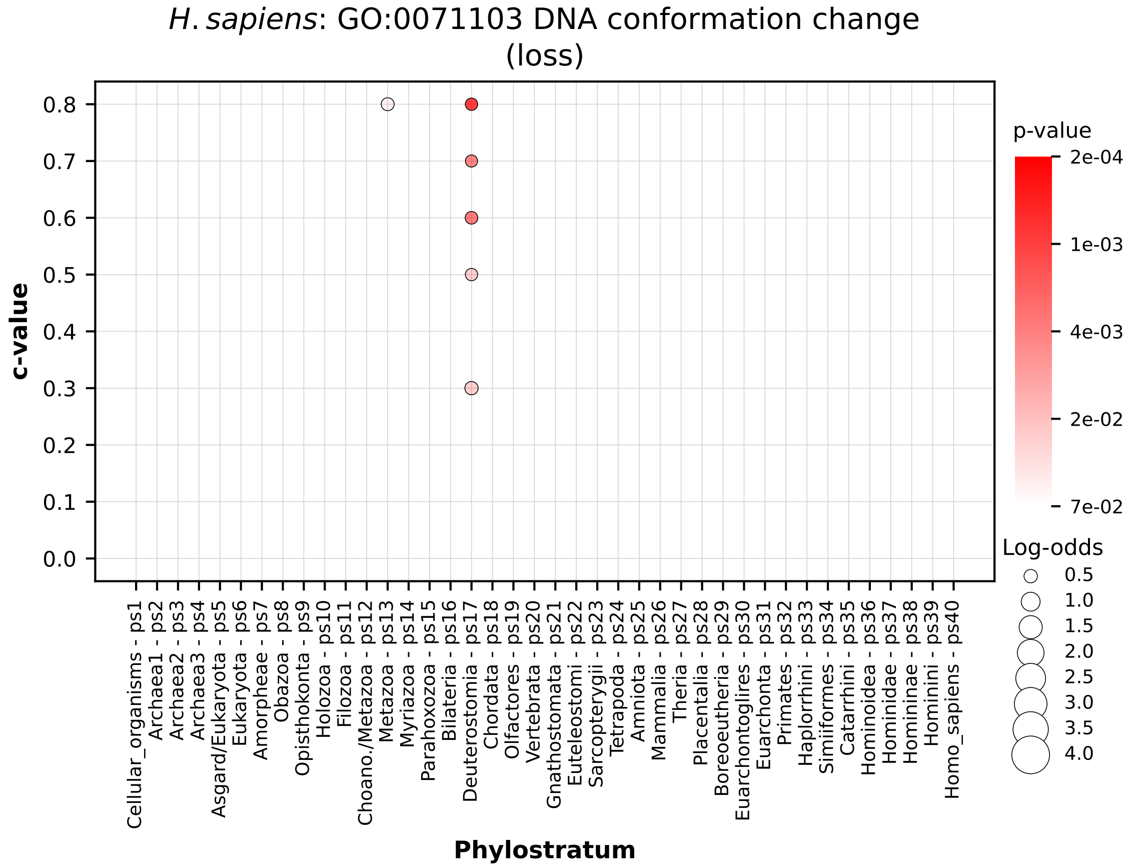

H. sapiens: GO:0071103 DNA conformation change
(loss)
c-value
0.0
0.1
0.2
0.3
0.4
0.5
0.6
0.7
0.8
Phylostratum
Cellular_organisms - ps1
Archaea1 - ps2
Archaea2 - ps3
Archaea3 - ps4
Asgard/Eukaryota - ps5
Eukaryota - ps6
Amorpheae - ps7
Obazoa - ps8
Opisthokonta - ps9
Holozoa - ps10
Filozoa - ps11
Choano./Metazoa - ps12
Metazoa - ps13
Myriazoa - ps14
Parahoxozoa - ps15
Bilateria - ps16
Deuterostomia - ps17
Chordata - ps18
Olfactores - ps19
Vertebrata - ps20
Gnathostomata - ps21
Euteleostomi - ps22
Sarcopterygii - ps23
Tetrapoda - ps24
Amniota - ps25
Mammalia - ps26
Theria - ps27
Placentalia - ps28
Boreoeutheria - ps29
Euarchontoglires - ps30
Euarchonta - ps31
Primates - ps32
Haplorrhini - ps33
Simiiformes - ps34
Catarrhini - ps35
Hominoidea - ps36
Hominidae - ps37
Homininae - ps38
Hominini - ps39
Homo_sapiens - ps40
p-value
2e-04
1e-03
4e-03
2e-02
7e-02
Log-odds
0.5
1.0
1.5
2.0
2.5
3.0
3.5
4.0

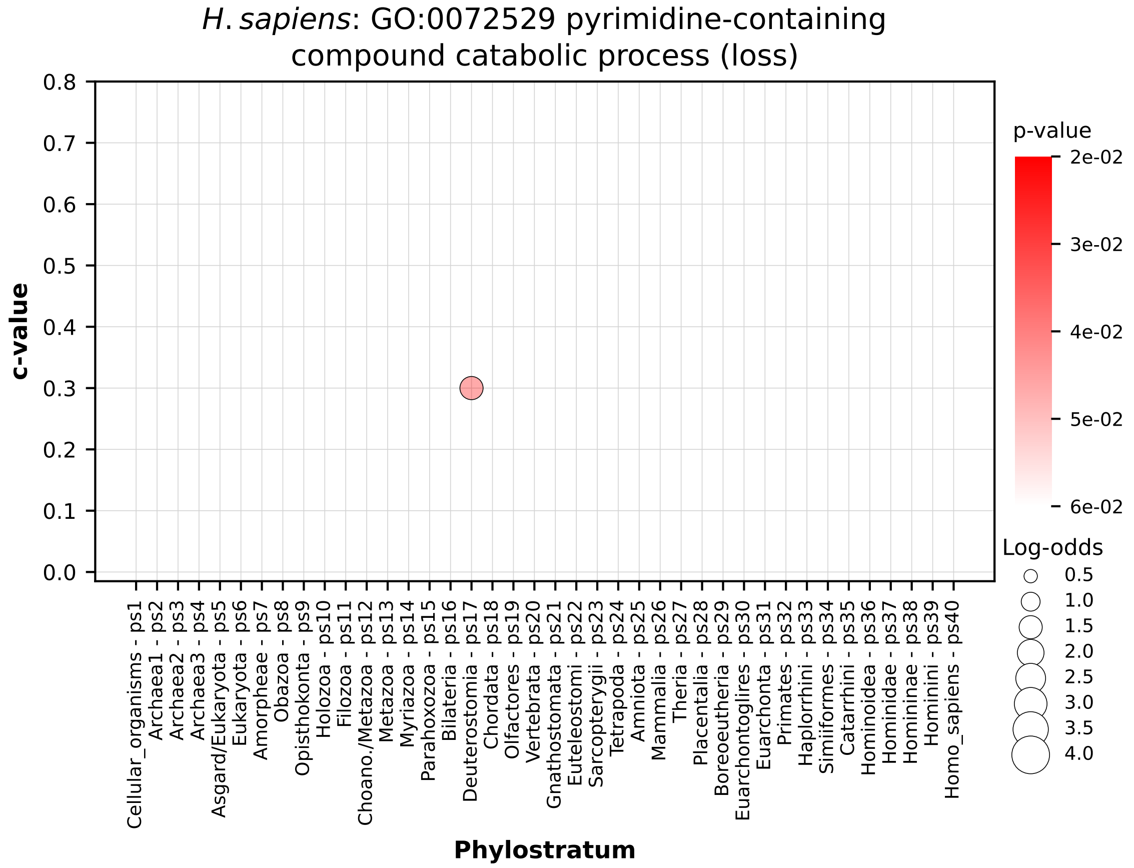
H. sapiens: GO:0072529 pyrimidine-containing compound catabolic process (loss)
c-value
0.8
0.7
0.6
0.5
0.4
0.3
0.2
0.1
0.0
Phylostratum
Cellular_organisms - ps1
Archaea1 - ps2
Archaea2 - ps3
Archaea3 - ps4
Asgard/Eukaryota - ps5
Eukaryota - ps6
Amorpheae - ps7
Obazoa - ps8
Opisthokonta - ps9
Holozoa - ps10
Filozoa - ps11
Choano./Metazoa - ps12
Metazoa - ps13
Myriazoa - ps14
Parahoxozoa - ps15
Bilateria - ps16
Deuterostomia - ps17
Chordata - ps18
Olfactores - ps19
Vertebrata - ps20
Gnathostomata - ps21
Euteleostomi - ps22
Sarcopterygii - ps23
Tetrapoda - ps24
Amniota - ps25
Mammalia - ps26
Theria - ps27
Placentalia - ps28
Boreoeutheria - ps29
Euarchontoglires - ps30
Euarchonta - ps31
Primates - ps32
Haplorrhini - ps33
Simiiformes - ps34
Catarrhini - ps35
Hominoidea - ps36
Hominidae - ps37
Homininae - ps38
Hominini - ps39
Homo_sapiens - ps40
p-value
2e-02
3e-02
4e-02
5e-02
6e-02
Log-odds
0.5
1.0
1.5
2.0
2.5
3.0
3.5
4.0

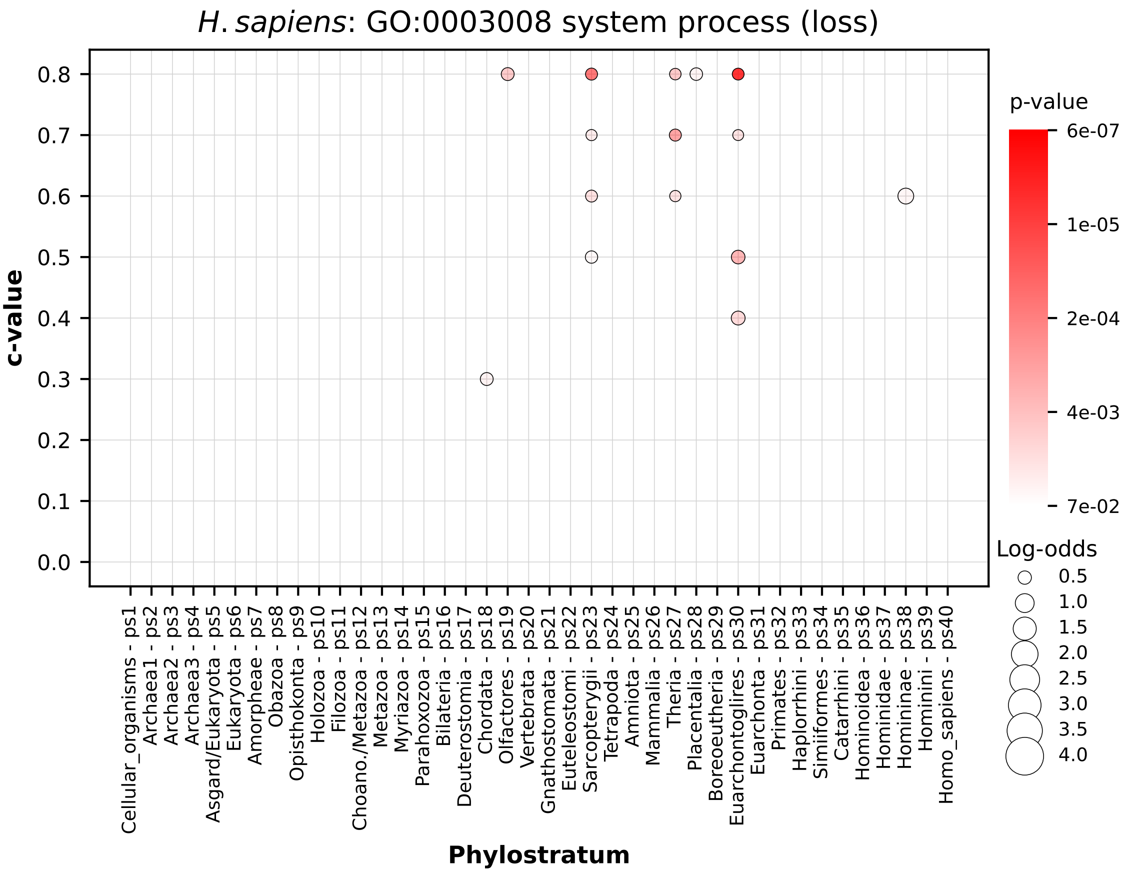
H. sapiens: GO:0003008 system process (loss)
c-value
Phylostratum
p-value
6e-07
1e-05
2e-04
4e-03
7e-02
Log-odds
0.5
1.0
1.5
2.0
2.5
3.0
3.5
4.0
Cellular_organisms - ps1
Archaea1 - ps2
Archaea2 - ps3
Archaea3 - ps4
Asgard/Eukaryota - ps5
Eukaryota - ps6
Amorpheae - ps7
Obazoa - ps8
Opisthokonta - ps9
Holozoa - ps10
Filozoa - ps11
Choano./Metazoa - ps12
Metazoa - ps13
Myriazoa - ps14
Parahoxozoa - ps15
Bilateria - ps16
Deuterostomia - ps17
Chordata - ps18
Olfactores - ps19
Vertebrata - ps20
Gnathostomata - ps21
Euteleostomi - ps22
Sarcopterygii - ps23
Tetrapoda - ps24
Amniota - ps25
Mammalia - ps26
Theria - ps27
Placentalia - ps28
Boreoeutheria - ps29
Euarchontoglires - ps30
Euarchonta - ps31
Primates - ps32
Haplorrhini - ps33
Simiiformes - ps34
Catarrhini - ps35
Hominoidea - ps36
Hominidae - ps37
Homininae - ps38
Hominini - ps39
Homo_sapiens - ps40

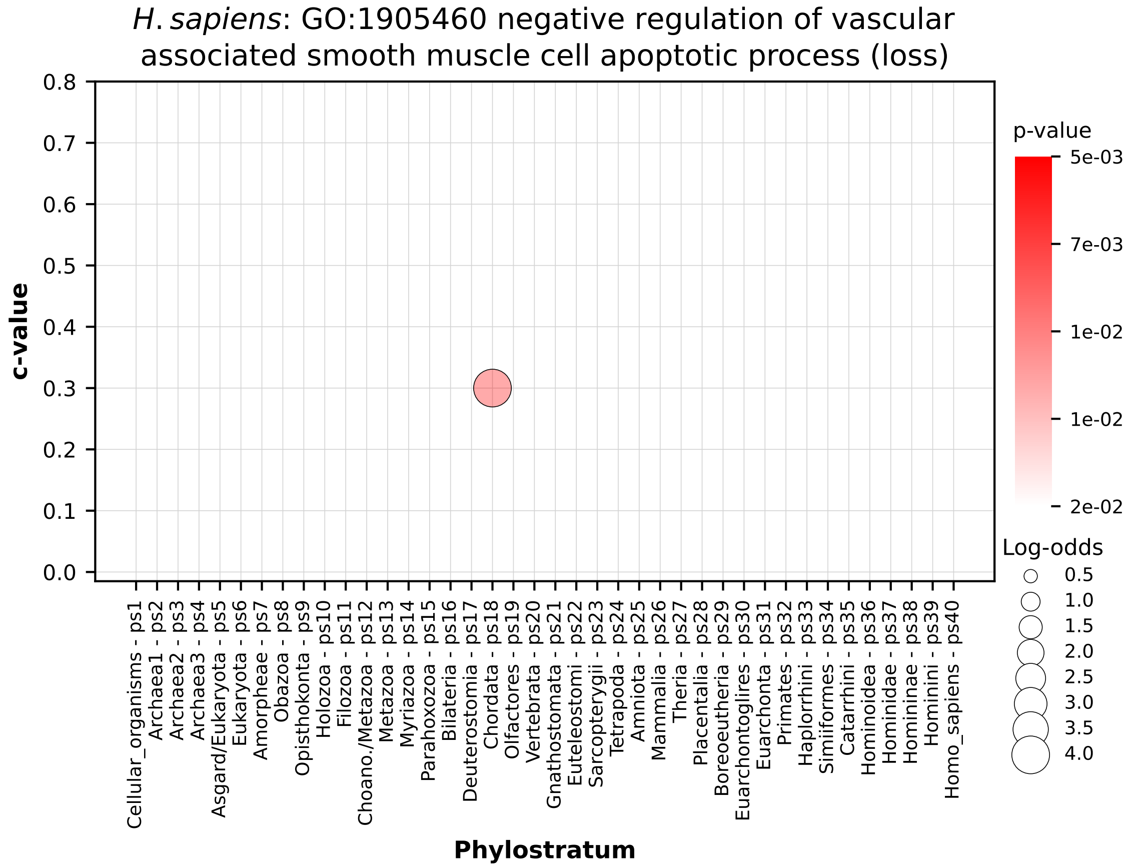

H. sapiens: GO:1905460 negative regulation of vascular associated smooth muscle cell apoptotic process (loss)
c-value
0.8
0.7
0.6
0.5
0.4
0.3
0.2
0.1
0.0
Phylostratum
Cellular_organisms - ps1
Archaea1 - ps2
Archaea2 - ps3
Archaea3 - ps4
Asgard/Eukaryota - ps5
Eukaryota - ps6
Amorpheae - ps7
Obazoa - ps8
Opisthokonta - ps9
Holozoa - ps10
Filozoa - ps11
Choano./Metazoa - ps12
Metazoa - ps13
Myriazoa - ps14
Parahoxozoa - ps15
Bilateria - ps16
Deuterostomia - ps17
Chordata - ps18
Olfactores - ps19
Vertebrata - ps20
Gnathostomata - ps21
Euteleostomi - ps22
Sarcopterygii - ps23
Tetrapoda - ps24
Amniota - ps25
Mammalia - ps26
Theria - ps27
Placentalia - ps28
Boreoeutheria - ps29
Euarchontoglires - ps30
Euarchonta - ps31
Primates - ps32
Haplorrhini - ps33
Simiiformes - ps34
Catarrhini - ps35
Hominoidea - ps36
Hominidae - ps37
Homininae - ps38
Hominini - ps39
Homo_sapiens - ps40
p-value
5e-03
7e-03
1e-02
1e-02
2e-02
Log-odds
0.5
1.0
1.5
2.0
2.5
3.0
3.5
4.0

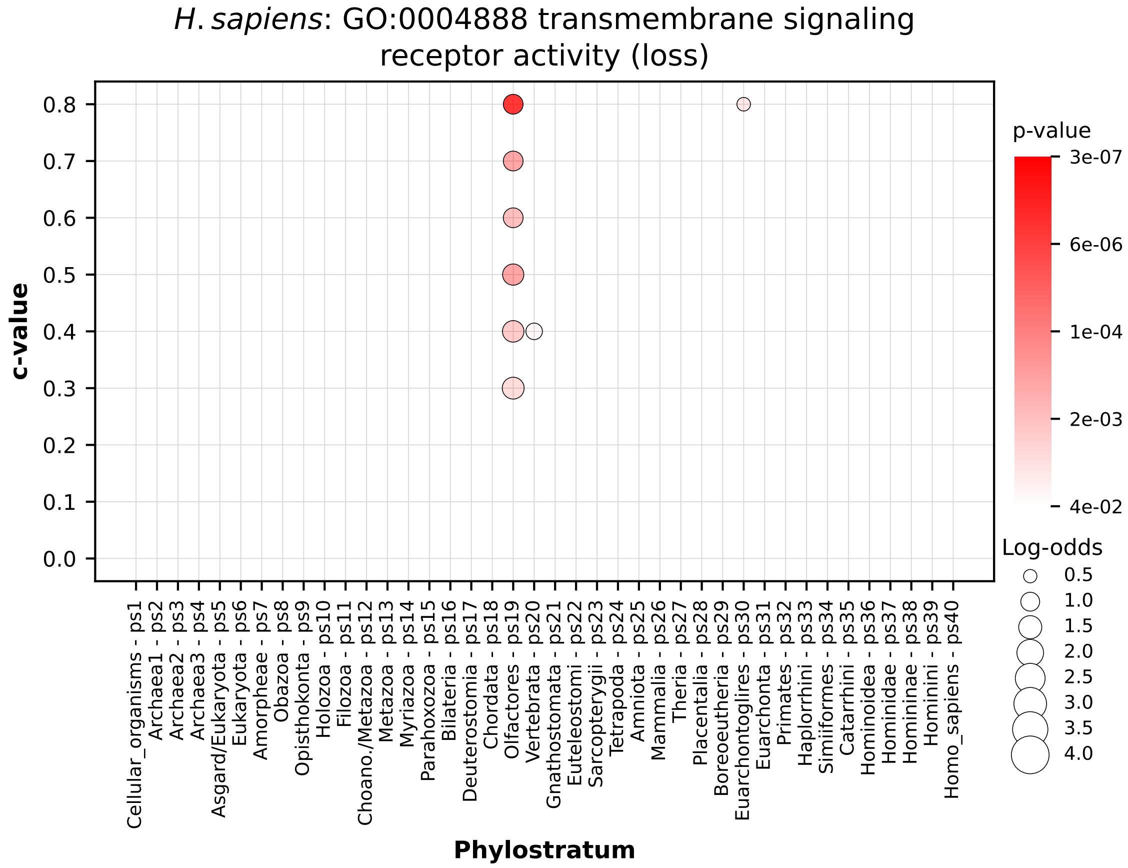
H. sapiens: GO:0004888 transmembrane signaling receptor activity (loss)
c-value
0.8
0.7
0.6
0.5
0.4
0.3
0.2
0.1
0.0
Phylostratum
Cellular_organisms - ps1
Archaea1 - ps2
Archaea2 - ps3
Archaea3 - ps4
Asgard/Eukaryota - ps5
Eukaryota - ps6
Amorpheae - ps7
Obazoa - ps8
Opisthokonta - ps9
Holozoa - ps10
Filozoa - ps11
Choano./Metazoa - ps12
Metazoa - ps13
Myriazoa - ps14
Parahoxozoa - ps15
Bilateria - ps16
Deuterostomia - ps17
Chordata - ps18
Olfactores - ps19
Vertebrata - ps20
Gnathostomata - ps21
Euteleostomi - ps22
Sarcopterygii - ps23
Tetrapoda - ps24
Amniota - ps25
Mammalia - ps26
Theria - ps27
Placentalia - ps28
Boreoeutheria - ps29
Euarchontoglires - ps30
Euarchonta - ps31
Primates - ps32
Haplorrhini - ps33
Simiiformes - ps34
Catarrhini - ps35
Hominoidea - ps36
Hominidae - ps37
Homininae - ps38
Hominini - ps39
Homo_sapiens - ps40
p-value
3e-07
6e-06
1e-04
2e-03
4e-02
Log-odds
0.5
1.0
1.5
2.0
2.5
3.0
3.5
4.0

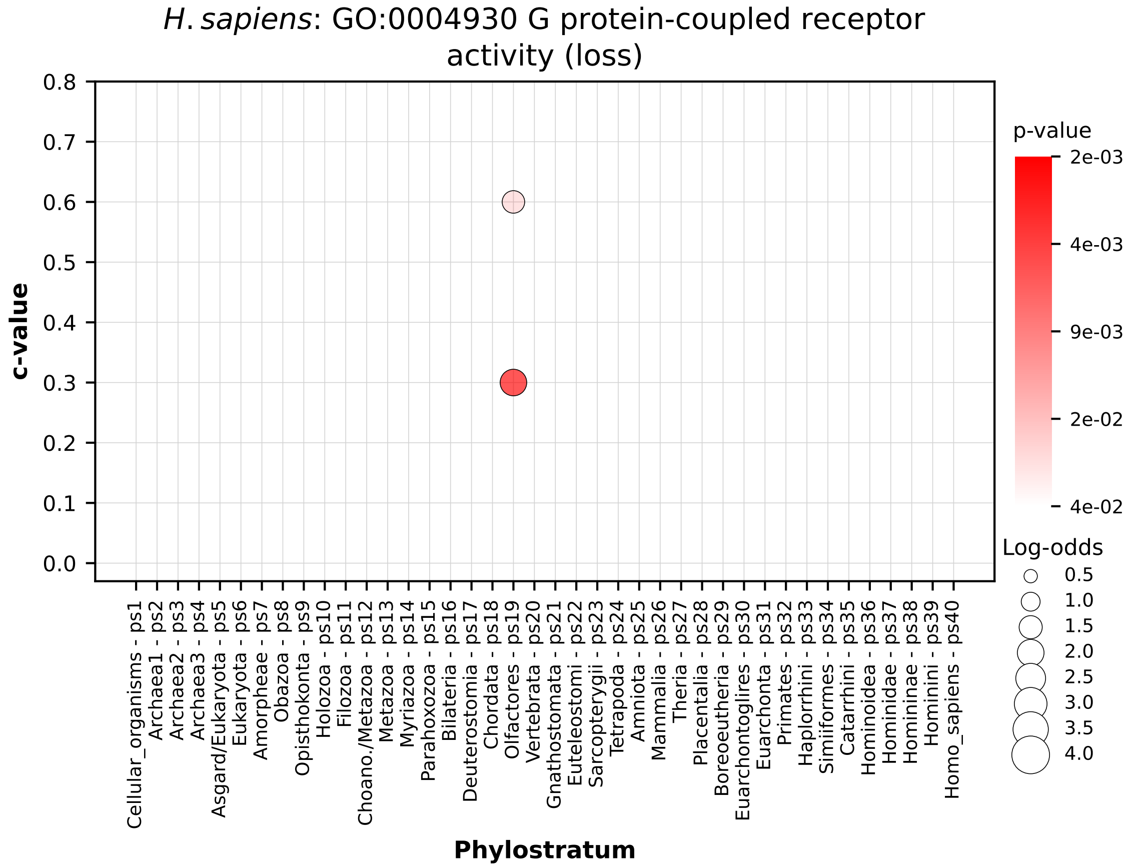

H. sapiens: GO:0004930 G protein-coupled receptor activity (loss)
c-value
0.8
0.7
0.6
0.5
0.4
0.3
0.2
0.1
0.0
p-value
2e-03
4e-03
9e-03
2e-02
4e-02
Log-odds
0.5
1.0
1.5
2.0
2.5
3.0
3.5
4.0
Cellular_organisms - ps1
Archaea1 - ps2
Archaea2 - ps3
Archaea3 - ps4
Asgard/Eukaryota - ps5
Eukaryota - ps6
Amorpheae - ps7
Obazoa - ps8
Opisthokonta - ps9
Holozoa - ps10
Filozoa - ps11
Choano./Metazoa - ps12
Metazoa - ps13
Myriazoa - ps14
Parahoxozoa - ps15
Bilateria - ps16
Deuterostomia - ps17
Chordata - ps18
Olfactores - ps19
Vertebrata - ps20
Gnathostomata - ps21
Euteleostomi - ps22
Sarcopterygii - ps23
Tetrapoda - ps24
Amniota - ps25
Mammalia - ps26
Theria - ps27
Placentalia - ps28
Boreoeutheria - ps29
Euarchontoglires - ps30
Euarchonta - ps31
Primates - ps32
Haplorrhini - ps33
Simiiformes - ps34
Catarrhini - ps35
Hominoidea - ps36
Hominidae - ps37
Homininae - ps38
Hominini - ps39
Homo_sapiens - ps40
Phylostratum

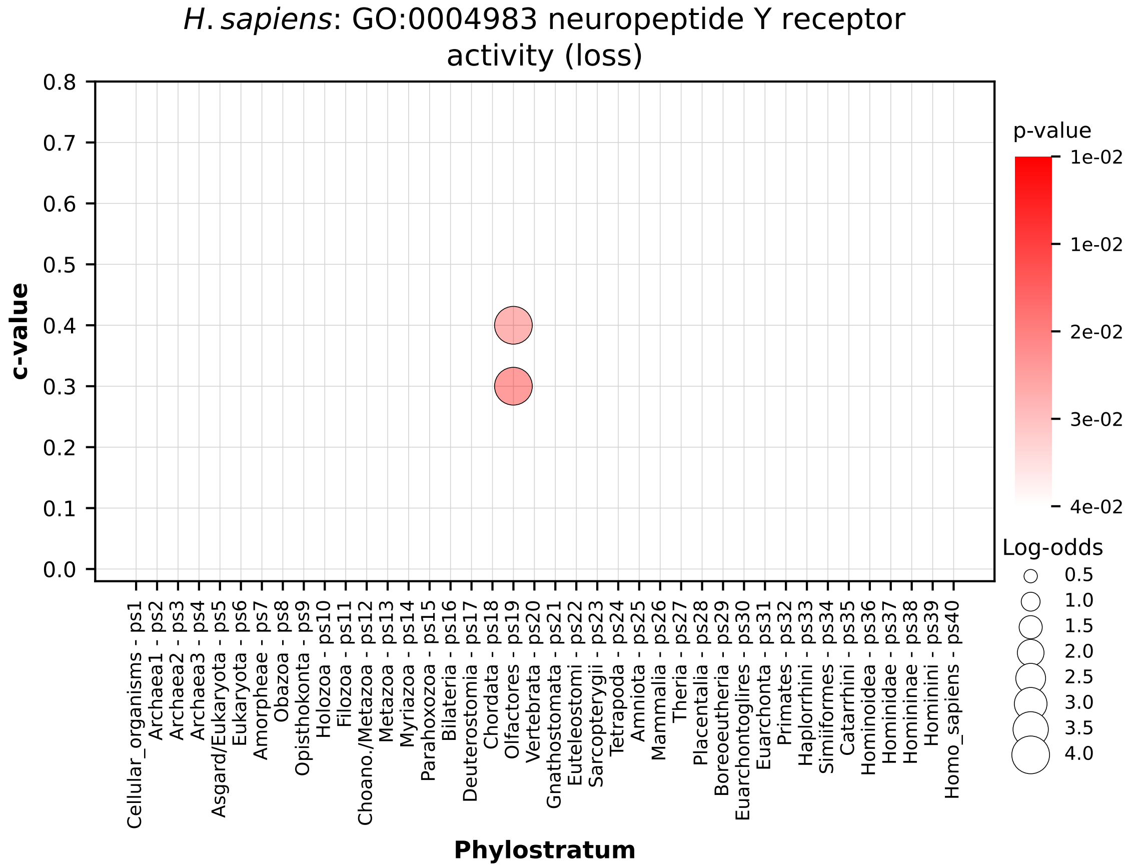

H. sapiens: GO:0004983 neuropeptide Y receptor activity (loss)
c-value
0.0
0.1
0.2
0.3
0.4
0.5
0.6
0.7
0.8
Phylostratum
Cellular_organisms - ps1
Archaea1 - ps2
Archaea2 - ps3
Archaea3 - ps4
Asgard/Eukaryota - ps5
Eukaryota - ps6
Amorpheae - ps7
Obazoa - ps8
Opisthokonta - ps9
Holozoa - ps10
Filozoa - ps11
Choano./Metazoa - ps12
Metazoa - ps13
Myriazoa - ps14
Parahoxozoa - ps15
Bilateria - ps16
Deuterostomia - ps17
Chordata - ps18
Olfactores - ps19
Vertebrata - ps20
Gnathostomata - ps21
Euteleostomi - ps22
Sarcopterygii - ps23
Tetrapoda - ps24
Amniota - ps25
Mammalia - ps26
Theria - ps27
Placentalia - ps28
Boreoeutheria - ps29
Euarchontoglires - ps30
Euarchonta - ps31
Primates - ps32
Haplorrhini - ps33
Simiiformes - ps34
Catarrhini - ps35
Hominoidea - ps36
Hominidae - ps37
Homininae - ps38
Hominini - ps39
Homo_sapiens - ps40
p-value
1e-02
1e-02
2e-02
3e-02
4e-02
Log-odds
0.5
1.0
1.5
2.0
2.5
3.0
3.5
4.0

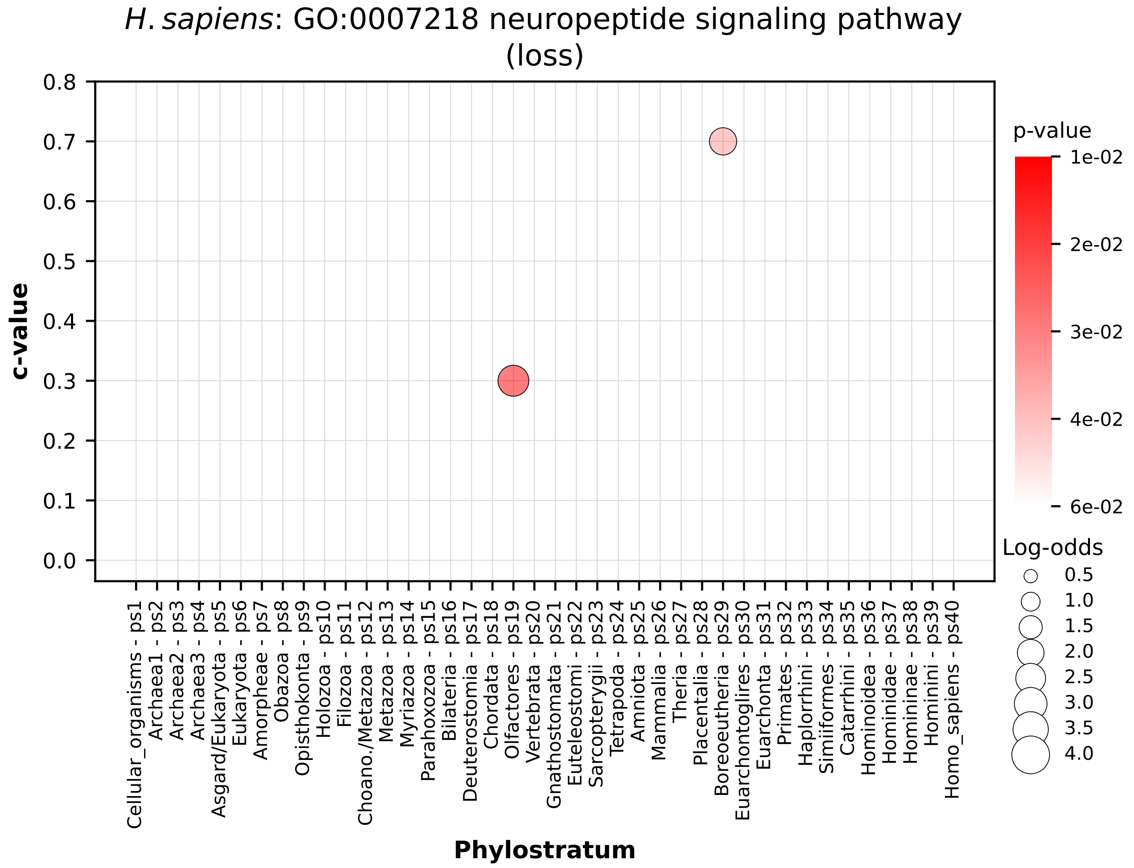

H. sapiens: GO:0007218 neuropeptide signaling pathway (loss)
c-value
0.0
0.1
0.2
0.3
0.4
0.5
0.6
0.7
0.8
Phylostratum
Cellular_organisms - ps1
Archaea1 - ps2
Archaea2 - ps3
Archaea3 - ps4
Asgard/Eukaryota - ps5
Eukaryota - ps6
Amorpheae - ps7
Obazoa - ps8
Opisthokonta - ps9
Holozoa - ps10
Filozoa - ps11
Choano./Metazoa - ps12
Metazoa - ps13
Myriazoa - ps14
Parahoxozoa - ps15
Bilateria - ps16
Deuterostomia - ps17
Chordata - ps18
Olfactores - ps19
Vertebrata - ps20
Gnathostomata - ps21
Euteleostomi - ps22
Sarcopterygii - ps23
Tetrapoda - ps24
Amniota - ps25
Mammalia - ps26
Theria - ps27
Placentalia - ps28
Boreoeutheria - ps29
Euarchontoglires - ps30
Euarchonta - ps31
Primates - ps32
Haplorrhini - ps33
Simiiformes - ps34
Catarrhini - ps35
Hominoidea - ps36
Hominidae - ps37
Homininae - ps38
Hominini - ps39
Homo_sapiens - ps40
p-value
1e-02
2e-02
3e-02
4e-02
6e-02
Log-odds
0.5
1.0
1.5
2.0
2.5
3.0
3.5
4.0

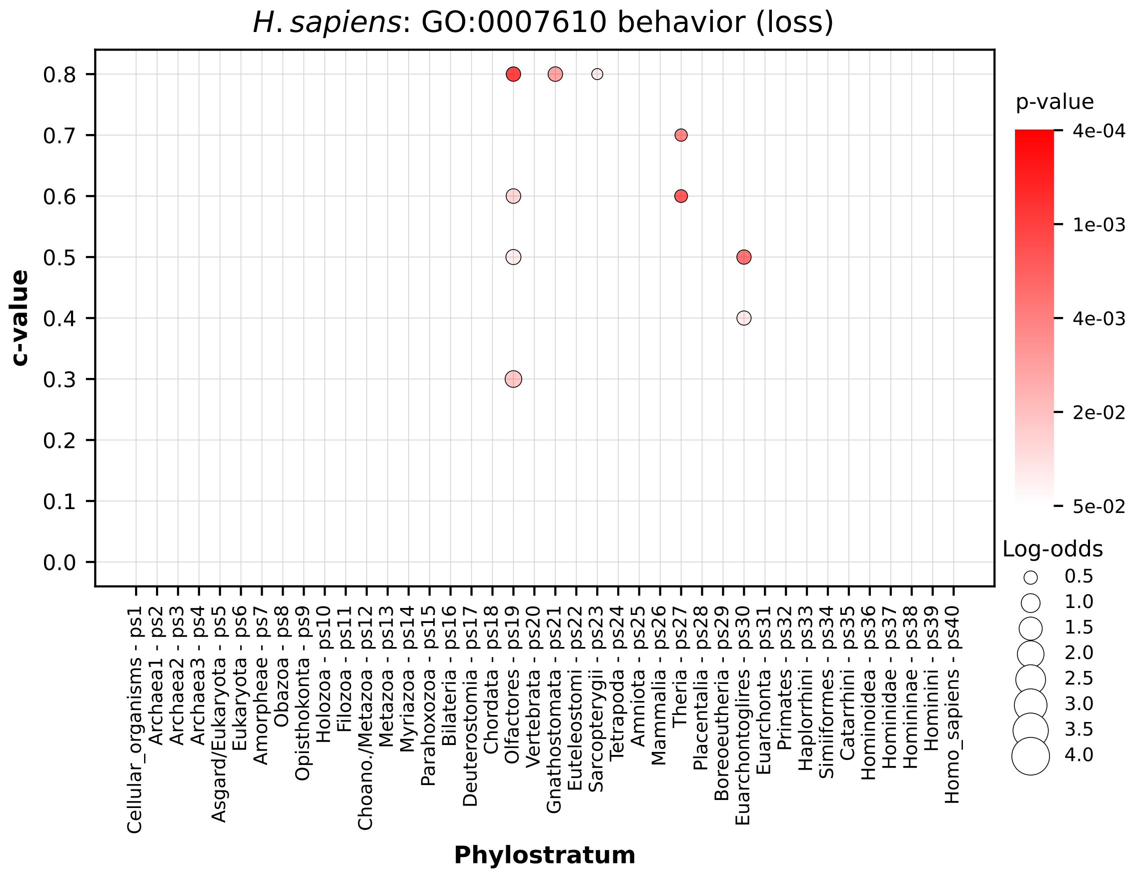

H. sapiens: GO:0007610 behavior (loss)
c-value
Phylostratum
p-value
4e-04
1e-03
4e-03
2e-02
5e-02
Log-odds
0.5
1.0
1.5
2.0
2.5
3.0
3.5
4.0
0.0
0.1
0.2
0.3
0.4
0.5
0.6
0.7
0.8
Cellular_organisms - ps1
Archaea1 - ps2
Archaea2 - ps3
Archaea3 - ps4
Asgard/Eukaryota - ps5
Eukaryota - ps6
Amorpheae - ps7
Obazoa - ps8
Opisthokonta - ps9
Holozoa - ps10
Filozoa - ps11
Choano./Metazoa - ps12
Metazoa - ps13
Myriazoa - ps14
Parahoxozoa - ps15
Bilateria - ps16
Deuterostomia - ps17
Chordata - ps18
Olfactores - ps19
Vertebrata - ps20
Gnathostomata - ps21
Euteleostomi - ps22
Sarcopterygii - ps23
Tetrapoda - ps24
Amniota - ps25
Mammalia - ps26
Theria - ps27
Placentalia - ps28
Boreoeutheria - ps29
Euarchontoglires - ps30
Euarchonta - ps31
Primates - ps32
Haplorrhini - ps33
Simiiformes - ps34
Catarrhini - ps35
Hominoidea - ps36
Hominidae - ps37
Homininae - ps38
Hominini - ps39
Homo_sapiens - ps40

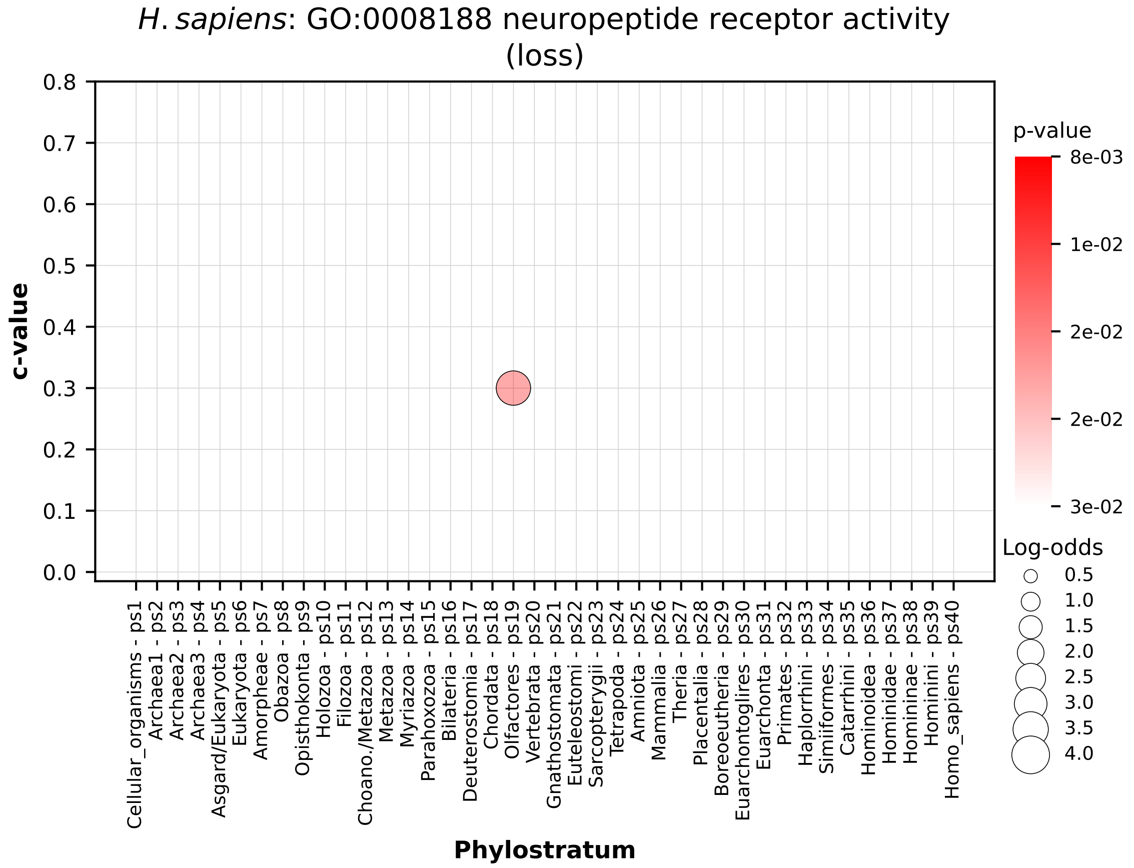

H. sapiens: GO:0008188 neuropeptide receptor activity
(loss)
c-value
0.0
0.1
0.2
0.3
0.4
0.5
0.6
0.7
0.8
Phylostratum
Cellular_organisms - ps1
Archaea1 - ps2
Archaea2 - ps3
Archaea3 - ps4
Asgard/Eukaryota - ps5
Eukaryota - ps6
Amorpheae - ps7
Obazoa - ps8
Opisthokonta - ps9
Holozoa - ps10
Filozoa - ps11
Choano./Metazoa - ps12
Metazoa - ps13
Myriazoa - ps14
Parahoxozoa - ps15
Bilateria - ps16
Deuterostomia - ps17
Chordata - ps18
Olfactores - ps19
Vertebrata - ps20
Gnathostomata - ps21
Euteleostomi - ps22
Sarcopterygii - ps23
Tetrapoda - ps24
Amniota - ps25
Mammalia - ps26
Theria - ps27
Placentalia - ps28
Boreoeutheria - ps29
Euarchontoglires - ps30
Euarchonta - ps31
Primates - ps32
Haplorrhini - ps33
Simiiformes - ps34
Catarrhini - ps35
Hominoidea - ps36
Hominidae - ps37
Homininae - ps38
Hominini - ps39
Homo_sapiens - ps40
p-value
8e-03
1e-02
2e-02
2e-02
3e-02
Log-odds
0.5
1.0
1.5
2.0
2.5
3.0
3.5
4.0

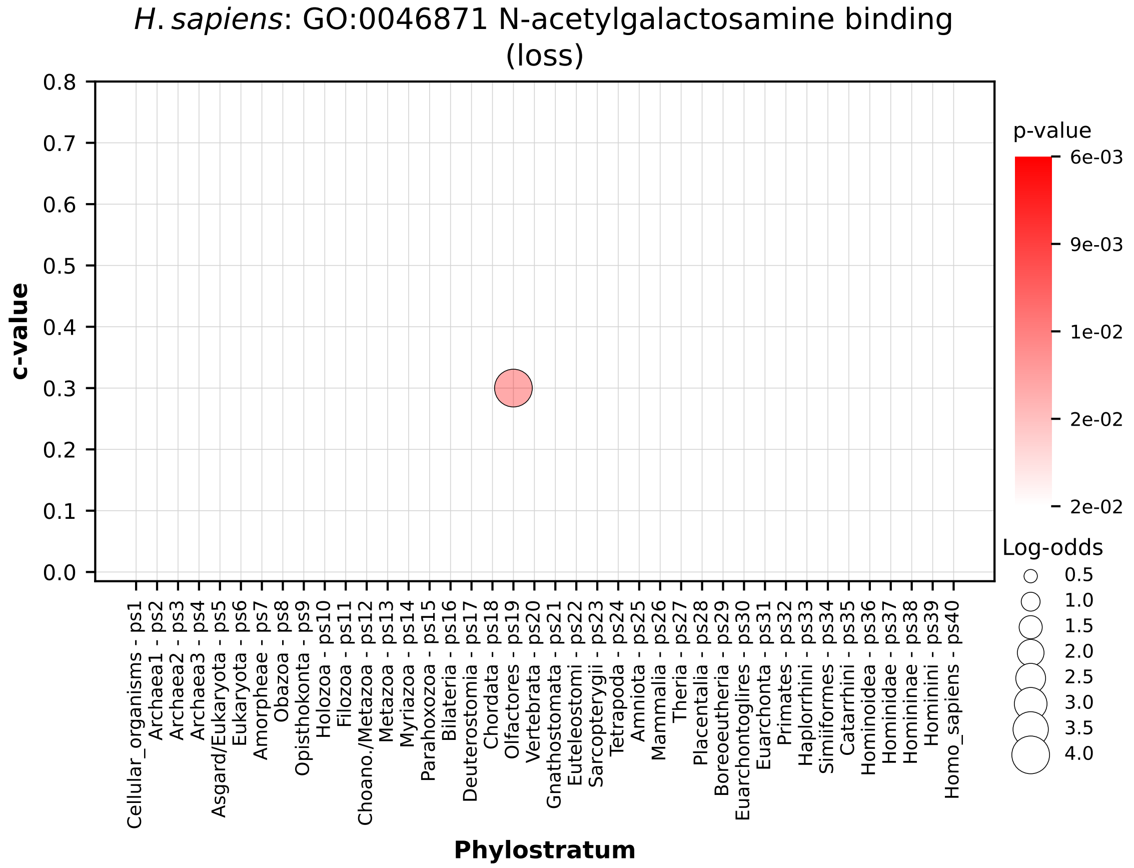

H. sapiens: GO:0046871 N-acetylgalactosamine binding (loss)
c-value
0.8
0.7
0.6
0.5
0.4
0.3
0.2
0.1
0.0
Phylostratum
Cellular_organisms - ps1
Archaea1 - ps2
Archaea2 - ps3
Archaea3 - ps4
Asgard/Eukaryota - ps5
Eukaryota - ps6
Amorpheae - ps7
Obazoa - ps8
Opisthokonta - ps9
Holozoa - ps10
Filozoa - ps11
Choano./Metazoa - ps12
Metazoa - ps13
Myriazoa - ps14
Parahoxozoa - ps15
Bilateria - ps16
Deuterostomia - ps17
Chordata - ps18
Olfactores - ps19
Vertebrata - ps20
Gnathostomata - ps21
Euteleostomi - ps22
Sarcopterygii - ps23
Tetrapoda - ps24
Amniota - ps25
Mammalia - ps26
Theria - ps27
Placentalia - ps28
Boreoeutheria - ps29
Euarchontoglires - ps30
Euarchonta - ps31
Primates - ps32
Haplorrhini - ps33
Simiiformes - ps34
Catarrhini - ps35
Hominoidea - ps36
Hominidae - ps37
Homininae - ps38
Hominini - ps39
Homo_sapiens - ps40
p-value
6e-03
9e-03
1e-02
2e-02
2e-02
Log-odds
0.5
1.0
1.5
2.0
2.5
3.0
3.5
4.0

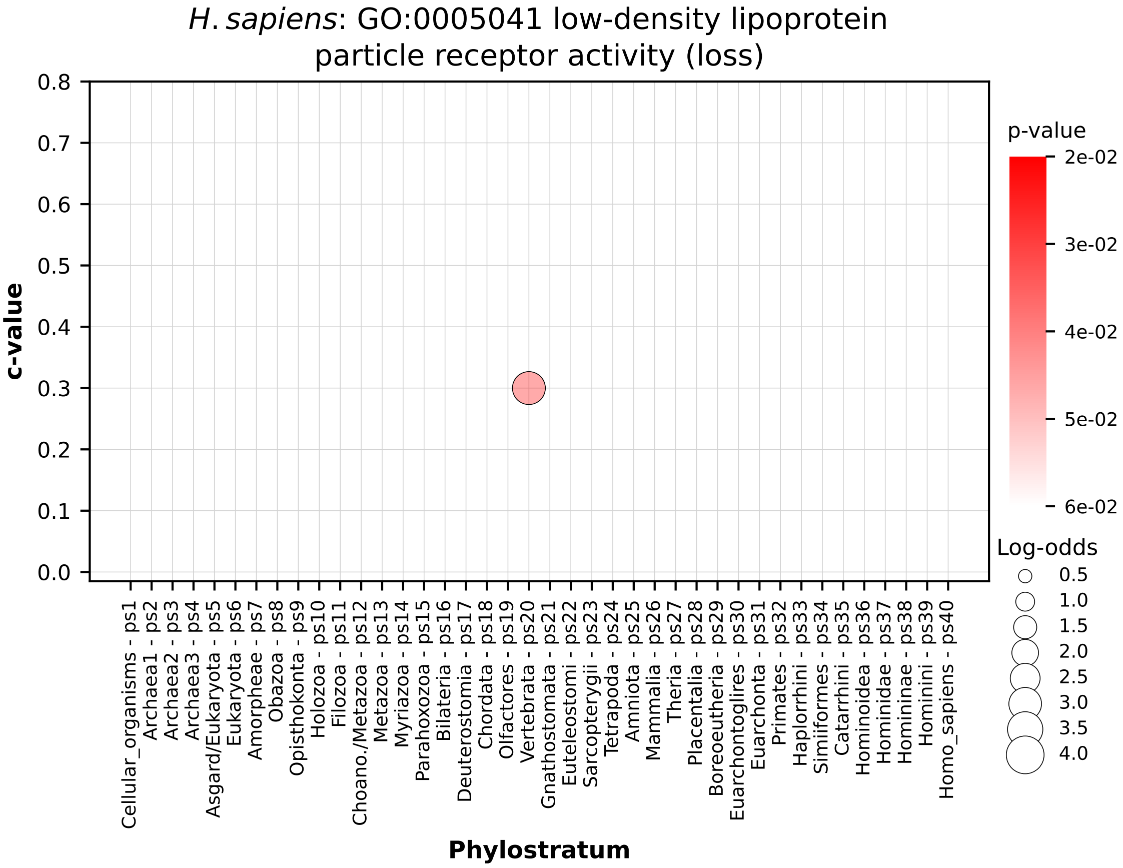

H. sapiens: GO:0005041 low-density lipoprotein particle receptor activity (loss)
c-value
0.0
0.1
0.2
0.3
0.4
0.5
0.6
0.7
0.8
Phylostratum
Cellular_organisms - ps1
Archaea1 - ps2
Archaea2 - ps3
Archaea3 - ps4
Asgard/Eukaryota - ps5
Eukaryota - ps6
Amorpheae - ps7
Obazoa - ps8
Opisthokonta - ps9
Holozoa - ps10
Filozoa - ps11
Choano./Metazoa - ps12
Metazoa - ps13
Myriazoa - ps14
Parahoxozoa - ps15
Bilateria - ps16
Deuterostomia - ps17
Chordata - ps18
Olfactores - ps19
Vertebrata - ps20
Gnathostomata - ps21
Euteleostomi - ps22
Sarcopterygii - ps23
Tetrapoda - ps24
Amniota - ps25
Mammalia - ps26
Theria - ps27
Placentalia - ps28
Boreoeutheria - ps29
Euarchontoglires - ps30
Euarchonta - ps31
Primates - ps32
Haplorrhini - ps33
Simiiformes - ps34
Catarrhini - ps35
Hominoidea - ps36
Hominidae - ps37
Homininae - ps38
Hominini - ps39
Homo_sapiens - ps40
p-value
2e-02
3e-02
4e-02
5e-02
6e-02
Log-odds
0.5
1.0
1.5
2.0
2.5
3.0
3.5
4.0

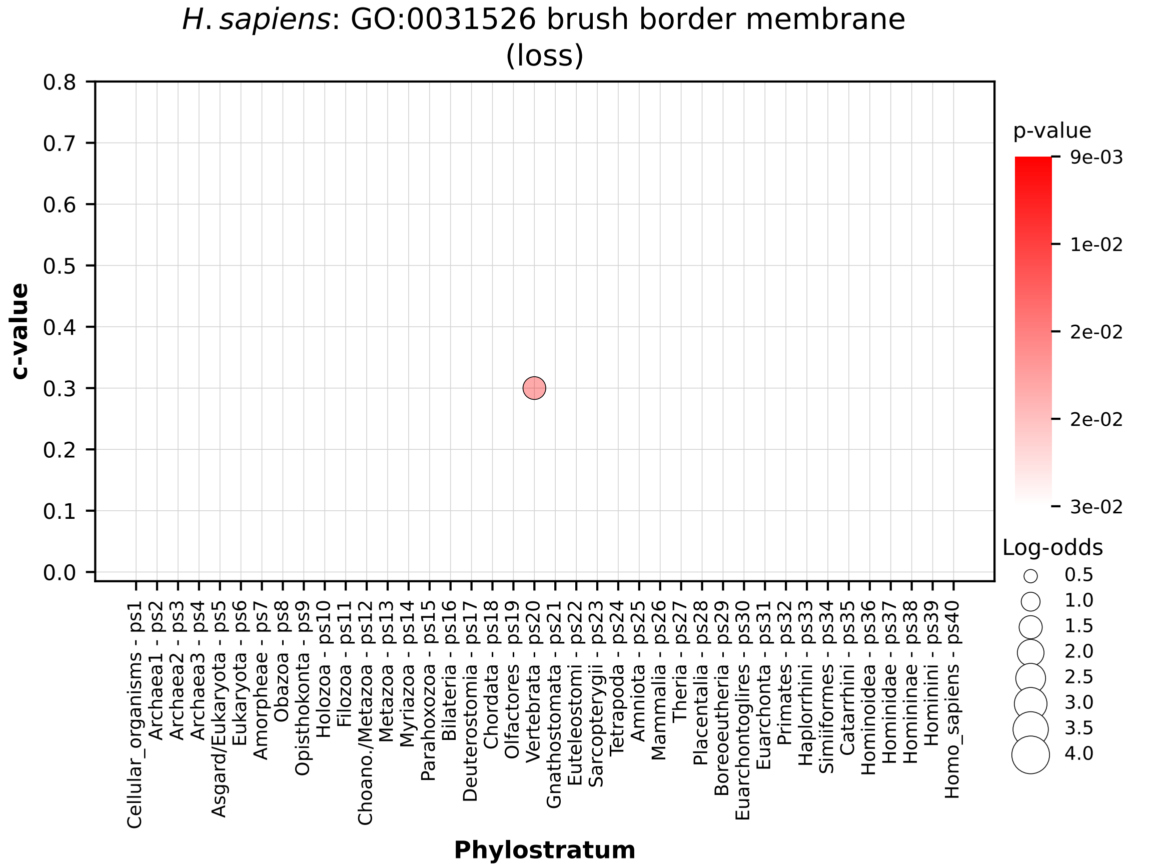

H. sapiens: GO:0031526 brush border membrane
(loss)
c-value
0.0
0.1
0.2
0.3
0.4
0.5
0.6
0.7
0.8
Phylostratum
Cellular_organisms - ps1
Archaea1 - ps2
Archaea2 - ps3
Archaea3 - ps4
Asgard/Eukaryota - ps5
Eukaryota - ps6
Amorpheae - ps7
Obazoa - ps8
Opisthokonta - ps9
Holozoa - ps10
Filozoa - ps11
Choano./Metazoa - ps12
Metazoa - ps13
Myriazoa - ps14
Parahoxozoa - ps15
Bilateria - ps16
Deuterostomia - ps17
Chordata - ps18
Olfactores - ps19
Vertebrata - ps20
Gnathostomata - ps21
Euteleostomi - ps22
Sarcopterygii - ps23
Tetrapoda - ps24
Amniota - ps25
Mammalia - ps26
Theria - ps27
Placentalia - ps28
Boreoeutheria - ps29
Euarchontoglires - ps30
Euarchonta - ps31
Primates - ps32
Haplorrhini - ps33
Simiiformes - ps34
Catarrhini - ps35
Hominoidea - ps36
Hominidae - ps37
Homininae - ps38
Hominini - ps39
Homo_sapiens - ps40
p-value
9e-03
1e-02
2e-02
2e-02
3e-02
Log-odds
0.5
1.0
1.5
2.0
2.5
3.0
3.5
4.0

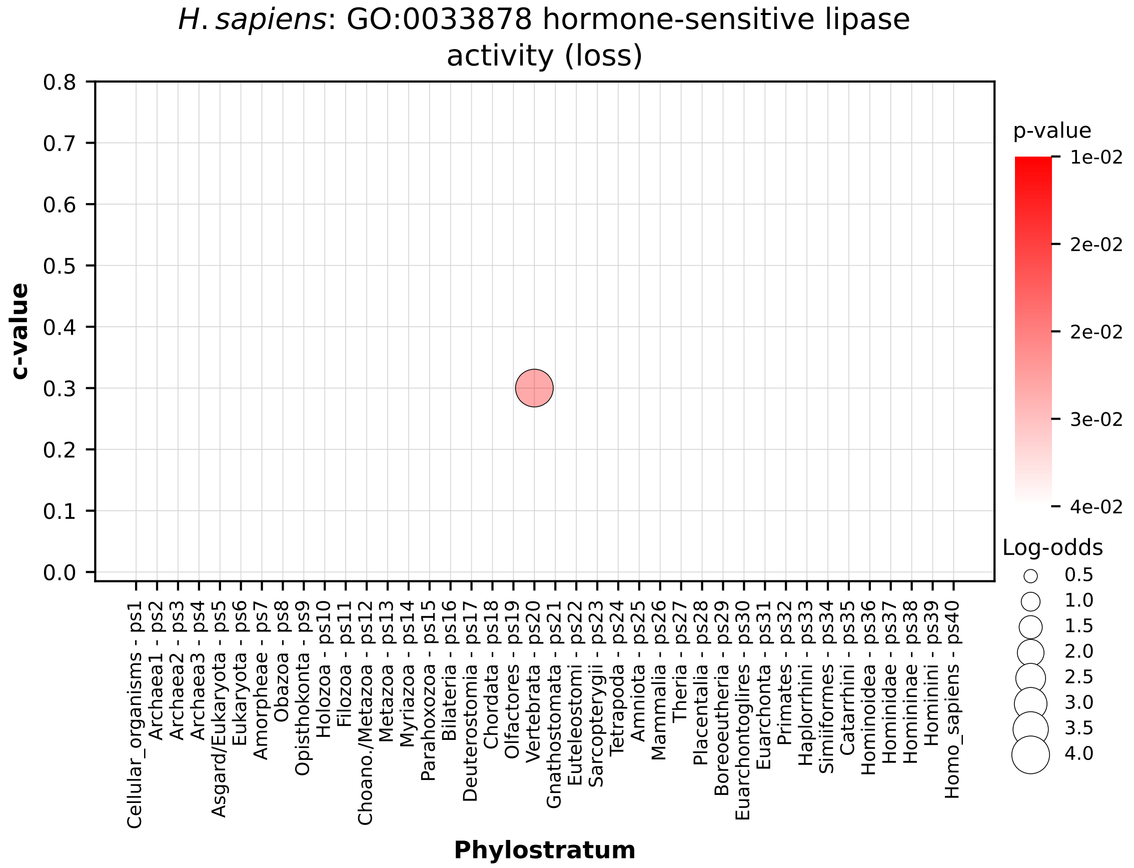

H. sapiens: GO:0033878 hormone-sensitive lipase activity (loss)
c-value
0.8
0.7
0.6
0.5
0.4
0.3
0.2
0.1
0.0
p-value
1e-02
2e-02
2e-02
3e-02
4e-02
Log-odds
0.5
1.0
1.5
2.0
2.5
3.0
3.5
4.0
Cellular_organisms - ps1
Archaea1 - ps2
Archaea2 - ps3
Archaea3 - ps4
Asgard/Eukaryota - ps5
Eukaryota - ps6
Amorpheae - ps7
Obazoa - ps8
Opisthokonta - ps9
Holozoa - ps10
Filozoa - ps11
Choano./Metazoa - ps12
Metazoa - ps13
Myriazoa - ps14
Parahoxozoa - ps15
Bilateria - ps16
Deuterostomia - ps17
Chordata - ps18
Olfactores - ps19
Vertebrata - ps20
Gnathostomata - ps21
Euteleostomi - ps22
Sarcopterygii - ps23
Tetrapoda - ps24
Amniota - ps25
Mammalia - ps26
Theria - ps27
Placentalia - ps28
Boreoeutheria - ps29
Euarchontoglires - ps30
Euarchonta - ps31
Primates - ps32
Haplorrhini - ps33
Simiiformes - ps34
Catarrhini - ps35
Hominoidea - ps36
Hominidae - ps37
Homininae - ps38
Hominini - ps39
Homo_sapiens - ps40
Phylostratum

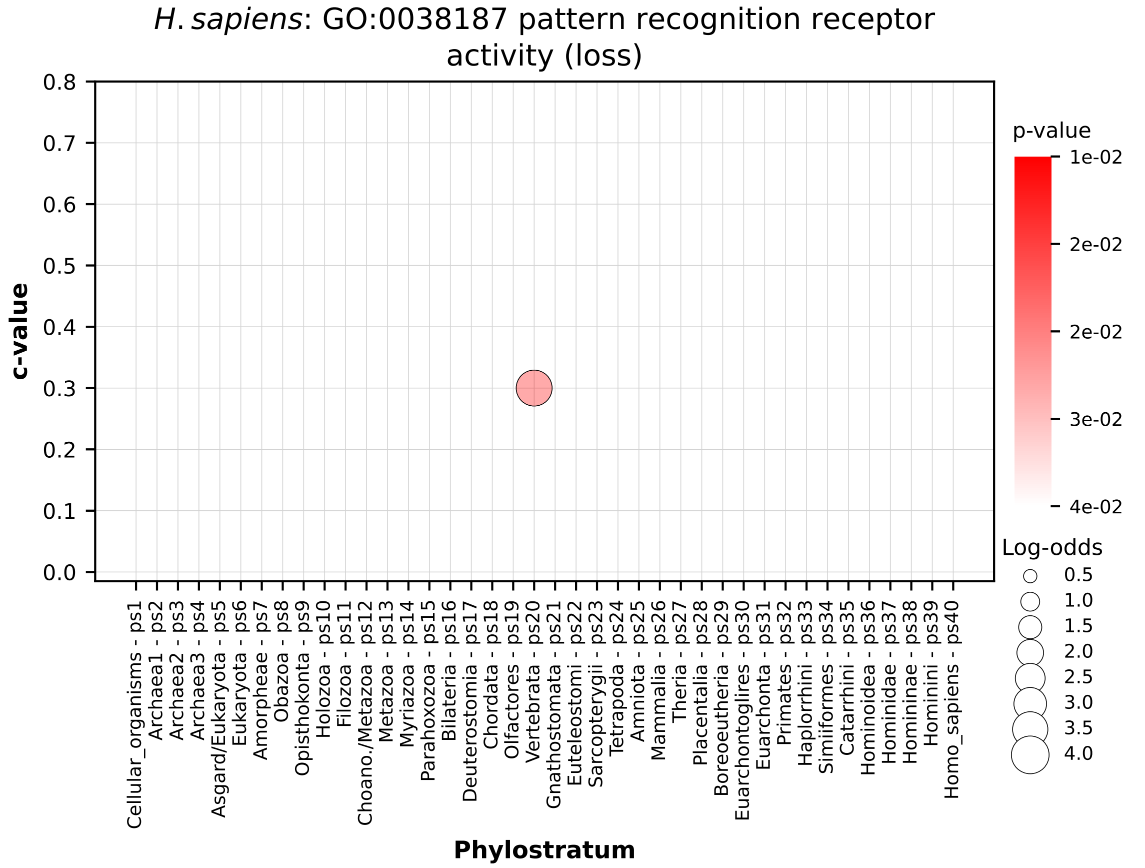

H. sapiens: GO:0038187 pattern recognition receptor activity (loss)
c-value
0.0
0.1
0.2
0.3
0.4
0.5
0.6
0.7
0.8
Phylostratum
Cellular_organisms - ps1
Archaea1 - ps2
Archaea2 - ps3
Archaea3 - ps4
Asgard/Eukaryota - ps5
Eukaryota - ps6
Amorpheae - ps7
Obazoa - ps8
Opisthokonta - ps9
Holozoa - ps10
Filozoa - ps11
Choano./Metazoa - ps12
Metazoa - ps13
Myriazoa - ps14
Parahoxozoa - ps15
Bilateria - ps16
Deuterostomia - ps17
Chordata - ps18
Olfactores - ps19
Vertebrata - ps20
Gnathostomata - ps21
Euteleostomi - ps22
Sarcopterygii - ps23
Tetrapoda - ps24
Amniota - ps25
Mammalia - ps26
Theria - ps27
Placentalia - ps28
Boreoeutheria - ps29
Euarchontoglires - ps30
Euarchonta - ps31
Primates - ps32
Haplorrhini - ps33
Simiiformes - ps34
Catarrhini - ps35
Hominoidea - ps36
Hominidae - ps37
Homininae - ps38
Hominini - ps39
Homo_sapiens - ps40
p-value
1e-02
2e-02
2e-02
3e-02
4e-02
Log-odds
0.5
1.0
1.5
2.0
2.5
3.0
3.5
4.0

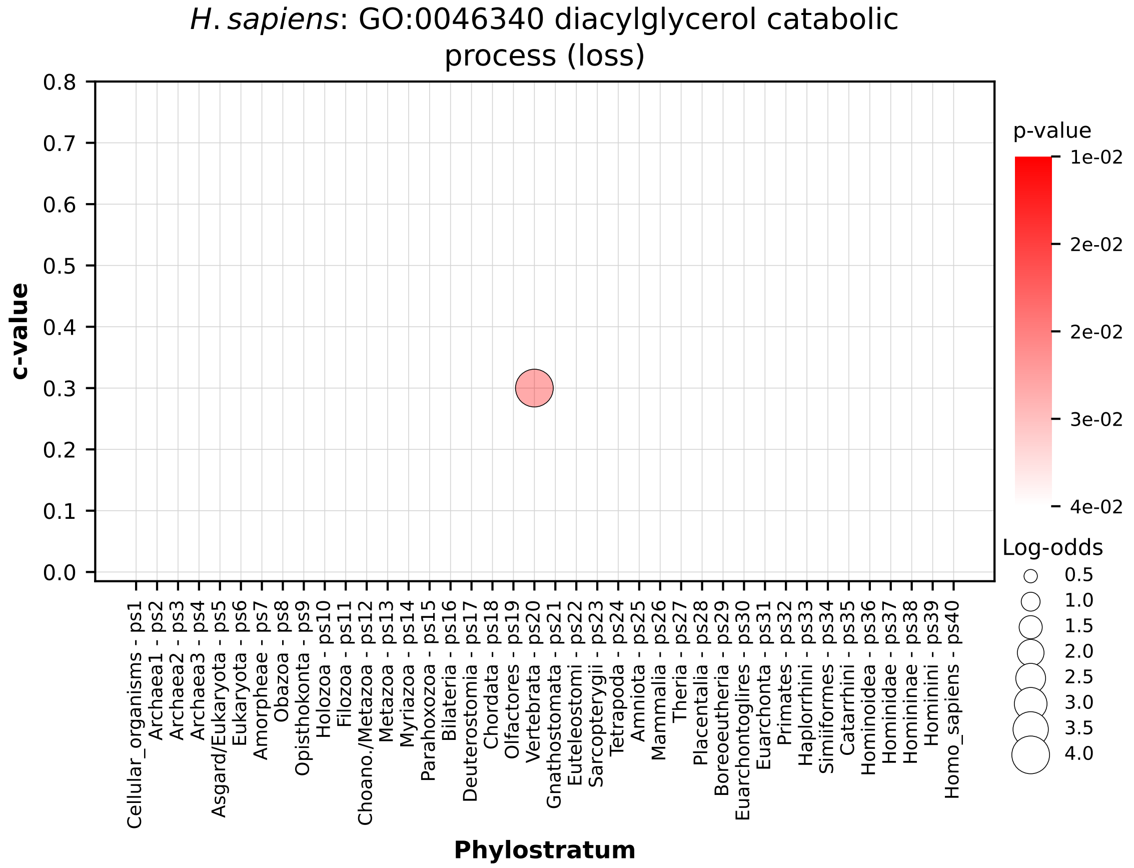

H. sapiens: GO:0046340 diacylglycerol catabolic process (loss)
c-value
0.8
0.7
0.6
0.5
0.4
0.3
0.2
0.1
0.0
Phylostratum
Cellular_organisms - ps1
Archaea1 - ps2
Archaea2 - ps3
Archaea3 - ps4
Asgard/Eukaryota - ps5
Eukaryota - ps6
Amorpheae - ps7
Obazoa - ps8
Opisthokonta - ps9
Holozoa - ps10
Filozoa - ps11
Choano./Metazoa - ps12
Metazoa - ps13
Myriazoa - ps14
Parahoxozoa - ps15
Bilateria - ps16
Deuterostomia - ps17
Chordata - ps18
Olfactores - ps19
Vertebrata - ps20
Gnathostomata - ps21
Euteleostomi - ps22
Sarcopterygii - ps23
Tetrapoda - ps24
Amniota - ps25
Mammalia - ps26
Theria - ps27
Placentalia - ps28
Boreoeutheria - ps29
Euarchontoglires - ps30
Euarchonta - ps31
Primates - ps32
Haplorrhini - ps33
Simiiformes - ps34
Catarrhini - ps35
Hominoidea - ps36
Hominidae - ps37
Homininae - ps38
Hominini - ps39
Homo_sapiens - ps40
p-value
1e-02
2e-02
2e-02
3e-02
4e-02
Log-odds
0.5
1.0
1.5
2.0
2.5
3.0
3.5
4.0

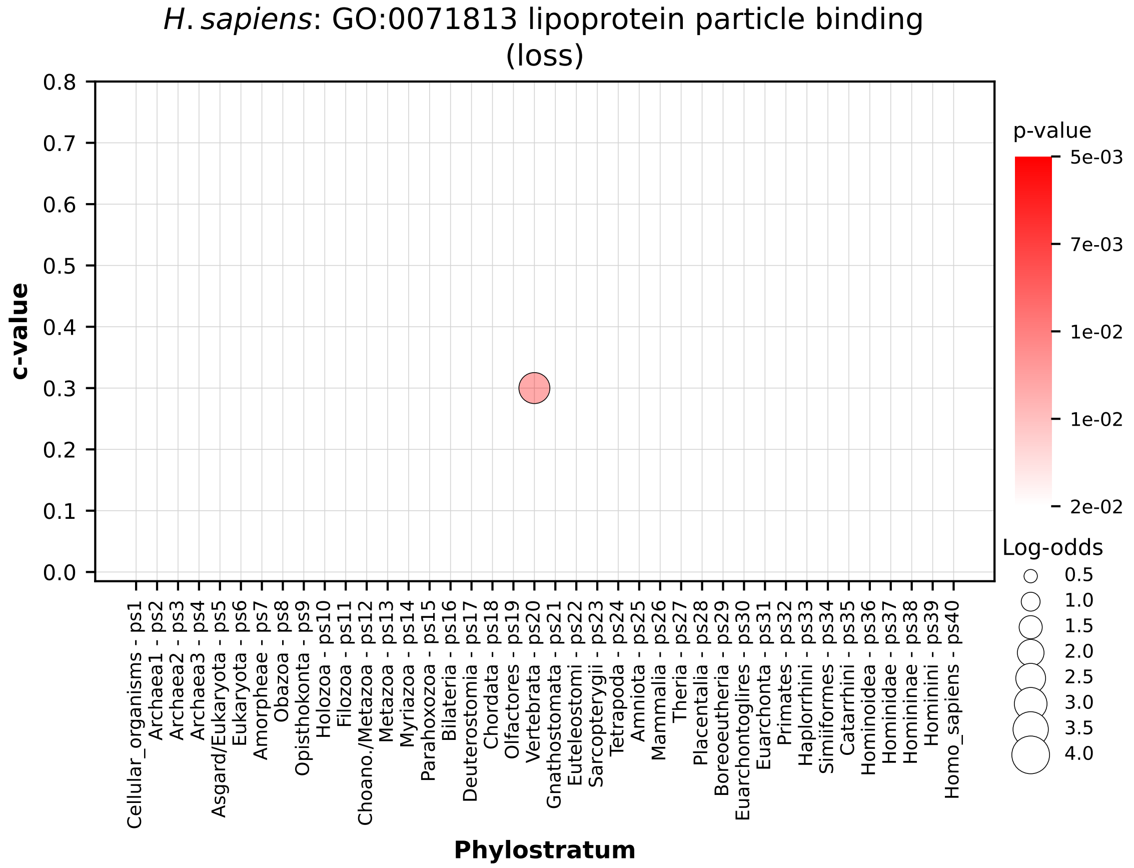

H. sapiens: GO:0071813 lipoprotein particle binding (loss)
c-value
0.8
0.7
0.6
0.5
0.4
0.3
0.2
0.1
0.0
p-value
5e-03
7e-03
1e-02
1e-02
2e-02
Log-odds
0.5
1.0
1.5
2.0
2.5
3.0
3.5
4.0
Cellular_organisms - ps1
Archaea1 - ps2
Archaea2 - ps3
Archaea3 - ps4
Asgard/Eukaryota - ps5
Eukaryota - ps6
Amorpheae - ps7
Obazoa - ps8
Opisthokonta - ps9
Holozoa - ps10
Filozoa - ps11
Choano./Metazoa - ps12
Metazoa - ps13
Myriazoa - ps14
Parahoxozoa - ps15
Bilateria - ps16
Deuterostomia - ps17
Chordata - ps18
Olfactores - ps19
Vertebrata - ps20
Gnathostomata - ps21
Euteleostomi - ps22
Sarcopterygii - ps23
Tetrapoda - ps24
Amniota - ps25
Mammalia - ps26
Theria - ps27
Placentalia - ps28
Boreoeutheria - ps29
Euarchontoglires - ps30
Euarchonta - ps31
Primates - ps32
Haplorrhini - ps33
Simiiformes - ps34
Catarrhini - ps35
Hominoidea - ps36
Hominidae - ps37
Homininae - ps38
Hominini - ps39
Homo_sapiens - ps40
Phylostratum

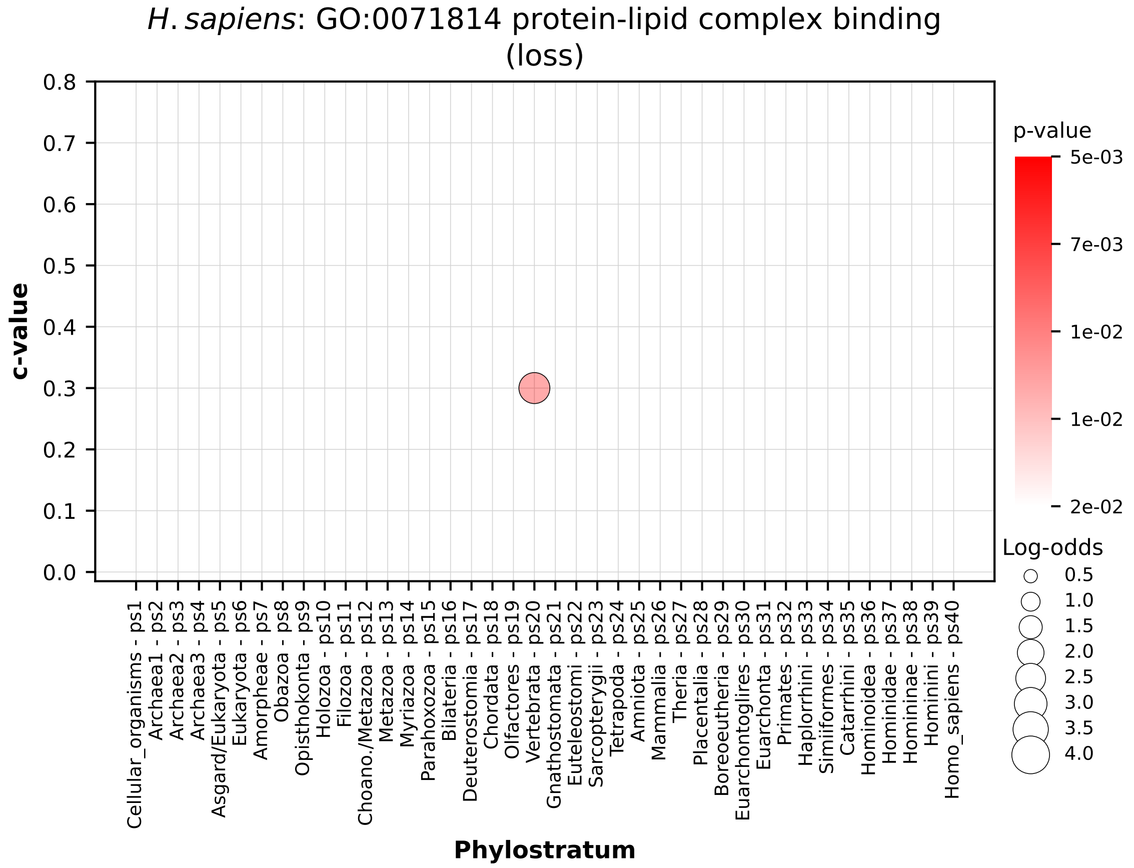
H. sapiens: GO:0071814 protein-lipid complex binding (loss)
c-value
0.0
0.1
0.2
0.3
0.4
0.5
0.6
0.7
0.8
Phylostratum
Cellular_organisms - ps1
Archaea1 - ps2
Archaea2 - ps3
Archaea3 - ps4
Asgard/Eukaryota - ps5
Eukaryota - ps6
Amorpheae - ps7
Obazoa - ps8
Opisthokonta - ps9
Holozoa - ps10
Filozoa - ps11
Choano./Metazoa - ps12
Metazoa - ps13
Myriazoa - ps14
Parahoxozoa - ps15
Bilateria - ps16
Deuterostomia - ps17
Chordata - ps18
Olfactores - ps19
Vertebrata - ps20
Gnathostomata - ps21
Euteleostomi - ps22
Sarcopterygii - ps23
Tetrapoda - ps24
Amniota - ps25
Mammalia - ps26
Theria - ps27
Placentalia - ps28
Boreoeutheria - ps29
Euarchontoglires - ps30
Euarchonta - ps31
Primates - ps32
Haplorrhini - ps33
Simiiformes - ps34
Catarrhini - ps35
Hominoidea - ps36
Hominidae - ps37
Homininae - ps38
Hominini - ps39
Homo_sapiens - ps40
p-value
5e-03
7e-03
1e-02
1e-02
2e-02
Log-odds
0.5
1.0
1.5
2.0
2.5
3.0
3.5
4.0

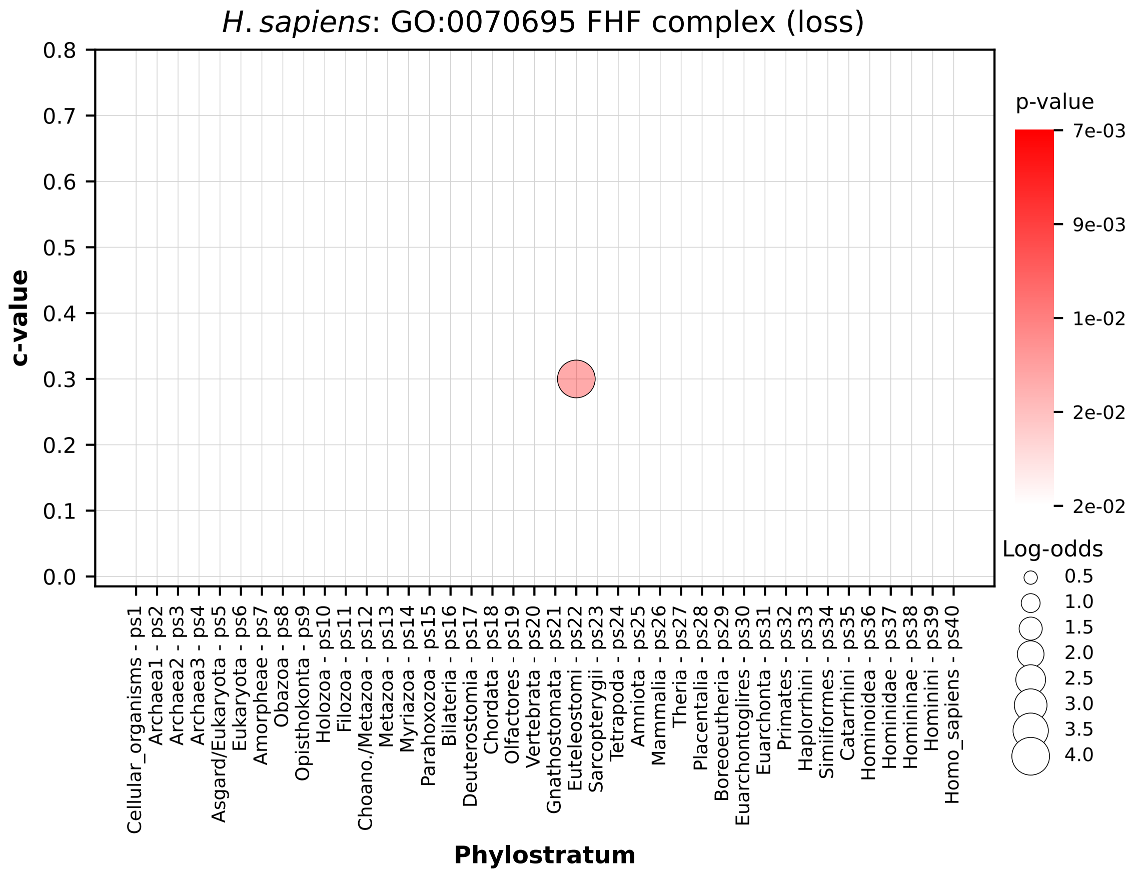

H. sapiens: GO:0070695 FHF complex (loss)
c-value
0.8
0.7
0.6
0.5
0.4
0.3
0.2
0.1
0.0
p-value
7e-03
9e-03
1e-02
2e-02
2e-02
Log-odds
0.5
1.0
1.5
2.0
2.5
3.0
3.5
4.0
Cellular_organisms - ps1
Archaea1 - ps2
Archaea2 - ps3
Archaea3 - ps4
Asgard/Eukaryota - ps5
Eukaryota - ps6
Amorpheae - ps7
Obazoa - ps8
Opisthokonta - ps9
Holozoa - ps10
Filozoa - ps11
Choano./Metazoa - ps12
Metazoa - ps13
Myriazoa - ps14
Parahoxozoa - ps15
Bilateria - ps16
Deuterostomia - ps17
Chordata - ps18
Olfactores - ps19
Vertebrata - ps20
Gnathostomata - ps21
Euteleostomi - ps22
Sarcopterygii - ps23
Tetrapoda - ps24
Amniota - ps25
Mammalia - ps26
Theria - ps27
Placentalia - ps28
Boreoeutheria - ps29
Euarchontoglires - ps30
Euarchonta - ps31
Primates - ps32
Haplorrhini - ps33
Simiiformes - ps34
Catarrhini - ps35
Hominoidea - ps36
Hominidae - ps37
Homininae - ps38
Hominini - ps39
Homo_sapiens - ps40
Phylostratum

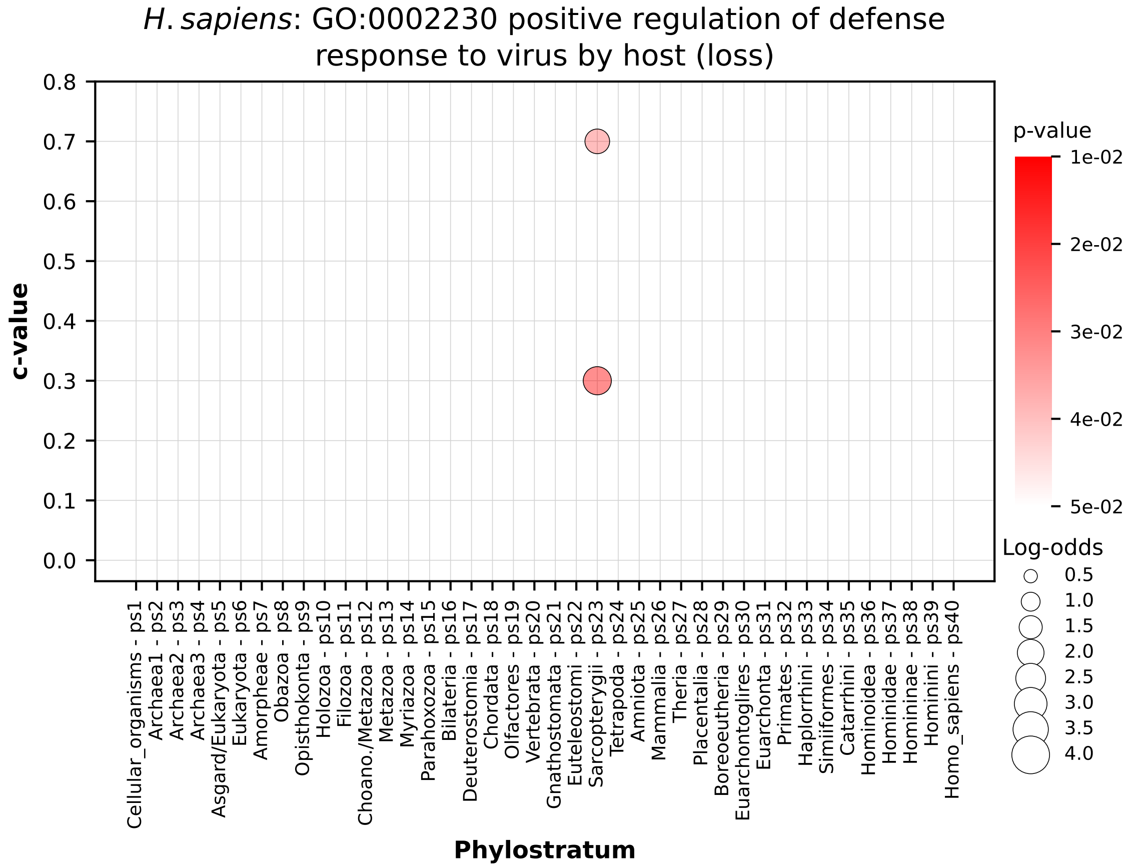

H. sapiens: GO:0002230 positive regulation of defense response to virus by host (loss)
c-value
0.0
0.1
0.2
0.3
0.4
0.5
0.6
0.7
0.8
Phylostratum
Cellular_organisms - ps1
Archaea1 - ps2
Archaea2 - ps3
Archaea3 - ps4
Asgard/Eukaryota - ps5
Eukaryota - ps6
Amorpheae - ps7
Obazoa - ps8
Opisthokonta - ps9
Holozoa - ps10
Filozoa - ps11
Choano./Metazoa - ps12
Metazoa - ps13
Myriazoa - ps14
Parahoxozoa - ps15
Bilateria - ps16
Deuterostomia - ps17
Chordata - ps18
Olfactores - ps19
Vertebrata - ps20
Gnathostomata - ps21
Euteleostomi - ps22
Sarcopterygii - ps23
Tetrapoda - ps24
Amniota - ps25
Mammalia - ps26
Theria - ps27
Placentalia - ps28
Boreoeutheria - ps29
Euarchontoglires - ps30
Euarchonta - ps31
Primates - ps32
Haplorrhini - ps33
Simiiformes - ps34
Catarrhini - ps35
Hominoidea - ps36
Hominidae - ps37
Homininae - ps38
Hominini - ps39
Homo_sapiens - ps40
p-value
1e-02
2e-02
3e-02
4e-02
5e-02
Log-odds
0.5
1.0
1.5
2.0
2.5
3.0
3.5
4.0

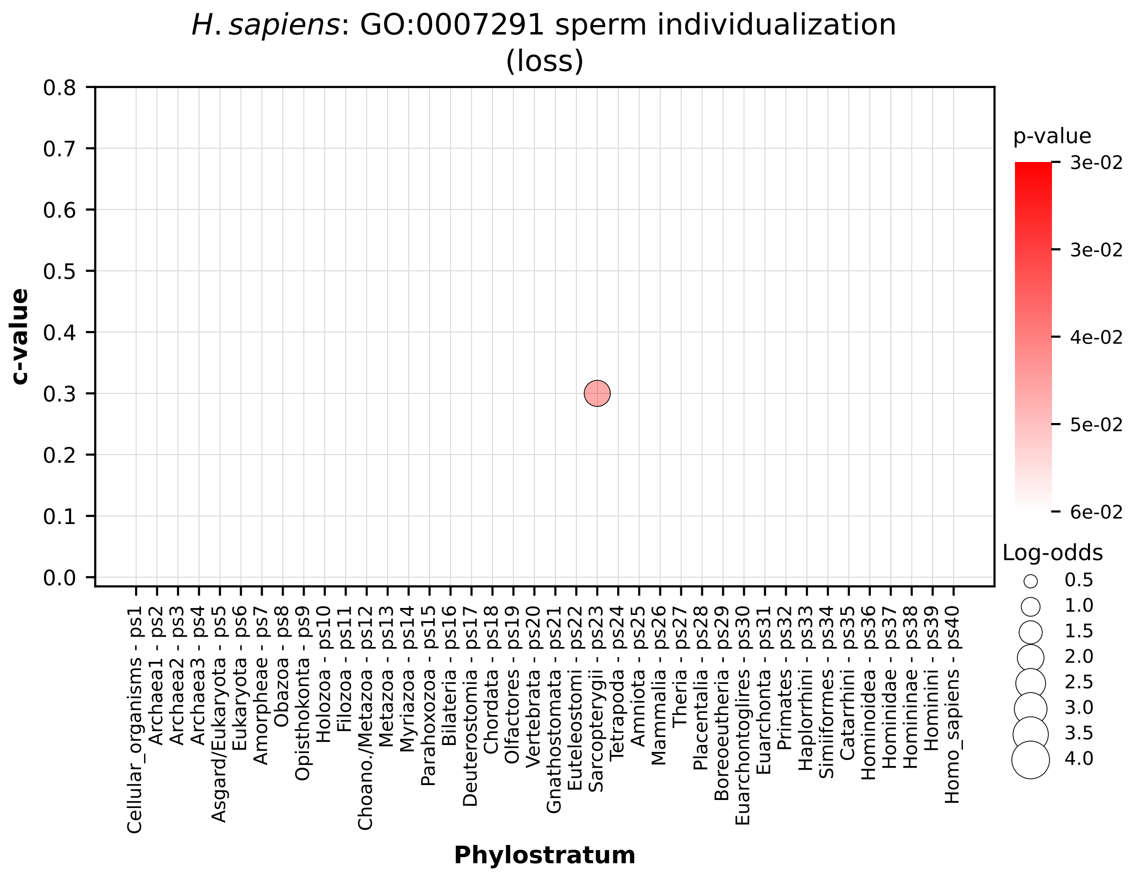

H. sapiens: GO:0007291 sperm individualization (loss)
c-value
0.8
0.7
0.6
0.5
0.4
0.3
0.2
0.1
0.0
Phylostratum
Cellular_organisms - ps1
Archaea1 - ps2
Archaea2 - ps3
Archaea3 - ps4
Asgard/Eukaryota - ps5
Eukaryota - ps6
Amorpheae - ps7
Obazoa - ps8
Opisthokonta - ps9
Holozoa - ps10
Filozoa - ps11
Choano./Metazoa - ps12
Metazoa - ps13
Myriazoa - ps14
Parahoxozoa - ps15
Bilateria - ps16
Deuterostomia - ps17
Chordata - ps18
Olfactores - ps19
Vertebrata - ps20
Gnathostomata - ps21
Euteleostomi - ps22
Sarcopterygii - ps23
Tetrapoda - ps24
Amniota - ps25
Mammalia - ps26
Theria - ps27
Placentalia - ps28
Boreoeutheria - ps29
Euarchontoglires - ps30
Euarchonta - ps31
Primates - ps32
Haplorrhini - ps33
Simiiformes - ps34
Catarrhini - ps35
Hominoidea - ps36
Hominidae - ps37
Homininae - ps38
Hominini - ps39
Homo_sapiens - ps40
p-value
3e-02
3e-02
4e-02
5e-02
6e-02
Log-odds
0.5
1.0
1.5
2.0
2.5
3.0
3.5
4.0

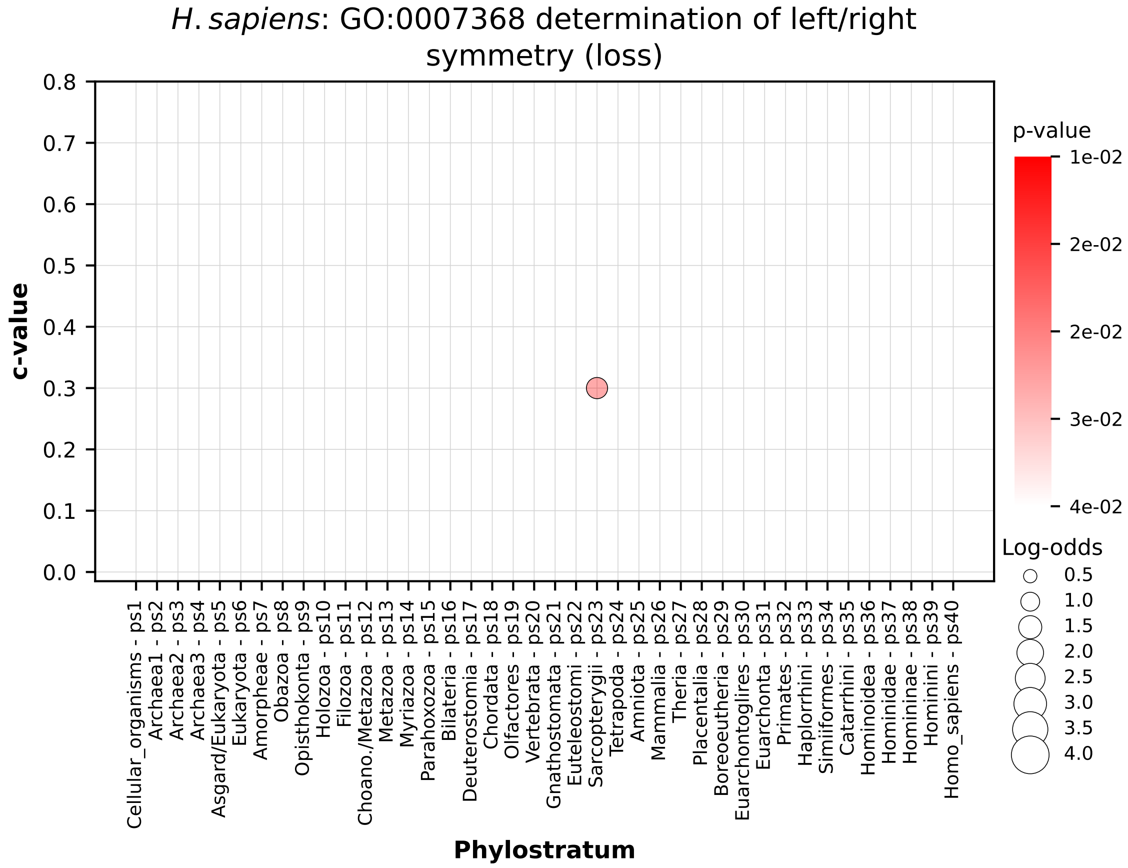

H. sapiens: GO:0007368 determination of left/right symmetry (loss)
c-value
0.8
0.7
0.6
0.5
0.4
0.3
0.2
0.1
0.0
Phylostratum
Cellular_organisms - ps1
Archaea1 - ps2
Archaea2 - ps3
Archaea3 - ps4
Asgard/Eukaryota - ps5
Eukaryota - ps6
Amorpheae - ps7
Obazoa - ps8
Opisthokonta - ps9
Holozoa - ps10
Filozoa - ps11
Choano./Metazoa - ps12
Metazoa - ps13
Myriazoa - ps14
Parahoxozoa - ps15
Bilateria - ps16
Deuterostomia - ps17
Chordata - ps18
Olfactores - ps19
Vertebrata - ps20
Gnathostomata - ps21
Euteleostomi - ps22
Sarcopterygii - ps23
Tetrapoda - ps24
Amniota - ps25
Mammalia - ps26
Theria - ps27
Placentalia - ps28
Boreoeutheria - ps29
Euarchontoglires - ps30
Euarchonta - ps31
Primates - ps32
Haplorrhini - ps33
Simiiformes - ps34
Catarrhini - ps35
Hominoidea - ps36
Hominidae - ps37
Homininae - ps38
Hominini - ps39
Homo_sapiens - ps40
p-value
1e-02
2e-02
2e-02
3e-02
4e-02
Log-odds
0.5
1.0
1.5
2.0
2.5
3.0
3.5
4.0

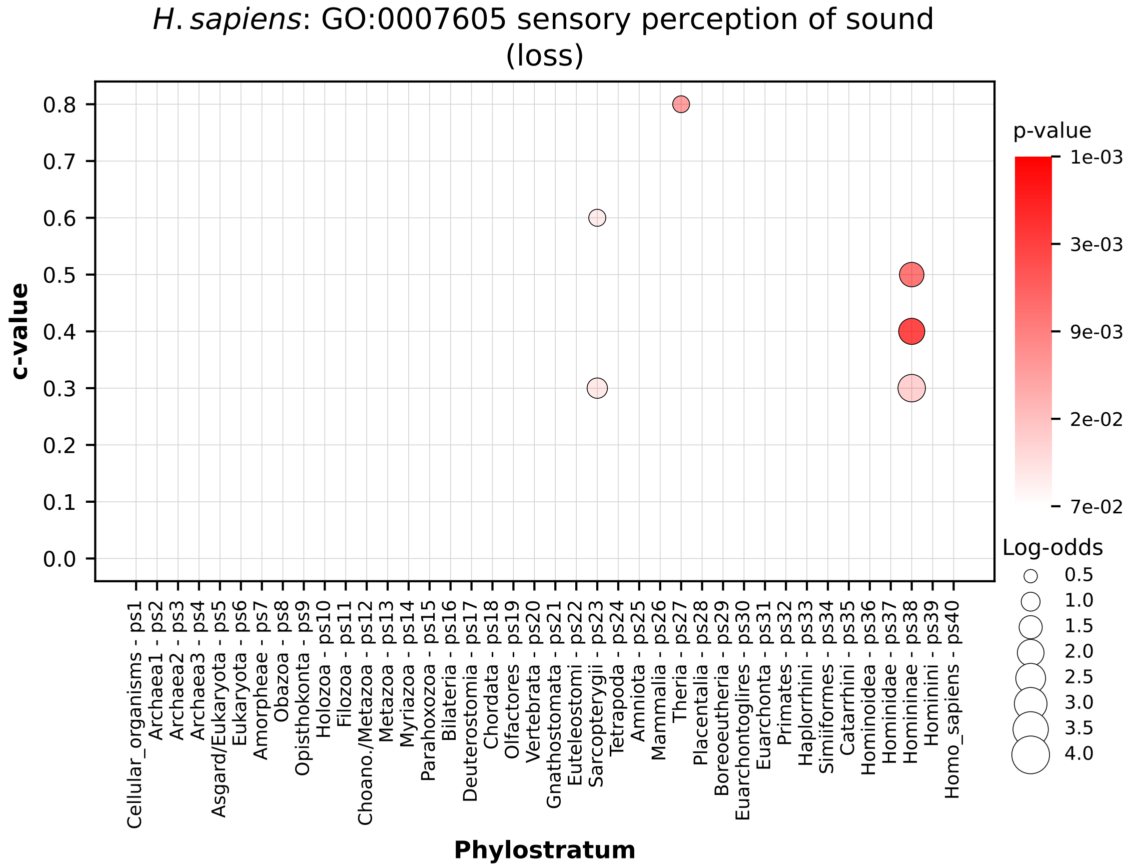

H. sapiens: GO:0007605 sensory perception of sound
(loss)
c-value
0.8
0.7
0.6
0.5
0.4
0.3
0.2
0.1
0.0
p-value
1e-03
3e-03
9e-03
2e-02
7e-02
Log-odds
0.5
1.0
1.5
2.0
2.5
3.0
3.5
4.0
Cellular_organisms - ps1
Archaea1 - ps2
Archaea2 - ps3
Archaea3 - ps4
Asgard/Eukaryota - ps5
Eukaryota - ps6
Amorpheae - ps7
Obazoa - ps8
Opisthokonta - ps9
Holozoa - ps10
Filozoa - ps11
Choano./Metazoa - ps12
Metazoa - ps13
Myriazoa - ps14
Parahoxozoa - ps15
Bilateria - ps16
Deuterostomia - ps17
Chordata - ps18
Olfactores - ps19
Vertebrata - ps20
Gnathostomata - ps21
Euteleostomi - ps22
Sarcopterygii - ps23
Tetrapoda - ps24
Amniota - ps25
Mammalia - ps26
Theria - ps27
Placentalia - ps28
Boreoeutheria - ps29
Euarchontoglires - ps30
Euarchonta - ps31
Primates - ps32
Haplorrhini - ps33
Simiiformes - ps34
Catarrhini - ps35
Hominoidea - ps36
Hominidae - ps37
Homininae - ps38
Hominini - ps39
Homo_sapiens - ps40
Phylostratum

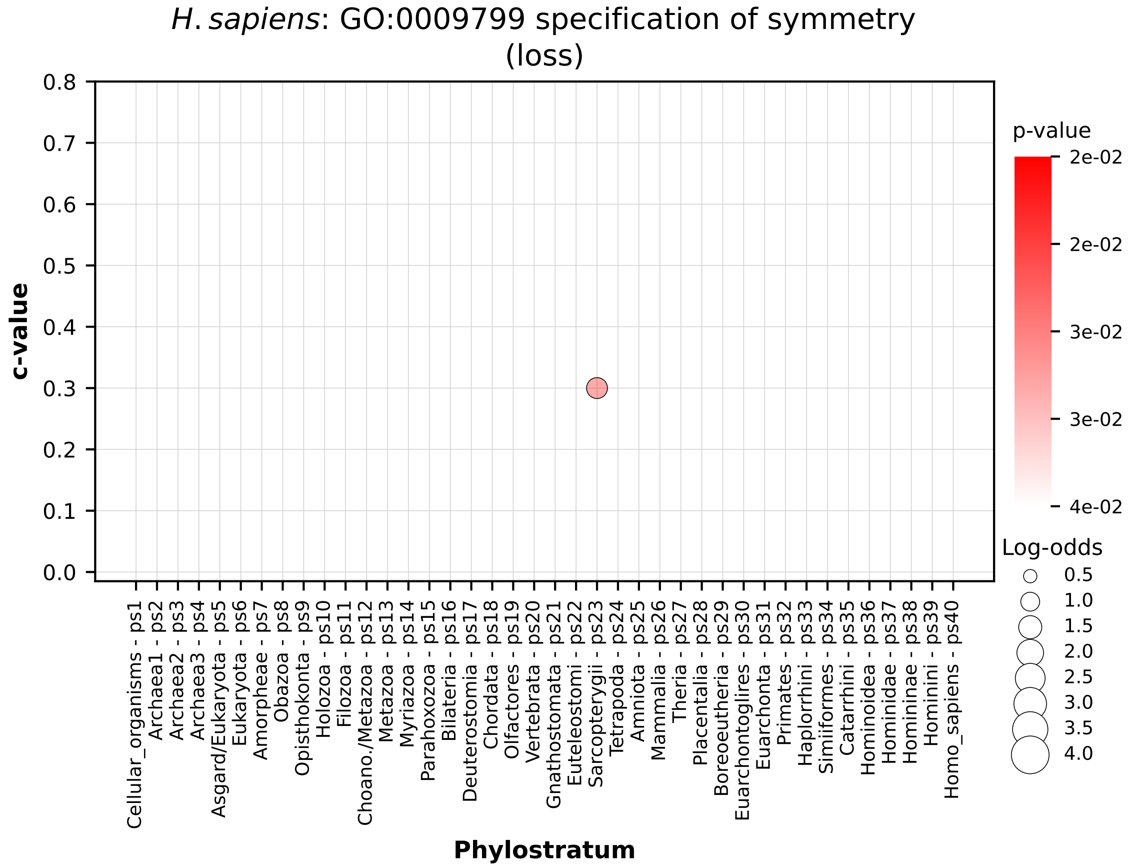

H. sapiens: GO:0009799 specification of symmetry (loss)
c-value
0.8
0.7
0.6
0.5
0.4
0.3
0.2
0.1
0.0
Phylostratum
Cellular_organisms - ps1
Archaea1 - ps2
Archaea2 - ps3
Archaea3 - ps4
Asgard/Eukaryota - ps5
Eukaryota - ps6
Amorpheae - ps7
Obazoa - ps8
Opisthokonta - ps9
Holozoa - ps10
Filozoa - ps11
Choano./Metazoa - ps12
Metazoa - ps13
Myriazoa - ps14
Parahoxozoa - ps15
Bilateria - ps16
Deuterostomia - ps17
Chordata - ps18
Olfactores - ps19
Vertebrata - ps20
Gnathostomata - ps21
Euteleostomi - ps22
Sarcopterygii - ps23
Tetrapoda - ps24
Amniota - ps25
Mammalia - ps26
Theria - ps27
Placentalia - ps28
Boreoeutheria - ps29
Euarchontoglires - ps30
Euarchonta - ps31
Primates - ps32
Haplorrhini - ps33
Simiiformes - ps34
Catarrhini - ps35
Hominoidea - ps36
Hominidae - ps37
Homininae - ps38
Hominini - ps39
Homo_sapiens - ps40
p-value
2e-02
2e-02
3e-02
3e-02
4e-02
Log-odds
0.5
1.0
1.5
2.0
2.5
3.0
3.5
4.0

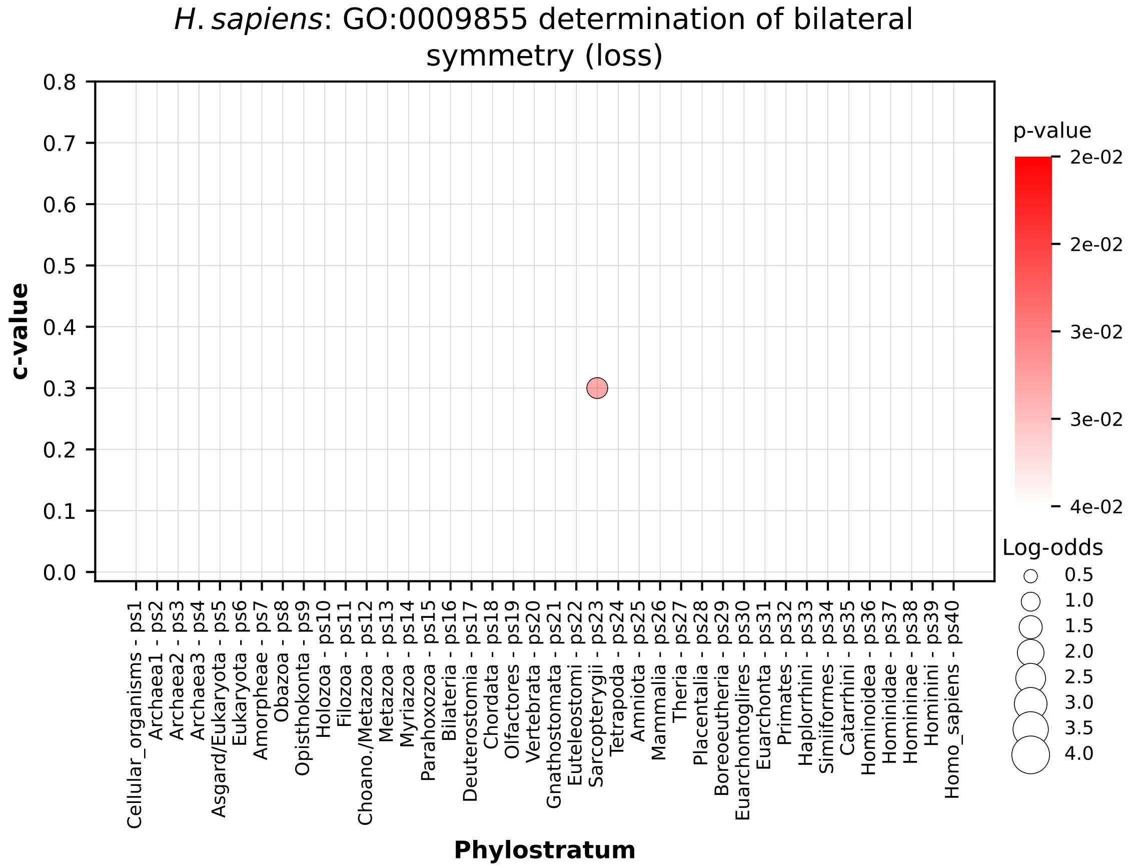

H. sapiens: GO:0009855 determination of bilateral symmetry (loss)
c-value
0.8
0.7
0.6
0.5
0.4
0.3
0.2
0.1
0.0
Phylostratum
Cellular_organisms - ps1
Archaea1 - ps2
Archaea2 - ps3
Archaea3 - ps4
Asgard/Eukaryota - ps5
Eukaryota - ps6
Amorpheae - ps7
Obazoa - ps8
Opisthokonta - ps9
Holozoa - ps10
Filozoa - ps11
Choano./Metazoa - ps12
Metazoa - ps13
Myriazoa - ps14
Parahoxozoa - ps15
Bilateria - ps16
Deuterostomia - ps17
Chordata - ps18
Olfactores - ps19
Vertebrata - ps20
Gnathostomata - ps21
Euteleostomi - ps22
Sarcopterygii - ps23
Tetrapoda - ps24
Amniota - ps25
Mammalia - ps26
Theria - ps27
Placentalia - ps28
Boreoeutheria - ps29
Euarchontoglires - ps30
Euarchonta - ps31
Primates - ps32
Haplorrhini - ps33
Simiiformes - ps34
Catarrhini - ps35
Hominoidea - ps36
Hominidae - ps37
Homininae - ps38
Hominini - ps39
Homo_sapiens - ps40
p-value
2e-02
2e-02
3e-02
3e-02
4e-02
Log-odds
0.5
1.0
1.5
2.0
2.5
3.0
3.5
4.0

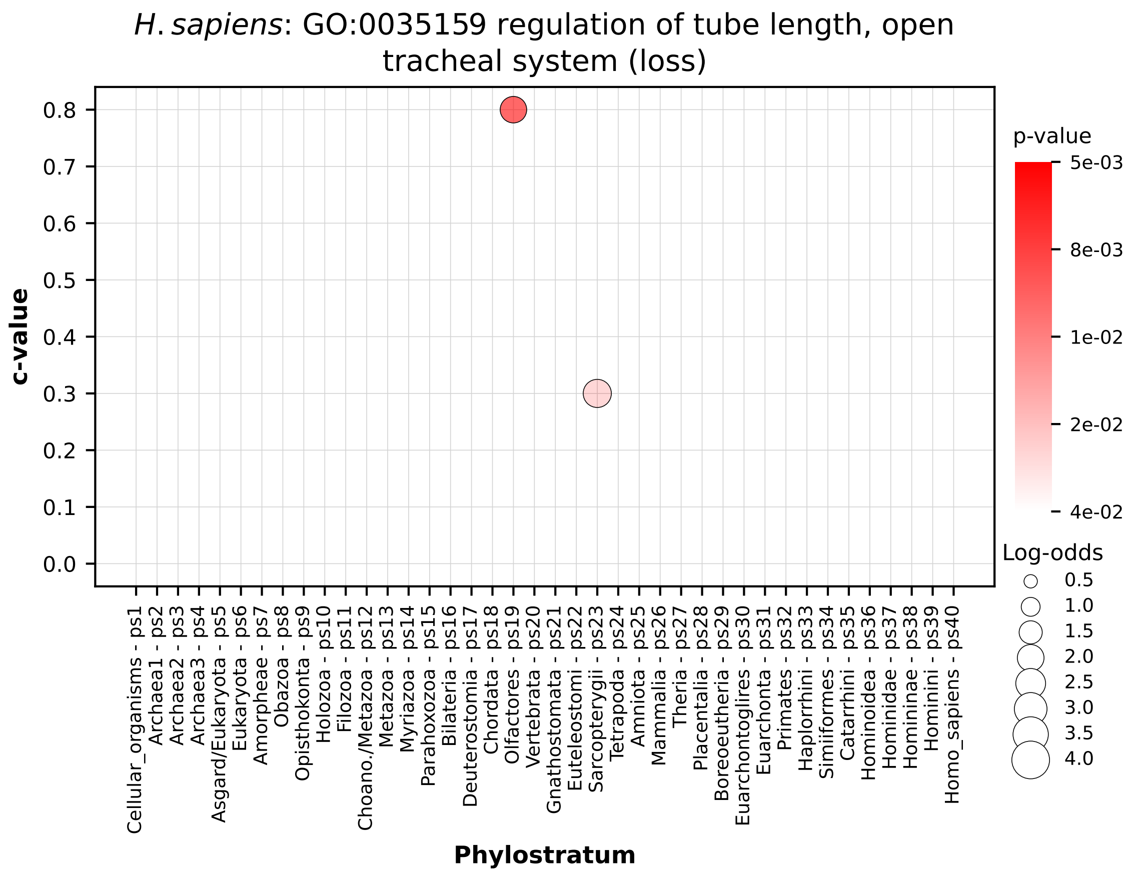

H. sapiens: GO:0035159 regulation of tube length, open tracheal system (loss)
c-value
0.0
0.1
0.2
0.3
0.4
0.5
0.6
0.7
0.8
p-value
5e-03
8e-03
1e-02
2e-02
4e-02
Log-odds
0.5
1.0
1.5
2.0
2.5
3.0
3.5
4.0
Cellular_organisms - ps1
Archaea1 - ps2
Archaea2 - ps3
Archaea3 - ps4
Asgard/Eukaryota - ps5
Eukaryota - ps6
Amorpheae - ps7
Obazoa - ps8
Opisthokonta - ps9
Holozoa - ps10
Filozoa - ps11
Choano./Metazoa - ps12
Metazoa - ps13
Myriazoa - ps14
Parahoxozoa - ps15
Bilateria - ps16
Deuterostomia - ps17
Chordata - ps18
Olfactores - ps19
Vertebrata - ps20
Gnathostomata - ps21
Euteleostomi - ps22
Sarcopterygii - ps23
Tetrapoda - ps24
Amniota - ps25
Mammalia - ps26
Theria - ps27
Placentalia - ps28
Boreoeutheria - ps29
Euarchontoglires - ps30
Euarchonta - ps31
Primates - ps32
Haplorrhini - ps33
Simiiformes - ps34
Catarrhini - ps35
Hominoidea - ps36
Hominidae - ps37
Homininae - ps38
Hominini - ps39
Homo_sapiens - ps40
Phylostratum

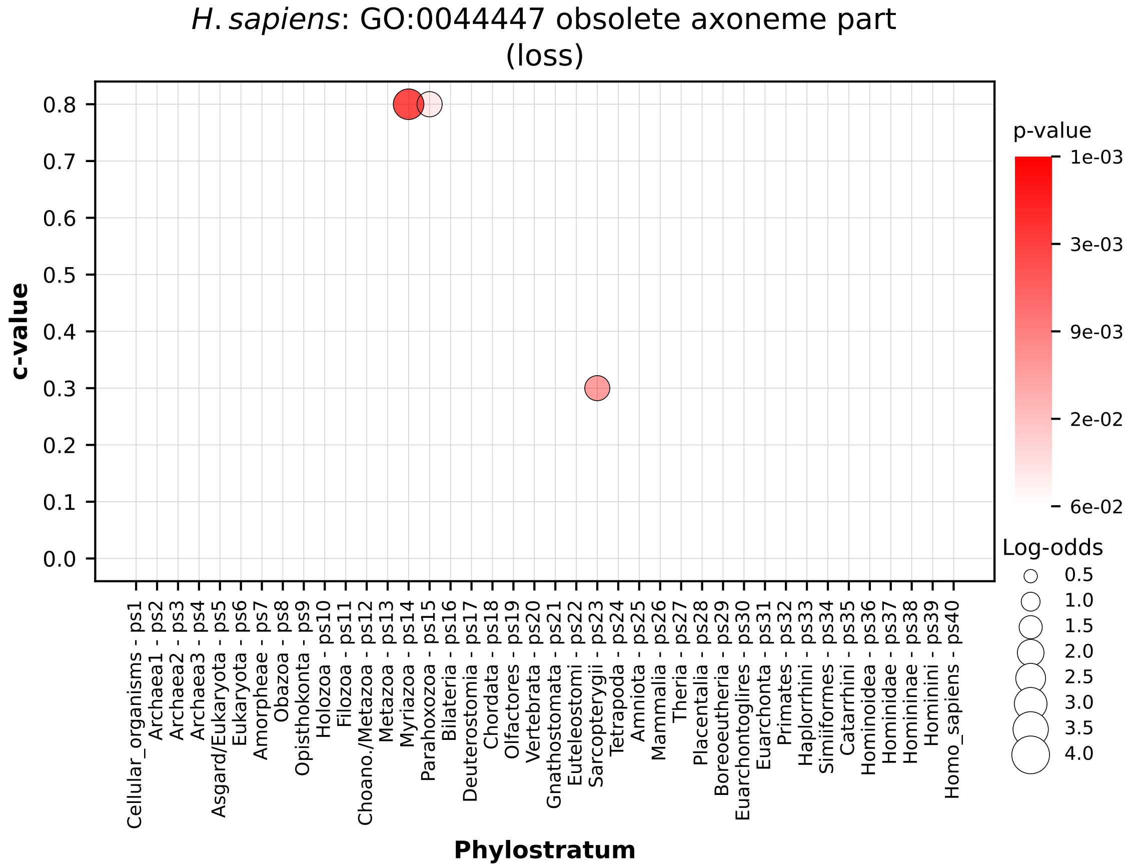

H. sapiens: GO:0044447 obsolete axoneme part
(loss)
c-value
0.8
0.7
0.6
0.5
0.4
0.3
0.2
0.1
0.0
p-value
1e-03
3e-03
9e-03
2e-02
6e-02
Log-odds
0.5
1.0
1.5
2.0
2.5
3.0
3.5
4.0
Cellular_organisms - ps1
Archaea1 - ps2
Archaea2 - ps3
Archaea3 - ps4
Asgard/Eukaryota - ps5
Eukaryota - ps6
Amorpheae - ps7
Obazoa - ps8
Opisthokonta - ps9
Holozoa - ps10
Filozoa - ps11
Choano./Metazoa - ps12
Metazoa - ps13
Myriazoa - ps14
Parahoxozoa - ps15
Bilateria - ps16
Deuterostomia - ps17
Chordata - ps18
Olfactores - ps19
Vertebrata - ps20
Gnathostomata - ps21
Euteleostomi - ps22
Sarcopterygii - ps23
Tetrapoda - ps24
Amniota - ps25
Mammalia - ps26
Theria - ps27
Placentalia - ps28
Boreoeutheria - ps29
Euarchontoglires - ps30
Euarchonta - ps31
Primates - ps32
Haplorrhini - ps33
Simiiformes - ps34
Catarrhini - ps35
Hominoidea - ps36
Hominidae - ps37
Homininae - ps38
Hominini - ps39
Homo_sapiens - ps40
Phylostratum

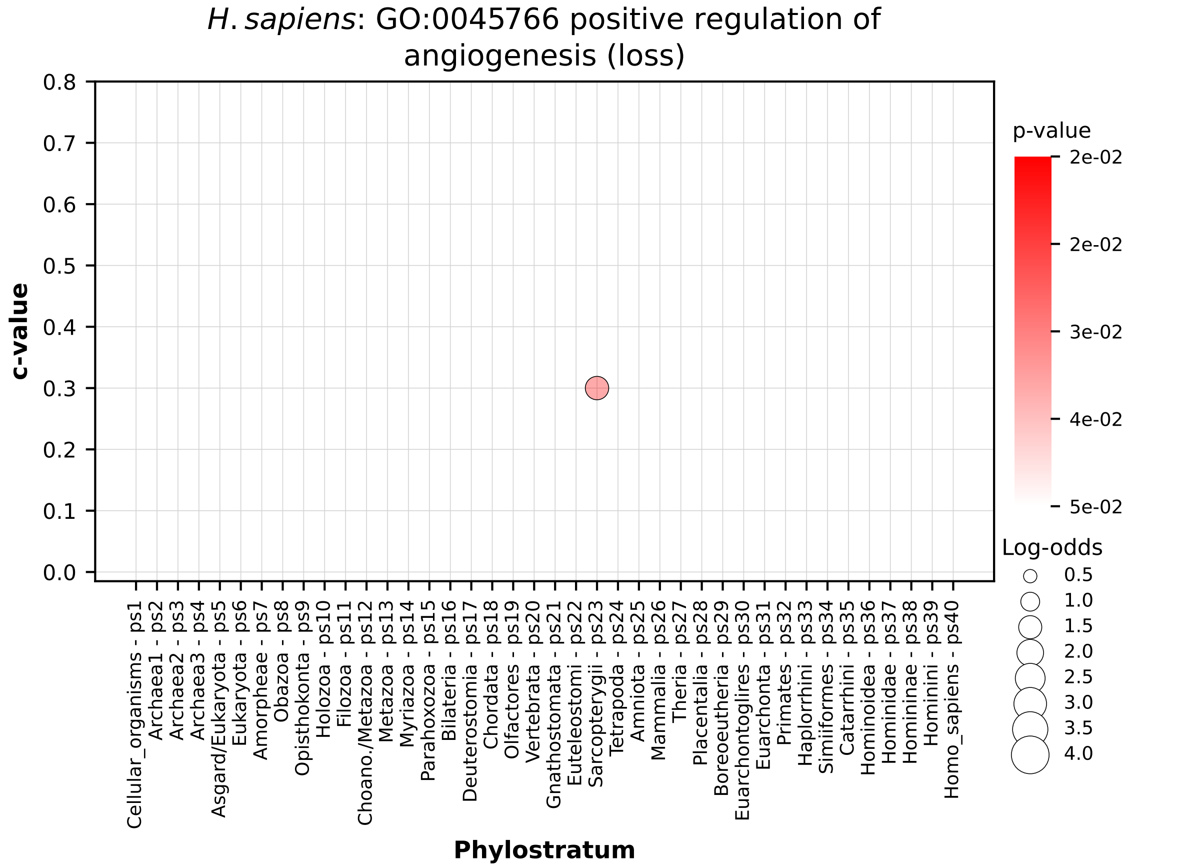

H. sapiens: GO:0045766 positive regulation of angiogenesis (loss)
c-value
0.8
0.7
0.6
0.5
0.4
0.3
0.2
0.1
0.0
p-value
2e-02
2e-02
3e-02
4e-02
5e-02
Log-odds
0.5
1.0
1.5
2.0
2.5
3.0
3.5
4.0
Cellular_organisms - ps1
Archaea1 - ps2
Archaea2 - ps3
Archaea3 - ps4
Asgard/Eukaryota - ps5
Eukaryota - ps6
Amorpheae - ps7
Obazoa - ps8
Opisthokonta - ps9
Holozoa - ps10
Filozoa - ps11
Choano./Metazoa - ps12
Metazoa - ps13
Myriazoa - ps14
Parahoxozoa - ps15
Bilateria - ps16
Deuterostomia - ps17
Chordata - ps18
Olfactores - ps19
Vertebrata - ps20
Gnathostomata - ps21
Euteleostomi - ps22
Sarcopterygii - ps23
Tetrapoda - ps24
Amniota - ps25
Mammalia - ps26
Theria - ps27
Placentalia - ps28
Boreoeutheria - ps29
Euarchontoglires - ps30
Euarchonta - ps31
Primates - ps32
Haplorrhini - ps33
Simiiformes - ps34
Catarrhini - ps35
Hominoidea - ps36
Hominidae - ps37
Homininae - ps38
Hominini - ps39
Homo_sapiens - ps40
Phylostratum

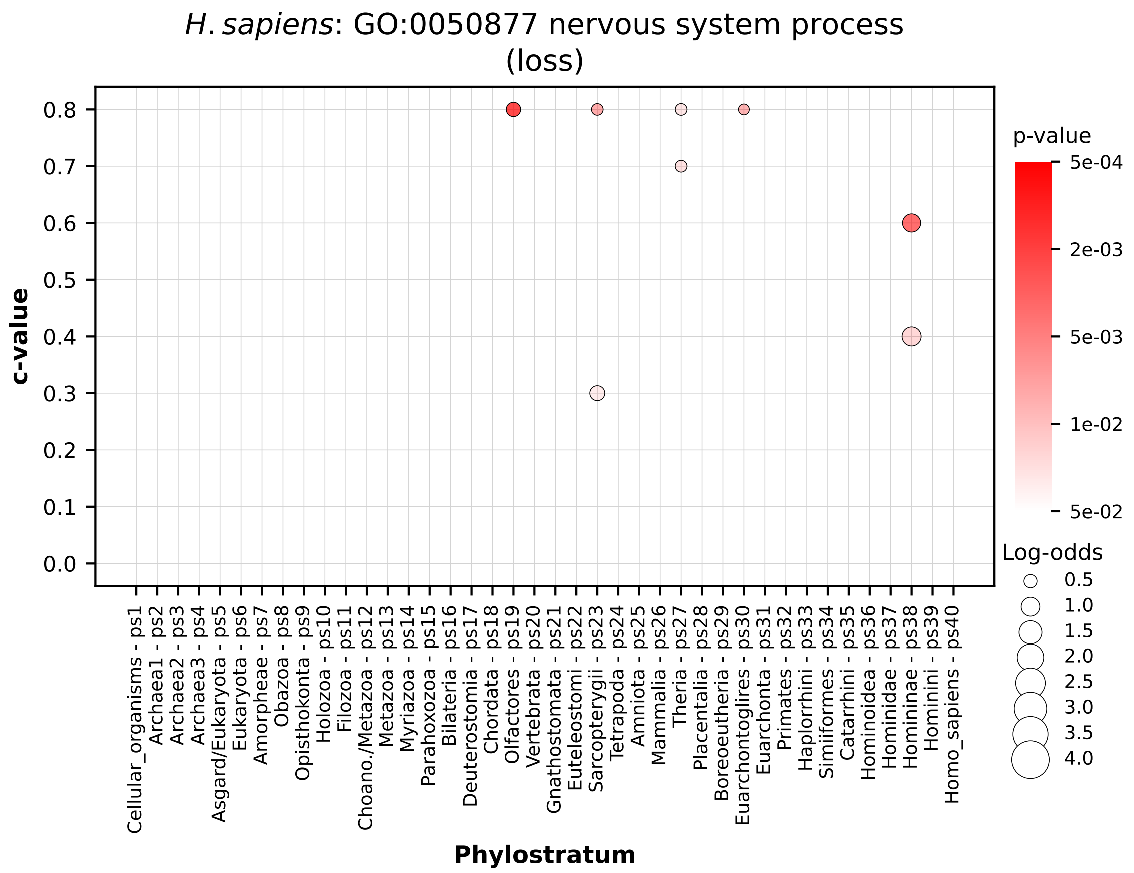

H. sapiens: GO:0050877 nervous system process
(loss)
c-value
0.0
0.1
0.2
0.3
0.4
0.5
0.6
0.7
0.8
Phylostratum
Cellular_organisms - ps1
Archaea1 - ps2
Archaea2 - ps3
Archaea3 - ps4
Asgard/Eukaryota - ps5
Eukaryota - ps6
Amorpheae - ps7
Obazoa - ps8
Opisthokonta - ps9
Holozoa - ps10
Filozoa - ps11
Choano./Metazoa - ps12
Metazoa - ps13
Myriazoa - ps14
Parahoxozoa - ps15
Bilateria - ps16
Deuterostomia - ps17
Chordata - ps18
Olfactores - ps19
Vertebrata - ps20
Gnathostomata - ps21
Euteleostomi - ps22
Sarcopterygii - ps23
Tetrapoda - ps24
Amniota - ps25
Mammalia - ps26
Theria - ps27
Placentalia - ps28
Boreoeutheria - ps29
Euarchontoglires - ps30
Euarchonta - ps31
Primates - ps32
Haplorrhini - ps33
Simiiformes - ps34
Catarrhini - ps35
Hominoidea - ps36
Hominidae - ps37
Homininae - ps38
Hominini - ps39
Homo_sapiens - ps40
p-value
5e-04
2e-03
5e-03
1e-02
5e-02
Log-odds
0.5
1.0
1.5
2.0
2.5
3.0
3.5
4.0

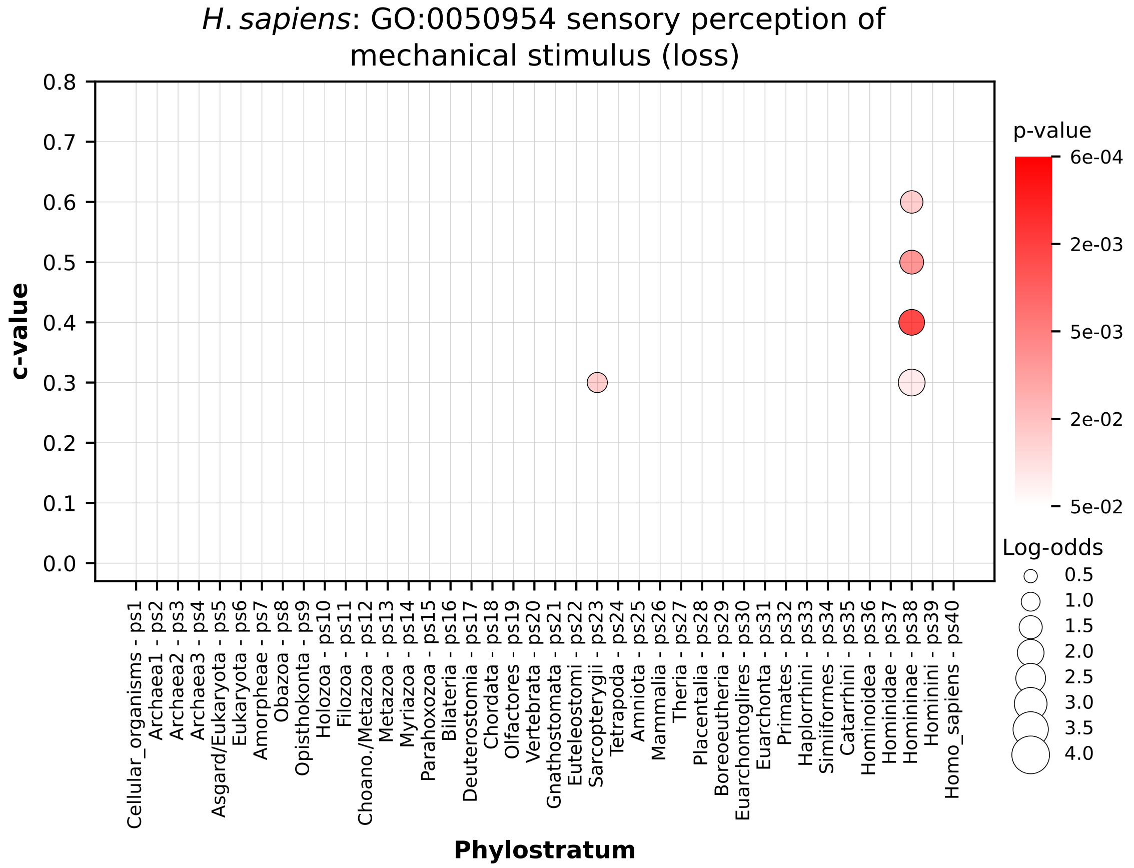

H. sapiens: GO:0050954 sensory perception of mechanical stimulus (loss)
c-value
0.0
0.1
0.2
0.3
0.4
0.5
0.6
0.7
0.8
Phylostratum
Cellular_organisms - ps1
Archaea1 - ps2
Archaea2 - ps3
Archaea3 - ps4
Asgard/Eukaryota - ps5
Eukaryota - ps6
Amorpheae - ps7
Obazoa - ps8
Opisthokonta - ps9
Holozoa - ps10
Filozoa - ps11
Choano./Metazoa - ps12
Metazoa - ps13
Myriazoa - ps14
Parahoxozoa - ps15
Bilateria - ps16
Deuterostomia - ps17
Chordata - ps18
Olfactores - ps19
Vertebrata - ps20
Gnathostomata - ps21
Euteleostomi - ps22
Sarcopterygii - ps23
Tetrapoda - ps24
Amniota - ps25
Mammalia - ps26
Theria - ps27
Placentalia - ps28
Boreoeutheria - ps29
Euarchontoglires - ps30
Euarchonta - ps31
Primates - ps32
Haplorrhini - ps33
Simiiformes - ps34
Catarrhini - ps35
Hominoidea - ps36
Hominidae - ps37
Homininae - ps38
Hominini - ps39
Homo_sapiens - ps40
p-value
6e-04
2e-03
5e-03
2e-02
5e-02
Log-odds
0.5
1.0
1.5
2.0
2.5
3.0
3.5
4.0

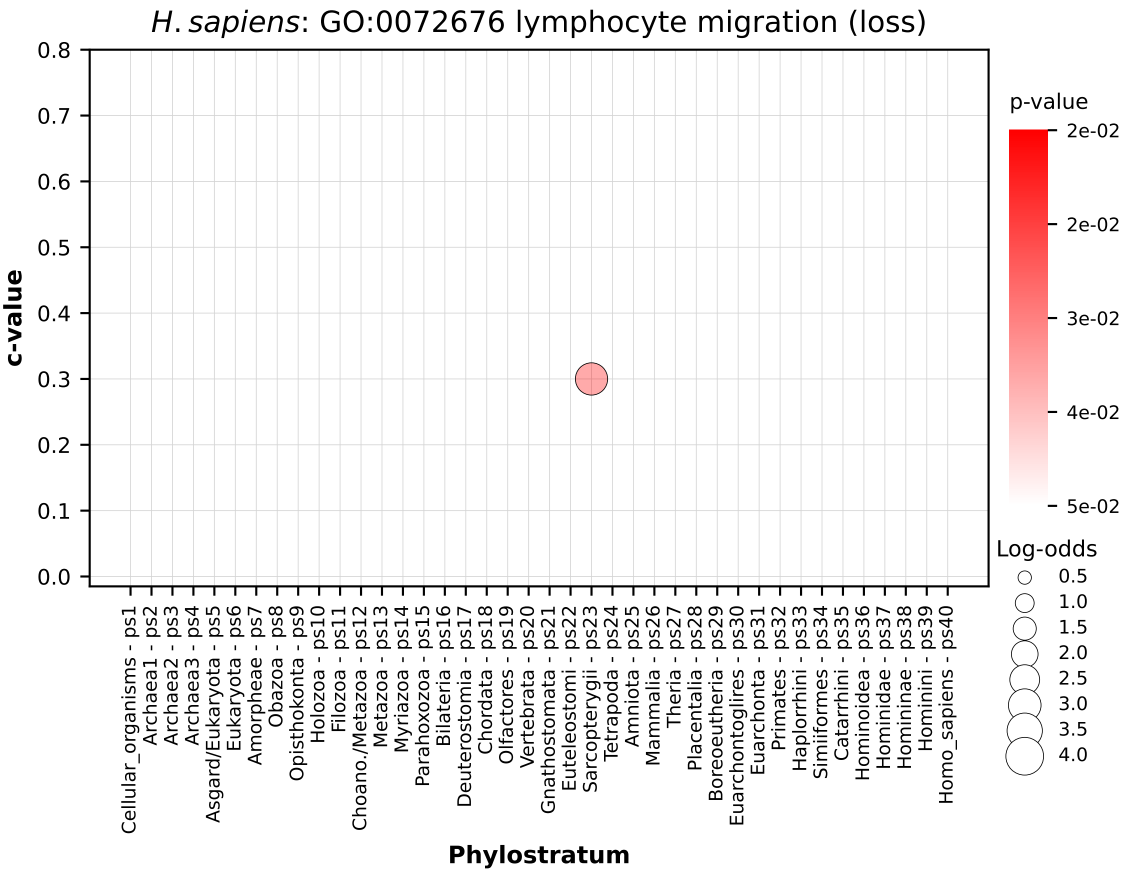

H. sapiens: GO:0072676 lymphocyte migration (loss)
c-value
0.8
0.7
0.6
0.5
0.4
0.3
0.2
0.1
0.0
Phylostratum
Cellular_organisms - ps1
Archaea1 - ps2
Archaea2 - ps3
Archaea3 - ps4
Asgard/Eukaryota - ps5
Eukaryota - ps6
Amorpheae - ps7
Obazoa - ps8
Opisthokonta - ps9
Holozoa - ps10
Filozoa - ps11
Choano./Metazoa - ps12
Metazoa - ps13
Myriazoa - ps14
Parahoxozoa - ps15
Bilateria - ps16
Deuterostomia - ps17
Chordata - ps18
Olfactores - ps19
Vertebrata - ps20
Gnathostomata - ps21
Euteleostomi - ps22
Sarcopterygii - ps23
Tetrapoda - ps24
Amniota - ps25
Mammalia - ps26
Theria - ps27
Placentalia - ps28
Boreoeutheria - ps29
Euarchontoglires - ps30
Euarchonta - ps31
Primates - ps32
Haplorrhini - ps33
Simiiformes - ps34
Catarrhini - ps35
Hominoidea - ps36
Hominidae - ps37
Homininae - ps38
Hominini - ps39
Homo_sapiens - ps40
p-value
2e-02
2e-02
3e-02
4e-02
5e-02
Log-odds
0.5
1.0
1.5
2.0
2.5
3.0
3.5
4.0

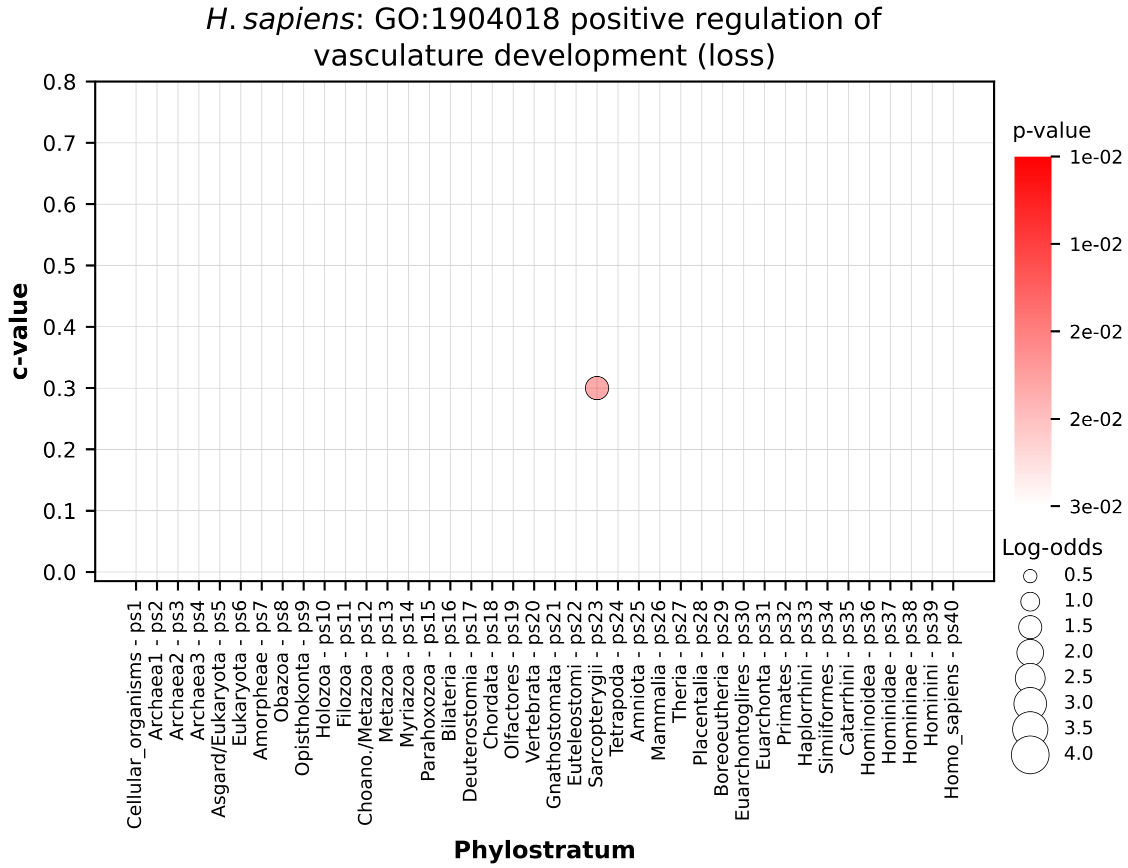
H. sapiens: GO:1904018 positive regulation of vasculature development (loss)
c-value
0.8
0.7
0.6
0.5
0.4
0.3
0.2
0.1
0.0
p-value
1e-02
1e-02
2e-02
2e-02
3e-02
Log-odds
0.5
1.0
1.5
2.0
2.5
3.0
3.5
4.0
Cellular_organisms - ps1
Archaea1 - ps2
Archaea2 - ps3
Archaea3 - ps4
Asgard/Eukaryota - ps5
Eukaryota - ps6
Amorpheae - ps7
Obazoa - ps8
Opisthokonta - ps9
Holozoa - ps10
Filozoa - ps11
Choano./Metazoa - ps12
Metazoa - ps13
Myriazoa - ps14
Parahoxozoa - ps15
Bilateria - ps16
Deuterostomia - ps17
Chordata - ps18
Olfactores - ps19
Vertebrata - ps20
Gnathostomata - ps21
Euteleostomi - ps22
Sarcopterygii - ps23
Tetrapoda - ps24
Amniota - ps25
Mammalia - ps26
Theria - ps27
Placentalia - ps28
Boreoeutheria - ps29
Euarchontoglires - ps30
Euarchonta - ps31
Primates - ps32
Haplorrhini - ps33
Simiiformes - ps34
Catarrhini - ps35
Hominoidea - ps36
Hominidae - ps37
Homininae - ps38
Hominini - ps39
Homo_sapiens - ps40
Phylostratum

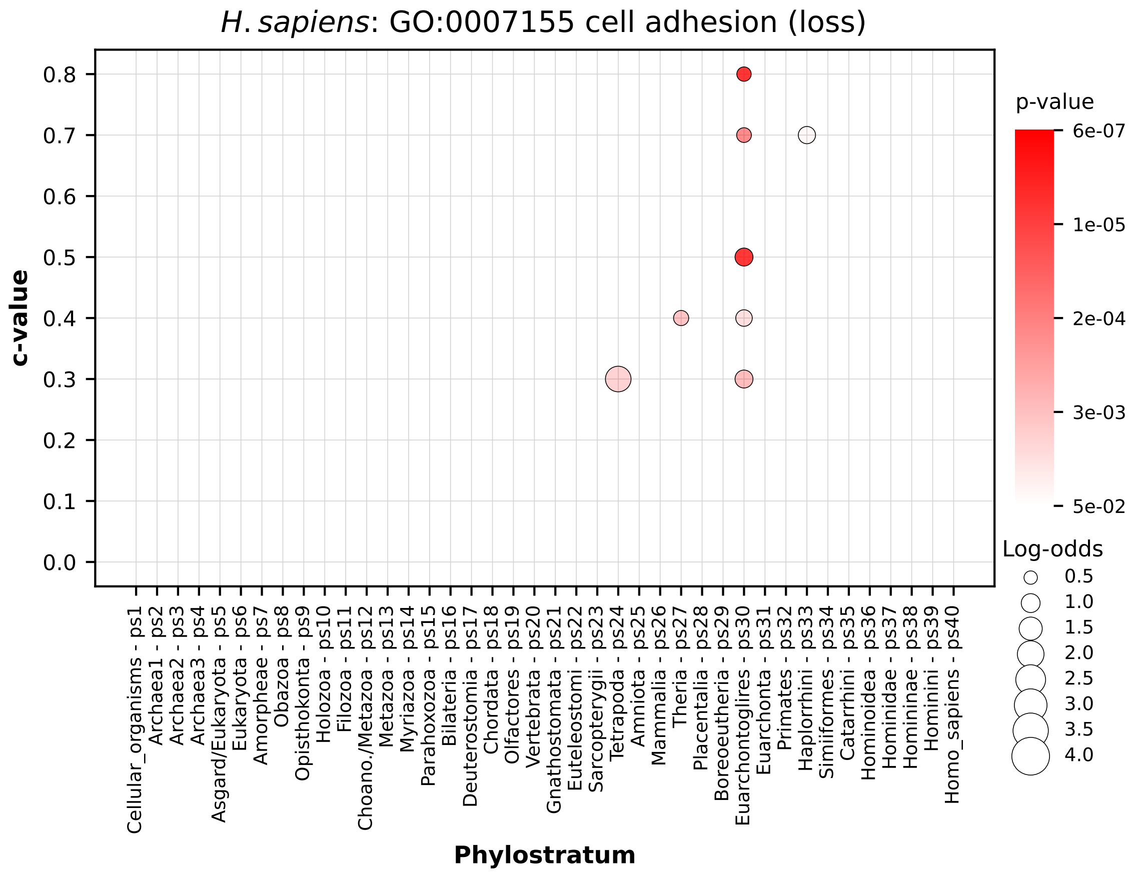
H. sapiens: GO:0007155 cell adhesion (loss)
c-value
0.8
0.7
0.6
0.5
0.4
0.3
0.2
0.1
0.0
Phylostratum
Cellular_organisms - ps1
Archaea1 - ps2
Archaea2 - ps3
Archaea3 - ps4
Asgard/Eukaryota - ps5
Eukaryota - ps6
Amorpheae - ps7
Obazoa - ps8
Opisthokonta - ps9
Holozoa - ps10
Filozoa - ps11
Choano./Metazoa - ps12
Metazoa - ps13
Myriazoa - ps14
Parahoxozoa - ps15
Bilateria - ps16
Deuterostomia - ps17
Chordata - ps18
Olfactores - ps19
Vertebrata - ps20
Gnathostomata - ps21
Euteleostomi - ps22
Sarcopterygii - ps23
Tetrapoda - ps24
Amniota - ps25
Mammalia - ps26
Theria - ps27
Placentalia - ps28
Boreoeutheria - ps29
Euarchontoglires - ps30
Euarchonta - ps31
Primates - ps32
Haplorrhini - ps33
Simiiformes - ps34
Catarrhini - ps35
Hominoidea - ps36
Hominidae - ps37
Homininae - ps38
Hominini - ps39
Homo_sapiens - ps40
p-value
6e-07
1e-05
2e-04
3e-03
5e-02
Log-odds
0.5
1.0
1.5
2.0
2.5
3.0
3.5
4.0

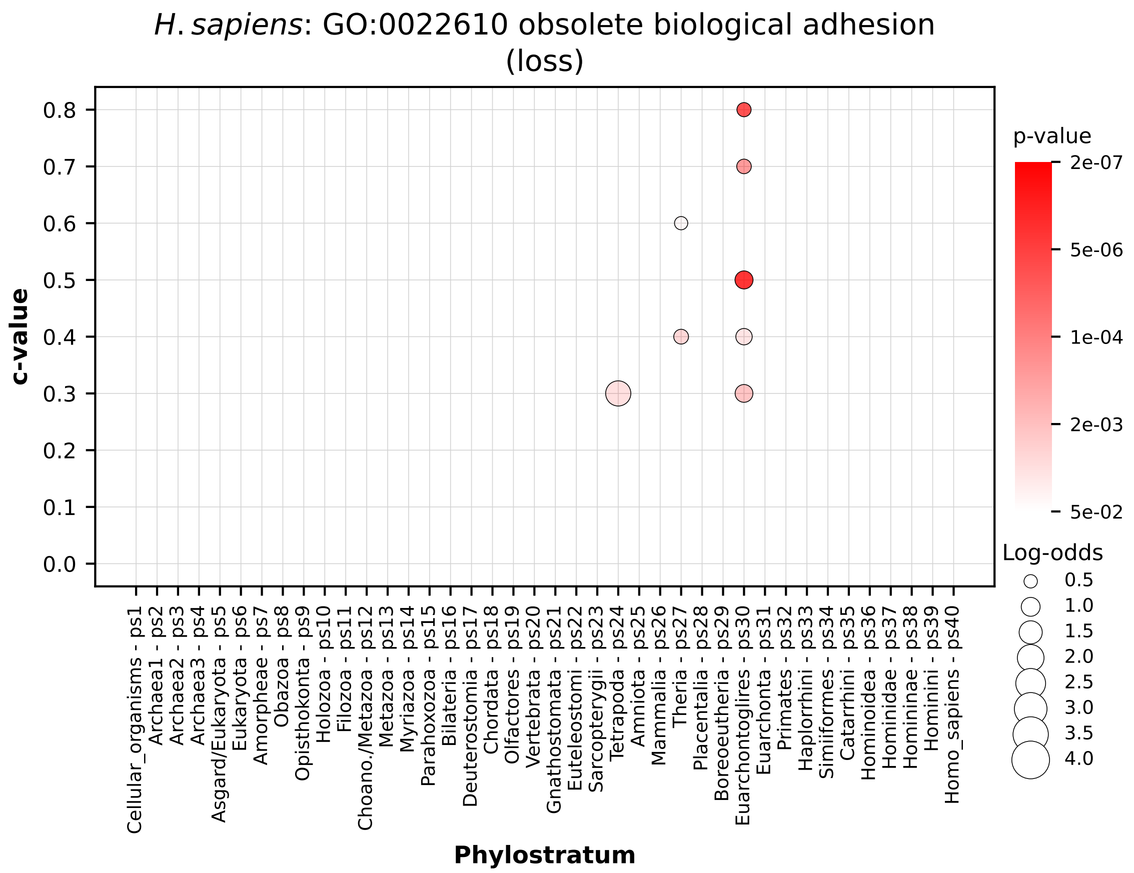

H. sapiens: GO:0022610 obsolete biological adhesion
(loss)
c-value
0.8
0.7
0.6
0.5
0.4
0.3
0.2
0.1
0.0
Phylostratum
Cellular_organisms - ps1
Archaea1 - ps2
Archaea2 - ps3
Archaea3 - ps4
Asgard/Eukaryota - ps5
Eukaryota - ps6
Amorpheae - ps7
Obazoa - ps8
Opisthokonta - ps9
Holozoa - ps10
Filozoa - ps11
Choano./Metazoa - ps12
Metazoa - ps13
Myriazoa - ps14
Parahoxozoa - ps15
Bilateria - ps16
Deuterostomia - ps17
Chordata - ps18
Olfactores - ps19
Vertebrata - ps20
Gnathostomata - ps21
Euteleostomi - ps22
Sarcopterygii - ps23
Tetrapoda - ps24
Amniota - ps25
Mammalia - ps26
Theria - ps27
Placentalia - ps28
Boreoeutheria - ps29
Euarchontoglires - ps30
Euarchonta - ps31
Primates - ps32
Haplorrhini - ps33
Simiiformes - ps34
Catarrhini - ps35
Hominoidea - ps36
Hominidae - ps37
Homininae - ps38
Hominini - ps39
Homo_sapiens - ps40
p-value
2e-07
5e-06
1e-04
2e-03
5e-02
Log-odds
0.5
1.0
1.5
2.0
2.5
3.0
3.5
4.0

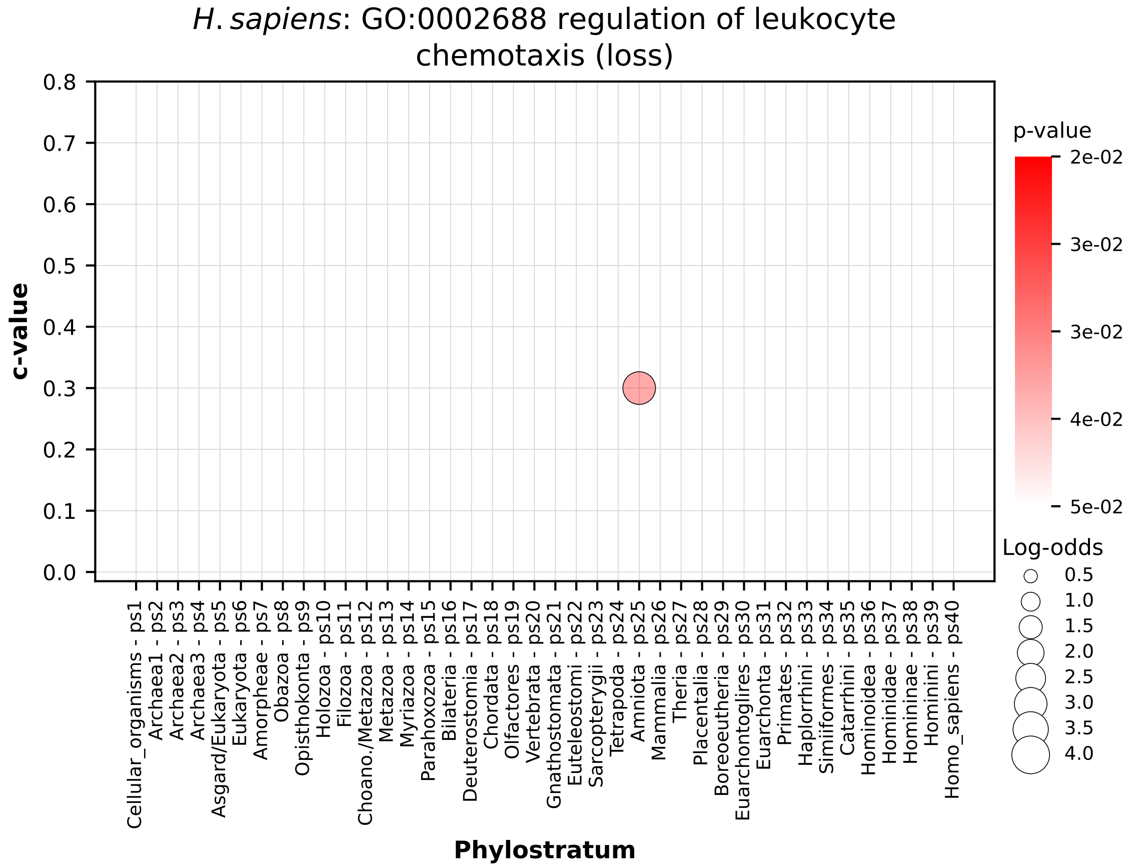

H. sapiens: GO:0002688 regulation of leukocyte chemotaxis (loss)
c-value
0.0
0.1
0.2
0.3
0.4
0.5
0.6
0.7
0.8
Phylostratum
Cellular_organisms - ps1
Archaea1 - ps2
Archaea2 - ps3
Archaea3 - ps4
Asgard/Eukaryota - ps5
Eukaryota - ps6
Amorpheae - ps7
Obazoa - ps8
Opisthokonta - ps9
Holozoa - ps10
Filozoa - ps11
Choano./Metazoa - ps12
Metazoa - ps13
Myriazoa - ps14
Parahoxozoa - ps15
Bilateria - ps16
Deuterostomia - ps17
Chordata - ps18
Olfactores - ps19
Vertebrata - ps20
Gnathostomata - ps21
Euteleostomi - ps22
Sarcopterygii - ps23
Tetrapoda - ps24
Amniota - ps25
Mammalia - ps26
Theria - ps27
Placentalia - ps28
Boreoeutheria - ps29
Euarchontoglires - ps30
Euarchonta - ps31
Primates - ps32
Haplorrhini - ps33
Simiiformes - ps34
Catarrhini - ps35
Hominoidea - ps36
Hominidae - ps37
Homininae - ps38
Hominini - ps39
Homo_sapiens - ps40
p-value
2e-02
3e-02
3e-02
4e-02
5e-02
Log-odds
0.5
1.0
1.5
2.0
2.5
3.0
3.5
4.0

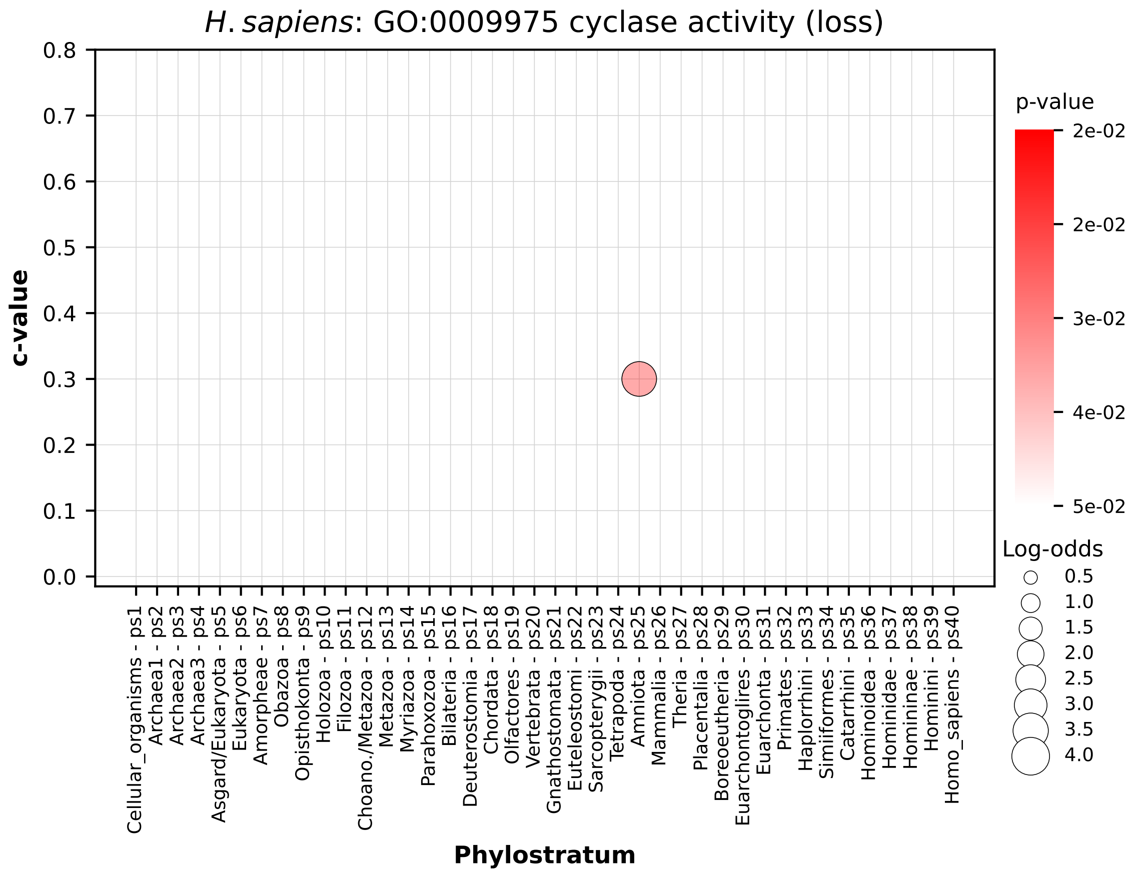
H. sapiens: GO:0009975 cyclase activity (loss)
c-value
0.0
0.1
0.2
0.3
0.4
0.5
0.6
0.7
0.8
Phylostratum
Cellular_organisms - ps1
Archaea1 - ps2
Archaea2 - ps3
Archaea3 - ps4
Asgard/Eukaryota - ps5
Eukaryota - ps6
Amorpheae - ps7
Obazoa - ps8
Opisthokonta - ps9
Holozoa - ps10
Filozoa - ps11
Choano./Metazoa - ps12
Metazoa - ps13
Myriazoa - ps14
Parahoxozoa - ps15
Bilateria - ps16
Deuterostomia - ps17
Chordata - ps18
Olfactores - ps19
Vertebrata - ps20
Gnathostomata - ps21
Euteleostomi - ps22
Sarcopterygii - ps23
Tetrapoda - ps24
Amniota - ps25
Mammalia - ps26
Theria - ps27
Placentalia - ps28
Boreoeutheria - ps29
Euarchontoglires - ps30
Euarchonta - ps31
Primates - ps32
Haplorrhini - ps33
Simiiformes - ps34
Catarrhini - ps35
Hominoidea - ps36
Hominidae - ps37
Homininae - ps38
Hominini - ps39
Homo_sapiens - ps40
p-value
2e-02
2e-02
3e-02
4e-02
5e-02
Log-odds
0.5
1.0
1.5
2.0
2.5
3.0
3.5
4.0

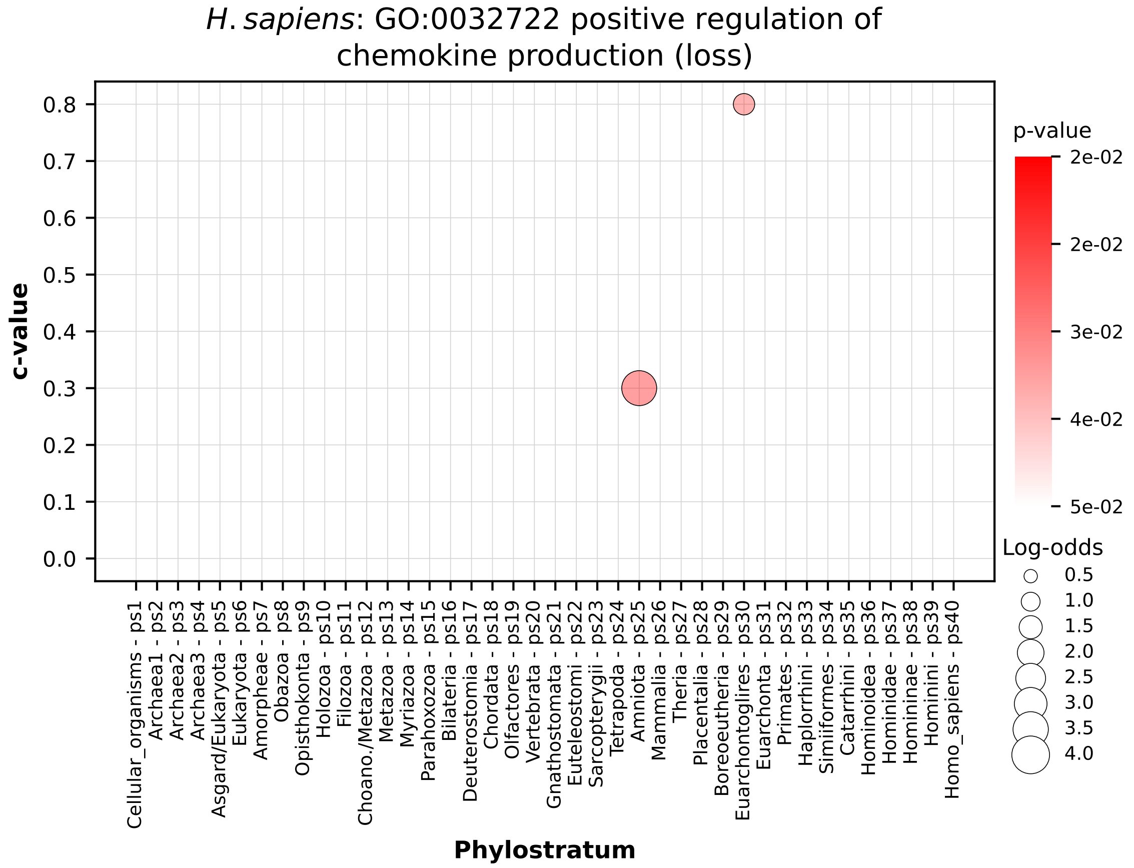

H. sapiens: GO:0032722 positive regulation of chemokine production (loss)
c-value
0.8
0.7
0.6
0.5
0.4
0.3
0.2
0.1
0.0
p-value
2e-02
2e-02
3e-02
4e-02
5e-02
Log-odds
0.5
1.0
1.5
2.0
2.5
3.0
3.5
4.0
Cellular_organisms - ps1
Archaea1 - ps2
Archaea2 - ps3
Archaea3 - ps4
Asgard/Eukaryota - ps5
Eukaryota - ps6
Amorpheae - ps7
Obazoa - ps8
Opisthokonta - ps9
Holozoa - ps10
Filozoa - ps11
Choano./Metazoa - ps12
Metazoa - ps13
Myriazoa - ps14
Parahoxozoa - ps15
Bilateria - ps16
Deuterostomia - ps17
Chordata - ps18
Olfactores - ps19
Vertebrata - ps20
Gnathostomata - ps21
Euteleostomi - ps22
Sarcopterygii - ps23
Tetrapoda - ps24
Amniota - ps25
Mammalia - ps26
Theria - ps27
Placentalia - ps28
Boreoeutheria - ps29
Euarchontoglires - ps30
Euarchonta - ps31
Primates - ps32
Haplorrhini - ps33
Simiiformes - ps34
Catarrhini - ps35
Hominoidea - ps36
Hominidae - ps37
Homininae - ps38
Hominini - ps39
Homo_sapiens - ps40
Phylostratum

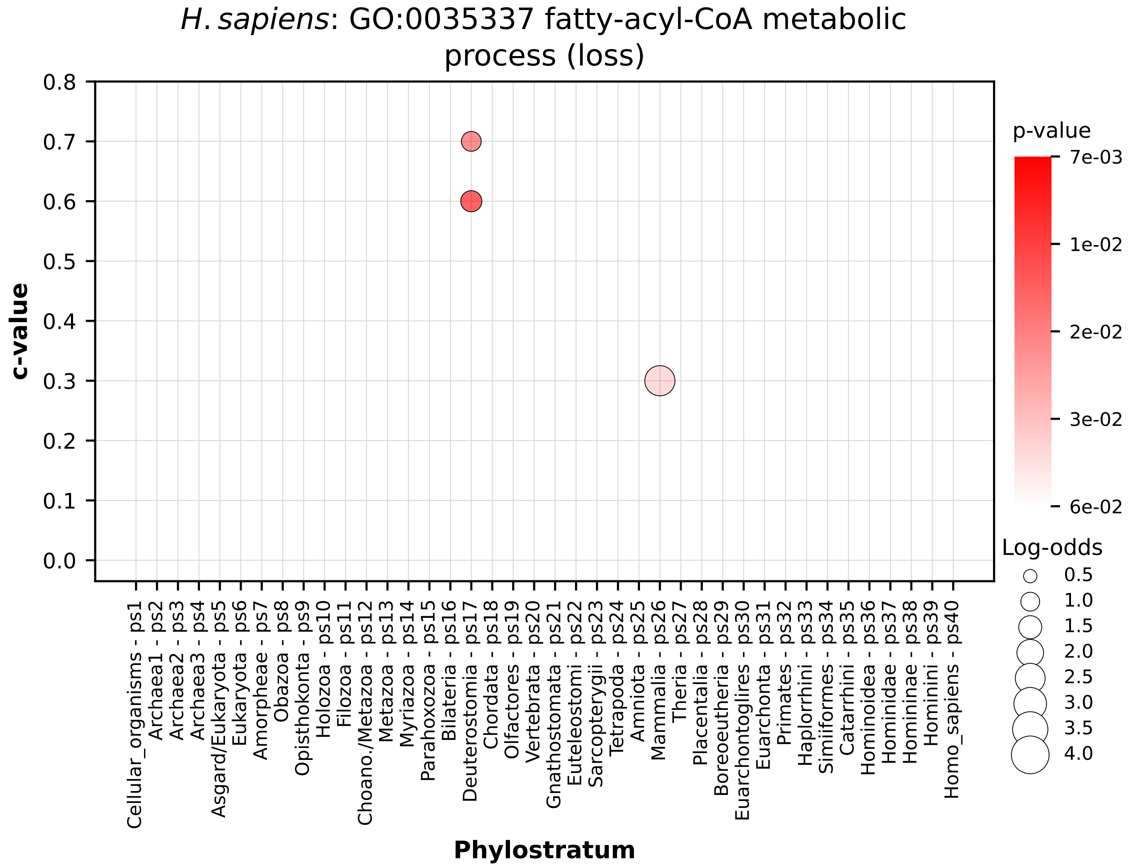

H. sapiens: GO:0035337 fatty-acyl-CoA metabolic process (loss)
c-value
0.8
0.7
0.6
0.5
0.4
0.3
0.2
0.1
0.0
p-value
7e-03
1e-02
2e-02
3e-02
6e-02
Log-odds
0.5
1.0
1.5
2.0
2.5
3.0
3.5
4.0
Cellular_organisms - ps1
Archaea1 - ps2
Archaea2 - ps3
Archaea3 - ps4
Asgard/Eukaryota - ps5
Eukaryota - ps6
Amorpheae - ps7
Obazoa - ps8
Opisthokonta - ps9
Holozoa - ps10
Filozoa - ps11
Choano./Metazoa - ps12
Metazoa - ps13
Myriazoa - ps14
Parahoxozoa - ps15
Bilateria - ps16
Deuterostomia - ps17
Chordata - ps18
Olfactores - ps19
Vertebrata - ps20
Gnathostomata - ps21
Euteleostomi - ps22
Sarcopterygii - ps23
Tetrapoda - ps24
Amniota - ps25
Mammalia - ps26
Theria - ps27
Placentalia - ps28
Boreoeutheria - ps29
Euarchontoglires - ps30
Euarchonta - ps31
Primates - ps32
Haplorrhini - ps33
Simiiformes - ps34
Catarrhini - ps35
Hominoidea - ps36
Hominidae - ps37
Homininae - ps38
Hominini - ps39
Homo_sapiens - ps40
Phylostratum

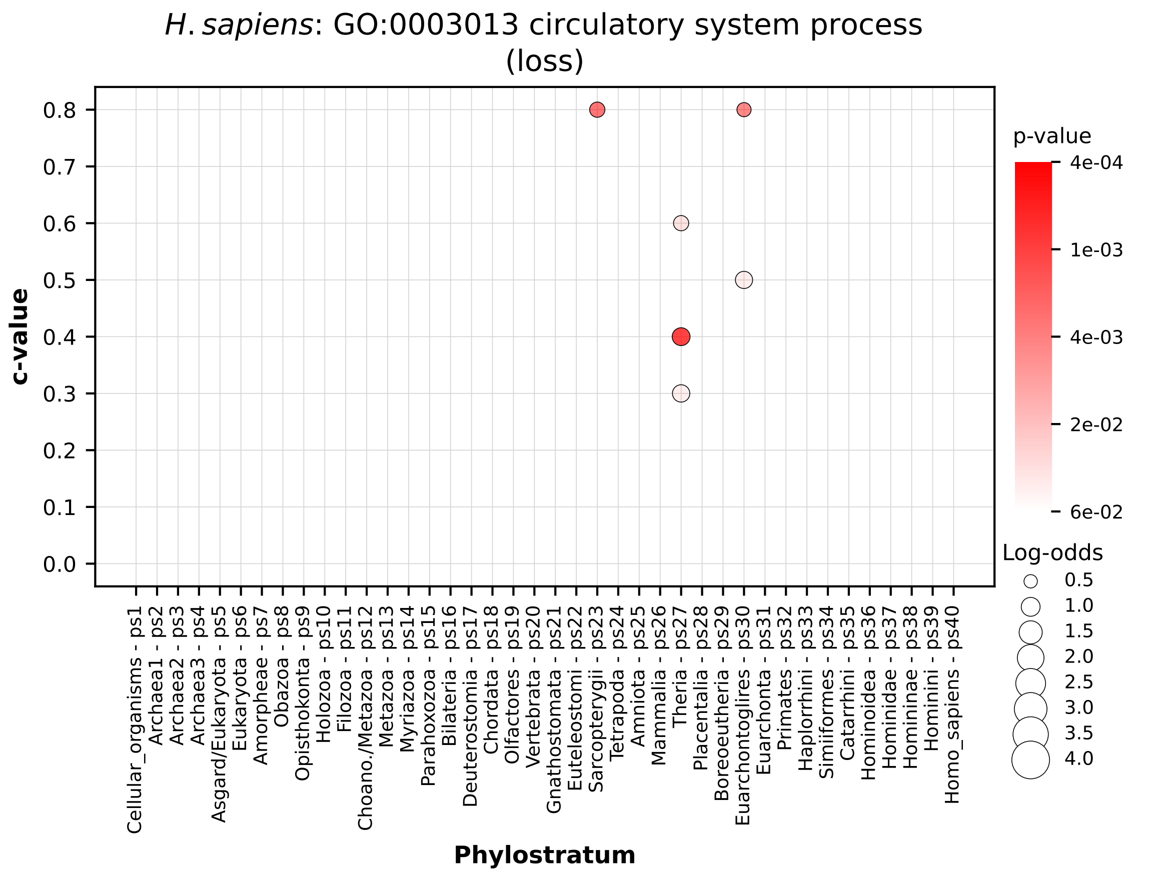
H. sapiens: GO:0003013 circulatory system process
(loss)
c-value
0.0
0.1
0.2
0.3
0.4
0.5
0.6
0.7
0.8
Phylostratum
Cellular_organisms - ps1
Archaea1 - ps2
Archaea2 - ps3
Archaea3 - ps4
Asgard/Eukaryota - ps5
Eukaryota - ps6
Amorpheae - ps7
Obazoa - ps8
Opisthokonta - ps9
Holozoa - ps10
Filozoa - ps11
Choano./Metazoa - ps12
Metazoa - ps13
Myriazoa - ps14
Parahoxozoa - ps15
Bilateria - ps16
Deuterostomia - ps17
Chordata - ps18
Olfactores - ps19
Vertebrata - ps20
Gnathostomata - ps21
Euteleostomi - ps22
Sarcopterygii - ps23
Tetrapoda - ps24
Amniota - ps25
Mammalia - ps26
Theria - ps27
Placentalia - ps28
Boreoeutheria - ps29
Euarchontoglires - ps30
Euarchonta - ps31
Primates - ps32
Haplorrhini - ps33
Simiiformes - ps34
Catarrhini - ps35
Hominoidea - ps36
Hominidae - ps37
Homininae - ps38
Hominini - ps39
Homo_sapiens - ps40
p-value
4e-04
1e-03
4e-03
2e-02
6e-02
Log-odds
0.5
1.0
1.5
2.0
2.5
3.0
3.5
4.0

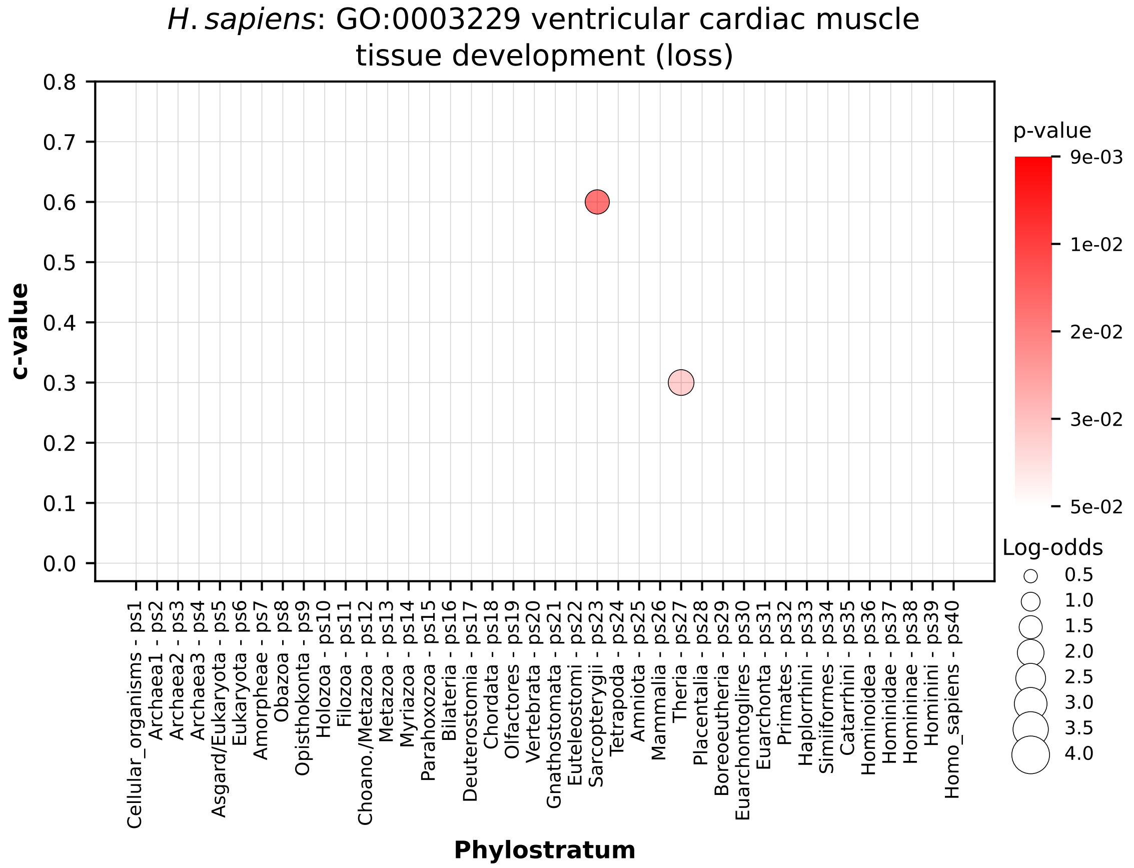

H. sapiens: GO:0003229 ventricular cardiac muscle tissue development (loss)
c-value
0.8
0.7
0.6
0.5
0.4
0.3
0.2
0.1
0.0
Phylostratum
Cellular_organisms - ps1
Archaea1 - ps2
Archaea2 - ps3
Archaea3 - ps4
Asgard/Eukaryota - ps5
Eukaryota - ps6
Amorpheae - ps7
Obazoa - ps8
Opisthokonta - ps9
Holozoa - ps10
Filozoa - ps11
Choano./Metazoa - ps12
Metazoa - ps13
Myriazoa - ps14
Parahoxozoa - ps15
Bilateria - ps16
Deuterostomia - ps17
Chordata - ps18
Olfactores - ps19
Vertebrata - ps20
Gnathostomata - ps21
Euteleostomi - ps22
Sarcopterygii - ps23
Tetrapoda - ps24
Amniota - ps25
Mammalia - ps26
Theria - ps27
Placentalia - ps28
Boreoeutheria - ps29
Euarchontoglires - ps30
Euarchonta - ps31
Primates - ps32
Haplorrhini - ps33
Simiiformes - ps34
Catarrhini - ps35
Hominoidea - ps36
Hominidae - ps37
Homininae - ps38
Hominini - ps39
Homo_sapiens - ps40
p-value
9e-03
1e-02
2e-02
3e-02
5e-02
Log-odds
0.5
1.0
1.5
2.0
2.5
3.0
3.5
4.0

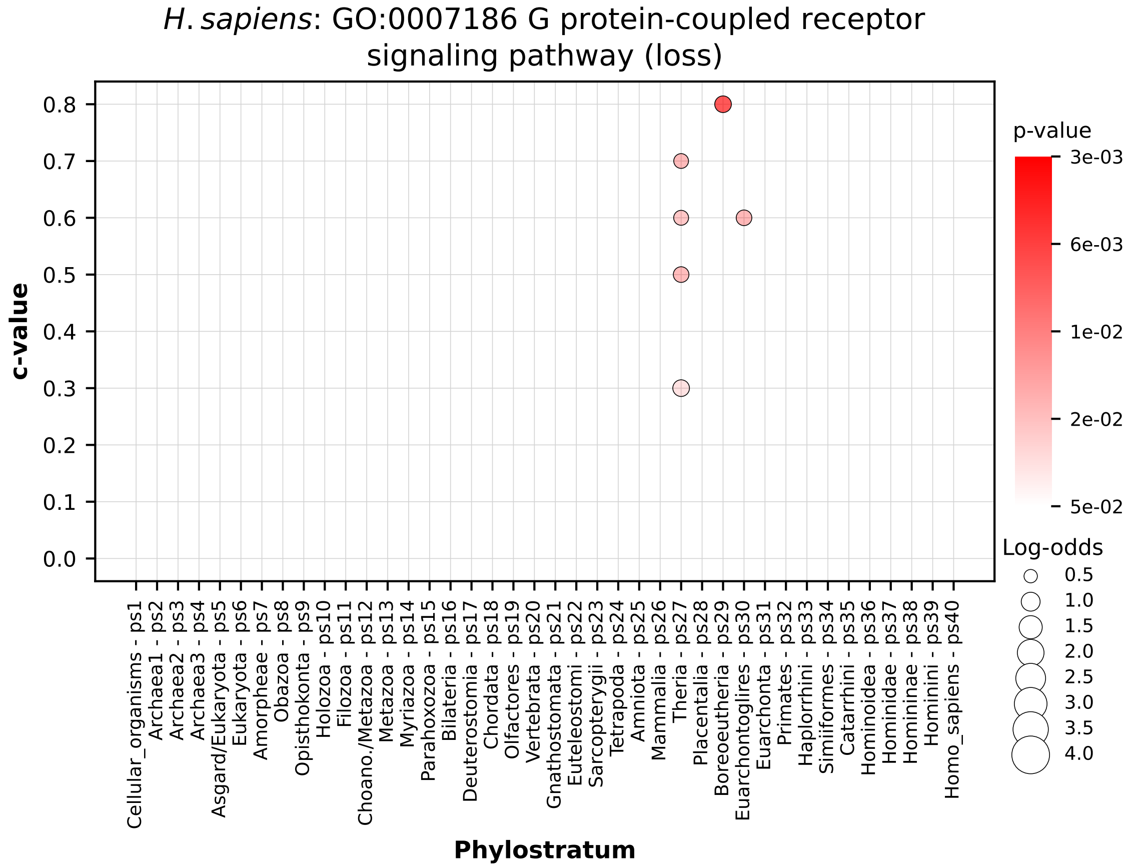

H. sapiens: GO:0007186 G protein-coupled receptor signaling pathway (loss)
c-value
0.0
0.1
0.2
0.3
0.4
0.5
0.6
0.7
0.8
Phylostratum
Cellular_organisms - ps1
Archaea1 - ps2
Archaea2 - ps3
Archaea3 - ps4
Asgard/Eukaryota - ps5
Eukaryota - ps6
Amorpheae - ps7
Obazoa - ps8
Opisthokonta - ps9
Holozoa - ps10
Filozoa - ps11
Choano./Metazoa - ps12
Metazoa - ps13
Myriazoa - ps14
Parahoxozoa - ps15
Bilateria - ps16
Deuterostomia - ps17
Chordata - ps18
Olfactores - ps19
Vertebrata - ps20
Gnathostomata - ps21
Euteleostomi - ps22
Sarcopterygii - ps23
Tetrapoda - ps24
Amniota - ps25
Mammalia - ps26
Theria - ps27
Placentalia - ps28
Boreoeutheria - ps29
Euarchontoglires - ps30
Euarchonta - ps31
Primates - ps32
Haplorrhini - ps33
Simiiformes - ps34
Catarrhini - ps35
Hominoidea - ps36
Hominidae - ps37
Homininae - ps38
Hominini - ps39
Homo_sapiens - ps40
p-value
3e-03
6e-03
1e-02
2e-02
5e-02
Log-odds
0.5
1.0
1.5
2.0
2.5
3.0
3.5
4.0

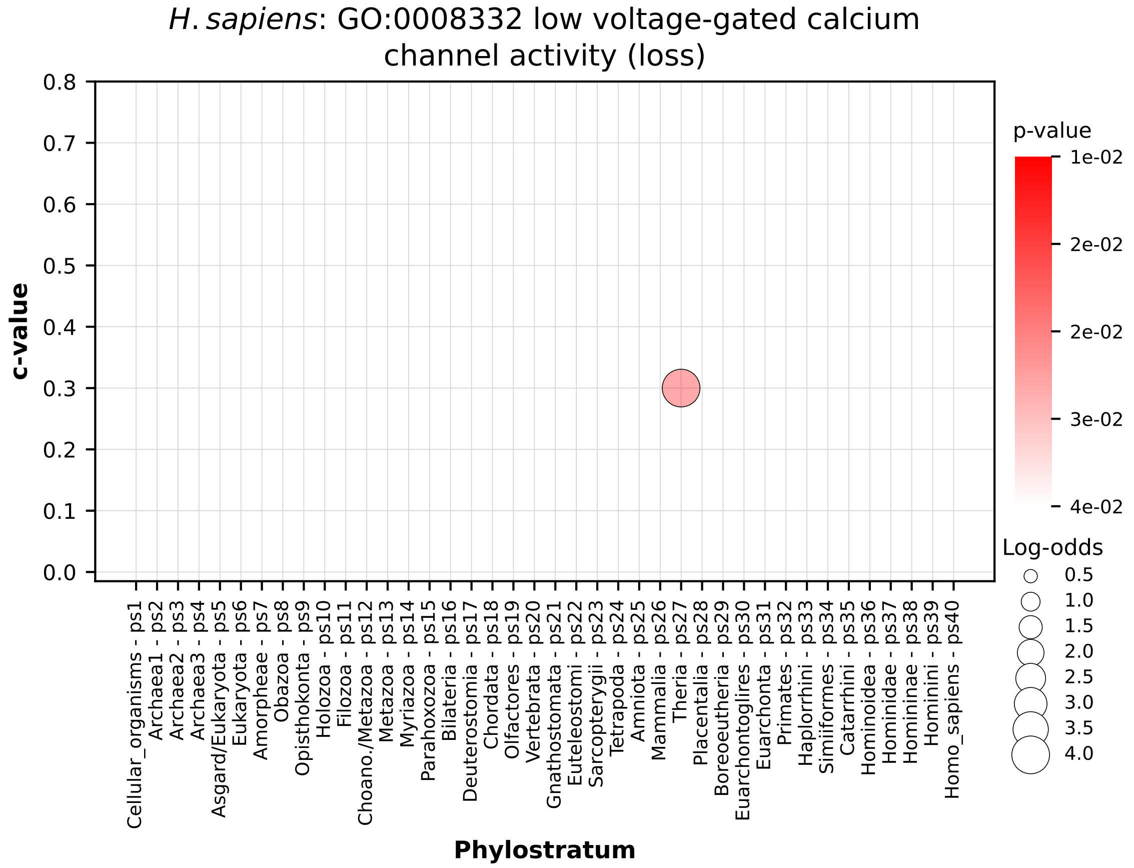

H. sapiens: GO:0008332 low voltage-gated calcium channel activity (loss)
c-value
0.0
0.1
0.2
0.3
0.4
0.5
0.6
0.7
0.8
Phylostratum
Cellular_organisms - ps1
Archaea1 - ps2
Archaea2 - ps3
Archaea3 - ps4
Asgard/Eukaryota - ps5
Eukaryota - ps6
Amorpheae - ps7
Obazoa - ps8
Opisthokonta - ps9
Holozoa - ps10
Filozoa - ps11
Choano./Metazoa - ps12
Metazoa - ps13
Myriazoa - ps14
Parahoxozoa - ps15
Bilateria - ps16
Deuterostomia - ps17
Chordata - ps18
Olfactores - ps19
Vertebrata - ps20
Gnathostomata - ps21
Euteleostomi - ps22
Sarcopterygii - ps23
Tetrapoda - ps24
Amniota - ps25
Mammalia - ps26
Theria - ps27
Placentalia - ps28
Boreoeutheria - ps29
Euarchontoglires - ps30
Euarchonta - ps31
Primates - ps32
Haplorrhini - ps33
Simiiformes - ps34
Catarrhini - ps35
Hominoidea - ps36
Hominidae - ps37
Homininae - ps38
Hominini - ps39
Homo_sapiens - ps40
p-value
1e-02
2e-02
2e-02
3e-02
4e-02
Log-odds
0.5
1.0
1.5
2.0
2.5
3.0
3.5
4.0

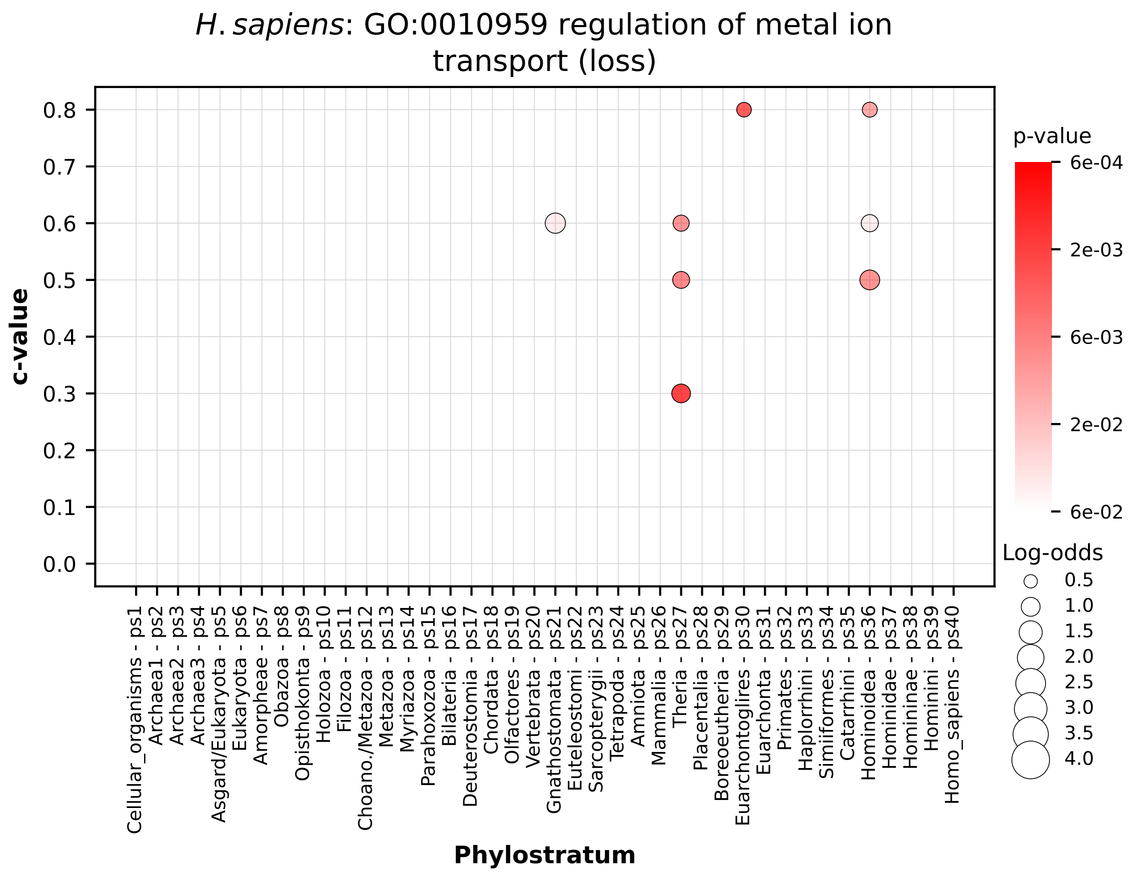

H. sapiens: GO:0010959 regulation of metal ion transport (loss)
c-value
0.0
0.1
0.2
0.3
0.4
0.5
0.6
0.7
0.8
p-value
6e-04
2e-03
6e-03
2e-02
6e-02
Log-odds
0.5
1.0
1.5
2.0
2.5
3.0
3.5
4.0
Cellular_organisms - ps1
Archaea1 - ps2
Archaea2 - ps3
Archaea3 - ps4
Asgard/Eukaryota - ps5
Eukaryota - ps6
Amorpheae - ps7
Obazoa - ps8
Opisthokonta - ps9
Holozoa - ps10
Filozoa - ps11
Choano./Metazoa - ps12
Metazoa - ps13
Myriazoa - ps14
Parahoxozoa - ps15
Bilateria - ps16
Deuterostomia - ps17
Chordata - ps18
Olfactores - ps19
Vertebrata - ps20
Gnathostomata - ps21
Euteleostomi - ps22
Sarcopterygii - ps23
Tetrapoda - ps24
Amniota - ps25
Mammalia - ps26
Theria - ps27
Placentalia - ps28
Boreoeutheria - ps29
Euarchontoglires - ps30
Euarchonta - ps31
Primates - ps32
Haplorrhini - ps33
Simiiformes - ps34
Catarrhini - ps35
Hominoidea - ps36
Hominidae - ps37
Homininae - ps38
Hominini - ps39
Homo_sapiens - ps40
Phylostratum

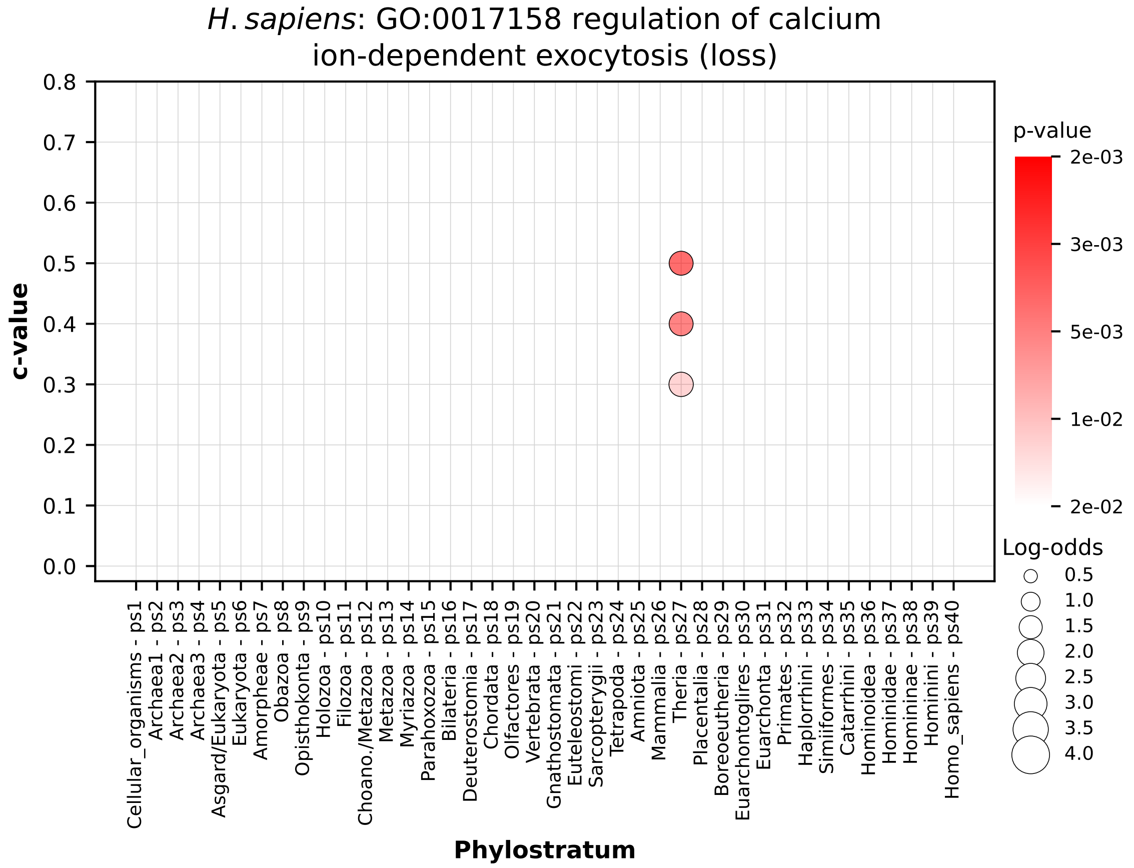

H. sapiens: GO:0017158 regulation of calcium ion-dependent exocytosis (loss)
c-value
0.8
0.7
0.6
0.5
0.4
0.3
0.2
0.1
0.0
p-value
2e-03
3e-03
5e-03
1e-02
2e-02
Log-odds
0.5
1.0
1.5
2.0
2.5
3.0
3.5
4.0
Cellular_organisms - ps1
Archaea1 - ps2
Archaea2 - ps3
Archaea3 - ps4
Asgard/Eukaryota - ps5
Eukaryota - ps6
Amorpheae - ps7
Obazoa - ps8
Opisthokonta - ps9
Holozoa - ps10
Filozoa - ps11
Choano./Metazoa - ps12
Metazoa - ps13
Myriazoa - ps14
Parahoxozoa - ps15
Bilateria - ps16
Deuterostomia - ps17
Chordata - ps18
Olfactores - ps19
Vertebrata - ps20
Gnathostomata - ps21
Euteleostomi - ps22
Sarcopterygii - ps23
Tetrapoda - ps24
Amniota - ps25
Mammalia - ps26
Theria - ps27
Placentalia - ps28
Boreoeutheria - ps29
Euarchontoglires - ps30
Euarchonta - ps31
Primates - ps32
Haplorrhini - ps33
Simiiformes - ps34
Catarrhini - ps35
Hominoidea - ps36
Hominidae - ps37
Homininae - ps38
Hominini - ps39
Homo_sapiens - ps40
Phylostratum

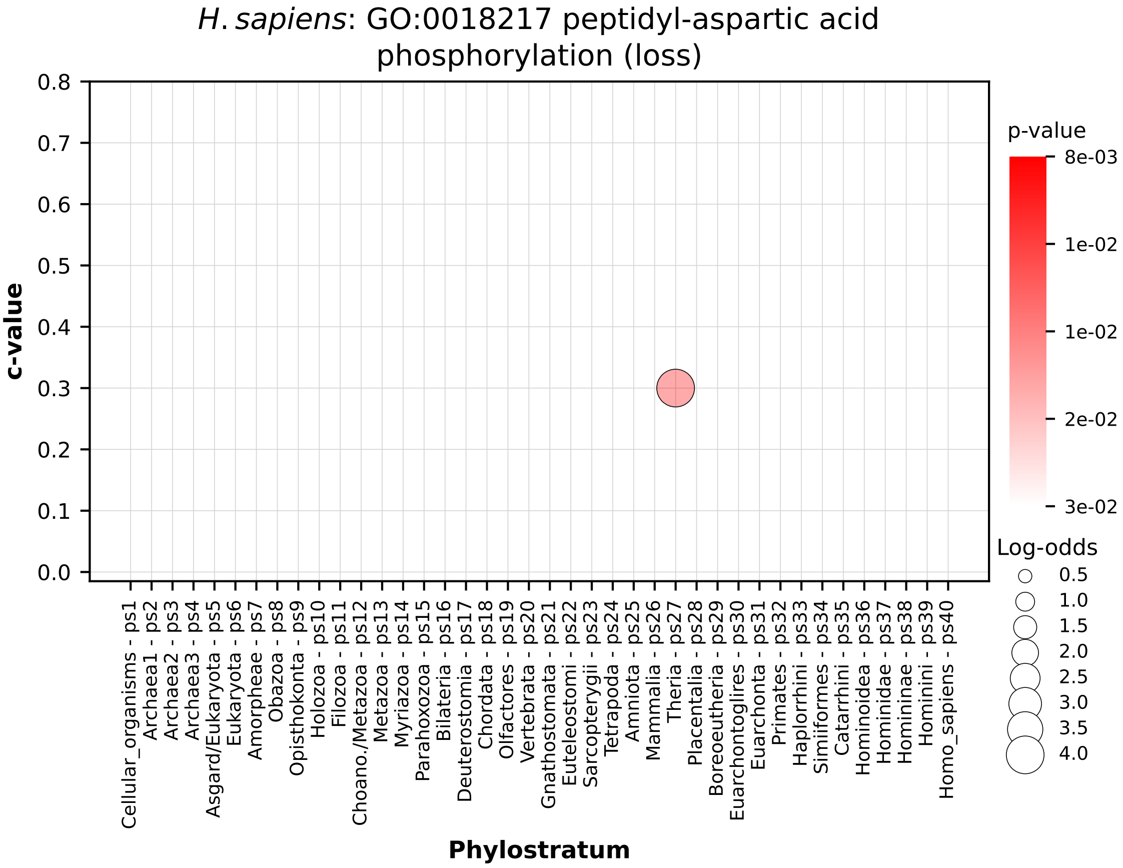

H. sapiens: GO:0018217 peptidyl-aspartic acid phosphorylation (loss)
c-value
0.8
0.7
0.6
0.5
0.4
0.3
0.2
0.1
0.0
p-value
8e-03
1e-02
1e-02
2e-02
3e-02
Log-odds
0.5
1.0
1.5
2.0
2.5
3.0
3.5
4.0
Cellular_organisms - ps1
Archaea1 - ps2
Archaea2 - ps3
Archaea3 - ps4
Asgard/Eukaryota - ps5
Eukaryota - ps6
Amorpheae - ps7
Obazoa - ps8
Opisthokonta - ps9
Holozoa - ps10
Filozoa - ps11
Choano./Metazoa - ps12
Metazoa - ps13
Myriazoa - ps14
Parahoxozoa - ps15
Bilateria - ps16
Deuterostomia - ps17
Chordata - ps18
Olfactores - ps19
Vertebrata - ps20
Gnathostomata - ps21
Euteleostomi - ps22
Sarcopterygii - ps23
Tetrapoda - ps24
Amniota - ps25
Mammalia - ps26
Theria - ps27
Placentalia - ps28
Boreoeutheria - ps29
Euarchontoglires - ps30
Euarchonta - ps31
Primates - ps32
Haplorrhini - ps33
Simiiformes - ps34
Catarrhini - ps35
Hominoidea - ps36
Hominidae - ps37
Homininae - ps38
Hominini - ps39
Homo_sapiens - ps40
Phylostratum

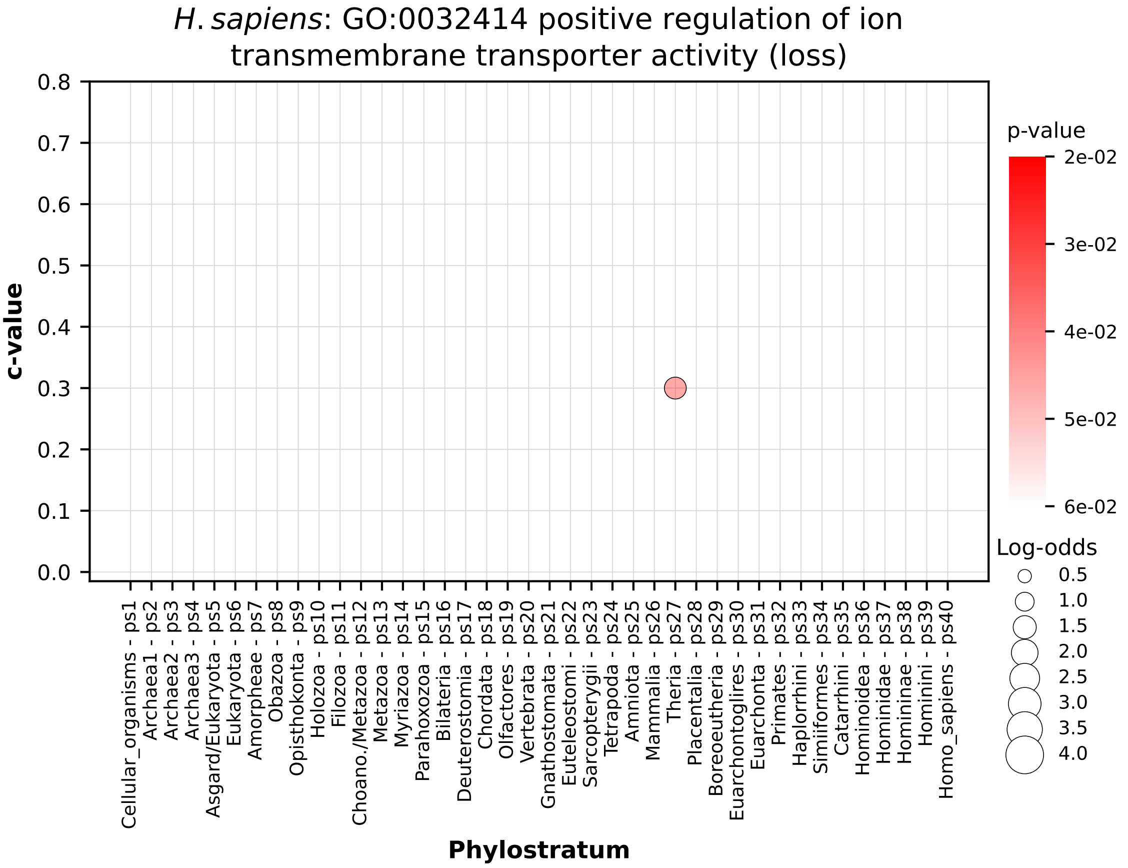

H. sapiens: GO:0032414 positive regulation of ion transmembrane transporter activity (loss)
c-value
0.8
0.7
0.6
0.5
0.4
0.3
0.2
0.1
0.0
Phylostratum
Cellular_organisms - ps1
Archaea1 - ps2
Archaea2 - ps3
Archaea3 - ps4
Asgard/Eukaryota - ps5
Eukaryota - ps6
Amorpheae - ps7
Obazoa - ps8
Opisthokonta - ps9
Holozoa - ps10
Filozoa - ps11
Choano./Metazoa - ps12
Metazoa - ps13
Myriazoa - ps14
Parahoxozoa - ps15
Bilateria - ps16
Deuterostomia - ps17
Chordata - ps18
Olfactores - ps19
Vertebrata - ps20
Gnathostomata - ps21
Euteleostomi - ps22
Sarcopterygii - ps23
Tetrapoda - ps24
Amniota - ps25
Mammalia - ps26
Theria - ps27
Placentalia - ps28
Boreoeutheria - ps29
Euarchontoglires - ps30
Euarchonta - ps31
Primates - ps32
Haplorrhini - ps33
Simiiformes - ps34
Catarrhini - ps35
Hominoidea - ps36
Hominidae - ps37
Homininae - ps38
Hominini - ps39
Homo_sapiens - ps40
p-value
2e-02
3e-02
4e-02
5e-02
6e-02
Log-odds
0.5
1.0
1.5
2.0
2.5
3.0
3.5
4.0

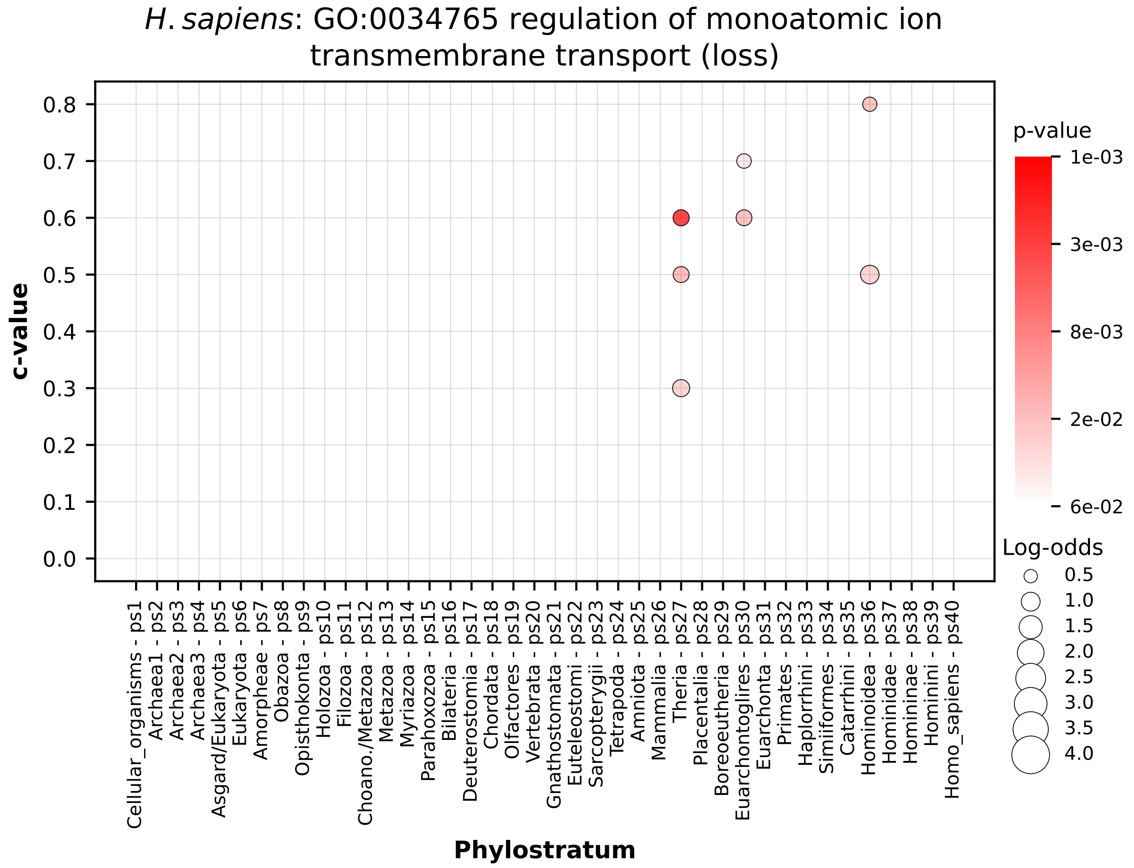

H. sapiens: GO:0034765 regulation of monoatomic ion transmembrane transport (loss)
c-value
0.8
0.7
0.6
0.5
0.4
0.3
0.2
0.1
0.0
p-value
1e-03
3e-03
8e-03
2e-02
6e-02
Log-odds
0.5
1.0
1.5
2.0
2.5
3.0
3.5
4.0
Cellular_organisms - ps1
Archaea1 - ps2
Archaea2 - ps3
Archaea3 - ps4
Asgard/Eukaryota - ps5
Eukaryota - ps6
Amorpheae - ps7
Obazoa - ps8
Opisthokonta - ps9
Holozoa - ps10
Filozoa - ps11
Choano./Metazoa - ps12
Metazoa - ps13
Myriazoa - ps14
Parahoxozoa - ps15
Bilateria - ps16
Deuterostomia - ps17
Chordata - ps18
Olfactores - ps19
Vertebrata - ps20
Gnathostomata - ps21
Euteleostomi - ps22
Sarcopterygii - ps23
Tetrapoda - ps24
Amniota - ps25
Mammalia - ps26
Theria - ps27
Placentalia - ps28
Boreoeutheria - ps29
Euarchontoglires - ps30
Euarchonta - ps31
Primates - ps32
Haplorrhini - ps33
Simiiformes - ps34
Catarrhini - ps35
Hominoidea - ps36
Hominidae - ps37
Homininae - ps38
Hominini - ps39
Homo_sapiens - ps40
Phylostratum

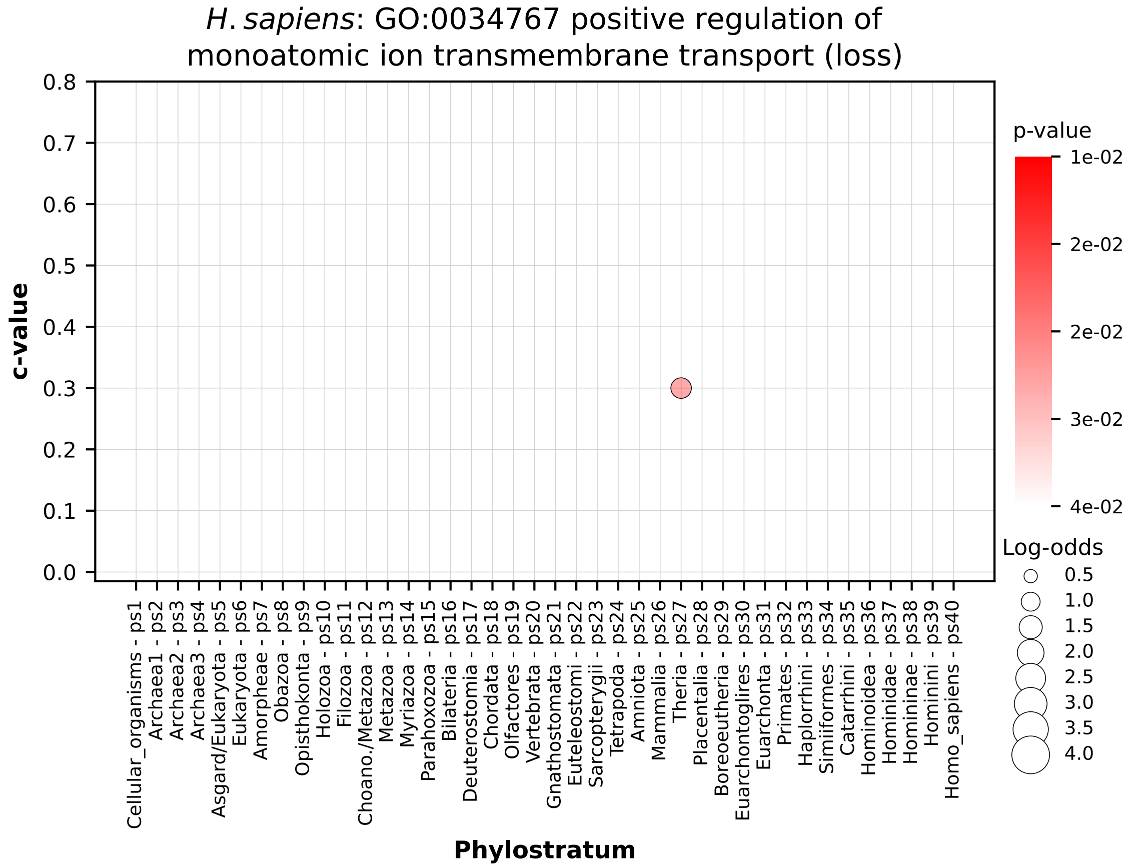
H. sapiens: GO:0034767 positive regulation of monoatomic ion transmembrane transport (loss)
c-value
0.8
0.7
0.6
0.5
0.4
0.3
0.2
0.1
0.0
Phylostratum
Cellular_organisms - ps1
Archaea1 - ps2
Archaea2 - ps3
Archaea3 - ps4
Asgard/Eukaryota - ps5
Eukaryota - ps6
Amorpheae - ps7
Obazoa - ps8
Opisthokonta - ps9
Holozoa - ps10
Filozoa - ps11
Choano./Metazoa - ps12
Metazoa - ps13
Myriazoa - ps14
Parahoxozoa - ps15
Bilateria - ps16
Deuterostomia - ps17
Chordata - ps18
Olfactores - ps19
Vertebrata - ps20
Gnathostomata - ps21
Euteleostomi - ps22
Sarcopterygii - ps23
Tetrapoda - ps24
Amniota - ps25
Mammalia - ps26
Theria - ps27
Placentalia - ps28
Boreoeutheria - ps29
Euarchontoglires - ps30
Euarchonta - ps31
Primates - ps32
Haplorrhini - ps33
Simiiformes - ps34
Catarrhini - ps35
Hominoidea - ps36
Hominidae - ps37
Homininae - ps38
Hominini - ps39
Homo_sapiens - ps40
p-value
1e-02
2e-02
2e-02
3e-02
4e-02
Log-odds
0.5
1.0
1.5
2.0
2.5
3.0
3.5
4.0

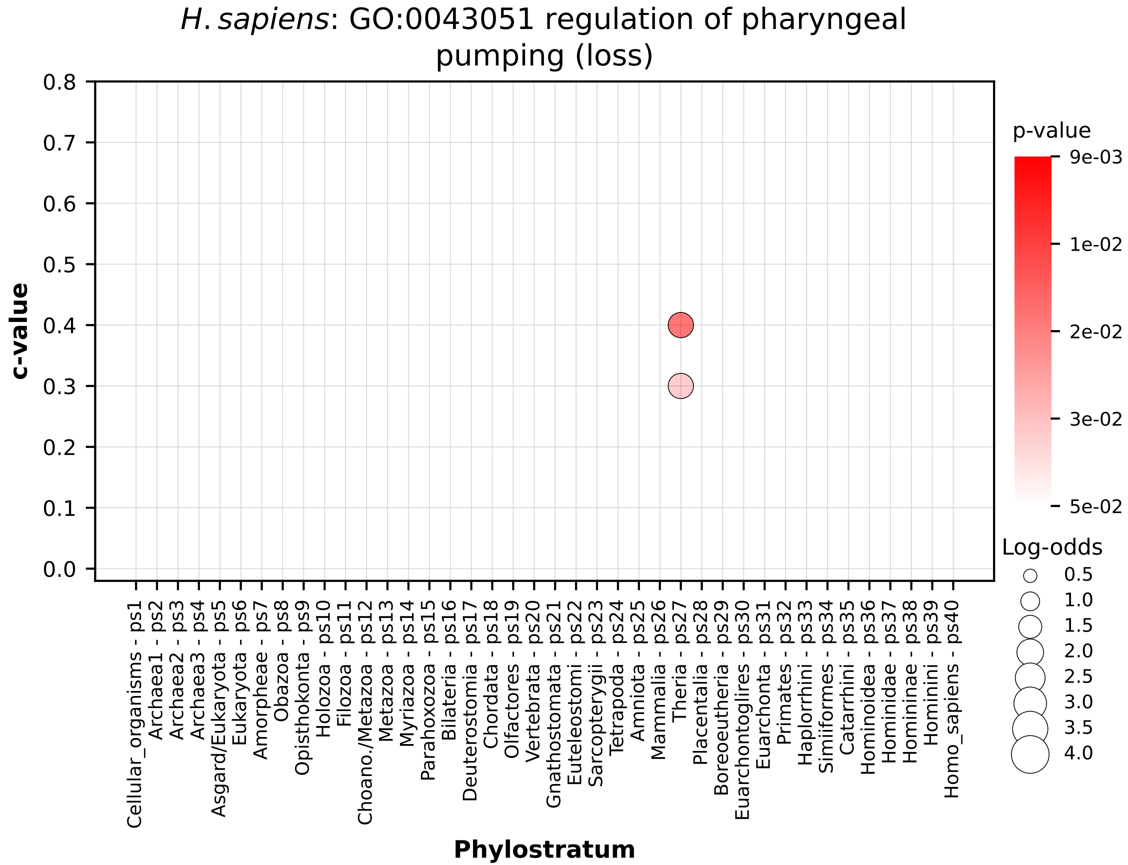

H. sapiens: GO:0043051 regulation of pharyngeal pumping (loss)
c-value
0.8
0.7
0.6
0.5
0.4
0.3
0.2
0.1
0.0
p-value
9e-03
1e-02
2e-02
3e-02
5e-02
Log-odds
0.5
1.0
1.5
2.0
2.5
3.0
3.5
4.0
Cellular_organisms - ps1
Archaea1 - ps2
Archaea2 - ps3
Archaea3 - ps4
Asgard/Eukaryota - ps5
Eukaryota - ps6
Amorpheae - ps7
Obazoa - ps8
Opisthokonta - ps9
Holozoa - ps10
Filozoa - ps11
Choano./Metazoa - ps12
Metazoa - ps13
Myriazoa - ps14
Parahoxozoa - ps15
Bilateria - ps16
Deuterostomia - ps17
Chordata - ps18
Olfactores - ps19
Vertebrata - ps20
Gnathostomata - ps21
Euteleostomi - ps22
Sarcopterygii - ps23
Tetrapoda - ps24
Amniota - ps25
Mammalia - ps26
Theria - ps27
Placentalia - ps28
Boreoeutheria - ps29
Euarchontoglires - ps30
Euarchonta - ps31
Primates - ps32
Haplorrhini - ps33
Simiiformes - ps34
Catarrhini - ps35
Hominoidea - ps36
Hominidae - ps37
Homininae - ps38
Hominini - ps39
Homo_sapiens - ps40
Phylostratum

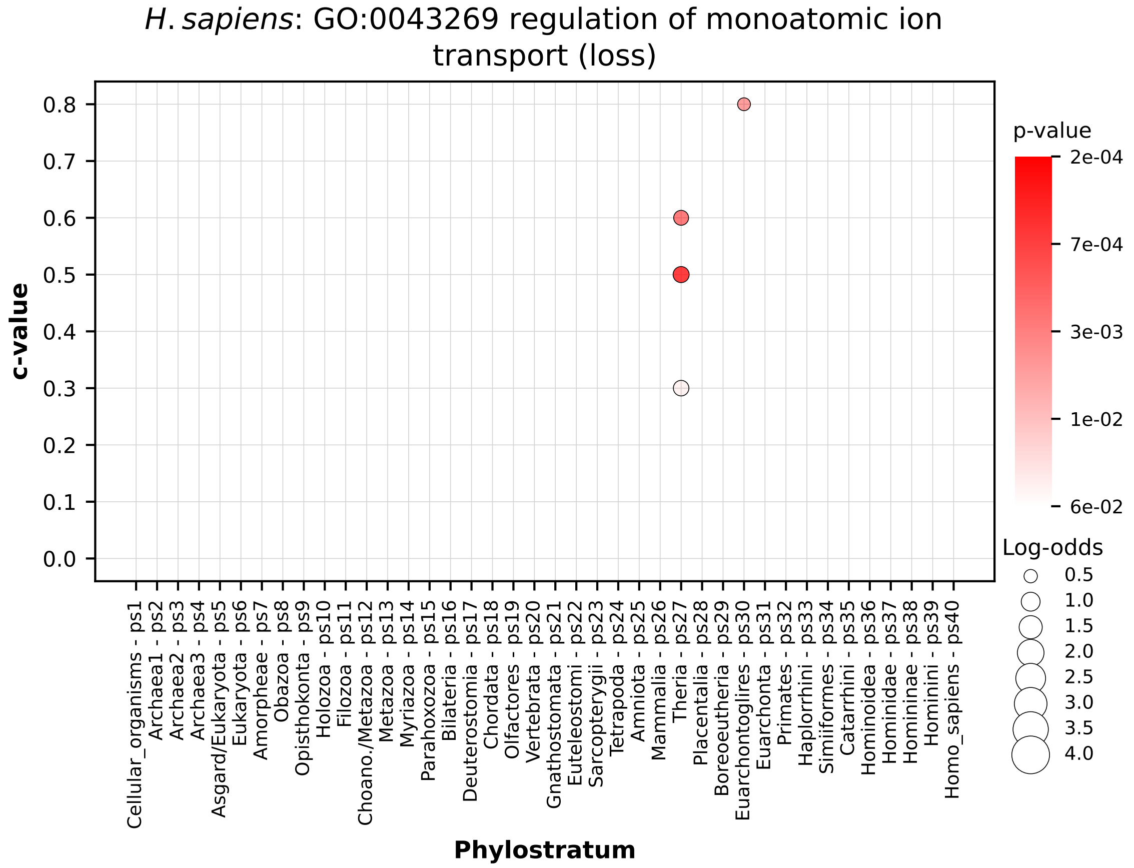

H. sapiens: GO:0043269 regulation of monoatomic ion transport (loss)
c-value
0.0
0.1
0.2
0.3
0.4
0.5
0.6
0.7
0.8
Phylostratum
Cellular_organisms - ps1
Archaea1 - ps2
Archaea2 - ps3
Archaea3 - ps4
Asgard/Eukaryota - ps5
Eukaryota - ps6
Amorpheae - ps7
Obazoa - ps8
Opisthokonta - ps9
Holozoa - ps10
Filozoa - ps11
Choano./Metazoa - ps12
Metazoa - ps13
Myriazoa - ps14
Parahoxozoa - ps15
Bilateria - ps16
Deuterostomia - ps17
Chordata - ps18
Olfactores - ps19
Vertebrata - ps20
Gnathostomata - ps21
Euteleostomi - ps22
Sarcopterygii - ps23
Tetrapoda - ps24
Amniota - ps25
Mammalia - ps26
Theria - ps27
Placentalia - ps28
Boreoeutheria - ps29
Euarchontoglires - ps30
Euarchonta - ps31
Primates - ps32
Haplorrhini - ps33
Simiiformes - ps34
Catarrhini - ps35
Hominoidea - ps36
Hominidae - ps37
Homininae - ps38
Hominini - ps39
Homo_sapiens - ps40
p-value
2e-04
7e-04
3e-03
1e-02
6e-02
Log-odds
0.5
1.0
1.5
2.0
2.5
3.0
3.5
4.0

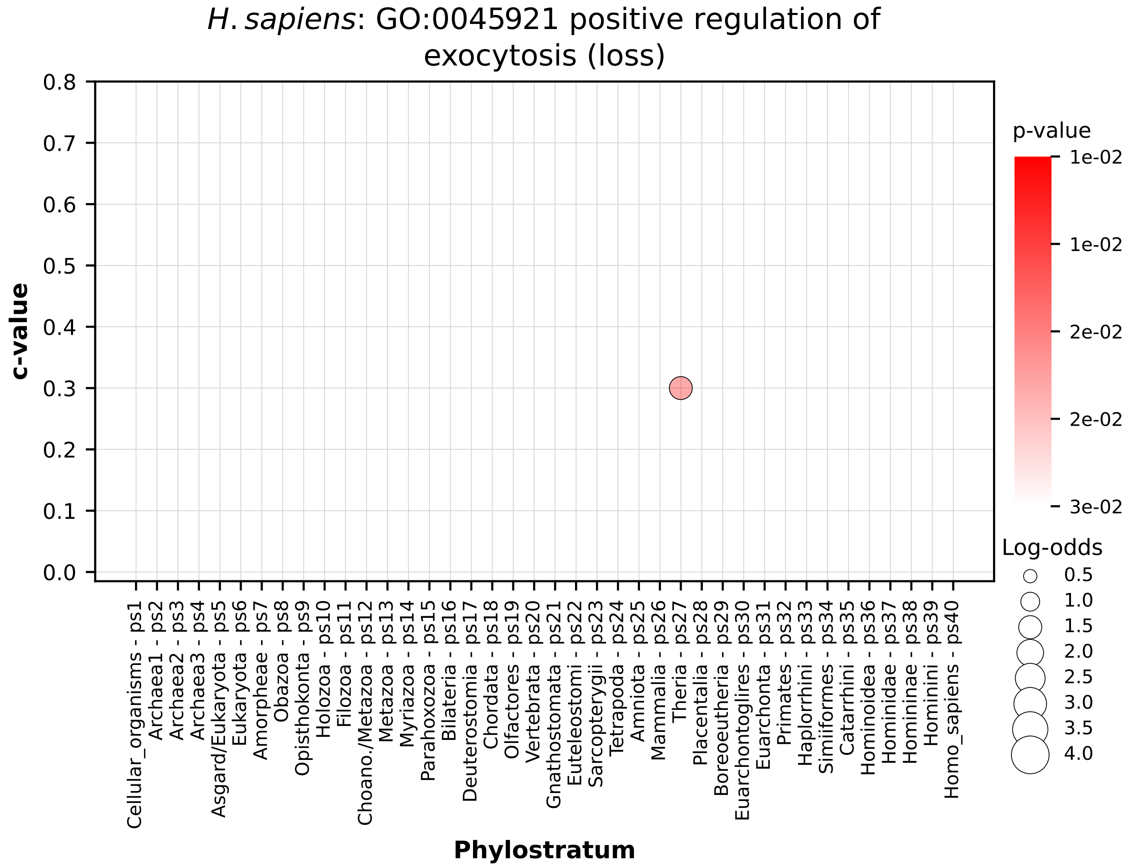

H. sapiens: GO:0045921 positive regulation of exocytosis (loss)
c-value
0.8
0.7
0.6
0.5
0.4
0.3
0.2
0.1
0.0
p-value
1e-02
1e-02
2e-02
2e-02
3e-02
Log-odds
0.5
1.0
1.5
2.0
2.5
3.0
3.5
4.0
Cellular_organisms - ps1
Archaea1 - ps2
Archaea2 - ps3
Archaea3 - ps4
Asgard/Eukaryota - ps5
Eukaryota - ps6
Amorpheae - ps7
Obazoa - ps8
Opisthokonta - ps9
Holozoa - ps10
Filozoa - ps11
Choano./Metazoa - ps12
Metazoa - ps13
Myriazoa - ps14
Parahoxozoa - ps15
Bilateria - ps16
Deuterostomia - ps17
Chordata - ps18
Olfactores - ps19
Vertebrata - ps20
Gnathostomata - ps21
Euteleostomi - ps22
Sarcopterygii - ps23
Tetrapoda - ps24
Amniota - ps25
Mammalia - ps26
Theria - ps27
Placentalia - ps28
Boreoeutheria - ps29
Euarchontoglires - ps30
Euarchonta - ps31
Primates - ps32
Haplorrhini - ps33
Simiiformes - ps34
Catarrhini - ps35
Hominoidea - ps36
Hominidae - ps37
Homininae - ps38
Hominini - ps39
Homo_sapiens - ps40
Phylostratum

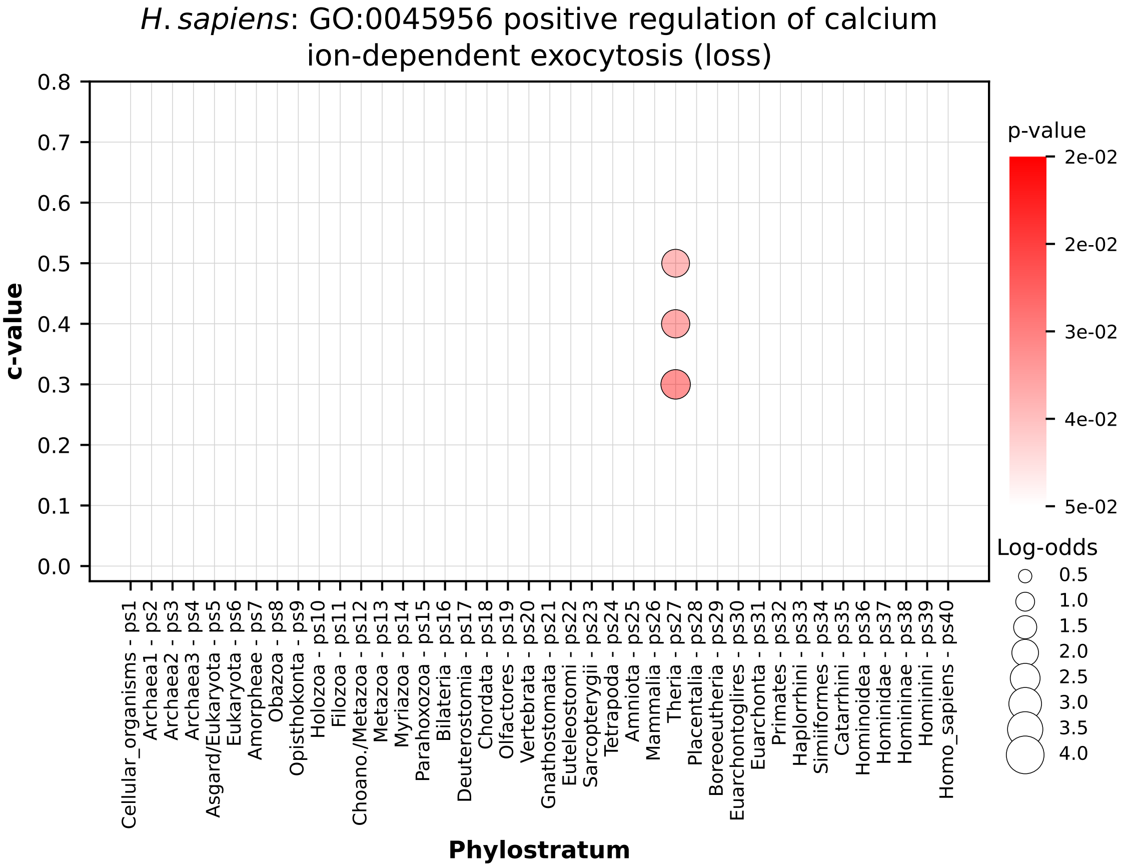

H. sapiens: GO:0045956 positive regulation of calcium ion-dependent exocytosis (loss)
c-value
0.0
0.1
0.2
0.3
0.4
0.5
0.6
0.7
0.8
Phylostratum
Cellular_organisms - ps1
Archaea1 - ps2
Archaea2 - ps3
Archaea3 - ps4
Asgard/Eukaryota - ps5
Eukaryota - ps6
Amorpheae - ps7
Obazoa - ps8
Opisthokonta - ps9
Holozoa - ps10
Filozoa - ps11
Choano./Metazoa - ps12
Metazoa - ps13
Myriazoa - ps14
Parahoxozoa - ps15
Bilateria - ps16
Deuterostomia - ps17
Chordata - ps18
Olfactores - ps19
Vertebrata - ps20
Gnathostomata - ps21
Euteleostomi - ps22
Sarcopterygii - ps23
Tetrapoda - ps24
Amniota - ps25
Mammalia - ps26
Theria - ps27
Placentalia - ps28
Boreoeutheria - ps29
Euarchontoglires - ps30
Euarchonta - ps31
Primates - ps32
Haplorrhini - ps33
Simiiformes - ps34
Catarrhini - ps35
Hominoidea - ps36
Hominidae - ps37
Homininae - ps38
Hominini - ps39
Homo_sapiens - ps40
p-value
2e-02
2e-02
3e-02
4e-02
5e-02
Log-odds
0.5
1.0
1.5
2.0
2.5
3.0
3.5
4.0

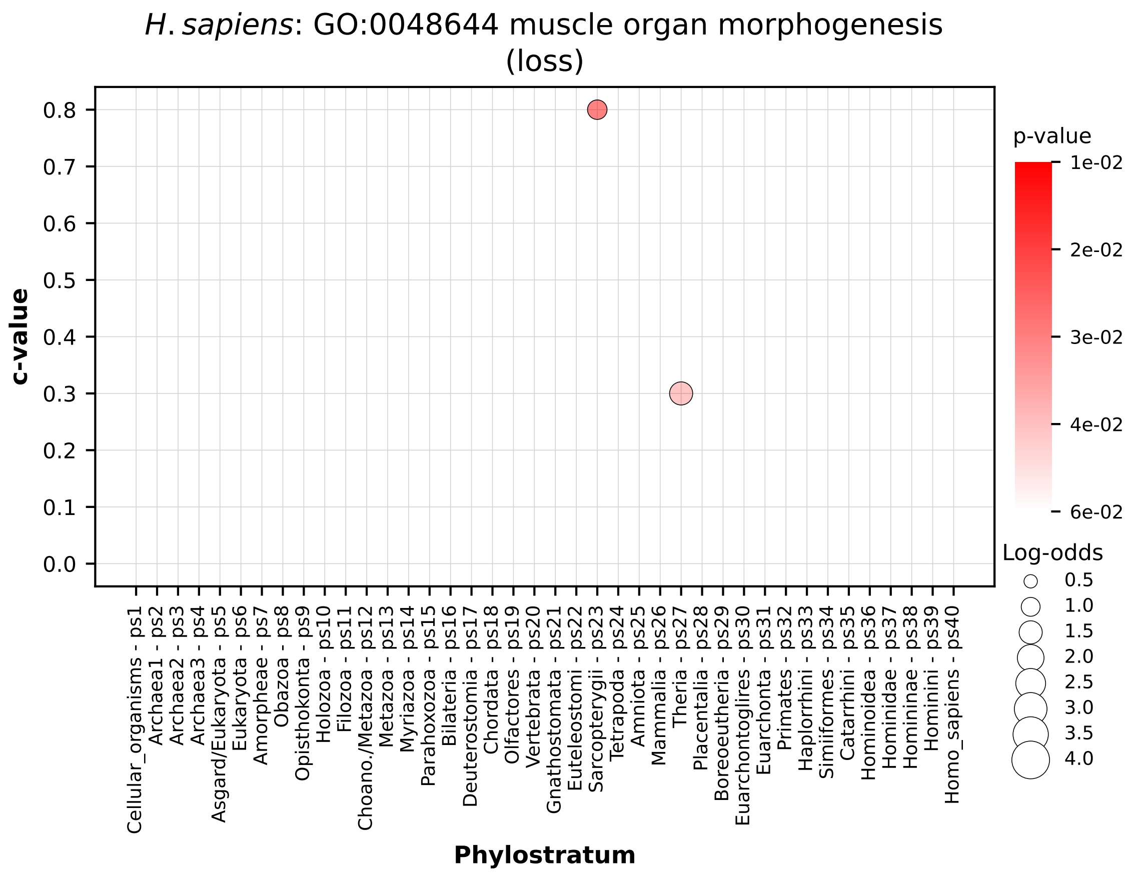
H. sapiens: GO:0048644 muscle organ morphogenesis
(loss)
c-value
0.0
0.1
0.2
0.3
0.4
0.5
0.6
0.7
0.8
p-value
1e-02
2e-02
3e-02
4e-02
6e-02
Log-odds
0.5
1.0
1.5
2.0
2.5
3.0
3.5
4.0
Cellular_organisms - ps1
Archaea1 - ps2
Archaea2 - ps3
Archaea3 - ps4
Asgard/Eukaryota - ps5
Eukaryota - ps6
Amorpheae - ps7
Obazoa - ps8
Opisthokonta - ps9
Holozoa - ps10
Filozoa - ps11
Choano./Metazoa - ps12
Metazoa - ps13
Myriazoa - ps14
Parahoxozoa - ps15
Bilateria - ps16
Deuterostomia - ps17
Chordata - ps18
Olfactores - ps19
Vertebrata - ps20
Gnathostomata - ps21
Euteleostomi - ps22
Sarcopterygii - ps23
Tetrapoda - ps24
Amniota - ps25
Mammalia - ps26
Theria - ps27
Placentalia - ps28
Boreoeutheria - ps29
Euarchontoglires - ps30
Euarchonta - ps31
Primates - ps32
Haplorrhini - ps33
Simiiformes - ps34
Catarrhini - ps35
Hominoidea - ps36
Hominidae - ps37
Homininae - ps38
Hominini - ps39
Homo_sapiens - ps40
Phylostratum

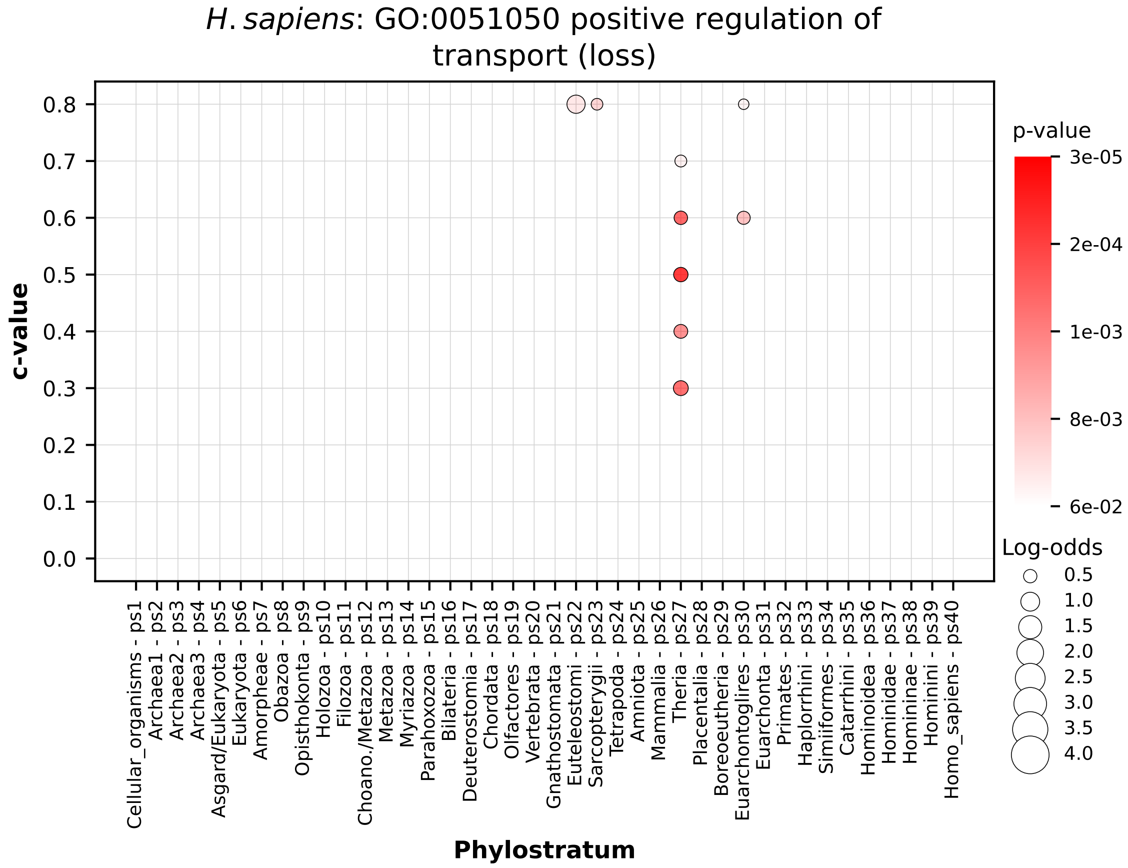

H. sapiens: GO:0051050 positive regulation of transport (loss)
c-value
0.8
0.7
0.6
0.5
0.4
0.3
0.2
0.1
0.0
Phylostratum
Cellular_organisms - ps1
Archaea1 - ps2
Archaea2 - ps3
Archaea3 - ps4
Asgard/Eukaryota - ps5
Eukaryota - ps6
Amorpheae - ps7
Obazoa - ps8
Opisthokonta - ps9
Holozoa - ps10
Filozoa - ps11
Choano./Metazoa - ps12
Metazoa - ps13
Myriazoa - ps14
Parahoxozoa - ps15
Bilateria - ps16
Deuterostomia - ps17
Chordata - ps18
Olfactores - ps19
Vertebrata - ps20
Gnathostomata - ps21
Euteleostomi - ps22
Sarcopterygii - ps23
Tetrapoda - ps24
Amniota - ps25
Mammalia - ps26
Theria - ps27
Placentalia - ps28
Boreoeutheria - ps29
Euarchontoglires - ps30
Euarchonta - ps31
Primates - ps32
Haplorrhini - ps33
Simiiformes - ps34
Catarrhini - ps35
Hominoidea - ps36
Hominidae - ps37
Homininae - ps38
Hominini - ps39
Homo_sapiens - ps40
p-value
3e-05
2e-04
1e-03
8e-03
6e-02
Log-odds
0.5
1.0
1.5
2.0
2.5
3.0
3.5
4.0

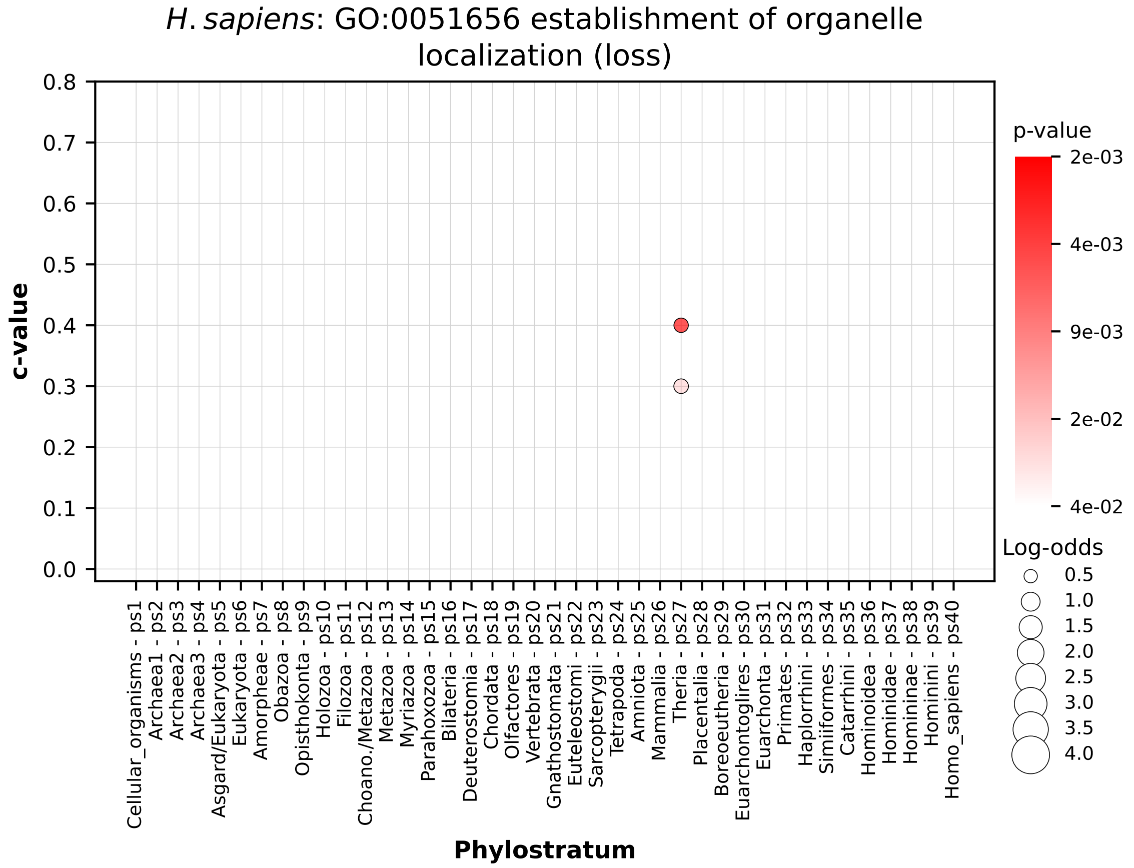
H. sapiens: GO:0051656 establishment of organelle localization (loss)
c-value
Phylostratum
p-value
2e-03
4e-03
9e-03
2e-02
4e-02
Log-odds
0.5
1.0
1.5
2.0
2.5
3.0
3.5
4.0
Cellular_organisms - ps1
Archaea1 - ps2
Archaea2 - ps3
Archaea3 - ps4
Asgard/Eukaryota - ps5
Eukaryota - ps6
Amorpheae - ps7
Obazoa - ps8
Opisthokonta - ps9
Holozoa - ps10
Filozoa - ps11
Choano./Metazoa - ps12
Metazoa - ps13
Myriazoa - ps14
Parahoxozoa - ps15
Bilateria - ps16
Deuterostomia - ps17
Chordata - ps18
Olfactores - ps19
Vertebrata - ps20
Gnathostomata - ps21
Euteleostomi - ps22
Sarcopterygii - ps23
Tetrapoda - ps24
Amniota - ps25
Mammalia - ps26
Theria - ps27
Placentalia - ps28
Boreoeutheria - ps29
Euarchontoglires - ps30
Euarchonta - ps31
Primates - ps32
Haplorrhini - ps33
Simiiformes - ps34
Catarrhini - ps35
Hominoidea - ps36
Hominidae - ps37
Homininae - ps38
Hominini - ps39
Homo_sapiens - ps40

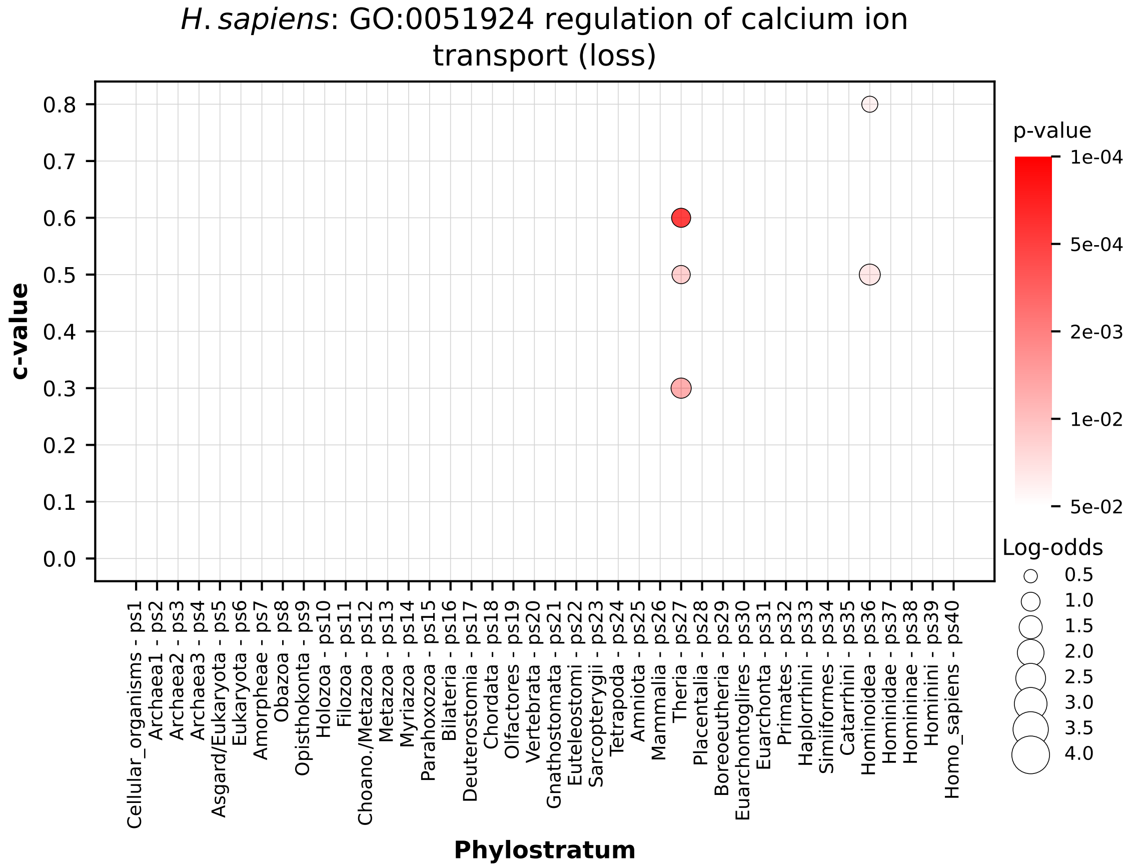

H. sapiens: GO:0051924 regulation of calcium ion transport (loss)
c-value
0.8
0.7
0.6
0.5
0.4
0.3
0.2
0.1
0.0
p-value
1e-04
5e-04
2e-03
1e-02
5e-02
Log-odds
0.5
1.0
1.5
2.0
2.5
3.0
3.5
4.0
Cellular_organisms - ps1
Archaea1 - ps2
Archaea2 - ps3
Archaea3 - ps4
Asgard/Eukaryota - ps5
Eukaryota - ps6
Amorpheae - ps7
Obazoa - ps8
Opisthokonta - ps9
Holozoa - ps10
Filozoa - ps11
Choano./Metazoa - ps12
Metazoa - ps13
Myriazoa - ps14
Parahoxozoa - ps15
Bilateria - ps16
Deuterostomia - ps17
Chordata - ps18
Olfactores - ps19
Vertebrata - ps20
Gnathostomata - ps21
Euteleostomi - ps22
Sarcopterygii - ps23
Tetrapoda - ps24
Amniota - ps25
Mammalia - ps26
Theria - ps27
Placentalia - ps28
Boreoeutheria - ps29
Euarchontoglires - ps30
Euarchonta - ps31
Primates - ps32
Haplorrhini - ps33
Simiiformes - ps34
Catarrhini - ps35
Hominoidea - ps36
Hominidae - ps37
Homininae - ps38
Hominini - ps39
Homo_sapiens - ps40
Phylostratum

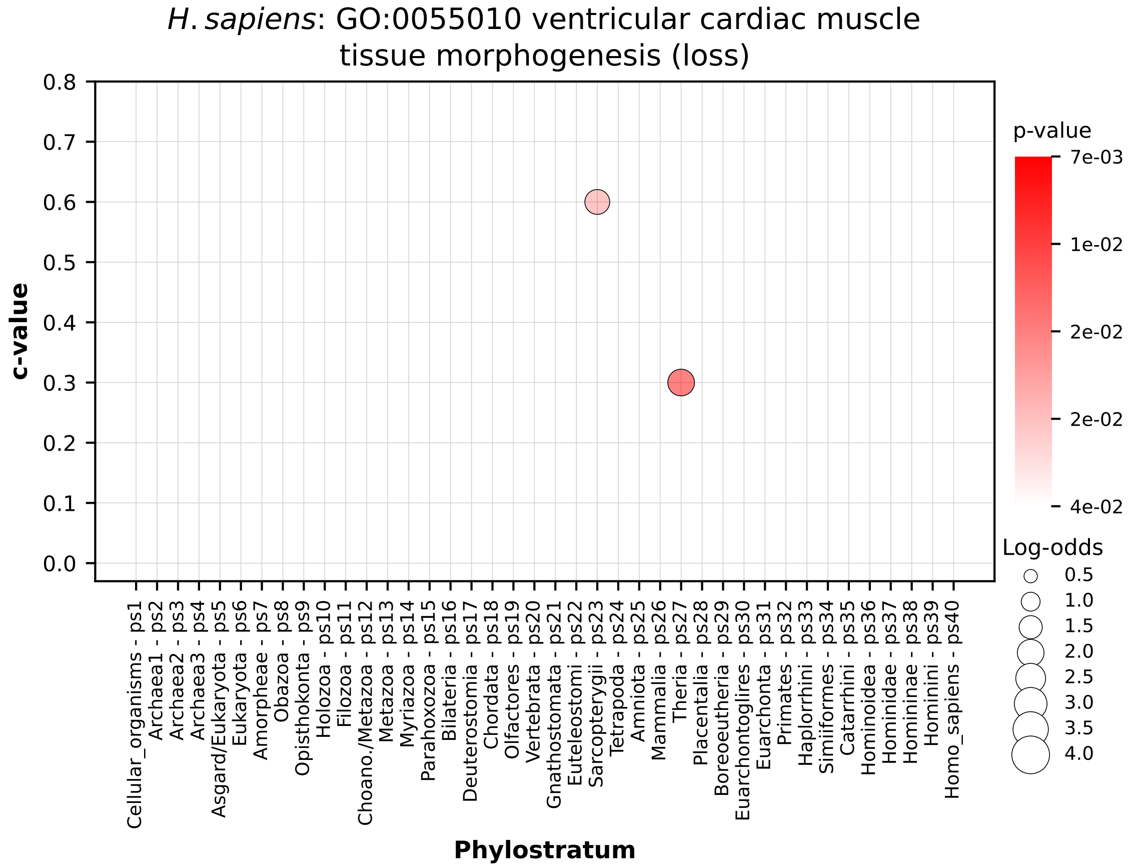

H. sapiens: GO:0055010 ventricular cardiac muscle tissue morphogenesis (loss)
c-value
0.0
0.1
0.2
0.3
0.4
0.5
0.6
0.7
0.8
Phylostratum
Cellular_organisms - ps1
Archaea1 - ps2
Archaea2 - ps3
Archaea3 - ps4
Asgard/Eukaryota - ps5
Eukaryota - ps6
Amorpheae - ps7
Obazoa - ps8
Opisthokonta - ps9
Holozoa - ps10
Filozoa - ps11
Choano./Metazoa - ps12
Metazoa - ps13
Myriazoa - ps14
Parahoxozoa - ps15
Bilateria - ps16
Deuterostomia - ps17
Chordata - ps18
Olfactores - ps19
Vertebrata - ps20
Gnathostomata - ps21
Euteleostomi - ps22
Sarcopterygii - ps23
Tetrapoda - ps24
Amniota - ps25
Mammalia - ps26
Theria - ps27
Placentalia - ps28
Boreoeutheria - ps29
Euarchontoglires - ps30
Euarchonta - ps31
Primates - ps32
Haplorrhini - ps33
Simiiformes - ps34
Catarrhini - ps35
Hominoidea - ps36
Hominidae - ps37
Homininae - ps38
Hominini - ps39
Homo_sapiens - ps40
p-value
7e-03
1e-02
2e-02
2e-02
4e-02
Log-odds
0.5
1.0
1.5
2.0
2.5
3.0
3.5
4.0

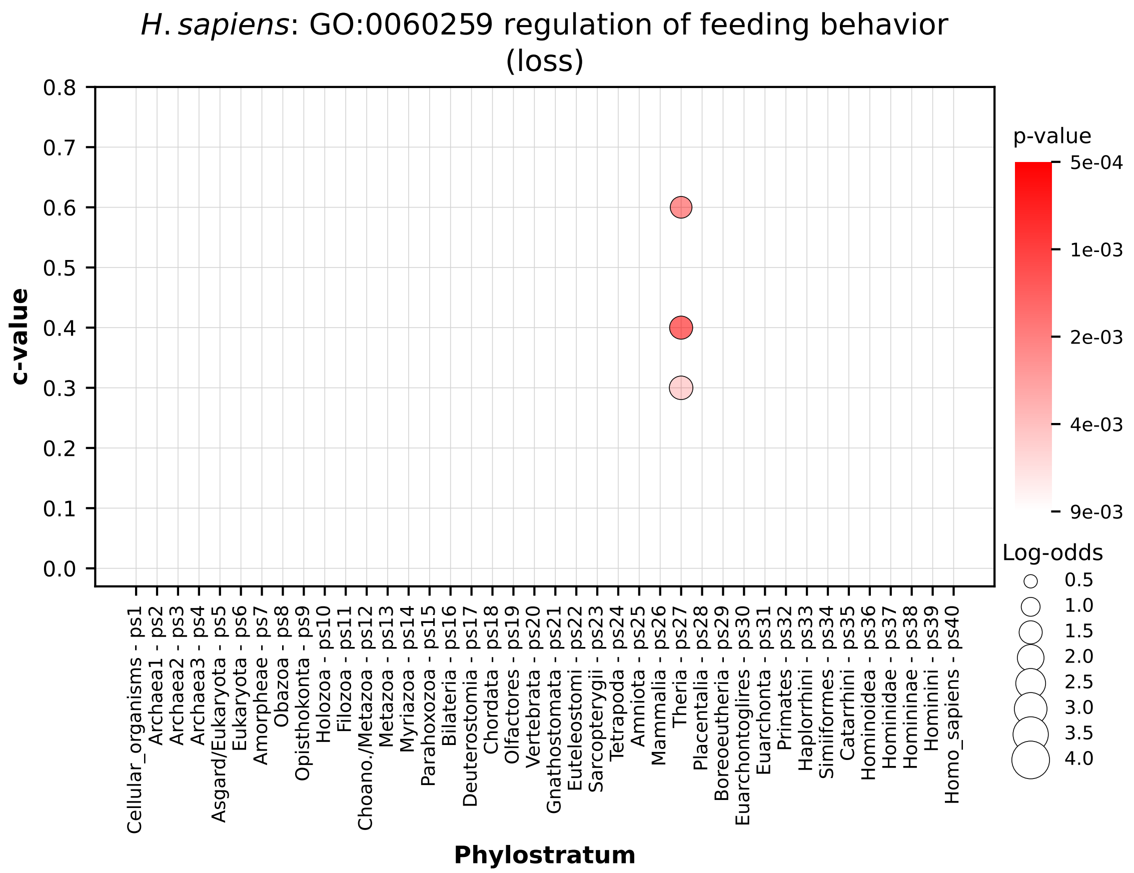

H. sapiens: GO:0060259 regulation of feeding behavior (loss)
c-value
0.8
0.7
0.6
0.5
0.4
0.3
0.2
0.1
0.0
p-value
5e-04
1e-03
2e-03
4e-03
9e-03
Log-odds
0.5
1.0
1.5
2.0
2.5
3.0
3.5
4.0
Cellular_organisms - ps1
Archaea1 - ps2
Archaea2 - ps3
Archaea3 - ps4
Asgard/Eukaryota - ps5
Eukaryota - ps6
Amorpheae - ps7
Obazoa - ps8
Opisthokonta - ps9
Holozoa - ps10
Filozoa - ps11
Choano./Metazoa - ps12
Metazoa - ps13
Myriazoa - ps14
Parahoxozoa - ps15
Bilateria - ps16
Deuterostomia - ps17
Chordata - ps18
Olfactores - ps19
Vertebrata - ps20
Gnathostomata - ps21
Euteleostomi - ps22
Sarcopterygii - ps23
Tetrapoda - ps24
Amniota - ps25
Mammalia - ps26
Theria - ps27
Placentalia - ps28
Boreoeutheria - ps29
Euarchontoglires - ps30
Euarchonta - ps31
Primates - ps32
Haplorrhini - ps33
Simiiformes - ps34
Catarrhini - ps35
Hominoidea - ps36
Hominidae - ps37
Homininae - ps38
Hominini - ps39
Homo_sapiens - ps40
Phylostratum

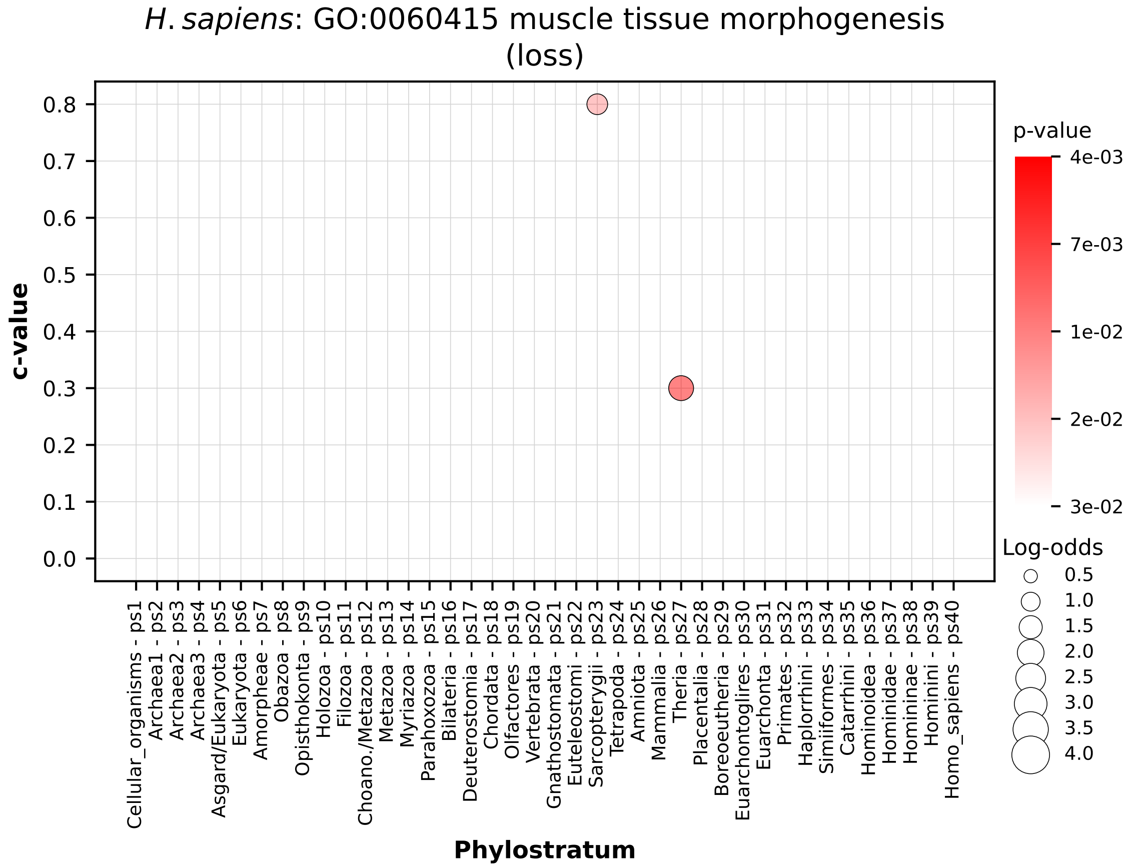

H. sapiens: GO:0060415 muscle tissue morphogenesis
(loss)
c-value
0.0
0.1
0.2
0.3
0.4
0.5
0.6
0.7
0.8
Phylostratum
Cellular_organisms - ps1
Archaea1 - ps2
Archaea2 - ps3
Archaea3 - ps4
Asgard/Eukaryota - ps5
Eukaryota - ps6
Amorpheae - ps7
Obazoa - ps8
Opisthokonta - ps9
Holozoa - ps10
Filozoa - ps11
Choano./Metazoa - ps12
Metazoa - ps13
Myriazoa - ps14
Parahoxozoa - ps15
Bilateria - ps16
Deuterostomia - ps17
Chordata - ps18
Olfactores - ps19
Vertebrata - ps20
Gnathostomata - ps21
Euteleostomi - ps22
Sarcopterygii - ps23
Tetrapoda - ps24
Amniota - ps25
Mammalia - ps26
Theria - ps27
Placentalia - ps28
Boreoeutheria - ps29
Euarchontoglires - ps30
Euarchonta - ps31
Primates - ps32
Haplorrhini - ps33
Simiiformes - ps34
Catarrhini - ps35
Hominoidea - ps36
Hominidae - ps37
Homininae - ps38
Hominini - ps39
Homo_sapiens - ps40
p-value
4e-03
7e-03
1e-02
2e-02
3e-02
Log-odds
0.5
1.0
1.5
2.0
2.5
3.0
3.5
4.0

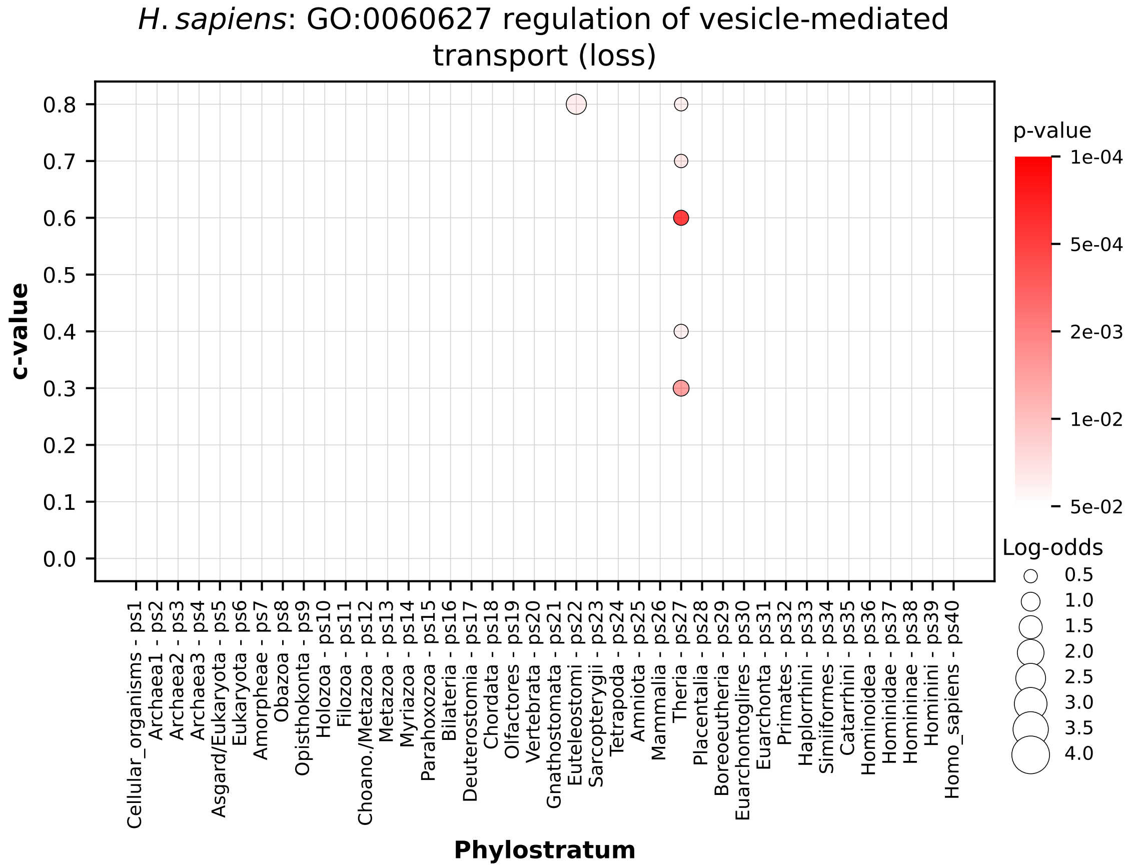
H. sapiens: GO:0060627 regulation of vesicle-mediated transport (loss)
c-value
0.0
0.1
0.2
0.3
0.4
0.5
0.6
0.7
0.8
Cellular_organisms - ps1
Archaea1 - ps2
Archaea2 - ps3
Archaea3 - ps4
Asgard/Eukaryota - ps5
Eukaryota - ps6
Amorpheae - ps7
Obazoa - ps8
Opisthokonta - ps9
Holozoa - ps10
Filozoa - ps11
Choano./Metazoa - ps12
Metazoa - ps13
Myriazoa - ps14
Parahoxozoa - ps15
Bilateria - ps16
Deuterostomia - ps17
Chordata - ps18
Olfactores - ps19
Vertebrata - ps20
Gnathostomata - ps21
Euteleostomi - ps22
Sarcopterygii - ps23
Tetrapoda - ps24
Amniota - ps25
Mammalia - ps26
Theria - ps27
Placentalia - ps28
Boreoeutheria - ps29
Euarchontoglires - ps30
Euarchonta - ps31
Primates - ps32
Haplorrhini - ps33
Simiiformes - ps34
Catarrhini - ps35
Hominoidea - ps36
Hominidae - ps37
Homininae - ps38
Hominini - ps39
Homo_sapiens - ps40
Phylostratum
p-value
1e-04
5e-04
2e-03
1e-02
5e-02
Log-odds
0.5
1.0
1.5
2.0
2.5
3.0
3.5
4.0

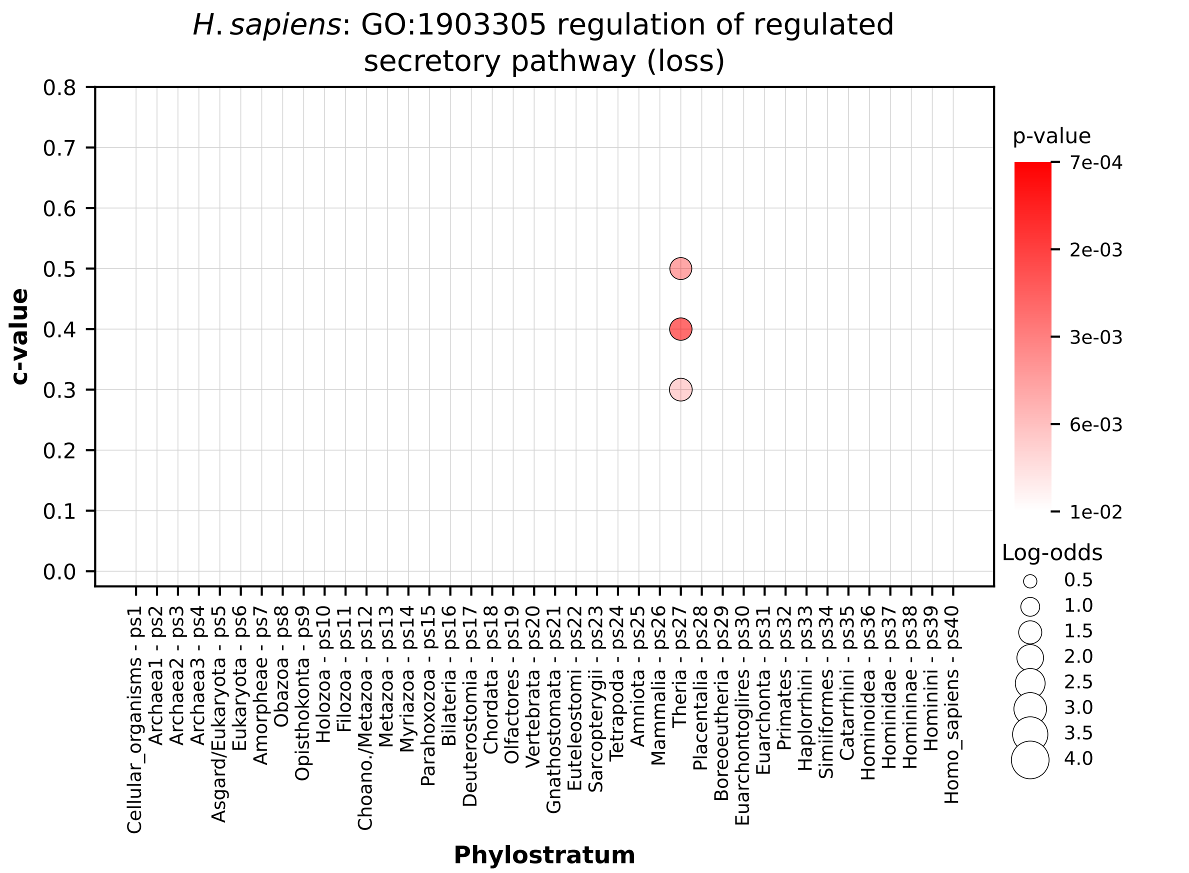

H. sapiens: GO:1903305 regulation of regulated secretory pathway (loss)
c-value
0.8
0.7
0.6
0.5
0.4
0.3
0.2
0.1
0.0
p-value
7e-04
2e-03
3e-03
6e-03
1e-02
Log-odds
0.5
1.0
1.5
2.0
2.5
3.0
3.5
4.0
Cellular_organisms - ps1
Archaea1 - ps2
Archaea2 - ps3
Archaea3 - ps4
Asgard/Eukaryota - ps5
Eukaryota - ps6
Amorpheae - ps7
Obazoa - ps8
Opisthokonta - ps9
Holozoa - ps10
Filozoa - ps11
Choano./Metazoa - ps12
Metazoa - ps13
Myriazoa - ps14
Parahoxozoa - ps15
Bilateria - ps16
Deuterostomia - ps17
Chordata - ps18
Olfactores - ps19
Vertebrata - ps20
Gnathostomata - ps21
Euteleostomi - ps22
Sarcopterygii - ps23
Tetrapoda - ps24
Amniota - ps25
Mammalia - ps26
Theria - ps27
Placentalia - ps28
Boreoeutheria - ps29
Euarchontoglires - ps30
Euarchonta - ps31
Primates - ps32
Haplorrhini - ps33
Simiiformes - ps34
Catarrhini - ps35
Hominoidea - ps36
Hominidae - ps37
Homininae - ps38
Hominini - ps39
Homo_sapiens - ps40
Phylostratum

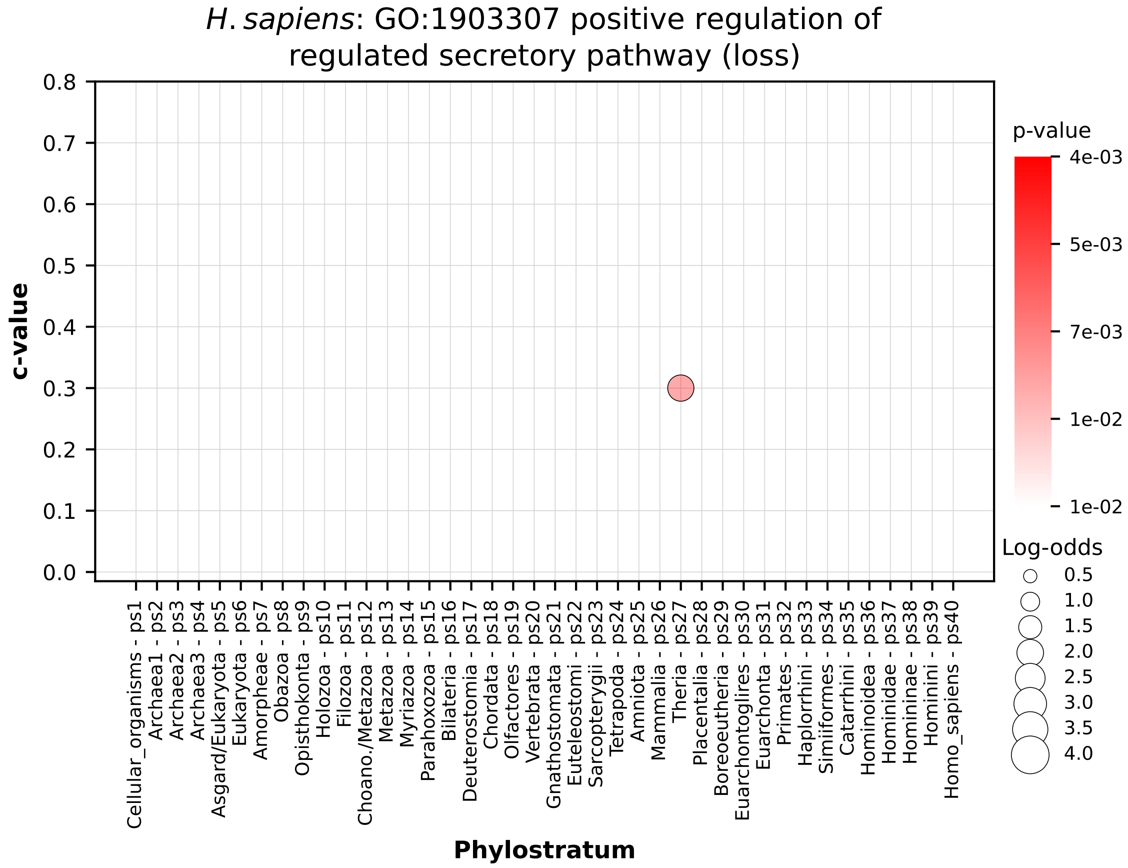

H. sapiens: GO:1903307 positive regulation of regulated secretory pathway (loss)
c-value
0.8
0.7
0.6
0.5
0.4
0.3
0.2
0.1
0.0
p-value
4e-03
5e-03
7e-03
1e-02
1e-02
Log-odds
0.5
1.0
1.5
2.0
2.5
3.0
3.5
4.0
Cellular_organisms - ps1
Archaea1 - ps2
Archaea2 - ps3
Archaea3 - ps4
Asgard/Eukaryota - ps5
Eukaryota - ps6
Amorpheae - ps7
Obazoa - ps8
Opisthokonta - ps9
Holozoa - ps10
Filozoa - ps11
Choano./Metazoa - ps12
Metazoa - ps13
Myriazoa - ps14
Parahoxozoa - ps15
Bilateria - ps16
Deuterostomia - ps17
Chordata - ps18
Olfactores - ps19
Vertebrata - ps20
Gnathostomata - ps21
Euteleostomi - ps22
Sarcopterygii - ps23
Tetrapoda - ps24
Amniota - ps25
Mammalia - ps26
Theria - ps27
Placentalia - ps28
Boreoeutheria - ps29
Euarchontoglires - ps30
Euarchonta - ps31
Primates - ps32
Haplorrhini - ps33
Simiiformes - ps34
Catarrhini - ps35
Hominoidea - ps36
Hominidae - ps37
Homininae - ps38
Hominini - ps39
Homo_sapiens - ps40
Phylostratum

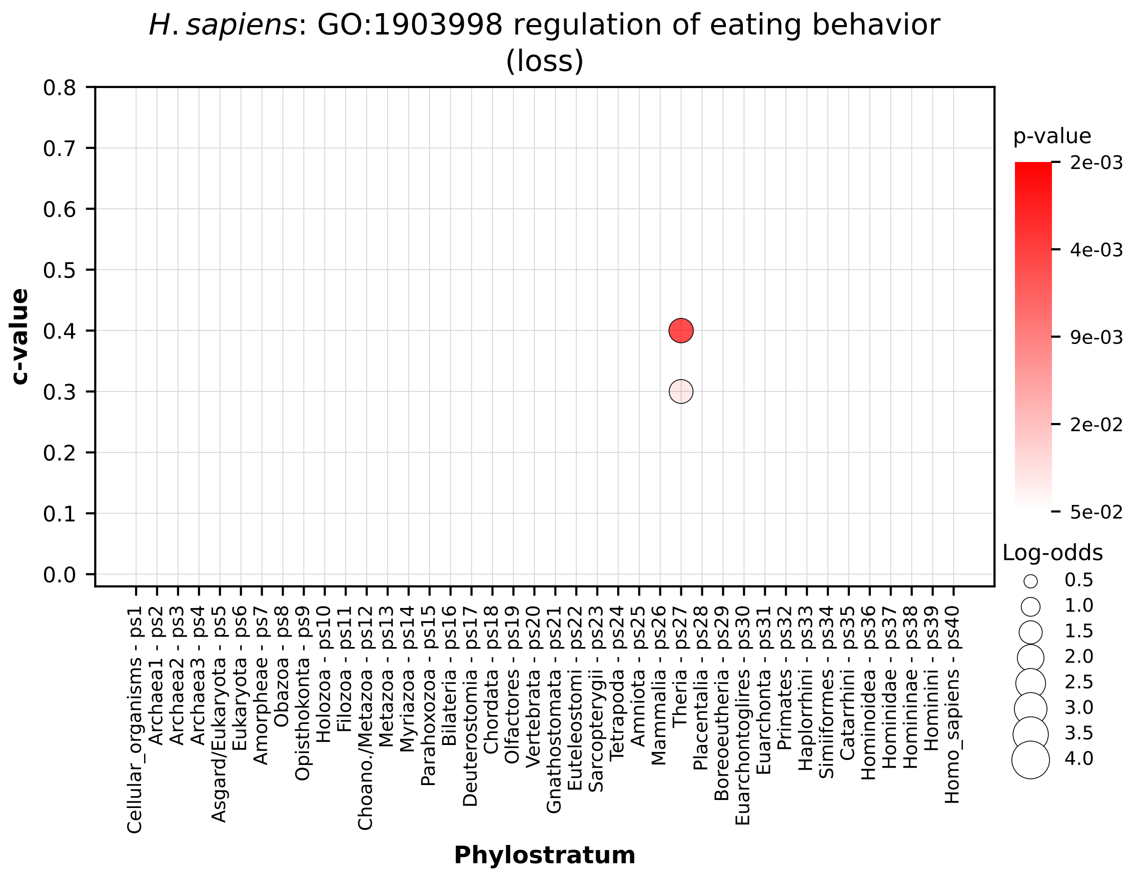

$H. sapiens$: GO:1903998 regulation of eating behavior
(loss)
c-value
0.0
0.1
0.2
0.3
0.4
0.5
0.6
0.7
0.8
Phylostratum
Cellular_organisms - ps1
Archaea1 - ps2
Archaea2 - ps3
Archaea3 - ps4
Asgard/Eukaryota - ps5
Eukaryota - ps6
Amorpheae - ps7
Obazoa - ps8
Opisthokonta - ps9
Holozoa - ps10
Filozoa - ps11
Choano./Metazoa - ps12
Metazoa - ps13
Myriazoa - ps14
Parahoxozoa - ps15
Bilateria - ps16
Deuterostomia - ps17
Chordata - ps18
Olfactores - ps19
Vertebrata - ps20
Gnathostomata - ps21
Euteleostomi - ps22
Sarcopterygii - ps23
Tetrapoda - ps24
Amniota - ps25
Mammalia - ps26
Theria - ps27
Placentalia - ps28
Boreoeutheria - ps29
Euarchontoglires - ps30
Euarchonta - ps31
Primates - ps32
Haplorrhini - ps33
Simiiformes - ps34
Catarrhini - ps35
Hominoidea - ps36
Hominidae - ps37
Homininae - ps38
Hominini - ps39
Homo_sapiens - ps40
p-value
2e-03
4e-03
9e-03
2e-02
5e-02
Log-odds
0.5
1.0
1.5
2.0
2.5
3.0
3.5
4.0

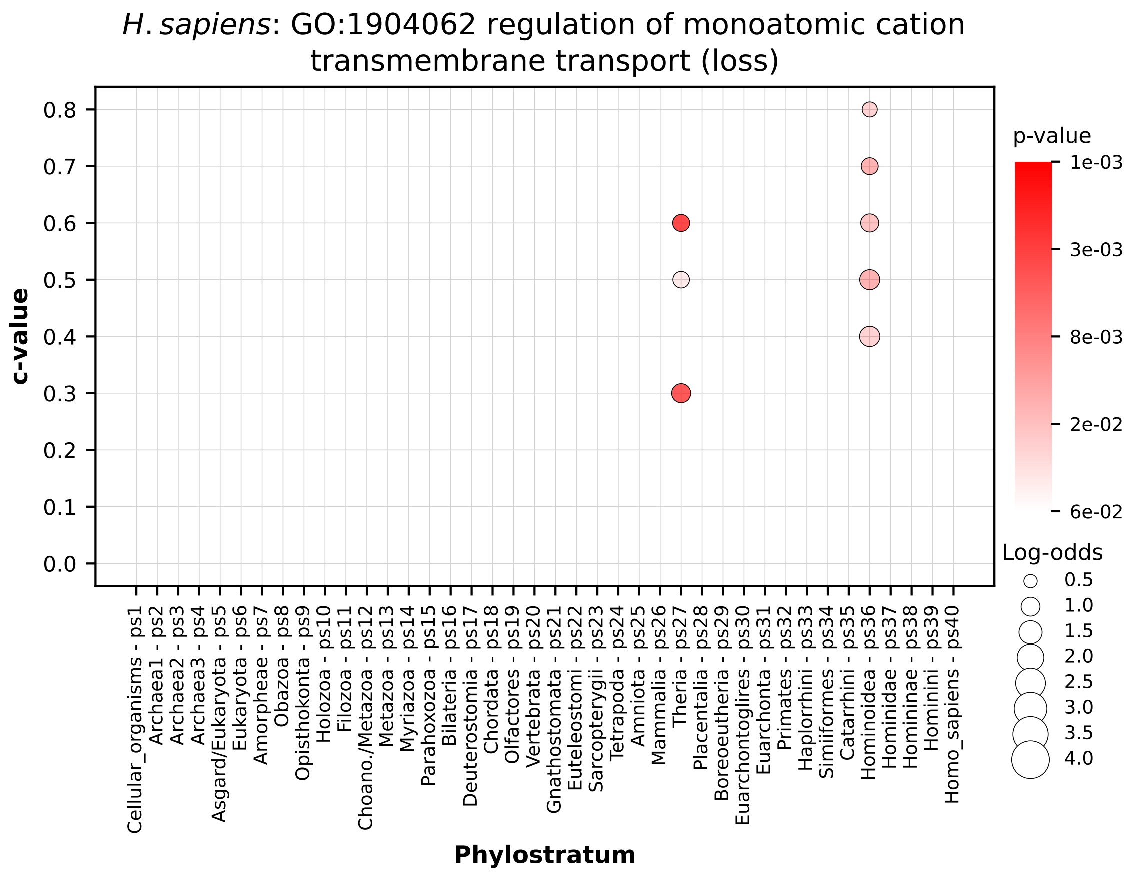
H. sapiens: GO:1904062 regulation of monoatomic cation transmembrane transport (loss)
c-value
Phylostratum
p-value
1e-03
3e-03
8e-03
2e-02
6e-02
Log-odds
0.5
1.0
1.5
2.0
2.5
3.0
3.5
4.0
Cellular_organisms - ps1
Archaea1 - ps2
Archaea2 - ps3
Archaea3 - ps4
Asgard/Eukaryota - ps5
Eukaryota - ps6
Amorpheae - ps7
Obazoa - ps8
Opisthokonta - ps9
Holozoa - ps10
Filozoa - ps11
Choano./Metazoa - ps12
Metazoa - ps13
Myriazoa - ps14
Parahoxozoa - ps15
Bilateria - ps16
Deuterostomia - ps17
Chordata - ps18
Olfactores - ps19
Vertebrata - ps20
Gnathostomata - ps21
Euteleostomi - ps22
Sarcopterygii - ps23
Tetrapoda - ps24
Amniota - ps25
Mammalia - ps26
Theria - ps27
Placentalia - ps28
Boreoeutheria - ps29
Euarchontoglires - ps30
Euarchonta - ps31
Primates - ps32
Haplorrhini - ps33
Simiiformes - ps34
Catarrhini - ps35
Hominoidea - ps36
Hominidae - ps37
Homininae - ps38
Hominini - ps39
Homo_sapiens - ps40

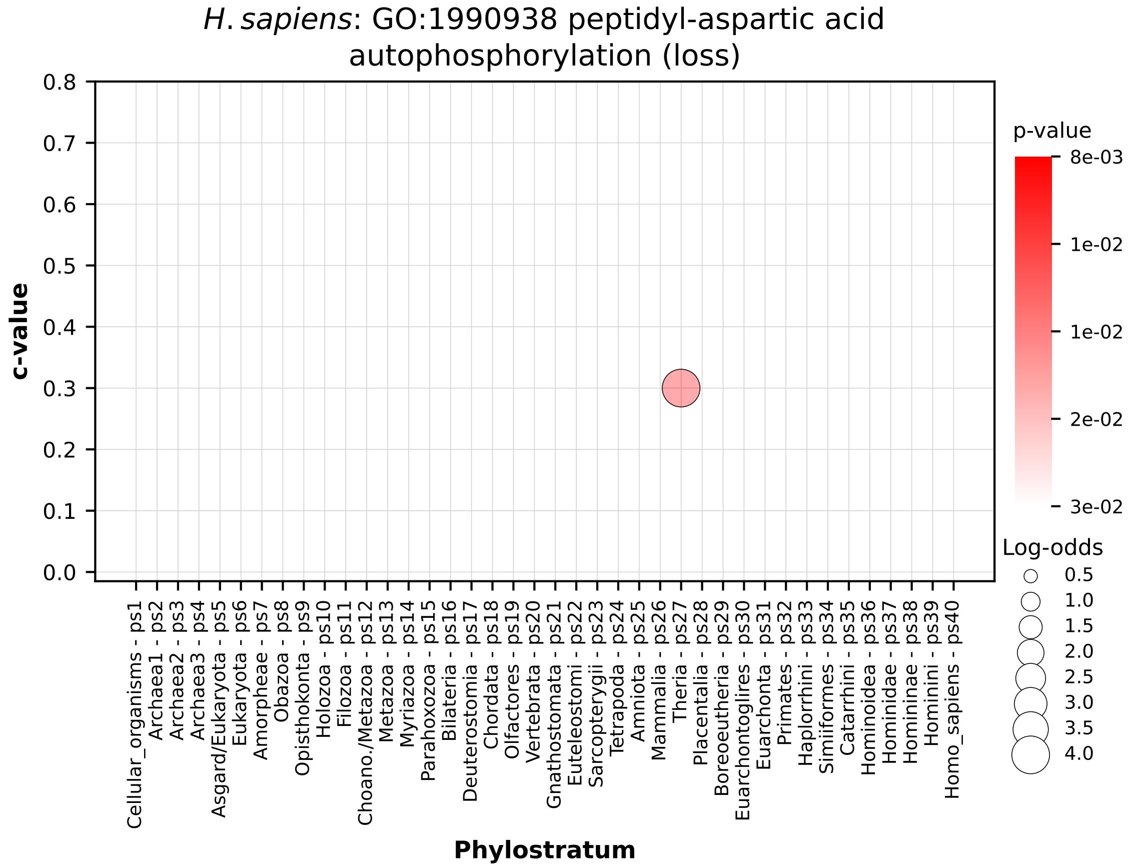

H. sapiens: GO:1990938 peptidyl-aspartic acid autophosphorylation (loss)
c-value
0.8
0.7
0.6
0.5
0.4
0.3
0.2
0.1
0.0
Phylostratum
Cellular_organisms - ps1
Archaea1 - ps2
Archaea2 - ps3
Archaea3 - ps4
Asgard/Eukaryota - ps5
Eukaryota - ps6
Amorpheae - ps7
Obazoa - ps8
Opisthokonta - ps9
Holozoa - ps10
Filozoa - ps11
Choano./Metazoa - ps12
Metazoa - ps13
Myriazoa - ps14
Parahoxozoa - ps15
Bilateria - ps16
Deuterostomia - ps17
Chordata - ps18
Olfactores - ps19
Vertebrata - ps20
Gnathostomata - ps21
Euteleostomi - ps22
Sarcopterygii - ps23
Tetrapoda - ps24
Amniota - ps25
Mammalia - ps26
Theria - ps27
Placentalia - ps28
Boreoeutheria - ps29
Euarchontoglires - ps30
Euarchonta - ps31
Primates - ps32
Haplorrhini - ps33
Simiiformes - ps34
Catarrhini - ps35
Hominoidea - ps36
Hominidae - ps37
Homininae - ps38
Hominini - ps39
Homo_sapiens - ps40
p-value
8e-03
1e-02
1e-02
2e-02
3e-02
Log-odds
0.5
1.0
1.5
2.0
2.5
3.0
3.5
4.0

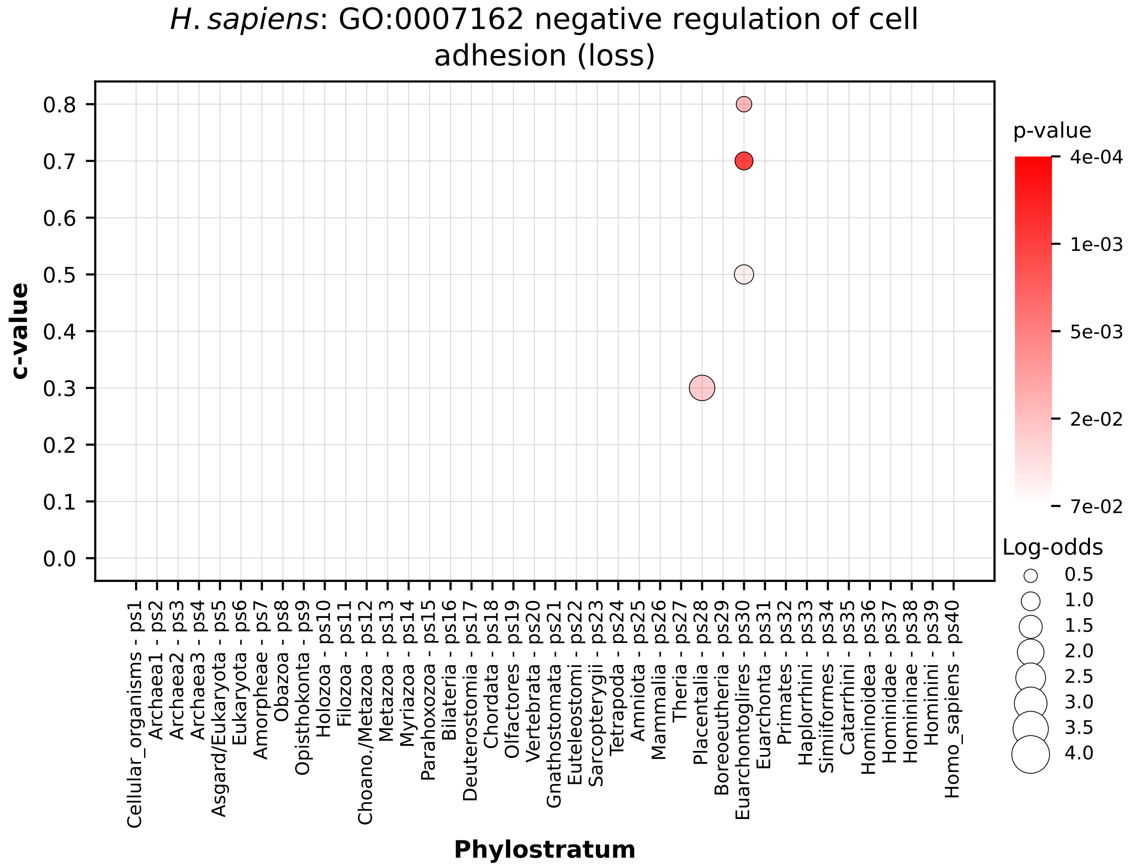

H. sapiens: GO:0007162 negative regulation of cell adhesion (loss)
c-value
0.8
0.7
0.6
0.5
0.4
0.3
0.2
0.1
0.0
Phylostratum
Cellular_organisms - ps1
Archaea1 - ps2
Archaea2 - ps3
Archaea3 - ps4
Asgard/Eukaryota - ps5
Eukaryota - ps6
Amorpheae - ps7
Obazoa - ps8
Opisthokonta - ps9
Holozoa - ps10
Filozoa - ps11
Choano./Metazoa - ps12
Metazoa - ps13
Myriazoa - ps14
Parahoxozoa - ps15
Bilateria - ps16
Deuterostomia - ps17
Chordata - ps18
Olfactores - ps19
Vertebrata - ps20
Gnathostomata - ps21
Euteleostomi - ps22
Sarcopterygii - ps23
Tetrapoda - ps24
Amniota - ps25
Mammalia - ps26
Theria - ps27
Placentalia - ps28
Boreoeutheria - ps29
Euarchontoglires - ps30
Euarchonta - ps31
Primates - ps32
Haplorrhini - ps33
Simiiformes - ps34
Catarrhini - ps35
Hominoidea - ps36
Hominidae - ps37
Homininae - ps38
Hominini - ps39
Homo_sapiens - ps40
p-value
4e-04
1e-03
5e-03
2e-02
7e-02
Log-odds
0.5
1.0
1.5
2.0
2.5
3.0
3.5
4.0

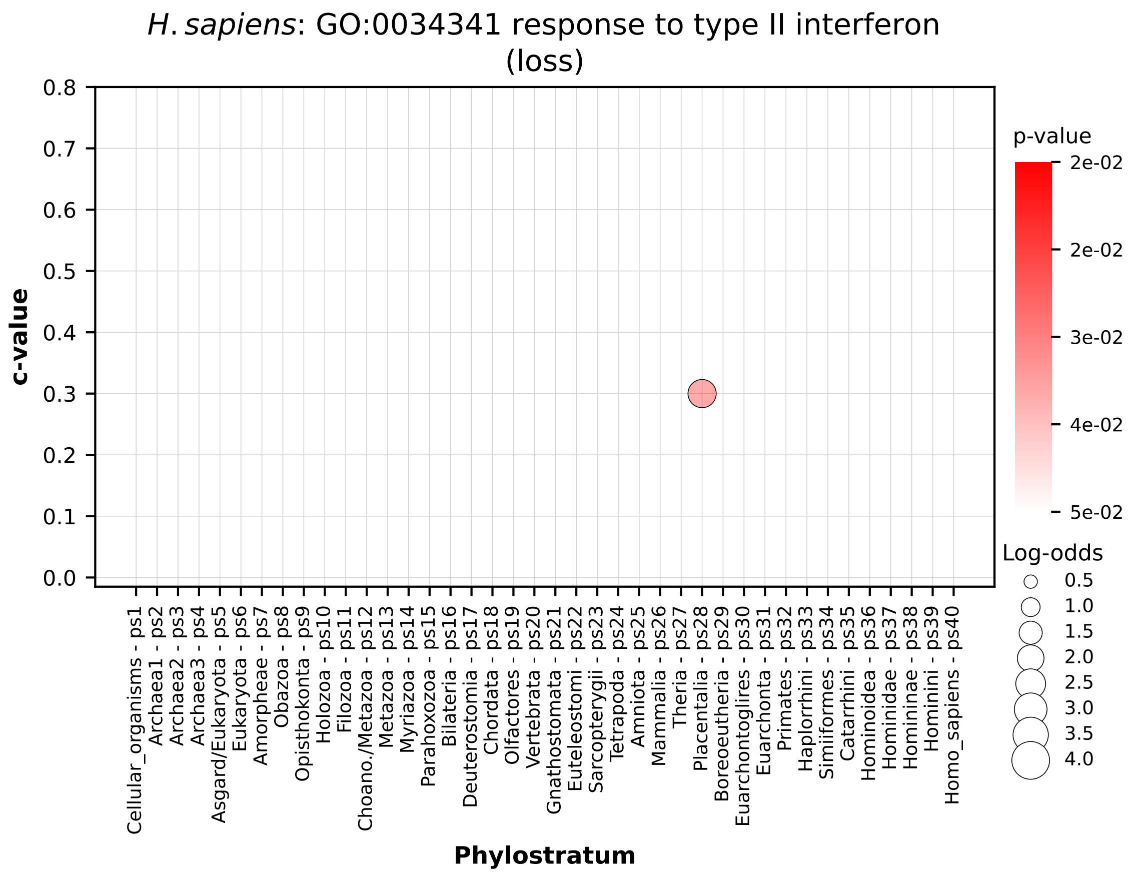

H. sapiens: GO:0034341 response to type II interferon (loss)
c-value
0.0
0.1
0.2
0.3
0.4
0.5
0.6
0.7
0.8
Phylostratum
Cellular_organisms - ps1
Archaea1 - ps2
Archaea2 - ps3
Archaea3 - ps4
Asgard/Eukaryota - ps5
Eukaryota - ps6
Amorpheae - ps7
Obazoa - ps8
Opisthokonta - ps9
Holozoa - ps10
Filozoa - ps11
Choano./Metazoa - ps12
Metazoa - ps13
Myriazoa - ps14
Parahoxozoa - ps15
Bilateria - ps16
Deuterostomia - ps17
Chordata - ps18
Olfactores - ps19
Vertebrata - ps20
Gnathostomata - ps21
Euteleostomi - ps22
Sarcopterygii - ps23
Tetrapoda - ps24
Amniota - ps25
Mammalia - ps26
Theria - ps27
Placentalia - ps28
Boreoeutheria - ps29
Euarchontoglires - ps30
Euarchonta - ps31
Primates - ps32
Haplorrhini - ps33
Simiiformes - ps34
Catarrhini - ps35
Hominoidea - ps36
Hominidae - ps37
Homininae - ps38
Hominini - ps39
Homo_sapiens - ps40
p-value
2e-02
2e-02
3e-02
4e-02
5e-02
Log-odds
0.5
1.0
1.5
2.0
2.5
3.0
3.5
4.0

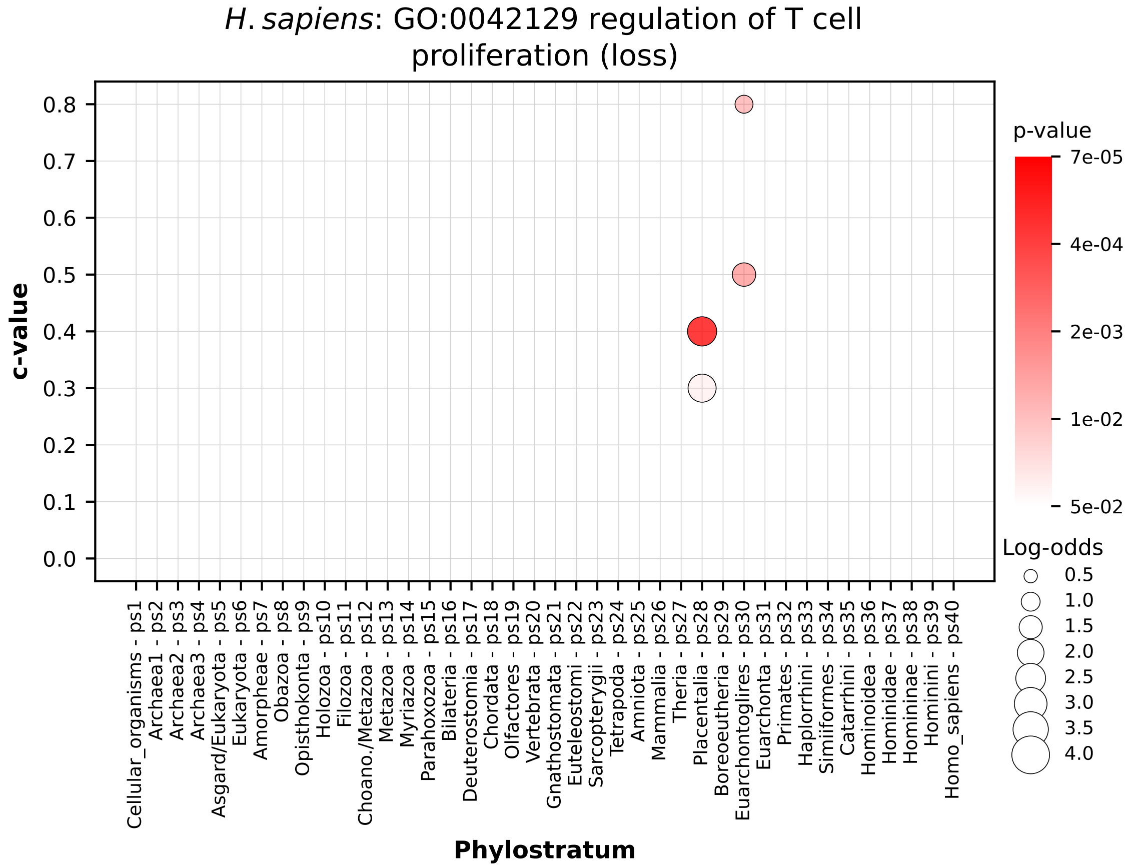

H. sapiens: GO:0042129 regulation of T cell proliferation (loss)
c-value
0.8
0.7
0.6
0.5
0.4
0.3
0.2
0.1
0.0
p-value
7e-05
4e-04
2e-03
1e-02
5e-02
Log-odds
0.5
1.0
1.5
2.0
2.5
3.0
3.5
4.0
Cellular_organisms - ps1
Archaea1 - ps2
Archaea2 - ps3
Archaea3 - ps4
Asgard/Eukaryota - ps5
Eukaryota - ps6
Amorpheae - ps7
Obazoa - ps8
Opisthokonta - ps9
Holozoa - ps10
Filozoa - ps11
Choano./Metazoa - ps12
Metazoa - ps13
Myriazoa - ps14
Parahoxozoa - ps15
Bilateria - ps16
Deuterostomia - ps17
Chordata - ps18
Olfactores - ps19
Vertebrata - ps20
Gnathostomata - ps21
Euteleostomi - ps22
Sarcopterygii - ps23
Tetrapoda - ps24
Amniota - ps25
Mammalia - ps26
Theria - ps27
Placentalia - ps28
Boreoeutheria - ps29
Euarchontoglires - ps30
Euarchonta - ps31
Primates - ps32
Haplorrhini - ps33
Simiiformes - ps34
Catarrhini - ps35
Hominoidea - ps36
Hominidae - ps37
Homininae - ps38
Hominini - ps39
Homo_sapiens - ps40
Phylostratum

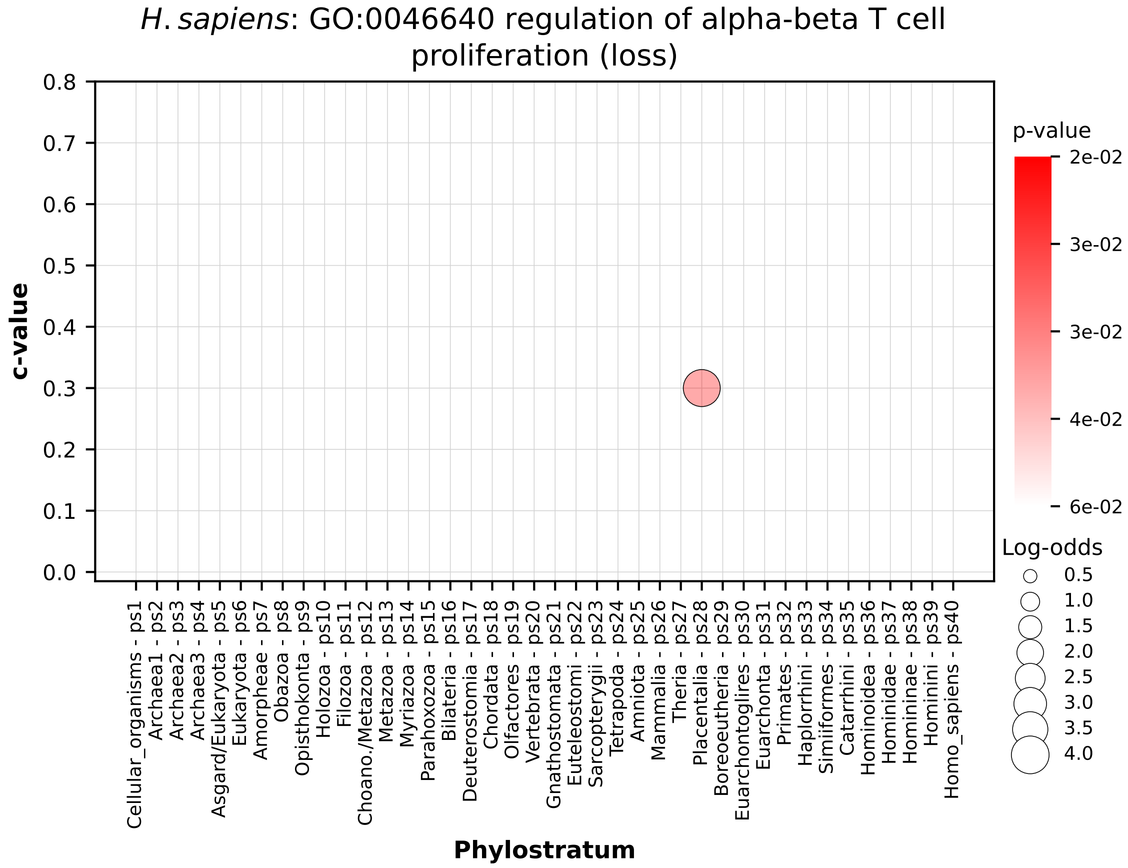
H. sapiens: GO:0046640 regulation of alpha-beta T cell proliferation (loss)
c-value
0.0
0.1
0.2
0.3
0.4
0.5
0.6
0.7
0.8
Phylostratum
Cellular_organisms - ps1
Archaea1 - ps2
Archaea2 - ps3
Archaea3 - ps4
Asgard/Eukaryota - ps5
Eukaryota - ps6
Amorpheae - ps7
Obazoa - ps8
Opisthokonta - ps9
Holozoa - ps10
Filozoa - ps11
Choano./Metazoa - ps12
Metazoa - ps13
Myriazoa - ps14
Parahoxozoa - ps15
Bilateria - ps16
Deuterostomia - ps17
Chordata - ps18
Olfactores - ps19
Vertebrata - ps20
Gnathostomata - ps21
Euteleostomi - ps22
Sarcopterygii - ps23
Tetrapoda - ps24
Amniota - ps25
Mammalia - ps26
Theria - ps27
Placentalia - ps28
Boreoeutheria - ps29
Euarchontoglires - ps30
Euarchonta - ps31
Primates - ps32
Haplorrhini - ps33
Simiiformes - ps34
Catarrhini - ps35
Hominoidea - ps36
Hominidae - ps37
Homininae - ps38
Hominini - ps39
Homo_sapiens - ps40
p-value
2e-02
3e-02
3e-02
4e-02
6e-02
Log-odds
0.5
1.0
1.5
2.0
2.5
3.0
3.5
4.0

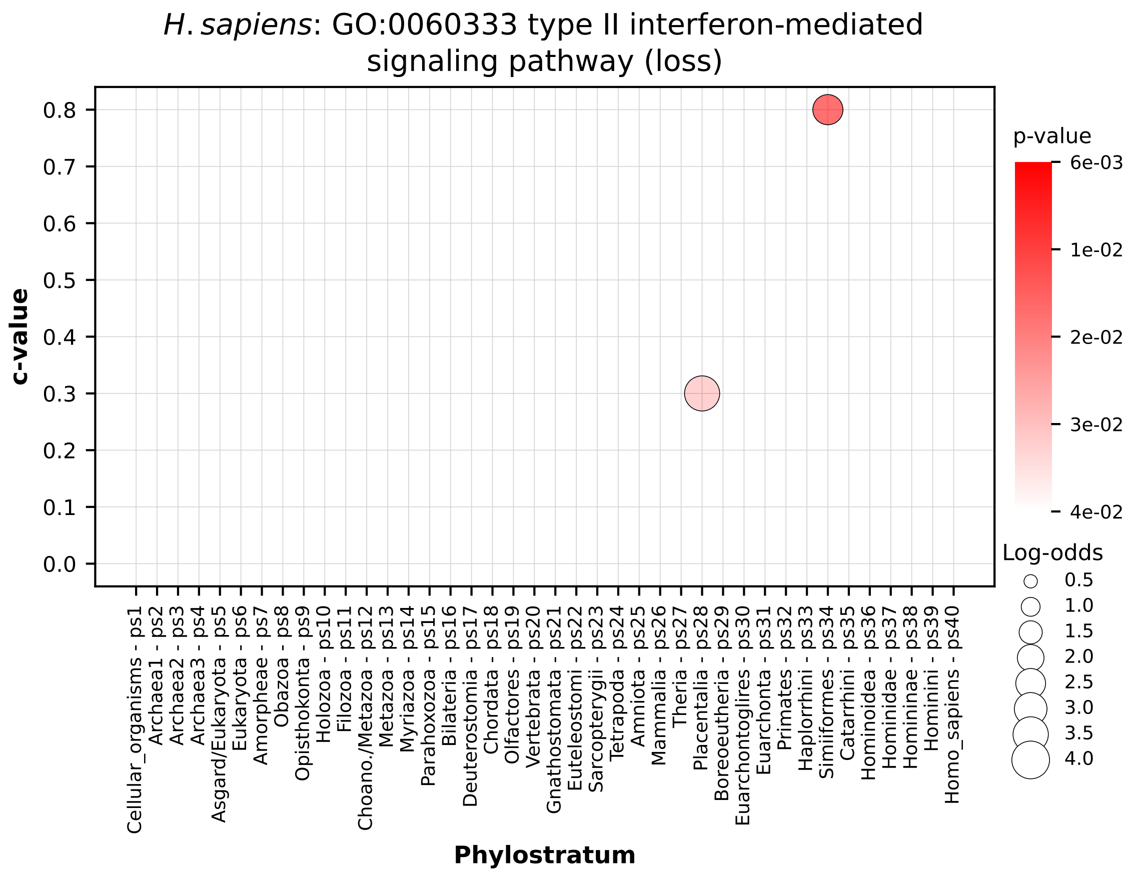

H. sapiens: GO:0060333 type II interferon-mediated signaling pathway (loss)
c-value
0.0
0.1
0.2
0.3
0.4
0.5
0.6
0.7
0.8
Phylostratum
Cellular_organisms - ps1
Archaea1 - ps2
Archaea2 - ps3
Archaea3 - ps4
Asgard/Eukaryota - ps5
Eukaryota - ps6
Amorpheae - ps7
Obazoa - ps8
Opisthokonta - ps9
Holozoa - ps10
Filozoa - ps11
Choano./Metazoa - ps12
Metazoa - ps13
Myriazoa - ps14
Parahoxozoa - ps15
Bilateria - ps16
Deuterostomia - ps17
Chordata - ps18
Olfactores - ps19
Vertebrata - ps20
Gnathostomata - ps21
Euteleostomi - ps22
Sarcopterygii - ps23
Tetrapoda - ps24
Amniota - ps25
Mammalia - ps26
Theria - ps27
Placentalia - ps28
Boreoeutheria - ps29
Euarchontoglires - ps30
Euarchonta - ps31
Primates - ps32
Haplorrhini - ps33
Simiiformes - ps34
Catarrhini - ps35
Hominoidea - ps36
Hominidae - ps37
Homininae - ps38
Hominini - ps39
Homo_sapiens - ps40
p-value
6e-03
1e-02
2e-02
3e-02
4e-02
Log-odds
0.5
1.0
1.5
2.0
2.5
3.0
3.5
4.0

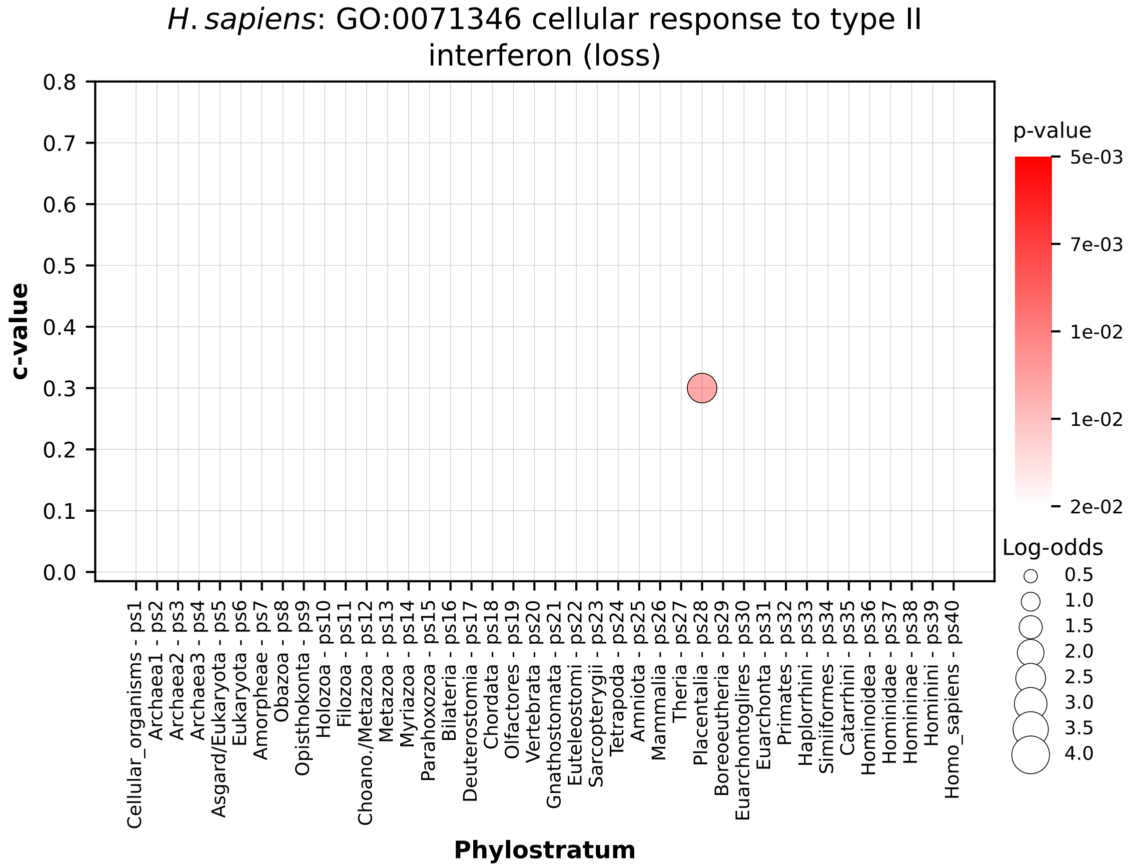

H. sapiens: GO:0071346 cellular response to type II interferon (loss)
c-value
0.8
0.7
0.6
0.5
0.4
0.3
0.2
0.1
0.0
p-value
5e-03
7e-03
1e-02
1e-02
2e-02
Log-odds
0.5
1.0
1.5
2.0
2.5
3.0
3.5
4.0
Cellular_organisms - ps1
Archaea1 - ps2
Archaea2 - ps3
Archaea3 - ps4
Asgard/Eukaryota - ps5
Eukaryota - ps6
Amorpheae - ps7
Obazoa - ps8
Opisthokonta - ps9
Holozoa - ps10
Filozoa - ps11
Choano./Metazoa - ps12
Metazoa - ps13
Myriazoa - ps14
Parahoxozoa - ps15
Bilateria - ps16
Deuterostomia - ps17
Chordata - ps18
Olfactores - ps19
Vertebrata - ps20
Gnathostomata - ps21
Euteleostomi - ps22
Sarcopterygii - ps23
Tetrapoda - ps24
Amniota - ps25
Mammalia - ps26
Theria - ps27
Placentalia - ps28
Boreoeutheria - ps29
Euarchontoglires - ps30
Euarchonta - ps31
Primates - ps32
Haplorrhini - ps33
Simiiformes - ps34
Catarrhini - ps35
Hominoidea - ps36
Hominidae - ps37
Homininae - ps38
Hominini - ps39
Homo_sapiens - ps40
Phylostratum

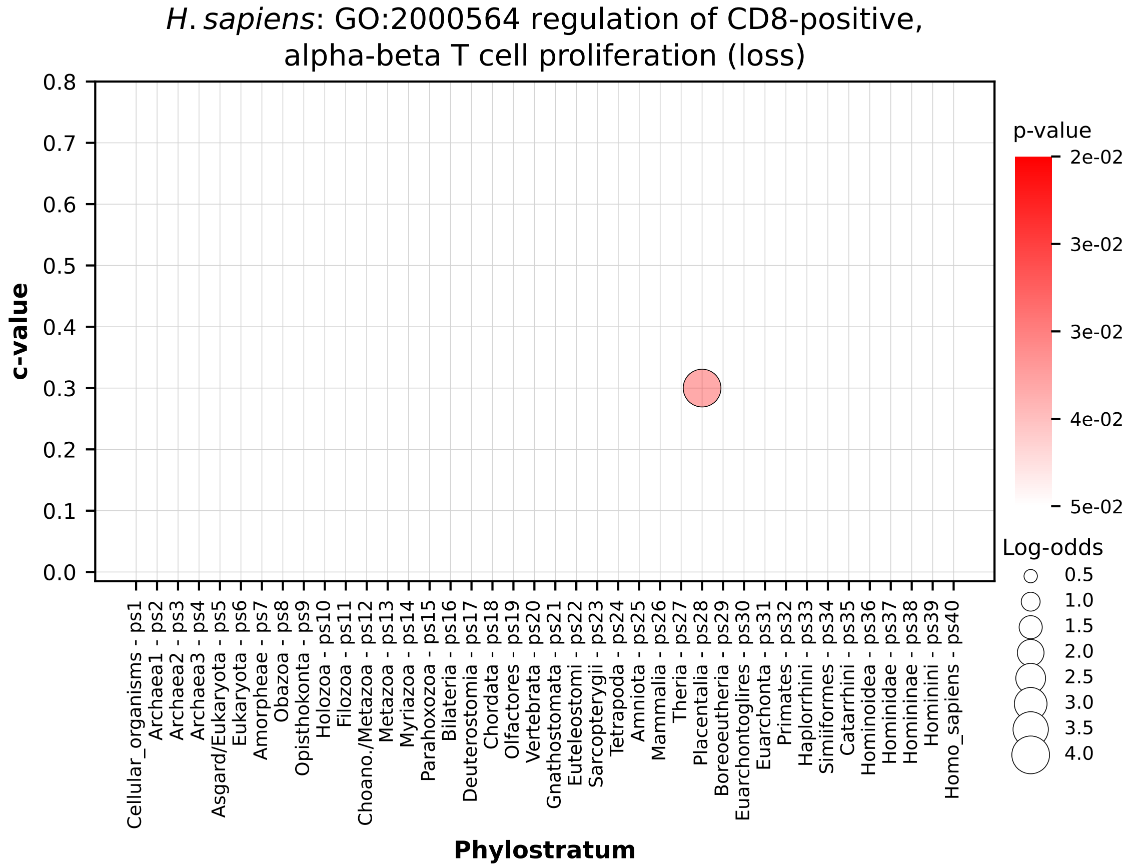

H. sapiens: GO:2000564 regulation of CD8-positive, alpha-beta T cell proliferation (loss)
c-value
0.0
0.1
0.2
0.3
0.4
0.5
0.6
0.7
0.8
Phylostratum
Cellular_organisms - ps1
Archaea1 - ps2
Archaea2 - ps3
Archaea3 - ps4
Asgard/Eukaryota - ps5
Eukaryota - ps6
Amorpheae - ps7
Obazoa - ps8
Opisthokonta - ps9
Holozoa - ps10
Filozoa - ps11
Choano./Metazoa - ps12
Metazoa - ps13
Myriazoa - ps14
Parahoxozoa - ps15
Bilateria - ps16
Deuterostomia - ps17
Chordata - ps18
Olfactores - ps19
Vertebrata - ps20
Gnathostomata - ps21
Euteleostomi - ps22
Sarcopterygii - ps23
Tetrapoda - ps24
Amniota - ps25
Mammalia - ps26
Theria - ps27
Placentalia - ps28
Boreoeutheria - ps29
Euarchontoglires - ps30
Euarchonta - ps31
Primates - ps32
Haplorrhini - ps33
Simiiformes - ps34
Catarrhini - ps35
Hominoidea - ps36
Hominidae - ps37
Homininae - ps38
Hominini - ps39
Homo_sapiens - ps40
p-value
2e-02
3e-02
3e-02
4e-02
5e-02
Log-odds
0.5
1.0
1.5
2.0
2.5
3.0
3.5
4.0

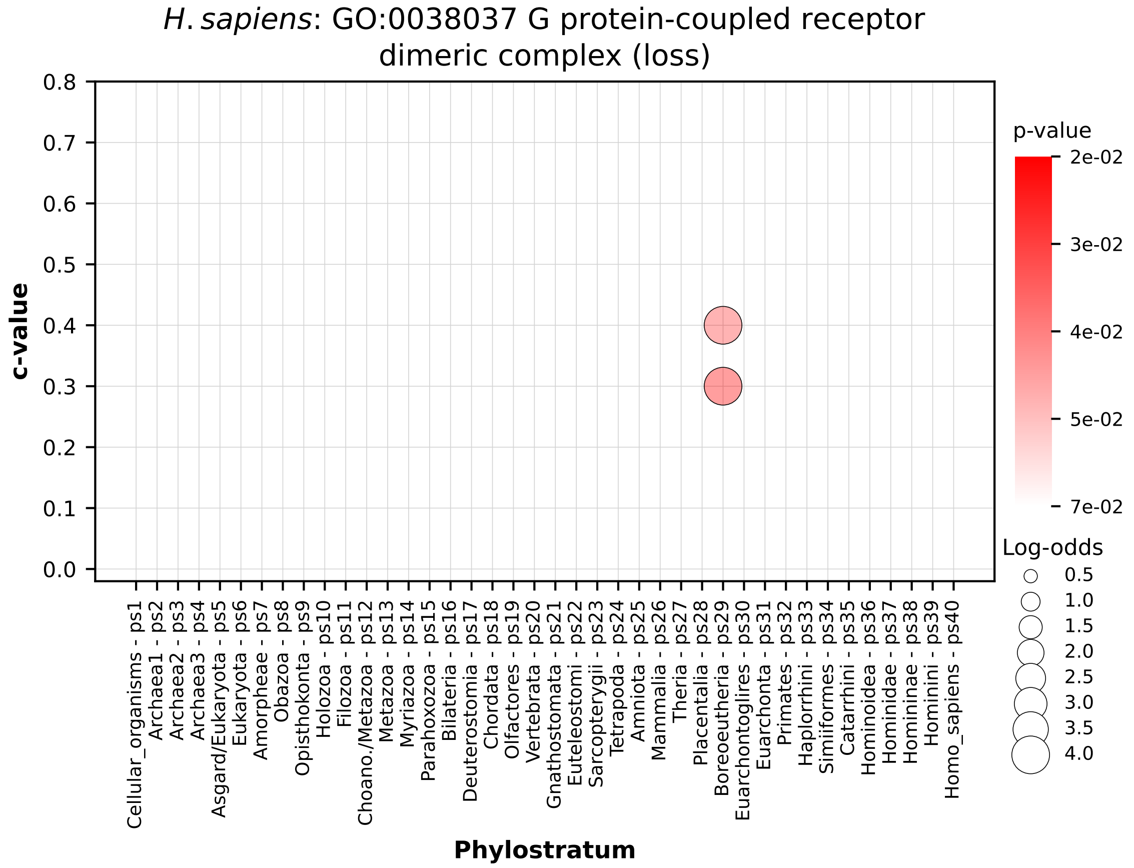
H. sapiens: GO:0038037 G protein-coupled receptor dimeric complex (loss)
c-value
0.0
0.1
0.2
0.3
0.4
0.5
0.6
0.7
0.8
Phylostratum
Cellular_organisms - ps1
Archaea1 - ps2
Archaea2 - ps3
Archaea3 - ps4
Asgard/Eukaryota - ps5
Eukaryota - ps6
Amorpheae - ps7
Obazoa - ps8
Opisthokonta - ps9
Holozoa - ps10
Filozoa - ps11
Choano./Metazoa - ps12
Metazoa - ps13
Myriazoa - ps14
Parahoxozoa - ps15
Bilateria - ps16
Deuterostomia - ps17
Chordata - ps18
Olfactores - ps19
Vertebrata - ps20
Gnathostomata - ps21
Euteleostomi - ps22
Sarcopterygii - ps23
Tetrapoda - ps24
Amniota - ps25
Mammalia - ps26
Theria - ps27
Placentalia - ps28
Boreoeutheria - ps29
Euarchontoglires - ps30
Euarchonta - ps31
Primates - ps32
Haplorrhini - ps33
Simiiformes - ps34
Catarrhini - ps35
Hominoidea - ps36
Hominidae - ps37
Homininae - ps38
Hominini - ps39
Homo_sapiens - ps40
p-value
2e-02
3e-02
4e-02
5e-02
7e-02
Log-odds
0.5
1.0
1.5
2.0
2.5
3.0
3.5
4.0

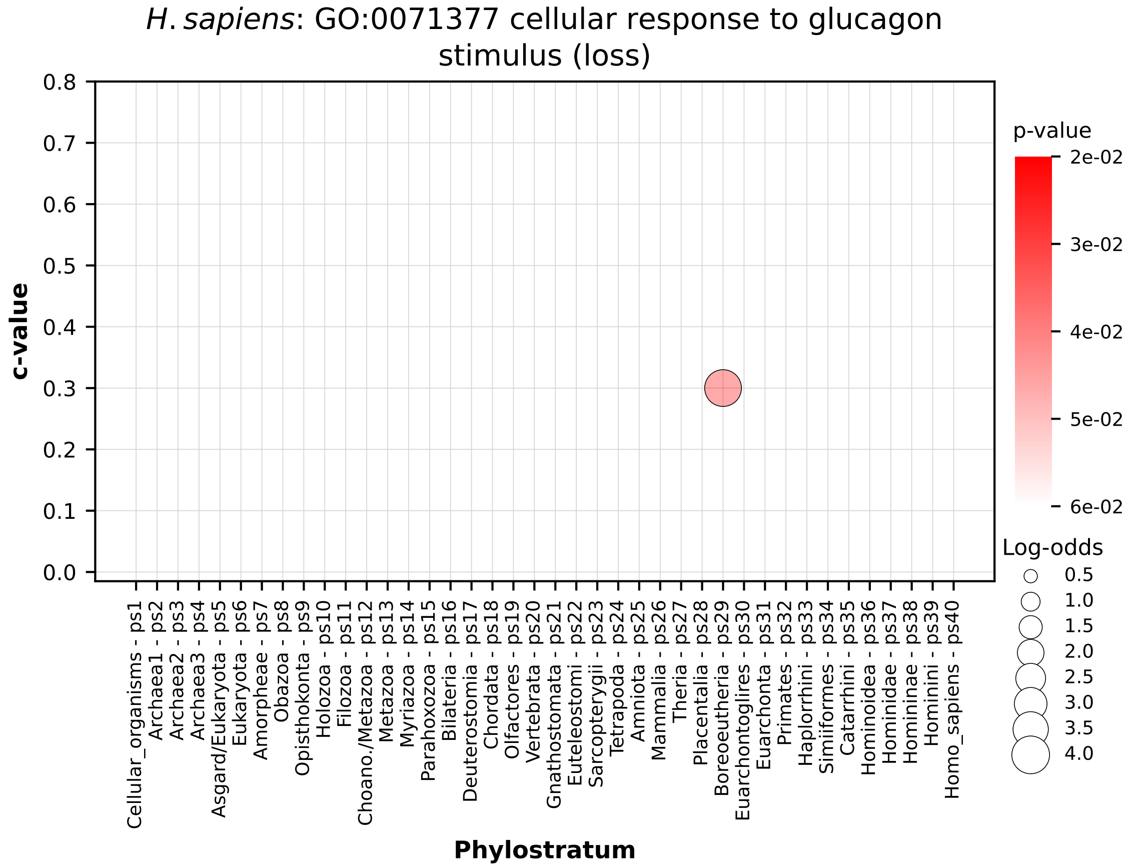
H. sapiens: GO:0071377 cellular response to glucagon stimulus (loss)
c-value
0.0
0.1
0.2
0.3
0.4
0.5
0.6
0.7
0.8
Phylostratum
Cellular_organisms - ps1
Archaea1 - ps2
Archaea2 - ps3
Archaea3 - ps4
Asgard/Eukaryota - ps5
Eukaryota - ps6
Amorpheae - ps7
Obazoa - ps8
Opisthokonta - ps9
Holozoa - ps10
Filozoa - ps11
Choano./Metazoa - ps12
Metazoa - ps13
Myriazoa - ps14
Parahoxozoa - ps15
Bilateria - ps16
Deuterostomia - ps17
Chordata - ps18
Olfactores - ps19
Vertebrata - ps20
Gnathostomata - ps21
Euteleostomi - ps22
Sarcopterygii - ps23
Tetrapoda - ps24
Amniota - ps25
Mammalia - ps26
Theria - ps27
Placentalia - ps28
Boreoeutheria - ps29
Euarchontoglires - ps30
Euarchonta - ps31
Primates - ps32
Haplorrhini - ps33
Simiiformes - ps34
Catarrhini - ps35
Hominoidea - ps36
Hominidae - ps37
Homininae - ps38
Hominini - ps39
Homo_sapiens - ps40
p-value
2e-02
3e-02
4e-02
5e-02
6e-02
Log-odds
0.5
1.0
1.5
2.0
2.5
3.0
3.5
4.0

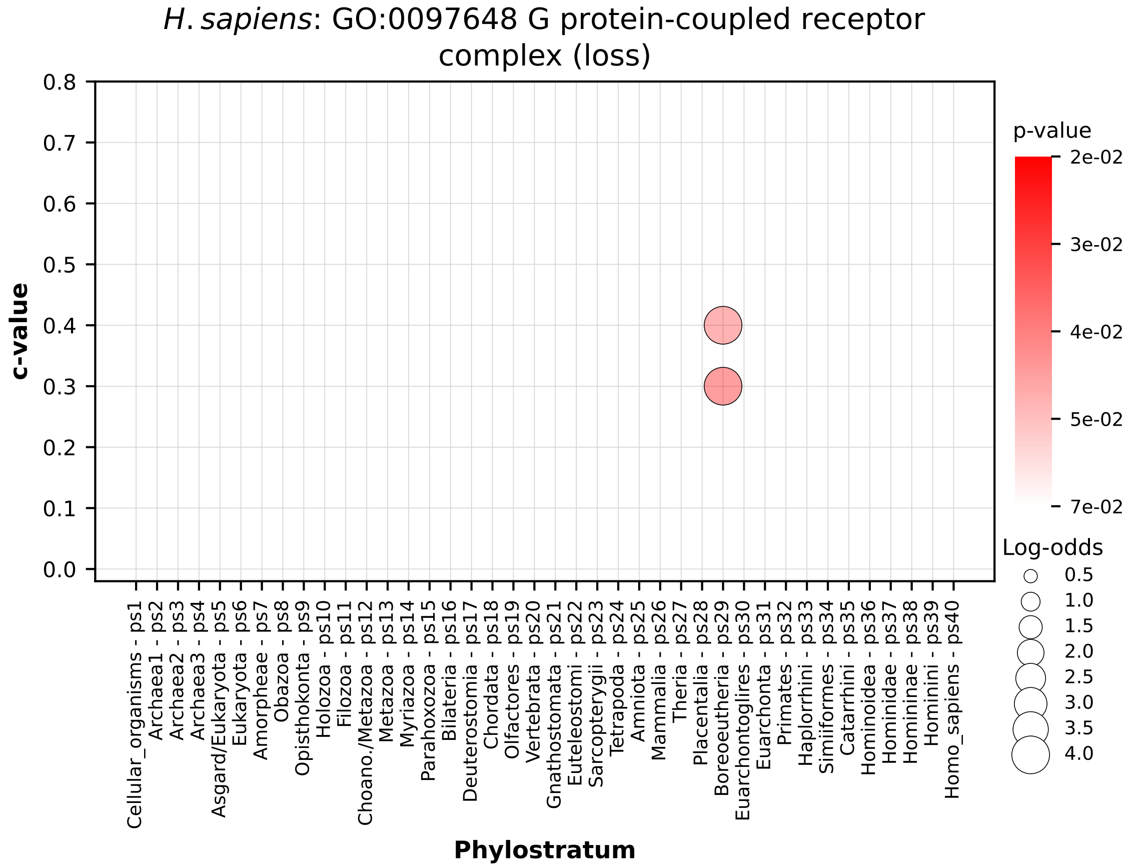

H. sapiens: GO:0097648 G protein-coupled receptor complex (loss)
c-value
0.0
0.1
0.2
0.3
0.4
0.5
0.6
0.7
0.8
Phylostratum
Cellular_organisms - ps1
Archaea1 - ps2
Archaea2 - ps3
Archaea3 - ps4
Asgard/Eukaryota - ps5
Eukaryota - ps6
Amorpheae - ps7
Obazoa - ps8
Opisthokonta - ps9
Holozoa - ps10
Filozoa - ps11
Choano./Metazoa - ps12
Metazoa - ps13
Myriazoa - ps14
Parahoxozoa - ps15
Bilateria - ps16
Deuterostomia - ps17
Chordata - ps18
Olfactores - ps19
Vertebrata - ps20
Gnathostomata - ps21
Euteleostomi - ps22
Sarcopterygii - ps23
Tetrapoda - ps24
Amniota - ps25
Mammalia - ps26
Theria - ps27
Placentalia - ps28
Boreoeutheria - ps29
Euarchontoglires - ps30
Euarchonta - ps31
Primates - ps32
Haplorrhini - ps33
Simiiformes - ps34
Catarrhini - ps35
Hominoidea - ps36
Hominidae - ps37
Homininae - ps38
Hominini - ps39
Homo_sapiens - ps40
p-value
2e-02
3e-02
4e-02
5e-02
7e-02
Log-odds
0.5
1.0
1.5
2.0
2.5
3.0
3.5
4.0

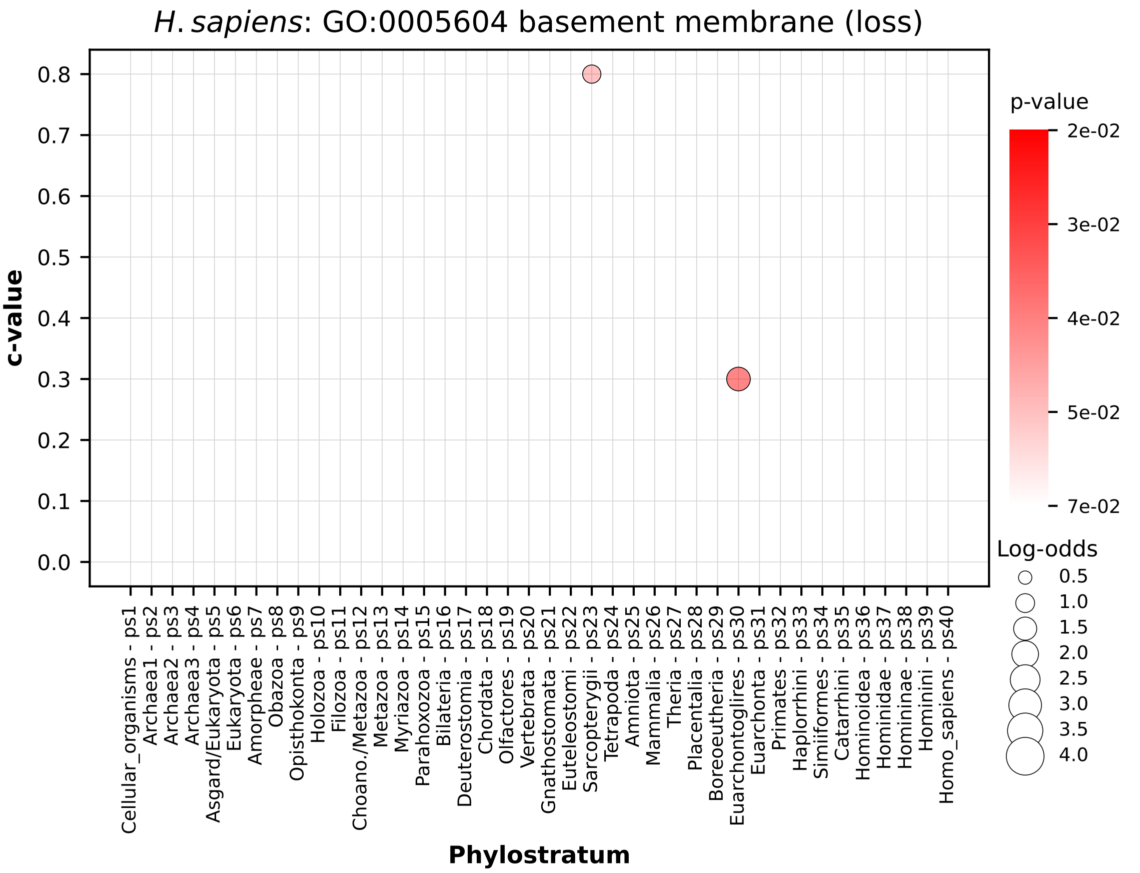
H. sapiens: GO:0005604 basement membrane (loss)
c-value
0.0
0.1
0.2
0.3
0.4
0.5
0.6
0.7
0.8
Phylostratum
Cellular_organisms - ps1
Archaea1 - ps2
Archaea2 - ps3
Archaea3 - ps4
Asgard/Eukaryota - ps5
Eukaryota - ps6
Amorpheae - ps7
Obazoa - ps8
Opisthokonta - ps9
Holozoa - ps10
Filozoa - ps11
Choano./Metazoa - ps12
Metazoa - ps13
Myriazoa - ps14
Parahoxozoa - ps15
Bilateria - ps16
Deuterostomia - ps17
Chordata - ps18
Olfactores - ps19
Vertebrata - ps20
Gnathostomata - ps21
Euteleostomi - ps22
Sarcopterygii - ps23
Tetrapoda - ps24
Amniota - ps25
Mammalia - ps26
Theria - ps27
Placentalia - ps28
Boreoeutheria - ps29
Euarchontoglires - ps30
Euarchonta - ps31
Primates - ps32
Haplorrhini - ps33
Simiiformes - ps34
Catarrhini - ps35
Hominoidea - ps36
Hominidae - ps37
Homininae - ps38
Hominini - ps39
Homo_sapiens - ps40
p-value
2e-02
3e-02
4e-02
5e-02
7e-02
Log-odds
0.5
1.0
1.5
2.0
2.5
3.0
3.5
4.0

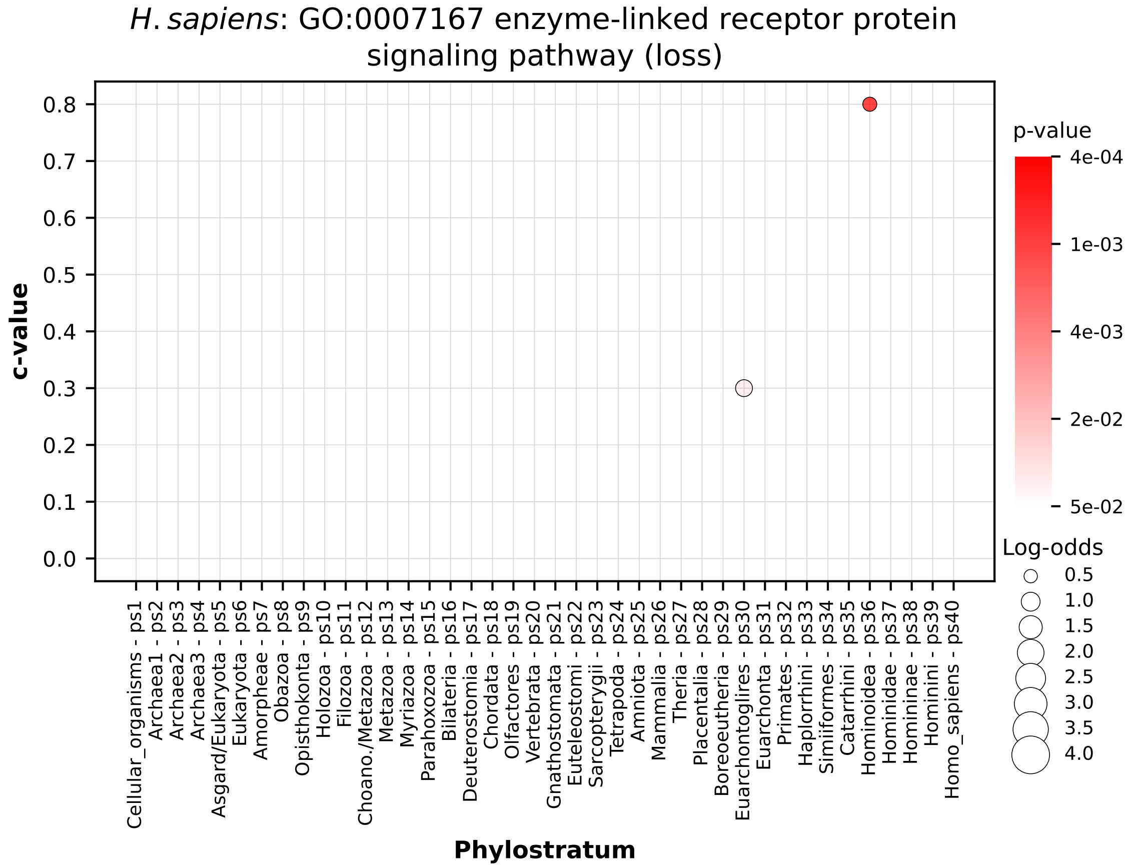
H. sapiens: GO:0007167 enzyme-linked receptor protein signaling pathway (loss)
c-value
Phylostratum
p-value
4e-04
1e-03
4e-03
2e-02
5e-02
Log-odds
0.5
1.0
1.5
2.0
2.5
3.0
3.5
4.0
Cellular_organisms - ps1
Archaea1 - ps2
Archaea2 - ps3
Archaea3 - ps4
Asgard/Eukaryota - ps5
Eukaryota - ps6
Amorpheae - ps7
Obazoa - ps8
Opisthokonta - ps9
Holozoa - ps10
Filozoa - ps11
Choano./Metazoa - ps12
Metazoa - ps13
Myriazoa - ps14
Parahoxozoa - ps15
Bilateria - ps16
Deuterostomia - ps17
Chordata - ps18
Olfactores - ps19
Vertebrata - ps20
Gnathostomata - ps21
Euteleostomi - ps22
Sarcopterygii - ps23
Tetrapoda - ps24
Amniota - ps25
Mammalia - ps26
Theria - ps27
Placentalia - ps28
Boreoeutheria - ps29
Euarchontoglires - ps30
Euarchonta - ps31
Primates - ps32
Haplorrhini - ps33
Simiiformes - ps34
Catarrhini - ps35
Hominoidea - ps36
Hominidae - ps37
Homininae - ps38
Hominini - ps39
Homo_sapiens - ps40

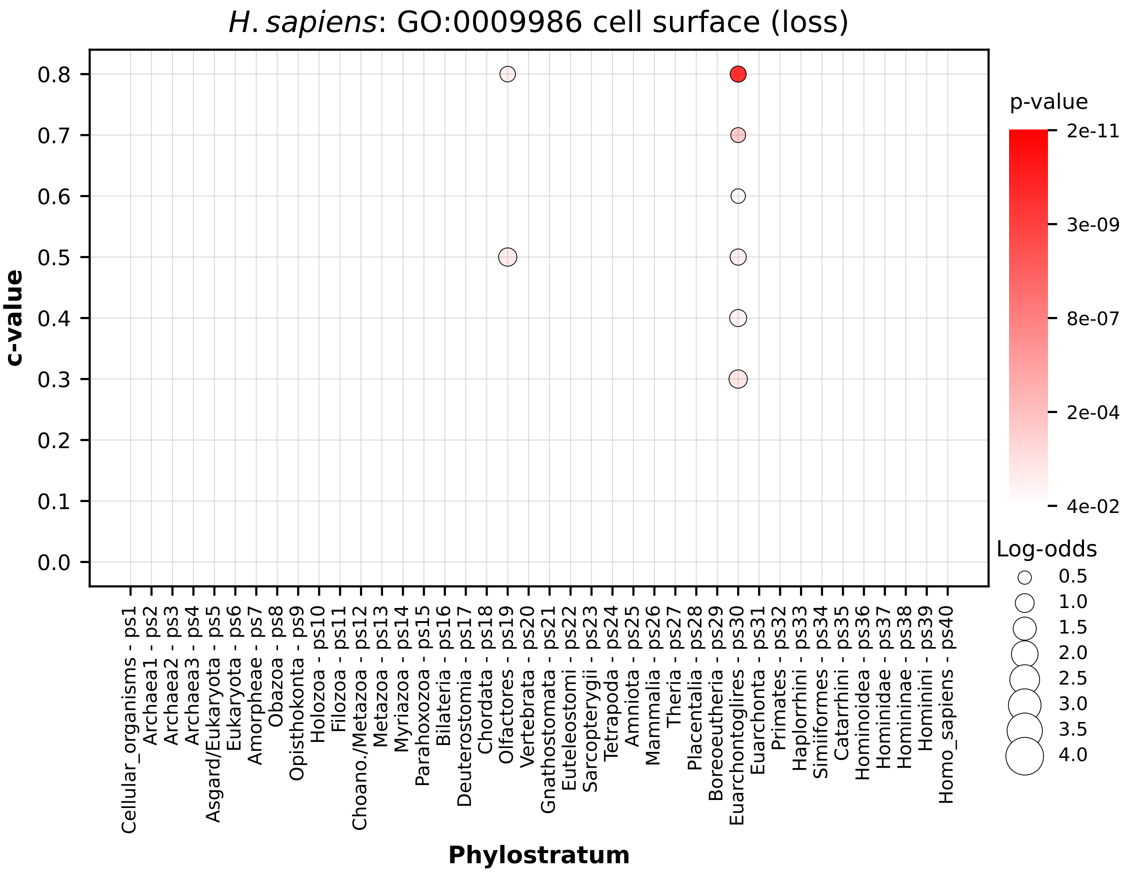

H. sapiens: GO:0009986 cell surface (loss)
c-value
0.0
0.1
0.2
0.3
0.4
0.5
0.6
0.7
0.8
p-value
2e-11
3e-09
8e-07
2e-04
4e-02
Log-odds
0.5
1.0
1.5
2.0
2.5
3.0
3.5
4.0
Cellular_organisms - ps1
Archaea1 - ps2
Archaea2 - ps3
Archaea3 - ps4
Asgard/Eukaryota - ps5
Eukaryota - ps6
Amorpheae - ps7
Obazoa - ps8
Opisthokonta - ps9
Holozoa - ps10
Filozoa - ps11
Choano./Metazoa - ps12
Metazoa - ps13
Myriazoa - ps14
Parahoxozoa - ps15
Bilateria - ps16
Deuterostomia - ps17
Chordata - ps18
Olfactores - ps19
Vertebrata - ps20
Gnathostomata - ps21
Euteleostomi - ps22
Sarcopterygii - ps23
Tetrapoda - ps24
Amniota - ps25
Mammalia - ps26
Theria - ps27
Placentalia - ps28
Boreoeutheria - ps29
Euarchontoglires - ps30
Euarchonta - ps31
Primates - ps32
Haplorrhini - ps33
Simiiformes - ps34
Catarrhini - ps35
Hominoidea - ps36
Hominidae - ps37
Homininae - ps38
Hominini - ps39
Homo_sapiens - ps40
Phylostratum

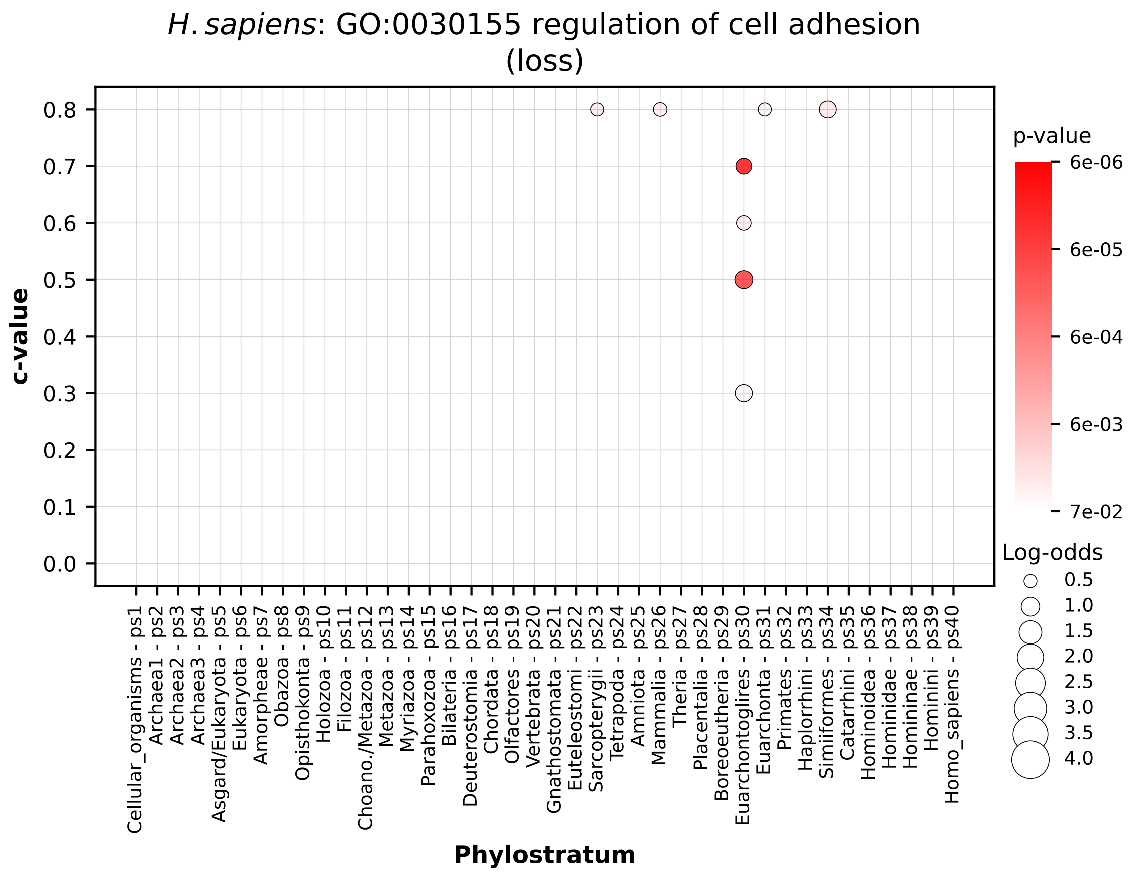

H. sapiens: GO:0030155 regulation of cell adhesion
(loss)
c-value
0.8
0.7
0.6
0.5
0.4
0.3
0.2
0.1
0.0
p-value
6e-06
6e-05
6e-04
6e-03
7e-02
Log-odds
0.5
1.0
1.5
2.0
2.5
3.0
3.5
4.0
Cellular_organisms - ps1
Archaea1 - ps2
Archaea2 - ps3
Archaea3 - ps4
Asgard/Eukaryota - ps5
Eukaryota - ps6
Amorpheae - ps7
Obazoa - ps8
Opisthokonta - ps9
Holozoa - ps10
Filozoa - ps11
Choano./Metazoa - ps12
Metazoa - ps13
Myriazoa - ps14
Parahoxozoa - ps15
Bilateria - ps16
Deuterostomia - ps17
Chordata - ps18
Olfactores - ps19
Vertebrata - ps20
Gnathostomata - ps21
Euteleostomi - ps22
Sarcopterygii - ps23
Tetrapoda - ps24
Amniota - ps25
Mammalia - ps26
Theria - ps27
Placentalia - ps28
Boreoeutheria - ps29
Euarchontoglires - ps30
Euarchonta - ps31
Primates - ps32
Haplorrhini - ps33
Simiiformes - ps34
Catarrhini - ps35
Hominoidea - ps36
Hominidae - ps37
Homininae - ps38
Hominini - ps39
Homo_sapiens - ps40
Phylostratum

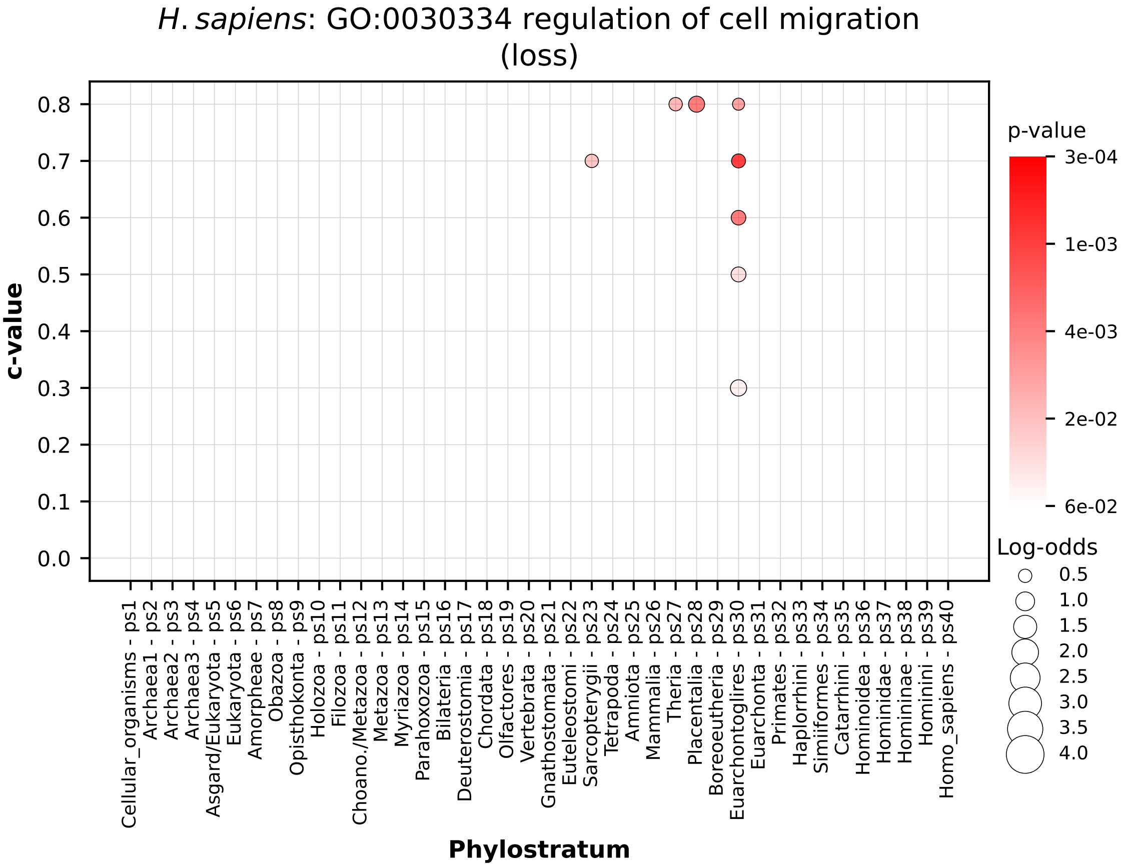

H. sapiens: GO:0030334 regulation of cell migration
(loss)
c-value
0.8
0.7
0.6
0.5
0.4
0.3
0.2
0.1
0.0
p-value
3e-04
1e-03
4e-03
2e-02
6e-02
Log-odds
0.5
1.0
1.5
2.0
2.5
3.0
3.5
4.0
Cellular_organisms - ps1
Archaea1 - ps2
Archaea2 - ps3
Archaea3 - ps4
Asgard/Eukaryota - ps5
Eukaryota - ps6
Amorpheae - ps7
Obazoa - ps8
Opisthokonta - ps9
Holozoa - ps10
Filozoa - ps11
Choano./Metazoa - ps12
Metazoa - ps13
Myriazoa - ps14
Parahoxozoa - ps15
Bilateria - ps16
Deuterostomia - ps17
Chordata - ps18
Olfactores - ps19
Vertebrata - ps20
Gnathostomata - ps21
Euteleostomi - ps22
Sarcopterygii - ps23
Tetrapoda - ps24
Amniota - ps25
Mammalia - ps26
Theria - ps27
Placentalia - ps28
Boreoeutheria - ps29
Euarchontoglires - ps30
Euarchonta - ps31
Primates - ps32
Haplorrhini - ps33
Simiiformes - ps34
Catarrhini - ps35
Hominoidea - ps36
Hominidae - ps37
Homininae - ps38
Hominini - ps39
Homo_sapiens - ps40
Phylostratum

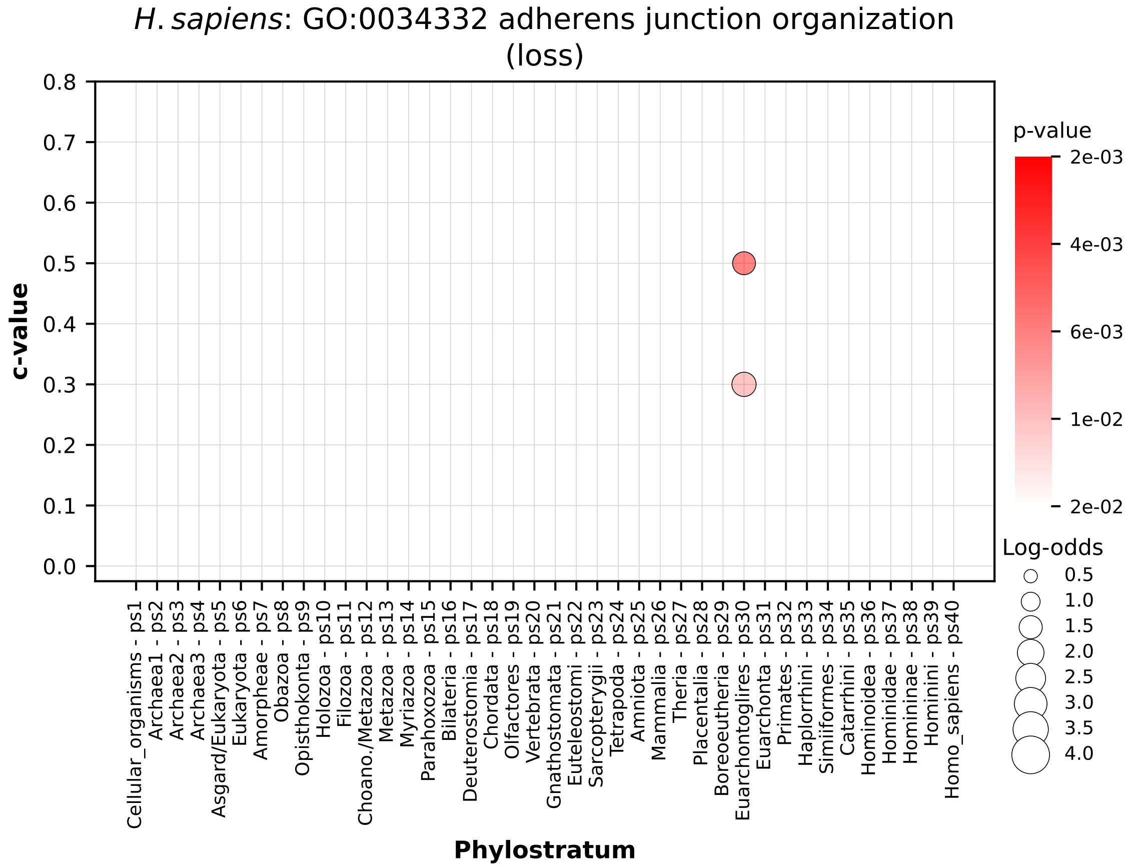

H. sapiens: GO:0034332 adherens junction organization (loss)
c-value
0.8
0.7
0.6
0.5
0.4
0.3
0.2
0.1
0.0
Phylostratum
Cellular_organisms - ps1
Archaea1 - ps2
Archaea2 - ps3
Archaea3 - ps4
Asgard/Eukaryota - ps5
Eukaryota - ps6
Amorpheae - ps7
Obazoa - ps8
Opisthokonta - ps9
Holozoa - ps10
Filozoa - ps11
Choano./Metazoa - ps12
Metazoa - ps13
Myriazoa - ps14
Parahoxozoa - ps15
Bilateria - ps16
Deuterostomia - ps17
Chordata - ps18
Olfactores - ps19
Vertebrata - ps20
Gnathostomata - ps21
Euteleostomi - ps22
Sarcopterygii - ps23
Tetrapoda - ps24
Amniota - ps25
Mammalia - ps26
Theria - ps27
Placentalia - ps28
Boreoeutheria - ps29
Euarchontoglires - ps30
Euarchonta - ps31
Primates - ps32
Haplorrhini - ps33
Simiiformes - ps34
Catarrhini - ps35
Hominoidea - ps36
Hominidae - ps37
Homininae - ps38
Hominini - ps39
Homo_sapiens - ps40
p-value
2e-03
4e-03
6e-03
1e-02
2e-02
Log-odds
0.5
1.0
1.5
2.0
2.5
3.0
3.5
4.0

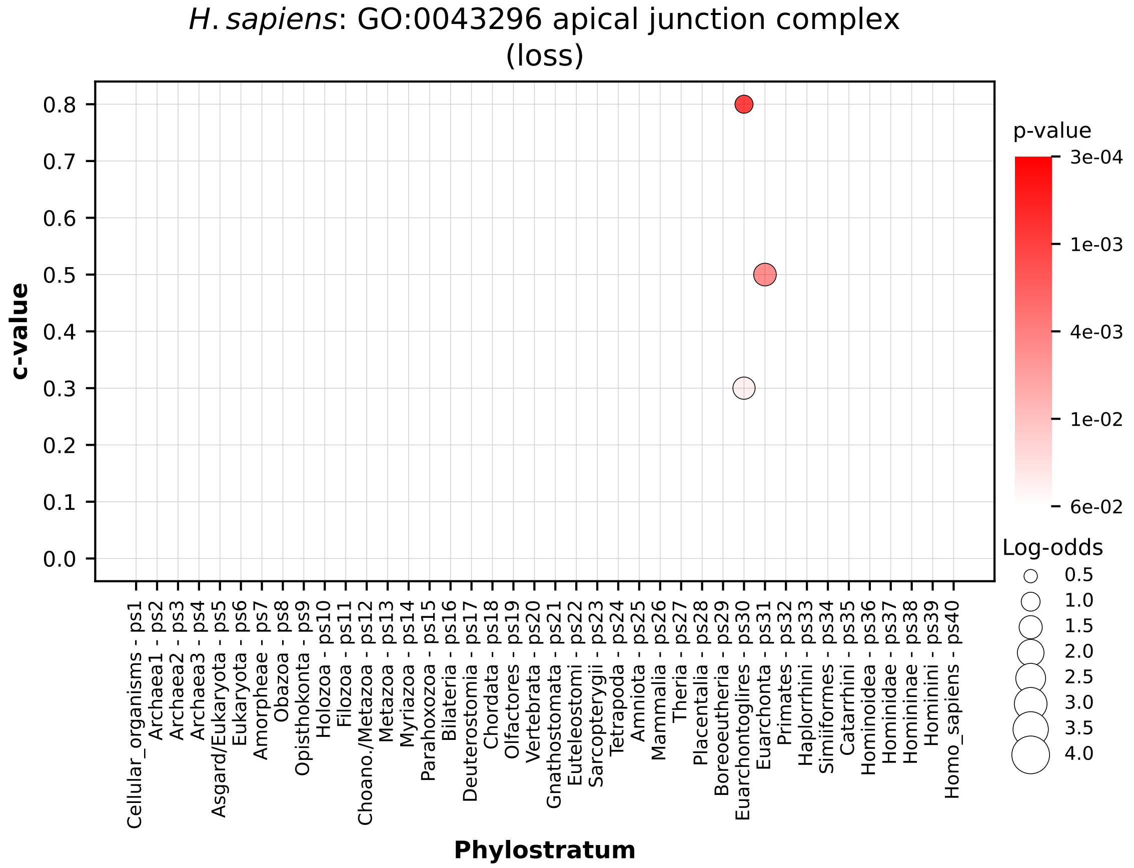

H. sapiens: GO:0043296 apical junction complex
(loss)
c-value
0.8
0.7
0.6
0.5
0.4
0.3
0.2
0.1
0.0
p-value
3e-04
1e-03
4e-03
1e-02
6e-02
Log-odds
0.5
1.0
1.5
2.0
2.5
3.0
3.5
4.0
Cellular_organisms - ps1
Archaea1 - ps2
Archaea2 - ps3
Archaea3 - ps4
Asgard/Eukaryota - ps5
Eukaryota - ps6
Amorpheae - ps7
Obazoa - ps8
Opisthokonta - ps9
Holozoa - ps10
Filozoa - ps11
Choano./Metazoa - ps12
Metazoa - ps13
Myriazoa - ps14
Parahoxozoa - ps15
Bilateria - ps16
Deuterostomia - ps17
Chordata - ps18
Olfactores - ps19
Vertebrata - ps20
Gnathostomata - ps21
Euteleostomi - ps22
Sarcopterygii - ps23
Tetrapoda - ps24
Amniota - ps25
Mammalia - ps26
Theria - ps27
Placentalia - ps28
Boreoeutheria - ps29
Euarchontoglires - ps30
Euarchonta - ps31
Primates - ps32
Haplorrhini - ps33
Simiiformes - ps34
Catarrhini - ps35
Hominoidea - ps36
Hominidae - ps37
Homininae - ps38
Hominini - ps39
Homo_sapiens - ps40
Phylostratum

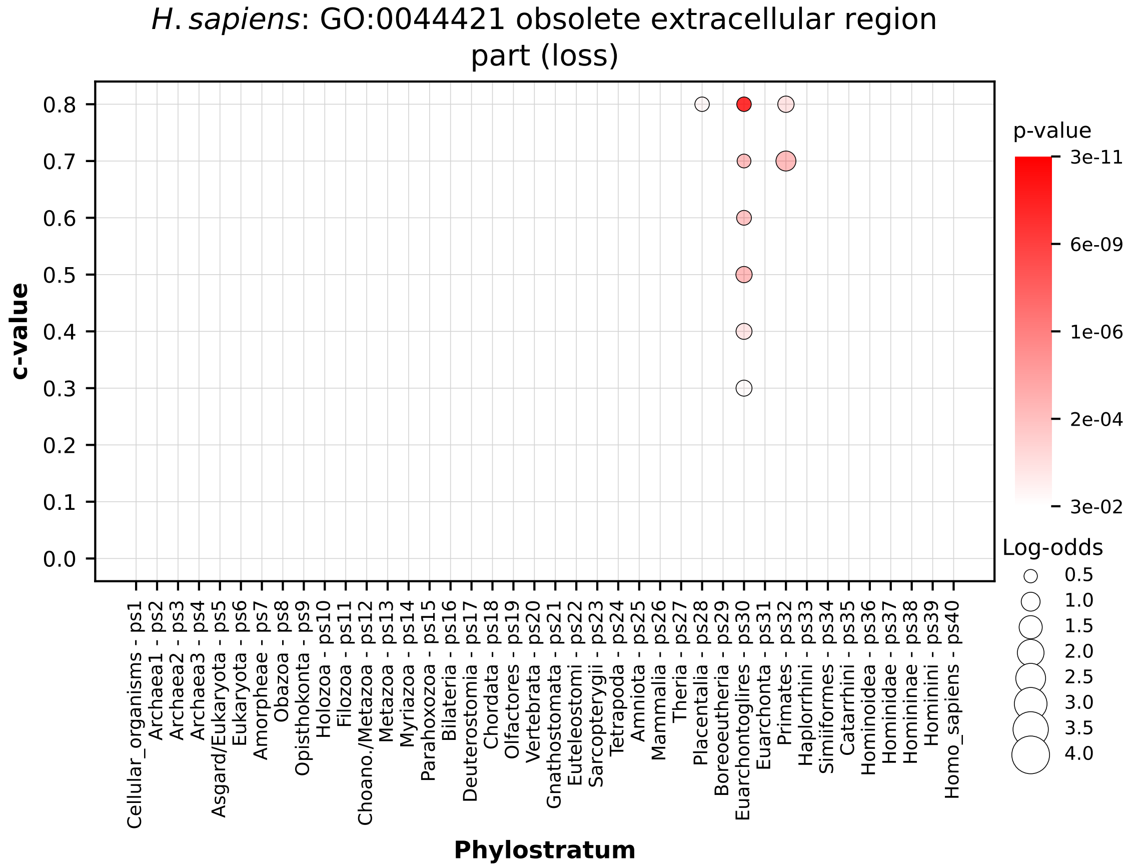
H. sapiens: GO:0044421 obsolete extracellular region part (loss)
c-value
0.0
0.1
0.2
0.3
0.4
0.5
0.6
0.7
0.8
Phylostratum
Cellular_organisms - ps1
Archaea1 - ps2
Archaea2 - ps3
Archaea3 - ps4
Asgard/Eukaryota - ps5
Eukaryota - ps6
Amorpheae - ps7
Obazoa - ps8
Opisthokonta - ps9
Holozoa - ps10
Filozoa - ps11
Choano./Metazoa - ps12
Metazoa - ps13
Myriazoa - ps14
Parahoxozoa - ps15
Bilateria - ps16
Deuterostomia - ps17
Chordata - ps18
Olfactores - ps19
Vertebrata - ps20
Gnathostomata - ps21
Euteleostomi - ps22
Sarcopterygii - ps23
Tetrapoda - ps24
Amniota - ps25
Mammalia - ps26
Theria - ps27
Placentalia - ps28
Boreoeutheria - ps29
Euarchontoglires - ps30
Euarchonta - ps31
Primates - ps32
Haplorrhini - ps33
Simiiformes - ps34
Catarrhini - ps35
Hominoidea - ps36
Hominidae - ps37
Homininae - ps38
Hominini - ps39
Homo_sapiens - ps40
p-value
3e-11
6e-09
1e-06
2e-04
3e-02
Log-odds
0.5
1.0
1.5
2.0
2.5
3.0
3.5
4.0

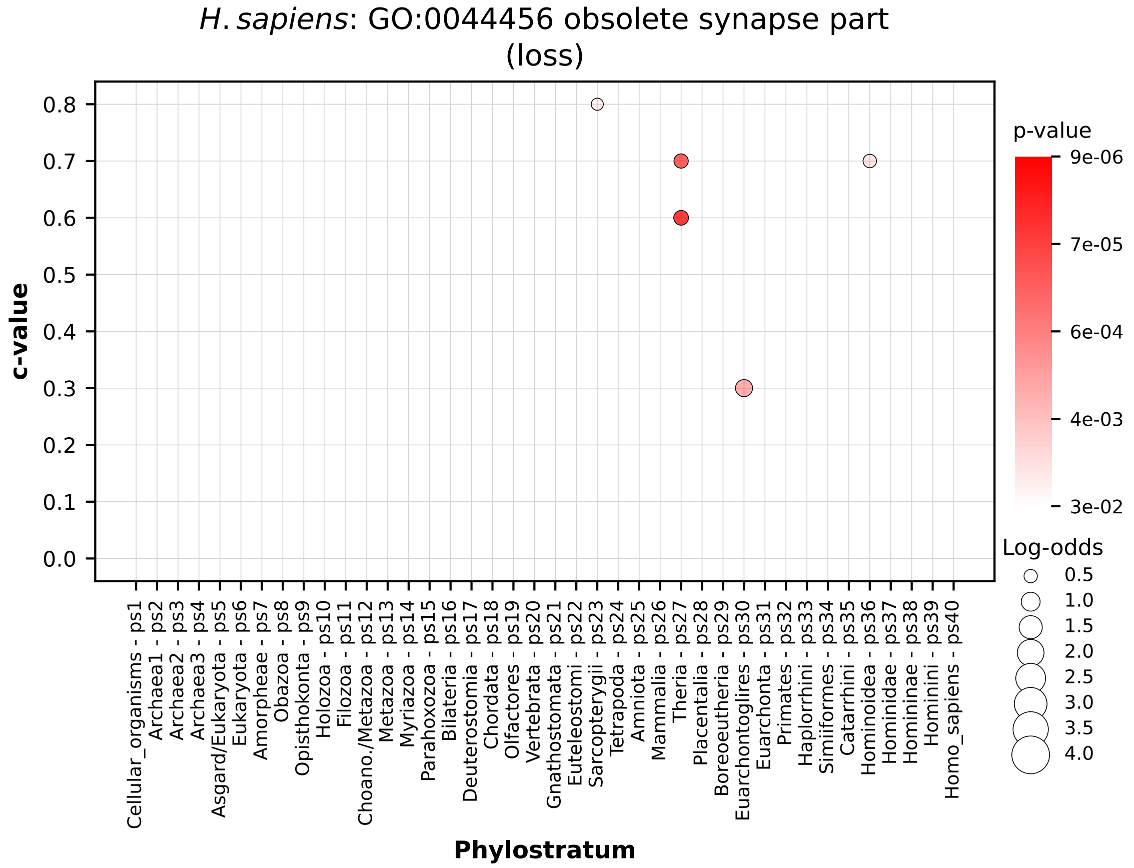

H. sapiens: GO:0044456 obsolete synapse part
(loss)
c-value
0.0
0.1
0.2
0.3
0.4
0.5
0.6
0.7
0.8
Phylostratum
Cellular_organisms - ps1
Archaea1 - ps2
Archaea2 - ps3
Archaea3 - ps4
Asgard/Eukaryota - ps5
Eukaryota - ps6
Amorpheae - ps7
Obazoa - ps8
Opisthokonta - ps9
Holozoa - ps10
Filozoa - ps11
Choano./Metazoa - ps12
Metazoa - ps13
Myriazoa - ps14
Parahoxozoa - ps15
Bilateria - ps16
Deuterostomia - ps17
Chordata - ps18
Olfactores - ps19
Vertebrata - ps20
Gnathostomata - ps21
Euteleostomi - ps22
Sarcopterygii - ps23
Tetrapoda - ps24
Amniota - ps25
Mammalia - ps26
Theria - ps27
Placentalia - ps28
Boreoeutheria - ps29
Euarchontoglires - ps30
Euarchonta - ps31
Primates - ps32
Haplorrhini - ps33
Simiiformes - ps34
Catarrhini - ps35
Hominoidea - ps36
Hominidae - ps37
Homininae - ps38
Hominini - ps39
Homo_sapiens - ps40
p-value
9e-06
7e-05
6e-04
4e-03
3e-02
Log-odds
0.5
1.0
1.5
2.0
2.5
3.0
3.5
4.0

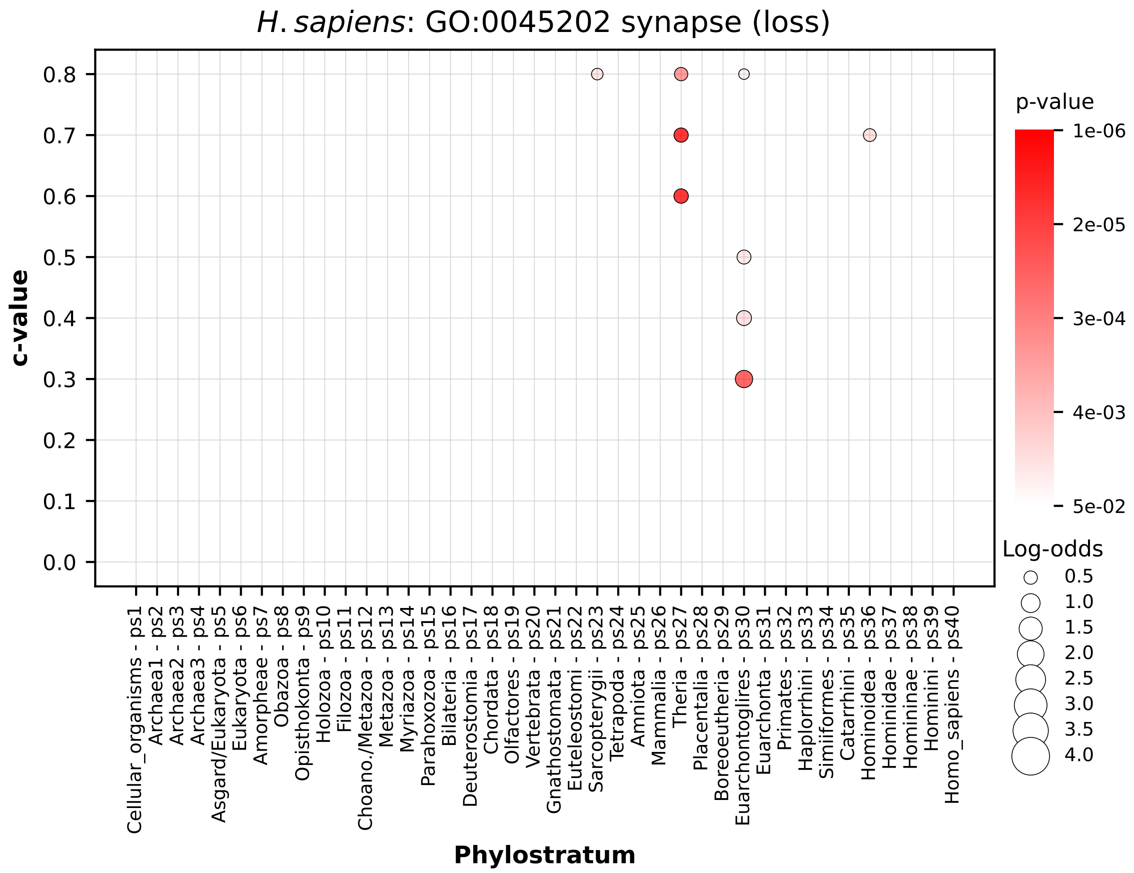

H. sapiens: GO:0045202 synapse (loss)
c-value
0.8
0.7
0.6
0.5
0.4
0.3
0.2
0.1
0.0
p-value
1e-06
2e-05
3e-04
4e-03
5e-02
Log-odds
0.5
1.0
1.5
2.0
2.5
3.0
3.5
4.0
Cellular_organisms - ps1
Archaea1 - ps2
Archaea2 - ps3
Archaea3 - ps4
Asgard/Eukaryota - ps5
Eukaryota - ps6
Amorpheae - ps7
Obazoa - ps8
Opisthokonta - ps9
Holozoa - ps10
Filozoa - ps11
Choano./Metazoa - ps12
Metazoa - ps13
Myriazoa - ps14
Parahoxozoa - ps15
Bilateria - ps16
Deuterostomia - ps17
Chordata - ps18
Olfactores - ps19
Vertebrata - ps20
Gnathostomata - ps21
Euteleostomi - ps22
Sarcopterygii - ps23
Tetrapoda - ps24
Amniota - ps25
Mammalia - ps26
Theria - ps27
Placentalia - ps28
Boreoeutheria - ps29
Euarchontoglires - ps30
Euarchonta - ps31
Primates - ps32
Haplorrhini - ps33
Simiiformes - ps34
Catarrhini - ps35
Hominoidea - ps36
Hominidae - ps37
Homininae - ps38
Hominini - ps39
Homo_sapiens - ps40
Phylostratum

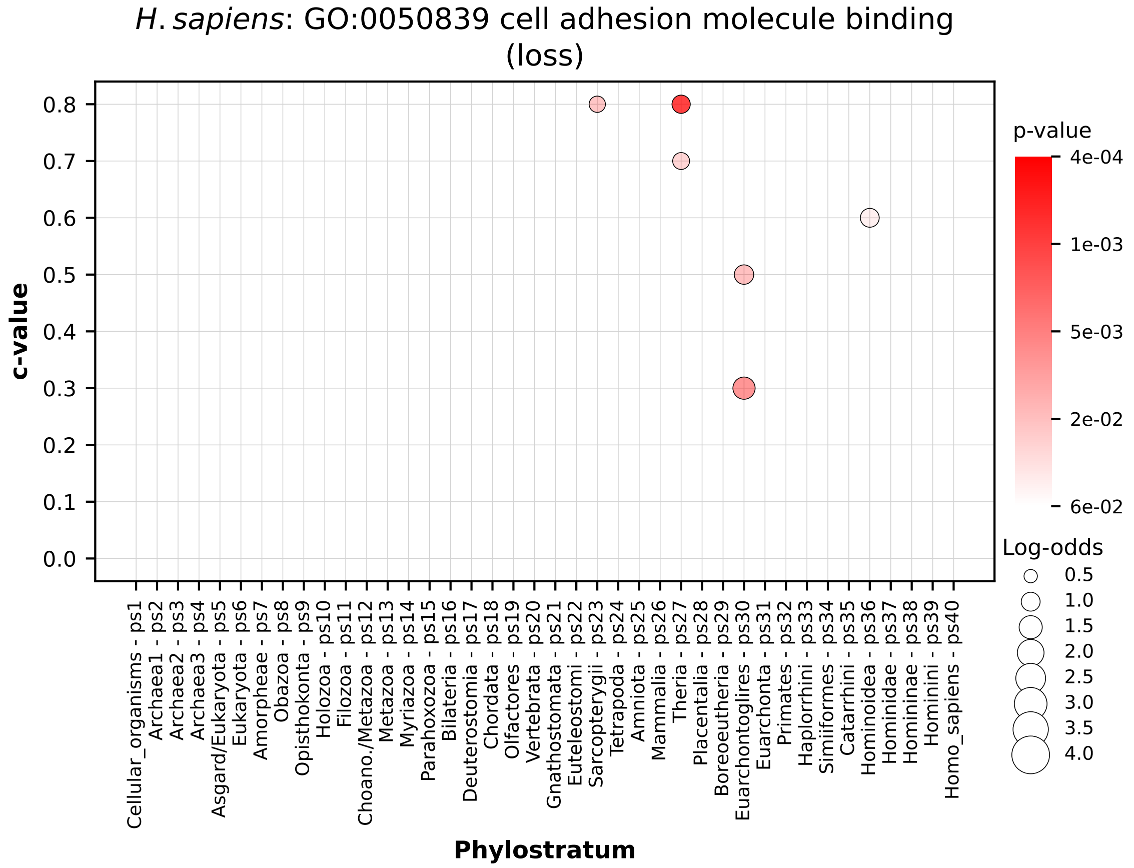
H. sapiens: GO:0050839 cell adhesion molecule binding
(loss)
c-value
0.0
0.1
0.2
0.3
0.4
0.5
0.6
0.7
0.8
Phylostratum
Cellular_organisms - ps1
Archaea1 - ps2
Archaea2 - ps3
Archaea3 - ps4
Asgard/Eukaryota - ps5
Eukaryota - ps6
Amorpheae - ps7
Obazoa - ps8
Opisthokonta - ps9
Holozoa - ps10
Filozoa - ps11
Choano./Metazoa - ps12
Metazoa - ps13
Myriazoa - ps14
Parahoxozoa - ps15
Bilateria - ps16
Deuterostomia - ps17
Chordata - ps18
Olfactores - ps19
Vertebrata - ps20
Gnathostomata - ps21
Euteleostomi - ps22
Sarcopterygii - ps23
Tetrapoda - ps24
Amniota - ps25
Mammalia - ps26
Theria - ps27
Placentalia - ps28
Boreoeutheria - ps29
Euarchontoglires - ps30
Euarchonta - ps31
Primates - ps32
Haplorrhini - ps33
Simiiformes - ps34
Catarrhini - ps35
Hominoidea - ps36
Hominidae - ps37
Homininae - ps38
Hominini - ps39
Homo_sapiens - ps40
p-value
4e-04
1e-03
5e-03
2e-02
6e-02
Log-odds
0.5
1.0
1.5
2.0
2.5
3.0
3.5
4.0

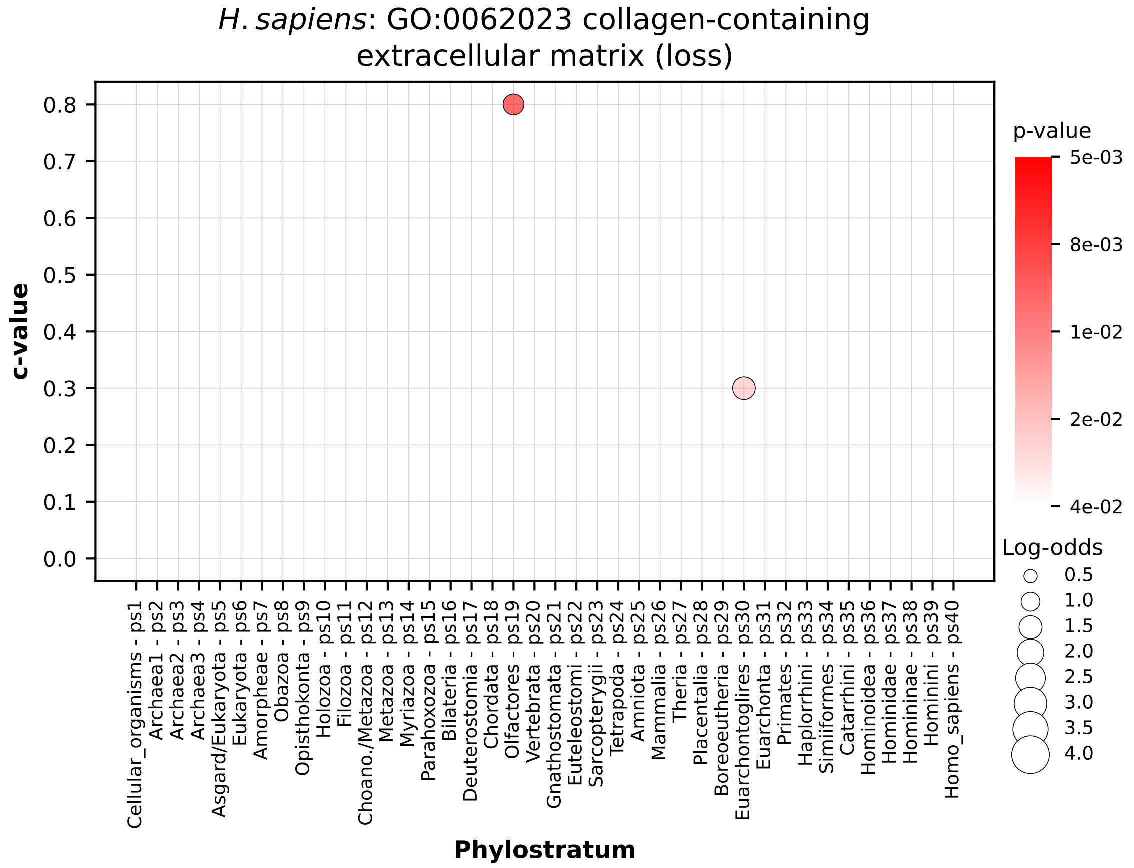

H. sapiens: GO:0062023 collagen-containing extracellular matrix (loss)
c-value
0.8
0.7
0.6
0.5
0.4
0.3
0.2
0.1
0.0
p-value
5e-03
8e-03
1e-02
2e-02
4e-02
Log-odds
0.5
1.0
1.5
2.0
2.5
3.0
3.5
4.0
Cellular_organisms - ps1
Archaea1 - ps2
Archaea2 - ps3
Archaea3 - ps4
Asgard/Eukaryota - ps5
Eukaryota - ps6
Amorpheae - ps7
Obazoa - ps8
Opisthokonta - ps9
Holozoa - ps10
Filozoa - ps11
Choano./Metazoa - ps12
Metazoa - ps13
Myriazoa - ps14
Parahoxozoa - ps15
Bilateria - ps16
Deuterostomia - ps17
Chordata - ps18
Olfactores - ps19
Vertebrata - ps20
Gnathostomata - ps21
Euteleostomi - ps22
Sarcopterygii - ps23
Tetrapoda - ps24
Amniota - ps25
Mammalia - ps26
Theria - ps27
Placentalia - ps28
Boreoeutheria - ps29
Euarchontoglires - ps30
Euarchonta - ps31
Primates - ps32
Haplorrhini - ps33
Simiiformes - ps34
Catarrhini - ps35
Hominoidea - ps36
Hominidae - ps37
Homininae - ps38
Hominini - ps39
Homo_sapiens - ps40
Phylostratum

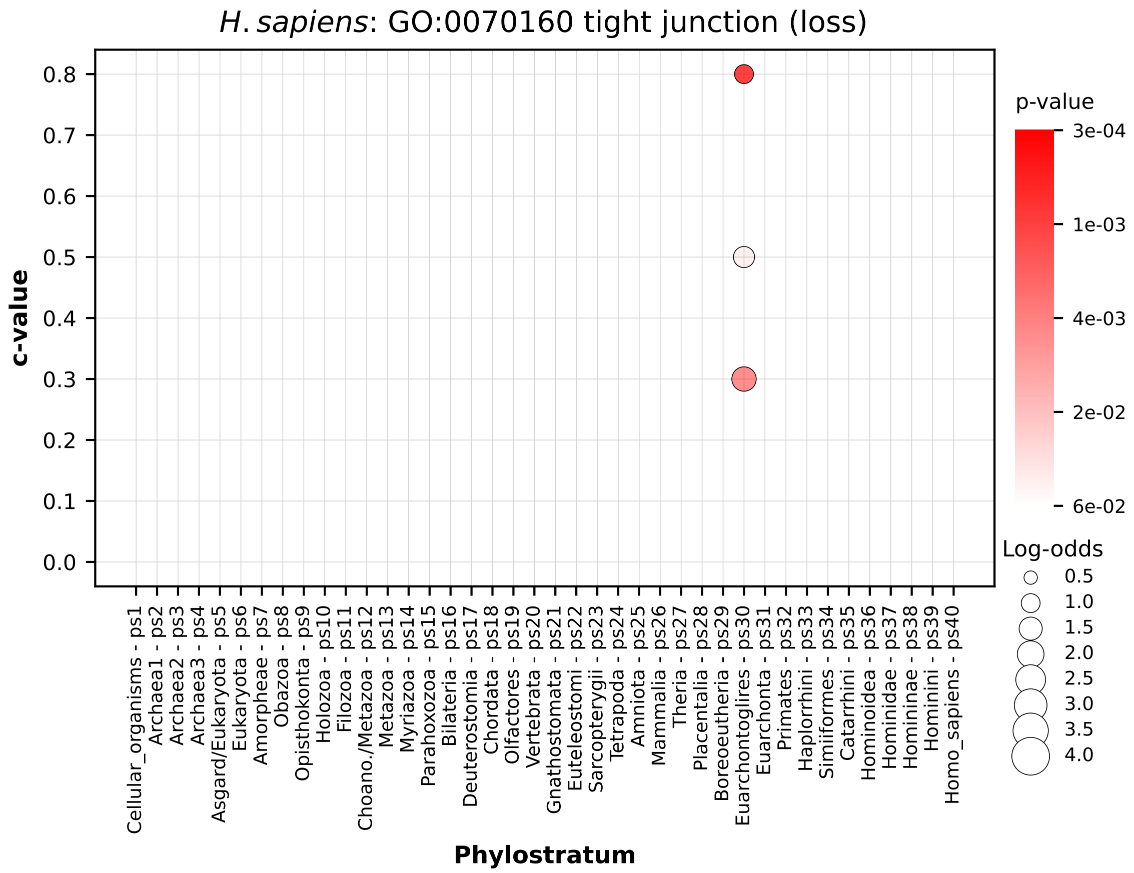

H. sapiens: GO:0070160 tight junction (loss)
c-value
0.0
0.1
0.2
0.3
0.4
0.5
0.6
0.7
0.8
p-value
3e-04
1e-03
4e-03
2e-02
6e-02
Log-odds
0.5
1.0
1.5
2.0
2.5
3.0
3.5
4.0
Cellular_organisms - ps1
Archaea1 - ps2
Archaea2 - ps3
Archaea3 - ps4
Asgard/Eukaryota - ps5
Eukaryota - ps6
Amorpheae - ps7
Obazoa - ps8
Opisthokonta - ps9
Holozoa - ps10
Filozoa - ps11
Choano./Metazoa - ps12
Metazoa - ps13
Myriazoa - ps14
Parahoxozoa - ps15
Bilateria - ps16
Deuterostomia - ps17
Chordata - ps18
Olfactores - ps19
Vertebrata - ps20
Gnathostomata - ps21
Euteleostomi - ps22
Sarcopterygii - ps23
Tetrapoda - ps24
Amniota - ps25
Mammalia - ps26
Theria - ps27
Placentalia - ps28
Boreoeutheria - ps29
Euarchontoglires - ps30
Euarchonta - ps31
Primates - ps32
Haplorrhini - ps33
Simiiformes - ps34
Catarrhini - ps35
Hominoidea - ps36
Hominidae - ps37
Homininae - ps38
Hominini - ps39
Homo_sapiens - ps40
Phylostratum

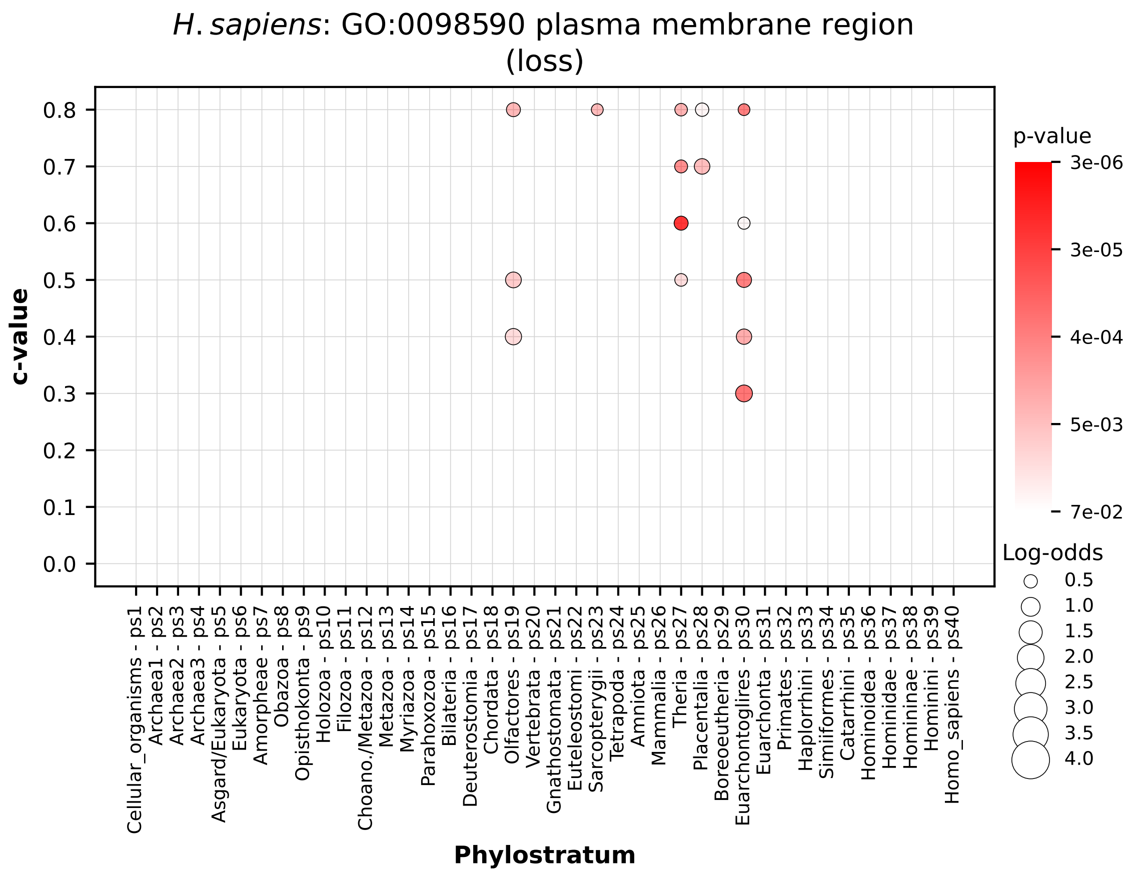

H. sapiens: GO:0098590 plasma membrane region
(loss)
c-value
0.8
0.7
0.6
0.5
0.4
0.3
0.2
0.1
0.0
Phylostratum
Cellular_organisms - ps1
Archaea1 - ps2
Archaea2 - ps3
Archaea3 - ps4
Asgard/Eukaryota - ps5
Eukaryota - ps6
Amorpheae - ps7
Obazoa - ps8
Opisthokonta - ps9
Holozoa - ps10
Filozoa - ps11
Choano./Metazoa - ps12
Metazoa - ps13
Myriazoa - ps14
Parahoxozoa - ps15
Bilateria - ps16
Deuterostomia - ps17
Chordata - ps18
Olfactores - ps19
Vertebrata - ps20
Gnathostomata - ps21
Euteleostomi - ps22
Sarcopterygii - ps23
Tetrapoda - ps24
Amniota - ps25
Mammalia - ps26
Theria - ps27
Placentalia - ps28
Boreoeutheria - ps29
Euarchontoglires - ps30
Euarchonta - ps31
Primates - ps32
Haplorrhini - ps33
Simiiformes - ps34
Catarrhini - ps35
Hominoidea - ps36
Hominidae - ps37
Homininae - ps38
Hominini - ps39
Homo_sapiens - ps40
p-value
3e-06
3e-05
4e-04
5e-03
7e-02
Log-odds
0.5
1.0
1.5
2.0
2.5
3.0
3.5
4.0

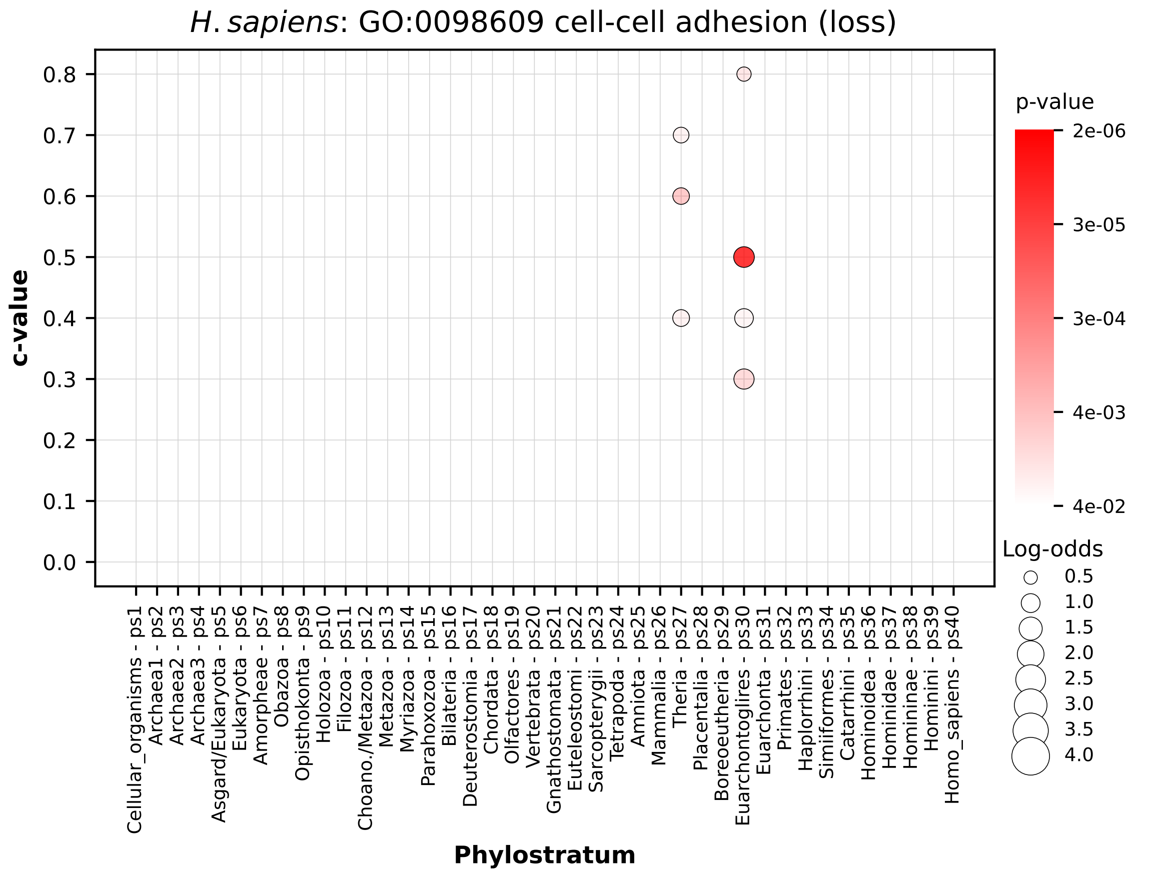

H. sapiens: GO:0098609 cell-cell adhesion (loss)
c-value
0.8
0.7
0.6
0.5
0.4
0.3
0.2
0.1
0.0
p-value
2e-06
3e-05
3e-04
4e-03
4e-02
Log-odds
0.5
1.0
1.5
2.0
2.5
3.0
3.5
4.0
Cellular_organisms - ps1
Archaea1 - ps2
Archaea2 - ps3
Archaea3 - ps4
Asgard/Eukaryota - ps5
Eukaryota - ps6
Amorpheae - ps7
Obazoa - ps8
Opisthokonta - ps9
Holozoa - ps10
Filozoa - ps11
Choano./Metazoa - ps12
Metazoa - ps13
Myriazoa - ps14
Parahoxozoa - ps15
Bilateria - ps16
Deuterostomia - ps17
Chordata - ps18
Olfactores - ps19
Vertebrata - ps20
Gnathostomata - ps21
Euteleostomi - ps22
Sarcopterygii - ps23
Tetrapoda - ps24
Amniota - ps25
Mammalia - ps26
Theria - ps27
Placentalia - ps28
Boreoeutheria - ps29
Euarchontoglires - ps30
Euarchonta - ps31
Primates - ps32
Haplorrhini - ps33
Simiiformes - ps34
Catarrhini - ps35
Hominoidea - ps36
Hominidae - ps37
Homininae - ps38
Hominini - ps39
Homo_sapiens - ps40
Phylostratum

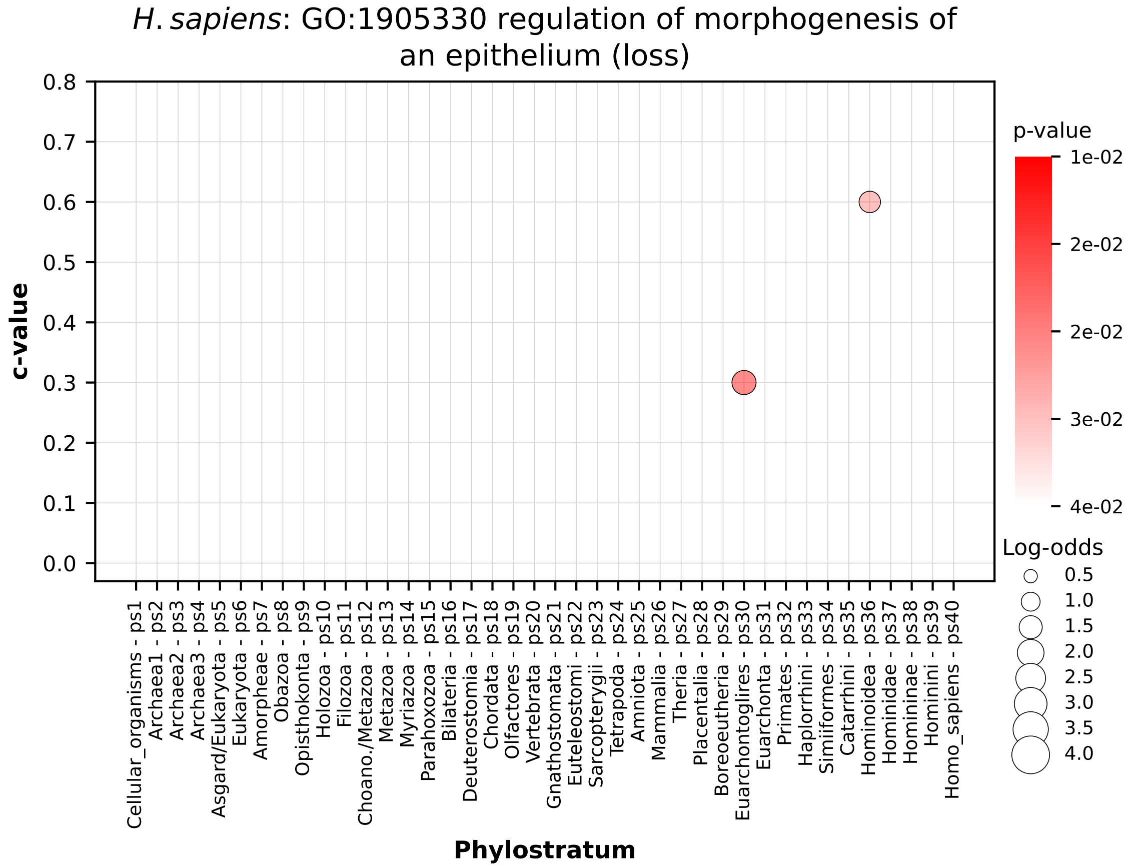
H. sapiens: GO:1905330 regulation of morphogenesis of an epithelium (loss)
c-value
0.8
0.7
0.6
0.5
0.4
0.3
0.2
0.1
0.0
Phylostratum
Cellular_organisms - ps1
Archaea1 - ps2
Archaea2 - ps3
Archaea3 - ps4
Asgard/Eukaryota - ps5
Eukaryota - ps6
Amorpheae - ps7
Obazoa - ps8
Opisthokonta - ps9
Holozoa - ps10
Filozoa - ps11
Choano./Metazoa - ps12
Metazoa - ps13
Myriazoa - ps14
Parahoxozoa - ps15
Bilateria - ps16
Deuterostomia - ps17
Chordata - ps18
Olfactores - ps19
Vertebrata - ps20
Gnathostomata - ps21
Euteleostomi - ps22
Sarcopterygii - ps23
Tetrapoda - ps24
Amniota - ps25
Mammalia - ps26
Theria - ps27
Placentalia - ps28
Boreoeutheria - ps29
Euarchontoglires - ps30
Euarchonta - ps31
Primates - ps32
Haplorrhini - ps33
Simiiformes - ps34
Catarrhini - ps35
Hominoidea - ps36
Hominidae - ps37
Homininae - ps38
Hominini - ps39
Homo_sapiens - ps40
p-value
1e-02
2e-02
2e-02
3e-02
4e-02
Log-odds
0.5
1.0
1.5
2.0
2.5
3.0
3.5
4.0

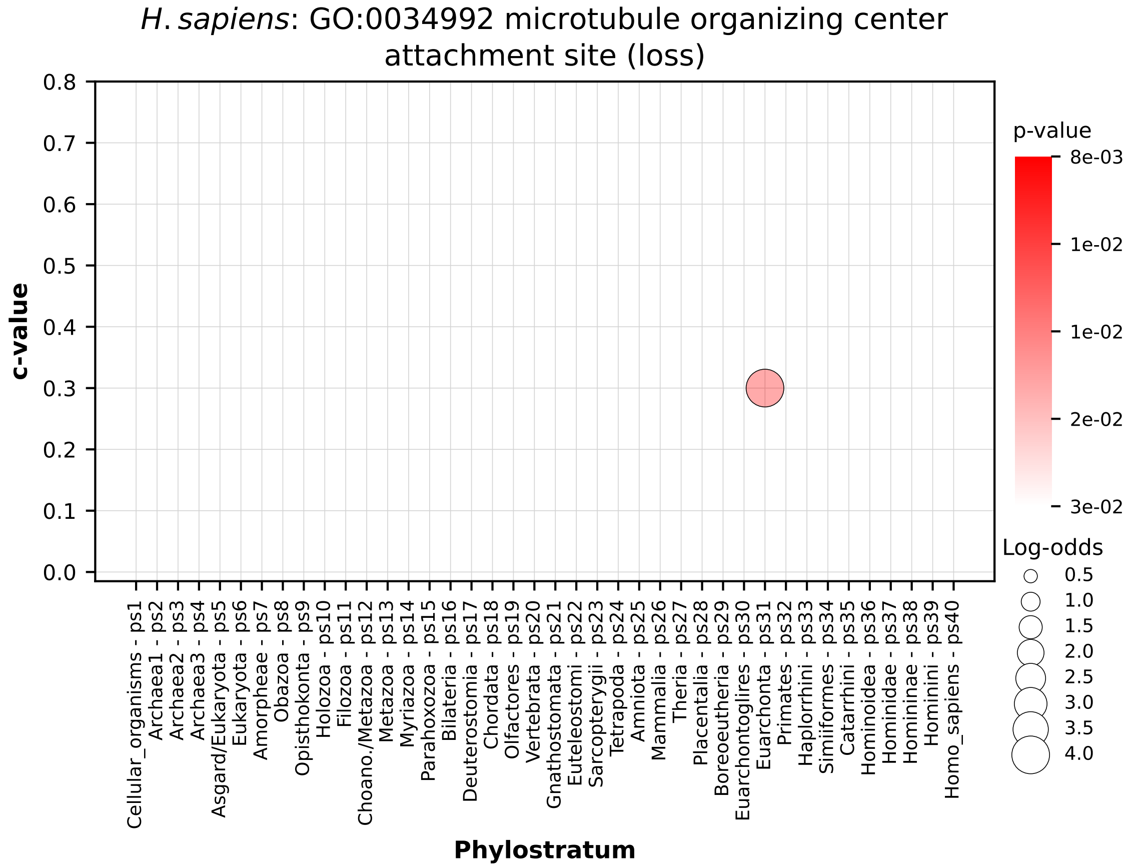

H. sapiens: GO:0034992 microtubule organizing center attachment site (loss)
c-value
0.0
0.1
0.2
0.3
0.4
0.5
0.6
0.7
0.8
p-value
8e-03
1e-02
1e-02
2e-02
3e-02
Log-odds
0.5
1.0
1.5
2.0
2.5
3.0
3.5
4.0
Cellular_organisms - ps1
Archaea1 - ps2
Archaea2 - ps3
Archaea3 - ps4
Asgard/Eukaryota - ps5
Eukaryota - ps6
Amorpheae - ps7
Obazoa - ps8
Opisthokonta - ps9
Holozoa - ps10
Filozoa - ps11
Choano./Metazoa - ps12
Metazoa - ps13
Myriazoa - ps14
Parahoxozoa - ps15
Bilateria - ps16
Deuterostomia - ps17
Chordata - ps18
Olfactores - ps19
Vertebrata - ps20
Gnathostomata - ps21
Euteleostomi - ps22
Sarcopterygii - ps23
Tetrapoda - ps24
Amniota - ps25
Mammalia - ps26
Theria - ps27
Placentalia - ps28
Boreoeutheria - ps29
Euarchontoglires - ps30
Euarchonta - ps31
Primates - ps32
Haplorrhini - ps33
Simiiformes - ps34
Catarrhini - ps35
Hominoidea - ps36
Hominidae - ps37
Homininae - ps38
Hominini - ps39
Homo_sapiens - ps40
Phylostratum

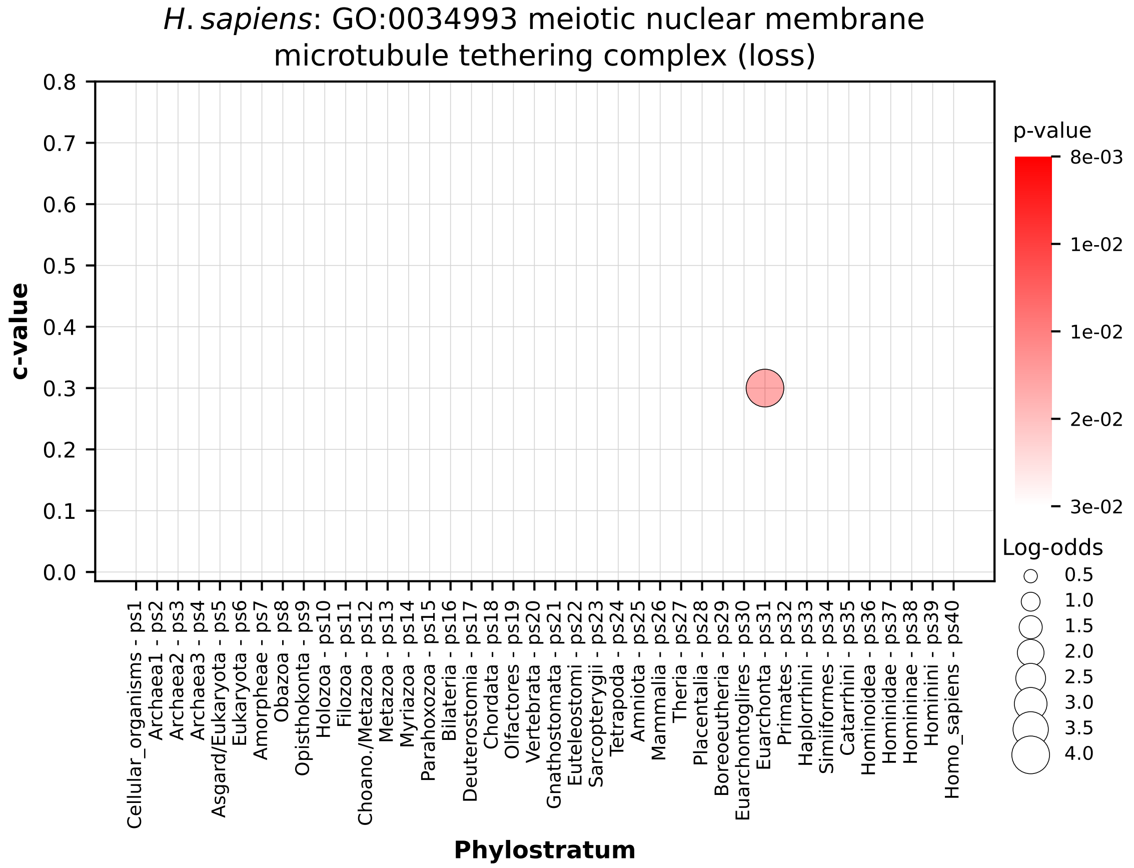

H. sapiens: GO:0034993 meiotic nuclear membrane microtubule tethering complex (loss)
c-value
0.0
0.1
0.2
0.3
0.4
0.5
0.6
0.7
0.8
Phylostratum
Cellular_organisms - ps1
Archaea1 - ps2
Archaea2 - ps3
Archaea3 - ps4
Asgard/Eukaryota - ps5
Eukaryota - ps6
Amorpheae - ps7
Obazoa - ps8
Opisthokonta - ps9
Holozoa - ps10
Filozoa - ps11
Choano./Metazoa - ps12
Metazoa - ps13
Myriazoa - ps14
Parahoxozoa - ps15
Bilateria - ps16
Deuterostomia - ps17
Chordata - ps18
Olfactores - ps19
Vertebrata - ps20
Gnathostomata - ps21
Euteleostomi - ps22
Sarcopterygii - ps23
Tetrapoda - ps24
Amniota - ps25
Mammalia - ps26
Theria - ps27
Placentalia - ps28
Boreoeutheria - ps29
Euarchontoglires - ps30
Euarchonta - ps31
Primates - ps32
Haplorrhini - ps33
Simiiformes - ps34
Catarrhini - ps35
Hominoidea - ps36
Hominidae - ps37
Homininae - ps38
Hominini - ps39
Homo_sapiens - ps40
p-value
8e-03
1e-02
1e-02
2e-02
3e-02
Log-odds
0.5
1.0
1.5
2.0
2.5
3.0
3.5
4.0

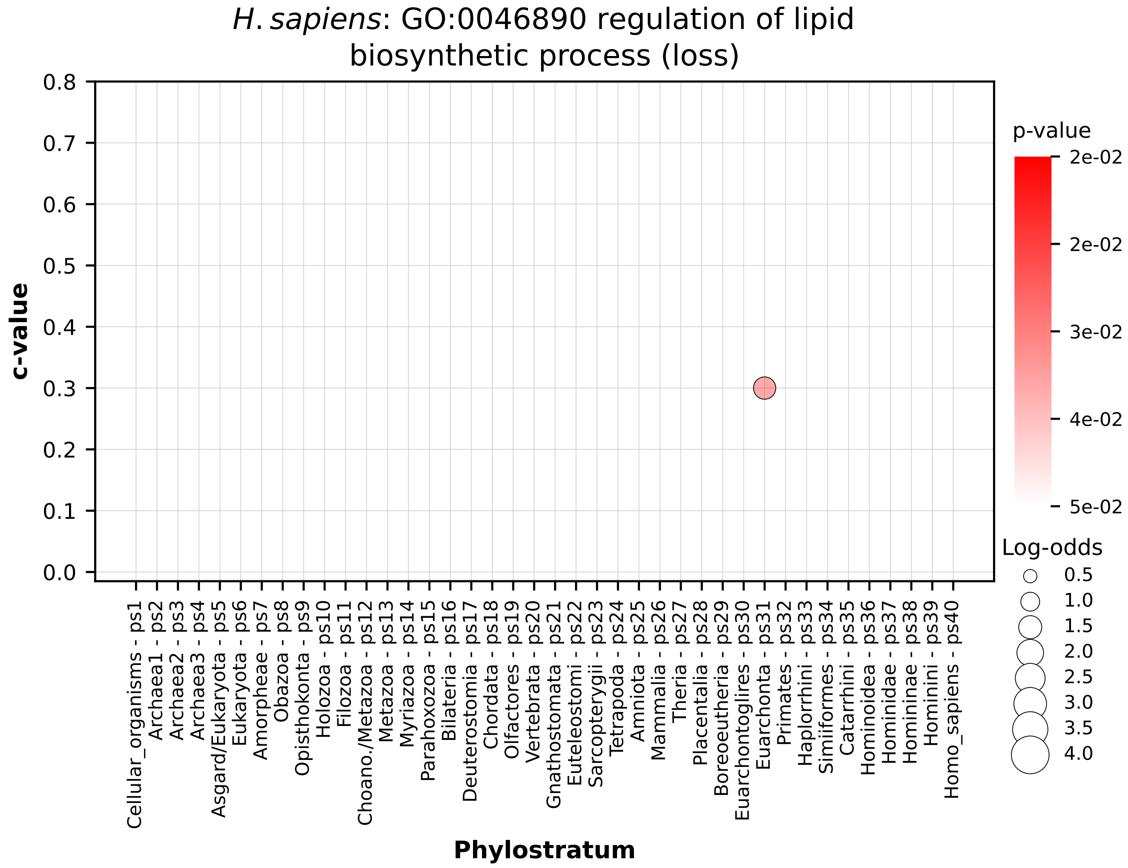
H. sapiens: GO:0046890 regulation of lipid biosynthetic process (loss)
c-value
0.8
0.7
0.6
0.5
0.4
0.3
0.2
0.1
0.0
Phylostratum
Cellular_organisms - ps1
Archaea1 - ps2
Archaea2 - ps3
Archaea3 - ps4
Asgard/Eukaryota - ps5
Eukaryota - ps6
Amorpheae - ps7
Obazoa - ps8
Opisthokonta - ps9
Holozoa - ps10
Filozoa - ps11
Choano./Metazoa - ps12
Metazoa - ps13
Myriazoa - ps14
Parahoxozoa - ps15
Bilateria - ps16
Deuterostomia - ps17
Chordata - ps18
Olfactores - ps19
Vertebrata - ps20
Gnathostomata - ps21
Euteleostomi - ps22
Sarcopterygii - ps23
Tetrapoda - ps24
Amniota - ps25
Mammalia - ps26
Theria - ps27
Placentalia - ps28
Boreoeutheria - ps29
Euarchontoglires - ps30
Euarchonta - ps31
Primates - ps32
Haplorrhini - ps33
Simiiformes - ps34
Catarrhini - ps35
Hominoidea - ps36
Hominidae - ps37
Homininae - ps38
Hominini - ps39
Homo_sapiens - ps40
p-value
2e-02
2e-02
3e-02
4e-02
5e-02
Log-odds
0.5
1.0
1.5
2.0
2.5
3.0
3.5
4.0

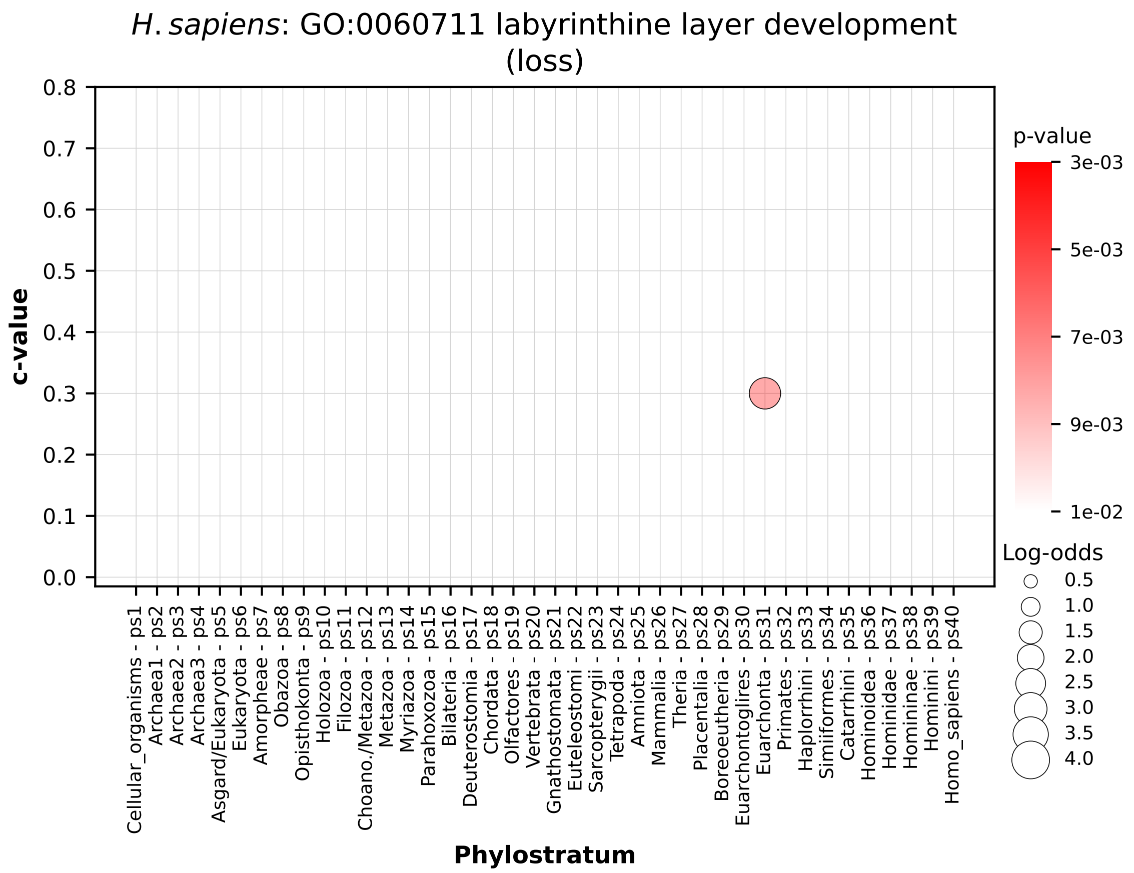

H. sapiens: GO:0060711 labyrinthine layer development (loss)
c-value
0.0
0.1
0.2
0.3
0.4
0.5
0.6
0.7
0.8
Phylostratum
Cellular_organisms - ps1
Archaea1 - ps2
Archaea2 - ps3
Archaea3 - ps4
Asgard/Eukaryota - ps5
Eukaryota - ps6
Amorpheae - ps7
Obazoa - ps8
Opisthokonta - ps9
Holozoa - ps10
Filozoa - ps11
Choano./Metazoa - ps12
Metazoa - ps13
Myriazoa - ps14
Parahoxozoa - ps15
Bilateria - ps16
Deuterostomia - ps17
Chordata - ps18
Olfactores - ps19
Vertebrata - ps20
Gnathostomata - ps21
Euteleostomi - ps22
Sarcopterygii - ps23
Tetrapoda - ps24
Amniota - ps25
Mammalia - ps26
Theria - ps27
Placentalia - ps28
Boreoeutheria - ps29
Euarchontoglires - ps30
Euarchonta - ps31
Primates - ps32
Haplorrhini - ps33
Simiiformes - ps34
Catarrhini - ps35
Hominoidea - ps36
Hominidae - ps37
Homininae - ps38
Hominini - ps39
Homo_sapiens - ps40
p-value
3e-03
5e-03
7e-03
9e-03
1e-02
Log-odds
0.5
1.0
1.5
2.0
2.5
3.0
3.5
4.0

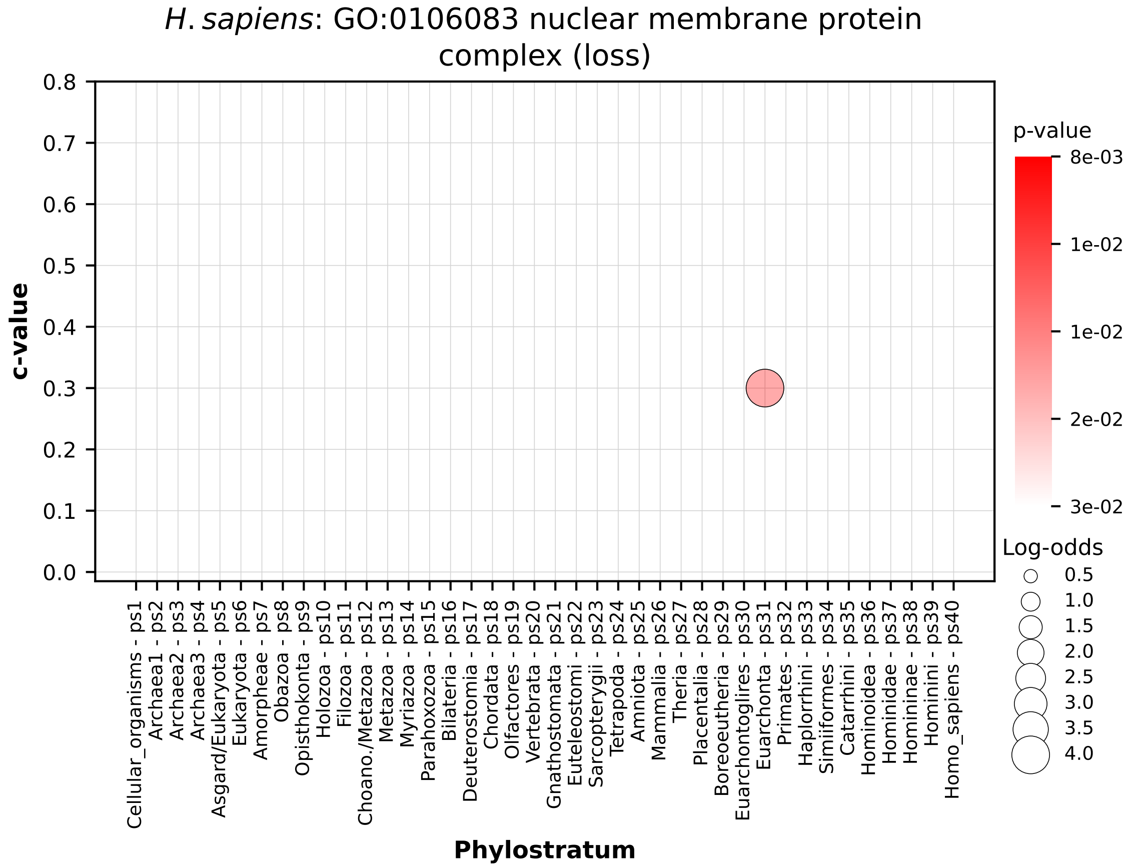

H. sapiens: GO:0106083 nuclear membrane protein complex (loss)
c-value
0.8
0.7
0.6
0.5
0.4
0.3
0.2
0.1
0.0
p-value
8e-03
1e-02
1e-02
2e-02
3e-02
Log-odds
0.5
1.0
1.5
2.0
2.5
3.0
3.5
4.0
Cellular_organisms - ps1
Archaea1 - ps2
Archaea2 - ps3
Archaea3 - ps4
Asgard/Eukaryota - ps5
Eukaryota - ps6
Amorpheae - ps7
Obazoa - ps8
Opisthokonta - ps9
Holozoa - ps10
Filozoa - ps11
Choano./Metazoa - ps12
Metazoa - ps13
Myriazoa - ps14
Parahoxozoa - ps15
Bilateria - ps16
Deuterostomia - ps17
Chordata - ps18
Olfactores - ps19
Vertebrata - ps20
Gnathostomata - ps21
Euteleostomi - ps22
Sarcopterygii - ps23
Tetrapoda - ps24
Amniota - ps25
Mammalia - ps26
Theria - ps27
Placentalia - ps28
Boreoeutheria - ps29
Euarchontoglires - ps30
Euarchonta - ps31
Primates - ps32
Haplorrhini - ps33
Simiiformes - ps34
Catarrhini - ps35
Hominoidea - ps36
Hominidae - ps37
Homininae - ps38
Hominini - ps39
Homo_sapiens - ps40
Phylostratum

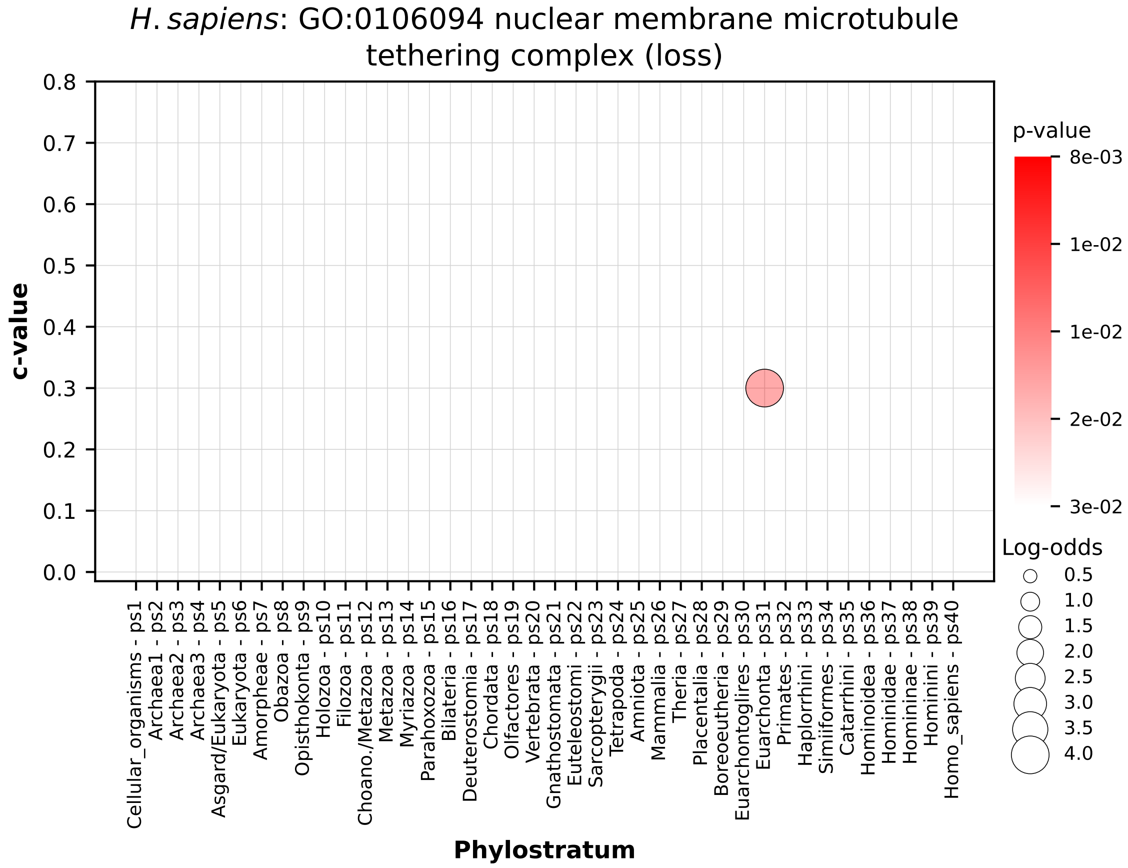

H. sapiens: GO:0106094 nuclear membrane microtubule tethering complex (loss)
c-value
0.0
0.1
0.2
0.3
0.4
0.5
0.6
0.7
0.8
Phylostratum
Cellular_organisms - ps1
Archaea1 - ps2
Archaea2 - ps3
Archaea3 - ps4
Asgard/Eukaryota - ps5
Eukaryota - ps6
Amorpheae - ps7
Obazoa - ps8
Opisthokonta - ps9
Holozoa - ps10
Filozoa - ps11
Choano./Metazoa - ps12
Metazoa - ps13
Myriazoa - ps14
Parahoxozoa - ps15
Bilateria - ps16
Deuterostomia - ps17
Chordata - ps18
Olfactores - ps19
Vertebrata - ps20
Gnathostomata - ps21
Euteleostomi - ps22
Sarcopterygii - ps23
Tetrapoda - ps24
Amniota - ps25
Mammalia - ps26
Theria - ps27
Placentalia - ps28
Boreoeutheria - ps29
Euarchontoglires - ps30
Euarchonta - ps31
Primates - ps32
Haplorrhini - ps33
Simiiformes - ps34
Catarrhini - ps35
Hominoidea - ps36
Hominidae - ps37
Homininae - ps38
Hominini - ps39
Homo_sapiens - ps40
p-value
8e-03
1e-02
1e-02
2e-02
3e-02
Log-odds
0.5
1.0
1.5
2.0
2.5
3.0
3.5
4.0

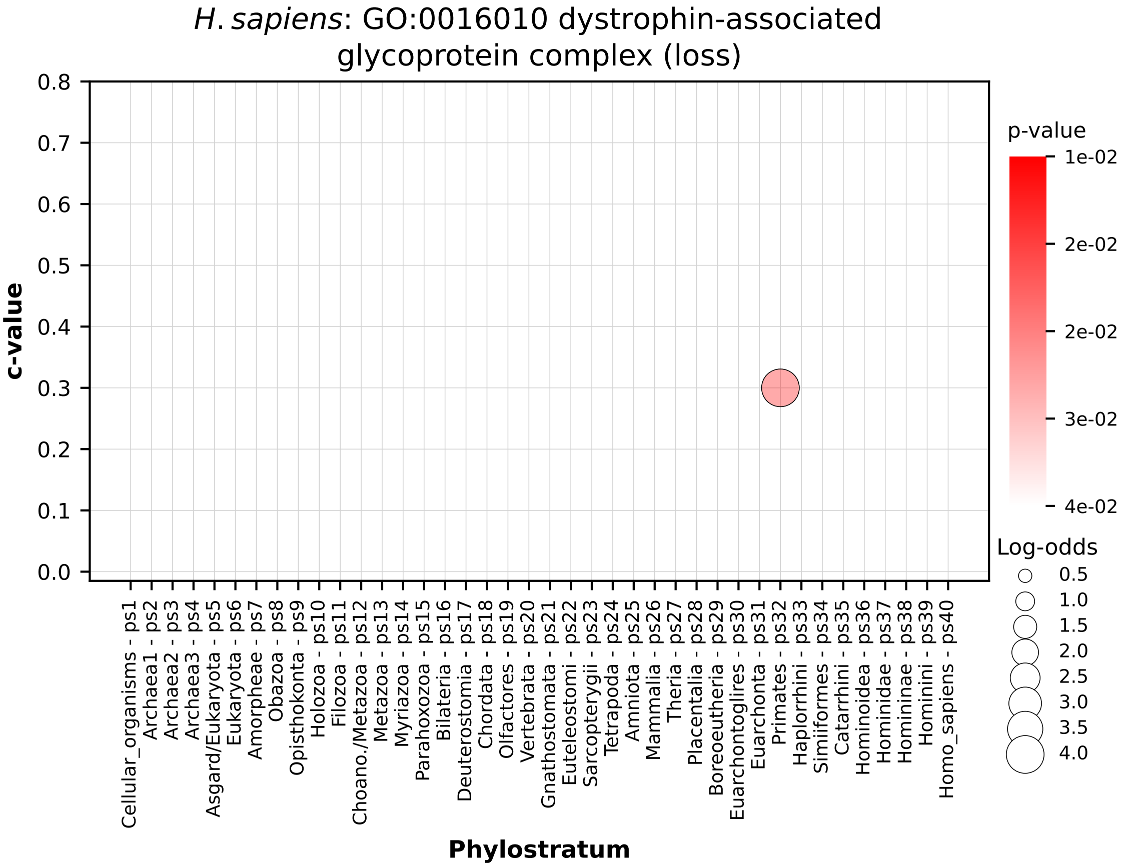

H. sapiens: GO:0016010 dystrophin-associated glycoprotein complex (loss)
c-value
0.0
0.1
0.2
0.3
0.4
0.5
0.6
0.7
0.8
Phylostratum
Cellular_organisms - ps1
Archaea1 - ps2
Archaea2 - ps3
Archaea3 - ps4
Asgard/Eukaryota - ps5
Eukaryota - ps6
Amorpheae - ps7
Obazoa - ps8
Opisthokonta - ps9
Holozoa - ps10
Filozoa - ps11
Choano./Metazoa - ps12
Metazoa - ps13
Myriazoa - ps14
Parahoxozoa - ps15
Bilateria - ps16
Deuterostomia - ps17
Chordata - ps18
Olfactores - ps19
Vertebrata - ps20
Gnathostomata - ps21
Euteleostomi - ps22
Sarcopterygii - ps23
Tetrapoda - ps24
Amniota - ps25
Mammalia - ps26
Theria - ps27
Placentalia - ps28
Boreoeutheria - ps29
Euarchontoglires - ps30
Euarchonta - ps31
Primates - ps32
Haplorrhini - ps33
Simiiformes - ps34
Catarrhini - ps35
Hominoidea - ps36
Hominidae - ps37
Homininae - ps38
Hominini - ps39
Homo_sapiens - ps40
p-value
1e-02
2e-02
2e-02
3e-02
4e-02
Log-odds
0.5
1.0
1.5
2.0
2.5
3.0
3.5
4.0

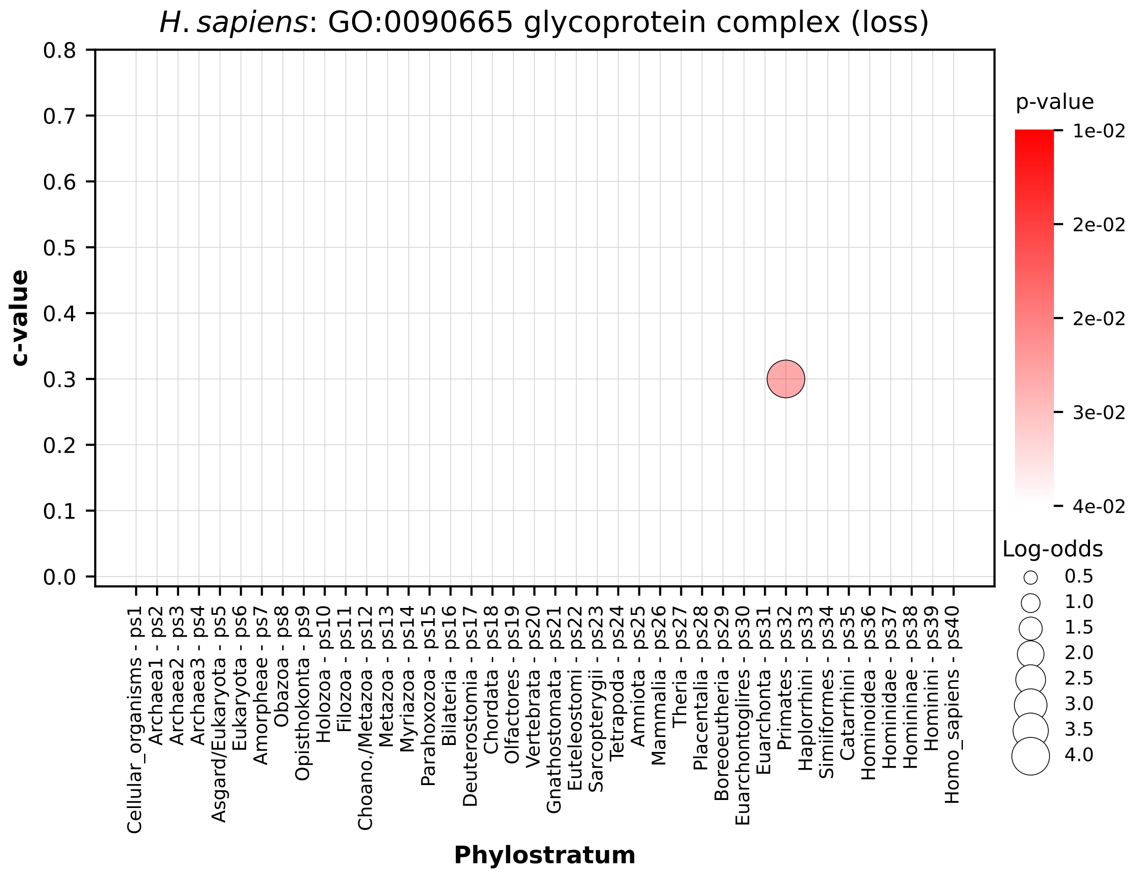
H. sapiens: GO:0090665 glycoprotein complex (loss)
c-value
0.0
0.1
0.2
0.3
0.4
0.5
0.6
0.7
0.8
Phylostratum
Cellular_organisms - ps1
Archaea1 - ps2
Archaea2 - ps3
Archaea3 - ps4
Asgard/Eukaryota - ps5
Eukaryota - ps6
Amorpheae - ps7
Obazoa - ps8
Opisthokonta - ps9
Holozoa - ps10
Filozoa - ps11
Choano./Metazoa - ps12
Metazoa - ps13
Myriazoa - ps14
Parahoxozoa - ps15
Bilateria - ps16
Deuterostomia - ps17
Chordata - ps18
Olfactores - ps19
Vertebrata - ps20
Gnathostomata - ps21
Euteleostomi - ps22
Sarcopterygii - ps23
Tetrapoda - ps24
Amniota - ps25
Mammalia - ps26
Theria - ps27
Placentalia - ps28
Boreoeutheria - ps29
Euarchontoglires - ps30
Euarchonta - ps31
Primates - ps32
Haplorrhini - ps33
Simiiformes - ps34
Catarrhini - ps35
Hominoidea - ps36
Hominidae - ps37
Homininae - ps38
Hominini - ps39
Homo_sapiens - ps40
p-value
1e-02
2e-02
2e-02
3e-02
4e-02
Log-odds
0.5
1.0
1.5
2.0
2.5
3.0
3.5
4.0

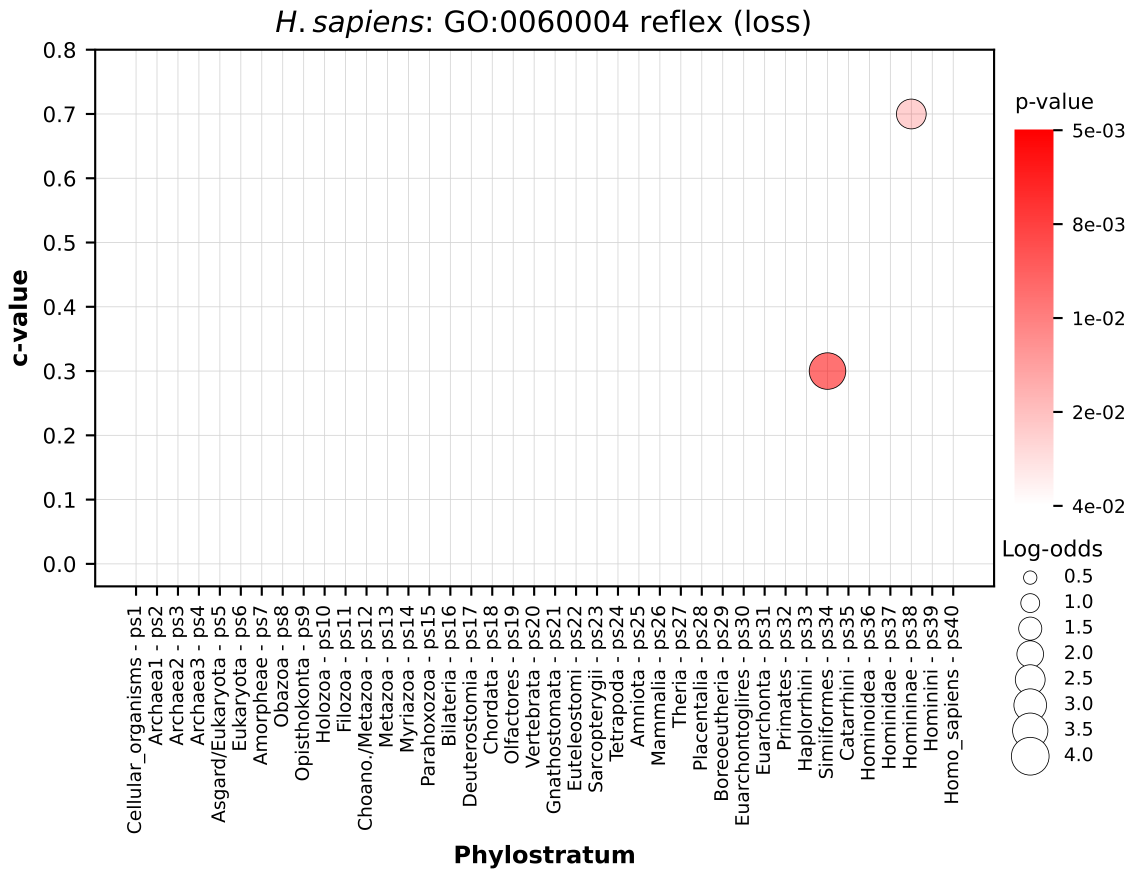

H. sapiens: GO:0060004 reflex (loss)
c-value
0.8
0.7
0.6
0.5
0.4
0.3
0.2
0.1
0.0
p-value
5e-03
8e-03
1e-02
2e-02
4e-02
Log-odds
0.5
1.0
1.5
2.0
2.5
3.0
3.5
4.0
Cellular_organisms - ps1
Archaea1 - ps2
Archaea2 - ps3
Archaea3 - ps4
Asgard/Eukaryota - ps5
Eukaryota - ps6
Amorpheae - ps7
Obazoa - ps8
Opisthokonta - ps9
Holozoa - ps10
Filozoa - ps11
Choano./Metazoa - ps12
Metazoa - ps13
Myriazoa - ps14
Parahoxozoa - ps15
Bilateria - ps16
Deuterostomia - ps17
Chordata - ps18
Olfactores - ps19
Vertebrata - ps20
Gnathostomata - ps21
Euteleostomi - ps22
Sarcopterygii - ps23
Tetrapoda - ps24
Amniota - ps25
Mammalia - ps26
Theria - ps27
Placentalia - ps28
Boreoeutheria - ps29
Euarchontoglires - ps30
Euarchonta - ps31
Primates - ps32
Haplorrhini - ps33
Simiiformes - ps34
Catarrhini - ps35
Hominoidea - ps36
Hominidae - ps37
Homininae - ps38
Hominini - ps39
Homo_sapiens - ps40
Phylostratum

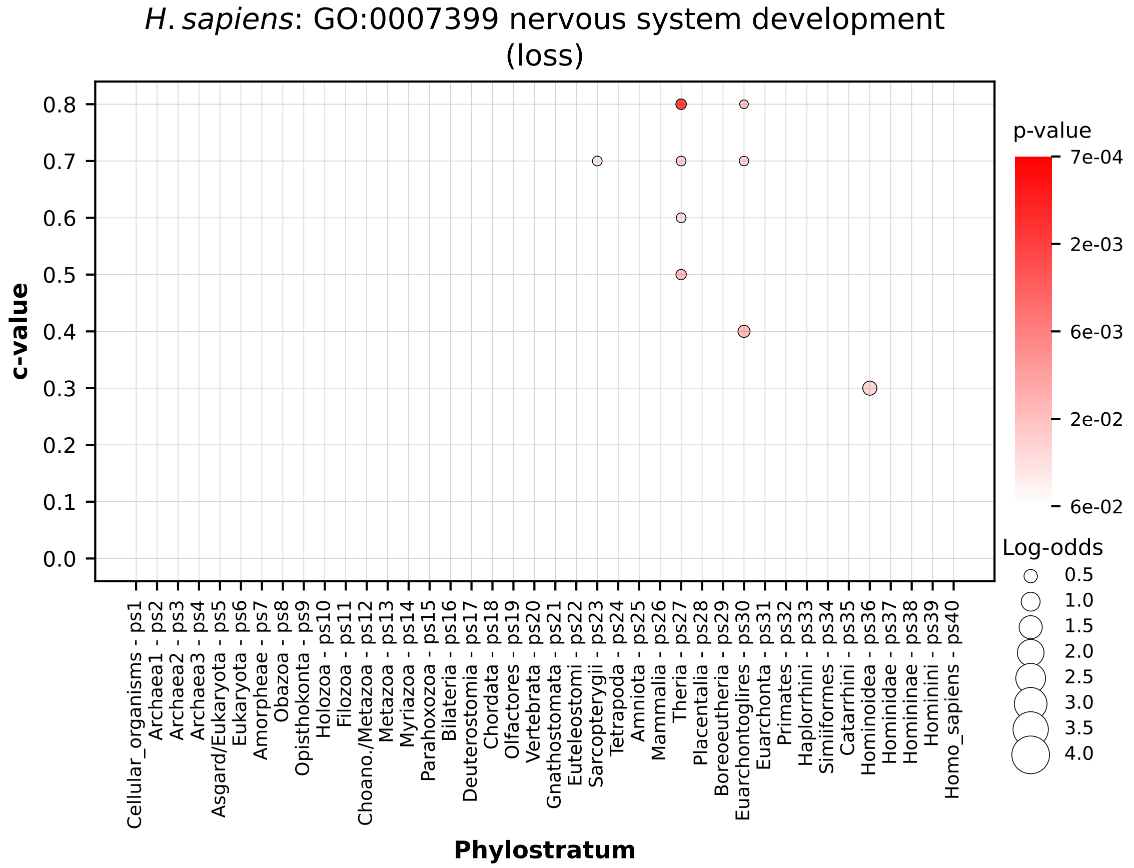

H. sapiens: GO:0007399 nervous system development (loss)
c-value
0.8
0.7
0.6
0.5
0.4
0.3
0.2
0.1
0.0
Phylostratum
Cellular_organisms - ps1
Archaea1 - ps2
Archaea2 - ps3
Archaea3 - ps4
Asgard/Eukaryota - ps5
Eukaryota - ps6
Amorpheae - ps7
Obazoa - ps8
Opisthokonta - ps9
Holozoa - ps10
Filozoa - ps11
Choano./Metazoa - ps12
Metazoa - ps13
Myriazoa - ps14
Parahoxozoa - ps15
Bilateria - ps16
Deuterostomia - ps17
Chordata - ps18
Olfactores - ps19
Vertebrata - ps20
Gnathostomata - ps21
Euteleostomi - ps22
Sarcopterygii - ps23
Tetrapoda - ps24
Amniota - ps25
Mammalia - ps26
Theria - ps27
Placentalia - ps28
Boreoeutheria - ps29
Euarchontoglires - ps30
Euarchonta - ps31
Primates - ps32
Haplorrhini - ps33
Simiiformes - ps34
Catarrhini - ps35
Hominoidea - ps36
Hominidae - ps37
Homininae - ps38
Hominini - ps39
Homo_sapiens - ps40
p-value
7e-04
2e-03
6e-03
2e-02
6e-02
Log-odds
0.5
1.0
1.5
2.0
2.5
3.0
3.5
4.0

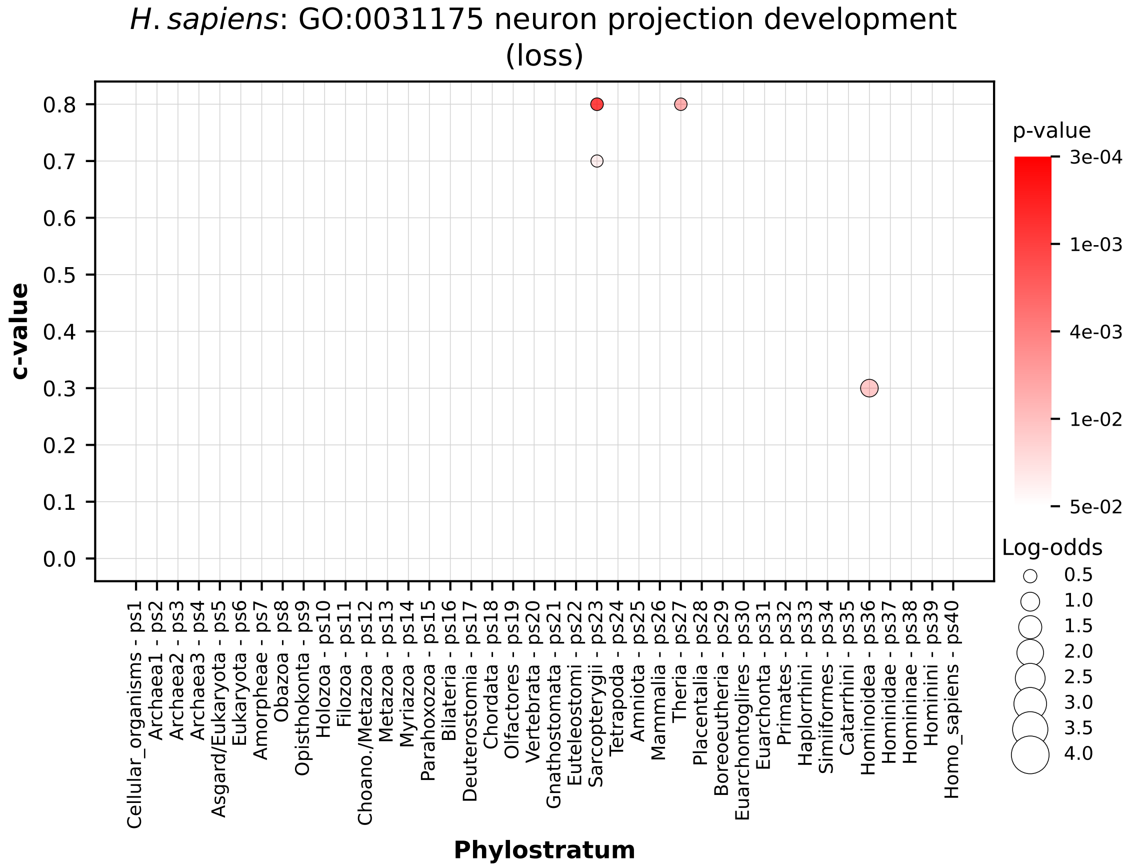

H. sapiens: GO:0031175 neuron projection development (loss)
c-value
0.0
0.1
0.2
0.3
0.4
0.5
0.6
0.7
0.8
Phylostratum
Cellular_organisms - ps1
Archaea1 - ps2
Archaea2 - ps3
Archaea3 - ps4
Asgard/Eukaryota - ps5
Eukaryota - ps6
Amorpheae - ps7
Obazoa - ps8
Opisthokonta - ps9
Holozoa - ps10
Filozoa - ps11
Choano./Metazoa - ps12
Metazoa - ps13
Myriazoa - ps14
Parahoxozoa - ps15
Bilateria - ps16
Deuterostomia - ps17
Chordata - ps18
Olfactores - ps19
Vertebrata - ps20
Gnathostomata - ps21
Euteleostomi - ps22
Sarcopterygii - ps23
Tetrapoda - ps24
Amniota - ps25
Mammalia - ps26
Theria - ps27
Placentalia - ps28
Boreoeutheria - ps29
Euarchontoglires - ps30
Euarchonta - ps31
Primates - ps32
Haplorrhini - ps33
Simiiformes - ps34
Catarrhini - ps35
Hominoidea - ps36
Hominidae - ps37
Homininae - ps38
Hominini - ps39
Homo_sapiens - ps40
p-value
3e-04
1e-03
4e-03
1e-02
5e-02
Log-odds
0.5
1.0
1.5
2.0
2.5
3.0
3.5
4.0

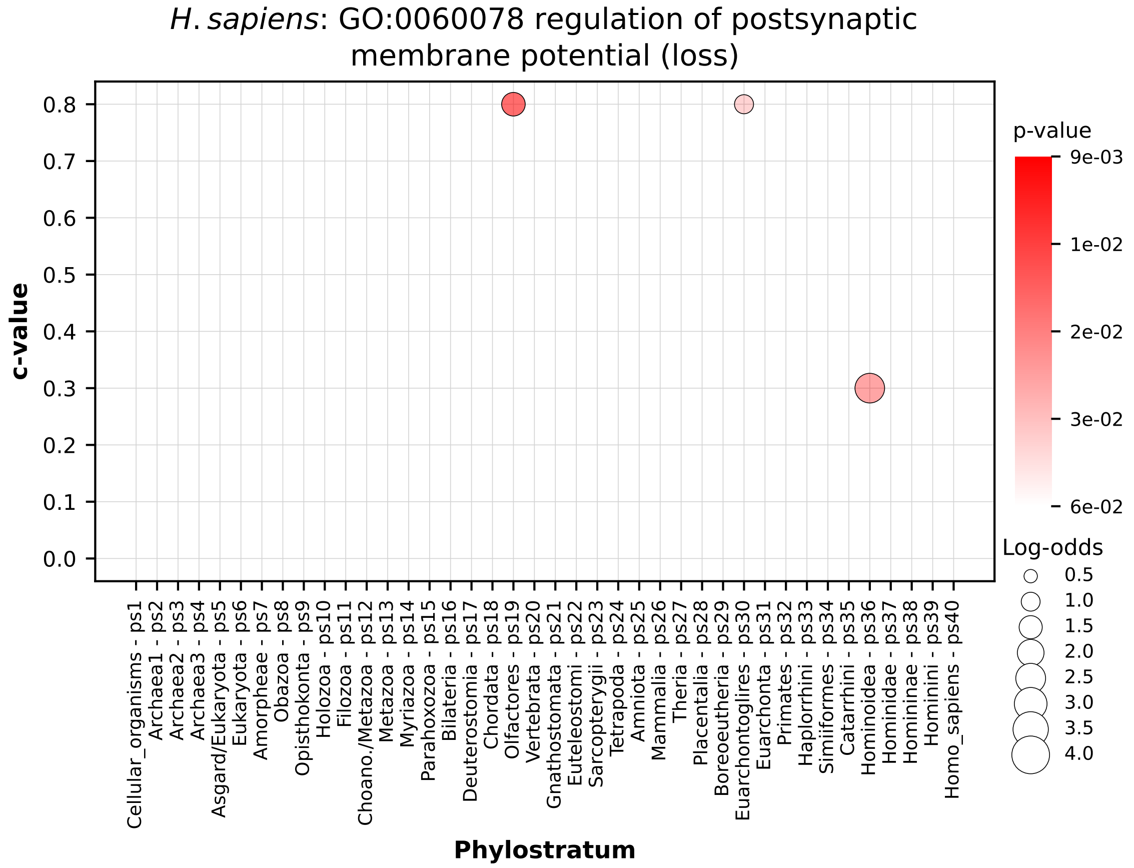
H. sapiens: GO:0060078 regulation of postsynaptic membrane potential (loss)
c-value
0.0
0.1
0.2
0.3
0.4
0.5
0.6
0.7
0.8
p-value
9e-03
1e-02
2e-02
3e-02
6e-02
Log-odds
0.5
1.0
1.5
2.0
2.5
3.0
3.5
4.0
Cellular_organisms - ps1
Archaea1 - ps2
Archaea2 - ps3
Archaea3 - ps4
Asgard/Eukaryota - ps5
Eukaryota - ps6
Amorpheae - ps7
Obazoa - ps8
Opisthokonta - ps9
Holozoa - ps10
Filozoa - ps11
Choano./Metazoa - ps12
Metazoa - ps13
Myriazoa - ps14
Parahoxozoa - ps15
Bilateria - ps16
Deuterostomia - ps17
Chordata - ps18
Olfactores - ps19
Vertebrata - ps20
Gnathostomata - ps21
Euteleostomi - ps22
Sarcopterygii - ps23
Tetrapoda - ps24
Amniota - ps25
Mammalia - ps26
Theria - ps27
Placentalia - ps28
Boreoeutheria - ps29
Euarchontoglires - ps30
Euarchonta - ps31
Primates - ps32
Haplorrhini - ps33
Simiiformes - ps34
Catarrhini - ps35
Hominoidea - ps36
Hominidae - ps37
Homininae - ps38
Hominini - ps39
Homo_sapiens - ps40
Phylostratum

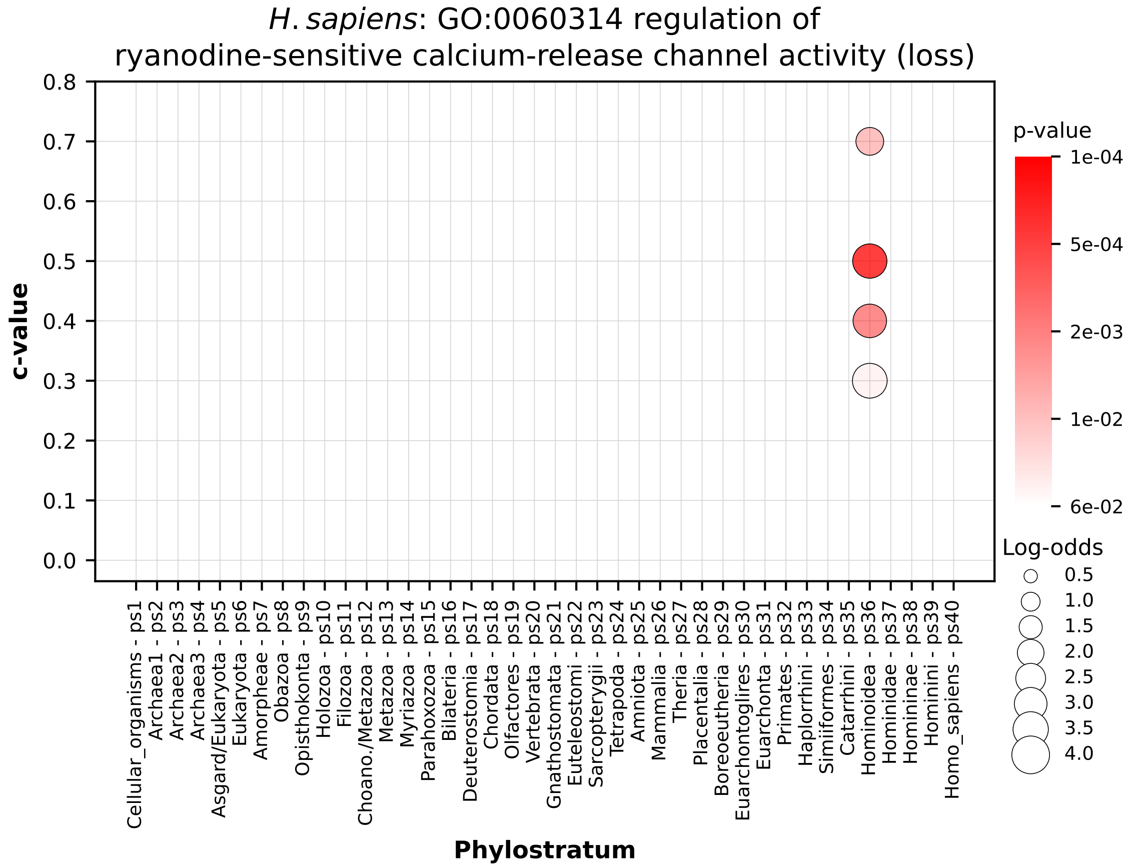

H. sapiens: GO:0060314 regulation of ryanodine-sensitive calcium-release channel activity (loss)
c-value
0.8
0.7
0.6
0.5
0.4
0.3
0.2
0.1
0.0
Phylostratum
Cellular_organisms - ps1
Archaea1 - ps2
Archaea2 - ps3
Archaea3 - ps4
Asgard/Eukaryota - ps5
Eukaryota - ps6
Amorpheae - ps7
Obazoa - ps8
Opisthokonta - ps9
Holozoa - ps10
Filozoa - ps11
Choano./Metazoa - ps12
Metazoa - ps13
Myriazoa - ps14
Parahoxozoa - ps15
Bilateria - ps16
Deuterostomia - ps17
Chordata - ps18
Olfactores - ps19
Vertebrata - ps20
Gnathostomata - ps21
Euteleostomi - ps22
Sarcopterygii - ps23
Tetrapoda - ps24
Amniota - ps25
Mammalia - ps26
Theria - ps27
Placentalia - ps28
Boreoeutheria - ps29
Euarchontoglires - ps30
Euarchonta - ps31
Primates - ps32
Haplorrhini - ps33
Simiiformes - ps34
Catarrhini - ps35
Hominoidea - ps36
Hominidae - ps37
Homininae - ps38
Hominini - ps39
Homo_sapiens - ps40
p-value
1e-04
5e-04
2e-03
1e-02
6e-02
Log-odds
0.5
1.0
1.5
2.0
2.5
3.0
3.5
4.0

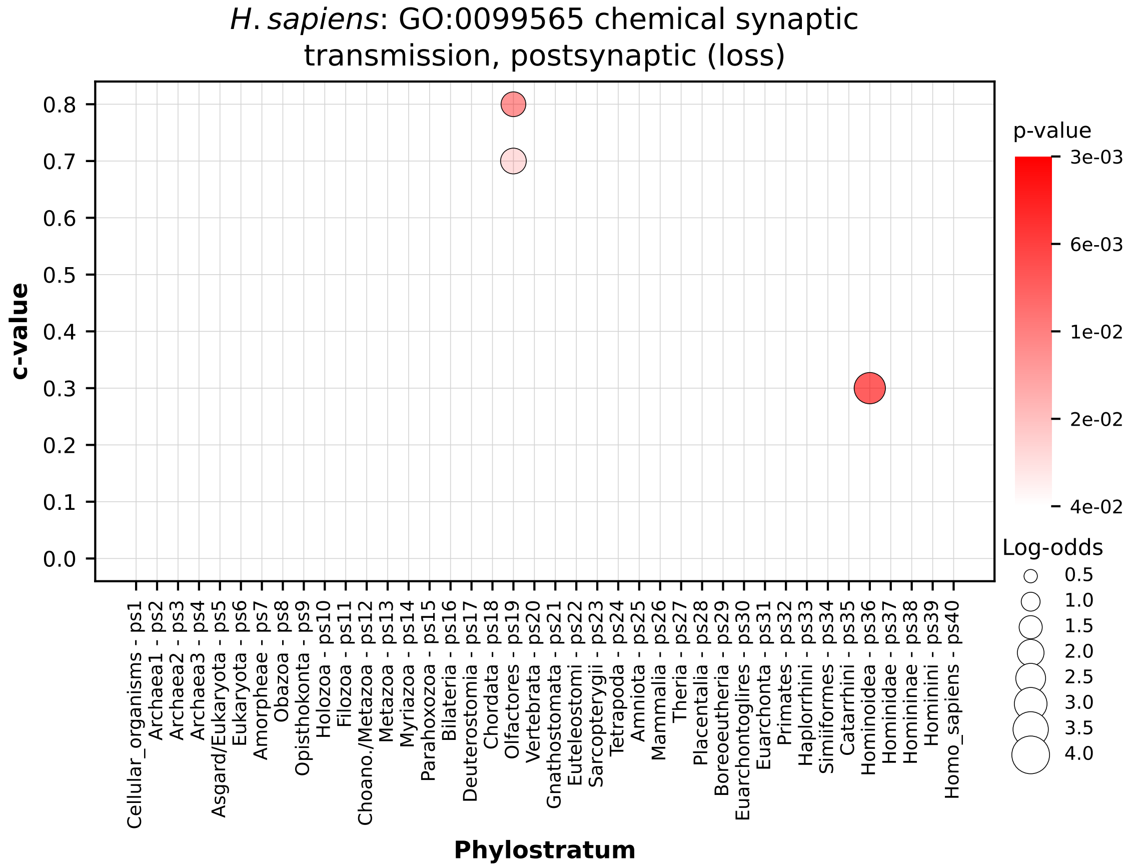

$H. sapiens$: GO:0099565 chemical synaptic transmission, postsynaptic (loss)
c-value
0.0
0.1
0.2
0.3
0.4
0.5
0.6
0.7
0.8
Phylostratum
Cellular_organisms - ps1
Archaea1 - ps2
Archaea2 - ps3
Archaea3 - ps4
Asgard/Eukaryota - ps5
Eukaryota - ps6
Amorpheae - ps7
Obazoa - ps8
Opisthokonta - ps9
Holozoa - ps10
Filozoa - ps11
Choano./Metazoa - ps12
Metazoa - ps13
Myriazoa - ps14
Parahoxozoa - ps15
Bilateria - ps16
Deuterostomia - ps17
Chordata - ps18
Olfactores - ps19
Vertebrata - ps20
Gnathostomata - ps21
Euteleostomi - ps22
Sarcopterygii - ps23
Tetrapoda - ps24
Amniota - ps25
Mammalia - ps26
Theria - ps27
Placentalia - ps28
Boreoeutheria - ps29
Euarchontoglires - ps30
Euarchonta - ps31
Primates - ps32
Haplorrhini - ps33
Simiiformes - ps34
Catarrhini - ps35
Hominoidea - ps36
Hominidae - ps37
Homininae - ps38
Hominini - ps39
Homo_sapiens - ps40
p-value
3e-03
6e-03
1e-02
2e-02
4e-02
Log-odds
0.5
1.0
1.5
2.0
2.5
3.0
3.5
4.0

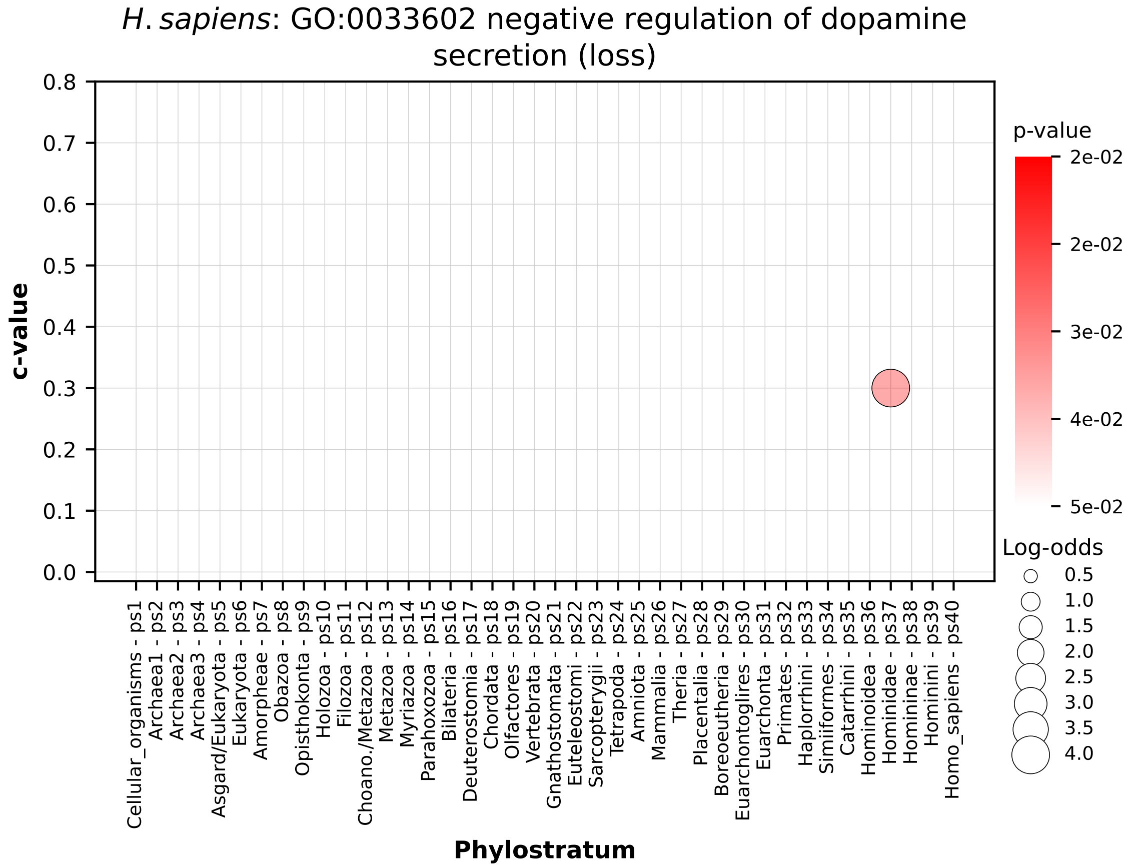
H. sapiens: GO:0033602 negative regulation of dopamine secretion (loss)
c-value
0.0
0.1
0.2
0.3
0.4
0.5
0.6
0.7
0.8
Phylostratum
Cellular_organisms - ps1
Archaea1 - ps2
Archaea2 - ps3
Archaea3 - ps4
Asgard/Eukaryota - ps5
Eukaryota - ps6
Amorpheae - ps7
Obazoa - ps8
Opisthokonta - ps9
Holozoa - ps10
Filozoa - ps11
Choano./Metazoa - ps12
Metazoa - ps13
Myriazoa - ps14
Parahoxozoa - ps15
Bilateria - ps16
Deuterostomia - ps17
Chordata - ps18
Olfactores - ps19
Vertebrata - ps20
Gnathostomata - ps21
Euteleostomi - ps22
Sarcopterygii - ps23
Tetrapoda - ps24
Amniota - ps25
Mammalia - ps26
Theria - ps27
Placentalia - ps28
Boreoeutheria - ps29
Euarchontoglires - ps30
Euarchonta - ps31
Primates - ps32
Haplorrhini - ps33
Simiiformes - ps34
Catarrhini - ps35
Hominoidea - ps36
Hominidae - ps37
Homininae - ps38
Hominini - ps39
Homo_sapiens - ps40
p-value
2e-02
2e-02
3e-02
4e-02
5e-02
Log-odds
0.5
1.0
1.5
2.0
2.5
3.0
3.5
4.0

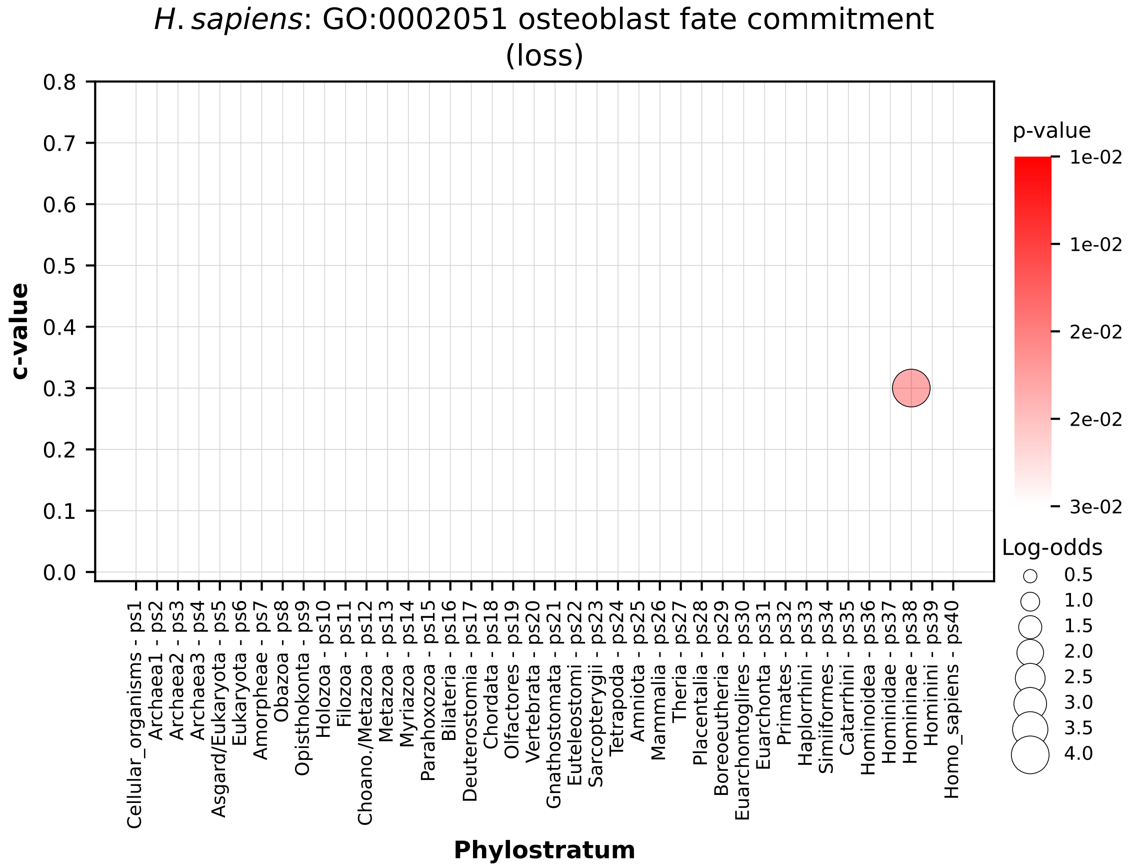
H. sapiens: GO:0002051 osteoblast fate commitment (loss)
c-value
Phylostratum
p-value
Log-odds
Cellular_organisms - ps1
Archaea1 - ps2
Archaea2 - ps3
Archaea3 - ps4
Asgard/Eukaryota - ps5
Eukaryota - ps6
Amorpheae - ps7
Obazoa - ps8
Opisthokonta - ps9
Holozoa - ps10
Filozoa - ps11
Choano./Metazoa - ps12
Metazoa - ps13
Myriazoa - ps14
Parahoxozoa - ps15
Bilateria - ps16
Deuterostomia - ps17
Chordata - ps18
Olfactores - ps19
Vertebrata - ps20
Gnathostomata - ps21
Euteleostomi - ps22
Sarcopterygii - ps23
Tetrapoda - ps24
Amniota - ps25
Mammalia - ps26
Theria - ps27
Placentalia - ps28
Boreoeutheria - ps29
Euarchontoglires - ps30
Euarchonta - ps31
Primates - ps32
Haplorrhini - ps33
Simiiformes - ps34
Catarrhini - ps35
Hominoidea - ps36
Hominidae - ps37
Homininae - ps38
Hominini - ps39
Homo_sapiens - ps40

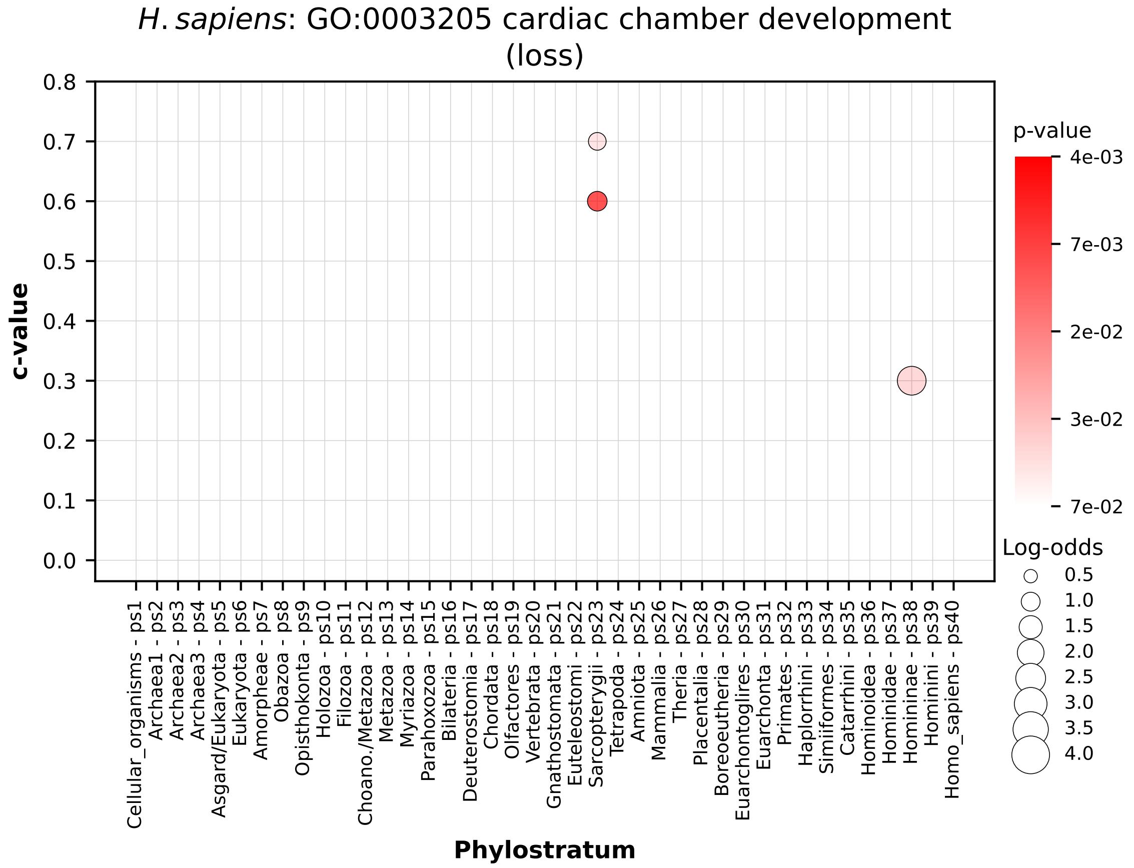

H. sapiens: GO:0003205 cardiac chamber development
(loss)
c-value
0.8
0.7
0.6
0.5
0.4
0.3
0.2
0.1
0.0
Phylostratum
Cellular_organisms - ps1
Archaea1 - ps2
Archaea2 - ps3
Archaea3 - ps4
Asgard/Eukaryota - ps5
Eukaryota - ps6
Amorpheae - ps7
Obazoa - ps8
Opisthokonta - ps9
Holozoa - ps10
Filozoa - ps11
Choano./Metazoa - ps12
Metazoa - ps13
Myriazoa - ps14
Parahoxozoa - ps15
Bilateria - ps16
Deuterostomia - ps17
Chordata - ps18
Olfactores - ps19
Vertebrata - ps20
Gnathostomata - ps21
Euteleostomi - ps22
Sarcopterygii - ps23
Tetrapoda - ps24
Amniota - ps25
Mammalia - ps26
Theria - ps27
Placentalia - ps28
Boreoeutheria - ps29
Euarchontoglires - ps30
Euarchonta - ps31
Primates - ps32
Haplorrhini - ps33
Simiiformes - ps34
Catarrhini - ps35
Hominoidea - ps36
Hominidae - ps37
Homininae - ps38
Hominini - ps39
Homo_sapiens - ps40
p-value
4e-03
7e-03
2e-02
3e-02
7e-02
Log-odds
0.5
1.0
1.5
2.0
2.5
3.0
3.5
4.0

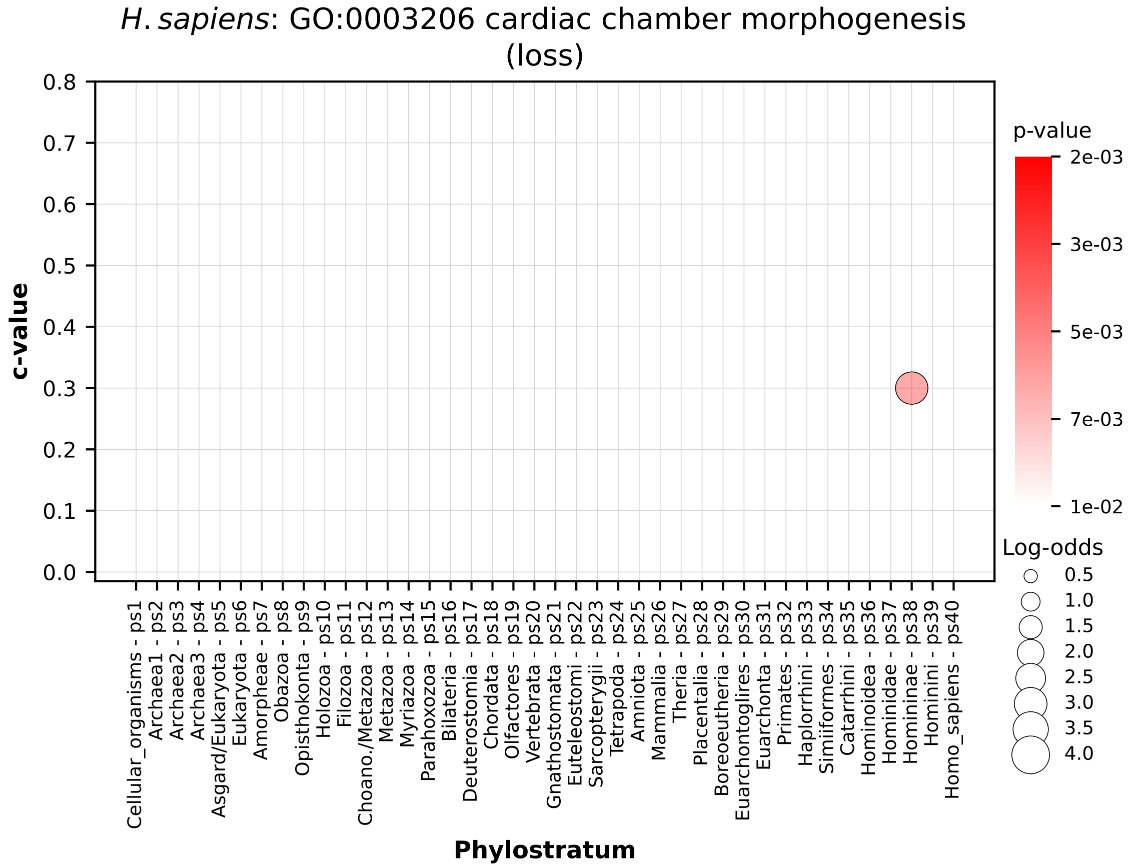
H. sapiens: GO:0003206 cardiac chamber morphogenesis
(loss)
c-value
0.8
0.7
0.6
0.5
0.4
0.3
0.2
0.1
0.0
Phylostratum
Cellular_organisms - ps1
Archaea1 - ps2
Archaea2 - ps3
Archaea3 - ps4
Asgard/Eukaryota - ps5
Eukaryota - ps6
Amorpheae - ps7
Obazoa - ps8
Opisthokonta - ps9
Holozoa - ps10
Filozoa - ps11
Choano./Metazoa - ps12
Metazoa - ps13
Myriazoa - ps14
Parahoxozoa - ps15
Bilateria - ps16
Deuterostomia - ps17
Chordata - ps18
Olfactores - ps19
Vertebrata - ps20
Gnathostomata - ps21
Euteleostomi - ps22
Sarcopterygii - ps23
Tetrapoda - ps24
Amniota - ps25
Mammalia - ps26
Theria - ps27
Placentalia - ps28
Boreoeutheria - ps29
Euarchontoglires - ps30
Euarchonta - ps31
Primates - ps32
Haplorrhini - ps33
Simiiformes - ps34
Catarrhini - ps35
Hominoidea - ps36
Hominidae - ps37
Homininae - ps38
Hominini - ps39
Homo_sapiens - ps40
p-value
2e-03
3e-03
5e-03
7e-03
1e-02
Log-odds
0.5
1.0
1.5
2.0
2.5
3.0
3.5
4.0

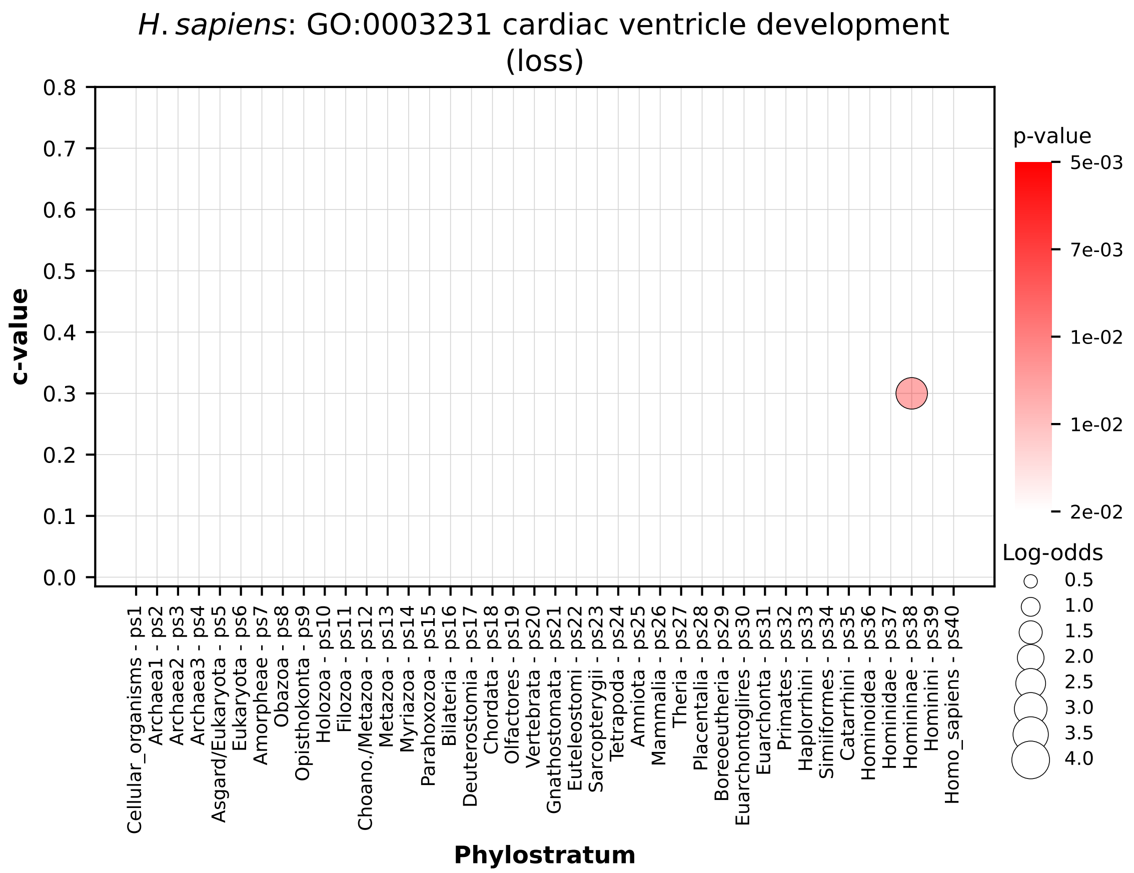
H. sapiens: GO:0003231 cardiac ventricle development
(loss)
c-value
0.8
0.7
0.6
0.5
0.4
0.3
0.2
0.1
0.0
Phylostratum
Cellular_organisms - ps1
Archaea1 - ps2
Archaea2 - ps3
Archaea3 - ps4
Asgard/Eukaryota - ps5
Eukaryota - ps6
Amorpheae - ps7
Obazoa - ps8
Opisthokonta - ps9
Holozoa - ps10
Filozoa - ps11
Choano./Metazoa - ps12
Metazoa - ps13
Myriazoa - ps14
Parahoxozoa - ps15
Bilateria - ps16
Deuterostomia - ps17
Chordata - ps18
Olfactores - ps19
Vertebrata - ps20
Gnathostomata - ps21
Euteleostomi - ps22
Sarcopterygii - ps23
Tetrapoda - ps24
Amniota - ps25
Mammalia - ps26
Theria - ps27
Placentalia - ps28
Boreoeutheria - ps29
Euarchontoglires - ps30
Euarchonta - ps31
Primates - ps32
Haplorrhini - ps33
Simiiformes - ps34
Catarrhini - ps35
Hominoidea - ps36
Hominidae - ps37
Homininae - ps38
Hominini - ps39
Homo_sapiens - ps40
p-value
5e-03
7e-03
1e-02
1e-02
2e-02
Log-odds
0.5
1.0
1.5
2.0
2.5
3.0
3.5
4.0

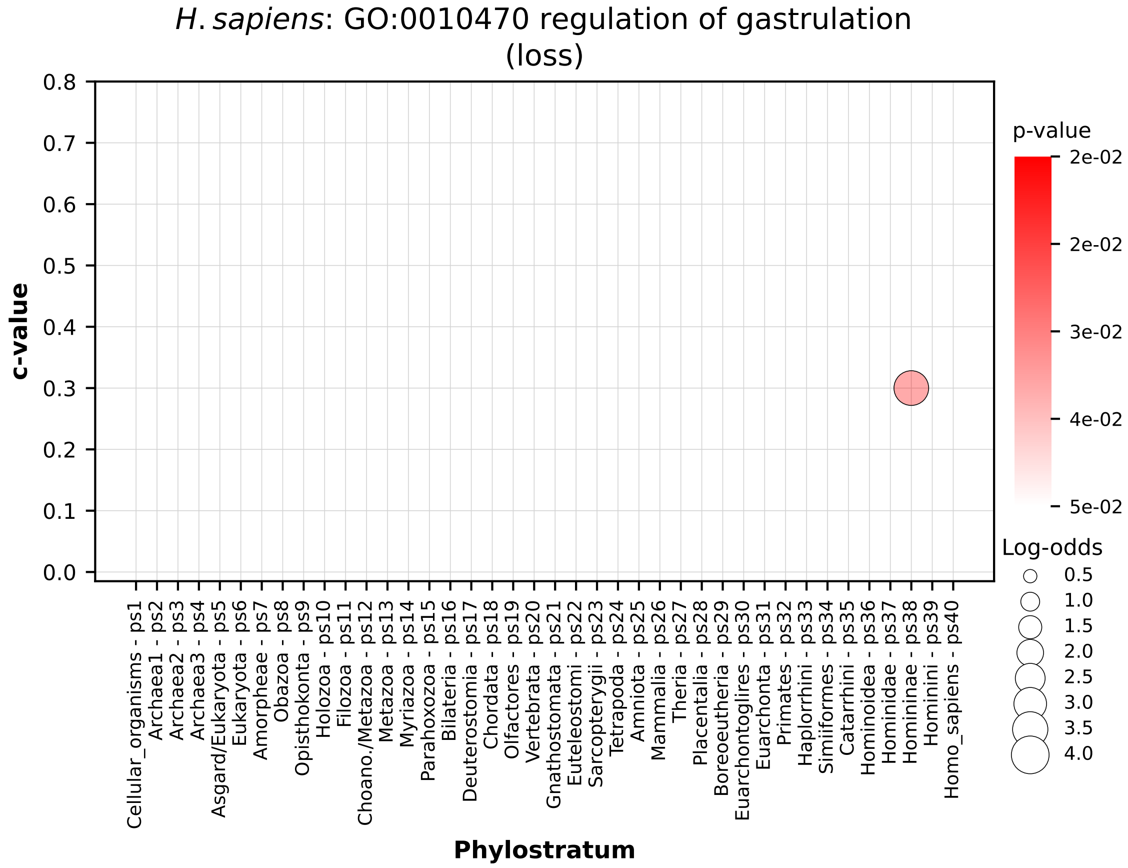

H. sapiens: GO:0010470 regulation of gastrulation
(loss)
c-value
0.8
0.7
0.6
0.5
0.4
0.3
0.2
0.1
0.0
p-value
2e-02
2e-02
3e-02
4e-02
5e-02
Log-odds
0.5
1.0
1.5
2.0
2.5
3.0
3.5
4.0
Cellular_organisms - ps1
Archaea1 - ps2
Archaea2 - ps3
Archaea3 - ps4
Asgard/Eukaryota - ps5
Eukaryota - ps6
Amorpheae - ps7
Obazoa - ps8
Opisthokonta - ps9
Holozoa - ps10
Filozoa - ps11
Choano./Metazoa - ps12
Metazoa - ps13
Myriazoa - ps14
Parahoxozoa - ps15
Bilateria - ps16
Deuterostomia - ps17
Chordata - ps18
Olfactores - ps19
Vertebrata - ps20
Gnathostomata - ps21
Euteleostomi - ps22
Sarcopterygii - ps23
Tetrapoda - ps24
Amniota - ps25
Mammalia - ps26
Theria - ps27
Placentalia - ps28
Boreoeutheria - ps29
Euarchontoglires - ps30
Euarchonta - ps31
Primates - ps32
Haplorrhini - ps33
Simiiformes - ps34
Catarrhini - ps35
Hominoidea - ps36
Hominidae - ps37
Homininae - ps38
Hominini - ps39
Homo_sapiens - ps40
Phylostratum

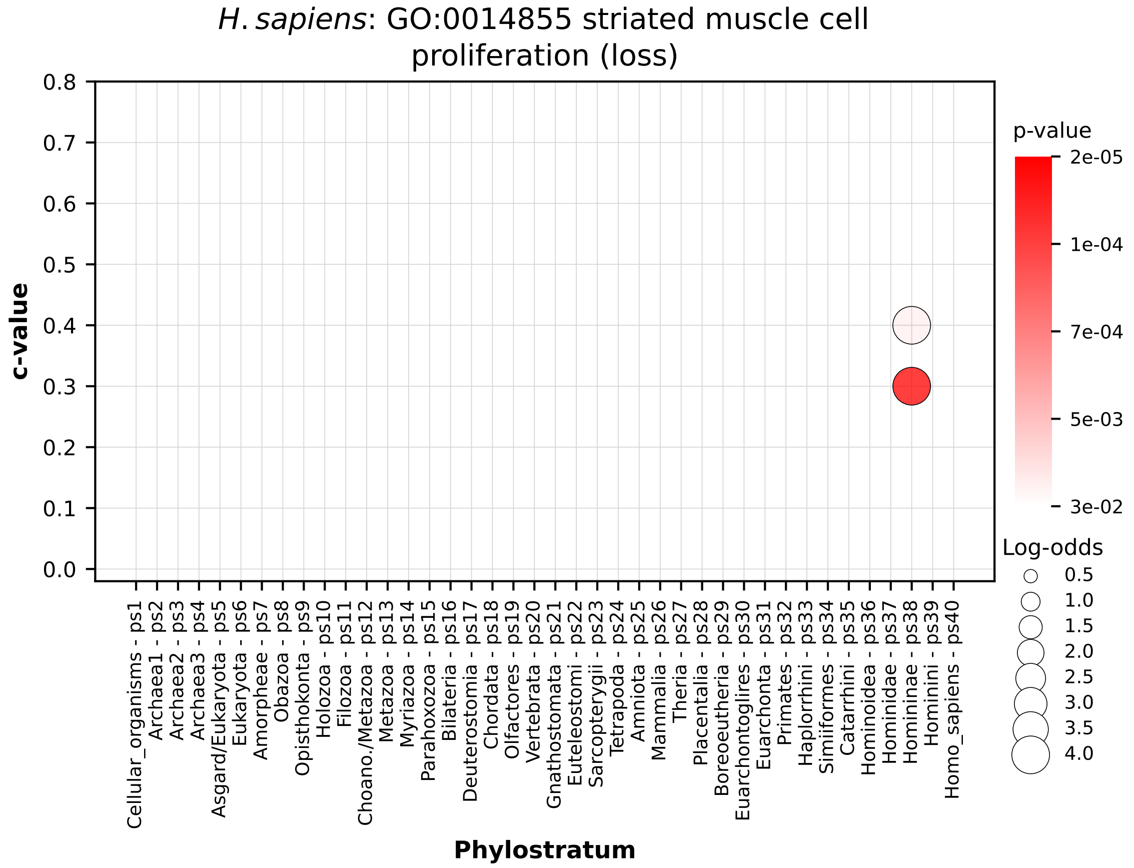

H. sapiens: GO:0014855 striated muscle cell proliferation (loss)
c-value
0.0
0.1
0.2
0.3
0.4
0.5
0.6
0.7
0.8
Phylostratum
Cellular_organisms - ps1
Archaea1 - ps2
Archaea2 - ps3
Archaea3 - ps4
Asgard/Eukaryota - ps5
Eukaryota - ps6
Amorpheae - ps7
Obazoa - ps8
Opisthokonta - ps9
Holozoa - ps10
Filozoa - ps11
Choano./Metazoa - ps12
Metazoa - ps13
Myriazoa - ps14
Parahoxozoa - ps15
Bilateria - ps16
Deuterostomia - ps17
Chordata - ps18
Olfactores - ps19
Vertebrata - ps20
Gnathostomata - ps21
Euteleostomi - ps22
Sarcopterygii - ps23
Tetrapoda - ps24
Amniota - ps25
Mammalia - ps26
Theria - ps27
Placentalia - ps28
Boreoeutheria - ps29
Euarchontoglires - ps30
Euarchonta - ps31
Primates - ps32
Haplorrhini - ps33
Simiiformes - ps34
Catarrhini - ps35
Hominoidea - ps36
Hominidae - ps37
Homininae - ps38
Hominini - ps39
Homo_sapiens - ps40
p-value
2e-05
1e-04
7e-04
5e-03
3e-02
Log-odds
0.5
1.0
1.5
2.0
2.5
3.0
3.5
4.0

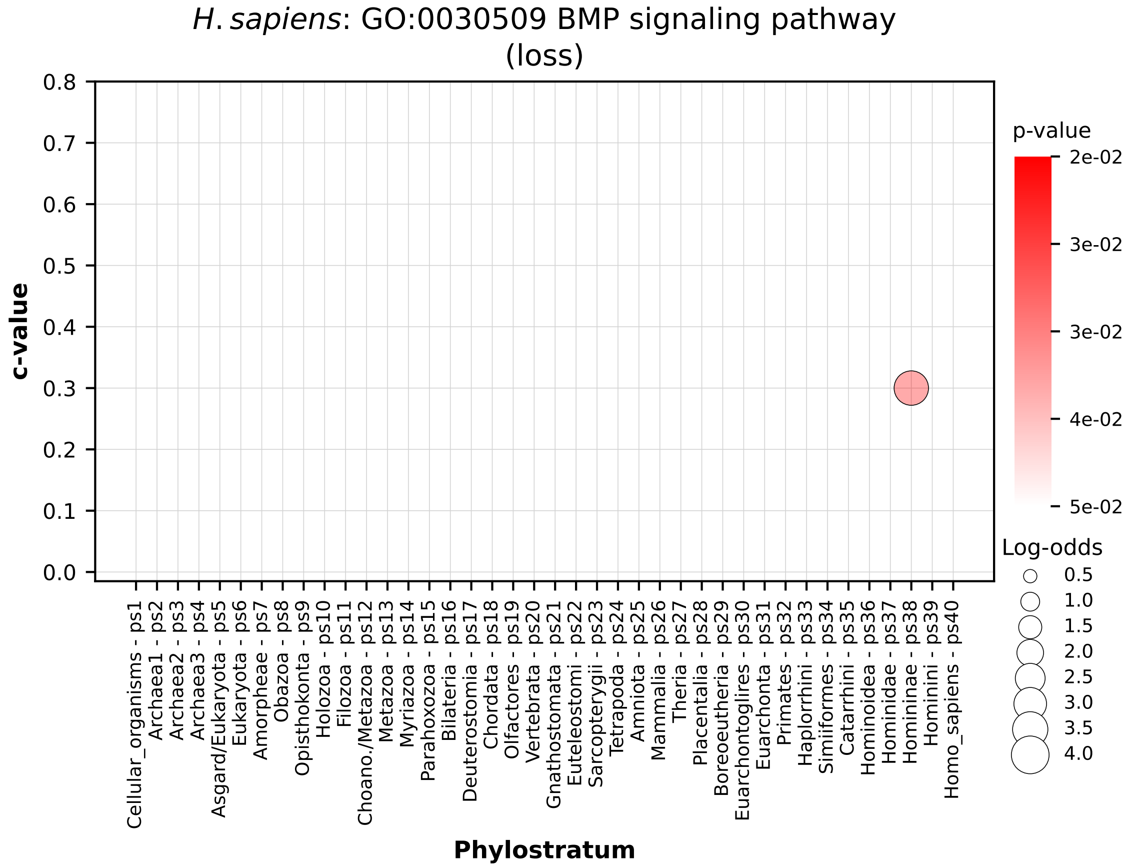

H. sapiens: GO:0030509 BMP signaling pathway
(loss)
c-value
0.8
0.7
0.6
0.5
0.4
0.3
0.2
0.1
0.0
p-value
2e-02
3e-02
3e-02
4e-02
5e-02
Log-odds
0.5
1.0
1.5
2.0
2.5
3.0
3.5
4.0
Cellular_organisms - ps1
Archaea1 - ps2
Archaea2 - ps3
Archaea3 - ps4
Asgard/Eukaryota - ps5
Eukaryota - ps6
Amorpheae - ps7
Obazoa - ps8
Opisthokonta - ps9
Holozoa - ps10
Filozoa - ps11
Choano./Metazoa - ps12
Metazoa - ps13
Myriazoa - ps14
Parahoxozoa - ps15
Bilateria - ps16
Deuterostomia - ps17
Chordata - ps18
Olfactores - ps19
Vertebrata - ps20
Gnathostomata - ps21
Euteleostomi - ps22
Sarcopterygii - ps23
Tetrapoda - ps24
Amniota - ps25
Mammalia - ps26
Theria - ps27
Placentalia - ps28
Boreoeutheria - ps29
Euarchontoglires - ps30
Euarchonta - ps31
Primates - ps32
Haplorrhini - ps33
Simiiformes - ps34
Catarrhini - ps35
Hominoidea - ps36
Hominidae - ps37
Homininae - ps38
Hominini - ps39
Homo_sapiens - ps40
Phylostratum

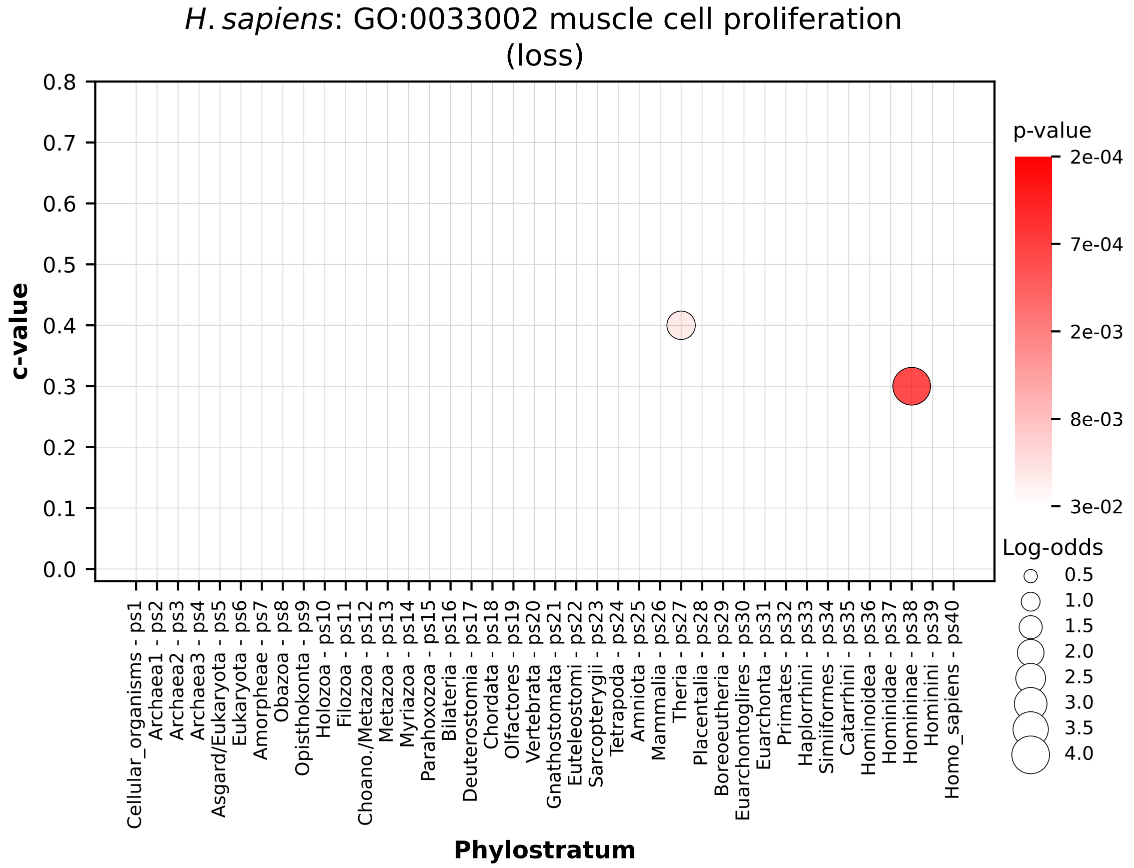
H. sapiens: GO:0033002 muscle cell proliferation (loss)
c-value
0.8
0.7
0.6
0.5
0.4
0.3
0.2
0.1
0.0
p-value
2e-04
7e-04
2e-03
8e-03
3e-02
Log-odds
0.5
1.0
1.5
2.0
2.5
3.0
3.5
4.0
Cellular_organisms - ps1
Archaea1 - ps2
Archaea2 - ps3
Archaea3 - ps4
Asgard/Eukaryota - ps5
Eukaryota - ps6
Amorpheae - ps7
Obazoa - ps8
Opisthokonta - ps9
Holozoa - ps10
Filozoa - ps11
Choano./Metazoa - ps12
Metazoa - ps13
Myriazoa - ps14
Parahoxozoa - ps15
Bilateria - ps16
Deuterostomia - ps17
Chordata - ps18
Olfactores - ps19
Vertebrata - ps20
Gnathostomata - ps21
Euteleostomi - ps22
Sarcopterygii - ps23
Tetrapoda - ps24
Amniota - ps25
Mammalia - ps26
Theria - ps27
Placentalia - ps28
Boreoeutheria - ps29
Euarchontoglires - ps30
Euarchonta - ps31
Primates - ps32
Haplorrhini - ps33
Simiiformes - ps34
Catarrhini - ps35
Hominoidea - ps36
Hominidae - ps37
Homininae - ps38
Hominini - ps39
Homo_sapiens - ps40
Phylostratum

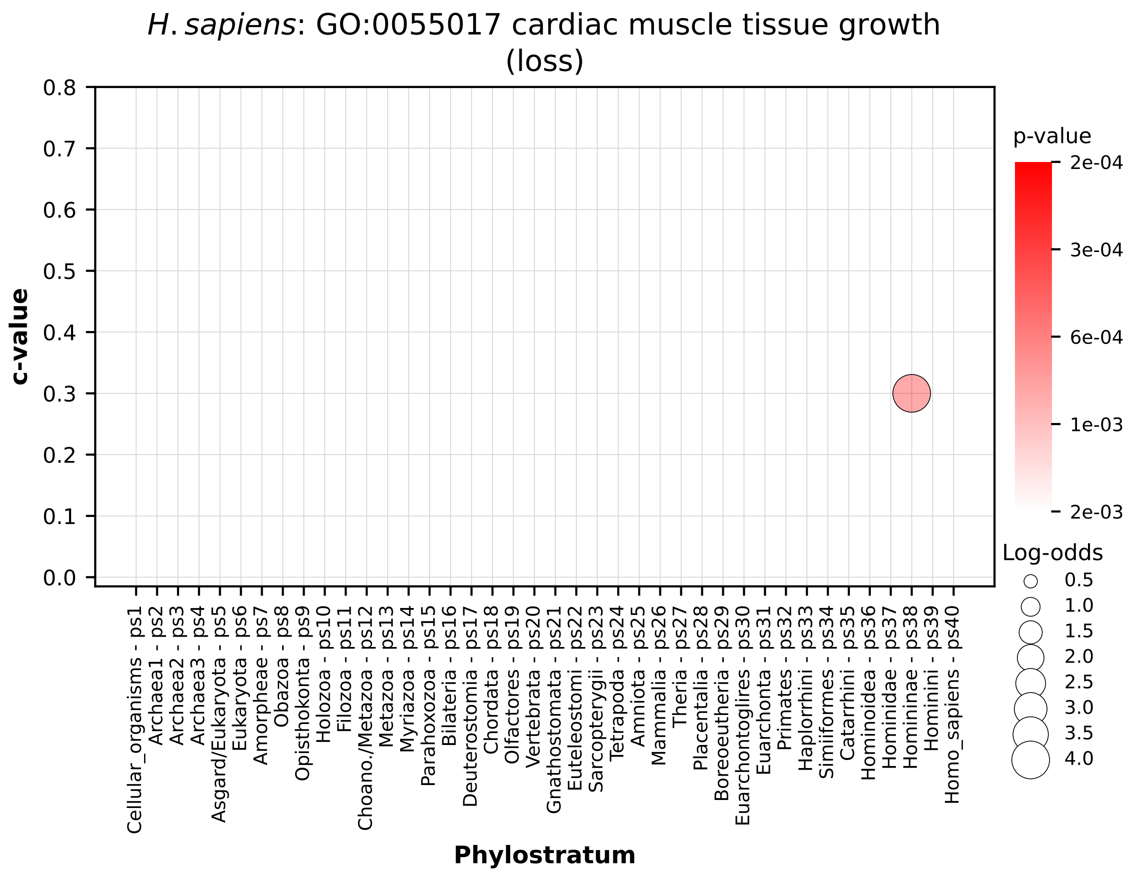

H. sapiens: GO:0055017 cardiac muscle tissue growth (loss)
c-value
0.0
0.1
0.2
0.3
0.4
0.5
0.6
0.7
0.8
Phylostratum
Cellular_organisms - ps1
Archaea1 - ps2
Archaea2 - ps3
Archaea3 - ps4
Asgard/Eukaryota - ps5
Eukaryota - ps6
Amorpheae - ps7
Obazoa - ps8
Opisthokonta - ps9
Holozoa - ps10
Filozoa - ps11
Choano./Metazoa - ps12
Metazoa - ps13
Myriazoa - ps14
Parahoxozoa - ps15
Bilateria - ps16
Deuterostomia - ps17
Chordata - ps18
Olfactores - ps19
Vertebrata - ps20
Gnathostomata - ps21
Euteleostomi - ps22
Sarcopterygii - ps23
Tetrapoda - ps24
Amniota - ps25
Mammalia - ps26
Theria - ps27
Placentalia - ps28
Boreoeutheria - ps29
Euarchontoglires - ps30
Euarchonta - ps31
Primates - ps32
Haplorrhini - ps33
Simiiformes - ps34
Catarrhini - ps35
Hominoidea - ps36
Hominidae - ps37
Homininae - ps38
Hominini - ps39
Homo_sapiens - ps40
p-value
2e-04
3e-04
6e-04
1e-03
2e-03
Log-odds
0.5
1.0
1.5
2.0
2.5
3.0
3.5
4.0

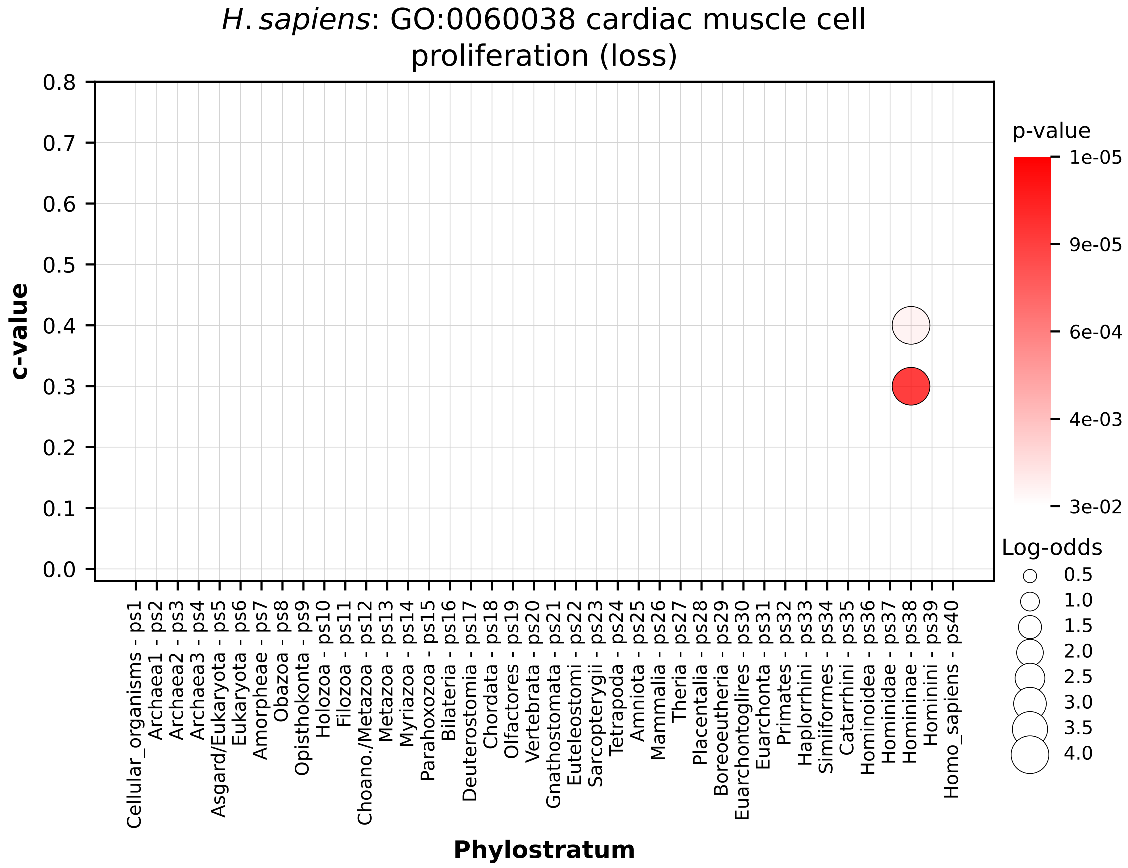

H. sapiens: GO:0060038 cardiac muscle cell proliferation (loss)
c-value
0.8
0.7
0.6
0.5
0.4
0.3
0.2
0.1
0.0
p-value
1e-05
9e-05
6e-04
4e-03
3e-02
Log-odds
0.5
1.0
1.5
2.0
2.5
3.0
3.5
4.0
Cellular_organisms - ps1
Archaea1 - ps2
Archaea2 - ps3
Archaea3 - ps4
Asgard/Eukaryota - ps5
Eukaryota - ps6
Amorpheae - ps7
Obazoa - ps8
Opisthokonta - ps9
Holozoa - ps10
Filozoa - ps11
Choano./Metazoa - ps12
Metazoa - ps13
Myriazoa - ps14
Parahoxozoa - ps15
Bilateria - ps16
Deuterostomia - ps17
Chordata - ps18
Olfactores - ps19
Vertebrata - ps20
Gnathostomata - ps21
Euteleostomi - ps22
Sarcopterygii - ps23
Tetrapoda - ps24
Amniota - ps25
Mammalia - ps26
Theria - ps27
Placentalia - ps28
Boreoeutheria - ps29
Euarchontoglires - ps30
Euarchonta - ps31
Primates - ps32
Haplorrhini - ps33
Simiiformes - ps34
Catarrhini - ps35
Hominoidea - ps36
Hominidae - ps37
Homininae - ps38
Hominini - ps39
Homo_sapiens - ps40
Phylostratum

H. sapiens: GO:0060411 cardiac septum morphogenesis (loss)

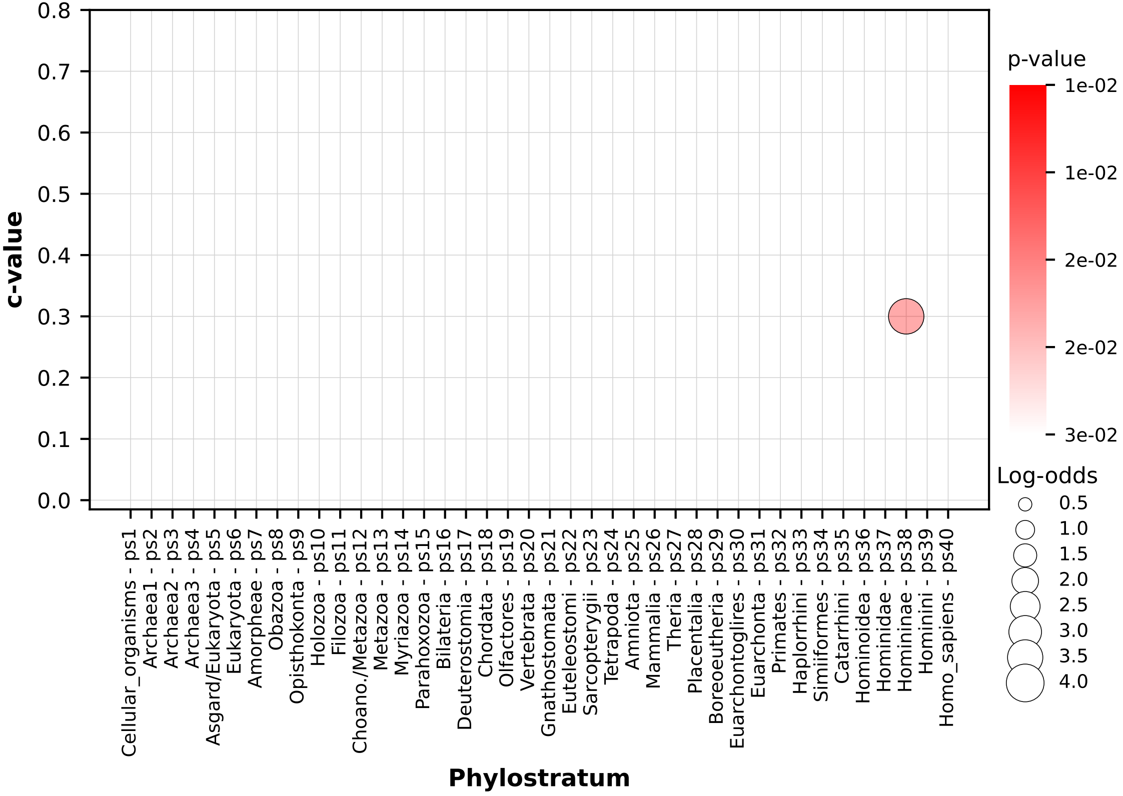

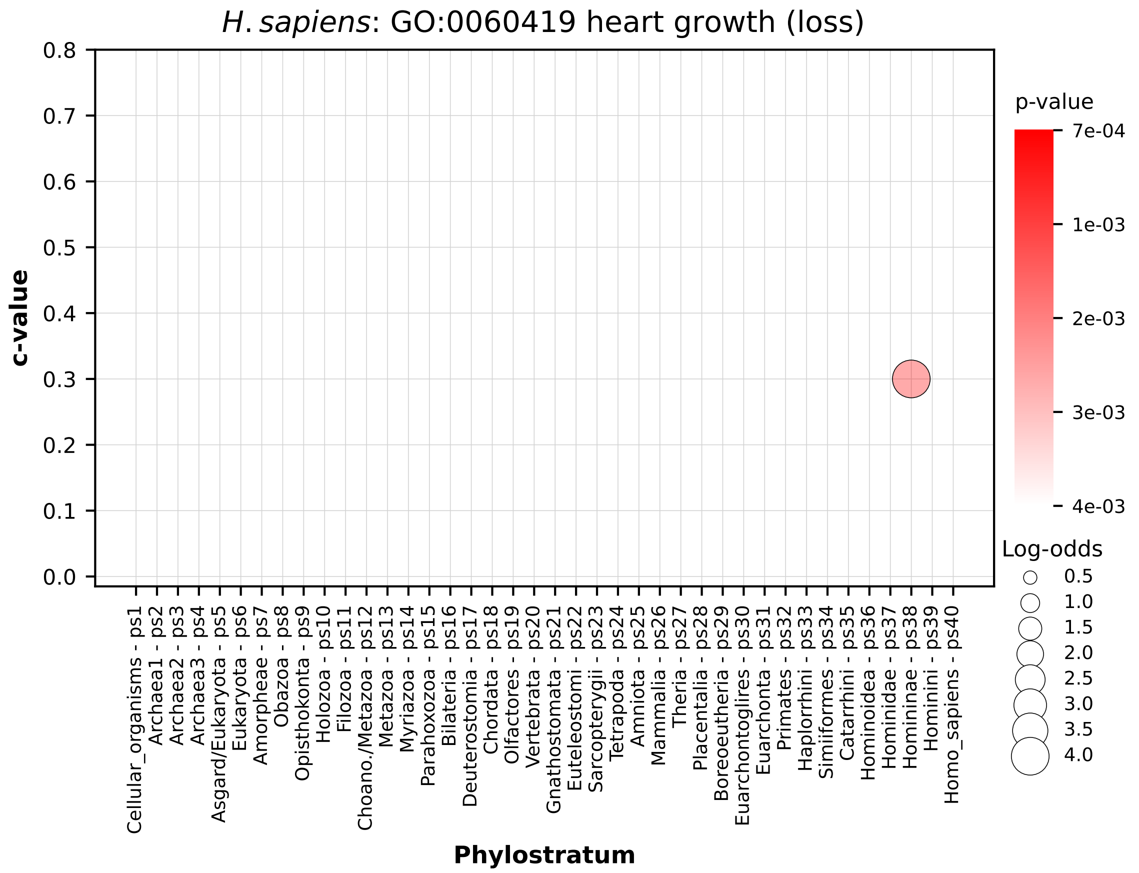

H. sapiens: GO:0060419 heart growth (loss)
c-value
0.0
0.1
0.2
0.3
0.4
0.5
0.6
0.7
0.8
Phylostratum
Cellular_organisms - ps1
Archaea1 - ps2
Archaea2 - ps3
Archaea3 - ps4
Asgard/Eukaryota - ps5
Eukaryota - ps6
Amorpheae - ps7
Obazoa - ps8
Opisthokonta - ps9
Holozoa - ps10
Filozoa - ps11
Choano./Metazoa - ps12
Metazoa - ps13
Myriazoa - ps14
Parahoxozoa - ps15
Bilateria - ps16
Deuterostomia - ps17
Chordata - ps18
Olfactores - ps19
Vertebrata - ps20
Gnathostomata - ps21
Euteleostomi - ps22
Sarcopterygii - ps23
Tetrapoda - ps24
Amniota - ps25
Mammalia - ps26
Theria - ps27
Placentalia - ps28
Boreoeutheria - ps29
Euarchontoglires - ps30
Euarchonta - ps31
Primates - ps32
Haplorrhini - ps33
Simiiformes - ps34
Catarrhini - ps35
Hominoidea - ps36
Hominidae - ps37
Homininae - ps38
Hominini - ps39
Homo_sapiens - ps40
p-value
7e-04
1e-03
2e-03
3e-03
4e-03
Log-odds
0.5
1.0
1.5
2.0
2.5
3.0
3.5
4.0

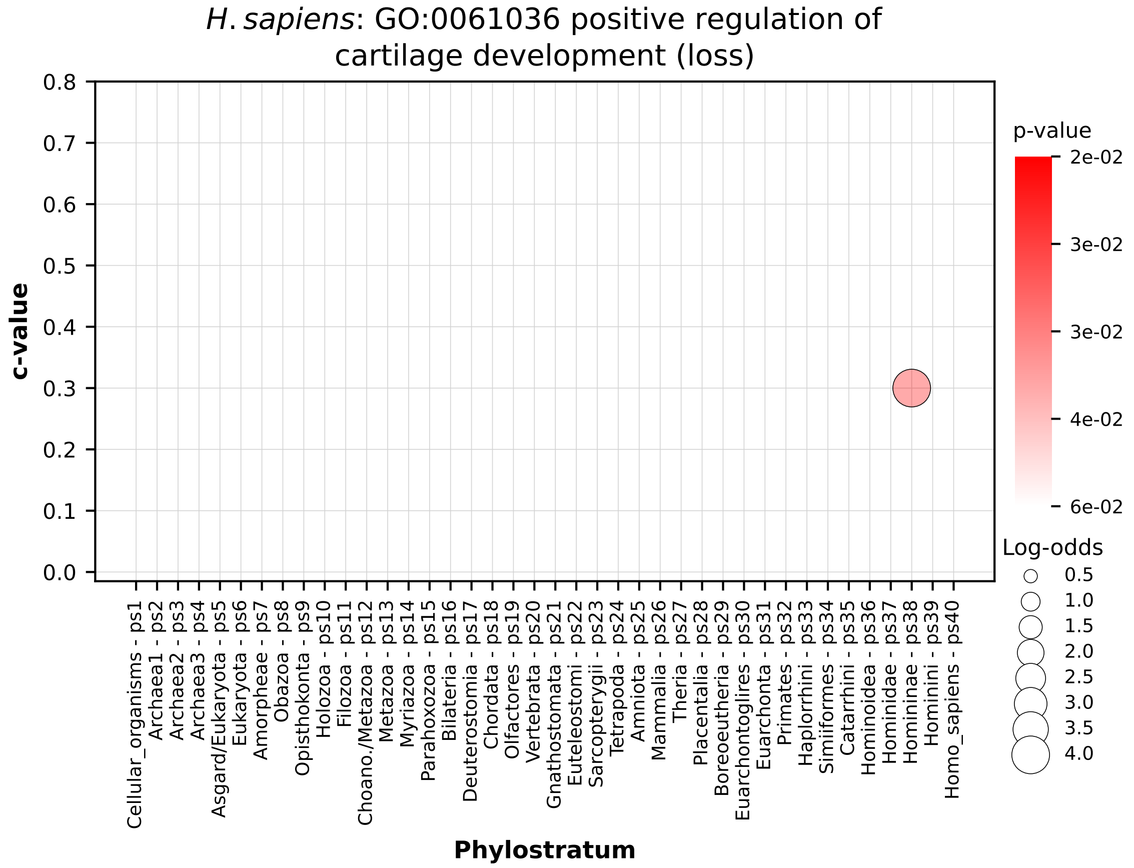

H. sapiens: GO:0061036 positive regulation of cartilage development (loss)
c-value
0.0
0.1
0.2
0.3
0.4
0.5
0.6
0.7
0.8
p-value
2e-02
3e-02
3e-02
4e-02
6e-02
Log-odds
0.5
1.0
1.5
2.0
2.5
3.0
3.5
4.0
Cellular_organisms - ps1
Archaea1 - ps2
Archaea2 - ps3
Archaea3 - ps4
Asgard/Eukaryota - ps5
Eukaryota - ps6
Amorpheae - ps7
Obazoa - ps8
Opisthokonta - ps9
Holozoa - ps10
Filozoa - ps11
Choano./Metazoa - ps12
Metazoa - ps13
Myriazoa - ps14
Parahoxozoa - ps15
Bilateria - ps16
Deuterostomia - ps17
Chordata - ps18
Olfactores - ps19
Vertebrata - ps20
Gnathostomata - ps21
Euteleostomi - ps22
Sarcopterygii - ps23
Tetrapoda - ps24
Amniota - ps25
Mammalia - ps26
Theria - ps27
Placentalia - ps28
Boreoeutheria - ps29
Euarchontoglires - ps30
Euarchonta - ps31
Primates - ps32
Haplorrhini - ps33
Simiiformes - ps34
Catarrhini - ps35
Hominoidea - ps36
Hominidae - ps37
Homininae - ps38
Hominini - ps39
Homo_sapiens - ps40
Phylostratum

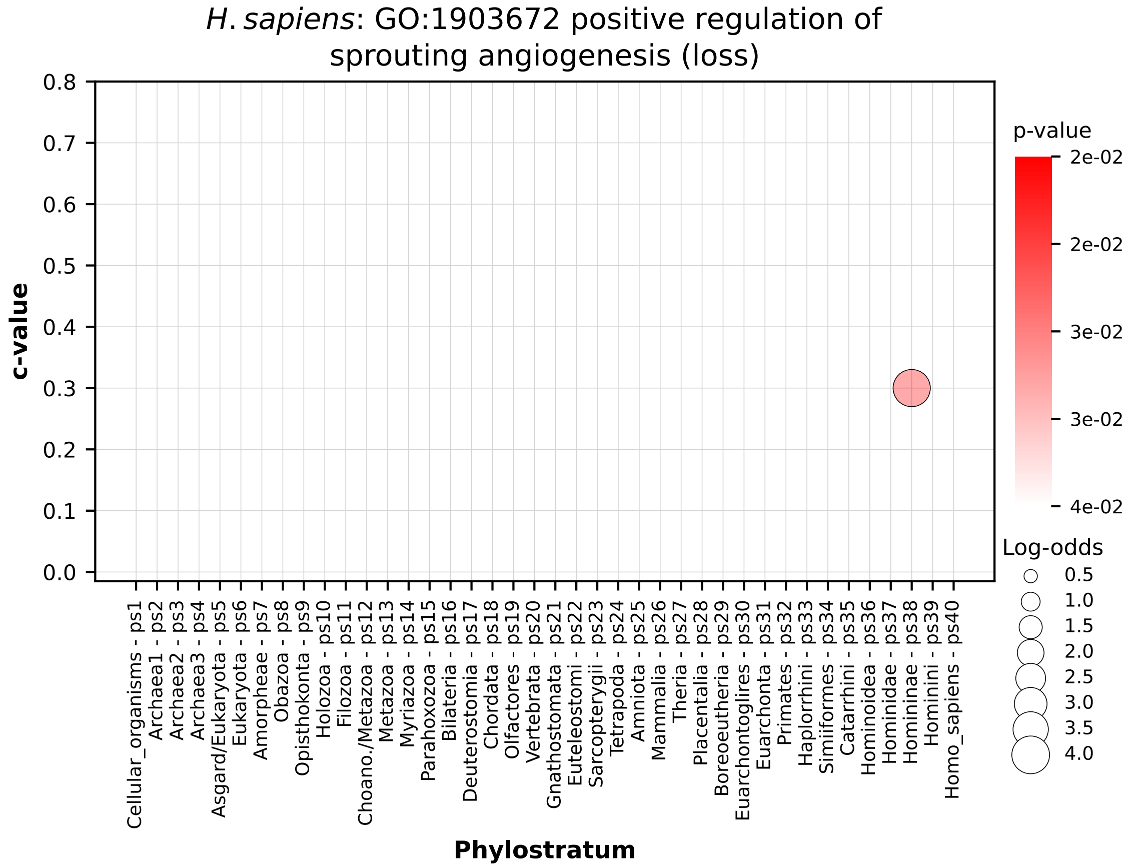

H. sapiens: GO:1903672 positive regulation of sprouting angiogenesis (loss)
c-value
0.8
0.7
0.6
0.5
0.4
0.3
0.2
0.1
0.0
Phylostratum
Cellular_organisms - ps1
Archaea1 - ps2
Archaea2 - ps3
Archaea3 - ps4
Asgard/Eukaryota - ps5
Eukaryota - ps6
Amorpheae - ps7
Obazoa - ps8
Opisthokonta - ps9
Holozoa - ps10
Filozoa - ps11
Choano./Metazoa - ps12
Metazoa - ps13
Myriazoa - ps14
Parahoxozoa - ps15
Bilateria - ps16
Deuterostomia - ps17
Chordata - ps18
Olfactores - ps19
Vertebrata - ps20
Gnathostomata - ps21
Euteleostomi - ps22
Sarcopterygii - ps23
Tetrapoda - ps24
Amniota - ps25
Mammalia - ps26
Theria - ps27
Placentalia - ps28
Boreoeutheria - ps29
Euarchontoglires - ps30
Euarchonta - ps31
Primates - ps32
Haplorrhini - ps33
Simiiformes - ps34
Catarrhini - ps35
Hominoidea - ps36
Hominidae - ps37
Homininae - ps38
Hominini - ps39
Homo_sapiens - ps40
p-value
2e-02
2e-02
3e-02
3e-02
4e-02
Log-odds
0.5
1.0
1.5
2.0
2.5
3.0
3.5
4.0

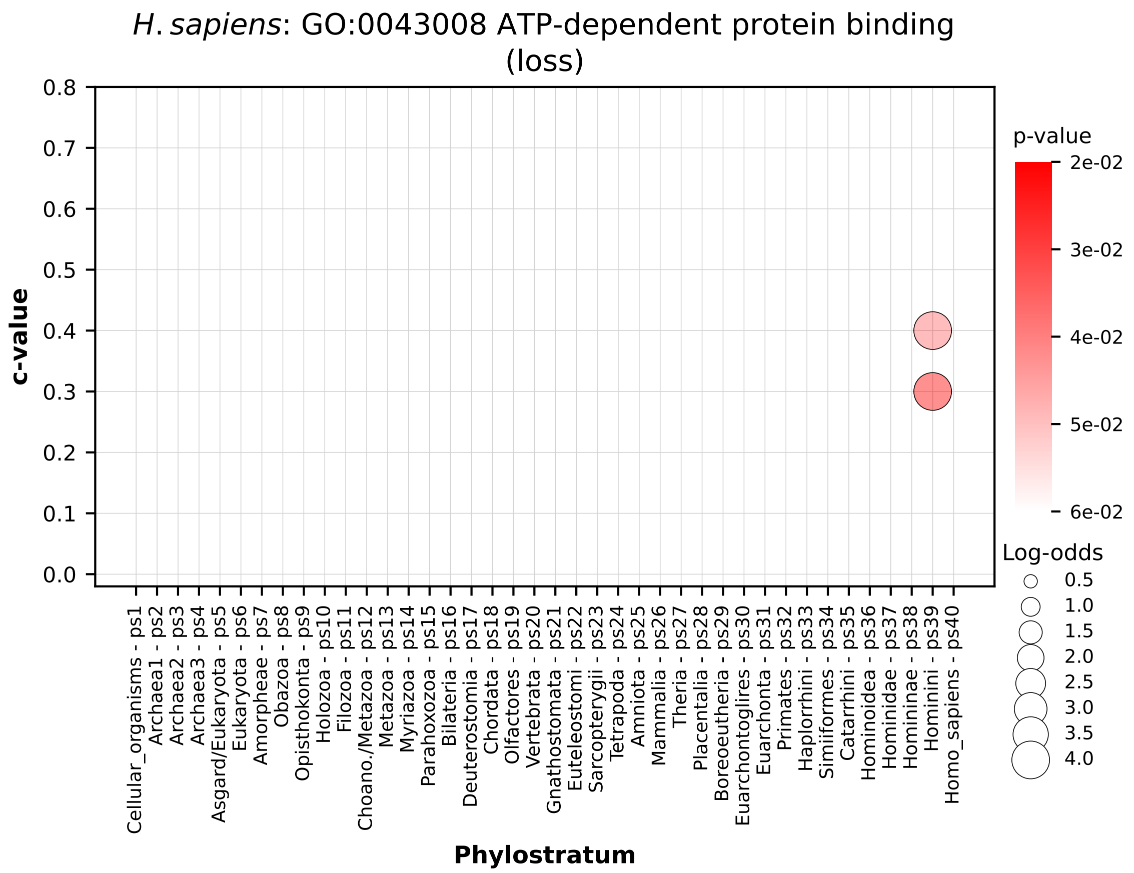

H. sapiens: GO:0043008 ATP-dependent protein binding (loss)
c-value
0.8
0.7
0.6
0.5
0.4
0.3
0.2
0.1
0.0
Phylostratum
Cellular_organisms - ps1
Archaea1 - ps2
Archaea2 - ps3
Archaea3 - ps4
Asgard/Eukaryota - ps5
Eukaryota - ps6
Amorpheae - ps7
Obazoa - ps8
Opisthokonta - ps9
Holozoa - ps10
Filozoa - ps11
Choano./Metazoa - ps12
Metazoa - ps13
Myriazoa - ps14
Parahoxozoa - ps15
Bilateria - ps16
Deuterostomia - ps17
Chordata - ps18
Olfactores - ps19
Vertebrata - ps20
Gnathostomata - ps21
Euteleostomi - ps22
Sarcopterygii - ps23
Tetrapoda - ps24
Amniota - ps25
Mammalia - ps26
Theria - ps27
Placentalia - ps28
Boreoeutheria - ps29
Euarchontoglires - ps30
Euarchonta - ps31
Primates - ps32
Haplorrhini - ps33
Simiiformes - ps34
Catarrhini - ps35
Hominoidea - ps36
Hominidae - ps37
Homininae - ps38
Hominini - ps39
Homo_sapiens - ps40
p-value
2e-02
3e-02
4e-02
5e-02
6e-02
Log-odds
0.5
1.0
1.5
2.0
2.5
3.0
3.5
4.0

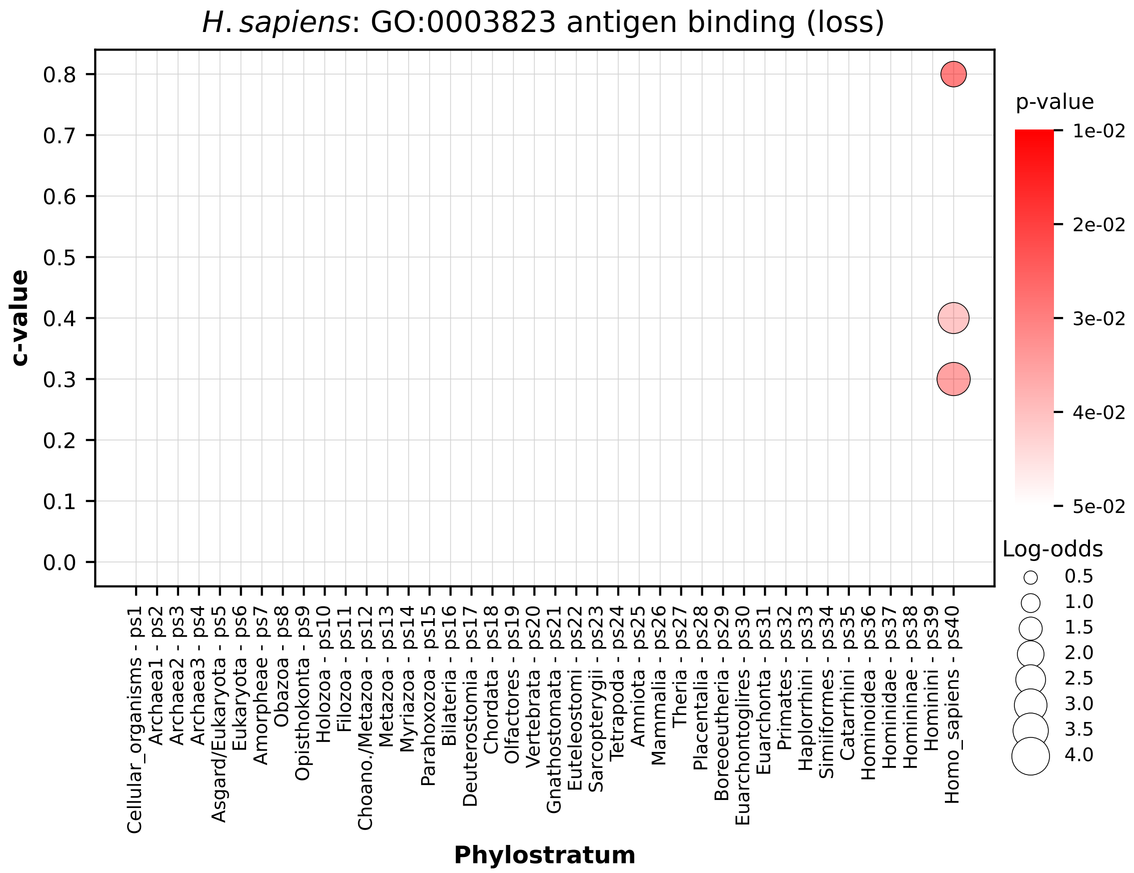
H. sapiens: GO:0003823 antigen binding (loss)
c-value
0.8
0.7
0.6
0.5
0.4
0.3
0.2
0.1
0.0
Phylostratum
Cellular_organisms - ps1
Archaea1 - ps2
Archaea2 - ps3
Archaea3 - ps4
Asgard/Eukaryota - ps5
Eukaryota - ps6
Amorpheae - ps7
Obazoa - ps8
Opisthokonta - ps9
Holozoa - ps10
Filozoa - ps11
Choano./Metazoa - ps12
Metazoa - ps13
Myriazoa - ps14
Parahoxozoa - ps15
Bilateria - ps16
Deuterostomia - ps17
Chordata - ps18
Olfactores - ps19
Vertebrata - ps20
Gnathostomata - ps21
Euteleostomi - ps22
Sarcopterygii - ps23
Tetrapoda - ps24
Amniota - ps25
Mammalia - ps26
Theria - ps27
Placentalia - ps28
Boreoeutheria - ps29
Euarchontoglires - ps30
Euarchonta - ps31
Primates - ps32
Haplorrhini - ps33
Simiiformes - ps34
Catarrhini - ps35
Hominoidea - ps36
Hominidae - ps37
Homininae - ps38
Hominini - ps39
Homo_sapiens - ps40
p-value
1e-02
2e-02
3e-02
4e-02
5e-02
Log-odds
0.5
1.0
1.5
2.0
2.5
3.0
3.5
4.0

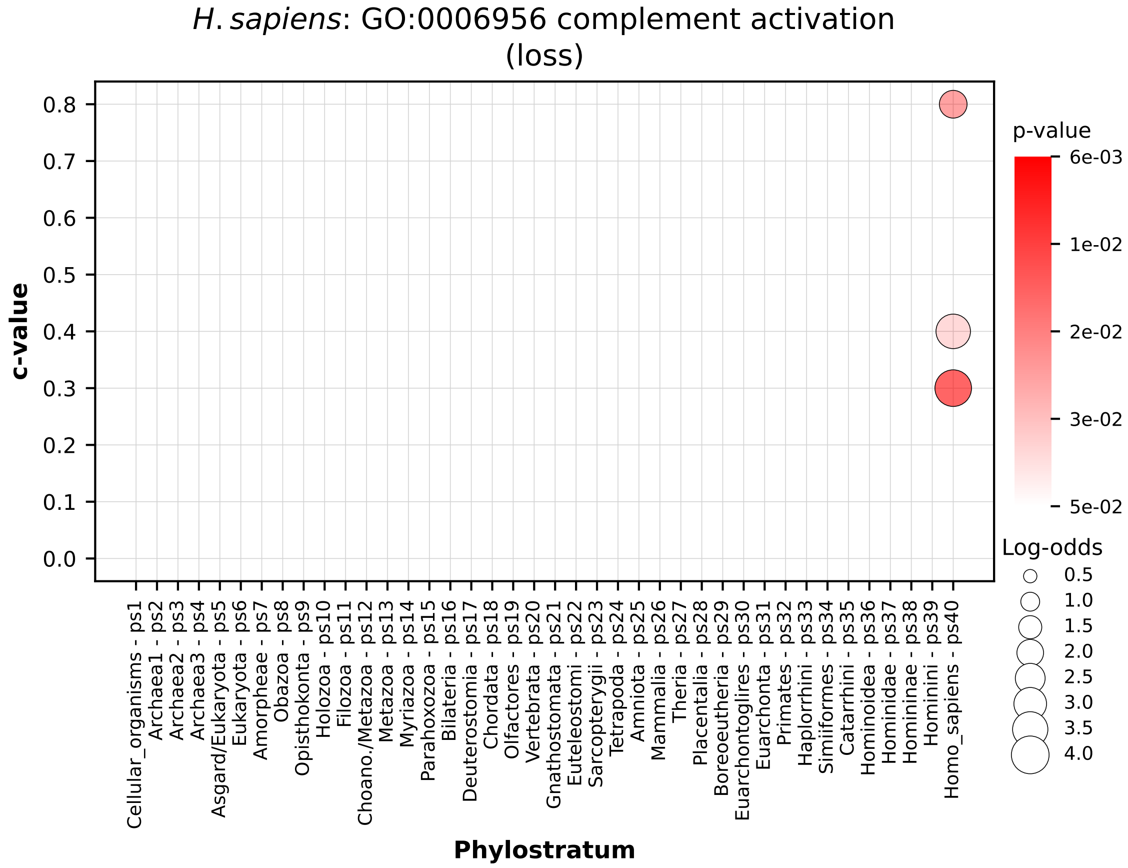

H. sapiens: GO:0006956 complement activation
(loss)
c-value
0.8
0.7
0.6
0.5
0.4
0.3
0.2
0.1
0.0
Phylostratum
Cellular_organisms - ps1
Archaea1 - ps2
Archaea2 - ps3
Archaea3 - ps4
Asgard/Eukaryota - ps5
Eukaryota - ps6
Amorpheae - ps7
Obazoa - ps8
Opisthokonta - ps9
Holozoa - ps10
Filozoa - ps11
Choano./Metazoa - ps12
Metazoa - ps13
Myriazoa - ps14
Parahoxozoa - ps15
Bilateria - ps16
Deuterostomia - ps17
Chordata - ps18
Olfactores - ps19
Vertebrata - ps20
Gnathostomata - ps21
Euteleostomi - ps22
Sarcopterygii - ps23
Tetrapoda - ps24
Amniota - ps25
Mammalia - ps26
Theria - ps27
Placentalia - ps28
Boreoeutheria - ps29
Euarchontoglires - ps30
Euarchonta - ps31
Primates - ps32
Haplorrhini - ps33
Simiiformes - ps34
Catarrhini - ps35
Hominoidea - ps36
Hominidae - ps37
Homininae - ps38
Hominini - ps39
Homo_sapiens - ps40
p-value
6e-03
1e-02
2e-02
3e-02
5e-02
Log-odds
0.5
1.0
1.5
2.0
2.5
3.0
3.5
4.0

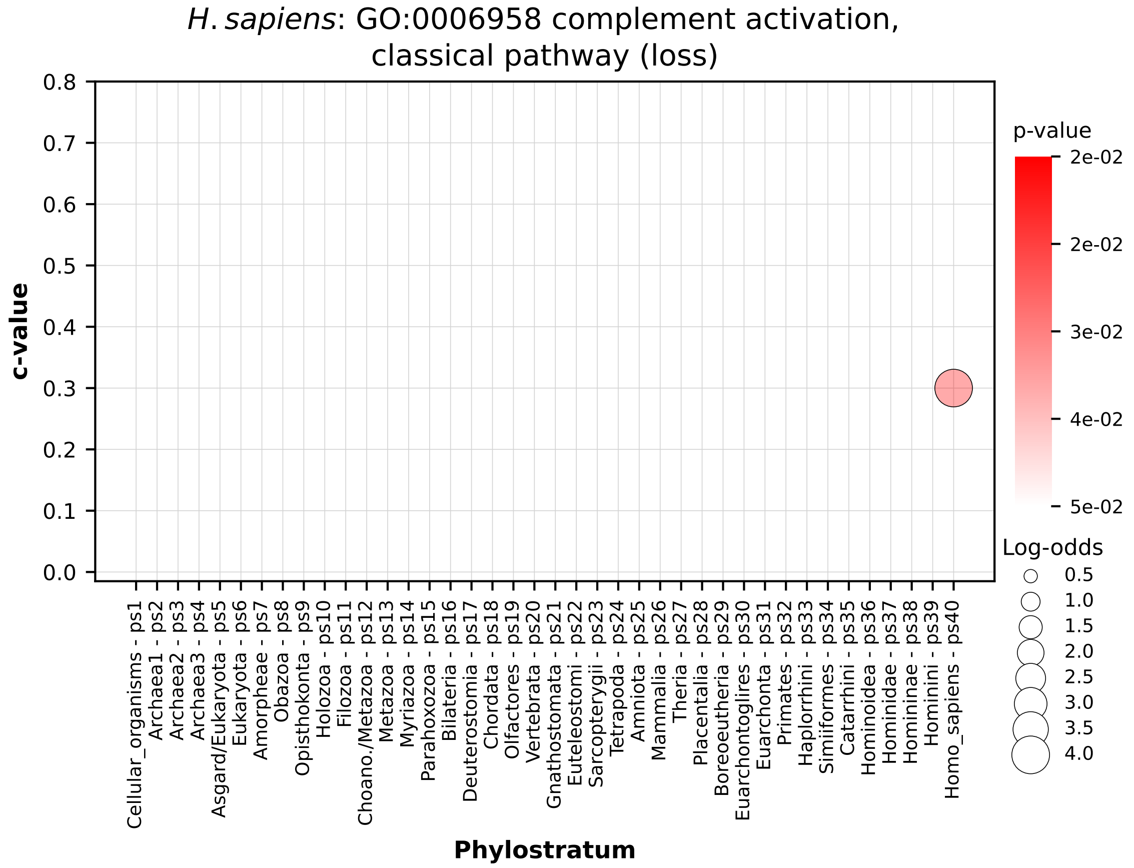

H. sapiens: GO:0006958 complement activation, classical pathway (loss)
c-value
0.8
0.7
0.6
0.5
0.4
0.3
0.2
0.1
0.0
p-value
2e-02
2e-02
3e-02
4e-02
5e-02
Log-odds
0.5
1.0
1.5
2.0
2.5
3.0
3.5
4.0
Cellular_organisms - ps1
Archaea1 - ps2
Archaea2 - ps3
Archaea3 - ps4
Asgard/Eukaryota - ps5
Eukaryota - ps6
Amorpheae - ps7
Obazoa - ps8
Opisthokonta - ps9
Holozoa - ps10
Filozoa - ps11
Choano./Metazoa - ps12
Metazoa - ps13
Myriazoa - ps14
Parahoxozoa - ps15
Bilateria - ps16
Deuterostomia - ps17
Chordata - ps18
Olfactores - ps19
Vertebrata - ps20
Gnathostomata - ps21
Euteleostomi - ps22
Sarcopterygii - ps23
Tetrapoda - ps24
Amniota - ps25
Mammalia - ps26
Theria - ps27
Placentalia - ps28
Boreoeutheria - ps29
Euarchontoglires - ps30
Euarchonta - ps31
Primates - ps32
Haplorrhini - ps33
Simiiformes - ps34
Catarrhini - ps35
Hominoidea - ps36
Hominidae - ps37
Homininae - ps38
Hominini - ps39
Homo_sapiens - ps40
Phylostratum

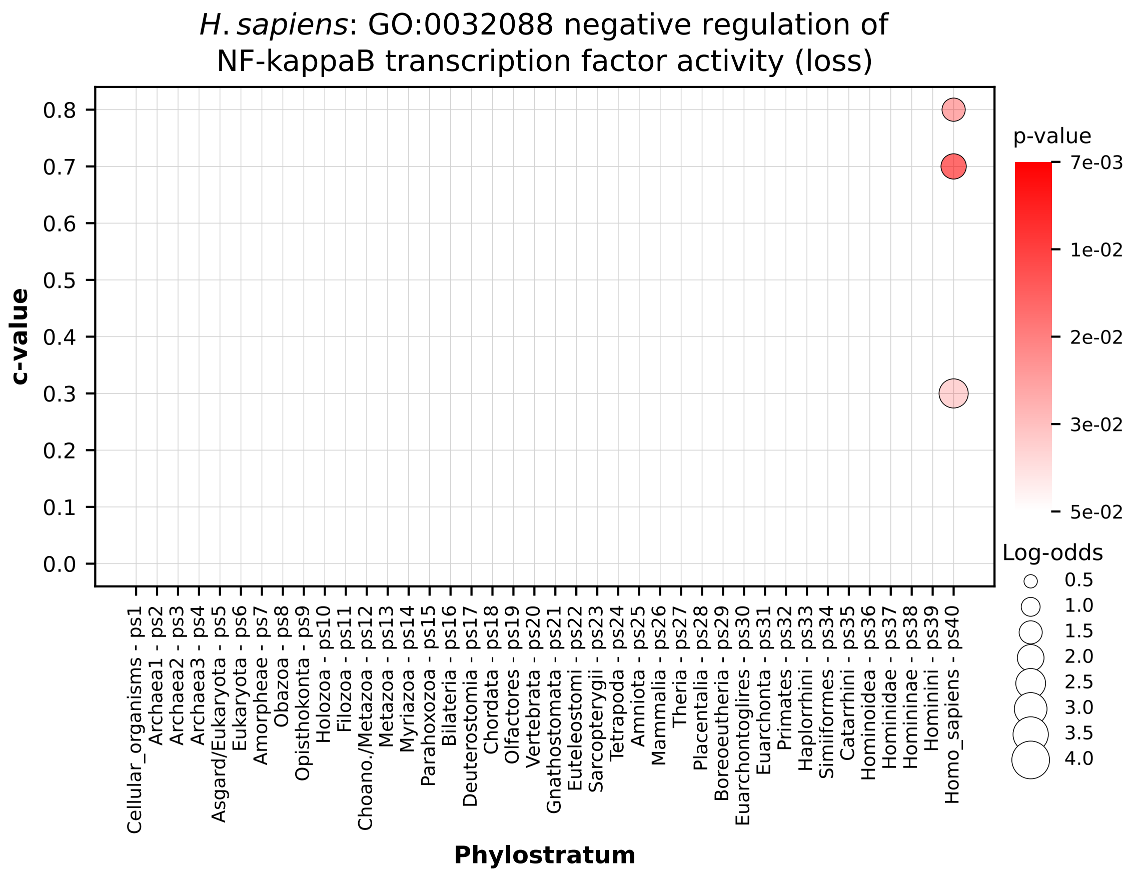

H. sapiens: GO:0032088 negative regulation of NF-kappaB transcription factor activity (loss)
c-value
0.0
0.1
0.2
0.3
0.4
0.5
0.6
0.7
0.8
Phylostratum
Cellular_organisms - ps1
Archaea1 - ps2
Archaea2 - ps3
Archaea3 - ps4
Asgard/Eukaryota - ps5
Eukaryota - ps6
Amorpheae - ps7
Obazoa - ps8
Opisthokonta - ps9
Holozoa - ps10
Filozoa - ps11
Choano./Metazoa - ps12
Metazoa - ps13
Myriazoa - ps14
Parahoxozoa - ps15
Bilateria - ps16
Deuterostomia - ps17
Chordata - ps18
Olfactores - ps19
Vertebrata - ps20
Gnathostomata - ps21
Euteleostomi - ps22
Sarcopterygii - ps23
Tetrapoda - ps24
Amniota - ps25
Mammalia - ps26
Theria - ps27
Placentalia - ps28
Boreoeutheria - ps29
Euarchontoglires - ps30
Euarchonta - ps31
Primates - ps32
Haplorrhini - ps33
Simiiformes - ps34
Catarrhini - ps35
Hominoidea - ps36
Hominidae - ps37
Homininae - ps38
Hominini - ps39
Homo_sapiens - ps40
p-value
7e-03
1e-02
2e-02
3e-02
5e-02
Log-odds
0.5
1.0
1.5
2.0
2.5
3.0
3.5
4.0

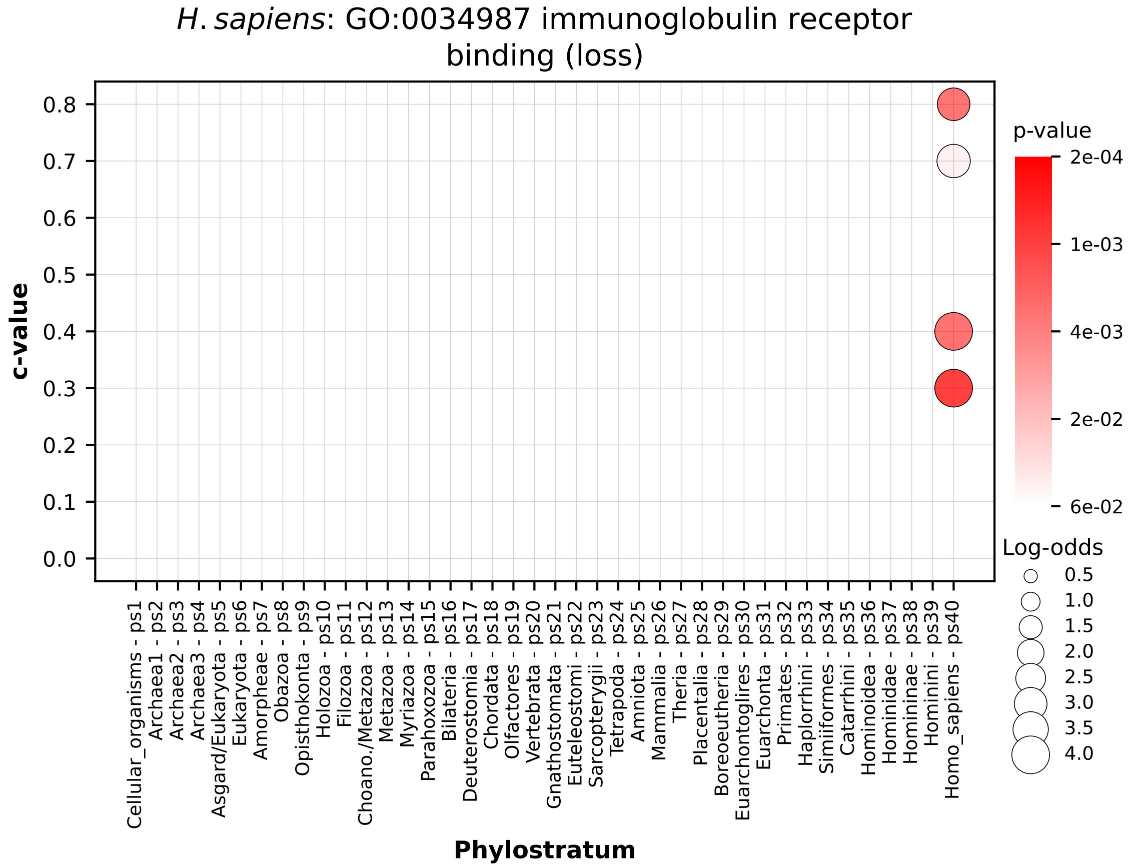

H. sapiens: GO:0034987 immunoglobulin receptor binding (loss)
c-value
0.0
0.1
0.2
0.3
0.4
0.5
0.6
0.7
0.8
p-value
2e-04
1e-03
4e-03
2e-02
6e-02
Log-odds
0.5
1.0
1.5
2.0
2.5
3.0
3.5
4.0
Cellular_organisms - ps1
Archaea1 - ps2
Archaea2 - ps3
Archaea3 - ps4
Asgard/Eukaryota - ps5
Eukaryota - ps6
Amorpheae - ps7
Obazoa - ps8
Opisthokonta - ps9
Holozoa - ps10
Filozoa - ps11
Choano./Metazoa - ps12
Metazoa - ps13
Myriazoa - ps14
Parahoxozoa - ps15
Bilateria - ps16
Deuterostomia - ps17
Chordata - ps18
Olfactores - ps19
Vertebrata - ps20
Gnathostomata - ps21
Euteleostomi - ps22
Sarcopterygii - ps23
Tetrapoda - ps24
Amniota - ps25
Mammalia - ps26
Theria - ps27
Placentalia - ps28
Boreoeutheria - ps29
Euarchontoglires - ps30
Euarchonta - ps31
Primates - ps32
Haplorrhini - ps33
Simiiformes - ps34
Catarrhini - ps35
Hominoidea - ps36
Hominidae - ps37
Homininae - ps38
Hominini - ps39
Homo_sapiens - ps40
Phylostratum

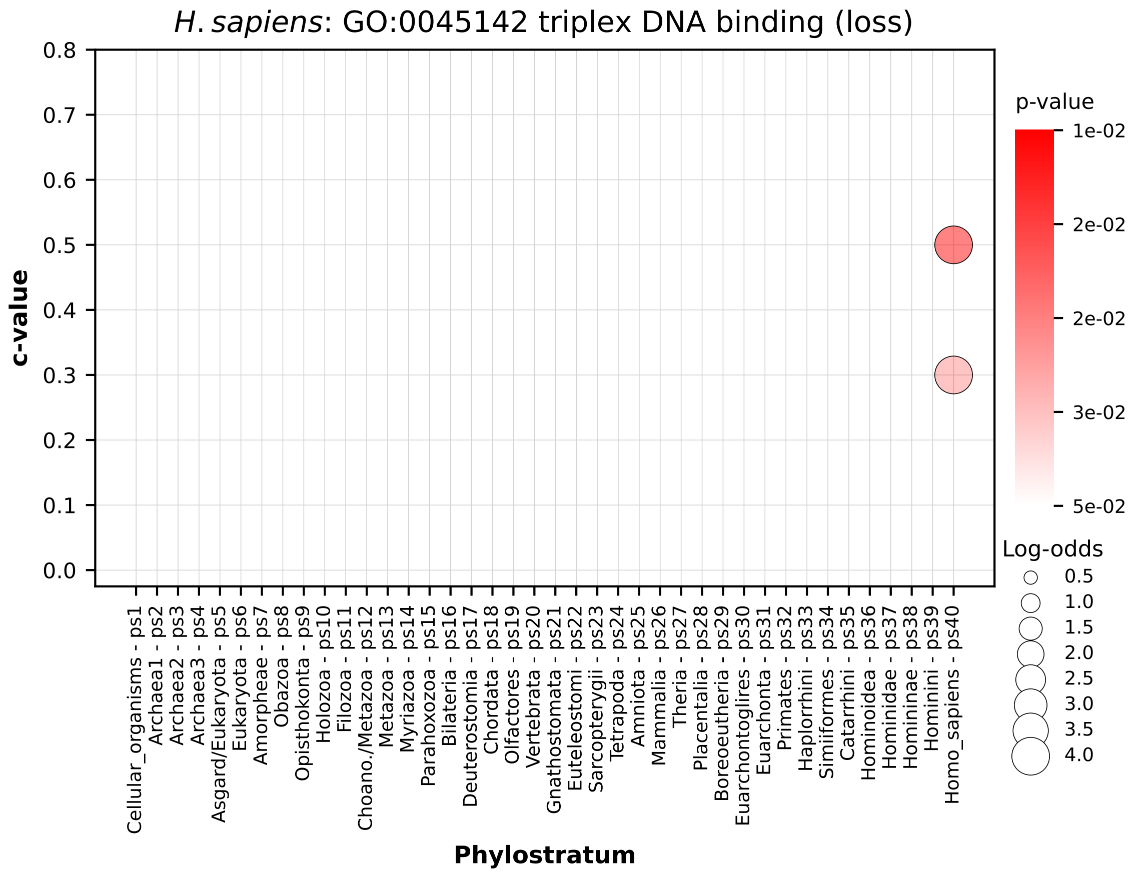

H. sapiens: GO:0045142 triplex DNA binding (loss)
c-value
0.8
0.7
0.6
0.5
0.4
0.3
0.2
0.1
0.0
Cellular_organisms - ps1
Archaea1 - ps2
Archaea2 - ps3
Archaea3 - ps4
Asgard/Eukaryota - ps5
Eukaryota - ps6
Amorpheae - ps7
Obazoa - ps8
Opisthokonta - ps9
Holozoa - ps10
Filozoa - ps11
Choano./Metazoa - ps12
Metazoa - ps13
Myriazoa - ps14
Parahoxozoa - ps15
Bilateria - ps16
Deuterostomia - ps17
Chordata - ps18
Olfactores - ps19
Vertebrata - ps20
Gnathostomata - ps21
Euteleostomi - ps22
Sarcopterygii - ps23
Tetrapoda - ps24
Amniota - ps25
Mammalia - ps26
Theria - ps27
Placentalia - ps28
Boreoeutheria - ps29
Euarchontoglires - ps30
Euarchonta - ps31
Primates - ps32
Haplorrhini - ps33
Simiiformes - ps34
Catarrhini - ps35
Hominoidea - ps36
Hominidae - ps37
Homininae - ps38
Hominini - ps39
Homo_sapiens - ps40
Phylostratum
p-value
1e-02
2e-02
2e-02
3e-02
5e-02
Log-odds
0.5
1.0
1.5
2.0
2.5
3.0
3.5
4.0

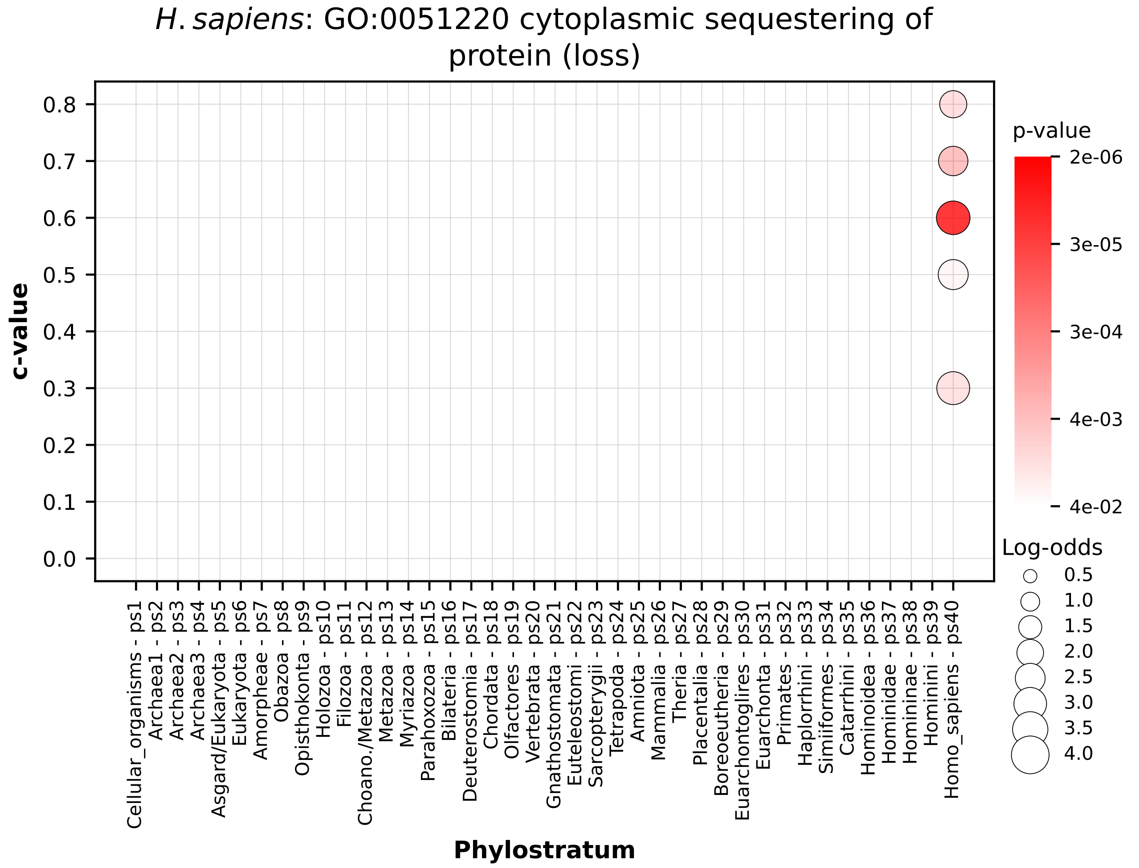

H. sapiens: GO:0051220 cytoplasmic sequestering of protein (loss)
c-value
0.8
0.7
0.6
0.5
0.4
0.3
0.2
0.1
0.0
p-value
2e-06
3e-05
3e-04
4e-03
4e-02
Log-odds
0.5
1.0
1.5
2.0
2.5
3.0
3.5
4.0
Cellular_organisms - ps1
Archaea1 - ps2
Archaea2 - ps3
Archaea3 - ps4
Asgard/Eukaryota - ps5
Eukaryota - ps6
Amorpheae - ps7
Obazoa - ps8
Opisthokonta - ps9
Holozoa - ps10
Filozoa - ps11
Choano./Metazoa - ps12
Metazoa - ps13
Myriazoa - ps14
Parahoxozoa - ps15
Bilateria - ps16
Deuterostomia - ps17
Chordata - ps18
Olfactores - ps19
Vertebrata - ps20
Gnathostomata - ps21
Euteleostomi - ps22
Sarcopterygii - ps23
Tetrapoda - ps24
Amniota - ps25
Mammalia - ps26
Theria - ps27
Placentalia - ps28
Boreoeutheria - ps29
Euarchontoglires - ps30
Euarchonta - ps31
Primates - ps32
Haplorrhini - ps33
Simiiformes - ps34
Catarrhini - ps35
Hominoidea - ps36
Hominidae - ps37
Homininae - ps38
Hominini - ps39
Homo_sapiens - ps40
Phylostratum

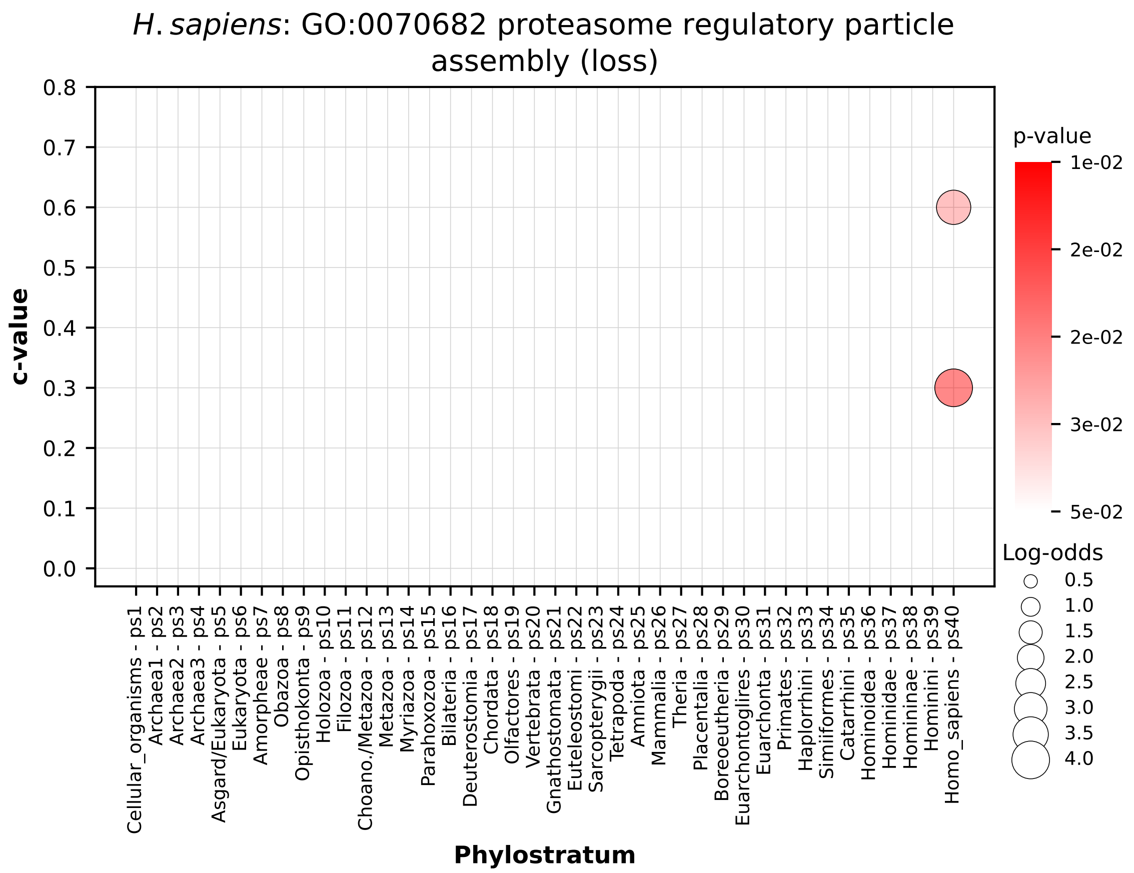

H. sapiens: GO:0070682 proteasome regulatory particle assembly (loss)
c-value
0.8
0.7
0.6
0.5
0.4
0.3
0.2
0.1
0.0
Phylostratum
Cellular_organisms - ps1
Archaea1 - ps2
Archaea2 - ps3
Archaea3 - ps4
Asgard/Eukaryota - ps5
Eukaryota - ps6
Amorpheae - ps7
Obazoa - ps8
Opisthokonta - ps9
Holozoa - ps10
Filozoa - ps11
Choano./Metazoa - ps12
Metazoa - ps13
Myriazoa - ps14
Parahoxozoa - ps15
Bilateria - ps16
Deuterostomia - ps17
Chordata - ps18
Olfactores - ps19
Vertebrata - ps20
Gnathostomata - ps21
Euteleostomi - ps22
Sarcopterygii - ps23
Tetrapoda - ps24
Amniota - ps25
Mammalia - ps26
Theria - ps27
Placentalia - ps28
Boreoeutheria - ps29
Euarchontoglires - ps30
Euarchonta - ps31
Primates - ps32
Haplorrhini - ps33
Simiiformes - ps34
Catarrhini - ps35
Hominoidea - ps36
Hominidae - ps37
Homininae - ps38
Hominini - ps39
Homo_sapiens - ps40
p-value
1e-02
2e-02
2e-02
3e-02
5e-02
Log-odds
0.5
1.0
1.5
2.0
2.5
3.0
3.5
4.0

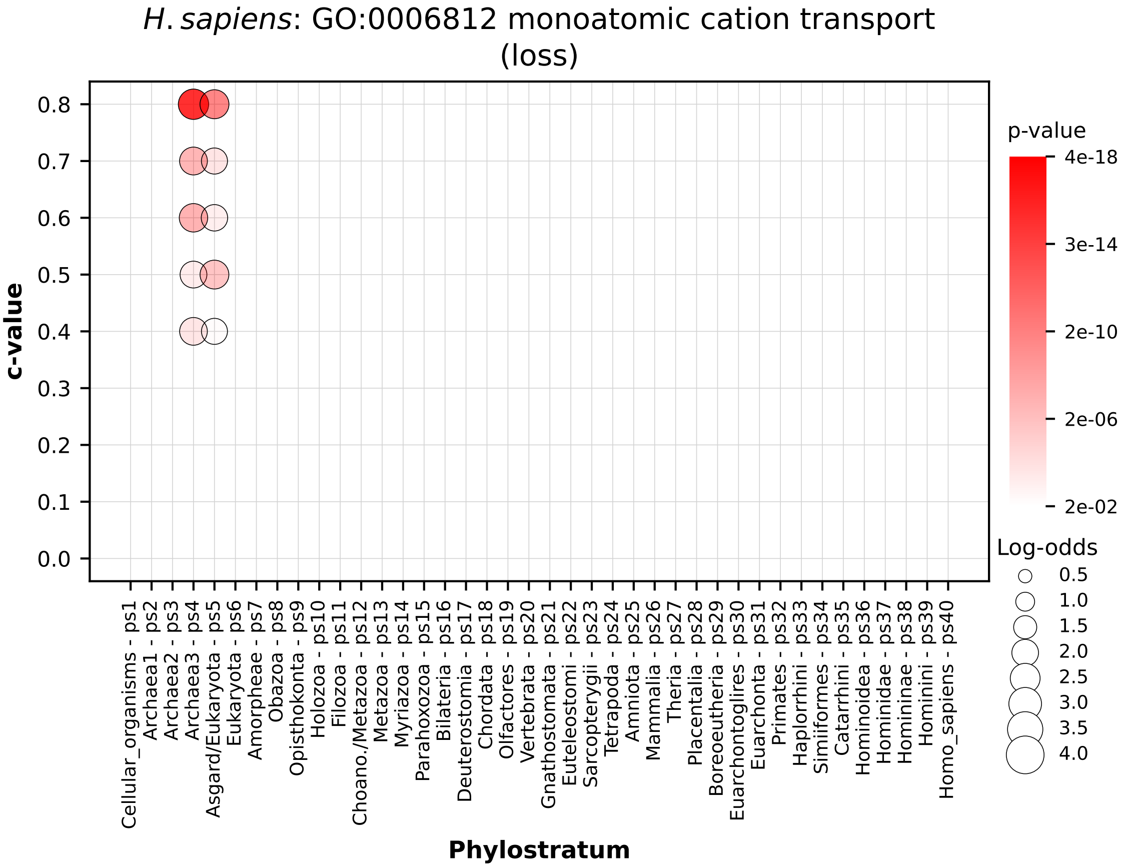

H. sapiens: GO:0006812 monoatomic cation transport
(loss)
c-value
0.8
0.7
0.6
0.5
0.4
0.3
0.2
0.1
0.0
p-value
4e-18
3e-14
2e-10
2e-06
2e-02
Log-odds
0.5
1.0
1.5
2.0
2.5
3.0
3.5
4.0
Cellular_organisms - ps1
Archaea1 - ps2
Archaea2 - ps3
Archaea3 - ps4
Asgard/Eukaryota - ps5
Eukaryota - ps6
Amorpheae - ps7
Obazoa - ps8
Opisthokonta - ps9
Holozoa - ps10
Filozoa - ps11
Choano./Metazoa - ps12
Metazoa - ps13
Myriazoa - ps14
Parahoxozoa - ps15
Bilateria - ps16
Deuterostomia - ps17
Chordata - ps18
Olfactores - ps19
Vertebrata - ps20
Gnathostomata - ps21
Euteleostomi - ps22
Sarcopterygii - ps23
Tetrapoda - ps24
Amniota - ps25
Mammalia - ps26
Theria - ps27
Placentalia - ps28
Boreoeutheria - ps29
Euarchontoglires - ps30
Euarchonta - ps31
Primates - ps32
Haplorrhini - ps33
Simiiformes - ps34
Catarrhini - ps35
Hominoidea - ps36
Hominidae - ps37
Homininae - ps38
Hominini - ps39
Homo_sapiens - ps40
Phylostratum

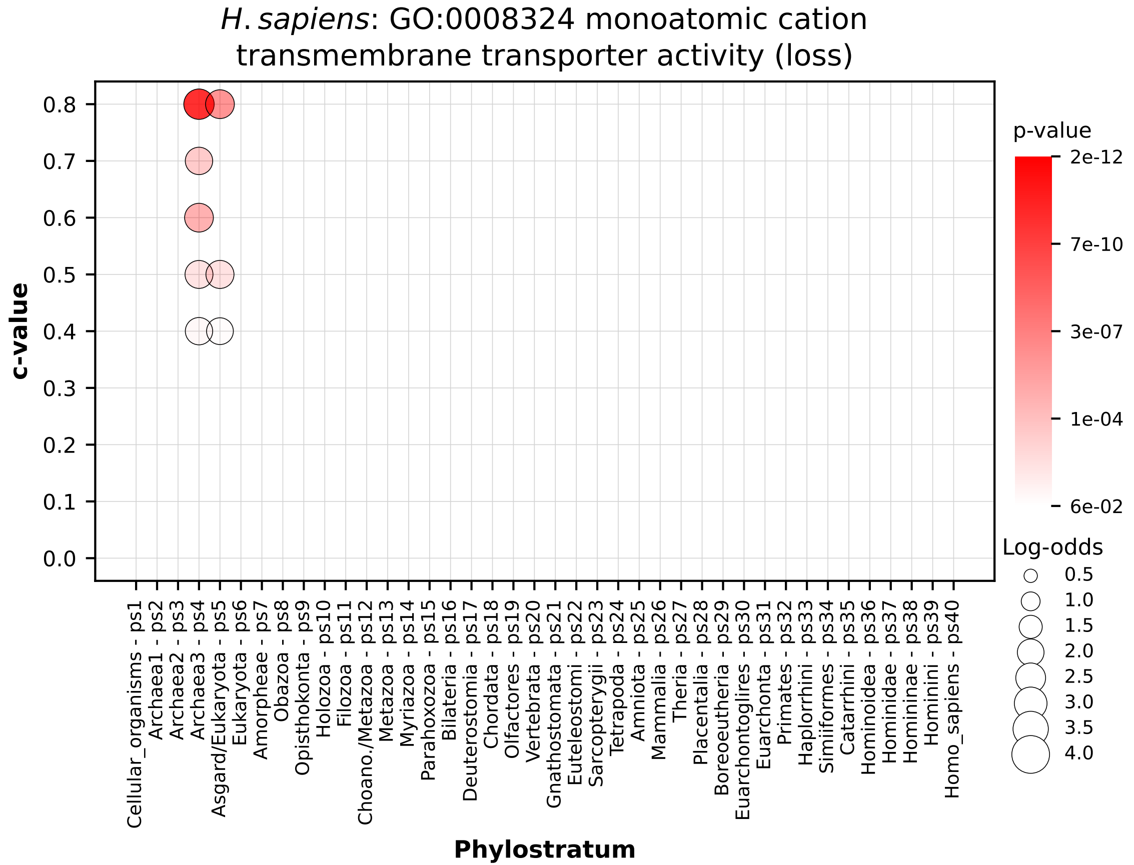

H. sapiens: GO:0008324 monoatomic cation transmembrane transporter activity (loss)
c-value
0.8
0.7
0.6
0.5
0.4
0.3
0.2
0.1
0.0
Phylostratum
Cellular_organisms - ps1
Archaea1 - ps2
Archaea2 - ps3
Archaea3 - ps4
Asgard/Eukaryota - ps5
Eukaryota - ps6
Amorpheae - ps7
Obazoa - ps8
Opisthokonta - ps9
Holozoa - ps10
Filozoa - ps11
Choano./Metazoa - ps12
Metazoa - ps13
Myriazoa - ps14
Parahoxozoa - ps15
Bilateria - ps16
Deuterostomia - ps17
Chordata - ps18
Olfactores - ps19
Vertebrata - ps20
Gnathostomata - ps21
Euteleostomi - ps22
Sarcopterygii - ps23
Tetrapoda - ps24
Amniota - ps25
Mammalia - ps26
Theria - ps27
Placentalia - ps28
Boreoeutheria - ps29
Euarchontoglires - ps30
Euarchonta - ps31
Primates - ps32
Haplorrhini - ps33
Simiiformes - ps34
Catarrhini - ps35
Hominoidea - ps36
Hominidae - ps37
Homininae - ps38
Hominini - ps39
Homo_sapiens - ps40
p-value
2e-12
7e-10
3e-07
1e-04
6e-02
Log-odds
0.5
1.0
1.5
2.0
2.5
3.0
3.5
4.0

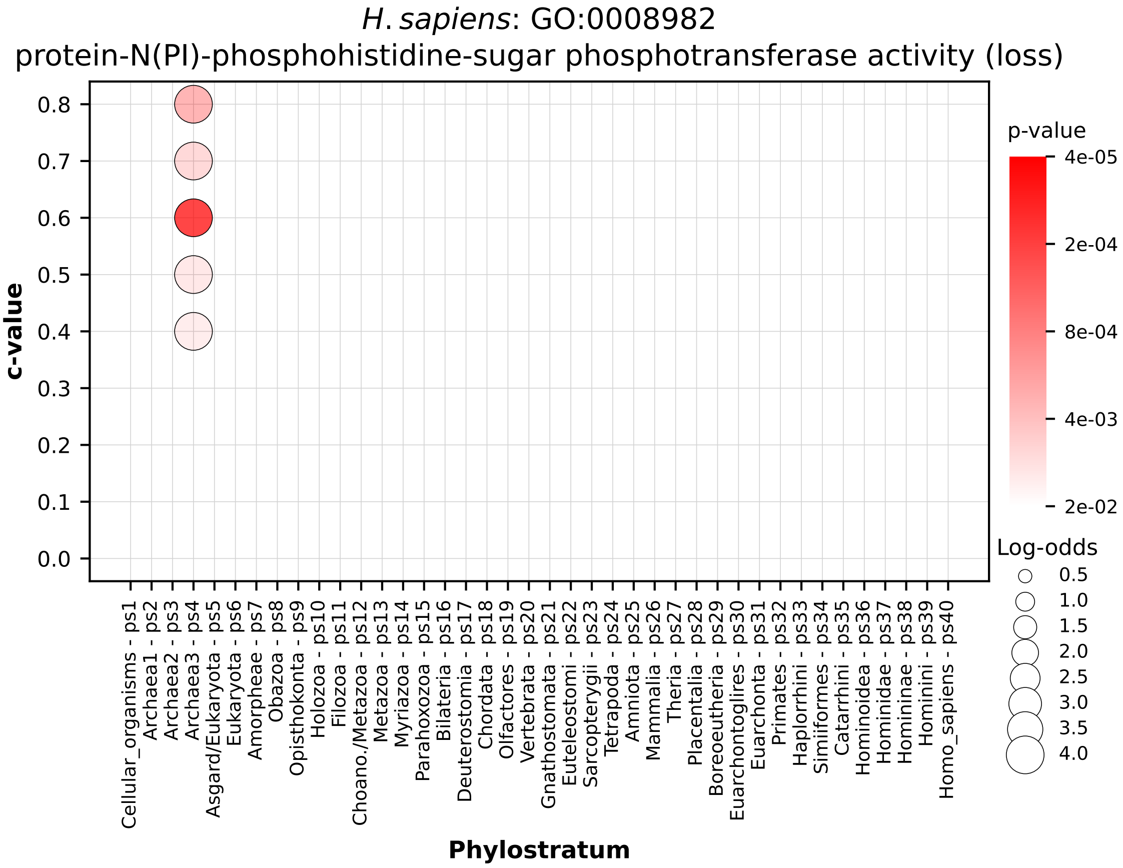

H. sapiens: GO:0008982
protein-N(PI)-phosphohistidine-sugar phosphotransferase activity (loss)
c-value
0.8
0.7
0.6
0.5
0.4
0.3
0.2
0.1
0.0
p-value
4e-05
2e-04
8e-04
4e-03
2e-02
Log-odds
0.5
1.0
1.5
2.0
2.5
3.0
3.5
4.0
Cellular_organisms - ps1
Archaea1 - ps2
Archaea2 - ps3
Archaea3 - ps4
Asgard/Eukaryota - ps5
Eukaryota - ps6
Amorpheae - ps7
Obazoa - ps8
Opisthokonta - ps9
Holozoa - ps10
Filozoa - ps11
Choano./Metazoa - ps12
Metazoa - ps13
Myriazoa - ps14
Parahoxozoa - ps15
Bilateria - ps16
Deuterostomia - ps17
Chordata - ps18
Olfactores - ps19
Vertebrata - ps20
Gnathostomata - ps21
Euteleostomi - ps22
Sarcopterygii - ps23
Tetrapoda - ps24
Amniota - ps25
Mammalia - ps26
Theria - ps27
Placentalia - ps28
Boreoeutheria - ps29
Euarchontoglires - ps30
Euarchonta - ps31
Primates - ps32
Haplorrhini - ps33
Simiiformes - ps34
Catarrhini - ps35
Hominoidea - ps36
Hominidae - ps37
Homininae - ps38
Hominini - ps39
Homo_sapiens - ps40
Phylostratum

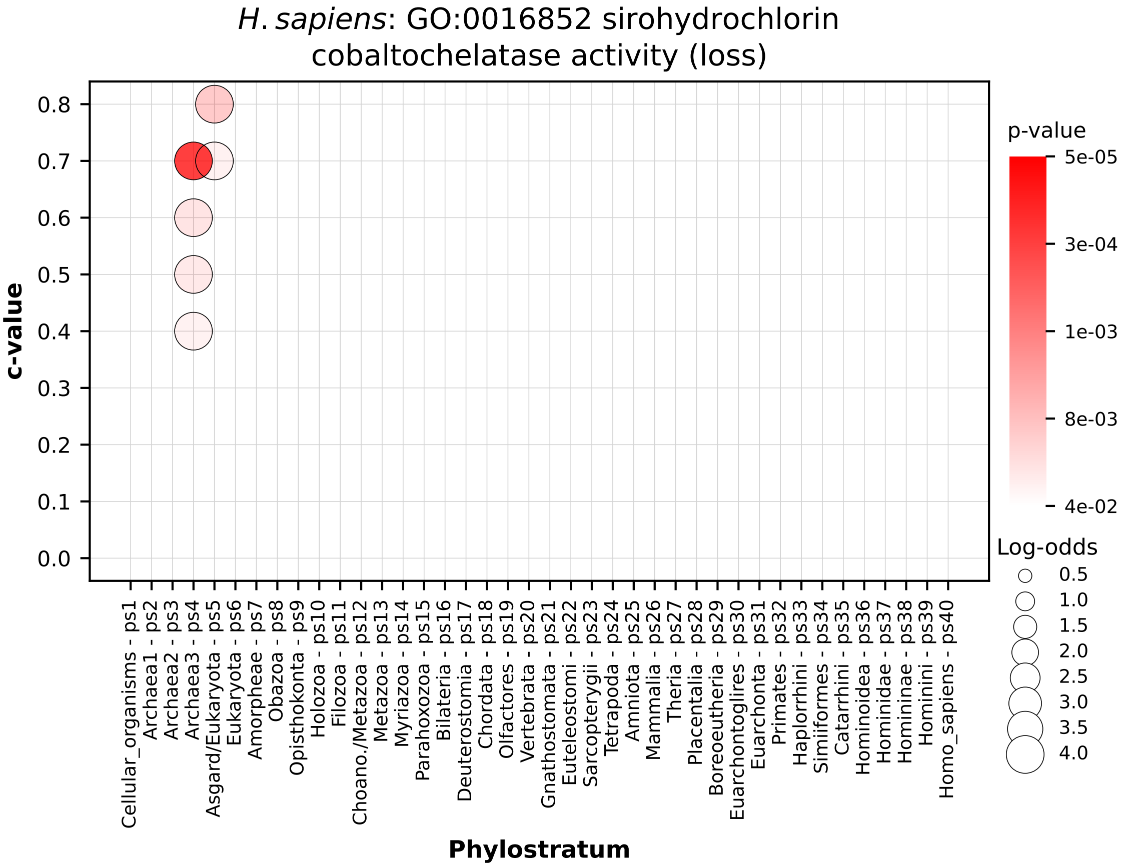

H. sapiens: GO:0016852 sirohydrochlorin cobaltochelatase activity (loss)
c-value
0.0
0.1
0.2
0.3
0.4
0.5
0.6
0.7
0.8
p-value
5e-05
3e-04
1e-03
8e-03
4e-02
Log-odds
0.5
1.0
1.5
2.0
2.5
3.0
3.5
4.0
Cellular_organisms - ps1
Archaea1 - ps2
Archaea2 - ps3
Archaea3 - ps4
Asgard/Eukaryota - ps5
Eukaryota - ps6
Amorpheae - ps7
Obazoa - ps8
Opisthokonta - ps9
Holozoa - ps10
Filozoa - ps11
Choano./Metazoa - ps12
Metazoa - ps13
Myriazoa - ps14
Parahoxozoa - ps15
Bilateria - ps16
Deuterostomia - ps17
Chordata - ps18
Olfactores - ps19
Vertebrata - ps20
Gnathostomata - ps21
Euteleostomi - ps22
Sarcopterygii - ps23
Tetrapoda - ps24
Amniota - ps25
Mammalia - ps26
Theria - ps27
Placentalia - ps28
Boreoeutheria - ps29
Euarchontoglires - ps30
Euarchonta - ps31
Primates - ps32
Haplorrhini - ps33
Simiiformes - ps34
Catarrhini - ps35
Hominoidea - ps36
Hominidae - ps37
Homininae - ps38
Hominini - ps39
Homo_sapiens - ps40
Phylostratum

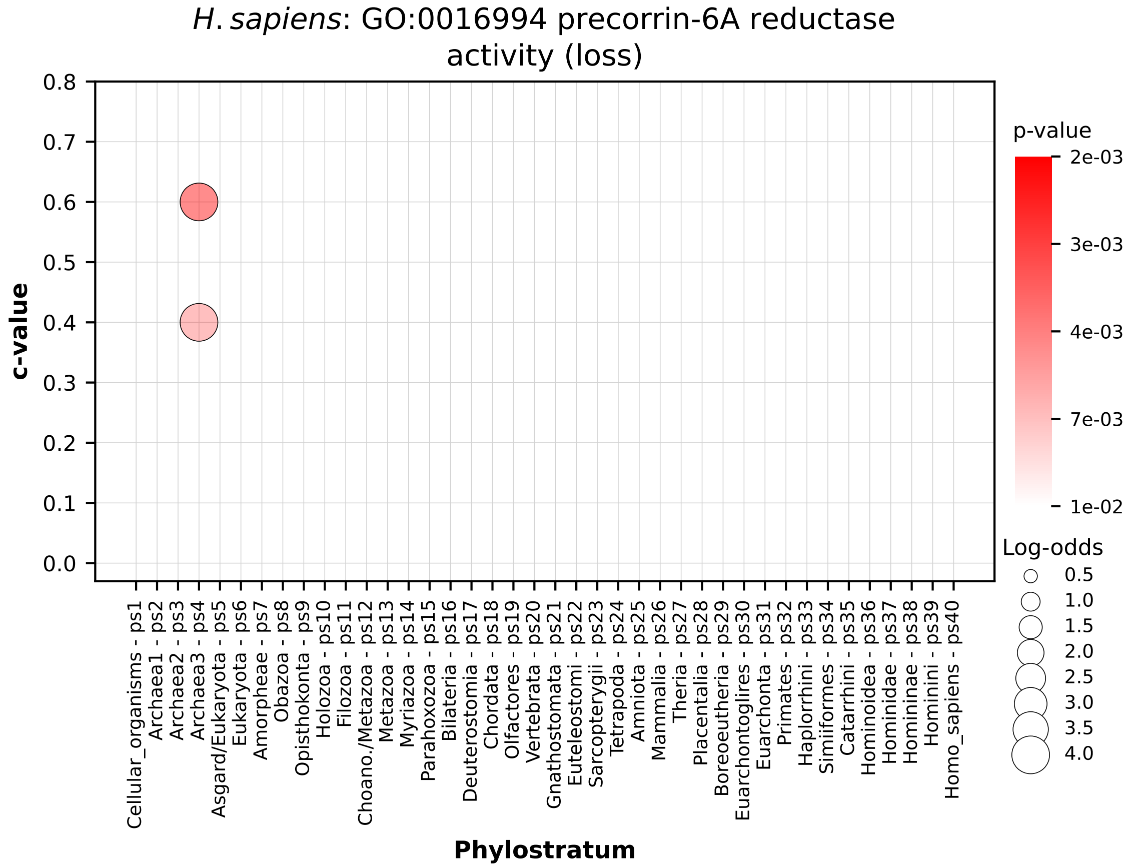

H. sapiens: GO:0016994 precorrin-6A reductase activity (loss)
c-value
0.0
0.1
0.2
0.3
0.4
0.5
0.6
0.7
0.8
Phylostratum
Cellular_organisms - ps1
Archaea1 - ps2
Archaea2 - ps3
Archaea3 - ps4
Asgard/Eukaryota - ps5
Eukaryota - ps6
Amorpheae - ps7
Obazoa - ps8
Opisthokonta - ps9
Holozoa - ps10
Filozoa - ps11
Choano./Metazoa - ps12
Metazoa - ps13
Myriazoa - ps14
Parahoxozoa - ps15
Bilateria - ps16
Deuterostomia - ps17
Chordata - ps18
Olfactores - ps19
Vertebrata - ps20
Gnathostomata - ps21
Euteleostomi - ps22
Sarcopterygii - ps23
Tetrapoda - ps24
Amniota - ps25
Mammalia - ps26
Theria - ps27
Placentalia - ps28
Boreoeutheria - ps29
Euarchontoglires - ps30
Euarchonta - ps31
Primates - ps32
Haplorrhini - ps33
Simiiformes - ps34
Catarrhini - ps35
Hominoidea - ps36
Hominidae - ps37
Homininae - ps38
Hominini - ps39
Homo_sapiens - ps40
p-value
2e-03
3e-03
4e-03
7e-03
1e-02
Log-odds
0.5
1.0
1.5
2.0
2.5
3.0
3.5
4.0

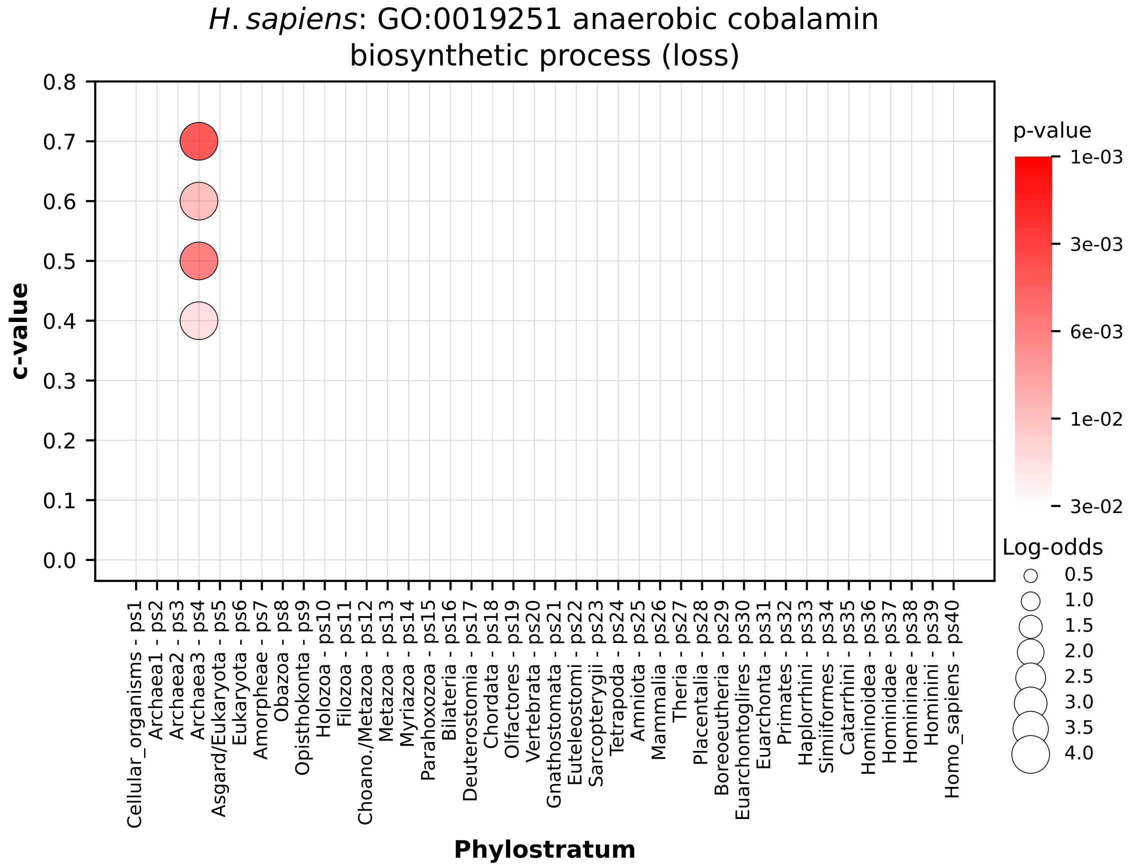

H. sapiens: GO:0019251 anaerobic cobalamin biosynthetic process (loss)
c-value
0.8
0.7
0.6
0.5
0.4
0.3
0.2
0.1
0.0
p-value
1e-03
3e-03
6e-03
1e-02
3e-02
Log-odds
0.5
1.0
1.5
2.0
2.5
3.0
3.5
4.0
Cellular_organisms - ps1
Archaea1 - ps2
Archaea2 - ps3
Archaea3 - ps4
Asgard/Eukaryota - ps5
Eukaryota - ps6
Amorpheae - ps7
Obazoa - ps8
Opisthokonta - ps9
Holozoa - ps10
Filozoa - ps11
Choano./Metazoa - ps12
Metazoa - ps13
Myriazoa - ps14
Parahoxozoa - ps15
Bilateria - ps16
Deuterostomia - ps17
Chordata - ps18
Olfactores - ps19
Vertebrata - ps20
Gnathostomata - ps21
Euteleostomi - ps22
Sarcopterygii - ps23
Tetrapoda - ps24
Amniota - ps25
Mammalia - ps26
Theria - ps27
Placentalia - ps28
Boreoeutheria - ps29
Euarchontoglires - ps30
Euarchonta - ps31
Primates - ps32
Haplorrhini - ps33
Simiiformes - ps34
Catarrhini - ps35
Hominoidea - ps36
Hominidae - ps37
Homininae - ps38
Hominini - ps39
Homo_sapiens - ps40
Phylostratum

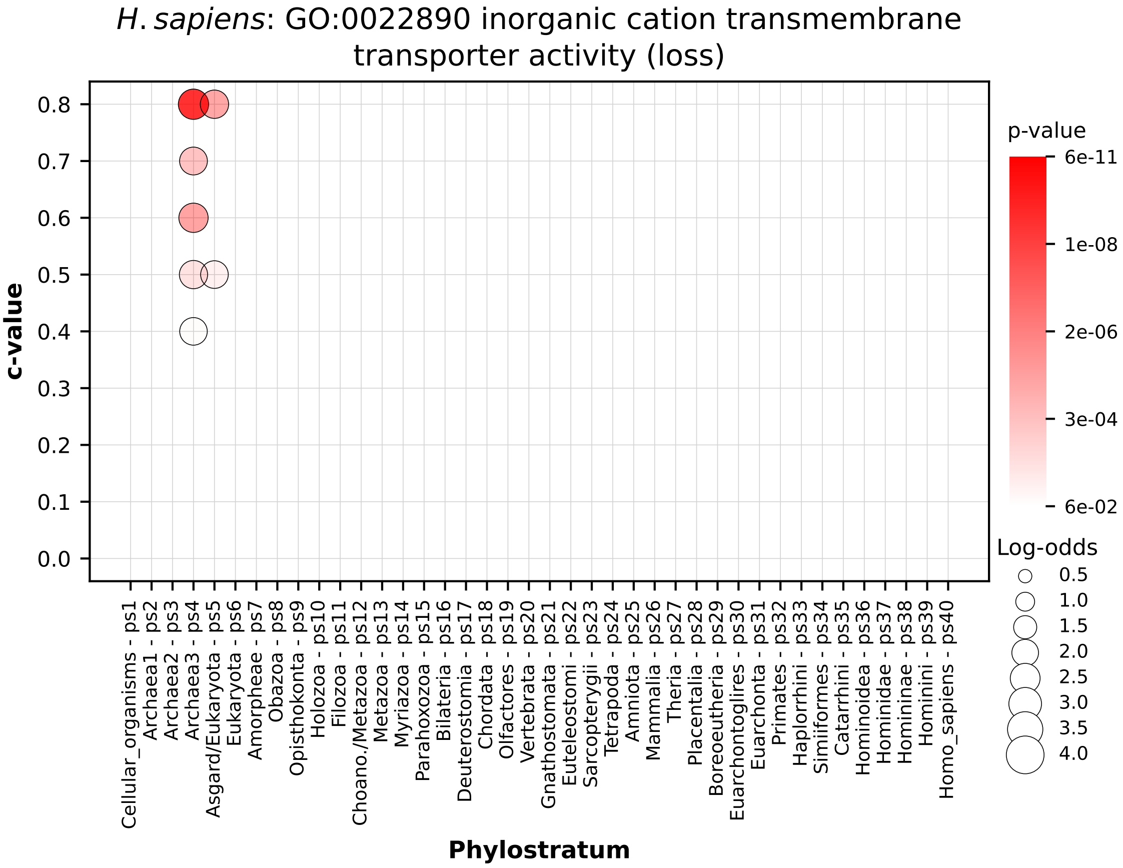

H. sapiens: GO:0022890 inorganic cation transmembrane transporter activity (loss)
c-value
0.0
0.1
0.2
0.3
0.4
0.5
0.6
0.7
0.8
Phylostratum
Cellular_organisms - ps1
Archaea1 - ps2
Archaea2 - ps3
Archaea3 - ps4
Asgard/Eukaryota - ps5
Eukaryota - ps6
Amorpheae - ps7
Obazoa - ps8
Opisthokonta - ps9
Holozoa - ps10
Filozoa - ps11
Choano./Metazoa - ps12
Metazoa - ps13
Myriazoa - ps14
Parahoxozoa - ps15
Bilateria - ps16
Deuterostomia - ps17
Chordata - ps18
Olfactores - ps19
Vertebrata - ps20
Gnathostomata - ps21
Euteleostomi - ps22
Sarcopterygii - ps23
Tetrapoda - ps24
Amniota - ps25
Mammalia - ps26
Theria - ps27
Placentalia - ps28
Boreoeutheria - ps29
Euarchontoglires - ps30
Euarchonta - ps31
Primates - ps32
Haplorrhini - ps33
Simiiformes - ps34
Catarrhini - ps35
Hominoidea - ps36
Hominidae - ps37
Homininae - ps38
Hominini - ps39
Homo_sapiens - ps40
p-value
6e-11
1e-08
2e-06
3e-04
6e-02
Log-odds
0.5
1.0
1.5
2.0
2.5
3.0
3.5
4.0

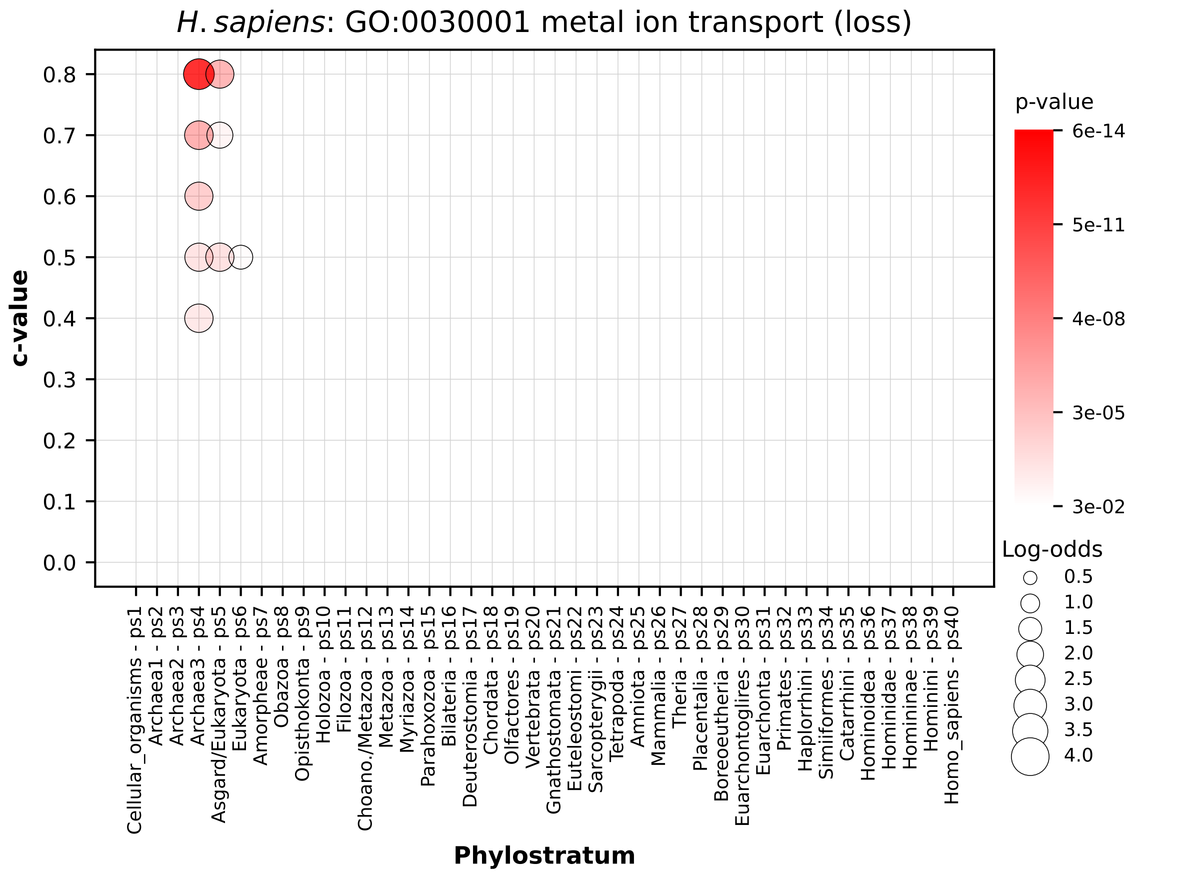

H. sapiens: GO:0030001 metal ion transport (loss)
c-value
0.0
0.1
0.2
0.3
0.4
0.5
0.6
0.7
0.8
Phylostratum
Cellular_organisms - ps1
Archaea1 - ps2
Archaea2 - ps3
Archaea3 - ps4
Asgard/Eukaryota - ps5
Eukaryota - ps6
Amorpheae - ps7
Obazoa - ps8
Opisthokonta - ps9
Holozoa - ps10
Filozoa - ps11
Choano./Metazoa - ps12
Metazoa - ps13
Myriazoa - ps14
Parahoxozoa - ps15
Bilateria - ps16
Deuterostomia - ps17
Chordata - ps18
Olfactores - ps19
Vertebrata - ps20
Gnathostomata - ps21
Euteleostomi - ps22
Sarcopterygii - ps23
Tetrapoda - ps24
Amniota - ps25
Mammalia - ps26
Theria - ps27
Placentalia - ps28
Boreoeutheria - ps29
Euarchontoglires - ps30
Euarchonta - ps31
Primates - ps32
Haplorrhini - ps33
Simiiformes - ps34
Catarrhini - ps35
Hominoidea - ps36
Hominidae - ps37
Homininae - ps38
Hominini - ps39
Homo_sapiens - ps40
p-value
6e-14
5e-11
4e-08
3e-05
3e-02
Log-odds
0.5
1.0
1.5
2.0
2.5
3.0
3.5
4.0

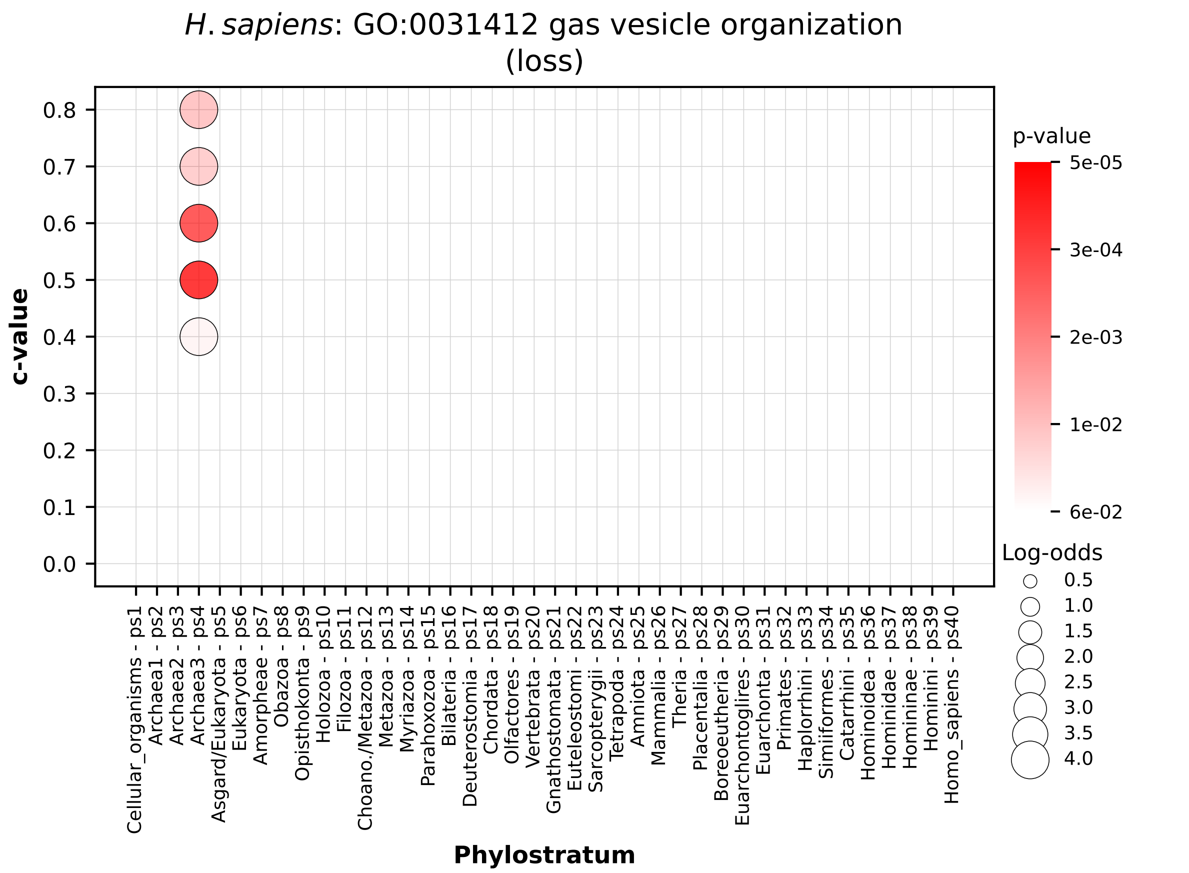
H. sapiens: GO:0031412 gas vesicle organization
(loss)
c-value
0.0
0.1
0.2
0.3
0.4
0.5
0.6
0.7
0.8
Phylostratum
Cellular_organisms - ps1
Archaea1 - ps2
Archaea2 - ps3
Archaea3 - ps4
Asgard/Eukaryota - ps5
Eukaryota - ps6
Amorpheae - ps7
Obazoa - ps8
Opisthokonta - ps9
Holozoa - ps10
Filozoa - ps11
Choano./Metazoa - ps12
Metazoa - ps13
Myriazoa - ps14
Parahoxozoa - ps15
Bilateria - ps16
Deuterostomia - ps17
Chordata - ps18
Olfactores - ps19
Vertebrata - ps20
Gnathostomata - ps21
Euteleostomi - ps22
Sarcopterygii - ps23
Tetrapoda - ps24
Amniota - ps25
Mammalia - ps26
Theria - ps27
Placentalia - ps28
Boreoeutheria - ps29
Euarchontoglires - ps30
Euarchonta - ps31
Primates - ps32
Haplorrhini - ps33
Simiiformes - ps34
Catarrhini - ps35
Hominoidea - ps36
Hominidae - ps37
Homininae - ps38
Hominini - ps39
Homo_sapiens - ps40
p-value
5e-05
3e-04
2e-03
1e-02
6e-02
Log-odds
0.5
1.0
1.5
2.0
2.5
3.0
3.5
4.0

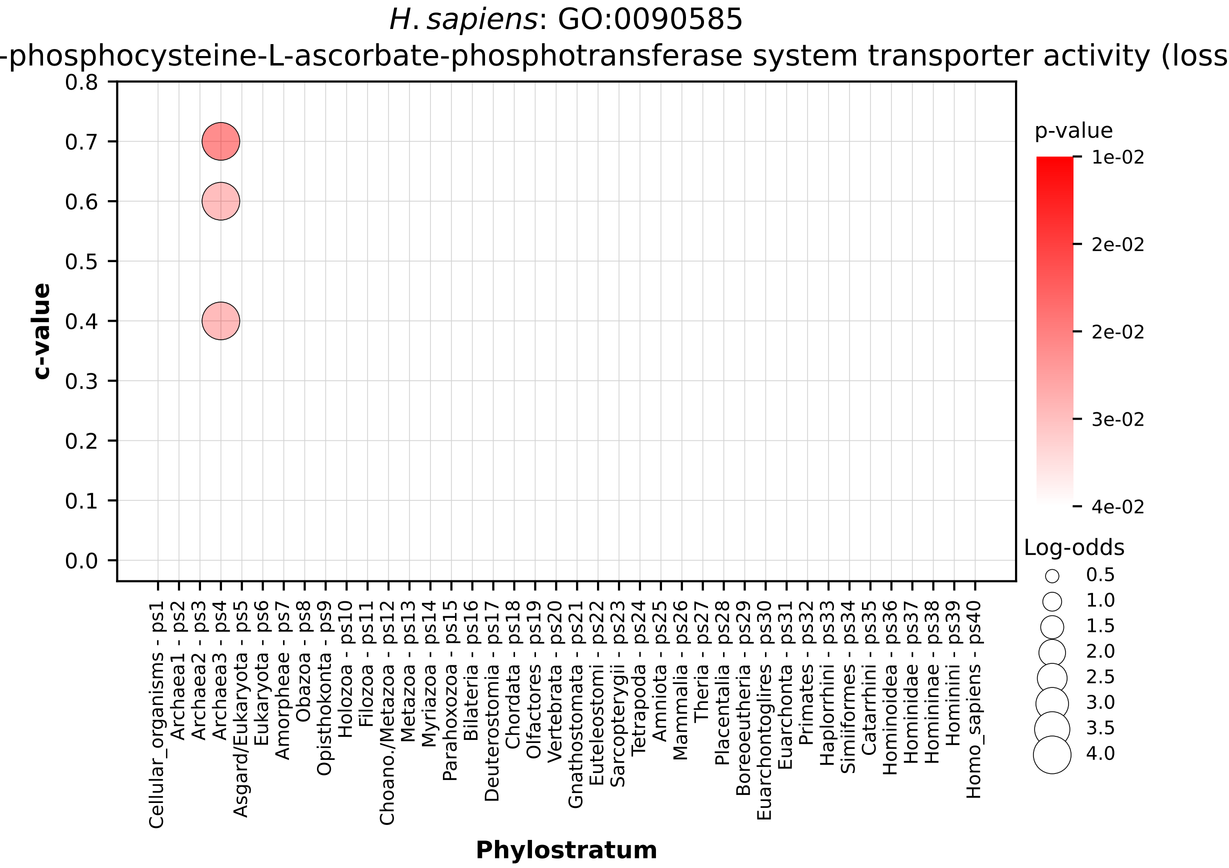

H. sapiens: GO:0090585
-phosphocysteine-L-ascorbate-phosphotransferase system transporter activity (loss
c-value
0.8
0.7
0.6
0.5
0.4
0.3
0.2
0.1
0.0
p-value
1e-02
2e-02
2e-02
3e-02
4e-02
Log-odds
0.5
1.0
1.5
2.0
2.5
3.0
3.5
4.0
Cellular_organisms - ps1
Archaea1 - ps2
Archaea2 - ps3
Archaea3 - ps4
Asgard/Eukaryota - ps5
Eukaryota - ps6
Amorpheae - ps7
Obazoa - ps8
Opisthokonta - ps9
Holozoa - ps10
Filozoa - ps11
Choano./Metazoa - ps12
Metazoa - ps13
Myriazoa - ps14
Parahoxozoa - ps15
Bilateria - ps16
Deuterostomia - ps17
Chordata - ps18
Olfactores - ps19
Vertebrata - ps20
Gnathostomata - ps21
Euteleostomi - ps22
Sarcopterygii - ps23
Tetrapoda - ps24
Amniota - ps25
Mammalia - ps26
Theria - ps27
Placentalia - ps28
Boreoeutheria - ps29
Euarchontoglires - ps30
Euarchonta - ps31
Primates - ps32
Haplorrhini - ps33
Simiiformes - ps34
Catarrhini - ps35
Hominoidea - ps36
Hominidae - ps37
Homininae - ps38
Hominini - ps39
Homo_sapiens - ps40
Phylostratum

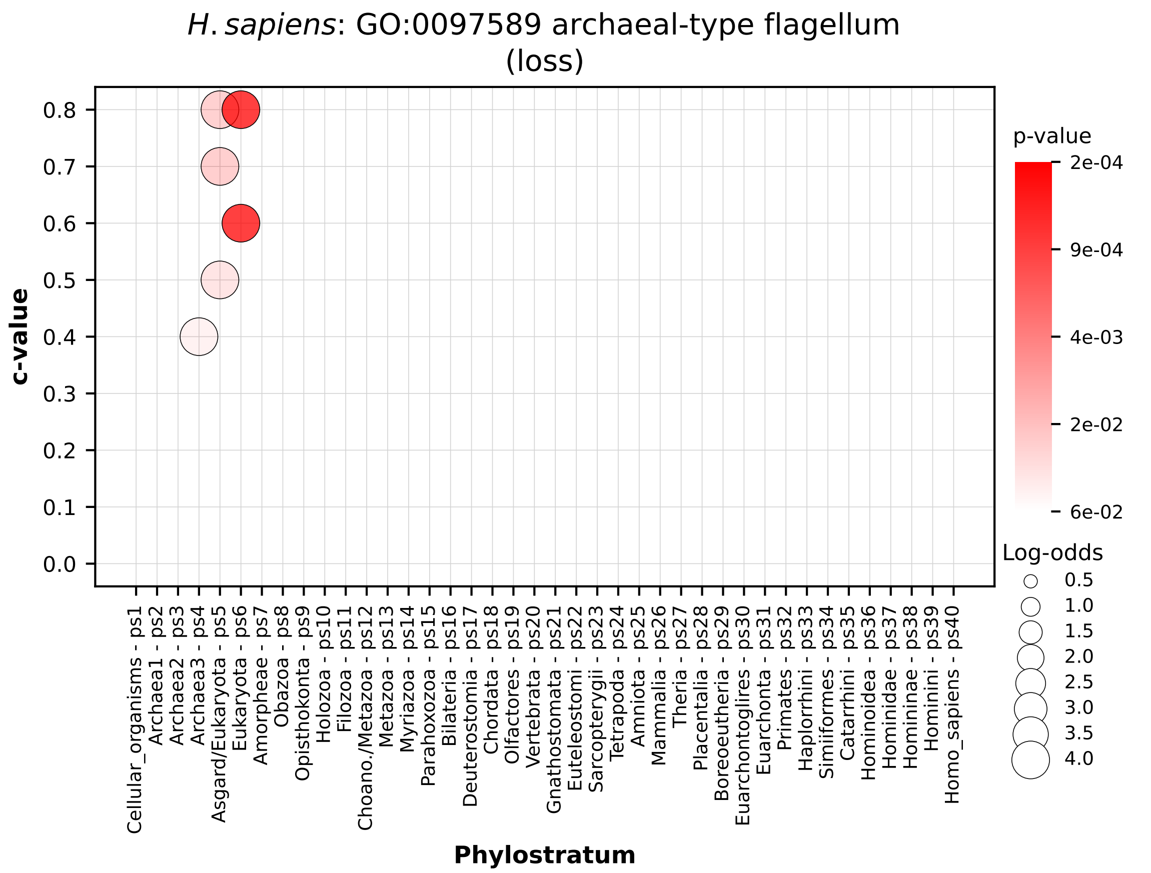

H. sapiens: GO:0097589 archaeal-type flagellum
(loss)
c-value
0.0
0.1
0.2
0.3
0.4
0.5
0.6
0.7
0.8
Phylostratum
Cellular_organisms - ps1
Archaea1 - ps2
Archaea2 - ps3
Archaea3 - ps4
Asgard/Eukaryota - ps5
Eukaryota - ps6
Amorpheae - ps7
Obazoa - ps8
Opisthokonta - ps9
Holozoa - ps10
Filozoa - ps11
Choano./Metazoa - ps12
Metazoa - ps13
Myriazoa - ps14
Parahoxozoa - ps15
Bilateria - ps16
Deuterostomia - ps17
Chordata - ps18
Olfactores - ps19
Vertebrata - ps20
Gnathostomata - ps21
Euteleostomi - ps22
Sarcopterygii - ps23
Tetrapoda - ps24
Amniota - ps25
Mammalia - ps26
Theria - ps27
Placentalia - ps28
Boreoeutheria - ps29
Euarchontoglires - ps30
Euarchonta - ps31
Primates - ps32
Haplorrhini - ps33
Simiiformes - ps34
Catarrhini - ps35
Hominoidea - ps36
Hominidae - ps37
Homininae - ps38
Hominini - ps39
Homo_sapiens - ps40
p-value
2e-04
9e-04
4e-03
2e-02
6e-02
Log-odds
0.5
1.0
1.5
2.0
2.5
3.0
3.5
4.0

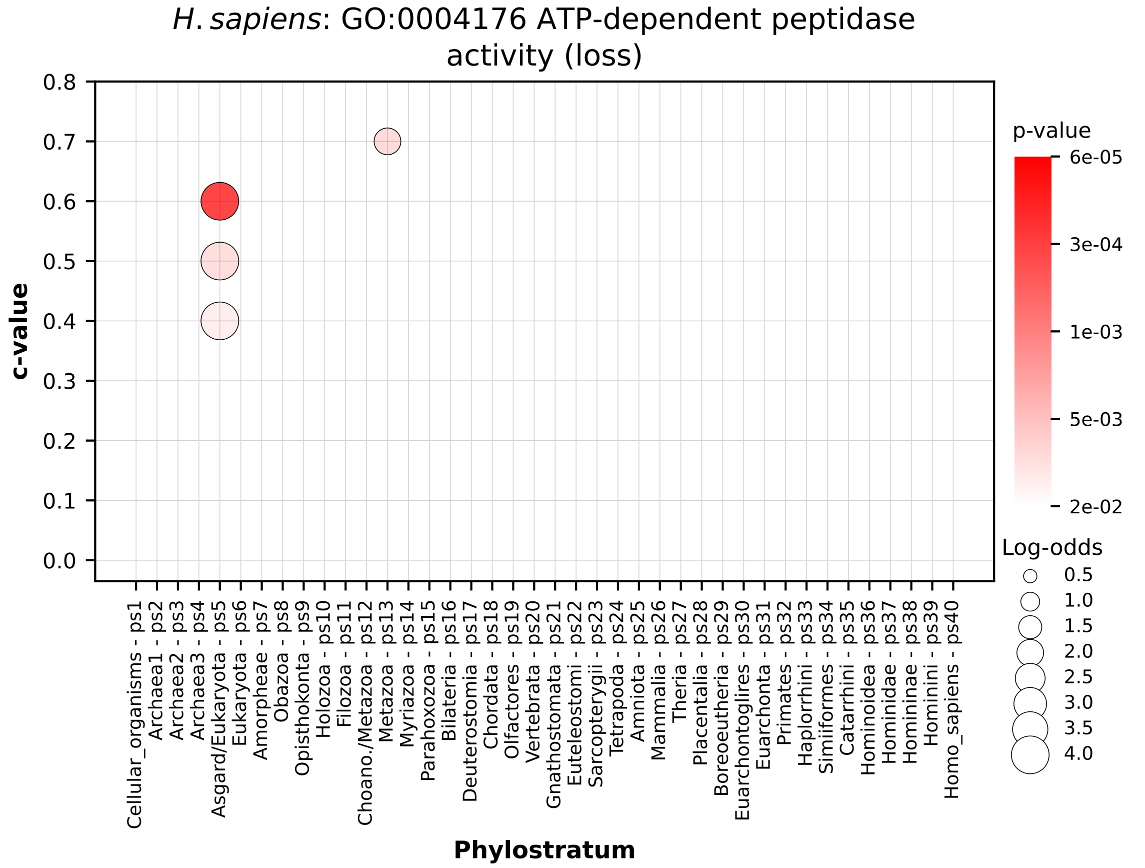

H. sapiens: GO:0004176 ATP-dependent peptidase activity (loss)
c-value
0.0
0.1
0.2
0.3
0.4
0.5
0.6
0.7
0.8
Phylostratum
Cellular_organisms - ps1
Archaea1 - ps2
Archaea2 - ps3
Archaea3 - ps4
Asgard/Eukaryota - ps5
Eukaryota - ps6
Amorpheae - ps7
Obazoa - ps8
Opisthokonta - ps9
Holozoa - ps10
Filozoa - ps11
Choano./Metazoa - ps12
Metazoa - ps13
Myriazoa - ps14
Parahoxozoa - ps15
Bilateria - ps16
Deuterostomia - ps17
Chordata - ps18
Olfactores - ps19
Vertebrata - ps20
Gnathostomata - ps21
Euteleostomi - ps22
Sarcopterygii - ps23
Tetrapoda - ps24
Amniota - ps25
Mammalia - ps26
Theria - ps27
Placentalia - ps28
Boreoeutheria - ps29
Euarchontoglires - ps30
Euarchonta - ps31
Primates - ps32
Haplorrhini - ps33
Simiiformes - ps34
Catarrhini - ps35
Hominoidea - ps36
Hominidae - ps37
Homininae - ps38
Hominini - ps39
Homo_sapiens - ps40
p-value
6e-05
3e-04
1e-03
5e-03
2e-02
Log-odds
0.5
1.0
1.5
2.0
2.5
3.0
3.5
4.0

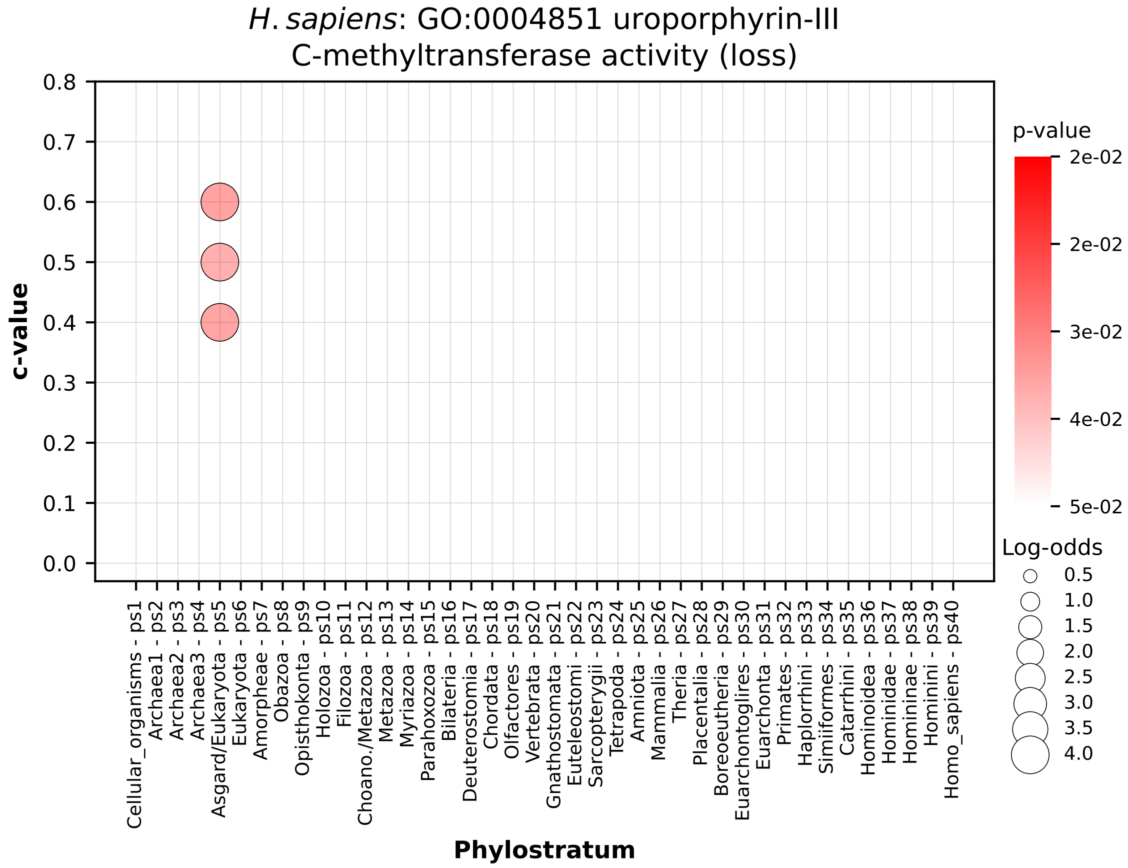

H. sapiens: GO:0004851 uroporphyrin-III C-methyltransferase activity (loss)
c-value
0.8
0.7
0.6
0.5
0.4
0.3
0.2
0.1
0.0
Phylostratum
Cellular_organisms - ps1
Archaea1 - ps2
Archaea2 - ps3
Archaea3 - ps4
Asgard/Eukaryota - ps5
Eukaryota - ps6
Amorpheae - ps7
Obazoa - ps8
Opisthokonta - ps9
Holozoa - ps10
Filozoa - ps11
Choano./Metazoa - ps12
Metazoa - ps13
Myriazoa - ps14
Parahoxozoa - ps15
Bilateria - ps16
Deuterostomia - ps17
Chordata - ps18
Olfactores - ps19
Vertebrata - ps20
Gnathostomata - ps21
Euteleostomi - ps22
Sarcopterygii - ps23
Tetrapoda - ps24
Amniota - ps25
Mammalia - ps26
Theria - ps27
Placentalia - ps28
Boreoeutheria - ps29
Euarchontoglires - ps30
Euarchonta - ps31
Primates - ps32
Haplorrhini - ps33
Simiiformes - ps34
Catarrhini - ps35
Hominoidea - ps36
Hominidae - ps37
Homininae - ps38
Hominini - ps39
Homo_sapiens - ps40
p-value
2e-02
2e-02
3e-02
4e-02
5e-02
Log-odds
0.5
1.0
1.5
2.0
2.5
3.0
3.5
4.0

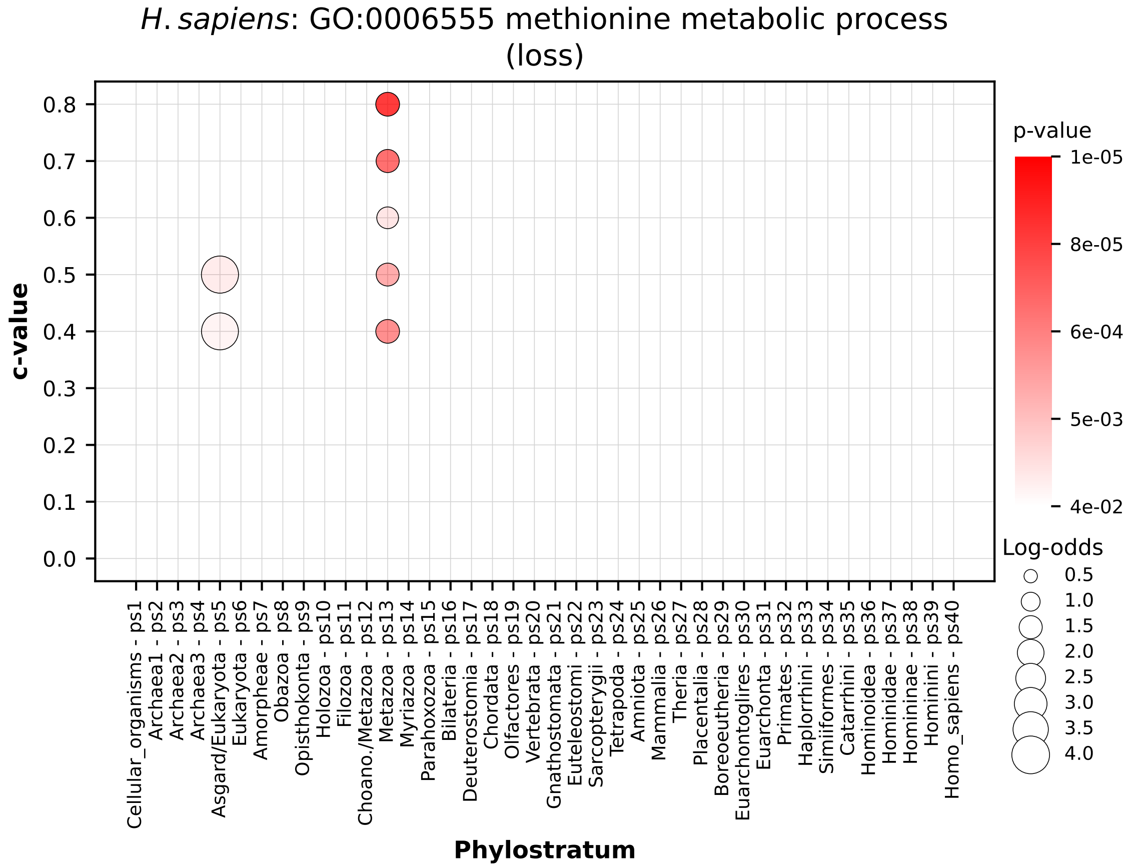

H. sapiens: GO:0006555 methionine metabolic process (loss)
c-value
0.0
0.1
0.2
0.3
0.4
0.5
0.6
0.7
0.8
p-value
1e-05
8e-05
6e-04
5e-03
4e-02
Log-odds
0.5
1.0
1.5
2.0
2.5
3.0
3.5
4.0
Cellular_organisms - ps1
Archaea1 - ps2
Archaea2 - ps3
Archaea3 - ps4
Asgard/Eukaryota - ps5
Eukaryota - ps6
Amorpheae - ps7
Obazoa - ps8
Opisthokonta - ps9
Holozoa - ps10
Filozoa - ps11
Choano./Metazoa - ps12
Metazoa - ps13
Myriazoa - ps14
Parahoxozoa - ps15
Bilateria - ps16
Deuterostomia - ps17
Chordata - ps18
Olfactores - ps19
Vertebrata - ps20
Gnathostomata - ps21
Euteleostomi - ps22
Sarcopterygii - ps23
Tetrapoda - ps24
Amniota - ps25
Mammalia - ps26
Theria - ps27
Placentalia - ps28
Boreoeutheria - ps29
Euarchontoglires - ps30
Euarchonta - ps31
Primates - ps32
Haplorrhini - ps33
Simiiformes - ps34
Catarrhini - ps35
Hominoidea - ps36
Hominidae - ps37
Homininae - ps38
Hominini - ps39
Homo_sapiens - ps40
Phylostratum

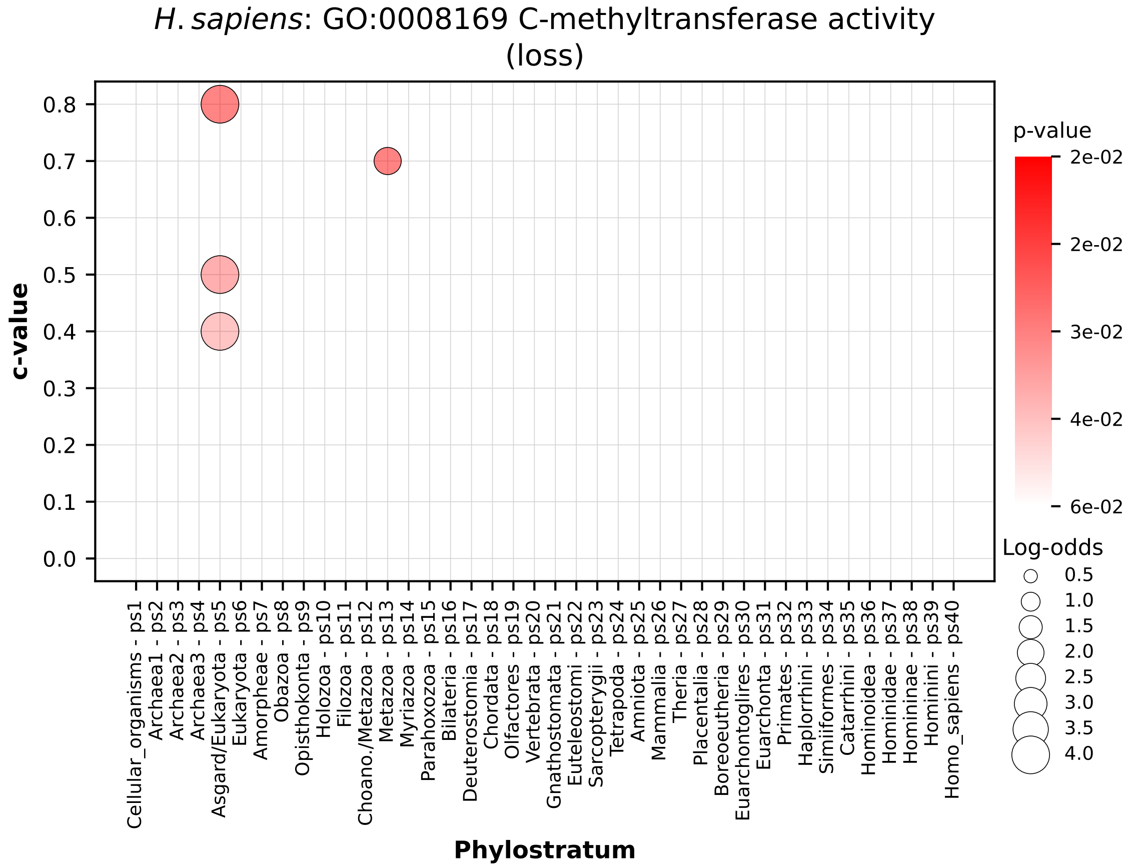

H. sapiens: GO:0008169 C-methyltransferase activity (loss)
c-value
0.0
0.1
0.2
0.3
0.4
0.5
0.6
0.7
0.8
Phylostratum
Cellular_organisms - ps1
Archaea1 - ps2
Archaea2 - ps3
Archaea3 - ps4
Asgard/Eukaryota - ps5
Eukaryota - ps6
Amorpheae - ps7
Obazoa - ps8
Opisthokonta - ps9
Holozoa - ps10
Filozoa - ps11
Choano./Metazoa - ps12
Metazoa - ps13
Myriazoa - ps14
Parahoxozoa - ps15
Bilateria - ps16
Deuterostomia - ps17
Chordata - ps18
Olfactores - ps19
Vertebrata - ps20
Gnathostomata - ps21
Euteleostomi - ps22
Sarcopterygii - ps23
Tetrapoda - ps24
Amniota - ps25
Mammalia - ps26
Theria - ps27
Placentalia - ps28
Boreoeutheria - ps29
Euarchontoglires - ps30
Euarchonta - ps31
Primates - ps32
Haplorrhini - ps33
Simiiformes - ps34
Catarrhini - ps35
Hominoidea - ps36
Hominidae - ps37
Homininae - ps38
Hominini - ps39
Homo_sapiens - ps40
p-value
2e-02
2e-02
3e-02
4e-02
6e-02
Log-odds
0.5
1.0
1.5
2.0
2.5
3.0
3.5
4.0

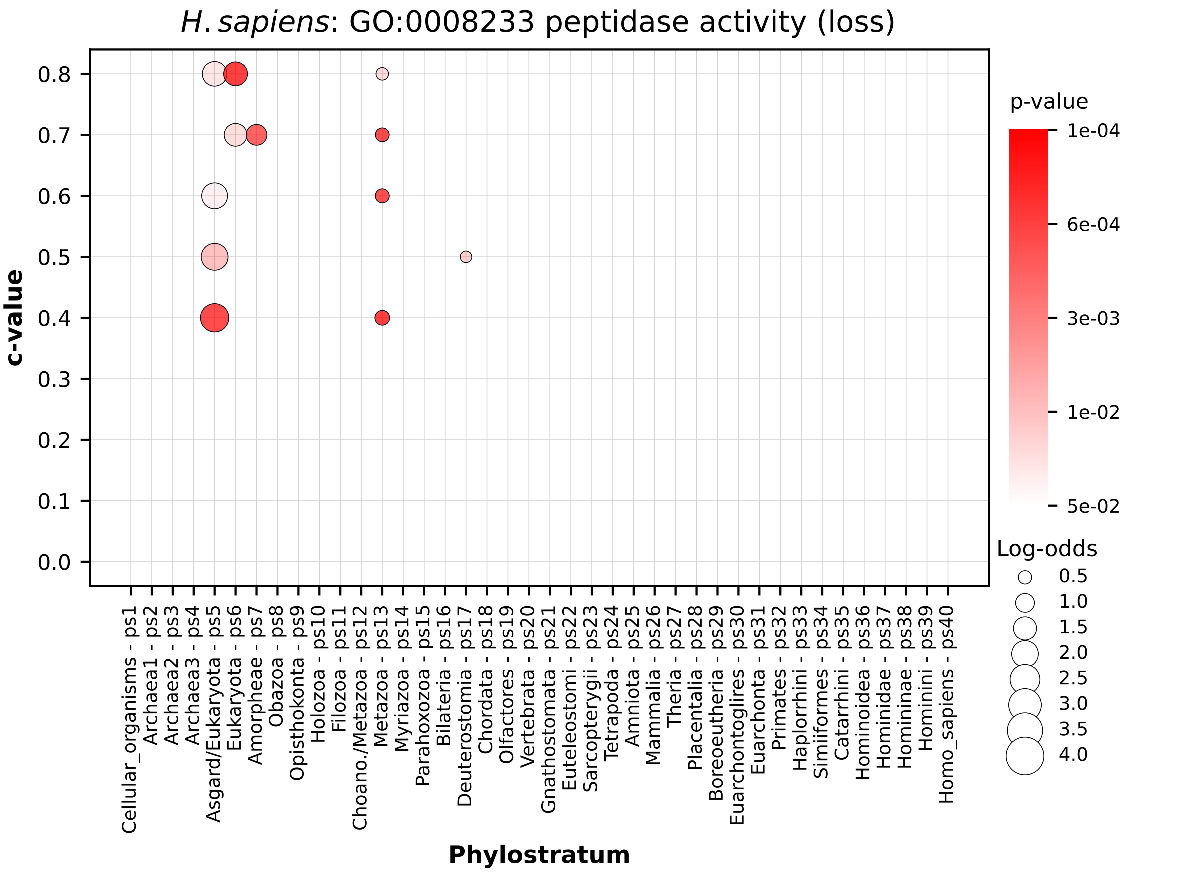

H. sapiens: GO:0008233 peptidase activity (loss)
c-value
0.0
0.1
0.2
0.3
0.4
0.5
0.6
0.7
0.8
p-value
1e-04
6e-04
3e-03
1e-02
5e-02
Log-odds
0.5
1.0
1.5
2.0
2.5
3.0
3.5
4.0
Cellular_organisms - ps1
Archaea1 - ps2
Archaea2 - ps3
Archaea3 - ps4
Asgard/Eukaryota - ps5
Eukaryota - ps6
Amorpheae - ps7
Obazoa - ps8
Opisthokonta - ps9
Holozoa - ps10
Filozoa - ps11
Choano./Metazoa - ps12
Metazoa - ps13
Myriazoa - ps14
Parahoxozoa - ps15
Bilateria - ps16
Deuterostomia - ps17
Chordata - ps18
Olfactores - ps19
Vertebrata - ps20
Gnathostomata - ps21
Euteleostomi - ps22
Sarcopterygii - ps23
Tetrapoda - ps24
Amniota - ps25
Mammalia - ps26
Theria - ps27
Placentalia - ps28
Boreoeutheria - ps29
Euarchontoglires - ps30
Euarchonta - ps31
Primates - ps32
Haplorrhini - ps33
Simiiformes - ps34
Catarrhini - ps35
Hominoidea - ps36
Hominidae - ps37
Homininae - ps38
Hominini - ps39
Homo_sapiens - ps40
Phylostratum

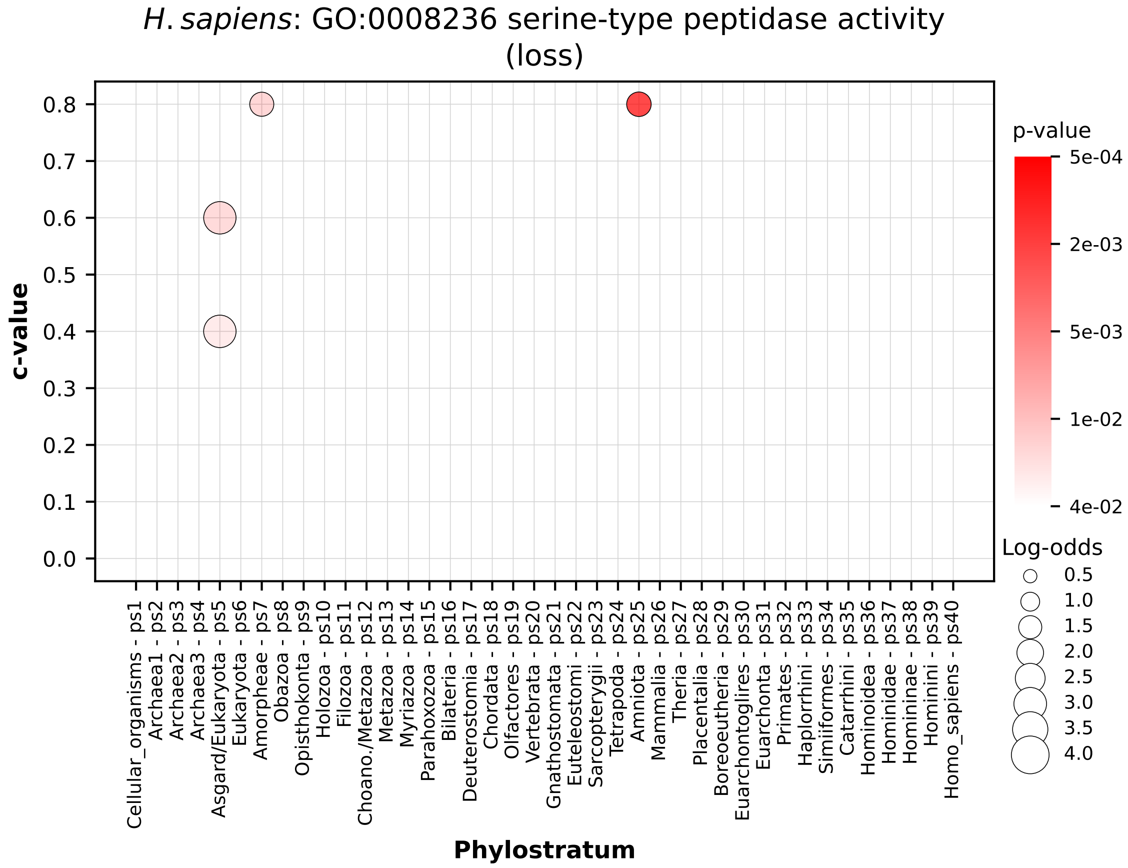
H. sapiens: GO:0008236 serine-type peptidase activity (loss)
c-value
0.0
0.1
0.2
0.3
0.4
0.5
0.6
0.7
0.8
Phylostratum
Cellular_organisms - ps1
Archaea1 - ps2
Archaea2 - ps3
Archaea3 - ps4
Asgard/Eukaryota - ps5
Eukaryota - ps6
Amorpheae - ps7
Obazoa - ps8
Opisthokonta - ps9
Holozoa - ps10
Filozoa - ps11
Choano./Metazoa - ps12
Metazoa - ps13
Myriazoa - ps14
Parahoxozoa - ps15
Bilateria - ps16
Deuterostomia - ps17
Chordata - ps18
Olfactores - ps19
Vertebrata - ps20
Gnathostomata - ps21
Euteleostomi - ps22
Sarcopterygii - ps23
Tetrapoda - ps24
Amniota - ps25
Mammalia - ps26
Theria - ps27
Placentalia - ps28
Boreoeutheria - ps29
Euarchontoglires - ps30
Euarchonta - ps31
Primates - ps32
Haplorrhini - ps33
Simiiformes - ps34
Catarrhini - ps35
Hominoidea - ps36
Hominidae - ps37
Homininae - ps38
Hominini - ps39
Homo_sapiens - ps40
p-value
5e-04
2e-03
5e-03
1e-02
4e-02
Log-odds
0.5
1.0
1.5
2.0
2.5
3.0
3.5
4.0

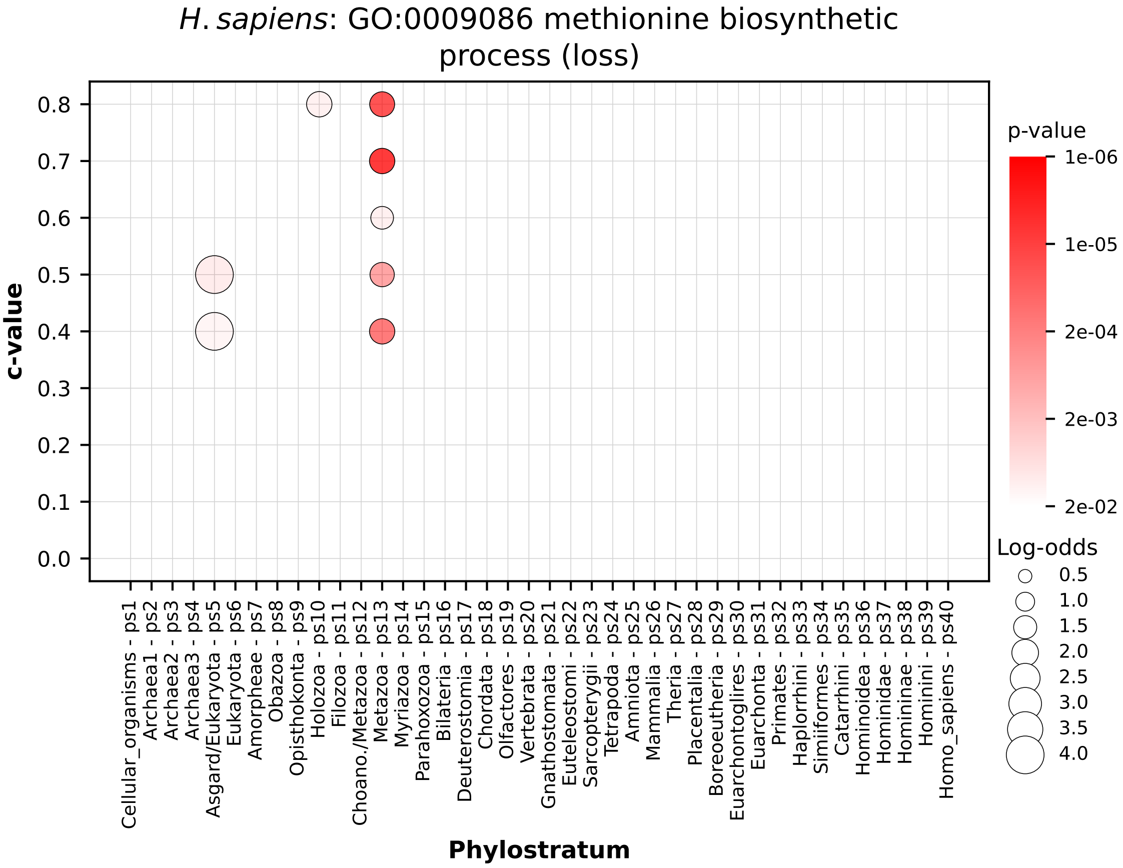
H. sapiens: GO:0009086 methionine biosynthetic process (loss)
c-value
0.0
0.1
0.2
0.3
0.4
0.5
0.6
0.7
0.8
Phylostratum
Cellular_organisms - ps1
Archaea1 - ps2
Archaea2 - ps3
Archaea3 - ps4
Asgard/Eukaryota - ps5
Eukaryota - ps6
Amorpheae - ps7
Obazoa - ps8
Opisthokonta - ps9
Holozoa - ps10
Filozoa - ps11
Choano./Metazoa - ps12
Metazoa - ps13
Myriazoa - ps14
Parahoxozoa - ps15
Bilateria - ps16
Deuterostomia - ps17
Chordata - ps18
Olfactores - ps19
Vertebrata - ps20
Gnathostomata - ps21
Euteleostomi - ps22
Sarcopterygii - ps23
Tetrapoda - ps24
Amniota - ps25
Mammalia - ps26
Theria - ps27
Placentalia - ps28
Boreoeutheria - ps29
Euarchontoglires - ps30
Euarchonta - ps31
Primates - ps32
Haplorrhini - ps33
Simiiformes - ps34
Catarrhini - ps35
Hominoidea - ps36
Hominidae - ps37
Homininae - ps38
Hominini - ps39
Homo_sapiens - ps40
p-value
1e-06
1e-05
2e-04
2e-03
2e-02
Log-odds
0.5
1.0
1.5
2.0
2.5
3.0
3.5
4.0

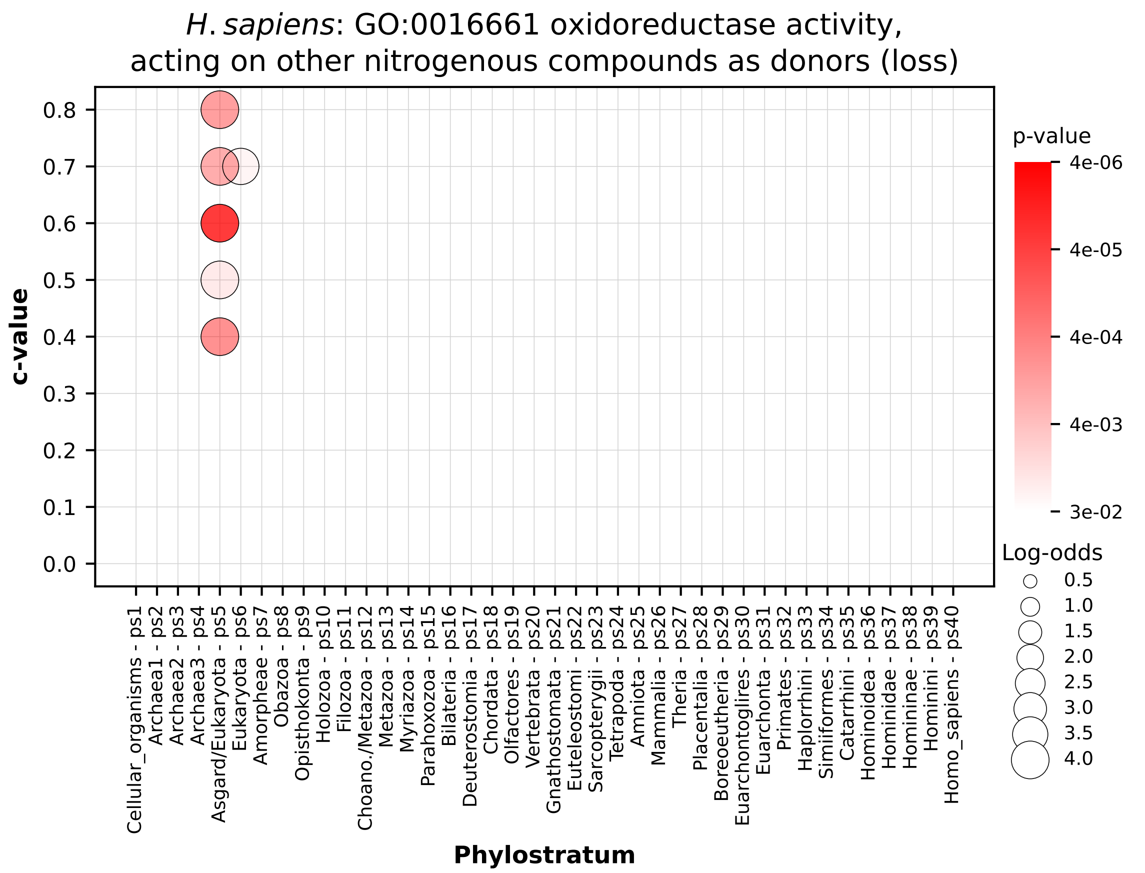
H. sapiens: GO:0016661 oxidoreductase activity, acting on other nitrogenous compounds as donors (loss)
c-value
0.0
0.1
0.2
0.3
0.4
0.5
0.6
0.7
0.8
Phylostratum
Cellular_organisms - ps1
Archaea1 - ps2
Archaea2 - ps3
Archaea3 - ps4
Asgard/Eukaryota - ps5
Eukaryota - ps6
Amorpheae - ps7
Obazoa - ps8
Opisthokonta - ps9
Holozoa - ps10
Filozoa - ps11
Choano./Metazoa - ps12
Metazoa - ps13
Myriazoa - ps14
Parahoxozoa - ps15
Bilateria - ps16
Deuterostomia - ps17
Chordata - ps18
Olfactores - ps19
Vertebrata - ps20
Gnathostomata - ps21
Euteleostomi - ps22
Sarcopterygii - ps23
Tetrapoda - ps24
Amniota - ps25
Mammalia - ps26
Theria - ps27
Placentalia - ps28
Boreoeutheria - ps29
Euarchontoglires - ps30
Euarchonta - ps31
Primates - ps32
Haplorrhini - ps33
Simiiformes - ps34
Catarrhini - ps35
Hominoidea - ps36
Hominidae - ps37
Homininae - ps38
Hominini - ps39
Homo_sapiens - ps40
p-value
4e-06
4e-05
4e-04
4e-03
3e-02
Log-odds
0.5
1.0
1.5
2.0
2.5
3.0
3.5
4.0

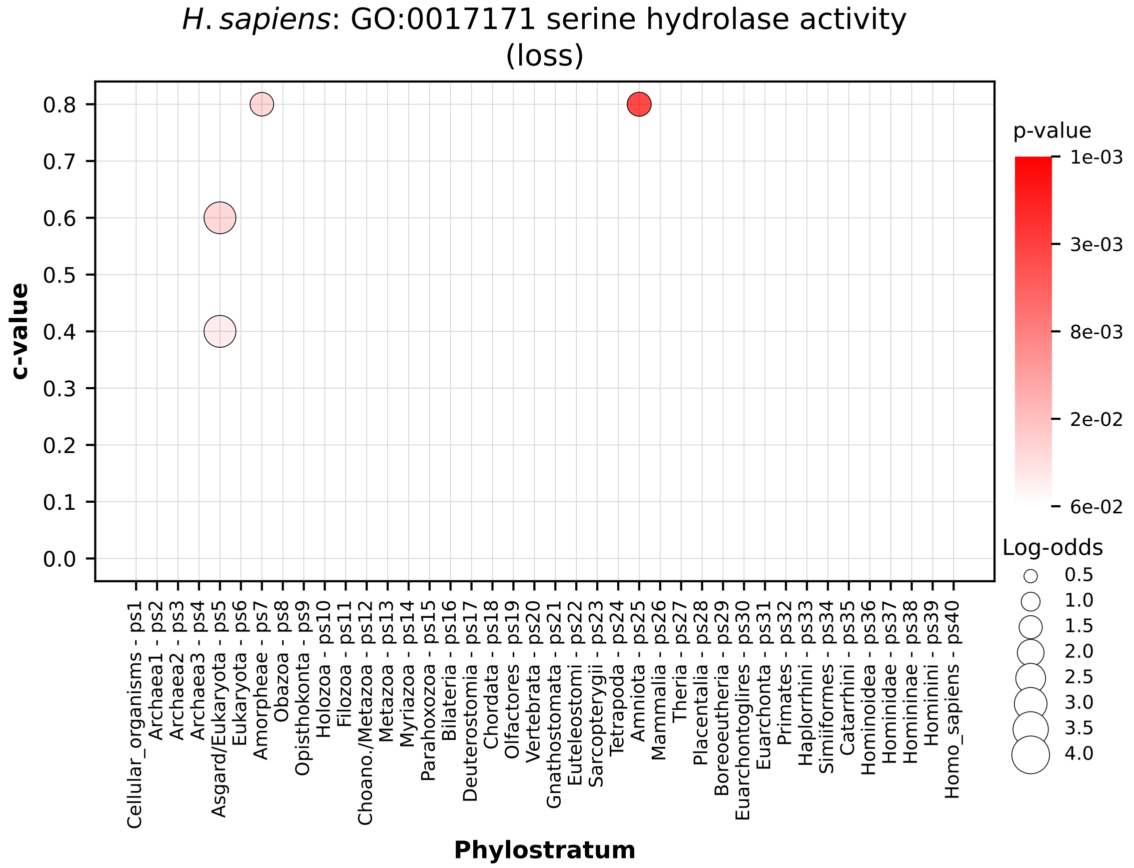
H. sapiens: GO:0017171 serine hydrolase activity (loss)
c-value
0.0
0.1
0.2
0.3
0.4
0.5
0.6
0.7
0.8
Phylostratum
Cellular_organisms - ps1
Archaea1 - ps2
Archaea2 - ps3
Archaea3 - ps4
Asgard/Eukaryota - ps5
Eukaryota - ps6
Amorpheae - ps7
Obazoa - ps8
Opisthokonta - ps9
Holozoa - ps10
Filozoa - ps11
Choano./Metazoa - ps12
Metazoa - ps13
Myriazoa - ps14
Parahoxozoa - ps15
Bilateria - ps16
Deuterostomia - ps17
Chordata - ps18
Olfactores - ps19
Vertebrata - ps20
Gnathostomata - ps21
Euteleostomi - ps22
Sarcopterygii - ps23
Tetrapoda - ps24
Amniota - ps25
Mammalia - ps26
Theria - ps27
Placentalia - ps28
Boreoeutheria - ps29
Euarchontoglires - ps30
Euarchonta - ps31
Primates - ps32
Haplorrhini - ps33
Simiiformes - ps34
Catarrhini - ps35
Hominoidea - ps36
Hominidae - ps37
Homininae - ps38
Hominini - ps39
Homo_sapiens - ps40
p-value
1e-03
3e-03
8e-03
2e-02
6e-02
Log-odds
0.5
1.0
1.5
2.0
2.5
3.0
3.5
4.0

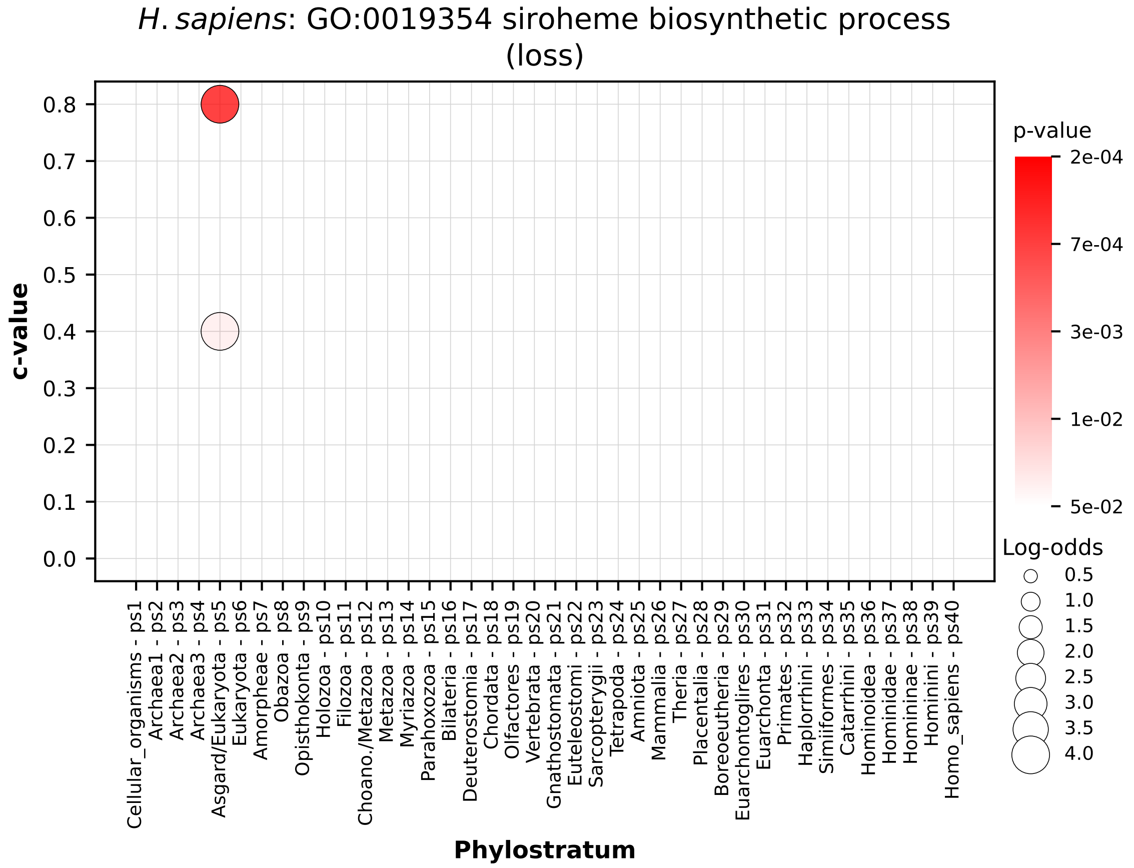
H. sapiens: GO:0019354 siroheme biosynthetic process
(loss)
c-value
0.0
0.1
0.2
0.3
0.4
0.5
0.6
0.7
0.8
Phylostratum
Cellular_organisms - ps1
Archaea1 - ps2
Archaea2 - ps3
Archaea3 - ps4
Asgard/Eukaryota - ps5
Eukaryota - ps6
Amorpheae - ps7
Obazoa - ps8
Opisthokonta - ps9
Holozoa - ps10
Filozoa - ps11
Choano./Metazoa - ps12
Metazoa - ps13
Myriazoa - ps14
Parahoxozoa - ps15
Bilateria - ps16
Deuterostomia - ps17
Chordata - ps18
Olfactores - ps19
Vertebrata - ps20
Gnathostomata - ps21
Euteleostomi - ps22
Sarcopterygii - ps23
Tetrapoda - ps24
Amniota - ps25
Mammalia - ps26
Theria - ps27
Placentalia - ps28
Boreoeutheria - ps29
Euarchontoglires - ps30
Euarchonta - ps31
Primates - ps32
Haplorrhini - ps33
Simiiformes - ps34
Catarrhini - ps35
Hominoidea - ps36
Hominidae - ps37
Homininae - ps38
Hominini - ps39
Homo_sapiens - ps40
p-value
2e-04
7e-04
3e-03
1e-02
5e-02
Log-odds
0.5
1.0
1.5
2.0
2.5
3.0
3.5
4.0

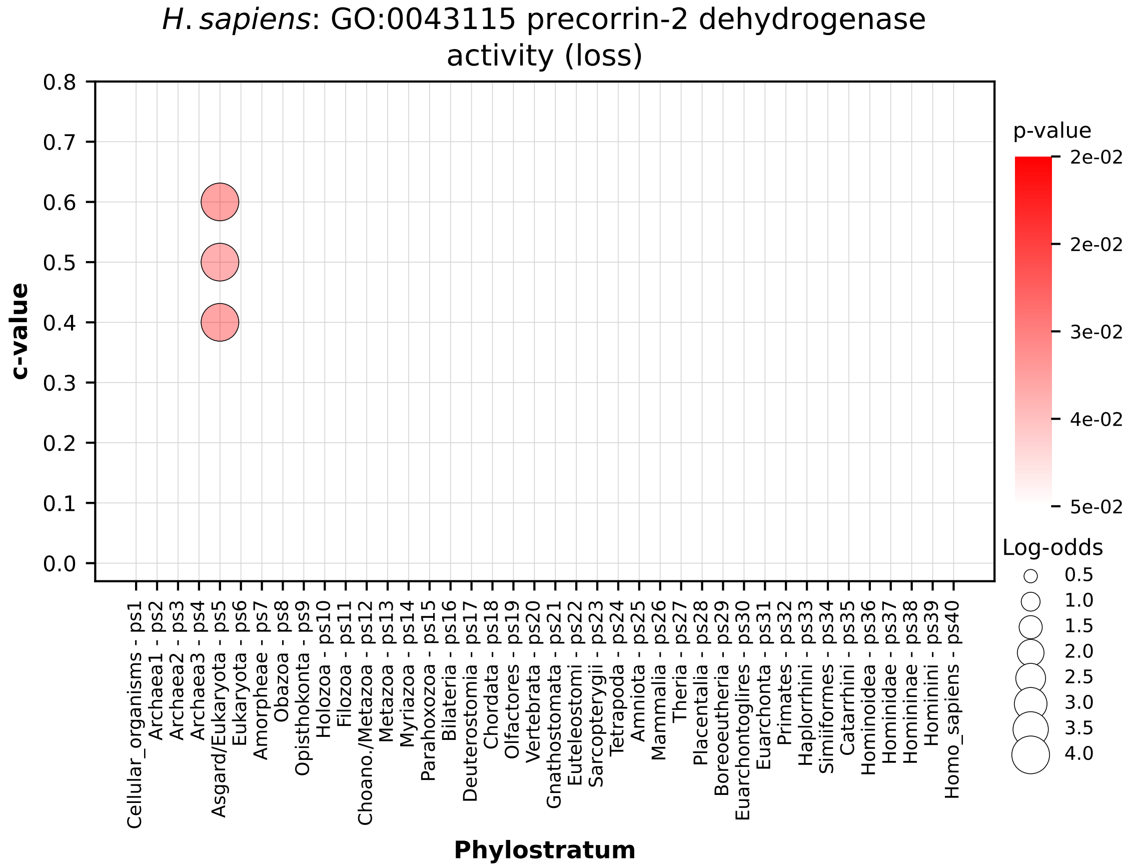

H. sapiens: GO:0043115 precorrin-2 dehydrogenase activity (loss)
c-value
0.8
0.7
0.6
0.5
0.4
0.3
0.2
0.1
0.0
Phylostratum
Cellular_organisms - ps1
Archaea1 - ps2
Archaea2 - ps3
Archaea3 - ps4
Asgard/Eukaryota - ps5
Eukaryota - ps6
Amorpheae - ps7
Obazoa - ps8
Opisthokonta - ps9
Holozoa - ps10
Filozoa - ps11
Choano./Metazoa - ps12
Metazoa - ps13
Myriazoa - ps14
Parahoxozoa - ps15
Bilateria - ps16
Deuterostomia - ps17
Chordata - ps18
Olfactores - ps19
Vertebrata - ps20
Gnathostomata - ps21
Euteleostomi - ps22
Sarcopterygii - ps23
Tetrapoda - ps24
Amniota - ps25
Mammalia - ps26
Theria - ps27
Placentalia - ps28
Boreoeutheria - ps29
Euarchontoglires - ps30
Euarchonta - ps31
Primates - ps32
Haplorrhini - ps33
Simiiformes - ps34
Catarrhini - ps35
Hominoidea - ps36
Hominidae - ps37
Homininae - ps38
Hominini - ps39
Homo_sapiens - ps40
p-value
2e-02
2e-02
3e-02
4e-02
5e-02
Log-odds
0.5
1.0
1.5
2.0
2.5
3.0
3.5
4.0

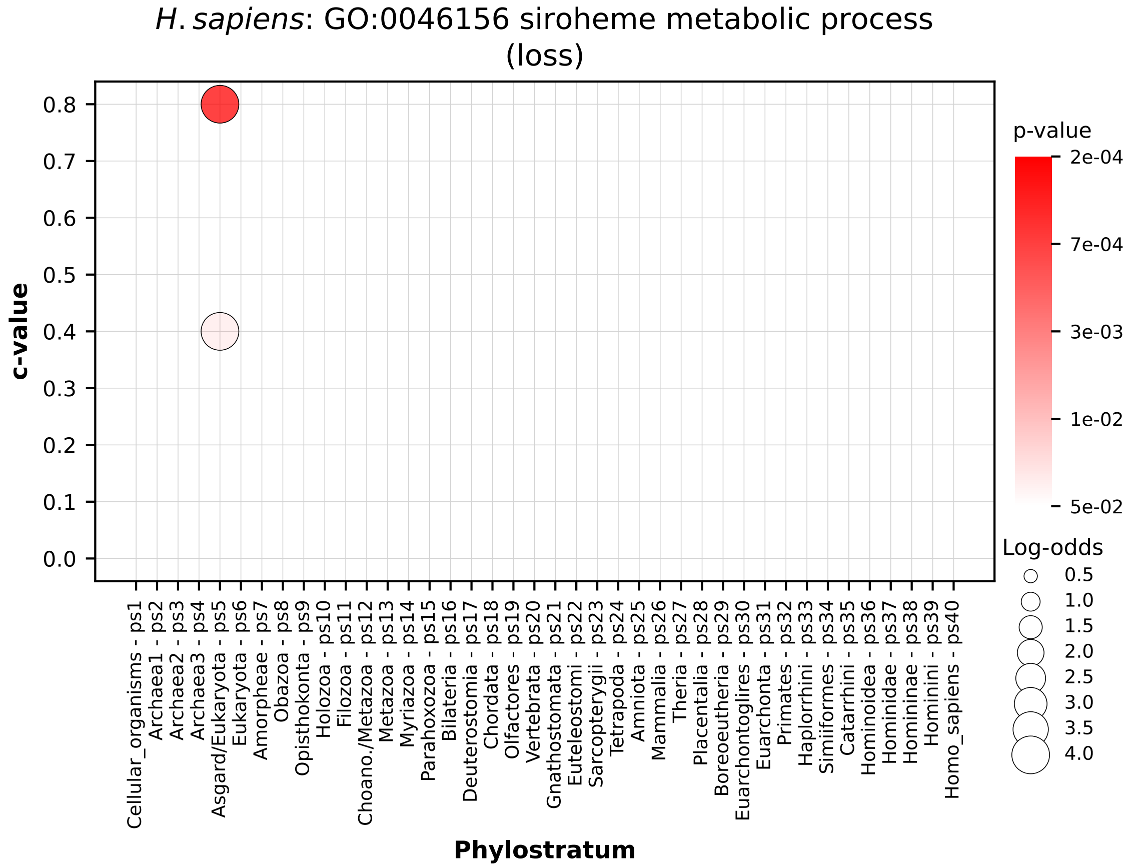

H. sapiens: GO:0046156 siroheme metabolic process
(loss)
c-value
0.0
0.1
0.2
0.3
0.4
0.5
0.6
0.7
0.8
Phylostratum
Cellular_organisms - ps1
Archaea1 - ps2
Archaea2 - ps3
Archaea3 - ps4
Asgard/Eukaryota - ps5
Eukaryota - ps6
Amorpheae - ps7
Obazoa - ps8
Opisthokonta - ps9
Holozoa - ps10
Filozoa - ps11
Choano./Metazoa - ps12
Metazoa - ps13
Myriazoa - ps14
Parahoxozoa - ps15
Bilateria - ps16
Deuterostomia - ps17
Chordata - ps18
Olfactores - ps19
Vertebrata - ps20
Gnathostomata - ps21
Euteleostomi - ps22
Sarcopterygii - ps23
Tetrapoda - ps24
Amniota - ps25
Mammalia - ps26
Theria - ps27
Placentalia - ps28
Boreoeutheria - ps29
Euarchontoglires - ps30
Euarchonta - ps31
Primates - ps32
Haplorrhini - ps33
Simiiformes - ps34
Catarrhini - ps35
Hominoidea - ps36
Hominidae - ps37
Homininae - ps38
Hominini - ps39
Homo_sapiens - ps40
p-value
2e-04
7e-04
3e-03
1e-02
5e-02
Log-odds
0.5
1.0
1.5
2.0
2.5
3.0
3.5
4.0

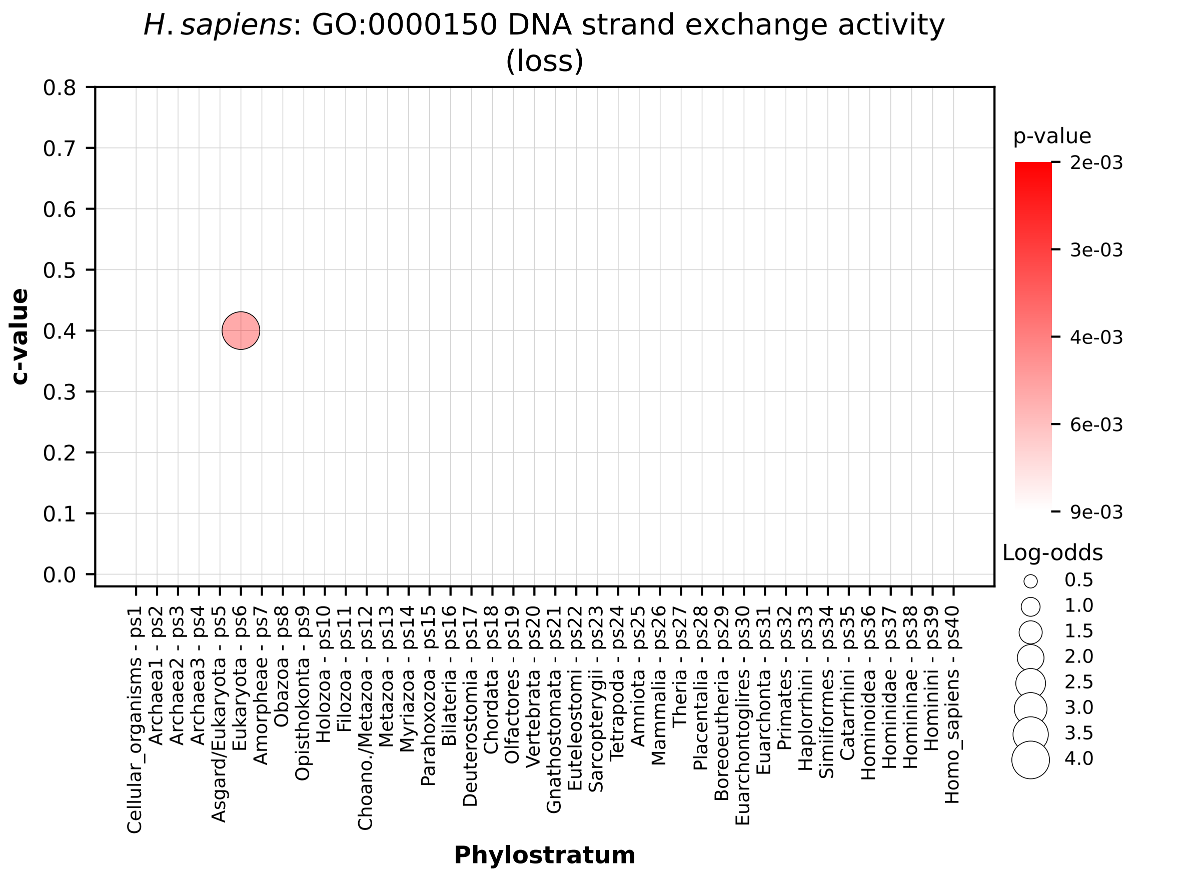
H. sapiens: GO:0000150 DNA strand exchange activity
(loss)
c-value
0.0
0.1
0.2
0.3
0.4
0.5
0.6
0.7
0.8
p-value
2e-03
3e-03
4e-03
6e-03
9e-03
Log-odds
0.5
1.0
1.5
2.0
2.5
3.0
3.5
4.0
Cellular_organisms - ps1
Archaea1 - ps2
Archaea2 - ps3
Archaea3 - ps4
Asgard/Eukaryota - ps5
Eukaryota - ps6
Amorpheae - ps7
Obazoa - ps8
Opisthokonta - ps9
Holozoa - ps10
Filozoa - ps11
Choano./Metazoa - ps12
Metazoa - ps13
Myriazoa - ps14
Parahoxozoa - ps15
Bilateria - ps16
Deuterostomia - ps17
Chordata - ps18
Olfactores - ps19
Vertebrata - ps20
Gnathostomata - ps21
Euteleostomi - ps22
Sarcopterygii - ps23
Tetrapoda - ps24
Amniota - ps25
Mammalia - ps26
Theria - ps27
Placentalia - ps28
Boreoeutheria - ps29
Euarchontoglires - ps30
Euarchonta - ps31
Primates - ps32
Haplorrhini - ps33
Simiiformes - ps34
Catarrhini - ps35
Hominoidea - ps36
Hominidae - ps37
Homininae - ps38
Hominini - ps39
Homo_sapiens - ps40
Phylostratum

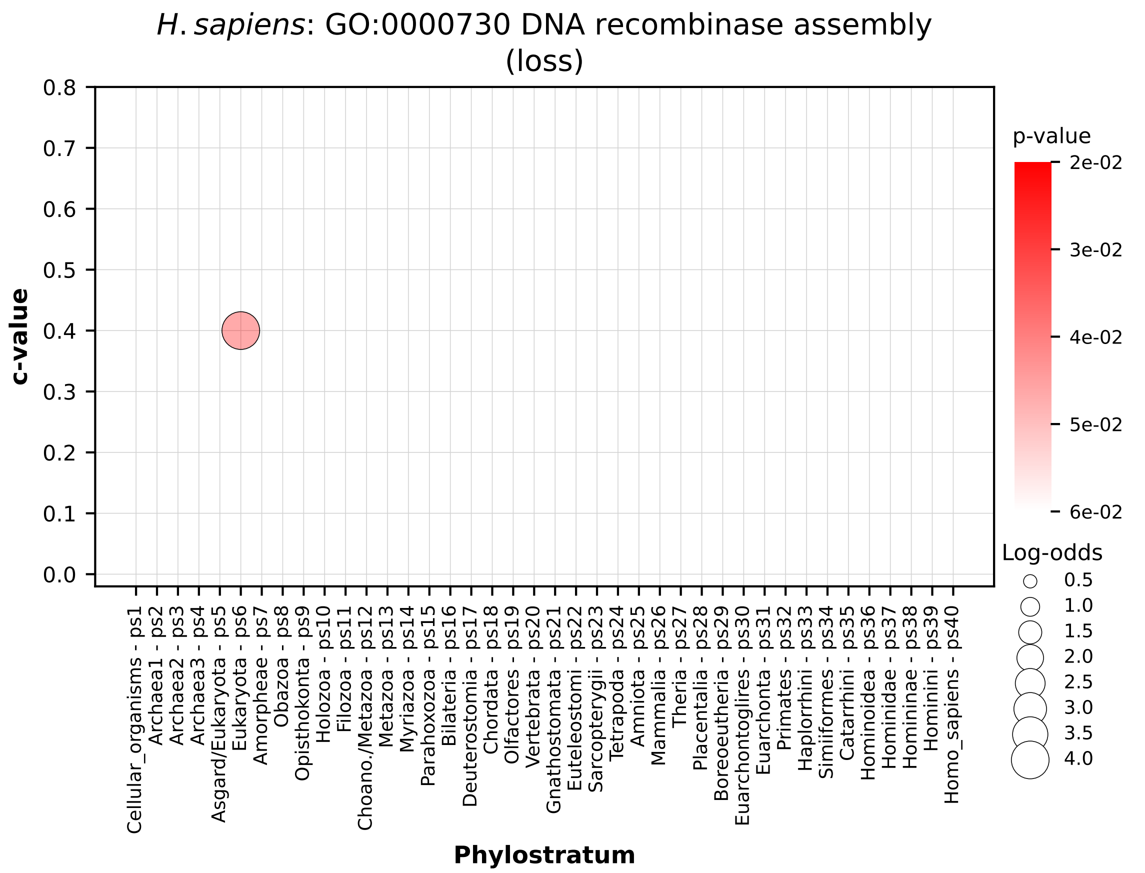

H. sapiens: GO:0000730 DNA recombinase assembly
(loss)
c-value
0.0
0.1
0.2
0.3
0.4
0.5
0.6
0.7
0.8
Phylostratum
Cellular_organisms - ps1
Archaea1 - ps2
Archaea2 - ps3
Archaea3 - ps4
Asgard/Eukaryota - ps5
Eukaryota - ps6
Amorpheae - ps7
Obazoa - ps8
Opisthokonta - ps9
Holozoa - ps10
Filozoa - ps11
Choano./Metazoa - ps12
Metazoa - ps13
Myriazoa - ps14
Parahoxozoa - ps15
Bilateria - ps16
Deuterostomia - ps17
Chordata - ps18
Olfactores - ps19
Vertebrata - ps20
Gnathostomata - ps21
Euteleostomi - ps22
Sarcopterygii - ps23
Tetrapoda - ps24
Amniota - ps25
Mammalia - ps26
Theria - ps27
Placentalia - ps28
Boreoeutheria - ps29
Euarchontoglires - ps30
Euarchonta - ps31
Primates - ps32
Haplorrhini - ps33
Simiiformes - ps34
Catarrhini - ps35
Hominoidea - ps36
Hominidae - ps37
Homininae - ps38
Hominini - ps39
Homo_sapiens - ps40
p-value
2e-02
3e-02
4e-02
5e-02
6e-02
Log-odds
0.5
1.0
1.5
2.0
2.5
3.0
3.5
4.0

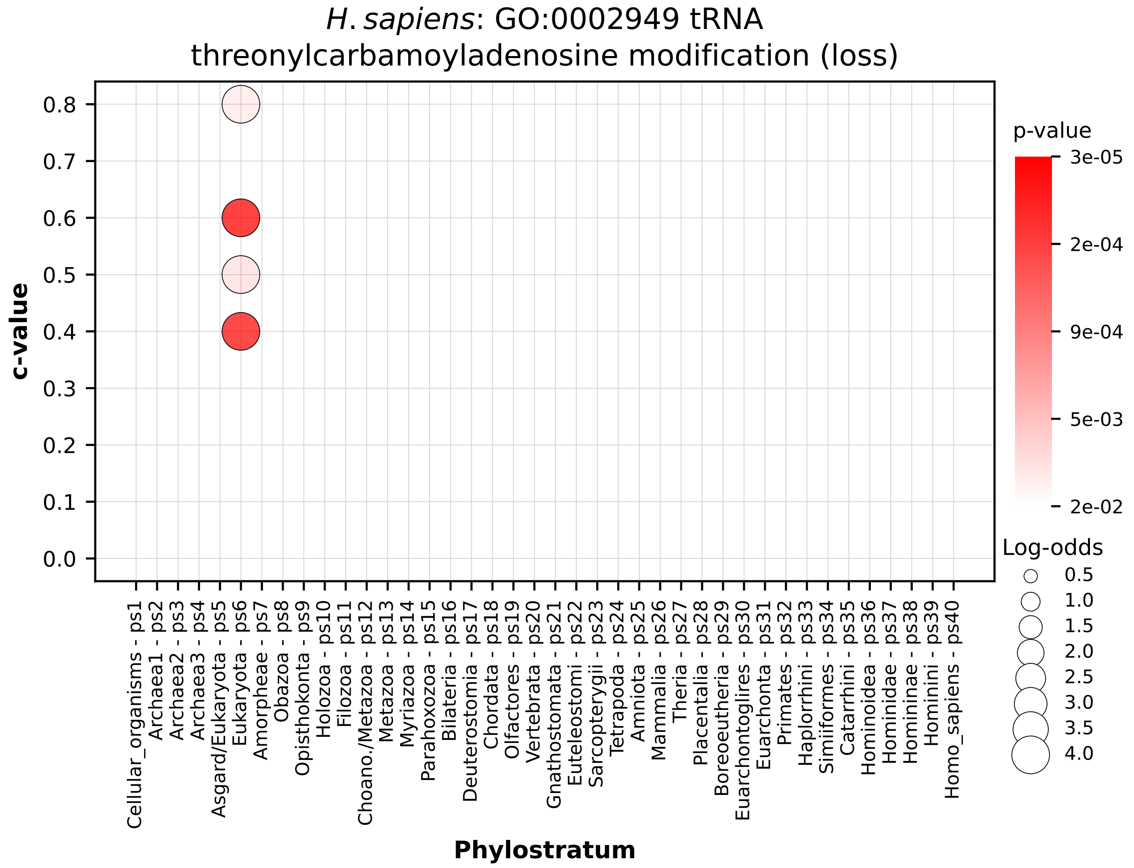

H. sapiens: GO:0002949 tRNA threonylcarbamoyladenosine modification (loss)
c-value
0.0
0.1
0.2
0.3
0.4
0.5
0.6
0.7
0.8
p-value
3e-05
2e-04
9e-04
5e-03
2e-02
Log-odds
0.5
1.0
1.5
2.0
2.5
3.0
3.5
4.0
Cellular_organisms - ps1
Archaea1 - ps2
Archaea2 - ps3
Archaea3 - ps4
Asgard/Eukaryota - ps5
Eukaryota - ps6
Amorpheae - ps7
Obazoa - ps8
Opisthokonta - ps9
Holozoa - ps10
Filozoa - ps11
Choano./Metazoa - ps12
Metazoa - ps13
Myriazoa - ps14
Parahoxozoa - ps15
Bilateria - ps16
Deuterostomia - ps17
Chordata - ps18
Olfactores - ps19
Vertebrata - ps20
Gnathostomata - ps21
Euteleostomi - ps22
Sarcopterygii - ps23
Tetrapoda - ps24
Amniota - ps25
Mammalia - ps26
Theria - ps27
Placentalia - ps28
Boreoeutheria - ps29
Euarchontoglires - ps30
Euarchonta - ps31
Primates - ps32
Haplorrhini - ps33
Simiiformes - ps34
Catarrhini - ps35
Hominoidea - ps36
Hominidae - ps37
Homininae - ps38
Hominini - ps39
Homo_sapiens - ps40
Phylostratum

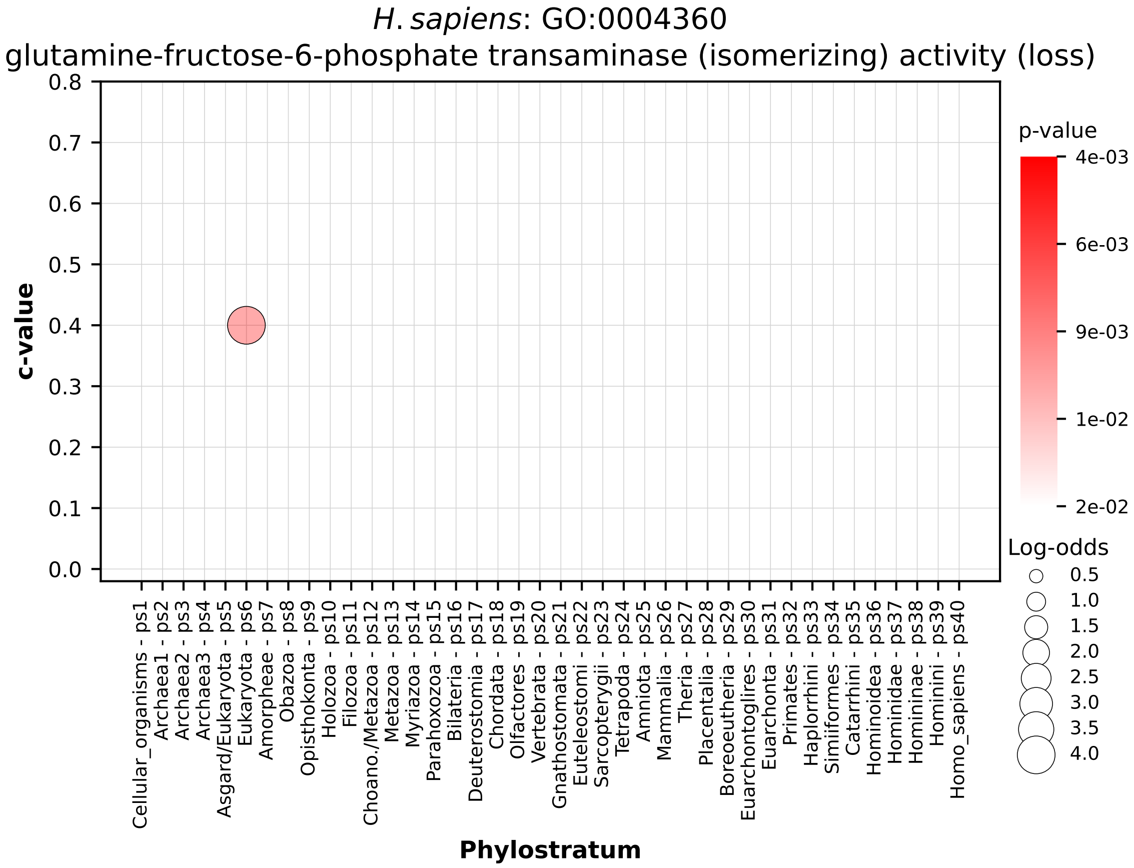
H. sapiens: GO:0004360
glutamine-fructose-6-phosphate transaminase (isomerizing) activity (loss)
c-value
0.0
0.1
0.2
0.3
0.4
0.5
0.6
0.7
0.8
Phylostratum
Cellular_organisms - ps1
Archaea1 - ps2
Archaea2 - ps3
Archaea3 - ps4
Asgard/Eukaryota - ps5
Eukaryota - ps6
Amorpheae - ps7
Obazoa - ps8
Opisthokonta - ps9
Holozoa - ps10
Filozoa - ps11
Choano./Metazoa - ps12
Metazoa - ps13
Myriazoa - ps14
Parahoxozoa - ps15
Bilateria - ps16
Deuterostomia - ps17
Chordata - ps18
Olfactores - ps19
Vertebrata - ps20
Gnathostomata - ps21
Euteleostomi - ps22
Sarcopterygii - ps23
Tetrapoda - ps24
Amniota - ps25
Mammalia - ps26
Theria - ps27
Placentalia - ps28
Boreoeutheria - ps29
Euarchontoglires - ps30
Euarchonta - ps31
Primates - ps32
Haplorrhini - ps33
Simiiformes - ps34
Catarrhini - ps35
Hominoidea - ps36
Hominidae - ps37
Homininae - ps38
Hominini - ps39
Homo_sapiens - ps40
p-value
4e-03
6e-03
9e-03
1e-02
2e-02
Log-odds
0.5
1.0
1.5
2.0
2.5
3.0
3.5
4.0

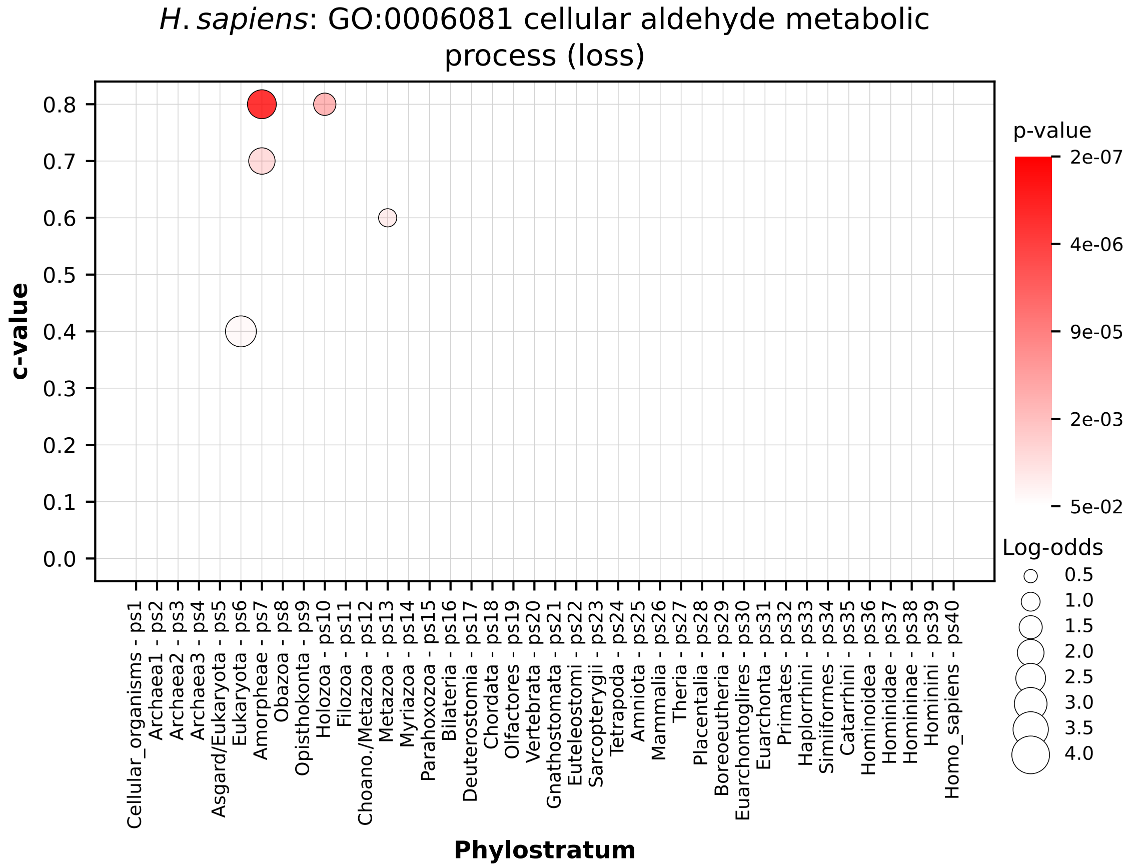
H. sapiens: GO:0006081 cellular aldehyde metabolic process (loss)
c-value
0.8
0.7
0.6
0.5
0.4
0.3
0.2
0.1
0.0
Phylostratum
Cellular_organisms - ps1
Archaea1 - ps2
Archaea2 - ps3
Archaea3 - ps4
Asgard/Eukaryota - ps5
Eukaryota - ps6
Amorpheae - ps7
Obazoa - ps8
Opisthokonta - ps9
Holozoa - ps10
Filozoa - ps11
Choano./Metazoa - ps12
Metazoa - ps13
Myriazoa - ps14
Parahoxozoa - ps15
Bilateria - ps16
Deuterostomia - ps17
Chordata - ps18
Olfactores - ps19
Vertebrata - ps20
Gnathostomata - ps21
Euteleostomi - ps22
Sarcopterygii - ps23
Tetrapoda - ps24
Amniota - ps25
Mammalia - ps26
Theria - ps27
Placentalia - ps28
Boreoeutheria - ps29
Euarchontoglires - ps30
Euarchonta - ps31
Primates - ps32
Haplorrhini - ps33
Simiiformes - ps34
Catarrhini - ps35
Hominoidea - ps36
Hominidae - ps37
Homininae - ps38
Hominini - ps39
Homo_sapiens - ps40
p-value
2e-07
4e-06
9e-05
2e-03
5e-02
Log-odds
0.5
1.0
1.5
2.0
2.5
3.0
3.5
4.0

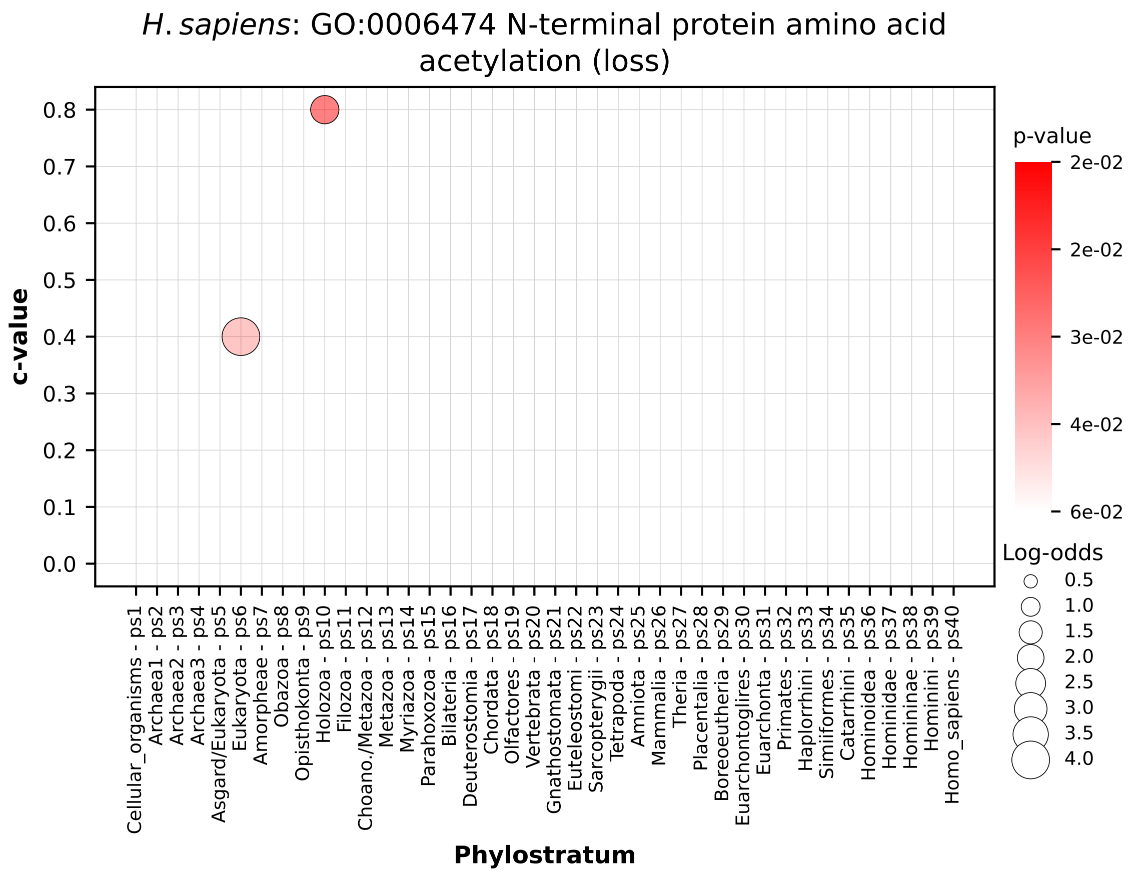

H. sapiens: GO:0006474 N-terminal protein amino acid acetylation (loss)
c-value
0.0
0.1
0.2
0.3
0.4
0.5
0.6
0.7
0.8
Phylostratum
Cellular_organisms - ps1
Archaea1 - ps2
Archaea2 - ps3
Archaea3 - ps4
Asgard/Eukaryota - ps5
Eukaryota - ps6
Amorpheae - ps7
Obazoa - ps8
Opisthokonta - ps9
Holozoa - ps10
Filozoa - ps11
Choano./Metazoa - ps12
Metazoa - ps13
Myriazoa - ps14
Parahoxozoa - ps15
Bilateria - ps16
Deuterostomia - ps17
Chordata - ps18
Olfactores - ps19
Vertebrata - ps20
Gnathostomata - ps21
Euteleostomi - ps22
Sarcopterygii - ps23
Tetrapoda - ps24
Amniota - ps25
Mammalia - ps26
Theria - ps27
Placentalia - ps28
Boreoeutheria - ps29
Euarchontoglires - ps30
Euarchonta - ps31
Primates - ps32
Haplorrhini - ps33
Simiiformes - ps34
Catarrhini - ps35
Hominoidea - ps36
Hominidae - ps37
Homininae - ps38
Hominini - ps39
Homo_sapiens - ps40
p-value
2e-02
2e-02
3e-02
4e-02
6e-02
Log-odds
0.5
1.0
1.5
2.0
2.5
3.0
3.5
4.0

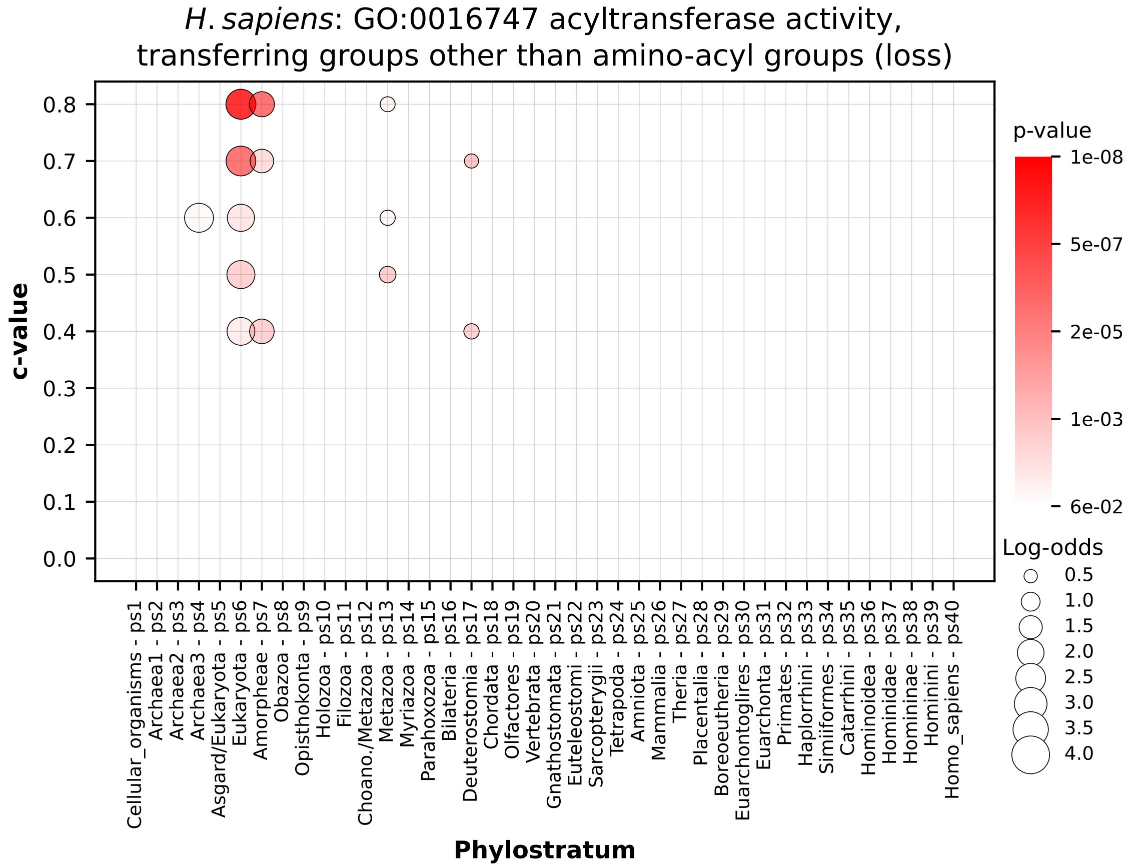
H. sapiens: GO:0016747 acyltransferase activity, transferring groups other than amino-acyl groups (loss)
c-value
0.0
0.1
0.2
0.3
0.4
0.5
0.6
0.7
0.8
p-value
1e-08
5e-07
2e-05
1e-03
6e-02
Log-odds
0.5
1.0
1.5
2.0
2.5
3.0
3.5
4.0
Cellular_organisms - ps1
Archaea1 - ps2
Archaea2 - ps3
Archaea3 - ps4
Asgard/Eukaryota - ps5
Eukaryota - ps6
Amorpheae - ps7
Obazoa - ps8
Opisthokonta - ps9
Holozoa - ps10
Filozoa - ps11
Choano./Metazoa - ps12
Metazoa - ps13
Myriazoa - ps14
Parahoxozoa - ps15
Bilateria - ps16
Deuterostomia - ps17
Chordata - ps18
Olfactores - ps19
Vertebrata - ps20
Gnathostomata - ps21
Euteleostomi - ps22
Sarcopterygii - ps23
Tetrapoda - ps24
Amniota - ps25
Mammalia - ps26
Theria - ps27
Placentalia - ps28
Boreoeutheria - ps29
Euarchontoglires - ps30
Euarchonta - ps31
Primates - ps32
Haplorrhini - ps33
Simiiformes - ps34
Catarrhini - ps35
Hominoidea - ps36
Hominidae - ps37
Homininae - ps38
Hominini - ps39
Homo_sapiens - ps40
Phylostratum

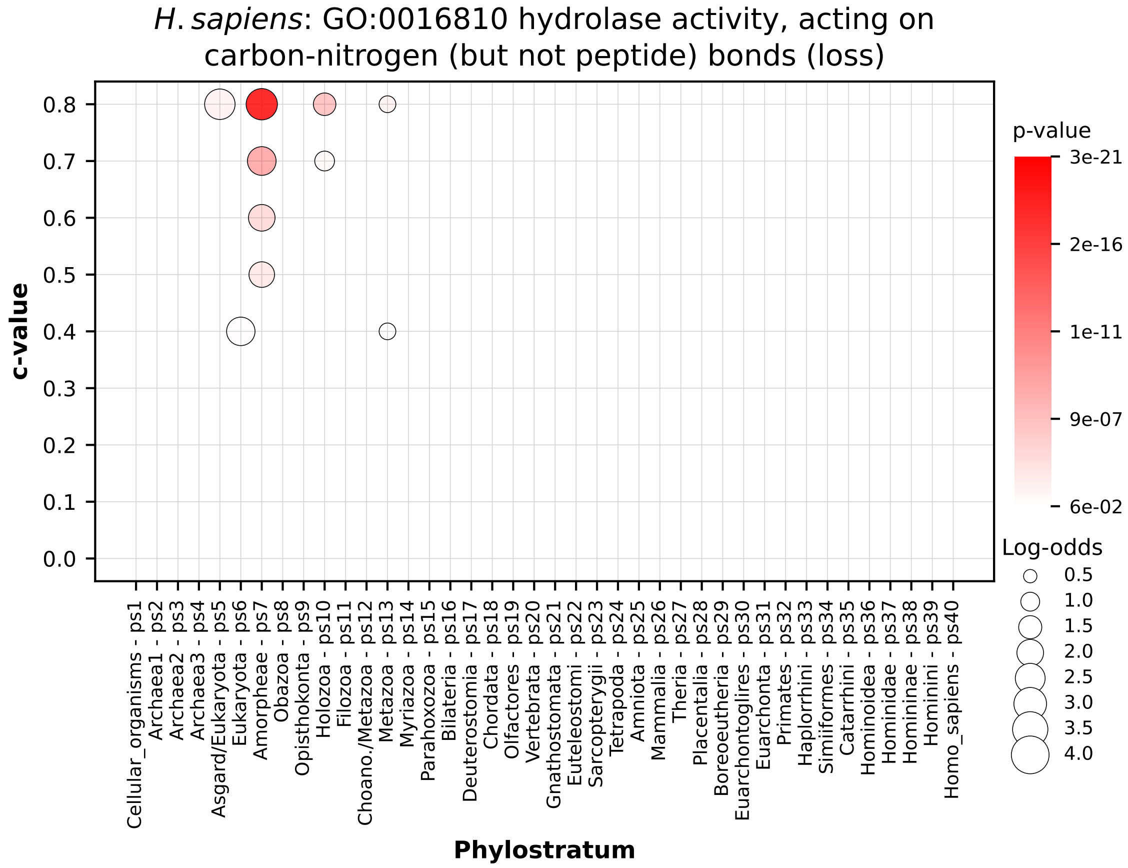

H. sapiens: GO:0016810 hydrolase activity, acting on carbon-nitrogen (but not peptide) bonds (loss)
c-value
0.8
0.7
0.6
0.5
0.4
0.3
0.2
0.1
0.0
Phylostratum
Cellular_organisms - ps1
Archaea1 - ps2
Archaea2 - ps3
Archaea3 - ps4
Asgard/Eukaryota - ps5
Eukaryota - ps6
Amorpheae - ps7
Obazoa - ps8
Opisthokonta - ps9
Holozoa - ps10
Filozoa - ps11
Choano./Metazoa - ps12
Metazoa - ps13
Myriazoa - ps14
Parahoxozoa - ps15
Bilateria - ps16
Deuterostomia - ps17
Chordata - ps18
Olfactores - ps19
Vertebrata - ps20
Gnathostomata - ps21
Euteleostomi - ps22
Sarcopterygii - ps23
Tetrapoda - ps24
Amniota - ps25
Mammalia - ps26
Theria - ps27
Placentalia - ps28
Boreoeutheria - ps29
Euarchontoglires - ps30
Euarchonta - ps31
Primates - ps32
Haplorrhini - ps33
Simiiformes - ps34
Catarrhini - ps35
Hominoidea - ps36
Hominidae - ps37
Homininae - ps38
Hominini - ps39
Homo_sapiens - ps40
p-value
3e-21
2e-16
1e-11
9e-07
6e-02
Log-odds
0.5
1.0
1.5
2.0
2.5
3.0
3.5
4.0

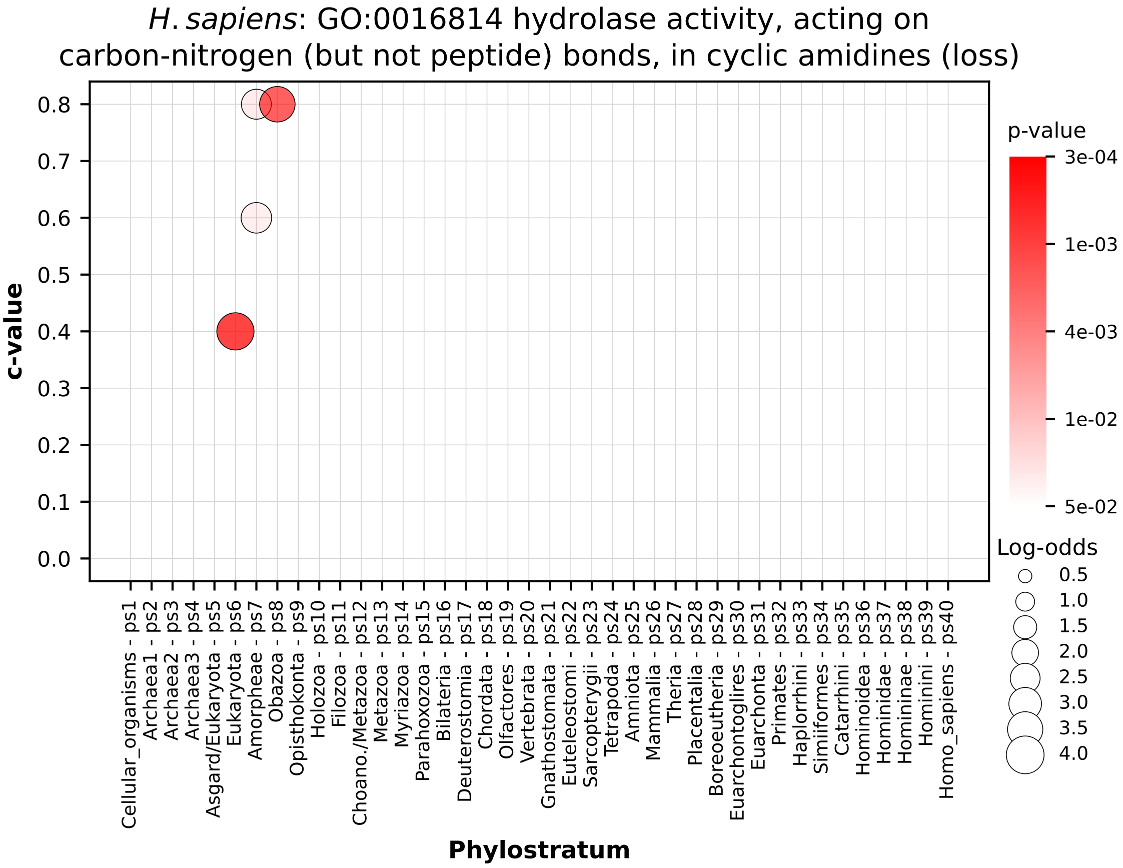

H. sapiens: GO:0016814 hydrolase activity, acting on carbon-nitrogen (but not peptide) bonds, in cyclic amidines (loss)
c-value
0.0
0.1
0.2
0.3
0.4
0.5
0.6
0.7
0.8
Phylostratum
Cellular_organisms - ps1
Archaea1 - ps2
Archaea2 - ps3
Archaea3 - ps4
Asgard/Eukaryota - ps5
Eukaryota - ps6
Amorpheae - ps7
Obazoa - ps8
Opisthokonta - ps9
Holozoa - ps10
Filozoa - ps11
Choano./Metazoa - ps12
Metazoa - ps13
Myriazoa - ps14
Parahoxozoa - ps15
Bilateria - ps16
Deuterostomia - ps17
Chordata - ps18
Olfactores - ps19
Vertebrata - ps20
Gnathostomata - ps21
Euteleostomi - ps22
Sarcopterygii - ps23
Tetrapoda - ps24
Amniota - ps25
Mammalia - ps26
Theria - ps27
Placentalia - ps28
Boreoeutheria - ps29
Euarchontoglires - ps30
Euarchonta - ps31
Primates - ps32
Haplorrhini - ps33
Simiiformes - ps34
Catarrhini - ps35
Hominoidea - ps36
Hominidae - ps37
Homininae - ps38
Hominini - ps39
Homo_sapiens - ps40
p-value
3e-04
1e-03
4e-03
1e-02
5e-02
Log-odds
0.5
1.0
1.5
2.0
2.5
3.0
3.5
4.0

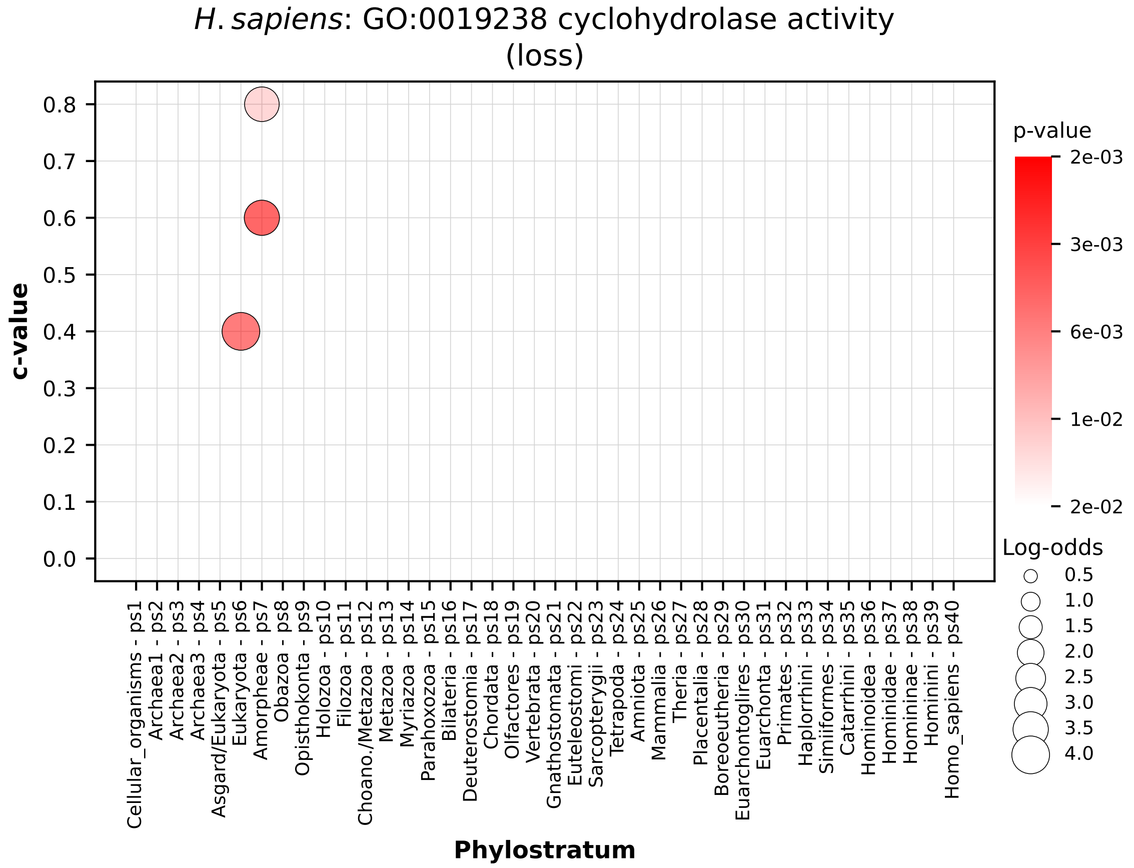

H. sapiens: GO:0019238 cyclohydrolase activity
(loss)
c-value
0.8
0.7
0.6
0.5
0.4
0.3
0.2
0.1
0.0
p-value
2e-03
3e-03
6e-03
1e-02
2e-02
Log-odds
0.5
1.0
1.5
2.0
2.5
3.0
3.5
4.0
Cellular_organisms - ps1
Archaea1 - ps2
Archaea2 - ps3
Archaea3 - ps4
Asgard/Eukaryota - ps5
Eukaryota - ps6
Amorpheae - ps7
Obazoa - ps8
Opisthokonta - ps9
Holozoa - ps10
Filozoa - ps11
Choano./Metazoa - ps12
Metazoa - ps13
Myriazoa - ps14
Parahoxozoa - ps15
Bilateria - ps16
Deuterostomia - ps17
Chordata - ps18
Olfactores - ps19
Vertebrata - ps20
Gnathostomata - ps21
Euteleostomi - ps22
Sarcopterygii - ps23
Tetrapoda - ps24
Amniota - ps25
Mammalia - ps26
Theria - ps27
Placentalia - ps28
Boreoeutheria - ps29
Euarchontoglires - ps30
Euarchonta - ps31
Primates - ps32
Haplorrhini - ps33
Simiiformes - ps34
Catarrhini - ps35
Hominoidea - ps36
Hominidae - ps37
Homininae - ps38
Hominini - ps39
Homo_sapiens - ps40
Phylostratum

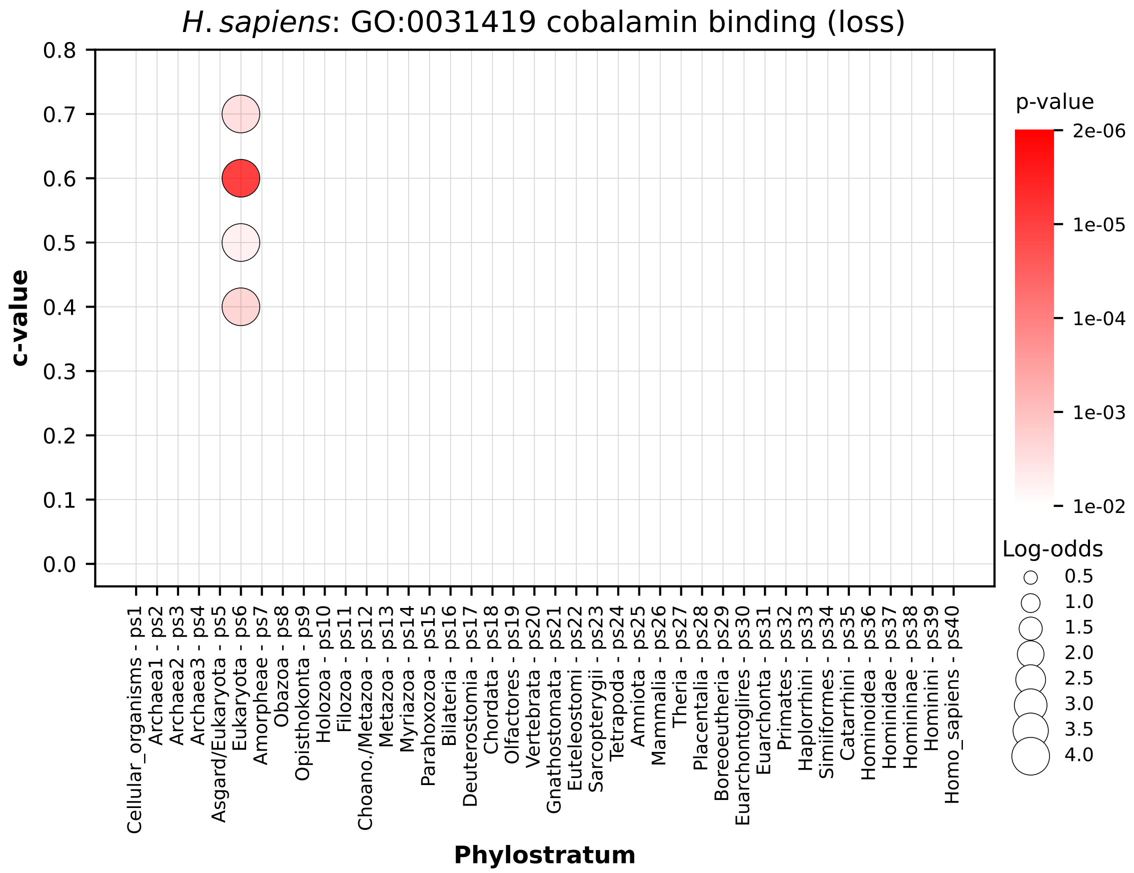

H. sapiens: GO:0031419 cobalamin binding (loss)
c-value
0.0
0.1
0.2
0.3
0.4
0.5
0.6
0.7
0.8
Phylostratum
Cellular_organisms - ps1
Archaea1 - ps2
Archaea2 - ps3
Archaea3 - ps4
Asgard/Eukaryota - ps5
Eukaryota - ps6
Amorpheae - ps7
Obazoa - ps8
Opisthokonta - ps9
Holozoa - ps10
Filozoa - ps11
Choano./Metazoa - ps12
Metazoa - ps13
Myriazoa - ps14
Parahoxozoa - ps15
Bilateria - ps16
Deuterostomia - ps17
Chordata - ps18
Olfactores - ps19
Vertebrata - ps20
Gnathostomata - ps21
Euteleostomi - ps22
Sarcopterygii - ps23
Tetrapoda - ps24
Amniota - ps25
Mammalia - ps26
Theria - ps27
Placentalia - ps28
Boreoeutheria - ps29
Euarchontoglires - ps30
Euarchonta - ps31
Primates - ps32
Haplorrhini - ps33
Simiiformes - ps34
Catarrhini - ps35
Hominoidea - ps36
Hominidae - ps37
Homininae - ps38
Hominini - ps39
Homo_sapiens - ps40
p-value
2e-06
1e-05
1e-04
1e-03
1e-02
Log-odds
0.5
1.0
1.5
2.0
2.5
3.0
3.5
4.0

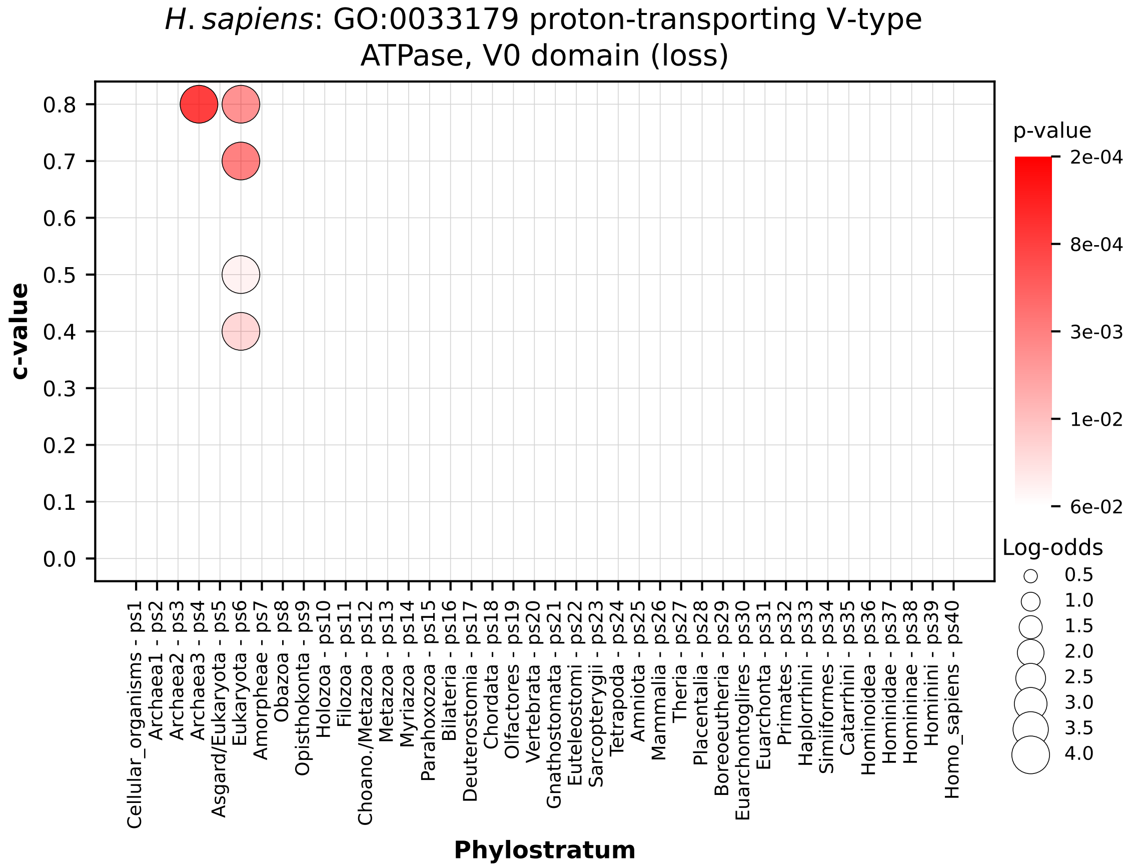
H. sapiens: GO:0033179 proton-transporting V-type ATPase, V0 domain (loss)
c-value
0.0
0.1
0.2
0.3
0.4
0.5
0.6
0.7
0.8
p-value
2e-04
8e-04
3e-03
1e-02
6e-02
Log-odds
0.5
1.0
1.5
2.0
2.5
3.0
3.5
4.0
Cellular_organisms - ps1
Archaea1 - ps2
Archaea2 - ps3
Archaea3 - ps4
Asgard/Eukaryota - ps5
Eukaryota - ps6
Amorpheae - ps7
Obazoa - ps8
Opisthokonta - ps9
Holozoa - ps10
Filozoa - ps11
Choano./Metazoa - ps12
Metazoa - ps13
Myriazoa - ps14
Parahoxozoa - ps15
Bilateria - ps16
Deuterostomia - ps17
Chordata - ps18
Olfactores - ps19
Vertebrata - ps20
Gnathostomata - ps21
Euteleostomi - ps22
Sarcopterygii - ps23
Tetrapoda - ps24
Amniota - ps25
Mammalia - ps26
Theria - ps27
Placentalia - ps28
Boreoeutheria - ps29
Euarchontoglires - ps30
Euarchonta - ps31
Primates - ps32
Haplorrhini - ps33
Simiiformes - ps34
Catarrhini - ps35
Hominoidea - ps36
Hominidae - ps37
Homininae - ps38
Hominini - ps39
Homo_sapiens - ps40
Phylostratum

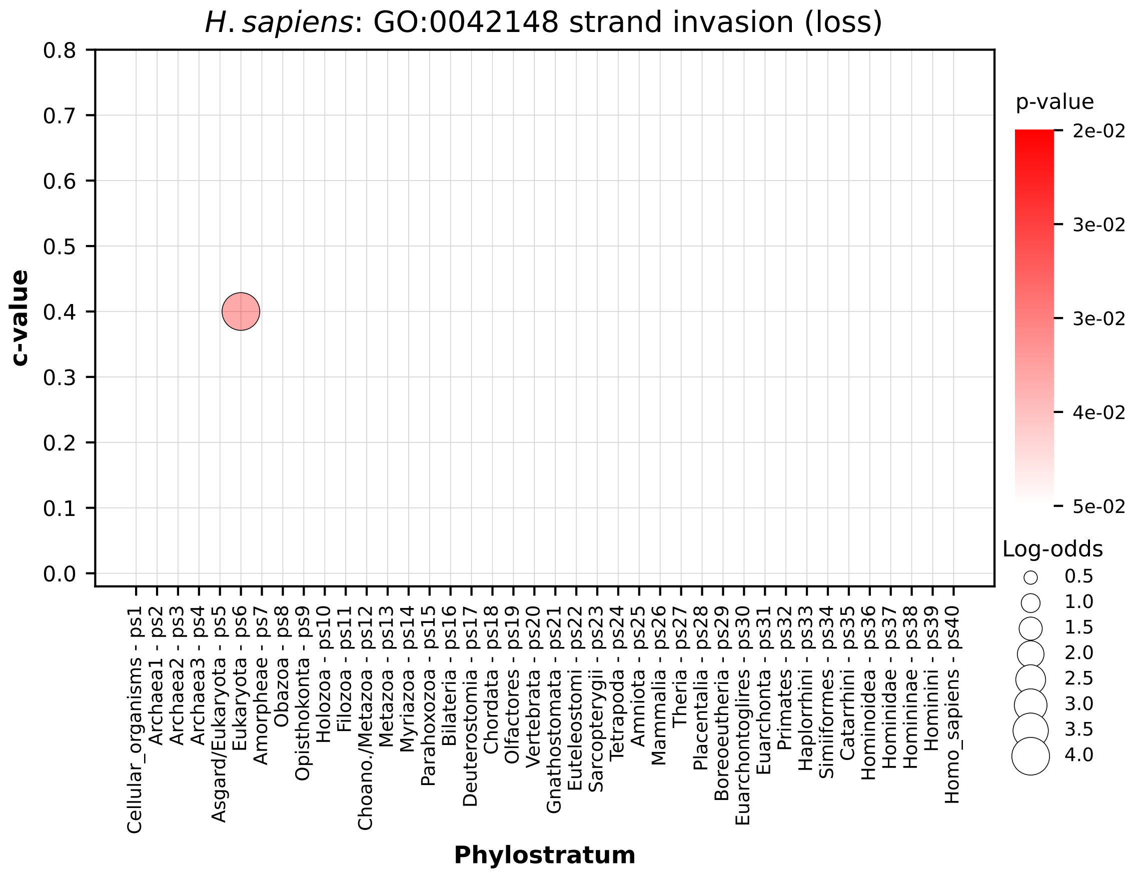

H. sapiens: GO:0042148 strand invasion (loss)
c-value
0.0
0.1
0.2
0.3
0.4
0.5
0.6
0.7
0.8
Phylostratum
Cellular_organisms - ps1
Archaea1 - ps2
Archaea2 - ps3
Archaea3 - ps4
Asgard/Eukaryota - ps5
Eukaryota - ps6
Amorpheae - ps7
Obazoa - ps8
Opisthokonta - ps9
Holozoa - ps10
Filozoa - ps11
Choano./Metazoa - ps12
Metazoa - ps13
Myriazoa - ps14
Parahoxozoa - ps15
Bilateria - ps16
Deuterostomia - ps17
Chordata - ps18
Olfactores - ps19
Vertebrata - ps20
Gnathostomata - ps21
Euteleostomi - ps22
Sarcopterygii - ps23
Tetrapoda - ps24
Amniota - ps25
Mammalia - ps26
Theria - ps27
Placentalia - ps28
Boreoeutheria - ps29
Euarchontoglires - ps30
Euarchonta - ps31
Primates - ps32
Haplorrhini - ps33
Simiiformes - ps34
Catarrhini - ps35
Hominoidea - ps36
Hominidae - ps37
Homininae - ps38
Hominini - ps39
Homo_sapiens - ps40
p-value
2e-02
3e-02
3e-02
4e-02
5e-02
Log-odds
0.5
1.0
1.5
2.0
2.5
3.0
3.5
4.0

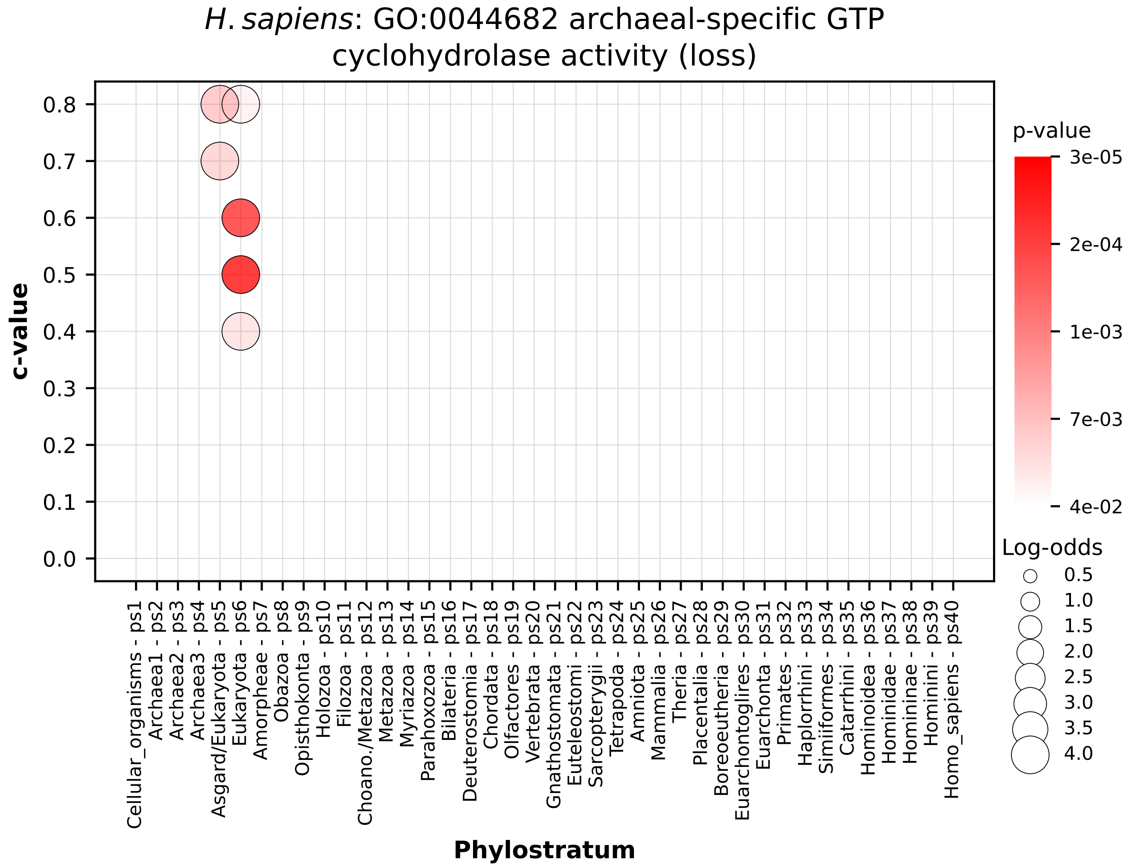
H. sapiens: GO:0044682 archaeal-specific GTP cyclohydrolase activity (loss)
c-value
0.0
0.1
0.2
0.3
0.4
0.5
0.6
0.7
0.8
p-value
3e-05
2e-04
1e-03
7e-03
4e-02
Log-odds
0.5
1.0
1.5
2.0
2.5
3.0
3.5
4.0
Cellular_organisms - ps1
Archaea1 - ps2
Archaea2 - ps3
Archaea3 - ps4
Asgard/Eukaryota - ps5
Eukaryota - ps6
Amorpheae - ps7
Obazoa - ps8
Opisthokonta - ps9
Holozoa - ps10
Filozoa - ps11
Choano./Metazoa - ps12
Metazoa - ps13
Myriazoa - ps14
Parahoxozoa - ps15
Bilateria - ps16
Deuterostomia - ps17
Chordata - ps18
Olfactores - ps19
Vertebrata - ps20
Gnathostomata - ps21
Euteleostomi - ps22
Sarcopterygii - ps23
Tetrapoda - ps24
Amniota - ps25
Mammalia - ps26
Theria - ps27
Placentalia - ps28
Boreoeutheria - ps29
Euarchontoglires - ps30
Euarchonta - ps31
Primates - ps32
Haplorrhini - ps33
Simiiformes - ps34
Catarrhini - ps35
Hominoidea - ps36
Hominidae - ps37
Homininae - ps38
Hominini - ps39
Homo_sapiens - ps40
Phylostratum

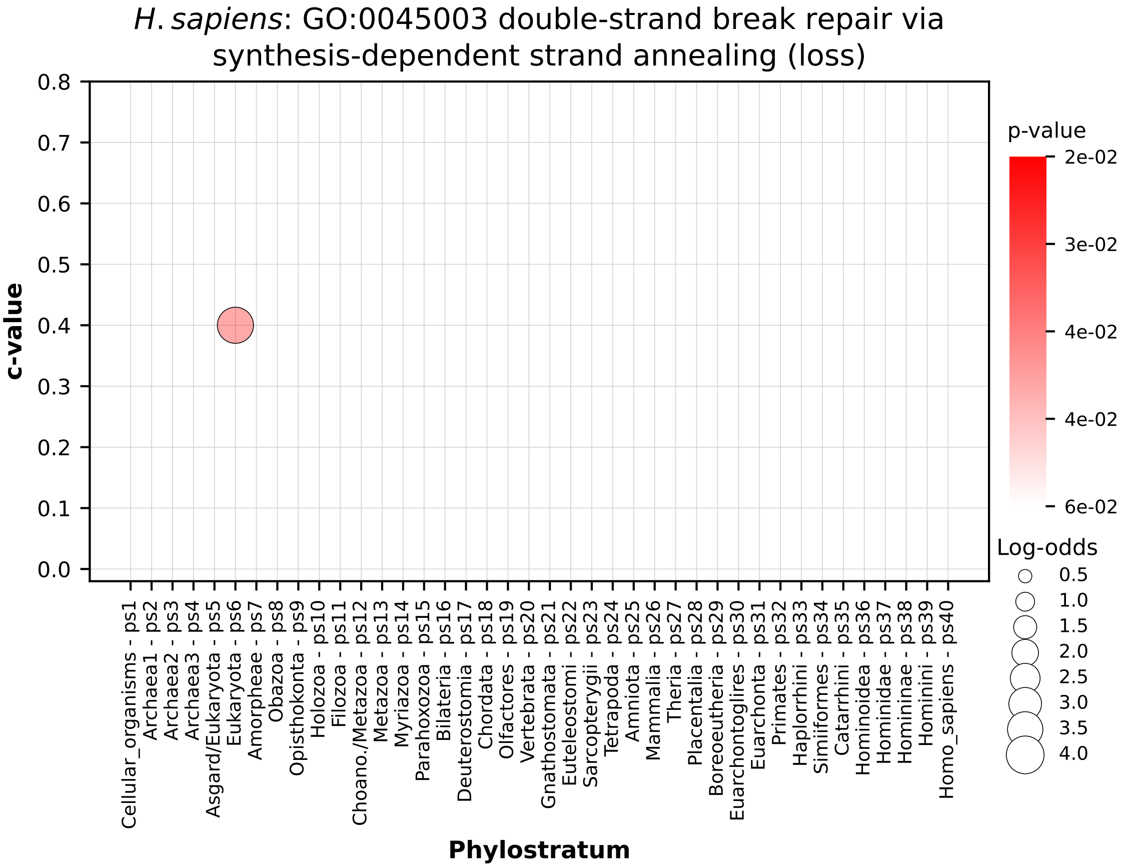

H. sapiens: GO:0045003 double-strand break repair via synthesis-dependent strand annealing (loss)
c-value
0.8
0.7
0.6
0.5
0.4
0.3
0.2
0.1
0.0
Phylostratum
Cellular_organisms - ps1
Archaea1 - ps2
Archaea2 - ps3
Archaea3 - ps4
Asgard/Eukaryota - ps5
Eukaryota - ps6
Amorpheae - ps7
Obazoa - ps8
Opisthokonta - ps9
Holozoa - ps10
Filozoa - ps11
Choano./Metazoa - ps12
Metazoa - ps13
Myriazoa - ps14
Parahoxozoa - ps15
Bilateria - ps16
Deuterostomia - ps17
Chordata - ps18
Olfactores - ps19
Vertebrata - ps20
Gnathostomata - ps21
Euteleostomi - ps22
Sarcopterygii - ps23
Tetrapoda - ps24
Amniota - ps25
Mammalia - ps26
Theria - ps27
Placentalia - ps28
Boreoeutheria - ps29
Euarchontoglires - ps30
Euarchonta - ps31
Primates - ps32
Haplorrhini - ps33
Simiiformes - ps34
Catarrhini - ps35
Hominoidea - ps36
Hominidae - ps37
Homininae - ps38
Hominini - ps39
Homo_sapiens - ps40
p-value
2e-02
3e-02
4e-02
4e-02
6e-02
Log-odds
0.5
1.0
1.5
2.0
2.5
3.0
3.5
4.0

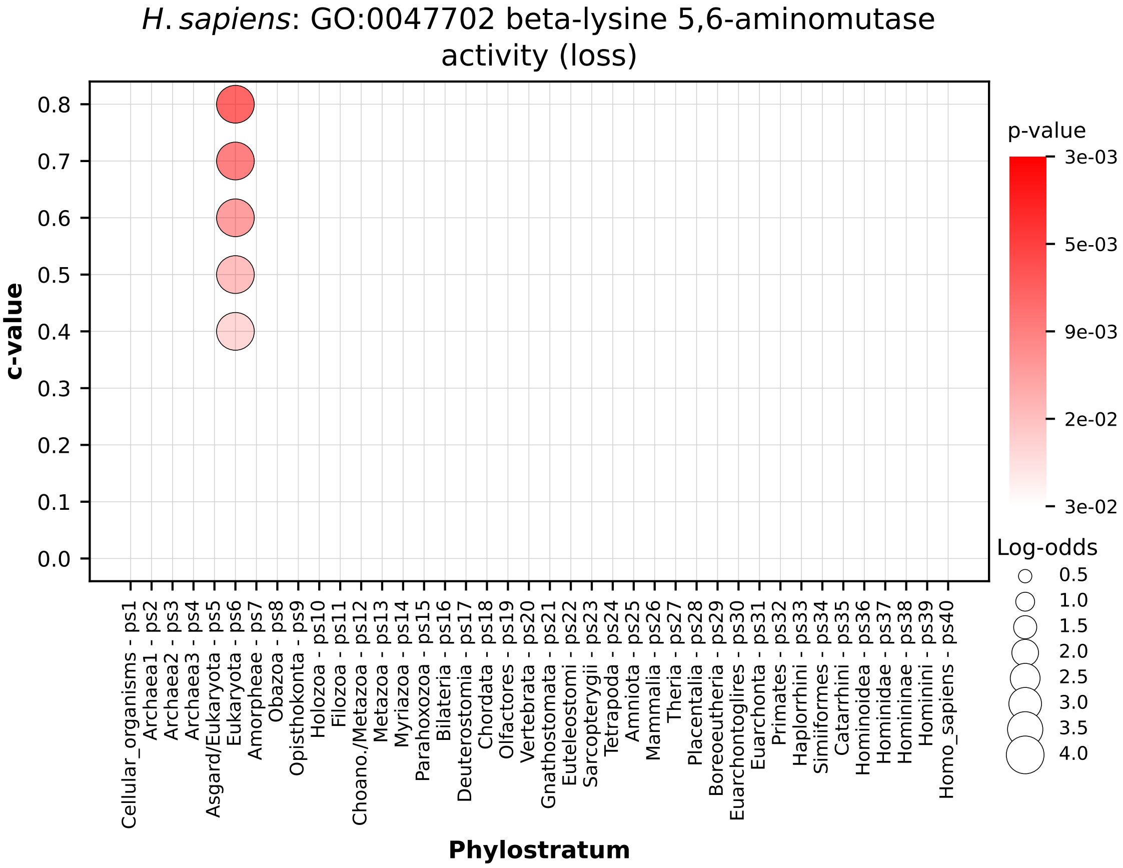

H. sapiens: GO:0047702 beta-lysine 5,6-aminomutase activity (loss)
c-value
0.0
0.1
0.2
0.3
0.4
0.5
0.6
0.7
0.8
Phylostratum
Cellular_organisms - ps1
Archaea1 - ps2
Archaea2 - ps3
Archaea3 - ps4
Asgard/Eukaryota - ps5
Eukaryota - ps6
Amorpheae - ps7
Obazoa - ps8
Opisthokonta - ps9
Holozoa - ps10
Filozoa - ps11
Choano./Metazoa - ps12
Metazoa - ps13
Myriazoa - ps14
Parahoxozoa - ps15
Bilateria - ps16
Deuterostomia - ps17
Chordata - ps18
Olfactores - ps19
Vertebrata - ps20
Gnathostomata - ps21
Euteleostomi - ps22
Sarcopterygii - ps23
Tetrapoda - ps24
Amniota - ps25
Mammalia - ps26
Theria - ps27
Placentalia - ps28
Boreoeutheria - ps29
Euarchontoglires - ps30
Euarchonta - ps31
Primates - ps32
Haplorrhini - ps33
Simiiformes - ps34
Catarrhini - ps35
Hominoidea - ps36
Hominidae - ps37
Homininae - ps38
Hominini - ps39
Homo_sapiens - ps40
p-value
3e-03
5e-03
9e-03
2e-02
3e-02
Log-odds
0.5
1.0
1.5
2.0
2.5
3.0
3.5
4.0

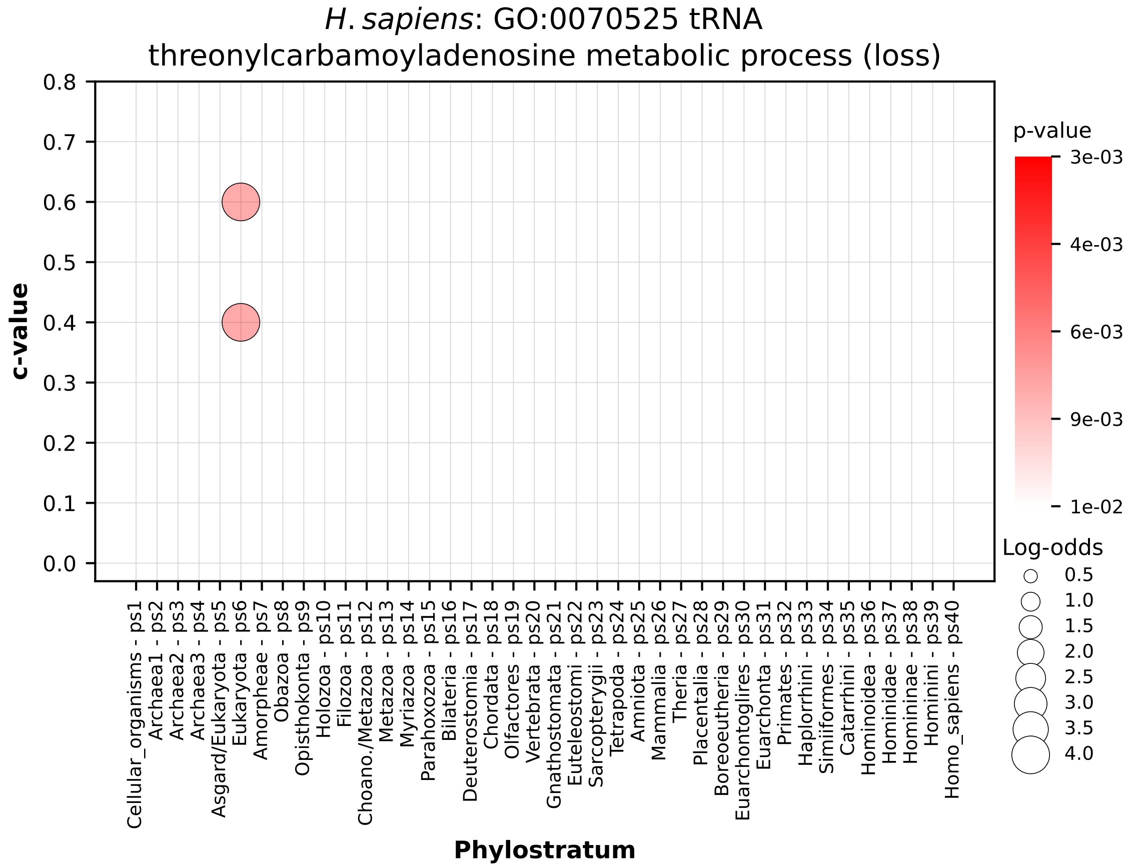

H. sapiens: GO:0070525 tRNA threonylcarbamoyladenosine metabolic process (loss)
c-value
Phylostratum
p-value
3e-03
4e-03
6e-03
9e-03
1e-02
Log-odds
0.5
1.0
1.5
2.0
2.5
3.0
3.5
4.0
Cellular_organisms - ps1
Archaea1 - ps2
Archaea2 - ps3
Archaea3 - ps4
Asgard/Eukaryota - ps5
Eukaryota - ps6
Amorpheae - ps7
Obazoa - ps8
Opisthokonta - ps9
Holozoa - ps10
Filozoa - ps11
Choano./Metazoa - ps12
Metazoa - ps13
Myriazoa - ps14
Parahoxozoa - ps15
Bilateria - ps16
Deuterostomia - ps17
Chordata - ps18
Olfactores - ps19
Vertebrata - ps20
Gnathostomata - ps21
Euteleostomi - ps22
Sarcopterygii - ps23
Tetrapoda - ps24
Amniota - ps25
Mammalia - ps26
Theria - ps27
Placentalia - ps28
Boreoeutheria - ps29
Euarchontoglires - ps30
Euarchonta - ps31
Primates - ps32
Haplorrhini - ps33
Simiiformes - ps34
Catarrhini - ps35
Hominoidea - ps36
Hominidae - ps37
Homininae - ps38
Hominini - ps39
Homo_sapiens - ps40

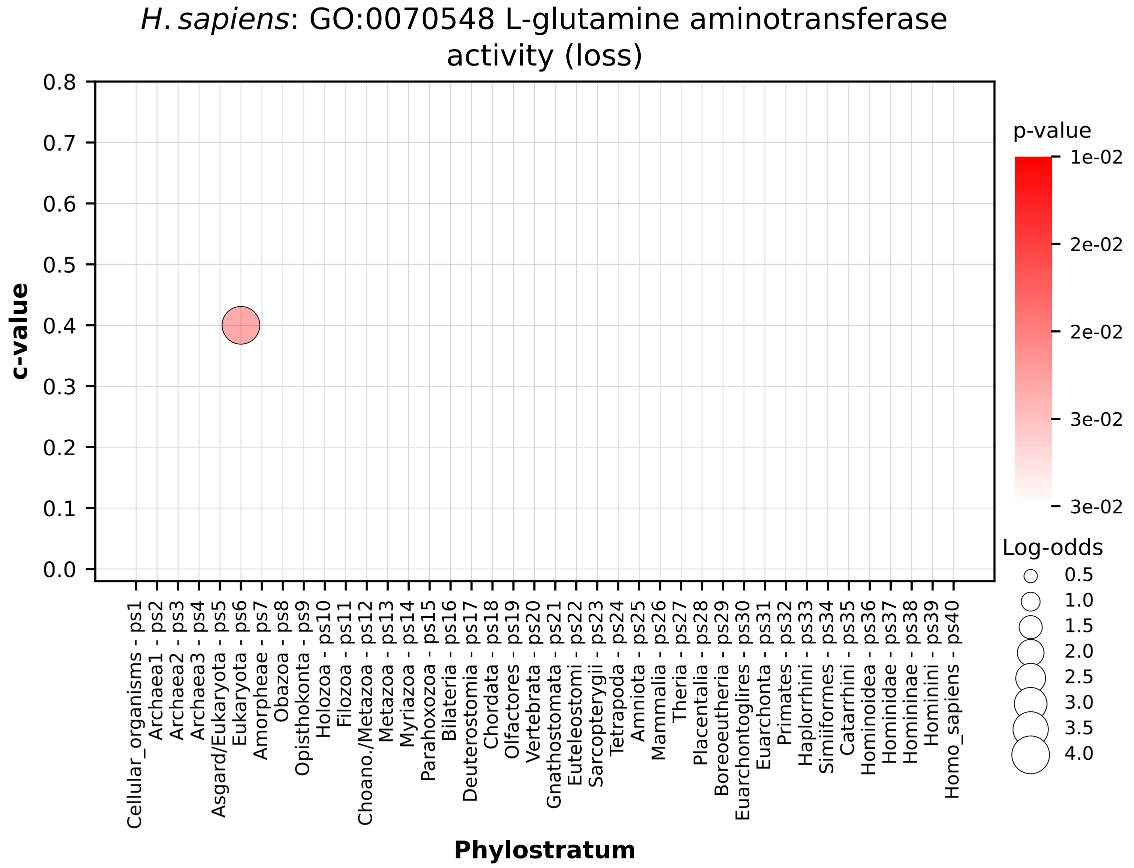

H. sapiens: GO:0070548 L-glutamine aminotransferase activity (loss)
c-value
0.0
0.1
0.2
0.3
0.4
0.5
0.6
0.7
0.8
Phylostratum
Cellular_organisms - ps1
Archaea1 - ps2
Archaea2 - ps3
Archaea3 - ps4
Asgard/Eukaryota - ps5
Eukaryota - ps6
Amorpheae - ps7
Obazoa - ps8
Opisthokonta - ps9
Holozoa - ps10
Filozoa - ps11
Choano./Metazoa - ps12
Metazoa - ps13
Myriazoa - ps14
Parahoxozoa - ps15
Bilateria - ps16
Deuterostomia - ps17
Chordata - ps18
Olfactores - ps19
Vertebrata - ps20
Gnathostomata - ps21
Euteleostomi - ps22
Sarcopterygii - ps23
Tetrapoda - ps24
Amniota - ps25
Mammalia - ps26
Theria - ps27
Placentalia - ps28
Boreoeutheria - ps29
Euarchontoglires - ps30
Euarchonta - ps31
Primates - ps32
Haplorrhini - ps33
Simiiformes - ps34
Catarrhini - ps35
Hominoidea - ps36
Hominidae - ps37
Homininae - ps38
Hominini - ps39
Homo_sapiens - ps40
p-value
1e-02
2e-02
2e-02
3e-02
3e-02
Log-odds
0.5
1.0
1.5
2.0
2.5
3.0
3.5
4.0

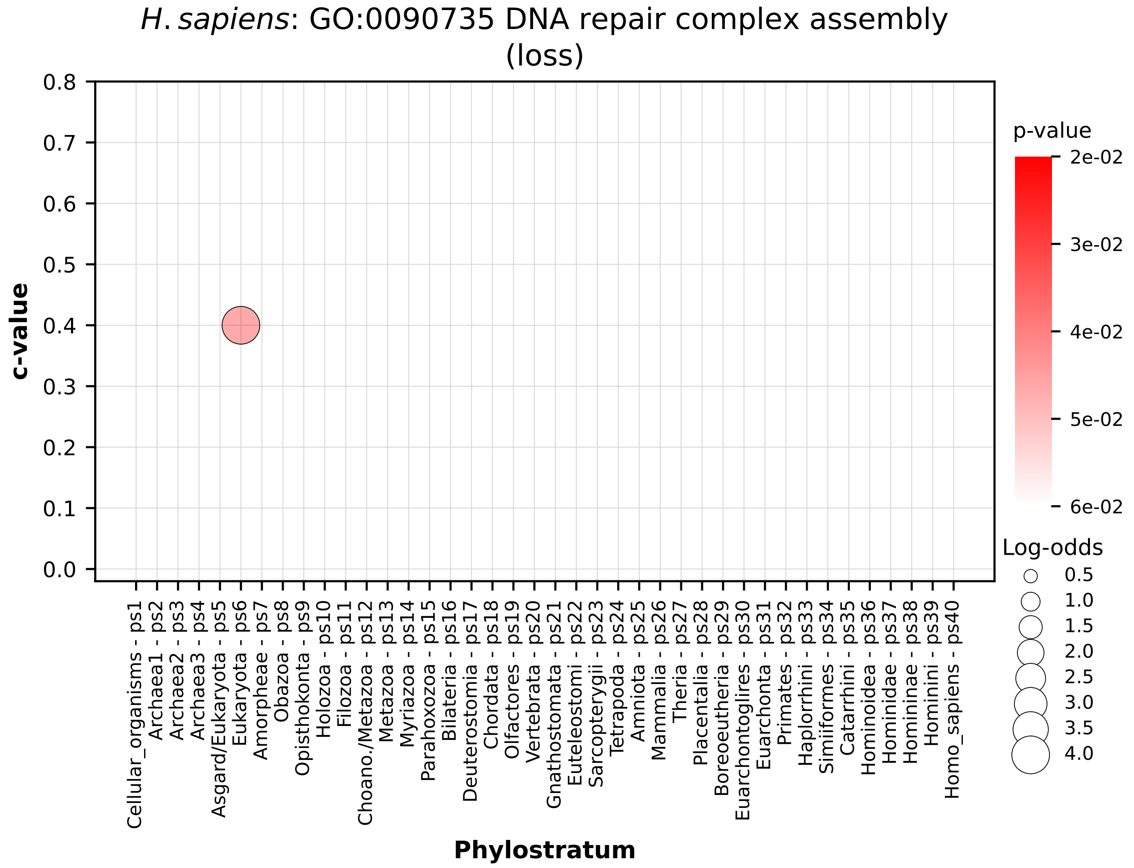

H. sapiens: GO:0090735 DNA repair complex assembly (loss)
c-value
0.8
0.7
0.6
0.5
0.4
0.3
0.2
0.1
0.0
Phylostratum
Cellular_organisms - ps1
Archaea1 - ps2
Archaea2 - ps3
Archaea3 - ps4
Asgard/Eukaryota - ps5
Eukaryota - ps6
Amorpheae - ps7
Obazoa - ps8
Opisthokonta - ps9
Holozoa - ps10
Filozoa - ps11
Choano./Metazoa - ps12
Metazoa - ps13
Myriazoa - ps14
Parahoxozoa - ps15
Bilateria - ps16
Deuterostomia - ps17
Chordata - ps18
Olfactores - ps19
Vertebrata - ps20
Gnathostomata - ps21
Euteleostomi - ps22
Sarcopterygii - ps23
Tetrapoda - ps24
Amniota - ps25
Mammalia - ps26
Theria - ps27
Placentalia - ps28
Boreoeutheria - ps29
Euarchontoglires - ps30
Euarchonta - ps31
Primates - ps32
Haplorrhini - ps33
Simiiformes - ps34
Catarrhini - ps35
Hominoidea - ps36
Hominidae - ps37
Homininae - ps38
Hominini - ps39
Homo_sapiens - ps40
p-value
2e-02
3e-02
4e-02
5e-02
6e-02
Log-odds
0.5
1.0
1.5
2.0
2.5
3.0
3.5
4.0

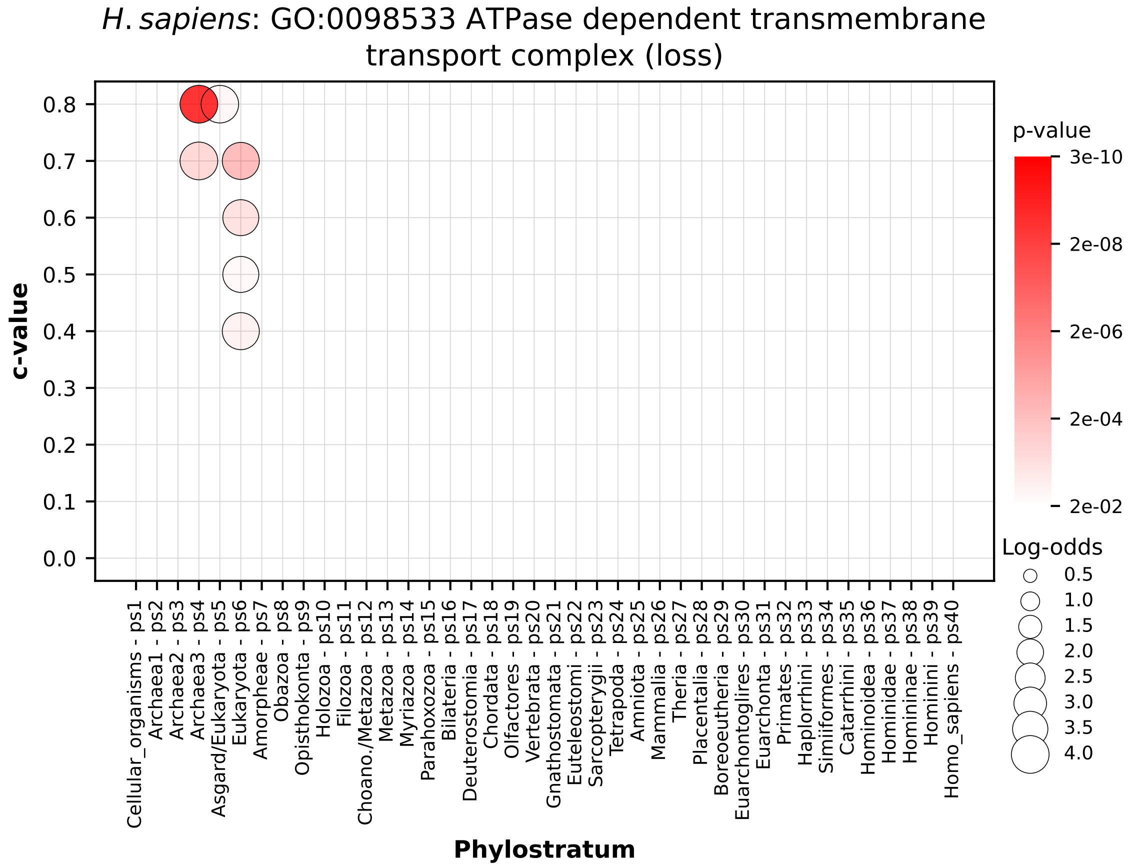

H. sapiens: GO:0098533 ATPase dependent transmembrane transport complex (loss)
c-value
0.8
0.7
0.6
0.5
0.4
0.3
0.2
0.1
0.0
p-value
3e-10
2e-08
2e-06
2e-04
2e-02
Log-odds
0.5
1.0
1.5
2.0
2.5
3.0
3.5
4.0
Cellular_organisms - ps1
Archaea1 - ps2
Archaea2 - ps3
Archaea3 - ps4
Asgard/Eukaryota - ps5
Eukaryota - ps6
Amorpheae - ps7
Obazoa - ps8
Opisthokonta - ps9
Holozoa - ps10
Filozoa - ps11
Choano./Metazoa - ps12
Metazoa - ps13
Myriazoa - ps14
Parahoxozoa - ps15
Bilateria - ps16
Deuterostomia - ps17
Chordata - ps18
Olfactores - ps19
Vertebrata - ps20
Gnathostomata - ps21
Euteleostomi - ps22
Sarcopterygii - ps23
Tetrapoda - ps24
Amniota - ps25
Mammalia - ps26
Theria - ps27
Placentalia - ps28
Boreoeutheria - ps29
Euarchontoglires - ps30
Euarchonta - ps31
Primates - ps32
Haplorrhini - ps33
Simiiformes - ps34
Catarrhini - ps35
Hominoidea - ps36
Hominidae - ps37
Homininae - ps38
Hominini - ps39
Homo_sapiens - ps40
Phylostratum

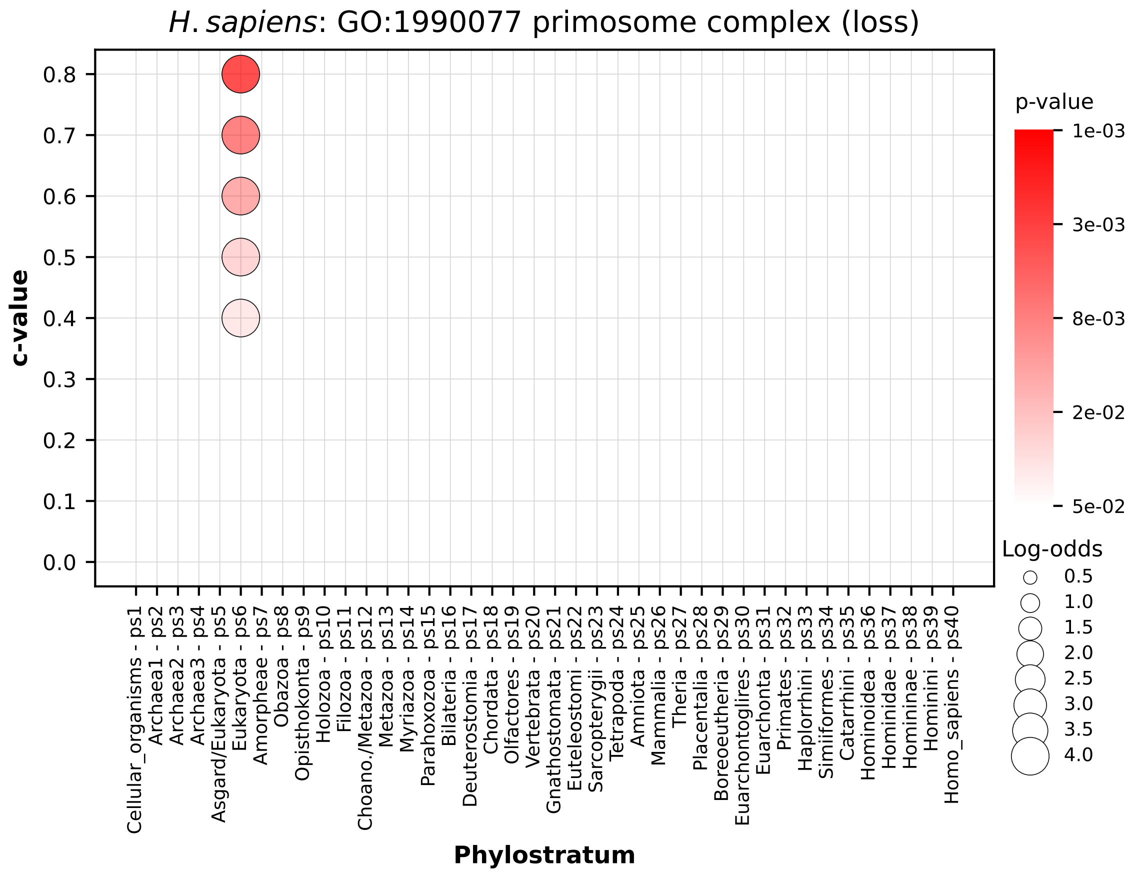

H. sapiens: GO:1990077 primosome complex (loss)
c-value
0.0
0.1
0.2
0.3
0.4
0.5
0.6
0.7
0.8
Phylostratum
Cellular_organisms - ps1
Archaea1 - ps2
Archaea2 - ps3
Archaea3 - ps4
Asgard/Eukaryota - ps5
Eukaryota - ps6
Amorpheae - ps7
Obazoa - ps8
Opisthokonta - ps9
Holozoa - ps10
Filozoa - ps11
Choano./Metazoa - ps12
Metazoa - ps13
Myriazoa - ps14
Parahoxozoa - ps15
Bilateria - ps16
Deuterostomia - ps17
Chordata - ps18
Olfactores - ps19
Vertebrata - ps20
Gnathostomata - ps21
Euteleostomi - ps22
Sarcopterygii - ps23
Tetrapoda - ps24
Amniota - ps25
Mammalia - ps26
Theria - ps27
Placentalia - ps28
Boreoeutheria - ps29
Euarchontoglires - ps30
Euarchonta - ps31
Primates - ps32
Haplorrhini - ps33
Simiiformes - ps34
Catarrhini - ps35
Hominoidea - ps36
Hominidae - ps37
Homininae - ps38
Hominini - ps39
Homo_sapiens - ps40
p-value
1e-03
3e-03
8e-03
2e-02
5e-02
Log-odds
0.5
1.0
1.5
2.0
2.5
3.0
3.5
4.0

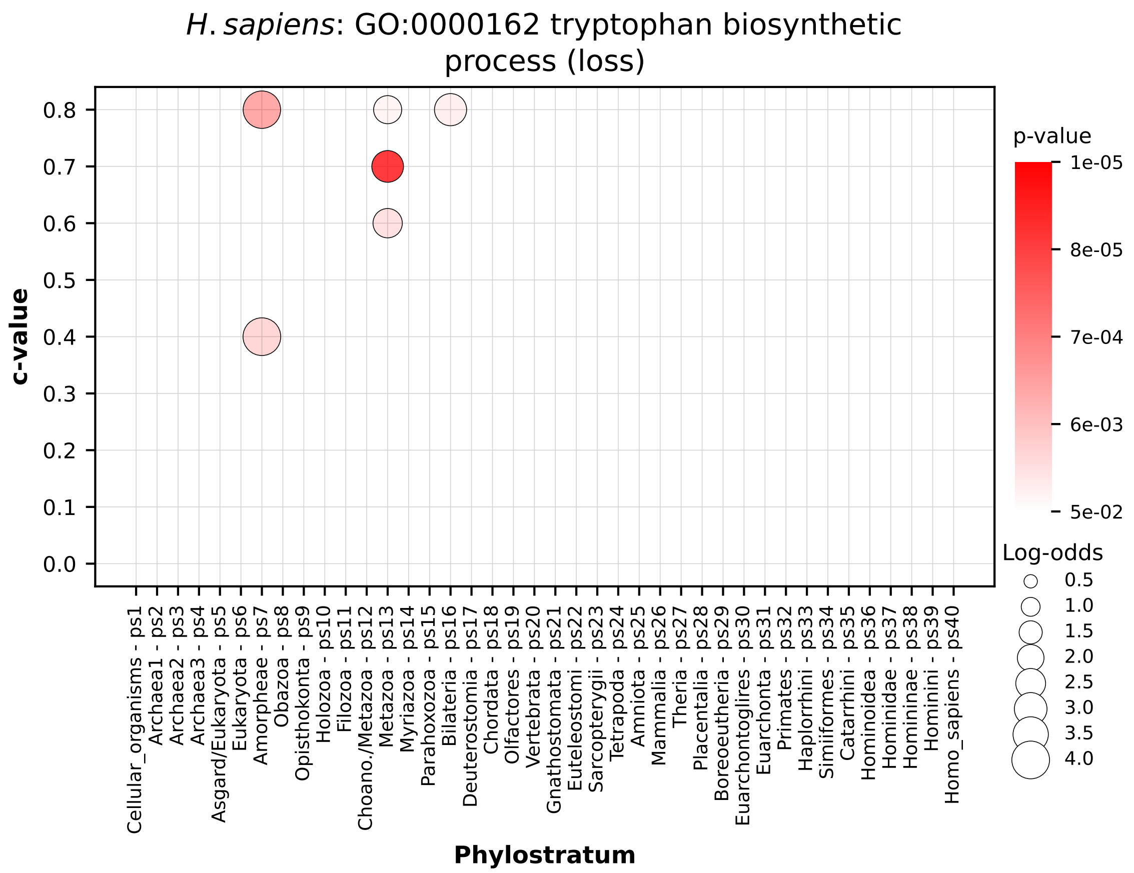
H. sapiens: GO:0000162 tryptophan biosynthetic process (loss)
c-value
0.0
0.1
0.2
0.3
0.4
0.5
0.6
0.7
0.8
Phylostratum
Cellular_organisms - ps1
Archaea1 - ps2
Archaea2 - ps3
Archaea3 - ps4
Asgard/Eukaryota - ps5
Eukaryota - ps6
Amorpheae - ps7
Obazoa - ps8
Opisthokonta - ps9
Holozoa - ps10
Filozoa - ps11
Choano./Metazoa - ps12
Metazoa - ps13
Myriazoa - ps14
Parahoxozoa - ps15
Bilateria - ps16
Deuterostomia - ps17
Chordata - ps18
Olfactores - ps19
Vertebrata - ps20
Gnathostomata - ps21
Euteleostomi - ps22
Sarcopterygii - ps23
Tetrapoda - ps24
Amniota - ps25
Mammalia - ps26
Theria - ps27
Placentalia - ps28
Boreoeutheria - ps29
Euarchontoglires - ps30
Euarchonta - ps31
Primates - ps32
Haplorrhini - ps33
Simiiformes - ps34
Catarrhini - ps35
Hominoidea - ps36
Hominidae - ps37
Homininae - ps38
Hominini - ps39
Homo_sapiens - ps40
p-value
1e-05
8e-05
7e-04
6e-03
5e-02
Log-odds
0.5
1.0
1.5
2.0
2.5
3.0
3.5
4.0

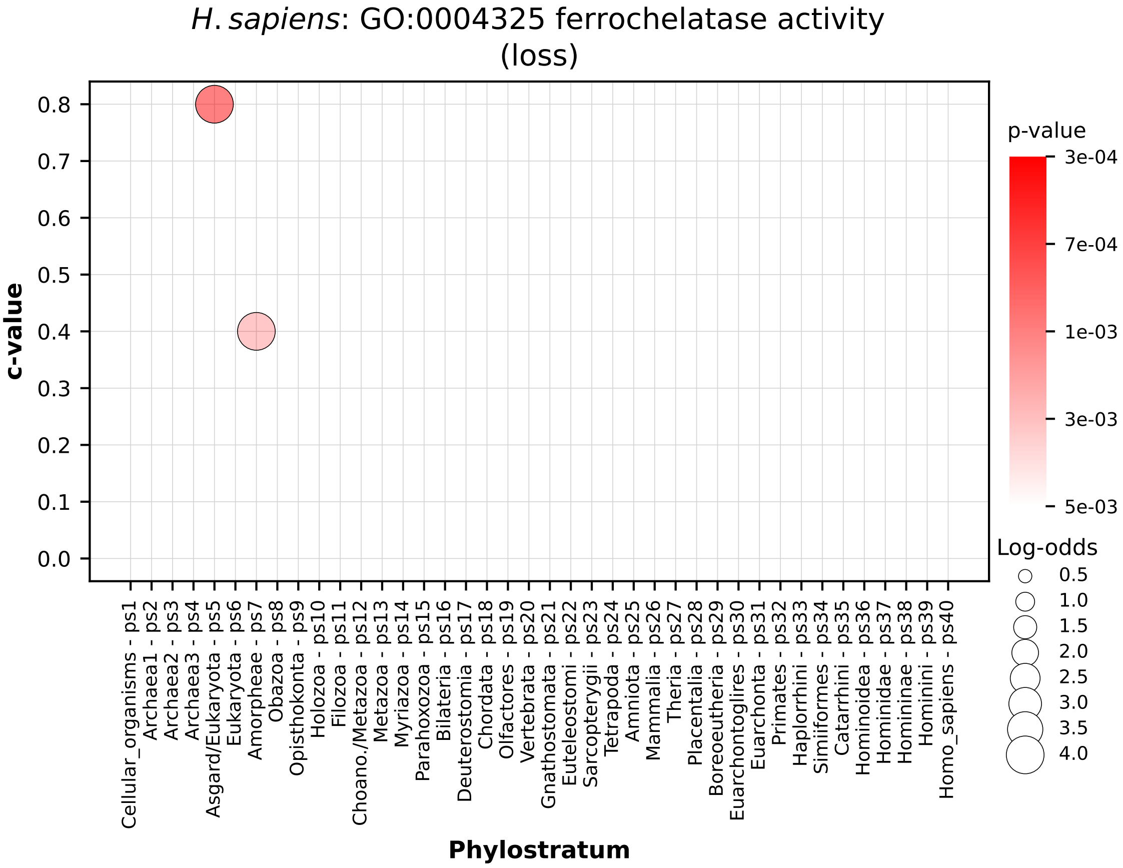

H. sapiens: GO:0004325 ferrochelatase activity (loss)
c-value
0.0
0.1
0.2
0.3
0.4
0.5
0.6
0.7
0.8
Phylostratum
Cellular_organisms - ps1
Archaea1 - ps2
Archaea2 - ps3
Archaea3 - ps4
Asgard/Eukaryota - ps5
Eukaryota - ps6
Amorpheae - ps7
Obazoa - ps8
Opisthokonta - ps9
Holozoa - ps10
Filozoa - ps11
Choano./Metazoa - ps12
Metazoa - ps13
Myriazoa - ps14
Parahoxozoa - ps15
Bilateria - ps16
Deuterostomia - ps17
Chordata - ps18
Olfactores - ps19
Vertebrata - ps20
Gnathostomata - ps21
Euteleostomi - ps22
Sarcopterygii - ps23
Tetrapoda - ps24
Amniota - ps25
Mammalia - ps26
Theria - ps27
Placentalia - ps28
Boreoeutheria - ps29
Euarchontoglires - ps30
Euarchonta - ps31
Primates - ps32
Haplorrhini - ps33
Simiiformes - ps34
Catarrhini - ps35
Hominoidea - ps36
Hominidae - ps37
Homininae - ps38
Hominini - ps39
Homo_sapiens - ps40
p-value
3e-04
7e-04
1e-03
3e-03
5e-03
Log-odds
0.5
1.0
1.5
2.0
2.5
3.0
3.5
4.0

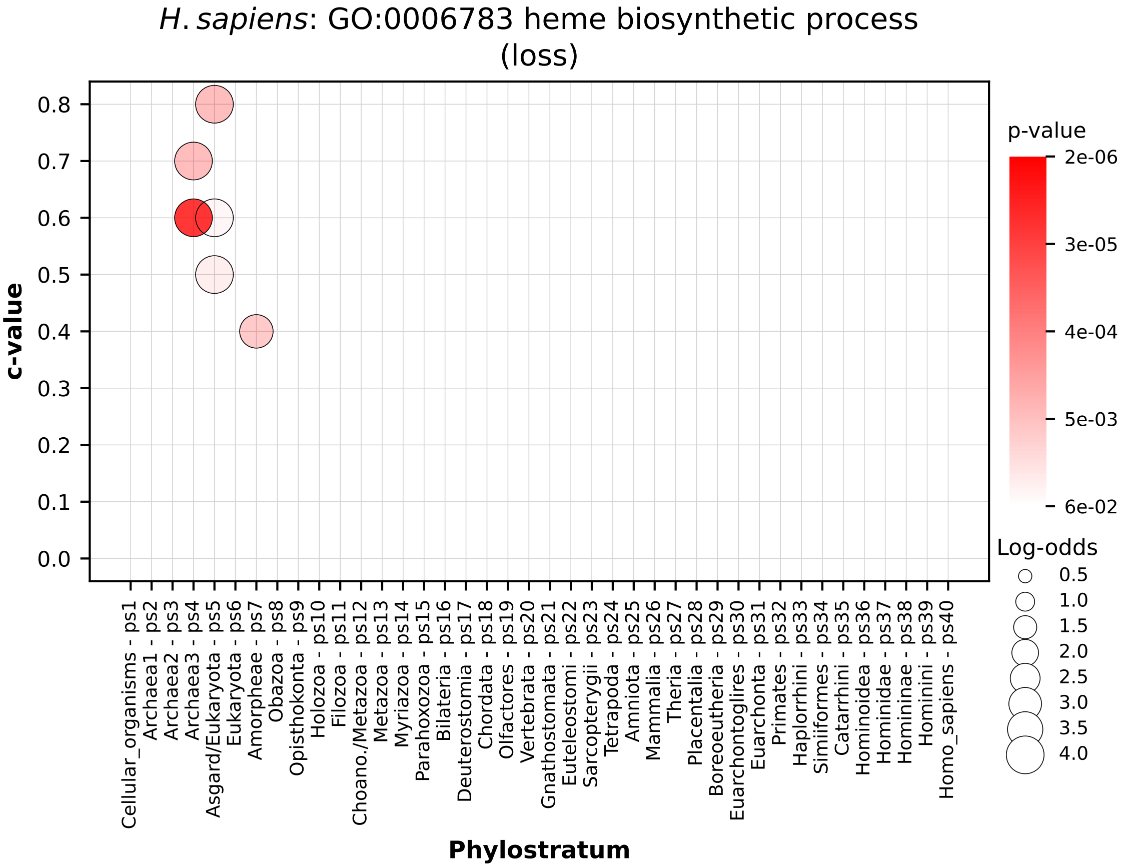

H. sapiens: GO:0006783 heme biosynthetic process
(loss)
c-value
0.8
0.7
0.6
0.5
0.4
0.3
0.2
0.1
0.0
Phylostratum
Cellular_organisms - ps1
Archaea1 - ps2
Archaea2 - ps3
Archaea3 - ps4
Asgard/Eukaryota - ps5
Eukaryota - ps6
Amorpheae - ps7
Obazoa - ps8
Opisthokonta - ps9
Holozoa - ps10
Filozoa - ps11
Choano./Metazoa - ps12
Metazoa - ps13
Myriazoa - ps14
Parahoxozoa - ps15
Bilateria - ps16
Deuterostomia - ps17
Chordata - ps18
Olfactores - ps19
Vertebrata - ps20
Gnathostomata - ps21
Euteleostomi - ps22
Sarcopterygii - ps23
Tetrapoda - ps24
Amniota - ps25
Mammalia - ps26
Theria - ps27
Placentalia - ps28
Boreoeutheria - ps29
Euarchontoglires - ps30
Euarchonta - ps31
Primates - ps32
Haplorrhini - ps33
Simiiformes - ps34
Catarrhini - ps35
Hominoidea - ps36
Hominidae - ps37
Homininae - ps38
Hominini - ps39
Homo_sapiens - ps40
p-value
2e-06
3e-05
4e-04
5e-03
6e-02
Log-odds
0.5
1.0
1.5
2.0
2.5
3.0
3.5
4.0

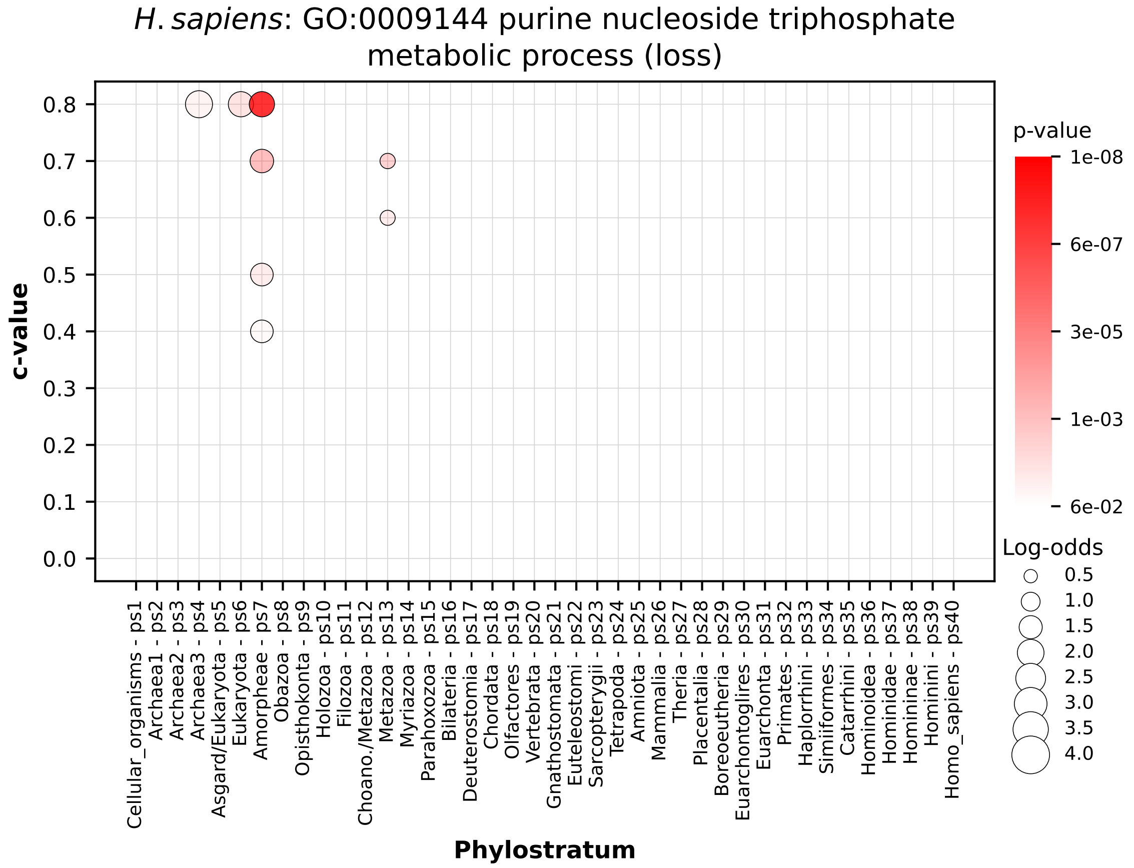

H. sapiens: GO:0009144 purine nucleoside triphosphate metabolic process (loss)
c-value
0.0
0.1
0.2
0.3
0.4
0.5
0.6
0.7
0.8
Phylostratum
Cellular_organisms - ps1
Archaea1 - ps2
Archaea2 - ps3
Archaea3 - ps4
Asgard/Eukaryota - ps5
Eukaryota - ps6
Amorpheae - ps7
Obazoa - ps8
Opisthokonta - ps9
Holozoa - ps10
Filozoa - ps11
Choano./Metazoa - ps12
Metazoa - ps13
Myriazoa - ps14
Parahoxozoa - ps15
Bilateria - ps16
Deuterostomia - ps17
Chordata - ps18
Olfactores - ps19
Vertebrata - ps20
Gnathostomata - ps21
Euteleostomi - ps22
Sarcopterygii - ps23
Tetrapoda - ps24
Amniota - ps25
Mammalia - ps26
Theria - ps27
Placentalia - ps28
Boreoeutheria - ps29
Euarchontoglires - ps30
Euarchonta - ps31
Primates - ps32
Haplorrhini - ps33
Simiiformes - ps34
Catarrhini - ps35
Hominoidea - ps36
Hominidae - ps37
Homininae - ps38
Hominini - ps39
Homo_sapiens - ps40
p-value
1e-08
6e-07
3e-05
1e-03
6e-02
Log-odds
0.5
1.0
1.5
2.0
2.5
3.0
3.5
4.0

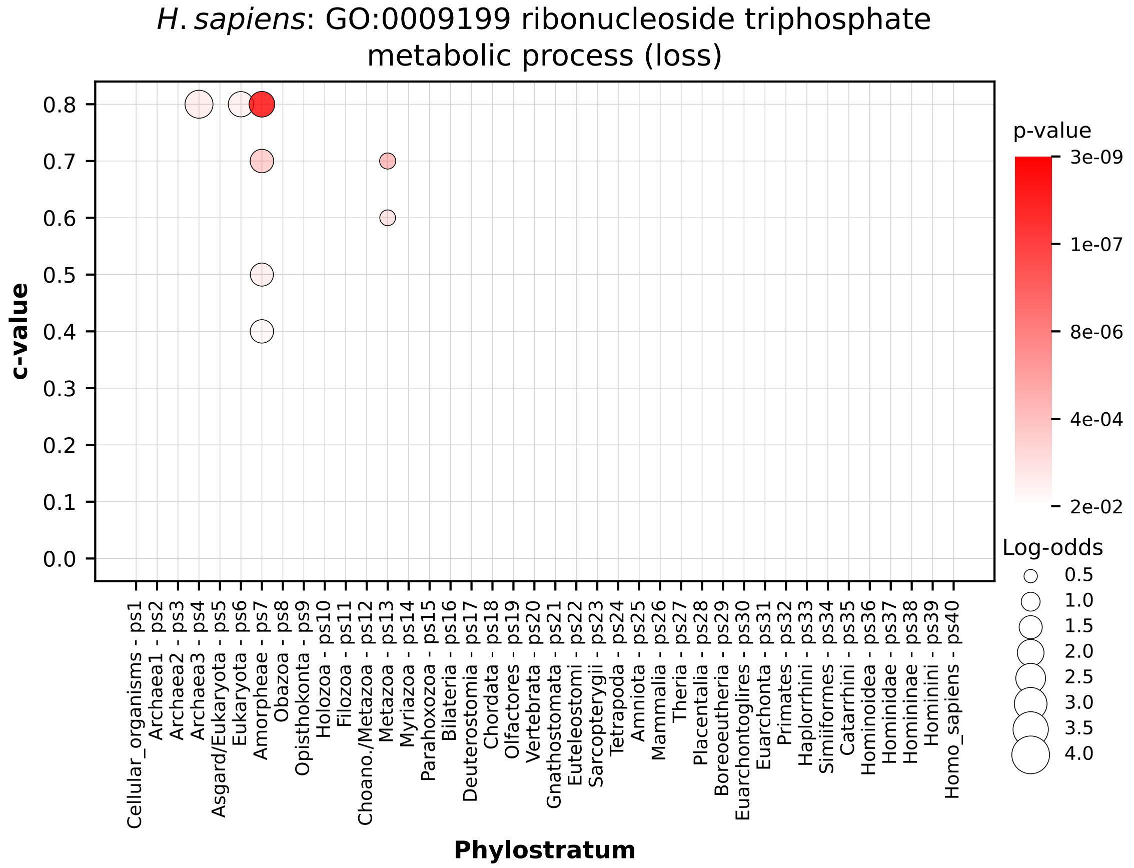

H. sapiens: GO:0009199 ribonucleoside triphosphate metabolic process (loss)
c-value
0.8
0.7
0.6
0.5
0.4
0.3
0.2
0.1
0.0
p-value
3e-09
1e-07
8e-06
4e-04
2e-02
Log-odds
0.5
1.0
1.5
2.0
2.5
3.0
3.5
4.0
Cellular_organisms - ps1
Archaea1 - ps2
Archaea2 - ps3
Archaea3 - ps4
Asgard/Eukaryota - ps5
Eukaryota - ps6
Amorpheae - ps7
Obazoa - ps8
Opisthokonta - ps9
Holozoa - ps10
Filozoa - ps11
Choano./Metazoa - ps12
Metazoa - ps13
Myriazoa - ps14
Parahoxozoa - ps15
Bilateria - ps16
Deuterostomia - ps17
Chordata - ps18
Olfactores - ps19
Vertebrata - ps20
Gnathostomata - ps21
Euteleostomi - ps22
Sarcopterygii - ps23
Tetrapoda - ps24
Amniota - ps25
Mammalia - ps26
Theria - ps27
Placentalia - ps28
Boreoeutheria - ps29
Euarchontoglires - ps30
Euarchonta - ps31
Primates - ps32
Haplorrhini - ps33
Simiiformes - ps34
Catarrhini - ps35
Hominoidea - ps36
Hominidae - ps37
Homininae - ps38
Hominini - ps39
Homo_sapiens - ps40
Phylostratum

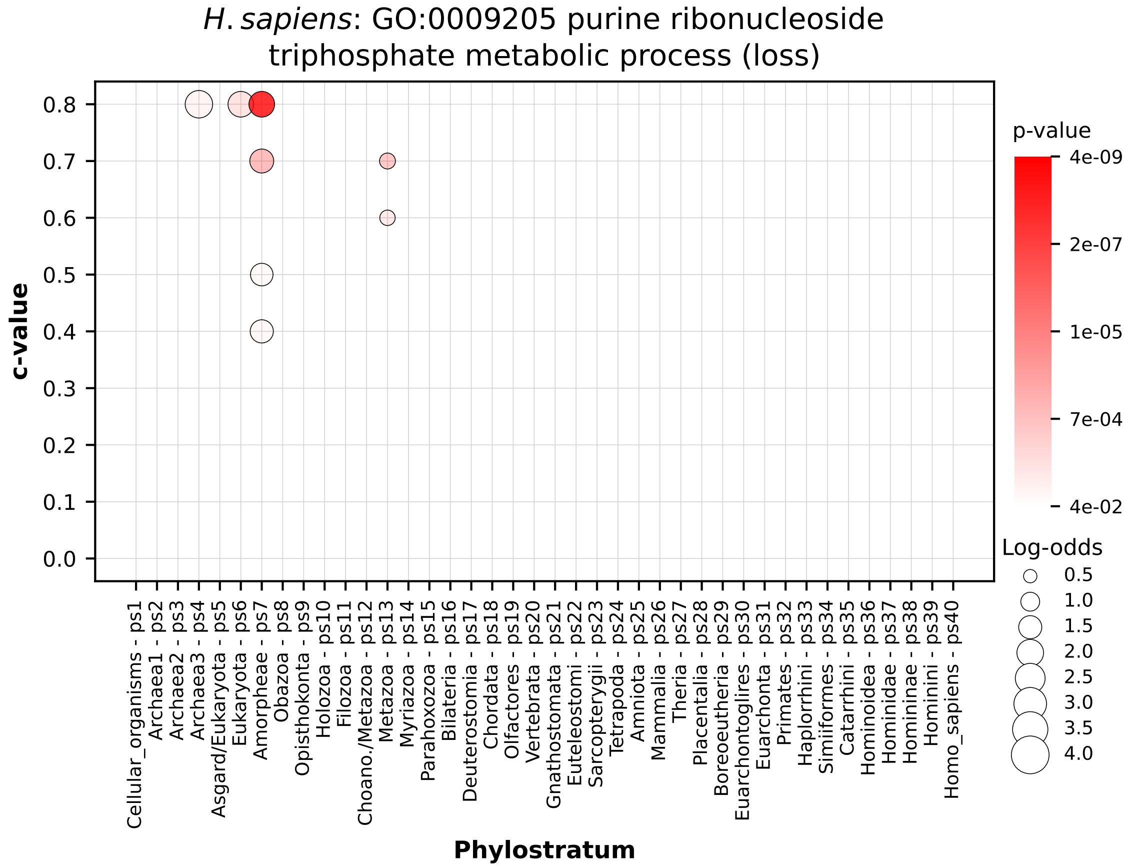

H. sapiens: GO:0009205 purine ribonucleoside triphosphate metabolic process (loss)
c-value
0.8
0.7
0.6
0.5
0.4
0.3
0.2
0.1
0.0
p-value
4e-09
2e-07
1e-05
7e-04
4e-02
Log-odds
0.5
1.0
1.5
2.0
2.5
3.0
3.5
4.0
Cellular_organisms - ps1
Archaea1 - ps2
Archaea2 - ps3
Archaea3 - ps4
Asgard/Eukaryota - ps5
Eukaryota - ps6
Amorpheae - ps7
Obazoa - ps8
Opisthokonta - ps9
Holozoa - ps10
Filozoa - ps11
Choano./Metazoa - ps12
Metazoa - ps13
Myriazoa - ps14
Parahoxozoa - ps15
Bilateria - ps16
Deuterostomia - ps17
Chordata - ps18
Olfactores - ps19
Vertebrata - ps20
Gnathostomata - ps21
Euteleostomi - ps22
Sarcopterygii - ps23
Tetrapoda - ps24
Amniota - ps25
Mammalia - ps26
Theria - ps27
Placentalia - ps28
Boreoeutheria - ps29
Euarchontoglires - ps30
Euarchonta - ps31
Primates - ps32
Haplorrhini - ps33
Simiiformes - ps34
Catarrhini - ps35
Hominoidea - ps36
Hominidae - ps37
Homininae - ps38
Hominini - ps39
Homo_sapiens - ps40
Phylostratum

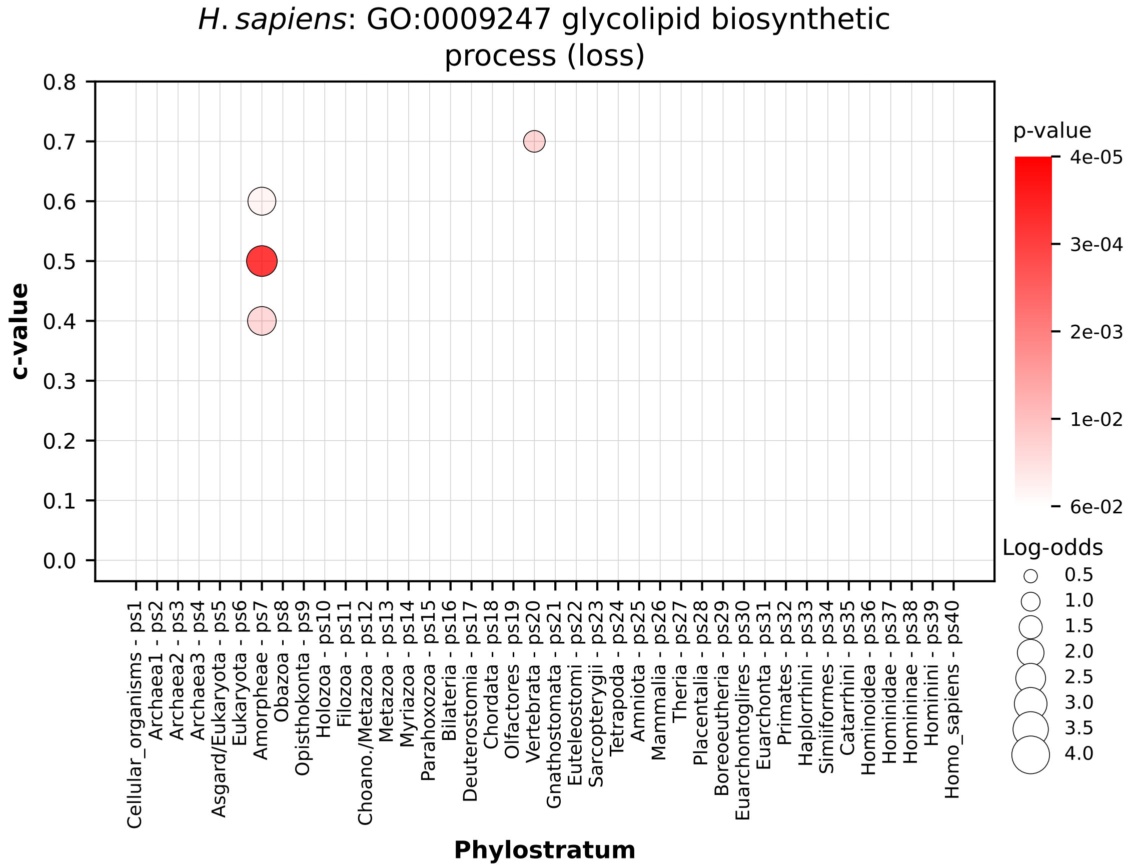

H. sapiens: GO:0009247 glycolipid biosynthetic process (loss)
c-value
0.8
0.7
0.6
0.5
0.4
0.3
0.2
0.1
0.0
p-value
4e-05
3e-04
2e-03
1e-02
6e-02
Log-odds
0.5
1.0
1.5
2.0
2.5
3.0
3.5
4.0
Cellular_organisms - ps1
Archaea1 - ps2
Archaea2 - ps3
Archaea3 - ps4
Asgard/Eukaryota - ps5
Eukaryota - ps6
Amorpheae - ps7
Obazoa - ps8
Opisthokonta - ps9
Holozoa - ps10
Filozoa - ps11
Choano./Metazoa - ps12
Metazoa - ps13
Myriazoa - ps14
Parahoxozoa - ps15
Bilateria - ps16
Deuterostomia - ps17
Chordata - ps18
Olfactores - ps19
Vertebrata - ps20
Gnathostomata - ps21
Euteleostomi - ps22
Sarcopterygii - ps23
Tetrapoda - ps24
Amniota - ps25
Mammalia - ps26
Theria - ps27
Placentalia - ps28
Boreoeutheria - ps29
Euarchontoglires - ps30
Euarchonta - ps31
Primates - ps32
Haplorrhini - ps33
Simiiformes - ps34
Catarrhini - ps35
Hominoidea - ps36
Hominidae - ps37
Homininae - ps38
Hominini - ps39
Homo_sapiens - ps40
Phylostratum

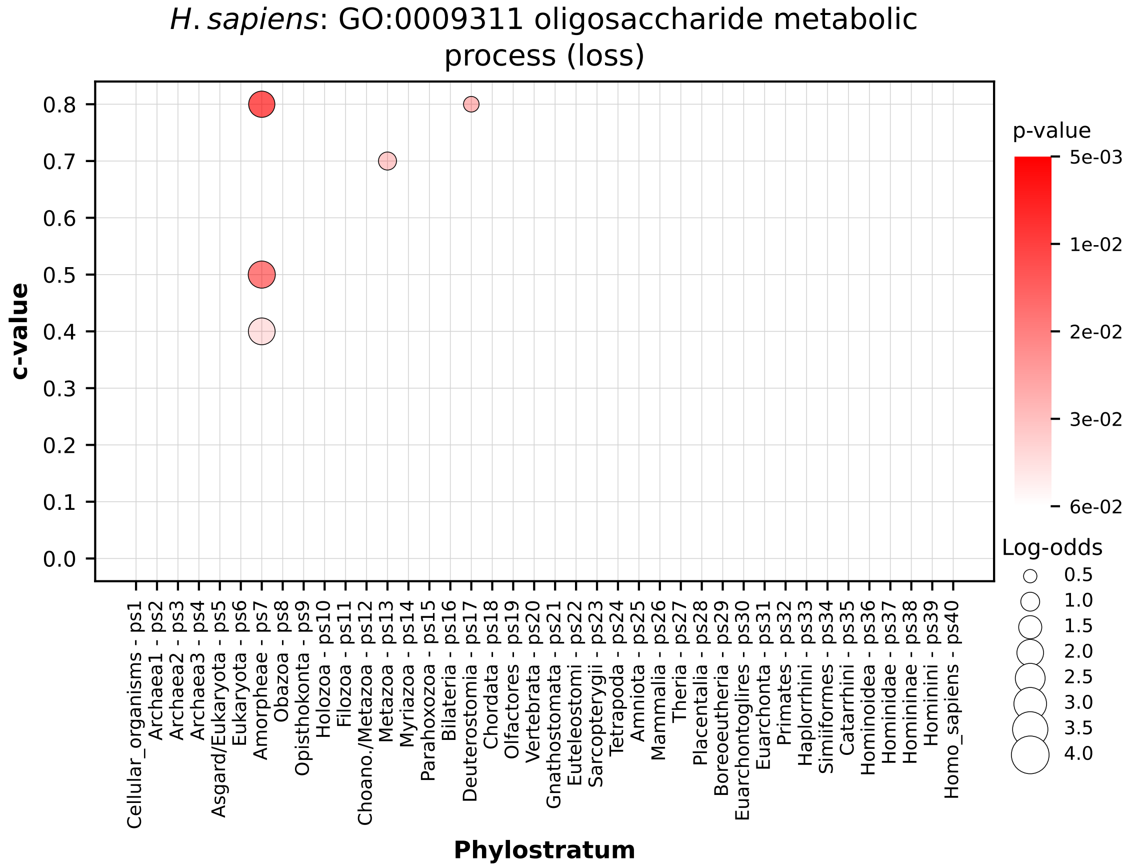

H. sapiens: GO:0009311 oligosaccharide metabolic process (loss)
c-value
0.0
0.1
0.2
0.3
0.4
0.5
0.6
0.7
0.8
Phylostratum
Cellular_organisms - ps1
Archaea1 - ps2
Archaea2 - ps3
Archaea3 - ps4
Asgard/Eukaryota - ps5
Eukaryota - ps6
Amorpheae - ps7
Obazoa - ps8
Opisthokonta - ps9
Holozoa - ps10
Filozoa - ps11
Choano./Metazoa - ps12
Metazoa - ps13
Myriazoa - ps14
Parahoxozoa - ps15
Bilateria - ps16
Deuterostomia - ps17
Chordata - ps18
Olfactores - ps19
Vertebrata - ps20
Gnathostomata - ps21
Euteleostomi - ps22
Sarcopterygii - ps23
Tetrapoda - ps24
Amniota - ps25
Mammalia - ps26
Theria - ps27
Placentalia - ps28
Boreoeutheria - ps29
Euarchontoglires - ps30
Euarchonta - ps31
Primates - ps32
Haplorrhini - ps33
Simiiformes - ps34
Catarrhini - ps35
Hominoidea - ps36
Hominidae - ps37
Homininae - ps38
Hominini - ps39
Homo_sapiens - ps40
p-value
5e-03
1e-02
2e-02
3e-02
6e-02
Log-odds
0.5
1.0
1.5
2.0
2.5
3.0
3.5
4.0

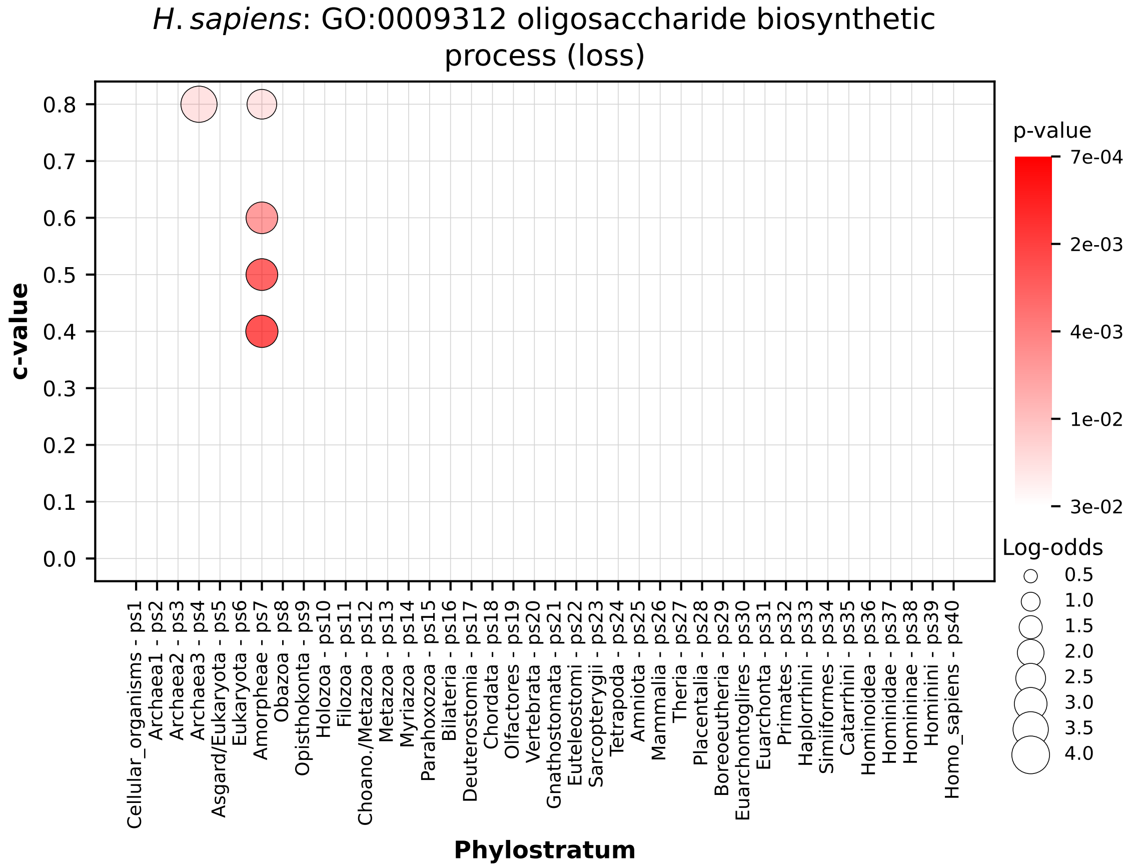

H. sapiens: GO:0009312 oligosaccharide biosynthetic process (loss)
c-value
0.8
0.7
0.6
0.5
0.4
0.3
0.2
0.1
0.0
p-value
7e-04
2e-03
4e-03
1e-02
3e-02
Log-odds
0.5
1.0
1.5
2.0
2.5
3.0
3.5
4.0
Cellular_organisms - ps1
Archaea1 - ps2
Archaea2 - ps3
Archaea3 - ps4
Asgard/Eukaryota - ps5
Eukaryota - ps6
Amorpheae - ps7
Obazoa - ps8
Opisthokonta - ps9
Holozoa - ps10
Filozoa - ps11
Choano./Metazoa - ps12
Metazoa - ps13
Myriazoa - ps14
Parahoxozoa - ps15
Bilateria - ps16
Deuterostomia - ps17
Chordata - ps18
Olfactores - ps19
Vertebrata - ps20
Gnathostomata - ps21
Euteleostomi - ps22
Sarcopterygii - ps23
Tetrapoda - ps24
Amniota - ps25
Mammalia - ps26
Theria - ps27
Placentalia - ps28
Boreoeutheria - ps29
Euarchontoglires - ps30
Euarchonta - ps31
Primates - ps32
Haplorrhini - ps33
Simiiformes - ps34
Catarrhini - ps35
Hominoidea - ps36
Hominidae - ps37
Homininae - ps38
Hominini - ps39
Homo_sapiens - ps40
Phylostratum

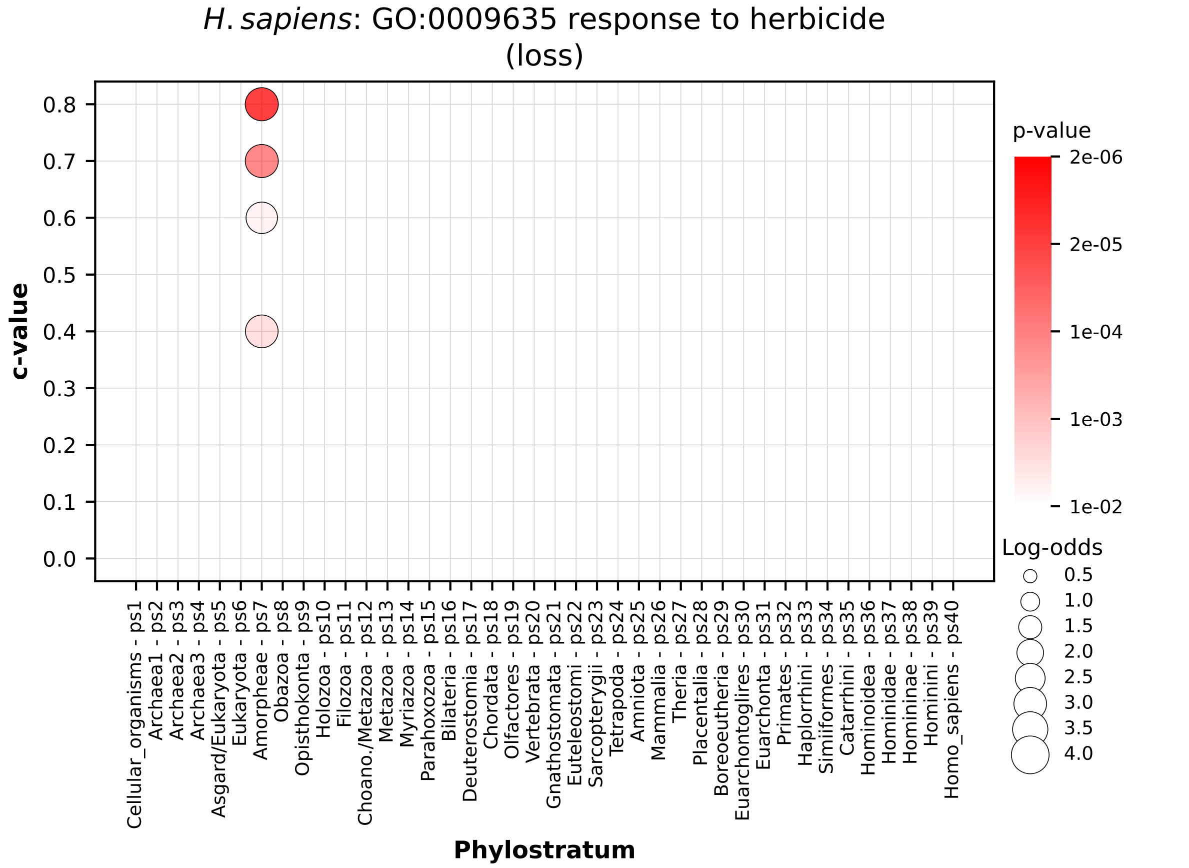

H. sapiens: GO:0009635 response to herbicide
(loss)
c-value
0.8
0.7
0.6
0.5
0.4
0.3
0.2
0.1
0.0
p-value
2e-06
2e-05
1e-04
1e-03
1e-02
Log-odds
0.5
1.0
1.5
2.0
2.5
3.0
3.5
4.0
Cellular_organisms - ps1
Archaea1 - ps2
Archaea2 - ps3
Archaea3 - ps4
Asgard/Eukaryota - ps5
Eukaryota - ps6
Amorpheae - ps7
Obazoa - ps8
Opisthokonta - ps9
Holozoa - ps10
Filozoa - ps11
Choano./Metazoa - ps12
Metazoa - ps13
Myriazoa - ps14
Parahoxozoa - ps15
Bilateria - ps16
Deuterostomia - ps17
Chordata - ps18
Olfactores - ps19
Vertebrata - ps20
Gnathostomata - ps21
Euteleostomi - ps22
Sarcopterygii - ps23
Tetrapoda - ps24
Amniota - ps25
Mammalia - ps26
Theria - ps27
Placentalia - ps28
Boreoeutheria - ps29
Euarchontoglires - ps30
Euarchonta - ps31
Primates - ps32
Haplorrhini - ps33
Simiiformes - ps34
Catarrhini - ps35
Hominoidea - ps36
Hominidae - ps37
Homininae - ps38
Hominini - ps39
Homo_sapiens - ps40
Phylostratum

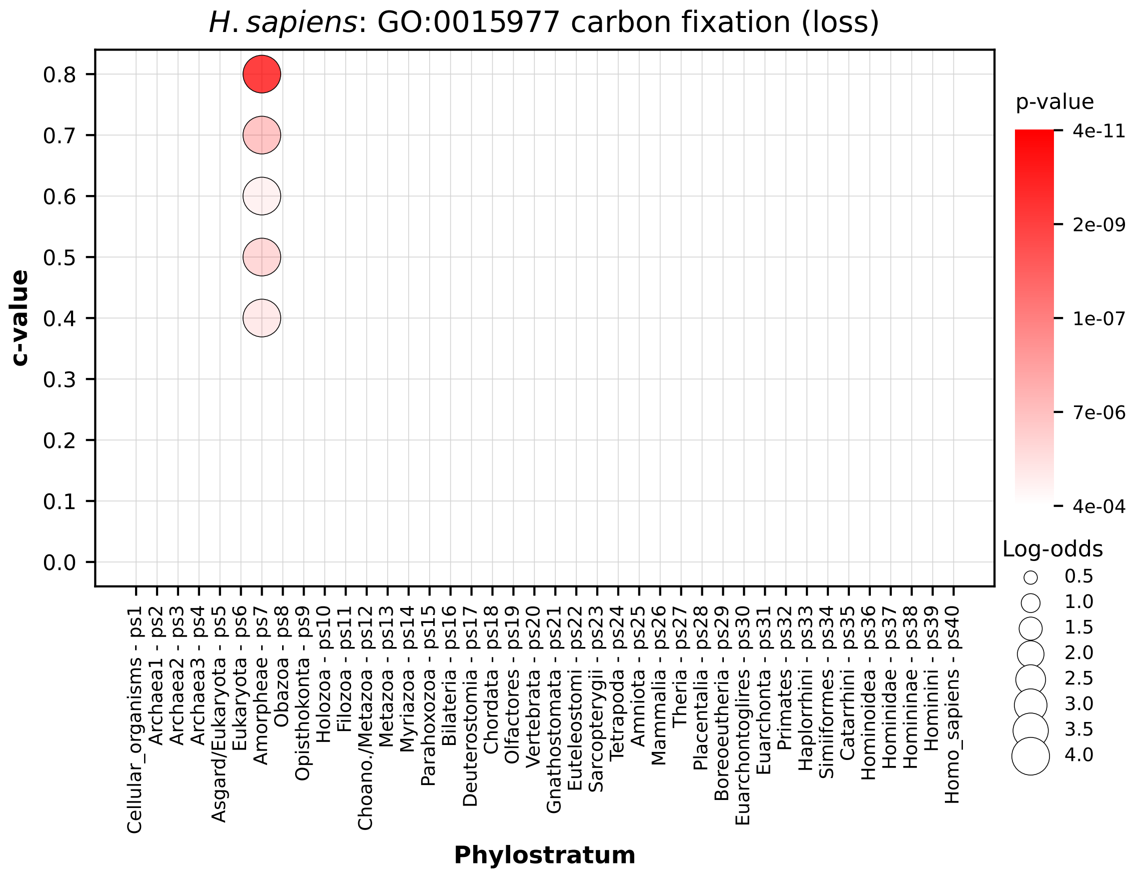

H. sapiens: GO:0015977 carbon fixation (loss)
c-value
0.0
0.1
0.2
0.3
0.4
0.5
0.6
0.7
0.8
p-value
4e-11
2e-09
1e-07
7e-06
4e-04
Log-odds
0.5
1.0
1.5
2.0
2.5
3.0
3.5
4.0
Cellular_organisms - ps1
Archaea1 - ps2
Archaea2 - ps3
Archaea3 - ps4
Asgard/Eukaryota - ps5
Eukaryota - ps6
Amorpheae - ps7
Obazoa - ps8
Opisthokonta - ps9
Holozoa - ps10
Filozoa - ps11
Choano./Metazoa - ps12
Metazoa - ps13
Myriazoa - ps14
Parahoxozoa - ps15
Bilateria - ps16
Deuterostomia - ps17
Chordata - ps18
Olfactores - ps19
Vertebrata - ps20
Gnathostomata - ps21
Euteleostomi - ps22
Sarcopterygii - ps23
Tetrapoda - ps24
Amniota - ps25
Mammalia - ps26
Theria - ps27
Placentalia - ps28
Boreoeutheria - ps29
Euarchontoglires - ps30
Euarchonta - ps31
Primates - ps32
Haplorrhini - ps33
Simiiformes - ps34
Catarrhini - ps35
Hominoidea - ps36
Hominidae - ps37
Homininae - ps38
Hominini - ps39
Homo_sapiens - ps40
Phylostratum

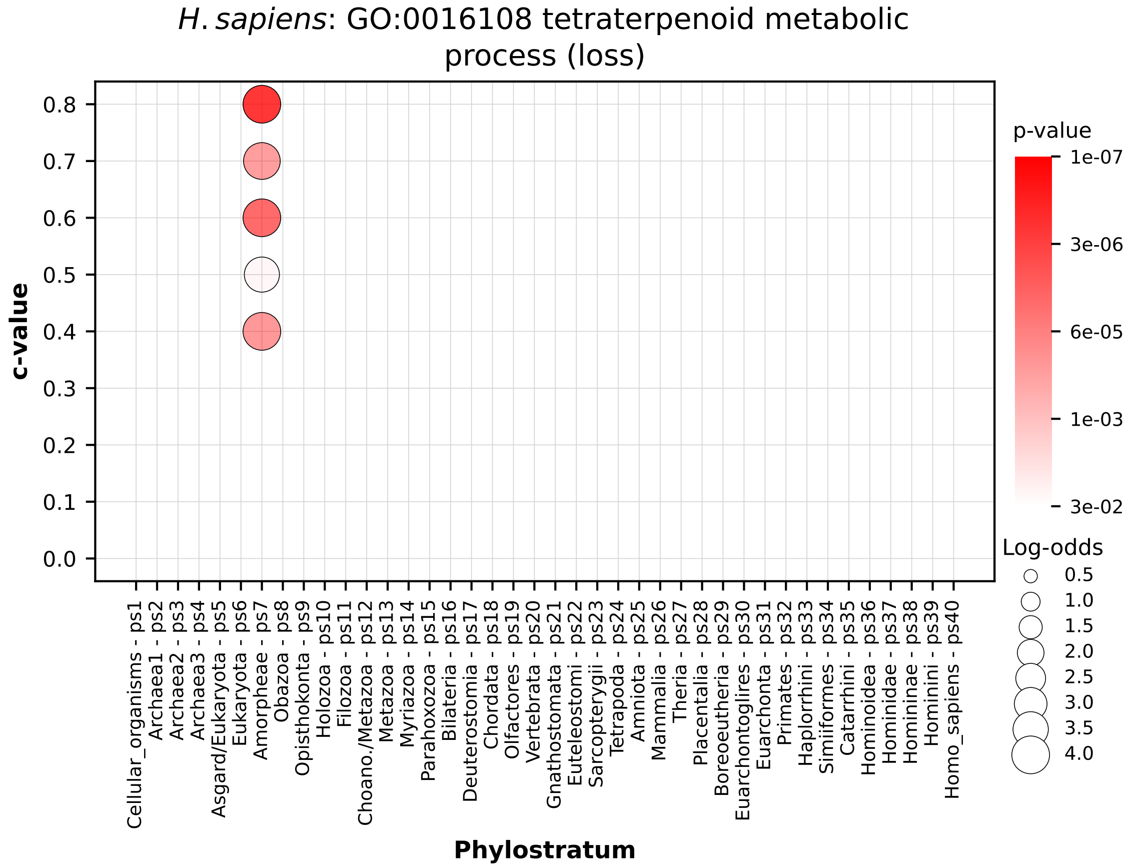
H. sapiens: GO:0016108 tetraterpenoid metabolic process (loss)
c-value
0.0
0.1
0.2
0.3
0.4
0.5
0.6
0.7
0.8
p-value
1e-07
3e-06
6e-05
1e-03
3e-02
Log-odds
0.5
1.0
1.5
2.0
2.5
3.0
3.5
4.0
Cellular_organisms - ps1
Archaea1 - ps2
Archaea2 - ps3
Archaea3 - ps4
Asgard/Eukaryota - ps5
Eukaryota - ps6
Amorpheae - ps7
Obazoa - ps8
Opisthokonta - ps9
Holozoa - ps10
Filozoa - ps11
Choano./Metazoa - ps12
Metazoa - ps13
Myriazoa - ps14
Parahoxozoa - ps15
Bilateria - ps16
Deuterostomia - ps17
Chordata - ps18
Olfactores - ps19
Vertebrata - ps20
Gnathostomata - ps21
Euteleostomi - ps22
Sarcopterygii - ps23
Tetrapoda - ps24
Amniota - ps25
Mammalia - ps26
Theria - ps27
Placentalia - ps28
Boreoeutheria - ps29
Euarchontoglires - ps30
Euarchonta - ps31
Primates - ps32
Haplorrhini - ps33
Simiiformes - ps34
Catarrhini - ps35
Hominoidea - ps36
Hominidae - ps37
Homininae - ps38
Hominini - ps39
Homo_sapiens - ps40
Phylostratum

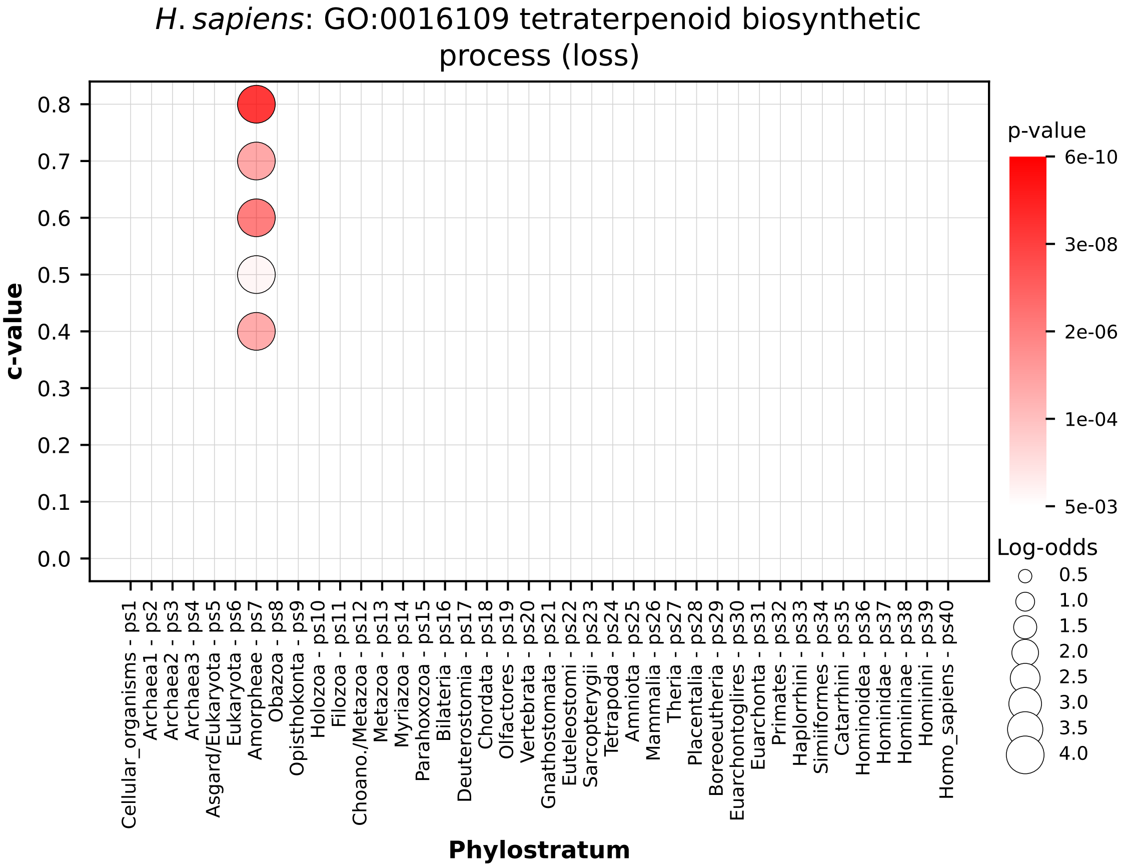

H. sapiens: GO:0016109 tetraterpenoid biosynthetic process (loss)
c-value
0.8
0.7
0.6
0.5
0.4
0.3
0.2
0.1
0.0
p-value
6e-10
3e-08
2e-06
1e-04
5e-03
Log-odds
0.5
1.0
1.5
2.0
2.5
3.0
3.5
4.0
Cellular_organisms - ps1
Archaea1 - ps2
Archaea2 - ps3
Archaea3 - ps4
Asgard/Eukaryota - ps5
Eukaryota - ps6
Amorpheae - ps7
Obazoa - ps8
Opisthokonta - ps9
Holozoa - ps10
Filozoa - ps11
Choano./Metazoa - ps12
Metazoa - ps13
Myriazoa - ps14
Parahoxozoa - ps15
Bilateria - ps16
Deuterostomia - ps17
Chordata - ps18
Olfactores - ps19
Vertebrata - ps20
Gnathostomata - ps21
Euteleostomi - ps22
Sarcopterygii - ps23
Tetrapoda - ps24
Amniota - ps25
Mammalia - ps26
Theria - ps27
Placentalia - ps28
Boreoeutheria - ps29
Euarchontoglires - ps30
Euarchonta - ps31
Primates - ps32
Haplorrhini - ps33
Simiiformes - ps34
Catarrhini - ps35
Hominoidea - ps36
Hominidae - ps37
Homininae - ps38
Hominini - ps39
Homo_sapiens - ps40
Phylostratum

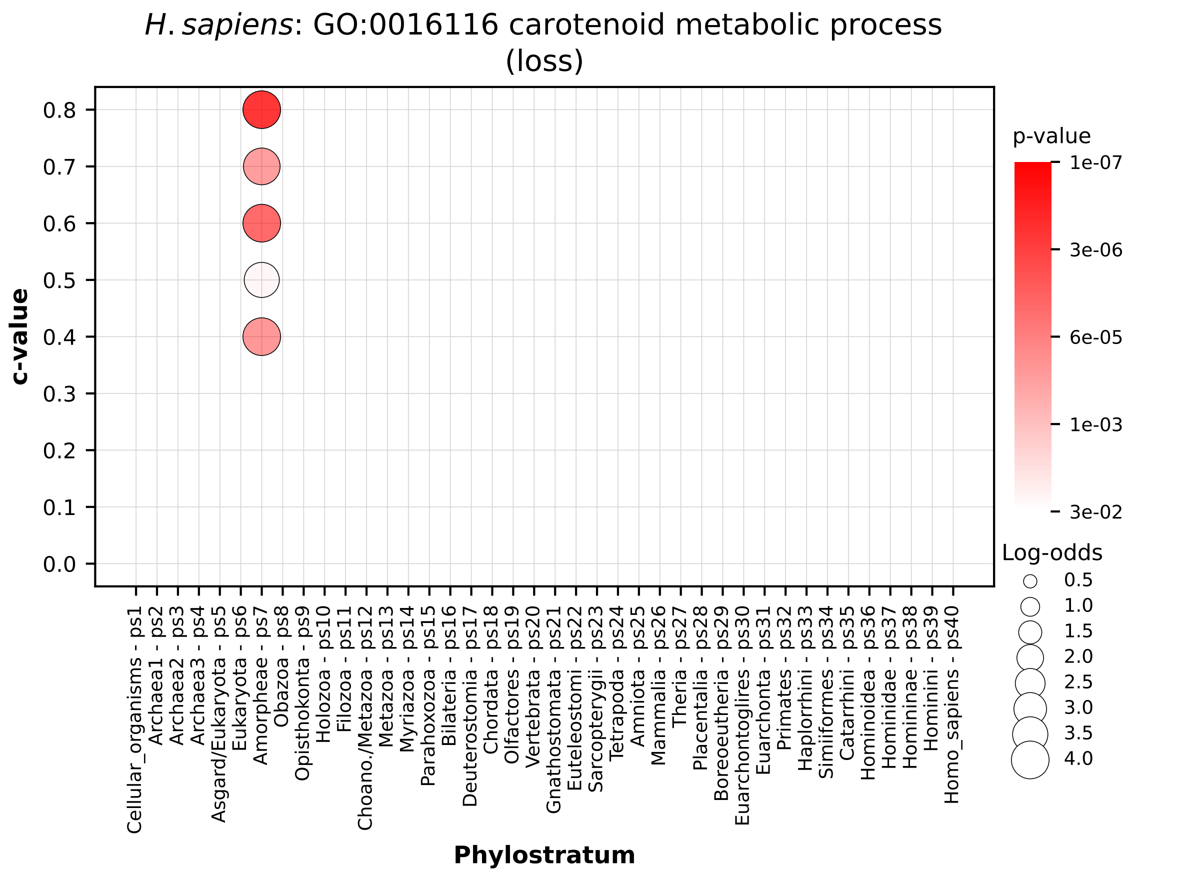

H. sapiens: GO:0016116 carotenoid metabolic process (loss)
c-value
0.0
0.1
0.2
0.3
0.4
0.5
0.6
0.7
0.8
Phylostratum
Cellular_organisms - ps1
Archaea1 - ps2
Archaea2 - ps3
Archaea3 - ps4
Asgard/Eukaryota - ps5
Eukaryota - ps6
Amorpheae - ps7
Obazoa - ps8
Opisthokonta - ps9
Holozoa - ps10
Filozoa - ps11
Choano./Metazoa - ps12
Metazoa - ps13
Myriazoa - ps14
Parahoxozoa - ps15
Bilateria - ps16
Deuterostomia - ps17
Chordata - ps18
Olfactores - ps19
Vertebrata - ps20
Gnathostomata - ps21
Euteleostomi - ps22
Sarcopterygii - ps23
Tetrapoda - ps24
Amniota - ps25
Mammalia - ps26
Theria - ps27
Placentalia - ps28
Boreoeutheria - ps29
Euarchontoglires - ps30
Euarchonta - ps31
Primates - ps32
Haplorrhini - ps33
Simiiformes - ps34
Catarrhini - ps35
Hominoidea - ps36
Hominidae - ps37
Homininae - ps38
Hominini - ps39
Homo_sapiens - ps40
p-value
1e-07
3e-06
6e-05
1e-03
3e-02
Log-odds
0.5
1.0
1.5
2.0
2.5
3.0
3.5
4.0

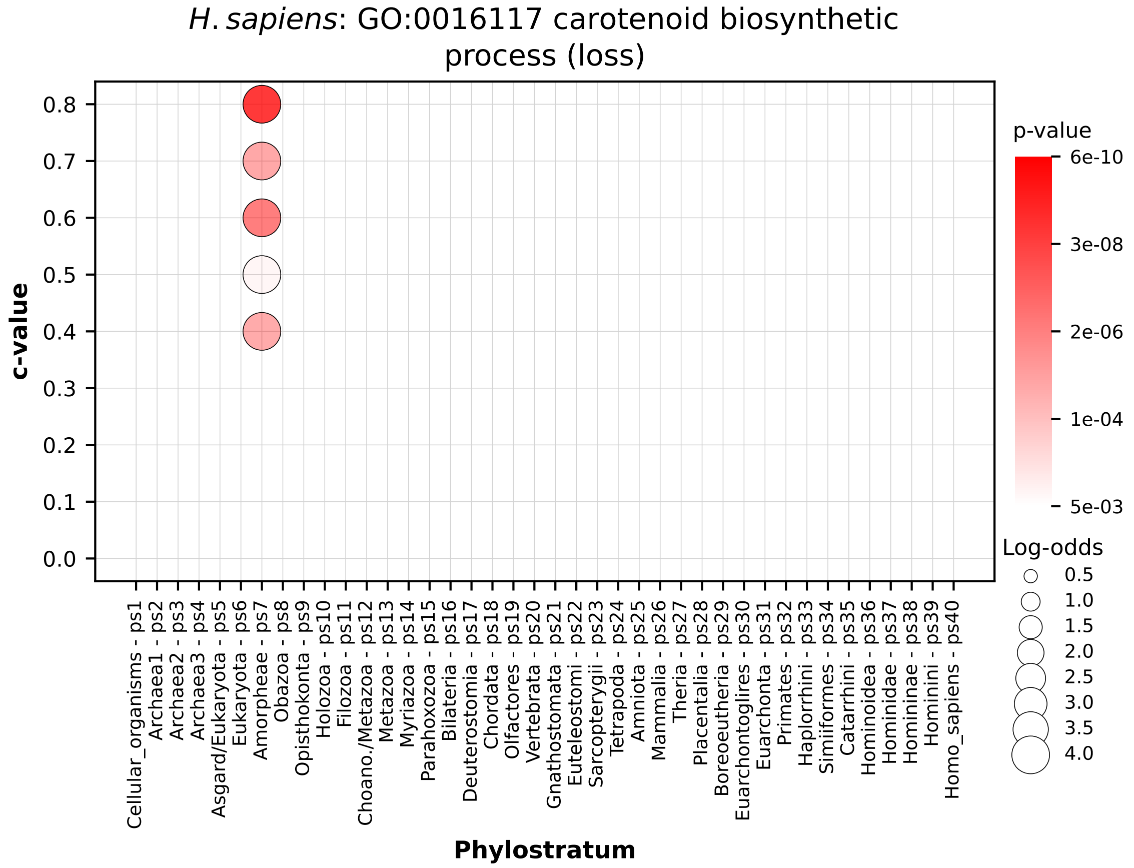

H. sapiens: GO:0016117 carotenoid biosynthetic process (loss)
c-value
0.0
0.1
0.2
0.3
0.4
0.5
0.6
0.7
0.8
Phylostratum
Cellular_organisms - ps1
Archaea1 - ps2
Archaea2 - ps3
Archaea3 - ps4
Asgard/Eukaryota - ps5
Eukaryota - ps6
Amorpheae - ps7
Obazoa - ps8
Opisthokonta - ps9
Holozoa - ps10
Filozoa - ps11
Choano./Metazoa - ps12
Metazoa - ps13
Myriazoa - ps14
Parahoxozoa - ps15
Bilateria - ps16
Deuterostomia - ps17
Chordata - ps18
Olfactores - ps19
Vertebrata - ps20
Gnathostomata - ps21
Euteleostomi - ps22
Sarcopterygii - ps23
Tetrapoda - ps24
Amniota - ps25
Mammalia - ps26
Theria - ps27
Placentalia - ps28
Boreoeutheria - ps29
Euarchontoglires - ps30
Euarchonta - ps31
Primates - ps32
Haplorrhini - ps33
Simiiformes - ps34
Catarrhini - ps35
Hominoidea - ps36
Hominidae - ps37
Homininae - ps38
Hominini - ps39
Homo_sapiens - ps40
p-value
6e-10
3e-08
2e-06
1e-04
5e-03
Log-odds
0.5
1.0
1.5
2.0
2.5
3.0
3.5
4.0

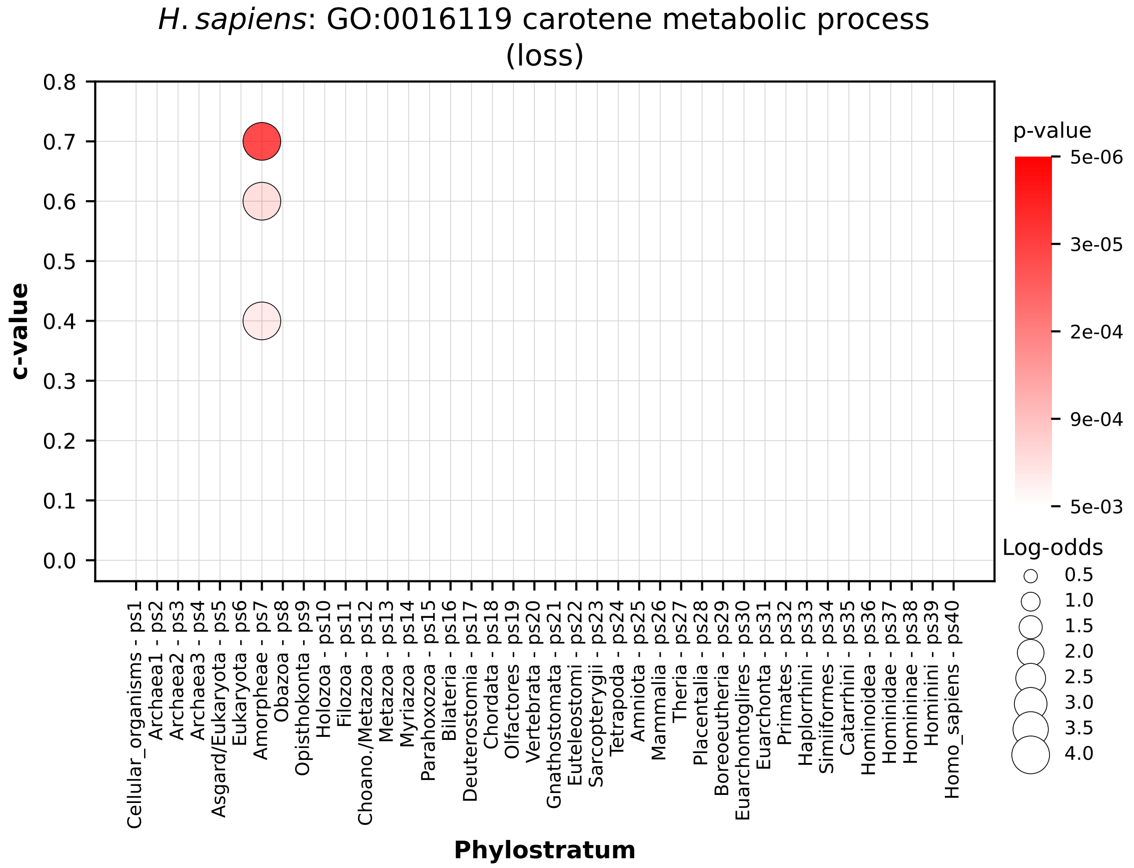

H. sapiens: GO:0016119 carotene metabolic process
(loss)
c-value
0.8
0.7
0.6
0.5
0.4
0.3
0.2
0.1
0.0
p-value
5e-06
3e-05
2e-04
9e-04
5e-03
Log-odds
0.5
1.0
1.5
2.0
2.5
3.0
3.5
4.0
Cellular_organisms - ps1
Archaea1 - ps2
Archaea2 - ps3
Archaea3 - ps4
Asgard/Eukaryota - ps5
Eukaryota - ps6
Amorpheae - ps7
Obazoa - ps8
Opisthokonta - ps9
Holozoa - ps10
Filozoa - ps11
Choano./Metazoa - ps12
Metazoa - ps13
Myriazoa - ps14
Parahoxozoa - ps15
Bilateria - ps16
Deuterostomia - ps17
Chordata - ps18
Olfactores - ps19
Vertebrata - ps20
Gnathostomata - ps21
Euteleostomi - ps22
Sarcopterygii - ps23
Tetrapoda - ps24
Amniota - ps25
Mammalia - ps26
Theria - ps27
Placentalia - ps28
Boreoeutheria - ps29
Euarchontoglires - ps30
Euarchonta - ps31
Primates - ps32
Haplorrhini - ps33
Simiiformes - ps34
Catarrhini - ps35
Hominoidea - ps36
Hominidae - ps37
Homininae - ps38
Hominini - ps39
Homo_sapiens - ps40
Phylostratum

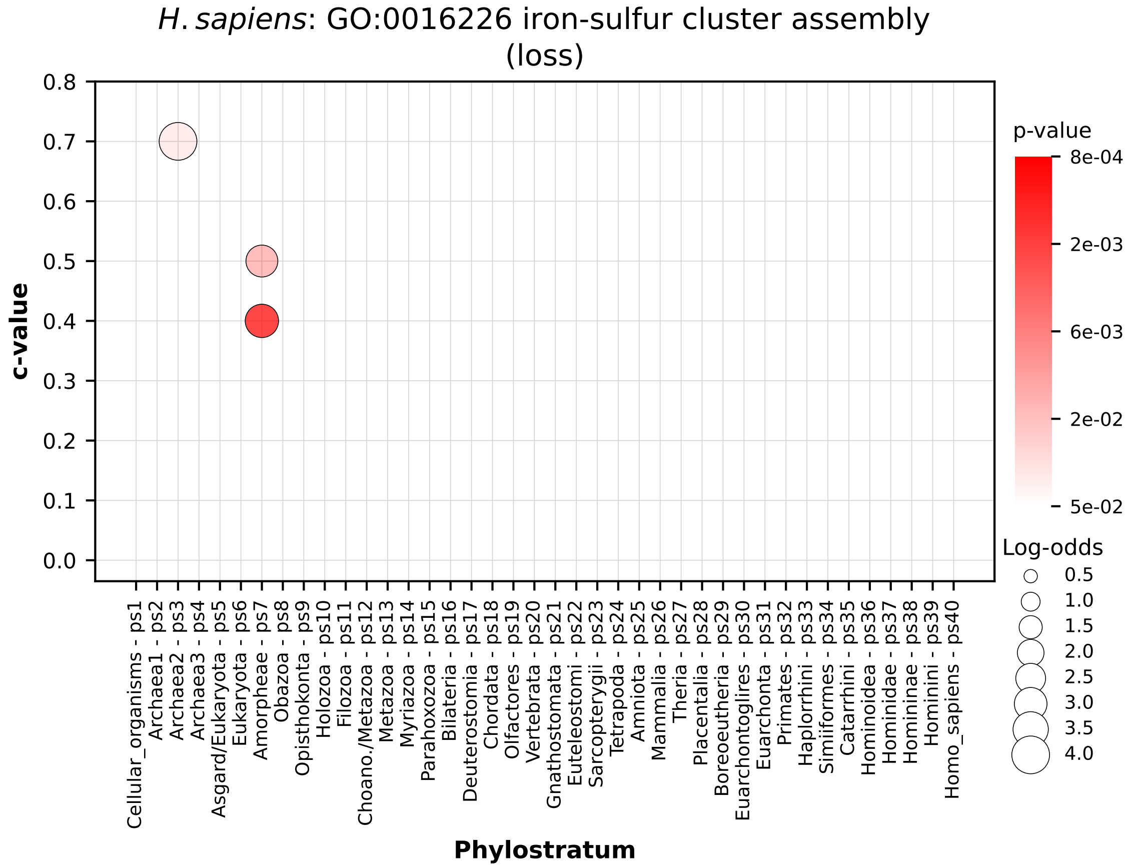
H. sapiens: GO:0016226 iron-sulfur cluster assembly (loss)
c-value
0.8
0.7
0.6
0.5
0.4
0.3
0.2
0.1
0.0
Phylostratum
Cellular_organisms - ps1
Archaea1 - ps2
Archaea2 - ps3
Archaea3 - ps4
Asgard/Eukaryota - ps5
Eukaryota - ps6
Amorpheae - ps7
Obazoa - ps8
Opisthokonta - ps9
Holozoa - ps10
Filozoa - ps11
Choano./Metazoa - ps12
Metazoa - ps13
Myriazoa - ps14
Parahoxozoa - ps15
Bilateria - ps16
Deuterostomia - ps17
Chordata - ps18
Olfactores - ps19
Vertebrata - ps20
Gnathostomata - ps21
Euteleostomi - ps22
Sarcopterygii - ps23
Tetrapoda - ps24
Amniota - ps25
Mammalia - ps26
Theria - ps27
Placentalia - ps28
Boreoeutheria - ps29
Euarchontoglires - ps30
Euarchonta - ps31
Primates - ps32
Haplorrhini - ps33
Simiiformes - ps34
Catarrhini - ps35
Hominoidea - ps36
Hominidae - ps37
Homininae - ps38
Hominini - ps39
Homo_sapiens - ps40
p-value
8e-04
2e-03
6e-03
2e-02
5e-02
Log-odds
0.5
1.0
1.5
2.0
2.5
3.0
3.5
4.0

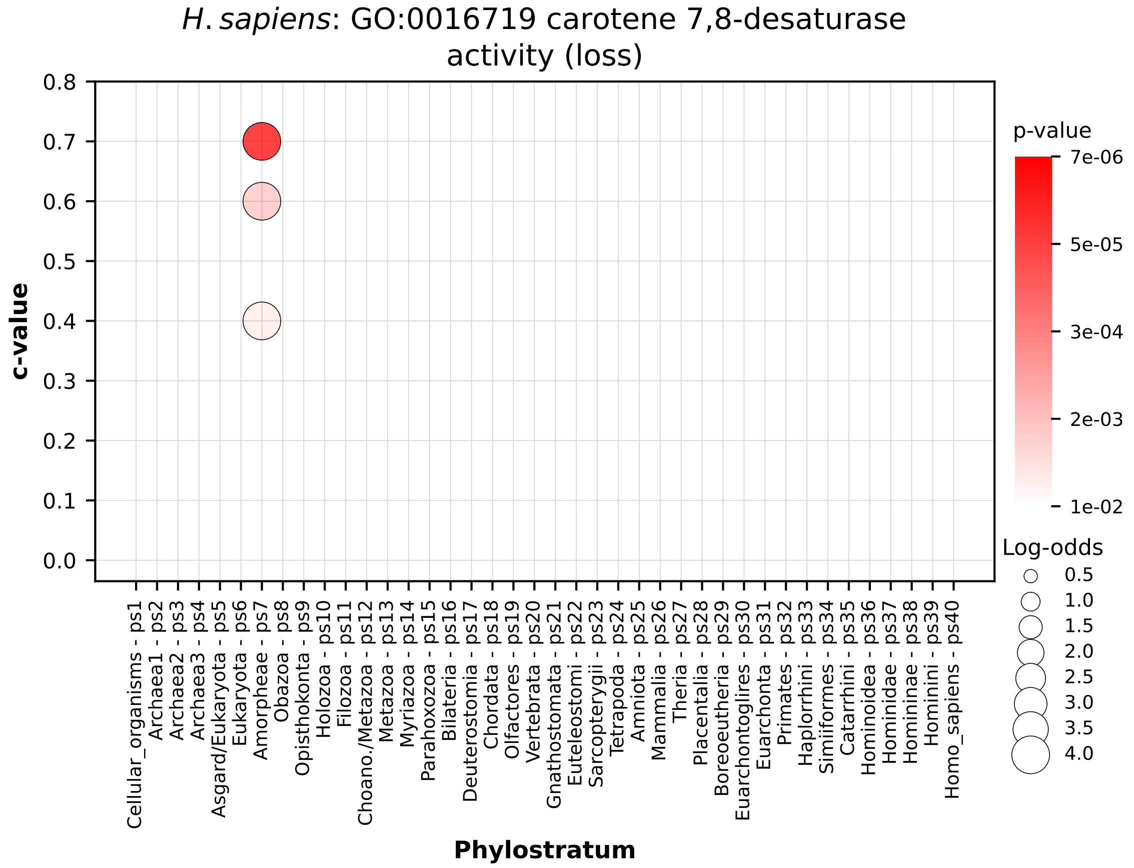
H. sapiens: GO:0016719 carotene 7,8-desaturase activity (loss)
c-value
0.8
0.7
0.6
0.5
0.4
0.3
0.2
0.1
0.0
Phylostratum
Cellular_organisms - ps1
Archaea1 - ps2
Archaea2 - ps3
Archaea3 - ps4
Asgard/Eukaryota - ps5
Eukaryota - ps6
Amorpheae - ps7
Obazoa - ps8
Opisthokonta - ps9
Holozoa - ps10
Filozoa - ps11
Choano./Metazoa - ps12
Metazoa - ps13
Myriazoa - ps14
Parahoxozoa - ps15
Bilateria - ps16
Deuterostomia - ps17
Chordata - ps18
Olfactores - ps19
Vertebrata - ps20
Gnathostomata - ps21
Euteleostomi - ps22
Sarcopterygii - ps23
Tetrapoda - ps24
Amniota - ps25
Mammalia - ps26
Theria - ps27
Placentalia - ps28
Boreoeutheria - ps29
Euarchontoglires - ps30
Euarchonta - ps31
Primates - ps32
Haplorrhini - ps33
Simiiformes - ps34
Catarrhini - ps35
Hominoidea - ps36
Hominidae - ps37
Homininae - ps38
Hominini - ps39
Homo_sapiens - ps40
p-value
7e-06
5e-05
3e-04
2e-03
1e-02
Log-odds
0.5
1.0
1.5
2.0
2.5
3.0
3.5
4.0

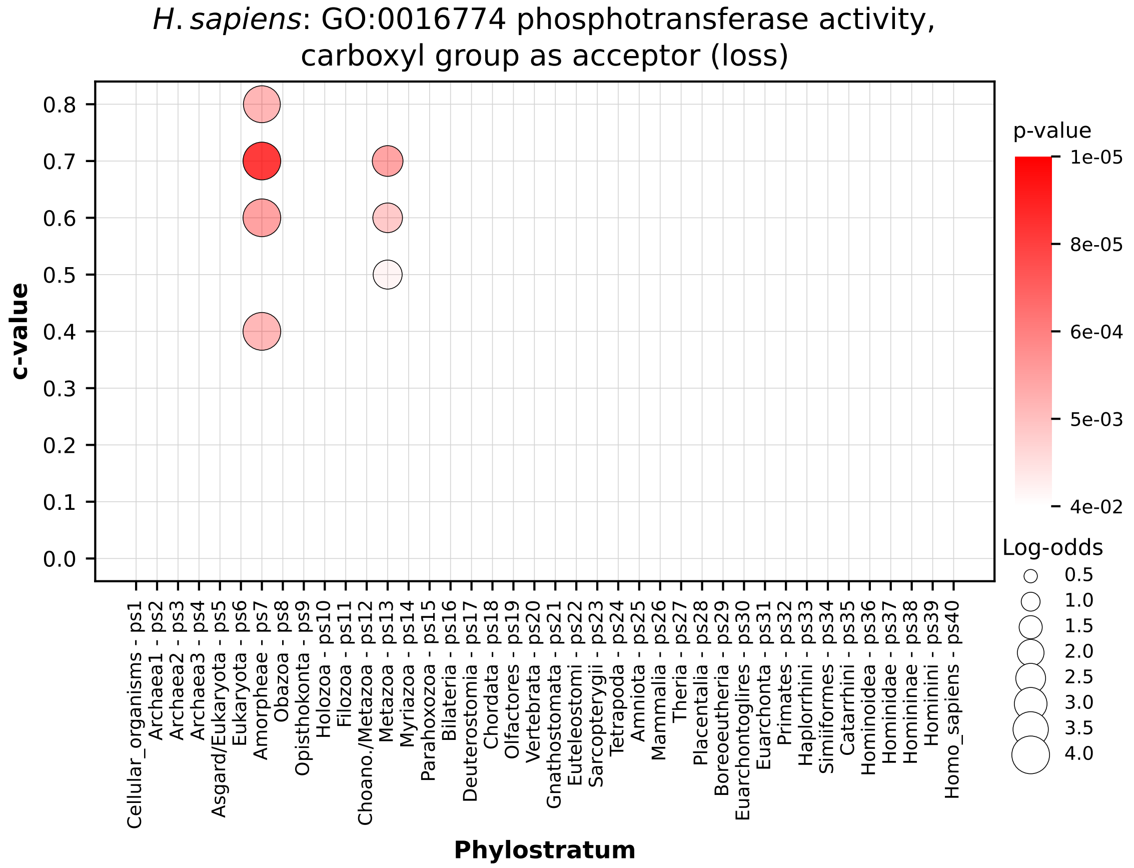

H. sapiens: GO:0016774 phosphotransferase activity, carboxyl group as acceptor (loss)
c-value
0.0
0.1
0.2
0.3
0.4
0.5
0.6
0.7
0.8
Phylostratum
Cellular_organisms - ps1
Archaea1 - ps2
Archaea2 - ps3
Archaea3 - ps4
Asgard/Eukaryota - ps5
Eukaryota - ps6
Amorpheae - ps7
Obazoa - ps8
Opisthokonta - ps9
Holozoa - ps10
Filozoa - ps11
Choano./Metazoa - ps12
Metazoa - ps13
Myriazoa - ps14
Parahoxozoa - ps15
Bilateria - ps16
Deuterostomia - ps17
Chordata - ps18
Olfactores - ps19
Vertebrata - ps20
Gnathostomata - ps21
Euteleostomi - ps22
Sarcopterygii - ps23
Tetrapoda - ps24
Amniota - ps25
Mammalia - ps26
Theria - ps27
Placentalia - ps28
Boreoeutheria - ps29
Euarchontoglires - ps30
Euarchonta - ps31
Primates - ps32
Haplorrhini - ps33
Simiiformes - ps34
Catarrhini - ps35
Hominoidea - ps36
Hominidae - ps37
Homininae - ps38
Hominini - ps39
Homo_sapiens - ps40
p-value
1e-05
8e-05
6e-04
5e-03
4e-02
Log-odds
0.5
1.0
1.5
2.0
2.5
3.0
3.5
4.0

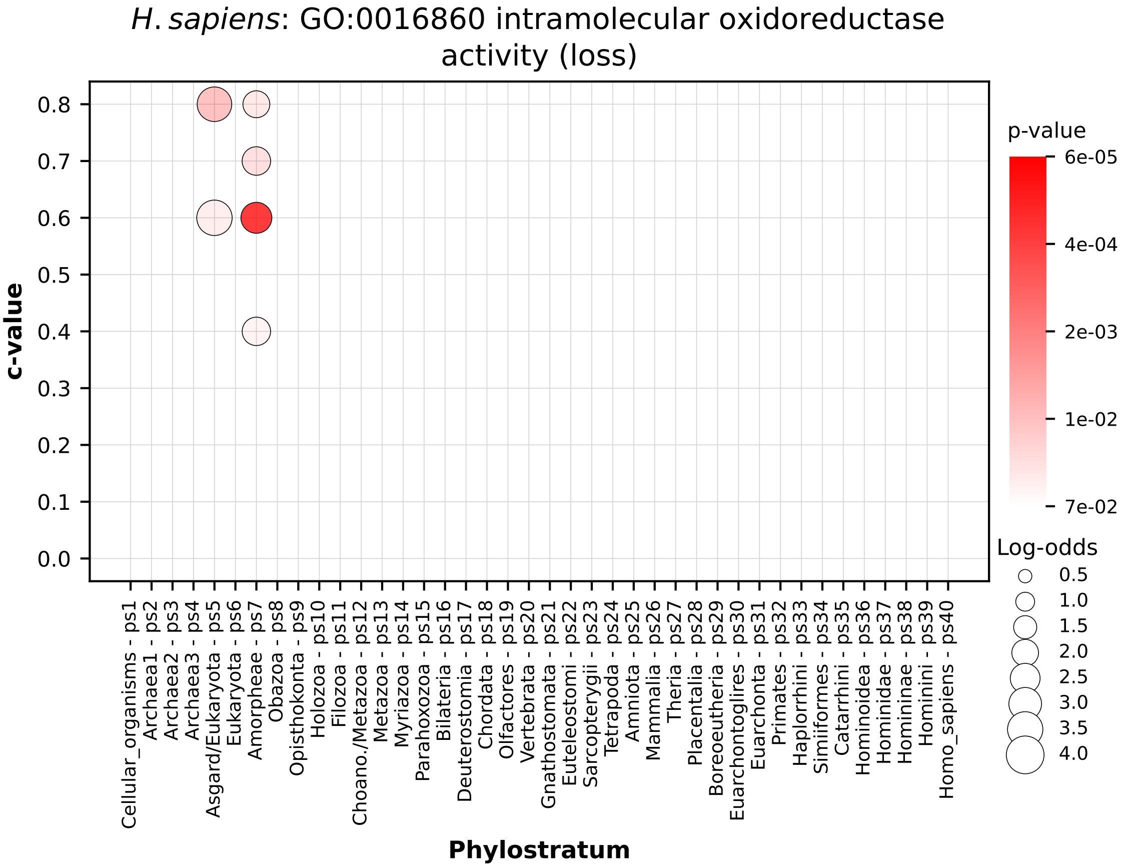

H. sapiens: GO:0016860 intramolecular oxidoreductase activity (loss)
c-value
0.0
0.1
0.2
0.3
0.4
0.5
0.6
0.7
0.8
Phylostratum
Cellular_organisms - ps1
Archaea1 - ps2
Archaea2 - ps3
Archaea3 - ps4
Asgard/Eukaryota - ps5
Eukaryota - ps6
Amorpheae - ps7
Obazoa - ps8
Opisthokonta - ps9
Holozoa - ps10
Filozoa - ps11
Choano./Metazoa - ps12
Metazoa - ps13
Myriazoa - ps14
Parahoxozoa - ps15
Bilateria - ps16
Deuterostomia - ps17
Chordata - ps18
Olfactores - ps19
Vertebrata - ps20
Gnathostomata - ps21
Euteleostomi - ps22
Sarcopterygii - ps23
Tetrapoda - ps24
Amniota - ps25
Mammalia - ps26
Theria - ps27
Placentalia - ps28
Boreoeutheria - ps29
Euarchontoglires - ps30
Euarchonta - ps31
Primates - ps32
Haplorrhini - ps33
Simiiformes - ps34
Catarrhini - ps35
Hominoidea - ps36
Hominidae - ps37
Homininae - ps38
Hominini - ps39
Homo_sapiens - ps40
p-value
6e-05
4e-04
2e-03
1e-02
7e-02
Log-odds
0.5
1.0
1.5
2.0
2.5
3.0
3.5
4.0

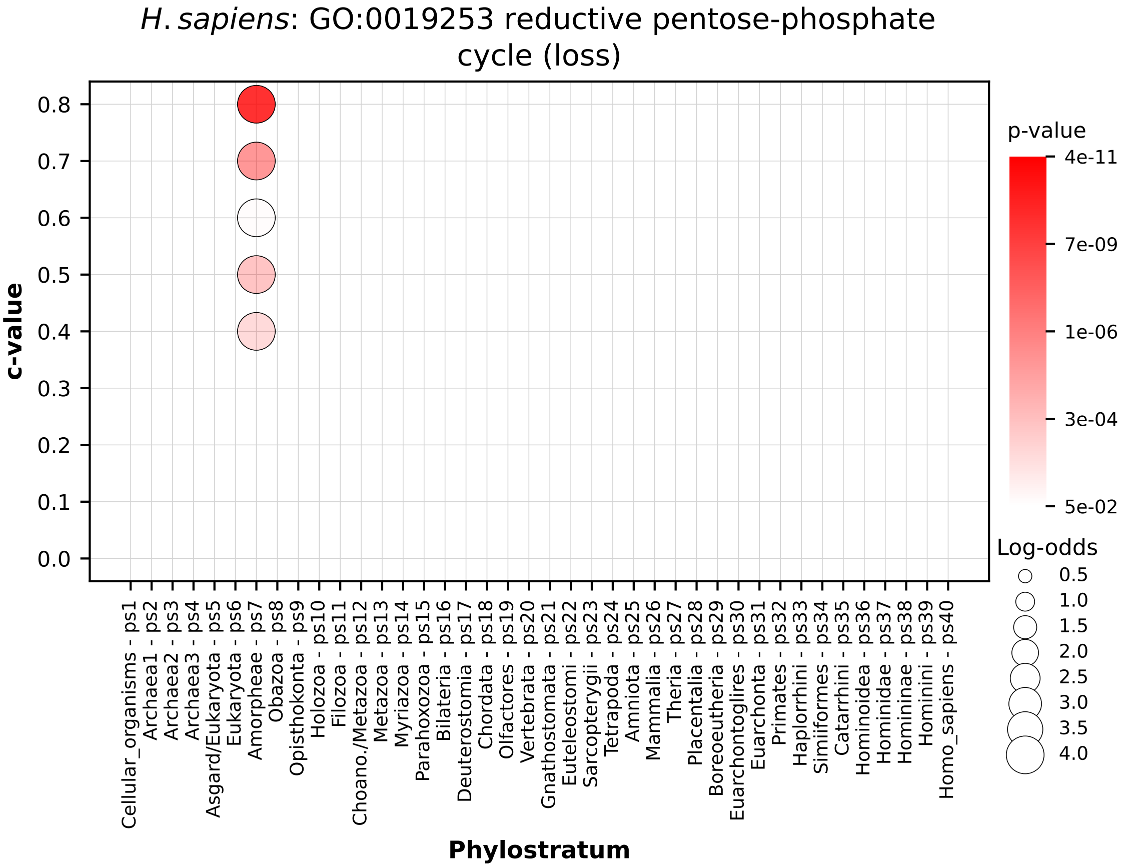
H. sapiens: GO:0019253 reductive pentose-phosphate cycle (loss)
c-value
0.0
0.1
0.2
0.3
0.4
0.5
0.6
0.7
0.8
p-value
4e-11
7e-09
1e-06
3e-04
5e-02
Log-odds
0.5
1.0
1.5
2.0
2.5
3.0
3.5
4.0
Cellular_organisms - ps1
Archaea1 - ps2
Archaea2 - ps3
Archaea3 - ps4
Asgard/Eukaryota - ps5
Eukaryota - ps6
Amorpheae - ps7
Obazoa - ps8
Opisthokonta - ps9
Holozoa - ps10
Filozoa - ps11
Choano./Metazoa - ps12
Metazoa - ps13
Myriazoa - ps14
Parahoxozoa - ps15
Bilateria - ps16
Deuterostomia - ps17
Chordata - ps18
Olfactores - ps19
Vertebrata - ps20
Gnathostomata - ps21
Euteleostomi - ps22
Sarcopterygii - ps23
Tetrapoda - ps24
Amniota - ps25
Mammalia - ps26
Theria - ps27
Placentalia - ps28
Boreoeutheria - ps29
Euarchontoglires - ps30
Euarchonta - ps31
Primates - ps32
Haplorrhini - ps33
Simiiformes - ps34
Catarrhini - ps35
Hominoidea - ps36
Hominidae - ps37
Homininae - ps38
Hominini - ps39
Homo_sapiens - ps40
Phylostratum

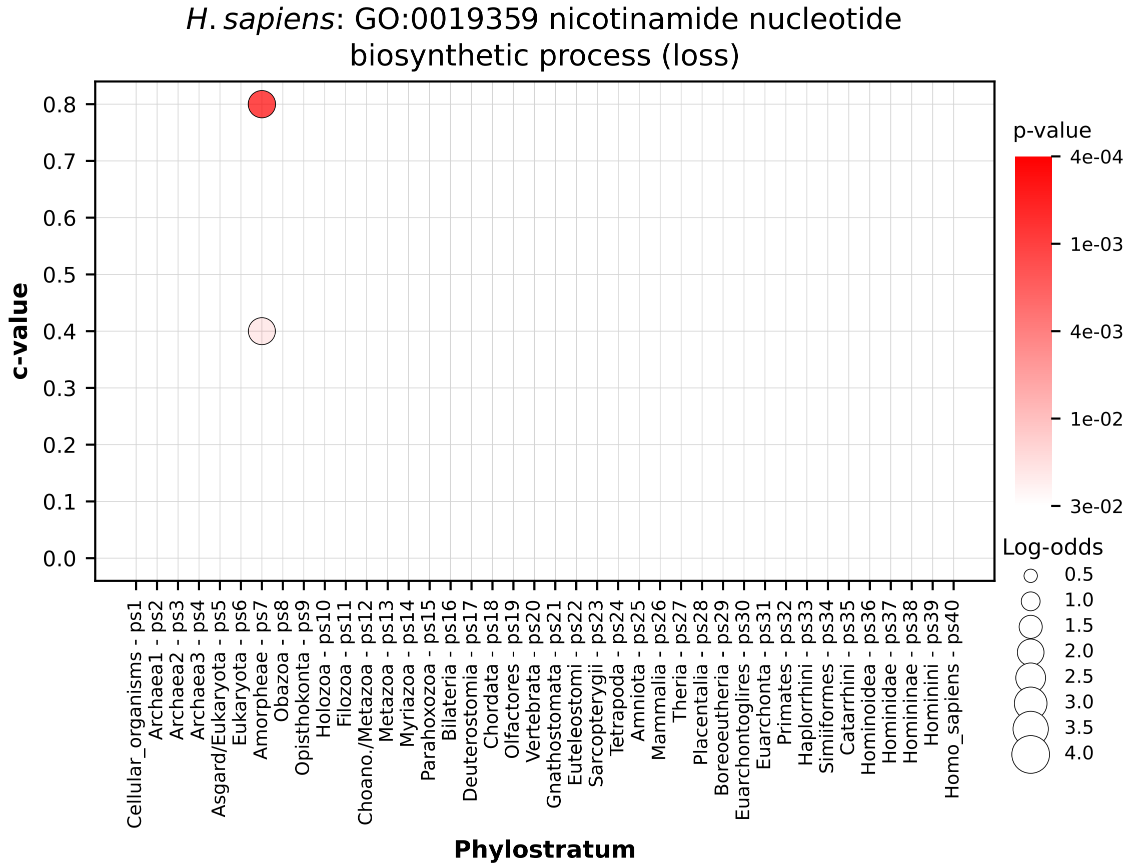

H. sapiens: GO:0019359 nicotinamide nucleotide biosynthetic process (loss)
c-value
Phylostratum
p-value
4e-04
1e-03
4e-03
1e-02
3e-02
Log-odds
0.5
1.0
1.5
2.0
2.5
3.0
3.5
4.0
Cellular_organisms - ps1
Archaea1 - ps2
Archaea2 - ps3
Archaea3 - ps4
Asgard/Eukaryota - ps5
Eukaryota - ps6
Amorpheae - ps7
Obazoa - ps8
Opisthokonta - ps9
Holozoa - ps10
Filozoa - ps11
Choano./Metazoa - ps12
Metazoa - ps13
Myriazoa - ps14
Parahoxozoa - ps15
Bilateria - ps16
Deuterostomia - ps17
Chordata - ps18
Olfactores - ps19
Vertebrata - ps20
Gnathostomata - ps21
Euteleostomi - ps22
Sarcopterygii - ps23
Tetrapoda - ps24
Amniota - ps25
Mammalia - ps26
Theria - ps27
Placentalia - ps28
Boreoeutheria - ps29
Euarchontoglires - ps30
Euarchonta - ps31
Primates - ps32
Haplorrhini - ps33
Simiiformes - ps34
Catarrhini - ps35
Hominoidea - ps36
Hominidae - ps37
Homininae - ps38
Hominini - ps39
Homo_sapiens - ps40

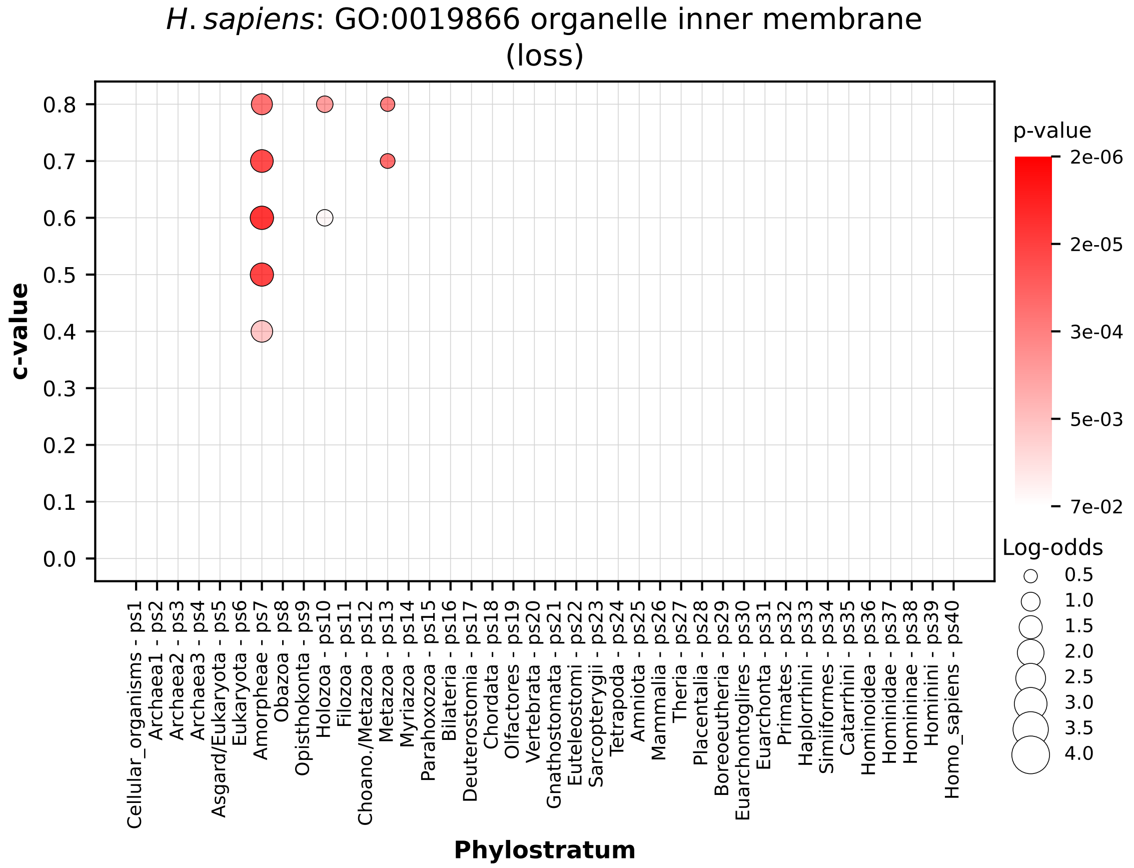
H. sapiens: GO:0019866 organelle inner membrane (loss)
c-value
0.8
0.7
0.6
0.5
0.4
0.3
0.2
0.1
0.0
Phylostratum
Cellular_organisms - ps1
Archaea1 - ps2
Archaea2 - ps3
Archaea3 - ps4
Asgard/Eukaryota - ps5
Eukaryota - ps6
Amorpheae - ps7
Obazoa - ps8
Opisthokonta - ps9
Holozoa - ps10
Filozoa - ps11
Choano./Metazoa - ps12
Metazoa - ps13
Myriazoa - ps14
Parahoxozoa - ps15
Bilateria - ps16
Deuterostomia - ps17
Chordata - ps18
Olfactores - ps19
Vertebrata - ps20
Gnathostomata - ps21
Euteleostomi - ps22
Sarcopterygii - ps23
Tetrapoda - ps24
Amniota - ps25
Mammalia - ps26
Theria - ps27
Placentalia - ps28
Boreoeutheria - ps29
Euarchontoglires - ps30
Euarchonta - ps31
Primates - ps32
Haplorrhini - ps33
Simiiformes - ps34
Catarrhini - ps35
Hominoidea - ps36
Hominidae - ps37
Homininae - ps38
Hominini - ps39
Homo_sapiens - ps40
p-value
2e-06
2e-05
3e-04
5e-03
7e-02
Log-odds
0.5
1.0
1.5
2.0
2.5
3.0
3.5
4.0

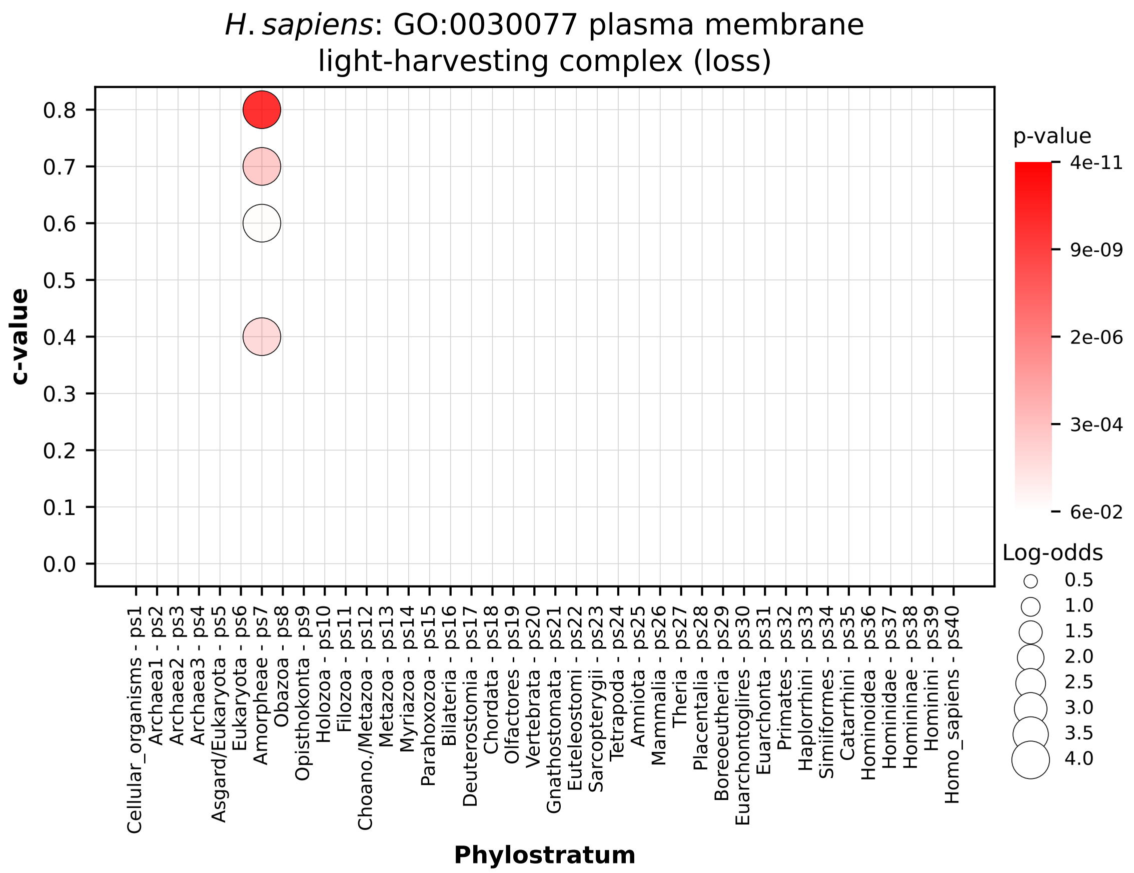

H. sapiens: GO:0030077 plasma membrane light-harvesting complex (loss)
c-value
0.0
0.1
0.2
0.3
0.4
0.5
0.6
0.7
0.8
Phylostratum
Cellular_organisms - ps1
Archaea1 - ps2
Archaea2 - ps3
Archaea3 - ps4
Asgard/Eukaryota - ps5
Eukaryota - ps6
Amorpheae - ps7
Obazoa - ps8
Opisthokonta - ps9
Holozoa - ps10
Filozoa - ps11
Choano./Metazoa - ps12
Metazoa - ps13
Myriazoa - ps14
Parahoxozoa - ps15
Bilateria - ps16
Deuterostomia - ps17
Chordata - ps18
Olfactores - ps19
Vertebrata - ps20
Gnathostomata - ps21
Euteleostomi - ps22
Sarcopterygii - ps23
Tetrapoda - ps24
Amniota - ps25
Mammalia - ps26
Theria - ps27
Placentalia - ps28
Boreoeutheria - ps29
Euarchontoglires - ps30
Euarchonta - ps31
Primates - ps32
Haplorrhini - ps33
Simiiformes - ps34
Catarrhini - ps35
Hominoidea - ps36
Hominidae - ps37
Homininae - ps38
Hominini - ps39
Homo_sapiens - ps40
p-value
4e-11
9e-09
2e-06
3e-04
6e-02
Log-odds
0.5
1.0
1.5
2.0
2.5
3.0
3.5
4.0

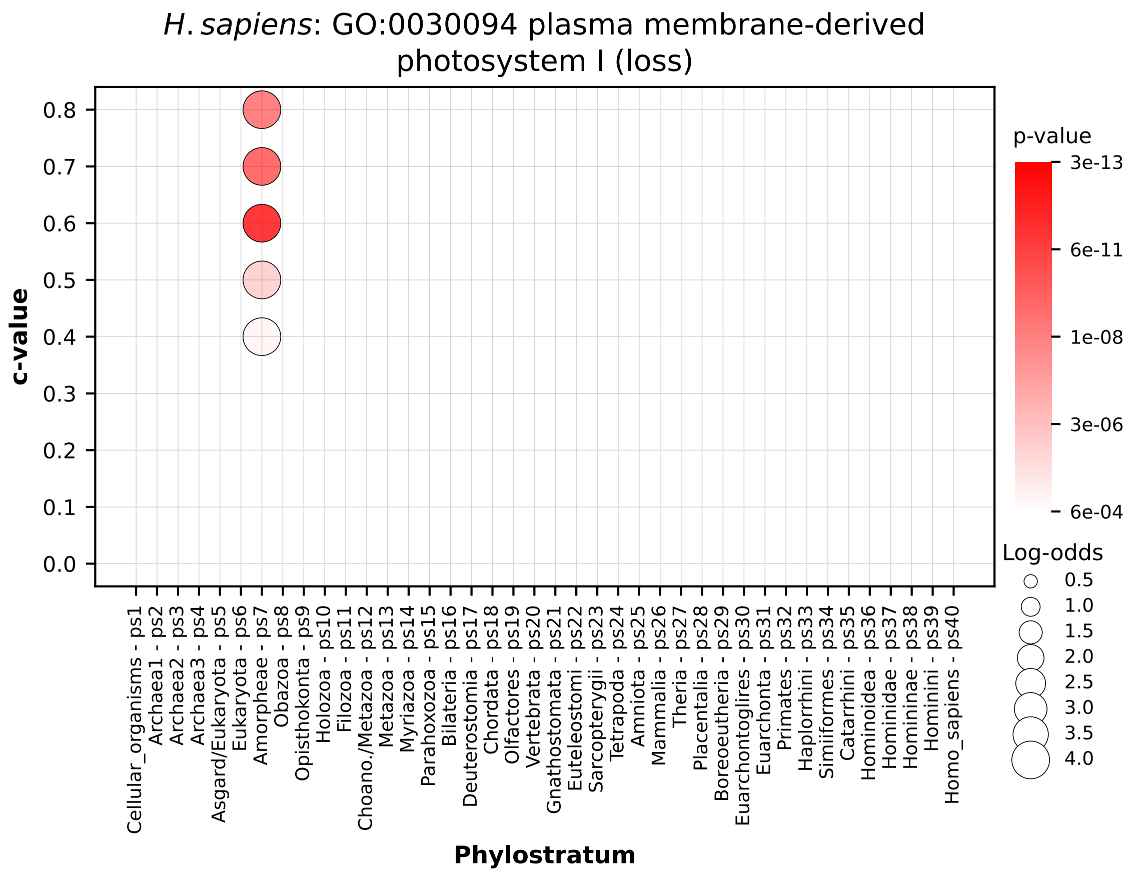

H. sapiens: GO:0030094 plasma membrane-derived photosystem I (loss)
c-value
0.0
0.1
0.2
0.3
0.4
0.5
0.6
0.7
0.8
Phylostratum
Cellular_organisms - ps1
Archaea1 - ps2
Archaea2 - ps3
Archaea3 - ps4
Asgard/Eukaryota - ps5
Eukaryota - ps6
Amorpheae - ps7
Obazoa - ps8
Opisthokonta - ps9
Holozoa - ps10
Filozoa - ps11
Choano./Metazoa - ps12
Metazoa - ps13
Myriazoa - ps14
Parahoxozoa - ps15
Bilateria - ps16
Deuterostomia - ps17
Chordata - ps18
Olfactores - ps19
Vertebrata - ps20
Gnathostomata - ps21
Euteleostomi - ps22
Sarcopterygii - ps23
Tetrapoda - ps24
Amniota - ps25
Mammalia - ps26
Theria - ps27
Placentalia - ps28
Boreoeutheria - ps29
Euarchontoglires - ps30
Euarchonta - ps31
Primates - ps32
Haplorrhini - ps33
Simiiformes - ps34
Catarrhini - ps35
Hominoidea - ps36
Hominidae - ps37
Homininae - ps38
Hominini - ps39
Homo_sapiens - ps40
p-value
3e-13
6e-11
1e-08
3e-06
6e-04
Log-odds
0.5
1.0
1.5
2.0
2.5
3.0
3.5
4.0

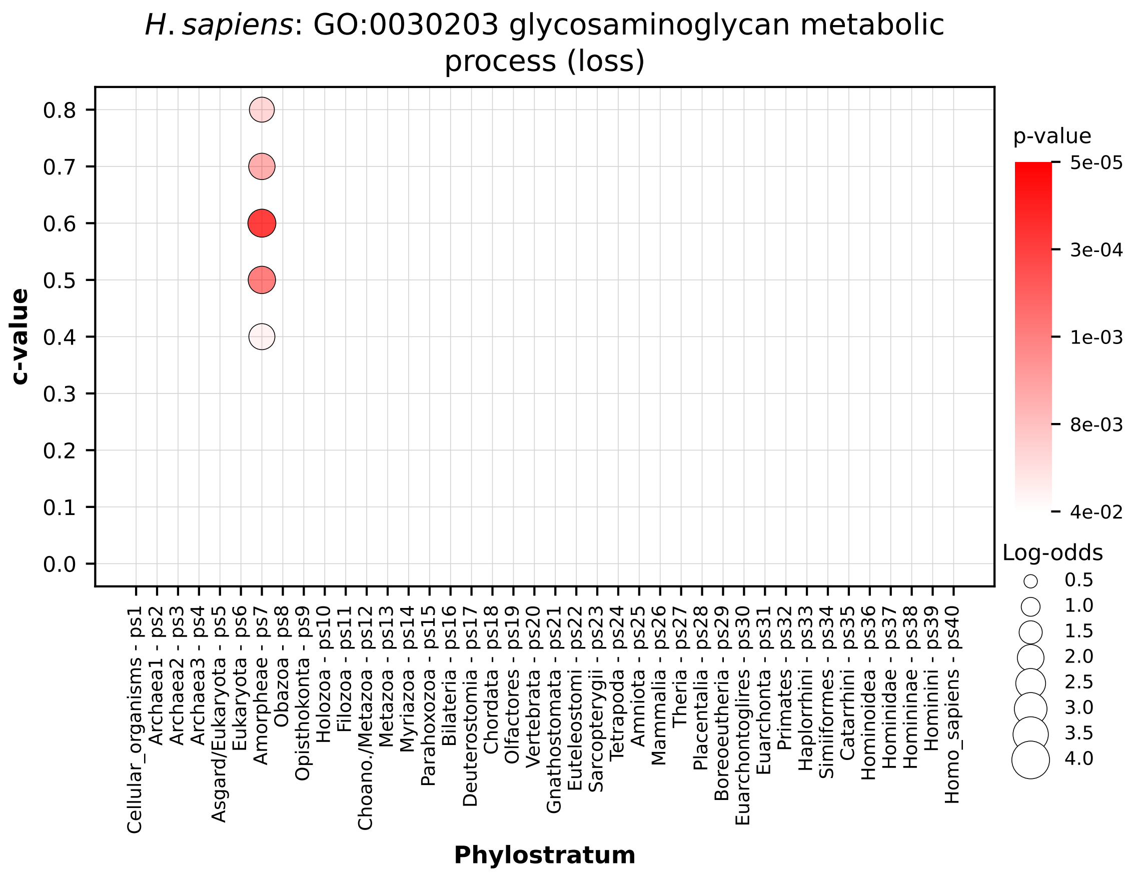

$H. sapiens$: GO:0030203 glycosaminoglycan metabolic process (loss)
c-value
0.0
0.1
0.2
0.3
0.4
0.5
0.6
0.7
0.8
Phylostratum
Cellular_organisms - ps1
Archaea1 - ps2
Archaea2 - ps3
Archaea3 - ps4
Asgard/Eukaryota - ps5
Eukaryota - ps6
Amorpheae - ps7
Obazoa - ps8
Opisthokonta - ps9
Holozoa - ps10
Filozoa - ps11
Choano./Metazoa - ps12
Metazoa - ps13
Myriazoa - ps14
Parahoxozoa - ps15
Bilateria - ps16
Deuterostomia - ps17
Chordata - ps18
Olfactores - ps19
Vertebrata - ps20
Gnathostomata - ps21
Euteleostomi - ps22
Sarcopterygii - ps23
Tetrapoda - ps24
Amniota - ps25
Mammalia - ps26
Theria - ps27
Placentalia - ps28
Boreoeutheria - ps29
Euarchontoglires - ps30
Euarchonta - ps31
Primates - ps32
Haplorrhini - ps33
Simiiformes - ps34
Catarrhini - ps35
Hominoidea - ps36
Hominidae - ps37
Homininae - ps38
Hominini - ps39
Homo_sapiens - ps40
p-value
5e-05
3e-04
1e-03
8e-03
4e-02
Log-odds
0.5
1.0
1.5
2.0
2.5
3.0
3.5
4.0

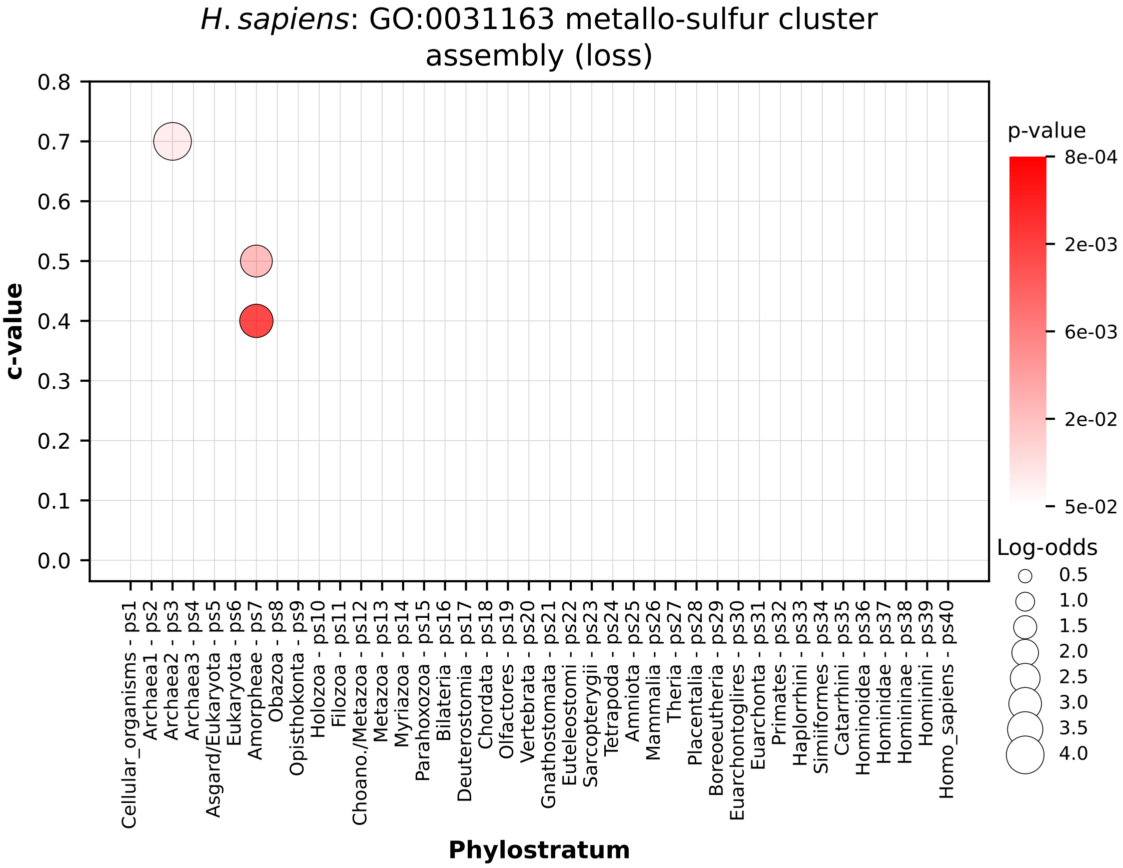

H. sapiens: GO:0031163 metallo-sulfur cluster assembly (loss)
c-value
0.8
0.7
0.6
0.5
0.4
0.3
0.2
0.1
0.0
p-value
8e-04
2e-03
6e-03
2e-02
5e-02
Log-odds
0.5
1.0
1.5
2.0
2.5
3.0
3.5
4.0
Cellular_organisms - ps1
Archaea1 - ps2
Archaea2 - ps3
Archaea3 - ps4
Asgard/Eukaryota - ps5
Eukaryota - ps6
Amorpheae - ps7
Obazoa - ps8
Opisthokonta - ps9
Holozoa - ps10
Filozoa - ps11
Choano./Metazoa - ps12
Metazoa - ps13
Myriazoa - ps14
Parahoxozoa - ps15
Bilateria - ps16
Deuterostomia - ps17
Chordata - ps18
Olfactores - ps19
Vertebrata - ps20
Gnathostomata - ps21
Euteleostomi - ps22
Sarcopterygii - ps23
Tetrapoda - ps24
Amniota - ps25
Mammalia - ps26
Theria - ps27
Placentalia - ps28
Boreoeutheria - ps29
Euarchontoglires - ps30
Euarchonta - ps31
Primates - ps32
Haplorrhini - ps33
Simiiformes - ps34
Catarrhini - ps35
Hominoidea - ps36
Hominidae - ps37
Homininae - ps38
Hominini - ps39
Homo_sapiens - ps40
Phylostratum

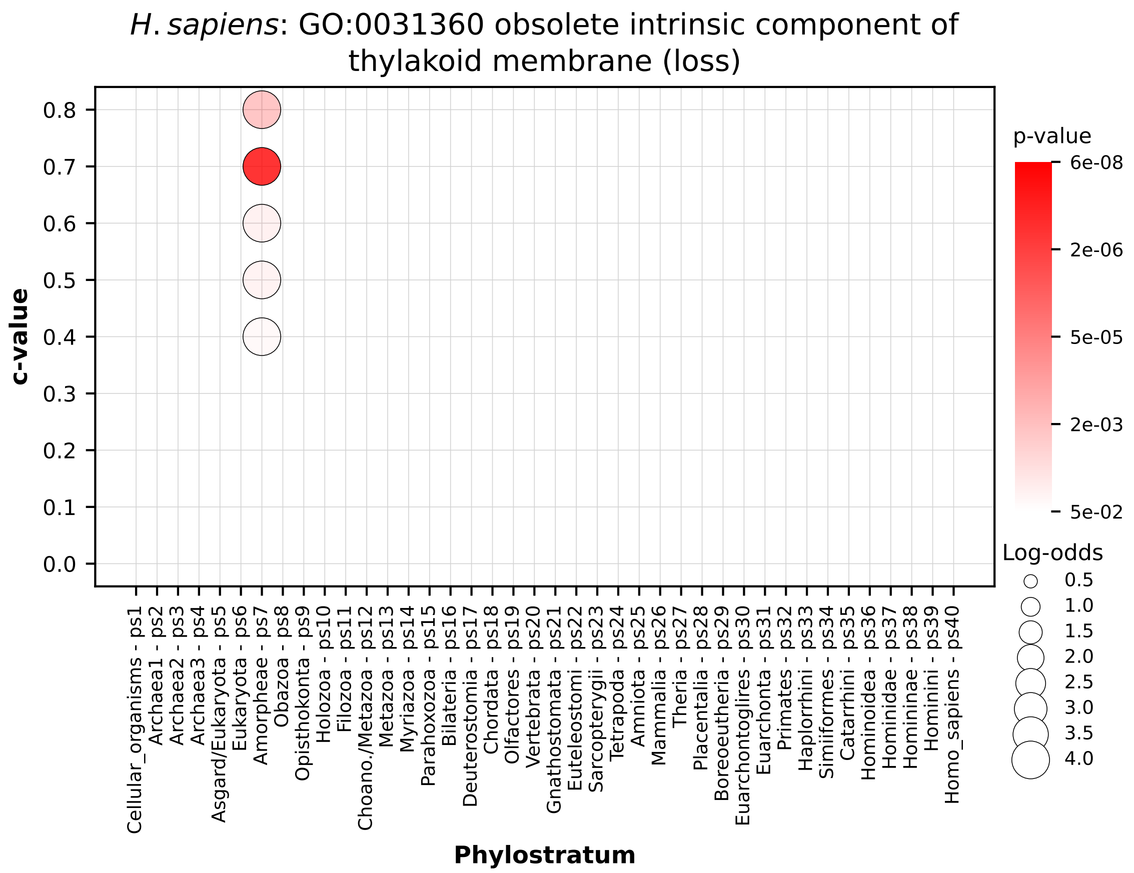
H. sapiens: GO:0031360 obsolete intrinsic component of thylakoid membrane (loss)
c-value
0.0
0.1
0.2
0.3
0.4
0.5
0.6
0.7
0.8
p-value
6e-08
2e-06
5e-05
2e-03
5e-02
Log-odds
0.5
1.0
1.5
2.0
2.5
3.0
3.5
4.0
Cellular_organisms - ps1
Archaea1 - ps2
Archaea2 - ps3
Archaea3 - ps4
Asgard/Eukaryota - ps5
Eukaryota - ps6
Amorpheae - ps7
Obazoa - ps8
Opisthokonta - ps9
Holozoa - ps10
Filozoa - ps11
Choano./Metazoa - ps12
Metazoa - ps13
Myriazoa - ps14
Parahoxozoa - ps15
Bilateria - ps16
Deuterostomia - ps17
Chordata - ps18
Olfactores - ps19
Vertebrata - ps20
Gnathostomata - ps21
Euteleostomi - ps22
Sarcopterygii - ps23
Tetrapoda - ps24
Amniota - ps25
Mammalia - ps26
Theria - ps27
Placentalia - ps28
Boreoeutheria - ps29
Euarchontoglires - ps30
Euarchonta - ps31
Primates - ps32
Haplorrhini - ps33
Simiiformes - ps34
Catarrhini - ps35
Hominoidea - ps36
Hominidae - ps37
Homininae - ps38
Hominini - ps39
Homo_sapiens - ps40
Phylostratum

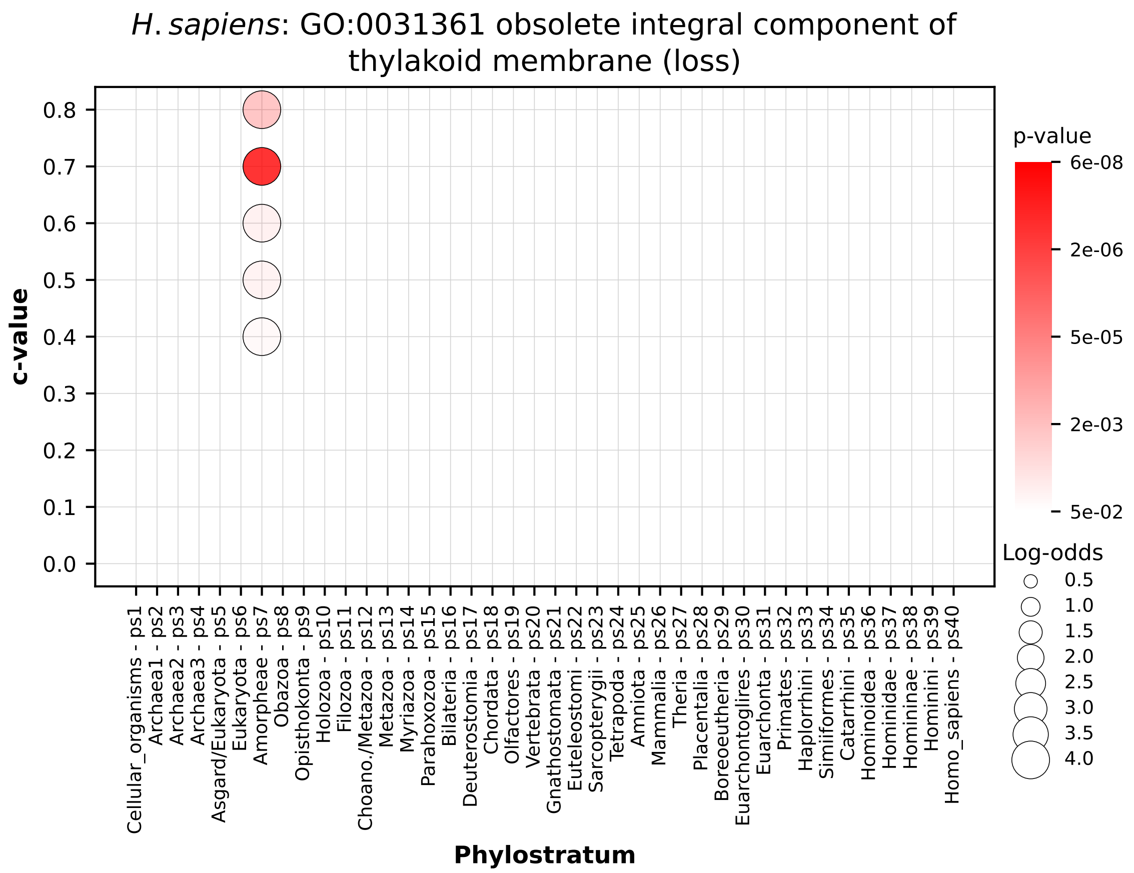

H. sapiens: GO:0031361 obsolete integral component of thylakoid membrane (loss)
c-value
0.0
0.1
0.2
0.3
0.4
0.5
0.6
0.7
0.8
p-value
6e-08
2e-06
5e-05
2e-03
5e-02
Log-odds
0.5
1.0
1.5
2.0
2.5
3.0
3.5
4.0
Cellular_organisms - ps1
Archaea1 - ps2
Archaea2 - ps3
Archaea3 - ps4
Asgard/Eukaryota - ps5
Eukaryota - ps6
Amorpheae - ps7
Obazoa - ps8
Opisthokonta - ps9
Holozoa - ps10
Filozoa - ps11
Choano./Metazoa - ps12
Metazoa - ps13
Myriazoa - ps14
Parahoxozoa - ps15
Bilateria - ps16
Deuterostomia - ps17
Chordata - ps18
Olfactores - ps19
Vertebrata - ps20
Gnathostomata - ps21
Euteleostomi - ps22
Sarcopterygii - ps23
Tetrapoda - ps24
Amniota - ps25
Mammalia - ps26
Theria - ps27
Placentalia - ps28
Boreoeutheria - ps29
Euarchontoglires - ps30
Euarchonta - ps31
Primates - ps32
Haplorrhini - ps33
Simiiformes - ps34
Catarrhini - ps35
Hominoidea - ps36
Hominidae - ps37
Homininae - ps38
Hominini - ps39
Homo_sapiens - ps40
Phylostratum

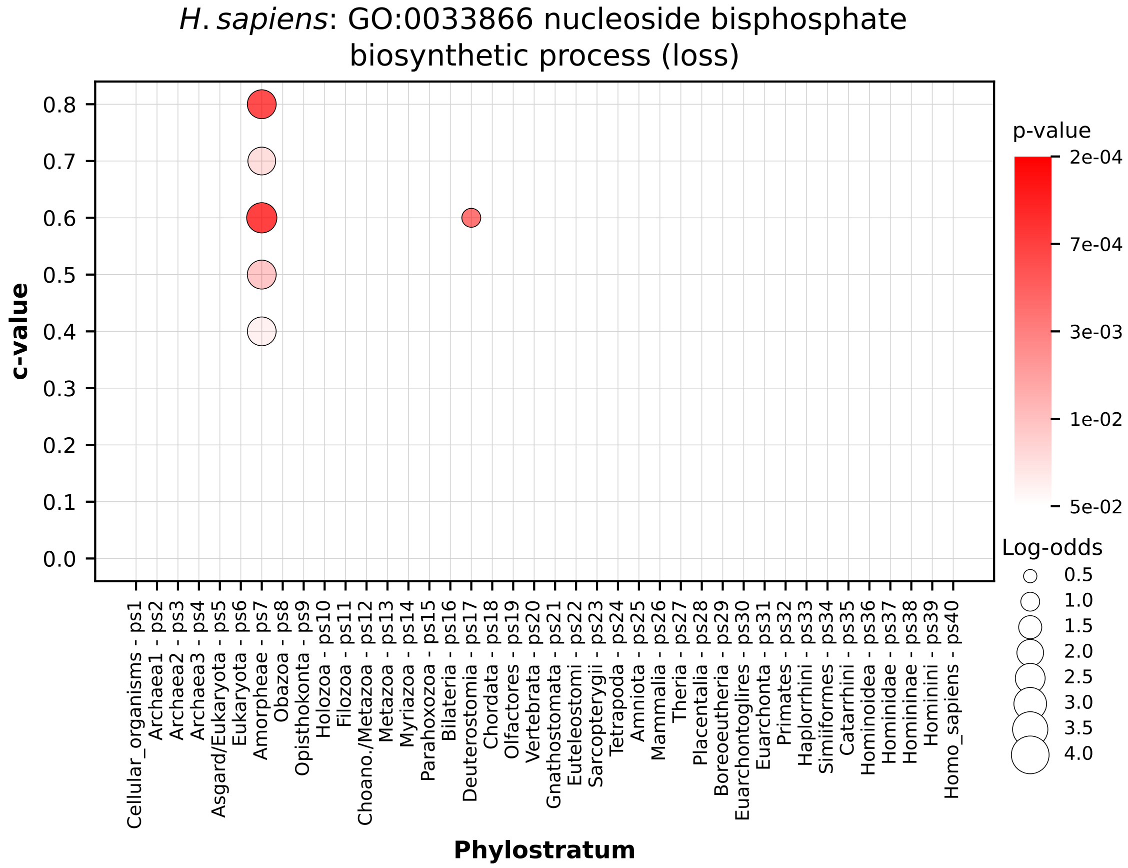

H. sapiens: GO:0033866 nucleoside bisphosphate biosynthetic process (loss)
c-value
0.8
0.7
0.6
0.5
0.4
0.3
0.2
0.1
0.0
p-value
2e-04
7e-04
3e-03
1e-02
5e-02
Log-odds
0.5
1.0
1.5
2.0
2.5
3.0
3.5
4.0
Cellular_organisms - ps1
Archaea1 - ps2
Archaea2 - ps3
Archaea3 - ps4
Asgard/Eukaryota - ps5
Eukaryota - ps6
Amorpheae - ps7
Obazoa - ps8
Opisthokonta - ps9
Holozoa - ps10
Filozoa - ps11
Choano./Metazoa - ps12
Metazoa - ps13
Myriazoa - ps14
Parahoxozoa - ps15
Bilateria - ps16
Deuterostomia - ps17
Chordata - ps18
Olfactores - ps19
Vertebrata - ps20
Gnathostomata - ps21
Euteleostomi - ps22
Sarcopterygii - ps23
Tetrapoda - ps24
Amniota - ps25
Mammalia - ps26
Theria - ps27
Placentalia - ps28
Boreoeutheria - ps29
Euarchontoglires - ps30
Euarchonta - ps31
Primates - ps32
Haplorrhini - ps33
Simiiformes - ps34
Catarrhini - ps35
Hominoidea - ps36
Hominidae - ps37
Homininae - ps38
Hominini - ps39
Homo_sapiens - ps40
Phylostratum

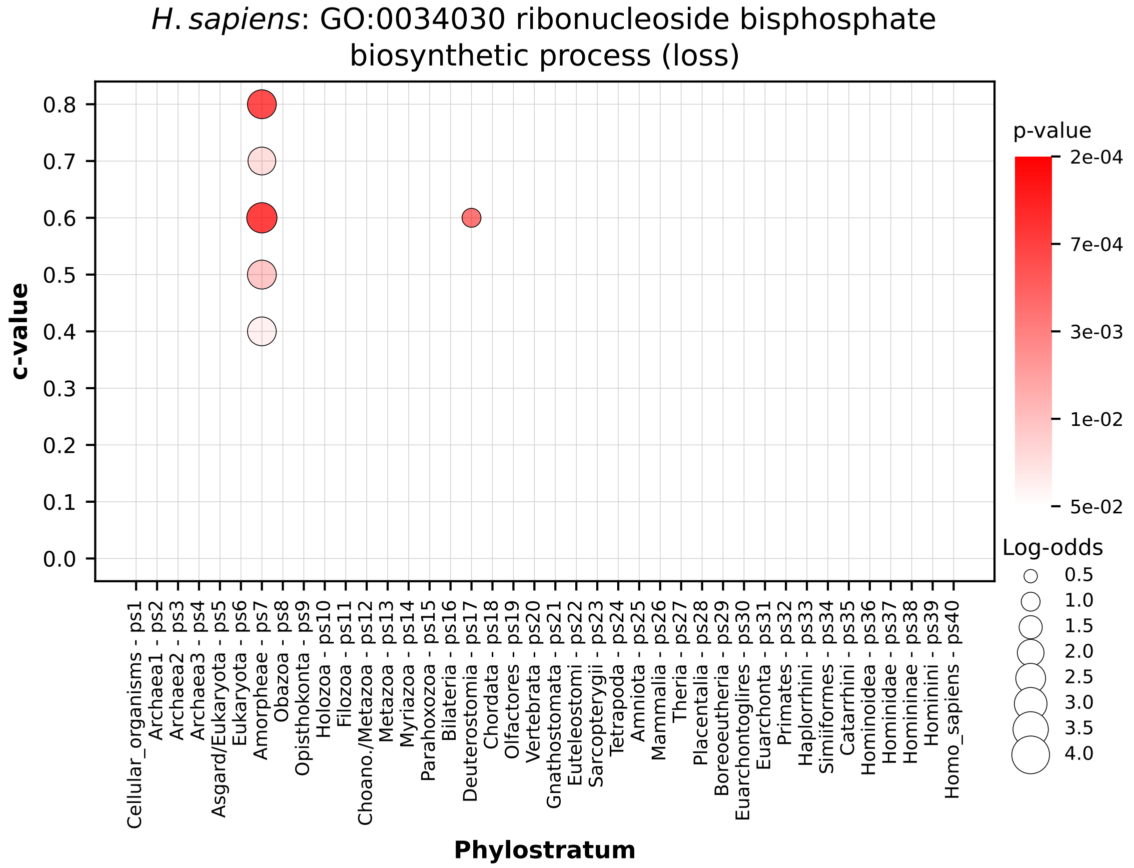

H. sapiens: GO:0034030 ribonucleoside bisphosphate biosynthetic process (loss)
c-value
0.8
0.7
0.6
0.5
0.4
0.3
0.2
0.1
0.0
p-value
2e-04
7e-04
3e-03
1e-02
5e-02
Log-odds
0.5
1.0
1.5
2.0
2.5
3.0
3.5
4.0
Cellular_organisms - ps1
Archaea1 - ps2
Archaea2 - ps3
Archaea3 - ps4
Asgard/Eukaryota - ps5
Eukaryota - ps6
Amorpheae - ps7
Obazoa - ps8
Opisthokonta - ps9
Holozoa - ps10
Filozoa - ps11
Choano./Metazoa - ps12
Metazoa - ps13
Myriazoa - ps14
Parahoxozoa - ps15
Bilateria - ps16
Deuterostomia - ps17
Chordata - ps18
Olfactores - ps19
Vertebrata - ps20
Gnathostomata - ps21
Euteleostomi - ps22
Sarcopterygii - ps23
Tetrapoda - ps24
Amniota - ps25
Mammalia - ps26
Theria - ps27
Placentalia - ps28
Boreoeutheria - ps29
Euarchontoglires - ps30
Euarchonta - ps31
Primates - ps32
Haplorrhini - ps33
Simiiformes - ps34
Catarrhini - ps35
Hominoidea - ps36
Hominidae - ps37
Homininae - ps38
Hominini - ps39
Homo_sapiens - ps40
Phylostratum

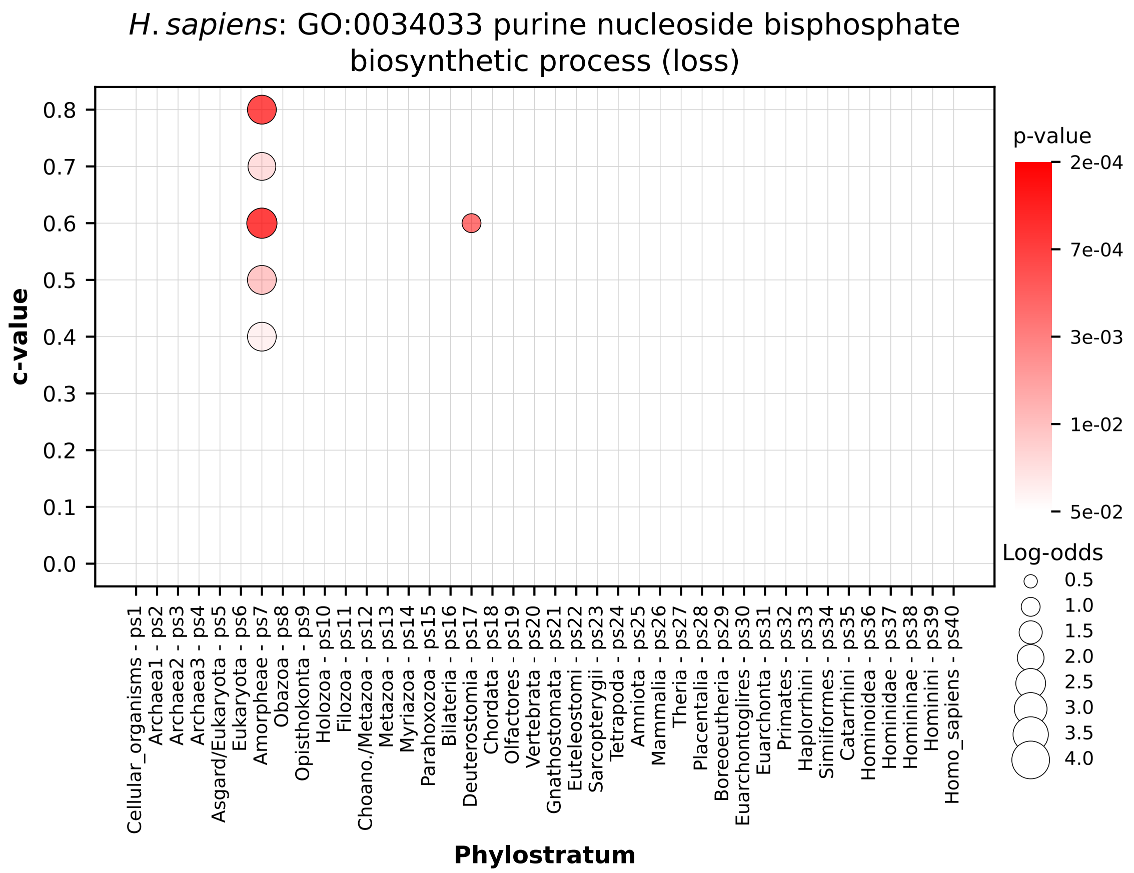

H. sapiens: GO:0034033 purine nucleoside bisphosphate biosynthetic process (loss)
c-value
0.0
0.1
0.2
0.3
0.4
0.5
0.6
0.7
0.8
Phylostratum
Cellular_organisms - ps1
Archaea1 - ps2
Archaea2 - ps3
Archaea3 - ps4
Asgard/Eukaryota - ps5
Eukaryota - ps6
Amorpheae - ps7
Obazoa - ps8
Opisthokonta - ps9
Holozoa - ps10
Filozoa - ps11
Choano./Metazoa - ps12
Metazoa - ps13
Myriazoa - ps14
Parahoxozoa - ps15
Bilateria - ps16
Deuterostomia - ps17
Chordata - ps18
Olfactores - ps19
Vertebrata - ps20
Gnathostomata - ps21
Euteleostomi - ps22
Sarcopterygii - ps23
Tetrapoda - ps24
Amniota - ps25
Mammalia - ps26
Theria - ps27
Placentalia - ps28
Boreoeutheria - ps29
Euarchontoglires - ps30
Euarchonta - ps31
Primates - ps32
Haplorrhini - ps33
Simiiformes - ps34
Catarrhini - ps35
Hominoidea - ps36
Hominidae - ps37
Homininae - ps38
Hominini - ps39
Homo_sapiens - ps40
p-value
2e-04
7e-04
3e-03
1e-02
5e-02
Log-odds
0.5
1.0
1.5
2.0
2.5
3.0
3.5
4.0

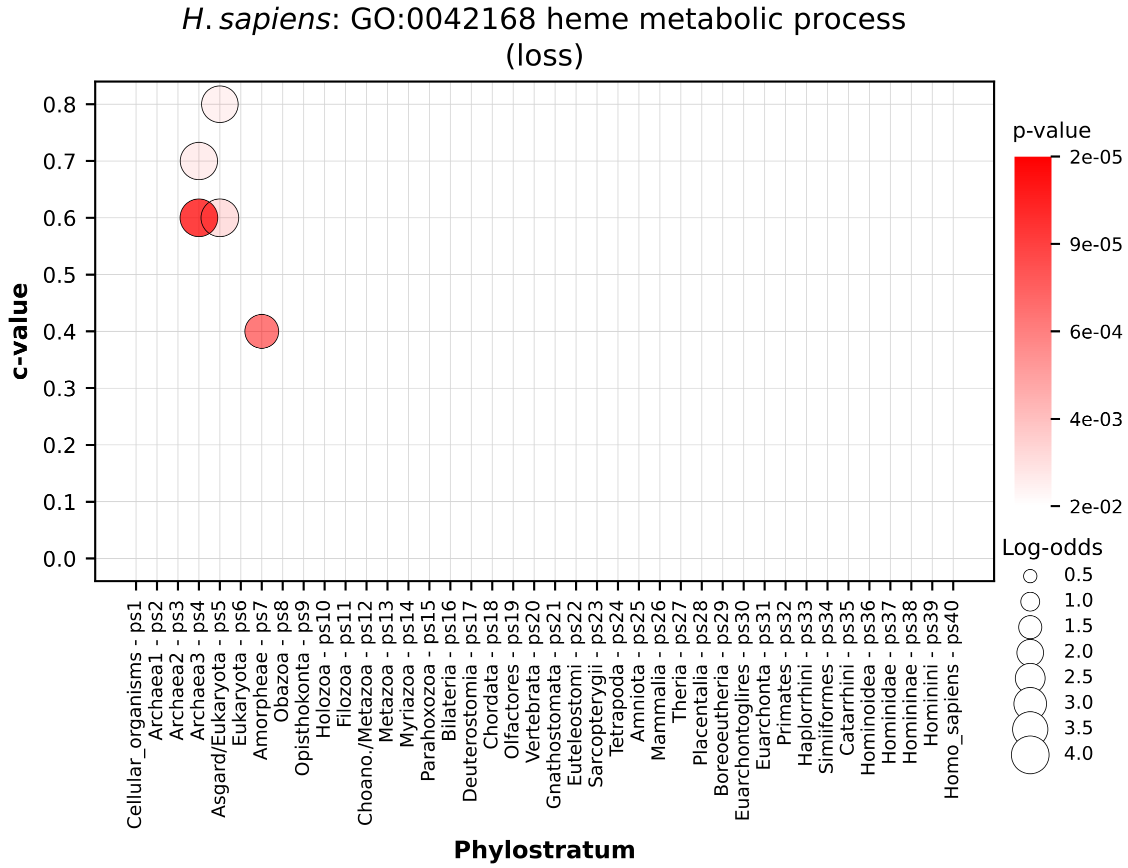

H. sapiens: GO:0042168 heme metabolic process
(loss)
c-value
0.8
0.7
0.6
0.5
0.4
0.3
0.2
0.1
0.0
p-value
2e-05
9e-05
6e-04
4e-03
2e-02
Log-odds
0.5
1.0
1.5
2.0
2.5
3.0
3.5
4.0
Cellular_organisms - ps1
Archaea1 - ps2
Archaea2 - ps3
Archaea3 - ps4
Asgard/Eukaryota - ps5
Eukaryota - ps6
Amorpheae - ps7
Obazoa - ps8
Opisthokonta - ps9
Holozoa - ps10
Filozoa - ps11
Choano./Metazoa - ps12
Metazoa - ps13
Myriazoa - ps14
Parahoxozoa - ps15
Bilateria - ps16
Deuterostomia - ps17
Chordata - ps18
Olfactores - ps19
Vertebrata - ps20
Gnathostomata - ps21
Euteleostomi - ps22
Sarcopterygii - ps23
Tetrapoda - ps24
Amniota - ps25
Mammalia - ps26
Theria - ps27
Placentalia - ps28
Boreoeutheria - ps29
Euarchontoglires - ps30
Euarchonta - ps31
Primates - ps32
Haplorrhini - ps33
Simiiformes - ps34
Catarrhini - ps35
Hominoidea - ps36
Hominidae - ps37
Homininae - ps38
Hominini - ps39
Homo_sapiens - ps40
Phylostratum

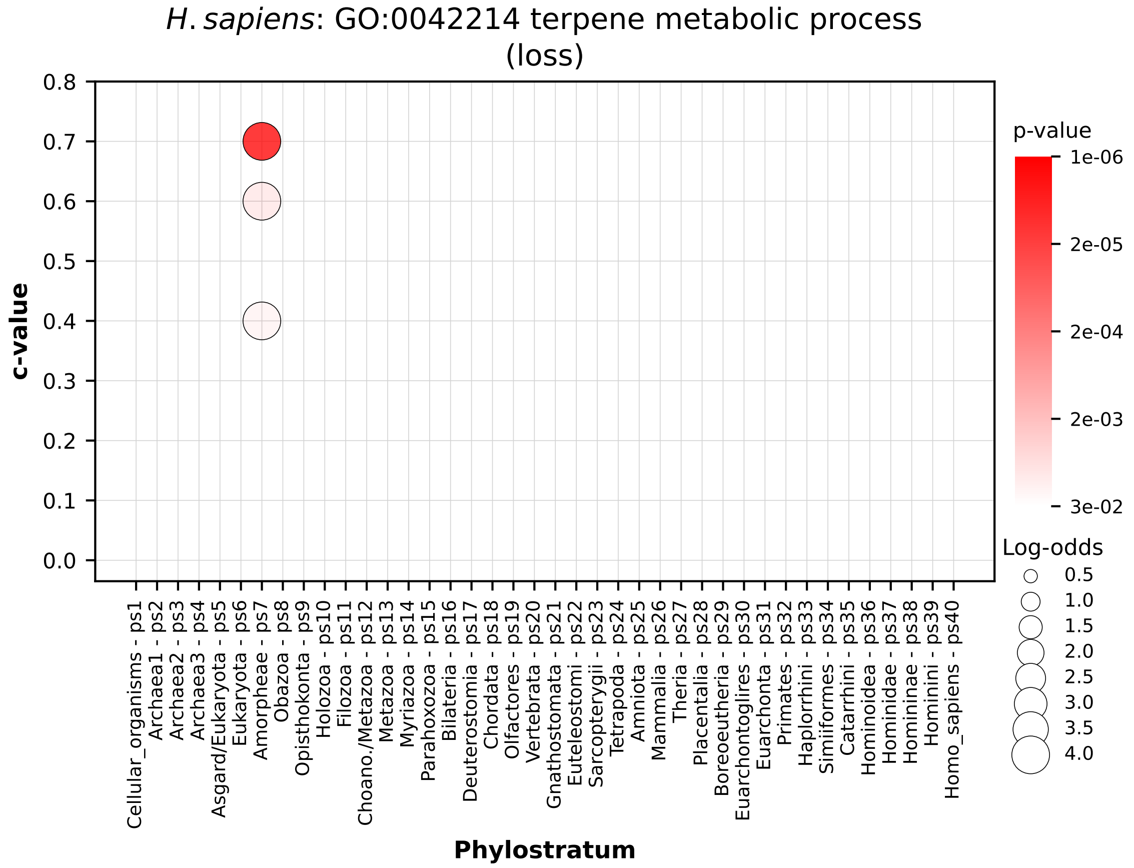

H. sapiens: GO:0042214 terpene metabolic process
(loss)
c-value
0.8
0.7
0.6
0.5
0.4
0.3
0.2
0.1
0.0
p-value
1e-06
2e-05
2e-04
2e-03
3e-02
Log-odds
0.5
1.0
1.5
2.0
2.5
3.0
3.5
4.0
Cellular_organisms - ps1
Archaea1 - ps2
Archaea2 - ps3
Archaea3 - ps4
Asgard/Eukaryota - ps5
Eukaryota - ps6
Amorpheae - ps7
Obazoa - ps8
Opisthokonta - ps9
Holozoa - ps10
Filozoa - ps11
Choano./Metazoa - ps12
Metazoa - ps13
Myriazoa - ps14
Parahoxozoa - ps15
Bilateria - ps16
Deuterostomia - ps17
Chordata - ps18
Olfactores - ps19
Vertebrata - ps20
Gnathostomata - ps21
Euteleostomi - ps22
Sarcopterygii - ps23
Tetrapoda - ps24
Amniota - ps25
Mammalia - ps26
Theria - ps27
Placentalia - ps28
Boreoeutheria - ps29
Euarchontoglires - ps30
Euarchonta - ps31
Primates - ps32
Haplorrhini - ps33
Simiiformes - ps34
Catarrhini - ps35
Hominoidea - ps36
Hominidae - ps37
Homininae - ps38
Hominini - ps39
Homo_sapiens - ps40
Phylostratum

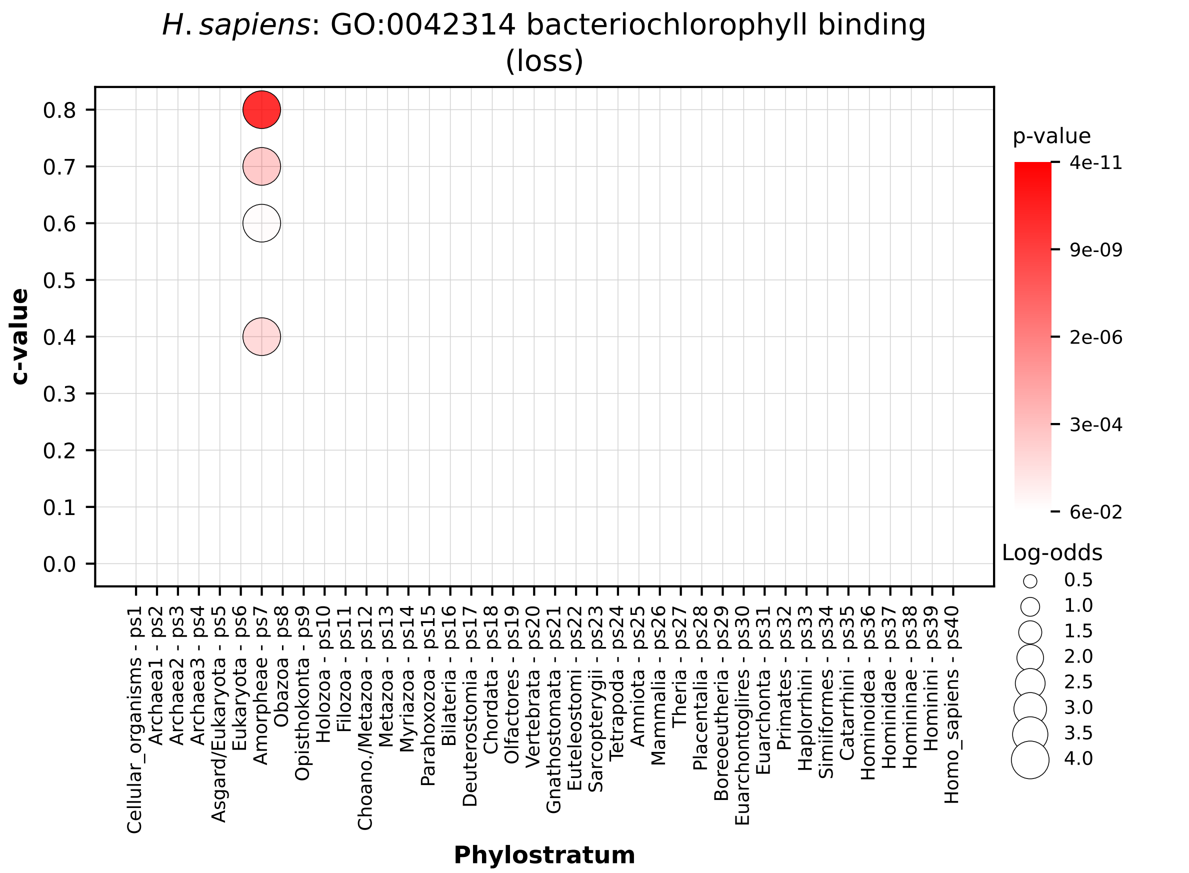

H. sapiens: GO:0042314 bacteriochlorophyll binding
(loss)
c-value
0.8
0.7
0.6
0.5
0.4
0.3
0.2
0.1
0.0
p-value
4e-11
9e-09
2e-06
3e-04
6e-02
Log-odds
0.5
1.0
1.5
2.0
2.5
3.0
3.5
4.0
Cellular_organisms - ps1
Archaea1 - ps2
Archaea2 - ps3
Archaea3 - ps4
Asgard/Eukaryota - ps5
Eukaryota - ps6
Amorpheae - ps7
Obazoa - ps8
Opisthokonta - ps9
Holozoa - ps10
Filozoa - ps11
Choano./Metazoa - ps12
Metazoa - ps13
Myriazoa - ps14
Parahoxozoa - ps15
Bilateria - ps16
Deuterostomia - ps17
Chordata - ps18
Olfactores - ps19
Vertebrata - ps20
Gnathostomata - ps21
Euteleostomi - ps22
Sarcopterygii - ps23
Tetrapoda - ps24
Amniota - ps25
Mammalia - ps26
Theria - ps27
Placentalia - ps28
Boreoeutheria - ps29
Euarchontoglires - ps30
Euarchonta - ps31
Primates - ps32
Haplorrhini - ps33
Simiiformes - ps34
Catarrhini - ps35
Hominoidea - ps36
Hominidae - ps37
Homininae - ps38
Hominini - ps39
Homo_sapiens - ps40
Phylostratum

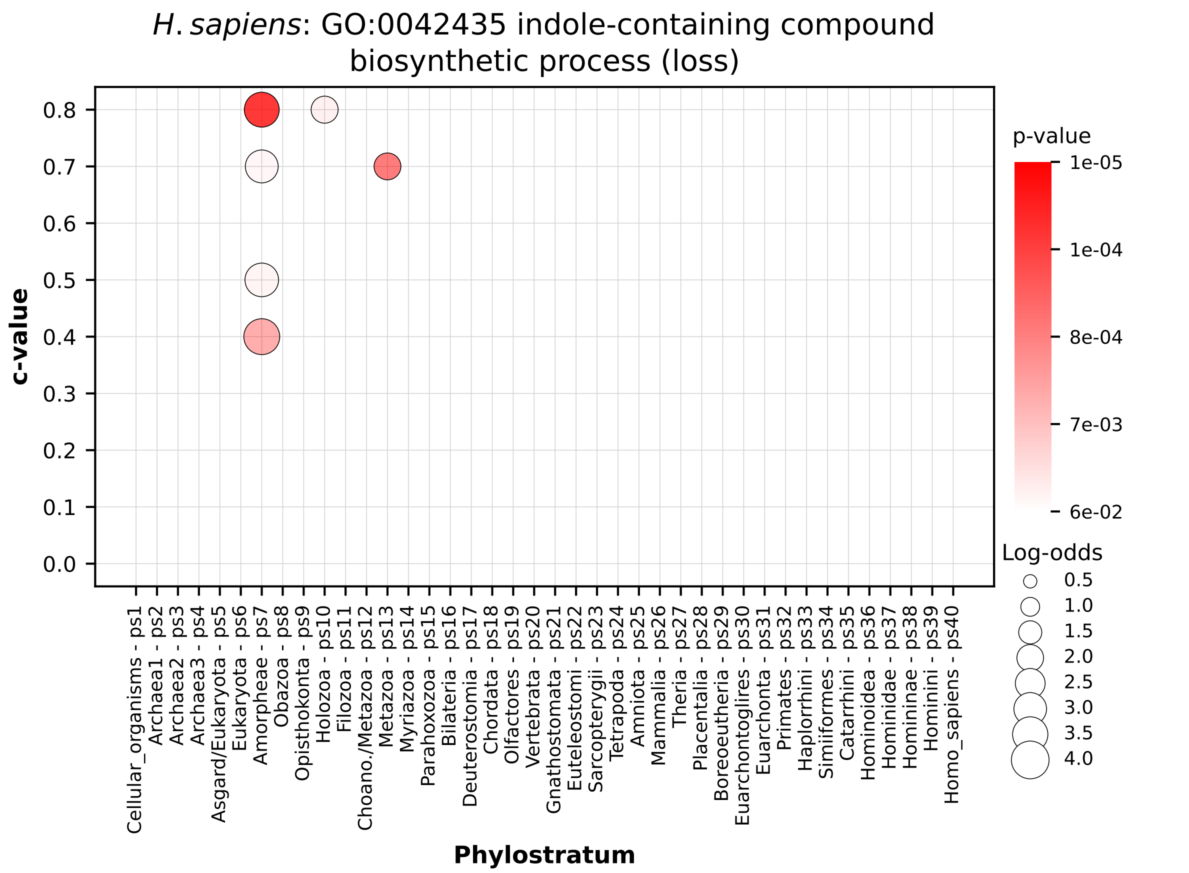

H. sapiens: GO:0042435 indole-containing compound biosynthetic process (loss)
c-value
0.0
0.1
0.2
0.3
0.4
0.5
0.6
0.7
0.8
p-value
1e-05
1e-04
8e-04
7e-03
6e-02
Log-odds
0.5
1.0
1.5
2.0
2.5
3.0
3.5
4.0
Cellular_organisms - ps1
Archaea1 - ps2
Archaea2 - ps3
Archaea3 - ps4
Asgard/Eukaryota - ps5
Eukaryota - ps6
Amorpheae - ps7
Obazoa - ps8
Opisthokonta - ps9
Holozoa - ps10
Filozoa - ps11
Choano./Metazoa - ps12
Metazoa - ps13
Myriazoa - ps14
Parahoxozoa - ps15
Bilateria - ps16
Deuterostomia - ps17
Chordata - ps18
Olfactores - ps19
Vertebrata - ps20
Gnathostomata - ps21
Euteleostomi - ps22
Sarcopterygii - ps23
Tetrapoda - ps24
Amniota - ps25
Mammalia - ps26
Theria - ps27
Placentalia - ps28
Boreoeutheria - ps29
Euarchontoglires - ps30
Euarchonta - ps31
Primates - ps32
Haplorrhini - ps33
Simiiformes - ps34
Catarrhini - ps35
Hominoidea - ps36
Hominidae - ps37
Homininae - ps38
Hominini - ps39
Homo_sapiens - ps40
Phylostratum

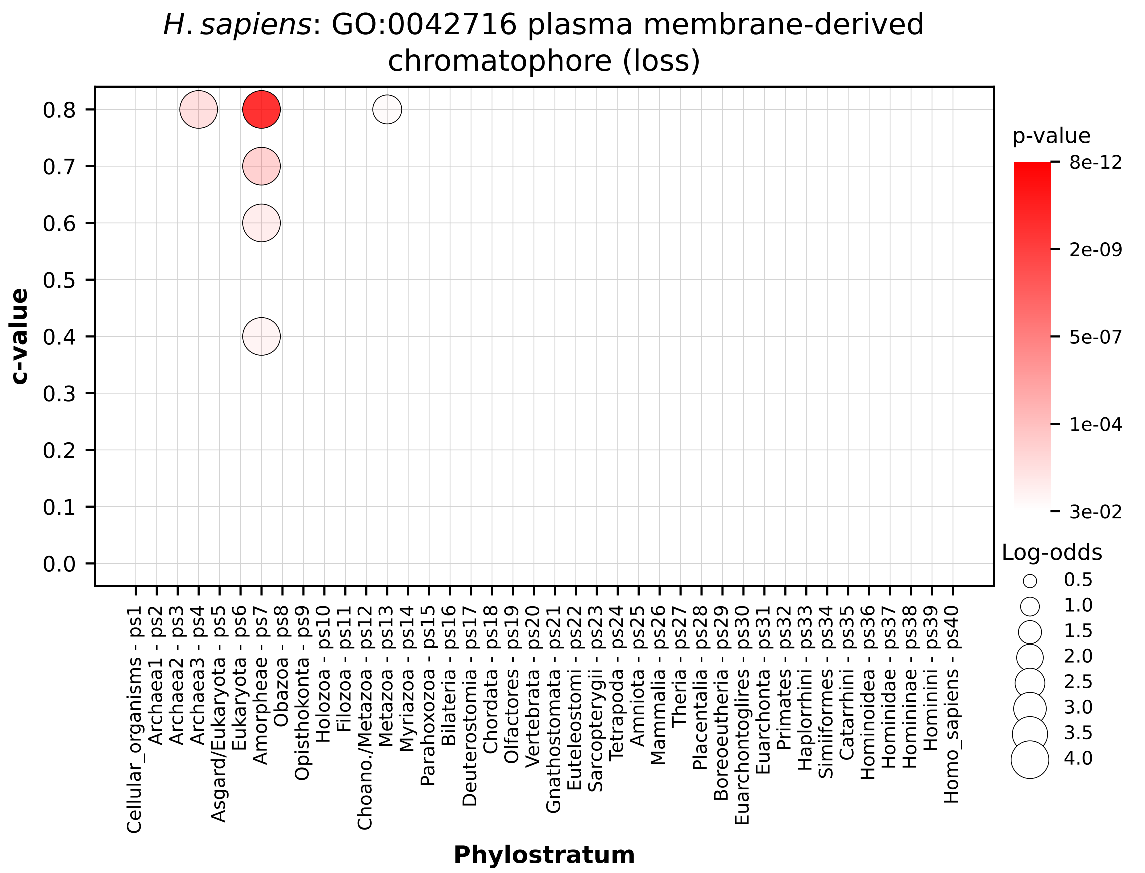
H. sapiens: GO:0042716 plasma membrane-derived chromatophore (loss)
c-value
0.8
0.7
0.6
0.5
0.4
0.3
0.2
0.1
0.0
p-value
8e-12
2e-09
5e-07
1e-04
3e-02
Log-odds
0.5
1.0
1.5
2.0
2.5
3.0
3.5
4.0
Cellular_organisms - ps1
Archaea1 - ps2
Archaea2 - ps3
Archaea3 - ps4
Asgard/Eukaryota - ps5
Eukaryota - ps6
Amorpheae - ps7
Obazoa - ps8
Opisthokonta - ps9
Holozoa - ps10
Filozoa - ps11
Choano./Metazoa - ps12
Metazoa - ps13
Myriazoa - ps14
Parahoxozoa - ps15
Bilateria - ps16
Deuterostomia - ps17
Chordata - ps18
Olfactores - ps19
Vertebrata - ps20
Gnathostomata - ps21
Euteleostomi - ps22
Sarcopterygii - ps23
Tetrapoda - ps24
Amniota - ps25
Mammalia - ps26
Theria - ps27
Placentalia - ps28
Boreoeutheria - ps29
Euarchontoglires - ps30
Euarchonta - ps31
Primates - ps32
Haplorrhini - ps33
Simiiformes - ps34
Catarrhini - ps35
Hominoidea - ps36
Hominidae - ps37
Homininae - ps38
Hominini - ps39
Homo_sapiens - ps40
Phylostratum

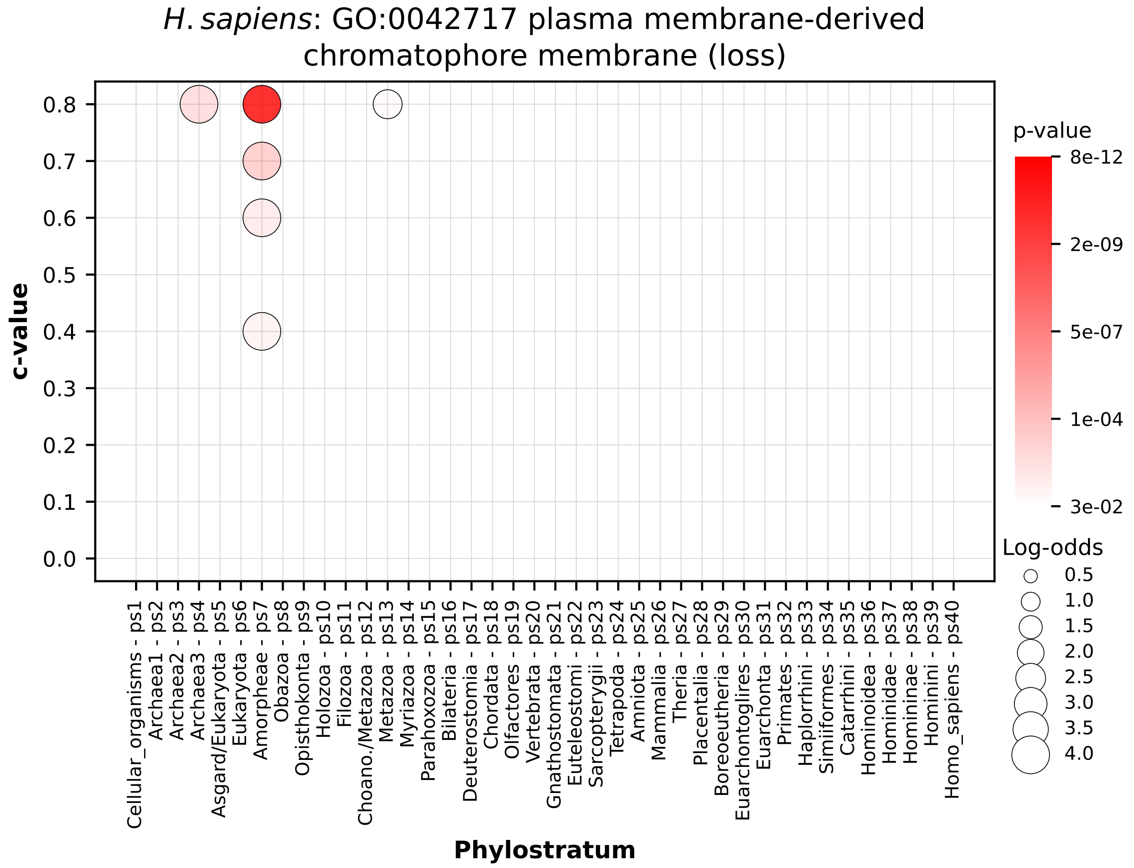
H. sapiens: GO:0042717 plasma membrane-derived chromatophore membrane (loss)
c-value
0.0
0.1
0.2
0.3
0.4
0.5
0.6
0.7
0.8
Phylostratum
Cellular_organisms - ps1
Archaea1 - ps2
Archaea2 - ps3
Archaea3 - ps4
Asgard/Eukaryota - ps5
Eukaryota - ps6
Amorpheae - ps7
Obazoa - ps8
Opisthokonta - ps9
Holozoa - ps10
Filozoa - ps11
Choano./Metazoa - ps12
Metazoa - ps13
Myriazoa - ps14
Parahoxozoa - ps15
Bilateria - ps16
Deuterostomia - ps17
Chordata - ps18
Olfactores - ps19
Vertebrata - ps20
Gnathostomata - ps21
Euteleostomi - ps22
Sarcopterygii - ps23
Tetrapoda - ps24
Amniota - ps25
Mammalia - ps26
Theria - ps27
Placentalia - ps28
Boreoeutheria - ps29
Euarchontoglires - ps30
Euarchonta - ps31
Primates - ps32
Haplorrhini - ps33
Simiiformes - ps34
Catarrhini - ps35
Hominoidea - ps36
Hominidae - ps37
Homininae - ps38
Hominini - ps39
Homo_sapiens - ps40
p-value
8e-12
2e-09
5e-07
1e-04
3e-02
Log-odds
0.5
1.0
1.5
2.0
2.5
3.0
3.5
4.0

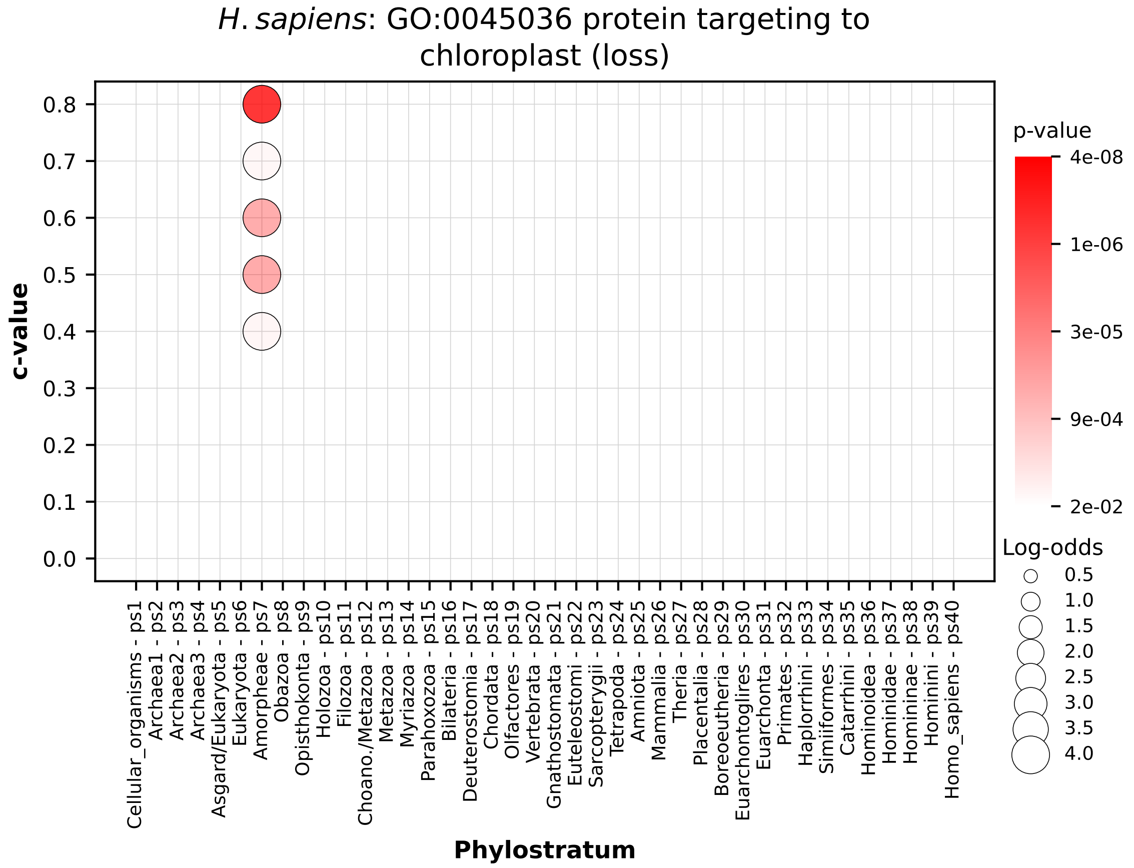
H. sapiens: GO:0045036 protein targeting to chloroplast (loss)
c-value
0.0
0.1
0.2
0.3
0.4
0.5
0.6
0.7
0.8
Phylostratum
Cellular_organisms - ps1
Archaea1 - ps2
Archaea2 - ps3
Archaea3 - ps4
Asgard/Eukaryota - ps5
Eukaryota - ps6
Amorpheae - ps7
Obazoa - ps8
Opisthokonta - ps9
Holozoa - ps10
Filozoa - ps11
Choano./Metazoa - ps12
Metazoa - ps13
Myriazoa - ps14
Parahoxozoa - ps15
Bilateria - ps16
Deuterostomia - ps17
Chordata - ps18
Olfactores - ps19
Vertebrata - ps20
Gnathostomata - ps21
Euteleostomi - ps22
Sarcopterygii - ps23
Tetrapoda - ps24
Amniota - ps25
Mammalia - ps26
Theria - ps27
Placentalia - ps28
Boreoeutheria - ps29
Euarchontoglires - ps30
Euarchonta - ps31
Primates - ps32
Haplorrhini - ps33
Simiiformes - ps34
Catarrhini - ps35
Hominoidea - ps36
Hominidae - ps37
Homininae - ps38
Hominini - ps39
Homo_sapiens - ps40
p-value
4e-08
1e-06
3e-05
9e-04
2e-02
Log-odds
0.5
1.0
1.5
2.0
2.5
3.0
3.5
4.0

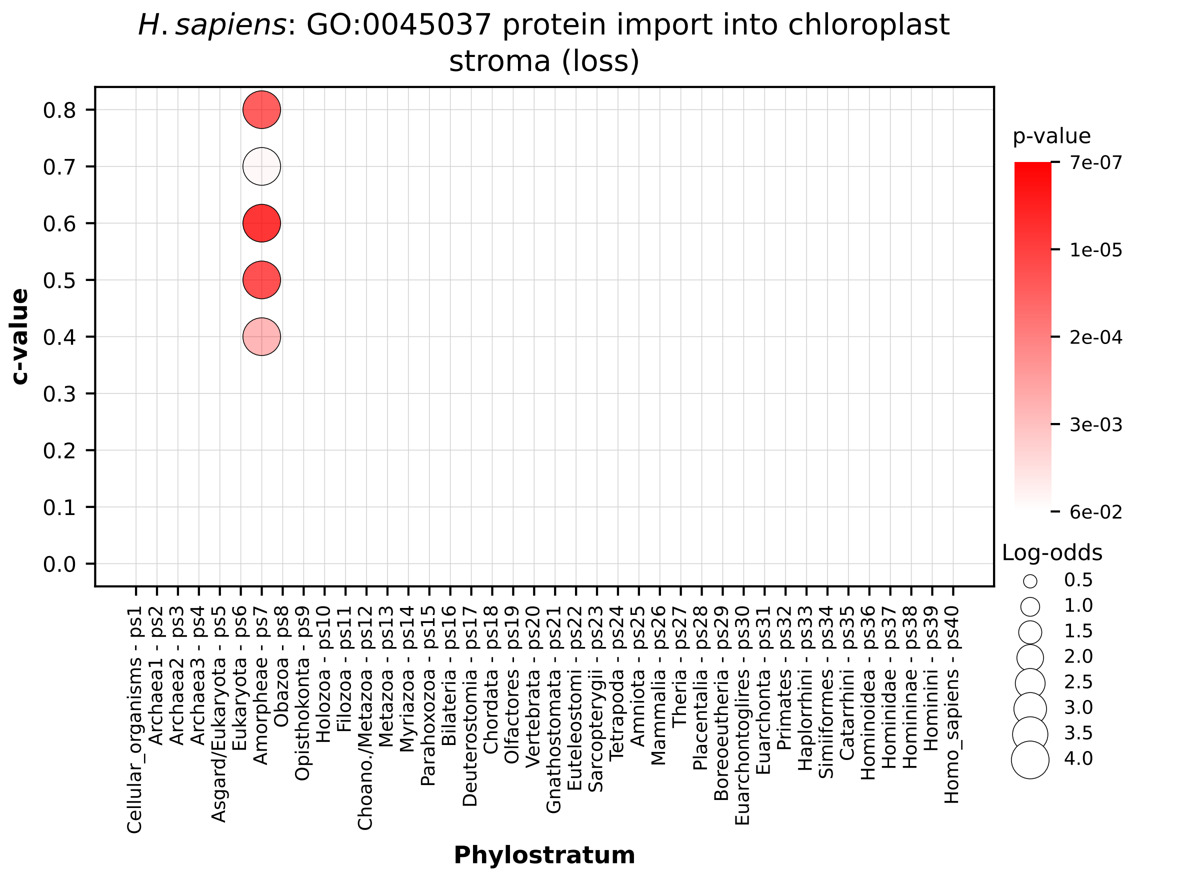
H. sapiens: GO:0045037 protein import into chloroplast stroma (loss)
c-value
0.8
0.7
0.6
0.5
0.4
0.3
0.2
0.1
0.0
p-value
7e-07
1e-05
2e-04
3e-03
6e-02
Log-odds
0.5
1.0
1.5
2.0
2.5
3.0
3.5
4.0
Cellular_organisms - ps1
Archaea1 - ps2
Archaea2 - ps3
Archaea3 - ps4
Asgard/Eukaryota - ps5
Eukaryota - ps6
Amorpheae - ps7
Obazoa - ps8
Opisthokonta - ps9
Holozoa - ps10
Filozoa - ps11
Choano./Metazoa - ps12
Metazoa - ps13
Myriazoa - ps14
Parahoxozoa - ps15
Bilateria - ps16
Deuterostomia - ps17
Chordata - ps18
Olfactores - ps19
Vertebrata - ps20
Gnathostomata - ps21
Euteleostomi - ps22
Sarcopterygii - ps23
Tetrapoda - ps24
Amniota - ps25
Mammalia - ps26
Theria - ps27
Placentalia - ps28
Boreoeutheria - ps29
Euarchontoglires - ps30
Euarchonta - ps31
Primates - ps32
Haplorrhini - ps33
Simiiformes - ps34
Catarrhini - ps35
Hominoidea - ps36
Hominidae - ps37
Homininae - ps38
Hominini - ps39
Homo_sapiens - ps40
Phylostratum

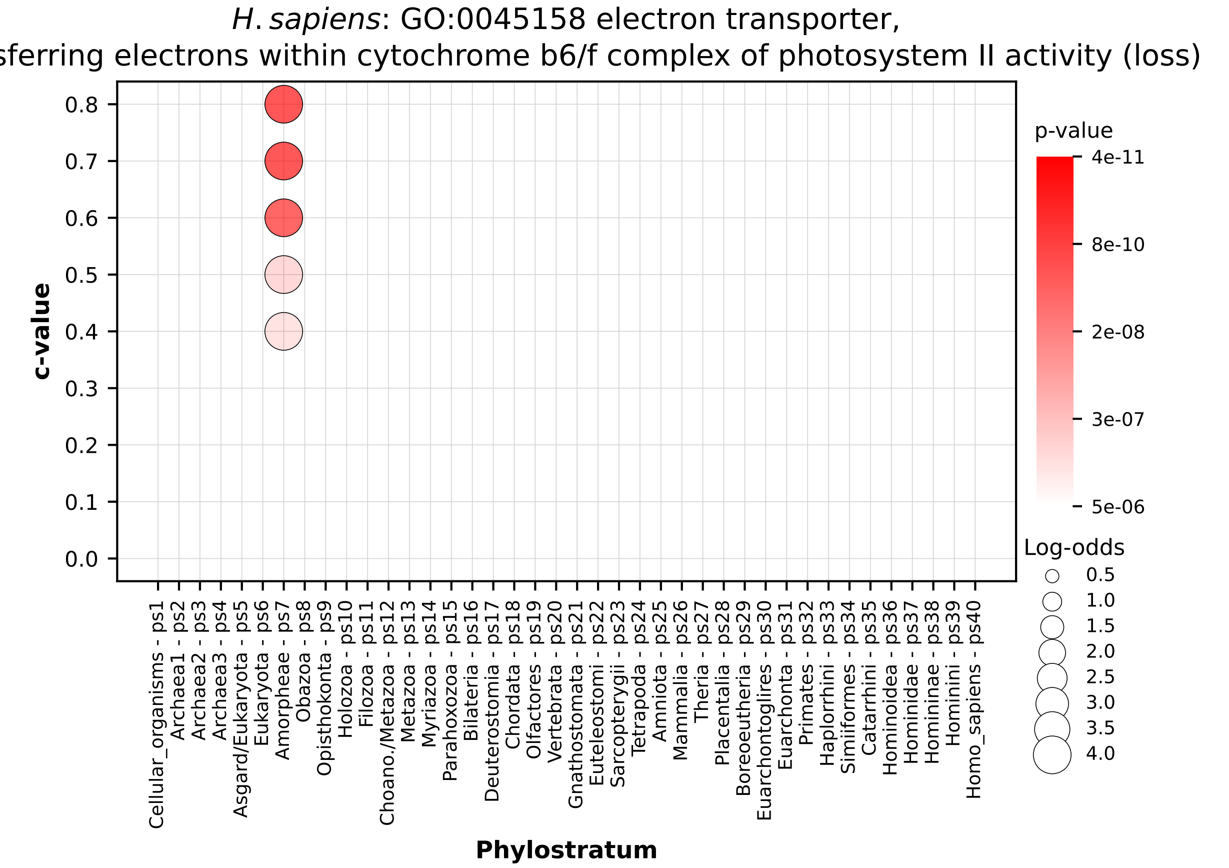

H. sapiens: GO:0045158 electron transporter,
sferring electrons within cytochrome b6/f complex of photosystem II activity (loss)
c-value
0.8
0.7
0.6
0.5
0.4
0.3
0.2
0.1
0.0
p-value
4e-11
8e-10
2e-08
3e-07
5e-06
Log-odds
0.5
1.0
1.5
2.0
2.5
3.0
3.5
4.0
Cellular_organisms - ps1
Archaea1 - ps2
Archaea2 - ps3
Archaea3 - ps4
Asgard/Eukaryota - ps5
Eukaryota - ps6
Amorpheae - ps7
Obazoa - ps8
Opisthokonta - ps9
Holozoa - ps10
Filozoa - ps11
Choano./Metazoa - ps12
Metazoa - ps13
Myriazoa - ps14
Parahoxozoa - ps15
Bilateria - ps16
Deuterostomia - ps17
Chordata - ps18
Olfactores - ps19
Vertebrata - ps20
Gnathostomata - ps21
Euteleostomi - ps22
Sarcopterygii - ps23
Tetrapoda - ps24
Amniota - ps25
Mammalia - ps26
Theria - ps27
Placentalia - ps28
Boreoeutheria - ps29
Euarchontoglires - ps30
Euarchonta - ps31
Primates - ps32
Haplorrhini - ps33
Simiiformes - ps34
Catarrhini - ps35
Hominoidea - ps36
Hominidae - ps37
Homininae - ps38
Hominini - ps39
Homo_sapiens - ps40
Phylostratum

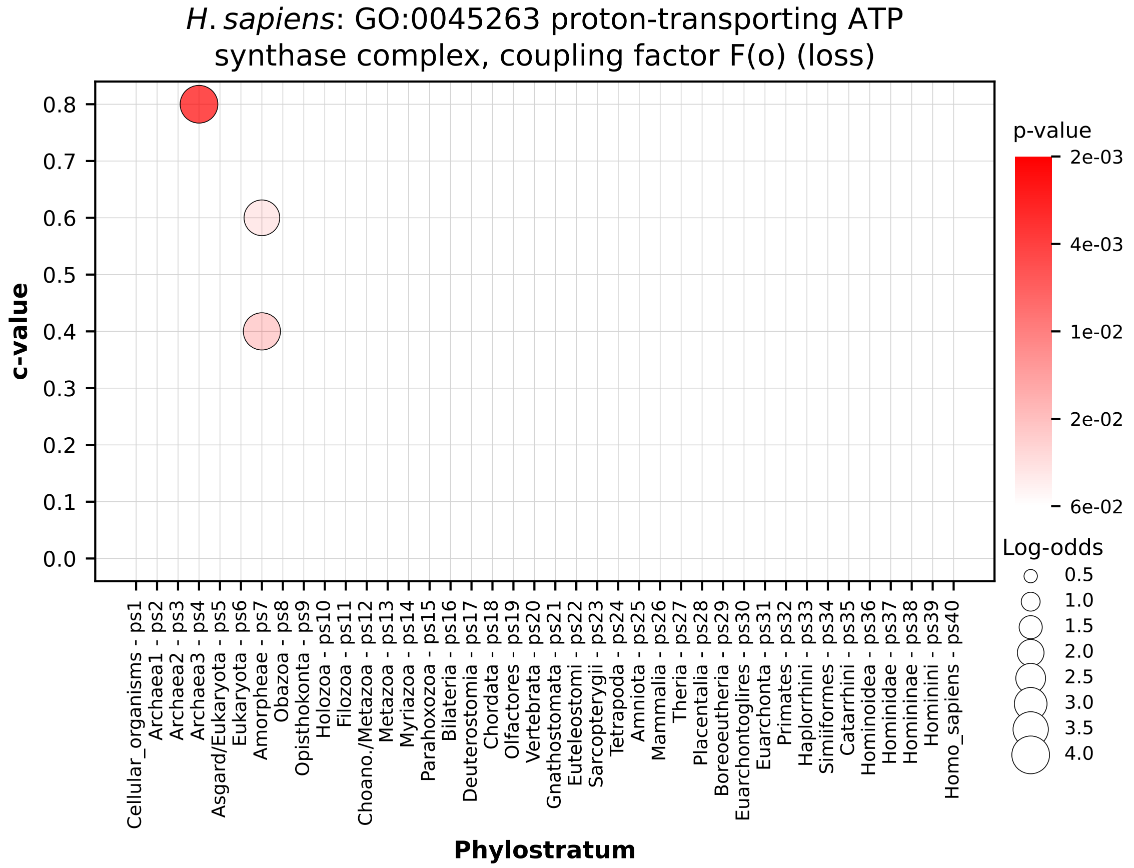

H. sapiens: GO:0045263 proton-transporting ATP synthase complex, coupling factor F(o) (loss)
c-value
0.8
0.7
0.6
0.5
0.4
0.3
0.2
0.1
0.0
p-value
2e-03
4e-03
1e-02
2e-02
6e-02
Log-odds
0.5
1.0
1.5
2.0
2.5
3.0
3.5
4.0
Cellular_organisms - ps1
Archaea1 - ps2
Archaea2 - ps3
Archaea3 - ps4
Asgard/Eukaryota - ps5
Eukaryota - ps6
Amorpheae - ps7
Obazoa - ps8
Opisthokonta - ps9
Holozoa - ps10
Filozoa - ps11
Choano./Metazoa - ps12
Metazoa - ps13
Myriazoa - ps14
Parahoxozoa - ps15
Bilateria - ps16
Deuterostomia - ps17
Chordata - ps18
Olfactores - ps19
Vertebrata - ps20
Gnathostomata - ps21
Euteleostomi - ps22
Sarcopterygii - ps23
Tetrapoda - ps24
Amniota - ps25
Mammalia - ps26
Theria - ps27
Placentalia - ps28
Boreoeutheria - ps29
Euarchontoglires - ps30
Euarchonta - ps31
Primates - ps32
Haplorrhini - ps33
Simiiformes - ps34
Catarrhini - ps35
Hominoidea - ps36
Hominidae - ps37
Homininae - ps38
Hominini - ps39
Homo_sapiens - ps40
Phylostratum

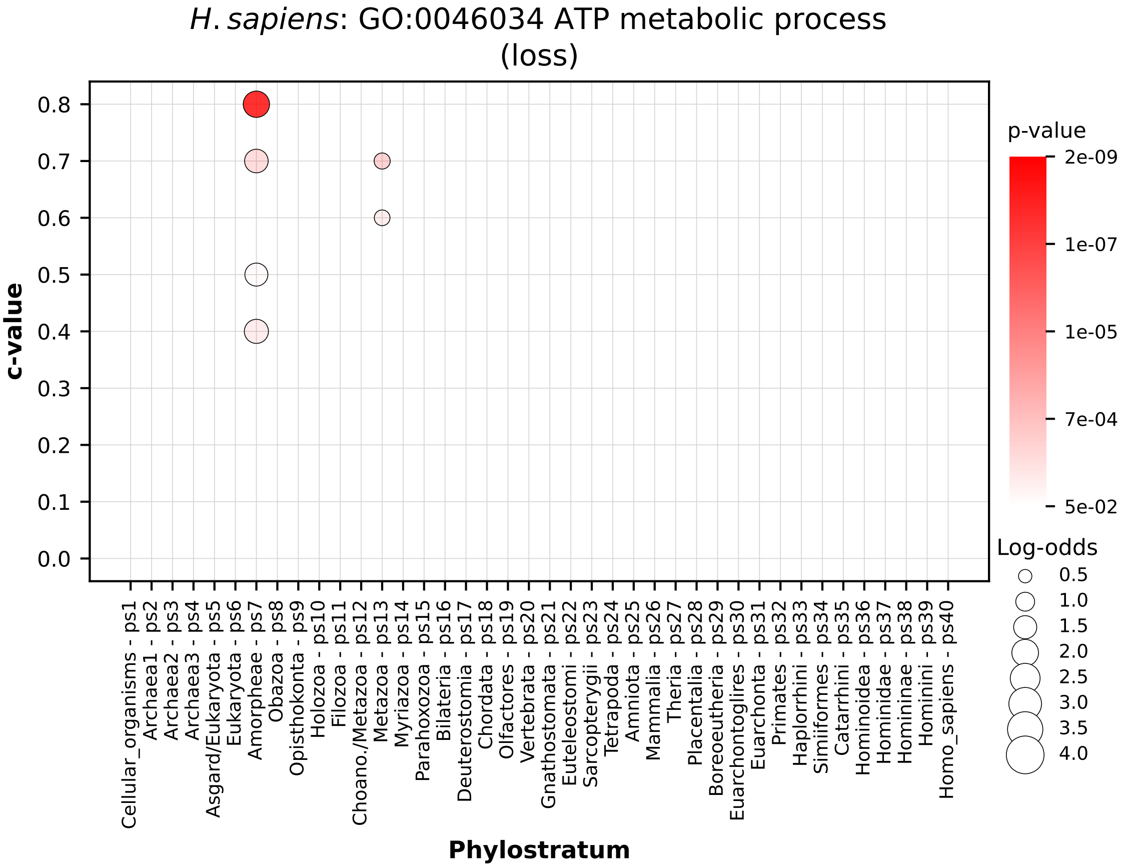

H. sapiens: GO:0046034 ATP metabolic process
(loss)
c-value
0.8
0.7
0.6
0.5
0.4
0.3
0.2
0.1
0.0
Phylostratum
Cellular_organisms - ps1
Archaea1 - ps2
Archaea2 - ps3
Archaea3 - ps4
Asgard/Eukaryota - ps5
Eukaryota - ps6
Amorpheae - ps7
Obazoa - ps8
Opisthokonta - ps9
Holozoa - ps10
Filozoa - ps11
Choano./Metazoa - ps12
Metazoa - ps13
Myriazoa - ps14
Parahoxozoa - ps15
Bilateria - ps16
Deuterostomia - ps17
Chordata - ps18
Olfactores - ps19
Vertebrata - ps20
Gnathostomata - ps21
Euteleostomi - ps22
Sarcopterygii - ps23
Tetrapoda - ps24
Amniota - ps25
Mammalia - ps26
Theria - ps27
Placentalia - ps28
Boreoeutheria - ps29
Euarchontoglires - ps30
Euarchonta - ps31
Primates - ps32
Haplorrhini - ps33
Simiiformes - ps34
Catarrhini - ps35
Hominoidea - ps36
Hominidae - ps37
Homininae - ps38
Hominini - ps39
Homo_sapiens - ps40
p-value
2e-09
1e-07
1e-05
7e-04
5e-02
Log-odds
0.5
1.0
1.5
2.0
2.5
3.0
3.5
4.0

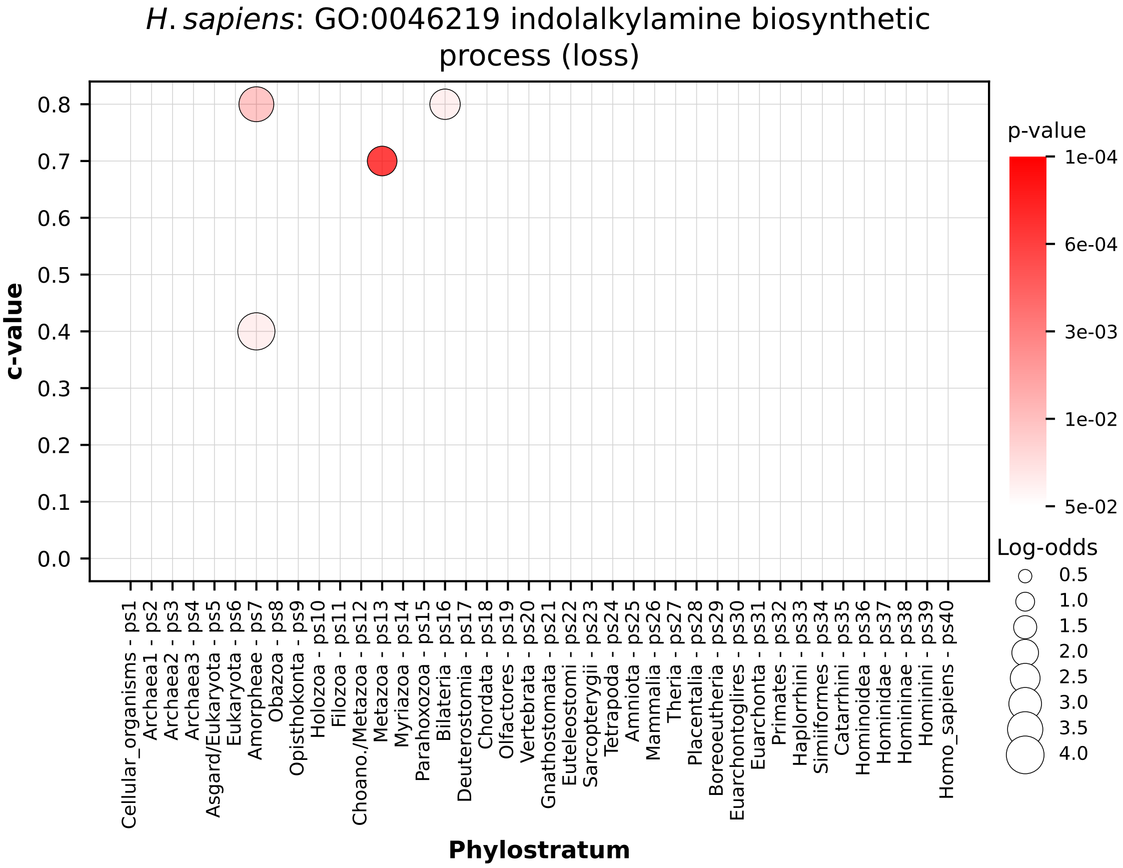

H. sapiens: GO:0046219 indolalkylamine biosynthetic process (loss)
c-value
0.0
0.1
0.2
0.3
0.4
0.5
0.6
0.7
0.8
Phylostratum
Cellular_organisms - ps1
Archaea1 - ps2
Archaea2 - ps3
Archaea3 - ps4
Asgard/Eukaryota - ps5
Eukaryota - ps6
Amorpheae - ps7
Obazoa - ps8
Opisthokonta - ps9
Holozoa - ps10
Filozoa - ps11
Choano./Metazoa - ps12
Metazoa - ps13
Myriazoa - ps14
Parahoxozoa - ps15
Bilateria - ps16
Deuterostomia - ps17
Chordata - ps18
Olfactores - ps19
Vertebrata - ps20
Gnathostomata - ps21
Euteleostomi - ps22
Sarcopterygii - ps23
Tetrapoda - ps24
Amniota - ps25
Mammalia - ps26
Theria - ps27
Placentalia - ps28
Boreoeutheria - ps29
Euarchontoglires - ps30
Euarchonta - ps31
Primates - ps32
Haplorrhini - ps33
Simiiformes - ps34
Catarrhini - ps35
Hominoidea - ps36
Hominidae - ps37
Homininae - ps38
Hominini - ps39
Homo_sapiens - ps40
p-value
1e-04
6e-04
3e-03
1e-02
5e-02
Log-odds
0.5
1.0
1.5
2.0
2.5
3.0
3.5
4.0

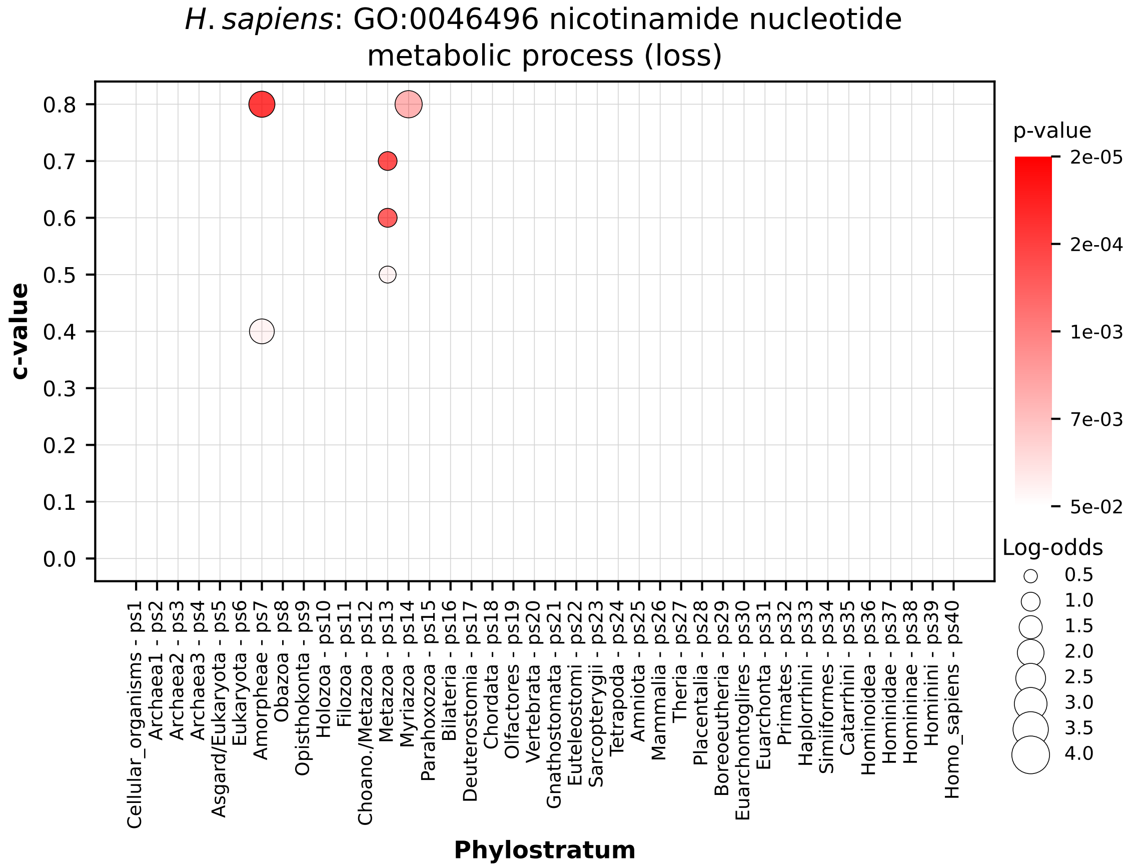

H. sapiens: GO:0046496 nicotinamide nucleotide metabolic process (loss)
c-value
0.0
0.1
0.2
0.3
0.4
0.5
0.6
0.7
0.8
Phylostratum
Cellular_organisms - ps1
Archaea1 - ps2
Archaea2 - ps3
Archaea3 - ps4
Asgard/Eukaryota - ps5
Eukaryota - ps6
Amorpheae - ps7
Obazoa - ps8
Opisthokonta - ps9
Holozoa - ps10
Filozoa - ps11
Choano./Metazoa - ps12
Metazoa - ps13
Myriazoa - ps14
Parahoxozoa - ps15
Bilateria - ps16
Deuterostomia - ps17
Chordata - ps18
Olfactores - ps19
Vertebrata - ps20
Gnathostomata - ps21
Euteleostomi - ps22
Sarcopterygii - ps23
Tetrapoda - ps24
Amniota - ps25
Mammalia - ps26
Theria - ps27
Placentalia - ps28
Boreoeutheria - ps29
Euarchontoglires - ps30
Euarchonta - ps31
Primates - ps32
Haplorrhini - ps33
Simiiformes - ps34
Catarrhini - ps35
Hominoidea - ps36
Hominidae - ps37
Homininae - ps38
Hominini - ps39
Homo_sapiens - ps40
p-value
2e-05
2e-04
1e-03
7e-03
5e-02
Log-odds
0.5
1.0
1.5
2.0
2.5
3.0
3.5
4.0

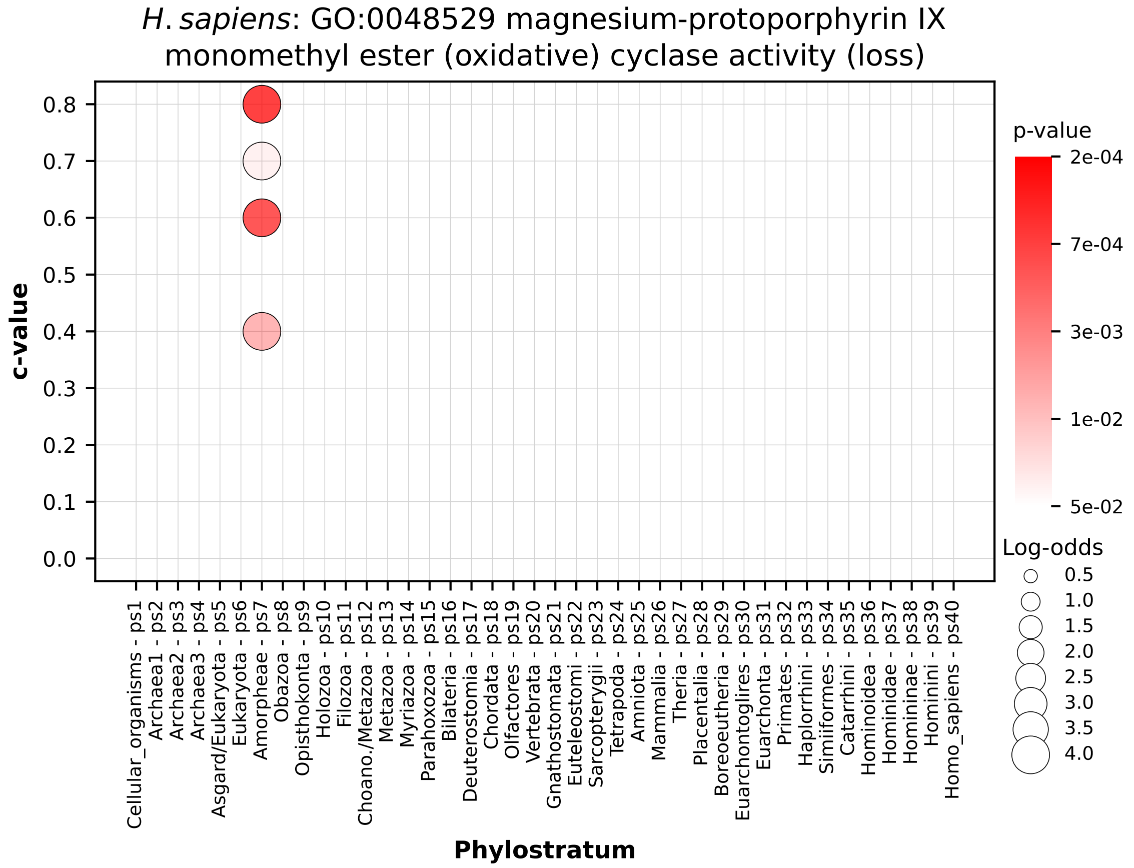
H. sapiens: GO:0048529 magnesium-protoporphyrin IX monomethyl ester (oxidative) cyclase activity (loss)
c-value
Phylostratum
p-value
2e-04
7e-04
3e-03
1e-02
5e-02
Log-odds
0.5
1.0
1.5
2.0
2.5
3.0
3.5
4.0
0.0
0.1
0.2
0.3
0.4
0.5
0.6
0.7
0.8
Cellular_organisms - ps1
Archaea1 - ps2
Archaea2 - ps3
Archaea3 - ps4
Asgard/Eukaryota - ps5
Eukaryota - ps6
Amorpheae - ps7
Obazoa - ps8
Opisthokonta - ps9
Holozoa - ps10
Filozoa - ps11
Choano./Metazoa - ps12
Metazoa - ps13
Myriazoa - ps14
Parahoxozoa - ps15
Bilateria - ps16
Deuterostomia - ps17
Chordata - ps18
Olfactores - ps19
Vertebrata - ps20
Gnathostomata - ps21
Euteleostomi - ps22
Sarcopterygii - ps23
Tetrapoda - ps24
Amniota - ps25
Mammalia - ps26
Theria - ps27
Placentalia - ps28
Boreoeutheria - ps29
Euarchontoglires - ps30
Euarchonta - ps31
Primates - ps32
Haplorrhini - ps33
Simiiformes - ps34
Catarrhini - ps35
Hominoidea - ps36
Hominidae - ps37
Homininae - ps38
Hominini - ps39
Homo_sapiens - ps40

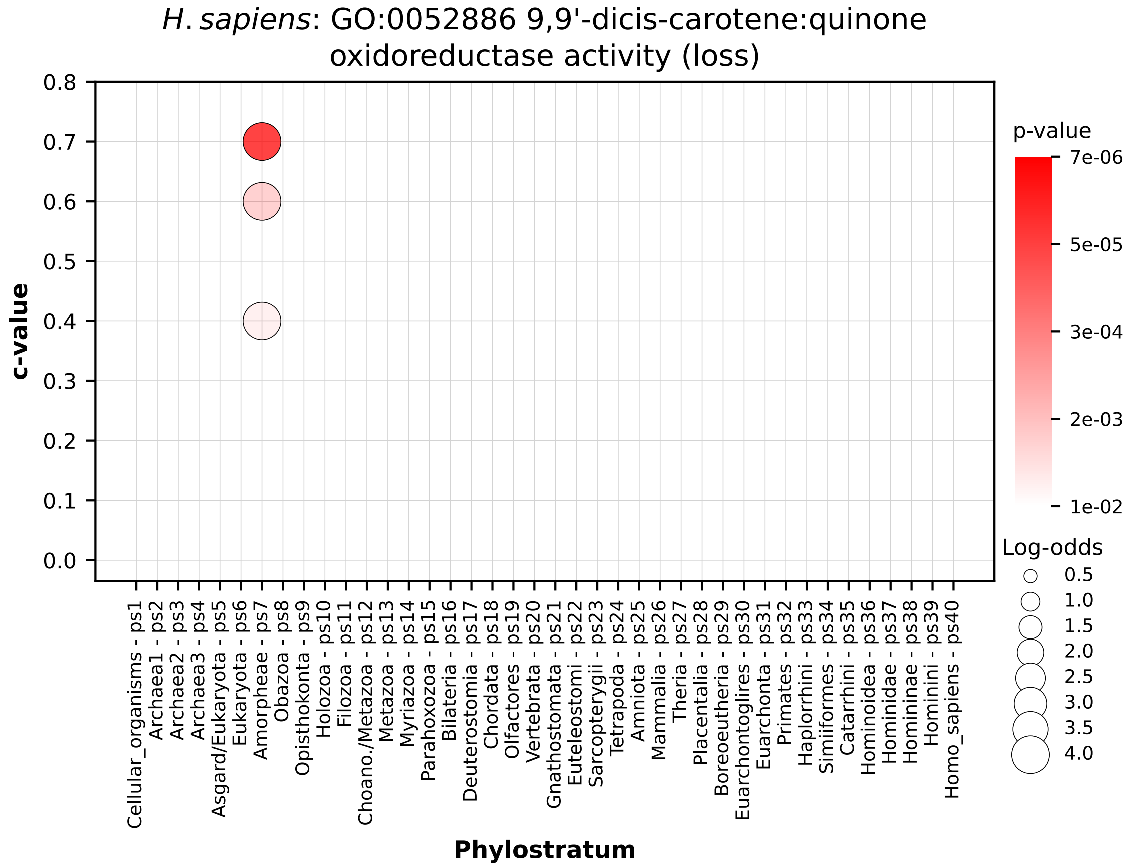

H. sapiens: GO:0052886 9,9'-dicis-carotene:quinone oxidoreductase activity (loss)
c-value
0.8
0.7
0.6
0.5
0.4
0.3
0.2
0.1
0.0
Phylostratum
Cellular_organisms - ps1
Archaea1 - ps2
Archaea2 - ps3
Archaea3 - ps4
Asgard/Eukaryota - ps5
Eukaryota - ps6
Amorpheae - ps7
Obazoa - ps8
Opisthokonta - ps9
Holozoa - ps10
Filozoa - ps11
Choano./Metazoa - ps12
Metazoa - ps13
Myriazoa - ps14
Parahoxozoa - ps15
Bilateria - ps16
Deuterostomia - ps17
Chordata - ps18
Olfactores - ps19
Vertebrata - ps20
Gnathostomata - ps21
Euteleostomi - ps22
Sarcopterygii - ps23
Tetrapoda - ps24
Amniota - ps25
Mammalia - ps26
Theria - ps27
Placentalia - ps28
Boreoeutheria - ps29
Euarchontoglires - ps30
Euarchonta - ps31
Primates - ps32
Haplorrhini - ps33
Simiiformes - ps34
Catarrhini - ps35
Hominoidea - ps36
Hominidae - ps37
Homininae - ps38
Hominini - ps39
Homo_sapiens - ps40
p-value
7e-06
5e-05
3e-04
2e-03
1e-02
Log-odds
0.5
1.0
1.5
2.0
2.5
3.0
3.5
4.0

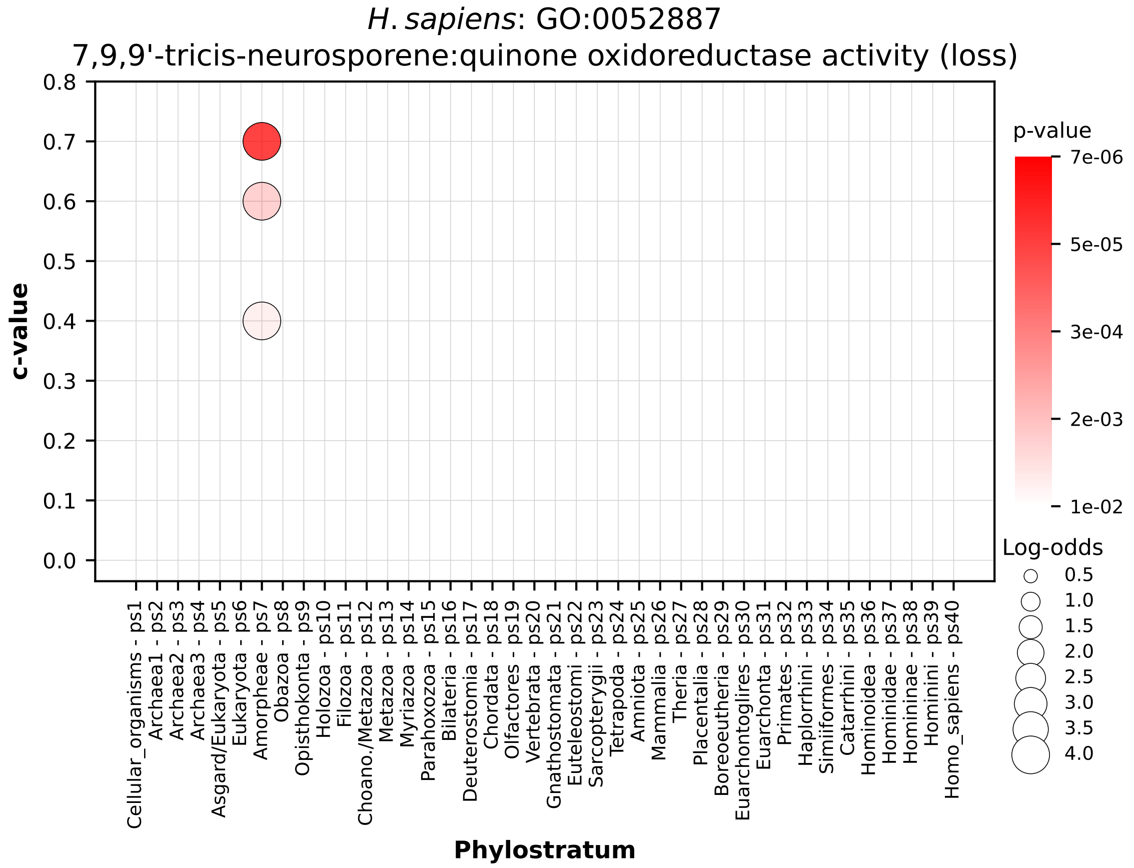
H. sapiens: GO:0052887
7,9,9'-tricis-neurosporene:quinone oxidoreductase activity (loss)
c-value
0.0
0.1
0.2
0.3
0.4
0.5
0.6
0.7
0.8
Phylostratum
Cellular_organisms - ps1
Archaea1 - ps2
Archaea2 - ps3
Archaea3 - ps4
Asgard/Eukaryota - ps5
Eukaryota - ps6
Amorpheae - ps7
Obazoa - ps8
Opisthokonta - ps9
Holozoa - ps10
Filozoa - ps11
Choano./Metazoa - ps12
Metazoa - ps13
Myriazoa - ps14
Parahoxozoa - ps15
Bilateria - ps16
Deuterostomia - ps17
Chordata - ps18
Olfactores - ps19
Vertebrata - ps20
Gnathostomata - ps21
Euteleostomi - ps22
Sarcopterygii - ps23
Tetrapoda - ps24
Amniota - ps25
Mammalia - ps26
Theria - ps27
Placentalia - ps28
Boreoeutheria - ps29
Euarchontoglires - ps30
Euarchonta - ps31
Primates - ps32
Haplorrhini - ps33
Simiiformes - ps34
Catarrhini - ps35
Hominoidea - ps36
Hominidae - ps37
Homininae - ps38
Hominini - ps39
Homo_sapiens - ps40
p-value
7e-06
5e-05
3e-04
2e-03
1e-02
Log-odds
0.5
1.0
1.5
2.0
2.5
3.0
3.5
4.0

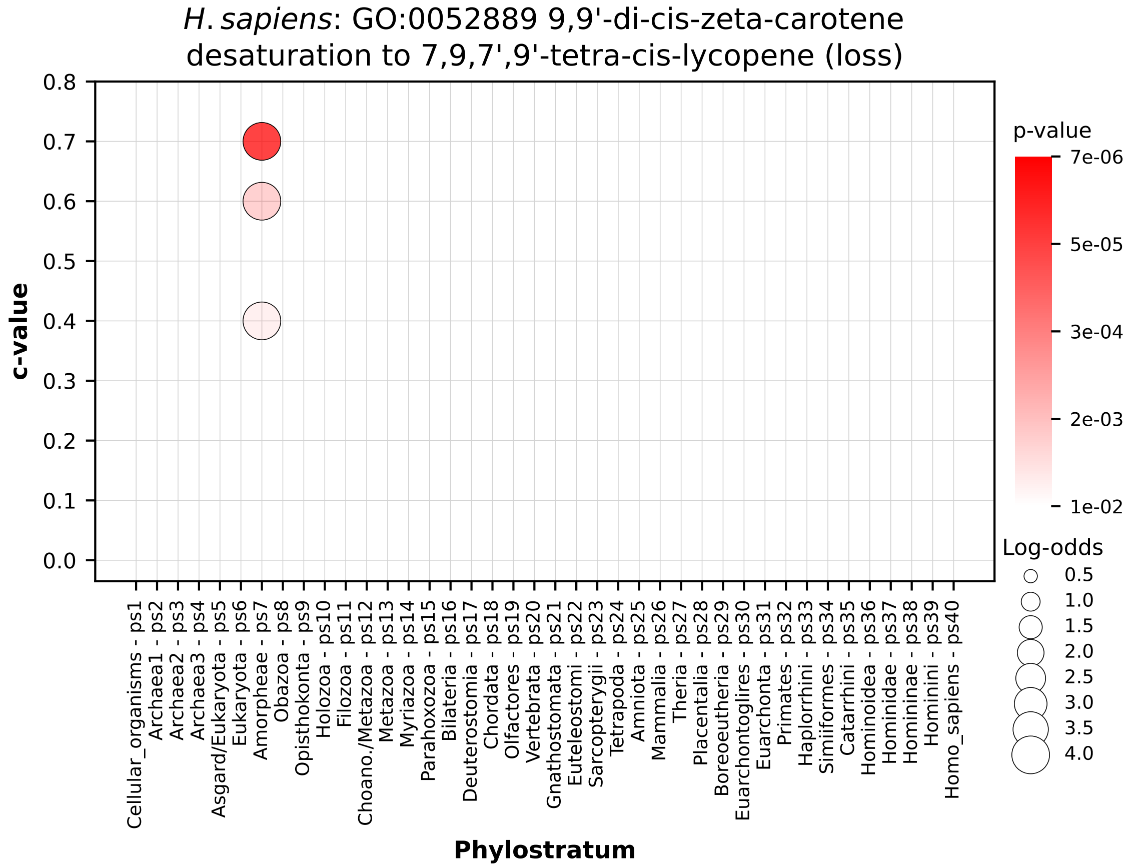

H. sapiens: GO:0052889 9,9'-di-cis-zeta-carotene desaturation to 7,9,7',9'-tetra-cis-lycopene (loss)
c-value
0.8
0.7
0.6
0.5
0.4
0.3
0.2
0.1
0.0
Phylostratum
Cellular_organisms - ps1
Archaea1 - ps2
Archaea2 - ps3
Archaea3 - ps4
Asgard/Eukaryota - ps5
Eukaryota - ps6
Amorpheae - ps7
Obazoa - ps8
Opisthokonta - ps9
Holozoa - ps10
Filozoa - ps11
Choano./Metazoa - ps12
Metazoa - ps13
Myriazoa - ps14
Parahoxozoa - ps15
Bilateria - ps16
Deuterostomia - ps17
Chordata - ps18
Olfactores - ps19
Vertebrata - ps20
Gnathostomata - ps21
Euteleostomi - ps22
Sarcopterygii - ps23
Tetrapoda - ps24
Amniota - ps25
Mammalia - ps26
Theria - ps27
Placentalia - ps28
Boreoeutheria - ps29
Euarchontoglires - ps30
Euarchonta - ps31
Primates - ps32
Haplorrhini - ps33
Simiiformes - ps34
Catarrhini - ps35
Hominoidea - ps36
Hominidae - ps37
Homininae - ps38
Hominini - ps39
Homo_sapiens - ps40
p-value
7e-06
5e-05
3e-04
2e-03
1e-02
Log-odds
0.5
1.0
1.5
2.0
2.5
3.0
3.5
4.0

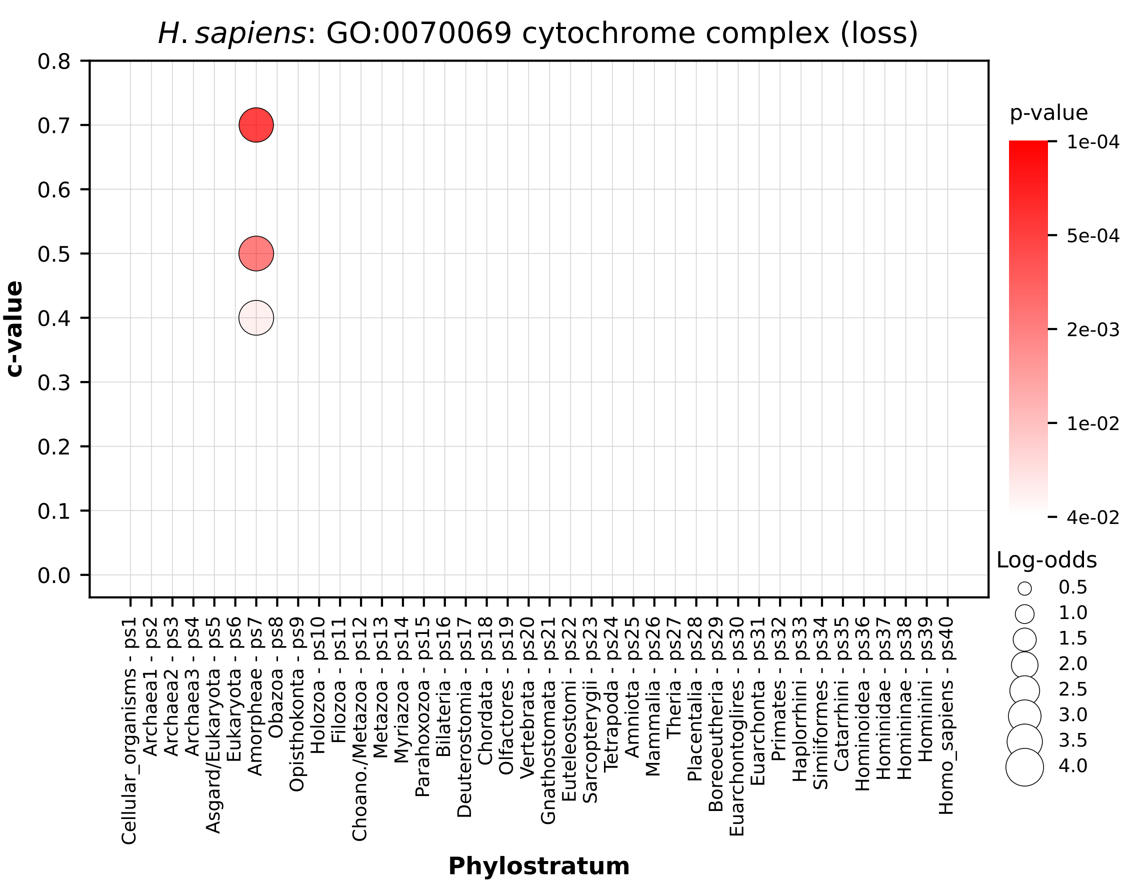

H. sapiens: GO:0070069 cytochrome complex (loss)
c-value
0.8
0.7
0.6
0.5
0.4
0.3
0.2
0.1
0.0
Phylostratum
Cellular_organisms - ps1
Archaea1 - ps2
Archaea2 - ps3
Archaea3 - ps4
Asgard/Eukaryota - ps5
Eukaryota - ps6
Amorpheae - ps7
Obazoa - ps8
Opisthokonta - ps9
Holozoa - ps10
Filozoa - ps11
Choano./Metazoa - ps12
Metazoa - ps13
Myriazoa - ps14
Parahoxozoa - ps15
Bilateria - ps16
Deuterostomia - ps17
Chordata - ps18
Olfactores - ps19
Vertebrata - ps20
Gnathostomata - ps21
Euteleostomi - ps22
Sarcopterygii - ps23
Tetrapoda - ps24
Amniota - ps25
Mammalia - ps26
Theria - ps27
Placentalia - ps28
Boreoeutheria - ps29
Euarchontoglires - ps30
Euarchonta - ps31
Primates - ps32
Haplorrhini - ps33
Simiiformes - ps34
Catarrhini - ps35
Hominoidea - ps36
Hominidae - ps37
Homininae - ps38
Hominini - ps39
Homo_sapiens - ps40
p-value
1e-04
5e-04
2e-03
1e-02
4e-02
Log-odds
0.5
1.0
1.5
2.0
2.5
3.0
3.5
4.0

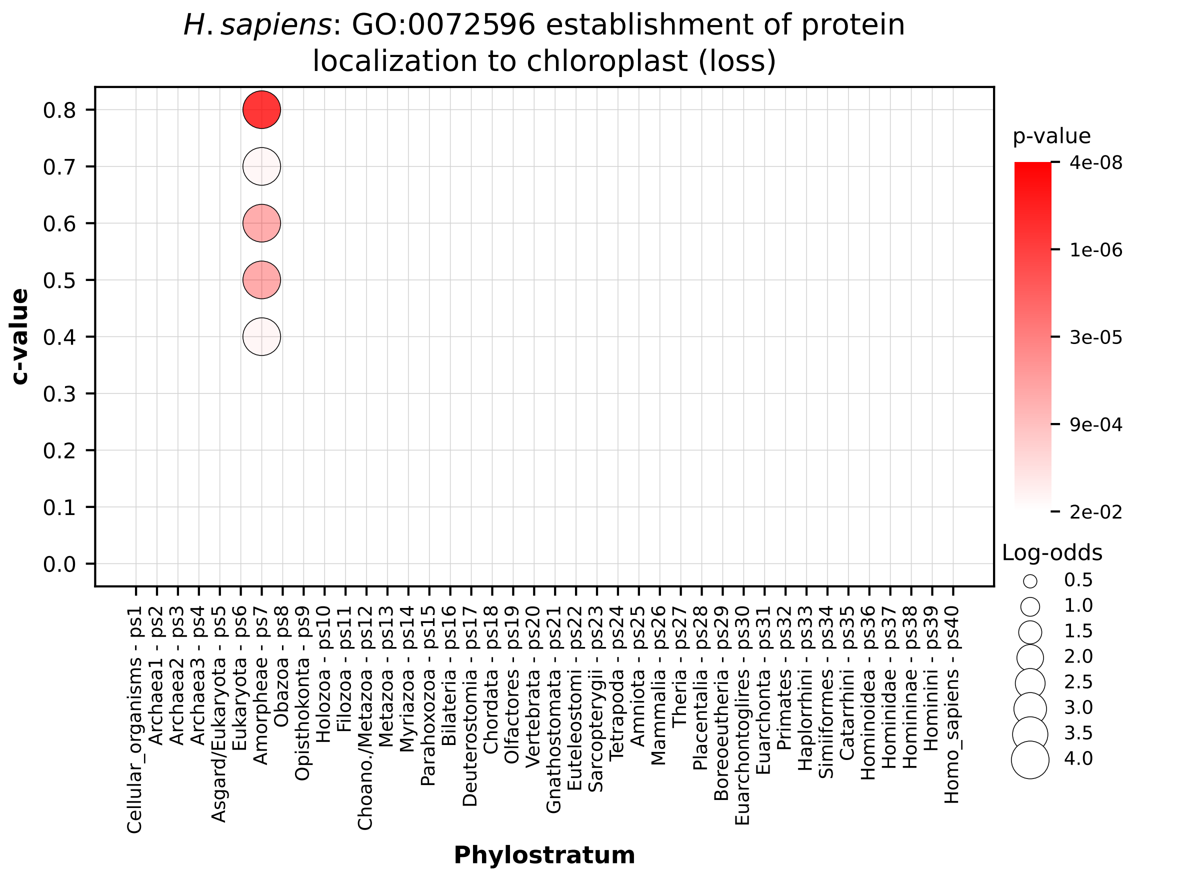

H. sapiens: GO:0072596 establishment of protein localization to chloroplast (loss)
c-value
0.8
0.7
0.6
0.5
0.4
0.3
0.2
0.1
0.0
Phylostratum
Cellular_organisms - ps1
Archaea1 - ps2
Archaea2 - ps3
Archaea3 - ps4
Asgard/Eukaryota - ps5
Eukaryota - ps6
Amorpheae - ps7
Obazoa - ps8
Opisthokonta - ps9
Holozoa - ps10
Filozoa - ps11
Choano./Metazoa - ps12
Metazoa - ps13
Myriazoa - ps14
Parahoxozoa - ps15
Bilateria - ps16
Deuterostomia - ps17
Chordata - ps18
Olfactores - ps19
Vertebrata - ps20
Gnathostomata - ps21
Euteleostomi - ps22
Sarcopterygii - ps23
Tetrapoda - ps24
Amniota - ps25
Mammalia - ps26
Theria - ps27
Placentalia - ps28
Boreoeutheria - ps29
Euarchontoglires - ps30
Euarchonta - ps31
Primates - ps32
Haplorrhini - ps33
Simiiformes - ps34
Catarrhini - ps35
Hominoidea - ps36
Hominidae - ps37
Homininae - ps38
Hominini - ps39
Homo_sapiens - ps40
p-value
4e-08
1e-06
3e-05
9e-04
2e-02
Log-odds
0.5
1.0
1.5
2.0
2.5
3.0
3.5
4.0

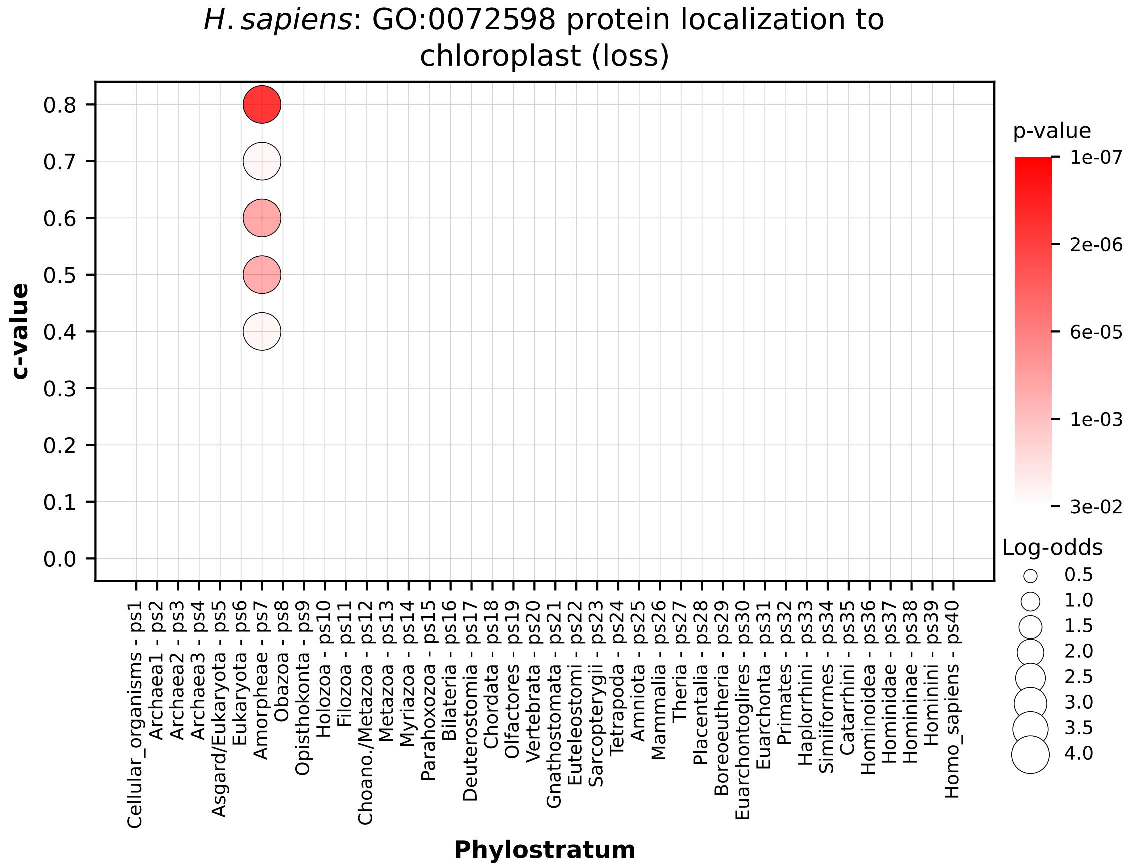

H. sapiens: GO:0072598 protein localization to chloroplast (loss)
c-value
0.8
0.7
0.6
0.5
0.4
0.3
0.2
0.1
0.0
Phylostratum
Cellular_organisms - ps1
Archaea1 - ps2
Archaea2 - ps3
Archaea3 - ps4
Asgard/Eukaryota - ps5
Eukaryota - ps6
Amorpheae - ps7
Obazoa - ps8
Opisthokonta - ps9
Holozoa - ps10
Filozoa - ps11
Choano./Metazoa - ps12
Metazoa - ps13
Myriazoa - ps14
Parahoxozoa - ps15
Bilateria - ps16
Deuterostomia - ps17
Chordata - ps18
Olfactores - ps19
Vertebrata - ps20
Gnathostomata - ps21
Euteleostomi - ps22
Sarcopterygii - ps23
Tetrapoda - ps24
Amniota - ps25
Mammalia - ps26
Theria - ps27
Placentalia - ps28
Boreoeutheria - ps29
Euarchontoglires - ps30
Euarchonta - ps31
Primates - ps32
Haplorrhini - ps33
Simiiformes - ps34
Catarrhini - ps35
Hominoidea - ps36
Hominidae - ps37
Homininae - ps38
Hominini - ps39
Homo_sapiens - ps40
p-value
1e-07
2e-06
6e-05
1e-03
3e-02
Log-odds
0.5
1.0
1.5
2.0
2.5
3.0
3.5
4.0

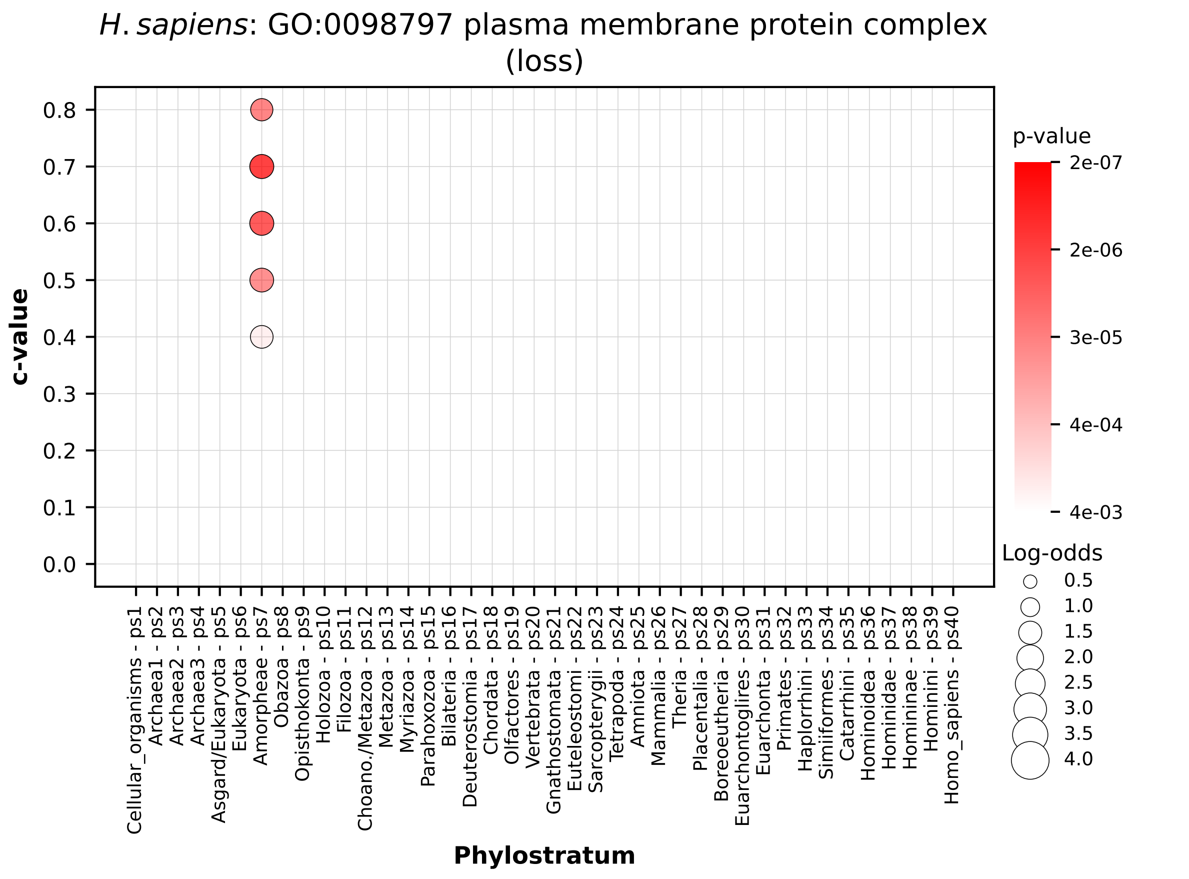

H. sapiens: GO:0098797 plasma membrane protein complex (loss)
c-value
0.0
0.1
0.2
0.3
0.4
0.5
0.6
0.7
0.8
p-value
2e-07
2e-06
3e-05
4e-04
4e-03
Log-odds
0.5
1.0
1.5
2.0
2.5
3.0
3.5
4.0
Cellular_organisms - ps1
Archaea1 - ps2
Archaea2 - ps3
Archaea3 - ps4
Asgard/Eukaryota - ps5
Eukaryota - ps6
Amorpheae - ps7
Obazoa - ps8
Opisthokonta - ps9
Holozoa - ps10
Filozoa - ps11
Choano./Metazoa - ps12
Metazoa - ps13
Myriazoa - ps14
Parahoxozoa - ps15
Bilateria - ps16
Deuterostomia - ps17
Chordata - ps18
Olfactores - ps19
Vertebrata - ps20
Gnathostomata - ps21
Euteleostomi - ps22
Sarcopterygii - ps23
Tetrapoda - ps24
Amniota - ps25
Mammalia - ps26
Theria - ps27
Placentalia - ps28
Boreoeutheria - ps29
Euarchontoglires - ps30
Euarchonta - ps31
Primates - ps32
Haplorrhini - ps33
Simiiformes - ps34
Catarrhini - ps35
Hominoidea - ps36
Hominidae - ps37
Homininae - ps38
Hominini - ps39
Homo_sapiens - ps40
Phylostratum

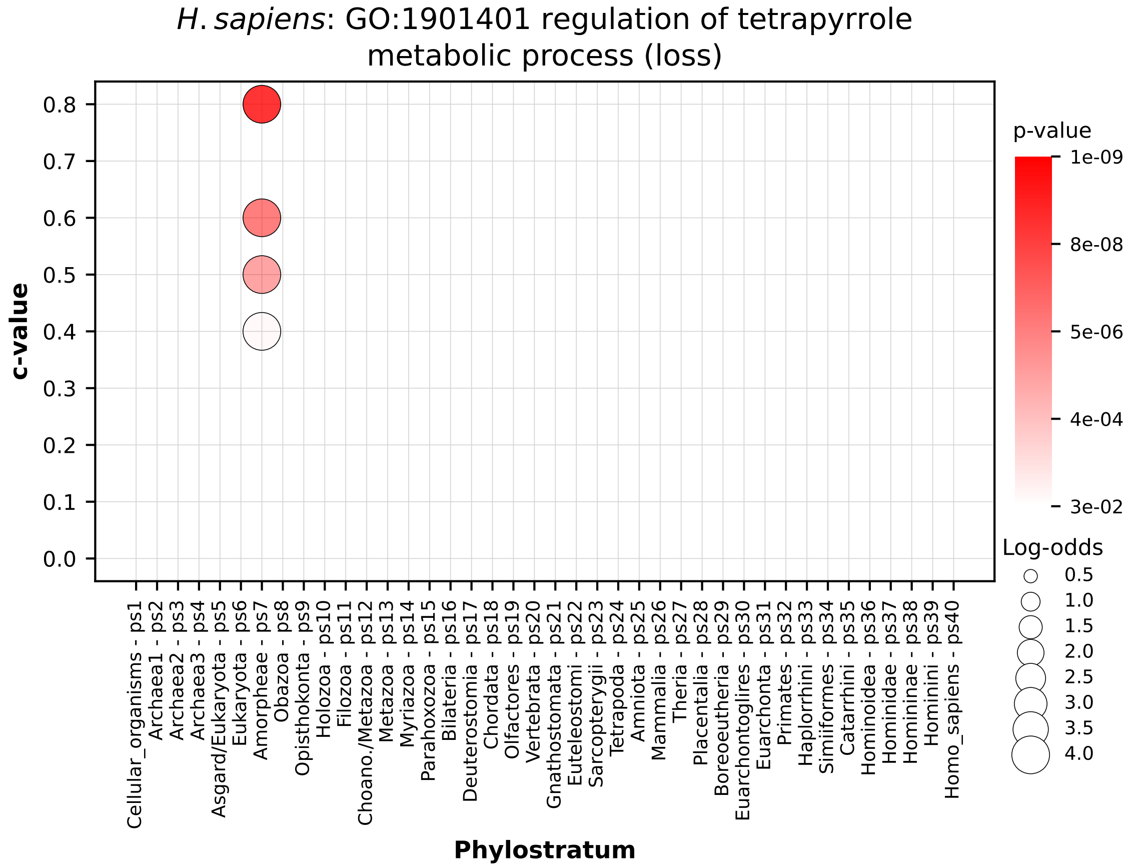

H. sapiens: GO:1901401 regulation of tetrapyrrole metabolic process (loss)
c-value
0.8
0.7
0.6
0.5
0.4
0.3
0.2
0.1
0.0
p-value
1e-09
8e-08
5e-06
4e-04
3e-02
Log-odds
0.5
1.0
1.5
2.0
2.5
3.0
3.5
4.0
Cellular_organisms - ps1
Archaea1 - ps2
Archaea2 - ps3
Archaea3 - ps4
Asgard/Eukaryota - ps5
Eukaryota - ps6
Amorpheae - ps7
Obazoa - ps8
Opisthokonta - ps9
Holozoa - ps10
Filozoa - ps11
Choano./Metazoa - ps12
Metazoa - ps13
Myriazoa - ps14
Parahoxozoa - ps15
Bilateria - ps16
Deuterostomia - ps17
Chordata - ps18
Olfactores - ps19
Vertebrata - ps20
Gnathostomata - ps21
Euteleostomi - ps22
Sarcopterygii - ps23
Tetrapoda - ps24
Amniota - ps25
Mammalia - ps26
Theria - ps27
Placentalia - ps28
Boreoeutheria - ps29
Euarchontoglires - ps30
Euarchonta - ps31
Primates - ps32
Haplorrhini - ps33
Simiiformes - ps34
Catarrhini - ps35
Hominoidea - ps36
Hominidae - ps37
Homininae - ps38
Hominini - ps39
Homo_sapiens - ps40
Phylostratum

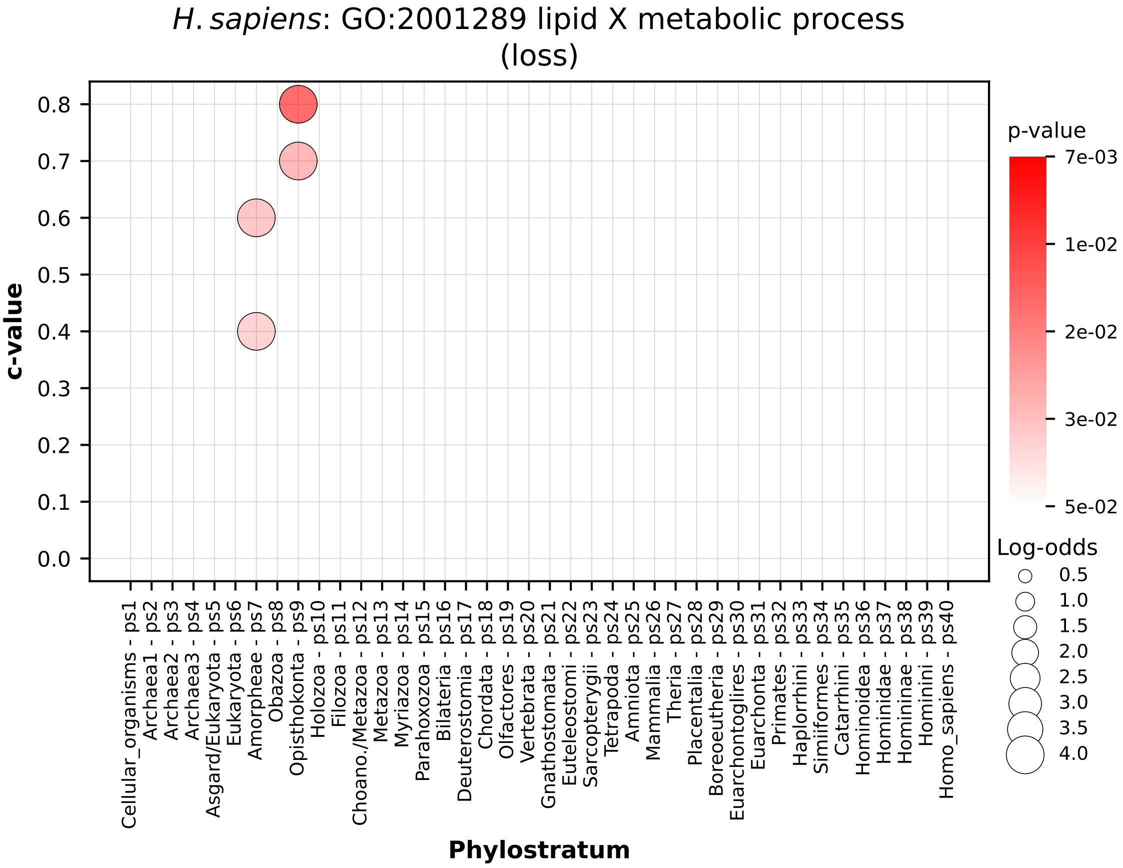
H. sapiens: GO:2001289 lipid X metabolic process
(loss)
c-value
0.8
0.7
0.6
0.5
0.4
0.3
0.2
0.1
0.0
p-value
7e-03
1e-02
2e-02
3e-02
5e-02
Log-odds
0.5
1.0
1.5
2.0
2.5
3.0
3.5
4.0
Cellular_organisms - ps1
Archaea1 - ps2
Archaea2 - ps3
Archaea3 - ps4
Asgard/Eukaryota - ps5
Eukaryota - ps6
Amorpheae - ps7
Obazoa - ps8
Opisthokonta - ps9
Holozoa - ps10
Filozoa - ps11
Choano./Metazoa - ps12
Metazoa - ps13
Myriazoa - ps14
Parahoxozoa - ps15
Bilateria - ps16
Deuterostomia - ps17
Chordata - ps18
Olfactores - ps19
Vertebrata - ps20
Gnathostomata - ps21
Euteleostomi - ps22
Sarcopterygii - ps23
Tetrapoda - ps24
Amniota - ps25
Mammalia - ps26
Theria - ps27
Placentalia - ps28
Boreoeutheria - ps29
Euarchontoglires - ps30
Euarchonta - ps31
Primates - ps32
Haplorrhini - ps33
Simiiformes - ps34
Catarrhini - ps35
Hominoidea - ps36
Hominidae - ps37
Homininae - ps38
Hominini - ps39
Homo_sapiens - ps40
Phylostratum

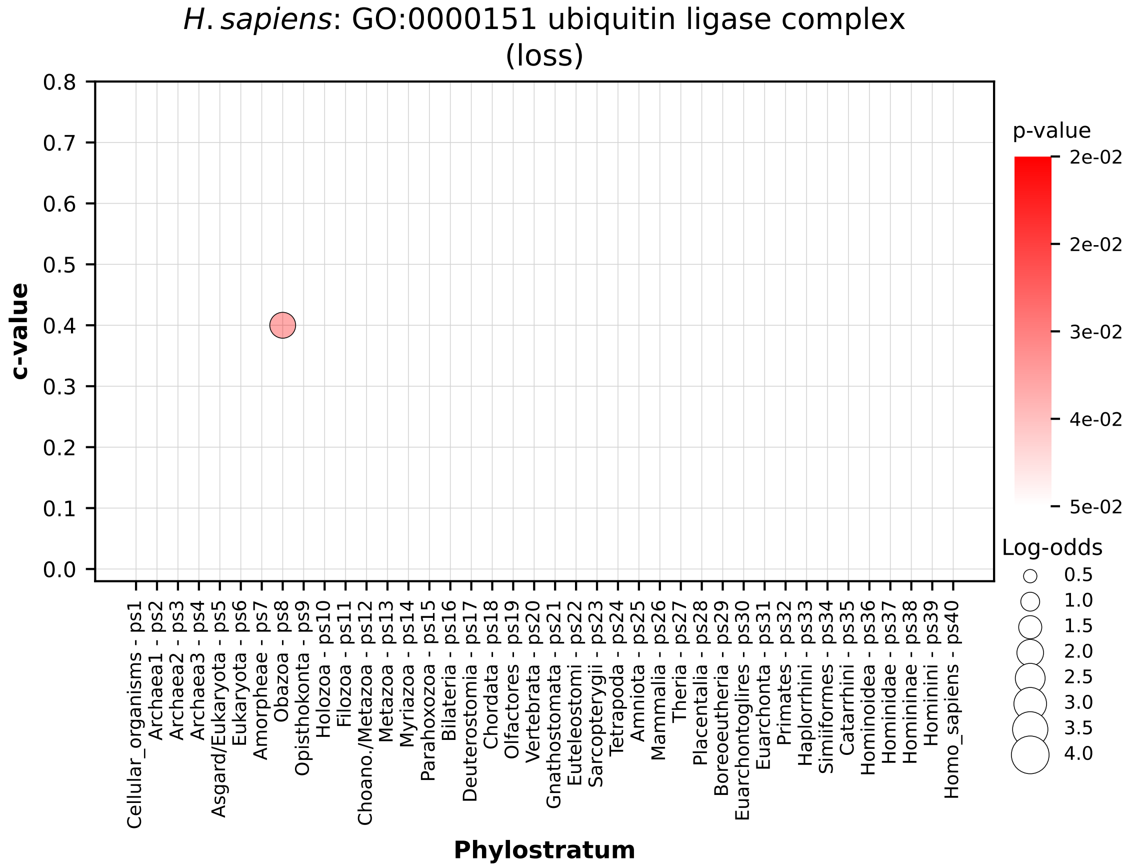

H. sapiens: GO:0000151 ubiquitin ligase complex
(loss)
c-value
0.8
0.7
0.6
0.5
0.4
0.3
0.2
0.1
0.0
Phylostratum
Cellular_organisms - ps1
Archaea1 - ps2
Archaea2 - ps3
Archaea3 - ps4
Asgard/Eukaryota - ps5
Eukaryota - ps6
Amorpheae - ps7
Obazoa - ps8
Opisthokonta - ps9
Holozoa - ps10
Filozoa - ps11
Choano./Metazoa - ps12
Metazoa - ps13
Myriazoa - ps14
Parahoxozoa - ps15
Bilateria - ps16
Deuterostomia - ps17
Chordata - ps18
Olfactores - ps19
Vertebrata - ps20
Gnathostomata - ps21
Euteleostomi - ps22
Sarcopterygii - ps23
Tetrapoda - ps24
Amniota - ps25
Mammalia - ps26
Theria - ps27
Placentalia - ps28
Boreoeutheria - ps29
Euarchontoglires - ps30
Euarchonta - ps31
Primates - ps32
Haplorrhini - ps33
Simiiformes - ps34
Catarrhini - ps35
Hominoidea - ps36
Hominidae - ps37
Homininae - ps38
Hominini - ps39
Homo_sapiens - ps40
p-value
2e-02
2e-02
3e-02
4e-02
5e-02
Log-odds
0.5
1.0
1.5
2.0
2.5
3.0
3.5
4.0

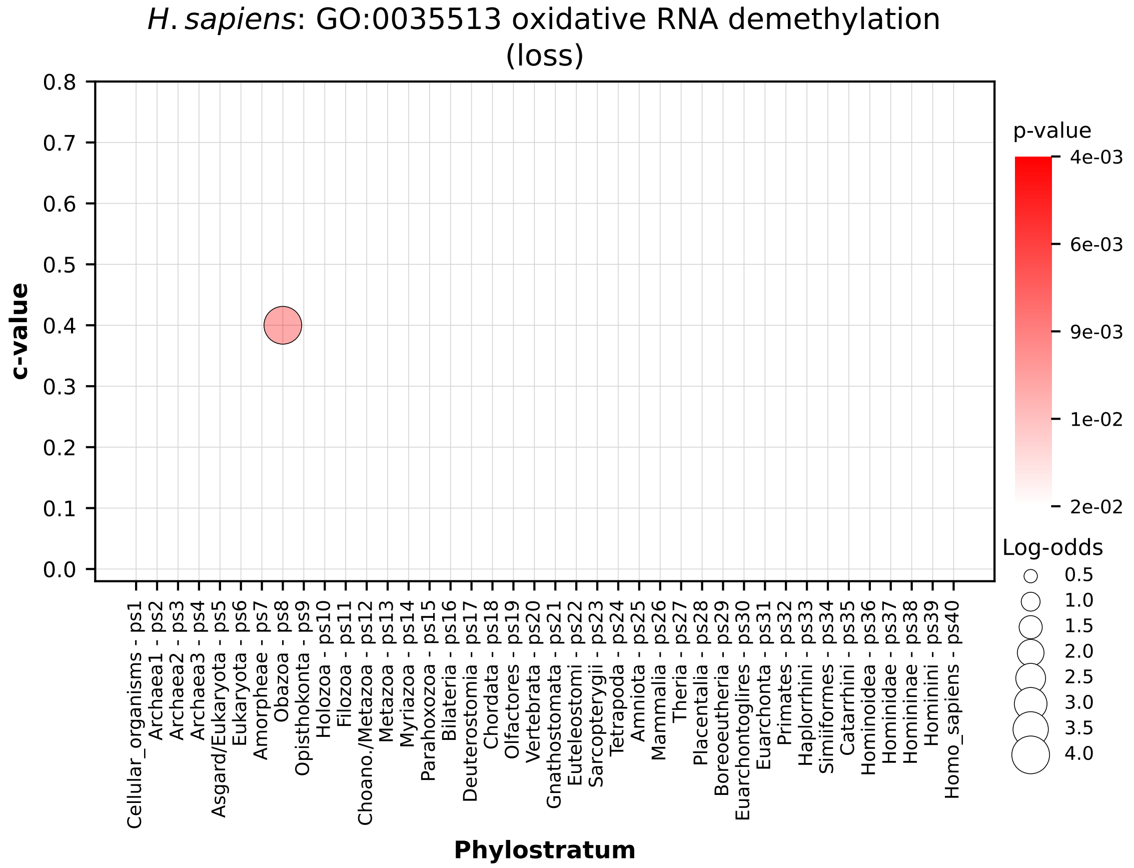

H. sapiens: GO:0035513 oxidative RNA demethylation
(loss)
c-value
0.0
0.1
0.2
0.3
0.4
0.5
0.6
0.7
0.8
Phylostratum
Cellular_organisms - ps1
Archaea1 - ps2
Archaea2 - ps3
Archaea3 - ps4
Asgard/Eukaryota - ps5
Eukaryota - ps6
Amorpheae - ps7
Obazoa - ps8
Opisthokonta - ps9
Holozoa - ps10
Filozoa - ps11
Choano./Metazoa - ps12
Metazoa - ps13
Myriazoa - ps14
Parahoxozoa - ps15
Bilateria - ps16
Deuterostomia - ps17
Chordata - ps18
Olfactores - ps19
Vertebrata - ps20
Gnathostomata - ps21
Euteleostomi - ps22
Sarcopterygii - ps23
Tetrapoda - ps24
Amniota - ps25
Mammalia - ps26
Theria - ps27
Placentalia - ps28
Boreoeutheria - ps29
Euarchontoglires - ps30
Euarchonta - ps31
Primates - ps32
Haplorrhini - ps33
Simiiformes - ps34
Catarrhini - ps35
Hominoidea - ps36
Hominidae - ps37
Homininae - ps38
Hominini - ps39
Homo_sapiens - ps40
p-value
4e-03
6e-03
9e-03
1e-02
2e-02
Log-odds
0.5
1.0
1.5
2.0
2.5
3.0
3.5
4.0

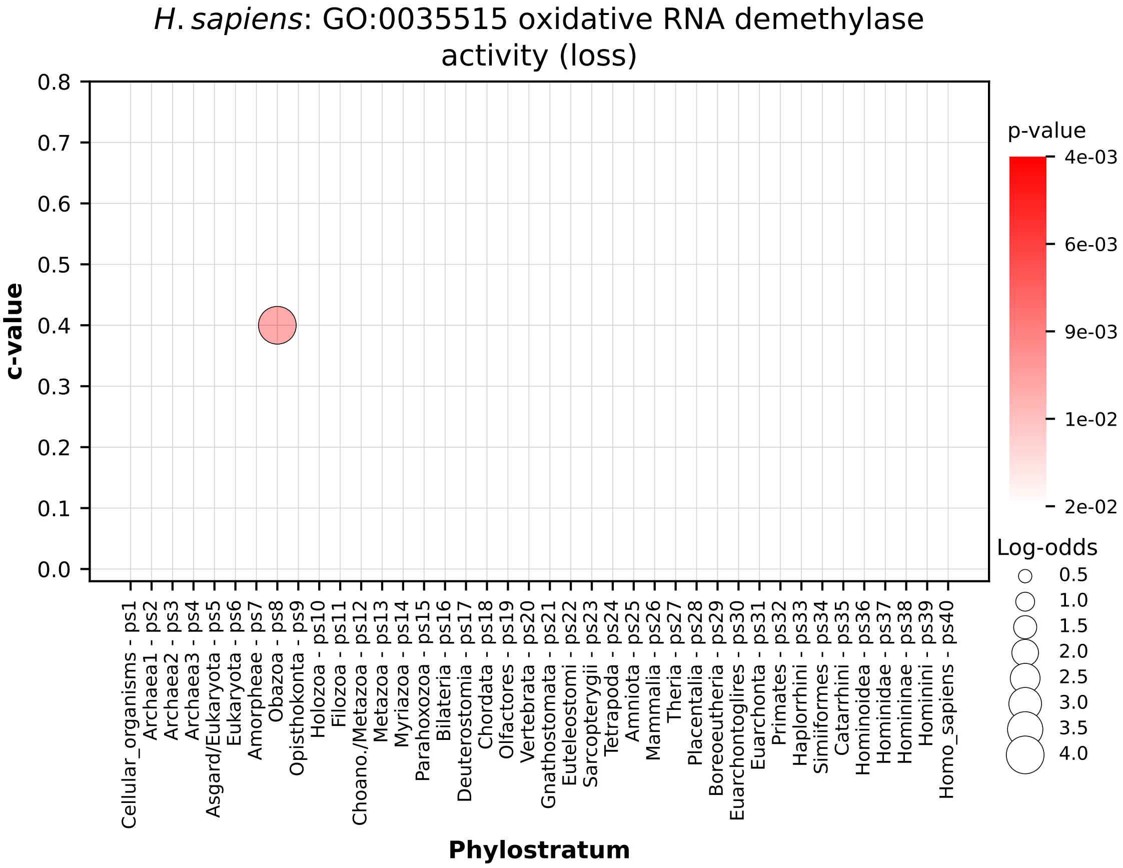

H. sapiens: GO:0035515 oxidative RNA demethylase activity (loss)
c-value
0.8
0.7
0.6
0.5
0.4
0.3
0.2
0.1
0.0
Phylostratum
Cellular_organisms - ps1
Archaea1 - ps2
Archaea2 - ps3
Archaea3 - ps4
Asgard/Eukaryota - ps5
Eukaryota - ps6
Amorpheae - ps7
Obazoa - ps8
Opisthokonta - ps9
Holozoa - ps10
Filozoa - ps11
Choano./Metazoa - ps12
Metazoa - ps13
Myriazoa - ps14
Parahoxozoa - ps15
Bilateria - ps16
Deuterostomia - ps17
Chordata - ps18
Olfactores - ps19
Vertebrata - ps20
Gnathostomata - ps21
Euteleostomi - ps22
Sarcopterygii - ps23
Tetrapoda - ps24
Amniota - ps25
Mammalia - ps26
Theria - ps27
Placentalia - ps28
Boreoeutheria - ps29
Euarchontoglires - ps30
Euarchonta - ps31
Primates - ps32
Haplorrhini - ps33
Simiiformes - ps34
Catarrhini - ps35
Hominoidea - ps36
Hominidae - ps37
Homininae - ps38
Hominini - ps39
Homo_sapiens - ps40
p-value
4e-03
6e-03
9e-03
1e-02
2e-02
Log-odds
0.5
1.0
1.5
2.0
2.5
3.0
3.5
4.0

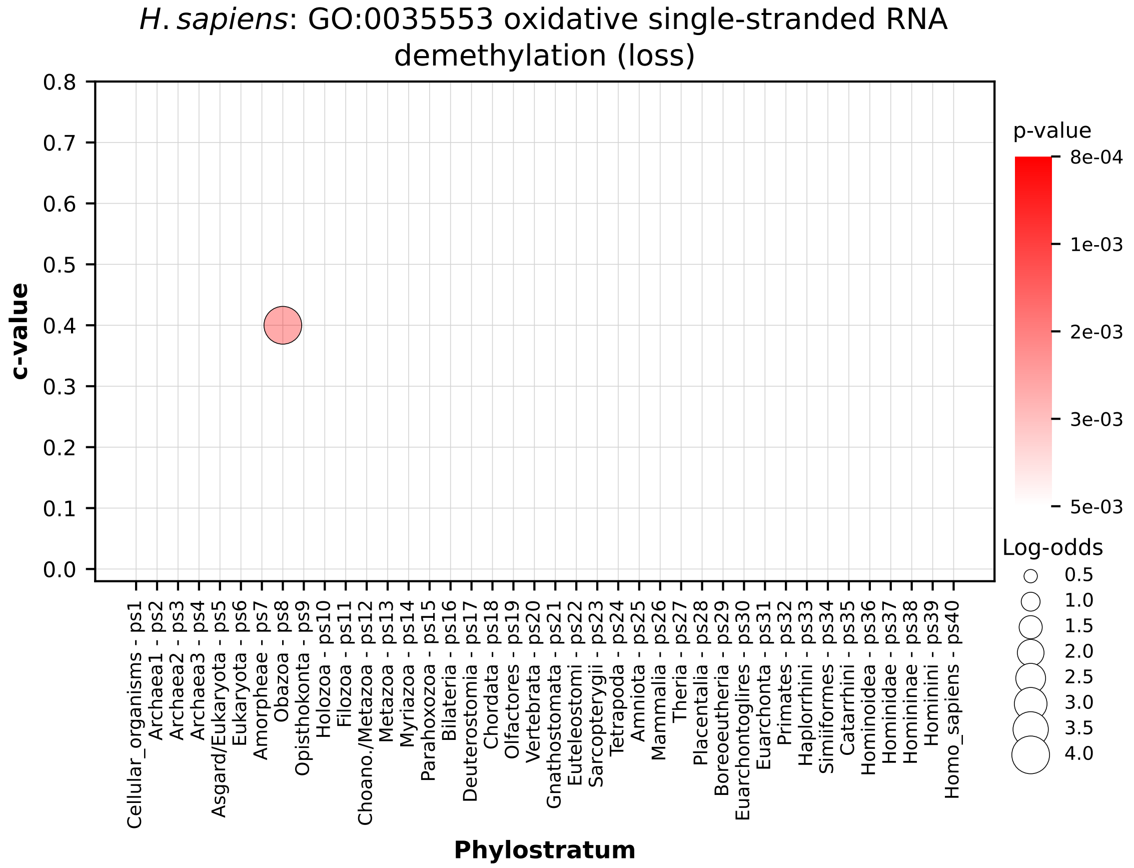
H. sapiens: GO:0035553 oxidative single-stranded RNA demethylation (loss)
c-value
0.0
0.1
0.2
0.3
0.4
0.5
0.6
0.7
0.8
Phylostratum
Cellular_organisms - ps1
Archaea1 - ps2
Archaea2 - ps3
Archaea3 - ps4
Asgard/Eukaryota - ps5
Eukaryota - ps6
Amorpheae - ps7
Obazoa - ps8
Opisthokonta - ps9
Holozoa - ps10
Filozoa - ps11
Choano./Metazoa - ps12
Metazoa - ps13
Myriazoa - ps14
Parahoxozoa - ps15
Bilateria - ps16
Deuterostomia - ps17
Chordata - ps18
Olfactores - ps19
Vertebrata - ps20
Gnathostomata - ps21
Euteleostomi - ps22
Sarcopterygii - ps23
Tetrapoda - ps24
Amniota - ps25
Mammalia - ps26
Theria - ps27
Placentalia - ps28
Boreoeutheria - ps29
Euarchontoglires - ps30
Euarchonta - ps31
Primates - ps32
Haplorrhini - ps33
Simiiformes - ps34
Catarrhini - ps35
Hominoidea - ps36
Hominidae - ps37
Homininae - ps38
Hominini - ps39
Homo_sapiens - ps40
p-value
8e-04
1e-03
2e-03
3e-03
5e-03
Log-odds
0.5
1.0
1.5
2.0
2.5
3.0
3.5
4.0

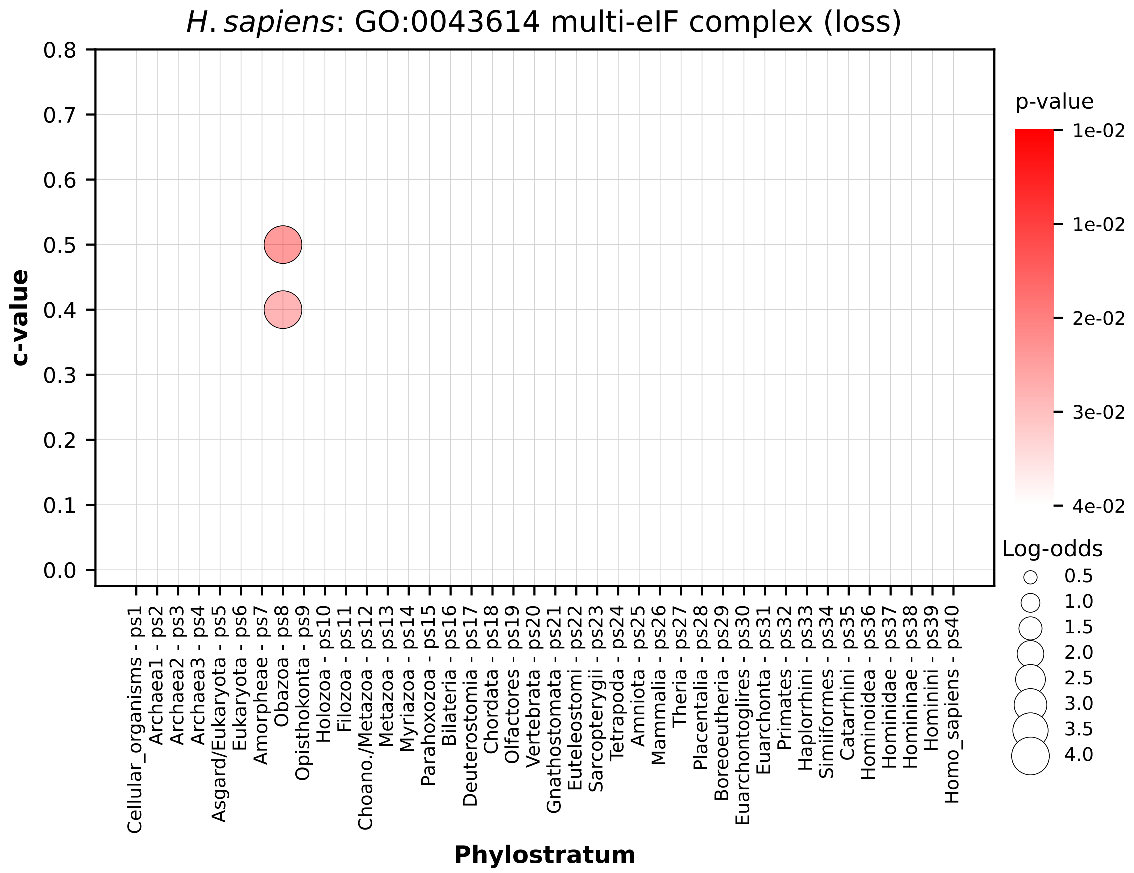

H. sapiens: GO:0043614 multi-eIF complex (loss)
c-value
0.0
0.1
0.2
0.3
0.4
0.5
0.6
0.7
0.8
Phylostratum
Cellular_organisms - ps1
Archaea1 - ps2
Archaea2 - ps3
Archaea3 - ps4
Asgard/Eukaryota - ps5
Eukaryota - ps6
Amorpheae - ps7
Obazoa - ps8
Opisthokonta - ps9
Holozoa - ps10
Filozoa - ps11
Choano./Metazoa - ps12
Metazoa - ps13
Myriazoa - ps14
Parahoxozoa - ps15
Bilateria - ps16
Deuterostomia - ps17
Chordata - ps18
Olfactores - ps19
Vertebrata - ps20
Gnathostomata - ps21
Euteleostomi - ps22
Sarcopterygii - ps23
Tetrapoda - ps24
Amniota - ps25
Mammalia - ps26
Theria - ps27
Placentalia - ps28
Boreoeutheria - ps29
Euarchontoglires - ps30
Euarchonta - ps31
Primates - ps32
Haplorrhini - ps33
Simiiformes - ps34
Catarrhini - ps35
Hominoidea - ps36
Hominidae - ps37
Homininae - ps38
Hominini - ps39
Homo_sapiens - ps40
p-value
1e-02
1e-02
2e-02
3e-02
4e-02
Log-odds
0.5
1.0
1.5
2.0
2.5
3.0
3.5
4.0

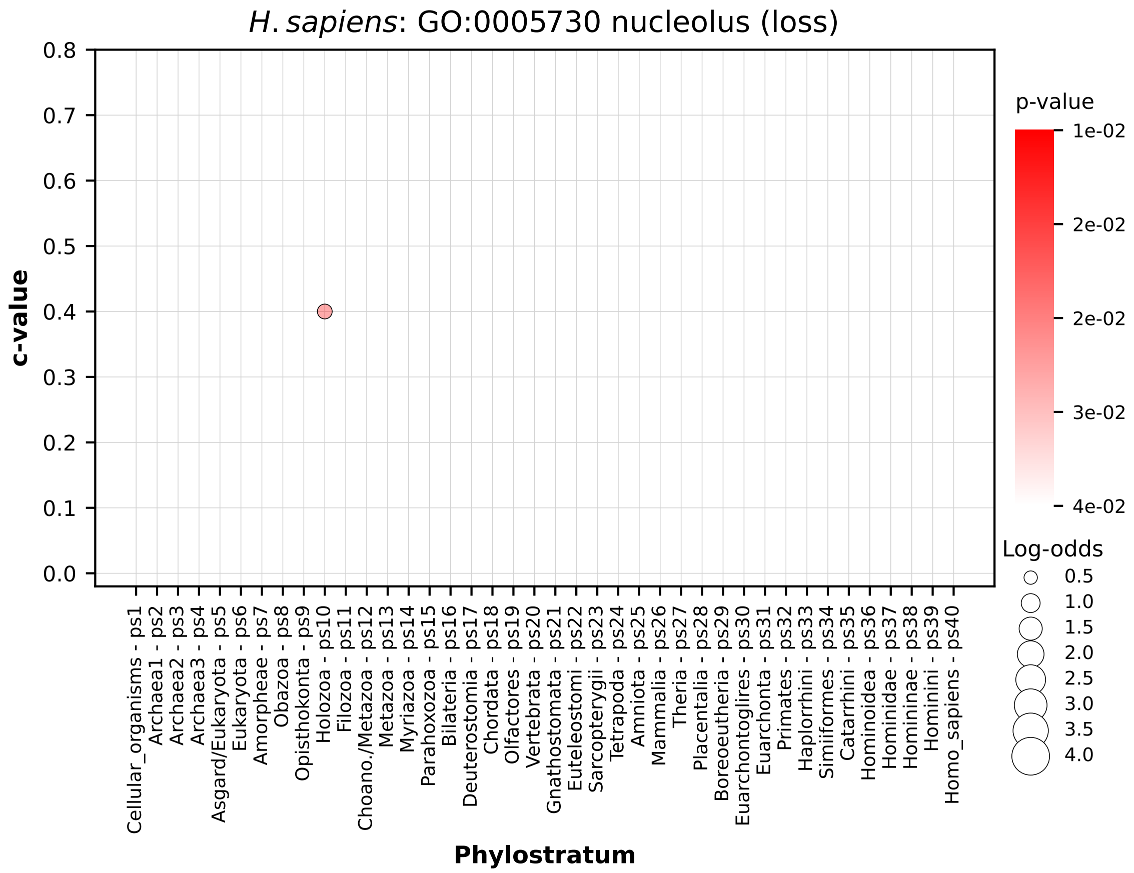

H. sapiens: GO:0005730 nucleolus (loss)
c-value
0.8
0.7
0.6
0.5
0.4
0.3
0.2
0.1
0.0
p-value
1e-02
2e-02
2e-02
3e-02
4e-02
Log-odds
0.5
1.0
1.5
2.0
2.5
3.0
3.5
4.0
Cellular_organisms - ps1
Archaea1 - ps2
Archaea2 - ps3
Archaea3 - ps4
Asgard/Eukaryota - ps5
Eukaryota - ps6
Amorpheae - ps7
Obazoa - ps8
Opisthokonta - ps9
Holozoa - ps10
Filozoa - ps11
Choano./Metazoa - ps12
Metazoa - ps13
Myriazoa - ps14
Parahoxozoa - ps15
Bilateria - ps16
Deuterostomia - ps17
Chordata - ps18
Olfactores - ps19
Vertebrata - ps20
Gnathostomata - ps21
Euteleostomi - ps22
Sarcopterygii - ps23
Tetrapoda - ps24
Amniota - ps25
Mammalia - ps26
Theria - ps27
Placentalia - ps28
Boreoeutheria - ps29
Euarchontoglires - ps30
Euarchonta - ps31
Primates - ps32
Haplorrhini - ps33
Simiiformes - ps34
Catarrhini - ps35
Hominoidea - ps36
Hominidae - ps37
Homininae - ps38
Hominini - ps39
Homo_sapiens - ps40
Phylostratum

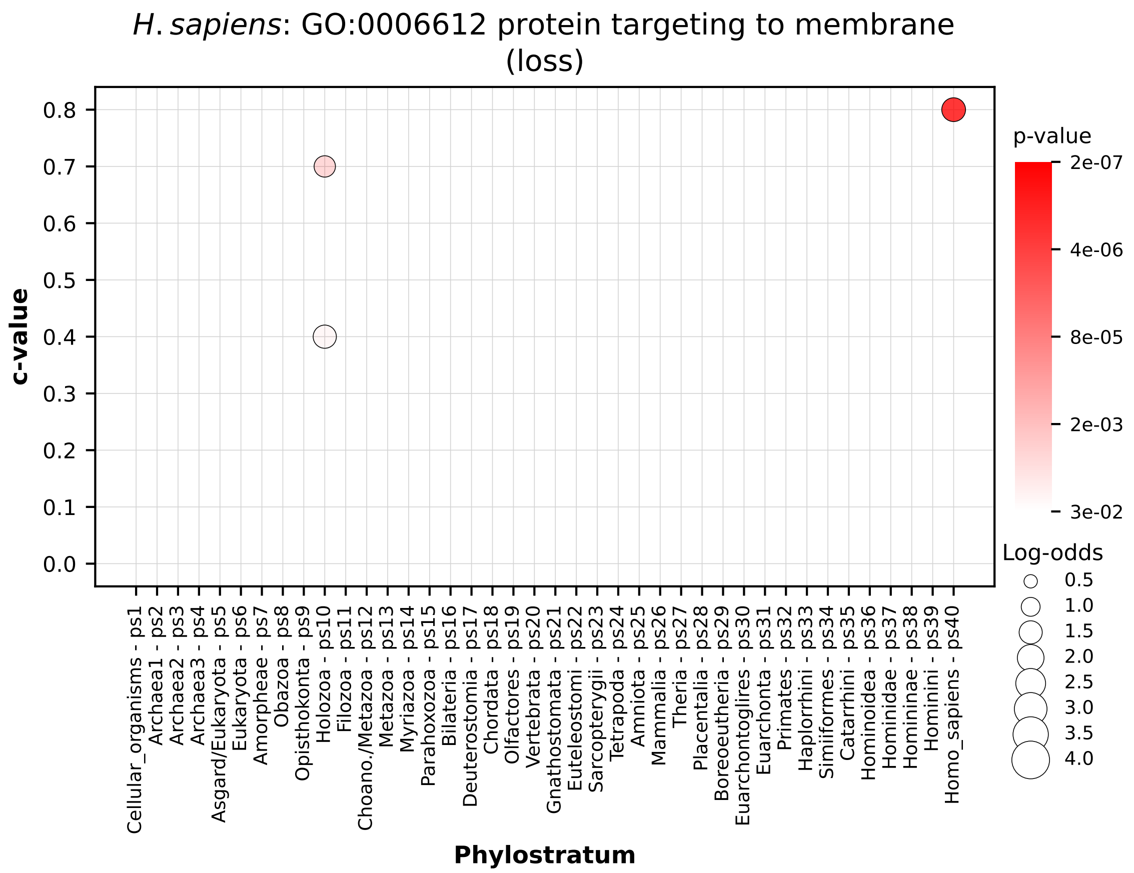

H. sapiens: GO:0006612 protein targeting to membrane (loss)
c-value
0.0
0.1
0.2
0.3
0.4
0.5
0.6
0.7
0.8
Phylostratum
Cellular_organisms - ps1
Archaea1 - ps2
Archaea2 - ps3
Archaea3 - ps4
Asgard/Eukaryota - ps5
Eukaryota - ps6
Amorpheae - ps7
Obazoa - ps8
Opisthokonta - ps9
Holozoa - ps10
Filozoa - ps11
Choano./Metazoa - ps12
Metazoa - ps13
Myriazoa - ps14
Parahoxozoa - ps15
Bilateria - ps16
Deuterostomia - ps17
Chordata - ps18
Olfactores - ps19
Vertebrata - ps20
Gnathostomata - ps21
Euteleostomi - ps22
Sarcopterygii - ps23
Tetrapoda - ps24
Amniota - ps25
Mammalia - ps26
Theria - ps27
Placentalia - ps28
Boreoeutheria - ps29
Euarchontoglires - ps30
Euarchonta - ps31
Primates - ps32
Haplorrhini - ps33
Simiiformes - ps34
Catarrhini - ps35
Hominoidea - ps36
Hominidae - ps37
Homininae - ps38
Hominini - ps39
Homo_sapiens - ps40
p-value
2e-07
4e-06
8e-05
2e-03
3e-02
Log-odds
0.5
1.0
1.5
2.0
2.5
3.0
3.5
4.0

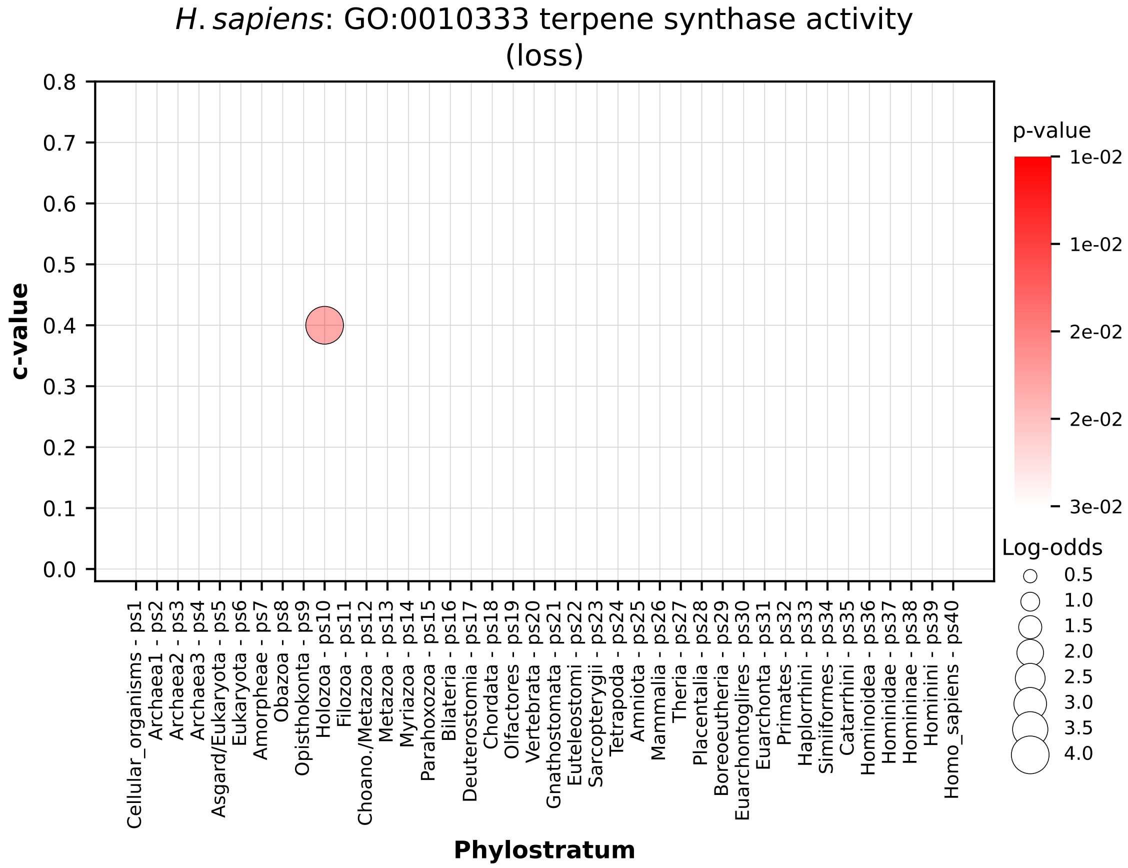

H. sapiens: GO:0010333 terpene synthase activity
(loss)
c-value
0.8
0.7
0.6
0.5
0.4
0.3
0.2
0.1
0.0
p-value
1e-02
1e-02
2e-02
2e-02
3e-02
Log-odds
0.5
1.0
1.5
2.0
2.5
3.0
3.5
4.0
Cellular_organisms - ps1
Archaea1 - ps2
Archaea2 - ps3
Archaea3 - ps4
Asgard/Eukaryota - ps5
Eukaryota - ps6
Amorpheae - ps7
Obazoa - ps8
Opisthokonta - ps9
Holozoa - ps10
Filozoa - ps11
Choano./Metazoa - ps12
Metazoa - ps13
Myriazoa - ps14
Parahoxozoa - ps15
Bilateria - ps16
Deuterostomia - ps17
Chordata - ps18
Olfactores - ps19
Vertebrata - ps20
Gnathostomata - ps21
Euteleostomi - ps22
Sarcopterygii - ps23
Tetrapoda - ps24
Amniota - ps25
Mammalia - ps26
Theria - ps27
Placentalia - ps28
Boreoeutheria - ps29
Euarchontoglires - ps30
Euarchonta - ps31
Primates - ps32
Haplorrhini - ps33
Simiiformes - ps34
Catarrhini - ps35
Hominoidea - ps36
Hominidae - ps37
Homininae - ps38
Hominini - ps39
Homo_sapiens - ps40
Phylostratum

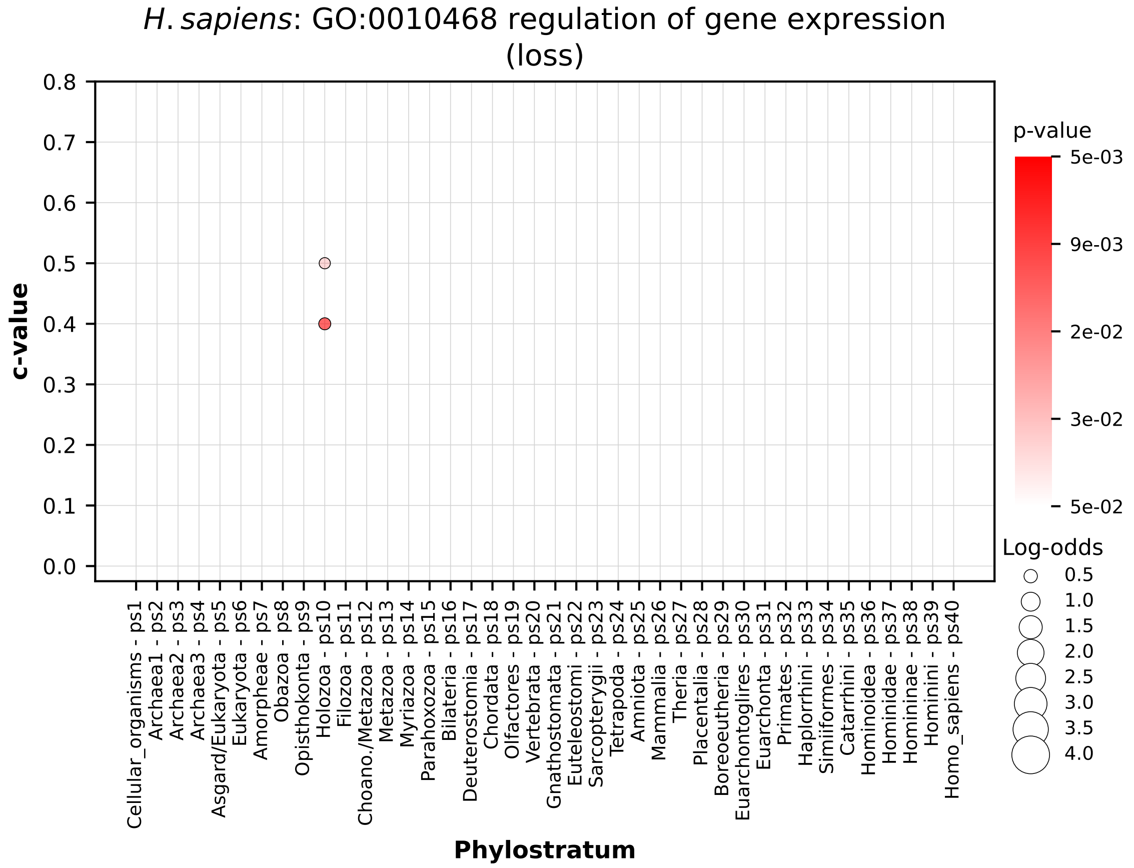

H. sapiens: GO:0010468 regulation of gene expression (loss)
c-value
0.0
0.1
0.2
0.3
0.4
0.5
0.6
0.7
0.8
Phylostratum
Cellular_organisms - ps1
Archaea1 - ps2
Archaea2 - ps3
Archaea3 - ps4
Asgard/Eukaryota - ps5
Eukaryota - ps6
Amorpheae - ps7
Obazoa - ps8
Opisthokonta - ps9
Holozoa - ps10
Filozoa - ps11
Choano./Metazoa - ps12
Metazoa - ps13
Myriazoa - ps14
Parahoxozoa - ps15
Bilateria - ps16
Deuterostomia - ps17
Chordata - ps18
Olfactores - ps19
Vertebrata - ps20
Gnathostomata - ps21
Euteleostomi - ps22
Sarcopterygii - ps23
Tetrapoda - ps24
Amniota - ps25
Mammalia - ps26
Theria - ps27
Placentalia - ps28
Boreoeutheria - ps29
Euarchontoglires - ps30
Euarchonta - ps31
Primates - ps32
Haplorrhini - ps33
Simiiformes - ps34
Catarrhini - ps35
Hominoidea - ps36
Hominidae - ps37
Homininae - ps38
Hominini - ps39
Homo_sapiens - ps40
p-value
5e-03
9e-03
2e-02
3e-02
5e-02
Log-odds
0.5
1.0
1.5
2.0
2.5
3.0
3.5
4.0

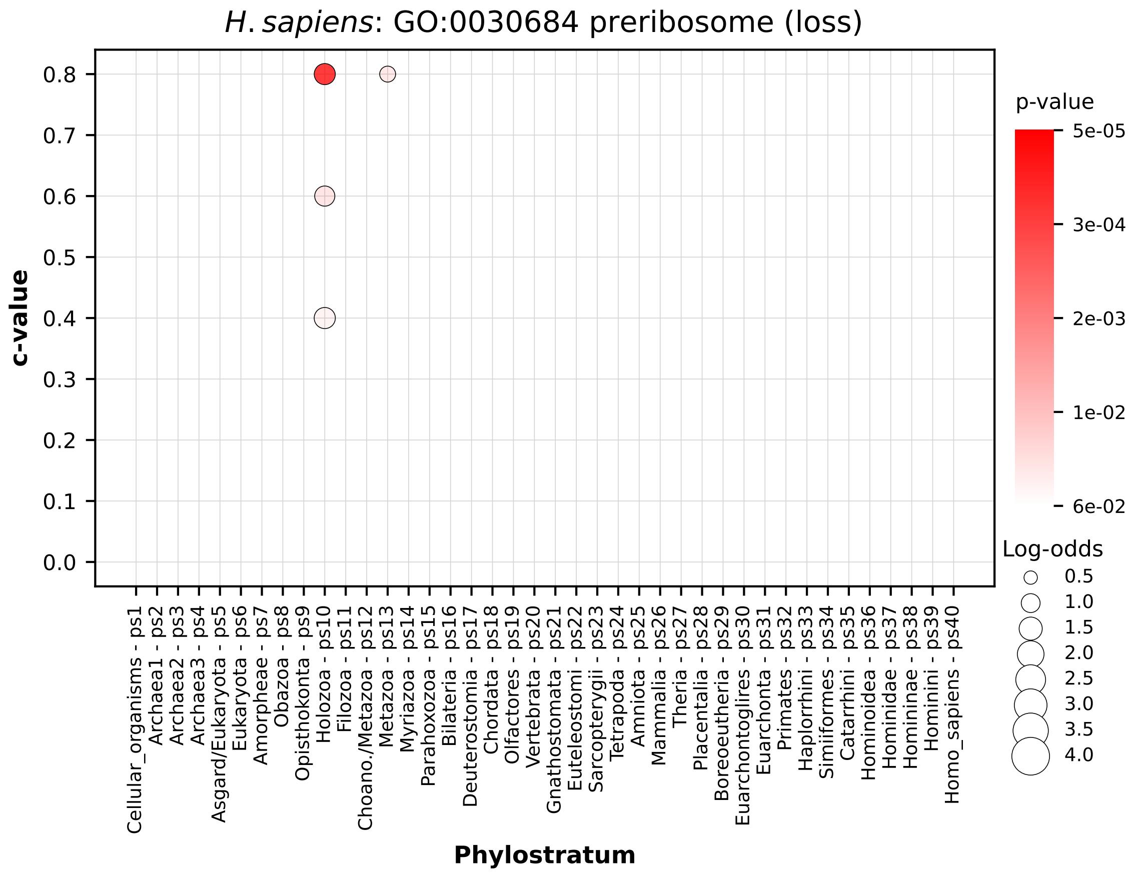

H. sapiens: GO:0030684 preribosome (loss)
c-value
0.0
0.1
0.2
0.3
0.4
0.5
0.6
0.7
0.8
Phylostratum
Cellular_organisms - ps1
Archaea1 - ps2
Archaea2 - ps3
Archaea3 - ps4
Asgard/Eukaryota - ps5
Eukaryota - ps6
Amorpheae - ps7
Obazoa - ps8
Opisthokonta - ps9
Holozoa - ps10
Filozoa - ps11
Choano./Metazoa - ps12
Metazoa - ps13
Myriazoa - ps14
Parahoxozoa - ps15
Bilateria - ps16
Deuterostomia - ps17
Chordata - ps18
Olfactores - ps19
Vertebrata - ps20
Gnathostomata - ps21
Euteleostomi - ps22
Sarcopterygii - ps23
Tetrapoda - ps24
Amniota - ps25
Mammalia - ps26
Theria - ps27
Placentalia - ps28
Boreoeutheria - ps29
Euarchontoglires - ps30
Euarchonta - ps31
Primates - ps32
Haplorrhini - ps33
Simiiformes - ps34
Catarrhini - ps35
Hominoidea - ps36
Hominidae - ps37
Homininae - ps38
Hominini - ps39
Homo_sapiens - ps40
p-value
5e-05
3e-04
2e-03
1e-02
6e-02
Log-odds
0.5
1.0
1.5
2.0
2.5
3.0
3.5
4.0

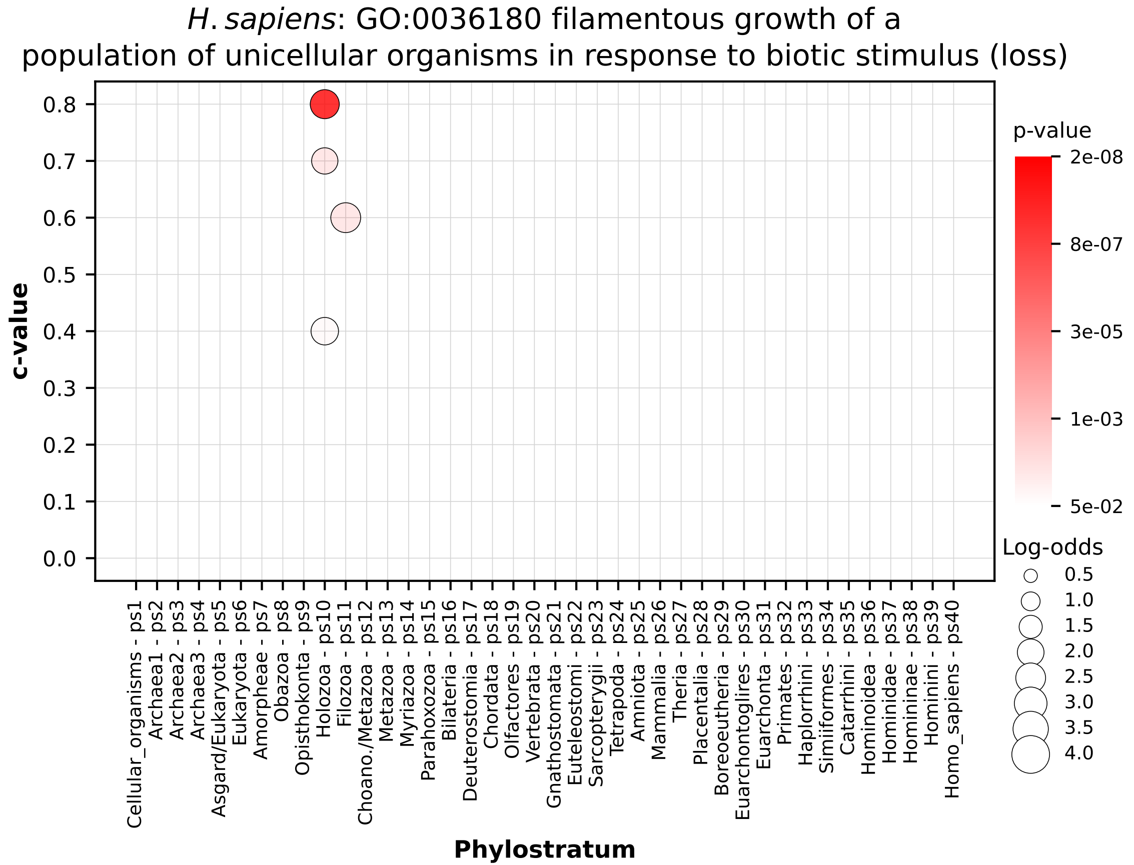

H. sapiens: GO:0036180 filamentous growth of a population of unicellular organisms in response to biotic stimulus (loss)
c-value
0.8
0.7
0.6
0.5
0.4
0.3
0.2
0.1
0.0
p-value
2e-08
8e-07
3e-05
1e-03
5e-02
Log-odds
0.5
1.0
1.5
2.0
2.5
3.0
3.5
4.0
Cellular_organisms - ps1
Archaea1 - ps2
Archaea2 - ps3
Archaea3 - ps4
Asgard/Eukaryota - ps5
Eukaryota - ps6
Amorpheae - ps7
Obazoa - ps8
Opisthokonta - ps9
Holozoa - ps10
Filozoa - ps11
Choano./Metazoa - ps12
Metazoa - ps13
Myriazoa - ps14
Parahoxozoa - ps15
Bilateria - ps16
Deuterostomia - ps17
Chordata - ps18
Olfactores - ps19
Vertebrata - ps20
Gnathostomata - ps21
Euteleostomi - ps22
Sarcopterygii - ps23
Tetrapoda - ps24
Amniota - ps25
Mammalia - ps26
Theria - ps27
Placentalia - ps28
Boreoeutheria - ps29
Euarchontoglires - ps30
Euarchonta - ps31
Primates - ps32
Haplorrhini - ps33
Simiiformes - ps34
Catarrhini - ps35
Hominoidea - ps36
Hominidae - ps37
Homininae - ps38
Hominini - ps39
Homo_sapiens - ps40
Phylostratum

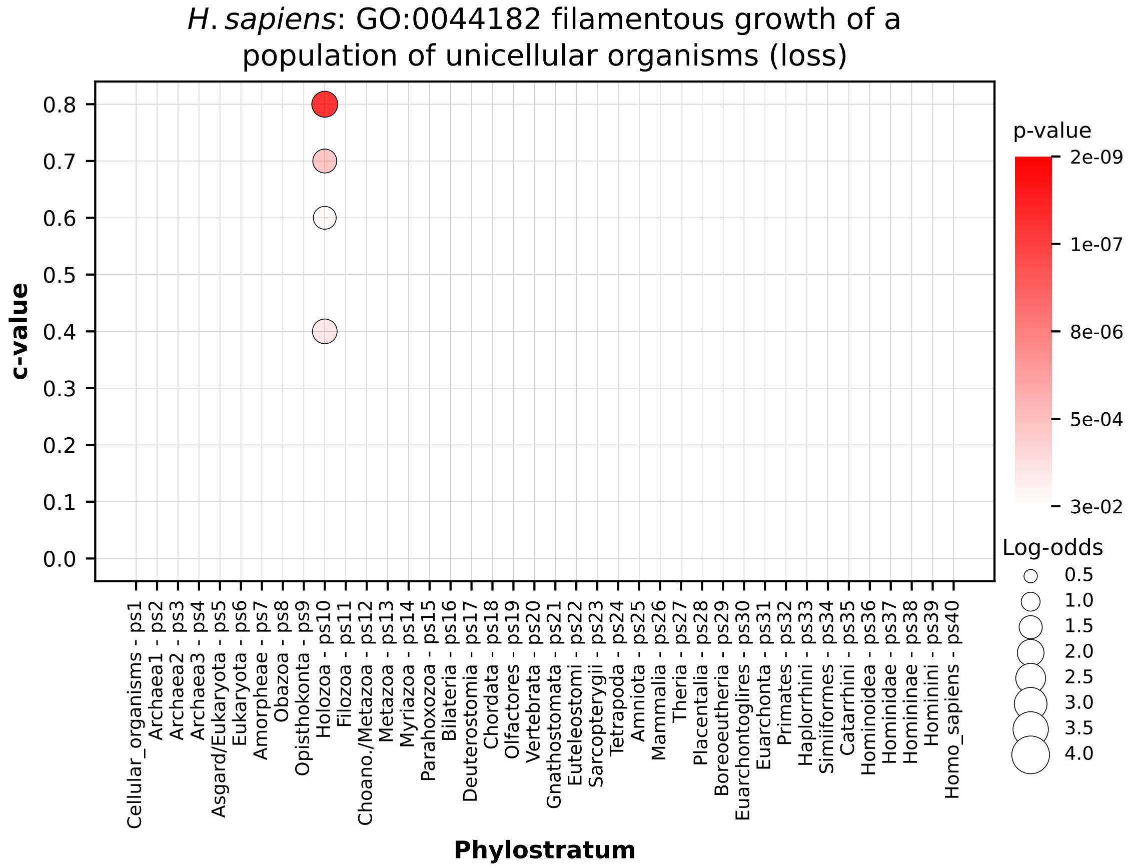

H. sapiens: GO:0044182 filamentous growth of a population of unicellular organisms (loss)
c-value
0.8
0.7
0.6
0.5
0.4
0.3
0.2
0.1
0.0
p-value
2e-09
1e-07
8e-06
5e-04
3e-02
Log-odds
0.5
1.0
1.5
2.0
2.5
3.0
3.5
4.0
Cellular_organisms - ps1
Archaea1 - ps2
Archaea2 - ps3
Archaea3 - ps4
Asgard/Eukaryota - ps5
Eukaryota - ps6
Amorpheae - ps7
Obazoa - ps8
Opisthokonta - ps9
Holozoa - ps10
Filozoa - ps11
Choano./Metazoa - ps12
Metazoa - ps13
Myriazoa - ps14
Parahoxozoa - ps15
Bilateria - ps16
Deuterostomia - ps17
Chordata - ps18
Olfactores - ps19
Vertebrata - ps20
Gnathostomata - ps21
Euteleostomi - ps22
Sarcopterygii - ps23
Tetrapoda - ps24
Amniota - ps25
Mammalia - ps26
Theria - ps27
Placentalia - ps28
Boreoeutheria - ps29
Euarchontoglires - ps30
Euarchonta - ps31
Primates - ps32
Haplorrhini - ps33
Simiiformes - ps34
Catarrhini - ps35
Hominoidea - ps36
Hominidae - ps37
Homininae - ps38
Hominini - ps39
Homo_sapiens - ps40
Phylostratum

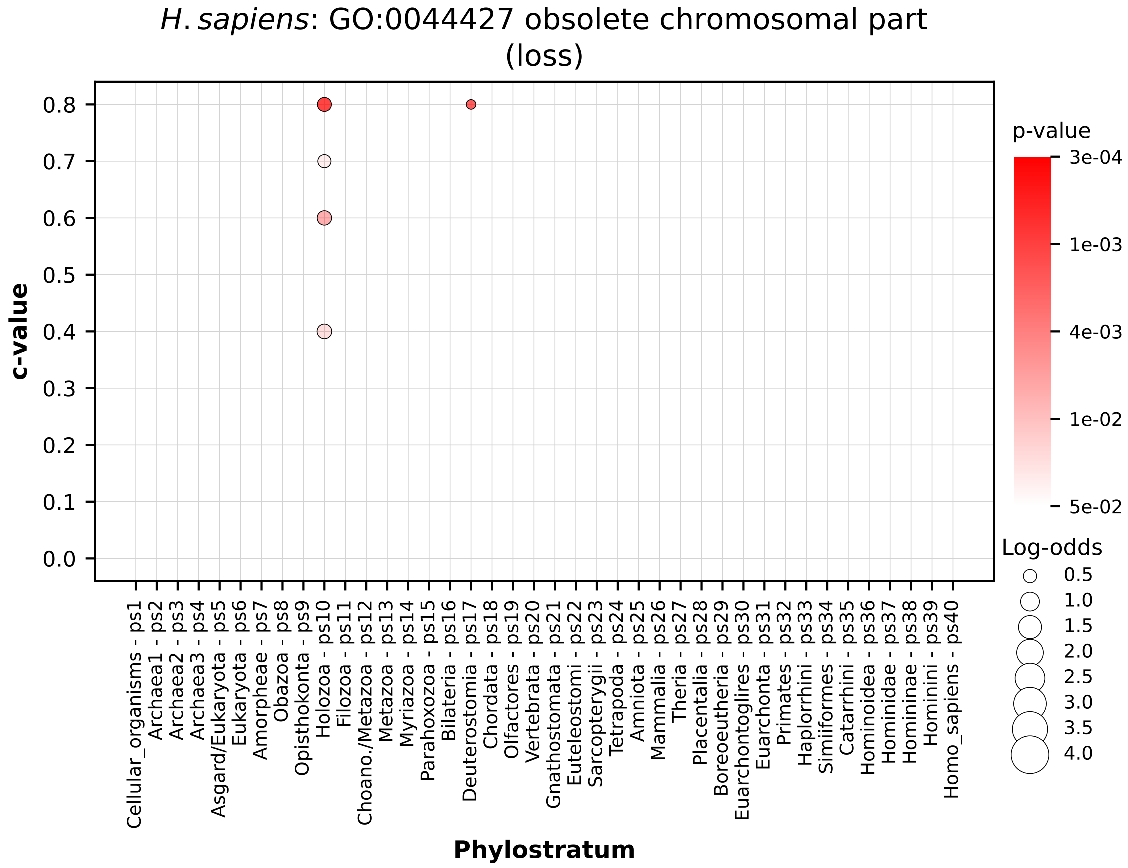

H. sapiens: GO:0044427 obsolete chromosomal part
(loss)
c-value
0.8
0.7
0.6
0.5
0.4
0.3
0.2
0.1
0.0
p-value
3e-04
1e-03
4e-03
1e-02
5e-02
Log-odds
0.5
1.0
1.5
2.0
2.5
3.0
3.5
4.0
Cellular_organisms - ps1
Archaea1 - ps2
Archaea2 - ps3
Archaea3 - ps4
Asgard/Eukaryota - ps5
Eukaryota - ps6
Amorpheae - ps7
Obazoa - ps8
Opisthokonta - ps9
Holozoa - ps10
Filozoa - ps11
Choano./Metazoa - ps12
Metazoa - ps13
Myriazoa - ps14
Parahoxozoa - ps15
Bilateria - ps16
Deuterostomia - ps17
Chordata - ps18
Olfactores - ps19
Vertebrata - ps20
Gnathostomata - ps21
Euteleostomi - ps22
Sarcopterygii - ps23
Tetrapoda - ps24
Amniota - ps25
Mammalia - ps26
Theria - ps27
Placentalia - ps28
Boreoeutheria - ps29
Euarchontoglires - ps30
Euarchonta - ps31
Primates - ps32
Haplorrhini - ps33
Simiiformes - ps34
Catarrhini - ps35
Hominoidea - ps36
Hominidae - ps37
Homininae - ps38
Hominini - ps39
Homo_sapiens - ps40
Phylostratum

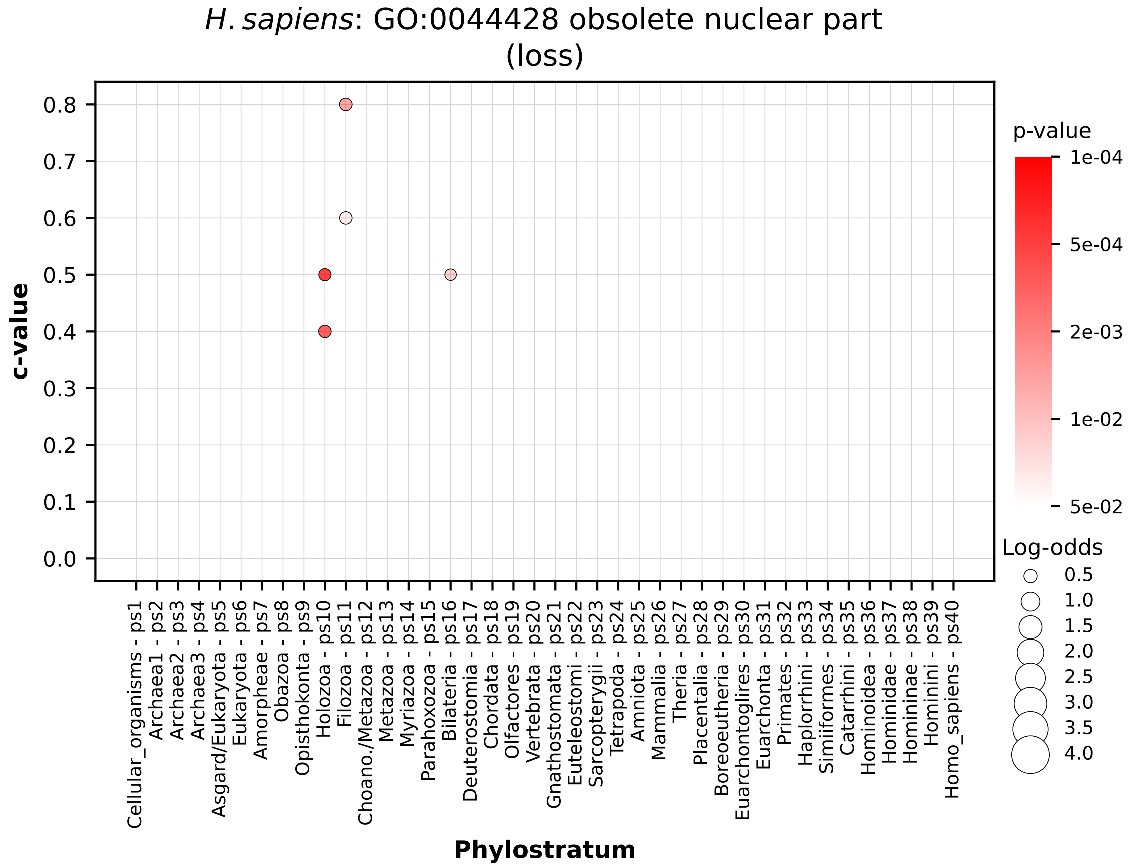

H. sapiens: GO:0044428 obsolete nuclear part
(loss)
c-value
0.8
0.7
0.6
0.5
0.4
0.3
0.2
0.1
0.0
p-value
1e-04
5e-04
2e-03
1e-02
5e-02
Log-odds
0.5
1.0
1.5
2.0
2.5
3.0
3.5
4.0
Cellular_organisms - ps1
Archaea1 - ps2
Archaea2 - ps3
Archaea3 - ps4
Asgard/Eukaryota - ps5
Eukaryota - ps6
Amorpheae - ps7
Obazoa - ps8
Opisthokonta - ps9
Holozoa - ps10
Filozoa - ps11
Choano./Metazoa - ps12
Metazoa - ps13
Myriazoa - ps14
Parahoxozoa - ps15
Bilateria - ps16
Deuterostomia - ps17
Chordata - ps18
Olfactores - ps19
Vertebrata - ps20
Gnathostomata - ps21
Euteleostomi - ps22
Sarcopterygii - ps23
Tetrapoda - ps24
Amniota - ps25
Mammalia - ps26
Theria - ps27
Placentalia - ps28
Boreoeutheria - ps29
Euarchontoglires - ps30
Euarchonta - ps31
Primates - ps32
Haplorrhini - ps33
Simiiformes - ps34
Catarrhini - ps35
Hominoidea - ps36
Hominidae - ps37
Homininae - ps38
Hominini - ps39
Homo_sapiens - ps40
Phylostratum

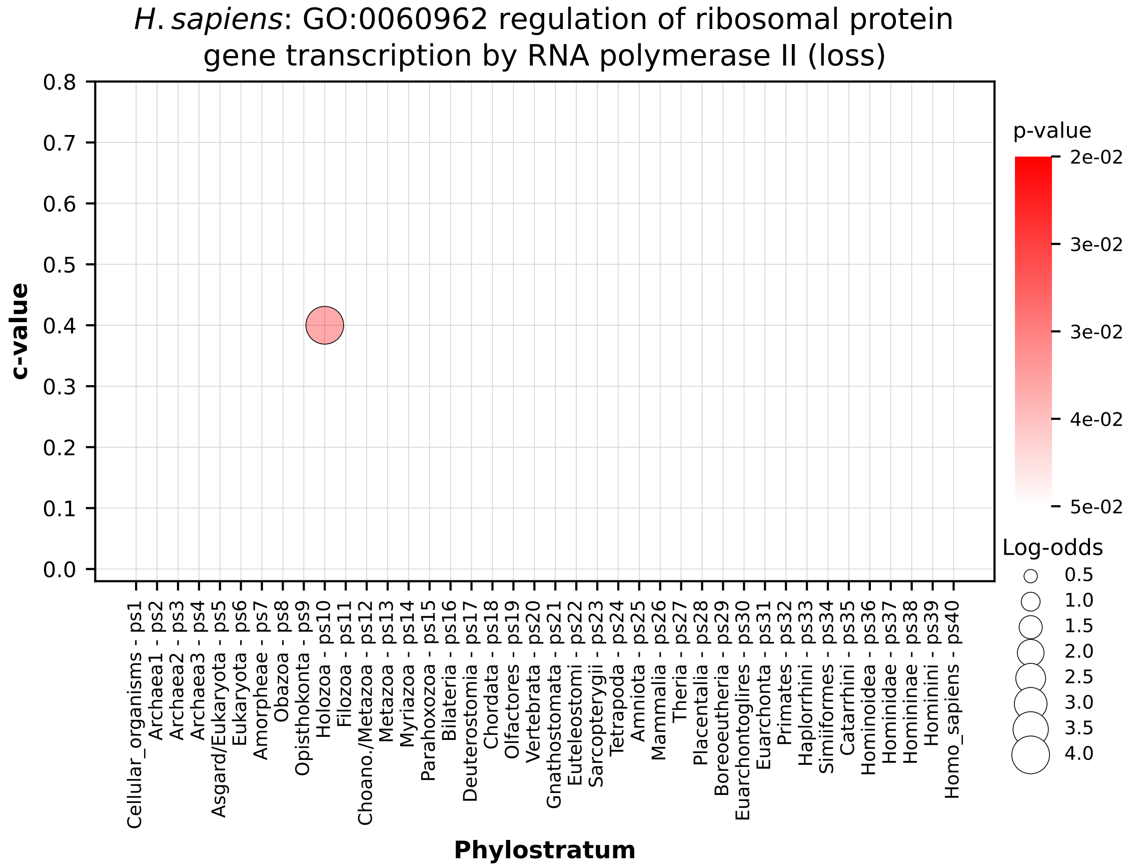
H. sapiens: GO:0060962 regulation of ribosomal protein gene transcription by RNA polymerase II (loss)
c-value
0.0
0.1
0.2
0.3
0.4
0.5
0.6
0.7
0.8
Phylostratum
Cellular_organisms - ps1
Archaea1 - ps2
Archaea2 - ps3
Archaea3 - ps4
Asgard/Eukaryota - ps5
Eukaryota - ps6
Amorpheae - ps7
Obazoa - ps8
Opisthokonta - ps9
Holozoa - ps10
Filozoa - ps11
Choano./Metazoa - ps12
Metazoa - ps13
Myriazoa - ps14
Parahoxozoa - ps15
Bilateria - ps16
Deuterostomia - ps17
Chordata - ps18
Olfactores - ps19
Vertebrata - ps20
Gnathostomata - ps21
Euteleostomi - ps22
Sarcopterygii - ps23
Tetrapoda - ps24
Amniota - ps25
Mammalia - ps26
Theria - ps27
Placentalia - ps28
Boreoeutheria - ps29
Euarchontoglires - ps30
Euarchonta - ps31
Primates - ps32
Haplorrhini - ps33
Simiiformes - ps34
Catarrhini - ps35
Hominoidea - ps36
Hominidae - ps37
Homininae - ps38
Hominini - ps39
Homo_sapiens - ps40
p-value
2e-02
3e-02
3e-02
4e-02
5e-02
Log-odds
0.5
1.0
1.5
2.0
2.5
3.0
3.5
4.0

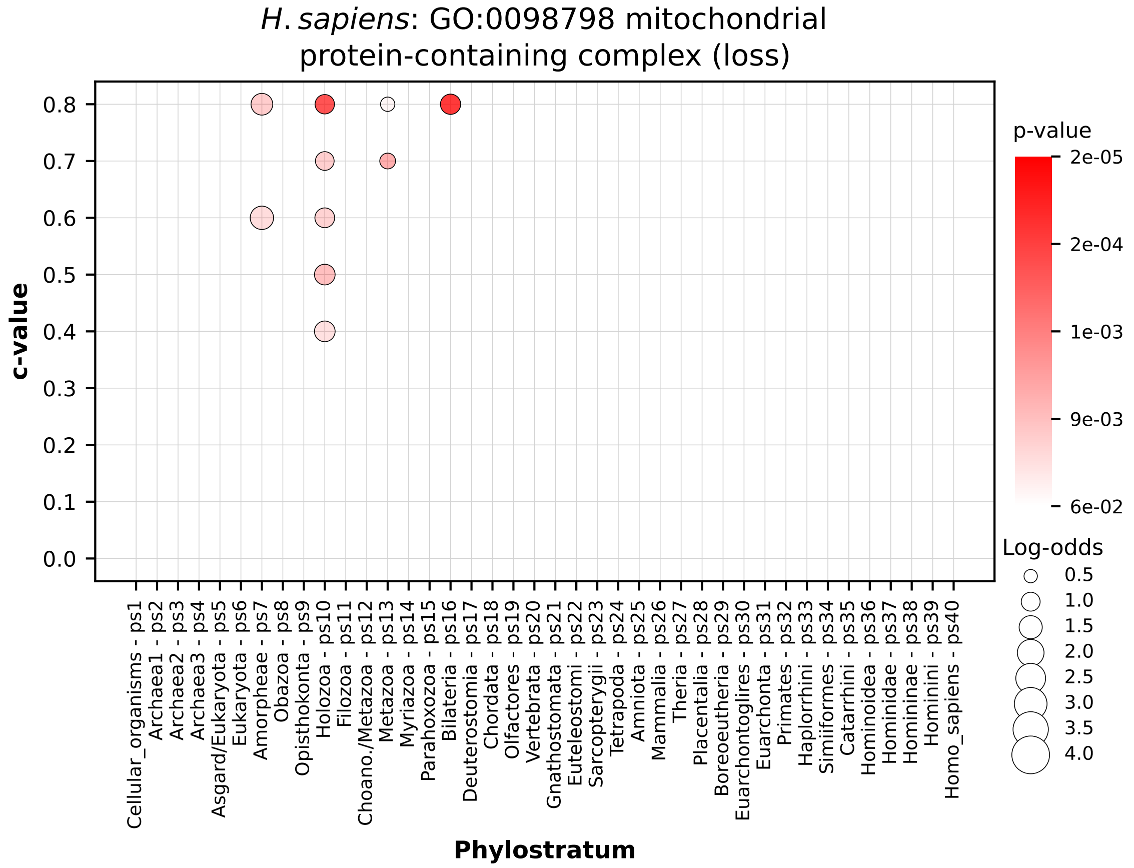

H. sapiens: GO:0098798 mitochondrial protein-containing complex (loss)
c-value
0.8
0.7
0.6
0.5
0.4
0.3
0.2
0.1
0.0
Phylostratum
Cellular_organisms - ps1
Archaea1 - ps2
Archaea2 - ps3
Archaea3 - ps4
Asgard/Eukaryota - ps5
Eukaryota - ps6
Amorpheae - ps7
Obazoa - ps8
Opisthokonta - ps9
Holozoa - ps10
Filozoa - ps11
Choano./Metazoa - ps12
Metazoa - ps13
Myriazoa - ps14
Parahoxozoa - ps15
Bilateria - ps16
Deuterostomia - ps17
Chordata - ps18
Olfactores - ps19
Vertebrata - ps20
Gnathostomata - ps21
Euteleostomi - ps22
Sarcopterygii - ps23
Tetrapoda - ps24
Amniota - ps25
Mammalia - ps26
Theria - ps27
Placentalia - ps28
Boreoeutheria - ps29
Euarchontoglires - ps30
Euarchonta - ps31
Primates - ps32
Haplorrhini - ps33
Simiiformes - ps34
Catarrhini - ps35
Hominoidea - ps36
Hominidae - ps37
Homininae - ps38
Hominini - ps39
Homo_sapiens - ps40
p-value
2e-05
2e-04
1e-03
9e-03
6e-02
Log-odds
0.5
1.0
1.5
2.0
2.5
3.0
3.5
4.0

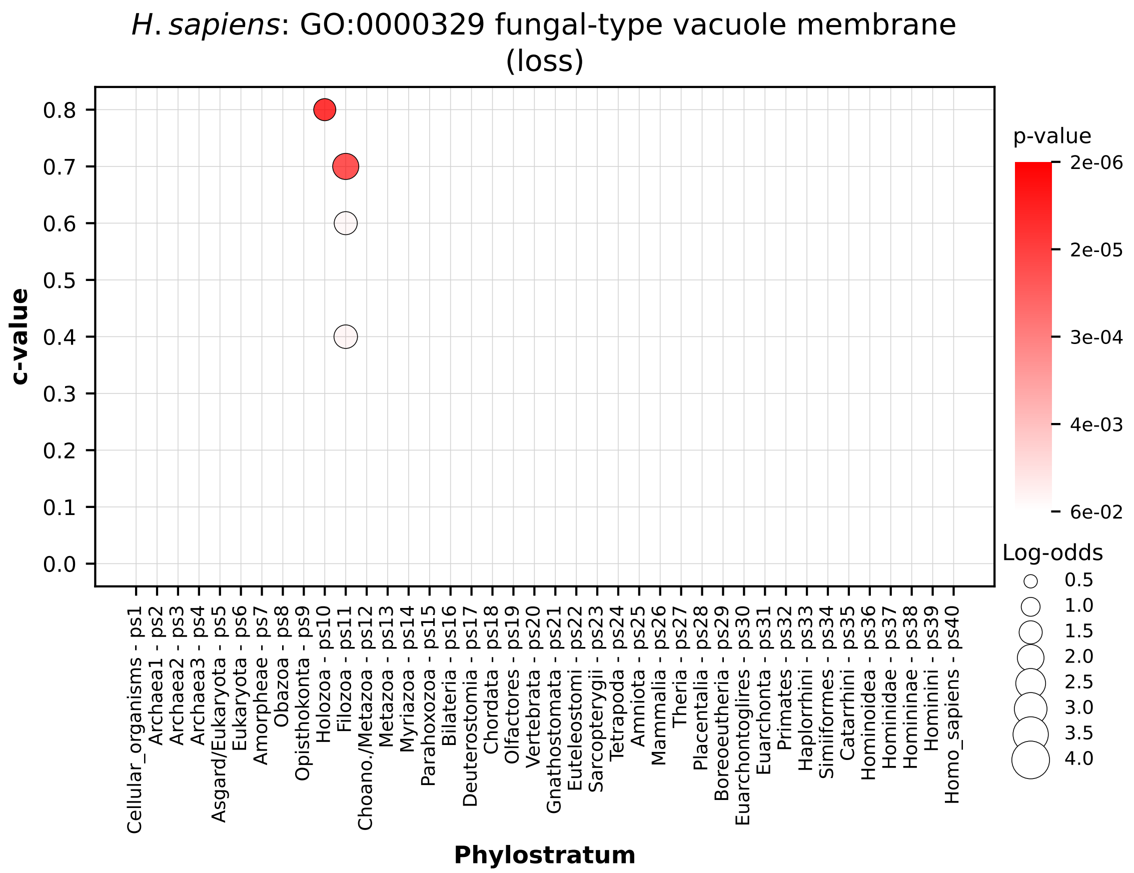

H. sapiens: GO:0000329 fungal-type vacuole membrane (loss)
c-value
0.0
0.1
0.2
0.3
0.4
0.5
0.6
0.7
0.8
Phylostratum
Cellular_organisms - ps1
Archaea1 - ps2
Archaea2 - ps3
Archaea3 - ps4
Asgard/Eukaryota - ps5
Eukaryota - ps6
Amorpheae - ps7
Obazoa - ps8
Opisthokonta - ps9
Holozoa - ps10
Filozoa - ps11
Choano./Metazoa - ps12
Metazoa - ps13
Myriazoa - ps14
Parahoxozoa - ps15
Bilateria - ps16
Deuterostomia - ps17
Chordata - ps18
Olfactores - ps19
Vertebrata - ps20
Gnathostomata - ps21
Euteleostomi - ps22
Sarcopterygii - ps23
Tetrapoda - ps24
Amniota - ps25
Mammalia - ps26
Theria - ps27
Placentalia - ps28
Boreoeutheria - ps29
Euarchontoglires - ps30
Euarchonta - ps31
Primates - ps32
Haplorrhini - ps33
Similformes - ps34
Catarrhini - ps35
Hominoidea - ps36
Hominidae - ps37
Homininae - ps38
Hominini - ps39
Homo_sapiens - ps40
p-value
2e-06
2e-05
3e-04
4e-03
6e-02
Log-odds
0.5
1.0
1.5
2.0
2.5
3.0
3.5
4.0

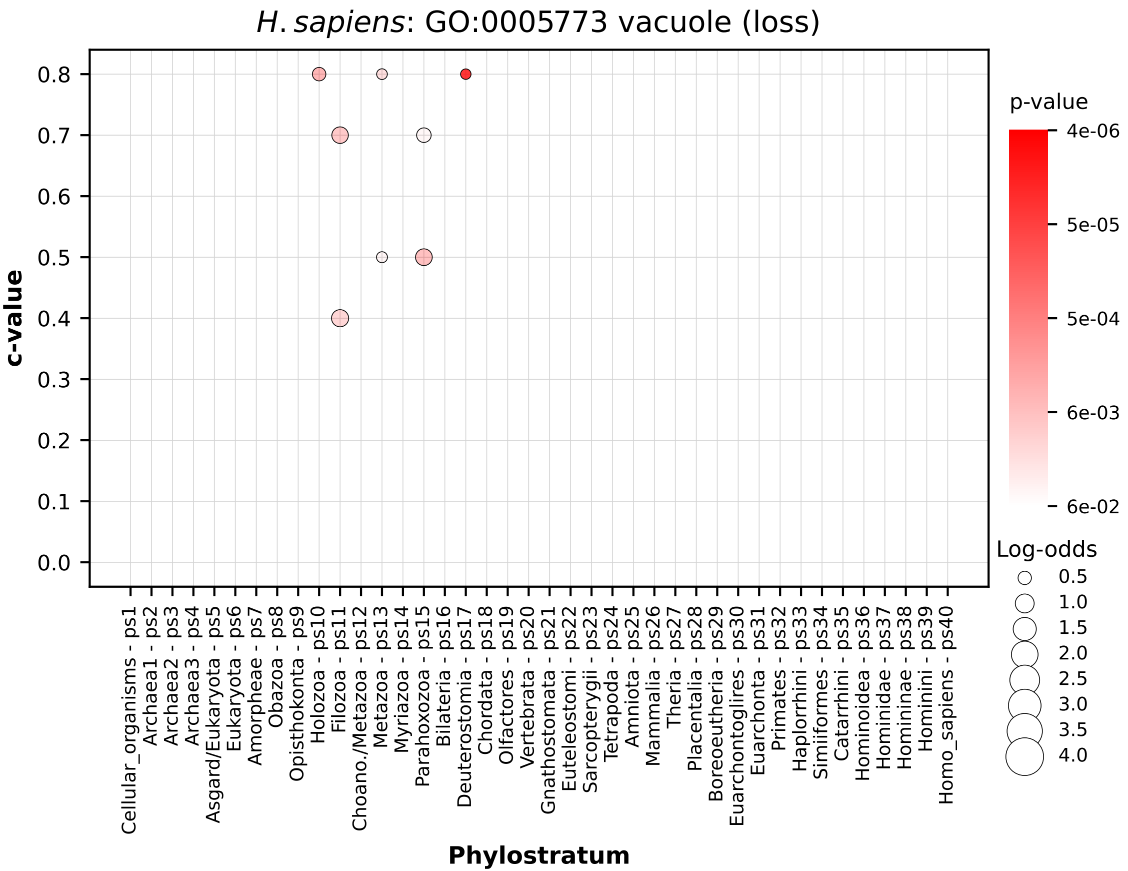

H. sapiens: GO:0005773 vacuole (loss)
c-value
0.8
0.7
0.6
0.5
0.4
0.3
0.2
0.1
0.0
Phylostratum
Cellular_organisms - ps1
Archaea1 - ps2
Archaea2 - ps3
Archaea3 - ps4
Asgard/Eukaryota - ps5
Eukaryota - ps6
Amorpheae - ps7
Obazoa - ps8
Opisthokonta - ps9
Holozoa - ps10
Filozoa - ps11
Choano./Metazoa - ps12
Metazoa - ps13
Myriazoa - ps14
Parahoxozoa - ps15
Bilateria - ps16
Deuterostomia - ps17
Chordata - ps18
Olfactores - ps19
Vertebrata - ps20
Gnathostomata - ps21
Euteleostomi - ps22
Sarcopterygii - ps23
Tetrapoda - ps24
Amniota - ps25
Mammalia - ps26
Theria - ps27
Placentalia - ps28
Boreoeutheria - ps29
Euarchontoglires - ps30
Euarchonta - ps31
Primates - ps32
Haplorrhini - ps33
Simiiformes - ps34
Catarrhini - ps35
Hominoidea - ps36
Hominidae - ps37
Homininae - ps38
Hominini - ps39
Homo_sapiens - ps40
p-value
4e-06
5e-05
5e-04
6e-03
6e-02
Log-odds
0.5
1.0
1.5
2.0
2.5
3.0
3.5
4.0

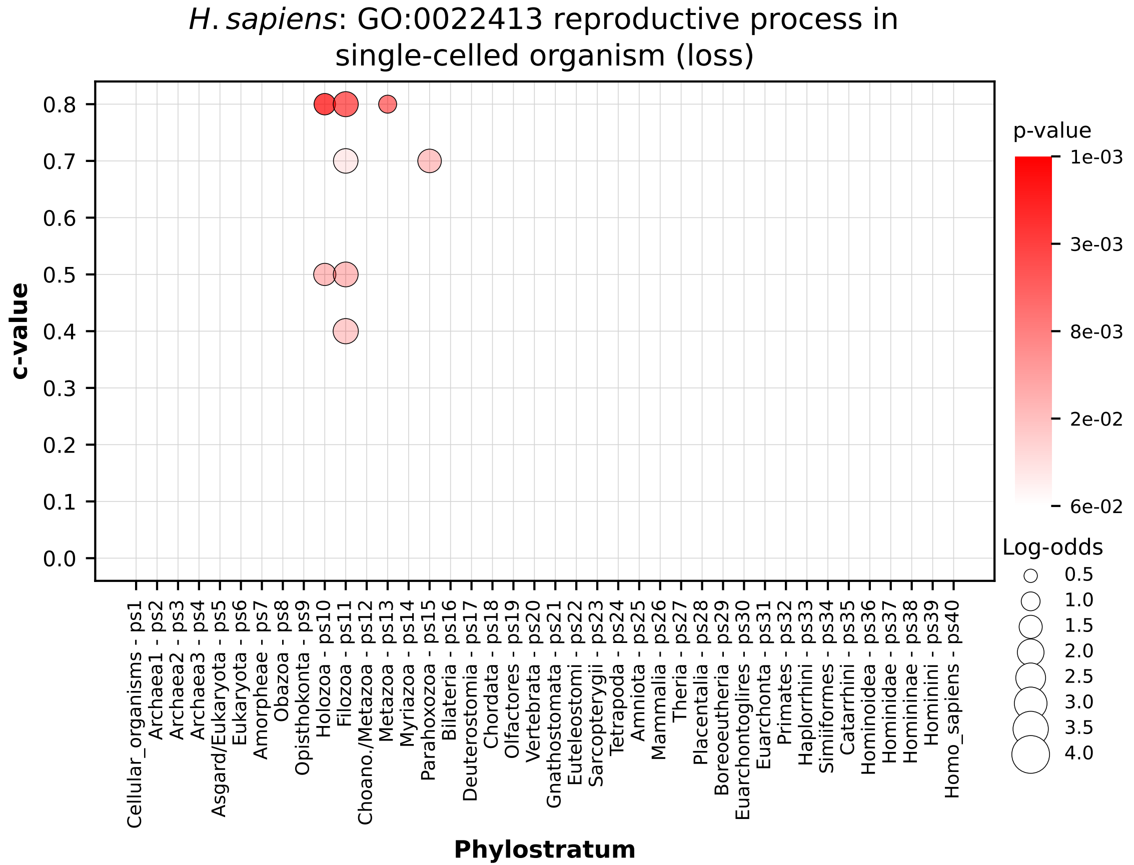
H. sapiens: GO:0022413 reproductive process in single-celled organism (loss)
c-value
0.0
0.1
0.2
0.3
0.4
0.5
0.6
0.7
0.8
Phylostratum
Cellular_organisms - ps1
Archaea1 - ps2
Archaea2 - ps3
Archaea3 - ps4
Asgard/Eukaryota - ps5
Eukaryota - ps6
Amorpheae - ps7
Obazoa - ps8
Opisthokonta - ps9
Holozoa - ps10
Filozoa - ps11
Choano./Metazoa - ps12
Metazoa - ps13
Myriazoa - ps14
Parahoxozoa - ps15
Bilateria - ps16
Deuterostomia - ps17
Chordata - ps18
Olfactores - ps19
Vertebrata - ps20
Gnathostomata - ps21
Euteleostomi - ps22
Sarcopterygii - ps23
Tetrapoda - ps24
Amniota - ps25
Mammalia - ps26
Theria - ps27
Placentalia - ps28
Boreoeutheria - ps29
Euarchontoglires - ps30
Euarchonta - ps31
Primates - ps32
Haplorrhini - ps33
Simiiformes - ps34
Catarrhini - ps35
Hominoidea - ps36
Hominidae - ps37
Homininae - ps38
Hominini - ps39
Homo_sapiens - ps40
p-value
1e-03
3e-03
8e-03
2e-02
6e-02
Log-odds
0.5
1.0
1.5
2.0
2.5
3.0
3.5
4.0

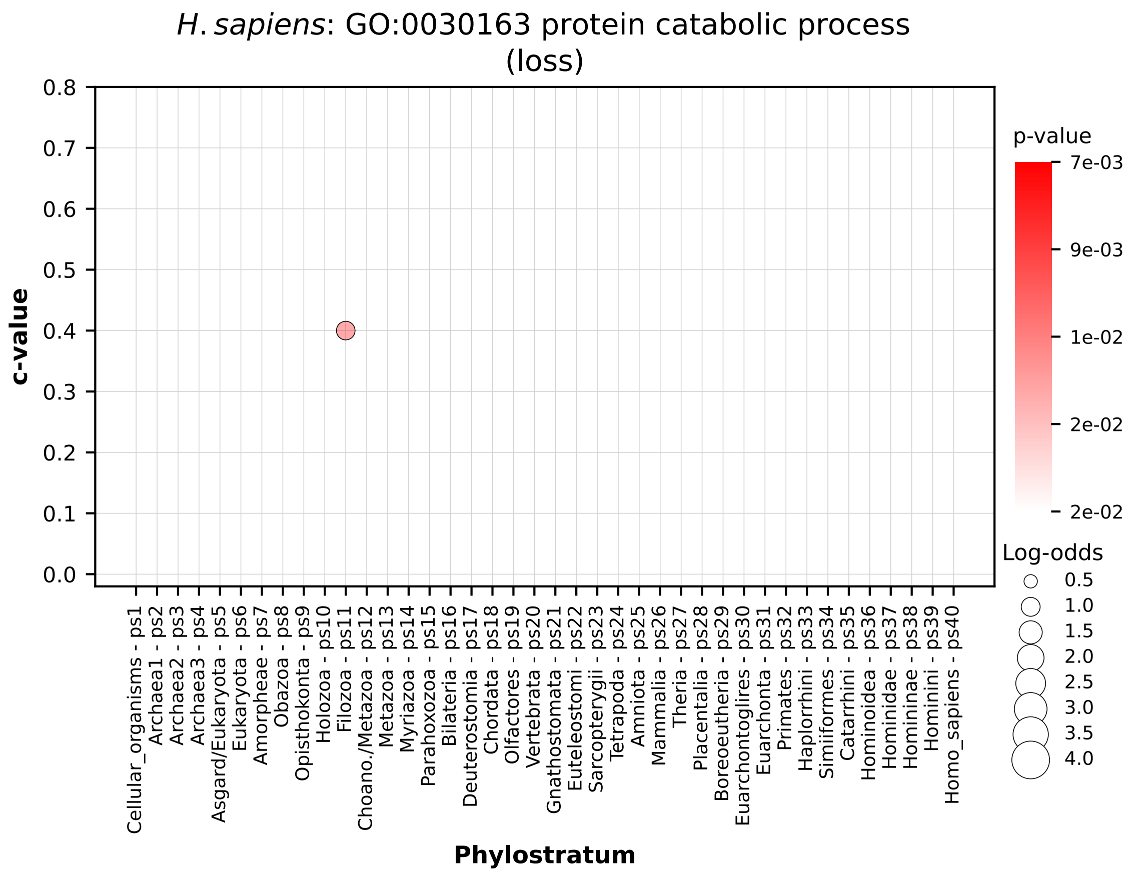
H. sapiens: GO:0030163 protein catabolic process
(loss)
c-value
0.8
0.7
0.6
0.5
0.4
0.3
0.2
0.1
0.0
p-value
7e-03
9e-03
1e-02
2e-02
2e-02
Log-odds
0.5
1.0
1.5
2.0
2.5
3.0
3.5
4.0
Cellular_organisms - ps1
Archaea1 - ps2
Archaea2 - ps3
Archaea3 - ps4
Asgard/Eukaryota - ps5
Eukaryota - ps6
Amorpheae - ps7
Obazoa - ps8
Opisthokonta - ps9
Holozoa - ps10
Filozoa - ps11
Choano./Metazoa - ps12
Metazoa - ps13
Myriazoa - ps14
Parahoxozoa - ps15
Bilateria - ps16
Deuterostomia - ps17
Chordata - ps18
Olfactores - ps19
Vertebrata - ps20
Gnathostomata - ps21
Euteleostomi - ps22
Sarcopterygii - ps23
Tetrapoda - ps24
Amniota - ps25
Mammalia - ps26
Theria - ps27
Placentalia - ps28
Boreoeutheria - ps29
Euarchontoglires - ps30
Euarchonta - ps31
Primates - ps32
Haplorrhini - ps33
Simiiformes - ps34
Catarrhini - ps35
Hominoidea - ps36
Hominidae - ps37
Homininae - ps38
Hominini - ps39
Homo_sapiens - ps40
Phylostratum

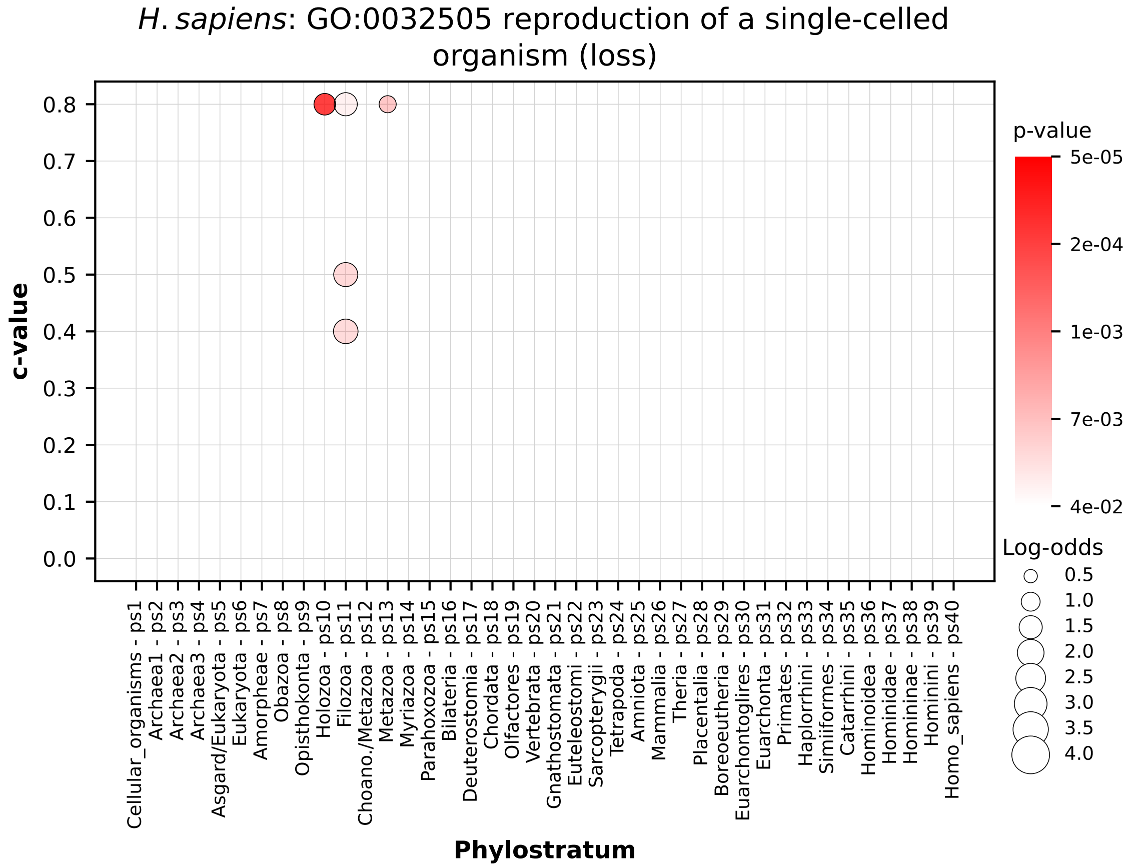

H. sapiens: GO:0032505 reproduction of a single-celled organism (loss)
c-value
0.0
0.1
0.2
0.3
0.4
0.5
0.6
0.7
0.8
Phylostratum
Cellular_organisms - ps1
Archaea1 - ps2
Archaea2 - ps3
Archaea3 - ps4
Asgard/Eukaryota - ps5
Eukaryota - ps6
Amorpheae - ps7
Obazoa - ps8
Opisthokonta - ps9
Holozoa - ps10
Filozoa - ps11
Choano./Metazoa - ps12
Metazoa - ps13
Myriazoa - ps14
Parahoxozoa - ps15
Bilateria - ps16
Deuterostomia - ps17
Chordata - ps18
Olfactores - ps19
Vertebrata - ps20
Gnathostomata - ps21
Euteleostomi - ps22
Sarcopterygii - ps23
Tetrapoda - ps24
Amniota - ps25
Mammalia - ps26
Theria - ps27
Placentalia - ps28
Boreoeutheria - ps29
Euarchontoglires - ps30
Euarchonta - ps31
Primates - ps32
Haplorrhini - ps33
Simiiformes - ps34
Catarrhini - ps35
Hominoidea - ps36
Hominidae - ps37
Homininae - ps38
Hominini - ps39
Homo_sapiens - ps40
p-value
5e-05
2e-04
1e-03
7e-03
4e-02
Log-odds
0.5
1.0
1.5
2.0
2.5
3.0
3.5
4.0

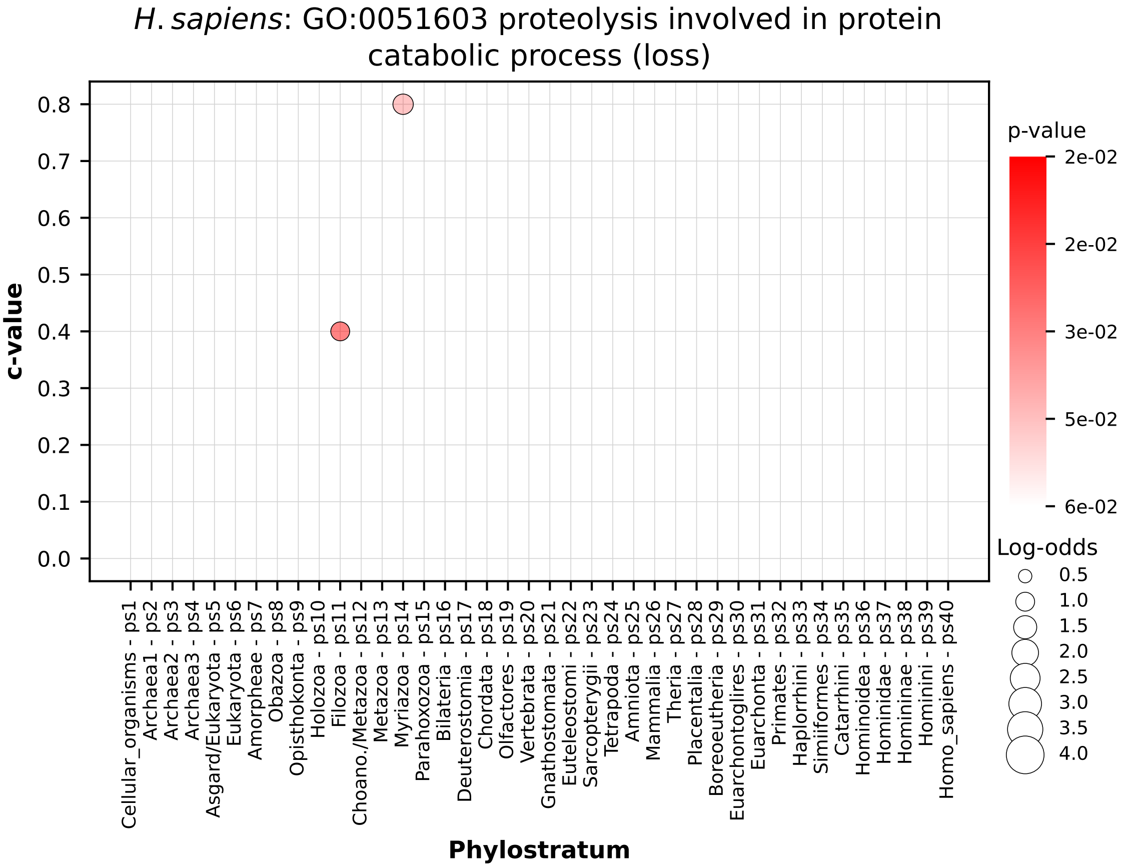

H. sapiens: GO:0051603 proteolysis involved in protein catabolic process (loss)
c-value
0.0
0.1
0.2
0.3
0.4
0.5
0.6
0.7
0.8
p-value
2e-02
2e-02
3e-02
5e-02
6e-02
Log-odds
0.5
1.0
1.5
2.0
2.5
3.0
3.5
4.0
Cellular_organisms - ps1
Archaea1 - ps2
Archaea2 - ps3
Archaea3 - ps4
Asgard/Eukaryota - ps5
Eukaryota - ps6
Amorpheae - ps7
Obazoa - ps8
Opisthokonta - ps9
Holozoa - ps10
Filozoa - ps11
Choano./Metazoa - ps12
Metazoa - ps13
Myriazoa - ps14
Parahoxozoa - ps15
Bilateria - ps16
Deuterostomia - ps17
Chordata - ps18
Olfactores - ps19
Vertebrata - ps20
Gnathostomata - ps21
Euteleostomi - ps22
Sarcopterygii - ps23
Tetrapoda - ps24
Amniota - ps25
Mammalia - ps26
Theria - ps27
Placentalia - ps28
Boreoeutheria - ps29
Euarchontoglires - ps30
Euarchonta - ps31
Primates - ps32
Haplorrhini - ps33
Simiiformes - ps34
Catarrhini - ps35
Hominoidea - ps36
Hominidae - ps37
Homininae - ps38
Hominini - ps39
Homo_sapiens - ps40
Phylostratum

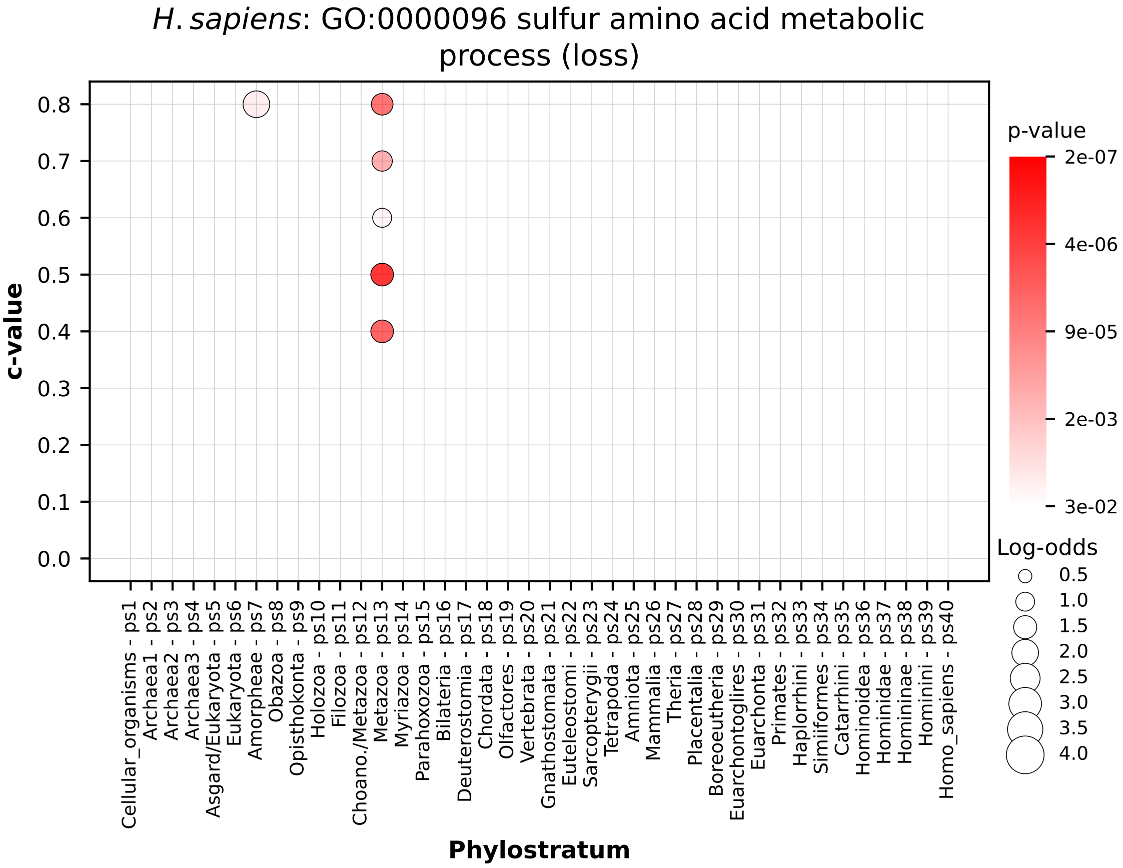

H. sapiens: GO:0000096 sulfur amino acid metabolic process (loss)
c-value
0.8
0.7
0.6
0.5
0.4
0.3
0.2
0.1
0.0
p-value
2e-07
4e-06
9e-05
2e-03
3e-02
Log-odds
0.5
1.0
1.5
2.0
2.5
3.0
3.5
4.0
Cellular_organisms - ps1
Archaea1 - ps2
Archaea2 - ps3
Archaea3 - ps4
Asgard/Eukaryota - ps5
Eukaryota - ps6
Amorpheae - ps7
Obazoa - ps8
Opisthokonta - ps9
Holozoa - ps10
Filozoa - ps11
Choano./Metazoa - ps12
Metazoa - ps13
Myriazoa - ps14
Parahoxozoa - ps15
Bilateria - ps16
Deuterostomia - ps17
Chordata - ps18
Olfactores - ps19
Vertebrata - ps20
Gnathostomata - ps21
Euteleostomi - ps22
Sarcopterygii - ps23
Tetrapoda - ps24
Amniota - ps25
Mammalia - ps26
Theria - ps27
Placentalia - ps28
Boreoeutheria - ps29
Euarchontoglires - ps30
Euarchonta - ps31
Primates - ps32
Haplorrhini - ps33
Simiiformes - ps34
Catarrhini - ps35
Hominoidea - ps36
Hominidae - ps37
Homininae - ps38
Hominini - ps39
Homo_sapiens - ps40
Phylostratum

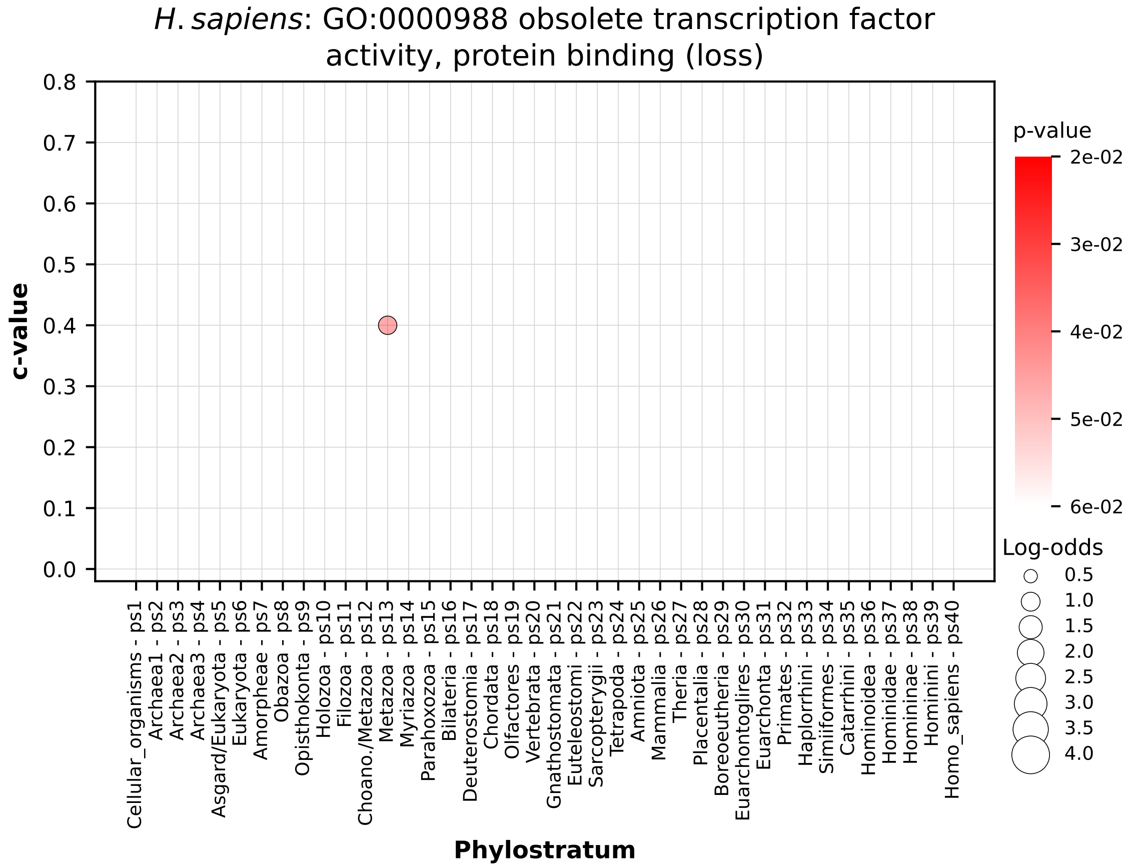
H. sapiens: GO:0000988 obsolete transcription factor activity, protein binding (loss)
c-value
0.8
0.7
0.6
0.5
0.4
0.3
0.2
0.1
0.0
Phylostratum
Cellular_organisms - ps1
Archaea1 - ps2
Archaea2 - ps3
Archaea3 - ps4
Asgard/Eukaryota - ps5
Eukaryota - ps6
Amorpheae - ps7
Obazoa - ps8
Opisthokonta - ps9
Holozoa - ps10
Filozoa - ps11
Choano./Metazoa - ps12
Metazoa - ps13
Myriazoa - ps14
Parahoxozoa - ps15
Bilateria - ps16
Deuterostomia - ps17
Chordata - ps18
Olfactores - ps19
Vertebrata - ps20
Gnathostomata - ps21
Euteleostomi - ps22
Sarcopterygii - ps23
Tetrapoda - ps24
Amniota - ps25
Mammalia - ps26
Theria - ps27
Placentalia - ps28
Boreoeutheria - ps29
Euarchontoglires - ps30
Euarchonta - ps31
Primates - ps32
Haplorrhini - ps33
Simiiformes - ps34
Catarrhini - ps35
Hominoidea - ps36
Hominidae - ps37
Homininae - ps38
Hominini - ps39
Homo_sapiens - ps40
p-value
2e-02
3e-02
4e-02
5e-02
6e-02
Log-odds
0.5
1.0
1.5
2.0
2.5
3.0
3.5
4.0

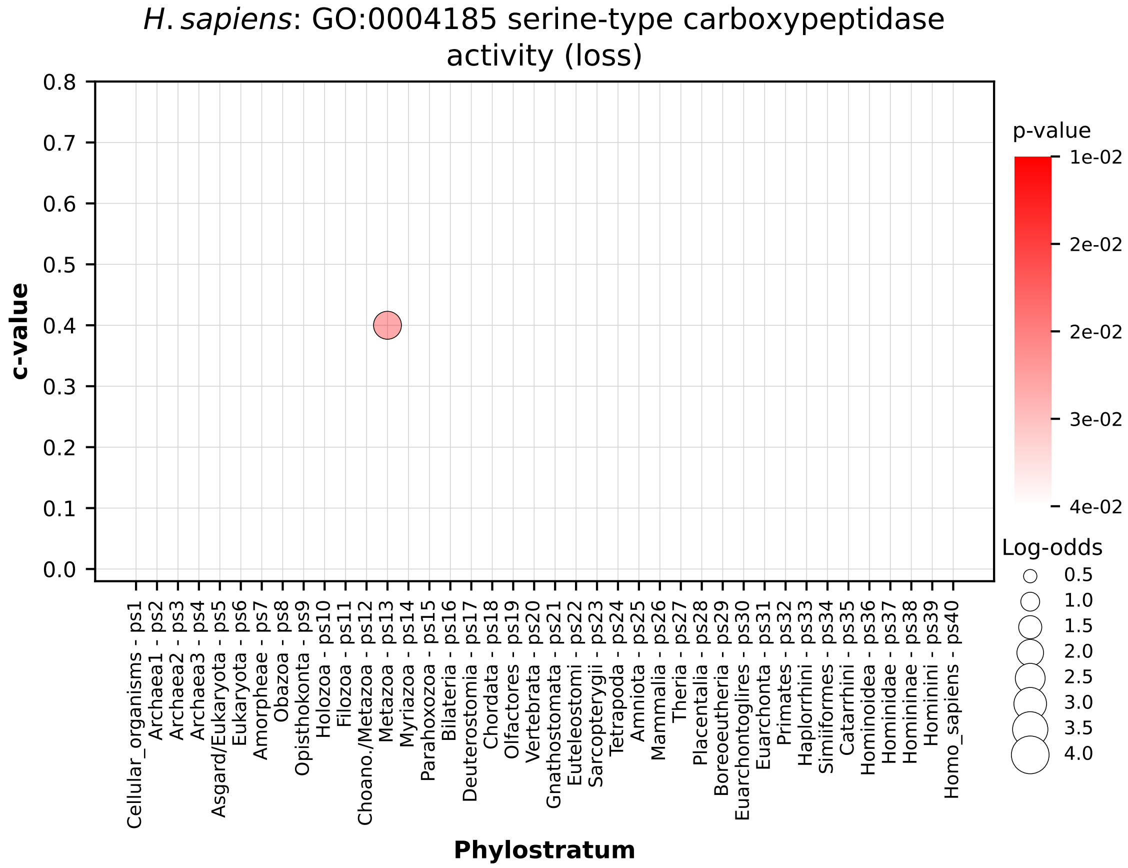

H. sapiens: GO:0004185 serine-type carboxypeptidase activity (loss)
c-value
0.0
0.1
0.2
0.3
0.4
0.5
0.6
0.7
0.8
Phylostratum
Cellular_organisms - ps1
Archaea1 - ps2
Archaea2 - ps3
Archaea3 - ps4
Asgard/Eukaryota - ps5
Eukaryota - ps6
Amorpheae - ps7
Obazoa - ps8
Opisthokonta - ps9
Holozoa - ps10
Filozoa - ps11
Choano./Metazoa - ps12
Metazoa - ps13
Myriazoa - ps14
Parahoxozoa - ps15
Bilateria - ps16
Deuterostomia - ps17
Chordata - ps18
Olfactores - ps19
Vertebrata - ps20
Gnathostomata - ps21
Euteleostomi - ps22
Sarcopterygii - ps23
Tetrapoda - ps24
Amniota - ps25
Mammalia - ps26
Theria - ps27
Placentalia - ps28
Boreoeutheria - ps29
Euarchontoglires - ps30
Euarchonta - ps31
Primates - ps32
Haplorrhini - ps33
Simiiformes - ps34
Catarrhini - ps35
Hominoidea - ps36
Hominidae - ps37
Homininae - ps38
Hominini - ps39
Homo_sapiens - ps40
p-value
1e-02
2e-02
2e-02
3e-02
4e-02
Log-odds
0.5
1.0
1.5
2.0
2.5
3.0
3.5
4.0

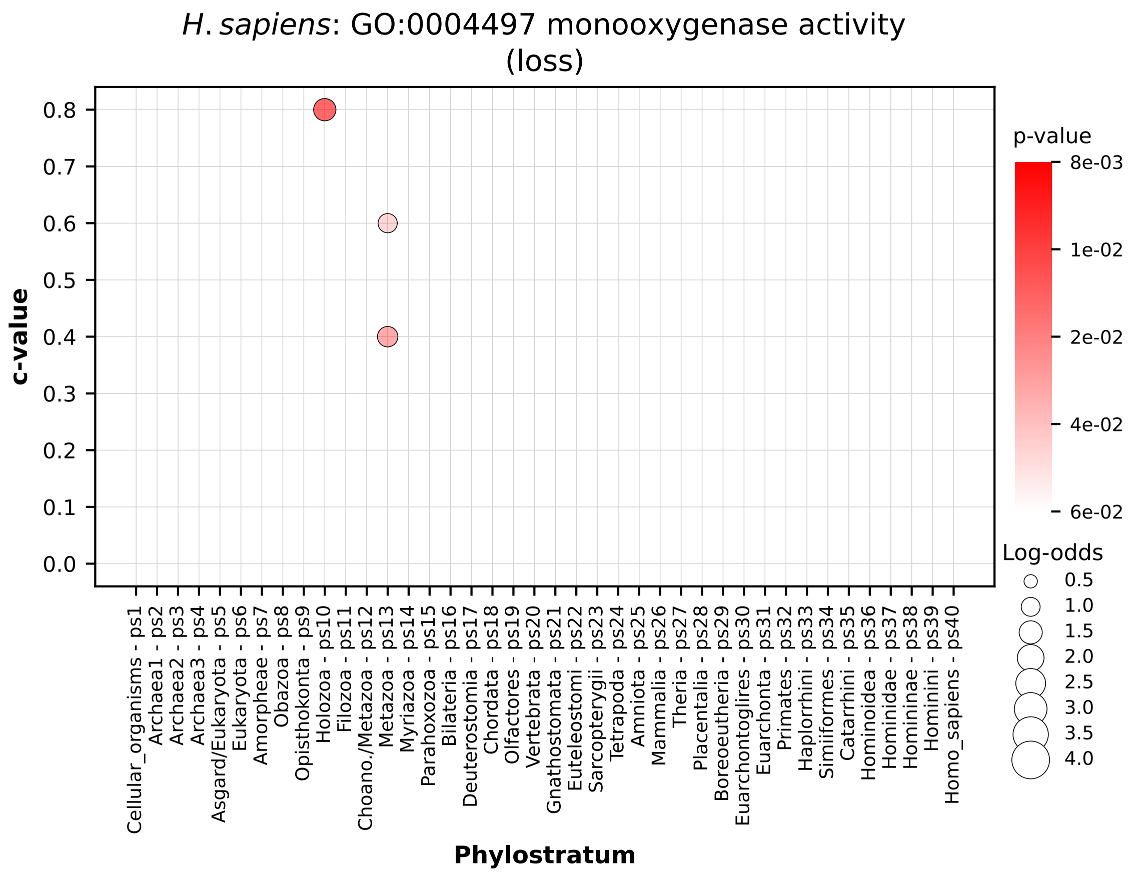

H. sapiens: GO:0004497 monooxygenase activity
(loss)
c-value
0.8
0.7
0.6
0.5
0.4
0.3
0.2
0.1
0.0
p-value
8e-03
1e-02
2e-02
4e-02
6e-02
Log-odds
0.5
1.0
1.5
2.0
2.5
3.0
3.5
4.0
Cellular_organisms - ps1
Archaea1 - ps2
Archaea2 - ps3
Archaea3 - ps4
Asgard/Eukaryota - ps5
Eukaryota - ps6
Amorpheae - ps7
Obazoa - ps8
Opisthokonta - ps9
Holozoa - ps10
Filozoa - ps11
Choano./Metazoa - ps12
Metazoa - ps13
Myriazoa - ps14
Parahoxozoa - ps15
Bilateria - ps16
Deuterostomia - ps17
Chordata - ps18
Olfactores - ps19
Vertebrata - ps20
Gnathostomata - ps21
Euteleostomi - ps22
Sarcopterygii - ps23
Tetrapoda - ps24
Amniota - ps25
Mammalia - ps26
Theria - ps27
Placentalia - ps28
Boreoeutheria - ps29
Euarchontoglires - ps30
Euarchonta - ps31
Primates - ps32
Haplorrhini - ps33
Simiiformes - ps34
Catarrhini - ps35
Hominoidea - ps36
Hominidae - ps37
Homininae - ps38
Hominini - ps39
Homo_sapiens - ps40
Phylostratum

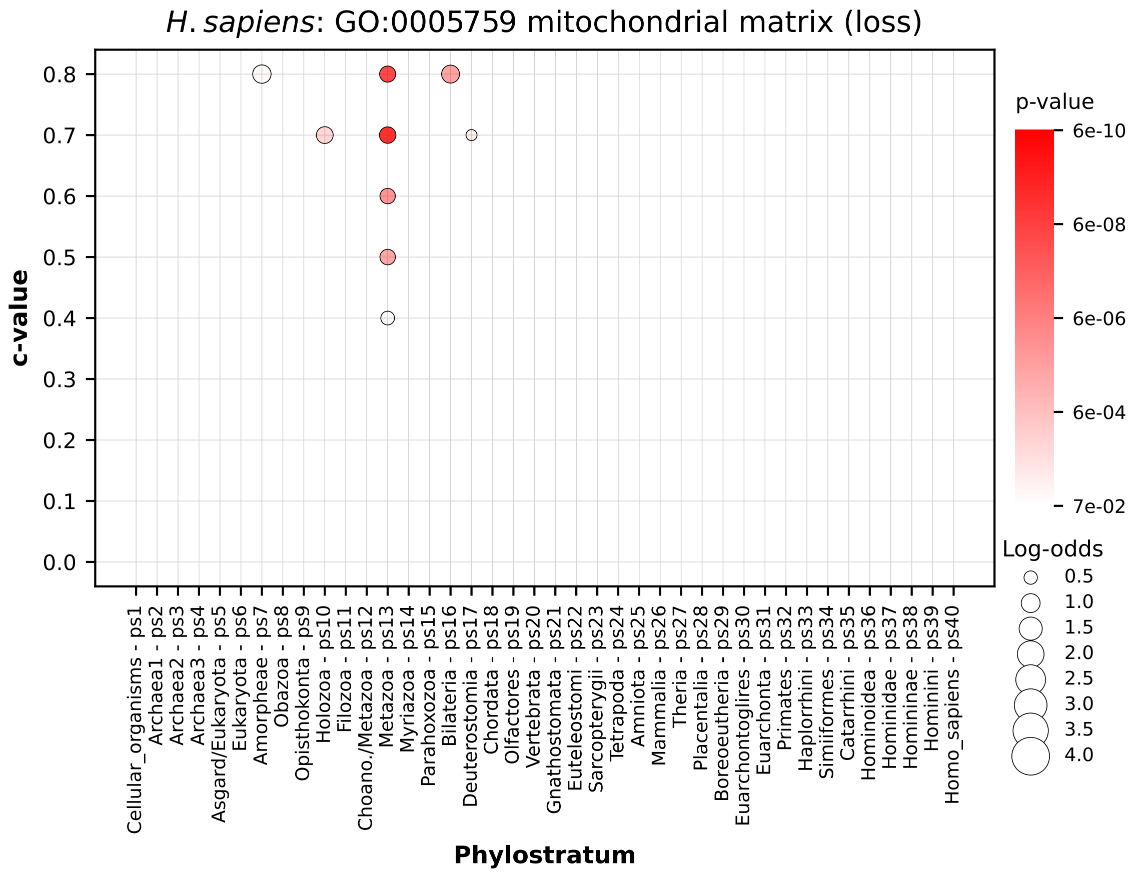

H. sapiens: GO:0005759 mitochondrial matrix (loss)
c-value
0.8
0.7
0.6
0.5
0.4
0.3
0.2
0.1
0.0
Phylostratum
Cellular_organisms - ps1
Archaea1 - ps2
Archaea2 - ps3
Archaea3 - ps4
Asgard/Eukaryota - ps5
Eukaryota - ps6
Amorpheae - ps7
Obazoa - ps8
Opisthokonta - ps9
Holozoa - ps10
Filozoa - ps11
Choano./Metazoa - ps12
Metazoa - ps13
Myriazoa - ps14
Parahoxozoa - ps15
Bilateria - ps16
Deuterostomia - ps17
Chordata - ps18
Olfactores - ps19
Vertebrata - ps20
Gnathostomata - ps21
Euteleostomi - ps22
Sarcopterygii - ps23
Tetrapoda - ps24
Amniota - ps25
Mammalia - ps26
Theria - ps27
Placentalia - ps28
Boreoeutheria - ps29
Euarchontoglires - ps30
Euarchonta - ps31
Primates - ps32
Haplorrhini - ps33
Simiiformes - ps34
Catarrhini - ps35
Hominoidea - ps36
Hominidae - ps37
Homininae - ps38
Hominini - ps39
Homo_sapiens - ps40
p-value
6e-10
6e-08
6e-06
6e-04
7e-02
Log-odds
0.5
1.0
1.5
2.0
2.5
3.0
3.5
4.0

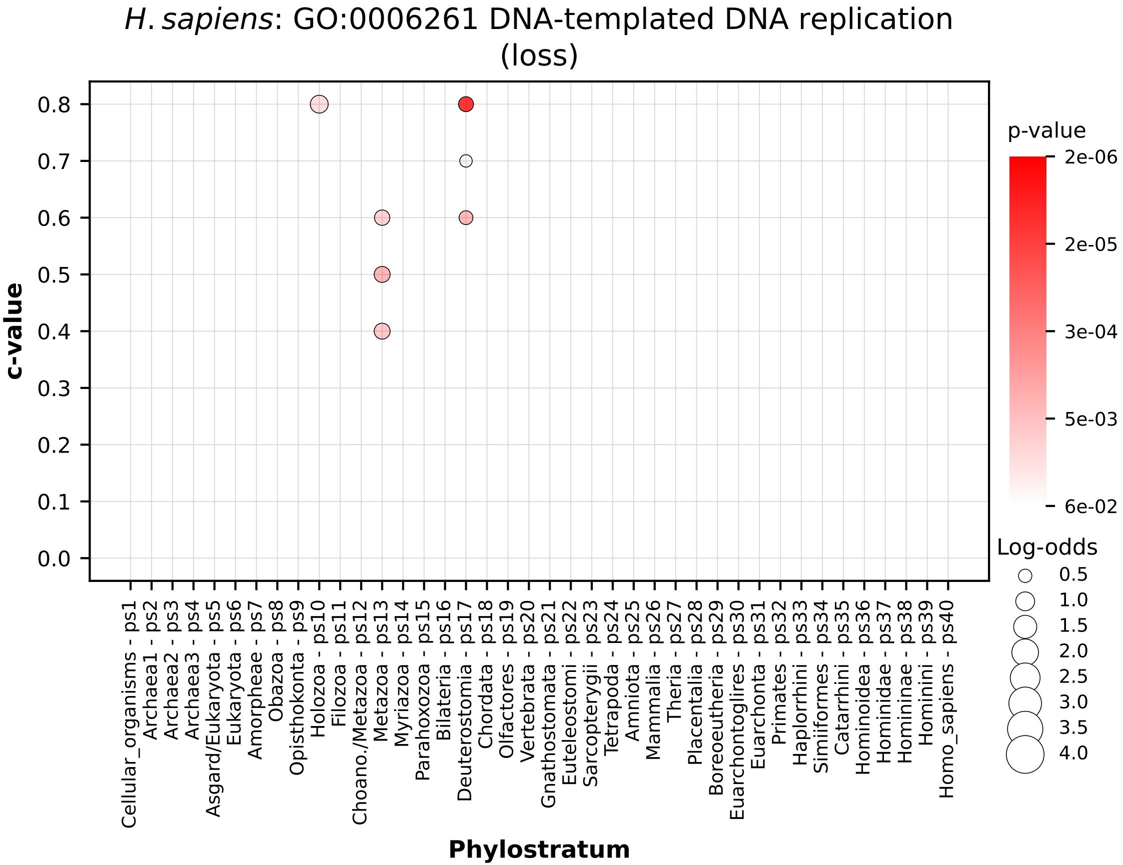

H. sapiens: GO:0006261 DNA-templated DNA replication (loss)
c-value
0.0
0.1
0.2
0.3
0.4
0.5
0.6
0.7
0.8
p-value
2e-06
2e-05
3e-04
5e-03
6e-02
Log-odds
0.5
1.0
1.5
2.0
2.5
3.0
3.5
4.0
Cellular_organisms - ps1
Archaea1 - ps2
Archaea2 - ps3
Archaea3 - ps4
Asgard/Eukaryota - ps5
Eukaryota - ps6
Amorpheae - ps7
Obazoa - ps8
Opisthokonta - ps9
Holozoa - ps10
Filozoa - ps11
Choano./Metazoa - ps12
Metazoa - ps13
Myriazoa - ps14
Parahoxozoa - ps15
Bilateria - ps16
Deuterostomia - ps17
Chordata - ps18
Olfactores - ps19
Vertebrata - ps20
Gnathostomata - ps21
Euteleostomi - ps22
Sarcopterygii - ps23
Tetrapoda - ps24
Amniota - ps25
Mammalia - ps26
Theria - ps27
Placentalia - ps28
Boreoeutheria - ps29
Euarchontoglires - ps30
Euarchonta - ps31
Primates - ps32
Haplorrhini - ps33
Simiiformes - ps34
Catarrhini - ps35
Hominoidea - ps36
Hominidae - ps37
Homininae - ps38
Hominini - ps39
Homo_sapiens - ps40
Phylostratum

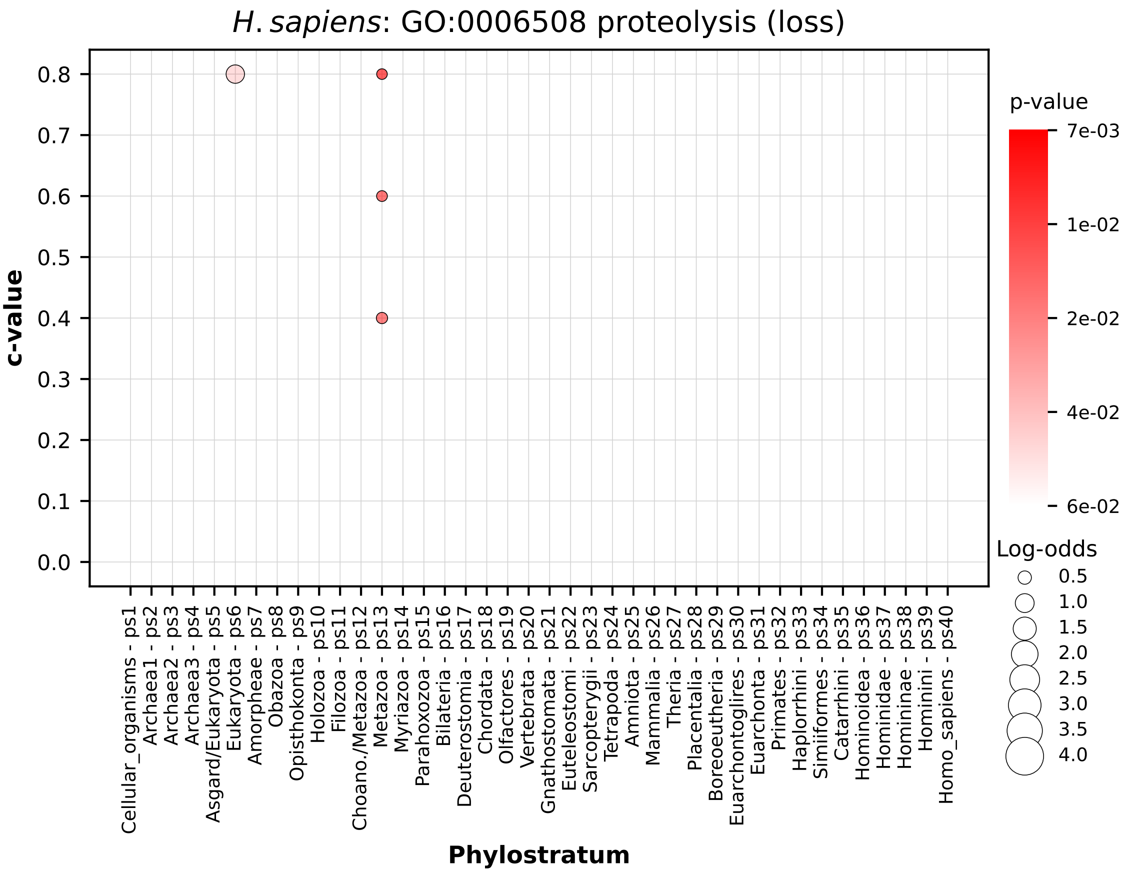

H. sapiens: GO:0006508 proteolysis (loss)
c-value
0.8
0.7
0.6
0.5
0.4
0.3
0.2
0.1
0.0
Phylostratum
Cellular_organisms - ps1
Archaea1 - ps2
Archaea2 - ps3
Archaea3 - ps4
Asgard/Eukaryota - ps5
Eukaryota - ps6
Amorpheae - ps7
Obazoa - ps8
Opisthokonta - ps9
Holozoa - ps10
Filozoa - ps11
Choano./Metazoa - ps12
Metazoa - ps13
Myriazoa - ps14
Parahoxozoa - ps15
Bilateria - ps16
Deuterostomia - ps17
Chordata - ps18
Olfactores - ps19
Vertebrata - ps20
Gnathostomata - ps21
Euteleostomi - ps22
Sarcopterygii - ps23
Tetrapoda - ps24
Amniota - ps25
Mammalia - ps26
Theria - ps27
Placentalia - ps28
Boreoeutheria - ps29
Euarchontoglires - ps30
Euarchonta - ps31
Primates - ps32
Haplorrhini - ps33
Simiiformes - ps34
Catarrhini - ps35
Hominoidea - ps36
Hominidae - ps37
Homininae - ps38
Hominini - ps39
Homo_sapiens - ps40
p-value
7e-03
1e-02
2e-02
4e-02
6e-02
Log-odds
0.5
1.0
1.5
2.0
2.5
3.0
3.5
4.0

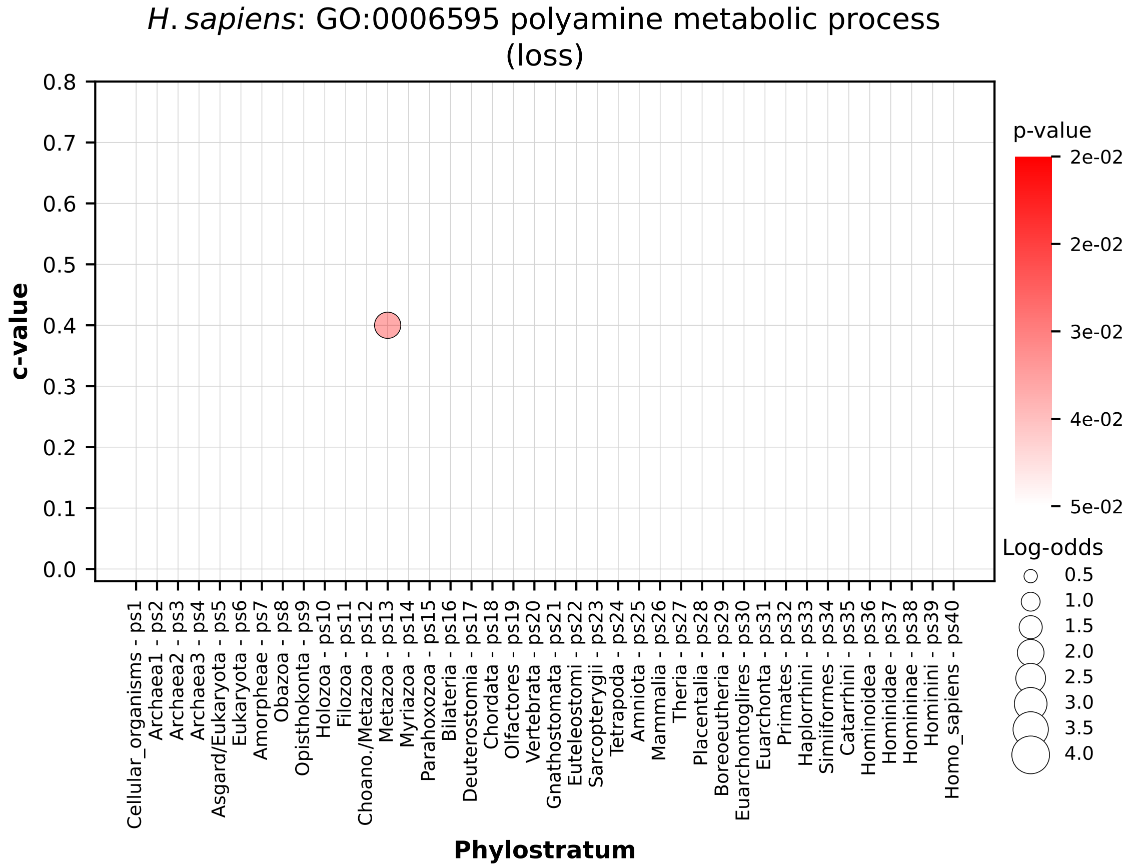

H. sapiens: GO:0006595 polyamine metabolic process (loss)
c-value
0.0
0.1
0.2
0.3
0.4
0.5
0.6
0.7
0.8
Phylostratum
Cellular_organisms - ps1
Archaea1 - ps2
Archaea2 - ps3
Archaea3 - ps4
Asgard/Eukaryota - ps5
Eukaryota - ps6
Amorpheae - ps7
Obazoa - ps8
Opisthokonta - ps9
Holozoa - ps10
Filozoa - ps11
Choano./Metazoa - ps12
Metazoa - ps13
Myriazoa - ps14
Parahoxozoa - ps15
Bilateria - ps16
Deuterostomia - ps17
Chordata - ps18
Olfactores - ps19
Vertebrata - ps20
Gnathostomata - ps21
Euteleostomi - ps22
Sarcopterygii - ps23
Tetrapoda - ps24
Amniota - ps25
Mammalia - ps26
Theria - ps27
Placentalia - ps28
Boreoeutheria - ps29
Euarchontoglires - ps30
Euarchonta - ps31
Primates - ps32
Haplorrhini - ps33
Simiiformes - ps34
Catarrhini - ps35
Hominoidea - ps36
Hominidae - ps37
Homininae - ps38
Hominini - ps39
Homo_sapiens - ps40
p-value
2e-02
2e-02
3e-02
4e-02
5e-02
Log-odds
0.5
1.0
1.5
2.0
2.5
3.0
3.5
4.0

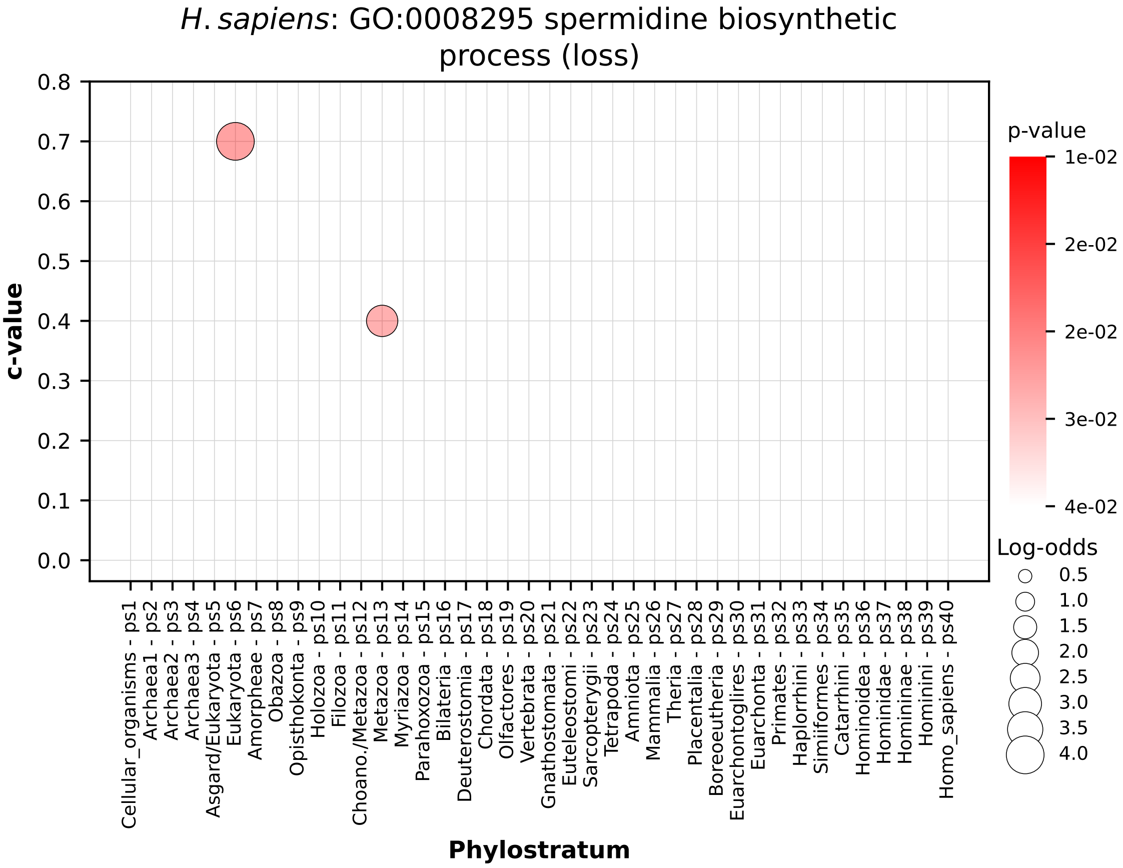

H. sapiens: GO:0008295 spermidine biosynthetic process (loss)
c-value
0.8
0.7
0.6
0.5
0.4
0.3
0.2
0.1
0.0
Phylostratum
Cellular_organisms - ps1
Archaea1 - ps2
Archaea2 - ps3
Archaea3 - ps4
Asgard/Eukaryota - ps5
Eukaryota - ps6
Amorpheae - ps7
Obazoa - ps8
Opisthokonta - ps9
Holozoa - ps10
Filozoa - ps11
Choano./Metazoa - ps12
Metazoa - ps13
Myriazoa - ps14
Parahoxozoa - ps15
Bilateria - ps16
Deuterostomia - ps17
Chordata - ps18
Olfactores - ps19
Vertebrata - ps20
Gnathostomata - ps21
Euteleostomi - ps22
Sarcopterygii - ps23
Tetrapoda - ps24
Amniota - ps25
Mammalia - ps26
Theria - ps27
Placentalia - ps28
Boreoeutheria - ps29
Euarchontoglires - ps30
Euarchonta - ps31
Primates - ps32
Haplorrhini - ps33
Simiiformes - ps34
Catarrhini - ps35
Hominoidea - ps36
Hominidae - ps37
Homininae - ps38
Hominini - ps39
Homo_sapiens - ps40
p-value
1e-02
2e-02
2e-02
3e-02
4e-02
Log-odds
0.5
1.0
1.5
2.0
2.5
3.0
3.5
4.0

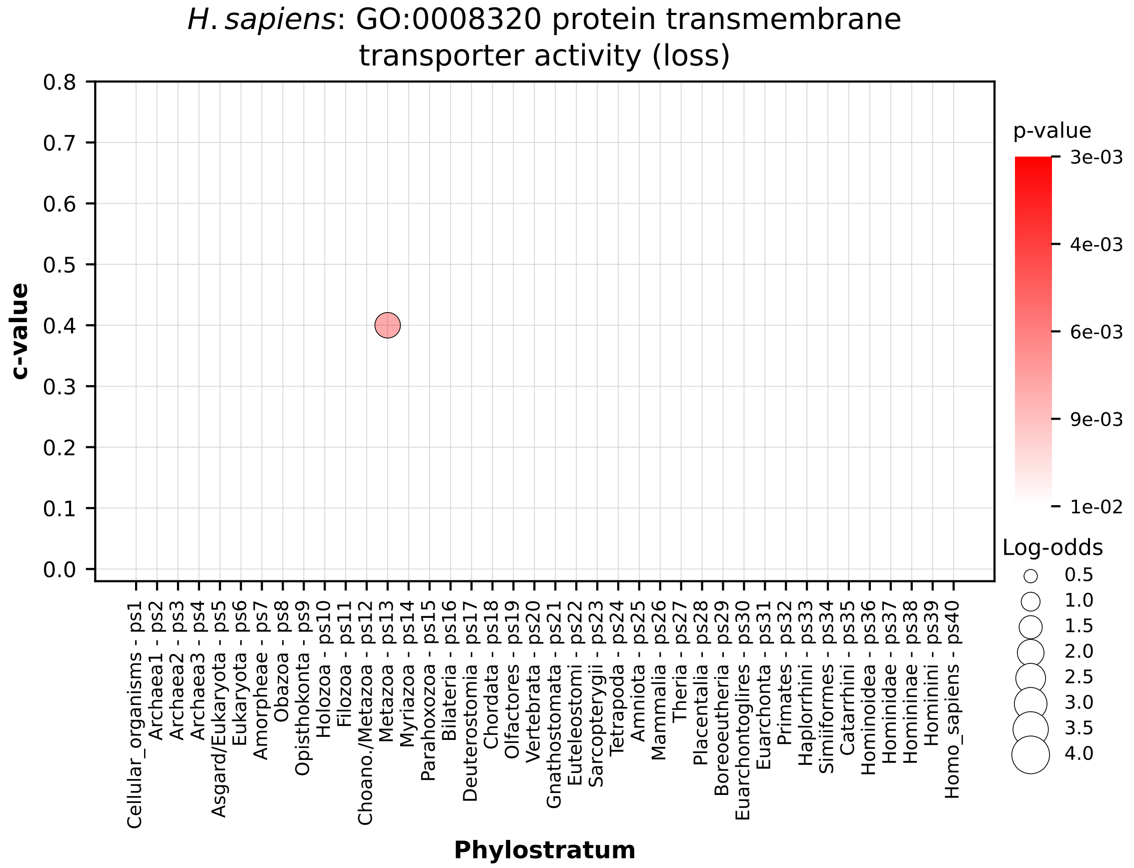
H. sapiens: GO:0008320 protein transmembrane transporter activity (loss)
c-value
0.0
0.1
0.2
0.3
0.4
0.5
0.6
0.7
0.8
Phylostratum
Cellular_organisms - ps1
Archaea1 - ps2
Archaea2 - ps3
Archaea3 - ps4
Asgard/Eukaryota - ps5
Eukaryota - ps6
Amorpheae - ps7
Obazoa - ps8
Opisthokonta - ps9
Holozoa - ps10
Filozoa - ps11
Choano./Metazoa - ps12
Metazoa - ps13
Myriazoa - ps14
Parahoxozoa - ps15
Bilateria - ps16
Deuterostomia - ps17
Chordata - ps18
Olfactores - ps19
Vertebrata - ps20
Gnathostomata - ps21
Euteleostomi - ps22
Sarcopterygii - ps23
Tetrapoda - ps24
Amniota - ps25
Mammalia - ps26
Theria - ps27
Placentalia - ps28
Boreoeutheria - ps29
Euarchontoglires - ps30
Euarchonta - ps31
Primates - ps32
Haplorrhini - ps33
Simiiformes - ps34
Catarrhini - ps35
Hominoidea - ps36
Hominidae - ps37
Homininae - ps38
Hominini - ps39
Homo_sapiens - ps40
p-value
3e-03
4e-03
6e-03
9e-03
1e-02
Log-odds
0.5
1.0
1.5
2.0
2.5
3.0
3.5
4.0

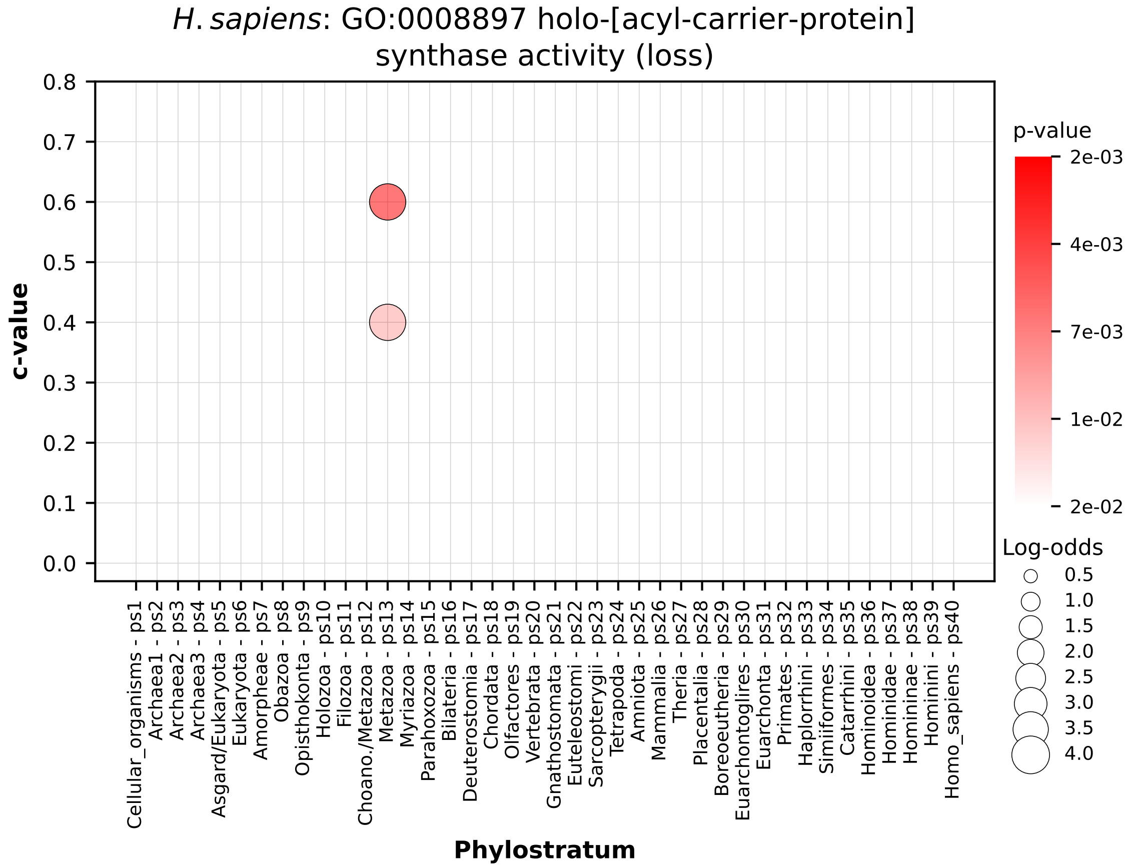

H. sapiens: GO:0008897 holo-[acyl-carrier-protein] synthase activity (loss)
c-value
0.8
0.7
0.6
0.5
0.4
0.3
0.2
0.1
0.0
p-value
2e-03
4e-03
7e-03
1e-02
2e-02
Log-odds
0.5
1.0
1.5
2.0
2.5
3.0
3.5
4.0
Cellular_organisms - ps1
Archaea1 - ps2
Archaea2 - ps3
Archaea3 - ps4
Asgard/Eukaryota - ps5
Eukaryota - ps6
Amorpheae - ps7
Obazoa - ps8
Opisthokonta - ps9
Holozoa - ps10
Filozoa - ps11
Choano./Metazoa - ps12
Metazoa - ps13
Myriazoa - ps14
Parahoxozoa - ps15
Bilateria - ps16
Deuterostomia - ps17
Chordata - ps18
Olfactores - ps19
Vertebrata - ps20
Gnathostomata - ps21
Euteleostomi - ps22
Sarcopterygii - ps23
Tetrapoda - ps24
Amniota - ps25
Mammalia - ps26
Theria - ps27
Placentalia - ps28
Boreoeutheria - ps29
Euarchontoglires - ps30
Euarchonta - ps31
Primates - ps32
Haplorrhini - ps33
Simiiformes - ps34
Catarrhini - ps35
Hominoidea - ps36
Hominidae - ps37
Homininae - ps38
Hominini - ps39
Homo_sapiens - ps40
Phylostratum

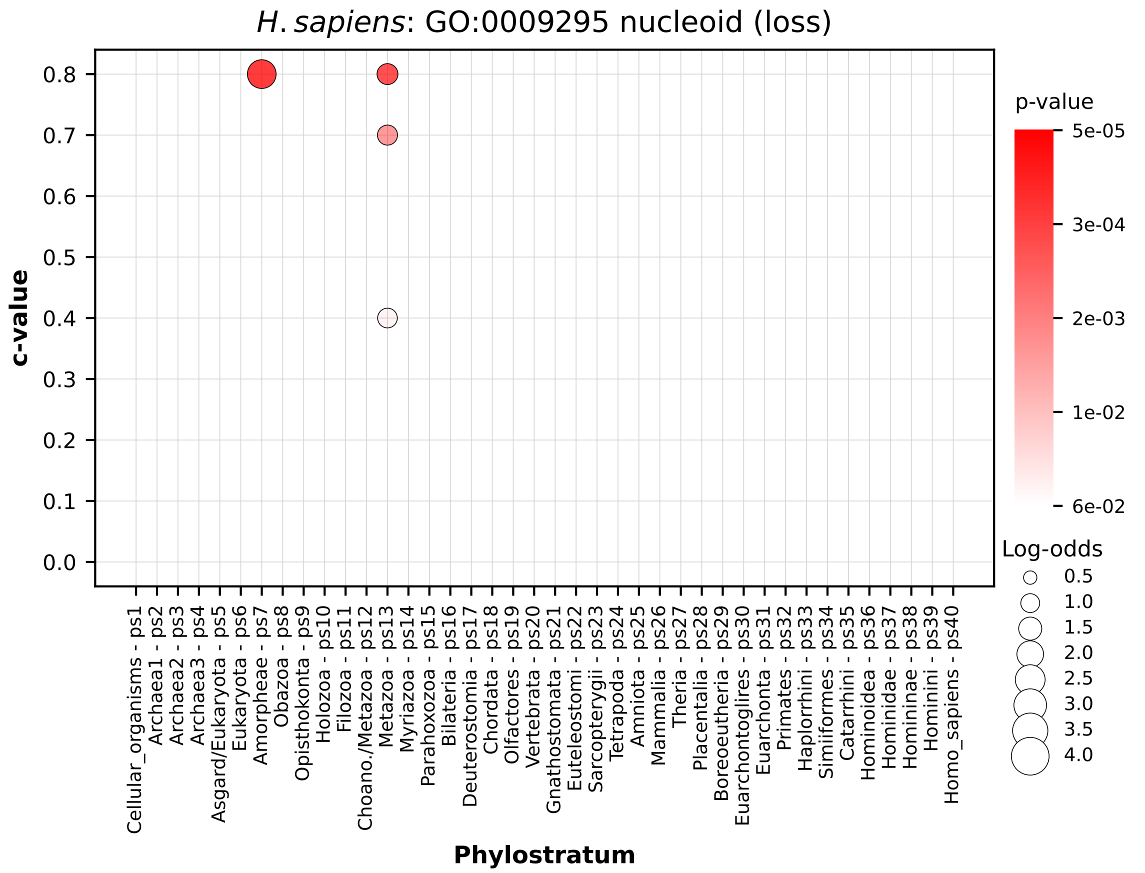

H. sapiens: GO:0009295 nucleoid (loss)
c-value
0.8
0.7
0.6
0.5
0.4
0.3
0.2
0.1
0.0
p-value
5e-05
3e-04
2e-03
1e-02
6e-02
Log-odds
0.5
1.0
1.5
2.0
2.5
3.0
3.5
4.0
Cellular_organisms - ps1
Archaea1 - ps2
Archaea2 - ps3
Archaea3 - ps4
Asgard/Eukaryota - ps5
Eukaryota - ps6
Amorpheae - ps7
Obazoa - ps8
Opisthokonta - ps9
Holozoa - ps10
Filozoa - ps11
Choano./Metazoa - ps12
Metazoa - ps13
Myriazoa - ps14
Parahoxozoa - ps15
Bilateria - ps16
Deuterostomia - ps17
Chordata - ps18
Olfactores - ps19
Vertebrata - ps20
Gnathostomata - ps21
Euteleostomi - ps22
Sarcopterygii - ps23
Tetrapoda - ps24
Amniota - ps25
Mammalia - ps26
Theria - ps27
Placentalia - ps28
Boreoeutheria - ps29
Euarchontoglires - ps30
Euarchonta - ps31
Primates - ps32
Haplorrhini - ps33
Simiiformes - ps34
Catarrhini - ps35
Hominoidea - ps36
Hominidae - ps37
Homininae - ps38
Hominini - ps39
Homo_sapiens - ps40
Phylostratum

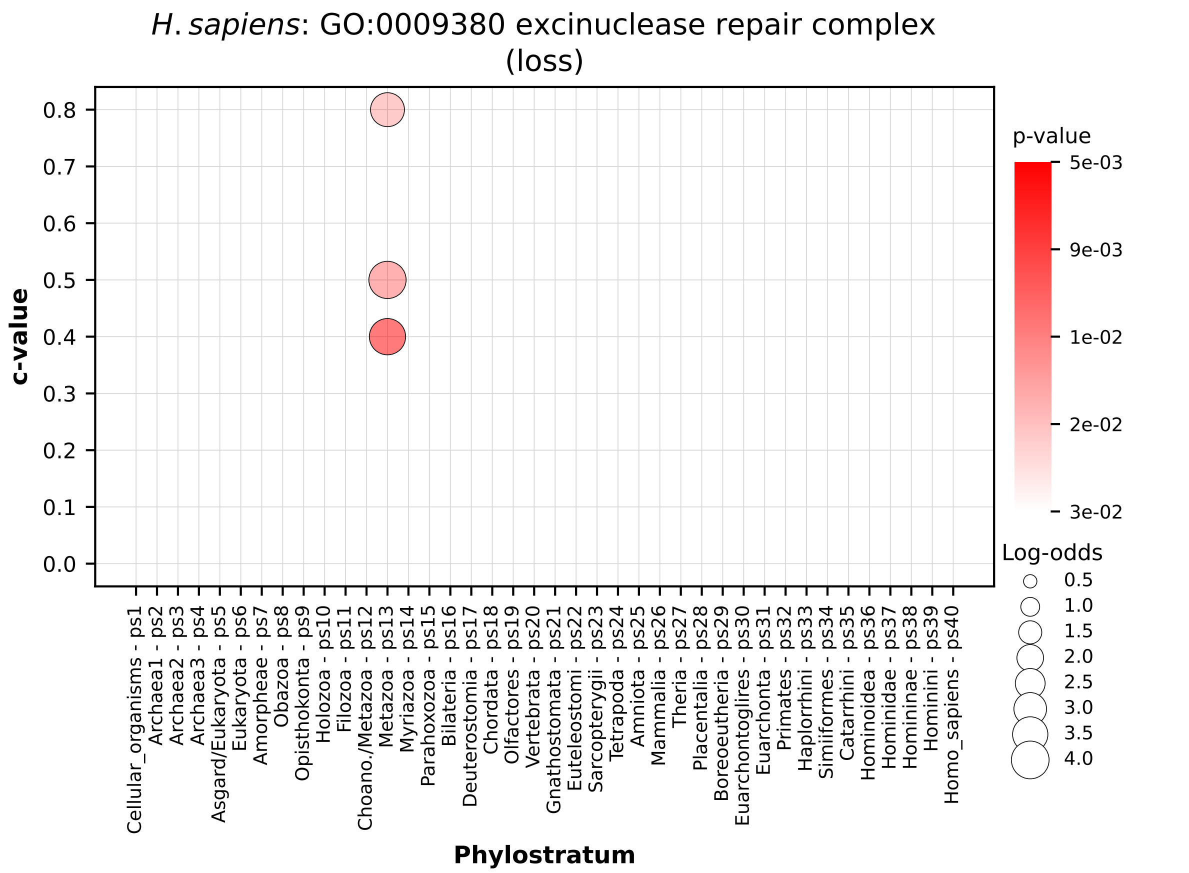

H. sapiens: GO:0009380 excinuclease repair complex
(loss)
c-value
0.0
0.1
0.2
0.3
0.4
0.5
0.6
0.7
0.8
p-value
5e-03
9e-03
1e-02
2e-02
3e-02
Log-odds
0.5
1.0
1.5
2.0
2.5
3.0
3.5
4.0
Cellular_organisms - ps1
Archaea1 - ps2
Archaea2 - ps3
Archaea3 - ps4
Asgard/Eukaryota - ps5
Eukaryota - ps6
Amorpheae - ps7
Obazoa - ps8
Opisthokonta - ps9
Holozoa - ps10
Filozoa - ps11
Choano./Metazoa - ps12
Metazoa - ps13
Myriazoa - ps14
Parahoxozoa - ps15
Bilateria - ps16
Deuterostomia - ps17
Chordata - ps18
Olfactores - ps19
Vertebrata - ps20
Gnathostomata - ps21
Euteleostomi - ps22
Sarcopterygii - ps23
Tetrapoda - ps24
Amniota - ps25
Mammalia - ps26
Theria - ps27
Placentalia - ps28
Boreoeutheria - ps29
Euarchontoglires - ps30
Euarchonta - ps31
Primates - ps32
Haplorrhini - ps33
Simiiformes - ps34
Catarrhini - ps35
Hominoidea - ps36
Hominidae - ps37
Homininae - ps38
Hominini - ps39
Homo_sapiens - ps40
Phylostratum

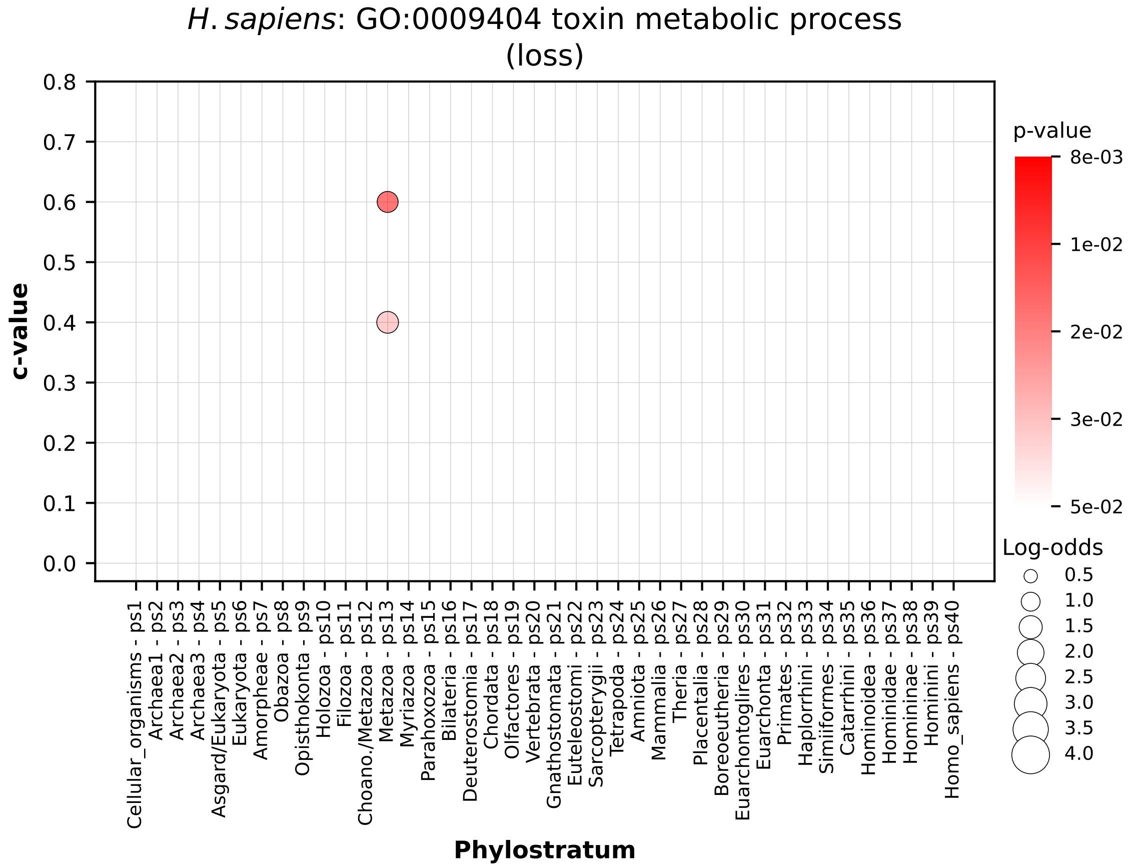
H. sapiens: GO:0009404 toxin metabolic process
(loss)
c-value
0.8
0.7
0.6
0.5
0.4
0.3
0.2
0.1
0.0
p-value
8e-03
1e-02
2e-02
3e-02
5e-02
Log-odds
0.5
1.0
1.5
2.0
2.5
3.0
3.5
4.0
Cellular_organisms - ps1
Archaea1 - ps2
Archaea2 - ps3
Archaea3 - ps4
Asgard/Eukaryota - ps5
Eukaryota - ps6
Amorpheae - ps7
Obazoa - ps8
Opisthokonta - ps9
Holozoa - ps10
Filozoa - ps11
Choano./Metazoa - ps12
Metazoa - ps13
Myriazoa - ps14
Parahoxozoa - ps15
Bilateria - ps16
Deuterostomia - ps17
Chordata - ps18
Olfactores - ps19
Vertebrata - ps20
Gnathostomata - ps21
Euteleostomi - ps22
Sarcopterygii - ps23
Tetrapoda - ps24
Amniota - ps25
Mammalia - ps26
Theria - ps27
Placentalia - ps28
Boreoeutheria - ps29
Euarchontoglires - ps30
Euarchonta - ps31
Primates - ps32
Haplorrhini - ps33
Simiiformes - ps34
Catarrhini - ps35
Hominoidea - ps36
Hominidae - ps37
Homininae - ps38
Hominini - ps39
Homo_sapiens - ps40
Phylostratum

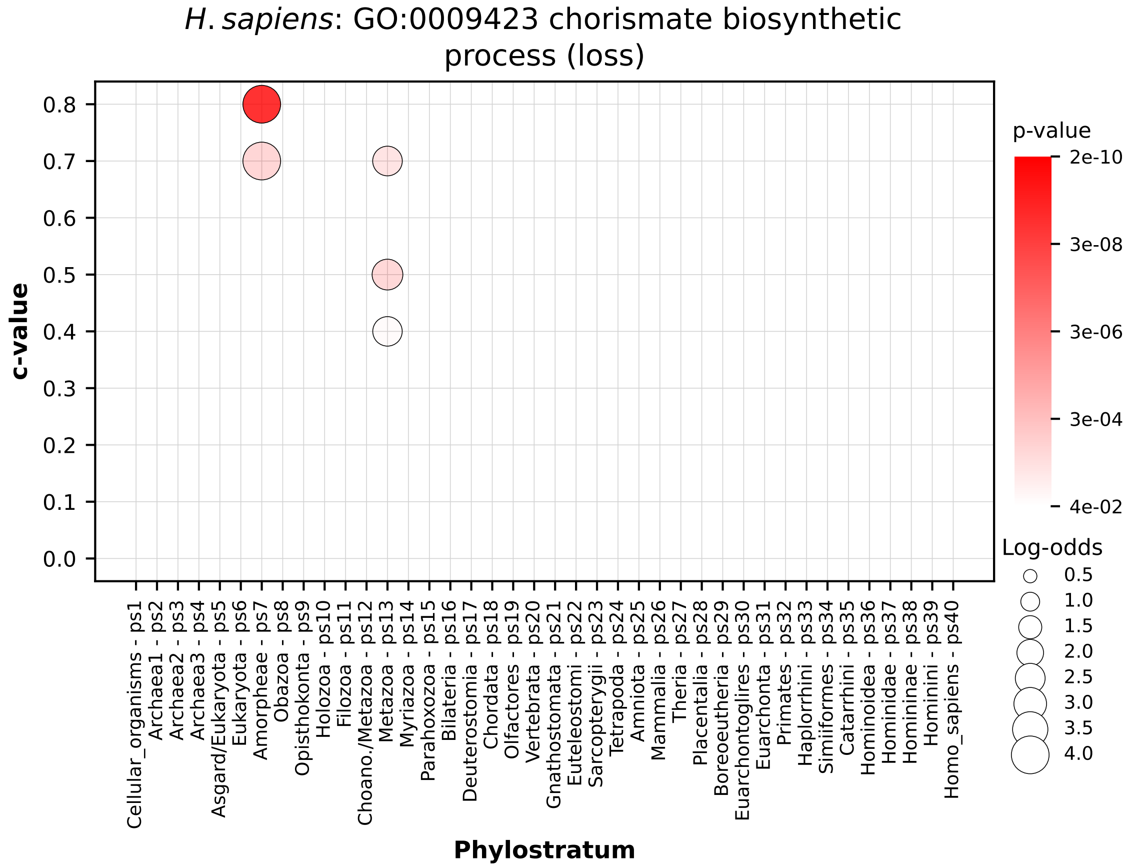

H. sapiens: GO:0009423 chorismate biosynthetic process (loss)
c-value
0.8
0.7
0.6
0.5
0.4
0.3
0.2
0.1
0.0
Phylostratum
Cellular_organisms - ps1
Archaea1 - ps2
Archaea2 - ps3
Archaea3 - ps4
Asgard/Eukaryota - ps5
Eukaryota - ps6
Amorpheae - ps7
Obazoa - ps8
Opisthokonta - ps9
Holozoa - ps10
Filozoa - ps11
Choano./Metazoa - ps12
Metazoa - ps13
Myriazoa - ps14
Parahoxozoa - ps15
Bilateria - ps16
Deuterostomia - ps17
Chordata - ps18
Olfactores - ps19
Vertebrata - ps20
Gnathostomata - ps21
Euteleostomi - ps22
Sarcopterygii - ps23
Tetrapoda - ps24
Amniota - ps25
Mammalia - ps26
Theria - ps27
Placentalia - ps28
Boreoeutheria - ps29
Euarchontoglires - ps30
Euarchonta - ps31
Primates - ps32
Haplorrhini - ps33
Simiiformes - ps34
Catarrhini - ps35
Hominoidea - ps36
Hominidae - ps37
Homininae - ps38
Hominini - ps39
Homo_sapiens - ps40
p-value
2e-10
3e-08
3e-06
3e-04
4e-02
Log-odds
0.5
1.0
1.5
2.0
2.5
3.0
3.5
4.0

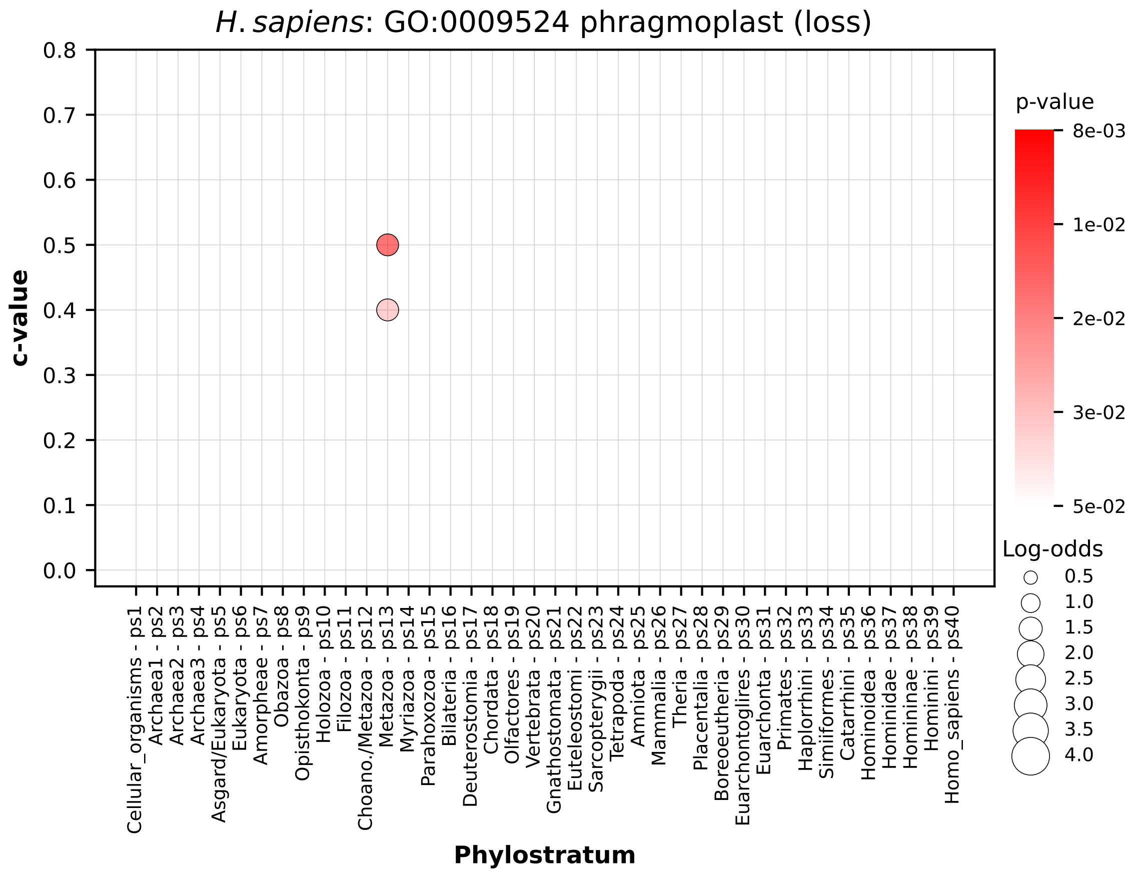
H. sapiens: GO:0009524 phragmoplast (loss)
c-value
0.8
0.7
0.6
0.5
0.4
0.3
0.2
0.1
0.0
Cellular_organisms - ps1
Archaea1 - ps2
Archaea2 - ps3
Archaea3 - ps4
Asgard/Eukaryota - ps5
Eukaryota - ps6
Amorpheae - ps7
Obazoa - ps8
Opisthokonta - ps9
Holozoa - ps10
Filozoa - ps11
Choano./Metazoa - ps12
Metazoa - ps13
Myriazoa - ps14
Parahoxozoa - ps15
Bilateria - ps16
Deuterostomia - ps17
Chordata - ps18
Olfactores - ps19
Vertebrata - ps20
Gnathostomata - ps21
Euteleostomi - ps22
Sarcopterygii - ps23
Tetrapoda - ps24
Amniota - ps25
Mammalia - ps26
Theria - ps27
Placentalia - ps28
Boreoeutheria - ps29
Euarchontoglires - ps30
Euarchonta - ps31
Primates - ps32
Haplorrhini - ps33
Simiiformes - ps34
Catarrhini - ps35
Hominoidea - ps36
Hominidae - ps37
Homininae - ps38
Hominini - ps39
Homo_sapiens - ps40
Phylostratum
p-value
8e-03
1e-02
2e-02
3e-02
5e-02
Log-odds
0.5
1.0
1.5
2.0
2.5
3.0
3.5
4.0

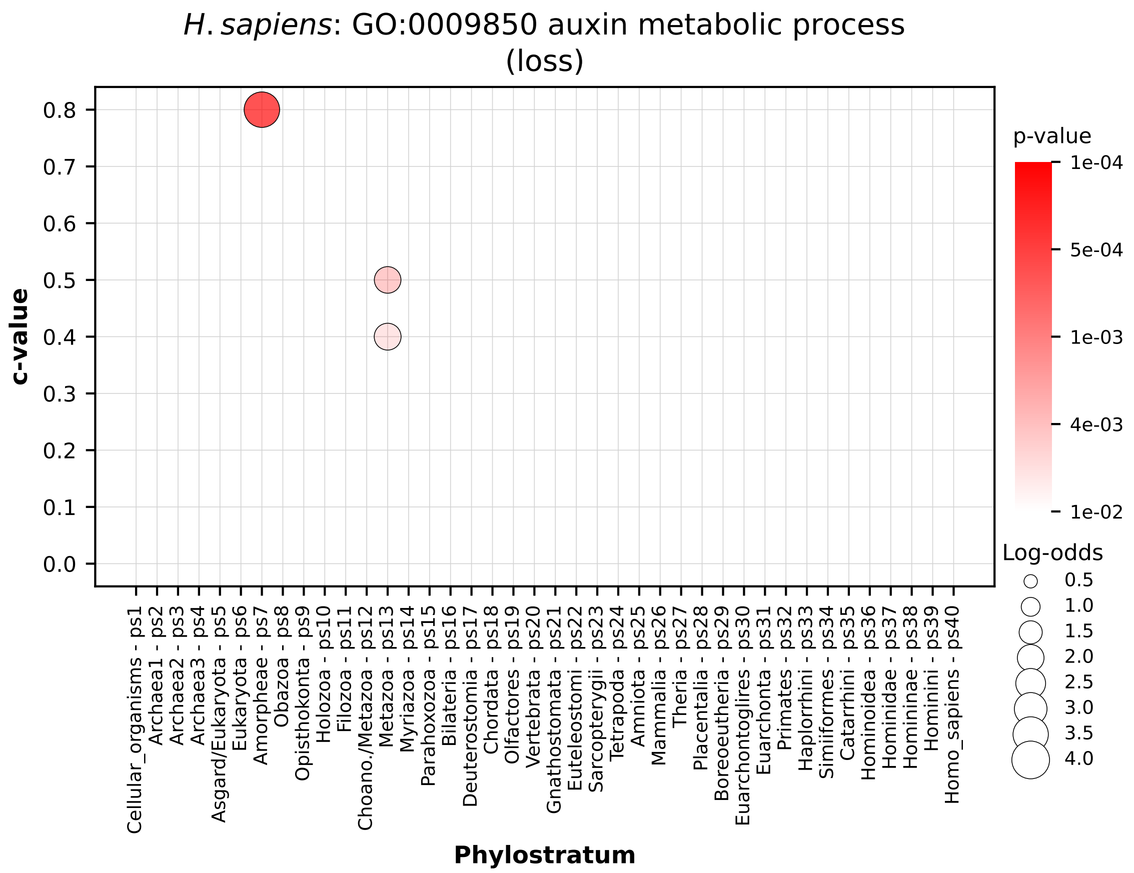

H. sapiens: GO:0009850 auxin metabolic process
(loss)
c-value
0.0
0.1
0.2
0.3
0.4
0.5
0.6
0.7
0.8
Phylostratum
Cellular_organisms - ps1
Archaea1 - ps2
Archaea2 - ps3
Archaea3 - ps4
Asgard/Eukaryota - ps5
Eukaryota - ps6
Amorpheae - ps7
Obazoa - ps8
Opisthokonta - ps9
Holozoa - ps10
Filozoa - ps11
Choano./Metazoa - ps12
Metazoa - ps13
Myriazoa - ps14
Parahoxozoa - ps15
Bilateria - ps16
Deuterostomia - ps17
Chordata - ps18
Olfactores - ps19
Vertebrata - ps20
Gnathostomata - ps21
Euteleostomi - ps22
Sarcopterygii - ps23
Tetrapoda - ps24
Amniota - ps25
Mammalia - ps26
Theria - ps27
Placentalia - ps28
Boreoeutheria - ps29
Euarchontoglires - ps30
Euarchonta - ps31
Primates - ps32
Haplorrhini - ps33
Simiiformes - ps34
Catarrhini - ps35
Hominoidea - ps36
Hominidae - ps37
Homininae - ps38
Hominini - ps39
Homo_sapiens - ps40
p-value
1e-04
5e-04
1e-03
4e-03
1e-02
Log-odds
0.5
1.0
1.5
2.0
2.5
3.0
3.5
4.0

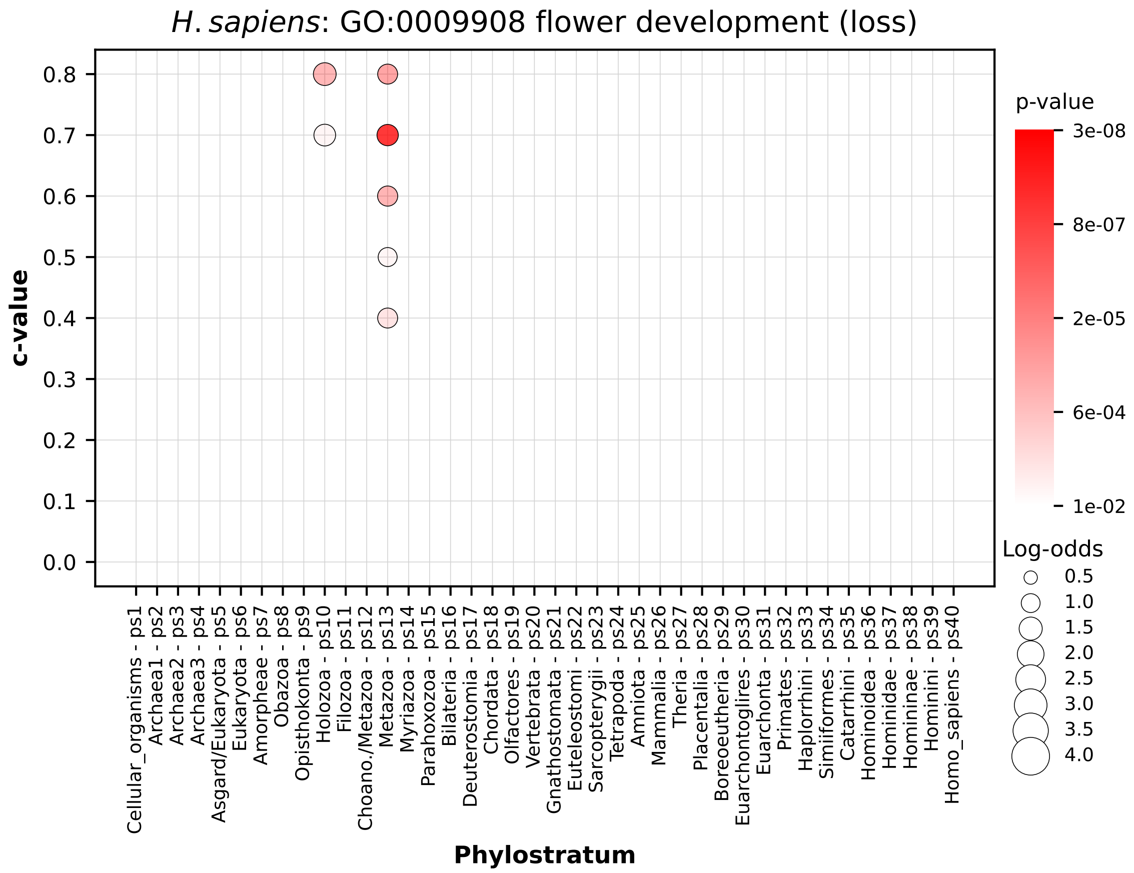

H. sapiens: GO:0009908 flower development (loss)
c-value
0.8
0.7
0.6
0.5
0.4
0.3
0.2
0.1
0.0
p-value
3e-08
8e-07
2e-05
6e-04
1e-02
Log-odds
0.5
1.0
1.5
2.0
2.5
3.0
3.5
4.0
Cellular_organisms - ps1
Archaea1 - ps2
Archaea2 - ps3
Archaea3 - ps4
Asgard/Eukaryota - ps5
Eukaryota - ps6
Amorpheae - ps7
Obazoa - ps8
Opisthokonta - ps9
Holozoa - ps10
Filozoa - ps11
Choano./Metazoa - ps12
Metazoa - ps13
Myriazoa - ps14
Parahoxozoa - ps15
Bilateria - ps16
Deuterostomia - ps17
Chordata - ps18
Olfactores - ps19
Vertebrata - ps20
Gnathostomata - ps21
Euteleostomi - ps22
Sarcopterygii - ps23
Tetrapoda - ps24
Amniota - ps25
Mammalia - ps26
Theria - ps27
Placentalia - ps28
Boreoeutheria - ps29
Euarchontoglires - ps30
Euarchonta - ps31
Primates - ps32
Haplorrhini - ps33
Simiiformes - ps34
Catarrhini - ps35
Hominoidea - ps36
Hominidae - ps37
Homininae - ps38
Hominini - ps39
Homo_sapiens - ps40
Phylostratum

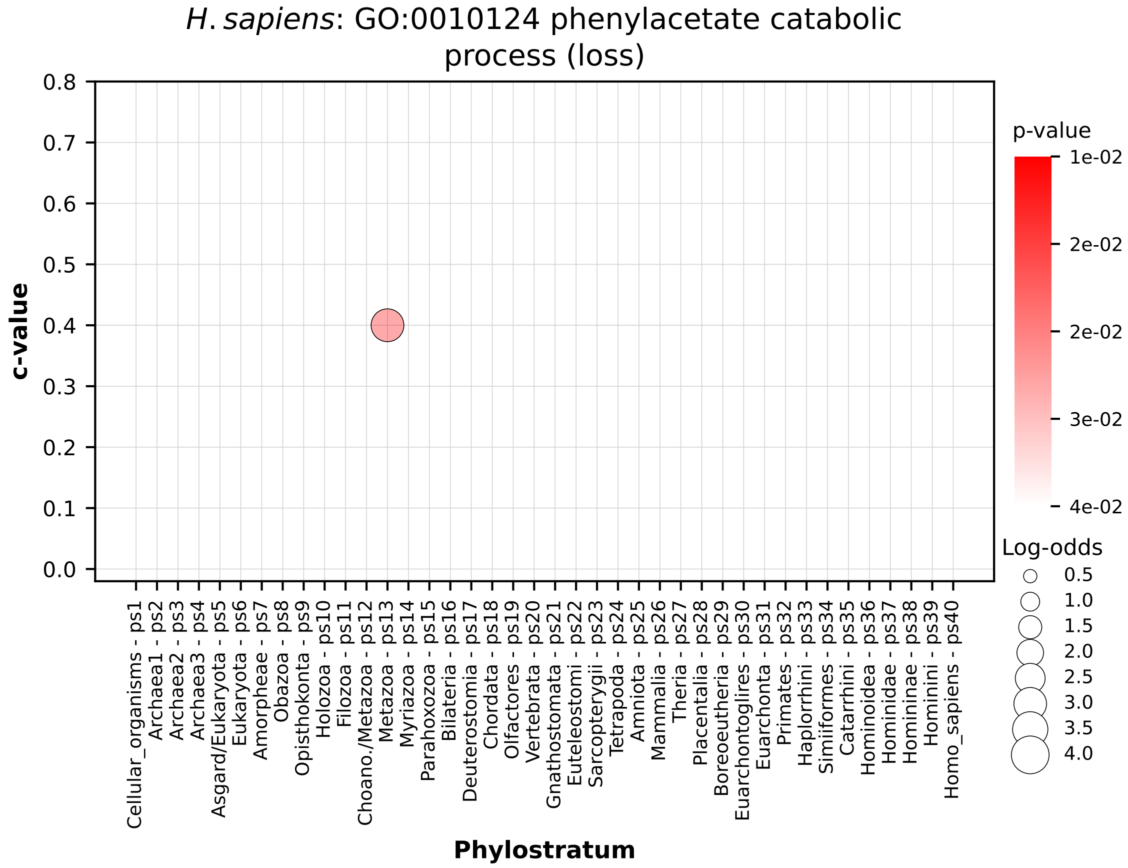

H. sapiens: GO:0010124 phenylacetate catabolic process (loss)
c-value
0.8
0.7
0.6
0.5
0.4
0.3
0.2
0.1
0.0
Phylostratum
Cellular_organisms - ps1
Archaea1 - ps2
Archaea2 - ps3
Archaea3 - ps4
Asgard/Eukaryota - ps5
Eukaryota - ps6
Amorpheae - ps7
Obazoa - ps8
Opisthokonta - ps9
Holozoa - ps10
Filozoa - ps11
Choano./Metazoa - ps12
Metazoa - ps13
Myriazoa - ps14
Parahoxozoa - ps15
Bilateria - ps16
Deuterostomia - ps17
Chordata - ps18
Olfactores - ps19
Vertebrata - ps20
Gnathostomata - ps21
Euteleostomi - ps22
Sarcopterygii - ps23
Tetrapoda - ps24
Amniota - ps25
Mammalia - ps26
Theria - ps27
Placentalia - ps28
Boreoeutheria - ps29
Euarchontoglires - ps30
Euarchonta - ps31
Primates - ps32
Haplorrhini - ps33
Simiiformes - ps34
Catarrhini - ps35
Hominoidea - ps36
Hominidae - ps37
Homininae - ps38
Hominini - ps39
Homo_sapiens - ps40
p-value
1e-02
2e-02
2e-02
3e-02
4e-02
Log-odds
0.5
1.0
1.5
2.0
2.5
3.0
3.5
4.0

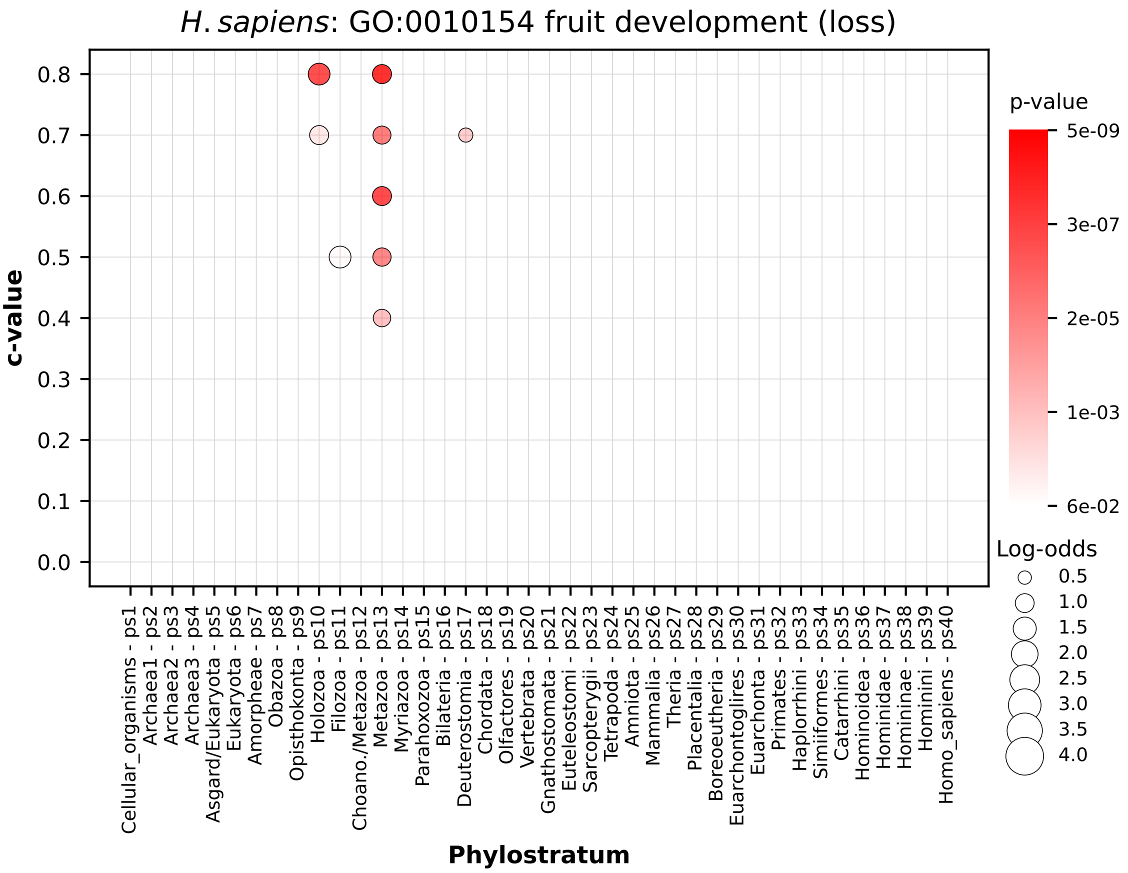

H. sapiens: GO:0010154 fruit development (loss)
c-value
0.0
0.1
0.2
0.3
0.4
0.5
0.6
0.7
0.8
Phylostratum
Cellular_organisms - ps1
Archaea1 - ps2
Archaea2 - ps3
Archaea3 - ps4
Asgard/Eukaryota - ps5
Eukaryota - ps6
Amorpheae - ps7
Obazoa - ps8
Opisthokonta - ps9
Holozoa - ps10
Filozoa - ps11
Choano./Metazoa - ps12
Metazoa - ps13
Myriazoa - ps14
Parahoxozoa - ps15
Bilateria - ps16
Deuterostomia - ps17
Chordata - ps18
Olfactores - ps19
Vertebrata - ps20
Gnathostomata - ps21
Euteleostomi - ps22
Sarcopterygii - ps23
Tetrapoda - ps24
Amniota - ps25
Mammalia - ps26
Theria - ps27
Placentalia - ps28
Boreoeutheria - ps29
Euarchontoglires - ps30
Euarchonta - ps31
Primates - ps32
Haplorrhini - ps33
Simiiformes - ps34
Catarrhini - ps35
Hominoidea - ps36
Hominidae - ps37
Homininae - ps38
Hominini - ps39
Homo_sapiens - ps40
p-value
5e-09
3e-07
2e-05
1e-03
6e-02
Log-odds
0.5
1.0
1.5
2.0
2.5
3.0
3.5
4.0

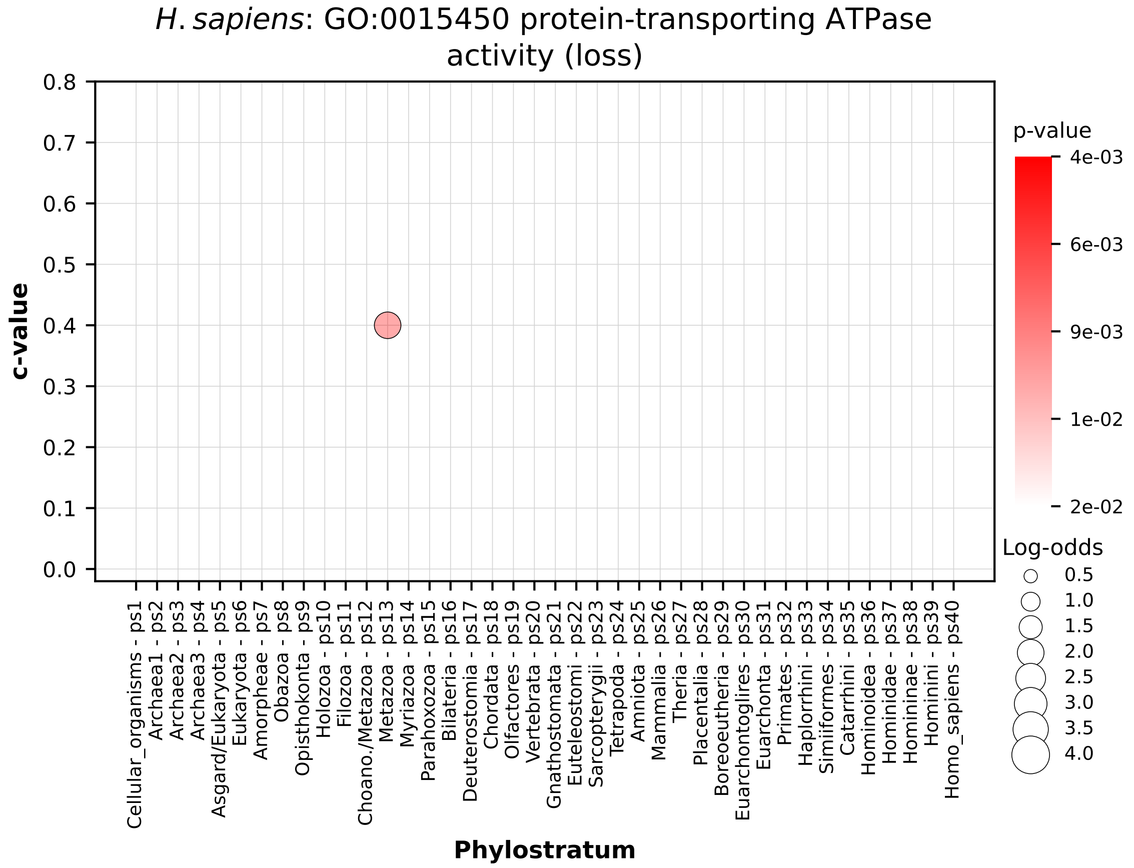
H. sapiens: GO:0015450 protein-transporting ATPase activity (loss)
c-value
0.0
0.1
0.2
0.3
0.4
0.5
0.6
0.7
0.8
Phylostratum
Cellular_organisms - ps1
Archaea1 - ps2
Archaea2 - ps3
Archaea3 - ps4
Asgard/Eukaryota - ps5
Eukaryota - ps6
Amorpheae - ps7
Obazoa - ps8
Opisthokonta - ps9
Holozoa - ps10
Filozoa - ps11
Choano./Metazoa - ps12
Metazoa - ps13
Myriazoa - ps14
Parahoxozoa - ps15
Bilateria - ps16
Deuterostomia - ps17
Chordata - ps18
Olfactores - ps19
Vertebrata - ps20
Gnathostomata - ps21
Euteleostomi - ps22
Sarcopterygii - ps23
Tetrapoda - ps24
Amniota - ps25
Mammalia - ps26
Theria - ps27
Placentalia - ps28
Boreoeutheria - ps29
Euarchontoglires - ps30
Euarchonta - ps31
Primates - ps32
Haplorrhini - ps33
Simiiformes - ps34
Catarrhini - ps35
Hominoidea - ps36
Hominidae - ps37
Homininae - ps38
Hominini - ps39
Homo_sapiens - ps40
p-value
4e-03
6e-03
9e-03
1e-02
2e-02
Log-odds
0.5
1.0
1.5
2.0
2.5
3.0
3.5
4.0

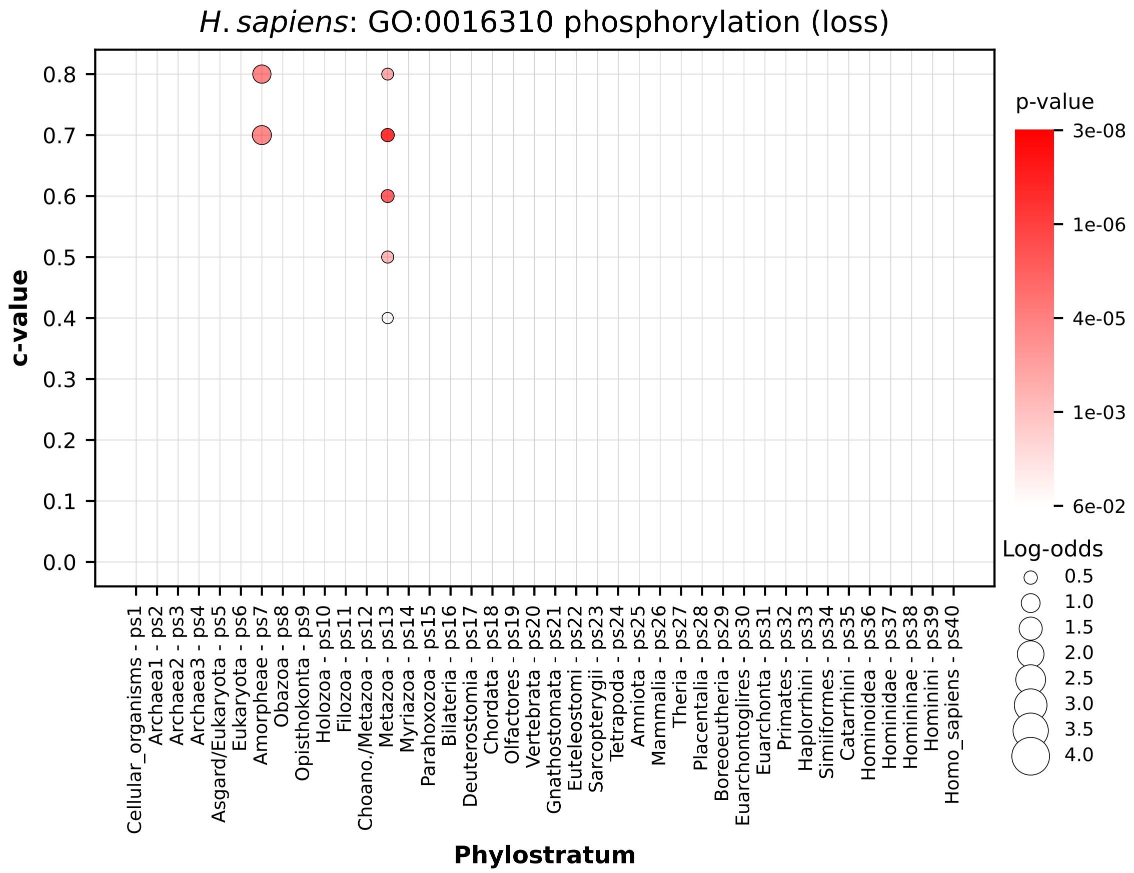

H. sapiens: GO:0016310 phosphorylation (loss)
c-value
0.0
0.1
0.2
0.3
0.4
0.5
0.6
0.7
0.8
Phylostratum
Cellular_organisms - ps1
Archaea1 - ps2
Archaea2 - ps3
Archaea3 - ps4
Asgard/Eukaryota - ps5
Eukaryota - ps6
Amorpheae - ps7
Obazoa - ps8
Opisthokonta - ps9
Holozoa - ps10
Filozoa - ps11
Choano./Metazoa - ps12
Metazoa - ps13
Myriazoa - ps14
Parahoxozoa - ps15
Bilateria - ps16
Deuterostomia - ps17
Chordata - ps18
Olfactores - ps19
Vertebrata - ps20
Gnathostomata - ps21
Euteleostomi - ps22
Sarcopterygii - ps23
Tetrapoda - ps24
Amniota - ps25
Mammalia - ps26
Theria - ps27
Placentalia - ps28
Boreoeutheria - ps29
Euarchontoglires - ps30
Euarchonta - ps31
Primates - ps32
Haplorrhini - ps33
Simiiformes - ps34
Catarrhini - ps35
Hominoidea - ps36
Hominidae - ps37
Homininae - ps38
Hominini - ps39
Homo_sapiens - ps40
p-value
3e-08
1e-06
4e-05
1e-03
6e-02
Log-odds
0.5
1.0
1.5
2.0
2.5
3.0
3.5
4.0

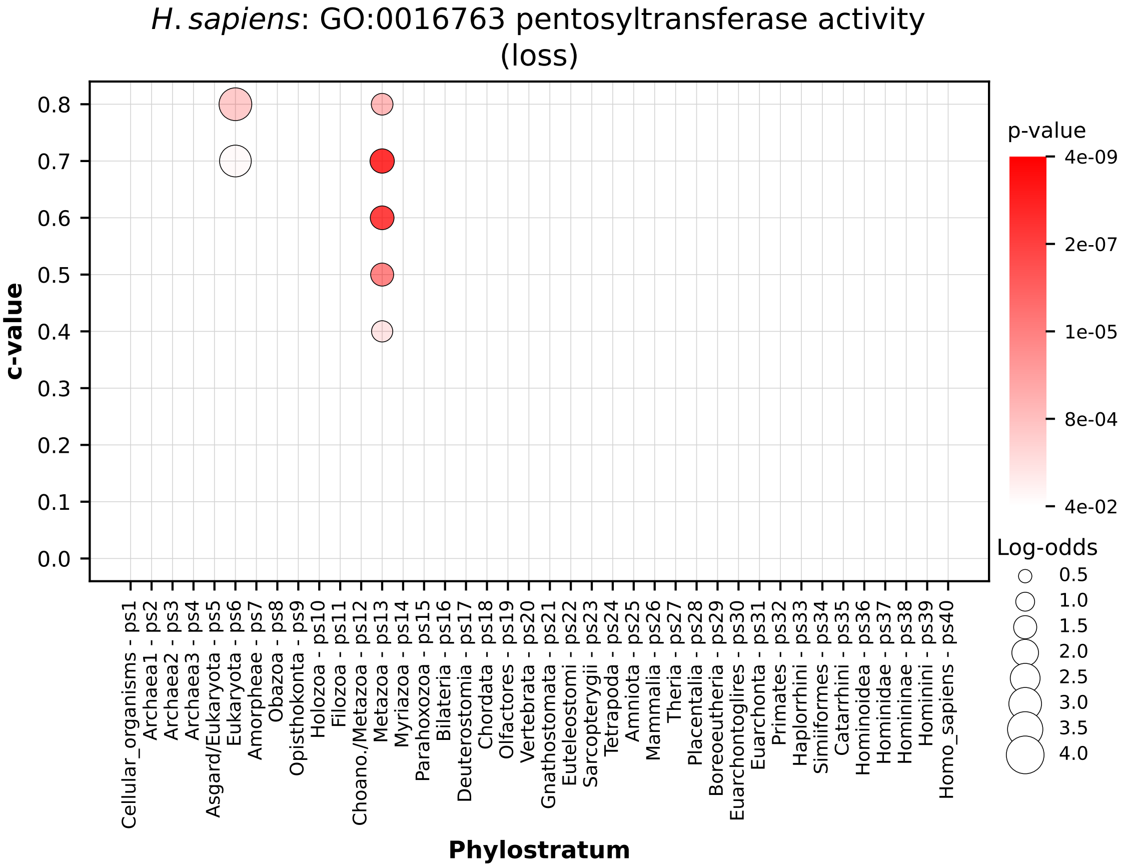

H. sapiens: GO:0016763 pentosyltransferase activity
(loss)
c-value
0.8
0.7
0.6
0.5
0.4
0.3
0.2
0.1
0.0
p-value
4e-09
2e-07
1e-05
8e-04
4e-02
Log-odds
0.5
1.0
1.5
2.0
2.5
3.0
3.5
4.0
Cellular_organisms - ps1
Archaea1 - ps2
Archaea2 - ps3
Archaea3 - ps4
Asgard/Eukaryota - ps5
Eukaryota - ps6
Amorpheae - ps7
Obazoa - ps8
Opisthokonta - ps9
Holozoa - ps10
Filozoa - ps11
Choano./Metazoa - ps12
Metazoa - ps13
Myriazoa - ps14
Parahoxozoa - ps15
Bilateria - ps16
Deuterostomia - ps17
Chordata - ps18
Olfactores - ps19
Vertebrata - ps20
Gnathostomata - ps21
Euteleostomi - ps22
Sarcopterygii - ps23
Tetrapoda - ps24
Amniota - ps25
Mammalia - ps26
Theria - ps27
Placentalia - ps28
Boreoeutheria - ps29
Euarchontoglires - ps30
Euarchonta - ps31
Primates - ps32
Haplorrhini - ps33
Simiiformes - ps34
Catarrhini - ps35
Hominoidea - ps36
Hominidae - ps37
Homininae - ps38
Hominini - ps39
Homo_sapiens - ps40
Phylostratum

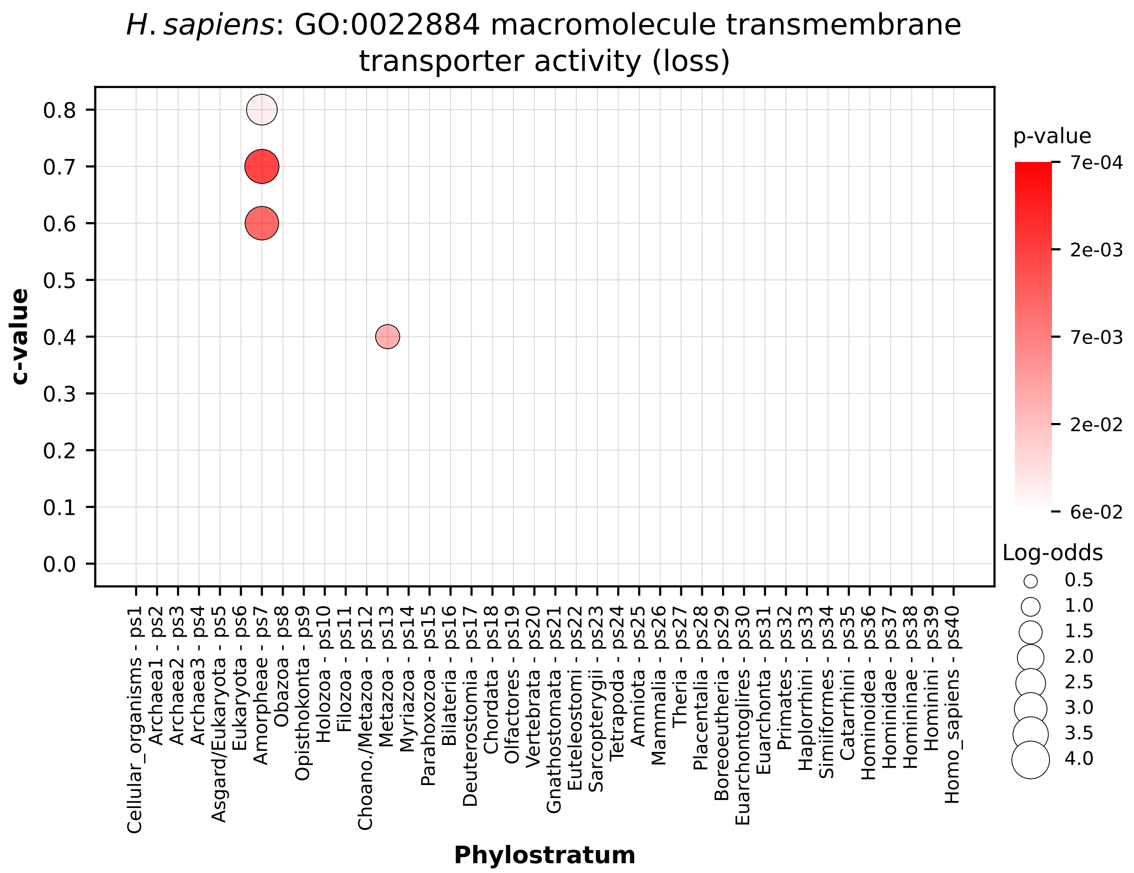
H. sapiens: GO:0022884 macromolecule transmembrane transporter activity (loss)
c-value
0.0
0.1
0.2
0.3
0.4
0.5
0.6
0.7
0.8
Phylostratum
Cellular_organisms - ps1
Archaea1 - ps2
Archaea2 - ps3
Archaea3 - ps4
Asgard/Eukaryota - ps5
Eukaryota - ps6
Amorpheae - ps7
Obazoa - ps8
Opisthokonta - ps9
Holozoa - ps10
Filozoa - ps11
Choano./Metazoa - ps12
Metazoa - ps13
Myriazoa - ps14
Parahoxozoa - ps15
Bilateria - ps16
Deuterostomia - ps17
Chordata - ps18
Olfactores - ps19
Vertebrata - ps20
Gnathostomata - ps21
Euteleostomi - ps22
Sarcopterygii - ps23
Tetrapoda - ps24
Amniota - ps25
Mammalia - ps26
Theria - ps27
Placentalia - ps28
Boreoeutheria - ps29
Euarchontoglires - ps30
Euarchonta - ps31
Primates - ps32
Haplorrhini - ps33
Simiiformes - ps34
Catarrhini - ps35
Hominoidea - ps36
Hominidae - ps37
Homininae - ps38
Hominini - ps39
Homo_sapiens - ps40
p-value
7e-04
2e-03
7e-03
2e-02
6e-02
Log-odds
0.5
1.0
1.5
2.0
2.5
3.0
3.5
4.0

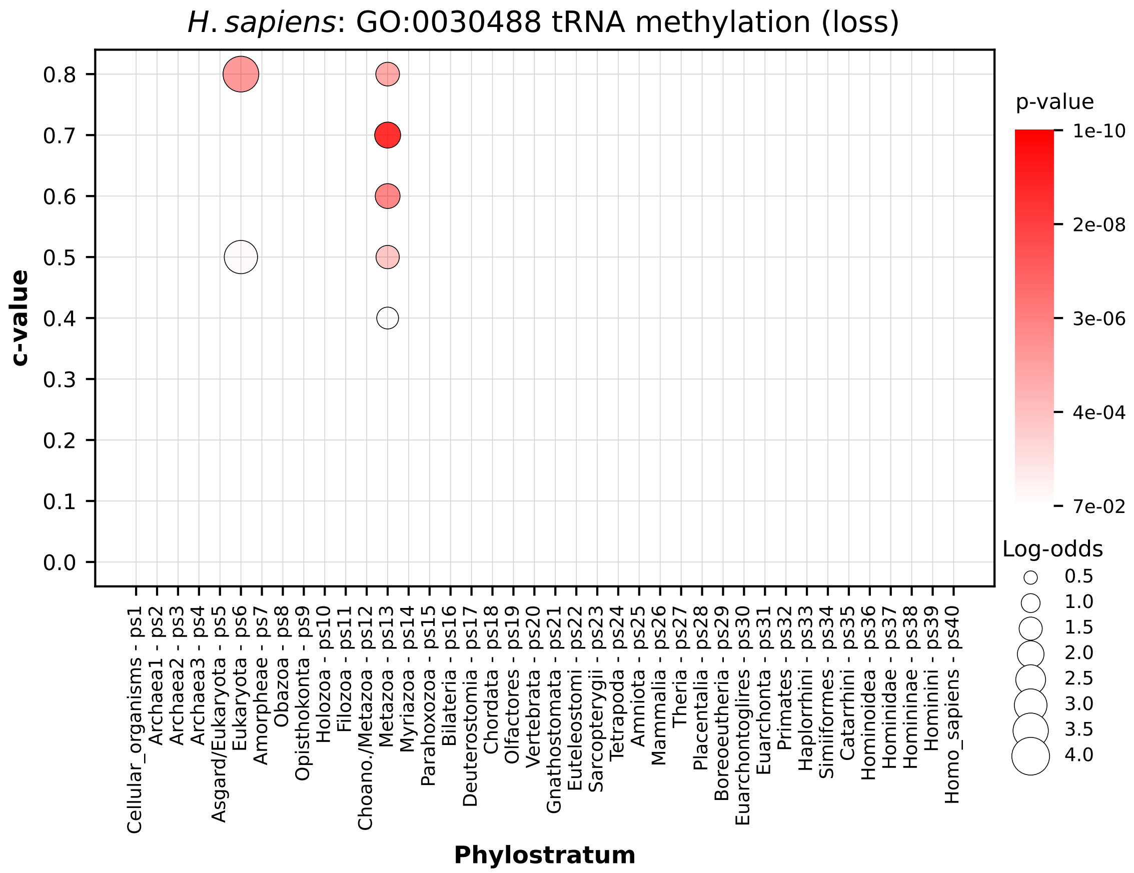

H. sapiens: GO:0030488 tRNA methylation (loss)
c-value
0.8
0.7
0.6
0.5
0.4
0.3
0.2
0.1
0.0
p-value
1e-10
2e-08
3e-06
4e-04
7e-02
Log-odds
0.5
1.0
1.5
2.0
2.5
3.0
3.5
4.0
Cellular_organisms - ps1
Archaea1 - ps2
Archaea2 - ps3
Archaea3 - ps4
Asgard/Eukaryota - ps5
Eukaryota - ps6
Amorpheae - ps7
Obazoa - ps8
Opisthokonta - ps9
Holozoa - ps10
Filozoa - ps11
Choano./Metazoa - ps12
Metazoa - ps13
Myriazoa - ps14
Parahoxozoa - ps15
Bilateria - ps16
Deuterostomia - ps17
Chordata - ps18
Olfactores - ps19
Vertebrata - ps20
Gnathostomata - ps21
Euteleostomi - ps22
Sarcopterygii - ps23
Tetrapoda - ps24
Amniota - ps25
Mammalia - ps26
Theria - ps27
Placentalia - ps28
Boreoeutheria - ps29
Euarchontoglires - ps30
Euarchonta - ps31
Primates - ps32
Haplorrhini - ps33
Simiiformes - ps34
Catarrhini - ps35
Hominoidea - ps36
Hominidae - ps37
Homininae - ps38
Hominini - ps39
Homo_sapiens - ps40
Phylostratum

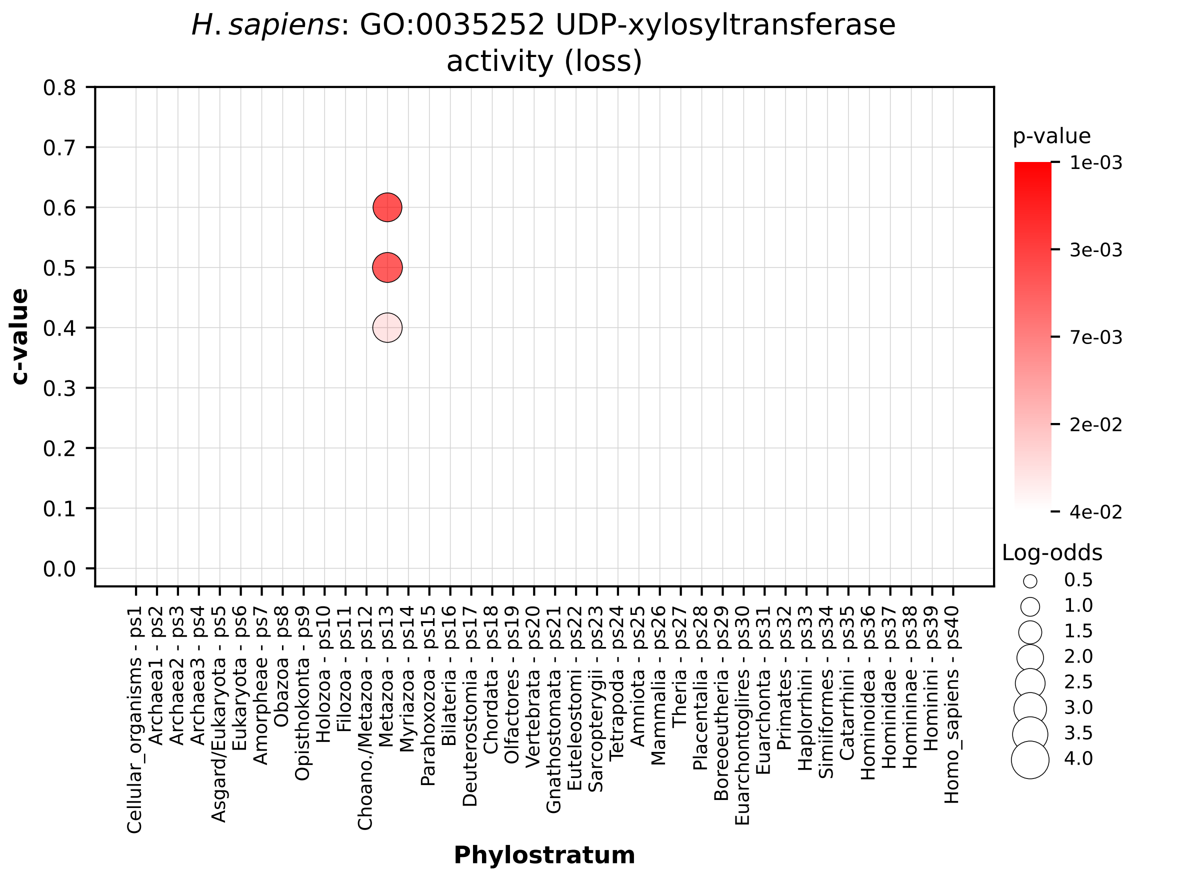

H. sapiens: GO:0035252 UDP-xylosyltransferase activity (loss)
c-value
0.8
0.7
0.6
0.5
0.4
0.3
0.2
0.1
0.0
p-value
1e-03
3e-03
7e-03
2e-02
4e-02
Log-odds
0.5
1.0
1.5
2.0
2.5
3.0
3.5
4.0
Cellular_organisms - ps1
Archaea1 - ps2
Archaea2 - ps3
Archaea3 - ps4
Asgard/Eukaryota - ps5
Eukaryota - ps6
Amorpheae - ps7
Obazoa - ps8
Opisthokonta - ps9
Holozoa - ps10
Filozoa - ps11
Choano./Metazoa - ps12
Metazoa - ps13
Myriazoa - ps14
Parahoxozoa - ps15
Bilateria - ps16
Deuterostomia - ps17
Chordata - ps18
Olfactores - ps19
Vertebrata - ps20
Gnathostomata - ps21
Euteleostomi - ps22
Sarcopterygii - ps23
Tetrapoda - ps24
Amniota - ps25
Mammalia - ps26
Theria - ps27
Placentalia - ps28
Boreoeutheria - ps29
Euarchontoglires - ps30
Euarchonta - ps31
Primates - ps32
Haplorrhini - ps33
Simiiformes - ps34
Catarrhini - ps35
Hominoidea - ps36
Hominidae - ps37
Homininae - ps38
Hominini - ps39
Homo_sapiens - ps40
Phylostratum

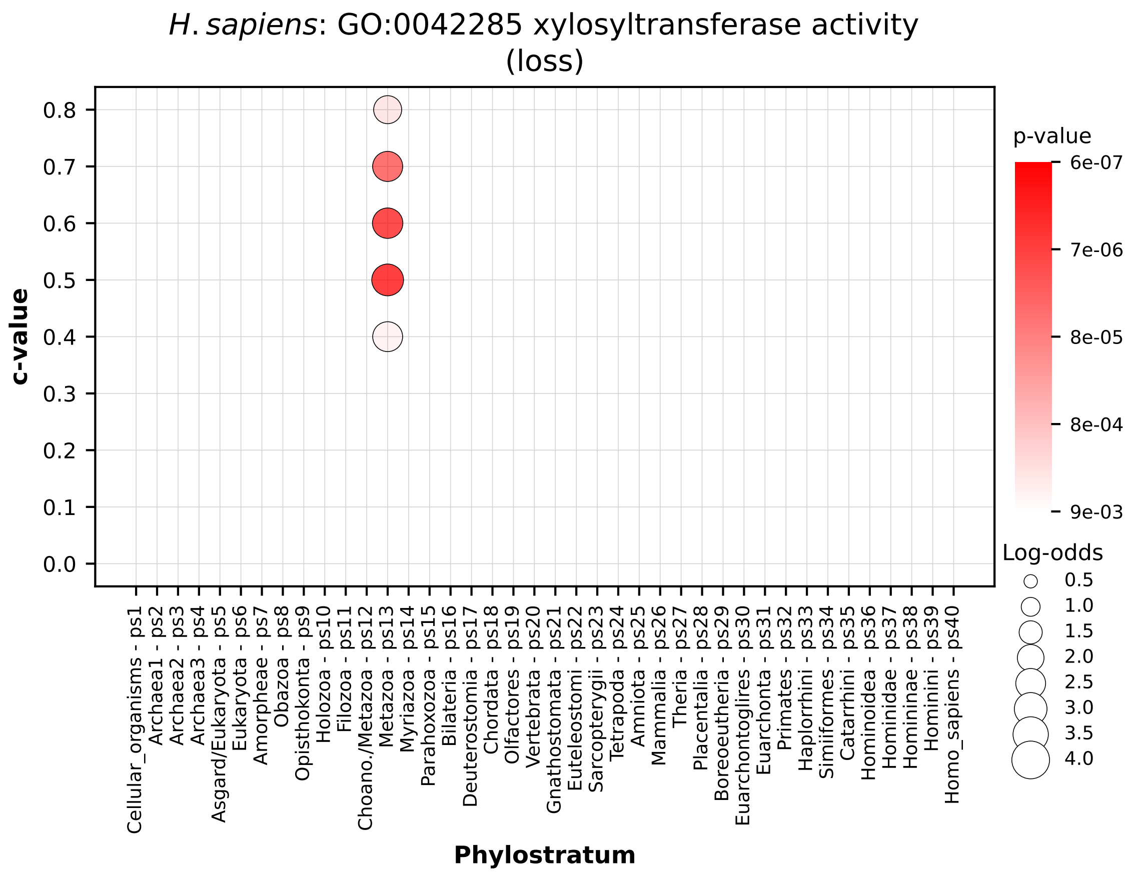

H. sapiens: GO:0042285 xylosyltransferase activity
(loss)
c-value
0.0
0.1
0.2
0.3
0.4
0.5
0.6
0.7
0.8
Phylostratum
Cellular_organisms - ps1
Archaea1 - ps2
Archaea2 - ps3
Archaea3 - ps4
Asgard/Eukaryota - ps5
Eukaryota - ps6
Amorpheae - ps7
Obazoa - ps8
Opisthokonta - ps9
Holozoa - ps10
Filozoa - ps11
Choano./Metazoa - ps12
Metazoa - ps13
Myriazoa - ps14
Parahoxozoa - ps15
Bilateria - ps16
Deuterostomia - ps17
Chordata - ps18
Olfactores - ps19
Vertebrata - ps20
Gnathostomata - ps21
Euteleostomi - ps22
Sarcopterygii - ps23
Tetrapoda - ps24
Amniota - ps25
Mammalia - ps26
Theria - ps27
Placentalia - ps28
Boreoeutheria - ps29
Euarchontoglires - ps30
Euarchonta - ps31
Primates - ps32
Haplorrhini - ps33
Simiiformes - ps34
Catarrhini - ps35
Hominoidea - ps36
Hominidae - ps37
Homininae - ps38
Hominini - ps39
Homo_sapiens - ps40
p-value
6e-07
7e-06
8e-05
8e-04
9e-03
Log-odds
0.5
1.0
1.5
2.0
2.5
3.0
3.5
4.0

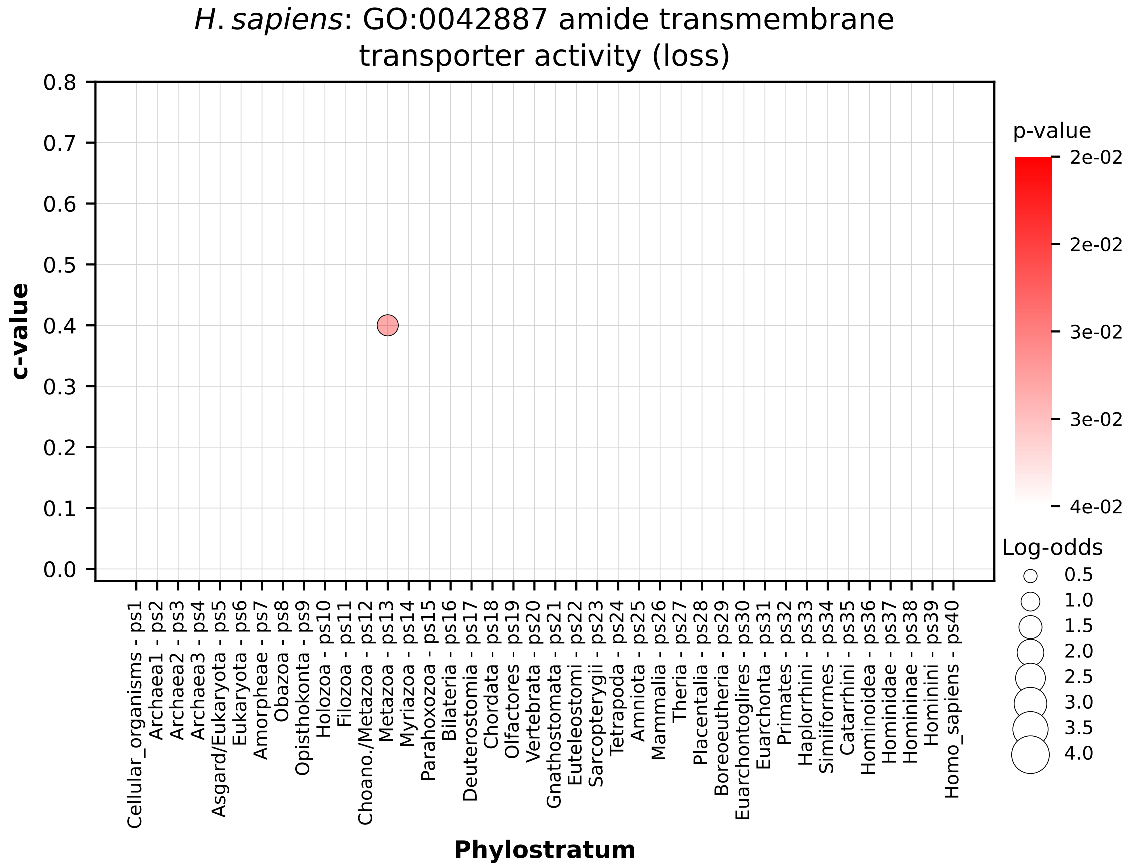

H. sapiens: GO:0042887 amide transmembrane transporter activity (loss)
c-value
0.8
0.7
0.6
0.5
0.4
0.3
0.2
0.1
0.0
p-value
2e-02
2e-02
3e-02
3e-02
4e-02
Log-odds
0.5
1.0
1.5
2.0
2.5
3.0
3.5
4.0
Cellular_organisms - ps1
Archaea1 - ps2
Archaea2 - ps3
Archaea3 - ps4
Asgard/Eukaryota - ps5
Eukaryota - ps6
Amorpheae - ps7
Obazoa - ps8
Opisthokonta - ps9
Holozoa - ps10
Filozoa - ps11
Choano./Metazoa - ps12
Metazoa - ps13
Myriazoa - ps14
Parahoxozoa - ps15
Bilateria - ps16
Deuterostomia - ps17
Chordata - ps18
Olfactores - ps19
Vertebrata - ps20
Gnathostomata - ps21
Euteleostomi - ps22
Sarcopterygii - ps23
Tetrapoda - ps24
Amniota - ps25
Mammalia - ps26
Theria - ps27
Placentalia - ps28
Boreoeutheria - ps29
Euarchontoglires - ps30
Euarchonta - ps31
Primates - ps32
Haplorrhini - ps33
Simiiformes - ps34
Catarrhini - ps35
Hominoidea - ps36
Hominidae - ps37
Homininae - ps38
Hominini - ps39
Homo_sapiens - ps40
Phylostratum

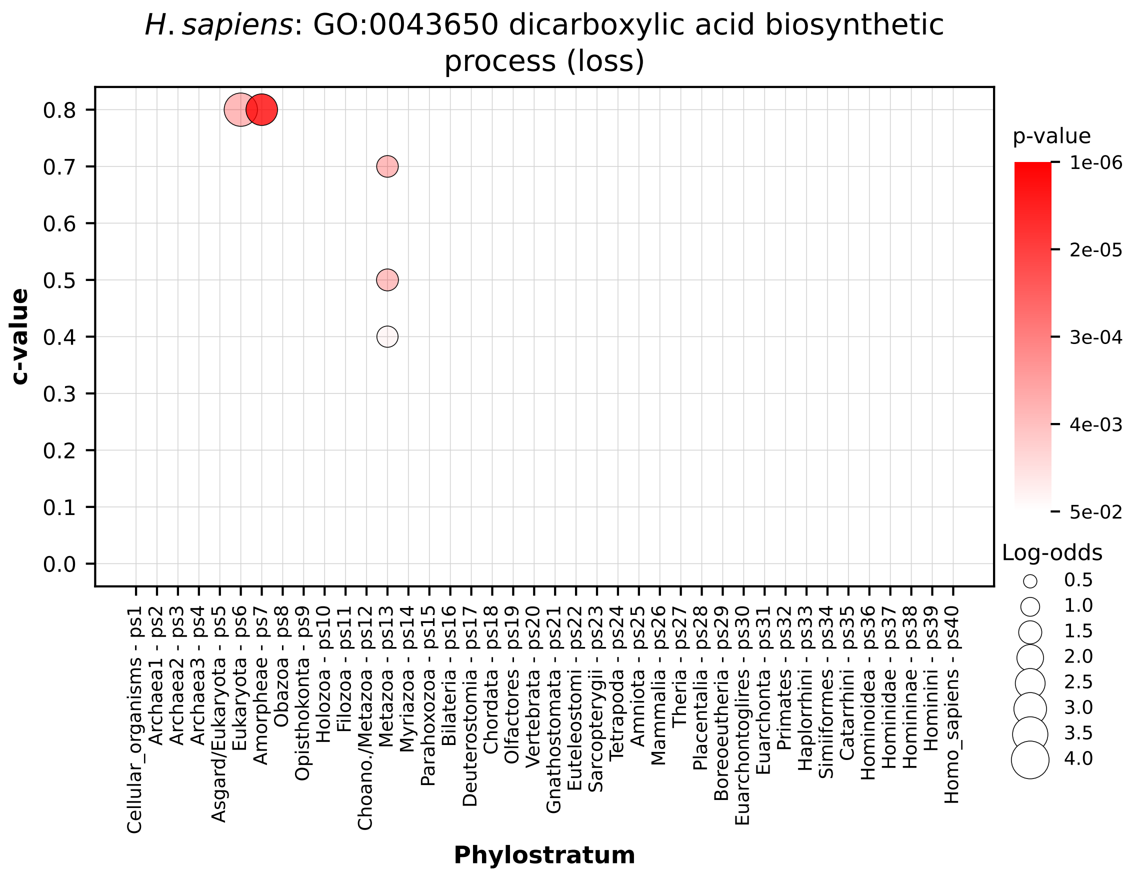

H. sapiens: GO:0043650 dicarboxylic acid biosynthetic process (loss)
c-value
0.0
0.1
0.2
0.3
0.4
0.5
0.6
0.7
0.8
Phylostratum
Cellular_organisms - ps1
Archaea1 - ps2
Archaea2 - ps3
Archaea3 - ps4
Asgard/Eukaryota - ps5
Eukaryota - ps6
Amorpheae - ps7
Obazoa - ps8
Opisthokonta - ps9
Holozoa - ps10
Filozoa - ps11
Choano./Metazoa - ps12
Metazoa - ps13
Myriazoa - ps14
Parahoxozoa - ps15
Bilateria - ps16
Deuterostomia - ps17
Chordata - ps18
Olfactores - ps19
Vertebrata - ps20
Gnathostomata - ps21
Euteleostomi - ps22
Sarcopterygii - ps23
Tetrapoda - ps24
Amniota - ps25
Mammalia - ps26
Theria - ps27
Placentalia - ps28
Boreoeutheria - ps29
Euarchontoglires - ps30
Euarchonta - ps31
Primates - ps32
Haplorrhini - ps33
Simiiformes - ps34
Catarrhini - ps35
Hominoidea - ps36
Hominidae - ps37
Homininae - ps38
Hominini - ps39
Homo_sapiens - ps40
p-value
1e-06
2e-05
3e-04
4e-03
5e-02
Log-odds
0.5
1.0
1.5
2.0
2.5
3.0
3.5
4.0

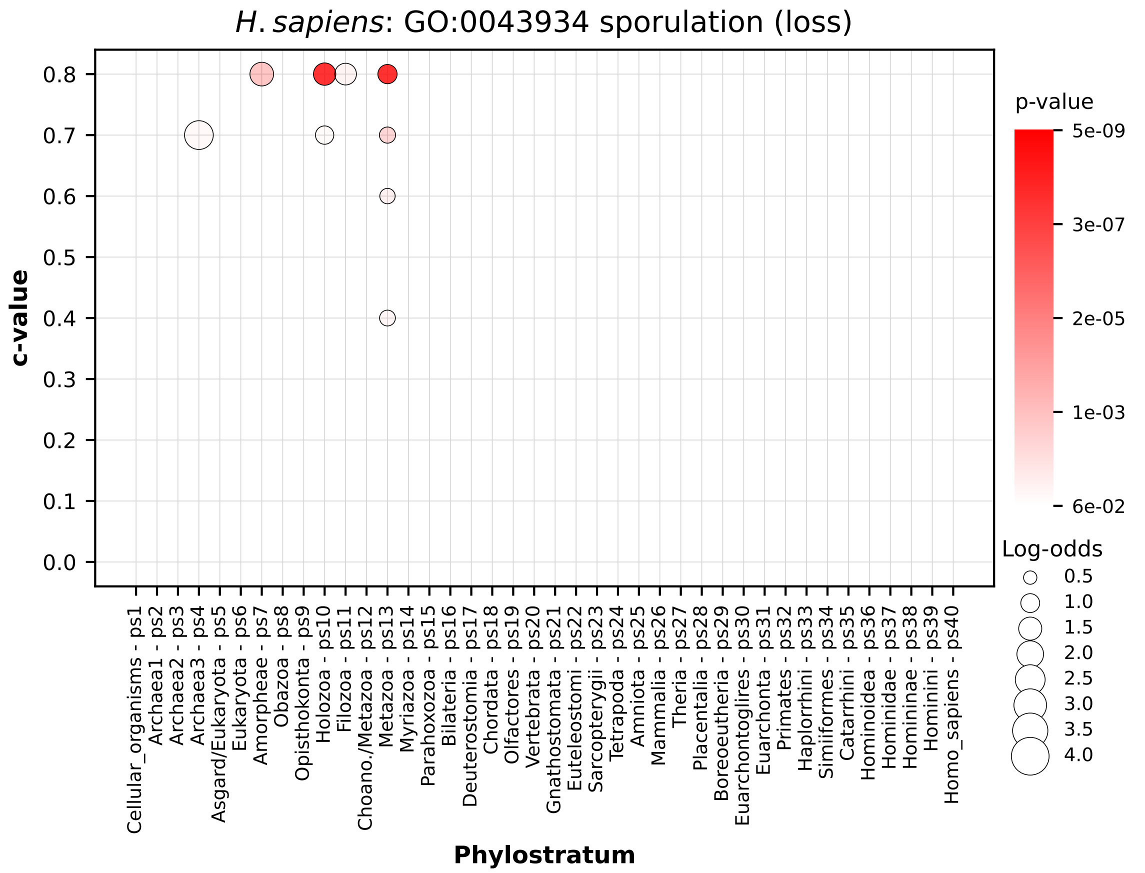

H. sapiens: GO:0043934 sporulation (loss)
c-value
0.0
0.1
0.2
0.3
0.4
0.5
0.6
0.7
0.8
Phylostratum
Cellular_organisms - ps1
Archaea1 - ps2
Archaea2 - ps3
Archaea3 - ps4
Asgard/Eukaryota - ps5
Eukaryota - ps6
Amorpheae - ps7
Obazoa - ps8
Opisthokonta - ps9
Holozoa - ps10
Filozoa - ps11
Choano./Metazoa - ps12
Metazoa - ps13
Myriazoa - ps14
Parahoxozoa - ps15
Bilateria - ps16
Deuterostomia - ps17
Chordata - ps18
Olfactores - ps19
Vertebrata - ps20
Gnathostomata - ps21
Euteleostomi - ps22
Sarcopterygii - ps23
Tetrapoda - ps24
Amniota - ps25
Mammalia - ps26
Theria - ps27
Placentalia - ps28
Boreoeutheria - ps29
Euarchontoglires - ps30
Euarchonta - ps31
Primates - ps32
Haplorrhini - ps33
Simiiformes - ps34
Catarrhini - ps35
Hominoidea - ps36
Hominidae - ps37
Homininae - ps38
Hominini - ps39
Homo_sapiens - ps40
p-value
5e-09
3e-07
2e-05
1e-03
6e-02
Log-odds
0.5
1.0
1.5
2.0
2.5
3.0
3.5
4.0

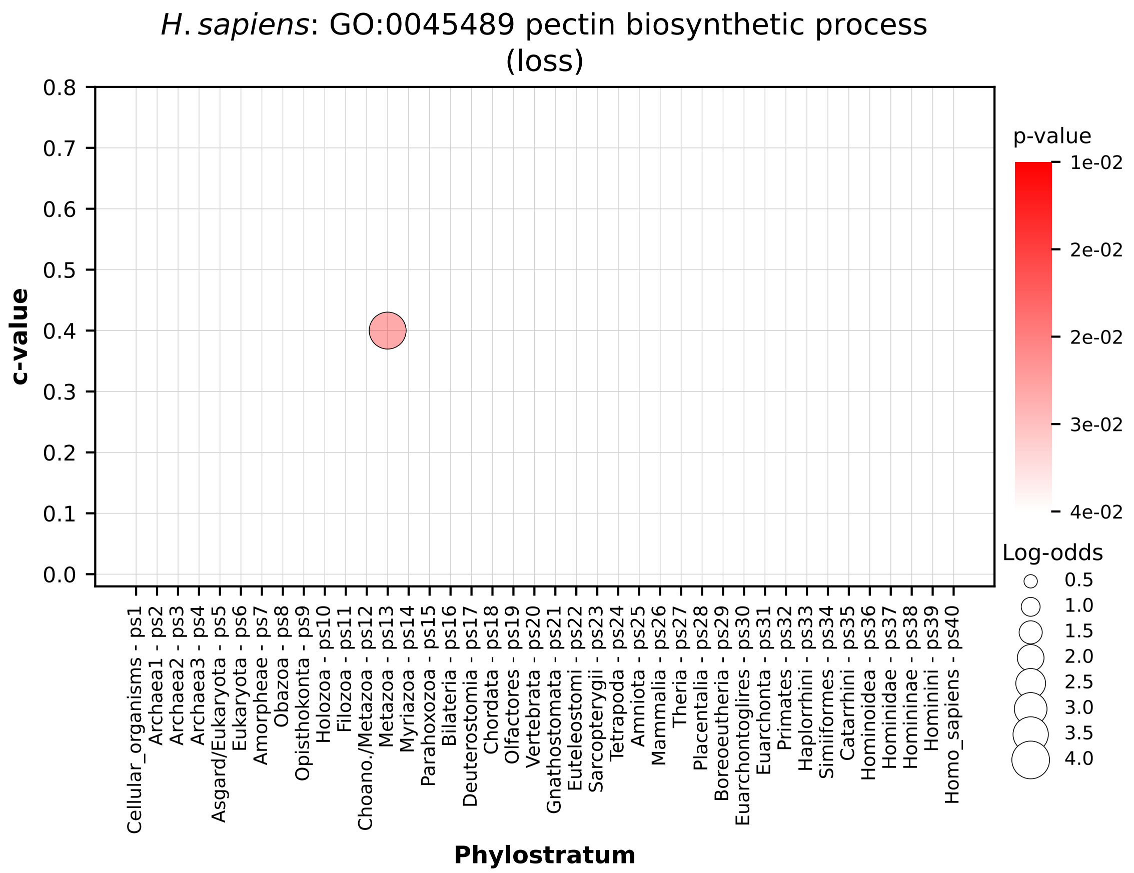

H. sapiens: GO:0045489 pectin biosynthetic process (loss)
c-value
0.0
0.1
0.2
0.3
0.4
0.5
0.6
0.7
0.8
Phylostratum
Cellular_organisms - ps1
Archaea1 - ps2
Archaea2 - ps3
Archaea3 - ps4
Asgard/Eukaryota - ps5
Eukaryota - ps6
Amorpheae - ps7
Obazoa - ps8
Opisthokonta - ps9
Holozoa - ps10
Filozoa - ps11
Choano./Metazoa - ps12
Metazoa - ps13
Myriazoa - ps14
Parahoxozoa - ps15
Bilateria - ps16
Deuterostomia - ps17
Chordata - ps18
Olfactores - ps19
Vertebrata - ps20
Gnathostomata - ps21
Euteleostomi - ps22
Sarcopterygii - ps23
Tetrapoda - ps24
Amniota - ps25
Mammalia - ps26
Theria - ps27
Placentalia - ps28
Boreoeutheria - ps29
Euarchontoglires - ps30
Euarchonta - ps31
Primates - ps32
Haplorrhini - ps33
Simiiformes - ps34
Catarrhini - ps35
Hominoidea - ps36
Hominidae - ps37
Homininae - ps38
Hominini - ps39
Homo_sapiens - ps40
p-value
1e-02
2e-02
2e-02
3e-02
4e-02
Log-odds
0.5
1.0
1.5
2.0
2.5
3.0
3.5
4.0

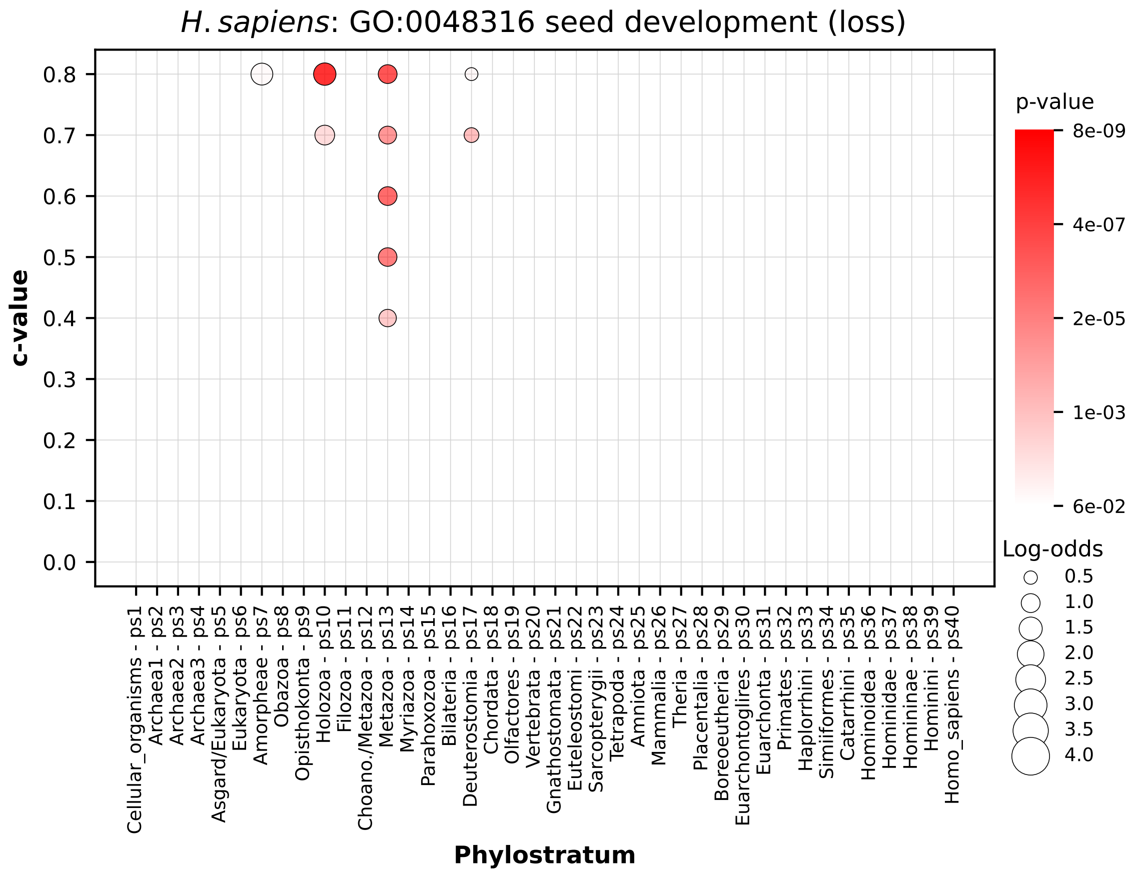

H. sapiens: GO:0048316 seed development (loss)
c-value
0.8
0.7
0.6
0.5
0.4
0.3
0.2
0.1
0.0
p-value
8e-09
4e-07
2e-05
1e-03
6e-02
Log-odds
0.5
1.0
1.5
2.0
2.5
3.0
3.5
4.0
Cellular_organisms - ps1
Archaea1 - ps2
Archaea2 - ps3
Archaea3 - ps4
Asgard/Eukaryota - ps5
Eukaryota - ps6
Amorpheae - ps7
Obazoa - ps8
Opisthokonta - ps9
Holozoa - ps10
Filozoa - ps11
Choano./Metazoa - ps12
Metazoa - ps13
Myriazoa - ps14
Parahoxozoa - ps15
Bilateria - ps16
Deuterostomia - ps17
Chordata - ps18
Olfactores - ps19
Vertebrata - ps20
Gnathostomata - ps21
Euteleostomi - ps22
Sarcopterygii - ps23
Tetrapoda - ps24
Amniota - ps25
Mammalia - ps26
Theria - ps27
Placentalia - ps28
Boreoeutheria - ps29
Euarchontoglires - ps30
Euarchonta - ps31
Primates - ps32
Haplorrhini - ps33
Simiiformes - ps34
Catarrhini - ps35
Hominoidea - ps36
Hominidae - ps37
Homininae - ps38
Hominini - ps39
Homo_sapiens - ps40
Phylostratum

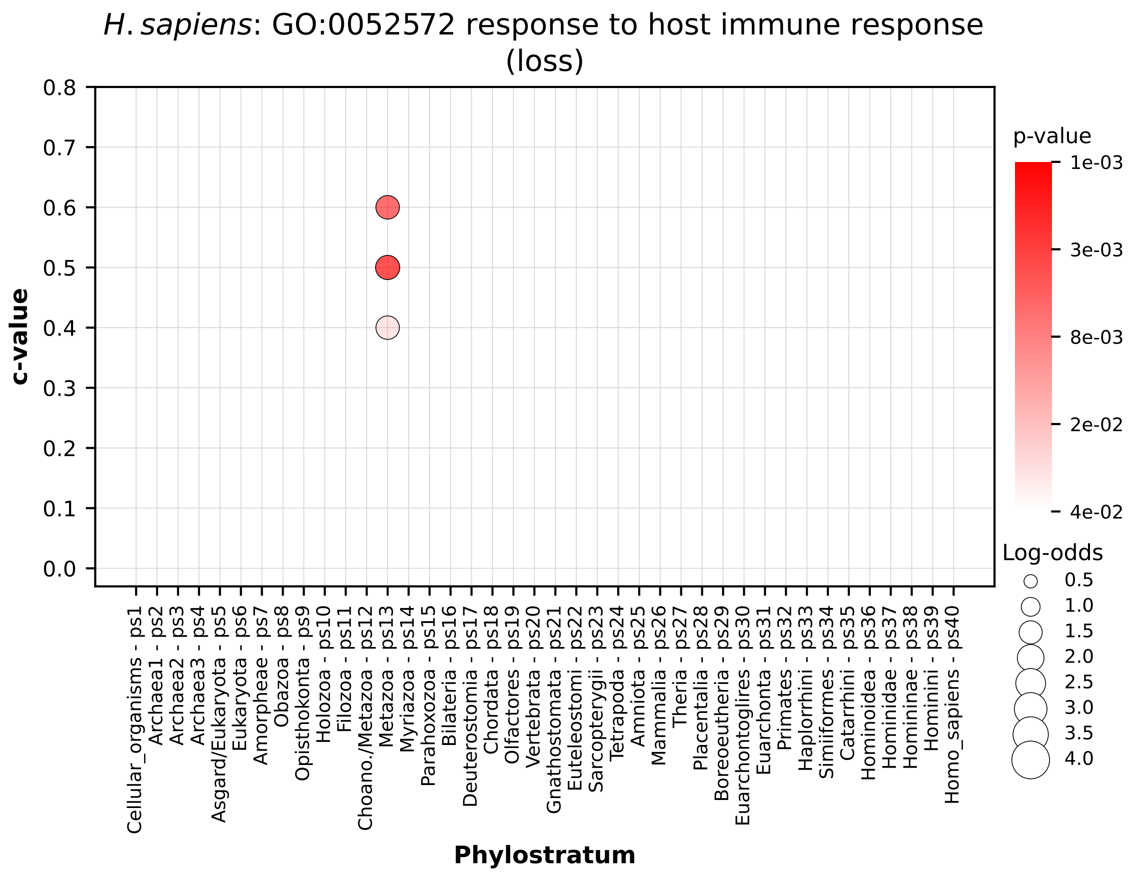

H. sapiens: GO:0052572 response to host immune response (loss)
c-value
0.8
0.7
0.6
0.5
0.4
0.3
0.2
0.1
0.0
p-value
1e-03
3e-03
8e-03
2e-02
4e-02
Log-odds
0.5
1.0
1.5
2.0
2.5
3.0
3.5
4.0
Cellular_organisms - ps1
Archaea1 - ps2
Archaea2 - ps3
Archaea3 - ps4
Asgard/Eukaryota - ps5
Eukaryota - ps6
Amorpheae - ps7
Obazoa - ps8
Opisthokonta - ps9
Holozoa - ps10
Filozoa - ps11
Choano./Metazoa - ps12
Metazoa - ps13
Myriazoa - ps14
Parahoxozoa - ps15
Bilateria - ps16
Deuterostomia - ps17
Chordata - ps18
Olfactores - ps19
Vertebrata - ps20
Gnathostomata - ps21
Euteleostomi - ps22
Sarcopterygii - ps23
Tetrapoda - ps24
Amniota - ps25
Mammalia - ps26
Theria - ps27
Placentalia - ps28
Boreoeutheria - ps29
Euarchontoglires - ps30
Euarchonta - ps31
Primates - ps32
Haplorrhini - ps33
Simiiformes - ps34
Catarrhini - ps35
Hominoidea - ps36
Hominidae - ps37
Homininae - ps38
Hominini - ps39
Homo_sapiens - ps40
Phylostratum

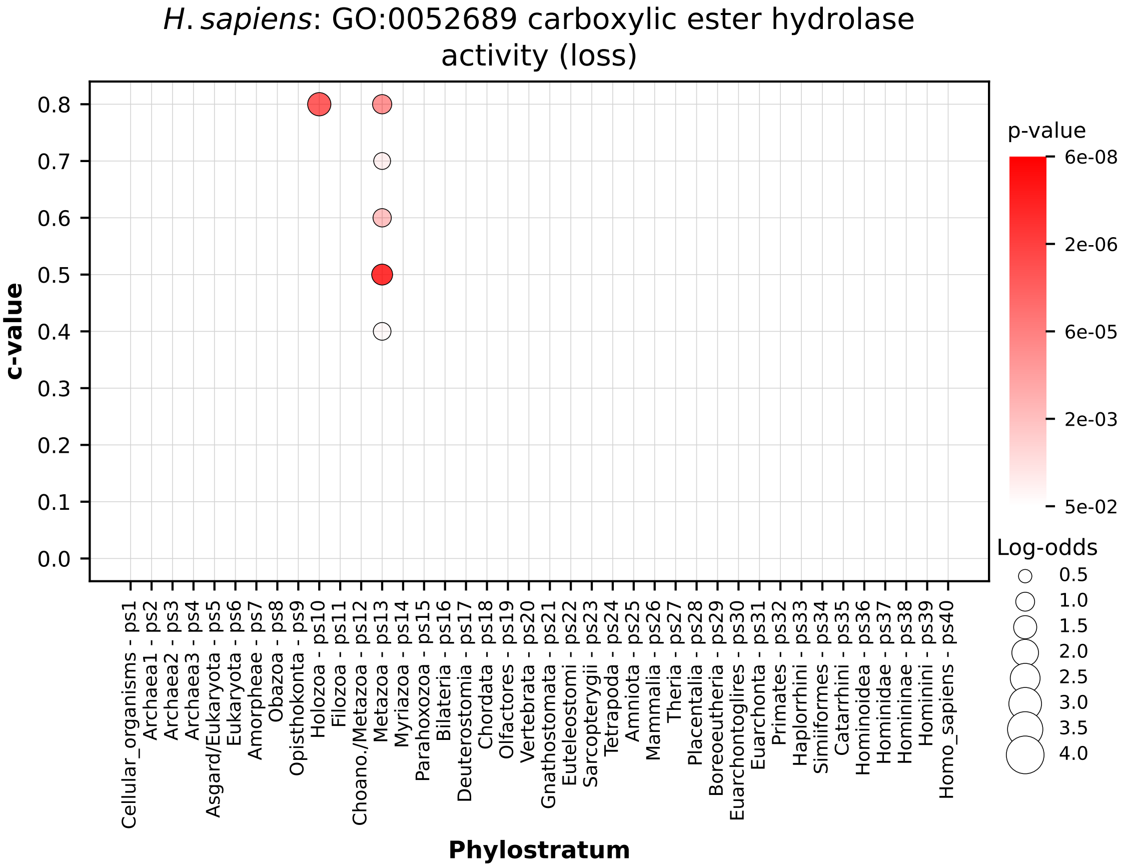

$H. sapiens$: GO:0052689 carboxylic ester hydrolase activity (loss)
c-value
0.0
0.1
0.2
0.3
0.4
0.5
0.6
0.7
0.8
Phylostratum
Cellular_organisms - ps1
Archaea1 - ps2
Archaea2 - ps3
Archaea3 - ps4
Asgard/Eukaryota - ps5
Eukaryota - ps6
Amorpheae - ps7
Obazoa - ps8
Opisthokonta - ps9
Holozoa - ps10
Filozoa - ps11
Choano./Metazoa - ps12
Metazoa - ps13
Myriazoa - ps14
Parahoxozoa - ps15
Bilateria - ps16
Deuterostomia - ps17
Chordata - ps18
Olfactores - ps19
Vertebrata - ps20
Gnathostomata - ps21
Euteleostomi - ps22
Sarcopterygii - ps23
Tetrapoda - ps24
Amniota - ps25
Mammalia - ps26
Theria - ps27
Placentalia - ps28
Boreoeutheria - ps29
Euarchontoglires - ps30
Euarchonta - ps31
Primates - ps32
Haplorrhini - ps33
Simiiformes - ps34
Catarrhini - ps35
Hominoidea - ps36
Hominidae - ps37
Homininae - ps38
Hominini - ps39
Homo_sapiens - ps40
p-value
6e-08
2e-06
6e-05
2e-03
5e-02
Log-odds
0.5
1.0
1.5
2.0
2.5
3.0
3.5
4.0

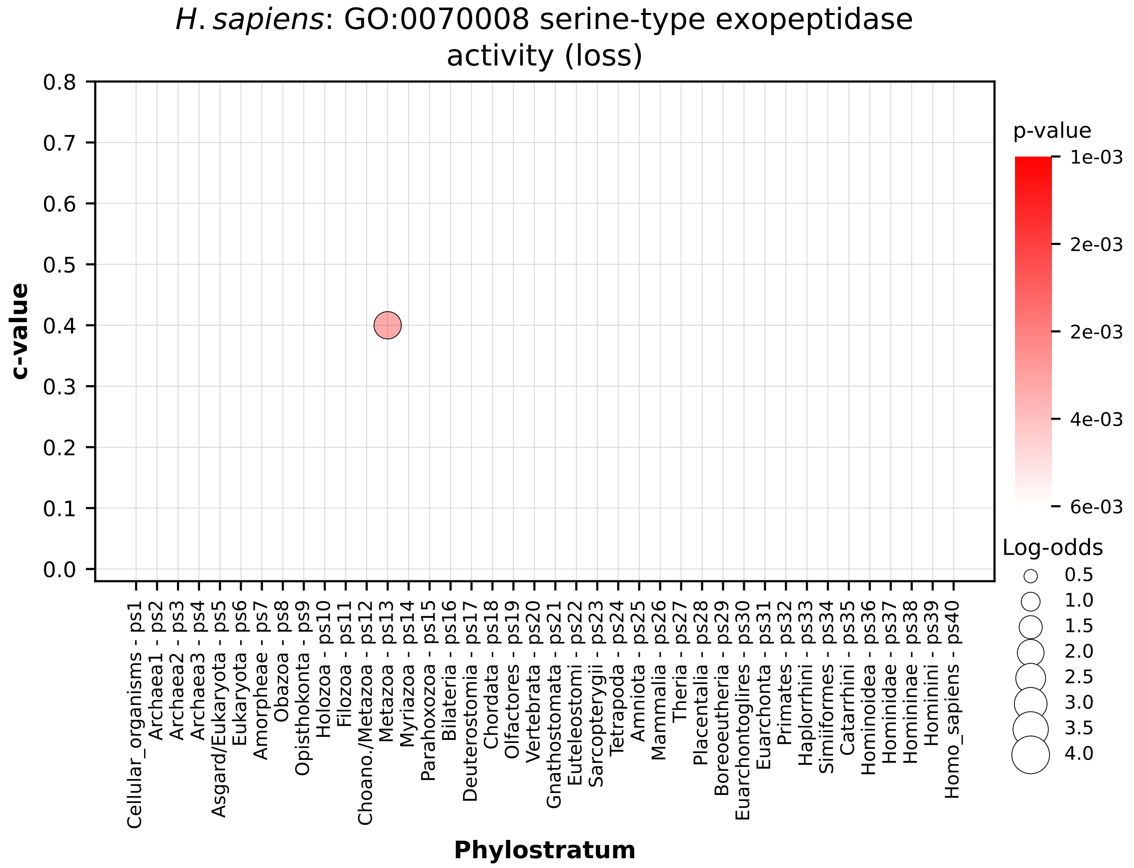

H. sapiens: GO:0070008 serine-type exopeptidase activity (loss)
c-value
0.0
0.1
0.2
0.3
0.4
0.5
0.6
0.7
0.8
Phylostratum
Cellular_organisms - ps1
Archaea1 - ps2
Archaea2 - ps3
Archaea3 - ps4
Asgard/Eukaryota - ps5
Eukaryota - ps6
Amorpheae - ps7
Obazoa - ps8
Opisthokonta - ps9
Holozoa - ps10
Filozoa - ps11
Choano./Metazoa - ps12
Metazoa - ps13
Myriazoa - ps14
Parahoxozoa - ps15
Bilateria - ps16
Deuterostomia - ps17
Chordata - ps18
Olfactores - ps19
Vertebrata - ps20
Gnathostomata - ps21
Euteleostomi - ps22
Sarcopterygii - ps23
Tetrapoda - ps24
Amniota - ps25
Mammalia - ps26
Theria - ps27
Placentalia - ps28
Boreoeutheria - ps29
Euarchontoglires - ps30
Euarchonta - ps31
Primates - ps32
Haplorrhini - ps33
Simiiformes - ps34
Catarrhini - ps35
Hominoidea - ps36
Hominidae - ps37
Homininae - ps38
Hominini - ps39
Homo_sapiens - ps40
p-value
1e-03
2e-03
2e-03
4e-03
6e-03
Log-odds
0.5
1.0
1.5
2.0
2.5
3.0
3.5
4.0

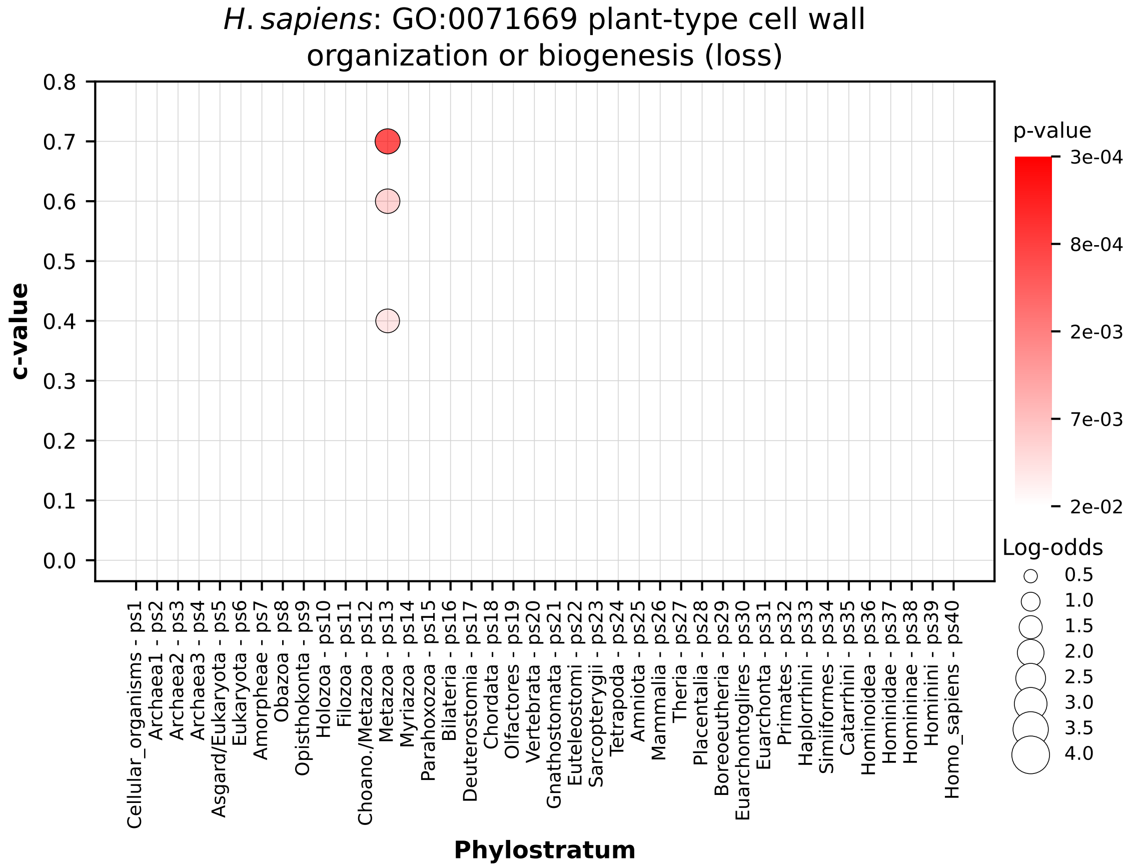
H. sapiens: GO:0071669 plant-type cell wall organization or biogenesis (loss)
c-value
0.8
0.7
0.6
0.5
0.4
0.3
0.2
0.1
0.0
Phylostratum
Cellular_organisms - ps1
Archaea1 - ps2
Archaea2 - ps3
Archaea3 - ps4
Asgard/Eukaryota - ps5
Eukaryota - ps6
Amorpheae - ps7
Obazoa - ps8
Opisthokonta - ps9
Holozoa - ps10
Filozoa - ps11
Choano./Metazoa - ps12
Metazoa - ps13
Myriazoa - ps14
Parahoxozoa - ps15
Bilateria - ps16
Deuterostomia - ps17
Chordata - ps18
Olfactores - ps19
Vertebrata - ps20
Gnathostomata - ps21
Euteleostomi - ps22
Sarcopterygii - ps23
Tetrapoda - ps24
Amniota - ps25
Mammalia - ps26
Theria - ps27
Placentalia - ps28
Boreoeutheria - ps29
Euarchontoglires - ps30
Euarchonta - ps31
Primates - ps32
Haplorrhini - ps33
Simiiformes - ps34
Catarrhini - ps35
Hominoidea - ps36
Hominidae - ps37
Homininae - ps38
Hominini - ps39
Homo_sapiens - ps40
p-value
3e-04
8e-04
2e-03
7e-03
2e-02
Log-odds
0.5
1.0
1.5
2.0
2.5
3.0
3.5
4.0

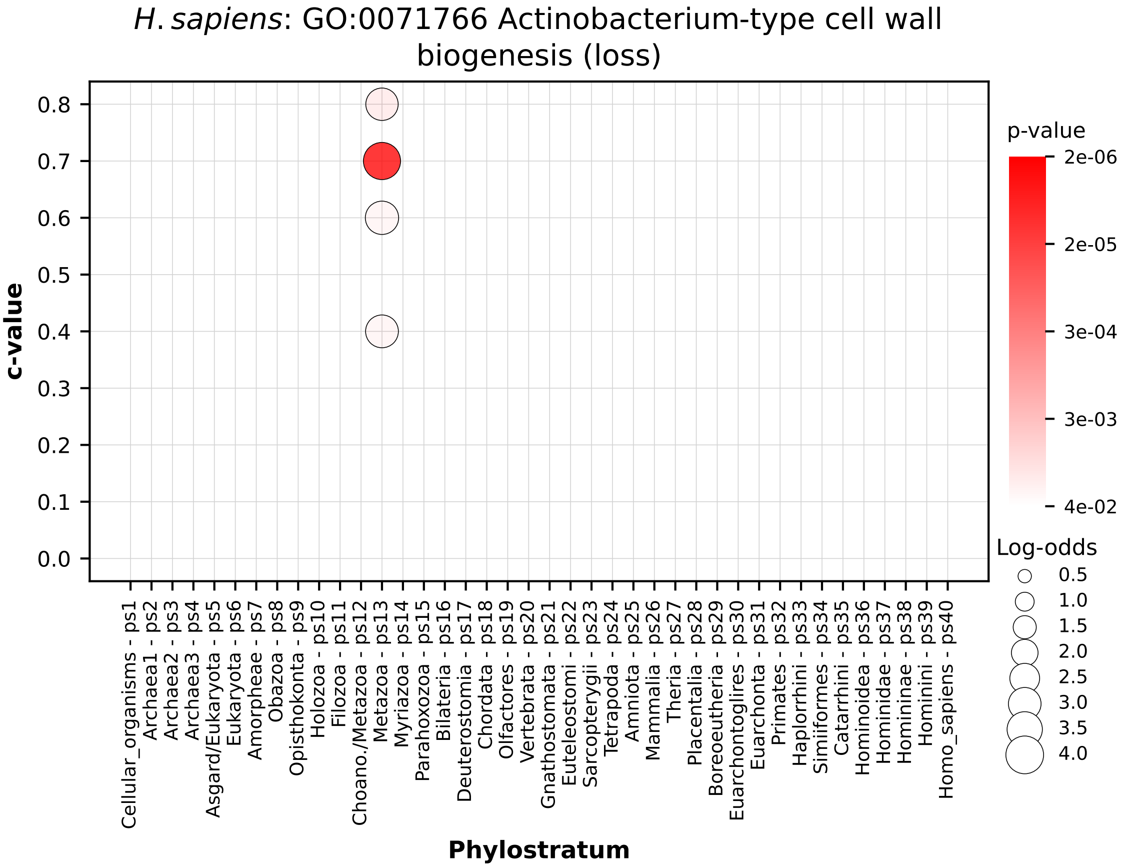
H. sapiens: GO:0071766 Actinobacterium-type cell wall biogenesis (loss)
c-value
0.0
0.1
0.2
0.3
0.4
0.5
0.6
0.7
0.8
p-value
2e-06
2e-05
3e-04
3e-03
4e-02
Log-odds
0.5
1.0
1.5
2.0
2.5
3.0
3.5
4.0
Cellular_organisms - ps1
Archaea1 - ps2
Archaea2 - ps3
Archaea3 - ps4
Asgard/Eukaryota - ps5
Eukaryota - ps6
Amorpheae - ps7
Obazoa - ps8
Opisthokonta - ps9
Holozoa - ps10
Filozoa - ps11
Choano./Metazoa - ps12
Metazoa - ps13
Myriazoa - ps14
Parahoxozoa - ps15
Bilateria - ps16
Deuterostomia - ps17
Chordata - ps18
Olfactores - ps19
Vertebrata - ps20
Gnathostomata - ps21
Euteleostomi - ps22
Sarcopterygii - ps23
Tetrapoda - ps24
Amniota - ps25
Mammalia - ps26
Theria - ps27
Placentalia - ps28
Boreoeutheria - ps29
Euarchontoglires - ps30
Euarchonta - ps31
Primates - ps32
Haplorrhini - ps33
Simiiformes - ps34
Catarrhini - ps35
Hominoidea - ps36
Hominidae - ps37
Homininae - ps38
Hominini - ps39
Homo_sapiens - ps40
Phylostratum

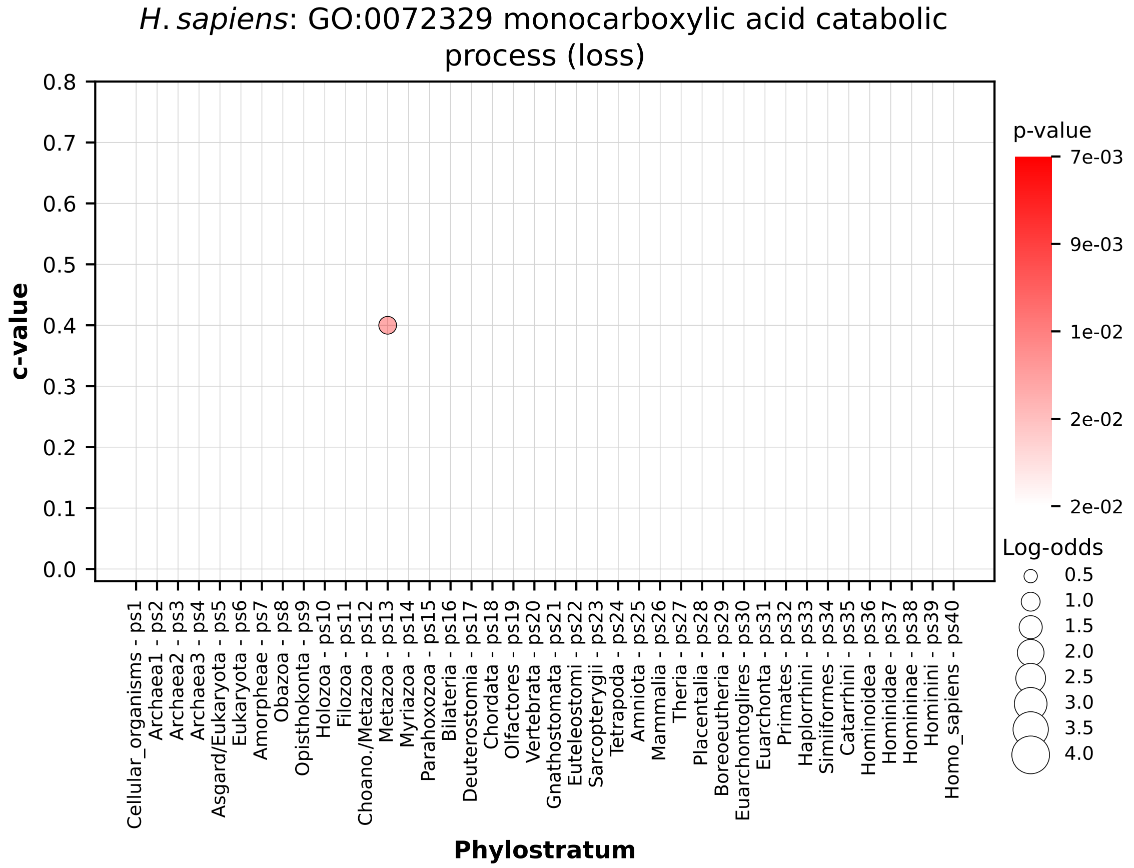
H. sapiens: GO:0072329 monocarboxylic acid catabolic process (loss)
c-value
Phylostratum
p-value
7e-03
9e-03
1e-02
2e-02
2e-02
Log-odds
0.5
1.0
1.5
2.0
2.5
3.0
3.5
4.0
Cellular_organisms - ps1
Archaea1 - ps2
Archaea2 - ps3
Archaea3 - ps4
Asgard/Eukaryota - ps5
Eukaryota - ps6
Amorpheae - ps7
Obazoa - ps8
Opisthokonta - ps9
Holozoa - ps10
Filozoa - ps11
Choano./Metazoa - ps12
Metazoa - ps13
Myriazoa - ps14
Parahoxozoa - ps15
Bilateria - ps16
Deuterostomia - ps17
Chordata - ps18
Olfactores - ps19
Vertebrata - ps20
Gnathostomata - ps21
Euteleostomi - ps22
Sarcopterygii - ps23
Tetrapoda - ps24
Amniota - ps25
Mammalia - ps26
Theria - ps27
Placentalia - ps28
Boreoeutheria - ps29
Euarchontoglires - ps30
Euarchonta - ps31
Primates - ps32
Haplorrhini - ps33
Simiiformes - ps34
Catarrhini - ps35
Hominoidea - ps36
Hominidae - ps37
Homininae - ps38
Hominini - ps39
Homo_sapiens - ps40

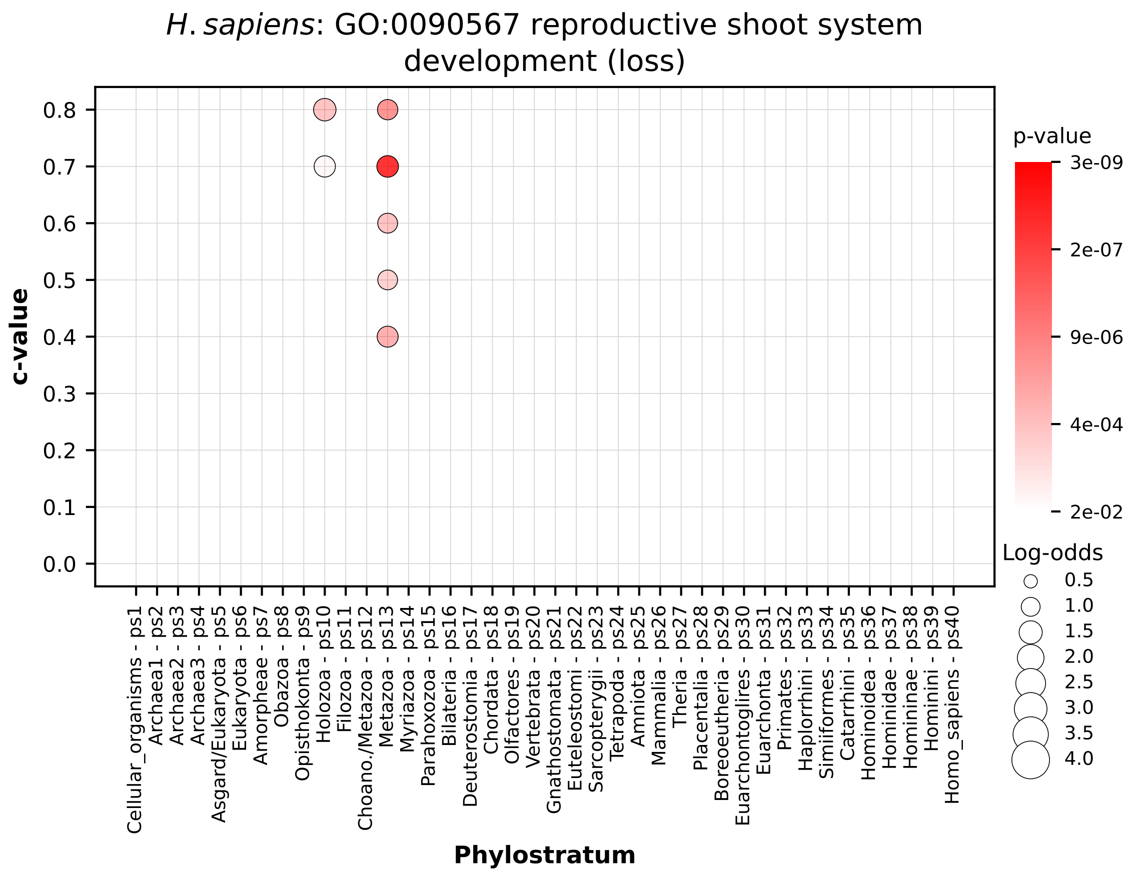

H. sapiens: GO:0090567 reproductive shoot system development (loss)
c-value
0.0
0.1
0.2
0.3
0.4
0.5
0.6
0.7
0.8
Phylostratum
Cellular_organisms - ps1
Archaea1 - ps2
Archaea2 - ps3
Archaea3 - ps4
Asgard/Eukaryota - ps5
Eukaryota - ps6
Amorpheae - ps7
Obazoa - ps8
Opisthokonta - ps9
Holozoa - ps10
Filozoa - ps11
Choano./Metazoa - ps12
Metazoa - ps13
Myriazoa - ps14
Parahoxozoa - ps15
Bilateria - ps16
Deuterostomia - ps17
Chordata - ps18
Olfactores - ps19
Vertebrata - ps20
Gnathostomata - ps21
Euteleostomi - ps22
Sarcopterygii - ps23
Tetrapoda - ps24
Amniota - ps25
Mammalia - ps26
Theria - ps27
Placentalia - ps28
Boreoeutheria - ps29
Euarchontoglires - ps30
Euarchonta - ps31
Primates - ps32
Haplorrhini - ps33
Simiiformes - ps34
Catarrhini - ps35
Hominoidea - ps36
Hominidae - ps37
Homininae - ps38
Hominini - ps39
Homo_sapiens - ps40
p-value
3e-09
2e-07
9e-06
4e-04
2e-02
Log-odds
0.5
1.0
1.5
2.0
2.5
3.0
3.5
4.0

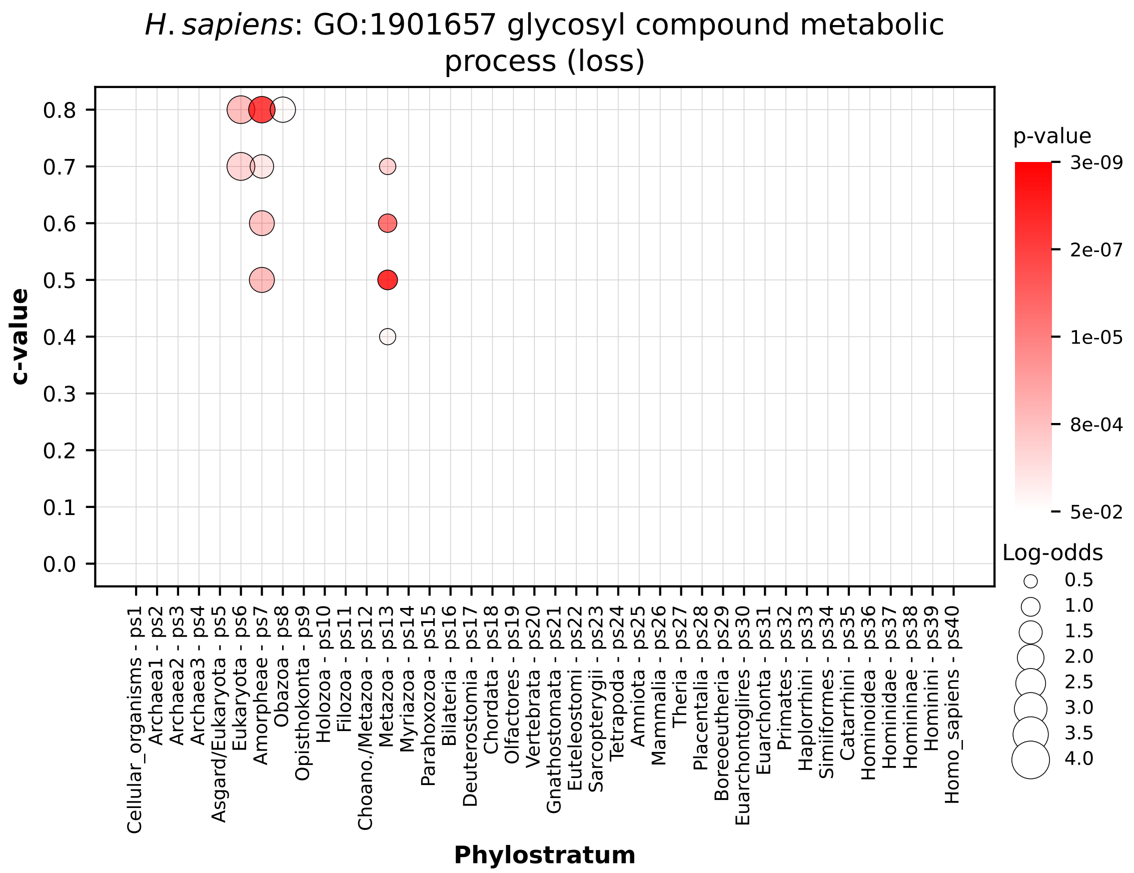

H. sapiens: GO:1901657 glycosyl compound metabolic process (loss)
c-value
0.8
0.7
0.6
0.5
0.4
0.3
0.2
0.1
0.0
p-value
3e-09
2e-07
1e-05
8e-04
5e-02
Log-odds
0.5
1.0
1.5
2.0
2.5
3.0
3.5
4.0
Cellular_organisms - ps1
Archaea1 - ps2
Archaea2 - ps3
Archaea3 - ps4
Asgard/Eukaryota - ps5
Eukaryota - ps6
Amorpheae - ps7
Obazoa - ps8
Opisthokonta - ps9
Holozoa - ps10
Filozoa - ps11
Choano./Metazoa - ps12
Metazoa - ps13
Myriazoa - ps14
Parahoxozoa - ps15
Bilateria - ps16
Deuterostomia - ps17
Chordata - ps18
Olfactores - ps19
Vertebrata - ps20
Gnathostomata - ps21
Euteleostomi - ps22
Sarcopterygii - ps23
Tetrapoda - ps24
Amniota - ps25
Mammalia - ps26
Theria - ps27
Placentalia - ps28
Boreoeutheria - ps29
Euarchontoglires - ps30
Euarchonta - ps31
Primates - ps32
Haplorrhini - ps33
Simiiformes - ps34
Catarrhini - ps35
Hominoidea - ps36
Hominidae - ps37
Homininae - ps38
Hominini - ps39
Homo_sapiens - ps40
Phylostratum

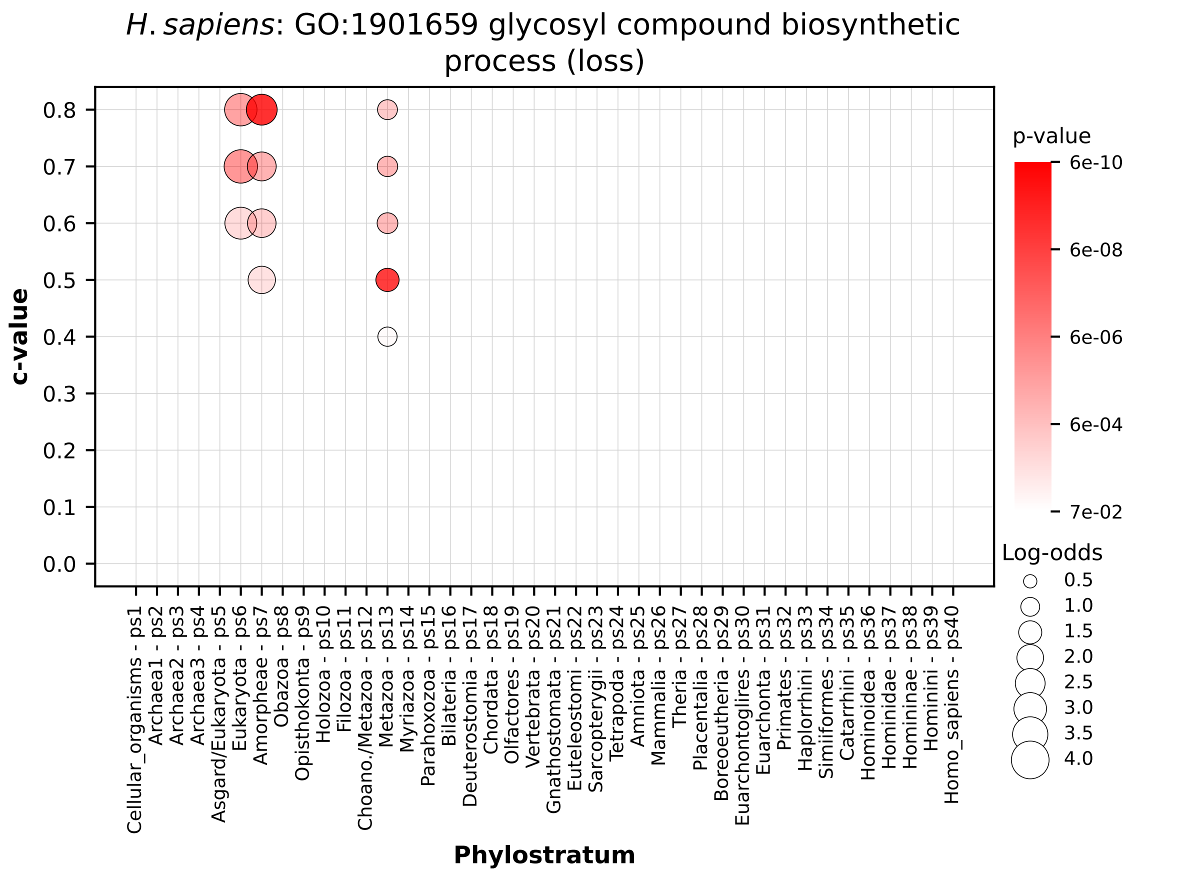

H. sapiens: GO:1901659 glycosyl compound biosynthetic process (loss)
c-value
0.8
0.7
0.6
0.5
0.4
0.3
0.2
0.1
0.0
p-value
6e-10
6e-08
6e-06
6e-04
7e-02
Log-odds
0.5
1.0
1.5
2.0
2.5
3.0
3.5
4.0
Cellular_organisms - ps1
Archaea1 - ps2
Archaea2 - ps3
Archaea3 - ps4
Asgard/Eukaryota - ps5
Eukaryota - ps6
Amorpheae - ps7
Obazoa - ps8
Opisthokonta - ps9
Holozoa - ps10
Filozoa - ps11
Choano./Metazoa - ps12
Metazoa - ps13
Myriazoa - ps14
Parahoxozoa - ps15
Bilateria - ps16
Deuterostomia - ps17
Chordata - ps18
Olfactores - ps19
Vertebrata - ps20
Gnathostomata - ps21
Euteleostomi - ps22
Sarcopterygii - ps23
Tetrapoda - ps24
Amniota - ps25
Mammalia - ps26
Theria - ps27
Placentalia - ps28
Boreoeutheria - ps29
Euarchontoglires - ps30
Euarchonta - ps31
Primates - ps32
Haplorrhini - ps33
Simiiformes - ps34
Catarrhini - ps35
Hominoidea - ps36
Hominidae - ps37
Homininae - ps38
Hominini - ps39
Homo_sapiens - ps40
Phylostratum

H. sapiens: GO:1904680 peptide transmembrane transporter activity (loss)

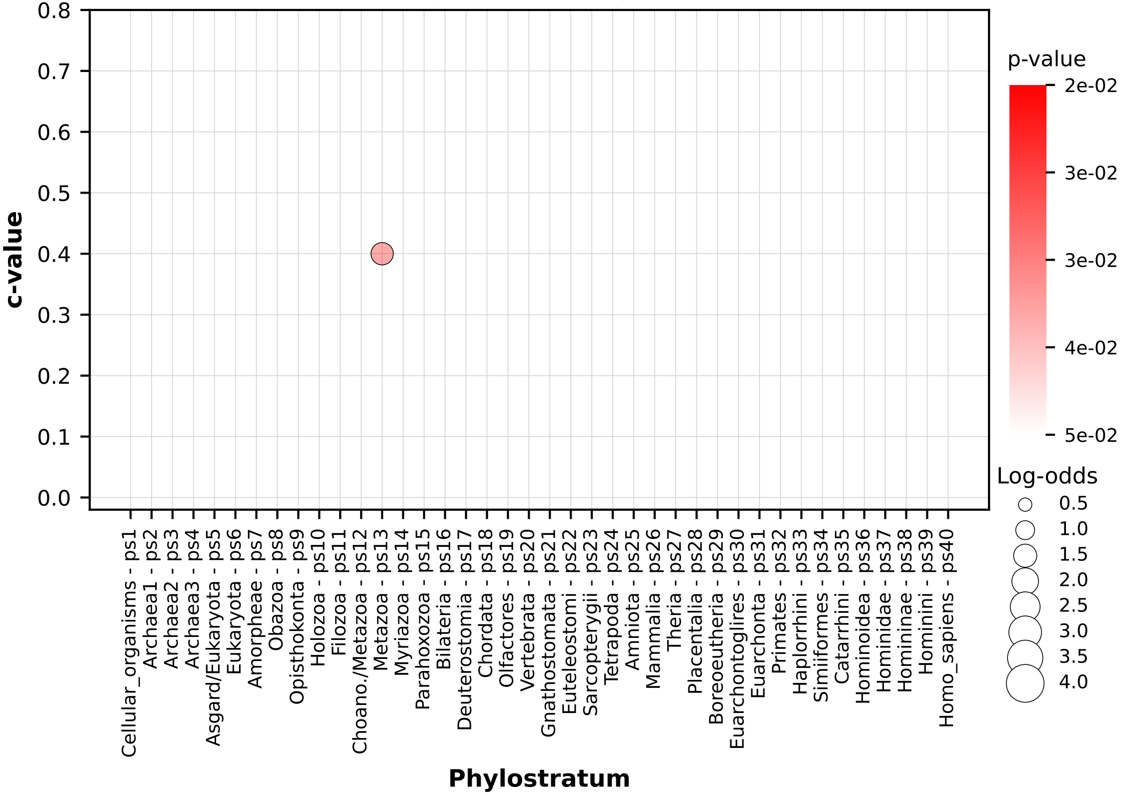

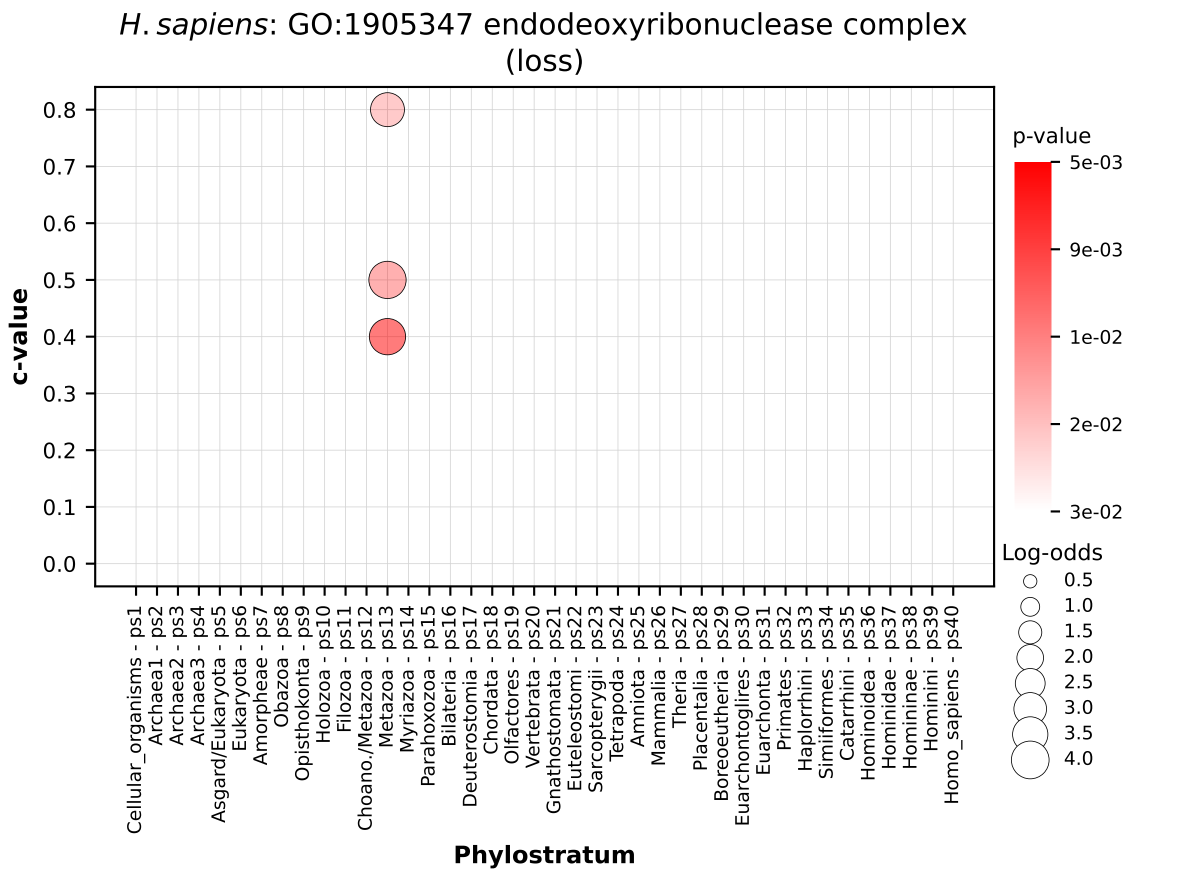

H. sapiens: GO:1905347 endodeoxyribonuclease complex (loss)
c-value
0.0
0.1
0.2
0.3
0.4
0.5
0.6
0.7
0.8
p-value
5e-03
9e-03
1e-02
2e-02
3e-02
Log-odds
0.5
1.0
1.5
2.0
2.5
3.0
3.5
4.0
Cellular_organisms - ps1
Archaea1 - ps2
Archaea2 - ps3
Archaea3 - ps4
Asgard/Eukaryota - ps5
Eukaryota - ps6
Amorpheae - ps7
Obazoa - ps8
Opisthokonta - ps9
Holozoa - ps10
Filozoa - ps11
Choano./Metazoa - ps12
Metazoa - ps13
Myriazoa - ps14
Parahoxozoa - ps15
Bilateria - ps16
Deuterostomia - ps17
Chordata - ps18
Olfactores - ps19
Vertebrata - ps20
Gnathostomata - ps21
Euteleostomi - ps22
Sarcopterygii - ps23
Tetrapoda - ps24
Amniota - ps25
Mammalia - ps26
Theria - ps27
Placentalia - ps28
Boreoeutheria - ps29
Euarchontoglires - ps30
Euarchonta - ps31
Primates - ps32
Haplorrhini - ps33
Simiiformes - ps34
Catarrhini - ps35
Hominoidea - ps36
Hominidae - ps37
Homininae - ps38
Hominini - ps39
Homo_sapiens - ps40
Phylostratum

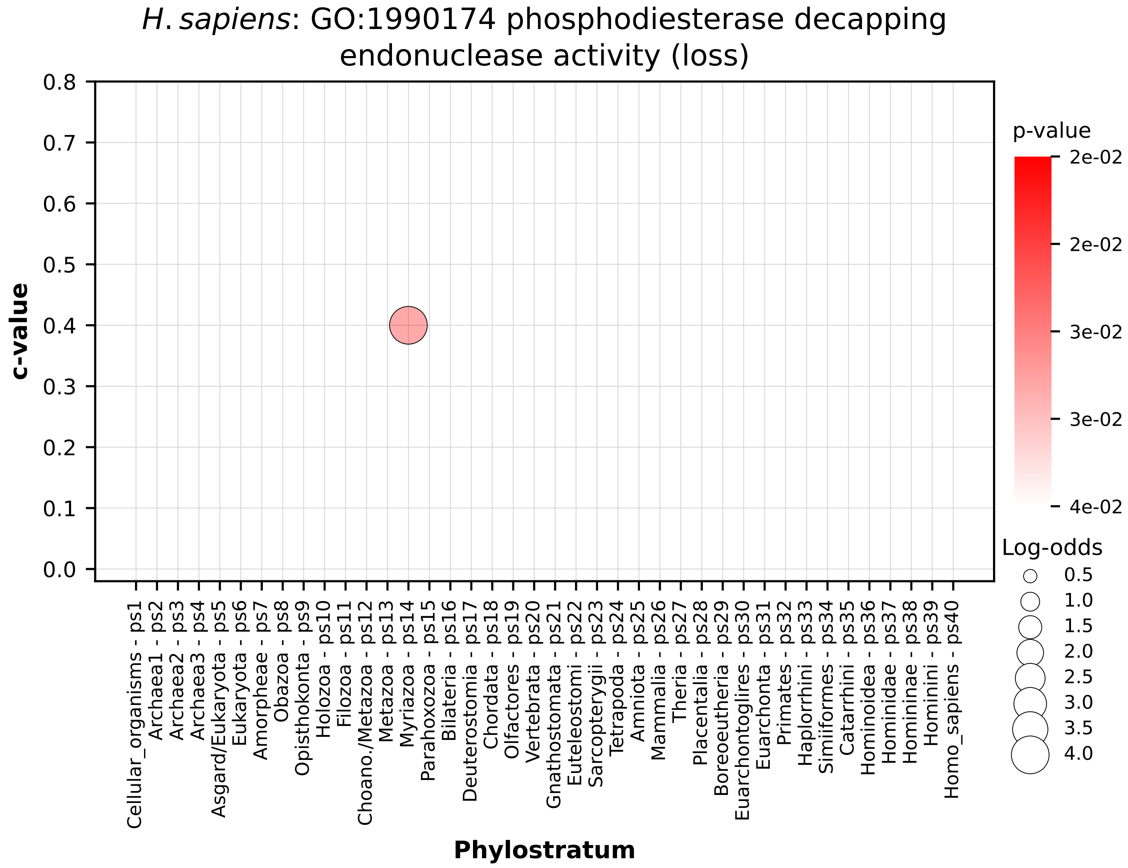

H. sapiens: GO:1990174 phosphodiesterase decapping endonuclease activity (loss)
c-value
0.0
0.1
0.2
0.3
0.4
0.5
0.6
0.7
0.8
Phylostratum
Cellular_organisms - ps1
Archaea1 - ps2
Archaea2 - ps3
Archaea3 - ps4
Asgard/Eukaryota - ps5
Eukaryota - ps6
Amorpheae - ps7
Obazoa - ps8
Opisthokonta - ps9
Holozoa - ps10
Filozoa - ps11
Choano./Metazoa - ps12
Metazoa - ps13
Myriazoa - ps14
Parahoxozoa - ps15
Bilateria - ps16
Deuterostomia - ps17
Chordata - ps18
Olfactores - ps19
Vertebrata - ps20
Gnathostomata - ps21
Euteleostomi - ps22
Sarcopterygii - ps23
Tetrapoda - ps24
Amniota - ps25
Mammalia - ps26
Theria - ps27
Placentalia - ps28
Boreoeutheria - ps29
Euarchontoglires - ps30
Euarchonta - ps31
Primates - ps32
Haplorrhini - ps33
Simiiformes - ps34
Catarrhini - ps35
Hominoidea - ps36
Hominidae - ps37
Homininae - ps38
Hominini - ps39
Homo_sapiens - ps40
p-value
2e-02
2e-02
3e-02
3e-02
4e-02
Log-odds
0.5
1.0
1.5
2.0
2.5
3.0
3.5
4.0

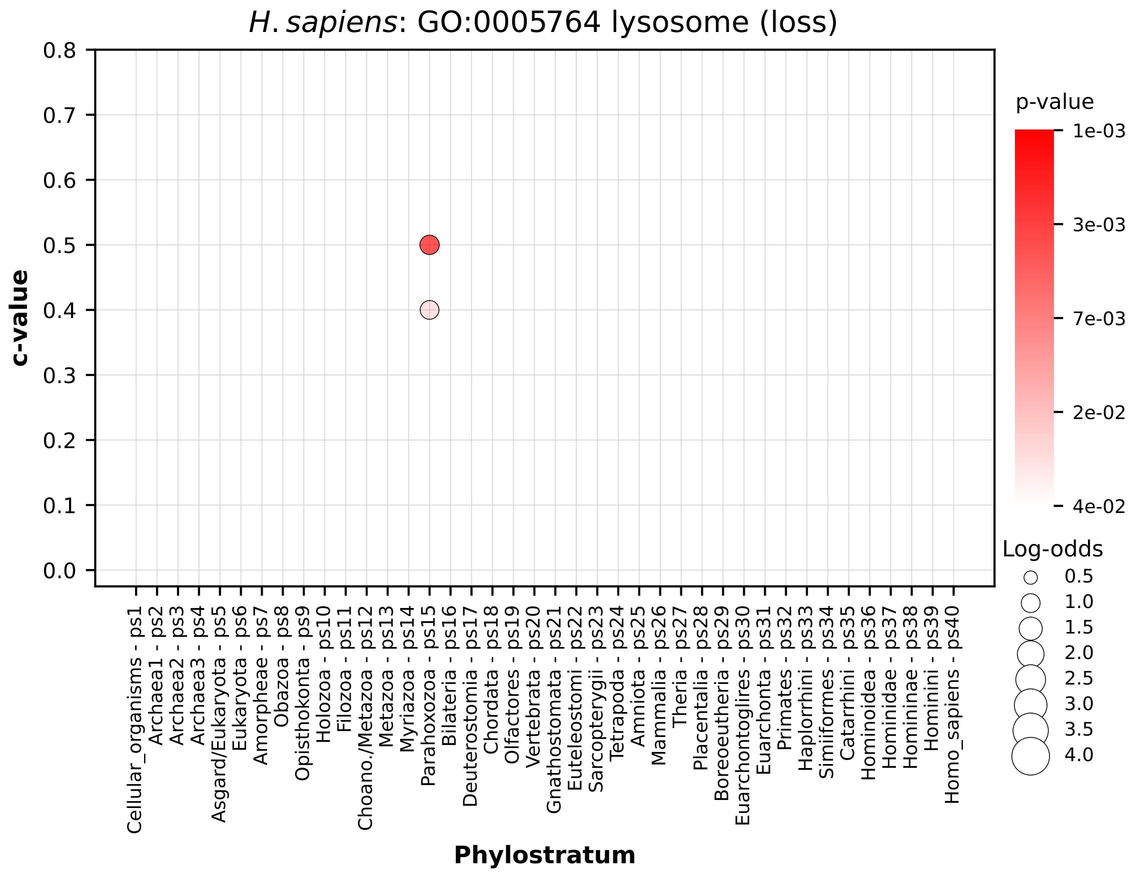

H. sapiens: GO:0005764 lysosome (loss)
c-value
0.0
0.1
0.2
0.3
0.4
0.5
0.6
0.7
0.8
Phylostratum
Cellular_organisms - ps1
Archaea1 - ps2
Archaea2 - ps3
Archaea3 - ps4
Asgard/Eukaryota - ps5
Eukaryota - ps6
Amorpheae - ps7
Obazoa - ps8
Opisthokonta - ps9
Holozoa - ps10
Filozoa - ps11
Choano./Metazoa - ps12
Metazoa - ps13
Myriazoa - ps14
Parahoxozoa - ps15
Bilateria - ps16
Deuterostomia - ps17
Chordata - ps18
Olfactores - ps19
Vertebrata - ps20
Gnathostomata - ps21
Euteleostomi - ps22
Sarcopterygii - ps23
Tetrapoda - ps24
Amniota - ps25
Mammalia - ps26
Theria - ps27
Placentalia - ps28
Boreoeutheria - ps29
Euarchontoglires - ps30
Euarchonta - ps31
Primates - ps32
Haplorrhini - ps33
Simiiformes - ps34
Catarrhini - ps35
Hominoidea - ps36
Hominidae - ps37
Homininae - ps38
Hominini - ps39
Homo_sapiens - ps40
p-value
1e-03
3e-03
7e-03
2e-02
4e-02
Log-odds
0.5
1.0
1.5
2.0
2.5
3.0
3.5
4.0

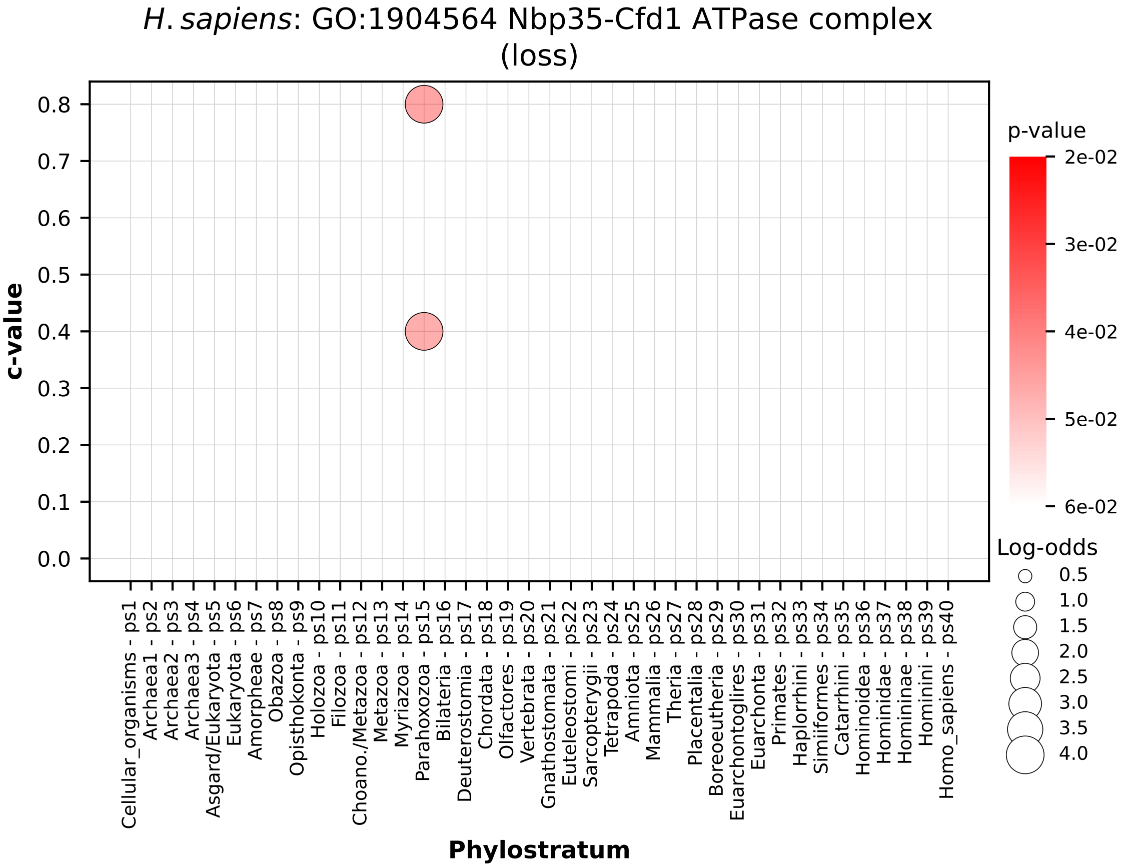

H. sapiens: GO:1904564 Nbp35-Cfd1 ATPase complex
(loss)
c-value
0.0
0.1
0.2
0.3
0.4
0.5
0.6
0.7
0.8
Phylostratum
Cellular_organisms - ps1
Archaea1 - ps2
Archaea2 - ps3
Archaea3 - ps4
Asgard/Eukaryota - ps5
Eukaryota - ps6
Amorpheae - ps7
Obazoa - ps8
Opisthokonta - ps9
Holozoa - ps10
Filozoa - ps11
Choano./Metazoa - ps12
Metazoa - ps13
Myriazoa - ps14
Parahoxozoa - ps15
Bilateria - ps16
Deuterostomia - ps17
Chordata - ps18
Olfactores - ps19
Vertebrata - ps20
Gnathostomata - ps21
Euteleostomi - ps22
Sarcopterygii - ps23
Tetrapoda - ps24
Amniota - ps25
Mammalia - ps26
Theria - ps27
Placentalia - ps28
Boreoeutheria - ps29
Euarchontoglires - ps30
Euarchonta - ps31
Primates - ps32
Haplorrhini - ps33
Simiiformes - ps34
Catarrhini - ps35
Hominoidea - ps36
Hominidae - ps37
Homininae - ps38
Hominini - ps39
Homo_sapiens - ps40
p-value
2e-02
3e-02
4e-02
5e-02
6e-02
Log-odds
0.5
1.0
1.5
2.0
2.5
3.0
3.5
4.0

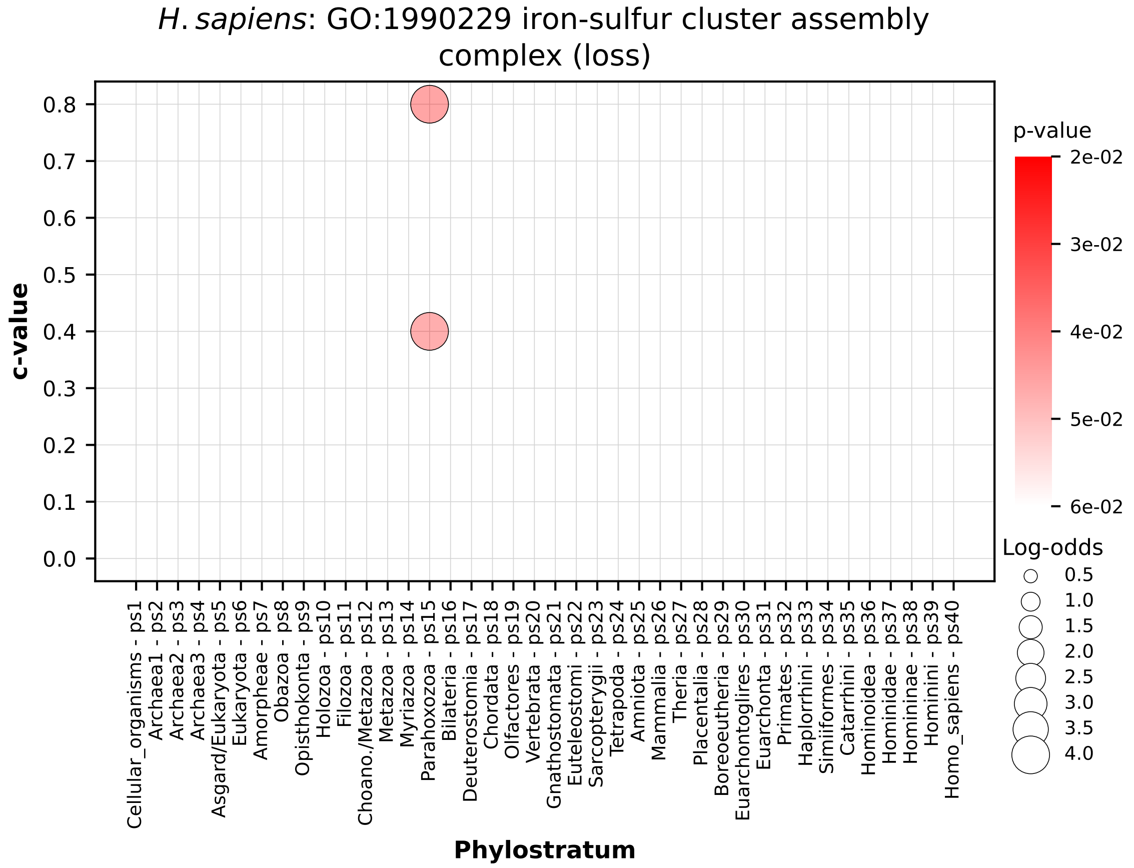

H. sapiens: GO:1990229 iron-sulfur cluster assembly complex (loss)
c-value
0.0
0.1
0.2
0.3
0.4
0.5
0.6
0.7
0.8
p-value
2e-02
3e-02
4e-02
5e-02
6e-02
Log-odds
0.5
1.0
1.5
2.0
2.5
3.0
3.5
4.0
Cellular_organisms - ps1
Archaea1 - ps2
Archaea2 - ps3
Archaea3 - ps4
Asgard/Eukaryota - ps5
Eukaryota - ps6
Amorpheae - ps7
Obazoa - ps8
Opisthokonta - ps9
Holozoa - ps10
Filozoa - ps11
Choano./Metazoa - ps12
Metazoa - ps13
Myriazoa - ps14
Parahoxozoa - ps15
Bilateria - ps16
Deuterostomia - ps17
Chordata - ps18
Olfactores - ps19
Vertebrata - ps20
Gnathostomata - ps21
Euteleostomi - ps22
Sarcopterygii - ps23
Tetrapoda - ps24
Amniota - ps25
Mammalia - ps26
Theria - ps27
Placentalia - ps28
Boreoeutheria - ps29
Euarchontoglires - ps30
Euarchonta - ps31
Primates - ps32
Haplorrhini - ps33
Simiiformes - ps34
Catarrhini - ps35
Hominoidea - ps36
Hominidae - ps37
Homininae - ps38
Hominini - ps39
Homo_sapiens - ps40
Phylostratum

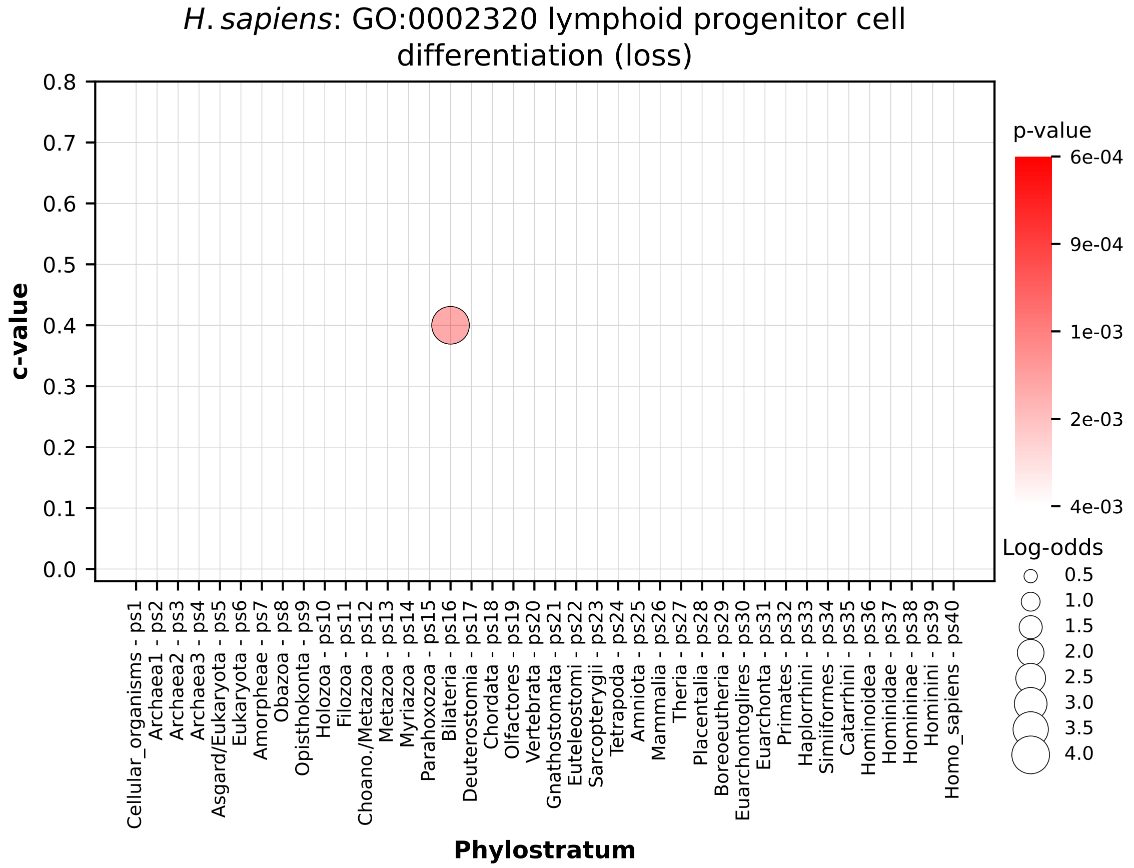

H. sapiens: GO:0002320 lymphoid progenitor cell differentiation (loss)
c-value
0.8
0.7
0.6
0.5
0.4
0.3
0.2
0.1
0.0
p-value
6e-04
9e-04
1e-03
2e-03
4e-03
Log-odds
0.5
1.0
1.5
2.0
2.5
3.0
3.5
4.0
Cellular_organisms - ps1
Archaea1 - ps2
Archaea2 - ps3
Archaea3 - ps4
Asgard/Eukaryota - ps5
Eukaryota - ps6
Amorpheae - ps7
Obazoa - ps8
Opisthokonta - ps9
Holozoa - ps10
Filozoa - ps11
Choano./Metazoa - ps12
Metazoa - ps13
Myriazoa - ps14
Parahoxozoa - ps15
Bilateria - ps16
Deuterostomia - ps17
Chordata - ps18
Olfactores - ps19
Vertebrata - ps20
Gnathostomata - ps21
Euteleostomi - ps22
Sarcopterygii - ps23
Tetrapoda - ps24
Amniota - ps25
Mammalia - ps26
Theria - ps27
Placentalia - ps28
Boreoeutheria - ps29
Euarchontoglires - ps30
Euarchonta - ps31
Primates - ps32
Haplorrhini - ps33
Simiiformes - ps34
Catarrhini - ps35
Hominoidea - ps36
Hominidae - ps37
Homininae - ps38
Hominini - ps39
Homo_sapiens - ps40
Phylostratum

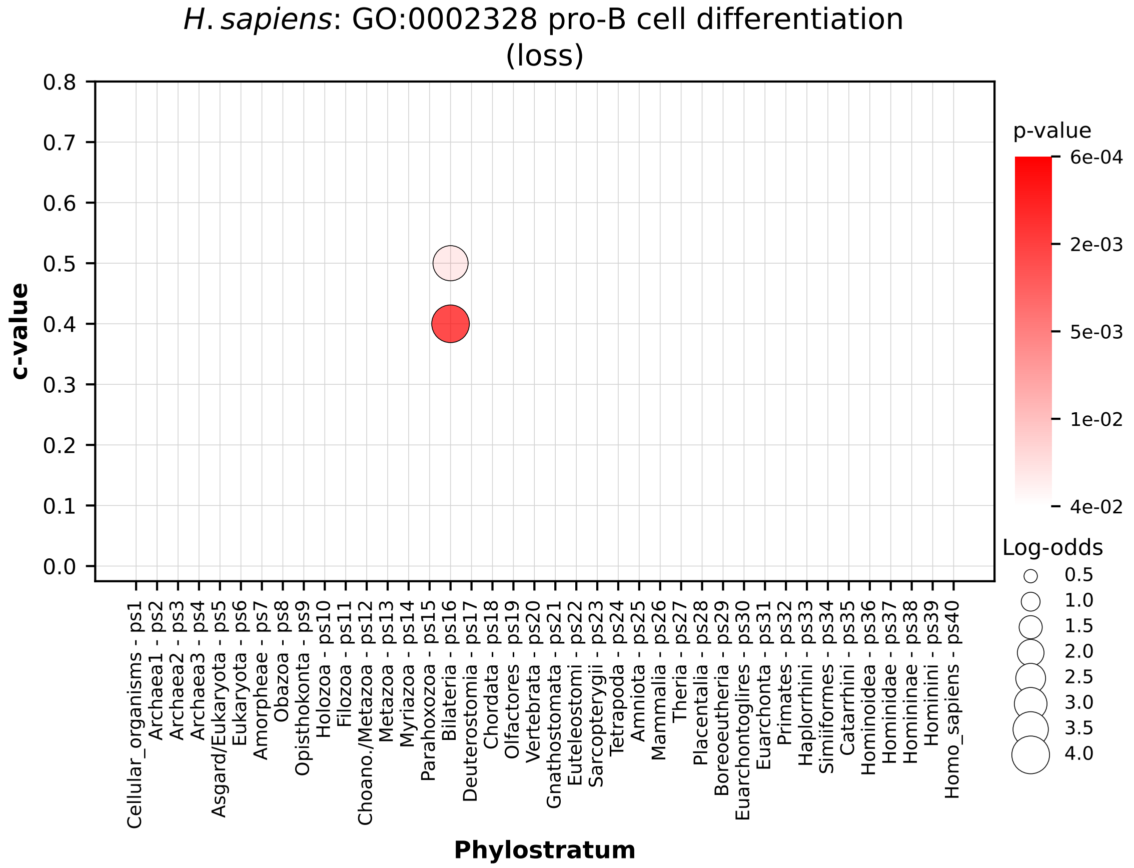

H. sapiens: GO:0002328 pro-B cell differentiation
(loss)
c-value
0.8
0.7
0.6
0.5
0.4
0.3
0.2
0.1
0.0
p-value
6e-04
2e-03
5e-03
1e-02
4e-02
Log-odds
0.5
1.0
1.5
2.0
2.5
3.0
3.5
4.0
Cellular_organisms - ps1
Archaea1 - ps2
Archaea2 - ps3
Archaea3 - ps4
Asgard/Eukaryota - ps5
Eukaryota - ps6
Amorpheae - ps7
Obazoa - ps8
Opisthokonta - ps9
Holozoa - ps10
Filozoa - ps11
Choano./Metazoa - ps12
Metazoa - ps13
Myriazoa - ps14
Parahoxozoa - ps15
Bilateria - ps16
Deuterostomia - ps17
Chordata - ps18
Olfactores - ps19
Vertebrata - ps20
Gnathostomata - ps21
Euteleostomi - ps22
Sarcopterygii - ps23
Tetrapoda - ps24
Amniota - ps25
Mammalia - ps26
Theria - ps27
Placentalia - ps28
Boreoeutheria - ps29
Euarchontoglires - ps30
Euarchonta - ps31
Primates - ps32
Haplorrhini - ps33
Simiiformes - ps34
Catarrhini - ps35
Hominoidea - ps36
Hominidae - ps37
Homininae - ps38
Hominini - ps39
Homo_sapiens - ps40
Phylostratum

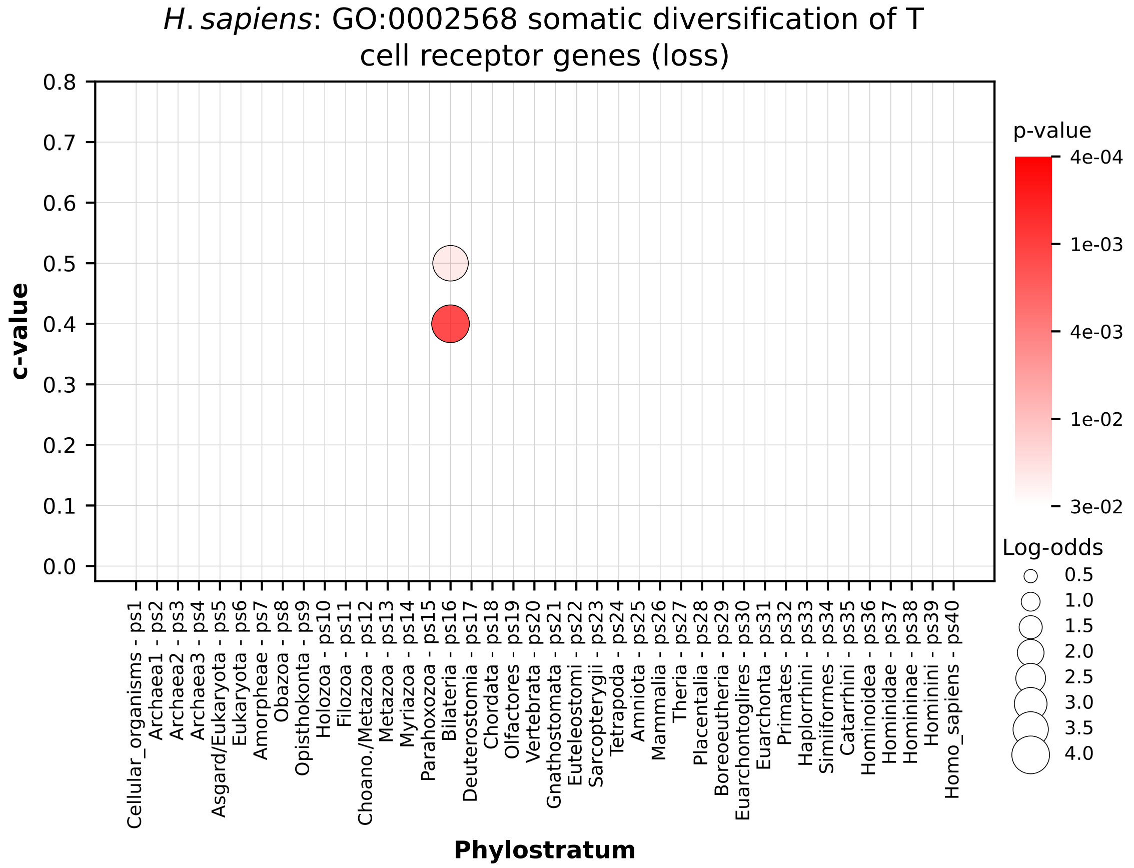

H. sapiens: GO:0002568 somatic diversification of T cell receptor genes (loss)
c-value
0.0
0.1
0.2
0.3
0.4
0.5
0.6
0.7
0.8
Phylostratum
Cellular_organisms - ps1
Archaea1 - ps2
Archaea2 - ps3
Archaea3 - ps4
Asgard/Eukaryota - ps5
Eukaryota - ps6
Amorpheae - ps7
Obazoa - ps8
Opisthokonta - ps9
Holozoa - ps10
Filozoa - ps11
Choano./Metazoa - ps12
Metazoa - ps13
Myriazoa - ps14
Parahoxozoa - ps15
Bilateria - ps16
Deuterostomia - ps17
Chordata - ps18
Olfactores - ps19
Vertebrata - ps20
Gnathostomata - ps21
Euteleostomi - ps22
Sarcopterygii - ps23
Tetrapoda - ps24
Amniota - ps25
Mammalia - ps26
Theria - ps27
Placentalia - ps28
Boreoeutheria - ps29
Euarchontoglires - ps30
Euarchonta - ps31
Primates - ps32
Haplorrhini - ps33
Simiiformes - ps34
Catarrhini - ps35
Hominoidea - ps36
Hominidae - ps37
Homininae - ps38
Hominini - ps39
Homo_sapiens - ps40
p-value
4e-04
1e-03
4e-03
1e-02
3e-02
Log-odds
0.5
1.0
1.5
2.0
2.5
3.0
3.5
4.0

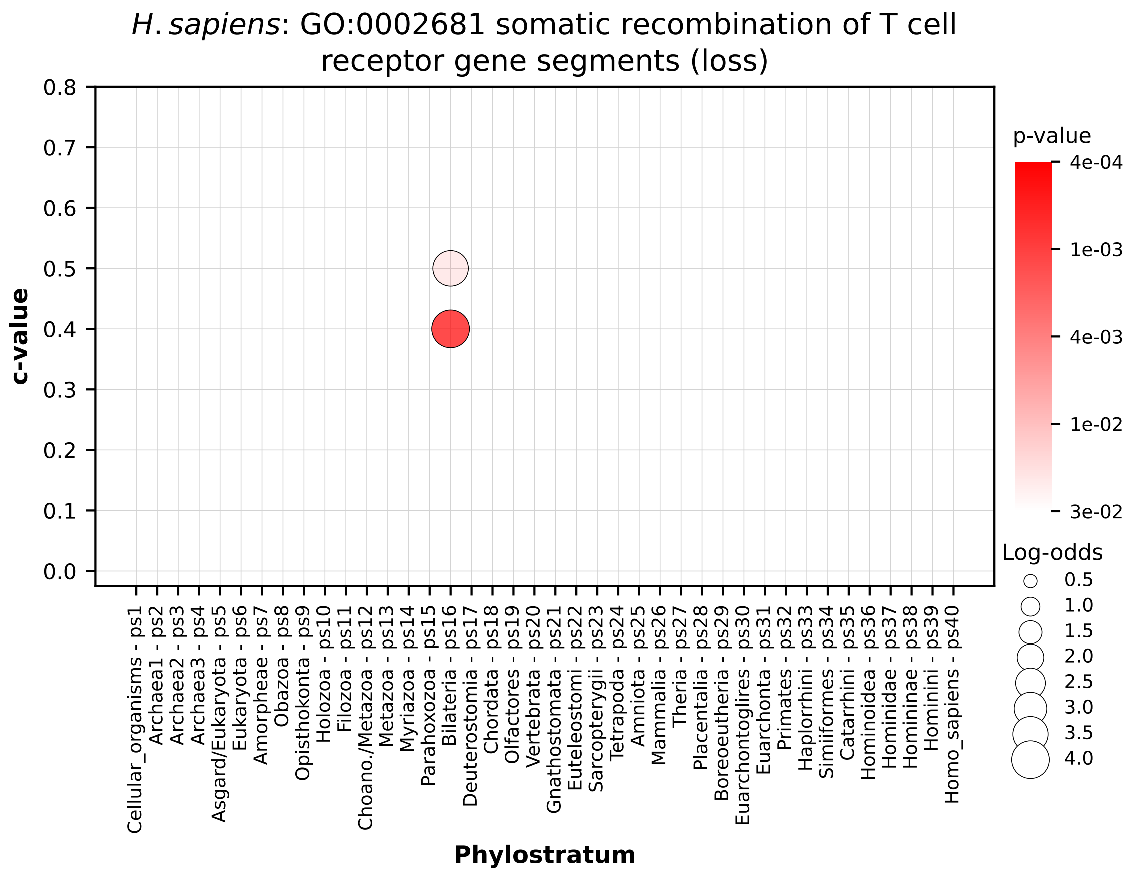

H. sapiens: GO:0002681 somatic recombination of T cell receptor gene segments (loss)
c-value
0.8
0.7
0.6
0.5
0.4
0.3
0.2
0.1
0.0
Phylostratum
Cellular_organisms - ps1
Archaea1 - ps2
Archaea2 - ps3
Archaea3 - ps4
Asgard/Eukaryota - ps5
Eukaryota - ps6
Amorpheae - ps7
Obazoa - ps8
Opisthokonta - ps9
Holozoa - ps10
Filozoa - ps11
Choano./Metazoa - ps12
Metazoa - ps13
Myriazoa - ps14
Parahoxozoa - ps15
Bilateria - ps16
Deuterostomia - ps17
Chordata - ps18
Olfactores - ps19
Vertebrata - ps20
Gnathostomata - ps21
Euteleostomi - ps22
Sarcopterygii - ps23
Tetrapoda - ps24
Amniota - ps25
Mammalia - ps26
Theria - ps27
Placentalia - ps28
Boreoeutheria - ps29
Euarchontoglires - ps30
Euarchonta - ps31
Primates - ps32
Haplorrhini - ps33
Simiiformes - ps34
Catarrhini - ps35
Hominoidea - ps36
Hominidae - ps37
Homininae - ps38
Hominini - ps39
Homo_sapiens - ps40
p-value
4e-04
1e-03
4e-03
1e-02
3e-02
Log-odds
0.5
1.0
1.5
2.0
2.5
3.0
3.5
4.0

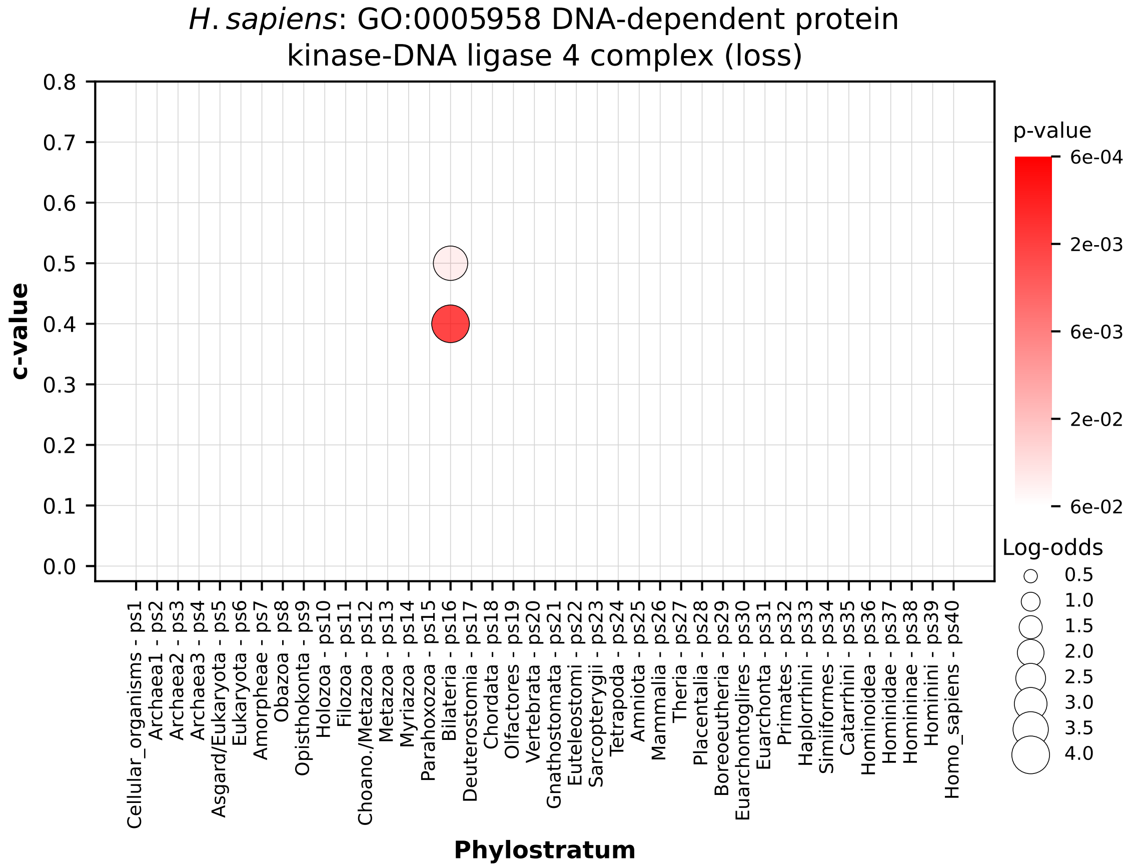

H. sapiens: GO:0005958 DNA-dependent protein kinase-DNA ligase 4 complex (loss)
c-value
0.8
0.7
0.6
0.5
0.4
0.3
0.2
0.1
0.0
Phylostratum
Cellular_organisms - ps1
Archaea1 - ps2
Archaea2 - ps3
Archaea3 - ps4
Asgard/Eukaryota - ps5
Eukaryota - ps6
Amorpheae - ps7
Obazoa - ps8
Opisthokonta - ps9
Holozoa - ps10
Filozoa - ps11
Choano./Metazoa - ps12
Metazoa - ps13
Myriazoa - ps14
Parahoxozoa - ps15
Bilateria - ps16
Deuterostomia - ps17
Chordata - ps18
Olfactores - ps19
Vertebrata - ps20
Gnathostomata - ps21
Euteleostomi - ps22
Sarcopterygii - ps23
Tetrapoda - ps24
Amniota - ps25
Mammalia - ps26
Theria - ps27
Placentalia - ps28
Boreoeutheria - ps29
Euarchontoglires - ps30
Euarchonta - ps31
Primates - ps32
Haplorrhini - ps33
Simiiformes - ps34
Catarrhini - ps35
Hominoidea - ps36
Hominidae - ps37
Homininae - ps38
Hominini - ps39
Homo_sapiens - ps40
p-value
6e-04
2e-03
6e-03
2e-02
6e-02
Log-odds
0.5
1.0
1.5
2.0
2.5
3.0
3.5
4.0

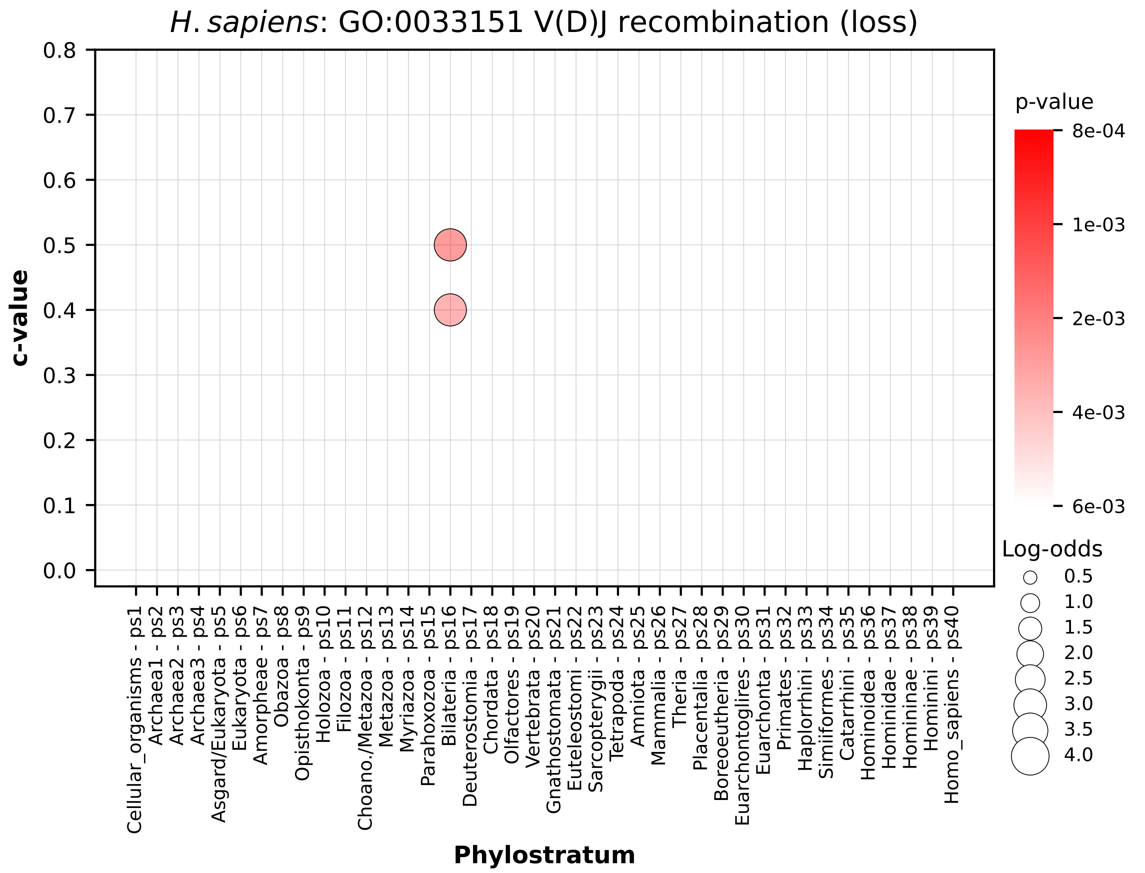

H. sapiens: GO:0033151 V(D)J recombination (loss)
c-value
0.0
0.1
0.2
0.3
0.4
0.5
0.6
0.7
0.8
Phylostratum
Cellular_organisms - ps1
Archaea1 - ps2
Archaea2 - ps3
Archaea3 - ps4
Asgard/Eukaryota - ps5
Eukaryota - ps6
Amorpheae - ps7
Obazoa - ps8
Opisthokonta - ps9
Holozoa - ps10
Filozoa - ps11
Choano./Metazoa - ps12
Metazoa - ps13
Myriazoa - ps14
Parahoxozoa - ps15
Bilateria - ps16
Deuterostomia - ps17
Chordata - ps18
Olfactores - ps19
Vertebrata - ps20
Gnathostomata - ps21
Euteleostomi - ps22
Sarcopterygii - ps23
Tetrapoda - ps24
Amniota - ps25
Mammalia - ps26
Theria - ps27
Placentalia - ps28
Boreoeutheria - ps29
Euarchontoglires - ps30
Euarchonta - ps31
Primates - ps32
Haplorrhini - ps33
Simiiformes - ps34
Catarrhini - ps35
Hominoidea - ps36
Hominidae - ps37
Homininae - ps38
Hominini - ps39
Homo_sapiens - ps40
p-value
8e-04
1e-03
2e-03
4e-03
6e-03
Log-odds
0.5
1.0
1.5
2.0
2.5
3.0
3.5
4.0

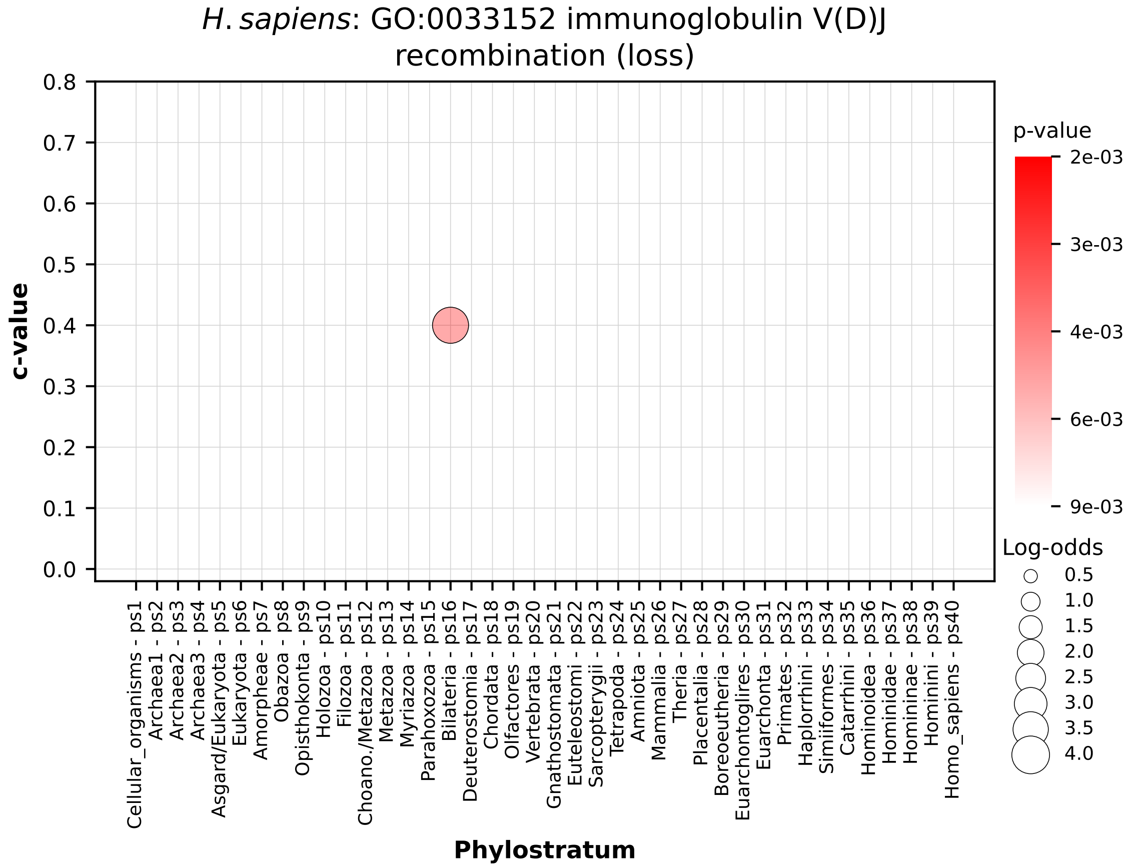

H. sapiens: GO:0033152 immunoglobulin V(D)J recombination (loss)
c-value
0.8
0.7
0.6
0.5
0.4
0.3
0.2
0.1
0.0
Phylostratum
Cellular_organisms - ps1
Archaea1 - ps2
Archaea2 - ps3
Archaea3 - ps4
Asgard/Eukaryota - ps5
Eukaryota - ps6
Amorpheae - ps7
Obazoa - ps8
Opisthokonta - ps9
Holozoa - ps10
Filozoa - ps11
Choano./Metazoa - ps12
Metazoa - ps13
Myriazoa - ps14
Parahoxozoa - ps15
Bilateria - ps16
Deuterostomia - ps17
Chordata - ps18
Olfactores - ps19
Vertebrata - ps20
Gnathostomata - ps21
Euteleostomi - ps22
Sarcopterygii - ps23
Tetrapoda - ps24
Amniota - ps25
Mammalia - ps26
Theria - ps27
Placentalia - ps28
Boreoeutheria - ps29
Euarchontoglires - ps30
Euarchonta - ps31
Primates - ps32
Haplorrhini - ps33
Simiiformes - ps34
Catarrhini - ps35
Hominoidea - ps36
Hominidae - ps37
Homininae - ps38
Hominini - ps39
Homo_sapiens - ps40
p-value
2e-03
3e-03
4e-03
6e-03
9e-03
Log-odds
0.5
1.0
1.5
2.0
2.5
3.0
3.5
4.0

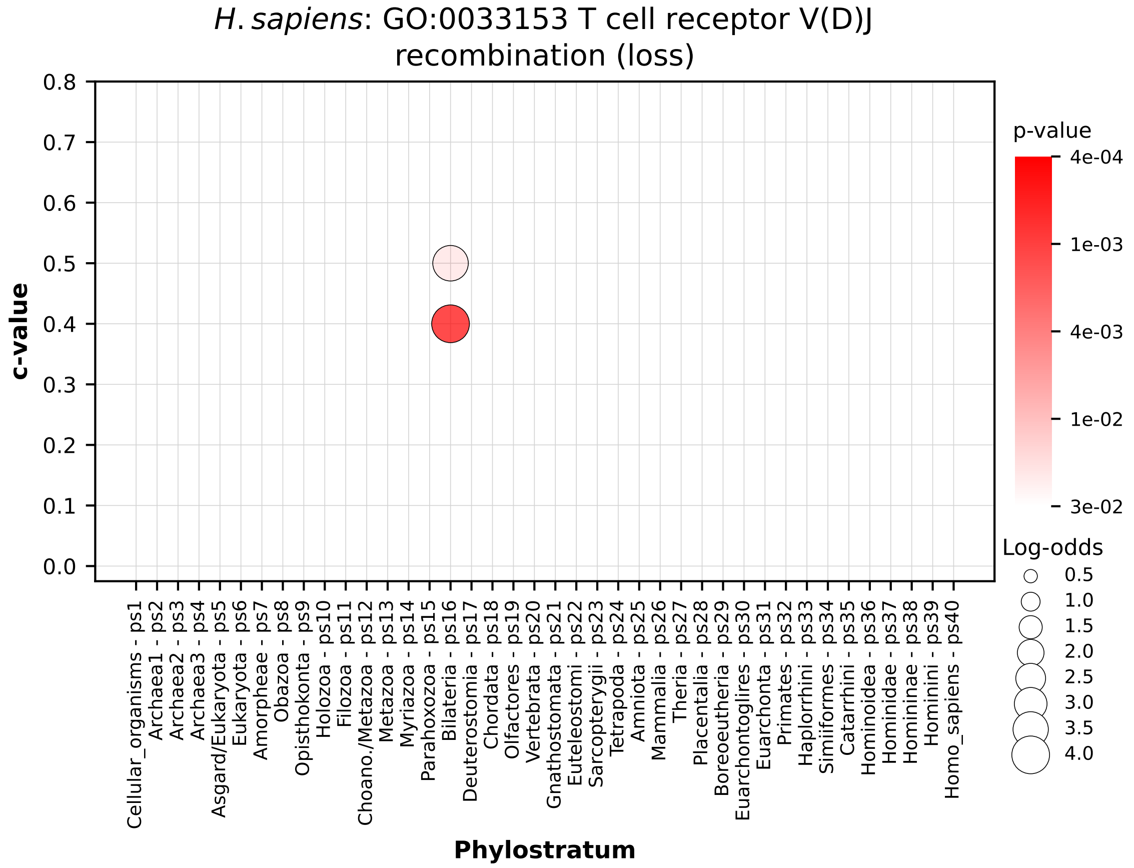

H. sapiens: GO:0033153 T cell receptor V(D)J recombination (loss)
c-value
0.8
0.7
0.6
0.5
0.4
0.3
0.2
0.1
0.0
p-value
4e-04
1e-03
4e-03
1e-02
3e-02
Log-odds
0.5
1.0
1.5
2.0
2.5
3.0
3.5
4.0
Cellular_organisms - ps1
Archaea1 - ps2
Archaea2 - ps3
Archaea3 - ps4
Asgard/Eukaryota - ps5
Eukaryota - ps6
Amorpheae - ps7
Obazoa - ps8
Opisthokonta - ps9
Holozoa - ps10
Filozoa - ps11
Choano./Metazoa - ps12
Metazoa - ps13
Myriazoa - ps14
Parahoxozoa - ps15
Bilateria - ps16
Deuterostomia - ps17
Chordata - ps18
Olfactores - ps19
Vertebrata - ps20
Gnathostomata - ps21
Euteleostomi - ps22
Sarcopterygii - ps23
Tetrapoda - ps24
Amniota - ps25
Mammalia - ps26
Theria - ps27
Placentalia - ps28
Boreoeutheria - ps29
Euarchontoglires - ps30
Euarchonta - ps31
Primates - ps32
Haplorrhini - ps33
Simiiformes - ps34
Catarrhini - ps35
Hominoidea - ps36
Hominidae - ps37
Homininae - ps38
Hominini - ps39
Homo_sapiens - ps40
Phylostratum

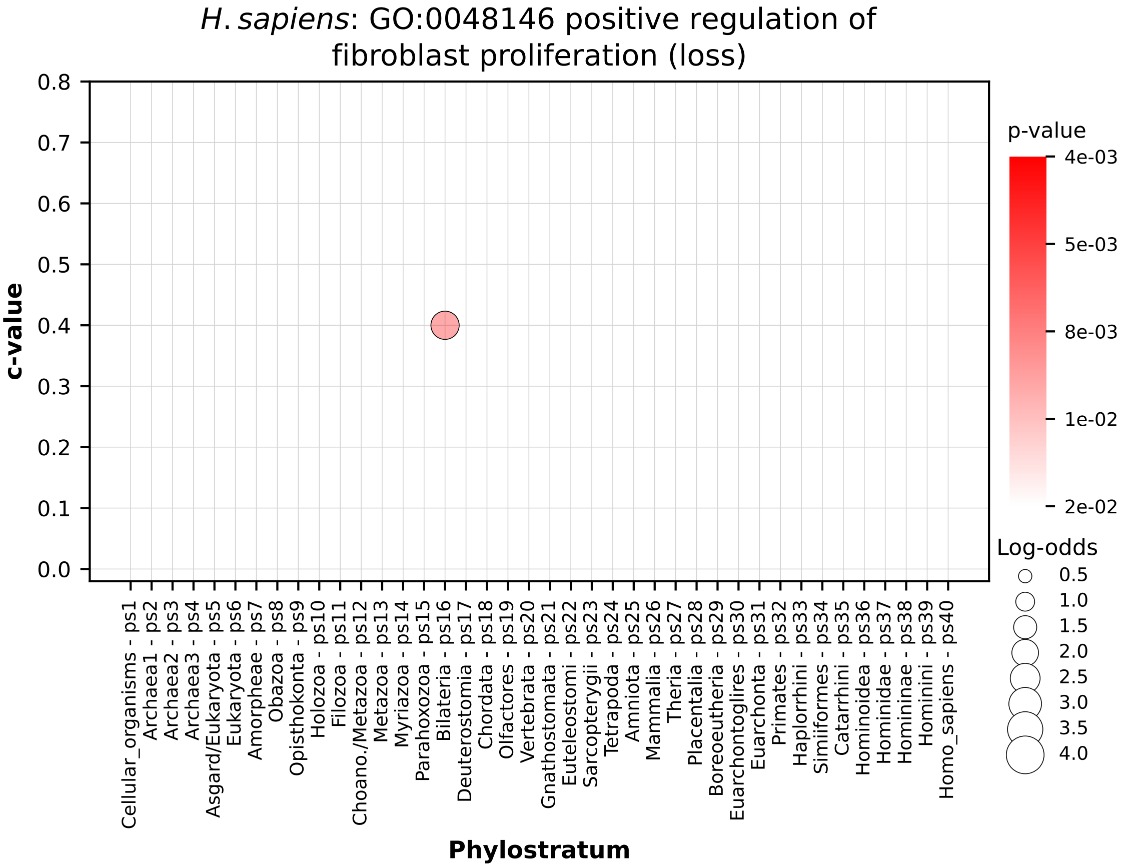
H. sapiens: GO:0048146 positive regulation of fibroblast proliferation (loss)
c-value
0.0
0.1
0.2
0.3
0.4
0.5
0.6
0.7
0.8
Phylostratum
Cellular_organisms - ps1
Archaea1 - ps2
Archaea2 - ps3
Archaea3 - ps4
Asgard/Eukaryota - ps5
Eukaryota - ps6
Amorpheae - ps7
Obazoa - ps8
Opisthokonta - ps9
Holozoa - ps10
Filozoa - ps11
Choano./Metazoa - ps12
Metazoa - ps13
Myriazoa - ps14
Parahoxozoa - ps15
Bilateria - ps16
Deuterostomia - ps17
Chordata - ps18
Olfactores - ps19
Vertebrata - ps20
Gnathostomata - ps21
Euteleostomi - ps22
Sarcopterygii - ps23
Tetrapoda - ps24
Amniota - ps25
Mammalia - ps26
Theria - ps27
Placentalia - ps28
Boreoeutheria - ps29
Euarchontoglires - ps30
Euarchonta - ps31
Primates - ps32
Haplorrhini - ps33
Simiiformes - ps34
Catarrhini - ps35
Hominoidea - ps36
Hominidae - ps37
Homininae - ps38
Hominini - ps39
Homo_sapiens - ps40
p-value
4e-03
5e-03
8e-03
1e-02
2e-02
Log-odds
0.5
1.0
1.5
2.0
2.5
3.0
3.5
4.0

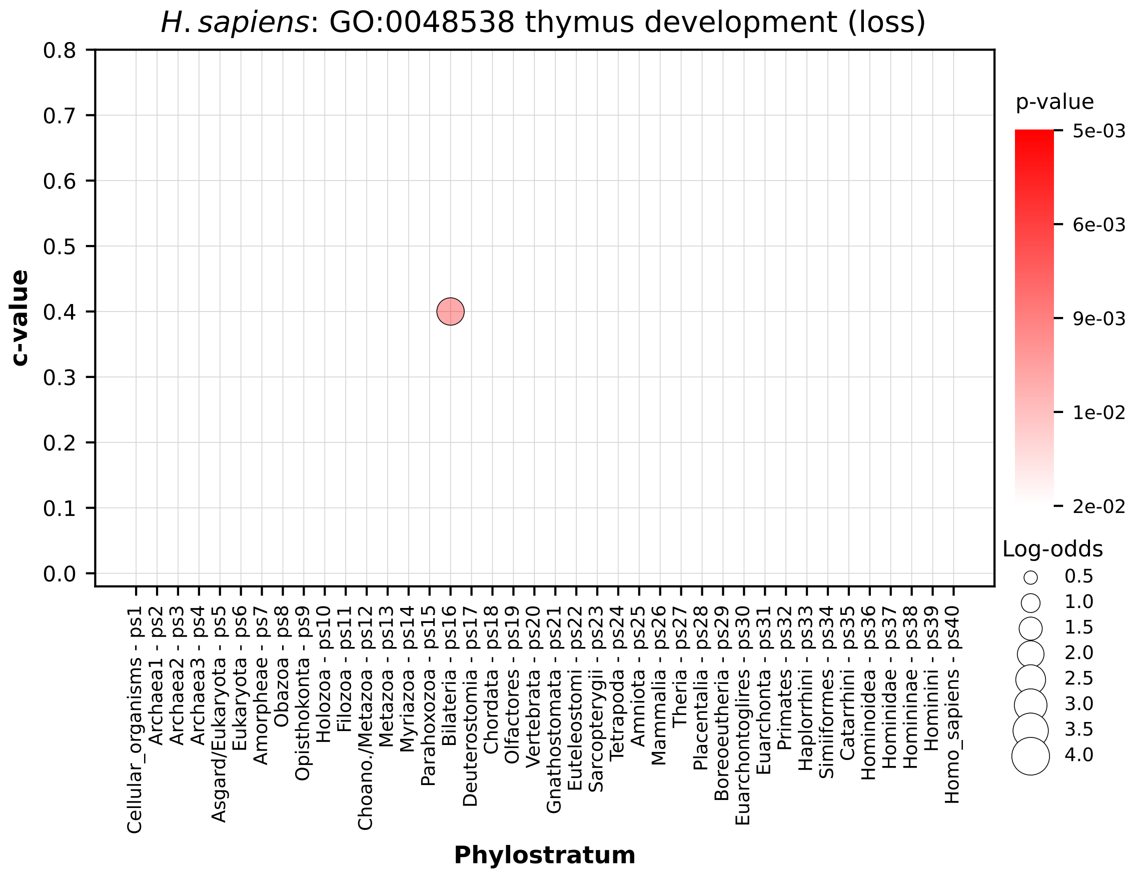

H. sapiens: GO:0048538 thymus development (loss)
c-value
0.0
0.1
0.2
0.3
0.4
0.5
0.6
0.7
0.8
Phylostratum
Cellular_organisms - ps1
Archaea1 - ps2
Archaea2 - ps3
Archaea3 - ps4
Asgard/Eukaryota - ps5
Eukaryota - ps6
Amorpheae - ps7
Obazoa - ps8
Opisthokonta - ps9
Holozoa - ps10
Filozoa - ps11
Choano./Metazoa - ps12
Metazoa - ps13
Myriazoa - ps14
Parahoxozoa - ps15
Bilateria - ps16
Deuterostomia - ps17
Chordata - ps18
Olfactores - ps19
Vertebrata - ps20
Gnathostomata - ps21
Euteleostomi - ps22
Sarcopterygii - ps23
Tetrapoda - ps24
Amniota - ps25
Mammalia - ps26
Theria - ps27
Placentalia - ps28
Boreoeutheria - ps29
Euarchontoglires - ps30
Euarchonta - ps31
Primates - ps32
Haplorrhini - ps33
Simiiformes - ps34
Catarrhini - ps35
Hominoidea - ps36
Hominidae - ps37
Homininae - ps38
Hominini - ps39
Homo_sapiens - ps40
p-value
5e-03
6e-03
9e-03
1e-02
2e-02
Log-odds
0.5
1.0
1.5
2.0
2.5
3.0
3.5
4.0

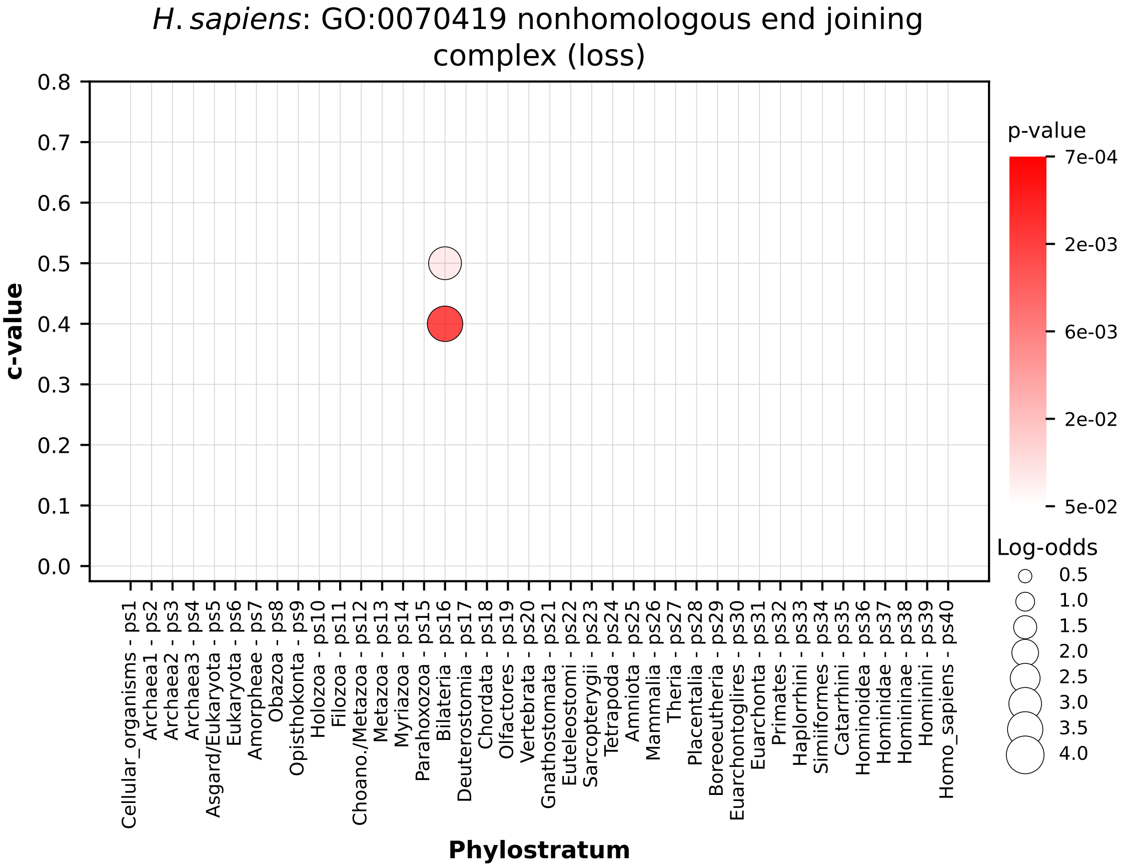

H. sapiens: GO:0070419 nonhomologous end joining complex (loss)
c-value
0.8
0.7
0.6
0.5
0.4
0.3
0.2
0.1
0.0
Phylostratum
Cellular_organisms - ps1
Archaea1 - ps2
Archaea2 - ps3
Archaea3 - ps4
Asgard/Eukaryota - ps5
Eukaryota - ps6
Amorpheae - ps7
Obazoa - ps8
Opisthokonta - ps9
Holozoa - ps10
Filozoa - ps11
Choano./Metazoa - ps12
Metazoa - ps13
Myriazoa - ps14
Parahoxozoa - ps15
Bilateria - ps16
Deuterostomia - ps17
Chordata - ps18
Olfactores - ps19
Vertebrata - ps20
Gnathostomata - ps21
Euteleostomi - ps22
Sarcopterygii - ps23
Tetrapoda - ps24
Amniota - ps25
Mammalia - ps26
Theria - ps27
Placentalia - ps28
Boreoeutheria - ps29
Euarchontoglires - ps30
Euarchonta - ps31
Primates - ps32
Haplorrhini - ps33
Simiiformes - ps34
Catarrhini - ps35
Hominoidea - ps36
Hominidae - ps37
Homininae - ps38
Hominini - ps39
Homo_sapiens - ps40
p-value
7e-04
2e-03
6e-03
2e-02
5e-02
Log-odds
0.5
1.0
1.5
2.0
2.5
3.0
3.5
4.0

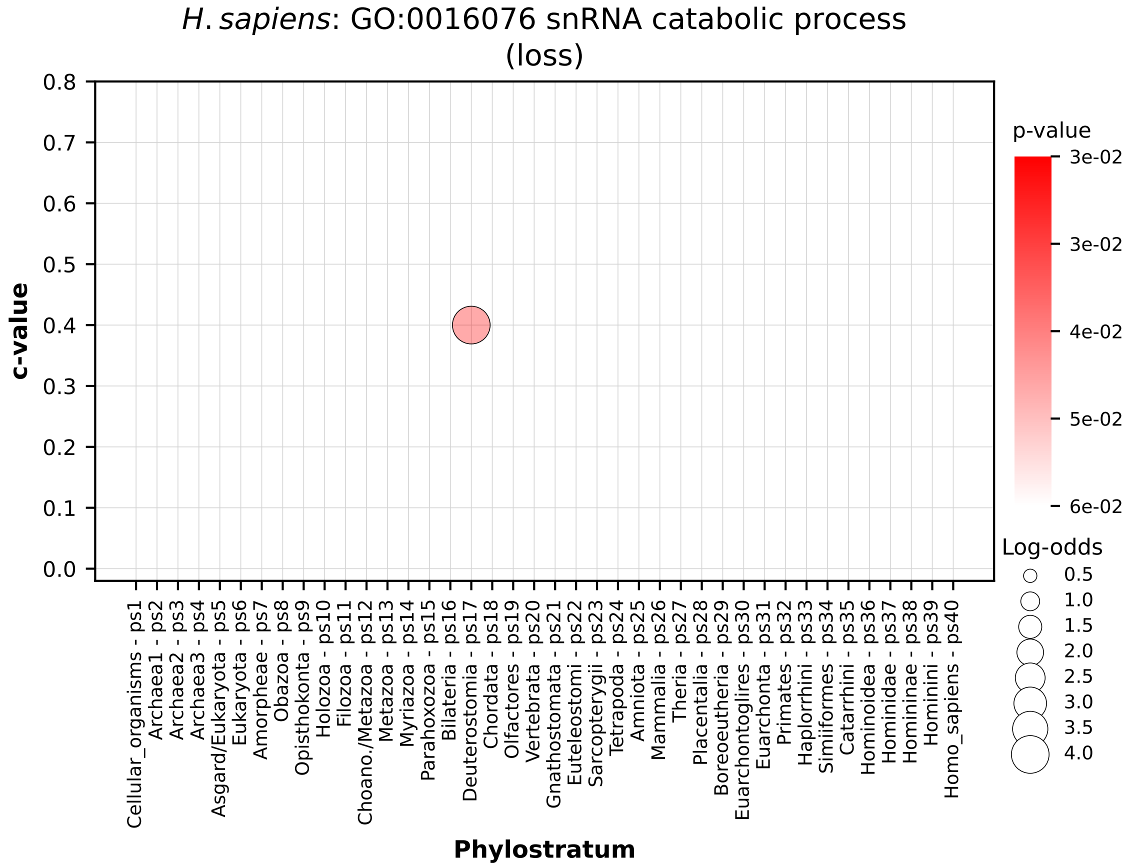

H. sapiens: GO:0016076 snRNA catabolic process
(loss)
c-value
0.0
0.1
0.2
0.3
0.4
0.5
0.6
0.7
0.8
Phylostratum
Cellular_organisms - ps1
Archaea1 - ps2
Archaea2 - ps3
Archaea3 - ps4
Asgard/Eukaryota - ps5
Eukaryota - ps6
Amorpheae - ps7
Obazoa - ps8
Opisthokonta - ps9
Holozoa - ps10
Filozoa - ps11
Choano./Metazoa - ps12
Metazoa - ps13
Myriazoa - ps14
Parahoxozoa - ps15
Bilateria - ps16
Deuterostomia - ps17
Chordata - ps18
Olfactores - ps19
Vertebrata - ps20
Gnathostomata - ps21
Euteleostomi - ps22
Sarcopterygii - ps23
Tetrapoda - ps24
Amniota - ps25
Mammalia - ps26
Theria - ps27
Placentalia - ps28
Boreoeutheria - ps29
Euarchontoglires - ps30
Euarchonta - ps31
Primates - ps32
Haplorrhini - ps33
Simiiformes - ps34
Catarrhini - ps35
Hominoidea - ps36
Hominidae - ps37
Homininae - ps38
Hominini - ps39
Homo_sapiens - ps40
p-value
3e-02
3e-02
4e-02
5e-02
6e-02
Log-odds
0.5
1.0
1.5
2.0
2.5
3.0
3.5
4.0

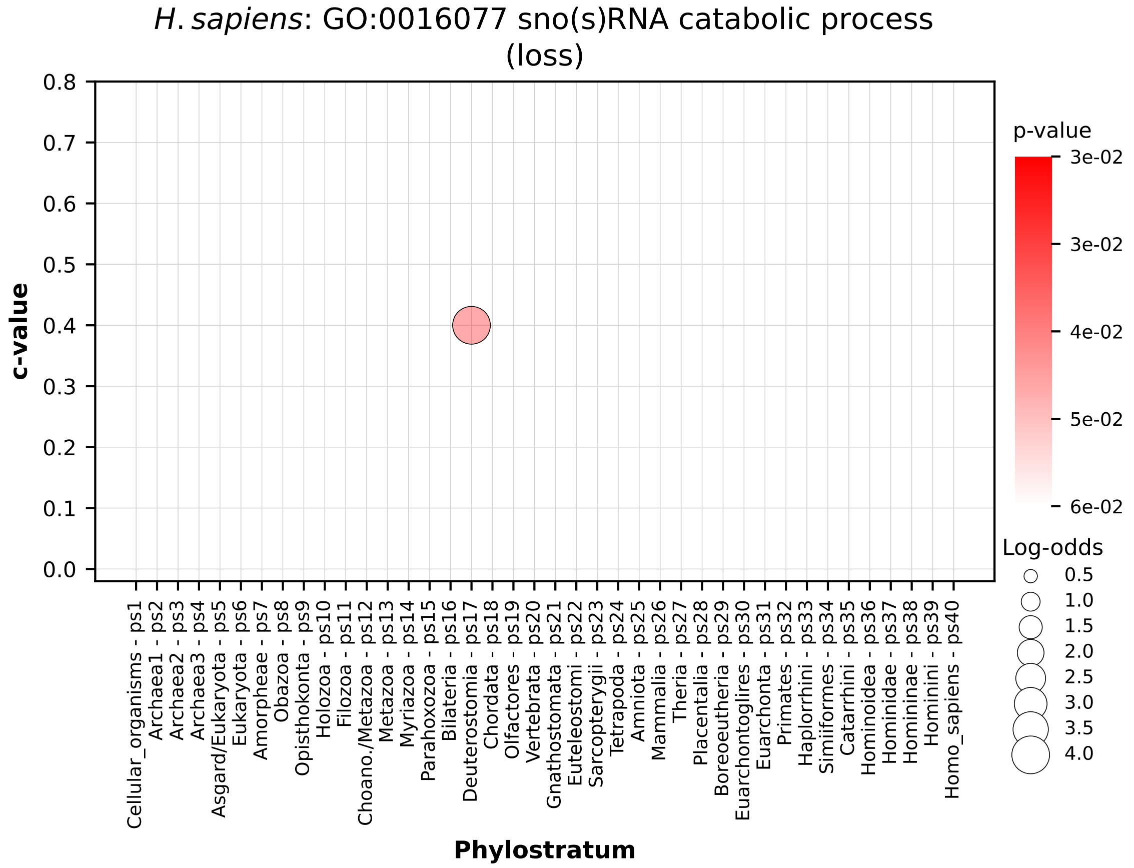

H. sapiens: GO:0016077 sno(s)RNA catabolic process
(loss)
c-value
0.0
0.1
0.2
0.3
0.4
0.5
0.6
0.7
0.8
Phylostratum
Cellular_organisms - ps1
Archaea1 - ps2
Archaea2 - ps3
Archaea3 - ps4
Asgard/Eukaryota - ps5
Eukaryota - ps6
Amorpheae - ps7
Obazoa - ps8
Opisthokonta - ps9
Holozoa - ps10
Filozoa - ps11
Choano./Metazoa - ps12
Metazoa - ps13
Myriazoa - ps14
Parahoxozoa - ps15
Bilateria - ps16
Deuterostomia - ps17
Chordata - ps18
Olfactores - ps19
Vertebrata - ps20
Gnathostomata - ps21
Euteleostomi - ps22
Sarcopterygii - ps23
Tetrapoda - ps24
Amniota - ps25
Mammalia - ps26
Theria - ps27
Placentalia - ps28
Boreoeutheria - ps29
Euarchontoglires - ps30
Euarchonta - ps31
Primates - ps32
Haplorrhini - ps33
Simiiformes - ps34
Catarrhini - ps35
Hominoidea - ps36
Hominidae - ps37
Homininae - ps38
Hominini - ps39
Homo_sapiens - ps40
p-value
3e-02
3e-02
4e-02
5e-02
6e-02
Log-odds
0.5
1.0
1.5
2.0
2.5
3.0
3.5
4.0

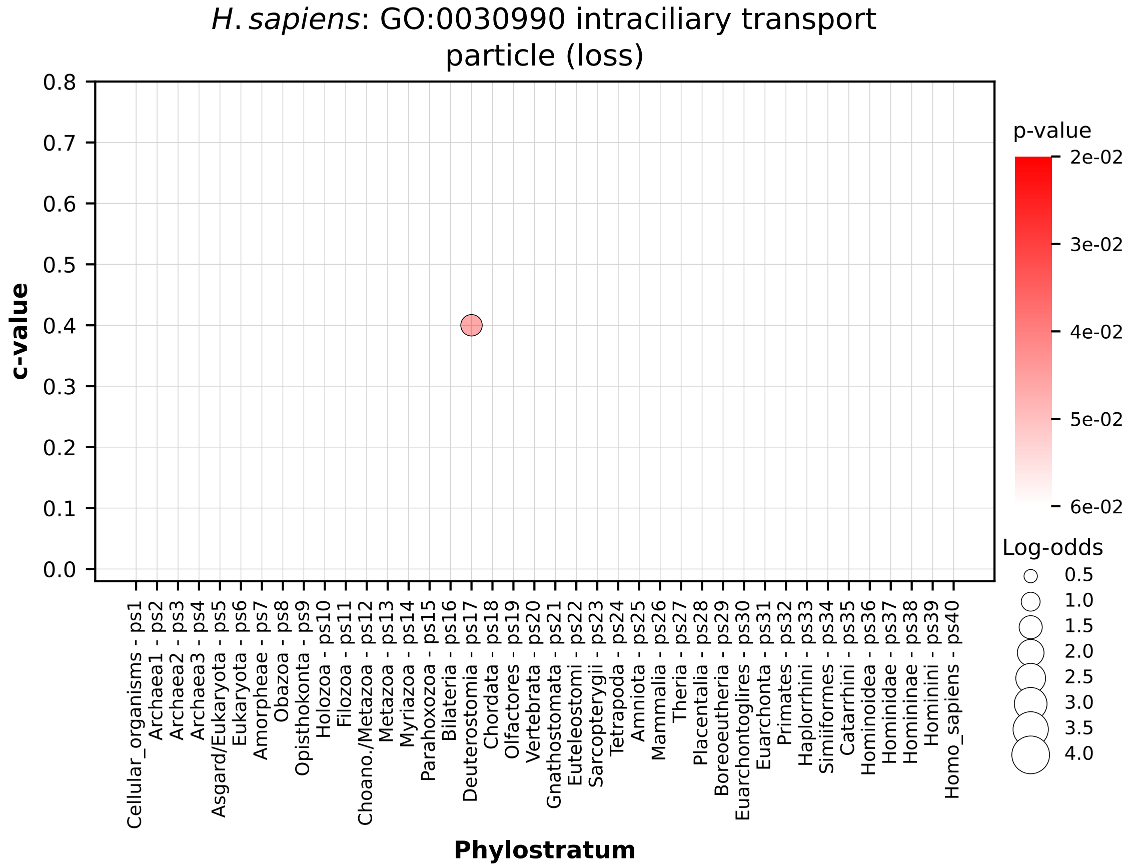

H. sapiens: GO:0030990 intraciliary transport particle (loss)
c-value
0.0
0.1
0.2
0.3
0.4
0.5
0.6
0.7
0.8
Phylostratum
Cellular_organisms - ps1
Archaea1 - ps2
Archaea2 - ps3
Archaea3 - ps4
Asgard/Eukaryota - ps5
Eukaryota - ps6
Amorpheae - ps7
Obazoa - ps8
Opisthokonta - ps9
Holozoa - ps10
Filozoa - ps11
Choano./Metazoa - ps12
Metazoa - ps13
Myriazoa - ps14
Parahoxozoa - ps15
Bilateria - ps16
Deuterostomia - ps17
Chordata - ps18
Olfactores - ps19
Vertebrata - ps20
Gnathostomata - ps21
Euteleostomi - ps22
Sarcopterygii - ps23
Tetrapoda - ps24
Amniota - ps25
Mammalia - ps26
Theria - ps27
Placentalia - ps28
Boreoeutheria - ps29
Euarchontoglires - ps30
Euarchonta - ps31
Primates - ps32
Haplorrhini - ps33
Simiiformes - ps34
Catarrhini - ps35
Hominoidea - ps36
Hominidae - ps37
Homininae - ps38
Hominini - ps39
Homo_sapiens - ps40
p-value
2e-02
3e-02
4e-02
5e-02
6e-02
Log-odds
0.5
1.0
1.5
2.0
2.5
3.0
3.5
4.0

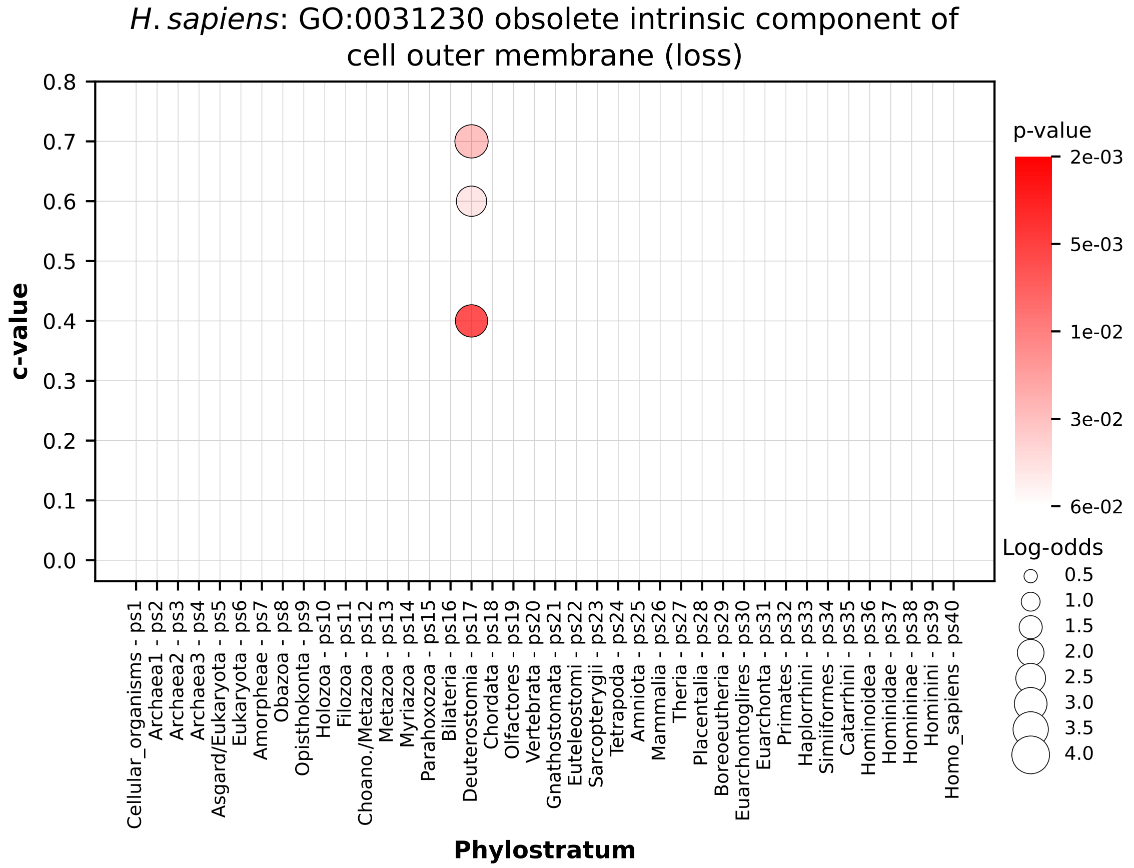

H. sapiens: GO:0031230 obsolete intrinsic component of cell outer membrane (loss)
c-value
0.0
0.1
0.2
0.3
0.4
0.5
0.6
0.7
0.8
p-value
2e-03
5e-03
1e-02
3e-02
6e-02
Log-odds
0.5
1.0
1.5
2.0
2.5
3.0
3.5
4.0
Cellular_organisms - ps1
Archaea1 - ps2
Archaea2 - ps3
Archaea3 - ps4
Asgard/Eukaryota - ps5
Eukaryota - ps6
Amorpheae - ps7
Obazoa - ps8
Opisthokonta - ps9
Holozoa - ps10
Filozoa - ps11
Choano./Metazoa - ps12
Metazoa - ps13
Myriazoa - ps14
Parahoxozoa - ps15
Bilateria - ps16
Deuterostomia - ps17
Chordata - ps18
Olfactores - ps19
Vertebrata - ps20
Gnathostomata - ps21
Euteleostomi - ps22
Sarcopterygii - ps23
Tetrapoda - ps24
Amniota - ps25
Mammalia - ps26
Theria - ps27
Placentalia - ps28
Boreoeutheria - ps29
Euarchontoglires - ps30
Euarchonta - ps31
Primates - ps32
Haplorrhini - ps33
Simiiformes - ps34
Catarrhini - ps35
Hominoidea - ps36
Hominidae - ps37
Homininae - ps38
Hominini - ps39
Homo_sapiens - ps40
Phylostratum

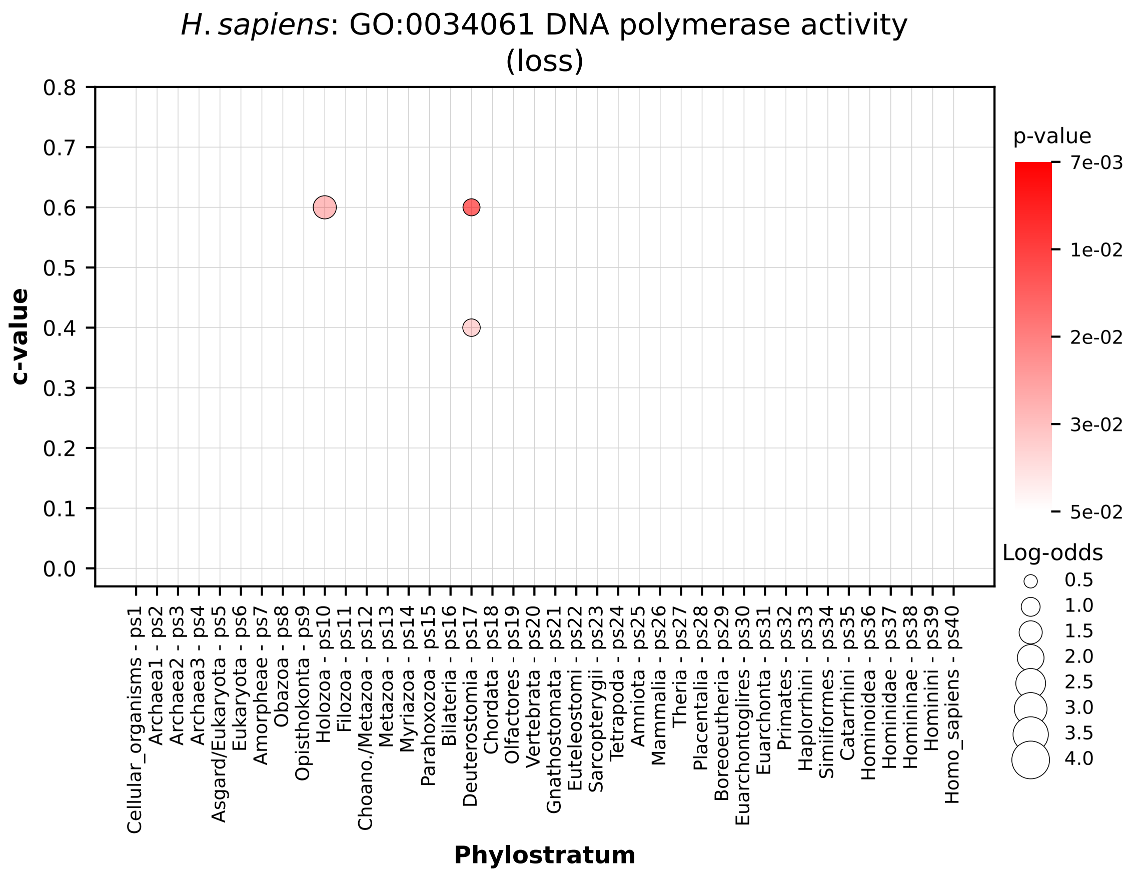

H. sapiens: GO:0034061 DNA polymerase activity (loss)
c-value
0.8
0.7
0.6
0.5
0.4
0.3
0.2
0.1
0.0
Phylostratum
Cellular_organisms - ps1
Archaea1 - ps2
Archaea2 - ps3
Archaea3 - ps4
Asgard/Eukaryota - ps5
Eukaryota - ps6
Amorpheae - ps7
Obazoa - ps8
Opisthokonta - ps9
Holozoa - ps10
Filozoa - ps11
Choano./Metazoa - ps12
Metazoa - ps13
Myriazoa - ps14
Parahoxozoa - ps15
Bilateria - ps16
Deuterostomia - ps17
Chordata - ps18
Olfactores - ps19
Vertebrata - ps20
Gnathostomata - ps21
Euteleostomi - ps22
Sarcopterygii - ps23
Tetrapoda - ps24
Amniota - ps25
Mammalia - ps26
Theria - ps27
Placentalia - ps28
Boreoeutheria - ps29
Euarchontoglires - ps30
Euarchonta - ps31
Primates - ps32
Haplorrhini - ps33
Simiiformes - ps34
Catarrhini - ps35
Hominoidea - ps36
Hominidae - ps37
Homininae - ps38
Hominini - ps39
Homo_sapiens - ps40
p-value
7e-03
1e-02
2e-02
3e-02
5e-02
Log-odds
0.5
1.0
1.5
2.0
2.5
3.0
3.5
4.0

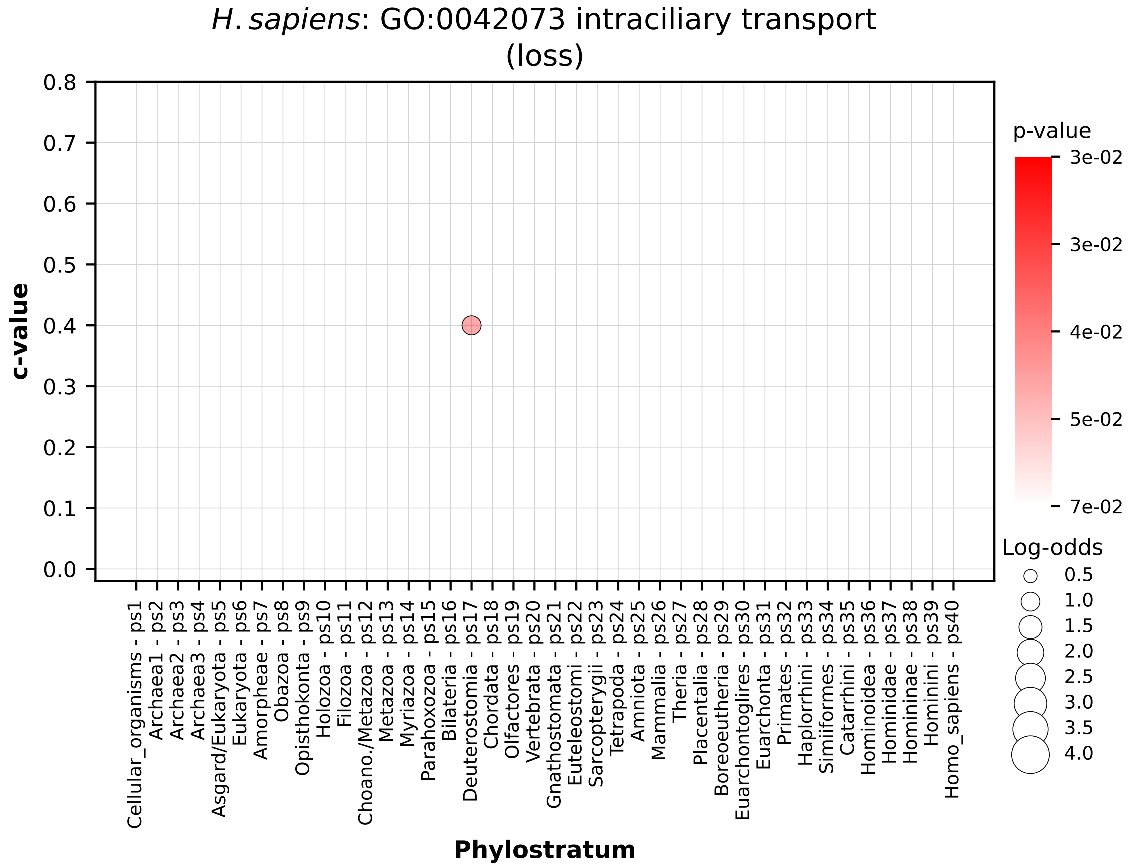
H. sapiens: GO:0042073 intraciliary transport
(loss)
c-value
0.8
0.7
0.6
0.5
0.4
0.3
0.2
0.1
0.0
p-value
3e-02
3e-02
4e-02
5e-02
7e-02
Log-odds
0.5
1.0
1.5
2.0
2.5
3.0
3.5
4.0
Cellular_organisms - ps1
Archaea1 - ps2
Archaea2 - ps3
Archaea3 - ps4
Asgard/Eukaryota - ps5
Eukaryota - ps6
Amorpheae - ps7
Obazoa - ps8
Opisthokonta - ps9
Holozoa - ps10
Filozoa - ps11
Choano./Metazoa - ps12
Metazoa - ps13
Myriazoa - ps14
Parahoxozoa - ps15
Bilateria - ps16
Deuterostomia - ps17
Chordata - ps18
Olfactores - ps19
Vertebrata - ps20
Gnathostomata - ps21
Euteleostomi - ps22
Sarcopterygii - ps23
Tetrapoda - ps24
Amniota - ps25
Mammalia - ps26
Theria - ps27
Placentalia - ps28
Boreoeutheria - ps29
Euarchontoglires - ps30
Euarchonta - ps31
Primates - ps32
Haplorrhini - ps33
Simiiformes - ps34
Catarrhini - ps35
Hominoidea - ps36
Hominidae - ps37
Homininae - ps38
Hominini - ps39
Homo_sapiens - ps40
Phylostratum

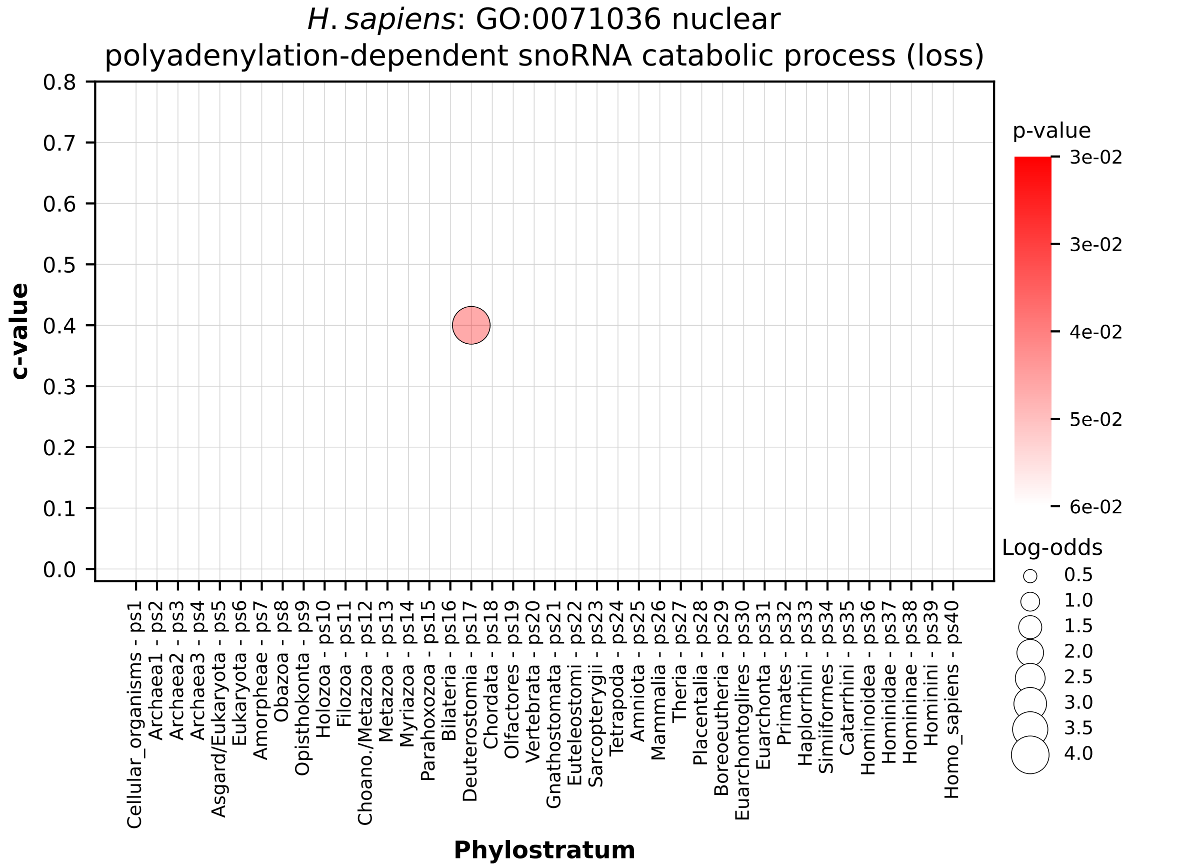

H. sapiens: GO:0071036 nuclear polyadenylation-dependent snoRNA catabolic process (loss)
c-value
0.8
0.7
0.6
0.5
0.4
0.3
0.2
0.1
0.0
p-value
3e-02
3e-02
4e-02
5e-02
6e-02
Log-odds
0.5
1.0
1.5
2.0
2.5
3.0
3.5
4.0
Cellular_organisms - ps1
Archaea1 - ps2
Archaea2 - ps3
Archaea3 - ps4
Asgard/Eukaryota - ps5
Eukaryota - ps6
Amorpheae - ps7
Obazoa - ps8
Opisthokonta - ps9
Holozoa - ps10
Filozoa - ps11
Choano./Metazoa - ps12
Metazoa - ps13
Myriazoa - ps14
Parahoxozoa - ps15
Bilateria - ps16
Deuterostomia - ps17
Chordata - ps18
Olfactores - ps19
Vertebrata - ps20
Gnathostomata - ps21
Euteleostomi - ps22
Sarcopterygii - ps23
Tetrapoda - ps24
Amniota - ps25
Mammalia - ps26
Theria - ps27
Placentalia - ps28
Boreoeutheria - ps29
Euarchontoglires - ps30
Euarchonta - ps31
Primates - ps32
Haplorrhini - ps33
Simiiformes - ps34
Catarrhini - ps35
Hominoidea - ps36
Hominidae - ps37
Homininae - ps38
Hominini - ps39
Homo_sapiens - ps40
Phylostratum

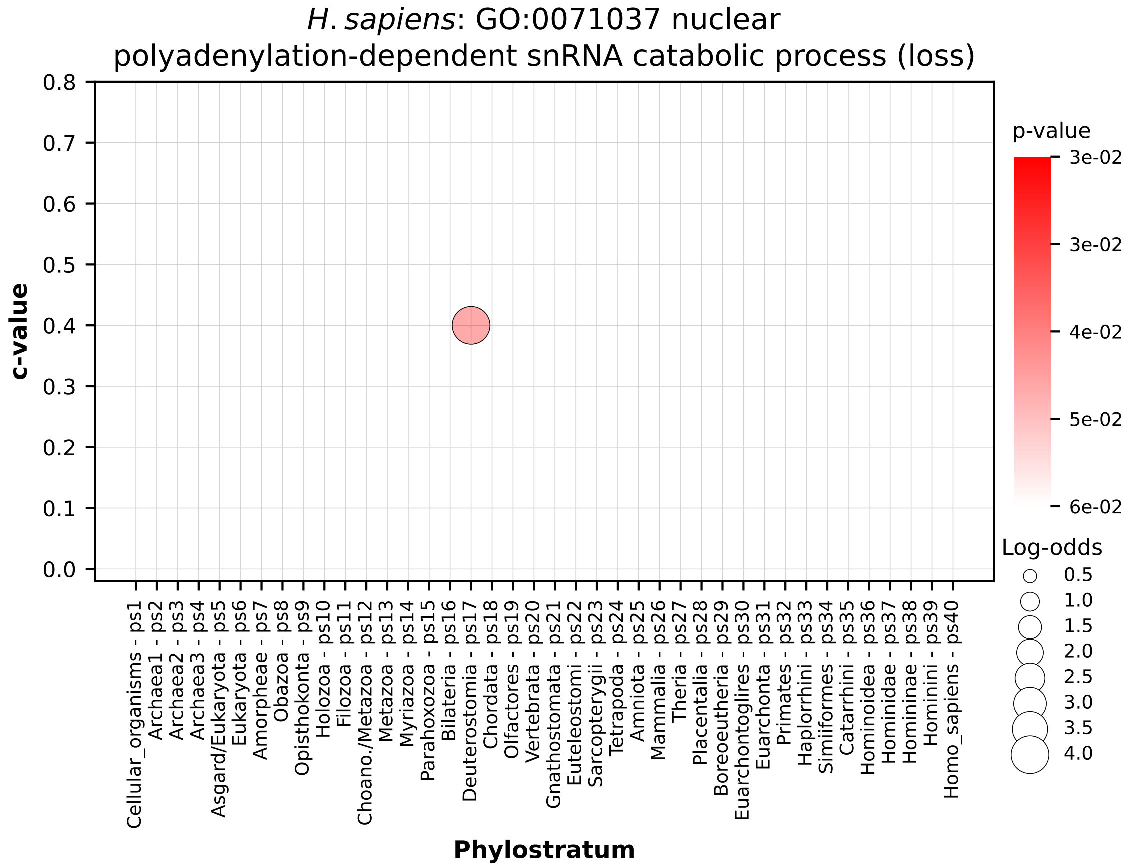

H. sapiens: GO:0071037 nuclear polyadenylation-dependent snRNA catabolic process (loss)
c-value
0.0
0.1
0.2
0.3
0.4
0.5
0.6
0.7
0.8
p-value
3e-02
3e-02
4e-02
5e-02
6e-02
Log-odds
0.5
1.0
1.5
2.0
2.5
3.0
3.5
4.0
Cellular_organisms - ps1
Archaea1 - ps2
Archaea2 - ps3
Archaea3 - ps4
Asgard/Eukaryota - ps5
Eukaryota - ps6
Amorpheae - ps7
Obazoa - ps8
Opisthokonta - ps9
Holozoa - ps10
Filozoa - ps11
Choano./Metazoa - ps12
Metazoa - ps13
Myriazoa - ps14
Parahoxozoa - ps15
Bilateria - ps16
Deuterostomia - ps17
Chordata - ps18
Olfactores - ps19
Vertebrata - ps20
Gnathostomata - ps21
Euteleostomi - ps22
Sarcopterygii - ps23
Tetrapoda - ps24
Amniota - ps25
Mammalia - ps26
Theria - ps27
Placentalia - ps28
Boreoeutheria - ps29
Euarchontoglires - ps30
Euarchonta - ps31
Primates - ps32
Haplorrhini - ps33
Simiiformes - ps34
Catarrhini - ps35
Hominoidea - ps36
Hominidae - ps37
Homininae - ps38
Hominini - ps39
Homo_sapiens - ps40
Phylostratum

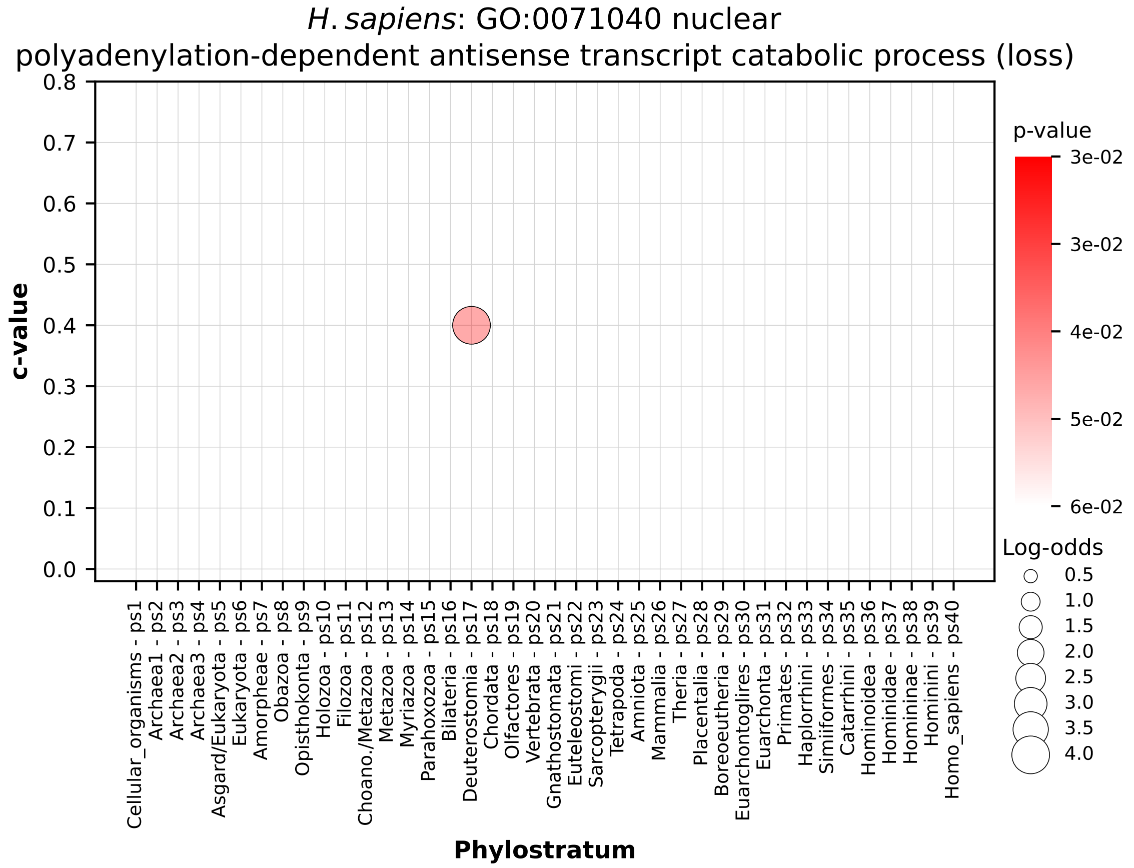

H. sapiens: GO:0071040 nuclear polyadenylation-dependent antisense transcript catabolic process (loss)
c-value
0.8
0.7
0.6
0.5
0.4
0.3
0.2
0.1
0.0
p-value
3e-02
3e-02
4e-02
5e-02
6e-02
Log-odds
0.5
1.0
1.5
2.0
2.5
3.0
3.5
4.0
Cellular_organisms - ps1
Archaea1 - ps2
Archaea2 - ps3
Archaea3 - ps4
Asgard/Eukaryota - ps5
Eukaryota - ps6
Amorpheae - ps7
Obazoa - ps8
Opisthokonta - ps9
Holozoa - ps10
Filozoa - ps11
Choano./Metazoa - ps12
Metazoa - ps13
Myriazoa - ps14
Parahoxozoa - ps15
Bilateria - ps16
Deuterostomia - ps17
Chordata - ps18
Olfactores - ps19
Vertebrata - ps20
Gnathostomata - ps21
Euteleostomi - ps22
Sarcopterygii - ps23
Tetrapoda - ps24
Amniota - ps25
Mammalia - ps26
Theria - ps27
Placentalia - ps28
Boreoeutheria - ps29
Euarchontoglires - ps30
Euarchonta - ps31
Primates - ps32
Haplorrhini - ps33
Simiiformes - ps34
Catarrhini - ps35
Hominoidea - ps36
Hominidae - ps37
Homininae - ps38
Hominini - ps39
Homo_sapiens - ps40
Phylostratum

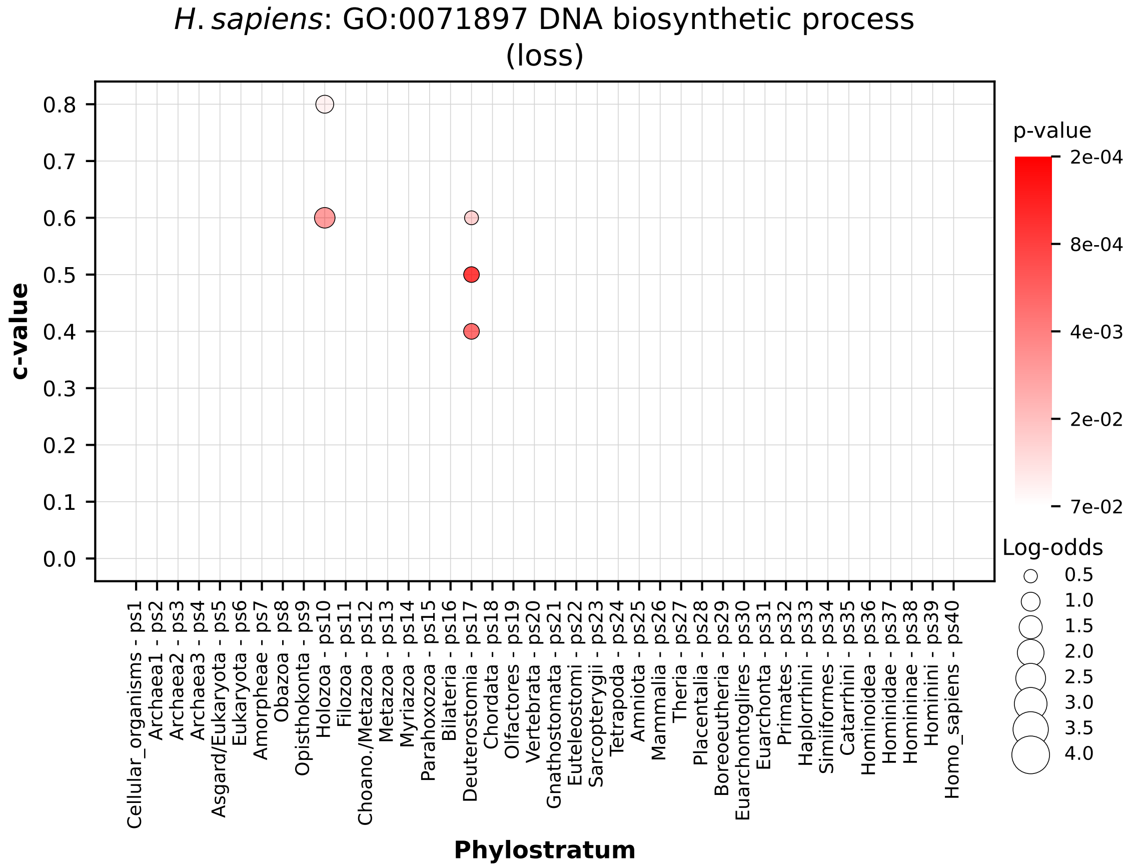

H. sapiens: GO:0071897 DNA biosynthetic process
(loss)
c-value
0.0
0.1
0.2
0.3
0.4
0.5
0.6
0.7
0.8
Phylostratum
Cellular_organisms - ps1
Archaea1 - ps2
Archaea2 - ps3
Archaea3 - ps4
Asgard/Eukaryota - ps5
Eukaryota - ps6
Amorpheae - ps7
Obazoa - ps8
Opisthokonta - ps9
Holozoa - ps10
Filozoa - ps11
Choano./Metazoa - ps12
Metazoa - ps13
Myriazoa - ps14
Parahoxozoa - ps15
Bilateria - ps16
Deuterostomia - ps17
Chordata - ps18
Olfactores - ps19
Vertebrata - ps20
Gnathostomata - ps21
Euteleostomi - ps22
Sarcopterygii - ps23
Tetrapoda - ps24
Amniota - ps25
Mammalia - ps26
Theria - ps27
Placentalia - ps28
Boreoeutheria - ps29
Euarchontoglires - ps30
Euarchonta - ps31
Primates - ps32
Haplorrhini - ps33
Simiiformes - ps34
Catarrhini - ps35
Hominoidea - ps36
Hominidae - ps37
Homininae - ps38
Hominini - ps39
Homo_sapiens - ps40
p-value
2e-04
8e-04
4e-03
2e-02
7e-02
Log-odds
0.5
1.0
1.5
2.0
2.5
3.0
3.5
4.0

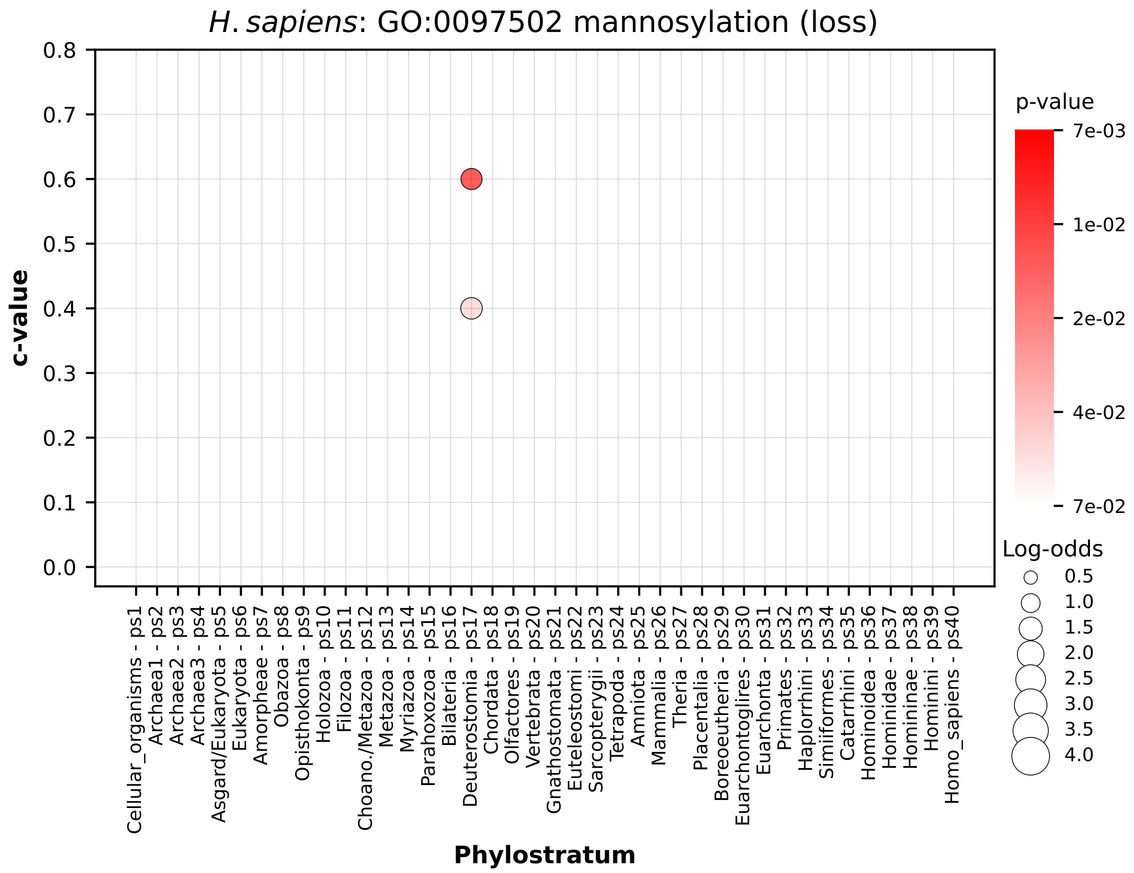

H. sapiens: GO:0097502 mannosylation (loss)
c-value
0.0
0.1
0.2
0.3
0.4
0.5
0.6
0.7
0.8
Phylostratum
Cellular_organisms - ps1
Archaea1 - ps2
Archaea2 - ps3
Archaea3 - ps4
Asgard/Eukaryota - ps5
Eukaryota - ps6
Amorpheae - ps7
Obazoa - ps8
Opisthokonta - ps9
Holozoa - ps10
Filozoa - ps11
Choano./Metazoa - ps12
Metazoa - ps13
Myriazoa - ps14
Parahoxozoa - ps15
Bilateria - ps16
Deuterostomia - ps17
Chordata - ps18
Olfactores - ps19
Vertebrata - ps20
Gnathostomata - ps21
Euteleostomi - ps22
Sarcopterygii - ps23
Tetrapoda - ps24
Amniota - ps25
Mammalia - ps26
Theria - ps27
Placentalia - ps28
Boreoeutheria - ps29
Euarchontoglires - ps30
Euarchonta - ps31
Primates - ps32
Haplorrhini - ps33
Simiiformes - ps34
Catarrhini - ps35
Hominoidea - ps36
Hominidae - ps37
Homininae - ps38
Hominini - ps39
Homo_sapiens - ps40
p-value
7e-03
1e-02
2e-02
4e-02
7e-02
Log-odds
0.5
1.0
1.5
2.0
2.5
3.0
3.5
4.0

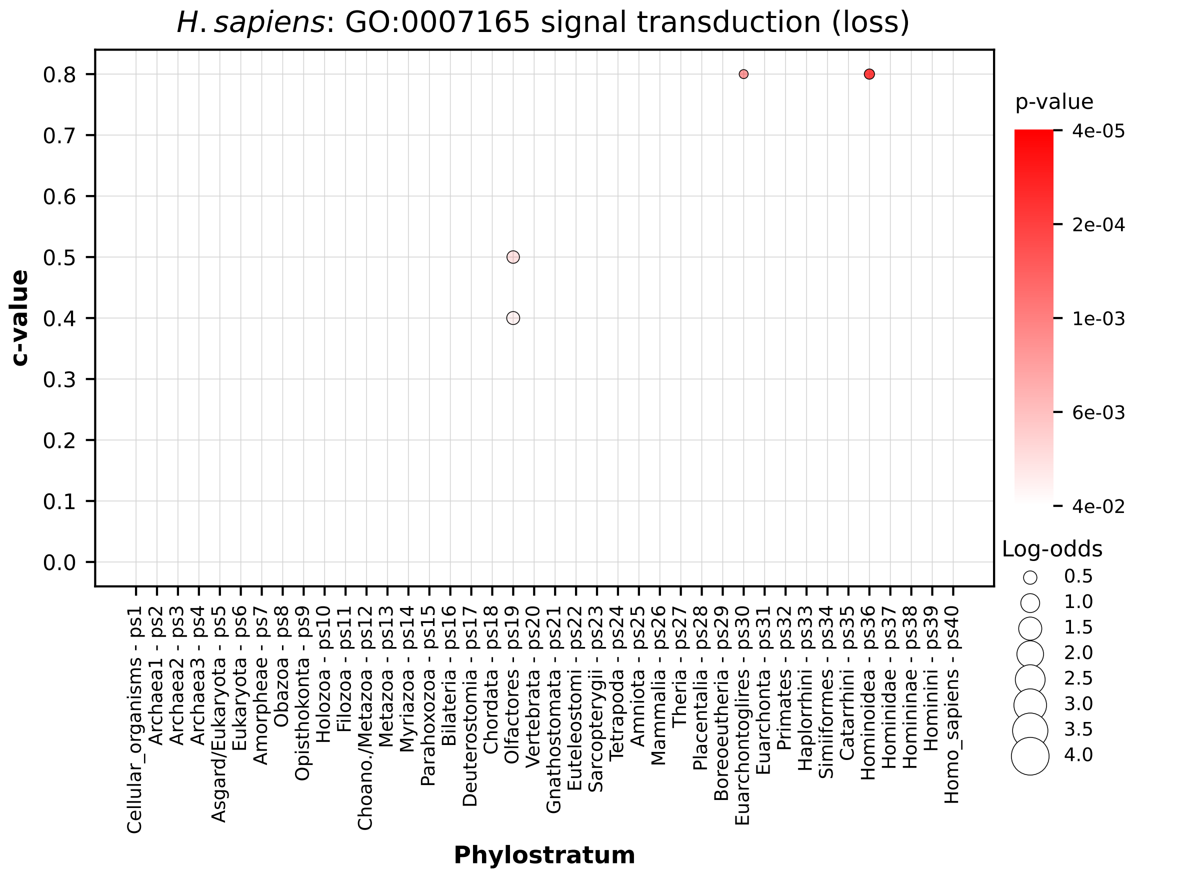

H. sapiens: GO:0007165 signal transduction (loss)
c-value
0.8
0.7
0.6
0.5
0.4
0.3
0.2
0.1
0.0
p-value
4e-05
2e-04
1e-03
6e-03
4e-02
Log-odds
0.5
1.0
1.5
2.0
2.5
3.0
3.5
4.0
Cellular_organisms - ps1
Archaea1 - ps2
Archaea2 - ps3
Archaea3 - ps4
Asgard/Eukaryota - ps5
Eukaryota - ps6
Amorpheae - ps7
Obazoa - ps8
Opisthokonta - ps9
Holozoa - ps10
Filozoa - ps11
Choano./Metazoa - ps12
Metazoa - ps13
Myriazoa - ps14
Parahoxozoa - ps15
Bilateria - ps16
Deuterostomia - ps17
Chordata - ps18
Olfactores - ps19
Vertebrata - ps20
Gnathostomata - ps21
Euteleostomi - ps22
Sarcopterygii - ps23
Tetrapoda - ps24
Amniota - ps25
Mammalia - ps26
Theria - ps27
Placentalia - ps28
Boreoeutheria - ps29
Euarchontoglires - ps30
Euarchonta - ps31
Primates - ps32
Haplorrhini - ps33
Simiiformes - ps34
Catarrhini - ps35
Hominoidea - ps36
Hominidae - ps37
Homininae - ps38
Hominini - ps39
Homo_sapiens - ps40
Phylostratum

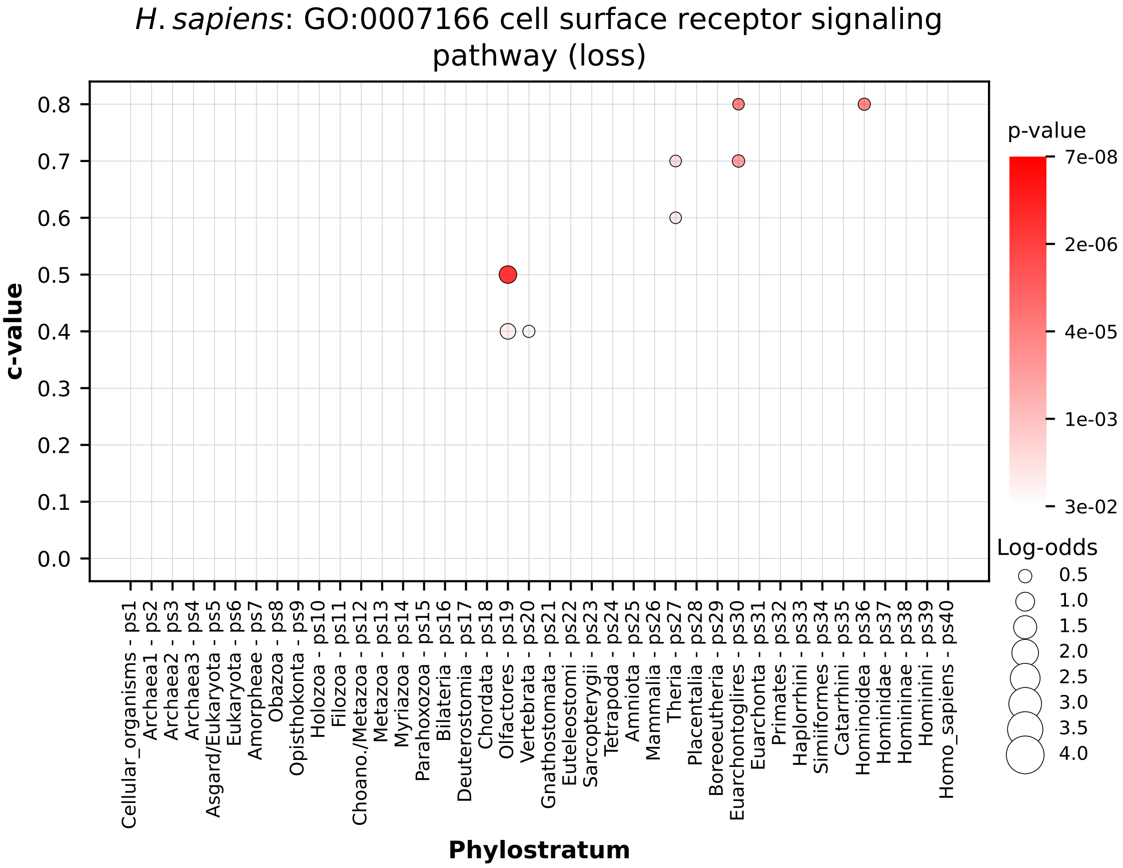

H. sapiens: GO:0007166 cell surface receptor signaling pathway (loss)
c-value
0.0
0.1
0.2
0.3
0.4
0.5
0.6
0.7
0.8
Phylostratum
Cellular_organisms - ps1
Archaea1 - ps2
Archaea2 - ps3
Archaea3 - ps4
Asgard/Eukaryota - ps5
Eukaryota - ps6
Amorpheae - ps7
Obazoa - ps8
Opisthokonta - ps9
Holozoa - ps10
Filozoa - ps11
Choano./Metazoa - ps12
Metazoa - ps13
Myriazoa - ps14
Parahoxozoa - ps15
Bilateria - ps16
Deuterostomia - ps17
Chordata - ps18
Olfactores - ps19
Vertebrata - ps20
Gnathostomata - ps21
Euteleostomi - ps22
Sarcopterygii - ps23
Tetrapoda - ps24
Amniota - ps25
Mammalia - ps26
Theria - ps27
Placentalia - ps28
Boreoeutheria - ps29
Euarchontoglires - ps30
Euarchonta - ps31
Primates - ps32
Haplorrhini - ps33
Simiiformes - ps34
Catarrhini - ps35
Hominoidea - ps36
Hominidae - ps37
Homininae - ps38
Hominini - ps39
Homo_sapiens - ps40
p-value
7e-08
2e-06
4e-05
1e-03
3e-02
Log-odds
0.5
1.0
1.5
2.0
2.5
3.0
3.5
4.0

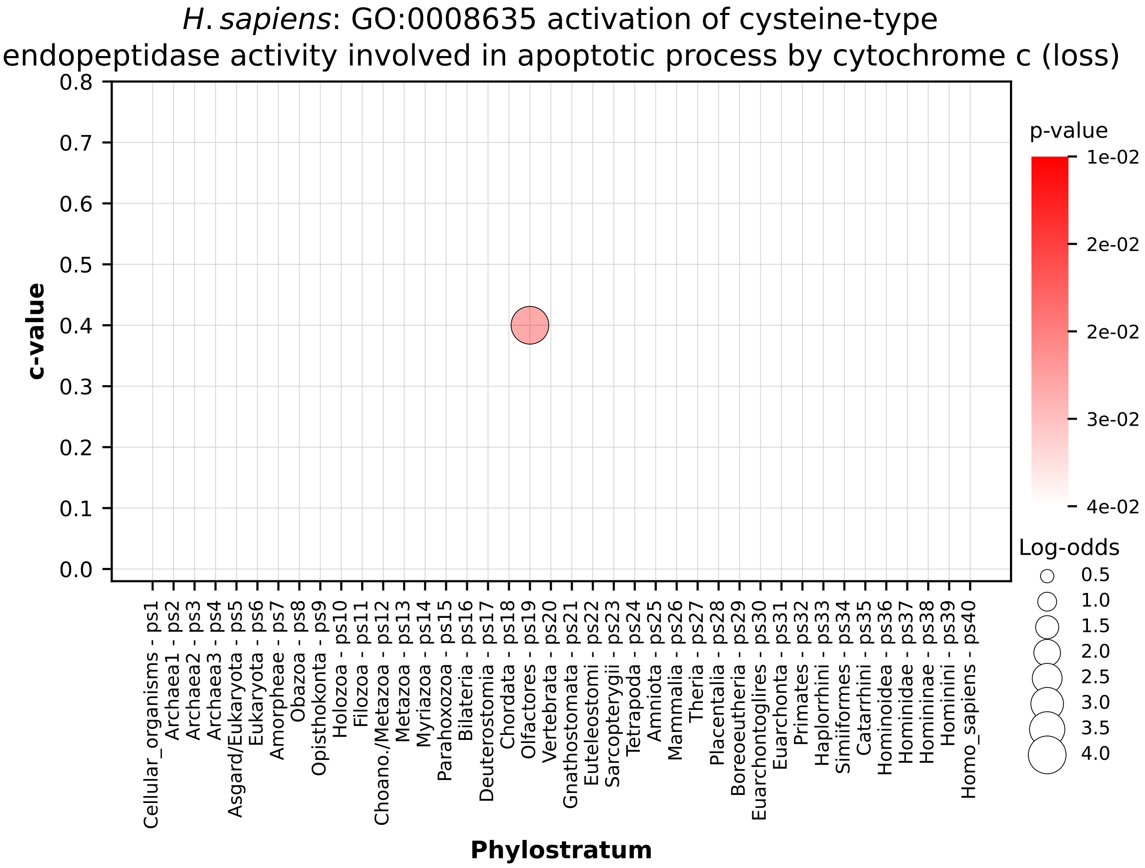
H. sapiens: GO:0008635 activation of cysteine-type endopeptidase activity involved in apoptotic process by cytochrome c (loss)
c-value
0.0
0.1
0.2
0.3
0.4
0.5
0.6
0.7
0.8
Phylostratum
Cellular_organisms - ps1
Archaea1 - ps2
Archaea2 - ps3
Archaea3 - ps4
Asgard/Eukaryota - ps5
Eukaryota - ps6
Amorpheae - ps7
Obazoa - ps8
Opisthokonta - ps9
Holozoa - ps10
Filozoa - ps11
Choano./Metazoa - ps12
Metazoa - ps13
Myriazoa - ps14
Parahoxozoa - ps15
Bilateria - ps16
Deuterostomia - ps17
Chordata - ps18
Olfactores - ps19
Vertebrata - ps20
Gnathostomata - ps21
Euteleostomi - ps22
Sarcopterygii - ps23
Tetrapoda - ps24
Amniota - ps25
Mammalia - ps26
Theria - ps27
Placentalia - ps28
Boreoeutheria - ps29
Euarchontoglires - ps30
Euarchonta - ps31
Primates - ps32
Haplorrhini - ps33
Simiiformes - ps34
Catarrhini - ps35
Hominoidea - ps36
Hominidae - ps37
Homininae - ps38
Hominini - ps39
Homo_sapiens - ps40
p-value
1e-02
2e-02
2e-02
3e-02
4e-02
Log-odds
0.5
1.0
1.5
2.0
2.5
3.0
3.5
4.0

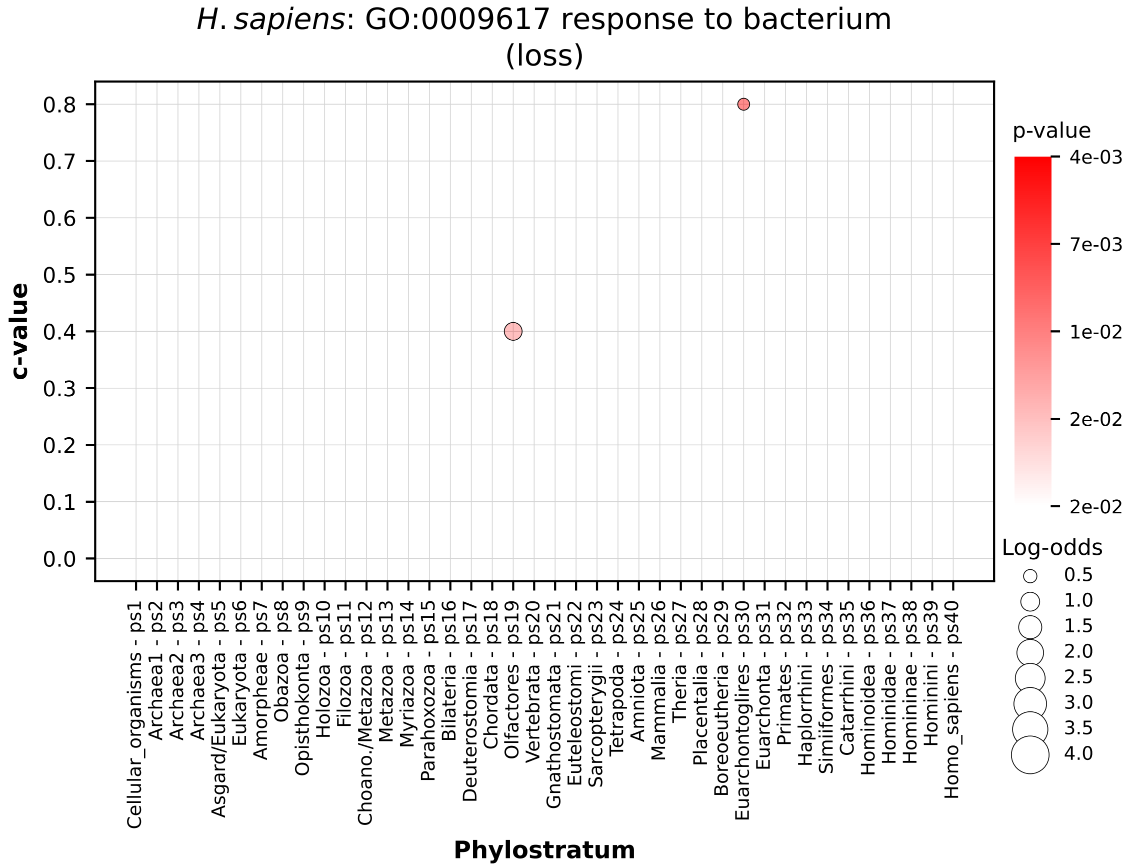

H. sapiens: GO:0009617 response to bacterium
(loss)
c-value
0.0
0.1
0.2
0.3
0.4
0.5
0.6
0.7
0.8
Phylostratum
Cellular_organisms - ps1
Archaea1 - ps2
Archaea2 - ps3
Archaea3 - ps4
Asgard/Eukaryota - ps5
Eukaryota - ps6
Amorpheae - ps7
Obazoa - ps8
Opisthokonta - ps9
Holozoa - ps10
Filozoa - ps11
Choano./Metazoa - ps12
Metazoa - ps13
Myriazoa - ps14
Parahoxozoa - ps15
Bilateria - ps16
Deuterostomia - ps17
Chordata - ps18
Olfactores - ps19
Vertebrata - ps20
Gnathostomata - ps21
Euteleostomi - ps22
Sarcopterygii - ps23
Tetrapoda - ps24
Amniota - ps25
Mammalia - ps26
Theria - ps27
Placentalia - ps28
Boreoeutheria - ps29
Euarchontoglires - ps30
Euarchonta - ps31
Primates - ps32
Haplorrhini - ps33
Simiiformes - ps34
Catarrhini - ps35
Hominoidea - ps36
Hominidae - ps37
Homininae - ps38
Hominini - ps39
Homo_sapiens - ps40
p-value
4e-03
7e-03
1e-02
2e-02
2e-02
Log-odds
0.5
1.0
1.5
2.0
2.5
3.0
3.5
4.0

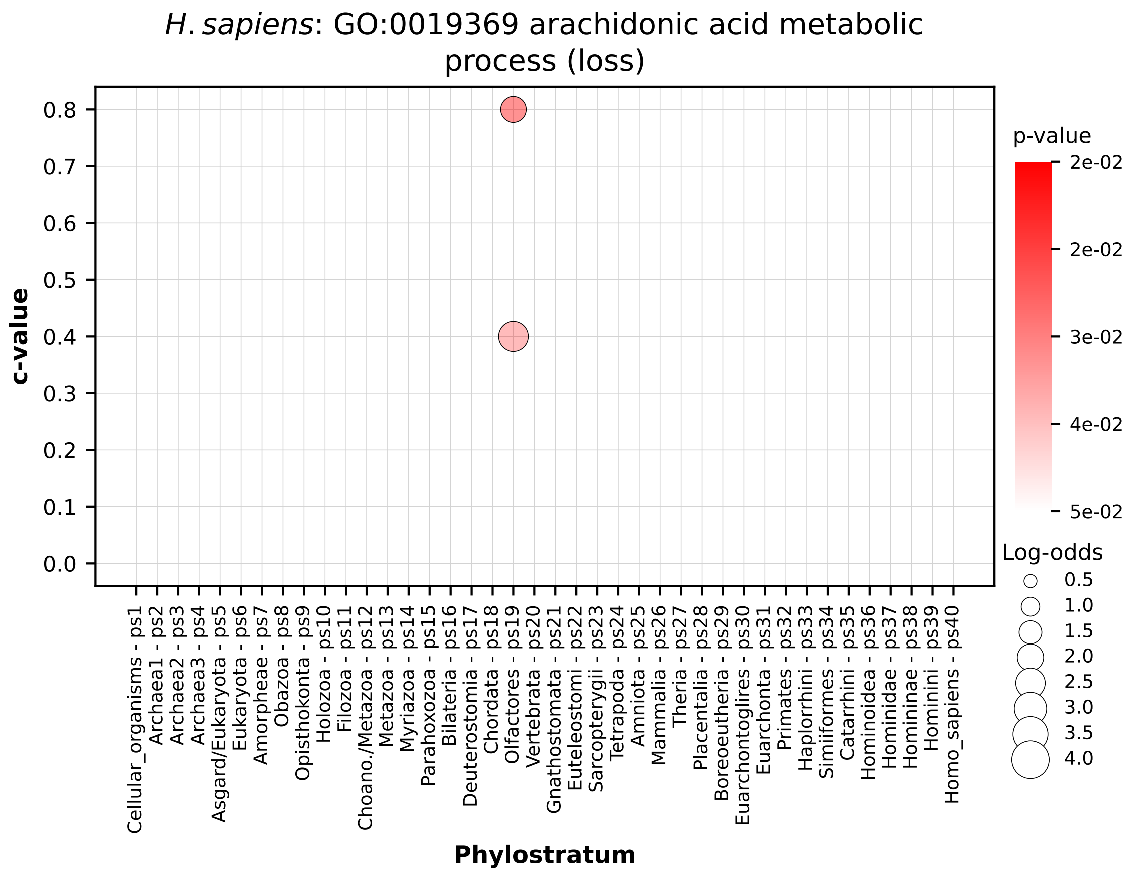

H. sapiens: GO:0019369 arachidonic acid metabolic process (loss)
c-value
0.8
0.7
0.6
0.5
0.4
0.3
0.2
0.1
0.0
p-value
2e-02
2e-02
3e-02
4e-02
5e-02
Log-odds
0.5
1.0
1.5
2.0
2.5
3.0
3.5
4.0
Cellular_organisms - ps1
Archaea1 - ps2
Archaea2 - ps3
Archaea3 - ps4
Asgard/Eukaryota - ps5
Eukaryota - ps6
Amorpheae - ps7
Obazoa - ps8
Opisthokonta - ps9
Holozoa - ps10
Filozoa - ps11
Choano./Metazoa - ps12
Metazoa - ps13
Myriazoa - ps14
Parahoxozoa - ps15
Bilateria - ps16
Deuterostomia - ps17
Chordata - ps18
Olfactores - ps19
Vertebrata - ps20
Gnathostomata - ps21
Euteleostomi - ps22
Sarcopterygii - ps23
Tetrapoda - ps24
Amniota - ps25
Mammalia - ps26
Theria - ps27
Placentalia - ps28
Boreoeutheria - ps29
Euarchontoglires - ps30
Euarchonta - ps31
Primates - ps32
Haplorrhini - ps33
Simiiformes - ps34
Catarrhini - ps35
Hominoidea - ps36
Hominidae - ps37
Homininae - ps38
Hominini - ps39
Homo_sapiens - ps40
Phylostratum

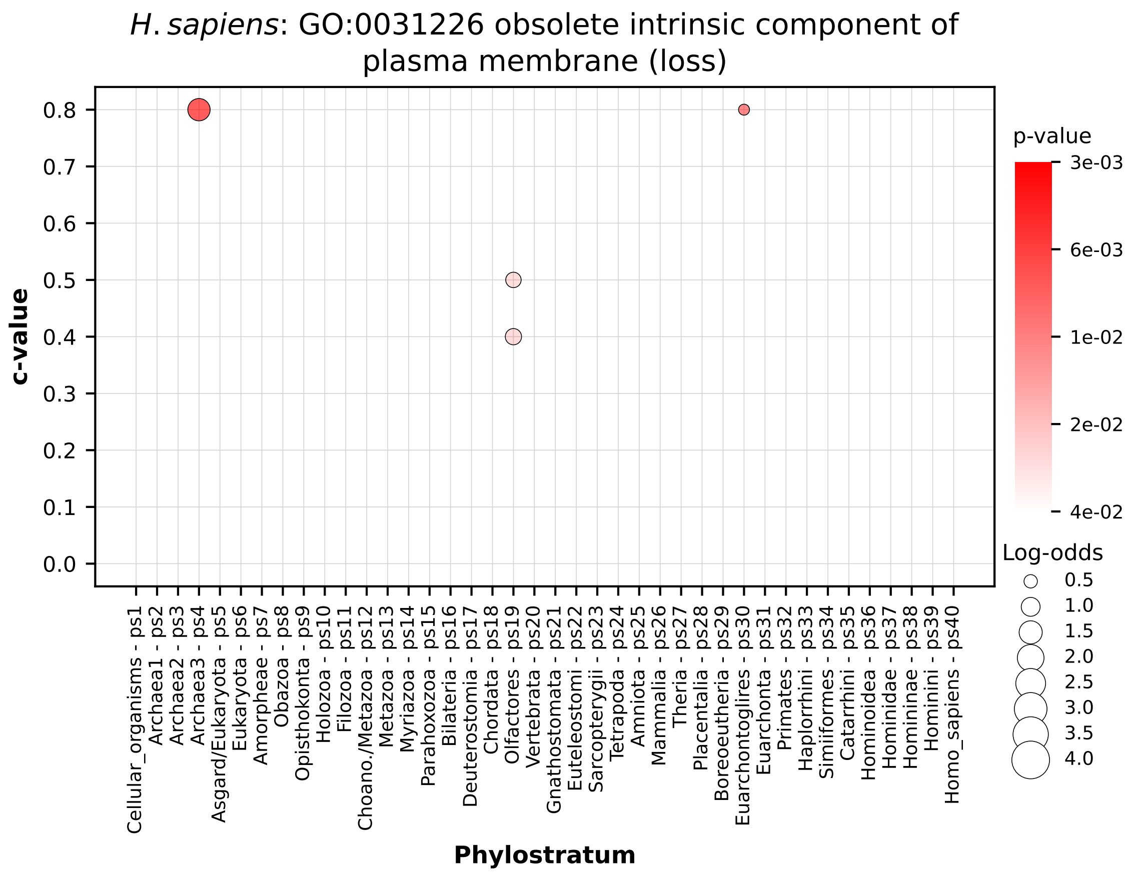

H. sapiens: GO:0031226 obsolete intrinsic component of plasma membrane (loss)
c-value
0.8
0.7
0.6
0.5
0.4
0.3
0.2
0.1
0.0
p-value
3e-03
6e-03
1e-02
2e-02
4e-02
Log-odds
0.5
1.0
1.5
2.0
2.5
3.0
3.5
4.0
Cellular_organisms - ps1
Archaea1 - ps2
Archaea2 - ps3
Archaea3 - ps4
Asgard/Eukaryota - ps5
Eukaryota - ps6
Amorpheae - ps7
Obazoa - ps8
Opisthokonta - ps9
Holozoa - ps10
Filozoa - ps11
Choano./Metazoa - ps12
Metazoa - ps13
Myriazoa - ps14
Parahoxozoa - ps15
Bilateria - ps16
Deuterostomia - ps17
Chordata - ps18
Olfactores - ps19
Vertebrata - ps20
Gnathostomata - ps21
Euteleostomi - ps22
Sarcopterygii - ps23
Tetrapoda - ps24
Amniota - ps25
Mammalia - ps26
Theria - ps27
Placentalia - ps28
Boreoeutheria - ps29
Euarchontoglires - ps30
Euarchonta - ps31
Primates - ps32
Haplorrhini - ps33
Simiiformes - ps34
Catarrhini - ps35
Hominoidea - ps36
Hominidae - ps37
Homininae - ps38
Hominini - ps39
Homo_sapiens - ps40
Phylostratum

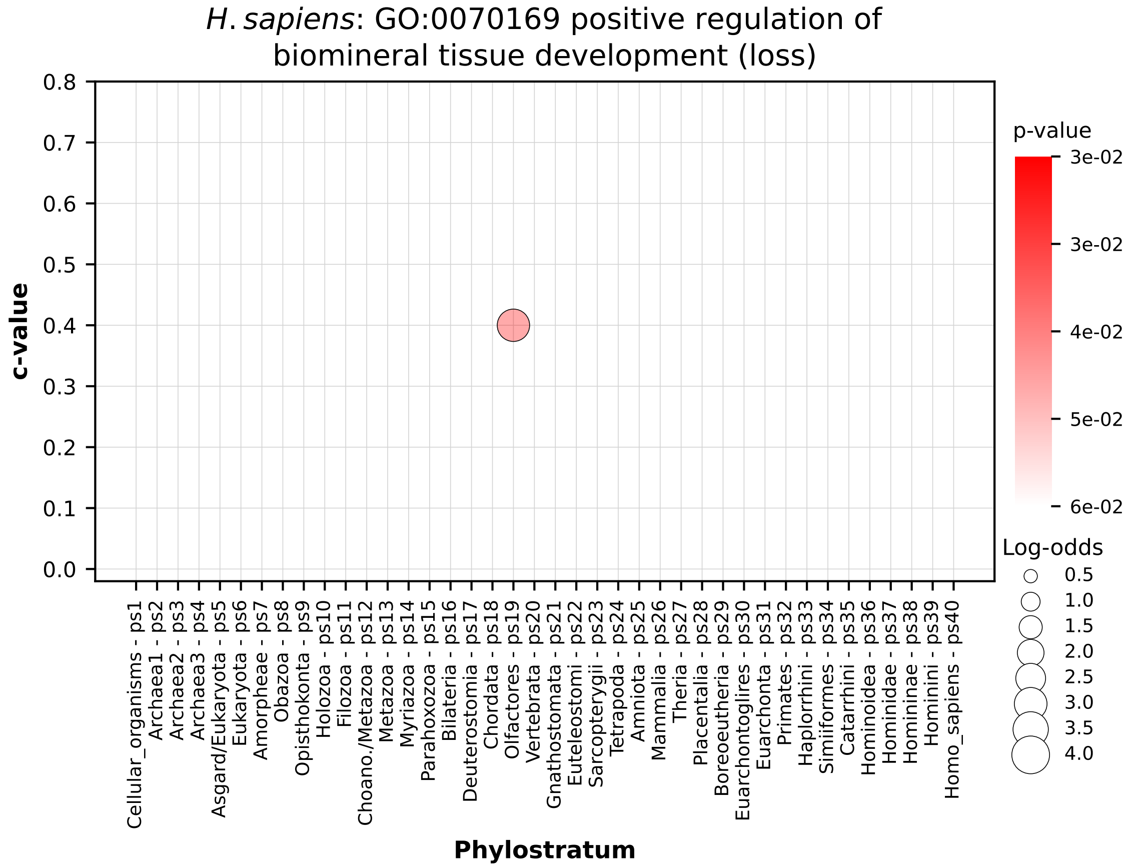
H. sapiens: GO:0070169 positive regulation of biomineral tissue development (loss)
c-value
0.8
0.7
0.6
0.5
0.4
0.3
0.2
0.1
0.0
Phylostratum
Cellular_organisms - ps1
Archaea1 - ps2
Archaea2 - ps3
Archaea3 - ps4
Asgard/Eukaryota - ps5
Eukaryota - ps6
Amorpheae - ps7
Obazoa - ps8
Opisthokonta - ps9
Holozoa - ps10
Filozoa - ps11
Choano./Metazoa - ps12
Metazoa - ps13
Myriazoa - ps14
Parahoxozoa - ps15
Bilateria - ps16
Deuterostomia - ps17
Chordata - ps18
Olfactores - ps19
Vertebrata - ps20
Gnathostomata - ps21
Euteleostomi - ps22
Sarcopterygii - ps23
Tetrapoda - ps24
Amniota - ps25
Mammalia - ps26
Theria - ps27
Placentalia - ps28
Boreoeutheria - ps29
Euarchontoglires - ps30
Euarchonta - ps31
Primates - ps32
Haplorrhini - ps33
Simiiformes - ps34
Catarrhini - ps35
Hominoidea - ps36
Hominidae - ps37
Homininae - ps38
Hominini - ps39
Homo_sapiens - ps40
p-value
3e-02
3e-02
4e-02
5e-02
6e-02
Log-odds
0.5
1.0
1.5
2.0
2.5
3.0
3.5
4.0

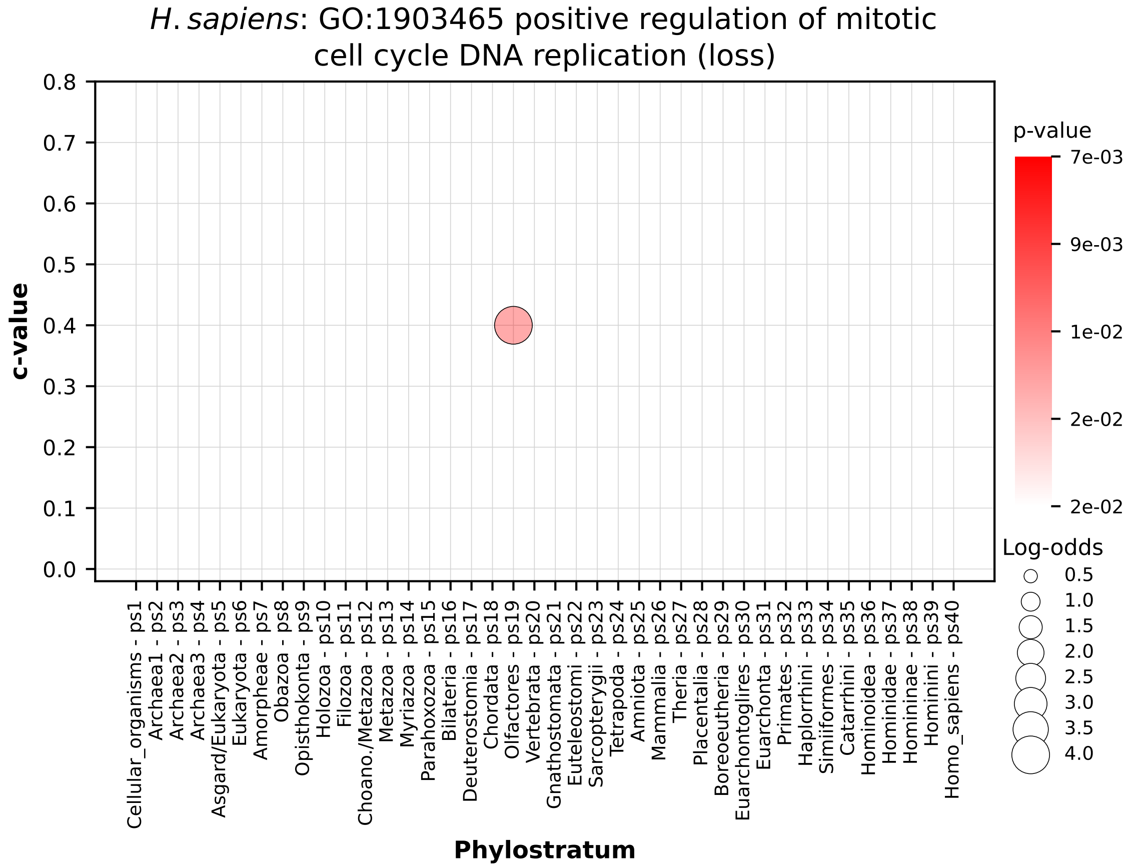
H. sapiens: GO:1903465 positive regulation of mitotic cell cycle DNA replication (loss)
c-value
0.8
0.7
0.6
0.5
0.4
0.3
0.2
0.1
0.0
Phylostratum
Cellular_organisms - ps1
Archaea1 - ps2
Archaea2 - ps3
Archaea3 - ps4
Asgard/Eukaryota - ps5
Eukaryota - ps6
Amorpheae - ps7
Obazoa - ps8
Opisthokonta - ps9
Holozoa - ps10
Filozoa - ps11
Choano./Metazoa - ps12
Metazoa - ps13
Myriazoa - ps14
Parahoxozoa - ps15
Bilateria - ps16
Deuterostomia - ps17
Chordata - ps18
Olfactores - ps19
Vertebrata - ps20
Gnathostomata - ps21
Euteleostomi - ps22
Sarcopterygii - ps23
Tetrapoda - ps24
Amniota - ps25
Mammalia - ps26
Theria - ps27
Placentalia - ps28
Boreoeutheria - ps29
Euarchontoglires - ps30
Euarchonta - ps31
Primates - ps32
Haplorrhini - ps33
Simiiformes - ps34
Catarrhini - ps35
Hominoidea - ps36
Hominidae - ps37
Homininae - ps38
Hominini - ps39
Homo_sapiens - ps40
p-value
7e-03
9e-03
1e-02
2e-02
2e-02
Log-odds
0.5
1.0
1.5
2.0
2.5
3.0
3.5
4.0

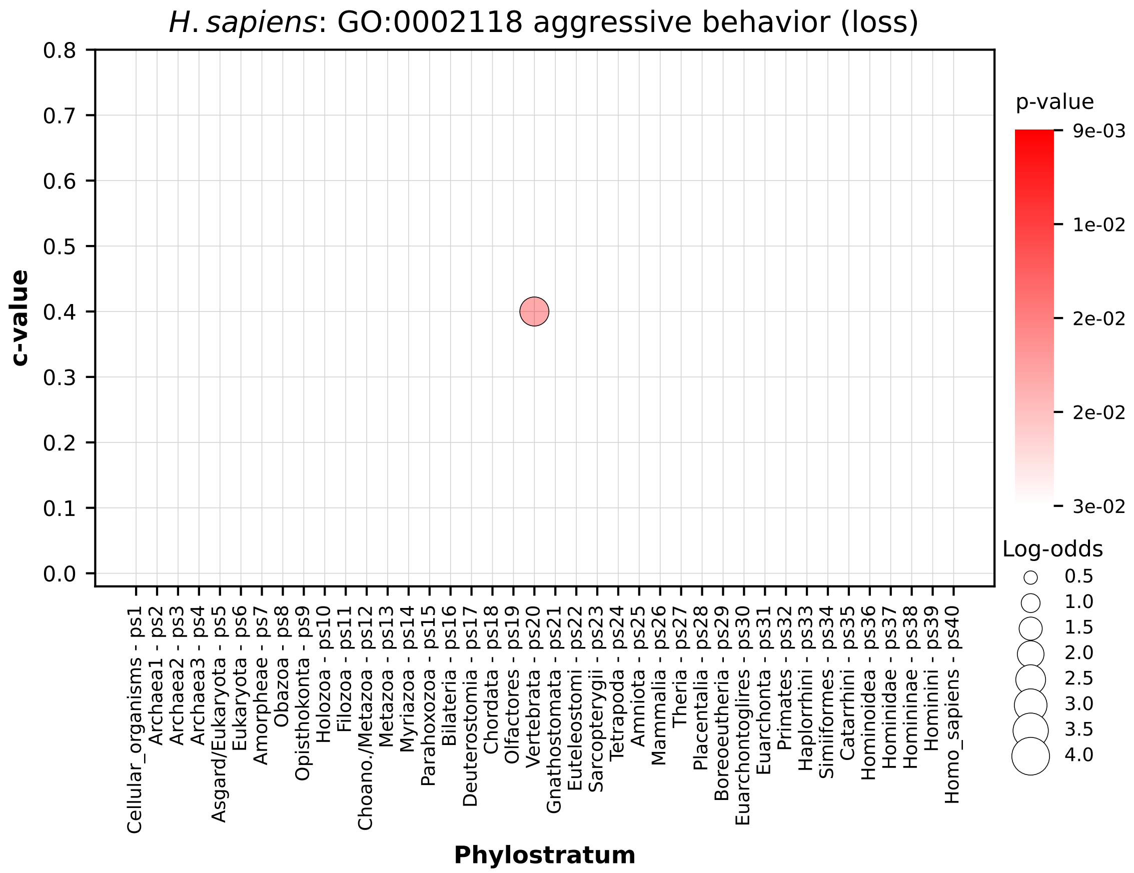

H. sapiens: GO:0002118 aggressive behavior (loss)
c-value
0.0
0.1
0.2
0.3
0.4
0.5
0.6
0.7
0.8
Phylostratum
Cellular_organisms - ps1
Archaea1 - ps2
Archaea2 - ps3
Archaea3 - ps4
Asgard/Eukaryota - ps5
Eukaryota - ps6
Amorpheae - ps7
Obazoa - ps8
Opisthokonta - ps9
Holozoa - ps10
Filozoa - ps11
Choano./Metazoa - ps12
Metazoa - ps13
Myriazoa - ps14
Parahoxozoa - ps15
Bilateria - ps16
Deuterostomia - ps17
Chordata - ps18
Olfactores - ps19
Vertebrata - ps20
Gnathostomata - ps21
Euteleostomi - ps22
Sarcopterygii - ps23
Tetrapoda - ps24
Amniota - ps25
Mammalia - ps26
Theria - ps27
Placentalia - ps28
Boreoeutheria - ps29
Euarchontoglires - ps30
Euarchonta - ps31
Primates - ps32
Haplorrhini - ps33
Simiiformes - ps34
Catarrhini - ps35
Hominoidea - ps36
Hominidae - ps37
Homininae - ps38
Hominini - ps39
Homo_sapiens - ps40
p-value
9e-03
1e-02
2e-02
2e-02
3e-02
Log-odds
0.5
1.0
1.5
2.0
2.5
3.0
3.5
4.0

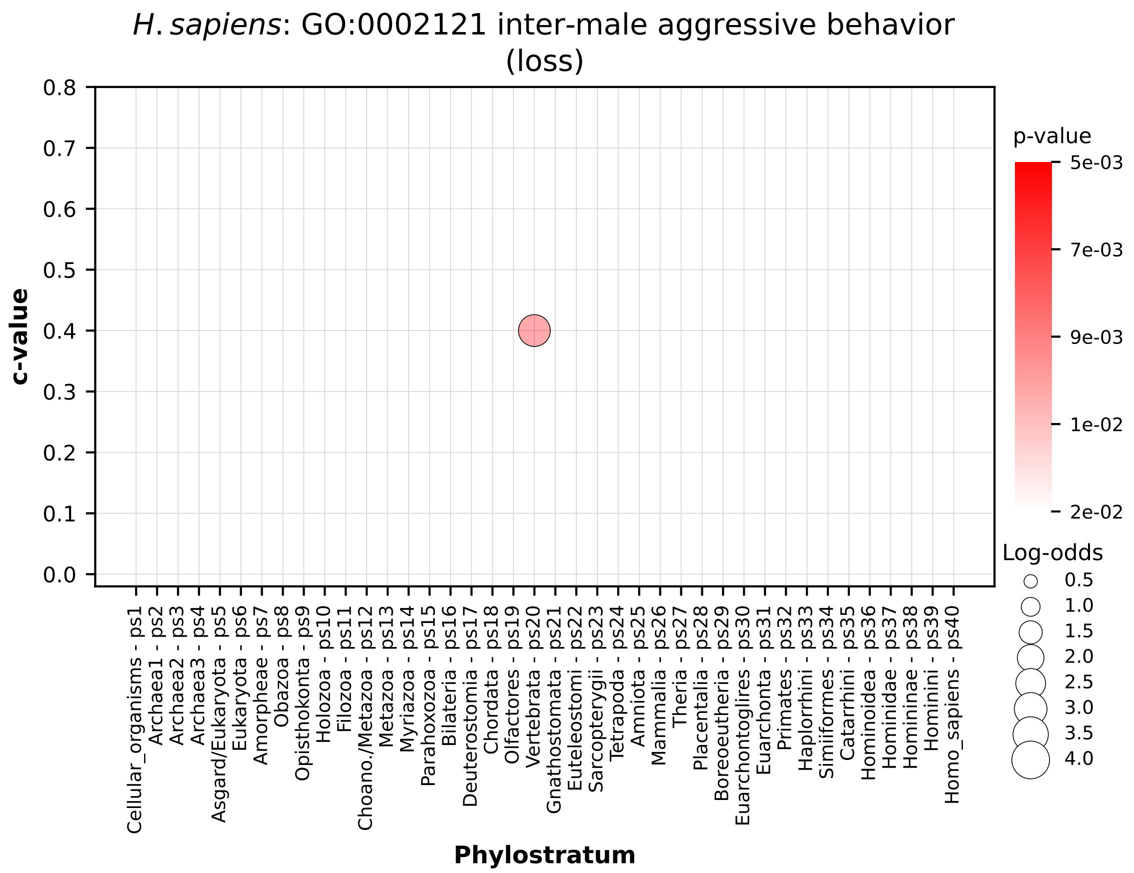

H. sapiens: GO:0002121 inter-male aggressive behavior
(loss)
c-value
0.8
0.7
0.6
0.5
0.4
0.3
0.2
0.1
0.0
Phylostratum
Cellular_organisms - ps1
Archaea1 - ps2
Archaea2 - ps3
Archaea3 - ps4
Asgard/Eukaryota - ps5
Eukaryota - ps6
Amorpheae - ps7
Obazoa - ps8
Opisthokonta - ps9
Holozoa - ps10
Filozoa - ps11
Choano./Metazoa - ps12
Metazoa - ps13
Myriazoa - ps14
Parahoxozoa - ps15
Bilateria - ps16
Deuterostomia - ps17
Chordata - ps18
Olfactores - ps19
Vertebrata - ps20
Gnathostomata - ps21
Euteleostomi - ps22
Sarcopterygii - ps23
Tetrapoda - ps24
Amniota - ps25
Mammalia - ps26
Theria - ps27
Placentalia - ps28
Boreoeutheria - ps29
Euarchontoglires - ps30
Euarchonta - ps31
Primates - ps32
Haplorrhini - ps33
Simiiformes - ps34
Catarrhini - ps35
Hominoidea - ps36
Hominidae - ps37
Homininae - ps38
Hominini - ps39
Homo_sapiens - ps40
p-value
5e-03
7e-03
9e-03
1e-02
2e-02
Log-odds
0.5
1.0
1.5
2.0
2.5
3.0
3.5
4.0

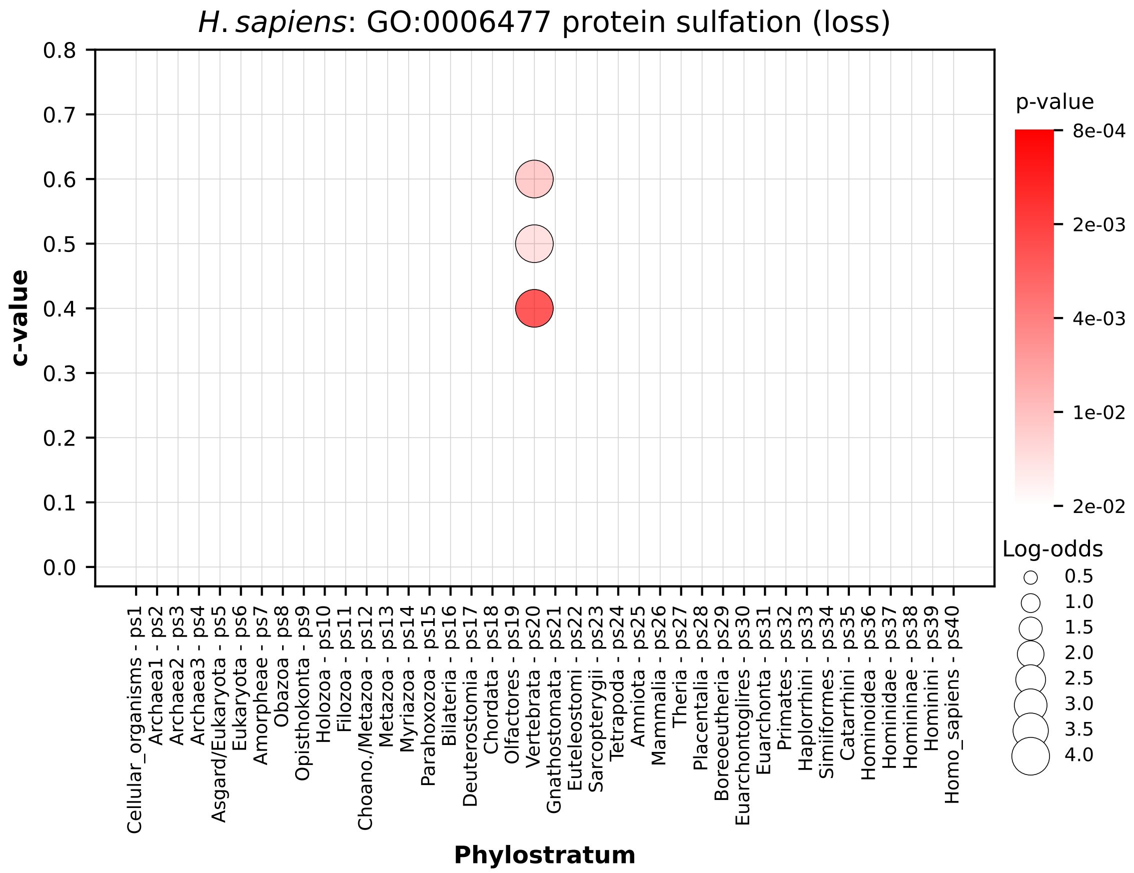

H. sapiens: GO:0006477 protein sulfation (loss)
c-value
0.8
0.7
0.6
0.5
0.4
0.3
0.2
0.1
0.0
Phylostratum
Cellular_organisms - ps1
Archaea1 - ps2
Archaea2 - ps3
Archaea3 - ps4
Asgard/Eukaryota - ps5
Eukaryota - ps6
Amorpheae - ps7
Obazoa - ps8
Opisthokonta - ps9
Holozoa - ps10
Filozoa - ps11
Choano./Metazoa - ps12
Metazoa - ps13
Myriazoa - ps14
Parahoxozoa - ps15
Bilateria - ps16
Deuterostomia - ps17
Chordata - ps18
Olfactores - ps19
Vertebrata - ps20
Gnathostomata - ps21
Euteleostomi - ps22
Sarcopterygii - ps23
Tetrapoda - ps24
Amniota - ps25
Mammalia - ps26
Theria - ps27
Placentalia - ps28
Boreoeutheria - ps29
Euarchontoglires - ps30
Euarchonta - ps31
Primates - ps32
Haplorrhini - ps33
Simiiformes - ps34
Catarrhini - ps35
Hominoidea - ps36
Hominidae - ps37
Homininae - ps38
Hominini - ps39
Homo_sapiens - ps40
p-value
8e-04
2e-03
4e-03
1e-02
2e-02
Log-odds
0.5
1.0
1.5
2.0
2.5
3.0
3.5
4.0

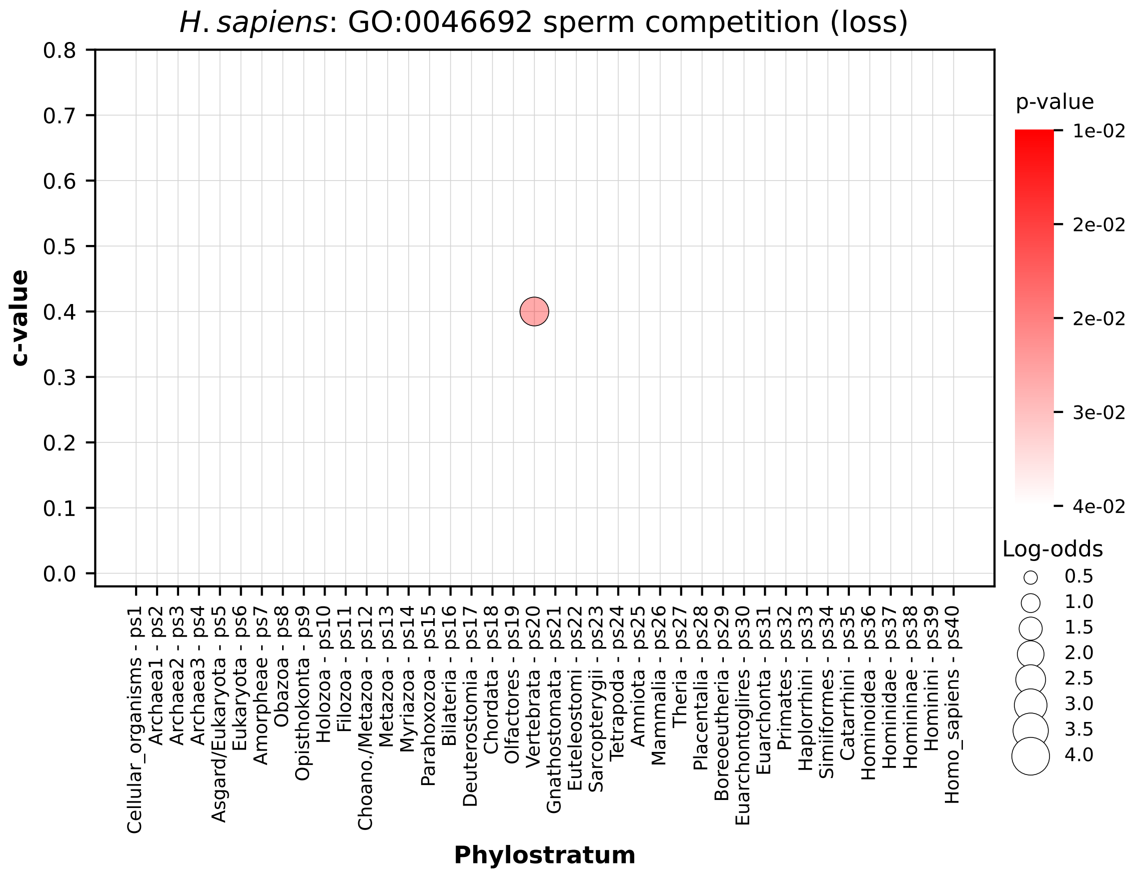

H. sapiens: GO:0046692 sperm competition (loss)
c-value
0.0
0.1
0.2
0.3
0.4
0.5
0.6
0.7
0.8
Phylostratum
Cellular_organisms - ps1
Archaea1 - ps2
Archaea2 - ps3
Archaea3 - ps4
Asgard/Eukaryota - ps5
Eukaryota - ps6
Amorpheae - ps7
Obazoa - ps8
Opisthokonta - ps9
Holozoa - ps10
Filozoa - ps11
Choano./Metazoa - ps12
Metazoa - ps13
Myriazoa - ps14
Parahoxozoa - ps15
Bilateria - ps16
Deuterostomia - ps17
Chordata - ps18
Olfactores - ps19
Vertebrata - ps20
Gnathostomata - ps21
Euteleostomi - ps22
Sarcopterygii - ps23
Tetrapoda - ps24
Amniota - ps25
Mammalia - ps26
Theria - ps27
Placentalia - ps28
Boreoeutheria - ps29
Euarchontoglires - ps30
Euarchonta - ps31
Primates - ps32
Haplorrhini - ps33
Simiiformes - ps34
Catarrhini - ps35
Hominoidea - ps36
Hominidae - ps37
Homininae - ps38
Hominini - ps39
Homo_sapiens - ps40
p-value
1e-02
2e-02
2e-02
3e-02
4e-02
Log-odds
0.5
1.0
1.5
2.0
2.5
3.0
3.5
4.0

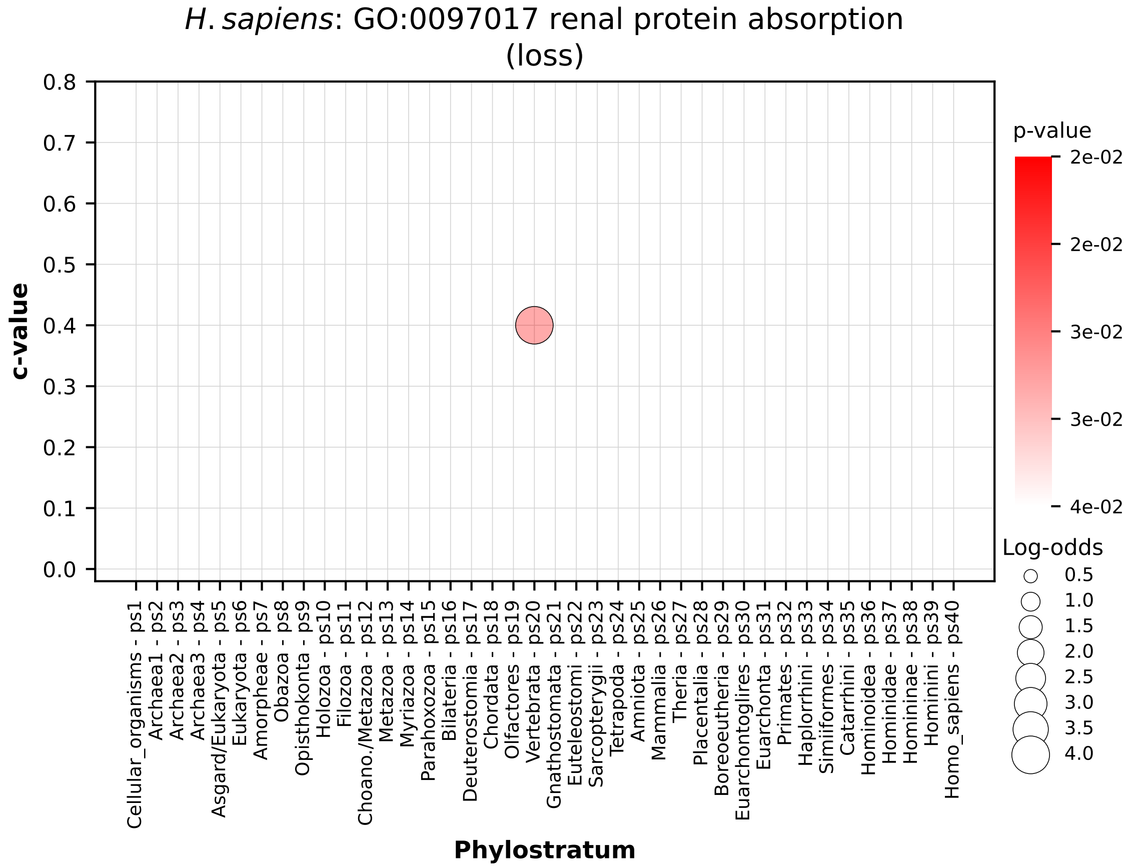

H. sapiens: GO:0097017 renal protein absorption
(loss)
c-value
0.8
0.7
0.6
0.5
0.4
0.3
0.2
0.1
0.0
Phylostratum
Cellular_organisms - ps1
Archaea1 - ps2
Archaea2 - ps3
Archaea3 - ps4
Asgard/Eukaryota - ps5
Eukaryota - ps6
Amorpheae - ps7
Obazoa - ps8
Opisthokonta - ps9
Holozoa - ps10
Filozoa - ps11
Choano./Metazoa - ps12
Metazoa - ps13
Myriazoa - ps14
Parahoxozoa - ps15
Bilateria - ps16
Deuterostomia - ps17
Chordata - ps18
Olfactores - ps19
Vertebrata - ps20
Gnathostomata - ps21
Euteleostomi - ps22
Sarcopterygii - ps23
Tetrapoda - ps24
Amniota - ps25
Mammalia - ps26
Theria - ps27
Placentalia - ps28
Boreoeutheria - ps29
Euarchontoglires - ps30
Euarchonta - ps31
Primates - ps32
Haplorrhini - ps33
Simiiformes - ps34
Catarrhini - ps35
Hominoidea - ps36
Hominidae - ps37
Homininae - ps38
Hominini - ps39
Homo_sapiens - ps40
p-value
2e-02
2e-02
3e-02
3e-02
4e-02
Log-odds
0.5
1.0
1.5
2.0
2.5
3.0
3.5
4.0

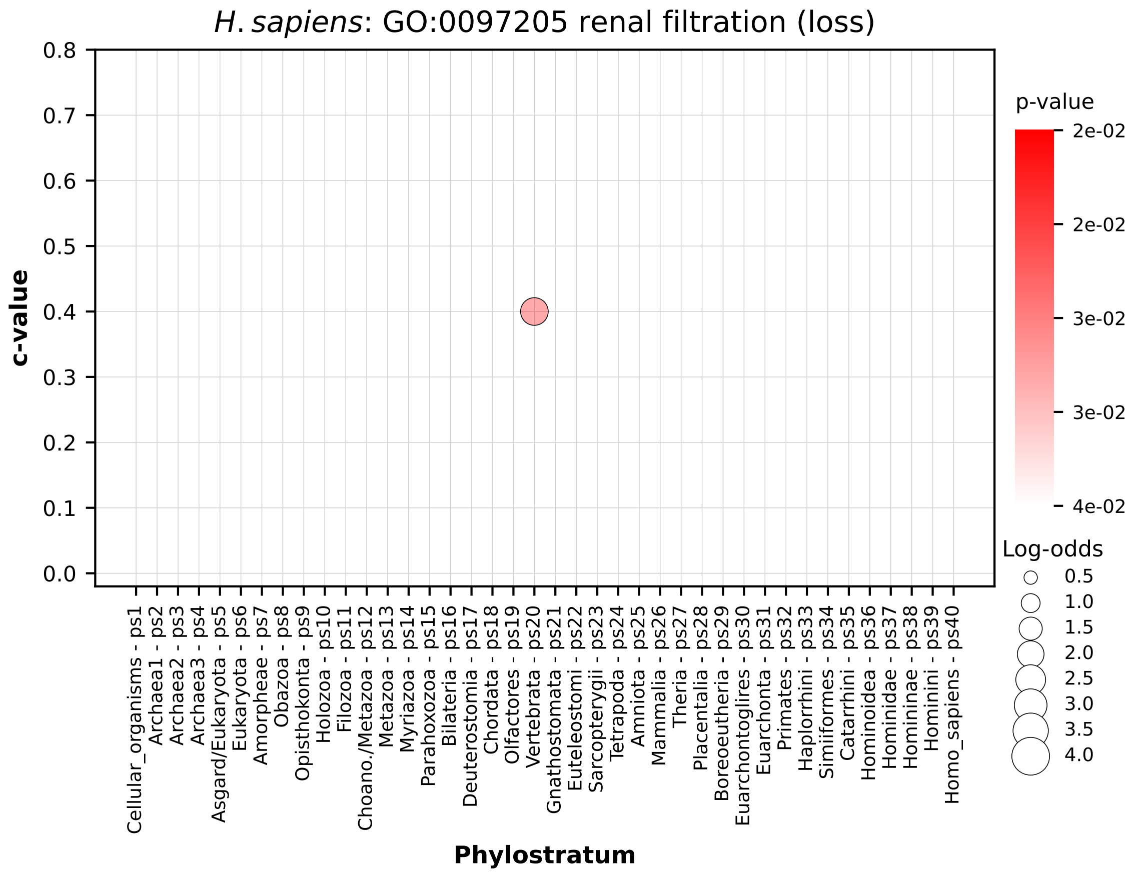
H. sapiens: GO:0097205 renal filtration (loss)
c-value
0.8
0.7
0.6
0.5
0.4
0.3
0.2
0.1
0.0
p-value
2e-02
2e-02
3e-02
3e-02
4e-02
Log-odds
0.5
1.0
1.5
2.0
2.5
3.0
3.5
4.0
Cellular_organisms - ps1
Archaea1 - ps2
Archaea2 - ps3
Archaea3 - ps4
Asgard/Eukaryota - ps5
Eukaryota - ps6
Amorpheae - ps7
Obazoa - ps8
Opisthokonta - ps9
Holozoa - ps10
Filozoa - ps11
Choano./Metazoa - ps12
Metazoa - ps13
Myriazoa - ps14
Parahoxozoa - ps15
Bilateria - ps16
Deuterostomia - ps17
Chordata - ps18
Olfactores - ps19
Vertebrata - ps20
Gnathostomata - ps21
Euteleostomi - ps22
Sarcopterygii - ps23
Tetrapoda - ps24
Amniota - ps25
Mammalia - ps26
Theria - ps27
Placentalia - ps28
Boreoeutheria - ps29
Euarchontoglires - ps30
Euarchonta - ps31
Primates - ps32
Haplorrhini - ps33
Simiiformes - ps34
Catarrhini - ps35
Hominoidea - ps36
Hominidae - ps37
Homininae - ps38
Hominini - ps39
Homo_sapiens - ps40
Phylostratum

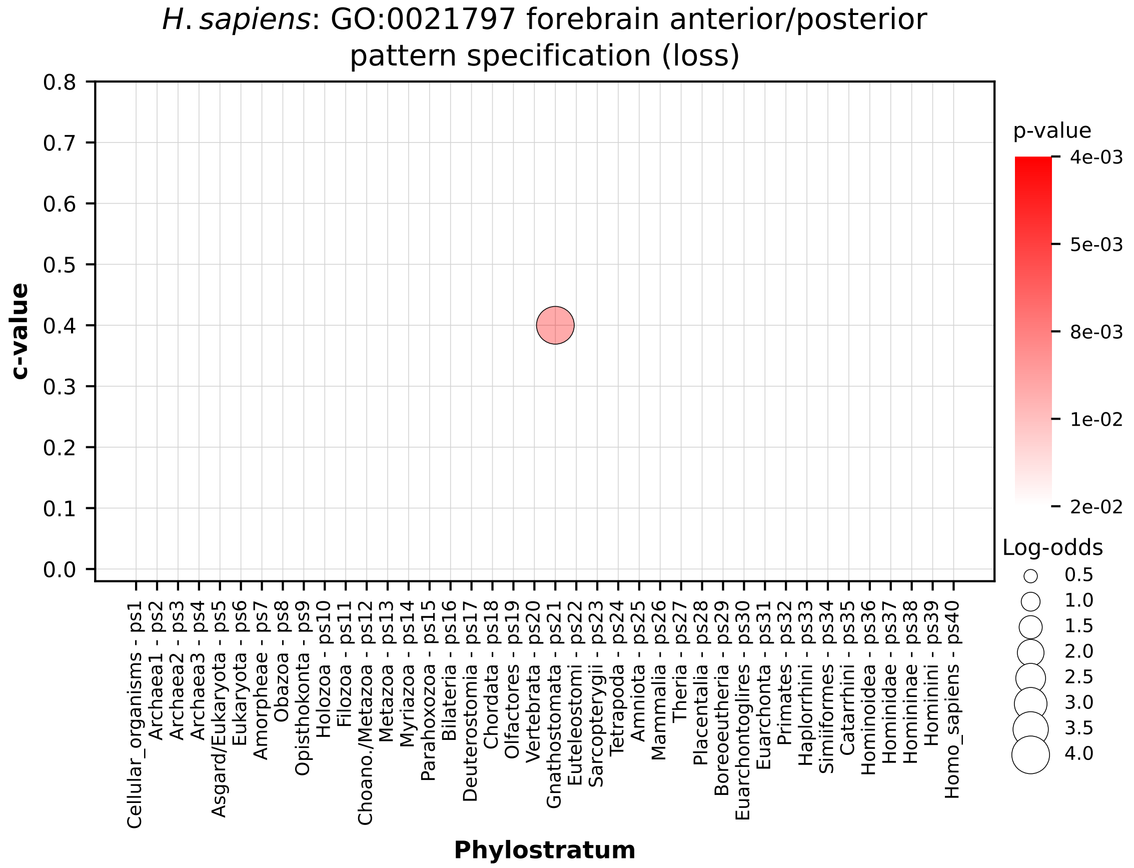
H. sapiens: GO:0021797 forebrain anterior/posterior pattern specification (loss)
c-value
0.8
0.7
0.6
0.5
0.4
0.3
0.2
0.1
0.0
Phylostratum
Cellular_organisms - ps1
Archaea1 - ps2
Archaea2 - ps3
Archaea3 - ps4
Asgard/Eukaryota - ps5
Eukaryota - ps6
Amorpheae - ps7
Obazoa - ps8
Opisthokonta - ps9
Holozoa - ps10
Filozoa - ps11
Choano./Metazoa - ps12
Metazoa - ps13
Myriazoa - ps14
Parahoxozoa - ps15
Bilateria - ps16
Deuterostomia - ps17
Chordata - ps18
Olfactores - ps19
Vertebrata - ps20
Gnathostomata - ps21
Euteleostomi - ps22
Sarcopterygii - ps23
Tetrapoda - ps24
Amniota - ps25
Mammalia - ps26
Theria - ps27
Placentalia - ps28
Boreoeutheria - ps29
Euarchontoglires - ps30
Euarchonta - ps31
Primates - ps32
Haplorrhini - ps33
Simiiformes - ps34
Catarrhini - ps35
Hominoidea - ps36
Hominidae - ps37
Homininae - ps38
Hominini - ps39
Homo_sapiens - ps40
p-value
4e-03
5e-03
8e-03
1e-02
2e-02
Log-odds
0.5
1.0
1.5
2.0
2.5
3.0
3.5
4.0

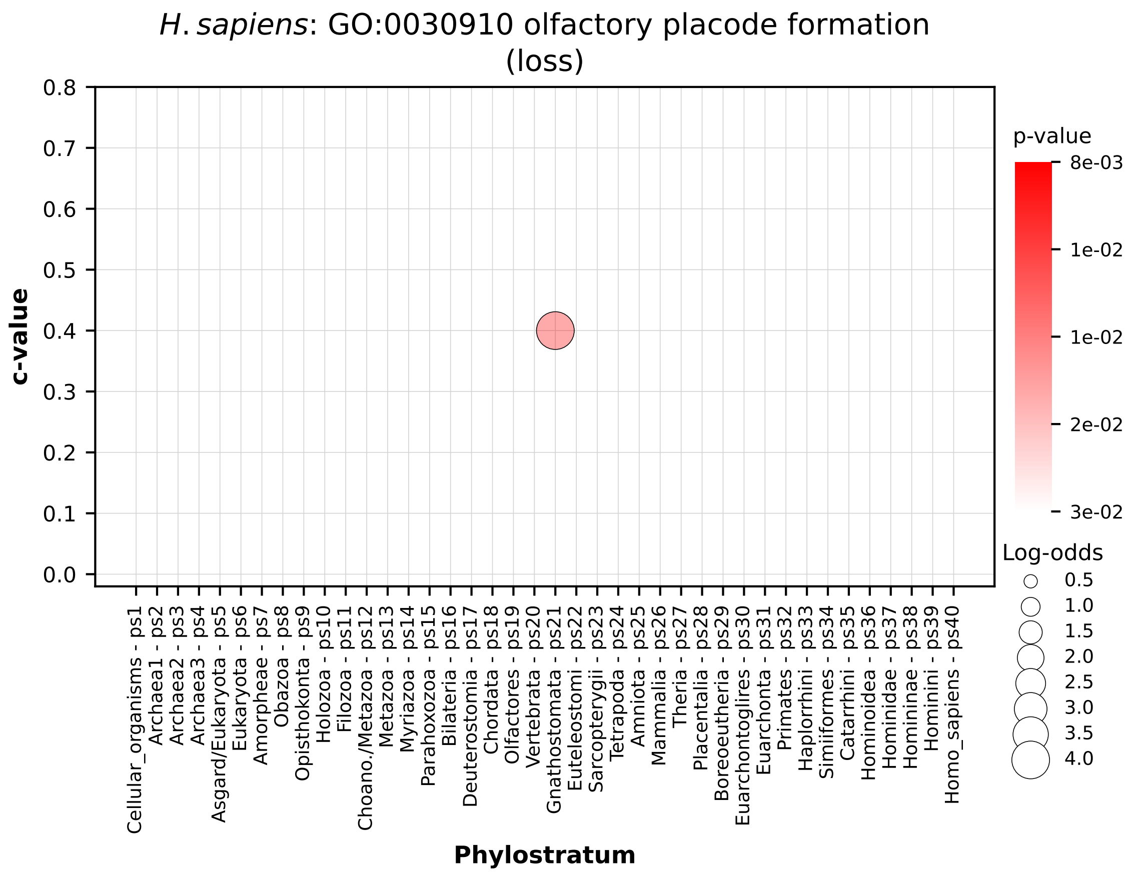

H. sapiens: GO:0030910 olfactory placode formation
(loss)
c-value
0.8
0.7
0.6
0.5
0.4
0.3
0.2
0.1
0.0
Phylostratum
Cellular_organisms - ps1
Archaea1 - ps2
Archaea2 - ps3
Archaea3 - ps4
Asgard/Eukaryota - ps5
Eukaryota - ps6
Amorpheae - ps7
Obazoa - ps8
Opisthokonta - ps9
Holozoa - ps10
Filozoa - ps11
Choano./Metazoa - ps12
Metazoa - ps13
Myriazoa - ps14
Parahoxozoa - ps15
Bilateria - ps16
Deuterostomia - ps17
Chordata - ps18
Olfactores - ps19
Vertebrata - ps20
Gnathostomata - ps21
Euteleostomi - ps22
Sarcopterygii - ps23
Tetrapoda - ps24
Amniota - ps25
Mammalia - ps26
Theria - ps27
Placentalia - ps28
Boreoeutheria - ps29
Euarchontoglires - ps30
Euarchonta - ps31
Primates - ps32
Haplorrhini - ps33
Simiiformes - ps34
Catarrhini - ps35
Hominoidea - ps36
Hominidae - ps37
Homininae - ps38
Hominini - ps39
Homo_sapiens - ps40
p-value
8e-03
1e-02
1e-02
2e-02
3e-02
Log-odds
0.5
1.0
1.5
2.0
2.5
3.0
3.5
4.0

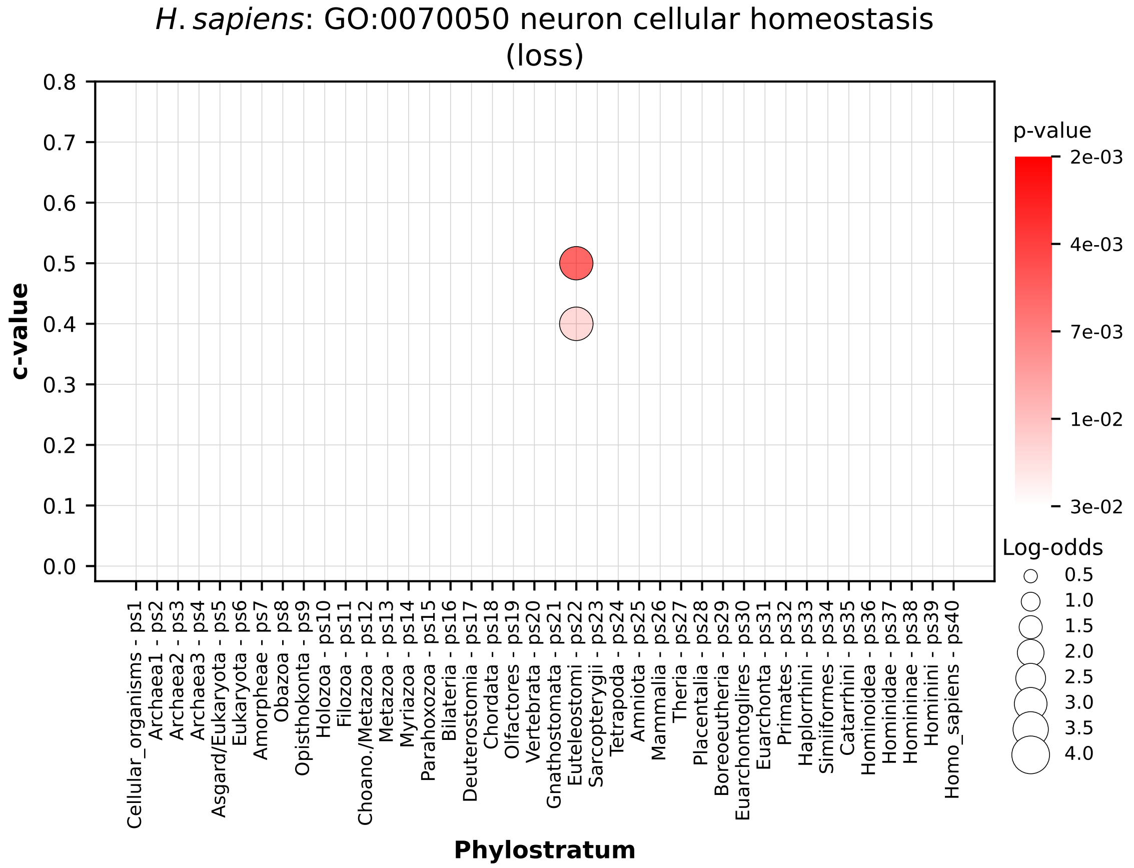

H. sapiens: GO:0070050 neuron cellular homeostasis (loss)
c-value
0.8
0.7
0.6
0.5
0.4
0.3
0.2
0.1
0.0
p-value
2e-03
4e-03
7e-03
1e-02
3e-02
Log-odds
0.5
1.0
1.5
2.0
2.5
3.0
3.5
4.0
Cellular_organisms - ps1
Archaea1 - ps2
Archaea2 - ps3
Archaea3 - ps4
Asgard/Eukaryota - ps5
Eukaryota - ps6
Amorpheae - ps7
Obazoa - ps8
Opisthokonta - ps9
Holozoa - ps10
Filozoa - ps11
Choano./Metazoa - ps12
Metazoa - ps13
Myriazoa - ps14
Parahoxozoa - ps15
Bilateria - ps16
Deuterostomia - ps17
Chordata - ps18
Olfactores - ps19
Vertebrata - ps20
Gnathostomata - ps21
Euteleostomi - ps22
Sarcopterygii - ps23
Tetrapoda - ps24
Amniota - ps25
Mammalia - ps26
Theria - ps27
Placentalia - ps28
Boreoeutheria - ps29
Euarchontoglires - ps30
Euarchonta - ps31
Primates - ps32
Haplorrhini - ps33
Simiiformes - ps34
Catarrhini - ps35
Hominoidea - ps36
Hominidae - ps37
Homininae - ps38
Hominini - ps39
Homo_sapiens - ps40
Phylostratum

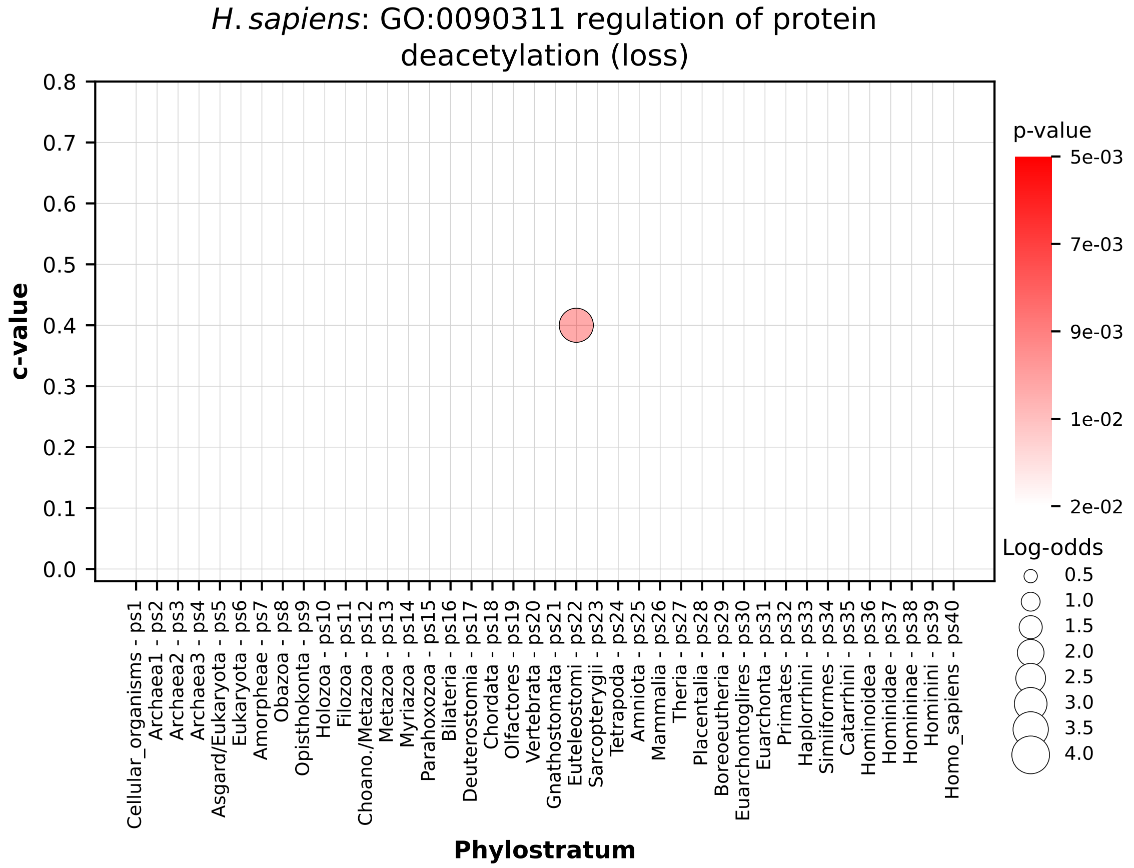
H. sapiens: GO:0090311 regulation of protein deacetylation (loss)
c-value
0.0
0.1
0.2
0.3
0.4
0.5
0.6
0.7
0.8
Phylostratum
Cellular_organisms - ps1
Archaea1 - ps2
Archaea2 - ps3
Archaea3 - ps4
Asgard/Eukaryota - ps5
Eukaryota - ps6
Amorpheae - ps7
Obazoa - ps8
Opisthokonta - ps9
Holozoa - ps10
Filozoa - ps11
Choano./Metazoa - ps12
Metazoa - ps13
Myriazoa - ps14
Parahoxozoa - ps15
Bilateria - ps16
Deuterostomia - ps17
Chordata - ps18
Olfactores - ps19
Vertebrata - ps20
Gnathostomata - ps21
Euteleostomi - ps22
Sarcopterygii - ps23
Tetrapoda - ps24
Amniota - ps25
Mammalia - ps26
Theria - ps27
Placentalia - ps28
Boreoeutheria - ps29
Euarchontoglires - ps30
Euarchonta - ps31
Primates - ps32
Haplorrhini - ps33
Simiiformes - ps34
Catarrhini - ps35
Hominoidea - ps36
Hominidae - ps37
Homininae - ps38
Hominini - ps39
Homo_sapiens - ps40
p-value
5e-03
7e-03
9e-03
1e-02
2e-02
Log-odds
0.5
1.0
1.5
2.0
2.5
3.0
3.5
4.0

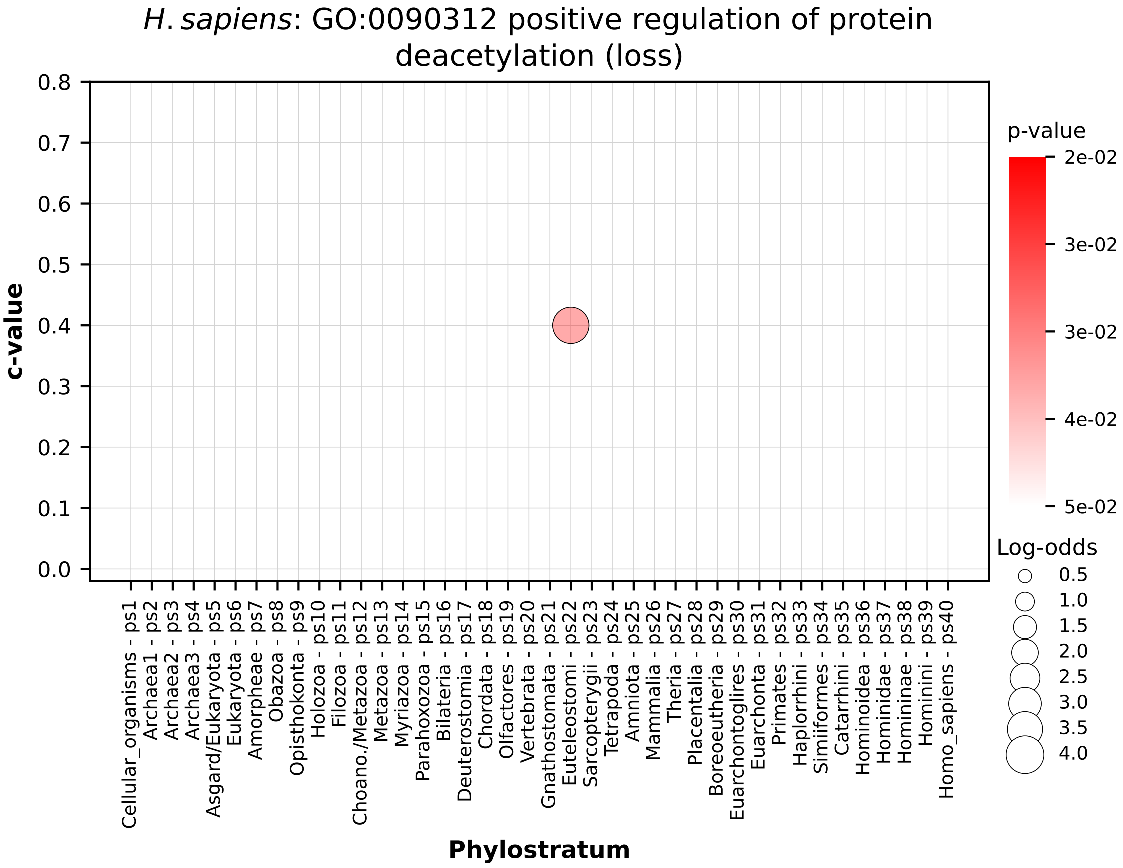

H. sapiens: GO:0090312 positive regulation of protein deacetylation (loss)
c-value
0.8
0.7
0.6
0.5
0.4
0.3
0.2
0.1
0.0
p-value
2e-02
3e-02
3e-02
4e-02
5e-02
Log-odds
0.5
1.0
1.5
2.0
2.5
3.0
3.5
4.0
Cellular_organisms - ps1
Archaea1 - ps2
Archaea2 - ps3
Archaea3 - ps4
Asgard/Eukaryota - ps5
Eukaryota - ps6
Amorpheae - ps7
Obazoa - ps8
Opisthokonta - ps9
Holozoa - ps10
Filozoa - ps11
Choano./Metazoa - ps12
Metazoa - ps13
Myriazoa - ps14
Parahoxozoa - ps15
Bilateria - ps16
Deuterostomia - ps17
Chordata - ps18
Olfactores - ps19
Vertebrata - ps20
Gnathostomata - ps21
Euteleostomi - ps22
Sarcopterygii - ps23
Tetrapoda - ps24
Amniota - ps25
Mammalia - ps26
Theria - ps27
Placentalia - ps28
Boreoeutheria - ps29
Euarchontoglires - ps30
Euarchonta - ps31
Primates - ps32
Haplorrhini - ps33
Simiiformes - ps34
Catarrhini - ps35
Hominoidea - ps36
Hominidae - ps37
Homininae - ps38
Hominini - ps39
Homo_sapiens - ps40
Phylostratum

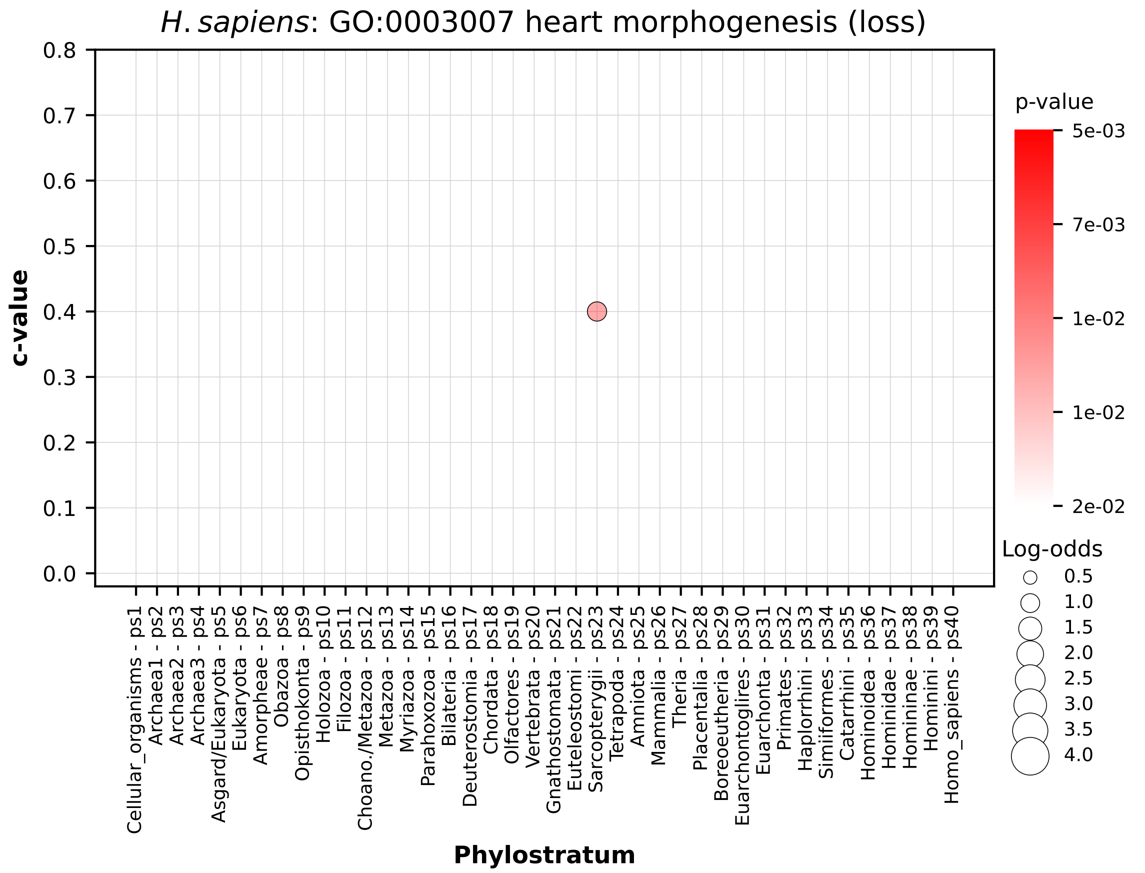

H. sapiens: GO:0003007 heart morphogenesis (loss)
c-value
0.8
0.7
0.6
0.5
0.4
0.3
0.2
0.1
0.0
Phylostratum
Cellular_organisms - ps1
Archaea1 - ps2
Archaea2 - ps3
Archaea3 - ps4
Asgard/Eukaryota - ps5
Eukaryota - ps6
Amorpheae - ps7
Obazoa - ps8
Opisthokonta - ps9
Holozoa - ps10
Filozoa - ps11
Choano./Metazoa - ps12
Metazoa - ps13
Myriazoa - ps14
Parahoxozoa - ps15
Bilateria - ps16
Deuterostomia - ps17
Chordata - ps18
Olfactores - ps19
Vertebrata - ps20
Gnathostomata - ps21
Euteleostomi - ps22
Sarcopterygii - ps23
Tetrapoda - ps24
Amniota - ps25
Mammalia - ps26
Theria - ps27
Placentalia - ps28
Boreoeutheria - ps29
Euarchontoglires - ps30
Euarchonta - ps31
Primates - ps32
Haplorrhini - ps33
Simiiformes - ps34
Catarrhini - ps35
Hominoidea - ps36
Hominidae - ps37
Homininae - ps38
Hominini - ps39
Homo_sapiens - ps40
p-value
5e-03
7e-03
1e-02
1e-02
2e-02
Log-odds
0.5
1.0
1.5
2.0
2.5
3.0
3.5
4.0

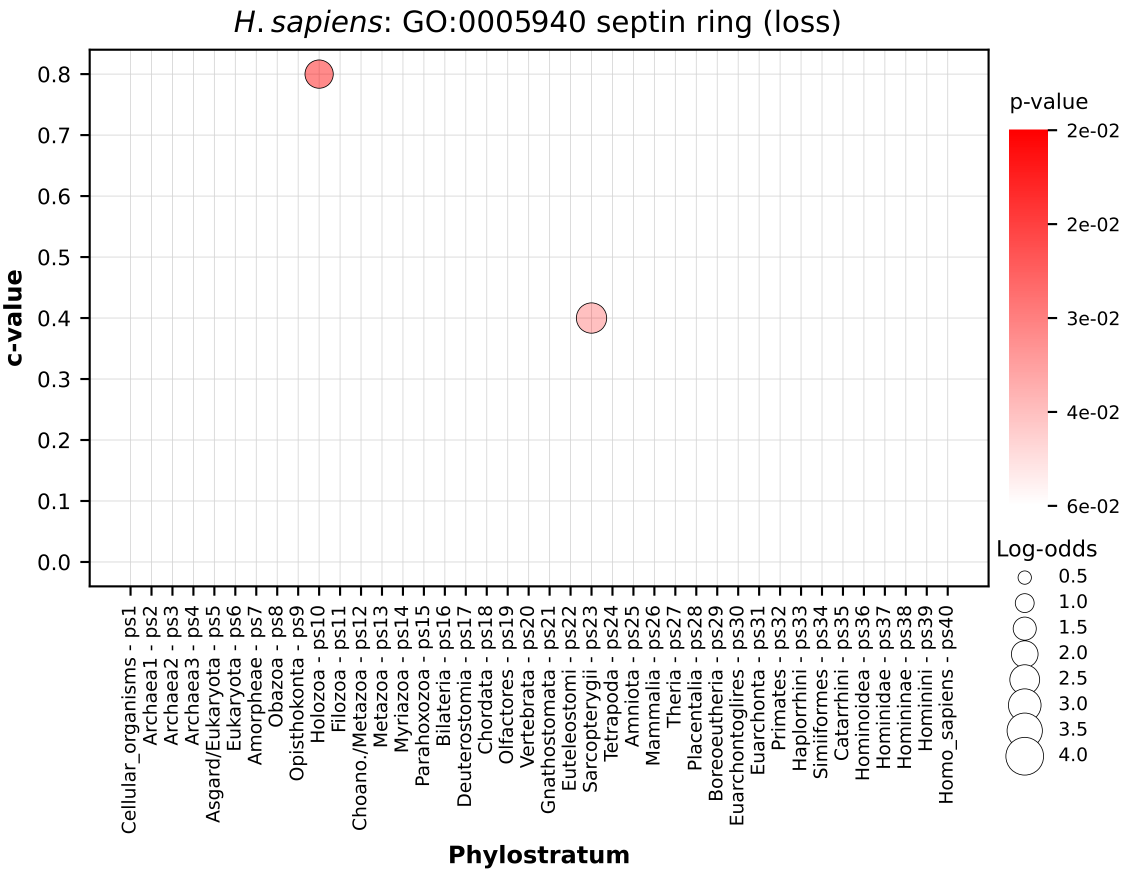

H. sapiens: GO:0005940 septin ring (loss)
c-value
0.8
0.7
0.6
0.5
0.4
0.3
0.2
0.1
0.0
p-value
2e-02
2e-02
3e-02
4e-02
6e-02
Log-odds
0.5
1.0
1.5
2.0
2.5
3.0
3.5
4.0
Cellular_organisms - ps1
Archaea1 - ps2
Archaea2 - ps3
Archaea3 - ps4
Asgard/Eukaryota - ps5
Eukaryota - ps6
Amorpheae - ps7
Obazoa - ps8
Opisthokonta - ps9
Holozoa - ps10
Filozoa - ps11
Choano./Metazoa - ps12
Metazoa - ps13
Myriazoa - ps14
Parahoxozoa - ps15
Bilateria - ps16
Deuterostomia - ps17
Chordata - ps18
Olfactores - ps19
Vertebrata - ps20
Gnathostomata - ps21
Euteleostomi - ps22
Sarcopterygii - ps23
Tetrapoda - ps24
Amniota - ps25
Mammalia - ps26
Theria - ps27
Placentalia - ps28
Boreoeutheria - ps29
Euarchontoglires - ps30
Euarchonta - ps31
Primates - ps32
Haplorrhini - ps33
Simiiformes - ps34
Catarrhini - ps35
Hominoidea - ps36
Hominidae - ps37
Homininae - ps38
Hominini - ps39
Homo_sapiens - ps40
Phylostratum

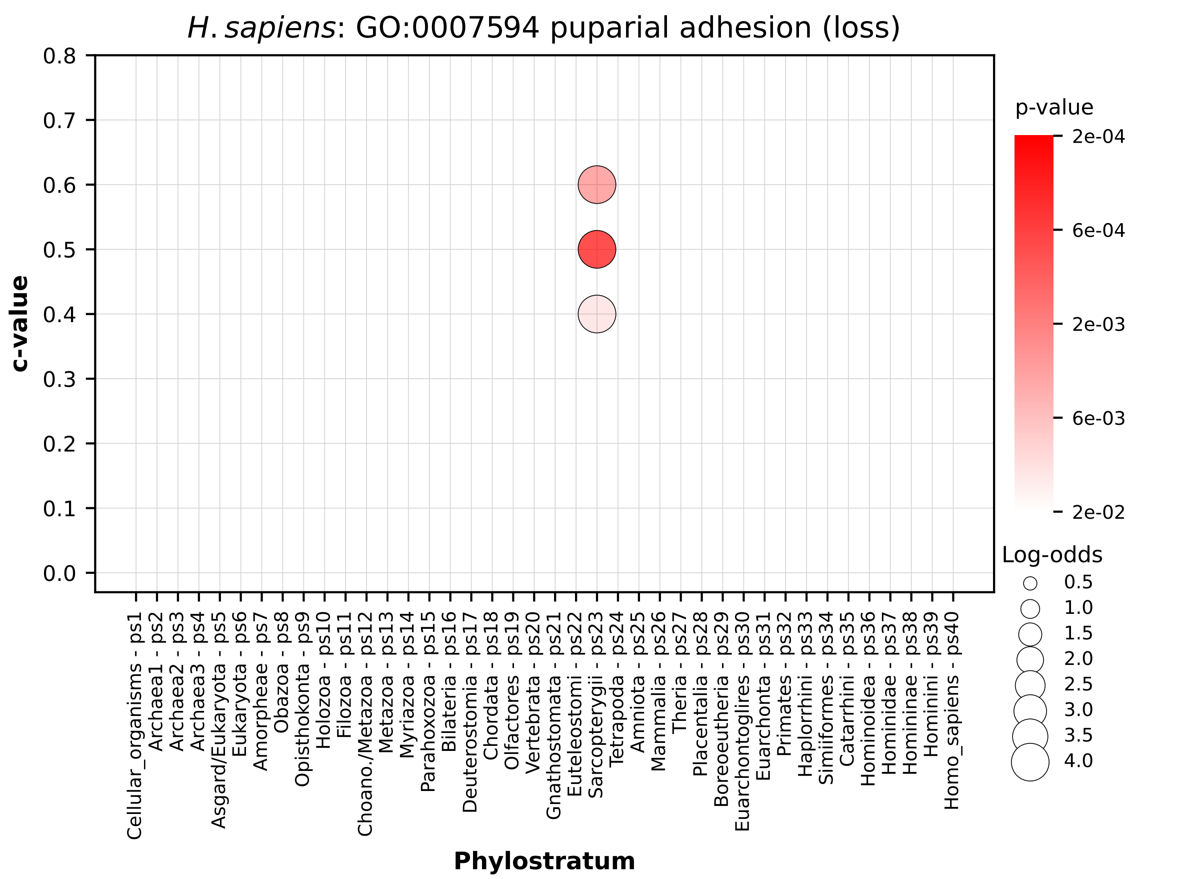
H. sapiens: GO:0007594 puparial adhesion (loss)
c-value
0.8
0.7
0.6
0.5
0.4
0.3
0.2
0.1
0.0
p-value
2e-04
6e-04
2e-03
6e-03
2e-02
Log-odds
0.5
1.0
1.5
2.0
2.5
3.0
3.5
4.0
Cellular_organisms - ps1
Archaea1 - ps2
Archaea2 - ps3
Archaea3 - ps4
Asgard/Eukaryota - ps5
Eukaryota - ps6
Amorpheae - ps7
Obazoa - ps8
Opisthokonta - ps9
Holozoa - ps10
Filozoa - ps11
Choano./Metazoa - ps12
Metazoa - ps13
Myriazoa - ps14
Parahoxozoa - ps15
Bilateria - ps16
Deuterostomia - ps17
Chordata - ps18
Olfactores - ps19
Vertebrata - ps20
Gnathostomata - ps21
Euteleostomi - ps22
Sarcopterygii - ps23
Tetrapoda - ps24
Amniota - ps25
Mammalia - ps26
Theria - ps27
Placentalia - ps28
Boreoeutheria - ps29
Euarchontoglires - ps30
Euarchonta - ps31
Primates - ps32
Haplorrhini - ps33
Simiiformes - ps34
Catarrhini - ps35
Hominoidea - ps36
Hominidae - ps37
Homininae - ps38
Hominini - ps39
Homo_sapiens - ps40
Phylostratum

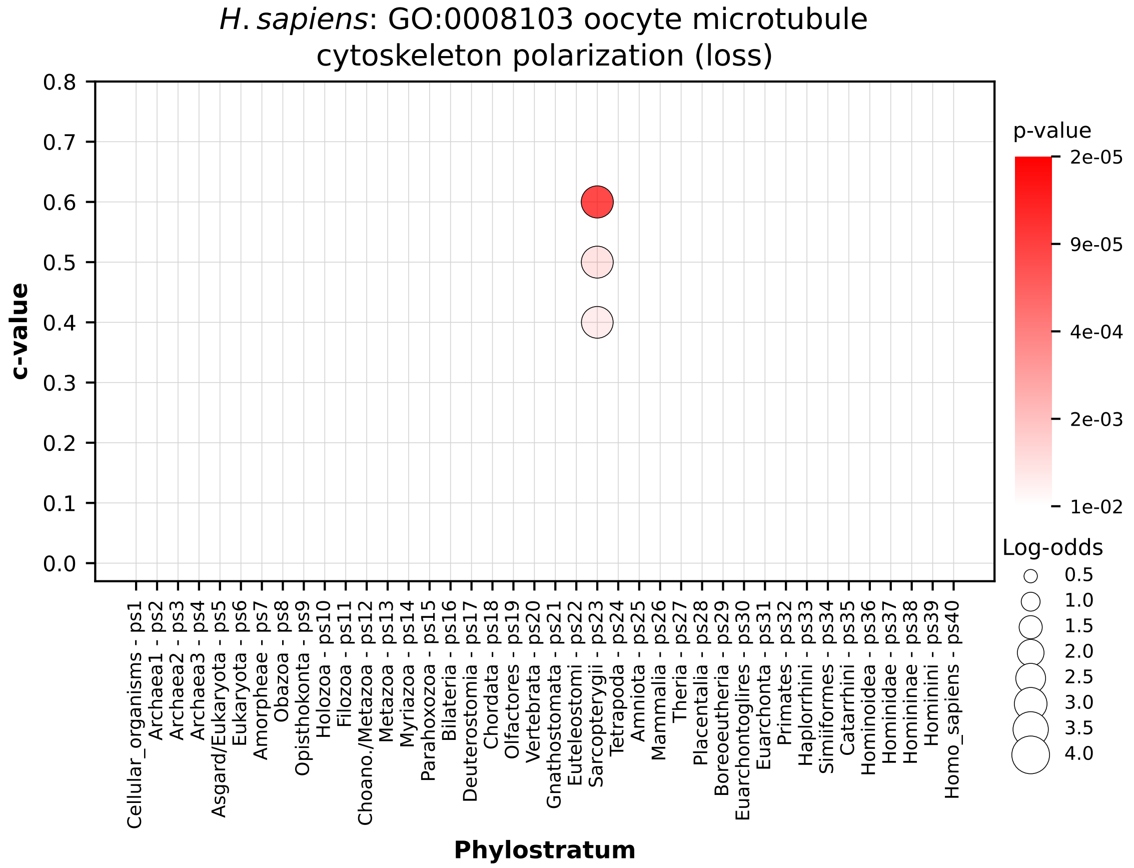

H. sapiens: GO:0008103 oocyte microtubule cytoskeleton polarization (loss)
c-value
0.8
0.7
0.6
0.5
0.4
0.3
0.2
0.1
0.0
Phylostratum
Cellular_organisms - ps1
Archaea1 - ps2
Archaea2 - ps3
Archaea3 - ps4
Asgard/Eukaryota - ps5
Eukaryota - ps6
Amorpheae - ps7
Obazoa - ps8
Opisthokonta - ps9
Holozoa - ps10
Filozoa - ps11
Choano./Metazoa - ps12
Metazoa - ps13
Myriazoa - ps14
Parahoxozoa - ps15
Bilateria - ps16
Deuterostomia - ps17
Chordata - ps18
Olfactores - ps19
Vertebrata - ps20
Gnathostomata - ps21
Euteleostomi - ps22
Sarcopterygii - ps23
Tetrapoda - ps24
Amniota - ps25
Mammalia - ps26
Theria - ps27
Placentalia - ps28
Boreoeutheria - ps29
Euarchontoglires - ps30
Euarchonta - ps31
Primates - ps32
Haplorrhini - ps33
Simiiformes - ps34
Catarrhini - ps35
Hominoidea - ps36
Hominidae - ps37
Homininae - ps38
Hominini - ps39
Homo_sapiens - ps40
p-value
2e-05
9e-05
4e-04
2e-03
1e-02
Log-odds
0.5
1.0
1.5
2.0
2.5
3.0
3.5
4.0

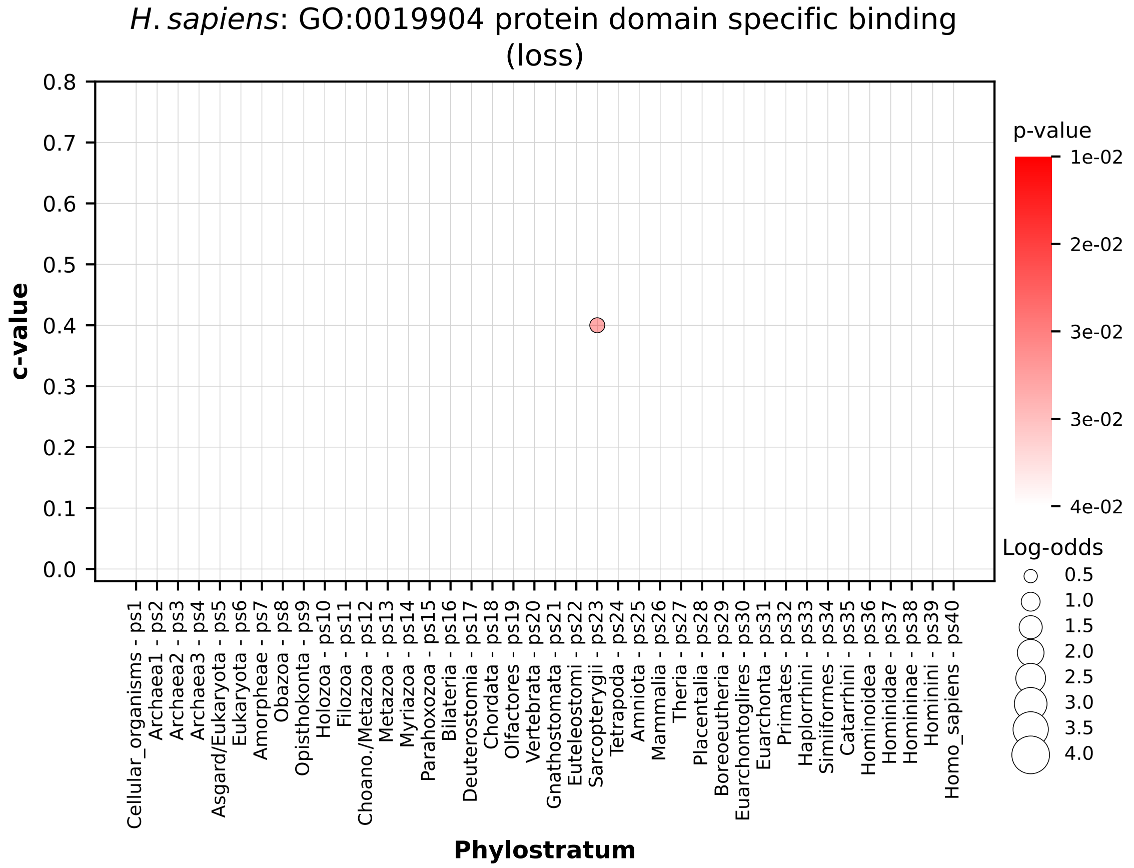
H. sapiens: GO:0019904 protein domain specific binding (loss)
c-value
0.0
0.1
0.2
0.3
0.4
0.5
0.6
0.7
0.8
Phylostratum
Cellular_organisms - ps1
Archaea1 - ps2
Archaea2 - ps3
Archaea3 - ps4
Asgard/Eukaryota - ps5
Eukaryota - ps6
Amorpheae - ps7
Obazoa - ps8
Opisthokonta - ps9
Holozoa - ps10
Filozoa - ps11
Choano./Metazoa - ps12
Metazoa - ps13
Myriazoa - ps14
Parahoxozoa - ps15
Bilateria - ps16
Deuterostomia - ps17
Chordata - ps18
Olfactores - ps19
Vertebrata - ps20
Gnathostomata - ps21
Euteleostomi - ps22
Sarcopterygii - ps23
Tetrapoda - ps24
Amniota - ps25
Mammalia - ps26
Theria - ps27
Placentalia - ps28
Boreoeutheria - ps29
Euarchontoglires - ps30
Euarchonta - ps31
Primates - ps32
Haplorrhini - ps33
Simiiformes - ps34
Catarrhini - ps35
Hominoidea - ps36
Hominidae - ps37
Homininae - ps38
Hominini - ps39
Homo_sapiens - ps40
p-value
1e-02
2e-02
3e-02
3e-02
4e-02
Log-odds
0.5
1.0
1.5
2.0
2.5
3.0
3.5
4.0

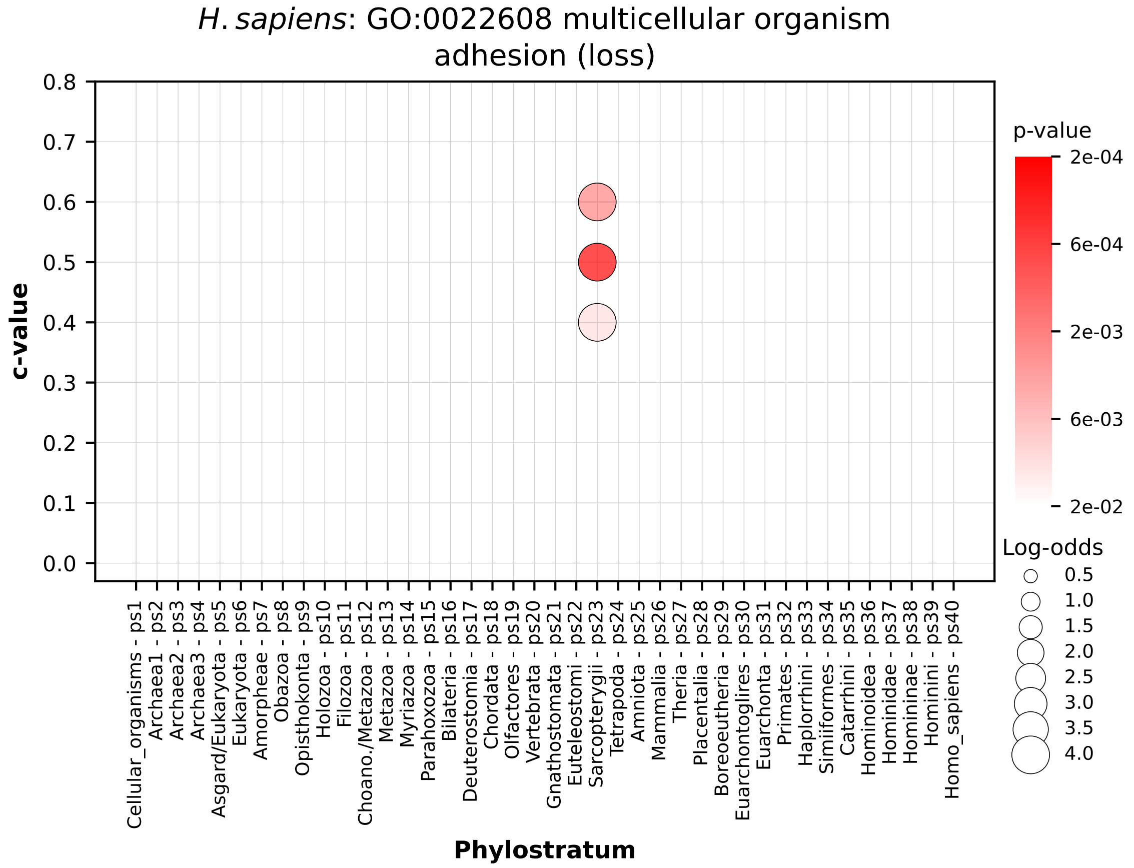

H. sapiens: GO:0022608 multicellular organism adhesion (loss)
c-value
0.8
0.7
0.6
0.5
0.4
0.3
0.2
0.1
0.0
p-value
2e-04
6e-04
2e-03
6e-03
2e-02
Log-odds
0.5
1.0
1.5
2.0
2.5
3.0
3.5
4.0
Cellular_organisms - ps1
Archaea1 - ps2
Archaea2 - ps3
Archaea3 - ps4
Asgard/Eukaryota - ps5
Eukaryota - ps6
Amorpheae - ps7
Obazoa - ps8
Opisthokonta - ps9
Holozoa - ps10
Filozoa - ps11
Choano./Metazoa - ps12
Metazoa - ps13
Myriazoa - ps14
Parahoxozoa - ps15
Bilateria - ps16
Deuterostomia - ps17
Chordata - ps18
Olfactores - ps19
Vertebrata - ps20
Gnathostomata - ps21
Euteleostomi - ps22
Sarcopterygii - ps23
Tetrapoda - ps24
Amniota - ps25
Mammalia - ps26
Theria - ps27
Placentalia - ps28
Boreoeutheria - ps29
Euarchontoglires - ps30
Euarchonta - ps31
Primates - ps32
Haplorrhini - ps33
Simiiformes - ps34
Catarrhini - ps35
Hominoidea - ps36
Hominidae - ps37
Homininae - ps38
Hominini - ps39
Homo_sapiens - ps40
Phylostratum

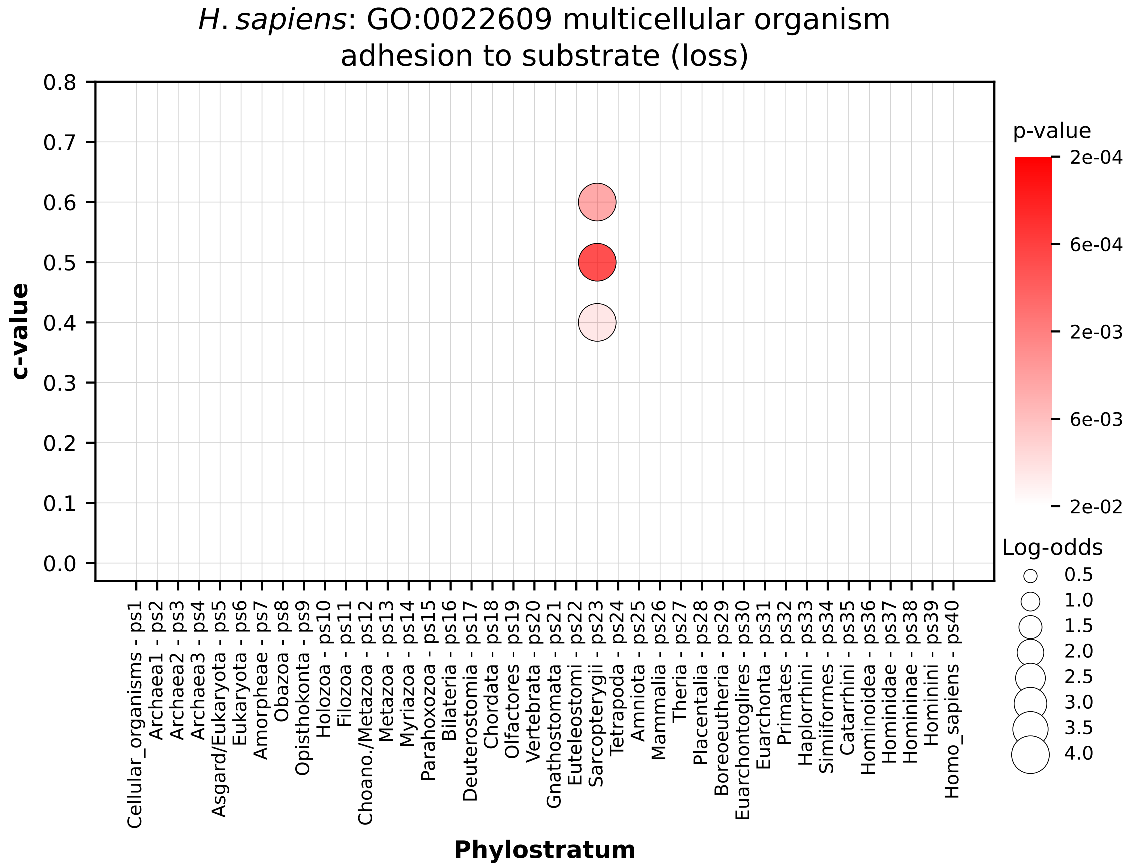

H. sapiens: GO:0022609 multicellular organism adhesion to substrate (loss)
c-value
0.0
0.1
0.2
0.3
0.4
0.5
0.6
0.7
0.8
Phylostratum
Cellular_organisms - ps1
Archaea1 - ps2
Archaea2 - ps3
Archaea3 - ps4
Asgard/Eukaryota - ps5
Eukaryota - ps6
Amorpheae - ps7
Obazoa - ps8
Opisthokonta - ps9
Holozoa - ps10
Filozoa - ps11
Choano./Metazoa - ps12
Metazoa - ps13
Myriazoa - ps14
Parahoxozoa - ps15
Bilateria - ps16
Deuterostomia - ps17
Chordata - ps18
Olfactores - ps19
Vertebrata - ps20
Gnathostomata - ps21
Euteleostomi - ps22
Sarcopterygii - ps23
Tetrapoda - ps24
Amniota - ps25
Mammalia - ps26
Theria - ps27
Placentalia - ps28
Boreoeutheria - ps29
Euarchontoglires - ps30
Euarchonta - ps31
Primates - ps32
Haplorrhini - ps33
Simiiformes - ps34
Catarrhini - ps35
Hominoidea - ps36
Hominidae - ps37
Homininae - ps38
Hominini - ps39
Homo_sapiens - ps40
p-value
2e-04
6e-04
2e-03
6e-03
2e-02
Log-odds
0.5
1.0
1.5
2.0
2.5
3.0
3.5
4.0

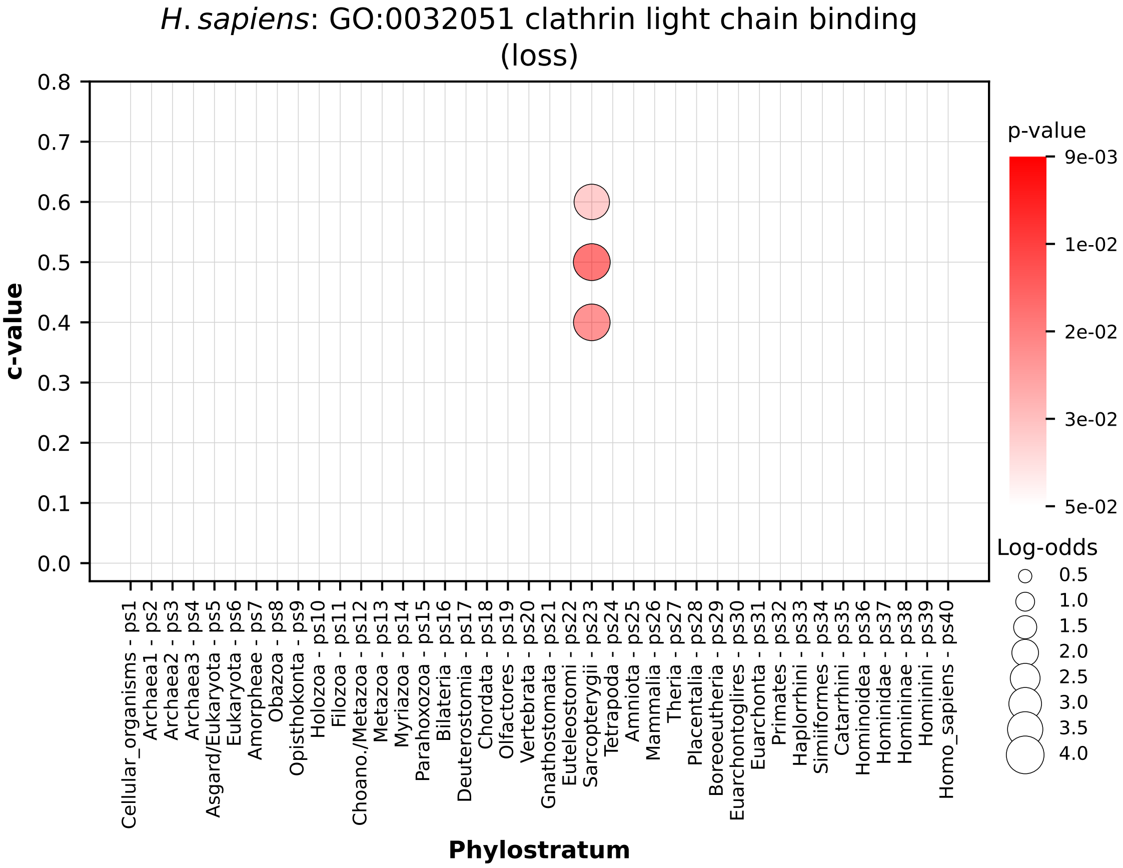

H. sapiens: GO:0032051 clathrin light chain binding
(loss)
c-value
0.8
0.7
0.6
0.5
0.4
0.3
0.2
0.1
0.0
p-value
9e-03
1e-02
2e-02
3e-02
5e-02
Log-odds
0.5
1.0
1.5
2.0
2.5
3.0
3.5
4.0
Cellular_organisms - ps1
Archaea1 - ps2
Archaea2 - ps3
Archaea3 - ps4
Asgard/Eukaryota - ps5
Eukaryota - ps6
Amorpheae - ps7
Obazoa - ps8
Opisthokonta - ps9
Holozoa - ps10
Filozoa - ps11
Choano./Metazoa - ps12
Metazoa - ps13
Myriazoa - ps14
Parahoxozoa - ps15
Bilateria - ps16
Deuterostomia - ps17
Chordata - ps18
Olfactores - ps19
Vertebrata - ps20
Gnathostomata - ps21
Euteleostomi - ps22
Sarcopterygii - ps23
Tetrapoda - ps24
Amniota - ps25
Mammalia - ps26
Theria - ps27
Placentalia - ps28
Boreoeutheria - ps29
Euarchontoglires - ps30
Euarchonta - ps31
Primates - ps32
Haplorrhini - ps33
Simiiformes - ps34
Catarrhini - ps35
Hominoidea - ps36
Hominidae - ps37
Homininae - ps38
Hominini - ps39
Homo_sapiens - ps40
Phylostratum

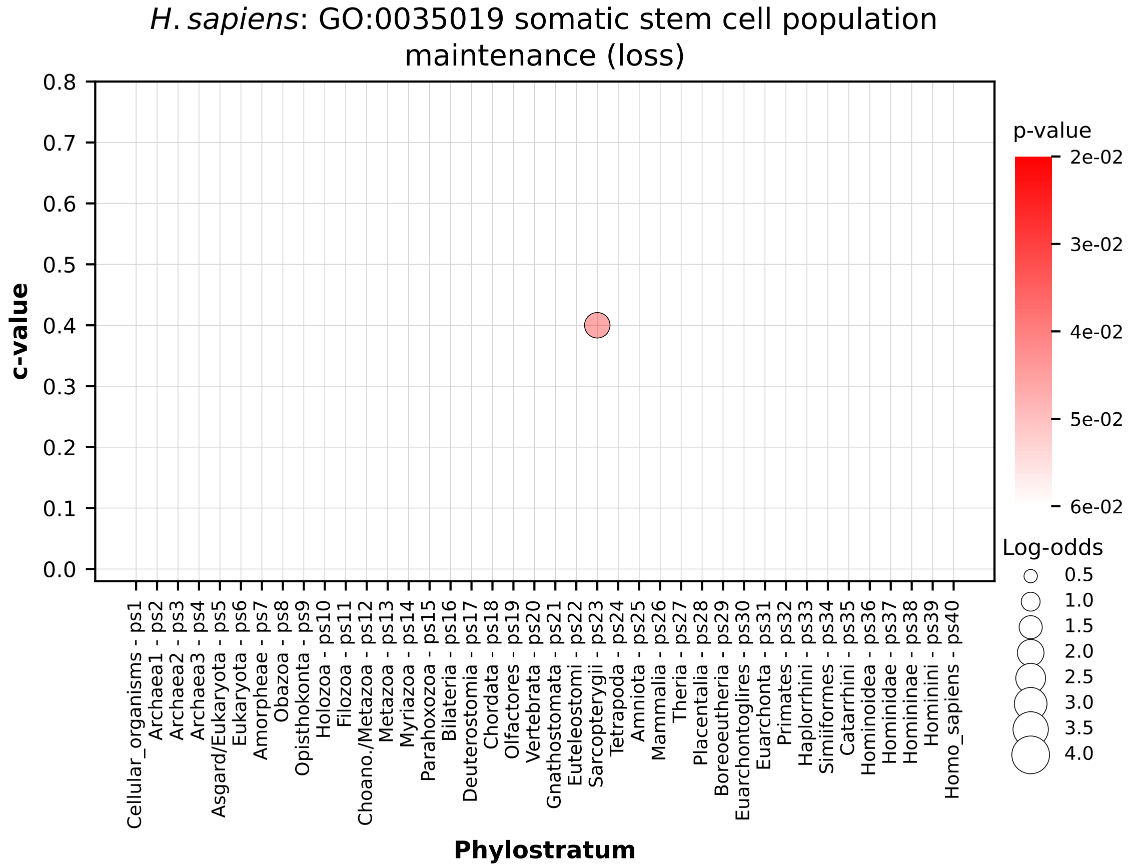

H. sapiens: GO:0035019 somatic stem cell population maintenance (loss)
c-value
0.0
0.1
0.2
0.3
0.4
0.5
0.6
0.7
0.8
Phylostratum
Cellular_organisms - ps1
Archaea1 - ps2
Archaea2 - ps3
Archaea3 - ps4
Asgard/Eukaryota - ps5
Eukaryota - ps6
Amorpheae - ps7
Obazoa - ps8
Opisthokonta - ps9
Holozoa - ps10
Filozoa - ps11
Choano./Metazoa - ps12
Metazoa - ps13
Myriazoa - ps14
Parahoxozoa - ps15
Bilateria - ps16
Deuterostomia - ps17
Chordata - ps18
Olfactores - ps19
Vertebrata - ps20
Gnathostomata - ps21
Euteleostomi - ps22
Sarcopterygii - ps23
Tetrapoda - ps24
Amniota - ps25
Mammalia - ps26
Theria - ps27
Placentalia - ps28
Boreoeutheria - ps29
Euarchontoglires - ps30
Euarchonta - ps31
Primates - ps32
Haplorrhini - ps33
Simiiformes - ps34
Catarrhini - ps35
Hominoidea - ps36
Hominidae - ps37
Homininae - ps38
Hominini - ps39
Homo_sapiens - ps40
p-value
2e-02
3e-02
4e-02
5e-02
6e-02
Log-odds
0.5
1.0
1.5
2.0
2.5
3.0
3.5
4.0

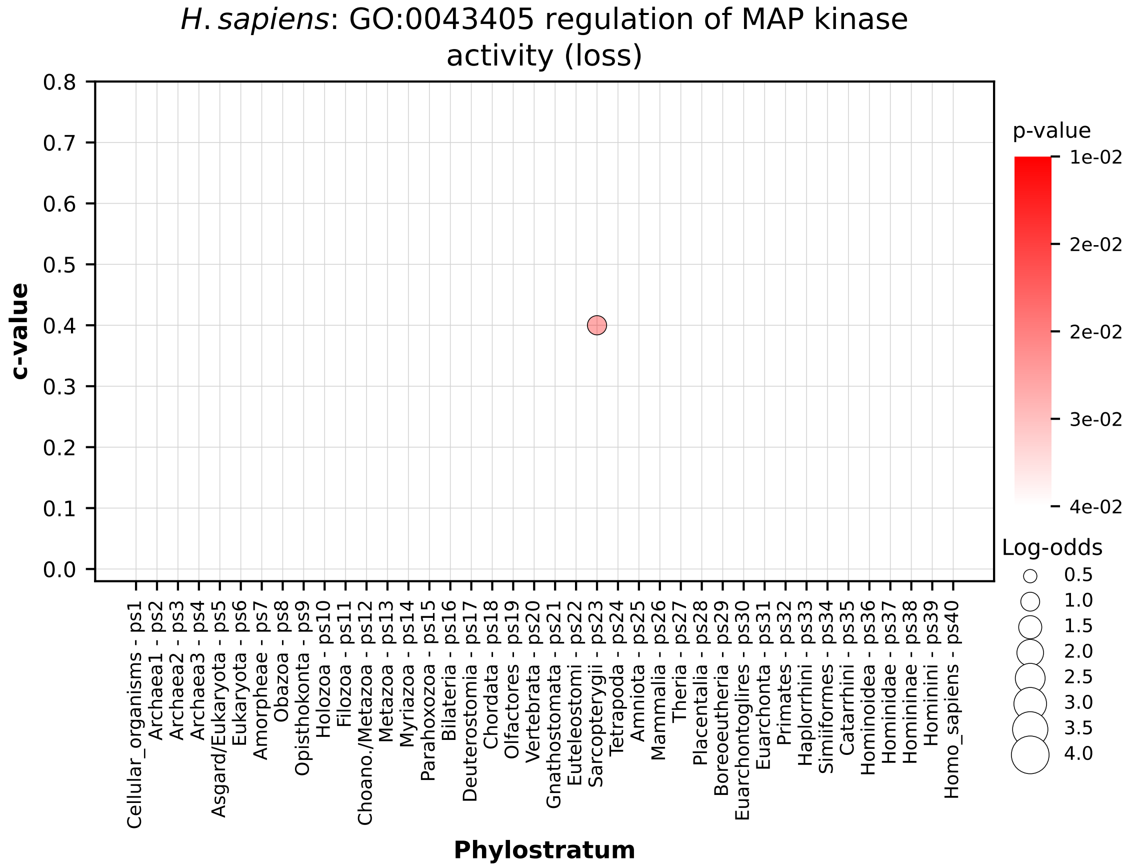

H. sapiens: GO:0043405 regulation of MAP kinase activity (loss)
c-value
0.8
0.7
0.6
0.5
0.4
0.3
0.2
0.1
0.0
Phylostratum
Cellular_organisms - ps1
Archaea1 - ps2
Archaea2 - ps3
Archaea3 - ps4
Asgard/Eukaryota - ps5
Eukaryota - ps6
Amorpheae - ps7
Obazoa - ps8
Opisthokonta - ps9
Holozoa - ps10
Filozoa - ps11
Choano./Metazoa - ps12
Metazoa - ps13
Myriazoa - ps14
Parahoxozoa - ps15
Bilateria - ps16
Deuterostomia - ps17
Chordata - ps18
Olfactores - ps19
Vertebrata - ps20
Gnathostomata - ps21
Euteleostomi - ps22
Sarcopterygii - ps23
Tetrapoda - ps24
Amniota - ps25
Mammalia - ps26
Theria - ps27
Placentalia - ps28
Boreoeutheria - ps29
Euarchontoglires - ps30
Euarchonta - ps31
Primates - ps32
Haplorrhini - ps33
Simiiformes - ps34
Catarrhini - ps35
Hominoidea - ps36
Hominidae - ps37
Homininae - ps38
Hominini - ps39
Homo_sapiens - ps40
p-value
1e-02
2e-02
2e-02
3e-02
4e-02
Log-odds
0.5
1.0
1.5
2.0
2.5
3.0
3.5
4.0

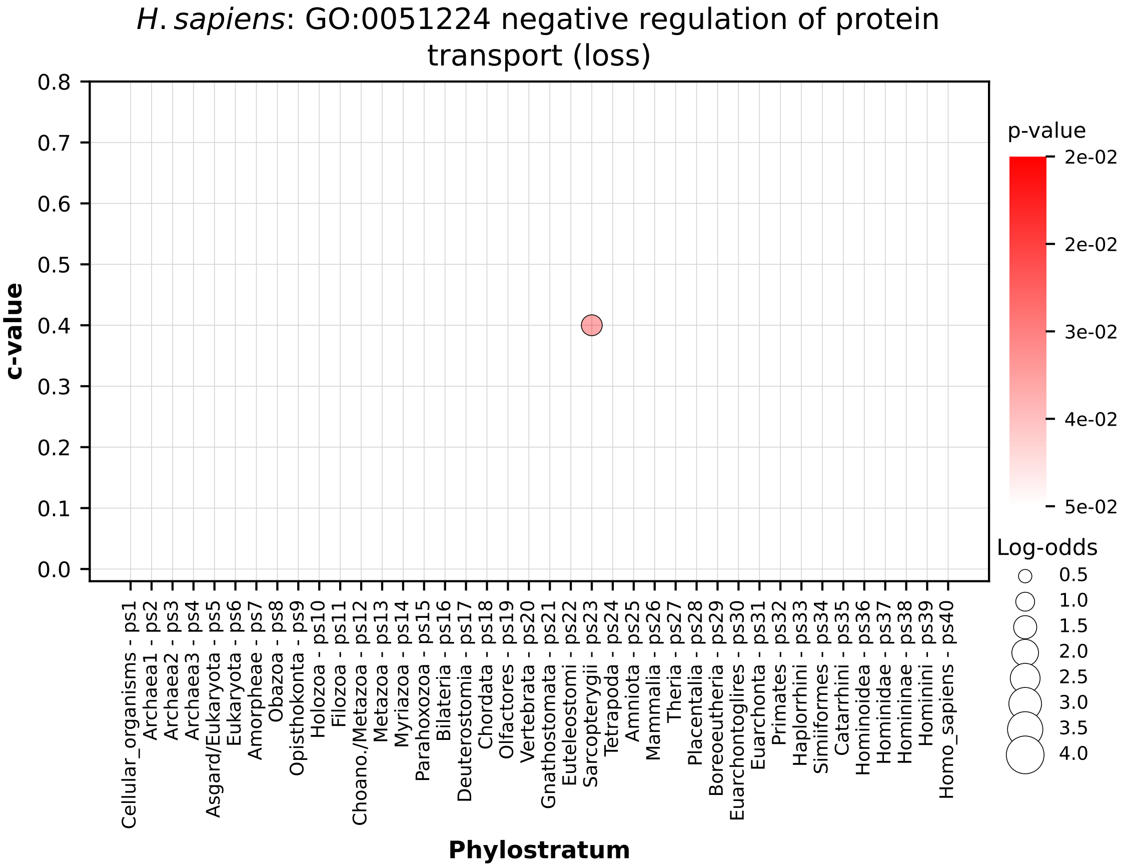
H. sapiens: GO:0051224 negative regulation of protein transport (loss)
c-value
0.8
0.7
0.6
0.5
0.4
0.3
0.2
0.1
0.0
p-value
2e-02
2e-02
3e-02
4e-02
5e-02
Log-odds
0.5
1.0
1.5
2.0
2.5
3.0
3.5
4.0
Cellular_organisms - ps1
Archaea1 - ps2
Archaea2 - ps3
Archaea3 - ps4
Asgard/Eukaryota - ps5
Eukaryota - ps6
Amorpheae - ps7
Obazoa - ps8
Opisthokonta - ps9
Holozoa - ps10
Filozoa - ps11
Choano./Metazoa - ps12
Metazoa - ps13
Myriazoa - ps14
Parahoxozoa - ps15
Bilateria - ps16
Deuterostomia - ps17
Chordata - ps18
Olfactores - ps19
Vertebrata - ps20
Gnathostomata - ps21
Euteleostomi - ps22
Sarcopterygii - ps23
Tetrapoda - ps24
Amniota - ps25
Mammalia - ps26
Theria - ps27
Placentalia - ps28
Boreoeutheria - ps29
Euarchontoglires - ps30
Euarchonta - ps31
Primates - ps32
Haplorrhini - ps33
Simiiformes - ps34
Catarrhini - ps35
Hominoidea - ps36
Hominidae - ps37
Homininae - ps38
Hominini - ps39
Homo_sapiens - ps40
Phylostratum

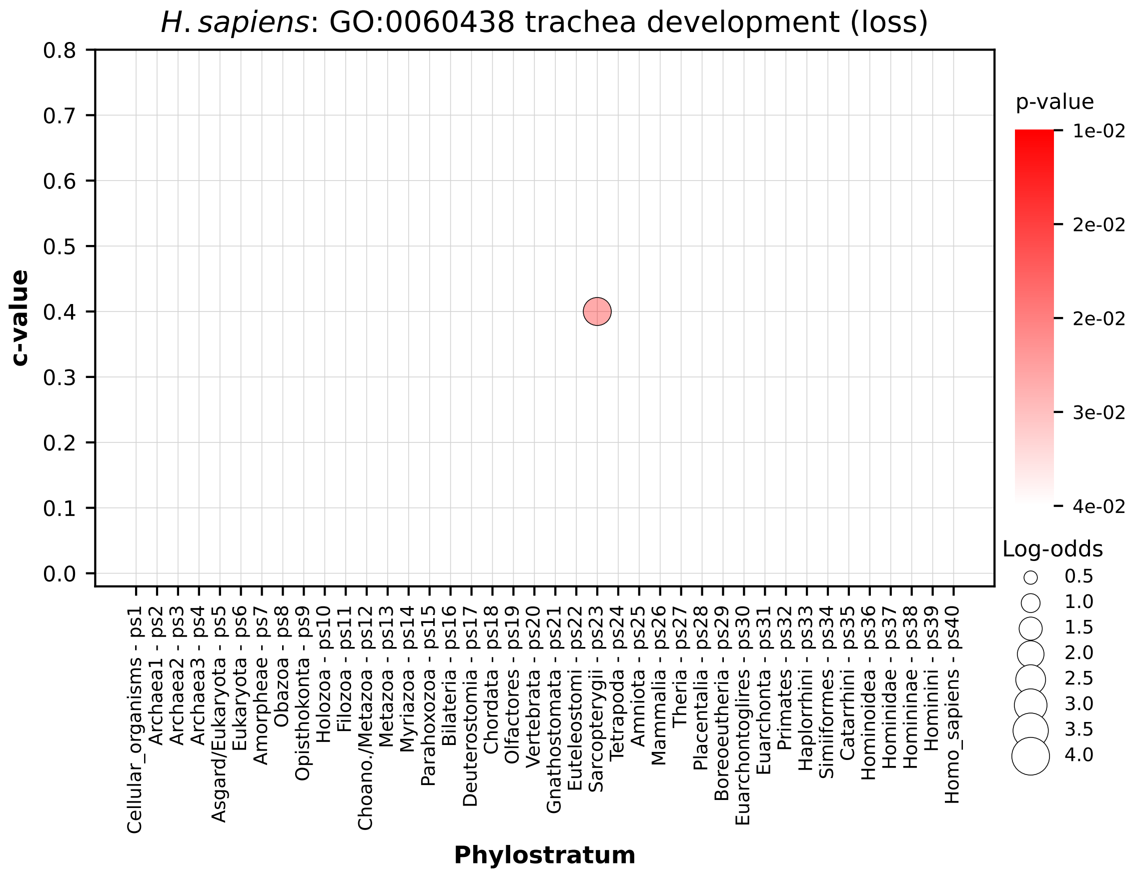

H. sapiens: GO:0060438 trachea development (loss)
c-value
0.0
0.1
0.2
0.3
0.4
0.5
0.6
0.7
0.8
Phylostratum
Cellular_organisms - ps1
Archaea1 - ps2
Archaea2 - ps3
Archaea3 - ps4
Asgard/Eukaryota - ps5
Eukaryota - ps6
Amorpheae - ps7
Obazoa - ps8
Opisthokonta - ps9
Holozoa - ps10
Filozoa - ps11
Choano./Metazoa - ps12
Metazoa - ps13
Myriazoa - ps14
Parahoxozoa - ps15
Bilateria - ps16
Deuterostomia - ps17
Chordata - ps18
Olfactores - ps19
Vertebrata - ps20
Gnathostomata - ps21
Euteleostomi - ps22
Sarcopterygii - ps23
Tetrapoda - ps24
Amniota - ps25
Mammalia - ps26
Theria - ps27
Placentalia - ps28
Boreoeutheria - ps29
Euarchontoglires - ps30
Euarchonta - ps31
Primates - ps32
Haplorrhini - ps33
Simiiformes - ps34
Catarrhini - ps35
Hominoidea - ps36
Hominidae - ps37
Homininae - ps38
Hominini - ps39
Homo_sapiens - ps40
p-value
1e-02
2e-02
2e-02
3e-02
4e-02
Log-odds
0.5
1.0
1.5
2.0
2.5
3.0
3.5
4.0

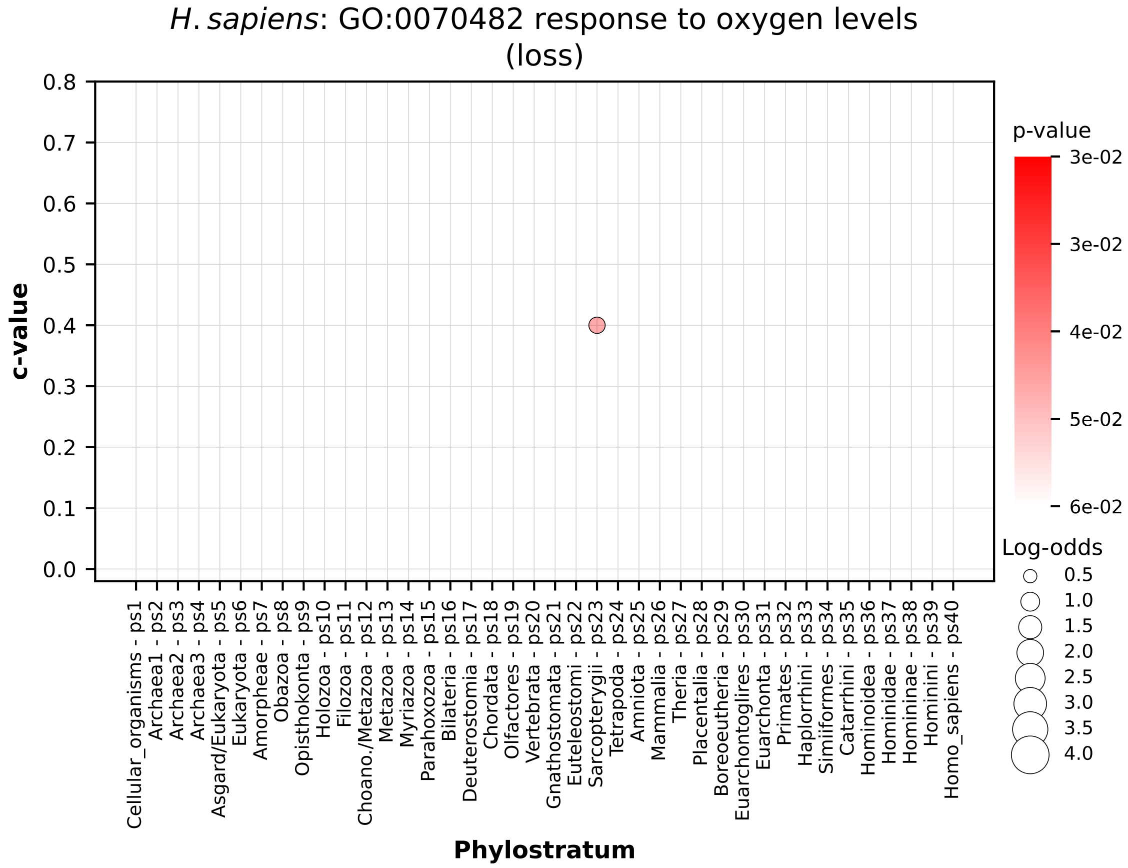
H. sapiens: GO:0070482 response to oxygen levels
(loss)
c-value
0.8
0.7
0.6
0.5
0.4
0.3
0.2
0.1
0.0
Phylostratum
Cellular_organisms - ps1
Archaea1 - ps2
Archaea2 - ps3
Archaea3 - ps4
Asgard/Eukaryota - ps5
Eukaryota - ps6
Amorpheae - ps7
Obazoa - ps8
Opisthokonta - ps9
Holozoa - ps10
Filozoa - ps11
Choano./Metazoa - ps12
Metazoa - ps13
Myriazoa - ps14
Parahoxozoa - ps15
Bilateria - ps16
Deuterostomia - ps17
Chordata - ps18
Olfactores - ps19
Vertebrata - ps20
Gnathostomata - ps21
Euteleostomi - ps22
Sarcopterygii - ps23
Tetrapoda - ps24
Amniota - ps25
Mammalia - ps26
Theria - ps27
Placentalia - ps28
Boreoeutheria - ps29
Euarchontoglires - ps30
Euarchonta - ps31
Primates - ps32
Haplorrhini - ps33
Simiiformes - ps34
Catarrhini - ps35
Hominoidea - ps36
Hominidae - ps37
Homininae - ps38
Hominini - ps39
Homo_sapiens - ps40
p-value
3e-02
3e-02
4e-02
5e-02
6e-02
Log-odds
0.5
1.0
1.5
2.0
2.5
3.0
3.5
4.0

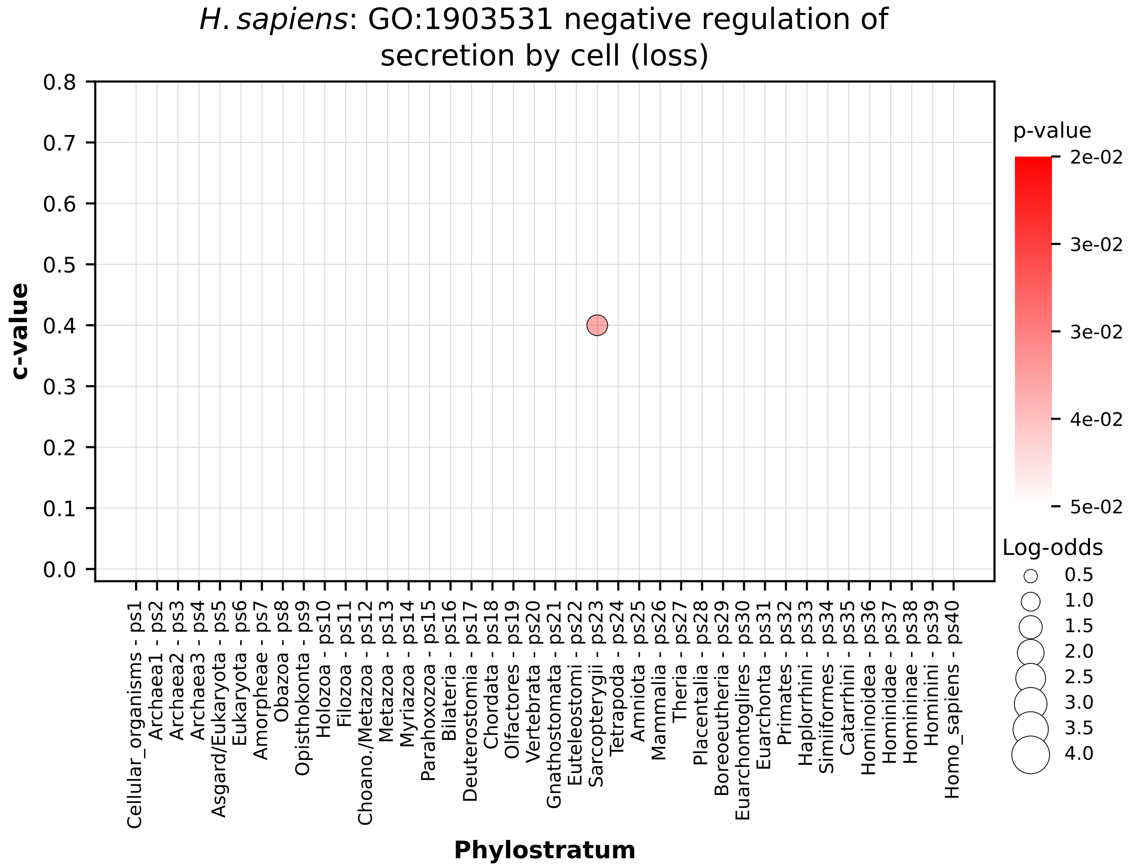

H. sapiens: GO:1903531 negative regulation of secretion by cell (loss)
c-value
0.0
0.1
0.2
0.3
0.4
0.5
0.6
0.7
0.8
Phylostratum
Cellular_organisms - ps1
Archaea1 - ps2
Archaea2 - ps3
Archaea3 - ps4
Asgard/Eukaryota - ps5
Eukaryota - ps6
Amorpheae - ps7
Obazoa - ps8
Opisthokonta - ps9
Holozoa - ps10
Filozoa - ps11
Choano./Metazoa - ps12
Metazoa - ps13
Myriazoa - ps14
Parahoxozoa - ps15
Bilateria - ps16
Deuterostomia - ps17
Chordata - ps18
Olfactores - ps19
Vertebrata - ps20
Gnathostomata - ps21
Euteleostomi - ps22
Sarcopterygii - ps23
Tetrapoda - ps24
Amniota - ps25
Mammalia - ps26
Theria - ps27
Placentalia - ps28
Boreoeutheria - ps29
Euarchontoglires - ps30
Euarchonta - ps31
Primates - ps32
Haplorrhini - ps33
Simiiformes - ps34
Catarrhini - ps35
Hominoidea - ps36
Hominidae - ps37
Homininae - ps38
Hominini - ps39
Homo_sapiens - ps40
p-value
2e-02
3e-02
3e-02
4e-02
5e-02
Log-odds
0.5
1.0
1.5
2.0
2.5
3.0
3.5
4.0

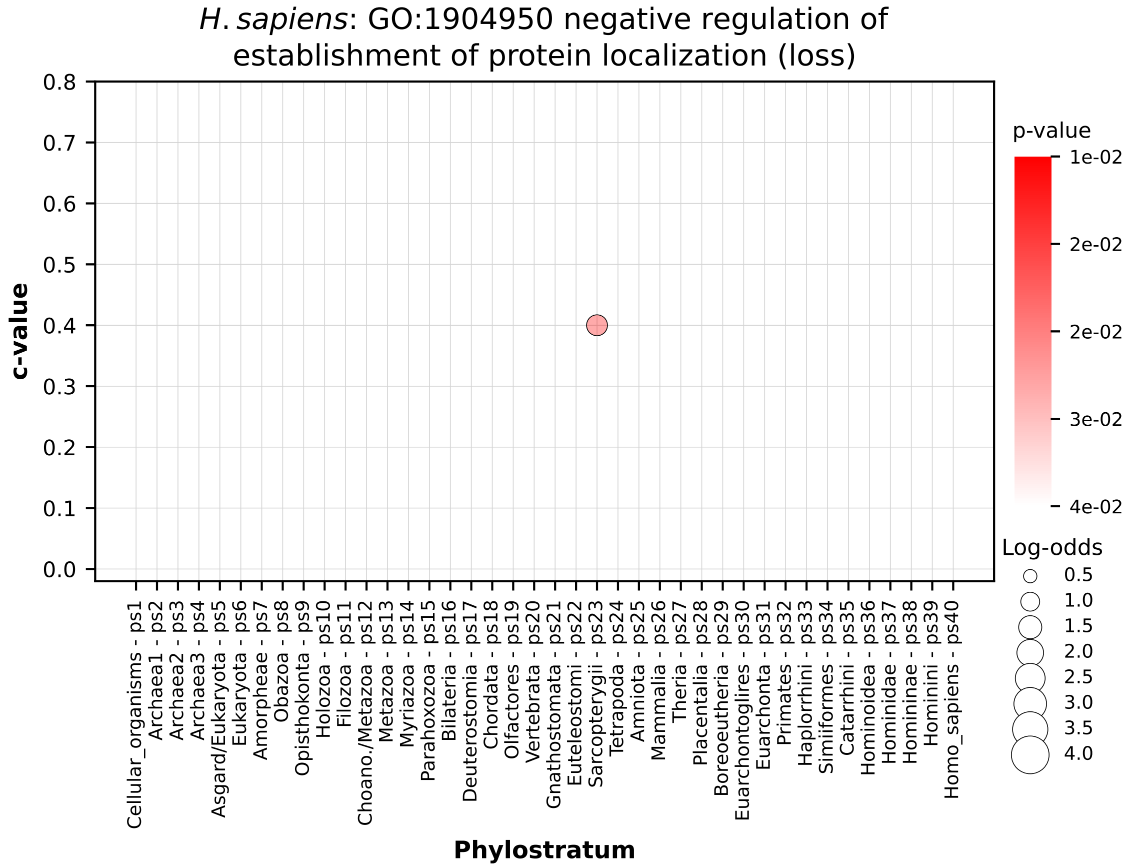

H. sapiens: GO:1904950 negative regulation of establishment of protein localization (loss)
c-value
0.8
0.7
0.6
0.5
0.4
0.3
0.2
0.1
0.0
p-value
1e-02
2e-02
2e-02
3e-02
4e-02
Log-odds
0.5
1.0
1.5
2.0
2.5
3.0
3.5
4.0
Cellular_organisms - ps1
Archaea1 - ps2
Archaea2 - ps3
Archaea3 - ps4
Asgard/Eukaryota - ps5
Eukaryota - ps6
Amorpheae - ps7
Obazoa - ps8
Opisthokonta - ps9
Holozoa - ps10
Filozoa - ps11
Choano./Metazoa - ps12
Metazoa - ps13
Myriazoa - ps14
Parahoxozoa - ps15
Bilateria - ps16
Deuterostomia - ps17
Chordata - ps18
Olfactores - ps19
Vertebrata - ps20
Gnathostomata - ps21
Euteleostomi - ps22
Sarcopterygii - ps23
Tetrapoda - ps24
Amniota - ps25
Mammalia - ps26
Theria - ps27
Placentalia - ps28
Boreoeutheria - ps29
Euarchontoglires - ps30
Euarchonta - ps31
Primates - ps32
Haplorrhini - ps33
Simiiformes - ps34
Catarrhini - ps35
Hominoidea - ps36
Hominidae - ps37
Homininae - ps38
Hominini - ps39
Homo_sapiens - ps40
Phylostratum

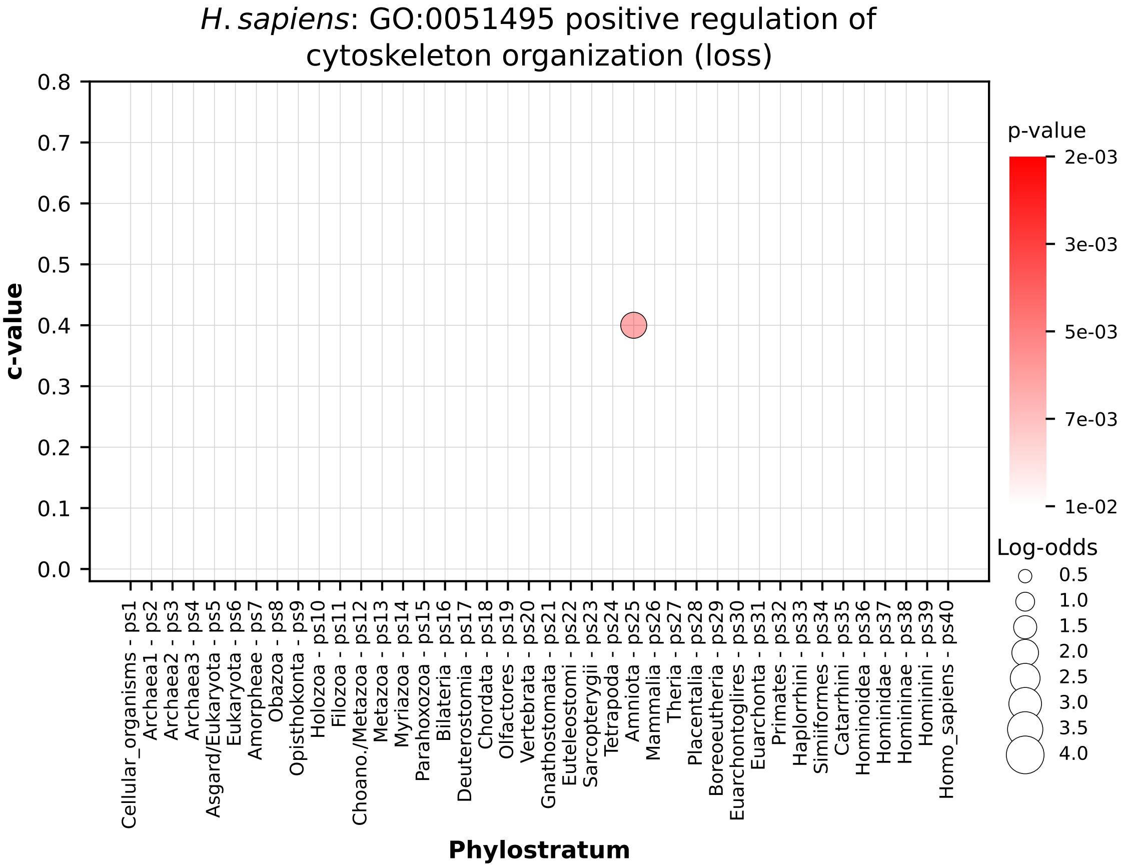
H. sapiens: GO:0051495 positive regulation of cytoskeleton organization (loss)
c-value
0.8
0.7
0.6
0.5
0.4
0.3
0.2
0.1
0.0
p-value
2e-03
3e-03
5e-03
7e-03
1e-02
Log-odds
0.5
1.0
1.5
2.0
2.5
3.0
3.5
4.0
Cellular_organisms - ps1
Archaea1 - ps2
Archaea2 - ps3
Archaea3 - ps4
Asgard/Eukaryota - ps5
Eukaryota - ps6
Amorpheae - ps7
Obazoa - ps8
Opisthokonta - ps9
Holozoa - ps10
Filozoa - ps11
Choano./Metazoa - ps12
Metazoa - ps13
Myriazoa - ps14
Parahoxozoa - ps15
Bilateria - ps16
Deuterostomia - ps17
Chordata - ps18
Olfactores - ps19
Vertebrata - ps20
Gnathostomata - ps21
Euteleostomi - ps22
Sarcopterygii - ps23
Tetrapoda - ps24
Amniota - ps25
Mammalia - ps26
Theria - ps27
Placentalia - ps28
Boreoeutheria - ps29
Euarchontoglires - ps30
Euarchonta - ps31
Primates - ps32
Haplorrhini - ps33
Simiiformes - ps34
Catarrhini - ps35
Hominoidea - ps36
Hominidae - ps37
Homininae - ps38
Hominini - ps39
Homo_sapiens - ps40
Phylostratum

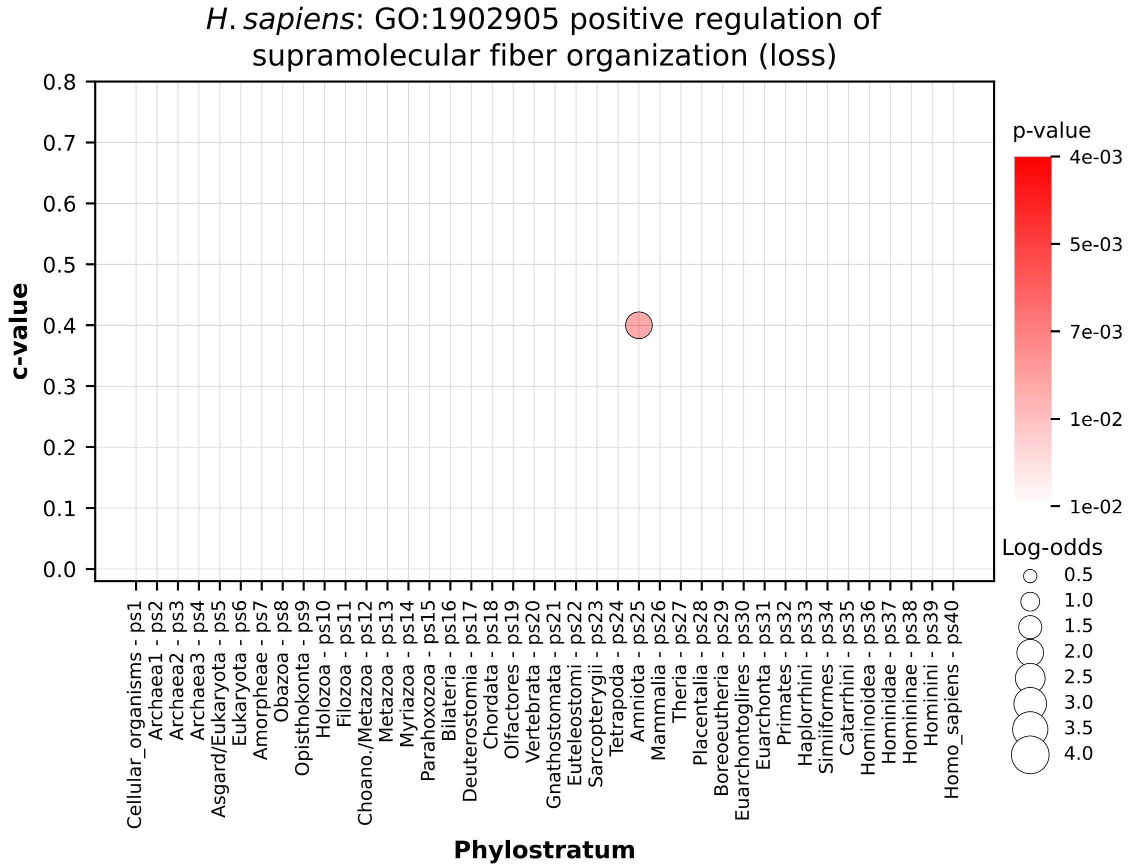
H. sapiens: GO:1902905 positive regulation of supramolecular fiber organization (loss)
c-value
0.0
0.1
0.2
0.3
0.4
0.5
0.6
0.7
0.8
Phylostratum
Cellular_organisms - ps1
Archaea1 - ps2
Archaea2 - ps3
Archaea3 - ps4
Asgard/Eukaryota - ps5
Eukaryota - ps6
Amorpheae - ps7
Obazoa - ps8
Opisthokonta - ps9
Holozoa - ps10
Filozoa - ps11
Choano./Metazoa - ps12
Metazoa - ps13
Myriazoa - ps14
Parahoxozoa - ps15
Bilateria - ps16
Deuterostomia - ps17
Chordata - ps18
Olfactores - ps19
Vertebrata - ps20
Gnathostomata - ps21
Euteleostomi - ps22
Sarcopterygii - ps23
Tetrapoda - ps24
Amniota - ps25
Mammalia - ps26
Theria - ps27
Placentalia - ps28
Boreoeutheria - ps29
Euarchontoglires - ps30
Euarchonta - ps31
Primates - ps32
Haplorrhini - ps33
Simiiformes - ps34
Catarrhini - ps35
Hominoidea - ps36
Hominidae - ps37
Homininae - ps38
Hominini - ps39
Homo_sapiens - ps40
p-value
4e-03
5e-03
7e-03
1e-02
1e-02
Log-odds
0.5
1.0
1.5
2.0
2.5
3.0
3.5
4.0

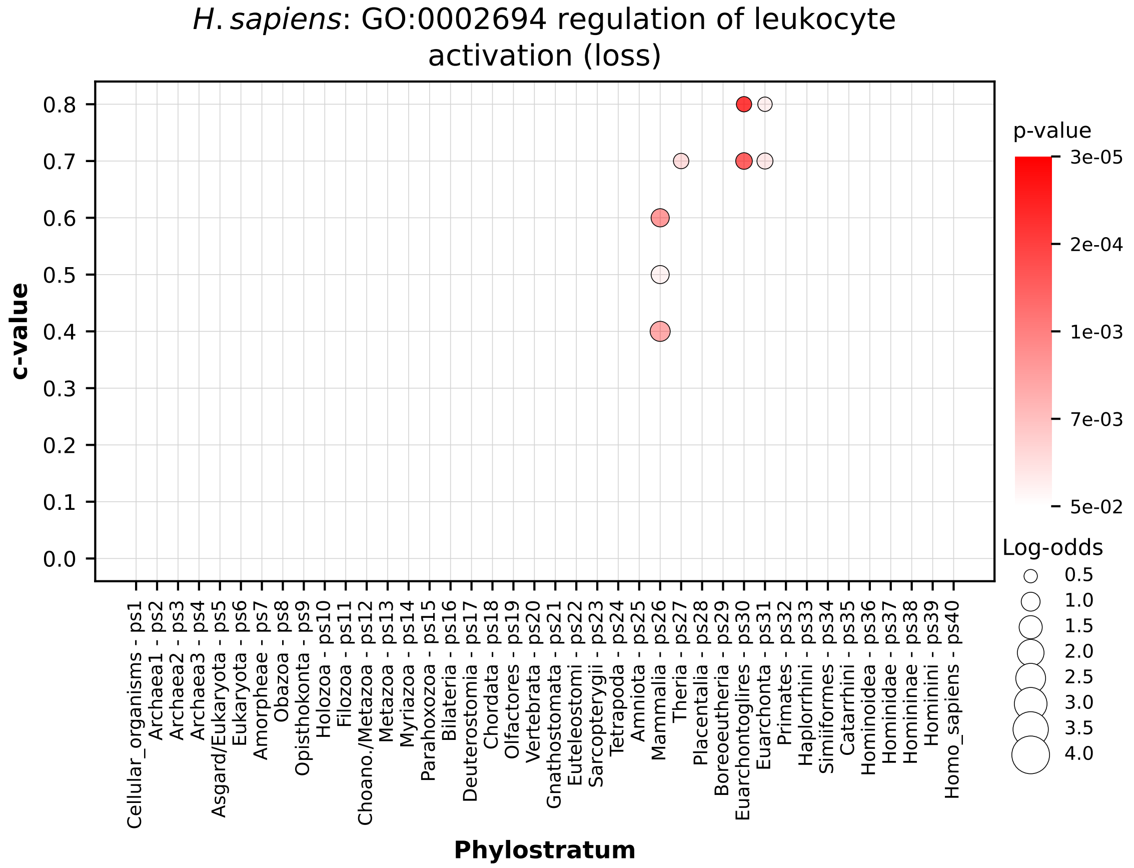

H. sapiens: GO:0002694 regulation of leukocyte activation (loss)
c-value
0.8
0.7
0.6
0.5
0.4
0.3
0.2
0.1
0.0
Phylostratum
Cellular_organisms - ps1
Archaea1 - ps2
Archaea2 - ps3
Archaea3 - ps4
Asgard/Eukaryota - ps5
Eukaryota - ps6
Amorpheae - ps7
Obazoa - ps8
Opisthokonta - ps9
Holozoa - ps10
Filozoa - ps11
Choano./Metazoa - ps12
Metazoa - ps13
Myriazoa - ps14
Parahoxozoa - ps15
Bilateria - ps16
Deuterostomia - ps17
Chordata - ps18
Olfactores - ps19
Vertebrata - ps20
Gnathostomata - ps21
Euteleostomi - ps22
Sarcopterygii - ps23
Tetrapoda - ps24
Amniota - ps25
Mammalia - ps26
Theria - ps27
Placentalia - ps28
Boreoeutheria - ps29
Euarchontoglires - ps30
Euarchonta - ps31
Primates - ps32
Haplorrhini - ps33
Simiiformes - ps34
Catarrhini - ps35
Hominoidea - ps36
Hominidae - ps37
Homininae - ps38
Hominini - ps39
Homo_sapiens - ps40
p-value
3e-05
2e-04
1e-03
7e-03
5e-02
Log-odds
0.5
1.0
1.5
2.0
2.5
3.0
3.5
4.0

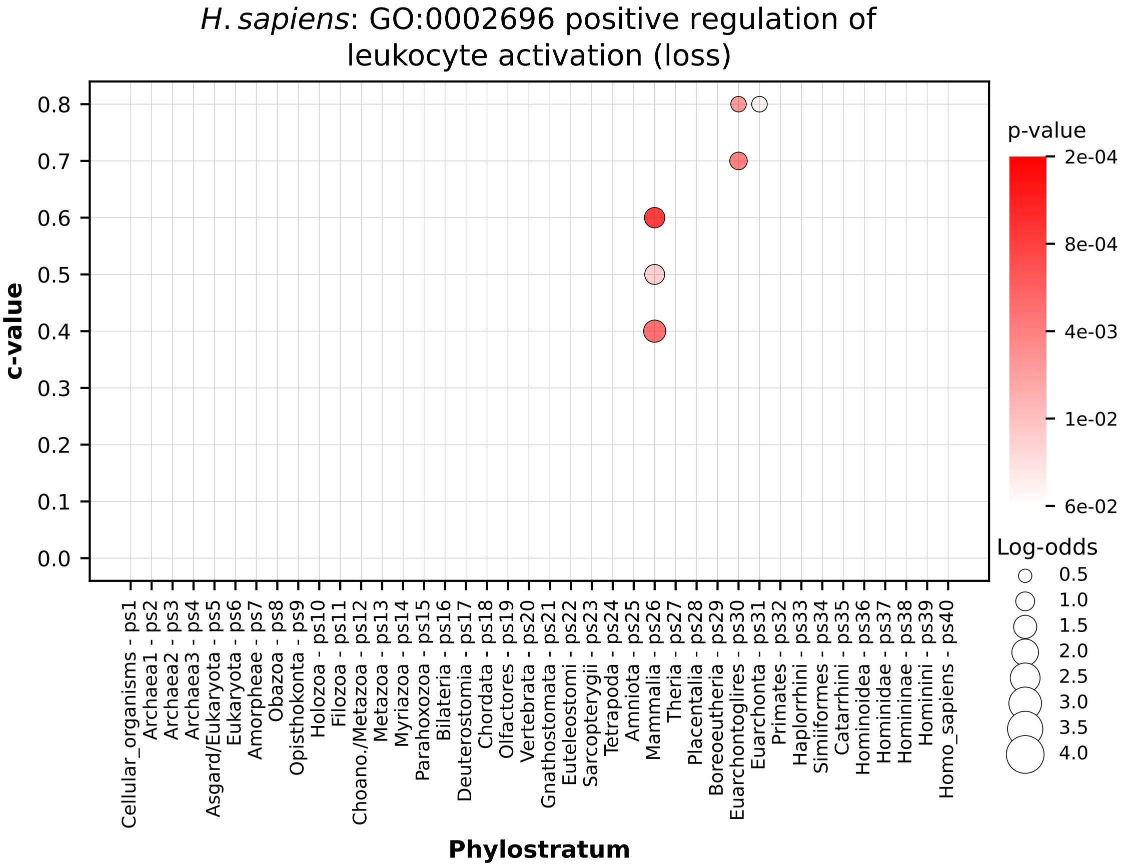
H. sapiens: GO:0002696 positive regulation of leukocyte activation (loss)
c-value
0.0
0.1
0.2
0.3
0.4
0.5
0.6
0.7
0.8
Phylostratum
Cellular_organisms - ps1
Archaea1 - ps2
Archaea2 - ps3
Archaea3 - ps4
Asgard/Eukaryota - ps5
Eukaryota - ps6
Amorpheae - ps7
Obazoa - ps8
Opisthokonta - ps9
Holozoa - ps10
Filozoa - ps11
Choano./Metazoa - ps12
Metazoa - ps13
Myriazoa - ps14
Parahoxozoa - ps15
Bilateria - ps16
Deuterostomia - ps17
Chordata - ps18
Olfactores - ps19
Vertebrata - ps20
Gnathostomata - ps21
Euteleostomi - ps22
Sarcopterygii - ps23
Tetrapoda - ps24
Amniota - ps25
Mammalia - ps26
Theria - ps27
Placentalia - ps28
Boreoeutheria - ps29
Euarchontoglires - ps30
Euarchonta - ps31
Primates - ps32
Haplorrhini - ps33
Simiiformes - ps34
Catarrhini - ps35
Hominoidea - ps36
Hominidae - ps37
Homininae - ps38
Hominini - ps39
Homo_sapiens - ps40
p-value
2e-04
8e-04
4e-03
1e-02
6e-02
Log-odds
0.5
1.0
1.5
2.0
2.5
3.0
3.5
4.0

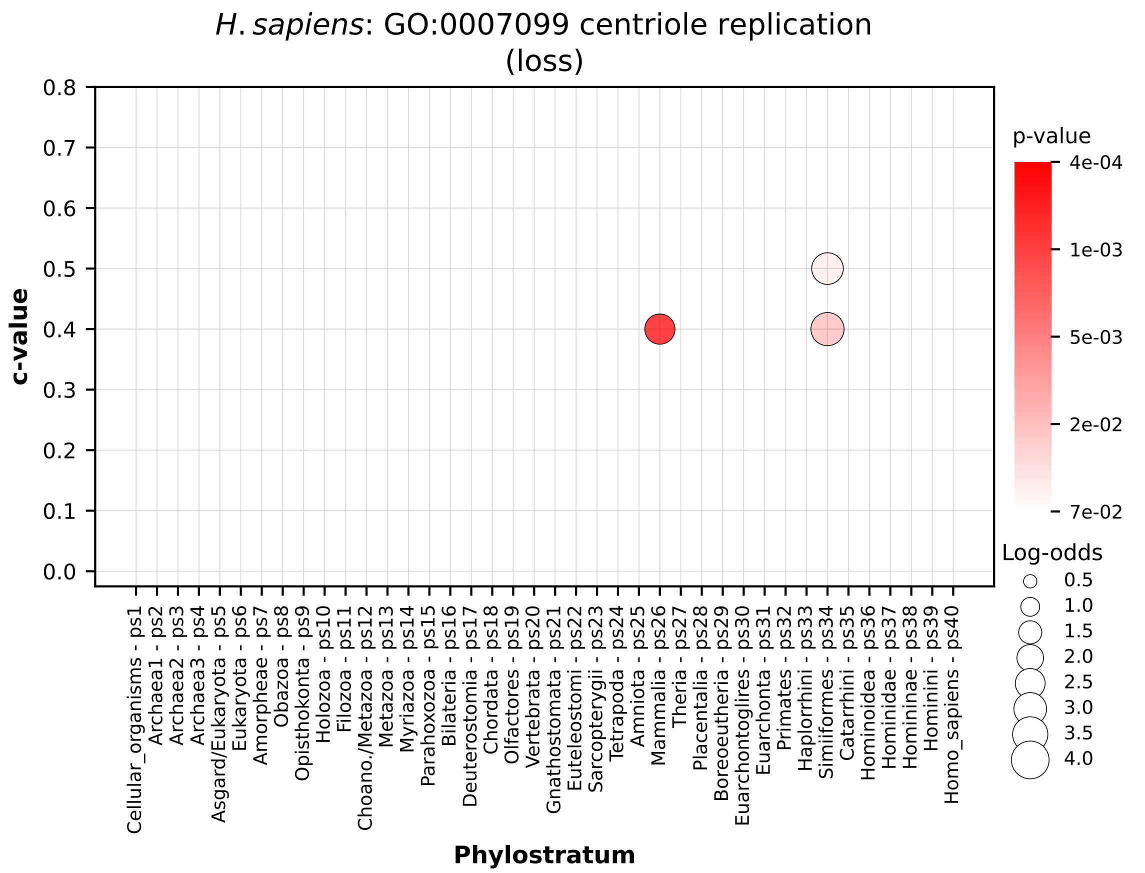

H. sapiens: GO:0007099 centriole replication
(loss)
c-value
0.8
0.7
0.6
0.5
0.4
0.3
0.2
0.1
0.0
p-value
4e-04
1e-03
5e-03
2e-02
7e-02
Log-odds
0.5
1.0
1.5
2.0
2.5
3.0
3.5
4.0
Cellular_organisms - ps1
Archaea1 - ps2
Archaea2 - ps3
Archaea3 - ps4
Asgard/Eukaryota - ps5
Eukaryota - ps6
Amorpheae - ps7
Obazoa - ps8
Opisthokonta - ps9
Holozoa - ps10
Filozoa - ps11
Choano./Metazoa - ps12
Metazoa - ps13
Myriazoa - ps14
Parahoxozoa - ps15
Bilateria - ps16
Deuterostomia - ps17
Chordata - ps18
Olfactores - ps19
Vertebrata - ps20
Gnathostomata - ps21
Euteleostomi - ps22
Sarcopterygii - ps23
Tetrapoda - ps24
Amniota - ps25
Mammalia - ps26
Theria - ps27
Placentalia - ps28
Boreoeutheria - ps29
Euarchontoglires - ps30
Euarchonta - ps31
Primates - ps32
Haplorrhini - ps33
Simiiformes - ps34
Catarrhini - ps35
Hominoidea - ps36
Hominidae - ps37
Homininae - ps38
Hominini - ps39
Homo_sapiens - ps40
Phylostratum

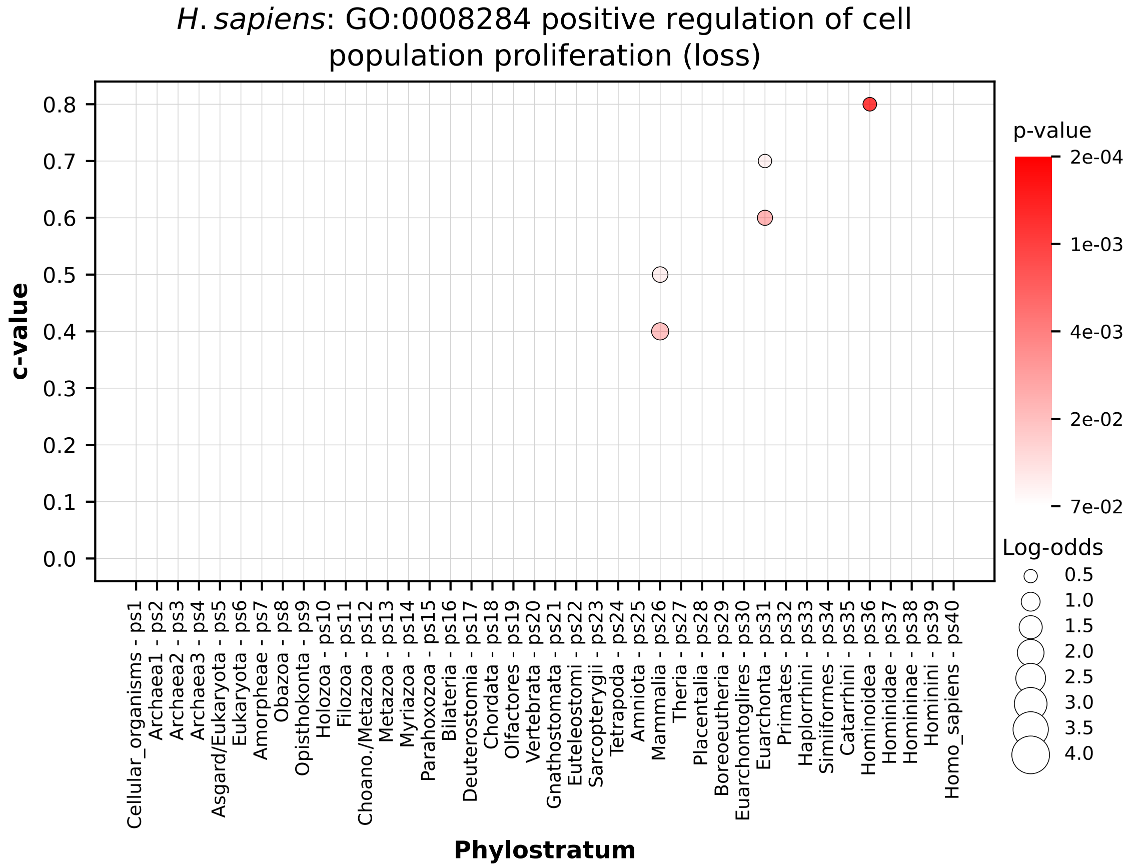
H. sapiens: GO:0008284 positive regulation of cell population proliferation (loss)
c-value
0.0
0.1
0.2
0.3
0.4
0.5
0.6
0.7
0.8
Phylostratum
Cellular_organisms - ps1
Archaea1 - ps2
Archaea2 - ps3
Archaea3 - ps4
Asgard/Eukaryota - ps5
Eukaryota - ps6
Amorpheae - ps7
Obazoa - ps8
Opisthokonta - ps9
Holozoa - ps10
Filozoa - ps11
Choano./Metazoa - ps12
Metazoa - ps13
Myriazoa - ps14
Parahoxozoa - ps15
Bilateria - ps16
Deuterostomia - ps17
Chordata - ps18
Olfactores - ps19
Vertebrata - ps20
Gnathostomata - ps21
Euteleostomi - ps22
Sarcopterygii - ps23
Tetrapoda - ps24
Amniota - ps25
Mammalia - ps26
Theria - ps27
Placentalia - ps28
Boreoeutheria - ps29
Euarchontoglires - ps30
Euarchonta - ps31
Primates - ps32
Haplorrhini - ps33
Simiiformes - ps34
Catarrhini - ps35
Hominoidea - ps36
Hominidae - ps37
Homininae - ps38
Hominini - ps39
Homo_sapiens - ps40
p-value
2e-04
1e-03
4e-03
2e-02
7e-02
Log-odds
0.5
1.0
1.5
2.0
2.5
3.0
3.5
4.0

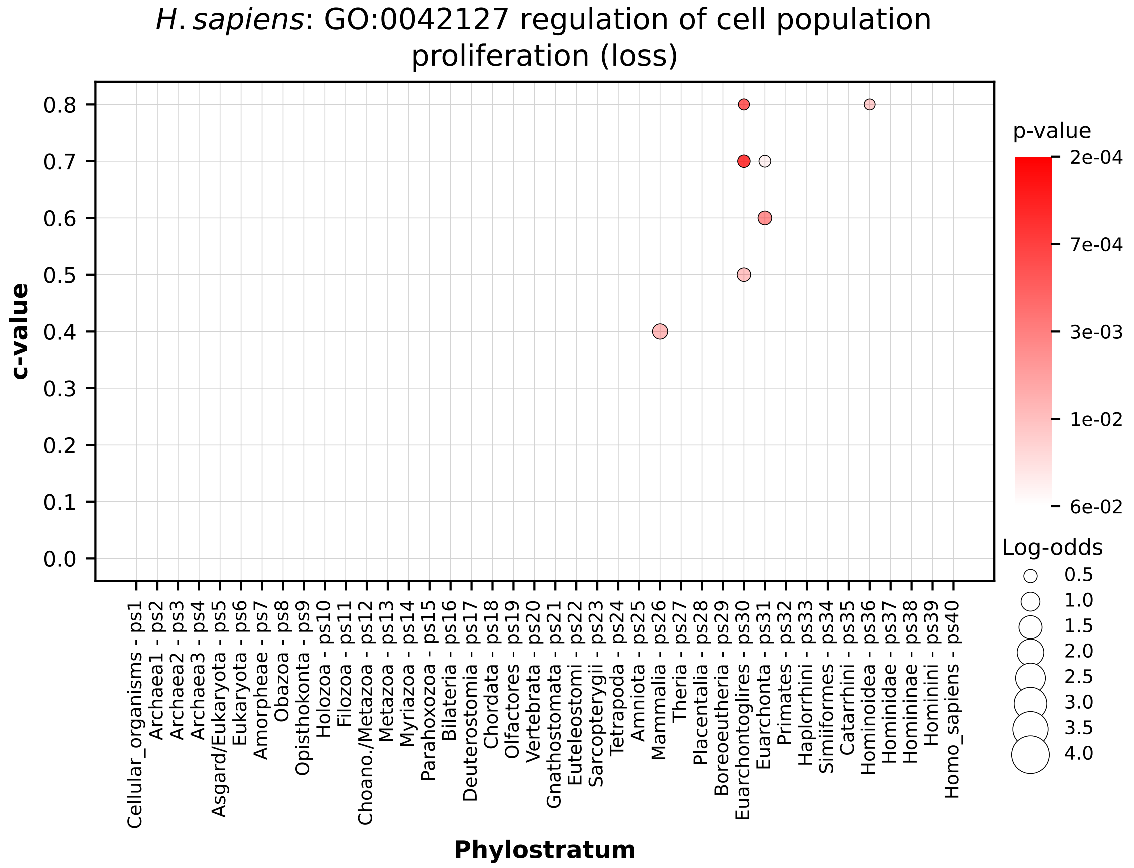
H. sapiens: GO:0042127 regulation of cell population proliferation (loss)
c-value
Phylostratum
p-value
2e-04
7e-04
3e-03
1e-02
6e-02
Log-odds
0.5
1.0
1.5
2.0
2.5
3.0
3.5
4.0
Cellular_organisms - ps1
Archaea1 - ps2
Archaea2 - ps3
Archaea3 - ps4
Asgard/Eukaryota - ps5
Eukaryota - ps6
Amorpheae - ps7
Obazoa - ps8
Opisthokonta - ps9
Holozoa - ps10
Filozoa - ps11
Choano./Metazoa - ps12
Metazoa - ps13
Myriazoa - ps14
Parahoxozoa - ps15
Bilateria - ps16
Deuterostomia - ps17
Chordata - ps18
Olfactores - ps19
Vertebrata - ps20
Gnathostomata - ps21
Euteleostomi - ps22
Sarcopterygii - ps23
Tetrapoda - ps24
Amniota - ps25
Mammalia - ps26
Theria - ps27
Placentalia - ps28
Boreoeutheria - ps29
Euarchontoglires - ps30
Euarchonta - ps31
Primates - ps32
Haplorrhini - ps33
Simiiformes - ps34
Catarrhini - ps35
Hominoidea - ps36
Hominidae - ps37
Homininae - ps38
Hominini - ps39
Homo_sapiens - ps40

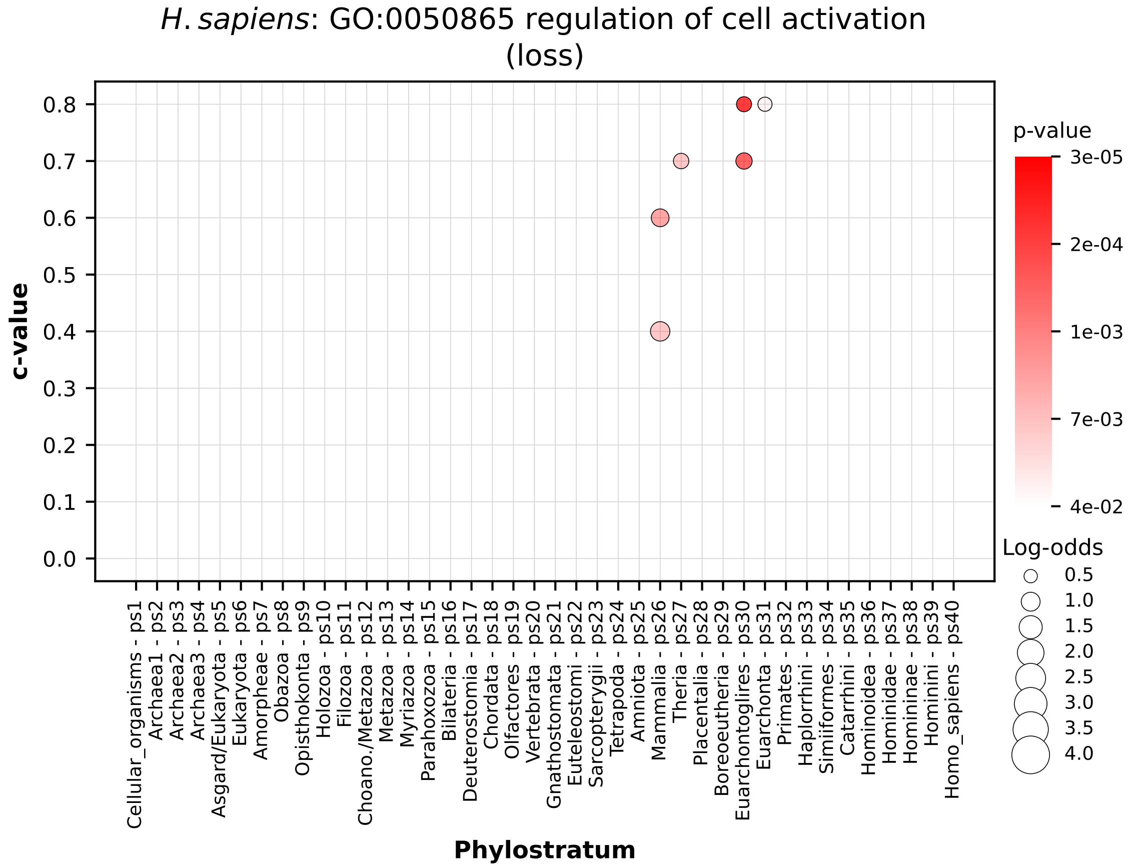

H. sapiens: GO:0050865 regulation of cell activation
(loss)
c-value
0.8
0.7
0.6
0.5
0.4
0.3
0.2
0.1
0.0
p-value
3e-05
2e-04
1e-03
7e-03
4e-02
Log-odds
0.5
1.0
1.5
2.0
2.5
3.0
3.5
4.0
Cellular_organisms - ps1
Archaea1 - ps2
Archaea2 - ps3
Archaea3 - ps4
Asgard/Eukaryota - ps5
Eukaryota - ps6
Amorpheae - ps7
Obazoa - ps8
Opisthokonta - ps9
Holozoa - ps10
Filozoa - ps11
Choano./Metazoa - ps12
Metazoa - ps13
Myriazoa - ps14
Parahoxozoa - ps15
Bilateria - ps16
Deuterostomia - ps17
Chordata - ps18
Olfactores - ps19
Vertebrata - ps20
Gnathostomata - ps21
Euteleostomi - ps22
Sarcopterygii - ps23
Tetrapoda - ps24
Amniota - ps25
Mammalia - ps26
Theria - ps27
Placentalia - ps28
Boreoeutheria - ps29
Euarchontoglires - ps30
Euarchonta - ps31
Primates - ps32
Haplorrhini - ps33
Simiiformes - ps34
Catarrhini - ps35
Hominoidea - ps36
Hominidae - ps37
Homininae - ps38
Hominini - ps39
Homo_sapiens - ps40
Phylostratum

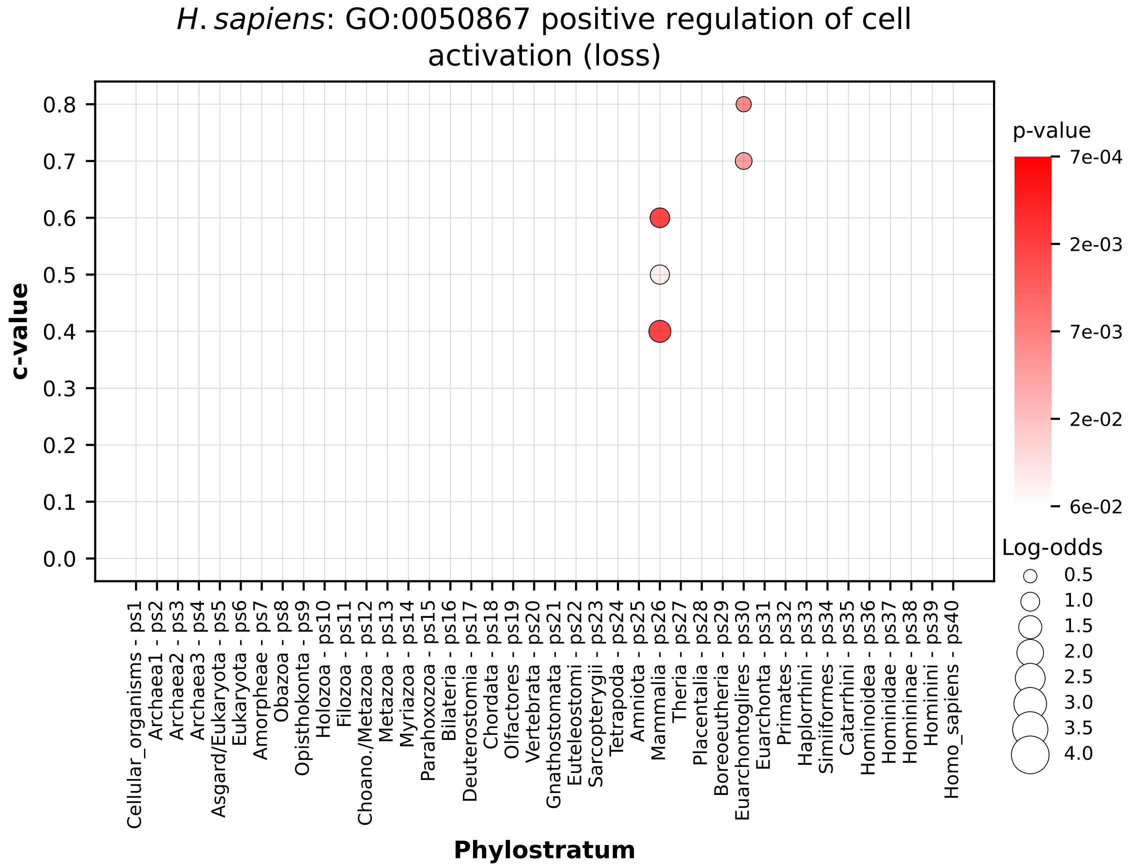

H. sapiens: GO:0050867 positive regulation of cell activation (loss)
c-value
0.8
0.7
0.6
0.5
0.4
0.3
0.2
0.1
0.0
p-value
7e-04
2e-03
7e-03
2e-02
6e-02
Log-odds
0.5
1.0
1.5
2.0
2.5
3.0
3.5
4.0
Cellular_organisms - ps1
Archaea1 - ps2
Archaea2 - ps3
Archaea3 - ps4
Asgard/Eukaryota - ps5
Eukaryota - ps6
Amorpheae - ps7
Obazoa - ps8
Opisthokonta - ps9
Holozoa - ps10
Filozoa - ps11
Choano./Metazoa - ps12
Metazoa - ps13
Myriazoa - ps14
Parahoxozoa - ps15
Bilateria - ps16
Deuterostomia - ps17
Chordata - ps18
Olfactores - ps19
Vertebrata - ps20
Gnathostomata - ps21
Euteleostomi - ps22
Sarcopterygii - ps23
Tetrapoda - ps24
Amniota - ps25
Mammalia - ps26
Theria - ps27
Placentalia - ps28
Boreoeutheria - ps29
Euarchontoglires - ps30
Euarchonta - ps31
Primates - ps32
Haplorrhini - ps33
Simiiformes - ps34
Catarrhini - ps35
Hominoidea - ps36
Hominidae - ps37
Homininae - ps38
Hominini - ps39
Homo_sapiens - ps40
Phylostratum

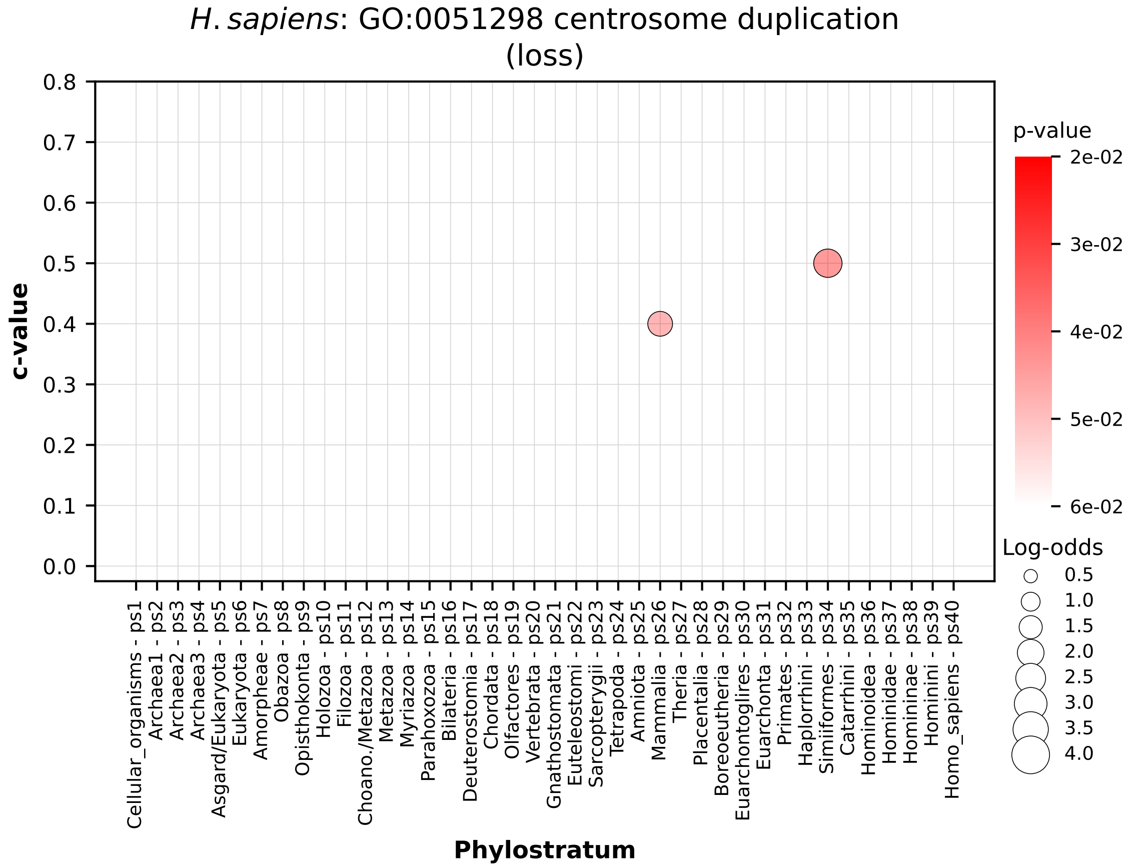

H. sapiens: GO:0051298 centrosome duplication (loss)
c-value
0.0
0.1
0.2
0.3
0.4
0.5
0.6
0.7
0.8
Phylostratum
Cellular_organisms - ps1
Archaea1 - ps2
Archaea2 - ps3
Archaea3 - ps4
Asgard/Eukaryota - ps5
Eukaryota - ps6
Amorpheae - ps7
Obazoa - ps8
Opisthokonta - ps9
Holozoa - ps10
Filozoa - ps11
Choano./Metazoa - ps12
Metazoa - ps13
Myriazoa - ps14
Parahoxozoa - ps15
Bilateria - ps16
Deuterostomia - ps17
Chordata - ps18
Olfactores - ps19
Vertebrata - ps20
Gnathostomata - ps21
Euteleostomi - ps22
Sarcopterygii - ps23
Tetrapoda - ps24
Amniota - ps25
Mammalia - ps26
Theria - ps27
Placentalia - ps28
Boreoeutheria - ps29
Euarchontoglires - ps30
Euarchonta - ps31
Primates - ps32
Haplorrhini - ps33
Simiiformes - ps34
Catarrhini - ps35
Hominoidea - ps36
Hominidae - ps37
Homininae - ps38
Hominini - ps39
Homo_sapiens - ps40
p-value
2e-02
3e-02
4e-02
5e-02
6e-02
Log-odds
0.5
1.0
1.5
2.0
2.5
3.0
3.5
4.0

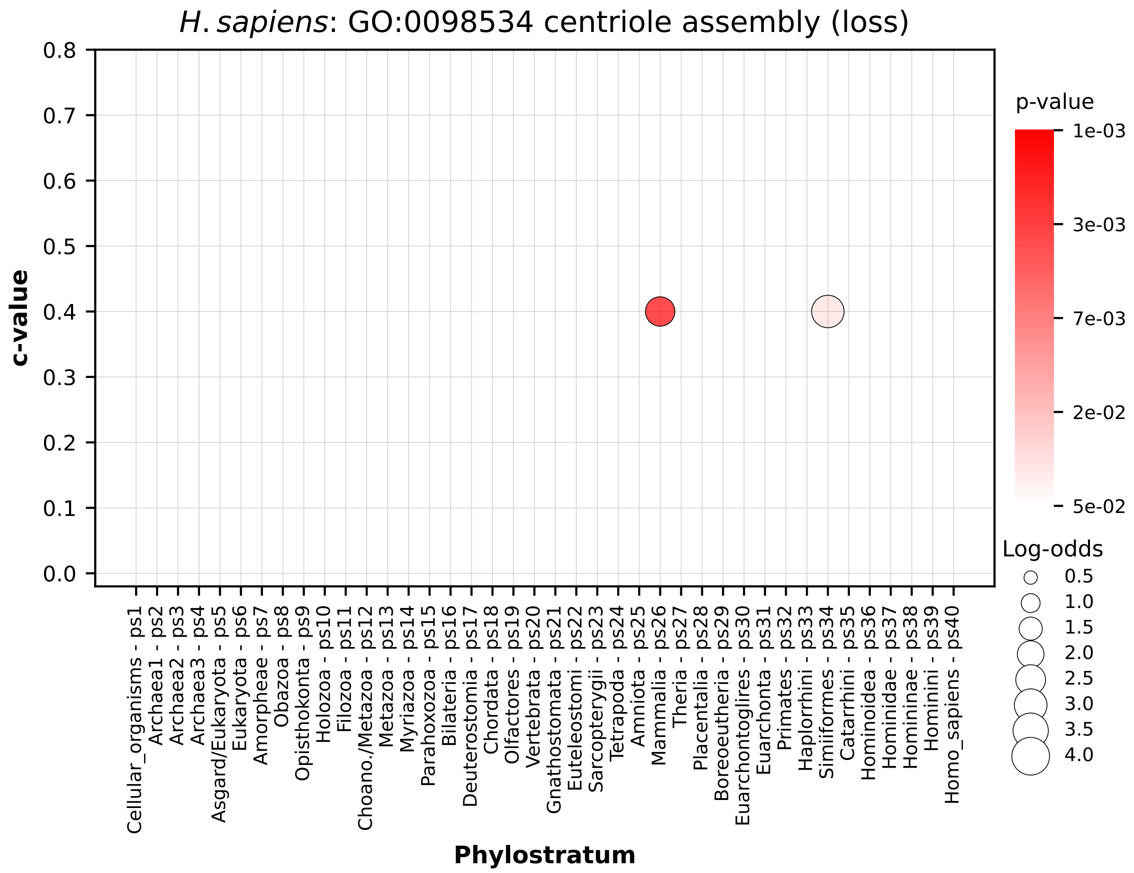
H. sapiens: GO:0098534 centriole assembly (loss)
c-value
0.0
0.1
0.2
0.3
0.4
0.5
0.6
0.7
0.8
Phylostratum
Cellular_organisms - ps1
Archaea1 - ps2
Archaea2 - ps3
Archaea3 - ps4
Asgard/Eukaryota - ps5
Eukaryota - ps6
Amorpheae - ps7
Obazoa - ps8
Opisthokonta - ps9
Holozoa - ps10
Filozoa - ps11
Choano./Metazoa - ps12
Metazoa - ps13
Myriazoa - ps14
Parahoxozoa - ps15
Bilateria - ps16
Deuterostomia - ps17
Chordata - ps18
Olfactores - ps19
Vertebrata - ps20
Gnathostomata - ps21
Euteleostomi - ps22
Sarcopterygii - ps23
Tetrapoda - ps24
Amniota - ps25
Mammalia - ps26
Theria - ps27
Placentalia - ps28
Boreoeutheria - ps29
Euarchontoglires - ps30
Euarchonta - ps31
Primates - ps32
Haplorrhini - ps33
Simiiformes - ps34
Catarrhini - ps35
Hominoidea - ps36
Hominidae - ps37
Homininae - ps38
Hominini - ps39
Homo_sapiens - ps40
p-value
1e-03
3e-03
7e-03
2e-02
5e-02
Log-odds
0.5
1.0
1.5
2.0
2.5
3.0
3.5
4.0

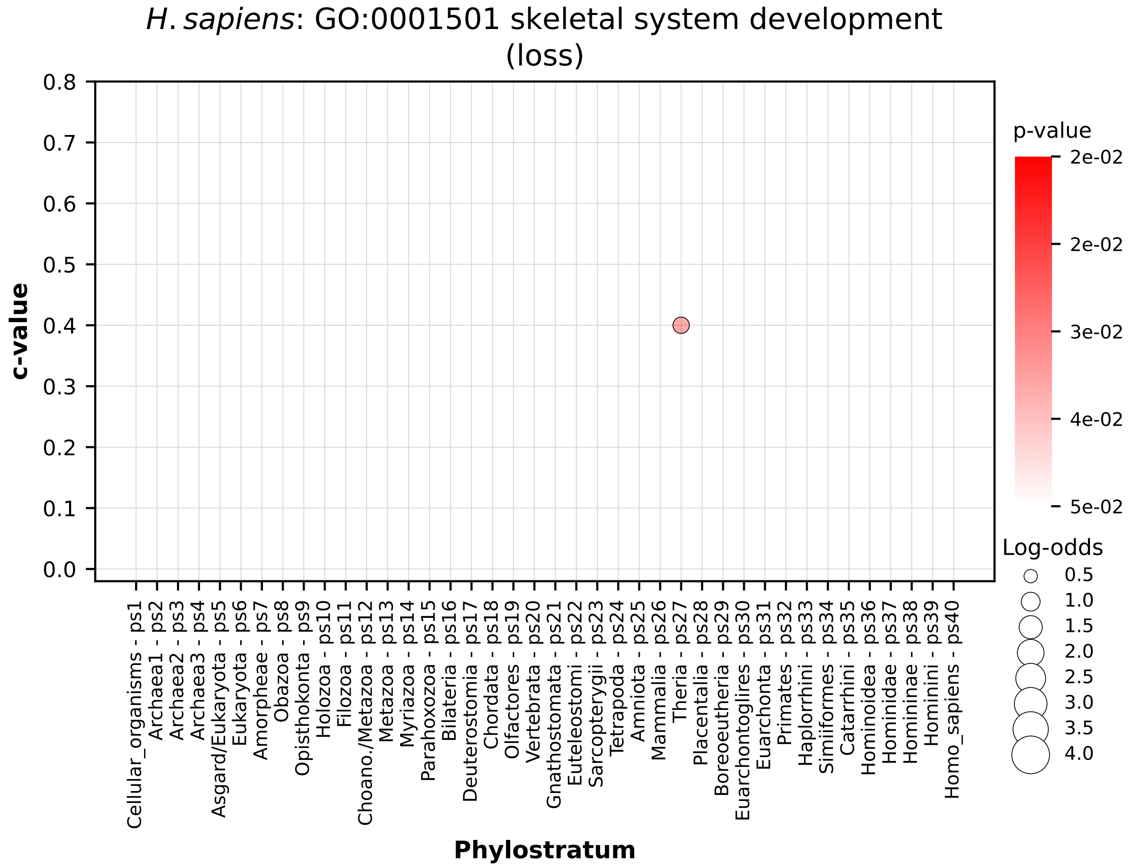

H. sapiens: GO:0001501 skeletal system development
(loss)
c-value
0.8
0.7
0.6
0.5
0.4
0.3
0.2
0.1
0.0
p-value
2e-02
2e-02
3e-02
4e-02
5e-02
Log-odds
0.5
1.0
1.5
2.0
2.5
3.0
3.5
4.0
Cellular_organisms - ps1
Archaea1 - ps2
Archaea2 - ps3
Archaea3 - ps4
Asgard/Eukaryota - ps5
Eukaryota - ps6
Amorpheae - ps7
Obazoa - ps8
Opisthokonta - ps9
Holozoa - ps10
Filozoa - ps11
Choano./Metazoa - ps12
Metazoa - ps13
Myriazoa - ps14
Parahoxozoa - ps15
Bilateria - ps16
Deuterostomia - ps17
Chordata - ps18
Olfactores - ps19
Vertebrata - ps20
Gnathostomata - ps21
Euteleostomi - ps22
Sarcopterygii - ps23
Tetrapoda - ps24
Amniota - ps25
Mammalia - ps26
Theria - ps27
Placentalia - ps28
Boreoeutheria - ps29
Euarchontoglires - ps30
Euarchonta - ps31
Primates - ps32
Haplorrhini - ps33
Simiiformes - ps34
Catarrhini - ps35
Hominoidea - ps36
Hominidae - ps37
Homininae - ps38
Hominini - ps39
Homo_sapiens - ps40
Phylostratum

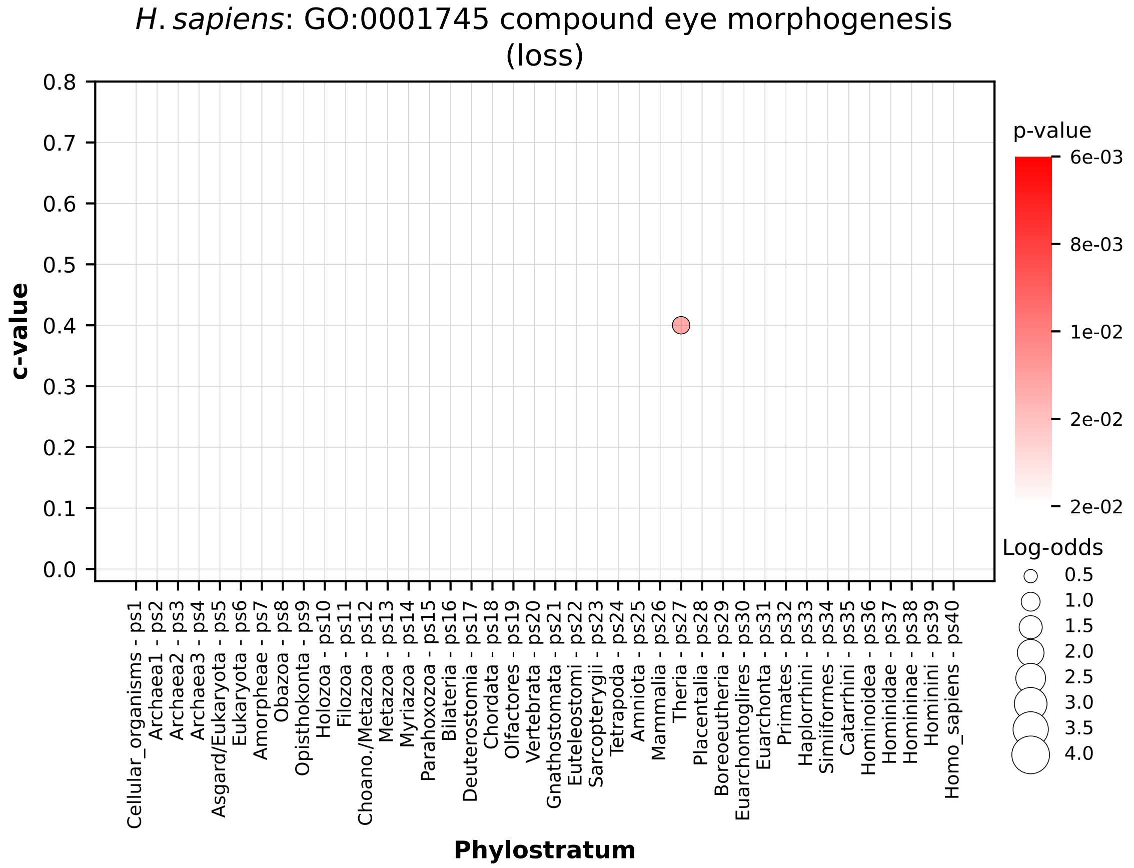
H. sapiens: GO:0001745 compound eye morphogenesis (loss)
c-value
0.8
0.7
0.6
0.5
0.4
0.3
0.2
0.1
0.0
Phylostratum
Cellular_organisms - ps1
Archaea1 - ps2
Archaea2 - ps3
Archaea3 - ps4
Asgard/Eukaryota - ps5
Eukaryota - ps6
Amorpheae - ps7
Obazoa - ps8
Opisthokonta - ps9
Holozoa - ps10
Filozoa - ps11
Choano./Metazoa - ps12
Metazoa - ps13
Myriazoa - ps14
Parahoxozoa - ps15
Bilateria - ps16
Deuterostomia - ps17
Chordata - ps18
Olfactores - ps19
Vertebrata - ps20
Gnathostomata - ps21
Euteleostomi - ps22
Sarcopterygii - ps23
Tetrapoda - ps24
Amniota - ps25
Mammalia - ps26
Theria - ps27
Placentalia - ps28
Boreoeutheria - ps29
Euarchontoglires - ps30
Euarchonta - ps31
Primates - ps32
Haplorrhini - ps33
Simiiformes - ps34
Catarrhini - ps35
Hominoidea - ps36
Hominidae - ps37
Homininae - ps38
Hominini - ps39
Homo_sapiens - ps40
p-value
6e-03
8e-03
1e-02
2e-02
2e-02
Log-odds
0.5
1.0
1.5
2.0
2.5
3.0
3.5
4.0

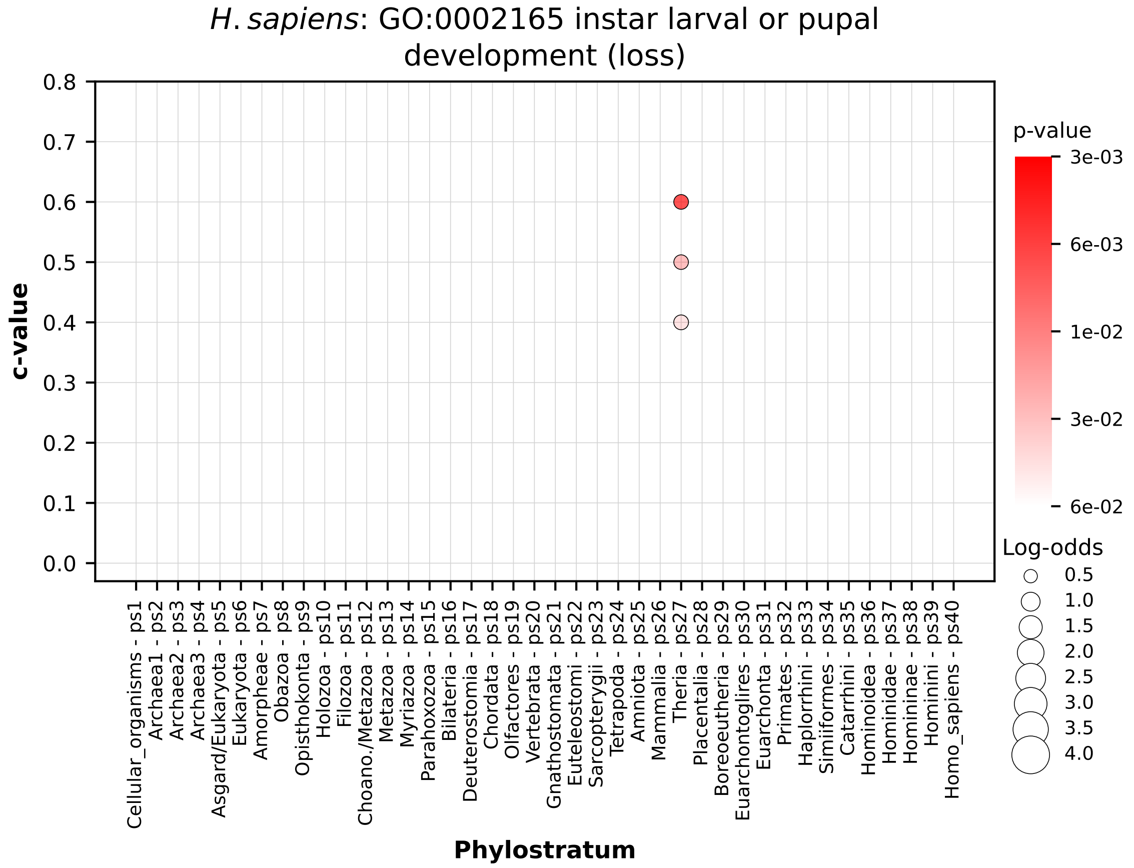

H. sapiens: GO:0002165 instar larval or pupal development (loss)
c-value
0.8
0.7
0.6
0.5
0.4
0.3
0.2
0.1
0.0
p-value
3e-03
6e-03
1e-02
3e-02
6e-02
Log-odds
0.5
1.0
1.5
2.0
2.5
3.0
3.5
4.0
Cellular_organisms - ps1
Archaea1 - ps2
Archaea2 - ps3
Archaea3 - ps4
Asgard/Eukaryota - ps5
Eukaryota - ps6
Amorpheae - ps7
Obazoa - ps8
Opisthokonta - ps9
Holozoa - ps10
Filozoa - ps11
Choano./Metazoa - ps12
Metazoa - ps13
Myriazoa - ps14
Parahoxozoa - ps15
Bilateria - ps16
Deuterostomia - ps17
Chordata - ps18
Olfactores - ps19
Vertebrata - ps20
Gnathostomata - ps21
Euteleostomi - ps22
Sarcopterygii - ps23
Tetrapoda - ps24
Amniota - ps25
Mammalia - ps26
Theria - ps27
Placentalia - ps28
Boreoeutheria - ps29
Euarchontoglires - ps30
Euarchonta - ps31
Primates - ps32
Haplorrhini - ps33
Simiiformes - ps34
Catarrhini - ps35
Hominoidea - ps36
Hominidae - ps37
Homininae - ps38
Hominini - ps39
Homo_sapiens - ps40
Phylostratum

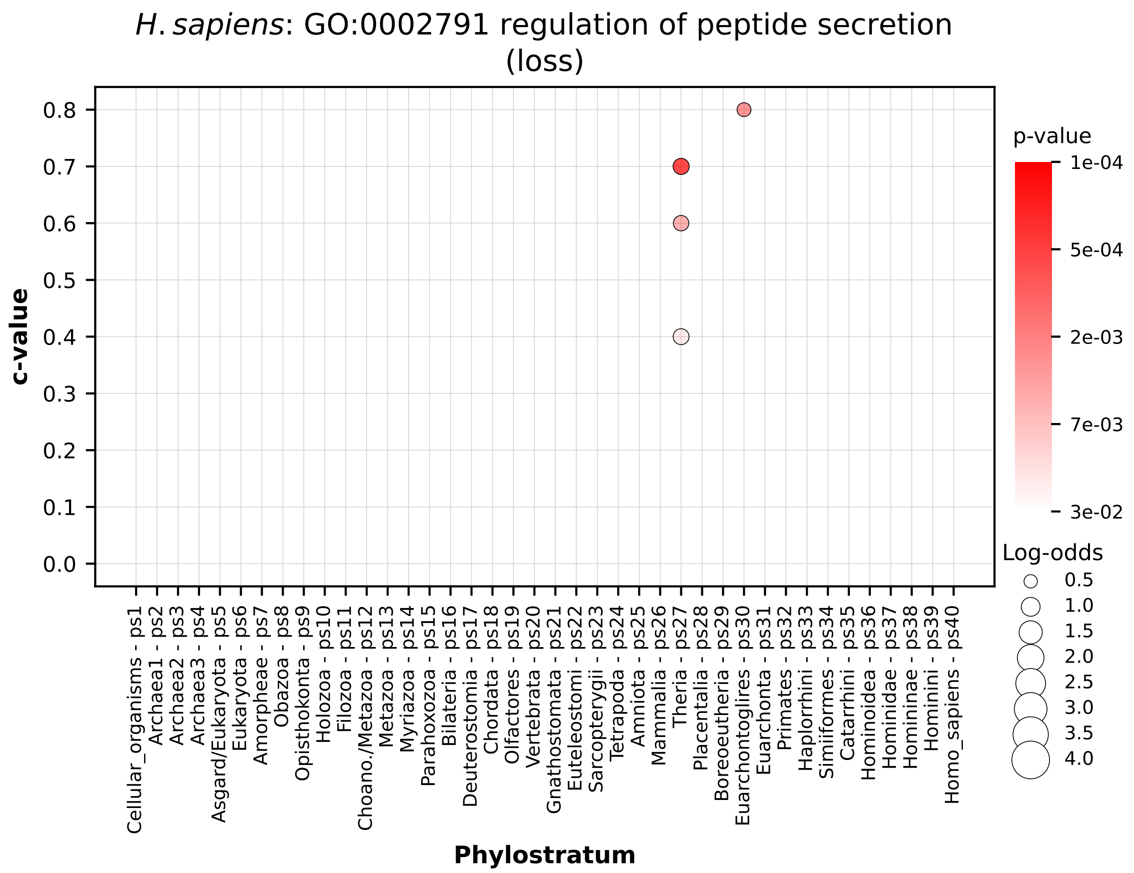

H. sapiens: GO:0002791 regulation of peptide secretion (loss)
c-value
0.0
0.1
0.2
0.3
0.4
0.5
0.6
0.7
0.8
p-value
1e-04
5e-04
2e-03
7e-03
3e-02
Log-odds
0.5
1.0
1.5
2.0
2.5
3.0
3.5
4.0
Phylostratum
Cellular_organisms - ps1
Archaea1 - ps2
Archaea2 - ps3
Archaea3 - ps4
Asgard/Eukaryota - ps5
Eukaryota - ps6
Amorpheae - ps7
Obazoa - ps8
Opisthokonta - ps9
Holozoa - ps10
Filozoa - ps11
Choano./Metazoa - ps12
Metazoa - ps13
Myriazoa - ps14
Parahoxozoa - ps15
Bilateria - ps16
Deuterostomia - ps17
Chordata - ps18
Olfactores - ps19
Vertebrata - ps20
Gnathostomata - ps21
Euteleostomi - ps22
Sarcopterygii - ps23
Tetrapoda - ps24
Amniota - ps25
Mammalia - ps26
Theria - ps27
Placentalia - ps28
Boreoeutheria - ps29
Euarchontoglires - ps30
Euarchonta - ps31
Primates - ps32
Haplorrhini - ps33
Simiiformes - ps34
Catarrhini - ps35
Hominoidea - ps36
Hominidae - ps37
Homininae - ps38
Hominini - ps39
Homo_sapiens - ps40

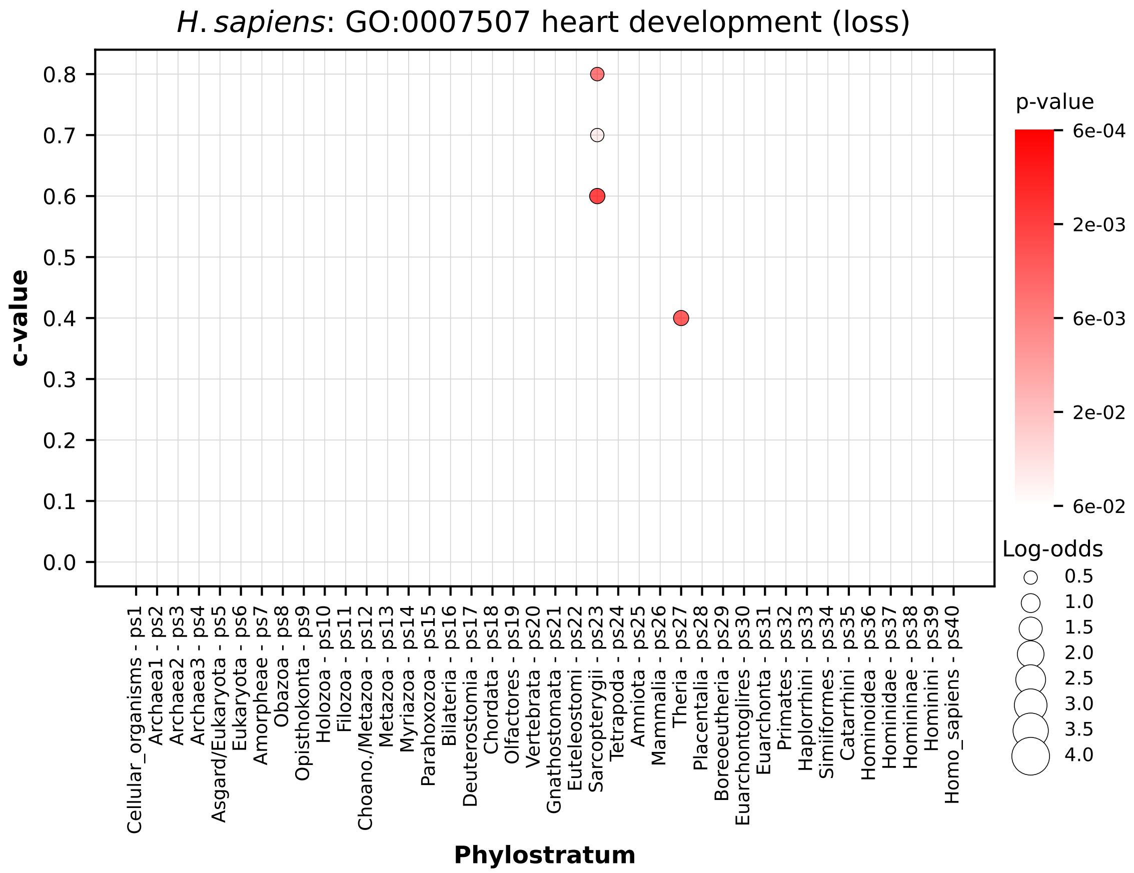

H. sapiens: GO:0007507 heart development (loss)
c-value
0.0
0.1
0.2
0.3
0.4
0.5
0.6
0.7
0.8
Phylostratum
Cellular_organisms - ps1
Archaea1 - ps2
Archaea2 - ps3
Archaea3 - ps4
Asgard/Eukaryota - ps5
Eukaryota - ps6
Amorpheae - ps7
Obazoa - ps8
Opisthokonta - ps9
Holozoa - ps10
Filozoa - ps11
Choano./Metazoa - ps12
Metazoa - ps13
Myriazoa - ps14
Parahoxozoa - ps15
Bilateria - ps16
Deuterostomia - ps17
Chordata - ps18
Olfactores - ps19
Vertebrata - ps20
Gnathostomata - ps21
Euteleostomi - ps22
Sarcopterygii - ps23
Tetrapoda - ps24
Amniota - ps25
Mammalia - ps26
Theria - ps27
Placentalia - ps28
Boreoeutheria - ps29
Euarchontoglires - ps30
Euarchonta - ps31
Primates - ps32
Haplorrhini - ps33
Simiiformes - ps34
Catarrhini - ps35
Hominoidea - ps36
Hominidae - ps37
Homininae - ps38
Hominini - ps39
Homo_sapiens - ps40
p-value
6e-04
2e-03
6e-03
2e-02
6e-02
Log-odds
0.5
1.0
1.5
2.0
2.5
3.0
3.5
4.0

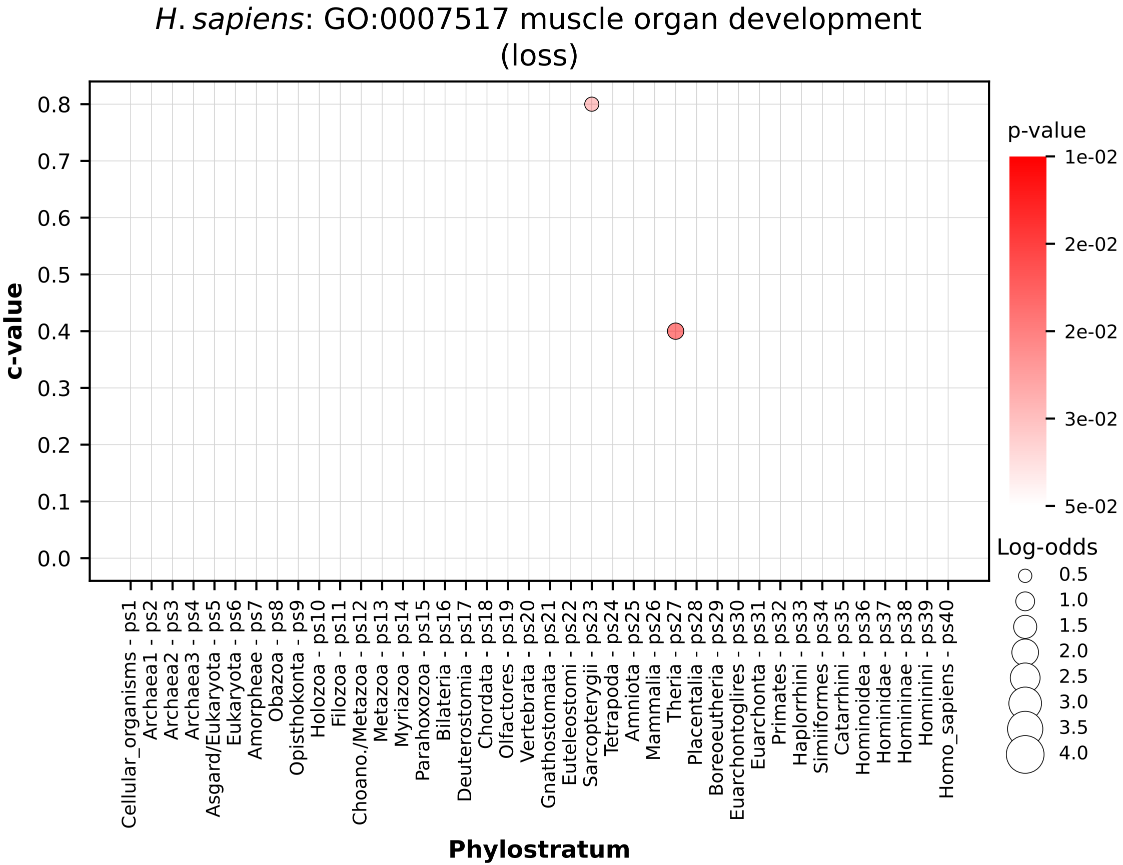

H. sapiens: GO:0007517 muscle organ development (loss)
c-value
0.8
0.7
0.6
0.5
0.4
0.3
0.2
0.1
0.0
p-value
1e-02
2e-02
2e-02
3e-02
5e-02
Log-odds
0.5
1.0
1.5
2.0
2.5
3.0
3.5
4.0
Cellular_organisms - ps1
Archaea1 - ps2
Archaea2 - ps3
Archaea3 - ps4
Asgard/Eukaryota - ps5
Eukaryota - ps6
Amorpheae - ps7
Obazoa - ps8
Opisthokonta - ps9
Holozoa - ps10
Filozoa - ps11
Choano./Metazoa - ps12
Metazoa - ps13
Myriazoa - ps14
Parahoxozoa - ps15
Bilateria - ps16
Deuterostomia - ps17
Chordata - ps18
Olfactores - ps19
Vertebrata - ps20
Gnathostomata - ps21
Euteleostomi - ps22
Sarcopterygii - ps23
Tetrapoda - ps24
Amniota - ps25
Mammalia - ps26
Theria - ps27
Placentalia - ps28
Boreoeutheria - ps29
Euarchontoglires - ps30
Euarchonta - ps31
Primates - ps32
Haplorrhini - ps33
Simiiformes - ps34
Catarrhini - ps35
Hominoidea - ps36
Hominidae - ps37
Homininae - ps38
Hominini - ps39
Homo_sapiens - ps40
Phylostratum

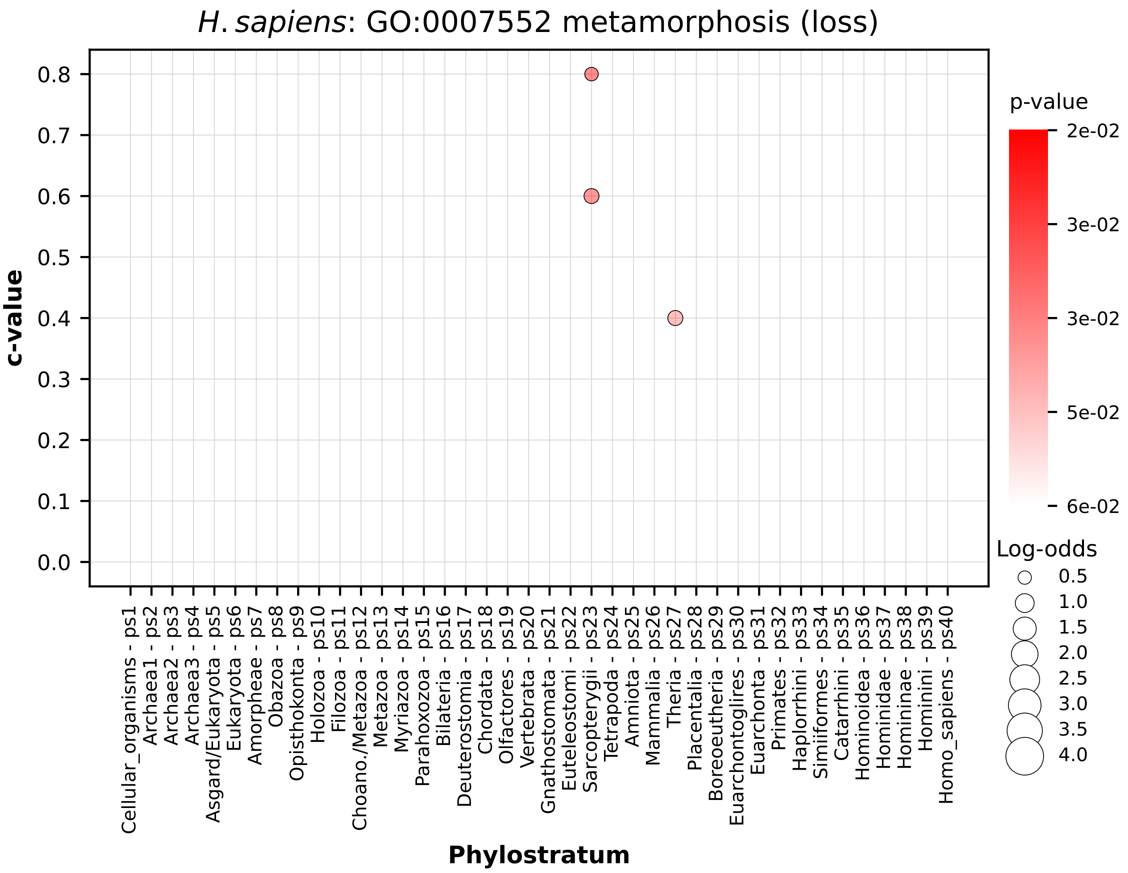

H. sapiens: GO:0007552 metamorphosis (loss)
c-value
0.8
0.7
0.6
0.5
0.4
0.3
0.2
0.1
0.0
p-value
2e-02
3e-02
3e-02
5e-02
6e-02
Log-odds
0.5
1.0
1.5
2.0
2.5
3.0
3.5
4.0
Cellular_organisms - ps1
Archaea1 - ps2
Archaea2 - ps3
Archaea3 - ps4
Asgard/Eukaryota - ps5
Eukaryota - ps6
Amorpheae - ps7
Obazoa - ps8
Opisthokonta - ps9
Holozoa - ps10
Filozoa - ps11
Choano./Metazoa - ps12
Metazoa - ps13
Myriazoa - ps14
Parahoxozoa - ps15
Bilateria - ps16
Deuterostomia - ps17
Chordata - ps18
Olfactores - ps19
Vertebrata - ps20
Gnathostomata - ps21
Euteleostomi - ps22
Sarcopterygii - ps23
Tetrapoda - ps24
Amniota - ps25
Mammalia - ps26
Theria - ps27
Placentalia - ps28
Boreoeutheria - ps29
Euarchontoglires - ps30
Euarchonta - ps31
Primates - ps32
Haplorrhini - ps33
Simiiformes - ps34
Catarrhini - ps35
Hominoidea - ps36
Hominidae - ps37
Homininae - ps38
Hominini - ps39
Homo_sapiens - ps40
Phylostratum

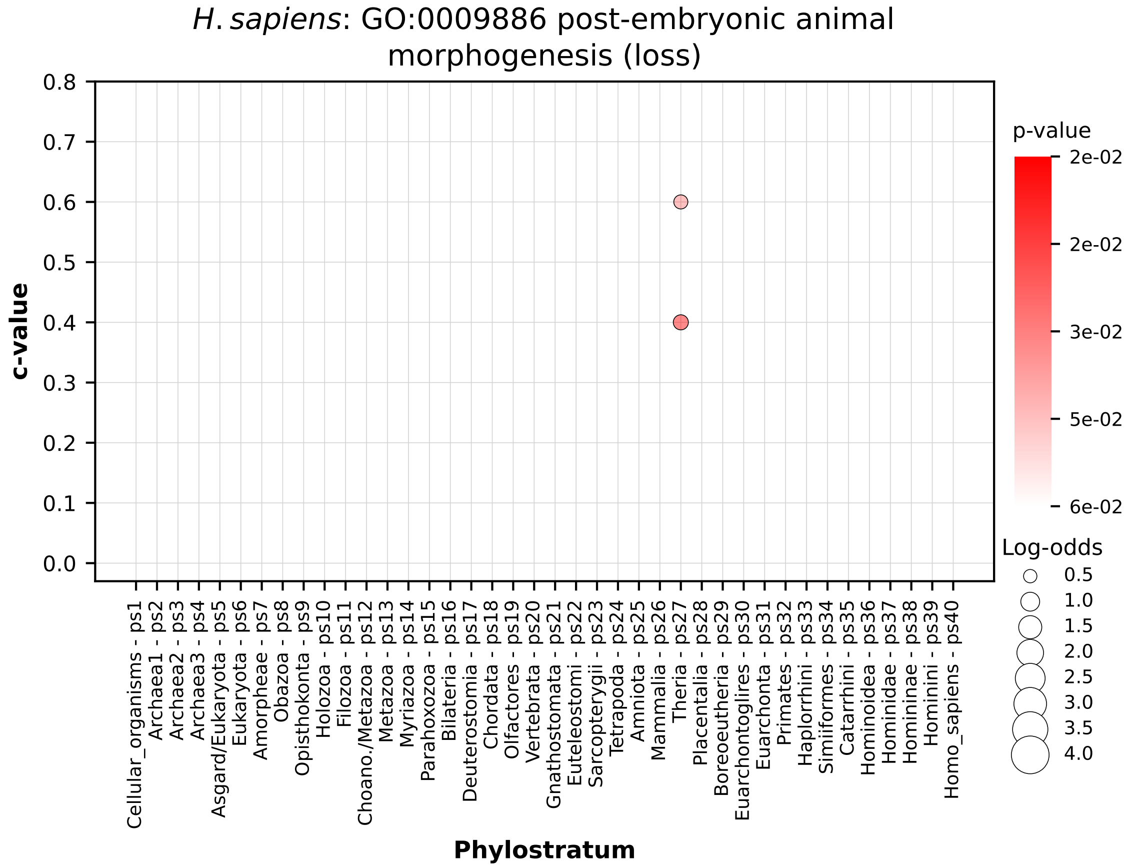

H. sapiens: GO:0009886 post-embryonic animal morphogenesis (loss)
c-value
Phylostratum
p-value
2e-02
2e-02
3e-02
5e-02
6e-02
Log-odds
0.5
1.0
1.5
2.0
2.5
3.0
3.5
4.0
Cellular_organisms - ps1
Archaea1 - ps2
Archaea2 - ps3
Archaea3 - ps4
Asgard/Eukaryota - ps5
Eukaryota - ps6
Amorpheae - ps7
Obazoa - ps8
Opisthokonta - ps9
Holozoa - ps10
Filozoa - ps11
Choano./Metazoa - ps12
Metazoa - ps13
Myriazoa - ps14
Parahoxozoa - ps15
Bilateria - ps16
Deuterostomia - ps17
Chordata - ps18
Olfactores - ps19
Vertebrata - ps20
Gnathostomata - ps21
Euteleostomi - ps22
Sarcopterygii - ps23
Tetrapoda - ps24
Amniota - ps25
Mammalia - ps26
Theria - ps27
Placentalia - ps28
Boreoeutheria - ps29
Euarchontoglires - ps30
Euarchonta - ps31
Primates - ps32
Haplorrhini - ps33
Simiiformes - ps34
Catarrhini - ps35
Hominoidea - ps36
Hominidae - ps37
Homininae - ps38
Hominini - ps39
Homo_sapiens - ps40

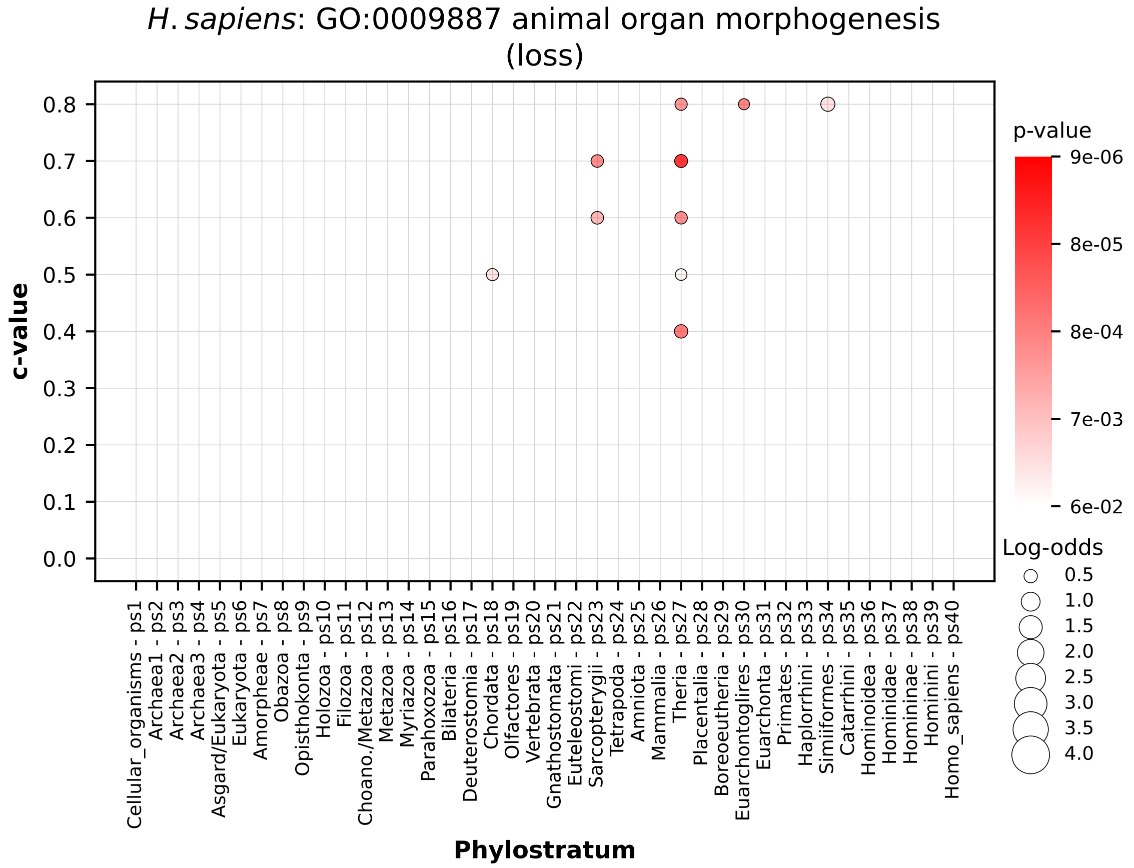
H. sapiens: GO:0009887 animal organ morphogenesis
(loss)
c-value
0.8
0.7
0.6
0.5
0.4
0.3
0.2
0.1
0.0
Phylostratum
Cellular_organisms - ps1
Archaea1 - ps2
Archaea2 - ps3
Archaea3 - ps4
Asgard/Eukaryota - ps5
Eukaryota - ps6
Amorpheae - ps7
Obazoa - ps8
Opisthokonta - ps9
Holozoa - ps10
Filozoa - ps11
Choano./Metazoa - ps12
Metazoa - ps13
Myriazoa - ps14
Parahoxozoa - ps15
Bilateria - ps16
Deuterostomia - ps17
Chordata - ps18
Olfactores - ps19
Vertebrata - ps20
Gnathostomata - ps21
Euteleostomi - ps22
Sarcopterygii - ps23
Tetrapoda - ps24
Amniota - ps25
Mammalia - ps26
Theria - ps27
Placentalia - ps28
Boreoeutheria - ps29
Euarchontoglires - ps30
Euarchonta - ps31
Primates - ps32
Haplorrhini - ps33
Simiiformes - ps34
Catarrhini - ps35
Hominoidea - ps36
Hominidae - ps37
Homininae - ps38
Hominini - ps39
Homo_sapiens - ps40
p-value
9e-06
8e-05
8e-04
7e-03
6e-02
Log-odds
0.5
1.0
1.5
2.0
2.5
3.0
3.5
4.0

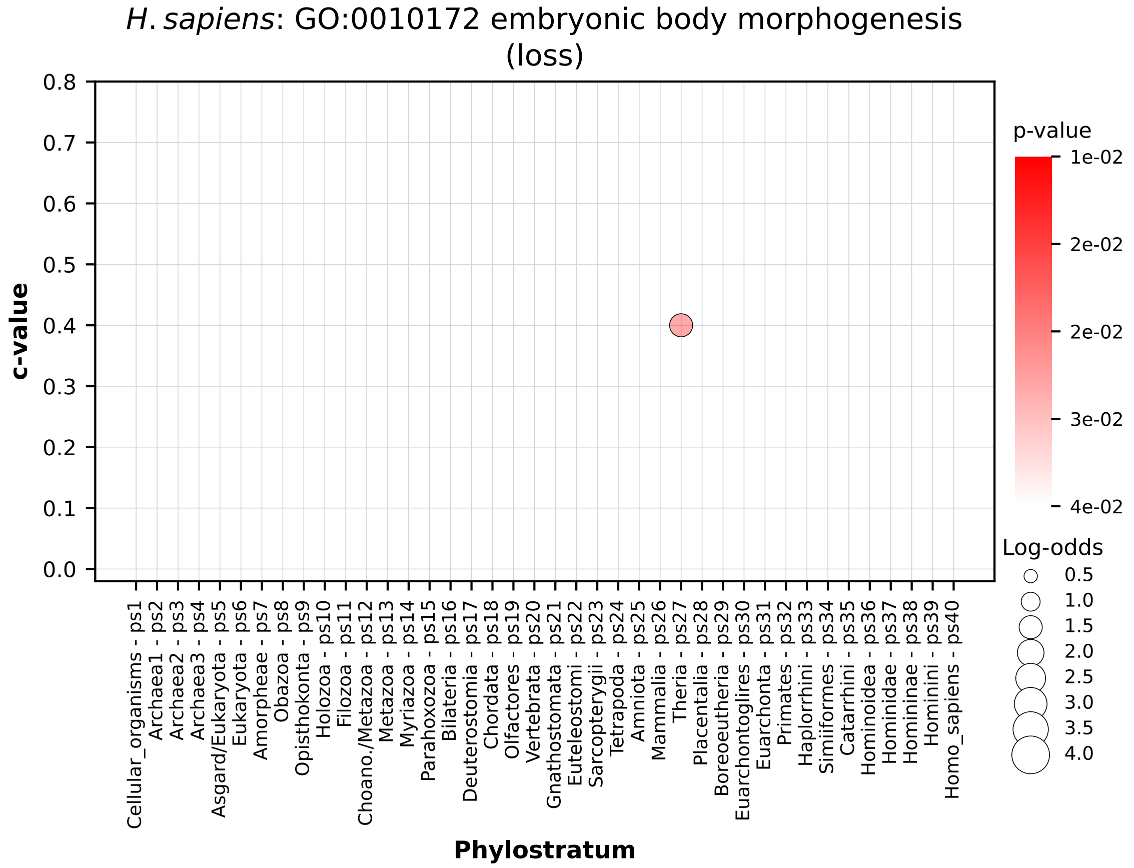

H. sapiens: GO:0010172 embryonic body morphogenesis (loss)
c-value
0.8
0.7
0.6
0.5
0.4
0.3
0.2
0.1
0.0
Phylostratum
Cellular_organisms - ps1
Archaea1 - ps2
Archaea2 - ps3
Archaea3 - ps4
Asgard/Eukaryota - ps5
Eukaryota - ps6
Amorpheae - ps7
Obazoa - ps8
Opisthokonta - ps9
Holozoa - ps10
Filozoa - ps11
Choano./Metazoa - ps12
Metazoa - ps13
Myriazoa - ps14
Parahoxozoa - ps15
Bilateria - ps16
Deuterostomia - ps17
Chordata - ps18
Olfactores - ps19
Vertebrata - ps20
Gnathostomata - ps21
Euteleostomi - ps22
Sarcopterygii - ps23
Tetrapoda - ps24
Amniota - ps25
Mammalia - ps26
Theria - ps27
Placentalia - ps28
Boreoeutheria - ps29
Euarchontoglires - ps30
Euarchonta - ps31
Primates - ps32
Haplorrhini - ps33
Simiiformes - ps34
Catarrhini - ps35
Hominoidea - ps36
Hominidae - ps37
Homininae - ps38
Hominini - ps39
Homo_sapiens - ps40
p-value
1e-02
2e-02
2e-02
3e-02
4e-02
Log-odds
0.5
1.0
1.5
2.0
2.5
3.0
3.5
4.0

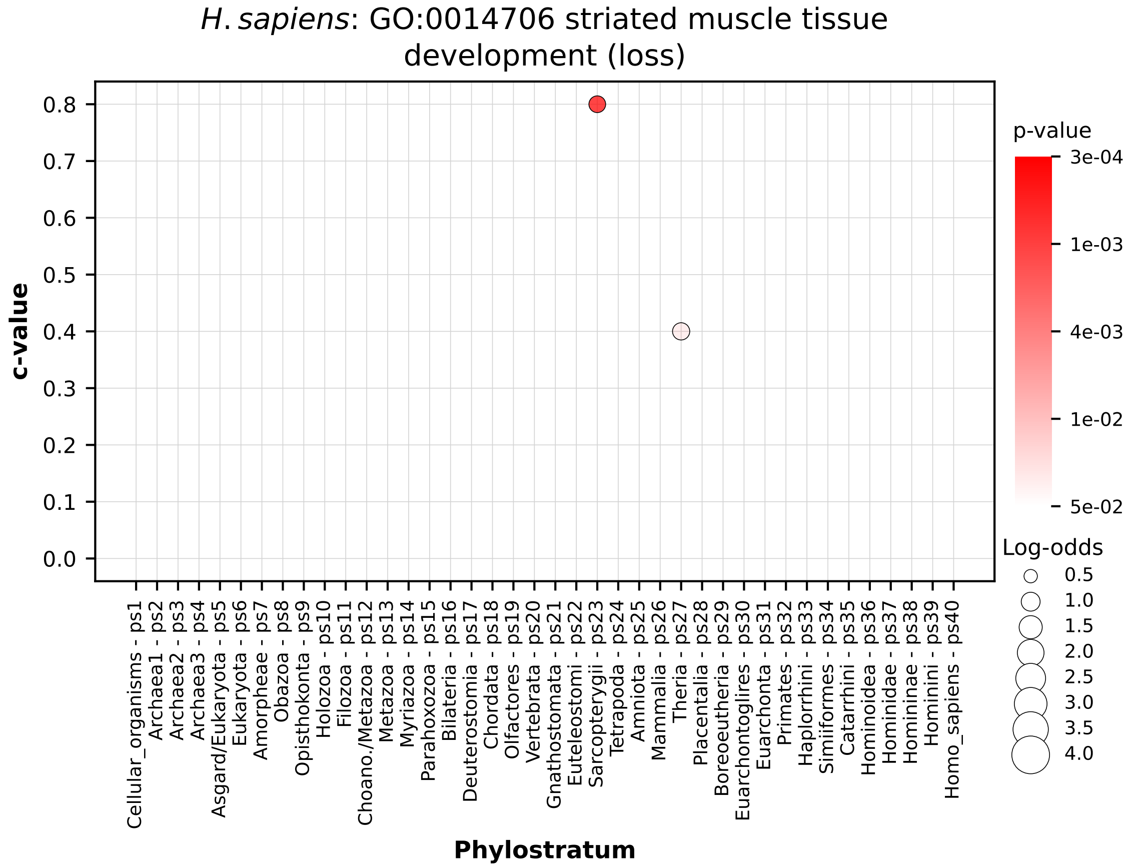

H. sapiens: GO:0014706 striated muscle tissue development (loss)
c-value
0.0
0.1
0.2
0.3
0.4
0.5
0.6
0.7
0.8
p-value
3e-04
1e-03
4e-03
1e-02
5e-02
Log-odds
0.5
1.0
1.5
2.0
2.5
3.0
3.5
4.0
Cellular_organisms - ps1
Archaea1 - ps2
Archaea2 - ps3
Archaea3 - ps4
Asgard/Eukaryota - ps5
Eukaryota - ps6
Amorpheae - ps7
Obazoa - ps8
Opisthokonta - ps9
Holozoa - ps10
Filozoa - ps11
Choano./Metazoa - ps12
Metazoa - ps13
Myriazoa - ps14
Parahoxozoa - ps15
Bilateria - ps16
Deuterostomia - ps17
Chordata - ps18
Olfactores - ps19
Vertebrata - ps20
Gnathostomata - ps21
Euteleostomi - ps22
Sarcopterygii - ps23
Tetrapoda - ps24
Amniota - ps25
Mammalia - ps26
Theria - ps27
Placentalia - ps28
Boreoeutheria - ps29
Euarchontoglires - ps30
Euarchonta - ps31
Primates - ps32
Haplorrhini - ps33
Simiiformes - ps34
Catarrhini - ps35
Hominoidea - ps36
Hominidae - ps37
Homininae - ps38
Hominini - ps39
Homo_sapiens - ps40
Phylostratum

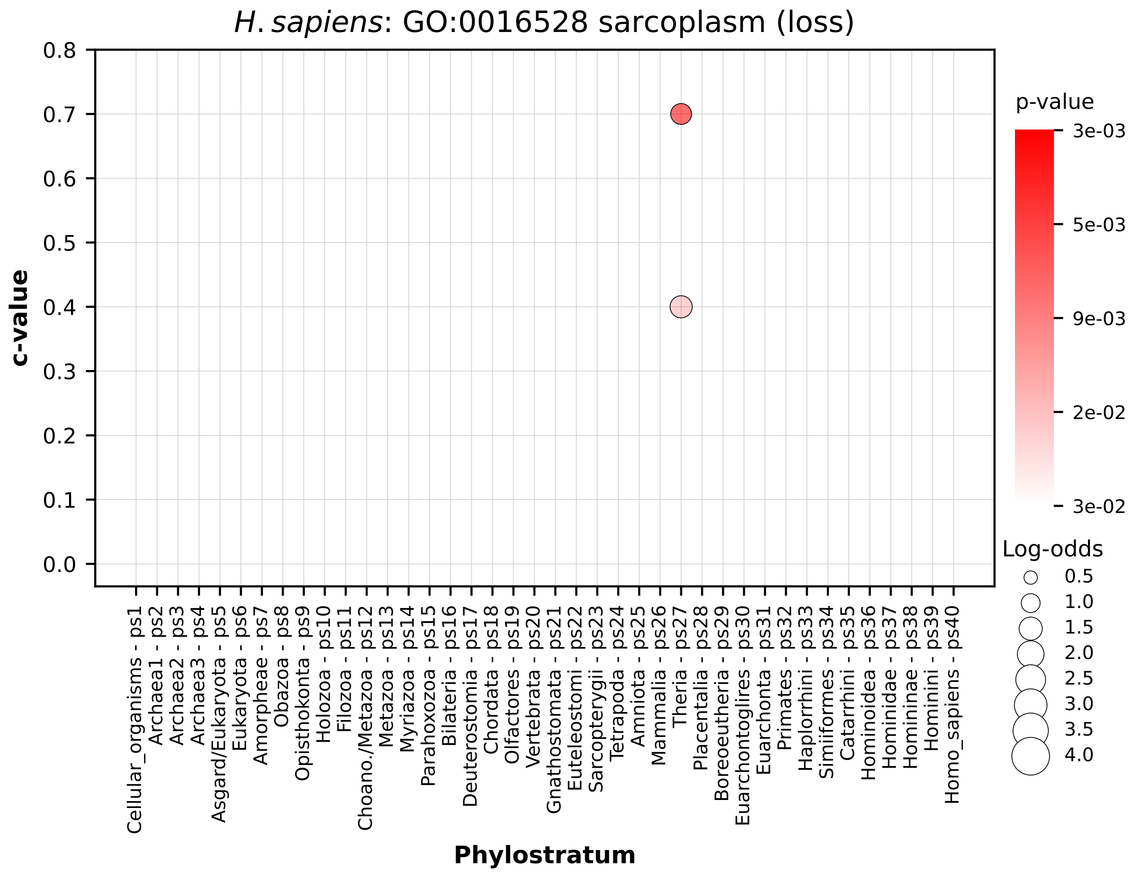
H. sapiens: GO:0016528 sarcoplasm (loss)
c-value
0.8
0.7
0.6
0.5
0.4
0.3
0.2
0.1
0.0
p-value
3e-03
5e-03
9e-03
2e-02
3e-02
Log-odds
0.5
1.0
1.5
2.0
2.5
3.0
3.5
4.0
Cellular_organisms - ps1
Archaea1 - ps2
Archaea2 - ps3
Archaea3 - ps4
Asgard/Eukaryota - ps5
Eukaryota - ps6
Amorpheae - ps7
Obazoa - ps8
Opisthokonta - ps9
Holozoa - ps10
Filozoa - ps11
Choano./Metazoa - ps12
Metazoa - ps13
Myriazoa - ps14
Parahoxozoa - ps15
Bilateria - ps16
Deuterostomia - ps17
Chordata - ps18
Olfactores - ps19
Vertebrata - ps20
Gnathostomata - ps21
Euteleostomi - ps22
Sarcopterygii - ps23
Tetrapoda - ps24
Amniota - ps25
Mammalia - ps26
Theria - ps27
Placentalia - ps28
Boreoeutheria - ps29
Euarchontoglires - ps30
Euarchonta - ps31
Primates - ps32
Haplorrhini - ps33
Simiiformes - ps34
Catarrhini - ps35
Hominoidea - ps36
Hominidae - ps37
Homininae - ps38
Hominini - ps39
Homo_sapiens - ps40
Phylostratum

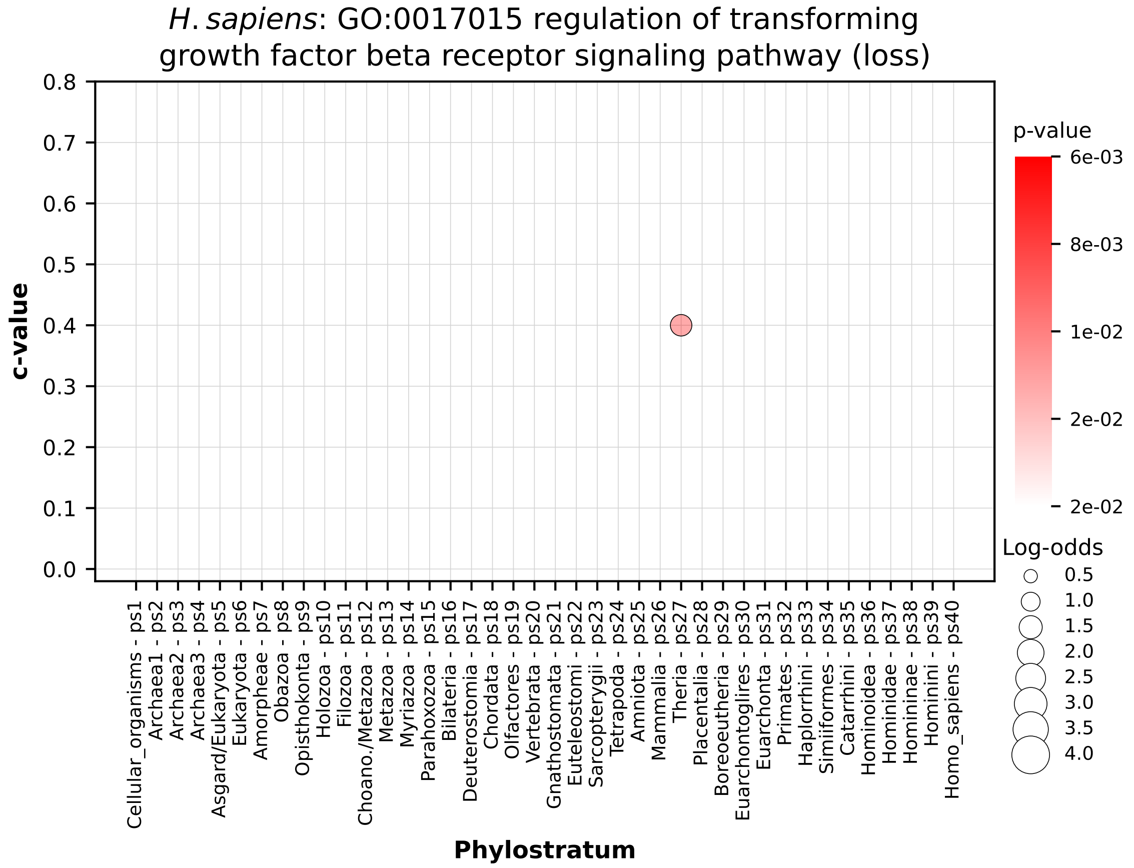

H. sapiens: GO:0017015 regulation of transforming growth factor beta receptor signaling pathway (loss)
c-value
0.0
0.1
0.2
0.3
0.4
0.5
0.6
0.7
0.8
Phylostratum
Cellular_organisms - ps1
Archaea1 - ps2
Archaea2 - ps3
Archaea3 - ps4
Asgard/Eukaryota - ps5
Eukaryota - ps6
Amorpheae - ps7
Obazoa - ps8
Opisthokonta - ps9
Holozoa - ps10
Filozoa - ps11
Choano./Metazoa - ps12
Metazoa - ps13
Myriazoa - ps14
Parahoxozoa - ps15
Bilateria - ps16
Deuterostomia - ps17
Chordata - ps18
Olfactores - ps19
Vertebrata - ps20
Gnathostomata - ps21
Euteleostomi - ps22
Sarcopterygii - ps23
Tetrapoda - ps24
Amniota - ps25
Mammalia - ps26
Theria - ps27
Placentalia - ps28
Boreoeutheria - ps29
Euarchontoglires - ps30
Euarchonta - ps31
Primates - ps32
Haplorrhini - ps33
Simiiformes - ps34
Catarrhini - ps35
Hominoidea - ps36
Hominidae - ps37
Homininae - ps38
Hominini - ps39
Homo_sapiens - ps40
p-value
6e-03
8e-03
1e-02
2e-02
2e-02
Log-odds
0.5
1.0
1.5
2.0
2.5
3.0
3.5
4.0

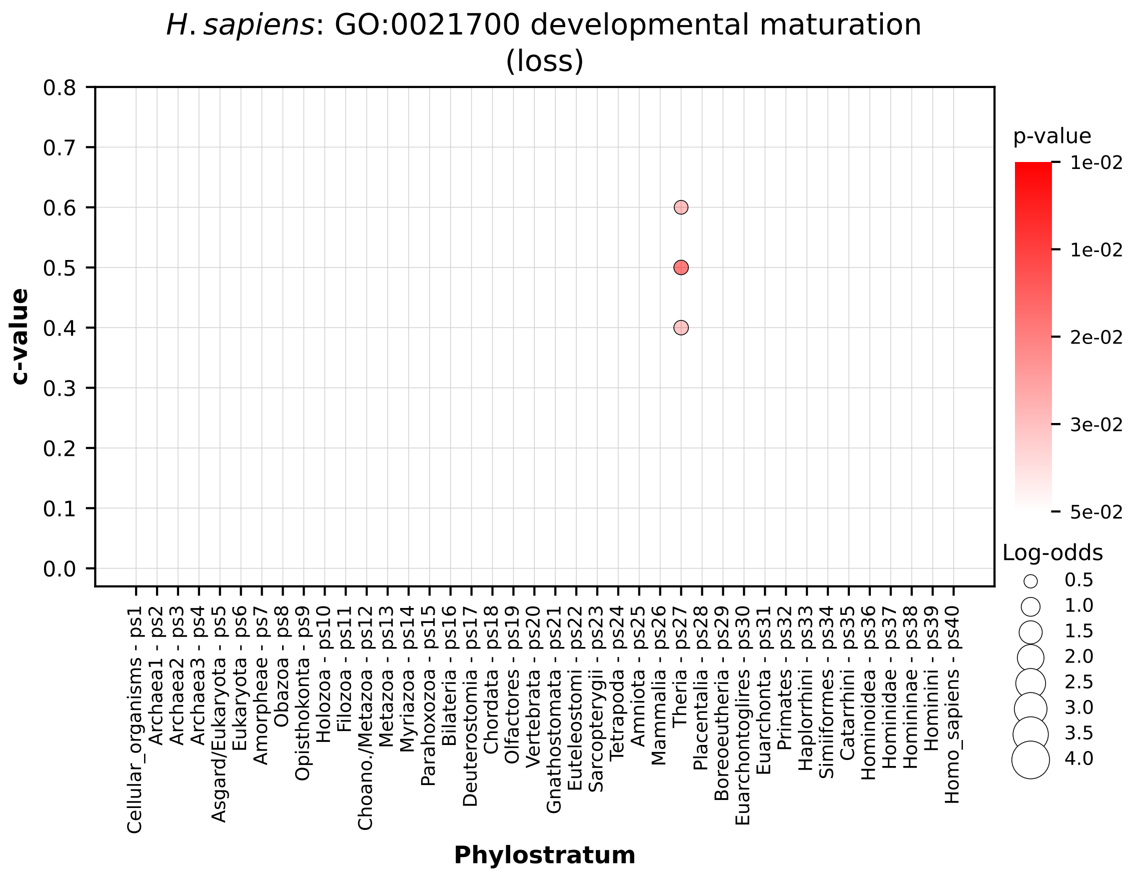

H. sapiens: GO:0021700 developmental maturation
(loss)
c-value
0.8
0.7
0.6
0.5
0.4
0.3
0.2
0.1
0.0
p-value
1e-02
1e-02
2e-02
3e-02
5e-02
Log-odds
0.5
1.0
1.5
2.0
2.5
3.0
3.5
4.0
Cellular_organisms - ps1
Archaea1 - ps2
Archaea2 - ps3
Archaea3 - ps4
Asgard/Eukaryota - ps5
Eukaryota - ps6
Amorpheae - ps7
Obazoa - ps8
Opisthokonta - ps9
Holozoa - ps10
Filozoa - ps11
Choano./Metazoa - ps12
Metazoa - ps13
Myriazoa - ps14
Parahoxozoa - ps15
Bilateria - ps16
Deuterostomia - ps17
Chordata - ps18
Olfactores - ps19
Vertebrata - ps20
Gnathostomata - ps21
Euteleostomi - ps22
Sarcopterygii - ps23
Tetrapoda - ps24
Amniota - ps25
Mammalia - ps26
Theria - ps27
Placentalia - ps28
Boreoeutheria - ps29
Euarchontoglires - ps30
Euarchonta - ps31
Primates - ps32
Haplorrhini - ps33
Similformes - ps34
Catarrhini - ps35
Hominoidea - ps36
Hominidae - ps37
Homininae - ps38
Hominini - ps39
Homo_sapiens - ps40
Phylostratum

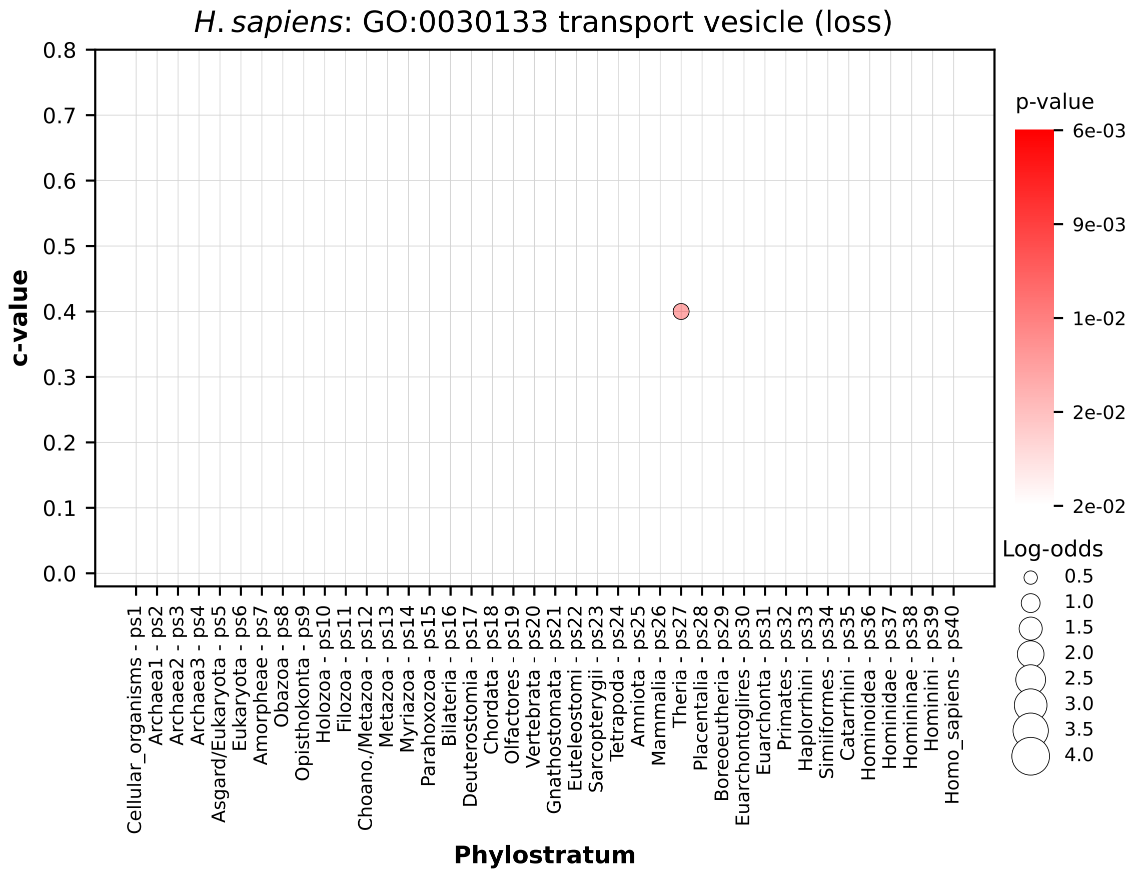
H. sapiens: GO:0030133 transport vesicle (loss)
c-value
0.8
0.7
0.6
0.5
0.4
0.3
0.2
0.1
0.0
Phylostratum
Cellular_organisms - ps1
Archaea1 - ps2
Archaea2 - ps3
Archaea3 - ps4
Asgard/Eukaryota - ps5
Eukaryota - ps6
Amorpheae - ps7
Obazoa - ps8
Opisthokonta - ps9
Holozoa - ps10
Filozoa - ps11
Choano./Metazoa - ps12
Metazoa - ps13
Myriazoa - ps14
Parahoxozoa - ps15
Bilateria - ps16
Deuterostomia - ps17
Chordata - ps18
Olfactores - ps19
Vertebrata - ps20
Gnathostomata - ps21
Euteleostomi - ps22
Sarcopterygii - ps23
Tetrapoda - ps24
Amniota - ps25
Mammalia - ps26
Theria - ps27
Placentalia - ps28
Boreoeutheria - ps29
Euarchontoglires - ps30
Euarchonta - ps31
Primates - ps32
Haplorrhini - ps33
Simiiformes - ps34
Catarrhini - ps35
Hominoidea - ps36
Hominidae - ps37
Homininae - ps38
Hominini - ps39
Homo_sapiens - ps40
p-value
6e-03
9e-03
1e-02
2e-02
2e-02
Log-odds
0.5
1.0
1.5
2.0
2.5
3.0
3.5
4.0

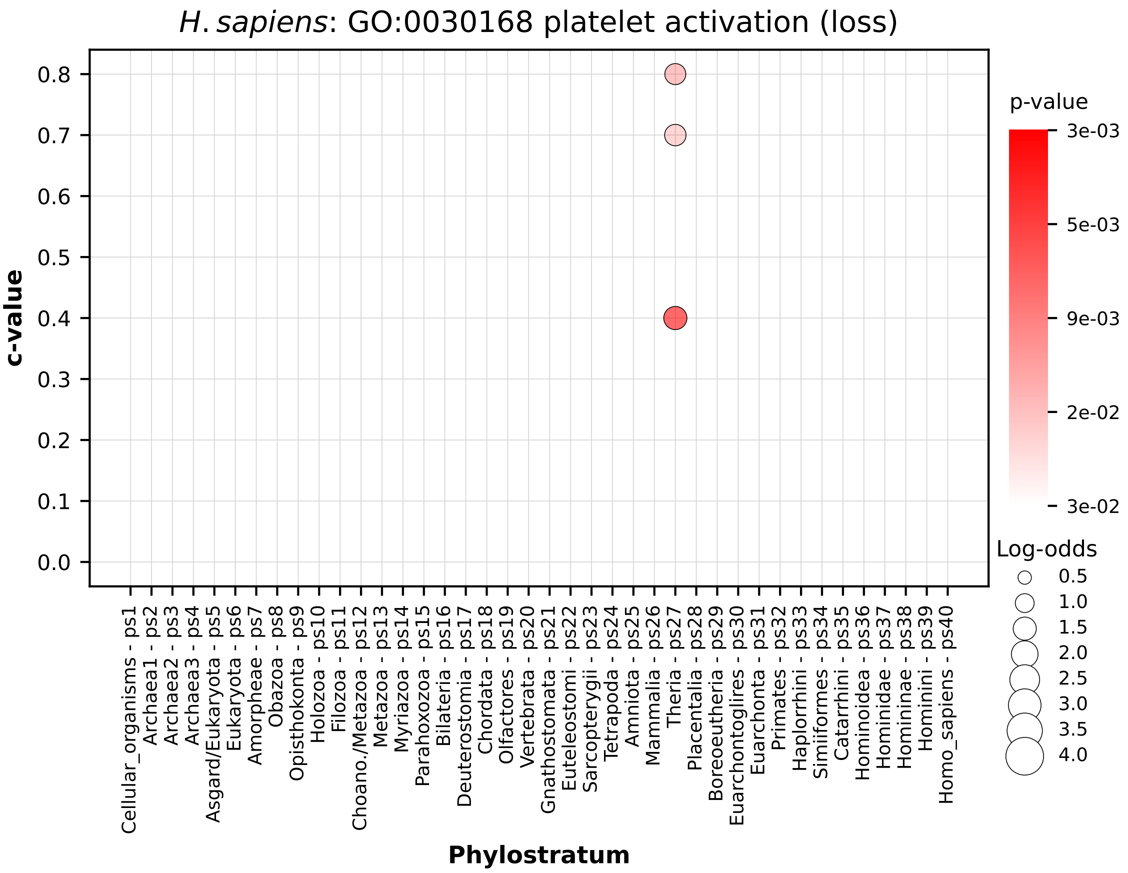

H. sapiens: GO:0030168 platelet activation (loss)
c-value
0.0
0.1
0.2
0.3
0.4
0.5
0.6
0.7
0.8
Phylostratum
Cellular_organisms - ps1
Archaea1 - ps2
Archaea2 - ps3
Archaea3 - ps4
Asgard/Eukaryota - ps5
Eukaryota - ps6
Amorpheae - ps7
Obazoa - ps8
Opisthokonta - ps9
Holozoa - ps10
Filozoa - ps11
Choano./Metazoa - ps12
Metazoa - ps13
Myriazoa - ps14
Parahoxozoa - ps15
Bilateria - ps16
Deuterostomia - ps17
Chordata - ps18
Olfactores - ps19
Vertebrata - ps20
Gnathostomata - ps21
Euteleostomi - ps22
Sarcopterygii - ps23
Tetrapoda - ps24
Amniota - ps25
Mammalia - ps26
Theria - ps27
Placentalia - ps28
Boreoeutheria - ps29
Euarchontoglires - ps30
Euarchonta - ps31
Primates - ps32
Haplorrhini - ps33
Simiiformes - ps34
Catarrhini - ps35
Hominoidea - ps36
Hominidae - ps37
Homininae - ps38
Hominini - ps39
Homo_sapiens - ps40
p-value
3e-03
5e-03
9e-03
2e-02
3e-02
Log-odds
0.5
1.0
1.5
2.0
2.5
3.0
3.5
4.0

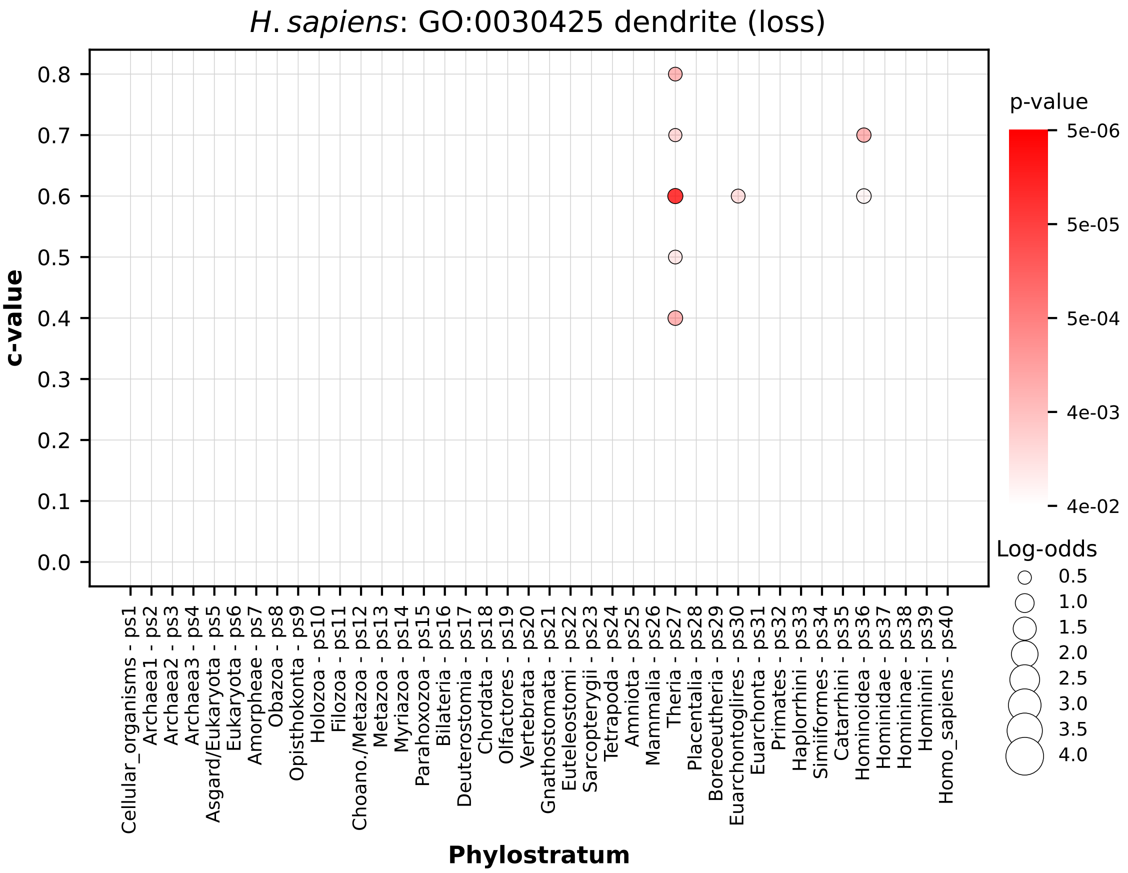
H. sapiens: GO:0030425 dendrite (loss)
c-value
0.8
0.7
0.6
0.5
0.4
0.3
0.2
0.1
0.0
p-value
5e-06
5e-05
5e-04
4e-03
4e-02
Log-odds
0.5
1.0
1.5
2.0
2.5
3.0
3.5
4.0
Cellular_organisms - ps1
Archaea1 - ps2
Archaea2 - ps3
Archaea3 - ps4
Asgard/Eukaryota - ps5
Eukaryota - ps6
Amorpheae - ps7
Obazoa - ps8
Opisthokonta - ps9
Holozoa - ps10
Filozoa - ps11
Choano./Metazoa - ps12
Metazoa - ps13
Myriazoa - ps14
Parahoxozoa - ps15
Bilateria - ps16
Deuterostomia - ps17
Chordata - ps18
Olfactores - ps19
Vertebrata - ps20
Gnathostomata - ps21
Euteleostomi - ps22
Sarcopterygii - ps23
Tetrapoda - ps24
Amniota - ps25
Mammalia - ps26
Theria - ps27
Placentalia - ps28
Boreoeutheria - ps29
Euarchontoglires - ps30
Euarchonta - ps31
Primates - ps32
Haplorrhini - ps33
Simiiformes - ps34
Catarrhini - ps35
Hominoidea - ps36
Hominidae - ps37
Homininae - ps38
Hominini - ps39
Homo_sapiens - ps40
Phylostratum

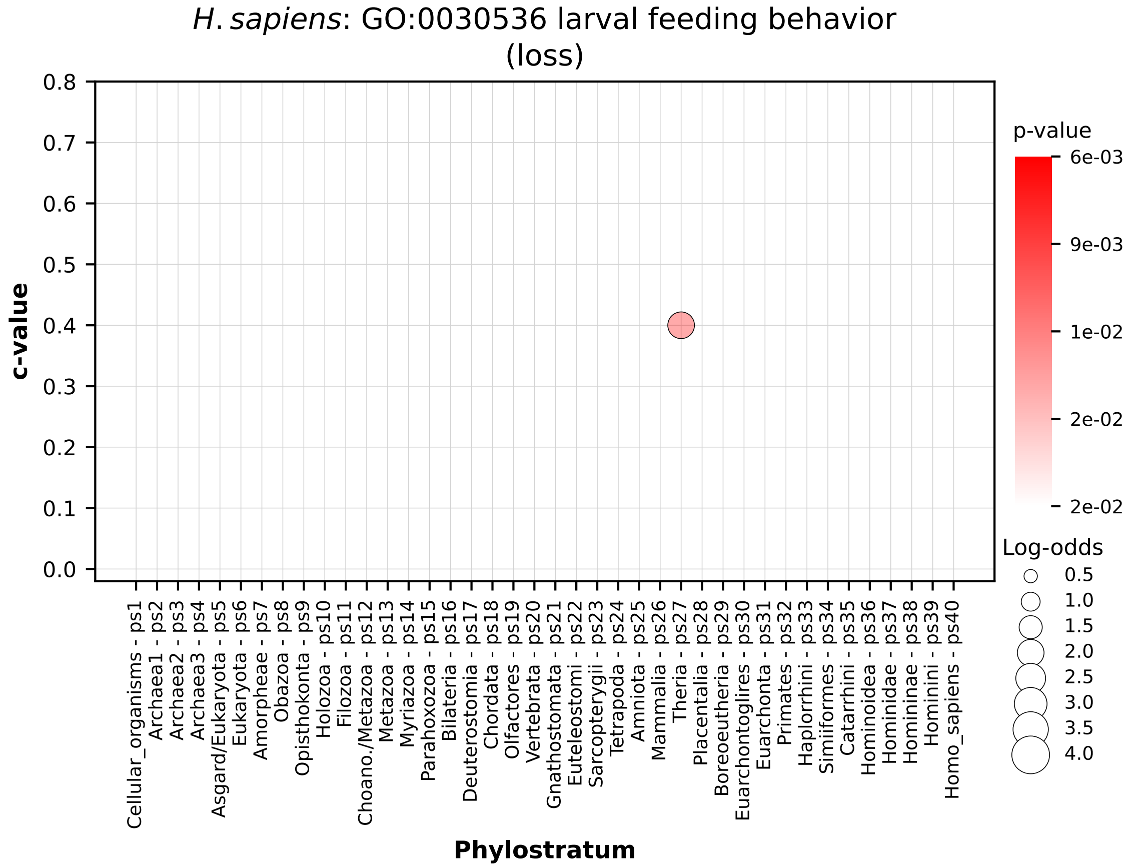

H. sapiens: GO:0030536 larval feeding behavior
(loss)
c-value
0.0
0.1
0.2
0.3
0.4
0.5
0.6
0.7
0.8
Phylostratum
Cellular_organisms - ps1
Archaea1 - ps2
Archaea2 - ps3
Archaea3 - ps4
Asgard/Eukaryota - ps5
Eukaryota - ps6
Amorpheae - ps7
Obazoa - ps8
Opisthokonta - ps9
Holozoa - ps10
Filozoa - ps11
Choano./Metazoa - ps12
Metazoa - ps13
Myriazoa - ps14
Parahoxozoa - ps15
Bilateria - ps16
Deuterostomia - ps17
Chordata - ps18
Olfactores - ps19
Vertebrata - ps20
Gnathostomata - ps21
Euteleostomi - ps22
Sarcopterygii - ps23
Tetrapoda - ps24
Amniota - ps25
Mammalia - ps26
Theria - ps27
Placentalia - ps28
Boreoeutheria - ps29
Euarchontoglires - ps30
Euarchonta - ps31
Primates - ps32
Haplorrhini - ps33
Simiiformes - ps34
Catarrhini - ps35
Hominoidea - ps36
Hominidae - ps37
Homininae - ps38
Hominini - ps39
Homo_sapiens - ps40
p-value
6e-03
9e-03
1e-02
2e-02
2e-02
Log-odds
0.5
1.0
1.5
2.0
2.5
3.0
3.5
4.0

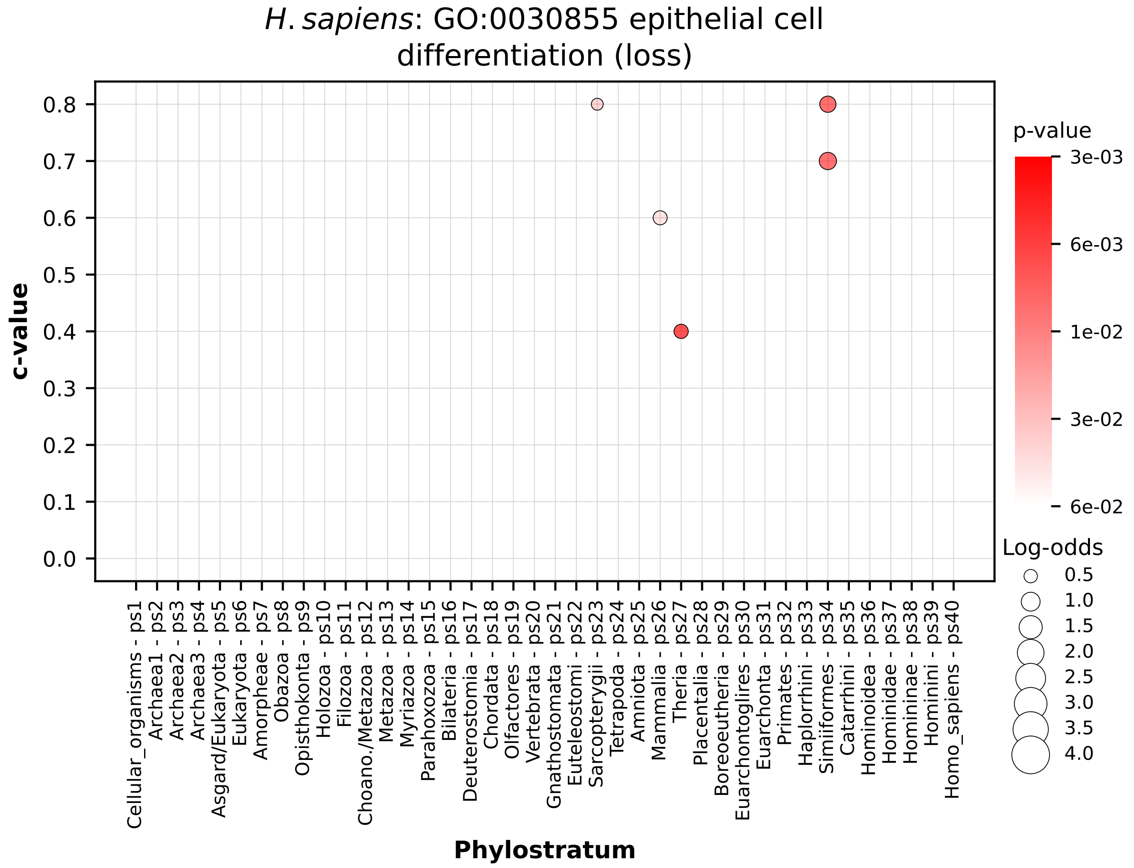

H. sapiens: GO:0030855 epithelial cell differentiation (loss)
c-value
0.8
0.7
0.6
0.5
0.4
0.3
0.2
0.1
0.0
p-value
3e-03
6e-03
1e-02
3e-02
6e-02
Log-odds
0.5
1.0
1.5
2.0
2.5
3.0
3.5
4.0
Cellular_organisms - ps1
Archaea1 - ps2
Archaea2 - ps3
Archaea3 - ps4
Asgard/Eukaryota - ps5
Eukaryota - ps6
Amorpheae - ps7
Obazoa - ps8
Opisthokonta - ps9
Holozoa - ps10
Filozoa - ps11
Choano./Metazoa - ps12
Metazoa - ps13
Myriazoa - ps14
Parahoxozoa - ps15
Bilateria - ps16
Deuterostomia - ps17
Chordata - ps18
Olfactores - ps19
Vertebrata - ps20
Gnathostomata - ps21
Euteleostomi - ps22
Sarcopterygii - ps23
Tetrapoda - ps24
Amniota - ps25
Mammalia - ps26
Theria - ps27
Placentalia - ps28
Boreoeutheria - ps29
Euarchontoglires - ps30
Euarchonta - ps31
Primates - ps32
Haplorrhini - ps33
Simiiformes - ps34
Catarrhini - ps35
Hominoidea - ps36
Hominidae - ps37
Homininae - ps38
Hominini - ps39
Homo_sapiens - ps40
Phylostratum

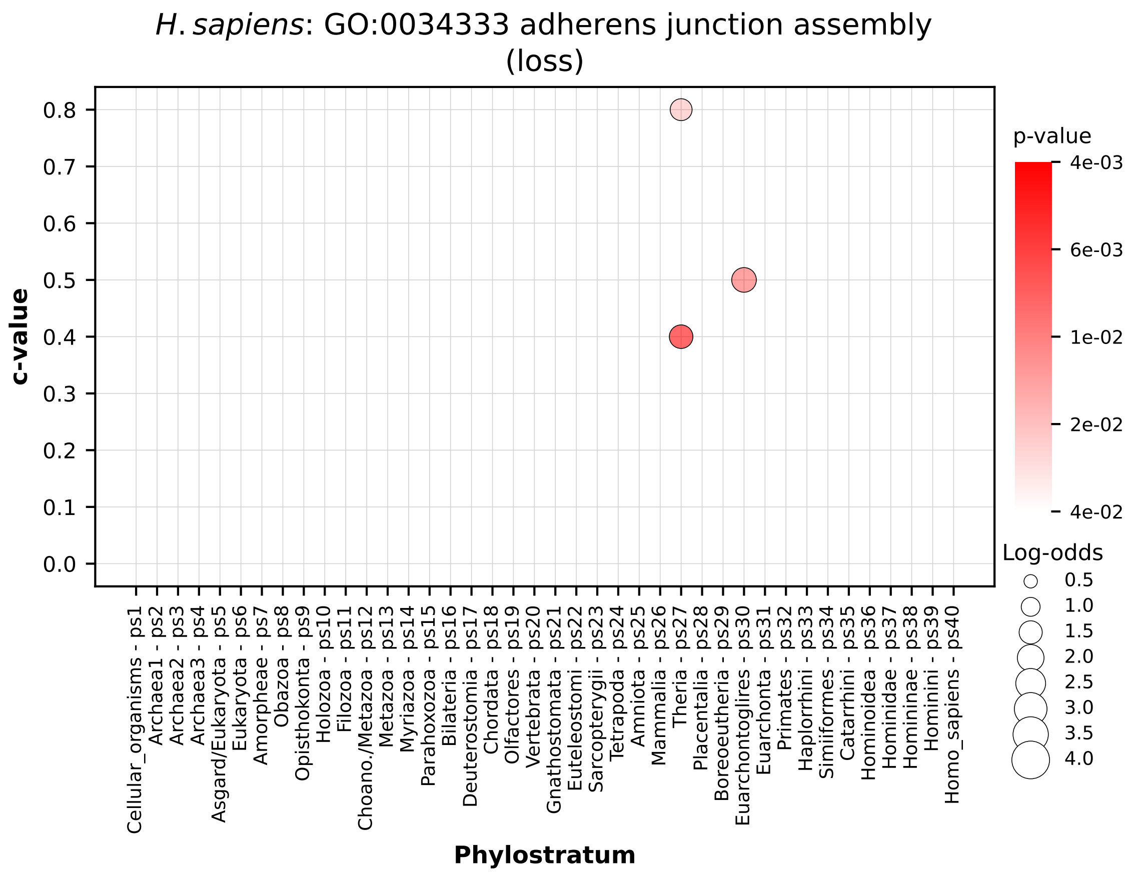

H. sapiens: GO:0034333 adherens junction assembly (loss)
c-value
0.0
0.1
0.2
0.3
0.4
0.5
0.6
0.7
0.8
Phylostratum
Cellular_organisms - ps1
Archaea1 - ps2
Archaea2 - ps3
Archaea3 - ps4
Asgard/Eukaryota - ps5
Eukaryota - ps6
Amorpheae - ps7
Obazoa - ps8
Opisthokonta - ps9
Holozoa - ps10
Filozoa - ps11
Choano./Metazoa - ps12
Metazoa - ps13
Myriazoa - ps14
Parahoxozoa - ps15
Bilateria - ps16
Deuterostomia - ps17
Chordata - ps18
Olfactores - ps19
Vertebrata - ps20
Gnathostomata - ps21
Euteleostomi - ps22
Sarcopterygii - ps23
Tetrapoda - ps24
Amniota - ps25
Mammalia - ps26
Theria - ps27
Placentalia - ps28
Boreoeutheria - ps29
Euarchontoglires - ps30
Euarchonta - ps31
Primates - ps32
Haplorrhini - ps33
Simiiformes - ps34
Catarrhini - ps35
Hominoidea - ps36
Hominidae - ps37
Homininae - ps38
Hominini - ps39
Homo_sapiens - ps40
p-value
4e-03
6e-03
1e-02
2e-02
4e-02
Log-odds
0.5
1.0
1.5
2.0
2.5
3.0
3.5
4.0

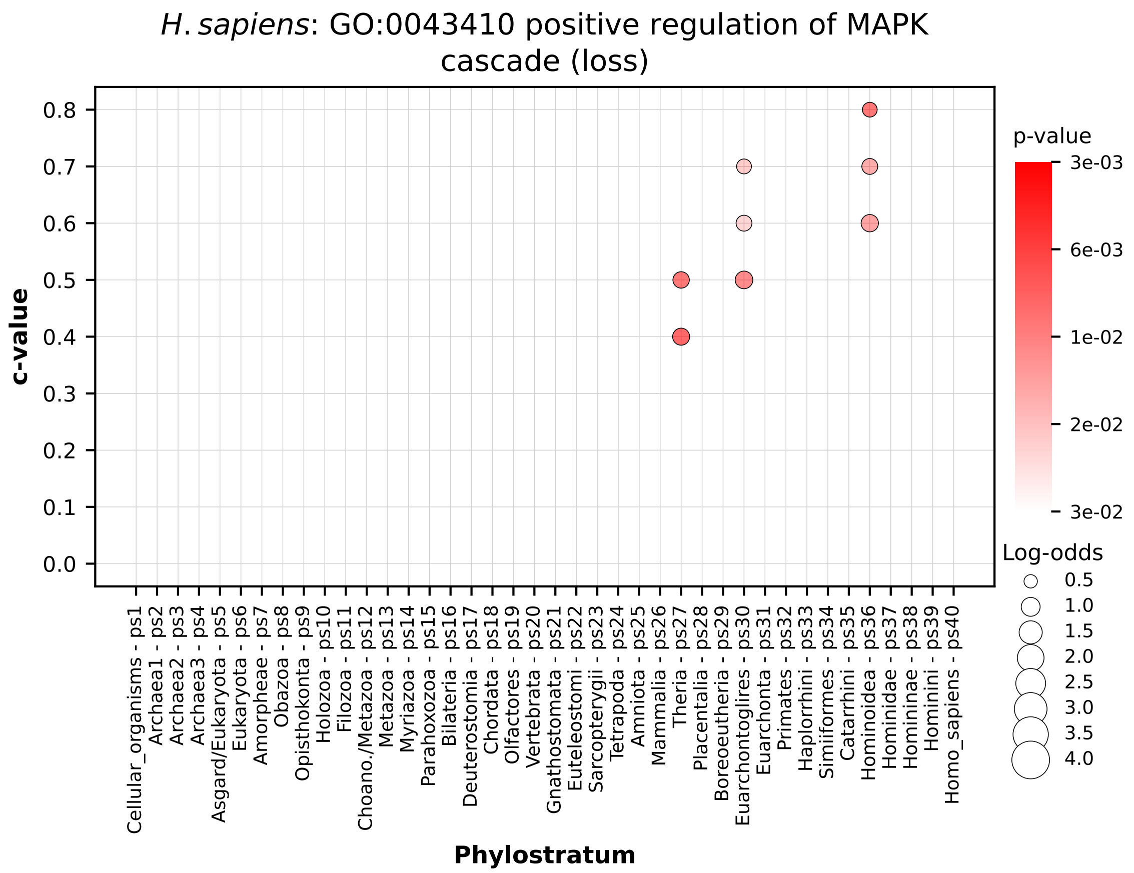

H. sapiens: GO:0043410 positive regulation of MAPK cascade (loss)
c-value
0.0
0.1
0.2
0.3
0.4
0.5
0.6
0.7
0.8
Phylostratum
Cellular_organisms - ps1
Archaea1 - ps2
Archaea2 - ps3
Archaea3 - ps4
Asgard/Eukaryota - ps5
Eukaryota - ps6
Amorpheae - ps7
Obazoa - ps8
Opisthokonta - ps9
Holozoa - ps10
Filozoa - ps11
Choano./Metazoa - ps12
Metazoa - ps13
Myriazoa - ps14
Parahoxozoa - ps15
Bilateria - ps16
Deuterostomia - ps17
Chordata - ps18
Olfactores - ps19
Vertebrata - ps20
Gnathostomata - ps21
Euteleostomi - ps22
Sarcopterygii - ps23
Tetrapoda - ps24
Amniota - ps25
Mammalia - ps26
Theria - ps27
Placentalia - ps28
Boreoeutheria - ps29
Euarchontoglires - ps30
Euarchonta - ps31
Primates - ps32
Haplorrhini - ps33
Simiiformes - ps34
Catarrhini - ps35
Hominoidea - ps36
Hominidae - ps37
Homininae - ps38
Hominini - ps39
Homo_sapiens - ps40
p-value
3e-03
6e-03
1e-02
2e-02
3e-02
Log-odds
0.5
1.0
1.5
2.0
2.5
3.0
3.5
4.0

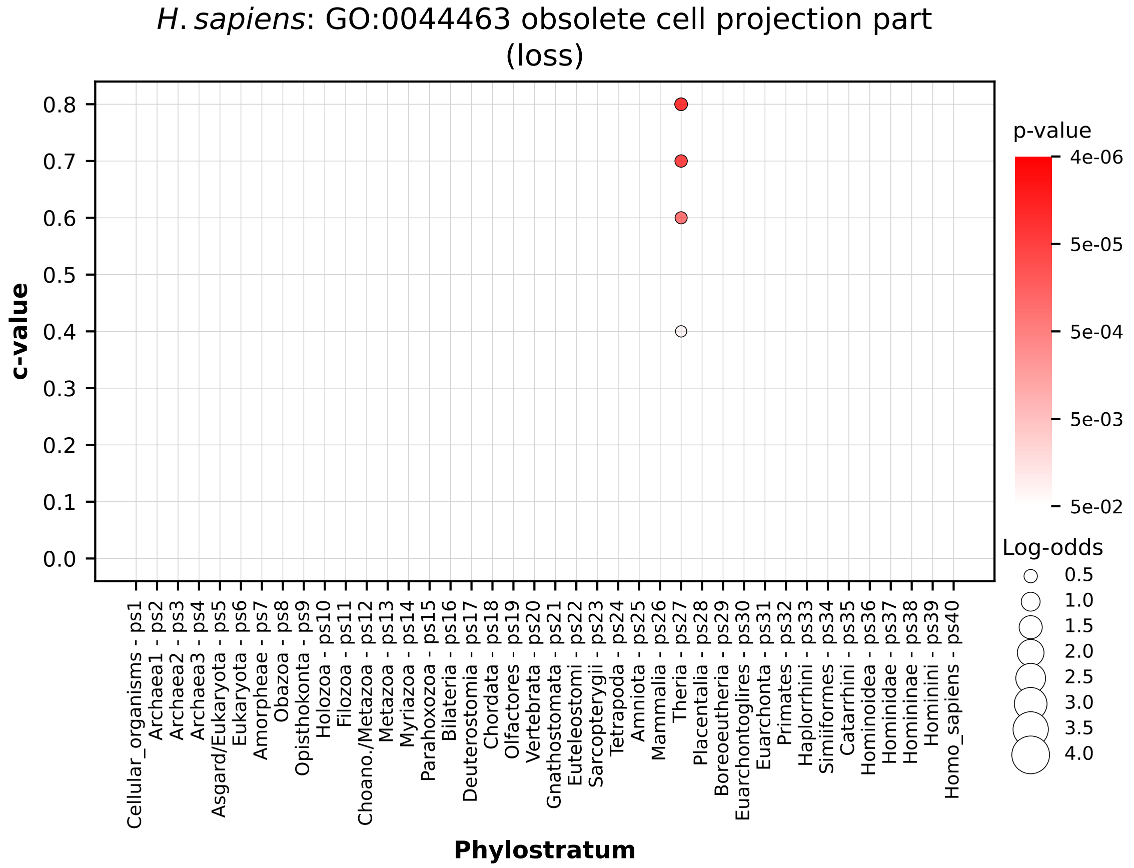
H. sapiens: GO:0044463 obsolete cell projection part
(loss)
c-value
0.8
0.7
0.6
0.5
0.4
0.3
0.2
0.1
0.0
p-value
4e-06
5e-05
5e-04
5e-03
5e-02
Log-odds
0.5
1.0
1.5
2.0
2.5
3.0
3.5
4.0
Cellular_organisms - ps1
Archaea1 - ps2
Archaea2 - ps3
Archaea3 - ps4
Asgard/Eukaryota - ps5
Eukaryota - ps6
Amorpheae - ps7
Obazoa - ps8
Opisthokonta - ps9
Holozoa - ps10
Filozoa - ps11
Choano./Metazoa - ps12
Metazoa - ps13
Myriazoa - ps14
Parahoxozoa - ps15
Bilateria - ps16
Deuterostomia - ps17
Chordata - ps18
Olfactores - ps19
Vertebrata - ps20
Gnathostomata - ps21
Euteleostomi - ps22
Sarcopterygii - ps23
Tetrapoda - ps24
Amniota - ps25
Mammalia - ps26
Theria - ps27
Placentalia - ps28
Boreoeutheria - ps29
Euarchontoglires - ps30
Euarchonta - ps31
Primates - ps32
Haplorrhini - ps33
Simiiformes - ps34
Catarrhini - ps35
Hominoidea - ps36
Hominidae - ps37
Homininae - ps38
Hominini - ps39
Homo_sapiens - ps40
Phylostratum

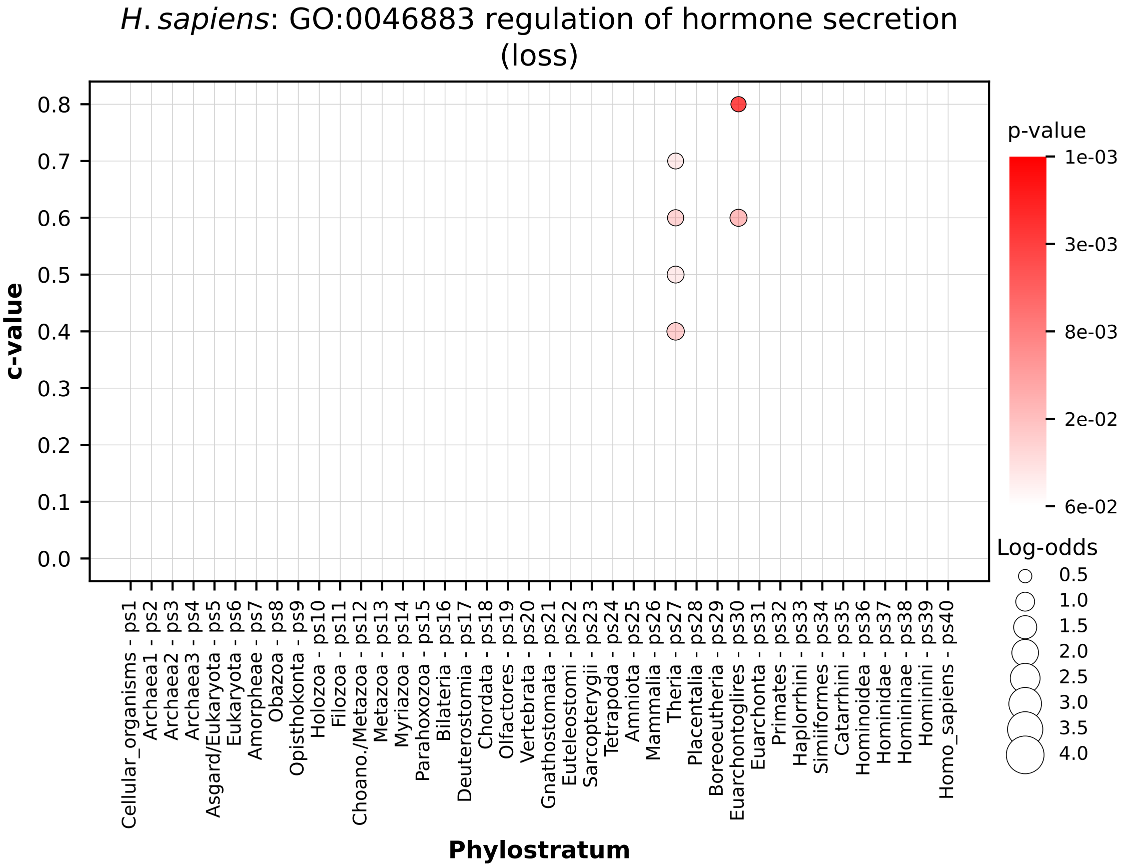

H. sapiens: GO:0046883 regulation of hormone secretion (loss)
c-value
0.0
0.1
0.2
0.3
0.4
0.5
0.6
0.7
0.8
p-value
1e-03
3e-03
8e-03
2e-02
6e-02
Log-odds
0.5
1.0
1.5
2.0
2.5
3.0
3.5
4.0
Cellular_organisms - ps1
Archaea1 - ps2
Archaea2 - ps3
Archaea3 - ps4
Asgard/Eukaryota - ps5
Eukaryota - ps6
Amorpheae - ps7
Obazoa - ps8
Opisthokonta - ps9
Holozoa - ps10
Filozoa - ps11
Choano./Metazoa - ps12
Metazoa - ps13
Myriazoa - ps14
Parahoxozoa - ps15
Bilateria - ps16
Deuterostomia - ps17
Chordata - ps18
Olfactores - ps19
Vertebrata - ps20
Gnathostomata - ps21
Euteleostomi - ps22
Sarcopterygii - ps23
Tetrapoda - ps24
Amniota - ps25
Mammalia - ps26
Theria - ps27
Placentalia - ps28
Boreoeutheria - ps29
Euarchontoglires - ps30
Euarchonta - ps31
Primates - ps32
Haplorrhini - ps33
Simiiformes - ps34
Catarrhini - ps35
Hominoidea - ps36
Hominidae - ps37
Homininae - ps38
Hominini - ps39
Homo_sapiens - ps40
Phylostratum

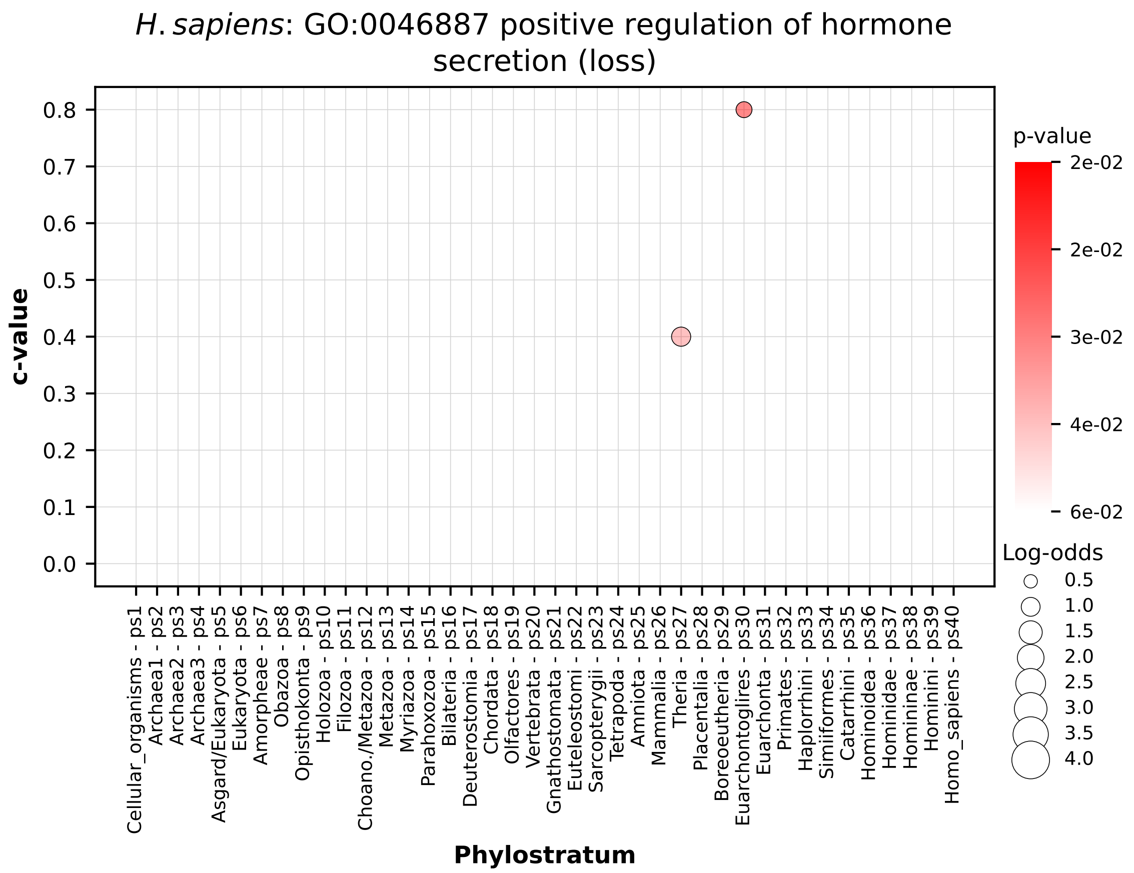

H. sapiens: GO:0046887 positive regulation of hormone secretion (loss)
c-value
0.0
0.1
0.2
0.3
0.4
0.5
0.6
0.7
0.8
Phylostratum
Cellular_organisms - ps1
Archaea1 - ps2
Archaea2 - ps3
Archaea3 - ps4
Asgard/Eukaryota - ps5
Eukaryota - ps6
Amorpheae - ps7
Obazoa - ps8
Opisthokonta - ps9
Holozoa - ps10
Filozoa - ps11
Choano./Metazoa - ps12
Metazoa - ps13
Myriazoa - ps14
Parahoxozoa - ps15
Bilateria - ps16
Deuterostomia - ps17
Chordata - ps18
Olfactores - ps19
Vertebrata - ps20
Gnathostomata - ps21
Euteleostomi - ps22
Sarcopterygii - ps23
Tetrapoda - ps24
Amniota - ps25
Mammalia - ps26
Theria - ps27
Placentalia - ps28
Boreoeutheria - ps29
Euarchontoglires - ps30
Euarchonta - ps31
Primates - ps32
Haplorrhini - ps33
Simiiformes - ps34
Catarrhini - ps35
Hominoidea - ps36
Hominidae - ps37
Homininae - ps38
Hominini - ps39
Homo_sapiens - ps40
p-value
2e-02
2e-02
3e-02
4e-02
6e-02
Log-odds
0.5
1.0
1.5
2.0
2.5
3.0
3.5
4.0

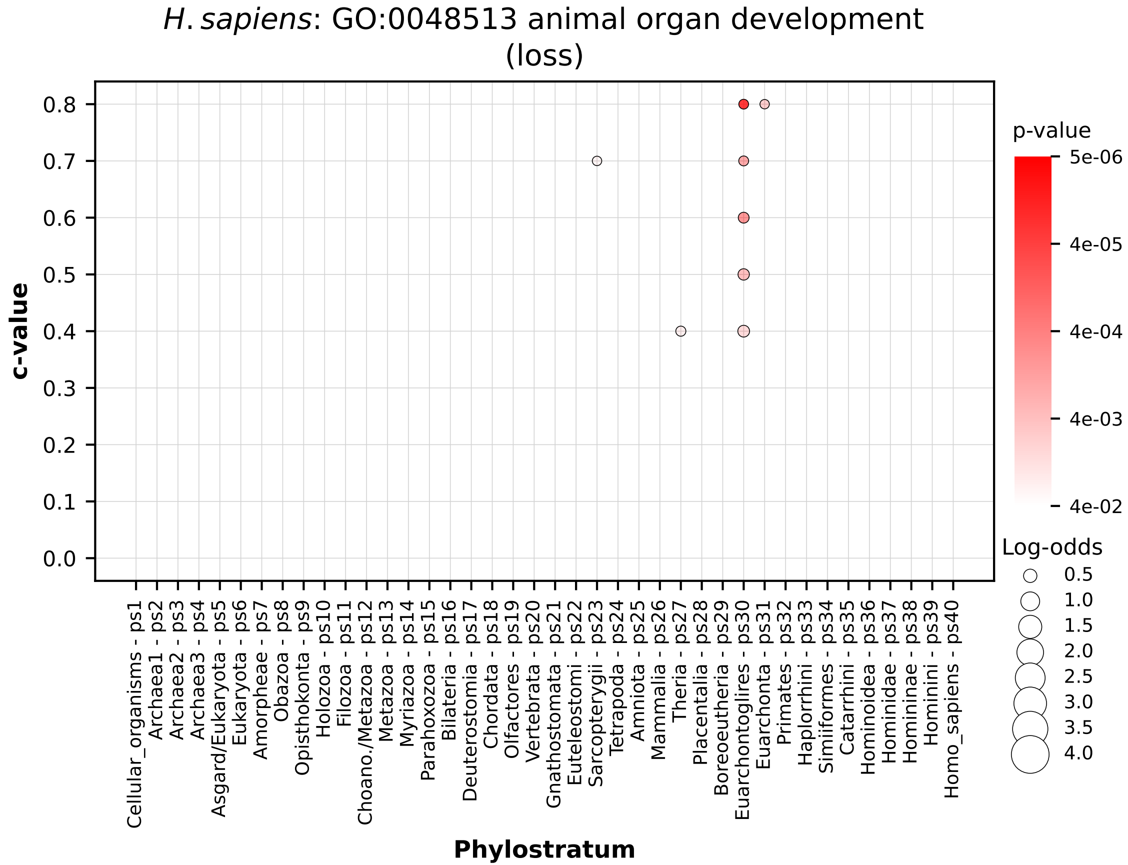

H. sapiens: GO:0048513 animal organ development
(loss)
c-value
0.8
0.7
0.6
0.5
0.4
0.3
0.2
0.1
0.0
p-value
5e-06
4e-05
4e-04
4e-03
4e-02
Log-odds
0.5
1.0
1.5
2.0
2.5
3.0
3.5
4.0
Cellular_organisms - ps1
Archaea1 - ps2
Archaea2 - ps3
Archaea3 - ps4
Asgard/Eukaryota - ps5
Eukaryota - ps6
Amorpheae - ps7
Obazoa - ps8
Opisthokonta - ps9
Holozoa - ps10
Filozoa - ps11
Choano./Metazoa - ps12
Metazoa - ps13
Myriazoa - ps14
Parahoxozoa - ps15
Bilateria - ps16
Deuterostomia - ps17
Chordata - ps18
Olfactores - ps19
Vertebrata - ps20
Gnathostomata - ps21
Euteleostomi - ps22
Sarcopterygii - ps23
Tetrapoda - ps24
Amniota - ps25
Mammalia - ps26
Theria - ps27
Placentalia - ps28
Boreoeutheria - ps29
Euarchontoglires - ps30
Euarchonta - ps31
Primates - ps32
Haplorrhini - ps33
Simiiformes - ps34
Catarrhini - ps35
Hominoidea - ps36
Hominidae - ps37
Homininae - ps38
Hominini - ps39
Homo_sapiens - ps40
Phylostratum

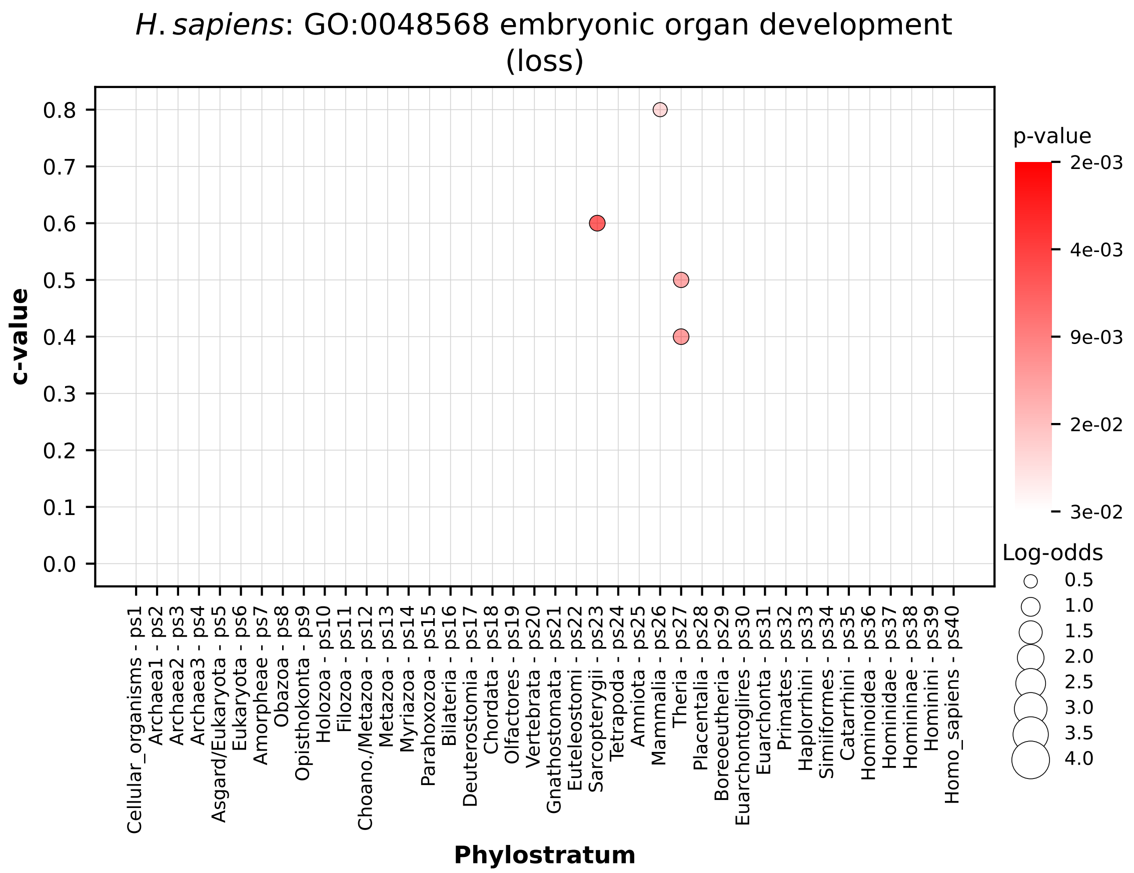

H. sapiens: GO:0048568 embryonic organ development
(loss)
c-value
0.0
0.1
0.2
0.3
0.4
0.5
0.6
0.7
0.8
p-value
2e-03
4e-03
9e-03
2e-02
3e-02
Log-odds
0.5
1.0
1.5
2.0
2.5
3.0
3.5
4.0
Cellular_organisms - ps1
Archaea1 - ps2
Archaea2 - ps3
Archaea3 - ps4
Asgard/Eukaryota - ps5
Eukaryota - ps6
Amorpheae - ps7
Obazoa - ps8
Opisthokonta - ps9
Holozoa - ps10
Filozoa - ps11
Choano./Metazoa - ps12
Metazoa - ps13
Myriazoa - ps14
Parahoxozoa - ps15
Bilateria - ps16
Deuterostomia - ps17
Chordata - ps18
Olfactores - ps19
Vertebrata - ps20
Gnathostomata - ps21
Euteleostomi - ps22
Sarcopterygii - ps23
Tetrapoda - ps24
Amniota - ps25
Mammalia - ps26
Theria - ps27
Placentalia - ps28
Boreoeutheria - ps29
Euarchontoglires - ps30
Euarchonta - ps31
Primates - ps32
Haplorrhini - ps33
Simiiformes - ps34
Catarrhini - ps35
Hominoidea - ps36
Hominidae - ps37
Homininae - ps38
Hominini - ps39
Homo_sapiens - ps40
Phylostratum

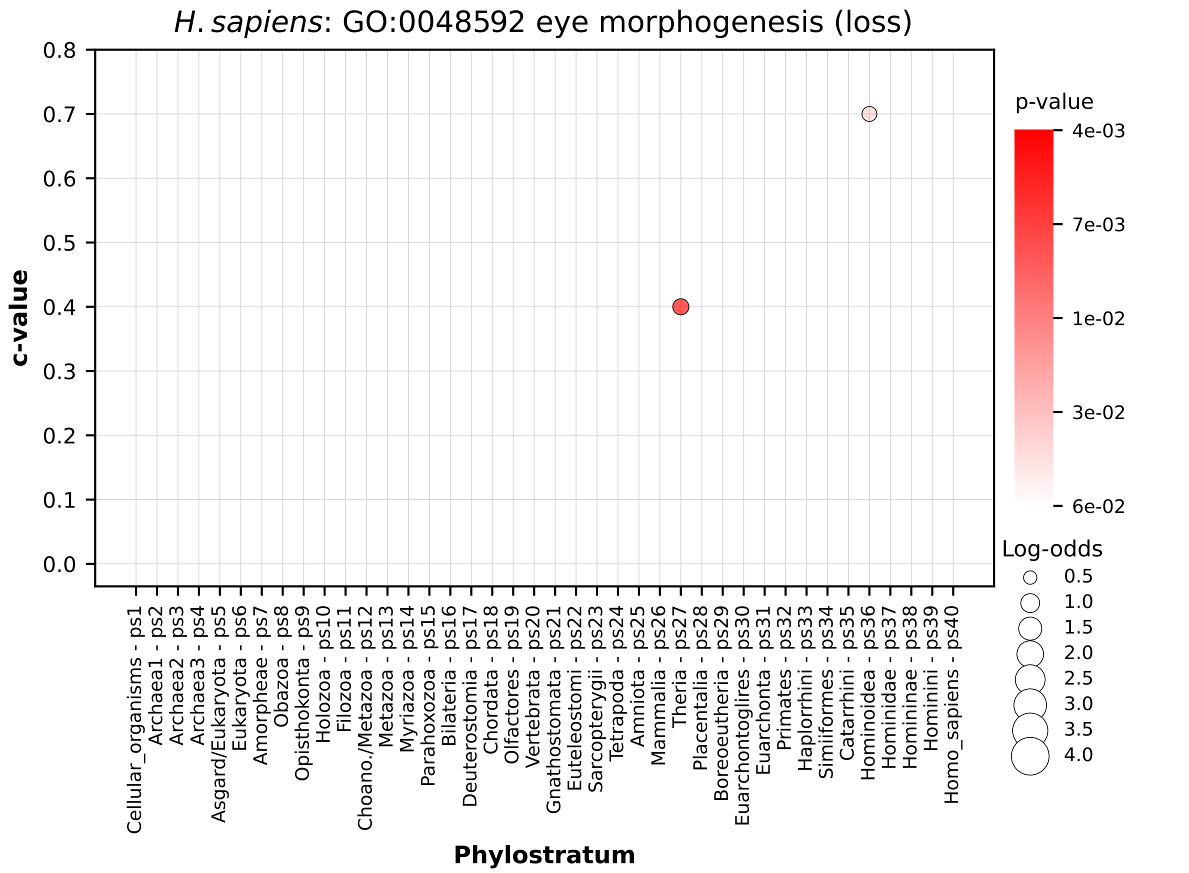

H. sapiens: GO:0048592 eye morphogenesis (loss)
c-value
0.0
0.1
0.2
0.3
0.4
0.5
0.6
0.7
0.8
Phylostratum
Cellular_organisms - ps1
Archaea1 - ps2
Archaea2 - ps3
Archaea3 - ps4
Asgard/Eukaryota - ps5
Eukaryota - ps6
Amorpheae - ps7
Obazoa - ps8
Opisthokonta - ps9
Holozoa - ps10
Filozoa - ps11
Choano./Metazoa - ps12
Metazoa - ps13
Myriazoa - ps14
Parahoxozoa - ps15
Bilateria - ps16
Deuterostomia - ps17
Chordata - ps18
Olfactores - ps19
Vertebrata - ps20
Gnathostomata - ps21
Euteleostomi - ps22
Sarcopterygii - ps23
Tetrapoda - ps24
Amniota - ps25
Mammalia - ps26
Theria - ps27
Placentalia - ps28
Boreoeutheria - ps29
Euarchontoglires - ps30
Euarchonta - ps31
Primates - ps32
Haplorrhini - ps33
Simiiformes - ps34
Catarrhini - ps35
Hominoidea - ps36
Hominidae - ps37
Homininae - ps38
Hominini - ps39
Homo_sapiens - ps40
p-value
4e-03
7e-03
1e-02
3e-02
6e-02
Log-odds
0.5
1.0
1.5
2.0
2.5
3.0
3.5
4.0

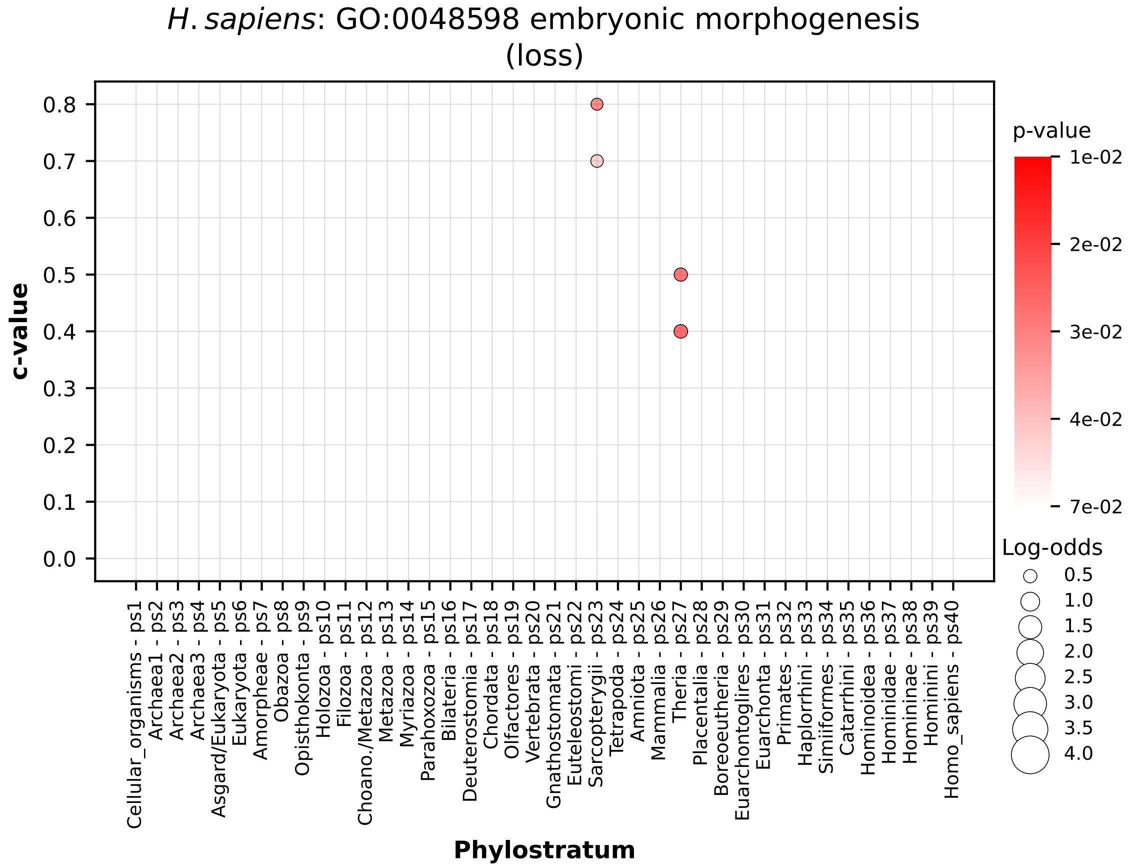
H. sapiens: GO:0048598 embryonic morphogenesis
(loss)
c-value
0.8
0.7
0.6
0.5
0.4
0.3
0.2
0.1
0.0
p-value
1e-02
2e-02
3e-02
4e-02
7e-02
Log-odds
0.5
1.0
1.5
2.0
2.5
3.0
3.5
4.0
Cellular_organisms - ps1
Archaea1 - ps2
Archaea2 - ps3
Archaea3 - ps4
Asgard/Eukaryota - ps5
Eukaryota - ps6
Amorpheae - ps7
Obazoa - ps8
Opisthokonta - ps9
Holozoa - ps10
Filozoa - ps11
Choano./Metazoa - ps12
Metazoa - ps13
Myriazoa - ps14
Parahoxozoa - ps15
Bilateria - ps16
Deuterostomia - ps17
Chordata - ps18
Olfactores - ps19
Vertebrata - ps20
Gnathostomata - ps21
Euteleostomi - ps22
Sarcopterygii - ps23
Tetrapoda - ps24
Amniota - ps25
Mammalia - ps26
Theria - ps27
Placentalia - ps28
Boreoeutheria - ps29
Euarchontoglires - ps30
Euarchonta - ps31
Primates - ps32
Haplorrhini - ps33
Simiiformes - ps34
Catarrhini - ps35
Hominoidea - ps36
Hominidae - ps37
Homininae - ps38
Hominini - ps39
Homo_sapiens - ps40
Phylostratum

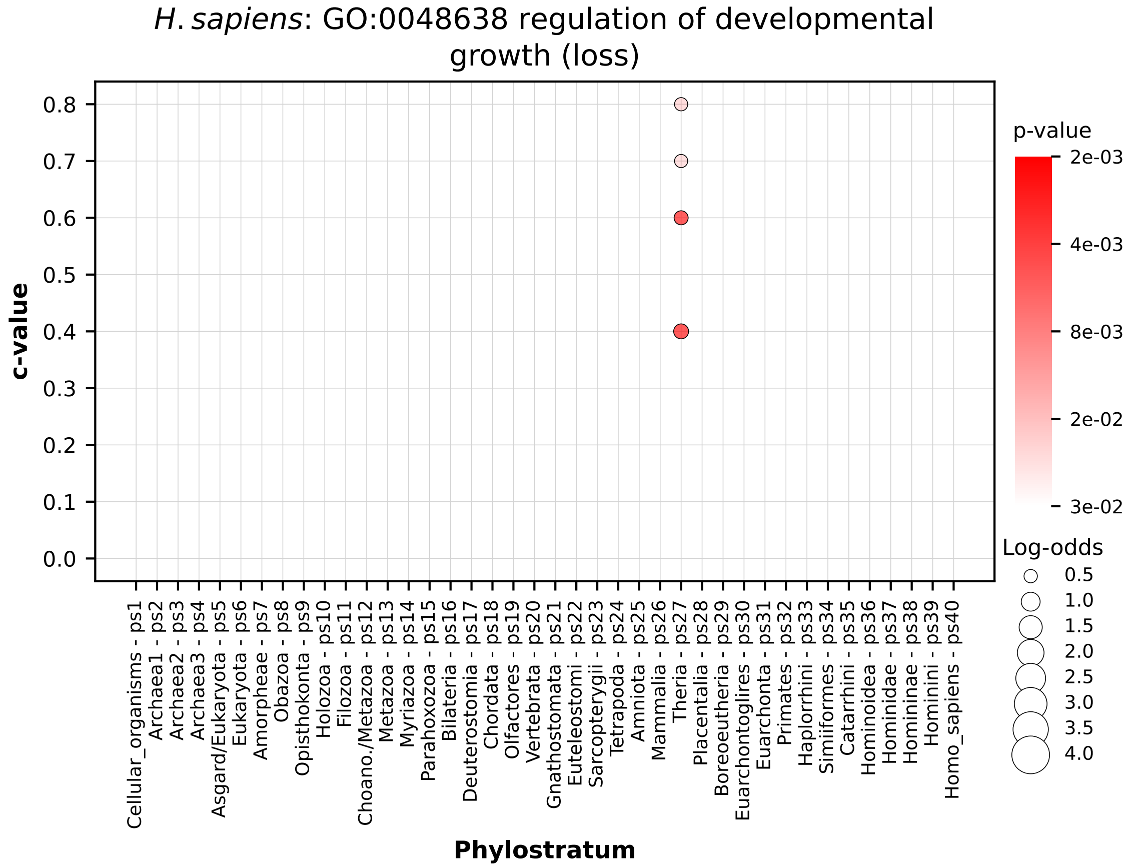
H. sapiens: GO:0048638 regulation of developmental growth (loss)
c-value
0.0
0.1
0.2
0.3
0.4
0.5
0.6
0.7
0.8
Phylostratum
Cellular_organisms - ps1
Archaea1 - ps2
Archaea2 - ps3
Archaea3 - ps4
Asgard/Eukaryota - ps5
Eukaryota - ps6
Amorpheae - ps7
Obazoa - ps8
Opisthokonta - ps9
Holozoa - ps10
Filozoa - ps11
Choano./Metazoa - ps12
Metazoa - ps13
Myriazoa - ps14
Parahoxozoa - ps15
Bilateria - ps16
Deuterostomia - ps17
Chordata - ps18
Olfactores - ps19
Vertebrata - ps20
Gnathostomata - ps21
Euteleostomi - ps22
Sarcopterygii - ps23
Tetrapoda - ps24
Amniota - ps25
Mammalia - ps26
Theria - ps27
Placentalia - ps28
Boreoeutheria - ps29
Euarchontoglires - ps30
Euarchonta - ps31
Primates - ps32
Haplorrhini - ps33
Simiiformes - ps34
Catarrhini - ps35
Hominoidea - ps36
Hominidae - ps37
Homininae - ps38
Hominini - ps39
Homo_sapiens - ps40
p-value
2e-03
4e-03
8e-03
2e-02
3e-02
Log-odds
0.5
1.0
1.5
2.0
2.5
3.0
3.5
4.0

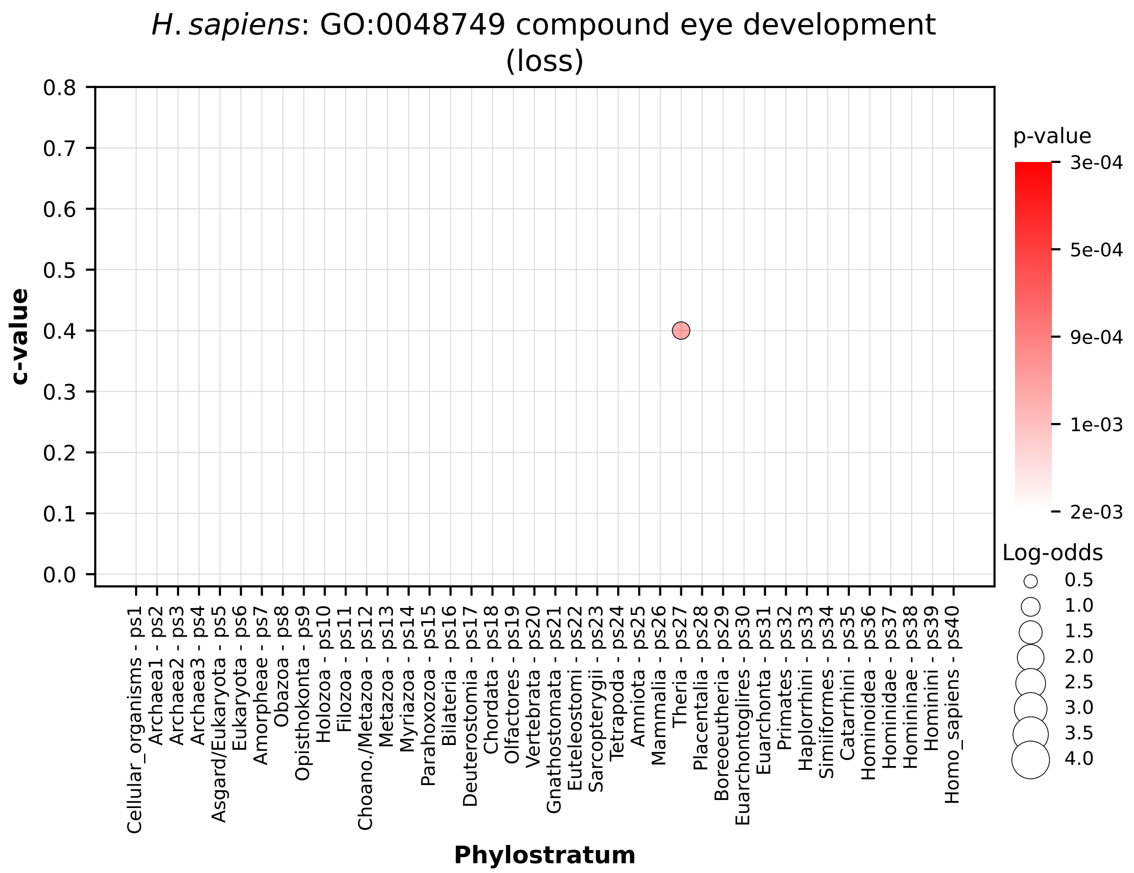

H. sapiens: GO:0048749 compound eye development
(loss)
c-value
0.0
0.1
0.2
0.3
0.4
0.5
0.6
0.7
0.8
Phylostratum
Cellular_organisms - ps1
Archaea1 - ps2
Archaea2 - ps3
Archaea3 - ps4
Asgard/Eukaryota - ps5
Eukaryota - ps6
Amorpheae - ps7
Obazoa - ps8
Opisthokonta - ps9
Holozoa - ps10
Filozoa - ps11
Choano./Metazoa - ps12
Metazoa - ps13
Myriazoa - ps14
Parahoxozoa - ps15
Bilateria - ps16
Deuterostomia - ps17
Chordata - ps18
Olfactores - ps19
Vertebrata - ps20
Gnathostomata - ps21
Euteleostomi - ps22
Sarcopterygii - ps23
Tetrapoda - ps24
Amniota - ps25
Mammalia - ps26
Theria - ps27
Placentalia - ps28
Boreoeutheria - ps29
Euarchontoglires - ps30
Euarchonta - ps31
Primates - ps32
Haplorrhini - ps33
Simiiformes - ps34
Catarrhini - ps35
Hominoidea - ps36
Hominidae - ps37
Homininae - ps38
Hominini - ps39
Homo_sapiens - ps40
p-value
3e-04
5e-04
9e-04
1e-03
2e-03
Log-odds
0.5
1.0
1.5
2.0
2.5
3.0
3.5
4.0

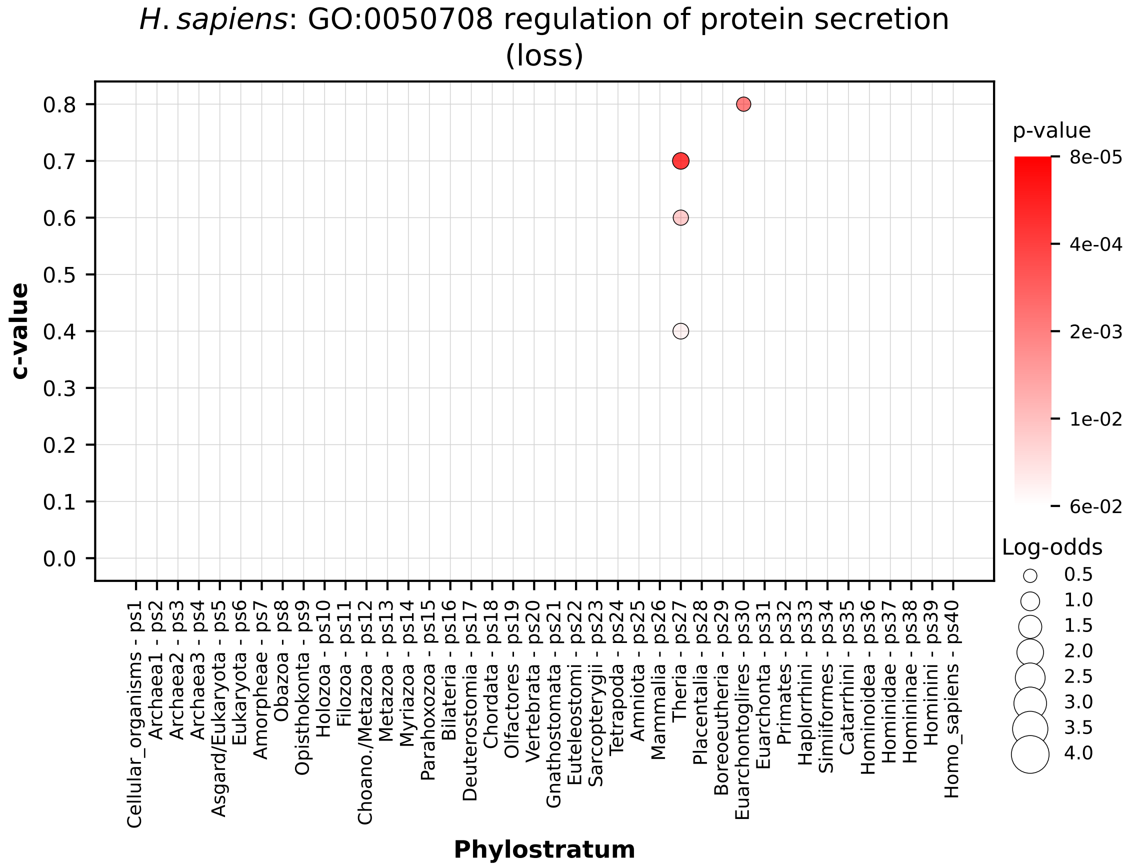

H. sapiens: GO:0050708 regulation of protein secretion (loss)
c-value
0.8
0.7
0.6
0.5
0.4
0.3
0.2
0.1
0.0
p-value
8e-05
4e-04
2e-03
1e-02
6e-02
Log-odds
0.5
1.0
1.5
2.0
2.5
3.0
3.5
4.0
Cellular_organisms - ps1
Archaea1 - ps2
Archaea2 - ps3
Archaea3 - ps4
Asgard/Eukaryota - ps5
Eukaryota - ps6
Amorpheae - ps7
Obazoa - ps8
Opisthokonta - ps9
Holozoa - ps10
Filozoa - ps11
Choano./Metazoa - ps12
Metazoa - ps13
Myriazoa - ps14
Parahoxozoa - ps15
Bilateria - ps16
Deuterostomia - ps17
Chordata - ps18
Olfactores - ps19
Vertebrata - ps20
Gnathostomata - ps21
Euteleostomi - ps22
Sarcopterygii - ps23
Tetrapoda - ps24
Amniota - ps25
Mammalia - ps26
Theria - ps27
Placentalia - ps28
Boreoeutheria - ps29
Euarchontoglires - ps30
Euarchonta - ps31
Primates - ps32
Haplorrhini - ps33
Simiiformes - ps34
Catarrhini - ps35
Hominoidea - ps36
Hominidae - ps37
Homininae - ps38
Hominini - ps39
Homo_sapiens - ps40
Phylostratum

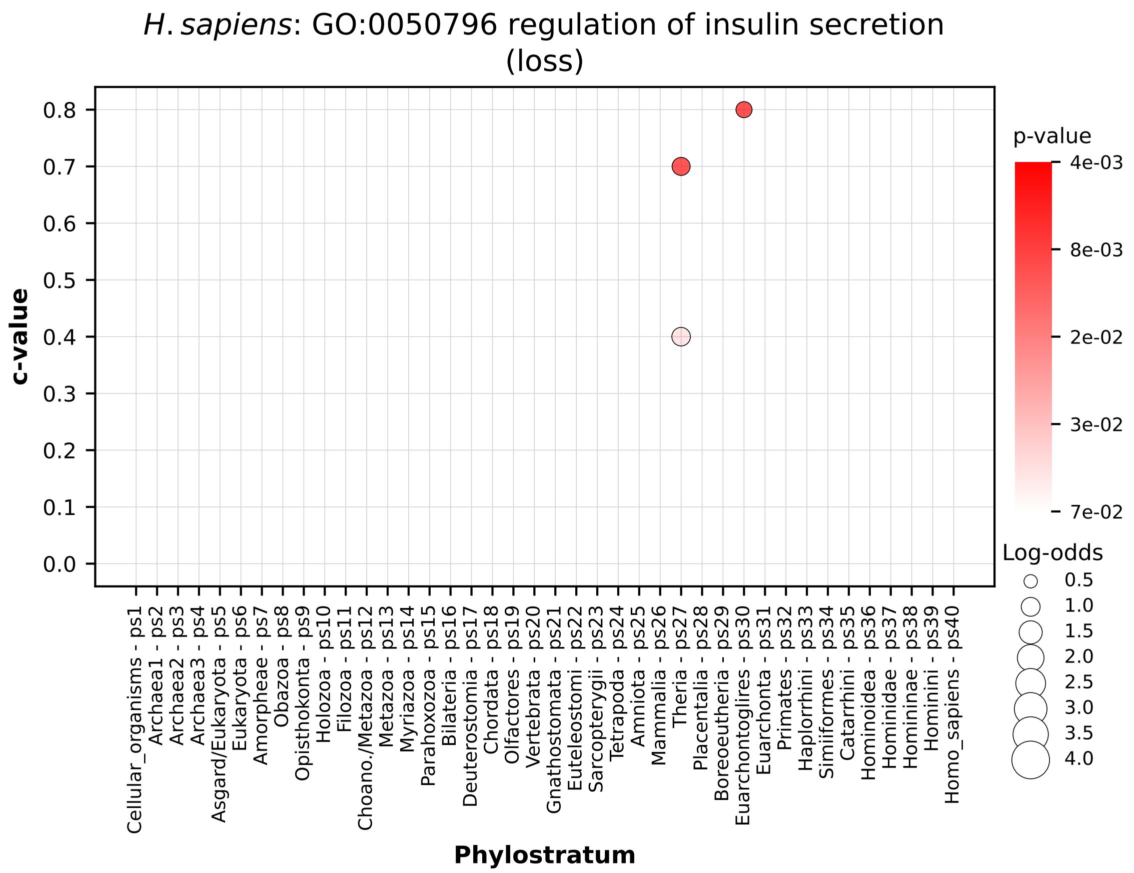

H. sapiens: GO:0050796 regulation of insulin secretion
(loss)
c-value
0.0
0.1
0.2
0.3
0.4
0.5
0.6
0.7
0.8
p-value
4e-03
8e-03
2e-02
3e-02
7e-02
Log-odds
0.5
1.0
1.5
2.0
2.5
3.0
3.5
4.0
Cellular_organisms - ps1
Archaea1 - ps2
Archaea2 - ps3
Archaea3 - ps4
Asgard/Eukaryota - ps5
Eukaryota - ps6
Amorpheae - ps7
Obazoa - ps8
Opisthokonta - ps9
Holozoa - ps10
Filozoa - ps11
Choano./Metazoa - ps12
Metazoa - ps13
Myriazoa - ps14
Parahoxozoa - ps15
Bilateria - ps16
Deuterostomia - ps17
Chordata - ps18
Olfactores - ps19
Vertebrata - ps20
Gnathostomata - ps21
Euteleostomi - ps22
Sarcopterygii - ps23
Tetrapoda - ps24
Amniota - ps25
Mammalia - ps26
Theria - ps27
Placentalia - ps28
Boreoeutheria - ps29
Euarchontoglires - ps30
Euarchonta - ps31
Primates - ps32
Haplorrhini - ps33
Simiiformes - ps34
Catarrhini - ps35
Hominoidea - ps36
Hominidae - ps37
Homininae - ps38
Hominini - ps39
Homo_sapiens - ps40
Phylostratum

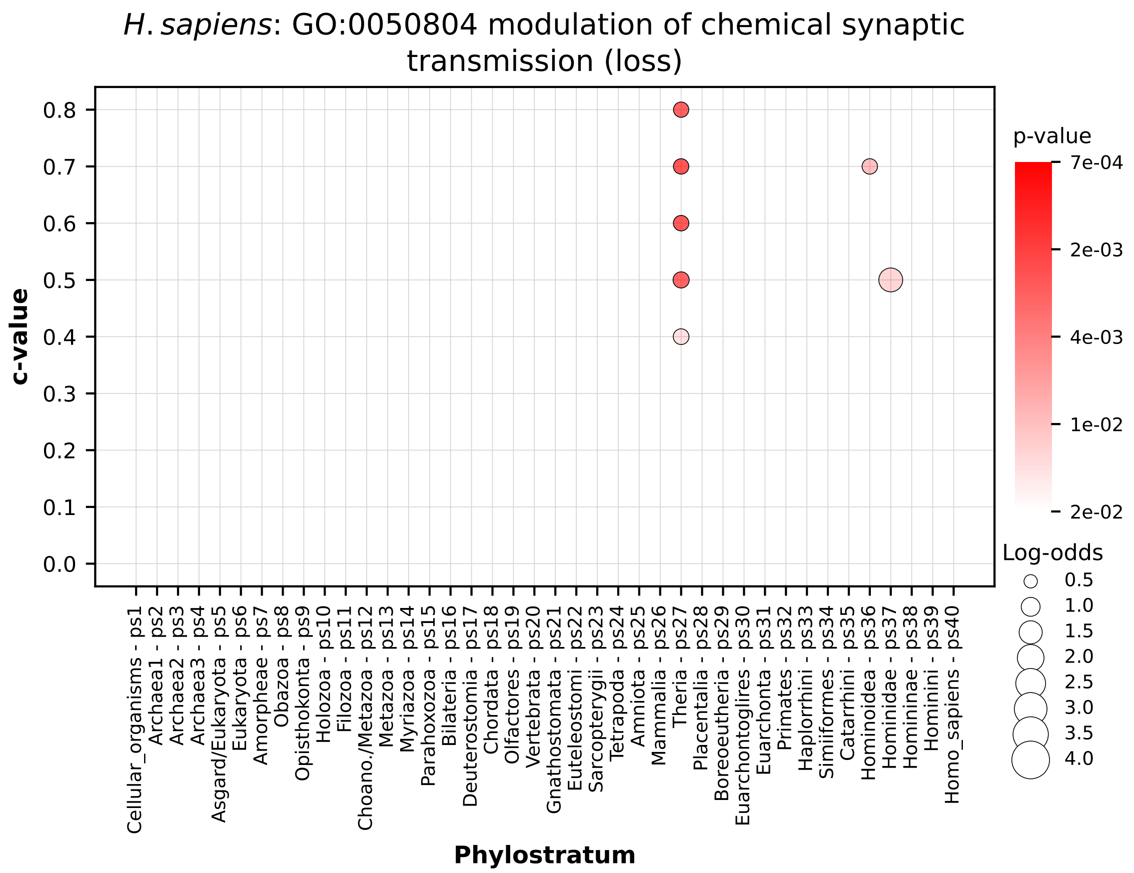

H. sapiens: GO:0050804 modulation of chemical synaptic transmission (loss)
c-value
Phylostratum
p-value
7e-04
2e-03
4e-03
1e-02
2e-02
Log-odds
0.5
1.0
1.5
2.0
2.5
3.0
3.5
4.0
Cellular_organisms - ps1
Archaea1 - ps2
Archaea2 - ps3
Archaea3 - ps4
Asgard/Eukaryota - ps5
Eukaryota - ps6
Amorpheae - ps7
Obazoa - ps8
Opisthokonta - ps9
Holozoa - ps10
Filozoa - ps11
Choano./Metazoa - ps12
Metazoa - ps13
Myriazoa - ps14
Parahoxozoa - ps15
Bilateria - ps16
Deuterostomia - ps17
Chordata - ps18
Olfactores - ps19
Vertebrata - ps20
Gnathostomata - ps21
Euteleostomi - ps22
Sarcopterygii - ps23
Tetrapoda - ps24
Amniota - ps25
Mammalia - ps26
Theria - ps27
Placentalia - ps28
Boreoeutheria - ps29
Euarchontoglires - ps30
Euarchonta - ps31
Primates - ps32
Haplorrhini - ps33
Simiiformes - ps34
Catarrhini - ps35
Hominoidea - ps36
Hominidae - ps37
Homininae - ps38
Hominini - ps39
Homo_sapiens - ps40

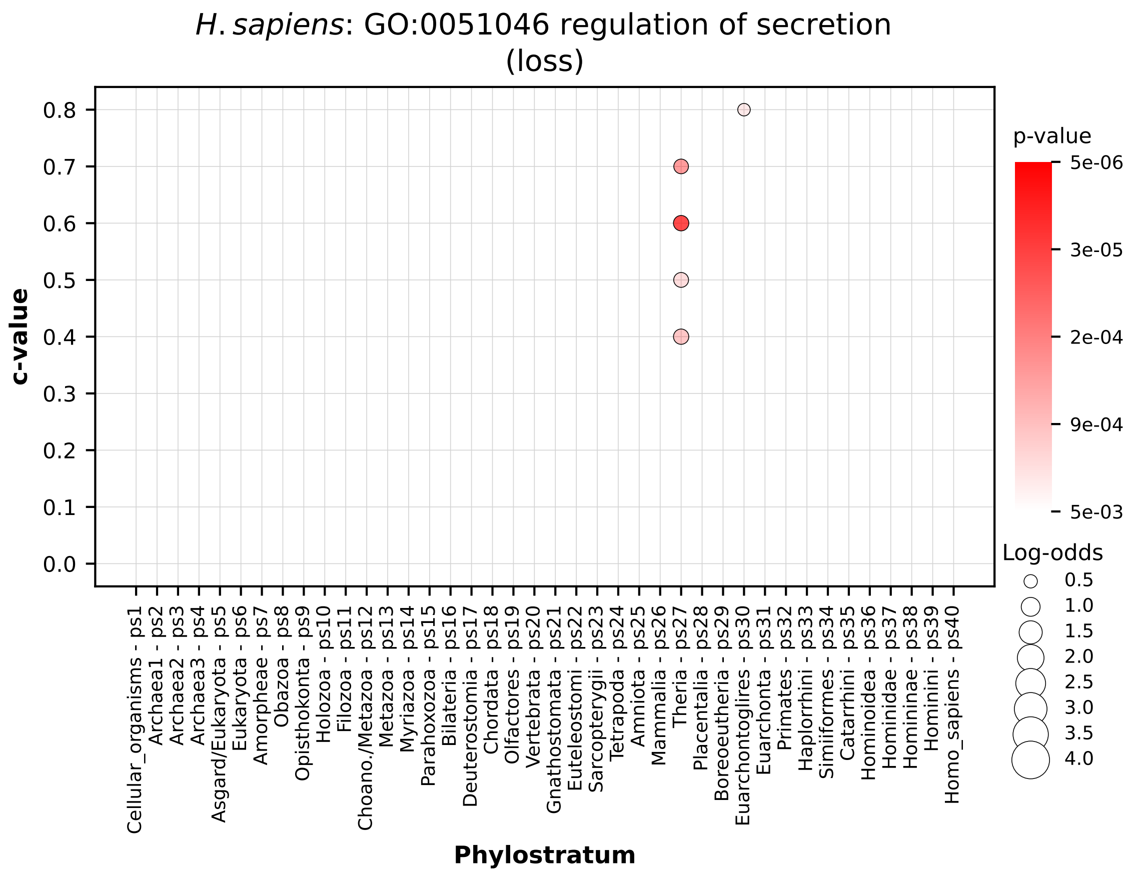

H. sapiens: GO:0051046 regulation of secretion
(loss)
c-value
0.0
0.1
0.2
0.3
0.4
0.5
0.6
0.7
0.8
Phylostratum
Cellular_organisms - ps1
Archaea1 - ps2
Archaea2 - ps3
Archaea3 - ps4
Asgard/Eukaryota - ps5
Eukaryota - ps6
Amorpheae - ps7
Obazoa - ps8
Opisthokonta - ps9
Holozoa - ps10
Filozoa - ps11
Choano./Metazoa - ps12
Metazoa - ps13
Myriazoa - ps14
Parahoxozoa - ps15
Bilateria - ps16
Deuterostomia - ps17
Chordata - ps18
Olfactores - ps19
Vertebrata - ps20
Gnathostomata - ps21
Euteleostomi - ps22
Sarcopterygii - ps23
Tetrapoda - ps24
Amniota - ps25
Mammalia - ps26
Theria - ps27
Placentalia - ps28
Boreoeutheria - ps29
Euarchontoglires - ps30
Euarchonta - ps31
Primates - ps32
Haplorrhini - ps33
Simiiformes - ps34
Catarrhini - ps35
Hominoidea - ps36
Hominidae - ps37
Homininae - ps38
Hominini - ps39
Homo_sapiens - ps40
p-value
5e-06
3e-05
2e-04
9e-04
5e-03
Log-odds
0.5
1.0
1.5
2.0
2.5
3.0
3.5
4.0

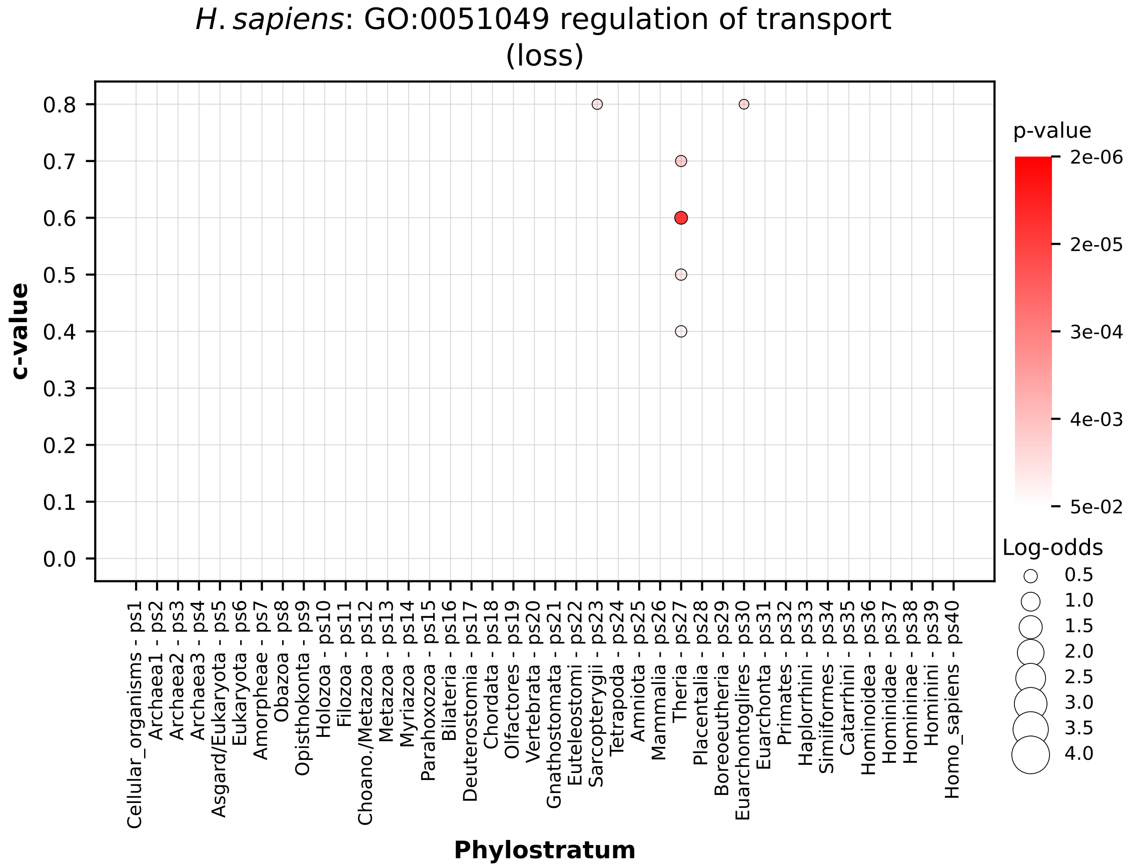
H. sapiens: GO:0051049 regulation of transport
(loss)
c-value
0.8
0.7
0.6
0.5
0.4
0.3
0.2
0.1
0.0
p-value
2e-06
2e-05
3e-04
4e-03
5e-02
Log-odds
0.5
1.0
1.5
2.0
2.5
3.0
3.5
4.0
Cellular_organisms - ps1
Archaea1 - ps2
Archaea2 - ps3
Archaea3 - ps4
Asgard/Eukaryota - ps5
Eukaryota - ps6
Amorpheae - ps7
Obazoa - ps8
Opisthokonta - ps9
Holozoa - ps10
Filozoa - ps11
Choano./Metazoa - ps12
Metazoa - ps13
Myriazoa - ps14
Parahoxozoa - ps15
Bilateria - ps16
Deuterostomia - ps17
Chordata - ps18
Olfactores - ps19
Vertebrata - ps20
Gnathostomata - ps21
Euteleostomi - ps22
Sarcopterygii - ps23
Tetrapoda - ps24
Amniota - ps25
Mammalia - ps26
Theria - ps27
Placentalia - ps28
Boreoeutheria - ps29
Euarchontoglires - ps30
Euarchonta - ps31
Primates - ps32
Haplorrhini - ps33
Simiiformes - ps34
Catarrhini - ps35
Hominoidea - ps36
Hominidae - ps37
Homininae - ps38
Hominini - ps39
Homo_sapiens - ps40
Phylostratum

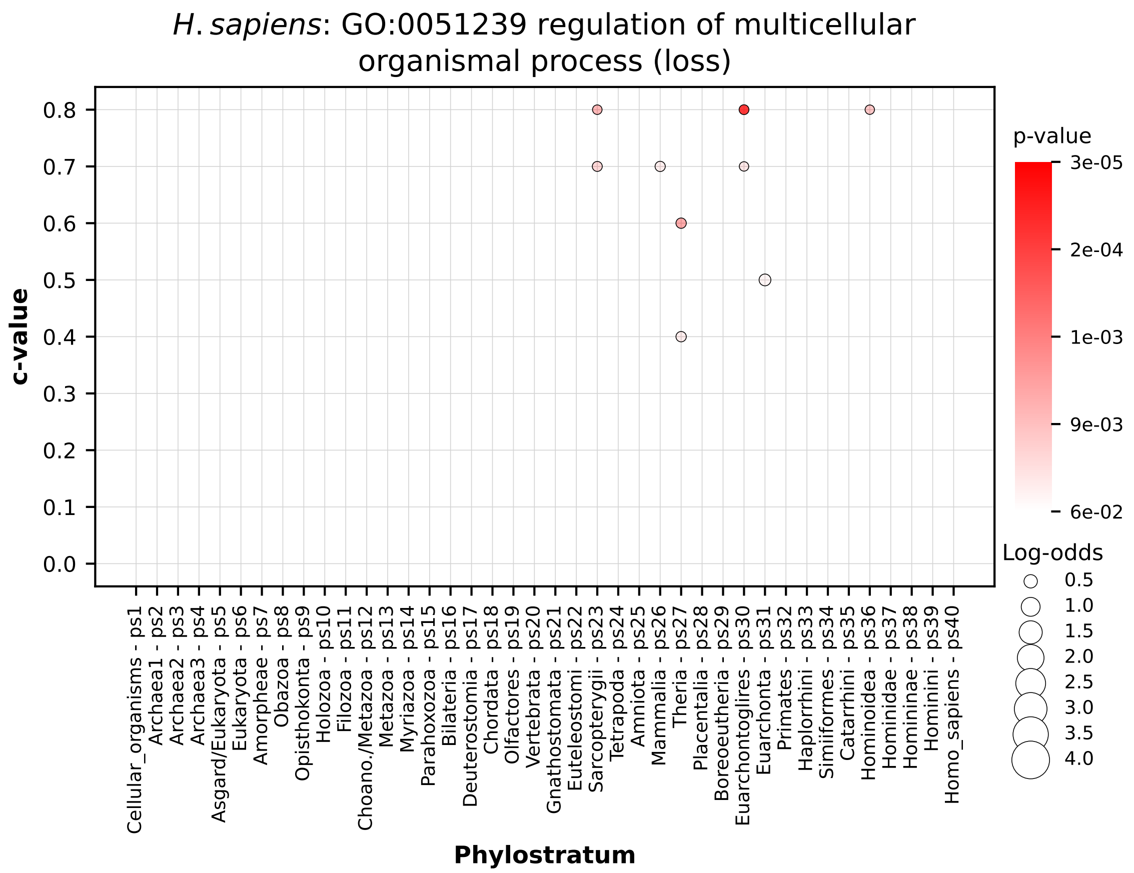

H. sapiens: GO:0051239 regulation of multicellular organismal process (loss)
c-value
0.0
0.1
0.2
0.3
0.4
0.5
0.6
0.7
0.8
Phylostratum
Cellular_organisms - ps1
Archaea1 - ps2
Archaea2 - ps3
Archaea3 - ps4
Asgard/Eukaryota - ps5
Eukaryota - ps6
Amorpheae - ps7
Obazoa - ps8
Opisthokonta - ps9
Holozoa - ps10
Filozoa - ps11
Choano./Metazoa - ps12
Metazoa - ps13
Myriazoa - ps14
Parahoxozoa - ps15
Bilateria - ps16
Deuterostomia - ps17
Chordata - ps18
Olfactores - ps19
Vertebrata - ps20
Gnathostomata - ps21
Euteleostomi - ps22
Sarcopterygii - ps23
Tetrapoda - ps24
Amniota - ps25
Mammalia - ps26
Theria - ps27
Placentalia - ps28
Boreoeutheria - ps29
Euarchontoglires - ps30
Euarchonta - ps31
Primates - ps32
Haplorrhini - ps33
Simiiformes - ps34
Catarrhini - ps35
Hominoidea - ps36
Hominidae - ps37
Homininae - ps38
Hominini - ps39
Homo_sapiens - ps40
p-value
3e-05
2e-04
1e-03
9e-03
6e-02
Log-odds
0.5
1.0
1.5
2.0
2.5
3.0
3.5
4.0

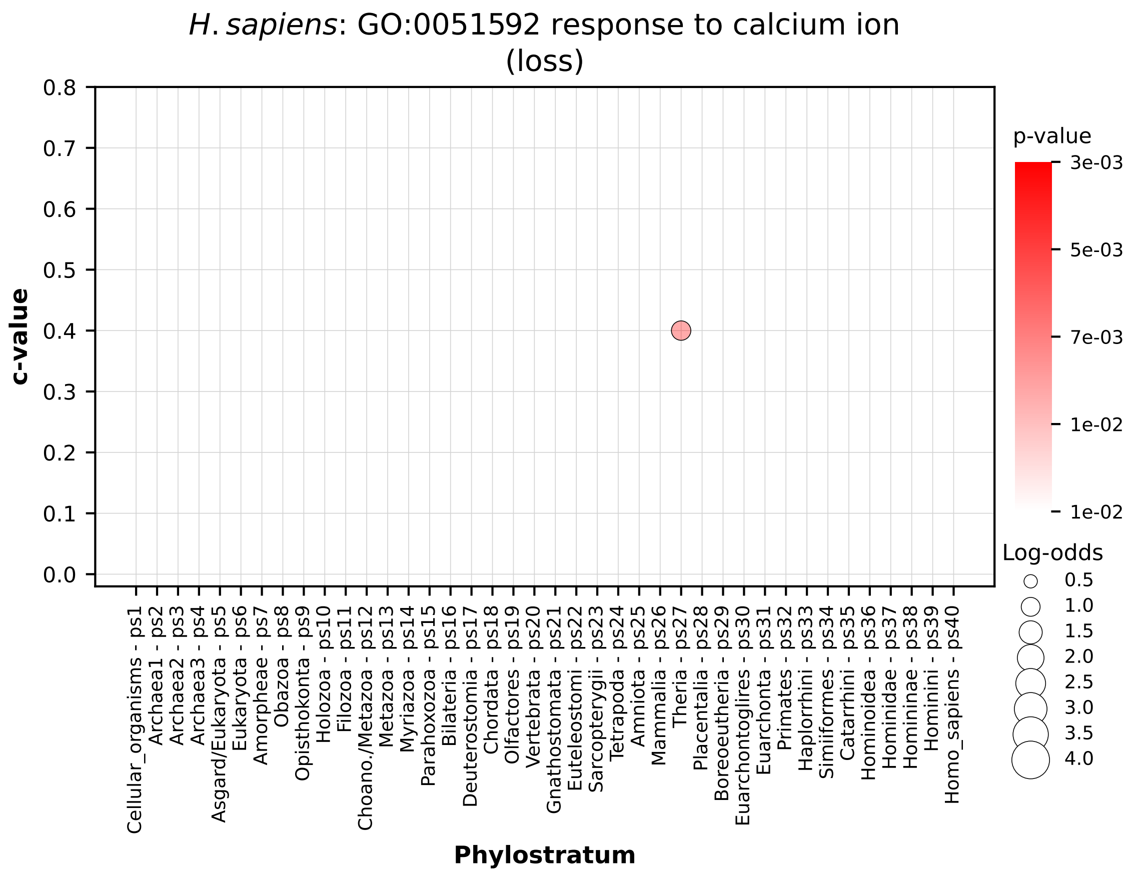

H. sapiens: GO:0051592 response to calcium ion
(loss)
c-value
0.0
0.1
0.2
0.3
0.4
0.5
0.6
0.7
0.8
Phylostratum
Cellular_organisms - ps1
Archaea1 - ps2
Archaea2 - ps3
Archaea3 - ps4
Asgard/Eukaryota - ps5
Eukaryota - ps6
Amorpheae - ps7
Obazoa - ps8
Opisthokonta - ps9
Holozoa - ps10
Filozoa - ps11
Choano./Metazoa - ps12
Metazoa - ps13
Myriazoa - ps14
Parahoxozoa - ps15
Bilateria - ps16
Deuterostomia - ps17
Chordata - ps18
Olfactores - ps19
Vertebrata - ps20
Gnathostomata - ps21
Euteleostomi - ps22
Sarcopterygii - ps23
Tetrapoda - ps24
Amniota - ps25
Mammalia - ps26
Theria - ps27
Placentalia - ps28
Boreoeutheria - ps29
Euarchontoglires - ps30
Euarchonta - ps31
Primates - ps32
Haplorrhini - ps33
Simiiformes - ps34
Catarrhini - ps35
Hominoidea - ps36
Hominidae - ps37
Homininae - ps38
Hominini - ps39
Homo_sapiens - ps40
p-value
3e-03
5e-03
7e-03
1e-02
1e-02
Log-odds
0.5
1.0
1.5
2.0
2.5
3.0
3.5
4.0

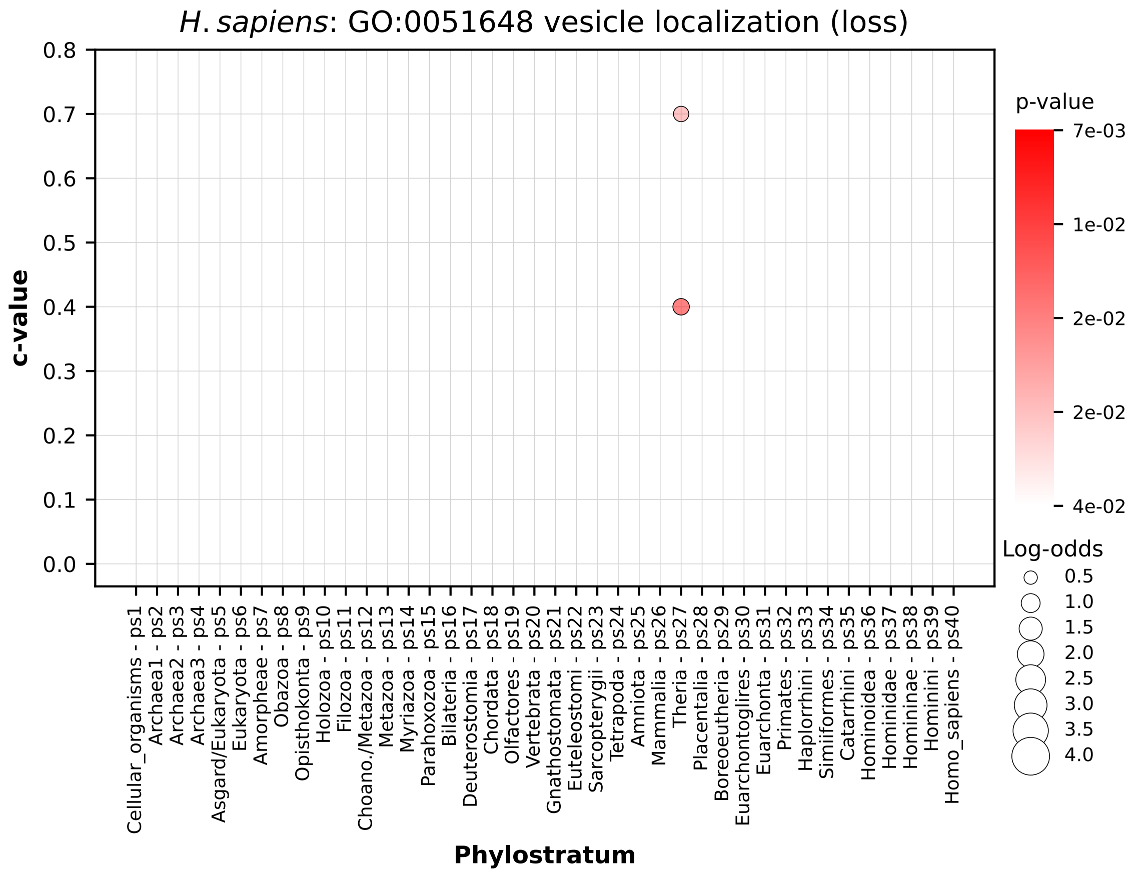

H. sapiens: GO:0051648 vesicle localization (loss)
c-value
0.8
0.7
0.6
0.5
0.4
0.3
0.2
0.1
0.0
Phylostratum
Cellular_organisms - ps1
Archaea1 - ps2
Archaea2 - ps3
Archaea3 - ps4
Asgard/Eukaryota - ps5
Eukaryota - ps6
Amorpheae - ps7
Obazoa - ps8
Opisthokonta - ps9
Holozoa - ps10
Filozoa - ps11
Choano./Metazoa - ps12
Metazoa - ps13
Myriazoa - ps14
Parahoxozoa - ps15
Bilateria - ps16
Deuterostomia - ps17
Chordata - ps18
Olfactores - ps19
Vertebrata - ps20
Gnathostomata - ps21
Euteleostomi - ps22
Sarcopterygii - ps23
Tetrapoda - ps24
Amniota - ps25
Mammalia - ps26
Theria - ps27
Placentalia - ps28
Boreoeutheria - ps29
Euarchontoglires - ps30
Euarchonta - ps31
Primates - ps32
Haplorrhini - ps33
Simiiformes - ps34
Catarrhini - ps35
Hominoidea - ps36
Hominidae - ps37
Homininae - ps38
Hominini - ps39
Homo_sapiens - ps40
p-value
7e-03
1e-02
2e-02
2e-02
4e-02
Log-odds
0.5
1.0
1.5
2.0
2.5
3.0
3.5
4.0

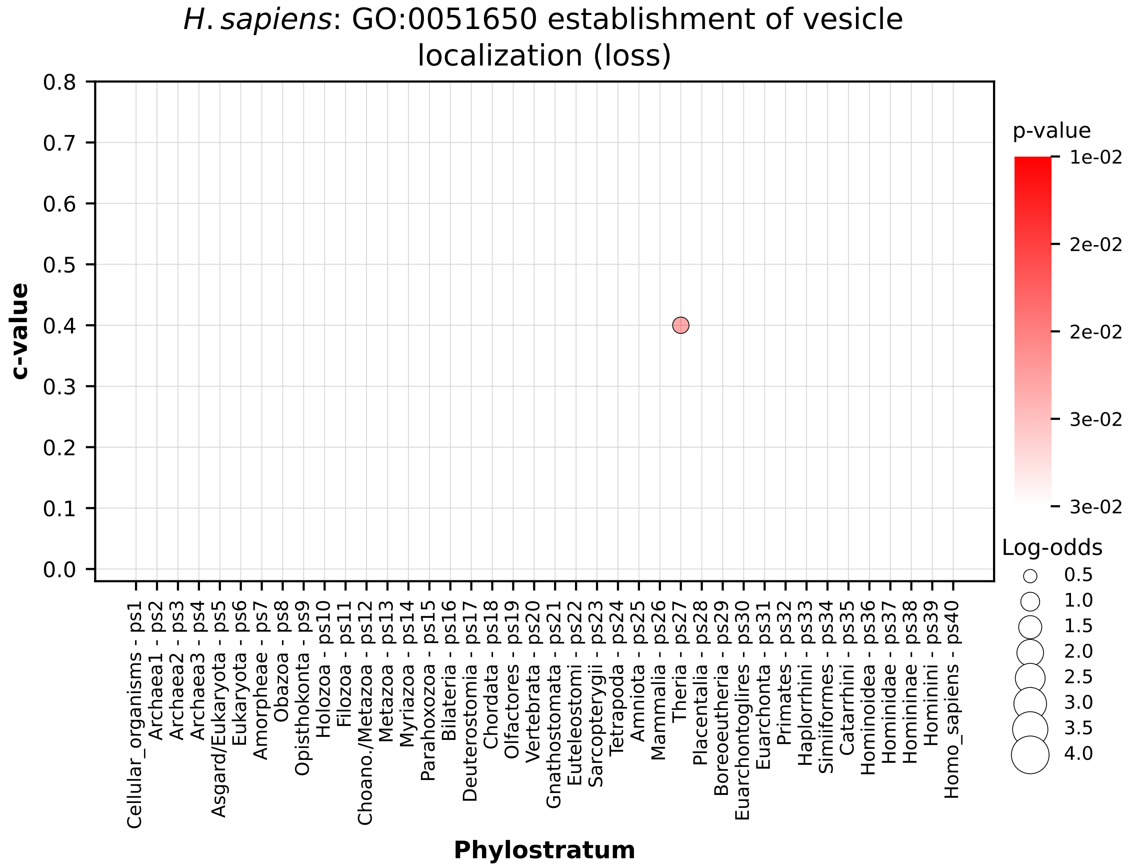

H. sapiens: GO:0051650 establishment of vesicle localization (loss)
c-value
0.8
0.7
0.6
0.5
0.4
0.3
0.2
0.1
0.0
p-value
1e-02
2e-02
2e-02
3e-02
3e-02
Log-odds
0.5
1.0
1.5
2.0
2.5
3.0
3.5
4.0
Cellular_organisms - ps1
Archaea1 - ps2
Archaea2 - ps3
Archaea3 - ps4
Asgard/Eukaryota - ps5
Eukaryota - ps6
Amorpheae - ps7
Obazoa - ps8
Opisthokonta - ps9
Holozoa - ps10
Filozoa - ps11
Choano./Metazoa - ps12
Metazoa - ps13
Myriazoa - ps14
Parahoxozoa - ps15
Bilateria - ps16
Deuterostomia - ps17
Chordata - ps18
Olfactores - ps19
Vertebrata - ps20
Gnathostomata - ps21
Euteleostomi - ps22
Sarcopterygii - ps23
Tetrapoda - ps24
Amniota - ps25
Mammalia - ps26
Theria - ps27
Placentalia - ps28
Boreoeutheria - ps29
Euarchontoglires - ps30
Euarchonta - ps31
Primates - ps32
Haplorrhini - ps33
Simiiformes - ps34
Catarrhini - ps35
Hominoidea - ps36
Hominidae - ps37
Homininae - ps38
Hominini - ps39
Homo_sapiens - ps40
Phylostratum

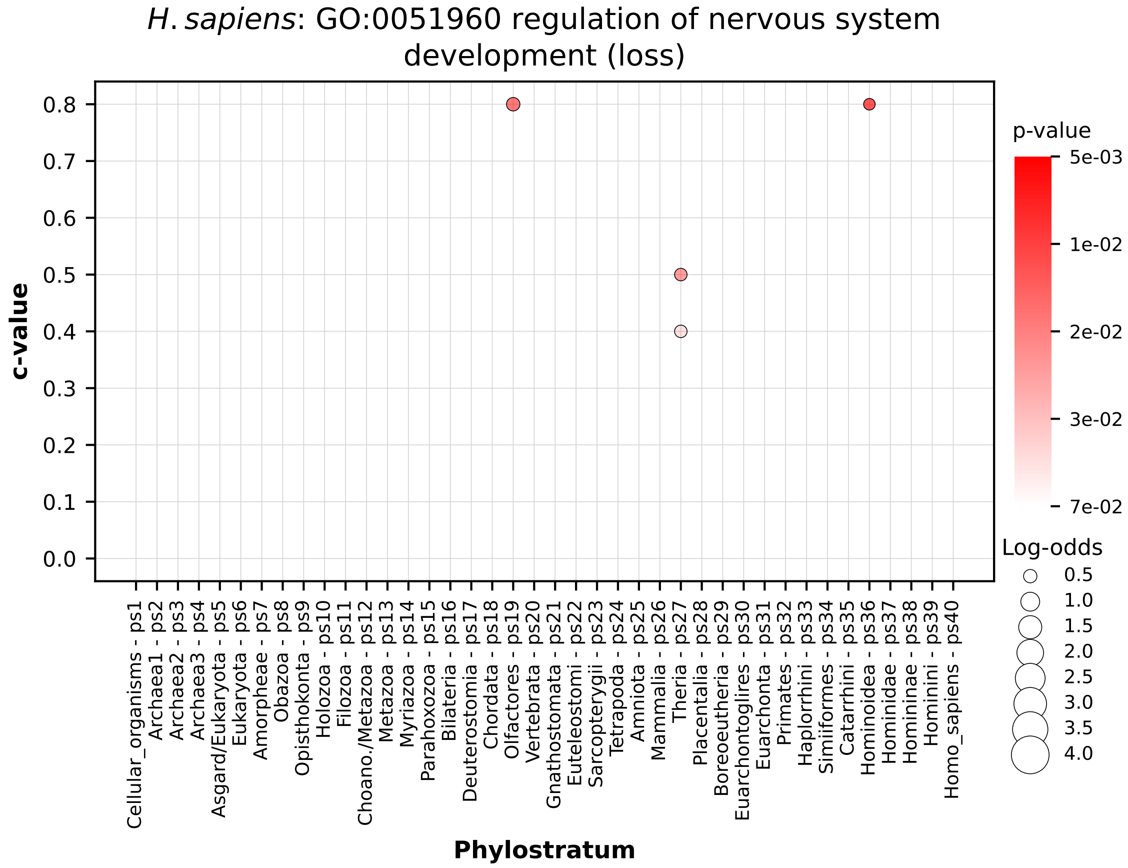

H. sapiens: GO:0051960 regulation of nervous system development (loss)
c-value
0.0
0.1
0.2
0.3
0.4
0.5
0.6
0.7
0.8
Phylostratum
Cellular_organisms - ps1
Archaea1 - ps2
Archaea2 - ps3
Archaea3 - ps4
Asgard/Eukaryota - ps5
Eukaryota - ps6
Amorpheae - ps7
Obazoa - ps8
Opisthokonta - ps9
Holozoa - ps10
Filozoa - ps11
Choano./Metazoa - ps12
Metazoa - ps13
Myriazoa - ps14
Parahoxozoa - ps15
Bilateria - ps16
Deuterostomia - ps17
Chordata - ps18
Olfactores - ps19
Vertebrata - ps20
Gnathostomata - ps21
Euteleostomi - ps22
Sarcopterygii - ps23
Tetrapoda - ps24
Amniota - ps25
Mammalia - ps26
Theria - ps27
Placentalia - ps28
Boreoeutheria - ps29
Euarchontoglires - ps30
Euarchonta - ps31
Primates - ps32
Haplorrhini - ps33
Simiiformes - ps34
Catarrhini - ps35
Hominoidea - ps36
Hominidae - ps37
Homininae - ps38
Hominini - ps39
Homo_sapiens - ps40
p-value
5e-03
1e-02
2e-02
3e-02
7e-02
Log-odds
0.5
1.0
1.5
2.0
2.5
3.0
3.5
4.0

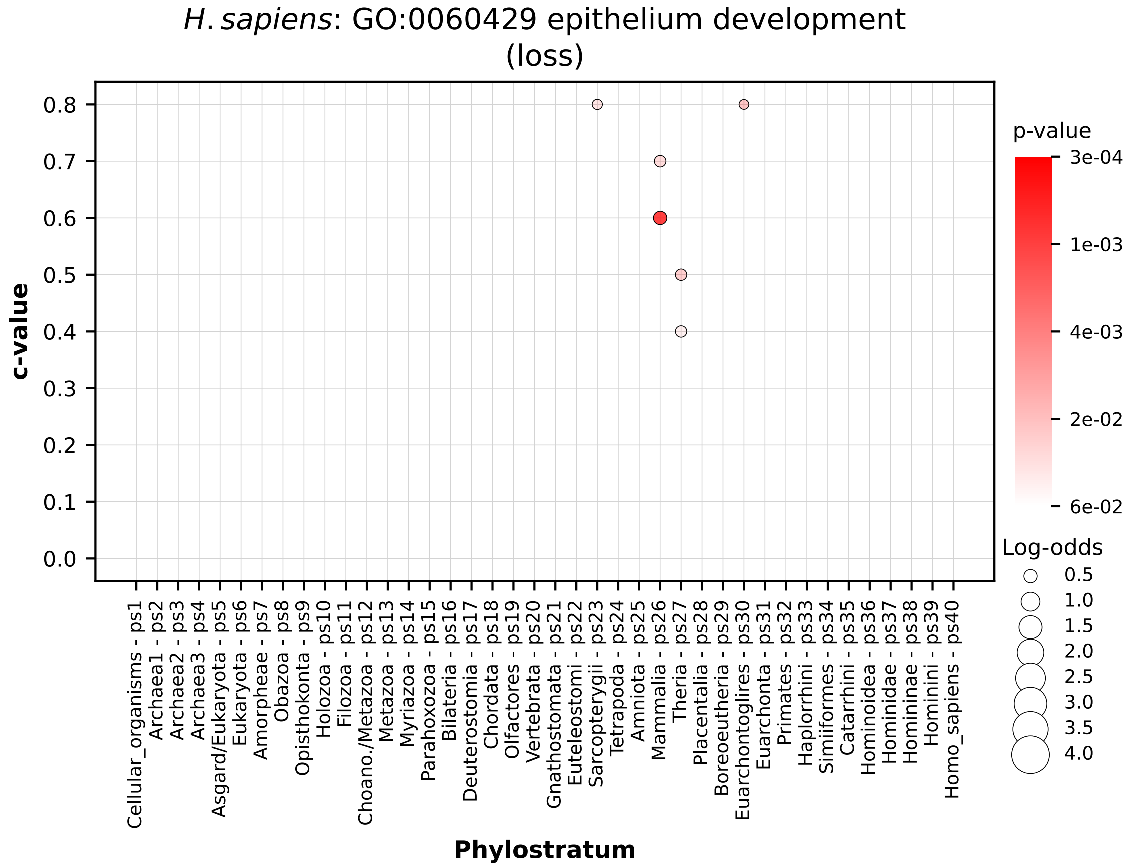

H. sapiens: GO:0060429 epithelium development
(loss)
c-value
0.0
0.1
0.2
0.3
0.4
0.5
0.6
0.7
0.8
Phylostratum
Cellular_organisms - ps1
Archaea1 - ps2
Archaea2 - ps3
Archaea3 - ps4
Asgard/Eukaryota - ps5
Eukaryota - ps6
Amorpheae - ps7
Obazoa - ps8
Opisthokonta - ps9
Holozoa - ps10
Filozoa - ps11
Choano./Metazoa - ps12
Metazoa - ps13
Myriazoa - ps14
Parahoxozoa - ps15
Bilateria - ps16
Deuterostomia - ps17
Chordata - ps18
Olfactores - ps19
Vertebrata - ps20
Gnathostomata - ps21
Euteleostomi - ps22
Sarcopterygii - ps23
Tetrapoda - ps24
Amniota - ps25
Mammalia - ps26
Theria - ps27
Placentalia - ps28
Boreoeutheria - ps29
Euarchontoglires - ps30
Euarchonta - ps31
Primates - ps32
Haplorrhini - ps33
Simiiformes - ps34
Catarrhini - ps35
Hominoidea - ps36
Hominidae - ps37
Homininae - ps38
Hominini - ps39
Homo_sapiens - ps40
p-value
3e-04
1e-03
4e-03
2e-02
6e-02
Log-odds
0.5
1.0
1.5
2.0
2.5
3.0
3.5
4.0

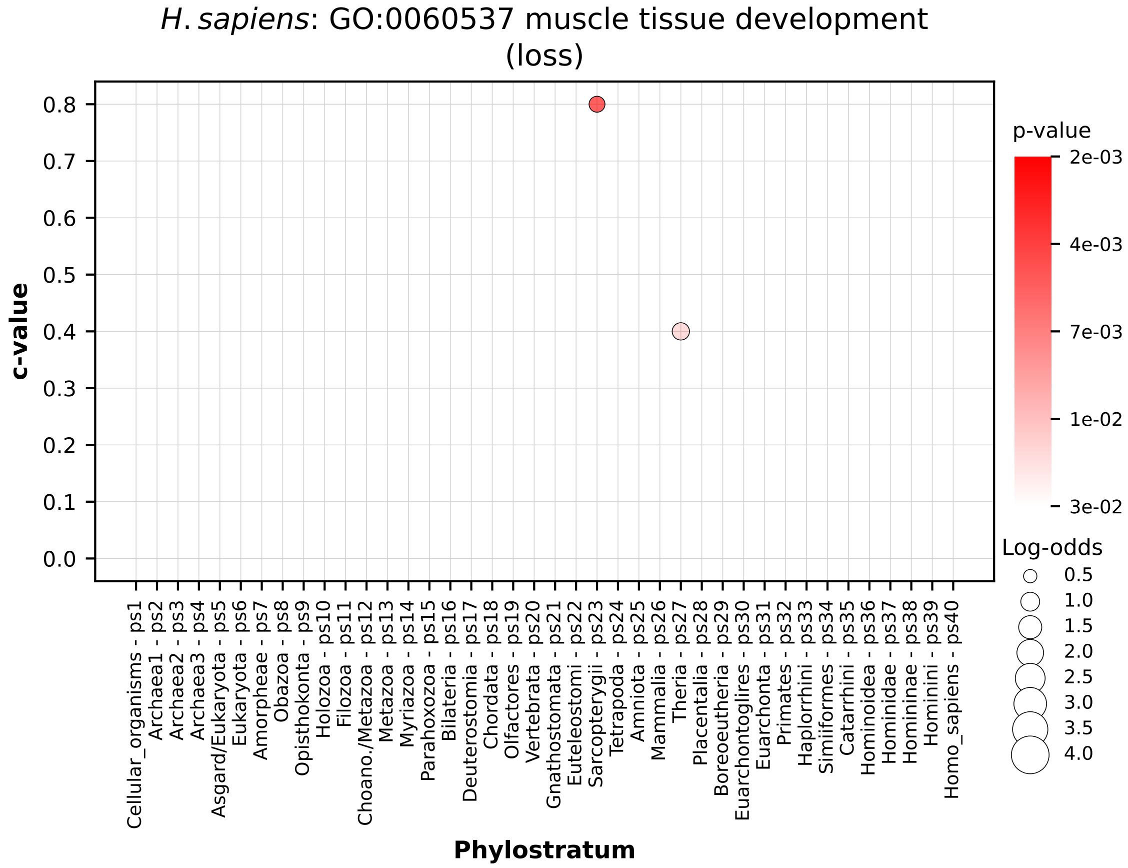

H. sapiens: GO:0060537 muscle tissue development (loss)
c-value
0.0
0.1
0.2
0.3
0.4
0.5
0.6
0.7
0.8
p-value
2e-03
4e-03
7e-03
1e-02
3e-02
Log-odds
0.5
1.0
1.5
2.0
2.5
3.0
3.5
4.0
Cellular_organisms - ps1
Archaea1 - ps2
Archaea2 - ps3
Archaea3 - ps4
Asgard/Eukaryota - ps5
Eukaryota - ps6
Amorpheae - ps7
Obazoa - ps8
Opisthokonta - ps9
Holozoa - ps10
Filozoa - ps11
Choano./Metazoa - ps12
Metazoa - ps13
Myriazoa - ps14
Parahoxozoa - ps15
Bilateria - ps16
Deuterostomia - ps17
Chordata - ps18
Olfactores - ps19
Vertebrata - ps20
Gnathostomata - ps21
Euteleostomi - ps22
Sarcopterygii - ps23
Tetrapoda - ps24
Amniota - ps25
Mammalia - ps26
Theria - ps27
Placentalia - ps28
Boreoeutheria - ps29
Euarchontoglires - ps30
Euarchonta - ps31
Primates - ps32
Haplorrhini - ps33
Simiiformes - ps34
Catarrhini - ps35
Hominoidea - ps36
Hominidae - ps37
Homininae - ps38
Hominini - ps39
Homo_sapiens - ps40
Phylostratum

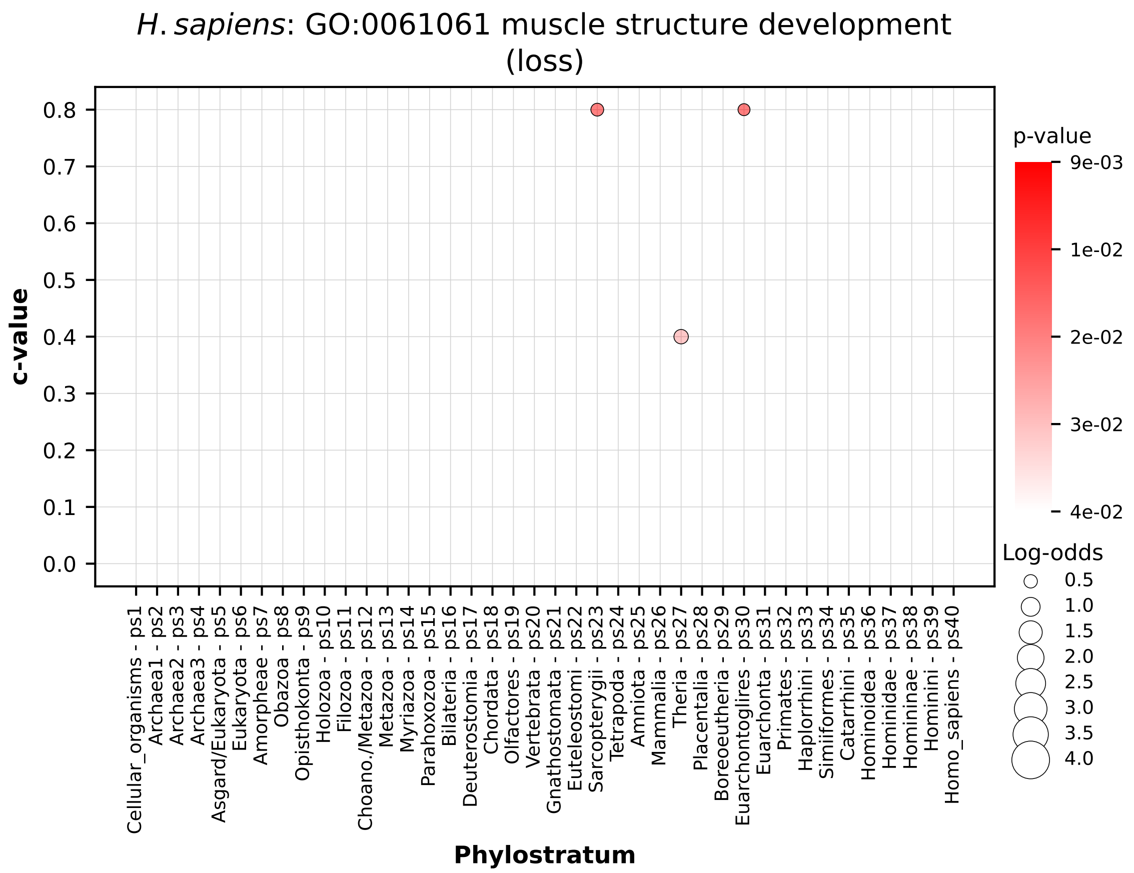

H. sapiens: GO:0061061 muscle structure development
(loss)
c-value
0.0
0.1
0.2
0.3
0.4
0.5
0.6
0.7
0.8
Phylostratum
Cellular_organisms - ps1
Archaea1 - ps2
Archaea2 - ps3
Archaea3 - ps4
Asgard/Eukaryota - ps5
Eukaryota - ps6
Amorpheae - ps7
Obazoa - ps8
Opisthokonta - ps9
Holozoa - ps10
Filozoa - ps11
Choano./Metazoa - ps12
Metazoa - ps13
Myriazoa - ps14
Parahoxozoa - ps15
Bilateria - ps16
Deuterostomia - ps17
Chordata - ps18
Olfactores - ps19
Vertebrata - ps20
Gnathostomata - ps21
Euteleostomi - ps22
Sarcopterygii - ps23
Tetrapoda - ps24
Amniota - ps25
Mammalia - ps26
Theria - ps27
Placentalia - ps28
Boreoeutheria - ps29
Euarchontoglires - ps30
Euarchonta - ps31
Primates - ps32
Haplorrhini - ps33
Simiiformes - ps34
Catarrhini - ps35
Hominoidea - ps36
Hominidae - ps37
Homininae - ps38
Hominini - ps39
Homo_sapiens - ps40
p-value
9e-03
1e-02
2e-02
3e-02
4e-02
Log-odds
0.5
1.0
1.5
2.0
2.5
3.0
3.5
4.0

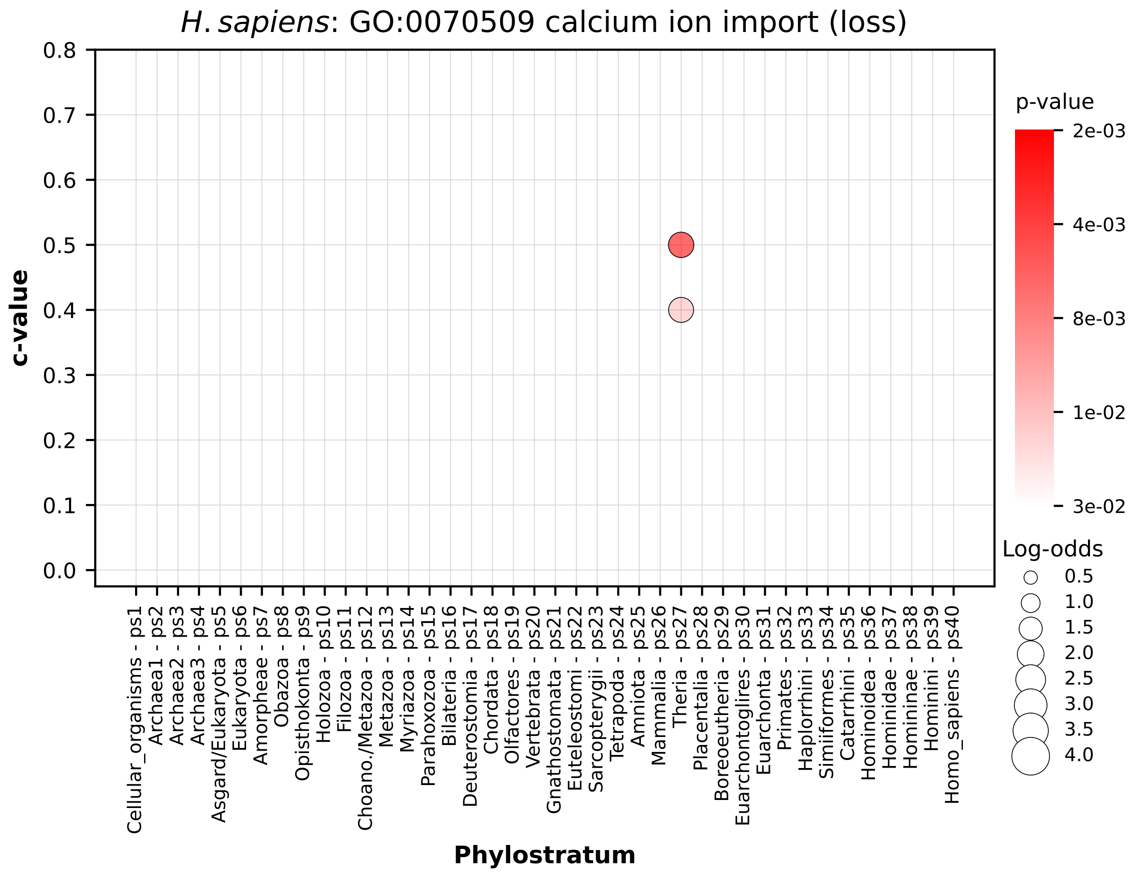

H. sapiens: GO:0070509 calcium ion import (loss)
c-value
0.0
0.1
0.2
0.3
0.4
0.5
0.6
0.7
0.8
Phylostratum
Cellular_organisms - ps1
Archaea1 - ps2
Archaea2 - ps3
Archaea3 - ps4
Asgard/Eukaryota - ps5
Eukaryota - ps6
Amorpheae - ps7
Obazoa - ps8
Opisthokonta - ps9
Holozoa - ps10
Filozoa - ps11
Choano./Metazoa - ps12
Metazoa - ps13
Myriazoa - ps14
Parahoxozoa - ps15
Bilateria - ps16
Deuterostomia - ps17
Chordata - ps18
Olfactores - ps19
Vertebrata - ps20
Gnathostomata - ps21
Euteleostomi - ps22
Sarcopterygii - ps23
Tetrapoda - ps24
Amniota - ps25
Mammalia - ps26
Theria - ps27
Placentalia - ps28
Boreoeutheria - ps29
Euarchontoglires - ps30
Euarchonta - ps31
Primates - ps32
Haplorrhini - ps33
Simiiformes - ps34
Catarrhini - ps35
Hominoidea - ps36
Hominidae - ps37
Homininae - ps38
Hominini - ps39
Homo_sapiens - ps40
p-value
2e-03
4e-03
8e-03
1e-02
3e-02
Log-odds
0.5
1.0
1.5
2.0
2.5
3.0
3.5
4.0

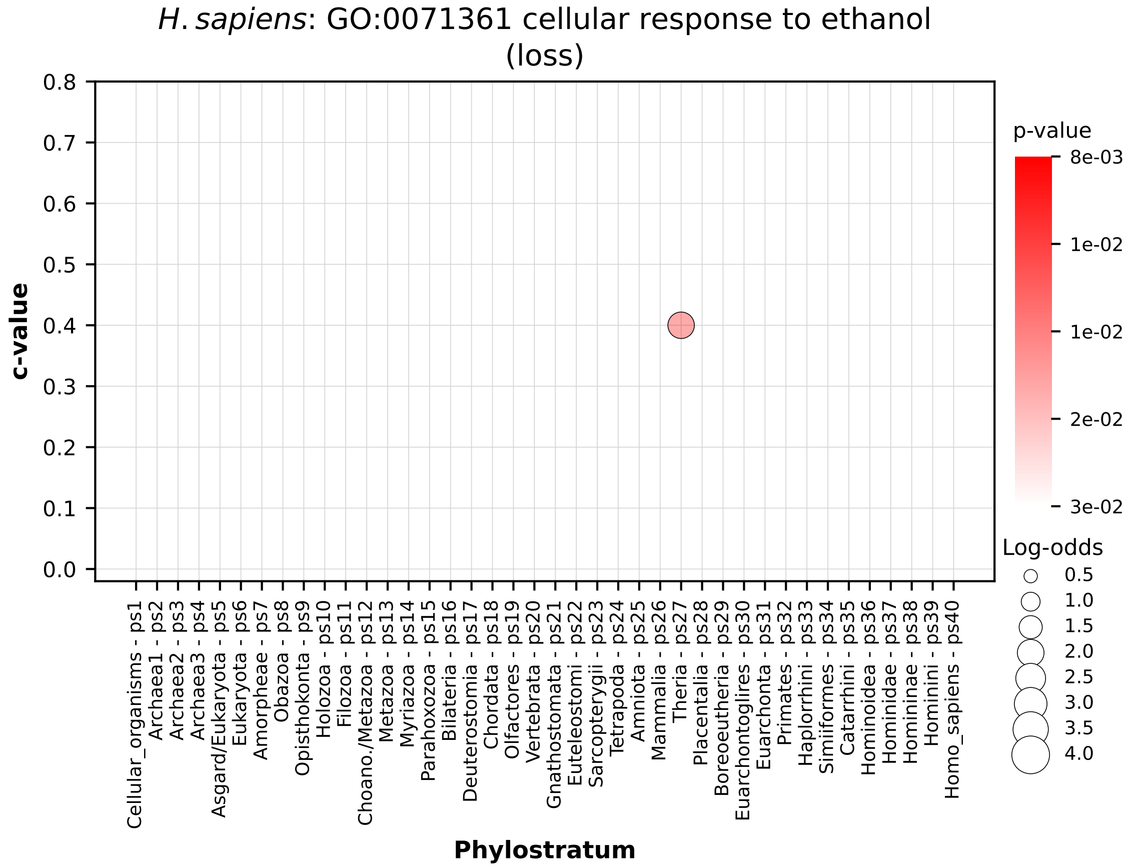

H. sapiens: GO:0071361 cellular response to ethanol (loss)
c-value
0.0
0.1
0.2
0.3
0.4
0.5
0.6
0.7
0.8
Phylostratum
Cellular_organisms - ps1
Archaea1 - ps2
Archaea2 - ps3
Archaea3 - ps4
Asgard/Eukaryota - ps5
Eukaryota - ps6
Amorpheae - ps7
Obazoa - ps8
Opisthokonta - ps9
Holozoa - ps10
Filozoa - ps11
Choano./Metazoa - ps12
Metazoa - ps13
Myriazoa - ps14
Parahoxozoa - ps15
Bilateria - ps16
Deuterostomia - ps17
Chordata - ps18
Olfactores - ps19
Vertebrata - ps20
Gnathostomata - ps21
Euteleostomi - ps22
Sarcopterygii - ps23
Tetrapoda - ps24
Amniota - ps25
Mammalia - ps26
Theria - ps27
Placentalia - ps28
Boreoeutheria - ps29
Euarchontoglires - ps30
Euarchonta - ps31
Primates - ps32
Haplorrhini - ps33
Simiiformes - ps34
Catarrhini - ps35
Hominoidea - ps36
Hominidae - ps37
Homininae - ps38
Hominini - ps39
Homo_sapiens - ps40
p-value
8e-03
1e-02
1e-02
2e-02
3e-02
Log-odds
0.5
1.0
1.5
2.0
2.5
3.0
3.5
4.0

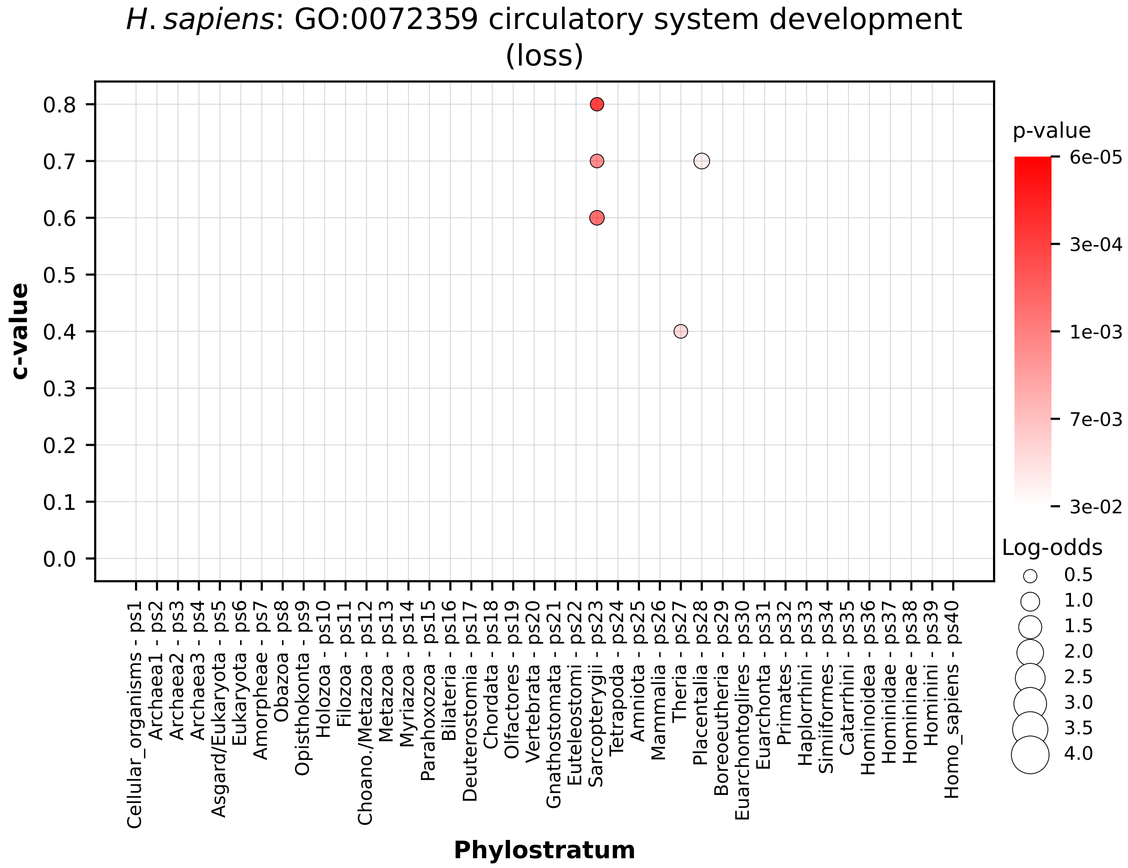

H. sapiens: GO:0072359 circulatory system development (loss)
c-value
0.0
0.1
0.2
0.3
0.4
0.5
0.6
0.7
0.8
Phylostratum
Cellular_organisms - ps1
Archaea1 - ps2
Archaea2 - ps3
Archaea3 - ps4
Asgard/Eukaryota - ps5
Eukaryota - ps6
Amorpheae - ps7
Obazoa - ps8
Opisthokonta - ps9
Holozoa - ps10
Filozoa - ps11
Choano./Metazoa - ps12
Metazoa - ps13
Myriazoa - ps14
Parahoxozoa - ps15
Bilateria - ps16
Deuterostomia - ps17
Chordata - ps18
Olfactores - ps19
Vertebrata - ps20
Gnathostomata - ps21
Euteleostomi - ps22
Sarcopterygii - ps23
Tetrapoda - ps24
Amniota - ps25
Mammalia - ps26
Theria - ps27
Placentalia - ps28
Boreoeutheria - ps29
Euarchontoglires - ps30
Euarchonta - ps31
Primates - ps32
Haplorrhini - ps33
Simiiformes - ps34
Catarrhini - ps35
Hominoidea - ps36
Hominidae - ps37
Homininae - ps38
Hominini - ps39
Homo_sapiens - ps40
p-value
6e-05
3e-04
1e-03
7e-03
3e-02
Log-odds
0.5
1.0
1.5
2.0
2.5
3.0
3.5
4.0

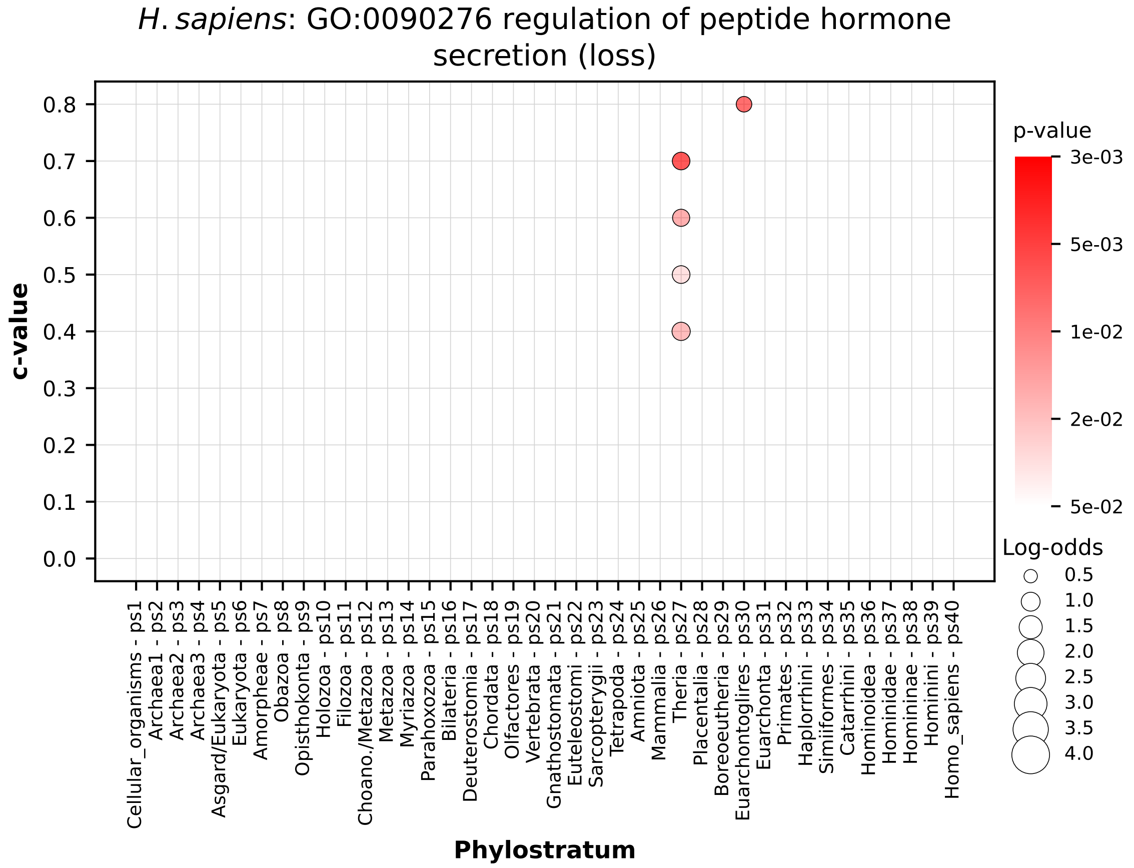
H. sapiens: GO:0090276 regulation of peptide hormone secretion (loss)
c-value
Phylostratum
p-value
3e-03
5e-03
1e-02
2e-02
5e-02
Log-odds
0.5
1.0
1.5
2.0
2.5
3.0
3.5
4.0
Cellular_organisms - ps1
Archaea1 - ps2
Archaea2 - ps3
Archaea3 - ps4
Asgard/Eukaryota - ps5
Eukaryota - ps6
Amorpheae - ps7
Obazoa - ps8
Opisthokonta - ps9
Holozoa - ps10
Filozoa - ps11
Choano./Metazoa - ps12
Metazoa - ps13
Myriazoa - ps14
Parahoxozoa - ps15
Bilateria - ps16
Deuterostomia - ps17
Chordata - ps18
Olfactores - ps19
Vertebrata - ps20
Gnathostomata - ps21
Euteleostomi - ps22
Sarcopterygii - ps23
Tetrapoda - ps24
Amniota - ps25
Mammalia - ps26
Theria - ps27
Placentalia - ps28
Boreoeutheria - ps29
Euarchontoglires - ps30
Euarchonta - ps31
Primates - ps32
Haplorrhini - ps33
Simiiformes - ps34
Catarrhini - ps35
Hominoidea - ps36
Hominidae - ps37
Homininae - ps38
Hominini - ps39
Homo_sapiens - ps40

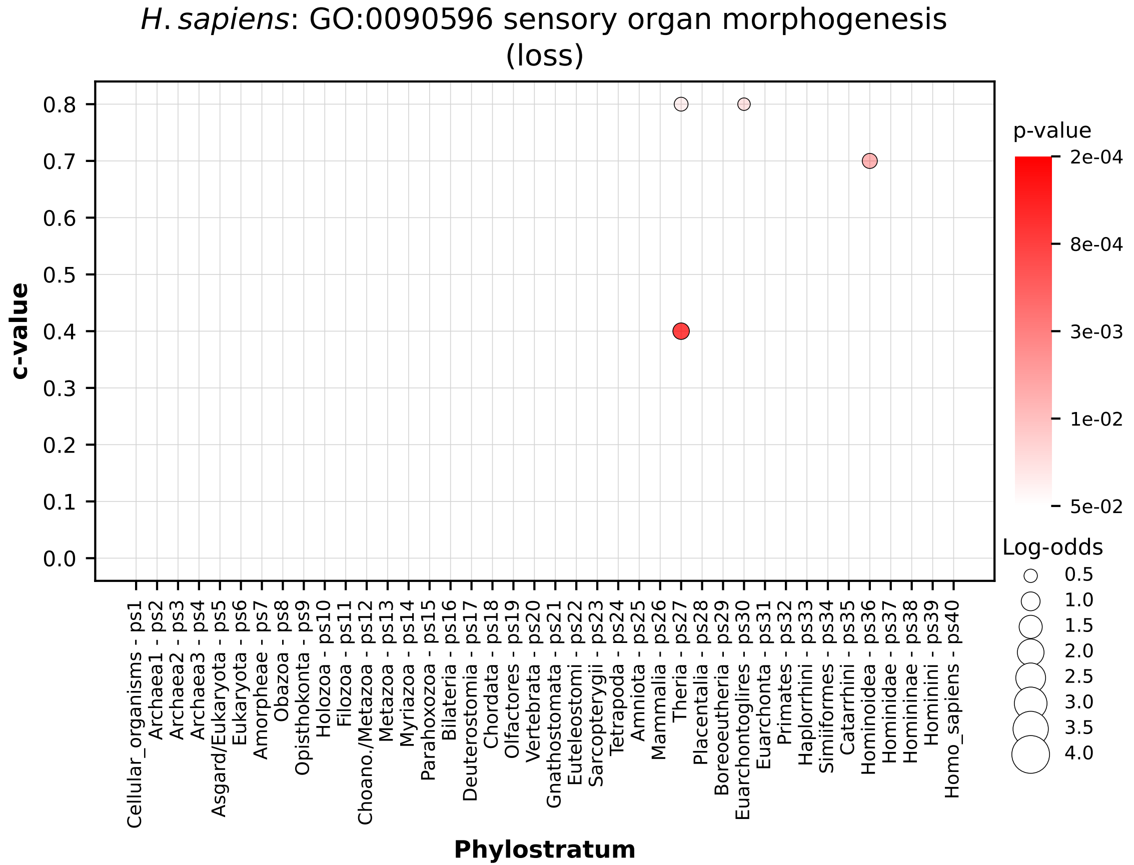
H. sapiens: GO:0090596 sensory organ morphogenesis
(loss)
c-value
0.0
0.1
0.2
0.3
0.4
0.5
0.6
0.7
0.8
p-value
2e-04
8e-04
3e-03
1e-02
5e-02
Log-odds
0.5
1.0
1.5
2.0
2.5
3.0
3.5
4.0
Cellular_organisms - ps1
Archaea1 - ps2
Archaea2 - ps3
Archaea3 - ps4
Asgard/Eukaryota - ps5
Eukaryota - ps6
Amorpheae - ps7
Obazoa - ps8
Opisthokonta - ps9
Holozoa - ps10
Filozoa - ps11
Choano./Metazoa - ps12
Metazoa - ps13
Myriazoa - ps14
Parahoxozoa - ps15
Bilateria - ps16
Deuterostomia - ps17
Chordata - ps18
Olfactores - ps19
Vertebrata - ps20
Gnathostomata - ps21
Euteleostomi - ps22
Sarcopterygii - ps23
Tetrapoda - ps24
Amniota - ps25
Mammalia - ps26
Theria - ps27
Placentalia - ps28
Boreoeutheria - ps29
Euarchontoglires - ps30
Euarchonta - ps31
Primates - ps32
Haplorrhini - ps33
Simiiformes - ps34
Catarrhini - ps35
Hominoidea - ps36
Hominidae - ps37
Homininae - ps38
Hominini - ps39
Homo_sapiens - ps40
Phylostratum

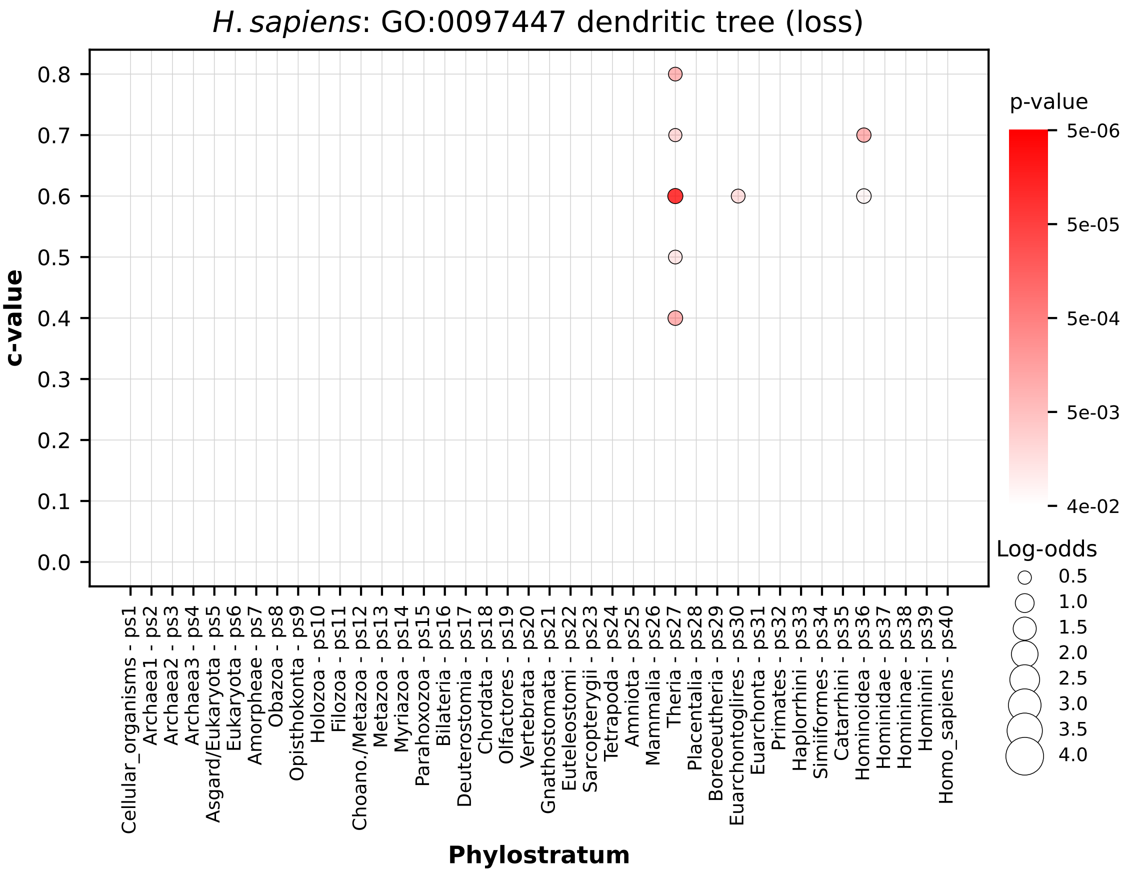

H. sapiens: GO:0097447 dendritic tree (loss)
c-value
0.8
0.7
0.6
0.5
0.4
0.3
0.2
0.1
0.0
Phylostratum
Cellular_organisms - ps1
Archaea1 - ps2
Archaea2 - ps3
Archaea3 - ps4
Asgard/Eukaryota - ps5
Eukaryota - ps6
Amorpheae - ps7
Obazoa - ps8
Opisthokonta - ps9
Holozoa - ps10
Filozoa - ps11
Choano./Metazoa - ps12
Metazoa - ps13
Myriazoa - ps14
Parahoxozoa - ps15
Bilateria - ps16
Deuterostomia - ps17
Chordata - ps18
Olfactores - ps19
Vertebrata - ps20
Gnathostomata - ps21
Euteleostomi - ps22
Sarcopterygii - ps23
Tetrapoda - ps24
Amniota - ps25
Mammalia - ps26
Theria - ps27
Placentalia - ps28
Boreoeutheria - ps29
Euarchontoglires - ps30
Euarchonta - ps31
Primates - ps32
Haplorrhini - ps33
Simiiformes - ps34
Catarrhini - ps35
Hominoidea - ps36
Hominidae - ps37
Homininae - ps38
Hominini - ps39
Homo_sapiens - ps40
p-value
5e-06
5e-05
5e-04
5e-03
4e-02
Log-odds
0.5
1.0
1.5
2.0
2.5
3.0
3.5
4.0

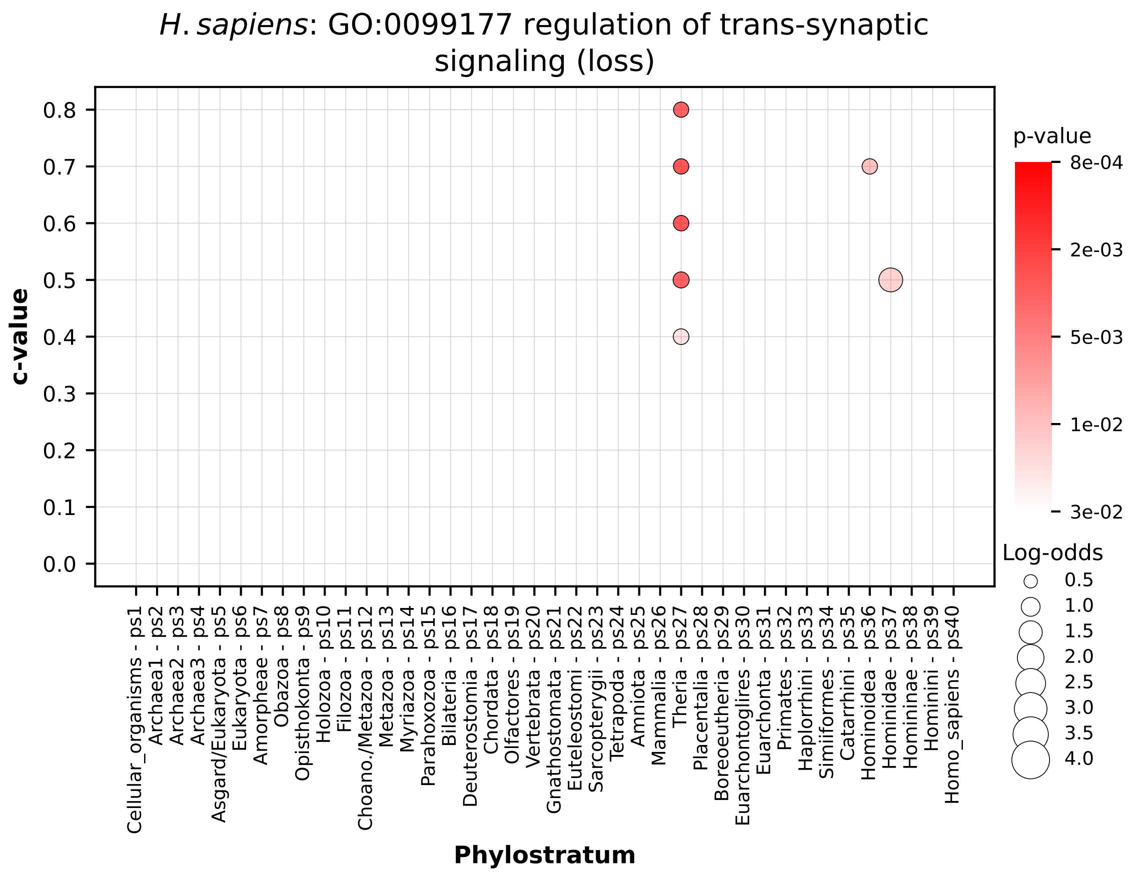
H. sapiens: GO:0099177 regulation of trans-synaptic signaling (loss)
c-value
0.8
0.7
0.6
0.5
0.4
0.3
0.2
0.1
0.0
Phylostratum
Cellular_organisms - ps1
Archaea1 - ps2
Archaea2 - ps3
Archaea3 - ps4
Asgard/Eukaryota - ps5
Eukaryota - ps6
Amorpheae - ps7
Obazoa - ps8
Opisthokonta - ps9
Holozoa - ps10
Filozoa - ps11
Choano./Metazoa - ps12
Metazoa - ps13
Myriazoa - ps14
Parahoxozoa - ps15
Bilateria - ps16
Deuterostomia - ps17
Chordata - ps18
Olfactores - ps19
Vertebrata - ps20
Gnathostomata - ps21
Euteleostomi - ps22
Sarcopterygii - ps23
Tetrapoda - ps24
Amniota - ps25
Mammalia - ps26
Theria - ps27
Placentalia - ps28
Boreoeutheria - ps29
Euarchontoglires - ps30
Euarchonta - ps31
Primates - ps32
Haplorrhini - ps33
Simiiformes - ps34
Catarrhini - ps35
Hominoidea - ps36
Hominidae - ps37
Homininae - ps38
Hominini - ps39
Homo_sapiens - ps40
p-value
8e-04
2e-03
5e-03
1e-02
3e-02
Log-odds
0.5
1.0
1.5
2.0
2.5
3.0
3.5
4.0

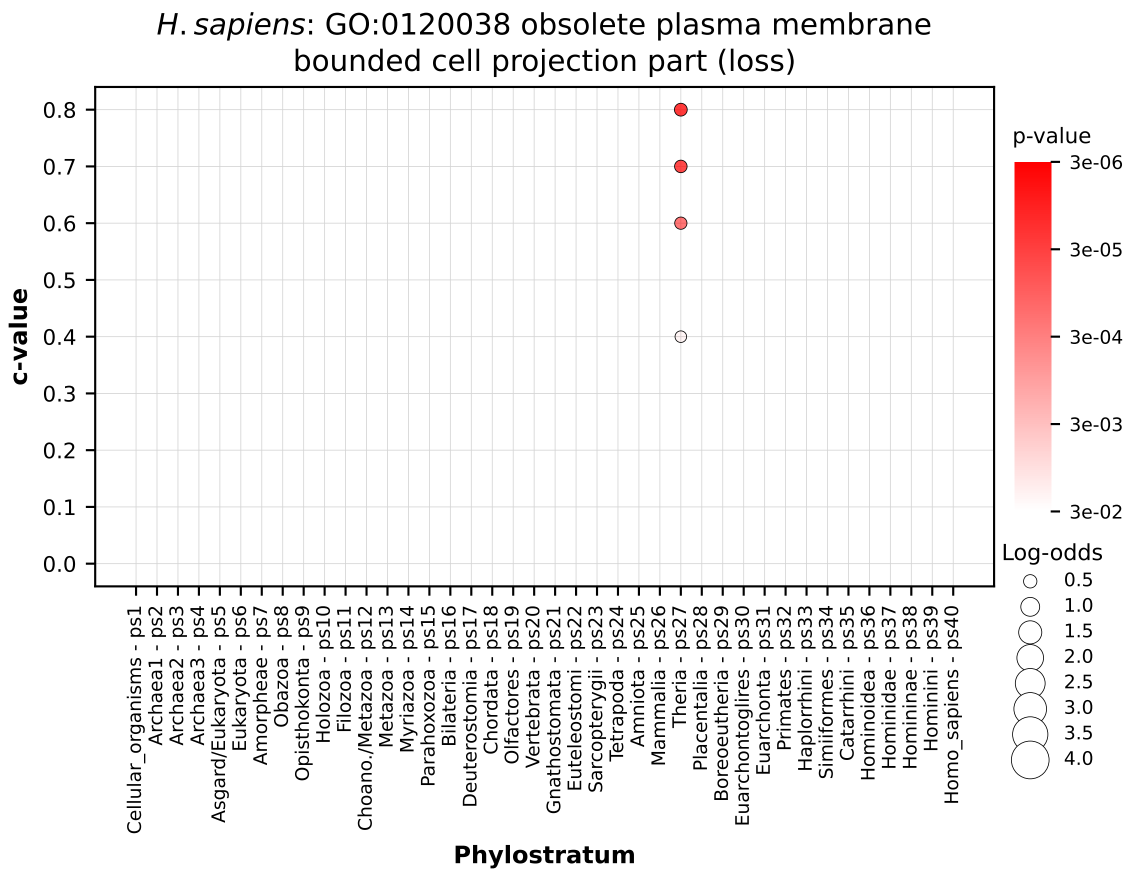

H. sapiens: GO:0120038 obsolete plasma membrane bounded cell projection part (loss)
c-value
0.0
0.1
0.2
0.3
0.4
0.5
0.6
0.7
0.8
Phylostratum
Cellular_organisms - ps1
Archaea1 - ps2
Archaea2 - ps3
Archaea3 - ps4
Asgard/Eukaryota - ps5
Eukaryota - ps6
Amorpheae - ps7
Obazoa - ps8
Opisthokonta - ps9
Holozoa - ps10
Filozoa - ps11
Choano./Metazoa - ps12
Metazoa - ps13
Myriazoa - ps14
Parahoxozoa - ps15
Bilateria - ps16
Deuterostomia - ps17
Chordata - ps18
Olfactores - ps19
Vertebrata - ps20
Gnathostomata - ps21
Euteleostomi - ps22
Sarcopterygii - ps23
Tetrapoda - ps24
Amniota - ps25
Mammalia - ps26
Theria - ps27
Placentalia - ps28
Boreoeutheria - ps29
Euarchontoglires - ps30
Euarchonta - ps31
Primates - ps32
Haplorrhini - ps33
Simiiformes - ps34
Catarrhini - ps35
Hominoidea - ps36
Hominidae - ps37
Homininae - ps38
Hominini - ps39
Homo_sapiens - ps40
p-value
3e-06
3e-05
3e-04
3e-03
3e-02
Log-odds
0.5
1.0
1.5
2.0
2.5
3.0
3.5
4.0

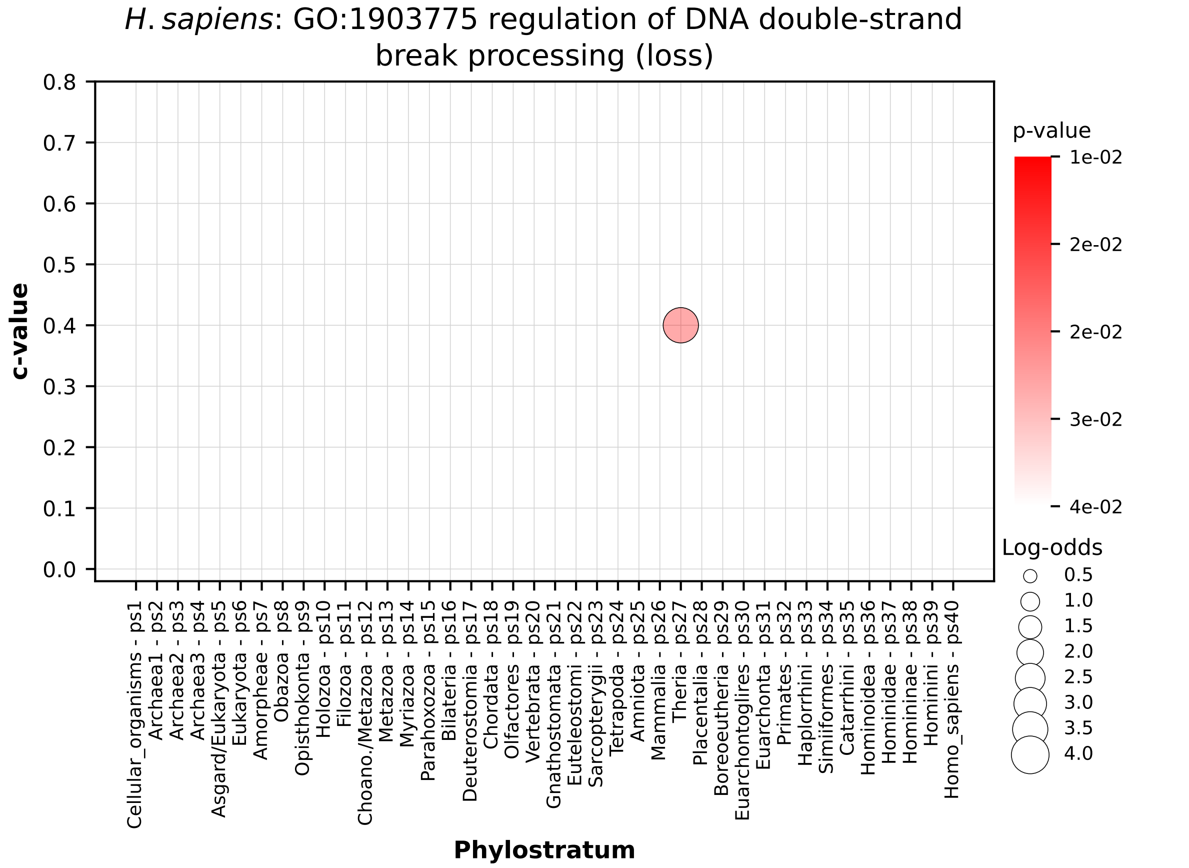

H. sapiens: GO:1903775 regulation of DNA double-strand break processing (loss)
c-value
0.8
0.7
0.6
0.5
0.4
0.3
0.2
0.1
0.0
Phylostratum
Cellular_organisms - ps1
Archaea1 - ps2
Archaea2 - ps3
Archaea3 - ps4
Asgard/Eukaryota - ps5
Eukaryota - ps6
Amorpheae - ps7
Obazoa - ps8
Opisthokonta - ps9
Holozoa - ps10
Filozoa - ps11
Choano./Metazoa - ps12
Metazoa - ps13
Myriazoa - ps14
Parahoxozoa - ps15
Bilateria - ps16
Deuterostomia - ps17
Chordata - ps18
Olfactores - ps19
Vertebrata - ps20
Gnathostomata - ps21
Euteleostomi - ps22
Sarcopterygii - ps23
Tetrapoda - ps24
Amniota - ps25
Mammalia - ps26
Theria - ps27
Placentalia - ps28
Boreoeutheria - ps29
Euarchontoglires - ps30
Euarchonta - ps31
Primates - ps32
Haplorrhini - ps33
Simiiformes - ps34
Catarrhini - ps35
Hominoidea - ps36
Hominidae - ps37
Homininae - ps38
Hominini - ps39
Homo_sapiens - ps40
p-value
1e-02
2e-02
2e-02
3e-02
4e-02
Log-odds
0.5
1.0
1.5
2.0
2.5
3.0
3.5
4.0

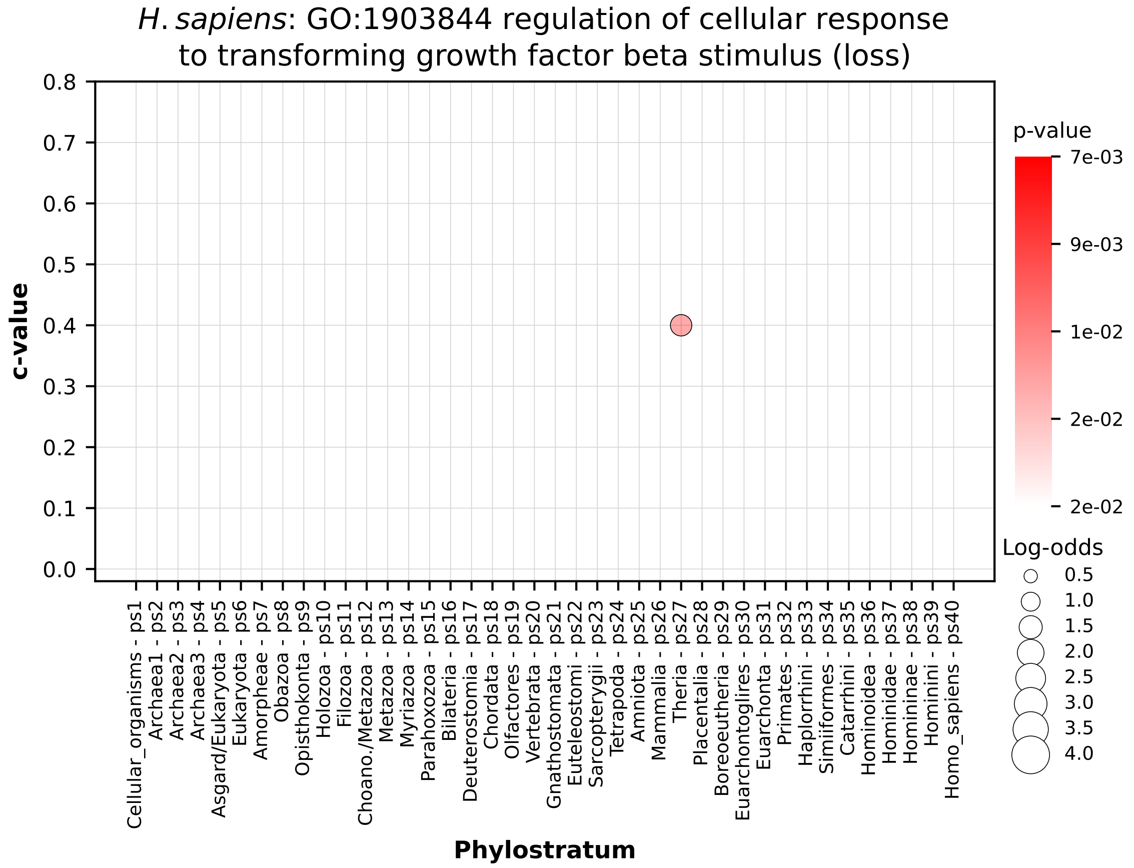

H. sapiens: GO:1903844 regulation of cellular response to transforming growth factor beta stimulus (loss)
c-value
0.0
0.1
0.2
0.3
0.4
0.5
0.6
0.7
0.8
Phylostratum
Cellular_organisms - ps1
Archaea1 - ps2
Archaea2 - ps3
Archaea3 - ps4
Asgard/Eukaryota - ps5
Eukaryota - ps6
Amorpheae - ps7
Obazoa - ps8
Opisthokonta - ps9
Holozoa - ps10
Filozoa - ps11
Choano./Metazoa - ps12
Metazoa - ps13
Myriazoa - ps14
Parahoxozoa - ps15
Bilateria - ps16
Deuterostomia - ps17
Chordata - ps18
Olfactores - ps19
Vertebrata - ps20
Gnathostomata - ps21
Euteleostomi - ps22
Sarcopterygii - ps23
Tetrapoda - ps24
Amniota - ps25
Mammalia - ps26
Theria - ps27
Placentalia - ps28
Boreoeutheria - ps29
Euarchontoglires - ps30
Euarchonta - ps31
Primates - ps32
Haplorrhini - ps33
Simiiformes - ps34
Catarrhini - ps35
Hominoidea - ps36
Hominidae - ps37
Homininae - ps38
Hominini - ps39
Homo_sapiens - ps40
p-value
7e-03
9e-03
1e-02
2e-02
2e-02
Log-odds
0.5
1.0
1.5
2.0
2.5
3.0
3.5
4.0

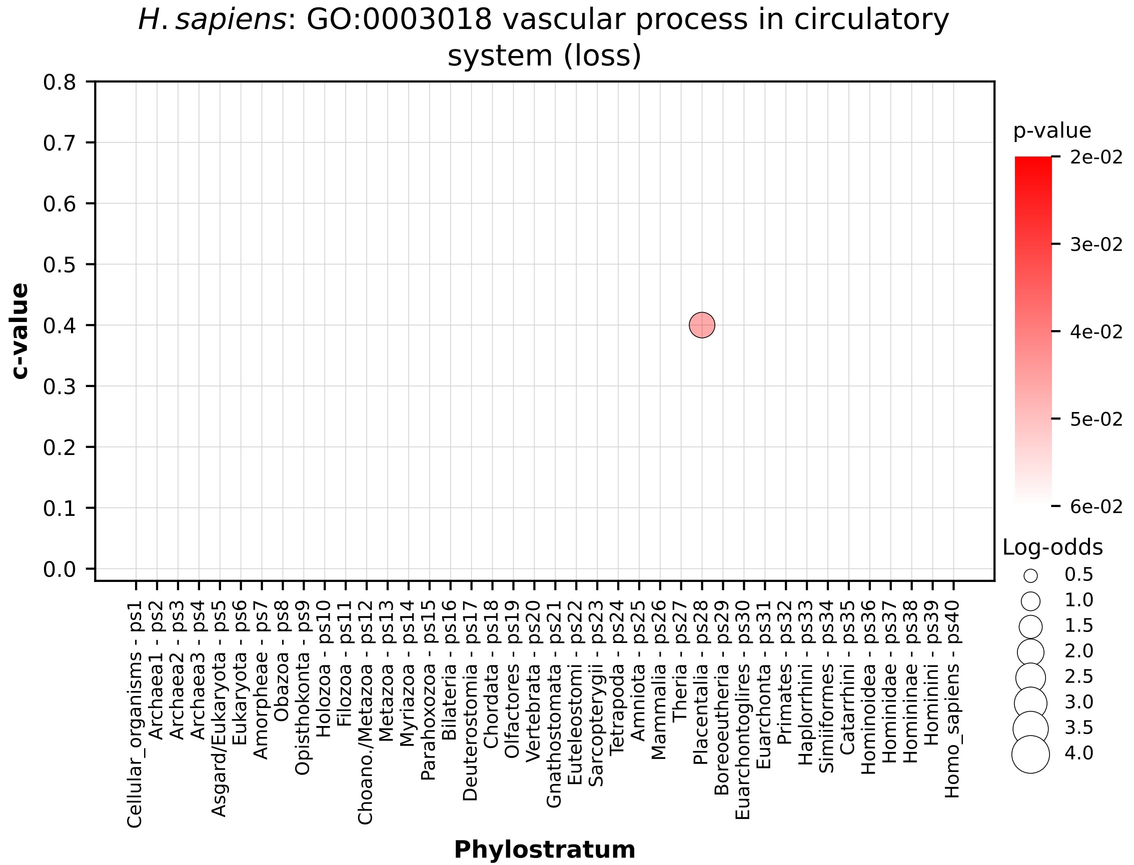

H. sapiens: GO:0003018 vascular process in circulatory system (loss)
c-value
0.0
0.1
0.2
0.3
0.4
0.5
0.6
0.7
0.8
Phylostratum
Cellular_organisms - ps1
Archaea1 - ps2
Archaea2 - ps3
Archaea3 - ps4
Asgard/Eukaryota - ps5
Eukaryota - ps6
Amorpheae - ps7
Obazoa - ps8
Opisthokonta - ps9
Holozoa - ps10
Filozoa - ps11
Choano./Metazoa - ps12
Metazoa - ps13
Myriazoa - ps14
Parahoxozoa - ps15
Bilateria - ps16
Deuterostomia - ps17
Chordata - ps18
Olfactores - ps19
Vertebrata - ps20
Gnathostomata - ps21
Euteleostomi - ps22
Sarcopterygii - ps23
Tetrapoda - ps24
Amniota - ps25
Mammalia - ps26
Theria - ps27
Placentalia - ps28
Boreoeutheria - ps29
Euarchontoglires - ps30
Euarchonta - ps31
Primates - ps32
Haplorrhini - ps33
Simiiformes - ps34
Catarrhini - ps35
Hominoidea - ps36
Hominidae - ps37
Homininae - ps38
Hominini - ps39
Homo_sapiens - ps40
p-value
2e-02
3e-02
4e-02
5e-02
6e-02
Log-odds
0.5
1.0
1.5
2.0
2.5
3.0
3.5
4.0

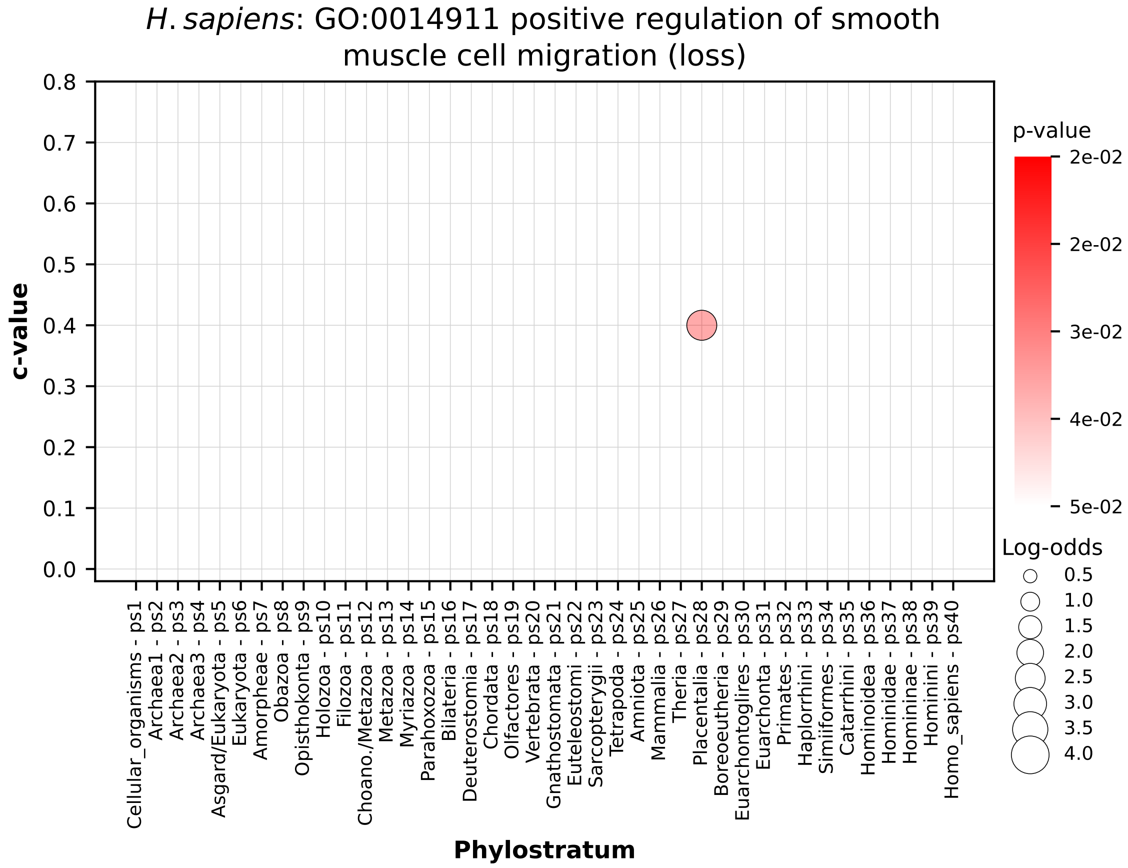
H. sapiens: GO:0014911 positive regulation of smooth muscle cell migration (loss)
c-value
0.0
0.1
0.2
0.3
0.4
0.5
0.6
0.7
0.8
Phylostratum
Cellular_organisms - ps1
Archaea1 - ps2
Archaea2 - ps3
Archaea3 - ps4
Asgard/Eukaryota - ps5
Eukaryota - ps6
Amorpheae - ps7
Obazoa - ps8
Opisthokonta - ps9
Holozoa - ps10
Filozoa - ps11
Choano./Metazoa - ps12
Metazoa - ps13
Myriazoa - ps14
Parahoxozoa - ps15
Bilateria - ps16
Deuterostomia - ps17
Chordata - ps18
Olfactores - ps19
Vertebrata - ps20
Gnathostomata - ps21
Euteleostomi - ps22
Sarcopterygii - ps23
Tetrapoda - ps24
Amniota - ps25
Mammalia - ps26
Theria - ps27
Placentalia - ps28
Boreoeutheria - ps29
Euarchontoglires - ps30
Euarchonta - ps31
Primates - ps32
Haplorrhini - ps33
Simiiformes - ps34
Catarrhini - ps35
Hominoidea - ps36
Hominidae - ps37
Homininae - ps38
Hominini - ps39
Homo_sapiens - ps40
p-value
2e-02
2e-02
3e-02
4e-02
5e-02
Log-odds
0.5
1.0
1.5
2.0
2.5
3.0
3.5
4.0

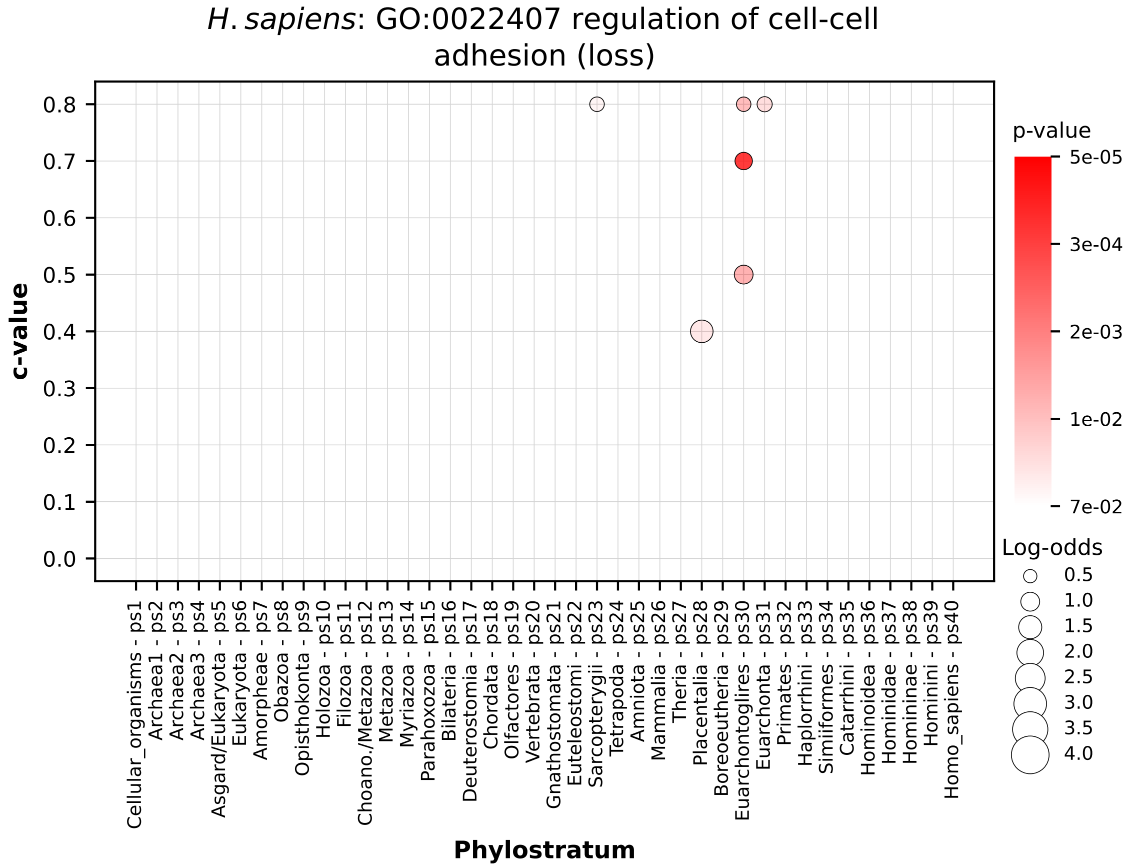

H. sapiens: GO:0022407 regulation of cell-cell adhesion (loss)
c-value
0.0
0.1
0.2
0.3
0.4
0.5
0.6
0.7
0.8
Phylostratum
Cellular_organisms - ps1
Archaea1 - ps2
Archaea2 - ps3
Archaea3 - ps4
Asgard/Eukaryota - ps5
Eukaryota - ps6
Amorpheae - ps7
Obazoa - ps8
Opisthokonta - ps9
Holozoa - ps10
Filozoa - ps11
Choano./Metazoa - ps12
Metazoa - ps13
Myriazoa - ps14
Parahoxozoa - ps15
Bilateria - ps16
Deuterostomia - ps17
Chordata - ps18
Olfactores - ps19
Vertebrata - ps20
Gnathostomata - ps21
Euteleostomi - ps22
Sarcopterygii - ps23
Tetrapoda - ps24
Amniota - ps25
Mammalia - ps26
Theria - ps27
Placentalia - ps28
Boreoeutheria - ps29
Euarchontoglires - ps30
Euarchonta - ps31
Primates - ps32
Haplorrhini - ps33
Simiiformes - ps34
Catarrhini - ps35
Hominoidea - ps36
Hominidae - ps37
Homininae - ps38
Hominini - ps39
Homo_sapiens - ps40
p-value
5e-05
3e-04
2e-03
1e-02
7e-02
Log-odds
0.5
1.0
1.5
2.0
2.5
3.0
3.5
4.0

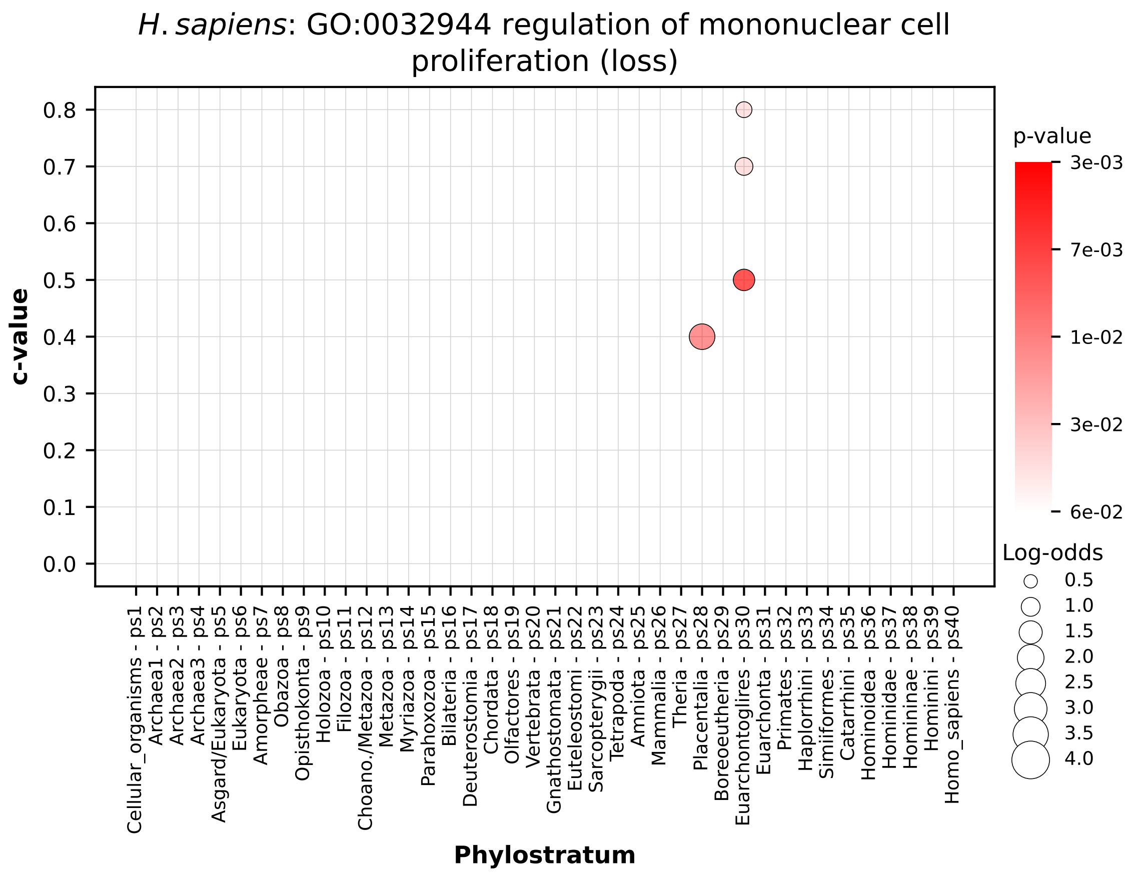

H. sapiens: GO:0032944 regulation of mononuclear cell proliferation (loss)
c-value
0.8
0.7
0.6
0.5
0.4
0.3
0.2
0.1
0.0
Phylostratum
Cellular_organisms - ps1
Archaea1 - ps2
Archaea2 - ps3
Archaea3 - ps4
Asgard/Eukaryota - ps5
Eukaryota - ps6
Amorpheae - ps7
Obazoa - ps8
Opisthokonta - ps9
Holozoa - ps10
Filozoa - ps11
Choano./Metazoa - ps12
Metazoa - ps13
Myriazoa - ps14
Parahoxozoa - ps15
Bilateria - ps16
Deuterostomia - ps17
Chordata - ps18
Olfactores - ps19
Vertebrata - ps20
Gnathostomata - ps21
Euteleostomi - ps22
Sarcopterygii - ps23
Tetrapoda - ps24
Amniota - ps25
Mammalia - ps26
Theria - ps27
Placentalia - ps28
Boreoeutheria - ps29
Euarchontoglires - ps30
Euarchonta - ps31
Primates - ps32
Haplorrhini - ps33
Simiiformes - ps34
Catarrhini - ps35
Hominoidea - ps36
Hominidae - ps37
Homininae - ps38
Hominini - ps39
Homo_sapiens - ps40
p-value
3e-03
7e-03
1e-02
3e-02
6e-02
Log-odds
0.5
1.0
1.5
2.0
2.5
3.0
3.5
4.0

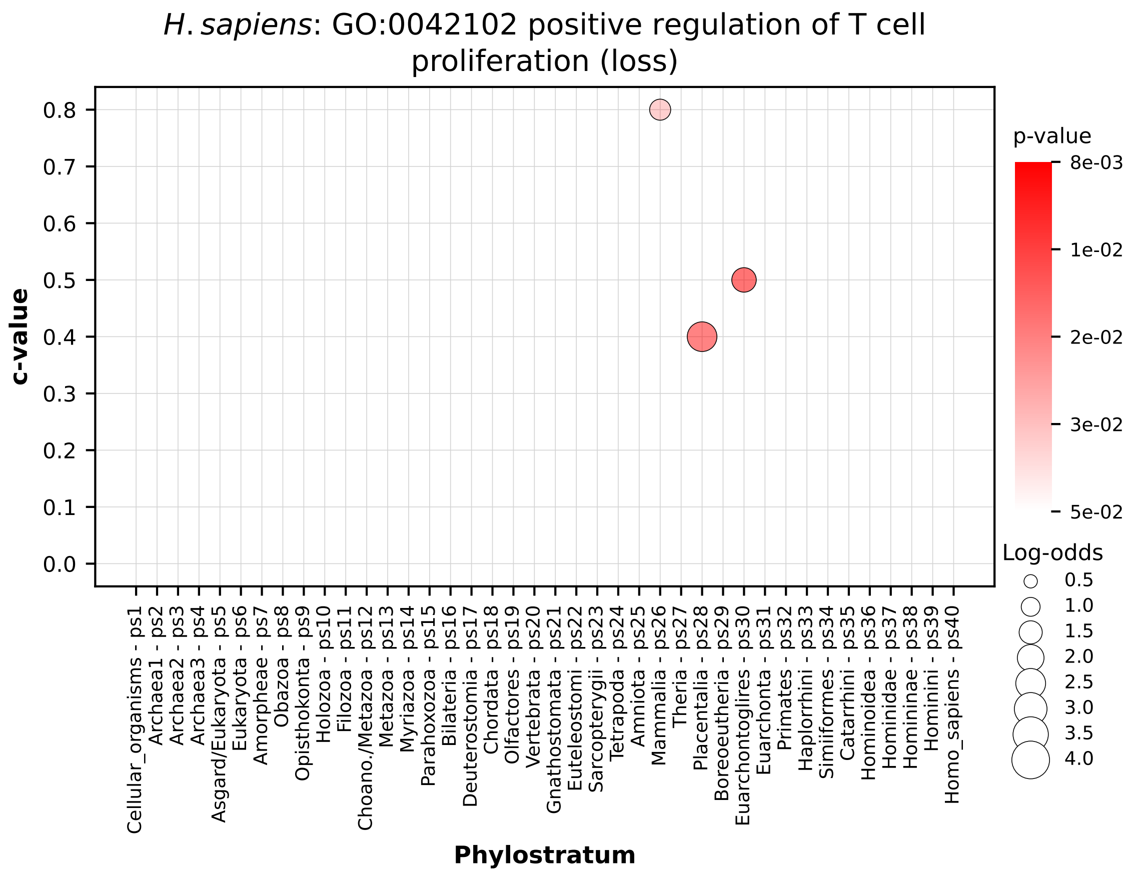

H. sapiens: GO:0042102 positive regulation of T cell proliferation (loss)
c-value
0.8
0.7
0.6
0.5
0.4
0.3
0.2
0.1
0.0
Phylostratum
Cellular_organisms - ps1
Archaea1 - ps2
Archaea2 - ps3
Archaea3 - ps4
Asgard/Eukaryota - ps5
Eukaryota - ps6
Amorpheae - ps7
Obazoa - ps8
Opisthokonta - ps9
Holozoa - ps10
Filozoa - ps11
Choano./Metazoa - ps12
Metazoa - ps13
Myriazoa - ps14
Parahoxozoa - ps15
Bilateria - ps16
Deuterostomia - ps17
Chordata - ps18
Olfactores - ps19
Vertebrata - ps20
Gnathostomata - ps21
Euteleostomi - ps22
Sarcopterygii - ps23
Tetrapoda - ps24
Amniota - ps25
Mammalia - ps26
Theria - ps27
Placentalia - ps28
Boreoeutheria - ps29
Euarchontoglires - ps30
Euarchonta - ps31
Primates - ps32
Haplorrhini - ps33
Simiiformes - ps34
Catarrhini - ps35
Hominoidea - ps36
Hominidae - ps37
Homininae - ps38
Hominini - ps39
Homo_sapiens - ps40
p-value
8e-03
1e-02
2e-02
3e-02
5e-02
Log-odds
0.5
1.0
1.5
2.0
2.5
3.0
3.5
4.0

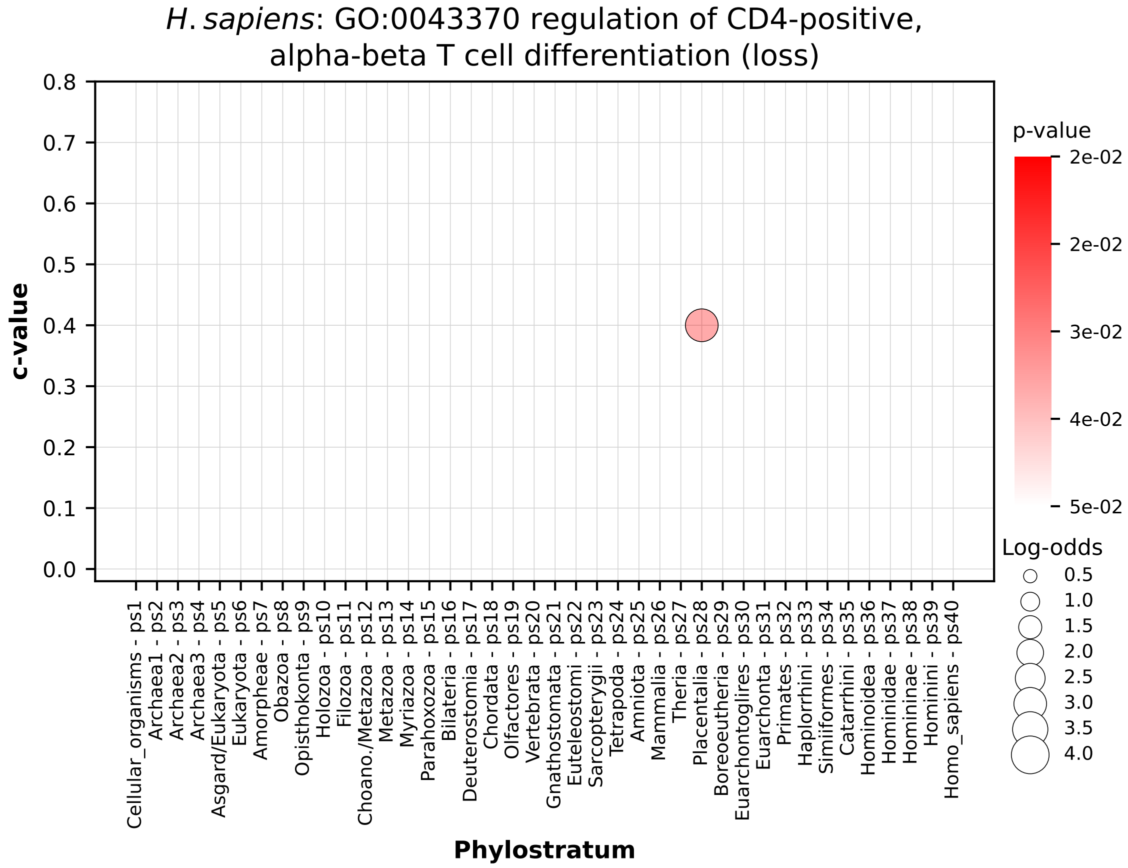
H. sapiens: GO:0043370 regulation of CD4-positive, alpha-beta T cell differentiation (loss)
c-value
0.0
0.1
0.2
0.3
0.4
0.5
0.6
0.7
0.8
p-value
2e-02
2e-02
3e-02
4e-02
5e-02
Log-odds
0.5
1.0
1.5
2.0
2.5
3.0
3.5
4.0
Cellular_organisms - ps1
Archaea1 - ps2
Archaea2 - ps3
Archaea3 - ps4
Asgard/Eukaryota - ps5
Eukaryota - ps6
Amorpheae - ps7
Obazoa - ps8
Opisthokonta - ps9
Holozoa - ps10
Filozoa - ps11
Choano./Metazoa - ps12
Metazoa - ps13
Myriazoa - ps14
Parahoxozoa - ps15
Bilateria - ps16
Deuterostomia - ps17
Chordata - ps18
Olfactores - ps19
Vertebrata - ps20
Gnathostomata - ps21
Euteleostomi - ps22
Sarcopterygii - ps23
Tetrapoda - ps24
Amniota - ps25
Mammalia - ps26
Theria - ps27
Placentalia - ps28
Boreoeutheria - ps29
Euarchontoglires - ps30
Euarchonta - ps31
Primates - ps32
Haplorrhini - ps33
Simiiformes - ps34
Catarrhini - ps35
Hominoidea - ps36
Hominidae - ps37
Homininae - ps38
Hominini - ps39
Homo_sapiens - ps40
Phylostratum

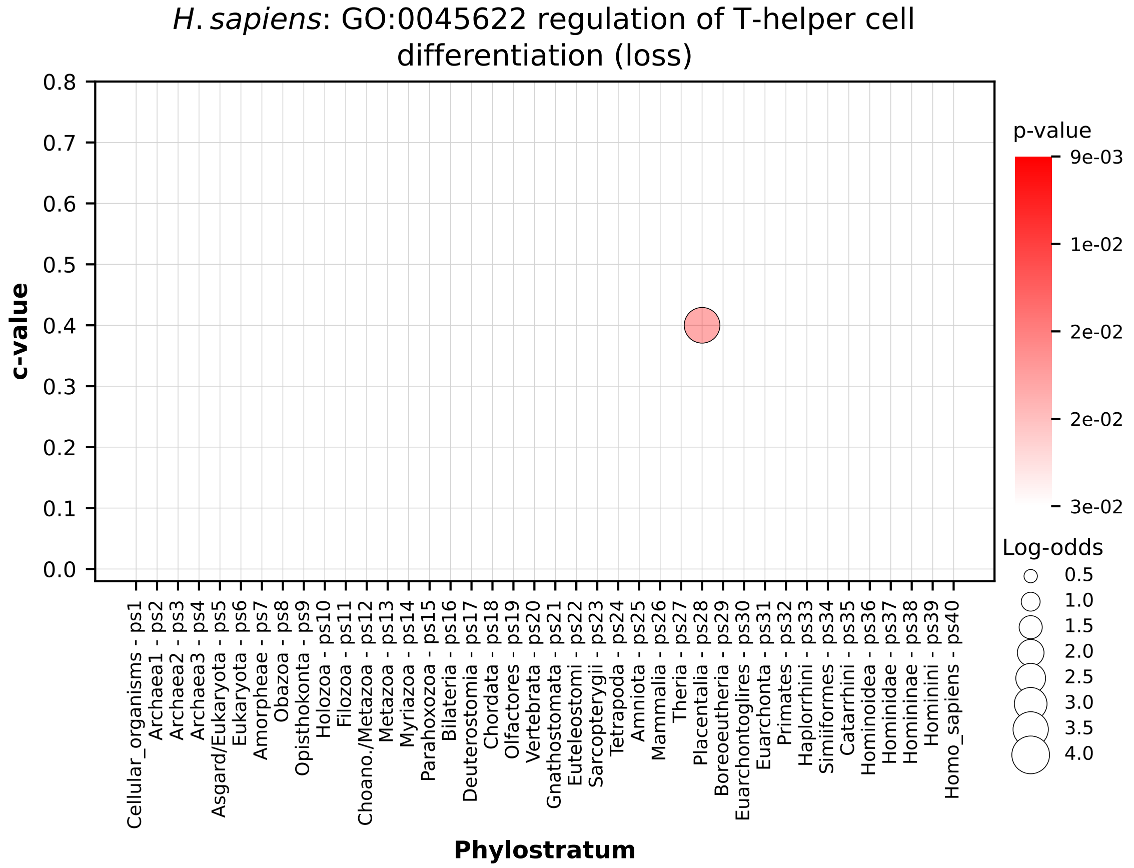
H. sapiens: GO:0045622 regulation of T-helper cell differentiation (loss)
c-value
0.8
0.7
0.6
0.5
0.4
0.3
0.2
0.1
0.0
p-value
9e-03
1e-02
2e-02
2e-02
3e-02
Log-odds
0.5
1.0
1.5
2.0
2.5
3.0
3.5
4.0
Cellular_organisms - ps1
Archaea1 - ps2
Archaea2 - ps3
Archaea3 - ps4
Asgard/Eukaryota - ps5
Eukaryota - ps6
Amorpheae - ps7
Obazoa - ps8
Opisthokonta - ps9
Holozoa - ps10
Filozoa - ps11
Choano./Metazoa - ps12
Metazoa - ps13
Myriazoa - ps14
Parahoxozoa - ps15
Bilateria - ps16
Deuterostomia - ps17
Chordata - ps18
Olfactores - ps19
Vertebrata - ps20
Gnathostomata - ps21
Euteleostomi - ps22
Sarcopterygii - ps23
Tetrapoda - ps24
Amniota - ps25
Mammalia - ps26
Theria - ps27
Placentalia - ps28
Boreoeutheria - ps29
Euarchontoglires - ps30
Euarchonta - ps31
Primates - ps32
Haplorrhini - ps33
Simiiformes - ps34
Catarrhini - ps35
Hominoidea - ps36
Hominidae - ps37
Homininae - ps38
Hominini - ps39
Homo_sapiens - ps40
Phylostratum

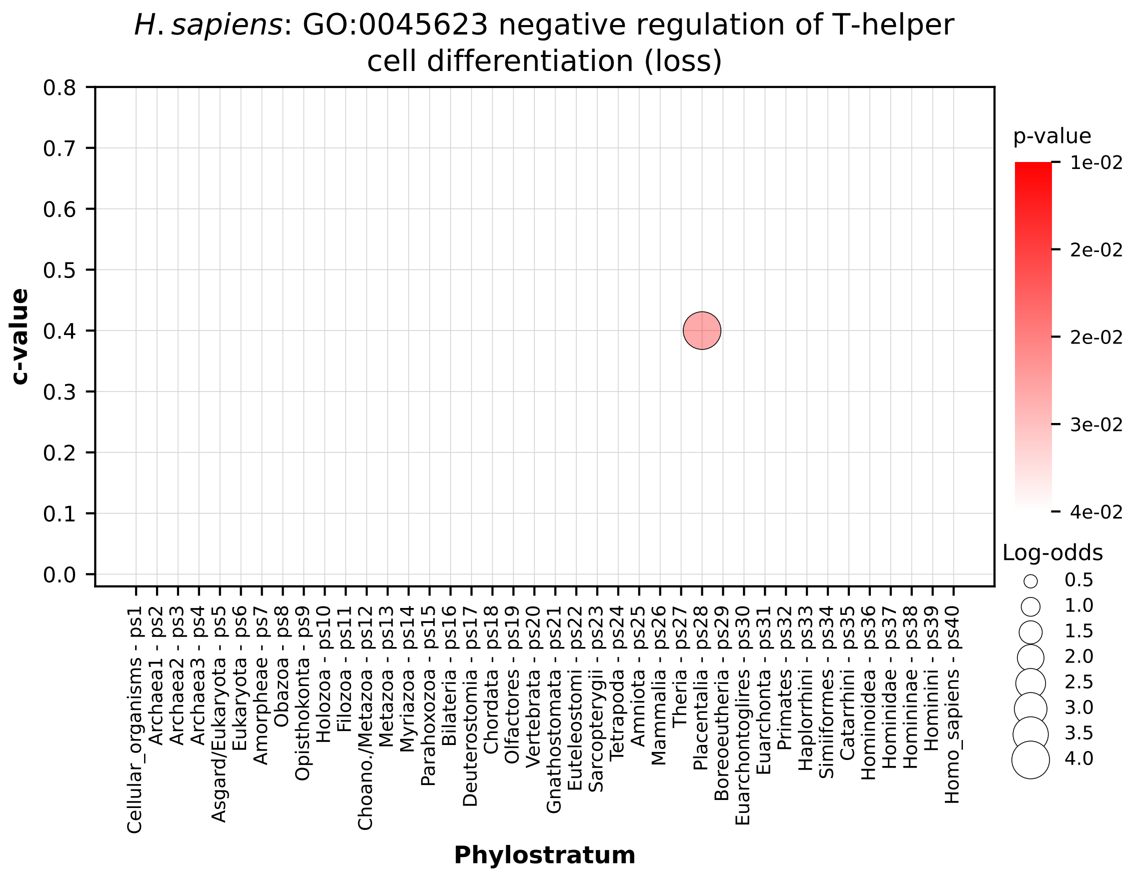

H. sapiens: GO:0045623 negative regulation of T-helper cell differentiation (loss)
c-value
0.8
0.7
0.6
0.5
0.4
0.3
0.2
0.1
0.0
Phylostratum
Cellular_organisms - ps1
Archaea1 - ps2
Archaea2 - ps3
Archaea3 - ps4
Asgard/Eukaryota - ps5
Eukaryota - ps6
Amorpheae - ps7
Obazoa - ps8
Opisthokonta - ps9
Holozoa - ps10
Filozoa - ps11
Choano./Metazoa - ps12
Metazoa - ps13
Myriazoa - ps14
Parahoxozoa - ps15
Bilateria - ps16
Deuterostomia - ps17
Chordata - ps18
Olfactores - ps19
Vertebrata - ps20
Gnathostomata - ps21
Euteleostomi - ps22
Sarcopterygii - ps23
Tetrapoda - ps24
Amniota - ps25
Mammalia - ps26
Theria - ps27
Placentalia - ps28
Boreoeutheria - ps29
Euarchontoglires - ps30
Euarchonta - ps31
Primates - ps32
Haplorrhini - ps33
Simiiformes - ps34
Catarrhini - ps35
Hominoidea - ps36
Hominidae - ps37
Homininae - ps38
Hominini - ps39
Homo_sapiens - ps40
p-value
1e-02
2e-02
2e-02
3e-02
4e-02
Log-odds
0.5
1.0
1.5
2.0
2.5
3.0
3.5
4.0

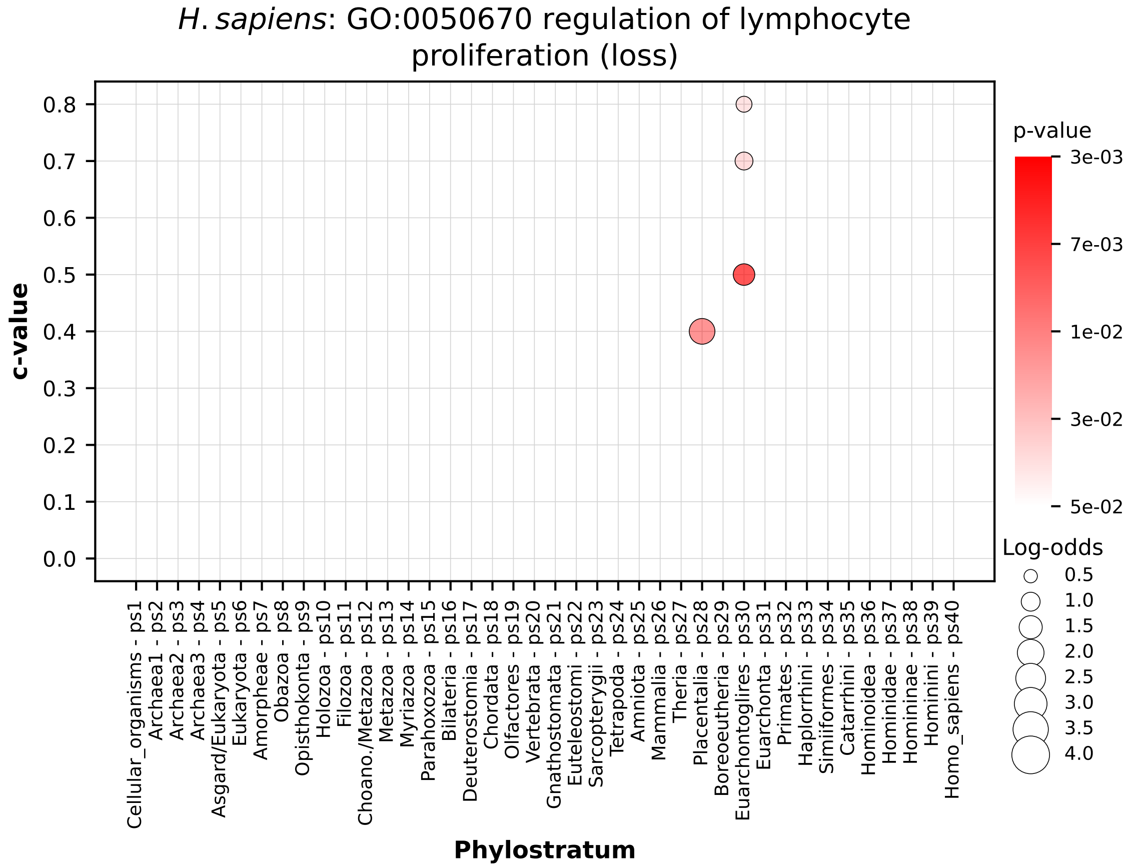

H. sapiens: GO:0050670 regulation of lymphocyte proliferation (loss)
c-value
0.8
0.7
0.6
0.5
0.4
0.3
0.2
0.1
0.0
p-value
3e-03
7e-03
1e-02
3e-02
5e-02
Log-odds
0.5
1.0
1.5
2.0
2.5
3.0
3.5
4.0
Cellular_organisms - ps1
Archaea1 - ps2
Archaea2 - ps3
Archaea3 - ps4
Asgard/Eukaryota - ps5
Eukaryota - ps6
Amorpheae - ps7
Obazoa - ps8
Opisthokonta - ps9
Holozoa - ps10
Filozoa - ps11
Choano./Metazoa - ps12
Metazoa - ps13
Myriazoa - ps14
Parahoxozoa - ps15
Bilateria - ps16
Deuterostomia - ps17
Chordata - ps18
Olfactores - ps19
Vertebrata - ps20
Gnathostomata - ps21
Euteleostomi - ps22
Sarcopterygii - ps23
Tetrapoda - ps24
Amniota - ps25
Mammalia - ps26
Theria - ps27
Placentalia - ps28
Boreoeutheria - ps29
Euarchontoglires - ps30
Euarchonta - ps31
Primates - ps32
Haplorrhini - ps33
Simiiformes - ps34
Catarrhini - ps35
Hominoidea - ps36
Hominidae - ps37
Homininae - ps38
Hominini - ps39
Homo_sapiens - ps40
Phylostratum

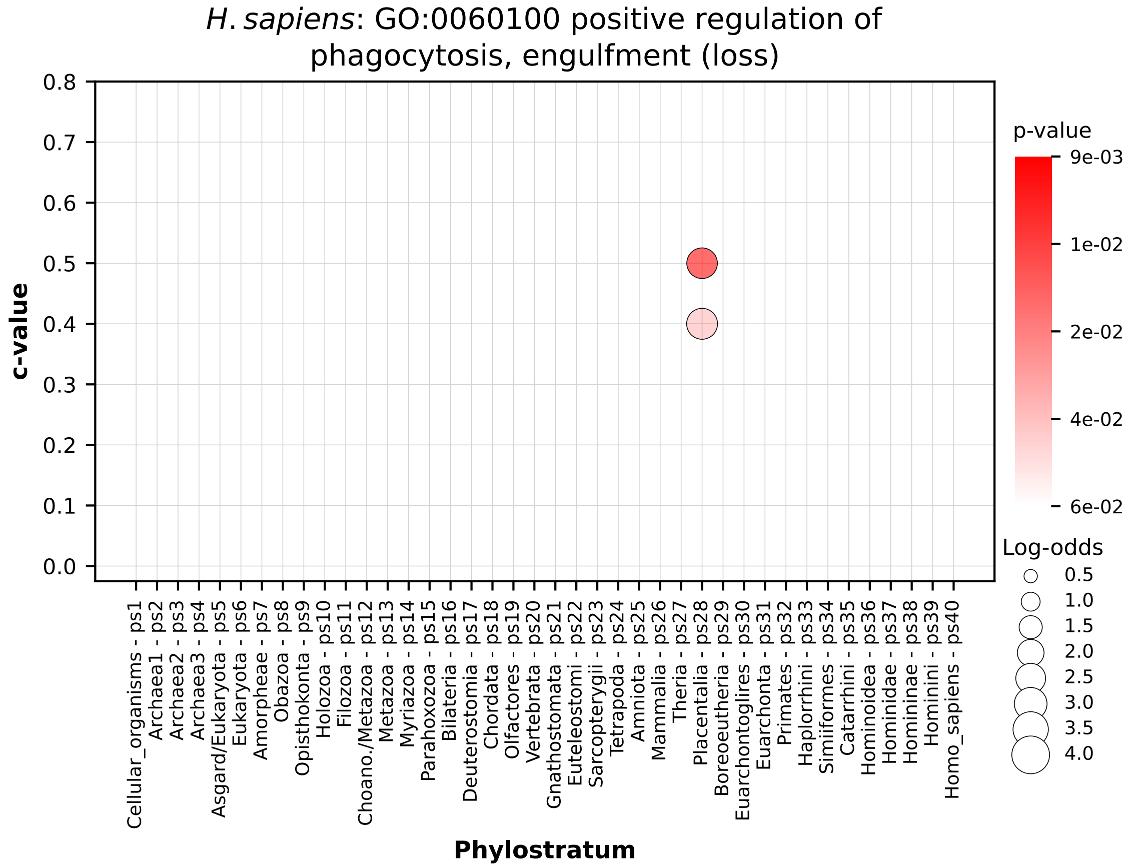

H. sapiens: GO:0060100 positive regulation of phagocytosis, engulfment (loss)
c-value
0.8
0.7
0.6
0.5
0.4
0.3
0.2
0.1
0.0
Phylostratum
Cellular_organisms - ps1
Archaea1 - ps2
Archaea2 - ps3
Archaea3 - ps4
Asgard/Eukaryota - ps5
Eukaryota - ps6
Amorpheae - ps7
Obazoa - ps8
Opisthokonta - ps9
Holozoa - ps10
Filozoa - ps11
Choano./Metazoa - ps12
Metazoa - ps13
Myriazoa - ps14
Parahoxozoa - ps15
Bilateria - ps16
Deuterostomia - ps17
Chordata - ps18
Olfactores - ps19
Vertebrata - ps20
Gnathostomata - ps21
Euteleostomi - ps22
Sarcopterygii - ps23
Tetrapoda - ps24
Amniota - ps25
Mammalia - ps26
Theria - ps27
Placentalia - ps28
Boreoeutheria - ps29
Euarchontoglires - ps30
Euarchonta - ps31
Primates - ps32
Haplorrhini - ps33
Simiiformes - ps34
Catarrhini - ps35
Hominoidea - ps36
Hominidae - ps37
Homininae - ps38
Hominini - ps39
Homo_sapiens - ps40
p-value
9e-03
1e-02
2e-02
4e-02
6e-02
Log-odds
0.5
1.0
1.5
2.0
2.5
3.0
3.5
4.0

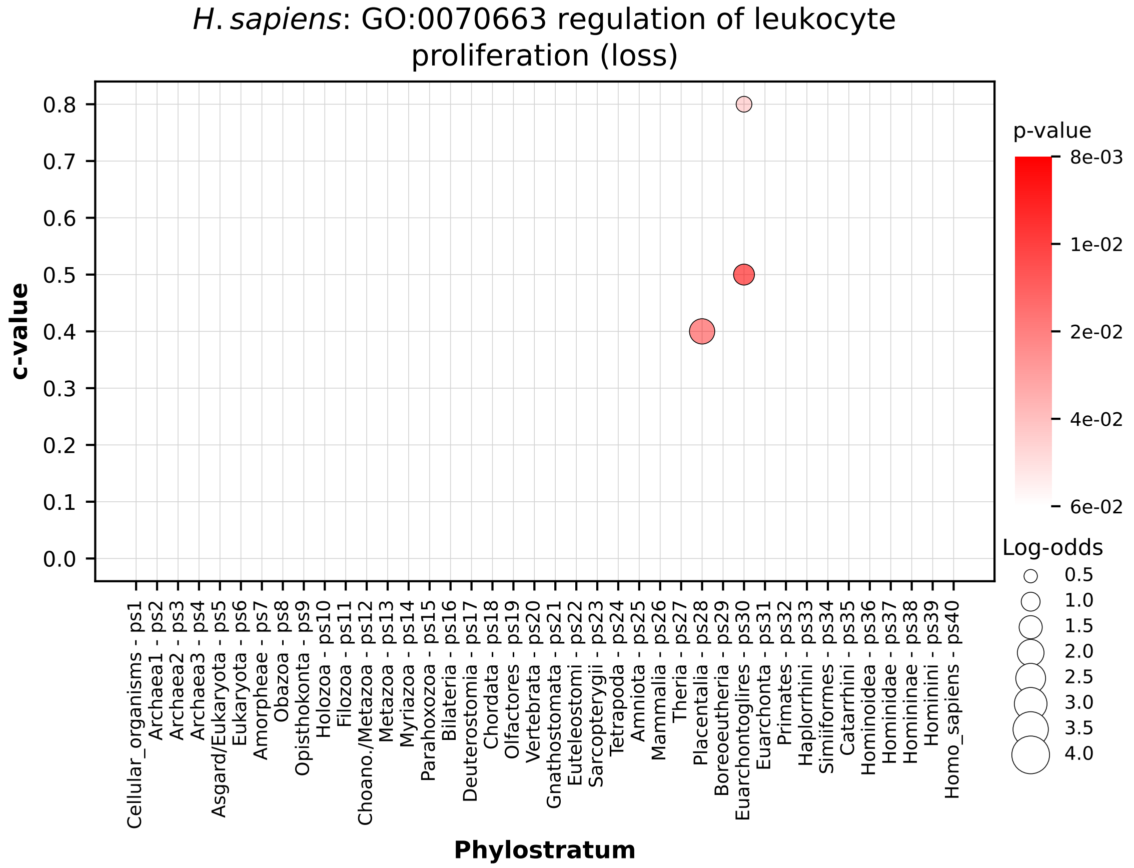

H. sapiens: GO:0070663 regulation of leukocyte proliferation (loss)
c-value
0.0
0.1
0.2
0.3
0.4
0.5
0.6
0.7
0.8
Phylostratum
Cellular_organisms - ps1
Archaea1 - ps2
Archaea2 - ps3
Archaea3 - ps4
Asgard/Eukaryota - ps5
Eukaryota - ps6
Amorpheae - ps7
Obazoa - ps8
Opisthokonta - ps9
Holozoa - ps10
Filozoa - ps11
Choano./Metazoa - ps12
Metazoa - ps13
Myriazoa - ps14
Parahoxozoa - ps15
Bilateria - ps16
Deuterostomia - ps17
Chordata - ps18
Olfactores - ps19
Vertebrata - ps20
Gnathostomata - ps21
Euteleostomi - ps22
Sarcopterygii - ps23
Tetrapoda - ps24
Amniota - ps25
Mammalia - ps26
Theria - ps27
Placentalia - ps28
Boreoeutheria - ps29
Euarchontoglires - ps30
Euarchonta - ps31
Primates - ps32
Haplorrhini - ps33
Simiiformes - ps34
Catarrhini - ps35
Hominoidea - ps36
Hominidae - ps37
Homininae - ps38
Hominini - ps39
Homo_sapiens - ps40
p-value
8e-03
1e-02
2e-02
4e-02
6e-02
Log-odds
0.5
1.0
1.5
2.0
2.5
3.0
3.5
4.0

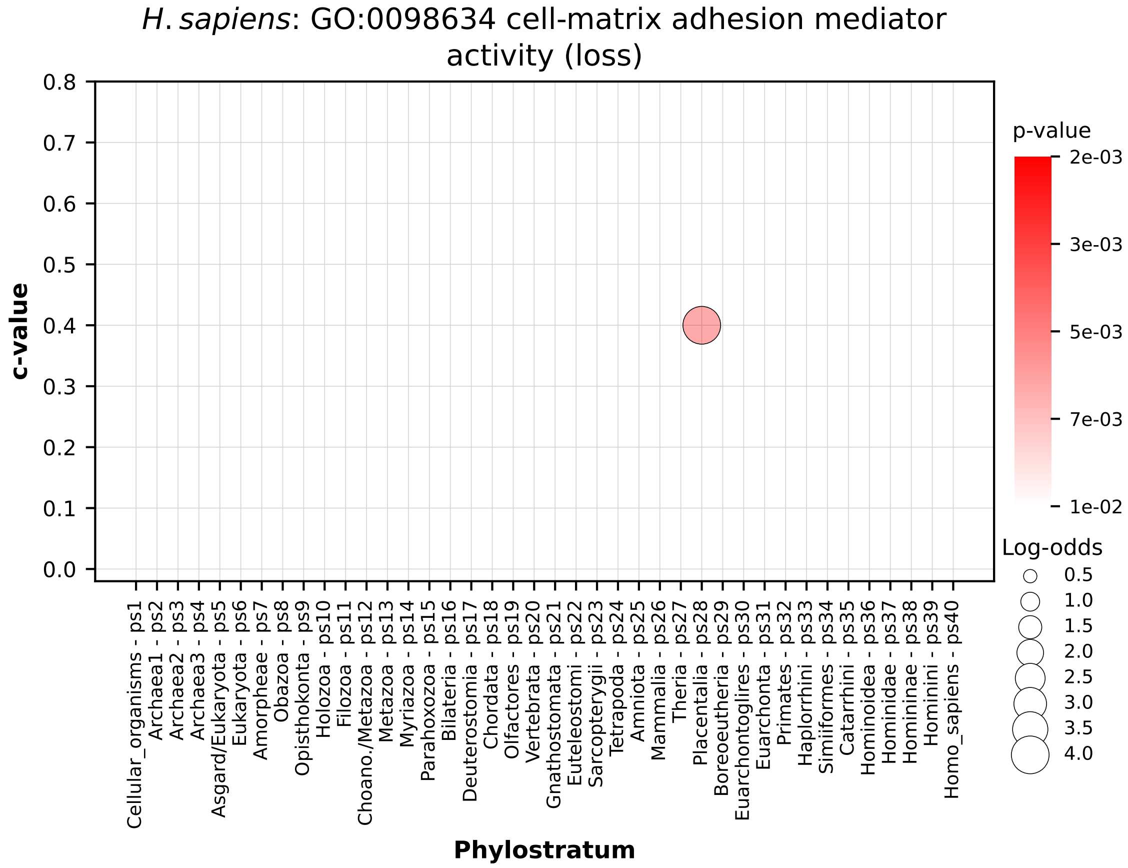

H. sapiens: GO:0098634 cell-matrix adhesion mediator activity (loss)
c-value
0.0
0.1
0.2
0.3
0.4
0.5
0.6
0.7
0.8
Phylostratum
Cellular_organisms - ps1
Archaea1 - ps2
Archaea2 - ps3
Archaea3 - ps4
Asgard/Eukaryota - ps5
Eukaryota - ps6
Amorpheae - ps7
Obazoa - ps8
Opisthokonta - ps9
Holozoa - ps10
Filozoa - ps11
Choano./Metazoa - ps12
Metazoa - ps13
Myriazoa - ps14
Parahoxozoa - ps15
Bilateria - ps16
Deuterostomia - ps17
Chordata - ps18
Olfactores - ps19
Vertebrata - ps20
Gnathostomata - ps21
Euteleostomi - ps22
Sarcopterygii - ps23
Tetrapoda - ps24
Amniota - ps25
Mammalia - ps26
Theria - ps27
Placentalia - ps28
Boreoeutheria - ps29
Euarchontoglires - ps30
Euarchonta - ps31
Primates - ps32
Haplorrhini - ps33
Simiiformes - ps34
Catarrhini - ps35
Hominoidea - ps36
Hominidae - ps37
Homininae - ps38
Hominini - ps39
Homo_sapiens - ps40
p-value
2e-03
3e-03
5e-03
7e-03
1e-02
Log-odds
0.5
1.0
1.5
2.0
2.5
3.0
3.5
4.0

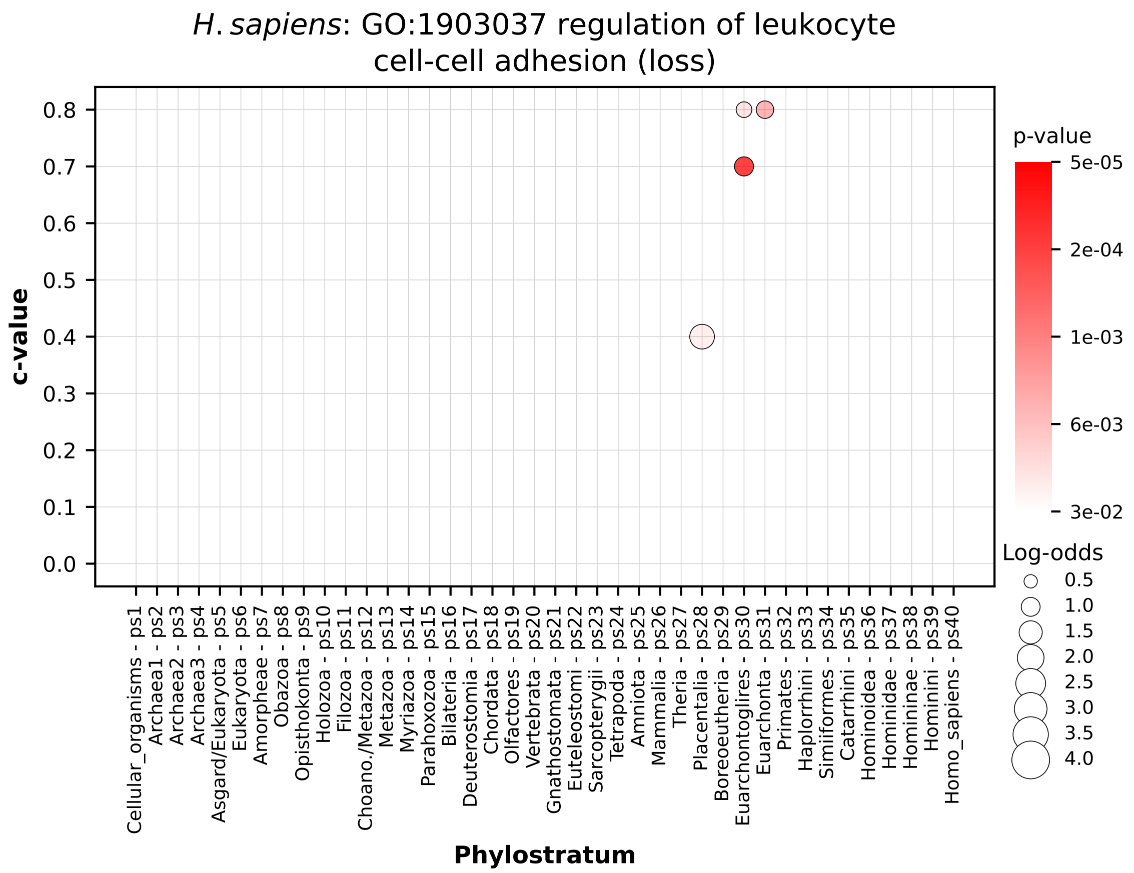
H. sapiens: GO:1903037 regulation of leukocyte cell-cell adhesion (loss)
c-value
Phylostratum
p-value
5e-05
2e-04
1e-03
6e-03
3e-02
Log-odds
0.5
1.0
1.5
2.0
2.5
3.0
3.5
4.0
Cellular_organisms - ps1
Archaea1 - ps2
Archaea2 - ps3
Archaea3 - ps4
Asgard/Eukaryota - ps5
Eukaryota - ps6
Amorpheae - ps7
Obazoa - ps8
Opisthokonta - ps9
Holozoa - ps10
Filozoa - ps11
Choano./Metazoa - ps12
Metazoa - ps13
Myriazoa - ps14
Parahoxozoa - ps15
Bilateria - ps16
Deuterostomia - ps17
Chordata - ps18
Olfactores - ps19
Vertebrata - ps20
Gnathostomata - ps21
Euteleostomi - ps22
Sarcopterygii - ps23
Tetrapoda - ps24
Amniota - ps25
Mammalia - ps26
Theria - ps27
Placentalia - ps28
Boreoeutheria - ps29
Euarchontoglires - ps30
Euarchonta - ps31
Primates - ps32
Haplorrhini - ps33
Simiiformes - ps34
Catarrhini - ps35
Hominoidea - ps36
Hominidae - ps37
Homininae - ps38
Hominini - ps39
Homo_sapiens - ps40

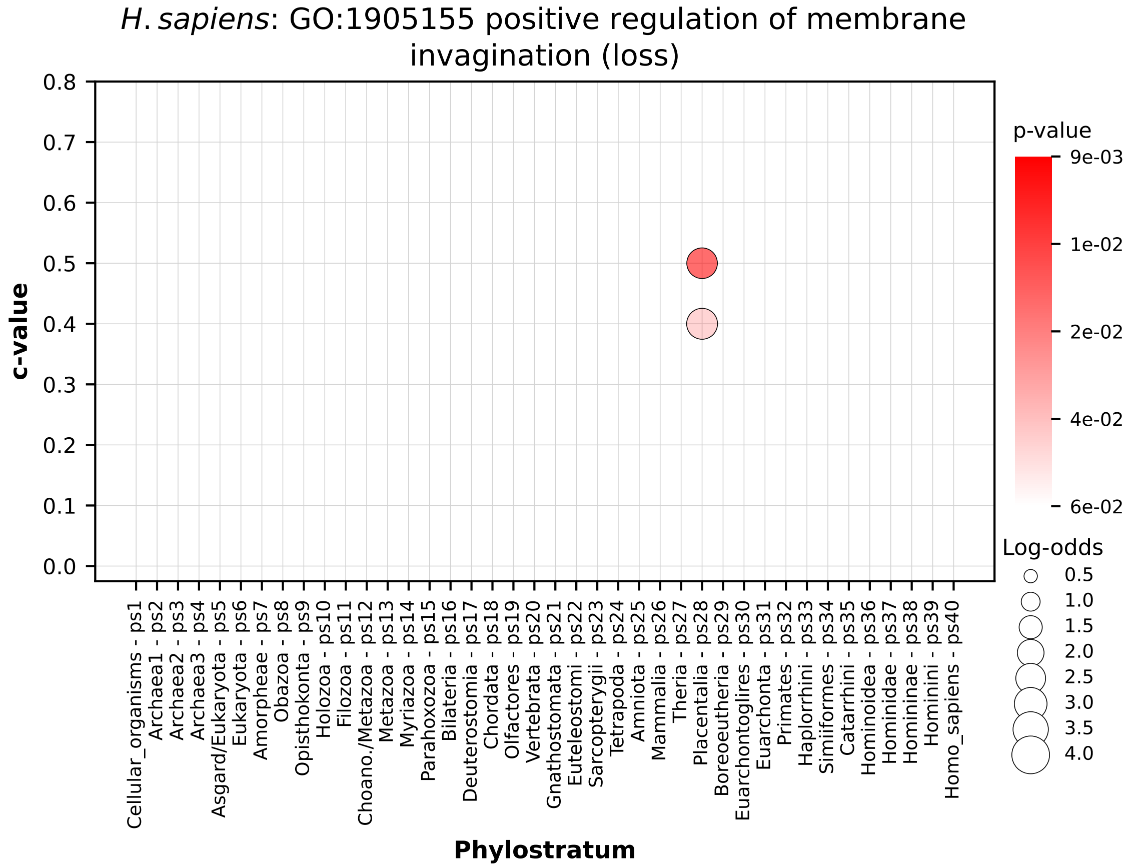
H. sapiens: GO:1905155 positive regulation of membrane invagination (loss)
c-value
0.0
0.1
0.2
0.3
0.4
0.5
0.6
0.7
0.8
Phylostratum
Cellular_organisms - ps1
Archaea1 - ps2
Archaea2 - ps3
Archaea3 - ps4
Asgard/Eukaryota - ps5
Eukaryota - ps6
Amorpheae - ps7
Obazoa - ps8
Opisthokonta - ps9
Holozoa - ps10
Filozoa - ps11
Choano./Metazoa - ps12
Metazoa - ps13
Myriazoa - ps14
Parahoxozoa - ps15
Bilateria - ps16
Deuterostomia - ps17
Chordata - ps18
Olfactores - ps19
Vertebrata - ps20
Gnathostomata - ps21
Euteleostomi - ps22
Sarcopterygii - ps23
Tetrapoda - ps24
Amniota - ps25
Mammalia - ps26
Theria - ps27
Placentalia - ps28
Boreoeutheria - ps29
Euarchontoglires - ps30
Euarchonta - ps31
Primates - ps32
Haplorrhini - ps33
Simiiformes - ps34
Catarrhini - ps35
Hominoidea - ps36
Hominidae - ps37
Homininae - ps38
Hominini - ps39
Homo_sapiens - ps40
p-value
9e-03
1e-02
2e-02
4e-02
6e-02
Log-odds
0.5
1.0
1.5
2.0
2.5
3.0
3.5
4.0

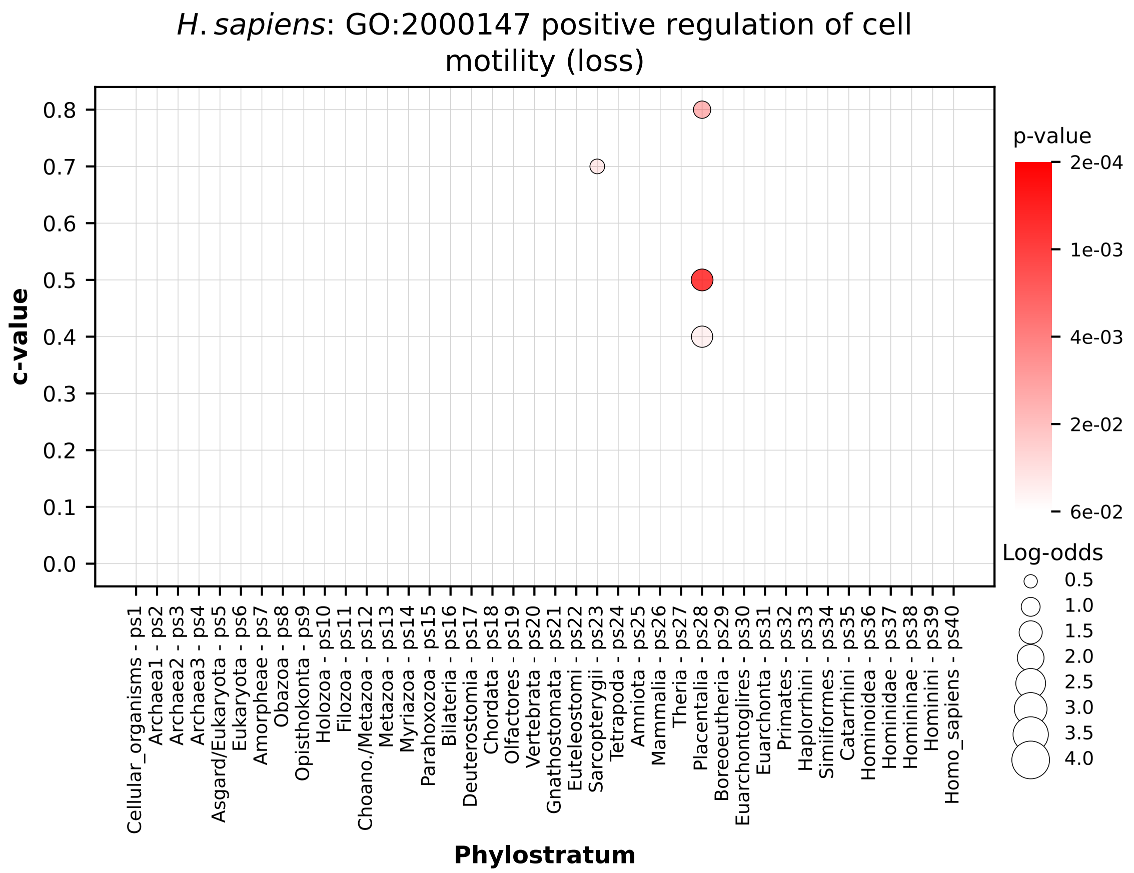

H. sapiens: GO:2000147 positive regulation of cell motility (loss)
c-value
0.8
0.7
0.6
0.5
0.4
0.3
0.2
0.1
0.0
Phylostratum
Cellular_organisms - ps1
Archaea1 - ps2
Archaea2 - ps3
Archaea3 - ps4
Asgard/Eukaryota - ps5
Eukaryota - ps6
Amorpheae - ps7
Obazoa - ps8
Opisthokonta - ps9
Holozoa - ps10
Filozoa - ps11
Choano./Metazoa - ps12
Metazoa - ps13
Myriazoa - ps14
Parahoxozoa - ps15
Bilateria - ps16
Deuterostomia - ps17
Chordata - ps18
Olfactores - ps19
Vertebrata - ps20
Gnathostomata - ps21
Euteleostomi - ps22
Sarcopterygii - ps23
Tetrapoda - ps24
Amniota - ps25
Mammalia - ps26
Theria - ps27
Placentalia - ps28
Boreoeutheria - ps29
Euarchontoglires - ps30
Euarchonta - ps31
Primates - ps32
Haplorrhini - ps33
Simiiformes - ps34
Catarrhini - ps35
Hominoidea - ps36
Hominidae - ps37
Homininae - ps38
Hominini - ps39
Homo_sapiens - ps40
p-value
2e-04
1e-03
4e-03
2e-02
6e-02
Log-odds
0.5
1.0
1.5
2.0
2.5
3.0
3.5
4.0

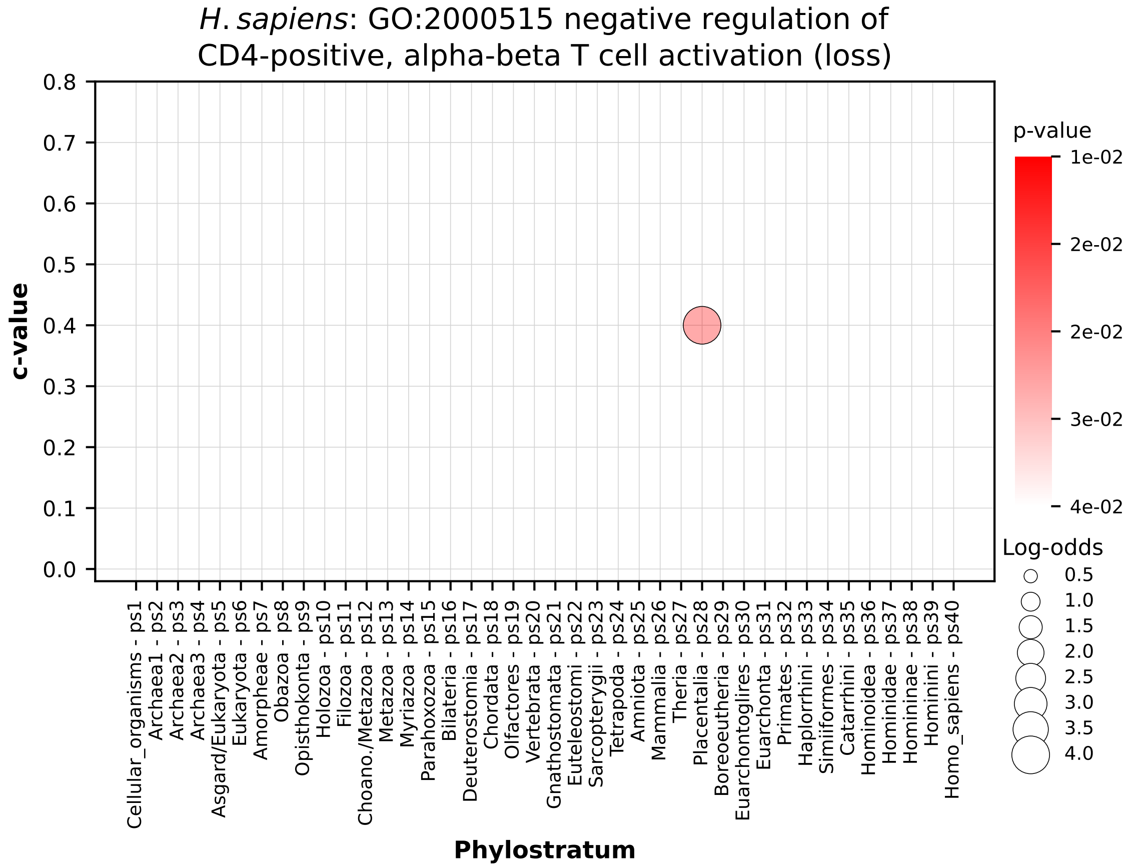

H. sapiens: GO:2000515 negative regulation of CD4-positive, alpha-beta T cell activation (loss)
c-value
0.0
0.1
0.2
0.3
0.4
0.5
0.6
0.7
0.8
Phylostratum
Cellular_organisms - ps1
Archaea1 - ps2
Archaea2 - ps3
Archaea3 - ps4
Asgard/Eukaryota - ps5
Eukaryota - ps6
Amorpheae - ps7
Obazoa - ps8
Opisthokonta - ps9
Holozoa - ps10
Filozoa - ps11
Choano./Metazoa - ps12
Metazoa - ps13
Myriazoa - ps14
Parahoxozoa - ps15
Bilateria - ps16
Deuterostomia - ps17
Chordata - ps18
Olfactores - ps19
Vertebrata - ps20
Gnathostomata - ps21
Euteleostomi - ps22
Sarcopterygii - ps23
Tetrapoda - ps24
Amniota - ps25
Mammalia - ps26
Theria - ps27
Placentalia - ps28
Boreoeutheria - ps29
Euarchontoglires - ps30
Euarchonta - ps31
Primates - ps32
Haplorrhini - ps33
Simiiformes - ps34
Catarrhini - ps35
Hominoidea - ps36
Hominidae - ps37
Homininae - ps38
Hominini - ps39
Homo_sapiens - ps40
p-value
1e-02
2e-02
2e-02
3e-02
4e-02
Log-odds
0.5
1.0
1.5
2.0
2.5
3.0
3.5
4.0

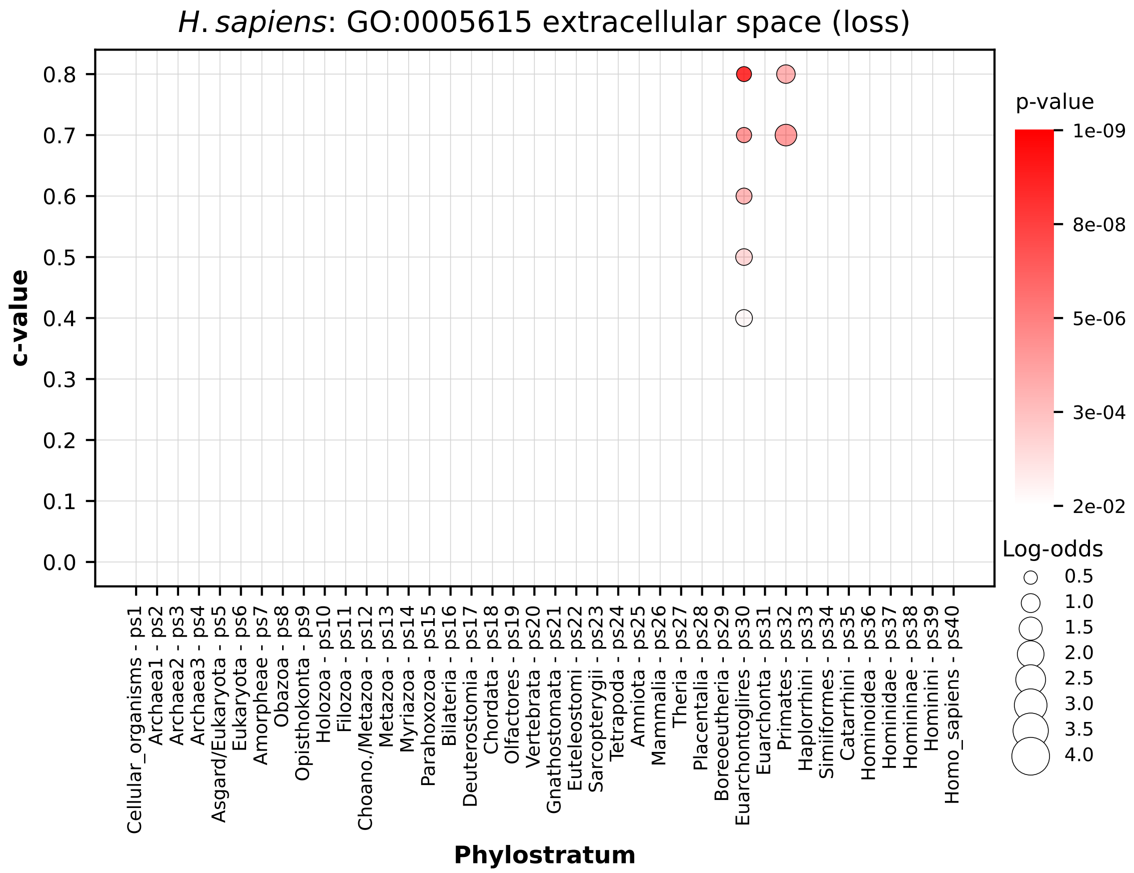

H. sapiens: GO:0005615 extracellular space (loss)
c-value
0.8
0.7
0.6
0.5
0.4
0.3
0.2
0.1
0.0
p-value
1e-09
8e-08
5e-06
3e-04
2e-02
Log-odds
0.5
1.0
1.5
2.0
2.5
3.0
3.5
4.0
Cellular_organisms - ps1
Archaea1 - ps2
Archaea2 - ps3
Archaea3 - ps4
Asgard/Eukaryota - ps5
Eukaryota - ps6
Amorpheae - ps7
Obazoa - ps8
Opisthokonta - ps9
Holozoa - ps10
Filozoa - ps11
Choano./Metazoa - ps12
Metazoa - ps13
Myriazoa - ps14
Parahoxozoa - ps15
Bilateria - ps16
Deuterostomia - ps17
Chordata - ps18
Olfactores - ps19
Vertebrata - ps20
Gnathostomata - ps21
Euteleostomi - ps22
Sarcopterygii - ps23
Tetrapoda - ps24
Amniota - ps25
Mammalia - ps26
Theria - ps27
Placentalia - ps28
Boreoeutheria - ps29
Euarchontoglires - ps30
Euarchonta - ps31
Primates - ps32
Haplorrhini - ps33
Simiiformes - ps34
Catarrhini - ps35
Hominoidea - ps36
Hominidae - ps37
Homininae - ps38
Hominini - ps39
Homo_sapiens - ps40
Phylostratum

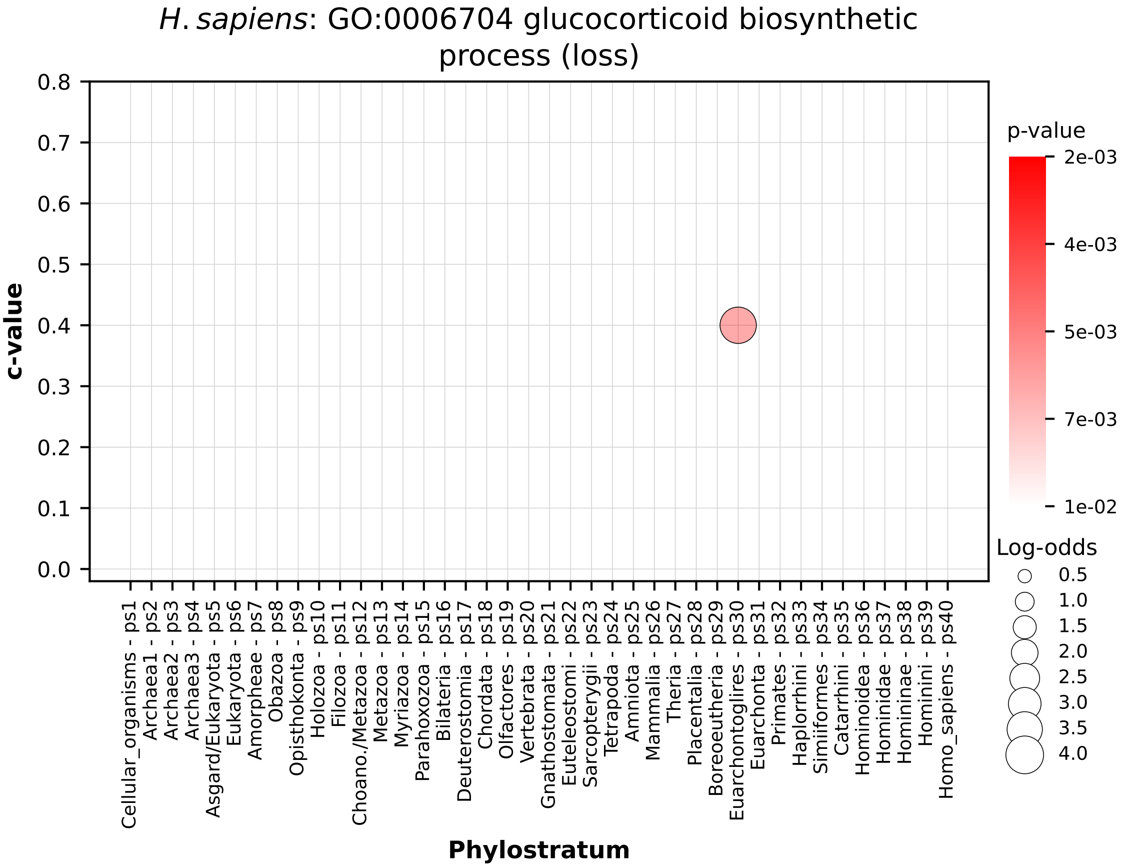
H. sapiens: GO:0006704 glucocorticoid biosynthetic process (loss)
c-value
0.8
0.7
0.6
0.5
0.4
0.3
0.2
0.1
0.0
Phylostratum
Cellular_organisms - ps1
Archaea1 - ps2
Archaea2 - ps3
Archaea3 - ps4
Asgard/Eukaryota - ps5
Eukaryota - ps6
Amorpheae - ps7
Obazoa - ps8
Opisthokonta - ps9
Holozoa - ps10
Filozoa - ps11
Choano./Metazoa - ps12
Metazoa - ps13
Myriazoa - ps14
Parahoxozoa - ps15
Bilateria - ps16
Deuterostomia - ps17
Chordata - ps18
Olfactores - ps19
Vertebrata - ps20
Gnathostomata - ps21
Euteleostomi - ps22
Sarcopterygii - ps23
Tetrapoda - ps24
Amniota - ps25
Mammalia - ps26
Theria - ps27
Placentalia - ps28
Boreoeutheria - ps29
Euarchontoglires - ps30
Euarchonta - ps31
Primates - ps32
Haplorrhini - ps33
Simiiformes - ps34
Catarrhini - ps35
Hominoidea - ps36
Hominidae - ps37
Homininae - ps38
Hominini - ps39
Homo_sapiens - ps40
p-value
2e-03
4e-03
5e-03
7e-03
1e-02
Log-odds
0.5
1.0
1.5
2.0
2.5
3.0
3.5
4.0

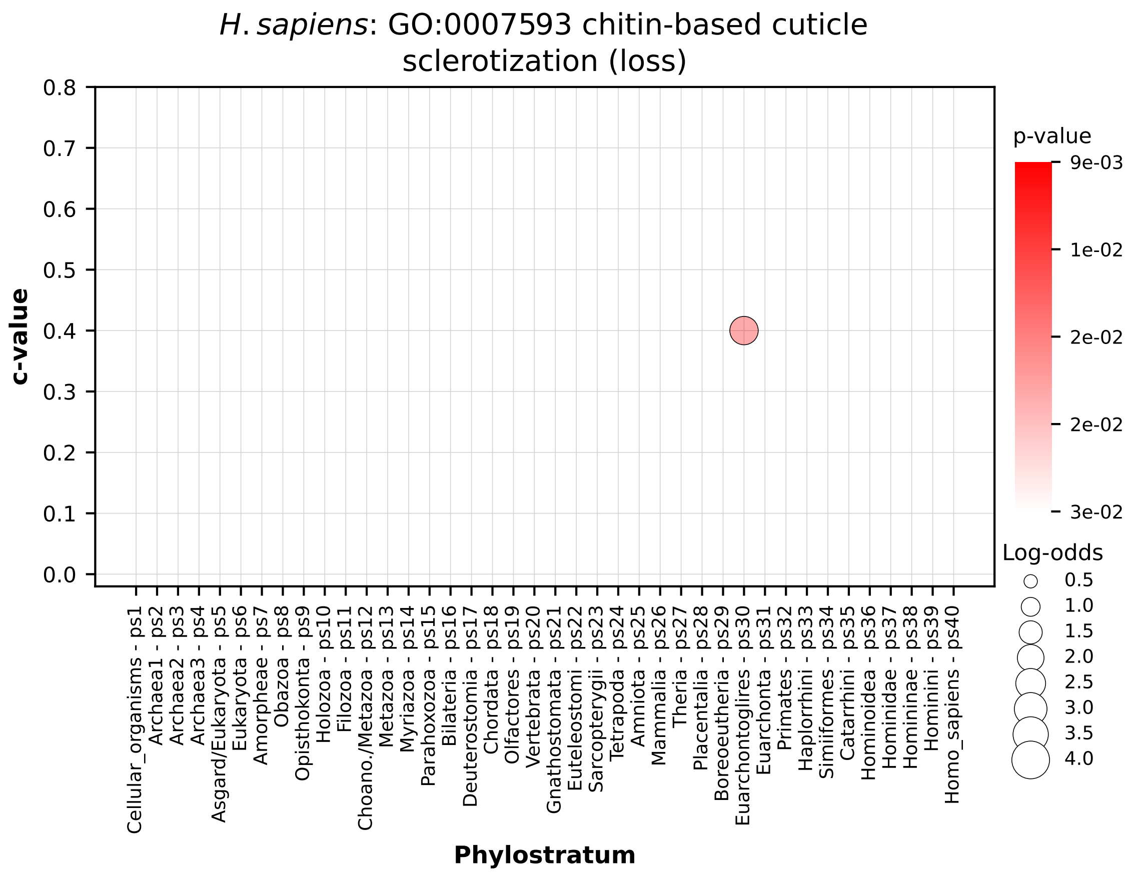

H. sapiens: GO:0007593 chitin-based cuticle sclerotization (loss)
c-value
0.8
0.7
0.6
0.5
0.4
0.3
0.2
0.1
0.0
p-value
9e-03
1e-02
2e-02
2e-02
3e-02
Log-odds
0.5
1.0
1.5
2.0
2.5
3.0
3.5
4.0
Cellular_organisms - ps1
Archaea1 - ps2
Archaea2 - ps3
Archaea3 - ps4
Asgard/Eukaryota - ps5
Eukaryota - ps6
Amorpheae - ps7
Obazoa - ps8
Opisthokonta - ps9
Holozoa - ps10
Filozoa - ps11
Choano./Metazoa - ps12
Metazoa - ps13
Myriazoa - ps14
Parahoxozoa - ps15
Bilateria - ps16
Deuterostomia - ps17
Chordata - ps18
Olfactores - ps19
Vertebrata - ps20
Gnathostomata - ps21
Euteleostomi - ps22
Sarcopterygii - ps23
Tetrapoda - ps24
Amniota - ps25
Mammalia - ps26
Theria - ps27
Placentalia - ps28
Boreoeutheria - ps29
Euarchontoglires - ps30
Euarchonta - ps31
Primates - ps32
Haplorrhini - ps33
Simiiformes - ps34
Catarrhini - ps35
Hominoidea - ps36
Hominidae - ps37
Homininae - ps38
Hominini - ps39
Homo_sapiens - ps40
Phylostratum

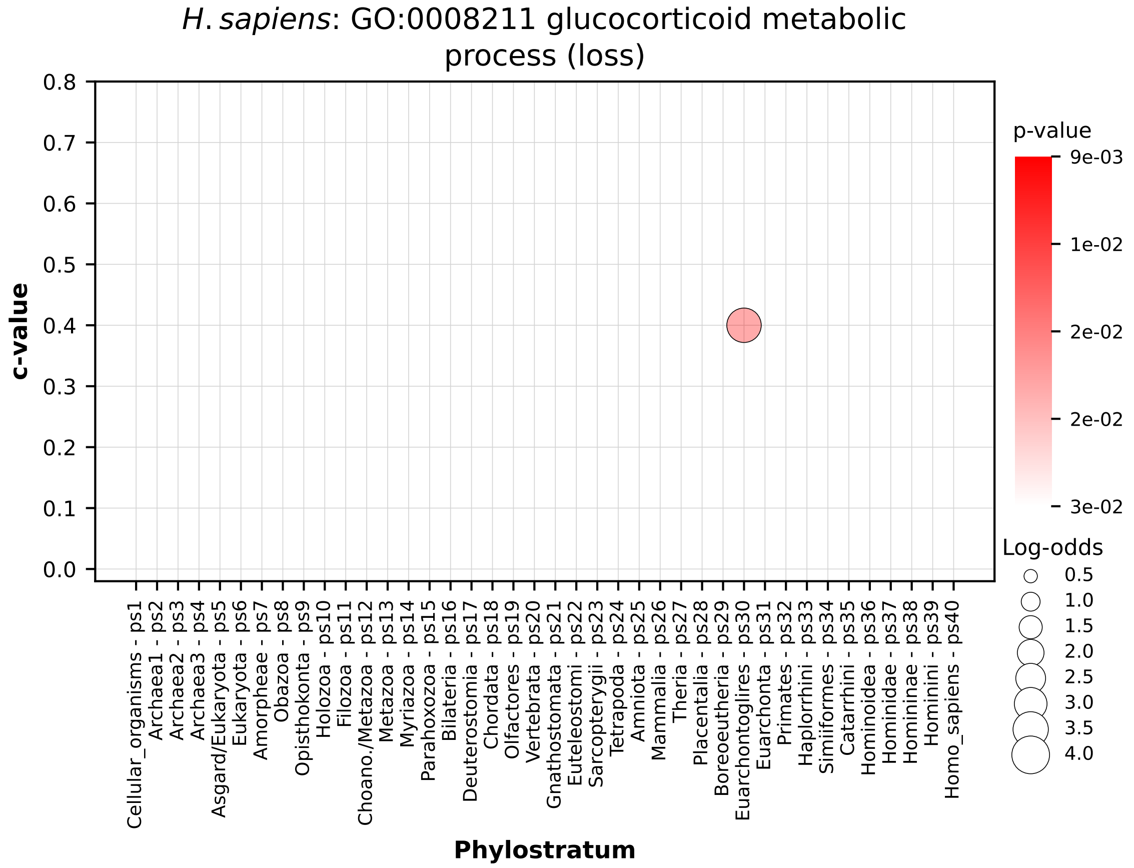

H. sapiens: GO:0008211 glucocorticoid metabolic process (loss)
c-value
0.0
0.1
0.2
0.3
0.4
0.5
0.6
0.7
0.8
Phylostratum
Cellular_organisms - ps1
Archaea1 - ps2
Archaea2 - ps3
Archaea3 - ps4
Asgard/Eukaryota - ps5
Eukaryota - ps6
Amorpheae - ps7
Obazoa - ps8
Opisthokonta - ps9
Holozoa - ps10
Filozoa - ps11
Choano./Metazoa - ps12
Metazoa - ps13
Myriazoa - ps14
Parahoxozoa - ps15
Bilateria - ps16
Deuterostomia - ps17
Chordata - ps18
Olfactores - ps19
Vertebrata - ps20
Gnathostomata - ps21
Euteleostomi - ps22
Sarcopterygii - ps23
Tetrapoda - ps24
Amniota - ps25
Mammalia - ps26
Theria - ps27
Placentalia - ps28
Boreoeutheria - ps29
Euarchontoglires - ps30
Euarchonta - ps31
Primates - ps32
Haplorrhini - ps33
Simiiformes - ps34
Catarrhini - ps35
Hominoidea - ps36
Hominidae - ps37
Homininae - ps38
Hominini - ps39
Homo_sapiens - ps40
p-value
9e-03
1e-02
2e-02
2e-02
3e-02
Log-odds
0.5
1.0
1.5
2.0
2.5
3.0
3.5
4.0

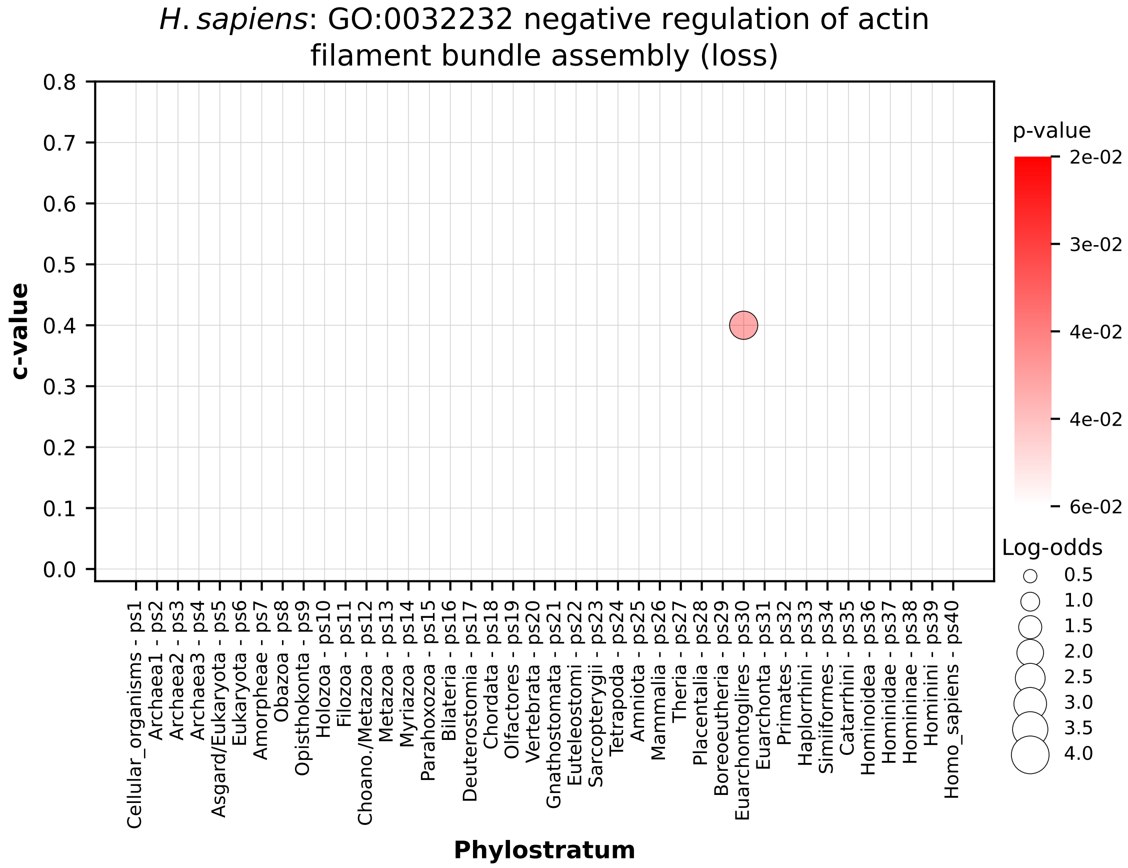

H. sapiens: GO:0032232 negative regulation of actin filament bundle assembly (loss)
c-value
0.8
0.7
0.6
0.5
0.4
0.3
0.2
0.1
0.0
Phylostratum
Cellular_organisms - ps1
Archaea1 - ps2
Archaea2 - ps3
Archaea3 - ps4
Asgard/Eukaryota - ps5
Eukaryota - ps6
Amorpheae - ps7
Obazoa - ps8
Opisthokonta - ps9
Holozoa - ps10
Filozoa - ps11
Choano./Metazoa - ps12
Metazoa - ps13
Myriazoa - ps14
Parahoxozoa - ps15
Bilateria - ps16
Deuterostomia - ps17
Chordata - ps18
Olfactores - ps19
Vertebrata - ps20
Gnathostomata - ps21
Euteleostomi - ps22
Sarcopterygii - ps23
Tetrapoda - ps24
Amniota - ps25
Mammalia - ps26
Theria - ps27
Placentalia - ps28
Boreoeutheria - ps29
Euarchontoglires - ps30
Euarchonta - ps31
Primates - ps32
Haplorrhini - ps33
Simiiformes - ps34
Catarrhini - ps35
Hominoidea - ps36
Hominidae - ps37
Homininae - ps38
Hominini - ps39
Homo_sapiens - ps40
p-value
2e-02
3e-02
4e-02
4e-02
6e-02
Log-odds
0.5
1.0
1.5
2.0
2.5
3.0
3.5
4.0

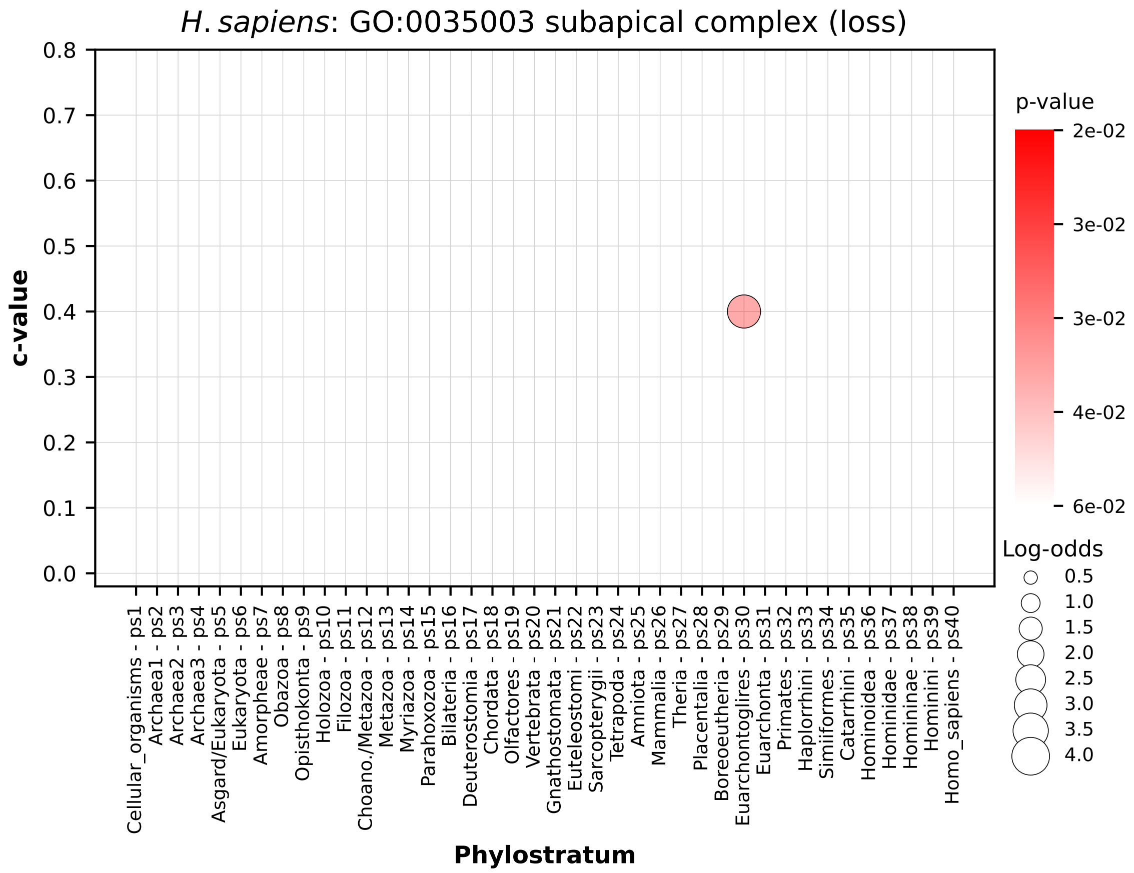

H. sapiens: GO:0035003 subapical complex (loss)
c-value
0.0
0.1
0.2
0.3
0.4
0.5
0.6
0.7
0.8
Phylostratum
Cellular_organisms - ps1
Archaea1 - ps2
Archaea2 - ps3
Archaea3 - ps4
Asgard/Eukaryota - ps5
Eukaryota - ps6
Amorpheae - ps7
Obazoa - ps8
Opisthokonta - ps9
Holozoa - ps10
Filozoa - ps11
Choano./Metazoa - ps12
Metazoa - ps13
Myriazoa - ps14
Parahoxozoa - ps15
Bilateria - ps16
Deuterostomia - ps17
Chordata - ps18
Olfactores - ps19
Vertebrata - ps20
Gnathostomata - ps21
Euteleostomi - ps22
Sarcopterygii - ps23
Tetrapoda - ps24
Amniota - ps25
Mammalia - ps26
Theria - ps27
Placentalia - ps28
Boreoeutheria - ps29
Euarchontoglires - ps30
Euarchonta - ps31
Primates - ps32
Haplorrhini - ps33
Simiiformes - ps34
Catarrhini - ps35
Hominoidea - ps36
Hominidae - ps37
Homininae - ps38
Hominini - ps39
Homo_sapiens - ps40
p-value
2e-02
3e-02
3e-02
4e-02
6e-02
Log-odds
0.5
1.0
1.5
2.0
2.5
3.0
3.5
4.0

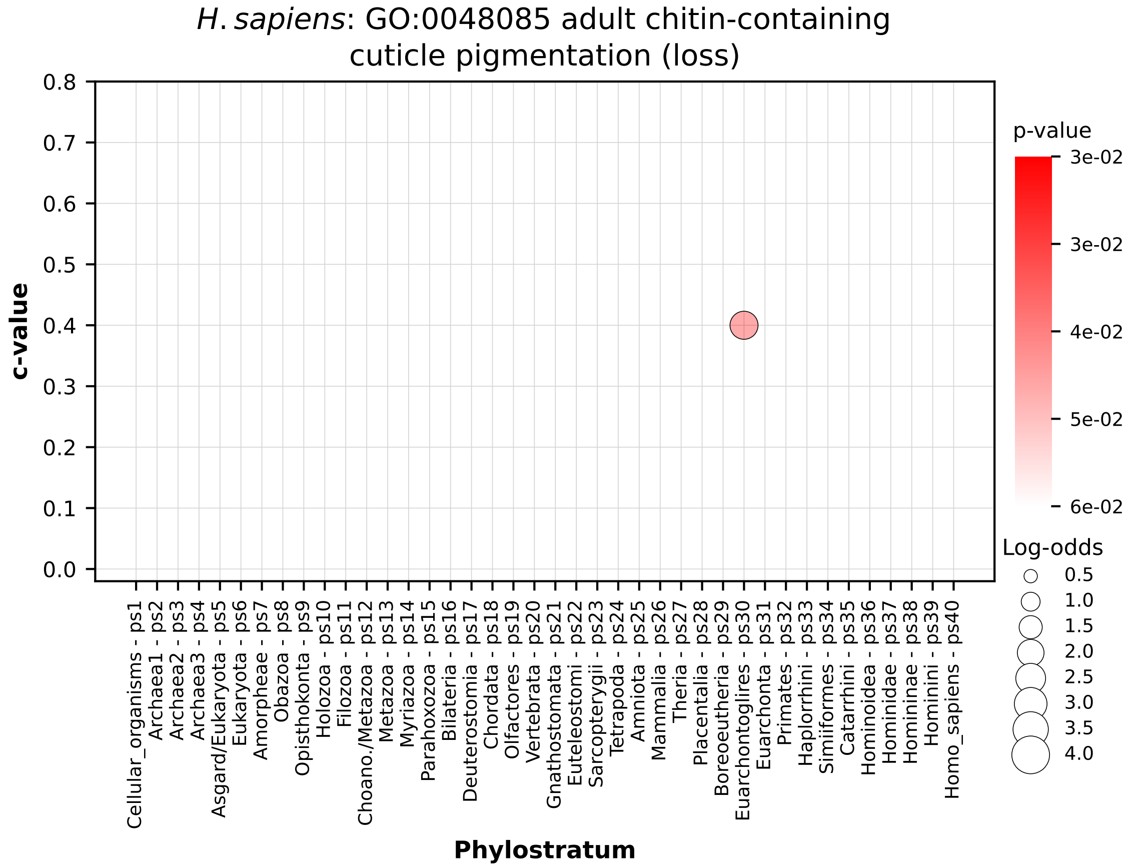

H. sapiens: GO:0048085 adult chitin-containing cuticle pigmentation (loss)
c-value
0.8
0.7
0.6
0.5
0.4
0.3
0.2
0.1
0.0
p-value
3e-02
3e-02
4e-02
5e-02
6e-02
Log-odds
0.5
1.0
1.5
2.0
2.5
3.0
3.5
4.0
Cellular_organisms - ps1
Archaea1 - ps2
Archaea2 - ps3
Archaea3 - ps4
Asgard/Eukaryota - ps5
Eukaryota - ps6
Amorpheae - ps7
Obazoa - ps8
Opisthokonta - ps9
Holozoa - ps10
Filozoa - ps11
Choano./Metazoa - ps12
Metazoa - ps13
Myriazoa - ps14
Parahoxozoa - ps15
Bilateria - ps16
Deuterostomia - ps17
Chordata - ps18
Olfactores - ps19
Vertebrata - ps20
Gnathostomata - ps21
Euteleostomi - ps22
Sarcopterygii - ps23
Tetrapoda - ps24
Amniota - ps25
Mammalia - ps26
Theria - ps27
Placentalia - ps28
Boreoeutheria - ps29
Euarchontoglires - ps30
Euarchonta - ps31
Primates - ps32
Haplorrhini - ps33
Simiiformes - ps34
Catarrhini - ps35
Hominoidea - ps36
Hominidae - ps37
Homininae - ps38
Hominini - ps39
Homo_sapiens - ps40
Phylostratum

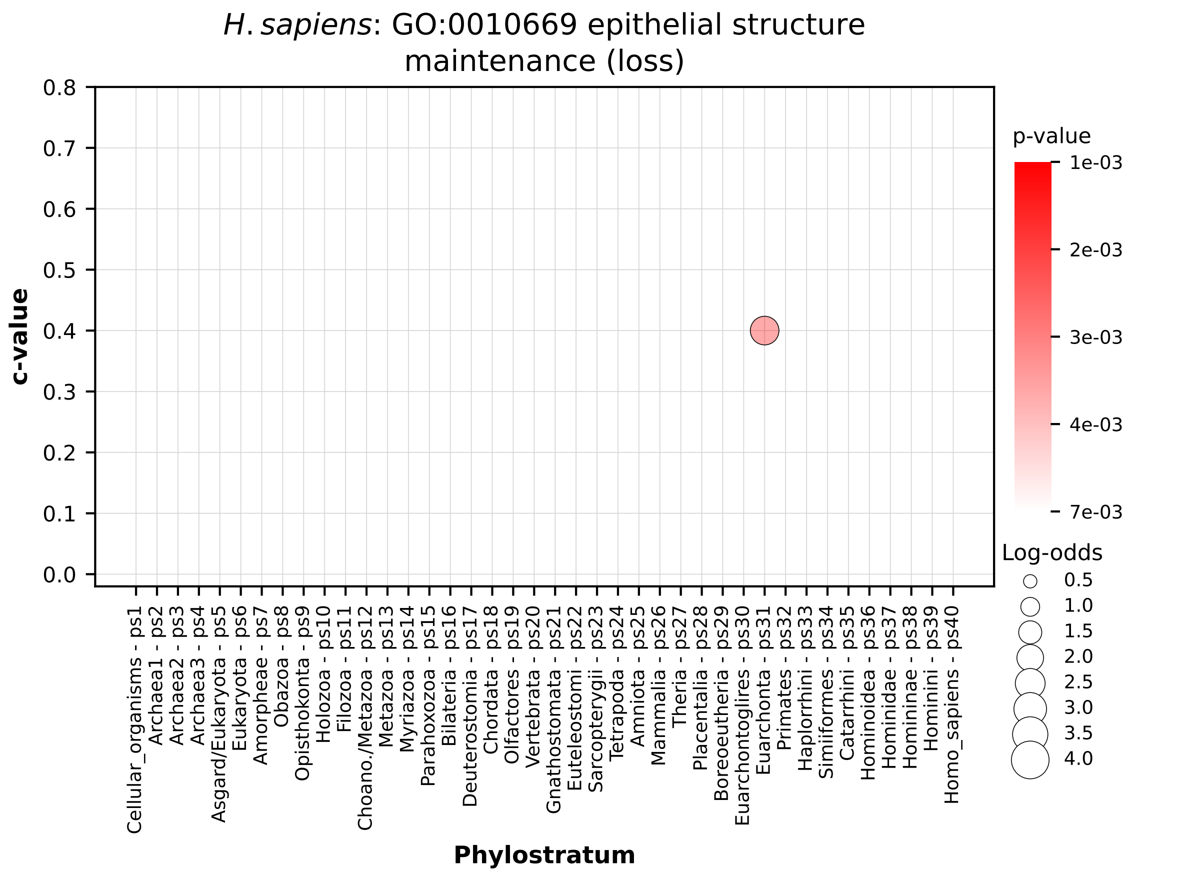

H. sapiens: GO:0010669 epithelial structure maintenance (loss)
c-value
0.8
0.7
0.6
0.5
0.4
0.3
0.2
0.1
0.0
p-value
1e-03
2e-03
3e-03
4e-03
7e-03
Log-odds
0.5
1.0
1.5
2.0
2.5
3.0
3.5
4.0
Cellular_organisms - ps1
Archaea1 - ps2
Archaea2 - ps3
Archaea3 - ps4
Asgard/Eukaryota - ps5
Eukaryota - ps6
Amorpheae - ps7
Obazoa - ps8
Opisthokonta - ps9
Holozoa - ps10
Filozoa - ps11
Choano./Metazoa - ps12
Metazoa - ps13
Myriazoa - ps14
Parahoxozoa - ps15
Bilateria - ps16
Deuterostomia - ps17
Chordata - ps18
Olfactores - ps19
Vertebrata - ps20
Gnathostomata - ps21
Euteleostomi - ps22
Sarcopterygii - ps23
Tetrapoda - ps24
Amniota - ps25
Mammalia - ps26
Theria - ps27
Placentalia - ps28
Boreoeutheria - ps29
Euarchontoglires - ps30
Euarchonta - ps31
Primates - ps32
Haplorrhini - ps33
Simiiformes - ps34
Catarrhini - ps35
Hominoidea - ps36
Hominidae - ps37
Homininae - ps38
Hominini - ps39
Homo_sapiens - ps40
Phylostratum

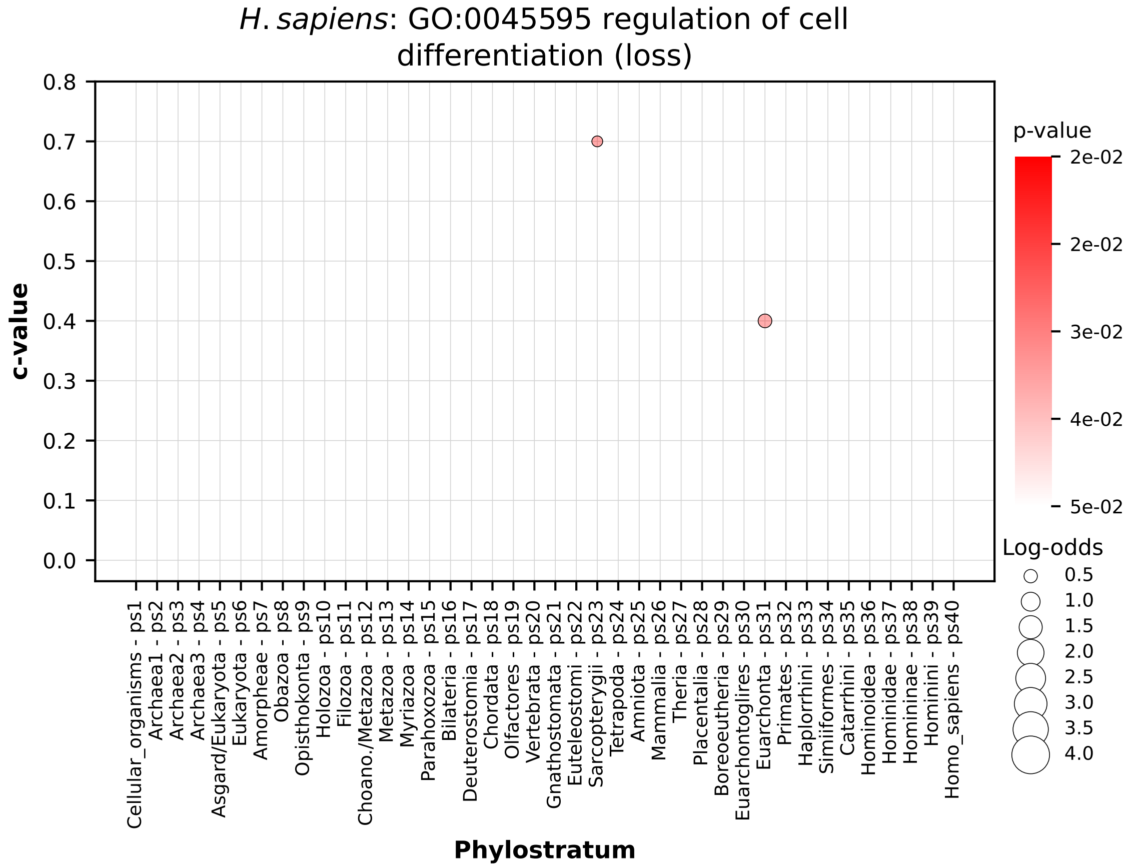

H. sapiens: GO:0045595 regulation of cell differentiation (loss)
c-value
0.8
0.7
0.6
0.5
0.4
0.3
0.2
0.1
0.0
Phylostratum
Cellular_organisms - ps1
Archaea1 - ps2
Archaea2 - ps3
Archaea3 - ps4
Asgard/Eukaryota - ps5
Eukaryota - ps6
Amorpheae - ps7
Obazoa - ps8
Opisthokonta - ps9
Holozoa - ps10
Filozoa - ps11
Choano./Metazoa - ps12
Metazoa - ps13
Myriazoa - ps14
Parahoxozoa - ps15
Bilateria - ps16
Deuterostomia - ps17
Chordata - ps18
Olfactores - ps19
Vertebrata - ps20
Gnathostomata - ps21
Euteleostomi - ps22
Sarcopterygii - ps23
Tetrapoda - ps24
Amniota - ps25
Mammalia - ps26
Theria - ps27
Placentalia - ps28
Boreoeutheria - ps29
Euarchontoglires - ps30
Euarchonta - ps31
Primates - ps32
Haplorrhini - ps33
Simiiformes - ps34
Catarrhini - ps35
Hominoidea - ps36
Hominidae - ps37
Homininae - ps38
Hominini - ps39
Homo_sapiens - ps40
p-value
2e-02
2e-02
3e-02
4e-02
5e-02
Log-odds
0.5
1.0
1.5
2.0
2.5
3.0
3.5
4.0

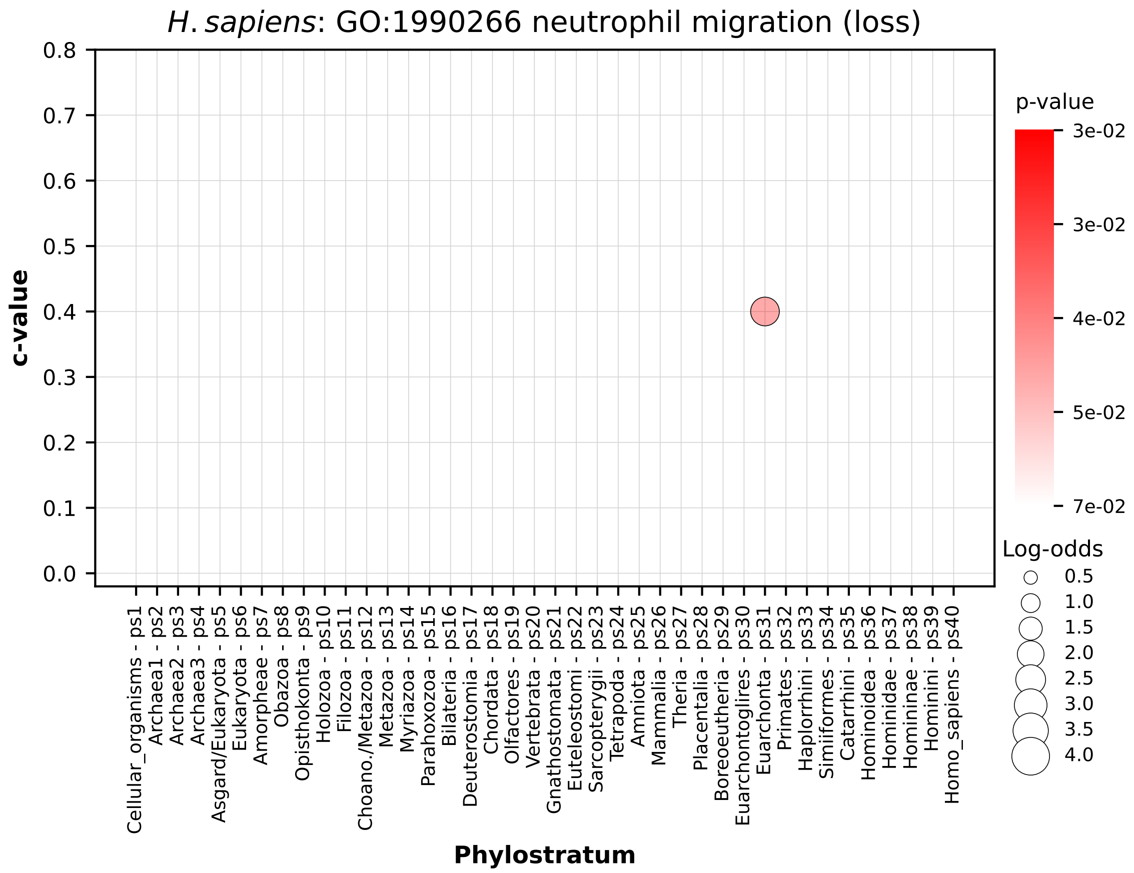

H. sapiens: GO:1990266 neutrophil migration (loss)
c-value
0.8
0.7
0.6
0.5
0.4
0.3
0.2
0.1
0.0
Phylostratum
Cellular_organisms - ps1
Archaea1 - ps2
Archaea2 - ps3
Archaea3 - ps4
Asgard/Eukaryota - ps5
Eukaryota - ps6
Amorpheae - ps7
Obazoa - ps8
Opisthokonta - ps9
Holozoa - ps10
Filozoa - ps11
Choano./Metazoa - ps12
Metazoa - ps13
Myriazoa - ps14
Parahoxozoa - ps15
Bilateria - ps16
Deuterostomia - ps17
Chordata - ps18
Olfactores - ps19
Vertebrata - ps20
Gnathostomata - ps21
Euteleostomi - ps22
Sarcopterygii - ps23
Tetrapoda - ps24
Amniota - ps25
Mammalia - ps26
Theria - ps27
Placentalia - ps28
Boreoeutheria - ps29
Euarchontoglires - ps30
Euarchonta - ps31
Primates - ps32
Haplorrhini - ps33
Simiiformes - ps34
Catarrhini - ps35
Hominoidea - ps36
Hominidae - ps37
Homininae - ps38
Hominini - ps39
Homo_sapiens - ps40
p-value
3e-02
3e-02
4e-02
5e-02
7e-02
Log-odds
0.5
1.0
1.5
2.0
2.5
3.0
3.5
4.0

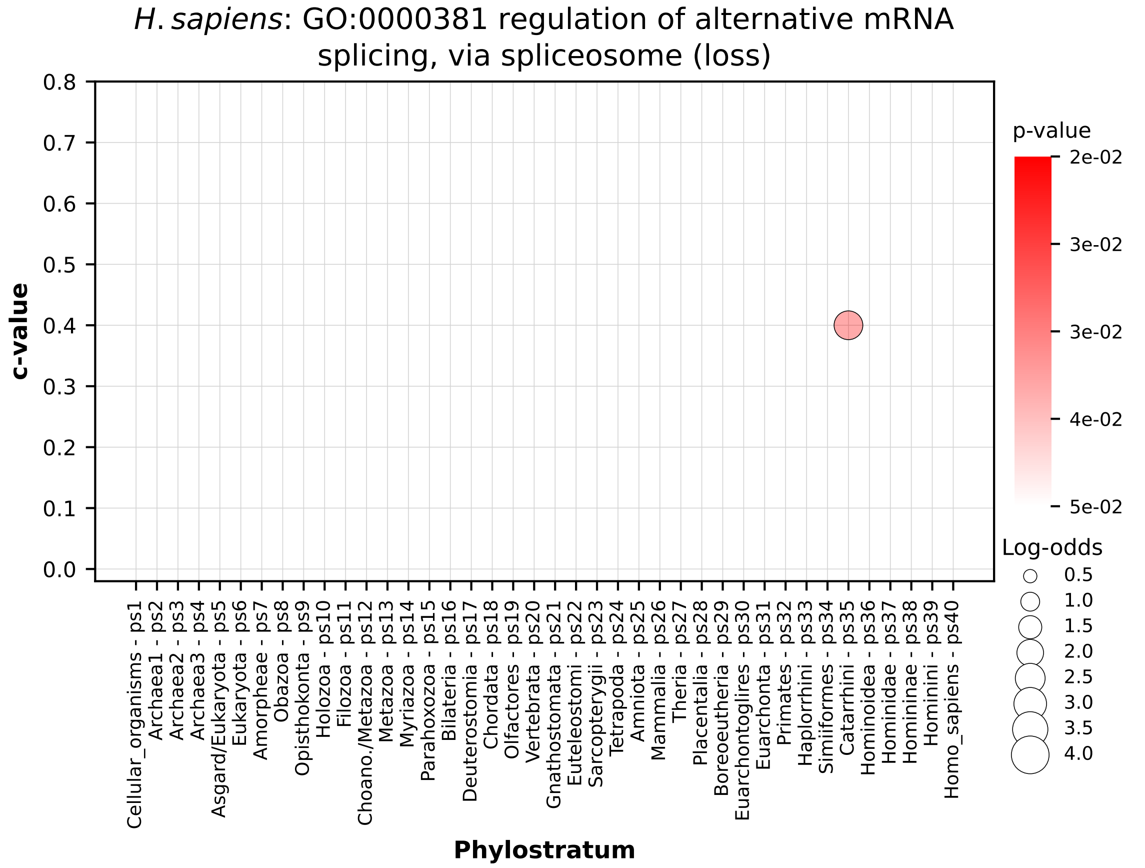
H. sapiens: GO:0000381 regulation of alternative mRNA splicing, via spliceosome (loss)
c-value
0.8
0.7
0.6
0.5
0.4
0.3
0.2
0.1
0.0
Phylostratum
Cellular_organisms - ps1
Archaea1 - ps2
Archaea2 - ps3
Archaea3 - ps4
Asgard/Eukaryota - ps5
Eukaryota - ps6
Amorpheae - ps7
Obazoa - ps8
Opisthokonta - ps9
Holozoa - ps10
Filozoa - ps11
Choano./Metazoa - ps12
Metazoa - ps13
Myriazoa - ps14
Parahoxozoa - ps15
Bilateria - ps16
Deuterostomia - ps17
Chordata - ps18
Olfactores - ps19
Vertebrata - ps20
Gnathostomata - ps21
Euteleostomi - ps22
Sarcopterygii - ps23
Tetrapoda - ps24
Amniota - ps25
Mammalia - ps26
Theria - ps27
Placentalia - ps28
Boreoeutheria - ps29
Euarchontoglires - ps30
Euarchonta - ps31
Primates - ps32
Haplorrhini - ps33
Simiiformes - ps34
Catarrhini - ps35
Hominoidea - ps36
Hominidae - ps37
Homininae - ps38
Hominini - ps39
Homo_sapiens - ps40
p-value
2e-02
3e-02
3e-02
4e-02
5e-02
Log-odds
0.5
1.0
1.5
2.0
2.5
3.0
3.5
4.0

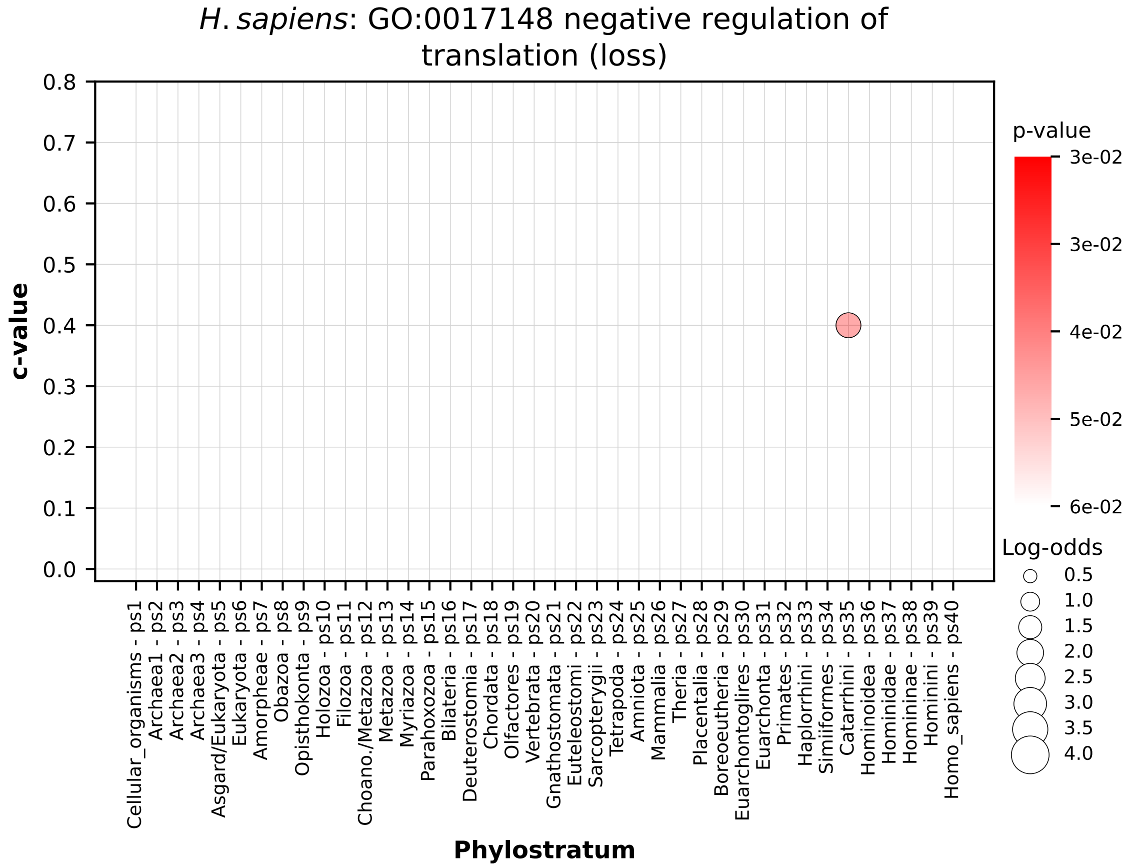

H. sapiens: GO:0017148 negative regulation of translation (loss)
c-value
0.8
0.7
0.6
0.5
0.4
0.3
0.2
0.1
0.0
Phylostratum
Cellular_organisms - ps1
Archaea1 - ps2
Archaea2 - ps3
Archaea3 - ps4
Asgard/Eukaryota - ps5
Eukaryota - ps6
Amorpheae - ps7
Obazoa - ps8
Opisthokonta - ps9
Holozoa - ps10
Filozoa - ps11
Choano./Metazoa - ps12
Metazoa - ps13
Myriazoa - ps14
Parahoxozoa - ps15
Bilateria - ps16
Deuterostomia - ps17
Chordata - ps18
Olfactores - ps19
Vertebrata - ps20
Gnathostomata - ps21
Euteleostomi - ps22
Sarcopterygii - ps23
Tetrapoda - ps24
Amniota - ps25
Mammalia - ps26
Theria - ps27
Placentalia - ps28
Boreoeutheria - ps29
Euarchontoglires - ps30
Euarchonta - ps31
Primates - ps32
Haplorrhini - ps33
Simiiformes - ps34
Catarrhini - ps35
Hominoidea - ps36
Hominidae - ps37
Homininae - ps38
Hominini - ps39
Homo_sapiens - ps40
p-value
3e-02
3e-02
4e-02
5e-02
6e-02
Log-odds
0.5
1.0
1.5
2.0
2.5
3.0
3.5
4.0

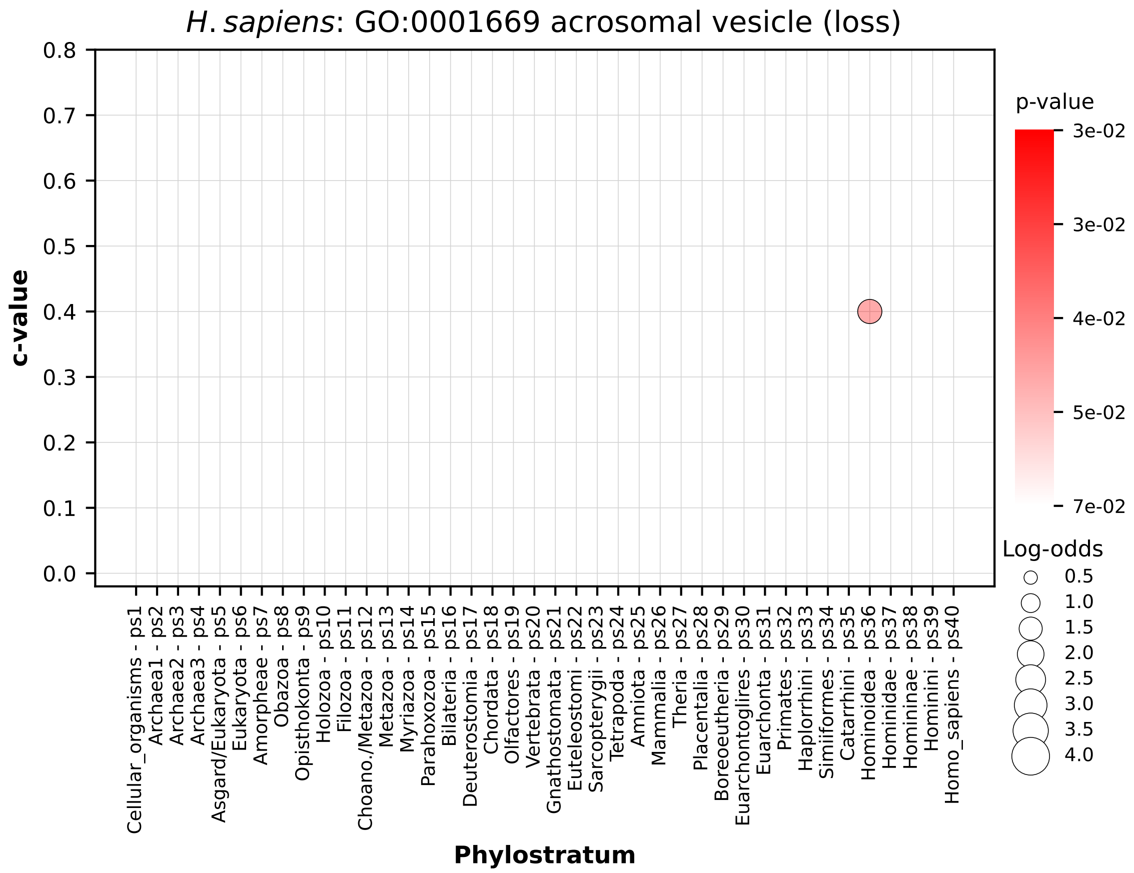

H. sapiens: GO:0001669 acrosomal vesicle (loss)
c-value
0.0
0.1
0.2
0.3
0.4
0.5
0.6
0.7
0.8
Phylostratum
Cellular_organisms - ps1
Archaea1 - ps2
Archaea2 - ps3
Archaea3 - ps4
Asgard/Eukaryota - ps5
Eukaryota - ps6
Amorpheae - ps7
Obazoa - ps8
Opisthokonta - ps9
Holozoa - ps10
Filozoa - ps11
Choano./Metazoa - ps12
Metazoa - ps13
Myriazoa - ps14
Parahoxozoa - ps15
Bilateria - ps16
Deuterostomia - ps17
Chordata - ps18
Olfactores - ps19
Vertebrata - ps20
Gnathostomata - ps21
Euteleostomi - ps22
Sarcopterygii - ps23
Tetrapoda - ps24
Amniota - ps25
Mammalia - ps26
Theria - ps27
Placentalia - ps28
Boreoeutheria - ps29
Euarchontoglires - ps30
Euarchonta - ps31
Primates - ps32
Haplorrhini - ps33
Simiiformes - ps34
Catarrhini - ps35
Hominoidea - ps36
Hominidae - ps37
Homininae - ps38
Hominini - ps39
Homo_sapiens - ps40
p-value
3e-02
3e-02
4e-02
5e-02
7e-02
Log-odds
0.5
1.0
1.5
2.0
2.5
3.0
3.5
4.0

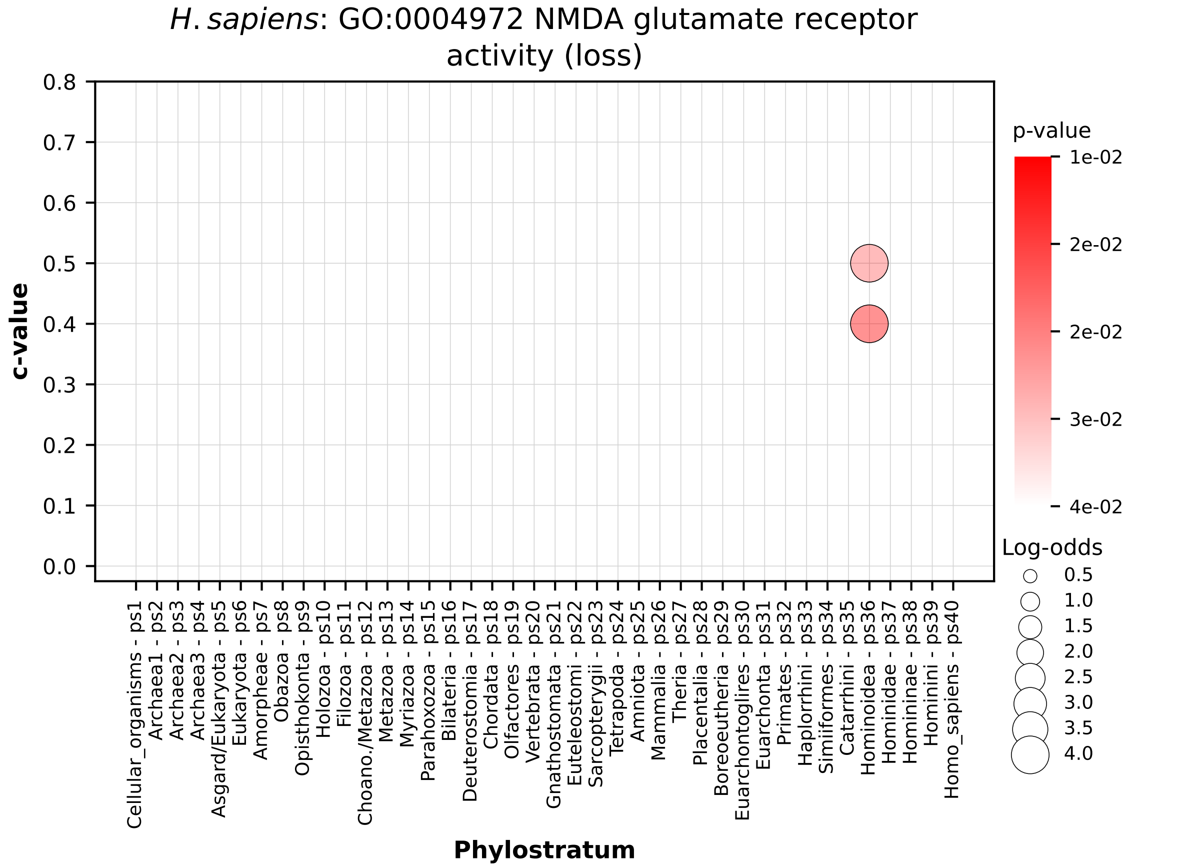

$H. sapiens$: GO:0004972 NMDA glutamate receptor activity (loss)
c-value
0.0
0.1
0.2
0.3
0.4
0.5
0.6
0.7
0.8
Phylostratum
Cellular_organisms - ps1
Archaea1 - ps2
Archaea2 - ps3
Archaea3 - ps4
Asgard/Eukaryota - ps5
Eukaryota - ps6
Amorpheae - ps7
Obazoa - ps8
Opisthokonta - ps9
Holozoa - ps10
Filozoa - ps11
Choano./Metazoa - ps12
Metazoa - ps13
Myriazoa - ps14
Parahoxozoa - ps15
Bilateria - ps16
Deuterostomia - ps17
Chordata - ps18
Olfactores - ps19
Vertebrata - ps20
Gnathostomata - ps21
Euteleostomi - ps22
Sarcopterygii - ps23
Tetrapoda - ps24
Amniota - ps25
Mammalia - ps26
Theria - ps27
Placentalia - ps28
Boreoeutheria - ps29
Euarchontoglires - ps30
Euarchonta - ps31
Primates - ps32
Haplorrhini - ps33
Simiiformes - ps34
Catarrhini - ps35
Hominoidea - ps36
Hominidae - ps37
Homininae - ps38
Hominini - ps39
Homo_sapiens - ps40
p-value
1e-02
2e-02
2e-02
3e-02
4e-02
Log-odds
0.5
1.0
1.5
2.0
2.5
3.0
3.5
4.0

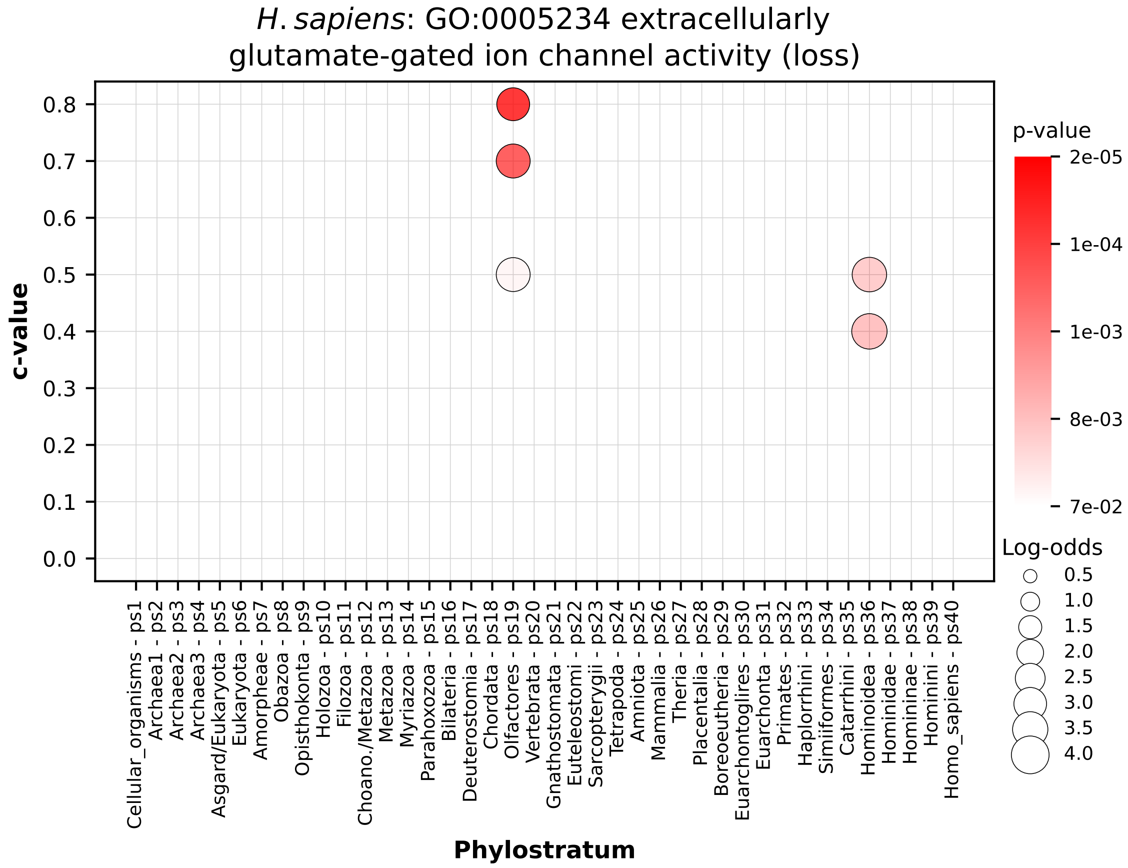

H. sapiens: GO:0005234 extracellularly glutamate-gated ion channel activity (loss)
c-value
0.8
0.7
0.6
0.5
0.4
0.3
0.2
0.1
0.0
p-value
2e-05
1e-04
1e-03
8e-03
7e-02
Log-odds
0.5
1.0
1.5
2.0
2.5
3.0
3.5
4.0
Cellular_organisms - ps1
Archaea1 - ps2
Archaea2 - ps3
Archaea3 - ps4
Asgard/Eukaryota - ps5
Eukaryota - ps6
Amorpheae - ps7
Obazoa - ps8
Opisthokonta - ps9
Holozoa - ps10
Filozoa - ps11
Choano./Metazoa - ps12
Metazoa - ps13
Myriazoa - ps14
Parahoxozoa - ps15
Bilateria - ps16
Deuterostomia - ps17
Chordata - ps18
Olfactores - ps19
Vertebrata - ps20
Gnathostomata - ps21
Euteleostomi - ps22
Sarcopterygii - ps23
Tetrapoda - ps24
Amniota - ps25
Mammalia - ps26
Theria - ps27
Placentalia - ps28
Boreoeutheria - ps29
Euarchontoglires - ps30
Euarchonta - ps31
Primates - ps32
Haplorrhini - ps33
Simiiformes - ps34
Catarrhini - ps35
Hominoidea - ps36
Hominidae - ps37
Homininae - ps38
Hominini - ps39
Homo_sapiens - ps40
Phylostratum

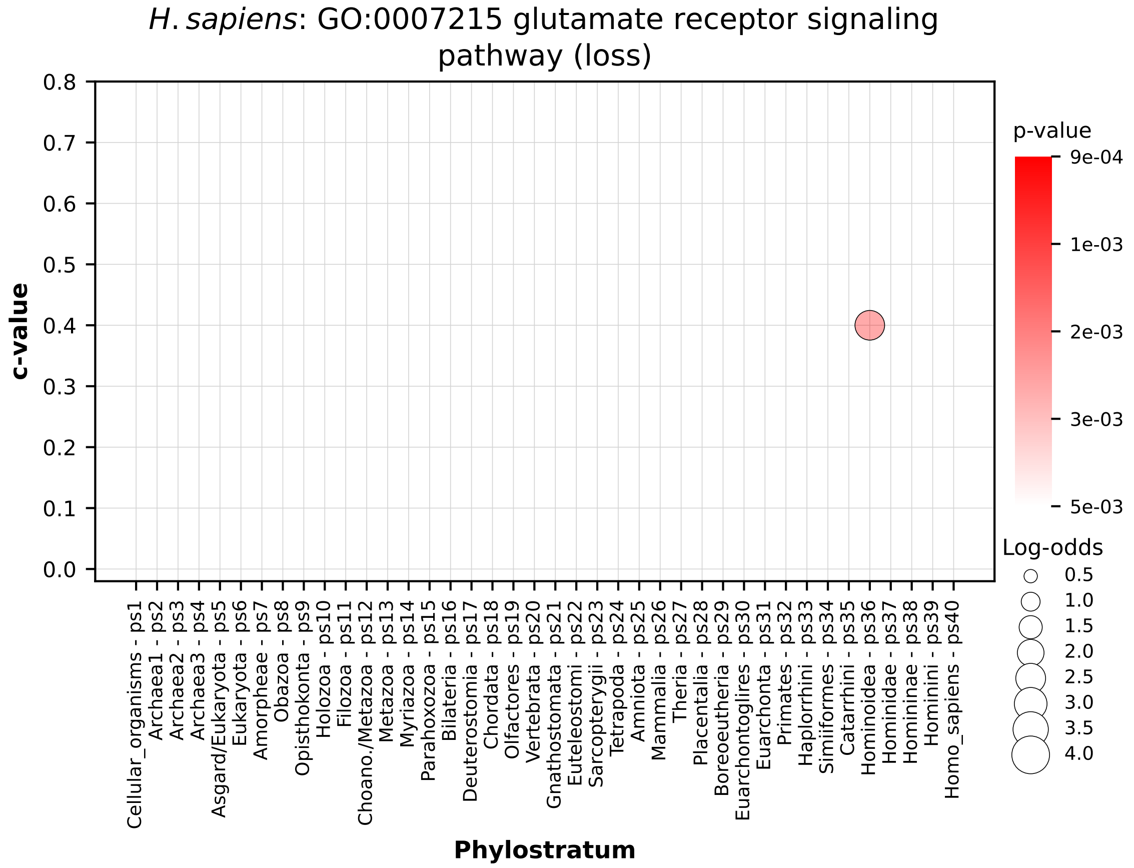

H. sapiens: GO:0007215 glutamate receptor signaling pathway (loss)
c-value
0.0
0.1
0.2
0.3
0.4
0.5
0.6
0.7
0.8
Phylostratum
Cellular_organisms - ps1
Archaea1 - ps2
Archaea2 - ps3
Archaea3 - ps4
Asgard/Eukaryota - ps5
Eukaryota - ps6
Amorpheae - ps7
Obazoa - ps8
Opisthokonta - ps9
Holozoa - ps10
Filozoa - ps11
Choano./Metazoa - ps12
Metazoa - ps13
Myriazoa - ps14
Parahoxozoa - ps15
Bilateria - ps16
Deuterostomia - ps17
Chordata - ps18
Olfactores - ps19
Vertebrata - ps20
Gnathostomata - ps21
Euteleostomi - ps22
Sarcopterygii - ps23
Tetrapoda - ps24
Amniota - ps25
Mammalia - ps26
Theria - ps27
Placentalia - ps28
Boreoeutheria - ps29
Euarchontoglires - ps30
Euarchonta - ps31
Primates - ps32
Haplorrhini - ps33
Simiiformes - ps34
Catarrhini - ps35
Hominoidea - ps36
Hominidae - ps37
Homininae - ps38
Hominini - ps39
Homo_sapiens - ps40
p-value
9e-04
1e-03
2e-03
3e-03
5e-03
Log-odds
0.5
1.0
1.5
2.0
2.5
3.0
3.5
4.0

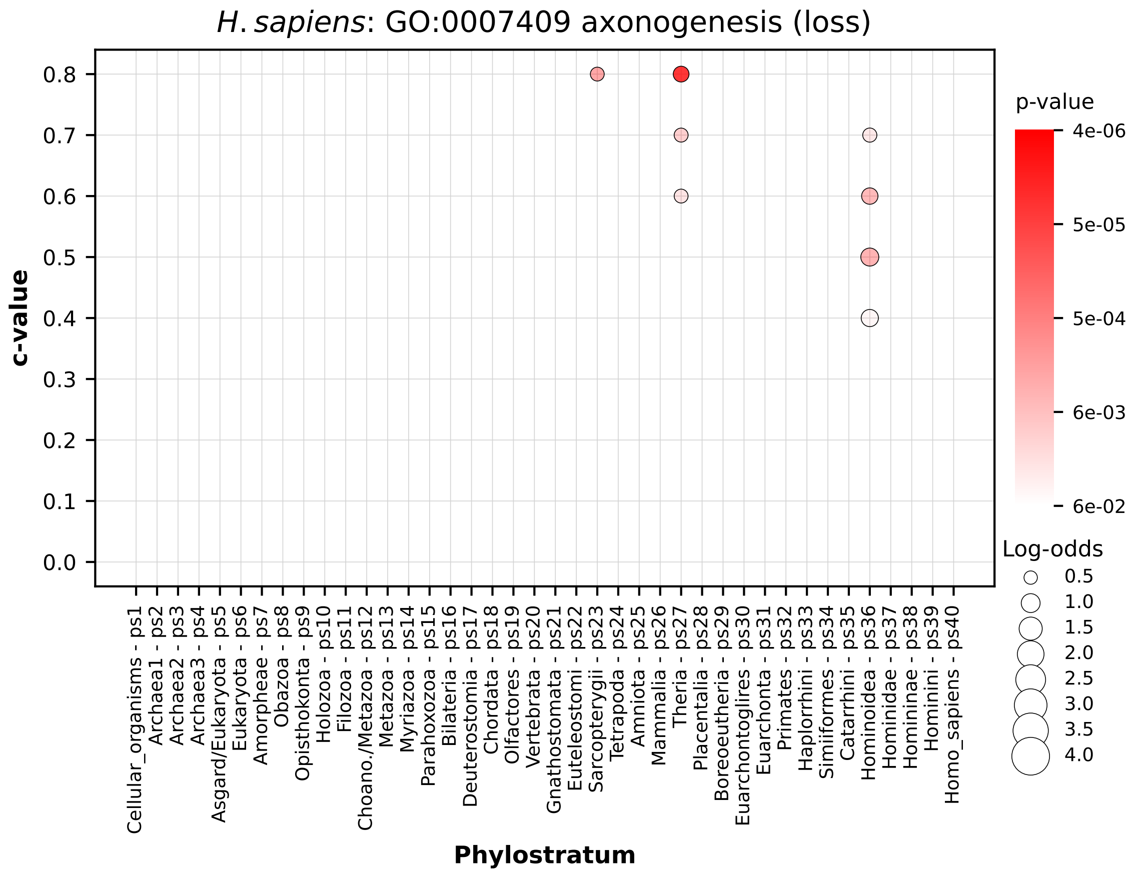

H. sapiens: GO:0007409 axonogenesis (loss)
c-value
0.0
0.1
0.2
0.3
0.4
0.5
0.6
0.7
0.8
Phylostratum
Cellular_organisms - ps1
Archaea1 - ps2
Archaea2 - ps3
Archaea3 - ps4
Asgard/Eukaryota - ps5
Eukaryota - ps6
Amorpheae - ps7
Obazoa - ps8
Opisthokonta - ps9
Holozoa - ps10
Filozoa - ps11
Choano./Metazoa - ps12
Metazoa - ps13
Myriazoa - ps14
Parahoxozoa - ps15
Bilateria - ps16
Deuterostomia - ps17
Chordata - ps18
Olfactores - ps19
Vertebrata - ps20
Gnathostomata - ps21
Euteleostomi - ps22
Sarcopterygii - ps23
Tetrapoda - ps24
Amniota - ps25
Mammalia - ps26
Theria - ps27
Placentalia - ps28
Boreoeutheria - ps29
Euarchontoglires - ps30
Euarchonta - ps31
Primates - ps32
Haplorrhini - ps33
Simiiformes - ps34
Catarrhini - ps35
Hominoidea - ps36
Hominidae - ps37
Homininae - ps38
Hominini - ps39
Homo_sapiens - ps40
p-value
4e-06
5e-05
5e-04
6e-03
6e-02
Log-odds
0.5
1.0
1.5
2.0
2.5
3.0
3.5
4.0

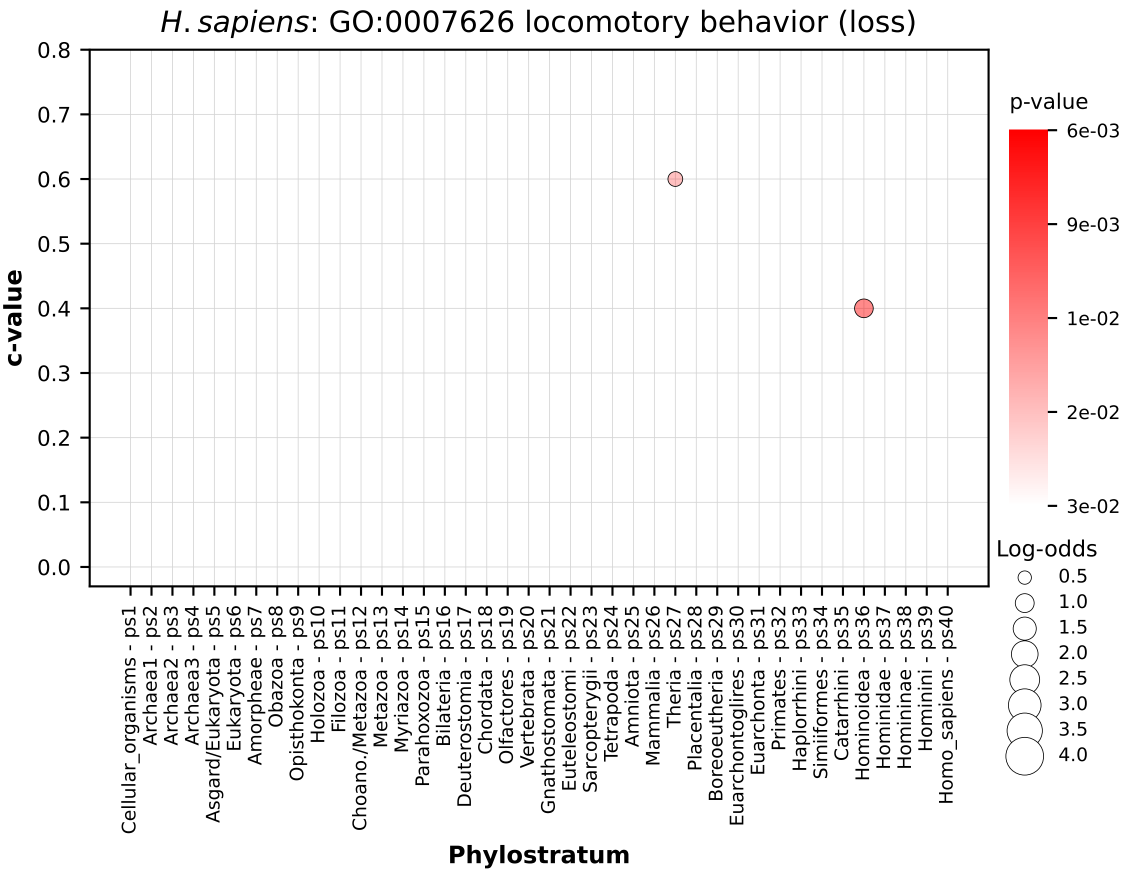

H. sapiens: GO:0007626 locomotory behavior (loss)
c-value
0.8
0.7
0.6
0.5
0.4
0.3
0.2
0.1
0.0
Phylostratum
Cellular_organisms - ps1
Archaea1 - ps2
Archaea2 - ps3
Archaea3 - ps4
Asgard/Eukaryota - ps5
Eukaryota - ps6
Amorpheae - ps7
Obazoa - ps8
Opisthokonta - ps9
Holozoa - ps10
Filozoa - ps11
Choano./Metazoa - ps12
Metazoa - ps13
Myriazoa - ps14
Parahoxozoa - ps15
Bilateria - ps16
Deuterostomia - ps17
Chordata - ps18
Olfactores - ps19
Vertebrata - ps20
Gnathostomata - ps21
Euteleostomi - ps22
Sarcopterygii - ps23
Tetrapoda - ps24
Amniota - ps25
Mammalia - ps26
Theria - ps27
Placentalia - ps28
Boreoeutheria - ps29
Euarchontoglires - ps30
Euarchonta - ps31
Primates - ps32
Haplorrhini - ps33
Simiiformes - ps34
Catarrhini - ps35
Hominoidea - ps36
Hominidae - ps37
Homininae - ps38
Hominini - ps39
Homo_sapiens - ps40
p-value
6e-03
9e-03
1e-02
2e-02
3e-02
Log-odds
0.5
1.0
1.5
2.0
2.5
3.0
3.5
4.0

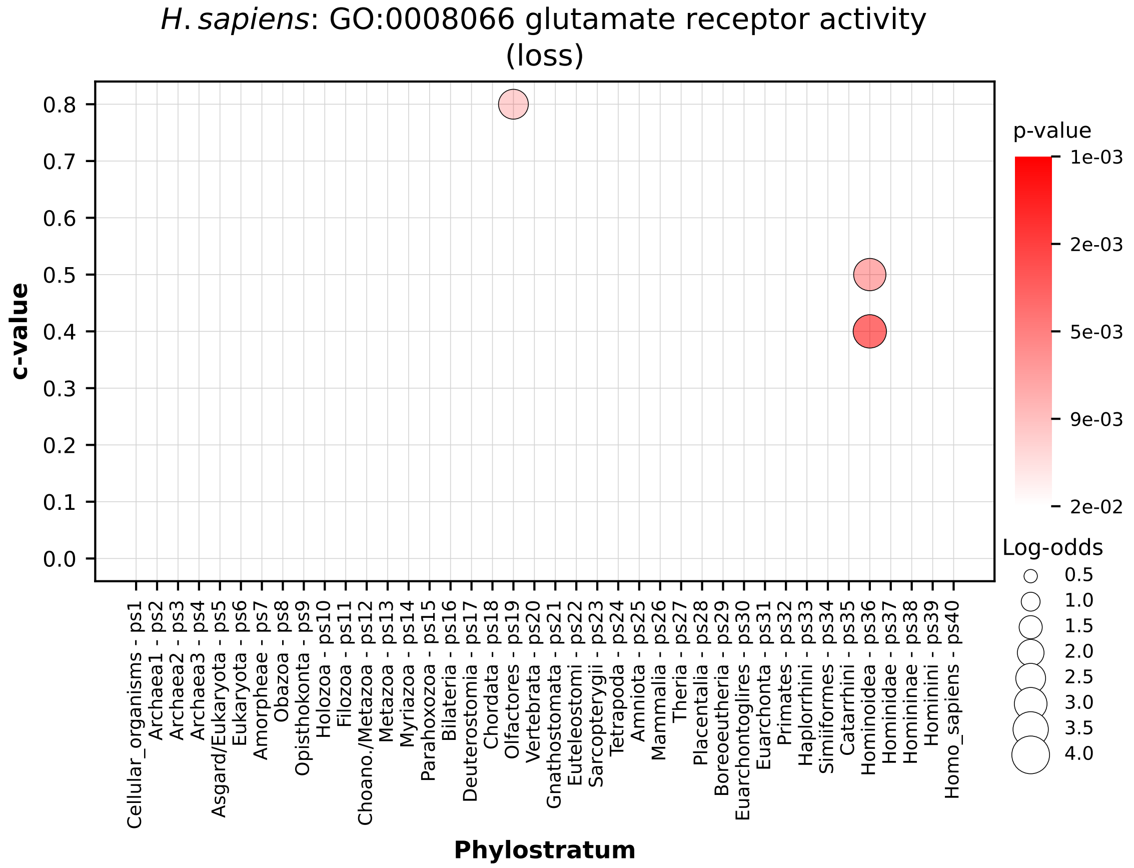

H. sapiens: GO:0008066 glutamate receptor activity
(loss)
c-value
0.0
0.1
0.2
0.3
0.4
0.5
0.6
0.7
0.8
p-value
1e-03
2e-03
5e-03
9e-03
2e-02
Log-odds
0.5
1.0
1.5
2.0
2.5
3.0
3.5
4.0
Cellular_organisms - ps1
Archaea1 - ps2
Archaea2 - ps3
Archaea3 - ps4
Asgard/Eukaryota - ps5
Eukaryota - ps6
Amorpheae - ps7
Obazoa - ps8
Opisthokonta - ps9
Holozoa - ps10
Filozoa - ps11
Choano./Metazoa - ps12
Metazoa - ps13
Myriazoa - ps14
Parahoxozoa - ps15
Bilateria - ps16
Deuterostomia - ps17
Chordata - ps18
Olfactores - ps19
Vertebrata - ps20
Gnathostomata - ps21
Euteleostomi - ps22
Sarcopterygii - ps23
Tetrapoda - ps24
Amniota - ps25
Mammalia - ps26
Theria - ps27
Placentalia - ps28
Boreoeutheria - ps29
Euarchontoglires - ps30
Euarchonta - ps31
Primates - ps32
Haplorrhini - ps33
Simiiformes - ps34
Catarrhini - ps35
Hominoidea - ps36
Hominidae - ps37
Homininae - ps38
Hominini - ps39
Homo_sapiens - ps40
Phylostratum

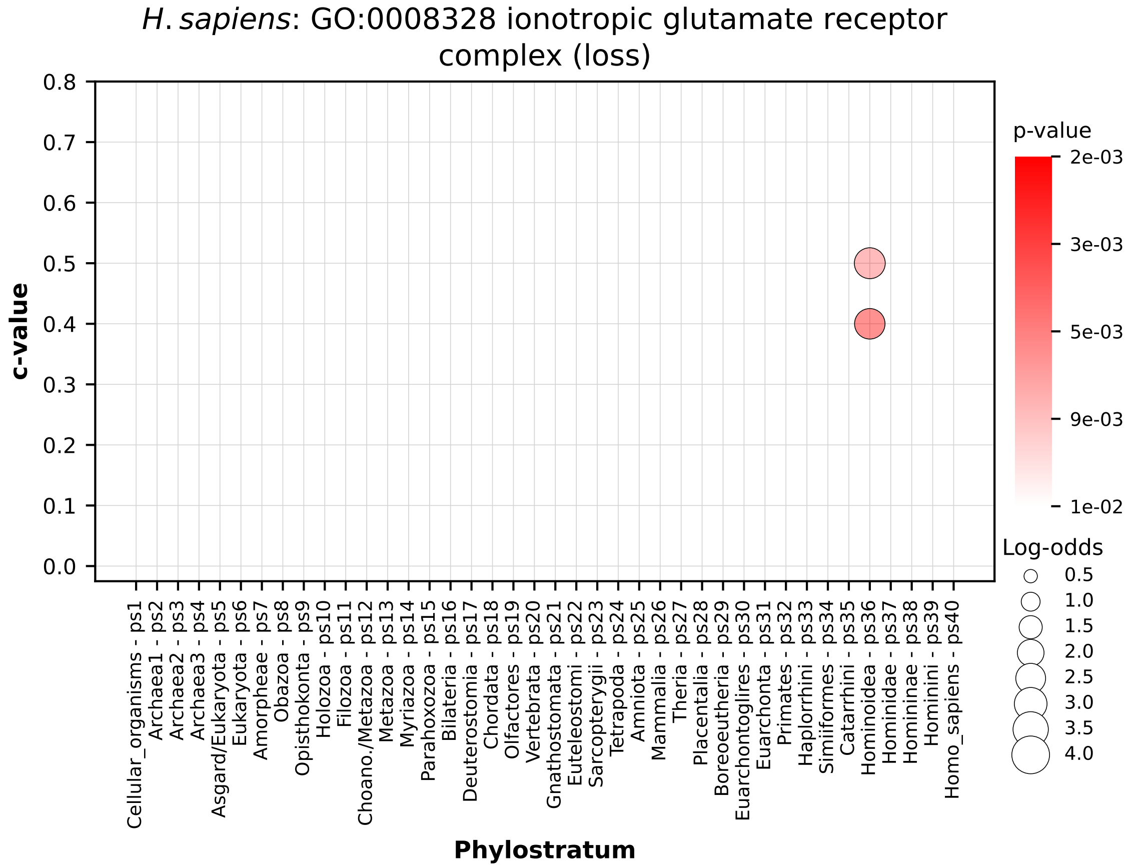

H. sapiens: GO:0008328 ionotropic glutamate receptor complex (loss)
c-value
0.8
0.7
0.6
0.5
0.4
0.3
0.2
0.1
0.0
Phylostratum
Cellular_organisms - ps1
Archaea1 - ps2
Archaea2 - ps3
Archaea3 - ps4
Asgard/Eukaryota - ps5
Eukaryota - ps6
Amorpheae - ps7
Obazoa - ps8
Opisthokonta - ps9
Holozoa - ps10
Filozoa - ps11
Choano./Metazoa - ps12
Metazoa - ps13
Myriazoa - ps14
Parahoxozoa - ps15
Bilateria - ps16
Deuterostomia - ps17
Chordata - ps18
Olfactores - ps19
Vertebrata - ps20
Gnathostomata - ps21
Euteleostomi - ps22
Sarcopterygii - ps23
Tetrapoda - ps24
Amniota - ps25
Mammalia - ps26
Theria - ps27
Placentalia - ps28
Boreoeutheria - ps29
Euarchontoglires - ps30
Euarchonta - ps31
Primates - ps32
Haplorrhini - ps33
Simiiformes - ps34
Catarrhini - ps35
Hominoidea - ps36
Hominidae - ps37
Homininae - ps38
Hominini - ps39
Homo_sapiens - ps40
p-value
2e-03
3e-03
5e-03
9e-03
1e-02
Log-odds
0.5
1.0
1.5
2.0
2.5
3.0
3.5
4.0

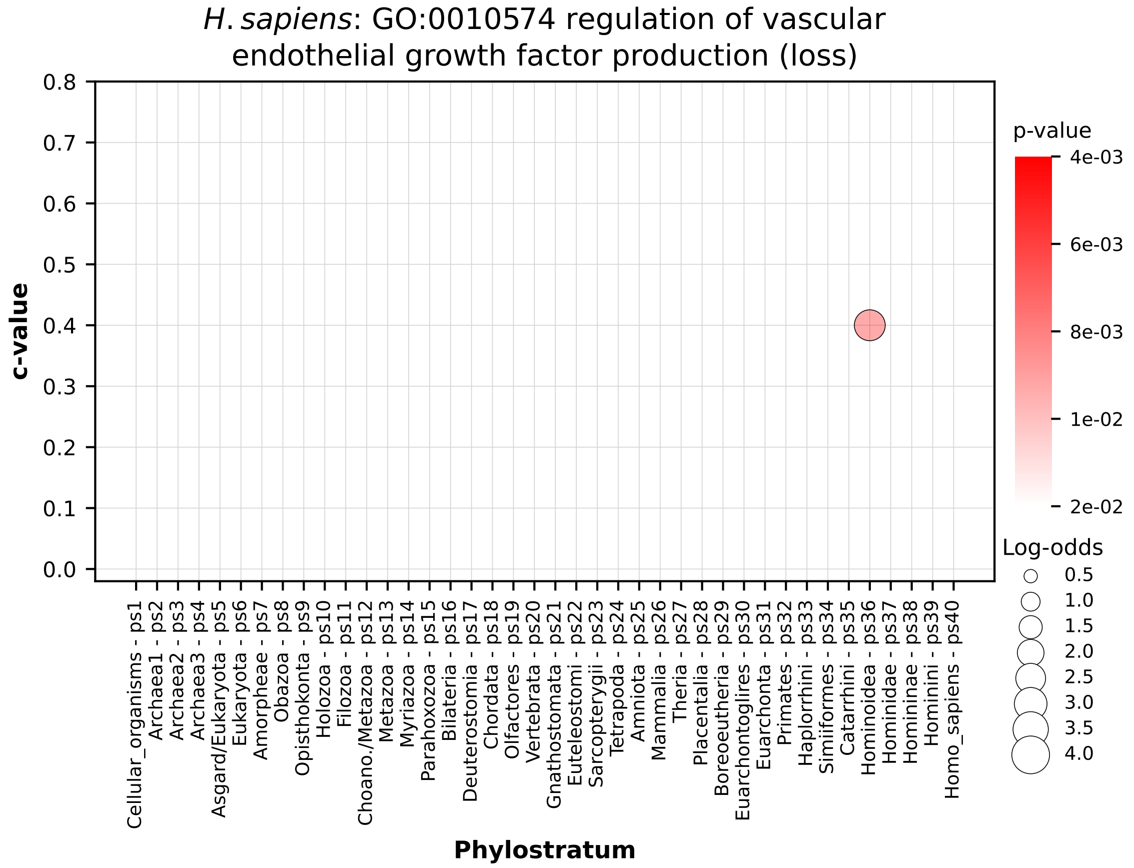
H. sapiens: GO:0010574 regulation of vascular endothelial growth factor production (loss)
c-value
0.8
0.7
0.6
0.5
0.4
0.3
0.2
0.1
0.0
Phylostratum
Cellular_organisms - ps1
Archaea1 - ps2
Archaea2 - ps3
Archaea3 - ps4
Asgard/Eukaryota - ps5
Eukaryota - ps6
Amorpheae - ps7
Obazoa - ps8
Opisthokonta - ps9
Holozoa - ps10
Filozoa - ps11
Choano./Metazoa - ps12
Metazoa - ps13
Myriazoa - ps14
Parahoxozoa - ps15
Bilateria - ps16
Deuterostomia - ps17
Chordata - ps18
Olfactores - ps19
Vertebrata - ps20
Gnathostomata - ps21
Euteleostomi - ps22
Sarcopterygii - ps23
Tetrapoda - ps24
Amniota - ps25
Mammalia - ps26
Theria - ps27
Placentalia - ps28
Boreoeutheria - ps29
Euarchontoglires - ps30
Euarchonta - ps31
Primates - ps32
Haplorrhini - ps33
Simiiformes - ps34
Catarrhini - ps35
Hominoidea - ps36
Hominidae - ps37
Homininae - ps38
Hominini - ps39
Homo_sapiens - ps40
p-value
4e-03
6e-03
8e-03
1e-02
2e-02
Log-odds
0.5
1.0
1.5
2.0
2.5
3.0
3.5
4.0

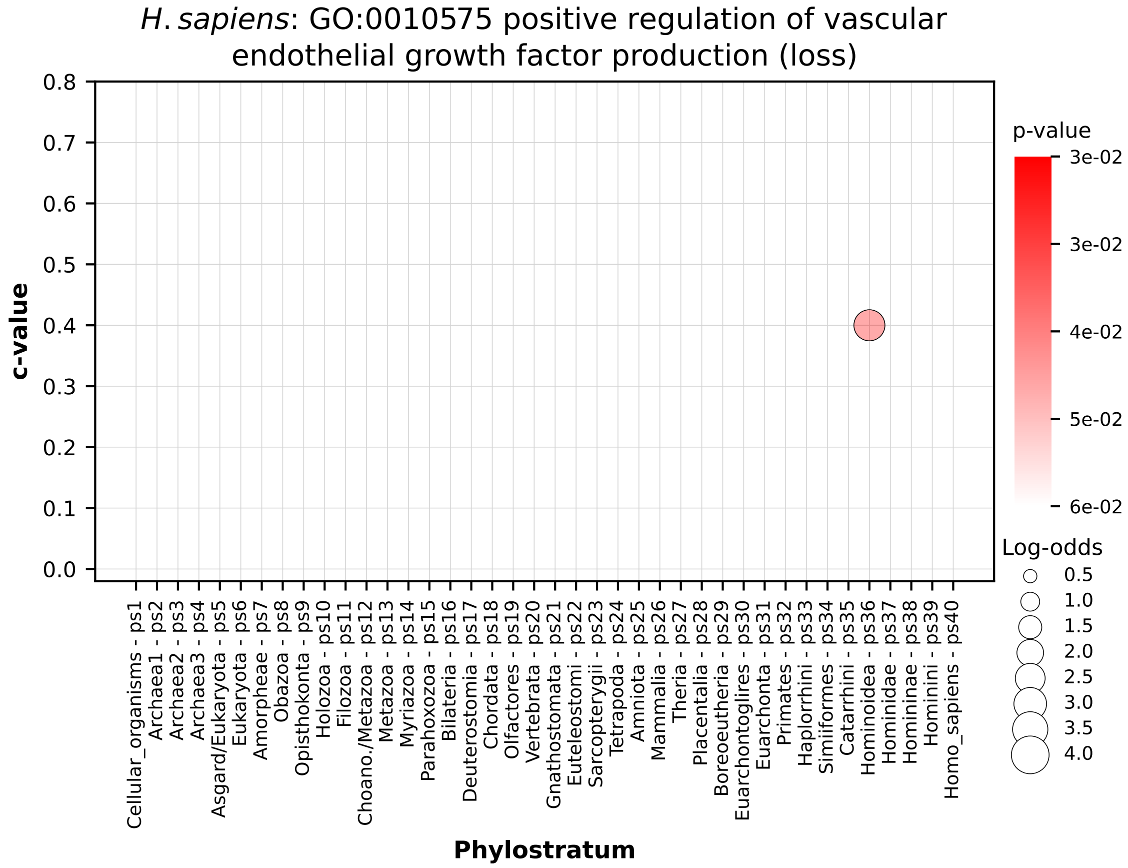

H. sapiens: GO:0010575 positive regulation of vascular endothelial growth factor production (loss)
c-value
0.8
0.7
0.6
0.5
0.4
0.3
0.2
0.1
0.0
Phylostratum
Cellular_organisms - ps1
Archaea1 - ps2
Archaea2 - ps3
Archaea3 - ps4
Asgard/Eukaryota - ps5
Eukaryota - ps6
Amorpheae - ps7
Obazoa - ps8
Opisthokonta - ps9
Holozoa - ps10
Filozoa - ps11
Choano./Metazoa - ps12
Metazoa - ps13
Myriazoa - ps14
Parahoxozoa - ps15
Bilateria - ps16
Deuterostomia - ps17
Chordata - ps18
Olfactores - ps19
Vertebrata - ps20
Gnathostomata - ps21
Euteleostomi - ps22
Sarcopterygii - ps23
Tetrapoda - ps24
Amniota - ps25
Mammalia - ps26
Theria - ps27
Placentalia - ps28
Boreoeutheria - ps29
Euarchontoglires - ps30
Euarchonta - ps31
Primates - ps32
Haplorrhini - ps33
Simiiformes - ps34
Catarrhini - ps35
Hominoidea - ps36
Hominidae - ps37
Homininae - ps38
Hominini - ps39
Homo_sapiens - ps40
p-value
3e-02
3e-02
4e-02
5e-02
6e-02
Log-odds
0.5
1.0
1.5
2.0
2.5
3.0
3.5
4.0

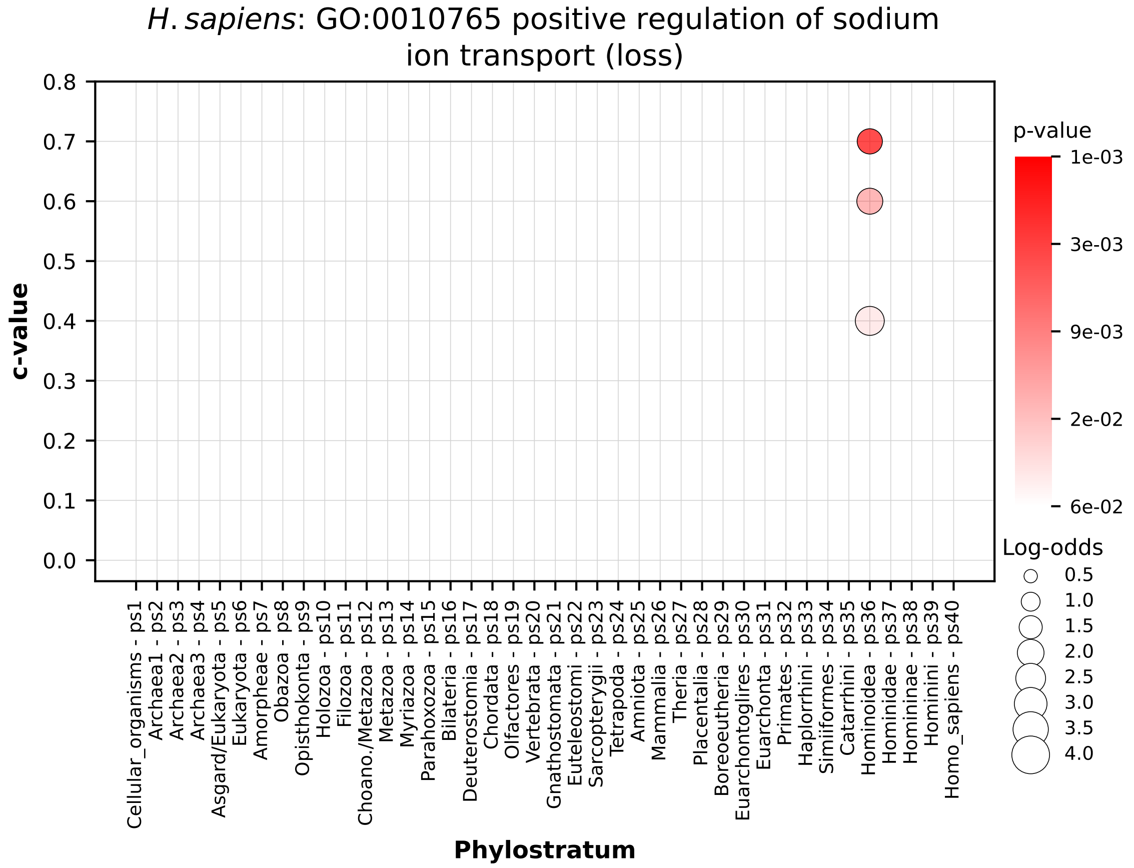

H. sapiens: GO:0010765 positive regulation of sodium ion transport (loss)
c-value
0.0
0.1
0.2
0.3
0.4
0.5
0.6
0.7
0.8
Phylostratum
Cellular_organisms - ps1
Archaea1 - ps2
Archaea2 - ps3
Archaea3 - ps4
Asgard/Eukaryota - ps5
Eukaryota - ps6
Amorpheae - ps7
Obazoa - ps8
Opisthokonta - ps9
Holozoa - ps10
Filozoa - ps11
Choano./Metazoa - ps12
Metazoa - ps13
Myriazoa - ps14
Parahoxozoa - ps15
Bilateria - ps16
Deuterostomia - ps17
Chordata - ps18
Olfactores - ps19
Vertebrata - ps20
Gnathostomata - ps21
Euteleostomi - ps22
Sarcopterygii - ps23
Tetrapoda - ps24
Amniota - ps25
Mammalia - ps26
Theria - ps27
Placentalia - ps28
Boreoeutheria - ps29
Euarchontoglires - ps30
Euarchonta - ps31
Primates - ps32
Haplorrhini - ps33
Simiiformes - ps34
Catarrhini - ps35
Hominoidea - ps36
Hominidae - ps37
Homininae - ps38
Hominini - ps39
Homo_sapiens - ps40
p-value
1e-03
3e-03
9e-03
2e-02
6e-02
Log-odds
0.5
1.0
1.5
2.0
2.5
3.0
3.5
4.0

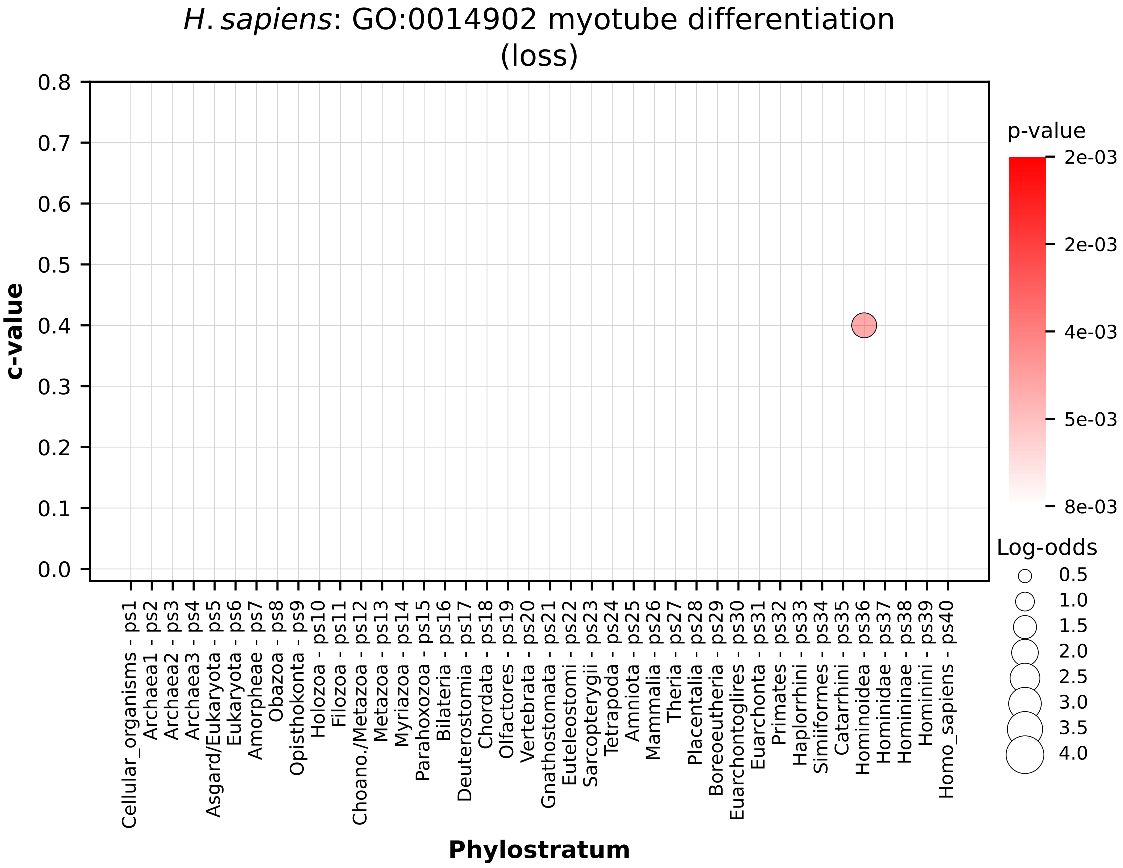

H. sapiens: GO:0014902 myotube differentiation (loss)
c-value
0.0
0.1
0.2
0.3
0.4
0.5
0.6
0.7
0.8
Phylostratum
Cellular_organisms - ps1
Archaea1 - ps2
Archaea2 - ps3
Archaea3 - ps4
Asgard/Eukaryota - ps5
Eukaryota - ps6
Amorpheae - ps7
Obazoa - ps8
Opisthokonta - ps9
Holozoa - ps10
Filozoa - ps11
Choano./Metazoa - ps12
Metazoa - ps13
Myriazoa - ps14
Parahoxozoa - ps15
Bilateria - ps16
Deuterostomia - ps17
Chordata - ps18
Olfactores - ps19
Vertebrata - ps20
Gnathostomata - ps21
Euteleostomi - ps22
Sarcopterygii - ps23
Tetrapoda - ps24
Amniota - ps25
Mammalia - ps26
Theria - ps27
Placentalia - ps28
Boreoeutheria - ps29
Euarchontoglires - ps30
Euarchonta - ps31
Primates - ps32
Haplorrhini - ps33
Simiiformes - ps34
Catarrhini - ps35
Hominoidea - ps36
Hominidae - ps37
Homininae - ps38
Hominini - ps39
Homo_sapiens - ps40
p-value
2e-03
2e-03
4e-03
5e-03
8e-03
Log-odds
0.5
1.0
1.5
2.0
2.5
3.0
3.5
4.0

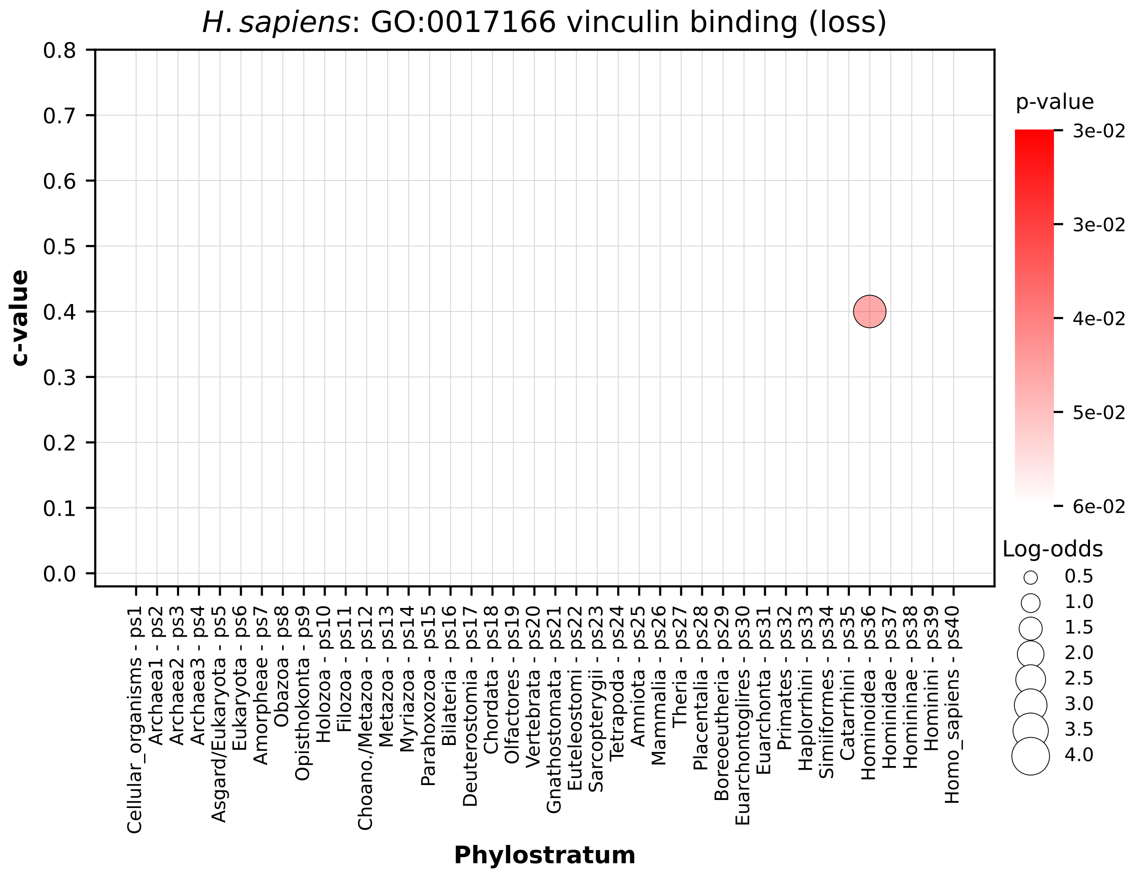

H. sapiens: GO:0017166 vinculin binding (loss)
c-value
0.0
0.1
0.2
0.3
0.4
0.5
0.6
0.7
0.8
Phylostratum
Cellular_organisms - ps1
Archaea1 - ps2
Archaea2 - ps3
Archaea3 - ps4
Asgard/Eukaryota - ps5
Eukaryota - ps6
Amorpheae - ps7
Obazoa - ps8
Opisthokonta - ps9
Holozoa - ps10
Filozoa - ps11
Choano./Metazoa - ps12
Metazoa - ps13
Myriazoa - ps14
Parahoxozoa - ps15
Bilateria - ps16
Deuterostomia - ps17
Chordata - ps18
Olfactores - ps19
Vertebrata - ps20
Gnathostomata - ps21
Euteleostomi - ps22
Sarcopterygii - ps23
Tetrapoda - ps24
Amniota - ps25
Mammalia - ps26
Theria - ps27
Placentalia - ps28
Boreoeutheria - ps29
Euarchontoglires - ps30
Euarchonta - ps31
Primates - ps32
Haplorrhini - ps33
Simiiformes - ps34
Catarrhini - ps35
Hominoidea - ps36
Hominidae - ps37
Homininae - ps38
Hominini - ps39
Homo_sapiens - ps40
p-value
3e-02
3e-02
4e-02
5e-02
6e-02
Log-odds
0.5
1.0
1.5
2.0
2.5
3.0
3.5
4.0

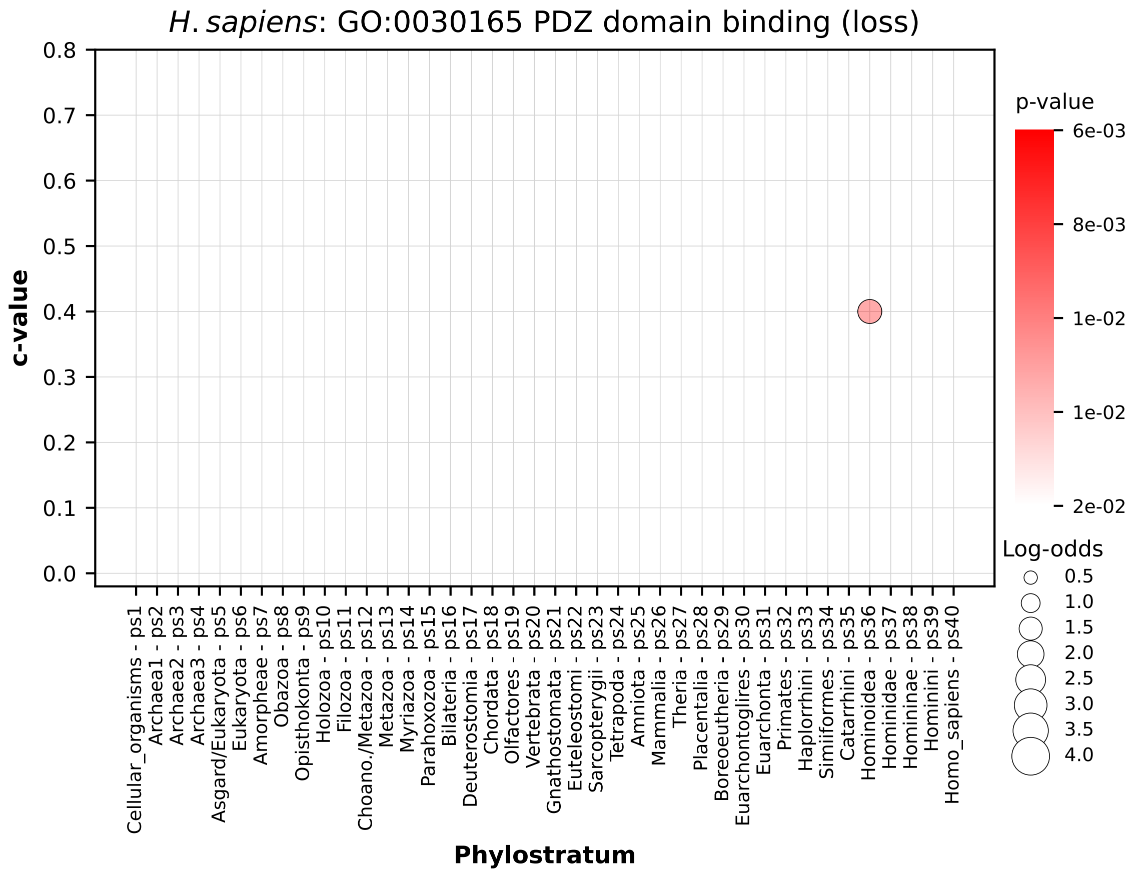

H. sapiens: GO:0030165 PDZ domain binding (loss)
c-value
0.8
0.7
0.6
0.5
0.4
0.3
0.2
0.1
0.0
p-value
6e-03
8e-03
1e-02
1e-02
2e-02
Log-odds
0.5
1.0
1.5
2.0
2.5
3.0
3.5
4.0
Cellular_organisms - ps1
Archaea1 - ps2
Archaea2 - ps3
Archaea3 - ps4
Asgard/Eukaryota - ps5
Eukaryota - ps6
Amorpheae - ps7
Obazoa - ps8
Opisthokonta - ps9
Holozoa - ps10
Filozoa - ps11
Choano./Metazoa - ps12
Metazoa - ps13
Myriazoa - ps14
Parahoxozoa - ps15
Bilateria - ps16
Deuterostomia - ps17
Chordata - ps18
Olfactores - ps19
Vertebrata - ps20
Gnathostomata - ps21
Euteleostomi - ps22
Sarcopterygii - ps23
Tetrapoda - ps24
Amniota - ps25
Mammalia - ps26
Theria - ps27
Placentalia - ps28
Boreoeutheria - ps29
Euarchontoglires - ps30
Euarchonta - ps31
Primates - ps32
Haplorrhini - ps33
Simiiformes - ps34
Catarrhini - ps35
Hominoidea - ps36
Hominidae - ps37
Homininae - ps38
Hominini - ps39
Homo_sapiens - ps40
Phylostratum

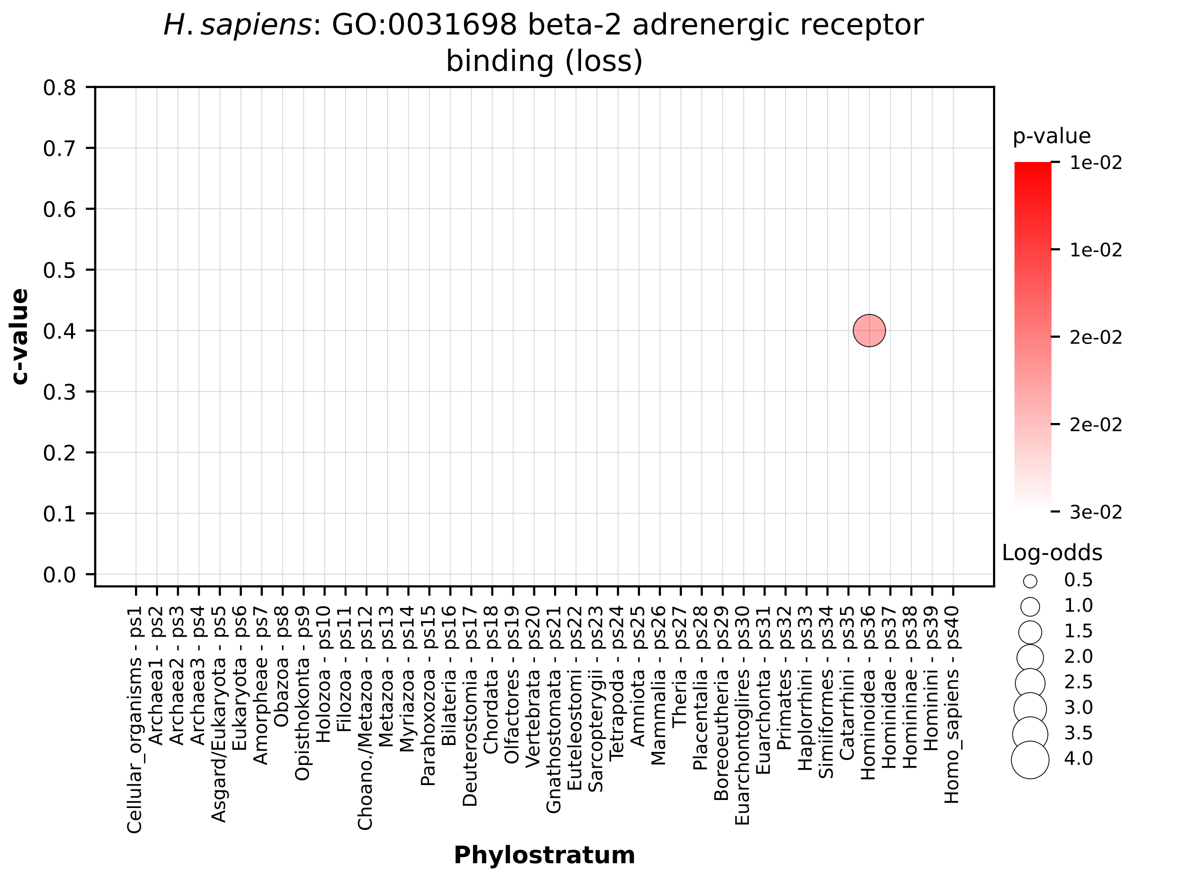

H. sapiens: GO:0031698 beta-2 adrenergic receptor binding (loss)
c-value
0.0
0.1
0.2
0.3
0.4
0.5
0.6
0.7
0.8
p-value
1e-02
1e-02
2e-02
2e-02
3e-02
Log-odds
0.5
1.0
1.5
2.0
2.5
3.0
3.5
4.0
Cellular_organisms - ps1
Archaea1 - ps2
Archaea2 - ps3
Archaea3 - ps4
Asgard/Eukaryota - ps5
Eukaryota - ps6
Amorpheae - ps7
Obazoa - ps8
Opisthokonta - ps9
Holozoa - ps10
Filozoa - ps11
Choano./Metazoa - ps12
Metazoa - ps13
Myriazoa - ps14
Parahoxozoa - ps15
Bilateria - ps16
Deuterostomia - ps17
Chordata - ps18
Olfactores - ps19
Vertebrata - ps20
Gnathostomata - ps21
Euteleostomi - ps22
Sarcopterygii - ps23
Tetrapoda - ps24
Amniota - ps25
Mammalia - ps26
Theria - ps27
Placentalia - ps28
Boreoeutheria - ps29
Euarchontoglires - ps30
Euarchonta - ps31
Primates - ps32
Haplorrhini - ps33
Simiiformes - ps34
Catarrhini - ps35
Hominoidea - ps36
Hominidae - ps37
Homininae - ps38
Hominini - ps39
Homo_sapiens - ps40
Phylostratum

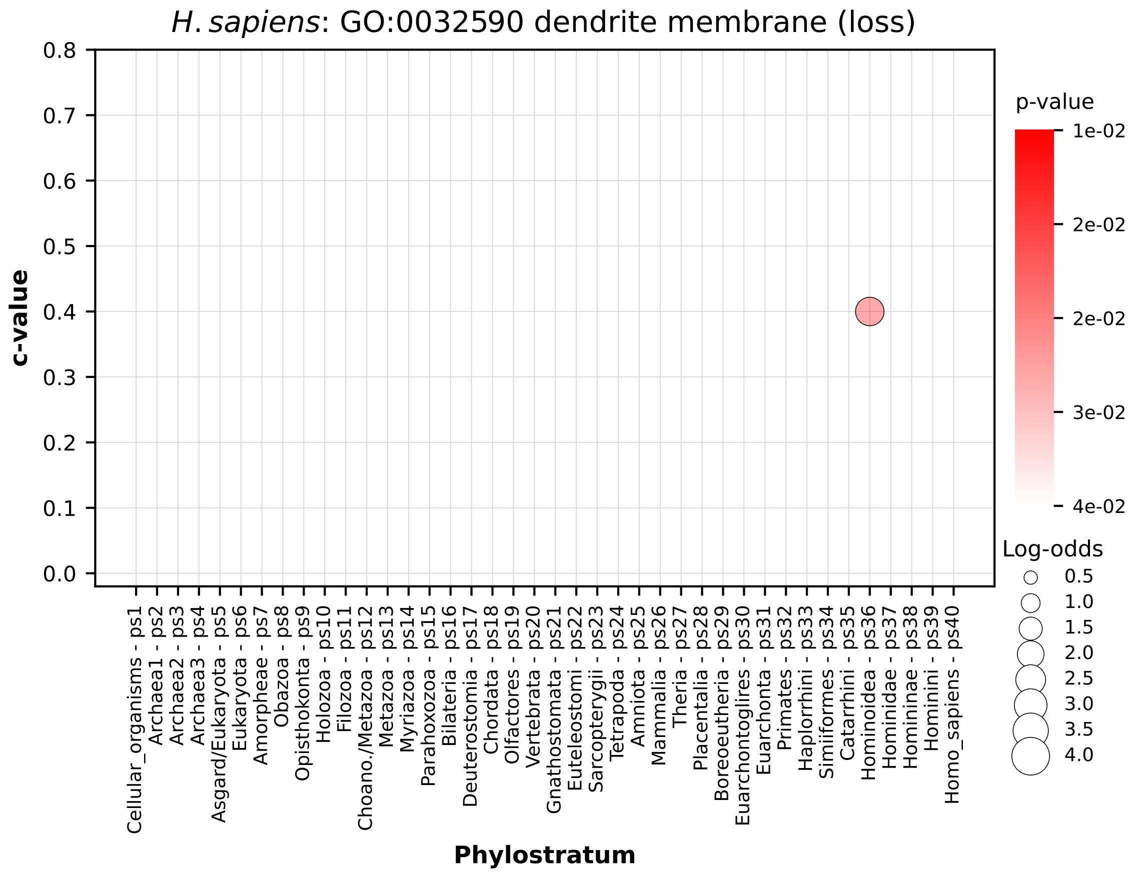

H. sapiens: GO:0032590 dendrite membrane (loss)
c-value
0.8
0.7
0.6
0.5
0.4
0.3
0.2
0.1
0.0
Phylostratum
Cellular_organisms - ps1
Archaea1 - ps2
Archaea2 - ps3
Archaea3 - ps4
Asgard/Eukaryota - ps5
Eukaryota - ps6
Amorpheae - ps7
Obazoa - ps8
Opisthokonta - ps9
Holozoa - ps10
Filozoa - ps11
Choano./Metazoa - ps12
Metazoa - ps13
Myriazoa - ps14
Parahoxozoa - ps15
Bilateria - ps16
Deuterostomia - ps17
Chordata - ps18
Olfactores - ps19
Vertebrata - ps20
Gnathostomata - ps21
Euteleostomi - ps22
Sarcopterygii - ps23
Tetrapoda - ps24
Amniota - ps25
Mammalia - ps26
Theria - ps27
Placentalia - ps28
Boreoeutheria - ps29
Euarchontoglires - ps30
Euarchonta - ps31
Primates - ps32
Haplorrhini - ps33
Simiiformes - ps34
Catarrhini - ps35
Hominoidea - ps36
Hominidae - ps37
Homininae - ps38
Hominini - ps39
Homo_sapiens - ps40
p-value
1e-02
2e-02
2e-02
3e-02
4e-02
Log-odds
0.5
1.0
1.5
2.0
2.5
3.0
3.5
4.0

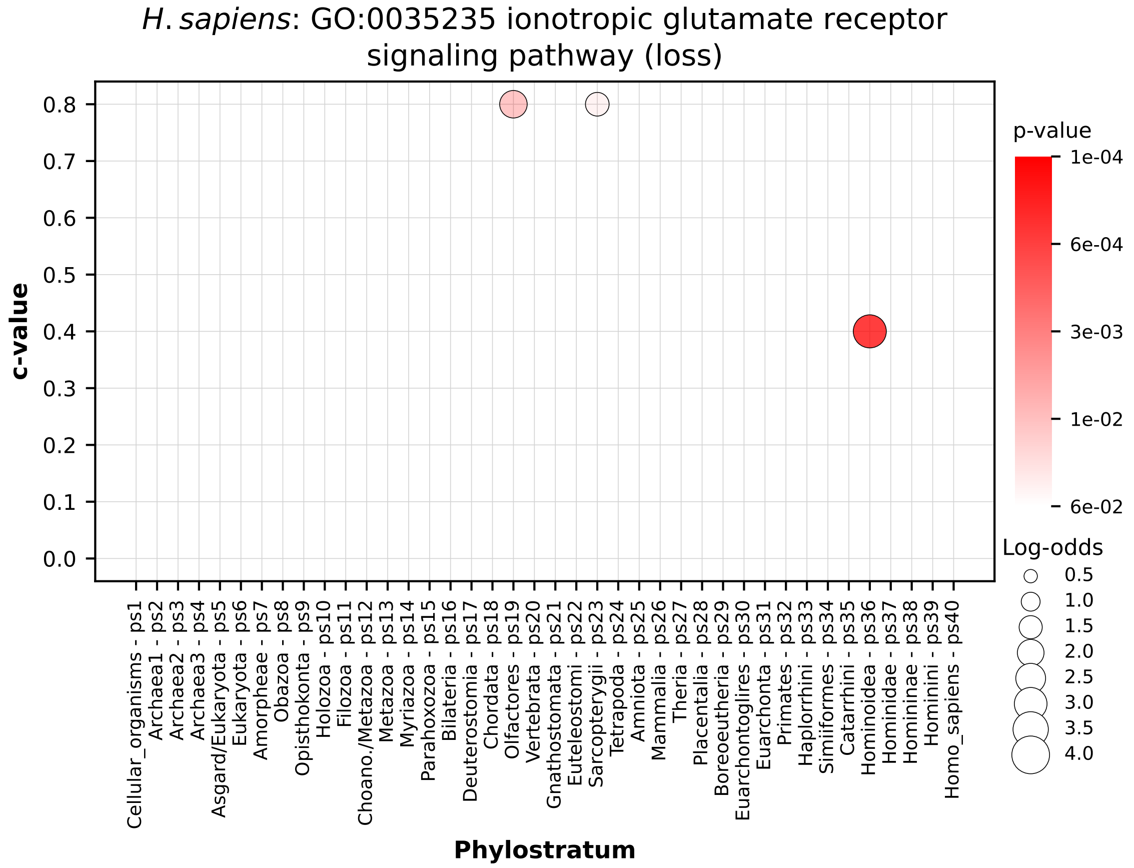

H. sapiens: GO:0035235 ionotropic glutamate receptor signaling pathway (loss)
c-value
0.0
0.1
0.2
0.3
0.4
0.5
0.6
0.7
0.8
Phylostratum
Cellular_organisms - ps1
Archaea1 - ps2
Archaea2 - ps3
Archaea3 - ps4
Asgard/Eukaryota - ps5
Eukaryota - ps6
Amorpheae - ps7
Obazoa - ps8
Opisthokonta - ps9
Holozoa - ps10
Filozoa - ps11
Choano./Metazoa - ps12
Metazoa - ps13
Myriazoa - ps14
Parahoxozoa - ps15
Bilateria - ps16
Deuterostomia - ps17
Chordata - ps18
Olfactores - ps19
Vertebrata - ps20
Gnathostomata - ps21
Euteleostomi - ps22
Sarcopterygii - ps23
Tetrapoda - ps24
Amniota - ps25
Mammalia - ps26
Theria - ps27
Placentalia - ps28
Boreoeutheria - ps29
Euarchontoglires - ps30
Euarchonta - ps31
Primates - ps32
Haplorrhini - ps33
Simiiformes - ps34
Catarrhini - ps35
Hominoidea - ps36
Hominidae - ps37
Homininae - ps38
Hominini - ps39
Homo_sapiens - ps40
p-value
1e-04
6e-04
3e-03
1e-02
6e-02
Log-odds
0.5
1.0
1.5
2.0
2.5
3.0
3.5
4.0

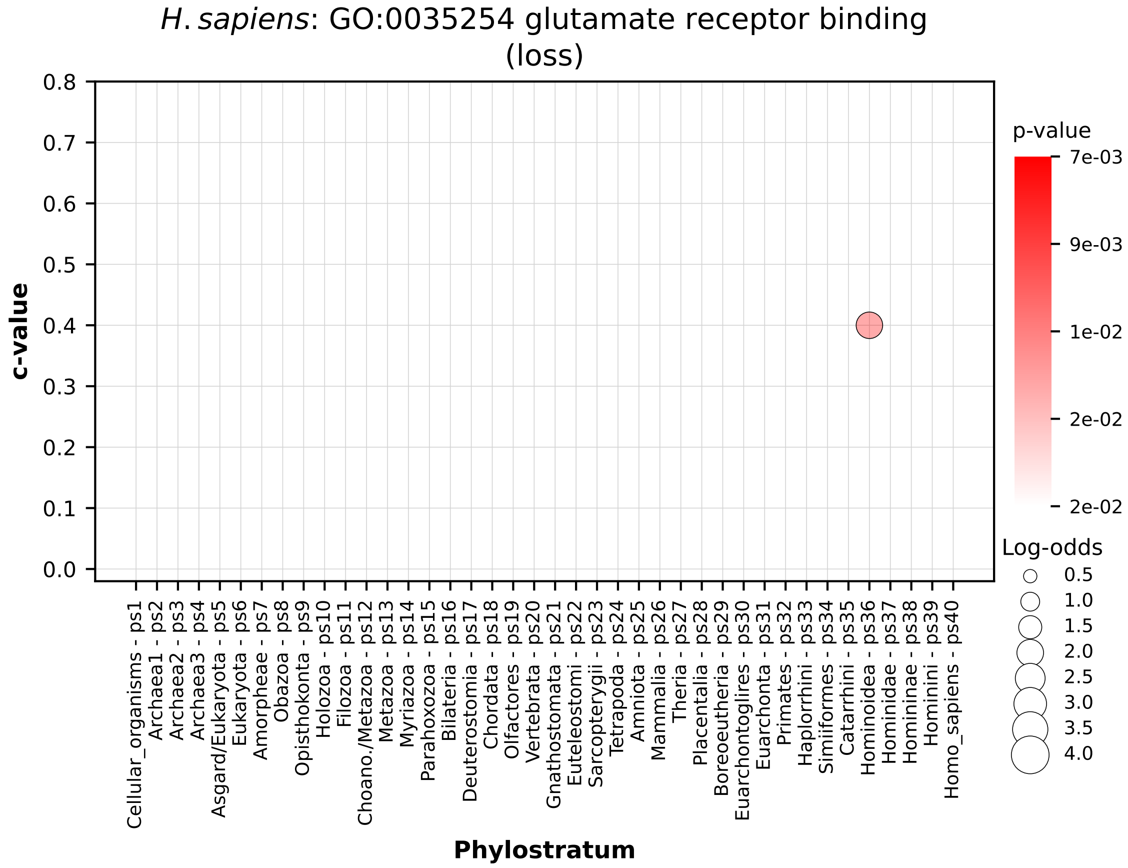

H. sapiens: GO:0035254 glutamate receptor binding
(loss)
c-value
0.8
0.7
0.6
0.5
0.4
0.3
0.2
0.1
0.0
Phylostratum
Cellular_organisms - ps1
Archaea1 - ps2
Archaea2 - ps3
Archaea3 - ps4
Asgard/Eukaryota - ps5
Eukaryota - ps6
Amorpheae - ps7
Obazoa - ps8
Opisthokonta - ps9
Holozoa - ps10
Filozoa - ps11
Choano./Metazoa - ps12
Metazoa - ps13
Myriazoa - ps14
Parahoxozoa - ps15
Bilateria - ps16
Deuterostomia - ps17
Chordata - ps18
Olfactores - ps19
Vertebrata - ps20
Gnathostomata - ps21
Euteleostomi - ps22
Sarcopterygii - ps23
Tetrapoda - ps24
Amniota - ps25
Mammalia - ps26
Theria - ps27
Placentalia - ps28
Boreoeutheria - ps29
Euarchontoglires - ps30
Euarchonta - ps31
Primates - ps32
Haplorrhini - ps33
Simiiformes - ps34
Catarrhini - ps35
Hominoidea - ps36
Hominidae - ps37
Homininae - ps38
Hominini - ps39
Homo_sapiens - ps40
p-value
7e-03
9e-03
1e-02
2e-02
2e-02
Log-odds
0.5
1.0
1.5
2.0
2.5
3.0
3.5
4.0

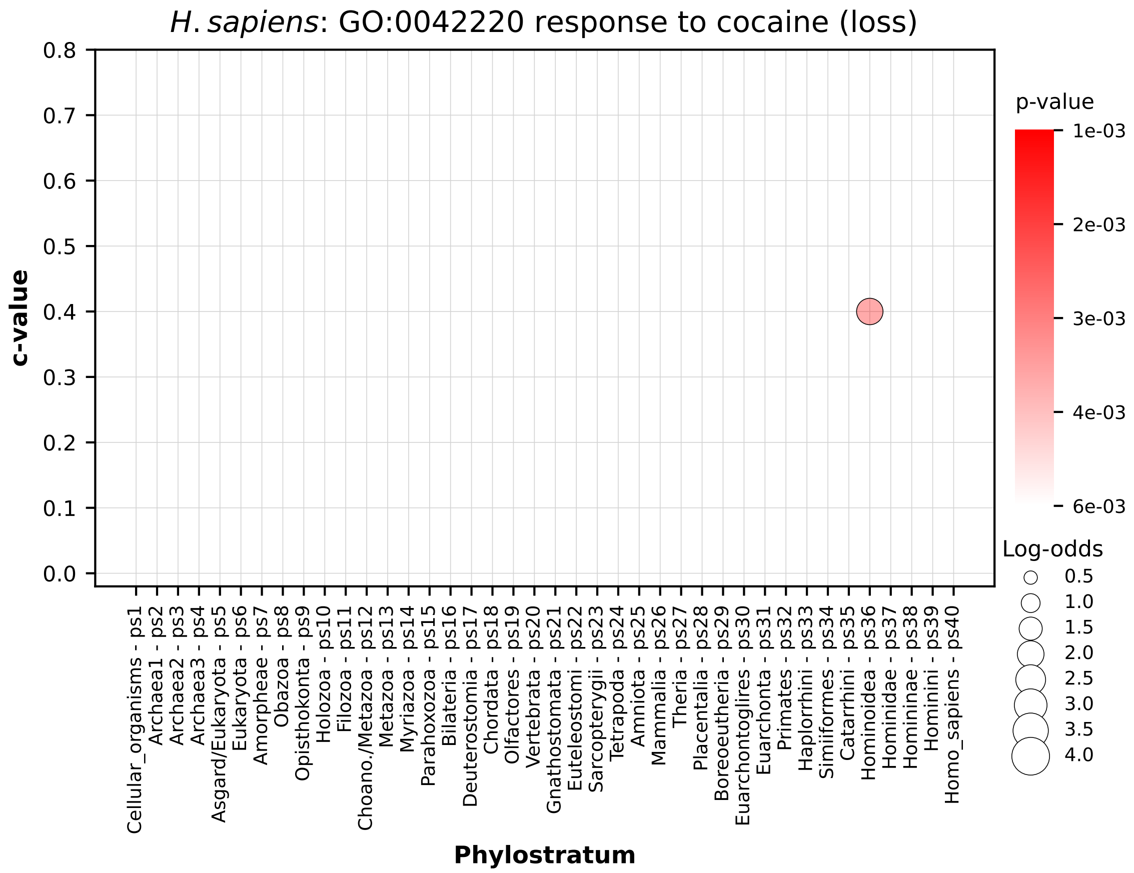

H. sapiens: GO:0042220 response to cocaine (loss)
c-value
0.8
0.7
0.6
0.5
0.4
0.3
0.2
0.1
0.0
Phylostratum
Cellular_organisms - ps1
Archaea1 - ps2
Archaea2 - ps3
Archaea3 - ps4
Asgard/Eukaryota - ps5
Eukaryota - ps6
Amorpheae - ps7
Obazoa - ps8
Opisthokonta - ps9
Holozoa - ps10
Filozoa - ps11
Choano./Metazoa - ps12
Metazoa - ps13
Myriazoa - ps14
Parahoxozoa - ps15
Bilateria - ps16
Deuterostomia - ps17
Chordata - ps18
Olfactores - ps19
Vertebrata - ps20
Gnathostomata - ps21
Euteleostomi - ps22
Sarcopterygii - ps23
Tetrapoda - ps24
Amniota - ps25
Mammalia - ps26
Theria - ps27
Placentalia - ps28
Boreoeutheria - ps29
Euarchontoglires - ps30
Euarchonta - ps31
Primates - ps32
Haplorrhini - ps33
Simiiformes - ps34
Catarrhini - ps35
Hominoidea - ps36
Hominidae - ps37
Homininae - ps38
Hominini - ps39
Homo_sapiens - ps40
p-value
1e-03
2e-03
3e-03
4e-03
6e-03
Log-odds
0.5
1.0
1.5
2.0
2.5
3.0
3.5
4.0

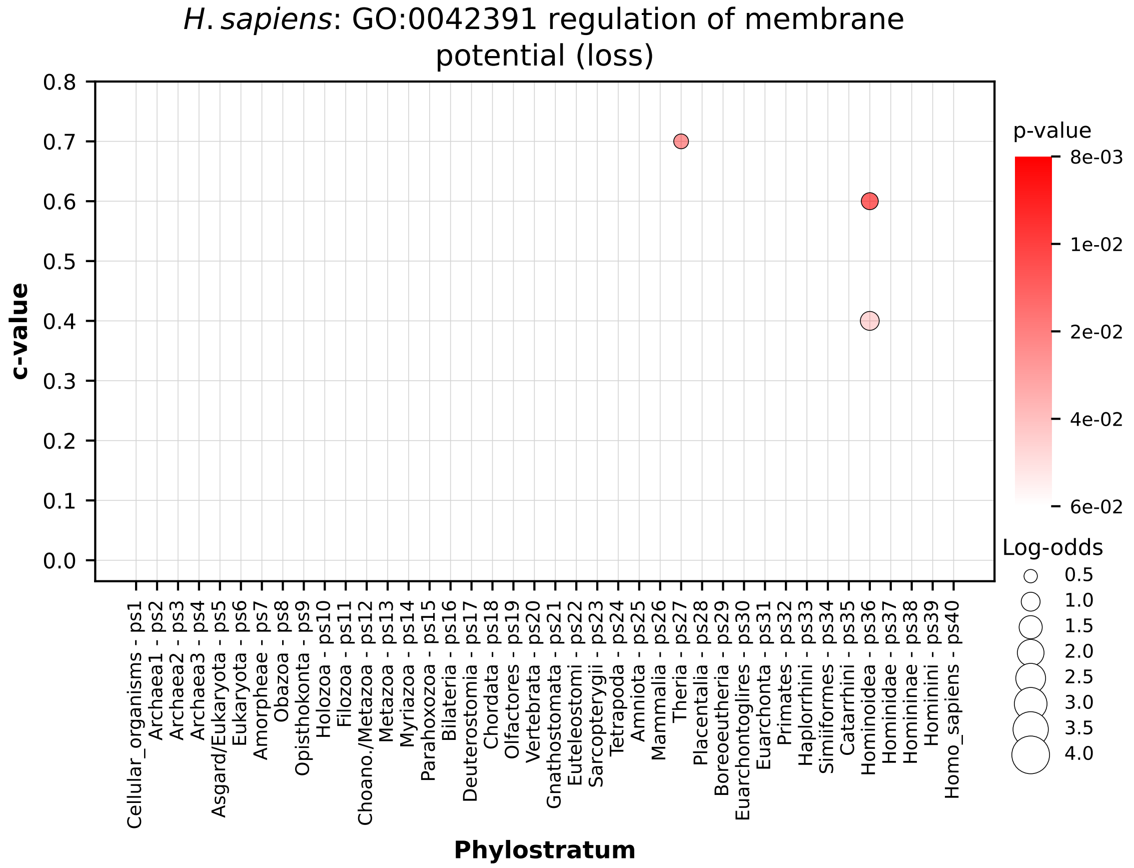

H. sapiens: GO:0042391 regulation of membrane potential (loss)
c-value
0.8
0.7
0.6
0.5
0.4
0.3
0.2
0.1
0.0
p-value
8e-03
1e-02
2e-02
4e-02
6e-02
Log-odds
0.5
1.0
1.5
2.0
2.5
3.0
3.5
4.0
Cellular_organisms - ps1
Archaea1 - ps2
Archaea2 - ps3
Archaea3 - ps4
Asgard/Eukaryota - ps5
Eukaryota - ps6
Amorpheae - ps7
Obazoa - ps8
Opisthokonta - ps9
Holozoa - ps10
Filozoa - ps11
Choano./Metazoa - ps12
Metazoa - ps13
Myriazoa - ps14
Parahoxozoa - ps15
Bilateria - ps16
Deuterostomia - ps17
Chordata - ps18
Olfactores - ps19
Vertebrata - ps20
Gnathostomata - ps21
Euteleostomi - ps22
Sarcopterygii - ps23
Tetrapoda - ps24
Amniota - ps25
Mammalia - ps26
Theria - ps27
Placentalia - ps28
Boreoeutheria - ps29
Euarchontoglires - ps30
Euarchonta - ps31
Primates - ps32
Haplorrhini - ps33
Simiiformes - ps34
Catarrhini - ps35
Hominoidea - ps36
Hominidae - ps37
Homininae - ps38
Hominini - ps39
Homo_sapiens - ps40
Phylostratum

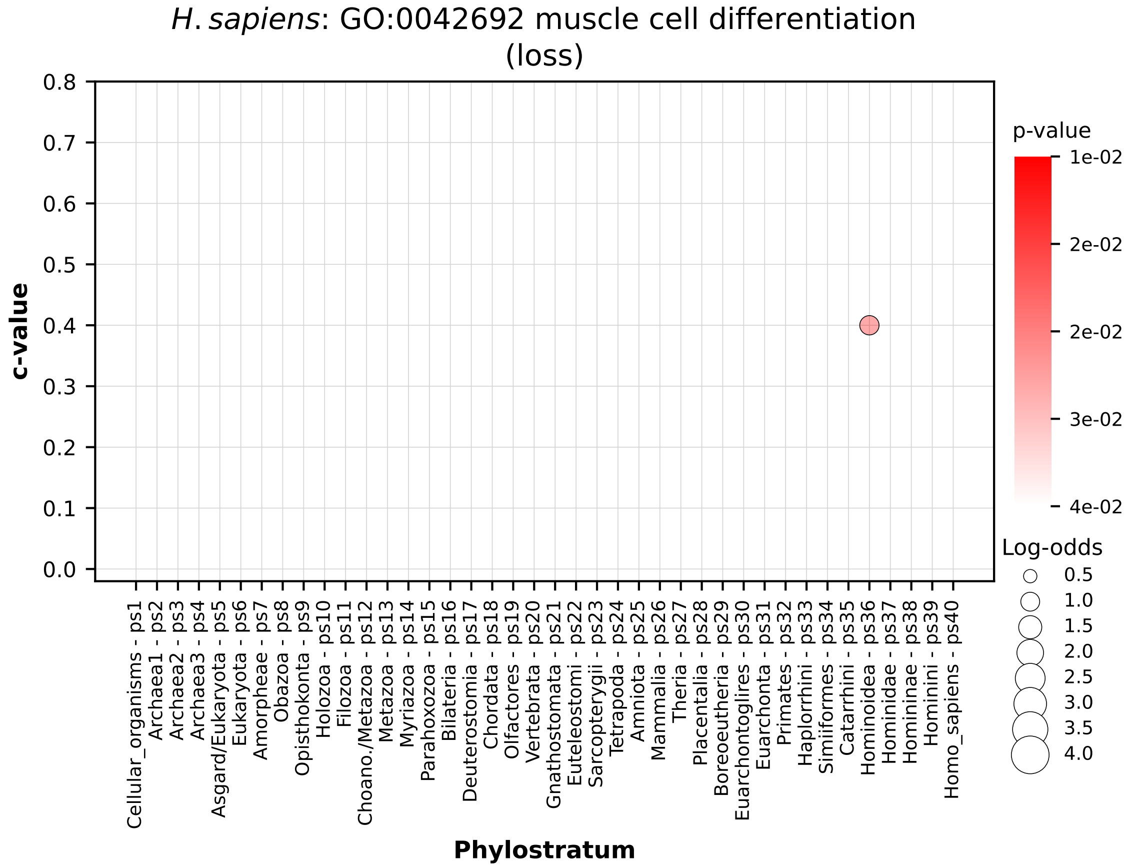

H. sapiens: GO:0042692 muscle cell differentiation (loss)
c-value
0.8
0.7
0.6
0.5
0.4
0.3
0.2
0.1
0.0
p-value
1e-02
2e-02
2e-02
3e-02
4e-02
Log-odds
0.5
1.0
1.5
2.0
2.5
3.0
3.5
4.0
Cellular_organisms - ps1
Archaea1 - ps2
Archaea2 - ps3
Archaea3 - ps4
Asgard/Eukaryota - ps5
Eukaryota - ps6
Amorpheae - ps7
Obazoa - ps8
Opisthokonta - ps9
Holozoa - ps10
Filozoa - ps11
Choano./Metazoa - ps12
Metazoa - ps13
Myriazoa - ps14
Parahoxozoa - ps15
Bilateria - ps16
Deuterostomia - ps17
Chordata - ps18
Olfactores - ps19
Vertebrata - ps20
Gnathostomata - ps21
Euteleostomi - ps22
Sarcopterygii - ps23
Tetrapoda - ps24
Amniota - ps25
Mammalia - ps26
Theria - ps27
Placentalia - ps28
Boreoeutheria - ps29
Euarchontoglires - ps30
Euarchonta - ps31
Primates - ps32
Haplorrhini - ps33
Simiiformes - ps34
Catarrhini - ps35
Hominoidea - ps36
Hominidae - ps37
Homininae - ps38
Hominini - ps39
Homo_sapiens - ps40
Phylostratum

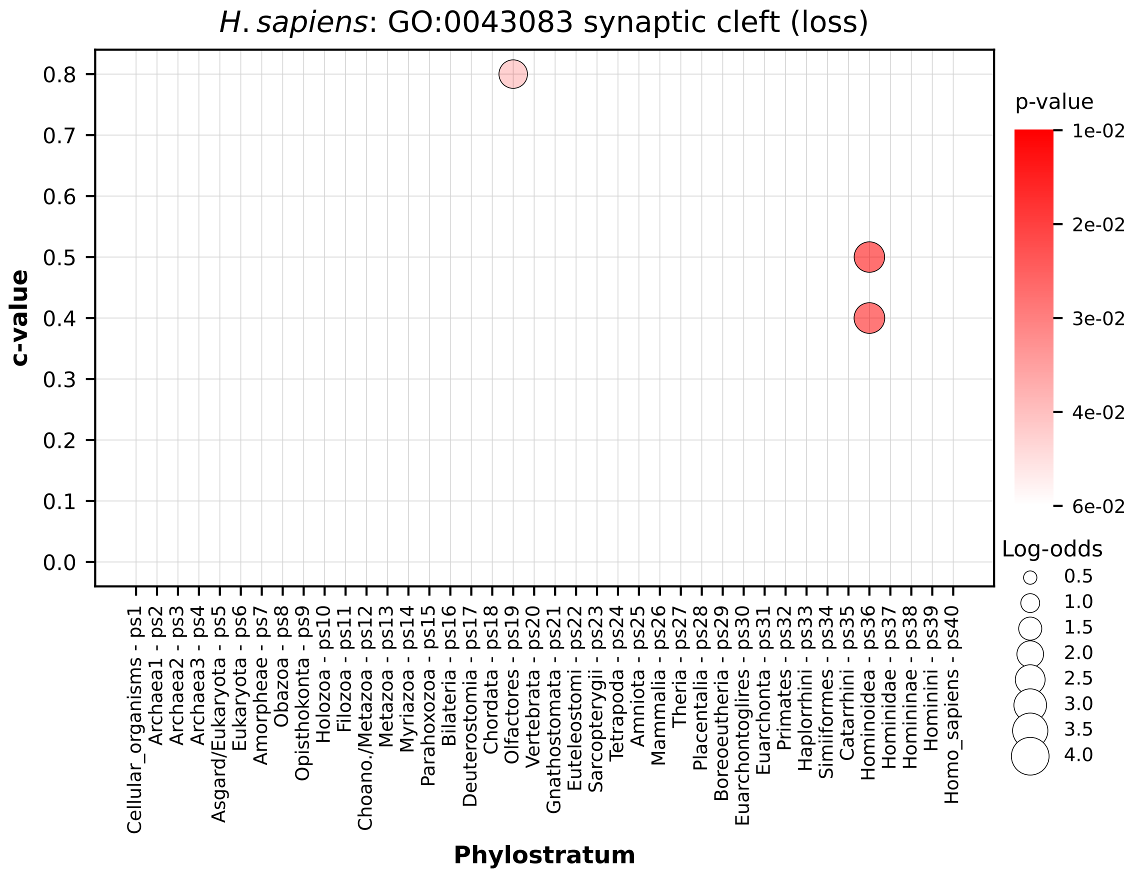

H. sapiens: GO:0043083 synaptic cleft (loss)
c-value
0.0
0.1
0.2
0.3
0.4
0.5
0.6
0.7
0.8
Phylostratum
Cellular_organisms - ps1
Archaea1 - ps2
Archaea2 - ps3
Archaea3 - ps4
Asgard/Eukaryota - ps5
Eukaryota - ps6
Amorpheae - ps7
Obazoa - ps8
Opisthokonta - ps9
Holozoa - ps10
Filozoa - ps11
Choano./Metazoa - ps12
Metazoa - ps13
Myriazoa - ps14
Parahoxozoa - ps15
Bilateria - ps16
Deuterostomia - ps17
Chordata - ps18
Olfactores - ps19
Vertebrata - ps20
Gnathostomata - ps21
Euteleostomi - ps22
Sarcopterygii - ps23
Tetrapoda - ps24
Amniota - ps25
Mammalia - ps26
Theria - ps27
Placentalia - ps28
Boreoeutheria - ps29
Euarchontoglires - ps30
Euarchonta - ps31
Primates - ps32
Haplorrhini - ps33
Simiiformes - ps34
Catarrhini - ps35
Hominoidea - ps36
Hominidae - ps37
Homininae - ps38
Hominini - ps39
Homo_sapiens - ps40
p-value
1e-02
2e-02
3e-02
4e-02
6e-02
Log-odds
0.5
1.0
1.5
2.0
2.5
3.0
3.5
4.0

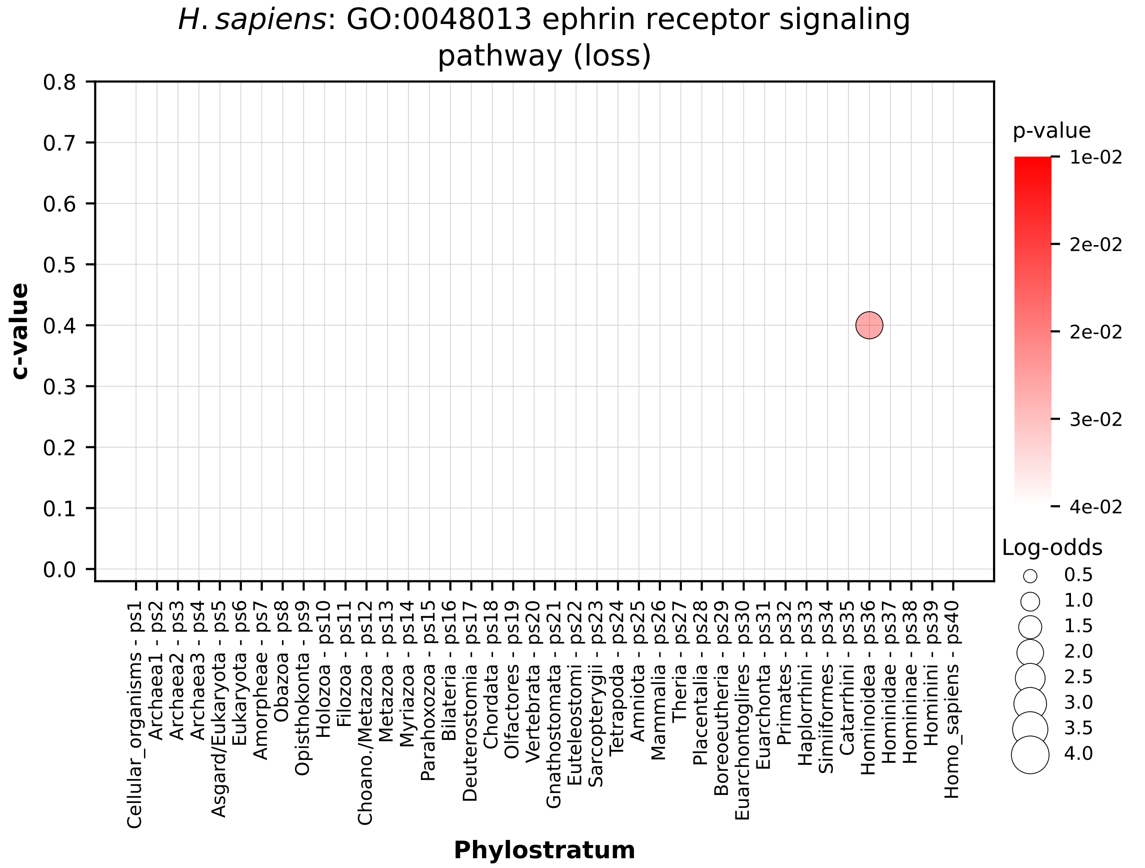

H. sapiens: GO:0048013 ephrin receptor signaling pathway (loss)
c-value
0.0
0.1
0.2
0.3
0.4
0.5
0.6
0.7
0.8
Phylostratum
Cellular_organisms - ps1
Archaea1 - ps2
Archaea2 - ps3
Archaea3 - ps4
Asgard/Eukaryota - ps5
Eukaryota - ps6
Amorpheae - ps7
Obazoa - ps8
Opisthokonta - ps9
Holozoa - ps10
Filozoa - ps11
Choano./Metazoa - ps12
Metazoa - ps13
Myriazoa - ps14
Parahoxozoa - ps15
Bilateria - ps16
Deuterostomia - ps17
Chordata - ps18
Olfactores - ps19
Vertebrata - ps20
Gnathostomata - ps21
Euteleostomi - ps22
Sarcopterygii - ps23
Tetrapoda - ps24
Amniota - ps25
Mammalia - ps26
Theria - ps27
Placentalia - ps28
Boreoeutheria - ps29
Euarchontoglires - ps30
Euarchonta - ps31
Primates - ps32
Haplorrhini - ps33
Simiiformes - ps34
Catarrhini - ps35
Hominoidea - ps36
Hominidae - ps37
Homininae - ps38
Hominini - ps39
Homo_sapiens - ps40
p-value
1e-02
2e-02
2e-02
3e-02
4e-02
Log-odds
0.5
1.0
1.5
2.0
2.5
3.0
3.5
4.0

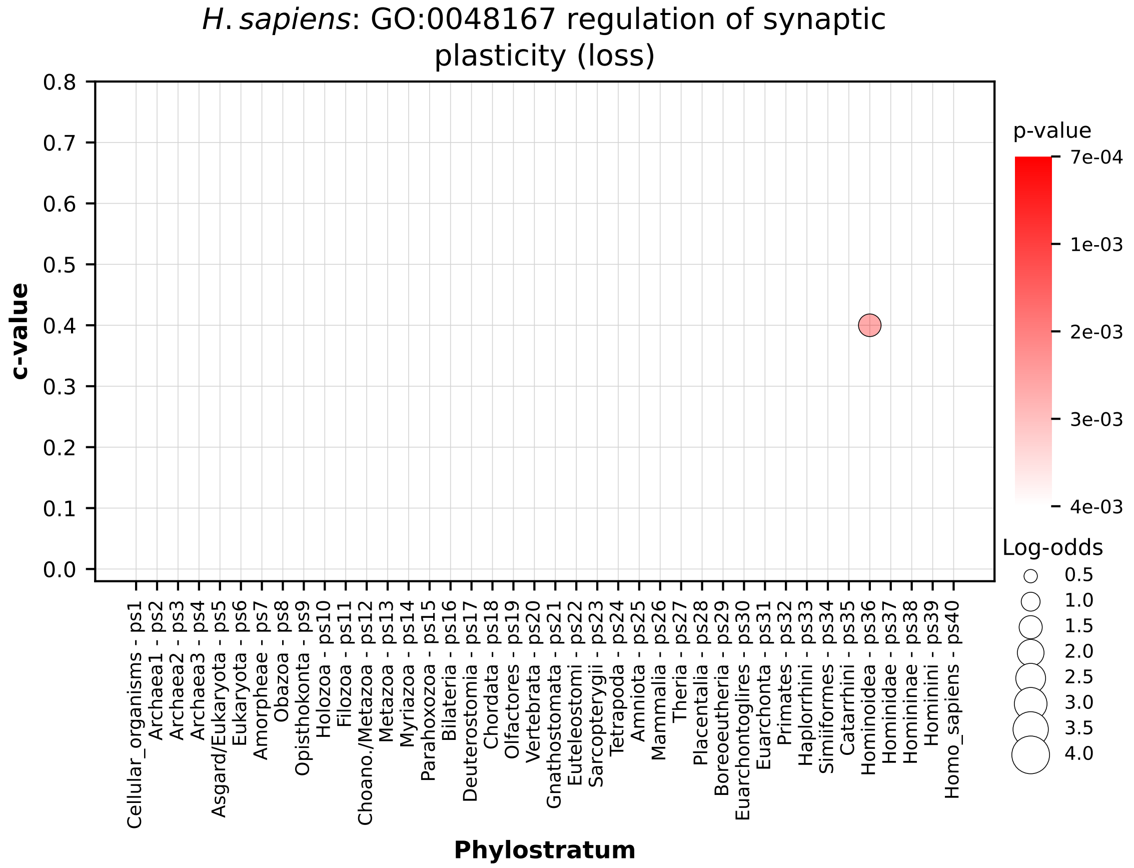

H. sapiens: GO:0048167 regulation of synaptic plasticity (loss)
c-value
0.8
0.7
0.6
0.5
0.4
0.3
0.2
0.1
0.0
Phylostratum
Cellular_organisms - ps1
Archaea1 - ps2
Archaea2 - ps3
Archaea3 - ps4
Asgard/Eukaryota - ps5
Eukaryota - ps6
Amorpheae - ps7
Obazoa - ps8
Opisthokonta - ps9
Holozoa - ps10
Filozoa - ps11
Choano./Metazoa - ps12
Metazoa - ps13
Myriazoa - ps14
Parahoxozoa - ps15
Bilateria - ps16
Deuterostomia - ps17
Chordata - ps18
Olfactores - ps19
Vertebrata - ps20
Gnathostomata - ps21
Euteleostomi - ps22
Sarcopterygii - ps23
Tetrapoda - ps24
Amniota - ps25
Mammalia - ps26
Theria - ps27
Placentalia - ps28
Boreoeutheria - ps29
Euarchontoglires - ps30
Euarchonta - ps31
Primates - ps32
Haplorrhini - ps33
Simiiformes - ps34
Catarrhini - ps35
Hominoidea - ps36
Hominidae - ps37
Homininae - ps38
Hominini - ps39
Homo_sapiens - ps40
p-value
7e-04
1e-03
2e-03
3e-03
4e-03
Log-odds
0.5
1.0
1.5
2.0
2.5
3.0
3.5
4.0

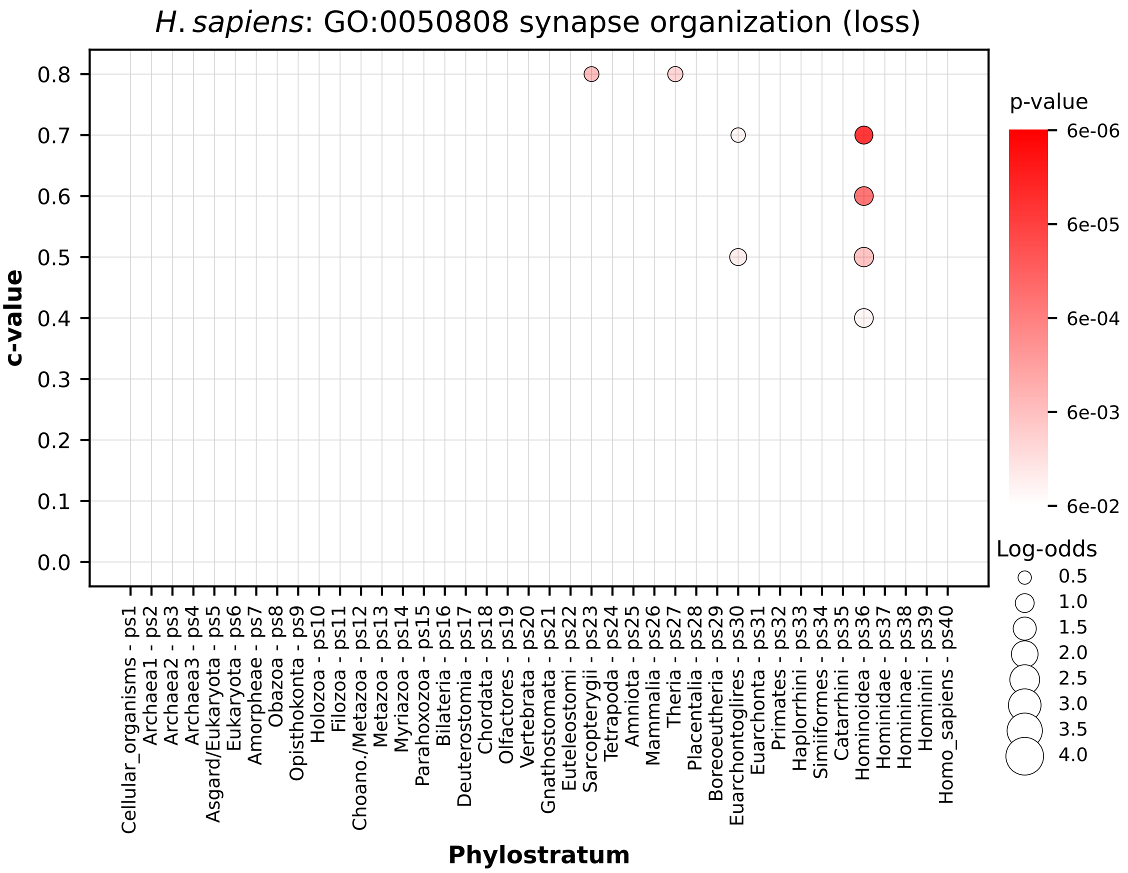

H. sapiens: GO:0050808 synapse organization (loss)
c-value
0.8
0.7
0.6
0.5
0.4
0.3
0.2
0.1
0.0
Phylostratum
Cellular_organisms - ps1
Archaea1 - ps2
Archaea2 - ps3
Archaea3 - ps4
Asgard/Eukaryota - ps5
Eukaryota - ps6
Amorpheae - ps7
Obazoa - ps8
Opisthokonta - ps9
Holozoa - ps10
Filozoa - ps11
Choano./Metazoa - ps12
Metazoa - ps13
Myriazoa - ps14
Parahoxozoa - ps15
Bilateria - ps16
Deuterostomia - ps17
Chordata - ps18
Olfactores - ps19
Vertebrata - ps20
Gnathostomata - ps21
Euteleostomi - ps22
Sarcopterygii - ps23
Tetrapoda - ps24
Amniota - ps25
Mammalia - ps26
Theria - ps27
Placentalia - ps28
Boreoeutheria - ps29
Euarchontoglires - ps30
Euarchonta - ps31
Primates - ps32
Haplorrhini - ps33
Simiiformes - ps34
Catarrhini - ps35
Hominoidea - ps36
Hominidae - ps37
Homininae - ps38
Hominini - ps39
Homo_sapiens - ps40
p-value
6e-06
6e-05
6e-04
6e-03
6e-02
Log-odds
0.5
1.0
1.5
2.0
2.5
3.0
3.5
4.0

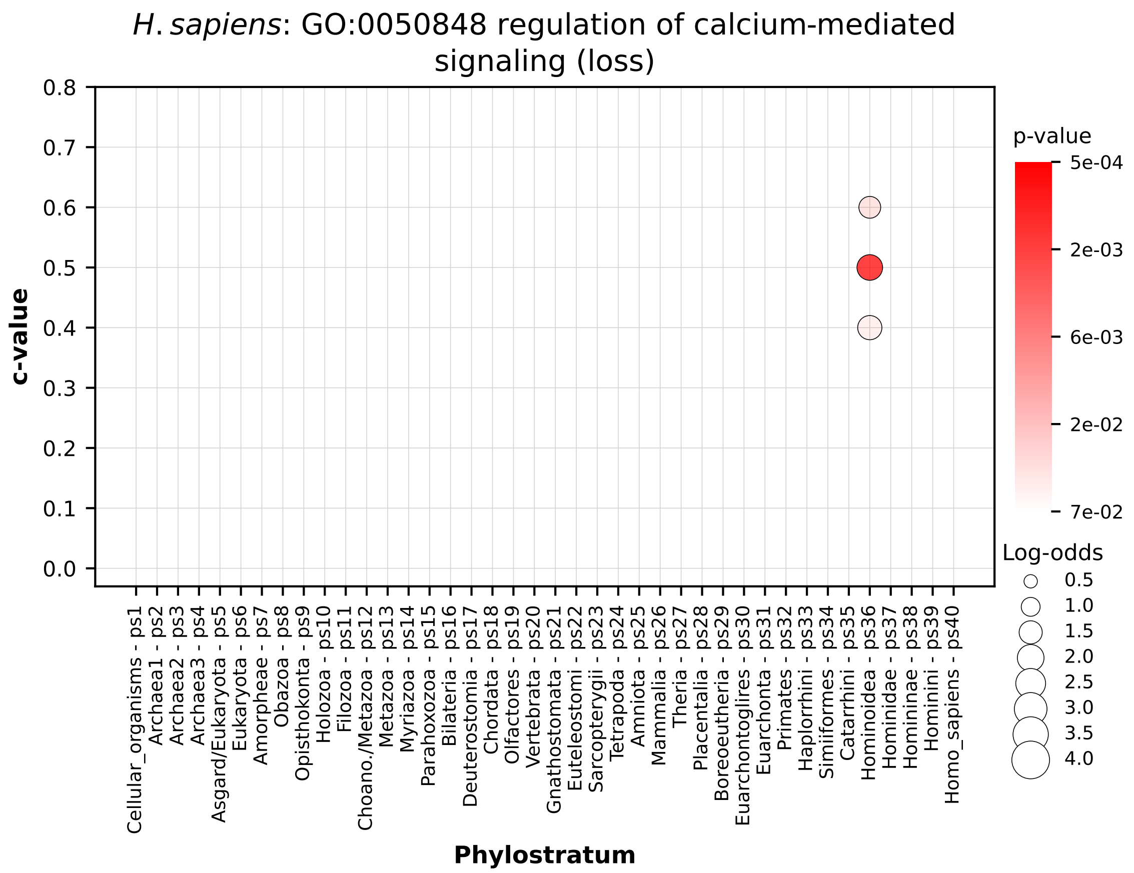

H. sapiens: GO:0050848 regulation of calcium-mediated signaling (loss)
c-value
0.8
0.7
0.6
0.5
0.4
0.3
0.2
0.1
0.0
Phylostratum
Cellular_organisms - ps1
Archaea1 - ps2
Archaea2 - ps3
Archaea3 - ps4
Asgard/Eukaryota - ps5
Eukaryota - ps6
Amorpheae - ps7
Obazoa - ps8
Opisthokonta - ps9
Holozoa - ps10
Filozoa - ps11
Choano./Metazoa - ps12
Metazoa - ps13
Myriazoa - ps14
Parahoxozoa - ps15
Bilateria - ps16
Deuterostomia - ps17
Chordata - ps18
Olfactores - ps19
Vertebrata - ps20
Gnathostomata - ps21
Euteleostomi - ps22
Sarcopterygii - ps23
Tetrapoda - ps24
Amniota - ps25
Mammalia - ps26
Theria - ps27
Placentalia - ps28
Boreoeutheria - ps29
Euarchontoglires - ps30
Euarchonta - ps31
Primates - ps32
Haplorrhini - ps33
Simiiformes - ps34
Catarrhini - ps35
Hominoidea - ps36
Hominidae - ps37
Homininae - ps38
Hominini - ps39
Homo_sapiens - ps40
p-value
5e-04
2e-03
6e-03
2e-02
7e-02
Log-odds
0.5
1.0
1.5
2.0
2.5
3.0
3.5
4.0

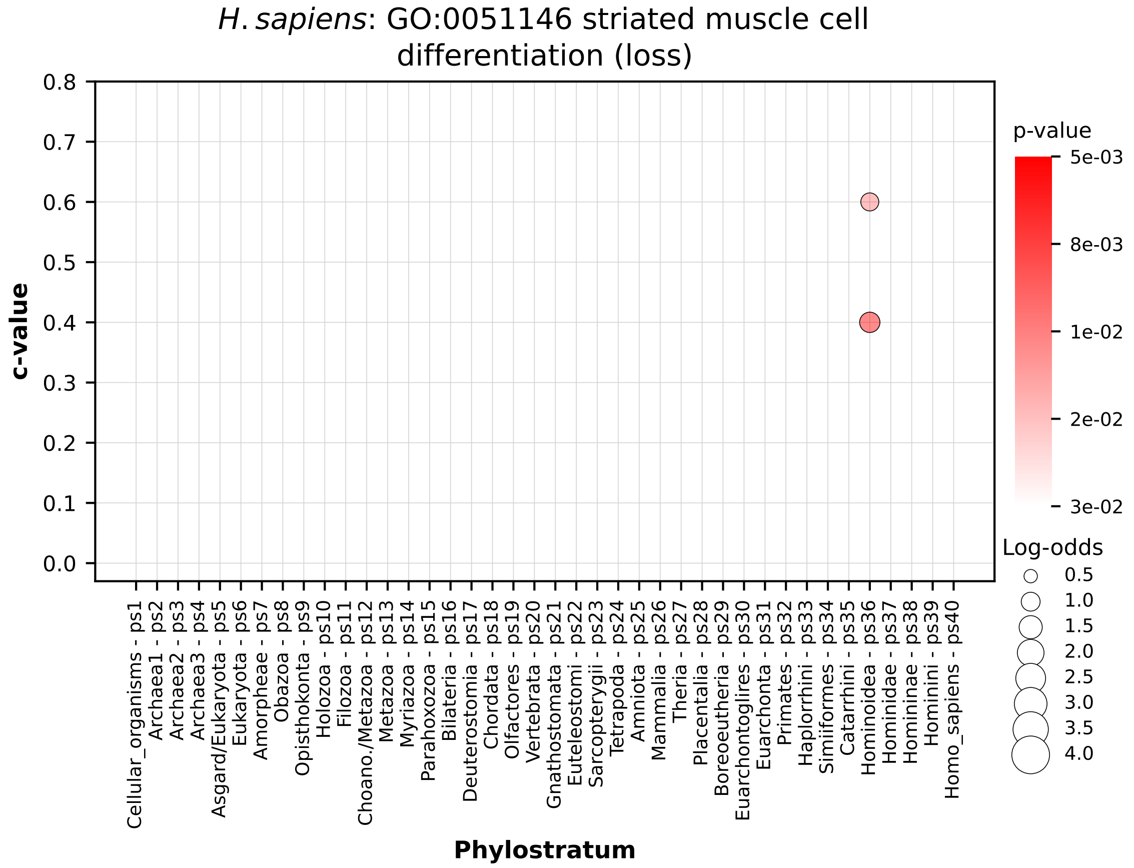
H. sapiens: GO:0051146 striated muscle cell differentiation (loss)
c-value
0.8
0.7
0.6
0.5
0.4
0.3
0.2
0.1
0.0
Phylostratum
Cellular_organisms - ps1
Archaea1 - ps2
Archaea2 - ps3
Archaea3 - ps4
Asgard/Eukaryota - ps5
Eukaryota - ps6
Amorpheae - ps7
Obazoa - ps8
Opisthokonta - ps9
Holozoa - ps10
Filozoa - ps11
Choano./Metazoa - ps12
Metazoa - ps13
Myriazoa - ps14
Parahoxozoa - ps15
Bilateria - ps16
Deuterostomia - ps17
Chordata - ps18
Olfactores - ps19
Vertebrata - ps20
Gnathostomata - ps21
Euteleostomi - ps22
Sarcopterygii - ps23
Tetrapoda - ps24
Amniota - ps25
Mammalia - ps26
Theria - ps27
Placentalia - ps28
Boreoeutheria - ps29
Euarchontoglires - ps30
Euarchonta - ps31
Primates - ps32
Haplorrhini - ps33
Simiiformes - ps34
Catarrhini - ps35
Hominoidea - ps36
Hominidae - ps37
Homininae - ps38
Hominini - ps39
Homo_sapiens - ps40
p-value
5e-03
8e-03
1e-02
2e-02
3e-02
Log-odds
0.5
1.0
1.5
2.0
2.5
3.0
3.5
4.0

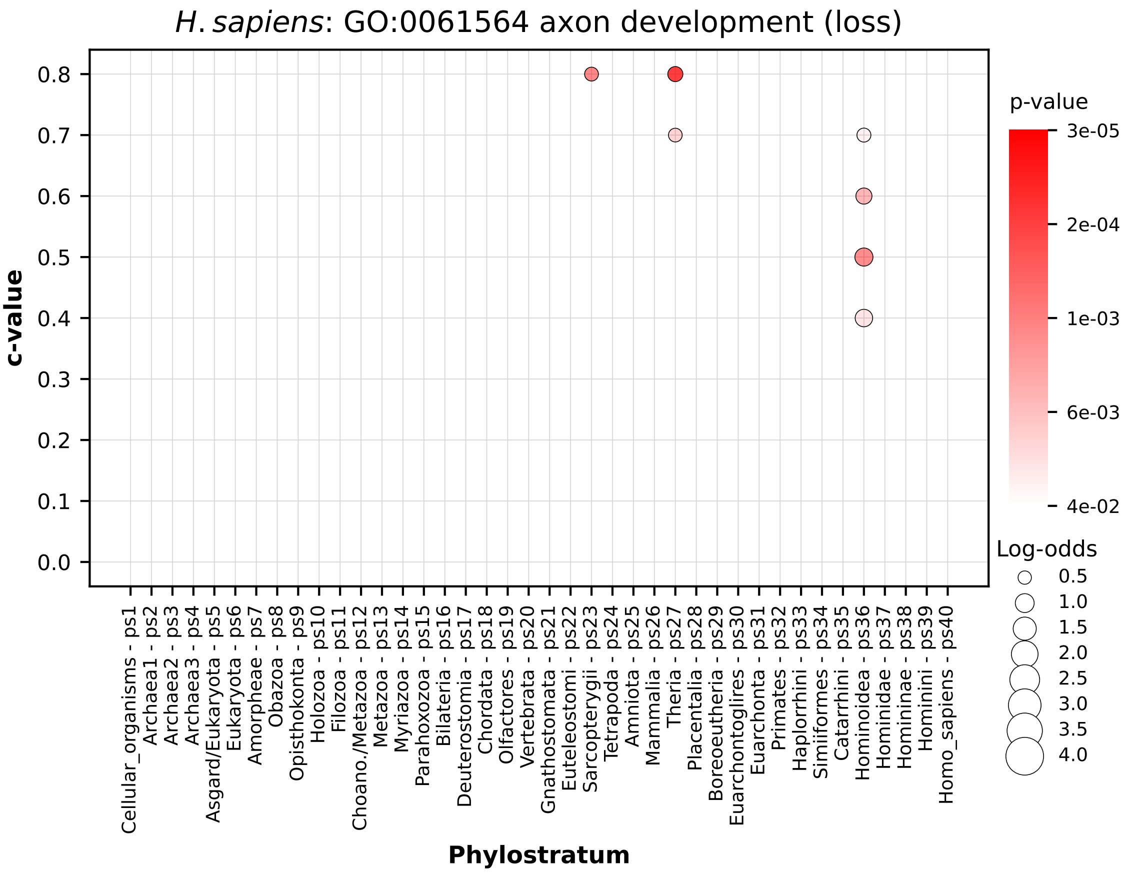

H. sapiens: GO:0061564 axon development (loss)
c-value
0.0
0.1
0.2
0.3
0.4
0.5
0.6
0.7
0.8
Phylostratum
Cellular_organisms - ps1
Archaea1 - ps2
Archaea2 - ps3
Archaea3 - ps4
Asgard/Eukaryota - ps5
Eukaryota - ps6
Amorpheae - ps7
Obazoa - ps8
Opisthokonta - ps9
Holozoa - ps10
Filozoa - ps11
Choano./Metazoa - ps12
Metazoa - ps13
Myriazoa - ps14
Parahoxozoa - ps15
Bilateria - ps16
Deuterostomia - ps17
Chordata - ps18
Olfactores - ps19
Vertebrata - ps20
Gnathostomata - ps21
Euteleostomi - ps22
Sarcopterygii - ps23
Tetrapoda - ps24
Amniota - ps25
Mammalia - ps26
Theria - ps27
Placentalia - ps28
Boreoeutheria - ps29
Euarchontoglires - ps30
Euarchonta - ps31
Primates - ps32
Haplorrhini - ps33
Simiiformes - ps34
Catarrhini - ps35
Hominoidea - ps36
Hominidae - ps37
Homininae - ps38
Hominini - ps39
Homo_sapiens - ps40
p-value
3e-05
2e-04
1e-03
6e-03
4e-02
Log-odds
0.5
1.0
1.5
2.0
2.5
3.0
3.5
4.0

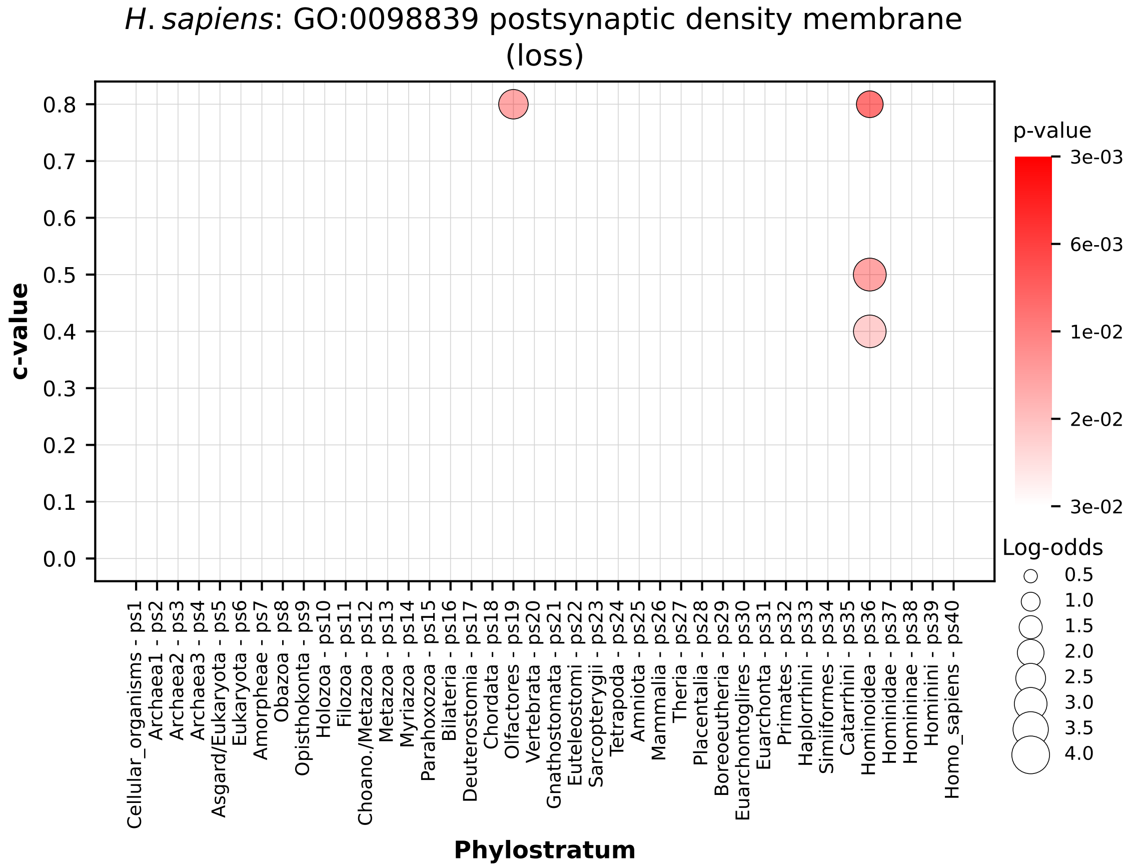

H. sapiens: GO:0098839 postsynaptic density membrane
(loss)
c-value
0.0
0.1
0.2
0.3
0.4
0.5
0.6
0.7
0.8
Phylostratum
Cellular_organisms - ps1
Archaea1 - ps2
Archaea2 - ps3
Archaea3 - ps4
Asgard/Eukaryota - ps5
Eukaryota - ps6
Amorpheae - ps7
Obazoa - ps8
Opisthokonta - ps9
Holozoa - ps10
Filozoa - ps11
Choano./Metazoa - ps12
Metazoa - ps13
Myriazoa - ps14
Parahoxozoa - ps15
Bilateria - ps16
Deuterostomia - ps17
Chordata - ps18
Olfactores - ps19
Vertebrata - ps20
Gnathostomata - ps21
Euteleostomi - ps22
Sarcopterygii - ps23
Tetrapoda - ps24
Amniota - ps25
Mammalia - ps26
Theria - ps27
Placentalia - ps28
Boreoeutheria - ps29
Euarchontoglires - ps30
Euarchonta - ps31
Primates - ps32
Haplorrhini - ps33
Simiiformes - ps34
Catarrhini - ps35
Hominoidea - ps36
Hominidae - ps37
Homininae - ps38
Hominini - ps39
Homo_sapiens - ps40
p-value
3e-03
6e-03
1e-02
2e-02
3e-02
Log-odds
0.5
1.0
1.5
2.0
2.5
3.0
3.5
4.0

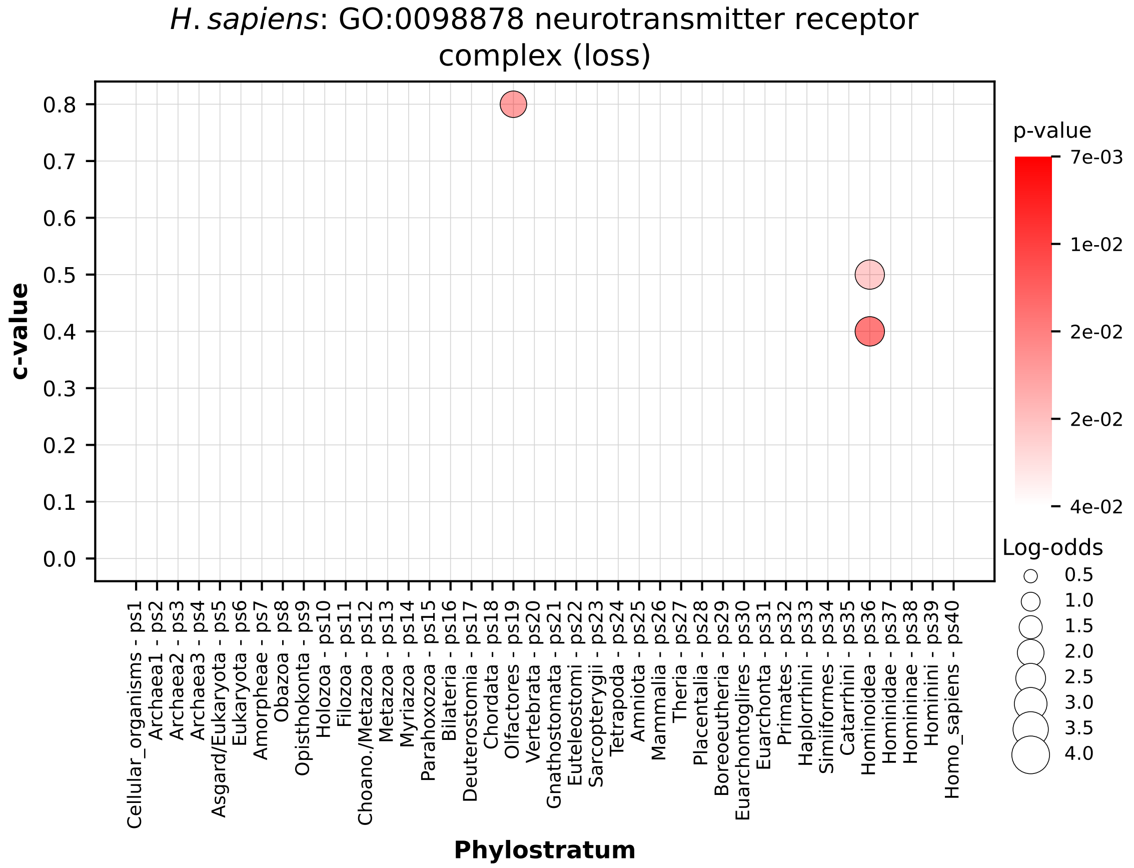

H. sapiens: GO:0098878 neurotransmitter receptor complex (loss)
c-value
0.8
0.7
0.6
0.5
0.4
0.3
0.2
0.1
0.0
p-value
7e-03
1e-02
2e-02
2e-02
4e-02
Log-odds
0.5
1.0
1.5
2.0
2.5
3.0
3.5
4.0
Cellular_organisms - ps1
Archaea1 - ps2
Archaea2 - ps3
Archaea3 - ps4
Asgard/Eukaryota - ps5
Eukaryota - ps6
Amorpheae - ps7
Obazoa - ps8
Opisthokonta - ps9
Holozoa - ps10
Filozoa - ps11
Choano./Metazoa - ps12
Metazoa - ps13
Myriazoa - ps14
Parahoxozoa - ps15
Bilateria - ps16
Deuterostomia - ps17
Chordata - ps18
Olfactores - ps19
Vertebrata - ps20
Gnathostomata - ps21
Euteleostomi - ps22
Sarcopterygii - ps23
Tetrapoda - ps24
Amniota - ps25
Mammalia - ps26
Theria - ps27
Placentalia - ps28
Boreoeutheria - ps29
Euarchontoglires - ps30
Euarchonta - ps31
Primates - ps32
Haplorrhini - ps33
Simiiformes - ps34
Catarrhini - ps35
Hominoidea - ps36
Hominidae - ps37
Homininae - ps38
Hominini - ps39
Homo_sapiens - ps40
Phylostratum

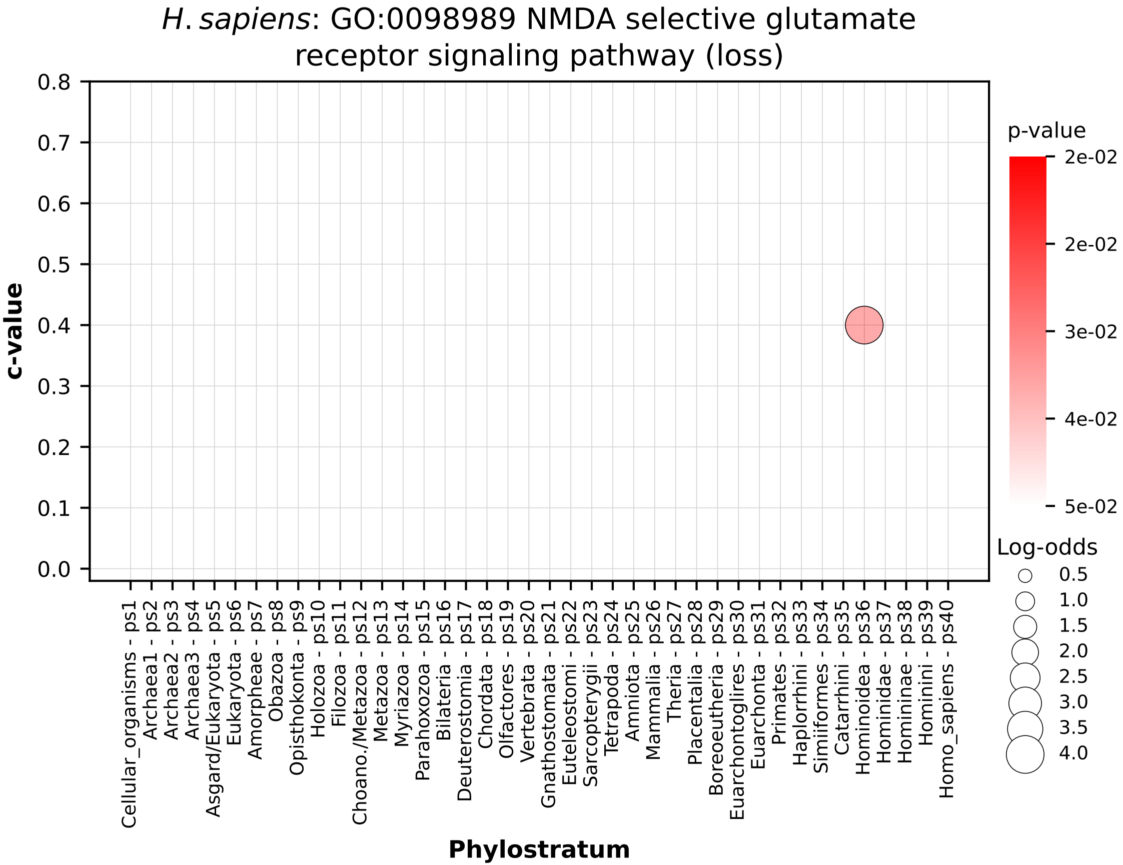

H. sapiens: GO:0098989 NMDA selective glutamate receptor signaling pathway (loss)
c-value
0.8
0.7
0.6
0.5
0.4
0.3
0.2
0.1
0.0
p-value
2e-02
2e-02
3e-02
4e-02
5e-02
Log-odds
0.5
1.0
1.5
2.0
2.5
3.0
3.5
4.0
Cellular_organisms - ps1
Archaea1 - ps2
Archaea2 - ps3
Archaea3 - ps4
Asgard/Eukaryota - ps5
Eukaryota - ps6
Amorpheae - ps7
Obazoa - ps8
Opisthokonta - ps9
Holozoa - ps10
Filozoa - ps11
Choano./Metazoa - ps12
Metazoa - ps13
Myriazoa - ps14
Parahoxozoa - ps15
Bilateria - ps16
Deuterostomia - ps17
Chordata - ps18
Olfactores - ps19
Vertebrata - ps20
Gnathostomata - ps21
Euteleostomi - ps22
Sarcopterygii - ps23
Tetrapoda - ps24
Amniota - ps25
Mammalia - ps26
Theria - ps27
Placentalia - ps28
Boreoeutheria - ps29
Euarchontoglires - ps30
Euarchonta - ps31
Primates - ps32
Haplorrhini - ps33
Simiiformes - ps34
Catarrhini - ps35
Hominoidea - ps36
Hominidae - ps37
Homininae - ps38
Hominini - ps39
Homo_sapiens - ps40
Phylostratum

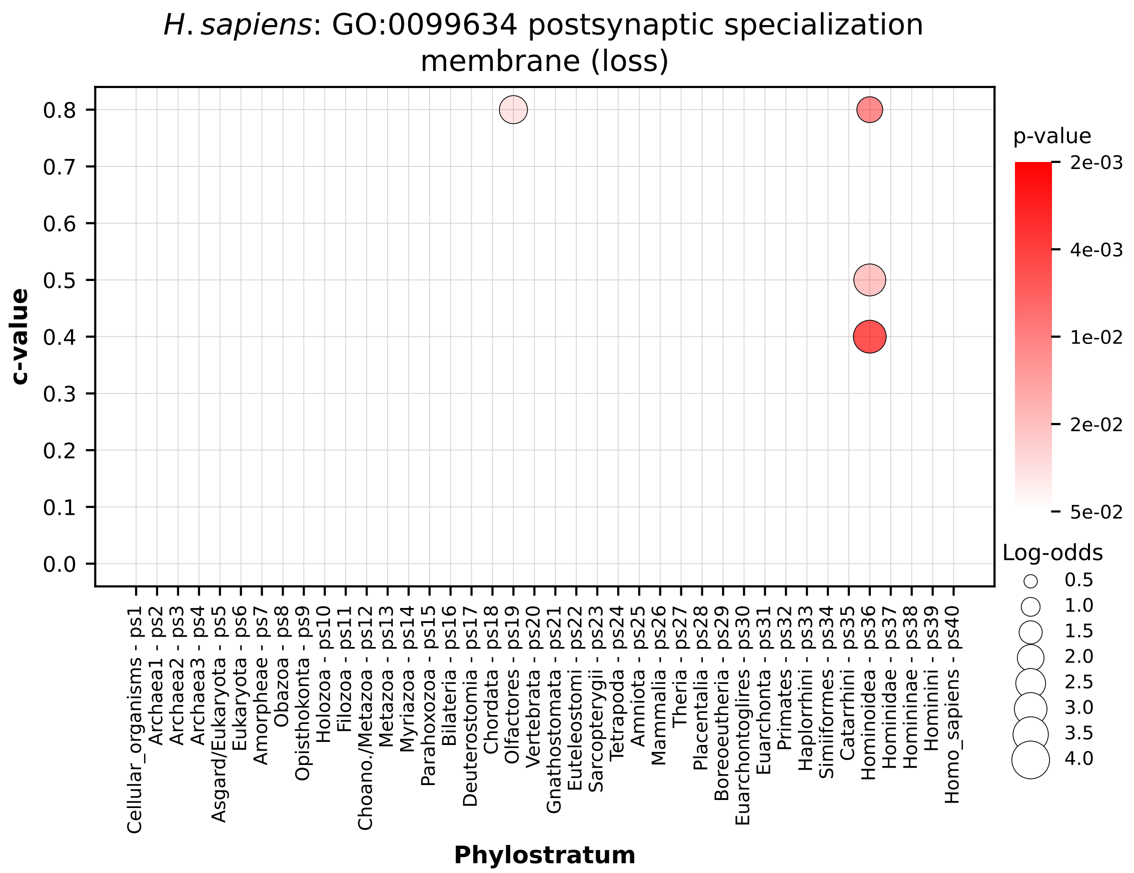
H. sapiens: GO:0099634 postsynaptic specialization membrane (loss)
c-value
0.0
0.1
0.2
0.3
0.4
0.5
0.6
0.7
0.8
p-value
2e-03
4e-03
1e-02
2e-02
5e-02
Log-odds
0.5
1.0
1.5
2.0
2.5
3.0
3.5
4.0
Cellular_organisms - ps1
Archaea1 - ps2
Archaea2 - ps3
Archaea3 - ps4
Asgard/Eukaryota - ps5
Eukaryota - ps6
Amorpheae - ps7
Obazoa - ps8
Opisthokonta - ps9
Holozoa - ps10
Filozoa - ps11
Choano./Metazoa - ps12
Metazoa - ps13
Myriazoa - ps14
Parahoxozoa - ps15
Bilateria - ps16
Deuterostomia - ps17
Chordata - ps18
Olfactores - ps19
Vertebrata - ps20
Gnathostomata - ps21
Euteleostomi - ps22
Sarcopterygii - ps23
Tetrapoda - ps24
Amniota - ps25
Mammalia - ps26
Theria - ps27
Placentalia - ps28
Boreoeutheria - ps29
Euarchontoglires - ps30
Euarchonta - ps31
Primates - ps32
Haplorrhini - ps33
Simiiformes - ps34
Catarrhini - ps35
Hominoidea - ps36
Hominidae - ps37
Homininae - ps38
Hominini - ps39
Homo_sapiens - ps40
Phylostratum

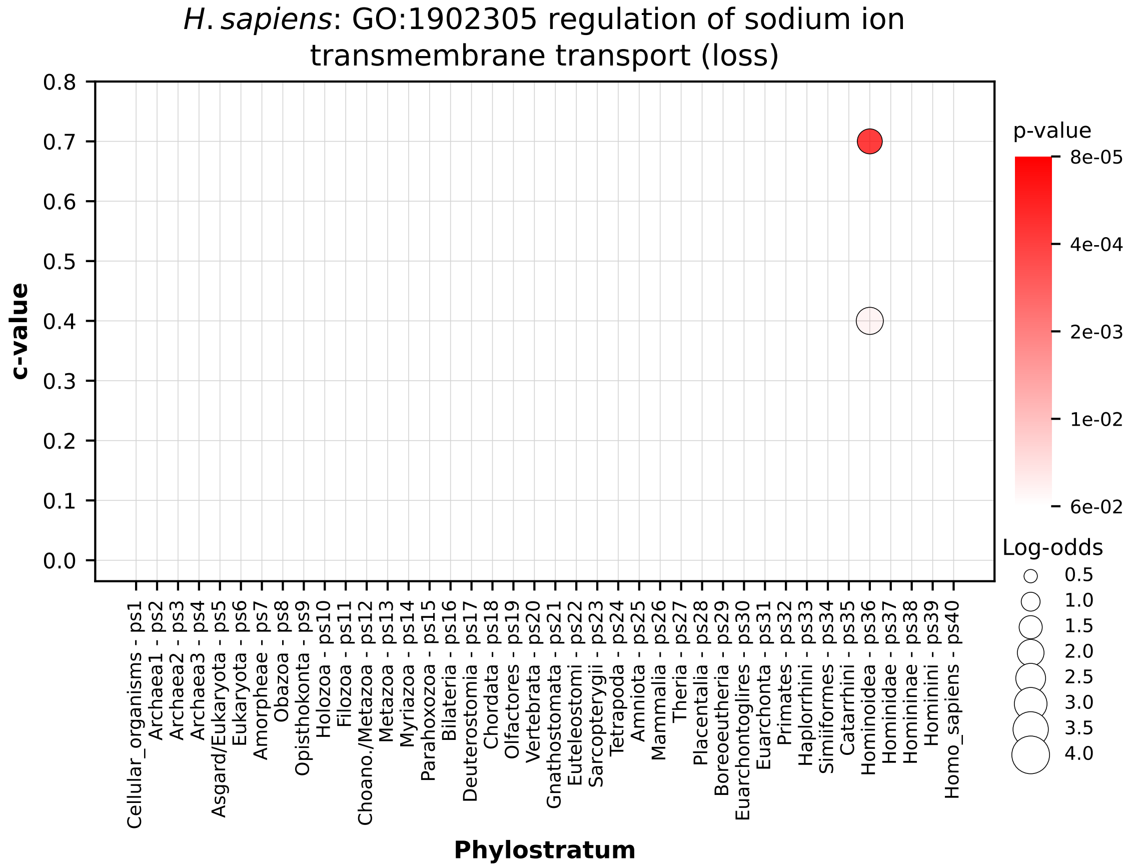

H. sapiens: GO:1902305 regulation of sodium ion transmembrane transport (loss)
c-value
0.8
0.7
0.6
0.5
0.4
0.3
0.2
0.1
0.0
p-value
8e-05
4e-04
2e-03
1e-02
6e-02
Log-odds
0.5
1.0
1.5
2.0
2.5
3.0
3.5
4.0
Cellular_organisms - ps1
Archaea1 - ps2
Archaea2 - ps3
Archaea3 - ps4
Asgard/Eukaryota - ps5
Eukaryota - ps6
Amorpheae - ps7
Obazoa - ps8
Opisthokonta - ps9
Holozoa - ps10
Filozoa - ps11
Choano./Metazoa - ps12
Metazoa - ps13
Myriazoa - ps14
Parahoxozoa - ps15
Bilateria - ps16
Deuterostomia - ps17
Chordata - ps18
Olfactores - ps19
Vertebrata - ps20
Gnathostomata - ps21
Euteleostomi - ps22
Sarcopterygii - ps23
Tetrapoda - ps24
Amniota - ps25
Mammalia - ps26
Theria - ps27
Placentalia - ps28
Boreoeutheria - ps29
Euarchontoglires - ps30
Euarchonta - ps31
Primates - ps32
Haplorrhini - ps33
Simiiformes - ps34
Catarrhini - ps35
Hominoidea - ps36
Hominidae - ps37
Homininae - ps38
Hominini - ps39
Homo_sapiens - ps40
Phylostratum

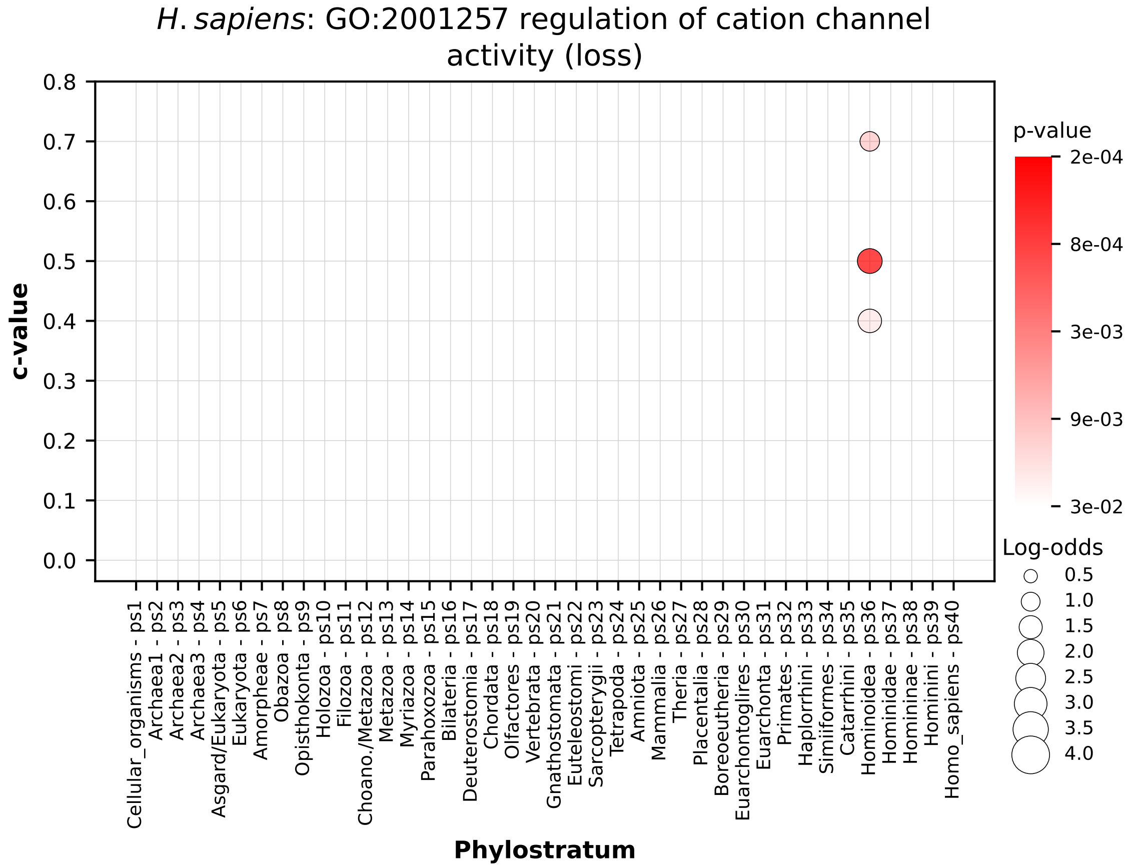

H. sapiens: GO:2001257 regulation of cation channel activity (loss)
c-value
Phylostratum
p-value
2e-04
8e-04
3e-03
9e-03
3e-02
Log-odds
0.5
1.0
1.5
2.0
2.5
3.0
3.5
4.0
Cellular_organisms - ps1
Archaea1 - ps2
Archaea2 - ps3
Archaea3 - ps4
Asgard/Eukaryota - ps5
Eukaryota - ps6
Amorpheae - ps7
Obazoa - ps8
Opisthokonta - ps9
Holozoa - ps10
Filozoa - ps11
Choano./Metazoa - ps12
Metazoa - ps13
Myriazoa - ps14
Parahoxozoa - ps15
Bilateria - ps16
Deuterostomia - ps17
Chordata - ps18
Olfactores - ps19
Vertebrata - ps20
Gnathostomata - ps21
Euteleostomi - ps22
Sarcopterygii - ps23
Tetrapoda - ps24
Amniota - ps25
Mammalia - ps26
Theria - ps27
Placentalia - ps28
Boreoeutheria - ps29
Euarchontoglires - ps30
Euarchonta - ps31
Primates - ps32
Haplorrhini - ps33
Simiiformes - ps34
Catarrhini - ps35
Hominoidea - ps36
Hominidae - ps37
Homininae - ps38
Hominini - ps39
Homo_sapiens - ps40

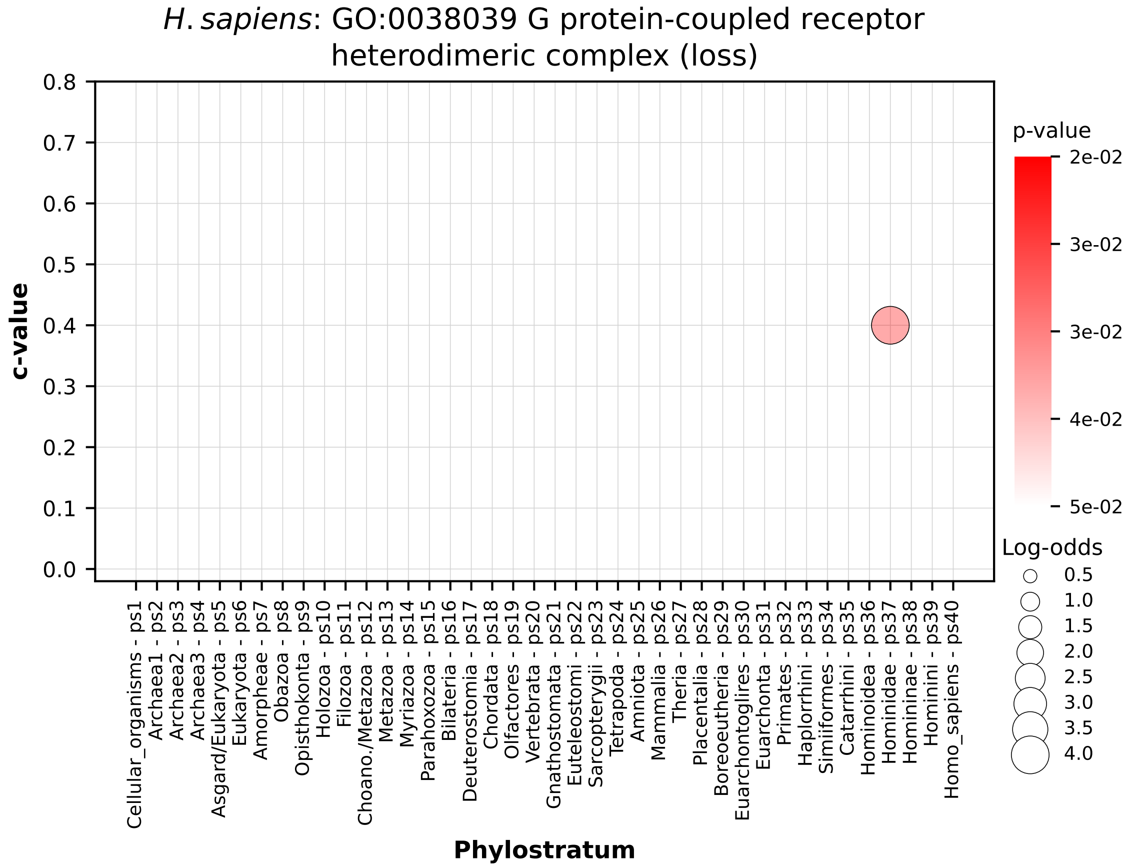

H. sapiens: GO:0038039 G protein-coupled receptor heterodimeric complex (loss)
c-value
0.0
0.1
0.2
0.3
0.4
0.5
0.6
0.7
0.8
Phylostratum
Cellular_organisms - ps1
Archaea1 - ps2
Archaea2 - ps3
Archaea3 - ps4
Asgard/Eukaryota - ps5
Eukaryota - ps6
Amorpheae - ps7
Obazoa - ps8
Opisthokonta - ps9
Holozoa - ps10
Filozoa - ps11
Choano./Metazoa - ps12
Metazoa - ps13
Myriazoa - ps14
Parahoxozoa - ps15
Bilateria - ps16
Deuterostomia - ps17
Chordata - ps18
Olfactores - ps19
Vertebrata - ps20
Gnathostomata - ps21
Euteleostomi - ps22
Sarcopterygii - ps23
Tetrapoda - ps24
Amniota - ps25
Mammalia - ps26
Theria - ps27
Placentalia - ps28
Boreoeutheria - ps29
Euarchontoglires - ps30
Euarchonta - ps31
Primates - ps32
Haplorrhini - ps33
Simiiformes - ps34
Catarrhini - ps35
Hominoidea - ps36
Hominidae - ps37
Homininae - ps38
Hominini - ps39
Homo_sapiens - ps40
p-value
2e-02
3e-02
3e-02
4e-02
5e-02
Log-odds
0.5
1.0
1.5
2.0
2.5
3.0
3.5
4.0

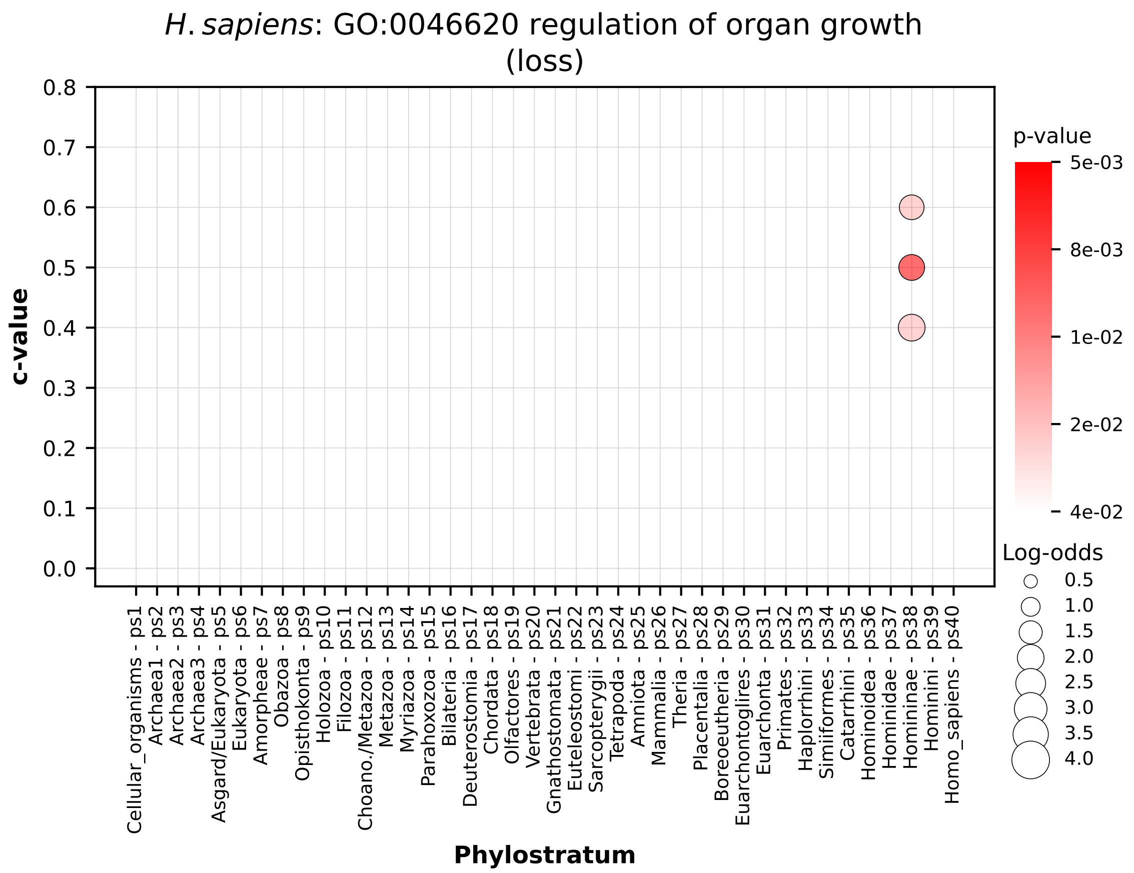

H. sapiens: GO:0046620 regulation of organ growth
(loss)
c-value
0.8
0.7
0.6
0.5
0.4
0.3
0.2
0.1
0.0
Phylostratum
Cellular_organisms - ps1
Archaea1 - ps2
Archaea2 - ps3
Archaea3 - ps4
Asgard/Eukaryota - ps5
Eukaryota - ps6
Amorpheae - ps7
Obazoa - ps8
Opisthokonta - ps9
Holozoa - ps10
Filozoa - ps11
Choano./Metazoa - ps12
Metazoa - ps13
Myriazoa - ps14
Parahoxozoa - ps15
Bilateria - ps16
Deuterostomia - ps17
Chordata - ps18
Olfactores - ps19
Vertebrata - ps20
Gnathostomata - ps21
Euteleostomi - ps22
Sarcopterygii - ps23
Tetrapoda - ps24
Amniota - ps25
Mammalia - ps26
Theria - ps27
Placentalia - ps28
Boreoeutheria - ps29
Euarchontoglires - ps30
Euarchonta - ps31
Primates - ps32
Haplorrhini - ps33
Simiiformes - ps34
Catarrhini - ps35
Hominoidea - ps36
Hominidae - ps37
Homininae - ps38
Hominini - ps39
Homo_sapiens - ps40
p-value
5e-03
8e-03
1e-02
2e-02
4e-02
Log-odds
0.5
1.0
1.5
2.0
2.5
3.0
3.5
4.0

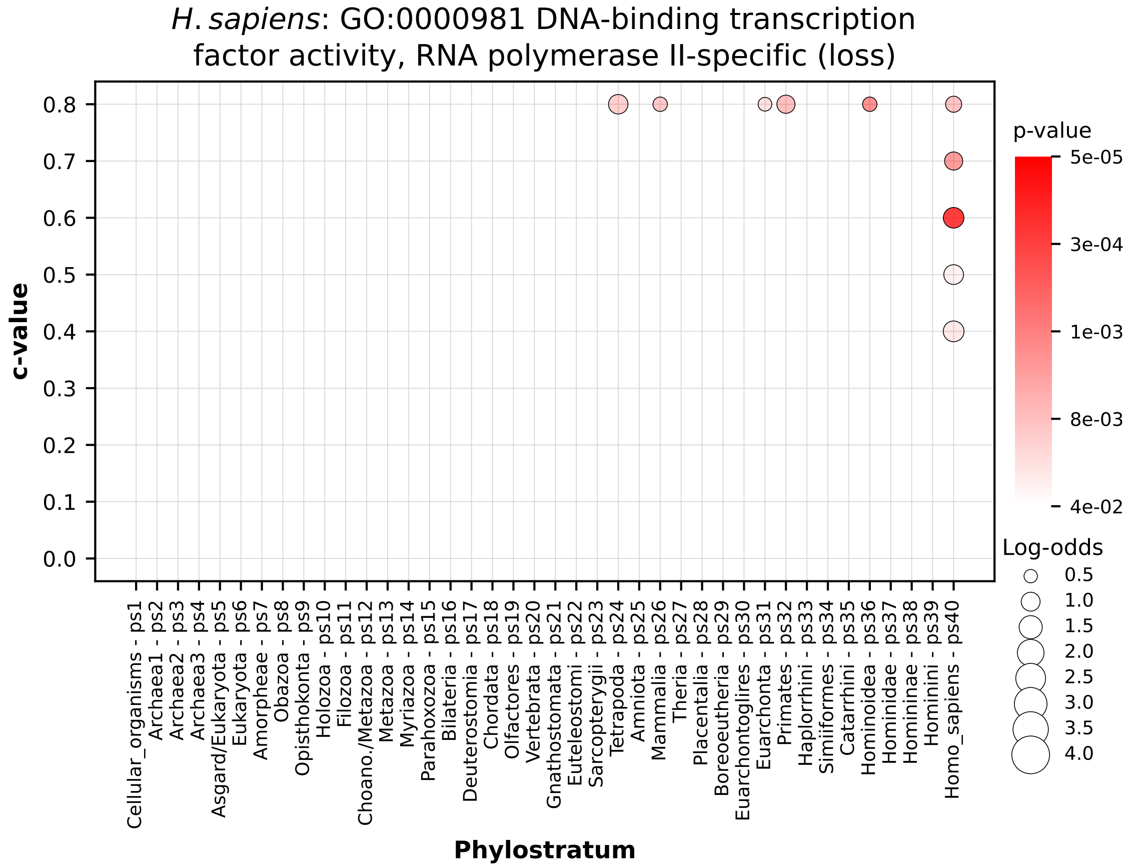

H. sapiens: GO:0000981 DNA-binding transcription factor activity, RNA polymerase II-specific (loss)
c-value
0.8
0.7
0.6
0.5
0.4
0.3
0.2
0.1
0.0
Phylostratum
Cellular_organisms - ps1
Archaea1 - ps2
Archaea2 - ps3
Archaea3 - ps4
Asgard/Eukaryota - ps5
Eukaryota - ps6
Amorpheae - ps7
Obazoa - ps8
Opisthokonta - ps9
Holozoa - ps10
Filozoa - ps11
Choano./Metazoa - ps12
Metazoa - ps13
Myriazoa - ps14
Parahoxozoa - ps15
Bilateria - ps16
Deuterostomia - ps17
Chordata - ps18
Olfactores - ps19
Vertebrata - ps20
Gnathostomata - ps21
Euteleostomi - ps22
Sarcopterygii - ps23
Tetrapoda - ps24
Amniota - ps25
Mammalia - ps26
Theria - ps27
Placentalia - ps28
Boreoeutheria - ps29
Euarchontoglires - ps30
Euarchonta - ps31
Primates - ps32
Haplorrhini - ps33
Simiiformes - ps34
Catarrhini - ps35
Hominoidea - ps36
Hominidae - ps37
Homininae - ps38
Hominini - ps39
Homo_sapiens - ps40
p-value
5e-05
3e-04
1e-03
8e-03
4e-02
Log-odds
0.5
1.0
1.5
2.0
2.5
3.0
3.5
4.0

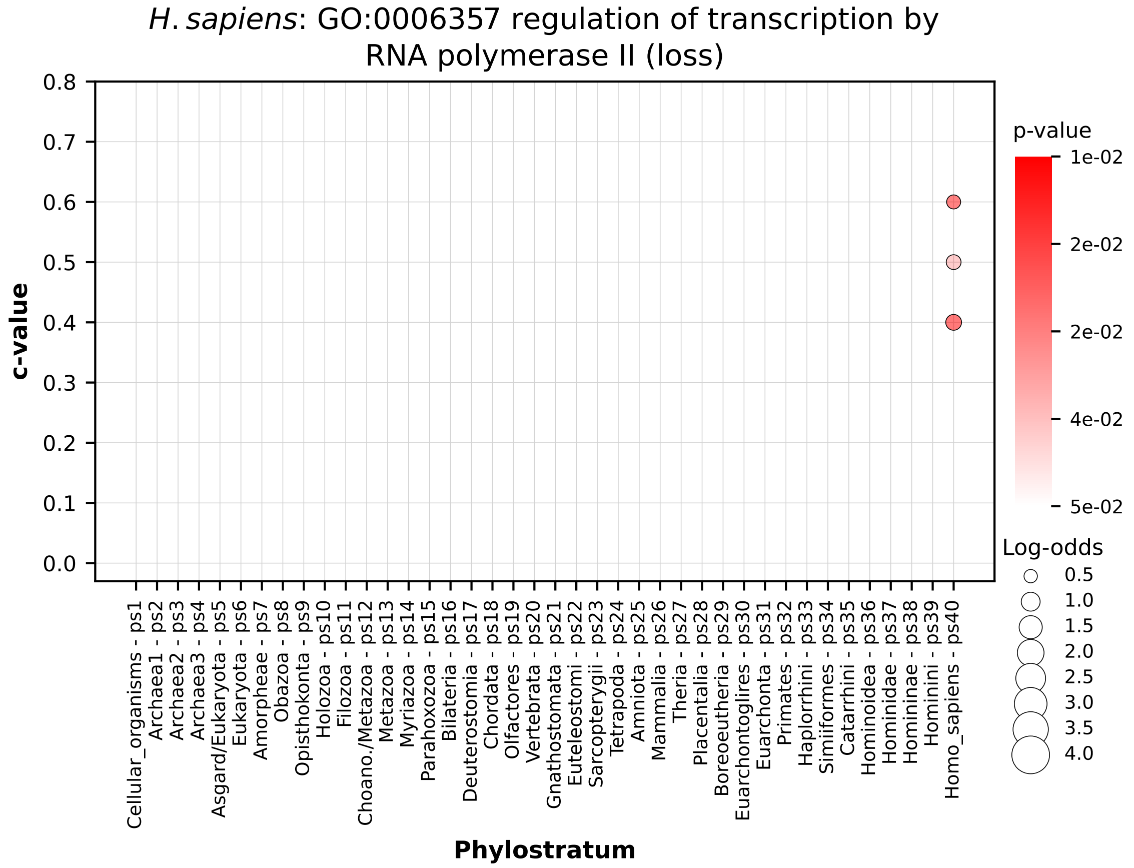

H. sapiens: GO:0006357 regulation of transcription by RNA polymerase II (loss)
c-value
0.0
0.1
0.2
0.3
0.4
0.5
0.6
0.7
0.8
Phylostratum
Cellular_organisms - ps1
Archaea1 - ps2
Archaea2 - ps3
Archaea3 - ps4
Asgard/Eukaryota - ps5
Eukaryota - ps6
Amorpheae - ps7
Obazoa - ps8
Opisthokonta - ps9
Holozoa - ps10
Filozoa - ps11
Choano./Metazoa - ps12
Metazoa - ps13
Myriazoa - ps14
Parahoxozoa - ps15
Bilateria - ps16
Deuterostomia - ps17
Chordata - ps18
Olfactores - ps19
Vertebrata - ps20
Gnathostomata - ps21
Euteleostomi - ps22
Sarcopterygii - ps23
Tetrapoda - ps24
Amniota - ps25
Mammalia - ps26
Theria - ps27
Placentalia - ps28
Boreoeutheria - ps29
Euarchontoglires - ps30
Euarchonta - ps31
Primates - ps32
Haplorrhini - ps33
Simiiformes - ps34
Catarrhini - ps35
Hominoidea - ps36
Hominidae - ps37
Homininae - ps38
Hominini - ps39
Homo_sapiens - ps40
p-value
1e-02
2e-02
2e-02
4e-02
5e-02
Log-odds
0.5
1.0
1.5
2.0
2.5
3.0
3.5
4.0

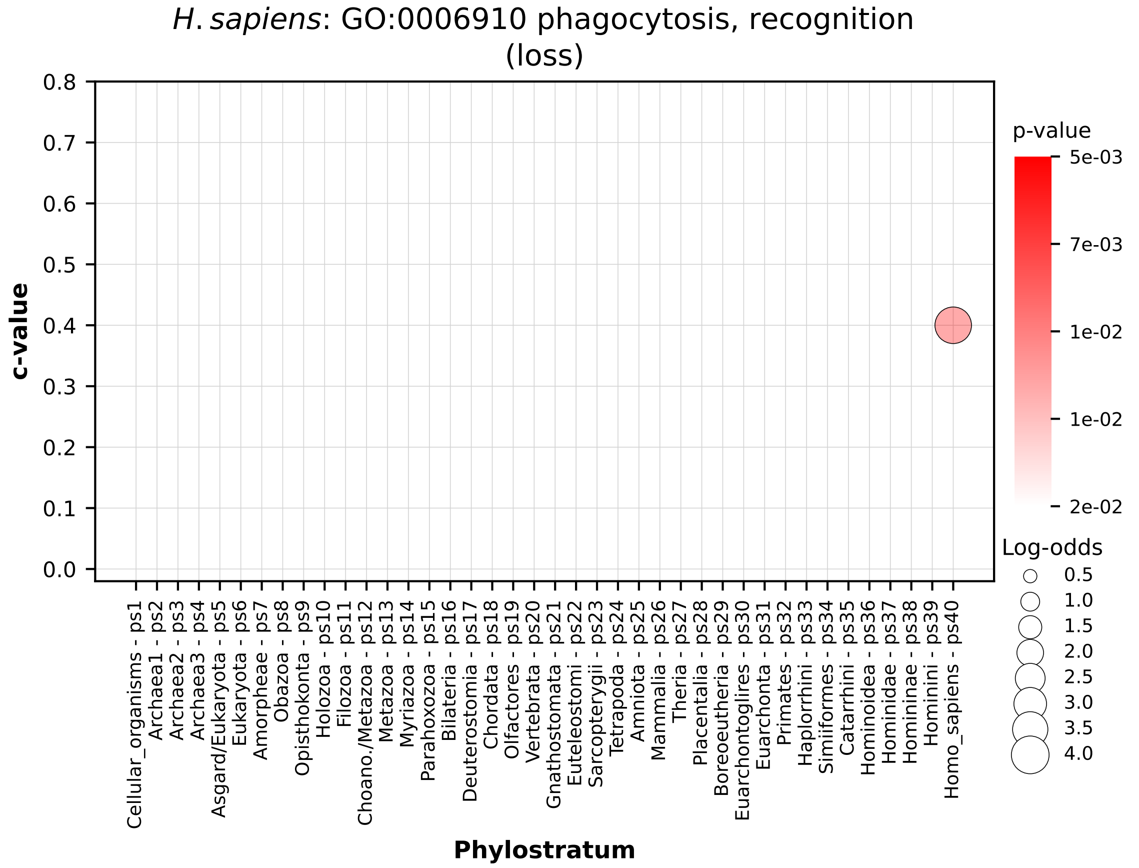

H. sapiens: GO:0006910 phagocytosis, recognition (loss)
c-value
0.8
0.7
0.6
0.5
0.4
0.3
0.2
0.1
0.0
Phylostratum
Cellular_organisms - ps1
Archaea1 - ps2
Archaea2 - ps3
Archaea3 - ps4
Asgard/Eukaryota - ps5
Eukaryota - ps6
Amorpheae - ps7
Obazoa - ps8
Opisthokonta - ps9
Holozoa - ps10
Filozoa - ps11
Choano./Metazoa - ps12
Metazoa - ps13
Myriazoa - ps14
Parahoxozoa - ps15
Bilateria - ps16
Deuterostomia - ps17
Chordata - ps18
Olfactores - ps19
Vertebrata - ps20
Gnathostomata - ps21
Euteleostomi - ps22
Sarcopterygii - ps23
Tetrapoda - ps24
Amniota - ps25
Mammalia - ps26
Theria - ps27
Placentalia - ps28
Boreoeutheria - ps29
Euarchontoglires - ps30
Euarchonta - ps31
Primates - ps32
Haplorrhini - ps33
Simiiformes - ps34
Catarrhini - ps35
Hominoidea - ps36
Hominidae - ps37
Homininae - ps38
Hominini - ps39
Homo_sapiens - ps40
p-value
5e-03
7e-03
1e-02
1e-02
2e-02
Log-odds
0.5
1.0
1.5
2.0
2.5
3.0
3.5
4.0

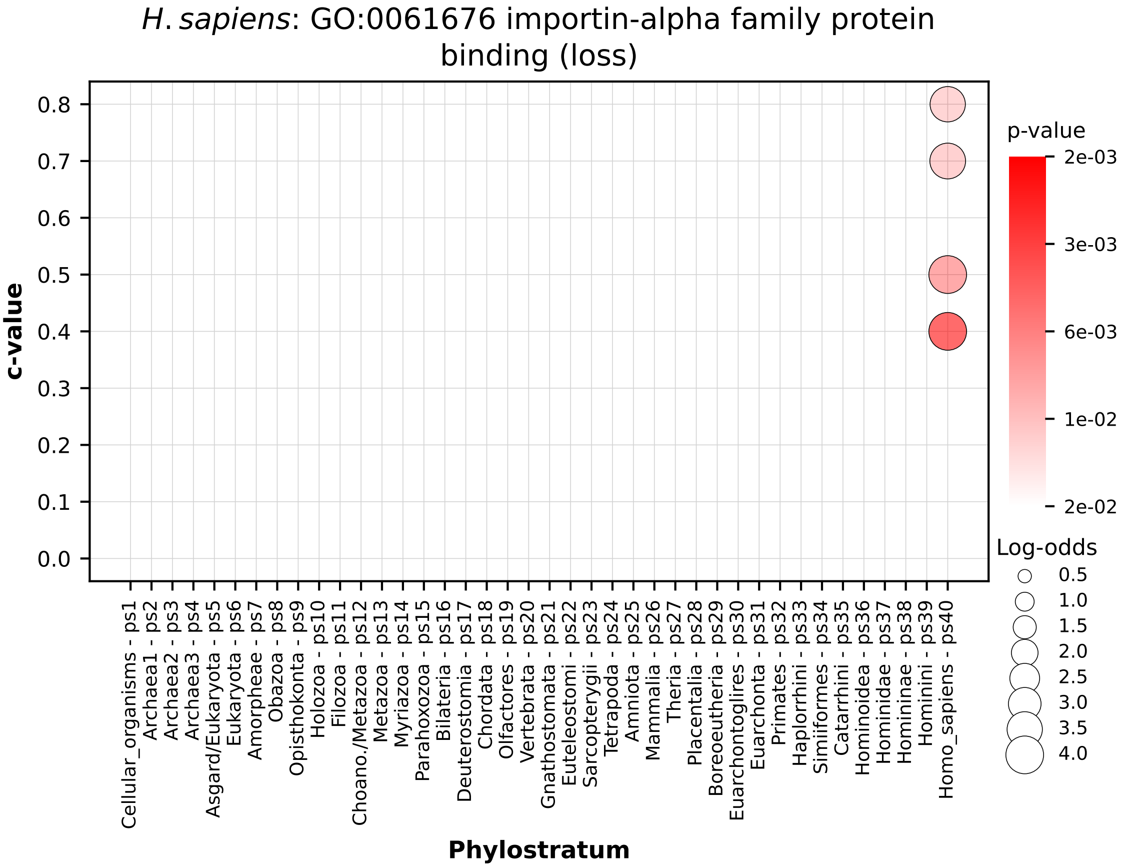

H. sapiens: GO:0061676 importin-alpha family protein binding (loss)
c-value
0.0
0.1
0.2
0.3
0.4
0.5
0.6
0.7
0.8
Phylostratum
Cellular_organisms - ps1
Archaea1 - ps2
Archaea2 - ps3
Archaea3 - ps4
Asgard/Eukaryota - ps5
Eukaryota - ps6
Amorpheae - ps7
Obazoa - ps8
Opisthokonta - ps9
Holozoa - ps10
Filozoa - ps11
Choano./Metazoa - ps12
Metazoa - ps13
Myriazoa - ps14
Parahoxozoa - ps15
Bilateria - ps16
Deuterostomia - ps17
Chordata - ps18
Olfactores - ps19
Vertebrata - ps20
Gnathostomata - ps21
Euteleostomi - ps22
Sarcopterygii - ps23
Tetrapoda - ps24
Amniota - ps25
Mammalia - ps26
Theria - ps27
Placentalia - ps28
Boreoeutheria - ps29
Euarchontoglires - ps30
Euarchonta - ps31
Primates - ps32
Haplorrhini - ps33
Simiiformes - ps34
Catarrhini - ps35
Hominoidea - ps36
Hominidae - ps37
Homininae - ps38
Hominini - ps39
Homo_sapiens - ps40
p-value
2e-03
3e-03
6e-03
1e-02
2e-02
Log-odds
0.5
1.0
1.5
2.0
2.5
3.0
3.5
4.0

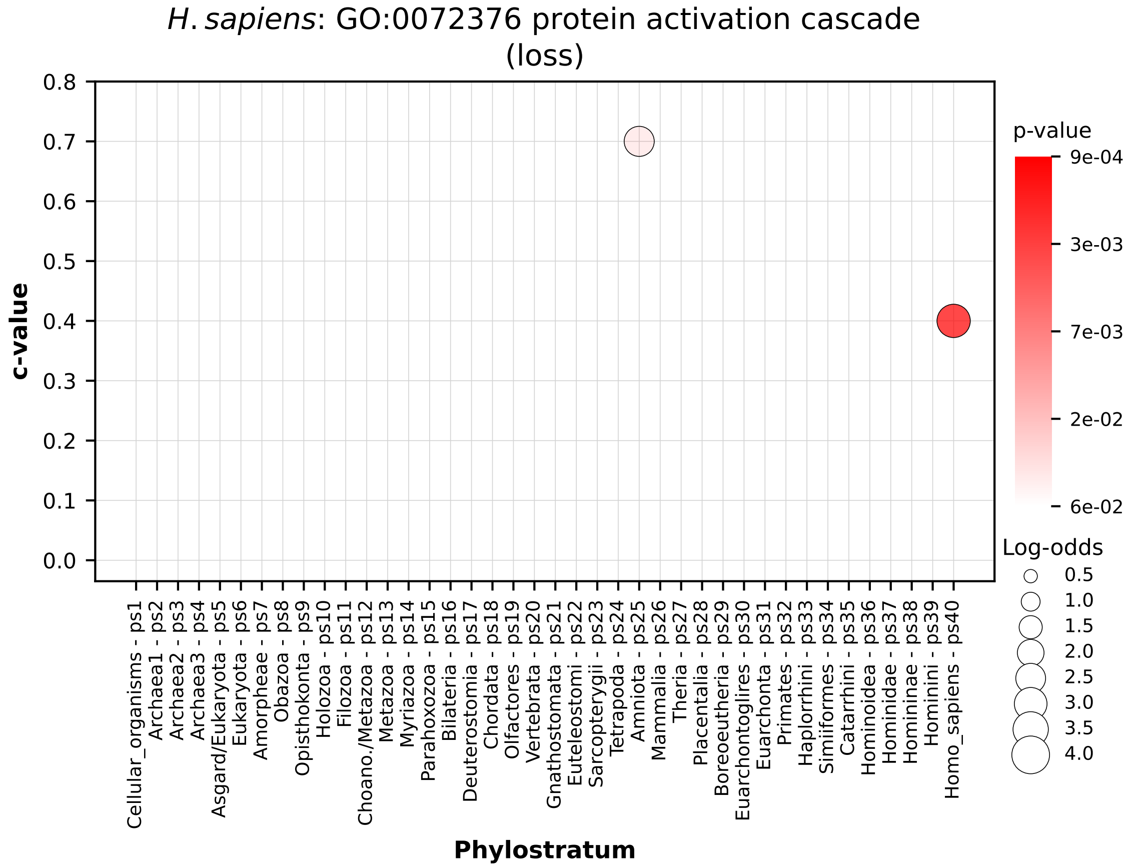

H. sapiens: GO:0072376 protein activation cascade (loss)
c-value
0.8
0.7
0.6
0.5
0.4
0.3
0.2
0.1
0.0
p-value
9e-04
3e-03
7e-03
2e-02
6e-02
Log-odds
0.5
1.0
1.5
2.0
2.5
3.0
3.5
4.0
Cellular_organisms - ps1
Archaea1 - ps2
Archaea2 - ps3
Archaea3 - ps4
Asgard/Eukaryota - ps5
Eukaryota - ps6
Amorpheae - ps7
Obazoa - ps8
Opisthokonta - ps9
Holozoa - ps10
Filozoa - ps11
Choano./Metazoa - ps12
Metazoa - ps13
Myriazoa - ps14
Parahoxozoa - ps15
Bilateria - ps16
Deuterostomia - ps17
Chordata - ps18
Olfactores - ps19
Vertebrata - ps20
Gnathostomata - ps21
Euteleostomi - ps22
Sarcopterygii - ps23
Tetrapoda - ps24
Amniota - ps25
Mammalia - ps26
Theria - ps27
Placentalia - ps28
Boreoeutheria - ps29
Euarchontoglires - ps30
Euarchonta - ps31
Primates - ps32
Haplorrhini - ps33
Simiiformes - ps34
Catarrhini - ps35
Hominoidea - ps36
Hominidae - ps37
Homininae - ps38
Hominini - ps39
Homo_sapiens - ps40
Phylostratum

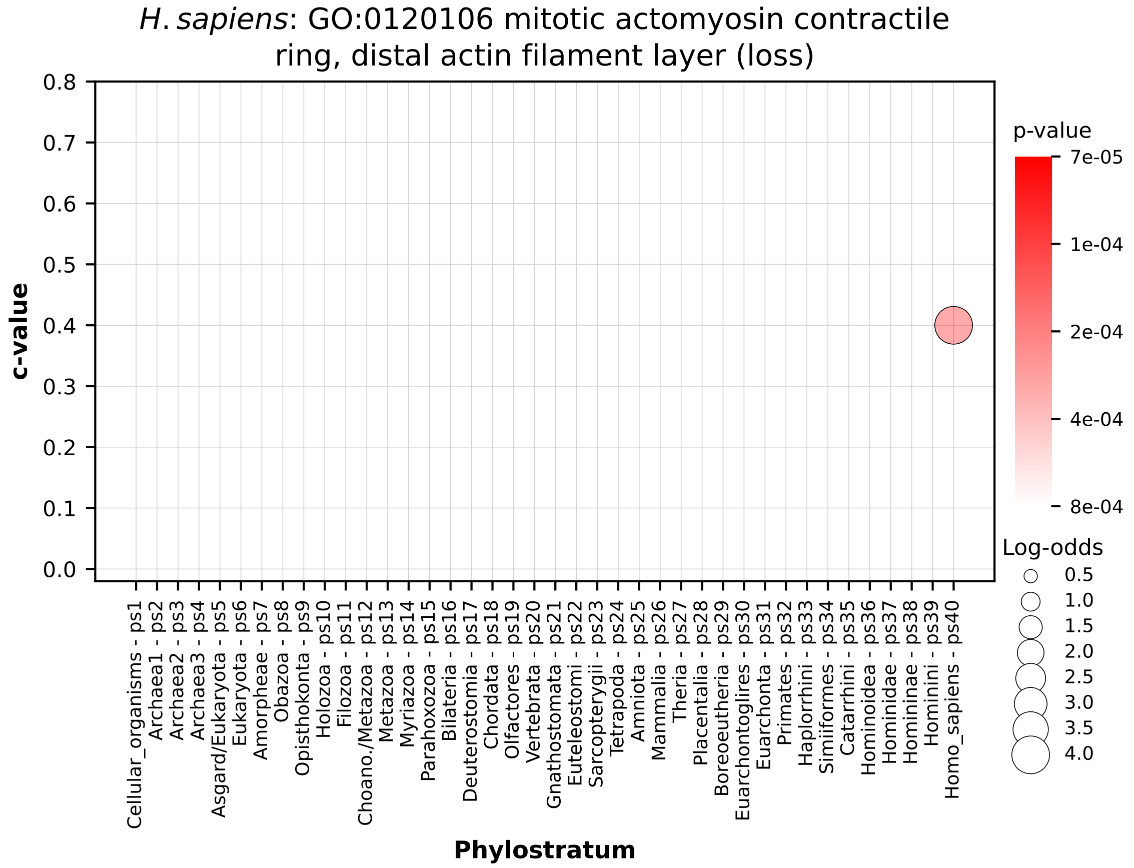
H. sapiens: GO:0120106 mitotic actomyosin contractile ring, distal actin filament layer (loss)
c-value
0.0
0.1
0.2
0.3
0.4
0.5
0.6
0.7
0.8
Phylostratum
Cellular_organisms - ps1
Archaea1 - ps2
Archaea2 - ps3
Archaea3 - ps4
Asgard/Eukaryota - ps5
Eukaryota - ps6
Amorpheae - ps7
Obazoa - ps8
Opisthokonta - ps9
Holozoa - ps10
Filozoa - ps11
Choano./Metazoa - ps12
Metazoa - ps13
Myriazoa - ps14
Parahoxozoa - ps15
Bilateria - ps16
Deuterostomia - ps17
Chordata - ps18
Olfactores - ps19
Vertebrata - ps20
Gnathostomata - ps21
Euteleostomi - ps22
Sarcopterygii - ps23
Tetrapoda - ps24
Amniota - ps25
Mammalia - ps26
Theria - ps27
Placentalia - ps28
Boreoeutheria - ps29
Euarchontoglires - ps30
Euarchonta - ps31
Primates - ps32
Haplorrhini - ps33
Simiiformes - ps34
Catarrhini - ps35
Hominoidea - ps36
Hominidae - ps37
Homininae - ps38
Hominini - ps39
Homo_sapiens - ps40
p-value
7e-05
1e-04
2e-04
4e-04
8e-04
Log-odds
0.5
1.0
1.5
2.0
2.5
3.0
3.5
4.0

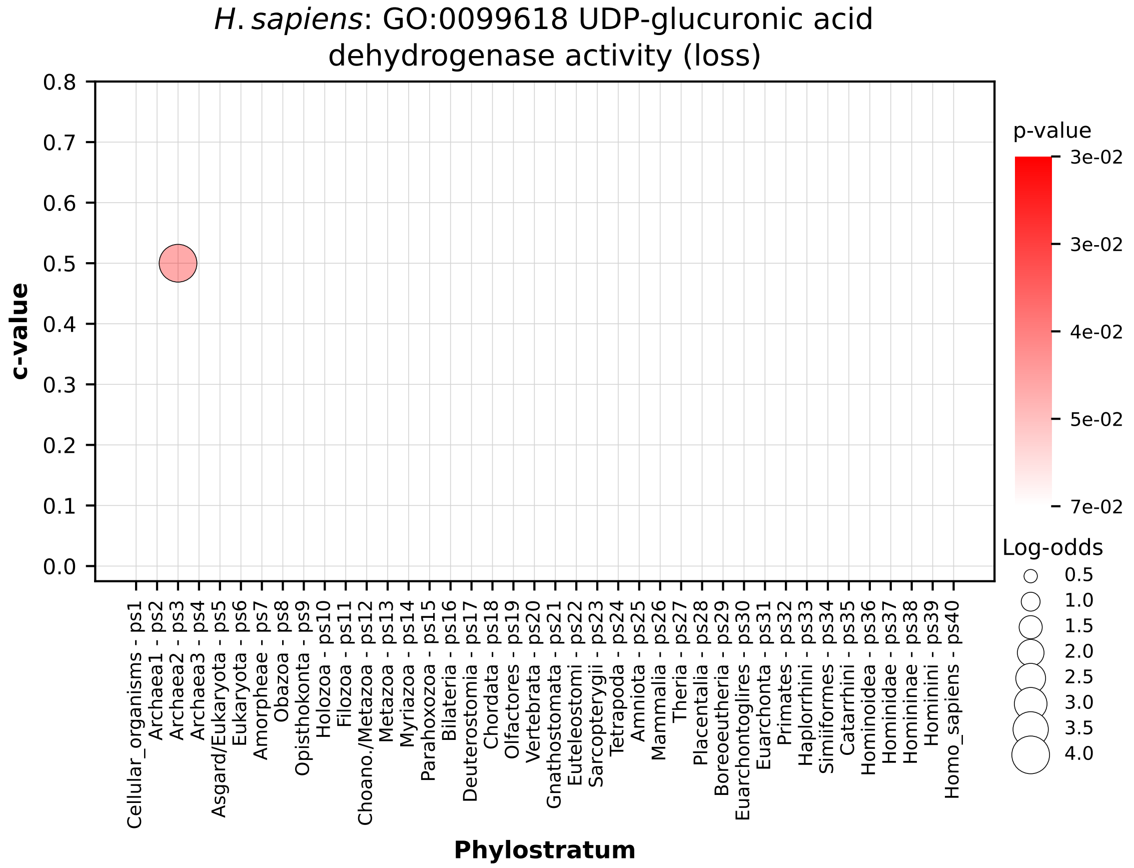

H. sapiens: GO:0099618 UDP-glucuronic acid dehydrogenase activity (loss)
c-value
0.8
0.7
0.6
0.5
0.4
0.3
0.2
0.1
0.0
Phylostratum
Cellular_organisms - ps1
Archaea1 - ps2
Archaea2 - ps3
Archaea3 - ps4
Asgard/Eukaryota - ps5
Eukaryota - ps6
Amorpheae - ps7
Obazoa - ps8
Opisthokonta - ps9
Holozoa - ps10
Filozoa - ps11
Choano./Metazoa - ps12
Metazoa - ps13
Myriazoa - ps14
Parahoxozoa - ps15
Bilateria - ps16
Deuterostomia - ps17
Chordata - ps18
Olfactores - ps19
Vertebrata - ps20
Gnathostomata - ps21
Euteleostomi - ps22
Sarcopterygii - ps23
Tetrapoda - ps24
Amniota - ps25
Mammalia - ps26
Theria - ps27
Placentalia - ps28
Boreoeutheria - ps29
Euarchontoglires - ps30
Euarchonta - ps31
Primates - ps32
Haplorrhini - ps33
Simiiformes - ps34
Catarrhini - ps35
Hominoidea - ps36
Hominidae - ps37
Homininae - ps38
Hominini - ps39
Homo_sapiens - ps40
p-value
3e-02
3e-02
4e-02
5e-02
7e-02
Log-odds
0.5
1.0
1.5
2.0
2.5
3.0
3.5
4.0

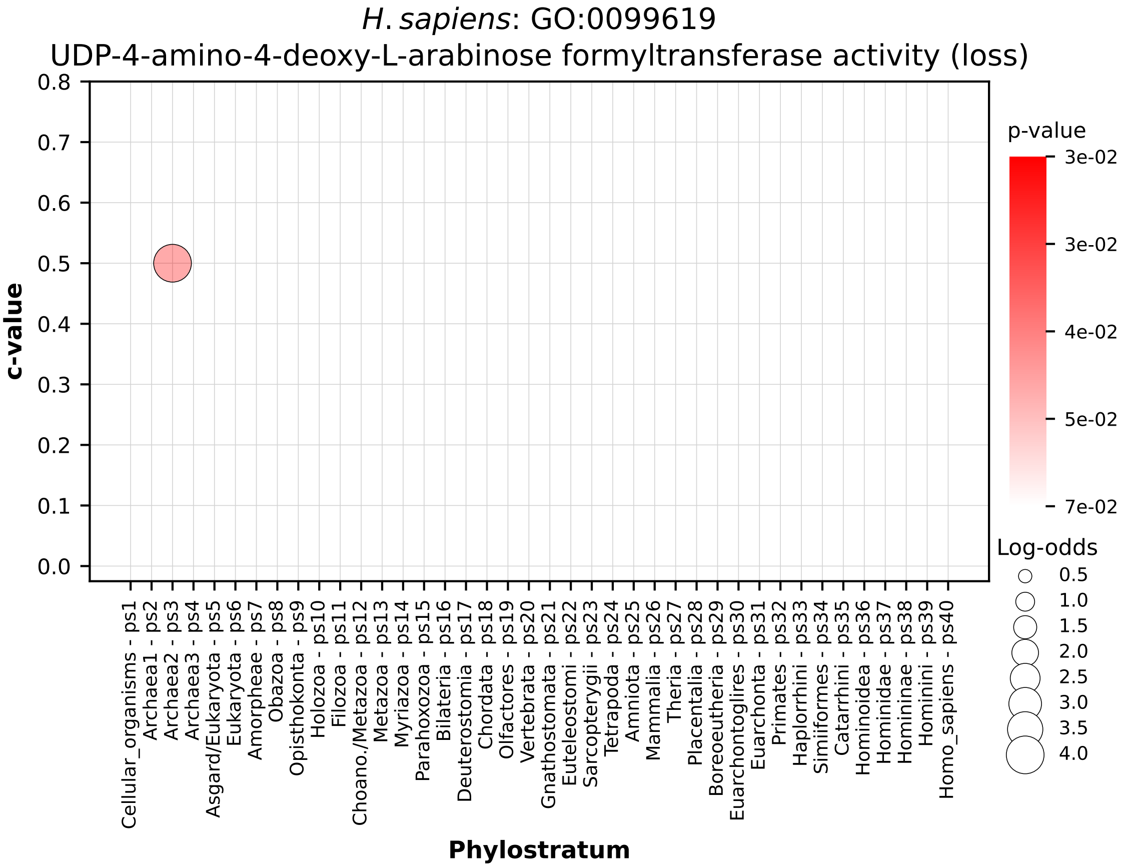

H. sapiens: GO:0099619
UDP-4-amino-4-deoxy-L-arabinose formyltransferase activity (loss)
c-value
Phylostratum
p-value
3e-02
3e-02
4e-02
5e-02
7e-02
Log-odds
0.5
1.0
1.5
2.0
2.5
3.0
3.5
4.0
Cellular_organisms - ps1
Archaea1 - ps2
Archaea2 - ps3
Archaea3 - ps4
Asgard/Eukaryota - ps5
Eukaryota - ps6
Amorpheae - ps7
Obazoa - ps8
Opisthokonta - ps9
Holozoa - ps10
Filozoa - ps11
Choano./Metazoa - ps12
Metazoa - ps13
Myriazoa - ps14
Parahoxozoa - ps15
Bilateria - ps16
Deuterostomia - ps17
Chordata - ps18
Olfactores - ps19
Vertebrata - ps20
Gnathostomata - ps21
Euteleostomi - ps22
Sarcopterygii - ps23
Tetrapoda - ps24
Amniota - ps25
Mammalia - ps26
Theria - ps27
Placentalia - ps28
Boreoeutheria - ps29
Euarchontoglires - ps30
Euarchonta - ps31
Primates - ps32
Haplorrhini - ps33
Simiiformes - ps34
Catarrhini - ps35
Hominoidea - ps36
Hominidae - ps37
Homininae - ps38
Hominini - ps39
Homo_sapiens - ps40

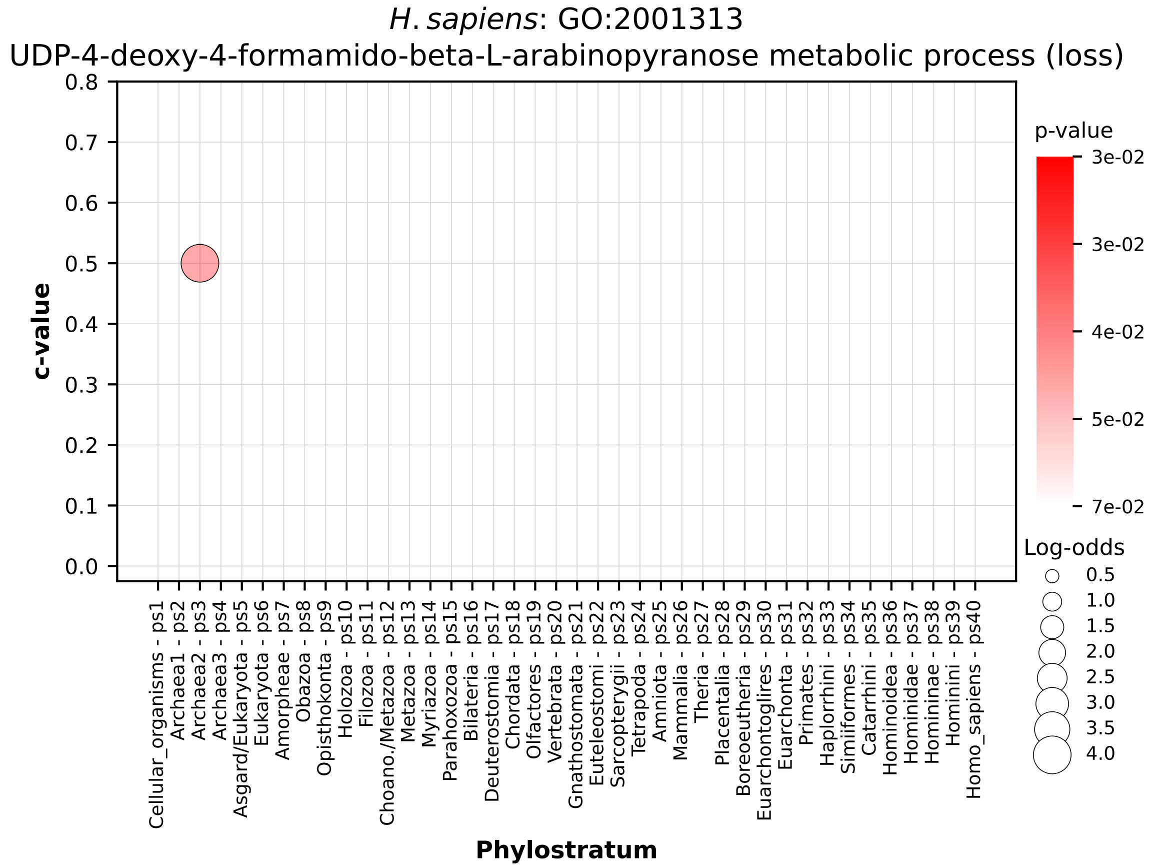

H. sapiens: GO:2001313
UDP-4-deoxy-4-formamido-beta-L-arabinopyranose metabolic process (loss)
c-value
0.8
0.7
0.6
0.5
0.4
0.3
0.2
0.1
0.0
p-value
3e-02
3e-02
4e-02
5e-02
7e-02
Log-odds
0.5
1.0
1.5
2.0
2.5
3.0
3.5
4.0
Cellular_organisms - ps1
Archaea1 - ps2
Archaea2 - ps3
Archaea3 - ps4
Asgard/Eukaryota - ps5
Eukaryota - ps6
Amorpheae - ps7
Obazoa - ps8
Opisthokonta - ps9
Holozoa - ps10
Filozoa - ps11
Choano./Metazoa - ps12
Metazoa - ps13
Myriazoa - ps14
Parahoxozoa - ps15
Bilateria - ps16
Deuterostomia - ps17
Chordata - ps18
Olfactores - ps19
Vertebrata - ps20
Gnathostomata - ps21
Euteleostomi - ps22
Sarcopterygii - ps23
Tetrapoda - ps24
Amniota - ps25
Mammalia - ps26
Theria - ps27
Placentalia - ps28
Boreoeutheria - ps29
Euarchontoglires - ps30
Euarchonta - ps31
Primates - ps32
Haplorrhini - ps33
Simiiformes - ps34
Catarrhini - ps35
Hominoidea - ps36
Hominidae - ps37
Homininae - ps38
Hominini - ps39
Homo_sapiens - ps40
Phylostratum

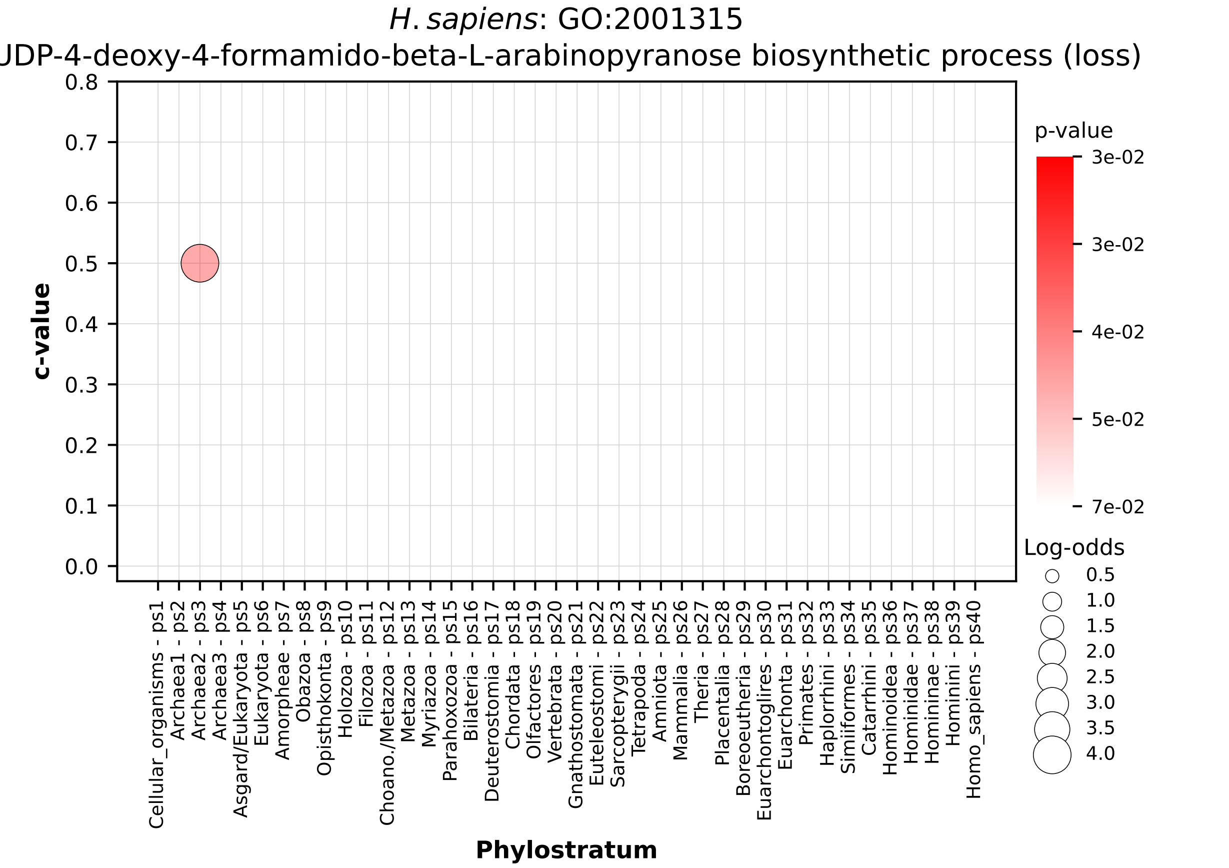

H. sapiens: GO:2001315
UDP-4-deoxy-4-formamido-beta-L-arabinopyranose biosynthetic process (loss)
c-value
Phylostratum
p-value
3e-02
3e-02
4e-02
5e-02
7e-02
Log-odds
0.5
1.0
1.5
2.0
2.5
3.0
3.5
4.0
0.0
0.1
0.2
0.3
0.4
0.5
0.6
0.7
0.8
Cellular_organisms - ps1
Archaea1 - ps2
Archaea2 - ps3
Archaea3 - ps4
Asgard/Eukaryota - ps5
Eukaryota - ps6
Amorpheae - ps7
Obazoa - ps8
Opisthokonta - ps9
Holozoa - ps10
Filozoa - ps11
Choano./Metazoa - ps12
Metazoa - ps13
Myriazoa - ps14
Parahoxozoa - ps15
Bilateria - ps16
Deuterostomia - ps17
Chordata - ps18
Olfactores - ps19
Vertebrata - ps20
Gnathostomata - ps21
Euteleostomi - ps22
Sarcopterygii - ps23
Tetrapoda - ps24
Amniota - ps25
Mammalia - ps26
Theria - ps27
Placentalia - ps28
Boreoeutheria - ps29
Euarchontoglires - ps30
Euarchonta - ps31
Primates - ps32
Haplorrhini - ps33
Simiiformes - ps34
Catarrhini - ps35
Hominoidea - ps36
Hominidae - ps37
Homininae - ps38
Hominini - ps39
Homo_sapiens - ps40

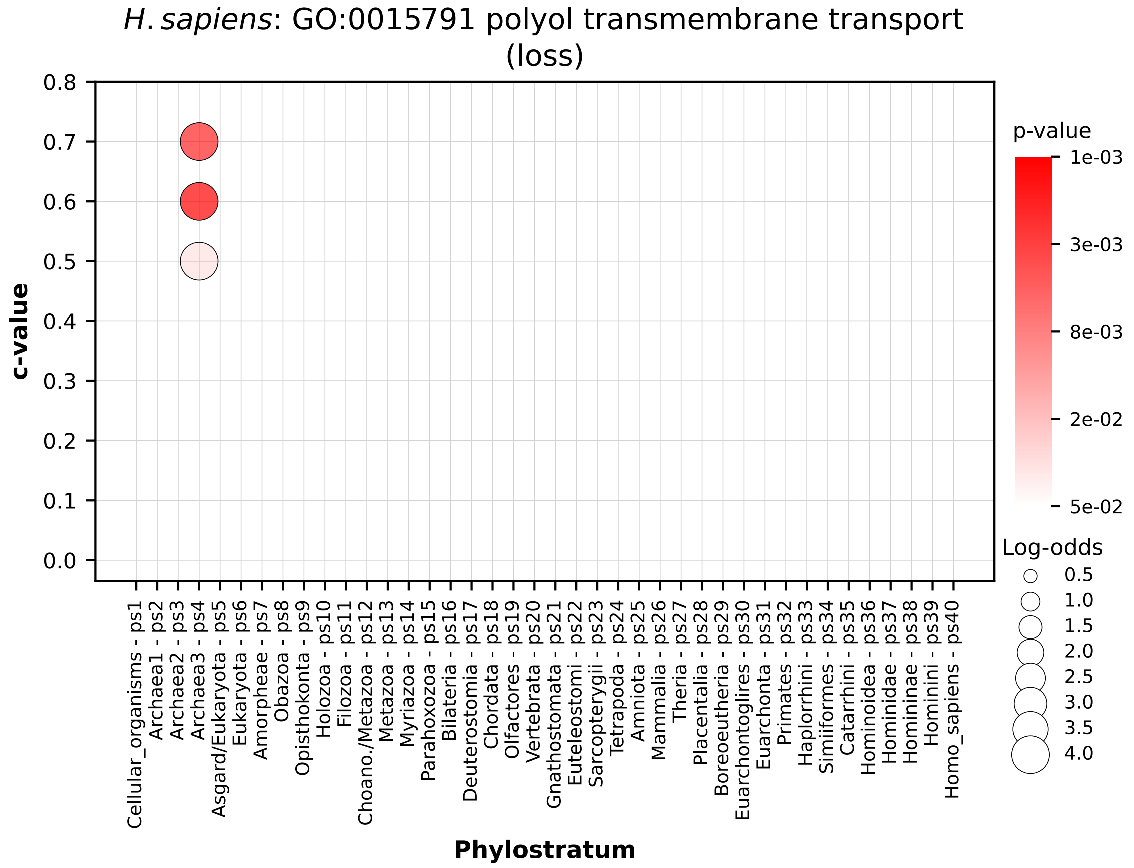

H. sapiens: GO:0015791 polyol transmembrane transport (loss)
c-value
0.8
0.7
0.6
0.5
0.4
0.3
0.2
0.1
0.0
Phylostratum
Cellular_organisms - ps1
Archaea1 - ps2
Archaea2 - ps3
Archaea3 - ps4
Asgard/Eukaryota - ps5
Eukaryota - ps6
Amorpheae - ps7
Obazoa - ps8
Opisthokonta - ps9
Holozoa - ps10
Filozoa - ps11
Choano./Metazoa - ps12
Metazoa - ps13
Myriazoa - ps14
Parahoxozoa - ps15
Bilateria - ps16
Deuterostomia - ps17
Chordata - ps18
Olfactores - ps19
Vertebrata - ps20
Gnathostomata - ps21
Euteleostomi - ps22
Sarcopterygii - ps23
Tetrapoda - ps24
Amniota - ps25
Mammalia - ps26
Theria - ps27
Placentalia - ps28
Boreoeutheria - ps29
Euarchontoglires - ps30
Euarchonta - ps31
Primates - ps32
Haplorrhini - ps33
Simiiformes - ps34
Catarrhini - ps35
Hominoidea - ps36
Hominidae - ps37
Homininae - ps38
Hominini - ps39
Homo_sapiens - ps40
p-value
1e-03
3e-03
8e-03
2e-02
5e-02
Log-odds
0.5
1.0
1.5
2.0
2.5
3.0
3.5
4.0

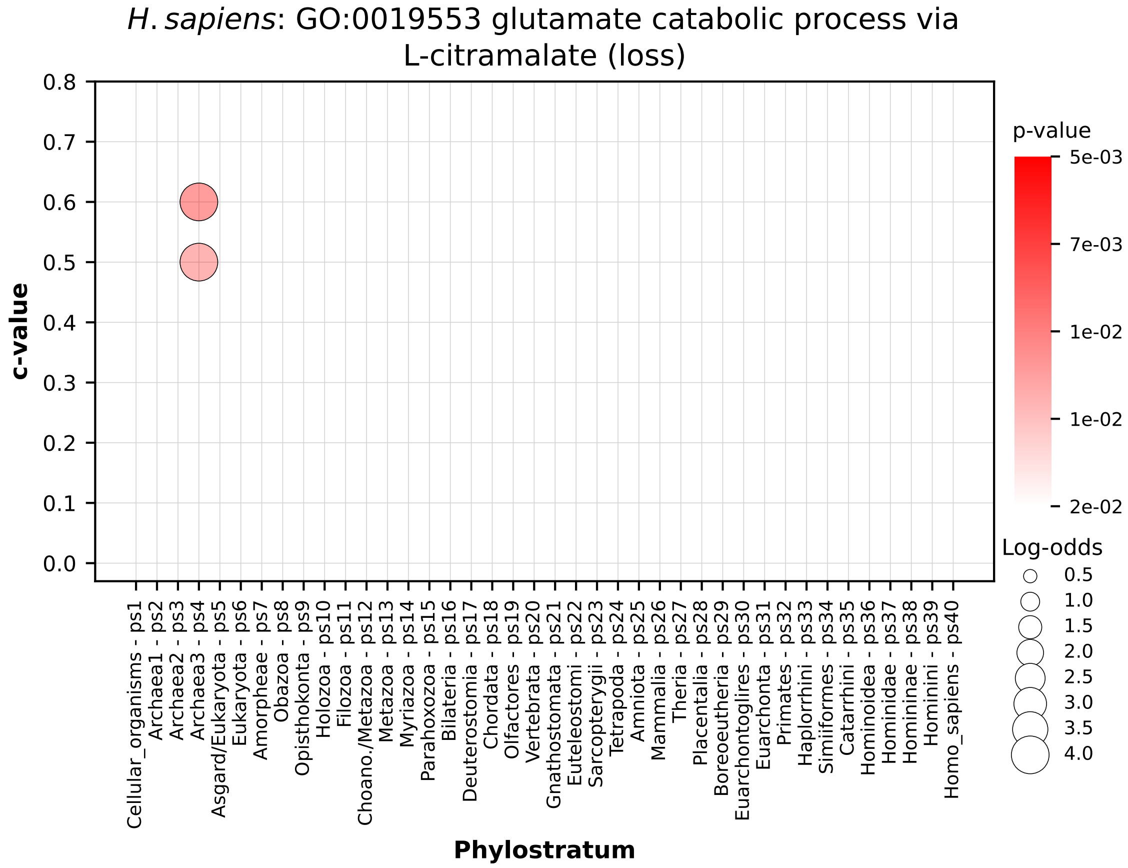

H. sapiens: GO:0019553 glutamate catabolic process via L-citramalate (loss)
c-value
0.8
0.7
0.6
0.5
0.4
0.3
0.2
0.1
0.0
Phylostratum
Cellular_organisms - ps1
Archaea1 - ps2
Archaea2 - ps3
Archaea3 - ps4
Asgard/Eukaryota - ps5
Eukaryota - ps6
Amorpheae - ps7
Obazoa - ps8
Opisthokonta - ps9
Holozoa - ps10
Filozoa - ps11
Choano./Metazoa - ps12
Metazoa - ps13
Myriazoa - ps14
Parahoxozoa - ps15
Bilateria - ps16
Deuterostomia - ps17
Chordata - ps18
Olfactores - ps19
Vertebrata - ps20
Gnathostomata - ps21
Euteleostomi - ps22
Sarcopterygii - ps23
Tetrapoda - ps24
Amniota - ps25
Mammalia - ps26
Theria - ps27
Placentalia - ps28
Boreoeutheria - ps29
Euarchontoglires - ps30
Euarchonta - ps31
Primates - ps32
Haplorrhini - ps33
Simiiformes - ps34
Catarrhini - ps35
Hominoidea - ps36
Hominidae - ps37
Homininae - ps38
Hominini - ps39
Homo_sapiens - ps40
p-value
5e-03
7e-03
1e-02
1e-02
2e-02
Log-odds
0.5
1.0
1.5
2.0
2.5
3.0
3.5
4.0

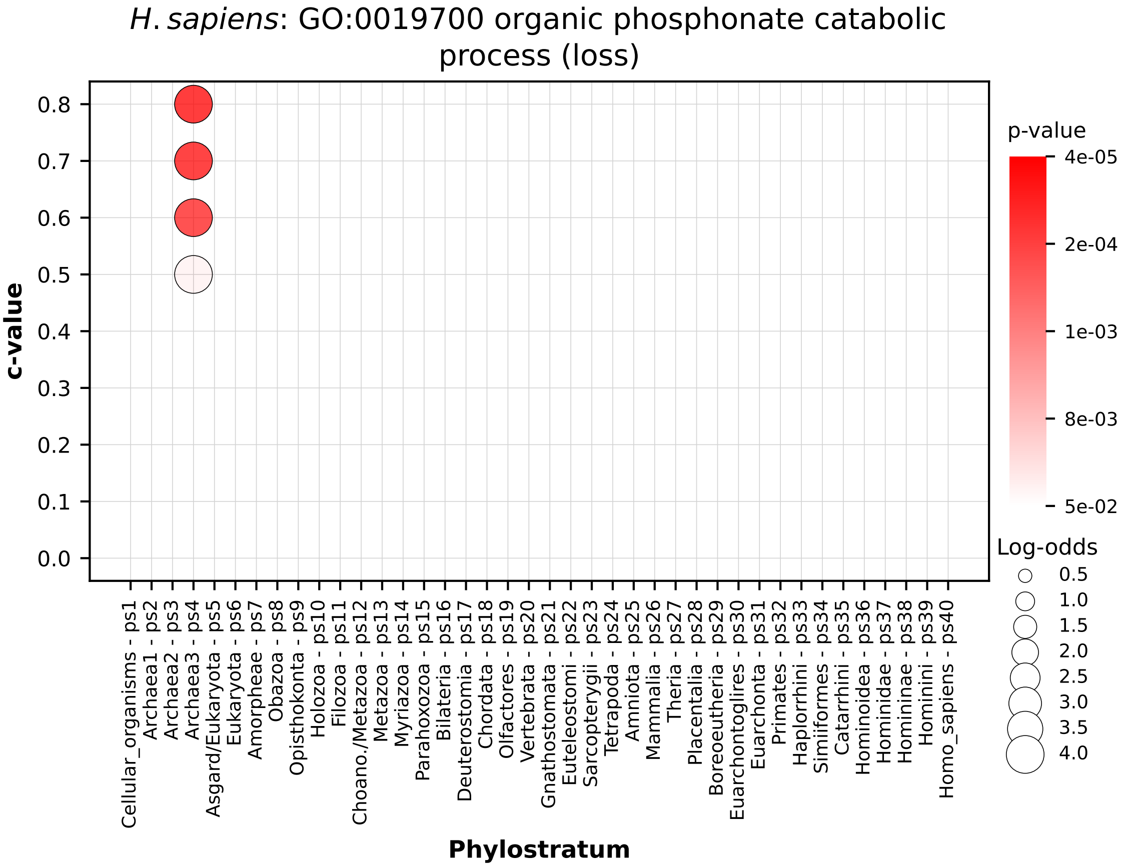

H. sapiens: GO:0019700 organic phosphonate catabolic process (loss)
c-value
0.8
0.7
0.6
0.5
0.4
0.3
0.2
0.1
0.0
Phylostratum
Cellular_organisms - ps1
Archaea1 - ps2
Archaea2 - ps3
Archaea3 - ps4
Asgard/Eukaryota - ps5
Eukaryota - ps6
Amorpheae - ps7
Obazoa - ps8
Opisthokonta - ps9
Holozoa - ps10
Filozoa - ps11
Choano./Metazoa - ps12
Metazoa - ps13
Myriazoa - ps14
Parahoxozoa - ps15
Bilateria - ps16
Deuterostomia - ps17
Chordata - ps18
Olfactores - ps19
Vertebrata - ps20
Gnathostomata - ps21
Euteleostomi - ps22
Sarcopterygii - ps23
Tetrapoda - ps24
Amniota - ps25
Mammalia - ps26
Theria - ps27
Placentalia - ps28
Boreoeutheria - ps29
Euarchontoglires - ps30
Euarchonta - ps31
Primates - ps32
Haplorrhini - ps33
Simiiformes - ps34
Catarrhini - ps35
Hominoidea - ps36
Hominidae - ps37
Homininae - ps38
Hominini - ps39
Homo_sapiens - ps40
p-value
4e-05
2e-04
1e-03
8e-03
5e-02
Log-odds
0.5
1.0
1.5
2.0
2.5
3.0
3.5
4.0

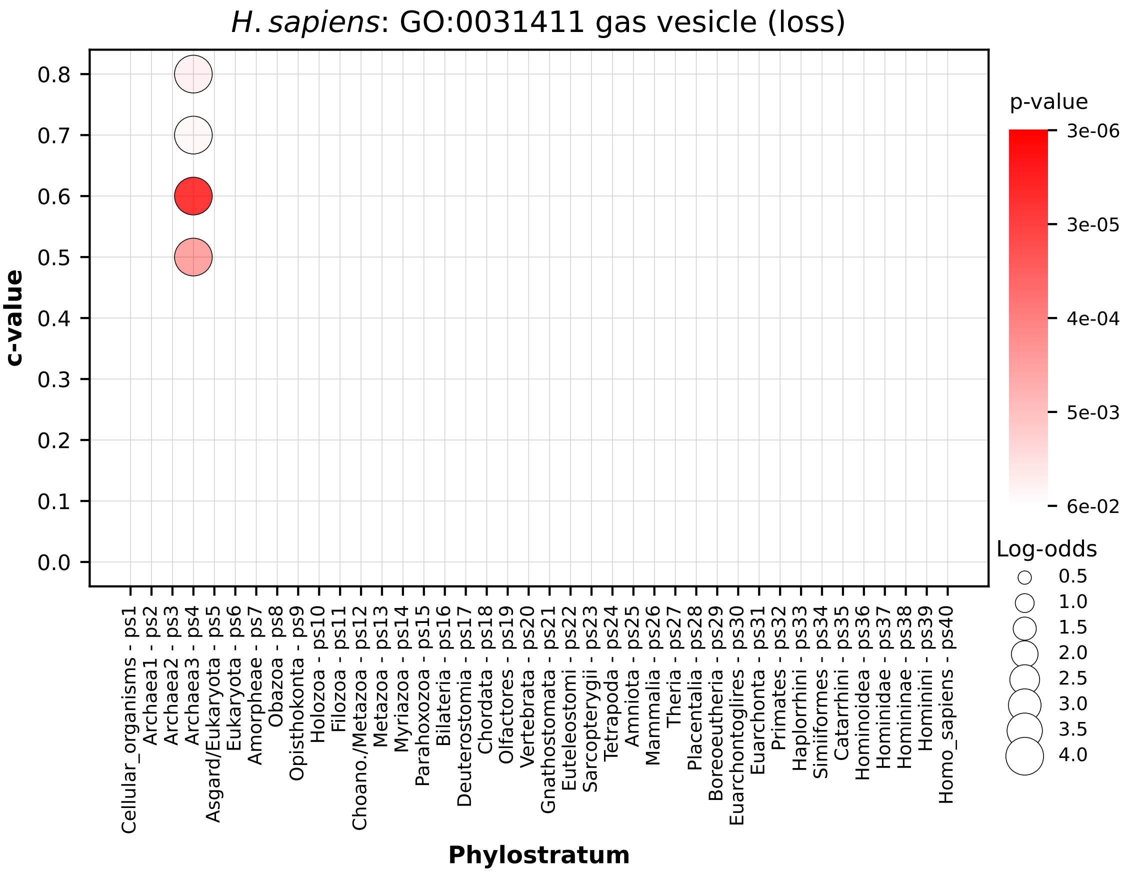
H. sapiens: GO:0031411 gas vesicle (loss)
c-value
0.8
0.7
0.6
0.5
0.4
0.3
0.2
0.1
0.0
Phylostratum
Cellular_organisms - ps1
Archaea1 - ps2
Archaea2 - ps3
Archaea3 - ps4
Asgard/Eukaryota - ps5
Eukaryota - ps6
Amorpheae - ps7
Obazoa - ps8
Opisthokonta - ps9
Holozoa - ps10
Filozoa - ps11
Choano./Metazoa - ps12
Metazoa - ps13
Myriazoa - ps14
Parahoxozoa - ps15
Bilateria - ps16
Deuterostomia - ps17
Chordata - ps18
Olfactores - ps19
Vertebrata - ps20
Gnathostomata - ps21
Euteleostomi - ps22
Sarcopterygii - ps23
Tetrapoda - ps24
Amniota - ps25
Mammalia - ps26
Theria - ps27
Placentalia - ps28
Boreoeutheria - ps29
Euarchontoglires - ps30
Euarchonta - ps31
Primates - ps32
Haplorrhini - ps33
Simiiformes - ps34
Catarrhini - ps35
Hominoidea - ps36
Hominidae - ps37
Homininae - ps38
Hominini - ps39
Homo_sapiens - ps40
p-value
3e-06
3e-05
4e-04
5e-03
6e-02
Log-odds
0.5
1.0
1.5
2.0
2.5
3.0
3.5
4.0

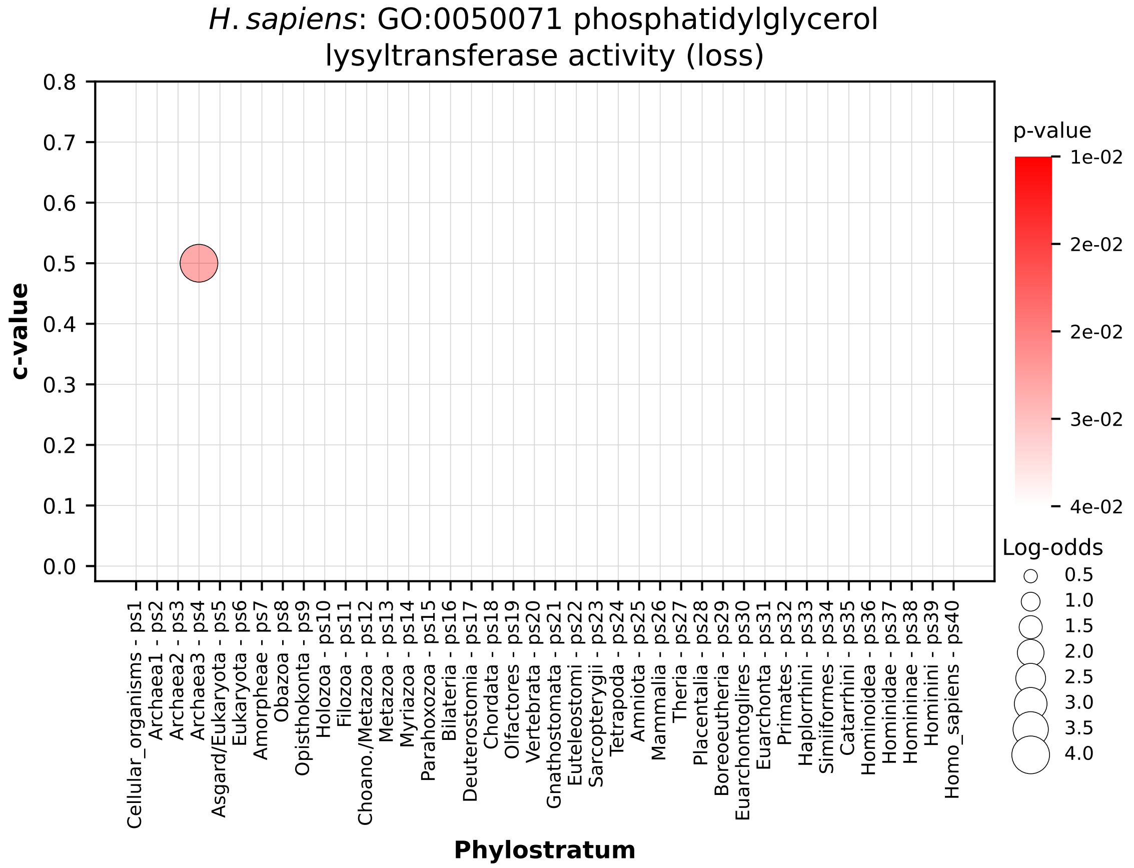

H. sapiens: GO:0050071 phosphatidylglycerol lysyltransferase activity (loss)
c-value
0.0
0.1
0.2
0.3
0.4
0.5
0.6
0.7
0.8
Phylostratum
Cellular_organisms - ps1
Archaea1 - ps2
Archaea2 - ps3
Archaea3 - ps4
Asgard/Eukaryota - ps5
Eukaryota - ps6
Amorpheae - ps7
Obazoa - ps8
Opisthokonta - ps9
Holozoa - ps10
Filozoa - ps11
Choano./Metazoa - ps12
Metazoa - ps13
Myriazoa - ps14
Parahoxozoa - ps15
Bilateria - ps16
Deuterostomia - ps17
Chordata - ps18
Olfactores - ps19
Vertebrata - ps20
Gnathostomata - ps21
Euteleostomi - ps22
Sarcopterygii - ps23
Tetrapoda - ps24
Amniota - ps25
Mammalia - ps26
Theria - ps27
Placentalia - ps28
Boreoeutheria - ps29
Euarchontoglires - ps30
Euarchonta - ps31
Primates - ps32
Haplorrhini - ps33
Simiiformes - ps34
Catarrhini - ps35
Hominoidea - ps36
Hominidae - ps37
Homininae - ps38
Hominini - ps39
Homo_sapiens - ps40
p-value
1e-02
2e-02
2e-02
3e-02
4e-02
Log-odds
0.5
1.0
1.5
2.0
2.5
3.0
3.5
4.0

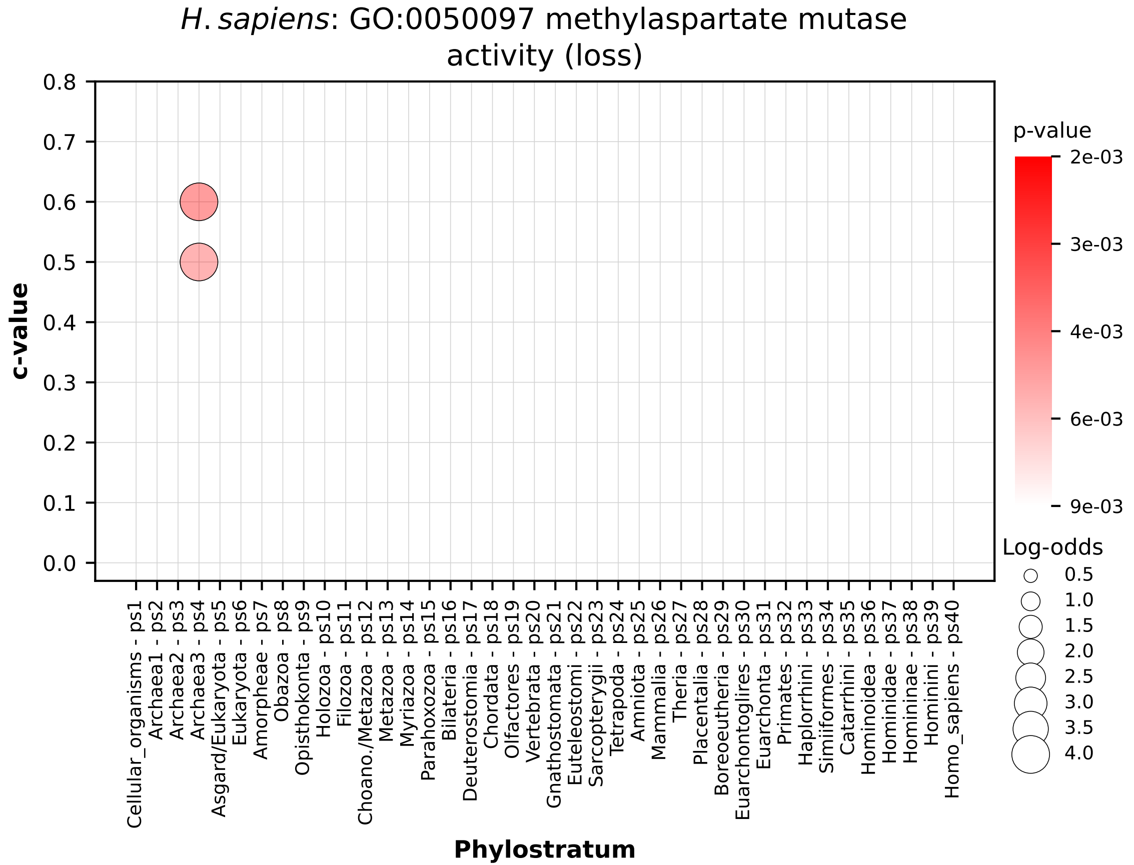

H. sapiens: GO:0050097 methylaspartate mutase activity (loss)
c-value
0.8
0.7
0.6
0.5
0.4
0.3
0.2
0.1
0.0
p-value
2e-03
3e-03
4e-03
6e-03
9e-03
Log-odds
0.5
1.0
1.5
2.0
2.5
3.0
3.5
4.0
Cellular_organisms - ps1
Archaea1 - ps2
Archaea2 - ps3
Archaea3 - ps4
Asgard/Eukaryota - ps5
Eukaryota - ps6
Amorpheae - ps7
Obazoa - ps8
Opisthokonta - ps9
Holozoa - ps10
Filozoa - ps11
Choano./Metazoa - ps12
Metazoa - ps13
Myriazoa - ps14
Parahoxozoa - ps15
Bilateria - ps16
Deuterostomia - ps17
Chordata - ps18
Olfactores - ps19
Vertebrata - ps20
Gnathostomata - ps21
Euteleostomi - ps22
Sarcopterygii - ps23
Tetrapoda - ps24
Amniota - ps25
Mammalia - ps26
Theria - ps27
Placentalia - ps28
Boreoeutheria - ps29
Euarchontoglires - ps30
Euarchonta - ps31
Primates - ps32
Haplorrhini - ps33
Simiiformes - ps34
Catarrhini - ps35
Hominoidea - ps36
Hominidae - ps37
Homininae - ps38
Hominini - ps39
Homo_sapiens - ps40
Phylostratum

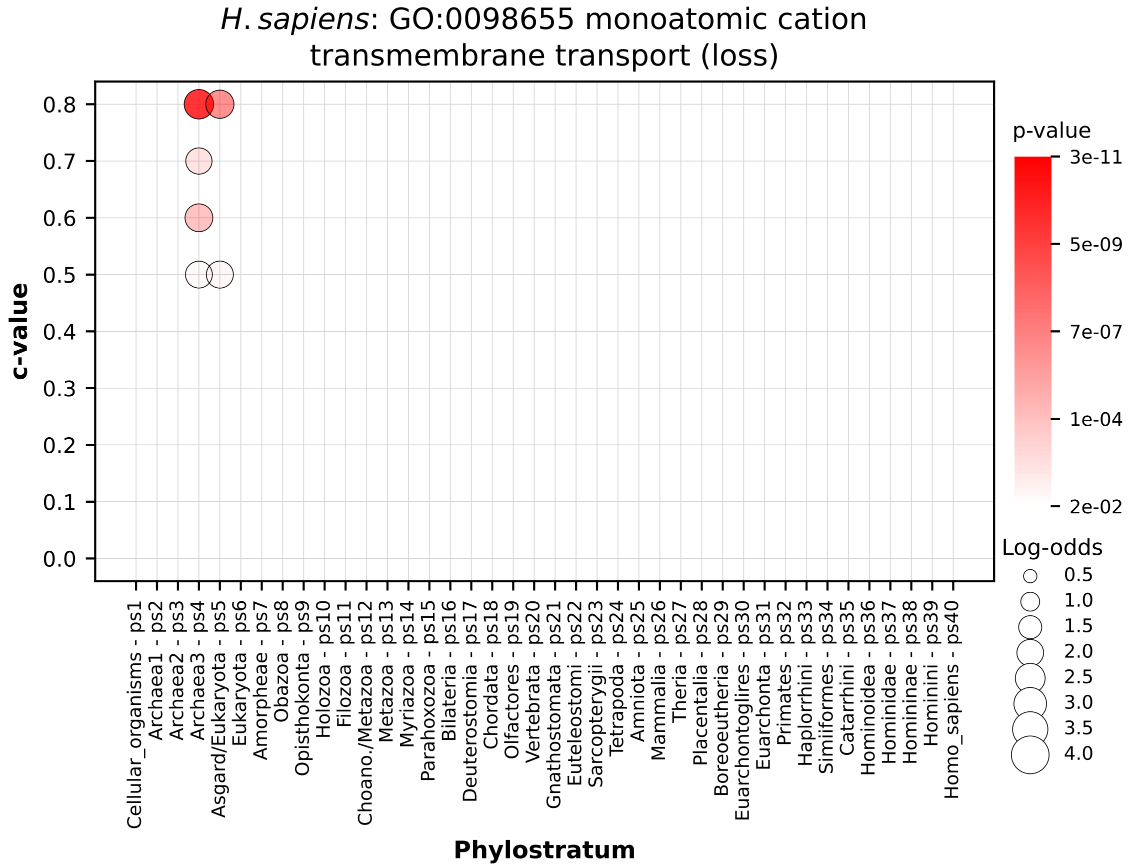

H. sapiens: GO:0098655 monoatomic cation transmembrane transport (loss)
c-value
0.8
0.7
0.6
0.5
0.4
0.3
0.2
0.1
0.0
p-value
3e-11
5e-09
7e-07
1e-04
2e-02
Log-odds
0.5
1.0
1.5
2.0
2.5
3.0
3.5
4.0
Cellular_organisms - ps1
Archaea1 - ps2
Archaea2 - ps3
Archaea3 - ps4
Asgard/Eukaryota - ps5
Eukaryota - ps6
Amorpheae - ps7
Obazoa - ps8
Opisthokonta - ps9
Holozoa - ps10
Filozoa - ps11
Choano./Metazoa - ps12
Metazoa - ps13
Myriazoa - ps14
Parahoxozoa - ps15
Bilateria - ps16
Deuterostomia - ps17
Chordata - ps18
Olfactores - ps19
Vertebrata - ps20
Gnathostomata - ps21
Euteleostomi - ps22
Sarcopterygii - ps23
Tetrapoda - ps24
Amniota - ps25
Mammalia - ps26
Theria - ps27
Placentalia - ps28
Boreoeutheria - ps29
Euarchontoglires - ps30
Euarchonta - ps31
Primates - ps32
Haplorrhini - ps33
Simiiformes - ps34
Catarrhini - ps35
Hominoidea - ps36
Hominidae - ps37
Homininae - ps38
Hominini - ps39
Homo_sapiens - ps40
Phylostratum

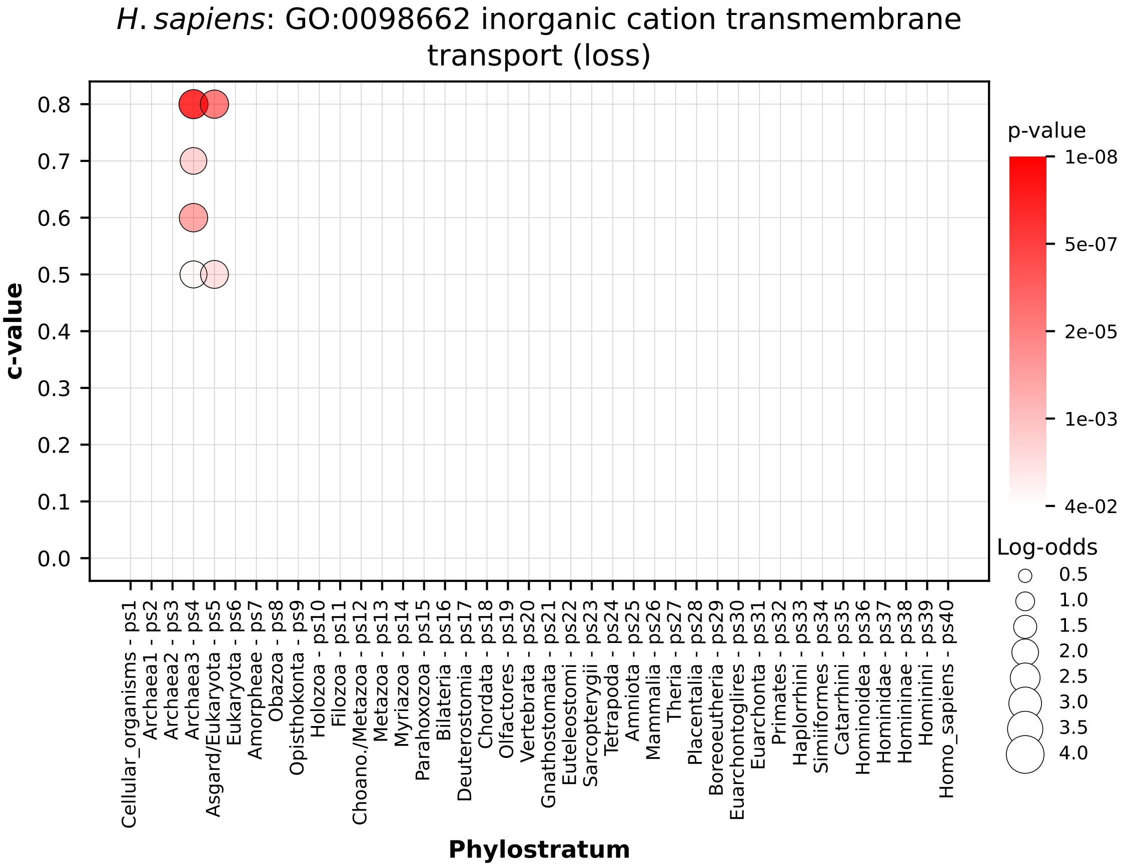

H. sapiens: GO:0098662 inorganic cation transmembrane transport (loss)
c-value
0.8
0.7
0.6
0.5
0.4
0.3
0.2
0.1
0.0
p-value
1e-08
5e-07
2e-05
1e-03
4e-02
Log-odds
0.5
1.0
1.5
2.0
2.5
3.0
3.5
4.0
Cellular_organisms - ps1
Archaea1 - ps2
Archaea2 - ps3
Archaea3 - ps4
Asgard/Eukaryota - ps5
Eukaryota - ps6
Amorpheae - ps7
Obazoa - ps8
Opisthokonta - ps9
Holozoa - ps10
Filozoa - ps11
Choano./Metazoa - ps12
Metazoa - ps13
Myriazoa - ps14
Parahoxozoa - ps15
Bilateria - ps16
Deuterostomia - ps17
Chordata - ps18
Olfactores - ps19
Vertebrata - ps20
Gnathostomata - ps21
Euteleostomi - ps22
Sarcopterygii - ps23
Tetrapoda - ps24
Amniota - ps25
Mammalia - ps26
Theria - ps27
Placentalia - ps28
Boreoeutheria - ps29
Euarchontoglires - ps30
Euarchonta - ps31
Primates - ps32
Haplorrhini - ps33
Simiiformes - ps34
Catarrhini - ps35
Hominoidea - ps36
Hominidae - ps37
Homininae - ps38
Hominini - ps39
Homo_sapiens - ps40
Phylostratum

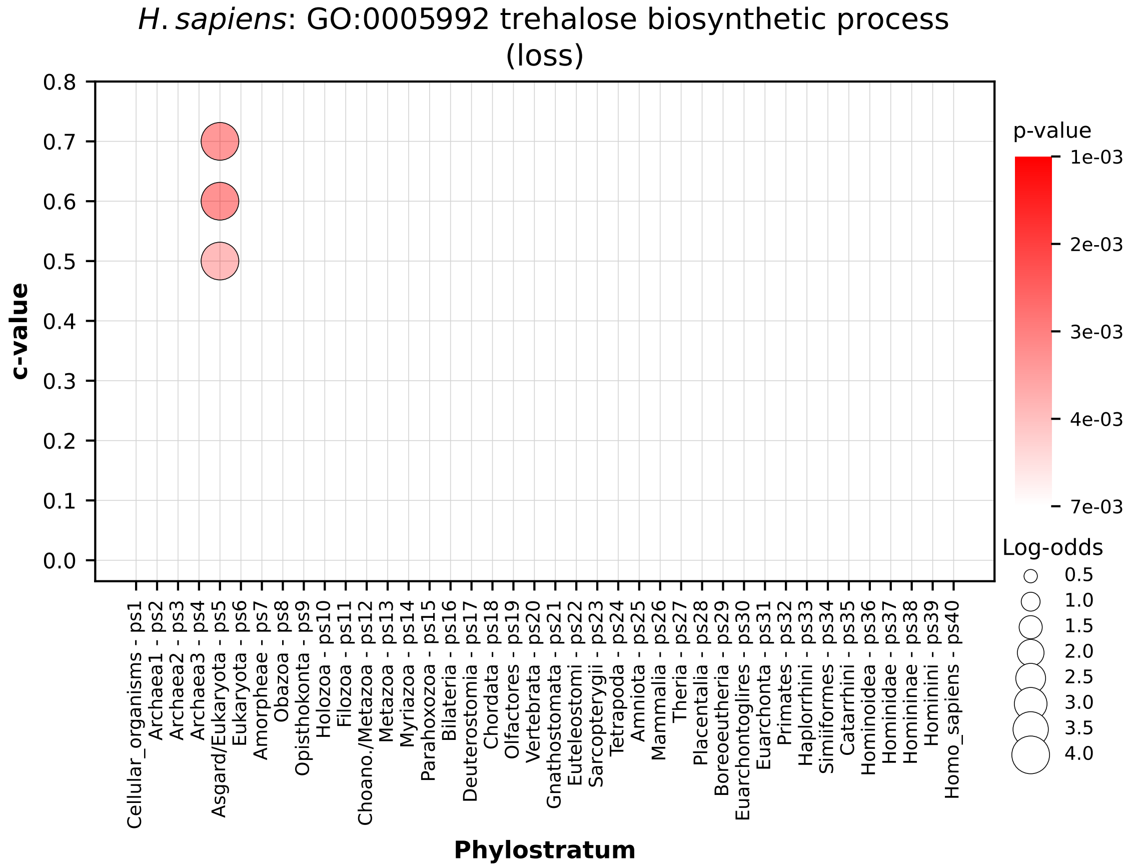
H. sapiens: GO:0005992 trehalose biosynthetic process (loss)
c-value
0.0
0.1
0.2
0.3
0.4
0.5
0.6
0.7
0.8
p-value
1e-03
2e-03
3e-03
4e-03
7e-03
Log-odds
0.5
1.0
1.5
2.0
2.5
3.0
3.5
4.0
Phylostratum
Cellular_organisms - ps1
Archaea1 - ps2
Archaea2 - ps3
Archaea3 - ps4
Asgard/Eukaryota - ps5
Eukaryota - ps6
Amorpheae - ps7
Obazoa - ps8
Opisthokonta - ps9
Holozoa - ps10
Filozoa - ps11
Choano./Metazoa - ps12
Metazoa - ps13
Myriazoa - ps14
Parahoxozoa - ps15
Bilateria - ps16
Deuterostomia - ps17
Chordata - ps18
Olfactores - ps19
Vertebrata - ps20
Gnathostomata - ps21
Euteleostomi - ps22
Sarcopterygii - ps23
Tetrapoda - ps24
Amniota - ps25
Mammalia - ps26
Theria - ps27
Placentalia - ps28
Boreoeutheria - ps29
Euarchontoglires - ps30
Euarchonta - ps31
Primates - ps32
Haplorrhini - ps33
Simiiformes - ps34
Catarrhini - ps35
Hominoidea - ps36
Hominidae - ps37
Homininae - ps38
Hominini - ps39
Homo_sapiens - ps40

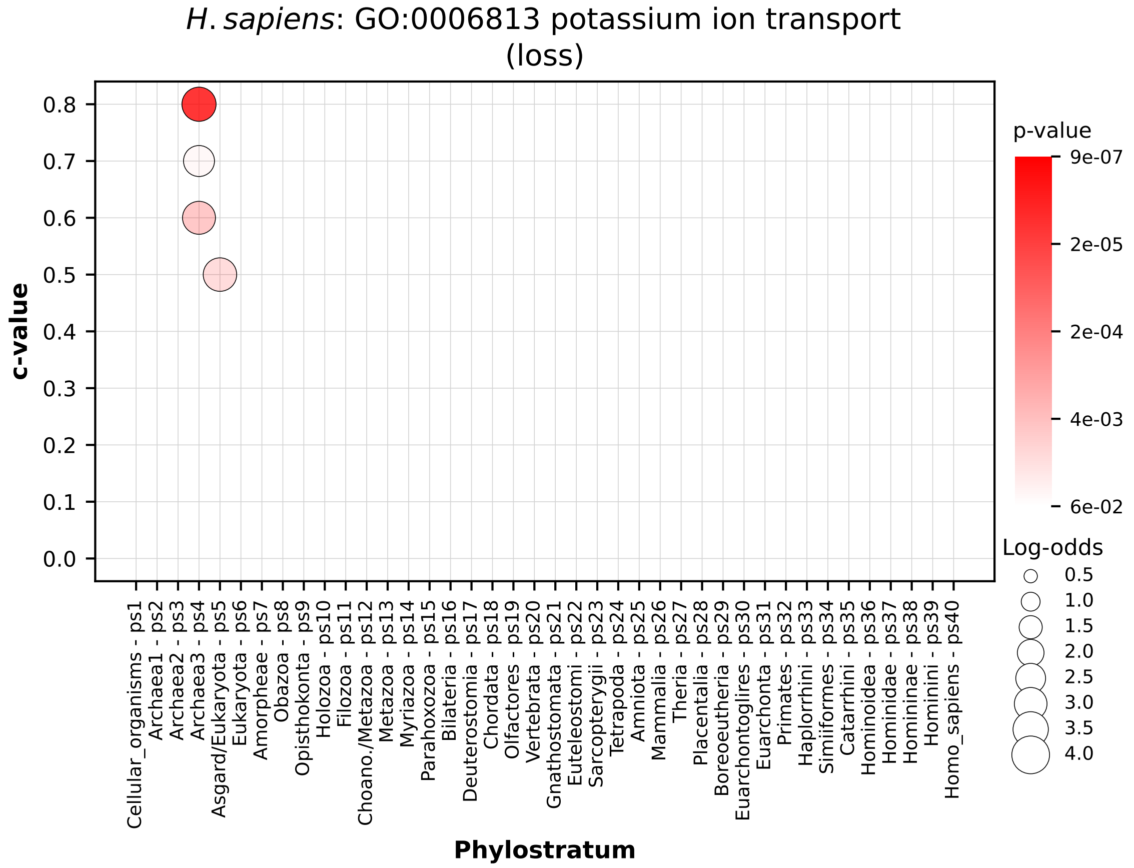

H. sapiens: GO:0006813 potassium ion transport
(loss)
c-value
0.0
0.1
0.2
0.3
0.4
0.5
0.6
0.7
0.8
p-value
9e-07
2e-05
2e-04
4e-03
6e-02
Log-odds
0.5
1.0
1.5
2.0
2.5
3.0
3.5
4.0
Cellular_organisms - ps1
Archaea1 - ps2
Archaea2 - ps3
Archaea3 - ps4
Asgard/Eukaryota - ps5
Eukaryota - ps6
Amorpheae - ps7
Obazoa - ps8
Opisthokonta - ps9
Holozoa - ps10
Filozoa - ps11
Choano./Metazoa - ps12
Metazoa - ps13
Myriazoa - ps14
Parahoxozoa - ps15
Bilateria - ps16
Deuterostomia - ps17
Chordata - ps18
Olfactores - ps19
Vertebrata - ps20
Gnathostomata - ps21
Euteleostomi - ps22
Sarcopterygii - ps23
Tetrapoda - ps24
Amniota - ps25
Mammalia - ps26
Theria - ps27
Placentalia - ps28
Boreoeutheria - ps29
Euarchontoglires - ps30
Euarchonta - ps31
Primates - ps32
Haplorrhini - ps33
Simiiformes - ps34
Catarrhini - ps35
Hominoidea - ps36
Hominidae - ps37
Homininae - ps38
Hominini - ps39
Homo_sapiens - ps40
Phylostratum

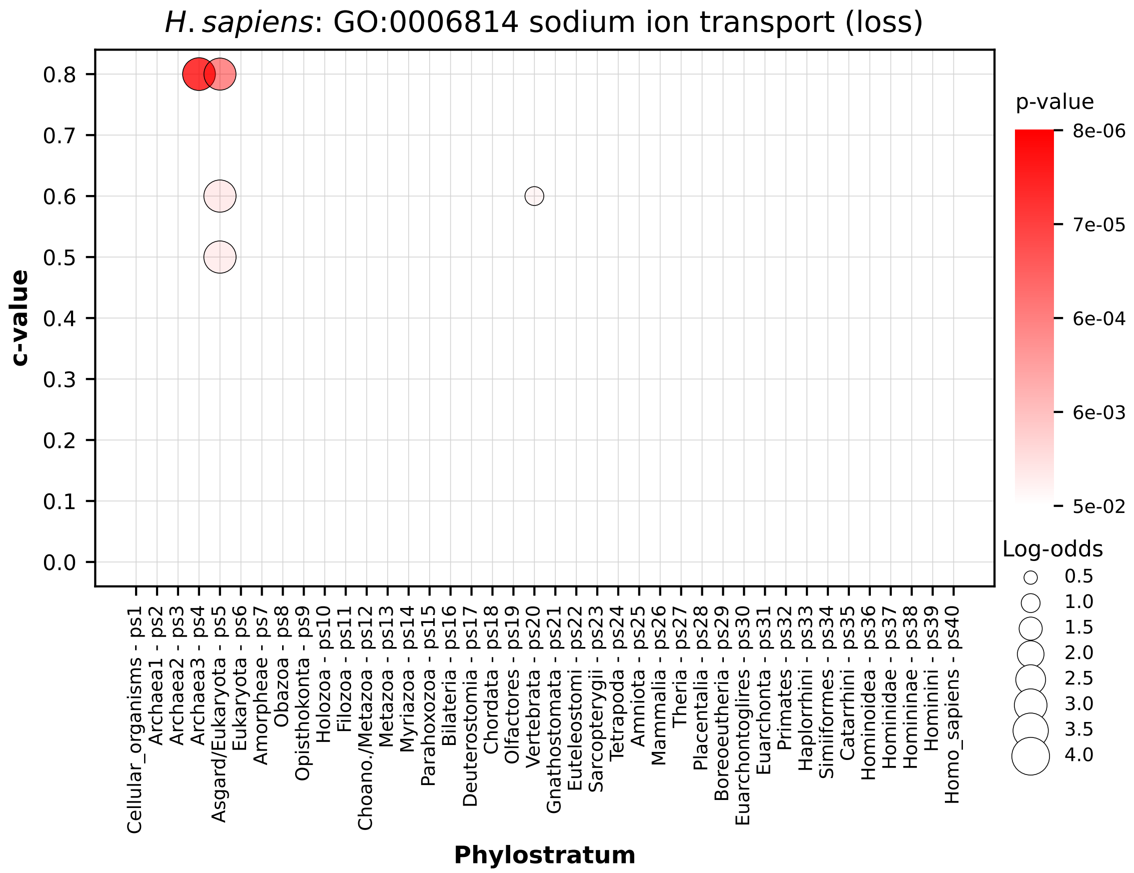

H. sapiens: GO:0006814 sodium ion transport (loss)
c-value
0.0
0.1
0.2
0.3
0.4
0.5
0.6
0.7
0.8
Phylostratum
Cellular_organisms - ps1
Archaea1 - ps2
Archaea2 - ps3
Archaea3 - ps4
Asgard/Eukaryota - ps5
Eukaryota - ps6
Amorpheae - ps7
Obazoa - ps8
Opisthokonta - ps9
Holozoa - ps10
Filozoa - ps11
Choano./Metazoa - ps12
Metazoa - ps13
Myriazoa - ps14
Parahoxozoa - ps15
Bilateria - ps16
Deuterostomia - ps17
Chordata - ps18
Olfactores - ps19
Vertebrata - ps20
Gnathostomata - ps21
Euteleostomi - ps22
Sarcopterygii - ps23
Tetrapoda - ps24
Amniota - ps25
Mammalia - ps26
Theria - ps27
Placentalia - ps28
Boreoeutheria - ps29
Euarchontoglires - ps30
Euarchonta - ps31
Primates - ps32
Haplorrhini - ps33
Simiiformes - ps34
Catarrhini - ps35
Hominoidea - ps36
Hominidae - ps37
Homininae - ps38
Hominini - ps39
Homo_sapiens - ps40
p-value
8e-06
7e-05
6e-04
6e-03
5e-02
Log-odds
0.5
1.0
1.5
2.0
2.5
3.0
3.5
4.0

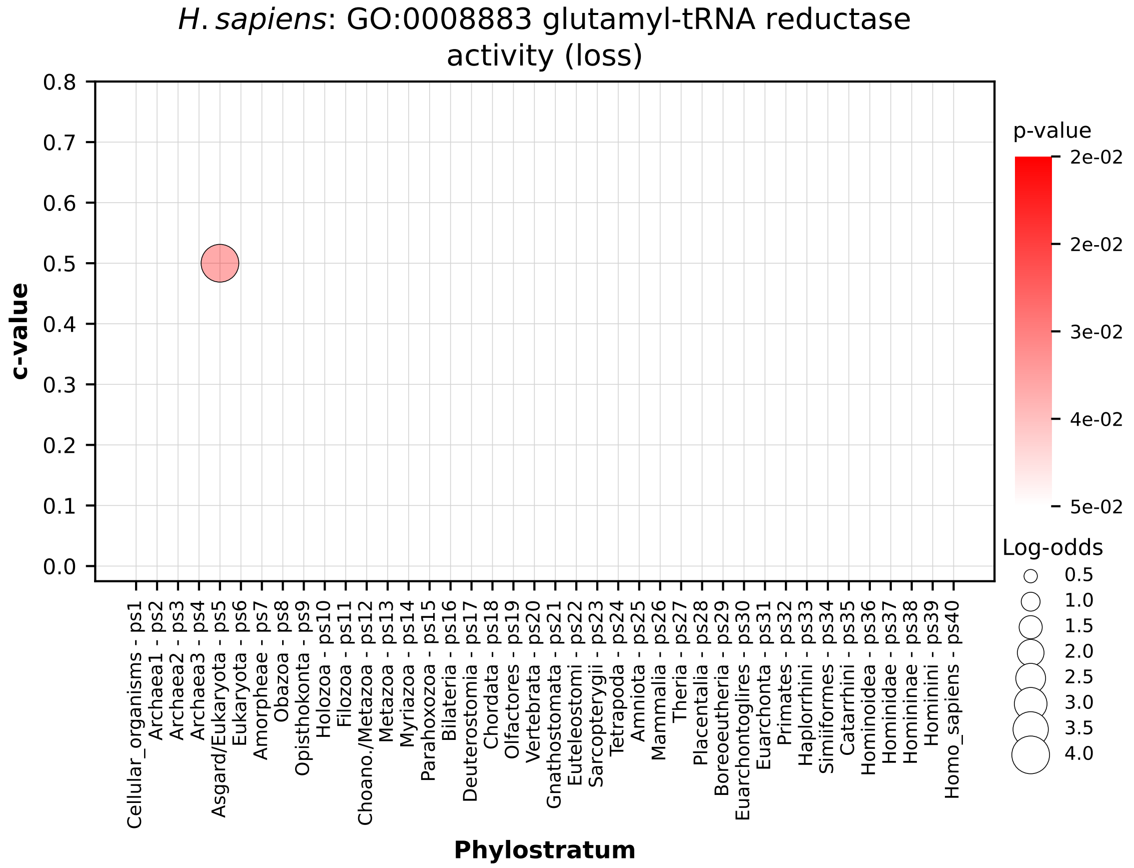
H. sapiens: GO:0008883 glutamyl-tRNA reductase activity (loss)
c-value
0.0
0.1
0.2
0.3
0.4
0.5
0.6
0.7
0.8
Phylostratum
Cellular_organisms - ps1
Archaea1 - ps2
Archaea2 - ps3
Archaea3 - ps4
Asgard/Eukaryota - ps5
Eukaryota - ps6
Amorpheae - ps7
Obazoa - ps8
Opisthokonta - ps9
Holozoa - ps10
Filozoa - ps11
Choano./Metazoa - ps12
Metazoa - ps13
Myriazoa - ps14
Parahoxozoa - ps15
Bilateria - ps16
Deuterostomia - ps17
Chordata - ps18
Olfactores - ps19
Vertebrata - ps20
Gnathostomata - ps21
Euteleostomi - ps22
Sarcopterygii - ps23
Tetrapoda - ps24
Amniota - ps25
Mammalia - ps26
Theria - ps27
Placentalia - ps28
Boreoeutheria - ps29
Euarchontoglires - ps30
Euarchonta - ps31
Primates - ps32
Haplorrhini - ps33
Simiiformes - ps34
Catarrhini - ps35
Hominoidea - ps36
Hominidae - ps37
Homininae - ps38
Hominini - ps39
Homo_sapiens - ps40
p-value
2e-02
2e-02
3e-02
4e-02
5e-02
Log-odds
0.5
1.0
1.5
2.0
2.5
3.0
3.5
4.0

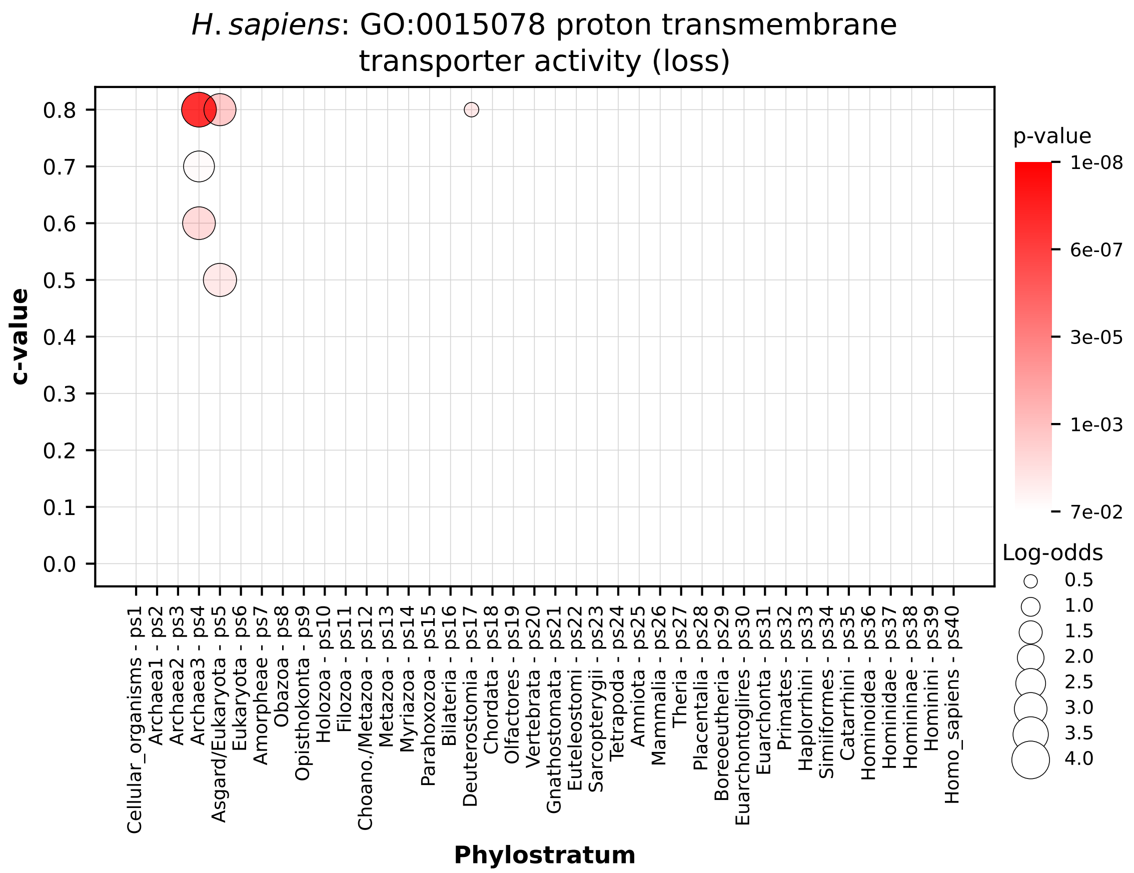

$H. sapiens$: GO:0015078 proton transmembrane transporter activity (loss)
c-value
0.0
0.1
0.2
0.3
0.4
0.5
0.6
0.7
0.8
Phylostratum
Cellular_organisms - ps1
Archaea1 - ps2
Archaea2 - ps3
Archaea3 - ps4
Asgard/Eukaryota - ps5
Eukaryota - ps6
Amorpheae - ps7
Obazoa - ps8
Opisthokonta - ps9
Holozoa - ps10
Filozoa - ps11
Choano./Metazoa - ps12
Metazoa - ps13
Myriazoa - ps14
Parahoxozoa - ps15
Bilateria - ps16
Deuterostomia - ps17
Chordata - ps18
Olfactores - ps19
Vertebrata - ps20
Gnathostomata - ps21
Euteleostomi - ps22
Sarcopterygii - ps23
Tetrapoda - ps24
Amniota - ps25
Mammalia - ps26
Theria - ps27
Placentalia - ps28
Boreoeutheria - ps29
Euarchontoglires - ps30
Euarchonta - ps31
Primates - ps32
Haplorrhini - ps33
Simiiformes - ps34
Catarrhini - ps35
Hominoidea - ps36
Hominidae - ps37
Homininae - ps38
Hominini - ps39
Homo_sapiens - ps40
p-value
1e-08
6e-07
3e-05
1e-03
7e-02
Log-odds
0.5
1.0
1.5
2.0
2.5
3.0
3.5
4.0

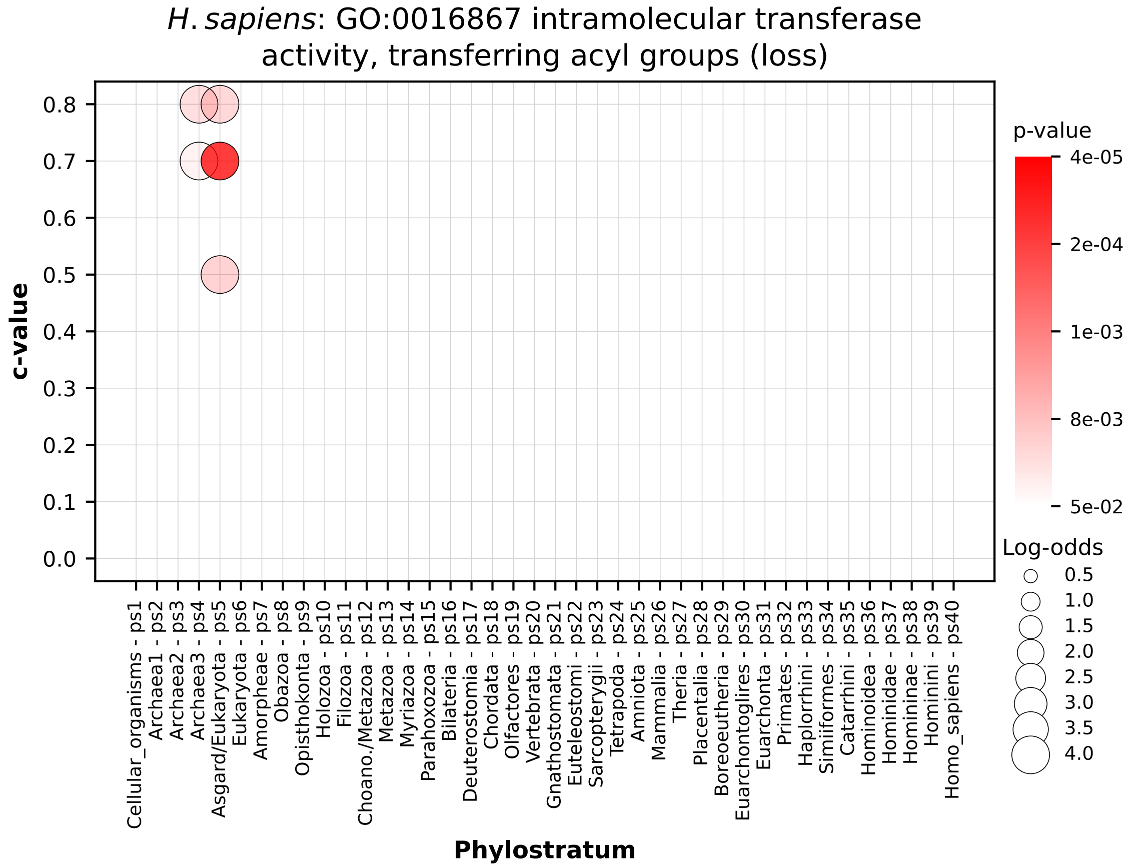

H. sapiens: GO:0016867 intramolecular transferase activity, transferring acyl groups (loss)
c-value
0.8
0.7
0.6
0.5
0.4
0.3
0.2
0.1
0.0
p-value
4e-05
2e-04
1e-03
8e-03
5e-02
Log-odds
0.5
1.0
1.5
2.0
2.5
3.0
3.5
4.0
Cellular_organisms - ps1
Archaea1 - ps2
Archaea2 - ps3
Archaea3 - ps4
Asgard/Eukaryota - ps5
Eukaryota - ps6
Amorpheae - ps7
Obazoa - ps8
Opisthokonta - ps9
Holozoa - ps10
Filozoa - ps11
Choano./Metazoa - ps12
Metazoa - ps13
Myriazoa - ps14
Parahoxozoa - ps15
Bilateria - ps16
Deuterostomia - ps17
Chordata - ps18
Olfactores - ps19
Vertebrata - ps20
Gnathostomata - ps21
Euteleostomi - ps22
Sarcopterygii - ps23
Tetrapoda - ps24
Amniota - ps25
Mammalia - ps26
Theria - ps27
Placentalia - ps28
Boreoeutheria - ps29
Euarchontoglires - ps30
Euarchonta - ps31
Primates - ps32
Haplorrhini - ps33
Simiiformes - ps34
Catarrhini - ps35
Hominoidea - ps36
Hominidae - ps37
Homininae - ps38
Hominini - ps39
Homo_sapiens - ps40
Phylostratum

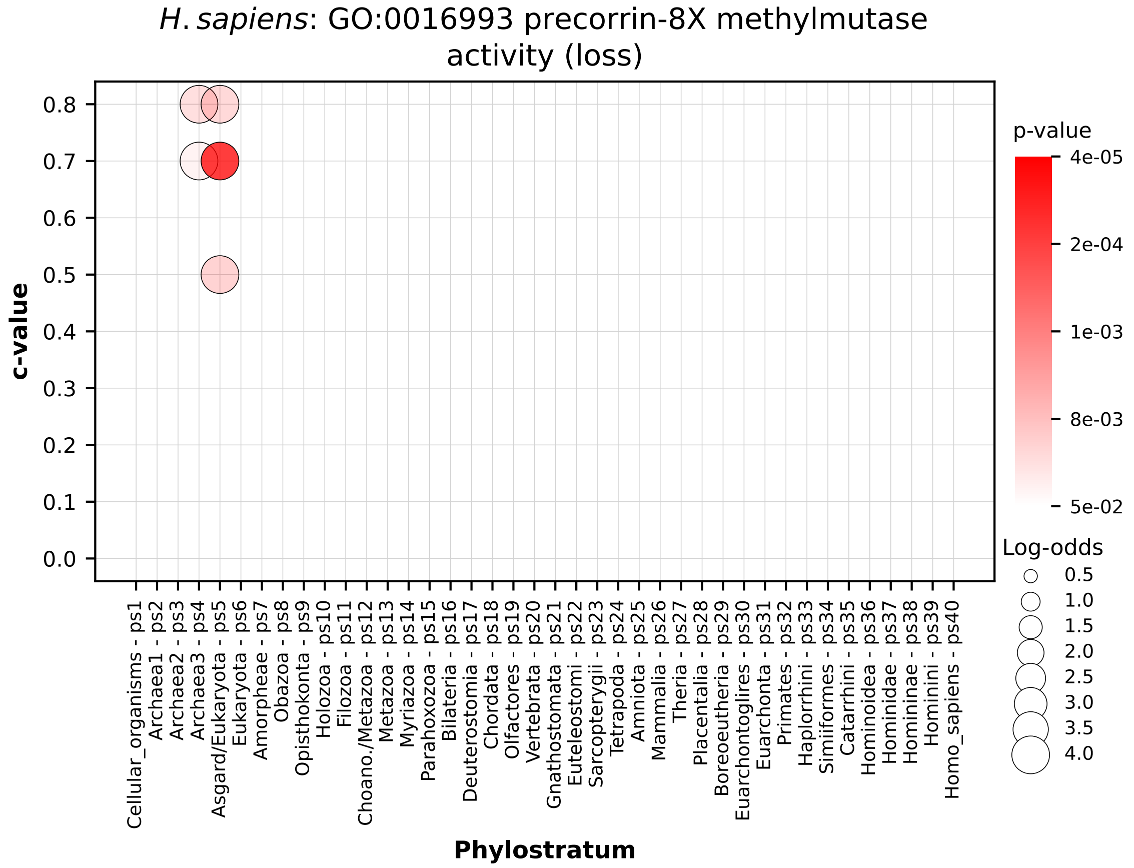
H. sapiens: GO:0016993 precorrin-8X methylmutase activity (loss)
c-value
0.8
0.7
0.6
0.5
0.4
0.3
0.2
0.1
0.0
p-value
4e-05
2e-04
1e-03
8e-03
5e-02
Log-odds
0.5
1.0
1.5
2.0
2.5
3.0
3.5
4.0
Cellular_organisms - ps1
Archaea1 - ps2
Archaea2 - ps3
Archaea3 - ps4
Asgard/Eukaryota - ps5
Eukaryota - ps6
Amorpheae - ps7
Obazoa - ps8
Opisthokonta - ps9
Holozoa - ps10
Filozoa - ps11
Choano./Metazoa - ps12
Metazoa - ps13
Myriazoa - ps14
Parahoxozoa - ps15
Bilateria - ps16
Deuterostomia - ps17
Chordata - ps18
Olfactores - ps19
Vertebrata - ps20
Gnathostomata - ps21
Euteleostomi - ps22
Sarcopterygii - ps23
Tetrapoda - ps24
Amniota - ps25
Mammalia - ps26
Theria - ps27
Placentalia - ps28
Boreoeutheria - ps29
Euarchontoglires - ps30
Euarchonta - ps31
Primates - ps32
Haplorrhini - ps33
Simiiformes - ps34
Catarrhini - ps35
Hominoidea - ps36
Hominidae - ps37
Homininae - ps38
Hominini - ps39
Homo_sapiens - ps40
Phylostratum

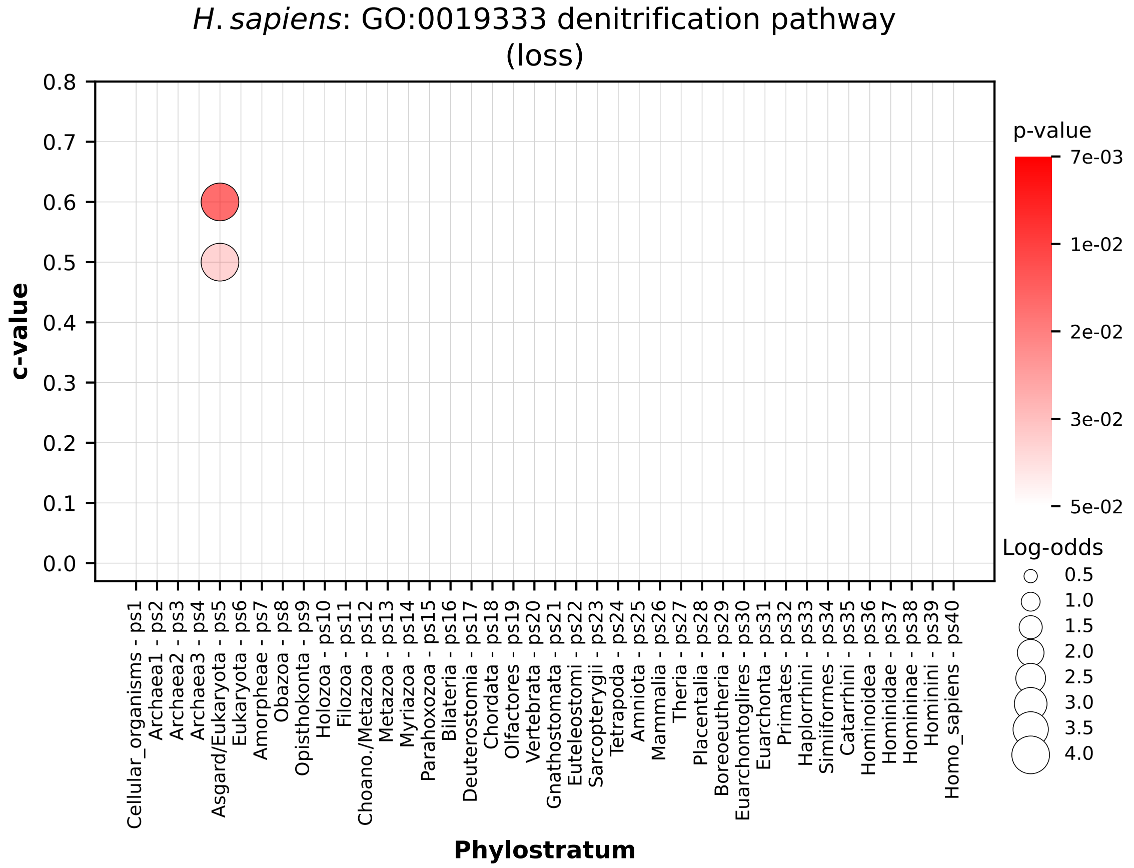

H. sapiens: GO:0019333 denitrification pathway
(loss)
c-value
0.8
0.7
0.6
0.5
0.4
0.3
0.2
0.1
0.0
p-value
7e-03
1e-02
2e-02
3e-02
5e-02
Log-odds
0.5
1.0
1.5
2.0
2.5
3.0
3.5
4.0
Cellular_organisms - ps1
Archaea1 - ps2
Archaea2 - ps3
Archaea3 - ps4
Asgard/Eukaryota - ps5
Eukaryota - ps6
Amorpheae - ps7
Obazoa - ps8
Opisthokonta - ps9
Holozoa - ps10
Filozoa - ps11
Choano./Metazoa - ps12
Metazoa - ps13
Myriazoa - ps14
Parahoxozoa - ps15
Bilateria - ps16
Deuterostomia - ps17
Chordata - ps18
Olfactores - ps19
Vertebrata - ps20
Gnathostomata - ps21
Euteleostomi - ps22
Sarcopterygii - ps23
Tetrapoda - ps24
Amniota - ps25
Mammalia - ps26
Theria - ps27
Placentalia - ps28
Boreoeutheria - ps29
Euarchontoglires - ps30
Euarchonta - ps31
Primates - ps32
Haplorrhini - ps33
Simiiformes - ps34
Catarrhini - ps35
Hominoidea - ps36
Hominidae - ps37
Homininae - ps38
Hominini - ps39
Homo_sapiens - ps40
Phylostratum

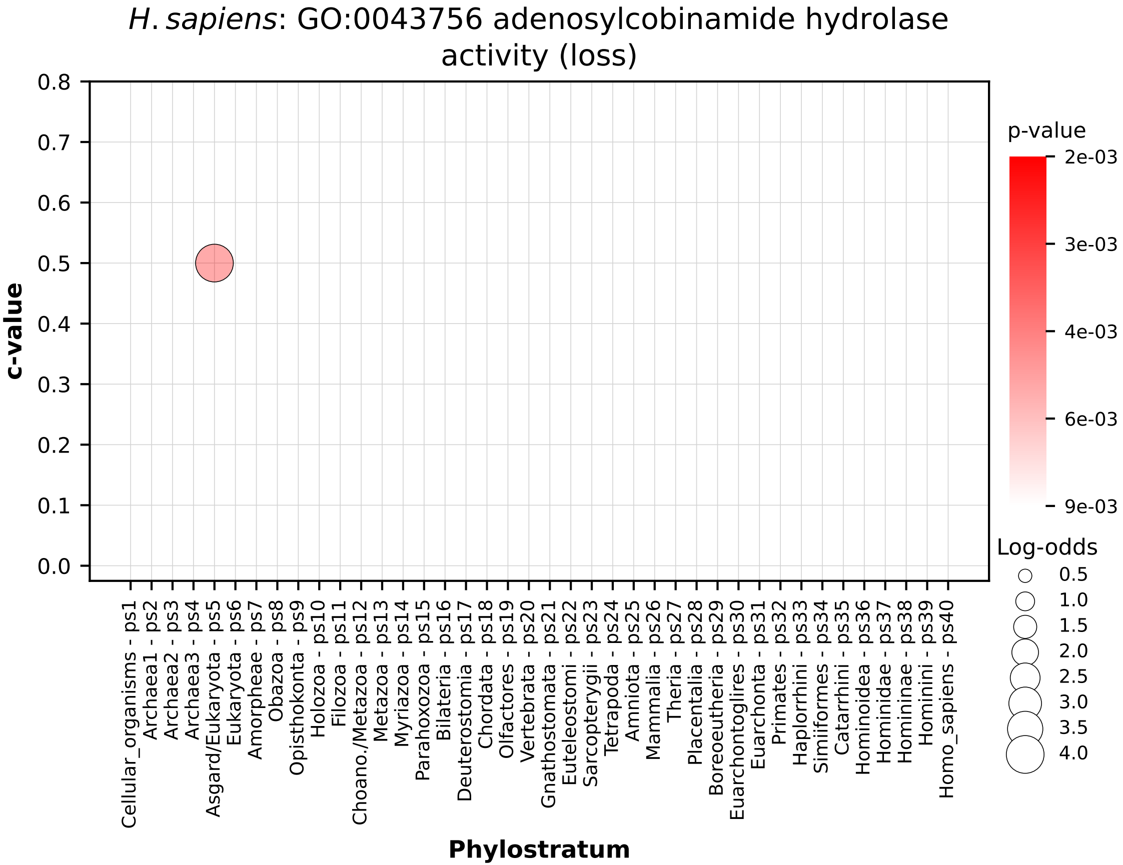

H. sapiens: GO:0043756 adenosylcobinamide hydrolase activity (loss)
c-value
0.0
0.1
0.2
0.3
0.4
0.5
0.6
0.7
0.8
Phylostratum
Cellular_organisms - ps1
Archaea1 - ps2
Archaea2 - ps3
Archaea3 - ps4
Asgard/Eukaryota - ps5
Eukaryota - ps6
Amorpheae - ps7
Obazoa - ps8
Opisthokonta - ps9
Holozoa - ps10
Filozoa - ps11
Choano./Metazoa - ps12
Metazoa - ps13
Myriazoa - ps14
Parahoxozoa - ps15
Bilateria - ps16
Deuterostomia - ps17
Chordata - ps18
Olfactores - ps19
Vertebrata - ps20
Gnathostomata - ps21
Euteleostomi - ps22
Sarcopterygii - ps23
Tetrapoda - ps24
Amniota - ps25
Mammalia - ps26
Theria - ps27
Placentalia - ps28
Boreoeutheria - ps29
Euarchontoglires - ps30
Euarchonta - ps31
Primates - ps32
Haplorrhini - ps33
Simiiformes - ps34
Catarrhini - ps35
Hominoidea - ps36
Hominidae - ps37
Homininae - ps38
Hominini - ps39
Homo_sapiens - ps40
p-value
2e-03
3e-03
4e-03
6e-03
9e-03
Log-odds
0.5
1.0
1.5
2.0
2.5
3.0
3.5
4.0

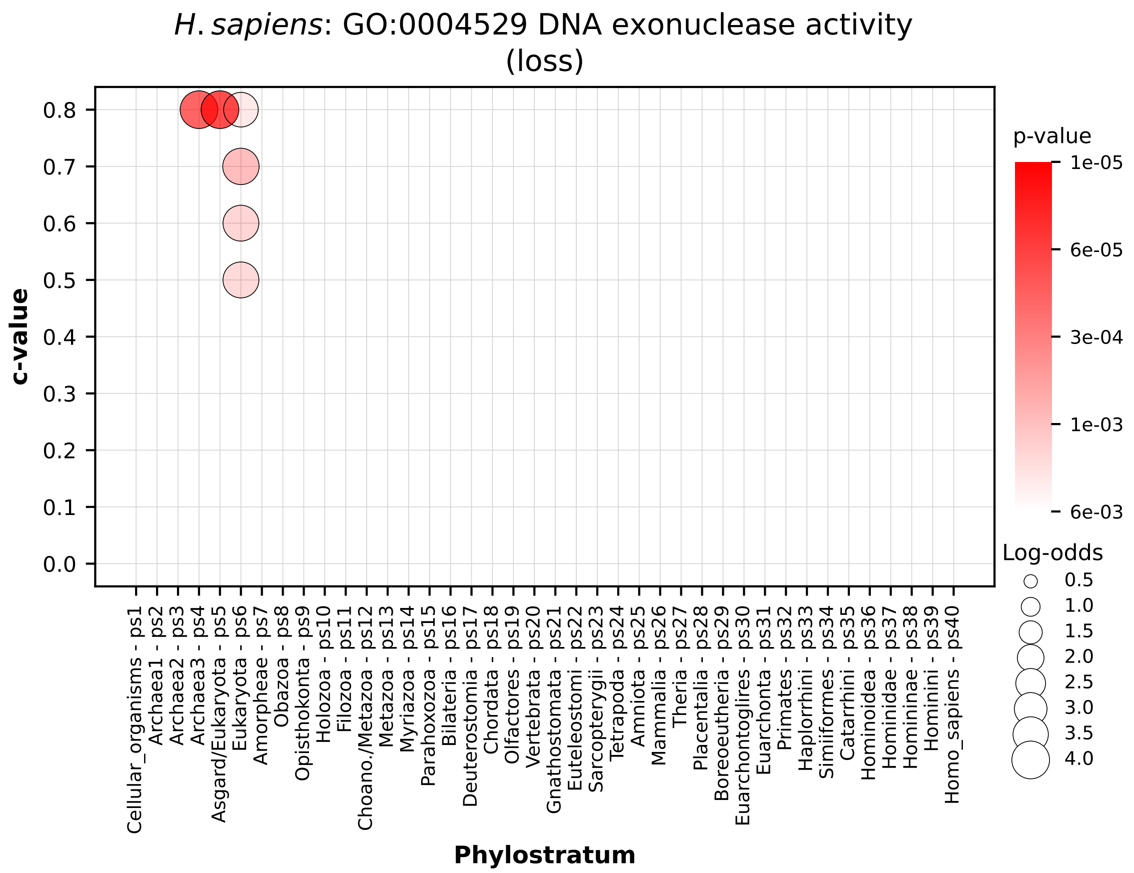

H. sapiens: GO:0004529 DNA exonuclease activity
(loss)
c-value
0.0
0.1
0.2
0.3
0.4
0.5
0.6
0.7
0.8
p-value
1e-05
6e-05
3e-04
1e-03
6e-03
Log-odds
0.5
1.0
1.5
2.0
2.5
3.0
3.5
4.0
Cellular_organisms - ps1
Archaea1 - ps2
Archaea2 - ps3
Archaea3 - ps4
Asgard/Eukaryota - ps5
Eukaryota - ps6
Amorpheae - ps7
Obazoa - ps8
Opisthokonta - ps9
Holozoa - ps10
Filozoa - ps11
Choano./Metazoa - ps12
Metazoa - ps13
Myriazoa - ps14
Parahoxozoa - ps15
Bilateria - ps16
Deuterostomia - ps17
Chordata - ps18
Olfactores - ps19
Vertebrata - ps20
Gnathostomata - ps21
Euteleostomi - ps22
Sarcopterygii - ps23
Tetrapoda - ps24
Amniota - ps25
Mammalia - ps26
Theria - ps27
Placentalia - ps28
Boreoeutheria - ps29
Euarchontoglires - ps30
Euarchonta - ps31
Primates - ps32
Haplorrhini - ps33
Simiiformes - ps34
Catarrhini - ps35
Hominoidea - ps36
Hominidae - ps37
Homininae - ps38
Hominini - ps39
Homo_sapiens - ps40
Phylostratum

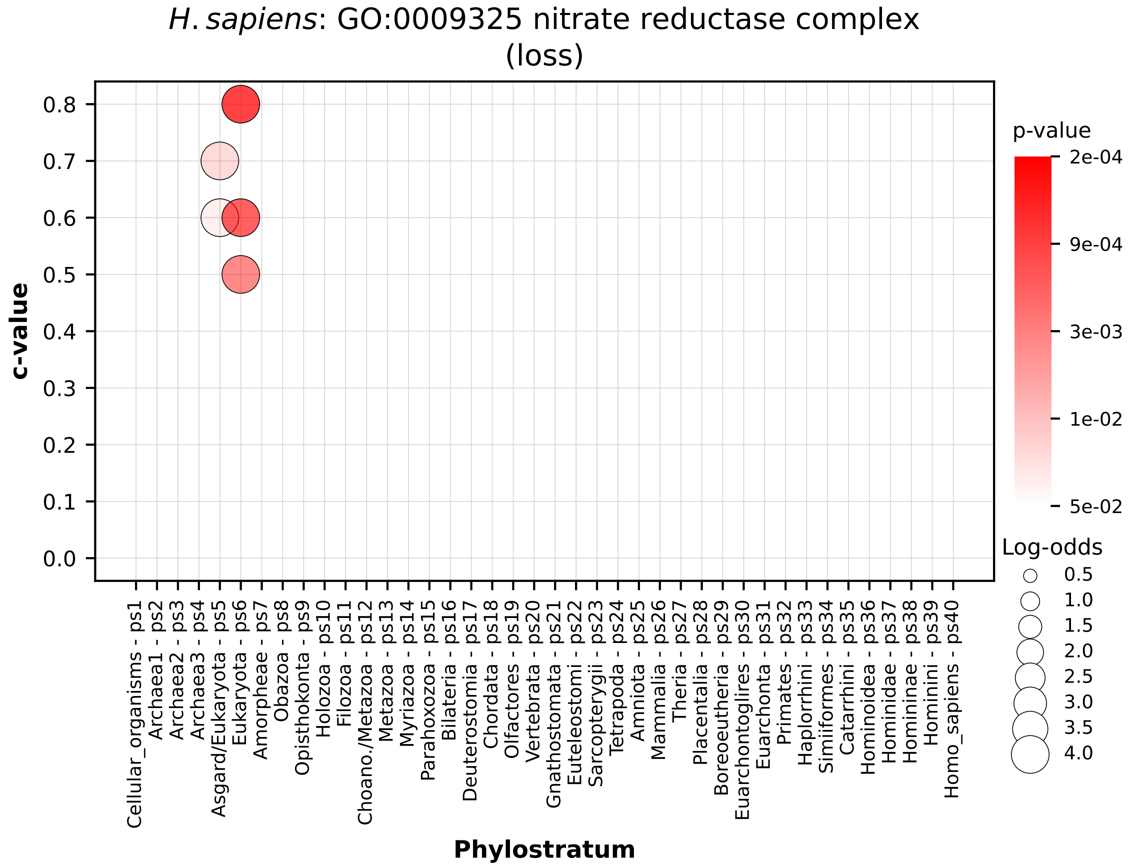

H. sapiens: GO:0009325 nitrate reductase complex
(loss)
c-value
0.8
0.7
0.6
0.5
0.4
0.3
0.2
0.1
0.0
p-value
2e-04
9e-04
3e-03
1e-02
5e-02
Log-odds
0.5
1.0
1.5
2.0
2.5
3.0
3.5
4.0
Cellular_organisms - ps1
Archaea1 - ps2
Archaea2 - ps3
Archaea3 - ps4
Asgard/Eukaryota - ps5
Eukaryota - ps6
Amorpheae - ps7
Obazoa - ps8
Opisthokonta - ps9
Holozoa - ps10
Filozoa - ps11
Choano./Metazoa - ps12
Metazoa - ps13
Myriazoa - ps14
Parahoxozoa - ps15
Bilateria - ps16
Deuterostomia - ps17
Chordata - ps18
Olfactores - ps19
Vertebrata - ps20
Gnathostomata - ps21
Euteleostomi - ps22
Sarcopterygii - ps23
Tetrapoda - ps24
Amniota - ps25
Mammalia - ps26
Theria - ps27
Placentalia - ps28
Boreoeutheria - ps29
Euarchontoglires - ps30
Euarchonta - ps31
Primates - ps32
Haplorrhini - ps33
Simiiformes - ps34
Catarrhini - ps35
Hominoidea - ps36
Hominidae - ps37
Homininae - ps38
Hominini - ps39
Homo_sapiens - ps40
Phylostratum

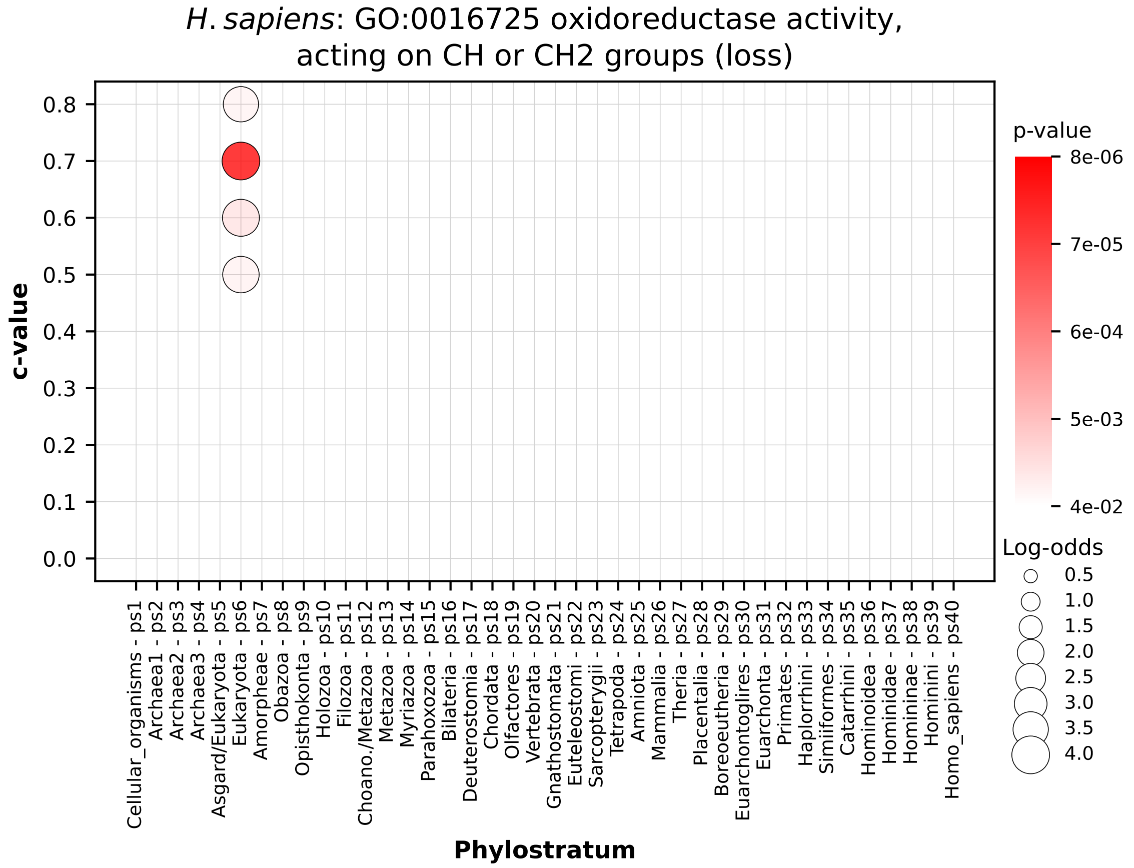
H. sapiens: GO:0016725 oxidoreductase activity, acting on CH or CH2 groups (loss)
c-value
0.0
0.1
0.2
0.3
0.4
0.5
0.6
0.7
0.8
p-value
8e-06
7e-05
6e-04
5e-03
4e-02
Log-odds
0.5
1.0
1.5
2.0
2.5
3.0
3.5
4.0
Cellular_organisms - ps1
Archaea1 - ps2
Archaea2 - ps3
Archaea3 - ps4
Asgard/Eukaryota - ps5
Eukaryota - ps6
Amorpheae - ps7
Obazoa - ps8
Opisthokonta - ps9
Holozoa - ps10
Filozoa - ps11
Choano./Metazoa - ps12
Metazoa - ps13
Myriazoa - ps14
Parahoxozoa - ps15
Bilateria - ps16
Deuterostomia - ps17
Chordata - ps18
Olfactores - ps19
Vertebrata - ps20
Gnathostomata - ps21
Euteleostomi - ps22
Sarcopterygii - ps23
Tetrapoda - ps24
Amniota - ps25
Mammalia - ps26
Theria - ps27
Placentalia - ps28
Boreoeutheria - ps29
Euarchontoglires - ps30
Euarchonta - ps31
Primates - ps32
Haplorrhini - ps33
Simiiformes - ps34
Catarrhini - ps35
Hominoidea - ps36
Hominidae - ps37
Homininae - ps38
Hominini - ps39
Homo_sapiens - ps40
Phylostratum

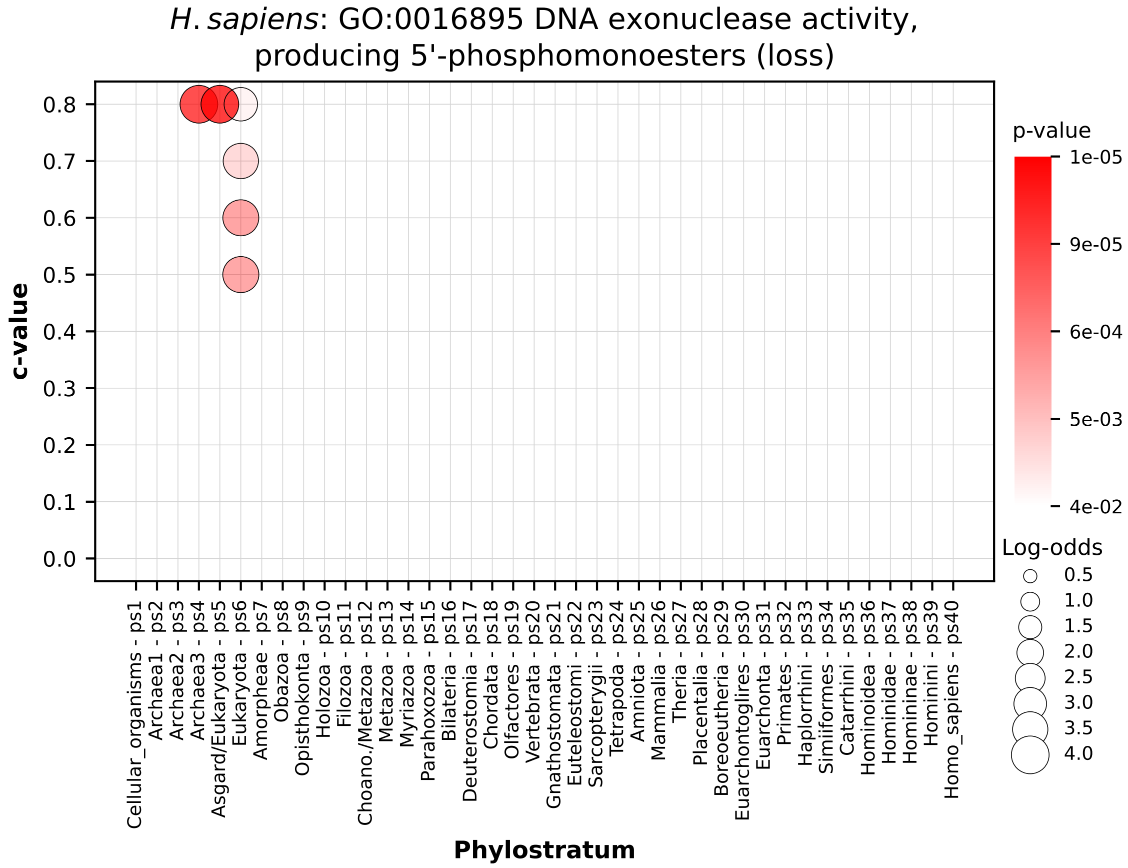

H. sapiens: GO:0016895 DNA exonuclease activity, producing 5'-phosphomonoesters (loss)
c-value
0.8
0.7
0.6
0.5
0.4
0.3
0.2
0.1
0.0
p-value
1e-05
9e-05
6e-04
5e-03
4e-02
Log-odds
0.5
1.0
1.5
2.0
2.5
3.0
3.5
4.0
Cellular_organisms - ps1
Archaea1 - ps2
Archaea2 - ps3
Archaea3 - ps4
Asgard/Eukaryota - ps5
Eukaryota - ps6
Amorpheae - ps7
Obazoa - ps8
Opisthokonta - ps9
Holozoa - ps10
Filozoa - ps11
Choano./Metazoa - ps12
Metazoa - ps13
Myriazoa - ps14
Parahoxozoa - ps15
Bilateria - ps16
Deuterostomia - ps17
Chordata - ps18
Olfactores - ps19
Vertebrata - ps20
Gnathostomata - ps21
Euteleostomi - ps22
Sarcopterygii - ps23
Tetrapoda - ps24
Amniota - ps25
Mammalia - ps26
Theria - ps27
Placentalia - ps28
Boreoeutheria - ps29
Euarchontoglires - ps30
Euarchonta - ps31
Primates - ps32
Haplorrhini - ps33
Simiiformes - ps34
Catarrhini - ps35
Hominoidea - ps36
Hominidae - ps37
Homininae - ps38
Hominini - ps39
Homo_sapiens - ps40
Phylostratum

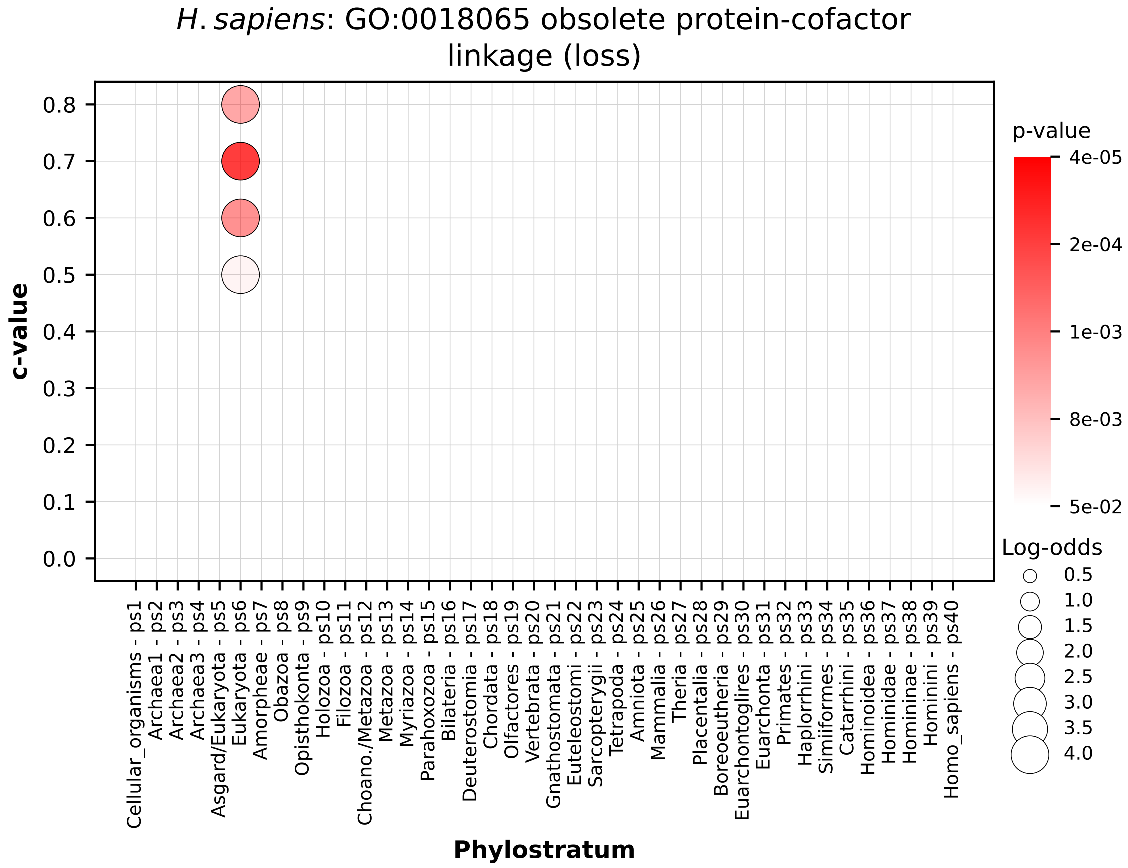

H. sapiens: GO:0018065 obsolete protein-cofactor linkage (loss)
c-value
0.8
0.7
0.6
0.5
0.4
0.3
0.2
0.1
0.0
p-value
4e-05
2e-04
1e-03
8e-03
5e-02
Log-odds
0.5
1.0
1.5
2.0
2.5
3.0
3.5
4.0
Cellular_organisms - ps1
Archaea1 - ps2
Archaea2 - ps3
Archaea3 - ps4
Asgard/Eukaryota - ps5
Eukaryota - ps6
Amorpheae - ps7
Obazoa - ps8
Opisthokonta - ps9
Holozoa - ps10
Filozoa - ps11
Choano./Metazoa - ps12
Metazoa - ps13
Myriazoa - ps14
Parahoxozoa - ps15
Bilateria - ps16
Deuterostomia - ps17
Chordata - ps18
Olfactores - ps19
Vertebrata - ps20
Gnathostomata - ps21
Euteleostomi - ps22
Sarcopterygii - ps23
Tetrapoda - ps24
Amniota - ps25
Mammalia - ps26
Theria - ps27
Placentalia - ps28
Boreoeutheria - ps29
Euarchontoglires - ps30
Euarchonta - ps31
Primates - ps32
Haplorrhini - ps33
Simiiformes - ps34
Catarrhini - ps35
Hominoidea - ps36
Hominidae - ps37
Homininae - ps38
Hominini - ps39
Homo_sapiens - ps40
Phylostratum

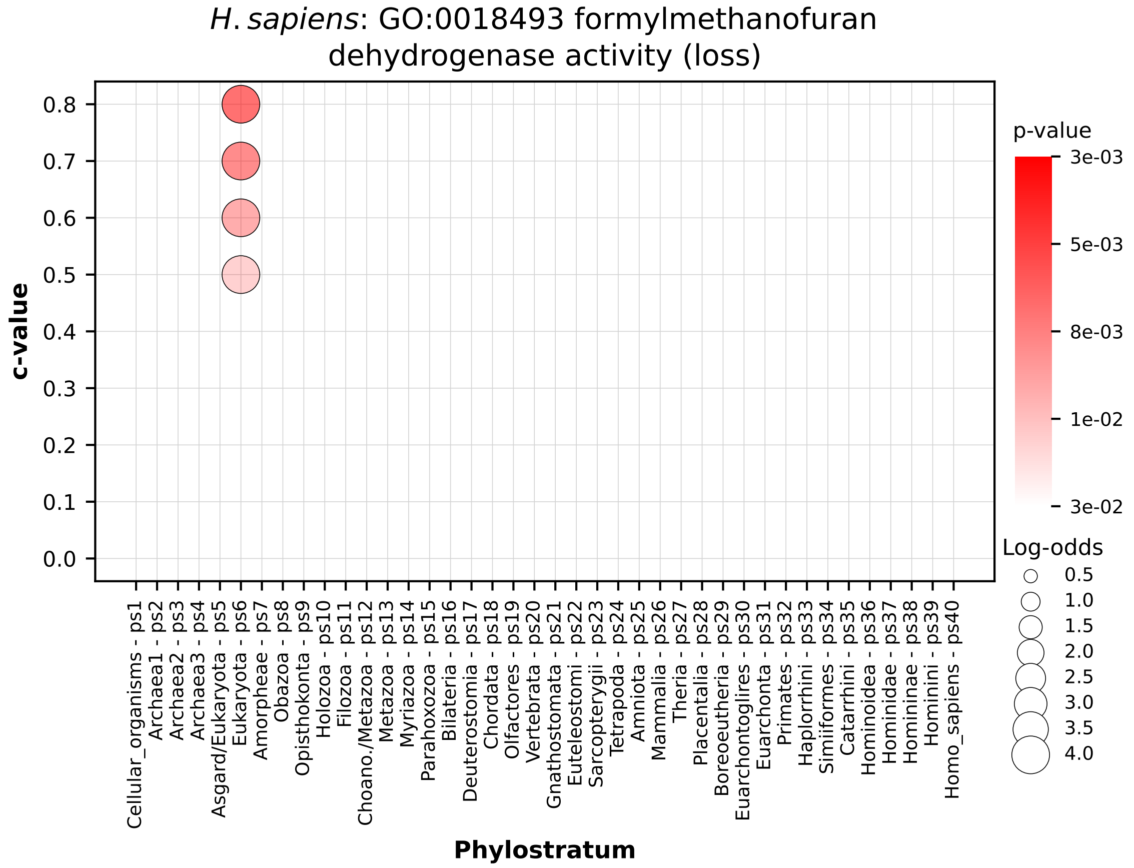

H. sapiens: GO:0018493 formylmethanofuran dehydrogenase activity (loss)
c-value
0.8
0.7
0.6
0.5
0.4
0.3
0.2
0.1
0.0
p-value
3e-03
5e-03
8e-03
1e-02
3e-02
Log-odds
0.5
1.0
1.5
2.0
2.5
3.0
3.5
4.0
Cellular_organisms - ps1
Archaea1 - ps2
Archaea2 - ps3
Archaea3 - ps4
Asgard/Eukaryota - ps5
Eukaryota - ps6
Amorpheae - ps7
Obazoa - ps8
Opisthokonta - ps9
Holozoa - ps10
Filozoa - ps11
Choano./Metazoa - ps12
Metazoa - ps13
Myriazoa - ps14
Parahoxozoa - ps15
Bilateria - ps16
Deuterostomia - ps17
Chordata - ps18
Olfactores - ps19
Vertebrata - ps20
Gnathostomata - ps21
Euteleostomi - ps22
Sarcopterygii - ps23
Tetrapoda - ps24
Amniota - ps25
Mammalia - ps26
Theria - ps27
Placentalia - ps28
Boreoeutheria - ps29
Euarchontoglires - ps30
Euarchonta - ps31
Primates - ps32
Haplorrhini - ps33
Simiiformes - ps34
Catarrhini - ps35
Hominoidea - ps36
Hominidae - ps37
Homininae - ps38
Hominini - ps39
Homo_sapiens - ps40
Phylostratum

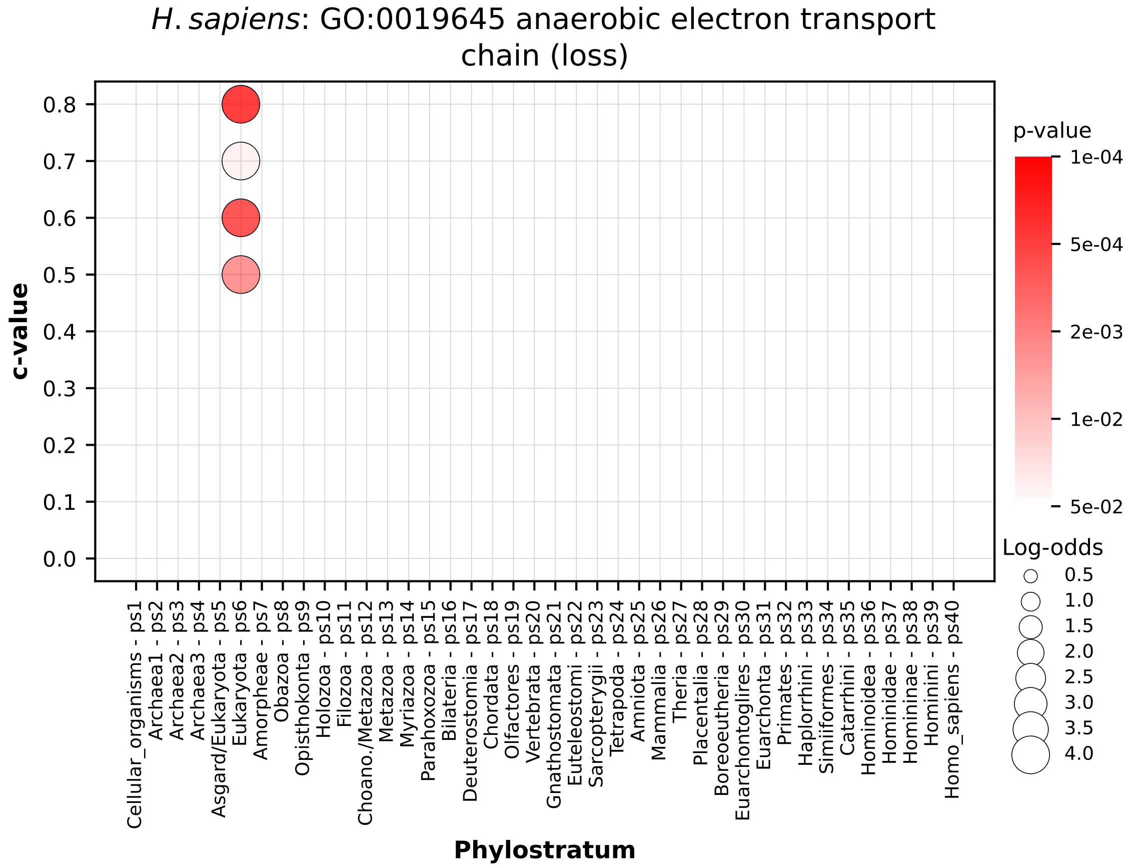
H. sapiens: GO:0019645 anaerobic electron transport chain (loss)
c-value
0.8
0.7
0.6
0.5
0.4
0.3
0.2
0.1
0.0
p-value
1e-04
5e-04
2e-03
1e-02
5e-02
Log-odds
0.5
1.0
1.5
2.0
2.5
3.0
3.5
4.0
Cellular_organisms - ps1
Archaea1 - ps2
Archaea2 - ps3
Archaea3 - ps4
Asgard/Eukaryota - ps5
Eukaryota - ps6
Amorpheae - ps7
Obazoa - ps8
Opisthokonta - ps9
Holozoa - ps10
Filozoa - ps11
Choano./Metazoa - ps12
Metazoa - ps13
Myriazoa - ps14
Parahoxozoa - ps15
Bilateria - ps16
Deuterostomia - ps17
Chordata - ps18
Olfactores - ps19
Vertebrata - ps20
Gnathostomata - ps21
Euteleostomi - ps22
Sarcopterygii - ps23
Tetrapoda - ps24
Amniota - ps25
Mammalia - ps26
Theria - ps27
Placentalia - ps28
Boreoeutheria - ps29
Euarchontoglires - ps30
Euarchonta - ps31
Primates - ps32
Haplorrhini - ps33
Simiiformes - ps34
Catarrhini - ps35
Hominoidea - ps36
Hominidae - ps37
Homininae - ps38
Hominini - ps39
Homo_sapiens - ps40
Phylostratum

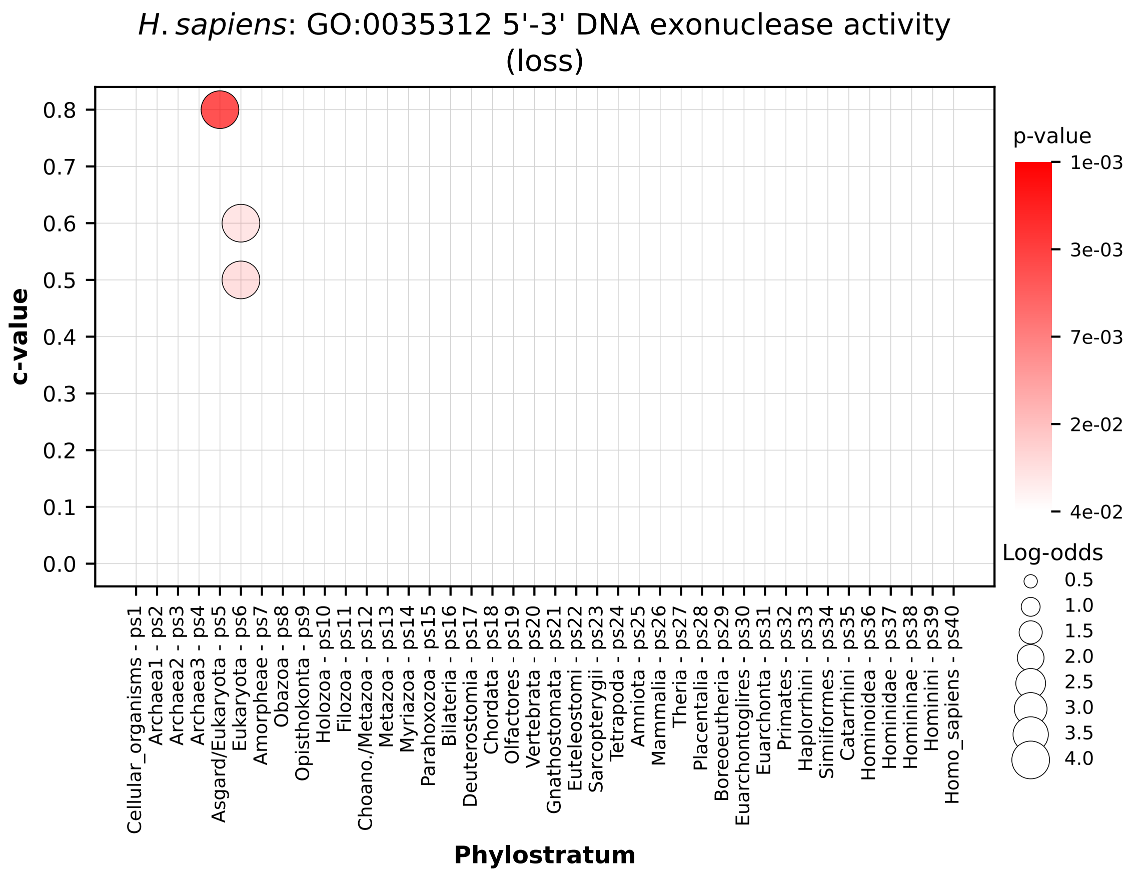

H. sapiens: GO:0035312 5'-3' DNA exonuclease activity
(loss)
c-value
0.8
0.7
0.6
0.5
0.4
0.3
0.2
0.1
0.0
p-value
1e-03
3e-03
7e-03
2e-02
4e-02
Log-odds
0.5
1.0
1.5
2.0
2.5
3.0
3.5
4.0
Cellular_organisms - ps1
Archaea1 - ps2
Archaea2 - ps3
Archaea3 - ps4
Asgard/Eukaryota - ps5
Eukaryota - ps6
Amorpheae - ps7
Obazoa - ps8
Opisthokonta - ps9
Holozoa - ps10
Filozoa - ps11
Choano./Metazoa - ps12
Metazoa - ps13
Myriazoa - ps14
Parahoxozoa - ps15
Bilateria - ps16
Deuterostomia - ps17
Chordata - ps18
Olfactores - ps19
Vertebrata - ps20
Gnathostomata - ps21
Euteleostomi - ps22
Sarcopterygii - ps23
Tetrapoda - ps24
Amniota - ps25
Mammalia - ps26
Theria - ps27
Placentalia - ps28
Boreoeutheria - ps29
Euarchontoglires - ps30
Euarchonta - ps31
Primates - ps32
Haplorrhini - ps33
Simiiformes - ps34
Catarrhini - ps35
Hominoidea - ps36
Hominidae - ps37
Homininae - ps38
Hominini - ps39
Homo_sapiens - ps40
Phylostratum

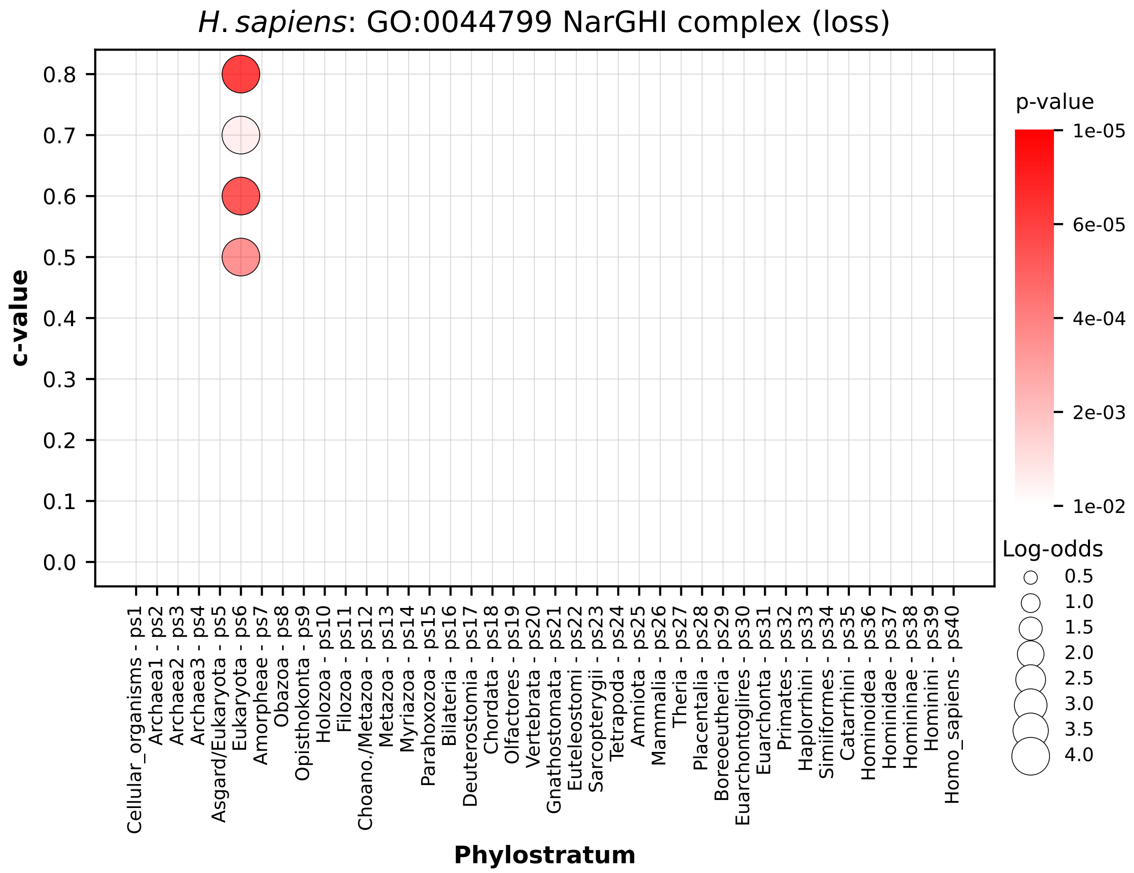
H. sapiens: GO:0044799 NarGHI complex (loss)
c-value
0.0
0.1
0.2
0.3
0.4
0.5
0.6
0.7
0.8
Phylostratum
Cellular_organisms - ps1
Archaea1 - ps2
Archaea2 - ps3
Archaea3 - ps4
Asgard/Eukaryota - ps5
Eukaryota - ps6
Amorpheae - ps7
Obazoa - ps8
Opisthokonta - ps9
Holozoa - ps10
Filozoa - ps11
Choano./Metazoa - ps12
Metazoa - ps13
Myriazoa - ps14
Parahoxozoa - ps15
Bilateria - ps16
Deuterostomia - ps17
Chordata - ps18
Olfactores - ps19
Vertebrata - ps20
Gnathostomata - ps21
Euteleostomi - ps22
Sarcopterygii - ps23
Tetrapoda - ps24
Amniota - ps25
Mammalia - ps26
Theria - ps27
Placentalia - ps28
Boreoeutheria - ps29
Euarchontoglires - ps30
Euarchonta - ps31
Primates - ps32
Haplorrhini - ps33
Simiiformes - ps34
Catarrhini - ps35
Hominoidea - ps36
Hominidae - ps37
Homininae - ps38
Hominini - ps39
Homo_sapiens - ps40
p-value
1e-05
6e-05
4e-04
2e-03
1e-02
Log-odds
0.5
1.0
1.5
2.0
2.5
3.0
3.5
4.0

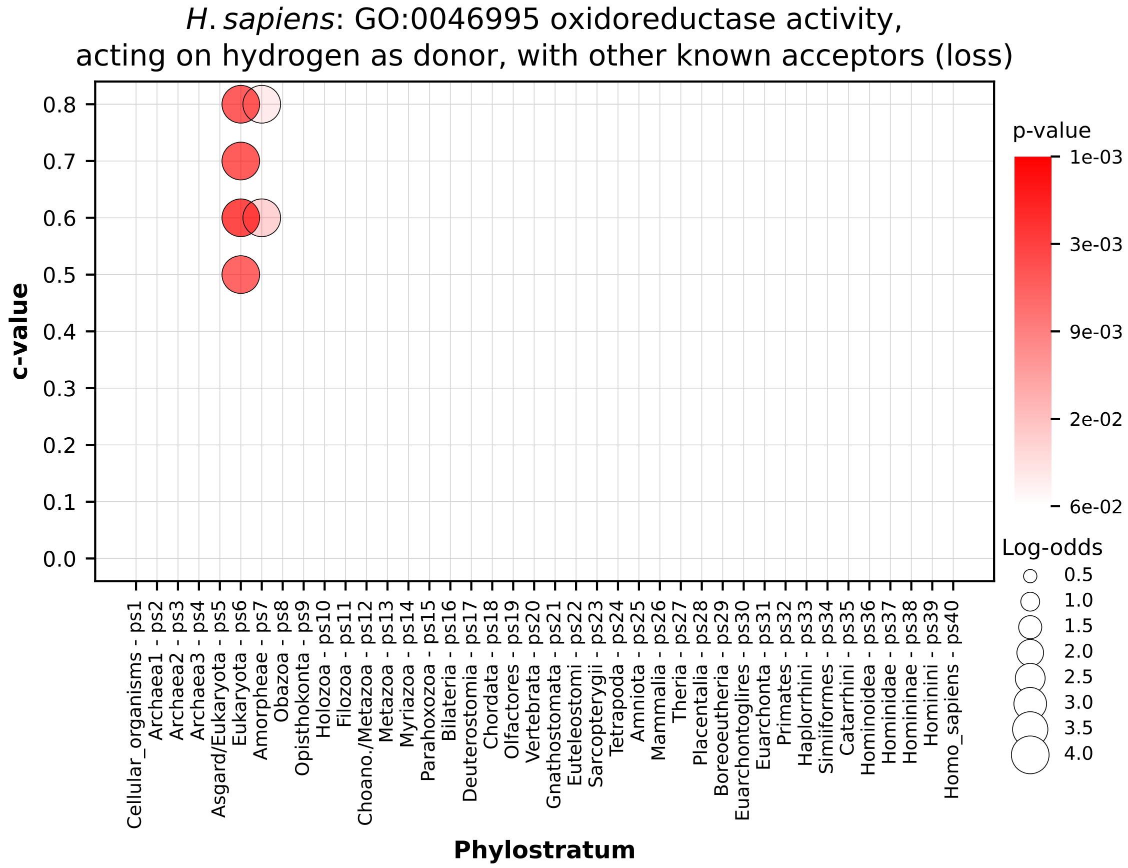

H. sapiens: GO:0046995 oxidoreductase activity,
acting on hydrogen as donor, with other known acceptors (loss)
c-value
0.0
0.1
0.2
0.3
0.4
0.5
0.6
0.7
0.8
p-value
1e-03
3e-03
9e-03
2e-02
6e-02
Log-odds
0.5
1.0
1.5
2.0
2.5
3.0
3.5
4.0
Cellular_organisms - ps1
Archaea1 - ps2
Archaea2 - ps3
Archaea3 - ps4
Asgard/Eukaryota - ps5
Eukaryota - ps6
Amorpheae - ps7
Obazoa - ps8
Opisthokonta - ps9
Holozoa - ps10
Filozoa - ps11
Choano./Metazoa - ps12
Metazoa - ps13
Myriazoa - ps14
Parahoxozoa - ps15
Bilateria - ps16
Deuterostomia - ps17
Chordata - ps18
Olfactores - ps19
Vertebrata - ps20
Gnathostomata - ps21
Euteleostomi - ps22
Sarcopterygii - ps23
Tetrapoda - ps24
Amniota - ps25
Mammalia - ps26
Theria - ps27
Placentalia - ps28
Boreoeutheria - ps29
Euarchontoglires - ps30
Euarchonta - ps31
Primates - ps32
Haplorrhini - ps33
Simiiformes - ps34
Catarrhini - ps35
Hominoidea - ps36
Hominidae - ps37
Homininae - ps38
Hominini - ps39
Homo_sapiens - ps40
Phylostratum

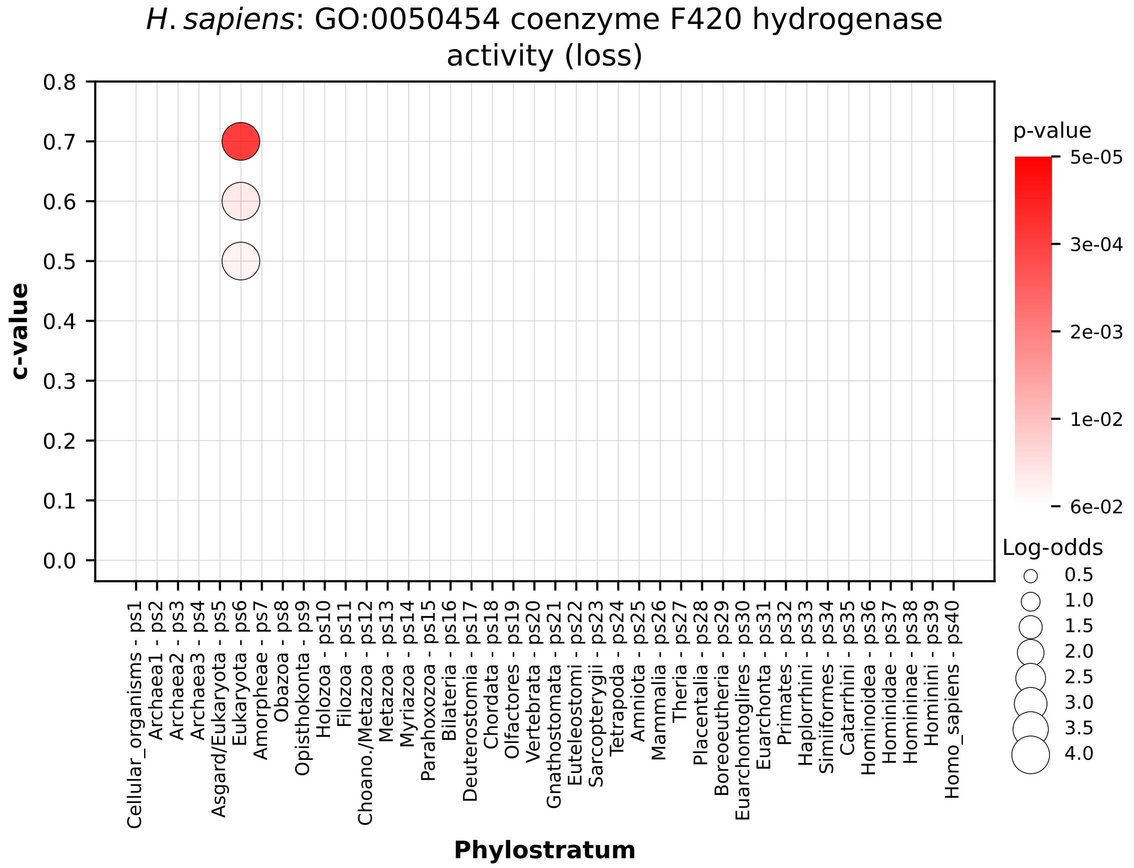

H. sapiens: GO:0050454 coenzyme F420 hydrogenase activity (loss)
c-value
0.8
0.7
0.6
0.5
0.4
0.3
0.2
0.1
0.0
p-value
5e-05
3e-04
2e-03
1e-02
6e-02
Log-odds
0.5
1.0
1.5
2.0
2.5
3.0
3.5
4.0
Cellular_organisms - ps1
Archaea1 - ps2
Archaea2 - ps3
Archaea3 - ps4
Asgard/Eukaryota - ps5
Eukaryota - ps6
Amorpheae - ps7
Obazoa - ps8
Opisthokonta - ps9
Holozoa - ps10
Filozoa - ps11
Choano./Metazoa - ps12
Metazoa - ps13
Myriazoa - ps14
Parahoxozoa - ps15
Bilateria - ps16
Deuterostomia - ps17
Chordata - ps18
Olfactores - ps19
Vertebrata - ps20
Gnathostomata - ps21
Euteleostomi - ps22
Sarcopterygii - ps23
Tetrapoda - ps24
Amniota - ps25
Mammalia - ps26
Theria - ps27
Placentalia - ps28
Boreoeutheria - ps29
Euarchontoglires - ps30
Euarchonta - ps31
Primates - ps32
Haplorrhini - ps33
Simiiformes - ps34
Catarrhini - ps35
Hominoidea - ps36
Hominidae - ps37
Homininae - ps38
Hominini - ps39
Homo_sapiens - ps40
Phylostratum

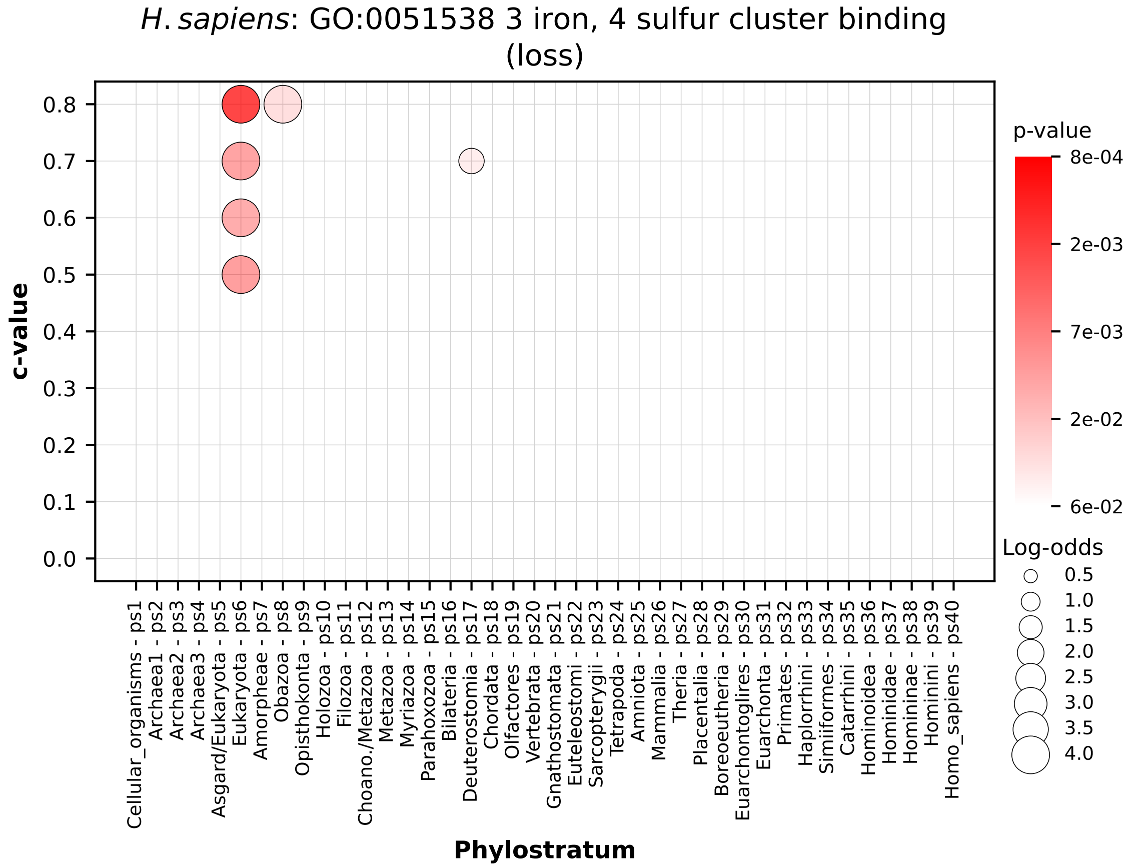

H. sapiens: GO:0051538 3 iron, 4 sulfur cluster binding (loss)
c-value
0.8
0.7
0.6
0.5
0.4
0.3
0.2
0.1
0.0
Phylostratum
Cellular_organisms - ps1
Archaea1 - ps2
Archaea2 - ps3
Archaea3 - ps4
Asgard/Eukaryota - ps5
Eukaryota - ps6
Amorpheae - ps7
Obazoa - ps8
Opisthokonta - ps9
Holozoa - ps10
Filozoa - ps11
Choano./Metazoa - ps12
Metazoa - ps13
Myriazoa - ps14
Parahoxozoa - ps15
Bilateria - ps16
Deuterostomia - ps17
Chordata - ps18
Olfactores - ps19
Vertebrata - ps20
Gnathostomata - ps21
Euteleostomi - ps22
Sarcopterygii - ps23
Tetrapoda - ps24
Amniota - ps25
Mammalia - ps26
Theria - ps27
Placentalia - ps28
Boreoeutheria - ps29
Euarchontoglires - ps30
Euarchonta - ps31
Primates - ps32
Haplorrhini - ps33
Simiiformes - ps34
Catarrhini - ps35
Hominoidea - ps36
Hominidae - ps37
Homininae - ps38
Hominini - ps39
Homo_sapiens - ps40
p-value
8e-04
2e-03
7e-03
2e-02
6e-02
Log-odds
0.5
1.0
1.5
2.0
2.5
3.0
3.5
4.0

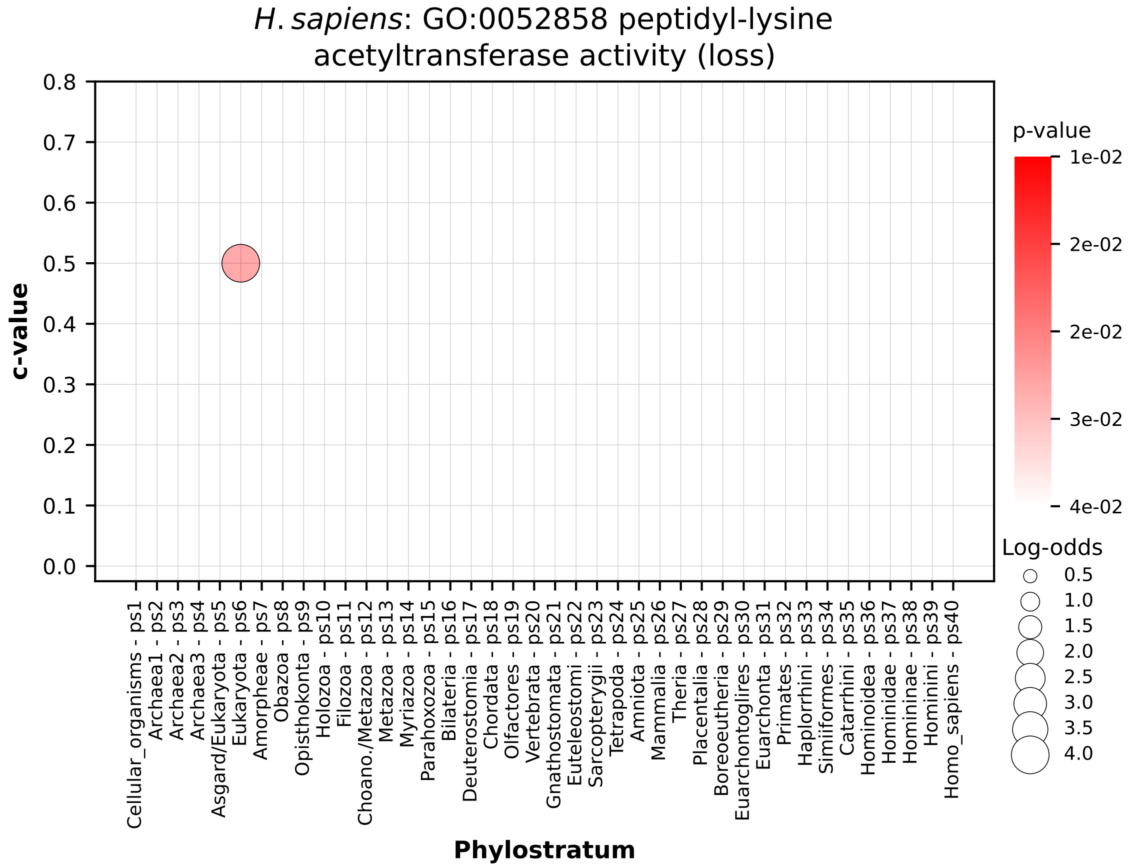

H. sapiens: GO:0052858 peptidyl-lysine acetyltransferase activity (loss)
c-value
0.0
0.1
0.2
0.3
0.4
0.5
0.6
0.7
0.8
Phylostratum
Cellular_organisms - ps1
Archaea1 - ps2
Archaea2 - ps3
Archaea3 - ps4
Asgard/Eukaryota - ps5
Eukaryota - ps6
Amorpheae - ps7
Obazoa - ps8
Opisthokonta - ps9
Holozoa - ps10
Filozoa - ps11
Choano./Metazoa - ps12
Metazoa - ps13
Myriazoa - ps14
Parahoxozoa - ps15
Bilateria - ps16
Deuterostomia - ps17
Chordata - ps18
Olfactores - ps19
Vertebrata - ps20
Gnathostomata - ps21
Euteleostomi - ps22
Sarcopterygii - ps23
Tetrapoda - ps24
Amniota - ps25
Mammalia - ps26
Theria - ps27
Placentalia - ps28
Boreoeutheria - ps29
Euarchontoglires - ps30
Euarchonta - ps31
Primates - ps32
Haplorrhini - ps33
Simiiformes - ps34
Catarrhini - ps35
Hominoidea - ps36
Hominidae - ps37
Homininae - ps38
Hominini - ps39
Homo_sapiens - ps40
p-value
1e-02
2e-02
2e-02
3e-02
4e-02
Log-odds
0.5
1.0
1.5
2.0
2.5
3.0
3.5
4.0

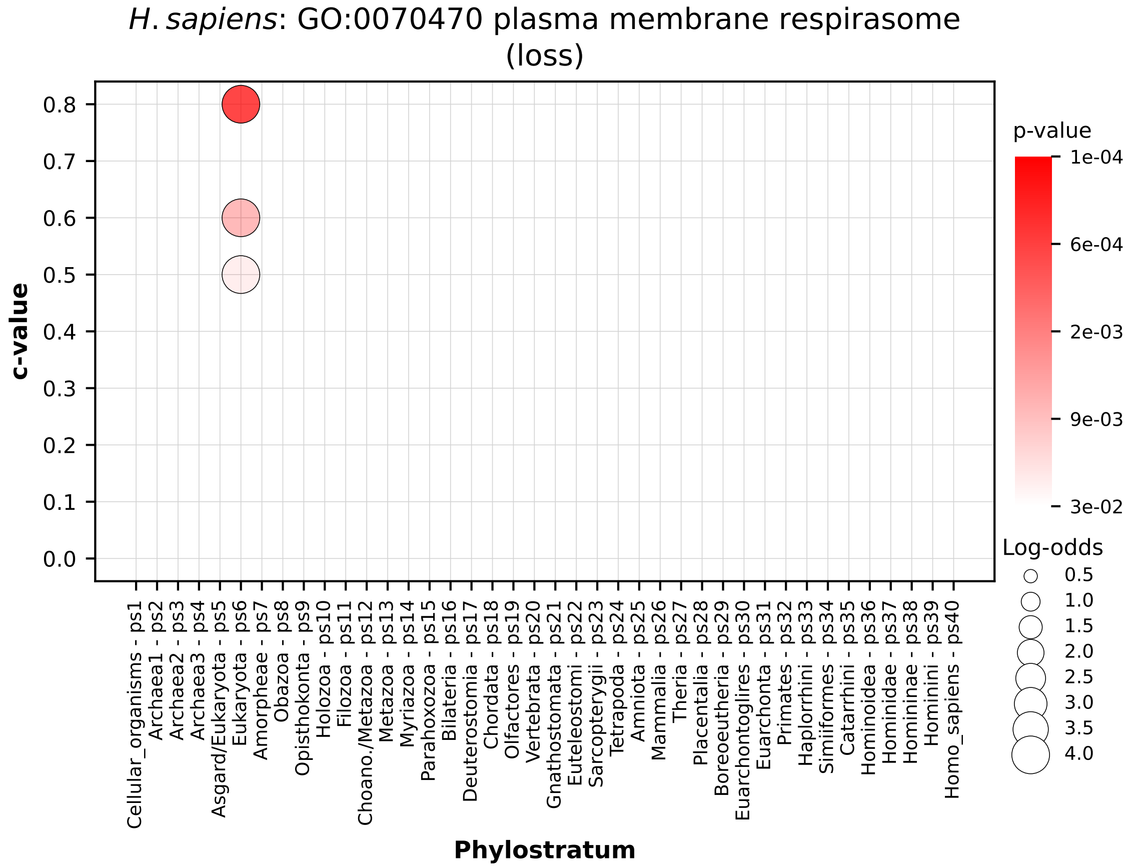

H. sapiens: GO:0070470 plasma membrane respirasome
(loss)
c-value
0.8
0.7
0.6
0.5
0.4
0.3
0.2
0.1
0.0
Phylostratum
Cellular_organisms - ps1
Archaea1 - ps2
Archaea2 - ps3
Archaea3 - ps4
Asgard/Eukaryota - ps5
Eukaryota - ps6
Amorpheae - ps7
Obazoa - ps8
Opisthokonta - ps9
Holozoa - ps10
Filozoa - ps11
Choano./Metazoa - ps12
Metazoa - ps13
Myriazoa - ps14
Parahoxozoa - ps15
Bilateria - ps16
Deuterostomia - ps17
Chordata - ps18
Olfactores - ps19
Vertebrata - ps20
Gnathostomata - ps21
Euteleostomi - ps22
Sarcopterygii - ps23
Tetrapoda - ps24
Amniota - ps25
Mammalia - ps26
Theria - ps27
Placentalia - ps28
Boreoeutheria - ps29
Euarchontoglires - ps30
Euarchonta - ps31
Primates - ps32
Haplorrhini - ps33
Simiiformes - ps34
Catarrhini - ps35
Hominoidea - ps36
Hominidae - ps37
Homininae - ps38
Hominini - ps39
Homo_sapiens - ps40
p-value
1e-04
6e-04
2e-03
9e-03
3e-02
Log-odds
0.5
1.0
1.5
2.0
2.5
3.0
3.5
4.0

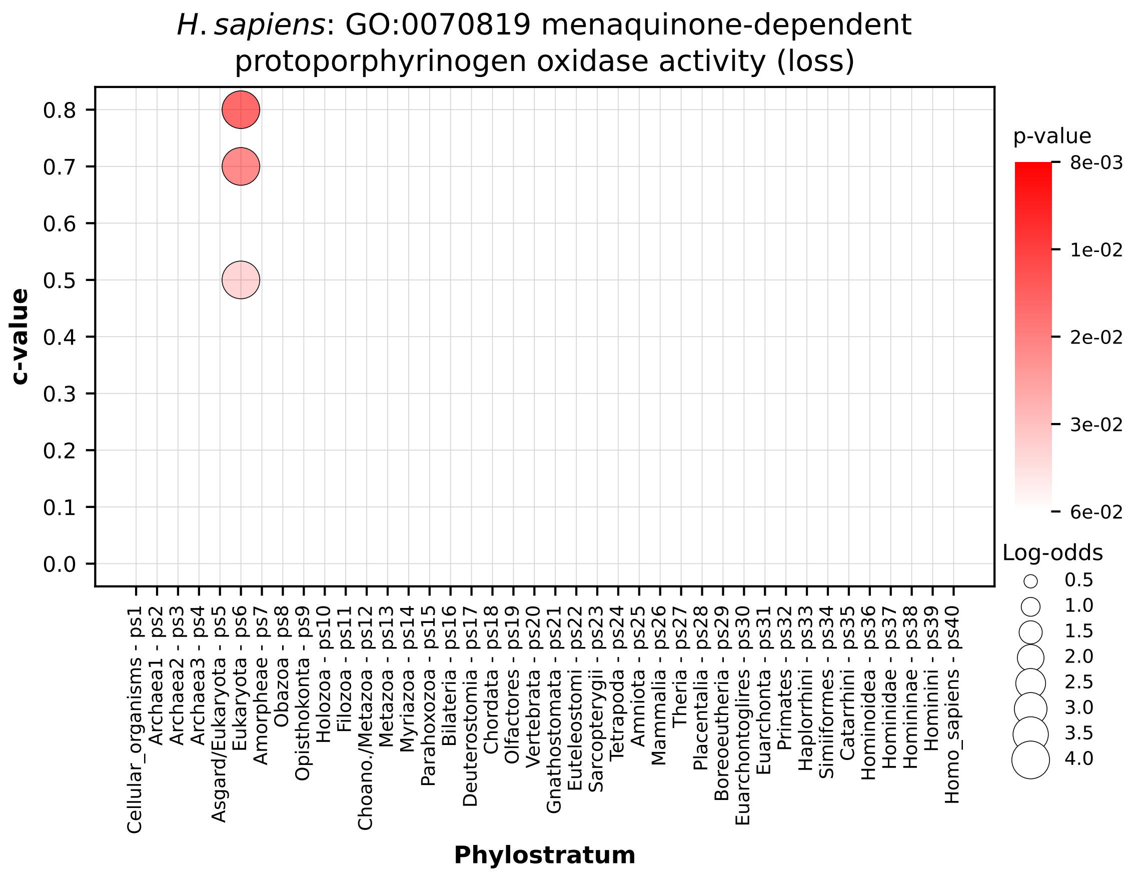

H. sapiens: GO:0070819 menaquinone-dependent protoporphyrinogen oxidase activity (loss)
c-value
0.8
0.7
0.6
0.5
0.4
0.3
0.2
0.1
0.0
Phylostratum
Cellular_organisms - ps1
Archaea1 - ps2
Archaea2 - ps3
Archaea3 - ps4
Asgard/Eukaryota - ps5
Eukaryota - ps6
Amorpheae - ps7
Obazoa - ps8
Opisthokonta - ps9
Holozoa - ps10
Filozoa - ps11
Choano./Metazoa - ps12
Metazoa - ps13
Myriazoa - ps14
Parahoxozoa - ps15
Bilateria - ps16
Deuterostomia - ps17
Chordata - ps18
Olfactores - ps19
Vertebrata - ps20
Gnathostomata - ps21
Euteleostomi - ps22
Sarcopterygii - ps23
Tetrapoda - ps24
Amniota - ps25
Mammalia - ps26
Theria - ps27
Placentalia - ps28
Boreoeutheria - ps29
Euarchontoglires - ps30
Euarchonta - ps31
Primates - ps32
Haplorrhini - ps33
Simiiformes - ps34
Catarrhini - ps35
Hominoidea - ps36
Hominidae - ps37
Homininae - ps38
Hominini - ps39
Homo_sapiens - ps40
p-value
8e-03
1e-02
2e-02
3e-02
6e-02
Log-odds
0.5
1.0
1.5
2.0
2.5
3.0
3.5
4.0

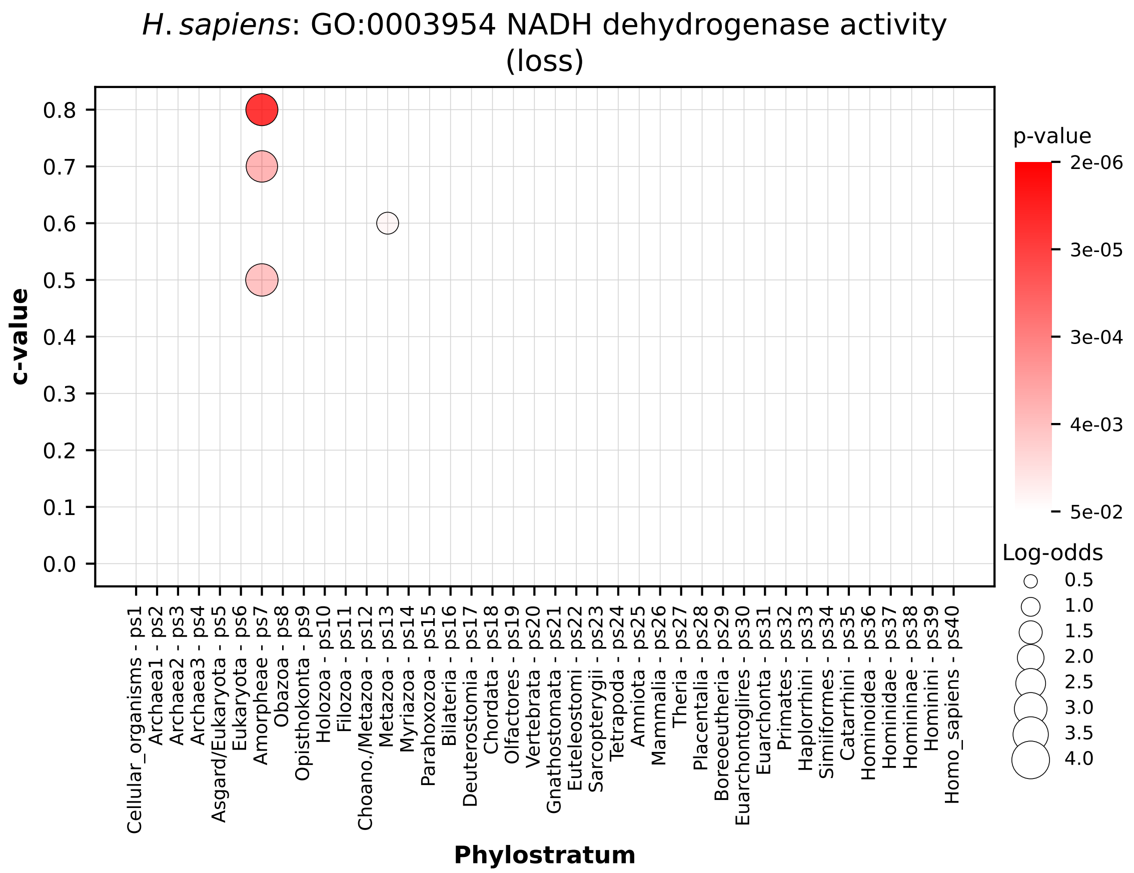

H. sapiens: GO:0003954 NADH dehydrogenase activity
(loss)
c-value
0.8
0.7
0.6
0.5
0.4
0.3
0.2
0.1
0.0
p-value
2e-06
3e-05
3e-04
4e-03
5e-02
Log-odds
0.5
1.0
1.5
2.0
2.5
3.0
3.5
4.0
Cellular_organisms - ps1
Archaea1 - ps2
Archaea2 - ps3
Archaea3 - ps4
Asgard/Eukaryota - ps5
Eukaryota - ps6
Amorpheae - ps7
Obazoa - ps8
Opisthokonta - ps9
Holozoa - ps10
Filozoa - ps11
Choano./Metazoa - ps12
Metazoa - ps13
Myriazoa - ps14
Parahoxozoa - ps15
Bilateria - ps16
Deuterostomia - ps17
Chordata - ps18
Olfactores - ps19
Vertebrata - ps20
Gnathostomata - ps21
Euteleostomi - ps22
Sarcopterygii - ps23
Tetrapoda - ps24
Amniota - ps25
Mammalia - ps26
Theria - ps27
Placentalia - ps28
Boreoeutheria - ps29
Euarchontoglires - ps30
Euarchonta - ps31
Primates - ps32
Haplorrhini - ps33
Simiiformes - ps34
Catarrhini - ps35
Hominoidea - ps36
Hominidae - ps37
Homininae - ps38
Hominini - ps39
Homo_sapiens - ps40
Phylostratum

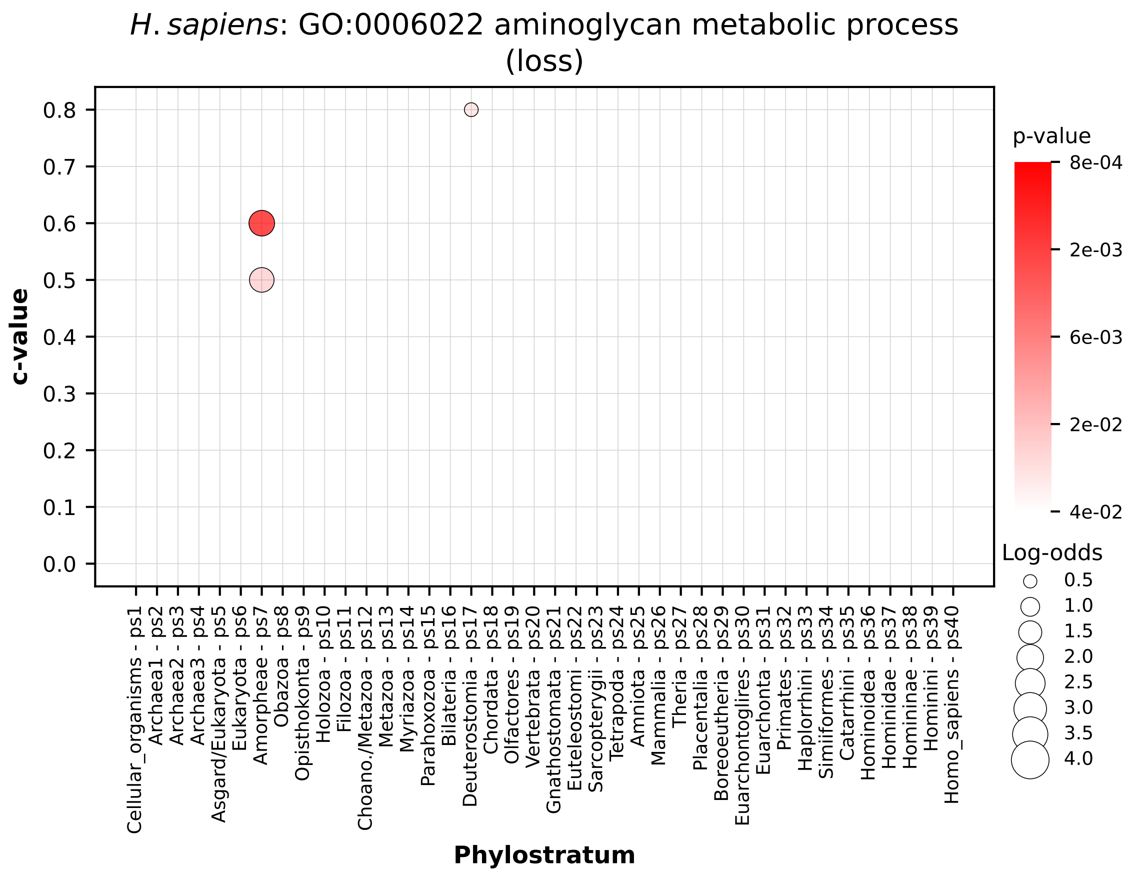

H. sapiens: GO:0006022 aminoglycan metabolic process
(loss)
c-value
0.0
0.1
0.2
0.3
0.4
0.5
0.6
0.7
0.8
Phylostratum
Cellular_organisms - ps1
Archaea1 - ps2
Archaea2 - ps3
Archaea3 - ps4
Asgard/Eukaryota - ps5
Eukaryota - ps6
Amorpheae - ps7
Obazoa - ps8
Opisthokonta - ps9
Holozoa - ps10
Filozoa - ps11
Choano./Metazoa - ps12
Metazoa - ps13
Myriazoa - ps14
Parahoxozoa - ps15
Bilateria - ps16
Deuterostomia - ps17
Chordata - ps18
Olfactores - ps19
Vertebrata - ps20
Gnathostomata - ps21
Euteleostomi - ps22
Sarcopterygii - ps23
Tetrapoda - ps24
Amniota - ps25
Mammalia - ps26
Theria - ps27
Placentalia - ps28
Boreoeutheria - ps29
Euarchontoglires - ps30
Euarchonta - ps31
Primates - ps32
Haplorrhini - ps33
Simiiformes - ps34
Catarrhini - ps35
Hominoidea - ps36
Hominidae - ps37
Homininae - ps38
Hominini - ps39
Homo_sapiens - ps40
p-value
8e-04
2e-03
6e-03
2e-02
4e-02
Log-odds
0.5
1.0
1.5
2.0
2.5
3.0
3.5
4.0

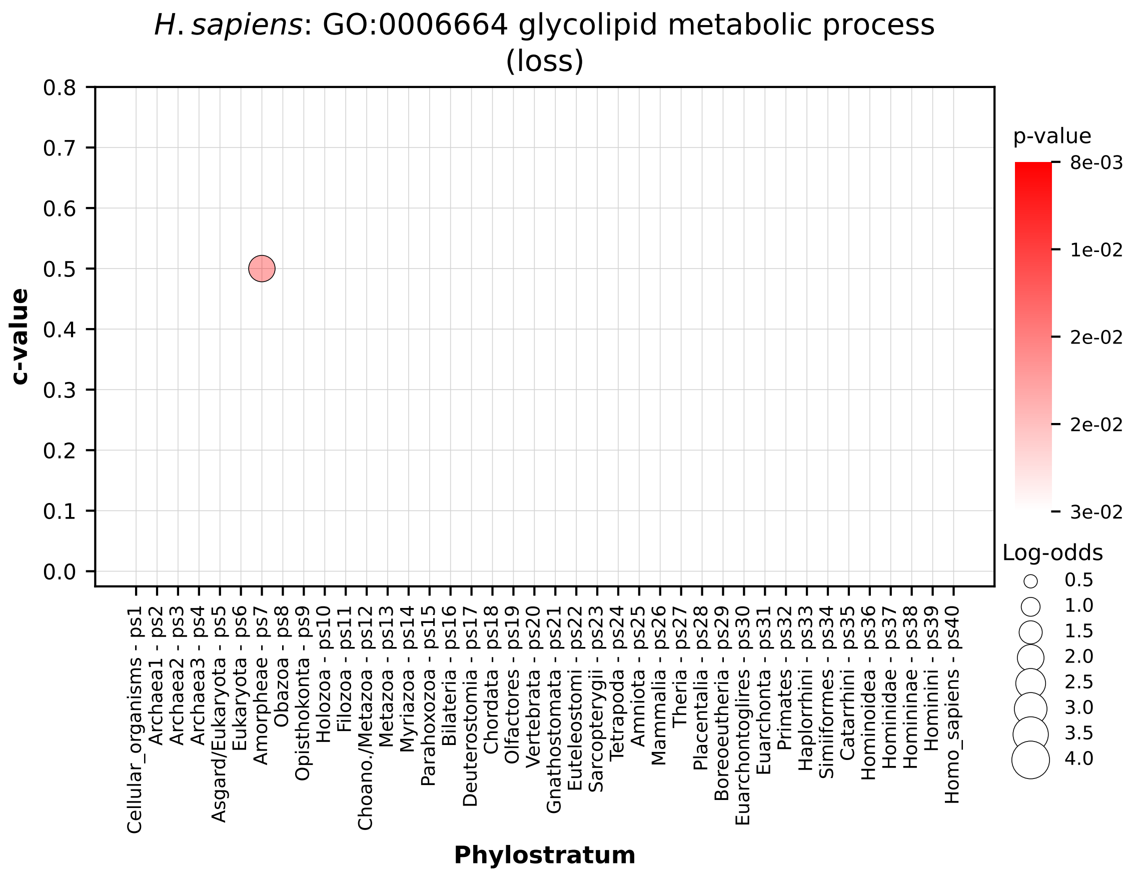

H. sapiens: GO:0006664 glycolipid metabolic process
(loss)
c-value
0.0
0.1
0.2
0.3
0.4
0.5
0.6
0.7
0.8
p-value
8e-03
1e-02
2e-02
2e-02
3e-02
Log-odds
0.5
1.0
1.5
2.0
2.5
3.0
3.5
4.0
Cellular_organisms - ps1
Archaea1 - ps2
Archaea2 - ps3
Archaea3 - ps4
Asgard/Eukaryota - ps5
Eukaryota - ps6
Amorpheae - ps7
Obazoa - ps8
Opisthokonta - ps9
Holozoa - ps10
Filozoa - ps11
Choano./Metazoa - ps12
Metazoa - ps13
Myriazoa - ps14
Parahoxozoa - ps15
Bilateria - ps16
Deuterostomia - ps17
Chordata - ps18
Olfactores - ps19
Vertebrata - ps20
Gnathostomata - ps21
Euteleostomi - ps22
Sarcopterygii - ps23
Tetrapoda - ps24
Amniota - ps25
Mammalia - ps26
Theria - ps27
Placentalia - ps28
Boreoeutheria - ps29
Euarchontoglires - ps30
Euarchonta - ps31
Primates - ps32
Haplorrhini - ps33
Simiiformes - ps34
Catarrhini - ps35
Hominoidea - ps36
Hominidae - ps37
Homininae - ps38
Hominini - ps39
Homo_sapiens - ps40
Phylostratum

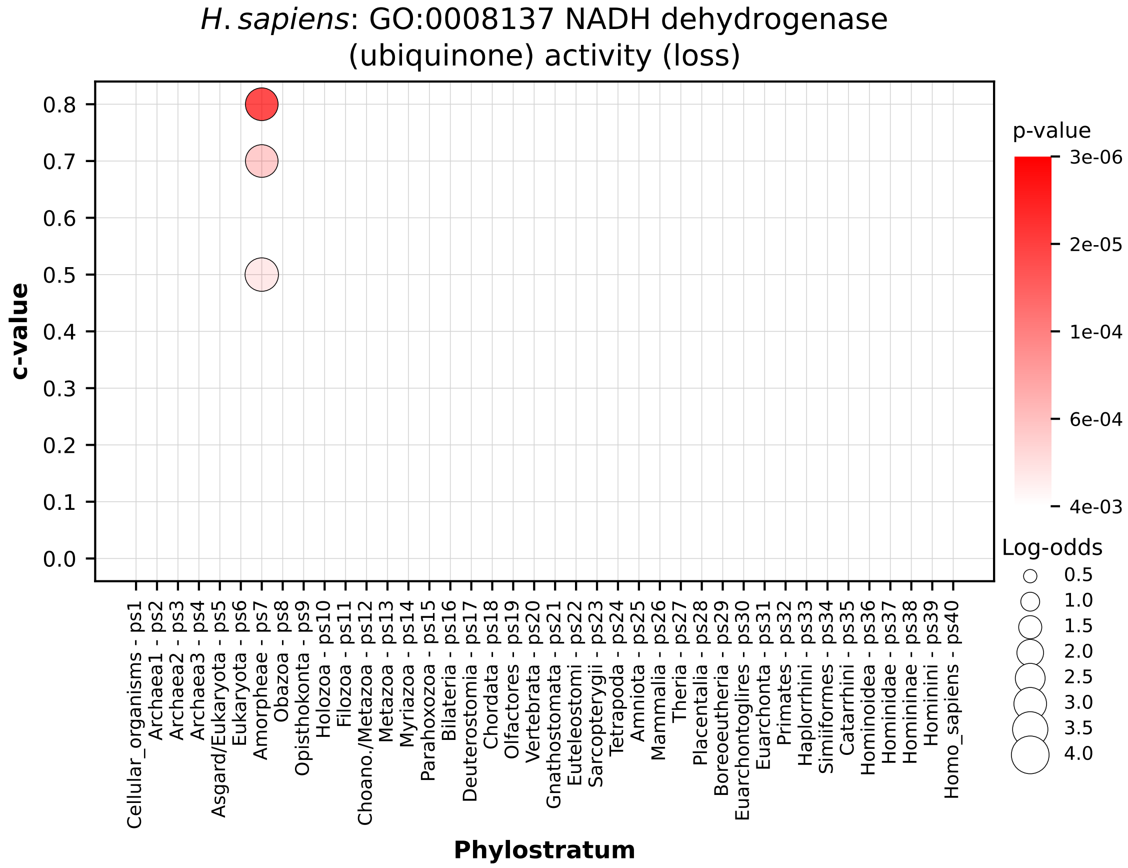

H. sapiens: GO:0008137 NADH dehydrogenase (ubiquinone) activity (loss)
c-value
0.0
0.1
0.2
0.3
0.4
0.5
0.6
0.7
0.8
Phylostratum
Cellular_organisms - ps1
Archaea1 - ps2
Archaea2 - ps3
Archaea3 - ps4
Asgard/Eukaryota - ps5
Eukaryota - ps6
Amorpheae - ps7
Obazoa - ps8
Opisthokonta - ps9
Holozoa - ps10
Filozoa - ps11
Choano./Metazoa - ps12
Metazoa - ps13
Myriazoa - ps14
Parahoxozoa - ps15
Bilateria - ps16
Deuterostomia - ps17
Chordata - ps18
Olfactores - ps19
Vertebrata - ps20
Gnathostomata - ps21
Euteleostomi - ps22
Sarcopterygii - ps23
Tetrapoda - ps24
Amniota - ps25
Mammalia - ps26
Theria - ps27
Placentalia - ps28
Boreoeutheria - ps29
Euarchontoglires - ps30
Euarchonta - ps31
Primates - ps32
Haplorrhini - ps33
Simiiformes - ps34
Catarrhini - ps35
Hominoidea - ps36
Hominidae - ps37
Homininae - ps38
Hominini - ps39
Homo_sapiens - ps40
p-value
3e-06
2e-05
1e-04
6e-04
4e-03
Log-odds
0.5
1.0
1.5
2.0
2.5
3.0
3.5
4.0

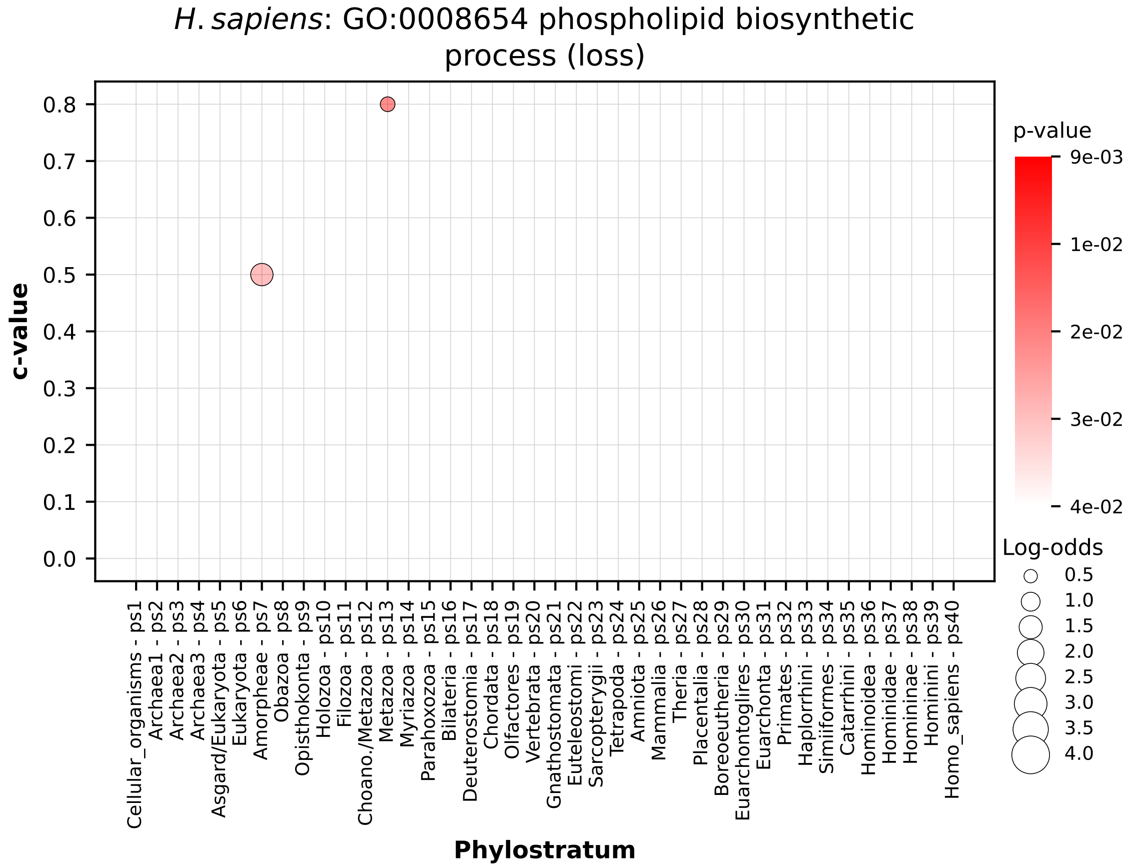
H. sapiens: GO:0008654 phospholipid biosynthetic process (loss)
c-value
0.0
0.1
0.2
0.3
0.4
0.5
0.6
0.7
0.8
p-value
9e-03
1e-02
2e-02
3e-02
4e-02
Log-odds
0.5
1.0
1.5
2.0
2.5
3.0
3.5
4.0
Cellular_organisms - ps1
Archaea1 - ps2
Archaea2 - ps3
Archaea3 - ps4
Asgard/Eukaryota - ps5
Eukaryota - ps6
Amorpheae - ps7
Obazoa - ps8
Opisthokonta - ps9
Holozoa - ps10
Filozoa - ps11
Choano./Metazoa - ps12
Metazoa - ps13
Myriazoa - ps14
Parahoxozoa - ps15
Bilateria - ps16
Deuterostomia - ps17
Chordata - ps18
Olfactores - ps19
Vertebrata - ps20
Gnathostomata - ps21
Euteleostomi - ps22
Sarcopterygii - ps23
Tetrapoda - ps24
Amniota - ps25
Mammalia - ps26
Theria - ps27
Placentalia - ps28
Boreoeutheria - ps29
Euarchontoglires - ps30
Euarchonta - ps31
Primates - ps32
Haplorrhini - ps33
Simiiformes - ps34
Catarrhini - ps35
Hominoidea - ps36
Hominidae - ps37
Homininae - ps38
Hominini - ps39
Homo_sapiens - ps40
Phylostratum

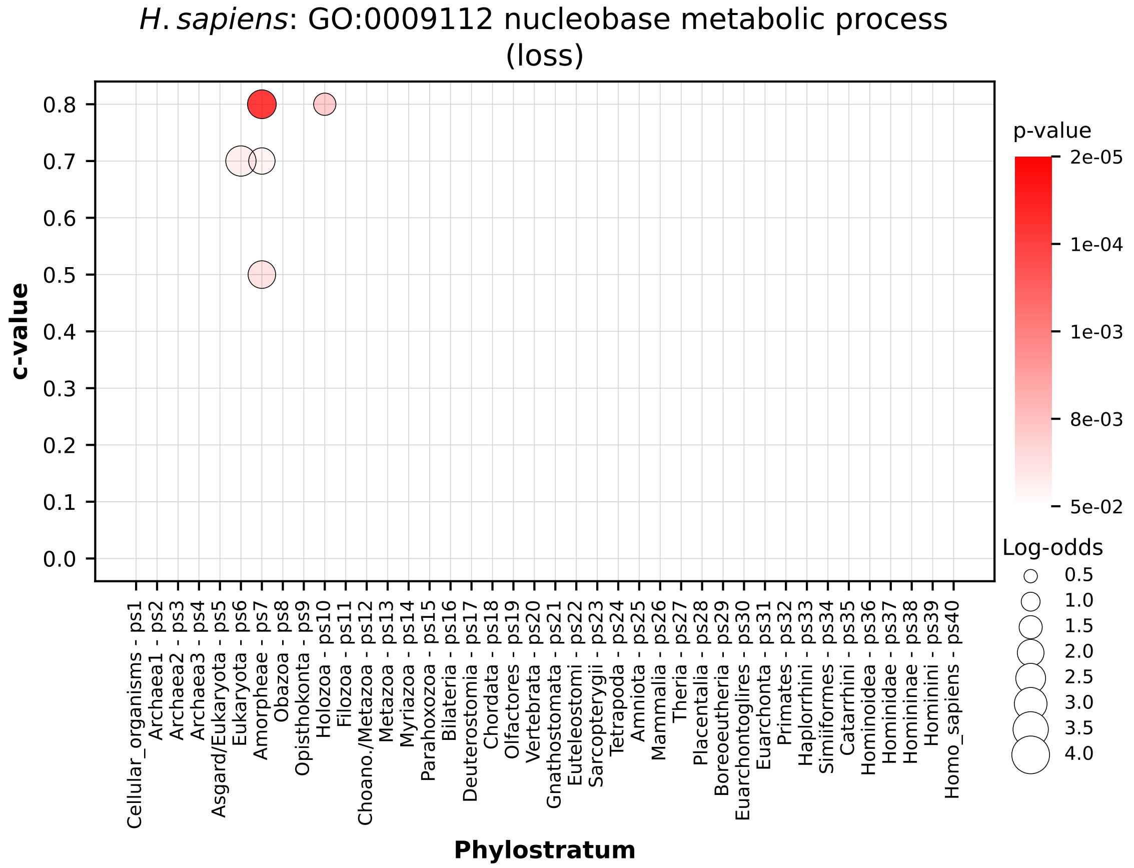

H. sapiens: GO:0009112 nucleobase metabolic process
(loss)
c-value
0.8
0.7
0.6
0.5
0.4
0.3
0.2
0.1
0.0
p-value
2e-05
1e-04
1e-03
8e-03
5e-02
Log-odds
0.5
1.0
1.5
2.0
2.5
3.0
3.5
4.0
Cellular_organisms - ps1
Archaea1 - ps2
Archaea2 - ps3
Archaea3 - ps4
Asgard/Eukaryota - ps5
Eukaryota - ps6
Amorpheae - ps7
Obazoa - ps8
Opisthokonta - ps9
Holozoa - ps10
Filozoa - ps11
Choano./Metazoa - ps12
Metazoa - ps13
Myriazoa - ps14
Parahoxozoa - ps15
Bilateria - ps16
Deuterostomia - ps17
Chordata - ps18
Olfactores - ps19
Vertebrata - ps20
Gnathostomata - ps21
Euteleostomi - ps22
Sarcopterygii - ps23
Tetrapoda - ps24
Amniota - ps25
Mammalia - ps26
Theria - ps27
Placentalia - ps28
Boreoeutheria - ps29
Euarchontoglires - ps30
Euarchonta - ps31
Primates - ps32
Haplorrhini - ps33
Simiiformes - ps34
Catarrhini - ps35
Hominoidea - ps36
Hominidae - ps37
Homininae - ps38
Hominini - ps39
Homo_sapiens - ps40
Phylostratum

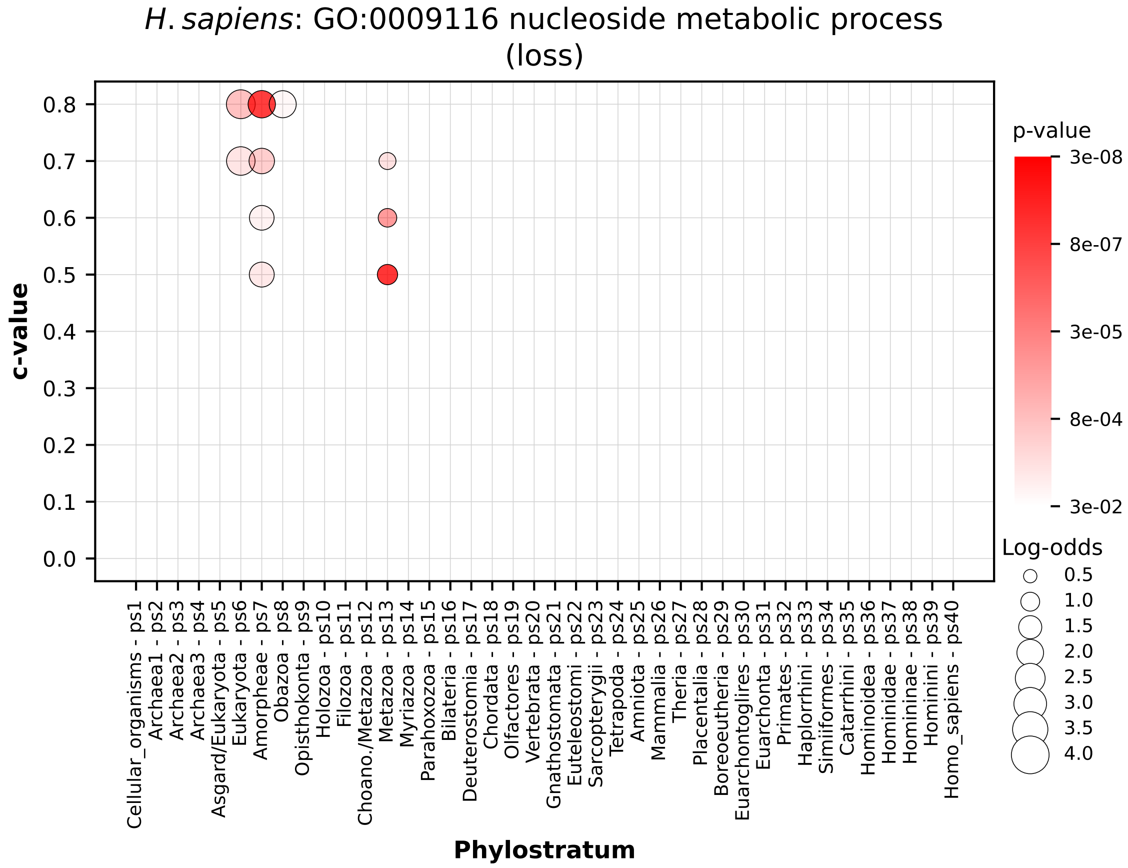
H. sapiens: GO:0009116 nucleoside metabolic process
(loss)
c-value
0.0
0.1
0.2
0.3
0.4
0.5
0.6
0.7
0.8
Phylostratum
Cellular_organisms - ps1
Archaea1 - ps2
Archaea2 - ps3
Archaea3 - ps4
Asgard/Eukaryota - ps5
Eukaryota - ps6
Amorpheae - ps7
Obazoa - ps8
Opisthokonta - ps9
Holozoa - ps10
Filozoa - ps11
Choano./Metazoa - ps12
Metazoa - ps13
Myriazoa - ps14
Parahoxozoa - ps15
Bilateria - ps16
Deuterostomia - ps17
Chordata - ps18
Olfactores - ps19
Vertebrata - ps20
Gnathostomata - ps21
Euteleostomi - ps22
Sarcopterygii - ps23
Tetrapoda - ps24
Amniota - ps25
Mammalia - ps26
Theria - ps27
Placentalia - ps28
Boreoeutheria - ps29
Euarchontoglires - ps30
Euarchonta - ps31
Primates - ps32
Haplorrhini - ps33
Simiiformes - ps34
Catarrhini - ps35
Hominoidea - ps36
Hominidae - ps37
Homininae - ps38
Hominini - ps39
Homo_sapiens - ps40
p-value
3e-08
8e-07
3e-05
8e-04
3e-02
Log-odds
0.5
1.0
1.5
2.0
2.5
3.0
3.5
4.0

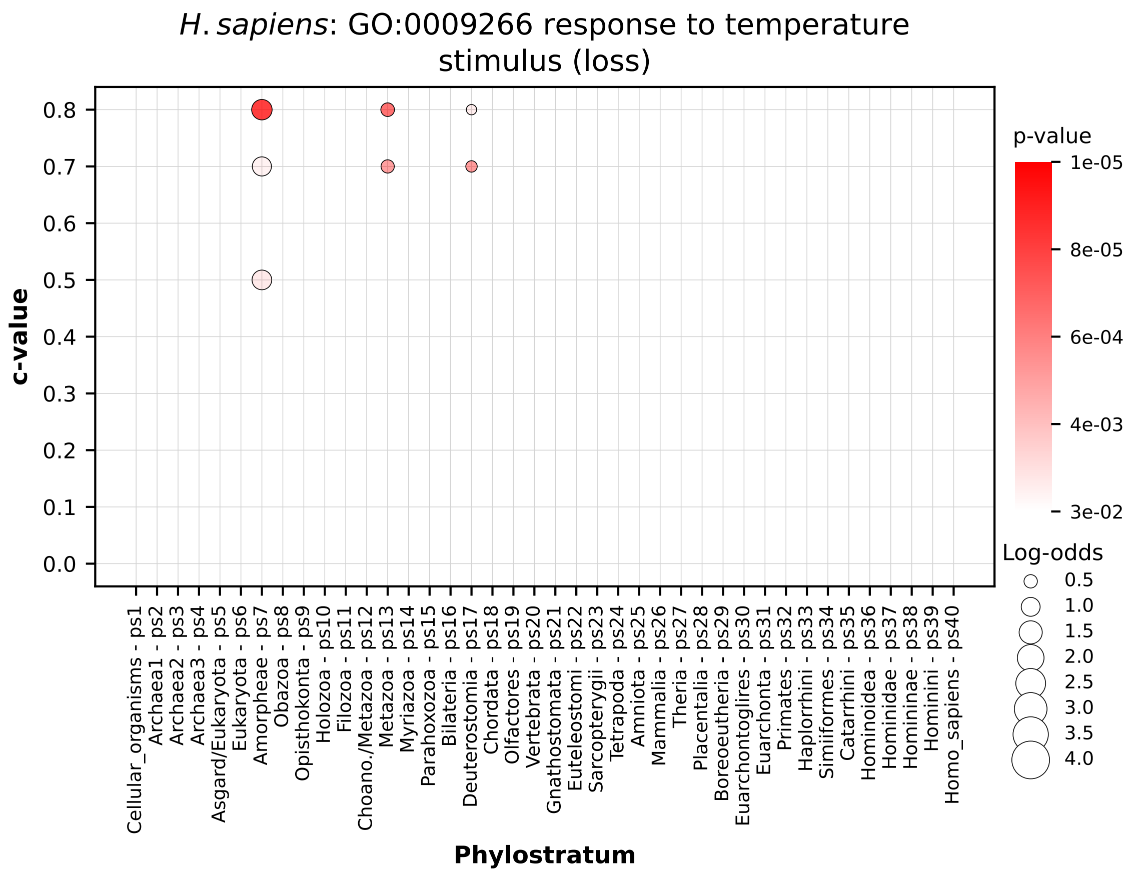

H. sapiens: GO:0009266 response to temperature stimulus (loss)
c-value
0.0
0.1
0.2
0.3
0.4
0.5
0.6
0.7
0.8
Phylostratum
Cellular_organisms - ps1
Archaea1 - ps2
Archaea2 - ps3
Archaea3 - ps4
Asgard/Eukaryota - ps5
Eukaryota - ps6
Amorpheae - ps7
Obazoa - ps8
Opisthokonta - ps9
Holozoa - ps10
Filozoa - ps11
Choano./Metazoa - ps12
Metazoa - ps13
Myriazoa - ps14
Parahoxozoa - ps15
Bilateria - ps16
Deuterostomia - ps17
Chordata - ps18
Olfactores - ps19
Vertebrata - ps20
Gnathostomata - ps21
Euteleostomi - ps22
Sarcopterygii - ps23
Tetrapoda - ps24
Amniota - ps25
Mammalia - ps26
Theria - ps27
Placentalia - ps28
Boreoeutheria - ps29
Euarchontoglires - ps30
Euarchonta - ps31
Primates - ps32
Haplorrhini - ps33
Simiiformes - ps34
Catarrhini - ps35
Hominoidea - ps36
Hominidae - ps37
Homininae - ps38
Hominini - ps39
Homo_sapiens - ps40
p-value
1e-05
8e-05
6e-04
4e-03
3e-02
Log-odds
0.5
1.0
1.5
2.0
2.5
3.0
3.5
4.0

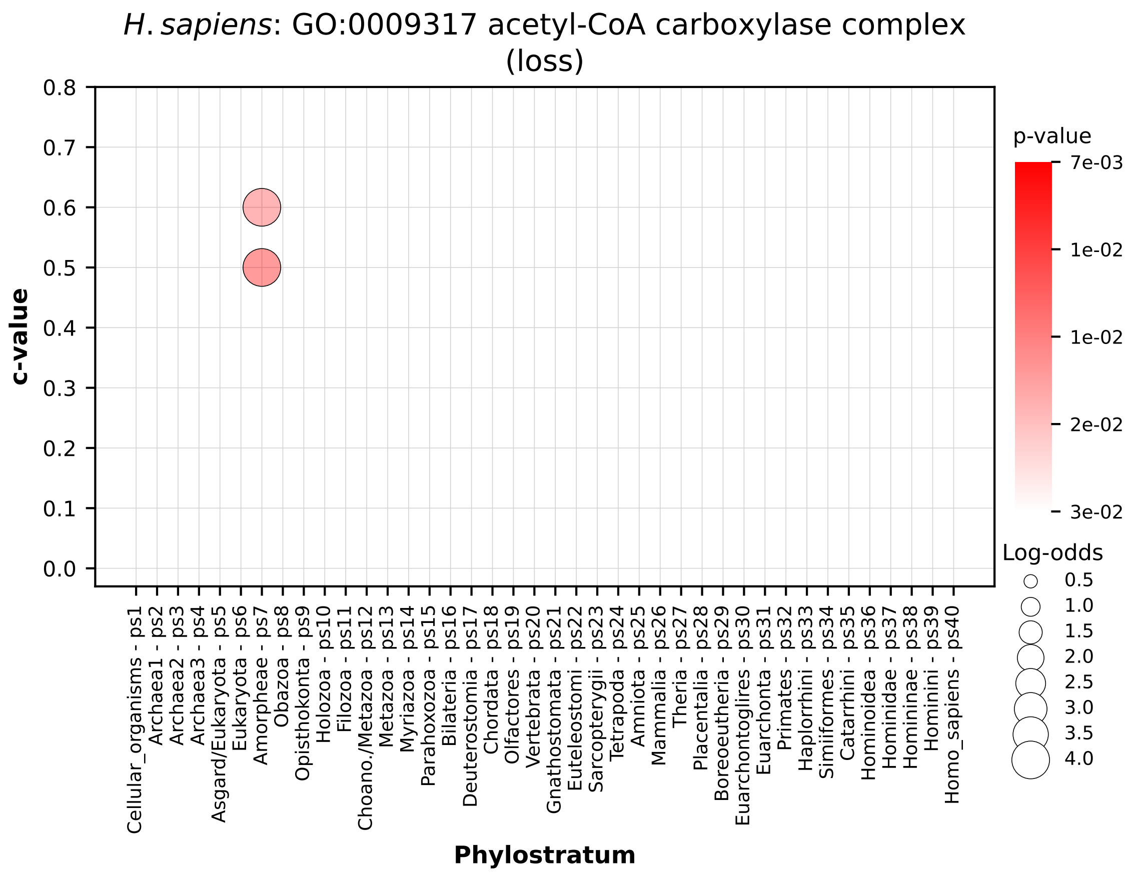
H. sapiens: GO:0009317 acetyl-CoA carboxylase complex (loss)
c-value
Phylostratum
p-value
7e-03
1e-02
1e-02
2e-02
3e-02
Log-odds
0.5
1.0
1.5
2.0
2.5
3.0
3.5
4.0
Cellular_organisms - ps1
Archaea1 - ps2
Archaea2 - ps3
Archaea3 - ps4
Asgard/Eukaryota - ps5
Eukaryota - ps6
Amorpheae - ps7
Obazoa - ps8
Opisthokonta - ps9
Holozoa - ps10
Filozoa - ps11
Choano./Metazoa - ps12
Metazoa - ps13
Myriazoa - ps14
Parahoxozoa - ps15
Bilateria - ps16
Deuterostomia - ps17
Chordata - ps18
Olfactores - ps19
Vertebrata - ps20
Gnathostomata - ps21
Euteleostomi - ps22
Sarcopterygii - ps23
Tetrapoda - ps24
Amniota - ps25
Mammalia - ps26
Theria - ps27
Placentalia - ps28
Boreoeutheria - ps29
Euarchontoglires - ps30
Euarchonta - ps31
Primates - ps32
Haplorrhini - ps33
Simiiformes - ps34
Catarrhini - ps35
Hominoidea - ps36
Hominidae - ps37
Homininae - ps38
Hominini - ps39
Homo_sapiens - ps40

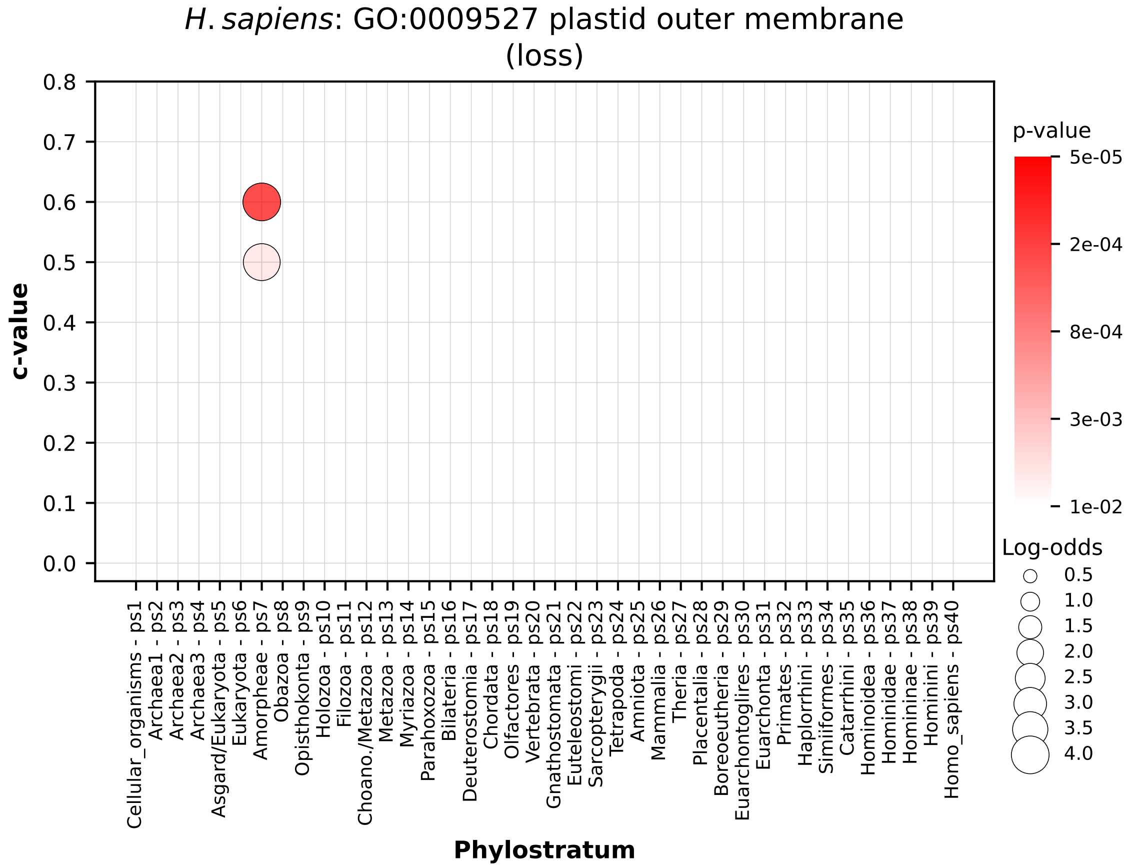

H. sapiens: GO:0009527 plastid outer membrane
(loss)
c-value
0.8
0.7
0.6
0.5
0.4
0.3
0.2
0.1
0.0
p-value
5e-05
2e-04
8e-04
3e-03
1e-02
Log-odds
0.5
1.0
1.5
2.0
2.5
3.0
3.5
4.0
Cellular_organisms - ps1
Archaea1 - ps2
Archaea2 - ps3
Archaea3 - ps4
Asgard/Eukaryota - ps5
Eukaryota - ps6
Amorpheae - ps7
Obazoa - ps8
Opisthokonta - ps9
Holozoa - ps10
Filozoa - ps11
Choano./Metazoa - ps12
Metazoa - ps13
Myriazoa - ps14
Parahoxozoa - ps15
Bilateria - ps16
Deuterostomia - ps17
Chordata - ps18
Olfactores - ps19
Vertebrata - ps20
Gnathostomata - ps21
Euteleostomi - ps22
Sarcopterygii - ps23
Tetrapoda - ps24
Amniota - ps25
Mammalia - ps26
Theria - ps27
Placentalia - ps28
Boreoeutheria - ps29
Euarchontoglires - ps30
Euarchonta - ps31
Primates - ps32
Haplorrhini - ps33
Simiiformes - ps34
Catarrhini - ps35
Hominoidea - ps36
Hominidae - ps37
Homininae - ps38
Hominini - ps39
Homo_sapiens - ps40
Phylostratum

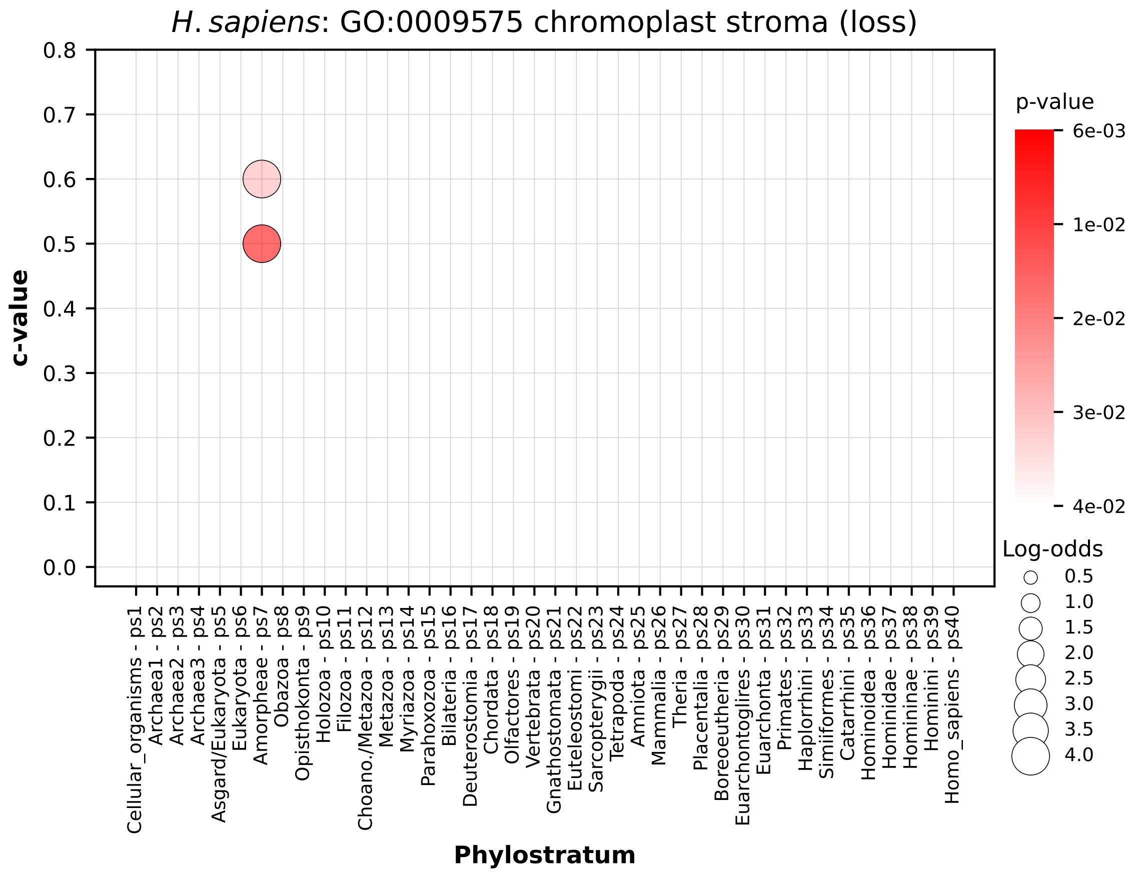

H. sapiens: GO:0009575 chromoplast stroma (loss)
c-value
0.0
0.1
0.2
0.3
0.4
0.5
0.6
0.7
0.8
Phylostratum
Cellular_organisms - ps1
Archaea1 - ps2
Archaea2 - ps3
Archaea3 - ps4
Asgard/Eukaryota - ps5
Eukaryota - ps6
Amorpheae - ps7
Obazoa - ps8
Opisthokonta - ps9
Holozoa - ps10
Filozoa - ps11
Choano./Metazoa - ps12
Metazoa - ps13
Myriazoa - ps14
Parahoxozoa - ps15
Bilateria - ps16
Deuterostomia - ps17
Chordata - ps18
Olfactores - ps19
Vertebrata - ps20
Gnathostomata - ps21
Euteleostomi - ps22
Sarcopterygii - ps23
Tetrapoda - ps24
Amniota - ps25
Mammalia - ps26
Theria - ps27
Placentalia - ps28
Boreoeutheria - ps29
Euarchontoglires - ps30
Euarchonta - ps31
Primates - ps32
Haplorrhini - ps33
Simiiformes - ps34
Catarrhini - ps35
Hominoidea - ps36
Hominidae - ps37
Homininae - ps38
Hominini - ps39
Homo_sapiens - ps40
p-value
6e-03
1e-02
2e-02
3e-02
4e-02
Log-odds
0.5
1.0
1.5
2.0
2.5
3.0
3.5
4.0

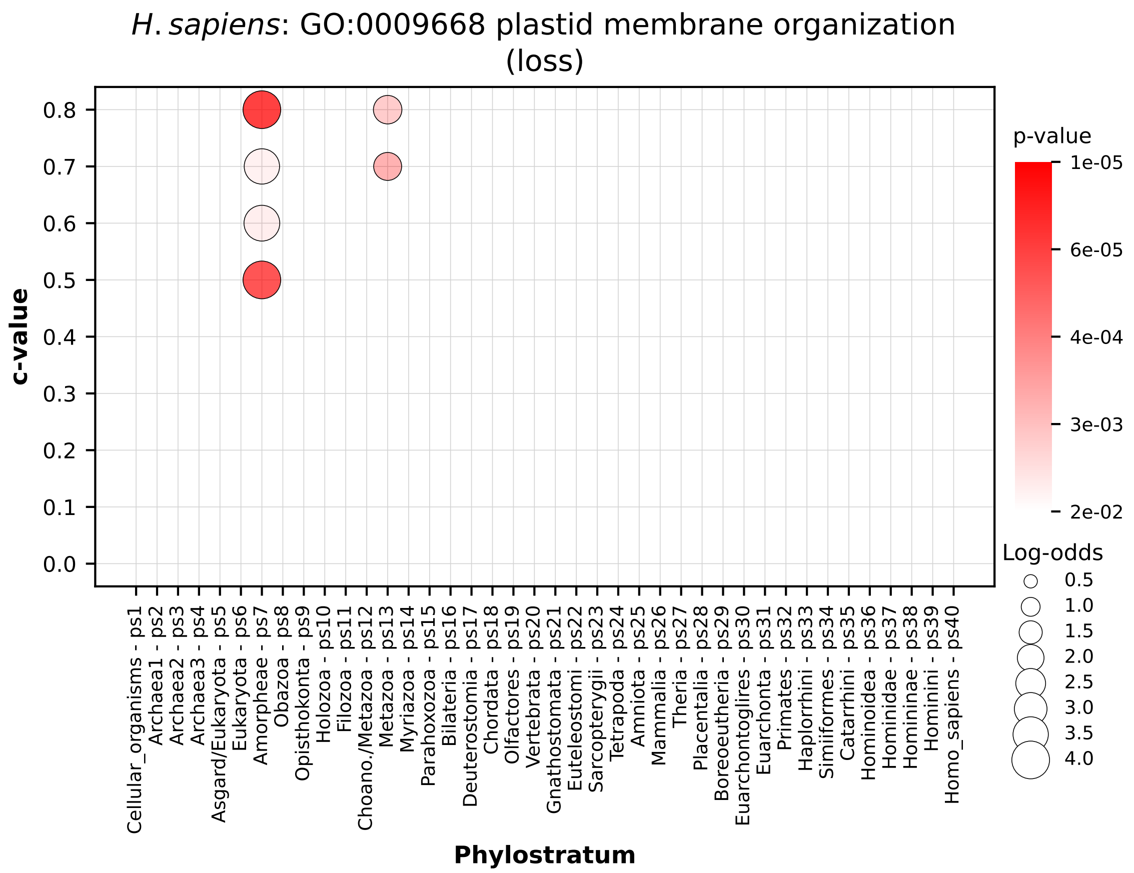
H. sapiens: GO:0009668 plastid membrane organization (loss)
c-value
0.0
0.1
0.2
0.3
0.4
0.5
0.6
0.7
0.8
Phylostratum
Cellular_organisms - ps1
Archaea1 - ps2
Archaea2 - ps3
Archaea3 - ps4
Asgard/Eukaryota - ps5
Eukaryota - ps6
Amorpheae - ps7
Obazoa - ps8
Opisthokonta - ps9
Holozoa - ps10
Filozoa - ps11
Choano./Metazoa - ps12
Metazoa - ps13
Myriazoa - ps14
Parahoxozoa - ps15
Bilateria - ps16
Deuterostomia - ps17
Chordata - ps18
Olfactores - ps19
Vertebrata - ps20
Gnathostomata - ps21
Euteleostomi - ps22
Sarcopterygii - ps23
Tetrapoda - ps24
Amniota - ps25
Mammalia - ps26
Theria - ps27
Placentalia - ps28
Boreoeutheria - ps29
Euarchontoglires - ps30
Euarchonta - ps31
Primates - ps32
Haplorrhini - ps33
Simiiformes - ps34
Catarrhini - ps35
Hominoidea - ps36
Hominidae - ps37
Homininae - ps38
Hominini - ps39
Homo_sapiens - ps40
p-value
1e-05
6e-05
4e-04
3e-03
2e-02
Log-odds
0.5
1.0
1.5
2.0
2.5
3.0
3.5
4.0

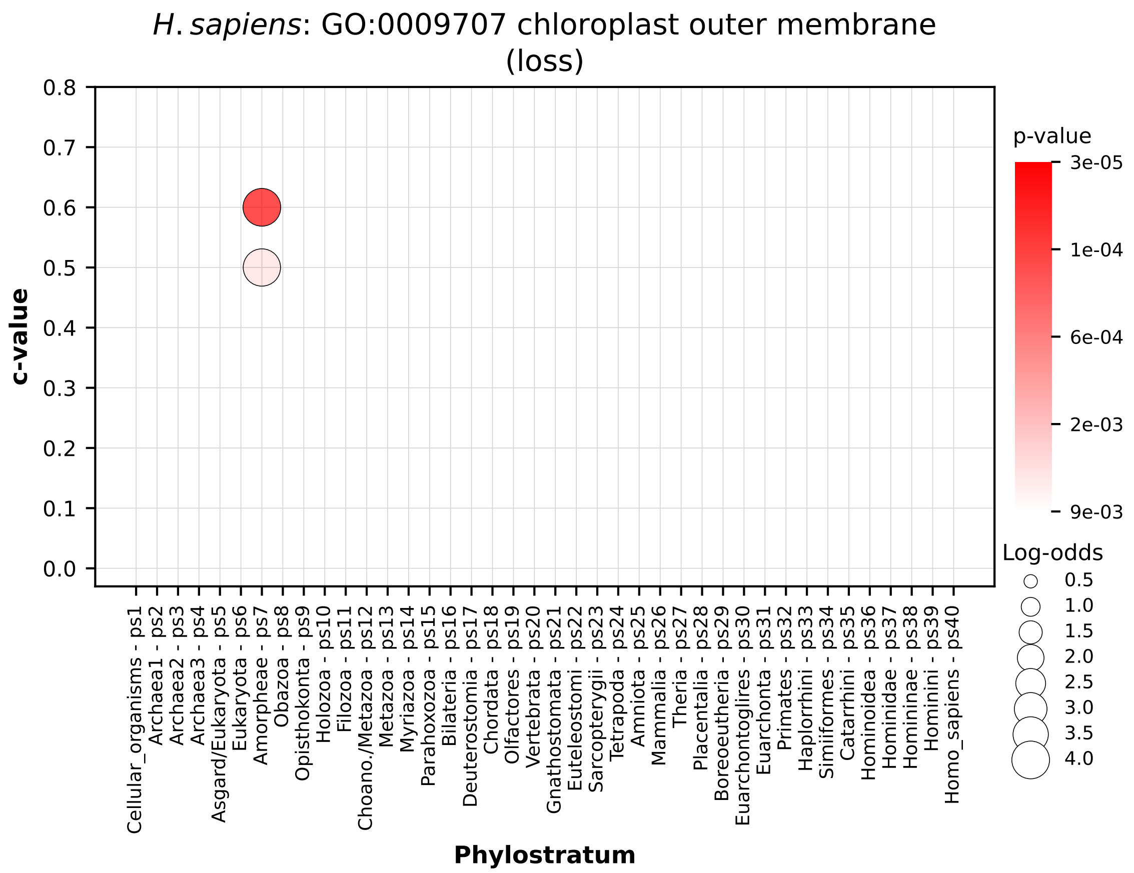

H. sapiens: GO:0009707 chloroplast outer membrane (loss)
c-value
0.8
0.7
0.6
0.5
0.4
0.3
0.2
0.1
0.0
Phylostratum
Cellular_organisms - ps1
Archaea1 - ps2
Archaea2 - ps3
Archaea3 - ps4
Asgard/Eukaryota - ps5
Eukaryota - ps6
Amorpheae - ps7
Obazoa - ps8
Opisthokonta - ps9
Holozoa - ps10
Filozoa - ps11
Choano./Metazoa - ps12
Metazoa - ps13
Myriazoa - ps14
Parahoxozoa - ps15
Bilateria - ps16
Deuterostomia - ps17
Chordata - ps18
Olfactores - ps19
Vertebrata - ps20
Gnathostomata - ps21
Euteleostomi - ps22
Sarcopterygii - ps23
Tetrapoda - ps24
Amniota - ps25
Mammalia - ps26
Theria - ps27
Placentalia - ps28
Boreoeutheria - ps29
Euarchontoglires - ps30
Euarchonta - ps31
Primates - ps32
Haplorrhini - ps33
Simiiformes - ps34
Catarrhini - ps35
Hominoidea - ps36
Hominidae - ps37
Homininae - ps38
Hominini - ps39
Homo_sapiens - ps40
p-value
3e-05
1e-04
6e-04
2e-03
9e-03
Log-odds
0.5
1.0
1.5
2.0
2.5
3.0
3.5
4.0

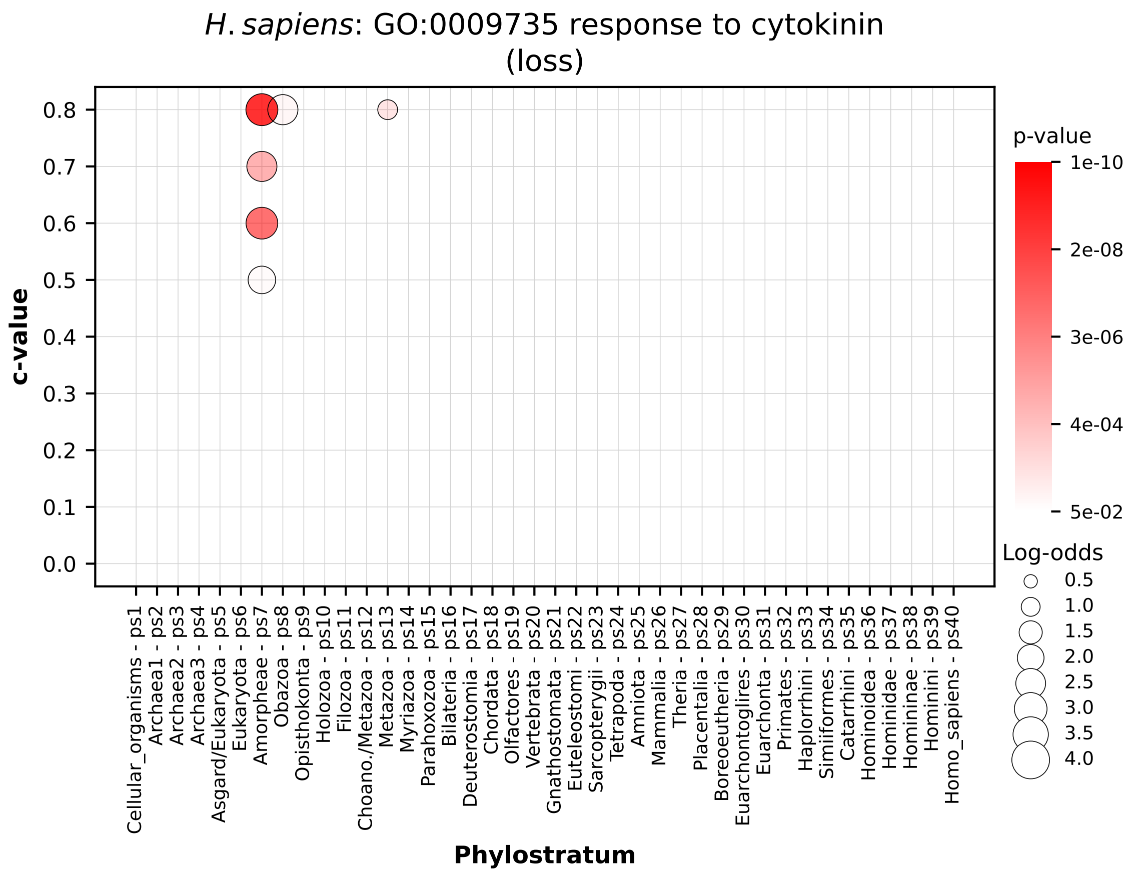

H. sapiens: GO:0009735 response to cytokinin
(loss)
c-value
0.8
0.7
0.6
0.5
0.4
0.3
0.2
0.1
0.0
p-value
1e-10
2e-08
3e-06
4e-04
5e-02
Log-odds
0.5
1.0
1.5
2.0
2.5
3.0
3.5
4.0
Cellular_organisms - ps1
Archaea1 - ps2
Archaea2 - ps3
Archaea3 - ps4
Asgard/Eukaryota - ps5
Eukaryota - ps6
Amorpheae - ps7
Obazoa - ps8
Opisthokonta - ps9
Holozoa - ps10
Filozoa - ps11
Choano./Metazoa - ps12
Metazoa - ps13
Myriazoa - ps14
Parahoxozoa - ps15
Bilateria - ps16
Deuterostomia - ps17
Chordata - ps18
Olfactores - ps19
Vertebrata - ps20
Gnathostomata - ps21
Euteleostomi - ps22
Sarcopterygii - ps23
Tetrapoda - ps24
Amniota - ps25
Mammalia - ps26
Theria - ps27
Placentalia - ps28
Boreoeutheria - ps29
Euarchontoglires - ps30
Euarchonta - ps31
Primates - ps32
Haplorrhini - ps33
Simiiformes - ps34
Catarrhini - ps35
Hominoidea - ps36
Hominidae - ps37
Homininae - ps38
Hominini - ps39
Homo_sapiens - ps40
Phylostratum

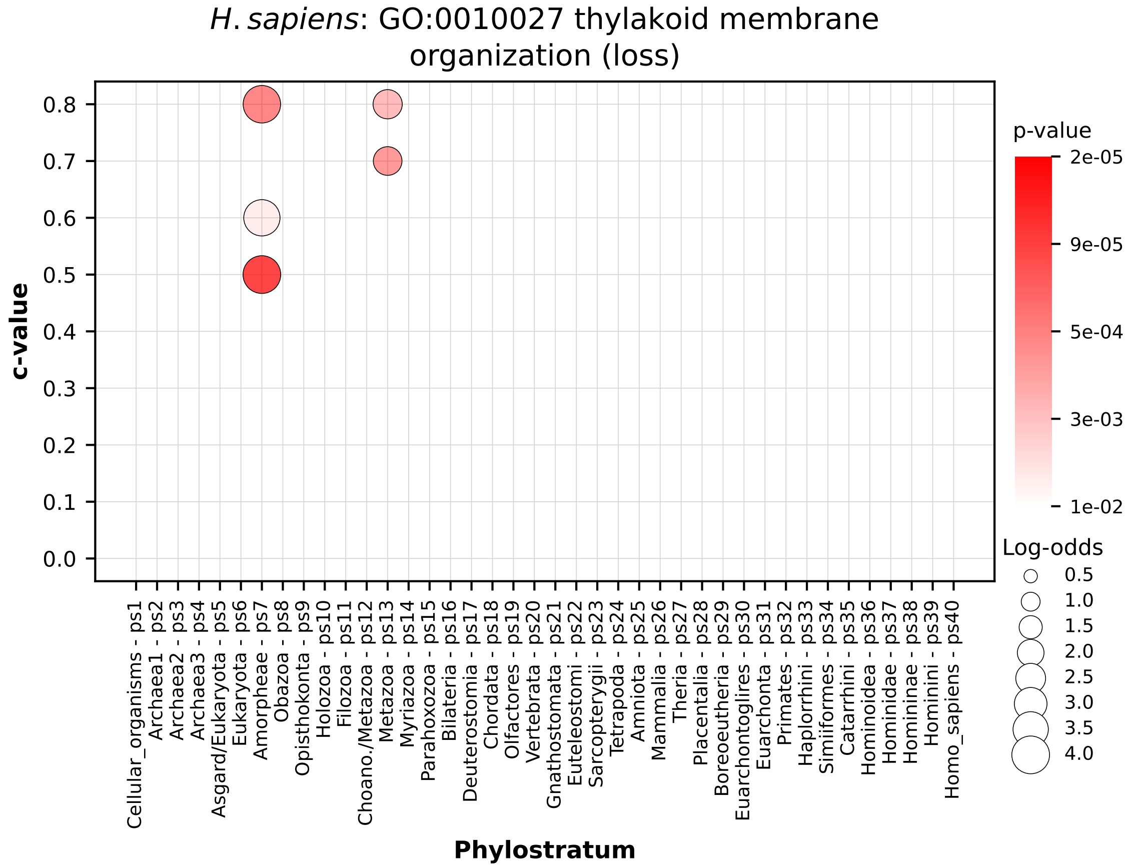
H. sapiens: GO:0010027 thylakoid membrane organization (loss)
c-value
0.0
0.1
0.2
0.3
0.4
0.5
0.6
0.7
0.8
Phylostratum
Cellular_organisms - ps1
Archaea1 - ps2
Archaea2 - ps3
Archaea3 - ps4
Asgard/Eukaryota - ps5
Eukaryota - ps6
Amorpheae - ps7
Obazoa - ps8
Opisthokonta - ps9
Holozoa - ps10
Filozoa - ps11
Choano./Metazoa - ps12
Metazoa - ps13
Myriazoa - ps14
Parahoxozoa - ps15
Bilateria - ps16
Deuterostomia - ps17
Chordata - ps18
Olfactores - ps19
Vertebrata - ps20
Gnathostomata - ps21
Euteleostomi - ps22
Sarcopterygii - ps23
Tetrapoda - ps24
Amniota - ps25
Mammalia - ps26
Theria - ps27
Placentalia - ps28
Boreoeutheria - ps29
Euarchontoglires - ps30
Euarchonta - ps31
Primates - ps32
Haplorrhini - ps33
Simiiformes - ps34
Catarrhini - ps35
Hominoidea - ps36
Hominidae - ps37
Homininae - ps38
Hominini - ps39
Homo_sapiens - ps40
p-value
2e-05
9e-05
5e-04
3e-03
1e-02
Log-odds
0.5
1.0
1.5
2.0
2.5
3.0
3.5
4.0

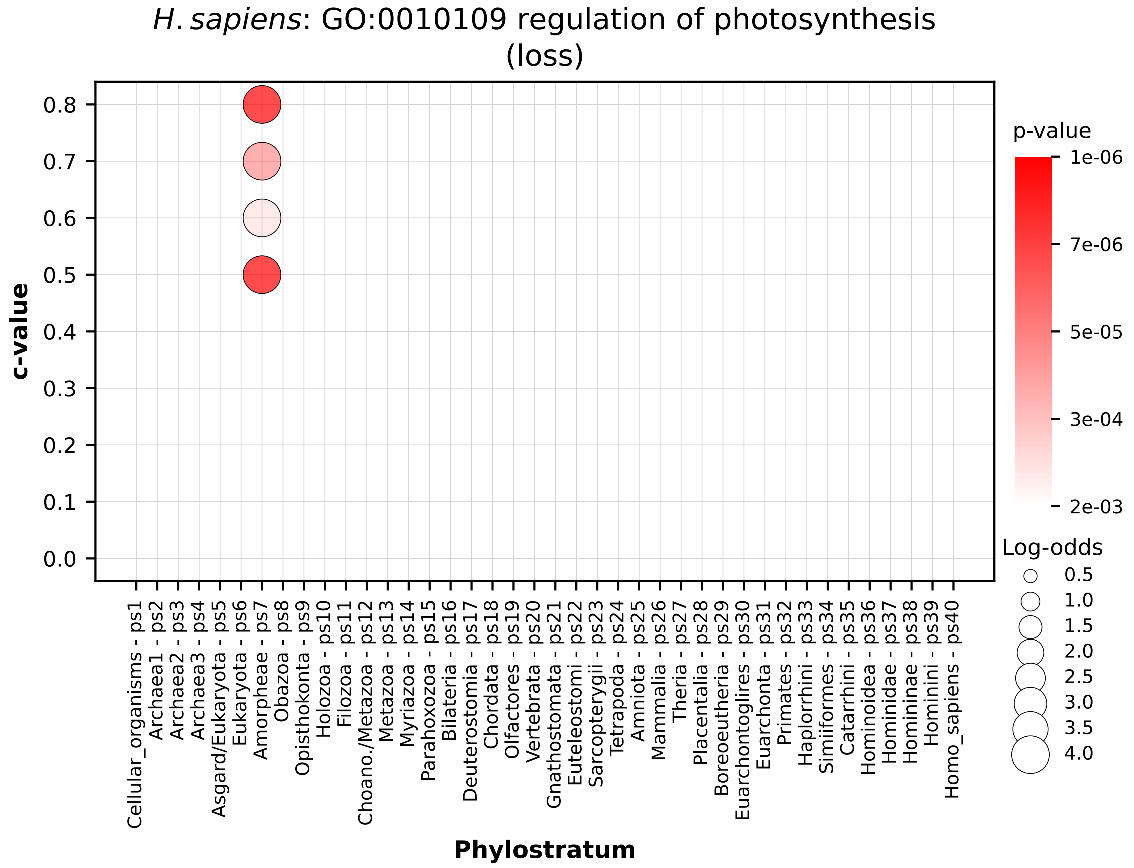
H. sapiens: GO:0010109 regulation of photosynthesis
(loss)
c-value
0.0
0.1
0.2
0.3
0.4
0.5
0.6
0.7
0.8
p-value
1e-06
7e-06
5e-05
3e-04
2e-03
Log-odds
0.5
1.0
1.5
2.0
2.5
3.0
3.5
4.0
Cellular_organisms - ps1
Archaea1 - ps2
Archaea2 - ps3
Archaea3 - ps4
Asgard/Eukaryota - ps5
Eukaryota - ps6
Amorpheae - ps7
Obazoa - ps8
Opisthokonta - ps9
Holozoa - ps10
Filozoa - ps11
Choano./Metazoa - ps12
Metazoa - ps13
Myriazoa - ps14
Parahoxozoa - ps15
Bilateria - ps16
Deuterostomia - ps17
Chordata - ps18
Olfactores - ps19
Vertebrata - ps20
Gnathostomata - ps21
Euteleostomi - ps22
Sarcopterygii - ps23
Tetrapoda - ps24
Amniota - ps25
Mammalia - ps26
Theria - ps27
Placentalia - ps28
Boreoeutheria - ps29
Euarchontoglires - ps30
Euarchonta - ps31
Primates - ps32
Haplorrhini - ps33
Simiiformes - ps34
Catarrhini - ps35
Hominoidea - ps36
Hominidae - ps37
Homininae - ps38
Hominini - ps39
Homo_sapiens - ps40
Phylostratum

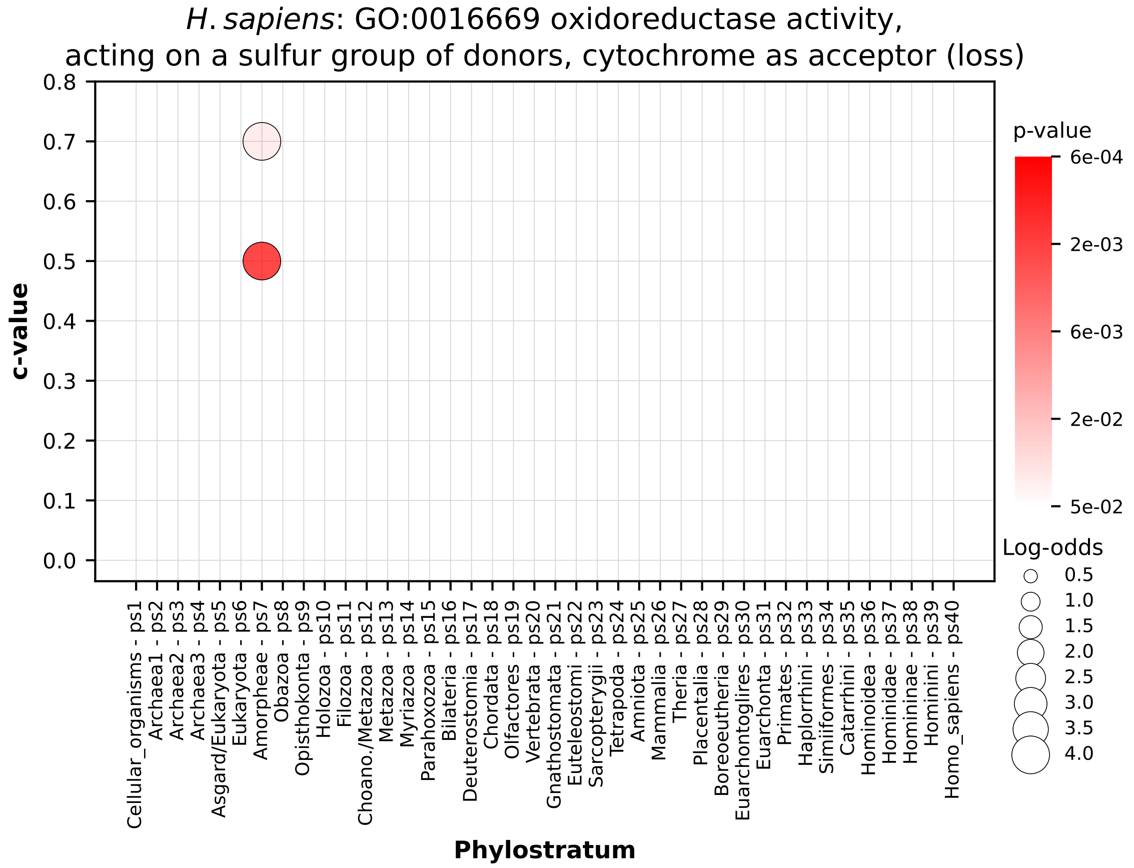

H. sapiens: GO:0016669 oxidoreductase activity,
acting on a sulfur group of donors, cytochrome as acceptor (loss)
c-value
0.8
0.7
0.6
0.5
0.4
0.3
0.2
0.1
0.0
Phylostratum
Cellular_organisms - ps1
Archaea1 - ps2
Archaea2 - ps3
Archaea3 - ps4
Asgard/Eukaryota - ps5
Eukaryota - ps6
Amorpheae - ps7
Obazoa - ps8
Opisthokonta - ps9
Holozoa - ps10
Filozoa - ps11
Choano./Metazoa - ps12
Metazoa - ps13
Myriazoa - ps14
Parahoxozoa - ps15
Bilateria - ps16
Deuterostomia - ps17
Chordata - ps18
Olfactores - ps19
Vertebrata - ps20
Gnathostomata - ps21
Euteleostomi - ps22
Sarcopterygii - ps23
Tetrapoda - ps24
Amniota - ps25
Mammalia - ps26
Theria - ps27
Placentalia - ps28
Boreoeutheria - ps29
Euarchontoglires - ps30
Euarchonta - ps31
Primates - ps32
Haplorrhini - ps33
Simiiformes - ps34
Catarrhini - ps35
Hominoidea - ps36
Hominidae - ps37
Homininae - ps38
Hominini - ps39
Homo_sapiens - ps40
p-value
6e-04
2e-03
6e-03
2e-02
5e-02
Log-odds
0.5
1.0
1.5
2.0
2.5
3.0
3.5
4.0

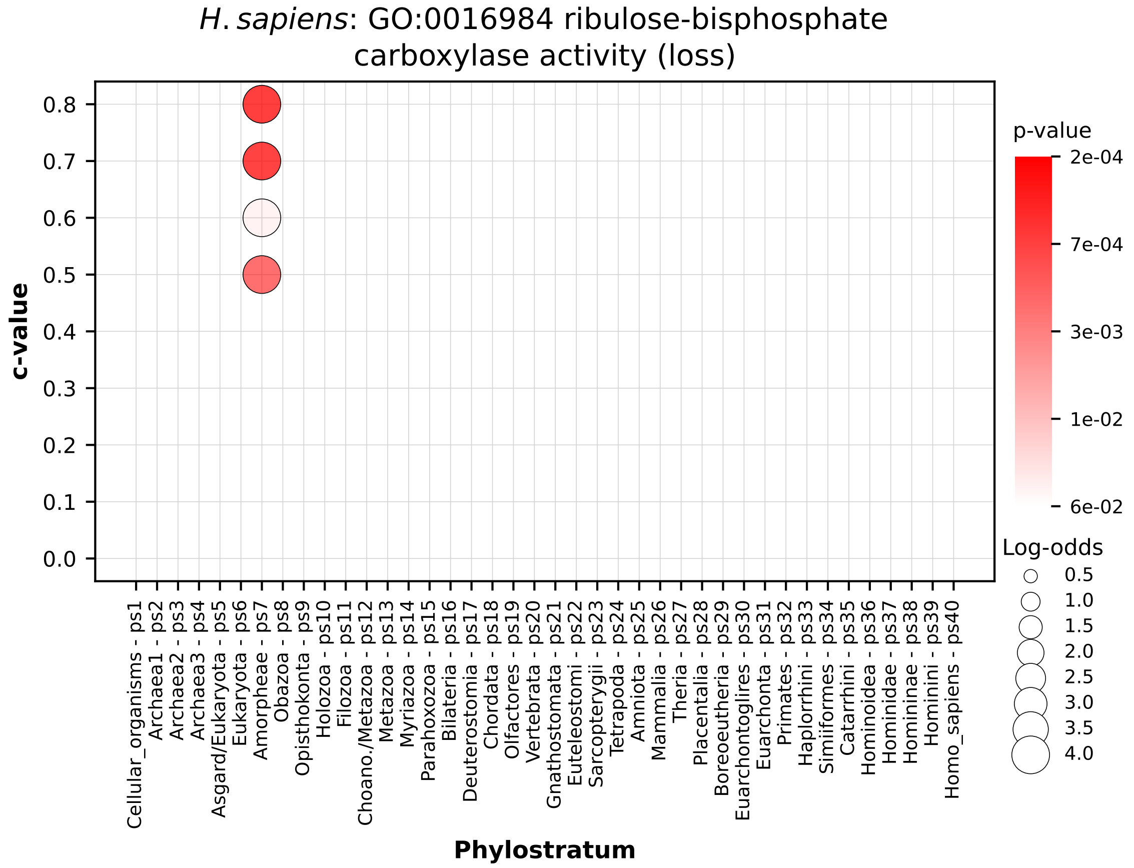

H. sapiens: GO:0016984 ribulose-bisphosphate carboxylase activity (loss)
c-value
0.8
0.7
0.6
0.5
0.4
0.3
0.2
0.1
0.0
Phylostratum
Cellular_organisms - ps1
Archaea1 - ps2
Archaea2 - ps3
Archaea3 - ps4
Asgard/Eukaryota - ps5
Eukaryota - ps6
Amorpheae - ps7
Obazoa - ps8
Opisthokonta - ps9
Holozoa - ps10
Filozoa - ps11
Choano./Metazoa - ps12
Metazoa - ps13
Myriazoa - ps14
Parahoxozoa - ps15
Bilateria - ps16
Deuterostomia - ps17
Chordata - ps18
Olfactores - ps19
Vertebrata - ps20
Gnathostomata - ps21
Euteleostomi - ps22
Sarcopterygii - ps23
Tetrapoda - ps24
Amniota - ps25
Mammalia - ps26
Theria - ps27
Placentalia - ps28
Boreoeutheria - ps29
Euarchontoglires - ps30
Euarchonta - ps31
Primates - ps32
Haplorrhini - ps33
Simiiformes - ps34
Catarrhini - ps35
Hominoidea - ps36
Hominidae - ps37
Homininae - ps38
Hominini - ps39
Homo_sapiens - ps40
p-value
2e-04
7e-04
3e-03
1e-02
6e-02
Log-odds
0.5
1.0
1.5
2.0
2.5
3.0
3.5
4.0

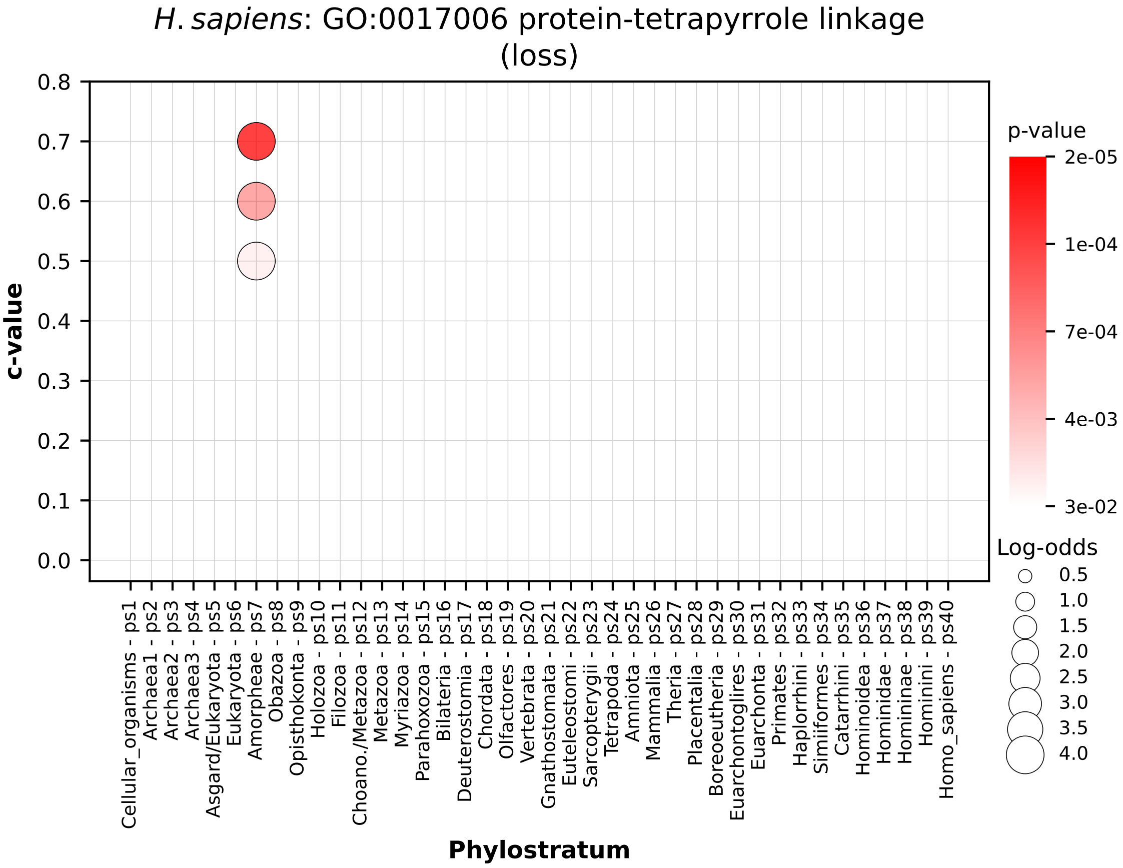

H. sapiens: GO:0017006 protein-tetrapyrrole linkage
(loss)
c-value
0.8
0.7
0.6
0.5
0.4
0.3
0.2
0.1
0.0
p-value
2e-05
1e-04
7e-04
4e-03
3e-02
Log-odds
0.5
1.0
1.5
2.0
2.5
3.0
3.5
4.0
Cellular_organisms - ps1
Archaea1 - ps2
Archaea2 - ps3
Archaea3 - ps4
Asgard/Eukaryota - ps5
Eukaryota - ps6
Amorpheae - ps7
Obazoa - ps8
Opisthokonta - ps9
Holozoa - ps10
Filozoa - ps11
Choano./Metazoa - ps12
Metazoa - ps13
Myriazoa - ps14
Parahoxozoa - ps15
Bilateria - ps16
Deuterostomia - ps17
Chordata - ps18
Olfactores - ps19
Vertebrata - ps20
Gnathostomata - ps21
Euteleostomi - ps22
Sarcopterygii - ps23
Tetrapoda - ps24
Amniota - ps25
Mammalia - ps26
Theria - ps27
Placentalia - ps28
Boreoeutheria - ps29
Euarchontoglires - ps30
Euarchonta - ps31
Primates - ps32
Haplorrhini - ps33
Simiiformes - ps34
Catarrhini - ps35
Hominoidea - ps36
Hominidae - ps37
Homininae - ps38
Hominini - ps39
Homo_sapiens - ps40
Phylostratum

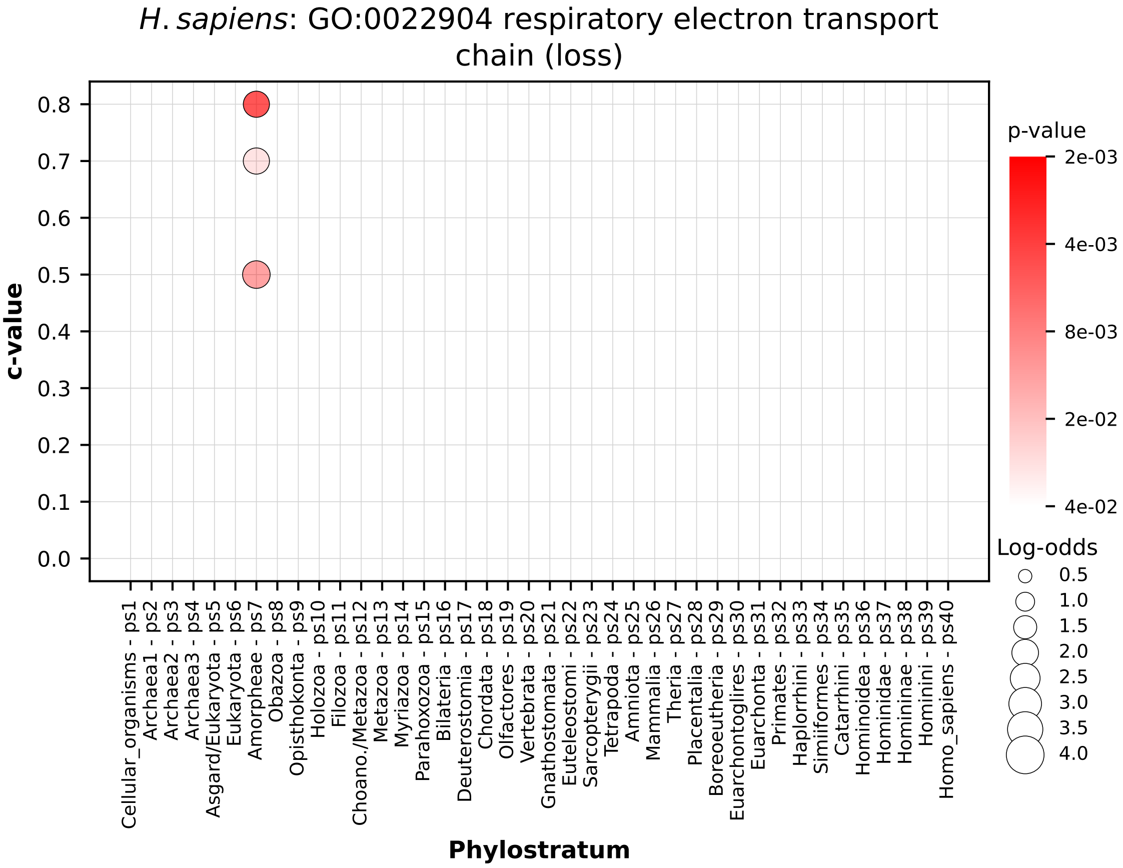

$H. sapiens$: GO:0022904 respiratory electron transport chain (loss)
c-value
0.8
0.7
0.6
0.5
0.4
0.3
0.2
0.1
0.0
p-value
2e-03
4e-03
8e-03
2e-02
4e-02
Log-odds
0.5
1.0
1.5
2.0
2.5
3.0
3.5
4.0
Cellular_organisms - ps1
Archaea1 - ps2
Archaea2 - ps3
Archaea3 - ps4
Asgard/Eukaryota - ps5
Eukaryota - ps6
Amorpheae - ps7
Obazoa - ps8
Opisthokonta - ps9
Holozoa - ps10
Filozoa - ps11
Choano./Metazoa - ps12
Metazoa - ps13
Myriazoa - ps14
Parahoxozoa - ps15
Bilateria - ps16
Deuterostomia - ps17
Chordata - ps18
Olfactores - ps19
Vertebrata - ps20
Gnathostomata - ps21
Euteleostomi - ps22
Sarcopterygii - ps23
Tetrapoda - ps24
Amniota - ps25
Mammalia - ps26
Theria - ps27
Placentalia - ps28
Boreoeutheria - ps29
Euarchontoglires - ps30
Euarchonta - ps31
Primates - ps32
Haplorrhini - ps33
Simiiformes - ps34
Catarrhini - ps35
Hominoidea - ps36
Hominidae - ps37
Homininae - ps38
Hominini - ps39
Homo_sapiens - ps40
Phylostratum

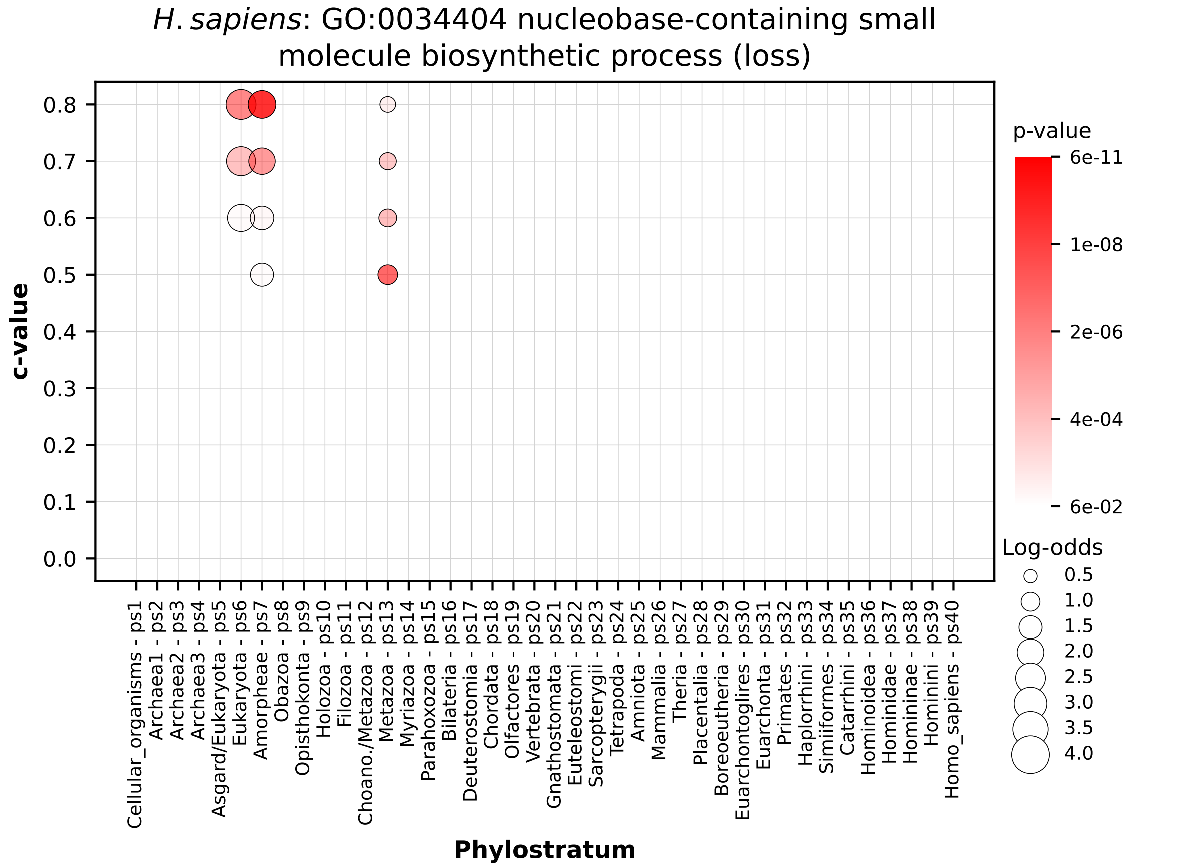
H. sapiens: GO:0034404 nucleobase-containing small molecule biosynthetic process (loss)
c-value
0.8
0.7
0.6
0.5
0.4
0.3
0.2
0.1
0.0
p-value
6e-11
1e-08
2e-06
4e-04
6e-02
Log-odds
0.5
1.0
1.5
2.0
2.5
3.0
3.5
4.0
Cellular_organisms - ps1
Archaea1 - ps2
Archaea2 - ps3
Archaea3 - ps4
Asgard/Eukaryota - ps5
Eukaryota - ps6
Amorpheae - ps7
Obazoa - ps8
Opisthokonta - ps9
Holozoa - ps10
Filozoa - ps11
Choano./Metazoa - ps12
Metazoa - ps13
Myriazoa - ps14
Parahoxozoa - ps15
Bilateria - ps16
Deuterostomia - ps17
Chordata - ps18
Olfactores - ps19
Vertebrata - ps20
Gnathostomata - ps21
Euteleostomi - ps22
Sarcopterygii - ps23
Tetrapoda - ps24
Amniota - ps25
Mammalia - ps26
Theria - ps27
Placentalia - ps28
Boreoeutheria - ps29
Euarchontoglires - ps30
Euarchonta - ps31
Primates - ps32
Haplorrhini - ps33
Simiiformes - ps34
Catarrhini - ps35
Hominoidea - ps36
Hominidae - ps37
Homininae - ps38
Hominini - ps39
Homo_sapiens - ps40
Phylostratum

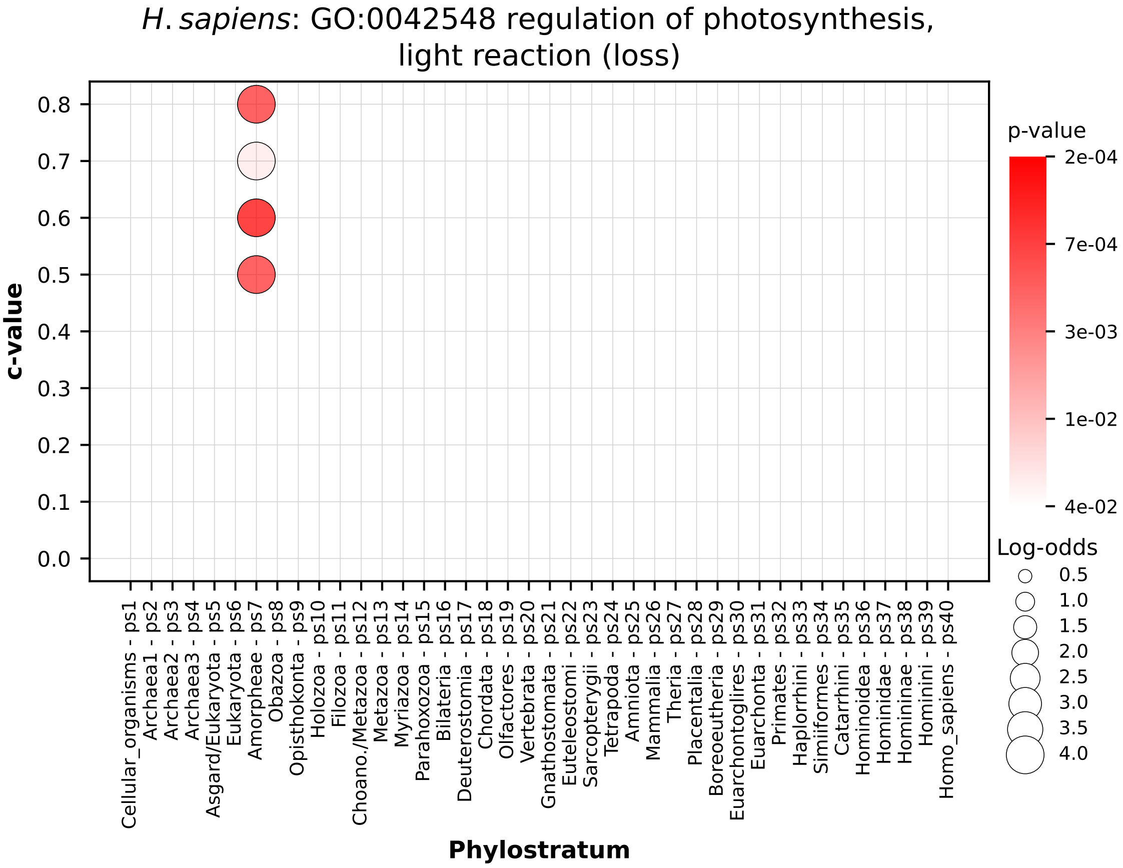

H. sapiens: GO:0042548 regulation of photosynthesis, light reaction (loss)
c-value
0.8
0.7
0.6
0.5
0.4
0.3
0.2
0.1
0.0
p-value
2e-04
7e-04
3e-03
1e-02
4e-02
Log-odds
0.5
1.0
1.5
2.0
2.5
3.0
3.5
4.0
Cellular_organisms - ps1
Archaea1 - ps2
Archaea2 - ps3
Archaea3 - ps4
Asgard/Eukaryota - ps5
Eukaryota - ps6
Amorpheae - ps7
Obazoa - ps8
Opisthokonta - ps9
Holozoa - ps10
Filozoa - ps11
Choano./Metazoa - ps12
Metazoa - ps13
Myriazoa - ps14
Parahoxozoa - ps15
Bilateria - ps16
Deuterostomia - ps17
Chordata - ps18
Olfactores - ps19
Vertebrata - ps20
Gnathostomata - ps21
Euteleostomi - ps22
Sarcopterygii - ps23
Tetrapoda - ps24
Amniota - ps25
Mammalia - ps26
Theria - ps27
Placentalia - ps28
Boreoeutheria - ps29
Euarchontoglires - ps30
Euarchonta - ps31
Primates - ps32
Haplorrhini - ps33
Simiiformes - ps34
Catarrhini - ps35
Hominoidea - ps36
Hominidae - ps37
Homininae - ps38
Hominini - ps39
Homo_sapiens - ps40
Phylostratum

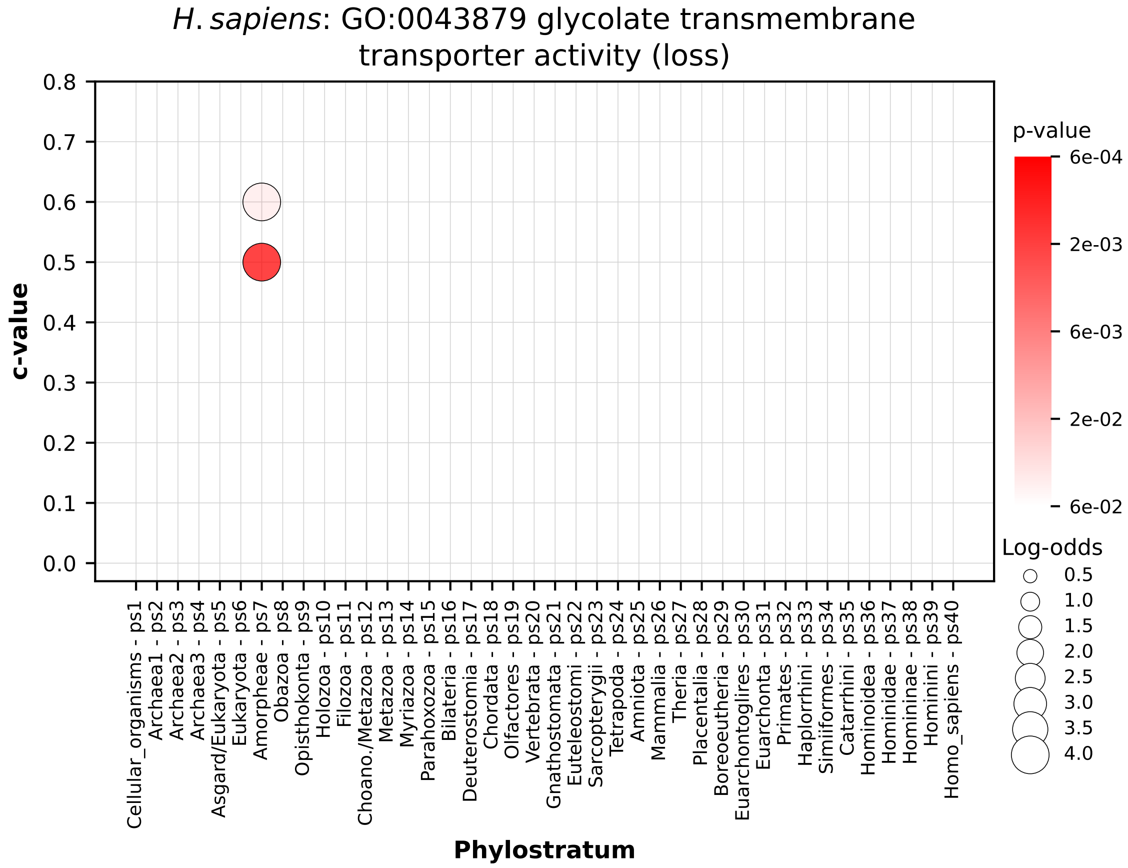
H. sapiens: GO:0043879 glycolate transmembrane transporter activity (loss)
c-value
0.0
0.1
0.2
0.3
0.4
0.5
0.6
0.7
0.8
Phylostratum
Cellular_organisms - ps1
Archaea1 - ps2
Archaea2 - ps3
Archaea3 - ps4
Asgard/Eukaryota - ps5
Eukaryota - ps6
Amorpheae - ps7
Obazoa - ps8
Opisthokonta - ps9
Holozoa - ps10
Filozoa - ps11
Choano./Metazoa - ps12
Metazoa - ps13
Myriazoa - ps14
Parahoxozoa - ps15
Bilateria - ps16
Deuterostomia - ps17
Chordata - ps18
Olfactores - ps19
Vertebrata - ps20
Gnathostomata - ps21
Euteleostomi - ps22
Sarcopterygii - ps23
Tetrapoda - ps24
Amniota - ps25
Mammalia - ps26
Theria - ps27
Placentalia - ps28
Boreoeutheria - ps29
Euarchontoglires - ps30
Euarchonta - ps31
Primates - ps32
Haplorrhini - ps33
Simiiformes - ps34
Catarrhini - ps35
Hominoidea - ps36
Hominidae - ps37
Homininae - ps38
Hominini - ps39
Homo_sapiens - ps40
p-value
6e-04
2e-03
6e-03
2e-02
6e-02
Log-odds
0.5
1.0
1.5
2.0
2.5
3.0
3.5
4.0

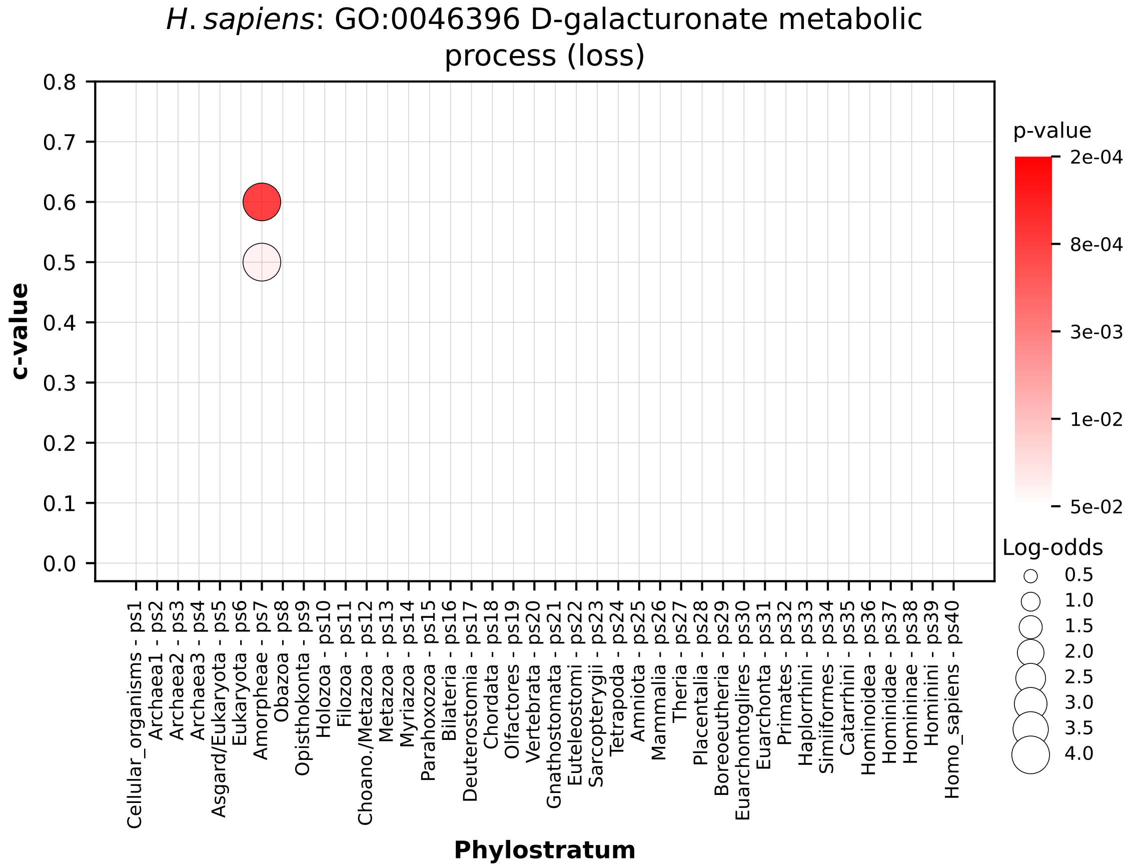

H. sapiens: GO:0046396 D-galacturonate metabolic process (loss)
c-value
Phylostratum
p-value
2e-04
8e-04
3e-03
1e-02
5e-02
Log-odds
0.5
1.0
1.5
2.0
2.5
3.0
3.5
4.0
0.0
0.1
0.2
0.3
0.4
0.5
0.6
0.7
0.8
Cellular_organisms - ps1
Archaea1 - ps2
Archaea2 - ps3
Archaea3 - ps4
Asgard/Eukaryota - ps5
Eukaryota - ps6
Amorpheae - ps7
Obazoa - ps8
Opisthokonta - ps9
Holozoa - ps10
Filozoa - ps11
Choano./Metazoa - ps12
Metazoa - ps13
Myriazoa - ps14
Parahoxozoa - ps15
Bilateria - ps16
Deuterostomia - ps17
Chordata - ps18
Olfactores - ps19
Vertebrata - ps20
Gnathostomata - ps21
Euteleostomi - ps22
Sarcopterygii - ps23
Tetrapoda - ps24
Amniota - ps25
Mammalia - ps26
Theria - ps27
Placentalia - ps28
Boreoeutheria - ps29
Euarchontoglires - ps30
Euarchonta - ps31
Primates - ps32
Haplorrhini - ps33
Simiiformes - ps34
Catarrhini - ps35
Hominoidea - ps36
Hominidae - ps37
Homininae - ps38
Hominini - ps39
Homo_sapiens - ps40

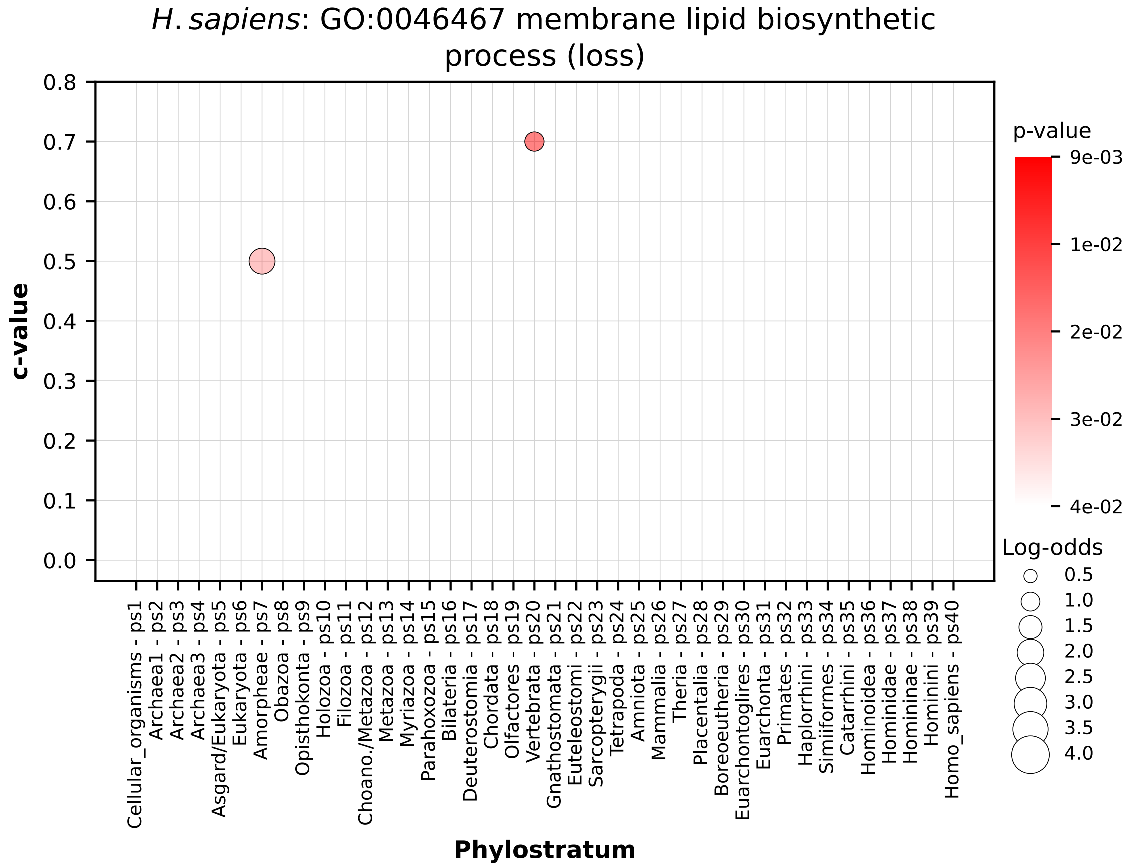
H. sapiens: GO:0046467 membrane lipid biosynthetic process (loss)
c-value
0.8
0.7
0.6
0.5
0.4
0.3
0.2
0.1
0.0
Phylostratum
Cellular_organisms - ps1
Archaea1 - ps2
Archaea2 - ps3
Archaea3 - ps4
Asgard/Eukaryota - ps5
Eukaryota - ps6
Amorpheae - ps7
Obazoa - ps8
Opisthokonta - ps9
Holozoa - ps10
Filozoa - ps11
Choano./Metazoa - ps12
Metazoa - ps13
Myriazoa - ps14
Parahoxozoa - ps15
Bilateria - ps16
Deuterostomia - ps17
Chordata - ps18
Olfactores - ps19
Vertebrata - ps20
Gnathostomata - ps21
Euteleostomi - ps22
Sarcopterygii - ps23
Tetrapoda - ps24
Amniota - ps25
Mammalia - ps26
Theria - ps27
Placentalia - ps28
Boreoeutheria - ps29
Euarchontoglires - ps30
Euarchonta - ps31
Primates - ps32
Haplorrhini - ps33
Simiiformes - ps34
Catarrhini - ps35
Hominoidea - ps36
Hominidae - ps37
Homininae - ps38
Hominini - ps39
Homo_sapiens - ps40
p-value
9e-03
1e-02
2e-02
3e-02
4e-02
Log-odds
0.5
1.0
1.5
2.0
2.5
3.0
3.5
4.0

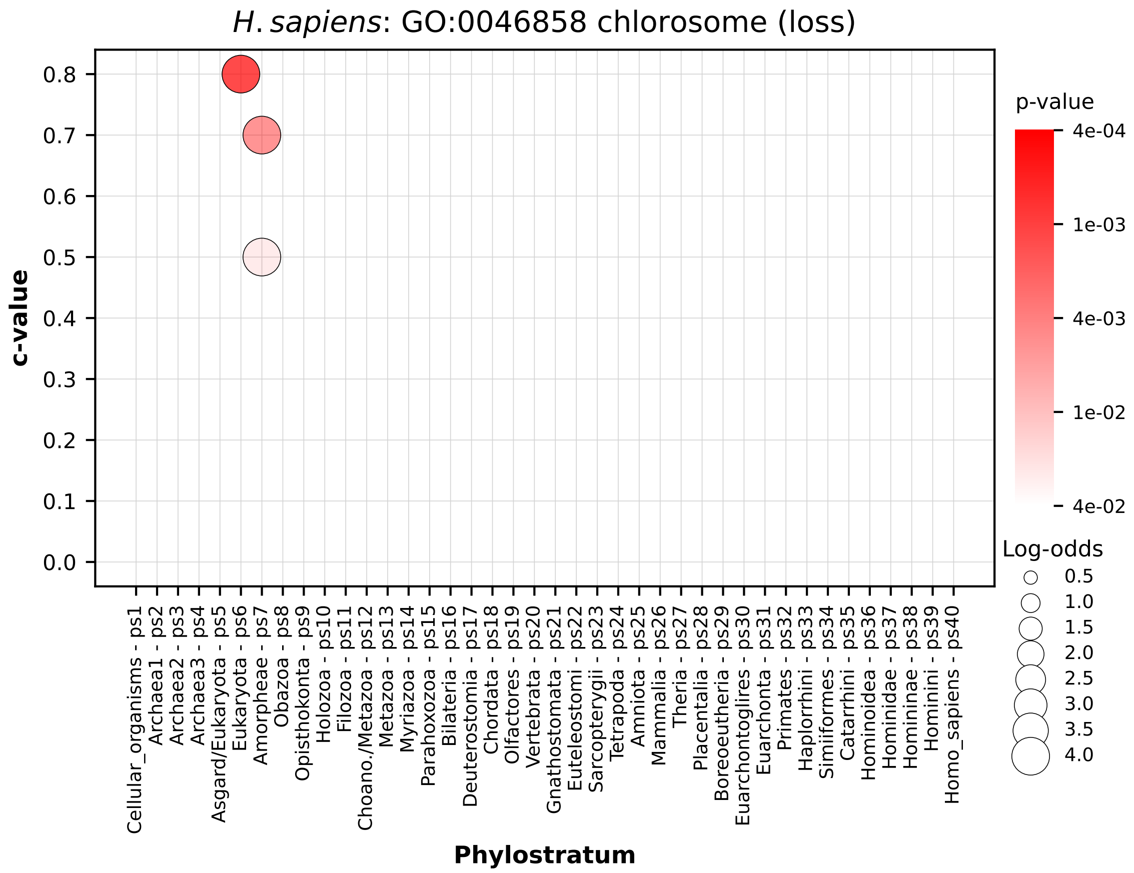

H. sapiens: GO:0046858 chlorosome (loss)
c-value
0.8
0.7
0.6
0.5
0.4
0.3
0.2
0.1
0.0
Phylostratum
Cellular_organisms - ps1
Archaea1 - ps2
Archaea2 - ps3
Archaea3 - ps4
Asgard/Eukaryota - ps5
Eukaryota - ps6
Amorpheae - ps7
Obazoa - ps8
Opisthokonta - ps9
Holozoa - ps10
Filozoa - ps11
Choano./Metazoa - ps12
Metazoa - ps13
Myriazoa - ps14
Parahoxozoa - ps15
Bilateria - ps16
Deuterostomia - ps17
Chordata - ps18
Olfactores - ps19
Vertebrata - ps20
Gnathostomata - ps21
Euteleostomi - ps22
Sarcopterygii - ps23
Tetrapoda - ps24
Amniota - ps25
Mammalia - ps26
Theria - ps27
Placentalia - ps28
Boreoeutheria - ps29
Euarchontoglires - ps30
Euarchonta - ps31
Primates - ps32
Haplorrhini - ps33
Simiiformes - ps34
Catarrhini - ps35
Hominoidea - ps36
Hominidae - ps37
Homininae - ps38
Hominini - ps39
Homo_sapiens - ps40
p-value
4e-04
1e-03
4e-03
1e-02
4e-02
Log-odds
0.5
1.0
1.5
2.0
2.5
3.0
3.5
4.0

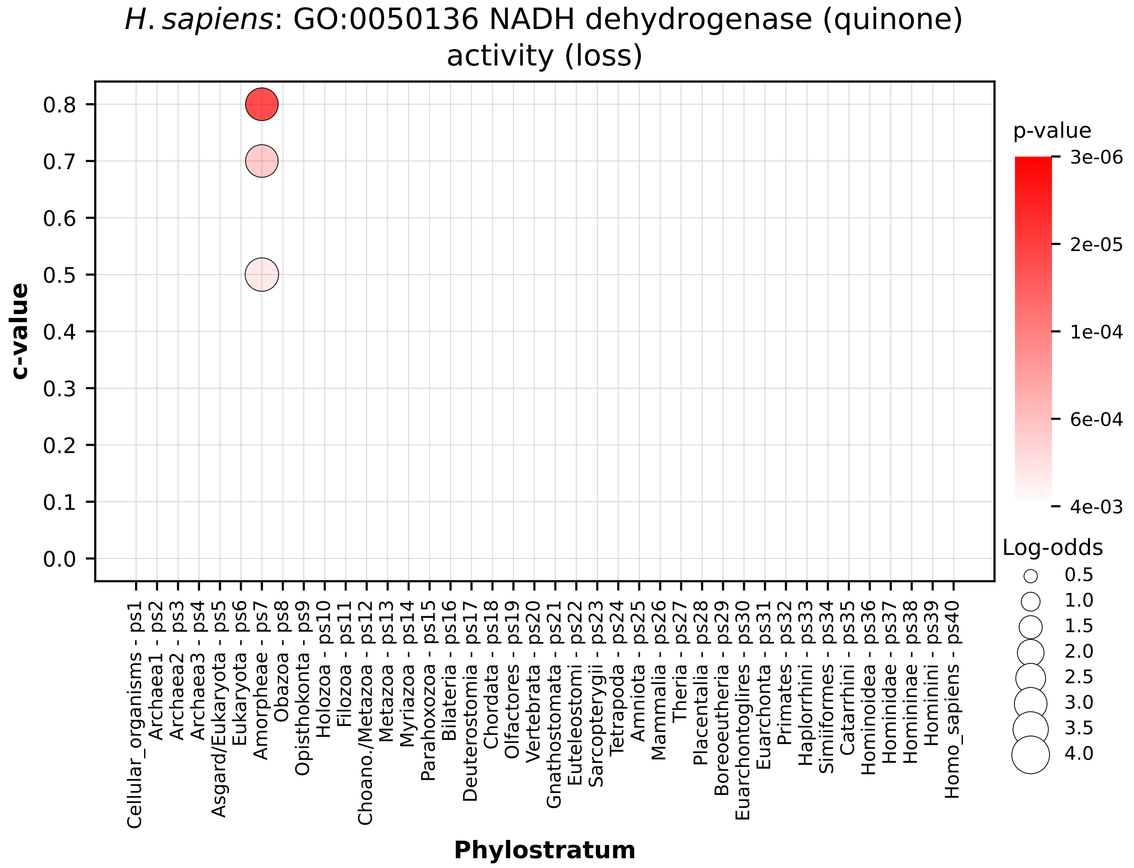

H. sapiens: GO:0050136 NADH dehydrogenase (quinone) activity (loss)
c-value
0.8
0.7
0.6
0.5
0.4
0.3
0.2
0.1
0.0
p-value
3e-06
2e-05
1e-04
6e-04
4e-03
Log-odds
0.5
1.0
1.5
2.0
2.5
3.0
3.5
4.0
Cellular_organisms - ps1
Archaea1 - ps2
Archaea2 - ps3
Archaea3 - ps4
Asgard/Eukaryota - ps5
Eukaryota - ps6
Amorpheae - ps7
Obazoa - ps8
Opisthokonta - ps9
Holozoa - ps10
Filozoa - ps11
Choano./Metazoa - ps12
Metazoa - ps13
Myriazoa - ps14
Parahoxozoa - ps15
Bilateria - ps16
Deuterostomia - ps17
Chordata - ps18
Olfactores - ps19
Vertebrata - ps20
Gnathostomata - ps21
Euteleostomi - ps22
Sarcopterygii - ps23
Tetrapoda - ps24
Amniota - ps25
Mammalia - ps26
Theria - ps27
Placentalia - ps28
Boreoeutheria - ps29
Euarchontoglires - ps30
Euarchonta - ps31
Primates - ps32
Haplorrhini - ps33
Simiiformes - ps34
Catarrhini - ps35
Hominoidea - ps36
Hominidae - ps37
Homininae - ps38
Hominini - ps39
Homo_sapiens - ps40
Phylostratum

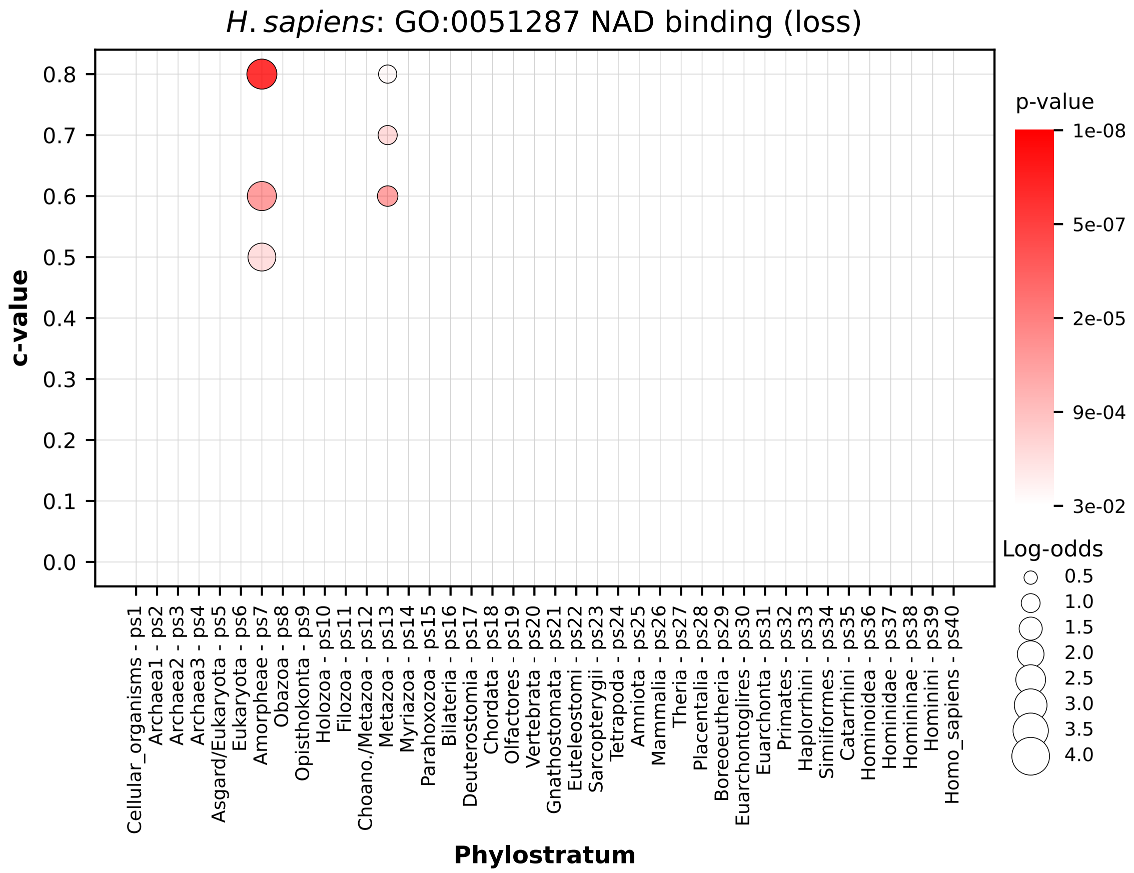

H. sapiens: GO:0051287 NAD binding (loss)
c-value
0.8
0.7
0.6
0.5
0.4
0.3
0.2
0.1
0.0
Phylostratum
Cellular_organisms - ps1
Archaea1 - ps2
Archaea2 - ps3
Archaea3 - ps4
Asgard/Eukaryota - ps5
Eukaryota - ps6
Amorpheae - ps7
Obazoa - ps8
Opisthokonta - ps9
Holozoa - ps10
Filozoa - ps11
Choano./Metazoa - ps12
Metazoa - ps13
Myriazoa - ps14
Parahoxozoa - ps15
Bilateria - ps16
Deuterostomia - ps17
Chordata - ps18
Olfactores - ps19
Vertebrata - ps20
Gnathostomata - ps21
Euteleostomi - ps22
Sarcopterygii - ps23
Tetrapoda - ps24
Amniota - ps25
Mammalia - ps26
Theria - ps27
Placentalia - ps28
Boreoeutheria - ps29
Euarchontoglires - ps30
Euarchonta - ps31
Primates - ps32
Haplorrhini - ps33
Simiiformes - ps34
Catarrhini - ps35
Hominoidea - ps36
Hominidae - ps37
Homininae - ps38
Hominini - ps39
Homo_sapiens - ps40
p-value
1e-08
5e-07
2e-05
9e-04
3e-02
Log-odds
0.5
1.0
1.5
2.0
2.5
3.0
3.5
4.0

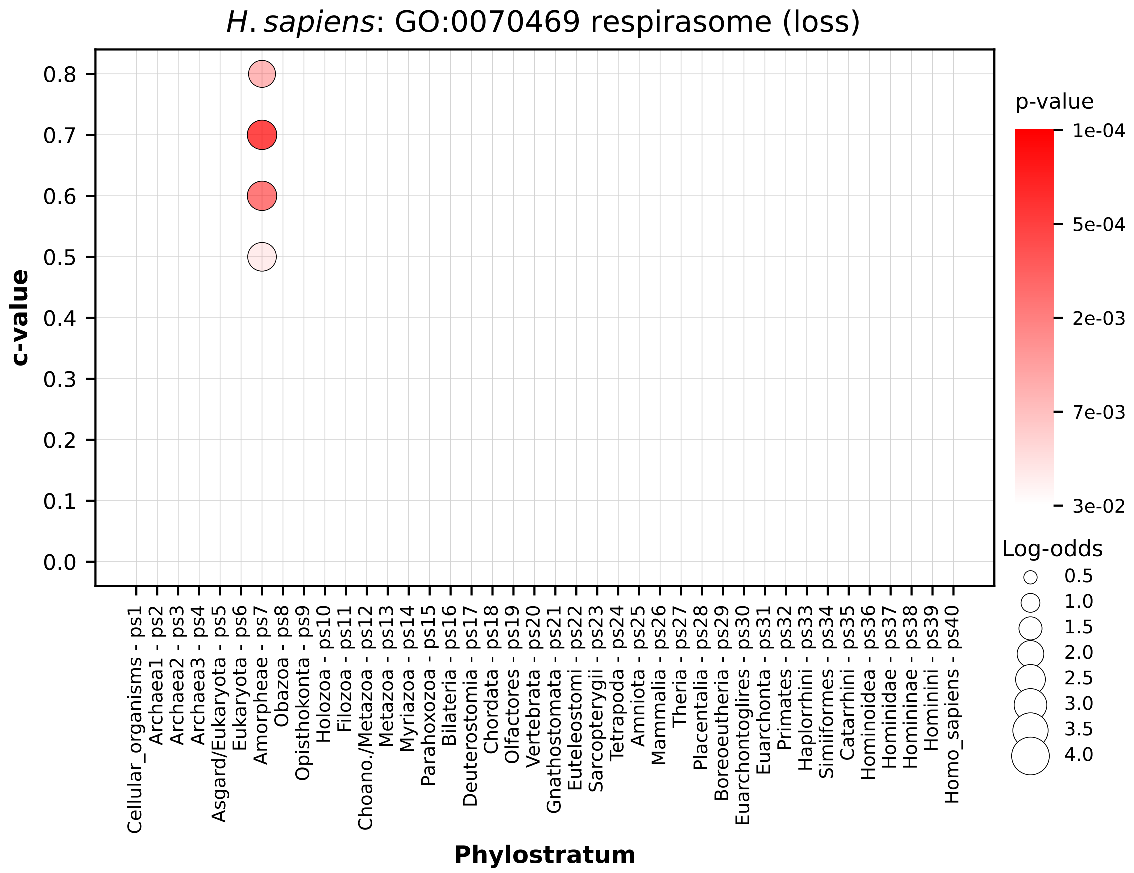

H. sapiens: GO:0070469 respirasome (loss)
c-value
0.8
0.7
0.6
0.5
0.4
0.3
0.2
0.1
0.0
p-value
1e-04
5e-04
2e-03
7e-03
3e-02
Log-odds
0.5
1.0
1.5
2.0
2.5
3.0
3.5
4.0
Cellular_organisms - ps1
Archaea1 - ps2
Archaea2 - ps3
Archaea3 - ps4
Asgard/Eukaryota - ps5
Eukaryota - ps6
Amorpheae - ps7
Obazoa - ps8
Opisthokonta - ps9
Holozoa - ps10
Filozoa - ps11
Choano./Metazoa - ps12
Metazoa - ps13
Myriazoa - ps14
Parahoxozoa - ps15
Bilateria - ps16
Deuterostomia - ps17
Chordata - ps18
Olfactores - ps19
Vertebrata - ps20
Gnathostomata - ps21
Euteleostomi - ps22
Sarcopterygii - ps23
Tetrapoda - ps24
Amniota - ps25
Mammalia - ps26
Theria - ps27
Placentalia - ps28
Boreoeutheria - ps29
Euarchontoglires - ps30
Euarchonta - ps31
Primates - ps32
Haplorrhini - ps33
Simiiformes - ps34
Catarrhini - ps35
Hominoidea - ps36
Hominidae - ps37
Homininae - ps38
Hominini - ps39
Homo_sapiens - ps40
Phylostratum

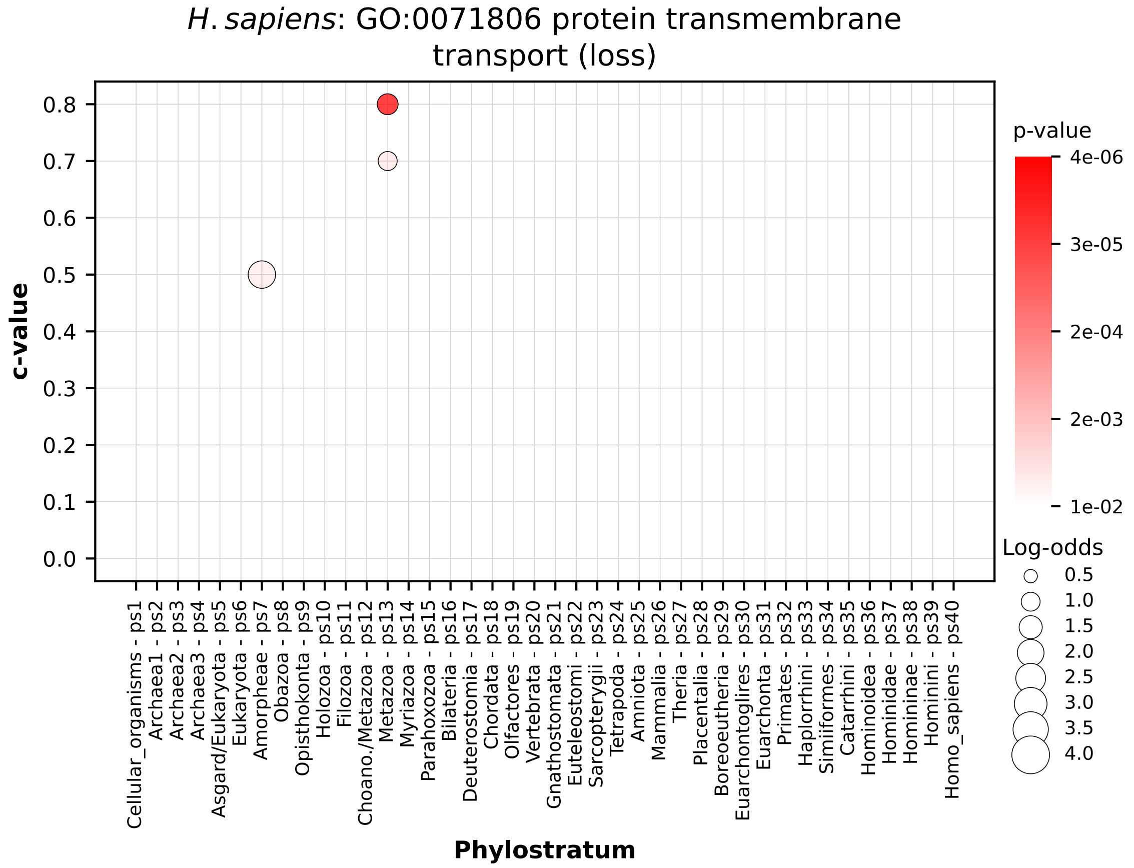

H. sapiens: GO:0071806 protein transmembrane transport (loss)
c-value
0.8
0.7
0.6
0.5
0.4
0.3
0.2
0.1
0.0
p-value
4e-06
3e-05
2e-04
2e-03
1e-02
Log-odds
0.5
1.0
1.5
2.0
2.5
3.0
3.5
4.0
Cellular_organisms - ps1
Archaea1 - ps2
Archaea2 - ps3
Archaea3 - ps4
Asgard/Eukaryota - ps5
Eukaryota - ps6
Amorpheae - ps7
Obazoa - ps8
Opisthokonta - ps9
Holozoa - ps10
Filozoa - ps11
Choano./Metazoa - ps12
Metazoa - ps13
Myriazoa - ps14
Parahoxozoa - ps15
Bilateria - ps16
Deuterostomia - ps17
Chordata - ps18
Olfactores - ps19
Vertebrata - ps20
Gnathostomata - ps21
Euteleostomi - ps22
Sarcopterygii - ps23
Tetrapoda - ps24
Amniota - ps25
Mammalia - ps26
Theria - ps27
Placentalia - ps28
Boreoeutheria - ps29
Euarchontoglires - ps30
Euarchonta - ps31
Primates - ps32
Haplorrhini - ps33
Simiiformes - ps34
Catarrhini - ps35
Hominoidea - ps36
Hominidae - ps37
Homininae - ps38
Hominini - ps39
Homo_sapiens - ps40
Phylostratum

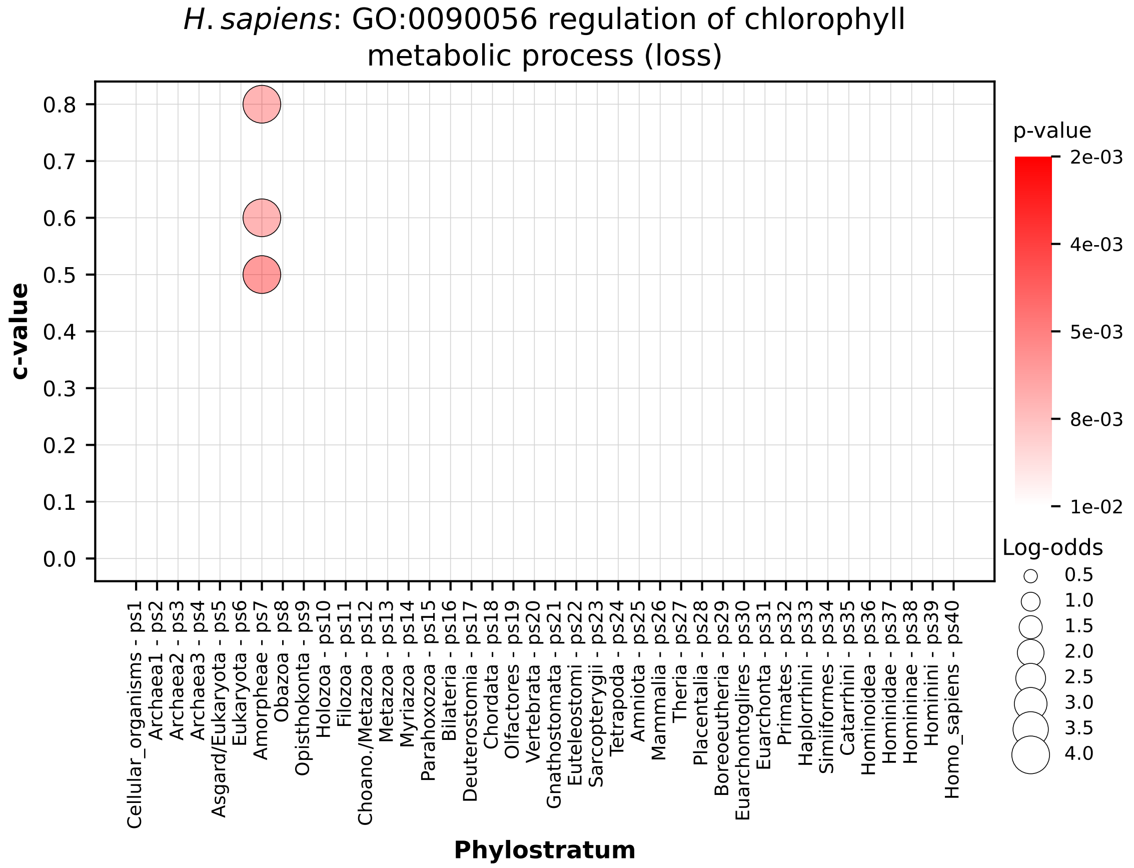

H. sapiens: GO:0090056 regulation of chlorophyll metabolic process (loss)
c-value
0.8
0.7
0.6
0.5
0.4
0.3
0.2
0.1
0.0
p-value
2e-03
4e-03
5e-03
8e-03
1e-02
Log-odds
0.5
1.0
1.5
2.0
2.5
3.0
3.5
4.0
Cellular_organisms - ps1
Archaea1 - ps2
Archaea2 - ps3
Archaea3 - ps4
Asgard/Eukaryota - ps5
Eukaryota - ps6
Amorpheae - ps7
Obazoa - ps8
Opisthokonta - ps9
Holozoa - ps10
Filozoa - ps11
Choano./Metazoa - ps12
Metazoa - ps13
Myriazoa - ps14
Parahoxozoa - ps15
Bilateria - ps16
Deuterostomia - ps17
Chordata - ps18
Olfactores - ps19
Vertebrata - ps20
Gnathostomata - ps21
Euteleostomi - ps22
Sarcopterygii - ps23
Tetrapoda - ps24
Amniota - ps25
Mammalia - ps26
Theria - ps27
Placentalia - ps28
Boreoeutheria - ps29
Euarchontoglires - ps30
Euarchonta - ps31
Primates - ps32
Haplorrhini - ps33
Simiiformes - ps34
Catarrhini - ps35
Hominoidea - ps36
Hominidae - ps37
Homininae - ps38
Hominini - ps39
Homo_sapiens - ps40
Phylostratum

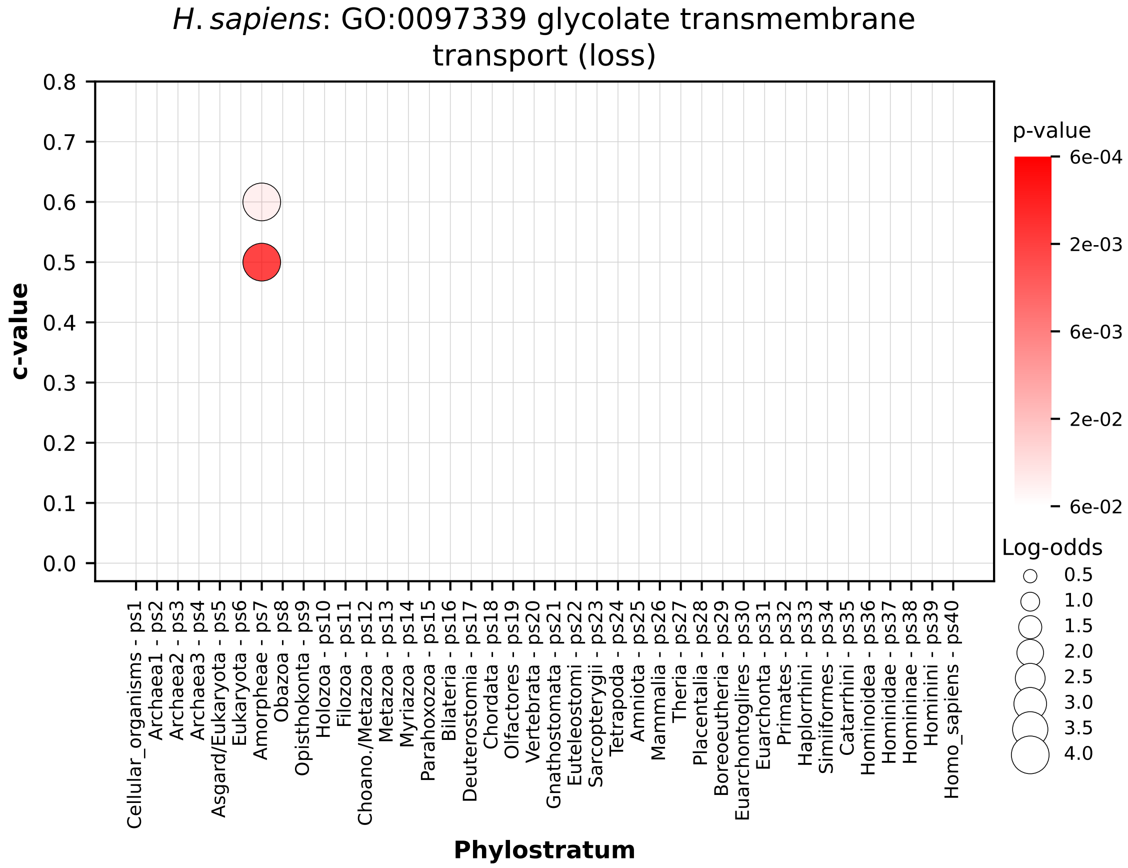

H. sapiens: GO:0097339 glycolate transmembrane transport (loss)
c-value
0.8
0.7
0.6
0.5
0.4
0.3
0.2
0.1
0.0
Phylostratum
Cellular_organisms - ps1
Archaea1 - ps2
Archaea2 - ps3
Archaea3 - ps4
Asgard/Eukaryota - ps5
Eukaryota - ps6
Amorpheae - ps7
Obazoa - ps8
Opisthokonta - ps9
Holozoa - ps10
Filozoa - ps11
Choano./Metazoa - ps12
Metazoa - ps13
Myriazoa - ps14
Parahoxozoa - ps15
Bilateria - ps16
Deuterostomia - ps17
Chordata - ps18
Olfactores - ps19
Vertebrata - ps20
Gnathostomata - ps21
Euteleostomi - ps22
Sarcopterygii - ps23
Tetrapoda - ps24
Amniota - ps25
Mammalia - ps26
Theria - ps27
Placentalia - ps28
Boreoeutheria - ps29
Euarchontoglires - ps30
Euarchonta - ps31
Primates - ps32
Haplorrhini - ps33
Simiiformes - ps34
Catarrhini - ps35
Hominoidea - ps36
Hominidae - ps37
Homininae - ps38
Hominini - ps39
Homo_sapiens - ps40
p-value
6e-04
2e-03
6e-03
2e-02
6e-02
Log-odds
0.5
1.0
1.5
2.0
2.5
3.0
3.5
4.0

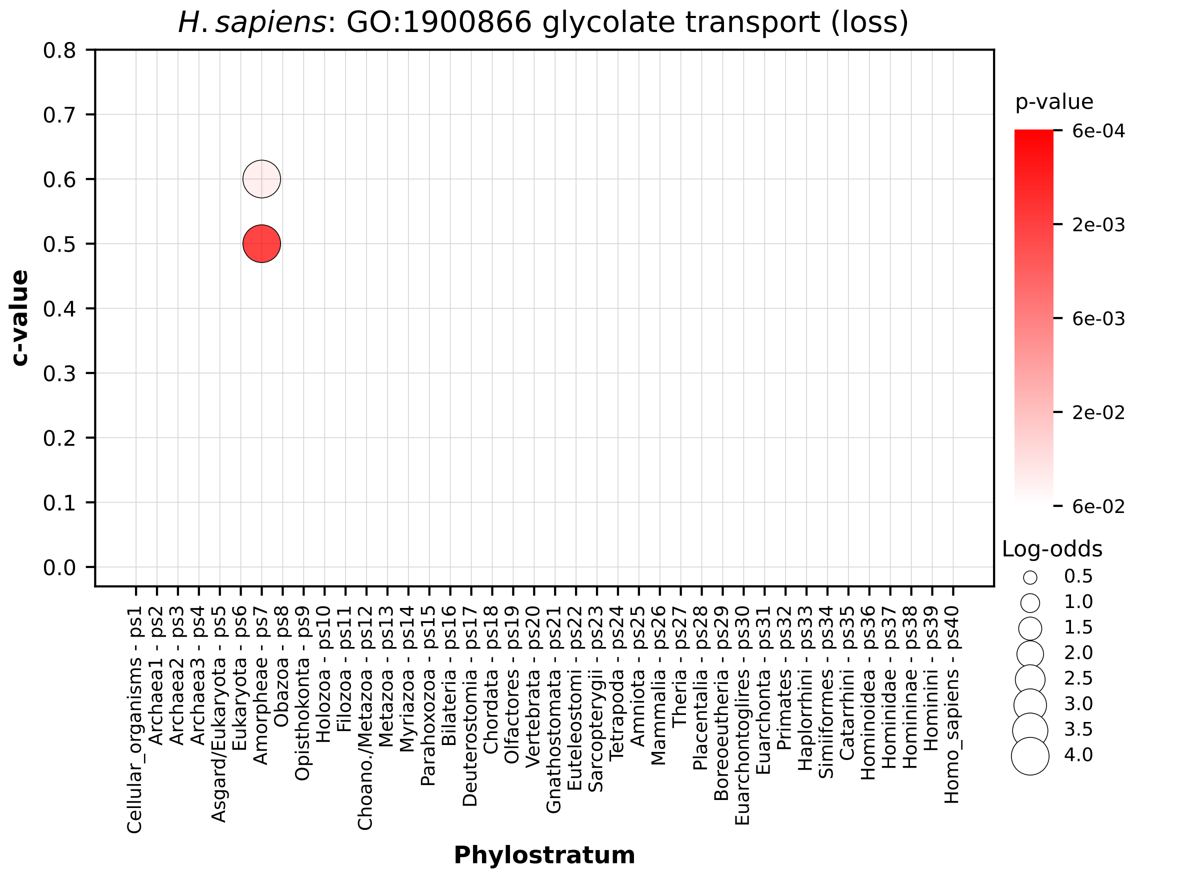

H. sapiens: GO:1900866 glycolate transport (loss)
c-value
0.0
0.1
0.2
0.3
0.4
0.5
0.6
0.7
0.8
Phylostratum
Cellular_organisms - ps1
Archaea1 - ps2
Archaea2 - ps3
Archaea3 - ps4
Asgard/Eukaryota - ps5
Eukaryota - ps6
Amorpheae - ps7
Obazoa - ps8
Opisthokonta - ps9
Holozoa - ps10
Filozoa - ps11
Choano./Metazoa - ps12
Metazoa - ps13
Myriazoa - ps14
Parahoxozoa - ps15
Bilateria - ps16
Deuterostomia - ps17
Chordata - ps18
Olfactores - ps19
Vertebrata - ps20
Gnathostomata - ps21
Euteleostomi - ps22
Sarcopterygii - ps23
Tetrapoda - ps24
Amniota - ps25
Mammalia - ps26
Theria - ps27
Placentalia - ps28
Boreoeutheria - ps29
Euarchontoglires - ps30
Euarchonta - ps31
Primates - ps32
Haplorrhini - ps33
Simiiformes - ps34
Catarrhini - ps35
Hominoidea - ps36
Hominidae - ps37
Homininae - ps38
Hominini - ps39
Homo_sapiens - ps40
p-value
6e-04
2e-03
6e-03
2e-02
6e-02
Log-odds
0.5
1.0
1.5
2.0
2.5
3.0
3.5
4.0

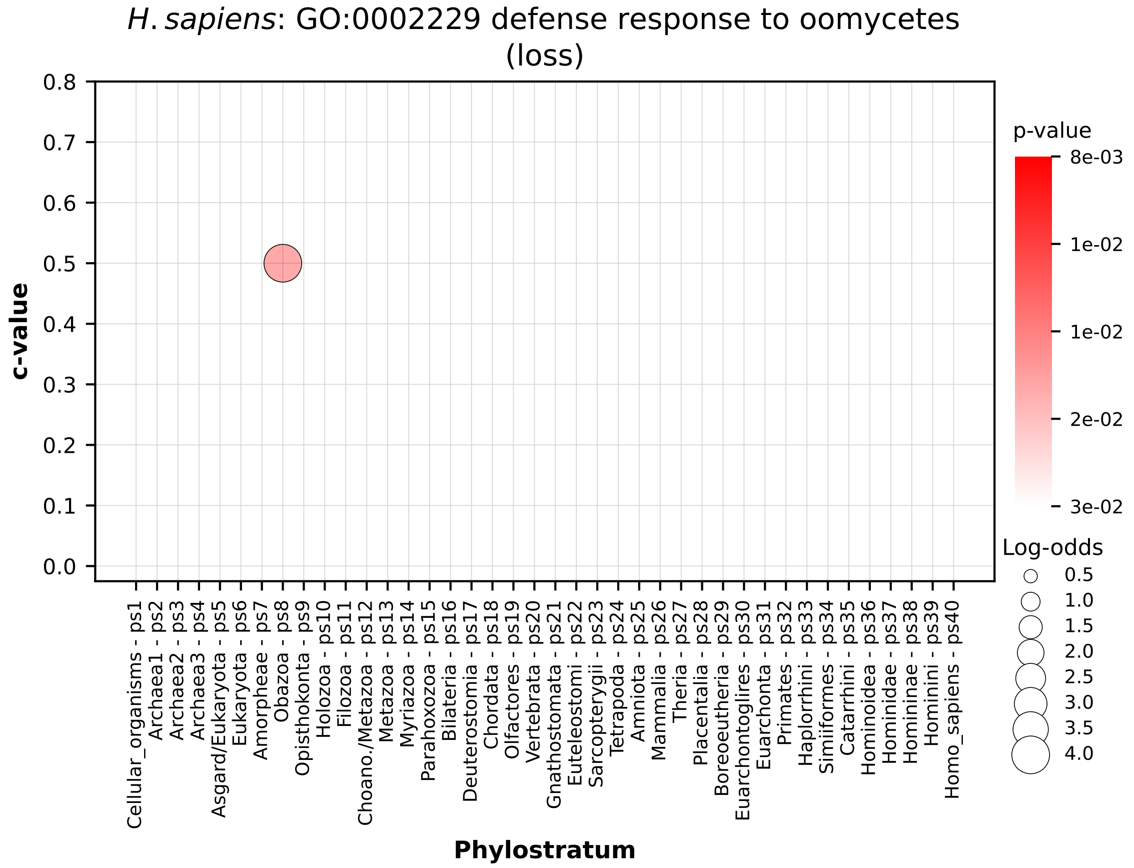

H. sapiens: GO:0002229 defense response to oomycetes (loss)
c-value
0.8
0.7
0.6
0.5
0.4
0.3
0.2
0.1
0.0
p-value
8e-03
1e-02
1e-02
2e-02
3e-02
Log-odds
0.5
1.0
1.5
2.0
2.5
3.0
3.5
4.0
Cellular_organisms - ps1
Archaea1 - ps2
Archaea2 - ps3
Archaea3 - ps4
Asgard/Eukaryota - ps5
Eukaryota - ps6
Amorpheae - ps7
Obazoa - ps8
Opisthokonta - ps9
Holozoa - ps10
Filozoa - ps11
Choano./Metazoa - ps12
Metazoa - ps13
Myriazoa - ps14
Parahoxozoa - ps15
Bilateria - ps16
Deuterostomia - ps17
Chordata - ps18
Olfactores - ps19
Vertebrata - ps20
Gnathostomata - ps21
Euteleostomi - ps22
Sarcopterygii - ps23
Tetrapoda - ps24
Amniota - ps25
Mammalia - ps26
Theria - ps27
Placentalia - ps28
Boreoeutheria - ps29
Euarchontoglires - ps30
Euarchonta - ps31
Primates - ps32
Haplorrhini - ps33
Simiiformes - ps34
Catarrhini - ps35
Hominoidea - ps36
Hominidae - ps37
Homininae - ps38
Hominini - ps39
Homo_sapiens - ps40
Phylostratum

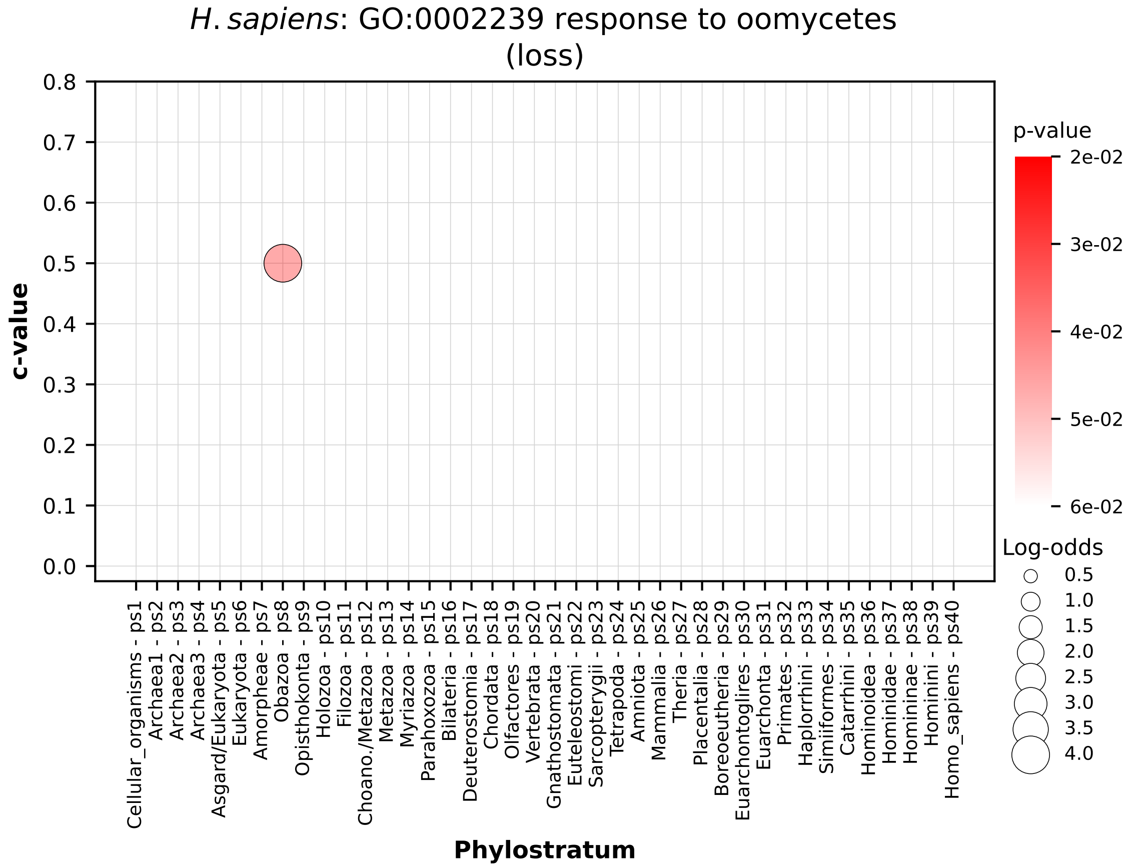

H. sapiens: GO:0002239 response to oomycetes
(loss)
c-value
0.8
0.7
0.6
0.5
0.4
0.3
0.2
0.1
0.0
Phylostratum
Cellular_organisms - ps1
Archaea1 - ps2
Archaea2 - ps3
Archaea3 - ps4
Asgard/Eukaryota - ps5
Eukaryota - ps6
Amorpheae - ps7
Obazoa - ps8
Opisthokonta - ps9
Holozoa - ps10
Filozoa - ps11
Choano./Metazoa - ps12
Metazoa - ps13
Myriazoa - ps14
Parahoxozoa - ps15
Bilateria - ps16
Deuterostomia - ps17
Chordata - ps18
Olfactores - ps19
Vertebrata - ps20
Gnathostomata - ps21
Euteleostomi - ps22
Sarcopterygii - ps23
Tetrapoda - ps24
Amniota - ps25
Mammalia - ps26
Theria - ps27
Placentalia - ps28
Boreoeutheria - ps29
Euarchontoglires - ps30
Euarchonta - ps31
Primates - ps32
Haplorrhini - ps33
Simiiformes - ps34
Catarrhini - ps35
Hominoidea - ps36
Hominidae - ps37
Homininae - ps38
Hominini - ps39
Homo_sapiens - ps40
p-value
2e-02
3e-02
4e-02
5e-02
6e-02
Log-odds
0.5
1.0
1.5
2.0
2.5
3.0
3.5
4.0

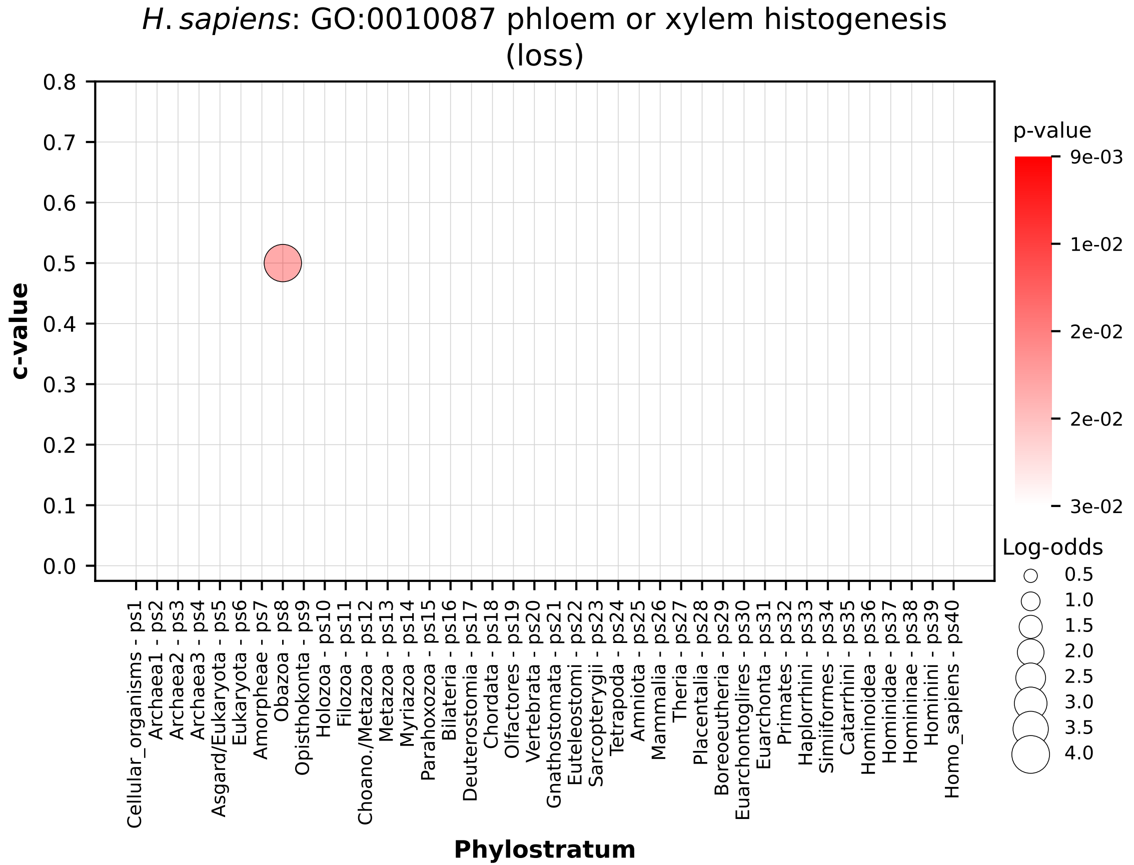

H. sapiens: GO:0010087 phloem or xylem histogenesis (loss)
c-value
0.0
0.1
0.2
0.3
0.4
0.5
0.6
0.7
0.8
Phylostratum
Cellular_organisms - ps1
Archaea1 - ps2
Archaea2 - ps3
Archaea3 - ps4
Asgard/Eukaryota - ps5
Eukaryota - ps6
Amorpheae - ps7
Obazoa - ps8
Opisthokonta - ps9
Holozoa - ps10
Filozoa - ps11
Choano./Metazoa - ps12
Metazoa - ps13
Myriazoa - ps14
Parahoxozoa - ps15
Bilateria - ps16
Deuterostomia - ps17
Chordata - ps18
Olfactores - ps19
Vertebrata - ps20
Gnathostomata - ps21
Euteleostomi - ps22
Sarcopterygii - ps23
Tetrapoda - ps24
Amniota - ps25
Mammalia - ps26
Theria - ps27
Placentalia - ps28
Boreoeutheria - ps29
Euarchontoglires - ps30
Euarchonta - ps31
Primates - ps32
Haplorrhini - ps33
Simiiformes - ps34
Catarrhini - ps35
Hominoidea - ps36
Hominidae - ps37
Homininae - ps38
Hominini - ps39
Homo_sapiens - ps40
p-value
9e-03
1e-02
2e-02
2e-02
3e-02
Log-odds
0.5
1.0
1.5
2.0
2.5
3.0
3.5
4.0

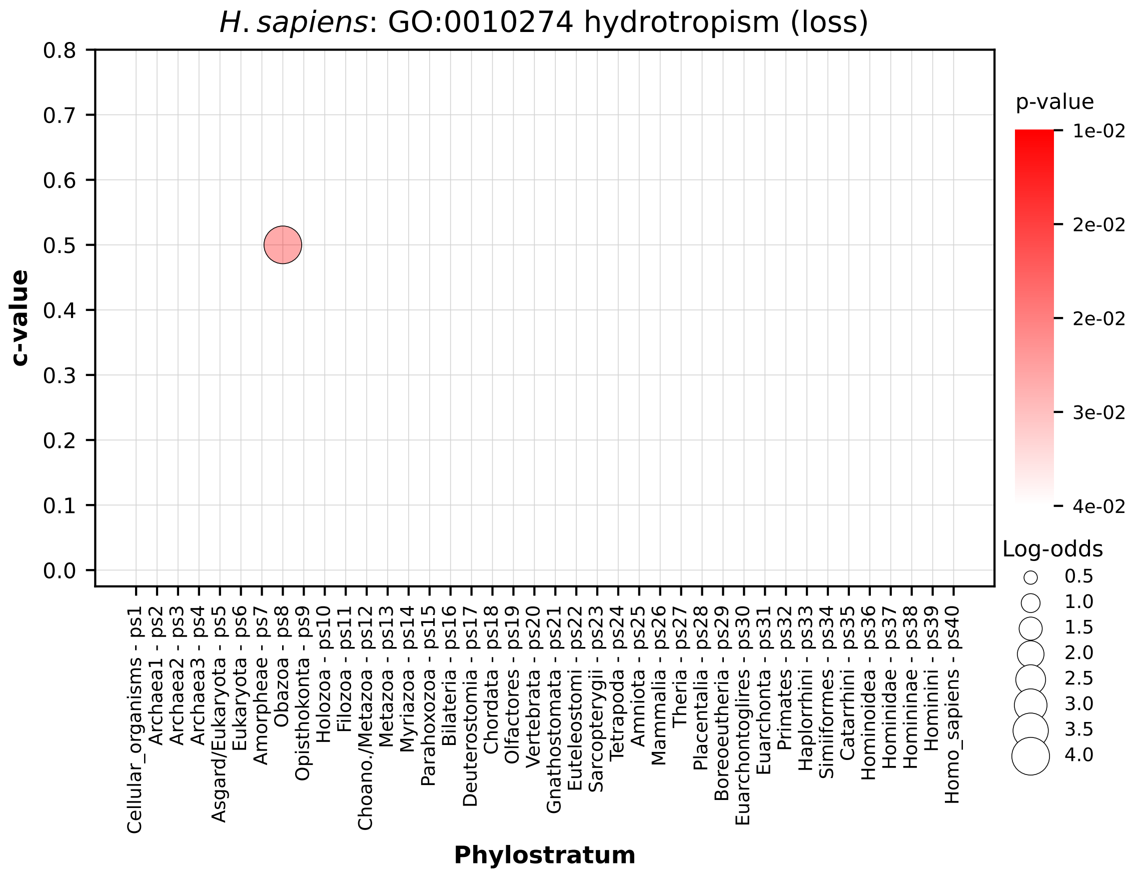
H. sapiens: GO:0010274 hydrotropism (loss)
c-value
0.8
0.7
0.6
0.5
0.4
0.3
0.2
0.1
0.0
Phylostratum
Cellular_organisms - ps1
Archaea1 - ps2
Archaea2 - ps3
Archaea3 - ps4
Asgard/Eukaryota - ps5
Eukaryota - ps6
Amorpheae - ps7
Obazoa - ps8
Opisthokonta - ps9
Holozoa - ps10
Filozoa - ps11
Choano./Metazoa - ps12
Metazoa - ps13
Myriazoa - ps14
Parahoxozoa - ps15
Bilateria - ps16
Deuterostomia - ps17
Chordata - ps18
Olfactores - ps19
Vertebrata - ps20
Gnathostomata - ps21
Euteleostomi - ps22
Sarcopterygii - ps23
Tetrapoda - ps24
Amniota - ps25
Mammalia - ps26
Theria - ps27
Placentalia - ps28
Boreoeutheria - ps29
Euarchontoglires - ps30
Euarchonta - ps31
Primates - ps32
Haplorrhini - ps33
Simiiformes - ps34
Catarrhini - ps35
Hominoidea - ps36
Hominidae - ps37
Homininae - ps38
Hominini - ps39
Homo_sapiens - ps40
p-value
1e-02
2e-02
2e-02
3e-02
4e-02
Log-odds
0.5
1.0
1.5
2.0
2.5
3.0
3.5
4.0

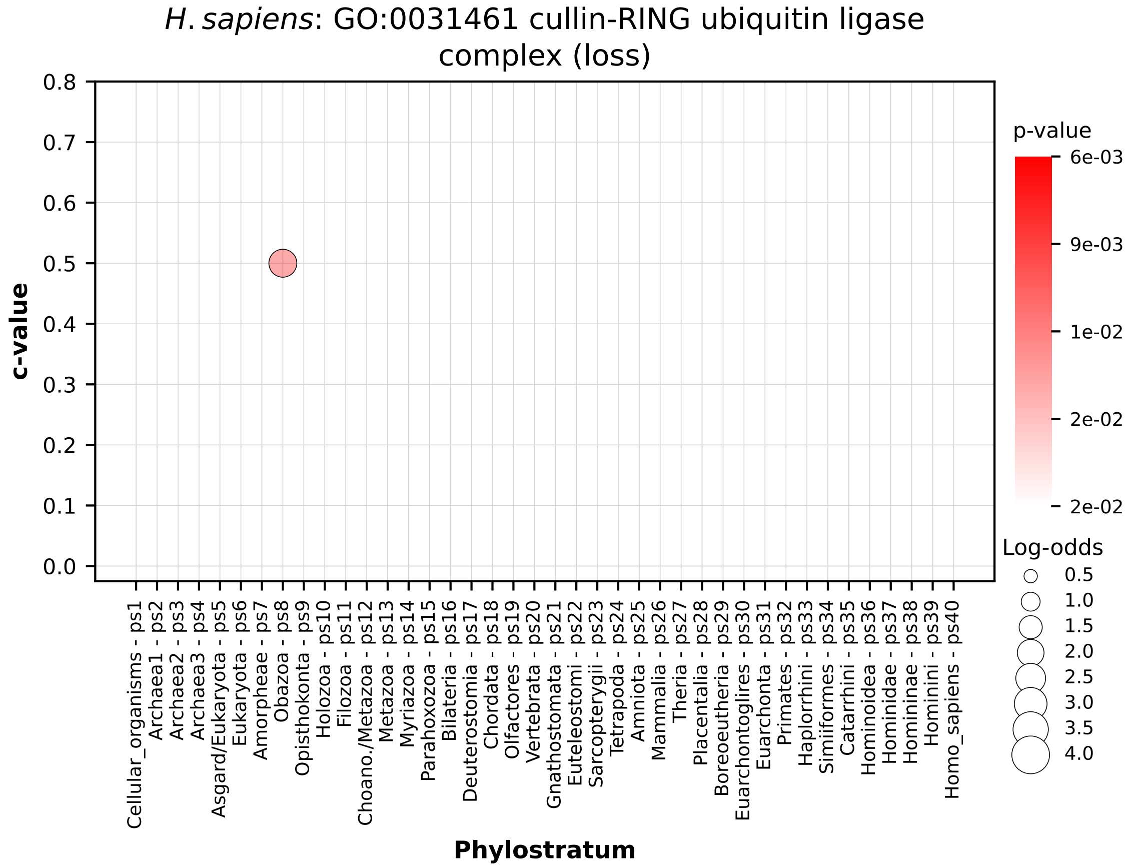

H. sapiens: GO:0031461 cullin-RING ubiquitin ligase complex (loss)
c-value
0.0
0.1
0.2
0.3
0.4
0.5
0.6
0.7
0.8
Phylostratum
Cellular_organisms - ps1
Archaea1 - ps2
Archaea2 - ps3
Archaea3 - ps4
Asgard/Eukaryota - ps5
Eukaryota - ps6
Amorpheae - ps7
Obazoa - ps8
Opisthokonta - ps9
Holozoa - ps10
Filozoa - ps11
Choano./Metazoa - ps12
Metazoa - ps13
Myriazoa - ps14
Parahoxozoa - ps15
Bilateria - ps16
Deuterostomia - ps17
Chordata - ps18
Olfactores - ps19
Vertebrata - ps20
Gnathostomata - ps21
Euteleostomi - ps22
Sarcopterygii - ps23
Tetrapoda - ps24
Amniota - ps25
Mammalia - ps26
Theria - ps27
Placentalia - ps28
Boreoeutheria - ps29
Euarchontoglires - ps30
Euarchonta - ps31
Primates - ps32
Haplorrhini - ps33
Simiiformes - ps34
Catarrhini - ps35
Hominoidea - ps36
Hominidae - ps37
Homininae - ps38
Hominini - ps39
Homo_sapiens - ps40
p-value
6e-03
9e-03
1e-02
2e-02
2e-02
Log-odds
0.5
1.0
1.5
2.0
2.5
3.0
3.5
4.0

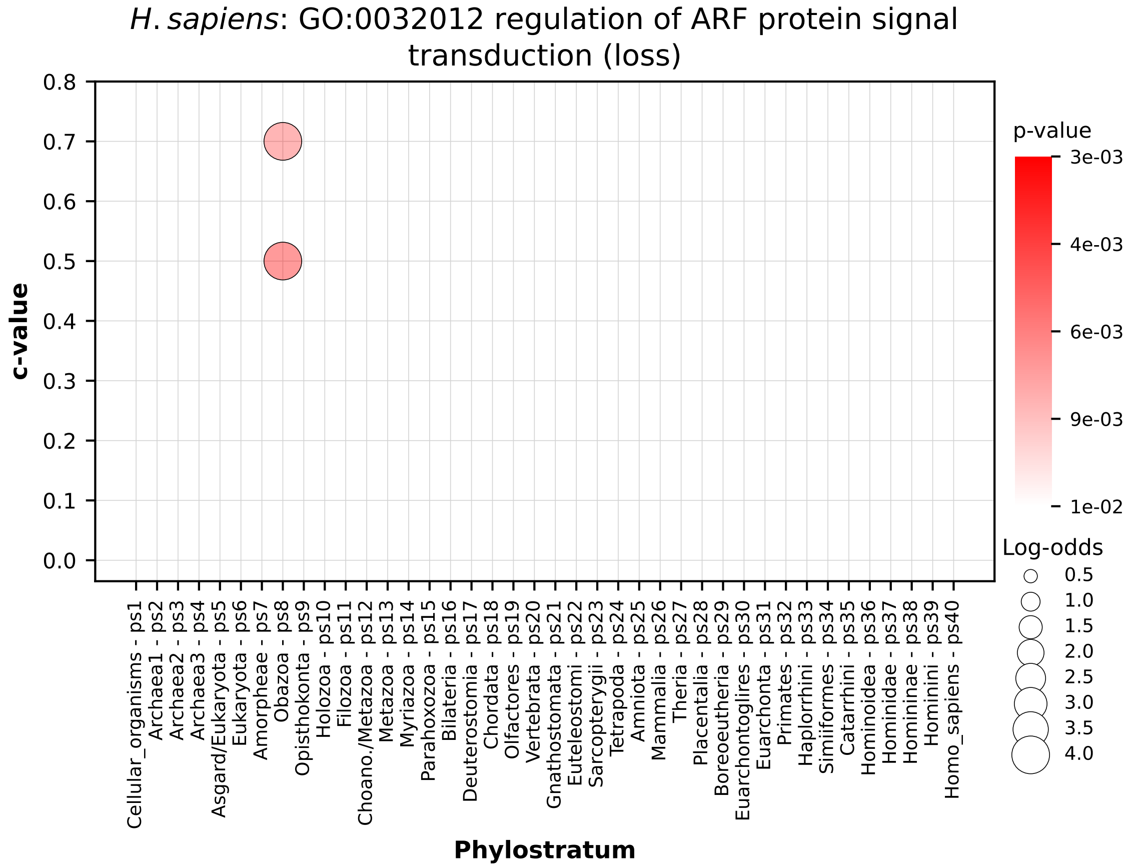

H. sapiens: GO:0032012 regulation of ARF protein signal transduction (loss)
c-value
0.8
0.7
0.6
0.5
0.4
0.3
0.2
0.1
0.0
Phylostratum
Cellular_organisms - ps1
Archaea1 - ps2
Archaea2 - ps3
Archaea3 - ps4
Asgard/Eukaryota - ps5
Eukaryota - ps6
Amorpheae - ps7
Obazoa - ps8
Opisthokonta - ps9
Holozoa - ps10
Filozoa - ps11
Choano./Metazoa - ps12
Metazoa - ps13
Myriazoa - ps14
Parahoxozoa - ps15
Bilateria - ps16
Deuterostomia - ps17
Chordata - ps18
Olfactores - ps19
Vertebrata - ps20
Gnathostomata - ps21
Euteleostomi - ps22
Sarcopterygii - ps23
Tetrapoda - ps24
Amniota - ps25
Mammalia - ps26
Theria - ps27
Placentalia - ps28
Boreoeutheria - ps29
Euarchontoglires - ps30
Euarchonta - ps31
Primates - ps32
Haplorrhini - ps33
Simiiformes - ps34
Catarrhini - ps35
Hominoidea - ps36
Hominidae - ps37
Homininae - ps38
Hominini - ps39
Homo_sapiens - ps40
p-value
3e-03
4e-03
6e-03
9e-03
1e-02
Log-odds
0.5
1.0
1.5
2.0
2.5
3.0
3.5
4.0

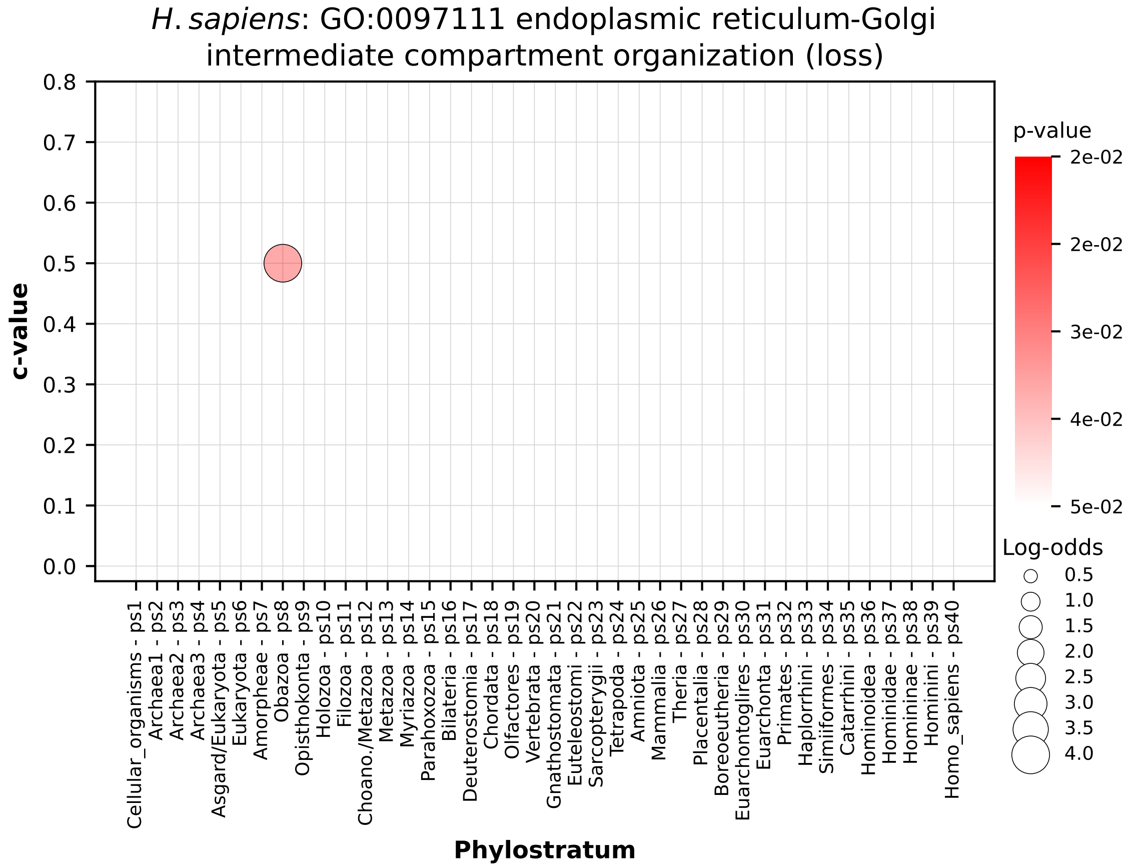

H. sapiens: GO:0097111 endoplasmic reticulum-Golgi intermediate compartment organization (loss)
c-value
0.8
0.7
0.6
0.5
0.4
0.3
0.2
0.1
0.0
Phylostratum
Cellular_organisms - ps1
Archaea1 - ps2
Archaea2 - ps3
Archaea3 - ps4
Asgard/Eukaryota - ps5
Eukaryota - ps6
Amorpheae - ps7
Obazoa - ps8
Opisthokonta - ps9
Holozoa - ps10
Filozoa - ps11
Choano./Metazoa - ps12
Metazoa - ps13
Myriazoa - ps14
Parahoxozoa - ps15
Bilateria - ps16
Deuterostomia - ps17
Chordata - ps18
Olfactores - ps19
Vertebrata - ps20
Gnathostomata - ps21
Euteleostomi - ps22
Sarcopterygii - ps23
Tetrapoda - ps24
Amniota - ps25
Mammalia - ps26
Theria - ps27
Placentalia - ps28
Boreoeutheria - ps29
Euarchontoglires - ps30
Euarchonta - ps31
Primates - ps32
Haplorrhini - ps33
Simiiformes - ps34
Catarrhini - ps35
Hominoidea - ps36
Hominidae - ps37
Homininae - ps38
Hominini - ps39
Homo_sapiens - ps40
p-value
2e-02
2e-02
3e-02
4e-02
5e-02
Log-odds
0.5
1.0
1.5
2.0
2.5
3.0
3.5
4.0

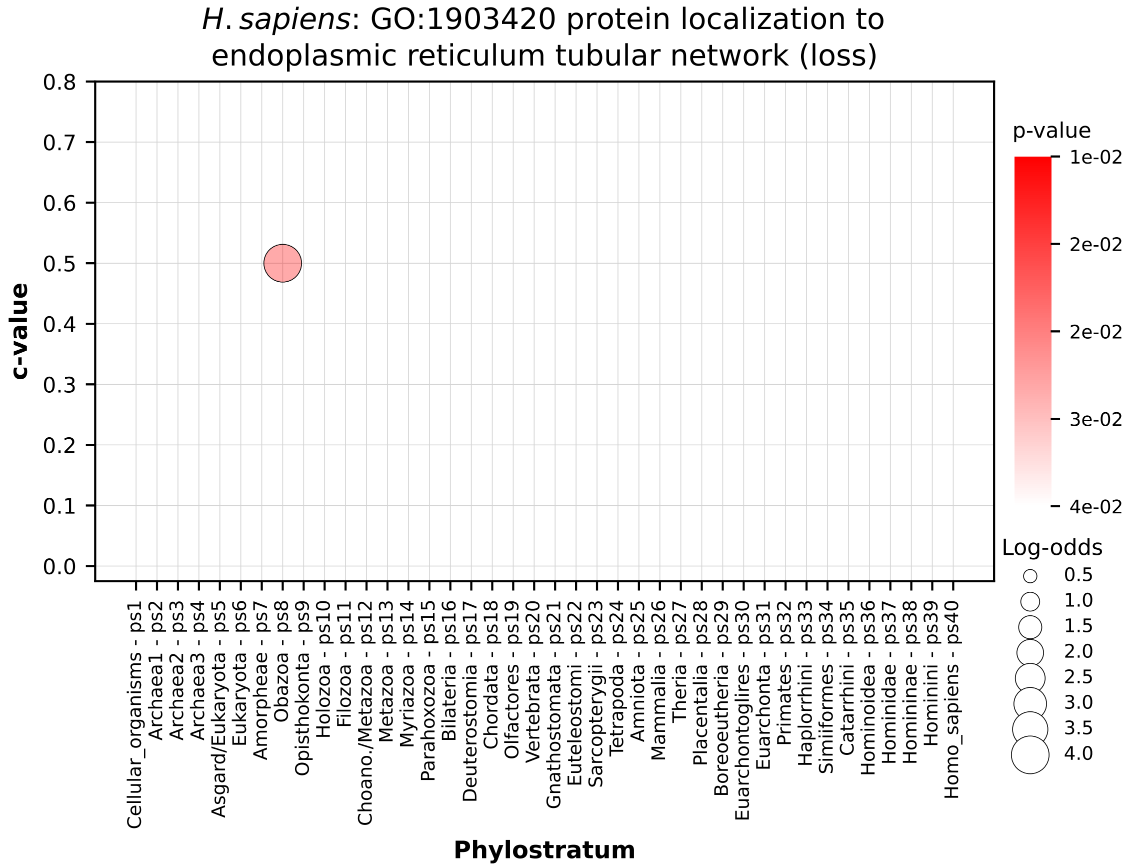

H. sapiens: GO:1903420 protein localization to endoplasmic reticulum tubular network (loss)
c-value
0.0
0.1
0.2
0.3
0.4
0.5
0.6
0.7
0.8
Phylostratum
Cellular_organisms - ps1
Archaea1 - ps2
Archaea2 - ps3
Archaea3 - ps4
Asgard/Eukaryota - ps5
Eukaryota - ps6
Amorpheae - ps7
Obazoa - ps8
Opisthokonta - ps9
Holozoa - ps10
Filozoa - ps11
Choano./Metazoa - ps12
Metazoa - ps13
Myriazoa - ps14
Parahoxozoa - ps15
Bilateria - ps16
Deuterostomia - ps17
Chordata - ps18
Olfactores - ps19
Vertebrata - ps20
Gnathostomata - ps21
Euteleostomi - ps22
Sarcopterygii - ps23
Tetrapoda - ps24
Amniota - ps25
Mammalia - ps26
Theria - ps27
Placentalia - ps28
Boreoeutheria - ps29
Euarchontoglires - ps30
Euarchonta - ps31
Primates - ps32
Haplorrhini - ps33
Simiiformes - ps34
Catarrhini - ps35
Hominoidea - ps36
Hominidae - ps37
Homininae - ps38
Hominini - ps39
Homo_sapiens - ps40
p-value
1e-02
2e-02
2e-02
3e-02
4e-02
Log-odds
0.5
1.0
1.5
2.0
2.5
3.0
3.5
4.0

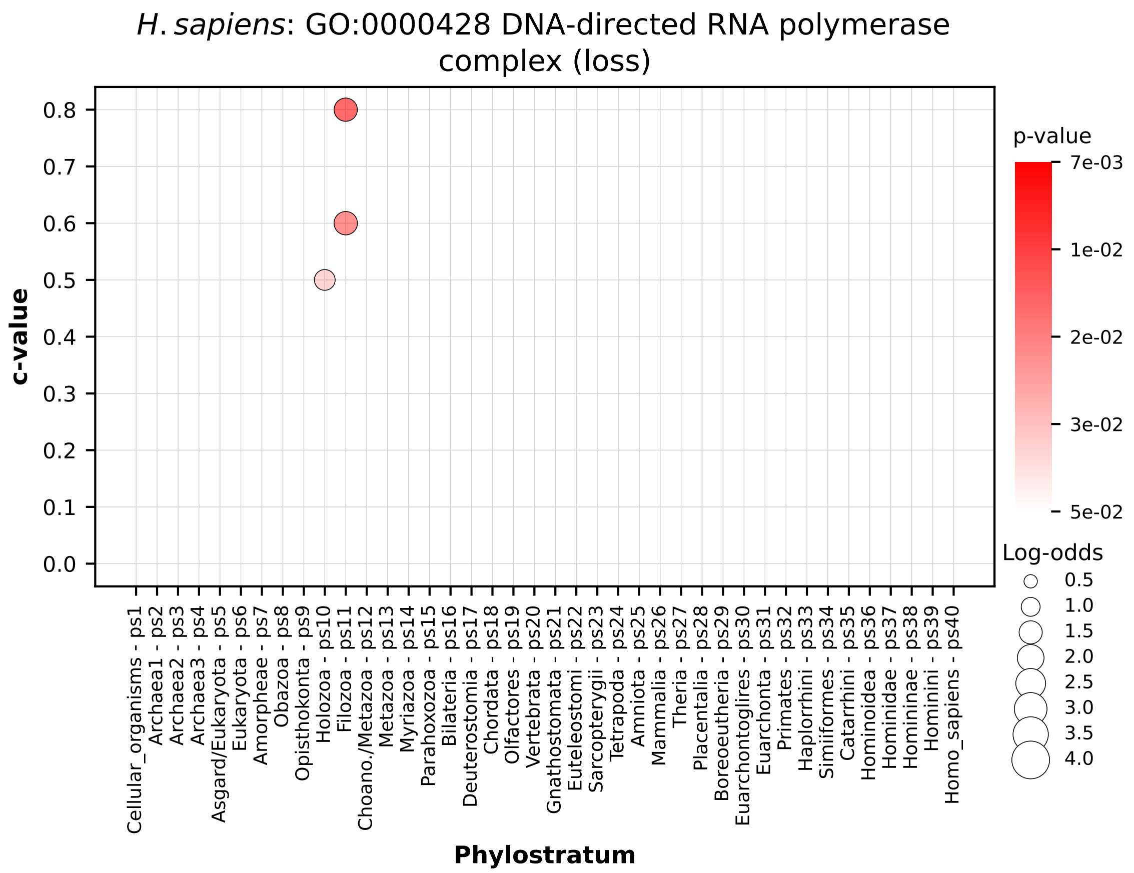
H. sapiens: GO:0000428 DNA-directed RNA polymerase complex (loss)
c-value
0.0
0.1
0.2
0.3
0.4
0.5
0.6
0.7
0.8
Phylostratum
Cellular_organisms - ps1
Archaea1 - ps2
Archaea2 - ps3
Archaea3 - ps4
Asgard/Eukaryota - ps5
Eukaryota - ps6
Amorpheae - ps7
Obazoa - ps8
Opisthokonta - ps9
Holozoa - ps10
Filozoa - ps11
Choano./Metazoa - ps12
Metazoa - ps13
Myriazoa - ps14
Parahoxozoa - ps15
Bilateria - ps16
Deuterostomia - ps17
Chordata - ps18
Olfactores - ps19
Vertebrata - ps20
Gnathostomata - ps21
Euteleostomi - ps22
Sarcopterygii - ps23
Tetrapoda - ps24
Amniota - ps25
Mammalia - ps26
Theria - ps27
Placentalia - ps28
Boreoeutheria - ps29
Euarchontoglires - ps30
Euarchonta - ps31
Primates - ps32
Haplorrhini - ps33
Simiiformes - ps34
Catarrhini - ps35
Hominoidea - ps36
Hominidae - ps37
Homininae - ps38
Hominini - ps39
Homo_sapiens - ps40
p-value
7e-03
1e-02
2e-02
3e-02
5e-02
Log-odds
0.5
1.0
1.5
2.0
2.5
3.0
3.5
4.0

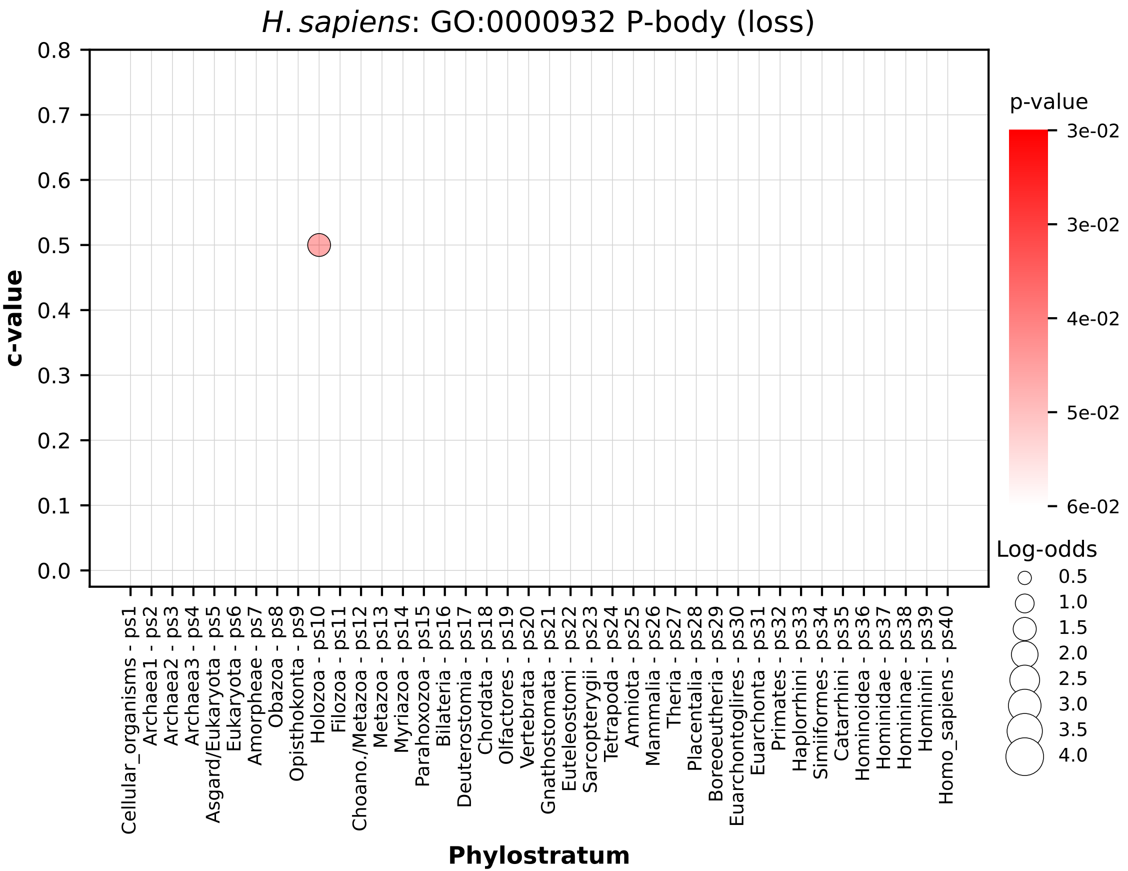

H. sapiens: GO:0000932 P-body (loss)
c-value
0.8
0.7
0.6
0.5
0.4
0.3
0.2
0.1
0.0
Phylostratum
Cellular_organisms - ps1
Archaea1 - ps2
Archaea2 - ps3
Archaea3 - ps4
Asgard/Eukaryota - ps5
Eukaryota - ps6
Amorpheae - ps7
Obazoa - ps8
Opisthokonta - ps9
Holozoa - ps10
Filozoa - ps11
Choano./Metazoa - ps12
Metazoa - ps13
Myriazoa - ps14
Parahoxozoa - ps15
Bilateria - ps16
Deuterostomia - ps17
Chordata - ps18
Olfactores - ps19
Vertebrata - ps20
Gnathostomata - ps21
Euteleostomi - ps22
Sarcopterygii - ps23
Tetrapoda - ps24
Amniota - ps25
Mammalia - ps26
Theria - ps27
Placentalia - ps28
Boreoeutheria - ps29
Euarchontoglires - ps30
Euarchonta - ps31
Primates - ps32
Haplorrhini - ps33
Simiiformes - ps34
Catarrhini - ps35
Hominoidea - ps36
Hominidae - ps37
Homininae - ps38
Hominini - ps39
Homo_sapiens - ps40
p-value
3e-02
3e-02
4e-02
5e-02
6e-02
Log-odds
0.5
1.0
1.5
2.0
2.5
3.0
3.5
4.0

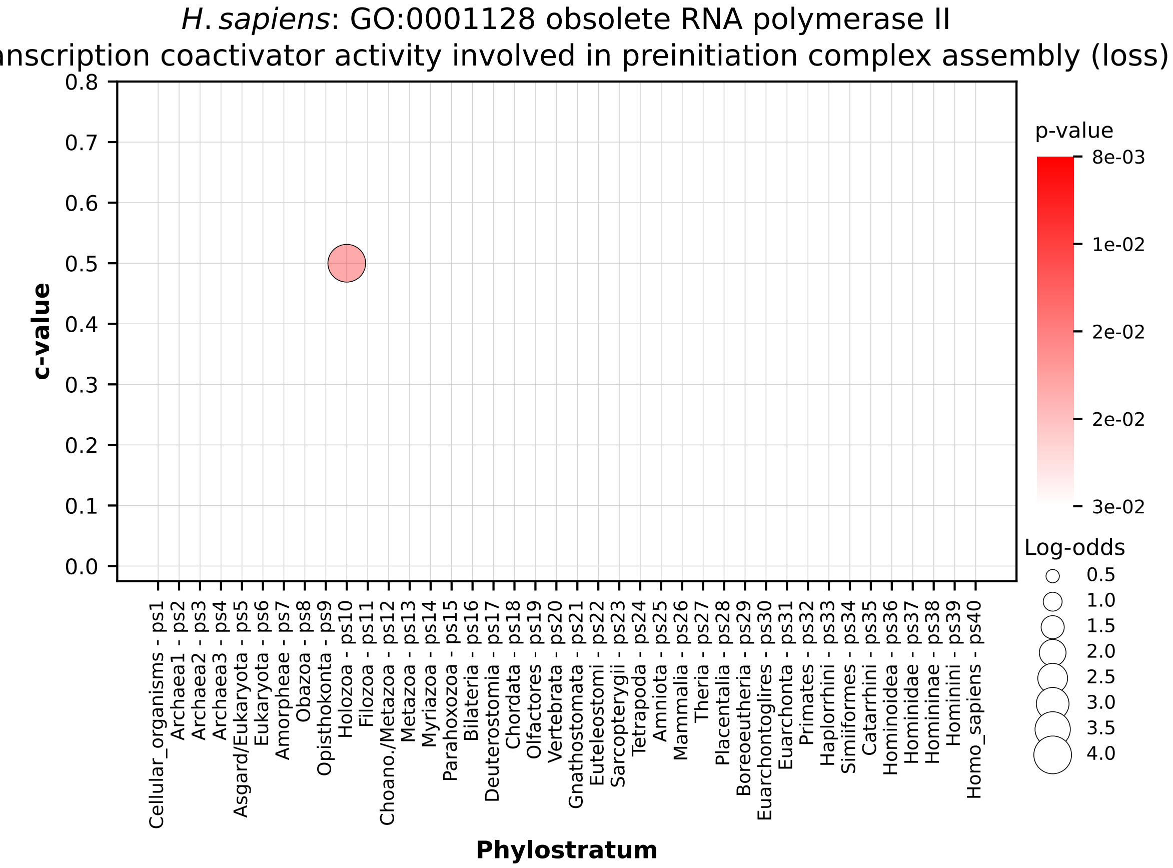

H. sapiens: GO:0001128 obsolete RNA polymerase II
anscription coactivator activity involved in preinitiation complex assembly (loss)
c-value
0.8
0.7
0.6
0.5
0.4
0.3
0.2
0.1
0.0
p-value
8e-03
1e-02
2e-02
2e-02
3e-02
Log-odds
0.5
1.0
1.5
2.0
2.5
3.0
3.5
4.0
Cellular_organisms - ps1
Archaea1 - ps2
Archaea2 - ps3
Archaea3 - ps4
Asgard/Eukaryota - ps5
Eukaryota - ps6
Amorpheae - ps7
Obazoa - ps8
Opisthokonta - ps9
Holozoa - ps10
Filozoa - ps11
Choano./Metazoa - ps12
Metazoa - ps13
Myriazoa - ps14
Parahoxozoa - ps15
Bilateria - ps16
Deuterostomia - ps17
Chordata - ps18
Olfactores - ps19
Vertebrata - ps20
Gnathostomata - ps21
Euteleostomi - ps22
Sarcopterygii - ps23
Tetrapoda - ps24
Amniota - ps25
Mammalia - ps26
Theria - ps27
Placentalia - ps28
Boreoeutheria - ps29
Euarchontoglires - ps30
Euarchonta - ps31
Primates - ps32
Haplorrhini - ps33
Simiiformes - ps34
Catarrhini - ps35
Hominoidea - ps36
Hominidae - ps37
Homininae - ps38
Hominini - ps39
Homo_sapiens - ps40
Phylostratum

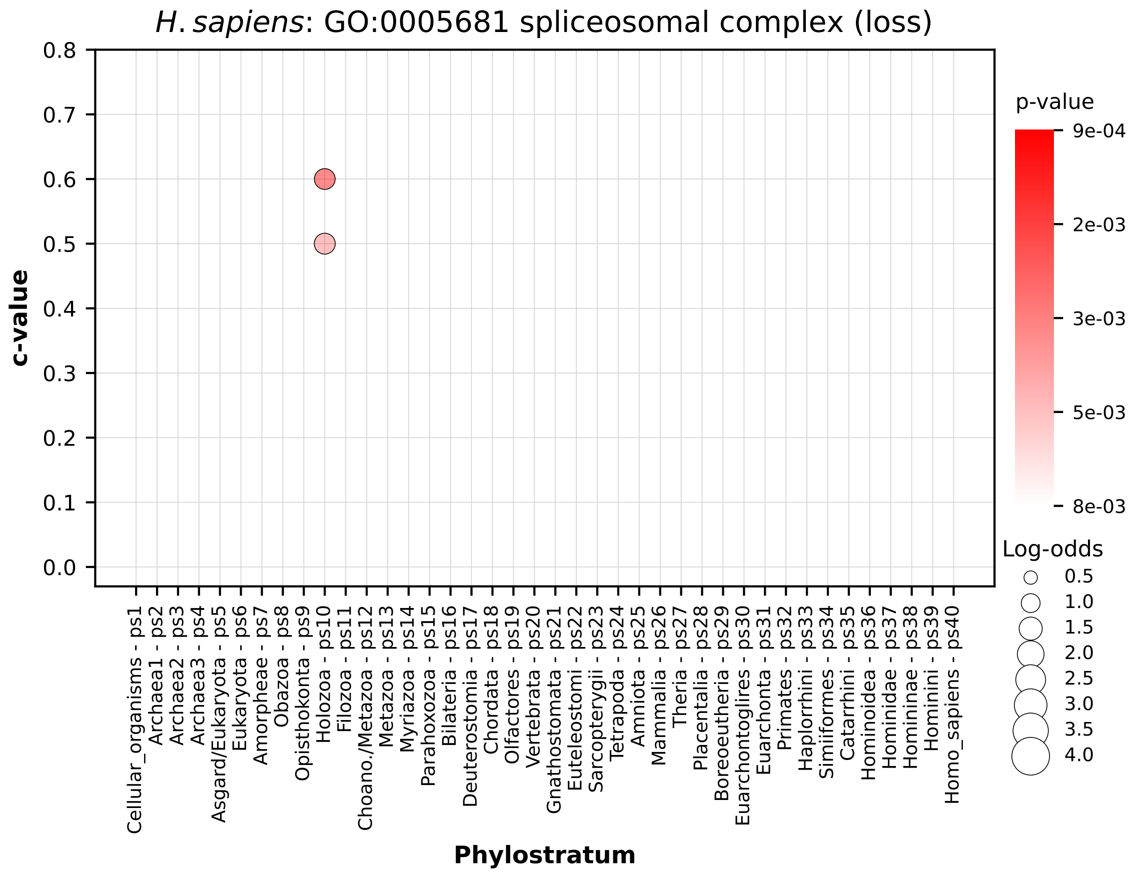

H. sapiens: GO:0005681 spliceosomal complex (loss)
c-value
0.8
0.7
0.6
0.5
0.4
0.3
0.2
0.1
0.0
Phylostratum
Cellular_organisms - ps1
Archaea1 - ps2
Archaea2 - ps3
Archaea3 - ps4
Asgard/Eukaryota - ps5
Eukaryota - ps6
Amorpheae - ps7
Obazoa - ps8
Opisthokonta - ps9
Holozoa - ps10
Filozoa - ps11
Choano./Metazoa - ps12
Metazoa - ps13
Myriazoa - ps14
Parahoxozoa - ps15
Bilateria - ps16
Deuterostomia - ps17
Chordata - ps18
Olfactores - ps19
Vertebrata - ps20
Gnathostomata - ps21
Euteleostomi - ps22
Sarcopterygii - ps23
Tetrapoda - ps24
Amniota - ps25
Mammalia - ps26
Theria - ps27
Placentalia - ps28
Boreoeutheria - ps29
Euarchontoglires - ps30
Euarchonta - ps31
Primates - ps32
Haplorrhini - ps33
Similformes - ps34
Catarrhini - ps35
Hominoidea - ps36
Hominidae - ps37
Homininae - ps38
Hominini - ps39
Homo_sapiens - ps40
p-value
9e-04
2e-03
3e-03
5e-03
8e-03
Log-odds
0.5
1.0
1.5
2.0
2.5
3.0
3.5
4.0

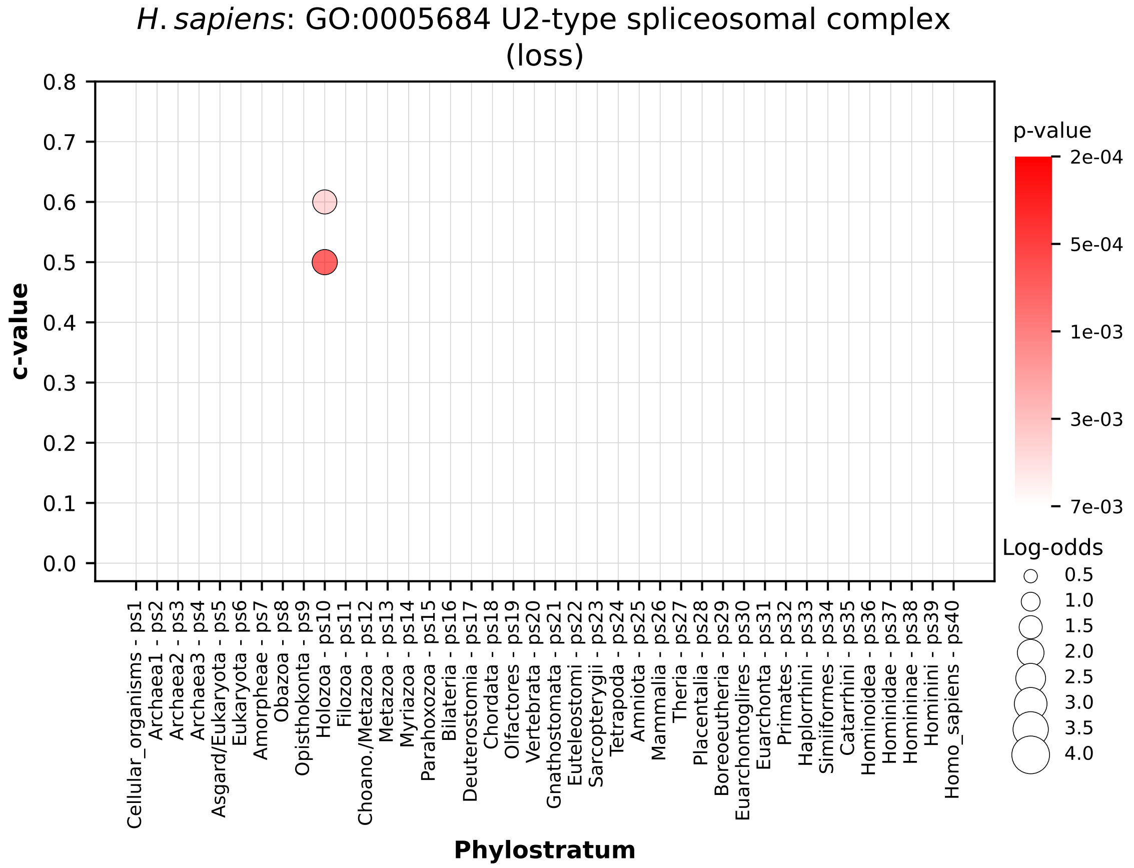
H. sapiens: GO:0005684 U2-type spliceosomal complex
(loss)
c-value
0.0
0.1
0.2
0.3
0.4
0.5
0.6
0.7
0.8
Phylostratum
Cellular_organisms - ps1
Archaea1 - ps2
Archaea2 - ps3
Archaea3 - ps4
Asgard/Eukaryota - ps5
Eukaryota - ps6
Amorpheae - ps7
Obazoa - ps8
Opisthokonta - ps9
Holozoa - ps10
Filozoa - ps11
Choano./Metazoa - ps12
Metazoa - ps13
Myriazoa - ps14
Parahoxozoa - ps15
Bilateria - ps16
Deuterostomia - ps17
Chordata - ps18
Olfactores - ps19
Vertebrata - ps20
Gnathostomata - ps21
Euteleostomi - ps22
Sarcopterygii - ps23
Tetrapoda - ps24
Amniota - ps25
Mammalia - ps26
Theria - ps27
Placentalia - ps28
Boreoeutheria - ps29
Euarchontoglires - ps30
Euarchonta - ps31
Primates - ps32
Haplorrhini - ps33
Simiiformes - ps34
Catarrhini - ps35
Hominoidea - ps36
Hominidae - ps37
Homininae - ps38
Hominini - ps39
Homo_sapiens - ps40
p-value
2e-04
5e-04
1e-03
3e-03
7e-03
Log-odds
0.5
1.0
1.5
2.0
2.5
3.0
3.5
4.0

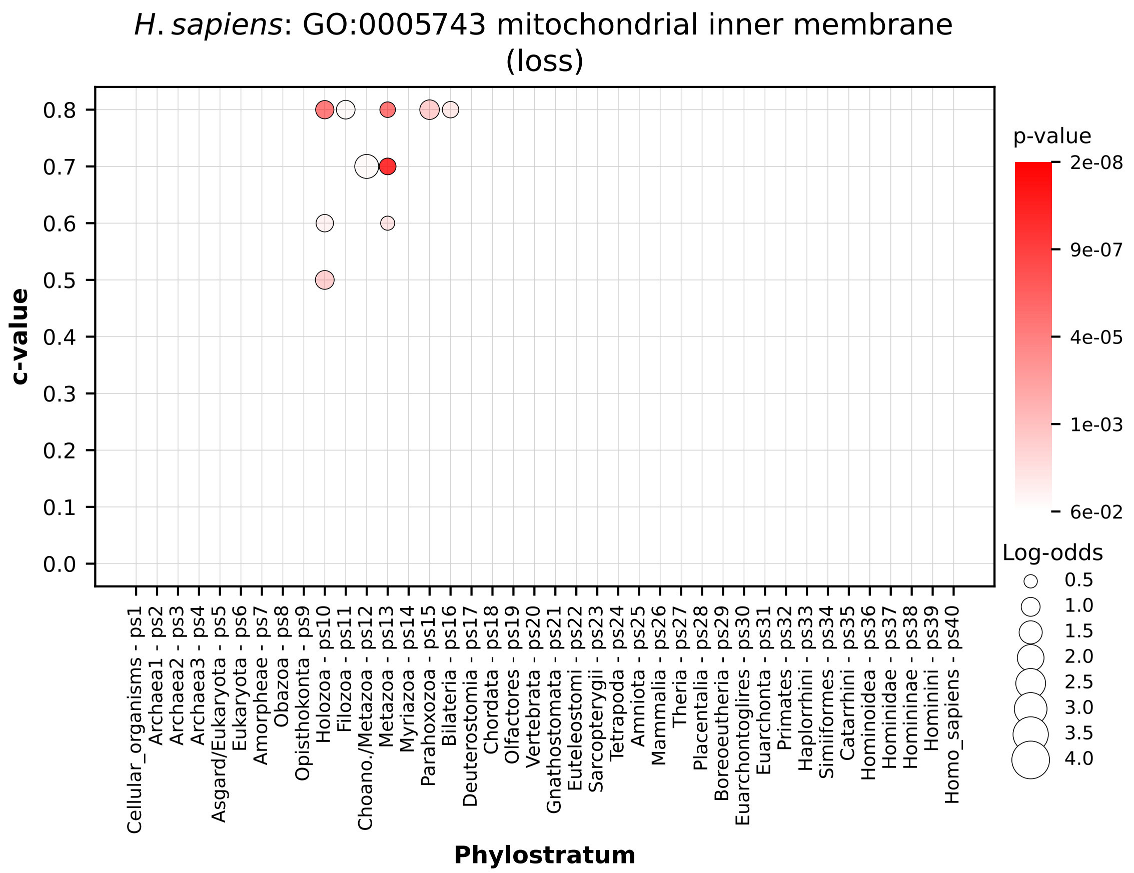

H. sapiens: GO:0005743 mitochondrial inner membrane
(loss)
c-value
0.8
0.7
0.6
0.5
0.4
0.3
0.2
0.1
0.0
p-value
2e-08
9e-07
4e-05
1e-03
6e-02
Log-odds
0.5
1.0
1.5
2.0
2.5
3.0
3.5
4.0
Cellular_organisms - ps1
Archaea1 - ps2
Archaea2 - ps3
Archaea3 - ps4
Asgard/Eukaryota - ps5
Eukaryota - ps6
Amorpheae - ps7
Obazoa - ps8
Opisthokonta - ps9
Holozoa - ps10
Filozoa - ps11
Choano./Metazoa - ps12
Metazoa - ps13
Myriazoa - ps14
Parahoxozoa - ps15
Bilateria - ps16
Deuterostomia - ps17
Chordata - ps18
Olfactores - ps19
Vertebrata - ps20
Gnathostomata - ps21
Euteleostomi - ps22
Sarcopterygii - ps23
Tetrapoda - ps24
Amniota - ps25
Mammalia - ps26
Theria - ps27
Placentalia - ps28
Boreoeutheria - ps29
Euarchontoglires - ps30
Euarchonta - ps31
Primates - ps32
Haplorrhini - ps33
Simiiformes - ps34
Catarrhini - ps35
Hominoidea - ps36
Hominidae - ps37
Homininae - ps38
Hominini - ps39
Homo_sapiens - ps40
Phylostratum

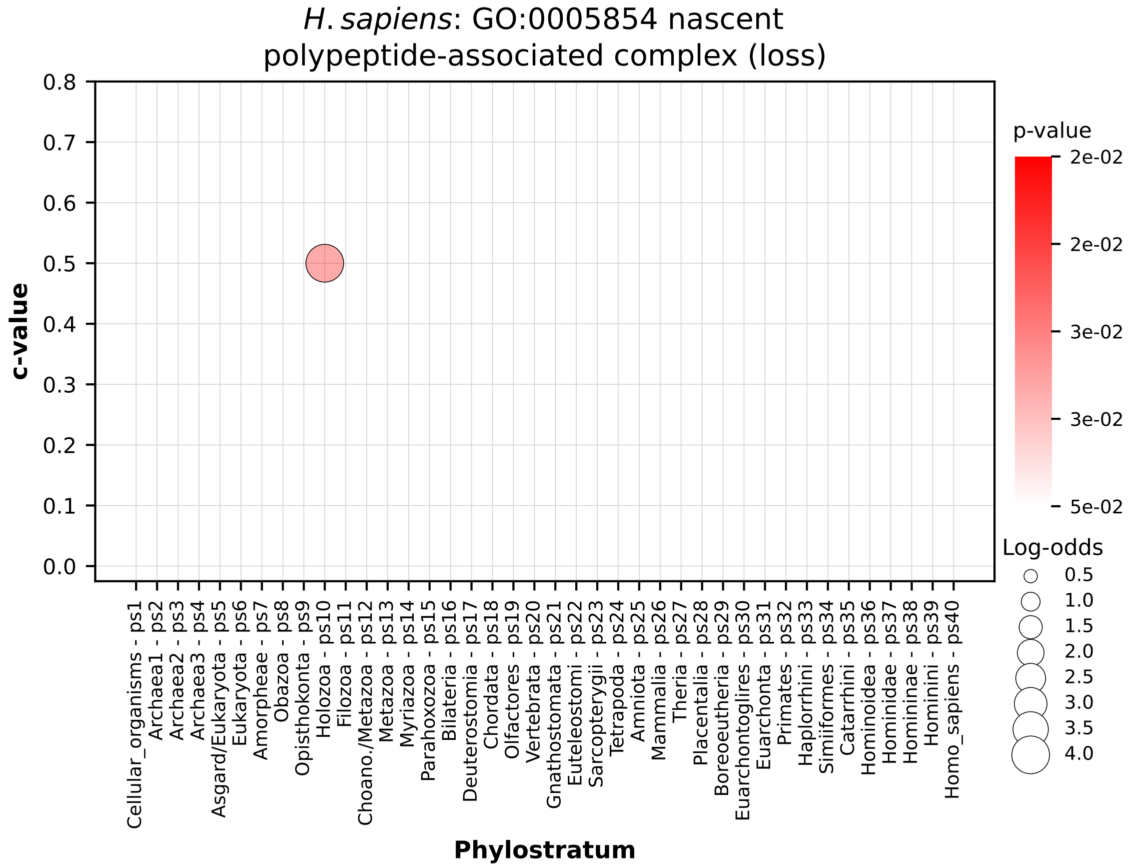

H. sapiens: GO:0005854 nascent polypeptide-associated complex (loss)
c-value
0.0
0.1
0.2
0.3
0.4
0.5
0.6
0.7
0.8
Phylostratum
Cellular_organisms - ps1
Archaea1 - ps2
Archaea2 - ps3
Archaea3 - ps4
Asgard/Eukaryota - ps5
Eukaryota - ps6
Amorpheae - ps7
Obazoa - ps8
Opisthokonta - ps9
Holozoa - ps10
Filozoa - ps11
Choano./Metazoa - ps12
Metazoa - ps13
Myriazoa - ps14
Parahoxozoa - ps15
Bilateria - ps16
Deuterostomia - ps17
Chordata - ps18
Olfactores - ps19
Vertebrata - ps20
Gnathostomata - ps21
Euteleostomi - ps22
Sarcopterygii - ps23
Tetrapoda - ps24
Amniota - ps25
Mammalia - ps26
Theria - ps27
Placentalia - ps28
Boreoeutheria - ps29
Euarchontoglires - ps30
Euarchonta - ps31
Primates - ps32
Haplorrhini - ps33
Simiiformes - ps34
Catarrhini - ps35
Hominoidea - ps36
Hominidae - ps37
Homininae - ps38
Hominini - ps39
Homo_sapiens - ps40
p-value
2e-02
2e-02
3e-02
3e-02
5e-02
Log-odds
0.5
1.0
1.5
2.0
2.5
3.0
3.5
4.0

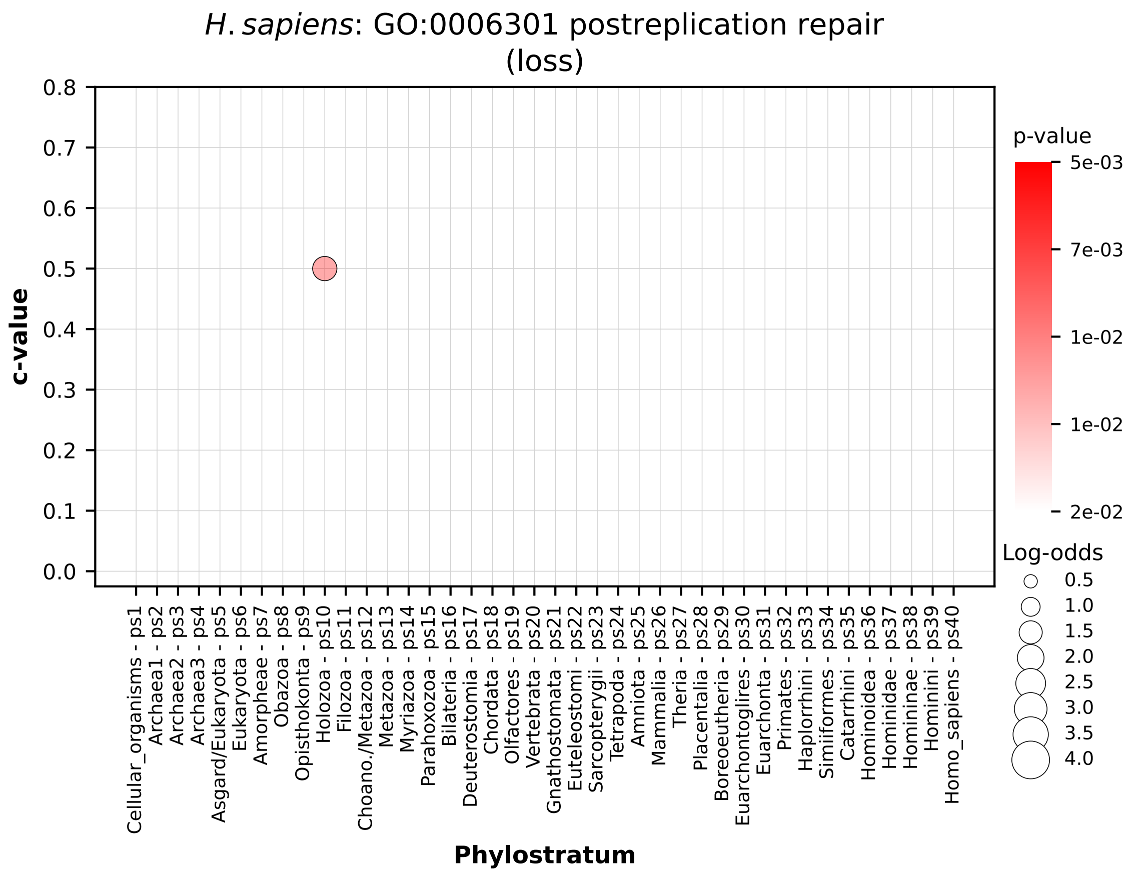

H. sapiens: GO:0006301 postreplication repair
(loss)
c-value
0.8
0.7
0.6
0.5
0.4
0.3
0.2
0.1
0.0
p-value
5e-03
7e-03
1e-02
1e-02
2e-02
Log-odds
0.5
1.0
1.5
2.0
2.5
3.0
3.5
4.0
Cellular_organisms - ps1
Archaea1 - ps2
Archaea2 - ps3
Archaea3 - ps4
Asgard/Eukaryota - ps5
Eukaryota - ps6
Amorpheae - ps7
Obazoa - ps8
Opisthokonta - ps9
Holozoa - ps10
Filozoa - ps11
Choano./Metazoa - ps12
Metazoa - ps13
Myriazoa - ps14
Parahoxozoa - ps15
Bilateria - ps16
Deuterostomia - ps17
Chordata - ps18
Olfactores - ps19
Vertebrata - ps20
Gnathostomata - ps21
Euteleostomi - ps22
Sarcopterygii - ps23
Tetrapoda - ps24
Amniota - ps25
Mammalia - ps26
Theria - ps27
Placentalia - ps28
Boreoeutheria - ps29
Euarchontoglires - ps30
Euarchonta - ps31
Primates - ps32
Haplorrhini - ps33
Simiiformes - ps34
Catarrhini - ps35
Hominoidea - ps36
Hominidae - ps37
Homininae - ps38
Hominini - ps39
Homo_sapiens - ps40
Phylostratum

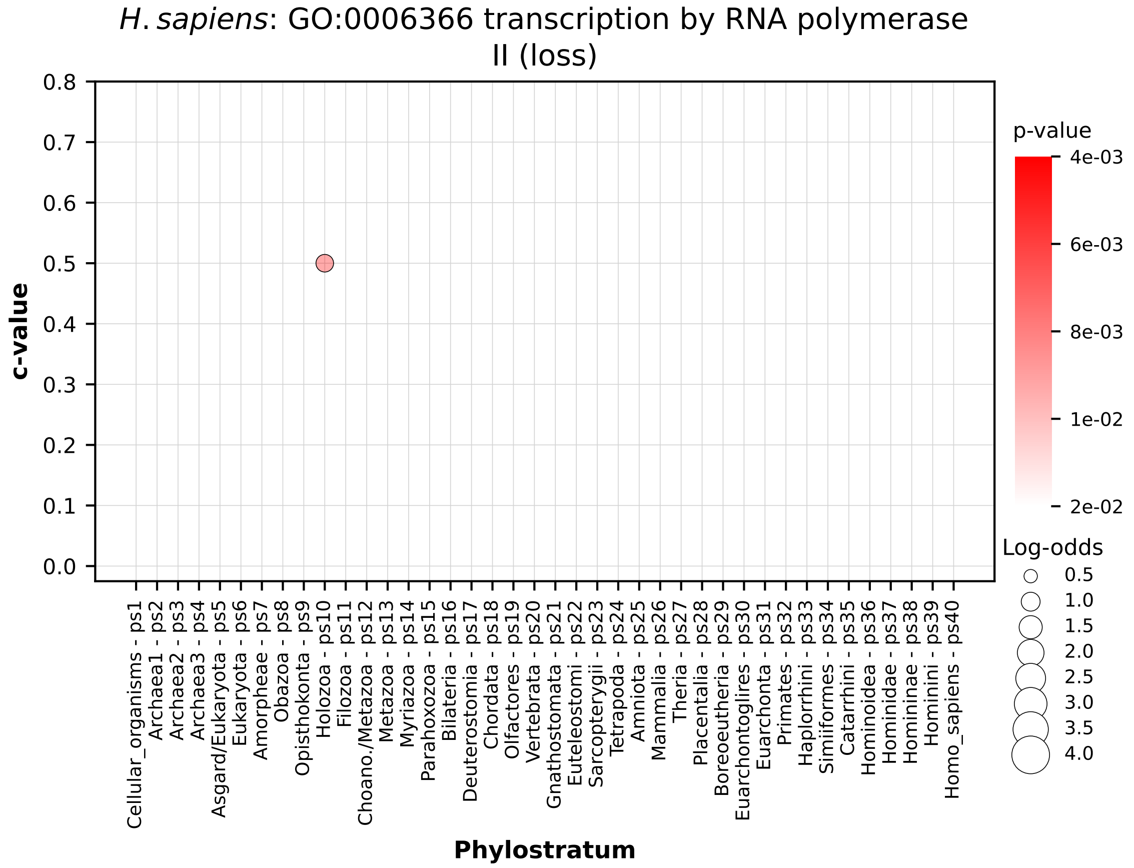

H. sapiens: GO:0006366 transcription by RNA polymerase II (loss)
c-value
0.8
0.7
0.6
0.5
0.4
0.3
0.2
0.1
0.0
p-value
4e-03
6e-03
8e-03
1e-02
2e-02
Log-odds
0.5
1.0
1.5
2.0
2.5
3.0
3.5
4.0
Cellular_organisms - ps1
Archaea1 - ps2
Archaea2 - ps3
Archaea3 - ps4
Asgard/Eukaryota - ps5
Eukaryota - ps6
Amorpheae - ps7
Obazoa - ps8
Opisthokonta - ps9
Holozoa - ps10
Filozoa - ps11
Choano./Metazoa - ps12
Metazoa - ps13
Myriazoa - ps14
Parahoxozoa - ps15
Bilateria - ps16
Deuterostomia - ps17
Chordata - ps18
Olfactores - ps19
Vertebrata - ps20
Gnathostomata - ps21
Euteleostomi - ps22
Sarcopterygii - ps23
Tetrapoda - ps24
Amniota - ps25
Mammalia - ps26
Theria - ps27
Placentalia - ps28
Boreoeutheria - ps29
Euarchontoglires - ps30
Euarchonta - ps31
Primates - ps32
Haplorrhini - ps33
Simiiformes - ps34
Catarrhini - ps35
Hominoidea - ps36
Hominidae - ps37
Homininae - ps38
Hominini - ps39
Homo_sapiens - ps40
Phylostratum

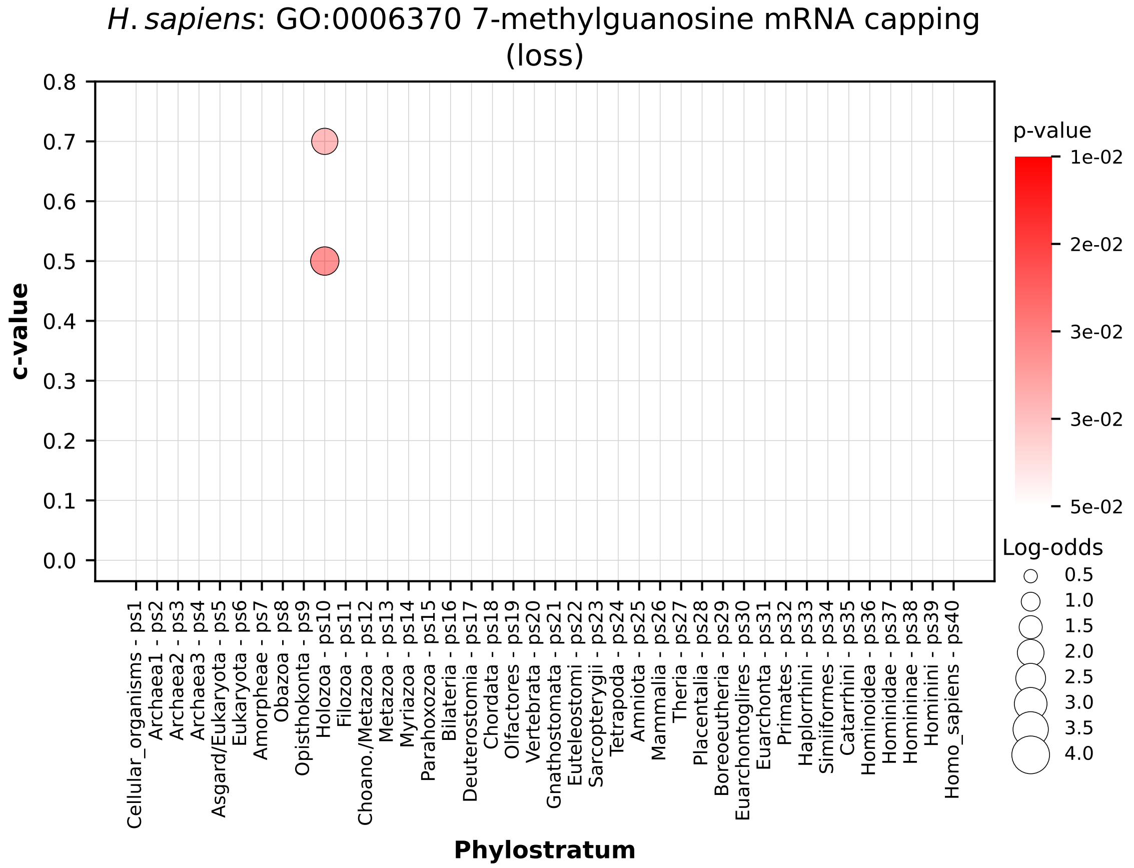

H. sapiens: GO:0006370 7-methylguanosine mRNA capping (loss)
c-value
0.8
0.7
0.6
0.5
0.4
0.3
0.2
0.1
0.0
p-value
1e-02
2e-02
3e-02
3e-02
5e-02
Log-odds
0.5
1.0
1.5
2.0
2.5
3.0
3.5
4.0
Cellular_organisms - ps1
Archaea1 - ps2
Archaea2 - ps3
Archaea3 - ps4
Asgard/Eukaryota - ps5
Eukaryota - ps6
Amorpheae - ps7
Obazoa - ps8
Opisthokonta - ps9
Holozoa - ps10
Filozoa - ps11
Choano./Metazoa - ps12
Metazoa - ps13
Myriazoa - ps14
Parahoxozoa - ps15
Bilateria - ps16
Deuterostomia - ps17
Chordata - ps18
Olfactores - ps19
Vertebrata - ps20
Gnathostomata - ps21
Euteleostomi - ps22
Sarcopterygii - ps23
Tetrapoda - ps24
Amniota - ps25
Mammalia - ps26
Theria - ps27
Placentalia - ps28
Boreoeutheria - ps29
Euarchontoglires - ps30
Euarchonta - ps31
Primates - ps32
Haplorrhini - ps33
Simiiformes - ps34
Catarrhini - ps35
Hominoidea - ps36
Hominidae - ps37
Homininae - ps38
Hominini - ps39
Homo_sapiens - ps40
Phylostratum

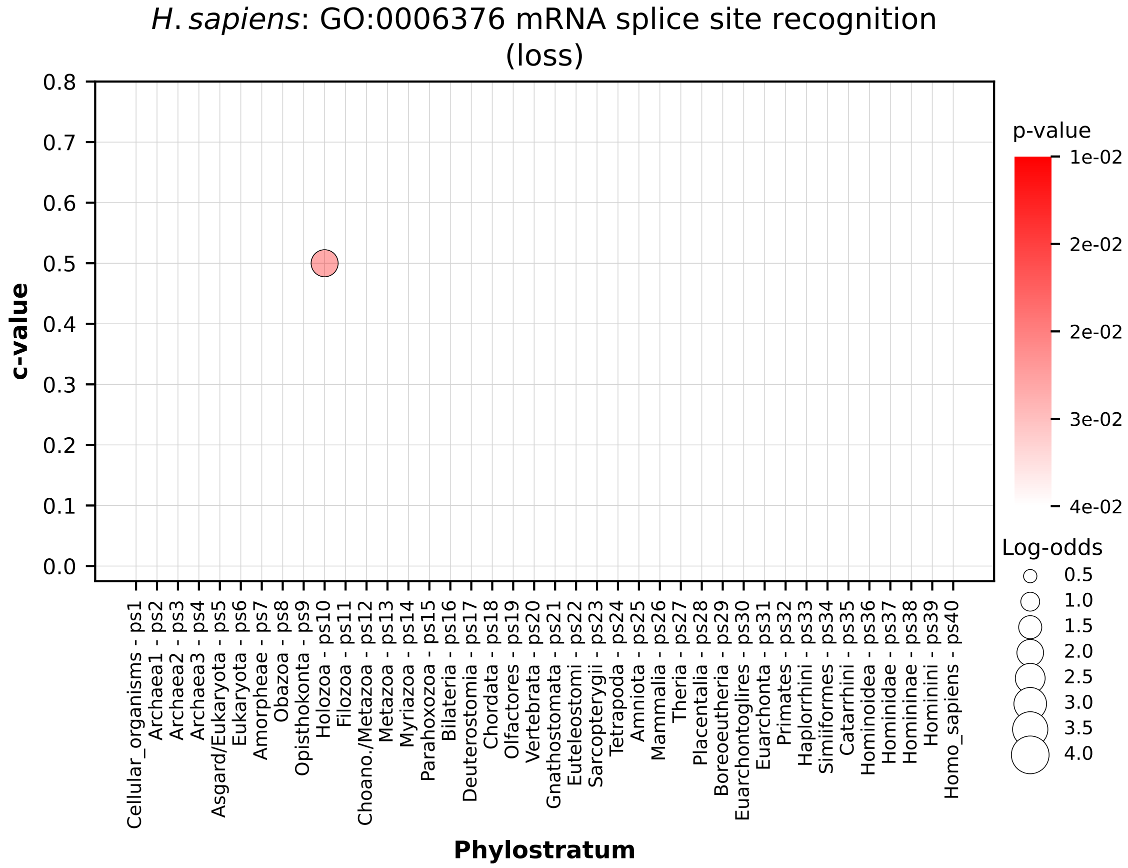
H. sapiens: GO:0006376 mRNA splice site recognition
(loss)
c-value
0.8
0.7
0.6
0.5
0.4
0.3
0.2
0.1
0.0
p-value
1e-02
2e-02
2e-02
3e-02
4e-02
Log-odds
0.5
1.0
1.5
2.0
2.5
3.0
3.5
4.0
Cellular_organisms - ps1
Archaea1 - ps2
Archaea2 - ps3
Archaea3 - ps4
Asgard/Eukaryota - ps5
Eukaryota - ps6
Amorpheae - ps7
Obazoa - ps8
Opisthokonta - ps9
Holozoa - ps10
Filozoa - ps11
Choano./Metazoa - ps12
Metazoa - ps13
Myriazoa - ps14
Parahoxozoa - ps15
Bilateria - ps16
Deuterostomia - ps17
Chordata - ps18
Olfactores - ps19
Vertebrata - ps20
Gnathostomata - ps21
Euteleostomi - ps22
Sarcopterygii - ps23
Tetrapoda - ps24
Amniota - ps25
Mammalia - ps26
Theria - ps27
Placentalia - ps28
Boreoeutheria - ps29
Euarchontoglires - ps30
Euarchonta - ps31
Primates - ps32
Haplorrhini - ps33
Simiiformes - ps34
Catarrhini - ps35
Hominoidea - ps36
Hominidae - ps37
Homininae - ps38
Hominini - ps39
Homo_sapiens - ps40
Phylostratum

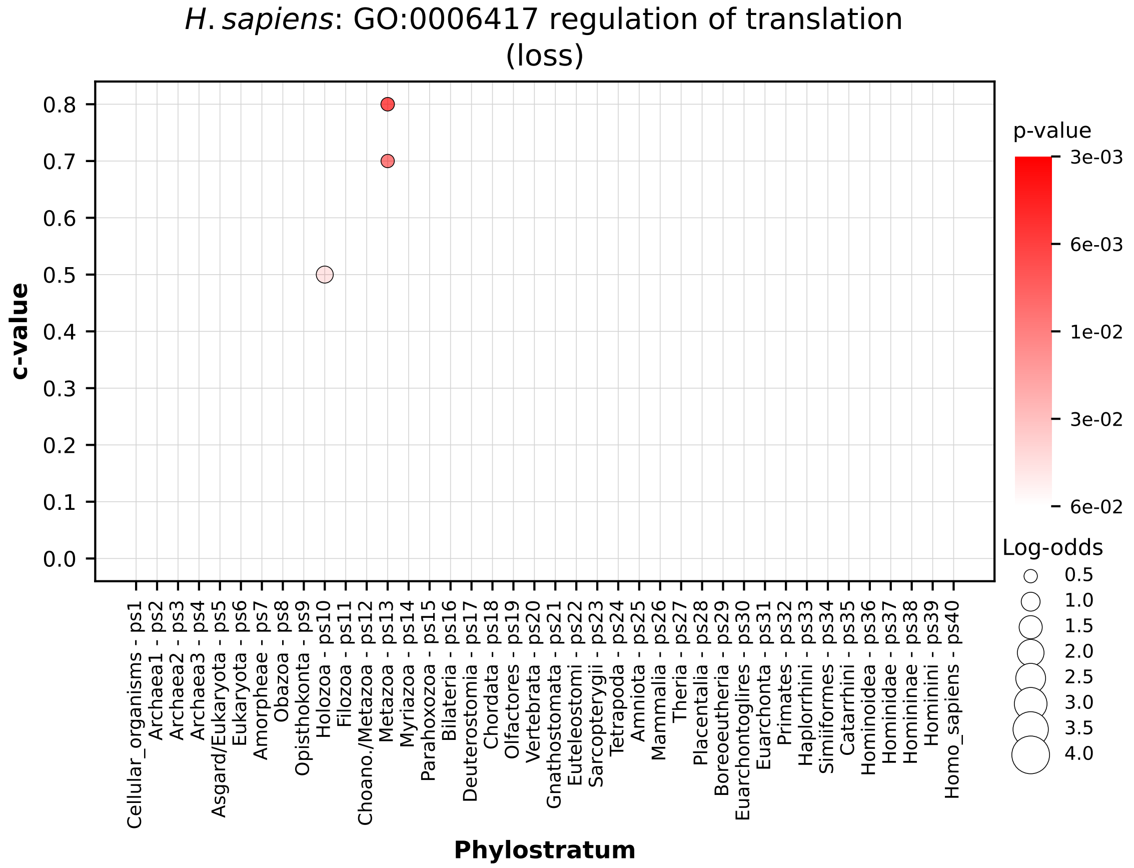

H. sapiens: GO:0006417 regulation of translation
(loss)
c-value
0.0
0.1
0.2
0.3
0.4
0.5
0.6
0.7
0.8
Phylostratum
Cellular_organisms - ps1
Archaea1 - ps2
Archaea2 - ps3
Archaea3 - ps4
Asgard/Eukaryota - ps5
Eukaryota - ps6
Amorpheae - ps7
Obazoa - ps8
Opisthokonta - ps9
Holozoa - ps10
Filozoa - ps11
Choano./Metazoa - ps12
Metazoa - ps13
Myriazoa - ps14
Parahoxozoa - ps15
Bilateria - ps16
Deuterostomia - ps17
Chordata - ps18
Olfactores - ps19
Vertebrata - ps20
Gnathostomata - ps21
Euteleostomi - ps22
Sarcopterygii - ps23
Tetrapoda - ps24
Amniota - ps25
Mammalia - ps26
Theria - ps27
Placentalia - ps28
Boreoeutheria - ps29
Euarchontoglires - ps30
Euarchonta - ps31
Primates - ps32
Haplorrhini - ps33
Simiiformes - ps34
Catarrhini - ps35
Hominoidea - ps36
Hominidae - ps37
Homininae - ps38
Hominini - ps39
Homo_sapiens - ps40
p-value
3e-03
6e-03
1e-02
3e-02
6e-02
Log-odds
0.5
1.0
1.5
2.0
2.5
3.0
3.5
4.0

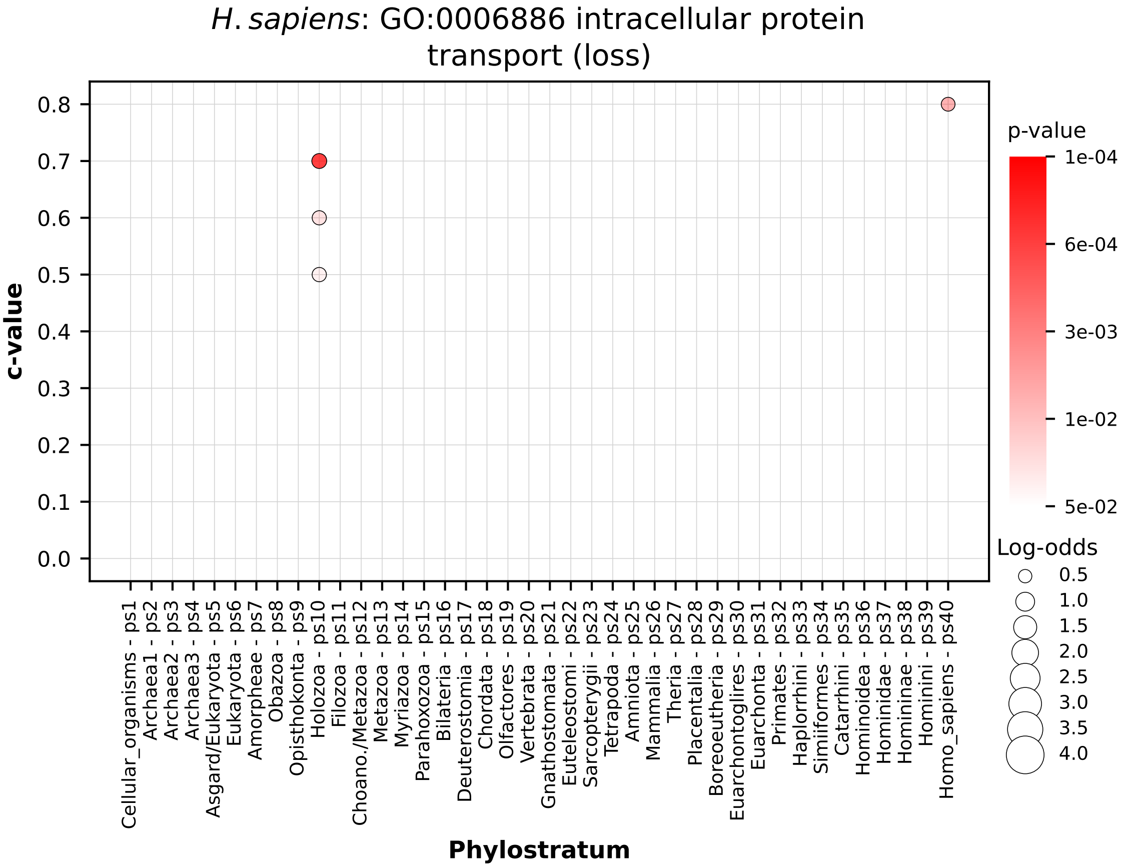

H. sapiens: GO:0006886 intracellular protein transport (loss)
c-value
0.0
0.1
0.2
0.3
0.4
0.5
0.6
0.7
0.8
Phylostratum
Cellular_organisms - ps1
Archaea1 - ps2
Archaea2 - ps3
Archaea3 - ps4
Asgard/Eukaryota - ps5
Eukaryota - ps6
Amorpheae - ps7
Obazoa - ps8
Opisthokonta - ps9
Holozoa - ps10
Filozoa - ps11
Choano./Metazoa - ps12
Metazoa - ps13
Myriazoa - ps14
Parahoxozoa - ps15
Bilateria - ps16
Deuterostomia - ps17
Chordata - ps18
Olfactores - ps19
Vertebrata - ps20
Gnathostomata - ps21
Euteleostomi - ps22
Sarcopterygii - ps23
Tetrapoda - ps24
Amniota - ps25
Mammalia - ps26
Theria - ps27
Placentalia - ps28
Boreoeutheria - ps29
Euarchontoglires - ps30
Euarchonta - ps31
Primates - ps32
Haplorrhini - ps33
Simiiformes - ps34
Catarrhini - ps35
Hominoidea - ps36
Hominidae - ps37
Homininae - ps38
Hominini - ps39
Homo_sapiens - ps40
p-value
1e-04
6e-04
3e-03
1e-02
5e-02
Log-odds
0.5
1.0
1.5
2.0
2.5
3.0
3.5
4.0

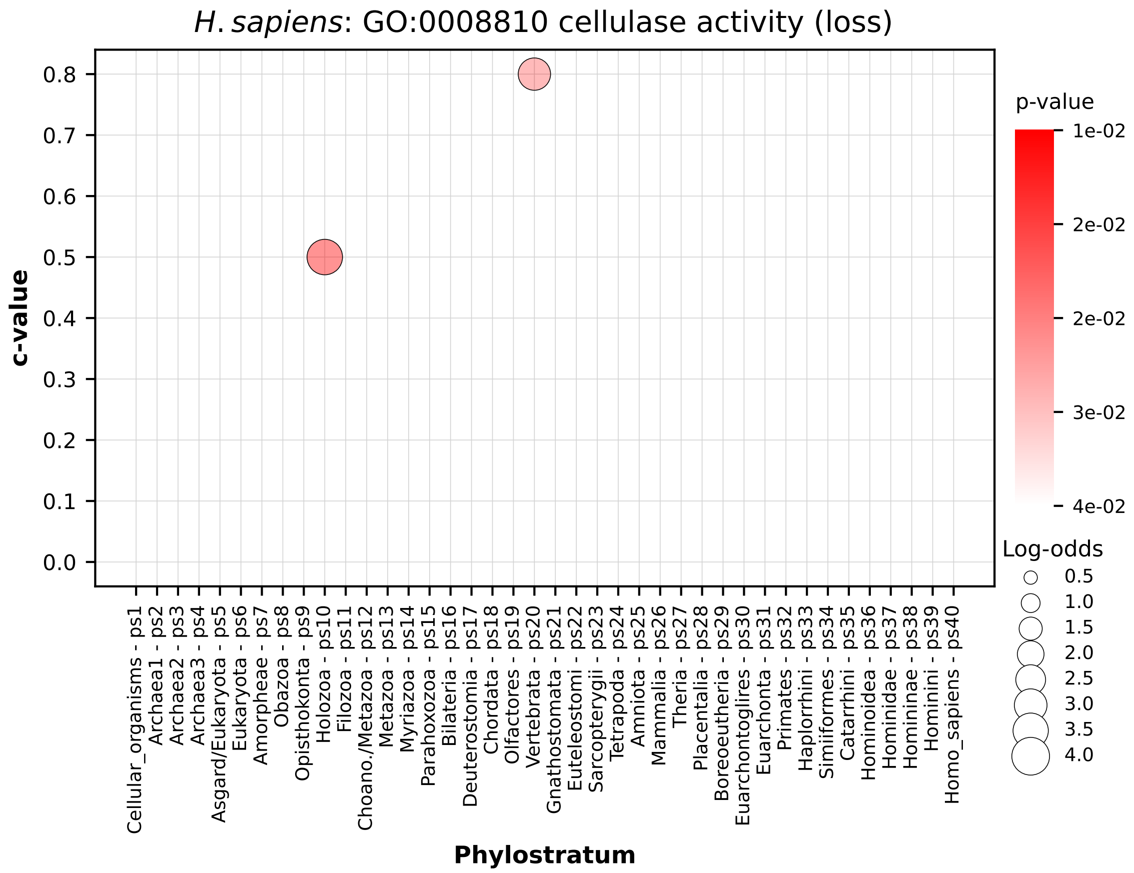

H. sapiens: GO:0008810 cellulase activity (loss)
c-value
0.8
0.7
0.6
0.5
0.4
0.3
0.2
0.1
0.0
p-value
1e-02
2e-02
2e-02
3e-02
4e-02
Log-odds
0.5
1.0
1.5
2.0
2.5
3.0
3.5
4.0
Cellular_organisms - ps1
Archaea1 - ps2
Archaea2 - ps3
Archaea3 - ps4
Asgard/Eukaryota - ps5
Eukaryota - ps6
Amorpheae - ps7
Obazoa - ps8
Opisthokonta - ps9
Holozoa - ps10
Filozoa - ps11
Choano./Metazoa - ps12
Metazoa - ps13
Myriazoa - ps14
Parahoxozoa - ps15
Bilateria - ps16
Deuterostomia - ps17
Chordata - ps18
Olfactores - ps19
Vertebrata - ps20
Gnathostomata - ps21
Euteleostomi - ps22
Sarcopterygii - ps23
Tetrapoda - ps24
Amniota - ps25
Mammalia - ps26
Theria - ps27
Placentalia - ps28
Boreoeutheria - ps29
Euarchontoglires - ps30
Euarchonta - ps31
Primates - ps32
Haplorrhini - ps33
Simiiformes - ps34
Catarrhini - ps35
Hominoidea - ps36
Hominidae - ps37
Homininae - ps38
Hominini - ps39
Homo_sapiens - ps40
Phylostratum

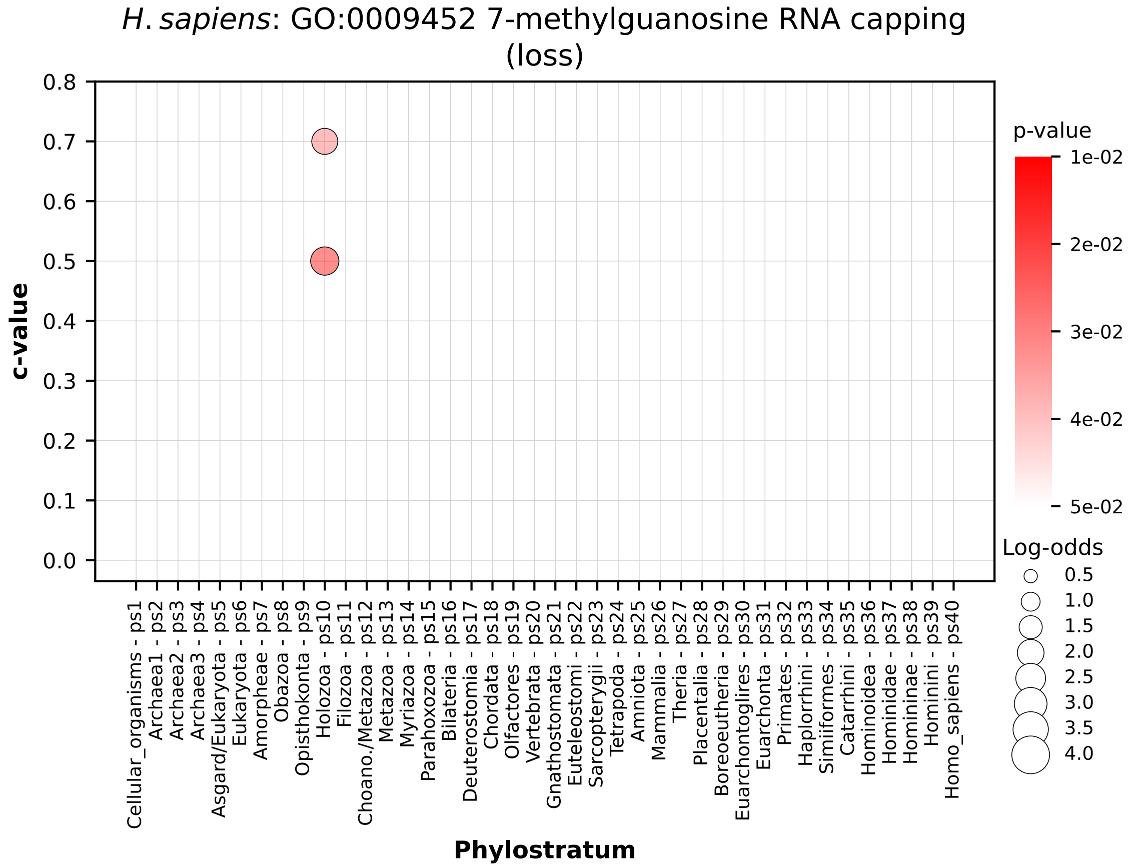

H. sapiens: GO:0009452 7-methylguanosine RNA capping (loss)
c-value
0.8
0.7
0.6
0.5
0.4
0.3
0.2
0.1
0.0
p-value
1e-02
2e-02
3e-02
4e-02
5e-02
Log-odds
0.5
1.0
1.5
2.0
2.5
3.0
3.5
4.0
Cellular_organisms - ps1
Archaea1 - ps2
Archaea2 - ps3
Archaea3 - ps4
Asgard/Eukaryota - ps5
Eukaryota - ps6
Amorpheae - ps7
Obazoa - ps8
Opisthokonta - ps9
Holozoa - ps10
Filozoa - ps11
Choano./Metazoa - ps12
Metazoa - ps13
Myriazoa - ps14
Parahoxozoa - ps15
Bilateria - ps16
Deuterostomia - ps17
Chordata - ps18
Olfactores - ps19
Vertebrata - ps20
Gnathostomata - ps21
Euteleostomi - ps22
Sarcopterygii - ps23
Tetrapoda - ps24
Amniota - ps25
Mammalia - ps26
Theria - ps27
Placentalia - ps28
Boreoeutheria - ps29
Euarchontoglires - ps30
Euarchonta - ps31
Primates - ps32
Haplorrhini - ps33
Simiiformes - ps34
Catarrhini - ps35
Hominoidea - ps36
Hominidae - ps37
Homininae - ps38
Hominini - ps39
Homo_sapiens - ps40
Phylostratum

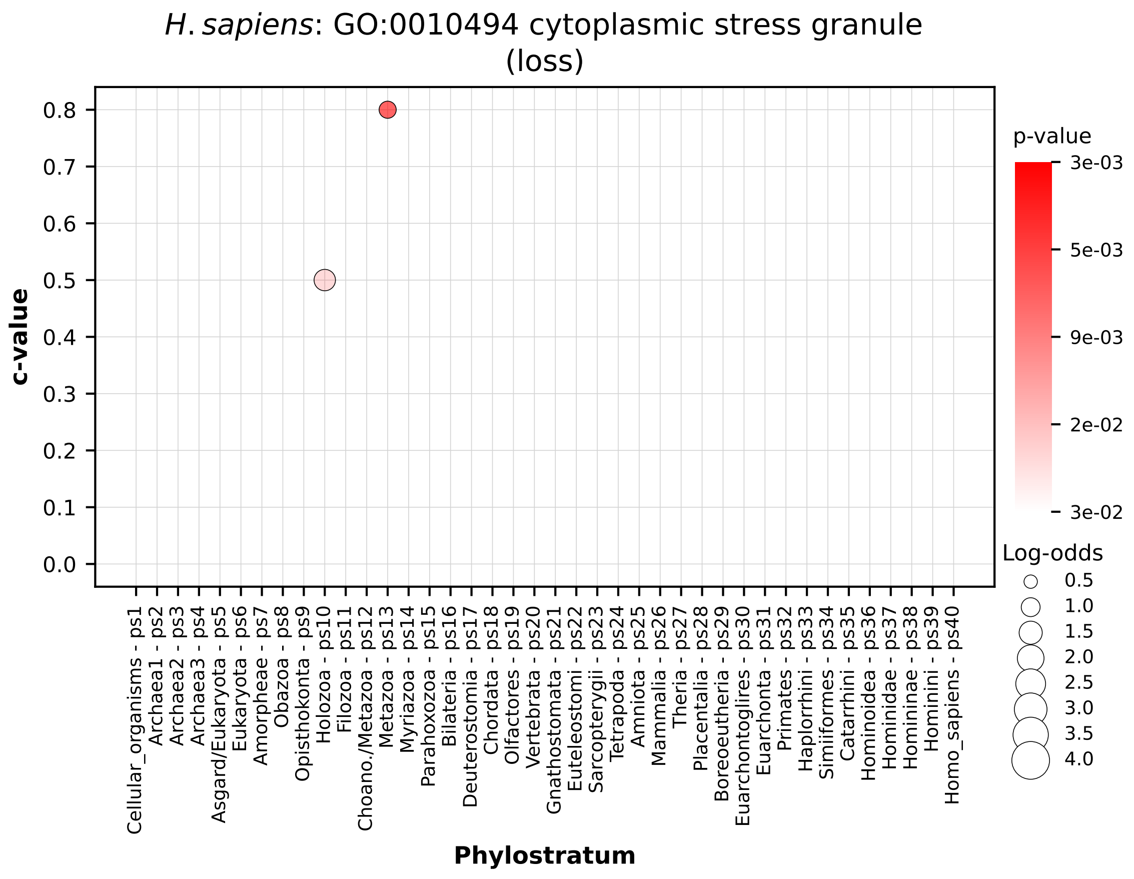

H. sapiens: GO:0010494 cytoplasmic stress granule (loss)
c-value
0.0
0.1
0.2
0.3
0.4
0.5
0.6
0.7
0.8
p-value
3e-03
5e-03
9e-03
2e-02
3e-02
Log-odds
0.5
1.0
1.5
2.0
2.5
3.0
3.5
4.0
Cellular_organisms - ps1
Archaea1 - ps2
Archaea2 - ps3
Archaea3 - ps4
Asgard/Eukaryota - ps5
Eukaryota - ps6
Amorpheae - ps7
Obazoa - ps8
Opisthokonta - ps9
Holozoa - ps10
Filozoa - ps11
Choano./Metazoa - ps12
Metazoa - ps13
Myriazoa - ps14
Parahoxozoa - ps15
Bilateria - ps16
Deuterostomia - ps17
Chordata - ps18
Olfactores - ps19
Vertebrata - ps20
Gnathostomata - ps21
Euteleostomi - ps22
Sarcopterygii - ps23
Tetrapoda - ps24
Amniota - ps25
Mammalia - ps26
Theria - ps27
Placentalia - ps28
Boreoeutheria - ps29
Euarchontoglires - ps30
Euarchonta - ps31
Primates - ps32
Haplorrhini - ps33
Simiiformes - ps34
Catarrhini - ps35
Hominoidea - ps36
Hominidae - ps37
Homininae - ps38
Hominini - ps39
Homo_sapiens - ps40
Phylostratum

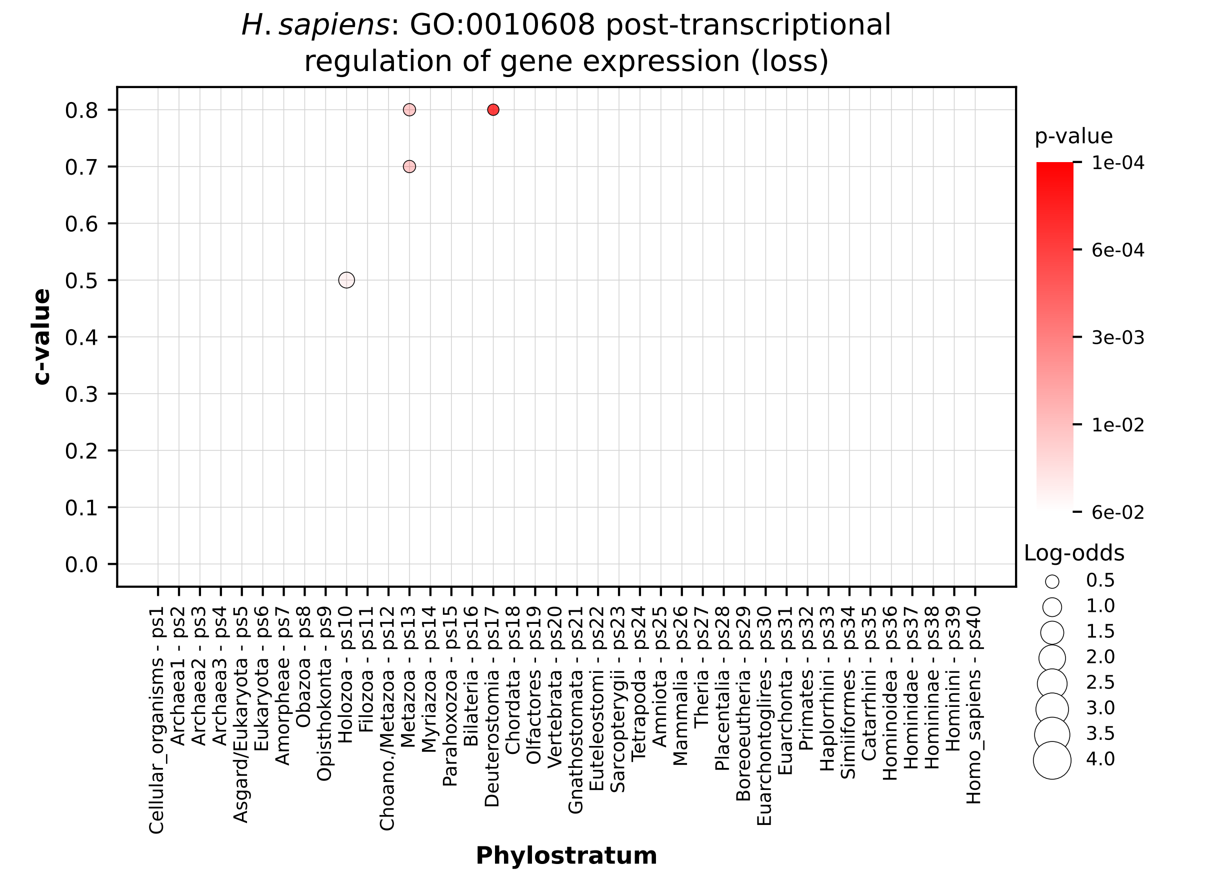

H. sapiens: GO:0010608 post-transcriptional regulation of gene expression (loss)
c-value
0.0
0.1
0.2
0.3
0.4
0.5
0.6
0.7
0.8
Phylostratum
Cellular_organisms - ps1
Archaea1 - ps2
Archaea2 - ps3
Archaea3 - ps4
Asgard/Eukaryota - ps5
Eukaryota - ps6
Amorpheae - ps7
Obazoa - ps8
Opisthokonta - ps9
Holozoa - ps10
Filozoa - ps11
Choano./Metazoa - ps12
Metazoa - ps13
Myriazoa - ps14
Parahoxozoa - ps15
Bilateria - ps16
Deuterostomia - ps17
Chordata - ps18
Olfactores - ps19
Vertebrata - ps20
Gnathostomata - ps21
Euteleostomi - ps22
Sarcopterygii - ps23
Tetrapoda - ps24
Amniota - ps25
Mammalia - ps26
Theria - ps27
Placentalia - ps28
Boreoeutheria - ps29
Euarchontoglires - ps30
Euarchonta - ps31
Primates - ps32
Haplorrhini - ps33
Simiiformes - ps34
Catarrhini - ps35
Hominoidea - ps36
Hominidae - ps37
Homininae - ps38
Hominini - ps39
Homo_sapiens - ps40
p-value
1e-04
6e-04
3e-03
1e-02
6e-02
Log-odds
0.5
1.0
1.5
2.0
2.5
3.0
3.5
4.0

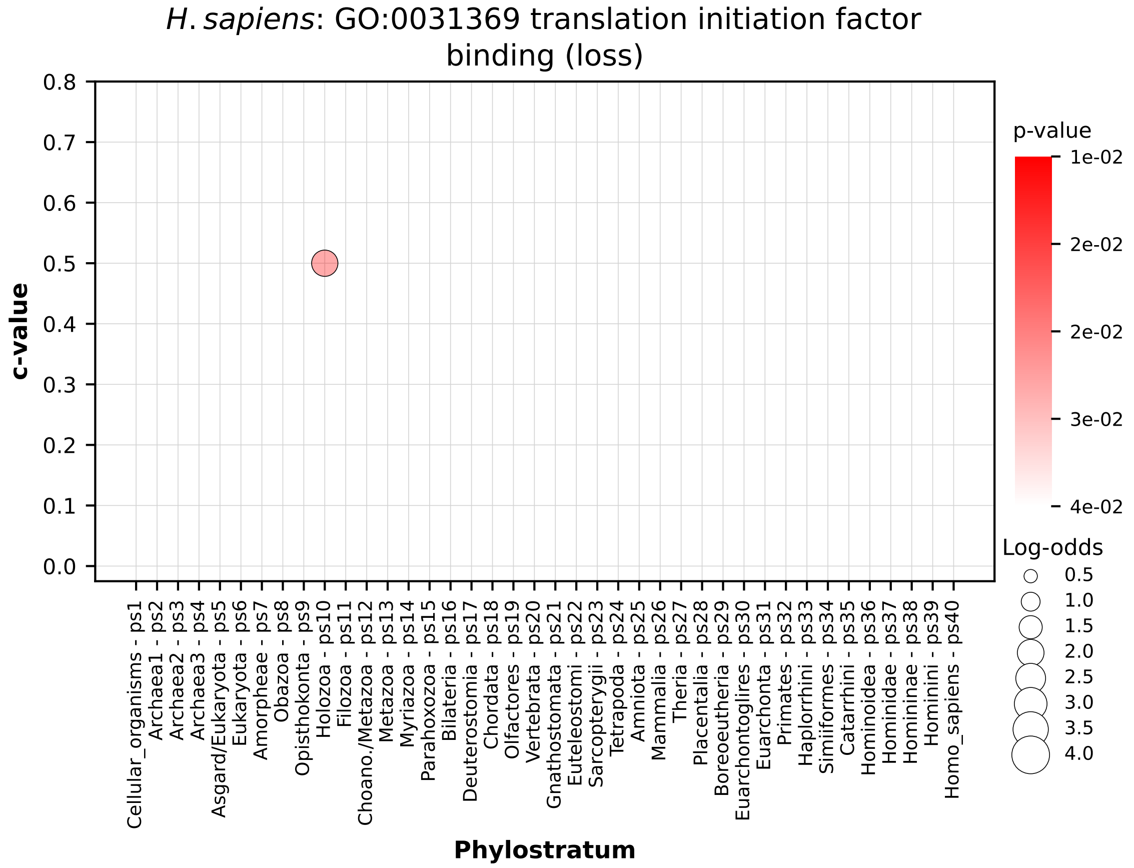

H. sapiens: GO:0031369 translation initiation factor binding (loss)
c-value
0.8
0.7
0.6
0.5
0.4
0.3
0.2
0.1
0.0
Phylostratum
Cellular_organisms - ps1
Archaea1 - ps2
Archaea2 - ps3
Archaea3 - ps4
Asgard/Eukaryota - ps5
Eukaryota - ps6
Amorpheae - ps7
Obazoa - ps8
Opisthokonta - ps9
Holozoa - ps10
Filozoa - ps11
Choano./Metazoa - ps12
Metazoa - ps13
Myriazoa - ps14
Parahoxozoa - ps15
Bilateria - ps16
Deuterostomia - ps17
Chordata - ps18
Olfactores - ps19
Vertebrata - ps20
Gnathostomata - ps21
Euteleostomi - ps22
Sarcopterygii - ps23
Tetrapoda - ps24
Amniota - ps25
Mammalia - ps26
Theria - ps27
Placentalia - ps28
Boreoeutheria - ps29
Euarchontoglires - ps30
Euarchonta - ps31
Primates - ps32
Haplorrhini - ps33
Simiiformes - ps34
Catarrhini - ps35
Hominoidea - ps36
Hominidae - ps37
Homininae - ps38
Hominini - ps39
Homo_sapiens - ps40
p-value
1e-02
2e-02
2e-02
3e-02
4e-02
Log-odds
0.5
1.0
1.5
2.0
2.5
3.0
3.5
4.0

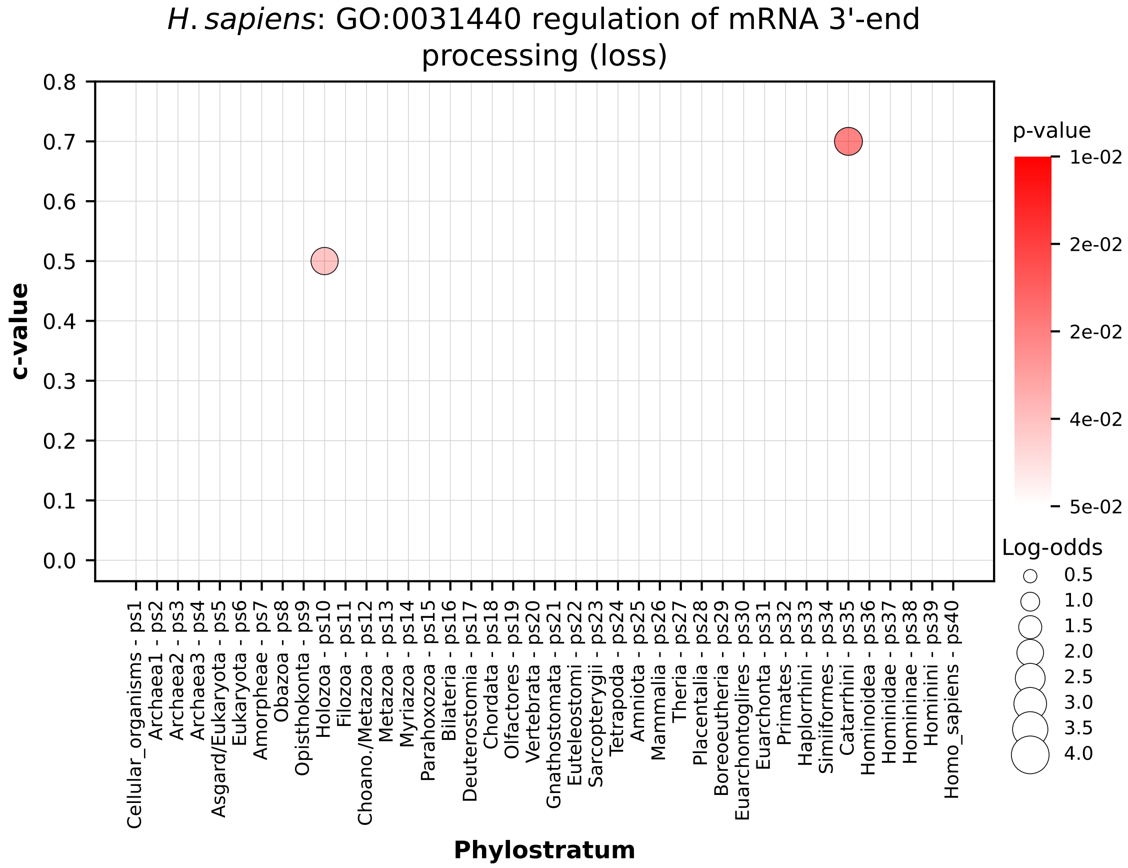
H. sapiens: GO:0031440 regulation of mRNA 3'-end processing (loss)
c-value
0.0
0.1
0.2
0.3
0.4
0.5
0.6
0.7
0.8
Phylostratum
Cellular_organisms - ps1
Archaea1 - ps2
Archaea2 - ps3
Archaea3 - ps4
Asgard/Eukaryota - ps5
Eukaryota - ps6
Amorpheae - ps7
Obazoa - ps8
Opisthokonta - ps9
Holozoa - ps10
Filozoa - ps11
Choano./Metazoa - ps12
Metazoa - ps13
Myriazoa - ps14
Parahoxozoa - ps15
Bilateria - ps16
Deuterostomia - ps17
Chordata - ps18
Olfactores - ps19
Vertebrata - ps20
Gnathostomata - ps21
Euteleostomi - ps22
Sarcopterygii - ps23
Tetrapoda - ps24
Amniota - ps25
Mammalia - ps26
Theria - ps27
Placentalia - ps28
Boreoeutheria - ps29
Euarchontoglires - ps30
Euarchonta - ps31
Primates - ps32
Haplorrhini - ps33
Simiiformes - ps34
Catarrhini - ps35
Hominoidea - ps36
Hominidae - ps37
Homininae - ps38
Hominini - ps39
Homo_sapiens - ps40
p-value
1e-02
2e-02
2e-02
4e-02
5e-02
Log-odds
0.5
1.0
1.5
2.0
2.5
3.0
3.5
4.0

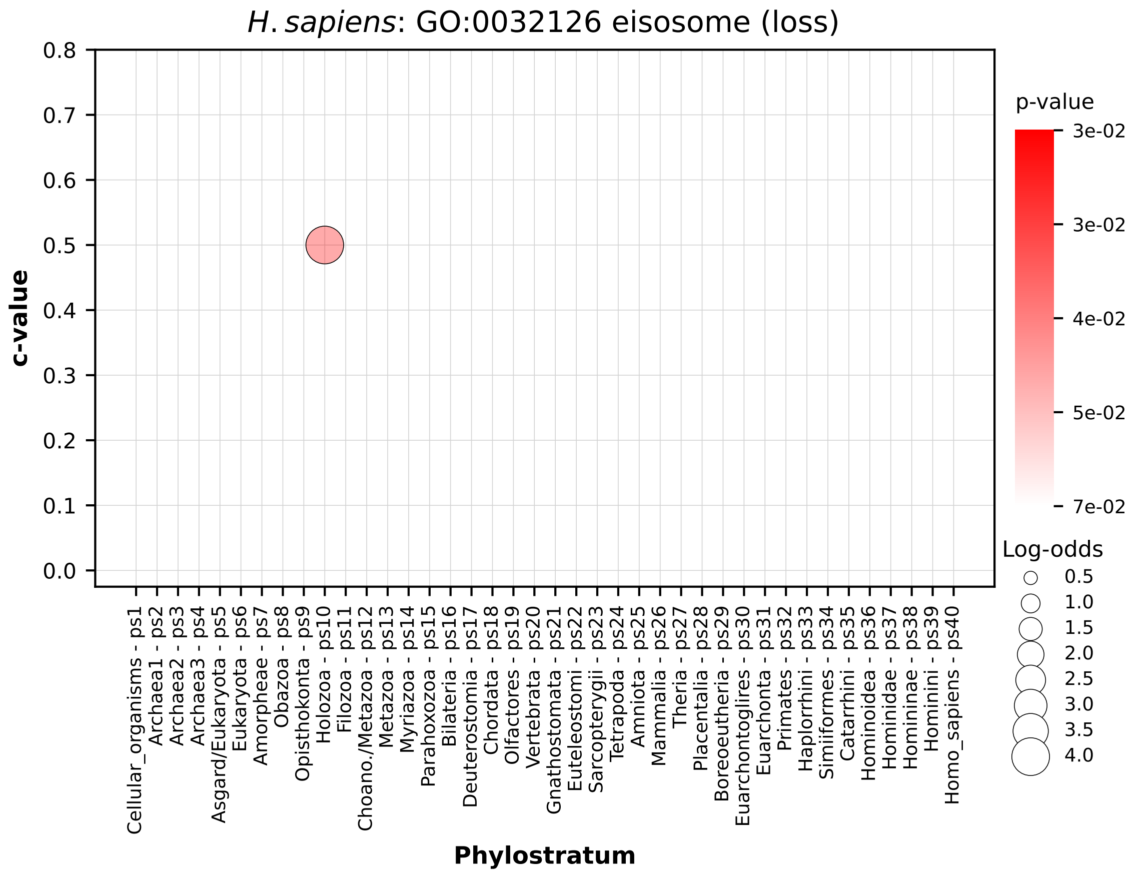

H. sapiens: GO:0032126 eisosome (loss)
c-value
0.8
0.7
0.6
0.5
0.4
0.3
0.2
0.1
0.0
Phylostratum
Cellular_organisms - ps1
Archaea1 - ps2
Archaea2 - ps3
Archaea3 - ps4
Asgard/Eukaryota - ps5
Eukaryota - ps6
Amorpheae - ps7
Obazoa - ps8
Opisthokonta - ps9
Holozoa - ps10
Filozoa - ps11
Choano./Metazoa - ps12
Metazoa - ps13
Myriazoa - ps14
Parahoxozoa - ps15
Bilateria - ps16
Deuterostomia - ps17
Chordata - ps18
Olfactores - ps19
Vertebrata - ps20
Gnathostomata - ps21
Euteleostomi - ps22
Sarcopterygii - ps23
Tetrapoda - ps24
Amniota - ps25
Mammalia - ps26
Theria - ps27
Placentalia - ps28
Boreoeutheria - ps29
Euarchontoglires - ps30
Euarchonta - ps31
Primates - ps32
Haplorrhini - ps33
Simiiformes - ps34
Catarrhini - ps35
Hominoidea - ps36
Hominidae - ps37
Homininae - ps38
Hominini - ps39
Homo_sapiens - ps40
p-value
3e-02
3e-02
4e-02
5e-02
7e-02
Log-odds
0.5
1.0
1.5
2.0
2.5
3.0
3.5
4.0

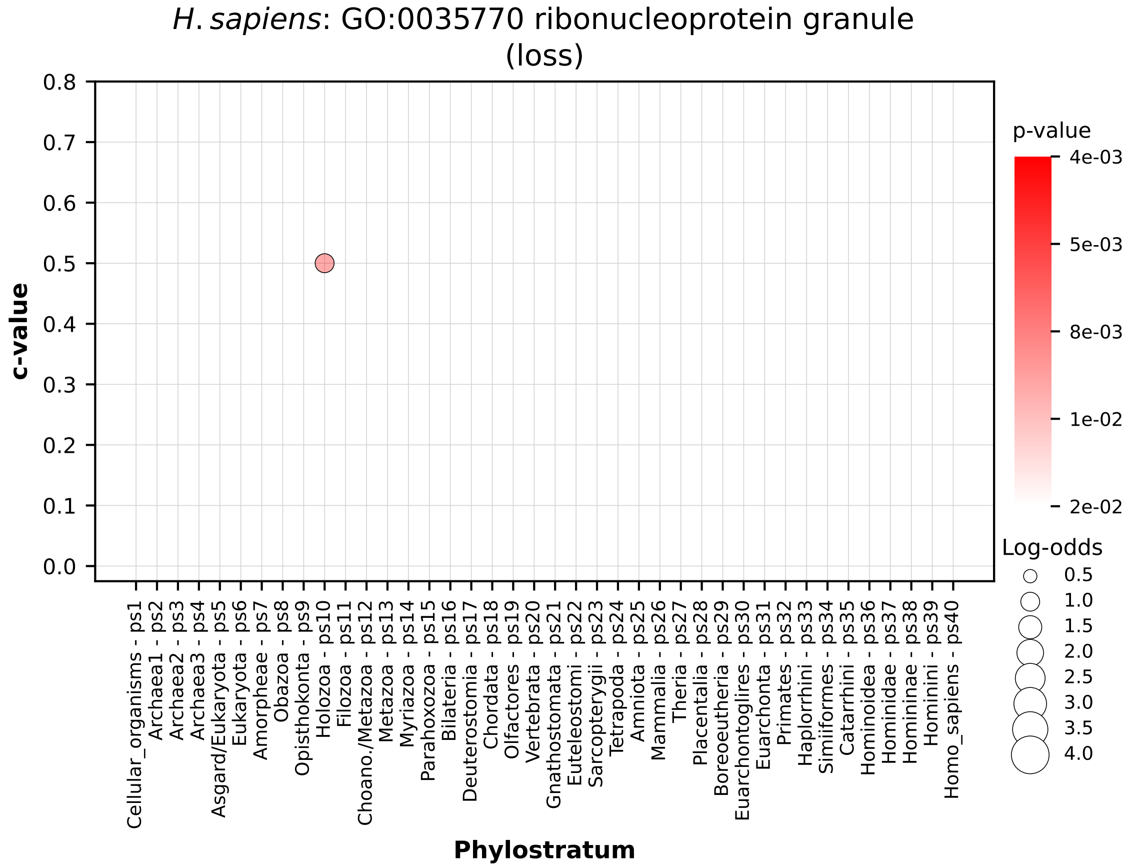

H. sapiens: GO:0035770 ribonucleoprotein granule
(loss)
c-value
0.8
0.7
0.6
0.5
0.4
0.3
0.2
0.1
0.0
Phylostratum
Cellular_organisms - ps1
Archaea1 - ps2
Archaea2 - ps3
Archaea3 - ps4
Asgard/Eukaryota - ps5
Eukaryota - ps6
Amorpheae - ps7
Obazoa - ps8
Opisthokonta - ps9
Holozoa - ps10
Filozoa - ps11
Choano./Metazoa - ps12
Metazoa - ps13
Myriazoa - ps14
Parahoxozoa - ps15
Bilateria - ps16
Deuterostomia - ps17
Chordata - ps18
Olfactores - ps19
Vertebrata - ps20
Gnathostomata - ps21
Euteleostomi - ps22
Sarcopterygii - ps23
Tetrapoda - ps24
Amniota - ps25
Mammalia - ps26
Theria - ps27
Placentalia - ps28
Boreoeutheria - ps29
Euarchontoglires - ps30
Euarchonta - ps31
Primates - ps32
Haplorrhini - ps33
Simiiformes - ps34
Catarrhini - ps35
Hominoidea - ps36
Hominidae - ps37
Homininae - ps38
Hominini - ps39
Homo_sapiens - ps40
p-value
4e-03
5e-03
8e-03
1e-02
2e-02
Log-odds
0.5
1.0
1.5
2.0
2.5
3.0
3.5
4.0

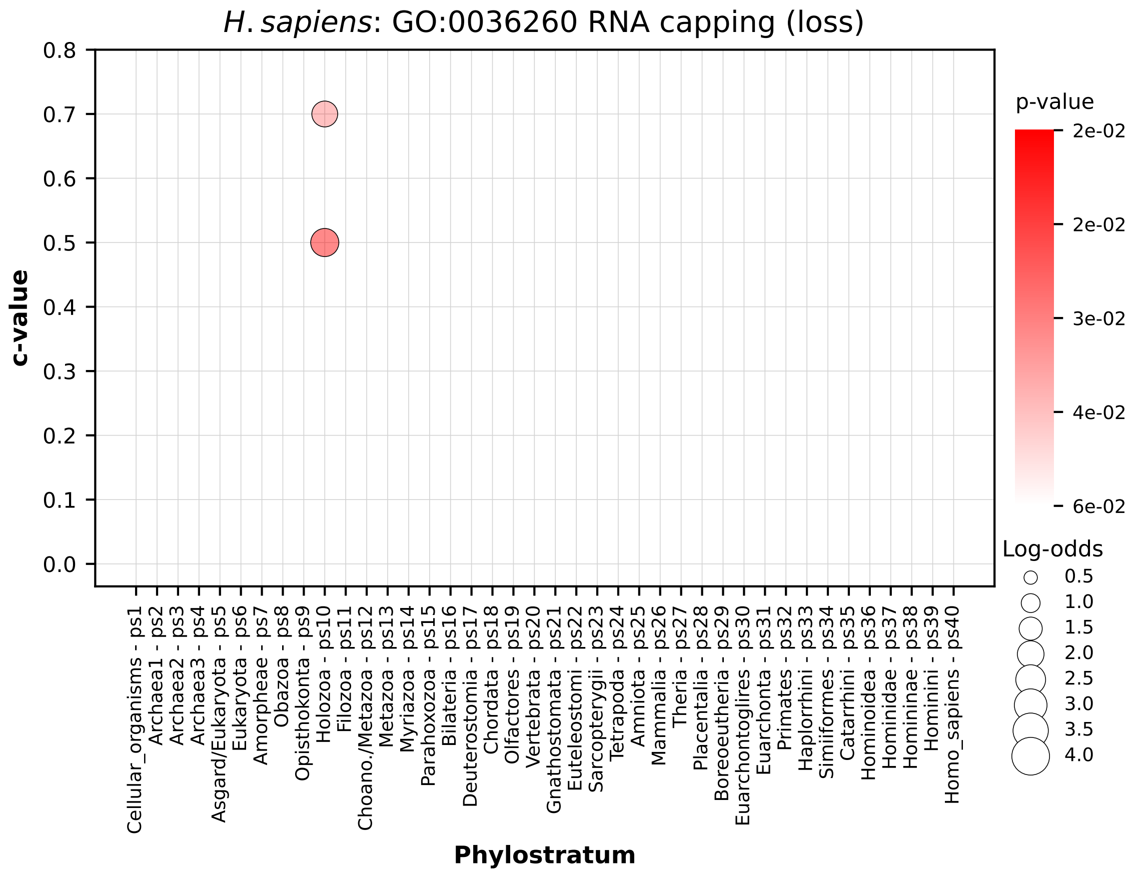

H. sapiens: GO:0036260 RNA capping (loss)
c-value
0.8
0.7
0.6
0.5
0.4
0.3
0.2
0.1
0.0
p-value
2e-02
2e-02
3e-02
4e-02
6e-02
Log-odds
0.5
1.0
1.5
2.0
2.5
3.0
3.5
4.0
Cellular_organisms - ps1
Archaea1 - ps2
Archaea2 - ps3
Archaea3 - ps4
Asgard/Eukaryota - ps5
Eukaryota - ps6
Amorpheae - ps7
Obazoa - ps8
Opisthokonta - ps9
Holozoa - ps10
Filozoa - ps11
Choano./Metazoa - ps12
Metazoa - ps13
Myriazoa - ps14
Parahoxozoa - ps15
Bilateria - ps16
Deuterostomia - ps17
Chordata - ps18
Olfactores - ps19
Vertebrata - ps20
Gnathostomata - ps21
Euteleostomi - ps22
Sarcopterygii - ps23
Tetrapoda - ps24
Amniota - ps25
Mammalia - ps26
Theria - ps27
Placentalia - ps28
Boreoeutheria - ps29
Euarchontoglires - ps30
Euarchonta - ps31
Primates - ps32
Haplorrhini - ps33
Simiiformes - ps34
Catarrhini - ps35
Hominoidea - ps36
Hominidae - ps37
Homininae - ps38
Hominini - ps39
Homo_sapiens - ps40
Phylostratum

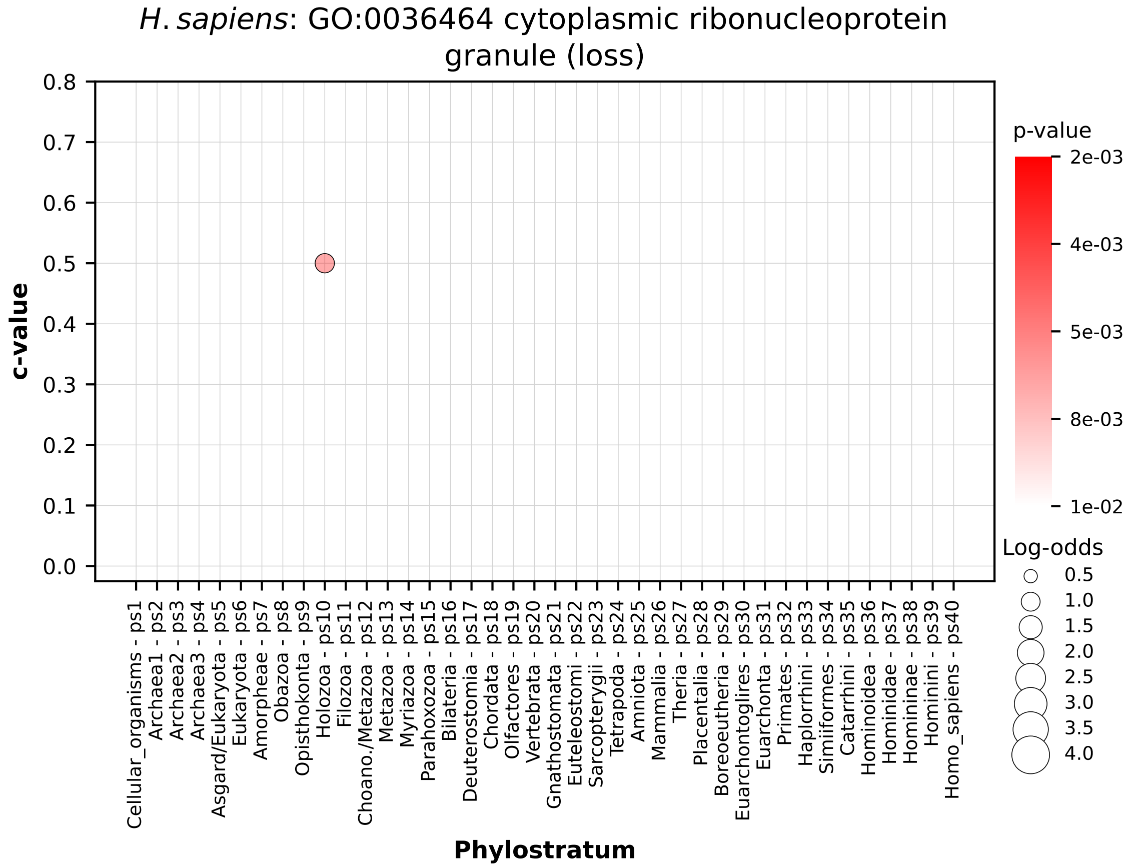
H. sapiens: GO:0036464 cytoplasmic ribonucleoprotein granule (loss)
c-value
0.8
0.7
0.6
0.5
0.4
0.3
0.2
0.1
0.0
Cellular_organisms - ps1
Archaea1 - ps2
Archaea2 - ps3
Archaea3 - ps4
Asgard/Eukaryota - ps5
Eukaryota - ps6
Amorpheae - ps7
Obazoa - ps8
Opisthokonta - ps9
Holozoa - ps10
Filozoa - ps11
Choano./Metazoa - ps12
Metazoa - ps13
Myriazoa - ps14
Parahoxozoa - ps15
Bilateria - ps16
Deuterostomia - ps17
Chordata - ps18
Olfactores - ps19
Vertebrata - ps20
Gnathostomata - ps21
Euteleostomi - ps22
Sarcopterygii - ps23
Tetrapoda - ps24
Amniota - ps25
Mammalia - ps26
Theria - ps27
Placentalia - ps28
Boreoeutheria - ps29
Euarchontoglires - ps30
Euarchonta - ps31
Primates - ps32
Haplorrhini - ps33
Simiiformes - ps34
Catarrhini - ps35
Hominoidea - ps36
Hominidae - ps37
Homininae - ps38
Hominini - ps39
Homo_sapiens - ps40
Phylostratum
p-value
2e-03
4e-03
5e-03
8e-03
1e-02
Log-odds
0.5
1.0
1.5
2.0
2.5
3.0
3.5
4.0

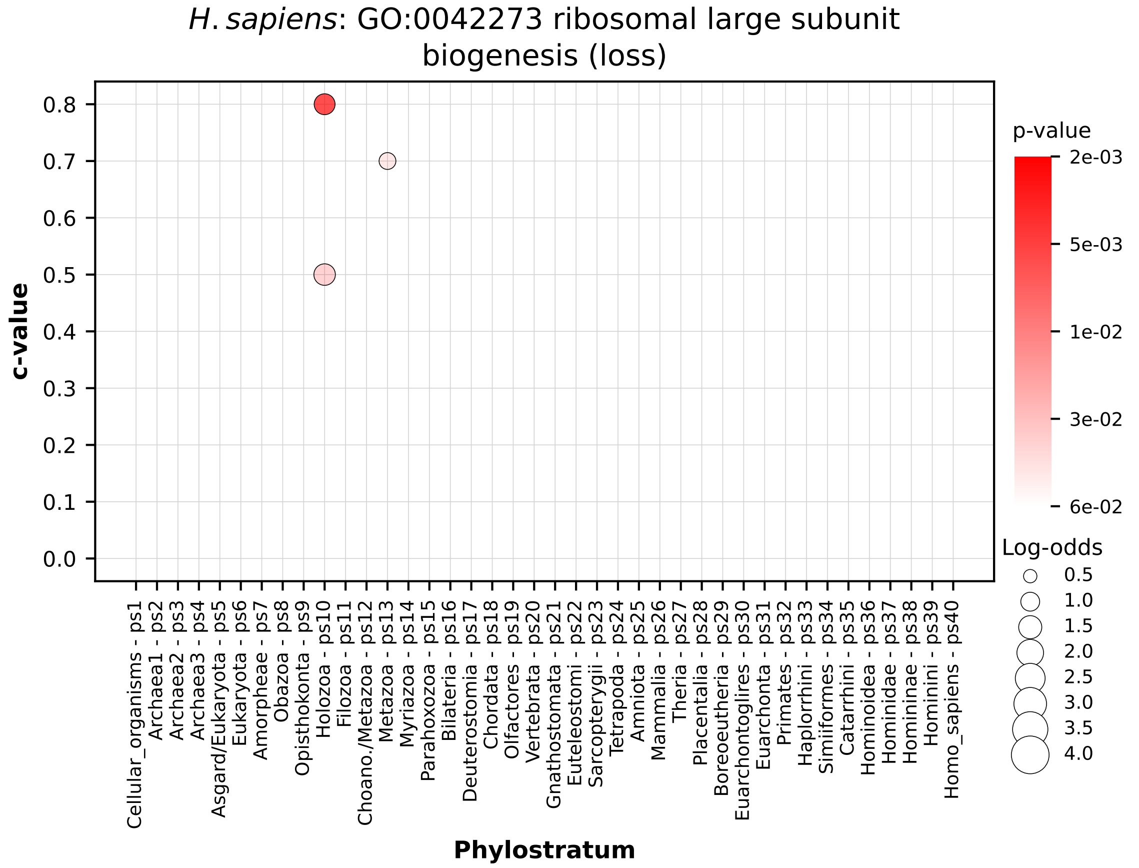

H. sapiens: GO:0042273 ribosomal large subunit biogenesis (loss)
c-value
0.8
0.7
0.6
0.5
0.4
0.3
0.2
0.1
0.0
p-value
2e-03
5e-03
1e-02
3e-02
6e-02
Log-odds
0.5
1.0
1.5
2.0
2.5
3.0
3.5
4.0
Cellular_organisms - ps1
Archaea1 - ps2
Archaea2 - ps3
Archaea3 - ps4
Asgard/Eukaryota - ps5
Eukaryota - ps6
Amorpheae - ps7
Obazoa - ps8
Opisthokonta - ps9
Holozoa - ps10
Filozoa - ps11
Choano./Metazoa - ps12
Metazoa - ps13
Myriazoa - ps14
Parahoxozoa - ps15
Bilateria - ps16
Deuterostomia - ps17
Chordata - ps18
Olfactores - ps19
Vertebrata - ps20
Gnathostomata - ps21
Euteleostomi - ps22
Sarcopterygii - ps23
Tetrapoda - ps24
Amniota - ps25
Mammalia - ps26
Theria - ps27
Placentalia - ps28
Boreoeutheria - ps29
Euarchontoglires - ps30
Euarchonta - ps31
Primates - ps32
Haplorrhini - ps33
Simiiformes - ps34
Catarrhini - ps35
Hominoidea - ps36
Hominidae - ps37
Homininae - ps38
Hominini - ps39
Homo_sapiens - ps40
Phylostratum

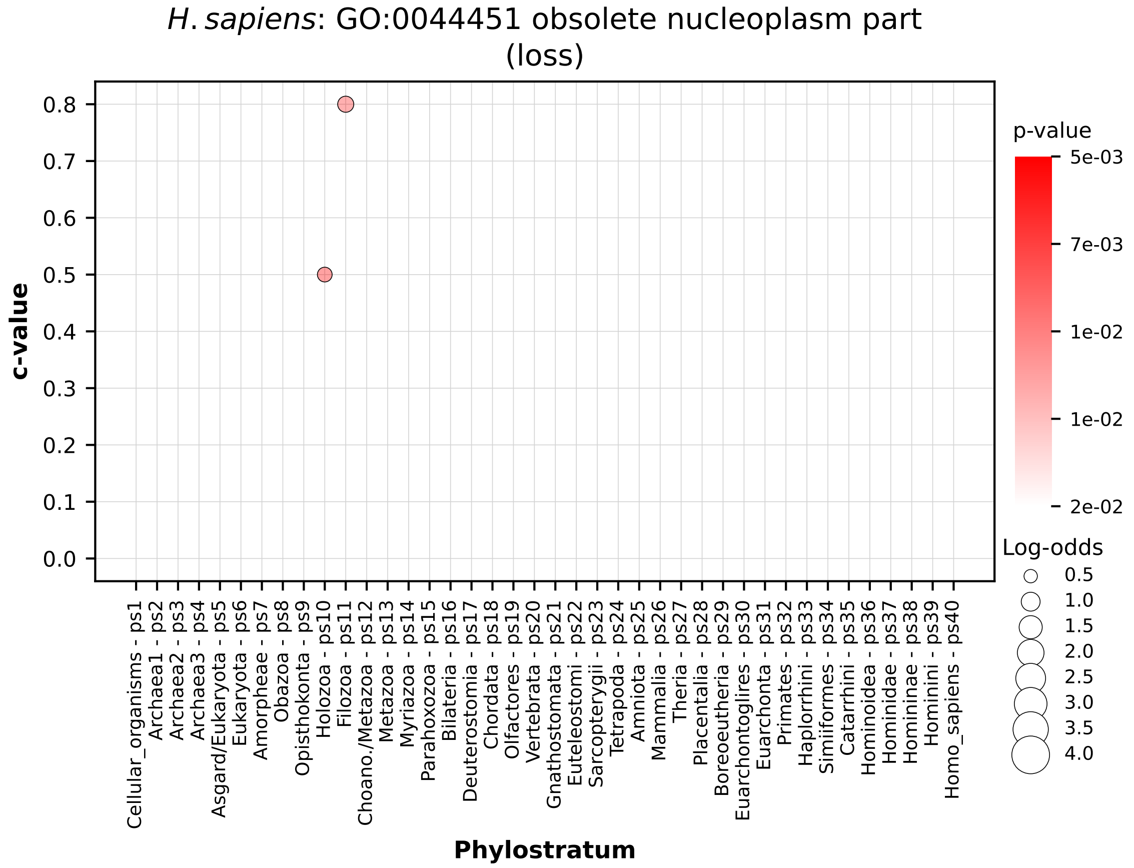

H. sapiens: GO:0044451 obsolete nucleoplasm part
(loss)
c-value
0.0
0.1
0.2
0.3
0.4
0.5
0.6
0.7
0.8
p-value
5e-03
7e-03
1e-02
1e-02
2e-02
Log-odds
0.5
1.0
1.5
2.0
2.5
3.0
3.5
4.0
Cellular_organisms - ps1
Archaea1 - ps2
Archaea2 - ps3
Archaea3 - ps4
Asgard/Eukaryota - ps5
Eukaryota - ps6
Amorpheae - ps7
Obazoa - ps8
Opisthokonta - ps9
Holozoa - ps10
Filozoa - ps11
Choano./Metazoa - ps12
Metazoa - ps13
Myriazoa - ps14
Parahoxozoa - ps15
Bilateria - ps16
Deuterostomia - ps17
Chordata - ps18
Olfactores - ps19
Vertebrata - ps20
Gnathostomata - ps21
Euteleostomi - ps22
Sarcopterygii - ps23
Tetrapoda - ps24
Amniota - ps25
Mammalia - ps26
Theria - ps27
Placentalia - ps28
Boreoeutheria - ps29
Euarchontoglires - ps30
Euarchonta - ps31
Primates - ps32
Haplorrhini - ps33
Simiiformes - ps34
Catarrhini - ps35
Hominoidea - ps36
Hominidae - ps37
Homininae - ps38
Hominini - ps39
Homo_sapiens - ps40
Phylostratum

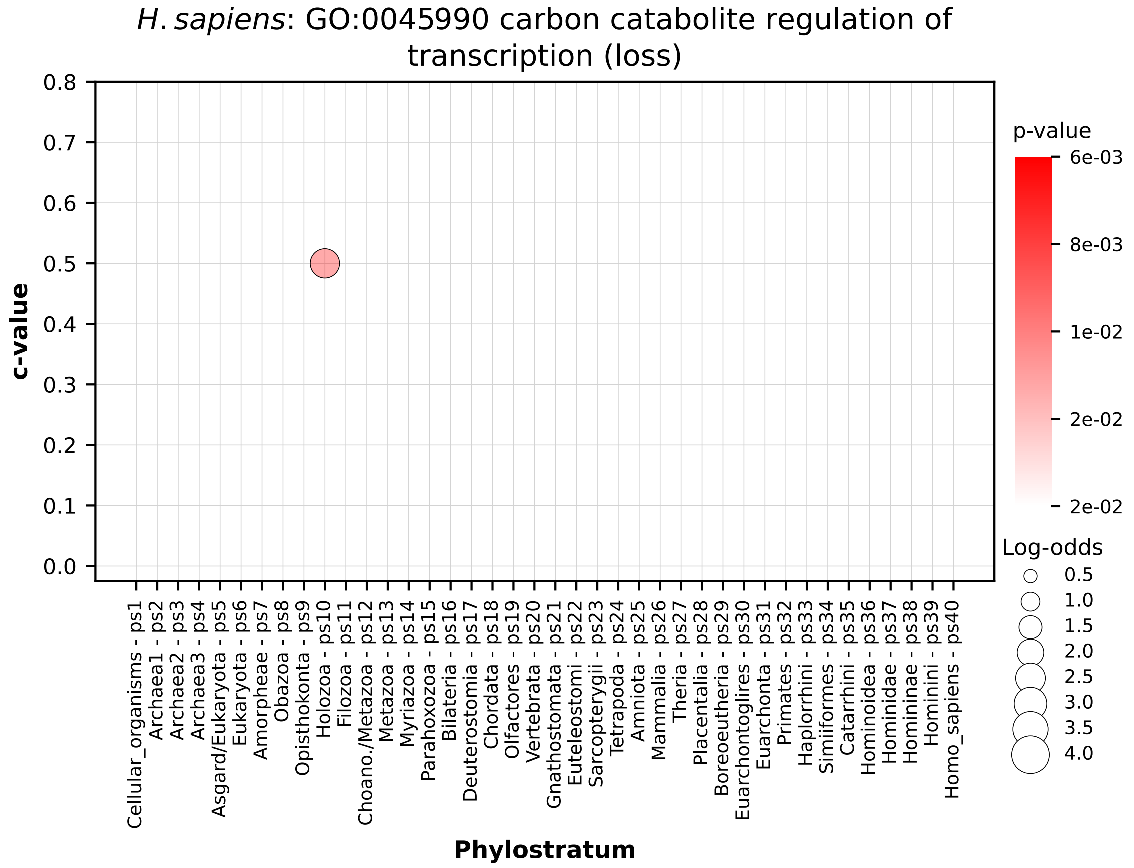

H. sapiens: GO:0045990 carbon catabolite regulation of transcription (loss)
c-value
0.8
0.7
0.6
0.5
0.4
0.3
0.2
0.1
0.0
p-value
6e-03
8e-03
1e-02
2e-02
2e-02
Log-odds
0.5
1.0
1.5
2.0
2.5
3.0
3.5
4.0
Cellular_organisms - ps1
Archaea1 - ps2
Archaea2 - ps3
Archaea3 - ps4
Asgard/Eukaryota - ps5
Eukaryota - ps6
Amorpheae - ps7
Obazoa - ps8
Opisthokonta - ps9
Holozoa - ps10
Filozoa - ps11
Choano./Metazoa - ps12
Metazoa - ps13
Myriazoa - ps14
Parahoxozoa - ps15
Bilateria - ps16
Deuterostomia - ps17
Chordata - ps18
Olfactores - ps19
Vertebrata - ps20
Gnathostomata - ps21
Euteleostomi - ps22
Sarcopterygii - ps23
Tetrapoda - ps24
Amniota - ps25
Mammalia - ps26
Theria - ps27
Placentalia - ps28
Boreoeutheria - ps29
Euarchontoglires - ps30
Euarchonta - ps31
Primates - ps32
Haplorrhini - ps33
Simiiformes - ps34
Catarrhini - ps35
Hominoidea - ps36
Hominidae - ps37
Homininae - ps38
Hominini - ps39
Homo_sapiens - ps40
Phylostratum

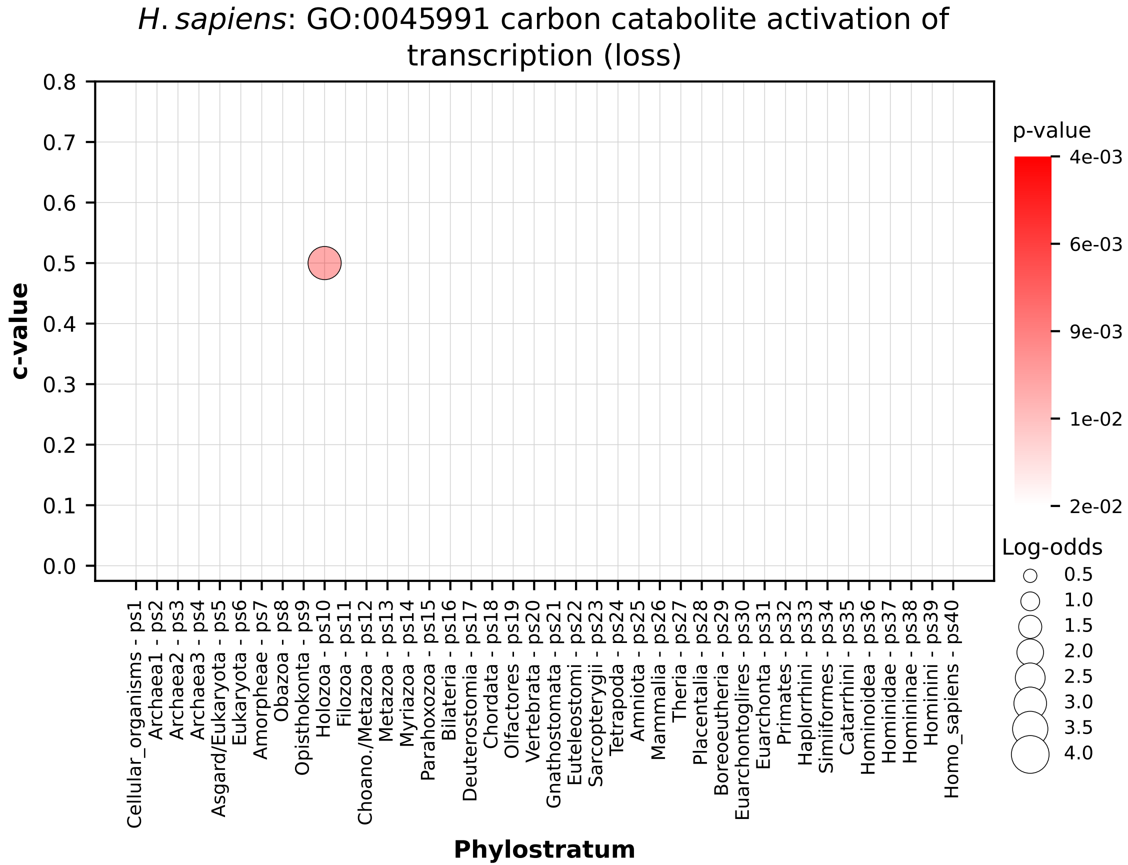

H. sapiens: GO:0045991 carbon catabolite activation of transcription (loss)
c-value
0.8
0.7
0.6
0.5
0.4
0.3
0.2
0.1
0.0
Phylostratum
Cellular_organisms - ps1
Archaea1 - ps2
Archaea2 - ps3
Archaea3 - ps4
Asgard/Eukaryota - ps5
Eukaryota - ps6
Amorpheae - ps7
Obazoa - ps8
Opisthokonta - ps9
Holozoa - ps10
Filozoa - ps11
Choano./Metazoa - ps12
Metazoa - ps13
Myriazoa - ps14
Parahoxozoa - ps15
Bilateria - ps16
Deuterostomia - ps17
Chordata - ps18
Olfactores - ps19
Vertebrata - ps20
Gnathostomata - ps21
Euteleostomi - ps22
Sarcopterygii - ps23
Tetrapoda - ps24
Amniota - ps25
Mammalia - ps26
Theria - ps27
Placentalia - ps28
Boreoeutheria - ps29
Euarchontoglires - ps30
Euarchonta - ps31
Primates - ps32
Haplorrhini - ps33
Simiiformes - ps34
Catarrhini - ps35
Hominoidea - ps36
Hominidae - ps37
Homininae - ps38
Hominini - ps39
Homo_sapiens - ps40
p-value
4e-03
6e-03
9e-03
1e-02
2e-02
Log-odds
0.5
1.0
1.5
2.0
2.5
3.0
3.5
4.0

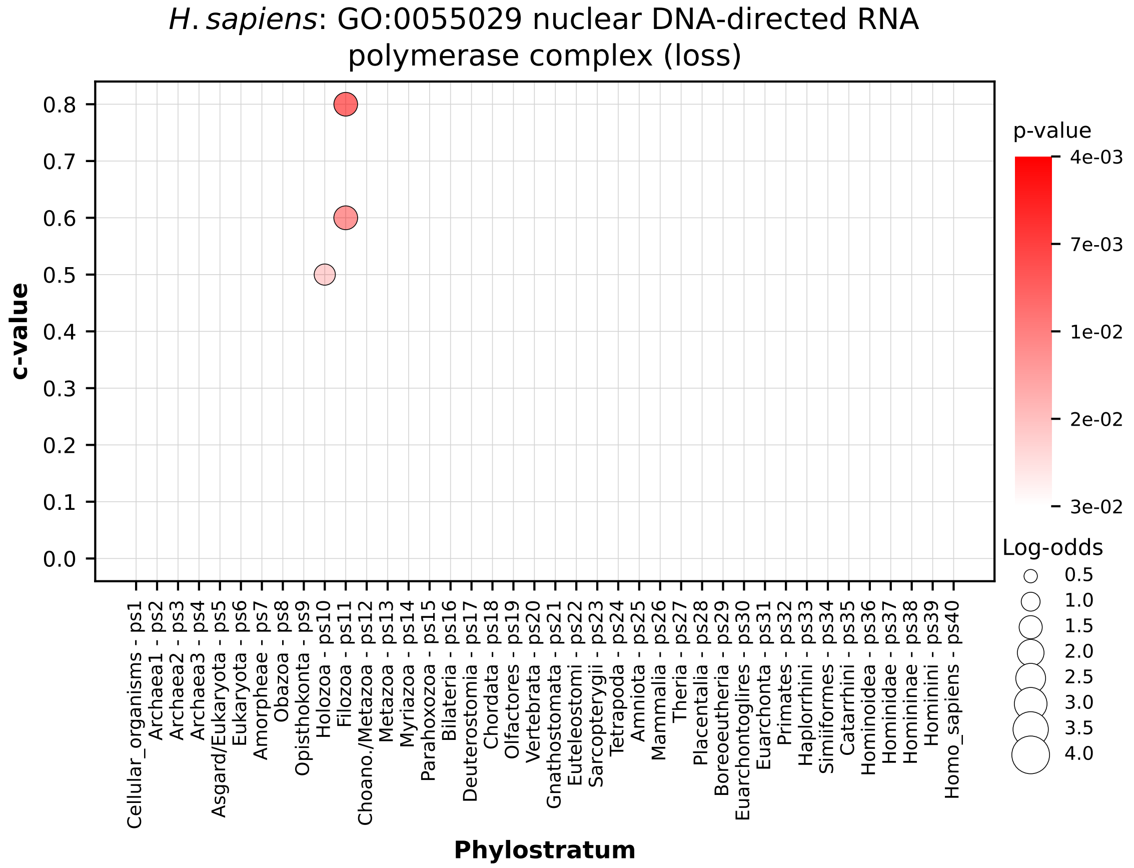

H. sapiens: GO:0055029 nuclear DNA-directed RNA polymerase complex (loss)
c-value
0.0
0.1
0.2
0.3
0.4
0.5
0.6
0.7
0.8
p-value
4e-03
7e-03
1e-02
2e-02
3e-02
Log-odds
0.5
1.0
1.5
2.0
2.5
3.0
3.5
4.0
Cellular_organisms - ps1
Archaea1 - ps2
Archaea2 - ps3
Archaea3 - ps4
Asgard/Eukaryota - ps5
Eukaryota - ps6
Amorpheae - ps7
Obazoa - ps8
Opisthokonta - ps9
Holozoa - ps10
Filozoa - ps11
Choano./Metazoa - ps12
Metazoa - ps13
Myriazoa - ps14
Parahoxozoa - ps15
Bilateria - ps16
Deuterostomia - ps17
Chordata - ps18
Olfactores - ps19
Vertebrata - ps20
Gnathostomata - ps21
Euteleostomi - ps22
Sarcopterygii - ps23
Tetrapoda - ps24
Amniota - ps25
Mammalia - ps26
Theria - ps27
Placentalia - ps28
Boreoeutheria - ps29
Euarchontoglires - ps30
Euarchonta - ps31
Primates - ps32
Haplorrhini - ps33
Simiiformes - ps34
Catarrhini - ps35
Hominoidea - ps36
Hominidae - ps37
Homininae - ps38
Hominini - ps39
Homo_sapiens - ps40
Phylostratum

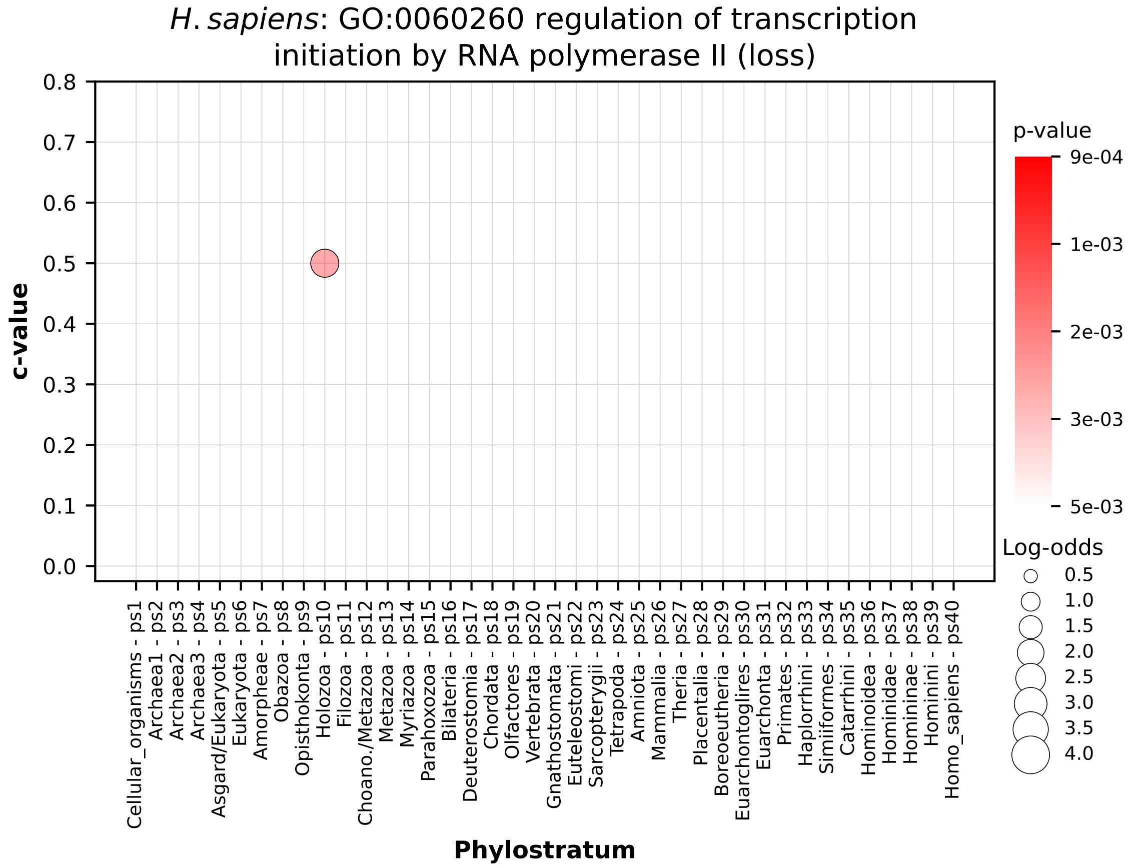

H. sapiens: GO:0060260 regulation of transcription initiation by RNA polymerase II (loss)
c-value
0.0
0.1
0.2
0.3
0.4
0.5
0.6
0.7
0.8
p-value
9e-04
1e-03
2e-03
3e-03
5e-03
Log-odds
0.5
1.0
1.5
2.0
2.5
3.0
3.5
4.0
Cellular_organisms - ps1
Archaea1 - ps2
Archaea2 - ps3
Archaea3 - ps4
Asgard/Eukaryota - ps5
Eukaryota - ps6
Amorpheae - ps7
Obazoa - ps8
Opisthokonta - ps9
Holozoa - ps10
Filozoa - ps11
Choano./Metazoa - ps12
Metazoa - ps13
Myriazoa - ps14
Parahoxozoa - ps15
Bilateria - ps16
Deuterostomia - ps17
Chordata - ps18
Olfactores - ps19
Vertebrata - ps20
Gnathostomata - ps21
Euteleostomi - ps22
Sarcopterygii - ps23
Tetrapoda - ps24
Amniota - ps25
Mammalia - ps26
Theria - ps27
Placentalia - ps28
Boreoeutheria - ps29
Euarchontoglires - ps30
Euarchonta - ps31
Primates - ps32
Haplorrhini - ps33
Simiiformes - ps34
Catarrhini - ps35
Hominoidea - ps36
Hominidae - ps37
Homininae - ps38
Hominini - ps39
Homo_sapiens - ps40
Phylostratum

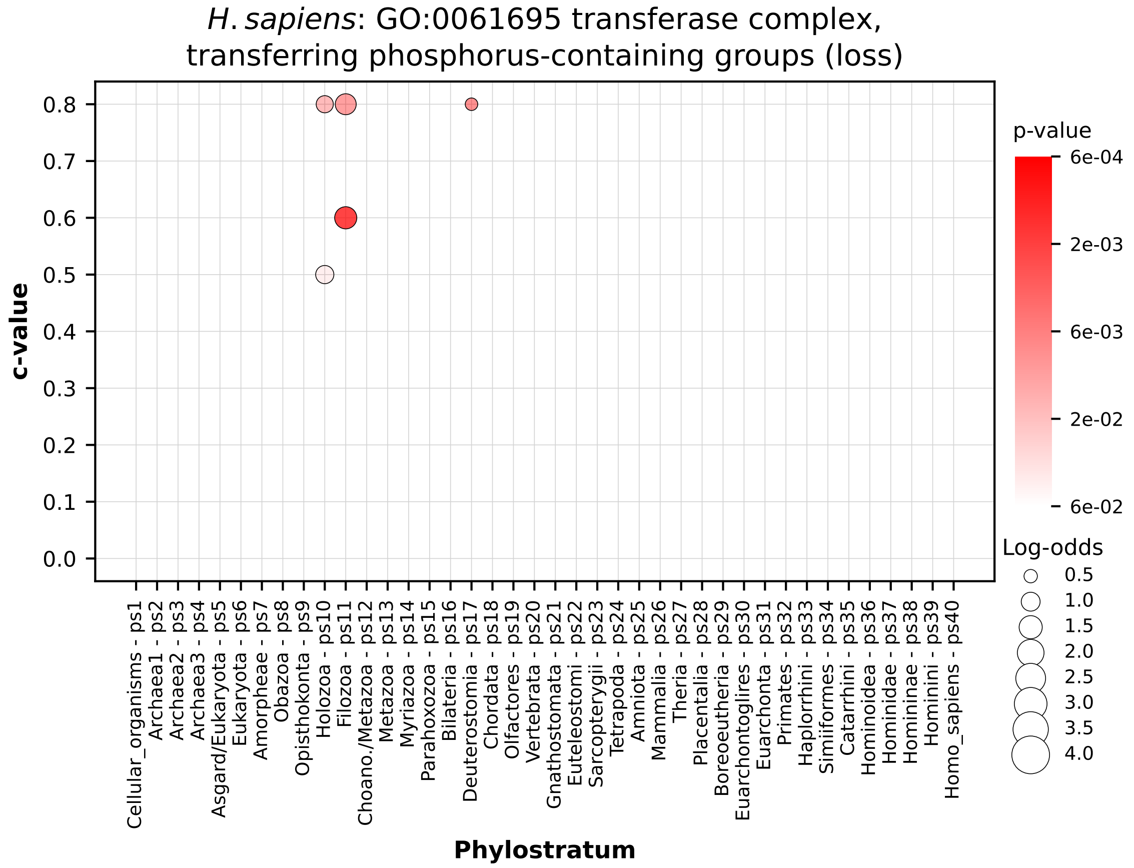

H. sapiens: GO:0061695 transferase complex, transferring phosphorus-containing groups (loss)
c-value
0.0
0.1
0.2
0.3
0.4
0.5
0.6
0.7
0.8
p-value
6e-04
2e-03
6e-03
2e-02
6e-02
Log-odds
0.5
1.0
1.5
2.0
2.5
3.0
3.5
4.0
Cellular_organisms - ps1
Archaea1 - ps2
Archaea2 - ps3
Archaea3 - ps4
Asgard/Eukaryota - ps5
Eukaryota - ps6
Amorpheae - ps7
Obazoa - ps8
Opisthokonta - ps9
Holozoa - ps10
Filozoa - ps11
Choano./Metazoa - ps12
Metazoa - ps13
Myriazoa - ps14
Parahoxozoa - ps15
Bilateria - ps16
Deuterostomia - ps17
Chordata - ps18
Olfactores - ps19
Vertebrata - ps20
Gnathostomata - ps21
Euteleostomi - ps22
Sarcopterygii - ps23
Tetrapoda - ps24
Amniota - ps25
Mammalia - ps26
Theria - ps27
Placentalia - ps28
Boreoeutheria - ps29
Euarchontoglires - ps30
Euarchonta - ps31
Primates - ps32
Haplorrhini - ps33
Simiiformes - ps34
Catarrhini - ps35
Hominoidea - ps36
Hominidae - ps37
Homininae - ps38
Hominini - ps39
Homo_sapiens - ps40
Phylostratum

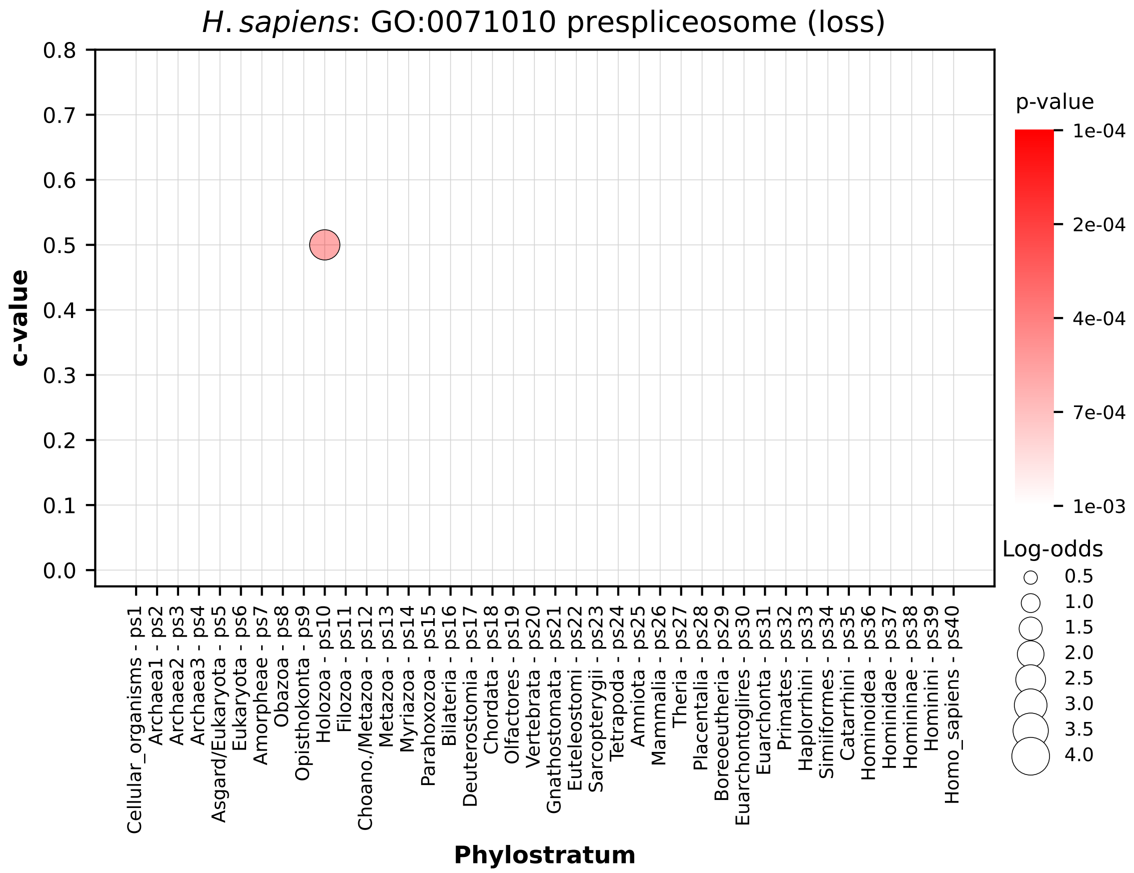

H. sapiens: GO:0071010 prespliceosome (loss)
c-value
0.8
0.7
0.6
0.5
0.4
0.3
0.2
0.1
0.0
p-value
1e-04
2e-04
4e-04
7e-04
1e-03
Log-odds
0.5
1.0
1.5
2.0
2.5
3.0
3.5
4.0
Cellular_organisms - ps1
Archaea1 - ps2
Archaea2 - ps3
Archaea3 - ps4
Asgard/Eukaryota - ps5
Eukaryota - ps6
Amorpheae - ps7
Obazoa - ps8
Opisthokonta - ps9
Holozoa - ps10
Filozoa - ps11
Choano./Metazoa - ps12
Metazoa - ps13
Myriazoa - ps14
Parahoxozoa - ps15
Bilateria - ps16
Deuterostomia - ps17
Chordata - ps18
Olfactores - ps19
Vertebrata - ps20
Gnathostomata - ps21
Euteleostomi - ps22
Sarcopterygii - ps23
Tetrapoda - ps24
Amniota - ps25
Mammalia - ps26
Theria - ps27
Placentalia - ps28
Boreoeutheria - ps29
Euarchontoglires - ps30
Euarchonta - ps31
Primates - ps32
Haplorrhini - ps33
Simiiformes - ps34
Catarrhini - ps35
Hominoidea - ps36
Hominidae - ps37
Homininae - ps38
Hominini - ps39
Homo_sapiens - ps40
Phylostratum

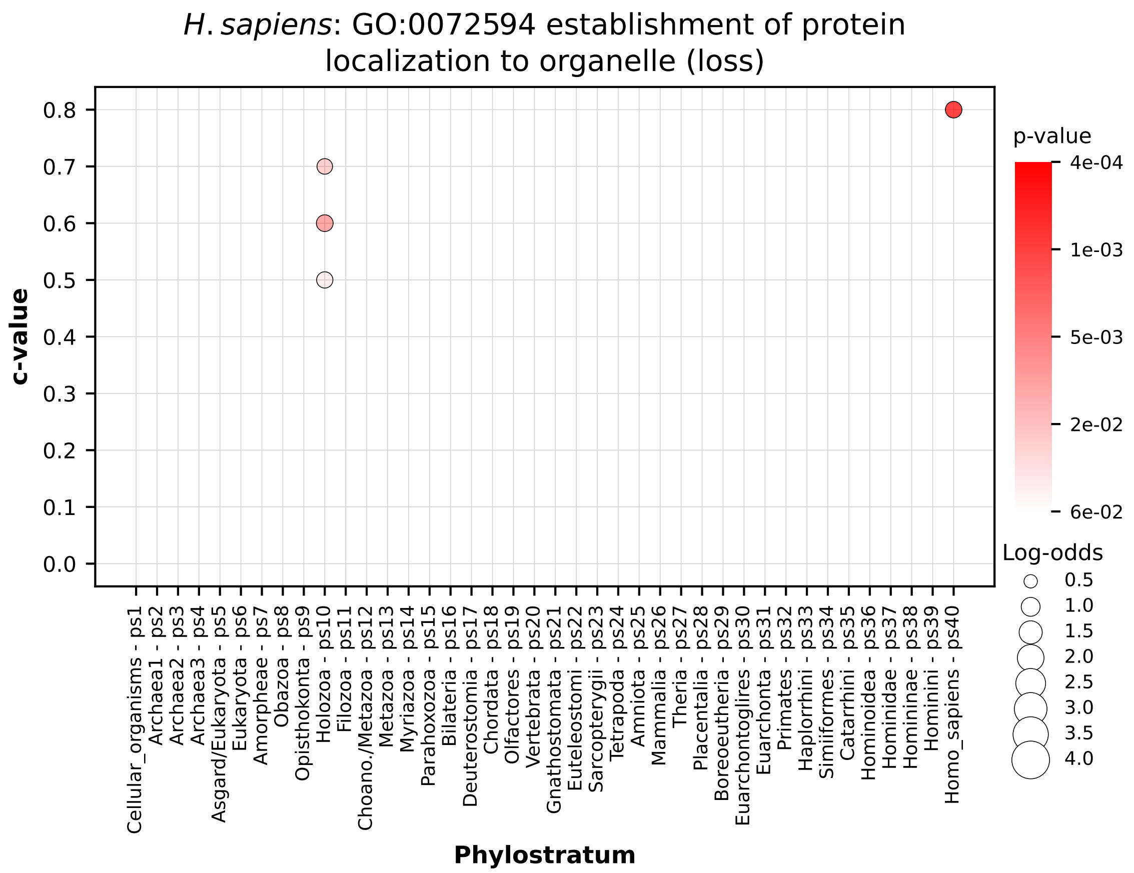

H. sapiens: GO:0072594 establishment of protein localization to organelle (loss)
c-value
0.8
0.7
0.6
0.5
0.4
0.3
0.2
0.1
0.0
p-value
4e-04
1e-03
5e-03
2e-02
6e-02
Log-odds
0.5
1.0
1.5
2.0
2.5
3.0
3.5
4.0
Cellular_organisms - ps1
Archaea1 - ps2
Archaea2 - ps3
Archaea3 - ps4
Asgard/Eukaryota - ps5
Eukaryota - ps6
Amorpheae - ps7
Obazoa - ps8
Opisthokonta - ps9
Holozoa - ps10
Filozoa - ps11
Choano./Metazoa - ps12
Metazoa - ps13
Myriazoa - ps14
Parahoxozoa - ps15
Bilateria - ps16
Deuterostomia - ps17
Chordata - ps18
Olfactores - ps19
Vertebrata - ps20
Gnathostomata - ps21
Euteleostomi - ps22
Sarcopterygii - ps23
Tetrapoda - ps24
Amniota - ps25
Mammalia - ps26
Theria - ps27
Placentalia - ps28
Boreoeutheria - ps29
Euarchontoglires - ps30
Euarchonta - ps31
Primates - ps32
Haplorrhini - ps33
Simiiformes - ps34
Catarrhini - ps35
Hominoidea - ps36
Hominidae - ps37
Homininae - ps38
Hominini - ps39
Homo_sapiens - ps40
Phylostratum

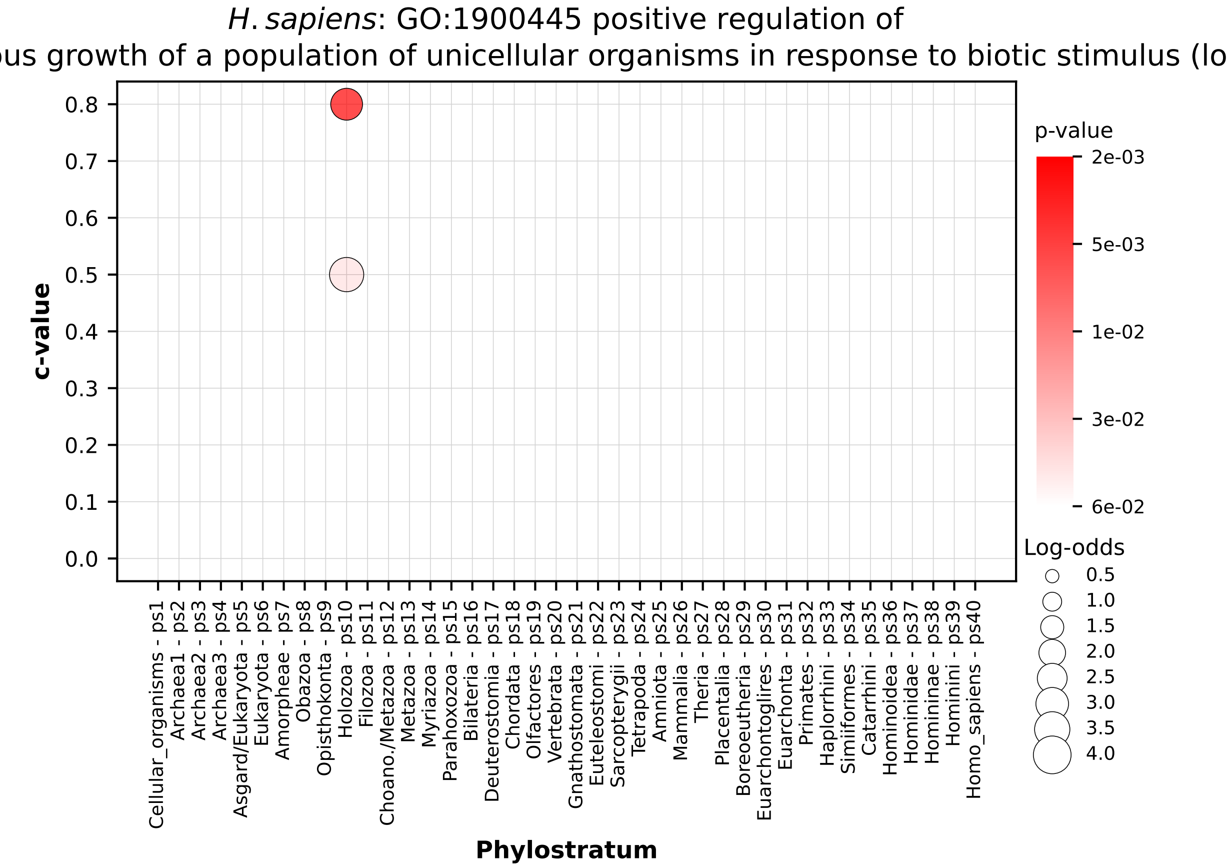

H. sapiens: GO:1900445 positive regulation of
ous growth of a population of unicellular organisms in response to biotic stimulus (lo
c-value
0.8
0.7
0.6
0.5
0.4
0.3
0.2
0.1
0.0
p-value
2e-03
5e-03
1e-02
3e-02
6e-02
Log-odds
0.5
1.0
1.5
2.0
2.5
3.0
3.5
4.0
Cellular_organisms - ps1
Archaea1 - ps2
Archaea2 - ps3
Archaea3 - ps4
Asgard/Eukaryota - ps5
Eukaryota - ps6
Amorpheae - ps7
Obazoa - ps8
Opisthokonta - ps9
Holozoa - ps10
Filozoa - ps11
Choano./Metazoa - ps12
Metazoa - ps13
Myriazoa - ps14
Parahoxozoa - ps15
Bilateria - ps16
Deuterostomia - ps17
Chordata - ps18
Olfactores - ps19
Vertebrata - ps20
Gnathostomata - ps21
Euteleostomi - ps22
Sarcopterygii - ps23
Tetrapoda - ps24
Amniota - ps25
Mammalia - ps26
Theria - ps27
Placentalia - ps28
Boreoeutheria - ps29
Euarchontoglires - ps30
Euarchonta - ps31
Primates - ps32
Haplorrhini - ps33
Simiiformes - ps34
Catarrhini - ps35
Hominoidea - ps36
Hominidae - ps37
Homininae - ps38
Hominini - ps39
Homo_sapiens - ps40
Phylostratum

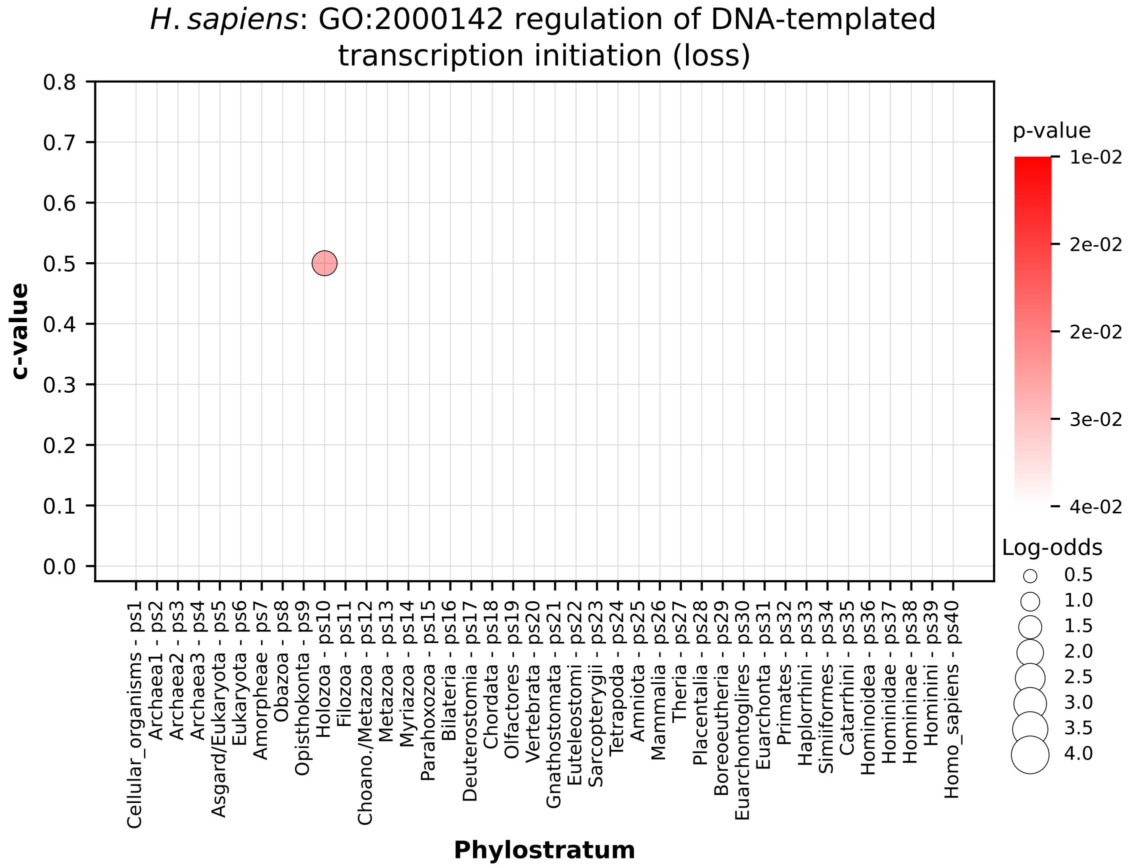

H. sapiens: GO:2000142 regulation of DNA-templated transcription initiation (loss)
c-value
0.8
0.7
0.6
0.5
0.4
0.3
0.2
0.1
0.0
Phylostratum
Cellular_organisms - ps1
Archaea1 - ps2
Archaea2 - ps3
Archaea3 - ps4
Asgard/Eukaryota - ps5
Eukaryota - ps6
Amorpheae - ps7
Obazoa - ps8
Opisthokonta - ps9
Holozoa - ps10
Filozoa - ps11
Choano./Metazoa - ps12
Metazoa - ps13
Myriazoa - ps14
Parahoxozoa - ps15
Bilateria - ps16
Deuterostomia - ps17
Chordata - ps18
Olfactores - ps19
Vertebrata - ps20
Gnathostomata - ps21
Euteleostomi - ps22
Sarcopterygii - ps23
Tetrapoda - ps24
Amniota - ps25
Mammalia - ps26
Theria - ps27
Placentalia - ps28
Boreoeutheria - ps29
Euarchontoglires - ps30
Euarchonta - ps31
Primates - ps32
Haplorrhini - ps33
Simiiformes - ps34
Catarrhini - ps35
Hominoidea - ps36
Hominidae - ps37
Homininae - ps38
Hominini - ps39
Homo_sapiens - ps40
p-value
1e-02
2e-02
2e-02
3e-02
4e-02
Log-odds
0.5
1.0
1.5
2.0
2.5
3.0
3.5
4.0

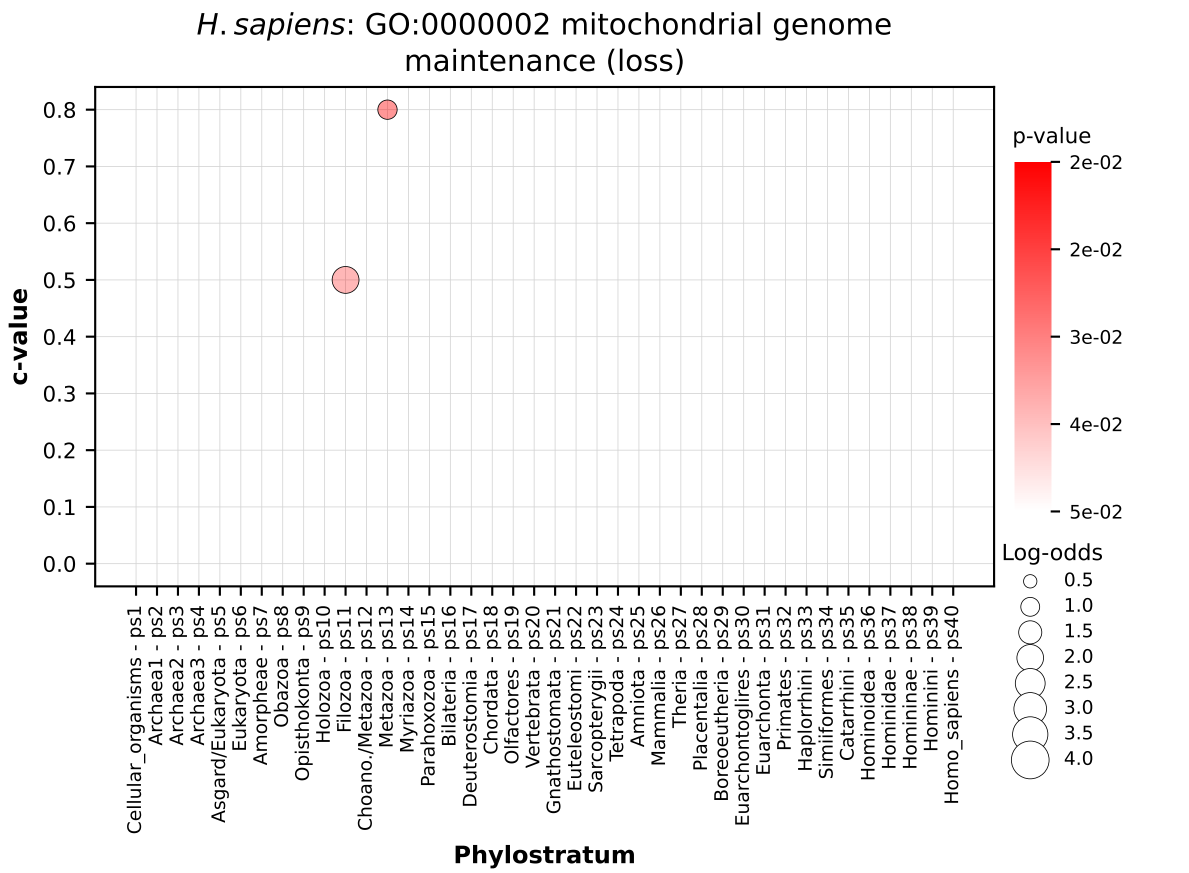
H. sapiens: GO:0000002 mitochondrial genome maintenance (loss)
c-value
0.0
0.1
0.2
0.3
0.4
0.5
0.6
0.7
0.8
Phylostratum
Cellular_organisms - ps1
Archaea1 - ps2
Archaea2 - ps3
Archaea3 - ps4
Asgard/Eukaryota - ps5
Eukaryota - ps6
Amorpheae - ps7
Obazoa - ps8
Opisthokonta - ps9
Holozoa - ps10
Filozoa - ps11
Choano./Metazoa - ps12
Metazoa - ps13
Myriazoa - ps14
Parahoxozoa - ps15
Bilateria - ps16
Deuterostomia - ps17
Chordata - ps18
Olfactores - ps19
Vertebrata - ps20
Gnathostomata - ps21
Euteleostomi - ps22
Sarcopterygii - ps23
Tetrapoda - ps24
Amniota - ps25
Mammalia - ps26
Theria - ps27
Placentalia - ps28
Boreoeutheria - ps29
Euarchontoglires - ps30
Euarchonta - ps31
Primates - ps32
Haplorrhini - ps33
Simiiformes - ps34
Catarrhini - ps35
Hominoidea - ps36
Hominidae - ps37
Homininae - ps38
Hominini - ps39
Homo_sapiens - ps40
p-value
2e-02
2e-02
3e-02
4e-02
5e-02
Log-odds
0.5
1.0
1.5
2.0
2.5
3.0
3.5
4.0

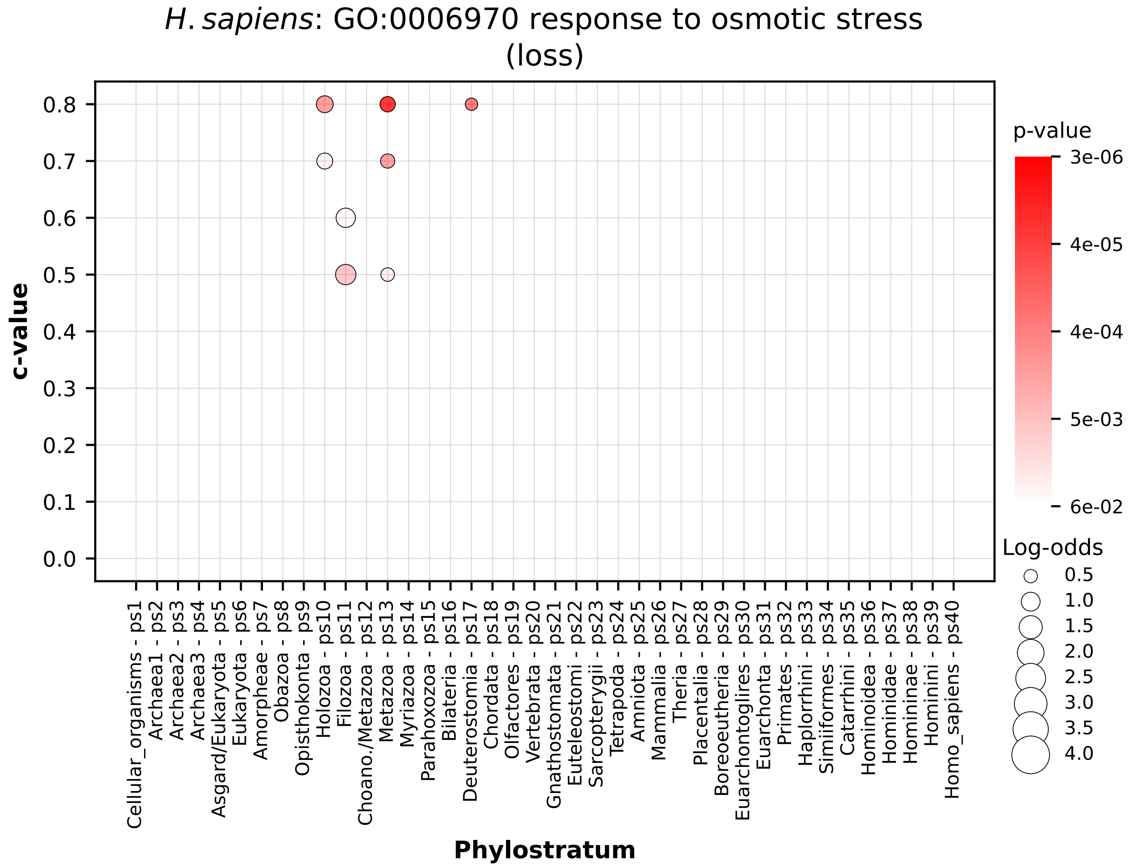
H. sapiens: GO:0006970 response to osmotic stress
(loss)
c-value
0.0
0.1
0.2
0.3
0.4
0.5
0.6
0.7
0.8
Phylostratum
Cellular_organisms - ps1
Archaea1 - ps2
Archaea2 - ps3
Archaea3 - ps4
Asgard/Eukaryota - ps5
Eukaryota - ps6
Amorpheae - ps7
Obazoa - ps8
Opisthokonta - ps9
Holozoa - ps10
Filozoa - ps11
Choano./Metazoa - ps12
Metazoa - ps13
Myriazoa - ps14
Parahoxozoa - ps15
Bilateria - ps16
Deuterostomia - ps17
Chordata - ps18
Olfactores - ps19
Vertebrata - ps20
Gnathostomata - ps21
Euteleostomi - ps22
Sarcopterygii - ps23
Tetrapoda - ps24
Amniota - ps25
Mammalia - ps26
Theria - ps27
Placentalia - ps28
Boreoeutheria - ps29
Euarchontoglires - ps30
Euarchonta - ps31
Primates - ps32
Haplorrhini - ps33
Simiiformes - ps34
Catarrhini - ps35
Hominoidea - ps36
Hominidae - ps37
Homininae - ps38
Hominini - ps39
Homo_sapiens - ps40
p-value
3e-06
4e-05
4e-04
5e-03
6e-02
Log-odds
0.5
1.0
1.5
2.0
2.5
3.0
3.5
4.0

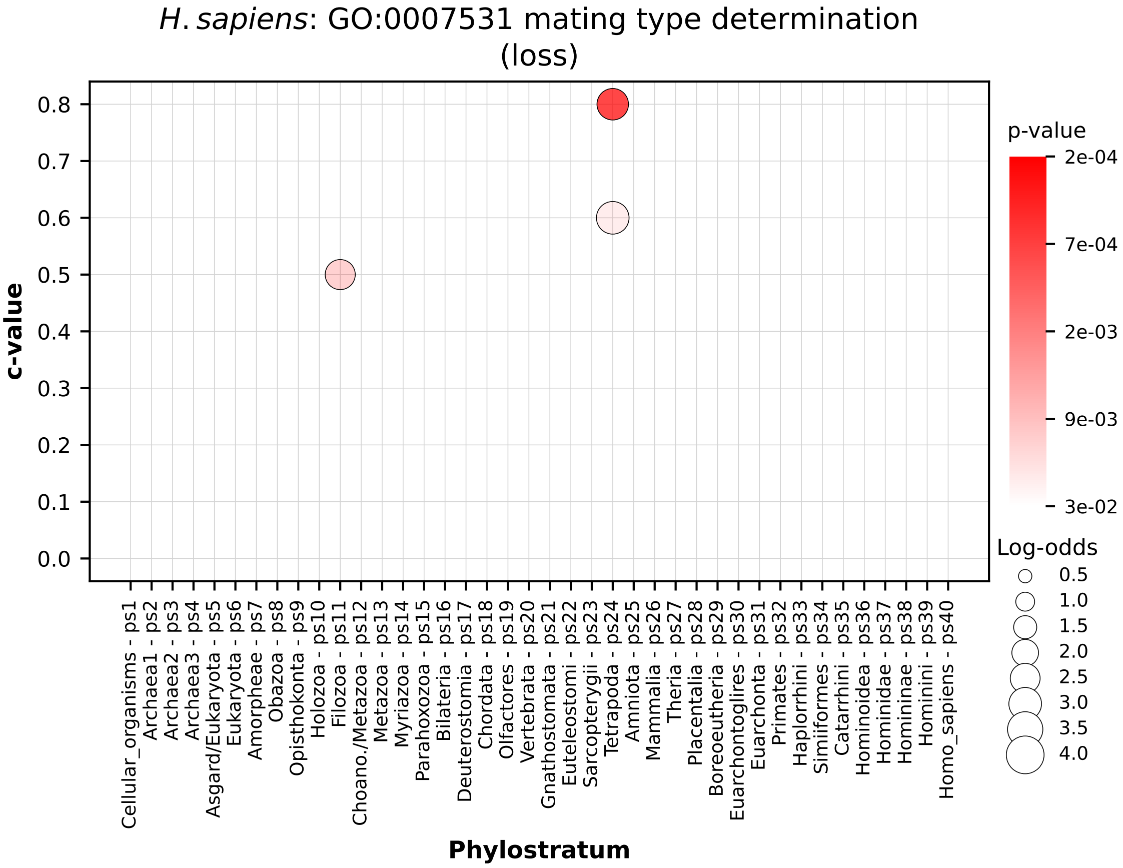

H. sapiens: GO:0007531 mating type determination
(loss)
c-value
0.0
0.1
0.2
0.3
0.4
0.5
0.6
0.7
0.8
Phylostratum
Cellular_organisms - ps1
Archaea1 - ps2
Archaea2 - ps3
Archaea3 - ps4
Asgard/Eukaryota - ps5
Eukaryota - ps6
Amorpheae - ps7
Obazoa - ps8
Opisthokonta - ps9
Holozoa - ps10
Filozoa - ps11
Choano./Metazoa - ps12
Metazoa - ps13
Myriazoa - ps14
Parahoxozoa - ps15
Bilateria - ps16
Deuterostomia - ps17
Chordata - ps18
Olfactores - ps19
Vertebrata - ps20
Gnathostomata - ps21
Euteleostomi - ps22
Sarcopterygii - ps23
Tetrapoda - ps24
Amniota - ps25
Mammalia - ps26
Theria - ps27
Placentalia - ps28
Boreoeutheria - ps29
Euarchontoglires - ps30
Euarchonta - ps31
Primates - ps32
Haplorrhini - ps33
Simiiformes - ps34
Catarrhini - ps35
Hominoidea - ps36
Hominidae - ps37
Homininae - ps38
Hominini - ps39
Homo_sapiens - ps40
p-value
2e-04
7e-04
2e-03
9e-03
3e-02
Log-odds
0.5
1.0
1.5
2.0
2.5
3.0
3.5
4.0

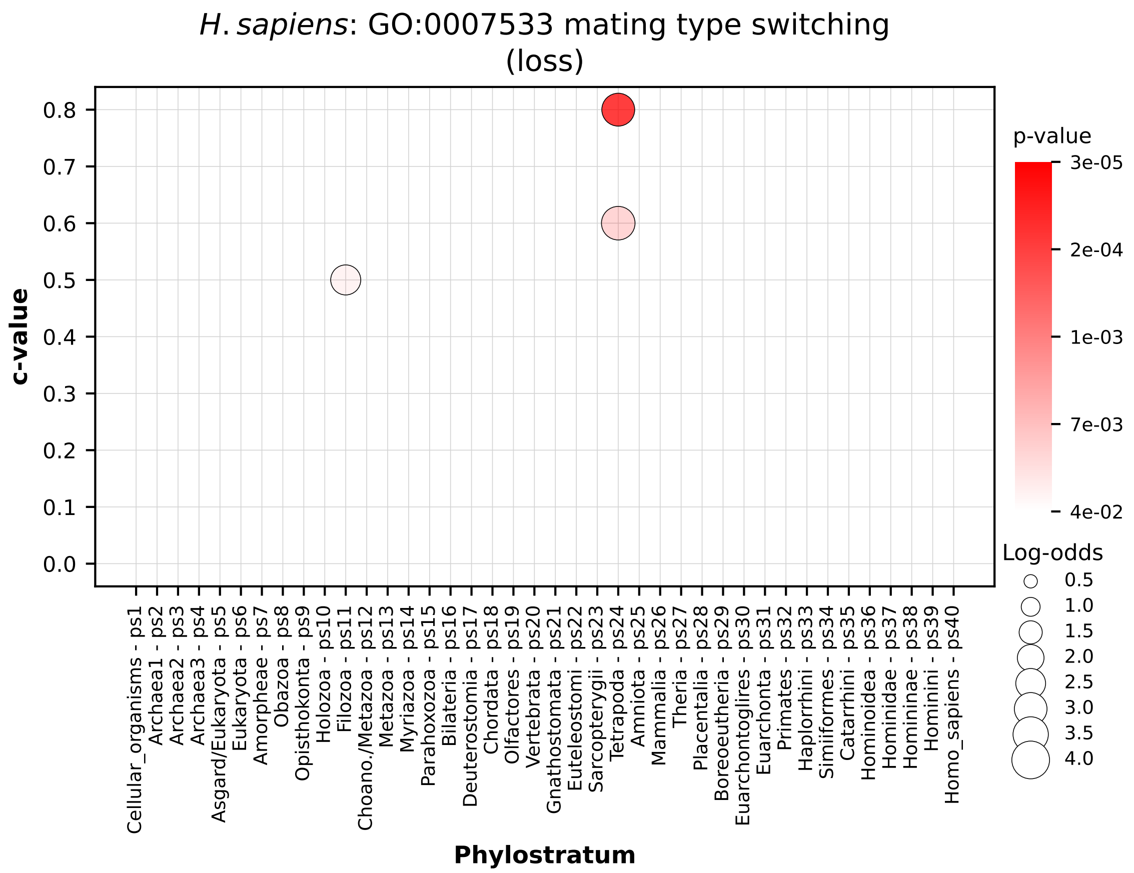

H. sapiens: GO:0007533 mating type switching
(loss)
c-value
0.0
0.1
0.2
0.3
0.4
0.5
0.6
0.7
0.8
Phylostratum
Cellular_organisms - ps1
Archaea1 - ps2
Archaea2 - ps3
Archaea3 - ps4
Asgard/Eukaryota - ps5
Eukaryota - ps6
Amorpheae - ps7
Obazoa - ps8
Opisthokonta - ps9
Holozoa - ps10
Filozoa - ps11
Choano./Metazoa - ps12
Metazoa - ps13
Myriazoa - ps14
Parahoxozoa - ps15
Bilateria - ps16
Deuterostomia - ps17
Chordata - ps18
Olfactores - ps19
Vertebrata - ps20
Gnathostomata - ps21
Euteleostomi - ps22
Sarcopterygii - ps23
Tetrapoda - ps24
Amniota - ps25
Mammalia - ps26
Theria - ps27
Placentalia - ps28
Boreoeutheria - ps29
Euarchontoglires - ps30
Euarchonta - ps31
Primates - ps32
Haplorrhini - ps33
Simiiformes - ps34
Catarrhini - ps35
Hominoidea - ps36
Hominidae - ps37
Homininae - ps38
Hominini - ps39
Homo_sapiens - ps40
p-value
3e-05
2e-04
1e-03
7e-03
4e-02
Log-odds
0.5
1.0
1.5
2.0
2.5
3.0
3.5
4.0

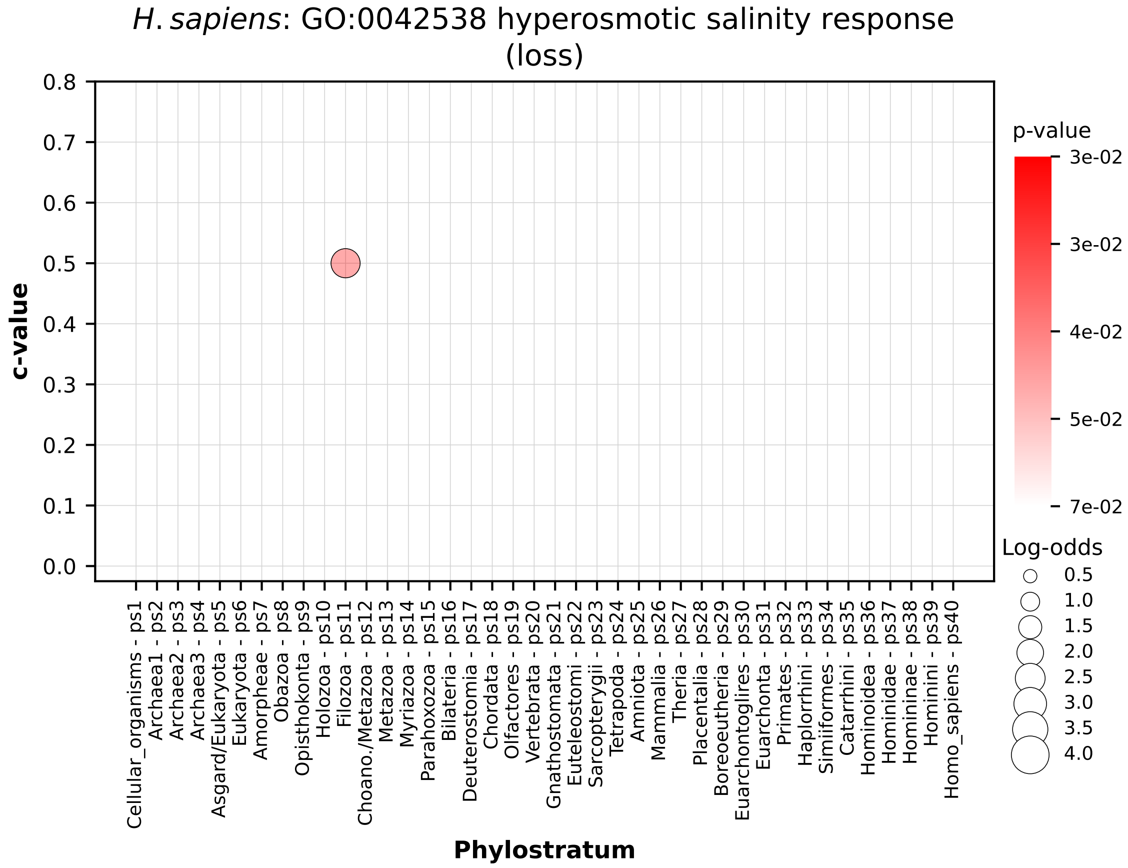

H. sapiens: GO:0042538 hyperosmotic salinity response (loss)
c-value
0.0
0.1
0.2
0.3
0.4
0.5
0.6
0.7
0.8
Phylostratum
Cellular_organisms - ps1
Archaea1 - ps2
Archaea2 - ps3
Archaea3 - ps4
Asgard/Eukaryota - ps5
Eukaryota - ps6
Amorpheae - ps7
Obazoa - ps8
Opisthokonta - ps9
Holozoa - ps10
Filozoa - ps11
Choano./Metazoa - ps12
Metazoa - ps13
Myriazoa - ps14
Parahoxozoa - ps15
Bilateria - ps16
Deuterostomia - ps17
Chordata - ps18
Olfactores - ps19
Vertebrata - ps20
Gnathostomata - ps21
Euteleostomi - ps22
Sarcopterygii - ps23
Tetrapoda - ps24
Amniota - ps25
Mammalia - ps26
Theria - ps27
Placentalia - ps28
Boreoeutheria - ps29
Euarchontoglires - ps30
Euarchonta - ps31
Primates - ps32
Haplorrhini - ps33
Simiiformes - ps34
Catarrhini - ps35
Hominoidea - ps36
Hominidae - ps37
Homininae - ps38
Hominini - ps39
Homo_sapiens - ps40
p-value
3e-02
3e-02
4e-02
5e-02
7e-02
Log-odds
0.5
1.0
1.5
2.0
2.5
3.0
3.5
4.0

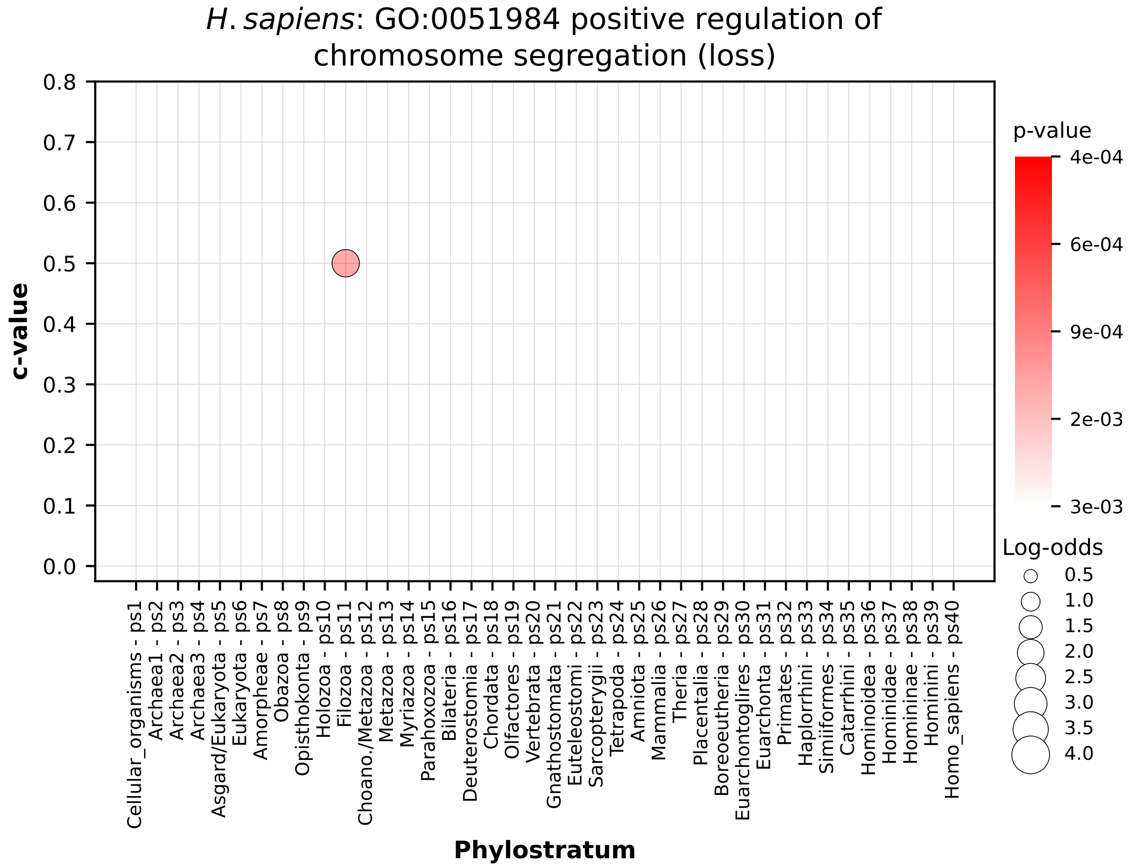

H. sapiens: GO:0051984 positive regulation of chromosome segregation (loss)
c-value
0.0
0.1
0.2
0.3
0.4
0.5
0.6
0.7
0.8
Phylostratum
Cellular_organisms - ps1
Archaea1 - ps2
Archaea2 - ps3
Archaea3 - ps4
Asgard/Eukaryota - ps5
Eukaryota - ps6
Amorpheae - ps7
Obazoa - ps8
Opisthokonta - ps9
Holozoa - ps10
Filozoa - ps11
Choano./Metazoa - ps12
Metazoa - ps13
Myriazoa - ps14
Parahoxozoa - ps15
Bilateria - ps16
Deuterostomia - ps17
Chordata - ps18
Olfactores - ps19
Vertebrata - ps20
Gnathostomata - ps21
Euteleostomi - ps22
Sarcopterygii - ps23
Tetrapoda - ps24
Amniota - ps25
Mammalia - ps26
Theria - ps27
Placentalia - ps28
Boreoeutheria - ps29
Euarchontoglires - ps30
Euarchonta - ps31
Primates - ps32
Haplorrhini - ps33
Simiiformes - ps34
Catarrhini - ps35
Hominoidea - ps36
Hominidae - ps37
Homininae - ps38
Hominini - ps39
Homo_sapiens - ps40
p-value
4e-04
6e-04
9e-04
2e-03
3e-03
Log-odds
0.5
1.0
1.5
2.0
2.5
3.0
3.5
4.0

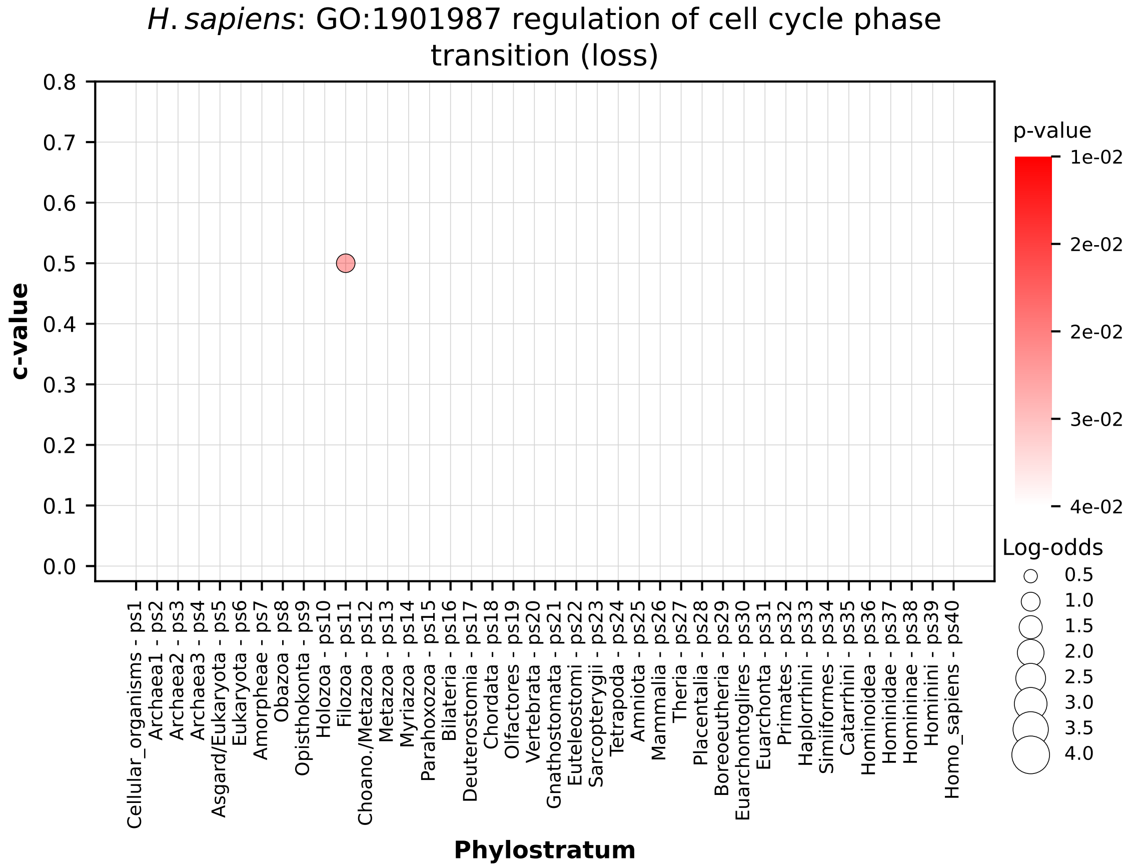
H. sapiens: GO:1901987 regulation of cell cycle phase transition (loss)
c-value
0.8
0.7
0.6
0.5
0.4
0.3
0.2
0.1
0.0
Phylostratum
Cellular_organisms - ps1
Archaea1 - ps2
Archaea2 - ps3
Archaea3 - ps4
Asgard/Eukaryota - ps5
Eukaryota - ps6
Amorpheae - ps7
Obazoa - ps8
Opisthokonta - ps9
Holozoa - ps10
Filozoa - ps11
Choano./Metazoa - ps12
Metazoa - ps13
Myriazoa - ps14
Parahoxozoa - ps15
Bilateria - ps16
Deuterostomia - ps17
Chordata - ps18
Olfactores - ps19
Vertebrata - ps20
Gnathostomata - ps21
Euteleostomi - ps22
Sarcopterygii - ps23
Tetrapoda - ps24
Amniota - ps25
Mammalia - ps26
Theria - ps27
Placentalia - ps28
Boreoeutheria - ps29
Euarchontoglires - ps30
Euarchonta - ps31
Primates - ps32
Haplorrhini - ps33
Simiiformes - ps34
Catarrhini - ps35
Hominoidea - ps36
Hominidae - ps37
Homininae - ps38
Hominini - ps39
Homo_sapiens - ps40
p-value
1e-02
2e-02
2e-02
3e-02
4e-02
Log-odds
0.5
1.0
1.5
2.0
2.5
3.0
3.5
4.0

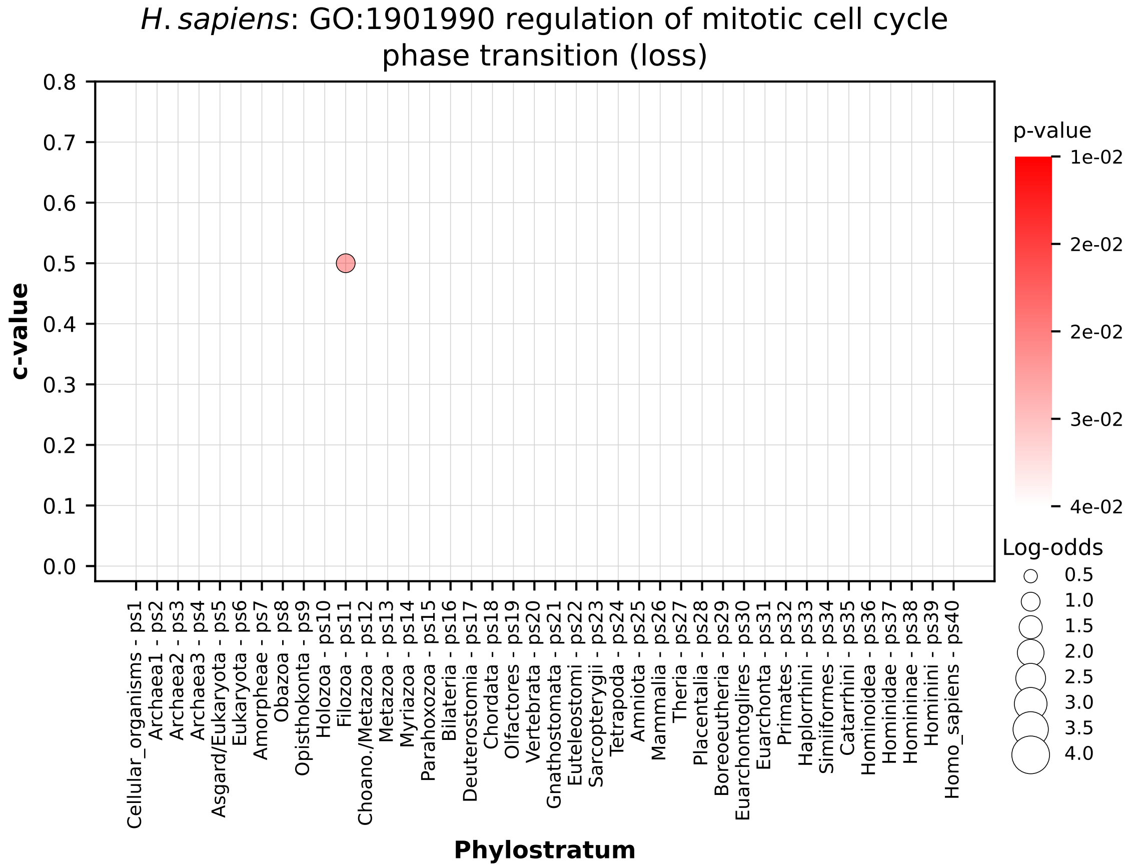

H. sapiens: GO:1901990 regulation of mitotic cell cycle phase transition (loss)
c-value
0.0
0.1
0.2
0.3
0.4
0.5
0.6
0.7
0.8
Phylostratum
Cellular_organisms - ps1
Archaea1 - ps2
Archaea2 - ps3
Archaea3 - ps4
Asgard/Eukaryota - ps5
Eukaryota - ps6
Amorpheae - ps7
Obazoa - ps8
Opisthokonta - ps9
Holozoa - ps10
Filozoa - ps11
Choano./Metazoa - ps12
Metazoa - ps13
Myriazoa - ps14
Parahoxozoa - ps15
Bilateria - ps16
Deuterostomia - ps17
Chordata - ps18
Olfactores - ps19
Vertebrata - ps20
Gnathostomata - ps21
Euteleostomi - ps22
Sarcopterygii - ps23
Tetrapoda - ps24
Amniota - ps25
Mammalia - ps26
Theria - ps27
Placentalia - ps28
Boreoeutheria - ps29
Euarchontoglires - ps30
Euarchonta - ps31
Primates - ps32
Haplorrhini - ps33
Simiiformes - ps34
Catarrhini - ps35
Hominoidea - ps36
Hominidae - ps37
Homininae - ps38
Hominini - ps39
Homo_sapiens - ps40
p-value
1e-02
2e-02
2e-02
3e-02
4e-02
Log-odds
0.5
1.0
1.5
2.0
2.5
3.0
3.5
4.0

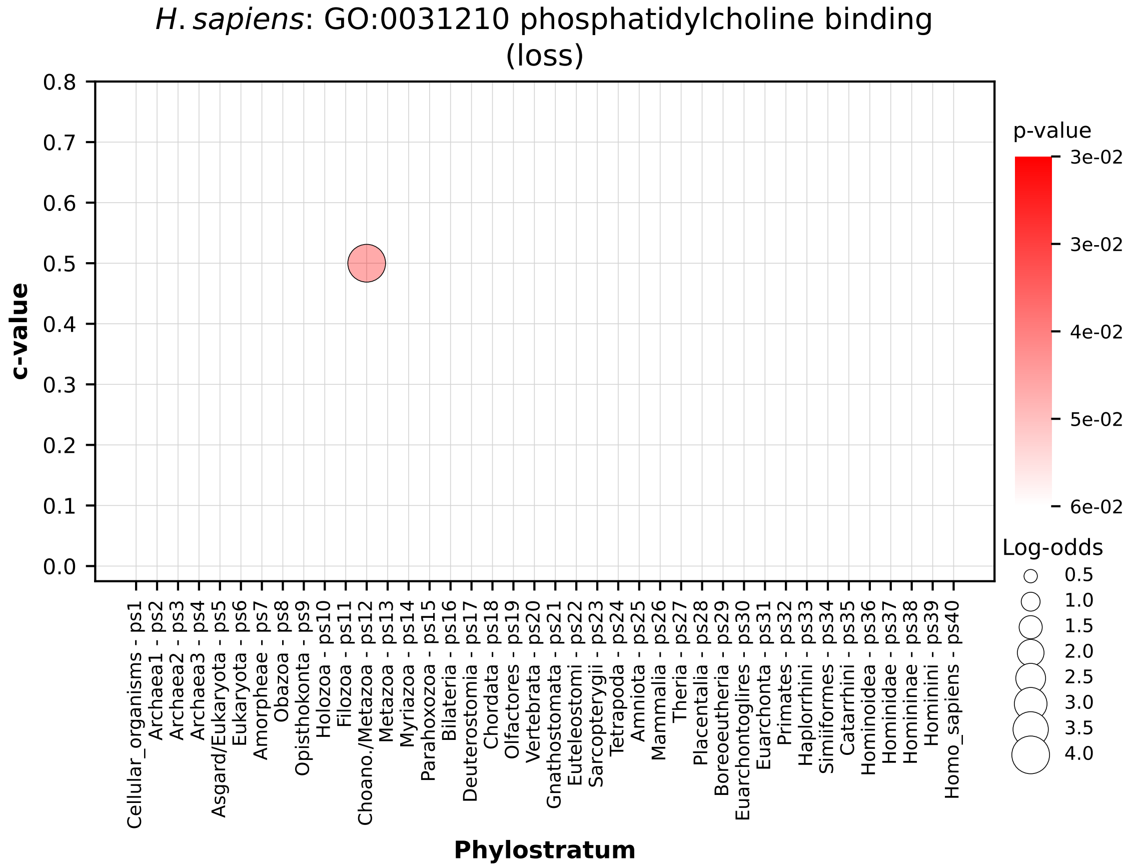

H. sapiens: GO:0031210 phosphatidylcholine binding
(loss)
c-value
0.0
0.1
0.2
0.3
0.4
0.5
0.6
0.7
0.8
Phylostratum
Cellular_organisms - ps1
Archaea1 - ps2
Archaea2 - ps3
Archaea3 - ps4
Asgard/Eukaryota - ps5
Eukaryota - ps6
Amorpheae - ps7
Obazoa - ps8
Opisthokonta - ps9
Holozoa - ps10
Filozoa - ps11
Choano./Metazoa - ps12
Metazoa - ps13
Myriazoa - ps14
Parahoxozoa - ps15
Bilateria - ps16
Deuterostomia - ps17
Chordata - ps18
Olfactores - ps19
Vertebrata - ps20
Gnathostomata - ps21
Euteleostomi - ps22
Sarcopterygii - ps23
Tetrapoda - ps24
Amniota - ps25
Mammalia - ps26
Theria - ps27
Placentalia - ps28
Boreoeutheria - ps29
Euarchontoglires - ps30
Euarchonta - ps31
Primates - ps32
Haplorrhini - ps33
Simiiformes - ps34
Catarrhini - ps35
Hominoidea - ps36
Hominidae - ps37
Homininae - ps38
Hominini - ps39
Homo_sapiens - ps40
p-value
3e-02
3e-02
4e-02
5e-02
6e-02
Log-odds
0.5
1.0
1.5
2.0
2.5
3.0
3.5
4.0

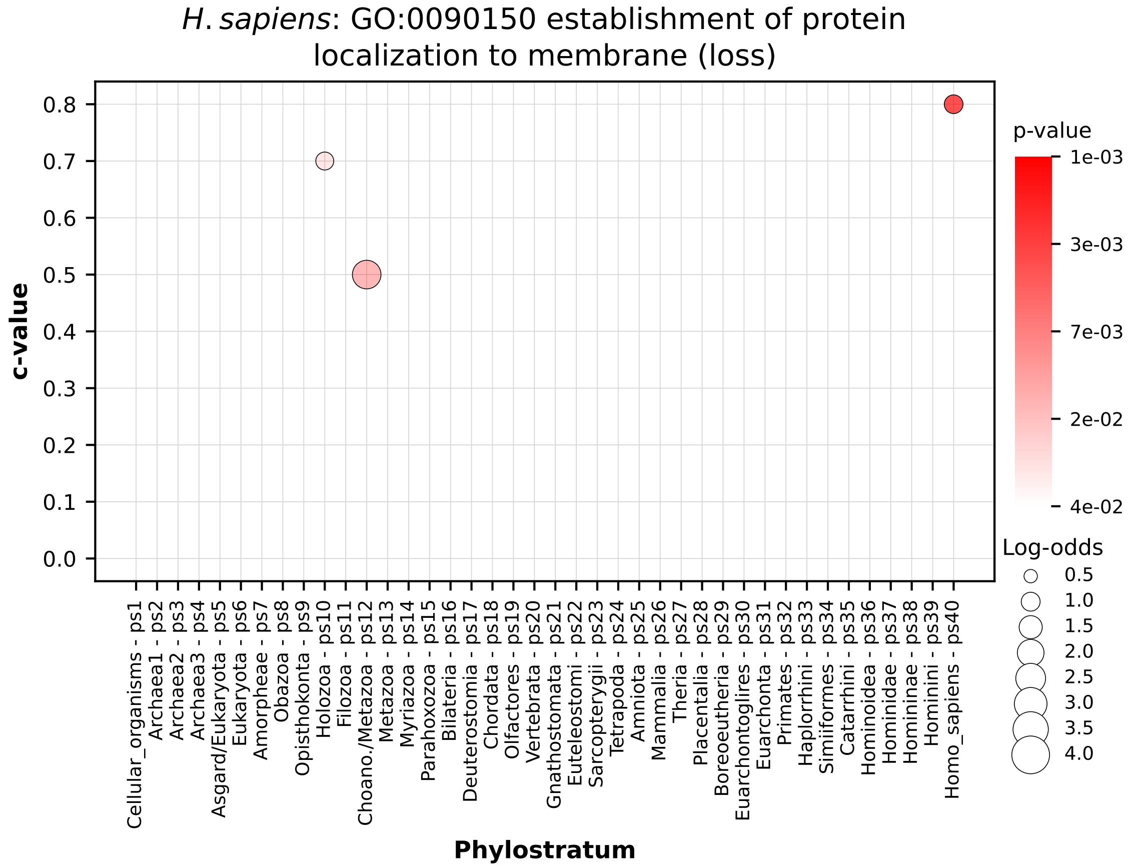

H. sapiens: GO:0090150 establishment of protein localization to membrane (loss)
c-value
0.0
0.1
0.2
0.3
0.4
0.5
0.6
0.7
0.8
p-value
1e-03
3e-03
7e-03
2e-02
4e-02
Log-odds
0.5
1.0
1.5
2.0
2.5
3.0
3.5
4.0
Cellular_organisms - ps1
Archaea1 - ps2
Archaea2 - ps3
Archaea3 - ps4
Asgard/Eukaryota - ps5
Eukaryota - ps6
Amorpheae - ps7
Obazoa - ps8
Opisthokonta - ps9
Holozoa - ps10
Filozoa - ps11
Choano./Metazoa - ps12
Metazoa - ps13
Myriazoa - ps14
Parahoxozoa - ps15
Bilateria - ps16
Deuterostomia - ps17
Chordata - ps18
Olfactores - ps19
Vertebrata - ps20
Gnathostomata - ps21
Euteleostomi - ps22
Sarcopterygii - ps23
Tetrapoda - ps24
Amniota - ps25
Mammalia - ps26
Theria - ps27
Placentalia - ps28
Boreoeutheria - ps29
Euarchontoglires - ps30
Euarchonta - ps31
Primates - ps32
Haplorrhini - ps33
Simiiformes - ps34
Catarrhini - ps35
Hominoidea - ps36
Hominidae - ps37
Homininae - ps38
Hominini - ps39
Homo_sapiens - ps40
Phylostratum

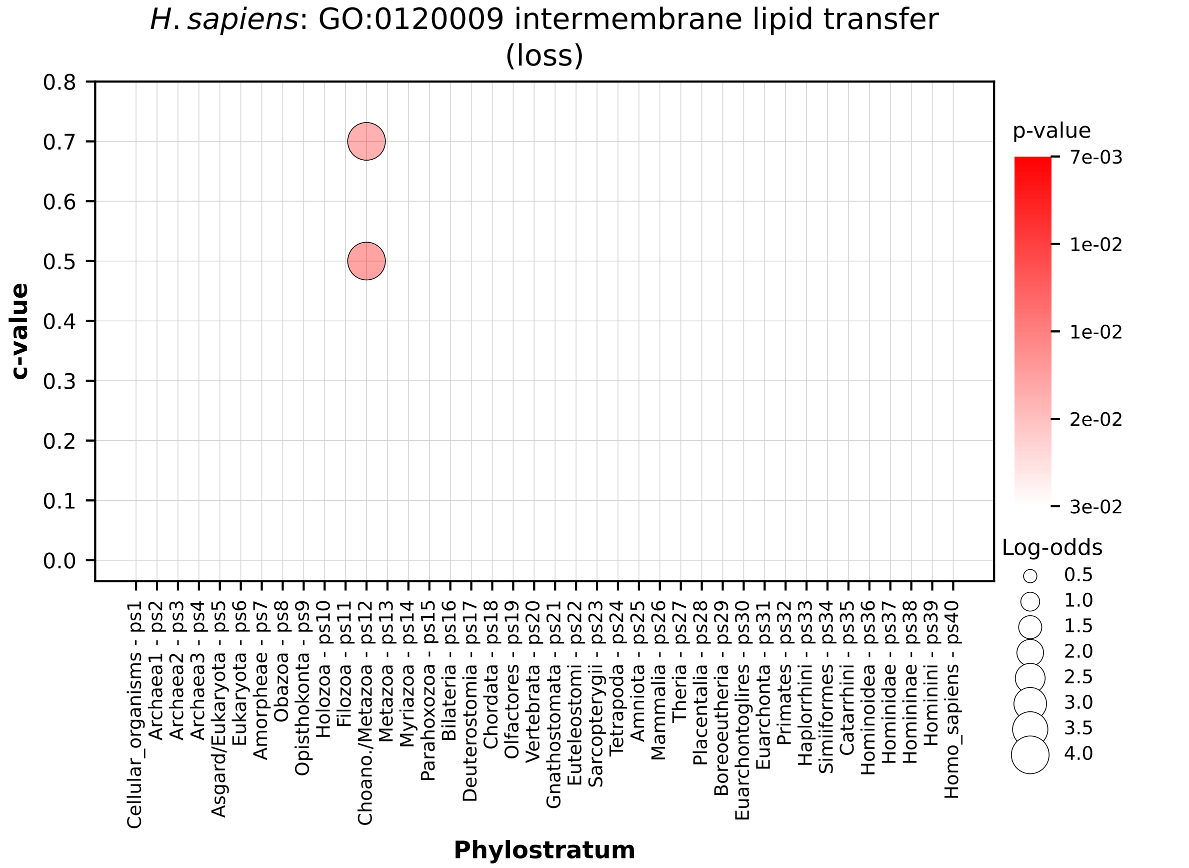

H. sapiens: GO:0120009 intermembrane lipid transfer
(loss)
c-value
0.8
0.7
0.6
0.5
0.4
0.3
0.2
0.1
0.0
Phylostratum
Cellular_organisms - ps1
Archaea1 - ps2
Archaea2 - ps3
Archaea3 - ps4
Asgard/Eukaryota - ps5
Eukaryota - ps6
Amorpheae - ps7
Obazoa - ps8
Opisthokonta - ps9
Holozoa - ps10
Filozoa - ps11
Choano./Metazoa - ps12
Metazoa - ps13
Myriazoa - ps14
Parahoxozoa - ps15
Bilateria - ps16
Deuterostomia - ps17
Chordata - ps18
Olfactores - ps19
Vertebrata - ps20
Gnathostomata - ps21
Euteleostomi - ps22
Sarcopterygii - ps23
Tetrapoda - ps24
Amniota - ps25
Mammalia - ps26
Theria - ps27
Placentalia - ps28
Boreoeutheria - ps29
Euarchontoglires - ps30
Euarchonta - ps31
Primates - ps32
Haplorrhini - ps33
Simiiformes - ps34
Catarrhini - ps35
Hominoidea - ps36
Hominidae - ps37
Homininae - ps38
Hominini - ps39
Homo_sapiens - ps40
p-value
7e-03
1e-02
1e-02
2e-02
3e-02
Log-odds
0.5
1.0
1.5
2.0
2.5
3.0
3.5
4.0

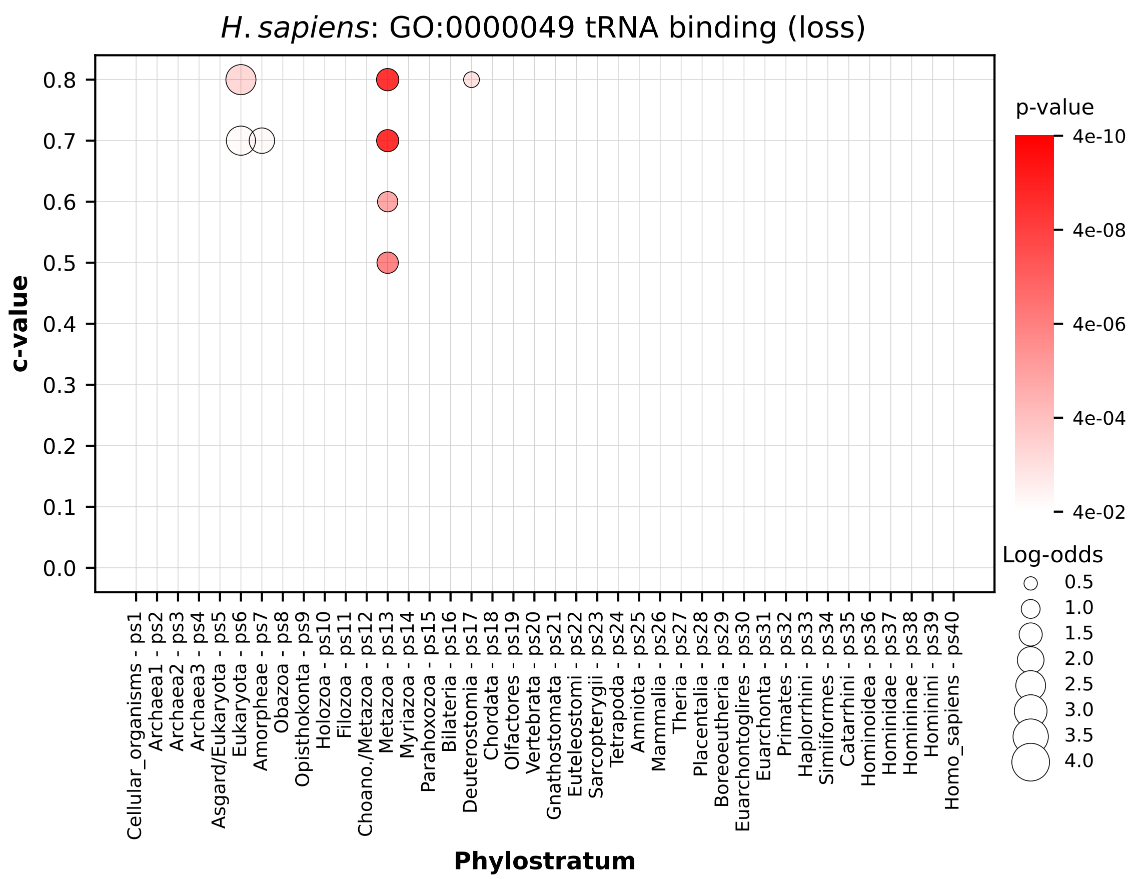

H. sapiens: GO:0000049 tRNA binding (loss)
c-value
0.8
0.7
0.6
0.5
0.4
0.3
0.2
0.1
0.0
p-value
4e-10
4e-08
4e-06
4e-04
4e-02
Log-odds
0.5
1.0
1.5
2.0
2.5
3.0
3.5
4.0
Cellular_organisms - ps1
Archaea1 - ps2
Archaea2 - ps3
Archaea3 - ps4
Asgard/Eukaryota - ps5
Eukaryota - ps6
Amorpheae - ps7
Obazoa - ps8
Opisthokonta - ps9
Holozoa - ps10
Filozoa - ps11
Choano./Metazoa - ps12
Metazoa - ps13
Myriazoa - ps14
Parahoxozoa - ps15
Bilateria - ps16
Deuterostomia - ps17
Chordata - ps18
Olfactores - ps19
Vertebrata - ps20
Gnathostomata - ps21
Euteleostomi - ps22
Sarcopterygii - ps23
Tetrapoda - ps24
Amniota - ps25
Mammalia - ps26
Theria - ps27
Placentalia - ps28
Boreoeutheria - ps29
Euarchontoglires - ps30
Euarchonta - ps31
Primates - ps32
Haplorrhini - ps33
Simiiformes - ps34
Catarrhini - ps35
Hominoidea - ps36
Hominidae - ps37
Homininae - ps38
Hominini - ps39
Homo_sapiens - ps40
Phylostratum

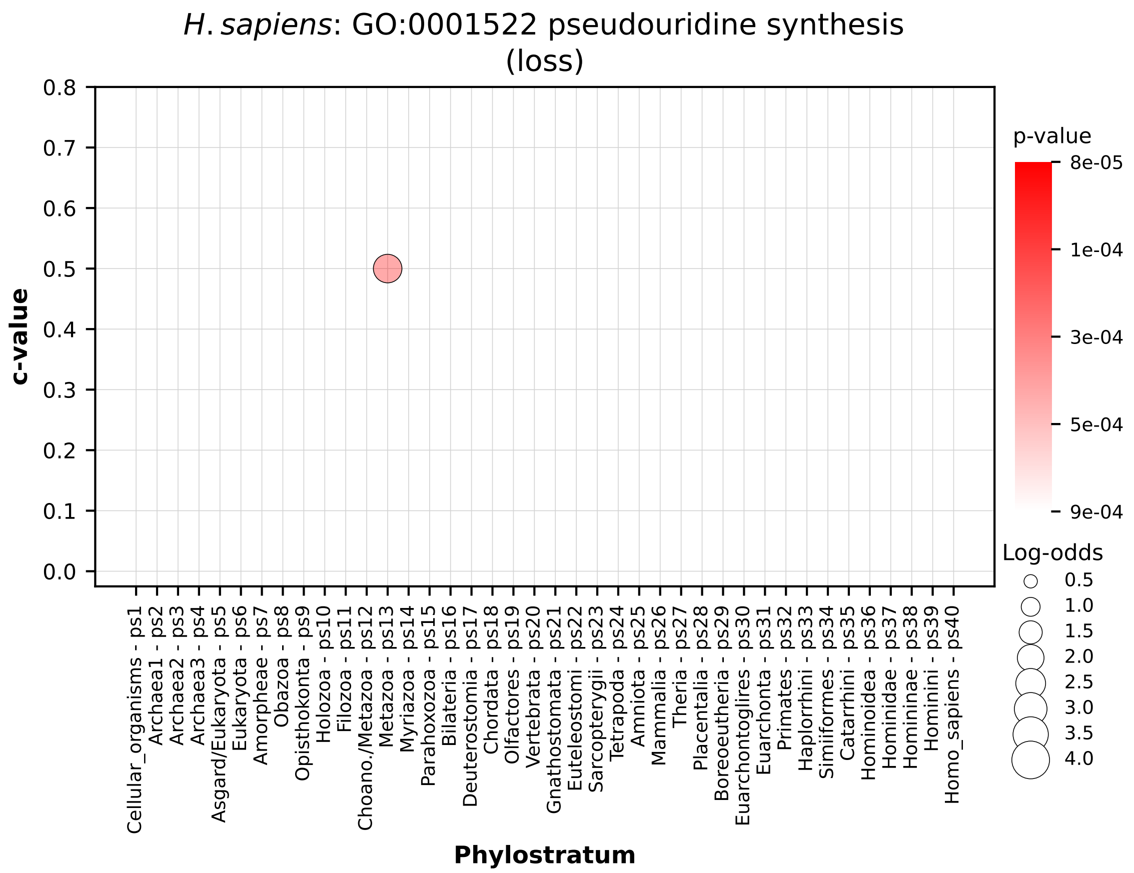
H. sapiens: GO:0001522 pseudouridine synthesis (loss)
c-value
0.0
0.1
0.2
0.3
0.4
0.5
0.6
0.7
0.8
Phylostratum
Cellular_organisms - ps1
Archaea1 - ps2
Archaea2 - ps3
Archaea3 - ps4
Asgard/Eukaryota - ps5
Eukaryota - ps6
Amorpheae - ps7
Obazoa - ps8
Opisthokonta - ps9
Holozoa - ps10
Filozoa - ps11
Choano./Metazoa - ps12
Metazoa - ps13
Myriazoa - ps14
Parahoxozoa - ps15
Bilateria - ps16
Deuterostomia - ps17
Chordata - ps18
Olfactores - ps19
Vertebrata - ps20
Gnathostomata - ps21
Euteleostomi - ps22
Sarcopterygii - ps23
Tetrapoda - ps24
Amniota - ps25
Mammalia - ps26
Theria - ps27
Placentalia - ps28
Boreoeutheria - ps29
Euarchontoglires - ps30
Euarchonta - ps31
Primates - ps32
Haplorrhini - ps33
Simiiformes - ps34
Catarrhini - ps35
Hominoidea - ps36
Hominidae - ps37
Homininae - ps38
Hominini - ps39
Homo_sapiens - ps40
p-value
8e-05
1e-04
3e-04
5e-04
9e-04
Log-odds
0.5
1.0
1.5
2.0
2.5
3.0
3.5
4.0

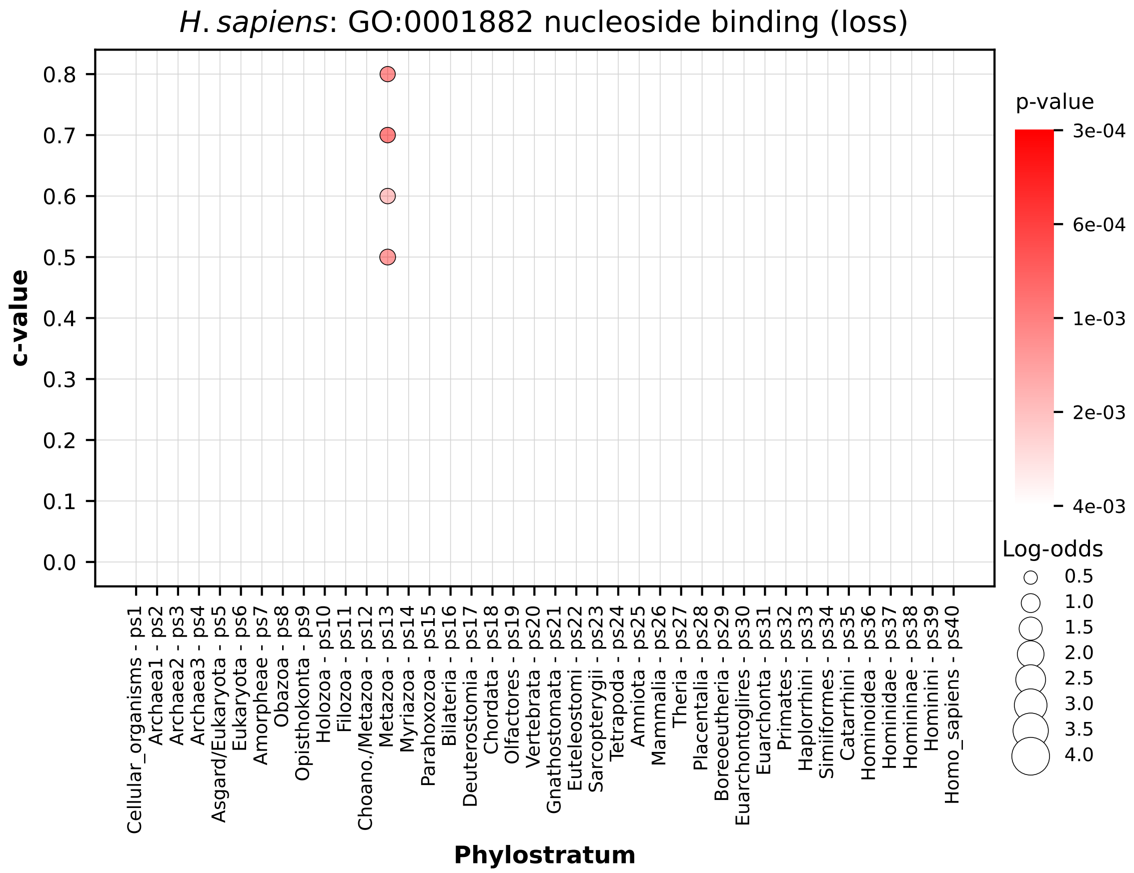

H. sapiens: GO:0001882 nucleoside binding (loss)
c-value
0.8
0.7
0.6
0.5
0.4
0.3
0.2
0.1
0.0
Phylostratum
Cellular_organisms - ps1
Archaea1 - ps2
Archaea2 - ps3
Archaea3 - ps4
Asgard/Eukaryota - ps5
Eukaryota - ps6
Amorpheae - ps7
Obazoa - ps8
Opisthokonta - ps9
Holozoa - ps10
Filozoa - ps11
Choano./Metazoa - ps12
Metazoa - ps13
Myriazoa - ps14
Parahoxozoa - ps15
Bilateria - ps16
Deuterostomia - ps17
Chordata - ps18
Olfactores - ps19
Vertebrata - ps20
Gnathostomata - ps21
Euteleostomi - ps22
Sarcopterygii - ps23
Tetrapoda - ps24
Amniota - ps25
Mammalia - ps26
Theria - ps27
Placentalia - ps28
Boreoeutheria - ps29
Euarchontoglires - ps30
Euarchonta - ps31
Primates - ps32
Haplorrhini - ps33
Simiiformes - ps34
Catarrhini - ps35
Hominoidea - ps36
Hominidae - ps37
Homininae - ps38
Hominini - ps39
Homo_sapiens - ps40
p-value
3e-04
6e-04
1e-03
2e-03
4e-03
Log-odds
0.5
1.0
1.5
2.0
2.5
3.0
3.5
4.0

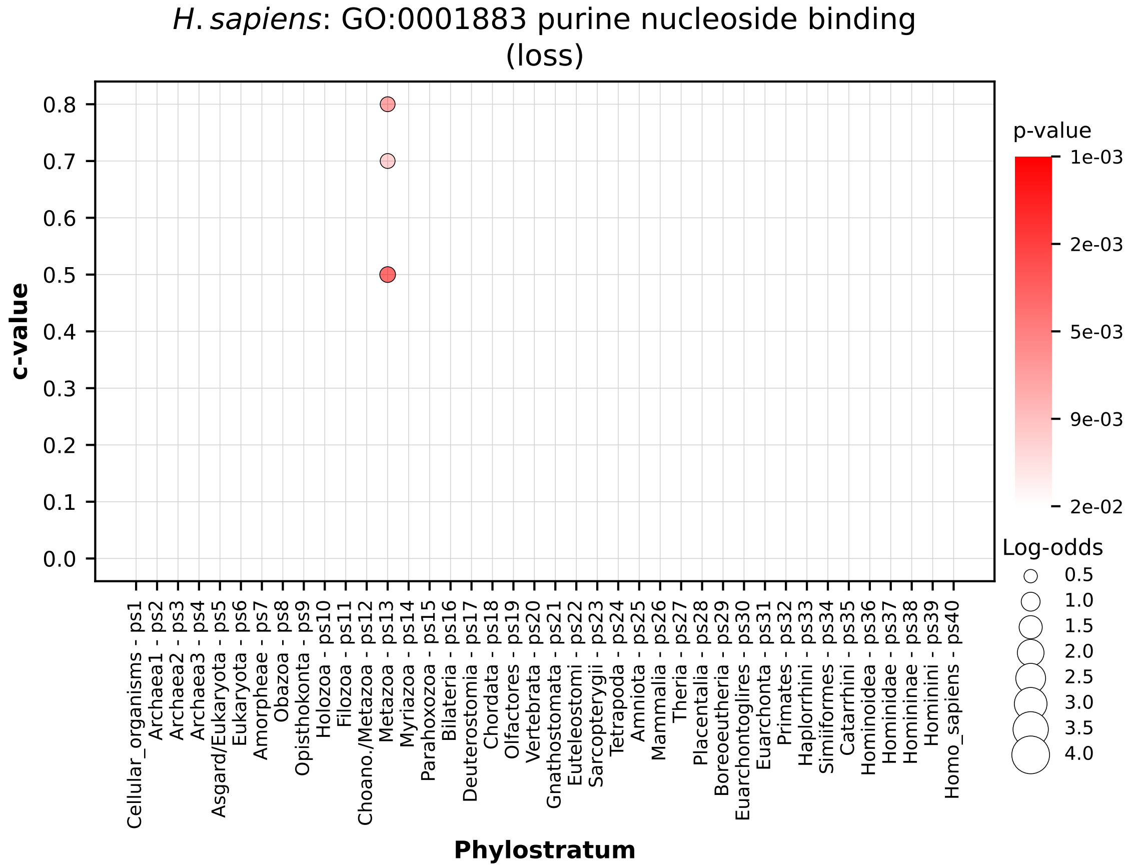

H. sapiens: GO:0001883 purine nucleoside binding
(loss)
c-value
0.8
0.7
0.6
0.5
0.4
0.3
0.2
0.1
0.0
p-value
1e-03
2e-03
5e-03
9e-03
2e-02
Log-odds
0.5
1.0
1.5
2.0
2.5
3.0
3.5
4.0
Cellular_organisms - ps1
Archaea1 - ps2
Archaea2 - ps3
Archaea3 - ps4
Asgard/Eukaryota - ps5
Eukaryota - ps6
Amorpheae - ps7
Obazoa - ps8
Opisthokonta - ps9
Holozoa - ps10
Filozoa - ps11
Choano./Metazoa - ps12
Metazoa - ps13
Myriazoa - ps14
Parahoxozoa - ps15
Bilateria - ps16
Deuterostomia - ps17
Chordata - ps18
Olfactores - ps19
Vertebrata - ps20
Gnathostomata - ps21
Euteleostomi - ps22
Sarcopterygii - ps23
Tetrapoda - ps24
Amniota - ps25
Mammalia - ps26
Theria - ps27
Placentalia - ps28
Boreoeutheria - ps29
Euarchontoglires - ps30
Euarchonta - ps31
Primates - ps32
Haplorrhini - ps33
Simiiformes - ps34
Catarrhini - ps35
Hominoidea - ps36
Hominidae - ps37
Homininae - ps38
Hominini - ps39
Homo_sapiens - ps40
Phylostratum

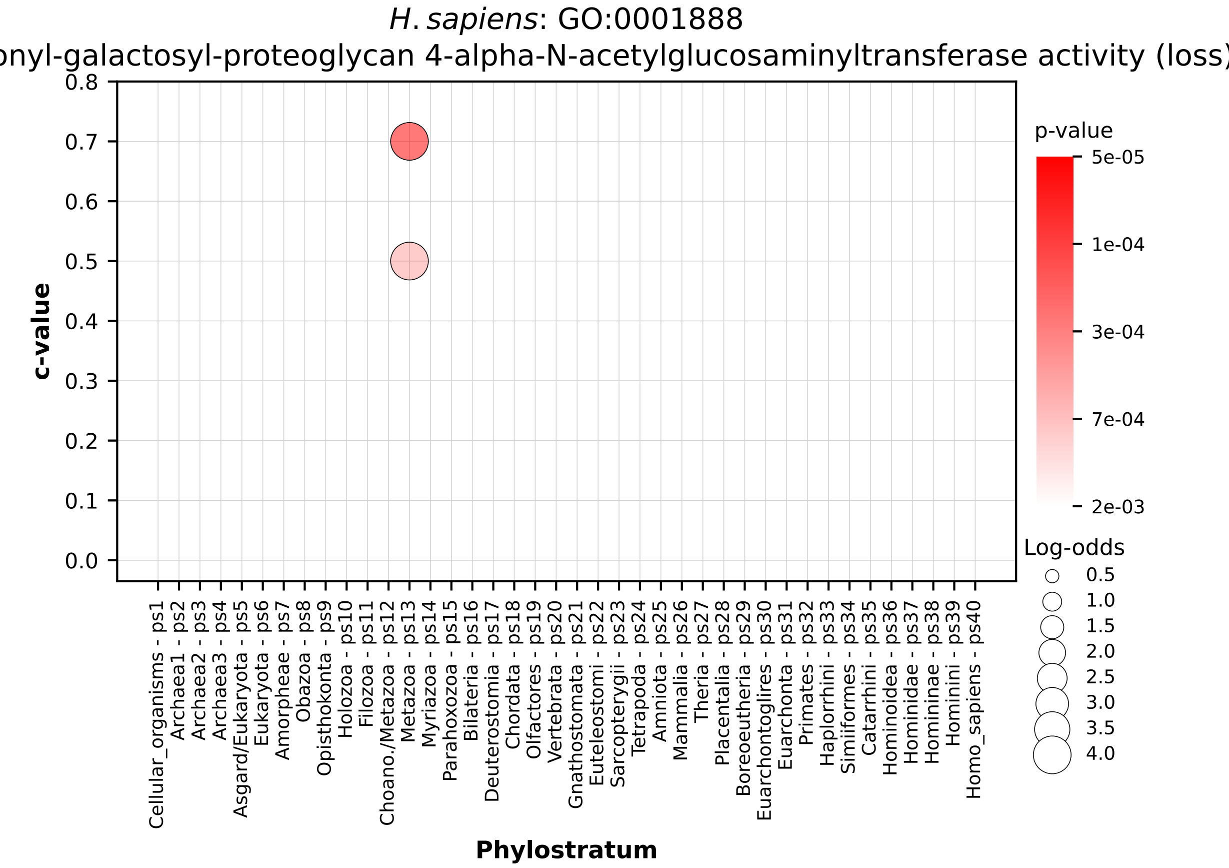
H. sapiens: GO:0001888
onyl-galactosyl-proteoglycan 4-alpha-N-acetylglucosaminyltransferase activity (loss)
c-value
0.8
0.7
0.6
0.5
0.4
0.3
0.2
0.1
0.0
p-value
5e-05
1e-04
3e-04
7e-04
2e-03
Log-odds
0.5
1.0
1.5
2.0
2.5
3.0
3.5
4.0
Cellular_organisms - ps1
Archaea1 - ps2
Archaea2 - ps3
Archaea3 - ps4
Asgard/Eukaryota - ps5
Eukaryota - ps6
Amorpheae - ps7
Obazoa - ps8
Opisthokonta - ps9
Holozoa - ps10
Filozoa - ps11
Choano./Metazoa - ps12
Metazoa - ps13
Myriazoa - ps14
Parahoxozoa - ps15
Bilateria - ps16
Deuterostomia - ps17
Chordata - ps18
Olfactores - ps19
Vertebrata - ps20
Gnathostomata - ps21
Euteleostomi - ps22
Sarcopterygii - ps23
Tetrapoda - ps24
Amniota - ps25
Mammalia - ps26
Theria - ps27
Placentalia - ps28
Boreoeutheria - ps29
Euarchontoglires - ps30
Euarchonta - ps31
Primates - ps32
Haplorrhini - ps33
Simiiformes - ps34
Catarrhini - ps35
Hominoidea - ps36
Hominidae - ps37
Homininae - ps38
Hominini - ps39
Homo_sapiens - ps40
Phylostratum

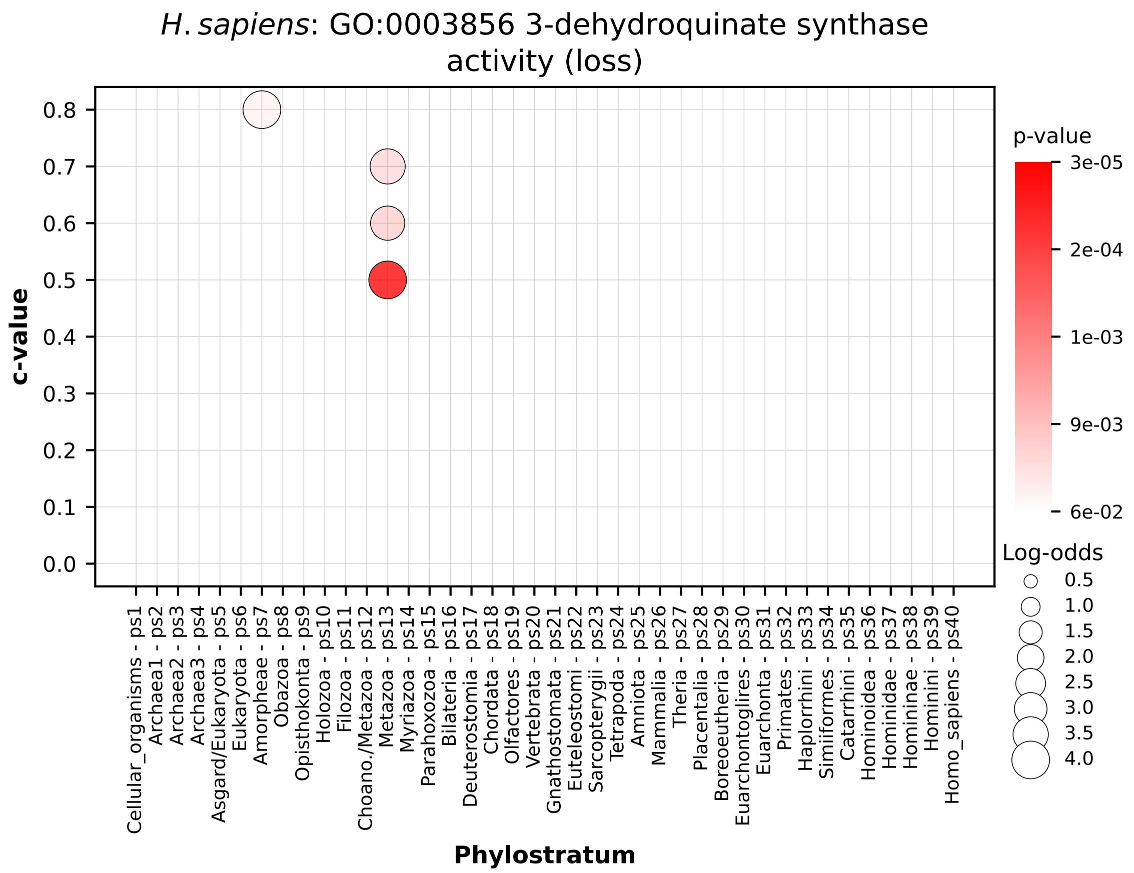
H. sapiens: GO:0003856 3-dehydroquinate synthase activity (loss)
c-value
0.0
0.1
0.2
0.3
0.4
0.5
0.6
0.7
0.8
Phylostratum
Cellular_organisms - ps1
Archaea1 - ps2
Archaea2 - ps3
Archaea3 - ps4
Asgard/Eukaryota - ps5
Eukaryota - ps6
Amorpheae - ps7
Obazoa - ps8
Opisthokonta - ps9
Holozoa - ps10
Filozoa - ps11
Choano./Metazoa - ps12
Metazoa - ps13
Myriazoa - ps14
Parahoxozoa - ps15
Bilateria - ps16
Deuterostomia - ps17
Chordata - ps18
Olfactores - ps19
Vertebrata - ps20
Gnathostomata - ps21
Euteleostomi - ps22
Sarcopterygii - ps23
Tetrapoda - ps24
Amniota - ps25
Mammalia - ps26
Theria - ps27
Placentalia - ps28
Boreoeutheria - ps29
Euarchontoglires - ps30
Euarchonta - ps31
Primates - ps32
Haplorrhini - ps33
Simiiformes - ps34
Catarrhini - ps35
Hominoidea - ps36
Hominidae - ps37
Homininae - ps38
Hominini - ps39
Homo_sapiens - ps40
p-value
3e-05
2e-04
1e-03
9e-03
6e-02
Log-odds
0.5
1.0
1.5
2.0
2.5
3.0
3.5
4.0

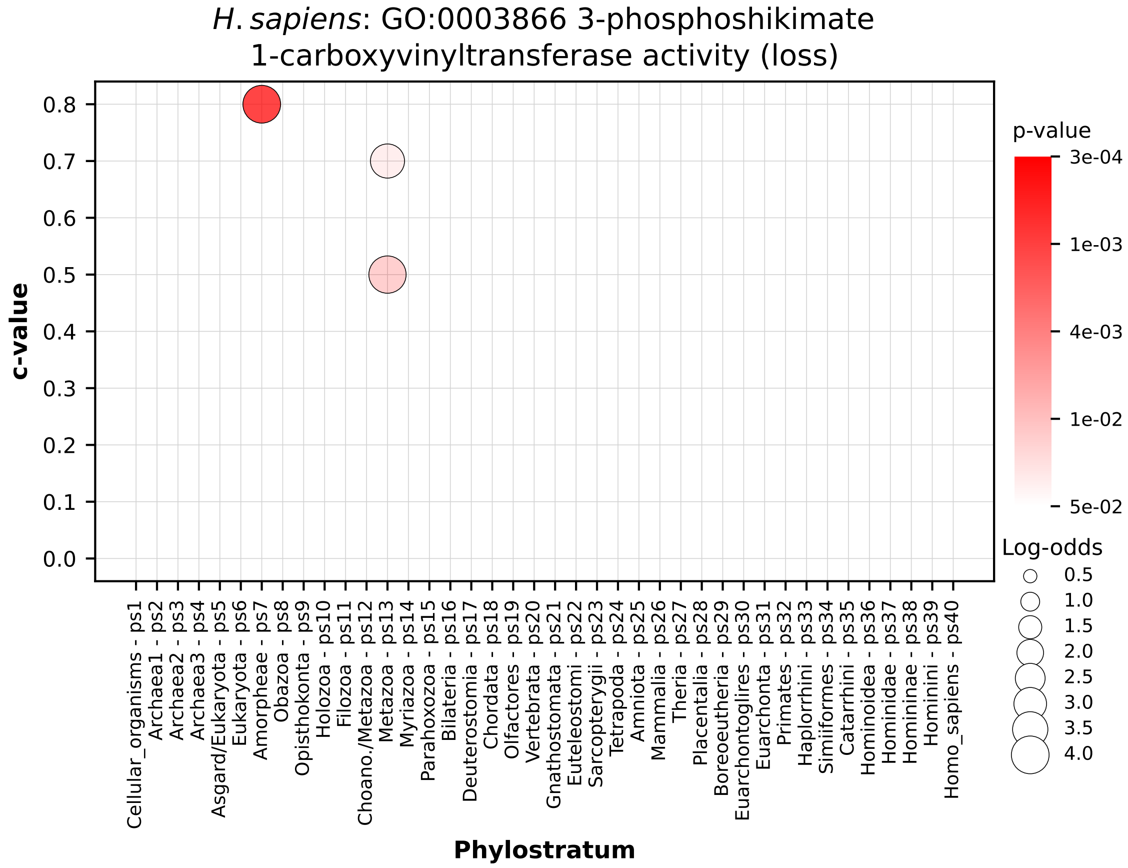

H. sapiens: GO:0003866 3-phosphoshikimate 1-carboxyvinyltransferase activity (loss)
c-value
0.8
0.7
0.6
0.5
0.4
0.3
0.2
0.1
0.0
Phylostratum
Cellular_organisms - ps1
Archaea1 - ps2
Archaea2 - ps3
Archaea3 - ps4
Asgard/Eukaryota - ps5
Eukaryota - ps6
Amorpheae - ps7
Obazoa - ps8
Opisthokonta - ps9
Holozoa - ps10
Filozoa - ps11
Choano./Metazoa - ps12
Metazoa - ps13
Myriazoa - ps14
Parahoxozoa - ps15
Bilateria - ps16
Deuterostomia - ps17
Chordata - ps18
Olfactores - ps19
Vertebrata - ps20
Gnathostomata - ps21
Euteleostomi - ps22
Sarcopterygii - ps23
Tetrapoda - ps24
Amniota - ps25
Mammalia - ps26
Theria - ps27
Placentalia - ps28
Boreoeutheria - ps29
Euarchontoglires - ps30
Euarchonta - ps31
Primates - ps32
Haplorrhini - ps33
Simiiformes - ps34
Catarrhini - ps35
Hominoidea - ps36
Hominidae - ps37
Homininae - ps38
Hominini - ps39
Homo_sapiens - ps40
p-value
3e-04
1e-03
4e-03
1e-02
5e-02
Log-odds
0.5
1.0
1.5
2.0
2.5
3.0
3.5
4.0

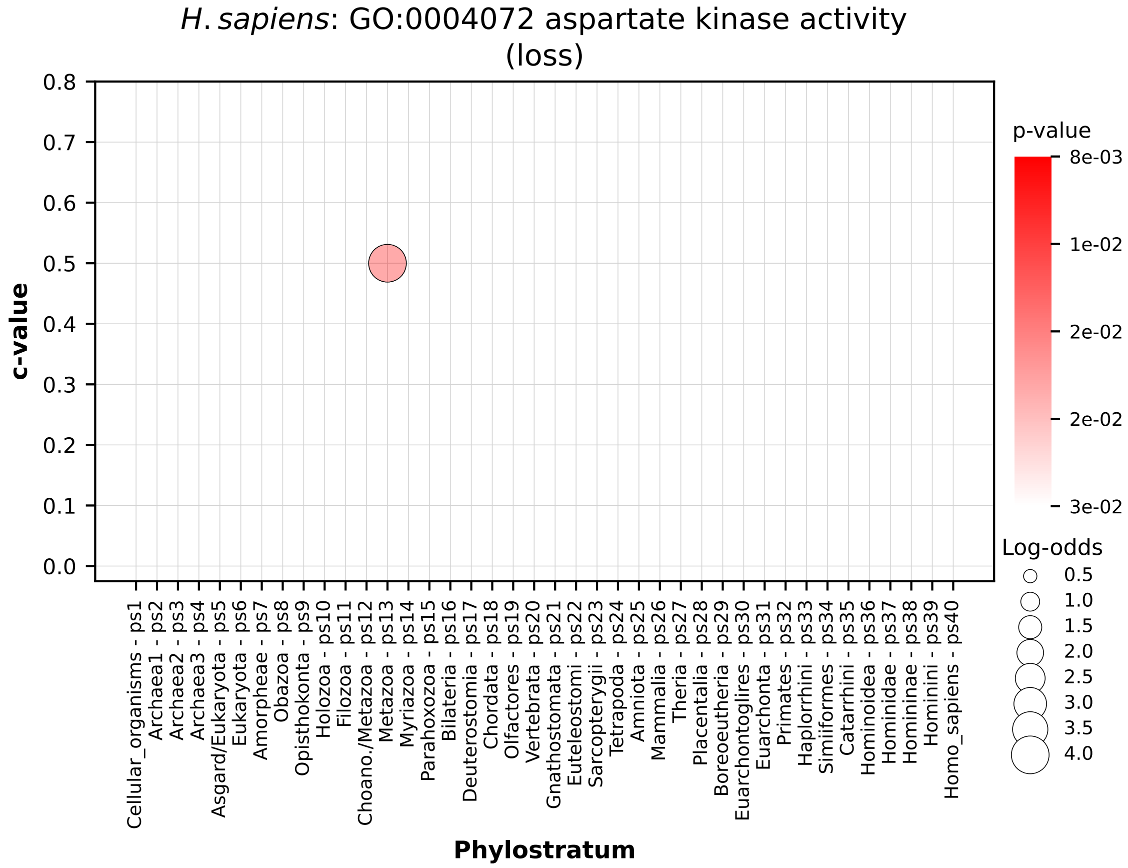

H. sapiens: GO:0004072 aspartate kinase activity (loss)
c-value
0.0
0.1
0.2
0.3
0.4
0.5
0.6
0.7
0.8
Phylostratum
Cellular_organisms - ps1
Archaea1 - ps2
Archaea2 - ps3
Archaea3 - ps4
Asgard/Eukaryota - ps5
Eukaryota - ps6
Amorpheae - ps7
Obazoa - ps8
Opisthokonta - ps9
Holozoa - ps10
Filozoa - ps11
Choano./Metazoa - ps12
Metazoa - ps13
Myriazoa - ps14
Parahoxozoa - ps15
Bilateria - ps16
Deuterostomia - ps17
Chordata - ps18
Olfactores - ps19
Vertebrata - ps20
Gnathostomata - ps21
Euteleostomi - ps22
Sarcopterygii - ps23
Tetrapoda - ps24
Amniota - ps25
Mammalia - ps26
Theria - ps27
Placentalia - ps28
Boreoeutheria - ps29
Euarchontoglires - ps30
Euarchonta - ps31
Primates - ps32
Haplorrhini - ps33
Simiiformes - ps34
Catarrhini - ps35
Hominoidea - ps36
Hominidae - ps37
Homininae - ps38
Hominini - ps39
Homo_sapiens - ps40
p-value
8e-03
1e-02
2e-02
2e-02
3e-02
Log-odds
0.5
1.0
1.5
2.0
2.5
3.0
3.5
4.0

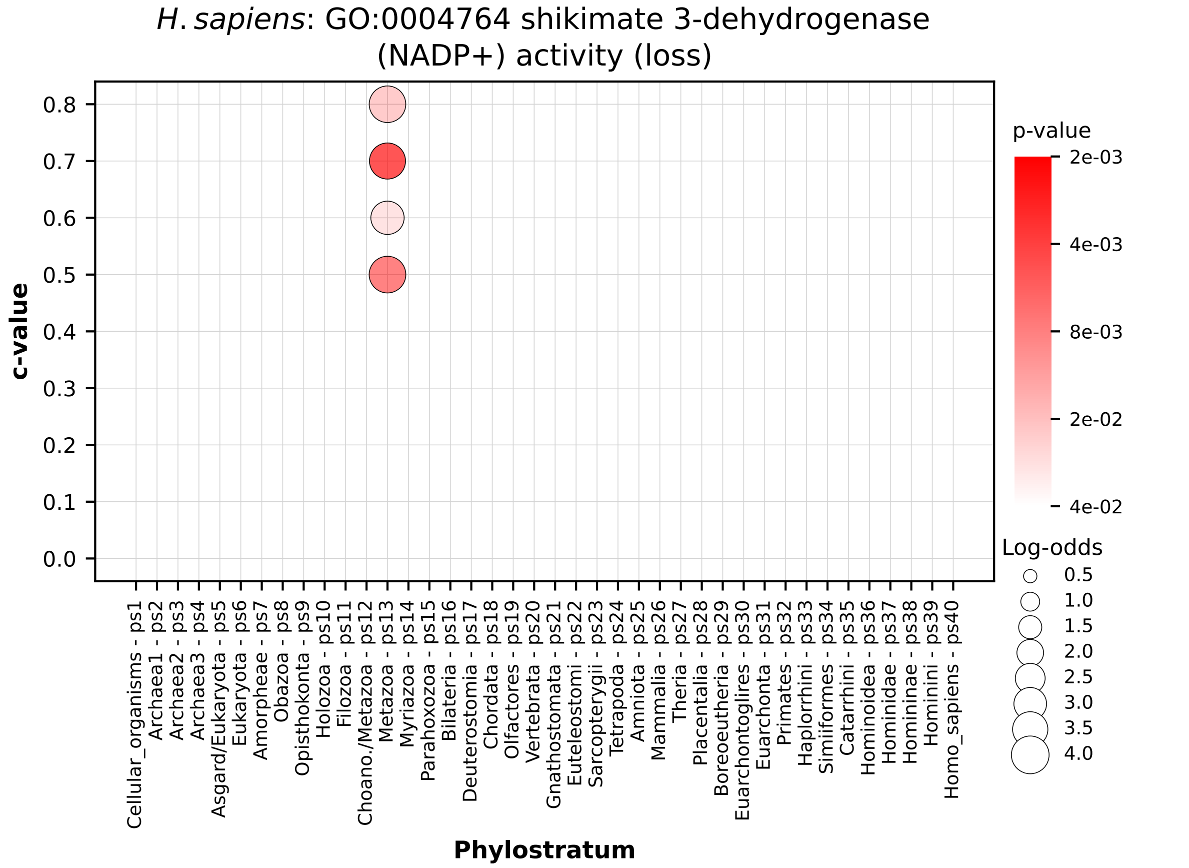
H. sapiens: GO:0004764 shikimate 3-dehydrogenase
(NADP+) activity (loss)
c-value
0.0
0.1
0.2
0.3
0.4
0.5
0.6
0.7
0.8
Phylostratum
Cellular_organisms - ps1
Archaea1 - ps2
Archaea2 - ps3
Archaea3 - ps4
Asgard/Eukaryota - ps5
Eukaryota - ps6
Amorpheae - ps7
Obazoa - ps8
Opisthokonta - ps9
Holozoa - ps10
Filozoa - ps11
Choano./Metazoa - ps12
Metazoa - ps13
Myriazoa - ps14
Parahoxozoa - ps15
Bilateria - ps16
Deuterostomia - ps17
Chordata - ps18
Olfactores - ps19
Vertebrata - ps20
Gnathostomata - ps21
Euteleostomi - ps22
Sarcopterygii - ps23
Tetrapoda - ps24
Amniota - ps25
Mammalia - ps26
Theria - ps27
Placentalia - ps28
Boreoeutheria - ps29
Euarchontoglires - ps30
Euarchonta - ps31
Primates - ps32
Haplorrhini - ps33
Simiiformes - ps34
Catarrhini - ps35
Hominoidea - ps36
Hominidae - ps37
Homininae - ps38
Hominini - ps39
Homo_sapiens - ps40
p-value
2e-03
4e-03
8e-03
2e-02
4e-02
Log-odds
0.5
1.0
1.5
2.0
2.5
3.0
3.5
4.0

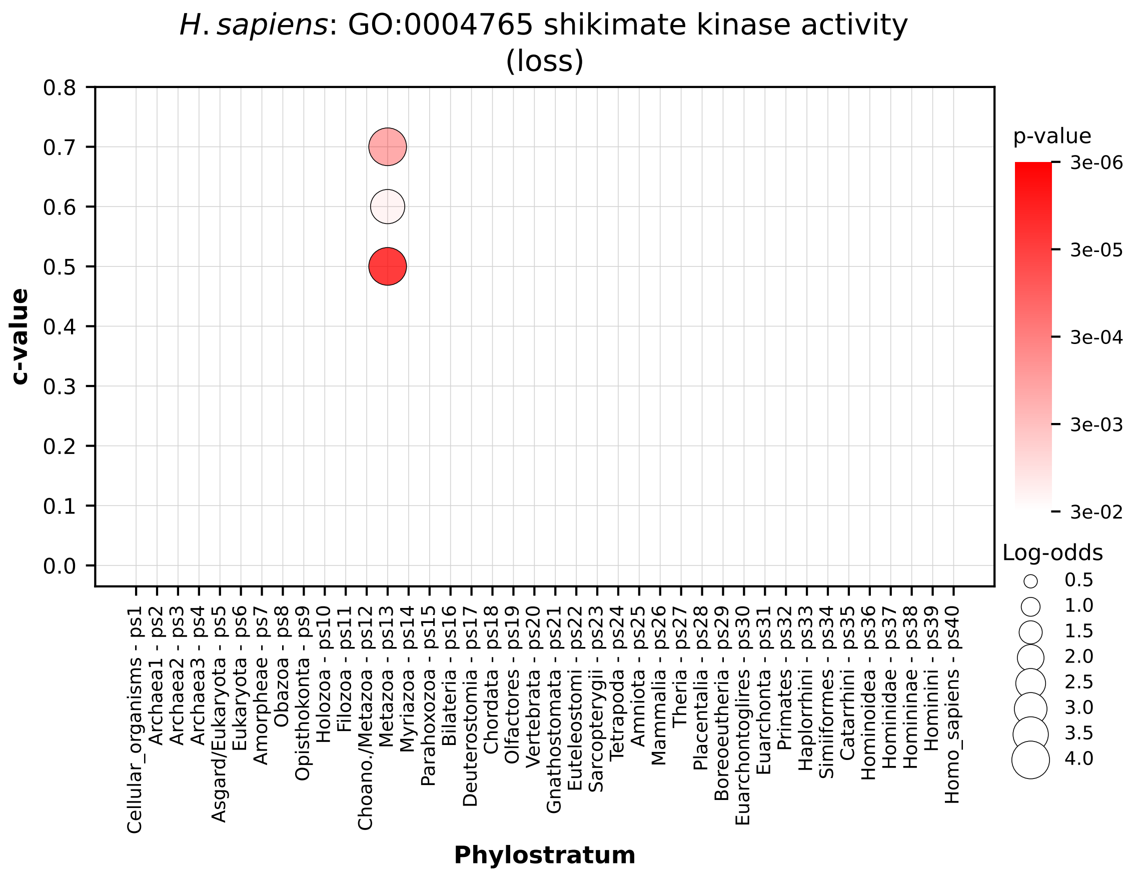

H. sapiens: GO:0004765 shikimate kinase activity
(loss)
c-value
0.0
0.1
0.2
0.3
0.4
0.5
0.6
0.7
0.8
Phylostratum
Cellular_organisms - ps1
Archaea1 - ps2
Archaea2 - ps3
Archaea3 - ps4
Asgard/Eukaryota - ps5
Eukaryota - ps6
Amorpheae - ps7
Obazoa - ps8
Opisthokonta - ps9
Holozoa - ps10
Filozoa - ps11
Choano./Metazoa - ps12
Metazoa - ps13
Myriazoa - ps14
Parahoxozoa - ps15
Bilateria - ps16
Deuterostomia - ps17
Chordata - ps18
Olfactores - ps19
Vertebrata - ps20
Gnathostomata - ps21
Euteleostomi - ps22
Sarcopterygii - ps23
Tetrapoda - ps24
Amniota - ps25
Mammalia - ps26
Theria - ps27
Placentalia - ps28
Boreoeutheria - ps29
Euarchontoglires - ps30
Euarchonta - ps31
Primates - ps32
Haplorrhini - ps33
Simiiformes - ps34
Catarrhini - ps35
Hominoidea - ps36
Hominidae - ps37
Homininae - ps38
Hominini - ps39
Homo_sapiens - ps40
p-value
3e-06
3e-05
3e-04
3e-03
3e-02
Log-odds
0.5
1.0
1.5
2.0
2.5
3.0
3.5
4.0

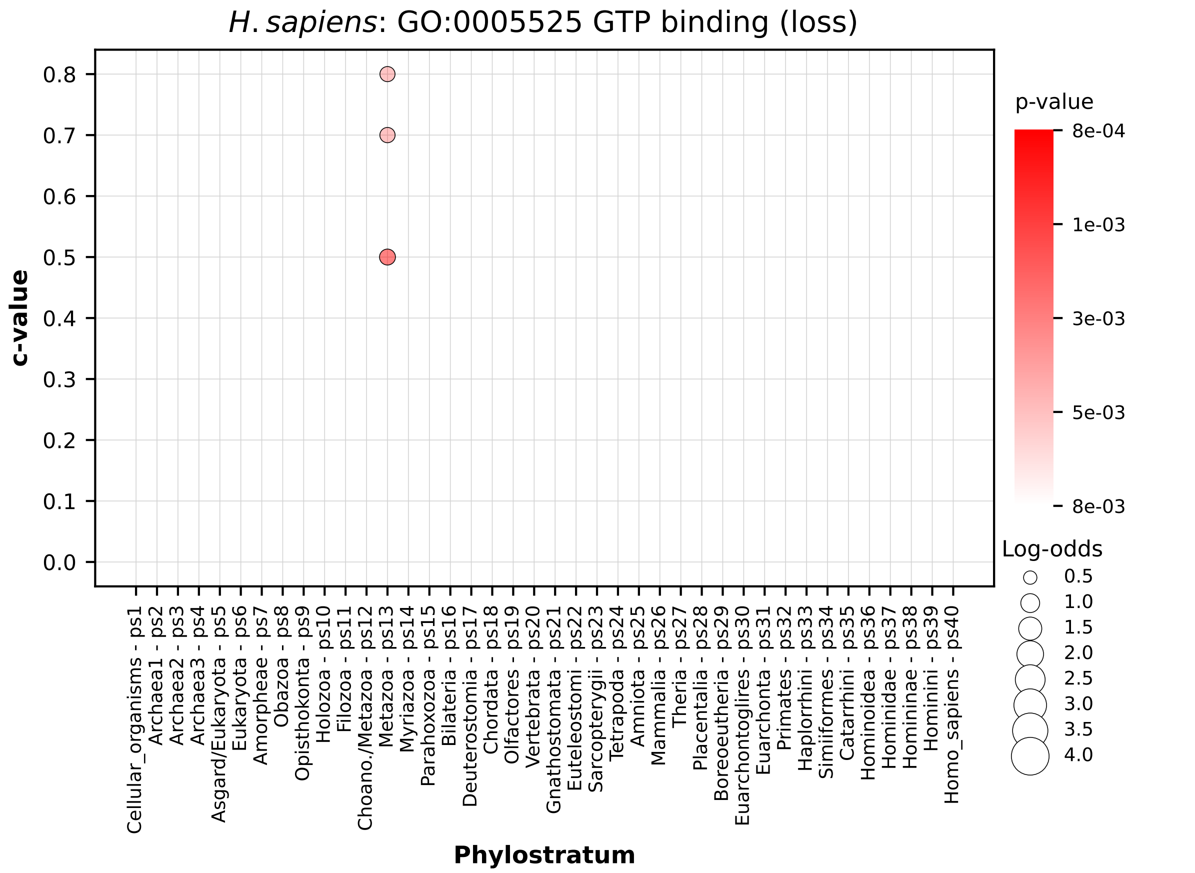

H. sapiens: GO:0005525 GTP binding (loss)
c-value
0.8
0.7
0.6
0.5
0.4
0.3
0.2
0.1
0.0
p-value
8e-04
1e-03
3e-03
5e-03
8e-03
Log-odds
0.5
1.0
1.5
2.0
2.5
3.0
3.5
4.0
Cellular_organisms - ps1
Archaea1 - ps2
Archaea2 - ps3
Archaea3 - ps4
Asgard/Eukaryota - ps5
Eukaryota - ps6
Amorpheae - ps7
Obazoa - ps8
Opisthokonta - ps9
Holozoa - ps10
Filozoa - ps11
Choano./Metazoa - ps12
Metazoa - ps13
Myriazoa - ps14
Parahoxozoa - ps15
Bilateria - ps16
Deuterostomia - ps17
Chordata - ps18
Olfactores - ps19
Vertebrata - ps20
Gnathostomata - ps21
Euteleostomi - ps22
Sarcopterygii - ps23
Tetrapoda - ps24
Amniota - ps25
Mammalia - ps26
Theria - ps27
Placentalia - ps28
Boreoeutheria - ps29
Euarchontoglires - ps30
Euarchonta - ps31
Primates - ps32
Haplorrhini - ps33
Simiiformes - ps34
Catarrhini - ps35
Hominoidea - ps36
Hominidae - ps37
Homininae - ps38
Hominini - ps39
Homo_sapiens - ps40
Phylostratum

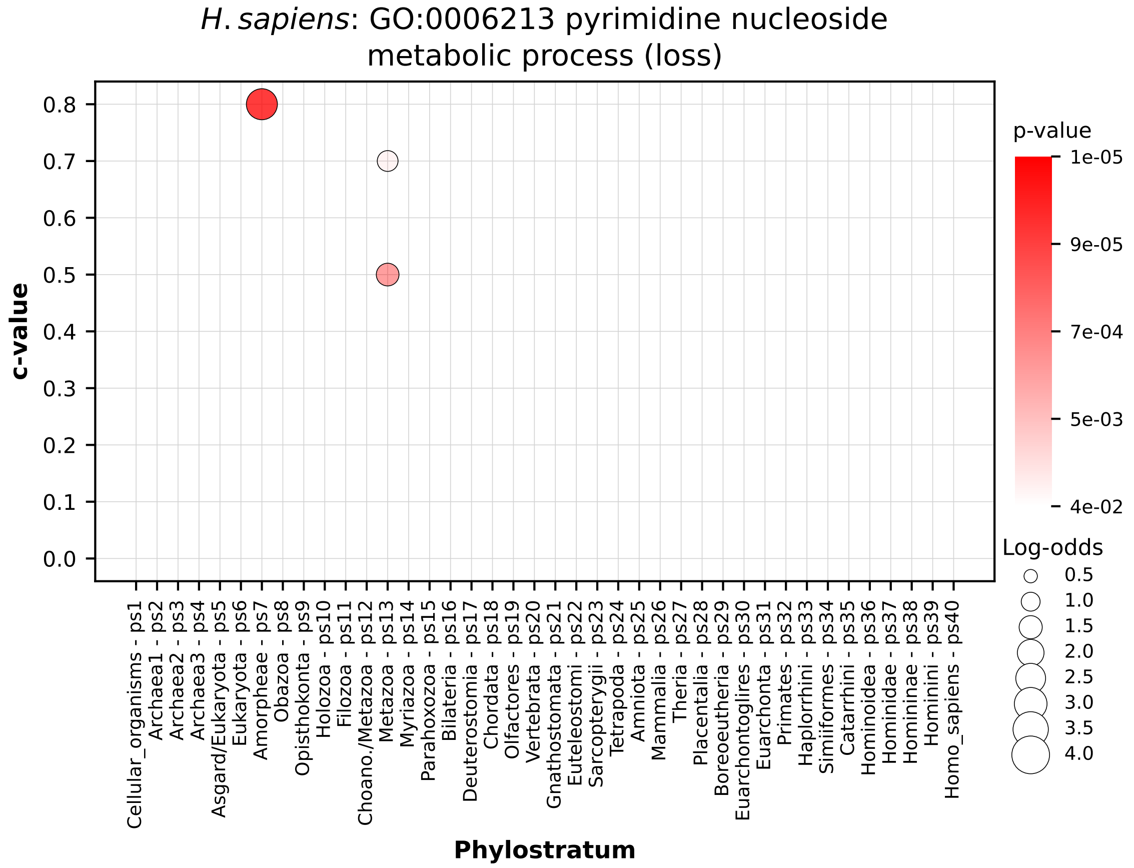

H. sapiens: GO:0006213 pyrimidine nucleoside metabolic process (loss)
c-value
0.0
0.1
0.2
0.3
0.4
0.5
0.6
0.7
0.8
Phylostratum
Cellular_organisms - ps1
Archaea1 - ps2
Archaea2 - ps3
Archaea3 - ps4
Asgard/Eukaryota - ps5
Eukaryota - ps6
Amorpheae - ps7
Obazoa - ps8
Opisthokonta - ps9
Holozoa - ps10
Filozoa - ps11
Choano./Metazoa - ps12
Metazoa - ps13
Myriazoa - ps14
Parahoxozoa - ps15
Bilateria - ps16
Deuterostomia - ps17
Chordata - ps18
Olfactores - ps19
Vertebrata - ps20
Gnathostomata - ps21
Euteleostomi - ps22
Sarcopterygii - ps23
Tetrapoda - ps24
Amniota - ps25
Mammalia - ps26
Theria - ps27
Placentalia - ps28
Boreoeutheria - ps29
Euarchontoglires - ps30
Euarchonta - ps31
Primates - ps32
Haplorrhini - ps33
Simiiformes - ps34
Catarrhini - ps35
Hominoidea - ps36
Hominidae - ps37
Homininae - ps38
Hominini - ps39
Homo_sapiens - ps40
p-value
1e-05
9e-05
7e-04
5e-03
4e-02
Log-odds
0.5
1.0
1.5
2.0
2.5
3.0
3.5
4.0

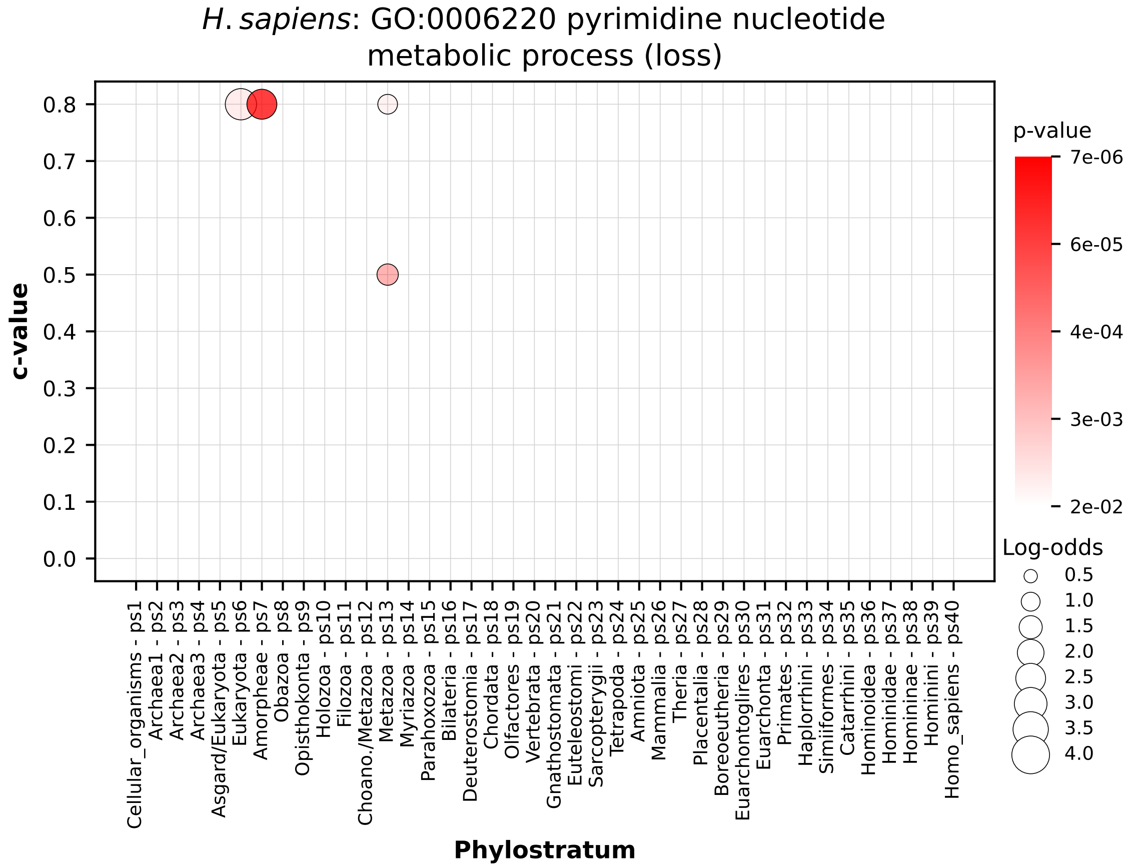

H. sapiens: GO:0006220 pyrimidine nucleotide metabolic process (loss)
c-value
0.8
0.7
0.6
0.5
0.4
0.3
0.2
0.1
0.0
Phylostratum
Cellular_organisms - ps1
Archaea1 - ps2
Archaea2 - ps3
Archaea3 - ps4
Asgard/Eukaryota - ps5
Eukaryota - ps6
Amorpheae - ps7
Obazoa - ps8
Opisthokonta - ps9
Holozoa - ps10
Filozoa - ps11
Choano./Metazoa - ps12
Metazoa - ps13
Myriazoa - ps14
Parahoxozoa - ps15
Bilateria - ps16
Deuterostomia - ps17
Chordata - ps18
Olfactores - ps19
Vertebrata - ps20
Gnathostomata - ps21
Euteleostomi - ps22
Sarcopterygii - ps23
Tetrapoda - ps24
Amniota - ps25
Mammalia - ps26
Theria - ps27
Placentalia - ps28
Boreoeutheria - ps29
Euarchontoglires - ps30
Euarchonta - ps31
Primates - ps32
Haplorrhini - ps33
Simiiformes - ps34
Catarrhini - ps35
Hominoidea - ps36
Hominidae - ps37
Homininae - ps38
Hominini - ps39
Homo_sapiens - ps40
p-value
7e-06
6e-05
4e-04
3e-03
2e-02
Log-odds
0.5
1.0
1.5
2.0
2.5
3.0
3.5
4.0

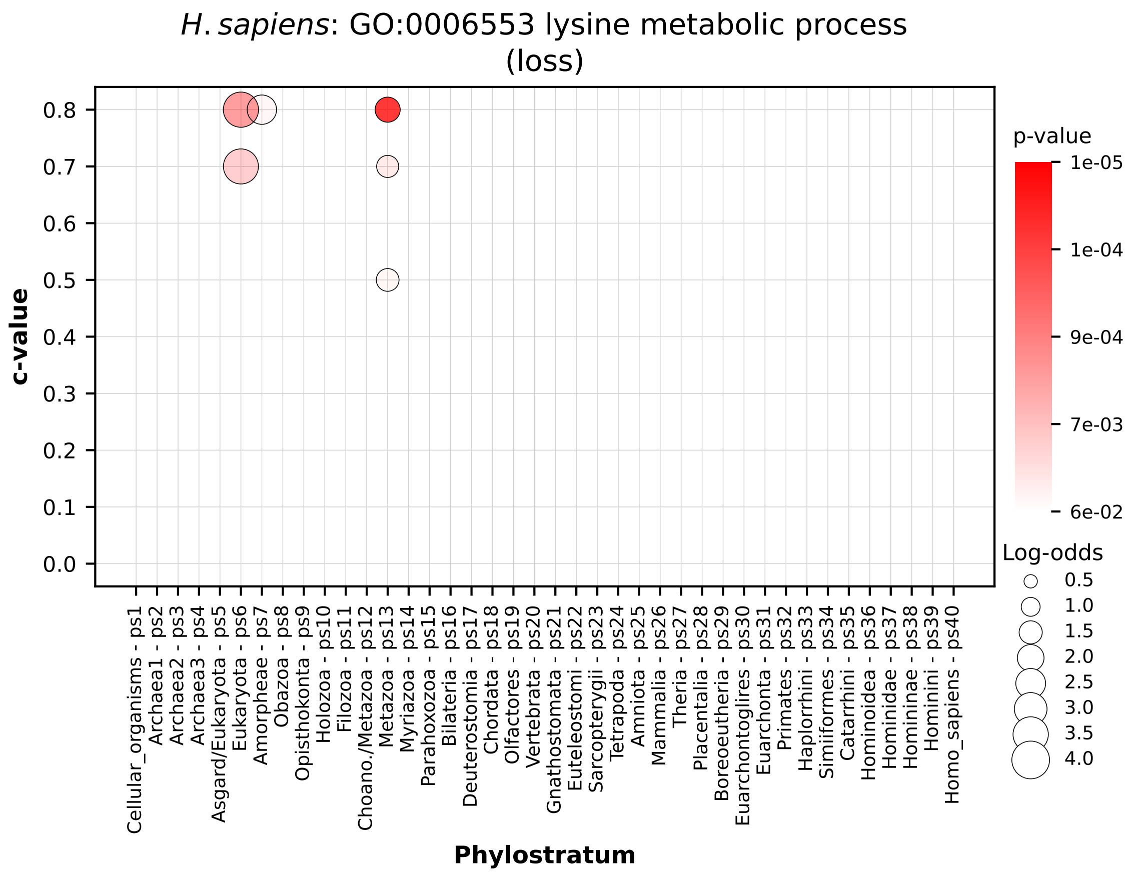
H. sapiens: GO:0006553 lysine metabolic process
(loss)
c-value
0.8
0.7
0.6
0.5
0.4
0.3
0.2
0.1
0.0
Phylostratum
Cellular_organisms - ps1
Archaea1 - ps2
Archaea2 - ps3
Archaea3 - ps4
Asgard/Eukaryota - ps5
Eukaryota - ps6
Amorpheae - ps7
Obazoa - ps8
Opisthokonta - ps9
Holozoa - ps10
Filozoa - ps11
Choano./Metazoa - ps12
Metazoa - ps13
Myriazoa - ps14
Parahoxozoa - ps15
Bilateria - ps16
Deuterostomia - ps17
Chordata - ps18
Olfactores - ps19
Vertebrata - ps20
Gnathostomata - ps21
Euteleostomi - ps22
Sarcopterygii - ps23
Tetrapoda - ps24
Amniota - ps25
Mammalia - ps26
Theria - ps27
Placentalia - ps28
Boreoeutheria - ps29
Euarchontoglires - ps30
Euarchonta - ps31
Primates - ps32
Haplorrhini - ps33
Simiiformes - ps34
Catarrhini - ps35
Hominoidea - ps36
Hominidae - ps37
Homininae - ps38
Hominini - ps39
Homo_sapiens - ps40
p-value
1e-05
1e-04
9e-04
7e-03
6e-02
Log-odds
0.5
1.0
1.5
2.0
2.5
3.0
3.5
4.0

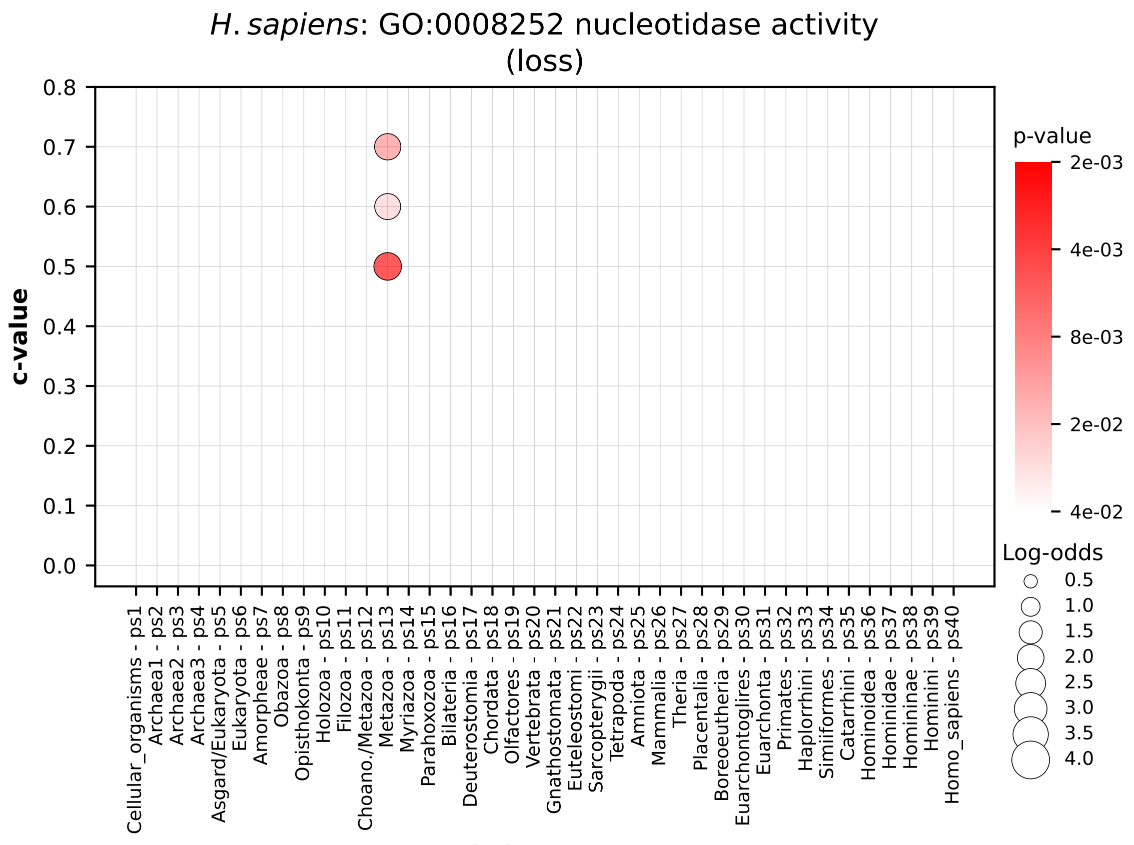
H. sapiens: GO:0008252 nucleotidase activity
(loss)
c-value
0.8
0.7
0.6
0.5
0.4
0.3
0.2
0.1
0.0
p-value
2e-03
4e-03
8e-03
2e-02
4e-02
Log-odds
0.5
1.0
1.5
2.0
2.5
3.0
3.5
4.0
Cellular_organisms - ps1
Archaea1 - ps2
Archaea2 - ps3
Archaea3 - ps4
Asgard/Eukaryota - ps5
Eukaryota - ps6
Amorpheae - ps7
Obazoa - ps8
Opisthokonta - ps9
Holozoa - ps10
Filozoa - ps11
Choano./Metazoa - ps12
Metazoa - ps13
Myriazoa - ps14
Parahoxozoa - ps15
Bilateria - ps16
Deuterostomia - ps17
Chordata - ps18
Olfactores - ps19
Vertebrata - ps20
Gnathostomata - ps21
Euteleostomi - ps22
Sarcopterygii - ps23
Tetrapoda - ps24
Amniota - ps25
Mammalia - ps26
Theria - ps27
Placentalia - ps28
Boreoeutheria - ps29
Euarchontoglires - ps30
Euarchonta - ps31
Primates - ps32
Haplorrhini - ps33
Simiiformes - ps34
Catarrhini - ps35
Hominoidea - ps36
Hominidae - ps37
Homininae - ps38
Hominini - ps39
Homo_sapiens - ps40
Phylostratum

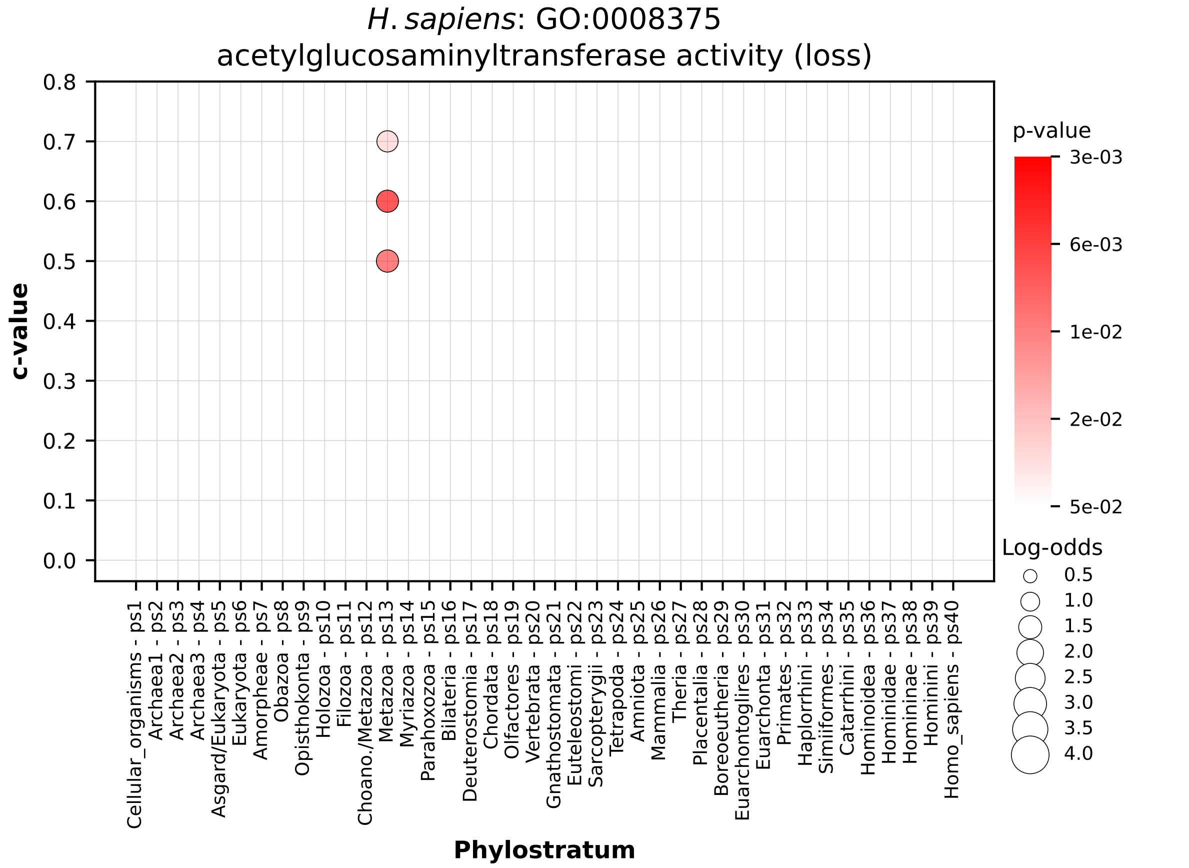
H. sapiens: GO:0008375
acetylglucosaminyltransferase activity (loss)
c-value
Phylostratum
p-value
3e-03
6e-03
1e-02
2e-02
5e-02
Log-odds
0.5
1.0
1.5
2.0
2.5
3.0
3.5
4.0
Cellular_organisms - ps1
Archaea1 - ps2
Archaea2 - ps3
Archaea3 - ps4
Asgard/Eukaryota - ps5
Eukaryota - ps6
Amorpheae - ps7
Obazoa - ps8
Opisthokonta - ps9
Holozoa - ps10
Filozoa - ps11
Choano./Metazoa - ps12
Metazoa - ps13
Myriazoa - ps14
Parahoxozoa - ps15
Bilateria - ps16
Deuterostomia - ps17
Chordata - ps18
Olfactores - ps19
Vertebrata - ps20
Gnathostomata - ps21
Euteleostomi - ps22
Sarcopterygii - ps23
Tetrapoda - ps24
Amniota - ps25
Mammalia - ps26
Theria - ps27
Placentalia - ps28
Boreoeutheria - ps29
Euarchontoglires - ps30
Euarchonta - ps31
Primates - ps32
Haplorrhini - ps33
Simiiformes - ps34
Catarrhini - ps35
Hominoidea - ps36
Hominidae - ps37
Homininae - ps38
Hominini - ps39
Homo_sapiens - ps40

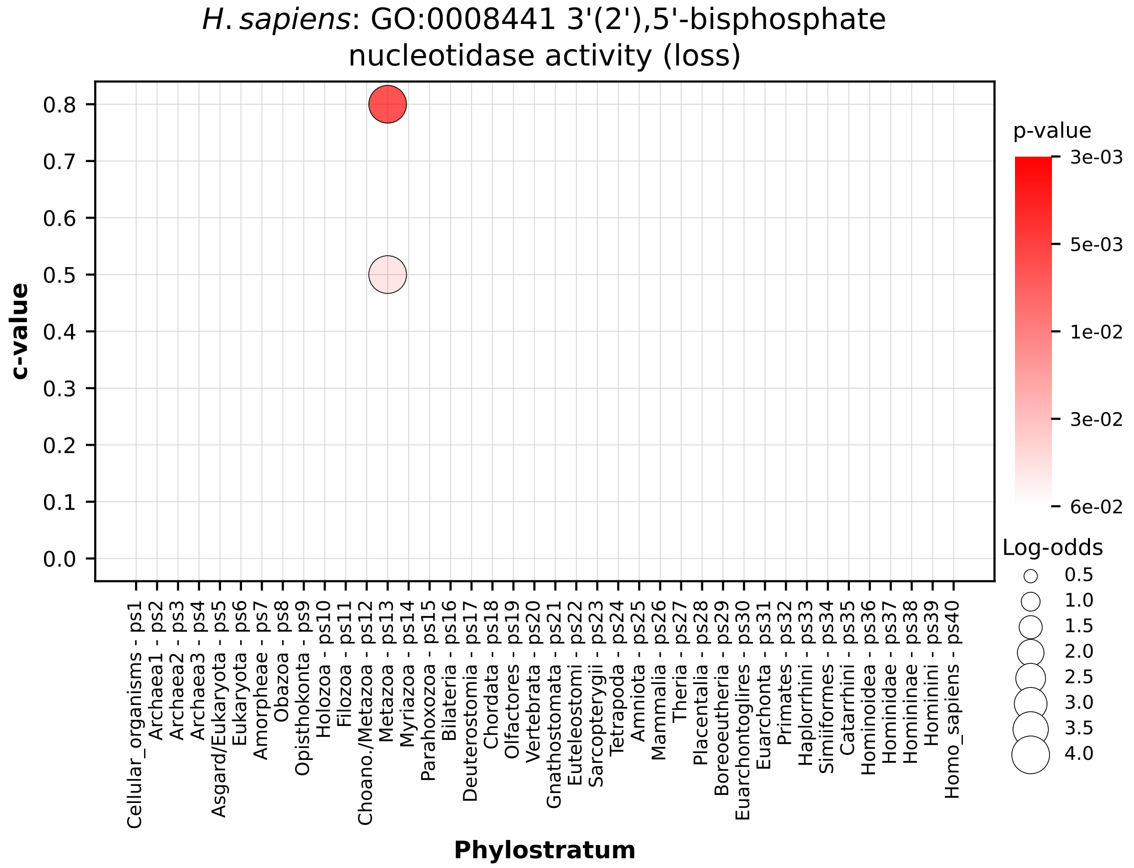

H. sapiens: GO:0008441 3'(2'),5'-bisphosphate nucleotidase activity (loss)
c-value
0.8
0.7
0.6
0.5
0.4
0.3
0.2
0.1
0.0
Phylostratum
Cellular_organisms - ps1
Archaea1 - ps2
Archaea2 - ps3
Archaea3 - ps4
Asgard/Eukaryota - ps5
Eukaryota - ps6
Amorpheae - ps7
Obazoa - ps8
Opisthokonta - ps9
Holozoa - ps10
Filozoa - ps11
Choano./Metazoa - ps12
Metazoa - ps13
Myriazoa - ps14
Parahoxozoa - ps15
Bilateria - ps16
Deuterostomia - ps17
Chordata - ps18
Olfactores - ps19
Vertebrata - ps20
Gnathostomata - ps21
Euteleostomi - ps22
Sarcopterygii - ps23
Tetrapoda - ps24
Amniota - ps25
Mammalia - ps26
Theria - ps27
Placentalia - ps28
Boreoeutheria - ps29
Euarchontoglires - ps30
Euarchonta - ps31
Primates - ps32
Haplorrhini - ps33
Simiiformes - ps34
Catarrhini - ps35
Hominoidea - ps36
Hominidae - ps37
Homininae - ps38
Hominini - ps39
Homo_sapiens - ps40
p-value
3e-03
5e-03
1e-02
3e-02
6e-02
Log-odds
0.5
1.0
1.5
2.0
2.5
3.0
3.5
4.0

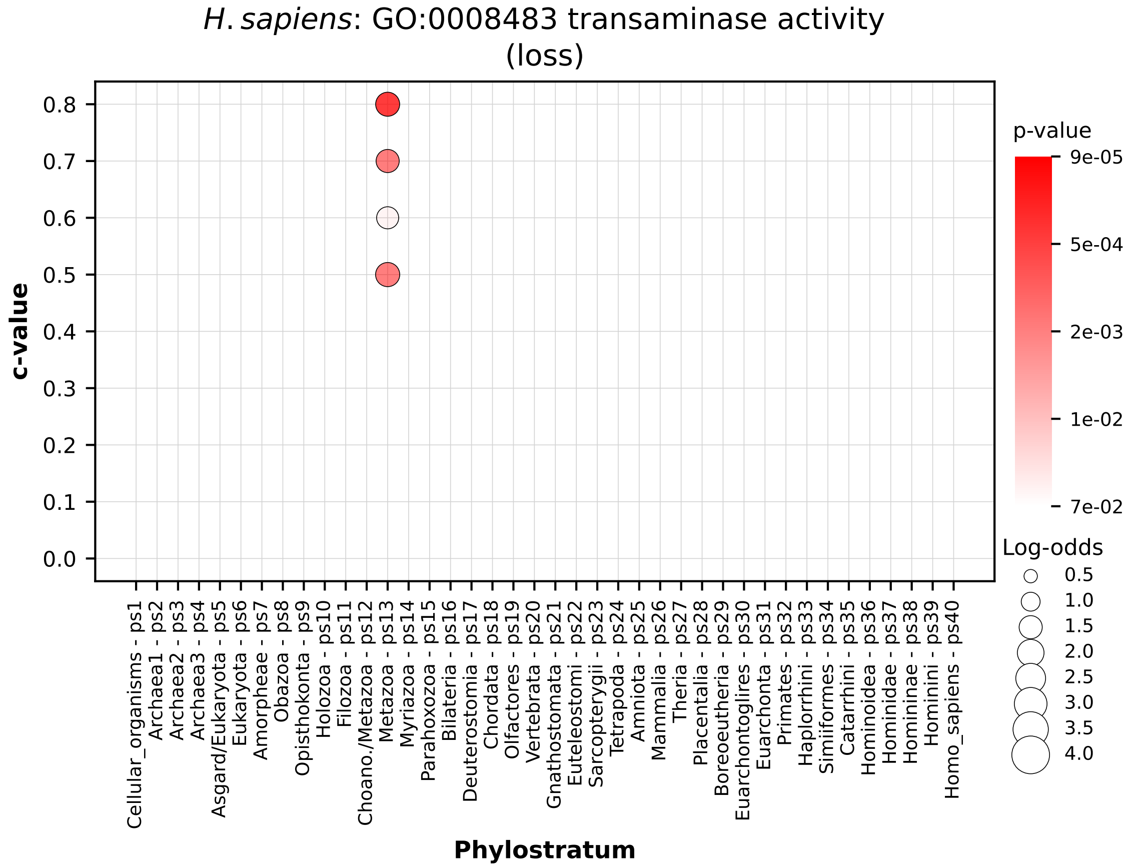

H. sapiens: GO:0008483 transaminase activity
(loss)
c-value
0.0
0.1
0.2
0.3
0.4
0.5
0.6
0.7
0.8
Phylostratum
Cellular_organisms - ps1
Archaea1 - ps2
Archaea2 - ps3
Archaea3 - ps4
Asgard/Eukaryota - ps5
Eukaryota - ps6
Amorpheae - ps7
Obazoa - ps8
Opisthokonta - ps9
Holozoa - ps10
Filozoa - ps11
Choano./Metazoa - ps12
Metazoa - ps13
Myriazoa - ps14
Parahoxozoa - ps15
Bilateria - ps16
Deuterostomia - ps17
Chordata - ps18
Olfactores - ps19
Vertebrata - ps20
Gnathostomata - ps21
Euteleostomi - ps22
Sarcopterygii - ps23
Tetrapoda - ps24
Amniota - ps25
Mammalia - ps26
Theria - ps27
Placentalia - ps28
Boreoeutheria - ps29
Euarchontoglires - ps30
Euarchonta - ps31
Primates - ps32
Haplorrhini - ps33
Simiiformes - ps34
Catarrhini - ps35
Hominoidea - ps36
Hominidae - ps37
Homininae - ps38
Hominini - ps39
Homo_sapiens - ps40
p-value
9e-05
5e-04
2e-03
1e-02
7e-02
Log-odds
0.5
1.0
1.5
2.0
2.5
3.0
3.5
4.0

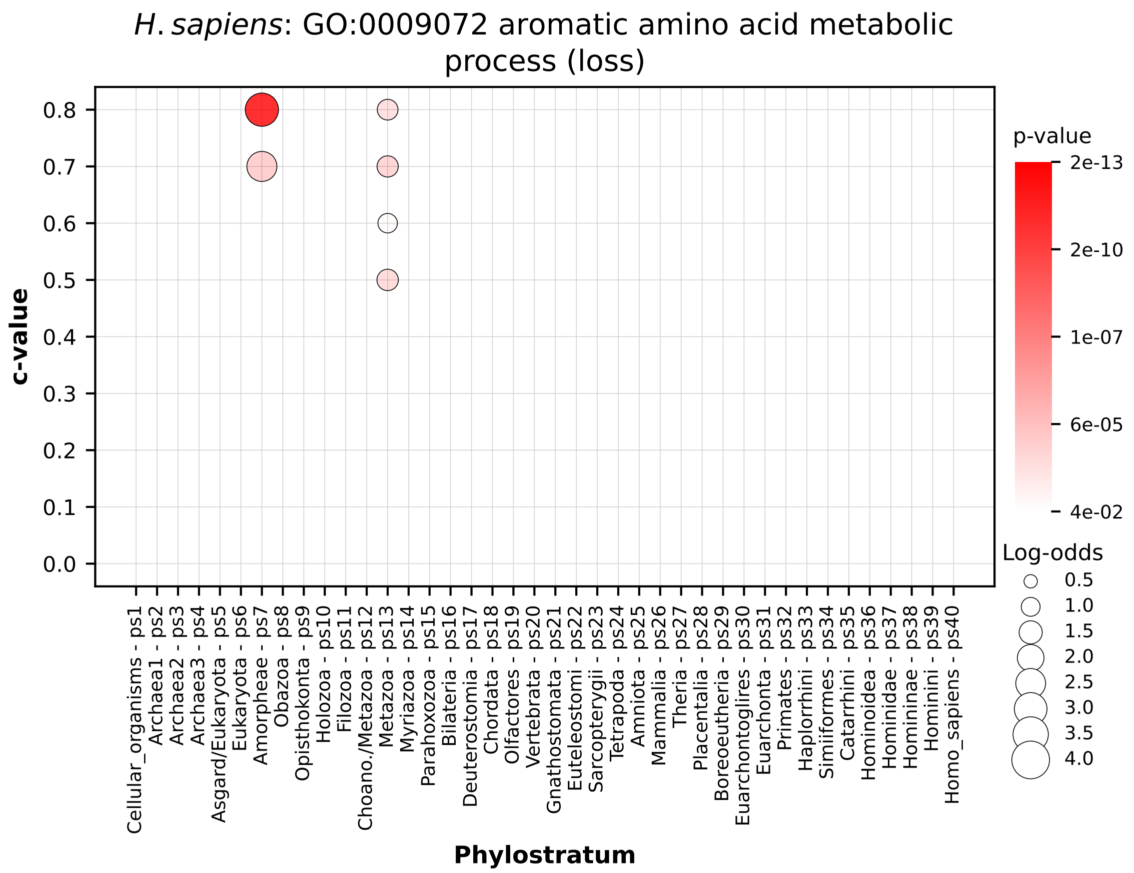
H. sapiens: GO:0009072 aromatic amino acid metabolic process (loss)
c-value
0.0
0.1
0.2
0.3
0.4
0.5
0.6
0.7
0.8
Phylostratum
Cellular_organisms - ps1
Archaea1 - ps2
Archaea2 - ps3
Archaea3 - ps4
Asgard/Eukaryota - ps5
Eukaryota - ps6
Amorpheae - ps7
Obazoa - ps8
Opisthokonta - ps9
Holozoa - ps10
Filozoa - ps11
Choano./Metazoa - ps12
Metazoa - ps13
Myriazoa - ps14
Parahoxozoa - ps15
Bilateria - ps16
Deuterostomia - ps17
Chordata - ps18
Olfactores - ps19
Vertebrata - ps20
Gnathostomata - ps21
Euteleostomi - ps22
Sarcopterygii - ps23
Tetrapoda - ps24
Amniota - ps25
Mammalia - ps26
Theria - ps27
Placentalia - ps28
Boreoeutheria - ps29
Euarchontoglires - ps30
Euarchonta - ps31
Primates - ps32
Haplorrhini - ps33
Simiiformes - ps34
Catarrhini - ps35
Hominoidea - ps36
Hominidae - ps37
Homininae - ps38
Hominini - ps39
Homo_sapiens - ps40
p-value
2e-13
2e-10
1e-07
6e-05
4e-02
Log-odds
0.5
1.0
1.5
2.0
2.5
3.0
3.5
4.0

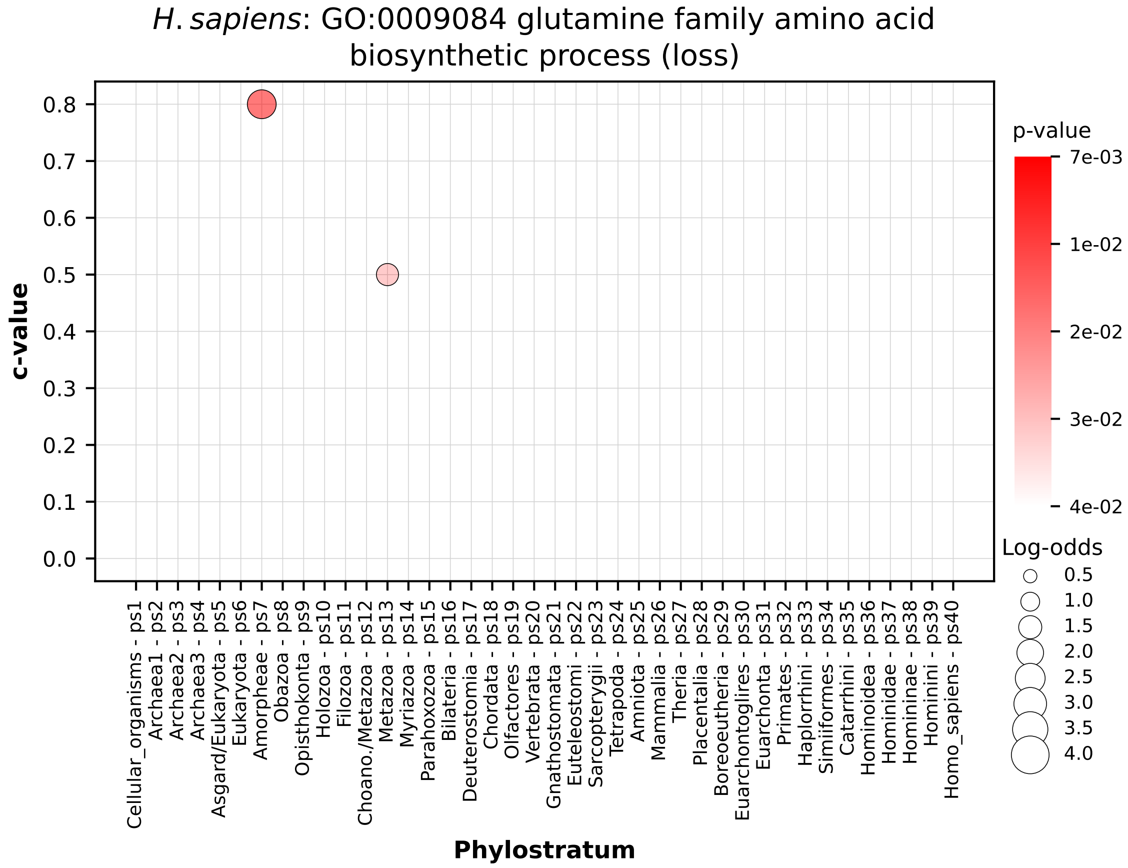

H. sapiens: GO:0009084 glutamine family amino acid biosynthetic process (loss)
c-value
0.0
0.1
0.2
0.3
0.4
0.5
0.6
0.7
0.8
Phylostratum
Cellular_organisms - ps1
Archaea1 - ps2
Archaea2 - ps3
Archaea3 - ps4
Asgard/Eukaryota - ps5
Eukaryota - ps6
Amorpheae - ps7
Obazoa - ps8
Opisthokonta - ps9
Holozoa - ps10
Filozoa - ps11
Choano./Metazoa - ps12
Metazoa - ps13
Myriazoa - ps14
Parahoxozoa - ps15
Bilateria - ps16
Deuterostomia - ps17
Chordata - ps18
Olfactores - ps19
Vertebrata - ps20
Gnathostomata - ps21
Euteleostomi - ps22
Sarcopterygii - ps23
Tetrapoda - ps24
Amniota - ps25
Mammalia - ps26
Theria - ps27
Placentalia - ps28
Boreoeutheria - ps29
Euarchontoglires - ps30
Euarchonta - ps31
Primates - ps32
Haplorrhini - ps33
Simiiformes - ps34
Catarrhini - ps35
Hominoidea - ps36
Hominidae - ps37
Homininae - ps38
Hominini - ps39
Homo_sapiens - ps40
p-value
7e-03
1e-02
2e-02
3e-02
4e-02
Log-odds
0.5
1.0
1.5
2.0
2.5
3.0
3.5
4.0

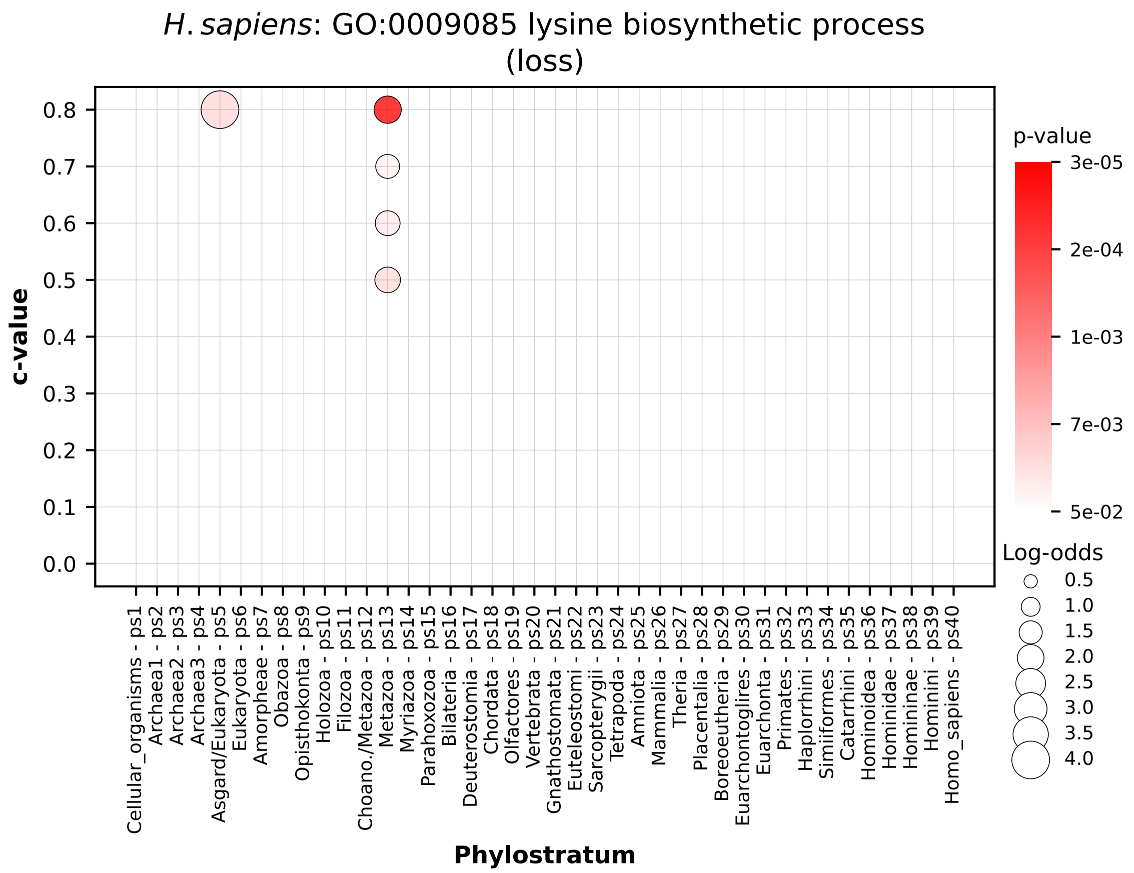
H. sapiens: GO:0009085 lysine biosynthetic process (loss)
c-value
0.0
0.1
0.2
0.3
0.4
0.5
0.6
0.7
0.8
Phylostratum
Cellular_organisms - ps1
Archaea1 - ps2
Archaea2 - ps3
Archaea3 - ps4
Asgard/Eukaryota - ps5
Eukaryota - ps6
Amorpheae - ps7
Obazoa - ps8
Opisthokonta - ps9
Holozoa - ps10
Filozoa - ps11
Choano./Metazoa - ps12
Metazoa - ps13
Myriazoa - ps14
Parahoxozoa - ps15
Bilateria - ps16
Deuterostomia - ps17
Chordata - ps18
Olfactores - ps19
Vertebrata - ps20
Gnathostomata - ps21
Euteleostomi - ps22
Sarcopterygii - ps23
Tetrapoda - ps24
Amniota - ps25
Mammalia - ps26
Theria - ps27
Placentalia - ps28
Boreoeutheria - ps29
Euarchontoglires - ps30
Euarchonta - ps31
Primates - ps32
Haplorrhini - ps33
Simiiformes - ps34
Catarrhini - ps35
Hominoidea - ps36
Hominidae - ps37
Homininae - ps38
Hominini - ps39
Homo_sapiens - ps40
p-value
3e-05
2e-04
1e-03
7e-03
5e-02
Log-odds
0.5
1.0
1.5
2.0
2.5
3.0
3.5
4.0

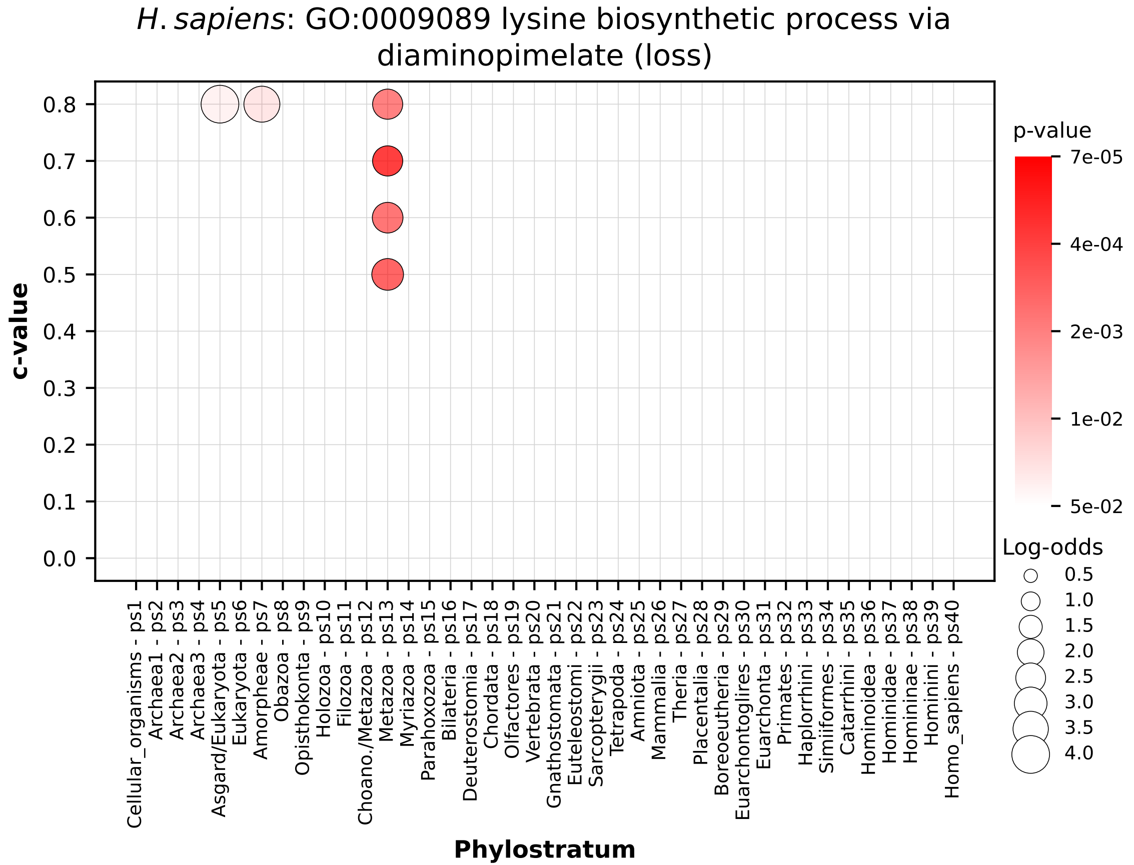

H. sapiens: GO:0009089 lysine biosynthetic process via diaminopimelate (loss)
c-value
0.0
0.1
0.2
0.3
0.4
0.5
0.6
0.7
0.8
Phylostratum
Cellular_organisms - ps1
Archaea1 - ps2
Archaea2 - ps3
Archaea3 - ps4
Asgard/Eukaryota - ps5
Eukaryota - ps6
Amorpheae - ps7
Obazoa - ps8
Opisthokonta - ps9
Holozoa - ps10
Filozoa - ps11
Choano./Metazoa - ps12
Metazoa - ps13
Myriazoa - ps14
Parahoxozoa - ps15
Bilateria - ps16
Deuterostomia - ps17
Chordata - ps18
Olfactores - ps19
Vertebrata - ps20
Gnathostomata - ps21
Euteleostomi - ps22
Sarcopterygii - ps23
Tetrapoda - ps24
Amniota - ps25
Mammalia - ps26
Theria - ps27
Placentalia - ps28
Boreoeutheria - ps29
Euarchontoglires - ps30
Euarchonta - ps31
Primates - ps32
Haplorrhini - ps33
Simiiformes - ps34
Catarrhini - ps35
Hominoidea - ps36
Hominidae - ps37
Homininae - ps38
Hominini - ps39
Homo_sapiens - ps40
p-value
7e-05
4e-04
2e-03
1e-02
5e-02
Log-odds
0.5
1.0
1.5
2.0
2.5
3.0
3.5
4.0

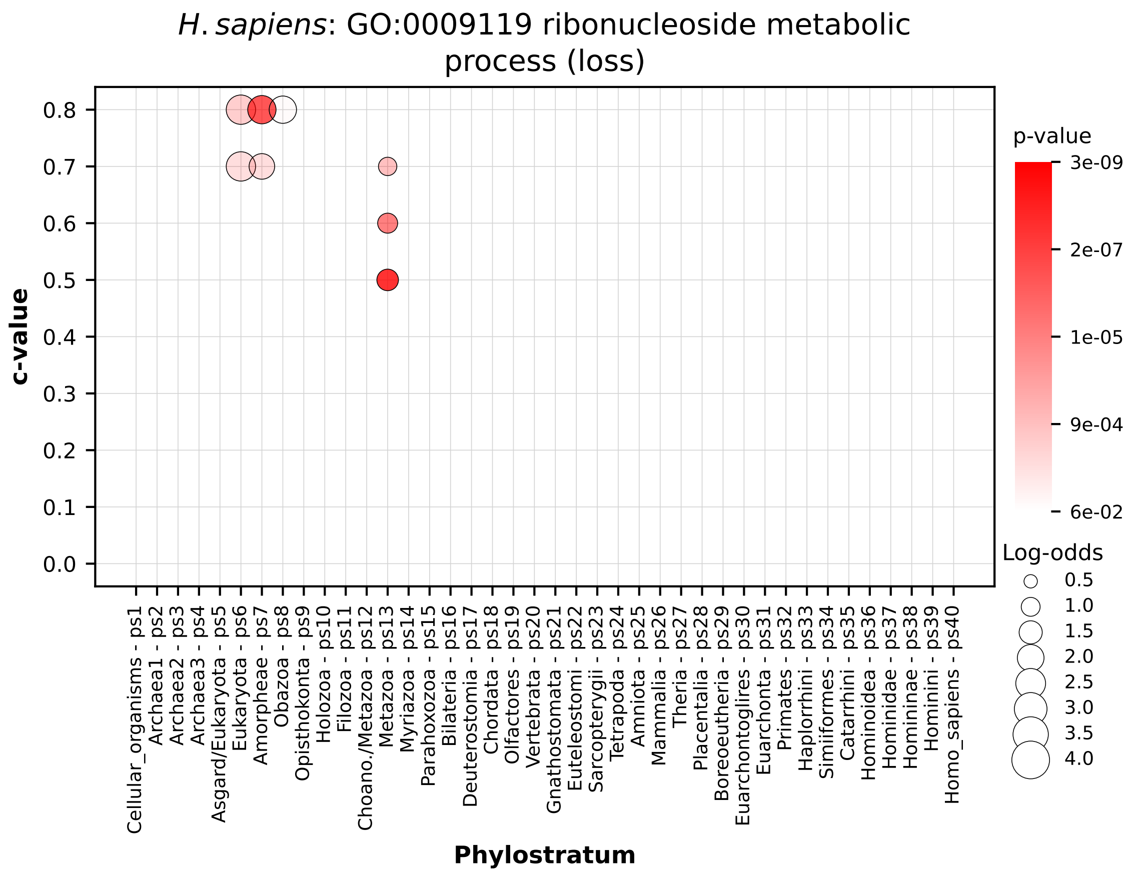
H. sapiens: GO:0009119 ribonucleoside metabolic process (loss)
c-value
Phylostratum
p-value
3e-09
2e-07
1e-05
9e-04
6e-02
Log-odds
0.5
1.0
1.5
2.0
2.5
3.0
3.5
4.0
Cellular_organisms - ps1
Archaea1 - ps2
Archaea2 - ps3
Archaea3 - ps4
Asgard/Eukaryota - ps5
Eukaryota - ps6
Amorpheae - ps7
Obazoa - ps8
Opisthokonta - ps9
Holozoa - ps10
Filozoa - ps11
Choano./Metazoa - ps12
Metazoa - ps13
Myriazoa - ps14
Parahoxozoa - ps15
Bilateria - ps16
Deuterostomia - ps17
Chordata - ps18
Olfactores - ps19
Vertebrata - ps20
Gnathostomata - ps21
Euteleostomi - ps22
Sarcopterygii - ps23
Tetrapoda - ps24
Amniota - ps25
Mammalia - ps26
Theria - ps27
Placentalia - ps28
Boreoeutheria - ps29
Euarchontoglires - ps30
Euarchonta - ps31
Primates - ps32
Haplorrhini - ps33
Simiiformes - ps34
Catarrhini - ps35
Hominoidea - ps36
Hominidae - ps37
Homininae - ps38
Hominini - ps39
Homo_sapiens - ps40

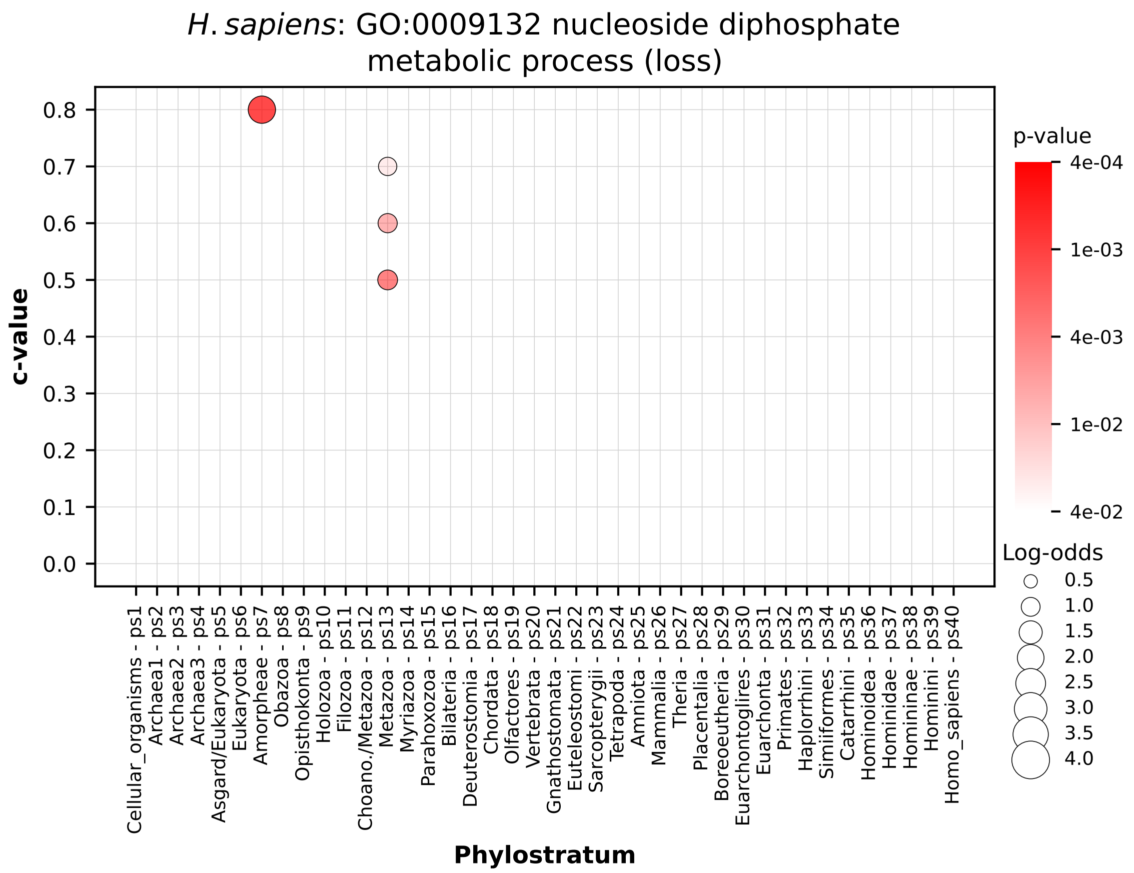

H. sapiens: GO:0009132 nucleoside diphosphate metabolic process (loss)
c-value
0.0
0.1
0.2
0.3
0.4
0.5
0.6
0.7
0.8
Phylostratum
Cellular_organisms - ps1
Archaea1 - ps2
Archaea2 - ps3
Archaea3 - ps4
Asgard/Eukaryota - ps5
Eukaryota - ps6
Amorpheae - ps7
Obazoa - ps8
Opisthokonta - ps9
Holozoa - ps10
Filozoa - ps11
Choano./Metazoa - ps12
Metazoa - ps13
Myriazoa - ps14
Parahoxozoa - ps15
Bilateria - ps16
Deuterostomia - ps17
Chordata - ps18
Olfactores - ps19
Vertebrata - ps20
Gnathostomata - ps21
Euteleostomi - ps22
Sarcopterygii - ps23
Tetrapoda - ps24
Amniota - ps25
Mammalia - ps26
Theria - ps27
Placentalia - ps28
Boreoeutheria - ps29
Euarchontoglires - ps30
Euarchonta - ps31
Primates - ps32
Haplorrhini - ps33
Simiiformes - ps34
Catarrhini - ps35
Hominoidea - ps36
Hominidae - ps37
Homininae - ps38
Hominini - ps39
Homo_sapiens - ps40
p-value
4e-04
1e-03
4e-03
1e-02
4e-02
Log-odds
0.5
1.0
1.5
2.0
2.5
3.0
3.5
4.0

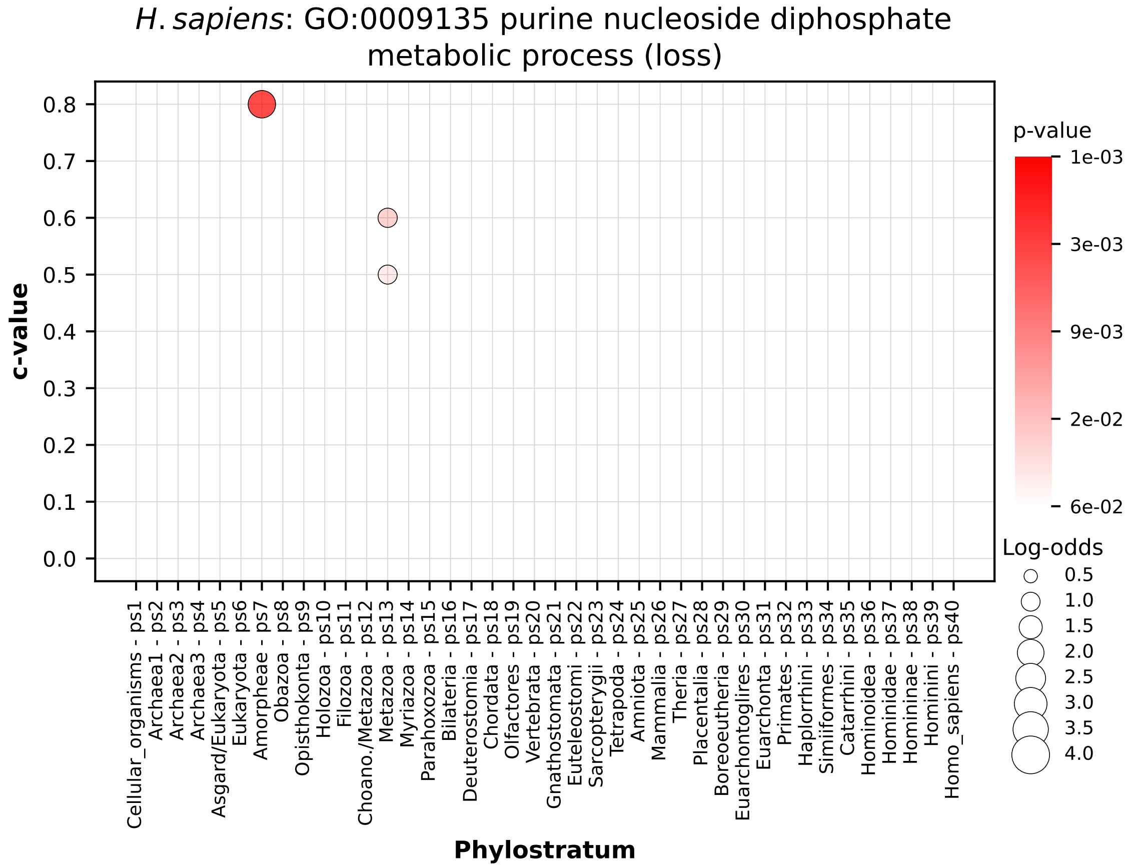

H. sapiens: GO:0009135 purine nucleoside diphosphate metabolic process (loss)
c-value
0.8
0.7
0.6
0.5
0.4
0.3
0.2
0.1
0.0
p-value
1e-03
3e-03
9e-03
2e-02
6e-02
Log-odds
0.5
1.0
1.5
2.0
2.5
3.0
3.5
4.0
Cellular_organisms - ps1
Archaea1 - ps2
Archaea2 - ps3
Archaea3 - ps4
Asgard/Eukaryota - ps5
Eukaryota - ps6
Amorpheae - ps7
Obazoa - ps8
Opisthokonta - ps9
Holozoa - ps10
Filozoa - ps11
Choano./Metazoa - ps12
Metazoa - ps13
Myriazoa - ps14
Parahoxozoa - ps15
Bilateria - ps16
Deuterostomia - ps17
Chordata - ps18
Olfactores - ps19
Vertebrata - ps20
Gnathostomata - ps21
Euteleostomi - ps22
Sarcopterygii - ps23
Tetrapoda - ps24
Amniota - ps25
Mammalia - ps26
Theria - ps27
Placentalia - ps28
Boreoeutheria - ps29
Euarchontoglires - ps30
Euarchonta - ps31
Primates - ps32
Haplorrhini - ps33
Simiiformes - ps34
Catarrhini - ps35
Hominoidea - ps36
Hominidae - ps37
Homininae - ps38
Hominini - ps39
Homo_sapiens - ps40
Phylostratum

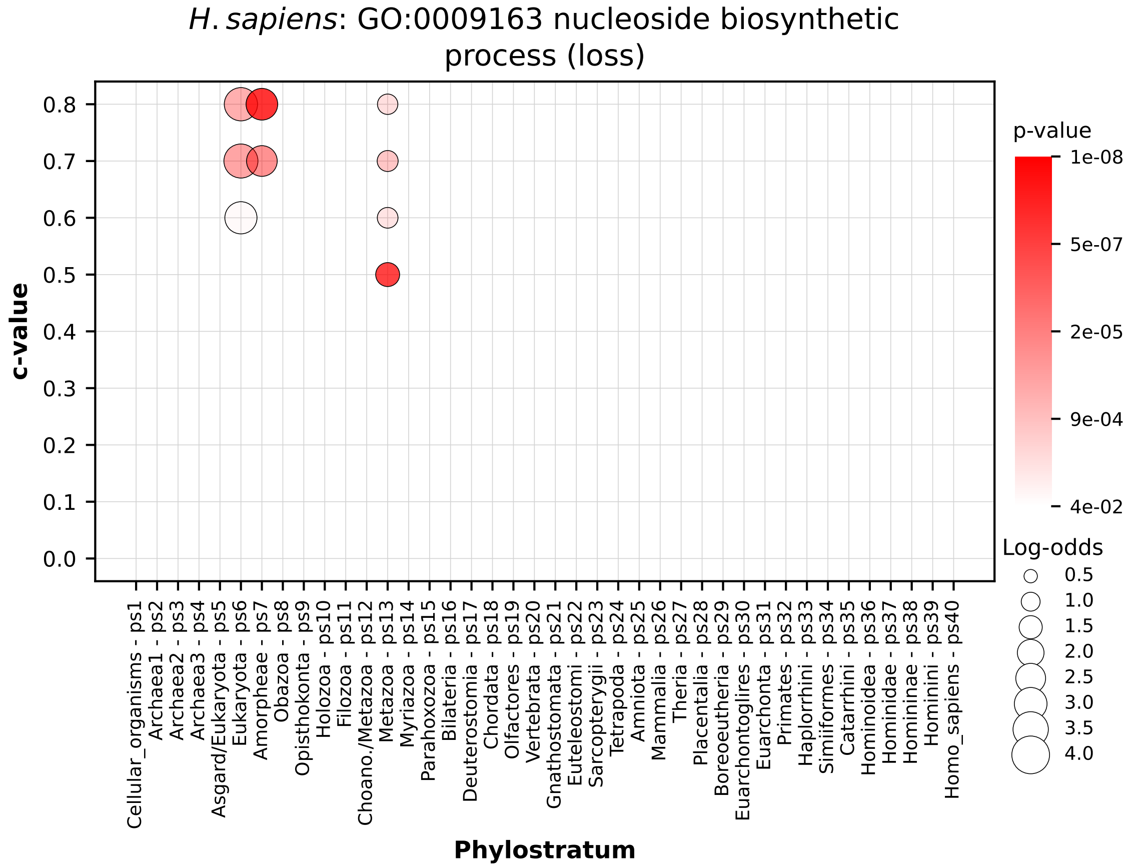

H. sapiens: GO:0009163 nucleoside biosynthetic process (loss)
c-value
0.8
0.7
0.6
0.5
0.4
0.3
0.2
0.1
0.0
Phylostratum
Cellular_organisms - ps1
Archaea1 - ps2
Archaea2 - ps3
Archaea3 - ps4
Asgard/Eukaryota - ps5
Eukaryota - ps6
Amorpheae - ps7
Obazoa - ps8
Opisthokonta - ps9
Holozoa - ps10
Filozoa - ps11
Choano./Metazoa - ps12
Metazoa - ps13
Myriazoa - ps14
Parahoxozoa - ps15
Bilateria - ps16
Deuterostomia - ps17
Chordata - ps18
Olfactores - ps19
Vertebrata - ps20
Gnathostomata - ps21
Euteleostomi - ps22
Sarcopterygii - ps23
Tetrapoda - ps24
Amniota - ps25
Mammalia - ps26
Theria - ps27
Placentalia - ps28
Boreoeutheria - ps29
Euarchontoglires - ps30
Euarchonta - ps31
Primates - ps32
Haplorrhini - ps33
Simiiformes - ps34
Catarrhini - ps35
Hominoidea - ps36
Hominidae - ps37
Homininae - ps38
Hominini - ps39
Homo_sapiens - ps40
p-value
1e-08
5e-07
2e-05
9e-04
4e-02
Log-odds
0.5
1.0
1.5
2.0
2.5
3.0
3.5
4.0

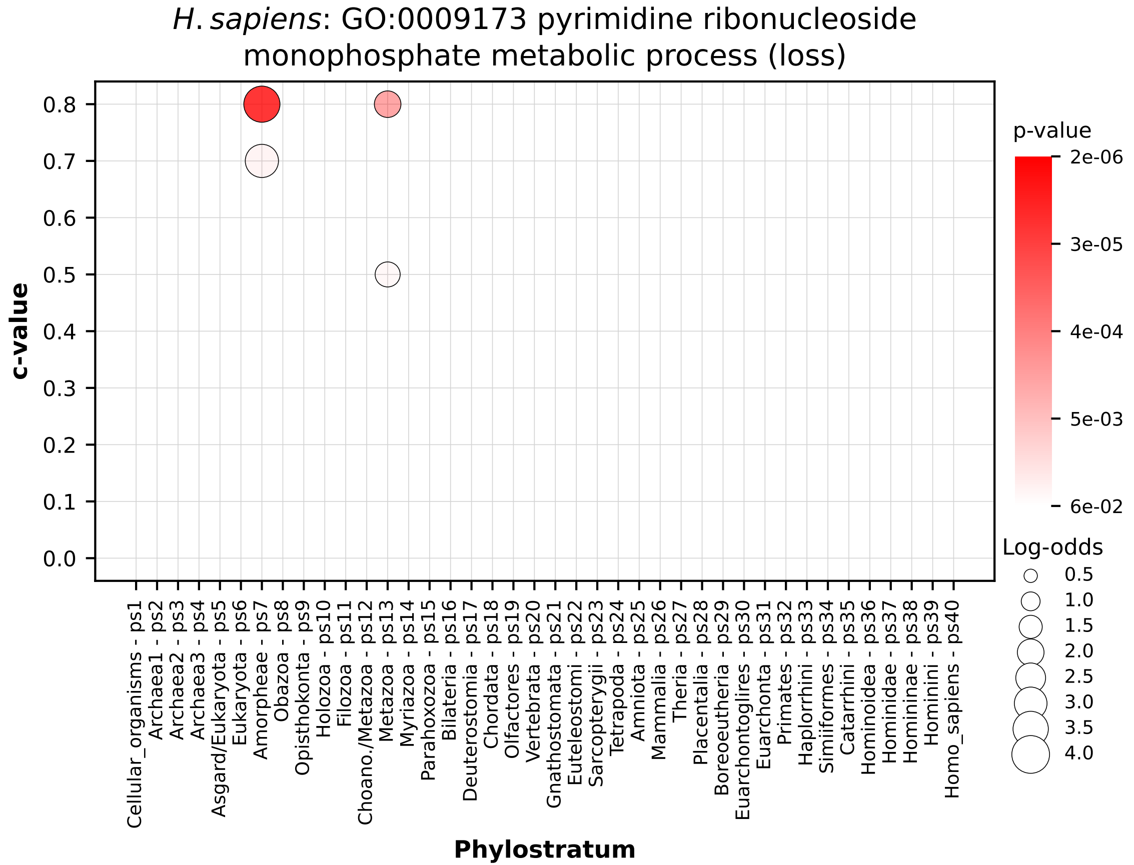

H. sapiens: GO:0009173 pyrimidine ribonucleoside monophosphate metabolic process (loss)
c-value
0.8
0.7
0.6
0.5
0.4
0.3
0.2
0.1
0.0
p-value
2e-06
3e-05
4e-04
5e-03
6e-02
Log-odds
0.5
1.0
1.5
2.0
2.5
3.0
3.5
4.0
Cellular_organisms - ps1
Archaea1 - ps2
Archaea2 - ps3
Archaea3 - ps4
Asgard/Eukaryota - ps5
Eukaryota - ps6
Amorpheae - ps7
Obazoa - ps8
Opisthokonta - ps9
Holozoa - ps10
Filozoa - ps11
Choano./Metazoa - ps12
Metazoa - ps13
Myriazoa - ps14
Parahoxozoa - ps15
Bilateria - ps16
Deuterostomia - ps17
Chordata - ps18
Olfactores - ps19
Vertebrata - ps20
Gnathostomata - ps21
Euteleostomi - ps22
Sarcopterygii - ps23
Tetrapoda - ps24
Amniota - ps25
Mammalia - ps26
Theria - ps27
Placentalia - ps28
Boreoeutheria - ps29
Euarchontoglires - ps30
Euarchonta - ps31
Primates - ps32
Haplorrhini - ps33
Simiiformes - ps34
Catarrhini - ps35
Hominoidea - ps36
Hominidae - ps37
Homininae - ps38
Hominini - ps39
Homo_sapiens - ps40
Phylostratum

H. sapiens: GO:0009179 purine ribonucleoside diphosphate metabolic process (loss)

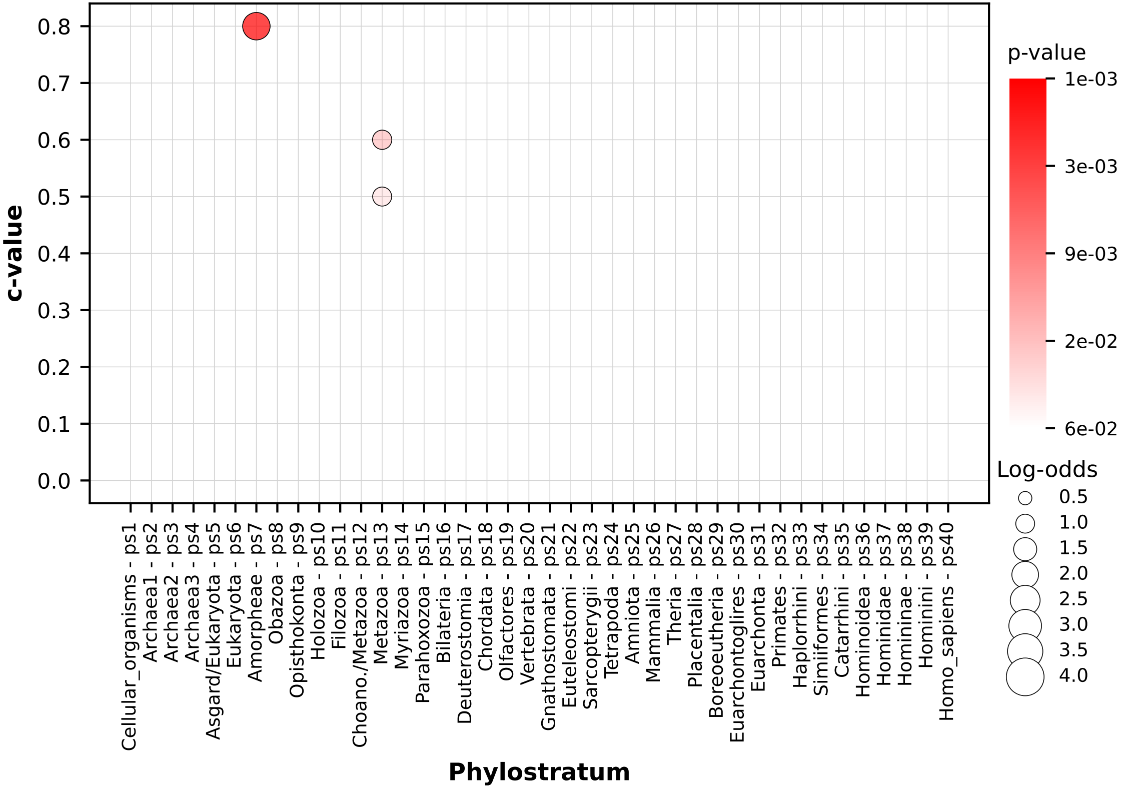

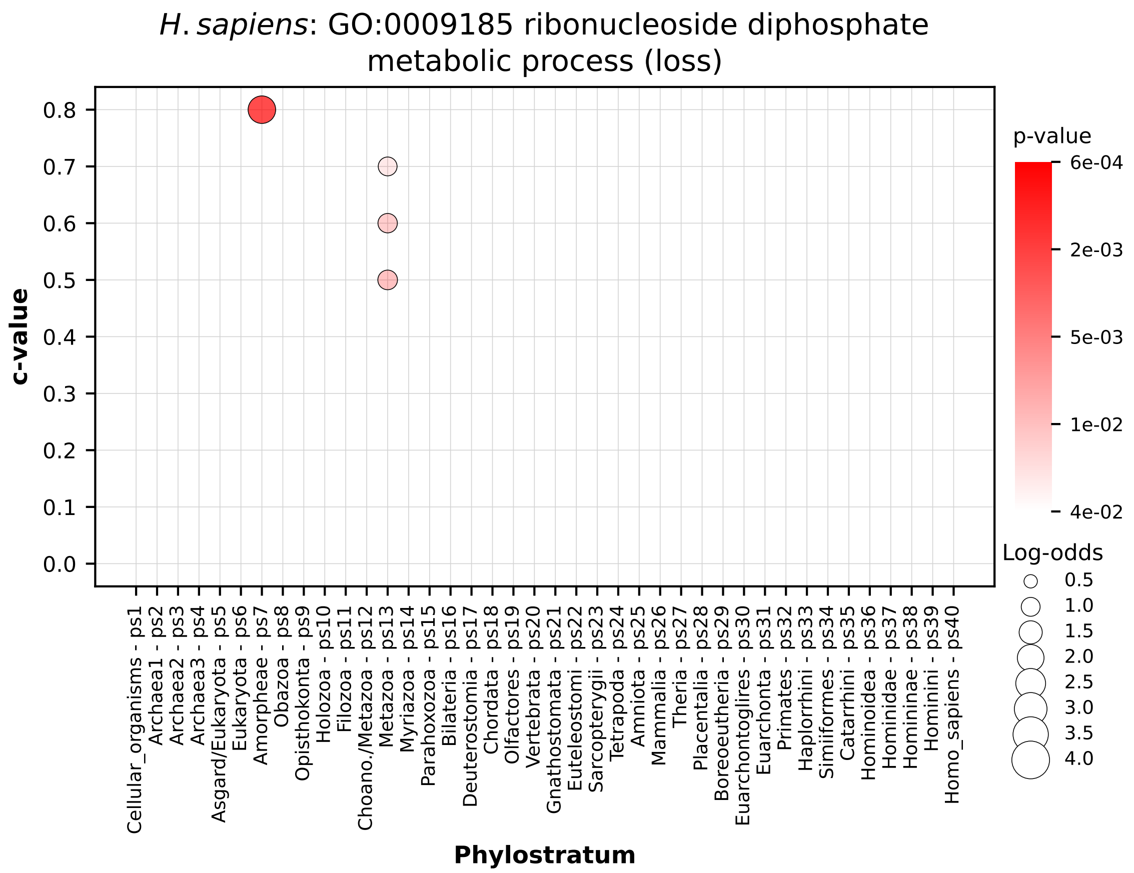

H. sapiens: GO:0009185 ribonucleoside diphosphate metabolic process (loss)
c-value
0.0
0.1
0.2
0.3
0.4
0.5
0.6
0.7
0.8
Phylostratum
Cellular_organisms - ps1
Archaea1 - ps2
Archaea2 - ps3
Archaea3 - ps4
Asgard/Eukaryota - ps5
Eukaryota - ps6
Amorpheae - ps7
Obazoa - ps8
Opisthokonta - ps9
Holozoa - ps10
Filozoa - ps11
Choano./Metazoa - ps12
Metazoa - ps13
Myriazoa - ps14
Parahoxozoa - ps15
Bilateria - ps16
Deuterostomia - ps17
Chordata - ps18
Olfactores - ps19
Vertebrata - ps20
Gnathostomata - ps21
Euteleostomi - ps22
Sarcopterygii - ps23
Tetrapoda - ps24
Amniota - ps25
Mammalia - ps26
Theria - ps27
Placentalia - ps28
Boreoeutheria - ps29
Euarchontoglires - ps30
Euarchonta - ps31
Primates - ps32
Haplorrhini - ps33
Simiiformes - ps34
Catarrhini - ps35
Hominoidea - ps36
Hominidae - ps37
Homininae - ps38
Hominini - ps39
Homo_sapiens - ps40
p-value
6e-04
2e-03
5e-03
1e-02
4e-02
Log-odds
0.5
1.0
1.5
2.0
2.5
3.0
3.5
4.0

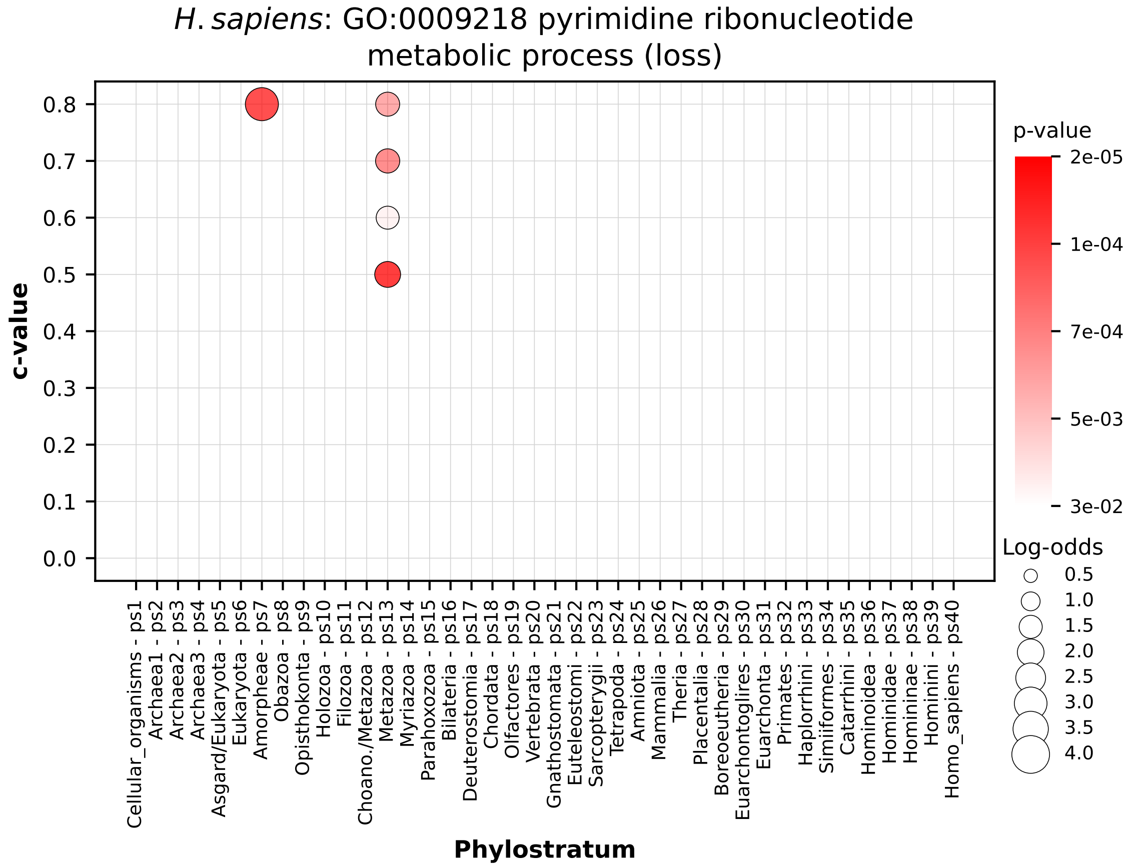
H. sapiens: GO:0009218 pyrimidine ribonucleotide metabolic process (loss)
c-value
0.0
0.1
0.2
0.3
0.4
0.5
0.6
0.7
0.8
p-value
2e-05
1e-04
7e-04
5e-03
3e-02
Log-odds
0.5
1.0
1.5
2.0
2.5
3.0
3.5
4.0
Phylostratum
Cellular_organisms - ps1
Archaea1 - ps2
Archaea2 - ps3
Archaea3 - ps4
Asgard/Eukaryota - ps5
Eukaryota - ps6
Amorpheae - ps7
Obazoa - ps8
Opisthokonta - ps9
Holozoa - ps10
Filozoa - ps11
Choano./Metazoa - ps12
Metazoa - ps13
Myriazoa - ps14
Parahoxozoa - ps15
Bilateria - ps16
Deuterostomia - ps17
Chordata - ps18
Olfactores - ps19
Vertebrata - ps20
Gnathostomata - ps21
Euteleostomi - ps22
Sarcopterygii - ps23
Tetrapoda - ps24
Amniota - ps25
Mammalia - ps26
Theria - ps27
Placentalia - ps28
Boreoeutheria - ps29
Euarchontoglires - ps30
Euarchonta - ps31
Primates - ps32
Haplorrhini - ps33
Simiiformes - ps34
Catarrhini - ps35
Hominoidea - ps36
Hominidae - ps37
Homininae - ps38
Hominini - ps39
Homo_sapiens - ps40

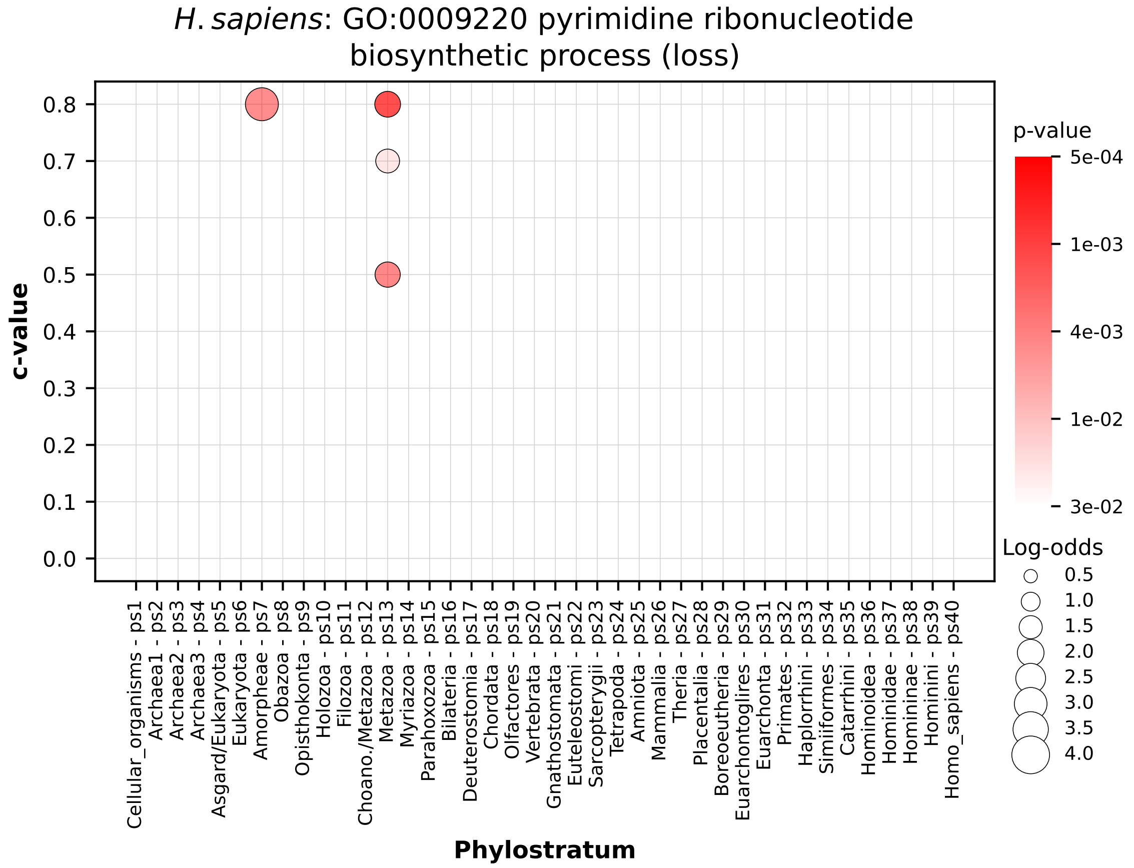
H. sapiens: GO:0009220 pyrimidine ribonucleotide biosynthetic process (loss)
c-value
0.0
0.1
0.2
0.3
0.4
0.5
0.6
0.7
0.8
p-value
5e-04
1e-03
4e-03
1e-02
3e-02
Log-odds
0.5
1.0
1.5
2.0
2.5
3.0
3.5
4.0
Cellular_organisms - ps1
Archaea1 - ps2
Archaea2 - ps3
Archaea3 - ps4
Asgard/Eukaryota - ps5
Eukaryota - ps6
Amorpheae - ps7
Obazoa - ps8
Opisthokonta - ps9
Holozoa - ps10
Filozoa - ps11
Choano./Metazoa - ps12
Metazoa - ps13
Myriazoa - ps14
Parahoxozoa - ps15
Bilateria - ps16
Deuterostomia - ps17
Chordata - ps18
Olfactores - ps19
Vertebrata - ps20
Gnathostomata - ps21
Euteleostomi - ps22
Sarcopterygii - ps23
Tetrapoda - ps24
Amniota - ps25
Mammalia - ps26
Theria - ps27
Placentalia - ps28
Boreoeutheria - ps29
Euarchontoglires - ps30
Euarchonta - ps31
Primates - ps32
Haplorrhini - ps33
Simiiformes - ps34
Catarrhini - ps35
Hominoidea - ps36
Hominidae - ps37
Homininae - ps38
Hominini - ps39
Homo_sapiens - ps40
Phylostratum

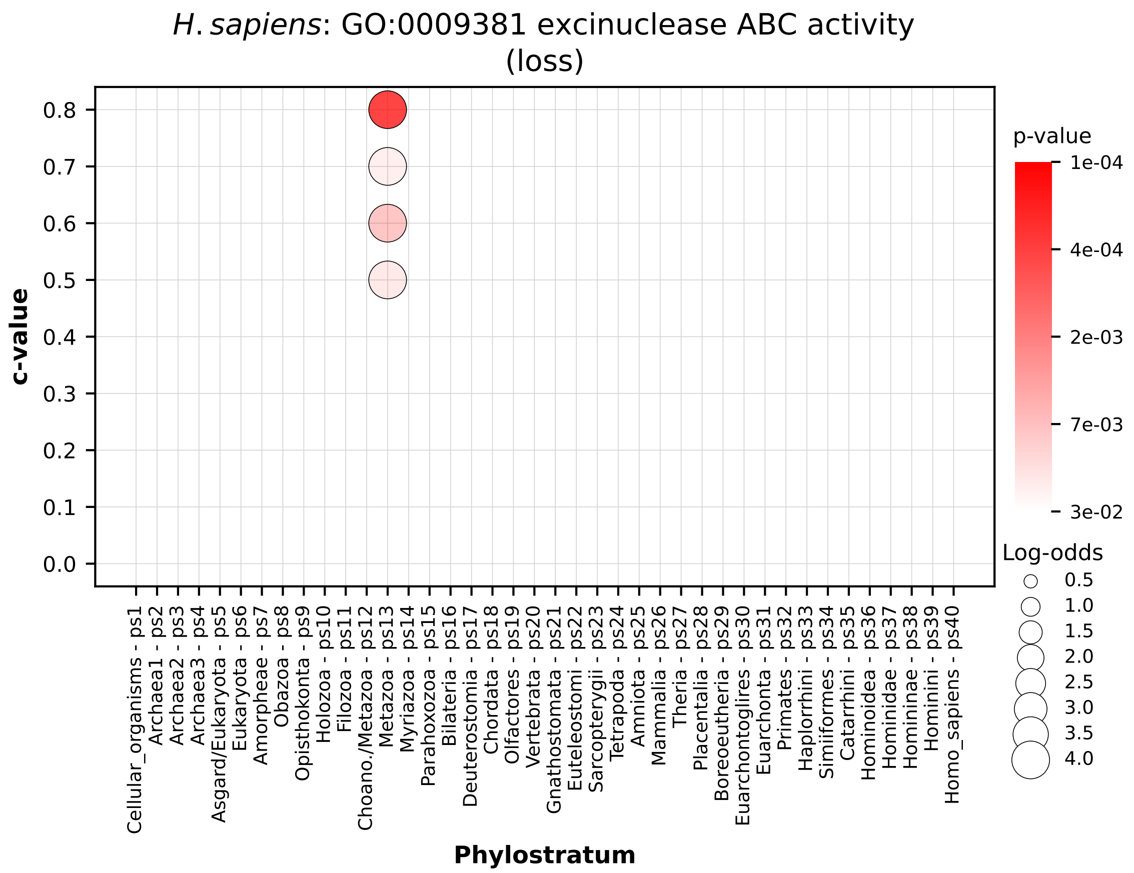

H. sapiens: GO:0009381 excinuclease ABC activity (loss)
c-value
0.0
0.1
0.2
0.3
0.4
0.5
0.6
0.7
0.8
p-value
1e-04
4e-04
2e-03
7e-03
3e-02
Log-odds
0.5
1.0
1.5
2.0
2.5
3.0
3.5
4.0
Cellular_organisms - ps1
Archaea1 - ps2
Archaea2 - ps3
Archaea3 - ps4
Asgard/Eukaryota - ps5
Eukaryota - ps6
Amorpheae - ps7
Obazoa - ps8
Opisthokonta - ps9
Holozoa - ps10
Filozoa - ps11
Choano./Metazoa - ps12
Metazoa - ps13
Myriazoa - ps14
Parahoxozoa - ps15
Bilateria - ps16
Deuterostomia - ps17
Chordata - ps18
Olfactores - ps19
Vertebrata - ps20
Gnathostomata - ps21
Euteleostomi - ps22
Sarcopterygii - ps23
Tetrapoda - ps24
Amniota - ps25
Mammalia - ps26
Theria - ps27
Placentalia - ps28
Boreoeutheria - ps29
Euarchontoglires - ps30
Euarchonta - ps31
Primates - ps32
Haplorrhini - ps33
Simiiformes - ps34
Catarrhini - ps35
Hominoidea - ps36
Hominidae - ps37
Homininae - ps38
Hominini - ps39
Homo_sapiens - ps40
Phylostratum

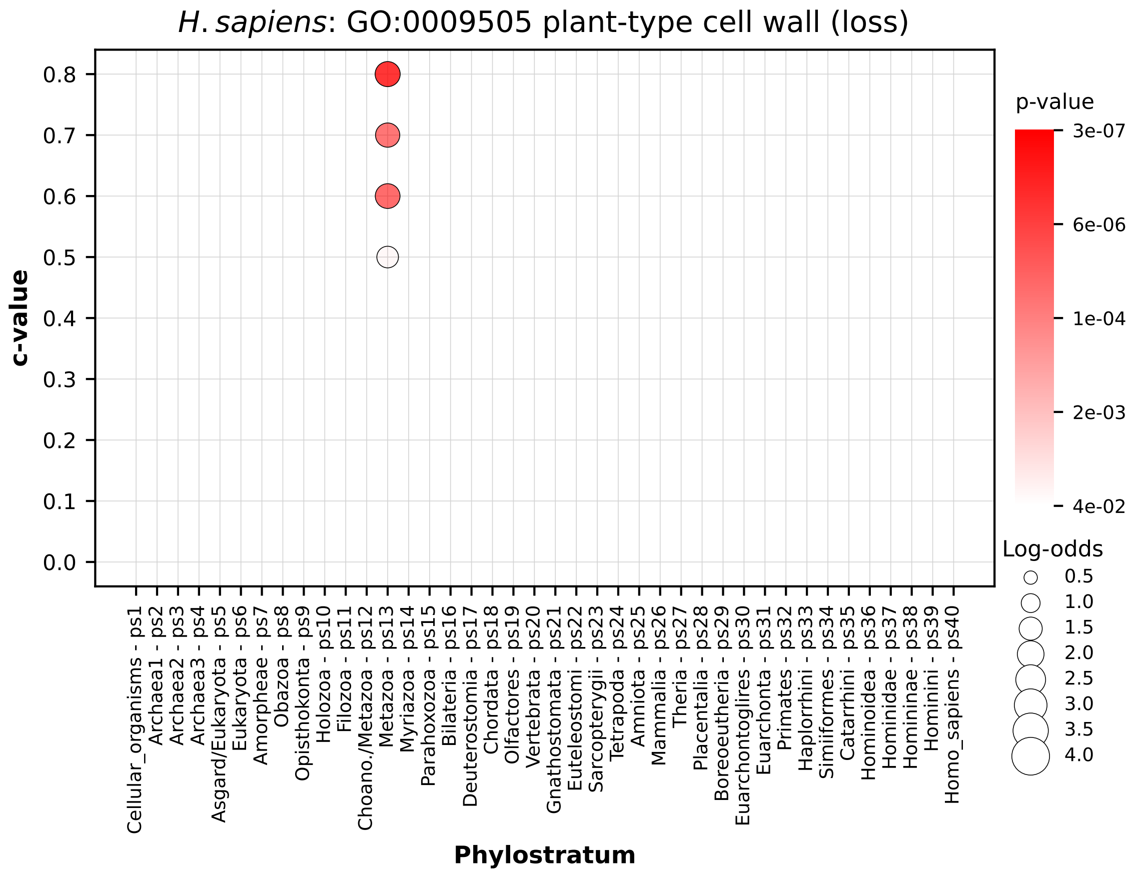

H. sapiens: GO:0009505 plant-type cell wall (loss)
c-value
0.8
0.7
0.6
0.5
0.4
0.3
0.2
0.1
0.0
p-value
3e-07
6e-06
1e-04
2e-03
4e-02
Log-odds
0.5
1.0
1.5
2.0
2.5
3.0
3.5
4.0
Cellular_organisms - ps1
Archaea1 - ps2
Archaea2 - ps3
Archaea3 - ps4
Asgard/Eukaryota - ps5
Eukaryota - ps6
Amorpheae - ps7
Obazoa - ps8
Opisthokonta - ps9
Holozoa - ps10
Filozoa - ps11
Choano./Metazoa - ps12
Metazoa - ps13
Myriazoa - ps14
Parahoxozoa - ps15
Bilateria - ps16
Deuterostomia - ps17
Chordata - ps18
Olfactores - ps19
Vertebrata - ps20
Gnathostomata - ps21
Euteleostomi - ps22
Sarcopterygii - ps23
Tetrapoda - ps24
Amniota - ps25
Mammalia - ps26
Theria - ps27
Placentalia - ps28
Boreoeutheria - ps29
Euarchontoglires - ps30
Euarchonta - ps31
Primates - ps32
Haplorrhini - ps33
Simiiformes - ps34
Catarrhini - ps35
Hominoidea - ps36
Hominidae - ps37
Homininae - ps38
Hominini - ps39
Homo_sapiens - ps40
Phylostratum

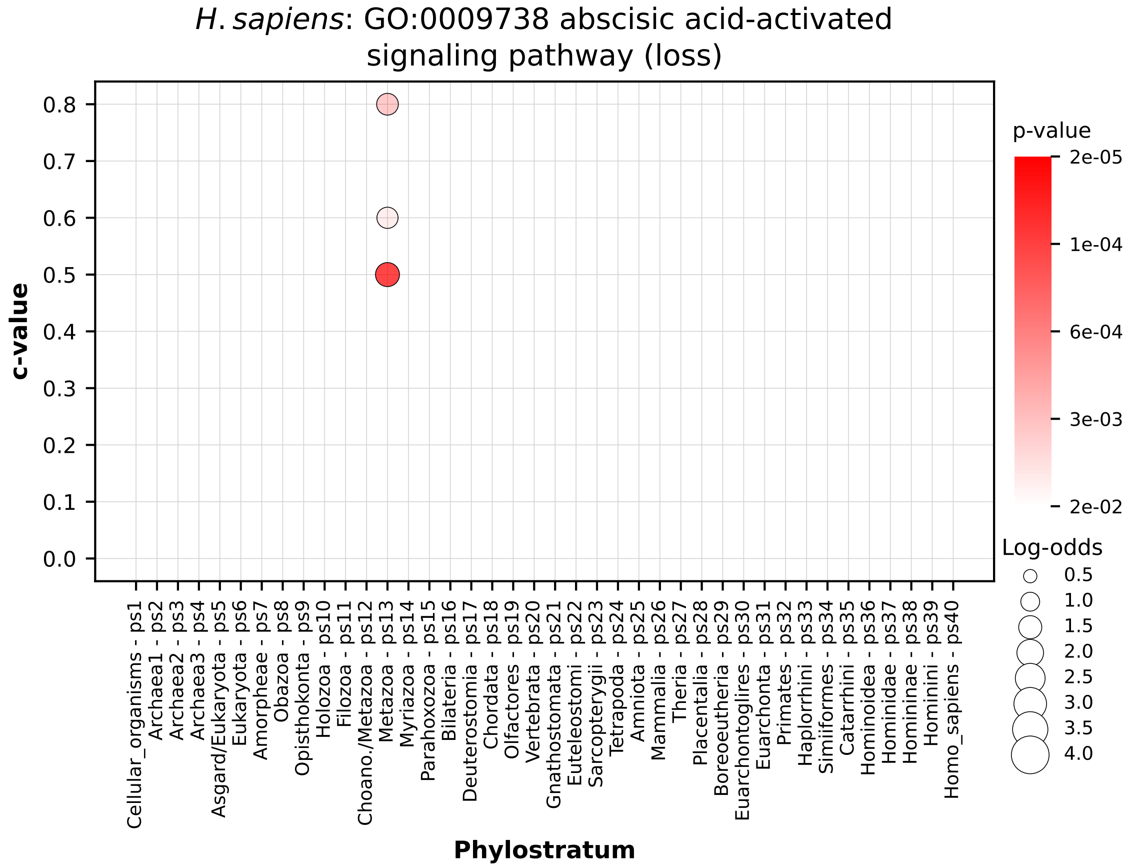

H. sapiens: GO:0009738 abscisic acid-activated signaling pathway (loss)
c-value
0.0
0.1
0.2
0.3
0.4
0.5
0.6
0.7
0.8
Phylostratum
Cellular_organisms - ps1
Archaea1 - ps2
Archaea2 - ps3
Archaea3 - ps4
Asgard/Eukaryota - ps5
Eukaryota - ps6
Amorpheae - ps7
Obazoa - ps8
Opisthokonta - ps9
Holozoa - ps10
Filozoa - ps11
Choano./Metazoa - ps12
Metazoa - ps13
Myriazoa - ps14
Parahoxozoa - ps15
Bilateria - ps16
Deuterostomia - ps17
Chordata - ps18
Olfactores - ps19
Vertebrata - ps20
Gnathostomata - ps21
Euteleostomi - ps22
Sarcopterygii - ps23
Tetrapoda - ps24
Amniota - ps25
Mammalia - ps26
Theria - ps27
Placentalia - ps28
Boreoeutheria - ps29
Euarchontoglires - ps30
Euarchonta - ps31
Primates - ps32
Haplorrhini - ps33
Simiiformes - ps34
Catarrhini - ps35
Hominoidea - ps36
Hominidae - ps37
Homininae - ps38
Hominini - ps39
Homo_sapiens - ps40
p-value
2e-05
1e-04
6e-04
3e-03
2e-02
Log-odds
0.5
1.0
1.5
2.0
2.5
3.0
3.5
4.0

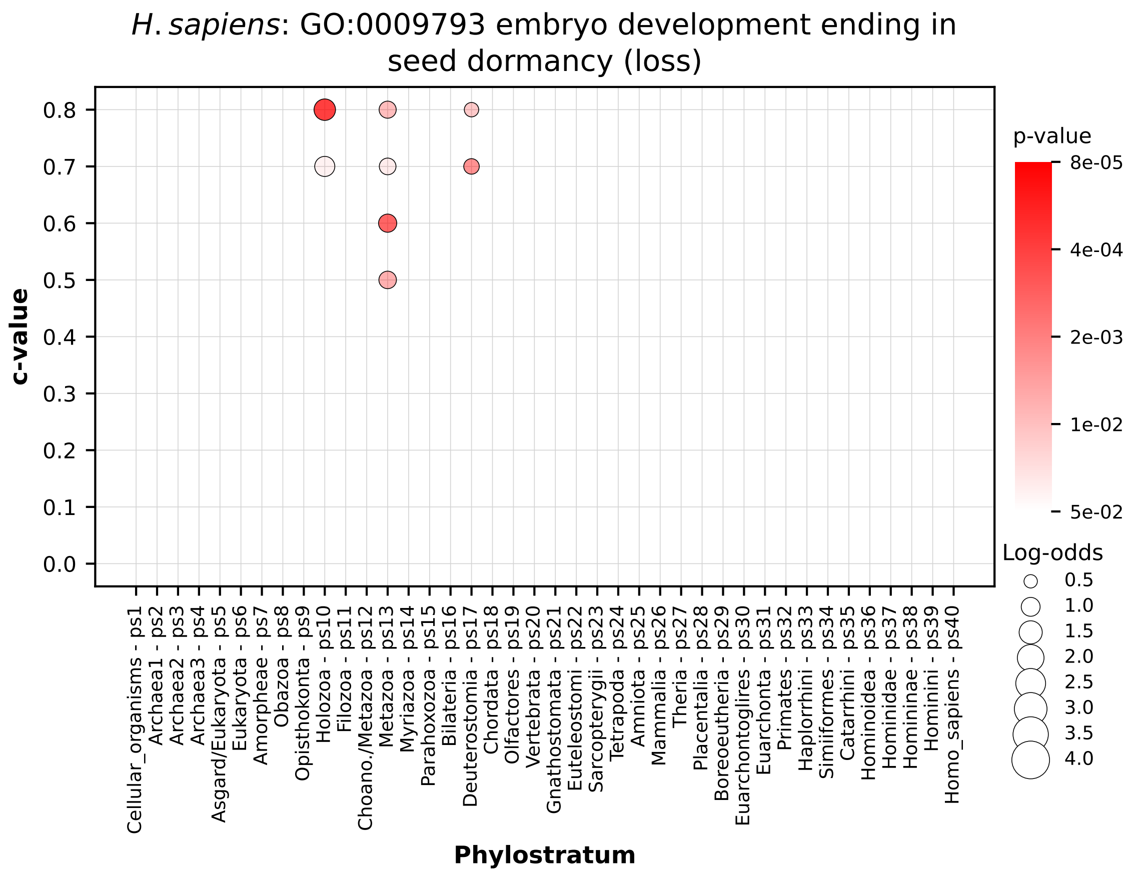
H. sapiens: GO:0009793 embryo development ending in seed dormancy (loss)
c-value
0.8
0.7
0.6
0.5
0.4
0.3
0.2
0.1
0.0
Phylostratum
Cellular_organisms - ps1
Archaea1 - ps2
Archaea2 - ps3
Archaea3 - ps4
Asgard/Eukaryota - ps5
Eukaryota - ps6
Amorpheae - ps7
Obazoa - ps8
Opisthokonta - ps9
Holozoa - ps10
Filozoa - ps11
Choano./Metazoa - ps12
Metazoa - ps13
Myriazoa - ps14
Parahoxozoa - ps15
Bilateria - ps16
Deuterostomia - ps17
Chordata - ps18
Olfactores - ps19
Vertebrata - ps20
Gnathostomata - ps21
Euteleostomi - ps22
Sarcopterygii - ps23
Tetrapoda - ps24
Amniota - ps25
Mammalia - ps26
Theria - ps27
Placentalia - ps28
Boreoeutheria - ps29
Euarchontoglires - ps30
Euarchonta - ps31
Primates - ps32
Haplorrhini - ps33
Simiiformes - ps34
Catarrhini - ps35
Hominoidea - ps36
Hominidae - ps37
Homininae - ps38
Hominini - ps39
Homo_sapiens - ps40
p-value
8e-05
4e-04
2e-03
1e-02
5e-02
Log-odds
0.5
1.0
1.5
2.0
2.5
3.0
3.5
4.0

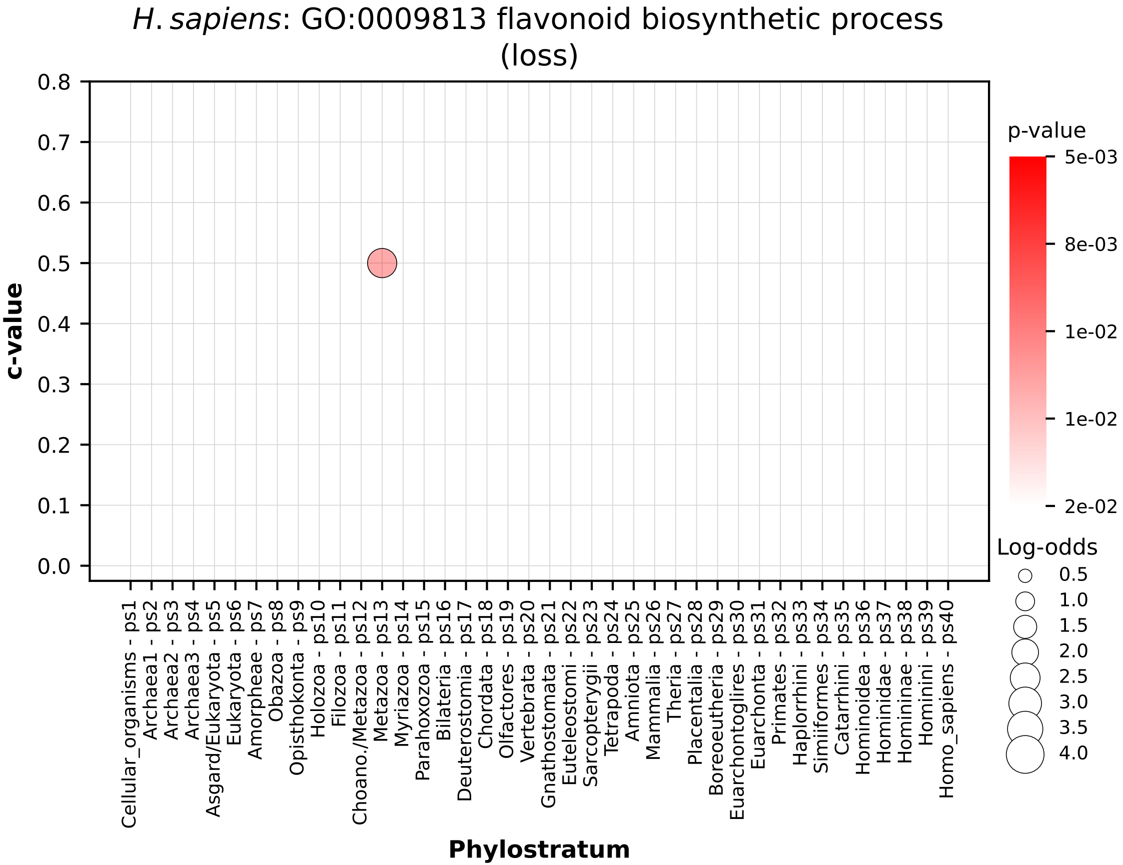

H. sapiens: GO:0009813 flavonoid biosynthetic process (loss)
c-value
0.8
0.7
0.6
0.5
0.4
0.3
0.2
0.1
0.0
p-value
5e-03
8e-03
1e-02
1e-02
2e-02
Log-odds
0.5
1.0
1.5
2.0
2.5
3.0
3.5
4.0
Cellular_organisms - ps1
Archaea1 - ps2
Archaea2 - ps3
Archaea3 - ps4
Asgard/Eukaryota - ps5
Eukaryota - ps6
Amorpheae - ps7
Obazoa - ps8
Opisthokonta - ps9
Holozoa - ps10
Filozoa - ps11
Choano./Metazoa - ps12
Metazoa - ps13
Myriazoa - ps14
Parahoxozoa - ps15
Bilateria - ps16
Deuterostomia - ps17
Chordata - ps18
Olfactores - ps19
Vertebrata - ps20
Gnathostomata - ps21
Euteleostomi - ps22
Sarcopterygii - ps23
Tetrapoda - ps24
Amniota - ps25
Mammalia - ps26
Theria - ps27
Placentalia - ps28
Boreoeutheria - ps29
Euarchontoglires - ps30
Euarchonta - ps31
Primates - ps32
Haplorrhini - ps33
Simiiformes - ps34
Catarrhini - ps35
Hominoidea - ps36
Hominidae - ps37
Homininae - ps38
Hominini - ps39
Homo_sapiens - ps40
Phylostratum

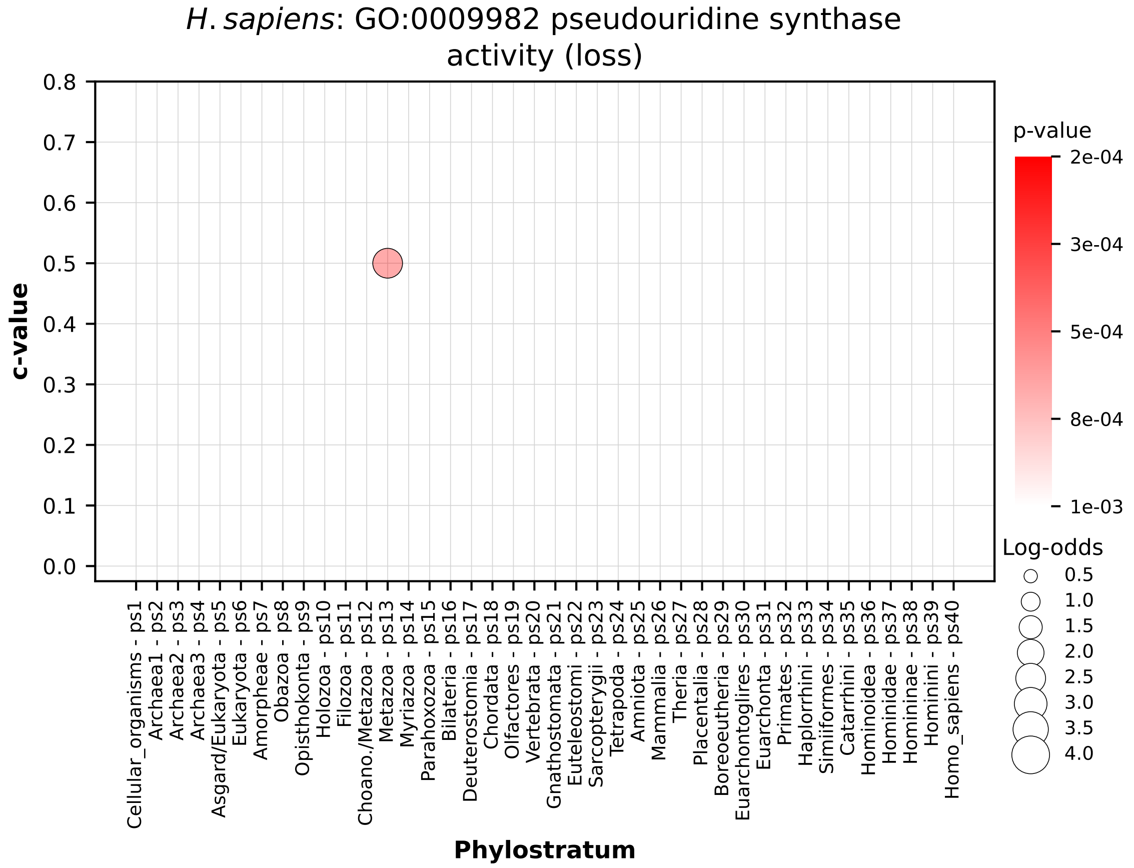

H. sapiens: GO:0009982 pseudouridine synthase activity (loss)
c-value
0.8
0.7
0.6
0.5
0.4
0.3
0.2
0.1
0.0
Phylostratum
Cellular_organisms - ps1
Archaea1 - ps2
Archaea2 - ps3
Archaea3 - ps4
Asgard/Eukaryota - ps5
Eukaryota - ps6
Amorpheae - ps7
Obazoa - ps8
Opisthokonta - ps9
Holozoa - ps10
Filozoa - ps11
Choano./Metazoa - ps12
Metazoa - ps13
Myriazoa - ps14
Parahoxozoa - ps15
Bilateria - ps16
Deuterostomia - ps17
Chordata - ps18
Olfactores - ps19
Vertebrata - ps20
Gnathostomata - ps21
Euteleostomi - ps22
Sarcopterygii - ps23
Tetrapoda - ps24
Amniota - ps25
Mammalia - ps26
Theria - ps27
Placentalia - ps28
Boreoeutheria - ps29
Euarchontoglires - ps30
Euarchonta - ps31
Primates - ps32
Haplorrhini - ps33
Simiiformes - ps34
Catarrhini - ps35
Hominoidea - ps36
Hominidae - ps37
Homininae - ps38
Hominini - ps39
Homo_sapiens - ps40
p-value
2e-04
3e-04
5e-04
8e-04
1e-03
Log-odds
0.5
1.0
1.5
2.0
2.5
3.0
3.5
4.0

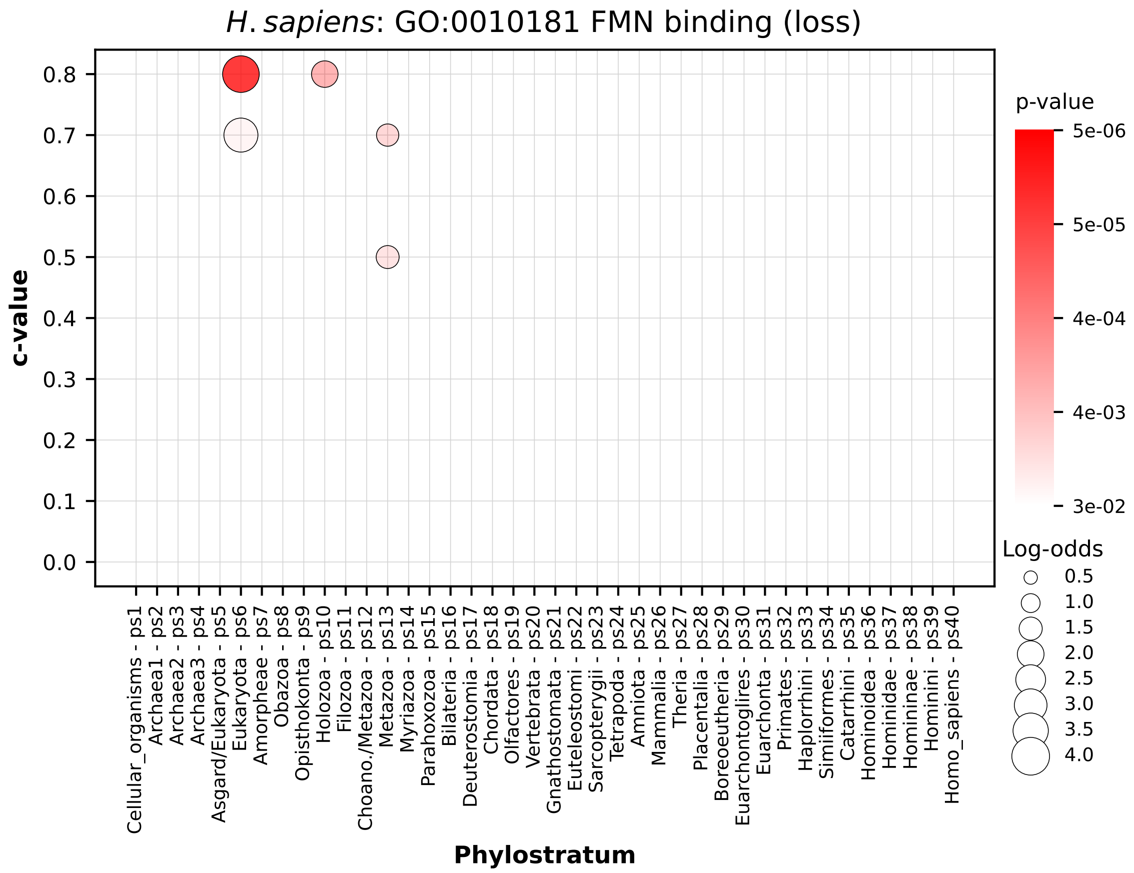

H. sapiens: GO:0010181 FMN binding (loss)
c-value
0.0
0.1
0.2
0.3
0.4
0.5
0.6
0.7
0.8
Phylostratum
Cellular_organisms - ps1
Archaea1 - ps2
Archaea2 - ps3
Archaea3 - ps4
Asgard/Eukaryota - ps5
Eukaryota - ps6
Amorpheae - ps7
Obazoa - ps8
Opisthokonta - ps9
Holozoa - ps10
Filozoa - ps11
Choano./Metazoa - ps12
Metazoa - ps13
Myriazoa - ps14
Parahoxozoa - ps15
Bilateria - ps16
Deuterostomia - ps17
Chordata - ps18
Olfactores - ps19
Vertebrata - ps20
Gnathostomata - ps21
Euteleostomi - ps22
Sarcopterygii - ps23
Tetrapoda - ps24
Amniota - ps25
Mammalia - ps26
Theria - ps27
Placentalia - ps28
Boreoeutheria - ps29
Euarchontoglires - ps30
Euarchonta - ps31
Primates - ps32
Haplorrhini - ps33
Simiiformes - ps34
Catarrhini - ps35
Hominoidea - ps36
Hominidae - ps37
Homininae - ps38
Hominini - ps39
Homo_sapiens - ps40
p-value
5e-06
5e-05
4e-04
4e-03
3e-02
Log-odds
0.5
1.0
1.5
2.0
2.5
3.0
3.5
4.0

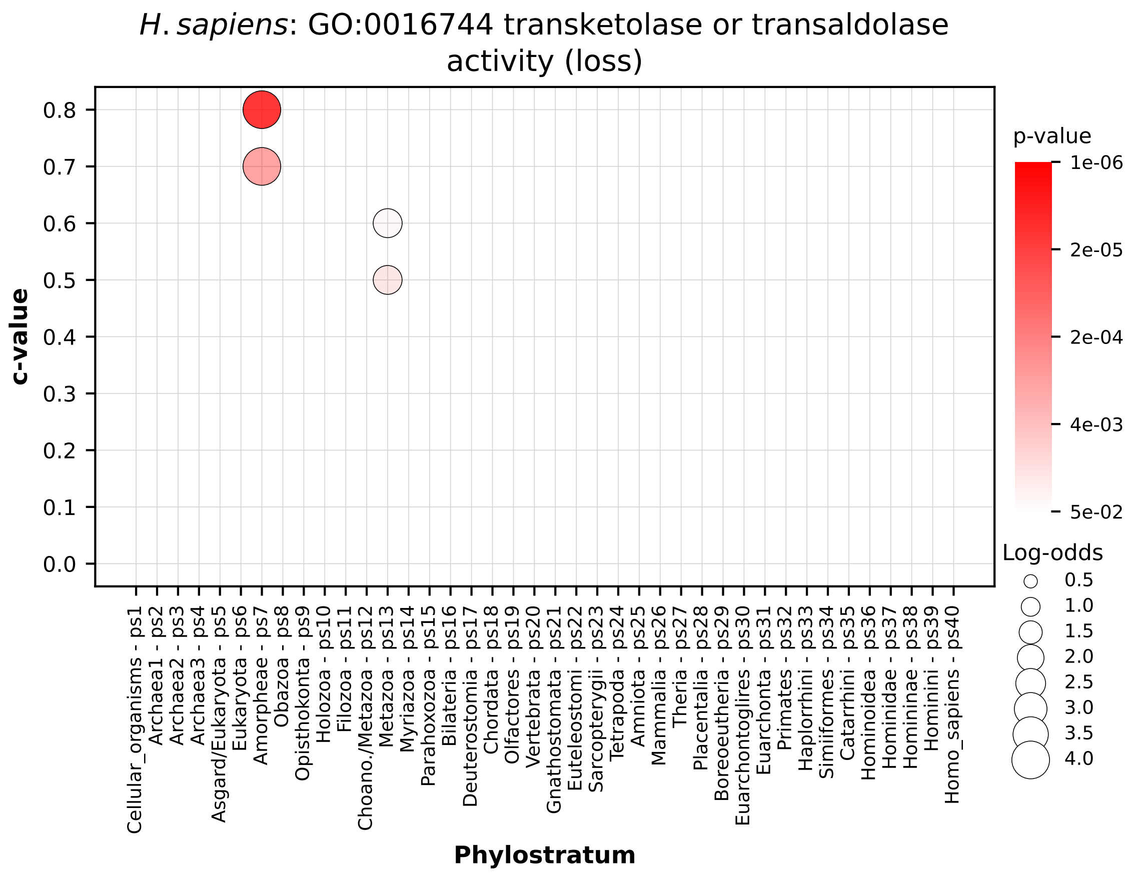
H. sapiens: GO:0016744 transketolase or transaldolase activity (loss)
c-value
0.0
0.1
0.2
0.3
0.4
0.5
0.6
0.7
0.8
p-value
1e-06
2e-05
2e-04
4e-03
5e-02
Log-odds
0.5
1.0
1.5
2.0
2.5
3.0
3.5
4.0
Cellular_organisms - ps1
Archaea1 - ps2
Archaea2 - ps3
Archaea3 - ps4
Asgard/Eukaryota - ps5
Eukaryota - ps6
Amorpheae - ps7
Obazoa - ps8
Opisthokonta - ps9
Holozoa - ps10
Filozoa - ps11
Choano./Metazoa - ps12
Metazoa - ps13
Myriazoa - ps14
Parahoxozoa - ps15
Bilateria - ps16
Deuterostomia - ps17
Chordata - ps18
Olfactores - ps19
Vertebrata - ps20
Gnathostomata - ps21
Euteleostomi - ps22
Sarcopterygii - ps23
Tetrapoda - ps24
Amniota - ps25
Mammalia - ps26
Theria - ps27
Placentalia - ps28
Boreoeutheria - ps29
Euarchontoglires - ps30
Euarchonta - ps31
Primates - ps32
Haplorrhini - ps33
Simiiformes - ps34
Catarrhini - ps35
Hominoidea - ps36
Hominidae - ps37
Homininae - ps38
Hominini - ps39
Homo_sapiens - ps40
Phylostratum

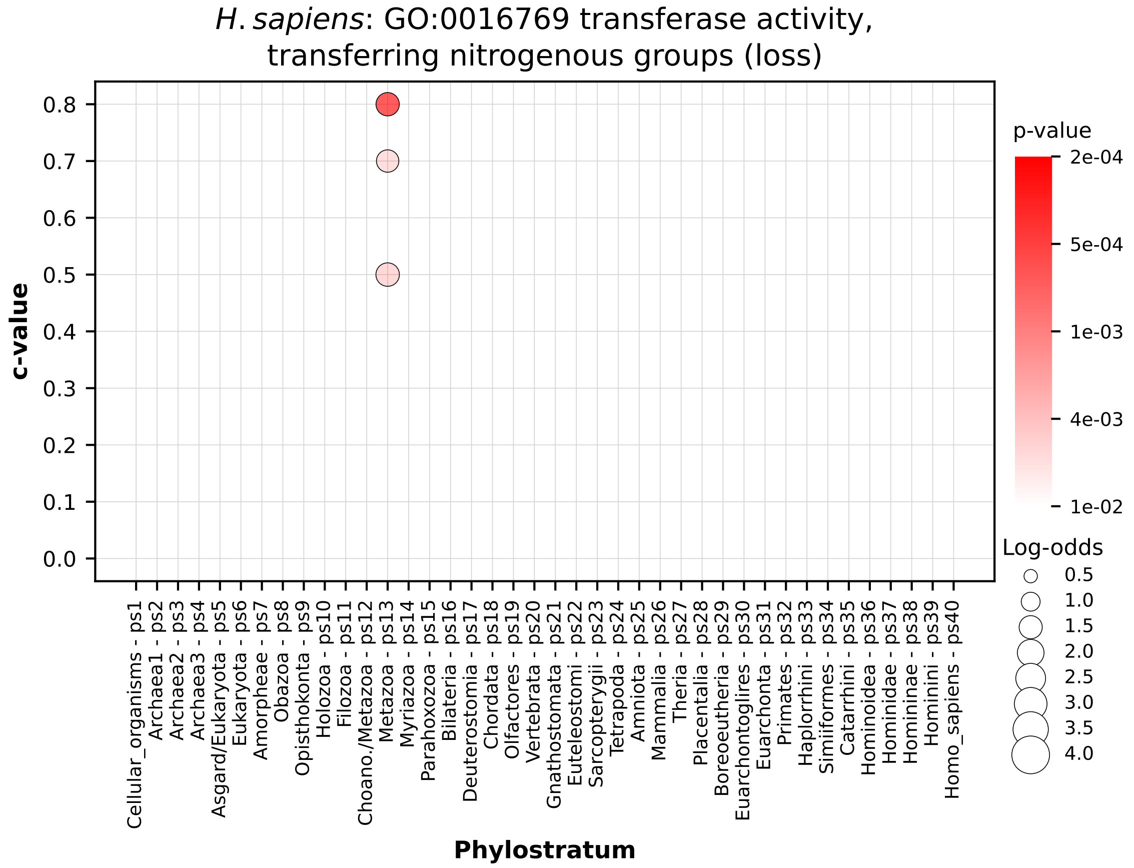

H. sapiens: GO:0016769 transferase activity, transferring nitrogenous groups (loss)
c-value
0.0
0.1
0.2
0.3
0.4
0.5
0.6
0.7
0.8
Phylostratum
Cellular_organisms - ps1
Archaea1 - ps2
Archaea2 - ps3
Archaea3 - ps4
Asgard/Eukaryota - ps5
Eukaryota - ps6
Amorpheae - ps7
Obazoa - ps8
Opisthokonta - ps9
Holozoa - ps10
Filozoa - ps11
Choano./Metazoa - ps12
Metazoa - ps13
Myriazoa - ps14
Parahoxozoa - ps15
Bilateria - ps16
Deuterostomia - ps17
Chordata - ps18
Olfactores - ps19
Vertebrata - ps20
Gnathostomata - ps21
Euteleostomi - ps22
Sarcopterygii - ps23
Tetrapoda - ps24
Amniota - ps25
Mammalia - ps26
Theria - ps27
Placentalia - ps28
Boreoeutheria - ps29
Euarchontoglires - ps30
Euarchonta - ps31
Primates - ps32
Haplorrhini - ps33
Simiiformes - ps34
Catarrhini - ps35
Hominoidea - ps36
Hominidae - ps37
Homininae - ps38
Hominini - ps39
Homo_sapiens - ps40
p-value
2e-04
5e-04
1e-03
4e-03
1e-02
Log-odds
0.5
1.0
1.5
2.0
2.5
3.0
3.5
4.0

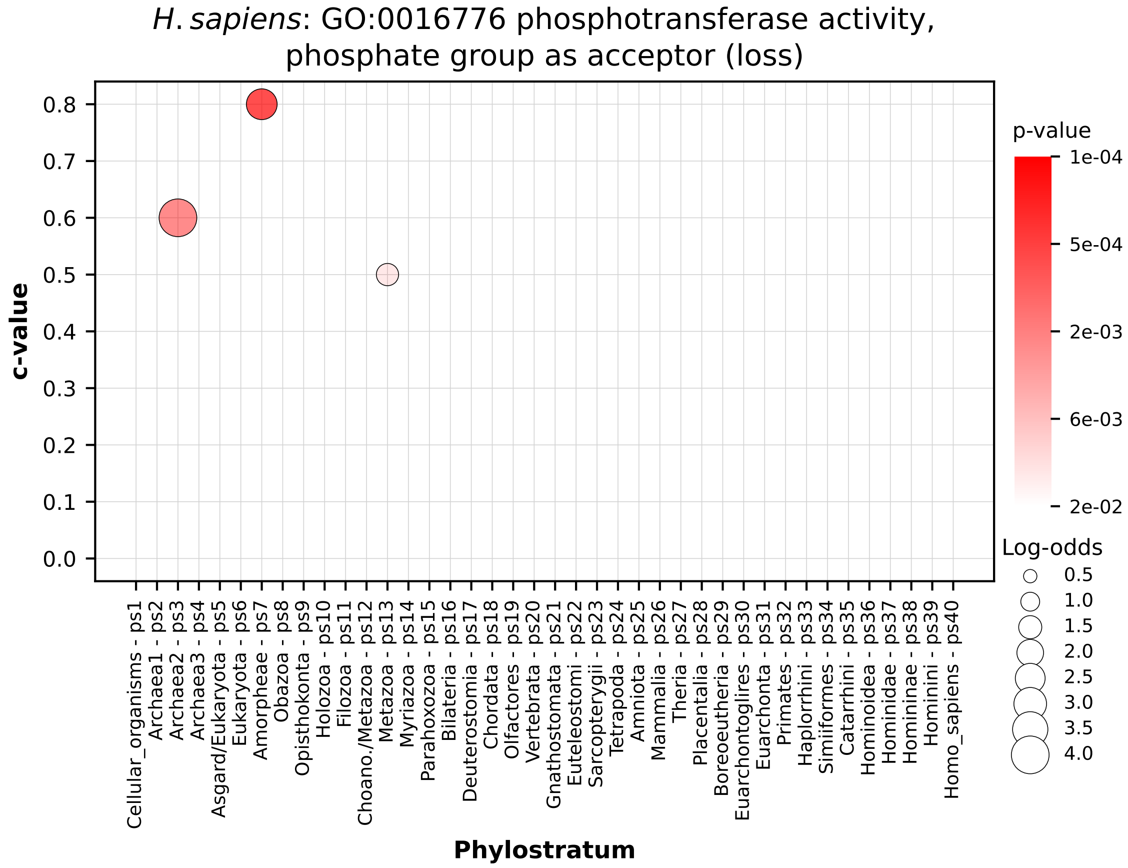

H. sapiens: GO:0016776 phosphotransferase activity, phosphate group as acceptor (loss)
c-value
0.0
0.1
0.2
0.3
0.4
0.5
0.6
0.7
0.8
p-value
1e-04
5e-04
2e-03
6e-03
2e-02
Log-odds
0.5
1.0
1.5
2.0
2.5
3.0
3.5
4.0
Cellular_organisms - ps1
Archaea1 - ps2
Archaea2 - ps3
Archaea3 - ps4
Asgard/Eukaryota - ps5
Eukaryota - ps6
Amorpheae - ps7
Obazoa - ps8
Opisthokonta - ps9
Holozoa - ps10
Filozoa - ps11
Choano./Metazoa - ps12
Metazoa - ps13
Myriazoa - ps14
Parahoxozoa - ps15
Bilateria - ps16
Deuterostomia - ps17
Chordata - ps18
Olfactores - ps19
Vertebrata - ps20
Gnathostomata - ps21
Euteleostomi - ps22
Sarcopterygii - ps23
Tetrapoda - ps24
Amniota - ps25
Mammalia - ps26
Theria - ps27
Placentalia - ps28
Boreoeutheria - ps29
Euarchontoglires - ps30
Euarchonta - ps31
Primates - ps32
Haplorrhini - ps33
Simiiformes - ps34
Catarrhini - ps35
Hominoidea - ps36
Hominidae - ps37
Homininae - ps38
Hominini - ps39
Homo_sapiens - ps40
Phylostratum

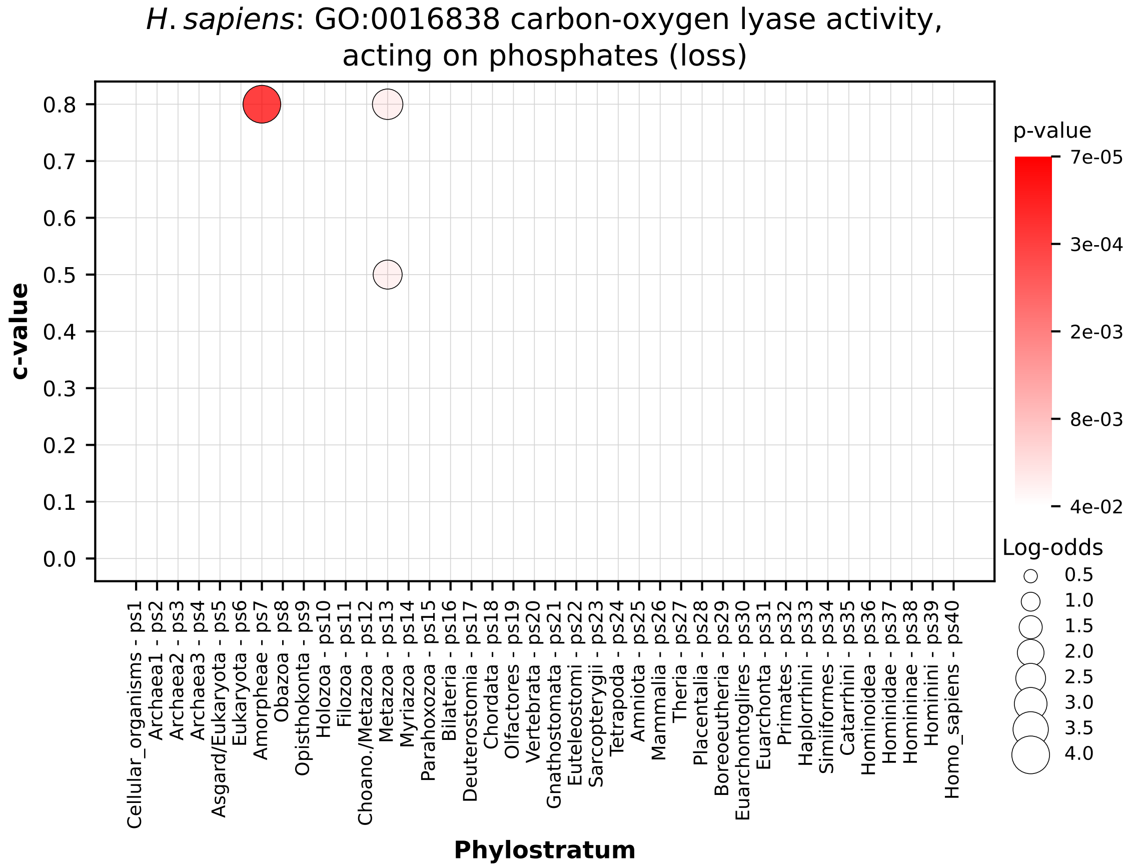
H. sapiens: GO:0016838 carbon-oxygen lyase activity, acting on phosphates (loss)
c-value
0.0
0.1
0.2
0.3
0.4
0.5
0.6
0.7
0.8
Phylostratum
Cellular_organisms - ps1
Archaea1 - ps2
Archaea2 - ps3
Archaea3 - ps4
Asgard/Eukaryota - ps5
Eukaryota - ps6
Amorpheae - ps7
Obazoa - ps8
Opisthokonta - ps9
Holozoa - ps10
Filozoa - ps11
Choano./Metazoa - ps12
Metazoa - ps13
Myriazoa - ps14
Parahoxozoa - ps15
Bilateria - ps16
Deuterostomia - ps17
Chordata - ps18
Olfactores - ps19
Vertebrata - ps20
Gnathostomata - ps21
Euteleostomi - ps22
Sarcopterygii - ps23
Tetrapoda - ps24
Amniota - ps25
Mammalia - ps26
Theria - ps27
Placentalia - ps28
Boreoeutheria - ps29
Euarchontoglires - ps30
Euarchonta - ps31
Primates - ps32
Haplorrhini - ps33
Simiiformes - ps34
Catarrhini - ps35
Hominoidea - ps36
Hominidae - ps37
Homininae - ps38
Hominini - ps39
Homo_sapiens - ps40
p-value
7e-05
3e-04
2e-03
8e-03
4e-02
Log-odds
0.5
1.0
1.5
2.0
2.5
3.0
3.5
4.0

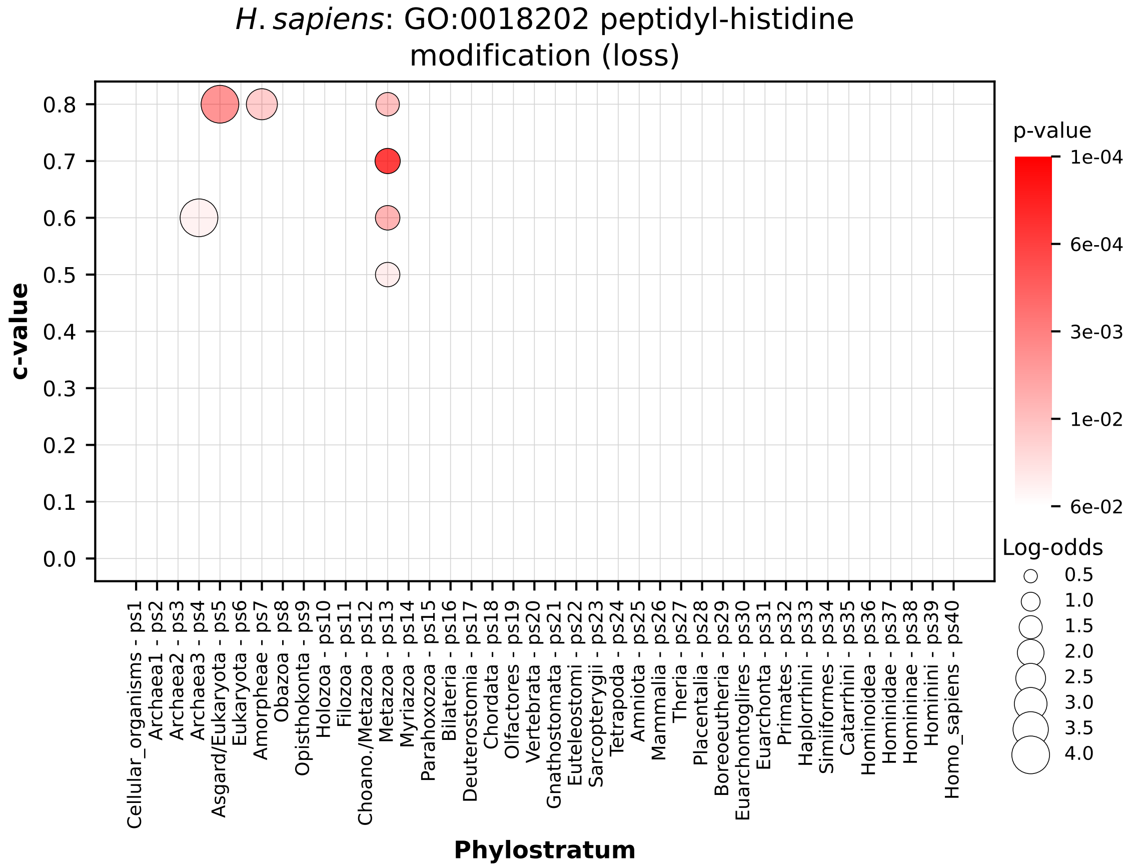

H. sapiens: GO:0018202 peptidyl-histidine modification (loss)
c-value
0.8
0.7
0.6
0.5
0.4
0.3
0.2
0.1
0.0
p-value
1e-04
6e-04
3e-03
1e-02
6e-02
Log-odds
0.5
1.0
1.5
2.0
2.5
3.0
3.5
4.0
Cellular_organisms - ps1
Archaea1 - ps2
Archaea2 - ps3
Archaea3 - ps4
Asgard/Eukaryota - ps5
Eukaryota - ps6
Amorpheae - ps7
Obazoa - ps8
Opisthokonta - ps9
Holozoa - ps10
Filozoa - ps11
Choano./Metazoa - ps12
Metazoa - ps13
Myriazoa - ps14
Parahoxozoa - ps15
Bilateria - ps16
Deuterostomia - ps17
Chordata - ps18
Olfactores - ps19
Vertebrata - ps20
Gnathostomata - ps21
Euteleostomi - ps22
Sarcopterygii - ps23
Tetrapoda - ps24
Amniota - ps25
Mammalia - ps26
Theria - ps27
Placentalia - ps28
Boreoeutheria - ps29
Euarchontoglires - ps30
Euarchonta - ps31
Primates - ps32
Haplorrhini - ps33
Simiiformes - ps34
Catarrhini - ps35
Hominoidea - ps36
Hominidae - ps37
Homininae - ps38
Hominini - ps39
Homo_sapiens - ps40
Phylostratum

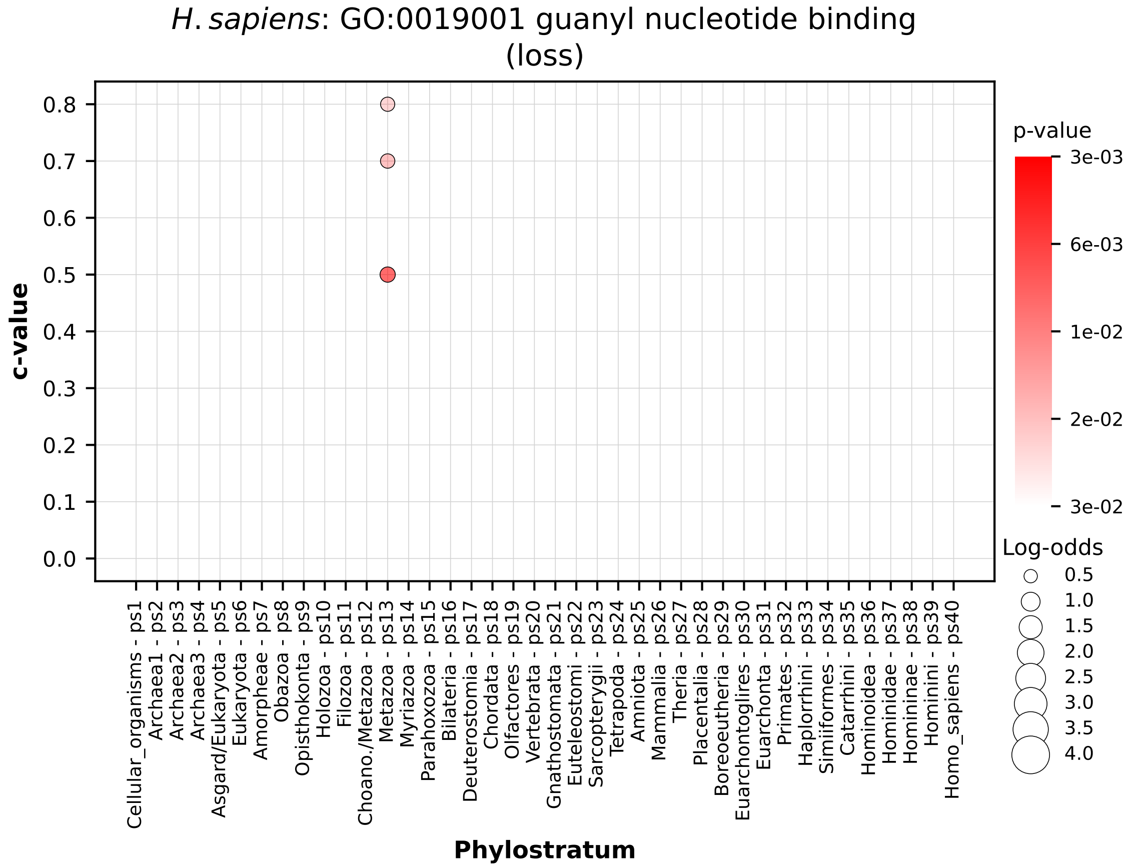
H. sapiens: GO:0019001 guanyl nucleotide binding (loss)
c-value
0.0
0.1
0.2
0.3
0.4
0.5
0.6
0.7
0.8
p-value
3e-03
6e-03
1e-02
2e-02
3e-02
Log-odds
0.5
1.0
1.5
2.0
2.5
3.0
3.5
4.0
Cellular_organisms - ps1
Archaea1 - ps2
Archaea2 - ps3
Archaea3 - ps4
Asgard/Eukaryota - ps5
Eukaryota - ps6
Amorpheae - ps7
Obazoa - ps8
Opisthokonta - ps9
Holozoa - ps10
Filozoa - ps11
Choano./Metazoa - ps12
Metazoa - ps13
Myriazoa - ps14
Parahoxozoa - ps15
Bilateria - ps16
Deuterostomia - ps17
Chordata - ps18
Olfactores - ps19
Vertebrata - ps20
Gnathostomata - ps21
Euteleostomi - ps22
Sarcopterygii - ps23
Tetrapoda - ps24
Amniota - ps25
Mammalia - ps26
Theria - ps27
Placentalia - ps28
Boreoeutheria - ps29
Euarchontoglires - ps30
Euarchonta - ps31
Primates - ps32
Haplorrhini - ps33
Simiiformes - ps34
Catarrhini - ps35
Hominoidea - ps36
Hominidae - ps37
Homininae - ps38
Hominini - ps39
Homo_sapiens - ps40
Phylostratum

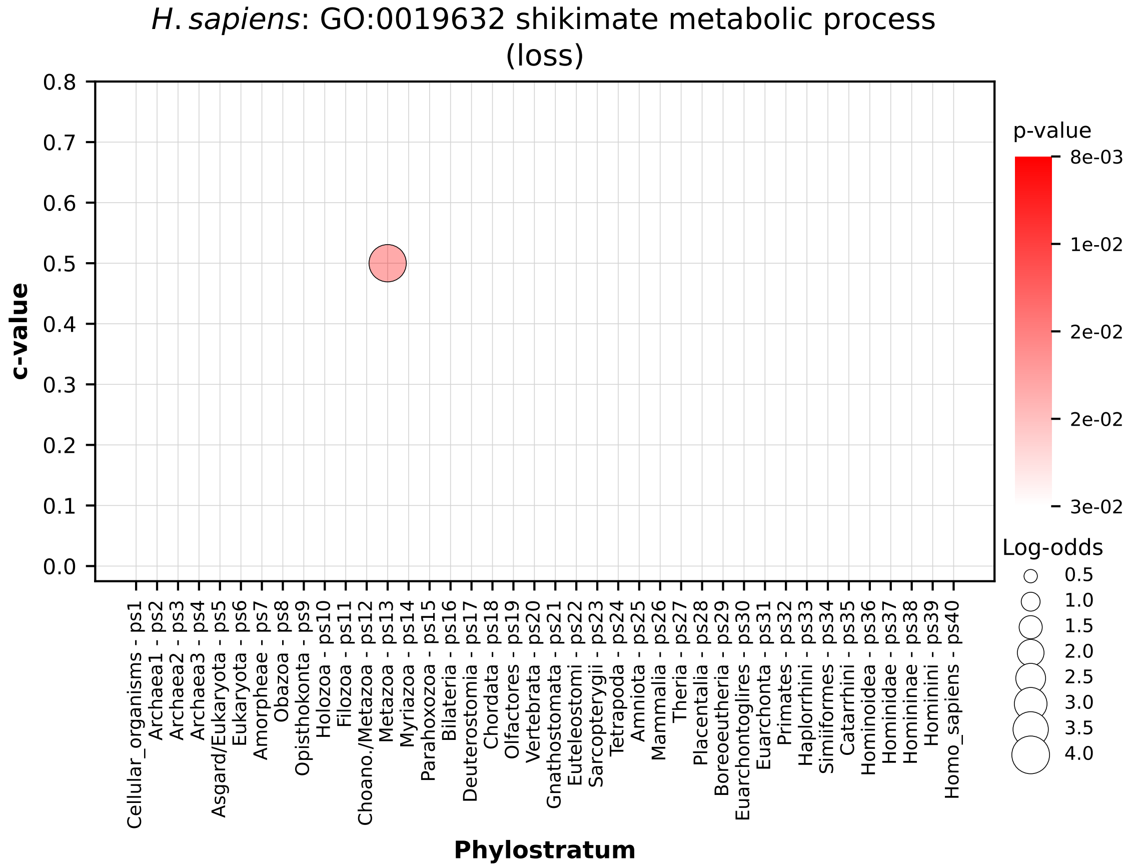

H. sapiens: GO:0019632 shikimate metabolic process
(loss)
c-value
0.0
0.1
0.2
0.3
0.4
0.5
0.6
0.7
0.8
p-value
8e-03
1e-02
2e-02
2e-02
3e-02
Log-odds
0.5
1.0
1.5
2.0
2.5
3.0
3.5
4.0
Cellular_organisms - ps1
Archaea1 - ps2
Archaea2 - ps3
Archaea3 - ps4
Asgard/Eukaryota - ps5
Eukaryota - ps6
Amorpheae - ps7
Obazoa - ps8
Opisthokonta - ps9
Holozoa - ps10
Filozoa - ps11
Choano./Metazoa - ps12
Metazoa - ps13
Myriazoa - ps14
Parahoxozoa - ps15
Bilateria - ps16
Deuterostomia - ps17
Chordata - ps18
Olfactores - ps19
Vertebrata - ps20
Gnathostomata - ps21
Euteleostomi - ps22
Sarcopterygii - ps23
Tetrapoda - ps24
Amniota - ps25
Mammalia - ps26
Theria - ps27
Placentalia - ps28
Boreoeutheria - ps29
Euarchontoglires - ps30
Euarchonta - ps31
Primates - ps32
Haplorrhini - ps33
Simiiformes - ps34
Catarrhini - ps35
Hominoidea - ps36
Hominidae - ps37
Homininae - ps38
Hominini - ps39
Homo_sapiens - ps40
Phylostratum

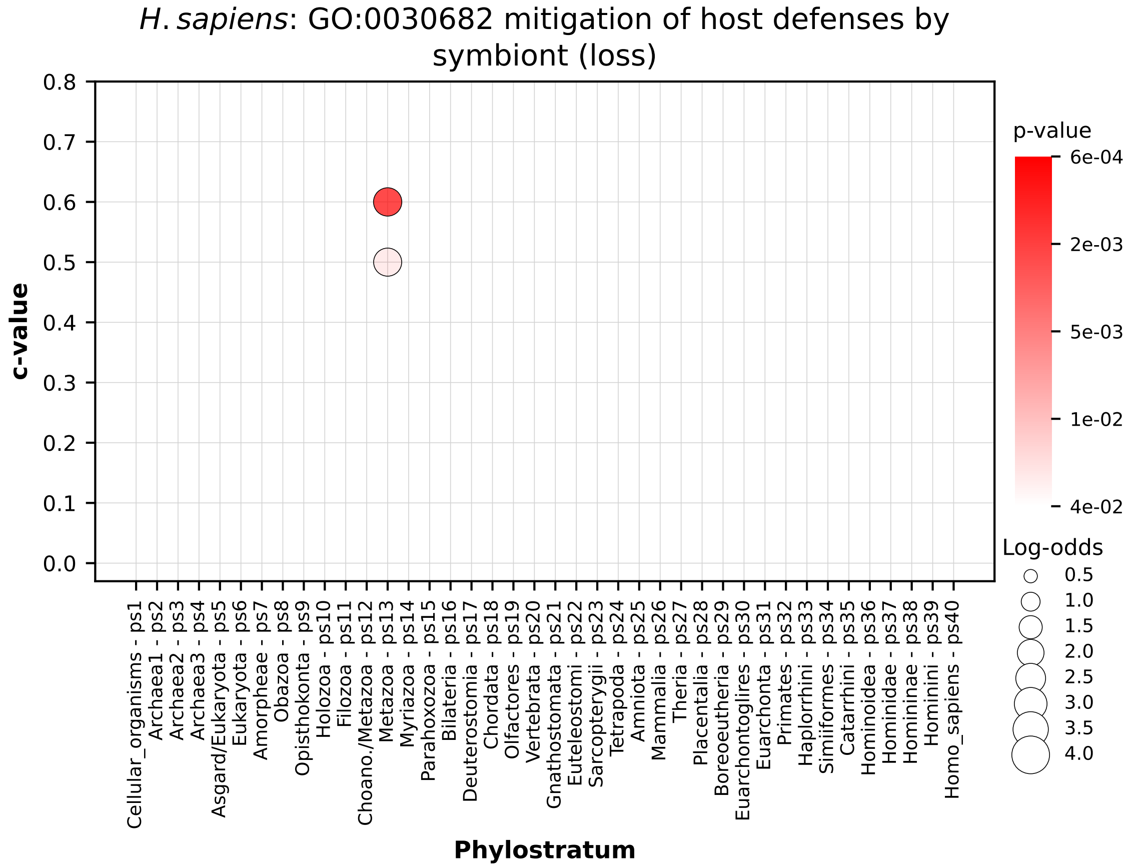
H. sapiens: GO:0030682 mitigation of host defenses by symbiont (loss)
c-value
0.8
0.7
0.6
0.5
0.4
0.3
0.2
0.1
0.0
p-value
6e-04
2e-03
5e-03
1e-02
4e-02
Log-odds
0.5
1.0
1.5
2.0
2.5
3.0
3.5
4.0
Cellular_organisms - ps1
Archaea1 - ps2
Archaea2 - ps3
Archaea3 - ps4
Asgard/Eukaryota - ps5
Eukaryota - ps6
Amorpheae - ps7
Obazoa - ps8
Opisthokonta - ps9
Holozoa - ps10
Filozoa - ps11
Choano./Metazoa - ps12
Metazoa - ps13
Myriazoa - ps14
Parahoxozoa - ps15
Bilateria - ps16
Deuterostomia - ps17
Chordata - ps18
Olfactores - ps19
Vertebrata - ps20
Gnathostomata - ps21
Euteleostomi - ps22
Sarcopterygii - ps23
Tetrapoda - ps24
Amniota - ps25
Mammalia - ps26
Theria - ps27
Placentalia - ps28
Boreoeutheria - ps29
Euarchontoglires - ps30
Euarchonta - ps31
Primates - ps32
Haplorrhini - ps33
Simiiformes - ps34
Catarrhini - ps35
Hominoidea - ps36
Hominidae - ps37
Homininae - ps38
Hominini - ps39
Homo_sapiens - ps40
Phylostratum

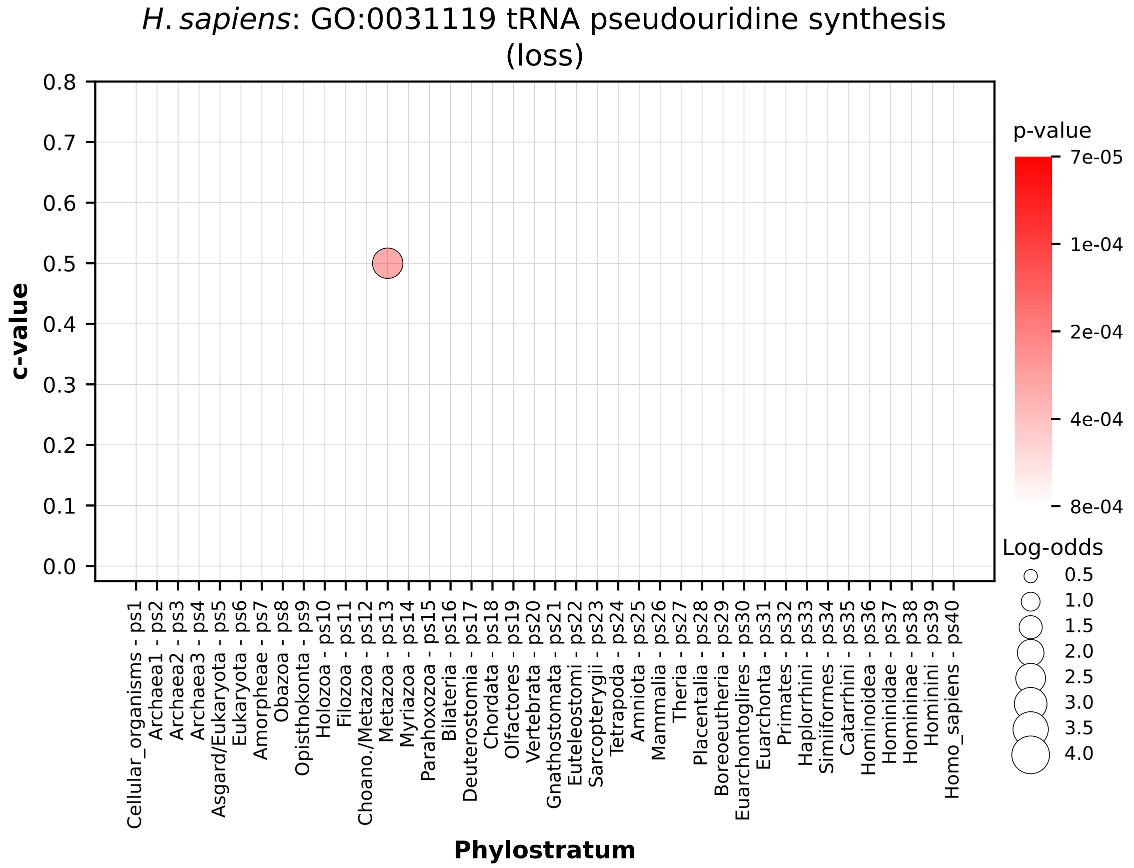

H. sapiens: GO:0031119 tRNA pseudouridine synthesis (loss)
c-value
0.8
0.7
0.6
0.5
0.4
0.3
0.2
0.1
0.0
Phylostratum
Cellular_organisms - ps1
Archaea1 - ps2
Archaea2 - ps3
Archaea3 - ps4
Asgard/Eukaryota - ps5
Eukaryota - ps6
Amorpheae - ps7
Obazoa - ps8
Opisthokonta - ps9
Holozoa - ps10
Filozoa - ps11
Choano./Metazoa - ps12
Metazoa - ps13
Myriazoa - ps14
Parahoxozoa - ps15
Bilateria - ps16
Deuterostomia - ps17
Chordata - ps18
Olfactores - ps19
Vertebrata - ps20
Gnathostomata - ps21
Euteleostomi - ps22
Sarcopterygii - ps23
Tetrapoda - ps24
Amniota - ps25
Mammalia - ps26
Theria - ps27
Placentalia - ps28
Boreoeutheria - ps29
Euarchontoglires - ps30
Euarchonta - ps31
Primates - ps32
Haplorrhini - ps33
Simiiformes - ps34
Catarrhini - ps35
Hominoidea - ps36
Hominidae - ps37
Homininae - ps38
Hominini - ps39
Homo_sapiens - ps40
p-value
7e-05
1e-04
2e-04
4e-04
8e-04
Log-odds
0.5
1.0
1.5
2.0
2.5
3.0
3.5
4.0

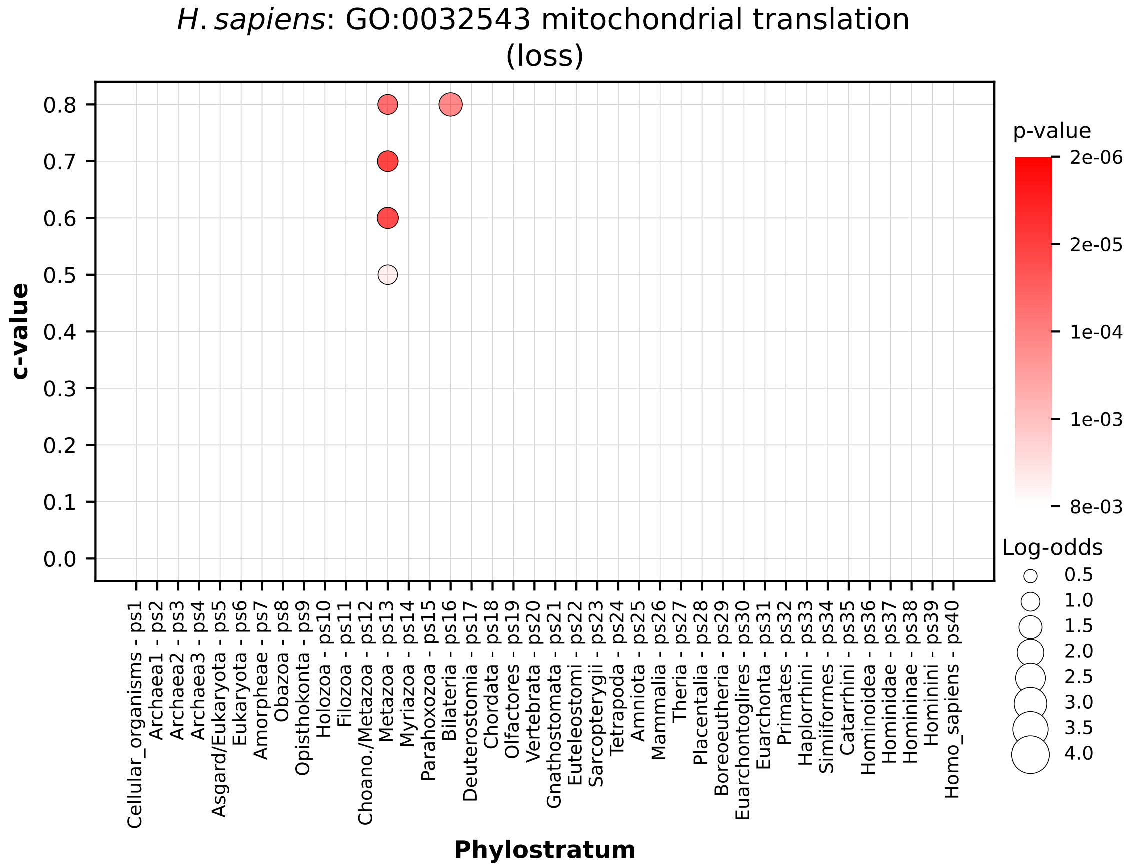

H. sapiens: GO:0032543 mitochondrial translation
(loss)
c-value
0.8
0.7
0.6
0.5
0.4
0.3
0.2
0.1
0.0
p-value
2e-06
2e-05
1e-04
1e-03
8e-03
Log-odds
0.5
1.0
1.5
2.0
2.5
3.0
3.5
4.0
Cellular_organisms - ps1
Archaea1 - ps2
Archaea2 - ps3
Archaea3 - ps4
Asgard/Eukaryota - ps5
Eukaryota - ps6
Amorpheae - ps7
Obazoa - ps8
Opisthokonta - ps9
Holozoa - ps10
Filozoa - ps11
Choano./Metazoa - ps12
Metazoa - ps13
Myriazoa - ps14
Parahoxozoa - ps15
Bilateria - ps16
Deuterostomia - ps17
Chordata - ps18
Olfactores - ps19
Vertebrata - ps20
Gnathostomata - ps21
Euteleostomi - ps22
Sarcopterygii - ps23
Tetrapoda - ps24
Amniota - ps25
Mammalia - ps26
Theria - ps27
Placentalia - ps28
Boreoeutheria - ps29
Euarchontoglires - ps30
Euarchonta - ps31
Primates - ps32
Haplorrhini - ps33
Simiiformes - ps34
Catarrhini - ps35
Hominoidea - ps36
Hominidae - ps37
Homininae - ps38
Hominini - ps39
Homo_sapiens - ps40
Phylostratum

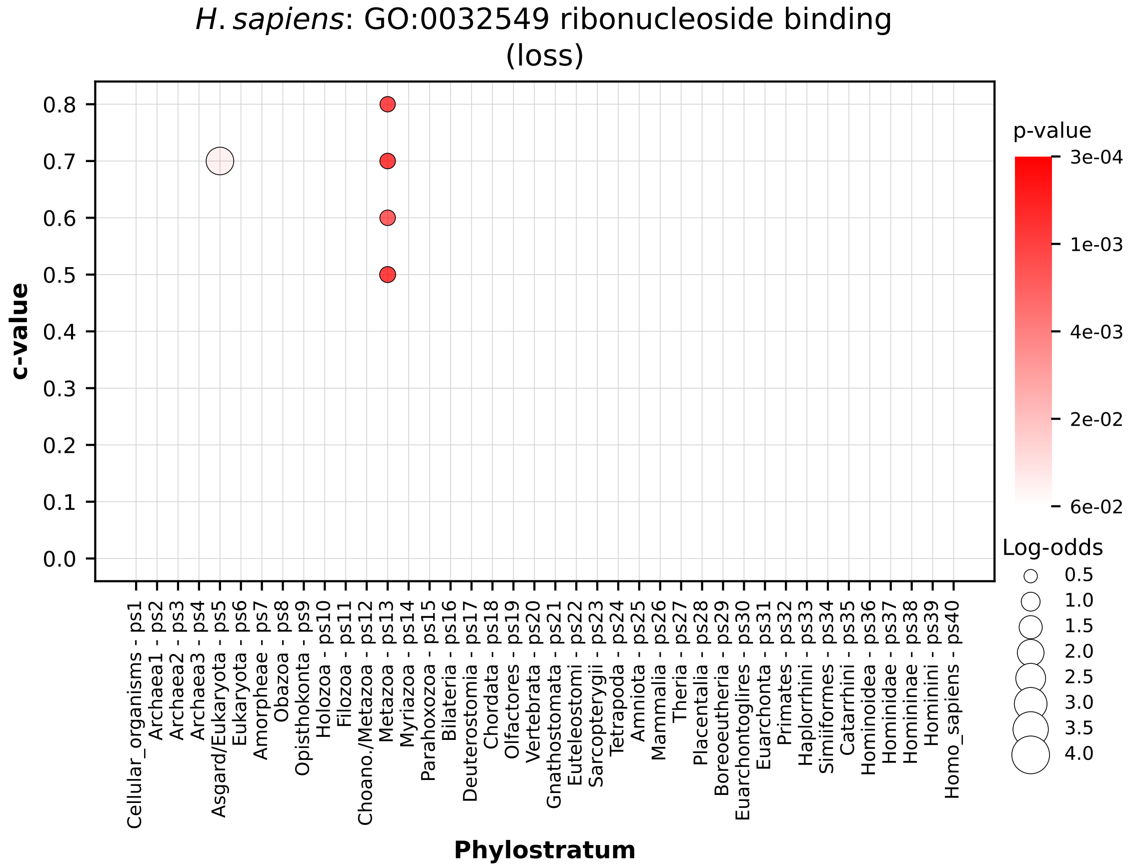

H. sapiens: GO:0032549 ribonucleoside binding (loss)
c-value
0.8
0.7
0.6
0.5
0.4
0.3
0.2
0.1
0.0
p-value
3e-04
1e-03
4e-03
2e-02
6e-02
Log-odds
0.5
1.0
1.5
2.0
2.5
3.0
3.5
4.0
Cellular_organisms - ps1
Archaea1 - ps2
Archaea2 - ps3
Archaea3 - ps4
Asgard/Eukaryota - ps5
Eukaryota - ps6
Amorpheae - ps7
Obazoa - ps8
Opisthokonta - ps9
Holozoa - ps10
Filozoa - ps11
Choano./Metazoa - ps12
Metazoa - ps13
Myriazoa - ps14
Parahoxozoa - ps15
Bilateria - ps16
Deuterostomia - ps17
Chordata - ps18
Olfactores - ps19
Vertebrata - ps20
Gnathostomata - ps21
Euteleostomi - ps22
Sarcopterygii - ps23
Tetrapoda - ps24
Amniota - ps25
Mammalia - ps26
Theria - ps27
Placentalia - ps28
Boreoeutheria - ps29
Euarchontoglires - ps30
Euarchonta - ps31
Primates - ps32
Haplorrhini - ps33
Simiiformes - ps34
Catarrhini - ps35
Hominoidea - ps36
Hominidae - ps37
Homininae - ps38
Hominini - ps39
Homo_sapiens - ps40
Phylostratum

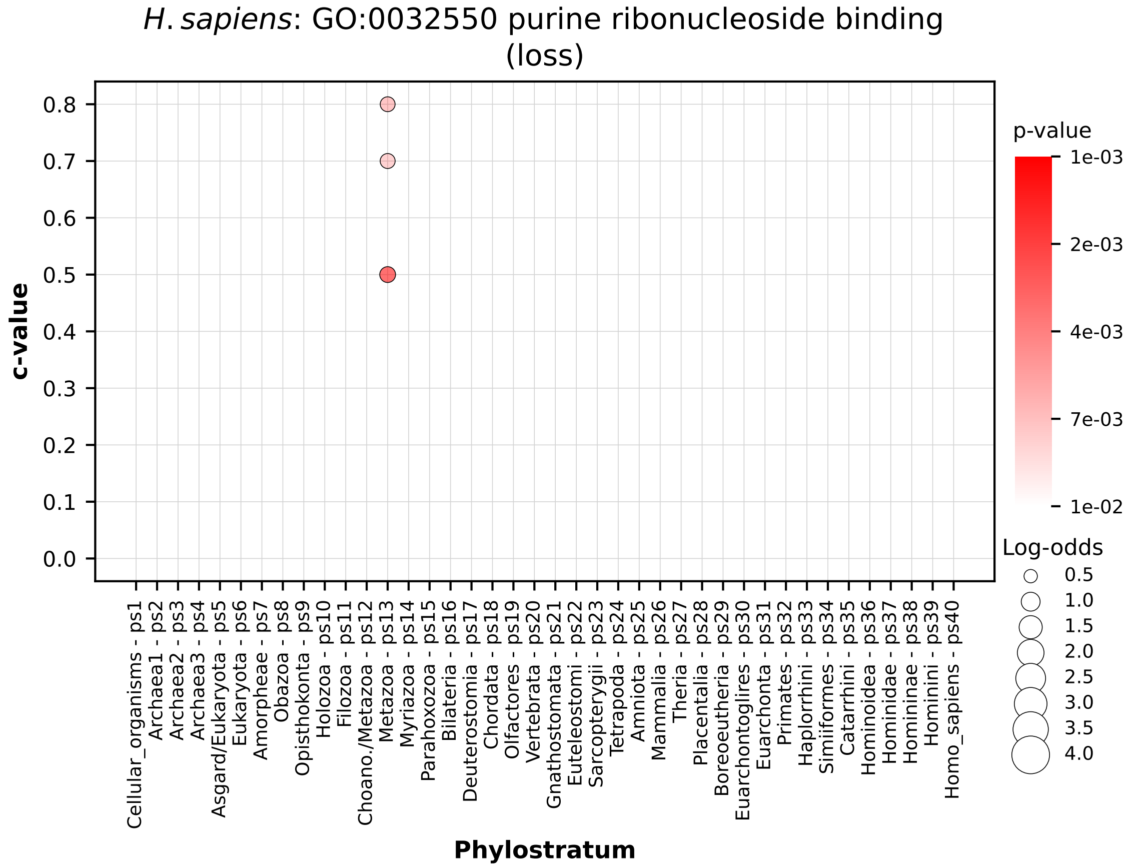

H. sapiens: GO:0032550 purine ribonucleoside binding
(loss)
c-value
0.8
0.7
0.6
0.5
0.4
0.3
0.2
0.1
0.0
Phylostratum
Cellular_organisms - ps1
Archaea1 - ps2
Archaea2 - ps3
Archaea3 - ps4
Asgard/Eukaryota - ps5
Eukaryota - ps6
Amorpheae - ps7
Obazoa - ps8
Opisthokonta - ps9
Holozoa - ps10
Filozoa - ps11
Choano./Metazoa - ps12
Metazoa - ps13
Myriazoa - ps14
Parahoxozoa - ps15
Bilateria - ps16
Deuterostomia - ps17
Chordata - ps18
Olfactores - ps19
Vertebrata - ps20
Gnathostomata - ps21
Euteleostomi - ps22
Sarcopterygii - ps23
Tetrapoda - ps24
Amniota - ps25
Mammalia - ps26
Theria - ps27
Placentalia - ps28
Boreoeutheria - ps29
Euarchontoglires - ps30
Euarchonta - ps31
Primates - ps32
Haplorrhini - ps33
Simiiformes - ps34
Catarrhini - ps35
Hominoidea - ps36
Hominidae - ps37
Homininae - ps38
Hominini - ps39
Homo_sapiens - ps40
p-value
1e-03
2e-03
4e-03
7e-03
1e-02
Log-odds
0.5
1.0
1.5
2.0
2.5
3.0
3.5
4.0

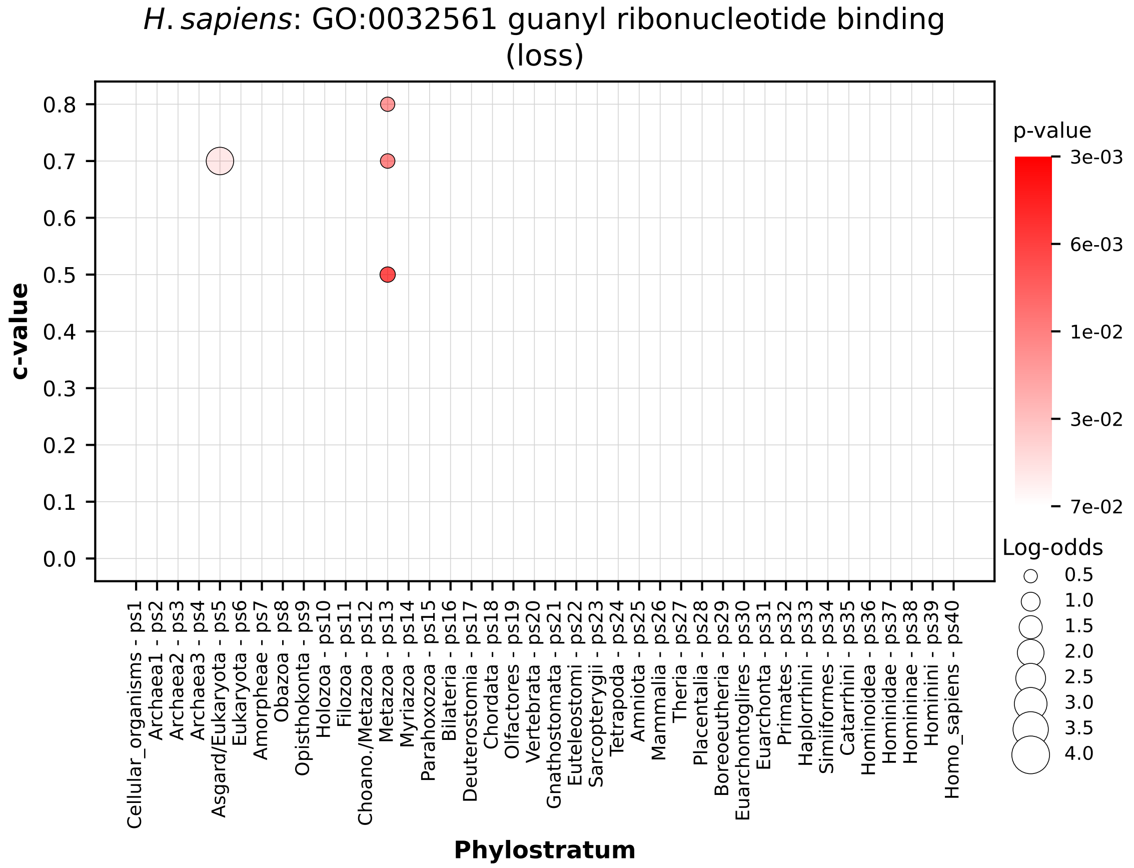

H. sapiens: GO:0032561 guanyl ribonucleotide binding
(loss)
c-value
0.0
0.1
0.2
0.3
0.4
0.5
0.6
0.7
0.8
Phylostratum
Cellular_organisms - ps1
Archaea1 - ps2
Archaea2 - ps3
Archaea3 - ps4
Asgard/Eukaryota - ps5
Eukaryota - ps6
Amorpheae - ps7
Obazoa - ps8
Opisthokonta - ps9
Holozoa - ps10
Filozoa - ps11
Choano./Metazoa - ps12
Metazoa - ps13
Myriazoa - ps14
Parahoxozoa - ps15
Bilateria - ps16
Deuterostomia - ps17
Chordata - ps18
Olfactores - ps19
Vertebrata - ps20
Gnathostomata - ps21
Euteleostomi - ps22
Sarcopterygii - ps23
Tetrapoda - ps24
Amniota - ps25
Mammalia - ps26
Theria - ps27
Placentalia - ps28
Boreoeutheria - ps29
Euarchontoglires - ps30
Euarchonta - ps31
Primates - ps32
Haplorrhini - ps33
Simiiformes - ps34
Catarrhini - ps35
Hominoidea - ps36
Hominidae - ps37
Homininae - ps38
Hominini - ps39
Homo_sapiens - ps40
p-value
3e-03
6e-03
1e-02
3e-02
7e-02
Log-odds
0.5
1.0
1.5
2.0
2.5
3.0
3.5
4.0

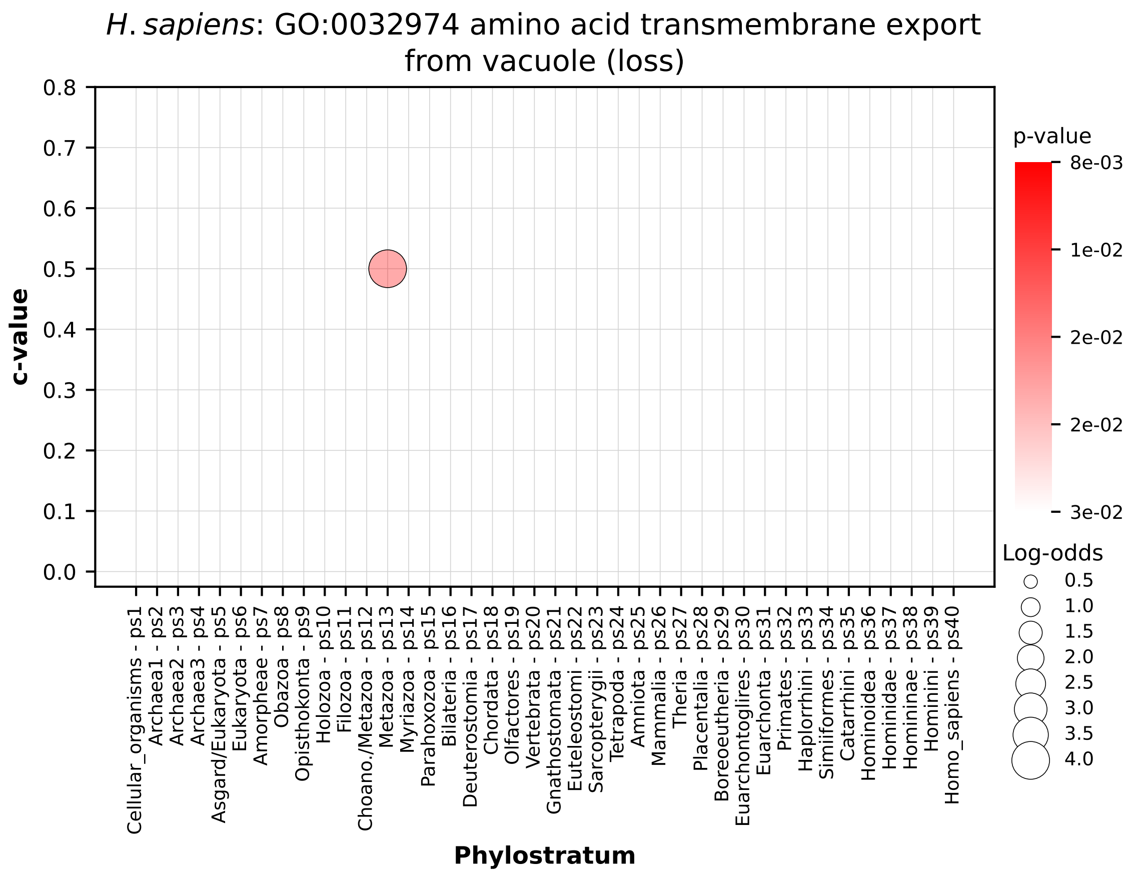

H. sapiens: GO:0032974 amino acid transmembrane export from vacuole (loss)
c-value
0.8
0.7
0.6
0.5
0.4
0.3
0.2
0.1
0.0
Phylostratum
Cellular_organisms - ps1
Archaea1 - ps2
Archaea2 - ps3
Archaea3 - ps4
Asgard/Eukaryota - ps5
Eukaryota - ps6
Amorpheae - ps7
Obazoa - ps8
Opisthokonta - ps9
Holozoa - ps10
Filozoa - ps11
Choano./Metazoa - ps12
Metazoa - ps13
Myriazoa - ps14
Parahoxozoa - ps15
Bilateria - ps16
Deuterostomia - ps17
Chordata - ps18
Olfactores - ps19
Vertebrata - ps20
Gnathostomata - ps21
Euteleostomi - ps22
Sarcopterygii - ps23
Tetrapoda - ps24
Amniota - ps25
Mammalia - ps26
Theria - ps27
Placentalia - ps28
Boreoeutheria - ps29
Euarchontoglires - ps30
Euarchonta - ps31
Primates - ps32
Haplorrhini - ps33
Simiiformes - ps34
Catarrhini - ps35
Hominoidea - ps36
Hominidae - ps37
Homininae - ps38
Hominini - ps39
Homo_sapiens - ps40
p-value
8e-03
1e-02
2e-02
2e-02
3e-02
Log-odds
0.5
1.0
1.5
2.0
2.5
3.0
3.5
4.0

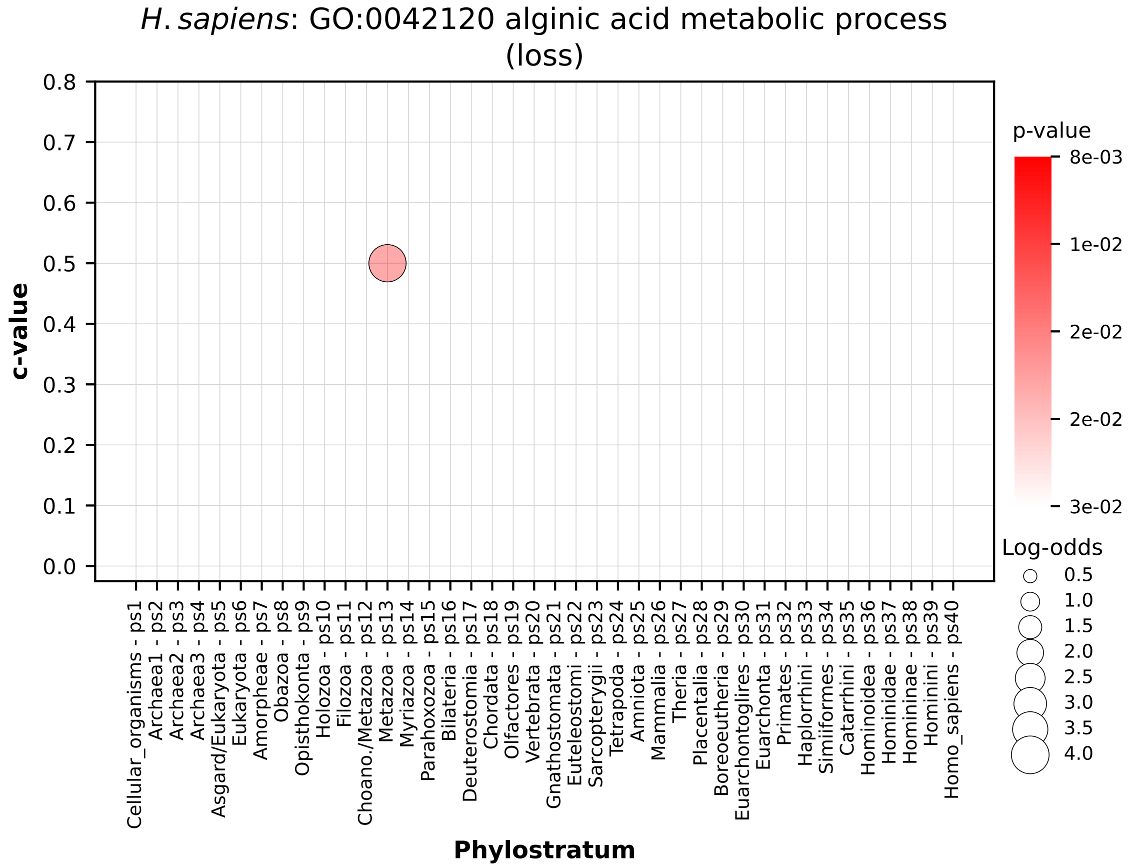

H. sapiens: GO:0042120 alginic acid metabolic process (loss)
c-value
0.8
0.7
0.6
0.5
0.4
0.3
0.2
0.1
0.0
Phylostratum
Cellular_organisms - ps1
Archaea1 - ps2
Archaea2 - ps3
Archaea3 - ps4
Asgard/Eukaryota - ps5
Eukaryota - ps6
Amorpheae - ps7
Obazoa - ps8
Opisthokonta - ps9
Holozoa - ps10
Filozoa - ps11
Choano./Metazoa - ps12
Metazoa - ps13
Myriazoa - ps14
Parahoxozoa - ps15
Bilateria - ps16
Deuterostomia - ps17
Chordata - ps18
Olfactores - ps19
Vertebrata - ps20
Gnathostomata - ps21
Euteleostomi - ps22
Sarcopterygii - ps23
Tetrapoda - ps24
Amniota - ps25
Mammalia - ps26
Theria - ps27
Placentalia - ps28
Boreoeutheria - ps29
Euarchontoglires - ps30
Euarchonta - ps31
Primates - ps32
Haplorrhini - ps33
Simiiformes - ps34
Catarrhini - ps35
Hominoidea - ps36
Hominidae - ps37
Homininae - ps38
Hominini - ps39
Homo_sapiens - ps40
p-value
8e-03
1e-02
2e-02
2e-02
3e-02
Log-odds
0.5
1.0
1.5
2.0
2.5
3.0
3.5
4.0

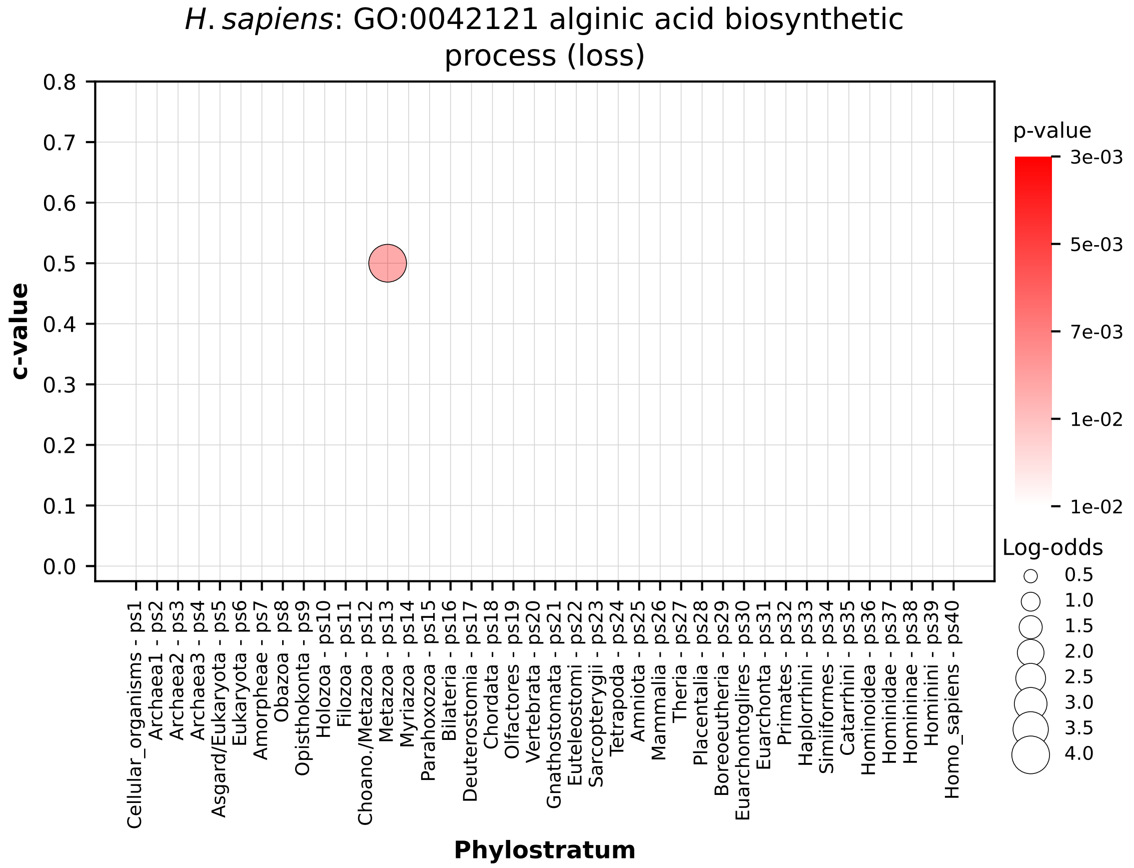

H. sapiens: GO:0042121 alginic acid biosynthetic process (loss)
c-value
0.8
0.7
0.6
0.5
0.4
0.3
0.2
0.1
0.0
p-value
3e-03
5e-03
7e-03
1e-02
1e-02
Log-odds
0.5
1.0
1.5
2.0
2.5
3.0
3.5
4.0
Cellular_organisms - ps1
Archaea1 - ps2
Archaea2 - ps3
Archaea3 - ps4
Asgard/Eukaryota - ps5
Eukaryota - ps6
Amorpheae - ps7
Obazoa - ps8
Opisthokonta - ps9
Holozoa - ps10
Filozoa - ps11
Choano./Metazoa - ps12
Metazoa - ps13
Myriazoa - ps14
Parahoxozoa - ps15
Bilateria - ps16
Deuterostomia - ps17
Chordata - ps18
Olfactores - ps19
Vertebrata - ps20
Gnathostomata - ps21
Euteleostomi - ps22
Sarcopterygii - ps23
Tetrapoda - ps24
Amniota - ps25
Mammalia - ps26
Theria - ps27
Placentalia - ps28
Boreoeutheria - ps29
Euarchontoglires - ps30
Euarchonta - ps31
Primates - ps32
Haplorrhini - ps33
Simiiformes - ps34
Catarrhini - ps35
Hominoidea - ps36
Hominidae - ps37
Homininae - ps38
Hominini - ps39
Homo_sapiens - ps40
Phylostratum

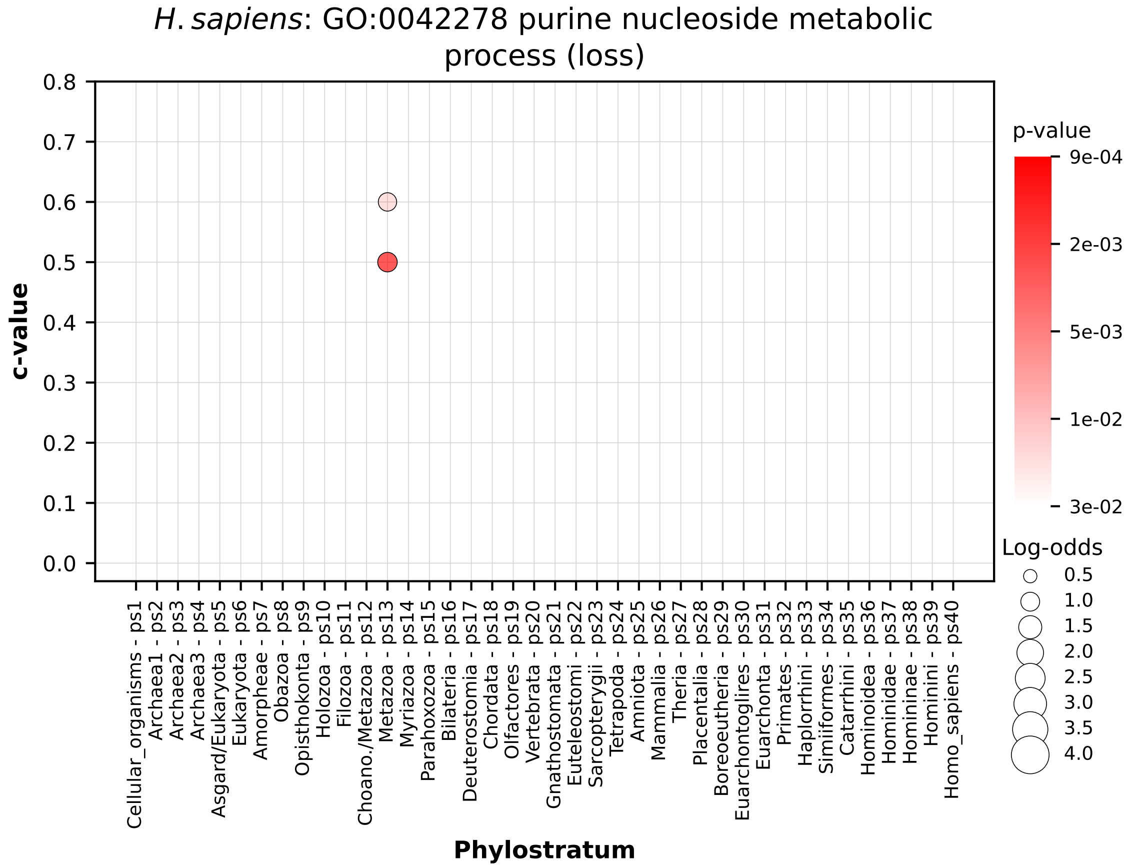

H. sapiens: GO:0042278 purine nucleoside metabolic process (loss)
c-value
0.8
0.7
0.6
0.5
0.4
0.3
0.2
0.1
0.0
p-value
9e-04
2e-03
5e-03
1e-02
3e-02
Log-odds
0.5
1.0
1.5
2.0
2.5
3.0
3.5
4.0
Cellular_organisms - ps1
Archaea1 - ps2
Archaea2 - ps3
Archaea3 - ps4
Asgard/Eukaryota - ps5
Eukaryota - ps6
Amorpheae - ps7
Obazoa - ps8
Opisthokonta - ps9
Holozoa - ps10
Filozoa - ps11
Choano./Metazoa - ps12
Metazoa - ps13
Myriazoa - ps14
Parahoxozoa - ps15
Bilateria - ps16
Deuterostomia - ps17
Chordata - ps18
Olfactores - ps19
Vertebrata - ps20
Gnathostomata - ps21
Euteleostomi - ps22
Sarcopterygii - ps23
Tetrapoda - ps24
Amniota - ps25
Mammalia - ps26
Theria - ps27
Placentalia - ps28
Boreoeutheria - ps29
Euarchontoglires - ps30
Euarchonta - ps31
Primates - ps32
Haplorrhini - ps33
Simiiformes - ps34
Catarrhini - ps35
Hominoidea - ps36
Hominidae - ps37
Homininae - ps38
Hominini - ps39
Homo_sapiens - ps40
Phylostratum

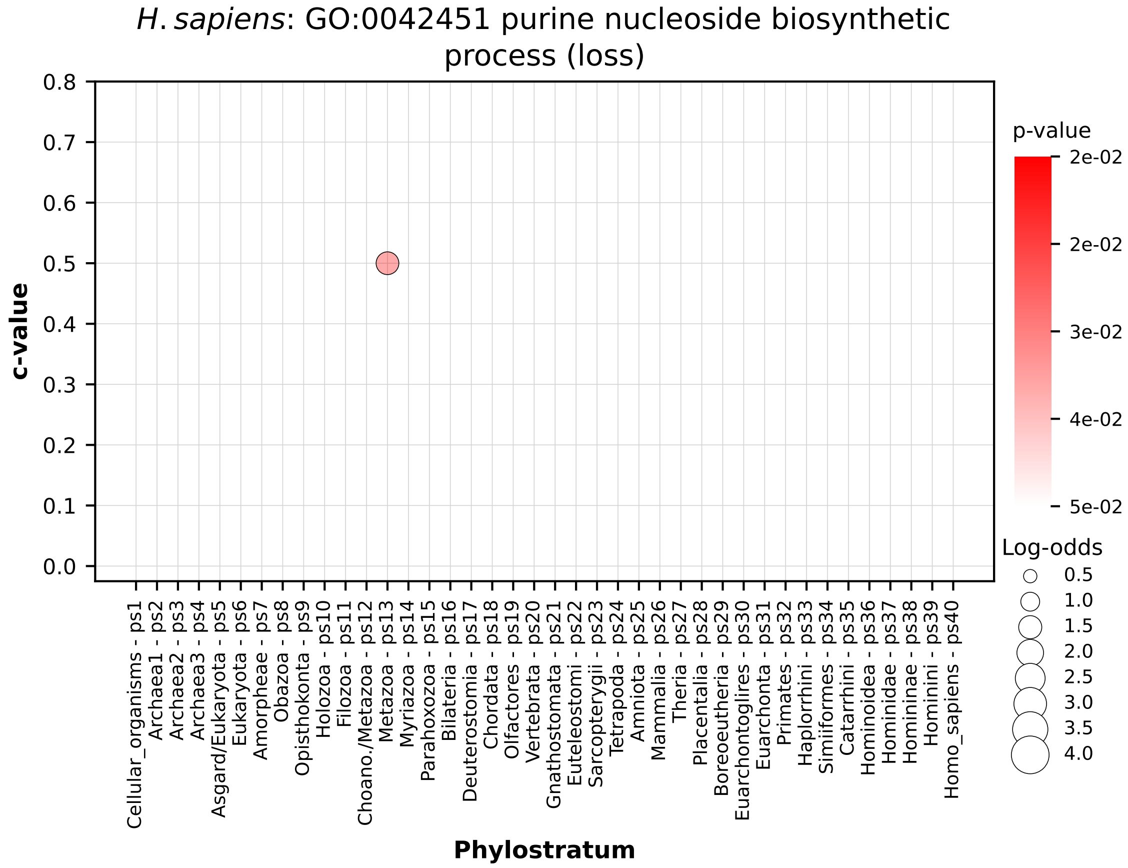

H. sapiens: GO:0042451 purine nucleoside biosynthetic process (loss)
c-value
0.8
0.7
0.6
0.5
0.4
0.3
0.2
0.1
0.0
Phylostratum
Cellular_organisms - ps1
Archaea1 - ps2
Archaea2 - ps3
Archaea3 - ps4
Asgard/Eukaryota - ps5
Eukaryota - ps6
Amorpheae - ps7
Obazoa - ps8
Opisthokonta - ps9
Holozoa - ps10
Filozoa - ps11
Choano./Metazoa - ps12
Metazoa - ps13
Myriazoa - ps14
Parahoxozoa - ps15
Bilateria - ps16
Deuterostomia - ps17
Chordata - ps18
Olfactores - ps19
Vertebrata - ps20
Gnathostomata - ps21
Euteleostomi - ps22
Sarcopterygii - ps23
Tetrapoda - ps24
Amniota - ps25
Mammalia - ps26
Theria - ps27
Placentalia - ps28
Boreoeutheria - ps29
Euarchontoglires - ps30
Euarchonta - ps31
Primates - ps32
Haplorrhini - ps33
Simiiformes - ps34
Catarrhini - ps35
Hominoidea - ps36
Hominidae - ps37
Homininae - ps38
Hominini - ps39
Homo_sapiens - ps40
p-value
2e-02
2e-02
3e-02
4e-02
5e-02
Log-odds
0.5
1.0
1.5
2.0
2.5
3.0
3.5
4.0

H. sapiens: GO:0042455 ribonucleoside biosynthetic process (loss)

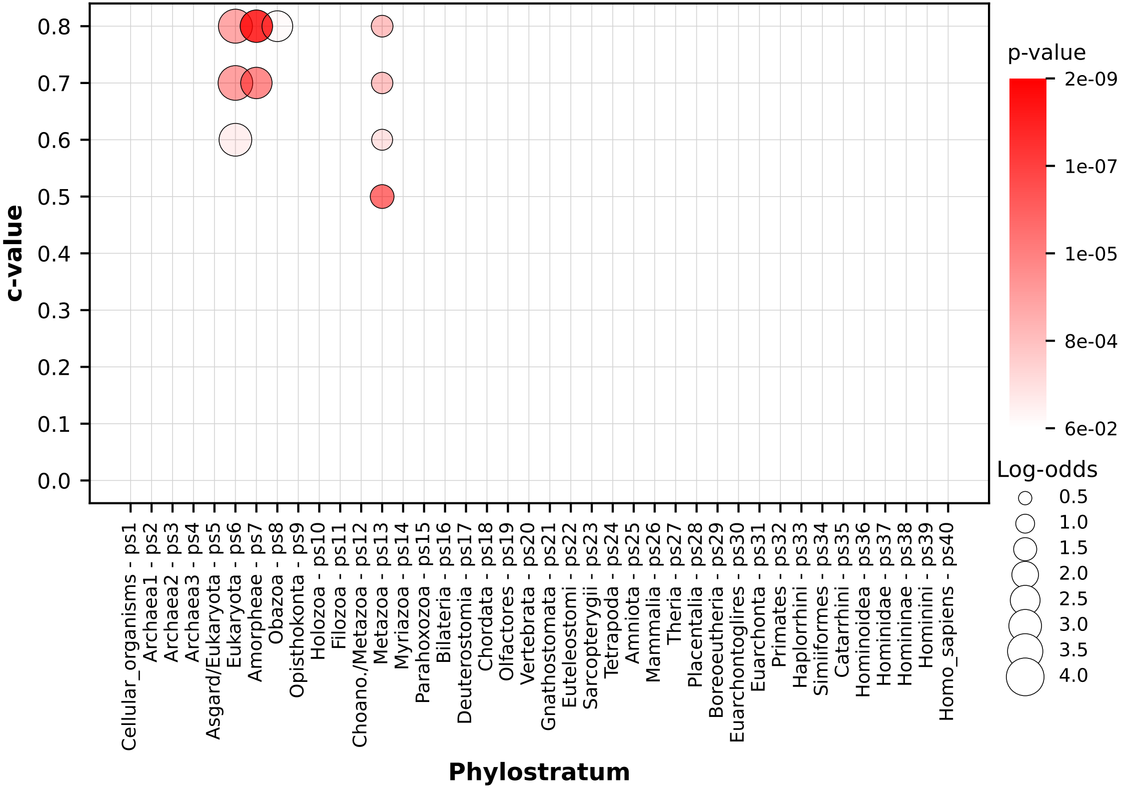

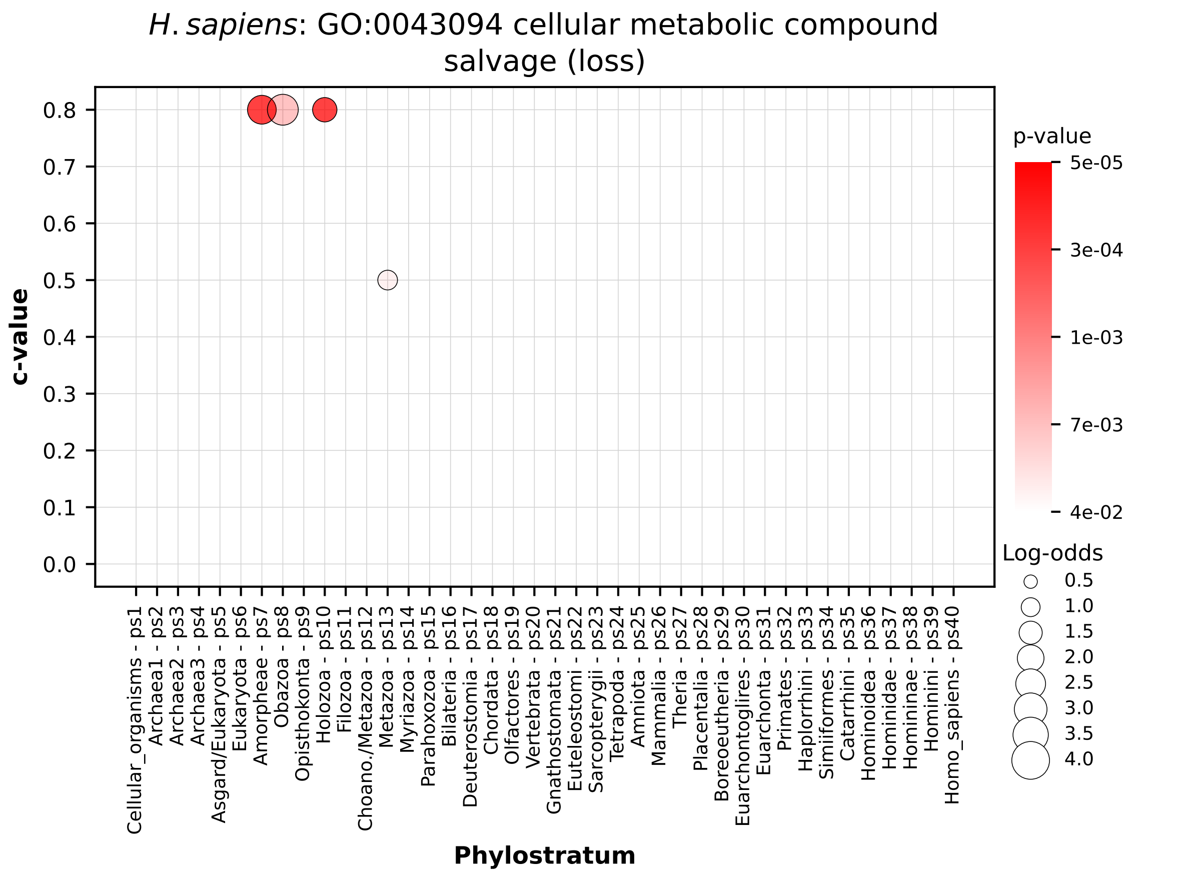

H. sapiens: GO:0043094 cellular metabolic compound salvage (loss)
c-value
0.0
0.1
0.2
0.3
0.4
0.5
0.6
0.7
0.8
Phylostratum
Cellular_organisms - ps1
Archaea1 - ps2
Archaea2 - ps3
Archaea3 - ps4
Asgard/Eukaryota - ps5
Eukaryota - ps6
Amorpheae - ps7
Obazoa - ps8
Opisthokonta - ps9
Holozoa - ps10
Filozoa - ps11
Choano./Metazoa - ps12
Metazoa - ps13
Myriazoa - ps14
Parahoxozoa - ps15
Bilateria - ps16
Deuterostomia - ps17
Chordata - ps18
Olfactores - ps19
Vertebrata - ps20
Gnathostomata - ps21
Euteleostomi - ps22
Sarcopterygii - ps23
Tetrapoda - ps24
Amniota - ps25
Mammalia - ps26
Theria - ps27
Placentalia - ps28
Boreoeutheria - ps29
Euarchontoglires - ps30
Euarchonta - ps31
Primates - ps32
Haplorrhini - ps33
Simiiformes - ps34
Catarrhini - ps35
Hominoidea - ps36
Hominidae - ps37
Homininae - ps38
Hominini - ps39
Homo_sapiens - ps40
p-value
5e-05
3e-04
1e-03
7e-03
4e-02
Log-odds
0.5
1.0
1.5
2.0
2.5
3.0
3.5
4.0

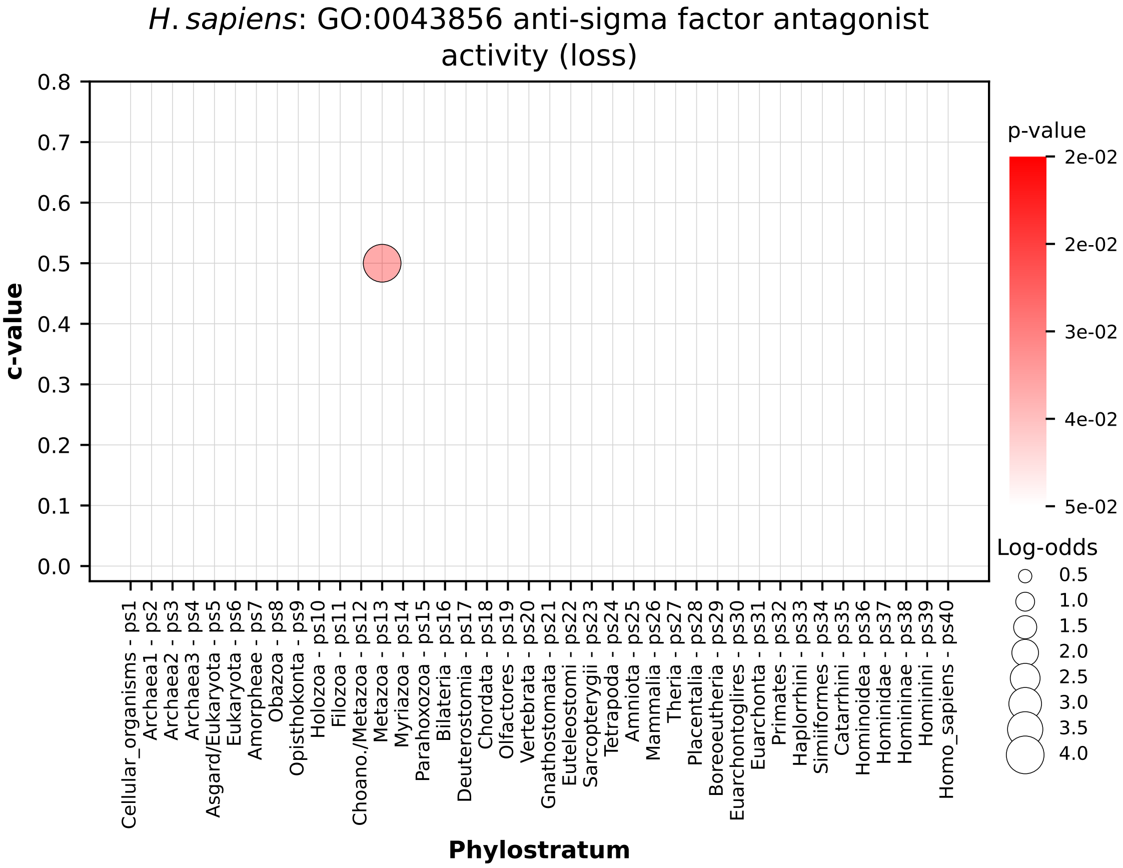

H. sapiens: GO:0043856 anti-sigma factor antagonist activity (loss)
c-value
0.8
0.7
0.6
0.5
0.4
0.3
0.2
0.1
0.0
Phylostratum
Cellular_organisms - ps1
Archaea1 - ps2
Archaea2 - ps3
Archaea3 - ps4
Asgard/Eukaryota - ps5
Eukaryota - ps6
Amorpheae - ps7
Obazoa - ps8
Opisthokonta - ps9
Holozoa - ps10
Filozoa - ps11
Choano./Metazoa - ps12
Metazoa - ps13
Myriazoa - ps14
Parahoxozoa - ps15
Bilateria - ps16
Deuterostomia - ps17
Chordata - ps18
Olfactores - ps19
Vertebrata - ps20
Gnathostomata - ps21
Euteleostomi - ps22
Sarcopterygii - ps23
Tetrapoda - ps24
Amniota - ps25
Mammalia - ps26
Theria - ps27
Placentalia - ps28
Boreoeutheria - ps29
Euarchontoglires - ps30
Euarchonta - ps31
Primates - ps32
Haplorrhini - ps33
Simiiformes - ps34
Catarrhini - ps35
Hominoidea - ps36
Hominidae - ps37
Homininae - ps38
Hominini - ps39
Homo_sapiens - ps40
p-value
2e-02
2e-02
3e-02
4e-02
5e-02
Log-odds
0.5
1.0
1.5
2.0
2.5
3.0
3.5
4.0

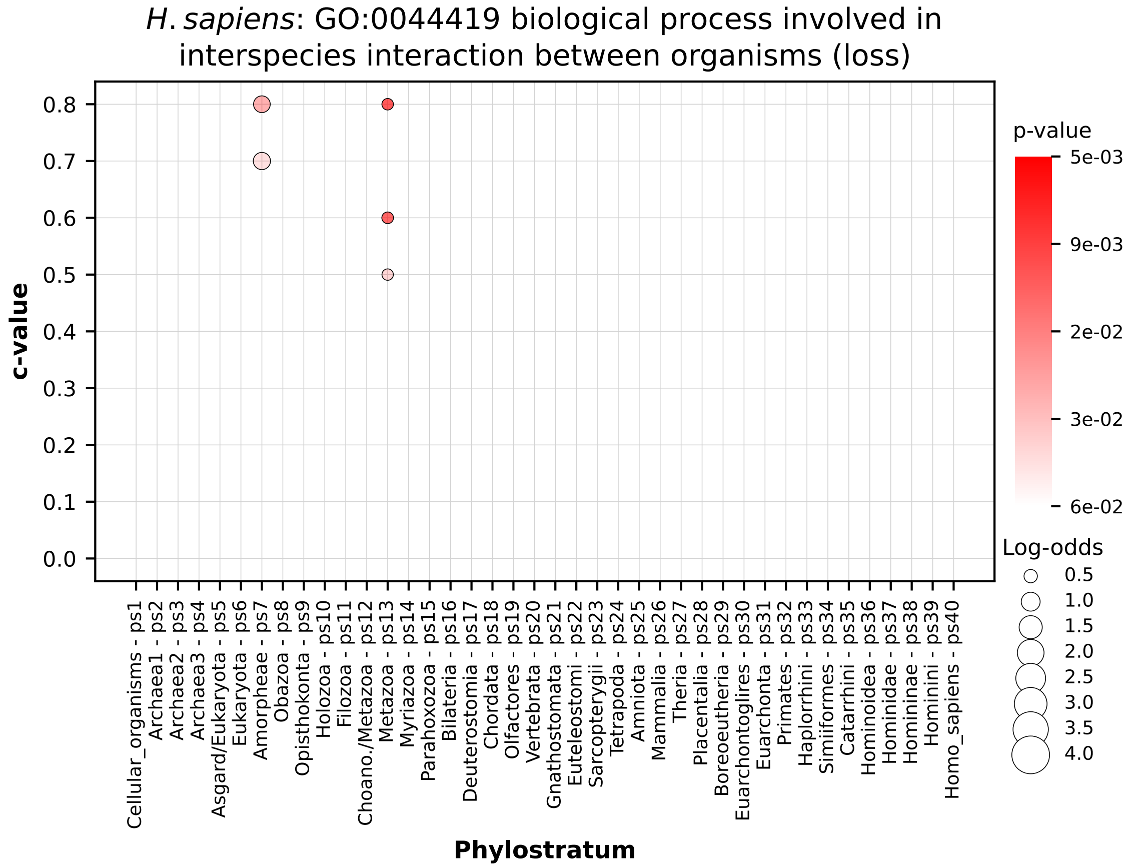
H. sapiens: GO:0044419 biological process involved in interspecies interaction between organisms (loss)
c-value
0.0
0.1
0.2
0.3
0.4
0.5
0.6
0.7
0.8
Phylostratum
Cellular_organisms - ps1
Archaea1 - ps2
Archaea2 - ps3
Archaea3 - ps4
Asgard/Eukaryota - ps5
Eukaryota - ps6
Amorpheae - ps7
Obazoa - ps8
Opisthokonta - ps9
Holozoa - ps10
Filozoa - ps11
Choano./Metazoa - ps12
Metazoa - ps13
Myriazoa - ps14
Parahoxozoa - ps15
Bilateria - ps16
Deuterostomia - ps17
Chordata - ps18
Olfactores - ps19
Vertebrata - ps20
Gnathostomata - ps21
Euteleostomi - ps22
Sarcopterygii - ps23
Tetrapoda - ps24
Amniota - ps25
Mammalia - ps26
Theria - ps27
Placentalia - ps28
Boreoeutheria - ps29
Euarchontoglires - ps30
Euarchonta - ps31
Primates - ps32
Haplorrhini - ps33
Simiiformes - ps34
Catarrhini - ps35
Hominoidea - ps36
Hominidae - ps37
Homininae - ps38
Hominini - ps39
Homo_sapiens - ps40
p-value
5e-03
9e-03
2e-02
3e-02
6e-02
Log-odds
0.5
1.0
1.5
2.0
2.5
3.0
3.5
4.0

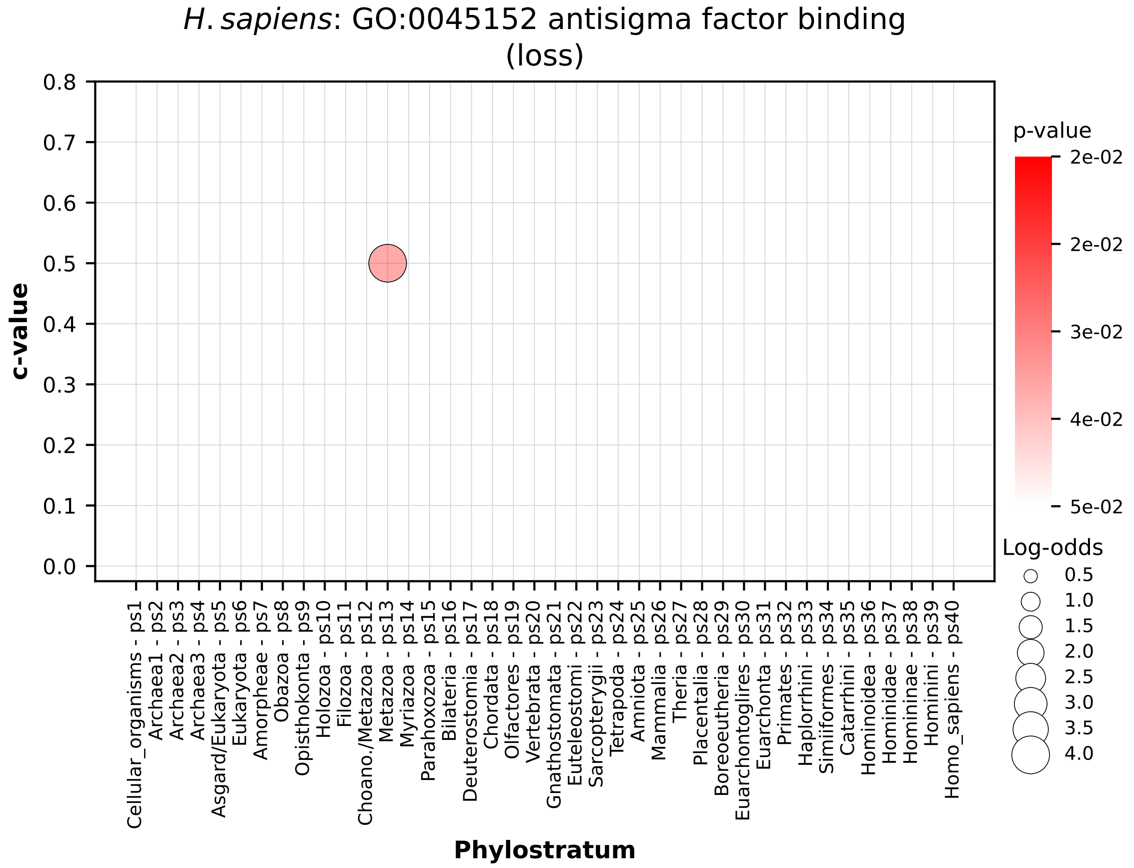

H. sapiens: GO:0045152 antisigma factor binding (loss)
c-value
0.8
0.7
0.6
0.5
0.4
0.3
0.2
0.1
0.0
p-value
2e-02
2e-02
3e-02
4e-02
5e-02
Log-odds
0.5
1.0
1.5
2.0
2.5
3.0
3.5
4.0
Cellular_organisms - ps1
Archaea1 - ps2
Archaea2 - ps3
Archaea3 - ps4
Asgard/Eukaryota - ps5
Eukaryota - ps6
Amorpheae - ps7
Obazoa - ps8
Opisthokonta - ps9
Holozoa - ps10
Filozoa - ps11
Choano./Metazoa - ps12
Metazoa - ps13
Myriazoa - ps14
Parahoxozoa - ps15
Bilateria - ps16
Deuterostomia - ps17
Chordata - ps18
Olfactores - ps19
Vertebrata - ps20
Gnathostomata - ps21
Euteleostomi - ps22
Sarcopterygii - ps23
Tetrapoda - ps24
Amniota - ps25
Mammalia - ps26
Theria - ps27
Placentalia - ps28
Boreoeutheria - ps29
Euarchontoglires - ps30
Euarchonta - ps31
Primates - ps32
Haplorrhini - ps33
Simiiformes - ps34
Catarrhini - ps35
Hominoidea - ps36
Hominidae - ps37
Homininae - ps38
Hominini - ps39
Homo_sapiens - ps40
Phylostratum

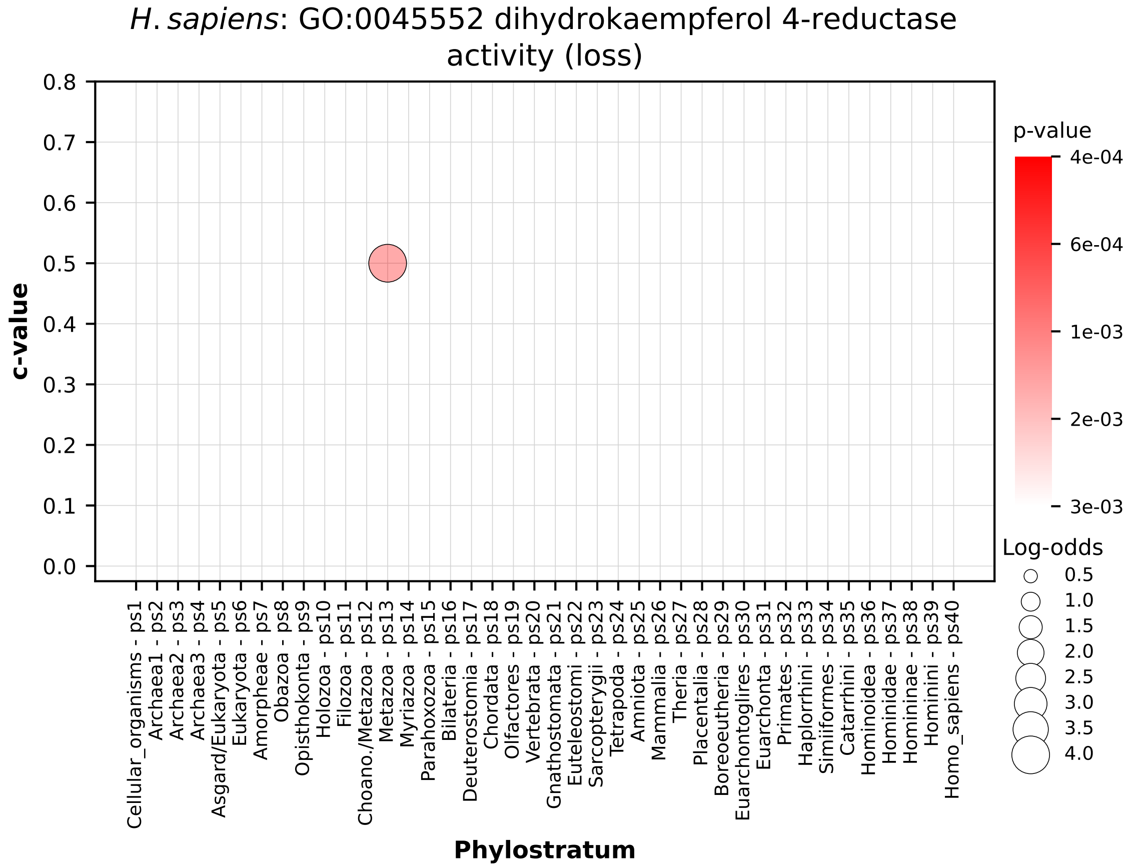

H. sapiens: GO:0045552 dihydrokaempferol 4-reductase activity (loss)
c-value
0.8
0.7
0.6
0.5
0.4
0.3
0.2
0.1
0.0
p-value
4e-04
6e-04
1e-03
2e-03
3e-03
Log-odds
0.5
1.0
1.5
2.0
2.5
3.0
3.5
4.0
Cellular_organisms - ps1
Archaea1 - ps2
Archaea2 - ps3
Archaea3 - ps4
Asgard/Eukaryota - ps5
Eukaryota - ps6
Amorpheae - ps7
Obazoa - ps8
Opisthokonta - ps9
Holozoa - ps10
Filozoa - ps11
Choano./Metazoa - ps12
Metazoa - ps13
Myriazoa - ps14
Parahoxozoa - ps15
Bilateria - ps16
Deuterostomia - ps17
Chordata - ps18
Olfactores - ps19
Vertebrata - ps20
Gnathostomata - ps21
Euteleostomi - ps22
Sarcopterygii - ps23
Tetrapoda - ps24
Amniota - ps25
Mammalia - ps26
Theria - ps27
Placentalia - ps28
Boreoeutheria - ps29
Euarchontoglires - ps30
Euarchonta - ps31
Primates - ps32
Haplorrhini - ps33
Simiiformes - ps34
Catarrhini - ps35
Hominoidea - ps36
Hominidae - ps37
Homininae - ps38
Hominini - ps39
Homo_sapiens - ps40
Phylostratum

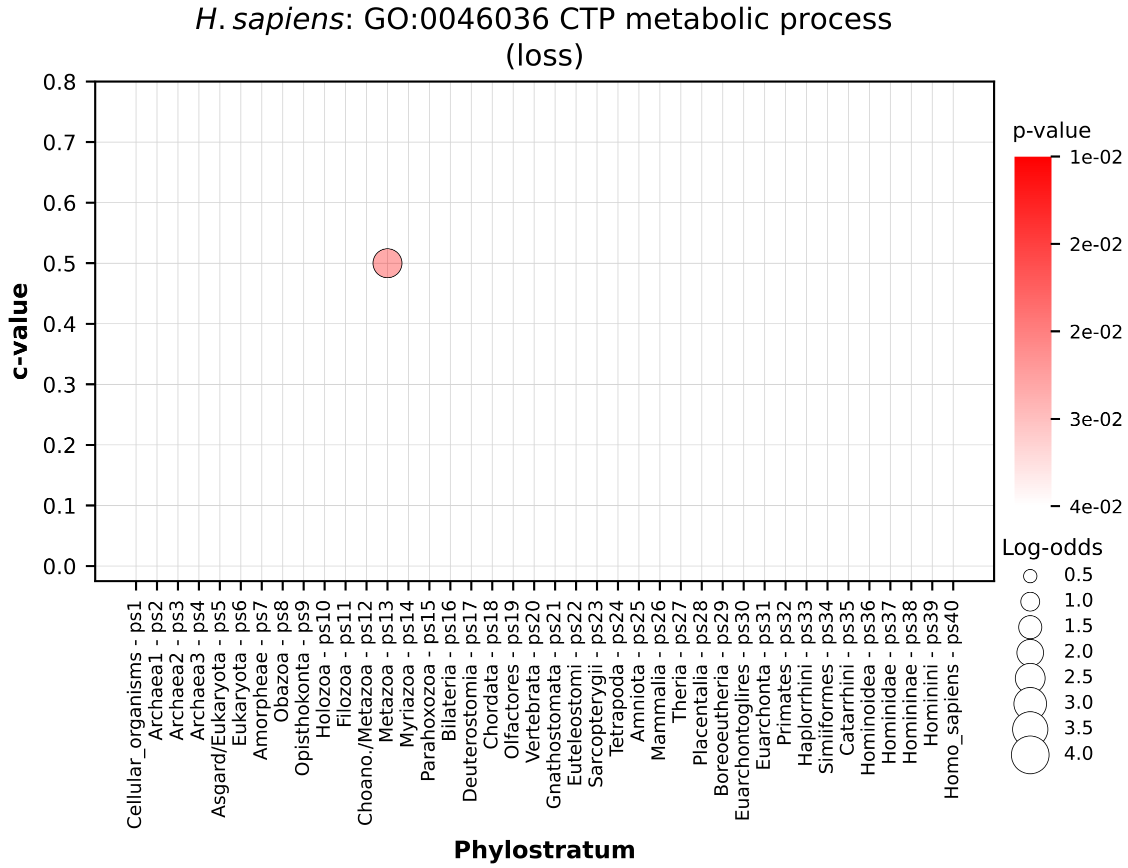

H. sapiens: GO:0046036 CTP metabolic process
(loss)
c-value
0.8
0.7
0.6
0.5
0.4
0.3
0.2
0.1
0.0
p-value
1e-02
2e-02
2e-02
3e-02
4e-02
Log-odds
0.5
1.0
1.5
2.0
2.5
3.0
3.5
4.0
Cellular_organisms - ps1
Archaea1 - ps2
Archaea2 - ps3
Archaea3 - ps4
Asgard/Eukaryota - ps5
Eukaryota - ps6
Amorpheae - ps7
Obazoa - ps8
Opisthokonta - ps9
Holozoa - ps10
Filozoa - ps11
Choano./Metazoa - ps12
Metazoa - ps13
Myriazoa - ps14
Parahoxozoa - ps15
Bilateria - ps16
Deuterostomia - ps17
Chordata - ps18
Olfactores - ps19
Vertebrata - ps20
Gnathostomata - ps21
Euteleostomi - ps22
Sarcopterygii - ps23
Tetrapoda - ps24
Amniota - ps25
Mammalia - ps26
Theria - ps27
Placentalia - ps28
Boreoeutheria - ps29
Euarchontoglires - ps30
Euarchonta - ps31
Primates - ps32
Haplorrhini - ps33
Simiiformes - ps34
Catarrhini - ps35
Hominoidea - ps36
Hominidae - ps37
Homininae - ps38
Hominini - ps39
Homo_sapiens - ps40
Phylostratum

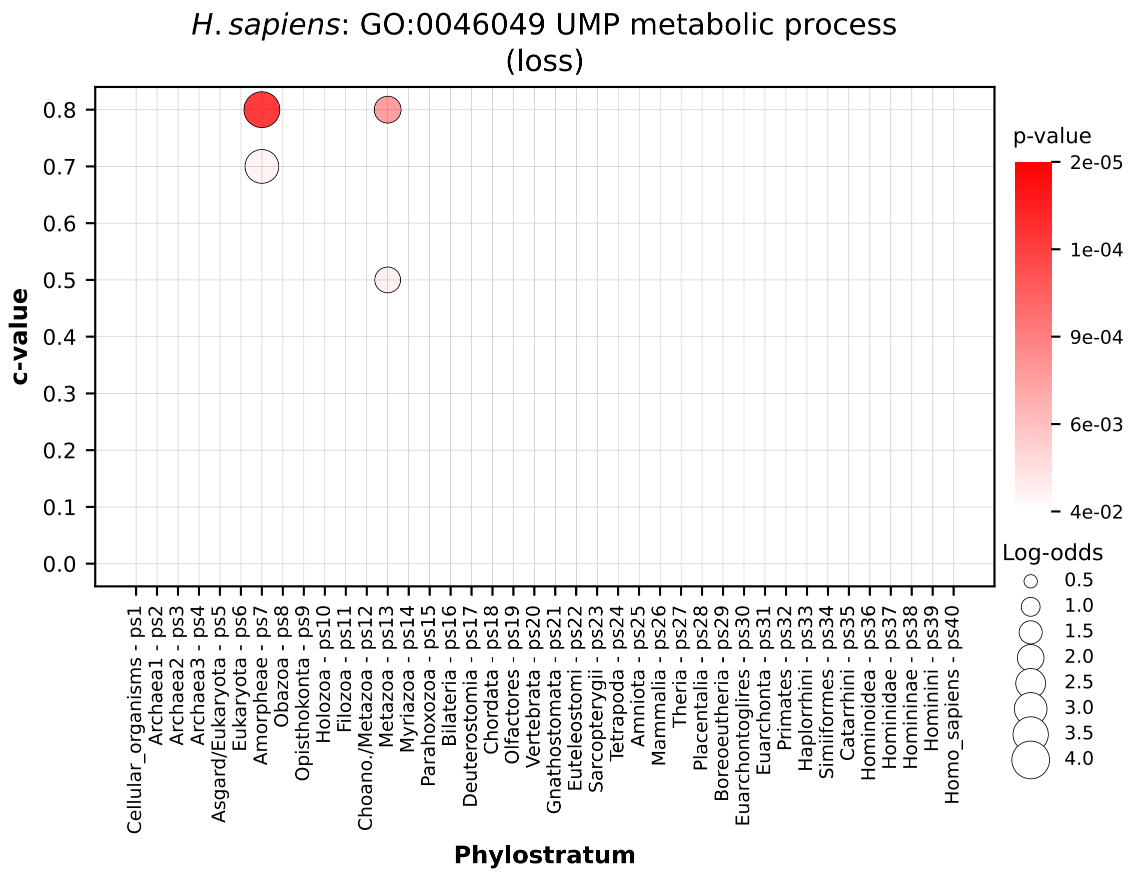

H. sapiens: GO:0046049 UMP metabolic process
(loss)
c-value
0.0
0.1
0.2
0.3
0.4
0.5
0.6
0.7
0.8
p-value
2e-05
1e-04
9e-04
6e-03
4e-02
Log-odds
0.5
1.0
1.5
2.0
2.5
3.0
3.5
4.0
Cellular_organisms - ps1
Archaea1 - ps2
Archaea2 - ps3
Archaea3 - ps4
Asgard/Eukaryota - ps5
Eukaryota - ps6
Amorpheae - ps7
Obazoa - ps8
Opisthokonta - ps9
Holozoa - ps10
Filozoa - ps11
Choano./Metazoa - ps12
Metazoa - ps13
Myriazoa - ps14
Parahoxozoa - ps15
Bilateria - ps16
Deuterostomia - ps17
Chordata - ps18
Olfactores - ps19
Vertebrata - ps20
Gnathostomata - ps21
Euteleostomi - ps22
Sarcopterygii - ps23
Tetrapoda - ps24
Amniota - ps25
Mammalia - ps26
Theria - ps27
Placentalia - ps28
Boreoeutheria - ps29
Euarchontoglires - ps30
Euarchonta - ps31
Primates - ps32
Haplorrhini - ps33
Simiiformes - ps34
Catarrhini - ps35
Hominoidea - ps36
Hominidae - ps37
Homininae - ps38
Hominini - ps39
Homo_sapiens - ps40
Phylostratum

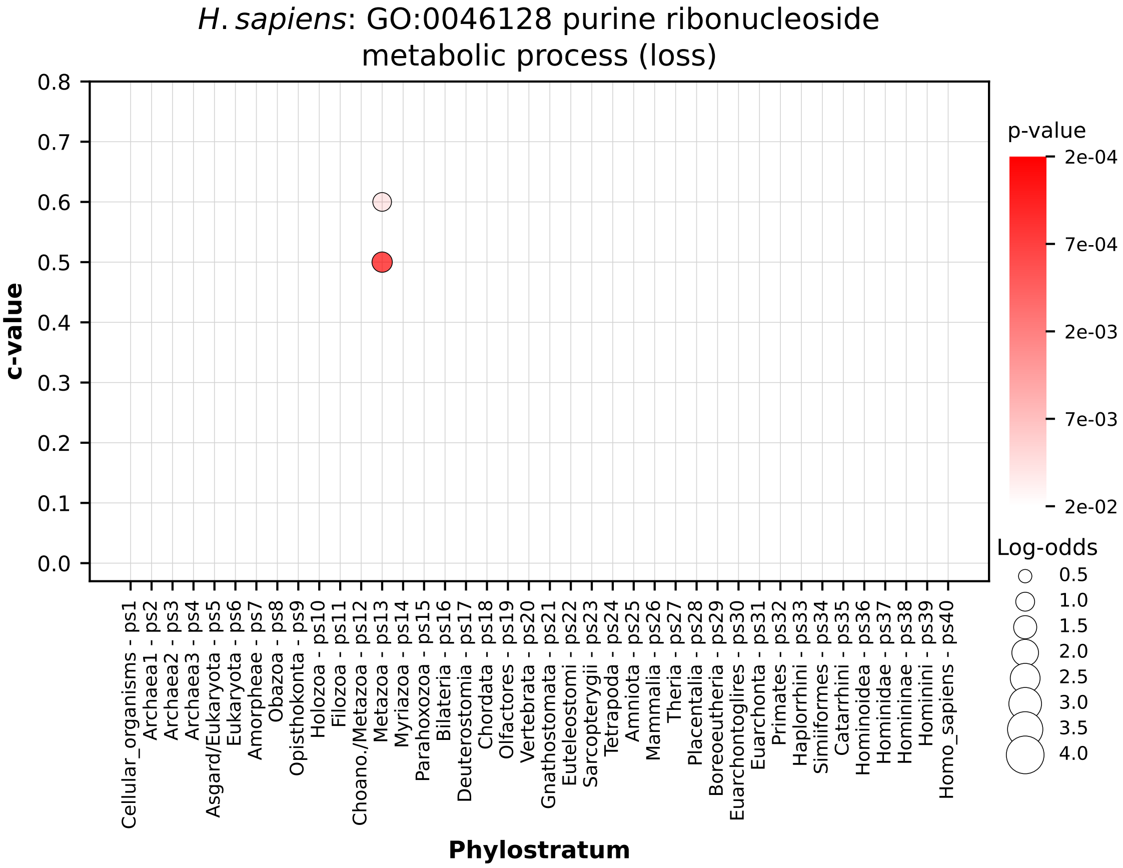
H. sapiens: GO:0046128 purine ribonucleoside metabolic process (loss)
c-value
0.8
0.7
0.6
0.5
0.4
0.3
0.2
0.1
0.0
Phylostratum
Cellular_organisms - ps1
Archaea1 - ps2
Archaea2 - ps3
Archaea3 - ps4
Asgard/Eukaryota - ps5
Eukaryota - ps6
Amorpheae - ps7
Obazoa - ps8
Opisthokonta - ps9
Holozoa - ps10
Filozoa - ps11
Choano./Metazoa - ps12
Metazoa - ps13
Myriazoa - ps14
Parahoxozoa - ps15
Bilateria - ps16
Deuterostomia - ps17
Chordata - ps18
Olfactores - ps19
Vertebrata - ps20
Gnathostomata - ps21
Euteleostomi - ps22
Sarcopterygii - ps23
Tetrapoda - ps24
Amniota - ps25
Mammalia - ps26
Theria - ps27
Placentalia - ps28
Boreoeutheria - ps29
Euarchontoglires - ps30
Euarchonta - ps31
Primates - ps32
Haplorrhini - ps33
Simiiformes - ps34
Catarrhini - ps35
Hominoidea - ps36
Hominidae - ps37
Homininae - ps38
Hominini - ps39
Homo_sapiens - ps40
p-value
2e-04
7e-04
2e-03
7e-03
2e-02
Log-odds
0.5
1.0
1.5
2.0
2.5
3.0
3.5
4.0

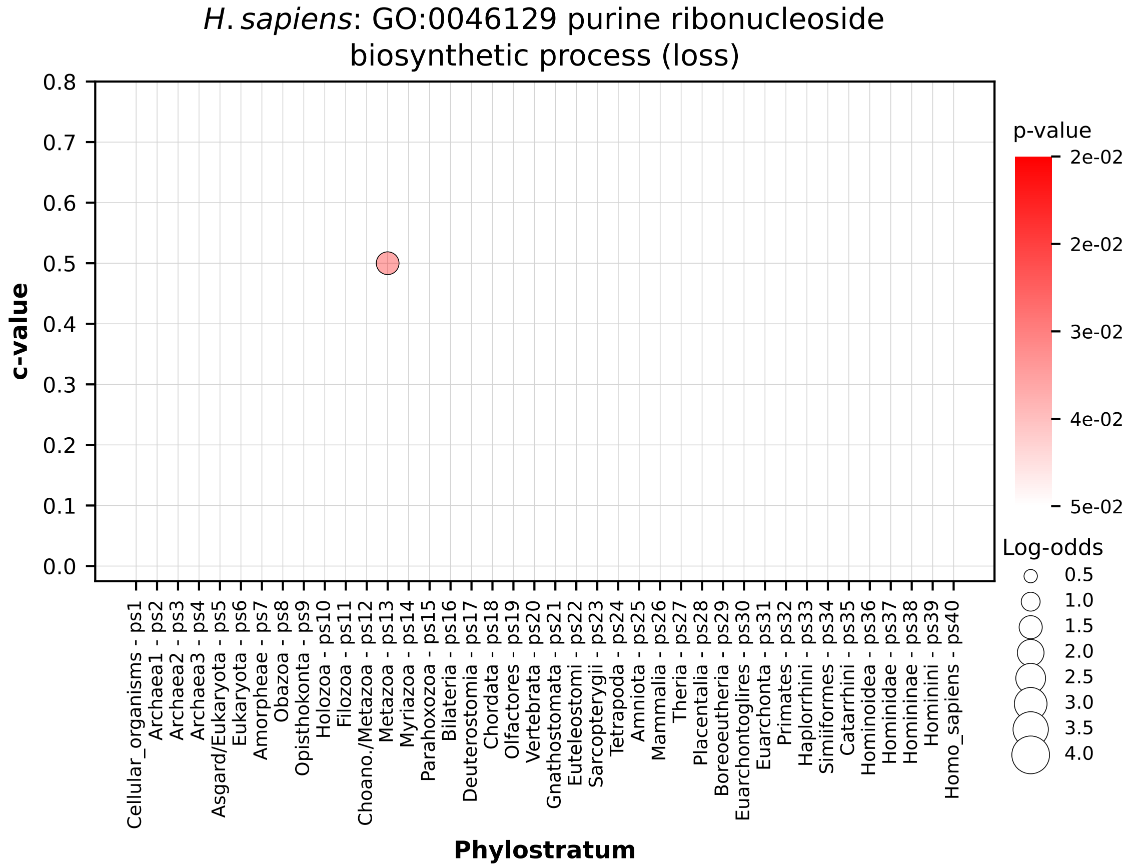

H. sapiens: GO:0046129 purine ribonucleoside biosynthetic process (loss)
c-value
0.8
0.7
0.6
0.5
0.4
0.3
0.2
0.1
0.0
p-value
2e-02
2e-02
3e-02
4e-02
5e-02
Log-odds
0.5
1.0
1.5
2.0
2.5
3.0
3.5
4.0
Cellular_organisms - ps1
Archaea1 - ps2
Archaea2 - ps3
Archaea3 - ps4
Asgard/Eukaryota - ps5
Eukaryota - ps6
Amorpheae - ps7
Obazoa - ps8
Opisthokonta - ps9
Holozoa - ps10
Filozoa - ps11
Choano./Metazoa - ps12
Metazoa - ps13
Myriazoa - ps14
Parahoxozoa - ps15
Bilateria - ps16
Deuterostomia - ps17
Chordata - ps18
Olfactores - ps19
Vertebrata - ps20
Gnathostomata - ps21
Euteleostomi - ps22
Sarcopterygii - ps23
Tetrapoda - ps24
Amniota - ps25
Mammalia - ps26
Theria - ps27
Placentalia - ps28
Boreoeutheria - ps29
Euarchontoglires - ps30
Euarchonta - ps31
Primates - ps32
Haplorrhini - ps33
Simiiformes - ps34
Catarrhini - ps35
Hominoidea - ps36
Hominidae - ps37
Homininae - ps38
Hominini - ps39
Homo_sapiens - ps40
Phylostratum

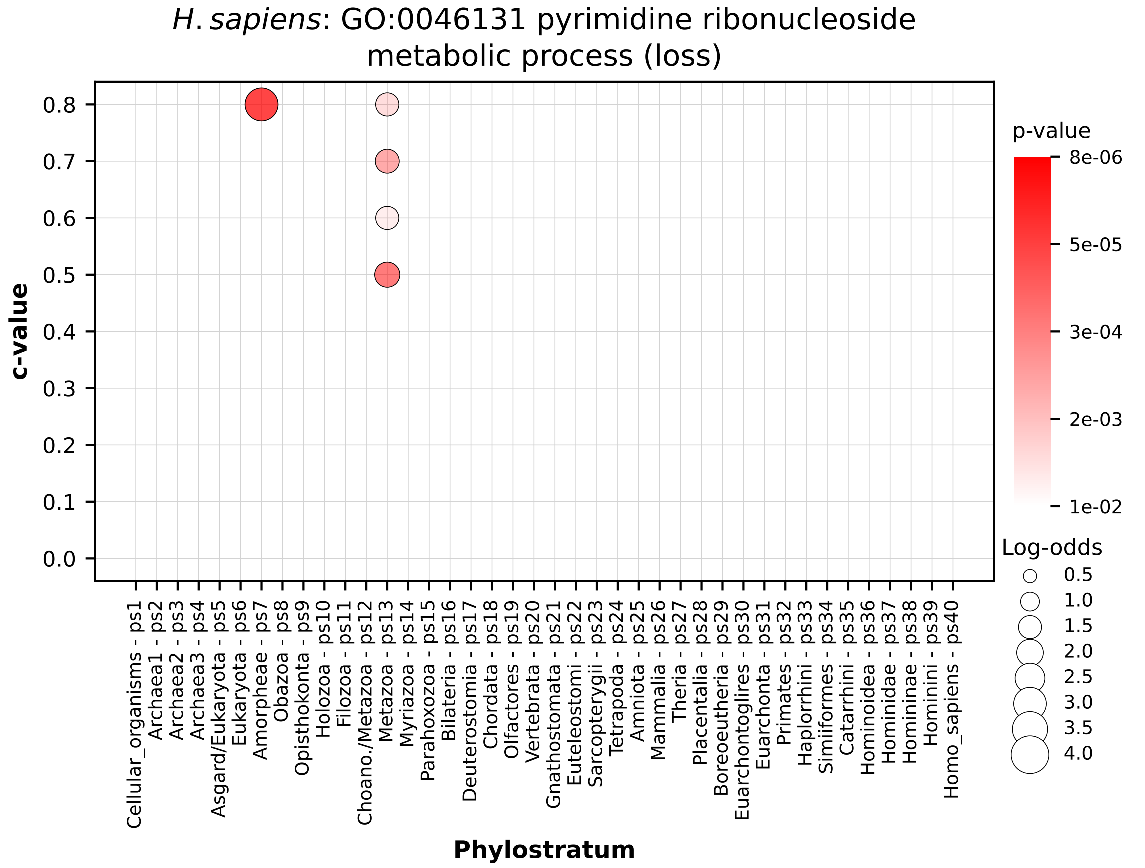

H. sapiens: GO:0046131 pyrimidine ribonucleoside metabolic process (loss)
c-value
0.0
0.1
0.2
0.3
0.4
0.5
0.6
0.7
0.8
Phylostratum
Cellular_organisms - ps1
Archaea1 - ps2
Archaea2 - ps3
Archaea3 - ps4
Asgard/Eukaryota - ps5
Eukaryota - ps6
Amorpheae - ps7
Obazoa - ps8
Opisthokonta - ps9
Holozoa - ps10
Filozoa - ps11
Choano./Metazoa - ps12
Metazoa - ps13
Myriazoa - ps14
Parahoxozoa - ps15
Bilateria - ps16
Deuterostomia - ps17
Chordata - ps18
Olfactores - ps19
Vertebrata - ps20
Gnathostomata - ps21
Euteleostomi - ps22
Sarcopterygii - ps23
Tetrapoda - ps24
Amniota - ps25
Mammalia - ps26
Theria - ps27
Placentalia - ps28
Boreoeutheria - ps29
Euarchontoglires - ps30
Euarchonta - ps31
Primates - ps32
Haplorrhini - ps33
Simiiformes - ps34
Catarrhini - ps35
Hominoidea - ps36
Hominidae - ps37
Homininae - ps38
Hominini - ps39
Homo_sapiens - ps40
p-value
8e-06
5e-05
3e-04
2e-03
1e-02
Log-odds
0.5
1.0
1.5
2.0
2.5
3.0
3.5
4.0

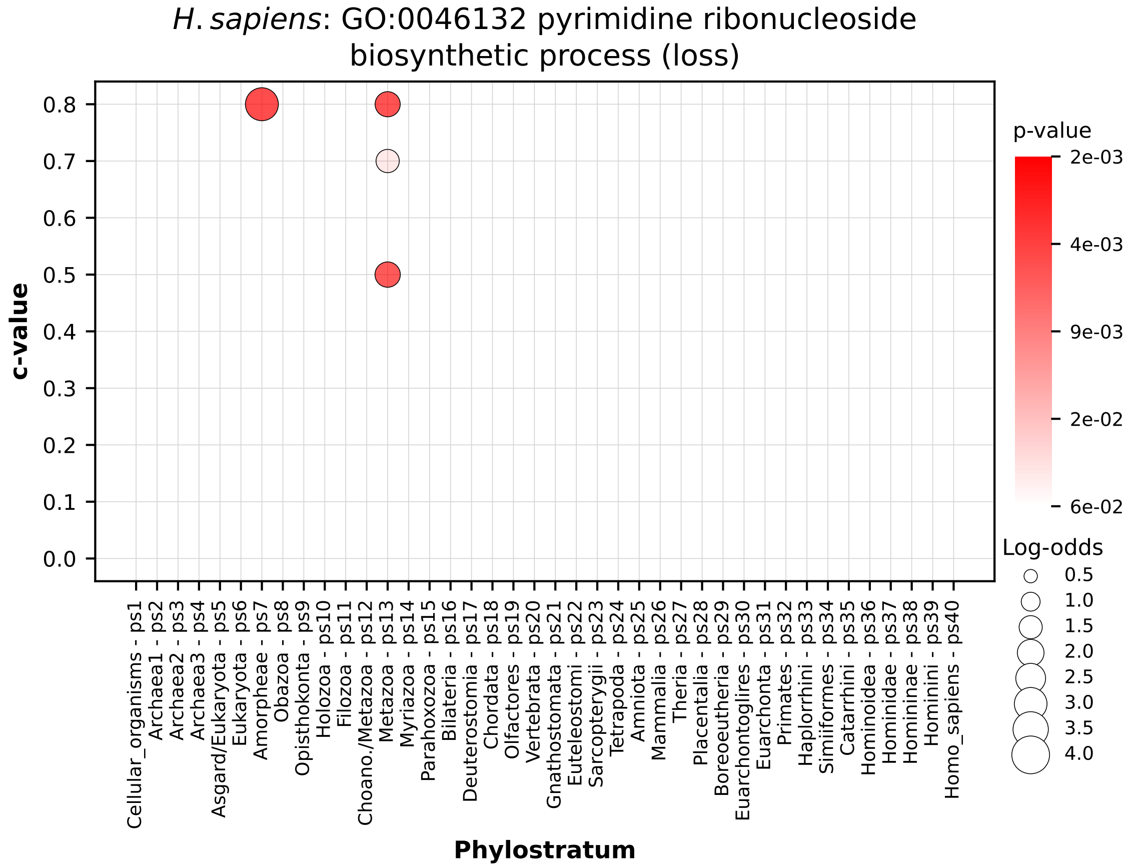
H. sapiens: GO:0046132 pyrimidine ribonucleoside biosynthetic process (loss)
c-value
0.0
0.1
0.2
0.3
0.4
0.5
0.6
0.7
0.8
Phylostratum
Cellular_organisms - ps1
Archaea1 - ps2
Archaea2 - ps3
Archaea3 - ps4
Asgard/Eukaryota - ps5
Eukaryota - ps6
Amorpheae - ps7
Obazoa - ps8
Opisthokonta - ps9
Holozoa - ps10
Filozoa - ps11
Choano./Metazoa - ps12
Metazoa - ps13
Myriazoa - ps14
Parahoxozoa - ps15
Bilateria - ps16
Deuterostomia - ps17
Chordata - ps18
Olfactores - ps19
Vertebrata - ps20
Gnathostomata - ps21
Euteleostomi - ps22
Sarcopterygii - ps23
Tetrapoda - ps24
Amniota - ps25
Mammalia - ps26
Theria - ps27
Placentalia - ps28
Boreoeutheria - ps29
Euarchontoglires - ps30
Euarchonta - ps31
Primates - ps32
Haplorrhini - ps33
Simiiformes - ps34
Catarrhini - ps35
Hominoidea - ps36
Hominidae - ps37
Homininae - ps38
Hominini - ps39
Homo_sapiens - ps40
p-value
2e-03
4e-03
9e-03
2e-02
6e-02
Log-odds
0.5
1.0
1.5
2.0
2.5
3.0
3.5
4.0

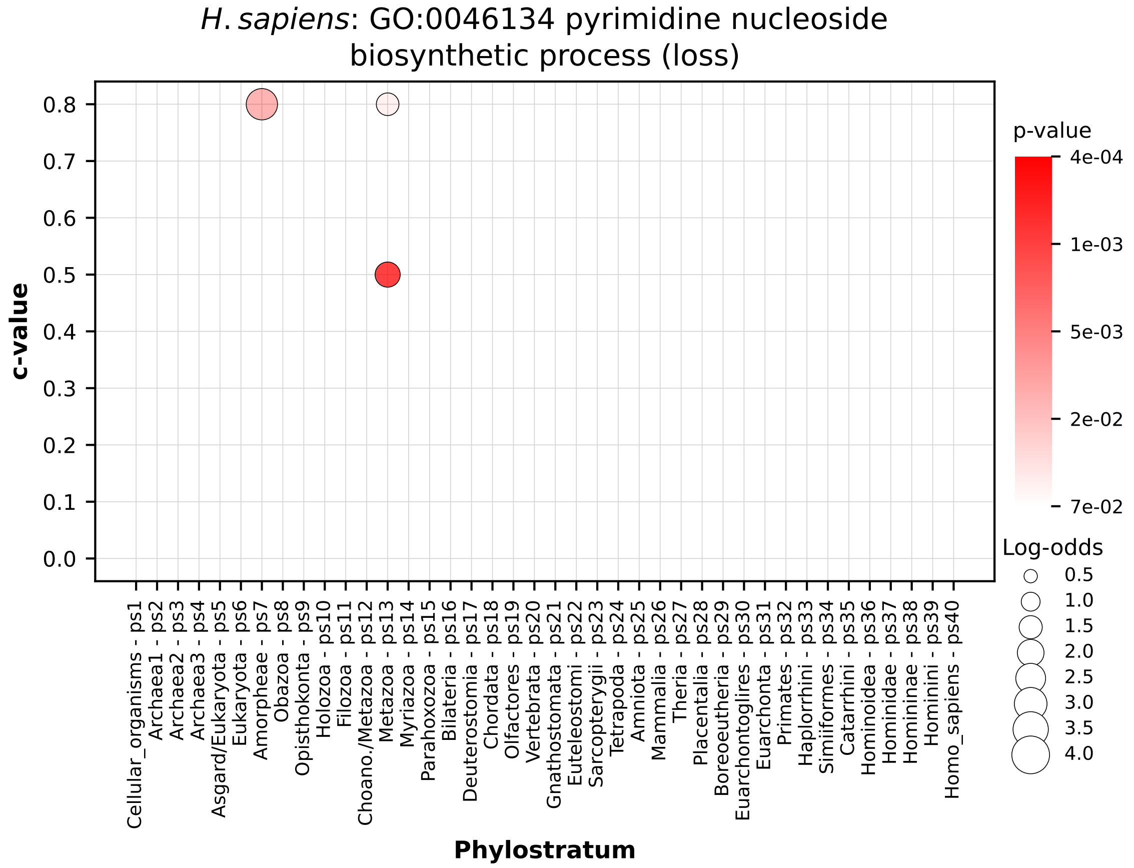

H. sapiens: GO:0046134 pyrimidine nucleoside biosynthetic process (loss)
c-value
0.0
0.1
0.2
0.3
0.4
0.5
0.6
0.7
0.8
Phylostratum
Cellular_organisms - ps1
Archaea1 - ps2
Archaea2 - ps3
Archaea3 - ps4
Asgard/Eukaryota - ps5
Eukaryota - ps6
Amorpheae - ps7
Obazoa - ps8
Opisthokonta - ps9
Holozoa - ps10
Filozoa - ps11
Choano./Metazoa - ps12
Metazoa - ps13
Myriazoa - ps14
Parahoxozoa - ps15
Bilateria - ps16
Deuterostomia - ps17
Chordata - ps18
Olfactores - ps19
Vertebrata - ps20
Gnathostomata - ps21
Euteleostomi - ps22
Sarcopterygii - ps23
Tetrapoda - ps24
Amniota - ps25
Mammalia - ps26
Theria - ps27
Placentalia - ps28
Boreoeutheria - ps29
Euarchontoglires - ps30
Euarchonta - ps31
Primates - ps32
Haplorrhini - ps33
Simiiformes - ps34
Catarrhini - ps35
Hominoidea - ps36
Hominidae - ps37
Homininae - ps38
Hominini - ps39
Homo_sapiens - ps40
p-value
4e-04
1e-03
5e-03
2e-02
7e-02
Log-odds
0.5
1.0
1.5
2.0
2.5
3.0
3.5
4.0

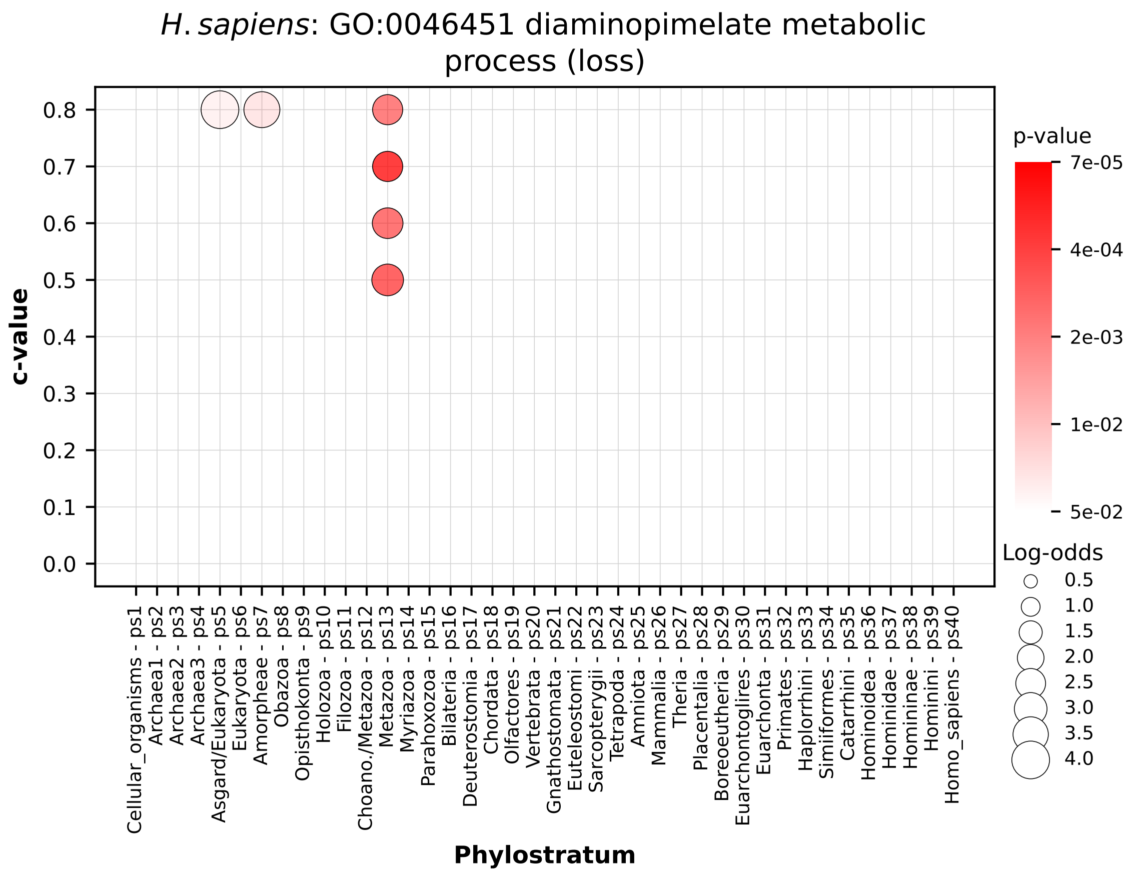
H. sapiens: GO:0046451 diaminopimelate metabolic process (loss)
c-value
0.0
0.1
0.2
0.3
0.4
0.5
0.6
0.7
0.8
p-value
7e-05
4e-04
2e-03
1e-02
5e-02
Log-odds
0.5
1.0
1.5
2.0
2.5
3.0
3.5
4.0
Cellular_organisms - ps1
Archaea1 - ps2
Archaea2 - ps3
Archaea3 - ps4
Asgard/Eukaryota - ps5
Eukaryota - ps6
Amorpheae - ps7
Obazoa - ps8
Opisthokonta - ps9
Holozoa - ps10
Filozoa - ps11
Choano./Metazoa - ps12
Metazoa - ps13
Myriazoa - ps14
Parahoxozoa - ps15
Bilateria - ps16
Deuterostomia - ps17
Chordata - ps18
Olfactores - ps19
Vertebrata - ps20
Gnathostomata - ps21
Euteleostomi - ps22
Sarcopterygii - ps23
Tetrapoda - ps24
Amniota - ps25
Mammalia - ps26
Theria - ps27
Placentalia - ps28
Boreoeutheria - ps29
Euarchontoglires - ps30
Euarchonta - ps31
Primates - ps32
Haplorrhini - ps33
Simiiformes - ps34
Catarrhini - ps35
Hominoidea - ps36
Hominidae - ps37
Homininae - ps38
Hominini - ps39
Homo_sapiens - ps40
Phylostratum

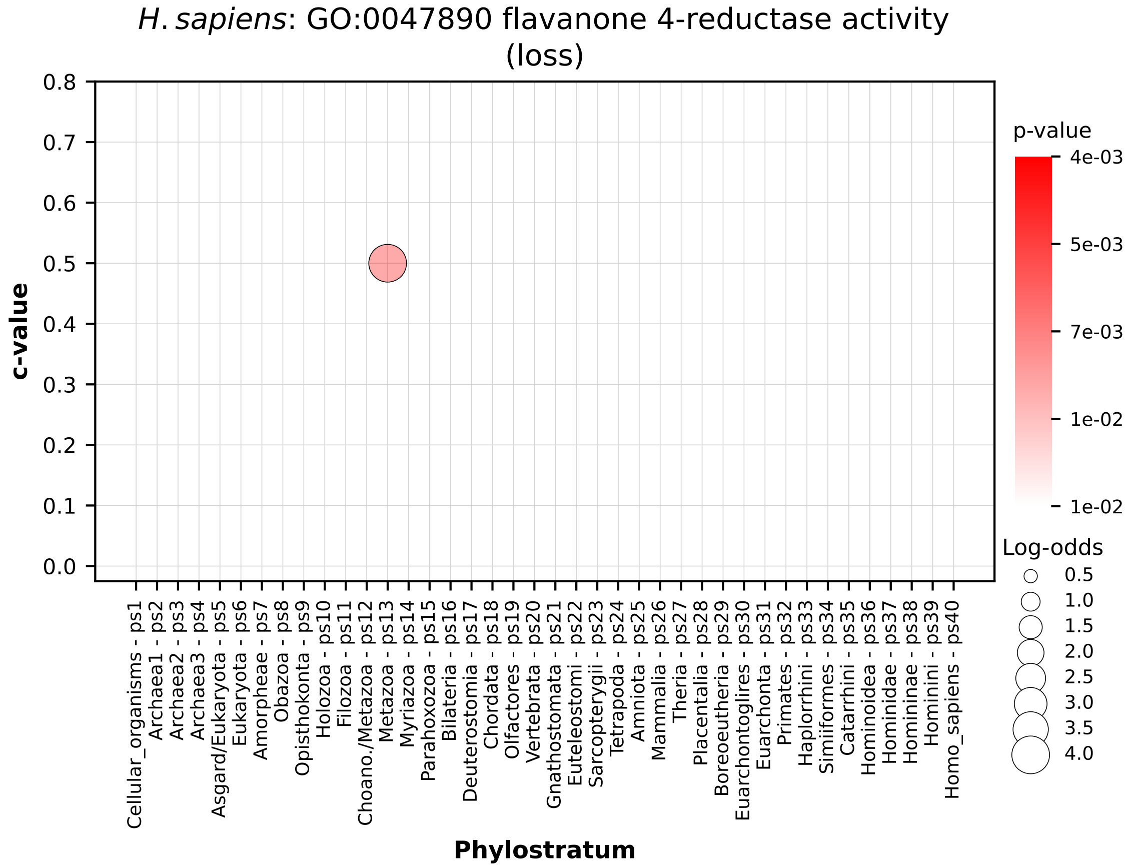

H. sapiens: GO:0047890 flavanone 4-reductase activity
(loss)
c-value
0.8
0.7
0.6
0.5
0.4
0.3
0.2
0.1
0.0
p-value
4e-03
5e-03
7e-03
1e-02
1e-02
Log-odds
0.5
1.0
1.5
2.0
2.5
3.0
3.5
4.0
Cellular_organisms - ps1
Archaea1 - ps2
Archaea2 - ps3
Archaea3 - ps4
Asgard/Eukaryota - ps5
Eukaryota - ps6
Amorpheae - ps7
Obazoa - ps8
Opisthokonta - ps9
Holozoa - ps10
Filozoa - ps11
Choano./Metazoa - ps12
Metazoa - ps13
Myriazoa - ps14
Parahoxozoa - ps15
Bilateria - ps16
Deuterostomia - ps17
Chordata - ps18
Olfactores - ps19
Vertebrata - ps20
Gnathostomata - ps21
Euteleostomi - ps22
Sarcopterygii - ps23
Tetrapoda - ps24
Amniota - ps25
Mammalia - ps26
Theria - ps27
Placentalia - ps28
Boreoeutheria - ps29
Euarchontoglires - ps30
Euarchonta - ps31
Primates - ps32
Haplorrhini - ps33
Simiiformes - ps34
Catarrhini - ps35
Hominoidea - ps36
Hominidae - ps37
Homininae - ps38
Hominini - ps39
Homo_sapiens - ps40
Phylostratum

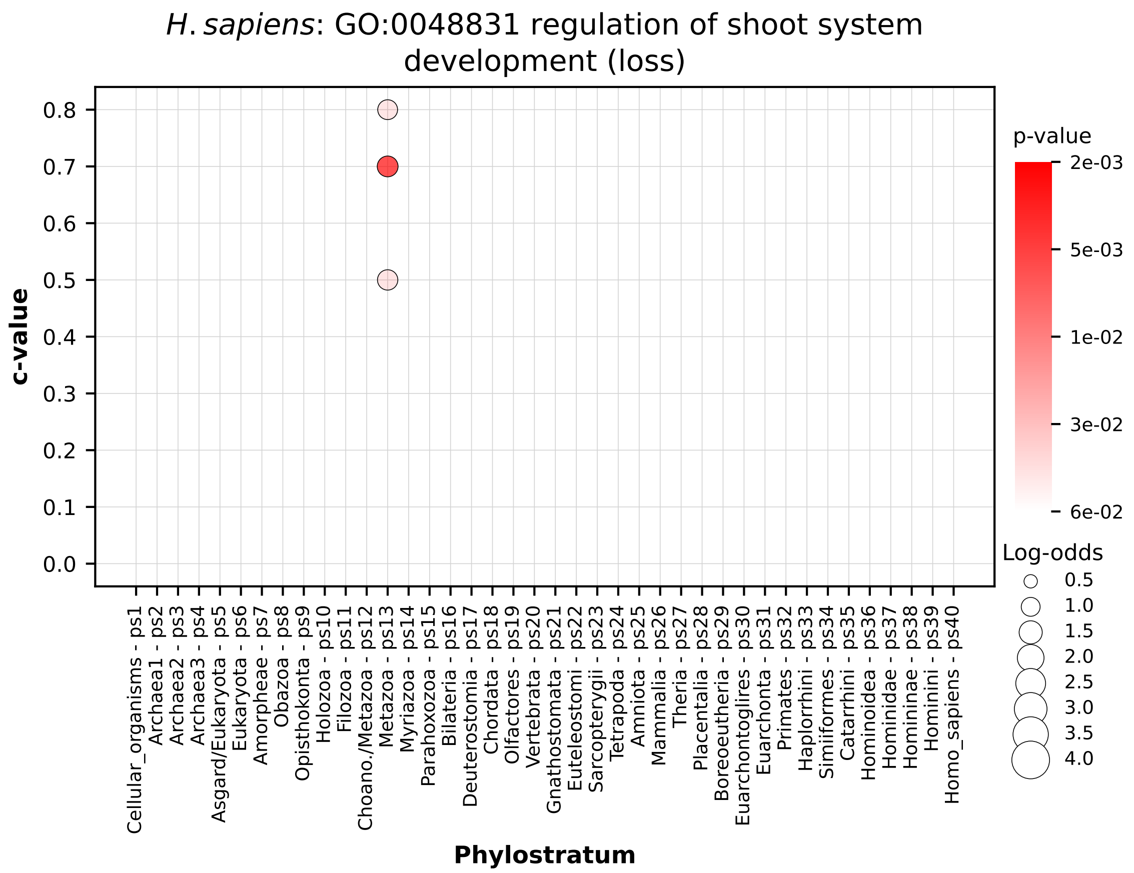
H. sapiens: GO:0048831 regulation of shoot system development (loss)
c-value
0.8
0.7
0.6
0.5
0.4
0.3
0.2
0.1
0.0
Phylostratum
Cellular_organisms - ps1
Archaea1 - ps2
Archaea2 - ps3
Archaea3 - ps4
Asgard/Eukaryota - ps5
Eukaryota - ps6
Amorpheae - ps7
Obazoa - ps8
Opisthokonta - ps9
Holozoa - ps10
Filozoa - ps11
Choano./Metazoa - ps12
Metazoa - ps13
Myriazoa - ps14
Parahoxozoa - ps15
Bilateria - ps16
Deuterostomia - ps17
Chordata - ps18
Olfactores - ps19
Vertebrata - ps20
Gnathostomata - ps21
Euteleostomi - ps22
Sarcopterygii - ps23
Tetrapoda - ps24
Amniota - ps25
Mammalia - ps26
Theria - ps27
Placentalia - ps28
Boreoeutheria - ps29
Euarchontoglires - ps30
Euarchonta - ps31
Primates - ps32
Haplorrhini - ps33
Simiiformes - ps34
Catarrhini - ps35
Hominoidea - ps36
Hominidae - ps37
Homininae - ps38
Hominini - ps39
Homo_sapiens - ps40
p-value
2e-03
5e-03
1e-02
3e-02
6e-02
Log-odds
0.5
1.0
1.5
2.0
2.5
3.0
3.5
4.0

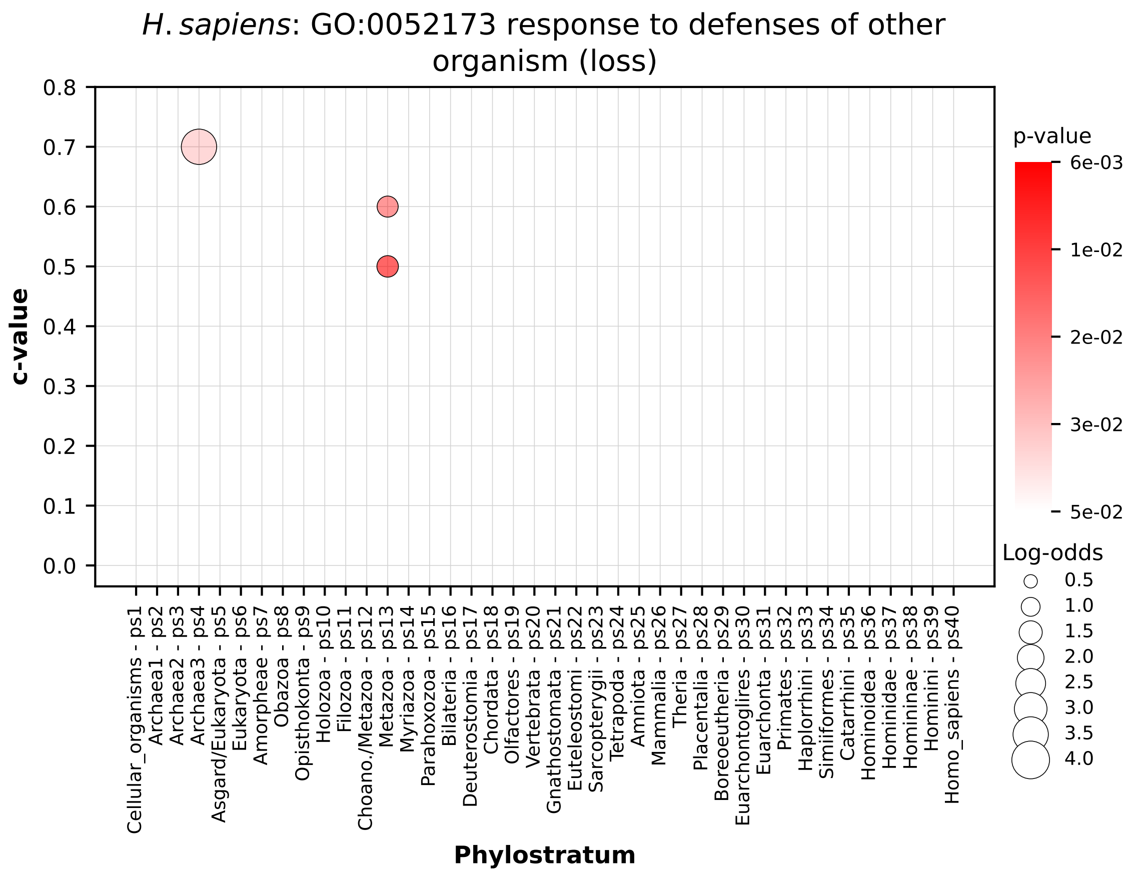

H. sapiens: GO:0052173 response to defenses of other organism (loss)
c-value
0.0
0.1
0.2
0.3
0.4
0.5
0.6
0.7
0.8
Phylostratum
Cellular_organisms - ps1
Archaea1 - ps2
Archaea2 - ps3
Archaea3 - ps4
Asgard/Eukaryota - ps5
Eukaryota - ps6
Amorpheae - ps7
Obazoa - ps8
Opisthokonta - ps9
Holozoa - ps10
Filozoa - ps11
Choano./Metazoa - ps12
Metazoa - ps13
Myriazoa - ps14
Parahoxozoa - ps15
Bilateria - ps16
Deuterostomia - ps17
Chordata - ps18
Olfactores - ps19
Vertebrata - ps20
Gnathostomata - ps21
Euteleostomi - ps22
Sarcopterygii - ps23
Tetrapoda - ps24
Amniota - ps25
Mammalia - ps26
Theria - ps27
Placentalia - ps28
Boreoeutheria - ps29
Euarchontoglires - ps30
Euarchonta - ps31
Primates - ps32
Haplorrhini - ps33
Simiiformes - ps34
Catarrhini - ps35
Hominoidea - ps36
Hominidae - ps37
Homininae - ps38
Hominini - ps39
Homo_sapiens - ps40
p-value
6e-03
1e-02
2e-02
3e-02
5e-02
Log-odds
0.5
1.0
1.5
2.0
2.5
3.0
3.5
4.0

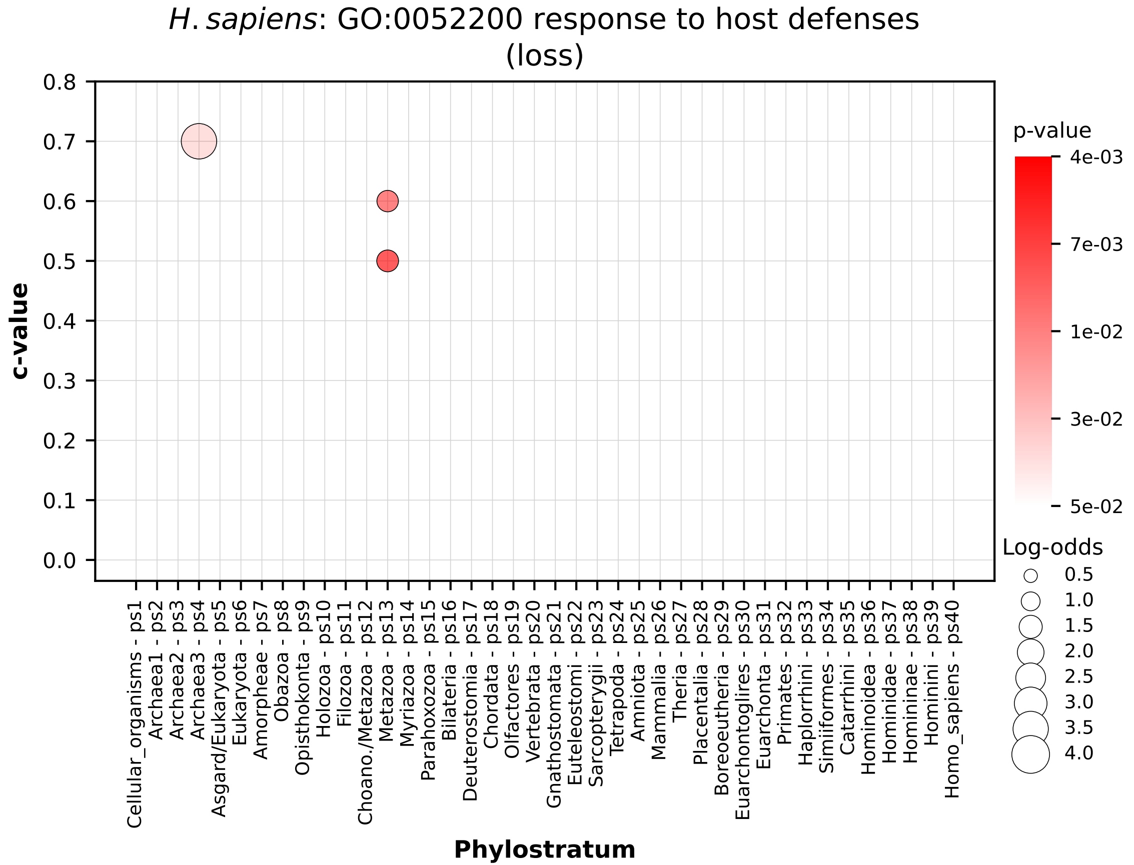

H. sapiens: GO:0052200 response to host defenses (loss)
c-value
0.0
0.1
0.2
0.3
0.4
0.5
0.6
0.7
0.8
Phylostratum
Cellular_organisms - ps1
Archaea1 - ps2
Archaea2 - ps3
Archaea3 - ps4
Asgard/Eukaryota - ps5
Eukaryota - ps6
Amorpheae - ps7
Obazoa - ps8
Opisthokonta - ps9
Holozoa - ps10
Filozoa - ps11
Choano./Metazoa - ps12
Metazoa - ps13
Myriazoa - ps14
Parahoxozoa - ps15
Bilateria - ps16
Deuterostomia - ps17
Chordata - ps18
Olfactores - ps19
Vertebrata - ps20
Gnathostomata - ps21
Euteleostomi - ps22
Sarcopterygii - ps23
Tetrapoda - ps24
Amniota - ps25
Mammalia - ps26
Theria - ps27
Placentalia - ps28
Boreoeutheria - ps29
Euarchontoglires - ps30
Euarchonta - ps31
Primates - ps32
Haplorrhini - ps33
Simiiformes - ps34
Catarrhini - ps35
Hominoidea - ps36
Hominidae - ps37
Homininae - ps38
Hominini - ps39
Homo_sapiens - ps40
p-value
4e-03
7e-03
1e-02
3e-02
5e-02
Log-odds
0.5
1.0
1.5
2.0
2.5
3.0
3.5
4.0

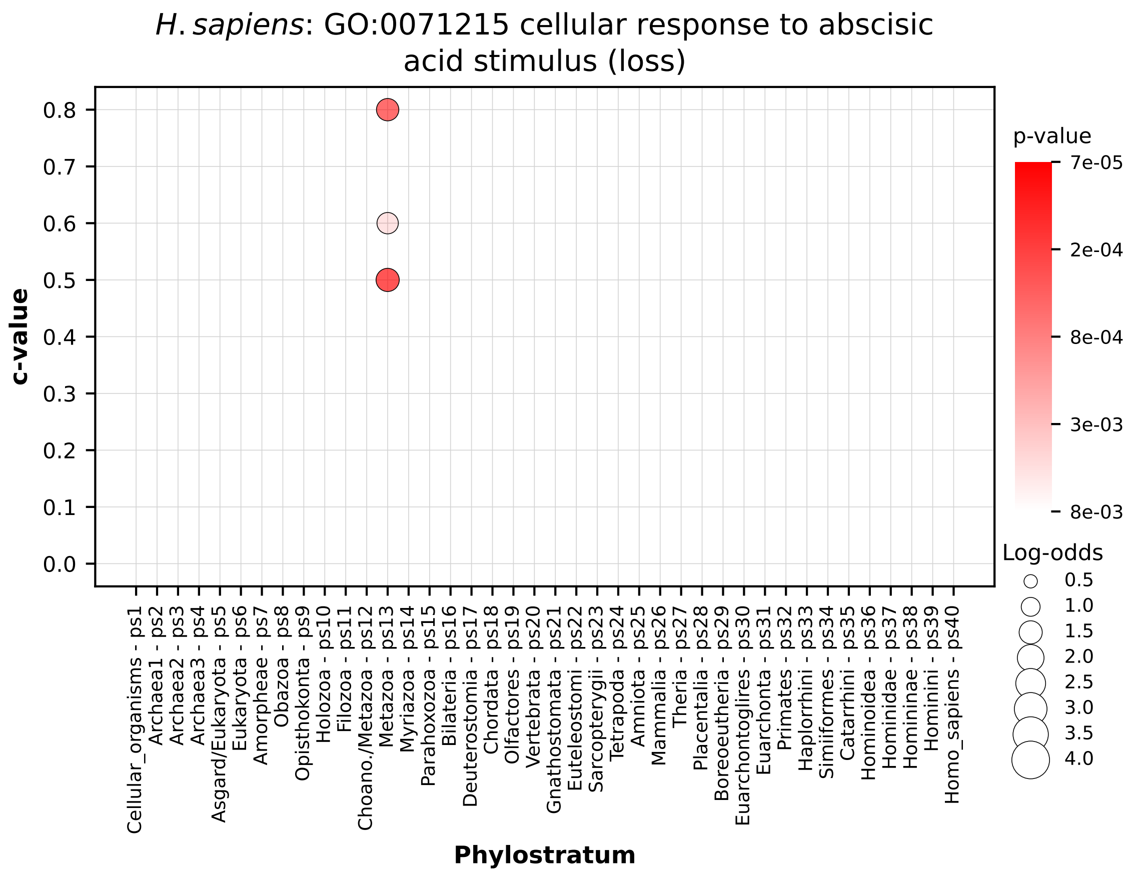

H. sapiens: GO:0071215 cellular response to abscisic acid stimulus (loss)
c-value
0.0
0.1
0.2
0.3
0.4
0.5
0.6
0.7
0.8
Phylostratum
Cellular_organisms - ps1
Archaea1 - ps2
Archaea2 - ps3
Archaea3 - ps4
Asgard/Eukaryota - ps5
Eukaryota - ps6
Amorpheae - ps7
Obazoa - ps8
Opisthokonta - ps9
Holozoa - ps10
Filozoa - ps11
Choano./Metazoa - ps12
Metazoa - ps13
Myriazoa - ps14
Parahoxozoa - ps15
Bilateria - ps16
Deuterostomia - ps17
Chordata - ps18
Olfactores - ps19
Vertebrata - ps20
Gnathostomata - ps21
Euteleostomi - ps22
Sarcopterygii - ps23
Tetrapoda - ps24
Amniota - ps25
Mammalia - ps26
Theria - ps27
Placentalia - ps28
Boreoeutheria - ps29
Euarchontoglires - ps30
Euarchonta - ps31
Primates - ps32
Haplorrhini - ps33
Simiiformes - ps34
Catarrhini - ps35
Hominoidea - ps36
Hominidae - ps37
Homininae - ps38
Hominini - ps39
Homo_sapiens - ps40
p-value
7e-05
2e-04
8e-04
3e-03
8e-03
Log-odds
0.5
1.0
1.5
2.0
2.5
3.0
3.5
4.0

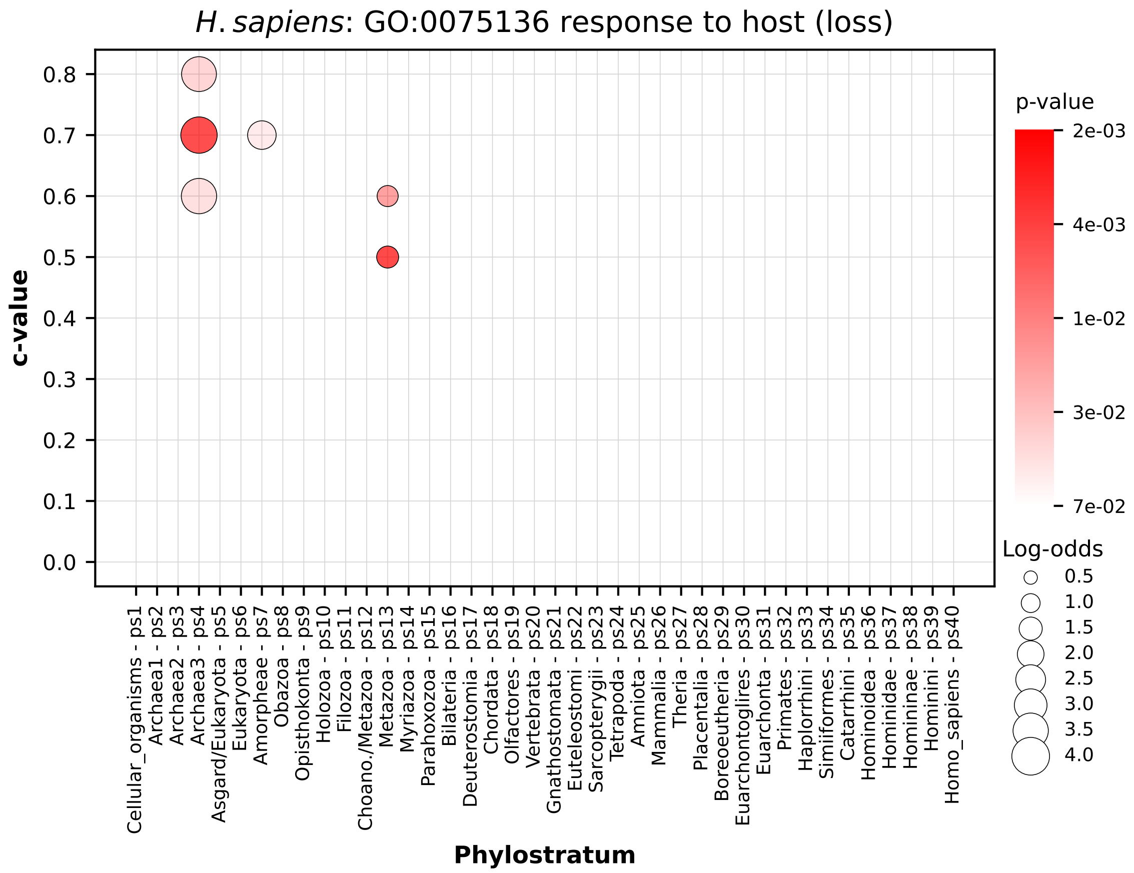

H. sapiens: GO:0075136 response to host (loss)
c-value
0.0
0.1
0.2
0.3
0.4
0.5
0.6
0.7
0.8
Phylostratum
Cellular_organisms - ps1
Archaea1 - ps2
Archaea2 - ps3
Archaea3 - ps4
Asgard/Eukaryota - ps5
Eukaryota - ps6
Amorpheae - ps7
Obazoa - ps8
Opisthokonta - ps9
Holozoa - ps10
Filozoa - ps11
Choano./Metazoa - ps12
Metazoa - ps13
Myriazoa - ps14
Parahoxozoa - ps15
Bilateria - ps16
Deuterostomia - ps17
Chordata - ps18
Olfactores - ps19
Vertebrata - ps20
Gnathostomata - ps21
Euteleostomi - ps22
Sarcopterygii - ps23
Tetrapoda - ps24
Amniota - ps25
Mammalia - ps26
Theria - ps27
Placentalia - ps28
Boreoeutheria - ps29
Euarchontoglires - ps30
Euarchonta - ps31
Primates - ps32
Haplorrhini - ps33
Simiiformes - ps34
Catarrhini - ps35
Hominoidea - ps36
Hominidae - ps37
Homininae - ps38
Hominini - ps39
Homo_sapiens - ps40
p-value
2e-03
4e-03
1e-02
3e-02
7e-02
Log-odds
0.5
1.0
1.5
2.0
2.5
3.0
3.5
4.0

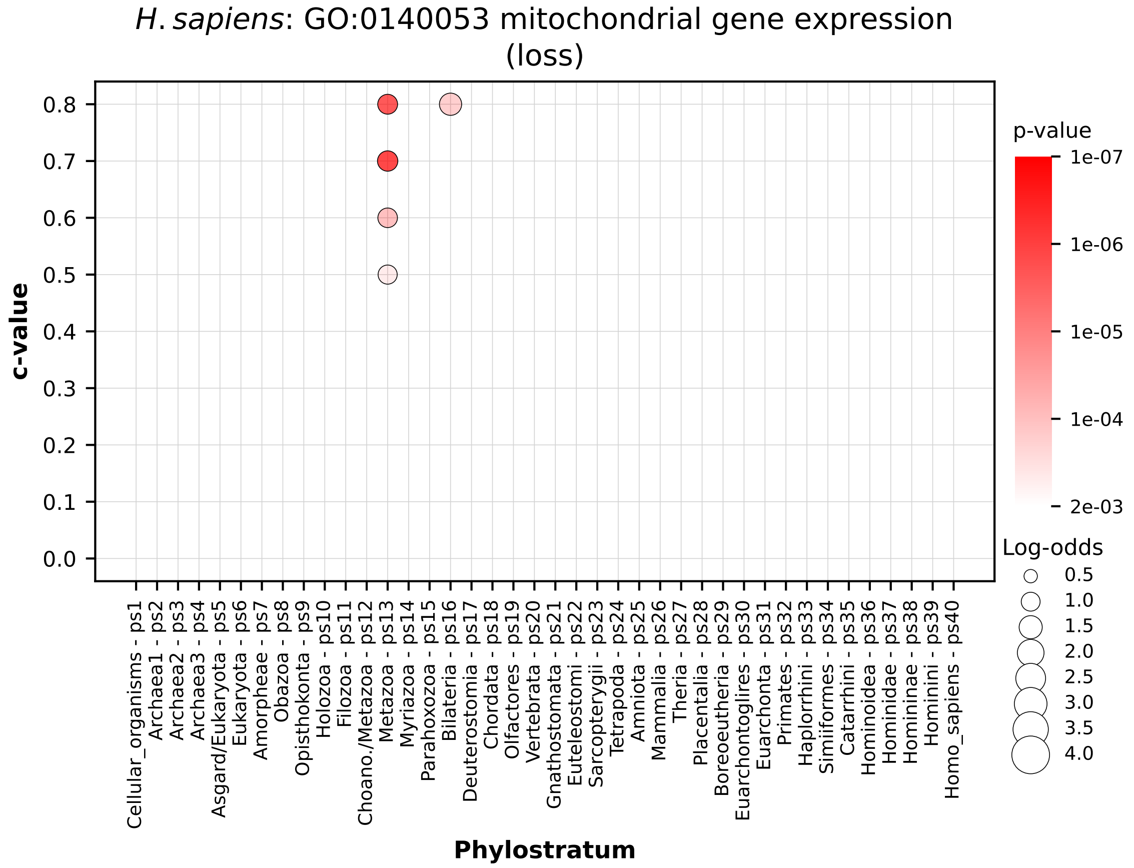

H. sapiens: GO:0140053 mitochondrial gene expression (loss)
c-value
0.0
0.1
0.2
0.3
0.4
0.5
0.6
0.7
0.8
p-value
1e-07
1e-06
1e-05
1e-04
2e-03
Log-odds
0.5
1.0
1.5
2.0
2.5
3.0
3.5
4.0
Cellular_organisms - ps1
Archaea1 - ps2
Archaea2 - ps3
Archaea3 - ps4
Asgard/Eukaryota - ps5
Eukaryota - ps6
Amorpheae - ps7
Obazoa - ps8
Opisthokonta - ps9
Holozoa - ps10
Filozoa - ps11
Choano./Metazoa - ps12
Metazoa - ps13
Myriazoa - ps14
Parahoxozoa - ps15
Bilateria - ps16
Deuterostomia - ps17
Chordata - ps18
Olfactores - ps19
Vertebrata - ps20
Gnathostomata - ps21
Euteleostomi - ps22
Sarcopterygii - ps23
Tetrapoda - ps24
Amniota - ps25
Mammalia - ps26
Theria - ps27
Placentalia - ps28
Boreoeutheria - ps29
Euarchontoglires - ps30
Euarchonta - ps31
Primates - ps32
Haplorrhini - ps33
Simiiformes - ps34
Catarrhini - ps35
Hominoidea - ps36
Hominidae - ps37
Homininae - ps38
Hominini - ps39
Homo_sapiens - ps40
Phylostratum

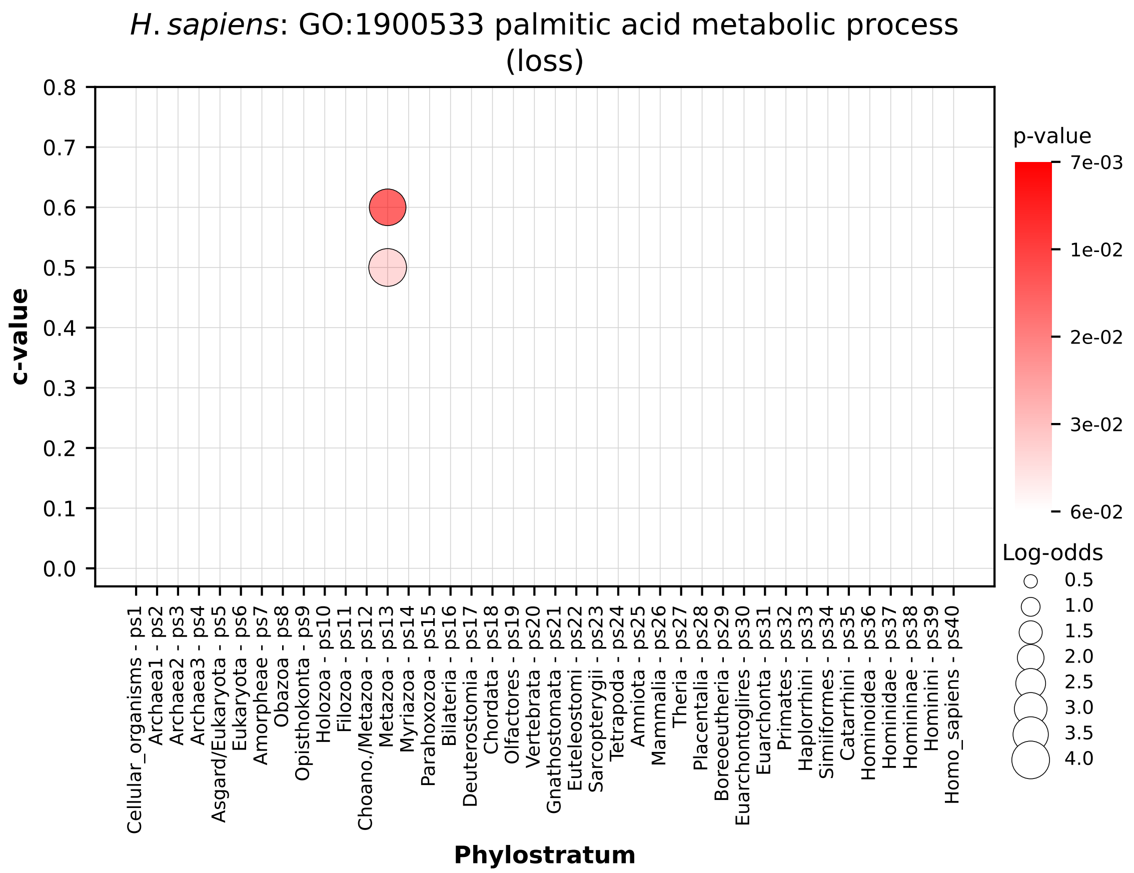

H. sapiens: GO:1900533 palmitic acid metabolic process
(loss)
c-value
0.0
0.1
0.2
0.3
0.4
0.5
0.6
0.7
0.8
Phylostratum
Cellular_organisms - ps1
Archaea1 - ps2
Archaea2 - ps3
Archaea3 - ps4
Asgard/Eukaryota - ps5
Eukaryota - ps6
Amorpheae - ps7
Obazoa - ps8
Opisthokonta - ps9
Holozoa - ps10
Filozoa - ps11
Choano./Metazoa - ps12
Metazoa - ps13
Myriazoa - ps14
Parahoxozoa - ps15
Bilateria - ps16
Deuterostomia - ps17
Chordata - ps18
Olfactores - ps19
Vertebrata - ps20
Gnathostomata - ps21
Euteleostomi - ps22
Sarcopterygii - ps23
Tetrapoda - ps24
Amniota - ps25
Mammalia - ps26
Theria - ps27
Placentalia - ps28
Boreoeutheria - ps29
Euarchontoglires - ps30
Euarchonta - ps31
Primates - ps32
Haplorrhini - ps33
Simiiformes - ps34
Catarrhini - ps35
Hominoidea - ps36
Hominidae - ps37
Homininae - ps38
Hominini - ps39
Homo_sapiens - ps40
p-value
7e-03
1e-02
2e-02
3e-02
6e-02
Log-odds
0.5
1.0
1.5
2.0
2.5
3.0
3.5
4.0

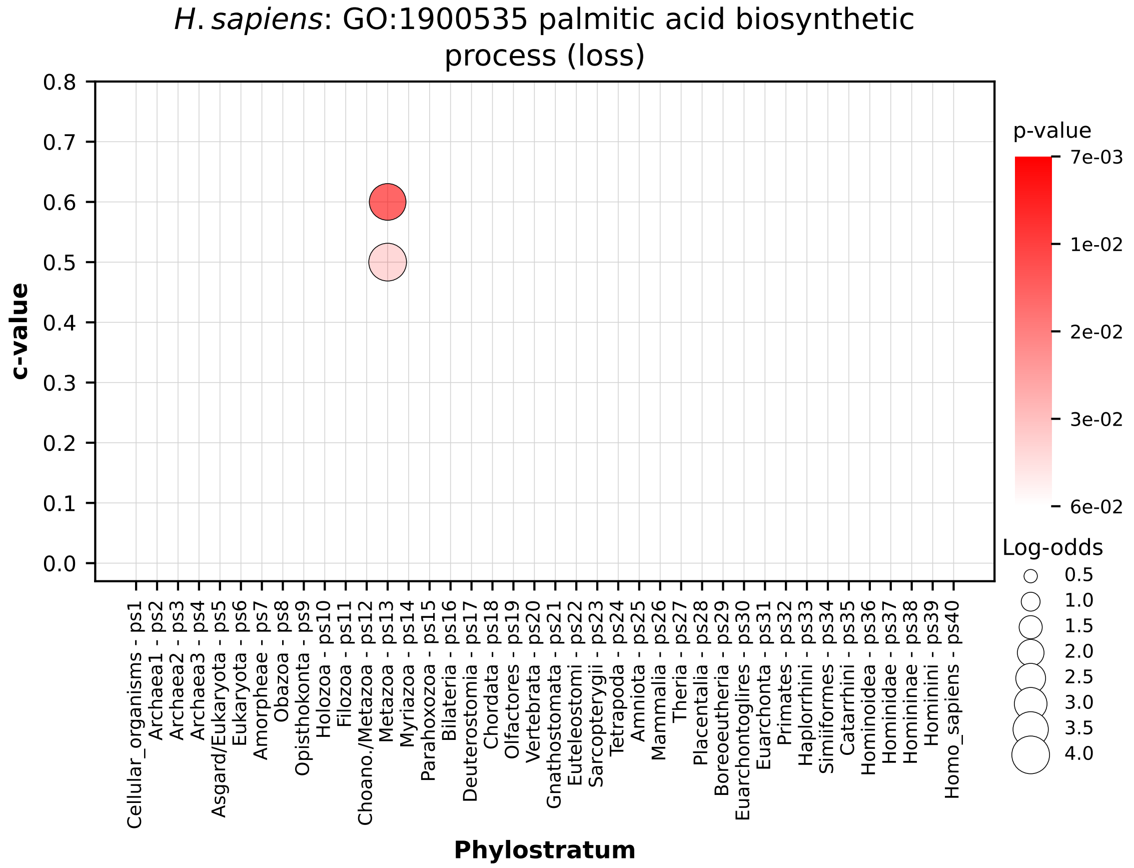

H. sapiens: GO:1900535 palmitic acid biosynthetic process (loss)
c-value
0.0
0.1
0.2
0.3
0.4
0.5
0.6
0.7
0.8
Phylostratum
Cellular_organisms - ps1
Archaea1 - ps2
Archaea2 - ps3
Archaea3 - ps4
Asgard/Eukaryota - ps5
Eukaryota - ps6
Amorpheae - ps7
Obazoa - ps8
Opisthokonta - ps9
Holozoa - ps10
Filozoa - ps11
Choano./Metazoa - ps12
Metazoa - ps13
Myriazoa - ps14
Parahoxozoa - ps15
Bilateria - ps16
Deuterostomia - ps17
Chordata - ps18
Olfactores - ps19
Vertebrata - ps20
Gnathostomata - ps21
Euteleostomi - ps22
Sarcopterygii - ps23
Tetrapoda - ps24
Amniota - ps25
Mammalia - ps26
Theria - ps27
Placentalia - ps28
Boreoeutheria - ps29
Euarchontoglires - ps30
Euarchonta - ps31
Primates - ps32
Haplorrhini - ps33
Simiiformes - ps34
Catarrhini - ps35
Hominoidea - ps36
Hominidae - ps37
Homininae - ps38
Hominini - ps39
Homo_sapiens - ps40
p-value
7e-03
1e-02
2e-02
3e-02
6e-02
Log-odds
0.5
1.0
1.5
2.0
2.5
3.0
3.5
4.0

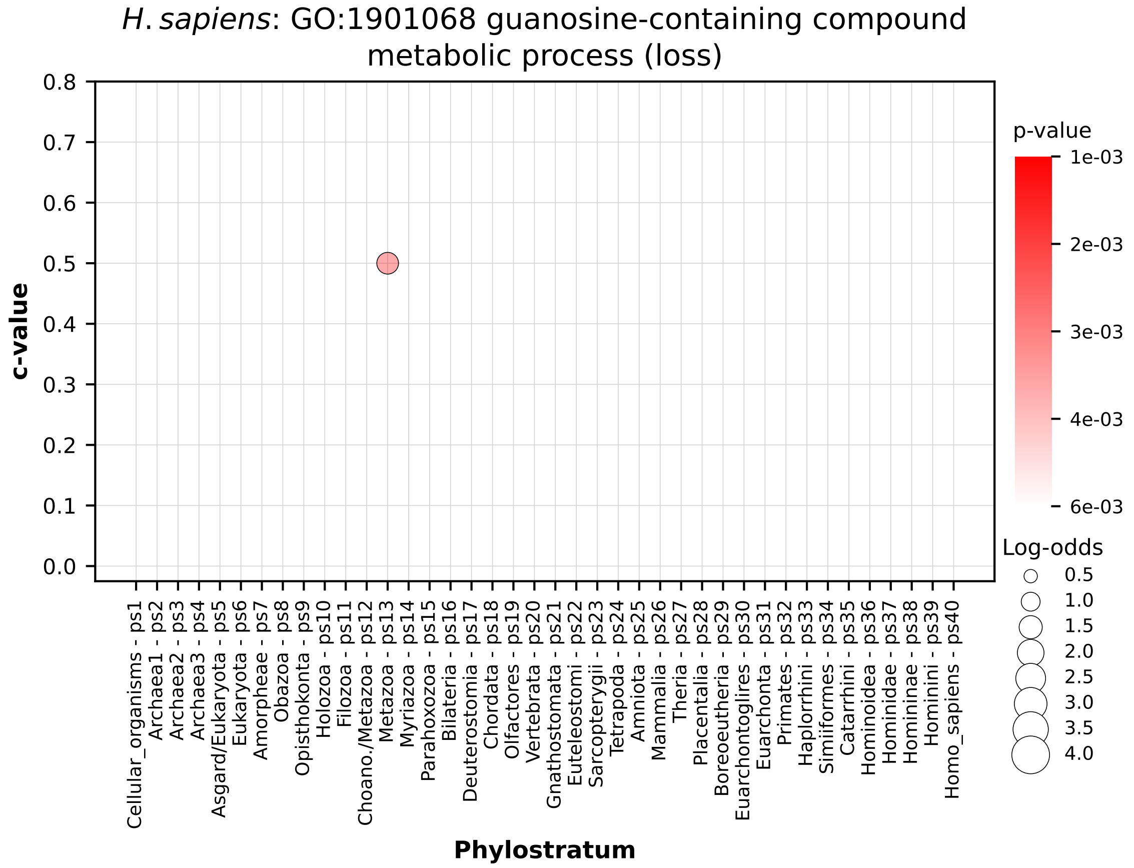
H. sapiens: GO:1901068 guanosine-containing compound metabolic process (loss)
c-value
0.8
0.7
0.6
0.5
0.4
0.3
0.2
0.1
0.0
Phylostratum
Cellular_organisms - ps1
Archaea1 - ps2
Archaea2 - ps3
Archaea3 - ps4
Asgard/Eukaryota - ps5
Eukaryota - ps6
Amorpheae - ps7
Obazoa - ps8
Opisthokonta - ps9
Holozoa - ps10
Filozoa - ps11
Choano./Metazoa - ps12
Metazoa - ps13
Myriazoa - ps14
Parahoxozoa - ps15
Bilateria - ps16
Deuterostomia - ps17
Chordata - ps18
Olfactores - ps19
Vertebrata - ps20
Gnathostomata - ps21
Euteleostomi - ps22
Sarcopterygii - ps23
Tetrapoda - ps24
Amniota - ps25
Mammalia - ps26
Theria - ps27
Placentalia - ps28
Boreoeutheria - ps29
Euarchontoglires - ps30
Euarchonta - ps31
Primates - ps32
Haplorrhini - ps33
Simiiformes - ps34
Catarrhini - ps35
Hominoidea - ps36
Hominidae - ps37
Homininae - ps38
Hominini - ps39
Homo_sapiens - ps40
p-value
1e-03
2e-03
3e-03
4e-03
6e-03
Log-odds
0.5
1.0
1.5
2.0
2.5
3.0
3.5
4.0

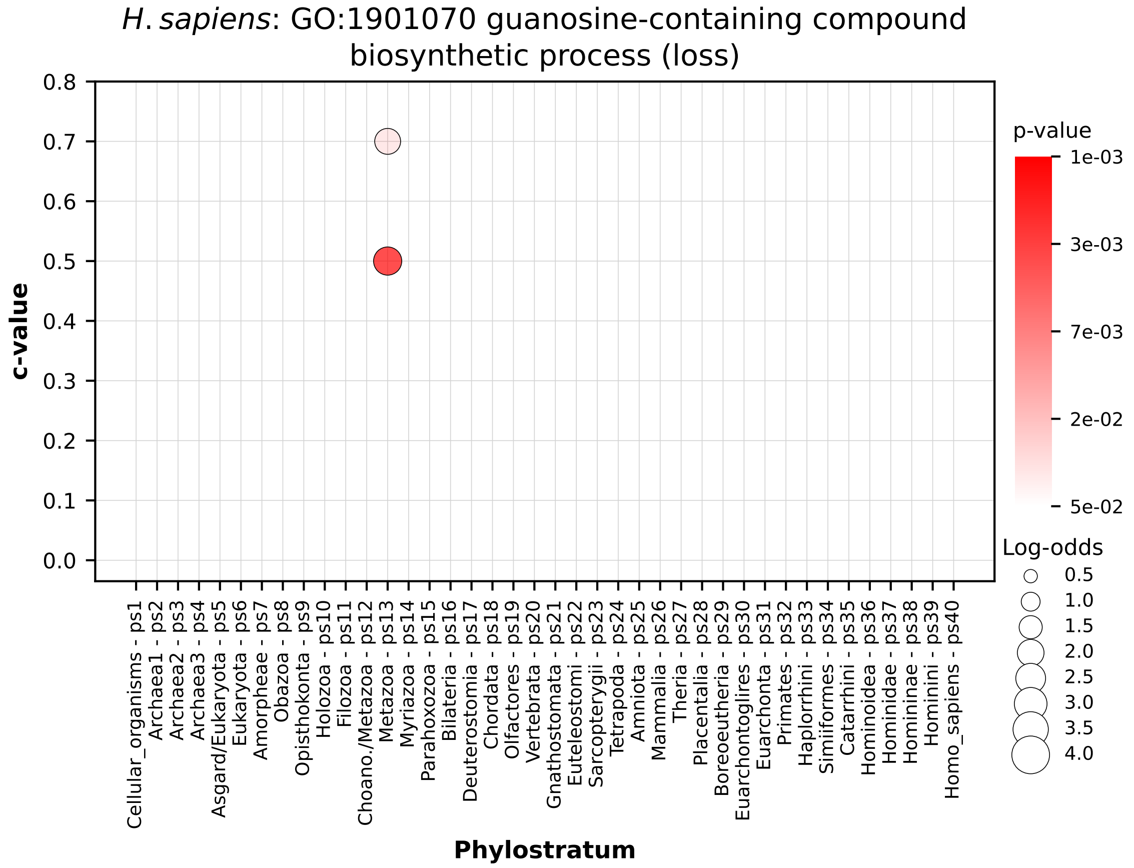

H. sapiens: GO:1901070 guanosine-containing compound biosynthetic process (loss)
c-value
0.8
0.7
0.6
0.5
0.4
0.3
0.2
0.1
0.0
Phylostratum
Cellular_organisms - ps1
Archaea1 - ps2
Archaea2 - ps3
Archaea3 - ps4
Asgard/Eukaryota - ps5
Eukaryota - ps6
Amorpheae - ps7
Obazoa - ps8
Opisthokonta - ps9
Holozoa - ps10
Filozoa - ps11
Choano./Metazoa - ps12
Metazoa - ps13
Myriazoa - ps14
Parahoxozoa - ps15
Bilateria - ps16
Deuterostomia - ps17
Chordata - ps18
Olfactores - ps19
Vertebrata - ps20
Gnathostomata - ps21
Euteleostomi - ps22
Sarcopterygii - ps23
Tetrapoda - ps24
Amniota - ps25
Mammalia - ps26
Theria - ps27
Placentalia - ps28
Boreoeutheria - ps29
Euarchontoglires - ps30
Euarchonta - ps31
Primates - ps32
Haplorrhini - ps33
Simiiformes - ps34
Catarrhini - ps35
Hominoidea - ps36
Hominidae - ps37
Homininae - ps38
Hominini - ps39
Homo_sapiens - ps40
p-value
1e-03
3e-03
7e-03
2e-02
5e-02
Log-odds
0.5
1.0
1.5
2.0
2.5
3.0
3.5
4.0

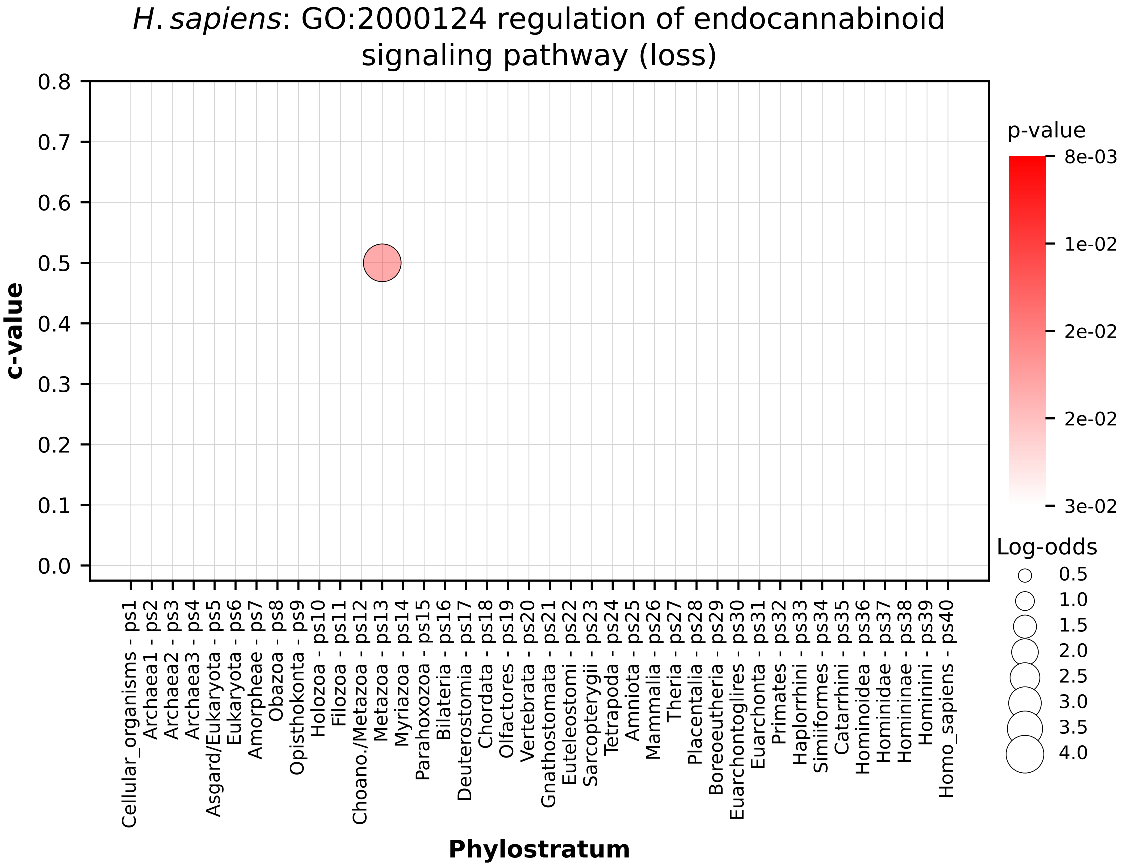

H. sapiens: GO:2000124 regulation of endocannabinoid signaling pathway (loss)
c-value
0.8
0.7
0.6
0.5
0.4
0.3
0.2
0.1
0.0
Phylostratum
Cellular_organisms - ps1
Archaea1 - ps2
Archaea2 - ps3
Archaea3 - ps4
Asgard/Eukaryota - ps5
Eukaryota - ps6
Amorpheae - ps7
Obazoa - ps8
Opisthokonta - ps9
Holozoa - ps10
Filozoa - ps11
Choano./Metazoa - ps12
Metazoa - ps13
Myriazoa - ps14
Parahoxozoa - ps15
Bilateria - ps16
Deuterostomia - ps17
Chordata - ps18
Olfactores - ps19
Vertebrata - ps20
Gnathostomata - ps21
Euteleostomi - ps22
Sarcopterygii - ps23
Tetrapoda - ps24
Amniota - ps25
Mammalia - ps26
Theria - ps27
Placentalia - ps28
Boreoeutheria - ps29
Euarchontoglires - ps30
Euarchonta - ps31
Primates - ps32
Haplorrhini - ps33
Simiiformes - ps34
Catarrhini - ps35
Hominoidea - ps36
Hominidae - ps37
Homininae - ps38
Hominini - ps39
Homo_sapiens - ps40
p-value
8e-03
1e-02
2e-02
2e-02
3e-02
Log-odds
0.5
1.0
1.5
2.0
2.5
3.0
3.5
4.0

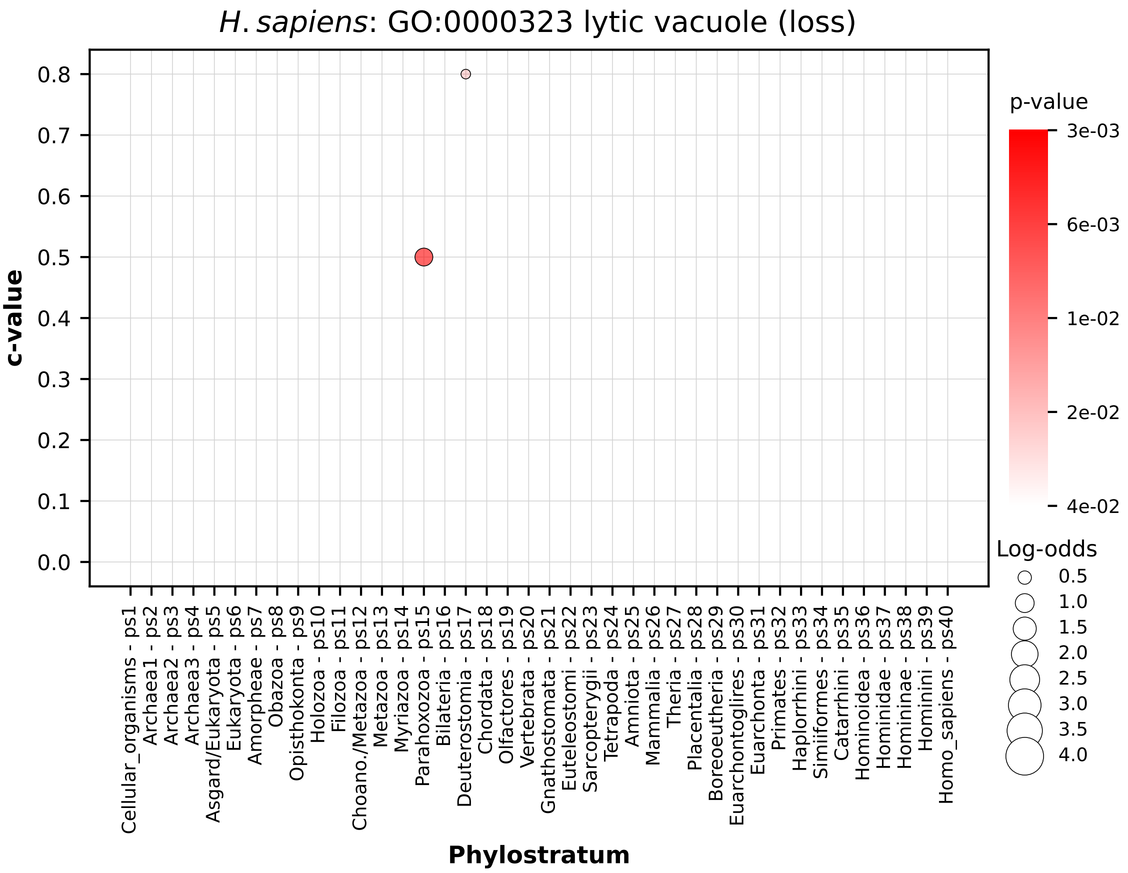
H. sapiens: GO:0000323 lytic vacuole (loss)
c-value
0.0
0.1
0.2
0.3
0.4
0.5
0.6
0.7
0.8
Phylostratum
Cellular_organisms - ps1
Archaea1 - ps2
Archaea2 - ps3
Archaea3 - ps4
Asgard/Eukaryota - ps5
Eukaryota - ps6
Amorpheae - ps7
Obazoa - ps8
Opisthokonta - ps9
Holozoa - ps10
Filozoa - ps11
Choano./Metazoa - ps12
Metazoa - ps13
Myriazoa - ps14
Parahoxozoa - ps15
Bilateria - ps16
Deuterostomia - ps17
Chordata - ps18
Olfactores - ps19
Vertebrata - ps20
Gnathostomata - ps21
Euteleostomi - ps22
Sarcopterygii - ps23
Tetrapoda - ps24
Amniota - ps25
Mammalia - ps26
Theria - ps27
Placentalia - ps28
Boreoeutheria - ps29
Euarchontoglires - ps30
Euarchonta - ps31
Primates - ps32
Haplorrhini - ps33
Simiiformes - ps34
Catarrhini - ps35
Hominoidea - ps36
Hominidae - ps37
Homininae - ps38
Hominini - ps39
Homo_sapiens - ps40
p-value
3e-03
6e-03
1e-02
2e-02
4e-02
Log-odds
0.5
1.0
1.5
2.0
2.5
3.0
3.5
4.0

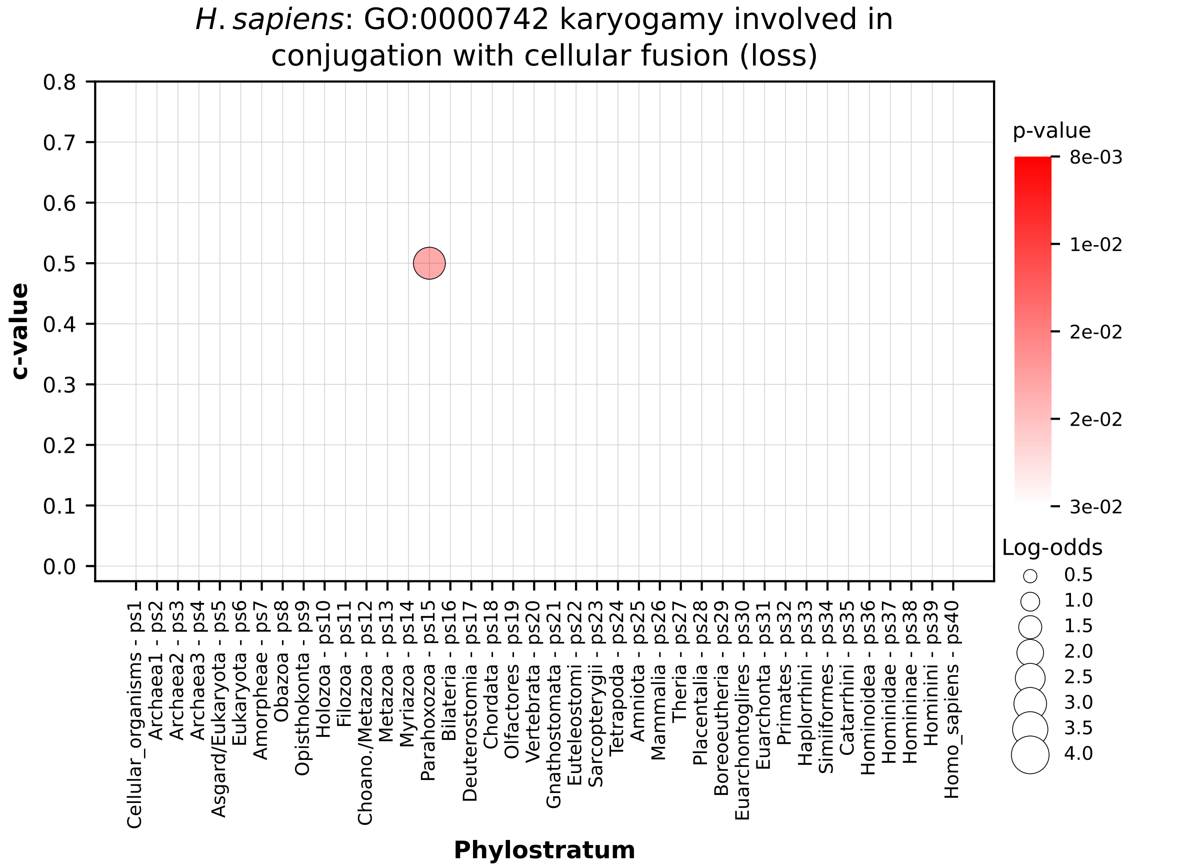
H. sapiens: GO:0000742 karyogamy involved in conjugation with cellular fusion (loss)
c-value
0.8
0.7
0.6
0.5
0.4
0.3
0.2
0.1
0.0
Phylostratum
Cellular_organisms - ps1
Archaea1 - ps2
Archaea2 - ps3
Archaea3 - ps4
Asgard/Eukaryota - ps5
Eukaryota - ps6
Amorpheae - ps7
Obazoa - ps8
Opisthokonta - ps9
Holozoa - ps10
Filozoa - ps11
Choano./Metazoa - ps12
Metazoa - ps13
Myriazoa - ps14
Parahoxozoa - ps15
Bilateria - ps16
Deuterostomia - ps17
Chordata - ps18
Olfactores - ps19
Vertebrata - ps20
Gnathostomata - ps21
Euteleostomi - ps22
Sarcopterygii - ps23
Tetrapoda - ps24
Amniota - ps25
Mammalia - ps26
Theria - ps27
Placentalia - ps28
Boreoeutheria - ps29
Euarchontoglires - ps30
Euarchonta - ps31
Primates - ps32
Haplorrhini - ps33
Simiiformes - ps34
Catarrhini - ps35
Hominoidea - ps36
Hominidae - ps37
Homininae - ps38
Hominini - ps39
Homo_sapiens - ps40
p-value
8e-03
1e-02
2e-02
2e-02
3e-02
Log-odds
0.5
1.0
1.5
2.0
2.5
3.0
3.5
4.0

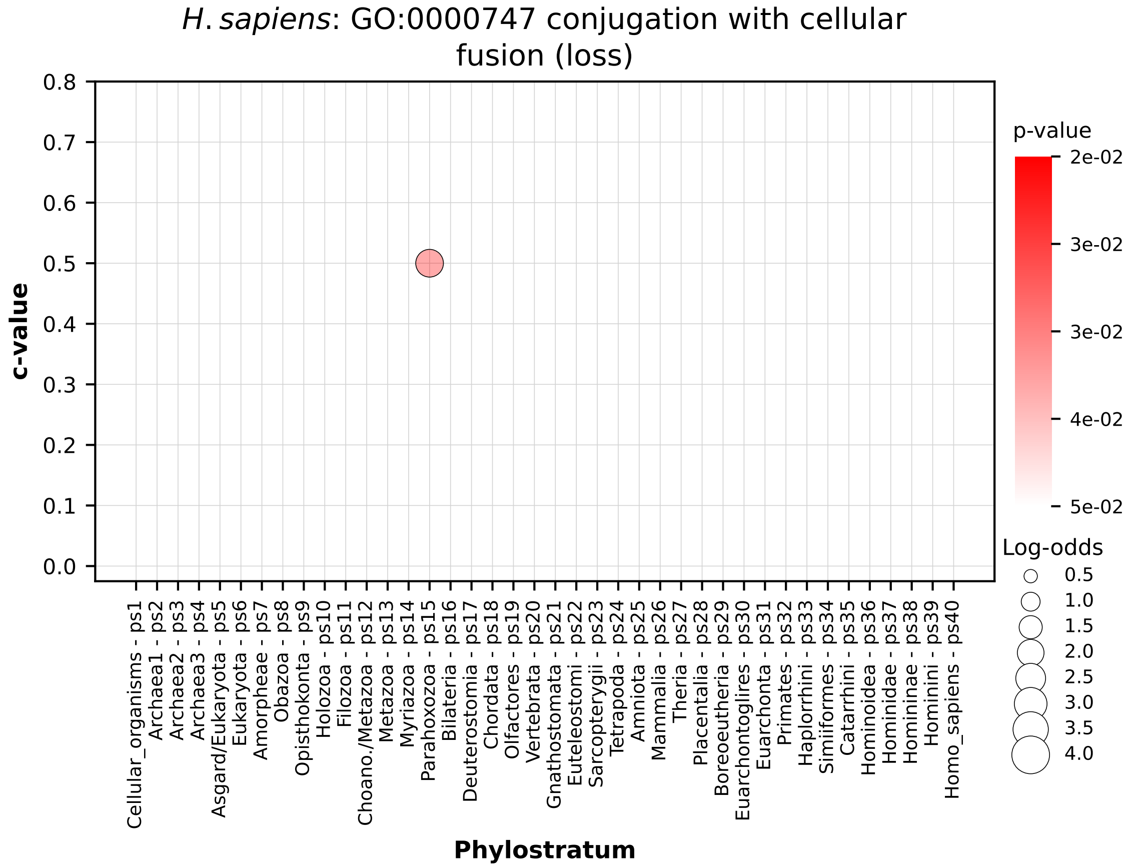

H. sapiens: GO:0000747 conjugation with cellular fusion (loss)
c-value
0.0
0.1
0.2
0.3
0.4
0.5
0.6
0.7
0.8
Phylostratum
Cellular_organisms - ps1
Archaea1 - ps2
Archaea2 - ps3
Archaea3 - ps4
Asgard/Eukaryota - ps5
Eukaryota - ps6
Amorpheae - ps7
Obazoa - ps8
Opisthokonta - ps9
Holozoa - ps10
Filozoa - ps11
Choano./Metazoa - ps12
Metazoa - ps13
Myriazoa - ps14
Parahoxozoa - ps15
Bilateria - ps16
Deuterostomia - ps17
Chordata - ps18
Olfactores - ps19
Vertebrata - ps20
Gnathostomata - ps21
Euteleostomi - ps22
Sarcopterygii - ps23
Tetrapoda - ps24
Amniota - ps25
Mammalia - ps26
Theria - ps27
Placentalia - ps28
Boreoeutheria - ps29
Euarchontoglires - ps30
Euarchonta - ps31
Primates - ps32
Haplorrhini - ps33
Simiiformes - ps34
Catarrhini - ps35
Hominoidea - ps36
Hominidae - ps37
Homininae - ps38
Hominini - ps39
Homo_sapiens - ps40
p-value
2e-02
3e-02
3e-02
4e-02
5e-02
Log-odds
0.5
1.0
1.5
2.0
2.5
3.0
3.5
4.0

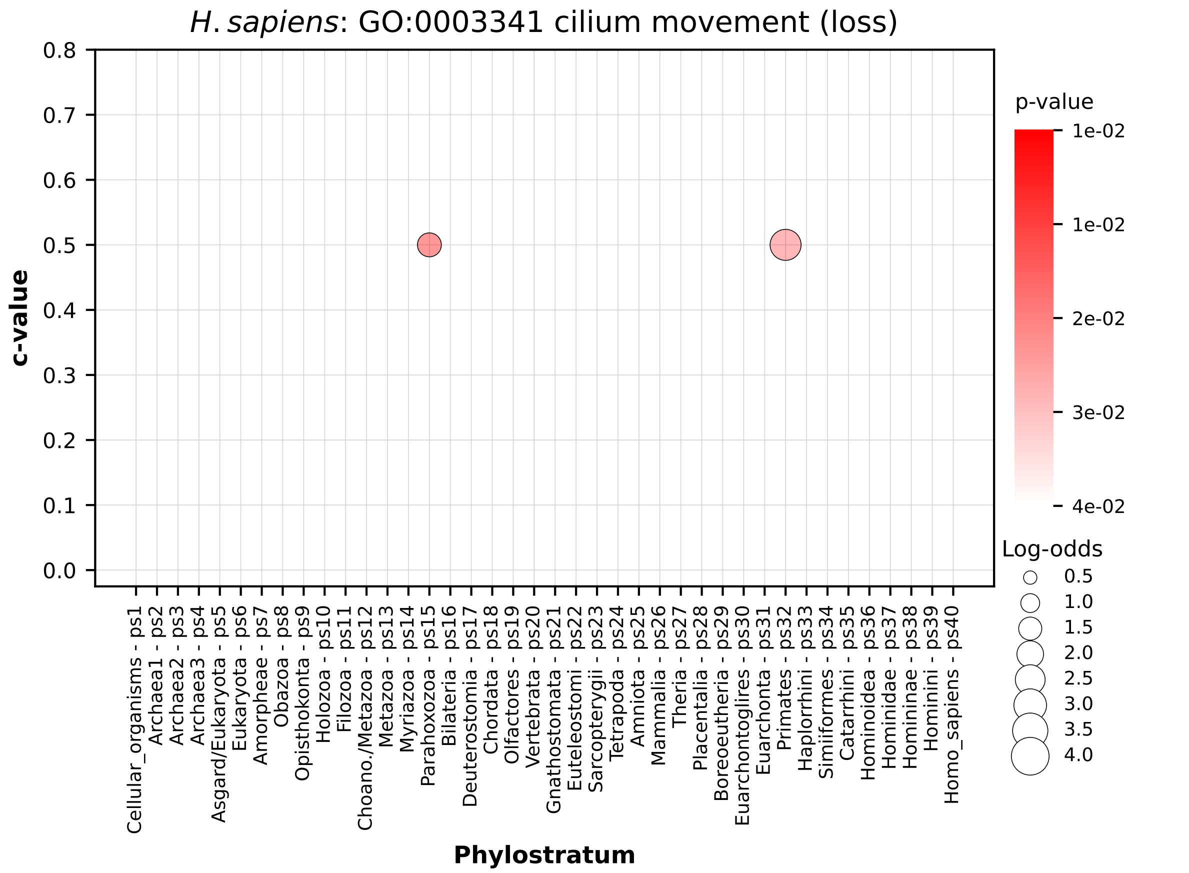
H. sapiens: GO:0003341 cilium movement (loss)
c-value
0.0
0.1
0.2
0.3
0.4
0.5
0.6
0.7
0.8
Phylostratum
Cellular_organisms - ps1
Archaea1 - ps2
Archaea2 - ps3
Archaea3 - ps4
Asgard/Eukaryota - ps5
Eukaryota - ps6
Amorpheae - ps7
Obazoa - ps8
Opisthokonta - ps9
Holozoa - ps10
Filozoa - ps11
Choano./Metazoa - ps12
Metazoa - ps13
Myriazoa - ps14
Parahoxozoa - ps15
Bilateria - ps16
Deuterostomia - ps17
Chordata - ps18
Olfactores - ps19
Vertebrata - ps20
Gnathostomata - ps21
Euteleostomi - ps22
Sarcopterygii - ps23
Tetrapoda - ps24
Amniota - ps25
Mammalia - ps26
Theria - ps27
Placentalia - ps28
Boreoeutheria - ps29
Euarchontoglires - ps30
Euarchonta - ps31
Primates - ps32
Haplorrhini - ps33
Simiiformes - ps34
Catarrhini - ps35
Hominoidea - ps36
Hominidae - ps37
Homininae - ps38
Hominini - ps39
Homo_sapiens - ps40
p-value
1e-02
1e-02
2e-02
3e-02
4e-02
Log-odds
0.5
1.0
1.5
2.0
2.5
3.0
3.5
4.0

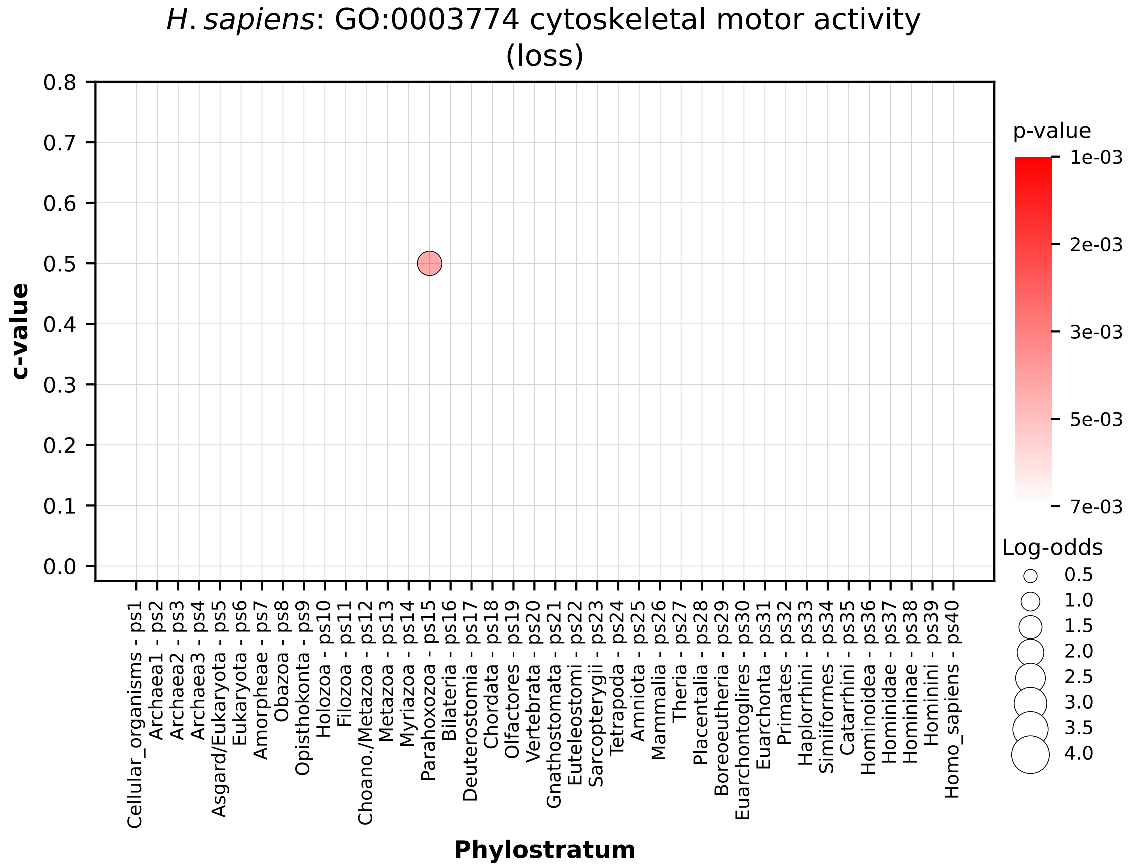

H. sapiens: GO:0003774 cytoskeletal motor activity (loss)
c-value
0.8
0.7
0.6
0.5
0.4
0.3
0.2
0.1
0.0
p-value
1e-03
2e-03
3e-03
5e-03
7e-03
Log-odds
0.5
1.0
1.5
2.0
2.5
3.0
3.5
4.0
Cellular_organisms - ps1
Archaea1 - ps2
Archaea2 - ps3
Archaea3 - ps4
Asgard/Eukaryota - ps5
Eukaryota - ps6
Amorpheae - ps7
Obazoa - ps8
Opisthokonta - ps9
Holozoa - ps10
Filozoa - ps11
Choano./Metazoa - ps12
Metazoa - ps13
Myriazoa - ps14
Parahoxozoa - ps15
Bilateria - ps16
Deuterostomia - ps17
Chordata - ps18
Olfactores - ps19
Vertebrata - ps20
Gnathostomata - ps21
Euteleostomi - ps22
Sarcopterygii - ps23
Tetrapoda - ps24
Amniota - ps25
Mammalia - ps26
Theria - ps27
Placentalia - ps28
Boreoeutheria - ps29
Euarchontoglires - ps30
Euarchonta - ps31
Primates - ps32
Haplorrhini - ps33
Simiiformes - ps34
Catarrhini - ps35
Hominoidea - ps36
Hominidae - ps37
Homininae - ps38
Hominini - ps39
Homo_sapiens - ps40
Phylostratum

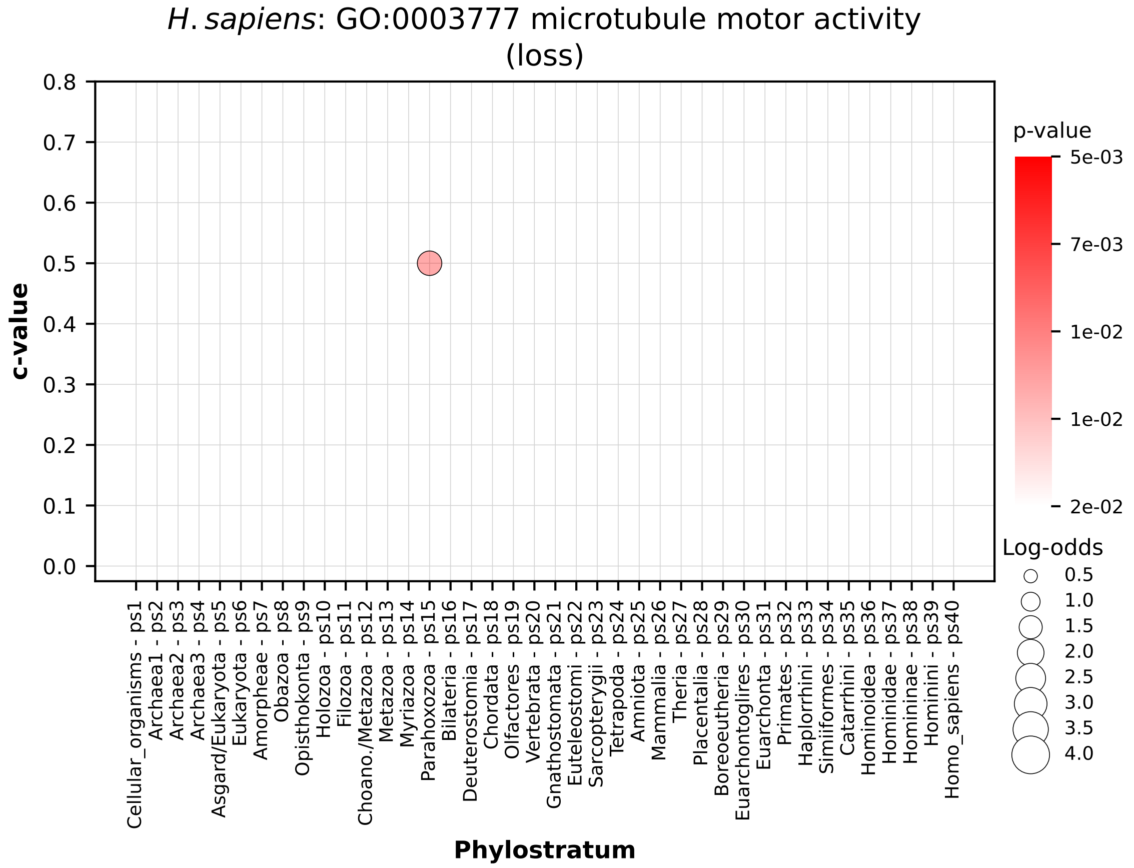

H. sapiens: GO:0003777 microtubule motor activity
(loss)
c-value
0.8
0.7
0.6
0.5
0.4
0.3
0.2
0.1
0.0
Phylostratum
Cellular_organisms - ps1
Archaea1 - ps2
Archaea2 - ps3
Archaea3 - ps4
Asgard/Eukaryota - ps5
Eukaryota - ps6
Amorpheae - ps7
Obazoa - ps8
Opisthokonta - ps9
Holozoa - ps10
Filozoa - ps11
Choano./Metazoa - ps12
Metazoa - ps13
Myriazoa - ps14
Parahoxozoa - ps15
Bilateria - ps16
Deuterostomia - ps17
Chordata - ps18
Olfactores - ps19
Vertebrata - ps20
Gnathostomata - ps21
Euteleostomi - ps22
Sarcopterygii - ps23
Tetrapoda - ps24
Amniota - ps25
Mammalia - ps26
Theria - ps27
Placentalia - ps28
Boreoeutheria - ps29
Euarchontoglires - ps30
Euarchonta - ps31
Primates - ps32
Haplorrhini - ps33
Simiiformes - ps34
Catarrhini - ps35
Hominoidea - ps36
Hominidae - ps37
Homininae - ps38
Hominini - ps39
Homo_sapiens - ps40
p-value
5e-03
7e-03
1e-02
1e-02
2e-02
Log-odds
0.5
1.0
1.5
2.0
2.5
3.0
3.5
4.0

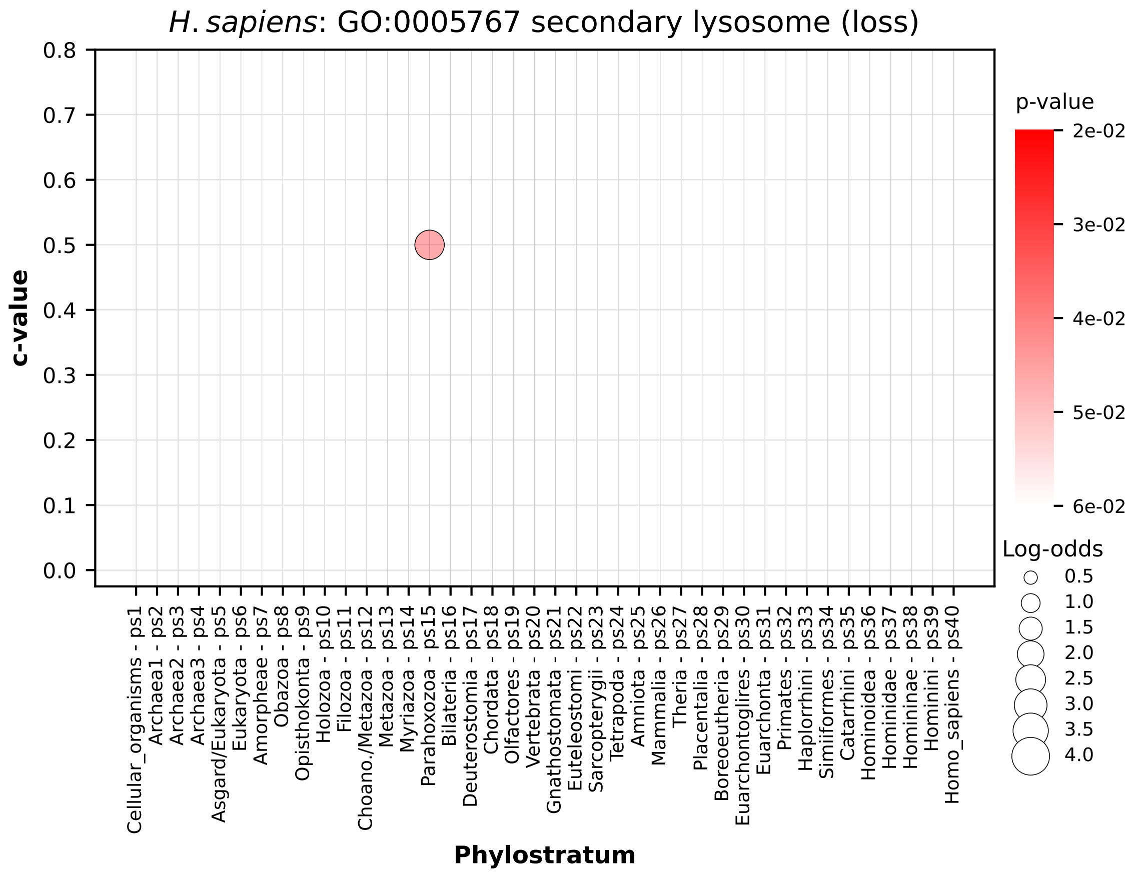

H. sapiens: GO:0005767 secondary lysosome (loss)
c-value
0.8
0.7
0.6
0.5
0.4
0.3
0.2
0.1
0.0
Phylostratum
Cellular_organisms - ps1
Archaea1 - ps2
Archaea2 - ps3
Archaea3 - ps4
Asgard/Eukaryota - ps5
Eukaryota - ps6
Amorpheae - ps7
Obazoa - ps8
Opisthokonta - ps9
Holozoa - ps10
Filozoa - ps11
Choano./Metazoa - ps12
Metazoa - ps13
Myriazoa - ps14
Parahoxozoa - ps15
Bilateria - ps16
Deuterostomia - ps17
Chordata - ps18
Olfactores - ps19
Vertebrata - ps20
Gnathostomata - ps21
Euteleostomi - ps22
Sarcopterygii - ps23
Tetrapoda - ps24
Amniota - ps25
Mammalia - ps26
Theria - ps27
Placentalia - ps28
Boreoeutheria - ps29
Euarchontoglires - ps30
Euarchonta - ps31
Primates - ps32
Haplorrhini - ps33
Simiiformes - ps34
Catarrhini - ps35
Hominoidea - ps36
Hominidae - ps37
Homininae - ps38
Hominini - ps39
Homo_sapiens - ps40
p-value
2e-02
3e-02
4e-02
5e-02
6e-02
Log-odds
0.5
1.0
1.5
2.0
2.5
3.0
3.5
4.0

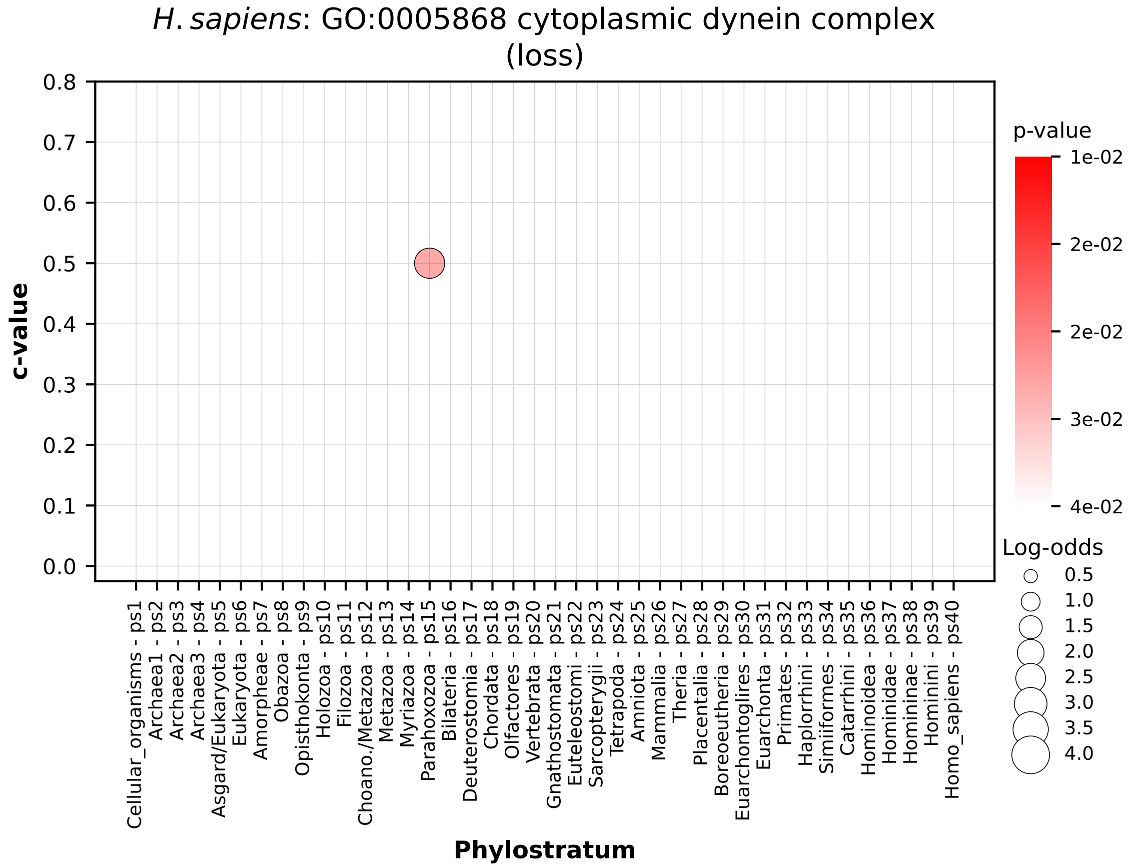

H. sapiens: GO:0005868 cytoplasmic dynein complex
(loss)
c-value
0.8
0.7
0.6
0.5
0.4
0.3
0.2
0.1
0.0
p-value
1e-02
2e-02
2e-02
3e-02
4e-02
Log-odds
0.5
1.0
1.5
2.0
2.5
3.0
3.5
4.0
Cellular_organisms - ps1
Archaea1 - ps2
Archaea2 - ps3
Archaea3 - ps4
Asgard/Eukaryota - ps5
Eukaryota - ps6
Amorpheae - ps7
Obazoa - ps8
Opisthokonta - ps9
Holozoa - ps10
Filozoa - ps11
Choano./Metazoa - ps12
Metazoa - ps13
Myriazoa - ps14
Parahoxozoa - ps15
Bilateria - ps16
Deuterostomia - ps17
Chordata - ps18
Olfactores - ps19
Vertebrata - ps20
Gnathostomata - ps21
Euteleostomi - ps22
Sarcopterygii - ps23
Tetrapoda - ps24
Amniota - ps25
Mammalia - ps26
Theria - ps27
Placentalia - ps28
Boreoeutheria - ps29
Euarchontoglires - ps30
Euarchonta - ps31
Primates - ps32
Haplorrhini - ps33
Simiiformes - ps34
Catarrhini - ps35
Hominoidea - ps36
Hominidae - ps37
Homininae - ps38
Hominini - ps39
Homo_sapiens - ps40
Phylostratum

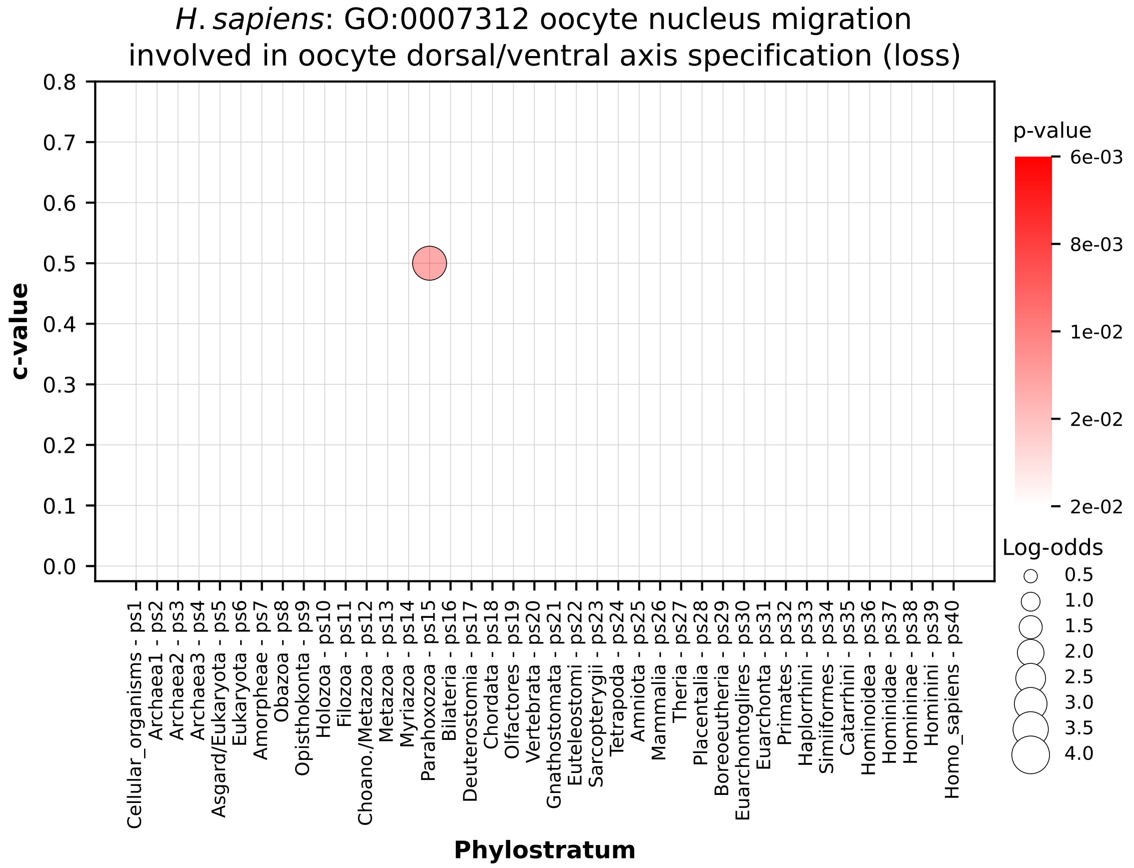
H. sapiens: GO:0007312 oocyte nucleus migration involved in oocyte dorsal/ventral axis specification (loss)
c-value
0.0
0.1
0.2
0.3
0.4
0.5
0.6
0.7
0.8
Phylostratum
Cellular_organisms - ps1
Archaea1 - ps2
Archaea2 - ps3
Archaea3 - ps4
Asgard/Eukaryota - ps5
Eukaryota - ps6
Amorpheae - ps7
Obazoa - ps8
Opisthokonta - ps9
Holozoa - ps10
Filozoa - ps11
Choano./Metazoa - ps12
Metazoa - ps13
Myriazoa - ps14
Parahoxozoa - ps15
Bilateria - ps16
Deuterostomia - ps17
Chordata - ps18
Olfactores - ps19
Vertebrata - ps20
Gnathostomata - ps21
Euteleostomi - ps22
Sarcopterygii - ps23
Tetrapoda - ps24
Amniota - ps25
Mammalia - ps26
Theria - ps27
Placentalia - ps28
Boreoeutheria - ps29
Euarchontoglires - ps30
Euarchonta - ps31
Primates - ps32
Haplorrhini - ps33
Simiiformes - ps34
Catarrhini - ps35
Hominoidea - ps36
Hominidae - ps37
Homininae - ps38
Hominini - ps39
Homo_sapiens - ps40
p-value
6e-03
8e-03
1e-02
2e-02
2e-02
Log-odds
0.5
1.0
1.5
2.0
2.5
3.0
3.5
4.0

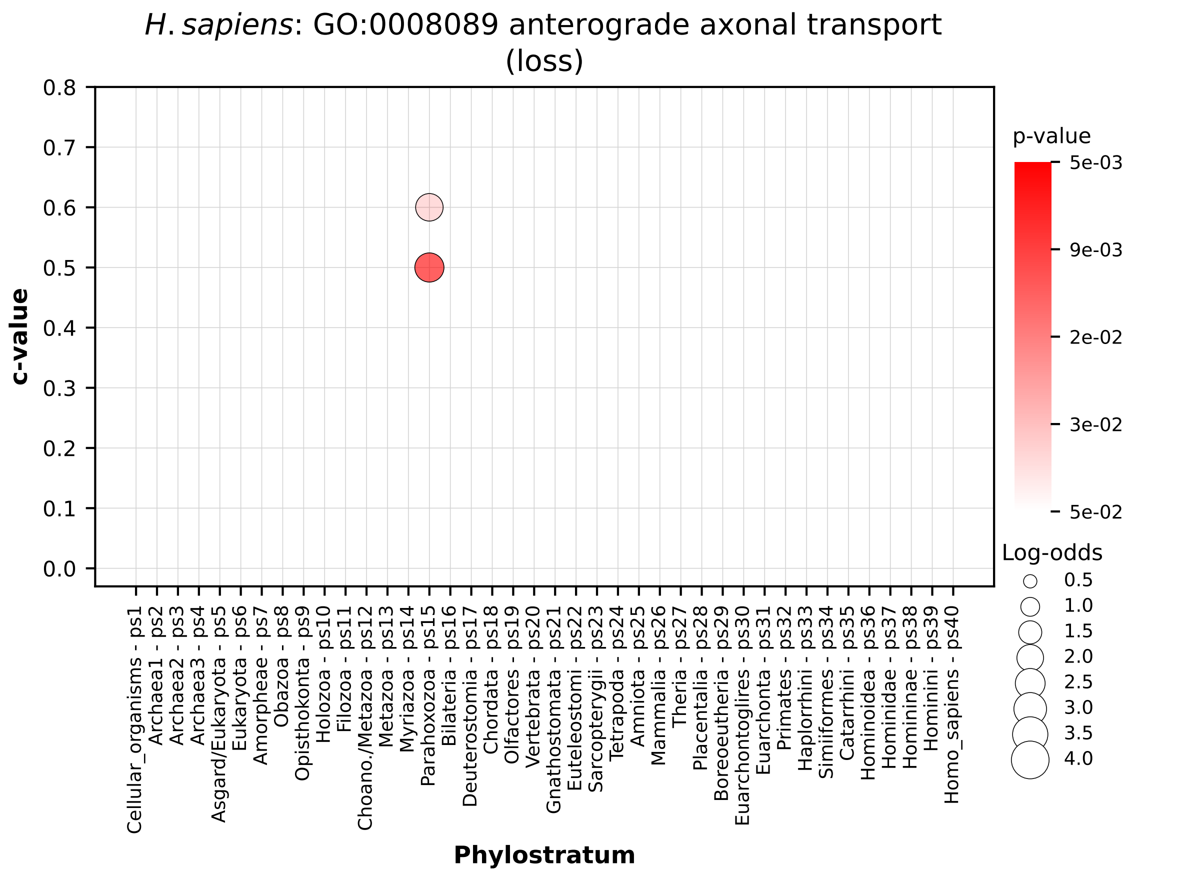

H. sapiens: GO:0008089 anterograde axonal transport
(loss)
c-value
0.8
0.7
0.6
0.5
0.4
0.3
0.2
0.1
0.0
p-value
5e-03
9e-03
2e-02
3e-02
5e-02
Log-odds
0.5
1.0
1.5
2.0
2.5
3.0
3.5
4.0
Cellular_organisms - ps1
Archaea1 - ps2
Archaea2 - ps3
Archaea3 - ps4
Asgard/Eukaryota - ps5
Eukaryota - ps6
Amorpheae - ps7
Obazoa - ps8
Opisthokonta - ps9
Holozoa - ps10
Filozoa - ps11
Choano./Metazoa - ps12
Metazoa - ps13
Myriazoa - ps14
Parahoxozoa - ps15
Bilateria - ps16
Deuterostomia - ps17
Chordata - ps18
Olfactores - ps19
Vertebrata - ps20
Gnathostomata - ps21
Euteleostomi - ps22
Sarcopterygii - ps23
Tetrapoda - ps24
Amniota - ps25
Mammalia - ps26
Theria - ps27
Placentalia - ps28
Boreoeutheria - ps29
Euarchontoglires - ps30
Euarchonta - ps31
Primates - ps32
Haplorrhini - ps33
Simiiformes - ps34
Catarrhini - ps35
Hominoidea - ps36
Hominidae - ps37
Homininae - ps38
Hominini - ps39
Homo_sapiens - ps40
Phylostratum

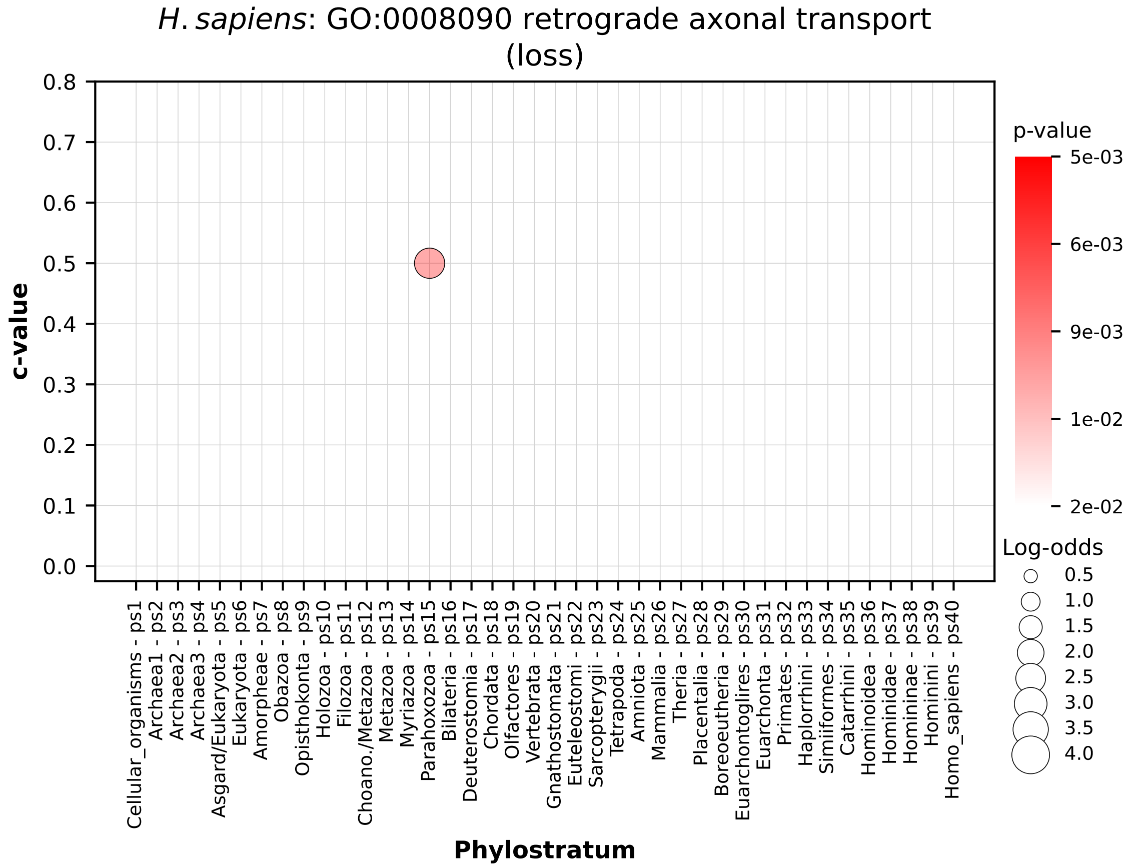
H. sapiens: GO:0008090 retrograde axonal transport
(loss)
c-value
0.8
0.7
0.6
0.5
0.4
0.3
0.2
0.1
0.0
p-value
5e-03
6e-03
9e-03
1e-02
2e-02
Log-odds
0.5
1.0
1.5
2.0
2.5
3.0
3.5
4.0
Cellular_organisms - ps1
Archaea1 - ps2
Archaea2 - ps3
Archaea3 - ps4
Asgard/Eukaryota - ps5
Eukaryota - ps6
Amorpheae - ps7
Obazoa - ps8
Opisthokonta - ps9
Holozoa - ps10
Filozoa - ps11
Choano./Metazoa - ps12
Metazoa - ps13
Myriazoa - ps14
Parahoxozoa - ps15
Bilateria - ps16
Deuterostomia - ps17
Chordata - ps18
Olfactores - ps19
Vertebrata - ps20
Gnathostomata - ps21
Euteleostomi - ps22
Sarcopterygii - ps23
Tetrapoda - ps24
Amniota - ps25
Mammalia - ps26
Theria - ps27
Placentalia - ps28
Boreoeutheria - ps29
Euarchontoglires - ps30
Euarchonta - ps31
Primates - ps32
Haplorrhini - ps33
Simiiformes - ps34
Catarrhini - ps35
Hominoidea - ps36
Hominidae - ps37
Homininae - ps38
Hominini - ps39
Homo_sapiens - ps40
Phylostratum

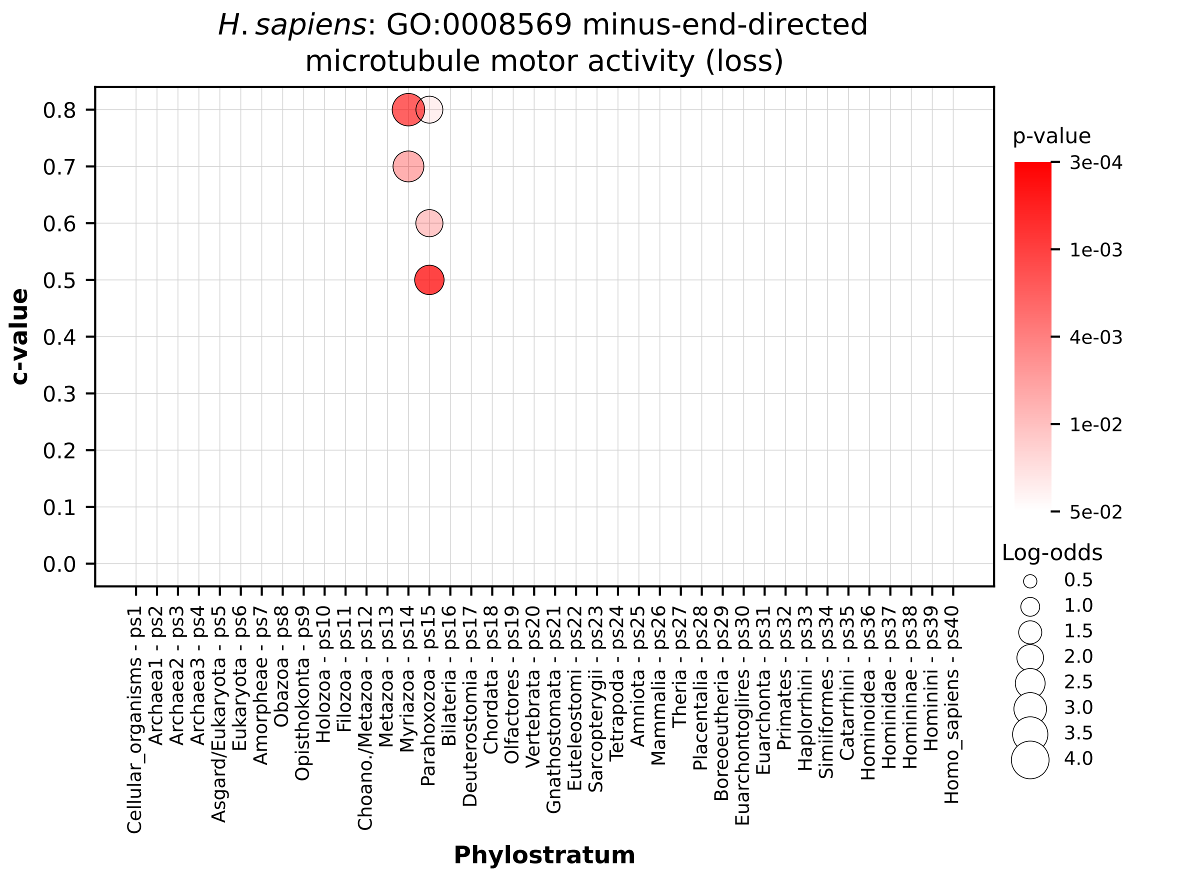

H. sapiens: GO:0008569 minus-end-directed microtubule motor activity (loss)
c-value
0.8
0.7
0.6
0.5
0.4
0.3
0.2
0.1
0.0
p-value
3e-04
1e-03
4e-03
1e-02
5e-02
Log-odds
0.5
1.0
1.5
2.0
2.5
3.0
3.5
4.0
Cellular_organisms - ps1
Archaea1 - ps2
Archaea2 - ps3
Archaea3 - ps4
Asgard/Eukaryota - ps5
Eukaryota - ps6
Amorpheae - ps7
Obazoa - ps8
Opisthokonta - ps9
Holozoa - ps10
Filozoa - ps11
Choano./Metazoa - ps12
Metazoa - ps13
Myriazoa - ps14
Parahoxozoa - ps15
Bilateria - ps16
Deuterostomia - ps17
Chordata - ps18
Olfactores - ps19
Vertebrata - ps20
Gnathostomata - ps21
Euteleostomi - ps22
Sarcopterygii - ps23
Tetrapoda - ps24
Amniota - ps25
Mammalia - ps26
Theria - ps27
Placentalia - ps28
Boreoeutheria - ps29
Euarchontoglires - ps30
Euarchonta - ps31
Primates - ps32
Haplorrhini - ps33
Simiiformes - ps34
Catarrhini - ps35
Hominoidea - ps36
Hominidae - ps37
Homininae - ps38
Hominini - ps39
Homo_sapiens - ps40
Phylostratum

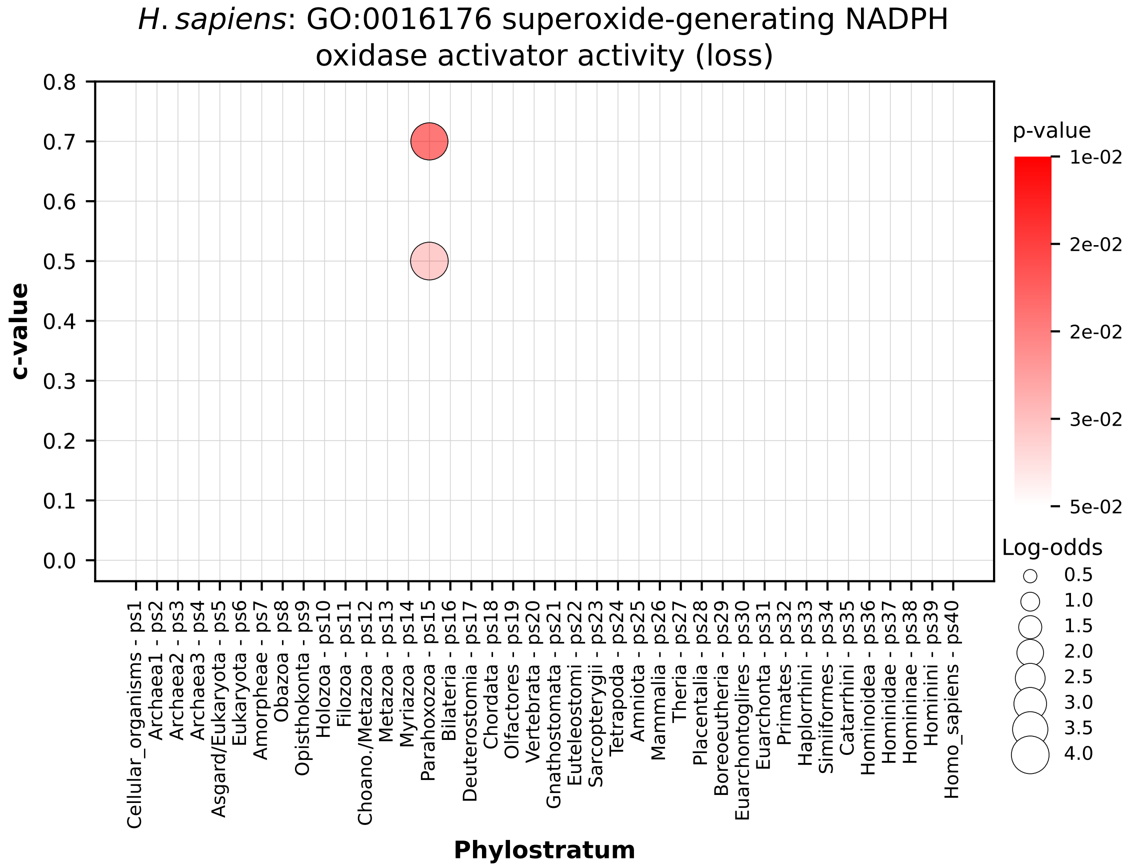
H. sapiens: GO:0016176 superoxide-generating NADPH oxidase activator activity (loss)
c-value
0.8
0.7
0.6
0.5
0.4
0.3
0.2
0.1
0.0
Phylostratum
Cellular_organisms - ps1
Archaea1 - ps2
Archaea2 - ps3
Archaea3 - ps4
Asgard/Eukaryota - ps5
Eukaryota - ps6
Amorpheae - ps7
Obazoa - ps8
Opisthokonta - ps9
Holozoa - ps10
Filozoa - ps11
Choano./Metazoa - ps12
Metazoa - ps13
Myriazoa - ps14
Parahoxozoa - ps15
Bilateria - ps16
Deuterostomia - ps17
Chordata - ps18
Olfactores - ps19
Vertebrata - ps20
Gnathostomata - ps21
Euteleostomi - ps22
Sarcopterygii - ps23
Tetrapoda - ps24
Amniota - ps25
Mammalia - ps26
Theria - ps27
Placentalia - ps28
Boreoeutheria - ps29
Euarchontoglires - ps30
Euarchonta - ps31
Primates - ps32
Haplorrhini - ps33
Simiiformes - ps34
Catarrhini - ps35
Hominoidea - ps36
Hominidae - ps37
Homininae - ps38
Hominini - ps39
Homo_sapiens - ps40
p-value
1e-02
2e-02
2e-02
3e-02
5e-02
Log-odds
0.5
1.0
1.5
2.0
2.5
3.0
3.5
4.0

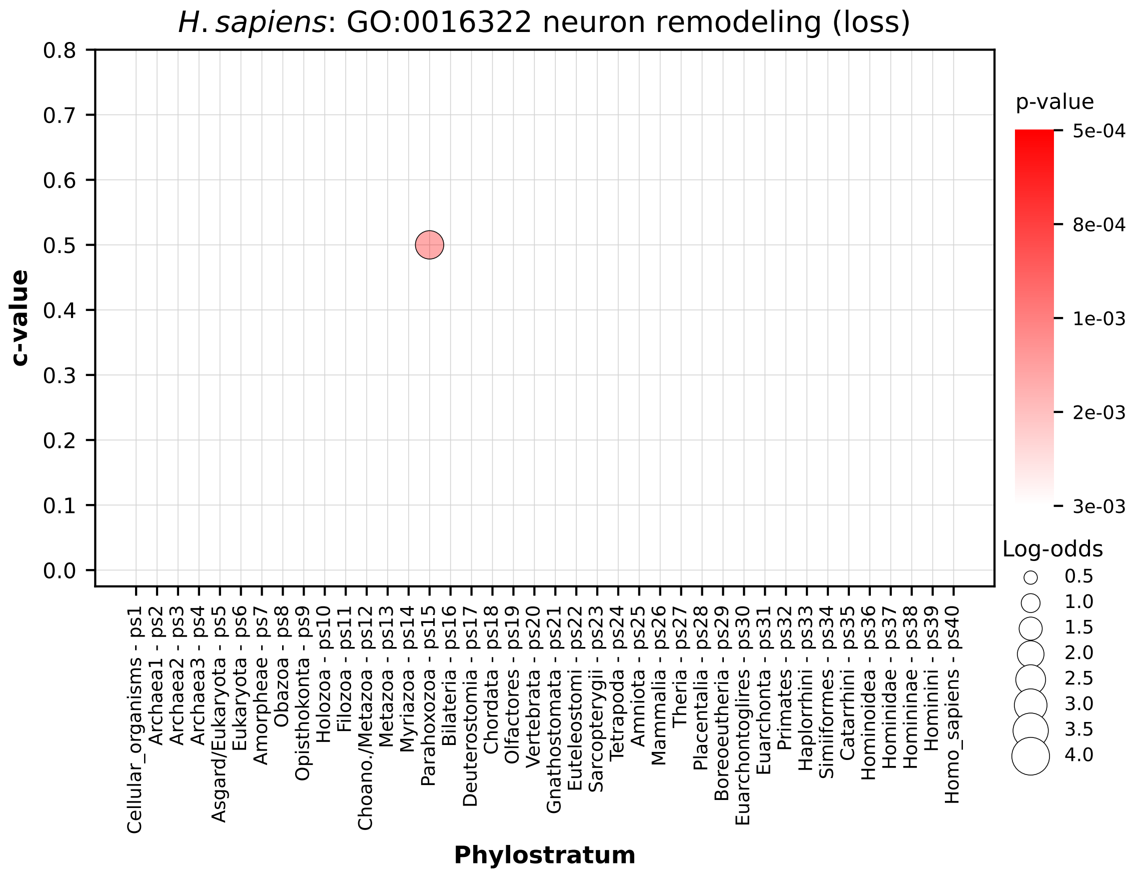

H. sapiens: GO:0016322 neuron remodeling (loss)
c-value
0.8
0.7
0.6
0.5
0.4
0.3
0.2
0.1
0.0
p-value
5e-04
8e-04
1e-03
2e-03
3e-03
Log-odds
0.5
1.0
1.5
2.0
2.5
3.0
3.5
4.0
Cellular_organisms - ps1
Archaea1 - ps2
Archaea2 - ps3
Archaea3 - ps4
Asgard/Eukaryota - ps5
Eukaryota - ps6
Amorpheae - ps7
Obazoa - ps8
Opisthokonta - ps9
Holozoa - ps10
Filozoa - ps11
Choano./Metazoa - ps12
Metazoa - ps13
Myriazoa - ps14
Parahoxozoa - ps15
Bilateria - ps16
Deuterostomia - ps17
Chordata - ps18
Olfactores - ps19
Vertebrata - ps20
Gnathostomata - ps21
Euteleostomi - ps22
Sarcopterygii - ps23
Tetrapoda - ps24
Amniota - ps25
Mammalia - ps26
Theria - ps27
Placentalia - ps28
Boreoeutheria - ps29
Euarchontoglires - ps30
Euarchonta - ps31
Primates - ps32
Haplorrhini - ps33
Simiiformes - ps34
Catarrhini - ps35
Hominoidea - ps36
Hominidae - ps37
Homininae - ps38
Hominini - ps39
Homo_sapiens - ps40
Phylostratum

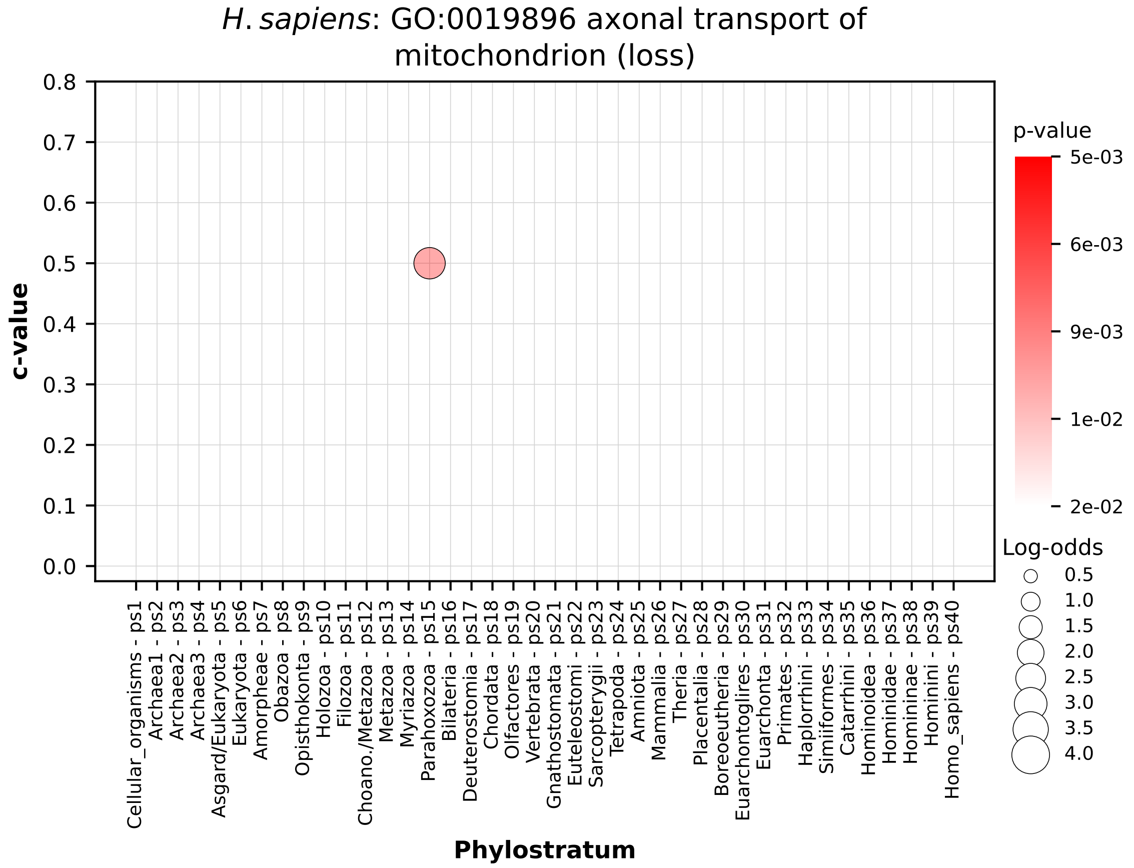

H. sapiens: GO:0019896 axonal transport of mitochondrion (loss)
c-value
0.8
0.7
0.6
0.5
0.4
0.3
0.2
0.1
0.0
p-value
5e-03
6e-03
9e-03
1e-02
2e-02
Log-odds
0.5
1.0
1.5
2.0
2.5
3.0
3.5
4.0
Cellular_organisms - ps1
Archaea1 - ps2
Archaea2 - ps3
Archaea3 - ps4
Asgard/Eukaryota - ps5
Eukaryota - ps6
Amorpheae - ps7
Obazoa - ps8
Opisthokonta - ps9
Holozoa - ps10
Filozoa - ps11
Choano./Metazoa - ps12
Metazoa - ps13
Myriazoa - ps14
Parahoxozoa - ps15
Bilateria - ps16
Deuterostomia - ps17
Chordata - ps18
Olfactores - ps19
Vertebrata - ps20
Gnathostomata - ps21
Euteleostomi - ps22
Sarcopterygii - ps23
Tetrapoda - ps24
Amniota - ps25
Mammalia - ps26
Theria - ps27
Placentalia - ps28
Boreoeutheria - ps29
Euarchontoglires - ps30
Euarchonta - ps31
Primates - ps32
Haplorrhini - ps33
Simiiformes - ps34
Catarrhini - ps35
Hominoidea - ps36
Hominidae - ps37
Homininae - ps38
Hominini - ps39
Homo_sapiens - ps40
Phylostratum

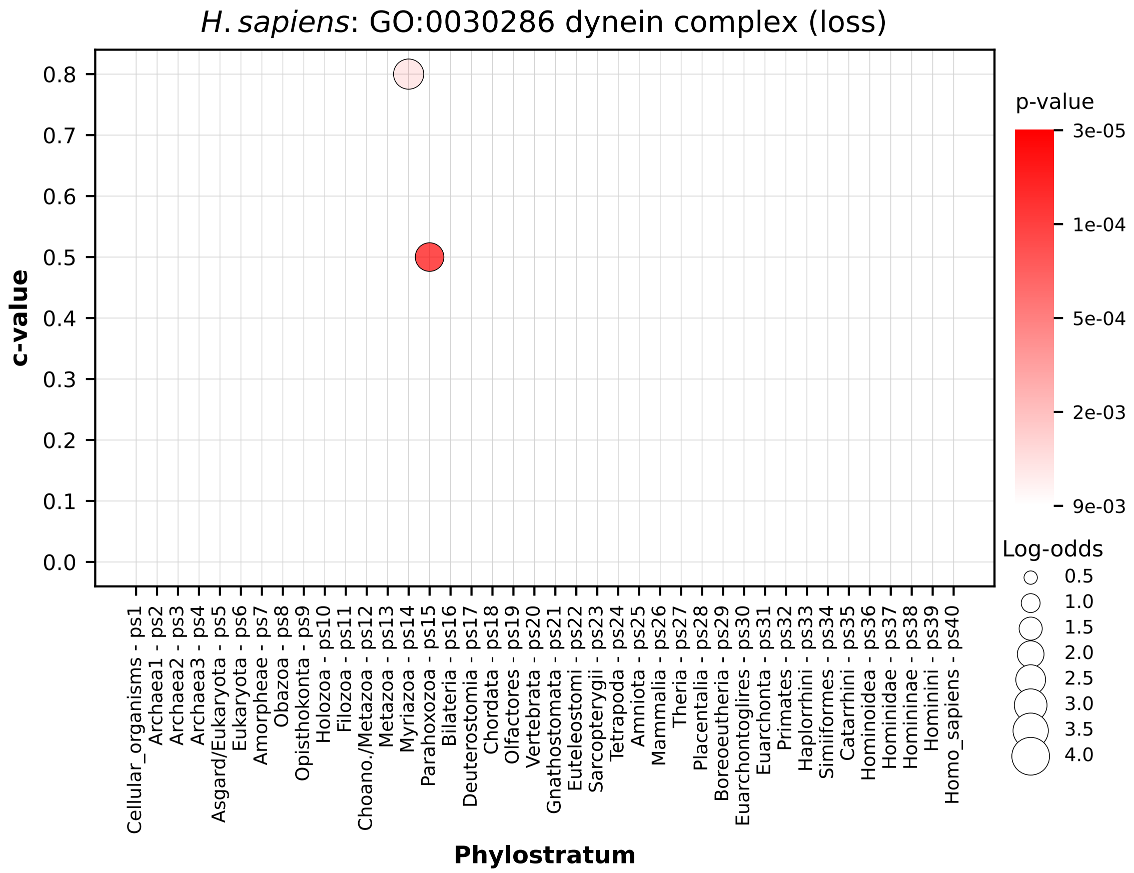

H. sapiens: GO:0030286 dynein complex (loss)
c-value
0.0
0.1
0.2
0.3
0.4
0.5
0.6
0.7
0.8
p-value
3e-05
1e-04
5e-04
2e-03
9e-03
Log-odds
0.5
1.0
1.5
2.0
2.5
3.0
3.5
4.0
Cellular_organisms - ps1
Archaea1 - ps2
Archaea2 - ps3
Archaea3 - ps4
Asgard/Eukaryota - ps5
Eukaryota - ps6
Amorpheae - ps7
Obazoa - ps8
Opisthokonta - ps9
Holozoa - ps10
Filozoa - ps11
Choano./Metazoa - ps12
Metazoa - ps13
Myriazoa - ps14
Parahoxozoa - ps15
Bilateria - ps16
Deuterostomia - ps17
Chordata - ps18
Olfactores - ps19
Vertebrata - ps20
Gnathostomata - ps21
Euteleostomi - ps22
Sarcopterygii - ps23
Tetrapoda - ps24
Amniota - ps25
Mammalia - ps26
Theria - ps27
Placentalia - ps28
Boreoeutheria - ps29
Euarchontoglires - ps30
Euarchonta - ps31
Primates - ps32
Haplorrhini - ps33
Simiiformes - ps34
Catarrhini - ps35
Hominoidea - ps36
Hominidae - ps37
Homininae - ps38
Hominini - ps39
Homo_sapiens - ps40
Phylostratum

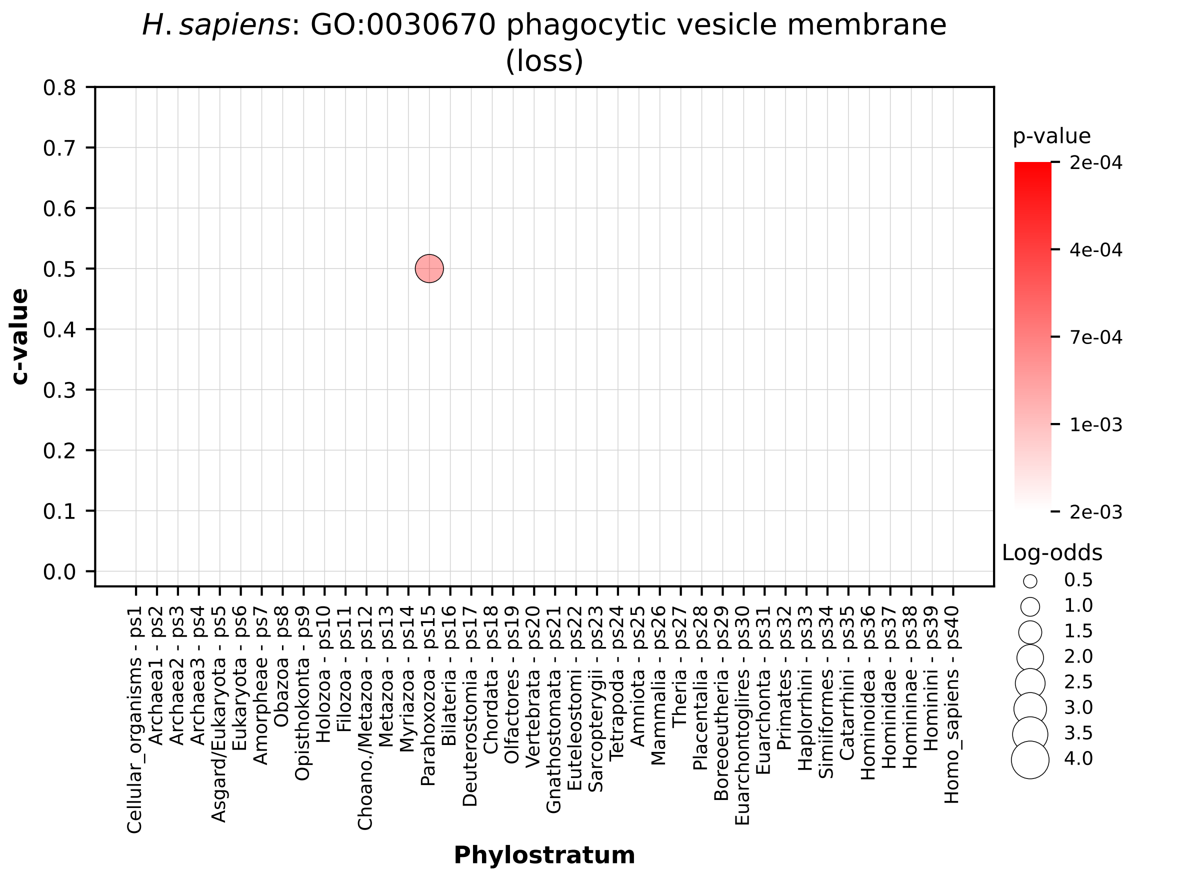

H. sapiens: GO:0030670 phagocytic vesicle membrane
(loss)
c-value
0.8
0.7
0.6
0.5
0.4
0.3
0.2
0.1
0.0
Cellular_organisms - ps1
Archaea1 - ps2
Archaea2 - ps3
Archaea3 - ps4
Asgard/Eukaryota - ps5
Eukaryota - ps6
Amorpheae - ps7
Obazoa - ps8
Opisthokonta - ps9
Holozoa - ps10
Filozoa - ps11
Choano./Metazoa - ps12
Metazoa - ps13
Myriazoa - ps14
Parahoxozoa - ps15
Bilateria - ps16
Deuterostomia - ps17
Chordata - ps18
Olfactores - ps19
Vertebrata - ps20
Gnathostomata - ps21
Euteleostomi - ps22
Sarcopterygii - ps23
Tetrapoda - ps24
Amniota - ps25
Mammalia - ps26
Theria - ps27
Placentalia - ps28
Boreoeutheria - ps29
Euarchontoglires - ps30
Euarchonta - ps31
Primates - ps32
Haplorrhini - ps33
Simiiformes - ps34
Catarrhini - ps35
Hominoidea - ps36
Hominidae - ps37
Homininae - ps38
Hominini - ps39
Homo_sapiens - ps40
Phylostratum
p-value
2e-04
4e-04
7e-04
1e-03
2e-03
Log-odds
0.5
1.0
1.5
2.0
2.5
3.0
3.5
4.0

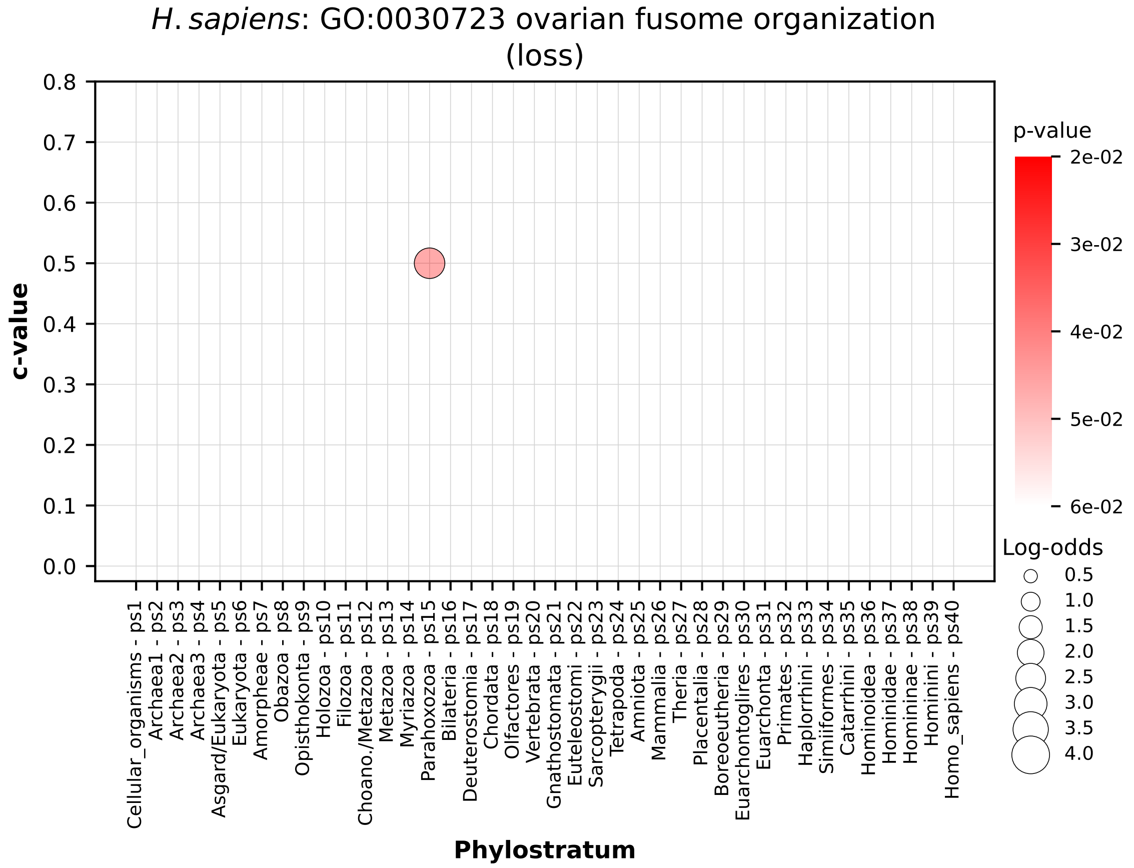

H. sapiens: GO:0030723 ovarian fusome organization
(loss)
c-value
0.8
0.7
0.6
0.5
0.4
0.3
0.2
0.1
0.0
p-value
2e-02
3e-02
4e-02
5e-02
6e-02
Log-odds
0.5
1.0
1.5
2.0
2.5
3.0
3.5
4.0
Cellular_organisms - ps1
Archaea1 - ps2
Archaea2 - ps3
Archaea3 - ps4
Asgard/Eukaryota - ps5
Eukaryota - ps6
Amorpheae - ps7
Obazoa - ps8
Opisthokonta - ps9
Holozoa - ps10
Filozoa - ps11
Choano./Metazoa - ps12
Metazoa - ps13
Myriazoa - ps14
Parahoxozoa - ps15
Bilateria - ps16
Deuterostomia - ps17
Chordata - ps18
Olfactores - ps19
Vertebrata - ps20
Gnathostomata - ps21
Euteleostomi - ps22
Sarcopterygii - ps23
Tetrapoda - ps24
Amniota - ps25
Mammalia - ps26
Theria - ps27
Placentalia - ps28
Boreoeutheria - ps29
Euarchontoglires - ps30
Euarchonta - ps31
Primates - ps32
Haplorrhini - ps33
Simiiformes - ps34
Catarrhini - ps35
Hominoidea - ps36
Hominidae - ps37
Homininae - ps38
Hominini - ps39
Homo_sapiens - ps40
Phylostratum

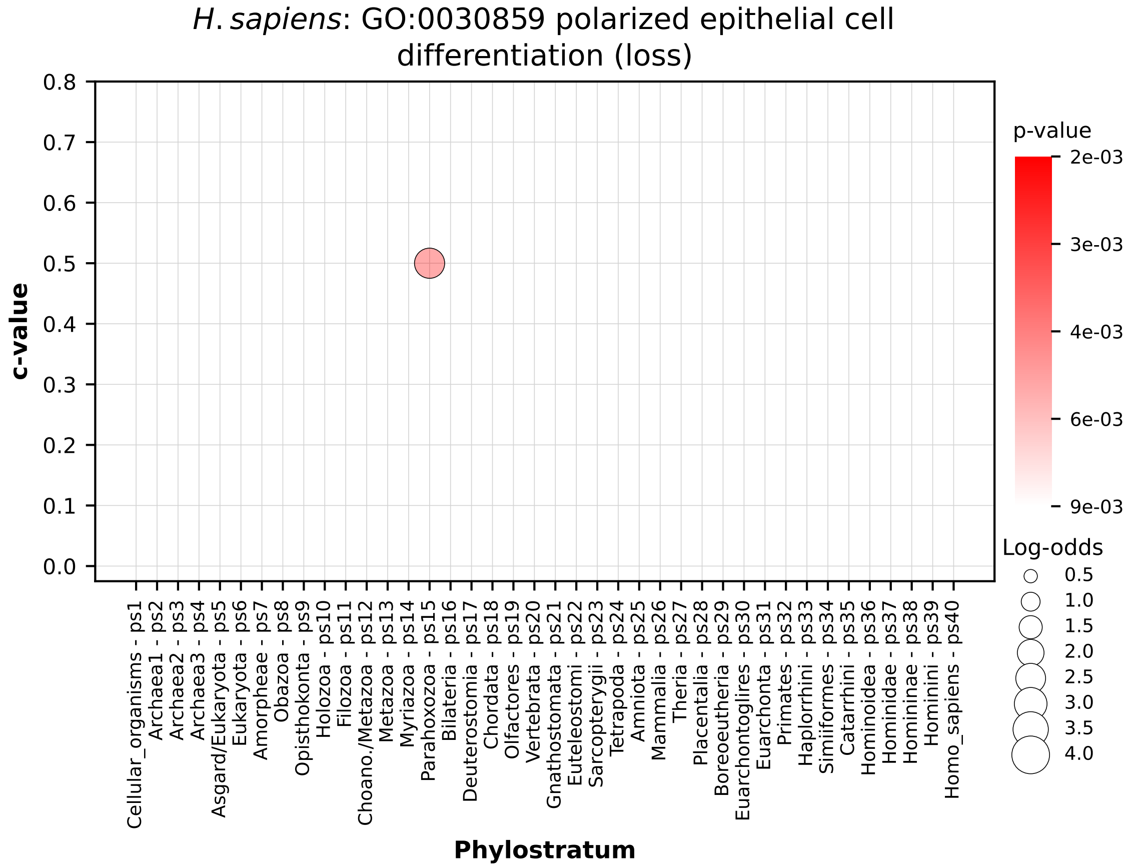

H. sapiens: GO:0030859 polarized epithelial cell differentiation (loss)
c-value
0.8
0.7
0.6
0.5
0.4
0.3
0.2
0.1
0.0
p-value
2e-03
3e-03
4e-03
6e-03
9e-03
Log-odds
0.5
1.0
1.5
2.0
2.5
3.0
3.5
4.0
Cellular_organisms - ps1
Archaea1 - ps2
Archaea2 - ps3
Archaea3 - ps4
Asgard/Eukaryota - ps5
Eukaryota - ps6
Amorpheae - ps7
Obazoa - ps8
Opisthokonta - ps9
Holozoa - ps10
Filozoa - ps11
Choano./Metazoa - ps12
Metazoa - ps13
Myriazoa - ps14
Parahoxozoa - ps15
Bilateria - ps16
Deuterostomia - ps17
Chordata - ps18
Olfactores - ps19
Vertebrata - ps20
Gnathostomata - ps21
Euteleostomi - ps22
Sarcopterygii - ps23
Tetrapoda - ps24
Amniota - ps25
Mammalia - ps26
Theria - ps27
Placentalia - ps28
Boreoeutheria - ps29
Euarchontoglires - ps30
Euarchonta - ps31
Primates - ps32
Haplorrhini - ps33
Simiiformes - ps34
Catarrhini - ps35
Hominoidea - ps36
Hominidae - ps37
Homininae - ps38
Hominini - ps39
Homo_sapiens - ps40
Phylostratum

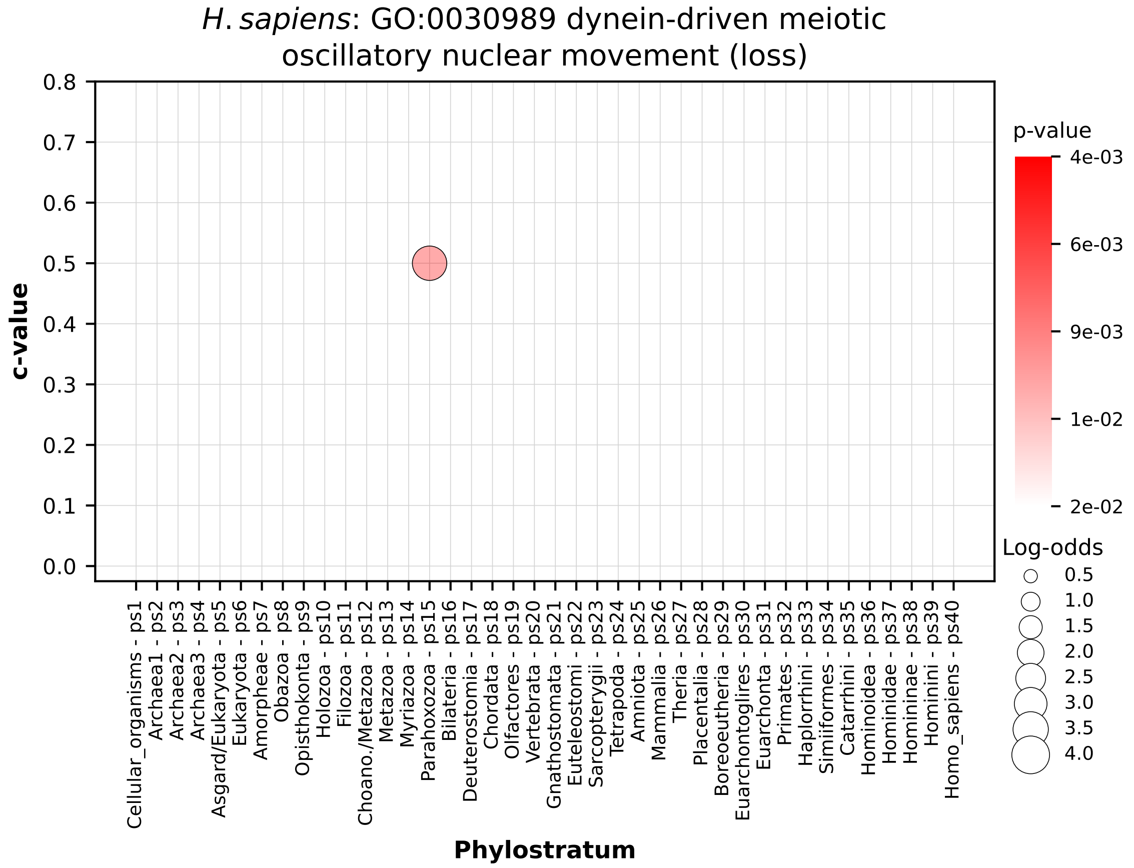

H. sapiens: GO:0030989 dynein-driven meiotic oscillatory nuclear movement (loss)
c-value
0.0
0.1
0.2
0.3
0.4
0.5
0.6
0.7
0.8
Phylostratum
Cellular_organisms - ps1
Archaea1 - ps2
Archaea2 - ps3
Archaea3 - ps4
Asgard/Eukaryota - ps5
Eukaryota - ps6
Amorpheae - ps7
Obazoa - ps8
Opisthokonta - ps9
Holozoa - ps10
Filozoa - ps11
Choano./Metazoa - ps12
Metazoa - ps13
Myriazoa - ps14
Parahoxozoa - ps15
Bilateria - ps16
Deuterostomia - ps17
Chordata - ps18
Olfactores - ps19
Vertebrata - ps20
Gnathostomata - ps21
Euteleostomi - ps22
Sarcopterygii - ps23
Tetrapoda - ps24
Amniota - ps25
Mammalia - ps26
Theria - ps27
Placentalia - ps28
Boreoeutheria - ps29
Euarchontoglires - ps30
Euarchonta - ps31
Primates - ps32
Haplorrhini - ps33
Simiiformes - ps34
Catarrhini - ps35
Hominoidea - ps36
Hominidae - ps37
Homininae - ps38
Hominini - ps39
Homo_sapiens - ps40
p-value
4e-03
6e-03
9e-03
1e-02
2e-02
Log-odds
0.5
1.0
1.5
2.0
2.5
3.0
3.5
4.0

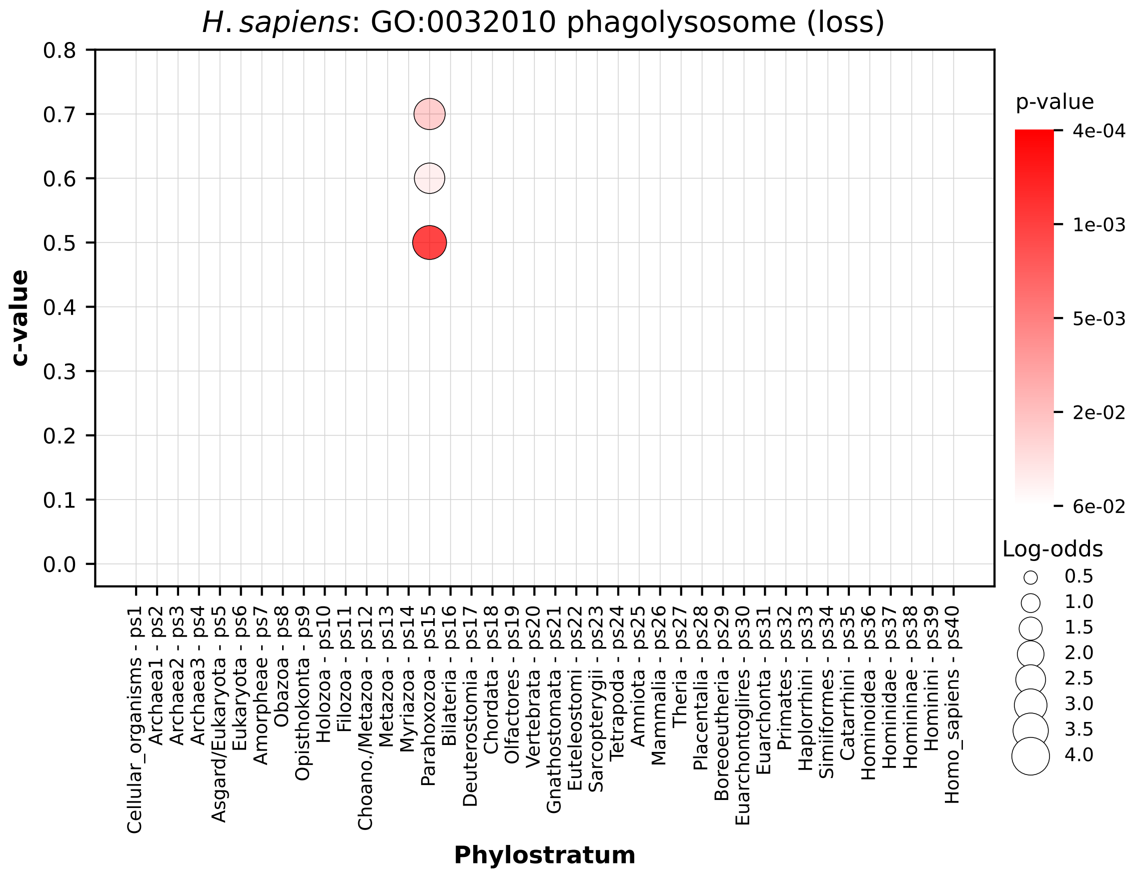
H. sapiens: GO:0032010 phagolysosome (loss)
c-value
0.8
0.7
0.6
0.5
0.4
0.3
0.2
0.1
0.0
p-value
4e-04
1e-03
5e-03
2e-02
6e-02
Log-odds
0.5
1.0
1.5
2.0
2.5
3.0
3.5
4.0
Cellular_organisms - ps1
Archaea1 - ps2
Archaea2 - ps3
Archaea3 - ps4
Asgard/Eukaryota - ps5
Eukaryota - ps6
Amorpheae - ps7
Obazoa - ps8
Opisthokonta - ps9
Holozoa - ps10
Filozoa - ps11
Choano./Metazoa - ps12
Metazoa - ps13
Myriazoa - ps14
Parahoxozoa - ps15
Bilateria - ps16
Deuterostomia - ps17
Chordata - ps18
Olfactores - ps19
Vertebrata - ps20
Gnathostomata - ps21
Euteleostomi - ps22
Sarcopterygii - ps23
Tetrapoda - ps24
Amniota - ps25
Mammalia - ps26
Theria - ps27
Placentalia - ps28
Boreoeutheria - ps29
Euarchontoglires - ps30
Euarchonta - ps31
Primates - ps32
Haplorrhini - ps33
Simiiformes - ps34
Catarrhini - ps35
Hominoidea - ps36
Hominidae - ps37
Homininae - ps38
Hominini - ps39
Homo_sapiens - ps40
Phylostratum

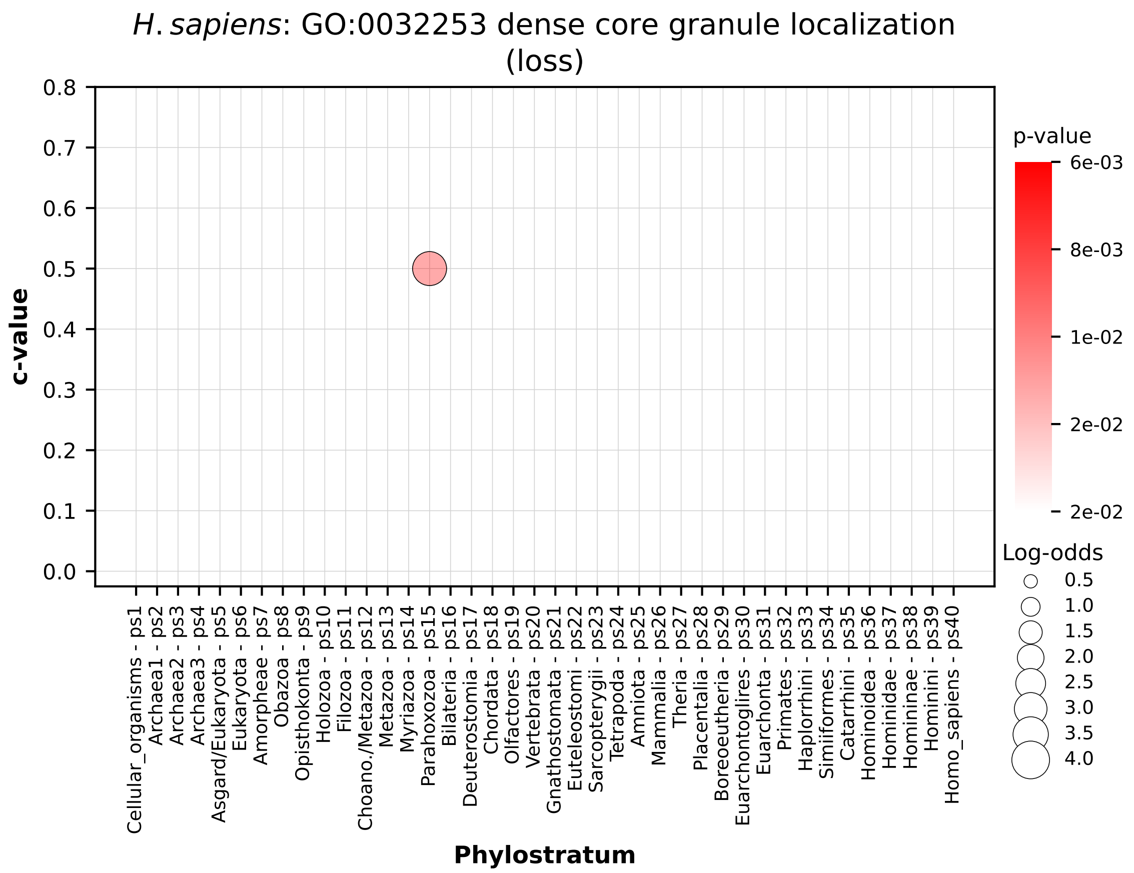

H. sapiens: GO:0032253 dense core granule localization (loss)
c-value
0.8
0.7
0.6
0.5
0.4
0.3
0.2
0.1
0.0
Phylostratum
Cellular_organisms - ps1
Archaea1 - ps2
Archaea2 - ps3
Archaea3 - ps4
Asgard/Eukaryota - ps5
Eukaryota - ps6
Amorpheae - ps7
Obazoa - ps8
Opisthokonta - ps9
Holozoa - ps10
Filozoa - ps11
Choano./Metazoa - ps12
Metazoa - ps13
Myriazoa - ps14
Parahoxozoa - ps15
Bilateria - ps16
Deuterostomia - ps17
Chordata - ps18
Olfactores - ps19
Vertebrata - ps20
Gnathostomata - ps21
Euteleostomi - ps22
Sarcopterygii - ps23
Tetrapoda - ps24
Amniota - ps25
Mammalia - ps26
Theria - ps27
Placentalia - ps28
Boreoeutheria - ps29
Euarchontoglires - ps30
Euarchonta - ps31
Primates - ps32
Haplorrhini - ps33
Simiiformes - ps34
Catarrhini - ps35
Hominoidea - ps36
Hominidae - ps37
Homininae - ps38
Hominini - ps39
Homo_sapiens - ps40
p-value
6e-03
8e-03
1e-02
2e-02
2e-02
Log-odds
0.5
1.0
1.5
2.0
2.5
3.0
3.5
4.0

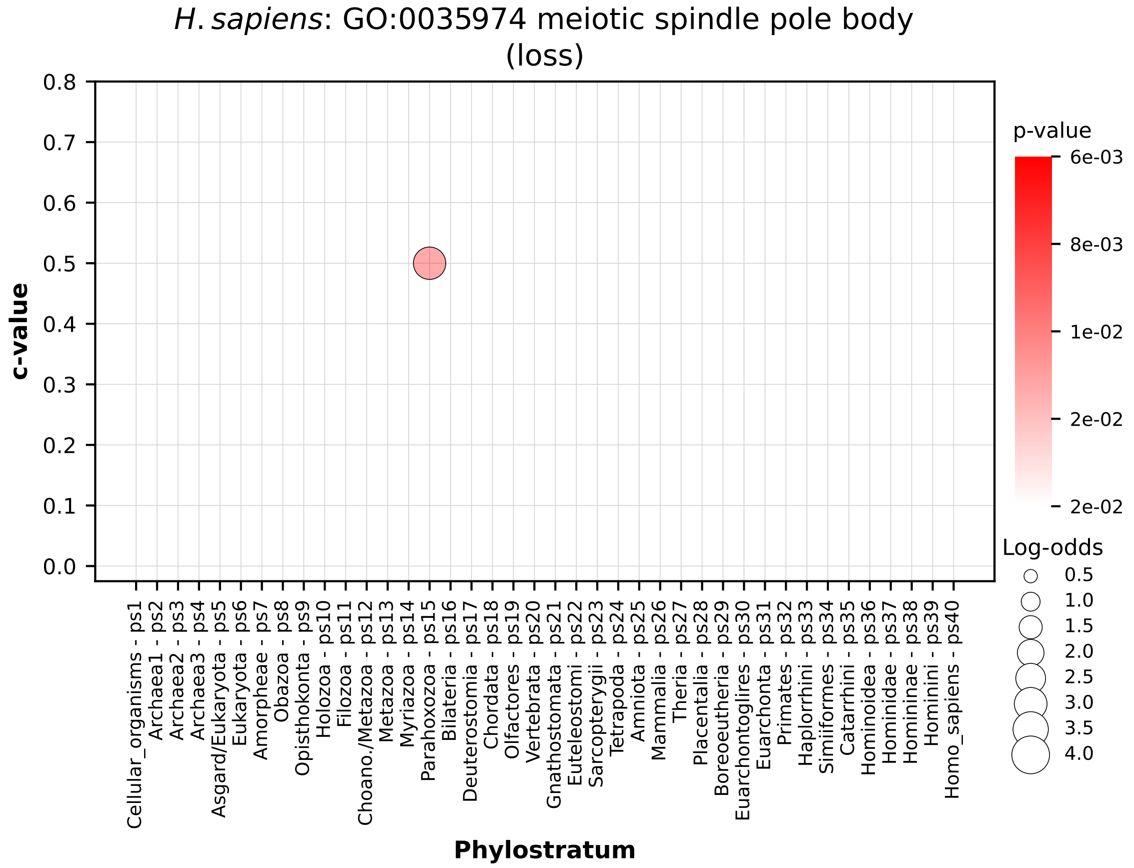

H. sapiens: GO:0035974 meiotic spindle pole body
(loss)
c-value
0.0
0.1
0.2
0.3
0.4
0.5
0.6
0.7
0.8
Phylostratum
Cellular_organisms - ps1
Archaea1 - ps2
Archaea2 - ps3
Archaea3 - ps4
Asgard/Eukaryota - ps5
Eukaryota - ps6
Amorpheae - ps7
Obazoa - ps8
Opisthokonta - ps9
Holozoa - ps10
Filozoa - ps11
Choano./Metazoa - ps12
Metazoa - ps13
Myriazoa - ps14
Parahoxozoa - ps15
Bilateria - ps16
Deuterostomia - ps17
Chordata - ps18
Olfactores - ps19
Vertebrata - ps20
Gnathostomata - ps21
Euteleostomi - ps22
Sarcopterygii - ps23
Tetrapoda - ps24
Amniota - ps25
Mammalia - ps26
Theria - ps27
Placentalia - ps28
Boreoeutheria - ps29
Euarchontoglires - ps30
Euarchonta - ps31
Primates - ps32
Haplorrhini - ps33
Simiiformes - ps34
Catarrhini - ps35
Hominoidea - ps36
Hominidae - ps37
Homininae - ps38
Hominini - ps39
Homo_sapiens - ps40
p-value
6e-03
8e-03
1e-02
2e-02
2e-02
Log-odds
0.5
1.0
1.5
2.0
2.5
3.0
3.5
4.0

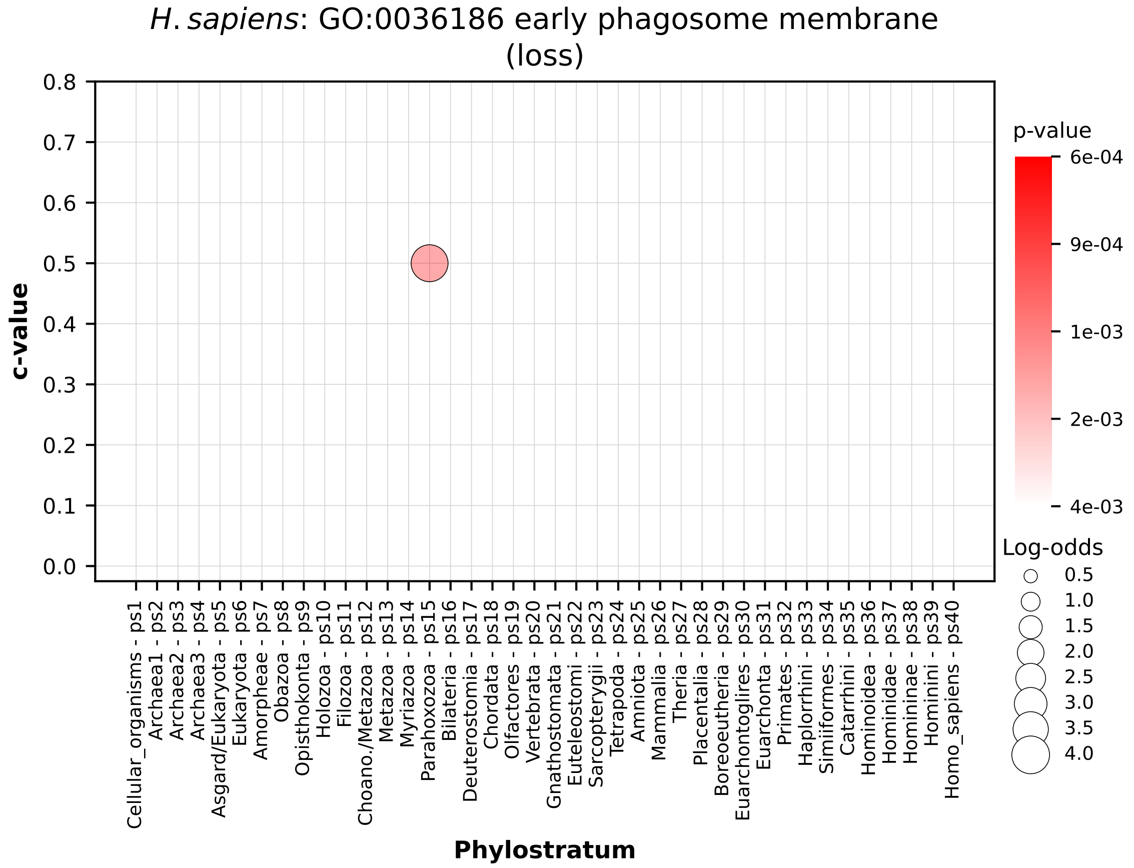
H. sapiens: GO:0036186 early phagosome membrane
(loss)
c-value
0.0
0.1
0.2
0.3
0.4
0.5
0.6
0.7
0.8
p-value
6e-04
9e-04
1e-03
2e-03
4e-03
Log-odds
0.5
1.0
1.5
2.0
2.5
3.0
3.5
4.0
Cellular_organisms - ps1
Archaea1 - ps2
Archaea2 - ps3
Archaea3 - ps4
Asgard/Eukaryota - ps5
Eukaryota - ps6
Amorpheae - ps7
Obazoa - ps8
Opisthokonta - ps9
Holozoa - ps10
Filozoa - ps11
Choano./Metazoa - ps12
Metazoa - ps13
Myriazoa - ps14
Parahoxozoa - ps15
Bilateria - ps16
Deuterostomia - ps17
Chordata - ps18
Olfactores - ps19
Vertebrata - ps20
Gnathostomata - ps21
Euteleostomi - ps22
Sarcopterygii - ps23
Tetrapoda - ps24
Amniota - ps25
Mammalia - ps26
Theria - ps27
Placentalia - ps28
Boreoeutheria - ps29
Euarchontoglires - ps30
Euarchonta - ps31
Primates - ps32
Haplorrhini - ps33
Simiiformes - ps34
Catarrhini - ps35
Hominoidea - ps36
Hominidae - ps37
Homininae - ps38
Hominini - ps39
Homo_sapiens - ps40
Phylostratum

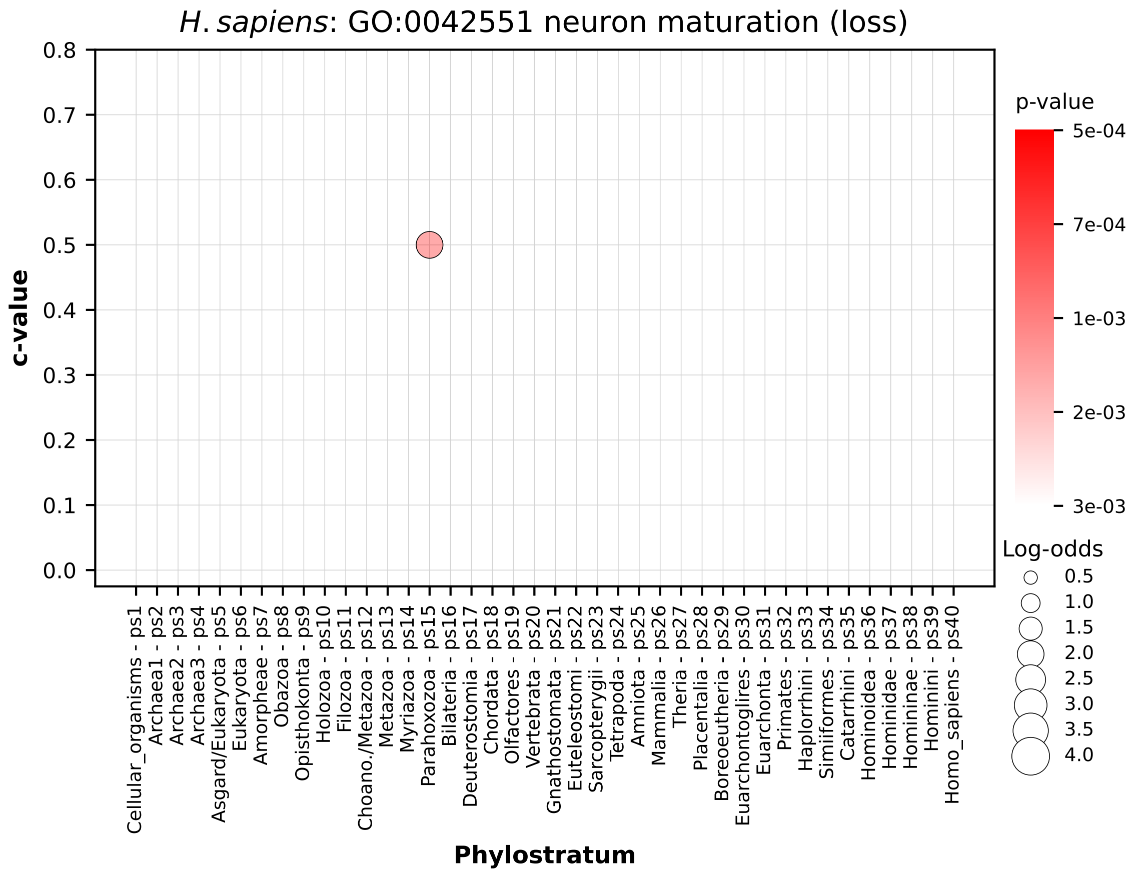

H. sapiens: GO:0042551 neuron maturation (loss)
c-value
0.8
0.7
0.6
0.5
0.4
0.3
0.2
0.1
0.0
Phylostratum
Cellular_organisms - ps1
Archaea1 - ps2
Archaea2 - ps3
Archaea3 - ps4
Asgard/Eukaryota - ps5
Eukaryota - ps6
Amorpheae - ps7
Obazoa - ps8
Opisthokonta - ps9
Holozoa - ps10
Filozoa - ps11
Choano./Metazoa - ps12
Metazoa - ps13
Myriazoa - ps14
Parahoxozoa - ps15
Bilateria - ps16
Deuterostomia - ps17
Chordata - ps18
Olfactores - ps19
Vertebrata - ps20
Gnathostomata - ps21
Euteleostomi - ps22
Sarcopterygii - ps23
Tetrapoda - ps24
Amniota - ps25
Mammalia - ps26
Theria - ps27
Placentalia - ps28
Boreoeutheria - ps29
Euarchontoglires - ps30
Euarchonta - ps31
Primates - ps32
Haplorrhini - ps33
Simiiformes - ps34
Catarrhini - ps35
Hominoidea - ps36
Hominidae - ps37
Homininae - ps38
Hominini - ps39
Homo_sapiens - ps40
p-value
5e-04
7e-04
1e-03
2e-03
3e-03
Log-odds
0.5
1.0
1.5
2.0
2.5
3.0
3.5
4.0

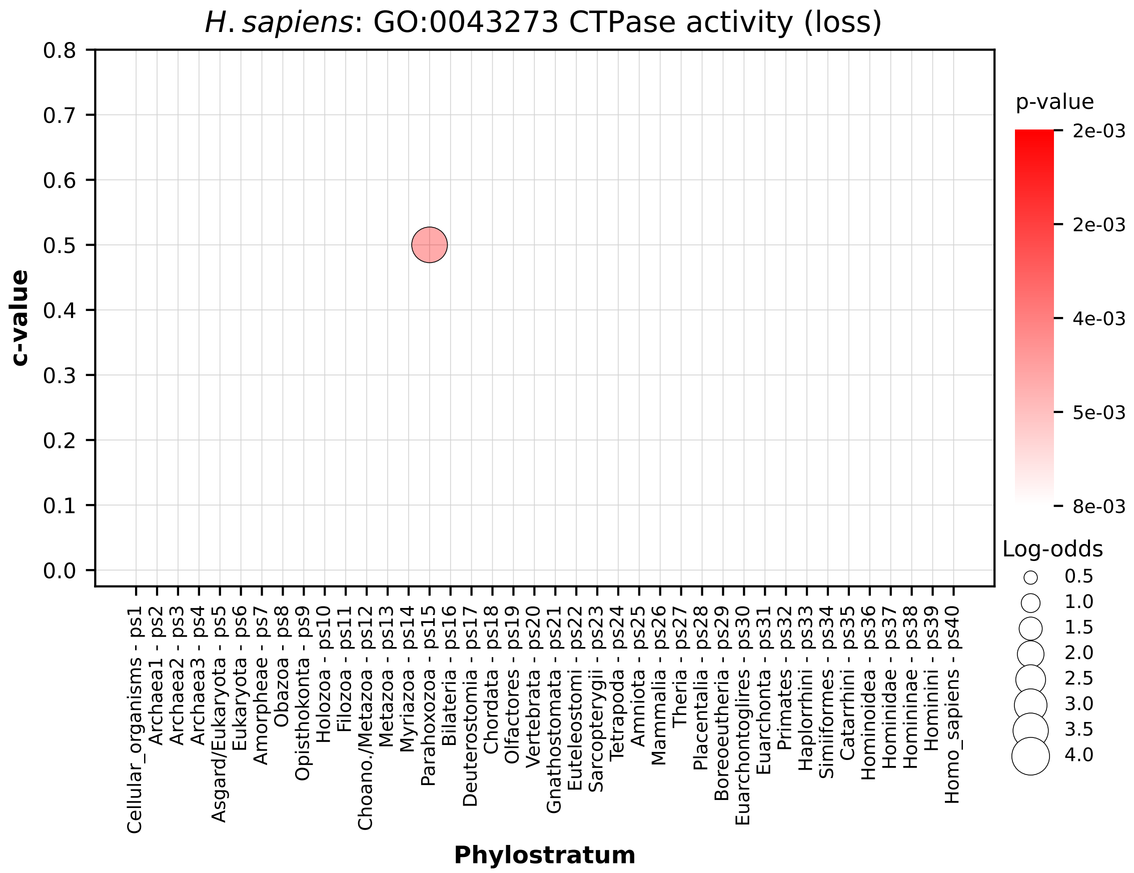

H. sapiens: GO:0043273 CTPase activity (loss)
c-value
0.8
0.7
0.6
0.5
0.4
0.3
0.2
0.1
0.0
Phylostratum
Cellular_organisms - ps1
Archaea1 - ps2
Archaea2 - ps3
Archaea3 - ps4
Asgard/Eukaryota - ps5
Eukaryota - ps6
Amorpheae - ps7
Obazoa - ps8
Opisthokonta - ps9
Holozoa - ps10
Filozoa - ps11
Choano./Metazoa - ps12
Metazoa - ps13
Myriazoa - ps14
Parahoxozoa - ps15
Bilateria - ps16
Deuterostomia - ps17
Chordata - ps18
Olfactores - ps19
Vertebrata - ps20
Gnathostomata - ps21
Euteleostomi - ps22
Sarcopterygii - ps23
Tetrapoda - ps24
Amniota - ps25
Mammalia - ps26
Theria - ps27
Placentalia - ps28
Boreoeutheria - ps29
Euarchontoglires - ps30
Euarchonta - ps31
Primates - ps32
Haplorrhini - ps33
Simiiformes - ps34
Catarrhini - ps35
Hominoidea - ps36
Hominidae - ps37
Homininae - ps38
Hominini - ps39
Homo_sapiens - ps40
p-value
2e-03
2e-03
4e-03
5e-03
8e-03
Log-odds
0.5
1.0
1.5
2.0
2.5
3.0
3.5
4.0

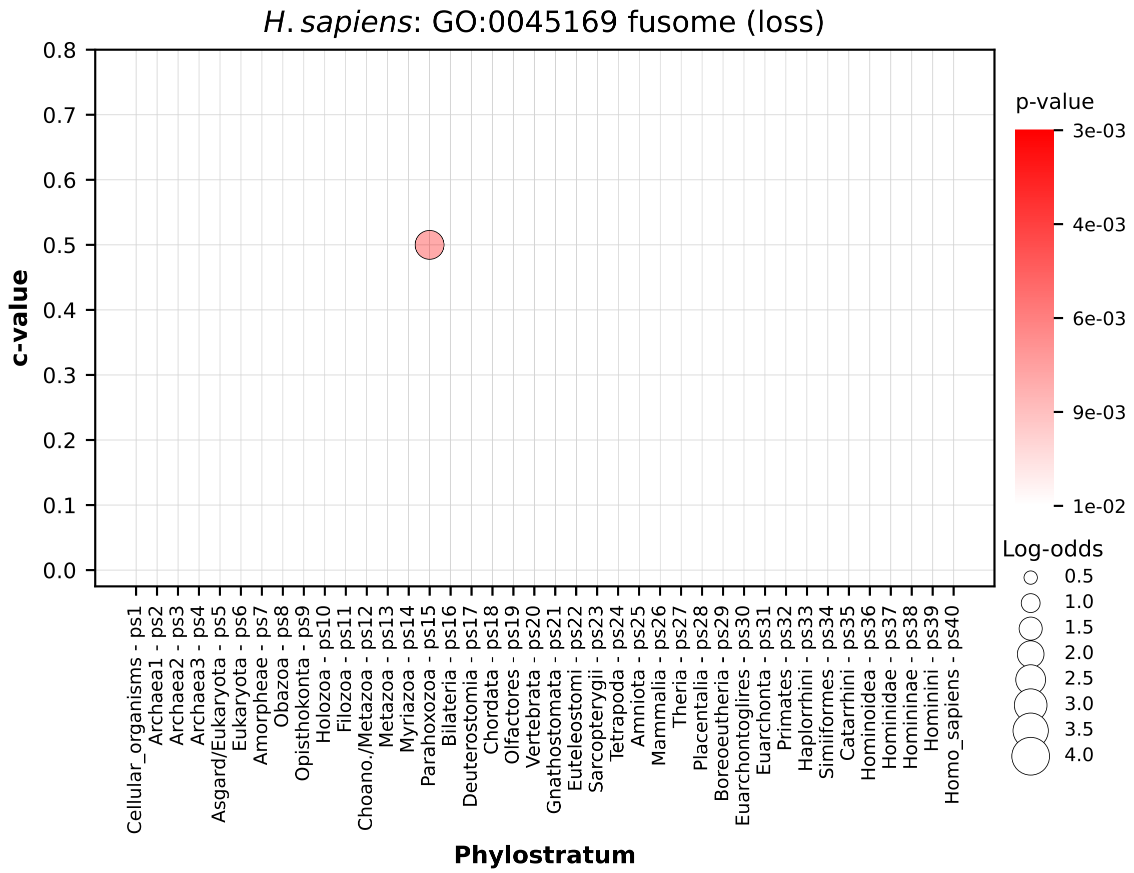

H. sapiens: GO:0045169 fusome (loss)
c-value
0.8
0.7
0.6
0.5
0.4
0.3
0.2
0.1
0.0
p-value
3e-03
4e-03
6e-03
9e-03
1e-02
Log-odds
0.5
1.0
1.5
2.0
2.5
3.0
3.5
4.0
Cellular_organisms - ps1
Archaea1 - ps2
Archaea2 - ps3
Archaea3 - ps4
Asgard/Eukaryota - ps5
Eukaryota - ps6
Amorpheae - ps7
Obazoa - ps8
Opisthokonta - ps9
Holozoa - ps10
Filozoa - ps11
Choano./Metazoa - ps12
Metazoa - ps13
Myriazoa - ps14
Parahoxozoa - ps15
Bilateria - ps16
Deuterostomia - ps17
Chordata - ps18
Olfactores - ps19
Vertebrata - ps20
Gnathostomata - ps21
Euteleostomi - ps22
Sarcopterygii - ps23
Tetrapoda - ps24
Amniota - ps25
Mammalia - ps26
Theria - ps27
Placentalia - ps28
Boreoeutheria - ps29
Euarchontoglires - ps30
Euarchonta - ps31
Primates - ps32
Haplorrhini - ps33
Simiiformes - ps34
Catarrhini - ps35
Hominoidea - ps36
Hominidae - ps37
Homininae - ps38
Hominini - ps39
Homo_sapiens - ps40
Phylostratum

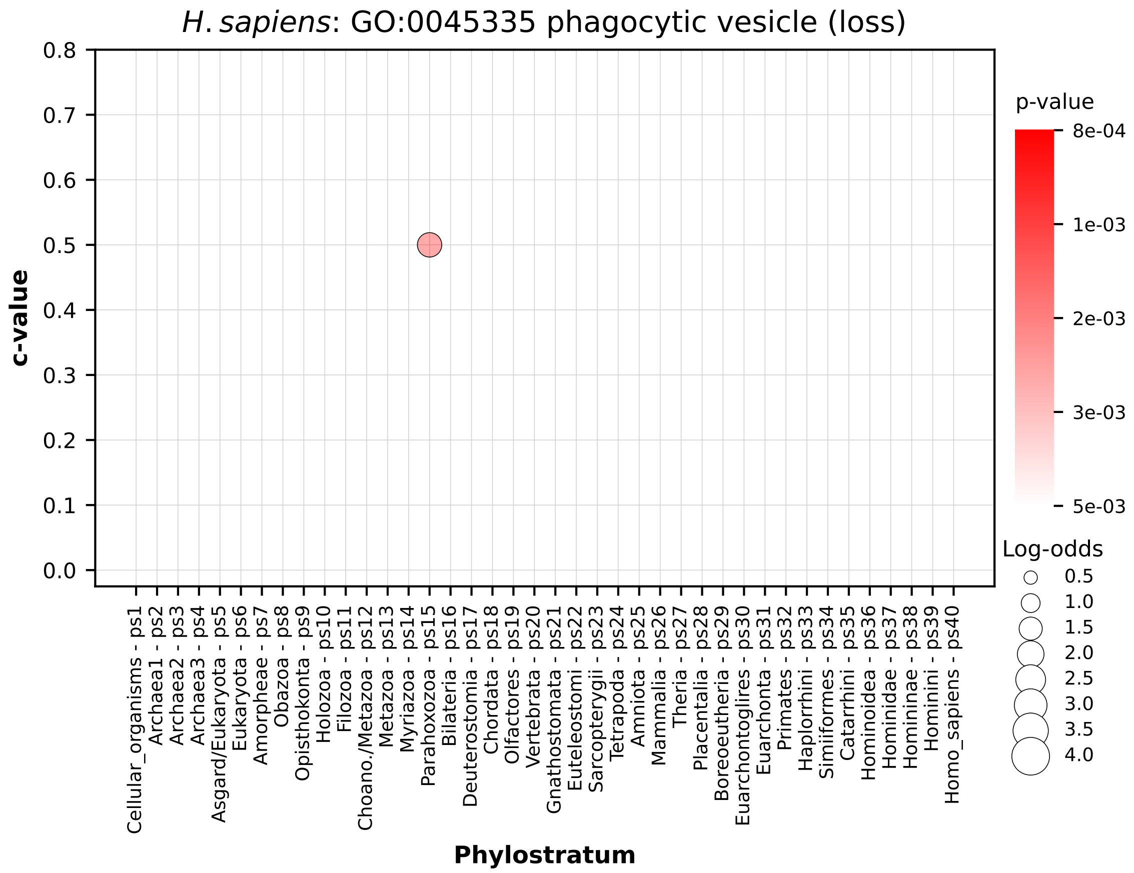
H. sapiens: GO:0045335 phagocytic vesicle (loss)
c-value
0.8
0.7
0.6
0.5
0.4
0.3
0.2
0.1
0.0
Phylostratum
Cellular_organisms - ps1
Archaea1 - ps2
Archaea2 - ps3
Archaea3 - ps4
Asgard/Eukaryota - ps5
Eukaryota - ps6
Amorpheae - ps7
Obazoa - ps8
Opisthokonta - ps9
Holozoa - ps10
Filozoa - ps11
Choano./Metazoa - ps12
Metazoa - ps13
Myriazoa - ps14
Parahoxozoa - ps15
Bilateria - ps16
Deuterostomia - ps17
Chordata - ps18
Olfactores - ps19
Vertebrata - ps20
Gnathostomata - ps21
Euteleostomi - ps22
Sarcopterygii - ps23
Tetrapoda - ps24
Amniota - ps25
Mammalia - ps26
Theria - ps27
Placentalia - ps28
Boreoeutheria - ps29
Euarchontoglires - ps30
Euarchonta - ps31
Primates - ps32
Haplorrhini - ps33
Simiiformes - ps34
Catarrhini - ps35
Hominoidea - ps36
Hominidae - ps37
Homininae - ps38
Hominini - ps39
Homo_sapiens - ps40
p-value
8e-04
1e-03
2e-03
3e-03
5e-03
Log-odds
0.5
1.0
1.5
2.0
2.5
3.0
3.5
4.0

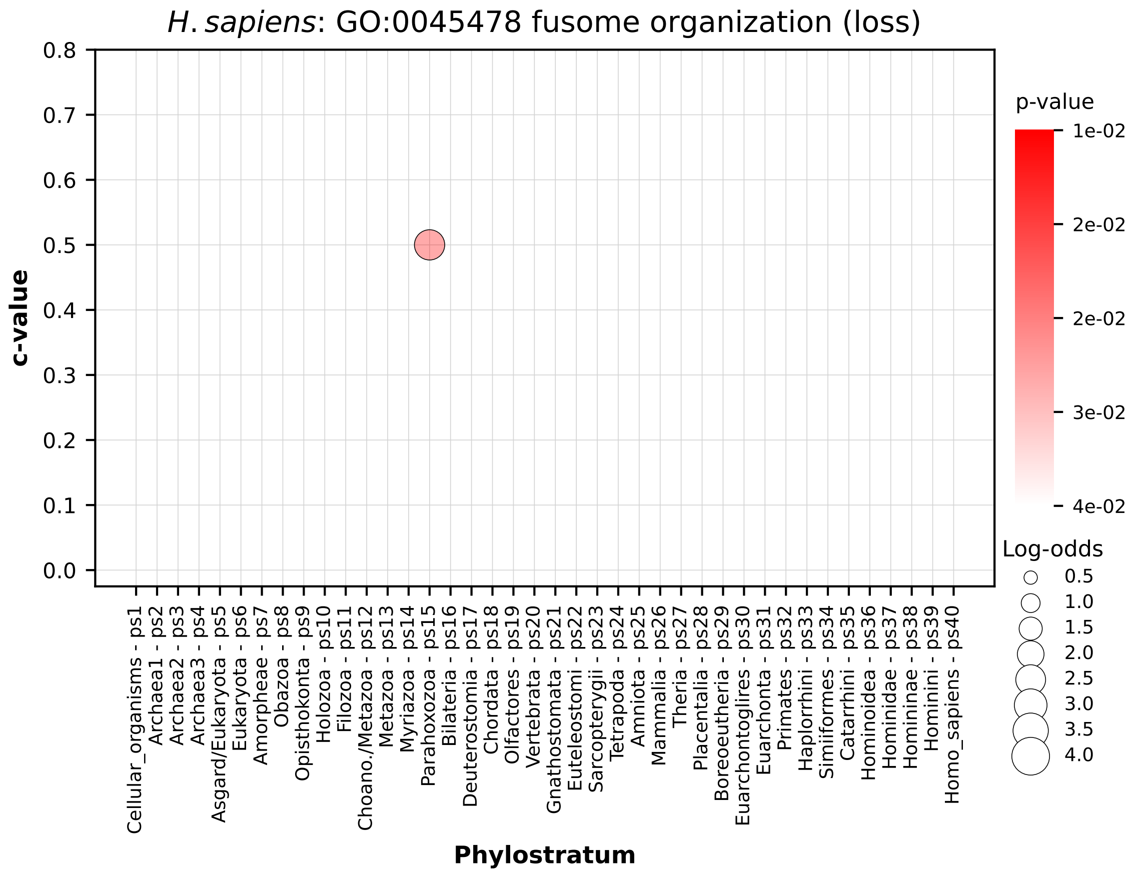

H. sapiens: GO:0045478 fusome organization (loss)
c-value
0.8
0.7
0.6
0.5
0.4
0.3
0.2
0.1
0.0
p-value
1e-02
2e-02
2e-02
3e-02
4e-02
Log-odds
0.5
1.0
1.5
2.0
2.5
3.0
3.5
4.0
Cellular_organisms - ps1
Archaea1 - ps2
Archaea2 - ps3
Archaea3 - ps4
Asgard/Eukaryota - ps5
Eukaryota - ps6
Amorpheae - ps7
Obazoa - ps8
Opisthokonta - ps9
Holozoa - ps10
Filozoa - ps11
Choano./Metazoa - ps12
Metazoa - ps13
Myriazoa - ps14
Parahoxozoa - ps15
Bilateria - ps16
Deuterostomia - ps17
Chordata - ps18
Olfactores - ps19
Vertebrata - ps20
Gnathostomata - ps21
Euteleostomi - ps22
Sarcopterygii - ps23
Tetrapoda - ps24
Amniota - ps25
Mammalia - ps26
Theria - ps27
Placentalia - ps28
Boreoeutheria - ps29
Euarchontoglires - ps30
Euarchonta - ps31
Primates - ps32
Haplorrhini - ps33
Simiiformes - ps34
Catarrhini - ps35
Hominoidea - ps36
Hominidae - ps37
Homininae - ps38
Hominini - ps39
Homo_sapiens - ps40
Phylostratum

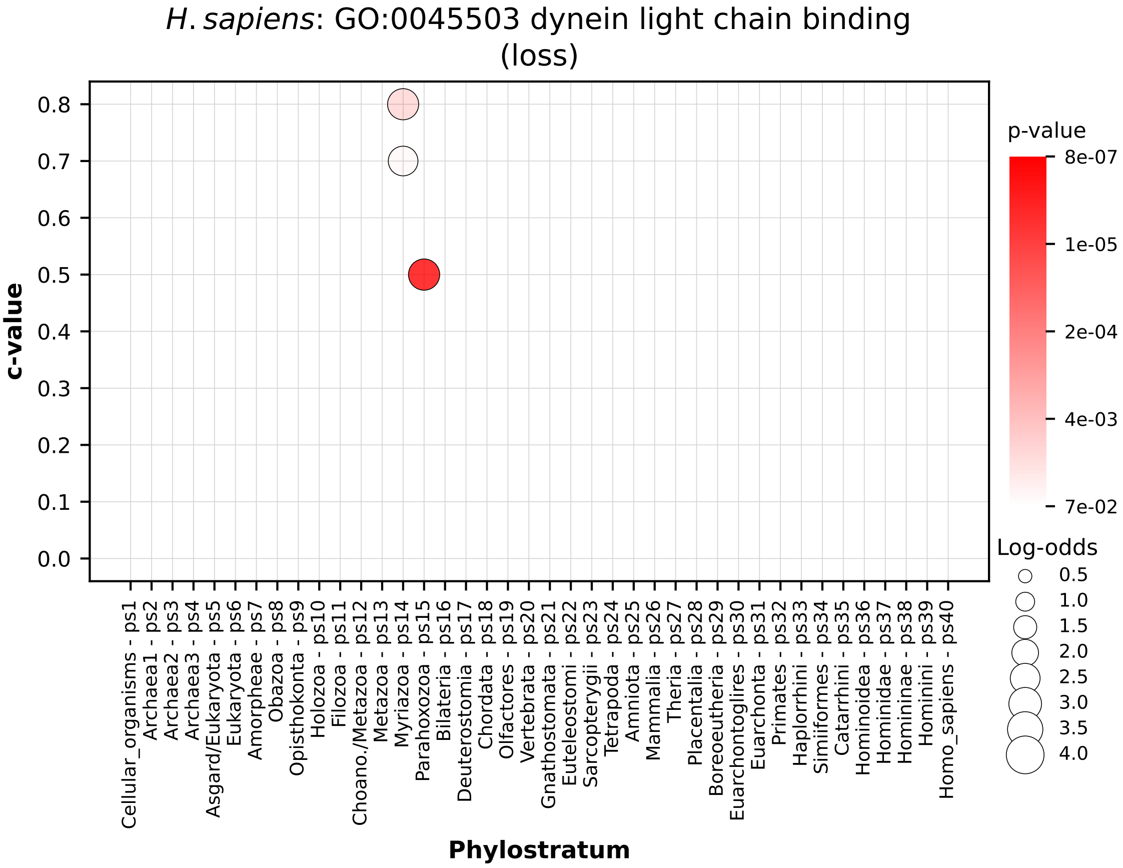
H. sapiens: GO:0045503 dynein light chain binding
(loss)
c-value
0.8
0.7
0.6
0.5
0.4
0.3
0.2
0.1
0.0
p-value
8e-07
1e-05
2e-04
4e-03
7e-02
Log-odds
0.5
1.0
1.5
2.0
2.5
3.0
3.5
4.0
Cellular_organisms - ps1
Archaea1 - ps2
Archaea2 - ps3
Archaea3 - ps4
Asgard/Eukaryota - ps5
Eukaryota - ps6
Amorpheae - ps7
Obazoa - ps8
Opisthokonta - ps9
Holozoa - ps10
Filozoa - ps11
Choano./Metazoa - ps12
Metazoa - ps13
Myriazoa - ps14
Parahoxozoa - ps15
Bilateria - ps16
Deuterostomia - ps17
Chordata - ps18
Olfactores - ps19
Vertebrata - ps20
Gnathostomata - ps21
Euteleostomi - ps22
Sarcopterygii - ps23
Tetrapoda - ps24
Amniota - ps25
Mammalia - ps26
Theria - ps27
Placentalia - ps28
Boreoeutheria - ps29
Euarchontoglires - ps30
Euarchonta - ps31
Primates - ps32
Haplorrhini - ps33
Simiiformes - ps34
Catarrhini - ps35
Hominoidea - ps36
Hominidae - ps37
Homininae - ps38
Hominini - ps39
Homo_sapiens - ps40
Phylostratum

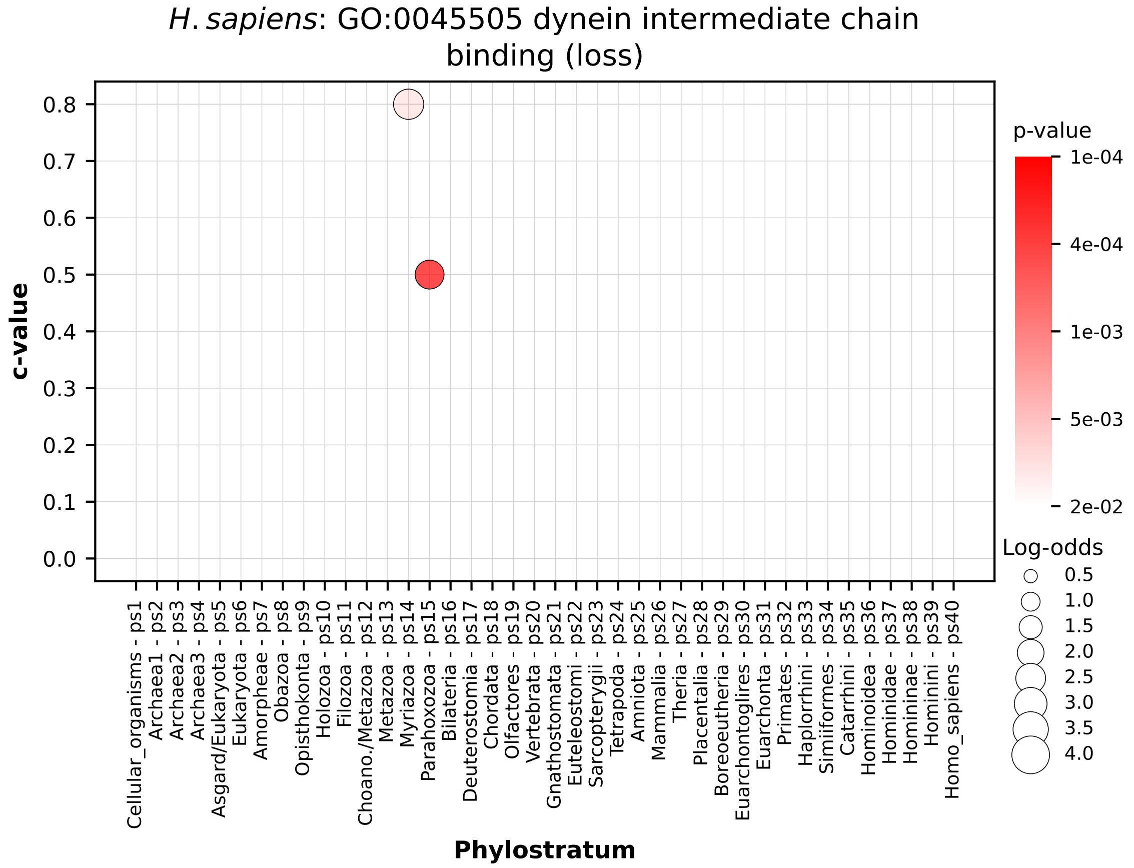
H. sapiens: GO:0045505 dynein intermediate chain binding (loss)
c-value
0.8
0.7
0.6
0.5
0.4
0.3
0.2
0.1
0.0
p-value
1e-04
4e-04
1e-03
5e-03
2e-02
Log-odds
0.5
1.0
1.5
2.0
2.5
3.0
3.5
4.0
Cellular_organisms - ps1
Archaea1 - ps2
Archaea2 - ps3
Archaea3 - ps4
Asgard/Eukaryota - ps5
Eukaryota - ps6
Amorpheae - ps7
Obazoa - ps8
Opisthokonta - ps9
Holozoa - ps10
Filozoa - ps11
Choano./Metazoa - ps12
Metazoa - ps13
Myriazoa - ps14
Parahoxozoa - ps15
Bilateria - ps16
Deuterostomia - ps17
Chordata - ps18
Olfactores - ps19
Vertebrata - ps20
Gnathostomata - ps21
Euteleostomi - ps22
Sarcopterygii - ps23
Tetrapoda - ps24
Amniota - ps25
Mammalia - ps26
Theria - ps27
Placentalia - ps28
Boreoeutheria - ps29
Euarchontoglires - ps30
Euarchonta - ps31
Primates - ps32
Haplorrhini - ps33
Simiiformes - ps34
Catarrhini - ps35
Hominoidea - ps36
Hominidae - ps37
Homininae - ps38
Hominini - ps39
Homo_sapiens - ps40
Phylostratum

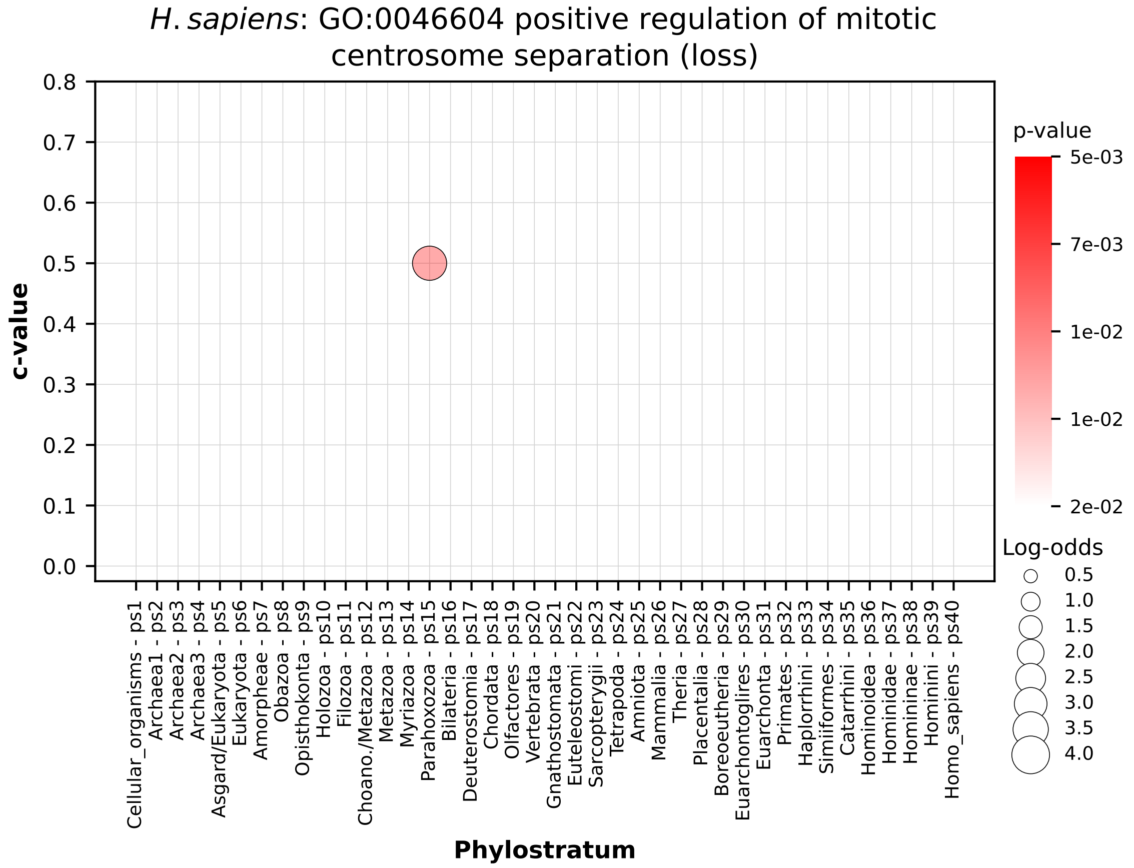

H. sapiens: GO:0046604 positive regulation of mitotic centrosome separation (loss)
c-value
0.8
0.7
0.6
0.5
0.4
0.3
0.2
0.1
0.0
Phylostratum
Cellular_organisms - ps1
Archaea1 - ps2
Archaea2 - ps3
Archaea3 - ps4
Asgard/Eukaryota - ps5
Eukaryota - ps6
Amorpheae - ps7
Obazoa - ps8
Opisthokonta - ps9
Holozoa - ps10
Filozoa - ps11
Choano./Metazoa - ps12
Metazoa - ps13
Myriazoa - ps14
Parahoxozoa - ps15
Bilateria - ps16
Deuterostomia - ps17
Chordata - ps18
Olfactores - ps19
Vertebrata - ps20
Gnathostomata - ps21
Euteleostomi - ps22
Sarcopterygii - ps23
Tetrapoda - ps24
Amniota - ps25
Mammalia - ps26
Theria - ps27
Placentalia - ps28
Boreoeutheria - ps29
Euarchontoglires - ps30
Euarchonta - ps31
Primates - ps32
Haplorrhini - ps33
Simiiformes - ps34
Catarrhini - ps35
Hominoidea - ps36
Hominidae - ps37
Homininae - ps38
Hominini - ps39
Homo_sapiens - ps40
p-value
5e-03
7e-03
1e-02
1e-02
2e-02
Log-odds
0.5
1.0
1.5
2.0
2.5
3.0
3.5
4.0

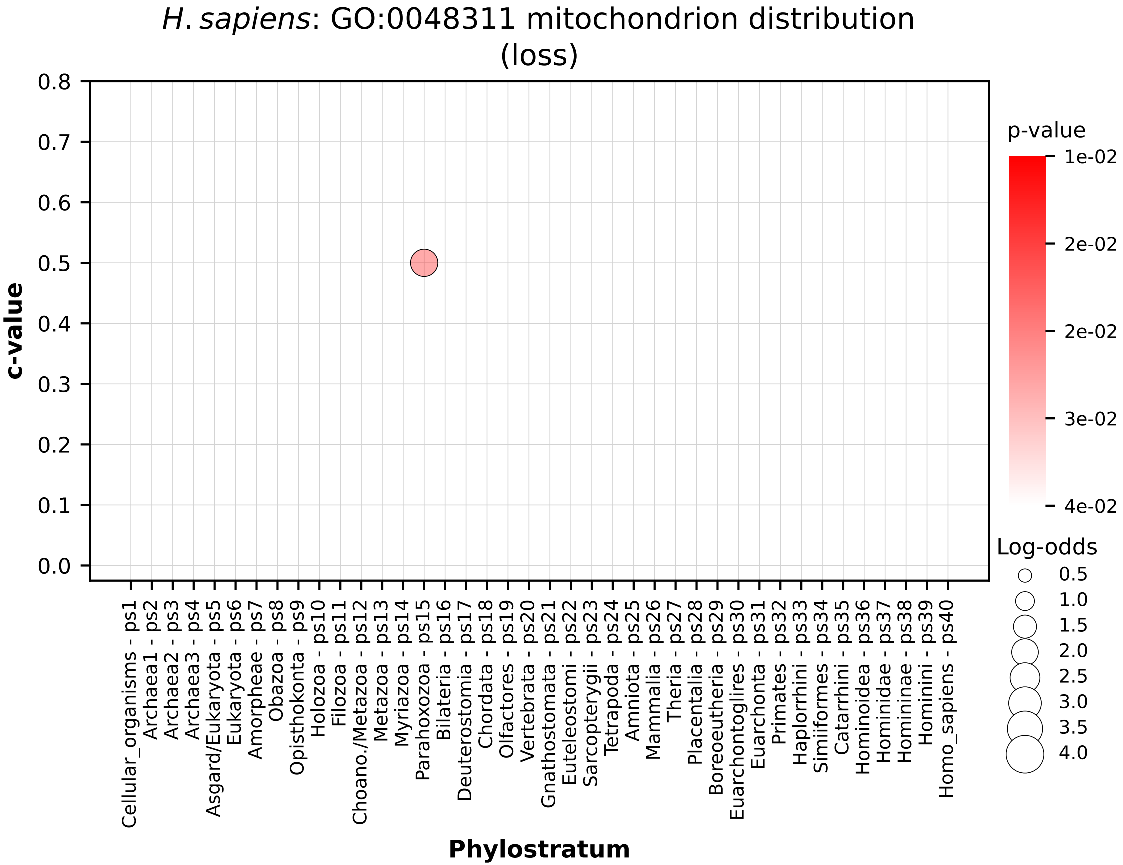
H. sapiens: GO:0048311 mitochondrion distribution
(loss)
c-value
0.8
0.7
0.6
0.5
0.4
0.3
0.2
0.1
0.0
Phylostratum
Cellular_organisms - ps1
Archaea1 - ps2
Archaea2 - ps3
Archaea3 - ps4
Asgard/Eukaryota - ps5
Eukaryota - ps6
Amorpheae - ps7
Obazoa - ps8
Opisthokonta - ps9
Holozoa - ps10
Filozoa - ps11
Choano./Metazoa - ps12
Metazoa - ps13
Myriazoa - ps14
Parahoxozoa - ps15
Bilateria - ps16
Deuterostomia - ps17
Chordata - ps18
Olfactores - ps19
Vertebrata - ps20
Gnathostomata - ps21
Euteleostomi - ps22
Sarcopterygii - ps23
Tetrapoda - ps24
Amniota - ps25
Mammalia - ps26
Theria - ps27
Placentalia - ps28
Boreoeutheria - ps29
Euarchontoglires - ps30
Euarchonta - ps31
Primates - ps32
Haplorrhini - ps33
Simiiformes - ps34
Catarrhini - ps35
Hominoidea - ps36
Hominidae - ps37
Homininae - ps38
Hominini - ps39
Homo_sapiens - ps40
p-value
1e-02
2e-02
2e-02
3e-02
4e-02
Log-odds
0.5
1.0
1.5
2.0
2.5
3.0
3.5
4.0

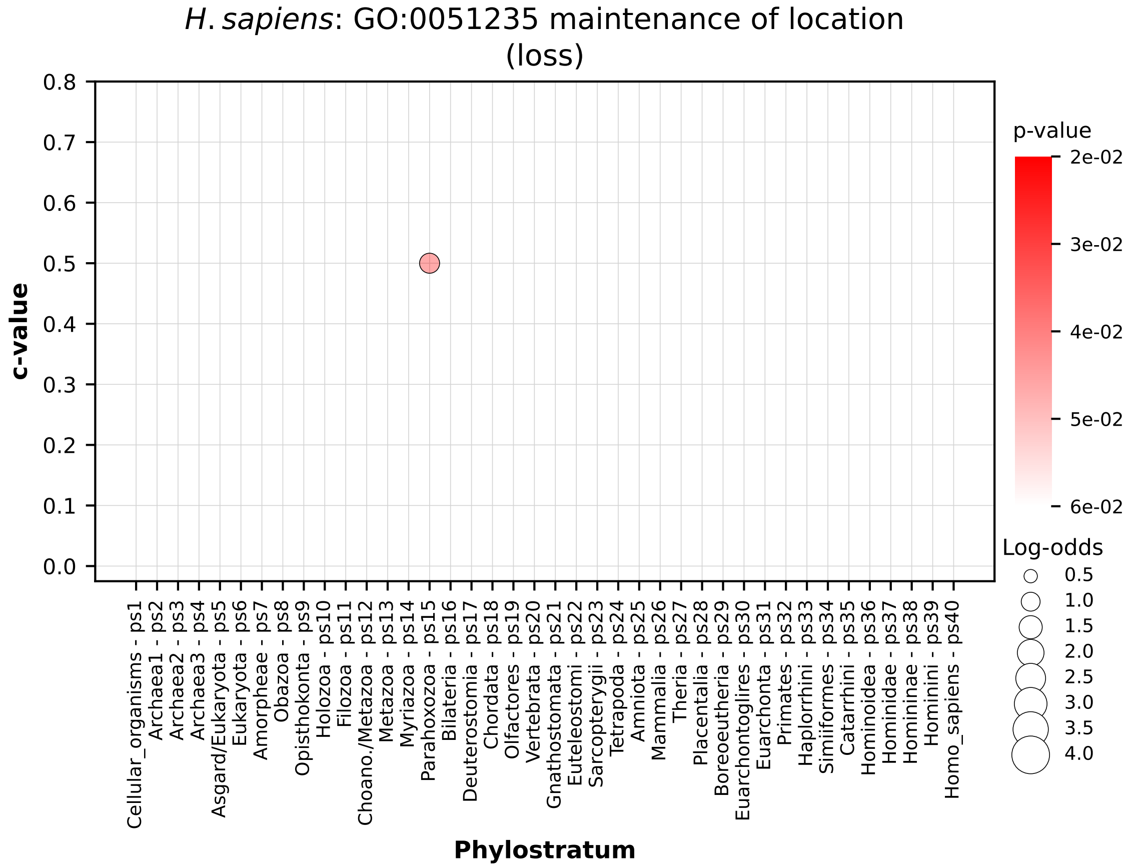

H. sapiens: GO:0051235 maintenance of location (loss)
c-value
0.0
0.1
0.2
0.3
0.4
0.5
0.6
0.7
0.8
Phylostratum
Cellular_organisms - ps1
Archaea1 - ps2
Archaea2 - ps3
Archaea3 - ps4
Asgard/Eukaryota - ps5
Eukaryota - ps6
Amorpheae - ps7
Obazoa - ps8
Opisthokonta - ps9
Holozoa - ps10
Filozoa - ps11
Choano./Metazoa - ps12
Metazoa - ps13
Myriazoa - ps14
Parahoxozoa - ps15
Bilateria - ps16
Deuterostomia - ps17
Chordata - ps18
Olfactores - ps19
Vertebrata - ps20
Gnathostomata - ps21
Euteleostomi - ps22
Sarcopterygii - ps23
Tetrapoda - ps24
Amniota - ps25
Mammalia - ps26
Theria - ps27
Placentalia - ps28
Boreoeutheria - ps29
Euarchontoglires - ps30
Euarchonta - ps31
Primates - ps32
Haplorrhini - ps33
Simiiformes - ps34
Catarrhini - ps35
Hominoidea - ps36
Hominidae - ps37
Homininae - ps38
Hominini - ps39
Homo_sapiens - ps40
p-value
2e-02
3e-02
4e-02
5e-02
6e-02
Log-odds
0.5
1.0
1.5
2.0
2.5
3.0
3.5
4.0

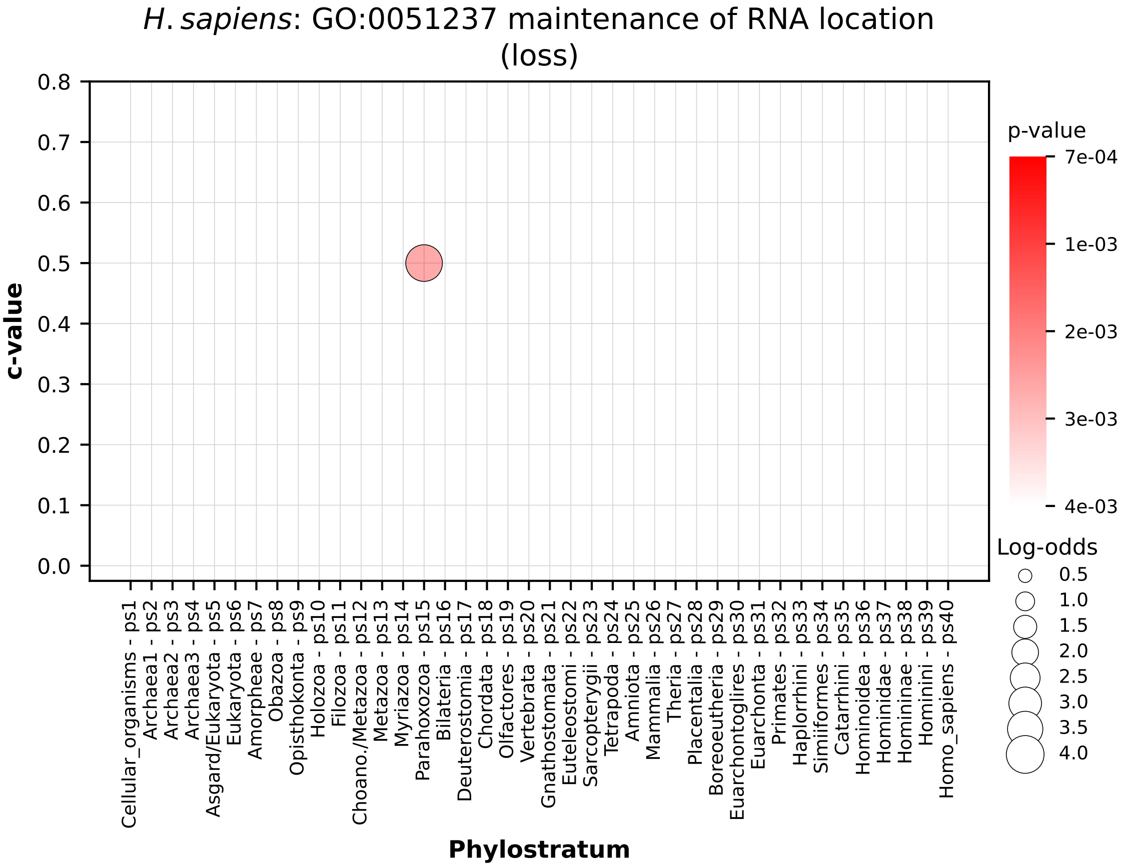

H. sapiens: GO:0051237 maintenance of RNA location (loss)
c-value
0.8
0.7
0.6
0.5
0.4
0.3
0.2
0.1
0.0
p-value
7e-04
1e-03
2e-03
3e-03
4e-03
Log-odds
0.5
1.0
1.5
2.0
2.5
3.0
3.5
4.0
Cellular_organisms - ps1
Archaea1 - ps2
Archaea2 - ps3
Archaea3 - ps4
Asgard/Eukaryota - ps5
Eukaryota - ps6
Amorpheae - ps7
Obazoa - ps8
Opisthokonta - ps9
Holozoa - ps10
Filozoa - ps11
Choano./Metazoa - ps12
Metazoa - ps13
Myriazoa - ps14
Parahoxozoa - ps15
Bilateria - ps16
Deuterostomia - ps17
Chordata - ps18
Olfactores - ps19
Vertebrata - ps20
Gnathostomata - ps21
Euteleostomi - ps22
Sarcopterygii - ps23
Tetrapoda - ps24
Amniota - ps25
Mammalia - ps26
Theria - ps27
Placentalia - ps28
Boreoeutheria - ps29
Euarchontoglires - ps30
Euarchonta - ps31
Primates - ps32
Haplorrhini - ps33
Simiiformes - ps34
Catarrhini - ps35
Hominoidea - ps36
Hominidae - ps37
Homininae - ps38
Hominini - ps39
Homo_sapiens - ps40
Phylostratum

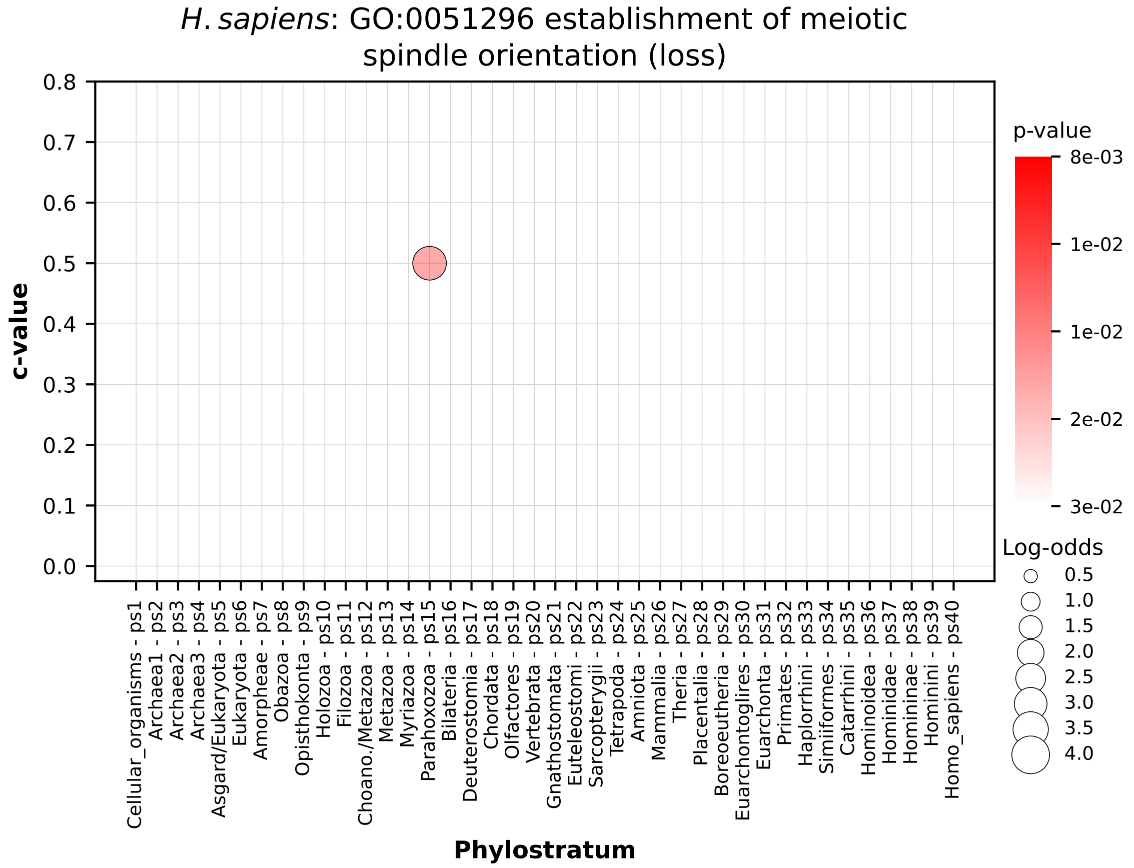

H. sapiens: GO:0051296 establishment of meiotic spindle orientation (loss)
c-value
0.8
0.7
0.6
0.5
0.4
0.3
0.2
0.1
0.0
Phylostratum
Cellular_organisms - ps1
Archaea1 - ps2
Archaea2 - ps3
Archaea3 - ps4
Asgard/Eukaryota - ps5
Eukaryota - ps6
Amorpheae - ps7
Obazoa - ps8
Opisthokonta - ps9
Holozoa - ps10
Filozoa - ps11
Choano./Metazoa - ps12
Metazoa - ps13
Myriazoa - ps14
Parahoxozoa - ps15
Bilateria - ps16
Deuterostomia - ps17
Chordata - ps18
Olfactores - ps19
Vertebrata - ps20
Gnathostomata - ps21
Euteleostomi - ps22
Sarcopterygii - ps23
Tetrapoda - ps24
Amniota - ps25
Mammalia - ps26
Theria - ps27
Placentalia - ps28
Boreoeutheria - ps29
Euarchontoglires - ps30
Euarchonta - ps31
Primates - ps32
Haplorrhini - ps33
Simiiformes - ps34
Catarrhini - ps35
Hominoidea - ps36
Hominidae - ps37
Homininae - ps38
Hominini - ps39
Homo_sapiens - ps40
p-value
8e-03
1e-02
1e-02
2e-02
3e-02
Log-odds
0.5
1.0
1.5
2.0
2.5
3.0
3.5
4.0

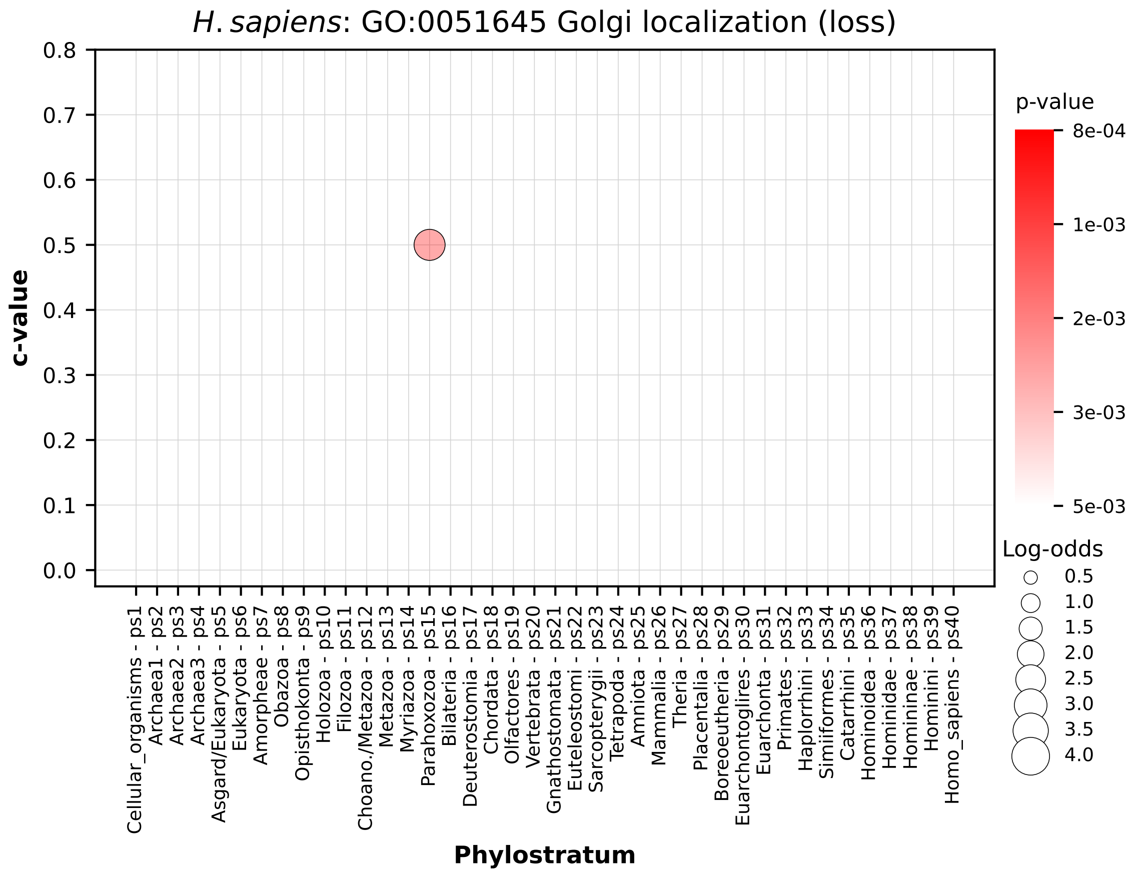

H. sapiens: GO:0051645 Golgi localization (loss)
c-value
0.8
0.7
0.6
0.5
0.4
0.3
0.2
0.1
0.0
p-value
8e-04
1e-03
2e-03
3e-03
5e-03
Log-odds
0.5
1.0
1.5
2.0
2.5
3.0
3.5
4.0
Cellular_organisms - ps1
Archaea1 - ps2
Archaea2 - ps3
Archaea3 - ps4
Asgard/Eukaryota - ps5
Eukaryota - ps6
Amorpheae - ps7
Obazoa - ps8
Opisthokonta - ps9
Holozoa - ps10
Filozoa - ps11
Choano./Metazoa - ps12
Metazoa - ps13
Myriazoa - ps14
Parahoxozoa - ps15
Bilateria - ps16
Deuterostomia - ps17
Chordata - ps18
Olfactores - ps19
Vertebrata - ps20
Gnathostomata - ps21
Euteleostomi - ps22
Sarcopterygii - ps23
Tetrapoda - ps24
Amniota - ps25
Mammalia - ps26
Theria - ps27
Placentalia - ps28
Boreoeutheria - ps29
Euarchontoglires - ps30
Euarchonta - ps31
Primates - ps32
Haplorrhini - ps33
Simiiformes - ps34
Catarrhini - ps35
Hominoidea - ps36
Hominidae - ps37
Homininae - ps38
Hominini - ps39
Homo_sapiens - ps40
Phylostratum

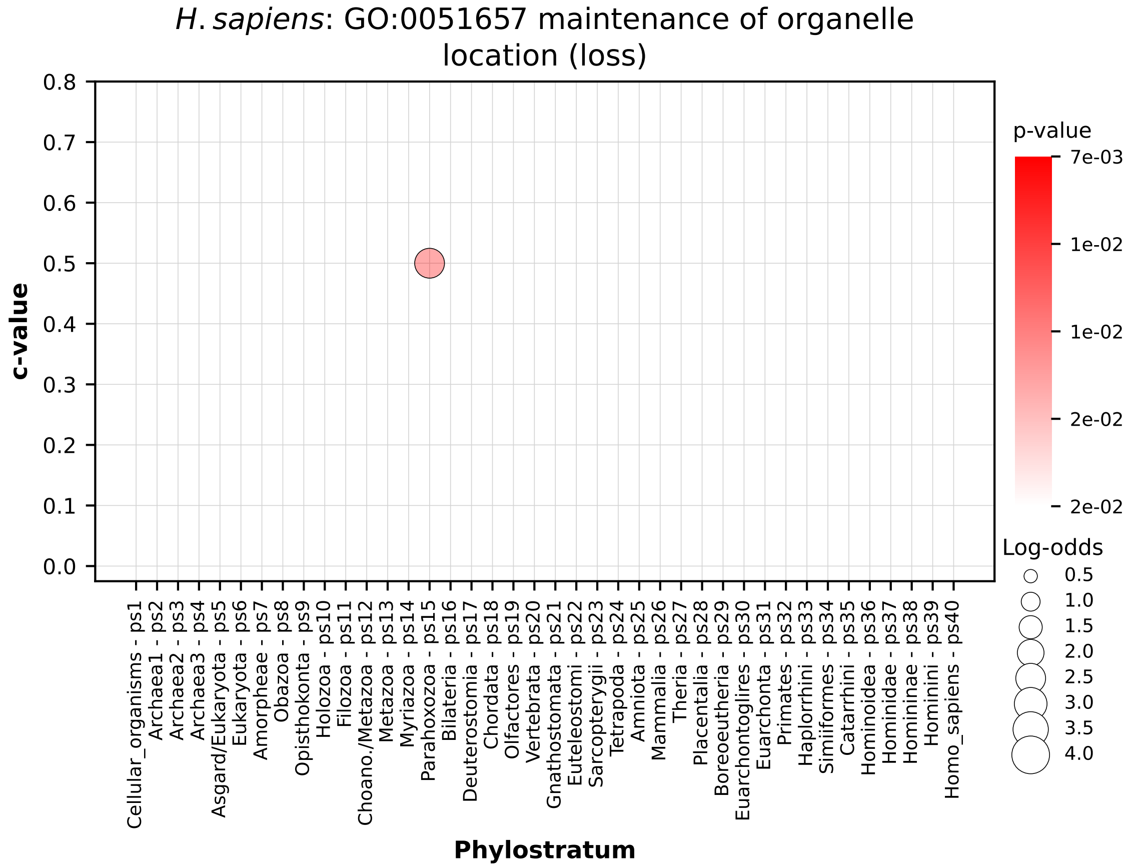

H. sapiens: GO:0051657 maintenance of organelle location (loss)
c-value
0.8
0.7
0.6
0.5
0.4
0.3
0.2
0.1
0.0
Phylostratum
Cellular_organisms - ps1
Archaea1 - ps2
Archaea2 - ps3
Archaea3 - ps4
Asgard/Eukaryota - ps5
Eukaryota - ps6
Amorpheae - ps7
Obazoa - ps8
Opisthokonta - ps9
Holozoa - ps10
Filozoa - ps11
Choano./Metazoa - ps12
Metazoa - ps13
Myriazoa - ps14
Parahoxozoa - ps15
Bilateria - ps16
Deuterostomia - ps17
Chordata - ps18
Olfactores - ps19
Vertebrata - ps20
Gnathostomata - ps21
Euteleostomi - ps22
Sarcopterygii - ps23
Tetrapoda - ps24
Amniota - ps25
Mammalia - ps26
Theria - ps27
Placentalia - ps28
Boreoeutheria - ps29
Euarchontoglires - ps30
Euarchonta - ps31
Primates - ps32
Haplorrhini - ps33
Simiiformes - ps34
Catarrhini - ps35
Hominoidea - ps36
Hominidae - ps37
Homininae - ps38
Hominini - ps39
Homo_sapiens - ps40
p-value
7e-03
1e-02
1e-02
2e-02
2e-02
Log-odds
0.5
1.0
1.5
2.0
2.5
3.0
3.5
4.0

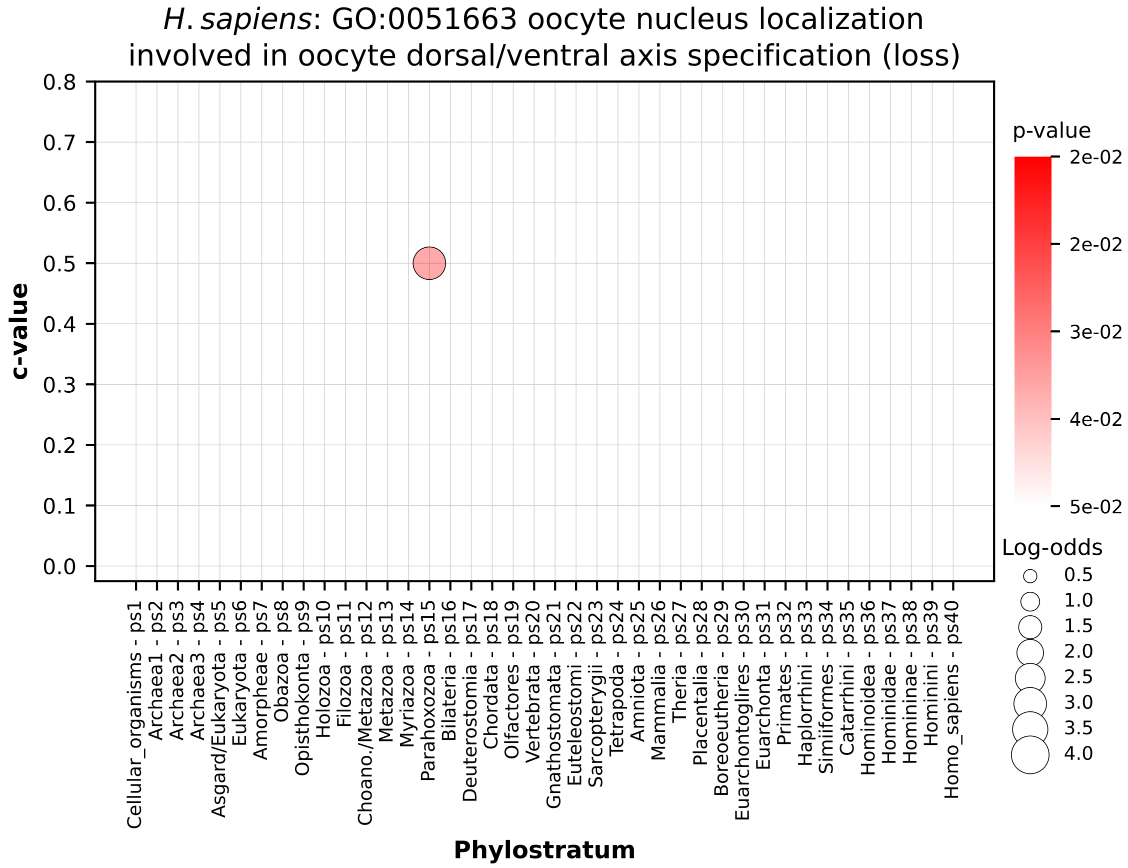

H. sapiens: GO:0051663 oocyte nucleus localization involved in oocyte dorsal/ventral axis specification (loss)
c-value
0.8
0.7
0.6
0.5
0.4
0.3
0.2
0.1
0.0
p-value
2e-02
2e-02
3e-02
4e-02
5e-02
Log-odds
0.5
1.0
1.5
2.0
2.5
3.0
3.5
4.0
Cellular_organisms - ps1
Archaea1 - ps2
Archaea2 - ps3
Archaea3 - ps4
Asgard/Eukaryota - ps5
Eukaryota - ps6
Amorpheae - ps7
Obazoa - ps8
Opisthokonta - ps9
Holozoa - ps10
Filozoa - ps11
Choano./Metazoa - ps12
Metazoa - ps13
Myriazoa - ps14
Parahoxozoa - ps15
Bilateria - ps16
Deuterostomia - ps17
Chordata - ps18
Olfactores - ps19
Vertebrata - ps20
Gnathostomata - ps21
Euteleostomi - ps22
Sarcopterygii - ps23
Tetrapoda - ps24
Amniota - ps25
Mammalia - ps26
Theria - ps27
Placentalia - ps28
Boreoeutheria - ps29
Euarchontoglires - ps30
Euarchonta - ps31
Primates - ps32
Haplorrhini - ps33
Simiiformes - ps34
Catarrhini - ps35
Hominoidea - ps36
Hominidae - ps37
Homininae - ps38
Hominini - ps39
Homo_sapiens - ps40
Phylostratum

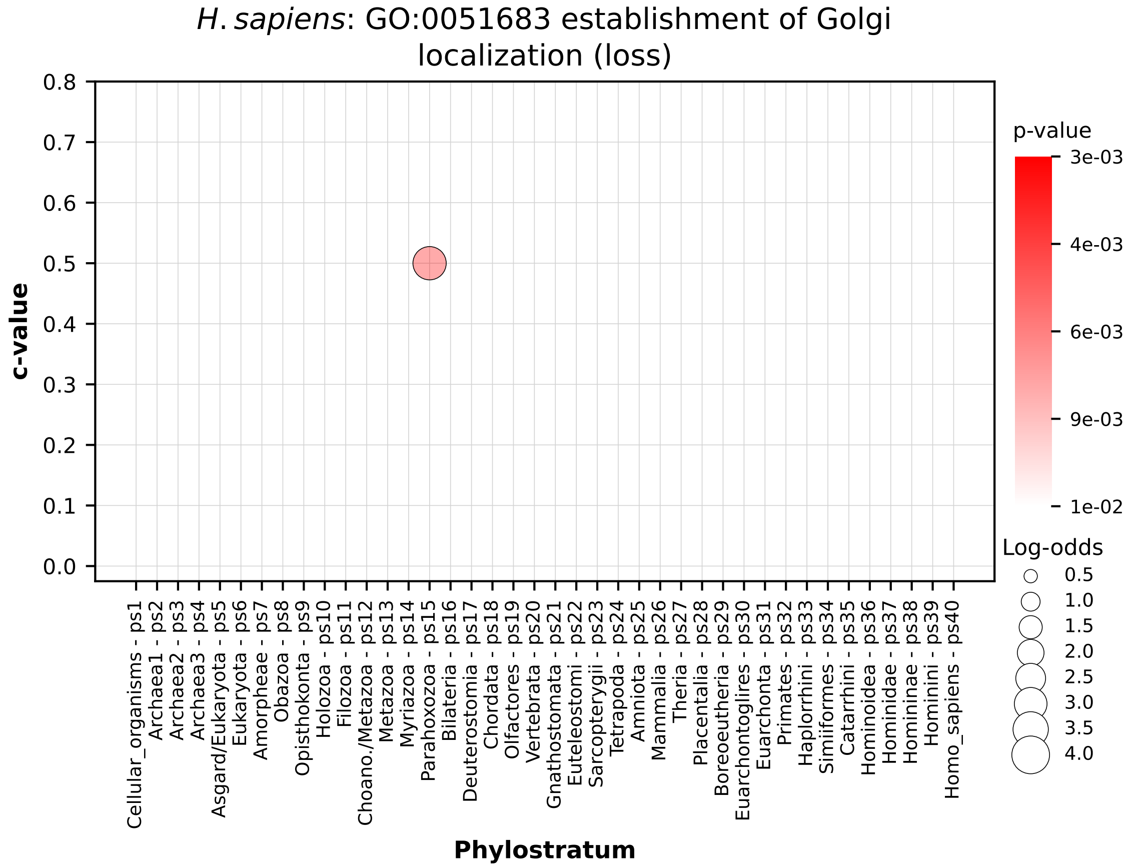
H. sapiens: GO:0051683 establishment of Golgi localization (loss)
c-value
0.8
0.7
0.6
0.5
0.4
0.3
0.2
0.1
0.0
p-value
3e-03
4e-03
6e-03
9e-03
1e-02
Log-odds
0.5
1.0
1.5
2.0
2.5
3.0
3.5
4.0
Cellular_organisms - ps1
Archaea1 - ps2
Archaea2 - ps3
Archaea3 - ps4
Asgard/Eukaryota - ps5
Eukaryota - ps6
Amorpheae - ps7
Obazoa - ps8
Opisthokonta - ps9
Holozoa - ps10
Filozoa - ps11
Choano./Metazoa - ps12
Metazoa - ps13
Myriazoa - ps14
Parahoxozoa - ps15
Bilateria - ps16
Deuterostomia - ps17
Chordata - ps18
Olfactores - ps19
Vertebrata - ps20
Gnathostomata - ps21
Euteleostomi - ps22
Sarcopterygii - ps23
Tetrapoda - ps24
Amniota - ps25
Mammalia - ps26
Theria - ps27
Placentalia - ps28
Boreoeutheria - ps29
Euarchontoglires - ps30
Euarchonta - ps31
Primates - ps32
Haplorrhini - ps33
Simiiformes - ps34
Catarrhini - ps35
Hominoidea - ps36
Hominidae - ps37
Homininae - ps38
Hominini - ps39
Homo_sapiens - ps40
Phylostratum

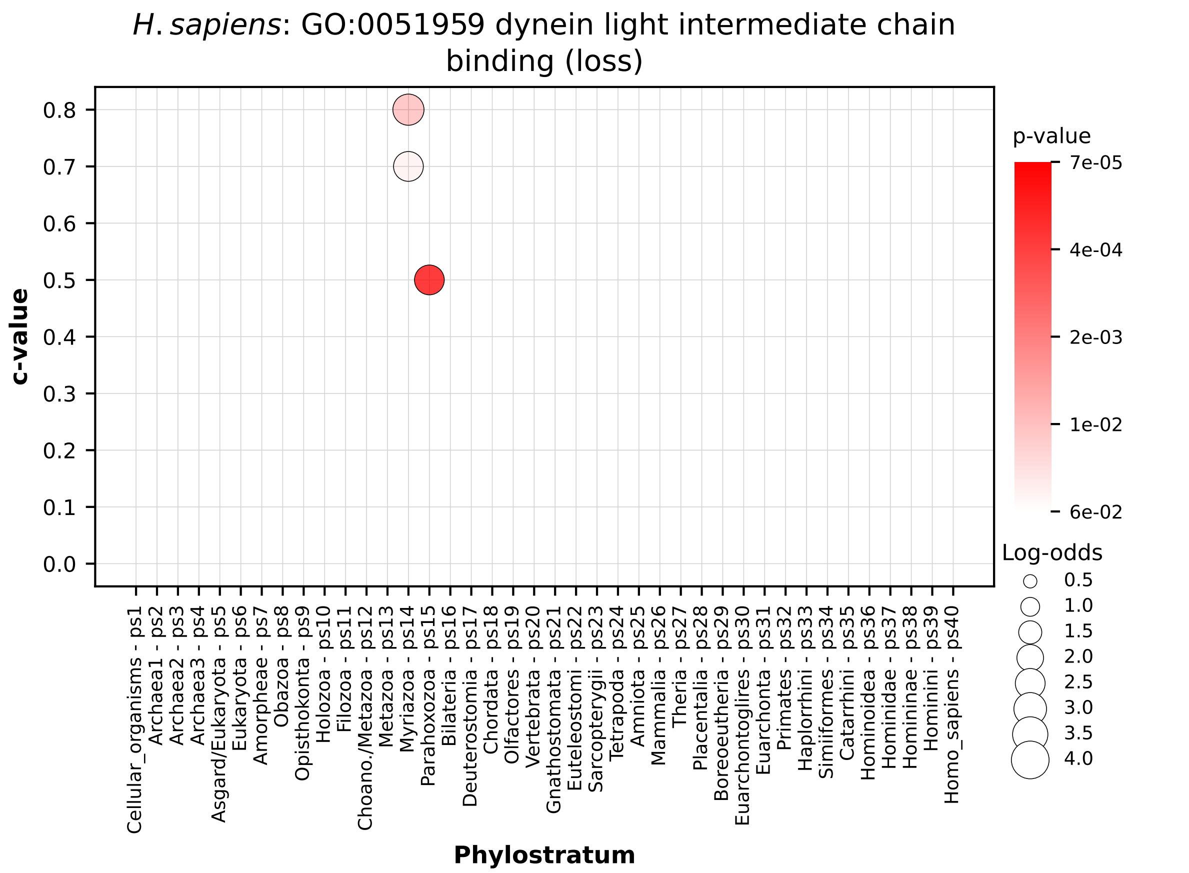

H. sapiens: GO:0051959 dynein light intermediate chain binding (loss)
c-value
0.0
0.1
0.2
0.3
0.4
0.5
0.6
0.7
0.8
p-value
7e-05
4e-04
2e-03
1e-02
6e-02
Log-odds
0.5
1.0
1.5
2.0
2.5
3.0
3.5
4.0
Cellular_organisms - ps1
Archaea1 - ps2
Archaea2 - ps3
Archaea3 - ps4
Asgard/Eukaryota - ps5
Eukaryota - ps6
Amorpheae - ps7
Obazoa - ps8
Opisthokonta - ps9
Holozoa - ps10
Filozoa - ps11
Choano./Metazoa - ps12
Metazoa - ps13
Myriazoa - ps14
Parahoxozoa - ps15
Bilateria - ps16
Deuterostomia - ps17
Chordata - ps18
Olfactores - ps19
Vertebrata - ps20
Gnathostomata - ps21
Euteleostomi - ps22
Sarcopterygii - ps23
Tetrapoda - ps24
Amniota - ps25
Mammalia - ps26
Theria - ps27
Placentalia - ps28
Boreoeutheria - ps29
Euarchontoglires - ps30
Euarchonta - ps31
Primates - ps32
Haplorrhini - ps33
Simiiformes - ps34
Catarrhini - ps35
Hominoidea - ps36
Hominidae - ps37
Homininae - ps38
Hominini - ps39
Homo_sapiens - ps40
Phylostratum

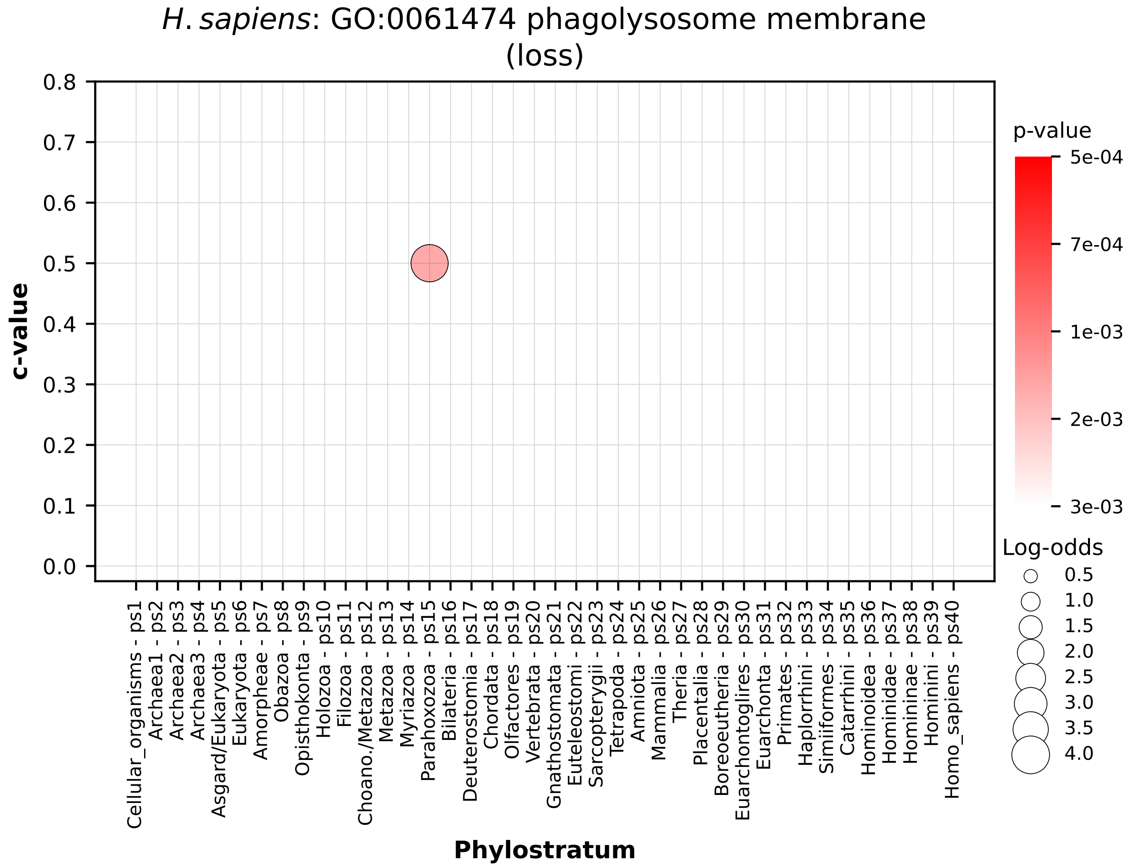
H. sapiens: GO:0061474 phagolysosome membrane
(loss)
c-value
0.0
0.1
0.2
0.3
0.4
0.5
0.6
0.7
0.8
Phylostratum
Cellular_organisms - ps1
Archaea1 - ps2
Archaea2 - ps3
Archaea3 - ps4
Asgard/Eukaryota - ps5
Eukaryota - ps6
Amorpheae - ps7
Obazoa - ps8
Opisthokonta - ps9
Holozoa - ps10
Filozoa - ps11
Choano./Metazoa - ps12
Metazoa - ps13
Myriazoa - ps14
Parahoxozoa - ps15
Bilateria - ps16
Deuterostomia - ps17
Chordata - ps18
Olfactores - ps19
Vertebrata - ps20
Gnathostomata - ps21
Euteleostomi - ps22
Sarcopterygii - ps23
Tetrapoda - ps24
Amniota - ps25
Mammalia - ps26
Theria - ps27
Placentalia - ps28
Boreoeutheria - ps29
Euarchontoglires - ps30
Euarchonta - ps31
Primates - ps32
Haplorrhini - ps33
Simiiformes - ps34
Catarrhini - ps35
Hominoidea - ps36
Hominidae - ps37
Homininae - ps38
Hominini - ps39
Homo_sapiens - ps40
p-value
5e-04
7e-04
1e-03
2e-03
3e-03
Log-odds
0.5
1.0
1.5
2.0
2.5
3.0
3.5
4.0

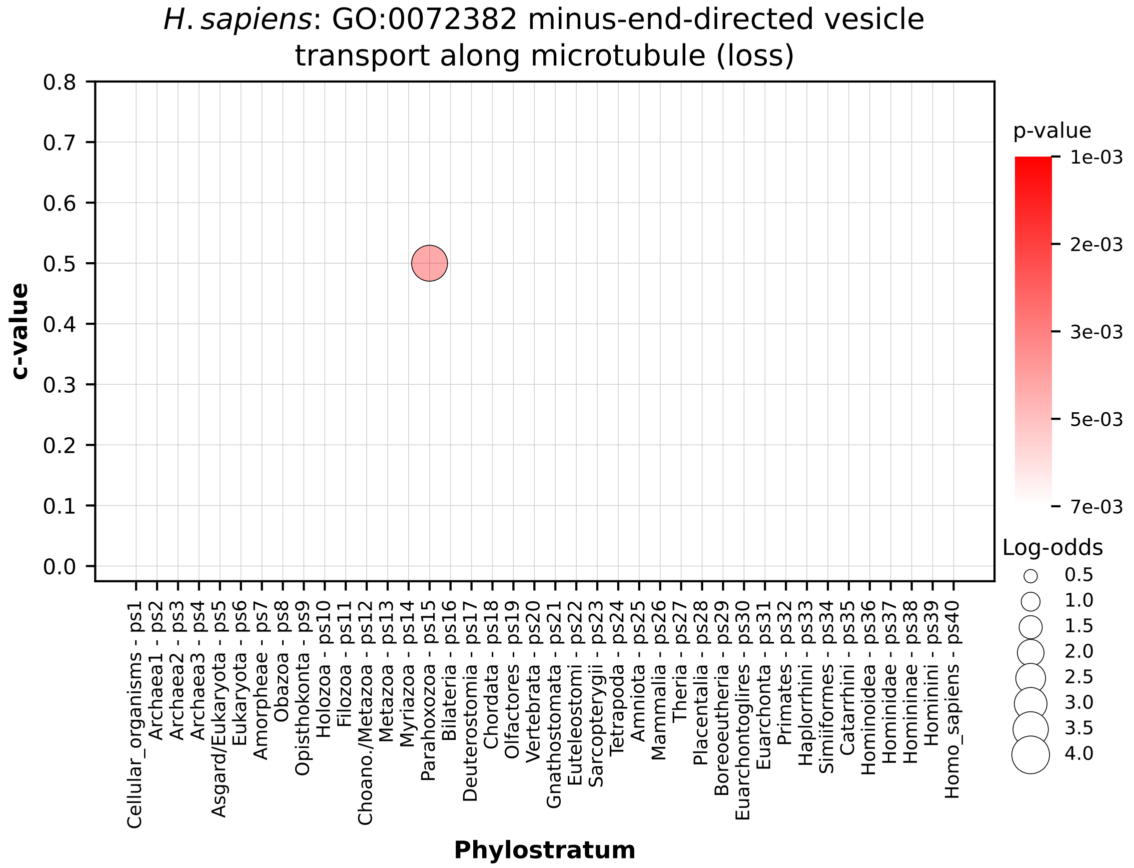

H. sapiens: GO:0072382 minus-end-directed vesicle transport along microtubule (loss)
c-value
0.8
0.7
0.6
0.5
0.4
0.3
0.2
0.1
0.0
Phylostratum
Cellular_organisms - ps1
Archaea1 - ps2
Archaea2 - ps3
Archaea3 - ps4
Asgard/Eukaryota - ps5
Eukaryota - ps6
Amorpheae - ps7
Obazoa - ps8
Opisthokonta - ps9
Holozoa - ps10
Filozoa - ps11
Choano./Metazoa - ps12
Metazoa - ps13
Myriazoa - ps14
Parahoxozoa - ps15
Bilateria - ps16
Deuterostomia - ps17
Chordata - ps18
Olfactores - ps19
Vertebrata - ps20
Gnathostomata - ps21
Euteleostomi - ps22
Sarcopterygii - ps23
Tetrapoda - ps24
Amniota - ps25
Mammalia - ps26
Theria - ps27
Placentalia - ps28
Boreoeutheria - ps29
Euarchontoglires - ps30
Euarchonta - ps31
Primates - ps32
Haplorrhini - ps33
Simiiformes - ps34
Catarrhini - ps35
Hominoidea - ps36
Hominidae - ps37
Homininae - ps38
Hominini - ps39
Homo_sapiens - ps40
p-value
1e-03
2e-03
3e-03
5e-03
7e-03
Log-odds
0.5
1.0
1.5
2.0
2.5
3.0
3.5
4.0

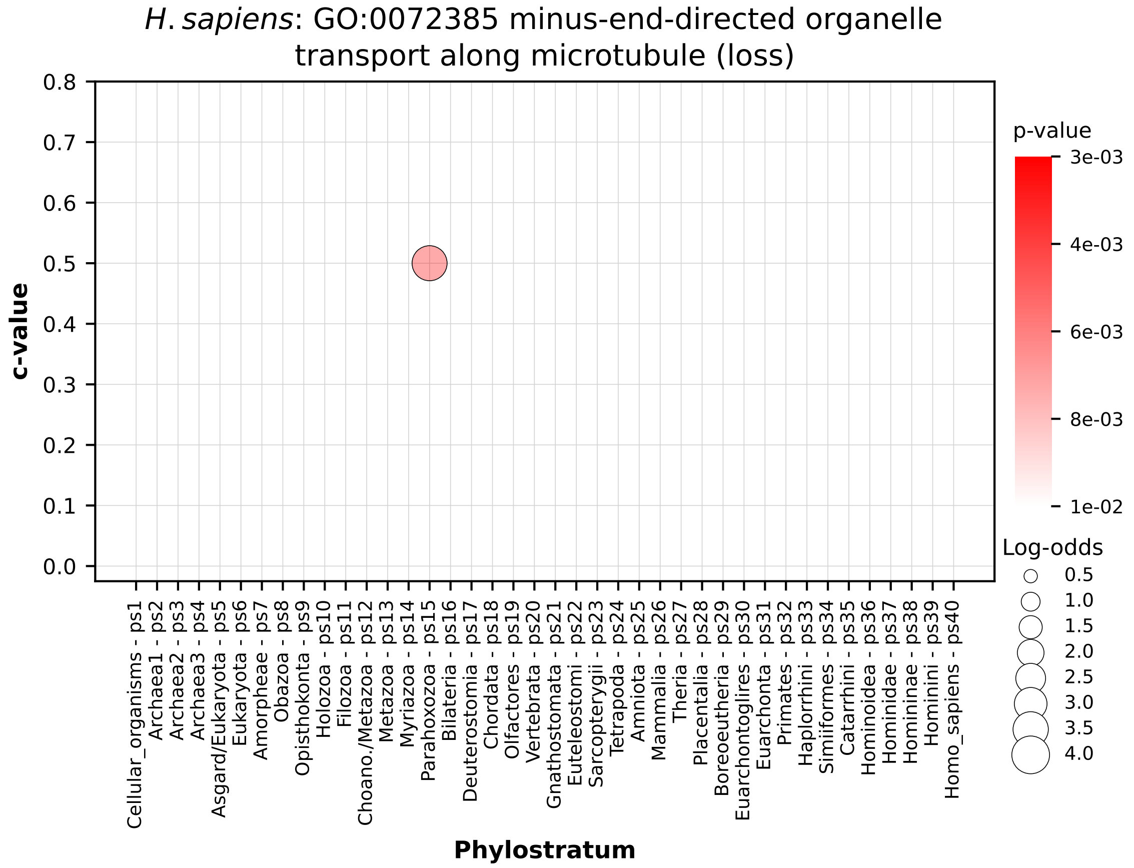
H. sapiens: GO:0072385 minus-end-directed organelle transport along microtubule (loss)
c-value
0.8
0.7
0.6
0.5
0.4
0.3
0.2
0.1
0.0
Phylostratum
Cellular_organisms - ps1
Archaea1 - ps2
Archaea2 - ps3
Archaea3 - ps4
Asgard/Eukaryota - ps5
Eukaryota - ps6
Amorpheae - ps7
Obazoa - ps8
Opisthokonta - ps9
Holozoa - ps10
Filozoa - ps11
Choano./Metazoa - ps12
Metazoa - ps13
Myriazoa - ps14
Parahoxozoa - ps15
Bilateria - ps16
Deuterostomia - ps17
Chordata - ps18
Olfactores - ps19
Vertebrata - ps20
Gnathostomata - ps21
Euteleostomi - ps22
Sarcopterygii - ps23
Tetrapoda - ps24
Amniota - ps25
Mammalia - ps26
Theria - ps27
Placentalia - ps28
Boreoeutheria - ps29
Euarchontoglires - ps30
Euarchonta - ps31
Primates - ps32
Haplorrhini - ps33
Simiiformes - ps34
Catarrhini - ps35
Hominoidea - ps36
Hominidae - ps37
Homininae - ps38
Hominini - ps39
Homo_sapiens - ps40
p-value
3e-03
4e-03
6e-03
8e-03
1e-02
Log-odds
0.5
1.0
1.5
2.0
2.5
3.0
3.5
4.0

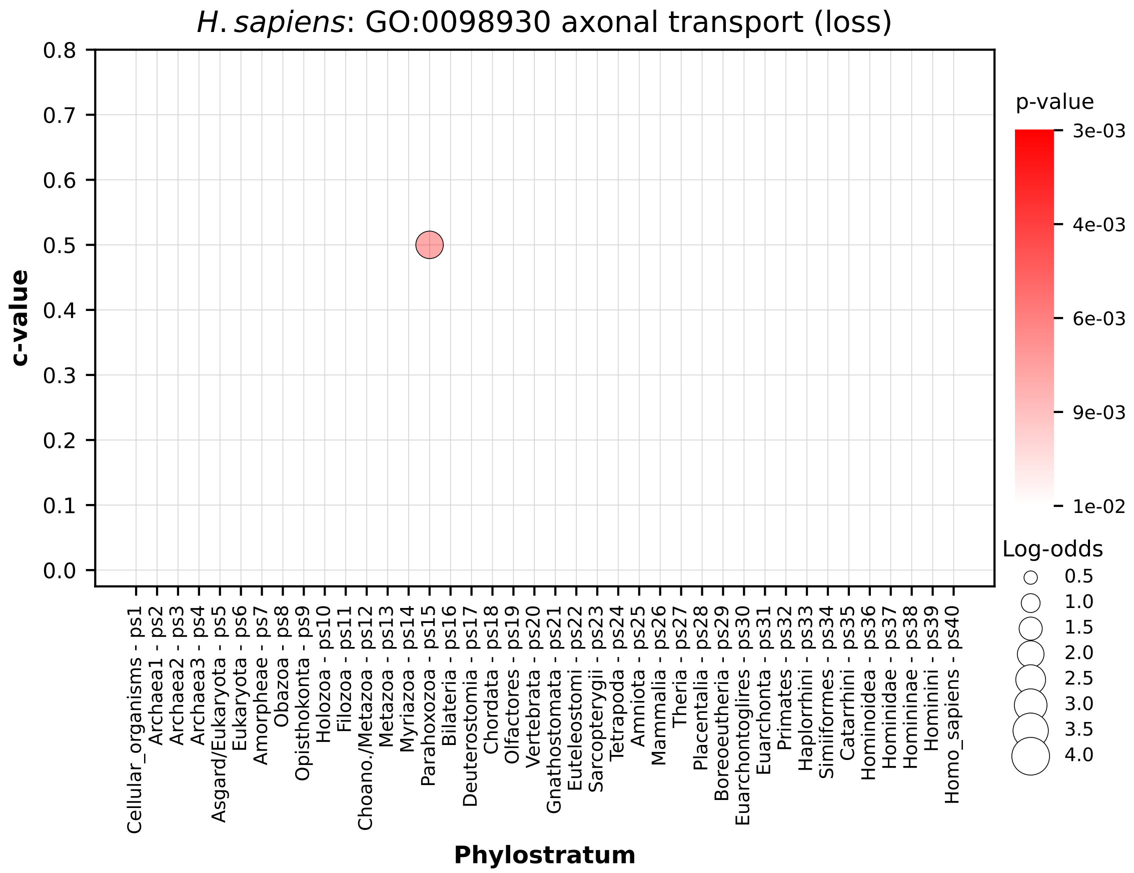

H. sapiens: GO:0098930 axonal transport (loss)
c-value
0.8
0.7
0.6
0.5
0.4
0.3
0.2
0.1
0.0
p-value
3e-03
4e-03
6e-03
9e-03
1e-02
Log-odds
0.5
1.0
1.5
2.0
2.5
3.0
3.5
4.0
Cellular_organisms - ps1
Archaea1 - ps2
Archaea2 - ps3
Archaea3 - ps4
Asgard/Eukaryota - ps5
Eukaryota - ps6
Amorpheae - ps7
Obazoa - ps8
Opisthokonta - ps9
Holozoa - ps10
Filozoa - ps11
Choano./Metazoa - ps12
Metazoa - ps13
Myriazoa - ps14
Parahoxozoa - ps15
Bilateria - ps16
Deuterostomia - ps17
Chordata - ps18
Olfactores - ps19
Vertebrata - ps20
Gnathostomata - ps21
Euteleostomi - ps22
Sarcopterygii - ps23
Tetrapoda - ps24
Amniota - ps25
Mammalia - ps26
Theria - ps27
Placentalia - ps28
Boreoeutheria - ps29
Euarchontoglires - ps30
Euarchonta - ps31
Primates - ps32
Haplorrhini - ps33
Simiiformes - ps34
Catarrhini - ps35
Hominoidea - ps36
Hominidae - ps37
Homininae - ps38
Hominini - ps39
Homo_sapiens - ps40
Phylostratum

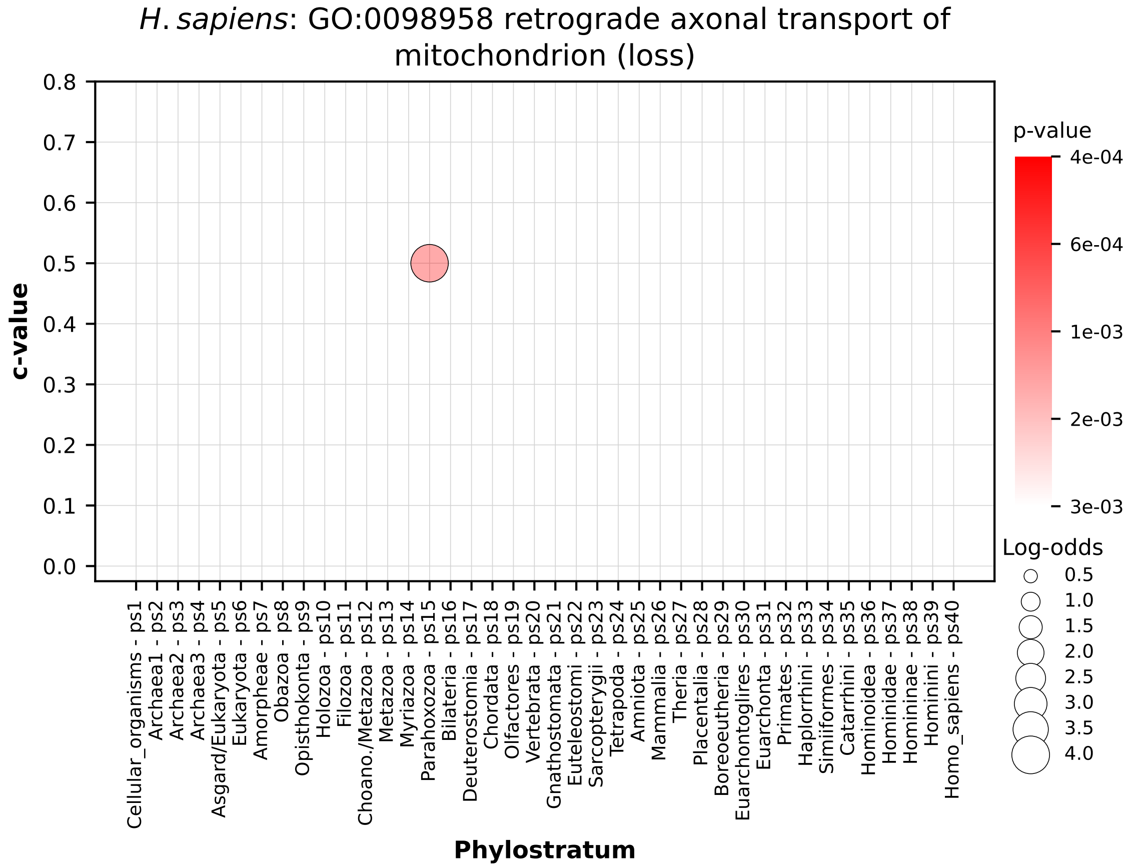
H. sapiens: GO:0098958 retrograde axonal transport of mitochondrion (loss)
c-value
0.8
0.7
0.6
0.5
0.4
0.3
0.2
0.1
0.0
Phylostratum
Cellular_organisms - ps1
Archaea1 - ps2
Archaea2 - ps3
Archaea3 - ps4
Asgard/Eukaryota - ps5
Eukaryota - ps6
Amorpheae - ps7
Obazoa - ps8
Opisthokonta - ps9
Holozoa - ps10
Filozoa - ps11
Choano./Metazoa - ps12
Metazoa - ps13
Myriazoa - ps14
Parahoxozoa - ps15
Bilateria - ps16
Deuterostomia - ps17
Chordata - ps18
Olfactores - ps19
Vertebrata - ps20
Gnathostomata - ps21
Euteleostomi - ps22
Sarcopterygii - ps23
Tetrapoda - ps24
Amniota - ps25
Mammalia - ps26
Theria - ps27
Placentalia - ps28
Boreoeutheria - ps29
Euarchontoglires - ps30
Euarchonta - ps31
Primates - ps32
Haplorrhini - ps33
Simiiformes - ps34
Catarrhini - ps35
Hominoidea - ps36
Hominidae - ps37
Homininae - ps38
Hominini - ps39
Homo_sapiens - ps40
p-value
4e-04
6e-04
1e-03
2e-03
3e-03
Log-odds
0.5
1.0
1.5
2.0
2.5
3.0
3.5
4.0

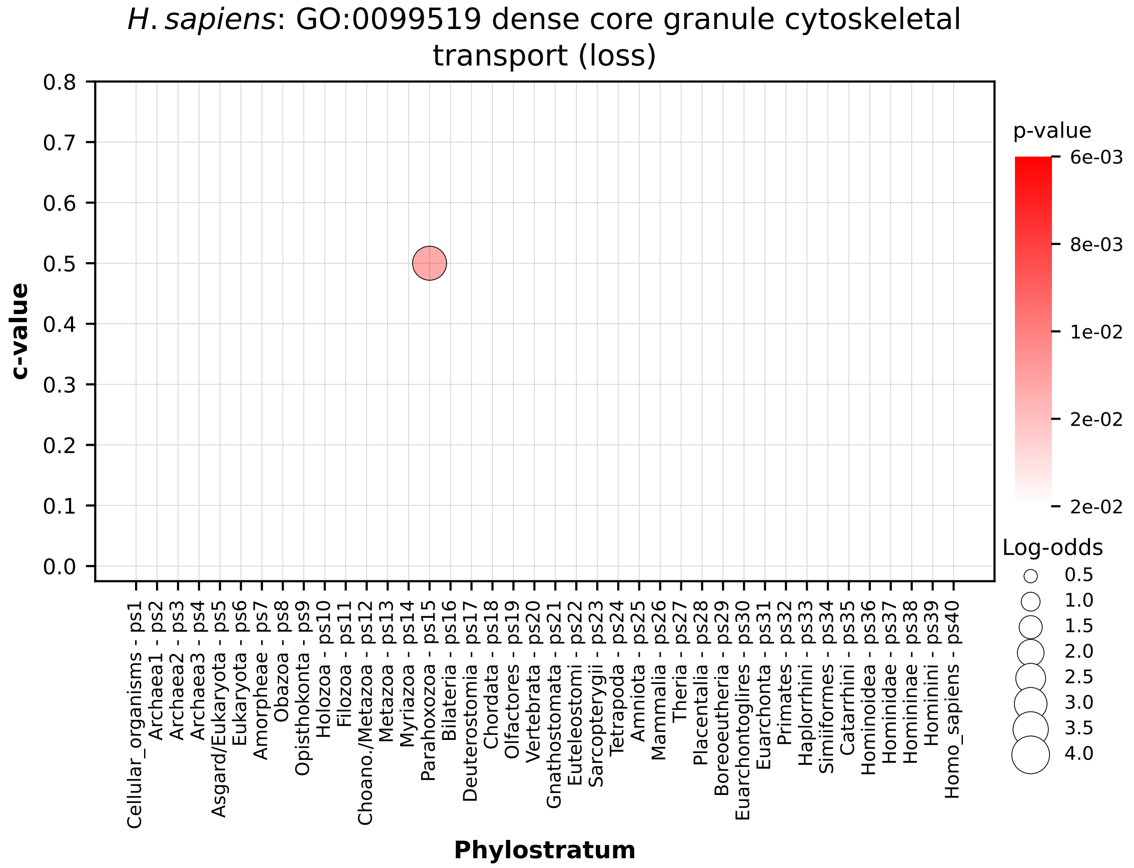

H. sapiens: GO:0099519 dense core granule cytoskeletal transport (loss)
c-value
0.8
0.7
0.6
0.5
0.4
0.3
0.2
0.1
0.0
Phylostratum
Cellular_organisms - ps1
Archaea1 - ps2
Archaea2 - ps3
Archaea3 - ps4
Asgard/Eukaryota - ps5
Eukaryota - ps6
Amorpheae - ps7
Obazoa - ps8
Opisthokonta - ps9
Holozoa - ps10
Filozoa - ps11
Choano./Metazoa - ps12
Metazoa - ps13
Myriazoa - ps14
Parahoxozoa - ps15
Bilateria - ps16
Deuterostomia - ps17
Chordata - ps18
Olfactores - ps19
Vertebrata - ps20
Gnathostomata - ps21
Euteleostomi - ps22
Sarcopterygii - ps23
Tetrapoda - ps24
Amniota - ps25
Mammalia - ps26
Theria - ps27
Placentalia - ps28
Boreoeutheria - ps29
Euarchontoglires - ps30
Euarchonta - ps31
Primates - ps32
Haplorrhini - ps33
Simiiformes - ps34
Catarrhini - ps35
Hominoidea - ps36
Hominidae - ps37
Homininae - ps38
Hominini - ps39
Homo_sapiens - ps40
p-value
6e-03
8e-03
1e-02
2e-02
2e-02
Log-odds
0.5
1.0
1.5
2.0
2.5
3.0
3.5
4.0

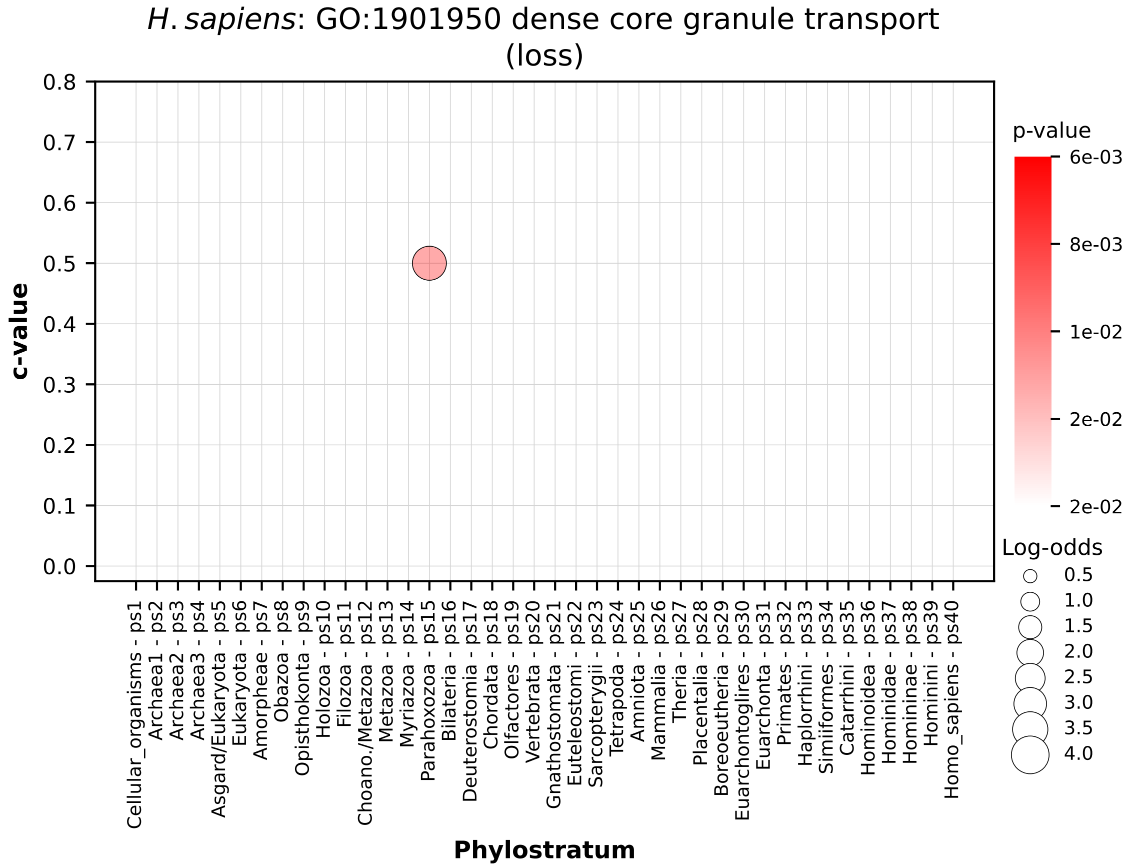

H. sapiens: GO:1901950 dense core granule transport (loss)
c-value
0.8
0.7
0.6
0.5
0.4
0.3
0.2
0.1
0.0
p-value
6e-03
8e-03
1e-02
2e-02
2e-02
Log-odds
0.5
1.0
1.5
2.0
2.5
3.0
3.5
4.0
Cellular_organisms - ps1
Archaea1 - ps2
Archaea2 - ps3
Archaea3 - ps4
Asgard/Eukaryota - ps5
Eukaryota - ps6
Amorpheae - ps7
Obazoa - ps8
Opisthokonta - ps9
Holozoa - ps10
Filozoa - ps11
Choano./Metazoa - ps12
Metazoa - ps13
Myriazoa - ps14
Parahoxozoa - ps15
Bilateria - ps16
Deuterostomia - ps17
Chordata - ps18
Olfactores - ps19
Vertebrata - ps20
Gnathostomata - ps21
Euteleostomi - ps22
Sarcopterygii - ps23
Tetrapoda - ps24
Amniota - ps25
Mammalia - ps26
Theria - ps27
Placentalia - ps28
Boreoeutheria - ps29
Euarchontoglires - ps30
Euarchonta - ps31
Primates - ps32
Haplorrhini - ps33
Simiiformes - ps34
Catarrhini - ps35
Hominoidea - ps36
Hominidae - ps37
Homininae - ps38
Hominini - ps39
Homo_sapiens - ps40
Phylostratum

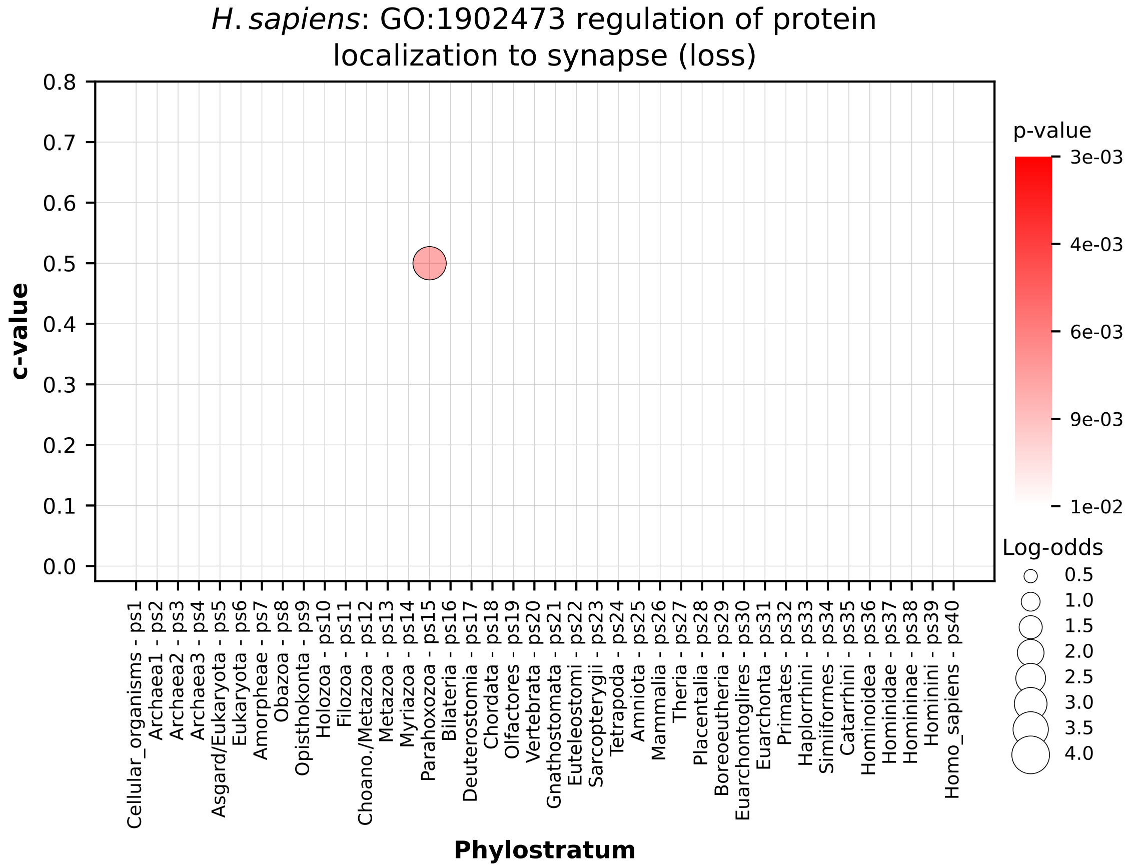
H. sapiens: GO:1902473 regulation of protein localization to synapse (loss)
c-value
0.8
0.7
0.6
0.5
0.4
0.3
0.2
0.1
0.0
p-value
3e-03
4e-03
6e-03
9e-03
1e-02
Log-odds
0.5
1.0
1.5
2.0
2.5
3.0
3.5
4.0
Cellular_organisms - ps1
Archaea1 - ps2
Archaea2 - ps3
Archaea3 - ps4
Asgard/Eukaryota - ps5
Eukaryota - ps6
Amorpheae - ps7
Obazoa - ps8
Opisthokonta - ps9
Holozoa - ps10
Filozoa - ps11
Choano./Metazoa - ps12
Metazoa - ps13
Myriazoa - ps14
Parahoxozoa - ps15
Bilateria - ps16
Deuterostomia - ps17
Chordata - ps18
Olfactores - ps19
Vertebrata - ps20
Gnathostomata - ps21
Euteleostomi - ps22
Sarcopterygii - ps23
Tetrapoda - ps24
Amniota - ps25
Mammalia - ps26
Theria - ps27
Placentalia - ps28
Boreoeutheria - ps29
Euarchontoglires - ps30
Euarchonta - ps31
Primates - ps32
Haplorrhini - ps33
Simiiformes - ps34
Catarrhini - ps35
Hominoidea - ps36
Hominidae - ps37
Homininae - ps38
Hominini - ps39
Homo_sapiens - ps40
Phylostratum

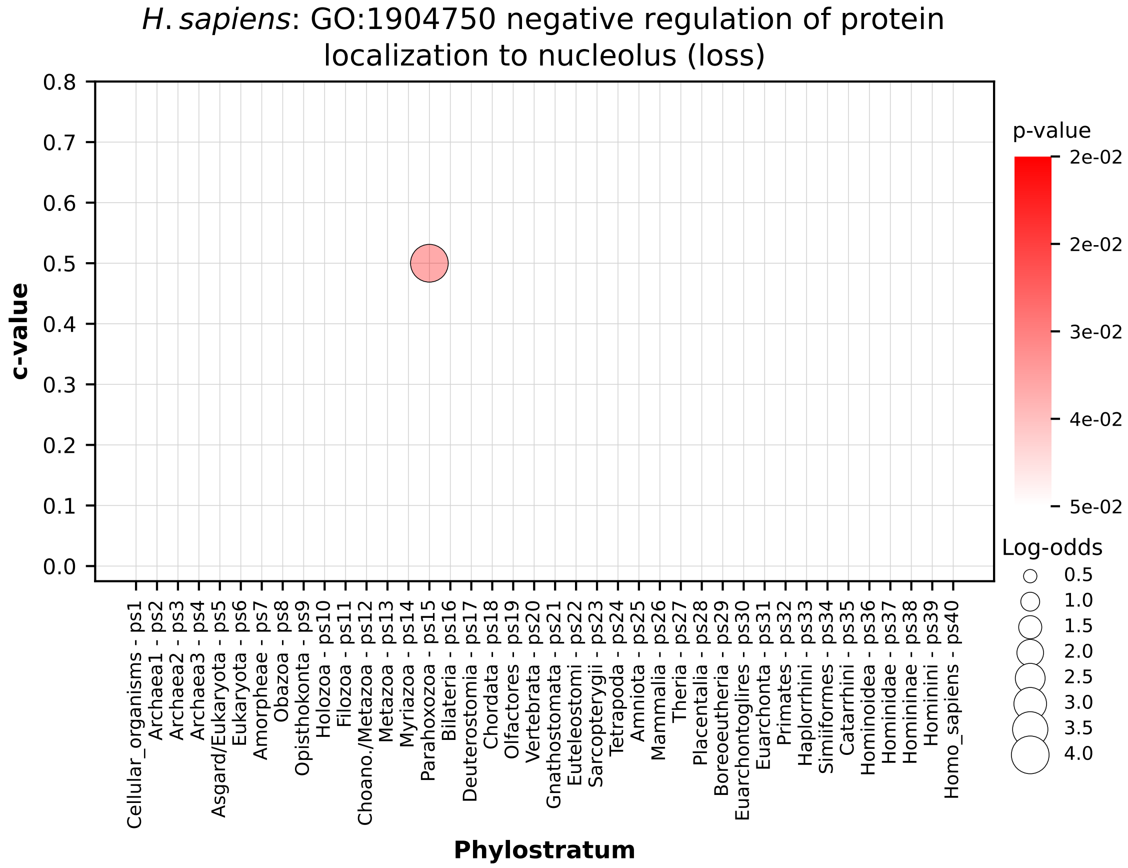
H. sapiens: GO:1904750 negative regulation of protein localization to nucleolus (loss)
c-value
0.8
0.7
0.6
0.5
0.4
0.3
0.2
0.1
0.0
Phylostratum
Cellular_organisms - ps1
Archaea1 - ps2
Archaea2 - ps3
Archaea3 - ps4
Asgard/Eukaryota - ps5
Eukaryota - ps6
Amorpheae - ps7
Obazoa - ps8
Opisthokonta - ps9
Holozoa - ps10
Filozoa - ps11
Choano./Metazoa - ps12
Metazoa - ps13
Myriazoa - ps14
Parahoxozoa - ps15
Bilateria - ps16
Deuterostomia - ps17
Chordata - ps18
Olfactores - ps19
Vertebrata - ps20
Gnathostomata - ps21
Euteleostomi - ps22
Sarcopterygii - ps23
Tetrapoda - ps24
Amniota - ps25
Mammalia - ps26
Theria - ps27
Placentalia - ps28
Boreoeutheria - ps29
Euarchontoglires - ps30
Euarchonta - ps31
Primates - ps32
Haplorrhini - ps33
Simiiformes - ps34
Catarrhini - ps35
Hominoidea - ps36
Hominidae - ps37
Homininae - ps38
Hominini - ps39
Homo_sapiens - ps40
p-value
2e-02
2e-02
3e-02
4e-02
5e-02
Log-odds
0.5
1.0
1.5
2.0
2.5
3.0
3.5
4.0

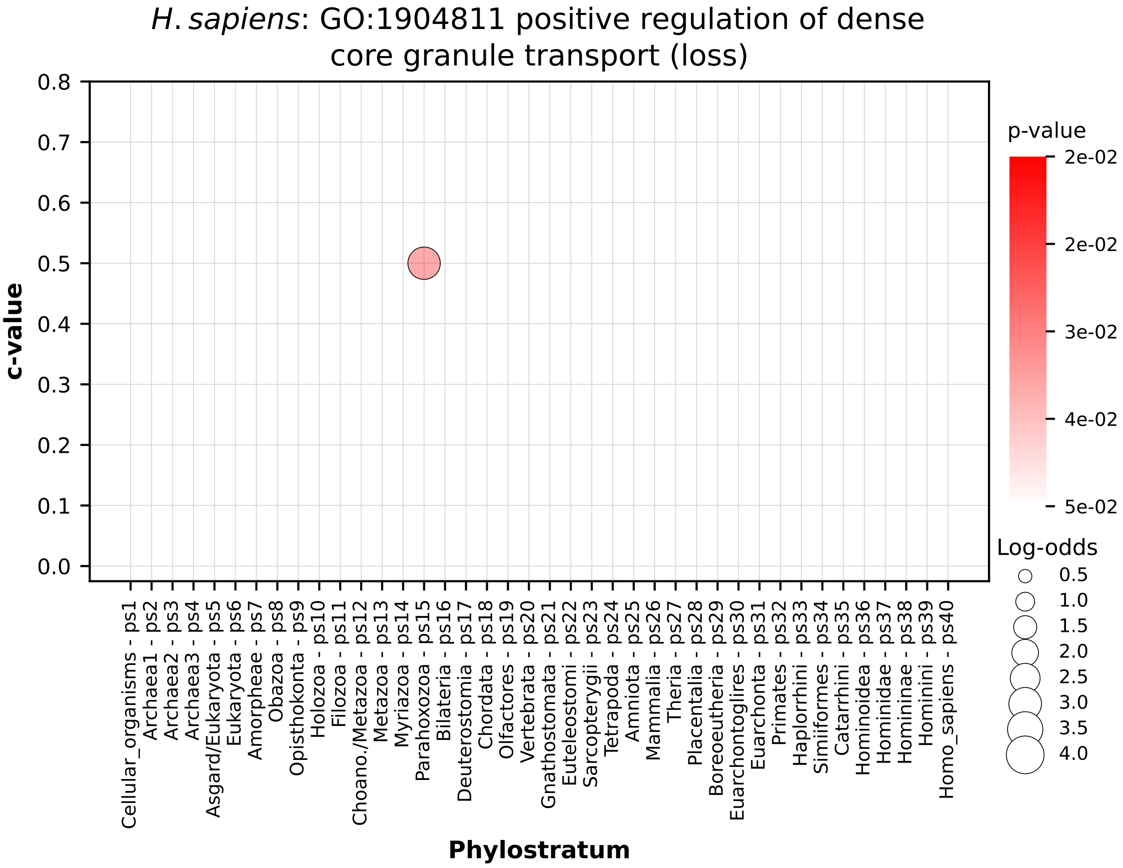

H. sapiens: GO:1904811 positive regulation of dense core granule transport (loss)
c-value
0.8
0.7
0.6
0.5
0.4
0.3
0.2
0.1
0.0
Phylostratum
Cellular_organisms - ps1
Archaea1 - ps2
Archaea2 - ps3
Archaea3 - ps4
Asgard/Eukaryota - ps5
Eukaryota - ps6
Amorpheae - ps7
Obazoa - ps8
Opisthokonta - ps9
Holozoa - ps10
Filozoa - ps11
Choano./Metazoa - ps12
Metazoa - ps13
Myriazoa - ps14
Parahoxozoa - ps15
Bilateria - ps16
Deuterostomia - ps17
Chordata - ps18
Olfactores - ps19
Vertebrata - ps20
Gnathostomata - ps21
Euteleostomi - ps22
Sarcopterygii - ps23
Tetrapoda - ps24
Amniota - ps25
Mammalia - ps26
Theria - ps27
Placentalia - ps28
Boreoeutheria - ps29
Euarchontoglires - ps30
Euarchonta - ps31
Primates - ps32
Haplorrhini - ps33
Simiiformes - ps34
Catarrhini - ps35
Hominoidea - ps36
Hominidae - ps37
Homininae - ps38
Hominini - ps39
Homo_sapiens - ps40
p-value
2e-02
2e-02
3e-02
4e-02
5e-02
Log-odds
0.5
1.0
1.5
2.0
2.5
3.0
3.5
4.0

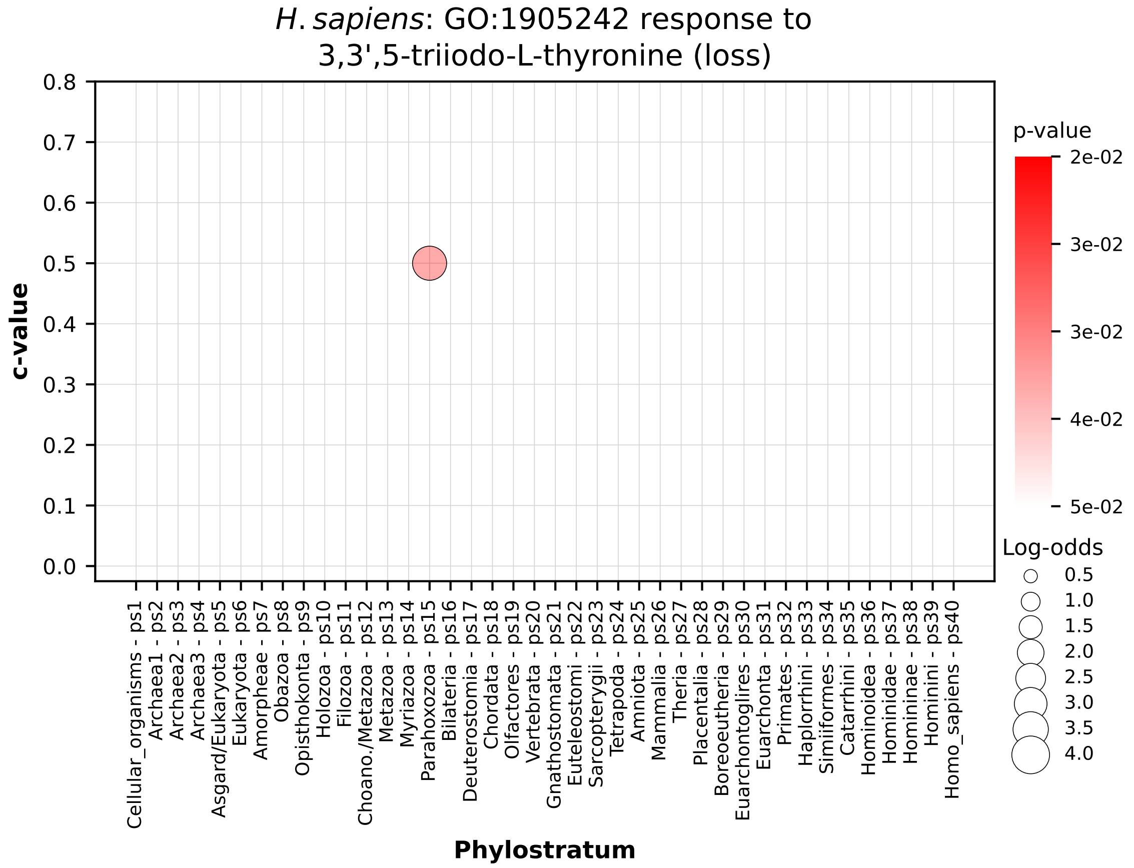

H. sapiens: GO:1905242 response to 3,3',5-triiodo-L-thyronine (loss)
c-value
0.0
0.1
0.2
0.3
0.4
0.5
0.6
0.7
0.8
Phylostratum
Cellular_organisms - ps1
Archaea1 - ps2
Archaea2 - ps3
Archaea3 - ps4
Asgard/Eukaryota - ps5
Eukaryota - ps6
Amorpheae - ps7
Obazoa - ps8
Opisthokonta - ps9
Holozoa - ps10
Filozoa - ps11
Choano./Metazoa - ps12
Metazoa - ps13
Myriazoa - ps14
Parahoxozoa - ps15
Bilateria - ps16
Deuterostomia - ps17
Chordata - ps18
Olfactores - ps19
Vertebrata - ps20
Gnathostomata - ps21
Euteleostomi - ps22
Sarcopterygii - ps23
Tetrapoda - ps24
Amniota - ps25
Mammalia - ps26
Theria - ps27
Placentalia - ps28
Boreoeutheria - ps29
Euarchontoglires - ps30
Euarchonta - ps31
Primates - ps32
Haplorrhini - ps33
Simiiformes - ps34
Catarrhini - ps35
Hominoidea - ps36
Hominidae - ps37
Homininae - ps38
Hominini - ps39
Homo_sapiens - ps40
p-value
2e-02
3e-02
3e-02
4e-02
5e-02
Log-odds
0.5
1.0
1.5
2.0
2.5
3.0
3.5
4.0

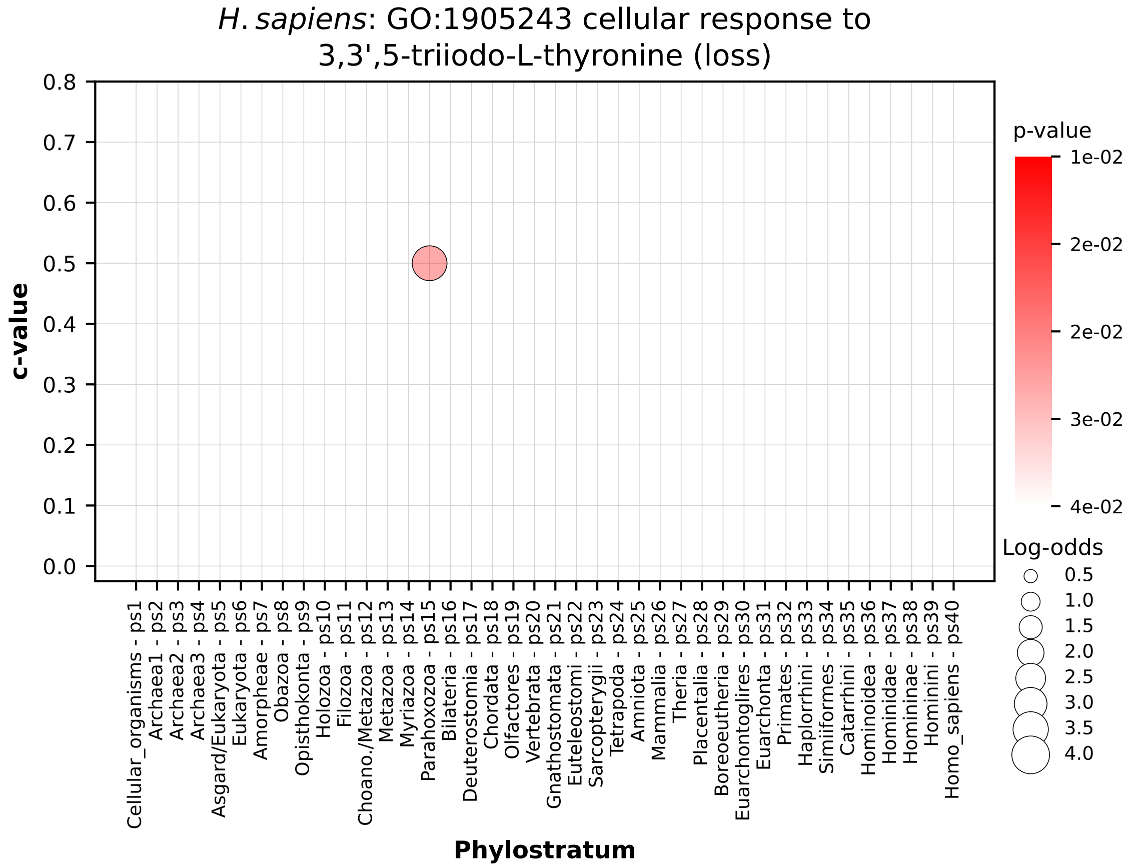

H. sapiens: GO:1905243 cellular response to 3,3',5-triiodo-L-thyronine (loss)
c-value
0.8
0.7
0.6
0.5
0.4
0.3
0.2
0.1
0.0
p-value
1e-02
2e-02
2e-02
3e-02
4e-02
Log-odds
0.5
1.0
1.5
2.0
2.5
3.0
3.5
4.0
Cellular_organisms - ps1
Archaea1 - ps2
Archaea2 - ps3
Archaea3 - ps4
Asgard/Eukaryota - ps5
Eukaryota - ps6
Amorpheae - ps7
Obazoa - ps8
Opisthokonta - ps9
Holozoa - ps10
Filozoa - ps11
Choano./Metazoa - ps12
Metazoa - ps13
Myriazoa - ps14
Parahoxozoa - ps15
Bilateria - ps16
Deuterostomia - ps17
Chordata - ps18
Olfactores - ps19
Vertebrata - ps20
Gnathostomata - ps21
Euteleostomi - ps22
Sarcopterygii - ps23
Tetrapoda - ps24
Amniota - ps25
Mammalia - ps26
Theria - ps27
Placentalia - ps28
Boreoeutheria - ps29
Euarchontoglires - ps30
Euarchonta - ps31
Primates - ps32
Haplorrhini - ps33
Simiiformes - ps34
Catarrhini - ps35
Hominoidea - ps36
Hominidae - ps37
Homininae - ps38
Hominini - ps39
Homo_sapiens - ps40
Phylostratum

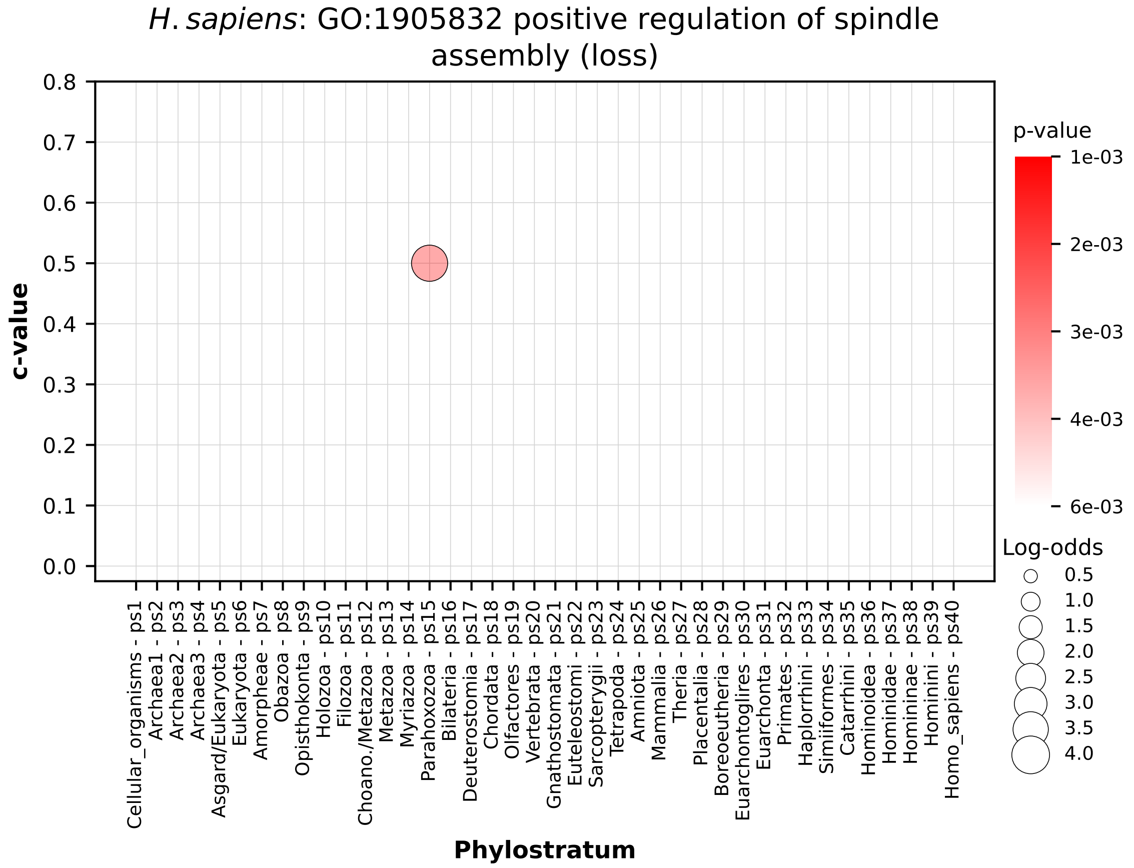
H. sapiens: GO:1905832 positive regulation of spindle assembly (loss)
c-value
0.8
0.7
0.6
0.5
0.4
0.3
0.2
0.1
0.0
Phylostratum
Cellular_organisms - ps1
Archaea1 - ps2
Archaea2 - ps3
Archaea3 - ps4
Asgard/Eukaryota - ps5
Eukaryota - ps6
Amorpheae - ps7
Obazoa - ps8
Opisthokonta - ps9
Holozoa - ps10
Filozoa - ps11
Choano./Metazoa - ps12
Metazoa - ps13
Myriazoa - ps14
Parahoxozoa - ps15
Bilateria - ps16
Deuterostomia - ps17
Chordata - ps18
Olfactores - ps19
Vertebrata - ps20
Gnathostomata - ps21
Euteleostomi - ps22
Sarcopterygii - ps23
Tetrapoda - ps24
Amniota - ps25
Mammalia - ps26
Theria - ps27
Placentalia - ps28
Boreoeutheria - ps29
Euarchontoglires - ps30
Euarchonta - ps31
Primates - ps32
Haplorrhini - ps33
Simiiformes - ps34
Catarrhini - ps35
Hominoidea - ps36
Hominidae - ps37
Homininae - ps38
Hominini - ps39
Homo_sapiens - ps40
p-value
1e-03
2e-03
3e-03
4e-03
6e-03
Log-odds
0.5
1.0
1.5
2.0
2.5
3.0
3.5
4.0

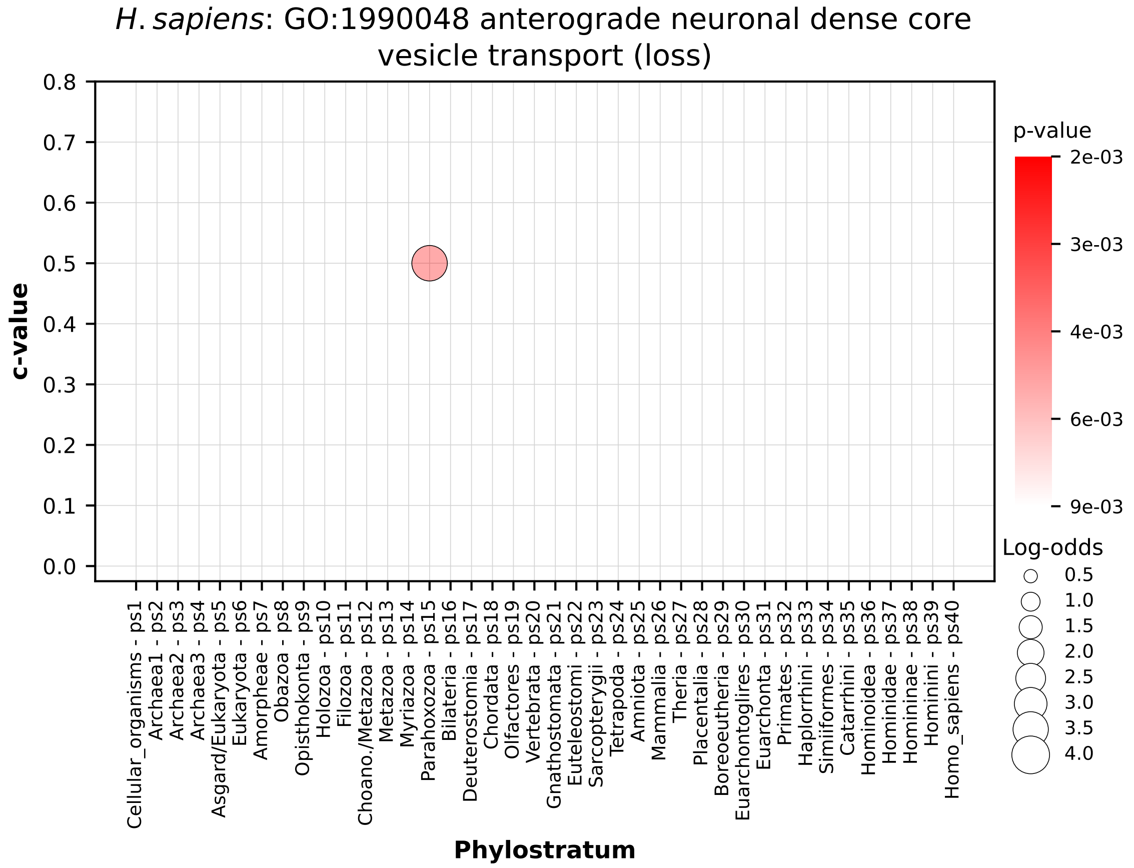

H. sapiens: GO:1990048 anterograde neuronal dense core vesicle transport (loss)
c-value
0.0
0.1
0.2
0.3
0.4
0.5
0.6
0.7
0.8
Phylostratum
Cellular_organisms - ps1
Archaea1 - ps2
Archaea2 - ps3
Archaea3 - ps4
Asgard/Eukaryota - ps5
Eukaryota - ps6
Amorpheae - ps7
Obazoa - ps8
Opisthokonta - ps9
Holozoa - ps10
Filozoa - ps11
Choano./Metazoa - ps12
Metazoa - ps13
Myriazoa - ps14
Parahoxozoa - ps15
Bilateria - ps16
Deuterostomia - ps17
Chordata - ps18
Olfactores - ps19
Vertebrata - ps20
Gnathostomata - ps21
Euteleostomi - ps22
Sarcopterygii - ps23
Tetrapoda - ps24
Amniota - ps25
Mammalia - ps26
Theria - ps27
Placentalia - ps28
Boreoeutheria - ps29
Euarchontoglires - ps30
Euarchonta - ps31
Primates - ps32
Haplorrhini - ps33
Simiiformes - ps34
Catarrhini - ps35
Hominoidea - ps36
Hominidae - ps37
Homininae - ps38
Hominini - ps39
Homo_sapiens - ps40
p-value
2e-03
3e-03
4e-03
6e-03
9e-03
Log-odds
0.5
1.0
1.5
2.0
2.5
3.0
3.5
4.0

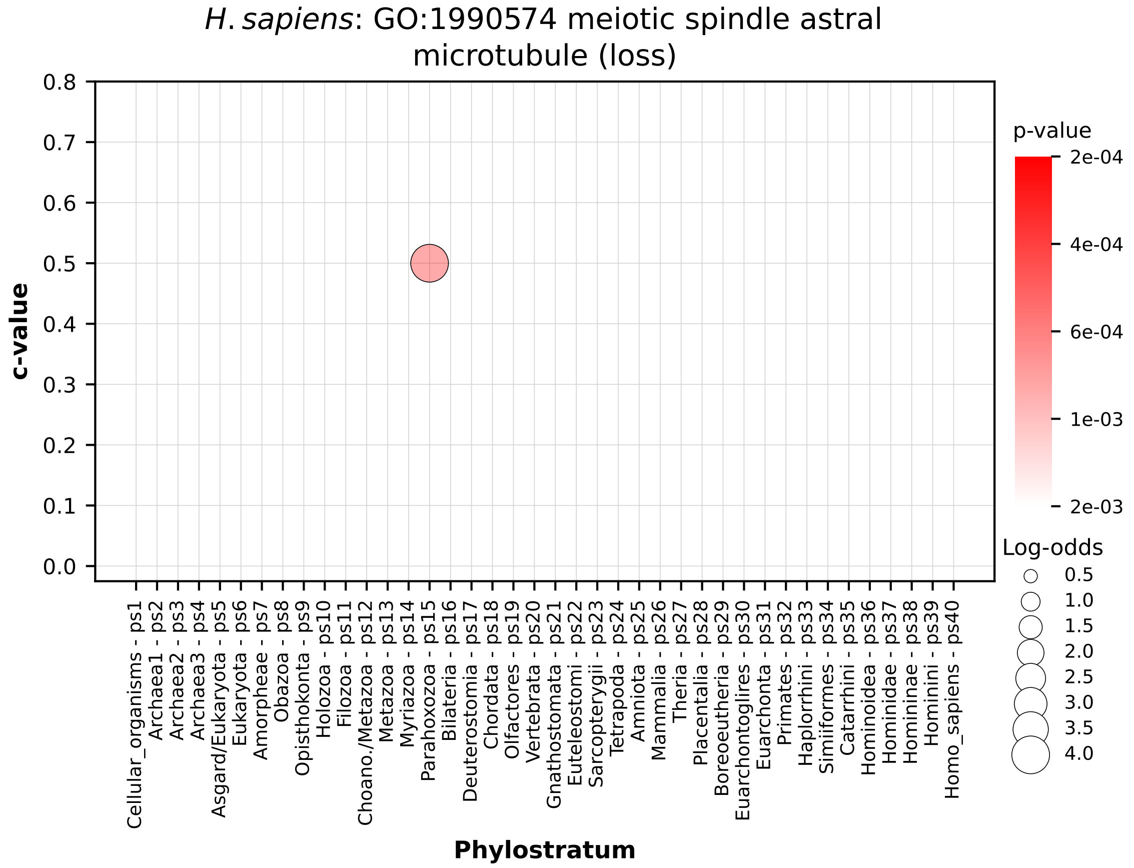

H. sapiens: GO:1990574 meiotic spindle astral microtubule (loss)
c-value
0.8
0.7
0.6
0.5
0.4
0.3
0.2
0.1
0.0
p-value
2e-04
4e-04
6e-04
1e-03
2e-03
Log-odds
0.5
1.0
1.5
2.0
2.5
3.0
3.5
4.0
Cellular_organisms - ps1
Archaea1 - ps2
Archaea2 - ps3
Archaea3 - ps4
Asgard/Eukaryota - ps5
Eukaryota - ps6
Amorpheae - ps7
Obazoa - ps8
Opisthokonta - ps9
Holozoa - ps10
Filozoa - ps11
Choano./Metazoa - ps12
Metazoa - ps13
Myriazoa - ps14
Parahoxozoa - ps15
Bilateria - ps16
Deuterostomia - ps17
Chordata - ps18
Olfactores - ps19
Vertebrata - ps20
Gnathostomata - ps21
Euteleostomi - ps22
Sarcopterygii - ps23
Tetrapoda - ps24
Amniota - ps25
Mammalia - ps26
Theria - ps27
Placentalia - ps28
Boreoeutheria - ps29
Euarchontoglires - ps30
Euarchonta - ps31
Primates - ps32
Haplorrhini - ps33
Simiiformes - ps34
Catarrhini - ps35
Hominoidea - ps36
Hominidae - ps37
Homininae - ps38
Hominini - ps39
Homo_sapiens - ps40
Phylostratum

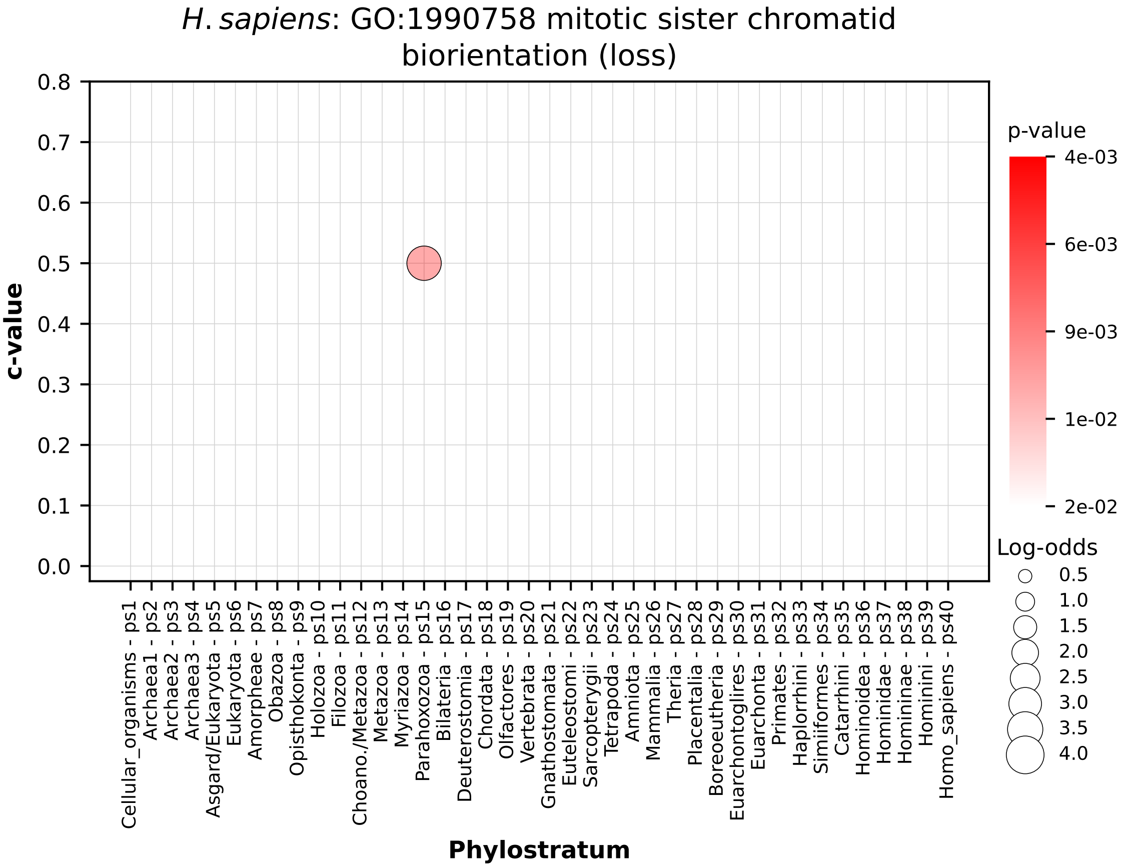

H. sapiens: GO:1990758 mitotic sister chromatid biorientation (loss)
c-value
0.8
0.7
0.6
0.5
0.4
0.3
0.2
0.1
0.0
p-value
4e-03
6e-03
9e-03
1e-02
2e-02
Log-odds
0.5
1.0
1.5
2.0
2.5
3.0
3.5
4.0
Cellular_organisms - ps1
Archaea1 - ps2
Archaea2 - ps3
Archaea3 - ps4
Asgard/Eukaryota - ps5
Eukaryota - ps6
Amorpheae - ps7
Obazoa - ps8
Opisthokonta - ps9
Holozoa - ps10
Filozoa - ps11
Choano./Metazoa - ps12
Metazoa - ps13
Myriazoa - ps14
Parahoxozoa - ps15
Bilateria - ps16
Deuterostomia - ps17
Chordata - ps18
Olfactores - ps19
Vertebrata - ps20
Gnathostomata - ps21
Euteleostomi - ps22
Sarcopterygii - ps23
Tetrapoda - ps24
Amniota - ps25
Mammalia - ps26
Theria - ps27
Placentalia - ps28
Boreoeutheria - ps29
Euarchontoglires - ps30
Euarchonta - ps31
Primates - ps32
Haplorrhini - ps33
Simiiformes - ps34
Catarrhini - ps35
Hominoidea - ps36
Hominidae - ps37
Homininae - ps38
Hominini - ps39
Homo_sapiens - ps40
Phylostratum

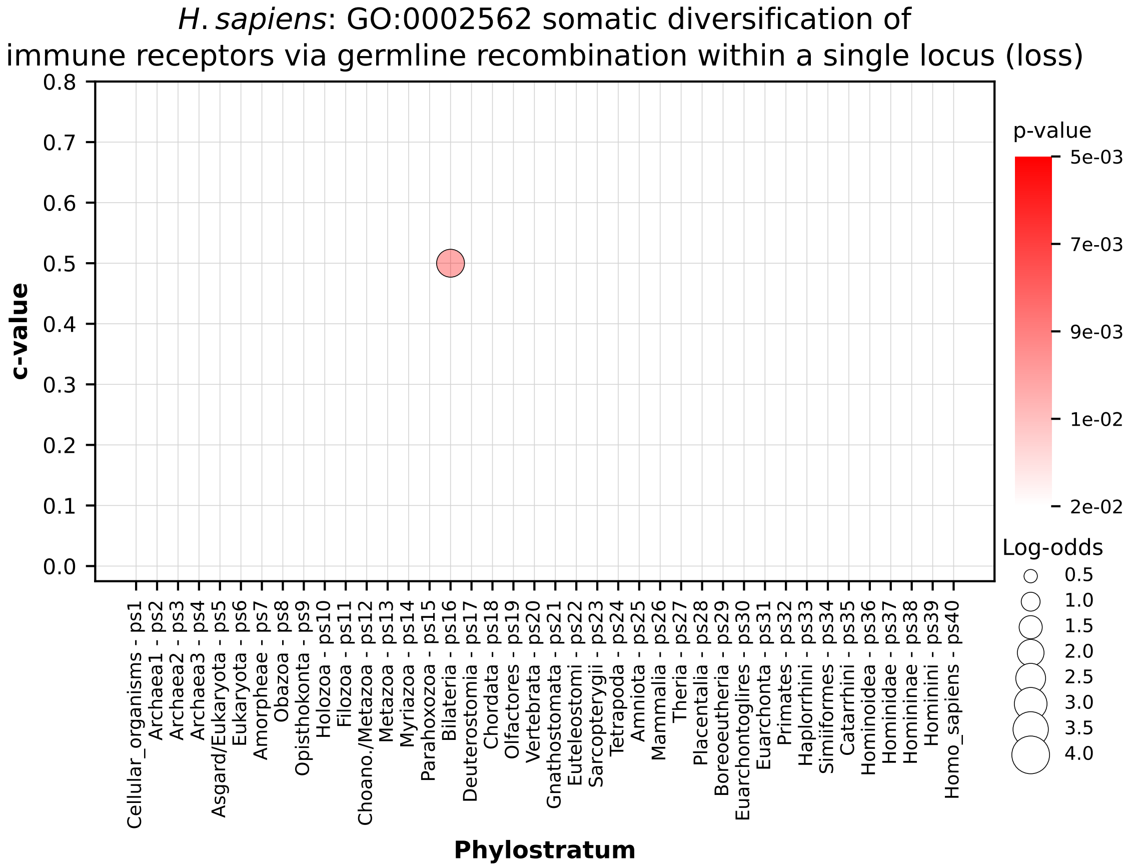

H. sapiens: GO:0002562 somatic diversification of immune receptors via germline recombination within a single locus (loss)
c-value
0.0
0.1
0.2
0.3
0.4
0.5
0.6
0.7
0.8
Phylostratum
Cellular_organisms - ps1
Archaea1 - ps2
Archaea2 - ps3
Archaea3 - ps4
Asgard/Eukaryota - ps5
Eukaryota - ps6
Amorpheae - ps7
Obazoa - ps8
Opisthokonta - ps9
Holozoa - ps10
Filozoa - ps11
Choano./Metazoa - ps12
Metazoa - ps13
Myriazoa - ps14
Parahoxozoa - ps15
Bilateria - ps16
Deuterostomia - ps17
Chordata - ps18
Olfactores - ps19
Vertebrata - ps20
Gnathostomata - ps21
Euteleostomi - ps22
Sarcopterygii - ps23
Tetrapoda - ps24
Amniota - ps25
Mammalia - ps26
Theria - ps27
Placentalia - ps28
Boreoeutheria - ps29
Euarchontoglires - ps30
Euarchonta - ps31
Primates - ps32
Haplorrhini - ps33
Simiiformes - ps34
Catarrhini - ps35
Hominoidea - ps36
Hominidae - ps37
Homininae - ps38
Hominini - ps39
Homo_sapiens - ps40
p-value
5e-03
7e-03
9e-03
1e-02
2e-02
Log-odds
0.5
1.0
1.5
2.0
2.5
3.0
3.5
4.0

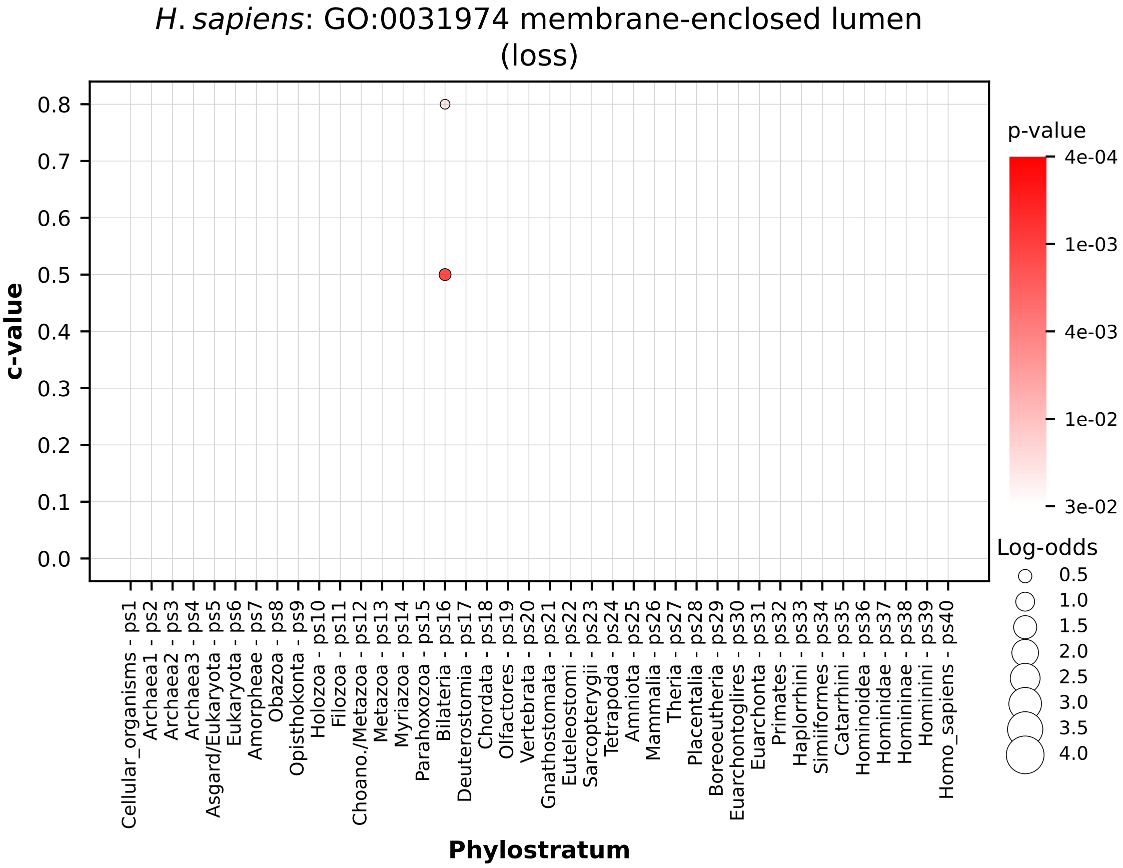

H. sapiens: GO:0031974 membrane-enclosed lumen
(loss)
c-value
0.0
0.1
0.2
0.3
0.4
0.5
0.6
0.7
0.8
Phylostratum
Cellular_organisms - ps1
Archaea1 - ps2
Archaea2 - ps3
Archaea3 - ps4
Asgard/Eukaryota - ps5
Eukaryota - ps6
Amorpheae - ps7
Obazoa - ps8
Opisthokonta - ps9
Holozoa - ps10
Filozoa - ps11
Choano./Metazoa - ps12
Metazoa - ps13
Myriazoa - ps14
Parahoxozoa - ps15
Bilateria - ps16
Deuterostomia - ps17
Chordata - ps18
Olfactores - ps19
Vertebrata - ps20
Gnathostomata - ps21
Euteleostomi - ps22
Sarcopterygii - ps23
Tetrapoda - ps24
Amniota - ps25
Mammalia - ps26
Theria - ps27
Placentalia - ps28
Boreoeutheria - ps29
Euarchontoglires - ps30
Euarchonta - ps31
Primates - ps32
Haplorrhini - ps33
Simiiformes - ps34
Catarrhini - ps35
Hominoidea - ps36
Hominidae - ps37
Homininae - ps38
Hominini - ps39
Homo_sapiens - ps40
p-value
4e-04
1e-03
4e-03
1e-02
3e-02
Log-odds
0.5
1.0
1.5
2.0
2.5
3.0
3.5
4.0

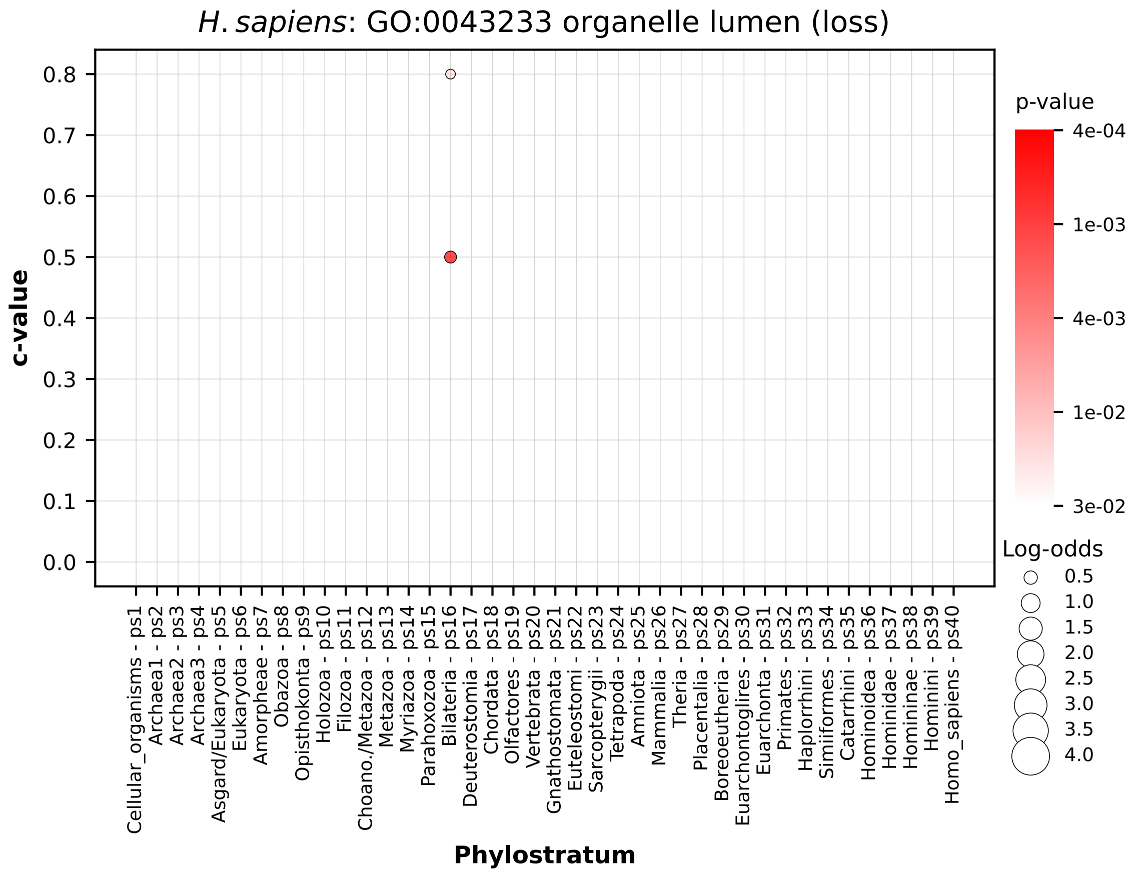

H. sapiens: GO:0043233 organelle lumen (loss)
c-value
0.8
0.7
0.6
0.5
0.4
0.3
0.2
0.1
0.0
Phylostratum
Cellular_organisms - ps1
Archaea1 - ps2
Archaea2 - ps3
Archaea3 - ps4
Asgard/Eukaryota - ps5
Eukaryota - ps6
Amorpheae - ps7
Obazoa - ps8
Opisthokonta - ps9
Holozoa - ps10
Filozoa - ps11
Choano./Metazoa - ps12
Metazoa - ps13
Myriazoa - ps14
Parahoxozoa - ps15
Bilateria - ps16
Deuterostomia - ps17
Chordata - ps18
Olfactores - ps19
Vertebrata - ps20
Gnathostomata - ps21
Euteleostomi - ps22
Sarcopterygii - ps23
Tetrapoda - ps24
Amniota - ps25
Mammalia - ps26
Theria - ps27
Placentalia - ps28
Boreoeutheria - ps29
Euarchontoglires - ps30
Euarchonta - ps31
Primates - ps32
Haplorrhini - ps33
Simiiformes - ps34
Catarrhini - ps35
Hominoidea - ps36
Hominidae - ps37
Homininae - ps38
Hominini - ps39
Homo_sapiens - ps40
p-value
4e-04
1e-03
4e-03
1e-02
3e-02
Log-odds
0.5
1.0
1.5
2.0
2.5
3.0
3.5
4.0

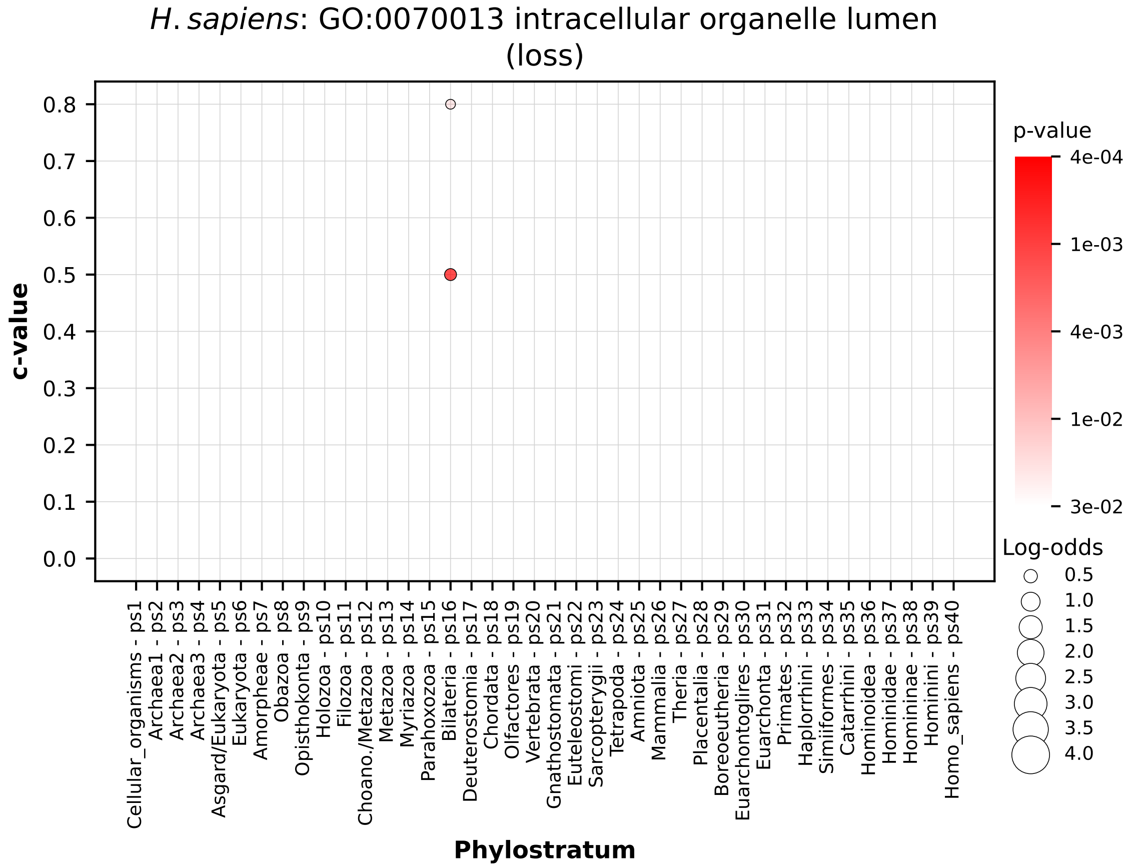

H. sapiens: GO:0070013 intracellular organelle lumen
(loss)
c-value
0.0
0.1
0.2
0.3
0.4
0.5
0.6
0.7
0.8
Phylostratum
Cellular_organisms - ps1
Archaea1 - ps2
Archaea2 - ps3
Archaea3 - ps4
Asgard/Eukaryota - ps5
Eukaryota - ps6
Amorpheae - ps7
Obazoa - ps8
Opisthokonta - ps9
Holozoa - ps10
Filozoa - ps11
Choano./Metazoa - ps12
Metazoa - ps13
Myriazoa - ps14
Parahoxozoa - ps15
Bilateria - ps16
Deuterostomia - ps17
Chordata - ps18
Olfactores - ps19
Vertebrata - ps20
Gnathostomata - ps21
Euteleostomi - ps22
Sarcopterygii - ps23
Tetrapoda - ps24
Amniota - ps25
Mammalia - ps26
Theria - ps27
Placentalia - ps28
Boreoeutheria - ps29
Euarchontoglires - ps30
Euarchonta - ps31
Primates - ps32
Haplorrhini - ps33
Simiiformes - ps34
Catarrhini - ps35
Hominoidea - ps36
Hominidae - ps37
Homininae - ps38
Hominini - ps39
Homo_sapiens - ps40
p-value
4e-04
1e-03
4e-03
1e-02
3e-02
Log-odds
0.5
1.0
1.5
2.0
2.5
3.0
3.5
4.0

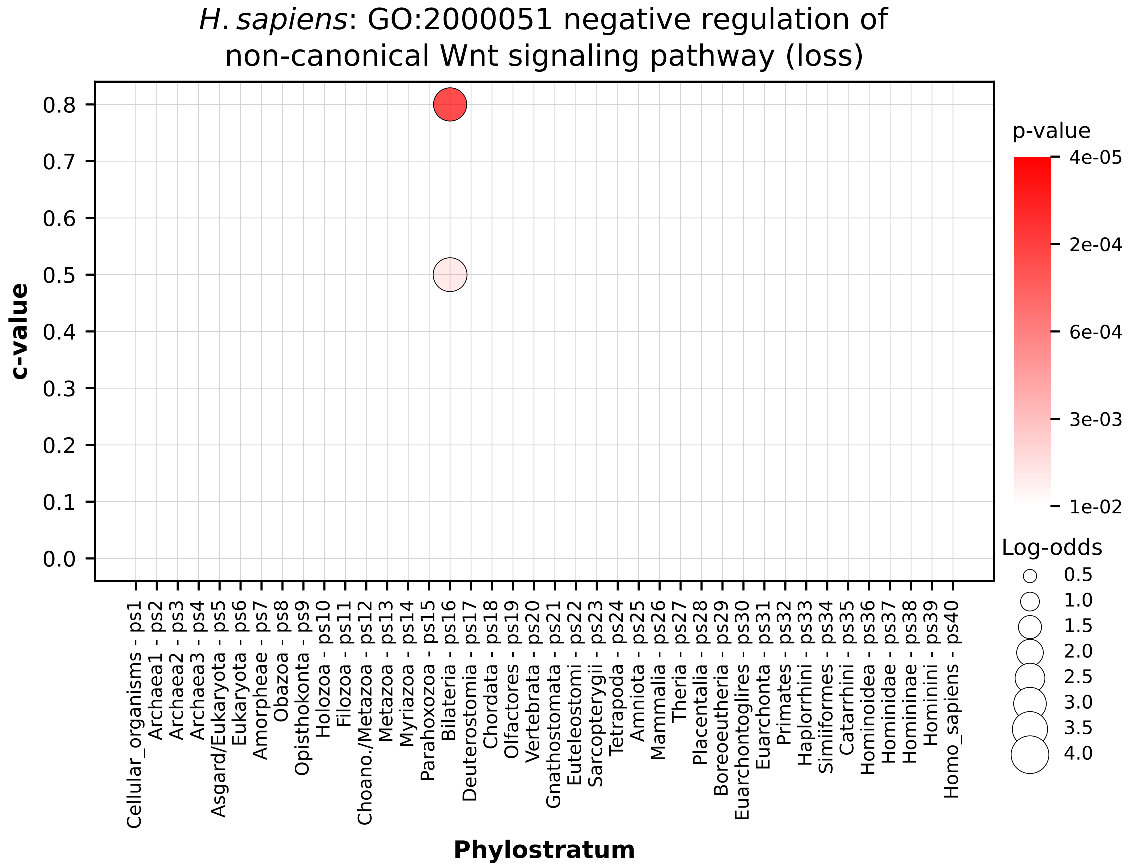
H. sapiens: GO:2000051 negative regulation of non-canonical Wnt signaling pathway (loss)
c-value
0.8
0.7
0.6
0.5
0.4
0.3
0.2
0.1
0.0
p-value
4e-05
2e-04
6e-04
3e-03
1e-02
Log-odds
0.5
1.0
1.5
2.0
2.5
3.0
3.5
4.0
Cellular_organisms - ps1
Archaea1 - ps2
Archaea2 - ps3
Archaea3 - ps4
Asgard/Eukaryota - ps5
Eukaryota - ps6
Amorpheae - ps7
Obazoa - ps8
Opisthokonta - ps9
Holozoa - ps10
Filozoa - ps11
Choano./Metazoa - ps12
Metazoa - ps13
Myriazoa - ps14
Parahoxozoa - ps15
Bilateria - ps16
Deuterostomia - ps17
Chordata - ps18
Olfactores - ps19
Vertebrata - ps20
Gnathostomata - ps21
Euteleostomi - ps22
Sarcopterygii - ps23
Tetrapoda - ps24
Amniota - ps25
Mammalia - ps26
Theria - ps27
Placentalia - ps28
Boreoeutheria - ps29
Euarchontoglires - ps30
Euarchonta - ps31
Primates - ps32
Haplorrhini - ps33
Simiiformes - ps34
Catarrhini - ps35
Hominoidea - ps36
Hominidae - ps37
Homininae - ps38
Hominini - ps39
Homo_sapiens - ps40
Phylostratum

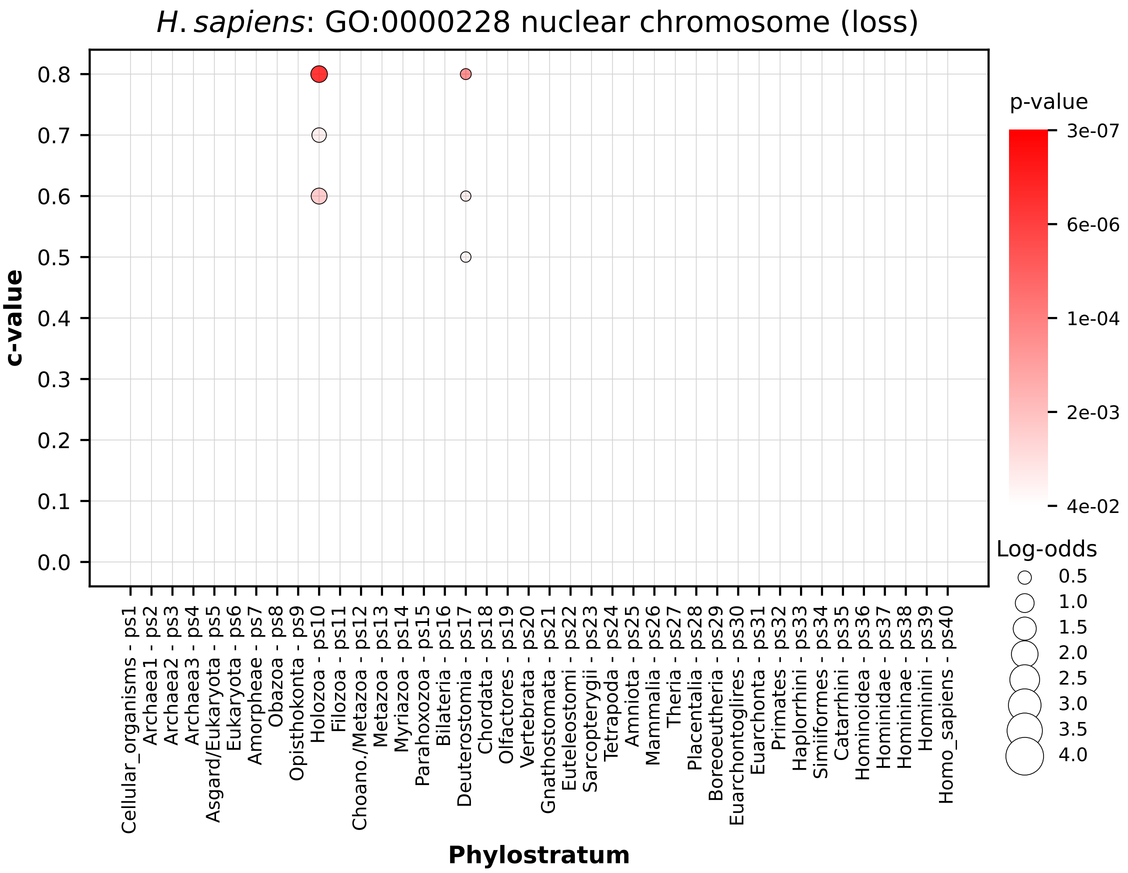

H. sapiens: GO:0000228 nuclear chromosome (loss)
c-value
0.0
0.1
0.2
0.3
0.4
0.5
0.6
0.7
0.8
Phylostratum
Cellular_organisms - ps1
Archaea1 - ps2
Archaea2 - ps3
Archaea3 - ps4
Asgard/Eukaryota - ps5
Eukaryota - ps6
Amorpheae - ps7
Obazoa - ps8
Opisthokonta - ps9
Holozoa - ps10
Filozoa - ps11
Choano./Metazoa - ps12
Metazoa - ps13
Myriazoa - ps14
Parahoxozoa - ps15
Bilateria - ps16
Deuterostomia - ps17
Chordata - ps18
Olfactores - ps19
Vertebrata - ps20
Gnathostomata - ps21
Euteleostomi - ps22
Sarcopterygii - ps23
Tetrapoda - ps24
Amniota - ps25
Mammalia - ps26
Theria - ps27
Placentalia - ps28
Boreoeutheria - ps29
Euarchontoglires - ps30
Euarchonta - ps31
Primates - ps32
Haplorrhini - ps33
Simiiformes - ps34
Catarrhini - ps35
Hominoidea - ps36
Hominidae - ps37
Homininae - ps38
Hominini - ps39
Homo_sapiens - ps40
p-value
3e-07
6e-06
1e-04
2e-03
4e-02
Log-odds
0.5
1.0
1.5
2.0
2.5
3.0
3.5
4.0

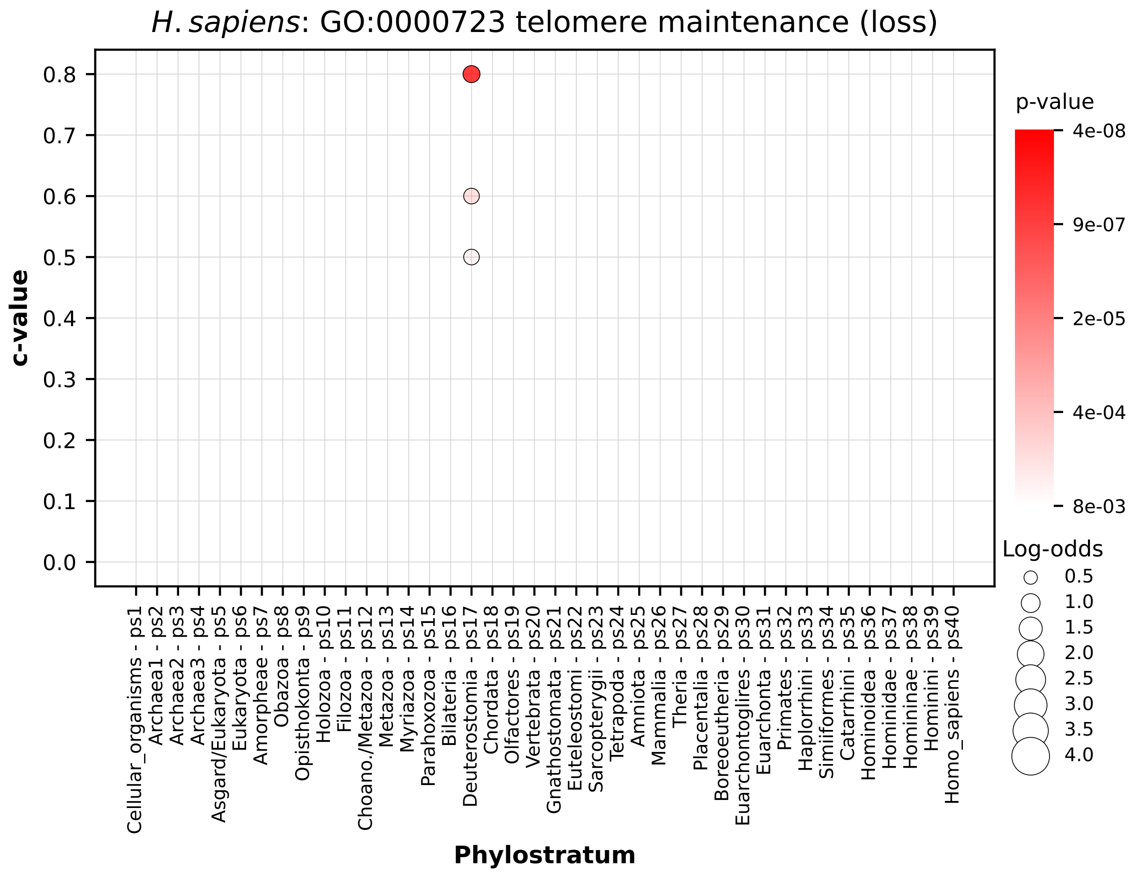

H. sapiens: GO:0000723 telomere maintenance (loss)
c-value
0.0
0.1
0.2
0.3
0.4
0.5
0.6
0.7
0.8
p-value
4e-08
9e-07
2e-05
4e-04
8e-03
Log-odds
0.5
1.0
1.5
2.0
2.5
3.0
3.5
4.0
Cellular_organisms - ps1
Archaea1 - ps2
Archaea2 - ps3
Archaea3 - ps4
Asgard/Eukaryota - ps5
Eukaryota - ps6
Amorpheae - ps7
Obazoa - ps8
Opisthokonta - ps9
Holozoa - ps10
Filozoa - ps11
Choano./Metazoa - ps12
Metazoa - ps13
Myriazoa - ps14
Parahoxozoa - ps15
Bilateria - ps16
Deuterostomia - ps17
Chordata - ps18
Olfactores - ps19
Vertebrata - ps20
Gnathostomata - ps21
Euteleostomi - ps22
Sarcopterygii - ps23
Tetrapoda - ps24
Amniota - ps25
Mammalia - ps26
Theria - ps27
Placentalia - ps28
Boreoeutheria - ps29
Euarchontoglires - ps30
Euarchonta - ps31
Primates - ps32
Haplorrhini - ps33
Simiiformes - ps34
Catarrhini - ps35
Hominoidea - ps36
Hominidae - ps37
Homininae - ps38
Hominini - ps39
Homo_sapiens - ps40
Phylostratum

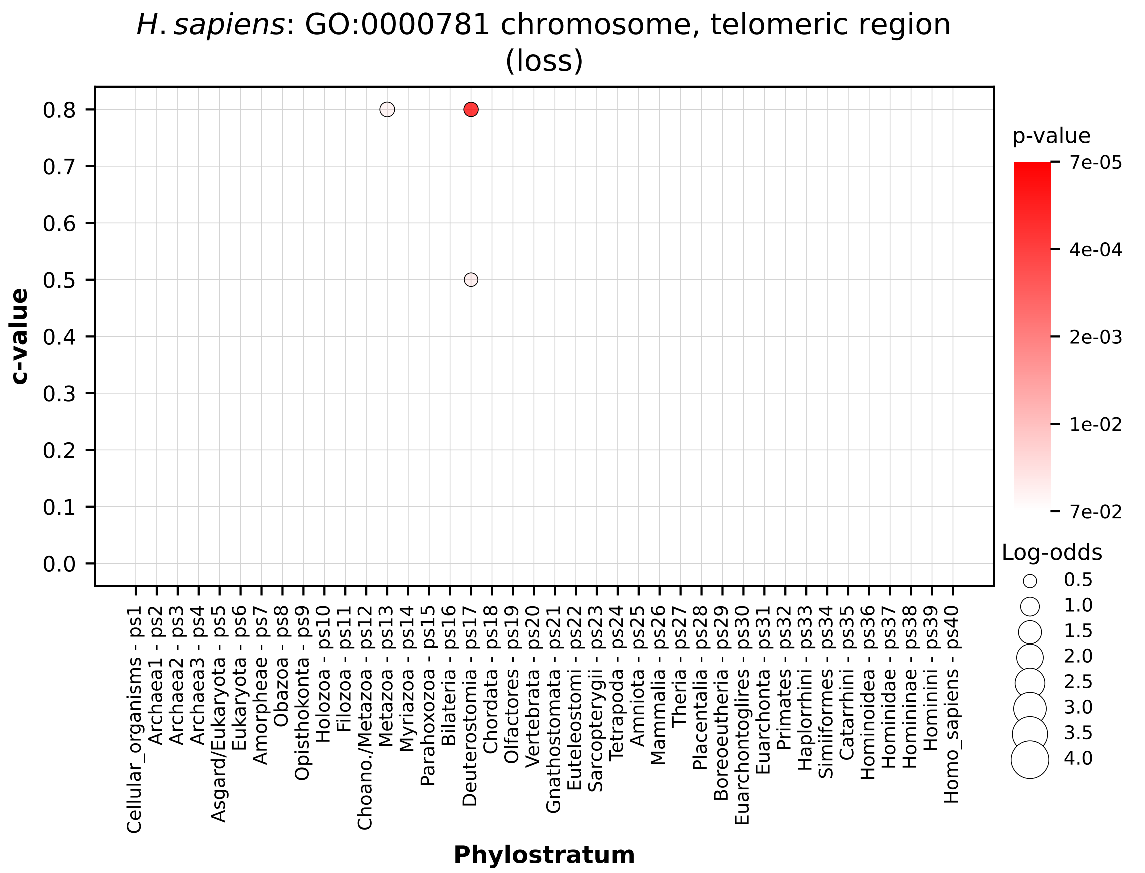

H. sapiens: GO:0000781 chromosome, telomeric region (loss)
c-value
0.8
0.7
0.6
0.5
0.4
0.3
0.2
0.1
0.0
Phylostratum
Cellular_organisms - ps1
Archaea1 - ps2
Archaea2 - ps3
Archaea3 - ps4
Asgard/Eukaryota - ps5
Eukaryota - ps6
Amorpheae - ps7
Obazoa - ps8
Opisthokonta - ps9
Holozoa - ps10
Filozoa - ps11
Choano./Metazoa - ps12
Metazoa - ps13
Myriazoa - ps14
Parahoxozoa - ps15
Bilateria - ps16
Deuterostomia - ps17
Chordata - ps18
Olfactores - ps19
Vertebrata - ps20
Gnathostomata - ps21
Euteleostomi - ps22
Sarcopterygii - ps23
Tetrapoda - ps24
Amniota - ps25
Mammalia - ps26
Theria - ps27
Placentalia - ps28
Boreoeutheria - ps29
Euarchontoglires - ps30
Euarchonta - ps31
Primates - ps32
Haplorrhini - ps33
Simiiformes - ps34
Catarrhini - ps35
Hominoidea - ps36
Hominidae - ps37
Homininae - ps38
Hominini - ps39
Homo_sapiens - ps40
p-value
7e-05
4e-04
2e-03
1e-02
7e-02
Log-odds
0.5
1.0
1.5
2.0
2.5
3.0
3.5
4.0

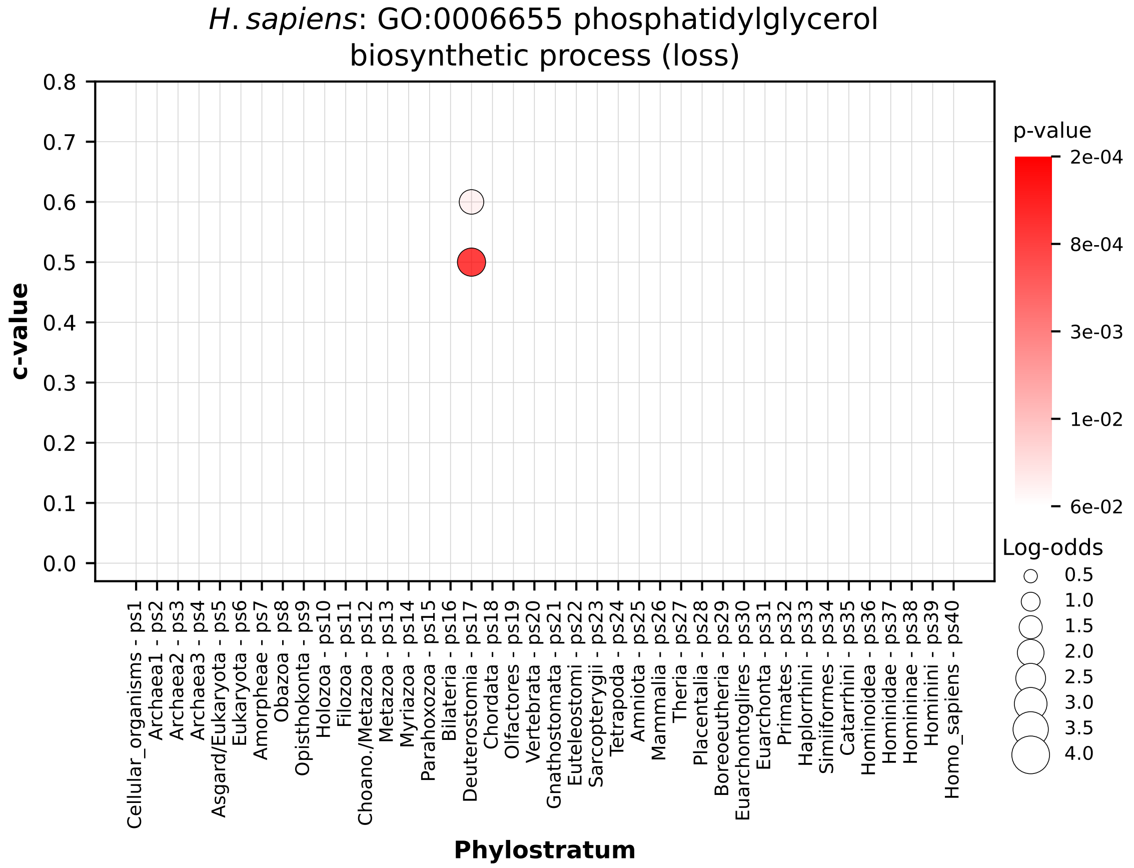
H. sapiens: GO:0006655 phosphatidylglycerol biosynthetic process (loss)
c-value
0.8
0.7
0.6
0.5
0.4
0.3
0.2
0.1
0.0
Phylostratum
Cellular_organisms - ps1
Archaea1 - ps2
Archaea2 - ps3
Archaea3 - ps4
Asgard/Eukaryota - ps5
Eukaryota - ps6
Amorpheae - ps7
Obazoa - ps8
Opisthokonta - ps9
Holozoa - ps10
Filozoa - ps11
Choano./Metazoa - ps12
Metazoa - ps13
Myriazoa - ps14
Parahoxozoa - ps15
Bilateria - ps16
Deuterostomia - ps17
Chordata - ps18
Olfactores - ps19
Vertebrata - ps20
Gnathostomata - ps21
Euteleostomi - ps22
Sarcopterygii - ps23
Tetrapoda - ps24
Amniota - ps25
Mammalia - ps26
Theria - ps27
Placentalia - ps28
Boreoeutheria - ps29
Euarchontoglires - ps30
Euarchonta - ps31
Primates - ps32
Haplorrhini - ps33
Simiiformes - ps34
Catarrhini - ps35
Hominoidea - ps36
Hominidae - ps37
Homininae - ps38
Hominini - ps39
Homo_sapiens - ps40
p-value
2e-04
8e-04
3e-03
1e-02
6e-02
Log-odds
0.5
1.0
1.5
2.0
2.5
3.0
3.5
4.0

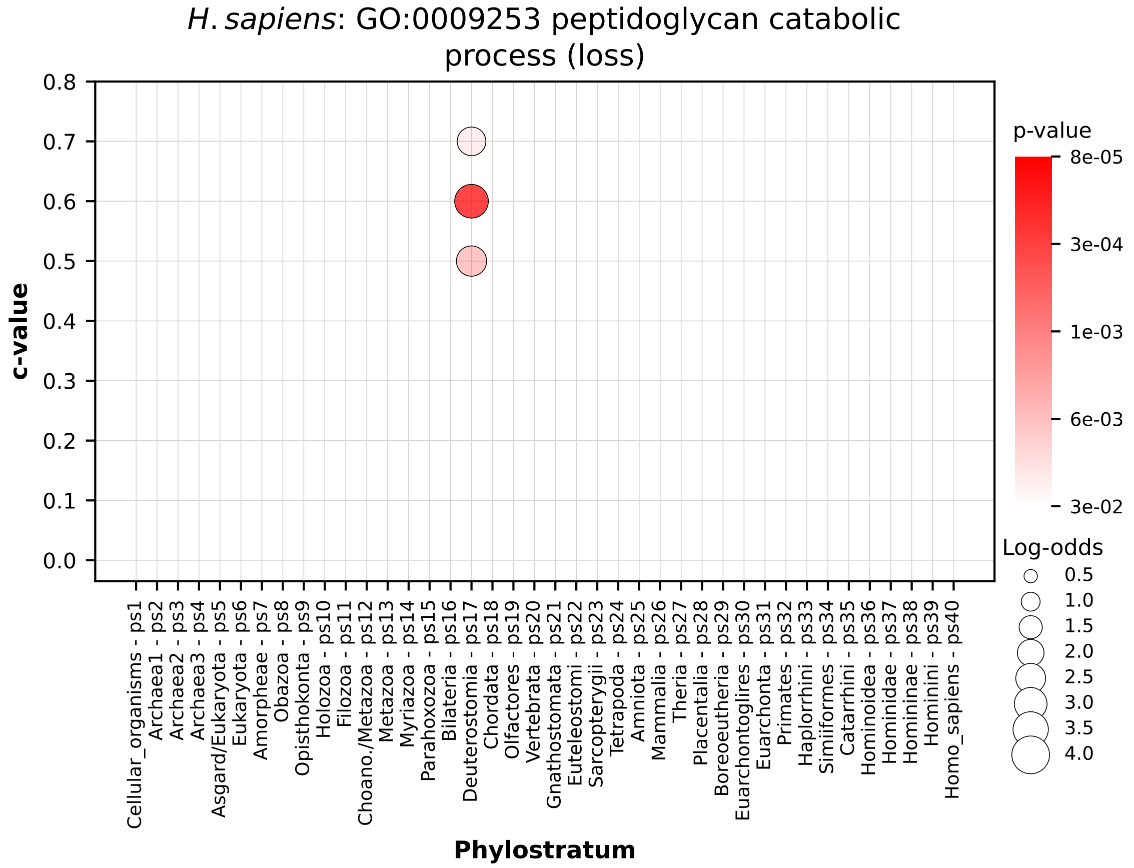

H. sapiens: GO:0009253 peptidoglycan catabolic process (loss)
c-value
0.8
0.7
0.6
0.5
0.4
0.3
0.2
0.1
0.0
Phylostratum
Cellular_organisms - ps1
Archaea1 - ps2
Archaea2 - ps3
Archaea3 - ps4
Asgard/Eukaryota - ps5
Eukaryota - ps6
Amorpheae - ps7
Obazoa - ps8
Opisthokonta - ps9
Holozoa - ps10
Filozoa - ps11
Choano./Metazoa - ps12
Metazoa - ps13
Myriazoa - ps14
Parahoxozoa - ps15
Bilateria - ps16
Deuterostomia - ps17
Chordata - ps18
Olfactores - ps19
Vertebrata - ps20
Gnathostomata - ps21
Euteleostomi - ps22
Sarcopterygii - ps23
Tetrapoda - ps24
Amniota - ps25
Mammalia - ps26
Theria - ps27
Placentalia - ps28
Boreoeutheria - ps29
Euarchontoglires - ps30
Euarchonta - ps31
Primates - ps32
Haplorrhini - ps33
Simiiformes - ps34
Catarrhini - ps35
Hominoidea - ps36
Hominidae - ps37
Homininae - ps38
Hominini - ps39
Homo_sapiens - ps40
p-value
8e-05
3e-04
1e-03
6e-03
3e-02
Log-odds
0.5
1.0
1.5
2.0
2.5
3.0
3.5
4.0

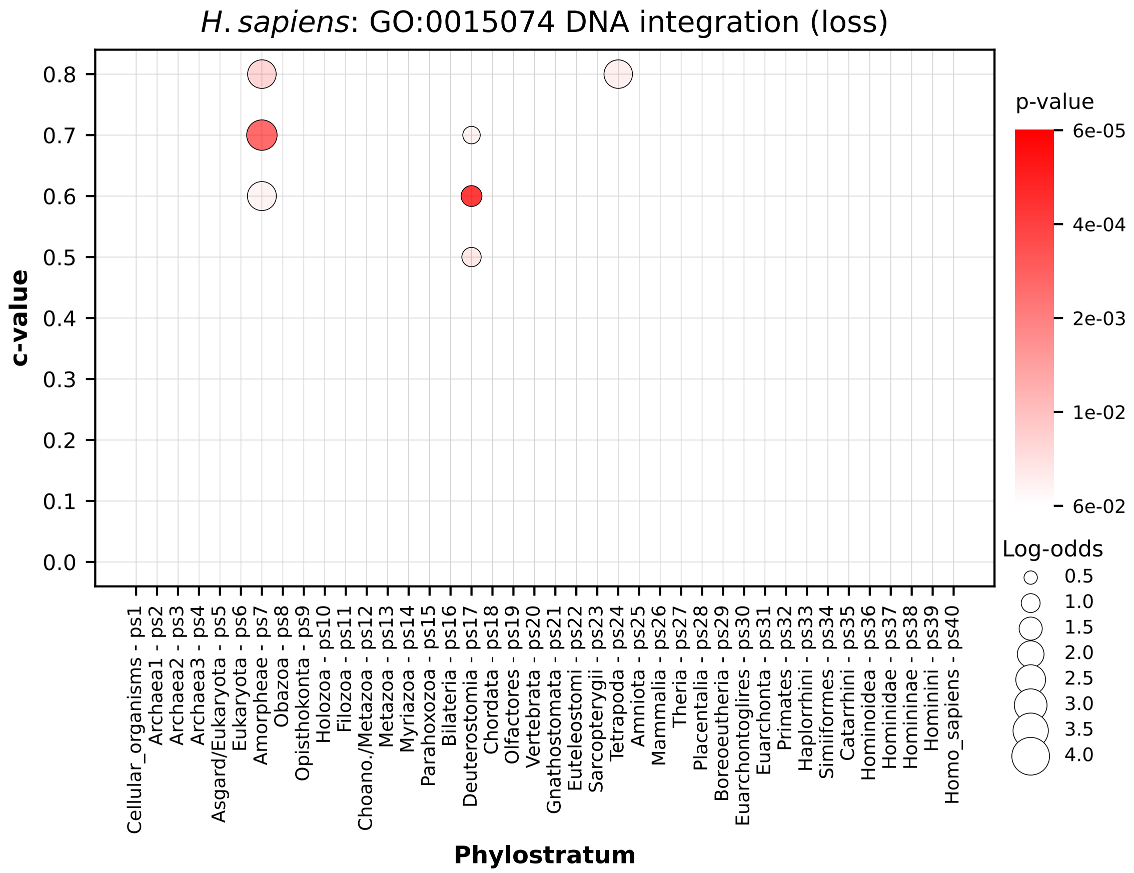

H. sapiens: GO:0015074 DNA integration (loss)
c-value
0.0
0.1
0.2
0.3
0.4
0.5
0.6
0.7
0.8
Phylostratum
Cellular_organisms - ps1
Archaea1 - ps2
Archaea2 - ps3
Archaea3 - ps4
Asgard/Eukaryota - ps5
Eukaryota - ps6
Amorpheae - ps7
Obazoa - ps8
Opisthokonta - ps9
Holozoa - ps10
Filozoa - ps11
Choano./Metazoa - ps12
Metazoa - ps13
Myriazoa - ps14
Parahoxozoa - ps15
Bilateria - ps16
Deuterostomia - ps17
Chordata - ps18
Olfactores - ps19
Vertebrata - ps20
Gnathostomata - ps21
Euteleostomi - ps22
Sarcopterygii - ps23
Tetrapoda - ps24
Amniota - ps25
Mammalia - ps26
Theria - ps27
Placentalia - ps28
Boreoeutheria - ps29
Euarchontoglires - ps30
Euarchonta - ps31
Primates - ps32
Haplorrhini - ps33
Simiiformes - ps34
Catarrhini - ps35
Hominoidea - ps36
Hominidae - ps37
Homininae - ps38
Hominini - ps39
Homo_sapiens - ps40
p-value
6e-05
4e-04
2e-03
1e-02
6e-02
Log-odds
0.5
1.0
1.5
2.0
2.5
3.0
3.5
4.0

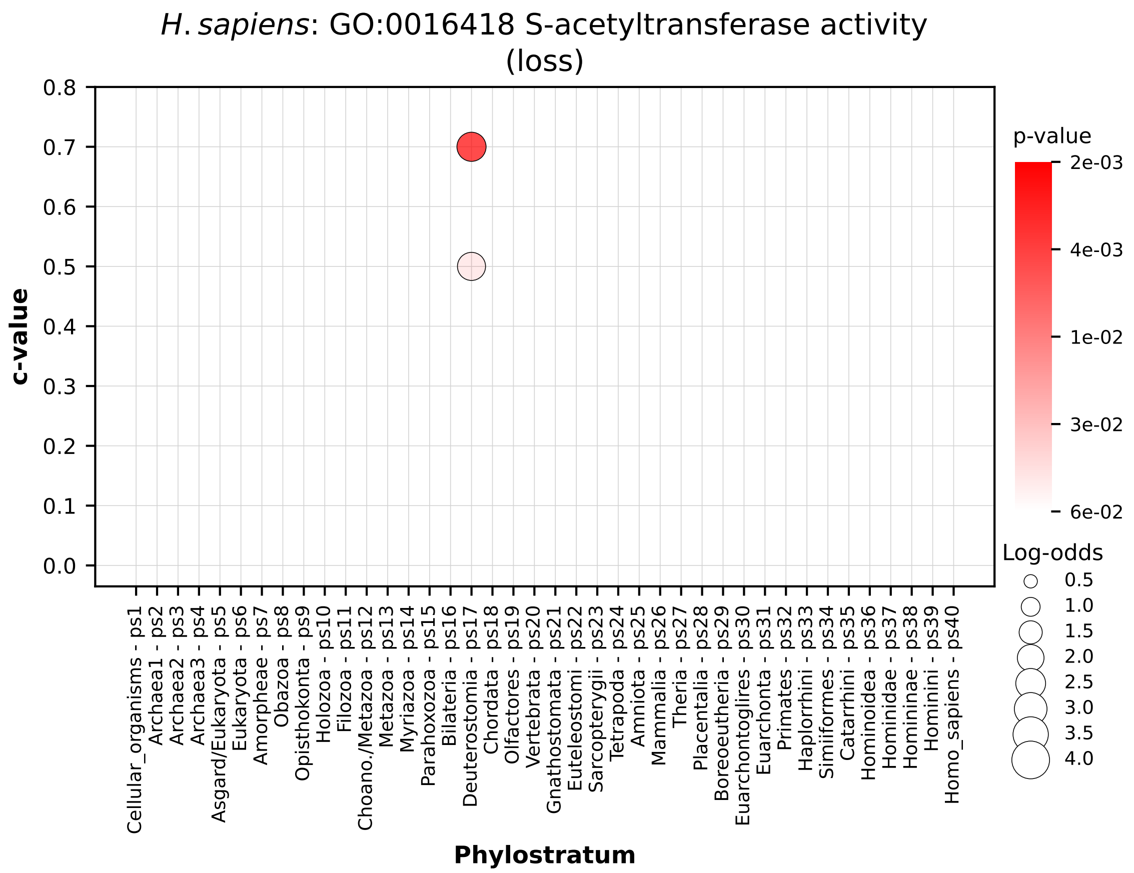

H. sapiens: GO:0016418 S-acetyltransferase activity
(loss)
c-value
0.8
0.7
0.6
0.5
0.4
0.3
0.2
0.1
0.0
p-value
2e-03
4e-03
1e-02
3e-02
6e-02
Log-odds
0.5
1.0
1.5
2.0
2.5
3.0
3.5
4.0
Cellular_organisms - ps1
Archaea1 - ps2
Archaea2 - ps3
Archaea3 - ps4
Asgard/Eukaryota - ps5
Eukaryota - ps6
Amorpheae - ps7
Obazoa - ps8
Opisthokonta - ps9
Holozoa - ps10
Filozoa - ps11
Choano./Metazoa - ps12
Metazoa - ps13
Myriazoa - ps14
Parahoxozoa - ps15
Bilateria - ps16
Deuterostomia - ps17
Chordata - ps18
Olfactores - ps19
Vertebrata - ps20
Gnathostomata - ps21
Euteleostomi - ps22
Sarcopterygii - ps23
Tetrapoda - ps24
Amniota - ps25
Mammalia - ps26
Theria - ps27
Placentalia - ps28
Boreoeutheria - ps29
Euarchontoglires - ps30
Euarchonta - ps31
Primates - ps32
Haplorrhini - ps33
Simiiformes - ps34
Catarrhini - ps35
Hominoidea - ps36
Hominidae - ps37
Homininae - ps38
Hominini - ps39
Homo_sapiens - ps40
Phylostratum

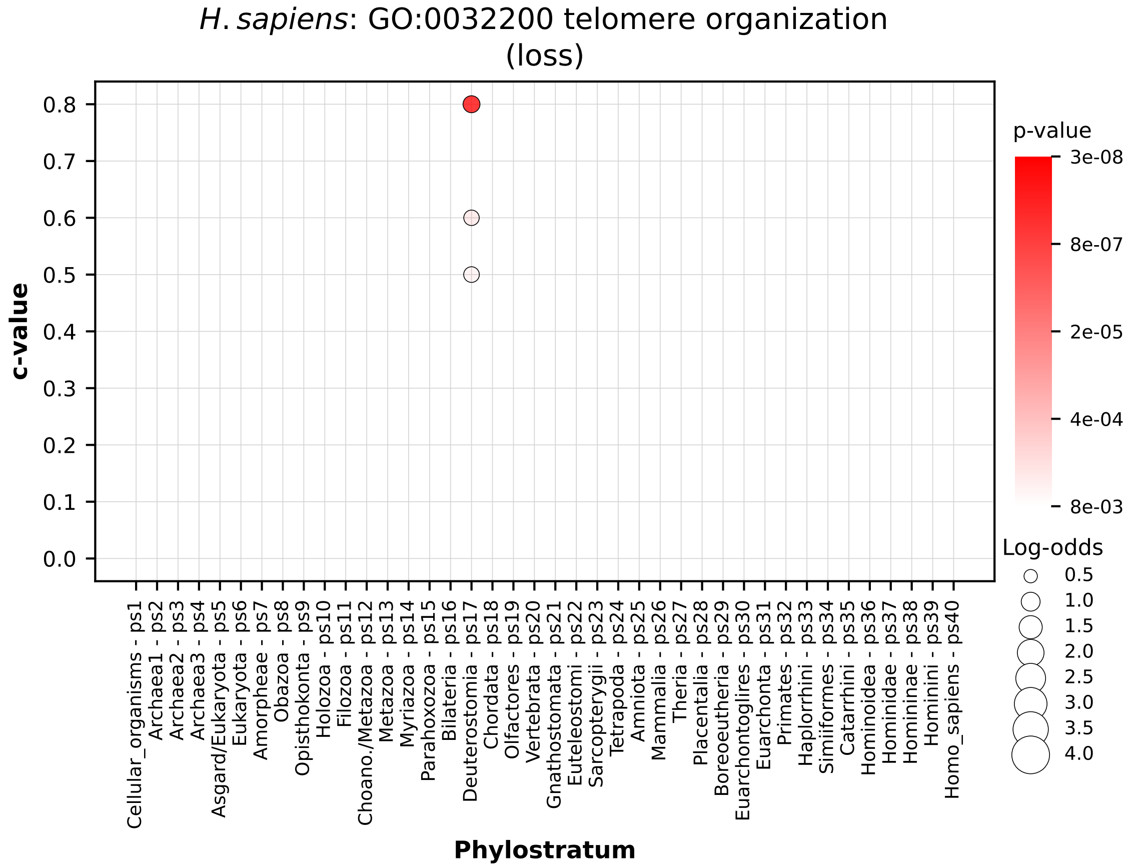

H. sapiens: GO:0032200 telomere organization
(loss)
c-value
0.0
0.1
0.2
0.3
0.4
0.5
0.6
0.7
0.8
p-value
3e-08
8e-07
2e-05
4e-04
8e-03
Log-odds
0.5
1.0
1.5
2.0
2.5
3.0
3.5
4.0
Cellular_organisms - ps1
Archaea1 - ps2
Archaea2 - ps3
Archaea3 - ps4
Asgard/Eukaryota - ps5
Eukaryota - ps6
Amorpheae - ps7
Obazoa - ps8
Opisthokonta - ps9
Holozoa - ps10
Filozoa - ps11
Choano./Metazoa - ps12
Metazoa - ps13
Myriazoa - ps14
Parahoxozoa - ps15
Bilateria - ps16
Deuterostomia - ps17
Chordata - ps18
Olfactores - ps19
Vertebrata - ps20
Gnathostomata - ps21
Euteleostomi - ps22
Sarcopterygii - ps23
Tetrapoda - ps24
Amniota - ps25
Mammalia - ps26
Theria - ps27
Placentalia - ps28
Boreoeutheria - ps29
Euarchontoglires - ps30
Euarchonta - ps31
Primates - ps32
Haplorrhini - ps33
Simiiformes - ps34
Catarrhini - ps35
Hominoidea - ps36
Hominidae - ps37
Homininae - ps38
Hominini - ps39
Homo_sapiens - ps40
Phylostratum

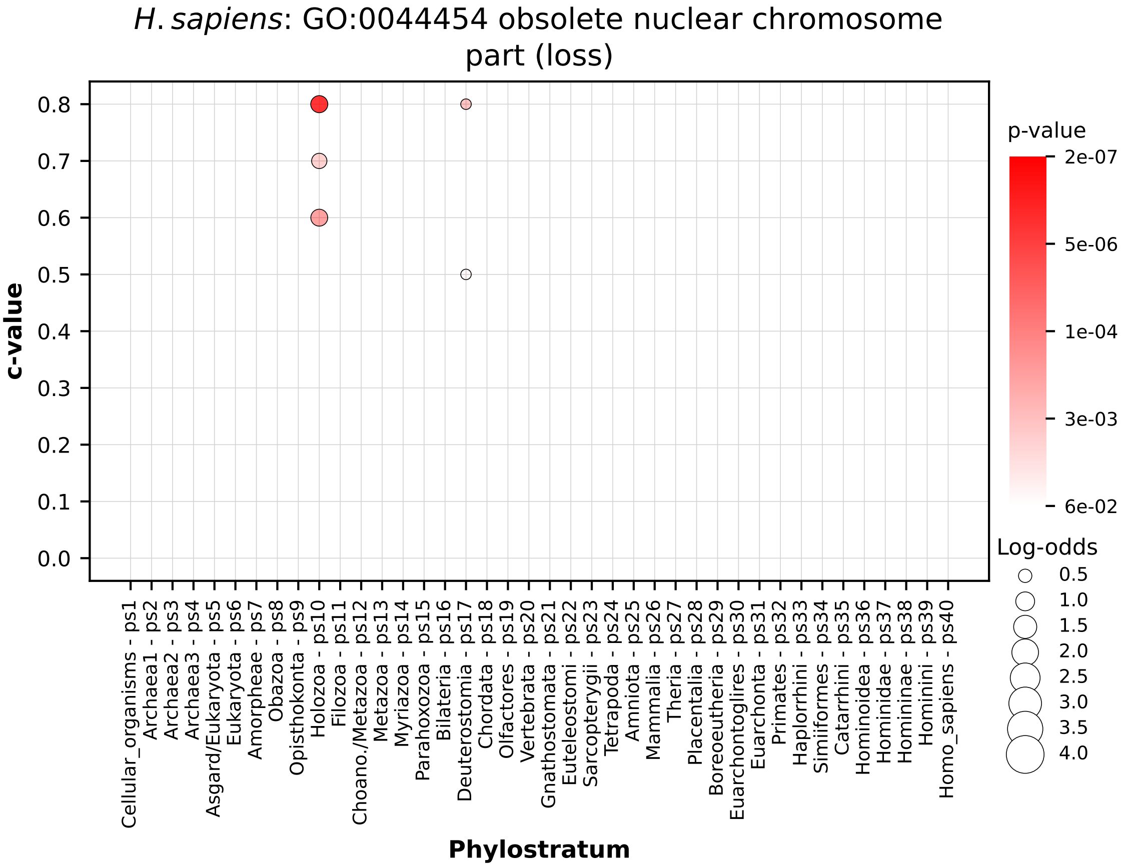
H. sapiens: GO:0044454 obsolete nuclear chromosome part (loss)
c-value
0.8
0.7
0.6
0.5
0.4
0.3
0.2
0.1
0.0
Phylostratum
Cellular_organisms - ps1
Archaea1 - ps2
Archaea2 - ps3
Archaea3 - ps4
Asgard/Eukaryota - ps5
Eukaryota - ps6
Amorpheae - ps7
Obazoa - ps8
Opisthokonta - ps9
Holozoa - ps10
Filozoa - ps11
Choano./Metazoa - ps12
Metazoa - ps13
Myriazoa - ps14
Parahoxozoa - ps15
Bilateria - ps16
Deuterostomia - ps17
Chordata - ps18
Olfactores - ps19
Vertebrata - ps20
Gnathostomata - ps21
Euteleostomi - ps22
Sarcopterygii - ps23
Tetrapoda - ps24
Amniota - ps25
Mammalia - ps26
Theria - ps27
Placentalia - ps28
Boreoeutheria - ps29
Euarchontoglires - ps30
Euarchonta - ps31
Primates - ps32
Haplorrhini - ps33
Simiiformes - ps34
Catarrhini - ps35
Hominoidea - ps36
Hominidae - ps37
Homininae - ps38
Hominini - ps39
Homo_sapiens - ps40
p-value
2e-07
5e-06
1e-04
3e-03
6e-02
Log-odds
0.5
1.0
1.5
2.0
2.5
3.0
3.5
4.0

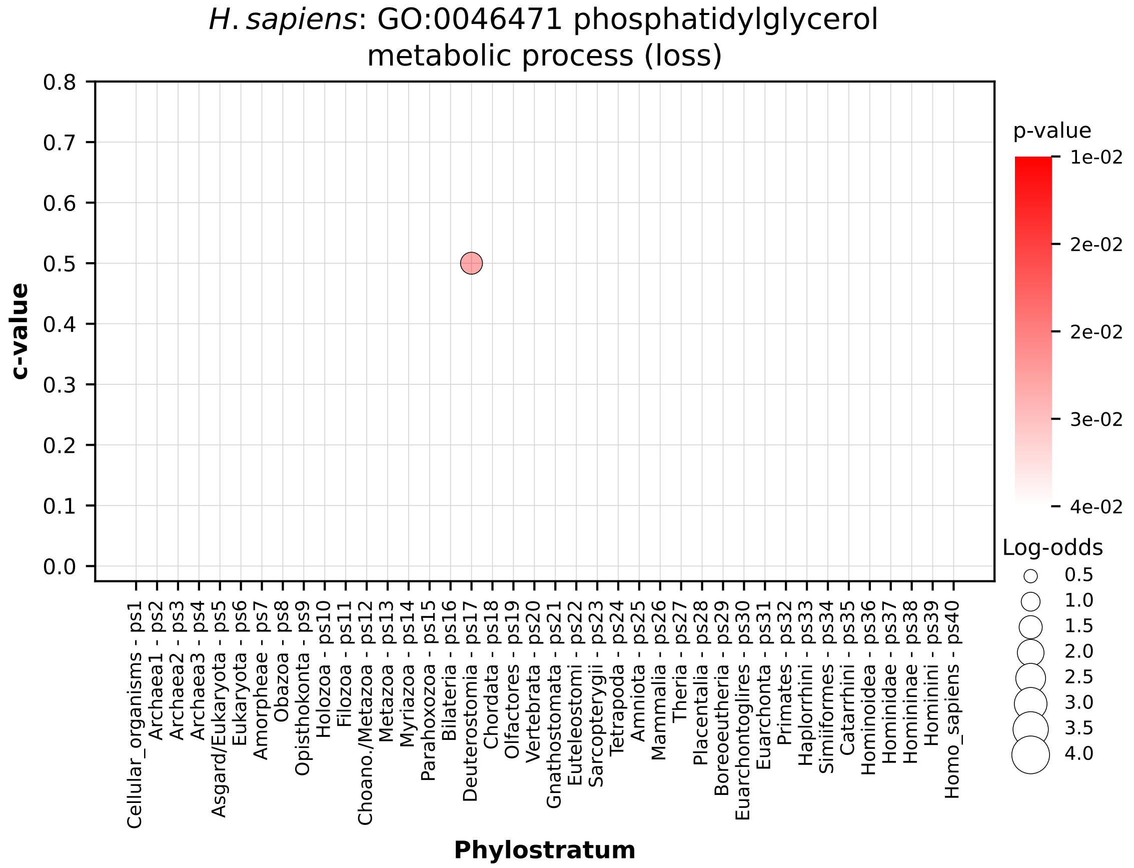

H. sapiens: GO:0046471 phosphatidylglycerol metabolic process (loss)
c-value
0.0
0.1
0.2
0.3
0.4
0.5
0.6
0.7
0.8
Phylostratum
Cellular_organisms - ps1
Archaea1 - ps2
Archaea2 - ps3
Archaea3 - ps4
Asgard/Eukaryota - ps5
Eukaryota - ps6
Amorpheae - ps7
Obazoa - ps8
Opisthokonta - ps9
Holozoa - ps10
Filozoa - ps11
Choano./Metazoa - ps12
Metazoa - ps13
Myriazoa - ps14
Parahoxozoa - ps15
Bilateria - ps16
Deuterostomia - ps17
Chordata - ps18
Olfactores - ps19
Vertebrata - ps20
Gnathostomata - ps21
Euteleostomi - ps22
Sarcopterygii - ps23
Tetrapoda - ps24
Amniota - ps25
Mammalia - ps26
Theria - ps27
Placentalia - ps28
Boreoeutheria - ps29
Euarchontoglires - ps30
Euarchonta - ps31
Primates - ps32
Haplorrhini - ps33
Simiiformes - ps34
Catarrhini - ps35
Hominoidea - ps36
Hominidae - ps37
Homininae - ps38
Hominini - ps39
Homo_sapiens - ps40
p-value
1e-02
2e-02
2e-02
3e-02
4e-02
Log-odds
0.5
1.0
1.5
2.0
2.5
3.0
3.5
4.0

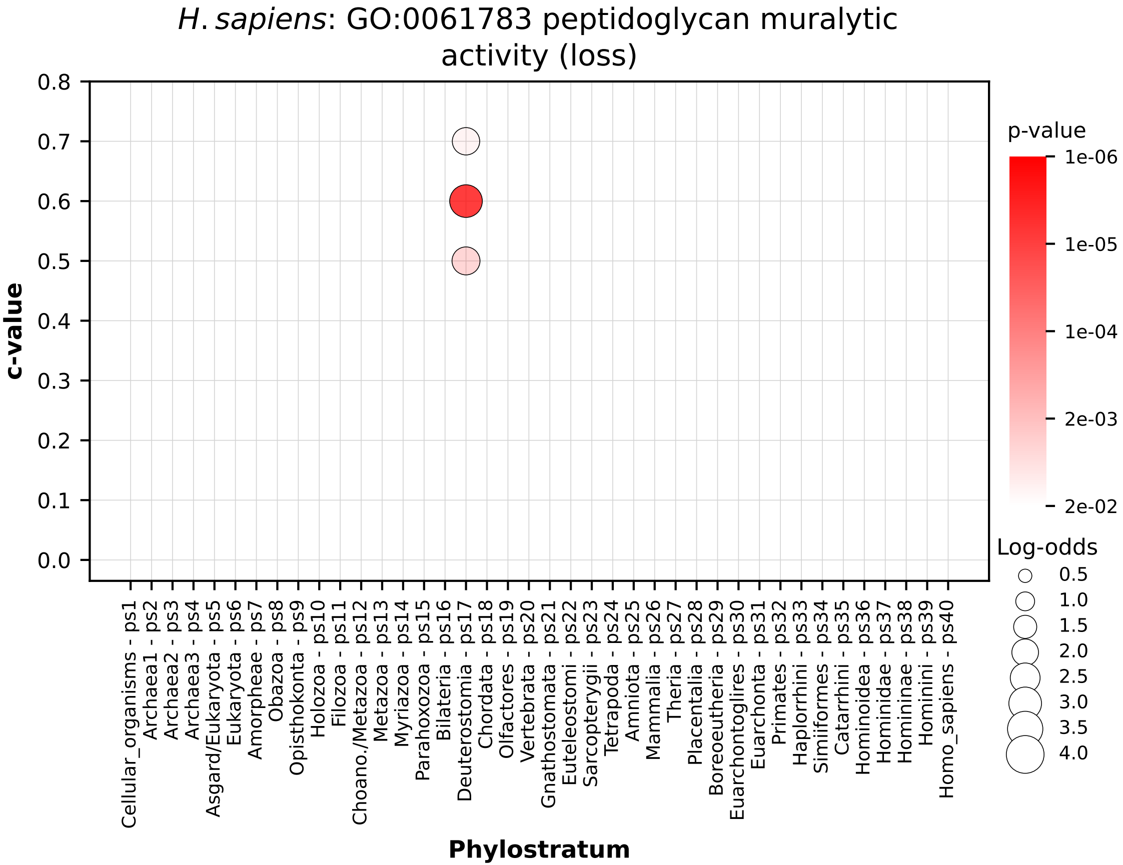

H. sapiens: GO:0061783 peptidoglycan muralytic activity (loss)
c-value
0.8
0.7
0.6
0.5
0.4
0.3
0.2
0.1
0.0
p-value
1e-06
1e-05
1e-04
2e-03
2e-02
Log-odds
0.5
1.0
1.5
2.0
2.5
3.0
3.5
4.0
Cellular_organisms - ps1
Archaea1 - ps2
Archaea2 - ps3
Archaea3 - ps4
Asgard/Eukaryota - ps5
Eukaryota - ps6
Amorpheae - ps7
Obazoa - ps8
Opisthokonta - ps9
Holozoa - ps10
Filozoa - ps11
Choano./Metazoa - ps12
Metazoa - ps13
Myriazoa - ps14
Parahoxozoa - ps15
Bilateria - ps16
Deuterostomia - ps17
Chordata - ps18
Olfactores - ps19
Vertebrata - ps20
Gnathostomata - ps21
Euteleostomi - ps22
Sarcopterygii - ps23
Tetrapoda - ps24
Amniota - ps25
Mammalia - ps26
Theria - ps27
Placentalia - ps28
Boreoeutheria - ps29
Euarchontoglires - ps30
Euarchonta - ps31
Primates - ps32
Haplorrhini - ps33
Simiiformes - ps34
Catarrhini - ps35
Hominoidea - ps36
Hominidae - ps37
Homininae - ps38
Hominini - ps39
Homo_sapiens - ps40
Phylostratum

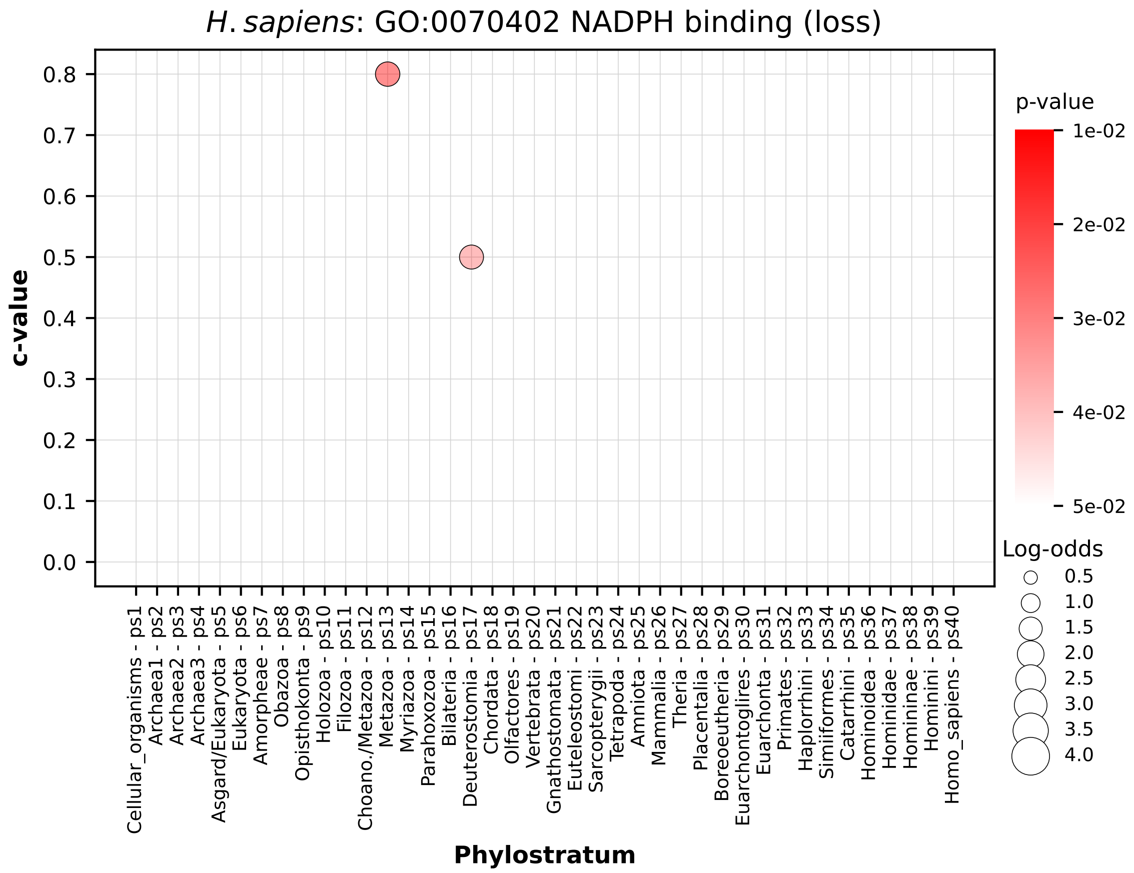

H. sapiens: GO:0070402 NADPH binding (loss)
c-value
0.0
0.1
0.2
0.3
0.4
0.5
0.6
0.7
0.8
Phylostratum
Cellular_organisms - ps1
Archaea1 - ps2
Archaea2 - ps3
Archaea3 - ps4
Asgard/Eukaryota - ps5
Eukaryota - ps6
Amorpheae - ps7
Obazoa - ps8
Opisthokonta - ps9
Holozoa - ps10
Filozoa - ps11
Choano./Metazoa - ps12
Metazoa - ps13
Myriazoa - ps14
Parahoxozoa - ps15
Bilateria - ps16
Deuterostomia - ps17
Chordata - ps18
Olfactores - ps19
Vertebrata - ps20
Gnathostomata - ps21
Euteleostomi - ps22
Sarcopterygii - ps23
Tetrapoda - ps24
Amniota - ps25
Mammalia - ps26
Theria - ps27
Placentalia - ps28
Boreoeutheria - ps29
Euarchontoglires - ps30
Euarchonta - ps31
Primates - ps32
Haplorrhini - ps33
Simiiformes - ps34
Catarrhini - ps35
Hominoidea - ps36
Hominidae - ps37
Homininae - ps38
Hominini - ps39
Homo_sapiens - ps40
p-value
1e-02
2e-02
3e-02
4e-02
5e-02
Log-odds
0.5
1.0
1.5
2.0
2.5
3.0
3.5
4.0

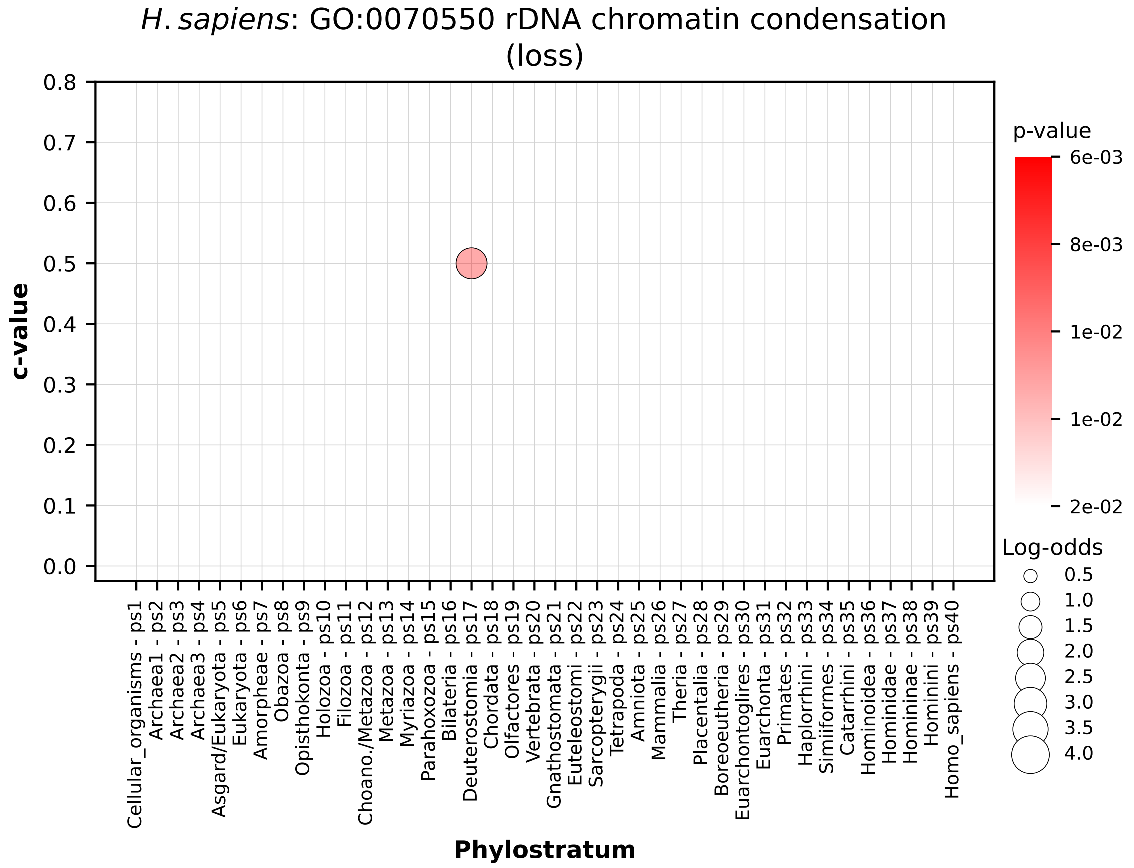

H. sapiens: GO:0070550 rDNA chromatin condensation
(loss)
c-value
0.8
0.7
0.6
0.5
0.4
0.3
0.2
0.1
0.0
Phylostratum
Cellular_organisms - ps1
Archaea1 - ps2
Archaea2 - ps3
Archaea3 - ps4
Asgard/Eukaryota - ps5
Eukaryota - ps6
Amorpheae - ps7
Obazoa - ps8
Opisthokonta - ps9
Holozoa - ps10
Filozoa - ps11
Choano./Metazoa - ps12
Metazoa - ps13
Myriazoa - ps14
Parahoxozoa - ps15
Bilateria - ps16
Deuterostomia - ps17
Chordata - ps18
Olfactores - ps19
Vertebrata - ps20
Gnathostomata - ps21
Euteleostomi - ps22
Sarcopterygii - ps23
Tetrapoda - ps24
Amniota - ps25
Mammalia - ps26
Theria - ps27
Placentalia - ps28
Boreoeutheria - ps29
Euarchontoglires - ps30
Euarchonta - ps31
Primates - ps32
Haplorrhini - ps33
Simiiformes - ps34
Catarrhini - ps35
Hominoidea - ps36
Hominidae - ps37
Homininae - ps38
Hominini - ps39
Homo_sapiens - ps40
p-value
6e-03
8e-03
1e-02
1e-02
2e-02
Log-odds
0.5
1.0
1.5
2.0
2.5
3.0
3.5
4.0

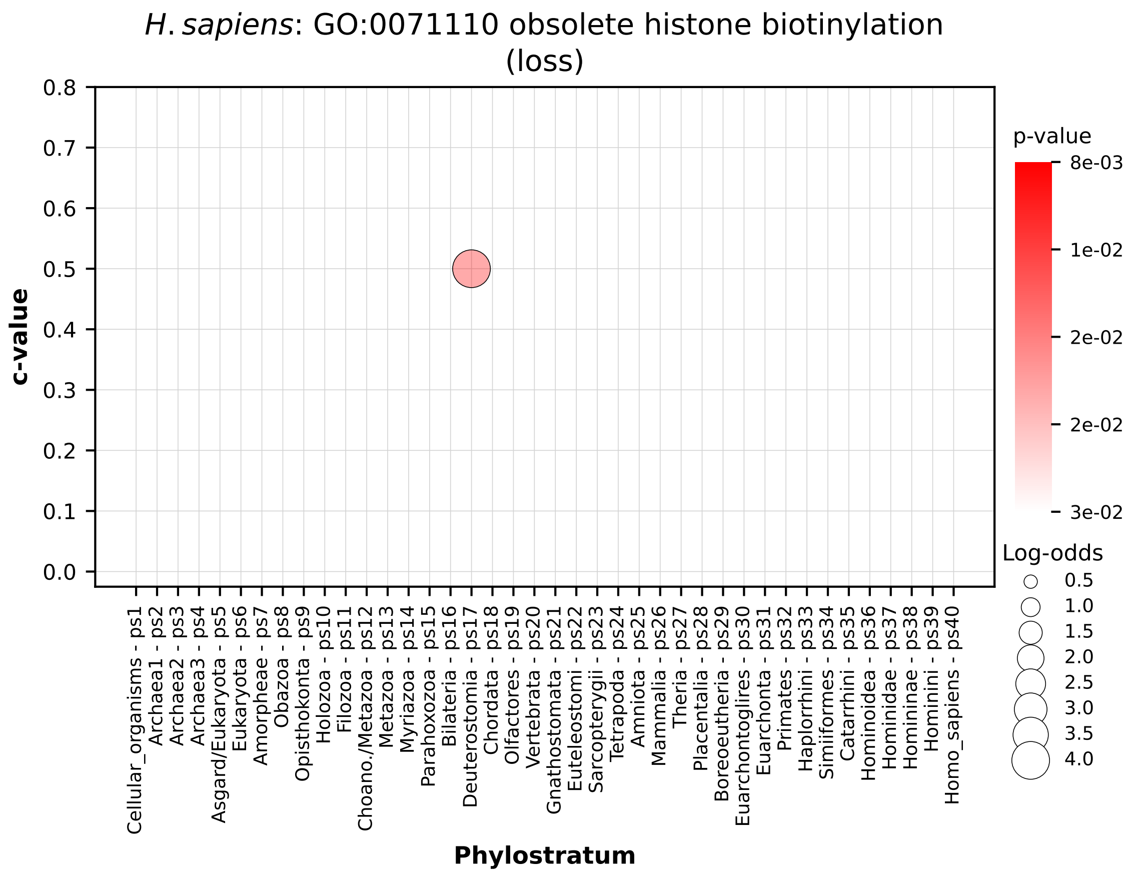

H. sapiens: GO:0071110 obsolete histone biotinylation (loss)
c-value
0.8
0.7
0.6
0.5
0.4
0.3
0.2
0.1
0.0
Phylostratum
Cellular_organisms - ps1
Archaea1 - ps2
Archaea2 - ps3
Archaea3 - ps4
Asgard/Eukaryota - ps5
Eukaryota - ps6
Amorpheae - ps7
Obazoa - ps8
Opisthokonta - ps9
Holozoa - ps10
Filozoa - ps11
Choano./Metazoa - ps12
Metazoa - ps13
Myriazoa - ps14
Parahoxozoa - ps15
Bilateria - ps16
Deuterostomia - ps17
Chordata - ps18
Olfactores - ps19
Vertebrata - ps20
Gnathostomata - ps21
Euteleostomi - ps22
Sarcopterygii - ps23
Tetrapoda - ps24
Amniota - ps25
Mammalia - ps26
Theria - ps27
Placentalia - ps28
Boreoeutheria - ps29
Euarchontoglires - ps30
Euarchonta - ps31
Primates - ps32
Haplorrhini - ps33
Simiiformes - ps34
Catarrhini - ps35
Hominoidea - ps36
Hominidae - ps37
Homininae - ps38
Hominini - ps39
Homo_sapiens - ps40
p-value
8e-03
1e-02
2e-02
2e-02
3e-02
Log-odds
0.5
1.0
1.5
2.0
2.5
3.0
3.5
4.0

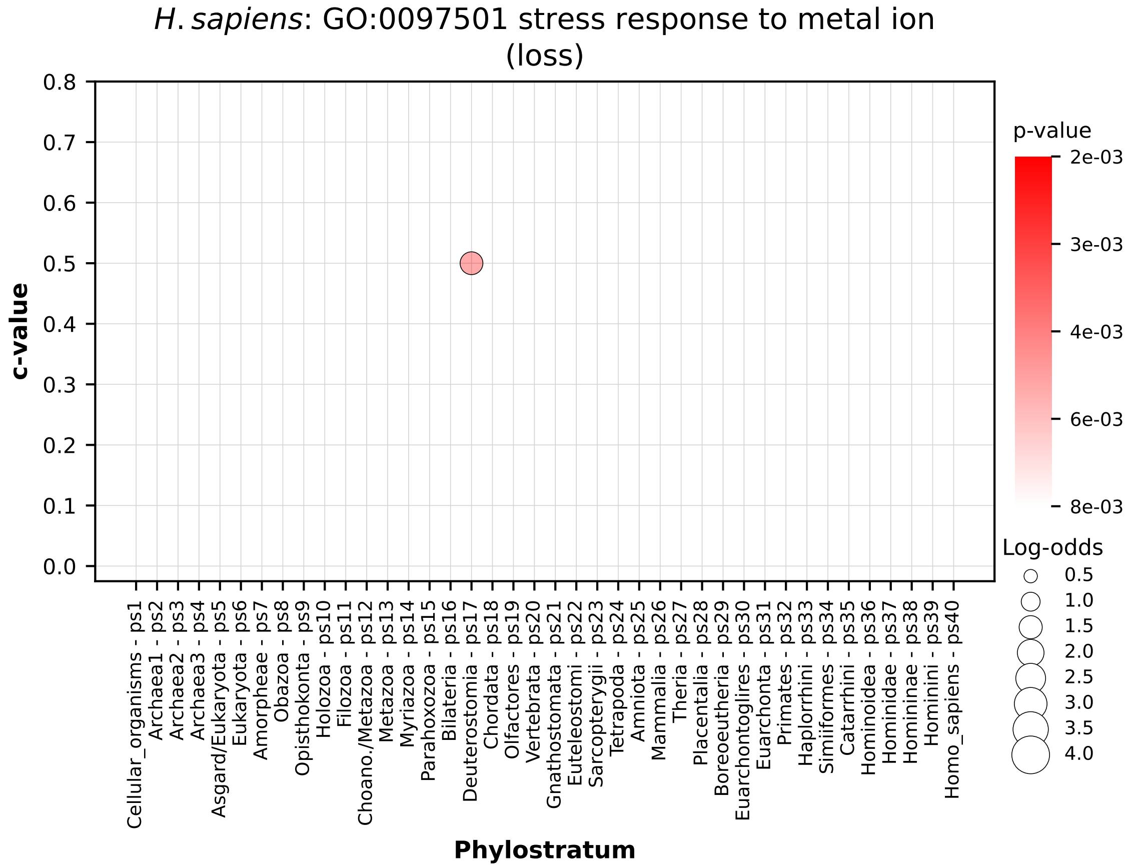
H. sapiens: GO:0097501 stress response to metal ion (loss)
c-value
0.0
0.1
0.2
0.3
0.4
0.5
0.6
0.7
0.8
Phylostratum
Cellular_organisms - ps1
Archaea1 - ps2
Archaea2 - ps3
Archaea3 - ps4
Asgard/Eukaryota - ps5
Eukaryota - ps6
Amorpheae - ps7
Obazoa - ps8
Opisthokonta - ps9
Holozoa - ps10
Filozoa - ps11
Choano./Metazoa - ps12
Metazoa - ps13
Myriazoa - ps14
Parahoxozoa - ps15
Bilateria - ps16
Deuterostomia - ps17
Chordata - ps18
Olfactores - ps19
Vertebrata - ps20
Gnathostomata - ps21
Euteleostomi - ps22
Sarcopterygii - ps23
Tetrapoda - ps24
Amniota - ps25
Mammalia - ps26
Theria - ps27
Placentalia - ps28
Boreoeutheria - ps29
Euarchontoglires - ps30
Euarchonta - ps31
Primates - ps32
Haplorrhini - ps33
Simiiformes - ps34
Catarrhini - ps35
Hominoidea - ps36
Hominidae - ps37
Homininae - ps38
Hominini - ps39
Homo_sapiens - ps40
p-value
2e-03
3e-03
4e-03
6e-03
8e-03
Log-odds
0.5
1.0
1.5
2.0
2.5
3.0
3.5
4.0

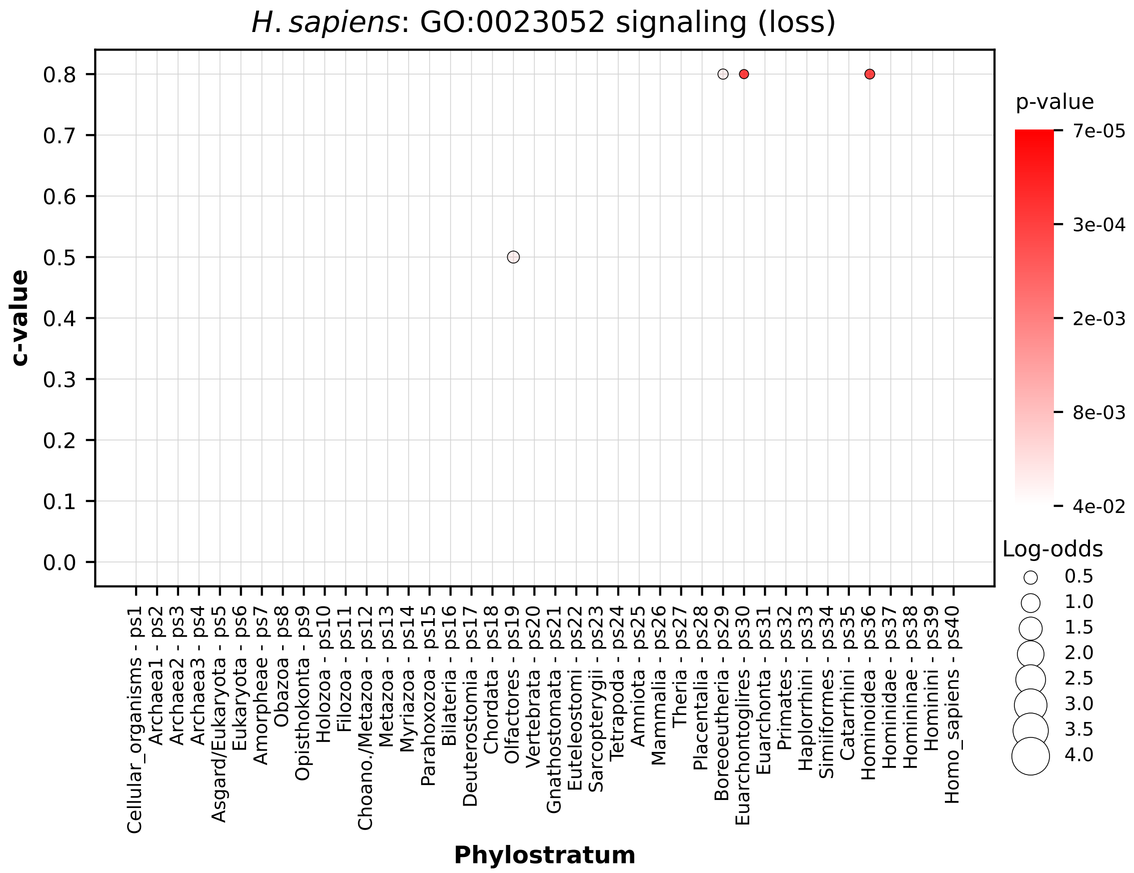

H. sapiens: GO:0023052 signaling (loss)
c-value
0.0
0.1
0.2
0.3
0.4
0.5
0.6
0.7
0.8
Phylostratum
Cellular_organisms - ps1
Archaea1 - ps2
Archaea2 - ps3
Archaea3 - ps4
Asgard/Eukaryota - ps5
Eukaryota - ps6
Amorpheae - ps7
Obazoa - ps8
Opisthokonta - ps9
Holozoa - ps10
Filozoa - ps11
Choano./Metazoa - ps12
Metazoa - ps13
Myriazoa - ps14
Parahoxozoa - ps15
Bilateria - ps16
Deuterostomia - ps17
Chordata - ps18
Olfactores - ps19
Vertebrata - ps20
Gnathostomata - ps21
Euteleostomi - ps22
Sarcopterygii - ps23
Tetrapoda - ps24
Amniota - ps25
Mammalia - ps26
Theria - ps27
Placentalia - ps28
Boreoeutheria - ps29
Euarchontoglires - ps30
Euarchonta - ps31
Primates - ps32
Haplorrhini - ps33
Simiiformes - ps34
Catarrhini - ps35
Hominoidea - ps36
Hominidae - ps37
Homininae - ps38
Hominini - ps39
Homo_sapiens - ps40
p-value
7e-05
3e-04
2e-03
8e-03
4e-02
Log-odds
0.5
1.0
1.5
2.0
2.5
3.0
3.5
4.0

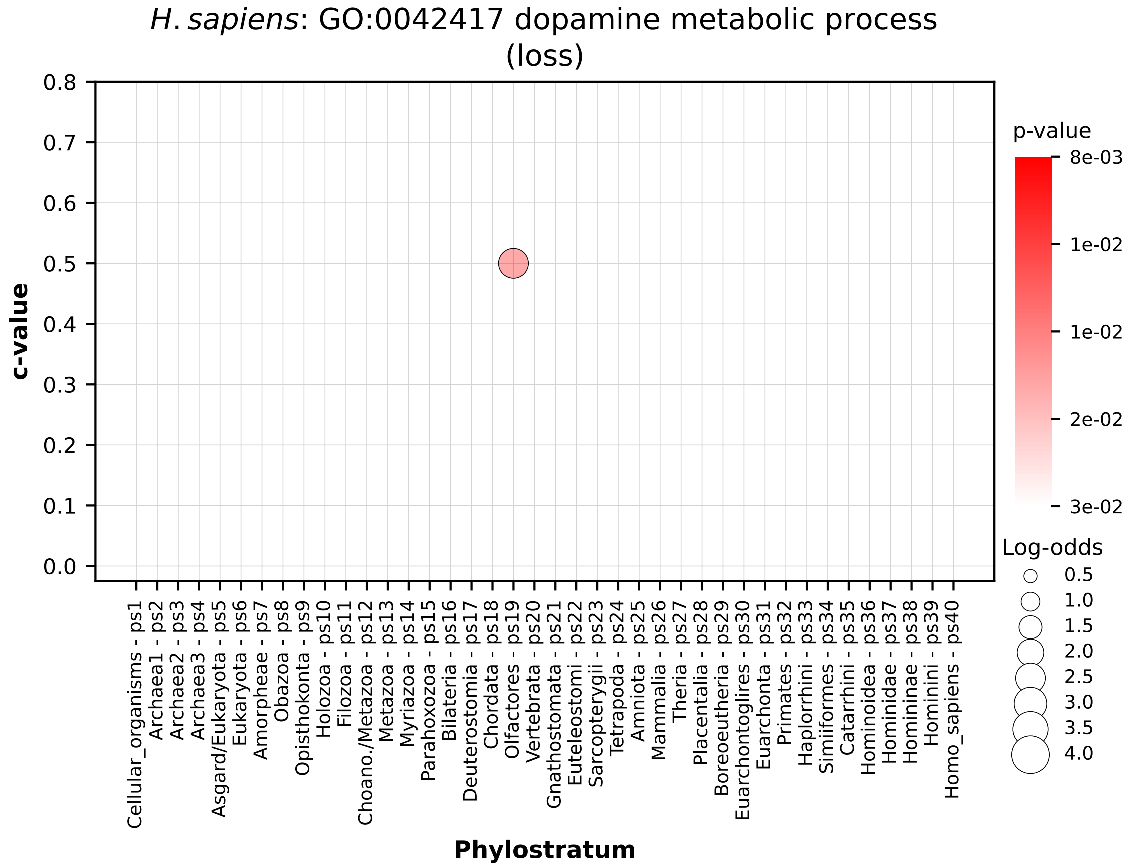
H. sapiens: GO:0042417 dopamine metabolic process (loss)
c-value
Phylostratum
p-value
8e-03
1e-02
1e-02
2e-02
3e-02
Log-odds
0.5
1.0
1.5
2.0
2.5
3.0
3.5
4.0
Cellular_organisms - ps1
Archaea1 - ps2
Archaea2 - ps3
Archaea3 - ps4
Asgard/Eukaryota - ps5
Eukaryota - ps6
Amorpheae - ps7
Obazoa - ps8
Opisthokonta - ps9
Holozoa - ps10
Filozoa - ps11
Choano./Metazoa - ps12
Metazoa - ps13
Myriazoa - ps14
Parahoxozoa - ps15
Bilateria - ps16
Deuterostomia - ps17
Chordata - ps18
Olfactores - ps19
Vertebrata - ps20
Gnathostomata - ps21
Euteleostomi - ps22
Sarcopterygii - ps23
Tetrapoda - ps24
Amniota - ps25
Mammalia - ps26
Theria - ps27
Placentalia - ps28
Boreoeutheria - ps29
Euarchontoglires - ps30
Euarchonta - ps31
Primates - ps32
Haplorrhini - ps33
Simiiformes - ps34
Catarrhini - ps35
Hominoidea - ps36
Hominidae - ps37
Homininae - ps38
Hominini - ps39
Homo_sapiens - ps40

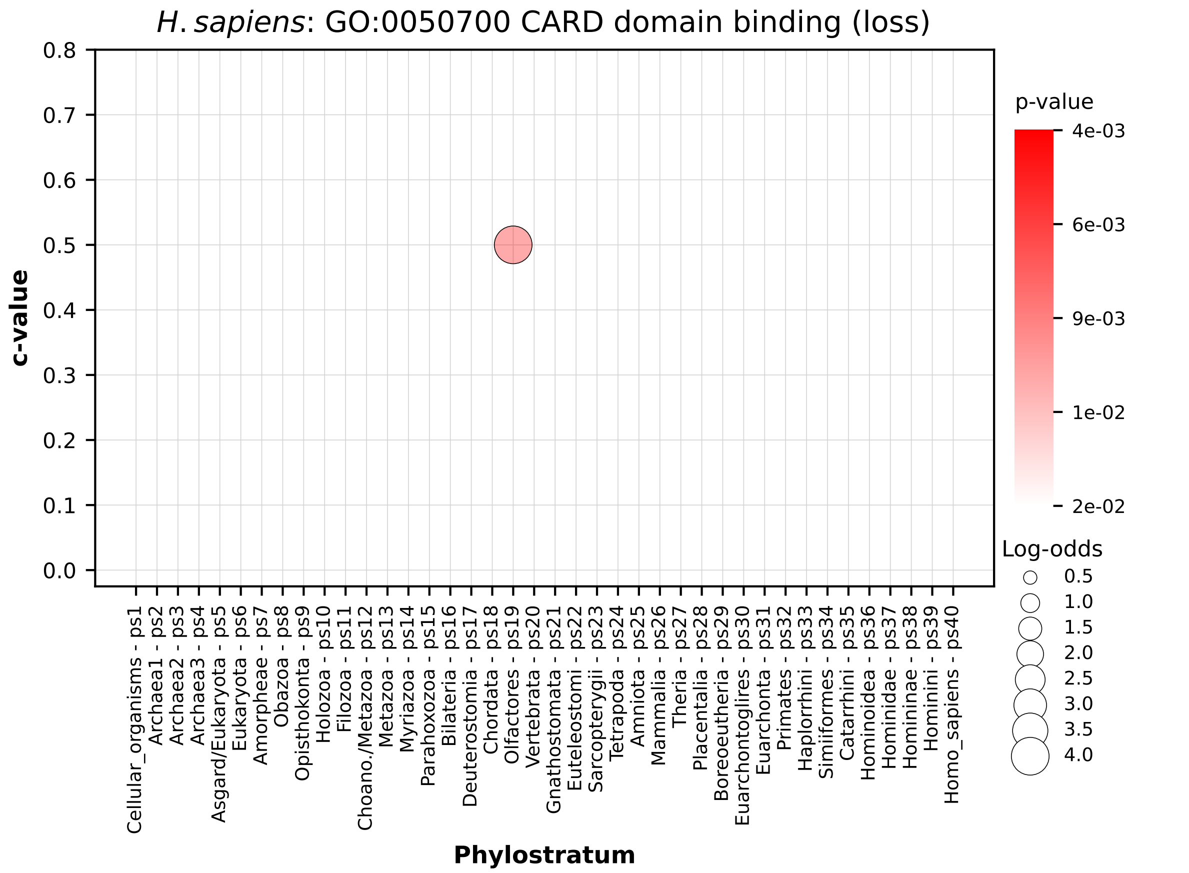

H. sapiens: GO:0050700 CARD domain binding (loss)
c-value
0.8
0.7
0.6
0.5
0.4
0.3
0.2
0.1
0.0
p-value
4e-03
6e-03
9e-03
1e-02
2e-02
Log-odds
0.5
1.0
1.5
2.0
2.5
3.0
3.5
4.0
Cellular_organisms - ps1
Archaea1 - ps2
Archaea2 - ps3
Archaea3 - ps4
Asgard/Eukaryota - ps5
Eukaryota - ps6
Amorpheae - ps7
Obazoa - ps8
Opisthokonta - ps9
Holozoa - ps10
Filozoa - ps11
Choano./Metazoa - ps12
Metazoa - ps13
Myriazoa - ps14
Parahoxozoa - ps15
Bilateria - ps16
Deuterostomia - ps17
Chordata - ps18
Olfactores - ps19
Vertebrata - ps20
Gnathostomata - ps21
Euteleostomi - ps22
Sarcopterygii - ps23
Tetrapoda - ps24
Amniota - ps25
Mammalia - ps26
Theria - ps27
Placentalia - ps28
Boreoeutheria - ps29
Euarchontoglires - ps30
Euarchonta - ps31
Primates - ps32
Haplorrhini - ps33
Simiiformes - ps34
Catarrhini - ps35
Hominoidea - ps36
Hominidae - ps37
Homininae - ps38
Hominini - ps39
Homo_sapiens - ps40
Phylostratum

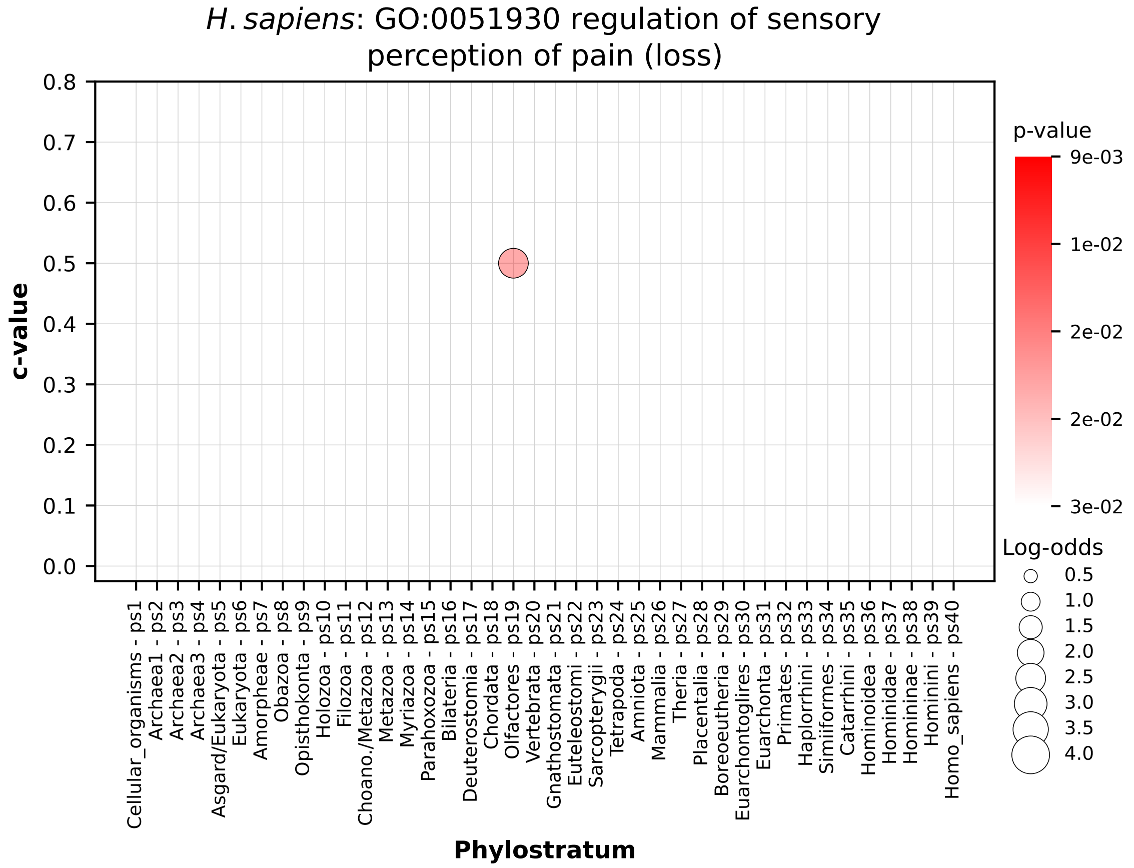
H. sapiens: GO:0051930 regulation of sensory perception of pain (loss)
c-value
0.8
0.7
0.6
0.5
0.4
0.3
0.2
0.1
0.0
Cellular_organisms - ps1
Archaea1 - ps2
Archaea2 - ps3
Archaea3 - ps4
Asgard/Eukaryota - ps5
Eukaryota - ps6
Amorpheae - ps7
Obazoa - ps8
Opisthokonta - ps9
Holozoa - ps10
Filozoa - ps11
Choano./Metazoa - ps12
Metazoa - ps13
Myriazoa - ps14
Parahoxozoa - ps15
Bilateria - ps16
Deuterostomia - ps17
Chordata - ps18
Olfactores - ps19
Vertebrata - ps20
Gnathostomata - ps21
Euteleostomi - ps22
Sarcopterygii - ps23
Tetrapoda - ps24
Amniota - ps25
Mammalia - ps26
Theria - ps27
Placentalia - ps28
Boreoeutheria - ps29
Euarchontoglires - ps30
Euarchonta - ps31
Primates - ps32
Haplorrhini - ps33
Simiiformes - ps34
Catarrhini - ps35
Hominoidea - ps36
Hominidae - ps37
Homininae - ps38
Hominini - ps39
Homo_sapiens - ps40
Phylostratum
p-value
9e-03
1e-02
2e-02
2e-02
3e-02
Log-odds
0.5
1.0
1.5
2.0
2.5
3.0
3.5
4.0

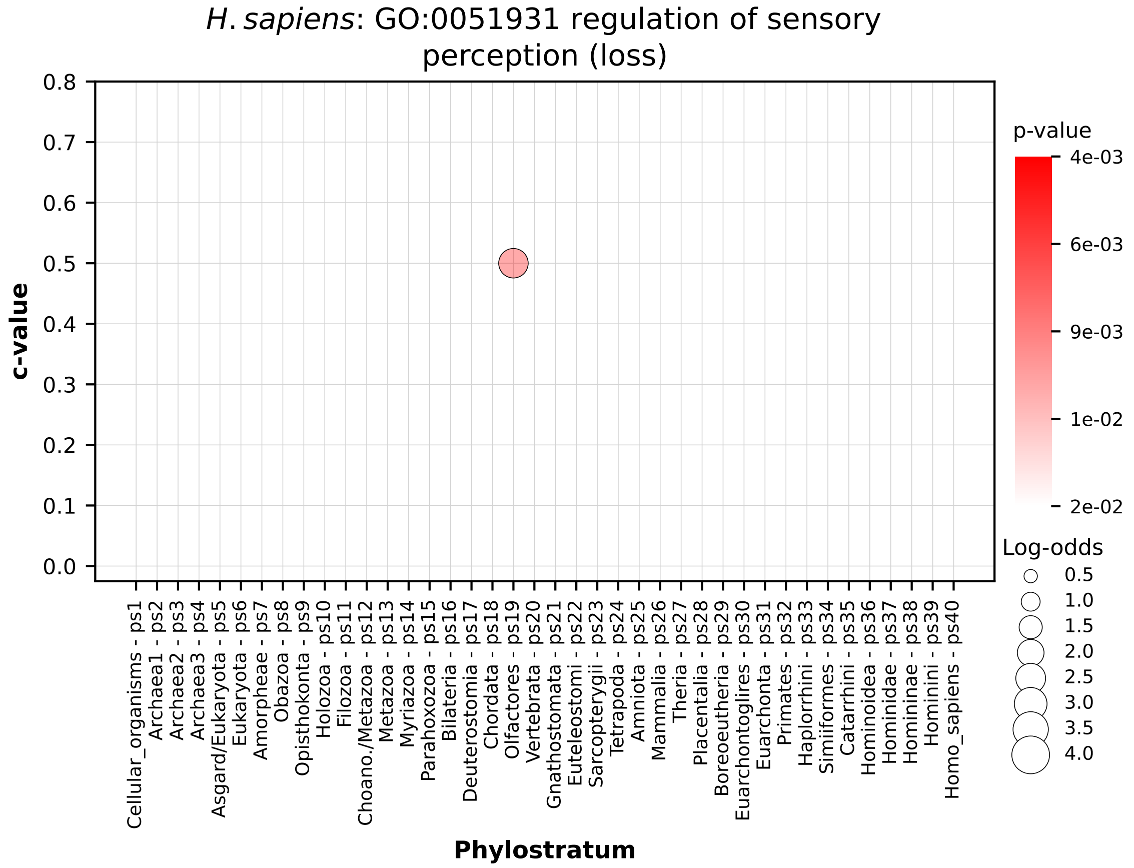

H. sapiens: GO:0051931 regulation of sensory perception (loss)
c-value
0.8
0.7
0.6
0.5
0.4
0.3
0.2
0.1
0.0
p-value
4e-03
6e-03
9e-03
1e-02
2e-02
Log-odds
0.5
1.0
1.5
2.0
2.5
3.0
3.5
4.0
Cellular_organisms - ps1
Archaea1 - ps2
Archaea2 - ps3
Archaea3 - ps4
Asgard/Eukaryota - ps5
Eukaryota - ps6
Amorpheae - ps7
Obazoa - ps8
Opisthokonta - ps9
Holozoa - ps10
Filozoa - ps11
Choano./Metazoa - ps12
Metazoa - ps13
Myriazoa - ps14
Parahoxozoa - ps15
Bilateria - ps16
Deuterostomia - ps17
Chordata - ps18
Olfactores - ps19
Vertebrata - ps20
Gnathostomata - ps21
Euteleostomi - ps22
Sarcopterygii - ps23
Tetrapoda - ps24
Amniota - ps25
Mammalia - ps26
Theria - ps27
Placentalia - ps28
Boreoeutheria - ps29
Euarchontoglires - ps30
Euarchonta - ps31
Primates - ps32
Haplorrhini - ps33
Simiiformes - ps34
Catarrhini - ps35
Hominoidea - ps36
Hominidae - ps37
Homininae - ps38
Hominini - ps39
Homo_sapiens - ps40
Phylostratum

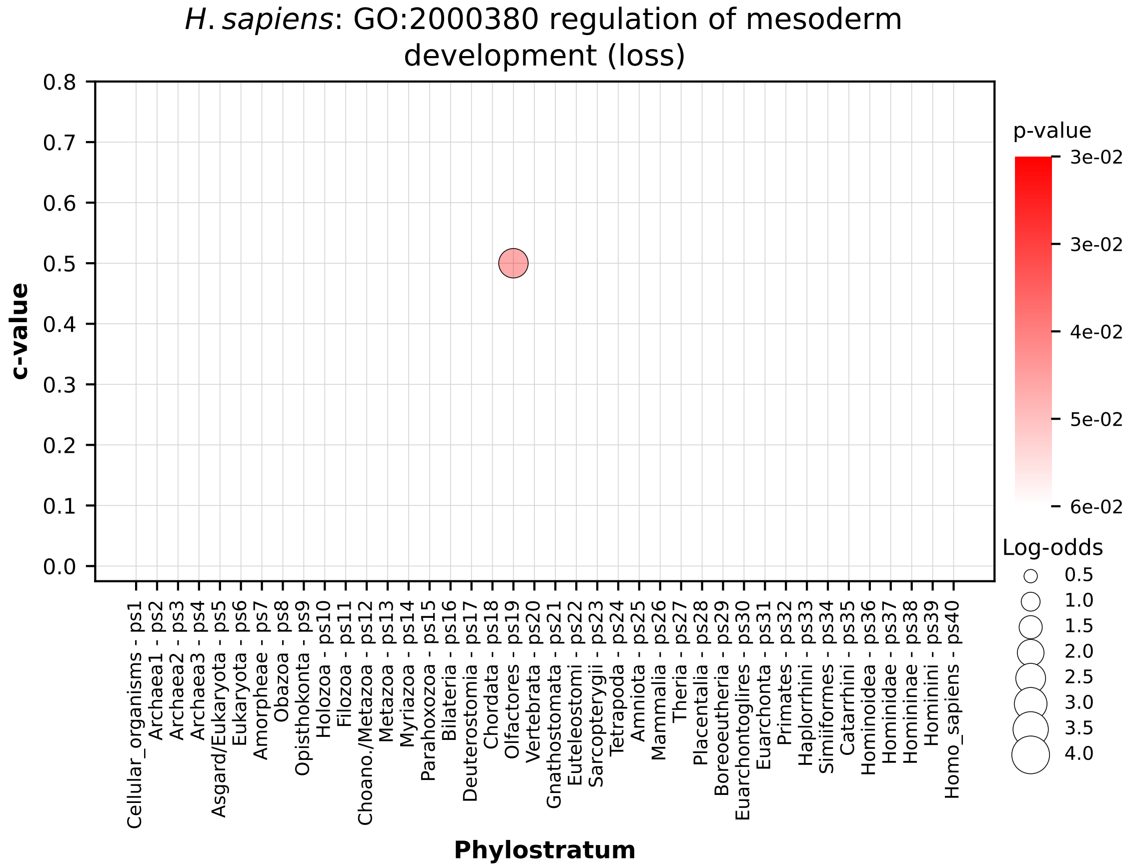

H. sapiens: GO:2000380 regulation of mesoderm development (loss)
c-value
0.0
0.1
0.2
0.3
0.4
0.5
0.6
0.7
0.8
Phylostratum
Cellular_organisms - ps1
Archaea1 - ps2
Archaea2 - ps3
Archaea3 - ps4
Asgard/Eukaryota - ps5
Eukaryota - ps6
Amorpheae - ps7
Obazoa - ps8
Opisthokonta - ps9
Holozoa - ps10
Filozoa - ps11
Choano./Metazoa - ps12
Metazoa - ps13
Myriazoa - ps14
Parahoxozoa - ps15
Bilateria - ps16
Deuterostomia - ps17
Chordata - ps18
Olfactores - ps19
Vertebrata - ps20
Gnathostomata - ps21
Euteleostomi - ps22
Sarcopterygii - ps23
Tetrapoda - ps24
Amniota - ps25
Mammalia - ps26
Theria - ps27
Placentalia - ps28
Boreoeutheria - ps29
Euarchontoglires - ps30
Euarchonta - ps31
Primates - ps32
Haplorrhini - ps33
Simiiformes - ps34
Catarrhini - ps35
Hominoidea - ps36
Hominidae - ps37
Homininae - ps38
Hominini - ps39
Homo_sapiens - ps40
p-value
3e-02
3e-02
4e-02
5e-02
6e-02
Log-odds
0.5
1.0
1.5
2.0
2.5
3.0
3.5
4.0

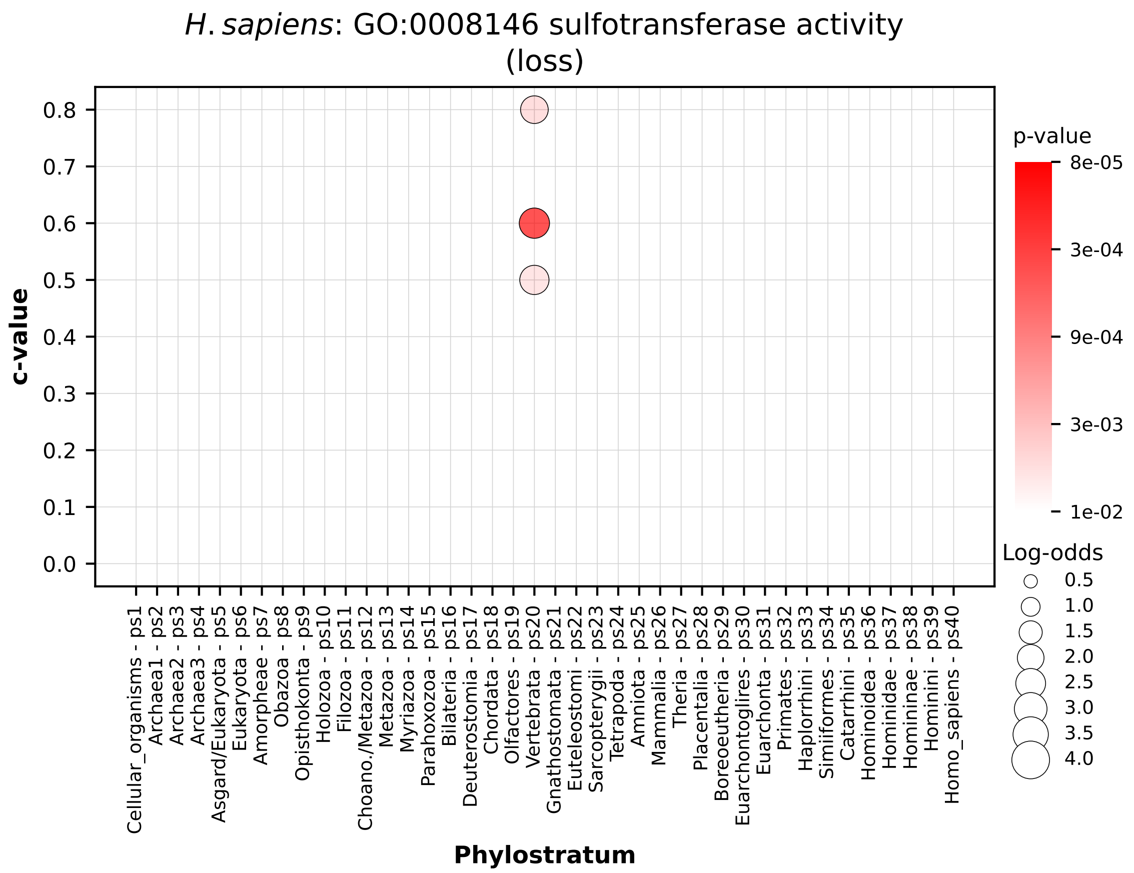

H. sapiens: GO:0008146 sulfotransferase activity (loss)
c-value
0.0
0.1
0.2
0.3
0.4
0.5
0.6
0.7
0.8
p-value
8e-05
3e-04
9e-04
3e-03
1e-02
Log-odds
0.5
1.0
1.5
2.0
2.5
3.0
3.5
4.0
Cellular_organisms - ps1
Archaea1 - ps2
Archaea2 - ps3
Archaea3 - ps4
Asgard/Eukaryota - ps5
Eukaryota - ps6
Amorpheae - ps7
Obazoa - ps8
Opisthokonta - ps9
Holozoa - ps10
Filozoa - ps11
Choano./Metazoa - ps12
Metazoa - ps13
Myriazoa - ps14
Parahoxozoa - ps15
Bilateria - ps16
Deuterostomia - ps17
Chordata - ps18
Olfactores - ps19
Vertebrata - ps20
Gnathostomata - ps21
Euteleostomi - ps22
Sarcopterygii - ps23
Tetrapoda - ps24
Amniota - ps25
Mammalia - ps26
Theria - ps27
Placentalia - ps28
Boreoeutheria - ps29
Euarchontoglires - ps30
Euarchonta - ps31
Primates - ps32
Haplorrhini - ps33
Simiiformes - ps34
Catarrhini - ps35
Hominoidea - ps36
Hominidae - ps37
Homininae - ps38
Hominini - ps39
Homo_sapiens - ps40
Phylostratum

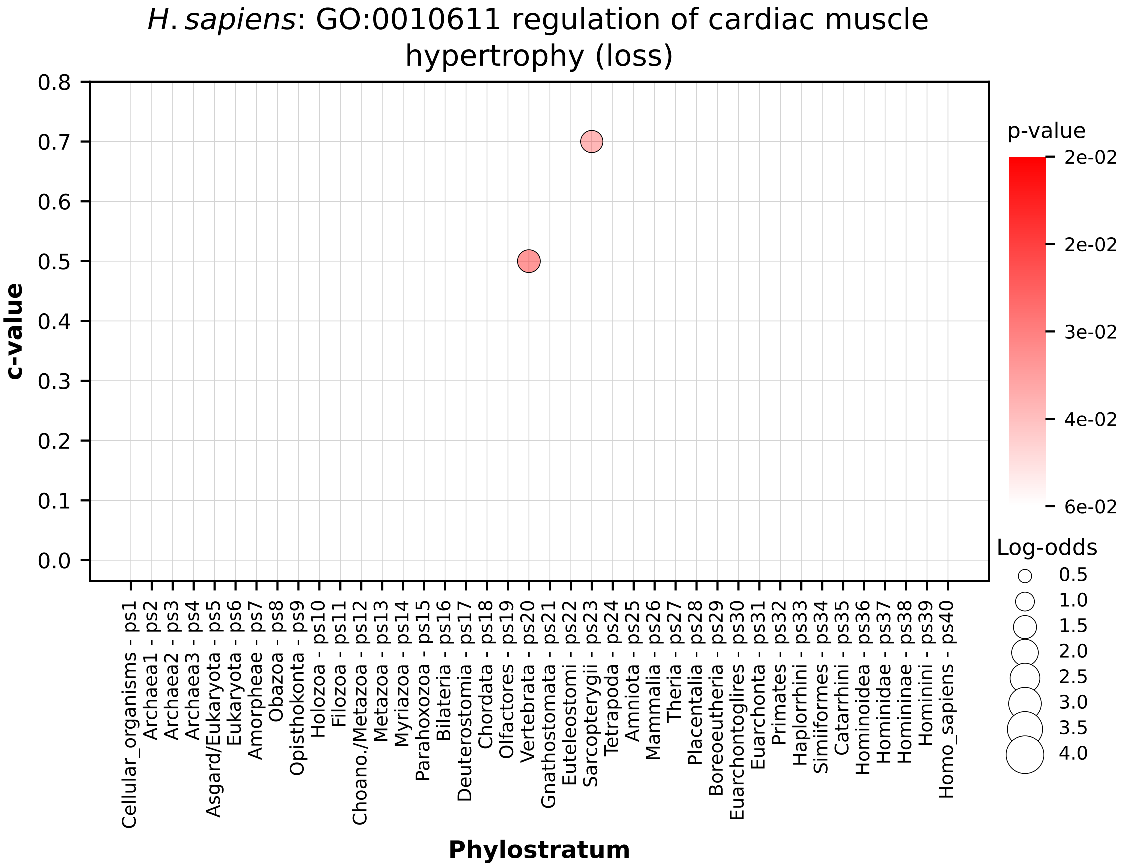

H. sapiens: GO:0010611 regulation of cardiac muscle hypertrophy (loss)
c-value
0.8
0.7
0.6
0.5
0.4
0.3
0.2
0.1
0.0
p-value
2e-02
2e-02
3e-02
4e-02
6e-02
Log-odds
0.5
1.0
1.5
2.0
2.5
3.0
3.5
4.0
Cellular_organisms - ps1
Archaea1 - ps2
Archaea2 - ps3
Archaea3 - ps4
Asgard/Eukaryota - ps5
Eukaryota - ps6
Amorpheae - ps7
Obazoa - ps8
Opisthokonta - ps9
Holozoa - ps10
Filozoa - ps11
Choano./Metazoa - ps12
Metazoa - ps13
Myriazoa - ps14
Parahoxozoa - ps15
Bilateria - ps16
Deuterostomia - ps17
Chordata - ps18
Olfactores - ps19
Vertebrata - ps20
Gnathostomata - ps21
Euteleostomi - ps22
Sarcopterygii - ps23
Tetrapoda - ps24
Amniota - ps25
Mammalia - ps26
Theria - ps27
Placentalia - ps28
Boreoeutheria - ps29
Euarchontoglires - ps30
Euarchonta - ps31
Primates - ps32
Haplorrhini - ps33
Simiiformes - ps34
Catarrhini - ps35
Hominoidea - ps36
Hominidae - ps37
Homininae - ps38
Hominini - ps39
Homo_sapiens - ps40
Phylostratum

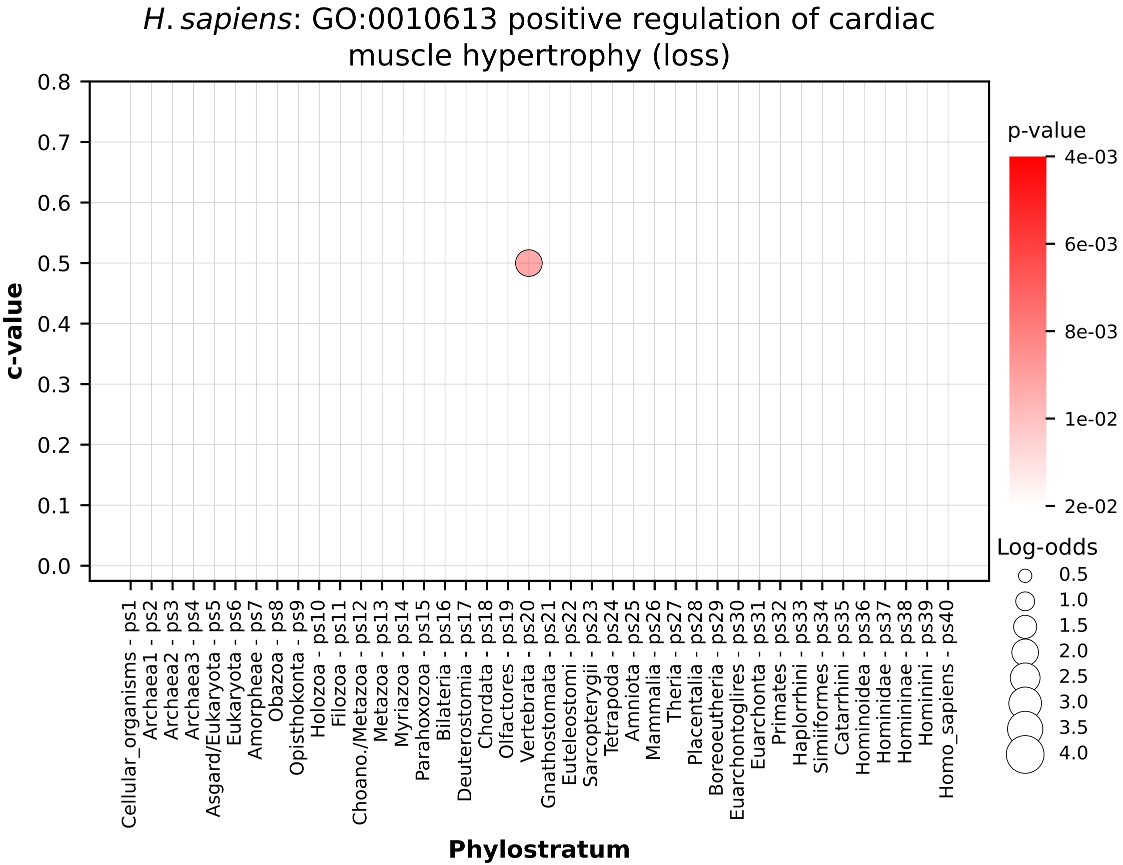
H. sapiens: GO:0010613 positive regulation of cardiac muscle hypertrophy (loss)
c-value
0.8
0.7
0.6
0.5
0.4
0.3
0.2
0.1
0.0
Phylostratum
Cellular_organisms - ps1
Archaea1 - ps2
Archaea2 - ps3
Archaea3 - ps4
Asgard/Eukaryota - ps5
Eukaryota - ps6
Amorpheae - ps7
Obazoa - ps8
Opisthokonta - ps9
Holozoa - ps10
Filozoa - ps11
Choano./Metazoa - ps12
Metazoa - ps13
Myriazoa - ps14
Parahoxozoa - ps15
Bilateria - ps16
Deuterostomia - ps17
Chordata - ps18
Olfactores - ps19
Vertebrata - ps20
Gnathostomata - ps21
Euteleostomi - ps22
Sarcopterygii - ps23
Tetrapoda - ps24
Amniota - ps25
Mammalia - ps26
Theria - ps27
Placentalia - ps28
Boreoeutheria - ps29
Euarchontoglires - ps30
Euarchonta - ps31
Primates - ps32
Haplorrhini - ps33
Simiiformes - ps34
Catarrhini - ps35
Hominoidea - ps36
Hominidae - ps37
Homininae - ps38
Hominini - ps39
Homo_sapiens - ps40
p-value
4e-03
6e-03
8e-03
1e-02
2e-02
Log-odds
0.5
1.0
1.5
2.0
2.5
3.0
3.5
4.0

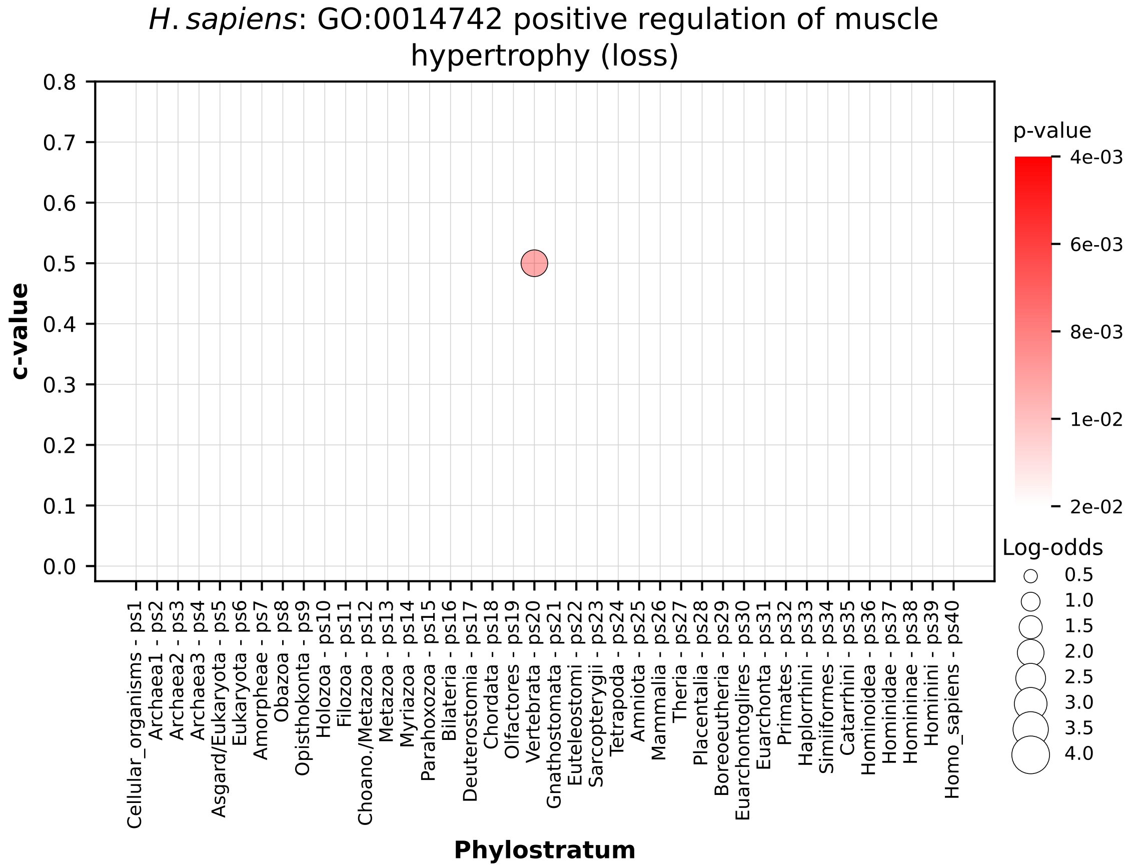

H. sapiens: GO:0014742 positive regulation of muscle hypertrophy (loss)
c-value
0.8
0.7
0.6
0.5
0.4
0.3
0.2
0.1
0.0
Phylostratum
Cellular_organisms - ps1
Archaea1 - ps2
Archaea2 - ps3
Archaea3 - ps4
Asgard/Eukaryota - ps5
Eukaryota - ps6
Amorpheae - ps7
Obazoa - ps8
Opisthokonta - ps9
Holozoa - ps10
Filozoa - ps11
Choano./Metazoa - ps12
Metazoa - ps13
Myriazoa - ps14
Parahoxozoa - ps15
Bilateria - ps16
Deuterostomia - ps17
Chordata - ps18
Olfactores - ps19
Vertebrata - ps20
Gnathostomata - ps21
Euteleostomi - ps22
Sarcopterygii - ps23
Tetrapoda - ps24
Amniota - ps25
Mammalia - ps26
Theria - ps27
Placentalia - ps28
Boreoeutheria - ps29
Euarchontoglires - ps30
Euarchonta - ps31
Primates - ps32
Haplorrhini - ps33
Simiiformes - ps34
Catarrhini - ps35
Hominoidea - ps36
Hominidae - ps37
Homininae - ps38
Hominini - ps39
Homo_sapiens - ps40
p-value
4e-03
6e-03
8e-03
1e-02
2e-02
Log-odds
0.5
1.0
1.5
2.0
2.5
3.0
3.5
4.0

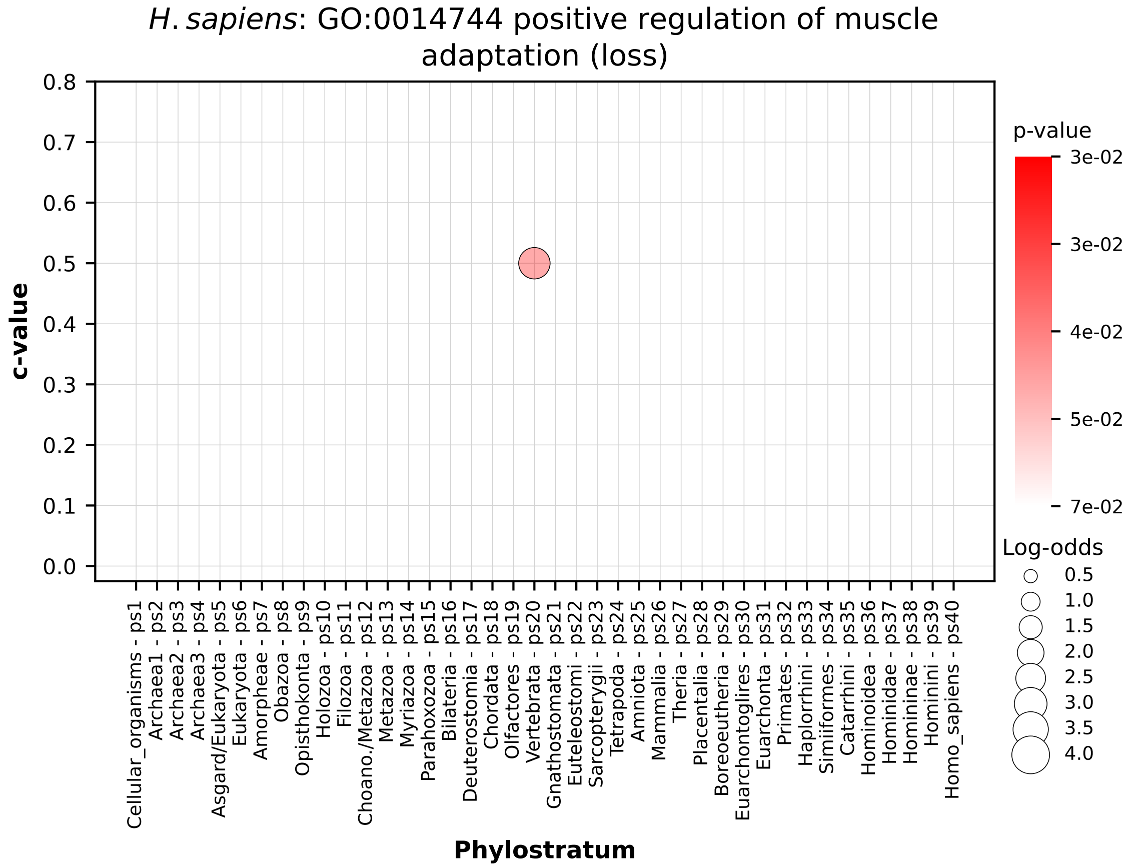

H. sapiens: GO:0014744 positive regulation of muscle adaptation (loss)
c-value
0.8
0.7
0.6
0.5
0.4
0.3
0.2
0.1
0.0
Phylostratum
Cellular_organisms - ps1
Archaea1 - ps2
Archaea2 - ps3
Archaea3 - ps4
Asgard/Eukaryota - ps5
Eukaryota - ps6
Amorpheae - ps7
Obazoa - ps8
Opisthokonta - ps9
Holozoa - ps10
Filozoa - ps11
Choano./Metazoa - ps12
Metazoa - ps13
Myriazoa - ps14
Parahoxozoa - ps15
Bilateria - ps16
Deuterostomia - ps17
Chordata - ps18
Olfactores - ps19
Vertebrata - ps20
Gnathostomata - ps21
Euteleostomi - ps22
Sarcopterygii - ps23
Tetrapoda - ps24
Amniota - ps25
Mammalia - ps26
Theria - ps27
Placentalia - ps28
Boreoeutheria - ps29
Euarchontoglires - ps30
Euarchonta - ps31
Primates - ps32
Haplorrhini - ps33
Simiiformes - ps34
Catarrhini - ps35
Hominoidea - ps36
Hominidae - ps37
Homininae - ps38
Hominini - ps39
Homo_sapiens - ps40
p-value
3e-02
3e-02
4e-02
5e-02
7e-02
Log-odds
0.5
1.0
1.5
2.0
2.5
3.0
3.5
4.0

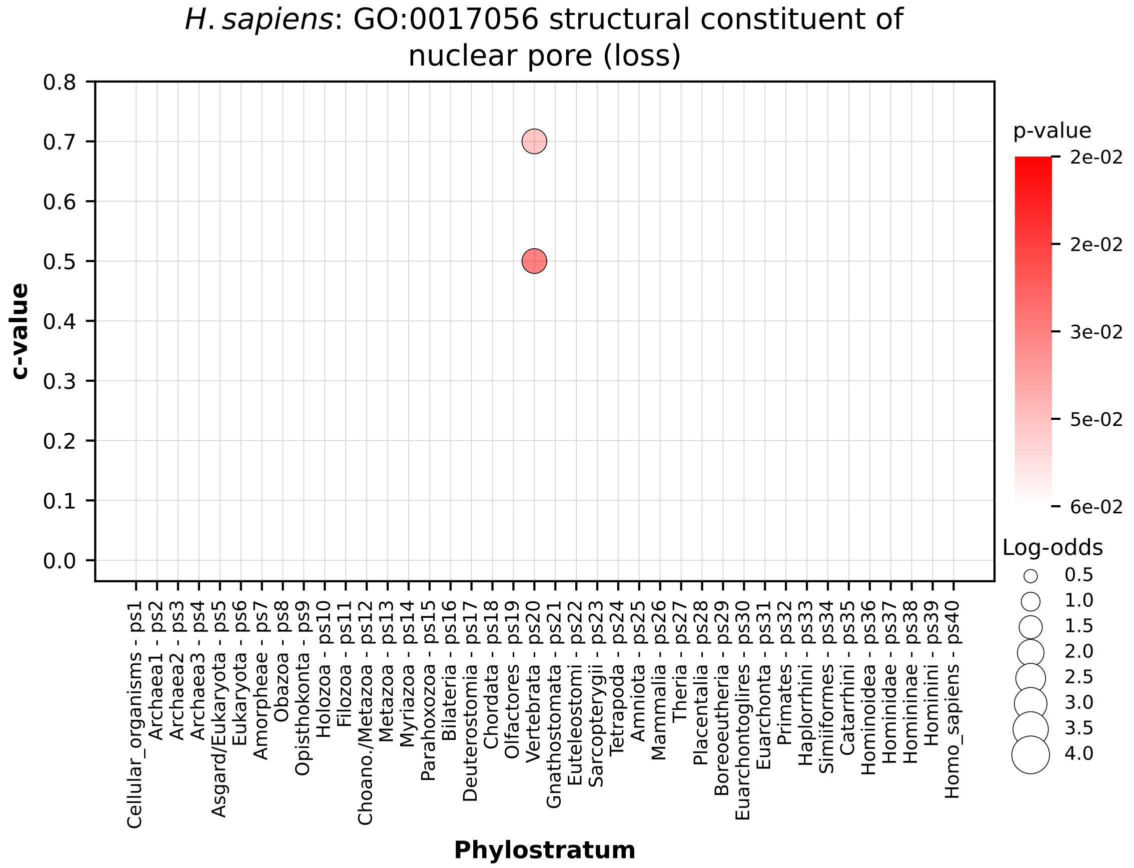

H. sapiens: GO:0017056 structural constituent of nuclear pore (loss)
c-value
0.8
0.7
0.6
0.5
0.4
0.3
0.2
0.1
0.0
p-value
2e-02
2e-02
3e-02
5e-02
6e-02
Log-odds
0.5
1.0
1.5
2.0
2.5
3.0
3.5
4.0
Cellular_organisms - ps1
Archaea1 - ps2
Archaea2 - ps3
Archaea3 - ps4
Asgard/Eukaryota - ps5
Eukaryota - ps6
Amorpheae - ps7
Obazoa - ps8
Opisthokonta - ps9
Holozoa - ps10
Filozoa - ps11
Choano./Metazoa - ps12
Metazoa - ps13
Myriazoa - ps14
Parahoxozoa - ps15
Bilateria - ps16
Deuterostomia - ps17
Chordata - ps18
Olfactores - ps19
Vertebrata - ps20
Gnathostomata - ps21
Euteleostomi - ps22
Sarcopterygii - ps23
Tetrapoda - ps24
Amniota - ps25
Mammalia - ps26
Theria - ps27
Placentalia - ps28
Boreoeutheria - ps29
Euarchontoglires - ps30
Euarchonta - ps31
Primates - ps32
Haplorrhini - ps33
Simiiformes - ps34
Catarrhini - ps35
Hominoidea - ps36
Hominidae - ps37
Homininae - ps38
Hominini - ps39
Homo_sapiens - ps40
Phylostratum

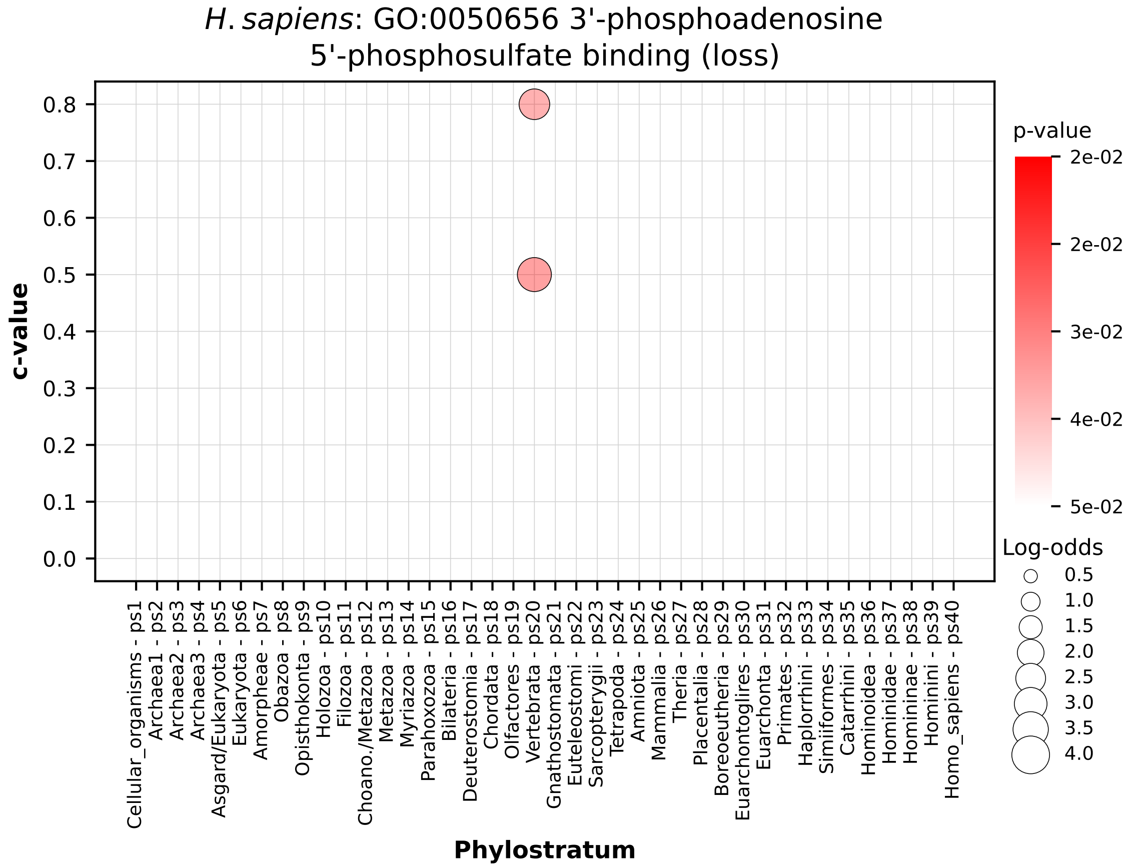

H. sapiens: GO:0050656 3'-phosphoadenosine 5'-phosphosulfate binding (loss)
c-value
0.8
0.7
0.6
0.5
0.4
0.3
0.2
0.1
0.0
p-value
2e-02
2e-02
3e-02
4e-02
5e-02
Log-odds
0.5
1.0
1.5
2.0
2.5
3.0
3.5
4.0
Cellular_organisms - ps1
Archaea1 - ps2
Archaea2 - ps3
Archaea3 - ps4
Asgard/Eukaryota - ps5
Eukaryota - ps6
Amorpheae - ps7
Obazoa - ps8
Opisthokonta - ps9
Holozoa - ps10
Filozoa - ps11
Choano./Metazoa - ps12
Metazoa - ps13
Myriazoa - ps14
Parahoxozoa - ps15
Bilateria - ps16
Deuterostomia - ps17
Chordata - ps18
Olfactores - ps19
Vertebrata - ps20
Gnathostomata - ps21
Euteleostomi - ps22
Sarcopterygii - ps23
Tetrapoda - ps24
Amniota - ps25
Mammalia - ps26
Theria - ps27
Placentalia - ps28
Boreoeutheria - ps29
Euarchontoglires - ps30
Euarchonta - ps31
Primates - ps32
Haplorrhini - ps33
Simiiformes - ps34
Catarrhini - ps35
Hominoidea - ps36
Hominidae - ps37
Homininae - ps38
Hominini - ps39
Homo_sapiens - ps40
Phylostratum

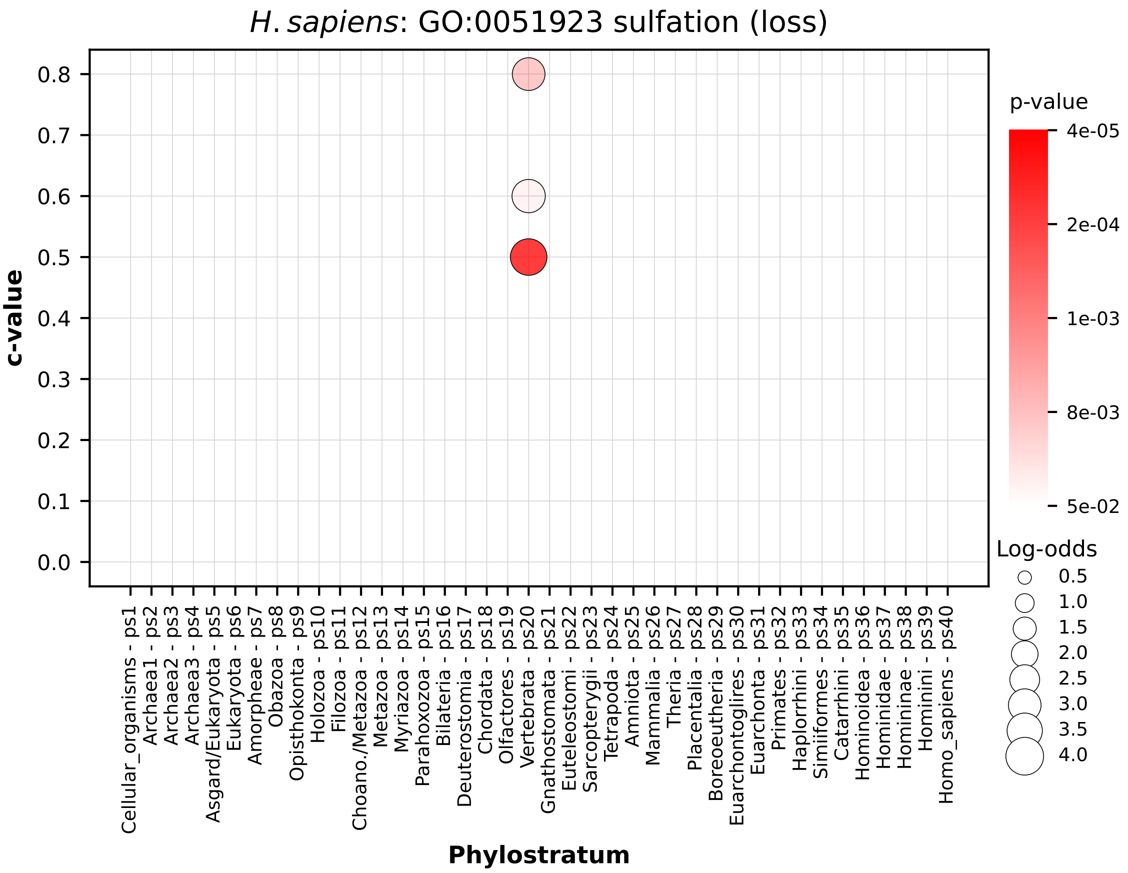

H. sapiens: GO:0051923 sulfation (loss)
c-value
0.0
0.1
0.2
0.3
0.4
0.5
0.6
0.7
0.8
Phylostratum
Cellular_organisms - ps1
Archaea1 - ps2
Archaea2 - ps3
Archaea3 - ps4
Asgard/Eukaryota - ps5
Eukaryota - ps6
Amorpheae - ps7
Obazoa - ps8
Opisthokonta - ps9
Holozoa - ps10
Filozoa - ps11
Choano./Metazoa - ps12
Metazoa - ps13
Myriazoa - ps14
Parahoxozoa - ps15
Bilateria - ps16
Deuterostomia - ps17
Chordata - ps18
Olfactores - ps19
Vertebrata - ps20
Gnathostomata - ps21
Euteleostomi - ps22
Sarcopterygii - ps23
Tetrapoda - ps24
Amniota - ps25
Mammalia - ps26
Theria - ps27
Placentalia - ps28
Boreoeutheria - ps29
Euarchontoglires - ps30
Euarchonta - ps31
Primates - ps32
Haplorrhini - ps33
Simiiformes - ps34
Catarrhini - ps35
Hominoidea - ps36
Hominidae - ps37
Homininae - ps38
Hominini - ps39
Homo_sapiens - ps40
p-value
4e-05
2e-04
1e-03
8e-03
5e-02
Log-odds
0.5
1.0
1.5
2.0
2.5
3.0
3.5
4.0

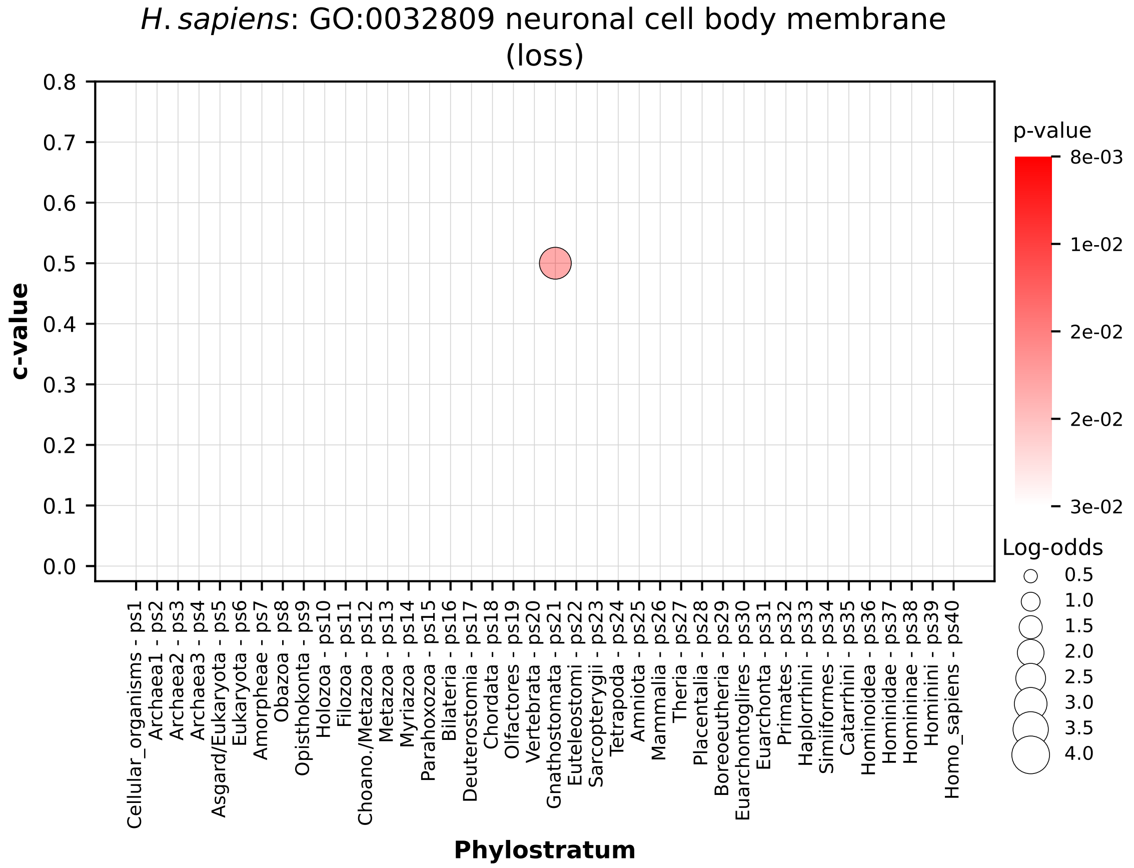

H. sapiens: GO:0032809 neuronal cell body membrane
(loss)
c-value
0.0
0.1
0.2
0.3
0.4
0.5
0.6
0.7
0.8
Phylostratum
Cellular_organisms - ps1
Archaea1 - ps2
Archaea2 - ps3
Archaea3 - ps4
Asgard/Eukaryota - ps5
Eukaryota - ps6
Amorpheae - ps7
Obazoa - ps8
Opisthokonta - ps9
Holozoa - ps10
Filozoa - ps11
Choano./Metazoa - ps12
Metazoa - ps13
Myriazoa - ps14
Parahoxozoa - ps15
Bilateria - ps16
Deuterostomia - ps17
Chordata - ps18
Olfactores - ps19
Vertebrata - ps20
Gnathostomata - ps21
Euteleostomi - ps22
Sarcopterygii - ps23
Tetrapoda - ps24
Amniota - ps25
Mammalia - ps26
Theria - ps27
Placentalia - ps28
Boreoeutheria - ps29
Euarchontoglires - ps30
Euarchonta - ps31
Primates - ps32
Haplorrhini - ps33
Simiiformes - ps34
Catarrhini - ps35
Hominoidea - ps36
Hominidae - ps37
Homininae - ps38
Hominini - ps39
Homo_sapiens - ps40
p-value
8e-03
1e-02
2e-02
2e-02
3e-02
Log-odds
0.5
1.0
1.5
2.0
2.5
3.0
3.5
4.0

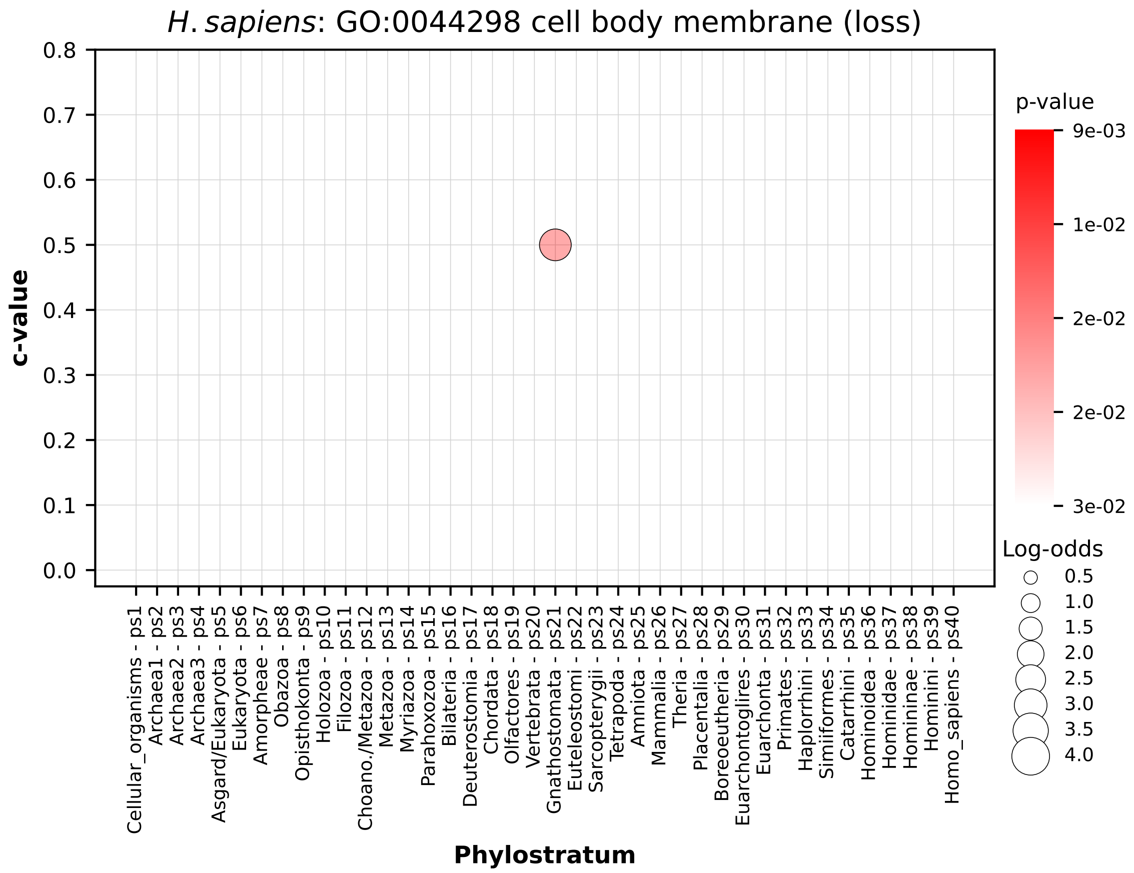
H. sapiens: GO:0044298 cell body membrane (loss)
c-value
0.0
0.1
0.2
0.3
0.4
0.5
0.6
0.7
0.8
Phylostratum
Cellular_organisms - ps1
Archaea1 - ps2
Archaea2 - ps3
Archaea3 - ps4
Asgard/Eukaryota - ps5
Eukaryota - ps6
Amorpheae - ps7
Obazoa - ps8
Opisthokonta - ps9
Holozoa - ps10
Filozoa - ps11
Choano./Metazoa - ps12
Metazoa - ps13
Myriazoa - ps14
Parahoxozoa - ps15
Bilateria - ps16
Deuterostomia - ps17
Chordata - ps18
Olfactores - ps19
Vertebrata - ps20
Gnathostomata - ps21
Euteleostomi - ps22
Sarcopterygii - ps23
Tetrapoda - ps24
Amniota - ps25
Mammalia - ps26
Theria - ps27
Placentalia - ps28
Boreoeutheria - ps29
Euarchontoglires - ps30
Euarchonta - ps31
Primates - ps32
Haplorrhini - ps33
Simiiformes - ps34
Catarrhini - ps35
Hominoidea - ps36
Hominidae - ps37
Homininae - ps38
Hominini - ps39
Homo_sapiens - ps40
p-value
9e-03
1e-02
2e-02
2e-02
3e-02
Log-odds
0.5
1.0
1.5
2.0
2.5
3.0
3.5
4.0

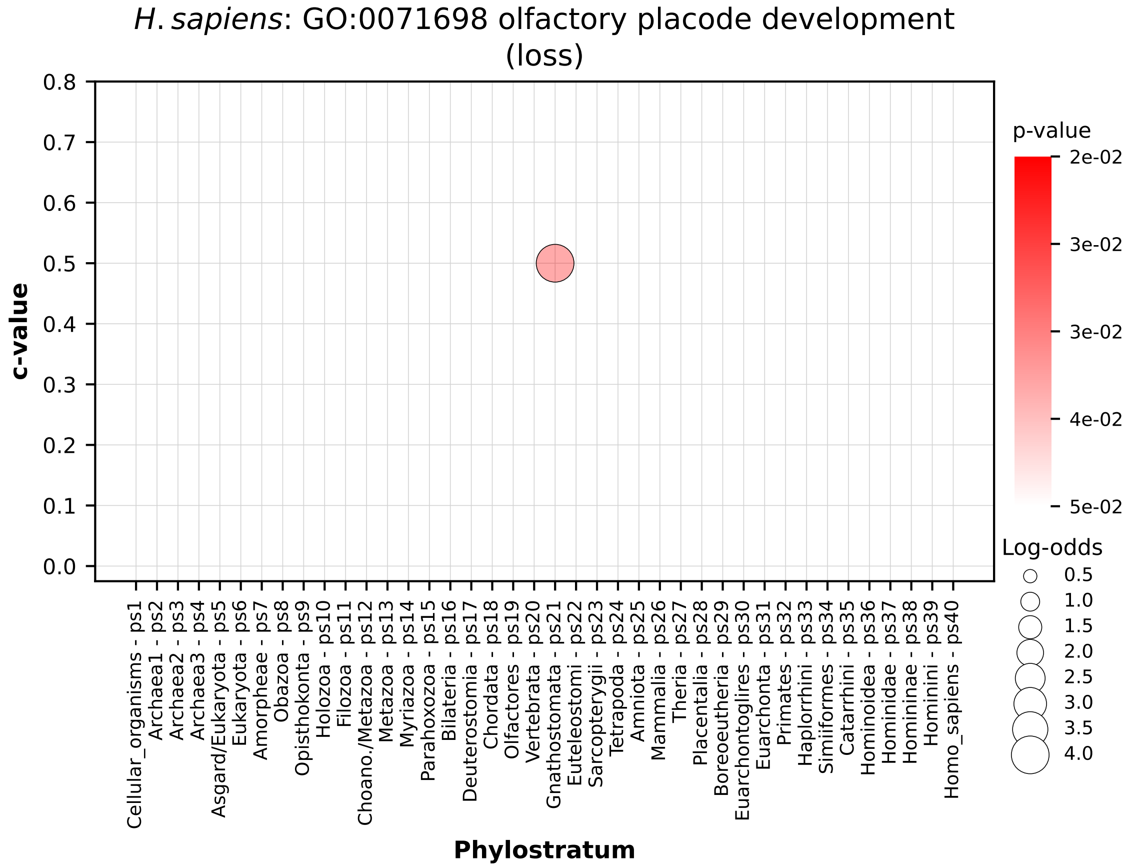

H. sapiens: GO:0071698 olfactory placode development
(loss)
c-value
0.0
0.1
0.2
0.3
0.4
0.5
0.6
0.7
0.8
Phylostratum
Cellular_organisms - ps1
Archaea1 - ps2
Archaea2 - ps3
Archaea3 - ps4
Asgard/Eukaryota - ps5
Eukaryota - ps6
Amorpheae - ps7
Obazoa - ps8
Opisthokonta - ps9
Holozoa - ps10
Filozoa - ps11
Choano./Metazoa - ps12
Metazoa - ps13
Myriazoa - ps14
Parahoxozoa - ps15
Bilateria - ps16
Deuterostomia - ps17
Chordata - ps18
Olfactores - ps19
Vertebrata - ps20
Gnathostomata - ps21
Euteleostomi - ps22
Sarcopterygii - ps23
Tetrapoda - ps24
Amniota - ps25
Mammalia - ps26
Theria - ps27
Placentalia - ps28
Boreoeutheria - ps29
Euarchontoglires - ps30
Euarchonta - ps31
Primates - ps32
Haplorrhini - ps33
Simiiformes - ps34
Catarrhini - ps35
Hominoidea - ps36
Hominidae - ps37
Homininae - ps38
Hominini - ps39
Homo_sapiens - ps40
p-value
2e-02
3e-02
3e-02
4e-02
5e-02
Log-odds
0.5
1.0
1.5
2.0
2.5
3.0
3.5
4.0

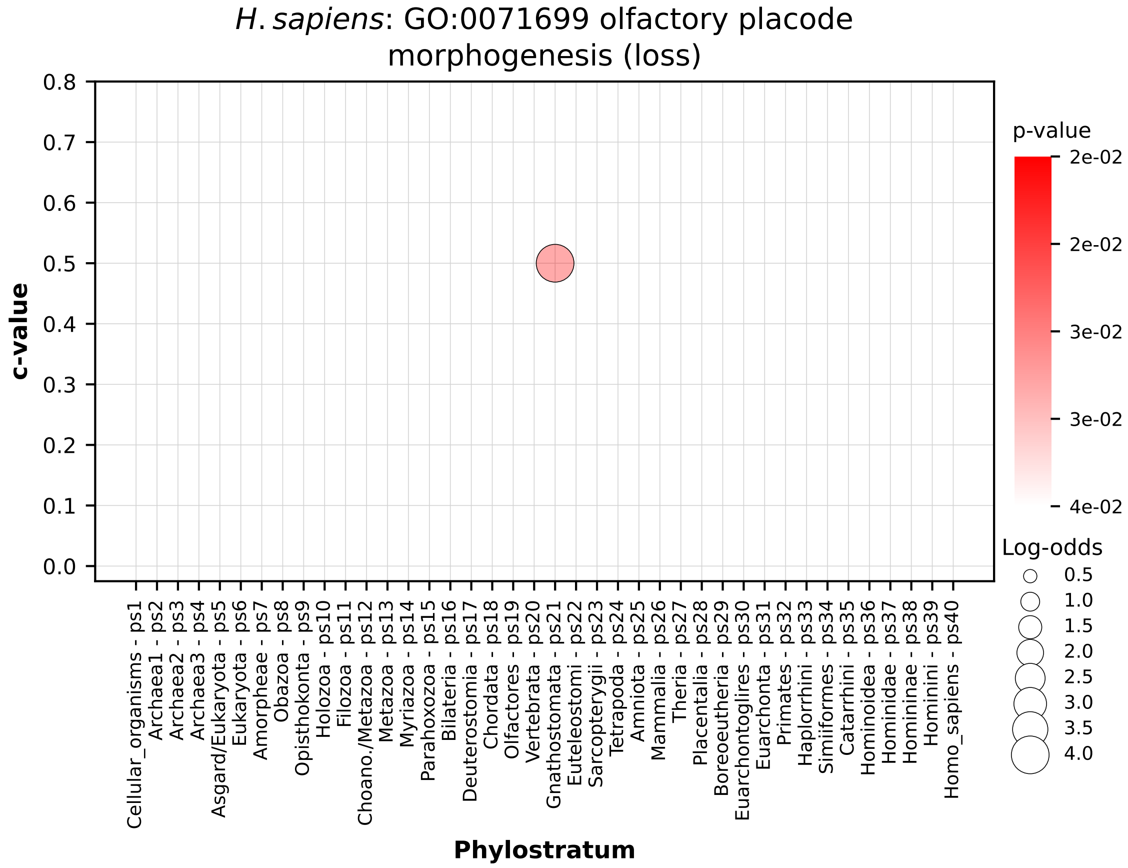

H. sapiens: GO:0071699 olfactory placode morphogenesis (loss)
c-value
0.8
0.7
0.6
0.5
0.4
0.3
0.2
0.1
0.0
p-value
2e-02
2e-02
3e-02
3e-02
4e-02
Log-odds
0.5
1.0
1.5
2.0
2.5
3.0
3.5
4.0
Cellular_organisms - ps1
Archaea1 - ps2
Archaea2 - ps3
Archaea3 - ps4
Asgard/Eukaryota - ps5
Eukaryota - ps6
Amorpheae - ps7
Obazoa - ps8
Opisthokonta - ps9
Holozoa - ps10
Filozoa - ps11
Choano./Metazoa - ps12
Metazoa - ps13
Myriazoa - ps14
Parahoxozoa - ps15
Bilateria - ps16
Deuterostomia - ps17
Chordata - ps18
Olfactores - ps19
Vertebrata - ps20
Gnathostomata - ps21
Euteleostomi - ps22
Sarcopterygii - ps23
Tetrapoda - ps24
Amniota - ps25
Mammalia - ps26
Theria - ps27
Placentalia - ps28
Boreoeutheria - ps29
Euarchontoglires - ps30
Euarchonta - ps31
Primates - ps32
Haplorrhini - ps33
Simiiformes - ps34
Catarrhini - ps35
Hominoidea - ps36
Hominidae - ps37
Homininae - ps38
Hominini - ps39
Homo_sapiens - ps40
Phylostratum

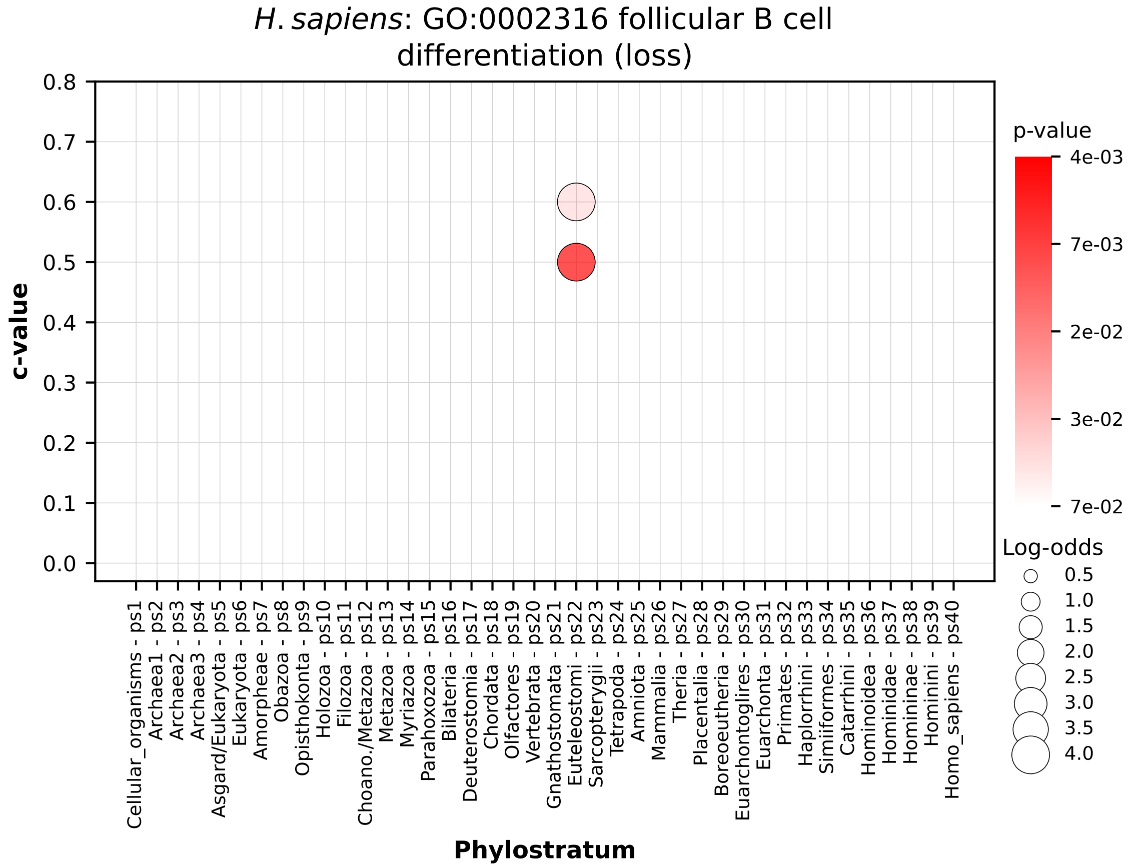

H. sapiens: GO:0002316 follicular B cell differentiation (loss)
c-value
0.8
0.7
0.6
0.5
0.4
0.3
0.2
0.1
0.0
p-value
4e-03
7e-03
2e-02
3e-02
7e-02
Log-odds
0.5
1.0
1.5
2.0
2.5
3.0
3.5
4.0
Cellular_organisms - ps1
Archaea1 - ps2
Archaea2 - ps3
Archaea3 - ps4
Asgard/Eukaryota - ps5
Eukaryota - ps6
Amorpheae - ps7
Obazoa - ps8
Opisthokonta - ps9
Holozoa - ps10
Filozoa - ps11
Choano./Metazoa - ps12
Metazoa - ps13
Myriazoa - ps14
Parahoxozoa - ps15
Bilateria - ps16
Deuterostomia - ps17
Chordata - ps18
Olfactores - ps19
Vertebrata - ps20
Gnathostomata - ps21
Euteleostomi - ps22
Sarcopterygii - ps23
Tetrapoda - ps24
Amniota - ps25
Mammalia - ps26
Theria - ps27
Placentalia - ps28
Boreoeutheria - ps29
Euarchontoglires - ps30
Euarchonta - ps31
Primates - ps32
Haplorrhini - ps33
Simiiformes - ps34
Catarrhini - ps35
Hominoidea - ps36
Hominidae - ps37
Homininae - ps38
Hominini - ps39
Homo_sapiens - ps40
Phylostratum

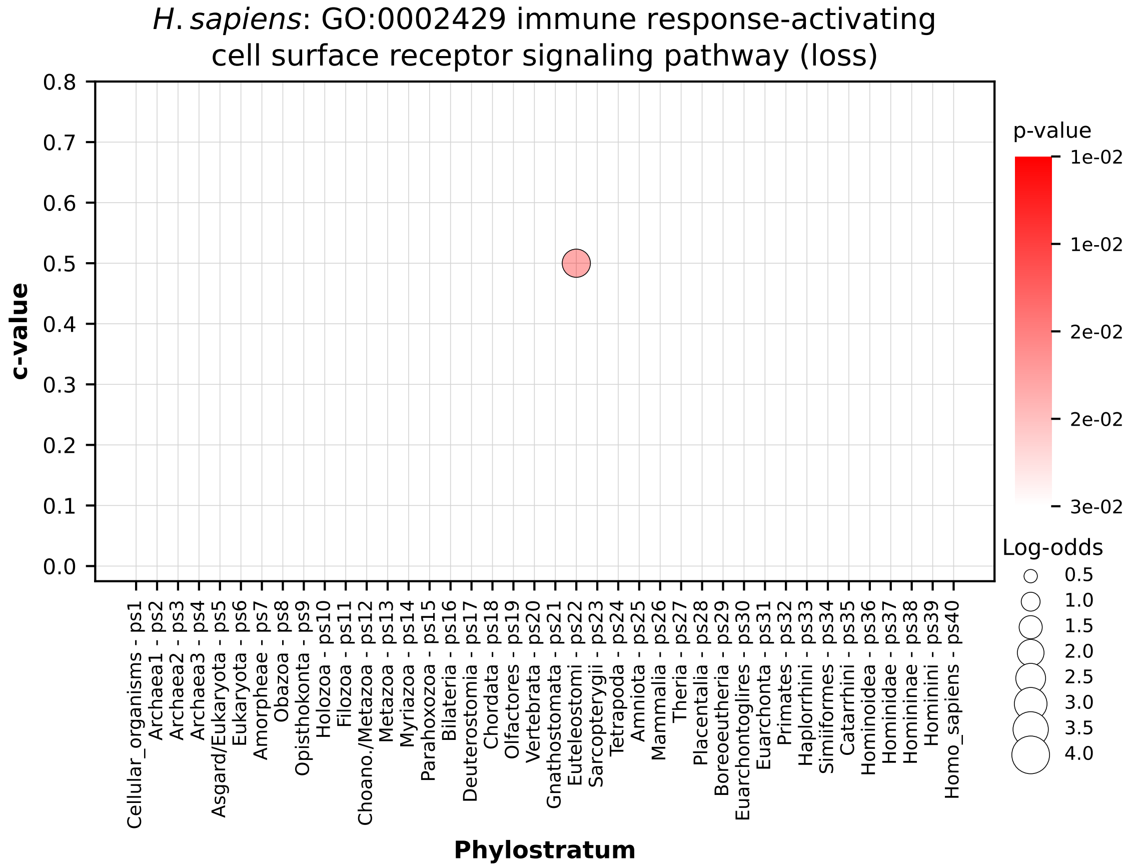

H. sapiens: GO:0002429 immune response-activating cell surface receptor signaling pathway (loss)
c-value
0.0
0.1
0.2
0.3
0.4
0.5
0.6
0.7
0.8
Phylostratum
Cellular_organisms - ps1
Archaea1 - ps2
Archaea2 - ps3
Archaea3 - ps4
Asgard/Eukaryota - ps5
Eukaryota - ps6
Amorpheae - ps7
Obazoa - ps8
Opisthokonta - ps9
Holozoa - ps10
Filozoa - ps11
Choano./Metazoa - ps12
Metazoa - ps13
Myriazoa - ps14
Parahoxozoa - ps15
Bilateria - ps16
Deuterostomia - ps17
Chordata - ps18
Olfactores - ps19
Vertebrata - ps20
Gnathostomata - ps21
Euteleostomi - ps22
Sarcopterygii - ps23
Tetrapoda - ps24
Amniota - ps25
Mammalia - ps26
Theria - ps27
Placentalia - ps28
Boreoeutheria - ps29
Euarchontoglires - ps30
Euarchonta - ps31
Primates - ps32
Haplorrhini - ps33
Simiiformes - ps34
Catarrhini - ps35
Hominoidea - ps36
Hominidae - ps37
Homininae - ps38
Hominini - ps39
Homo_sapiens - ps40
p-value
1e-02
1e-02
2e-02
2e-02
3e-02
Log-odds
0.5
1.0
1.5
2.0
2.5
3.0
3.5
4.0

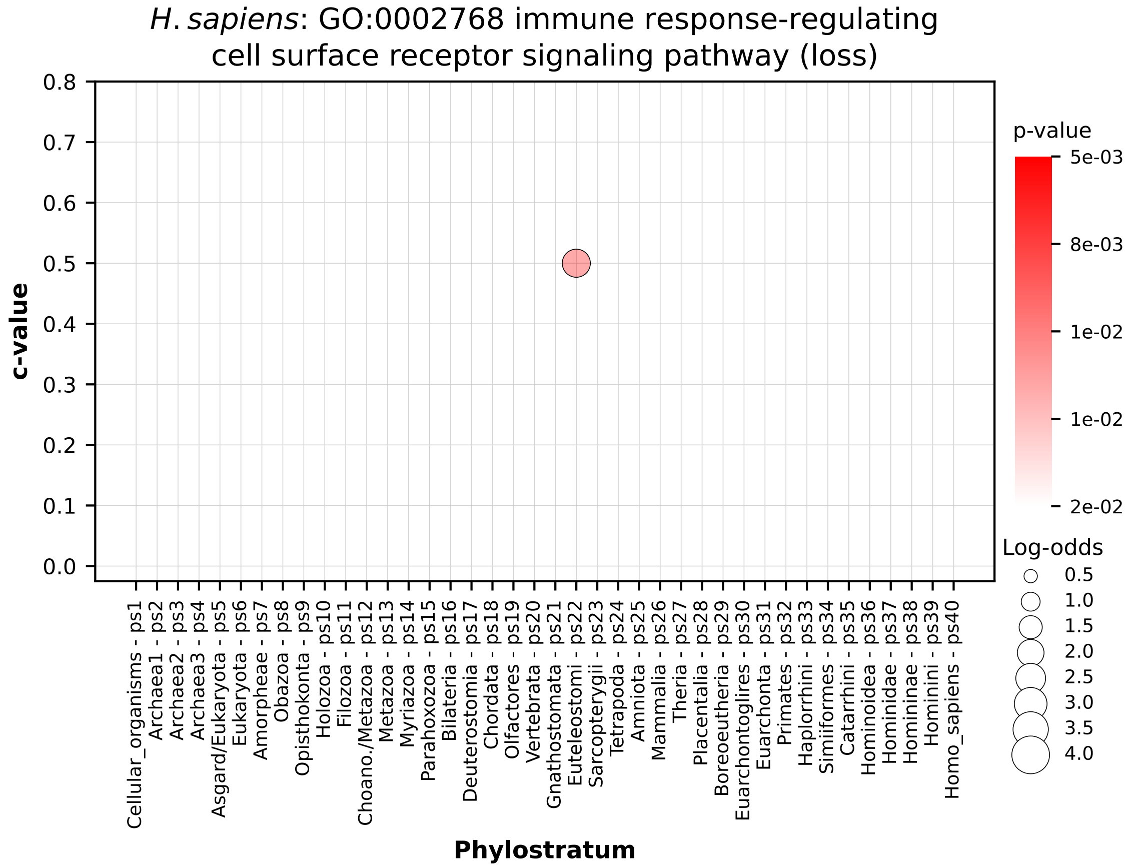

H. sapiens: GO:0002768 immune response-regulating cell surface receptor signaling pathway (loss)
c-value
0.0
0.1
0.2
0.3
0.4
0.5
0.6
0.7
0.8
Phylostratum
Cellular_organisms - ps1
Archaea1 - ps2
Archaea2 - ps3
Archaea3 - ps4
Asgard/Eukaryota - ps5
Eukaryota - ps6
Amorpheae - ps7
Obazoa - ps8
Opisthokonta - ps9
Holozoa - ps10
Filozoa - ps11
Choano./Metazoa - ps12
Metazoa - ps13
Myriazoa - ps14
Parahoxozoa - ps15
Bilateria - ps16
Deuterostomia - ps17
Chordata - ps18
Olfactores - ps19
Vertebrata - ps20
Gnathostomata - ps21
Euteleostomi - ps22
Sarcopterygii - ps23
Tetrapoda - ps24
Amniota - ps25
Mammalia - ps26
Theria - ps27
Placentalia - ps28
Boreoeutheria - ps29
Euarchontoglires - ps30
Euarchonta - ps31
Primates - ps32
Haplorrhini - ps33
Simiiformes - ps34
Catarrhini - ps35
Hominoidea - ps36
Hominidae - ps37
Homininae - ps38
Hominini - ps39
Homo_sapiens - ps40
p-value
5e-03
8e-03
1e-02
1e-02
2e-02
Log-odds
0.5
1.0
1.5
2.0
2.5
3.0
3.5
4.0

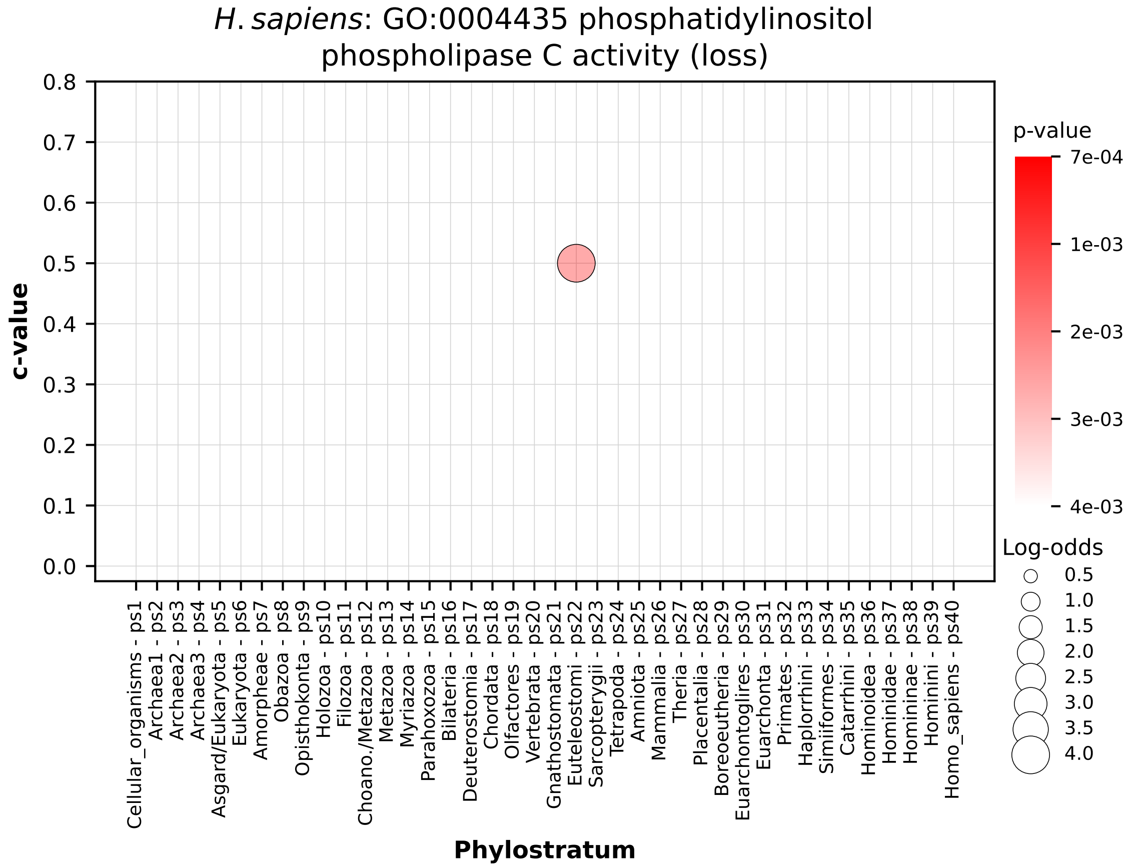

H. sapiens: GO:0004435 phosphatidylinositol phospholipase C activity (loss)
c-value
0.8
0.7
0.6
0.5
0.4
0.3
0.2
0.1
0.0
Phylostratum
Cellular_organisms - ps1
Archaea1 - ps2
Archaea2 - ps3
Archaea3 - ps4
Asgard/Eukaryota - ps5
Eukaryota - ps6
Amorpheae - ps7
Obazoa - ps8
Opisthokonta - ps9
Holozoa - ps10
Filozoa - ps11
Choano./Metazoa - ps12
Metazoa - ps13
Myriazoa - ps14
Parahoxozoa - ps15
Bilateria - ps16
Deuterostomia - ps17
Chordata - ps18
Olfactores - ps19
Vertebrata - ps20
Gnathostomata - ps21
Euteleostomi - ps22
Sarcopterygii - ps23
Tetrapoda - ps24
Amniota - ps25
Mammalia - ps26
Theria - ps27
Placentalia - ps28
Boreoeutheria - ps29
Euarchontoglires - ps30
Euarchonta - ps31
Primates - ps32
Haplorrhini - ps33
Simiiformes - ps34
Catarrhini - ps35
Hominoidea - ps36
Hominidae - ps37
Homininae - ps38
Hominini - ps39
Homo_sapiens - ps40
p-value
7e-04
1e-03
2e-03
3e-03
4e-03
Log-odds
0.5
1.0
1.5
2.0
2.5
3.0
3.5
4.0

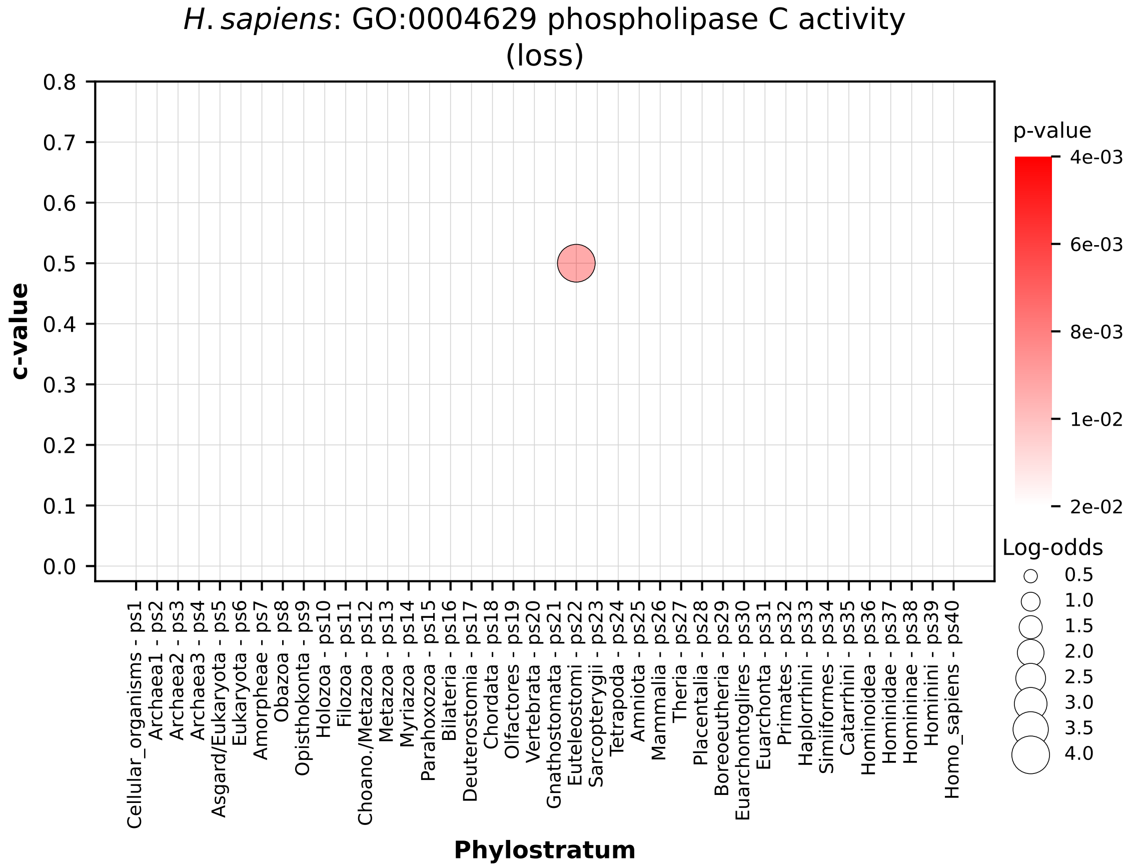
H. sapiens: GO:0004629 phospholipase C activity
(loss)
c-value
0.8
0.7
0.6
0.5
0.4
0.3
0.2
0.1
0.0
p-value
4e-03
6e-03
8e-03
1e-02
2e-02
Log-odds
0.5
1.0
1.5
2.0
2.5
3.0
3.5
4.0
Cellular_organisms - ps1
Archaea1 - ps2
Archaea2 - ps3
Archaea3 - ps4
Asgard/Eukaryota - ps5
Eukaryota - ps6
Amorpheae - ps7
Obazoa - ps8
Opisthokonta - ps9
Holozoa - ps10
Filozoa - ps11
Choano./Metazoa - ps12
Metazoa - ps13
Myriazoa - ps14
Parahoxozoa - ps15
Bilateria - ps16
Deuterostomia - ps17
Chordata - ps18
Olfactores - ps19
Vertebrata - ps20
Gnathostomata - ps21
Euteleostomi - ps22
Sarcopterygii - ps23
Tetrapoda - ps24
Amniota - ps25
Mammalia - ps26
Theria - ps27
Placentalia - ps28
Boreoeutheria - ps29
Euarchontoglires - ps30
Euarchonta - ps31
Primates - ps32
Haplorrhini - ps33
Simiiformes - ps34
Catarrhini - ps35
Hominoidea - ps36
Hominidae - ps37
Homininae - ps38
Hominini - ps39
Homo_sapiens - ps40
Phylostratum

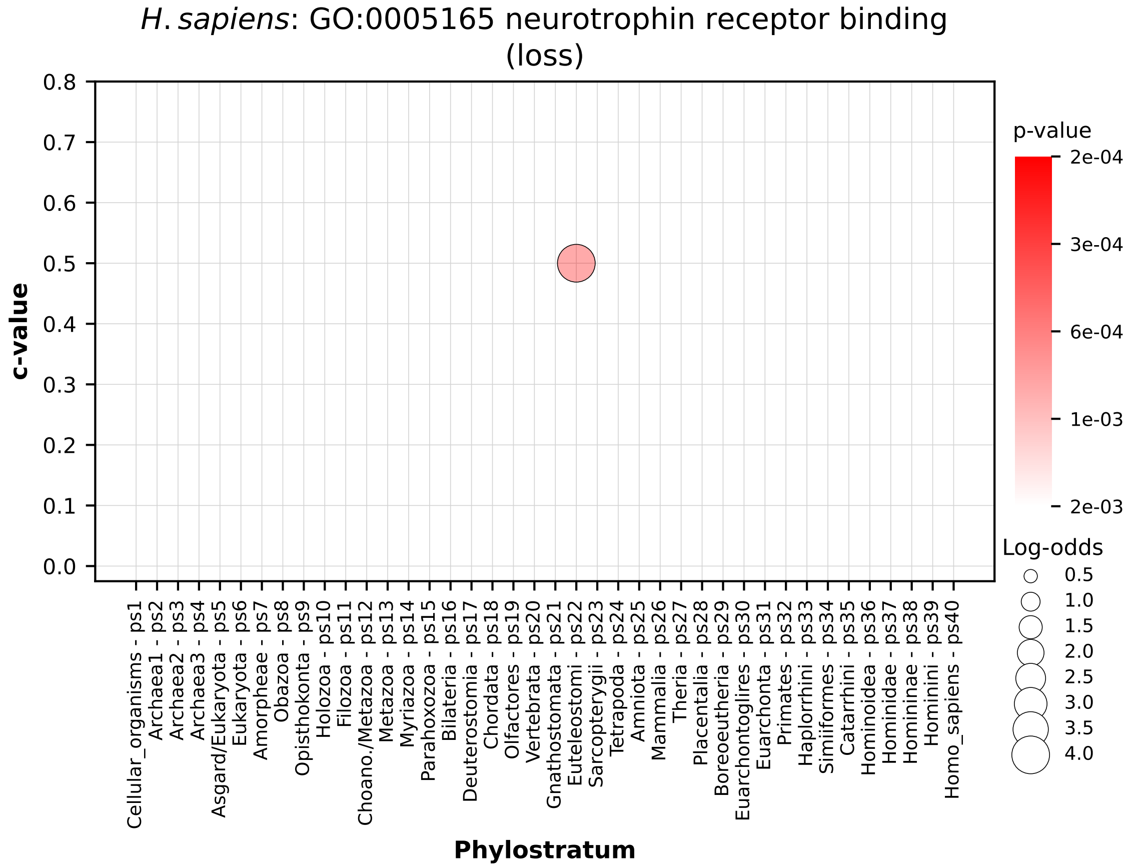

H. sapiens: GO:0005165 neurotrophin receptor binding
(loss)
c-value
0.8
0.7
0.6
0.5
0.4
0.3
0.2
0.1
0.0
Phylostratum
Cellular_organisms - ps1
Archaea1 - ps2
Archaea2 - ps3
Archaea3 - ps4
Asgard/Eukaryota - ps5
Eukaryota - ps6
Amorpheae - ps7
Obazoa - ps8
Opisthokonta - ps9
Holozoa - ps10
Filozoa - ps11
Choano./Metazoa - ps12
Metazoa - ps13
Myriazoa - ps14
Parahoxozoa - ps15
Bilateria - ps16
Deuterostomia - ps17
Chordata - ps18
Olfactores - ps19
Vertebrata - ps20
Gnathostomata - ps21
Euteleostomi - ps22
Sarcopterygii - ps23
Tetrapoda - ps24
Amniota - ps25
Mammalia - ps26
Theria - ps27
Placentalia - ps28
Boreoeutheria - ps29
Euarchontoglires - ps30
Euarchonta - ps31
Primates - ps32
Haplorrhini - ps33
Simiiformes - ps34
Catarrhini - ps35
Hominoidea - ps36
Hominidae - ps37
Homininae - ps38
Hominini - ps39
Homo_sapiens - ps40
p-value
2e-04
3e-04
6e-04
1e-03
2e-03
Log-odds
0.5
1.0
1.5
2.0
2.5
3.0
3.5
4.0

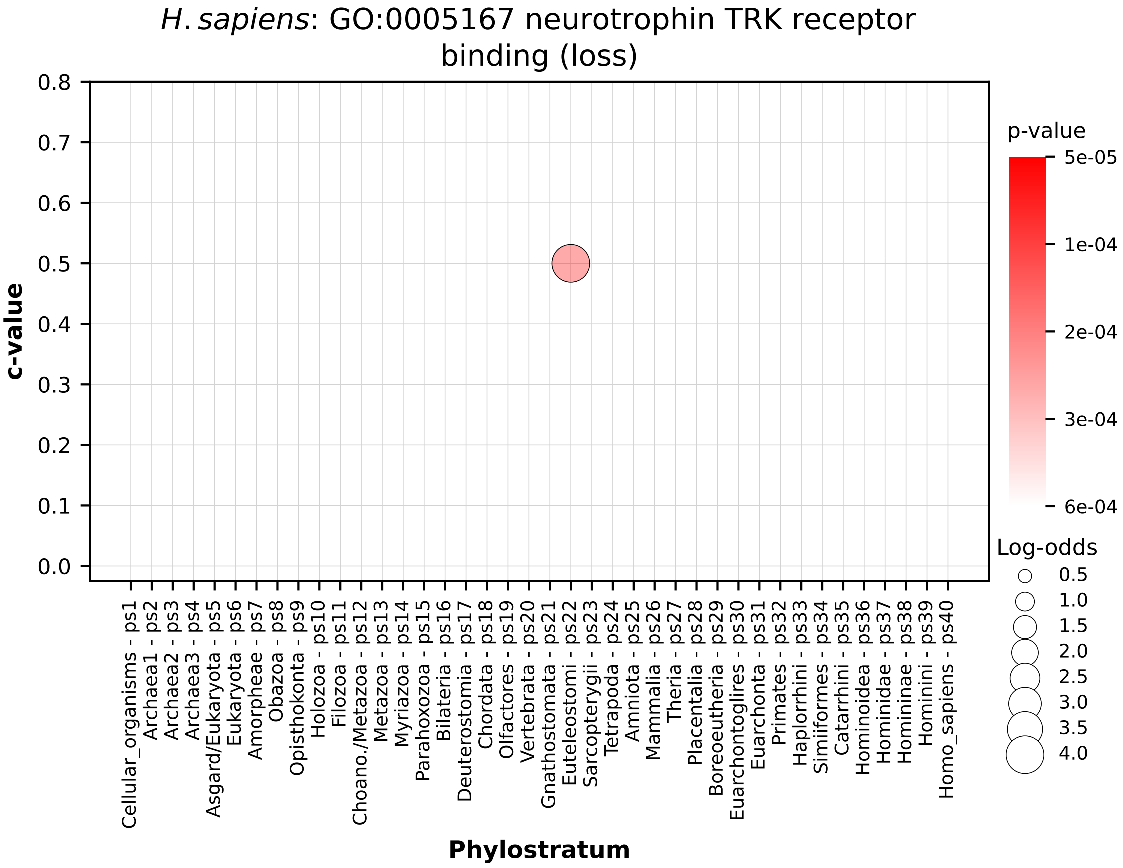

H. sapiens: GO:0005167 neurotrophin TRK receptor binding (loss)
c-value
0.0
0.1
0.2
0.3
0.4
0.5
0.6
0.7
0.8
Phylostratum
Cellular_organisms - ps1
Archaea1 - ps2
Archaea2 - ps3
Archaea3 - ps4
Asgard/Eukaryota - ps5
Eukaryota - ps6
Amorpheae - ps7
Obazoa - ps8
Opisthokonta - ps9
Holozoa - ps10
Filozoa - ps11
Choano./Metazoa - ps12
Metazoa - ps13
Myriazoa - ps14
Parahoxozoa - ps15
Bilateria - ps16
Deuterostomia - ps17
Chordata - ps18
Olfactores - ps19
Vertebrata - ps20
Gnathostomata - ps21
Euteleostomi - ps22
Sarcopterygii - ps23
Tetrapoda - ps24
Amniota - ps25
Mammalia - ps26
Theria - ps27
Placentalia - ps28
Boreoeutheria - ps29
Euarchontoglires - ps30
Euarchonta - ps31
Primates - ps32
Haplorrhini - ps33
Simiiformes - ps34
Catarrhini - ps35
Hominoidea - ps36
Hominidae - ps37
Homininae - ps38
Hominini - ps39
Homo_sapiens - ps40
p-value
5e-05
1e-04
2e-04
3e-04
6e-04
Log-odds
0.5
1.0
1.5
2.0
2.5
3.0
3.5
4.0

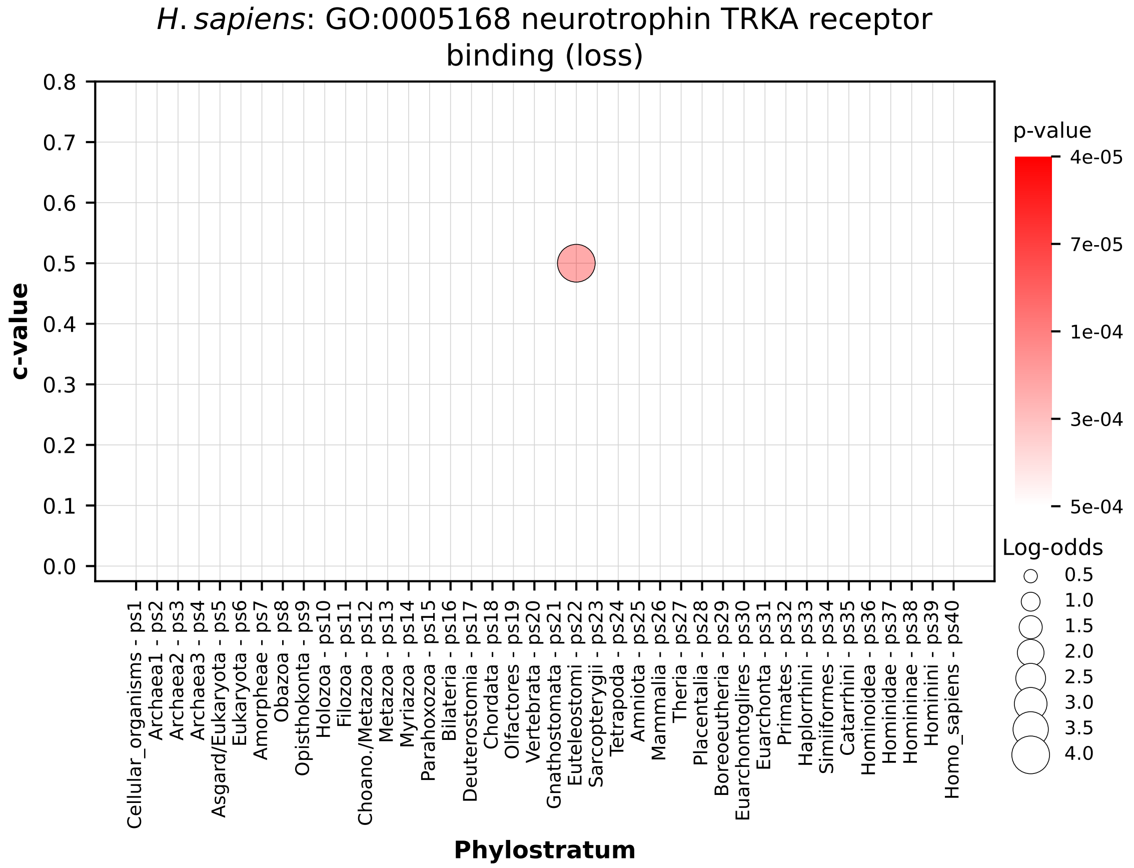

H. sapiens: GO:0005168 neurotrophin TRKA receptor binding (loss)
c-value
0.0
0.1
0.2
0.3
0.4
0.5
0.6
0.7
0.8
Phylostratum
Cellular_organisms - ps1
Archaea1 - ps2
Archaea2 - ps3
Archaea3 - ps4
Asgard/Eukaryota - ps5
Eukaryota - ps6
Amorpheae - ps7
Obazoa - ps8
Opisthokonta - ps9
Holozoa - ps10
Filozoa - ps11
Choano./Metazoa - ps12
Metazoa - ps13
Myriazoa - ps14
Parahoxozoa - ps15
Bilateria - ps16
Deuterostomia - ps17
Chordata - ps18
Olfactores - ps19
Vertebrata - ps20
Gnathostomata - ps21
Euteleostomi - ps22
Sarcopterygii - ps23
Tetrapoda - ps24
Amniota - ps25
Mammalia - ps26
Theria - ps27
Placentalia - ps28
Boreoeutheria - ps29
Euarchontoglires - ps30
Euarchonta - ps31
Primates - ps32
Haplorrhini - ps33
Simiiformes - ps34
Catarrhini - ps35
Hominoidea - ps36
Hominidae - ps37
Homininae - ps38
Hominini - ps39
Homo_sapiens - ps40
p-value
4e-05
7e-05
1e-04
3e-04
5e-04
Log-odds
0.5
1.0
1.5
2.0
2.5
3.0
3.5
4.0

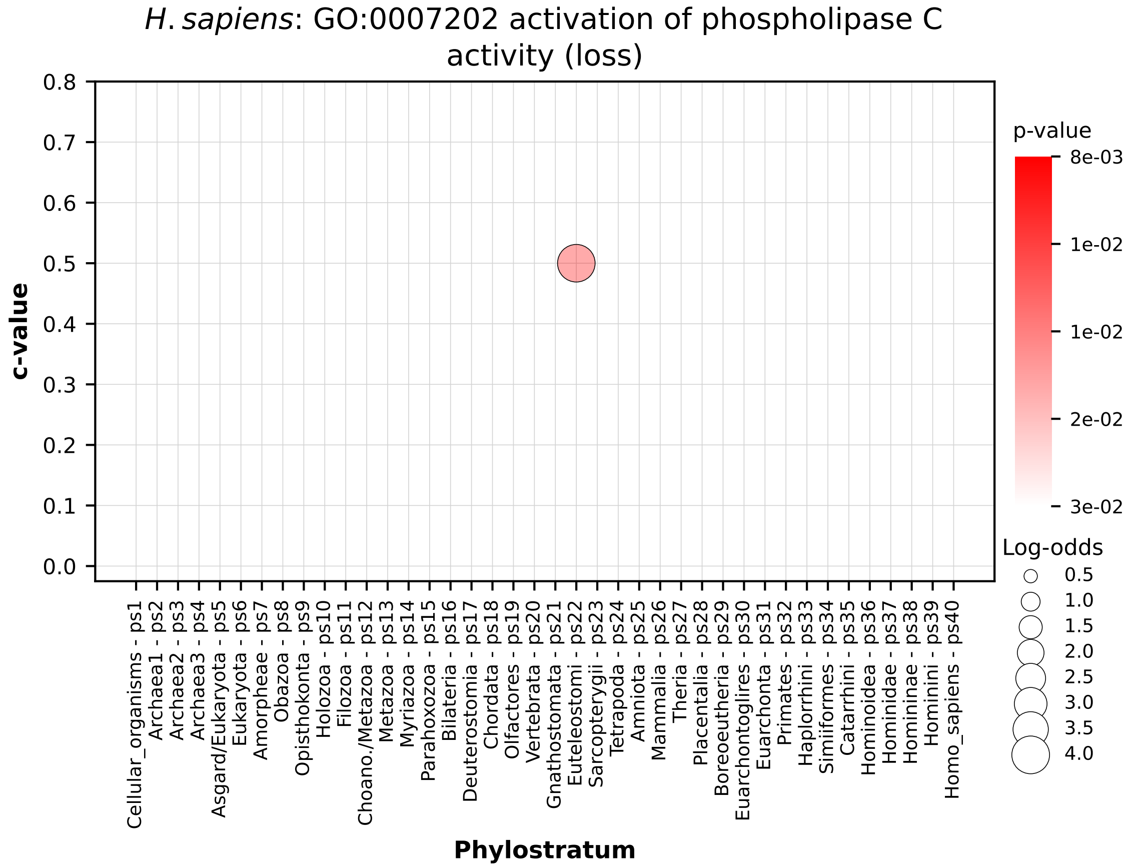

H. sapiens: GO:0007202 activation of phospholipase C activity (loss)
c-value
0.8
0.7
0.6
0.5
0.4
0.3
0.2
0.1
0.0
Phylostratum
Cellular_organisms - ps1
Archaea1 - ps2
Archaea2 - ps3
Archaea3 - ps4
Asgard/Eukaryota - ps5
Eukaryota - ps6
Amorpheae - ps7
Obazoa - ps8
Opisthokonta - ps9
Holozoa - ps10
Filozoa - ps11
Choano./Metazoa - ps12
Metazoa - ps13
Myriazoa - ps14
Parahoxozoa - ps15
Bilateria - ps16
Deuterostomia - ps17
Chordata - ps18
Olfactores - ps19
Vertebrata - ps20
Gnathostomata - ps21
Euteleostomi - ps22
Sarcopterygii - ps23
Tetrapoda - ps24
Amniota - ps25
Mammalia - ps26
Theria - ps27
Placentalia - ps28
Boreoeutheria - ps29
Euarchontoglires - ps30
Euarchonta - ps31
Primates - ps32
Haplorrhini - ps33
Simiiformes - ps34
Catarrhini - ps35
Hominoidea - ps36
Hominidae - ps37
Homininae - ps38
Hominini - ps39
Homo_sapiens - ps40
p-value
8e-03
1e-02
1e-02
2e-02
3e-02
Log-odds
0.5
1.0
1.5
2.0
2.5
3.0
3.5
4.0

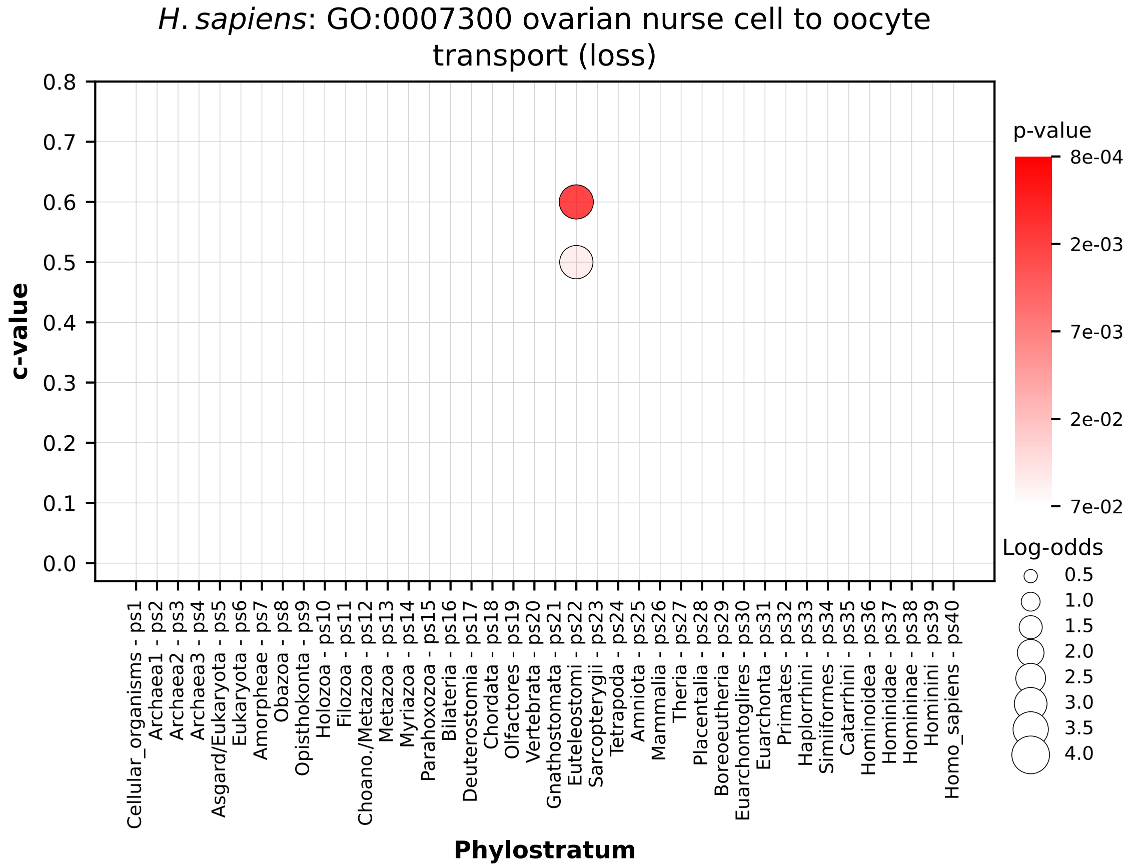

H. sapiens: GO:0007300 ovarian nurse cell to oocyte transport (loss)
c-value
0.0
0.1
0.2
0.3
0.4
0.5
0.6
0.7
0.8
Phylostratum
Cellular_organisms - ps1
Archaea1 - ps2
Archaea2 - ps3
Archaea3 - ps4
Asgard/Eukaryota - ps5
Eukaryota - ps6
Amorpheae - ps7
Obazoa - ps8
Opisthokonta - ps9
Holozoa - ps10
Filozoa - ps11
Choano./Metazoa - ps12
Metazoa - ps13
Myriazoa - ps14
Parahoxozoa - ps15
Bilateria - ps16
Deuterostomia - ps17
Chordata - ps18
Olfactores - ps19
Vertebrata - ps20
Gnathostomata - ps21
Euteleostomi - ps22
Sarcopterygii - ps23
Tetrapoda - ps24
Amniota - ps25
Mammalia - ps26
Theria - ps27
Placentalia - ps28
Boreoeutheria - ps29
Euarchontoglires - ps30
Euarchonta - ps31
Primates - ps32
Haplorrhini - ps33
Simiiformes - ps34
Catarrhini - ps35
Hominoidea - ps36
Hominidae - ps37
Homininae - ps38
Hominini - ps39
Homo_sapiens - ps40
p-value
8e-04
2e-03
7e-03
2e-02
7e-02
Log-odds
0.5
1.0
1.5
2.0
2.5
3.0
3.5
4.0

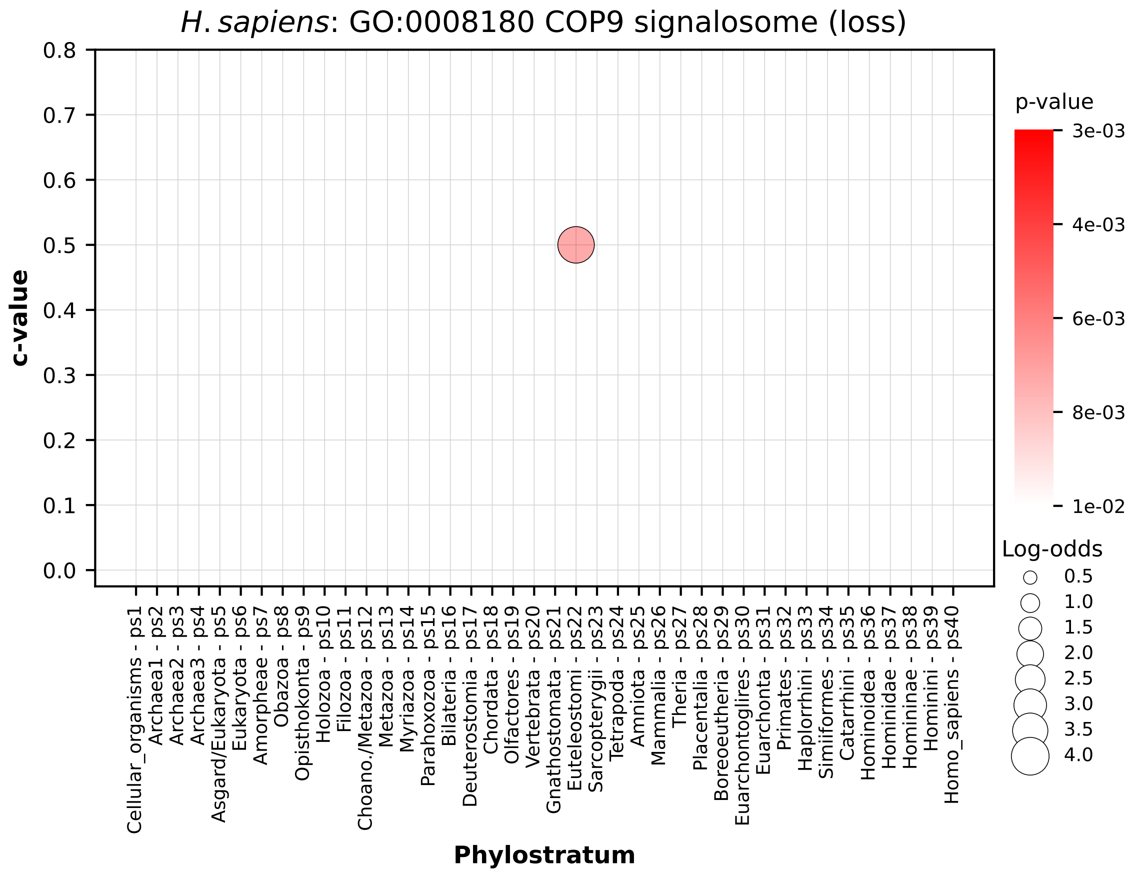
H. sapiens: GO:0008180 COP9 signalosome (loss)
c-value
0.8
0.7
0.6
0.5
0.4
0.3
0.2
0.1
0.0
Phylostratum
Cellular_organisms - ps1
Archaea1 - ps2
Archaea2 - ps3
Archaea3 - ps4
Asgard/Eukaryota - ps5
Eukaryota - ps6
Amorpheae - ps7
Obazoa - ps8
Opisthokonta - ps9
Holozoa - ps10
Filozoa - ps11
Choano./Metazoa - ps12
Metazoa - ps13
Myriazoa - ps14
Parahoxozoa - ps15
Bilateria - ps16
Deuterostomia - ps17
Chordata - ps18
Olfactores - ps19
Vertebrata - ps20
Gnathostomata - ps21
Euteleostomi - ps22
Sarcopterygii - ps23
Tetrapoda - ps24
Amniota - ps25
Mammalia - ps26
Theria - ps27
Placentalia - ps28
Boreoeutheria - ps29
Euarchontoglires - ps30
Euarchonta - ps31
Primates - ps32
Haplorrhini - ps33
Simiiformes - ps34
Catarrhini - ps35
Hominoidea - ps36
Hominidae - ps37
Homininae - ps38
Hominini - ps39
Homo_sapiens - ps40
p-value
3e-03
4e-03
6e-03
8e-03
1e-02
Log-odds
0.5
1.0
1.5
2.0
2.5
3.0
3.5
4.0

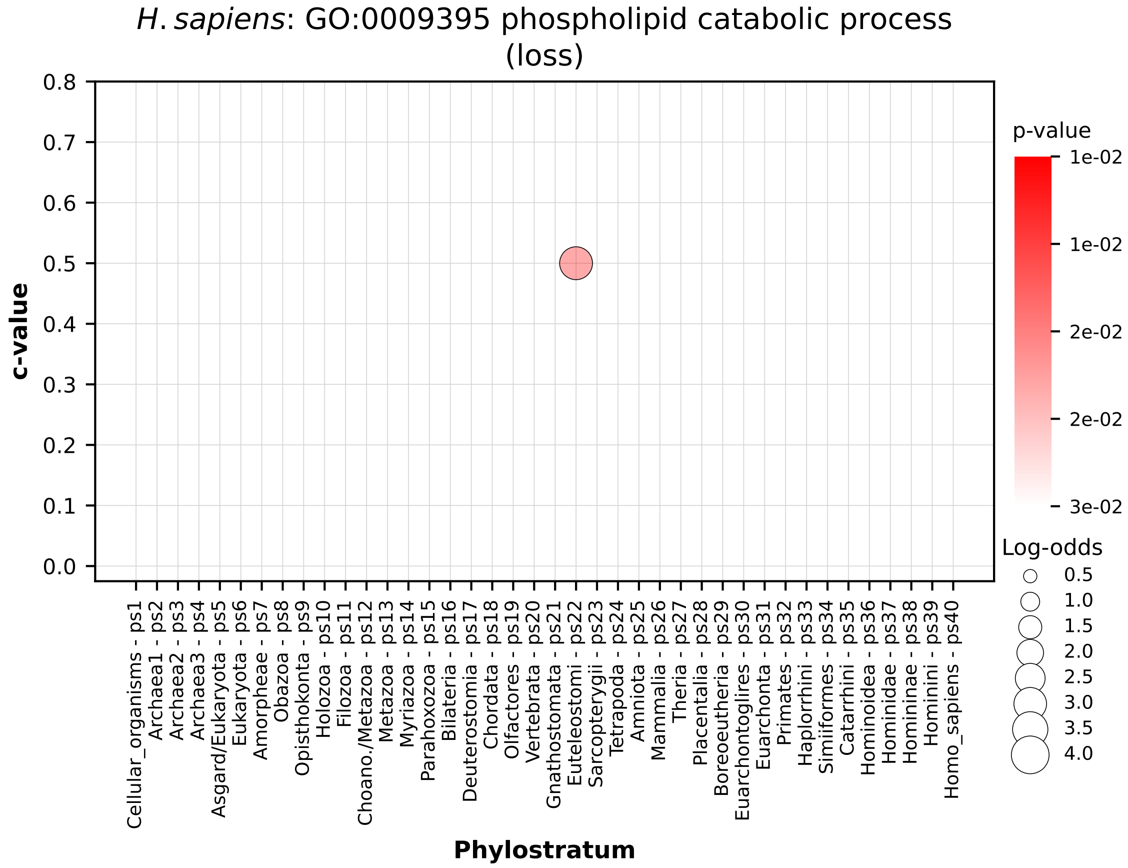

H. sapiens: GO:0009395 phospholipid catabolic process
(loss)
c-value
0.8
0.7
0.6
0.5
0.4
0.3
0.2
0.1
0.0
p-value
1e-02
1e-02
2e-02
2e-02
3e-02
Log-odds
0.5
1.0
1.5
2.0
2.5
3.0
3.5
4.0
Cellular_organisms - ps1
Archaea1 - ps2
Archaea2 - ps3
Archaea3 - ps4
Asgard/Eukaryota - ps5
Eukaryota - ps6
Amorpheae - ps7
Obazoa - ps8
Opisthokonta - ps9
Holozoa - ps10
Filozoa - ps11
Choano./Metazoa - ps12
Metazoa - ps13
Myriazoa - ps14
Parahoxozoa - ps15
Bilateria - ps16
Deuterostomia - ps17
Chordata - ps18
Olfactores - ps19
Vertebrata - ps20
Gnathostomata - ps21
Euteleostomi - ps22
Sarcopterygii - ps23
Tetrapoda - ps24
Amniota - ps25
Mammalia - ps26
Theria - ps27
Placentalia - ps28
Boreoeutheria - ps29
Euarchontoglires - ps30
Euarchonta - ps31
Primates - ps32
Haplorrhini - ps33
Simiiformes - ps34
Catarrhini - ps35
Hominoidea - ps36
Hominidae - ps37
Homininae - ps38
Hominini - ps39
Homo_sapiens - ps40
Phylostratum

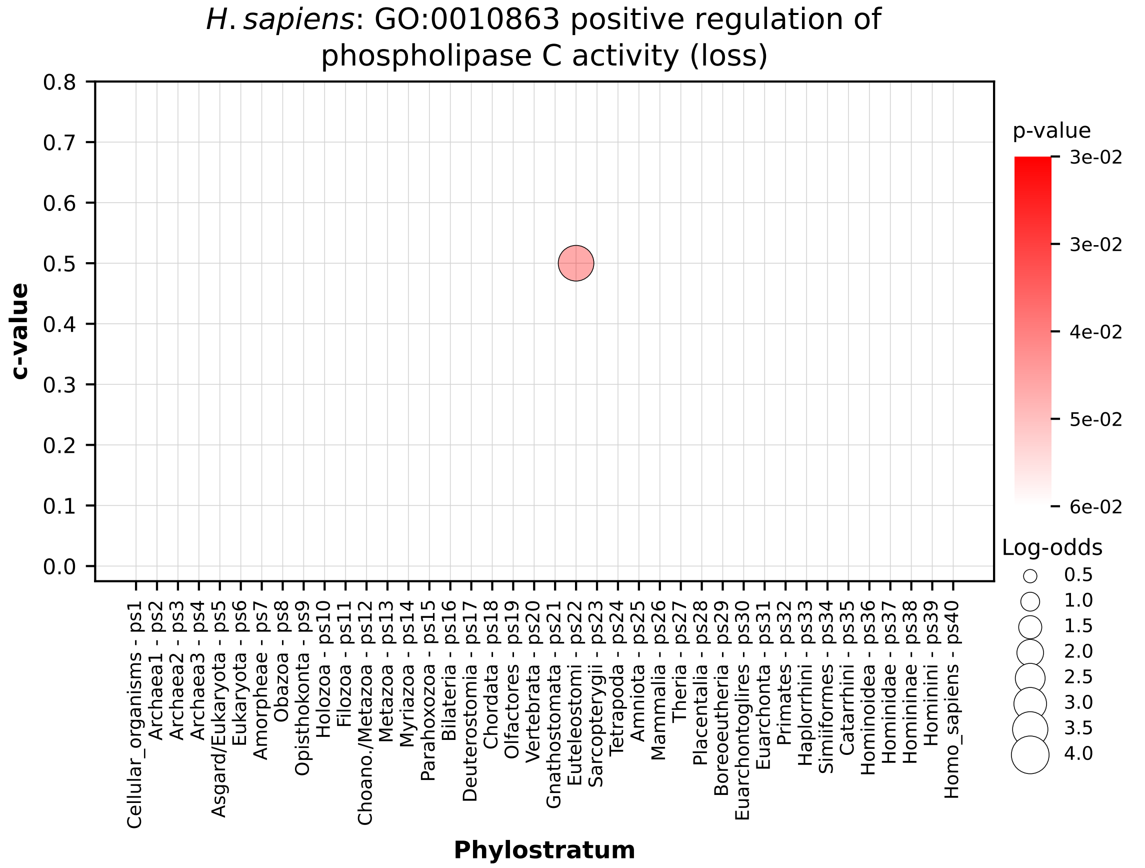

H. sapiens: GO:0010863 positive regulation of phospholipase C activity (loss)
c-value
0.8
0.7
0.6
0.5
0.4
0.3
0.2
0.1
0.0
Cellular_organisms - ps1
Archaea1 - ps2
Archaea2 - ps3
Archaea3 - ps4
Asgard/Eukaryota - ps5
Eukaryota - ps6
Amorpheae - ps7
Obazoa - ps8
Opisthokonta - ps9
Holozoa - ps10
Filozoa - ps11
Choano./Metazoa - ps12
Metazoa - ps13
Myriazoa - ps14
Parahoxozoa - ps15
Bilateria - ps16
Deuterostomia - ps17
Chordata - ps18
Olfactores - ps19
Vertebrata - ps20
Gnathostomata - ps21
Euteleostomi - ps22
Sarcopterygii - ps23
Tetrapoda - ps24
Amniota - ps25
Mammalia - ps26
Theria - ps27
Placentalia - ps28
Boreoeutheria - ps29
Euarchontoglires - ps30
Euarchonta - ps31
Primates - ps32
Haplorrhini - ps33
Simiiformes - ps34
Catarrhini - ps35
Hominoidea - ps36
Hominidae - ps37
Homininae - ps38
Hominini - ps39
Homo_sapiens - ps40
Phylostratum
p-value
3e-02
3e-02
4e-02
5e-02
6e-02
Log-odds
0.5
1.0
1.5
2.0
2.5
3.0
3.5
4.0

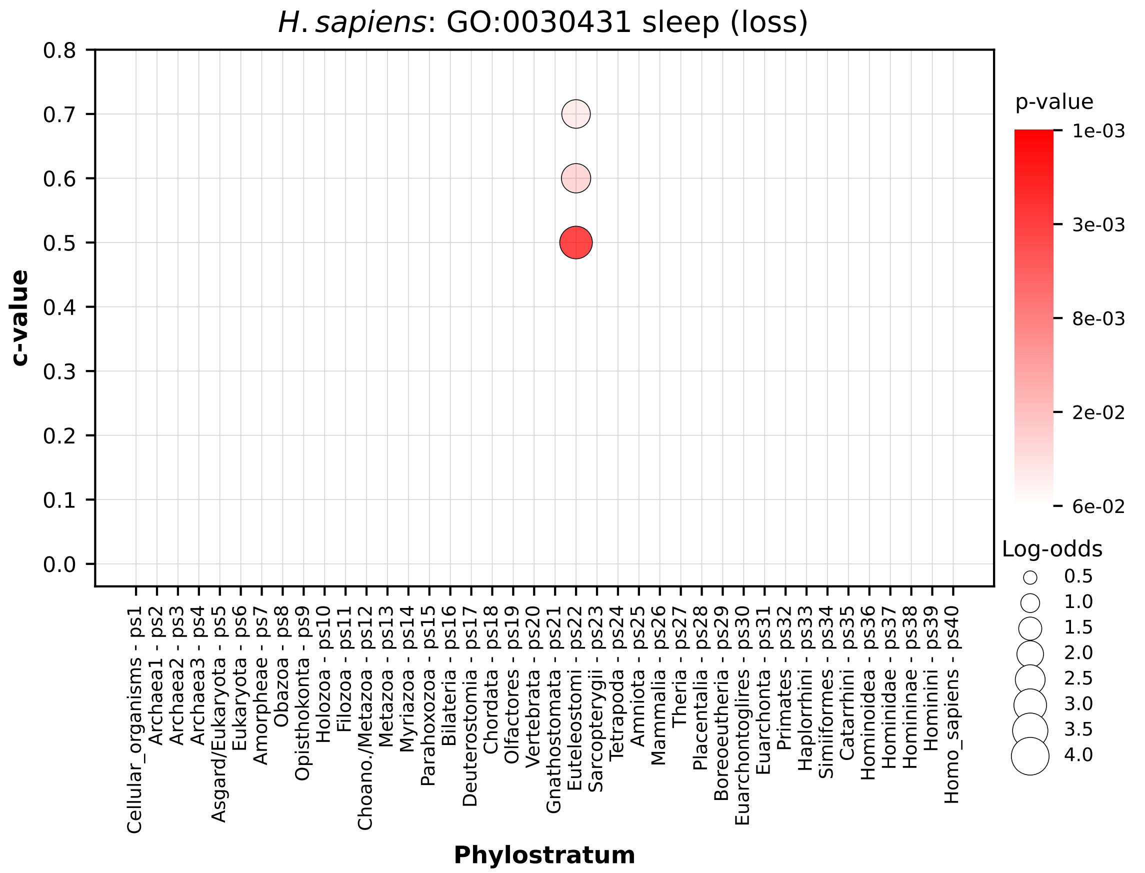

H. sapiens: GO:0030431 sleep (loss)
c-value
0.8
0.7
0.6
0.5
0.4
0.3
0.2
0.1
0.0
Phylostratum
Cellular_organisms - ps1
Archaea1 - ps2
Archaea2 - ps3
Archaea3 - ps4
Asgard/Eukaryota - ps5
Eukaryota - ps6
Amorpheae - ps7
Obazoa - ps8
Opisthokonta - ps9
Holozoa - ps10
Filozoa - ps11
Choano./Metazoa - ps12
Metazoa - ps13
Myriazoa - ps14
Parahoxozoa - ps15
Bilateria - ps16
Deuterostomia - ps17
Chordata - ps18
Olfactores - ps19
Vertebrata - ps20
Gnathostomata - ps21
Euteleostomi - ps22
Sarcopterygii - ps23
Tetrapoda - ps24
Amniota - ps25
Mammalia - ps26
Theria - ps27
Placentalia - ps28
Boreoeutheria - ps29
Euarchontoglires - ps30
Euarchonta - ps31
Primates - ps32
Haplorrhini - ps33
Simiiformes - ps34
Catarrhini - ps35
Hominoidea - ps36
Hominidae - ps37
Homininae - ps38
Hominini - ps39
Homo_sapiens - ps40
p-value
1e-03
3e-03
8e-03
2e-02
6e-02
Log-odds
0.5
1.0
1.5
2.0
2.5
3.0
3.5
4.0

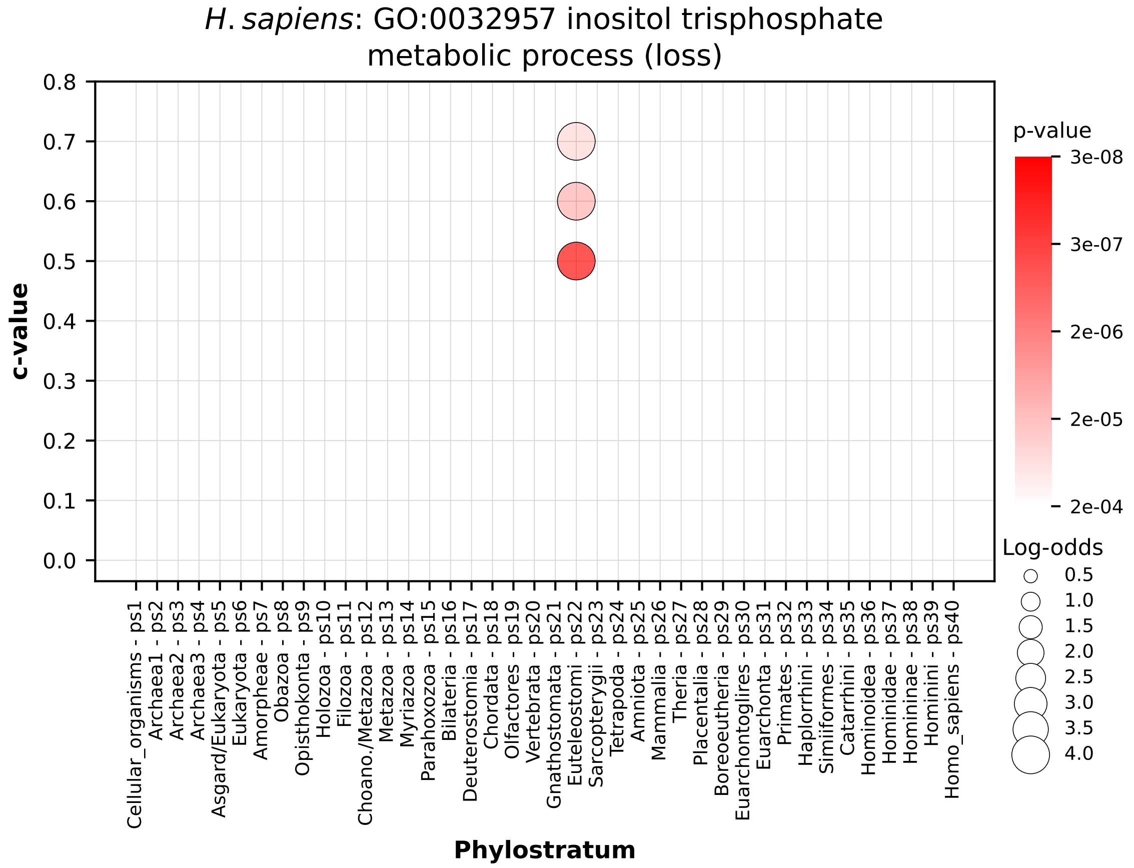
H. sapiens: GO:0032957 inositol trisphosphate metabolic process (loss)
c-value
0.0
0.1
0.2
0.3
0.4
0.5
0.6
0.7
0.8
Phylostratum
Cellular_organisms - ps1
Archaea1 - ps2
Archaea2 - ps3
Archaea3 - ps4
Asgard/Eukaryota - ps5
Eukaryota - ps6
Amorpheae - ps7
Obazoa - ps8
Opisthokonta - ps9
Holozoa - ps10
Filozoa - ps11
Choano./Metazoa - ps12
Metazoa - ps13
Myriazoa - ps14
Parahoxozoa - ps15
Bilateria - ps16
Deuterostomia - ps17
Chordata - ps18
Olfactores - ps19
Vertebrata - ps20
Gnathostomata - ps21
Euteleostomi - ps22
Sarcopterygii - ps23
Tetrapoda - ps24
Amniota - ps25
Mammalia - ps26
Theria - ps27
Placentalia - ps28
Boreoeutheria - ps29
Euarchontoglires - ps30
Euarchonta - ps31
Primates - ps32
Haplorrhini - ps33
Simiiformes - ps34
Catarrhini - ps35
Hominoidea - ps36
Hominidae - ps37
Homininae - ps38
Hominini - ps39
Homo_sapiens - ps40
p-value
3e-08
3e-07
2e-06
2e-05
2e-04
Log-odds
0.5
1.0
1.5
2.0
2.5
3.0
3.5
4.0

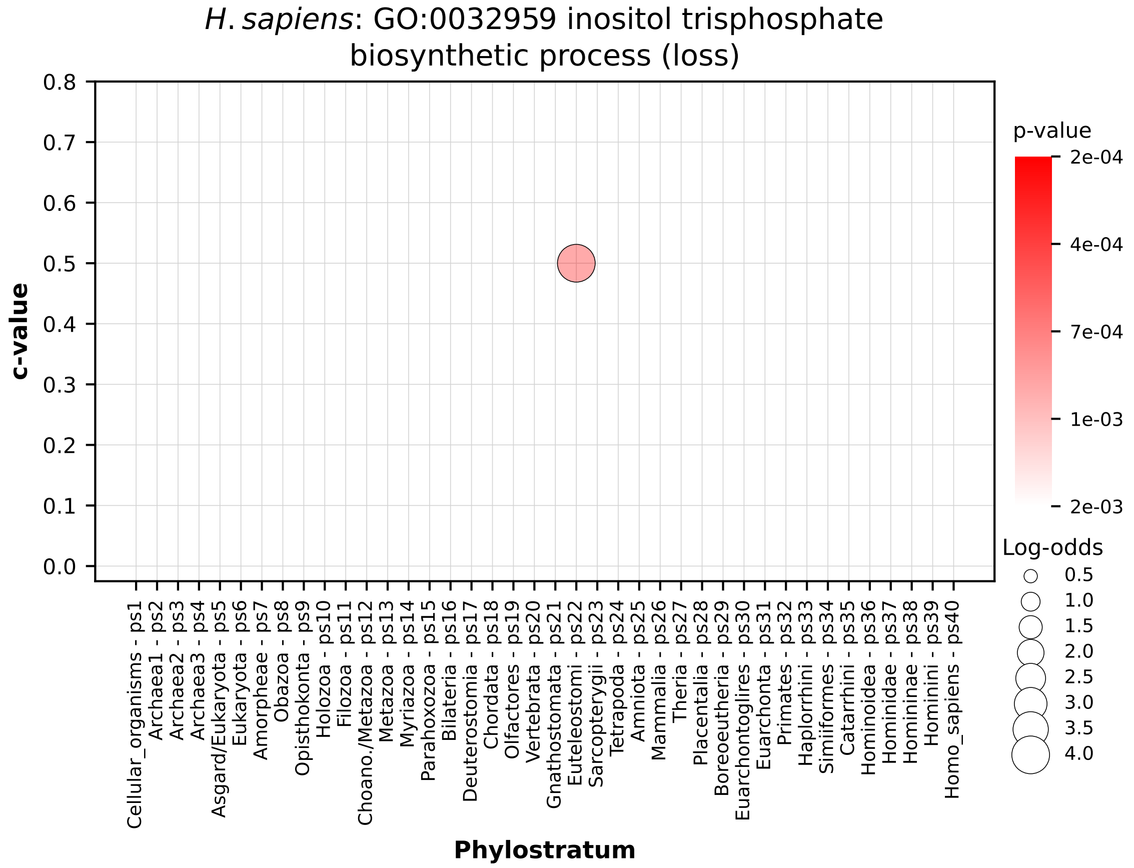

H. sapiens: GO:0032959 inositol trisphosphate biosynthetic process (loss)
c-value
0.8
0.7
0.6
0.5
0.4
0.3
0.2
0.1
0.0
Phylostratum
Cellular_organisms - ps1
Archaea1 - ps2
Archaea2 - ps3
Archaea3 - ps4
Asgard/Eukaryota - ps5
Eukaryota - ps6
Amorpheae - ps7
Obazoa - ps8
Opisthokonta - ps9
Holozoa - ps10
Filozoa - ps11
Choano./Metazoa - ps12
Metazoa - ps13
Myriazoa - ps14
Parahoxozoa - ps15
Bilateria - ps16
Deuterostomia - ps17
Chordata - ps18
Olfactores - ps19
Vertebrata - ps20
Gnathostomata - ps21
Euteleostomi - ps22
Sarcopterygii - ps23
Tetrapoda - ps24
Amniota - ps25
Mammalia - ps26
Theria - ps27
Placentalia - ps28
Boreoeutheria - ps29
Euarchontoglires - ps30
Euarchonta - ps31
Primates - ps32
Haplorrhini - ps33
Simiiformes - ps34
Catarrhini - ps35
Hominoidea - ps36
Hominidae - ps37
Homininae - ps38
Hominini - ps39
Homo_sapiens - ps40
p-value
2e-04
4e-04
7e-04
1e-03
2e-03
Log-odds
0.5
1.0
1.5
2.0
2.5
3.0
3.5
4.0

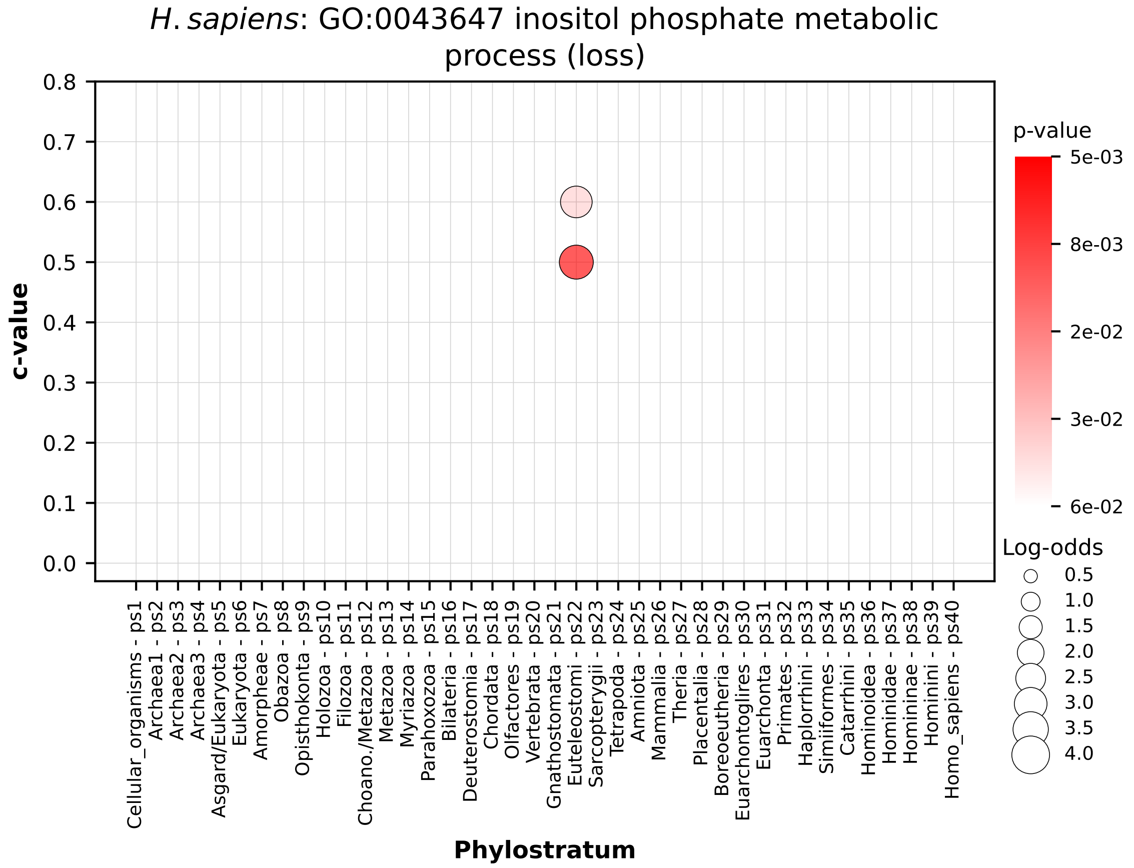

H. sapiens: GO:0043647 inositol phosphate metabolic process (loss)
c-value
0.8
0.7
0.6
0.5
0.4
0.3
0.2
0.1
0.0
Phylostratum
Cellular_organisms - ps1
Archaea1 - ps2
Archaea2 - ps3
Archaea3 - ps4
Asgard/Eukaryota - ps5
Eukaryota - ps6
Amorpheae - ps7
Obazoa - ps8
Opisthokonta - ps9
Holozoa - ps10
Filozoa - ps11
Choano./Metazoa - ps12
Metazoa - ps13
Myriazoa - ps14
Parahoxozoa - ps15
Bilateria - ps16
Deuterostomia - ps17
Chordata - ps18
Olfactores - ps19
Vertebrata - ps20
Gnathostomata - ps21
Euteleostomi - ps22
Sarcopterygii - ps23
Tetrapoda - ps24
Amniota - ps25
Mammalia - ps26
Theria - ps27
Placentalia - ps28
Boreoeutheria - ps29
Euarchontoglires - ps30
Euarchonta - ps31
Primates - ps32
Haplorrhini - ps33
Simiiformes - ps34
Catarrhini - ps35
Hominoidea - ps36
Hominidae - ps37
Homininae - ps38
Hominini - ps39
Homo_sapiens - ps40
p-value
5e-03
8e-03
2e-02
3e-02
6e-02
Log-odds
0.5
1.0
1.5
2.0
2.5
3.0
3.5
4.0

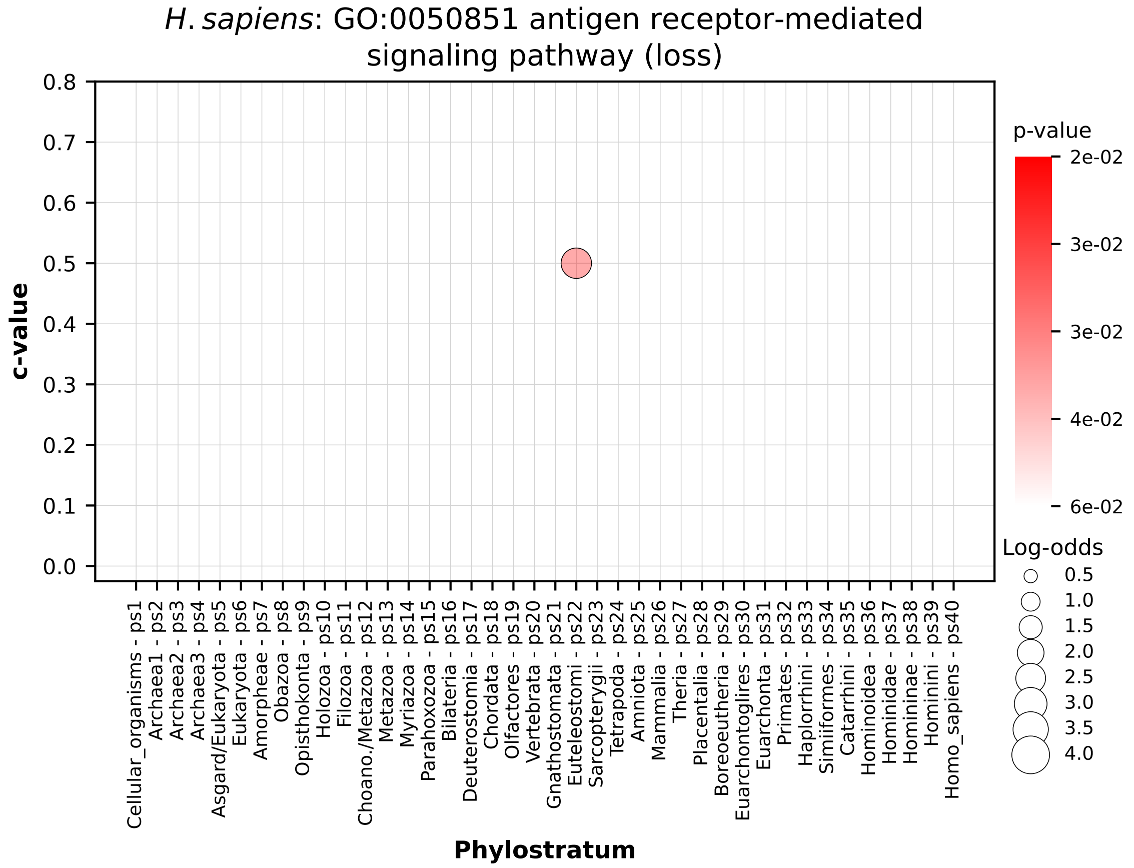

H. sapiens: GO:0050851 antigen receptor-mediated signaling pathway (loss)
c-value
0.0
0.1
0.2
0.3
0.4
0.5
0.6
0.7
0.8
Phylostratum
Cellular_organisms - ps1
Archaea1 - ps2
Archaea2 - ps3
Archaea3 - ps4
Asgard/Eukaryota - ps5
Eukaryota - ps6
Amorpheae - ps7
Obazoa - ps8
Opisthokonta - ps9
Holozoa - ps10
Filozoa - ps11
Choano./Metazoa - ps12
Metazoa - ps13
Myriazoa - ps14
Parahoxozoa - ps15
Bilateria - ps16
Deuterostomia - ps17
Chordata - ps18
Olfactores - ps19
Vertebrata - ps20
Gnathostomata - ps21
Euteleostomi - ps22
Sarcopterygii - ps23
Tetrapoda - ps24
Amniota - ps25
Mammalia - ps26
Theria - ps27
Placentalia - ps28
Boreoeutheria - ps29
Euarchontoglires - ps30
Euarchonta - ps31
Primates - ps32
Haplorrhini - ps33
Simiiformes - ps34
Catarrhini - ps35
Hominoidea - ps36
Hominidae - ps37
Homininae - ps38
Hominini - ps39
Homo_sapiens - ps40
p-value
2e-02
3e-02
3e-02
4e-02
6e-02
Log-odds
0.5
1.0
1.5
2.0
2.5
3.0
3.5
4.0

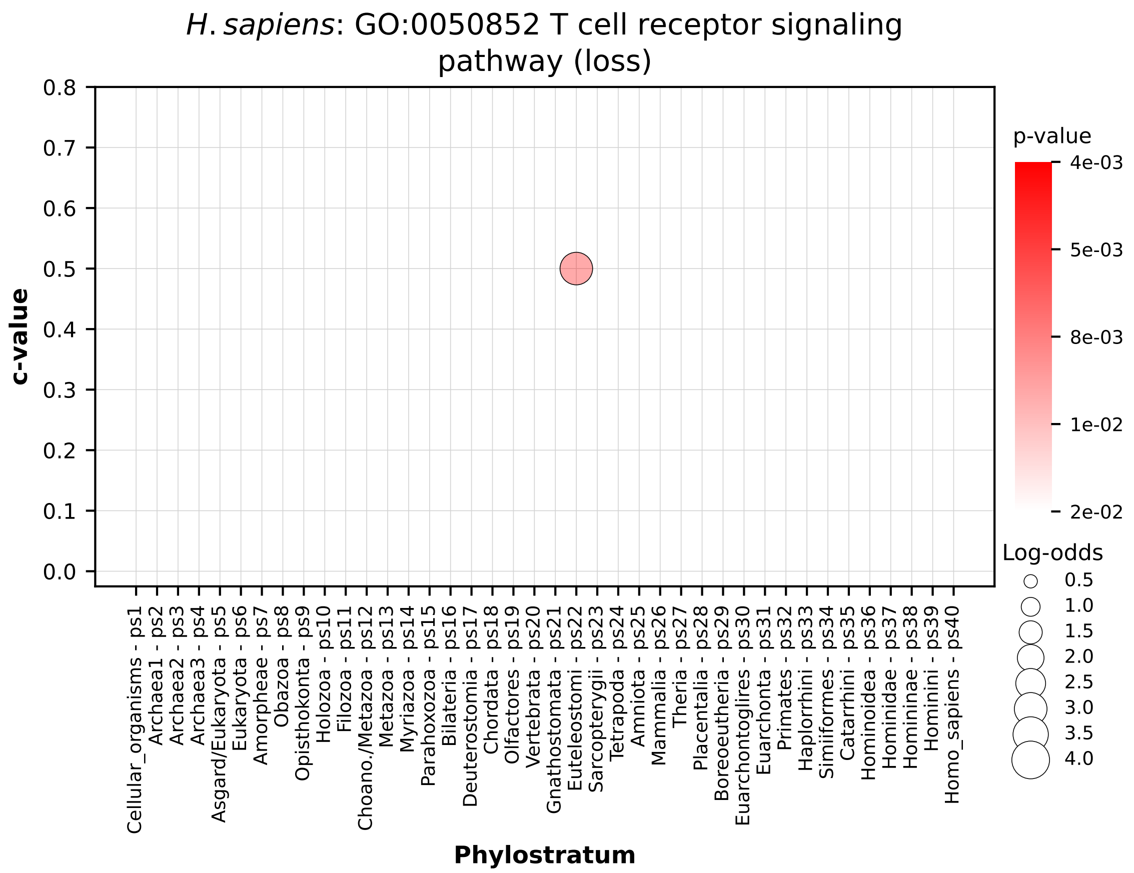
H. sapiens: GO:0050852 T cell receptor signaling pathway (loss)
c-value
0.0
0.1
0.2
0.3
0.4
0.5
0.6
0.7
0.8
p-value
4e-03
5e-03
8e-03
1e-02
2e-02
Log-odds
0.5
1.0
1.5
2.0
2.5
3.0
3.5
4.0
Cellular_organisms - ps1
Archaea1 - ps2
Archaea2 - ps3
Archaea3 - ps4
Asgard/Eukaryota - ps5
Eukaryota - ps6
Amorpheae - ps7
Obazoa - ps8
Opisthokonta - ps9
Holozoa - ps10
Filozoa - ps11
Choano./Metazoa - ps12
Metazoa - ps13
Myriazoa - ps14
Parahoxozoa - ps15
Bilateria - ps16
Deuterostomia - ps17
Chordata - ps18
Olfactores - ps19
Vertebrata - ps20
Gnathostomata - ps21
Euteleostomi - ps22
Sarcopterygii - ps23
Tetrapoda - ps24
Amniota - ps25
Mammalia - ps26
Theria - ps27
Placentalia - ps28
Boreoeutheria - ps29
Euarchontoglires - ps30
Euarchonta - ps31
Primates - ps32
Haplorrhini - ps33
Simiiformes - ps34
Catarrhini - ps35
Hominoidea - ps36
Hominidae - ps37
Homininae - ps38
Hominini - ps39
Homo_sapiens - ps40
Phylostratum

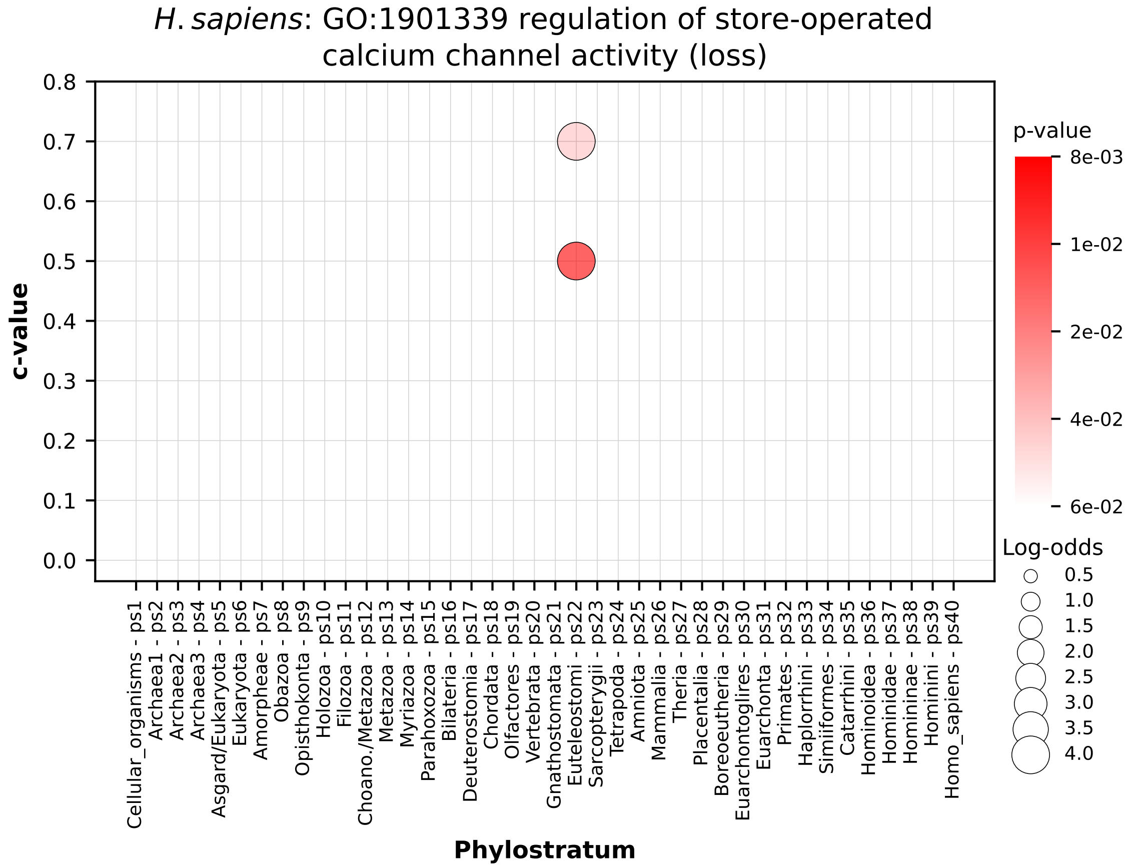

H. sapiens: GO:1901339 regulation of store-operated calcium channel activity (loss)
c-value
0.8
0.7
0.6
0.5
0.4
0.3
0.2
0.1
0.0
p-value
8e-03
1e-02
2e-02
4e-02
6e-02
Log-odds
0.5
1.0
1.5
2.0
2.5
3.0
3.5
4.0
Cellular_organisms - ps1
Archaea1 - ps2
Archaea2 - ps3
Archaea3 - ps4
Asgard/Eukaryota - ps5
Eukaryota - ps6
Amorpheae - ps7
Obazoa - ps8
Opisthokonta - ps9
Holozoa - ps10
Filozoa - ps11
Choano./Metazoa - ps12
Metazoa - ps13
Myriazoa - ps14
Parahoxozoa - ps15
Bilateria - ps16
Deuterostomia - ps17
Chordata - ps18
Olfactores - ps19
Vertebrata - ps20
Gnathostomata - ps21
Euteleostomi - ps22
Sarcopterygii - ps23
Tetrapoda - ps24
Amniota - ps25
Mammalia - ps26
Theria - ps27
Placentalia - ps28
Boreoeutheria - ps29
Euarchontoglires - ps30
Euarchonta - ps31
Primates - ps32
Haplorrhini - ps33
Simiiformes - ps34
Catarrhini - ps35
Hominoidea - ps36
Hominidae - ps37
Homininae - ps38
Hominini - ps39
Homo_sapiens - ps40
Phylostratum

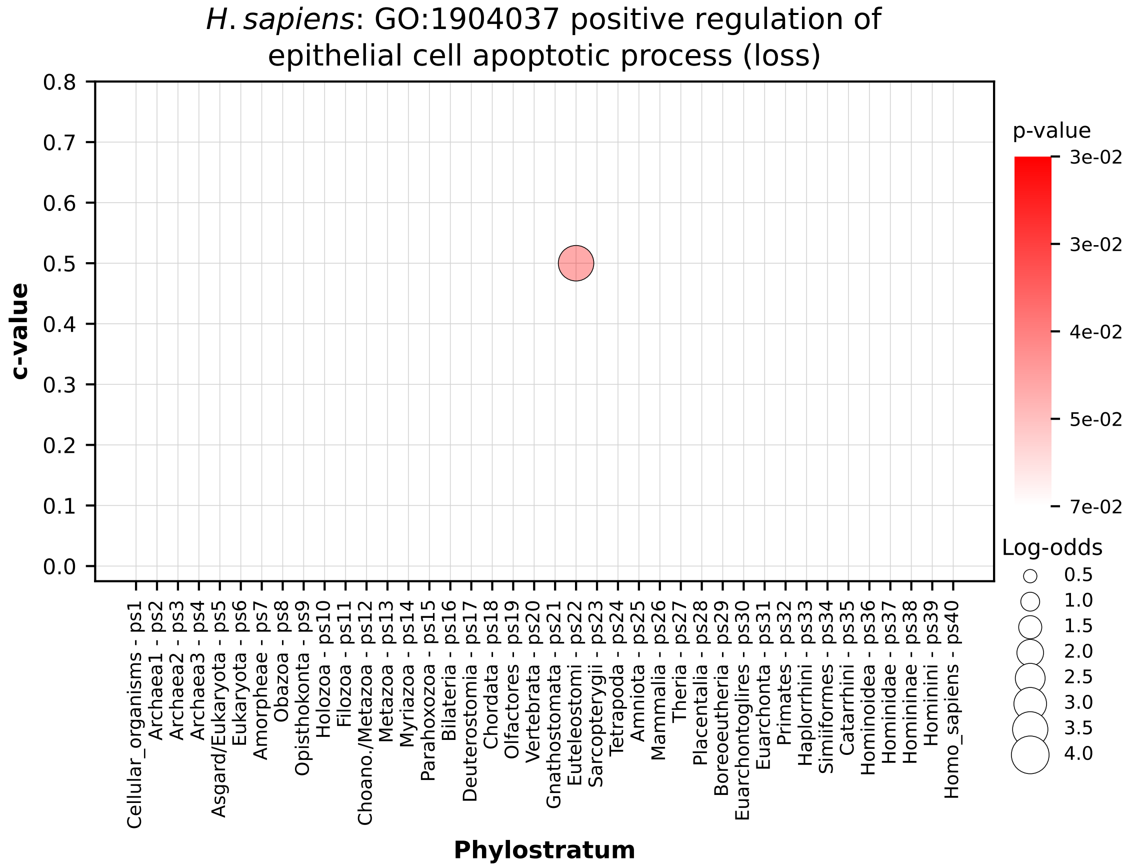

H. sapiens: GO:1904037 positive regulation of epithelial cell apoptotic process (loss)
c-value
0.8
0.7
0.6
0.5
0.4
0.3
0.2
0.1
0.0
p-value
3e-02
3e-02
4e-02
5e-02
7e-02
Log-odds
0.5
1.0
1.5
2.0
2.5
3.0
3.5
4.0
Cellular_organisms - ps1
Archaea1 - ps2
Archaea2 - ps3
Archaea3 - ps4
Asgard/Eukaryota - ps5
Eukaryota - ps6
Amorpheae - ps7
Obazoa - ps8
Opisthokonta - ps9
Holozoa - ps10
Filozoa - ps11
Choano./Metazoa - ps12
Metazoa - ps13
Myriazoa - ps14
Parahoxozoa - ps15
Bilateria - ps16
Deuterostomia - ps17
Chordata - ps18
Olfactores - ps19
Vertebrata - ps20
Gnathostomata - ps21
Euteleostomi - ps22
Sarcopterygii - ps23
Tetrapoda - ps24
Amniota - ps25
Mammalia - ps26
Theria - ps27
Placentalia - ps28
Boreoeutheria - ps29
Euarchontoglires - ps30
Euarchonta - ps31
Primates - ps32
Haplorrhini - ps33
Simiiformes - ps34
Catarrhini - ps35
Hominoidea - ps36
Hominidae - ps37
Homininae - ps38
Hominini - ps39
Homo_sapiens - ps40
Phylostratum

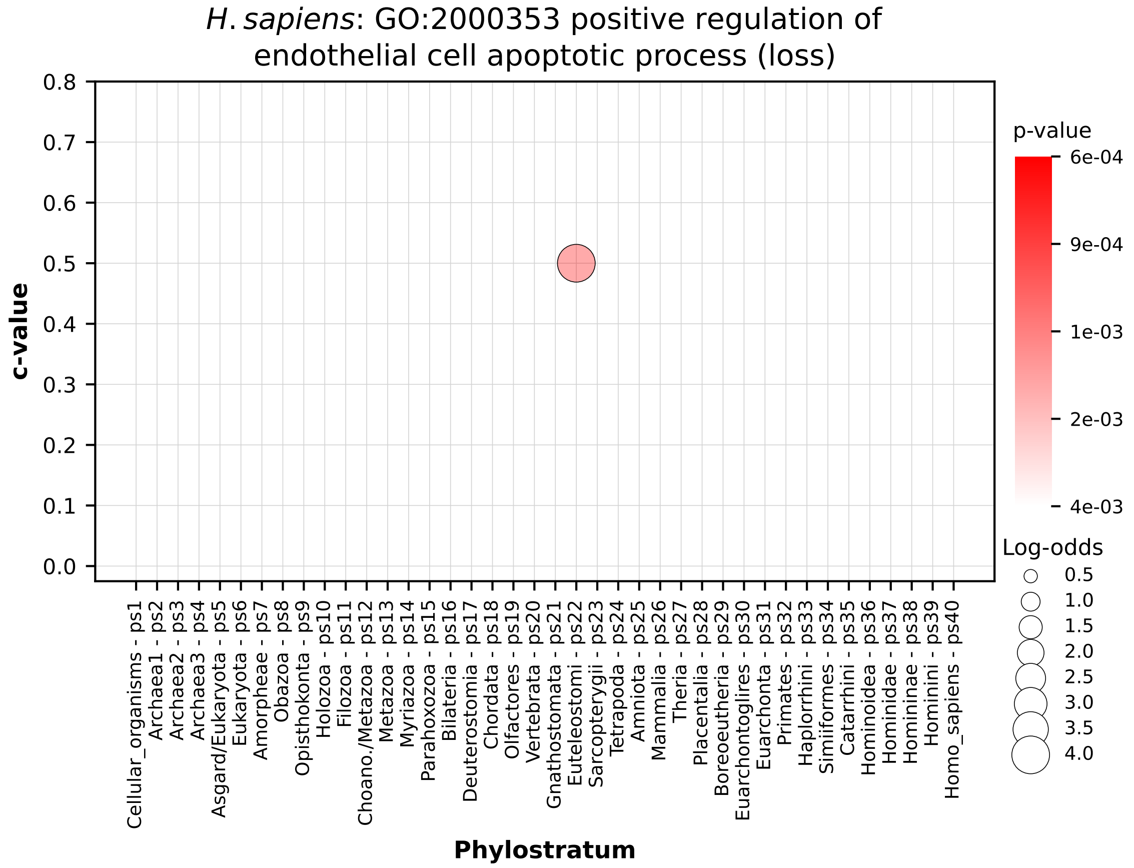
H. sapiens: GO:2000353 positive regulation of endothelial cell apoptotic process (loss)
c-value
0.8
0.7
0.6
0.5
0.4
0.3
0.2
0.1
0.0
Phylostratum
Cellular_organisms - ps1
Archaea1 - ps2
Archaea2 - ps3
Archaea3 - ps4
Asgard/Eukaryota - ps5
Eukaryota - ps6
Amorpheae - ps7
Obazoa - ps8
Opisthokonta - ps9
Holozoa - ps10
Filozoa - ps11
Choano./Metazoa - ps12
Metazoa - ps13
Myriazoa - ps14
Parahoxozoa - ps15
Bilateria - ps16
Deuterostomia - ps17
Chordata - ps18
Olfactores - ps19
Vertebrata - ps20
Gnathostomata - ps21
Euteleostomi - ps22
Sarcopterygii - ps23
Tetrapoda - ps24
Amniota - ps25
Mammalia - ps26
Theria - ps27
Placentalia - ps28
Boreoeutheria - ps29
Euarchontoglires - ps30
Euarchonta - ps31
Primates - ps32
Haplorrhini - ps33
Simiiformes - ps34
Catarrhini - ps35
Hominoidea - ps36
Hominidae - ps37
Homininae - ps38
Hominini - ps39
Homo_sapiens - ps40
p-value
6e-04
9e-04
1e-03
2e-03
4e-03
Log-odds
0.5
1.0
1.5
2.0
2.5
3.0
3.5
4.0

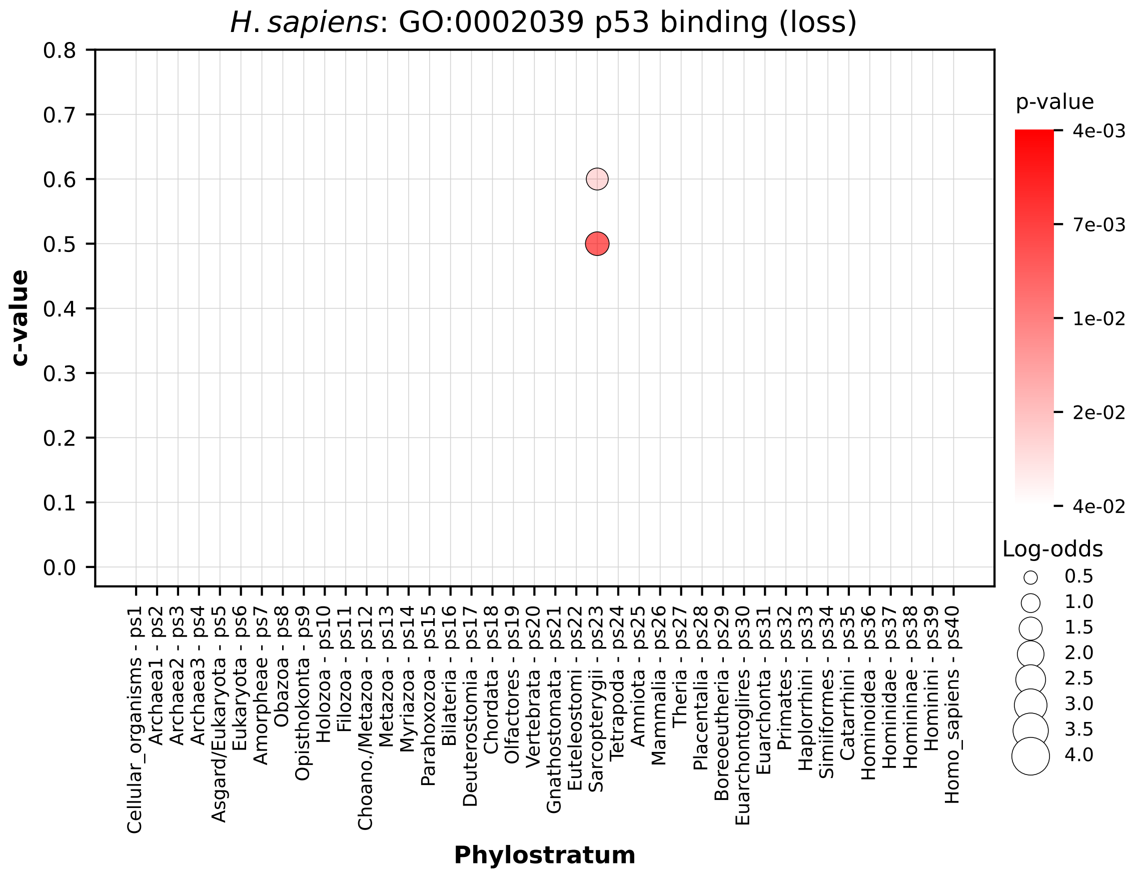

H. sapiens: GO:0002039 p53 binding (loss)
c-value
0.0
0.1
0.2
0.3
0.4
0.5
0.6
0.7
0.8
p-value
4e-03
7e-03
1e-02
2e-02
4e-02
Log-odds
0.5
1.0
1.5
2.0
2.5
3.0
3.5
4.0
Cellular_organisms - ps1
Archaea1 - ps2
Archaea2 - ps3
Archaea3 - ps4
Asgard/Eukaryota - ps5
Eukaryota - ps6
Amorpheae - ps7
Obazoa - ps8
Opisthokonta - ps9
Holozoa - ps10
Filozoa - ps11
Choano./Metazoa - ps12
Metazoa - ps13
Myriazoa - ps14
Parahoxozoa - ps15
Bilateria - ps16
Deuterostomia - ps17
Chordata - ps18
Olfactores - ps19
Vertebrata - ps20
Gnathostomata - ps21
Euteleostomi - ps22
Sarcopterygii - ps23
Tetrapoda - ps24
Amniota - ps25
Mammalia - ps26
Theria - ps27
Placentalia - ps28
Boreoeutheria - ps29
Euarchontoglires - ps30
Euarchonta - ps31
Primates - ps32
Haplorrhini - ps33
Simiiformes - ps34
Catarrhini - ps35
Hominoidea - ps36
Hominidae - ps37
Homininae - ps38
Hominini - ps39
Homo_sapiens - ps40
Phylostratum

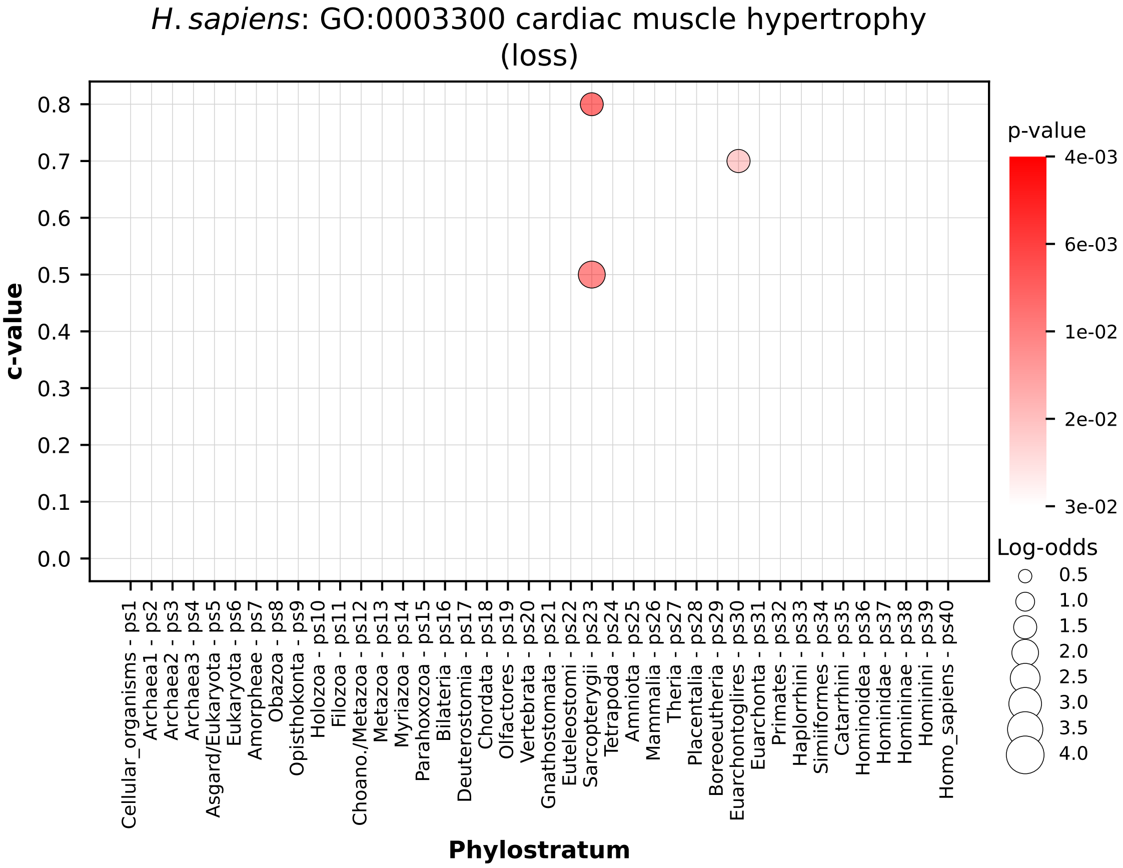

H. sapiens: GO:0003300 cardiac muscle hypertrophy
(loss)
c-value
0.0
0.1
0.2
0.3
0.4
0.5
0.6
0.7
0.8
Phylostratum
Cellular_organisms - ps1
Archaea1 - ps2
Archaea2 - ps3
Archaea3 - ps4
Asgard/Eukaryota - ps5
Eukaryota - ps6
Amorpheae - ps7
Obazoa - ps8
Opisthokonta - ps9
Holozoa - ps10
Filozoa - ps11
Choano./Metazoa - ps12
Metazoa - ps13
Myriazoa - ps14
Parahoxozoa - ps15
Bilateria - ps16
Deuterostomia - ps17
Chordata - ps18
Olfactores - ps19
Vertebrata - ps20
Gnathostomata - ps21
Euteleostomi - ps22
Sarcopterygii - ps23
Tetrapoda - ps24
Amniota - ps25
Mammalia - ps26
Theria - ps27
Placentalia - ps28
Boreoeutheria - ps29
Euarchontoglires - ps30
Euarchonta - ps31
Primates - ps32
Haplorrhini - ps33
Simiiformes - ps34
Catarrhini - ps35
Hominoidea - ps36
Hominidae - ps37
Homininae - ps38
Hominini - ps39
Homo_sapiens - ps40
p-value
4e-03
6e-03
1e-02
2e-02
3e-02
Log-odds
0.5
1.0
1.5
2.0
2.5
3.0
3.5
4.0

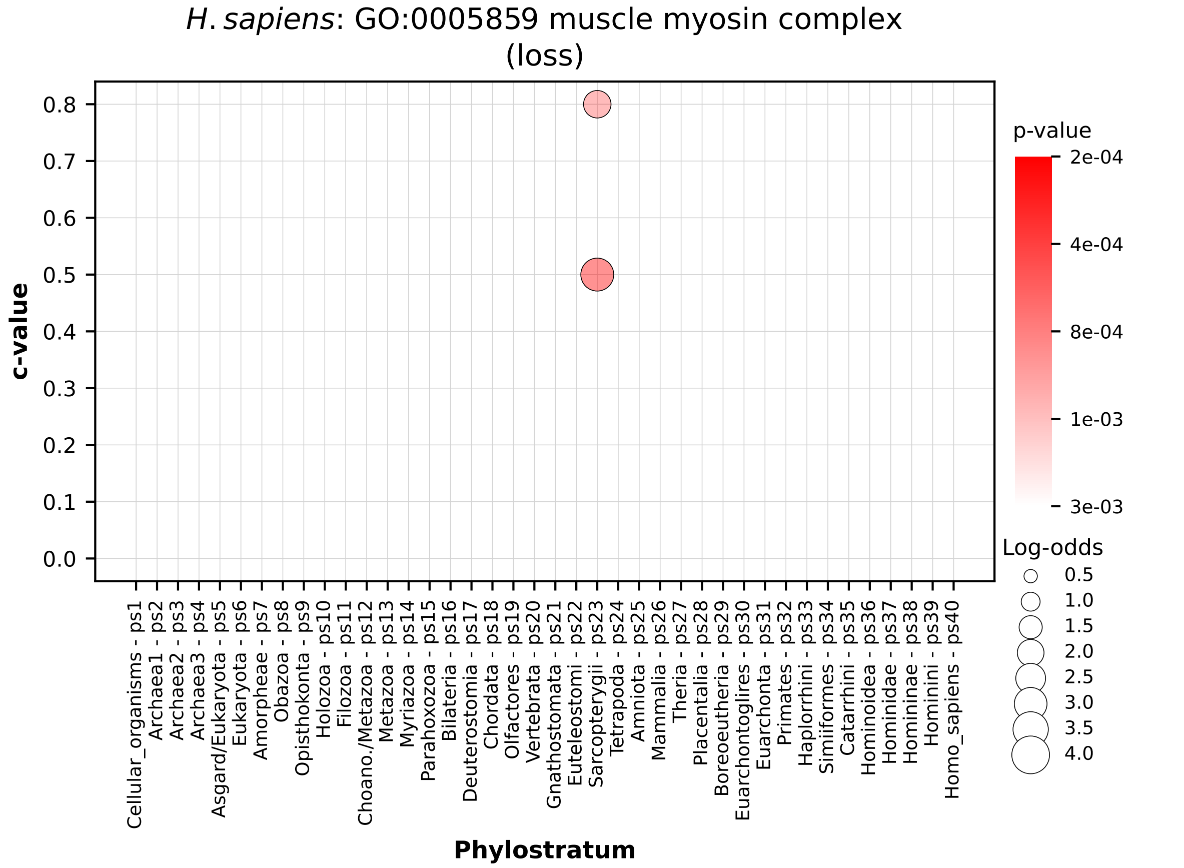

H. sapiens: GO:0005859 muscle myosin complex
(loss)
c-value
0.8
0.7
0.6
0.5
0.4
0.3
0.2
0.1
0.0
p-value
2e-04
4e-04
8e-04
1e-03
3e-03
Log-odds
0.5
1.0
1.5
2.0
2.5
3.0
3.5
4.0
Cellular_organisms - ps1
Archaea1 - ps2
Archaea2 - ps3
Archaea3 - ps4
Asgard/Eukaryota - ps5
Eukaryota - ps6
Amorpheae - ps7
Obazoa - ps8
Opisthokonta - ps9
Holozoa - ps10
Filozoa - ps11
Choano./Metazoa - ps12
Metazoa - ps13
Myriazoa - ps14
Parahoxozoa - ps15
Bilateria - ps16
Deuterostomia - ps17
Chordata - ps18
Olfactores - ps19
Vertebrata - ps20
Gnathostomata - ps21
Euteleostomi - ps22
Sarcopterygii - ps23
Tetrapoda - ps24
Amniota - ps25
Mammalia - ps26
Theria - ps27
Placentalia - ps28
Boreoeutheria - ps29
Euarchontoglires - ps30
Euarchonta - ps31
Primates - ps32
Haplorrhini - ps33
Simiiformes - ps34
Catarrhini - ps35
Hominoidea - ps36
Hominidae - ps37
Homininae - ps38
Hominini - ps39
Homo_sapiens - ps40
Phylostratum

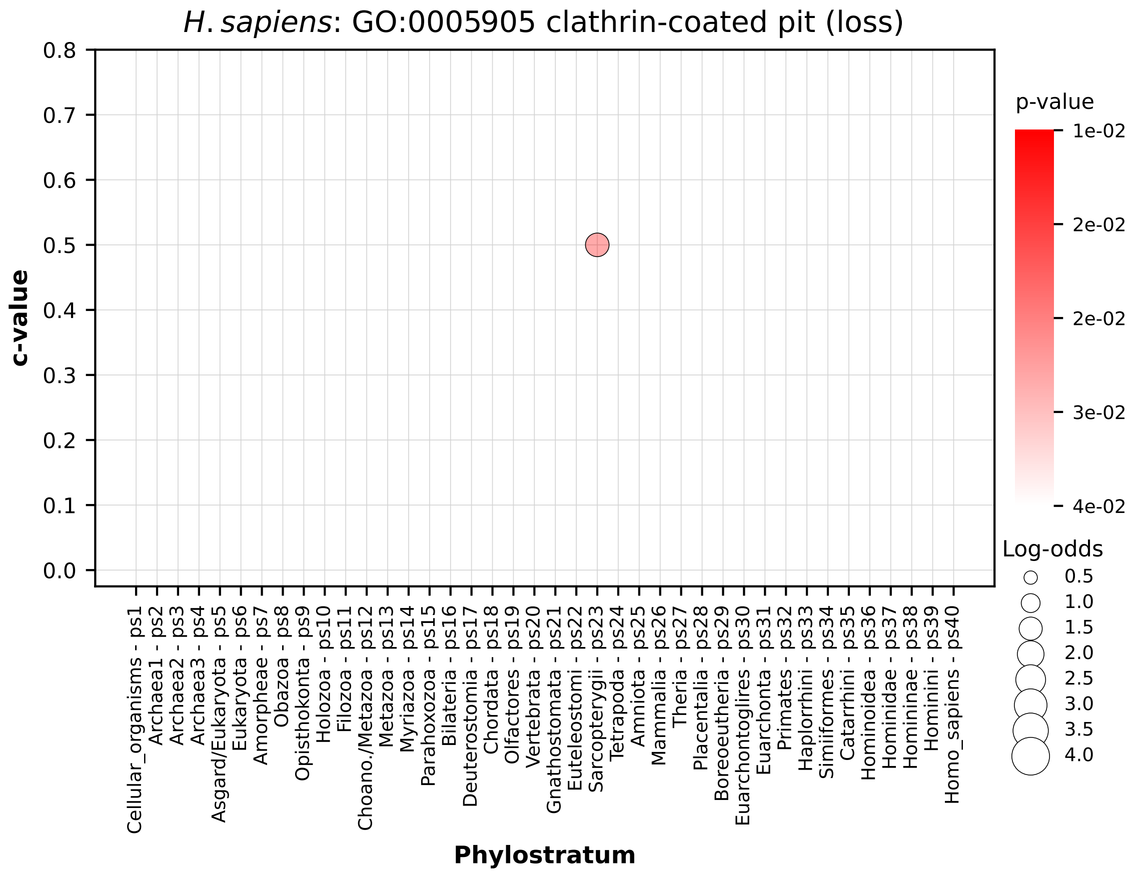

H. sapiens: GO:0005905 clathrin-coated pit (loss)
c-value
0.0
0.1
0.2
0.3
0.4
0.5
0.6
0.7
0.8
Phylostratum
Cellular_organisms - ps1
Archaea1 - ps2
Archaea2 - ps3
Archaea3 - ps4
Asgard/Eukaryota - ps5
Eukaryota - ps6
Amorpheae - ps7
Obazoa - ps8
Opisthokonta - ps9
Holozoa - ps10
Filozoa - ps11
Choano./Metazoa - ps12
Metazoa - ps13
Myriazoa - ps14
Parahoxozoa - ps15
Bilateria - ps16
Deuterostomia - ps17
Chordata - ps18
Olfactores - ps19
Vertebrata - ps20
Gnathostomata - ps21
Euteleostomi - ps22
Sarcopterygii - ps23
Tetrapoda - ps24
Amniota - ps25
Mammalia - ps26
Theria - ps27
Placentalia - ps28
Boreoeutheria - ps29
Euarchontoglires - ps30
Euarchonta - ps31
Primates - ps32
Haplorrhini - ps33
Simiiformes - ps34
Catarrhini - ps35
Hominoidea - ps36
Hominidae - ps37
Homininae - ps38
Hominini - ps39
Homo_sapiens - ps40
p-value
1e-02
2e-02
2e-02
3e-02
4e-02
Log-odds
0.5
1.0
1.5
2.0
2.5
3.0
3.5
4.0

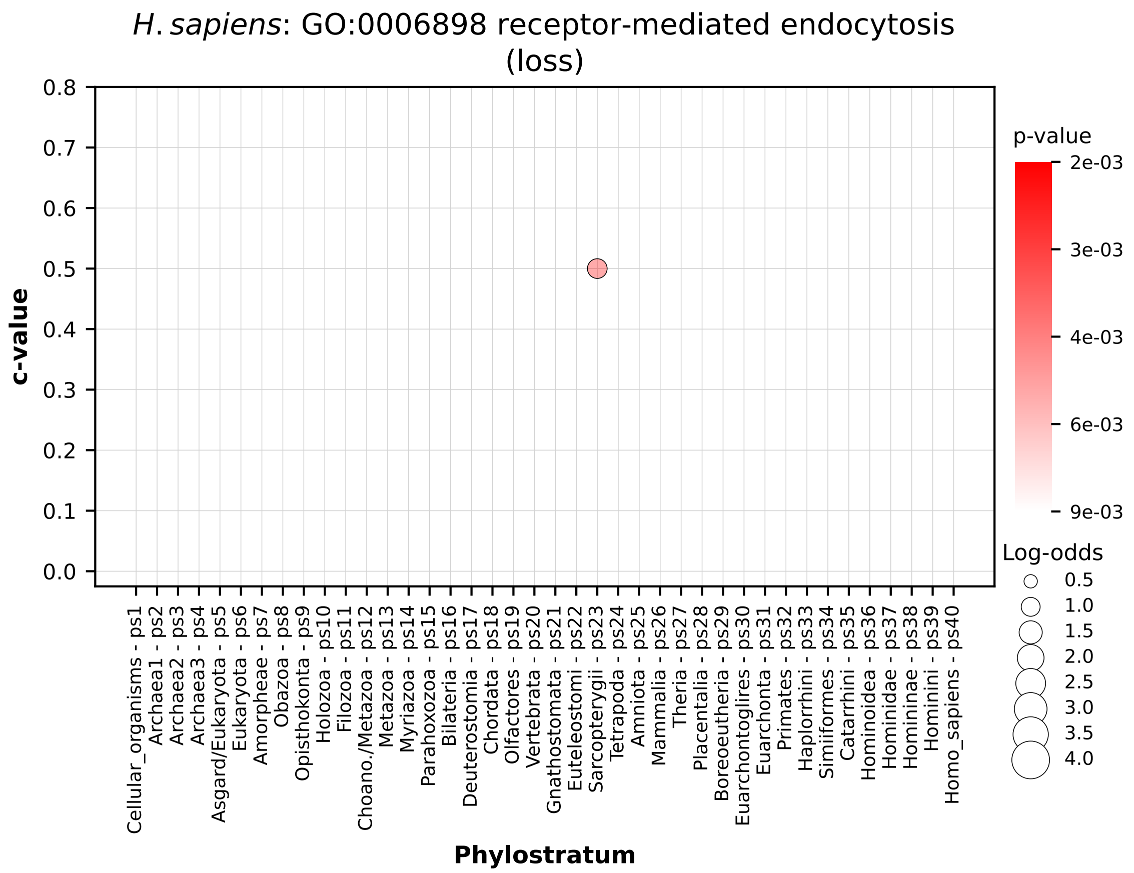

H. sapiens: GO:0006898 receptor-mediated endocytosis (loss)
c-value
0.8
0.7
0.6
0.5
0.4
0.3
0.2
0.1
0.0
Phylostratum
Cellular_organisms - ps1
Archaea1 - ps2
Archaea2 - ps3
Archaea3 - ps4
Asgard/Eukaryota - ps5
Eukaryota - ps6
Amorpheae - ps7
Obazoa - ps8
Opisthokonta - ps9
Holozoa - ps10
Filozoa - ps11
Choano./Metazoa - ps12
Metazoa - ps13
Myriazoa - ps14
Parahoxozoa - ps15
Bilateria - ps16
Deuterostomia - ps17
Chordata - ps18
Olfactores - ps19
Vertebrata - ps20
Gnathostomata - ps21
Euteleostomi - ps22
Sarcopterygii - ps23
Tetrapoda - ps24
Amniota - ps25
Mammalia - ps26
Theria - ps27
Placentalia - ps28
Boreoeutheria - ps29
Euarchontoglires - ps30
Euarchonta - ps31
Primates - ps32
Haplorrhini - ps33
Simiiformes - ps34
Catarrhini - ps35
Hominoidea - ps36
Hominidae - ps37
Homininae - ps38
Hominini - ps39
Homo_sapiens - ps40
p-value
2e-03
3e-03
4e-03
6e-03
9e-03
Log-odds
0.5
1.0
1.5
2.0
2.5
3.0
3.5
4.0

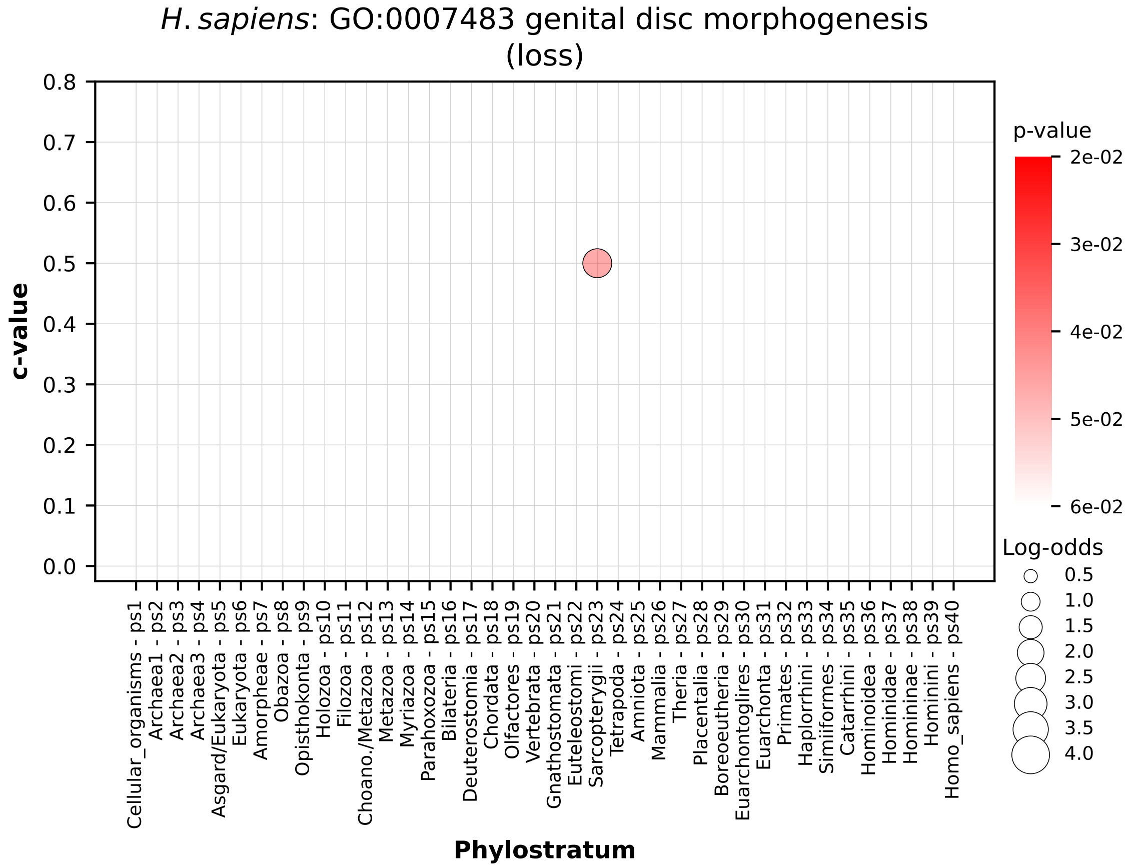

H. sapiens: GO:0007483 genital disc morphogenesis
(loss)
c-value
0.0
0.1
0.2
0.3
0.4
0.5
0.6
0.7
0.8
p-value
2e-02
3e-02
4e-02
5e-02
6e-02
Log-odds
0.5
1.0
1.5
2.0
2.5
3.0
3.5
4.0
Cellular_organisms - ps1
Archaea1 - ps2
Archaea2 - ps3
Archaea3 - ps4
Asgard/Eukaryota - ps5
Eukaryota - ps6
Amorpheae - ps7
Obazoa - ps8
Opisthokonta - ps9
Holozoa - ps10
Filozoa - ps11
Choano./Metazoa - ps12
Metazoa - ps13
Myriazoa - ps14
Parahoxozoa - ps15
Bilateria - ps16
Deuterostomia - ps17
Chordata - ps18
Olfactores - ps19
Vertebrata - ps20
Gnathostomata - ps21
Euteleostomi - ps22
Sarcopterygii - ps23
Tetrapoda - ps24
Amniota - ps25
Mammalia - ps26
Theria - ps27
Placentalia - ps28
Boreoeutheria - ps29
Euarchontoglires - ps30
Euarchonta - ps31
Primates - ps32
Haplorrhini - ps33
Simiiformes - ps34
Catarrhini - ps35
Hominoidea - ps36
Hominidae - ps37
Homininae - ps38
Hominini - ps39
Homo_sapiens - ps40
Phylostratum

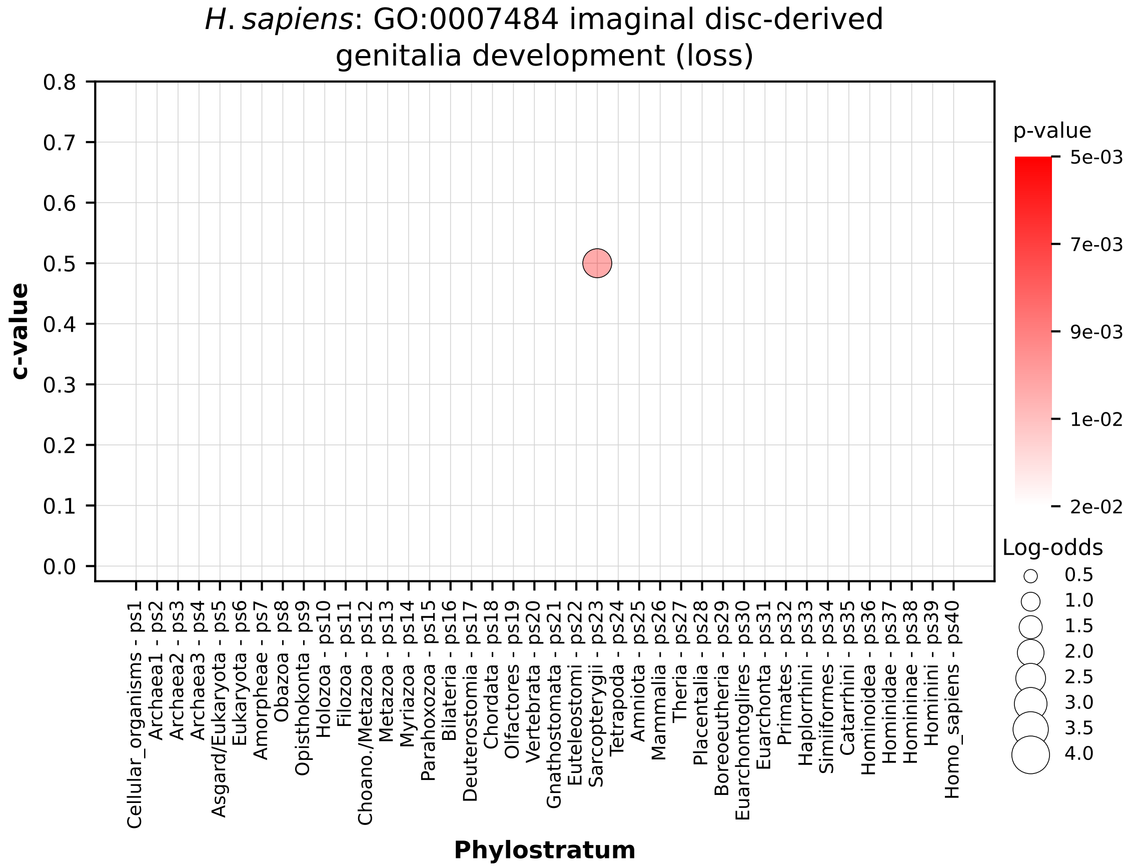

H. sapiens: GO:0007484 imaginal disc-derived genitalia development (loss)
c-value
0.8
0.7
0.6
0.5
0.4
0.3
0.2
0.1
0.0
p-value
5e-03
7e-03
9e-03
1e-02
2e-02
Log-odds
0.5
1.0
1.5
2.0
2.5
3.0
3.5
4.0
Cellular_organisms - ps1
Archaea1 - ps2
Archaea2 - ps3
Archaea3 - ps4
Asgard/Eukaryota - ps5
Eukaryota - ps6
Amorpheae - ps7
Obazoa - ps8
Opisthokonta - ps9
Holozoa - ps10
Filozoa - ps11
Choano./Metazoa - ps12
Metazoa - ps13
Myriazoa - ps14
Parahoxozoa - ps15
Bilateria - ps16
Deuterostomia - ps17
Chordata - ps18
Olfactores - ps19
Vertebrata - ps20
Gnathostomata - ps21
Euteleostomi - ps22
Sarcopterygii - ps23
Tetrapoda - ps24
Amniota - ps25
Mammalia - ps26
Theria - ps27
Placentalia - ps28
Boreoeutheria - ps29
Euarchontoglires - ps30
Euarchonta - ps31
Primates - ps32
Haplorrhini - ps33
Simiiformes - ps34
Catarrhini - ps35
Hominoidea - ps36
Hominidae - ps37
Homininae - ps38
Hominini - ps39
Homo_sapiens - ps40
Phylostratum

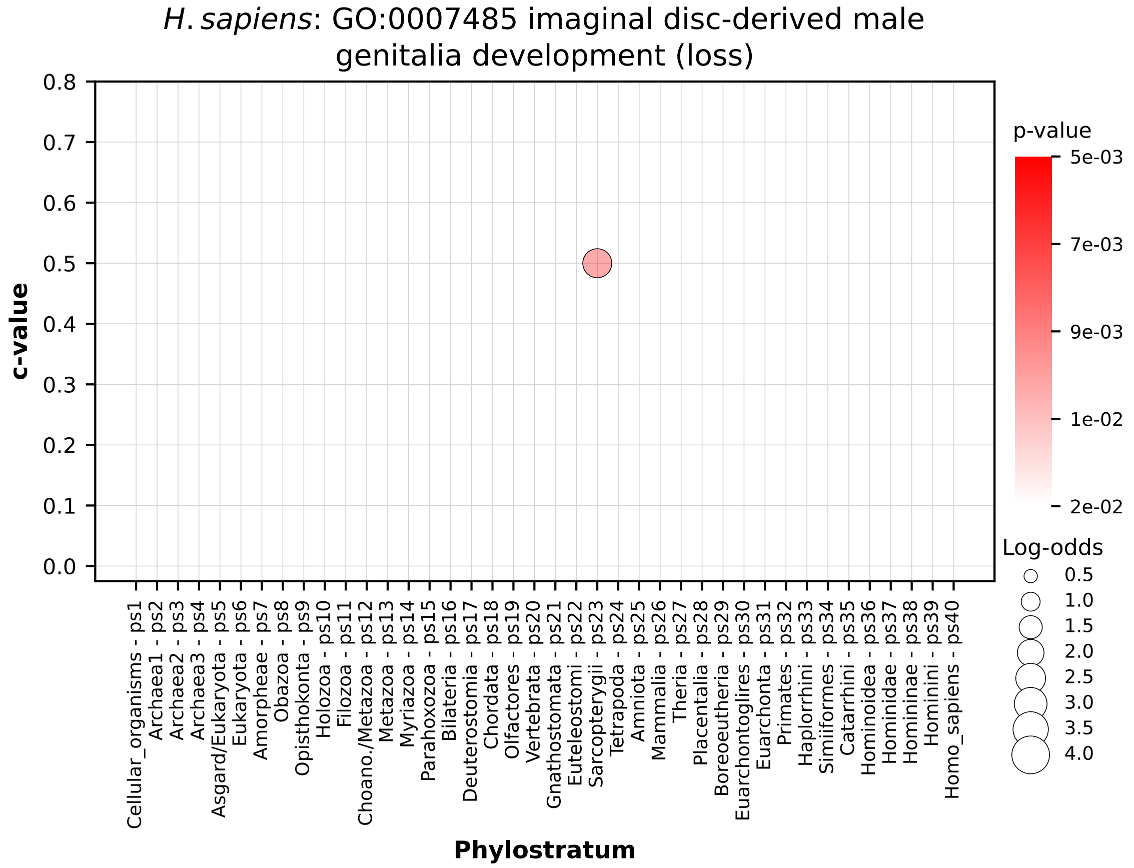
H. sapiens: GO:0007485 imaginal disc-derived male genitalia development (loss)
c-value
0.0
0.1
0.2
0.3
0.4
0.5
0.6
0.7
0.8
Phylostratum
Cellular_organisms - ps1
Archaea1 - ps2
Archaea2 - ps3
Archaea3 - ps4
Asgard/Eukaryota - ps5
Eukaryota - ps6
Amorpheae - ps7
Obazoa - ps8
Opisthokonta - ps9
Holozoa - ps10
Filozoa - ps11
Choano./Metazoa - ps12
Metazoa - ps13
Myriazoa - ps14
Parahoxozoa - ps15
Bilateria - ps16
Deuterostomia - ps17
Chordata - ps18
Olfactores - ps19
Vertebrata - ps20
Gnathostomata - ps21
Euteleostomi - ps22
Sarcopterygii - ps23
Tetrapoda - ps24
Amniota - ps25
Mammalia - ps26
Theria - ps27
Placentalia - ps28
Boreoeutheria - ps29
Euarchontoglires - ps30
Euarchonta - ps31
Primates - ps32
Haplorrhini - ps33
Simiiformes - ps34
Catarrhini - ps35
Hominoidea - ps36
Hominidae - ps37
Homininae - ps38
Hominini - ps39
Homo_sapiens - ps40
p-value
5e-03
7e-03
9e-03
1e-02
2e-02
Log-odds
0.5
1.0
1.5
2.0
2.5
3.0
3.5
4.0

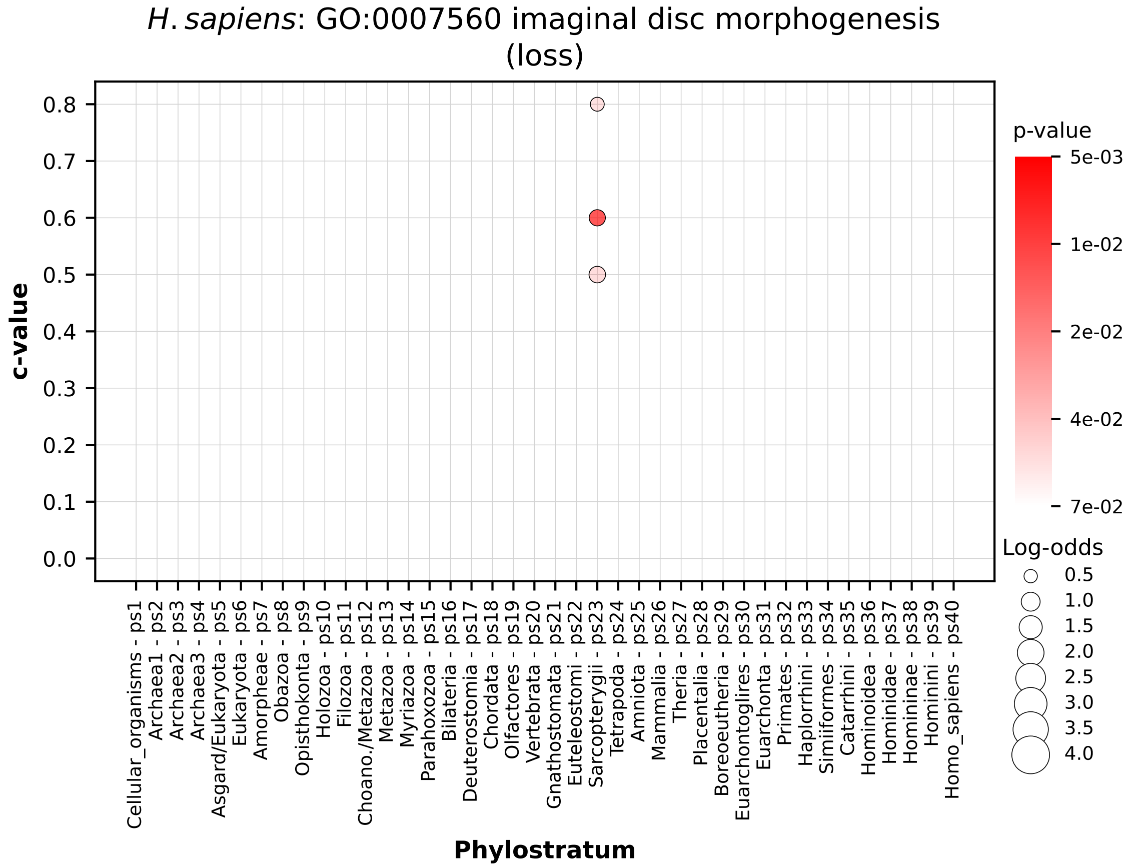

H. sapiens: GO:0007560 imaginal disc morphogenesis (loss)
c-value
0.8
0.7
0.6
0.5
0.4
0.3
0.2
0.1
0.0
Phylostratum
Cellular_organisms - ps1
Archaea1 - ps2
Archaea2 - ps3
Archaea3 - ps4
Asgard/Eukaryota - ps5
Eukaryota - ps6
Amorpheae - ps7
Obazoa - ps8
Opisthokonta - ps9
Holozoa - ps10
Filozoa - ps11
Choano./Metazoa - ps12
Metazoa - ps13
Myriazoa - ps14
Parahoxozoa - ps15
Bilateria - ps16
Deuterostomia - ps17
Chordata - ps18
Olfactores - ps19
Vertebrata - ps20
Gnathostomata - ps21
Euteleostomi - ps22
Sarcopterygii - ps23
Tetrapoda - ps24
Amniota - ps25
Mammalia - ps26
Theria - ps27
Placentalia - ps28
Boreoeutheria - ps29
Euarchontoglires - ps30
Euarchonta - ps31
Primates - ps32
Haplorrhini - ps33
Simiiformes - ps34
Catarrhini - ps35
Hominoidea - ps36
Hominidae - ps37
Homininae - ps38
Hominini - ps39
Homo_sapiens - ps40
p-value
5e-03
1e-02
2e-02
4e-02
7e-02
Log-odds
0.5
1.0
1.5
2.0
2.5
3.0
3.5
4.0

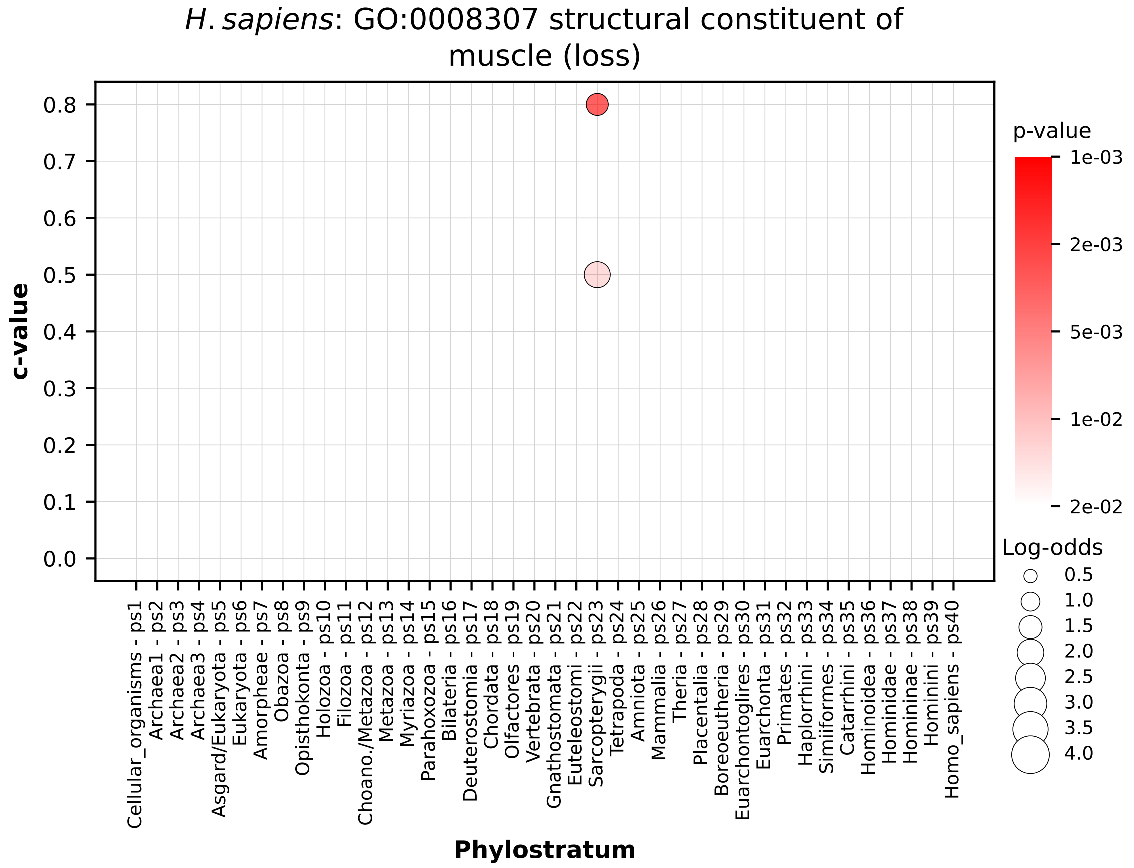

H. sapiens: GO:0008307 structural constituent of muscle (loss)
c-value
0.0
0.1
0.2
0.3
0.4
0.5
0.6
0.7
0.8
Phylostratum
Cellular_organisms - ps1
Archaea1 - ps2
Archaea2 - ps3
Archaea3 - ps4
Asgard/Eukaryota - ps5
Eukaryota - ps6
Amorpheae - ps7
Obazoa - ps8
Opisthokonta - ps9
Holozoa - ps10
Filozoa - ps11
Choano./Metazoa - ps12
Metazoa - ps13
Myriazoa - ps14
Parahoxozoa - ps15
Bilateria - ps16
Deuterostomia - ps17
Chordata - ps18
Olfactores - ps19
Vertebrata - ps20
Gnathostomata - ps21
Euteleostomi - ps22
Sarcopterygii - ps23
Tetrapoda - ps24
Amniota - ps25
Mammalia - ps26
Theria - ps27
Placentalia - ps28
Boreoeutheria - ps29
Euarchontoglires - ps30
Euarchonta - ps31
Primates - ps32
Haplorrhini - ps33
Simiiformes - ps34
Catarrhini - ps35
Hominoidea - ps36
Hominidae - ps37
Homininae - ps38
Hominini - ps39
Homo_sapiens - ps40
p-value
1e-03
2e-03
5e-03
1e-02
2e-02
Log-odds
0.5
1.0
1.5
2.0
2.5
3.0
3.5
4.0

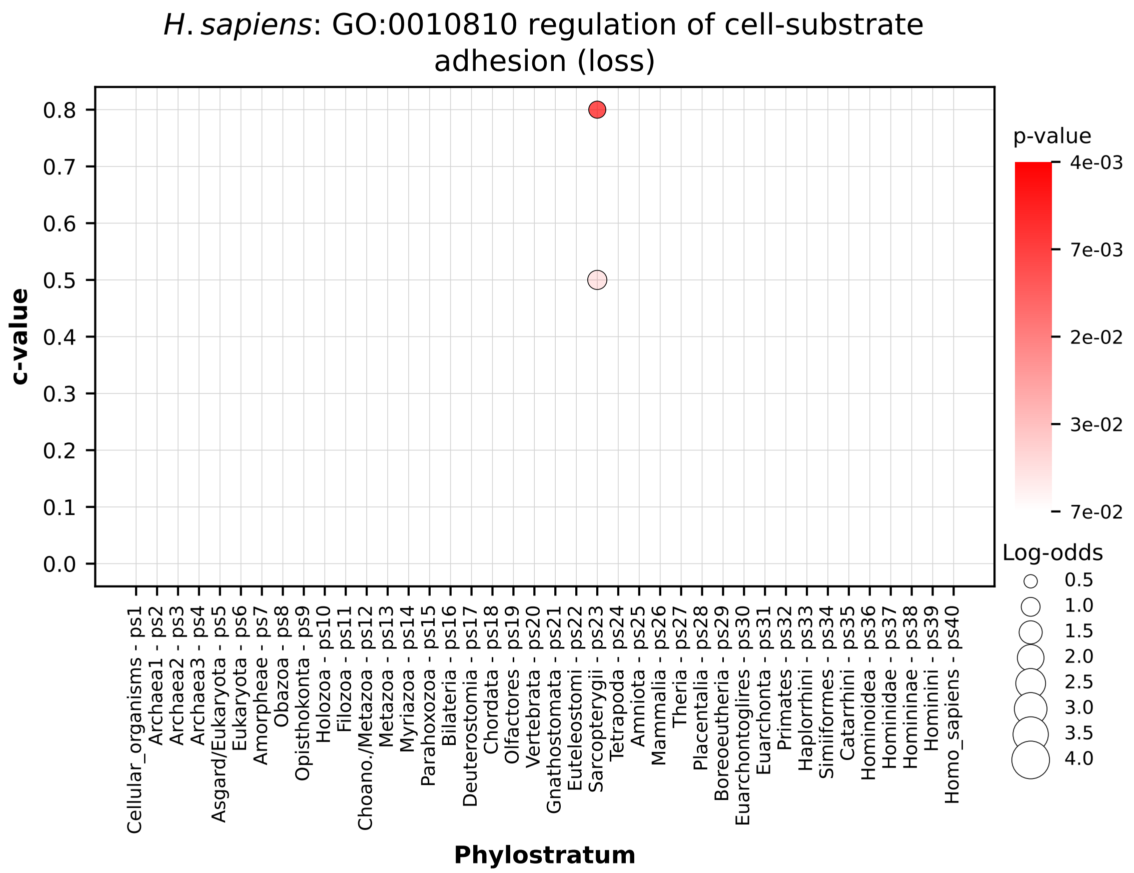

H. sapiens: GO:0010810 regulation of cell-substrate adhesion (loss)
c-value
0.8
0.7
0.6
0.5
0.4
0.3
0.2
0.1
0.0
p-value
4e-03
7e-03
2e-02
3e-02
7e-02
Log-odds
0.5
1.0
1.5
2.0
2.5
3.0
3.5
4.0
Cellular_organisms - ps1
Archaea1 - ps2
Archaea2 - ps3
Archaea3 - ps4
Asgard/Eukaryota - ps5
Eukaryota - ps6
Amorpheae - ps7
Obazoa - ps8
Opisthokonta - ps9
Holozoa - ps10
Filozoa - ps11
Choano./Metazoa - ps12
Metazoa - ps13
Myriazoa - ps14
Parahoxozoa - ps15
Bilateria - ps16
Deuterostomia - ps17
Chordata - ps18
Olfactores - ps19
Vertebrata - ps20
Gnathostomata - ps21
Euteleostomi - ps22
Sarcopterygii - ps23
Tetrapoda - ps24
Amniota - ps25
Mammalia - ps26
Theria - ps27
Placentalia - ps28
Boreoeutheria - ps29
Euarchontoglires - ps30
Euarchonta - ps31
Primates - ps32
Haplorrhini - ps33
Simiiformes - ps34
Catarrhini - ps35
Hominoidea - ps36
Hominidae - ps37
Homininae - ps38
Hominini - ps39
Homo_sapiens - ps40
Phylostratum

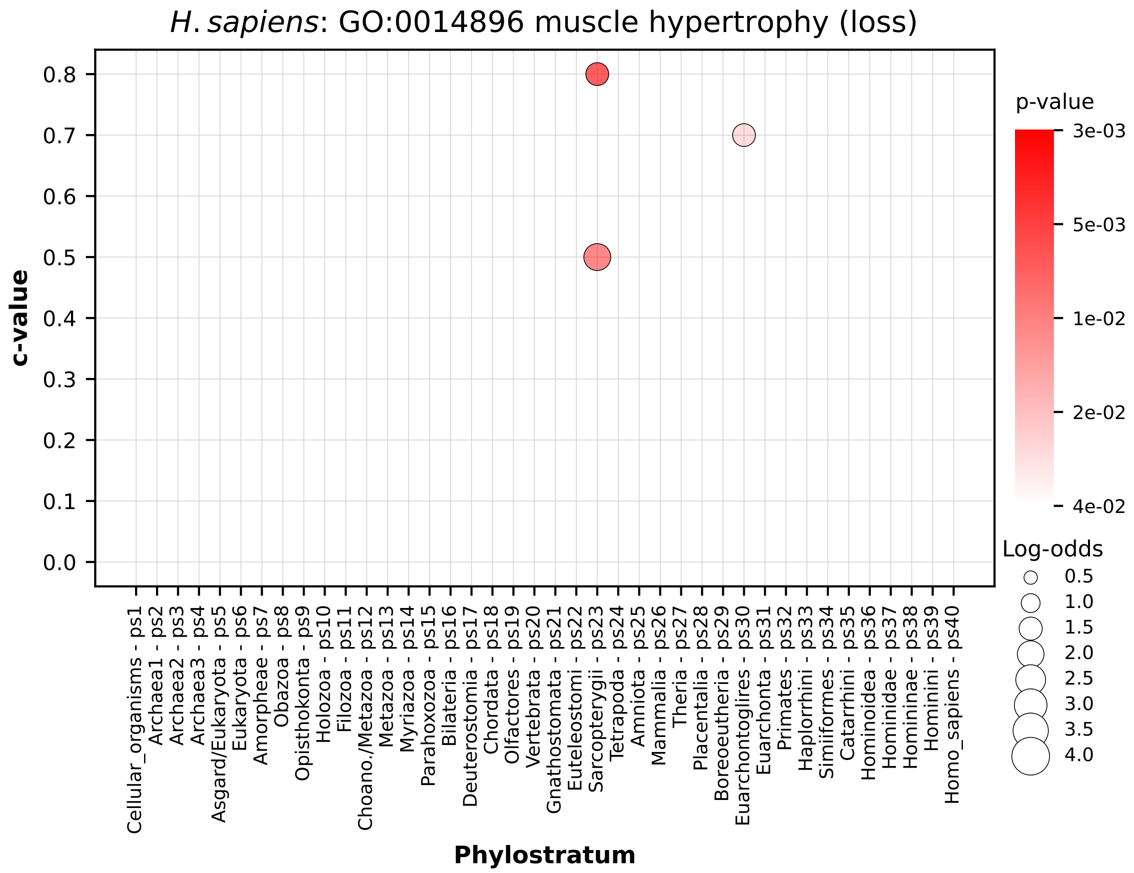

H. sapiens: GO:0014896 muscle hypertrophy (loss)
c-value
0.0
0.1
0.2
0.3
0.4
0.5
0.6
0.7
0.8
Phylostratum
Cellular_organisms - ps1
Archaea1 - ps2
Archaea2 - ps3
Archaea3 - ps4
Asgard/Eukaryota - ps5
Eukaryota - ps6
Amorpheae - ps7
Obazoa - ps8
Opisthokonta - ps9
Holozoa - ps10
Filozoa - ps11
Choano./Metazoa - ps12
Metazoa - ps13
Myriazoa - ps14
Parahoxozoa - ps15
Bilateria - ps16
Deuterostomia - ps17
Chordata - ps18
Olfactores - ps19
Vertebrata - ps20
Gnathostomata - ps21
Euteleostomi - ps22
Sarcopterygii - ps23
Tetrapoda - ps24
Amniota - ps25
Mammalia - ps26
Theria - ps27
Placentalia - ps28
Boreoeutheria - ps29
Euarchontoglires - ps30
Euarchonta - ps31
Primates - ps32
Haplorrhini - ps33
Simiiformes - ps34
Catarrhini - ps35
Hominoidea - ps36
Hominidae - ps37
Homininae - ps38
Hominini - ps39
Homo_sapiens - ps40
p-value
3e-03
5e-03
1e-02
2e-02
4e-02
Log-odds
0.5
1.0
1.5
2.0
2.5
3.0
3.5
4.0

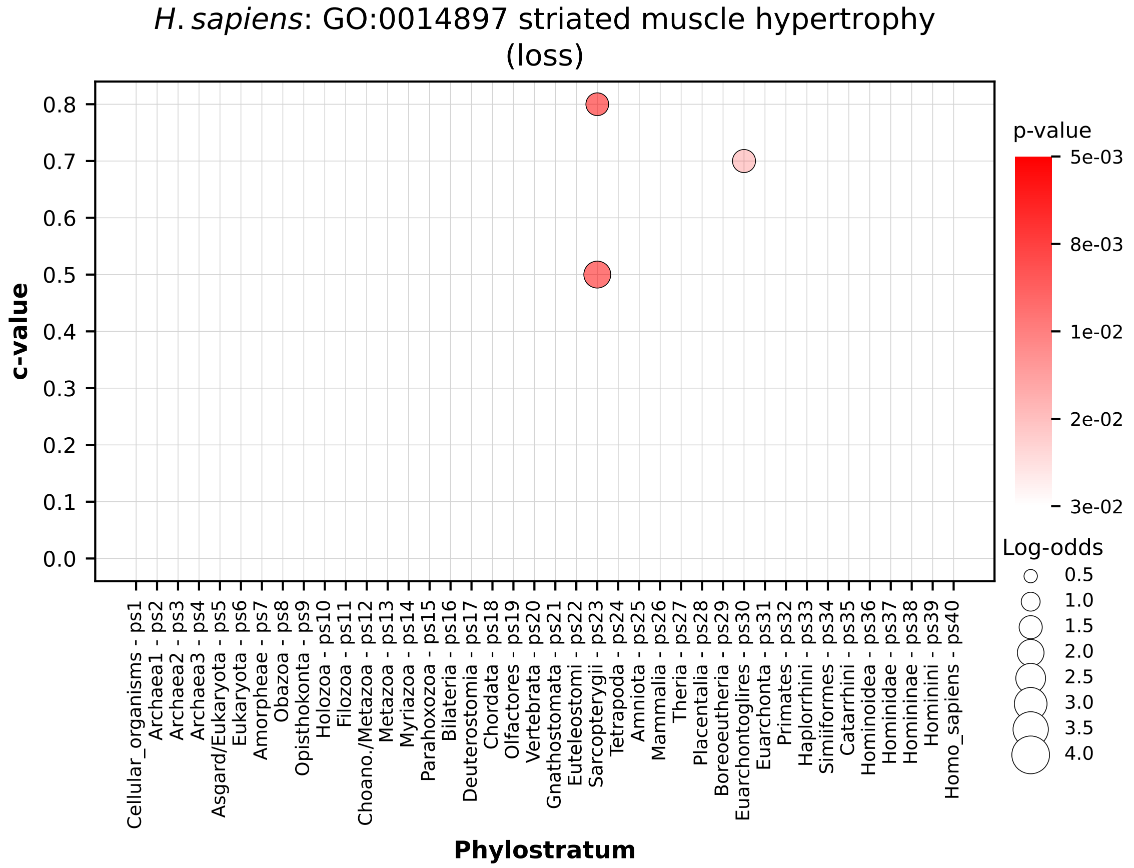
H. sapiens: GO:0014897 striated muscle hypertrophy (loss)
c-value
0.0
0.1
0.2
0.3
0.4
0.5
0.6
0.7
0.8
Phylostratum
Cellular_organisms - ps1
Archaea1 - ps2
Archaea2 - ps3
Archaea3 - ps4
Asgard/Eukaryota - ps5
Eukaryota - ps6
Amorpheae - ps7
Obazoa - ps8
Opisthokonta - ps9
Holozoa - ps10
Filozoa - ps11
Choano./Metazoa - ps12
Metazoa - ps13
Myriazoa - ps14
Parahoxozoa - ps15
Bilateria - ps16
Deuterostomia - ps17
Chordata - ps18
Olfactores - ps19
Vertebrata - ps20
Gnathostomata - ps21
Euteleostomi - ps22
Sarcopterygii - ps23
Tetrapoda - ps24
Amniota - ps25
Mammalia - ps26
Theria - ps27
Placentalia - ps28
Boreoeutheria - ps29
Euarchontoglires - ps30
Euarchonta - ps31
Primates - ps32
Haplorrhini - ps33
Simiiformes - ps34
Catarrhini - ps35
Hominoidea - ps36
Hominidae - ps37
Homininae - ps38
Hominini - ps39
Homo_sapiens - ps40
p-value
5e-03
8e-03
1e-02
2e-02
3e-02
Log-odds
0.5
1.0
1.5
2.0
2.5
3.0
3.5
4.0

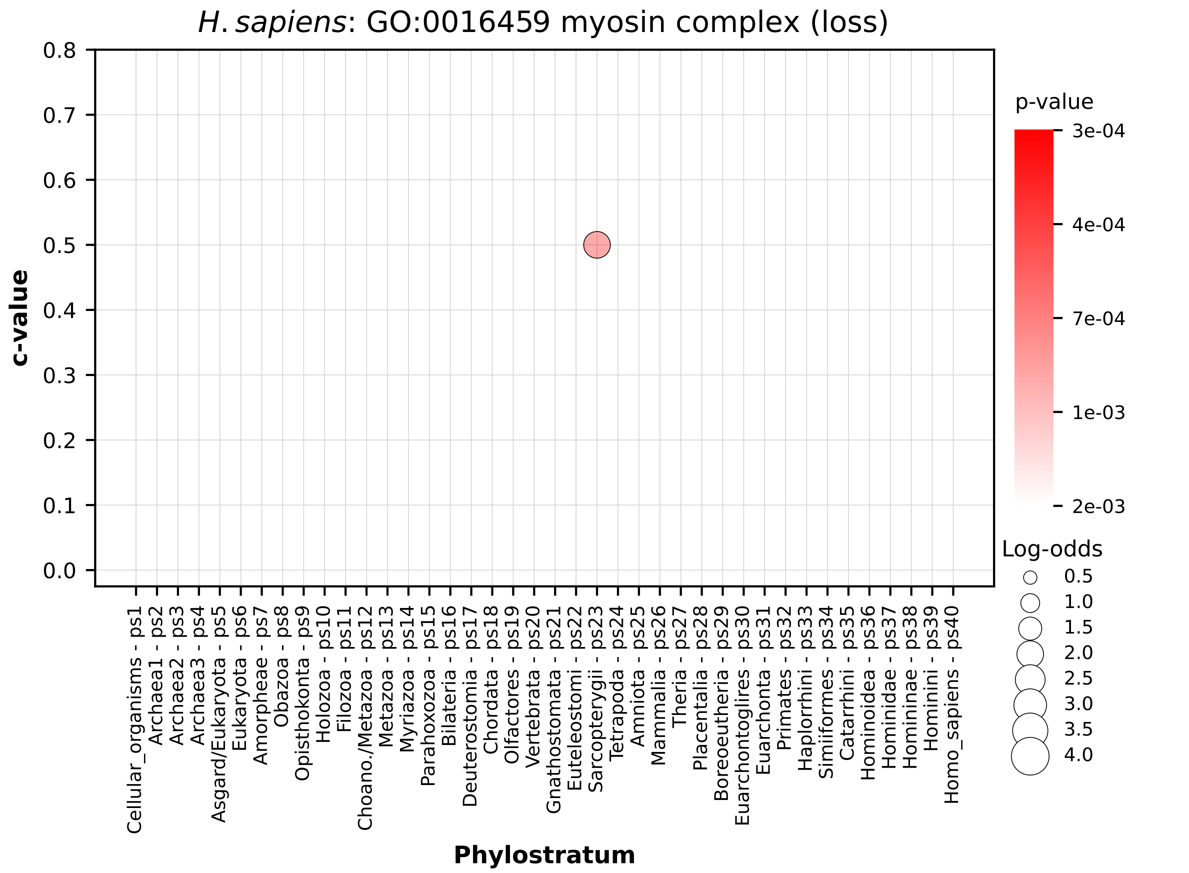

H. sapiens: GO:0016459 myosin complex (loss)
c-value
0.0
0.1
0.2
0.3
0.4
0.5
0.6
0.7
0.8
Phylostratum
Cellular_organisms - ps1
Archaea1 - ps2
Archaea2 - ps3
Archaea3 - ps4
Asgard/Eukaryota - ps5
Eukaryota - ps6
Amorpheae - ps7
Obazoa - ps8
Opisthokonta - ps9
Holozoa - ps10
Filozoa - ps11
Choano./Metazoa - ps12
Metazoa - ps13
Myriazoa - ps14
Parahoxozoa - ps15
Bilateria - ps16
Deuterostomia - ps17
Chordata - ps18
Olfactores - ps19
Vertebrata - ps20
Gnathostomata - ps21
Euteleostomi - ps22
Sarcopterygii - ps23
Tetrapoda - ps24
Amniota - ps25
Mammalia - ps26
Theria - ps27
Placentalia - ps28
Boreoeutheria - ps29
Euarchontoglires - ps30
Euarchonta - ps31
Primates - ps32
Haplorrhini - ps33
Simiiformes - ps34
Catarrhini - ps35
Hominoidea - ps36
Hominidae - ps37
Homininae - ps38
Hominini - ps39
Homo_sapiens - ps40
p-value
3e-04
4e-04
7e-04
1e-03
2e-03
Log-odds
0.5
1.0
1.5
2.0
2.5
3.0
3.5
4.0

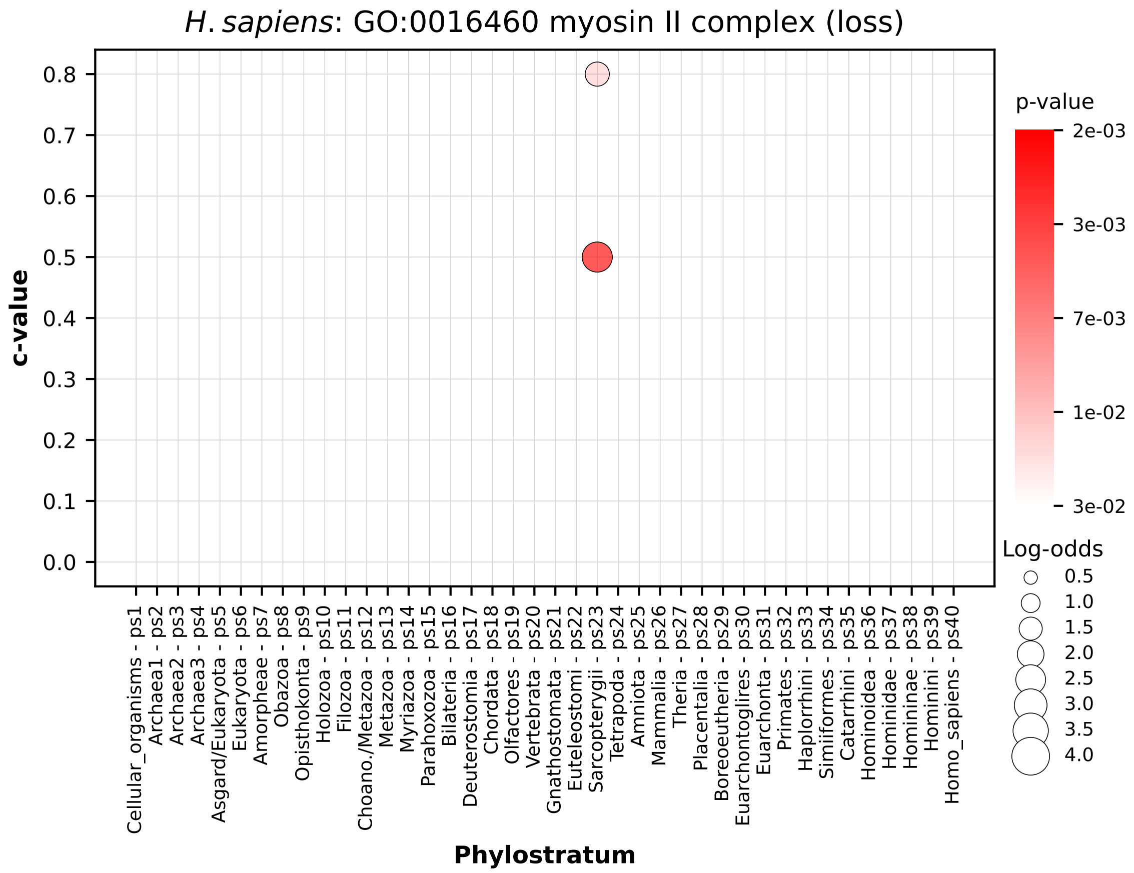

H. sapiens: GO:0016460 myosin II complex (loss)
c-value
0.8
0.7
0.6
0.5
0.4
0.3
0.2
0.1
0.0
Phylostratum
Cellular_organisms - ps1
Archaea1 - ps2
Archaea2 - ps3
Archaea3 - ps4
Asgard/Eukaryota - ps5
Eukaryota - ps6
Amorpheae - ps7
Obazoa - ps8
Opisthokonta - ps9
Holozoa - ps10
Filozoa - ps11
Choano./Metazoa - ps12
Metazoa - ps13
Myriazoa - ps14
Parahoxozoa - ps15
Bilateria - ps16
Deuterostomia - ps17
Chordata - ps18
Olfactores - ps19
Vertebrata - ps20
Gnathostomata - ps21
Euteleostomi - ps22
Sarcopterygii - ps23
Tetrapoda - ps24
Amniota - ps25
Mammalia - ps26
Theria - ps27
Placentalia - ps28
Boreoeutheria - ps29
Euarchontoglires - ps30
Euarchonta - ps31
Primates - ps32
Haplorrhini - ps33
Simiiformes - ps34
Catarrhini - ps35
Hominoidea - ps36
Hominidae - ps37
Homininae - ps38
Hominini - ps39
Homo_sapiens - ps40
p-value
2e-03
3e-03
7e-03
1e-02
3e-02
Log-odds
0.5
1.0
1.5
2.0
2.5
3.0
3.5
4.0

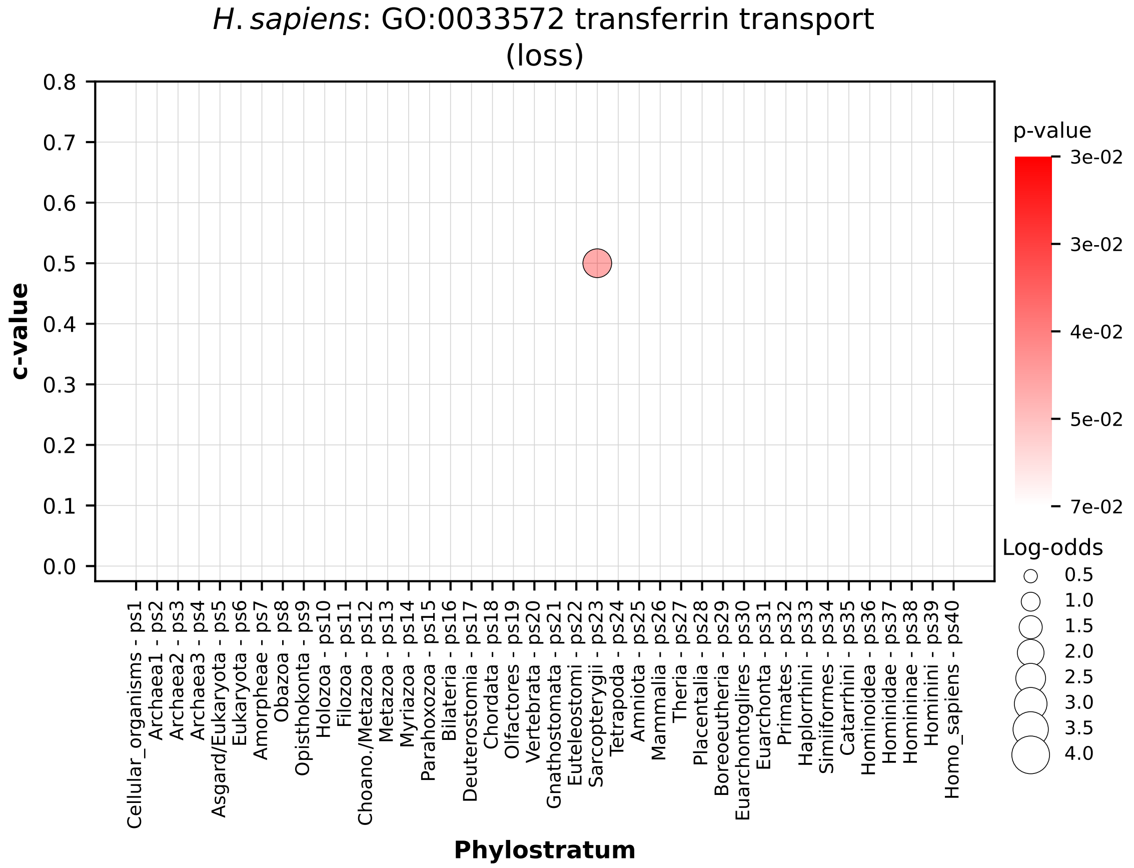

H. sapiens: GO:0033572 transferrin transport
(loss)
c-value
0.8
0.7
0.6
0.5
0.4
0.3
0.2
0.1
0.0
p-value
3e-02
3e-02
4e-02
5e-02
7e-02
Log-odds
0.5
1.0
1.5
2.0
2.5
3.0
3.5
4.0
Cellular_organisms - ps1
Archaea1 - ps2
Archaea2 - ps3
Archaea3 - ps4
Asgard/Eukaryota - ps5
Eukaryota - ps6
Amorpheae - ps7
Obazoa - ps8
Opisthokonta - ps9
Holozoa - ps10
Filozoa - ps11
Choano./Metazoa - ps12
Metazoa - ps13
Myriazoa - ps14
Parahoxozoa - ps15
Bilateria - ps16
Deuterostomia - ps17
Chordata - ps18
Olfactores - ps19
Vertebrata - ps20
Gnathostomata - ps21
Euteleostomi - ps22
Sarcopterygii - ps23
Tetrapoda - ps24
Amniota - ps25
Mammalia - ps26
Theria - ps27
Placentalia - ps28
Boreoeutheria - ps29
Euarchontoglires - ps30
Euarchonta - ps31
Primates - ps32
Haplorrhini - ps33
Simiiformes - ps34
Catarrhini - ps35
Hominoidea - ps36
Hominidae - ps37
Homininae - ps38
Hominini - ps39
Homo_sapiens - ps40
Phylostratum

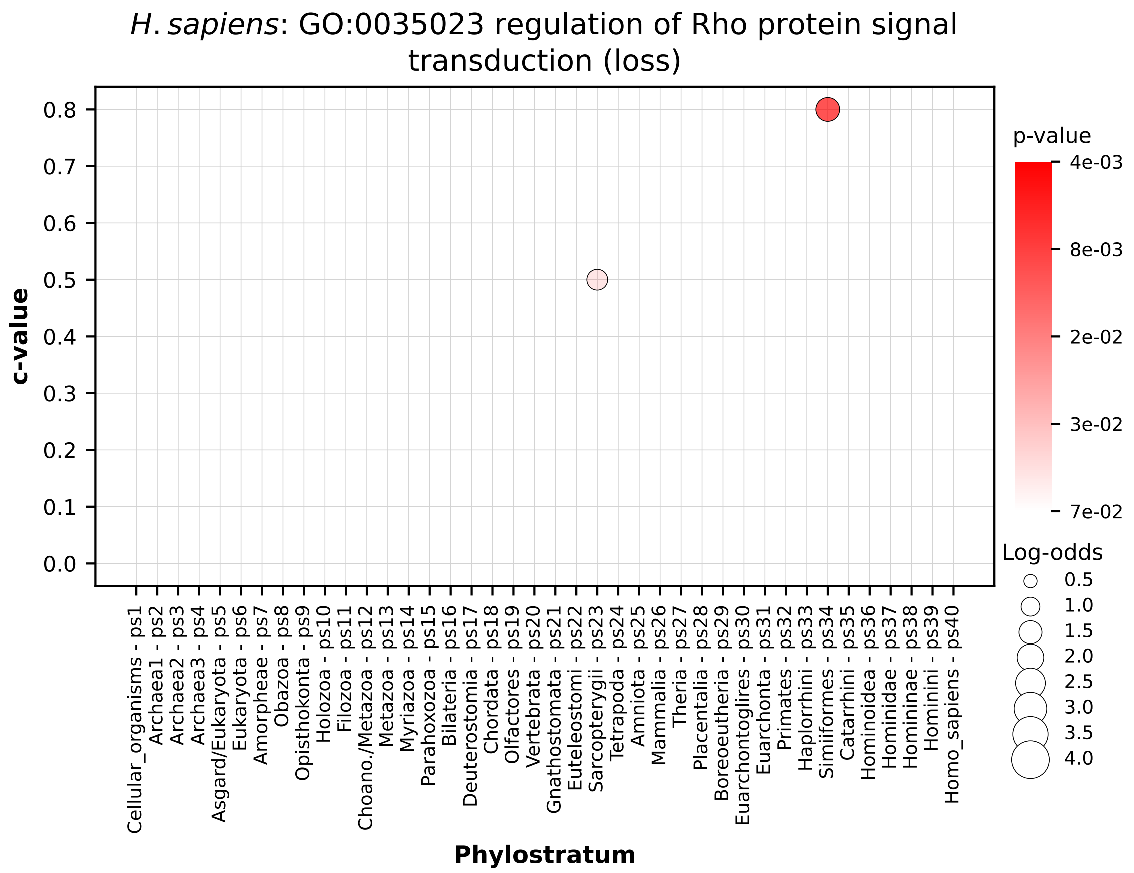

H. sapiens: GO:0035023 regulation of Rho protein signal transduction (loss)
c-value
0.8
0.7
0.6
0.5
0.4
0.3
0.2
0.1
0.0
Phylostratum
Cellular_organisms - ps1
Archaea1 - ps2
Archaea2 - ps3
Archaea3 - ps4
Asgard/Eukaryota - ps5
Eukaryota - ps6
Amorpheae - ps7
Obazoa - ps8
Opisthokonta - ps9
Holozoa - ps10
Filozoa - ps11
Choano./Metazoa - ps12
Metazoa - ps13
Myriazoa - ps14
Parahoxozoa - ps15
Bilateria - ps16
Deuterostomia - ps17
Chordata - ps18
Olfactores - ps19
Vertebrata - ps20
Gnathostomata - ps21
Euteleostomi - ps22
Sarcopterygii - ps23
Tetrapoda - ps24
Amniota - ps25
Mammalia - ps26
Theria - ps27
Placentalia - ps28
Boreoeutheria - ps29
Euarchontoglires - ps30
Euarchonta - ps31
Primates - ps32
Haplorrhini - ps33
Simiiformes - ps34
Catarrhini - ps35
Hominoidea - ps36
Hominidae - ps37
Homininae - ps38
Hominini - ps39
Homo_sapiens - ps40
p-value
4e-03
8e-03
2e-02
3e-02
7e-02
Log-odds
0.5
1.0
1.5
2.0
2.5
3.0
3.5
4.0

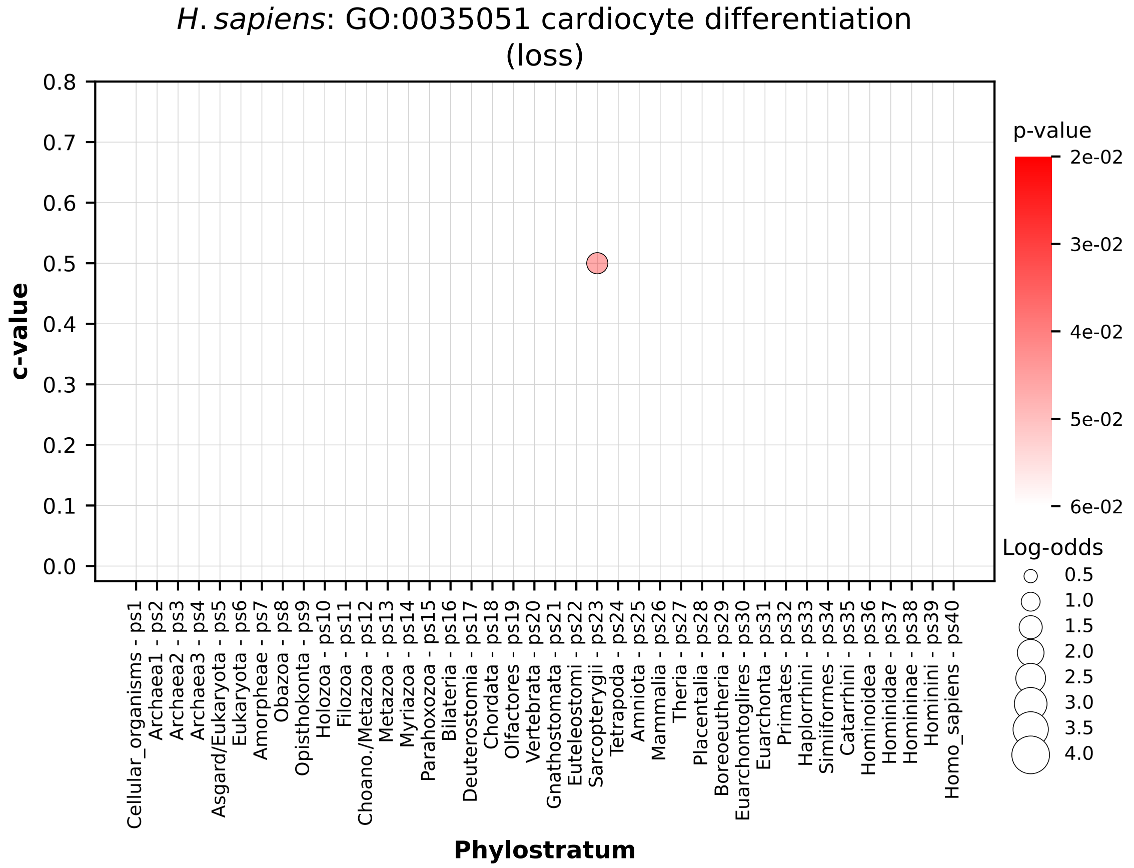
H. sapiens: GO:0035051 cardiocyte differentiation (loss)
c-value
0.8
0.7
0.6
0.5
0.4
0.3
0.2
0.1
0.0
p-value
2e-02
3e-02
4e-02
5e-02
6e-02
Log-odds
0.5
1.0
1.5
2.0
2.5
3.0
3.5
4.0
Cellular_organisms - ps1
Archaea1 - ps2
Archaea2 - ps3
Archaea3 - ps4
Asgard/Eukaryota - ps5
Eukaryota - ps6
Amorpheae - ps7
Obazoa - ps8
Opisthokonta - ps9
Holozoa - ps10
Filozoa - ps11
Choano./Metazoa - ps12
Metazoa - ps13
Myriazoa - ps14
Parahoxozoa - ps15
Bilateria - ps16
Deuterostomia - ps17
Chordata - ps18
Olfactores - ps19
Vertebrata - ps20
Gnathostomata - ps21
Euteleostomi - ps22
Sarcopterygii - ps23
Tetrapoda - ps24
Amniota - ps25
Mammalia - ps26
Theria - ps27
Placentalia - ps28
Boreoeutheria - ps29
Euarchontoglires - ps30
Euarchonta - ps31
Primates - ps32
Haplorrhini - ps33
Simiiformes - ps34
Catarrhini - ps35
Hominoidea - ps36
Hominidae - ps37
Homininae - ps38
Hominini - ps39
Homo_sapiens - ps40
Phylostratum

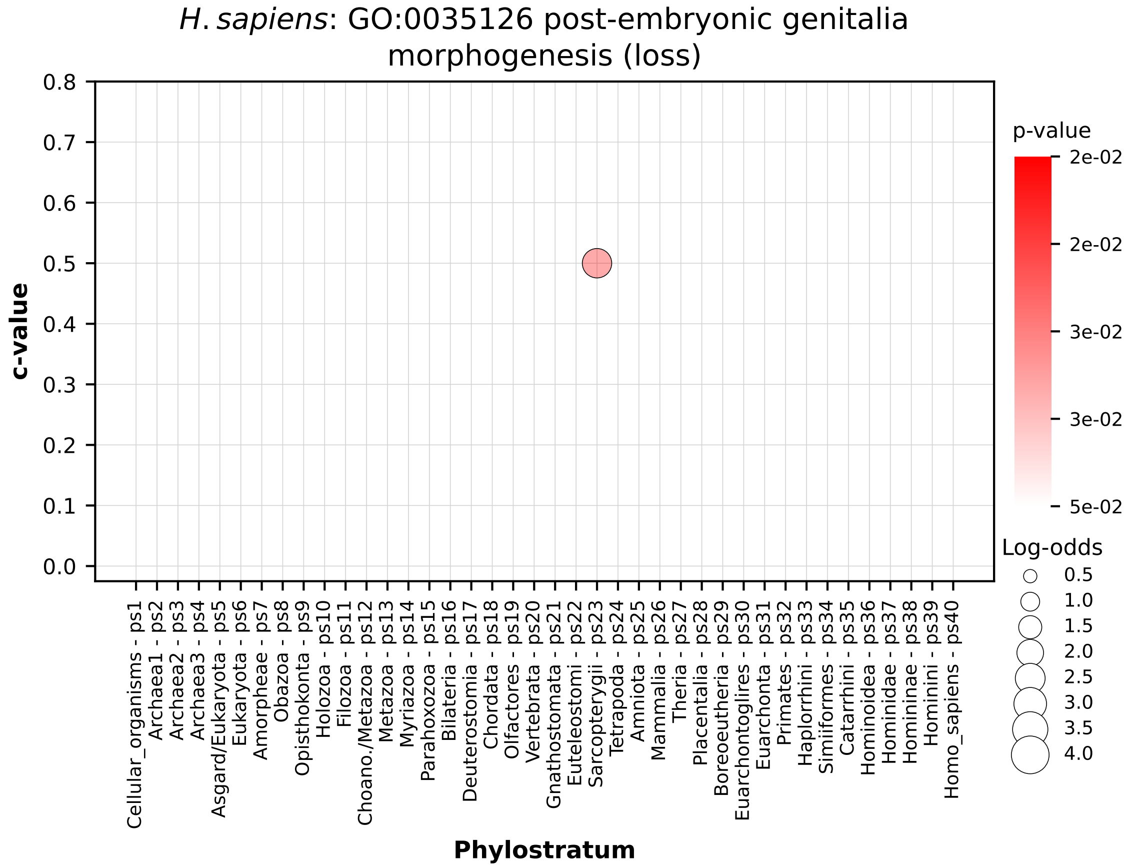

H. sapiens: GO:0035126 post-embryonic genitalia morphogenesis (loss)
c-value
0.8
0.7
0.6
0.5
0.4
0.3
0.2
0.1
0.0
Phylostratum
Cellular_organisms - ps1
Archaea1 - ps2
Archaea2 - ps3
Archaea3 - ps4
Asgard/Eukaryota - ps5
Eukaryota - ps6
Amorpheae - ps7
Obazoa - ps8
Opisthokonta - ps9
Holozoa - ps10
Filozoa - ps11
Choano./Metazoa - ps12
Metazoa - ps13
Myriazoa - ps14
Parahoxozoa - ps15
Bilateria - ps16
Deuterostomia - ps17
Chordata - ps18
Olfactores - ps19
Vertebrata - ps20
Gnathostomata - ps21
Euteleostomi - ps22
Sarcopterygii - ps23
Tetrapoda - ps24
Amniota - ps25
Mammalia - ps26
Theria - ps27
Placentalia - ps28
Boreoeutheria - ps29
Euarchontoglires - ps30
Euarchonta - ps31
Primates - ps32
Haplorrhini - ps33
Simiiformes - ps34
Catarrhini - ps35
Hominoidea - ps36
Hominidae - ps37
Homininae - ps38
Hominini - ps39
Homo_sapiens - ps40
p-value
2e-02
2e-02
3e-02
3e-02
5e-02
Log-odds
0.5
1.0
1.5
2.0
2.5
3.0
3.5
4.0

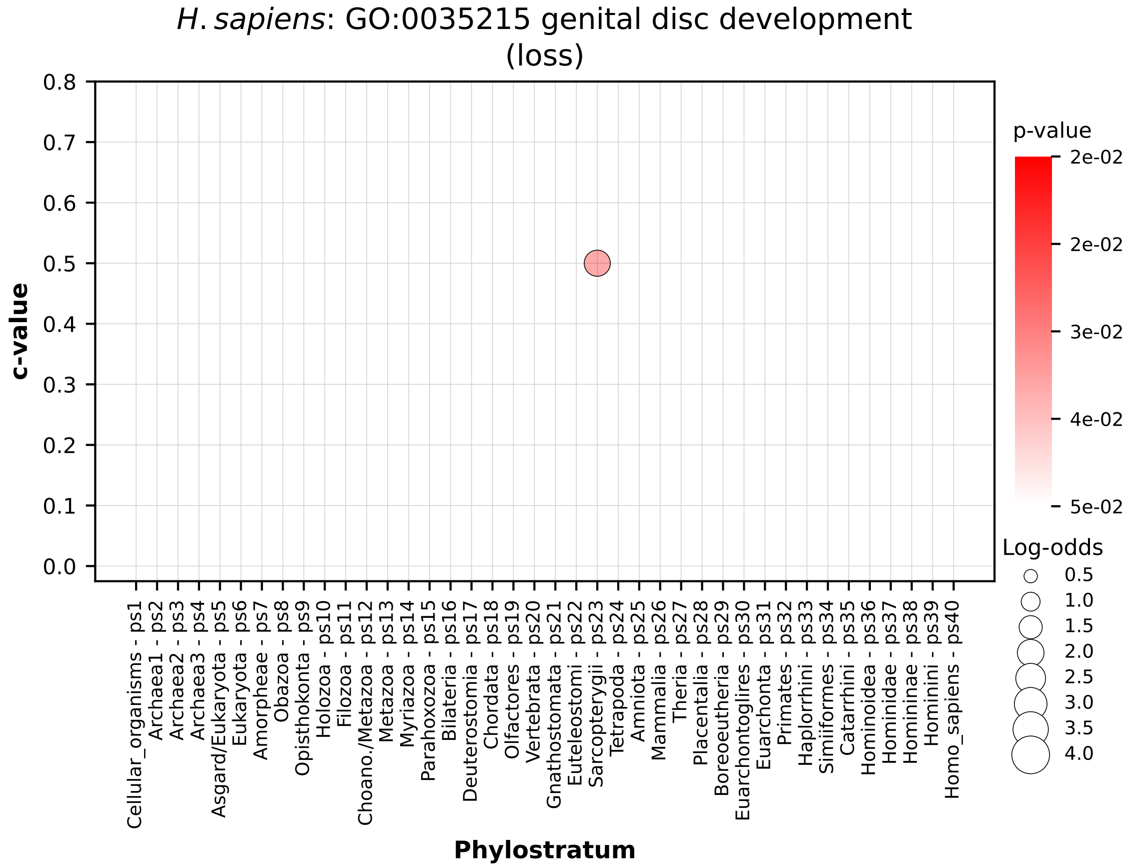

H. sapiens: GO:0035215 genital disc development
(loss)
c-value
0.8
0.7
0.6
0.5
0.4
0.3
0.2
0.1
0.0
p-value
2e-02
2e-02
3e-02
4e-02
5e-02
Log-odds
0.5
1.0
1.5
2.0
2.5
3.0
3.5
4.0
Cellular_organisms - ps1
Archaea1 - ps2
Archaea2 - ps3
Archaea3 - ps4
Asgard/Eukaryota - ps5
Eukaryota - ps6
Amorpheae - ps7
Obazoa - ps8
Opisthokonta - ps9
Holozoa - ps10
Filozoa - ps11
Choano./Metazoa - ps12
Metazoa - ps13
Myriazoa - ps14
Parahoxozoa - ps15
Bilateria - ps16
Deuterostomia - ps17
Chordata - ps18
Olfactores - ps19
Vertebrata - ps20
Gnathostomata - ps21
Euteleostomi - ps22
Sarcopterygii - ps23
Tetrapoda - ps24
Amniota - ps25
Mammalia - ps26
Theria - ps27
Placentalia - ps28
Boreoeutheria - ps29
Euarchontoglires - ps30
Euarchonta - ps31
Primates - ps32
Haplorrhini - ps33
Simiiformes - ps34
Catarrhini - ps35
Hominoidea - ps36
Hominidae - ps37
Homininae - ps38
Hominini - ps39
Homo_sapiens - ps40
Phylostratum

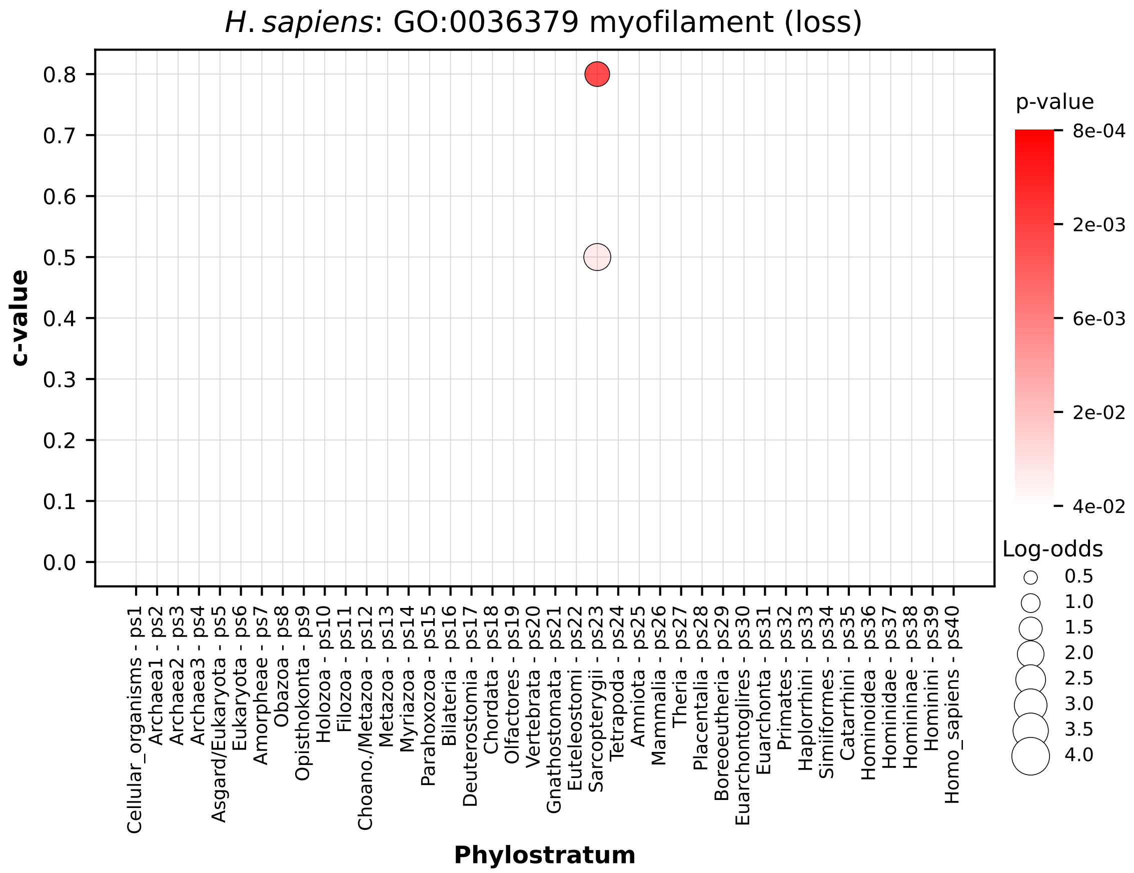
H. sapiens: GO:0036379 myofilament (loss)
c-value
0.8
0.7
0.6
0.5
0.4
0.3
0.2
0.1
0.0
p-value
8e-04
2e-03
6e-03
2e-02
4e-02
Log-odds
0.5
1.0
1.5
2.0
2.5
3.0
3.5
4.0
Cellular_organisms - ps1
Archaea1 - ps2
Archaea2 - ps3
Archaea3 - ps4
Asgard/Eukaryota - ps5
Eukaryota - ps6
Amorpheae - ps7
Obazoa - ps8
Opisthokonta - ps9
Holozoa - ps10
Filozoa - ps11
Choano./Metazoa - ps12
Metazoa - ps13
Myriazoa - ps14
Parahoxozoa - ps15
Bilateria - ps16
Deuterostomia - ps17
Chordata - ps18
Olfactores - ps19
Vertebrata - ps20
Gnathostomata - ps21
Euteleostomi - ps22
Sarcopterygii - ps23
Tetrapoda - ps24
Amniota - ps25
Mammalia - ps26
Theria - ps27
Placentalia - ps28
Boreoeutheria - ps29
Euarchontoglires - ps30
Euarchonta - ps31
Primates - ps32
Haplorrhini - ps33
Simiiformes - ps34
Catarrhini - ps35
Hominoidea - ps36
Hominidae - ps37
Homininae - ps38
Hominini - ps39
Homo_sapiens - ps40
Phylostratum

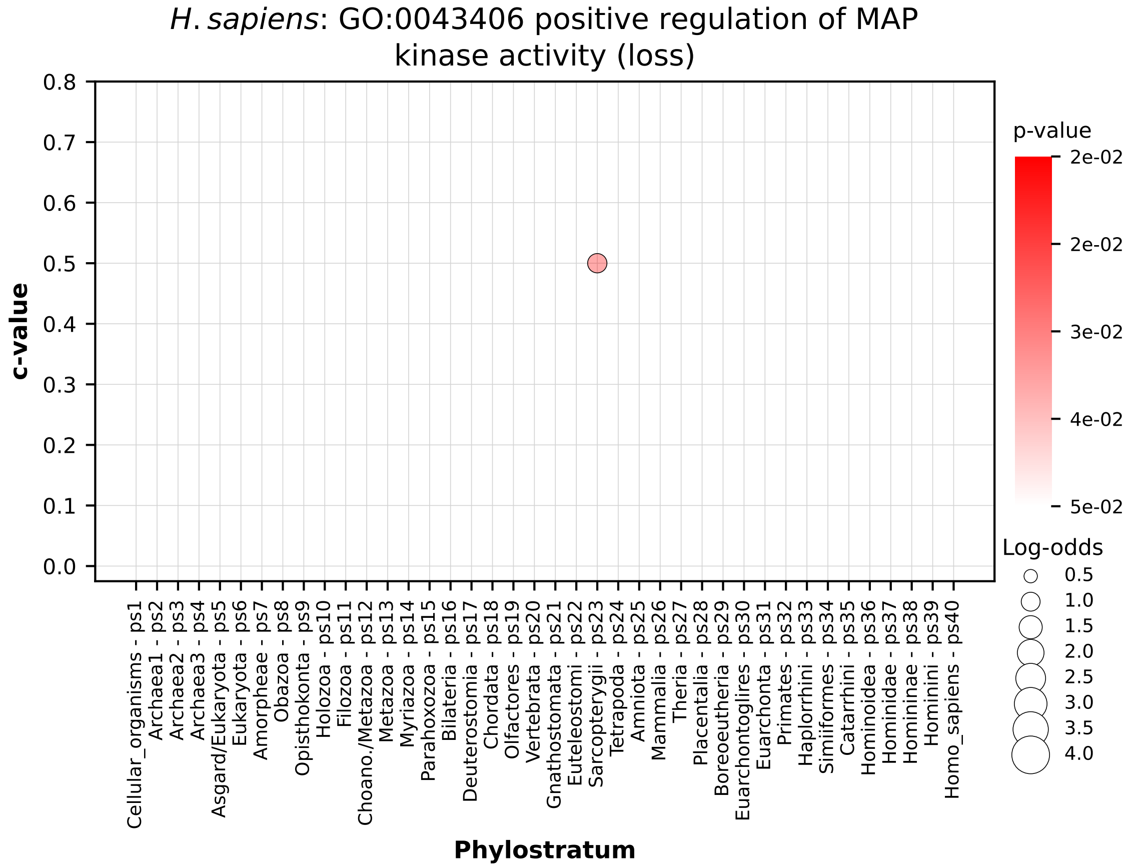

H. sapiens: GO:0043406 positive regulation of MAP kinase activity (loss)
c-value
0.8
0.7
0.6
0.5
0.4
0.3
0.2
0.1
0.0
Phylostratum
Cellular_organisms - ps1
Archaea1 - ps2
Archaea2 - ps3
Archaea3 - ps4
Asgard/Eukaryota - ps5
Eukaryota - ps6
Amorpheae - ps7
Obazoa - ps8
Opisthokonta - ps9
Holozoa - ps10
Filozoa - ps11
Choano./Metazoa - ps12
Metazoa - ps13
Myriazoa - ps14
Parahoxozoa - ps15
Bilateria - ps16
Deuterostomia - ps17
Chordata - ps18
Olfactores - ps19
Vertebrata - ps20
Gnathostomata - ps21
Euteleostomi - ps22
Sarcopterygii - ps23
Tetrapoda - ps24
Amniota - ps25
Mammalia - ps26
Theria - ps27
Placentalia - ps28
Boreoeutheria - ps29
Euarchontoglires - ps30
Euarchonta - ps31
Primates - ps32
Haplorrhini - ps33
Simiiformes - ps34
Catarrhini - ps35
Hominoidea - ps36
Hominidae - ps37
Homininae - ps38
Hominini - ps39
Homo_sapiens - ps40
p-value
2e-02
2e-02
3e-02
4e-02
5e-02
Log-odds
0.5
1.0
1.5
2.0
2.5
3.0
3.5
4.0

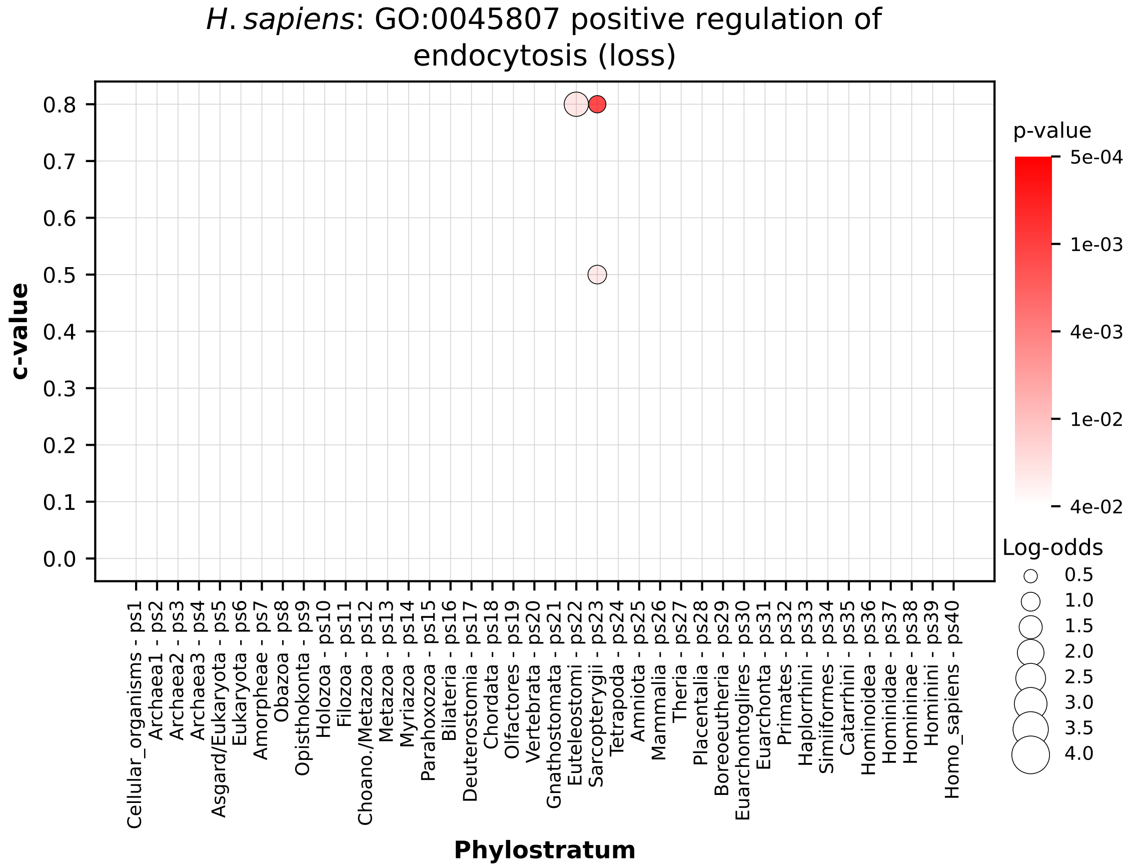

H. sapiens: GO:0045807 positive regulation of endocytosis (loss)
c-value
0.0
0.1
0.2
0.3
0.4
0.5
0.6
0.7
0.8
p-value
5e-04
1e-03
4e-03
1e-02
4e-02
Log-odds
0.5
1.0
1.5
2.0
2.5
3.0
3.5
4.0
Cellular_organisms - ps1
Archaea1 - ps2
Archaea2 - ps3
Archaea3 - ps4
Asgard/Eukaryota - ps5
Eukaryota - ps6
Amorpheae - ps7
Obazoa - ps8
Opisthokonta - ps9
Holozoa - ps10
Filozoa - ps11
Choano./Metazoa - ps12
Metazoa - ps13
Myriazoa - ps14
Parahoxozoa - ps15
Bilateria - ps16
Deuterostomia - ps17
Chordata - ps18
Olfactores - ps19
Vertebrata - ps20
Gnathostomata - ps21
Euteleostomi - ps22
Sarcopterygii - ps23
Tetrapoda - ps24
Amniota - ps25
Mammalia - ps26
Theria - ps27
Placentalia - ps28
Boreoeutheria - ps29
Euarchontoglires - ps30
Euarchonta - ps31
Primates - ps32
Haplorrhini - ps33
Simiiformes - ps34
Catarrhini - ps35
Hominoidea - ps36
Hominidae - ps37
Homininae - ps38
Hominini - ps39
Homo_sapiens - ps40
Phylostratum

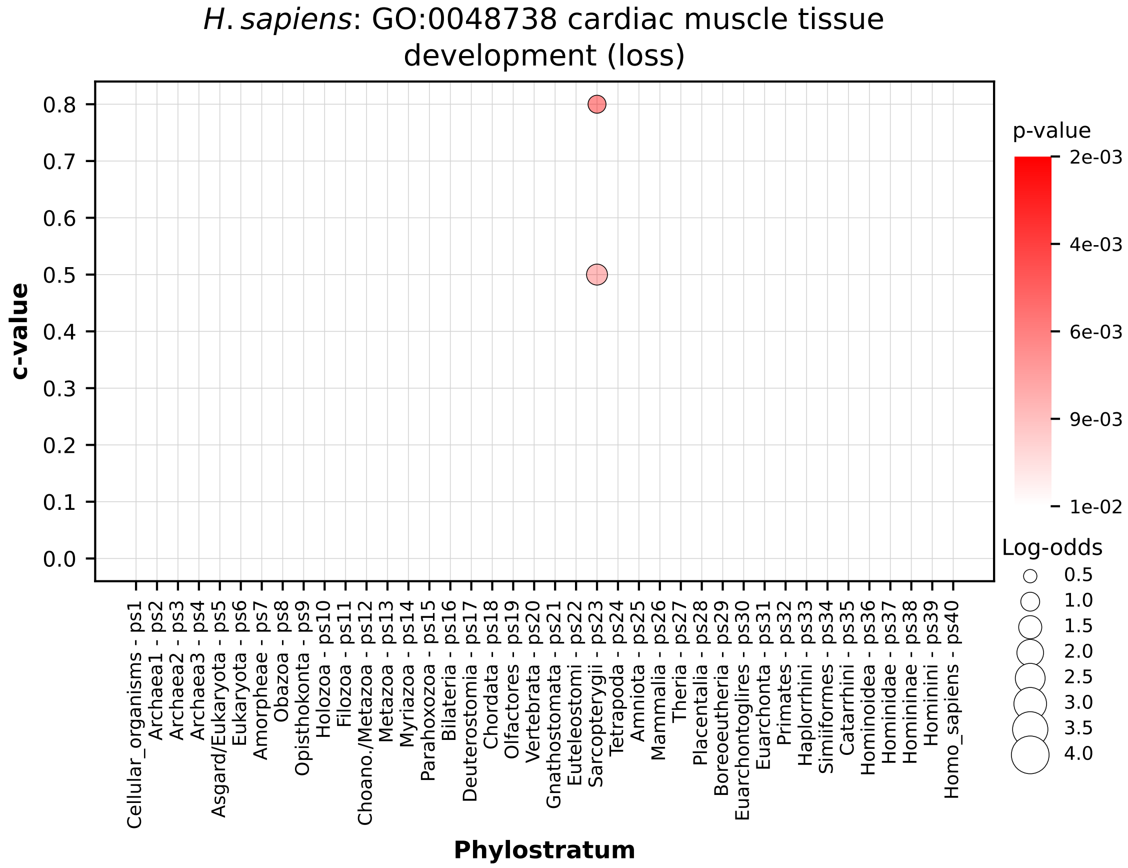

H. sapiens: GO:0048738 cardiac muscle tissue development (loss)
c-value
0.0
0.1
0.2
0.3
0.4
0.5
0.6
0.7
0.8
Phylostratum
Cellular_organisms - ps1
Archaea1 - ps2
Archaea2 - ps3
Archaea3 - ps4
Asgard/Eukaryota - ps5
Eukaryota - ps6
Amorpheae - ps7
Obazoa - ps8
Opisthokonta - ps9
Holozoa - ps10
Filozoa - ps11
Choano./Metazoa - ps12
Metazoa - ps13
Myriazoa - ps14
Parahoxozoa - ps15
Bilateria - ps16
Deuterostomia - ps17
Chordata - ps18
Olfactores - ps19
Vertebrata - ps20
Gnathostomata - ps21
Euteleostomi - ps22
Sarcopterygii - ps23
Tetrapoda - ps24
Amniota - ps25
Mammalia - ps26
Theria - ps27
Placentalia - ps28
Boreoeutheria - ps29
Euarchontoglires - ps30
Euarchonta - ps31
Primates - ps32
Haplorrhini - ps33
Simiiformes - ps34
Catarrhini - ps35
Hominoidea - ps36
Hominidae - ps37
Homininae - ps38
Hominini - ps39
Homo_sapiens - ps40
p-value
2e-03
4e-03
6e-03
9e-03
1e-02
Log-odds
0.5
1.0
1.5
2.0
2.5
3.0
3.5
4.0

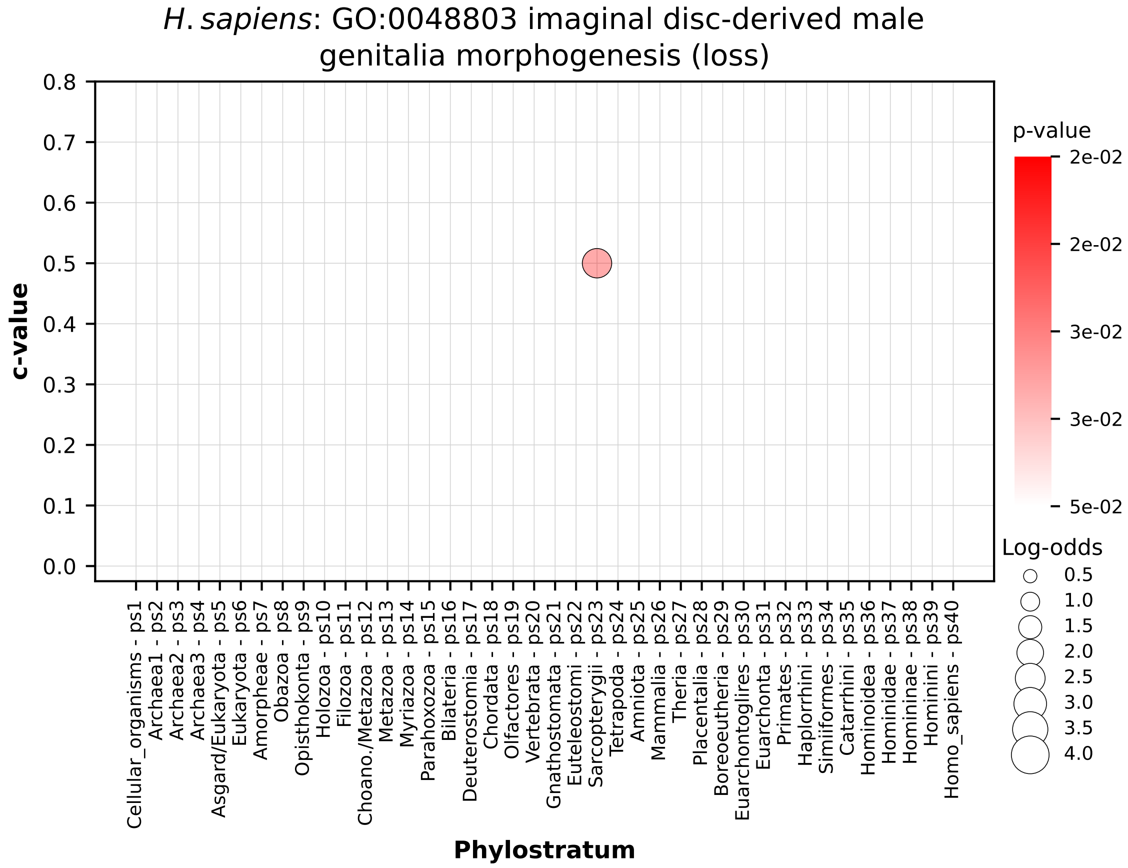

H. sapiens: GO:0048803 imaginal disc-derived male genitalia morphogenesis (loss)
c-value
0.8
0.7
0.6
0.5
0.4
0.3
0.2
0.1
0.0
p-value
2e-02
2e-02
3e-02
3e-02
5e-02
Log-odds
0.5
1.0
1.5
2.0
2.5
3.0
3.5
4.0
Cellular_organisms - ps1
Archaea1 - ps2
Archaea2 - ps3
Archaea3 - ps4
Asgard/Eukaryota - ps5
Eukaryota - ps6
Amorpheae - ps7
Obazoa - ps8
Opisthokonta - ps9
Holozoa - ps10
Filozoa - ps11
Choano./Metazoa - ps12
Metazoa - ps13
Myriazoa - ps14
Parahoxozoa - ps15
Bilateria - ps16
Deuterostomia - ps17
Chordata - ps18
Olfactores - ps19
Vertebrata - ps20
Gnathostomata - ps21
Euteleostomi - ps22
Sarcopterygii - ps23
Tetrapoda - ps24
Amniota - ps25
Mammalia - ps26
Theria - ps27
Placentalia - ps28
Boreoeutheria - ps29
Euarchontoglires - ps30
Euarchonta - ps31
Primates - ps32
Haplorrhini - ps33
Simiiformes - ps34
Catarrhini - ps35
Hominoidea - ps36
Hominidae - ps37
Homininae - ps38
Hominini - ps39
Homo_sapiens - ps40
Phylostratum

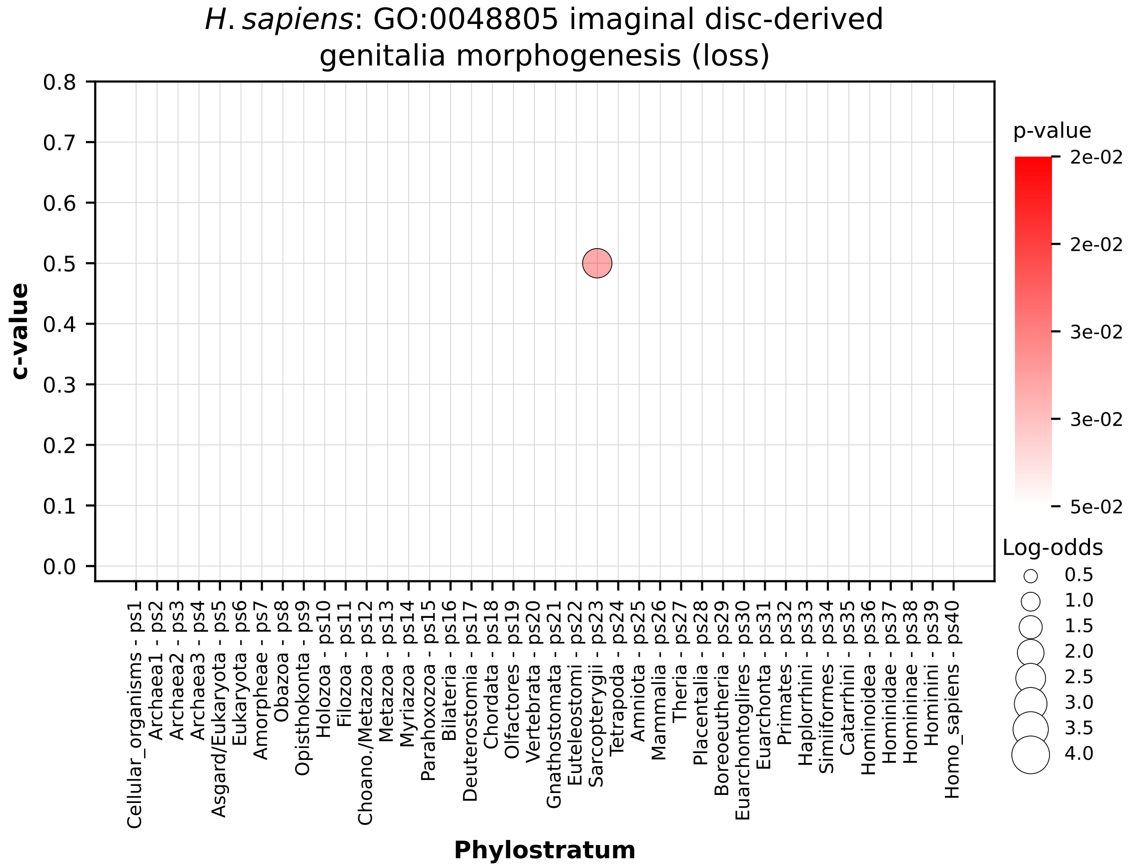

H. sapiens: GO:0048805 imaginal disc-derived genitalia morphogenesis (loss)
c-value
0.0
0.1
0.2
0.3
0.4
0.5
0.6
0.7
0.8
Phylostratum
Cellular_organisms - ps1
Archaea1 - ps2
Archaea2 - ps3
Archaea3 - ps4
Asgard/Eukaryota - ps5
Eukaryota - ps6
Amorpheae - ps7
Obazoa - ps8
Opisthokonta - ps9
Holozoa - ps10
Filozoa - ps11
Choano./Metazoa - ps12
Metazoa - ps13
Myriazoa - ps14
Parahoxozoa - ps15
Bilateria - ps16
Deuterostomia - ps17
Chordata - ps18
Olfactores - ps19
Vertebrata - ps20
Gnathostomata - ps21
Euteleostomi - ps22
Sarcopterygii - ps23
Tetrapoda - ps24
Amniota - ps25
Mammalia - ps26
Theria - ps27
Placentalia - ps28
Boreoeutheria - ps29
Euarchontoglires - ps30
Euarchonta - ps31
Primates - ps32
Haplorrhini - ps33
Simiiformes - ps34
Catarrhini - ps35
Hominoidea - ps36
Hominidae - ps37
Homininae - ps38
Hominini - ps39
Homo_sapiens - ps40
p-value
2e-02
2e-02
3e-02
3e-02
5e-02
Log-odds
0.5
1.0
1.5
2.0
2.5
3.0
3.5
4.0

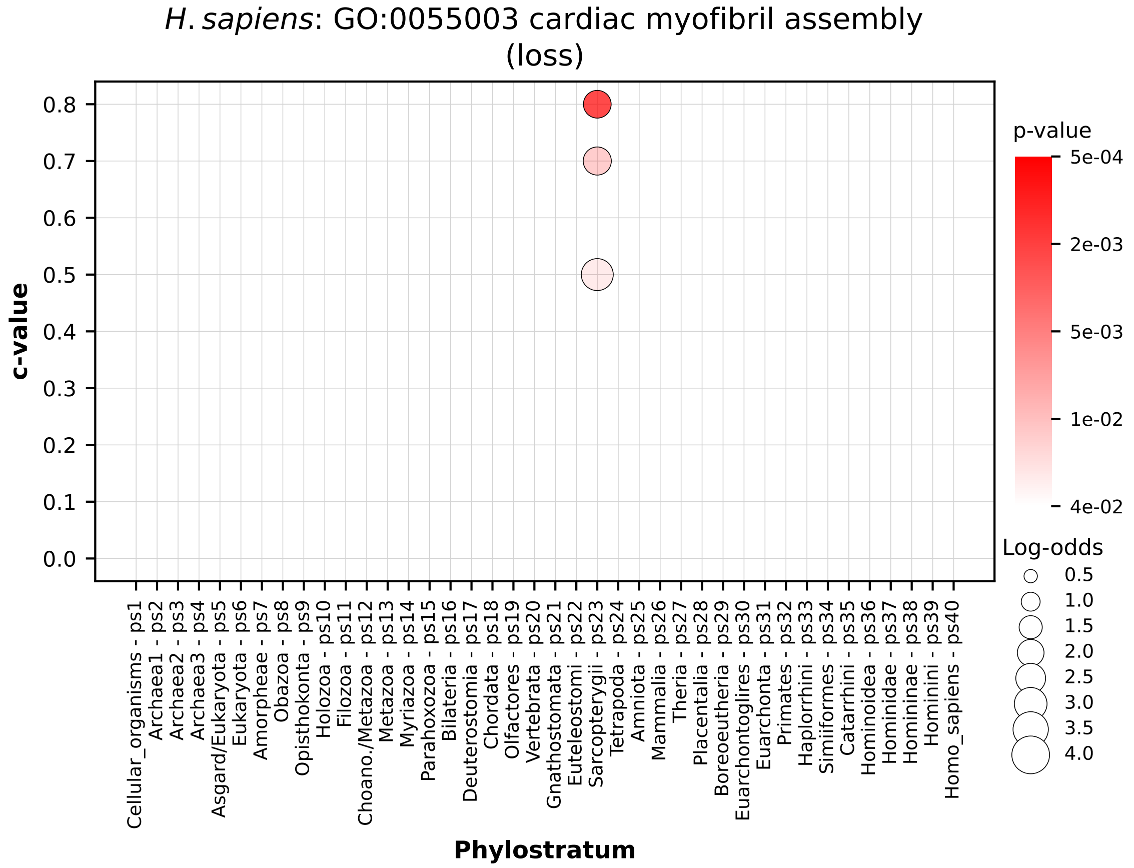

H. sapiens: GO:0055003 cardiac myofibril assembly (loss)
c-value
0.8
0.7
0.6
0.5
0.4
0.3
0.2
0.1
0.0
Phylostratum
Cellular_organisms - ps1
Archaea1 - ps2
Archaea2 - ps3
Archaea3 - ps4
Asgard/Eukaryota - ps5
Eukaryota - ps6
Amorpheae - ps7
Obazoa - ps8
Opisthokonta - ps9
Holozoa - ps10
Filozoa - ps11
Choano./Metazoa - ps12
Metazoa - ps13
Myriazoa - ps14
Parahoxozoa - ps15
Bilateria - ps16
Deuterostomia - ps17
Chordata - ps18
Olfactores - ps19
Vertebrata - ps20
Gnathostomata - ps21
Euteleostomi - ps22
Sarcopterygii - ps23
Tetrapoda - ps24
Amniota - ps25
Mammalia - ps26
Theria - ps27
Placentalia - ps28
Boreoeutheria - ps29
Euarchontoglires - ps30
Euarchonta - ps31
Primates - ps32
Haplorrhini - ps33
Simiiformes - ps34
Catarrhini - ps35
Hominoidea - ps36
Hominidae - ps37
Homininae - ps38
Hominini - ps39
Homo_sapiens - ps40
p-value
5e-04
2e-03
5e-03
1e-02
4e-02
Log-odds
0.5
1.0
1.5
2.0
2.5
3.0
3.5
4.0

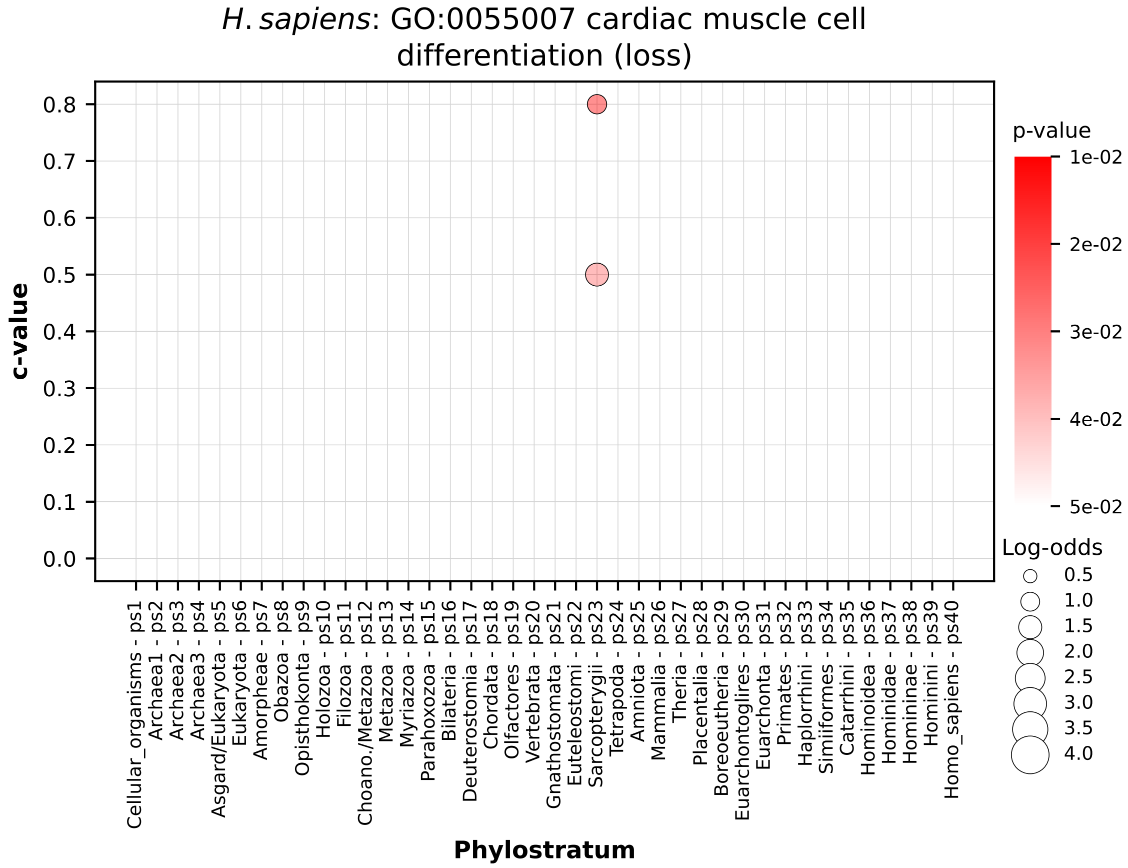

H. sapiens: GO:0055007 cardiac muscle cell differentiation (loss)
c-value
0.0
0.1
0.2
0.3
0.4
0.5
0.6
0.7
0.8
Phylostratum
Cellular_organisms - ps1
Archaea1 - ps2
Archaea2 - ps3
Archaea3 - ps4
Asgard/Eukaryota - ps5
Eukaryota - ps6
Amorpheae - ps7
Obazoa - ps8
Opisthokonta - ps9
Holozoa - ps10
Filozoa - ps11
Choano./Metazoa - ps12
Metazoa - ps13
Myriazoa - ps14
Parahoxozoa - ps15
Bilateria - ps16
Deuterostomia - ps17
Chordata - ps18
Olfactores - ps19
Vertebrata - ps20
Gnathostomata - ps21
Euteleostomi - ps22
Sarcopterygii - ps23
Tetrapoda - ps24
Amniota - ps25
Mammalia - ps26
Theria - ps27
Placentalia - ps28
Boreoeutheria - ps29
Euarchontoglires - ps30
Euarchonta - ps31
Primates - ps32
Haplorrhini - ps33
Simiiformes - ps34
Catarrhini - ps35
Hominoidea - ps36
Hominidae - ps37
Homininae - ps38
Hominini - ps39
Homo_sapiens - ps40
p-value
1e-02
2e-02
3e-02
4e-02
5e-02
Log-odds
0.5
1.0
1.5
2.0
2.5
3.0
3.5
4.0

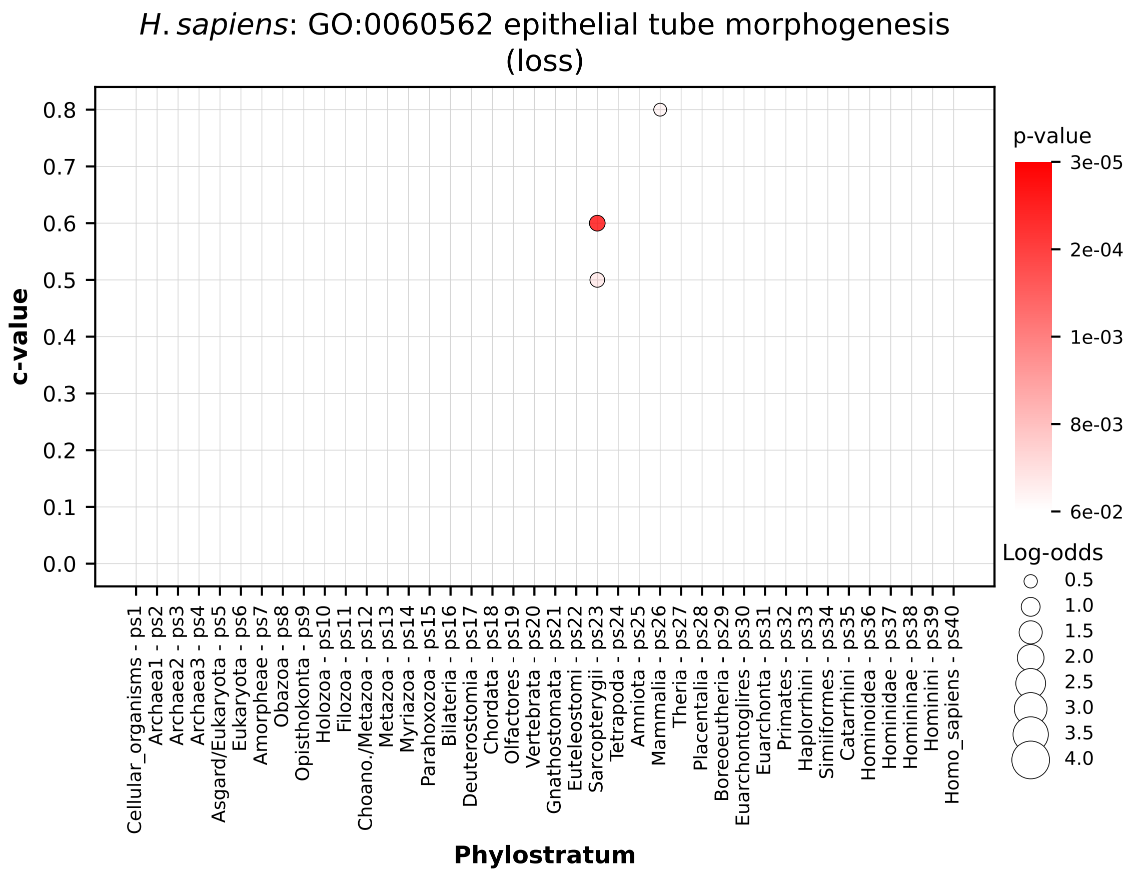

H. sapiens: GO:0060562 epithelial tube morphogenesis
(loss)
c-value
0.8
0.7
0.6
0.5
0.4
0.3
0.2
0.1
0.0
p-value
3e-05
2e-04
1e-03
8e-03
6e-02
Log-odds
0.5
1.0
1.5
2.0
2.5
3.0
3.5
4.0
Cellular_organisms - ps1
Archaea1 - ps2
Archaea2 - ps3
Archaea3 - ps4
Asgard/Eukaryota - ps5
Eukaryota - ps6
Amorpheae - ps7
Obazoa - ps8
Opisthokonta - ps9
Holozoa - ps10
Filozoa - ps11
Choano./Metazoa - ps12
Metazoa - ps13
Myriazoa - ps14
Parahoxozoa - ps15
Bilateria - ps16
Deuterostomia - ps17
Chordata - ps18
Olfactores - ps19
Vertebrata - ps20
Gnathostomata - ps21
Euteleostomi - ps22
Sarcopterygii - ps23
Tetrapoda - ps24
Amniota - ps25
Mammalia - ps26
Theria - ps27
Placentalia - ps28
Boreoeutheria - ps29
Euarchontoglires - ps30
Euarchonta - ps31
Primates - ps32
Haplorrhini - ps33
Simiiformes - ps34
Catarrhini - ps35
Hominoidea - ps36
Hominidae - ps37
Homininae - ps38
Hominini - ps39
Homo_sapiens - ps40
Phylostratum

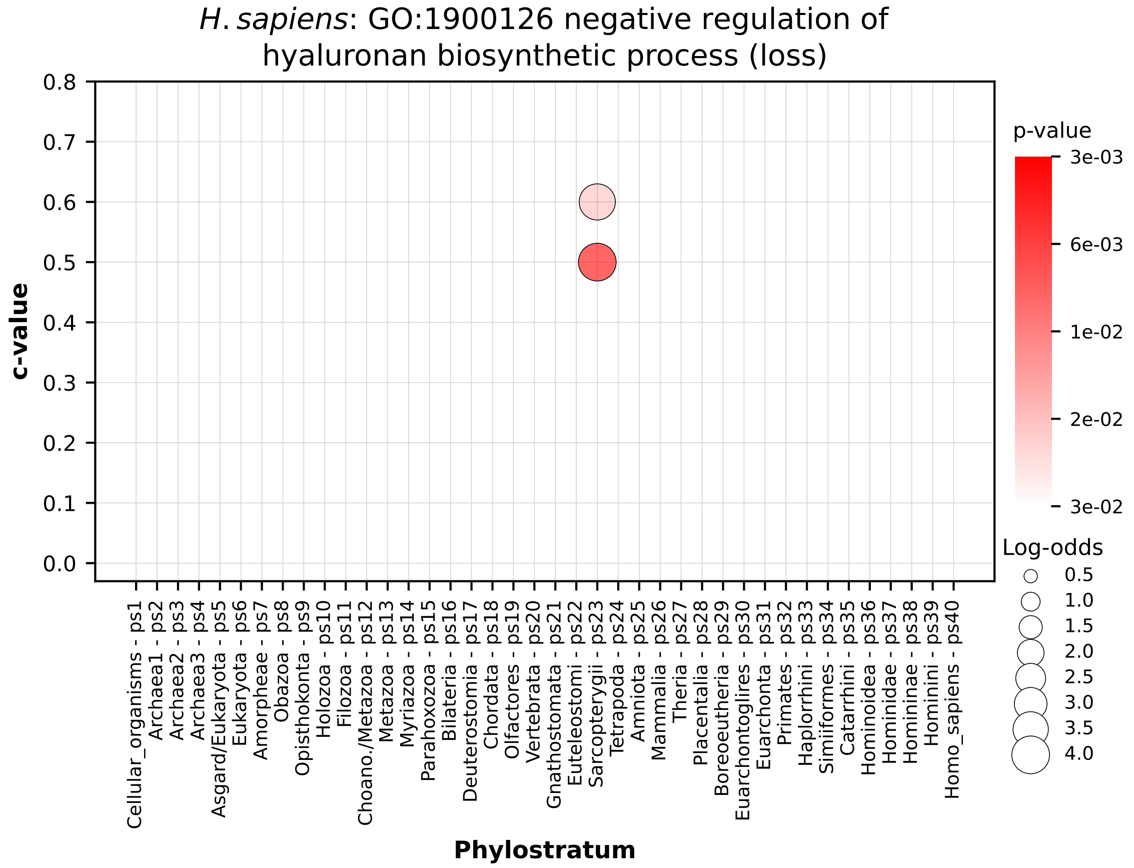

H. sapiens: GO:1900126 negative regulation of hyaluronan biosynthetic process (loss)
c-value
0.8
0.7
0.6
0.5
0.4
0.3
0.2
0.1
0.0
p-value
3e-03
6e-03
1e-02
2e-02
3e-02
Log-odds
0.5
1.0
1.5
2.0
2.5
3.0
3.5
4.0
Cellular_organisms - ps1
Archaea1 - ps2
Archaea2 - ps3
Archaea3 - ps4
Asgard/Eukaryota - ps5
Eukaryota - ps6
Amorpheae - ps7
Obazoa - ps8
Opisthokonta - ps9
Holozoa - ps10
Filozoa - ps11
Choano./Metazoa - ps12
Metazoa - ps13
Myriazoa - ps14
Parahoxozoa - ps15
Bilateria - ps16
Deuterostomia - ps17
Chordata - ps18
Olfactores - ps19
Vertebrata - ps20
Gnathostomata - ps21
Euteleostomi - ps22
Sarcopterygii - ps23
Tetrapoda - ps24
Amniota - ps25
Mammalia - ps26
Theria - ps27
Placentalia - ps28
Boreoeutheria - ps29
Euarchontoglires - ps30
Euarchonta - ps31
Primates - ps32
Haplorrhini - ps33
Simiiformes - ps34
Catarrhini - ps35
Hominoidea - ps36
Hominidae - ps37
Homininae - ps38
Hominini - ps39
Homo_sapiens - ps40
Phylostratum

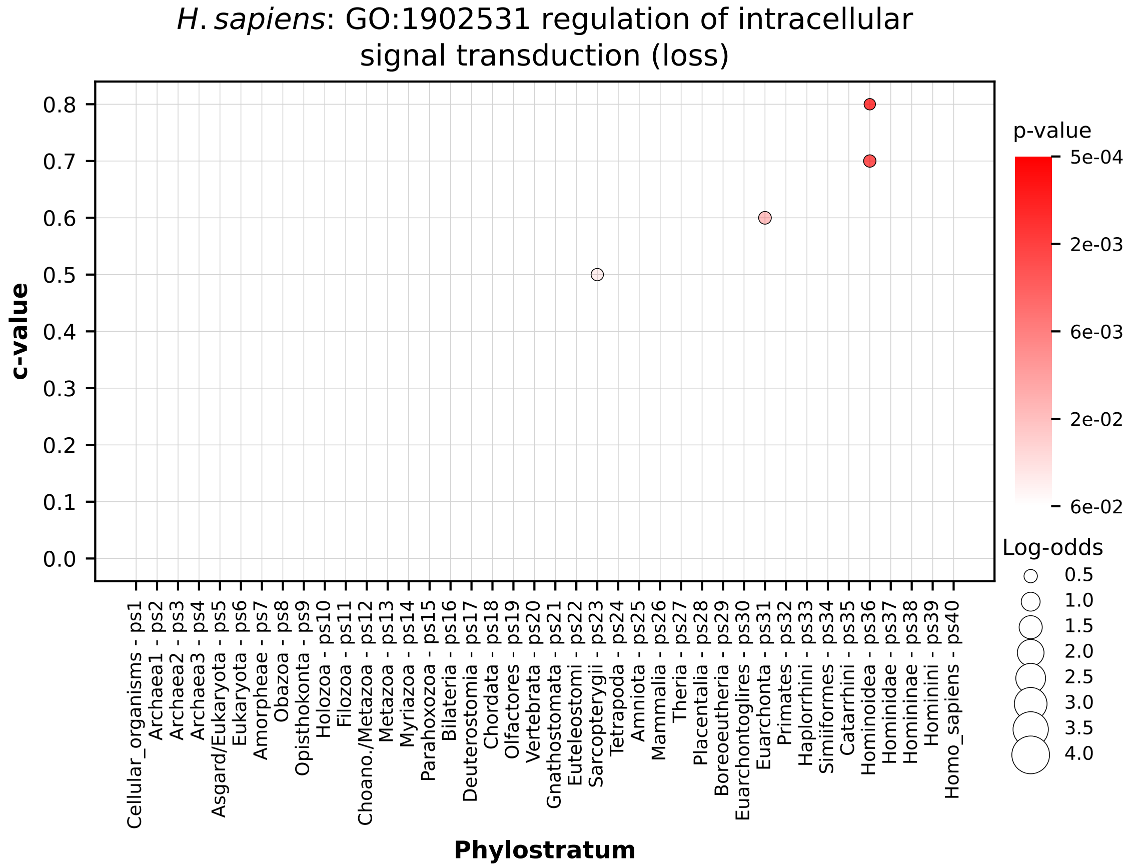

H. sapiens: GO:1902531 regulation of intracellular signal transduction (loss)
c-value
0.0
0.1
0.2
0.3
0.4
0.5
0.6
0.7
0.8
Phylostratum
Cellular_organisms - ps1
Archaea1 - ps2
Archaea2 - ps3
Archaea3 - ps4
Asgard/Eukaryota - ps5
Eukaryota - ps6
Amorpheae - ps7
Obazoa - ps8
Opisthokonta - ps9
Holozoa - ps10
Filozoa - ps11
Choano./Metazoa - ps12
Metazoa - ps13
Myriazoa - ps14
Parahoxozoa - ps15
Bilateria - ps16
Deuterostomia - ps17
Chordata - ps18
Olfactores - ps19
Vertebrata - ps20
Gnathostomata - ps21
Euteleostomi - ps22
Sarcopterygii - ps23
Tetrapoda - ps24
Amniota - ps25
Mammalia - ps26
Theria - ps27
Placentalia - ps28
Boreoeutheria - ps29
Euarchontoglires - ps30
Euarchonta - ps31
Primates - ps32
Haplorrhini - ps33
Simiiformes - ps34
Catarrhini - ps35
Hominoidea - ps36
Hominidae - ps37
Homininae - ps38
Hominini - ps39
Homo_sapiens - ps40
p-value
5e-04
2e-03
6e-03
2e-02
6e-02
Log-odds
0.5
1.0
1.5
2.0
2.5
3.0
3.5
4.0

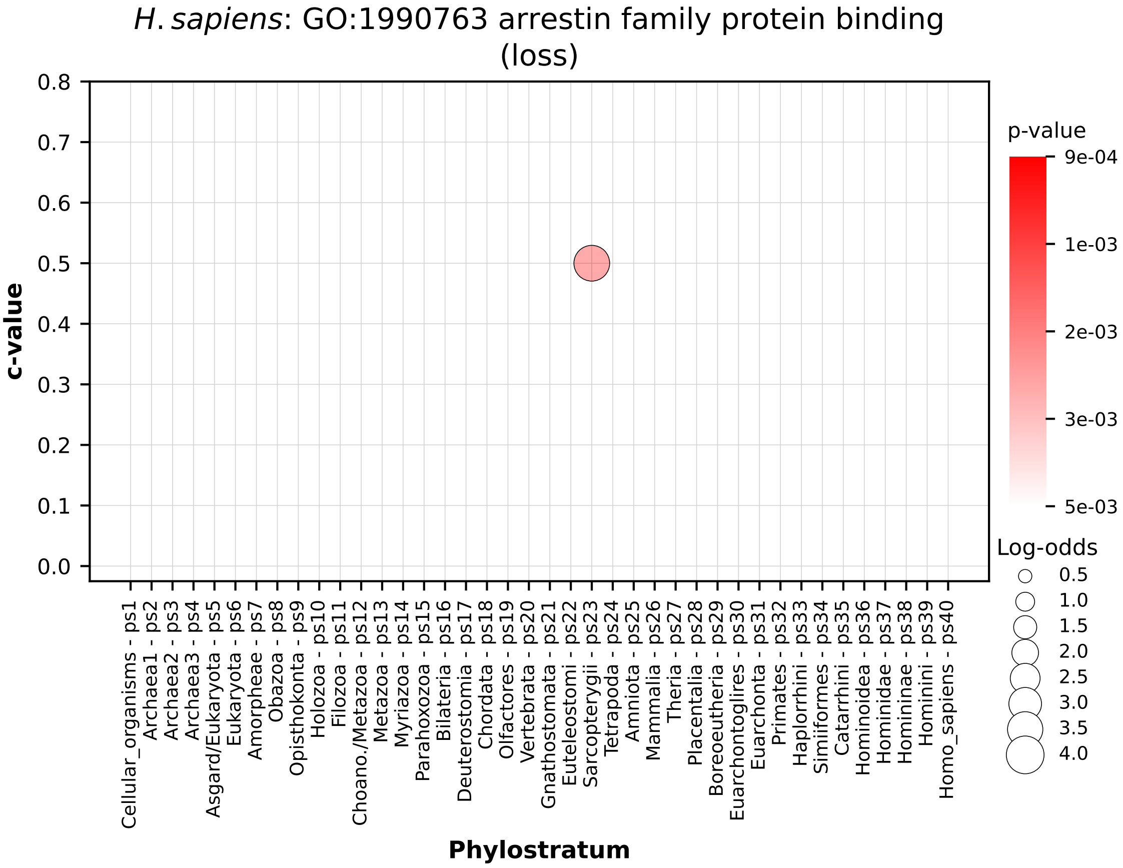

H. sapiens: GO:1990763 arrestin family protein binding (loss)
c-value
0.8
0.7
0.6
0.5
0.4
0.3
0.2
0.1
0.0
p-value
9e-04
1e-03
2e-03
3e-03
5e-03
Log-odds
0.5
1.0
1.5
2.0
2.5
3.0
3.5
4.0
Cellular_organisms - ps1
Archaea1 - ps2
Archaea2 - ps3
Archaea3 - ps4
Asgard/Eukaryota - ps5
Eukaryota - ps6
Amorpheae - ps7
Obazoa - ps8
Opisthokonta - ps9
Holozoa - ps10
Filozoa - ps11
Choano./Metazoa - ps12
Metazoa - ps13
Myriazoa - ps14
Parahoxozoa - ps15
Bilateria - ps16
Deuterostomia - ps17
Chordata - ps18
Olfactores - ps19
Vertebrata - ps20
Gnathostomata - ps21
Euteleostomi - ps22
Sarcopterygii - ps23
Tetrapoda - ps24
Amniota - ps25
Mammalia - ps26
Theria - ps27
Placentalia - ps28
Boreoeutheria - ps29
Euarchontoglires - ps30
Euarchonta - ps31
Primates - ps32
Haplorrhini - ps33
Simiiformes - ps34
Catarrhini - ps35
Hominoidea - ps36
Hominidae - ps37
Homininae - ps38
Hominini - ps39
Homo_sapiens - ps40
Phylostratum

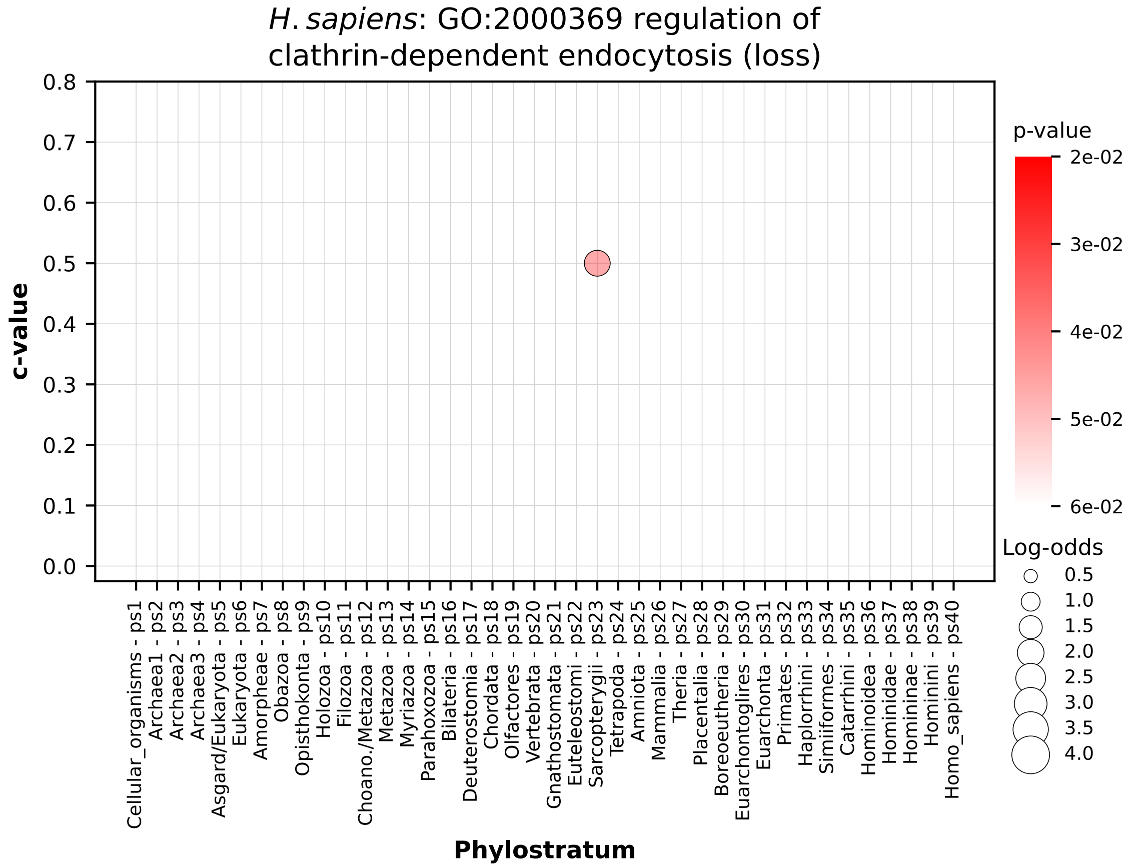

H. sapiens: GO:2000369 regulation of clathrin-dependent endocytosis (loss)
c-value
0.0
0.1
0.2
0.3
0.4
0.5
0.6
0.7
0.8
Phylostratum
Cellular_organisms - ps1
Archaea1 - ps2
Archaea2 - ps3
Archaea3 - ps4
Asgard/Eukaryota - ps5
Eukaryota - ps6
Amorpheae - ps7
Obazoa - ps8
Opisthokonta - ps9
Holozoa - ps10
Filozoa - ps11
Choano./Metazoa - ps12
Metazoa - ps13
Myriazoa - ps14
Parahoxozoa - ps15
Bilateria - ps16
Deuterostomia - ps17
Chordata - ps18
Olfactores - ps19
Vertebrata - ps20
Gnathostomata - ps21
Euteleostomi - ps22
Sarcopterygii - ps23
Tetrapoda - ps24
Amniota - ps25
Mammalia - ps26
Theria - ps27
Placentalia - ps28
Boreoeutheria - ps29
Euarchontoglires - ps30
Euarchonta - ps31
Primates - ps32
Haplorrhini - ps33
Simiiformes - ps34
Catarrhini - ps35
Hominoidea - ps36
Hominidae - ps37
Homininae - ps38
Hominini - ps39
Homo_sapiens - ps40
p-value
2e-02
3e-02
4e-02
5e-02
6e-02
Log-odds
0.5
1.0
1.5
2.0
2.5
3.0
3.5
4.0

H. sapiens: GO:2000370 positive regulation of clathrin-dependent endocytosis (loss)

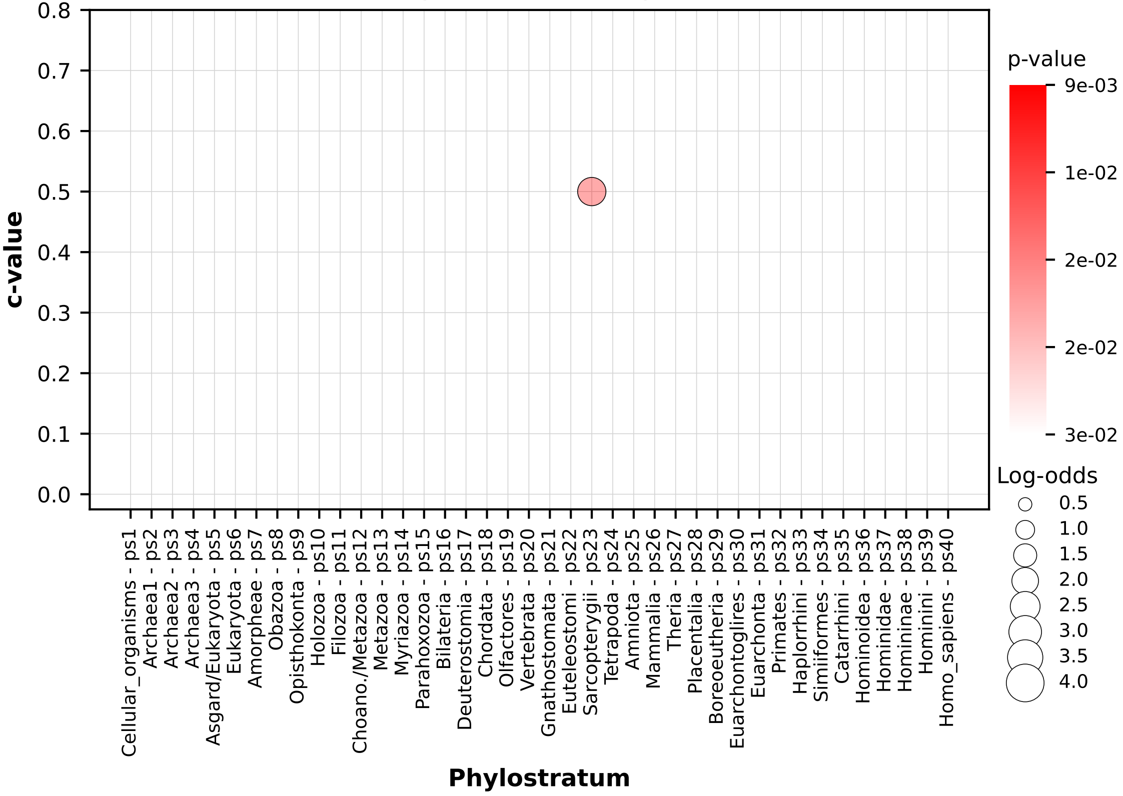

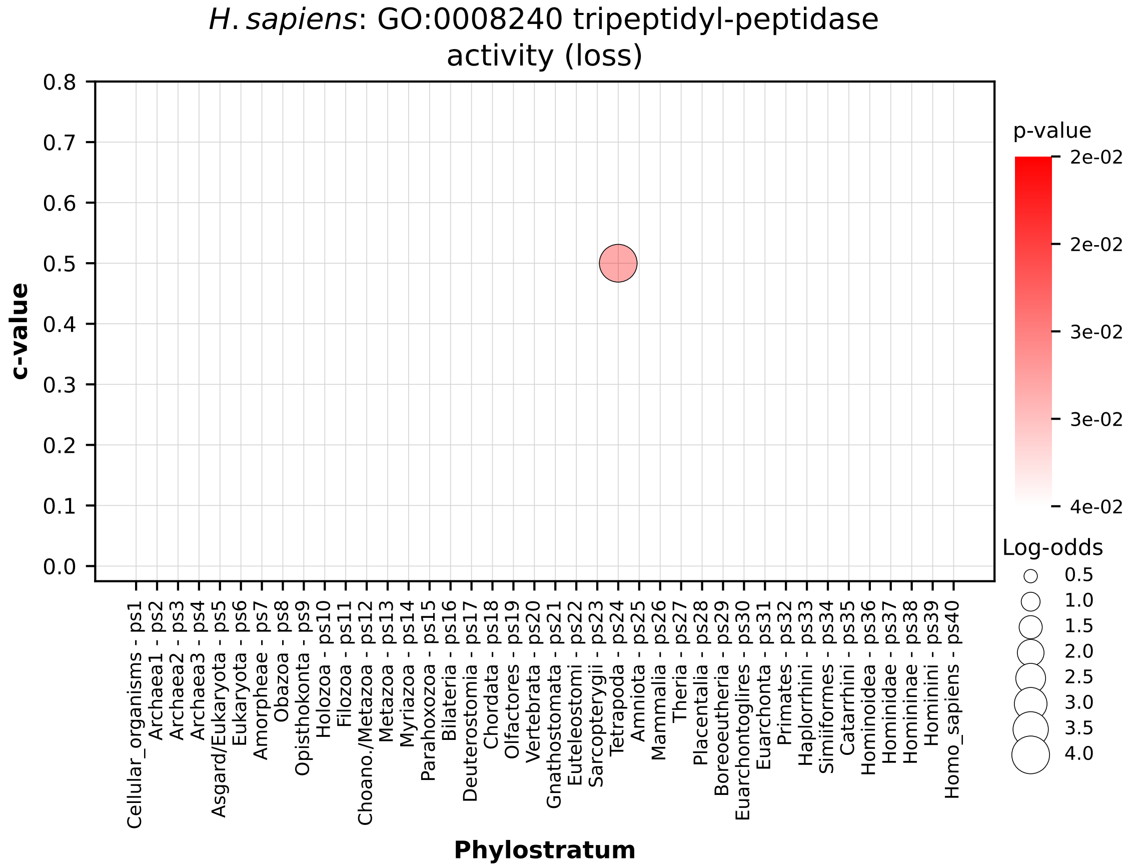

H. sapiens: GO:0008240 tripeptidyl-peptidase activity (loss)
c-value
0.8
0.7
0.6
0.5
0.4
0.3
0.2
0.1
0.0
Phylostratum
Cellular_organisms - ps1
Archaea1 - ps2
Archaea2 - ps3
Archaea3 - ps4
Asgard/Eukaryota - ps5
Eukaryota - ps6
Amorpheae - ps7
Obazoa - ps8
Opisthokonta - ps9
Holozoa - ps10
Filozoa - ps11
Choano./Metazoa - ps12
Metazoa - ps13
Myriazoa - ps14
Parahoxozoa - ps15
Bilateria - ps16
Deuterostomia - ps17
Chordata - ps18
Olfactores - ps19
Vertebrata - ps20
Gnathostomata - ps21
Euteleostomi - ps22
Sarcopterygii - ps23
Tetrapoda - ps24
Amniota - ps25
Mammalia - ps26
Theria - ps27
Placentalia - ps28
Boreoeutheria - ps29
Euarchontoglires - ps30
Euarchonta - ps31
Primates - ps32
Haplorrhini - ps33
Simiiformes - ps34
Catarrhini - ps35
Hominoidea - ps36
Hominidae - ps37
Homininae - ps38
Hominini - ps39
Homo_sapiens - ps40
p-value
2e-02
2e-02
3e-02
3e-02
4e-02
Log-odds
0.5
1.0
1.5
2.0
2.5
3.0
3.5
4.0

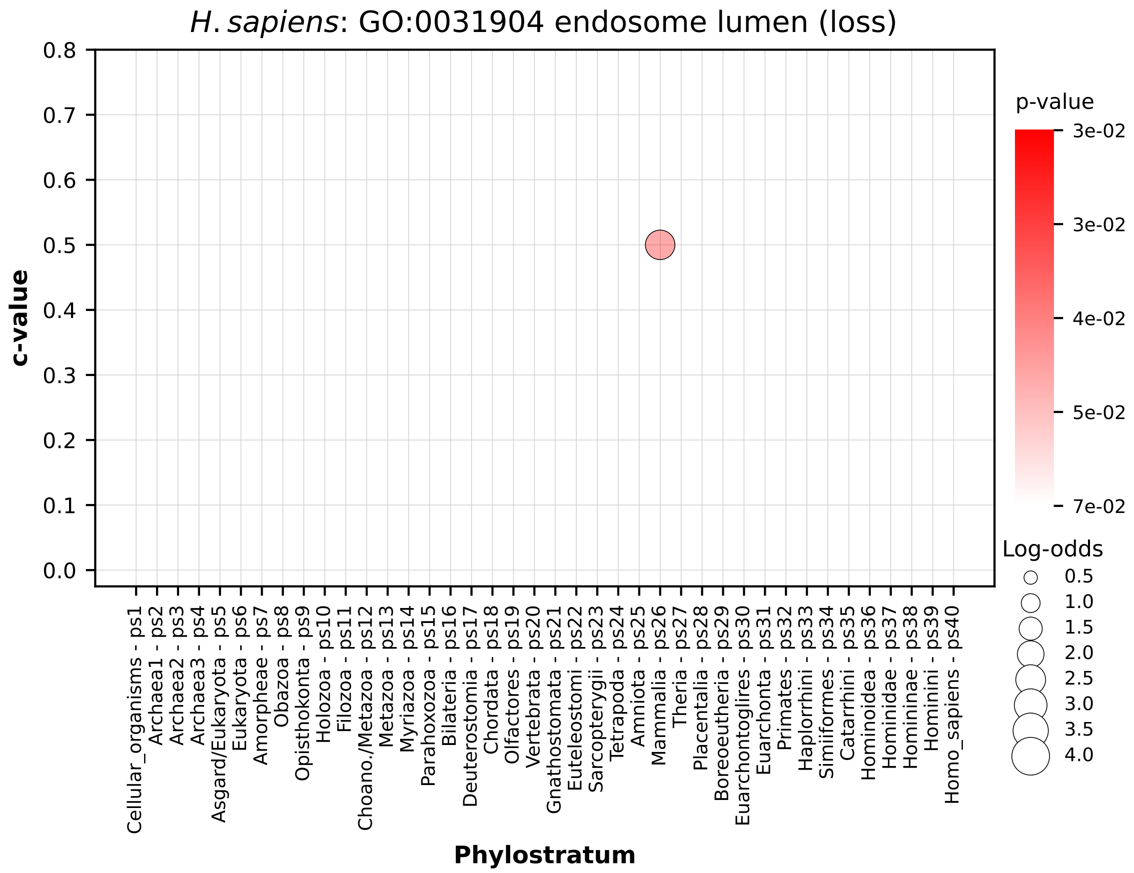

H. sapiens: GO:0031904 endosome lumen (loss)
c-value
0.8
0.7
0.6
0.5
0.4
0.3
0.2
0.1
0.0
p-value
3e-02
3e-02
4e-02
5e-02
7e-02
Log-odds
0.5
1.0
1.5
2.0
2.5
3.0
3.5
4.0
Cellular_organisms - ps1
Archaea1 - ps2
Archaea2 - ps3
Archaea3 - ps4
Asgard/Eukaryota - ps5
Eukaryota - ps6
Amorpheae - ps7
Obazoa - ps8
Opisthokonta - ps9
Holozoa - ps10
Filozoa - ps11
Choano./Metazoa - ps12
Metazoa - ps13
Myriazoa - ps14
Parahoxozoa - ps15
Bilateria - ps16
Deuterostomia - ps17
Chordata - ps18
Olfactores - ps19
Vertebrata - ps20
Gnathostomata - ps21
Euteleostomi - ps22
Sarcopterygii - ps23
Tetrapoda - ps24
Amniota - ps25
Mammalia - ps26
Theria - ps27
Placentalia - ps28
Boreoeutheria - ps29
Euarchontoglires - ps30
Euarchonta - ps31
Primates - ps32
Haplorrhini - ps33
Simiiformes - ps34
Catarrhini - ps35
Hominoidea - ps36
Hominidae - ps37
Homininae - ps38
Hominini - ps39
Homo_sapiens - ps40
Phylostratum

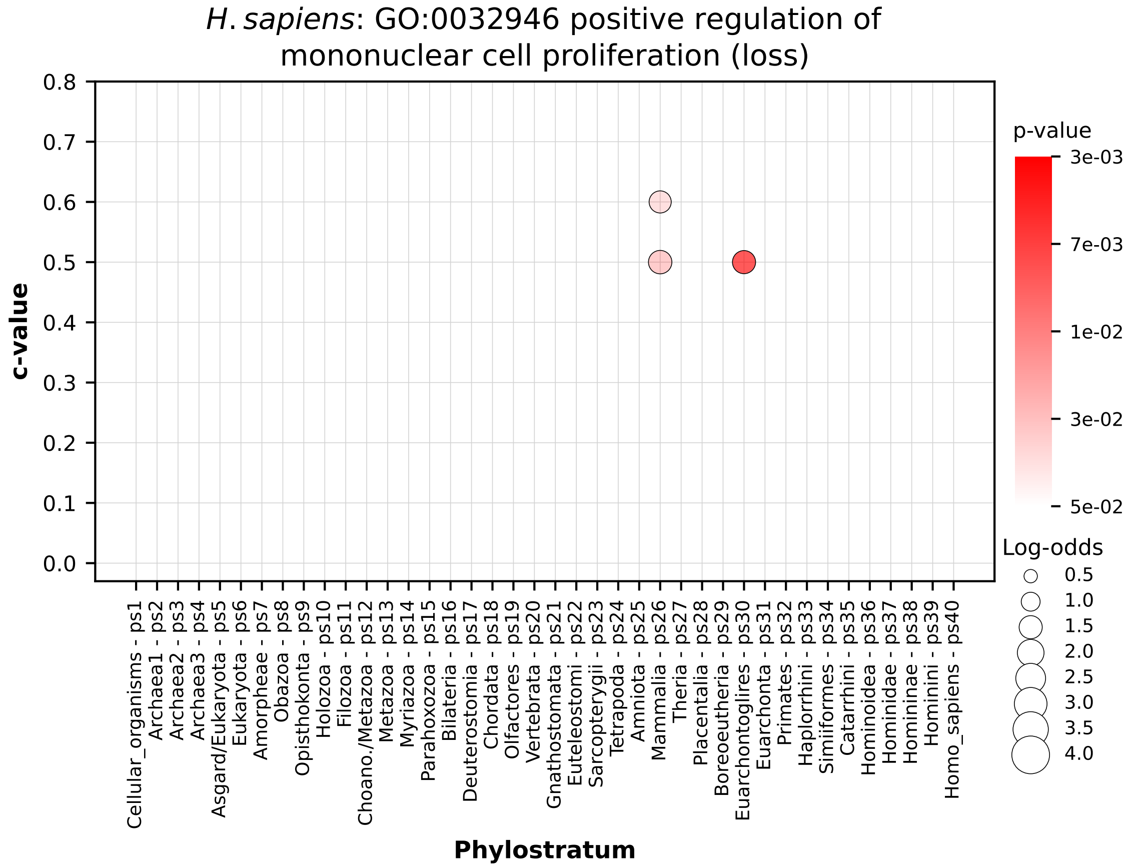

H. sapiens: GO:0032946 positive regulation of mononuclear cell proliferation (loss)
c-value
0.8
0.7
0.6
0.5
0.4
0.3
0.2
0.1
0.0
p-value
3e-03
7e-03
1e-02
3e-02
5e-02
Log-odds
0.5
1.0
1.5
2.0
2.5
3.0
3.5
4.0
Cellular_organisms - ps1
Archaea1 - ps2
Archaea2 - ps3
Archaea3 - ps4
Asgard/Eukaryota - ps5
Eukaryota - ps6
Amorpheae - ps7
Obazoa - ps8
Opisthokonta - ps9
Holozoa - ps10
Filozoa - ps11
Choano./Metazoa - ps12
Metazoa - ps13
Myriazoa - ps14
Parahoxozoa - ps15
Bilateria - ps16
Deuterostomia - ps17
Chordata - ps18
Olfactores - ps19
Vertebrata - ps20
Gnathostomata - ps21
Euteleostomi - ps22
Sarcopterygii - ps23
Tetrapoda - ps24
Amniota - ps25
Mammalia - ps26
Theria - ps27
Placentalia - ps28
Boreoeutheria - ps29
Euarchontoglires - ps30
Euarchonta - ps31
Primates - ps32
Haplorrhini - ps33
Simiiformes - ps34
Catarrhini - ps35
Hominoidea - ps36
Hominidae - ps37
Homininae - ps38
Hominini - ps39
Homo_sapiens - ps40
Phylostratum

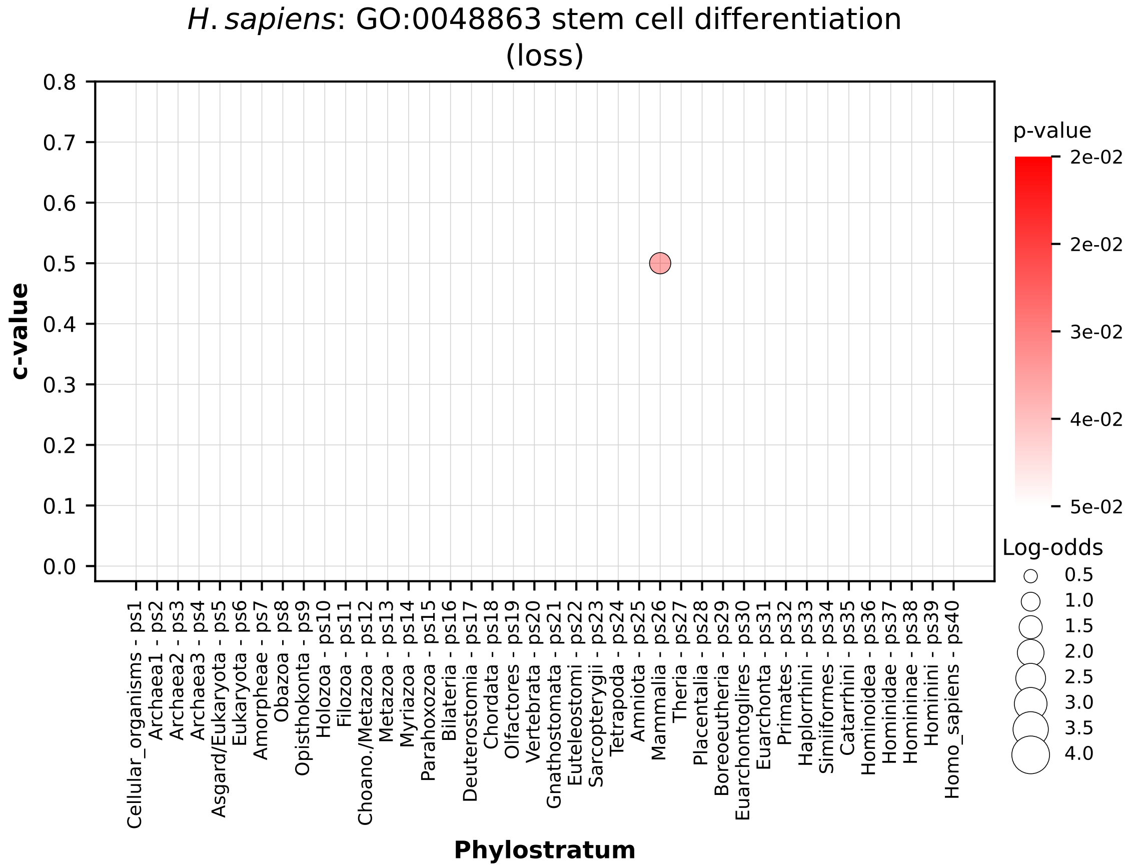

H. sapiens: GO:0048863 stem cell differentiation
(loss)
c-value
0.8
0.7
0.6
0.5
0.4
0.3
0.2
0.1
0.0
p-value
2e-02
2e-02
3e-02
4e-02
5e-02
Log-odds
0.5
1.0
1.5
2.0
2.5
3.0
3.5
4.0
Cellular_organisms - ps1
Archaea1 - ps2
Archaea2 - ps3
Archaea3 - ps4
Asgard/Eukaryota - ps5
Eukaryota - ps6
Amorpheae - ps7
Obazoa - ps8
Opisthokonta - ps9
Holozoa - ps10
Filozoa - ps11
Choano./Metazoa - ps12
Metazoa - ps13
Myriazoa - ps14
Parahoxozoa - ps15
Bilateria - ps16
Deuterostomia - ps17
Chordata - ps18
Olfactores - ps19
Vertebrata - ps20
Gnathostomata - ps21
Euteleostomi - ps22
Sarcopterygii - ps23
Tetrapoda - ps24
Amniota - ps25
Mammalia - ps26
Theria - ps27
Placentalia - ps28
Boreoeutheria - ps29
Euarchontoglires - ps30
Euarchonta - ps31
Primates - ps32
Haplorrhini - ps33
Simiiformes - ps34
Catarrhini - ps35
Hominoidea - ps36
Hominidae - ps37
Homininae - ps38
Hominini - ps39
Homo_sapiens - ps40
Phylostratum

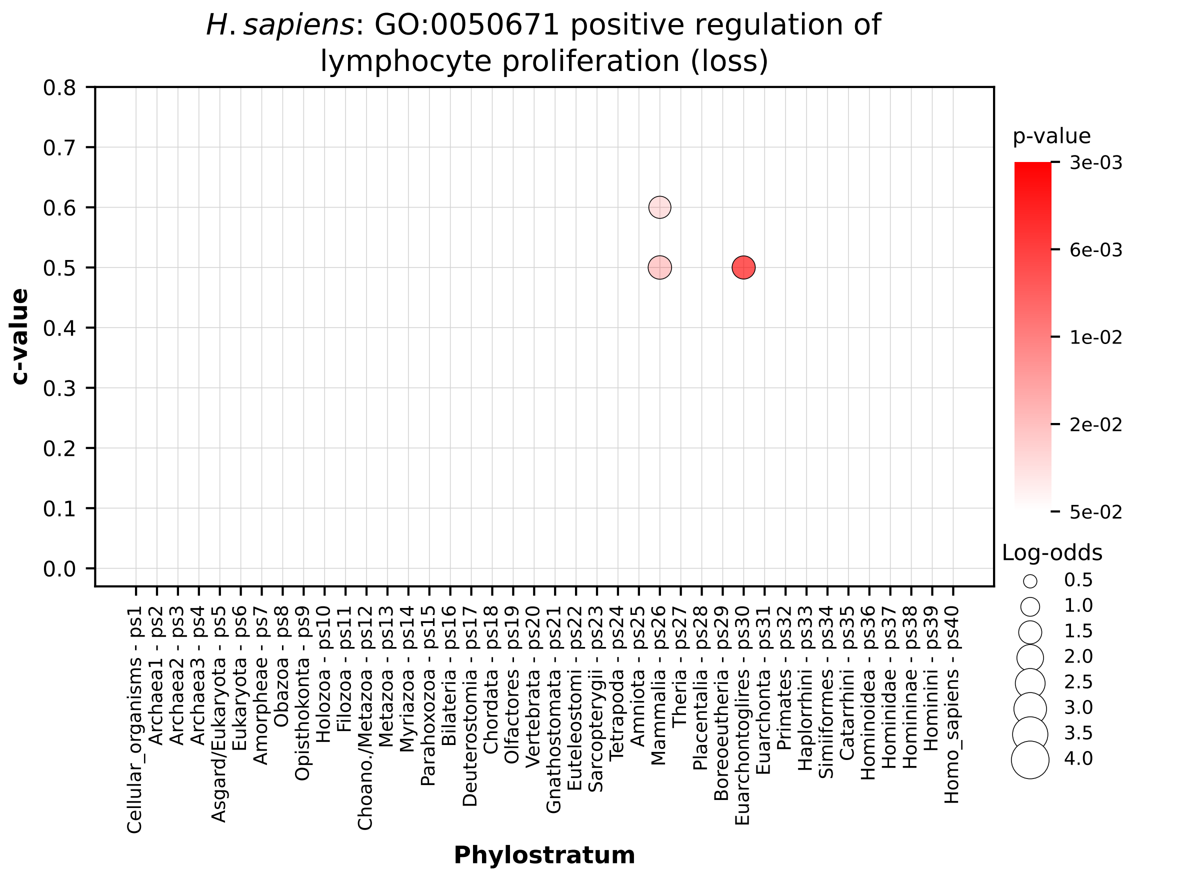
H. sapiens: GO:0050671 positive regulation of lymphocyte proliferation (loss)
c-value
0.8
0.7
0.6
0.5
0.4
0.3
0.2
0.1
0.0
Phylostratum
Cellular_organisms - ps1
Archaea1 - ps2
Archaea2 - ps3
Archaea3 - ps4
Asgard/Eukaryota - ps5
Eukaryota - ps6
Amorpheae - ps7
Obazoa - ps8
Opisthokonta - ps9
Holozoa - ps10
Filozoa - ps11
Choano./Metazoa - ps12
Metazoa - ps13
Myriazoa - ps14
Parahoxozoa - ps15
Bilateria - ps16
Deuterostomia - ps17
Chordata - ps18
Olfactores - ps19
Vertebrata - ps20
Gnathostomata - ps21
Euteleostomi - ps22
Sarcopterygii - ps23
Tetrapoda - ps24
Amniota - ps25
Mammalia - ps26
Theria - ps27
Placentalia - ps28
Boreoeutheria - ps29
Euarchontoglires - ps30
Euarchonta - ps31
Primates - ps32
Haplorrhini - ps33
Simiiformes - ps34
Catarrhini - ps35
Hominoidea - ps36
Hominidae - ps37
Homininae - ps38
Hominini - ps39
Homo_sapiens - ps40
p-value
3e-03
6e-03
1e-02
2e-02
5e-02
Log-odds
0.5
1.0
1.5
2.0
2.5
3.0
3.5
4.0

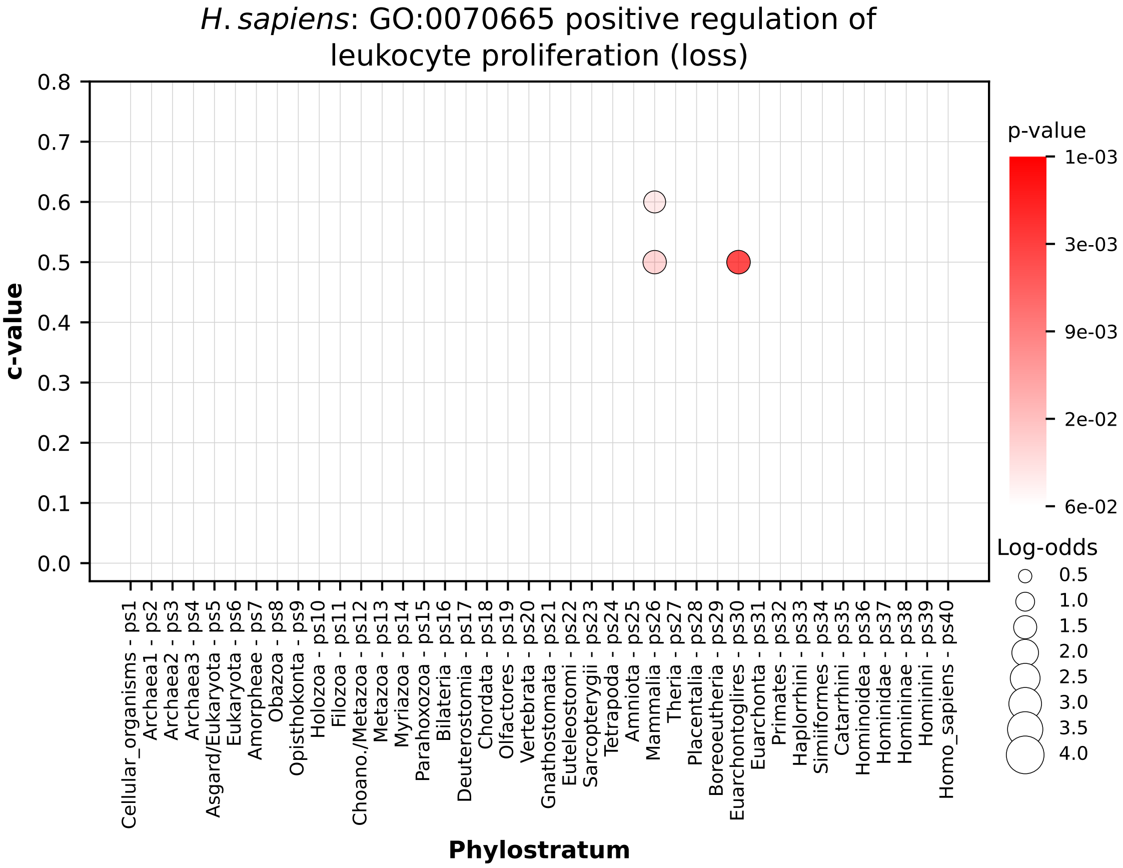

H. sapiens: GO:0070665 positive regulation of leukocyte proliferation (loss)
c-value
0.0
0.1
0.2
0.3
0.4
0.5
0.6
0.7
0.8
p-value
1e-03
3e-03
9e-03
2e-02
6e-02
Log-odds
0.5
1.0
1.5
2.0
2.5
3.0
3.5
4.0
Cellular_organisms - ps1
Archaea1 - ps2
Archaea2 - ps3
Archaea3 - ps4
Asgard/Eukaryota - ps5
Eukaryota - ps6
Amorpheae - ps7
Obazoa - ps8
Opisthokonta - ps9
Holozoa - ps10
Filozoa - ps11
Choano./Metazoa - ps12
Metazoa - ps13
Myriazoa - ps14
Parahoxozoa - ps15
Bilateria - ps16
Deuterostomia - ps17
Chordata - ps18
Olfactores - ps19
Vertebrata - ps20
Gnathostomata - ps21
Euteleostomi - ps22
Sarcopterygii - ps23
Tetrapoda - ps24
Amniota - ps25
Mammalia - ps26
Theria - ps27
Placentalia - ps28
Boreoeutheria - ps29
Euarchontoglires - ps30
Euarchonta - ps31
Primates - ps32
Haplorrhini - ps33
Simiiformes - ps34
Catarrhini - ps35
Hominoidea - ps36
Hominidae - ps37
Homininae - ps38
Hominini - ps39
Homo_sapiens - ps40
Phylostratum

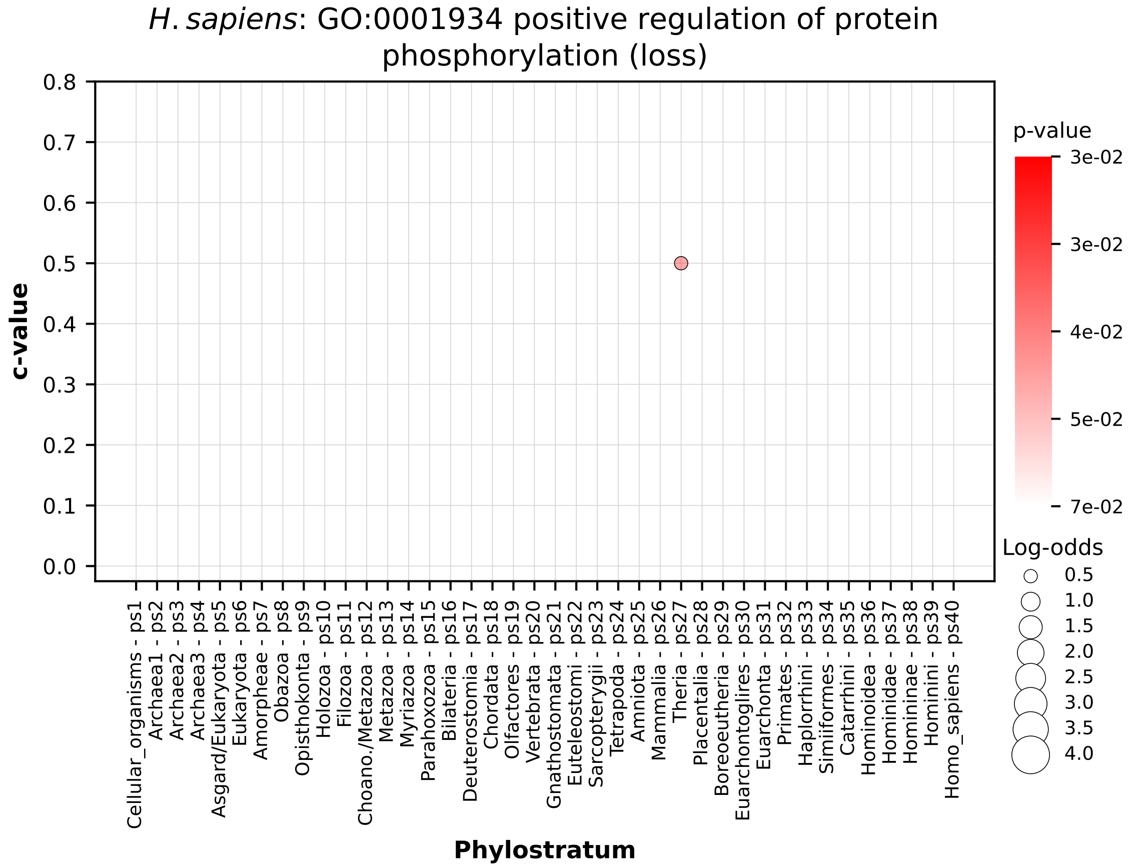
H. sapiens: GO:0001934 positive regulation of protein phosphorylation (loss)
c-value
0.8
0.7
0.6
0.5
0.4
0.3
0.2
0.1
0.0
Phylostratum
Cellular_organisms - ps1
Archaea1 - ps2
Archaea2 - ps3
Archaea3 - ps4
Asgard/Eukaryota - ps5
Eukaryota - ps6
Amorpheae - ps7
Obazoa - ps8
Opisthokonta - ps9
Holozoa - ps10
Filozoa - ps11
Choano./Metazoa - ps12
Metazoa - ps13
Myriazoa - ps14
Parahoxozoa - ps15
Bilateria - ps16
Deuterostomia - ps17
Chordata - ps18
Olfactores - ps19
Vertebrata - ps20
Gnathostomata - ps21
Euteleostomi - ps22
Sarcopterygii - ps23
Tetrapoda - ps24
Amniota - ps25
Mammalia - ps26
Theria - ps27
Placentalia - ps28
Boreoeutheria - ps29
Euarchontoglires - ps30
Euarchonta - ps31
Primates - ps32
Haplorrhini - ps33
Simiiformes - ps34
Catarrhini - ps35
Hominoidea - ps36
Hominidae - ps37
Homininae - ps38
Hominini - ps39
Homo_sapiens - ps40
p-value
3e-02
3e-02
4e-02
5e-02
7e-02
Log-odds
0.5
1.0
1.5
2.0
2.5
3.0
3.5
4.0

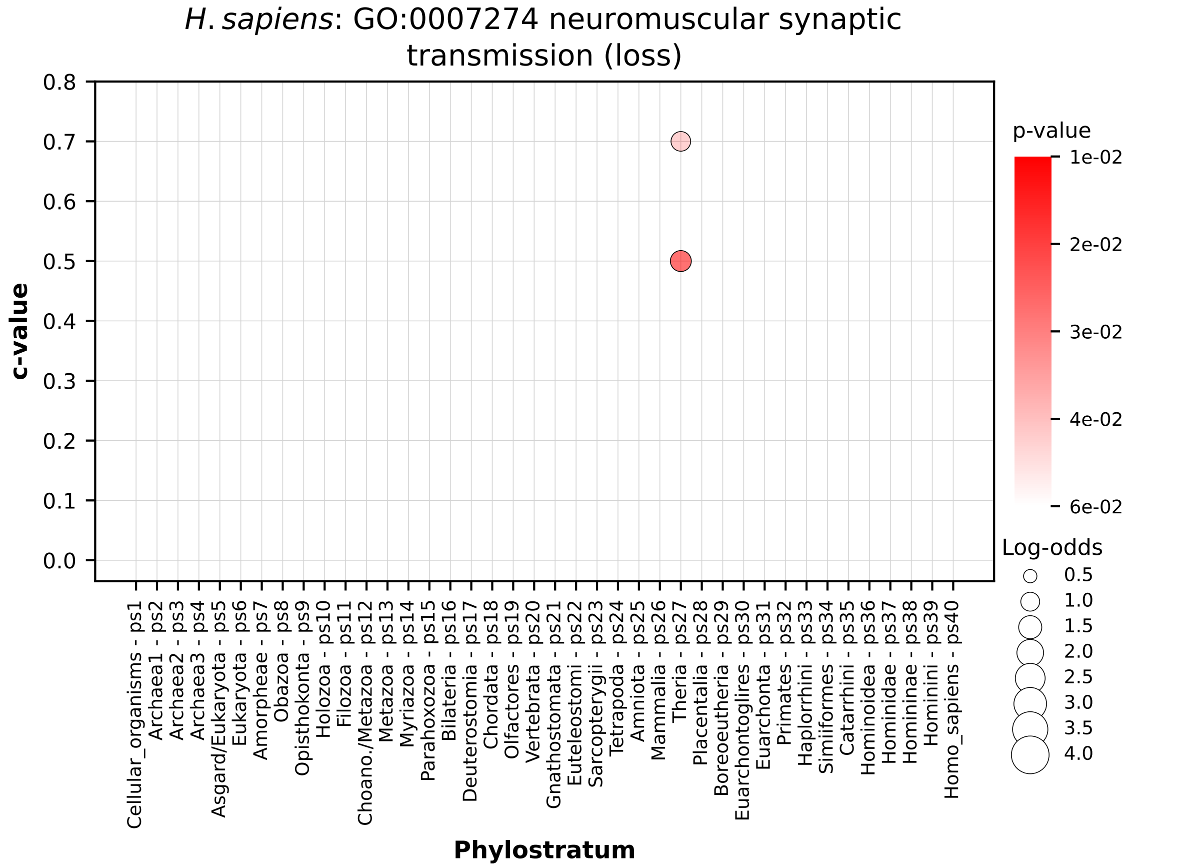

H. sapiens: GO:0007274 neuromuscular synaptic transmission (loss)
c-value
0.8
0.7
0.6
0.5
0.4
0.3
0.2
0.1
0.0
p-value
1e-02
2e-02
3e-02
4e-02
6e-02
Log-odds
0.5
1.0
1.5
2.0
2.5
3.0
3.5
4.0
Cellular_organisms - ps1
Archaea1 - ps2
Archaea2 - ps3
Archaea3 - ps4
Asgard/Eukaryota - ps5
Eukaryota - ps6
Amorpheae - ps7
Obazoa - ps8
Opisthokonta - ps9
Holozoa - ps10
Filozoa - ps11
Choano./Metazoa - ps12
Metazoa - ps13
Myriazoa - ps14
Parahoxozoa - ps15
Bilateria - ps16
Deuterostomia - ps17
Chordata - ps18
Olfactores - ps19
Vertebrata - ps20
Gnathostomata - ps21
Euteleostomi - ps22
Sarcopterygii - ps23
Tetrapoda - ps24
Amniota - ps25
Mammalia - ps26
Theria - ps27
Placentalia - ps28
Boreoeutheria - ps29
Euarchontoglires - ps30
Euarchonta - ps31
Primates - ps32
Haplorrhini - ps33
Simiiformes - ps34
Catarrhini - ps35
Hominoidea - ps36
Hominidae - ps37
Homininae - ps38
Hominini - ps39
Homo_sapiens - ps40
Phylostratum

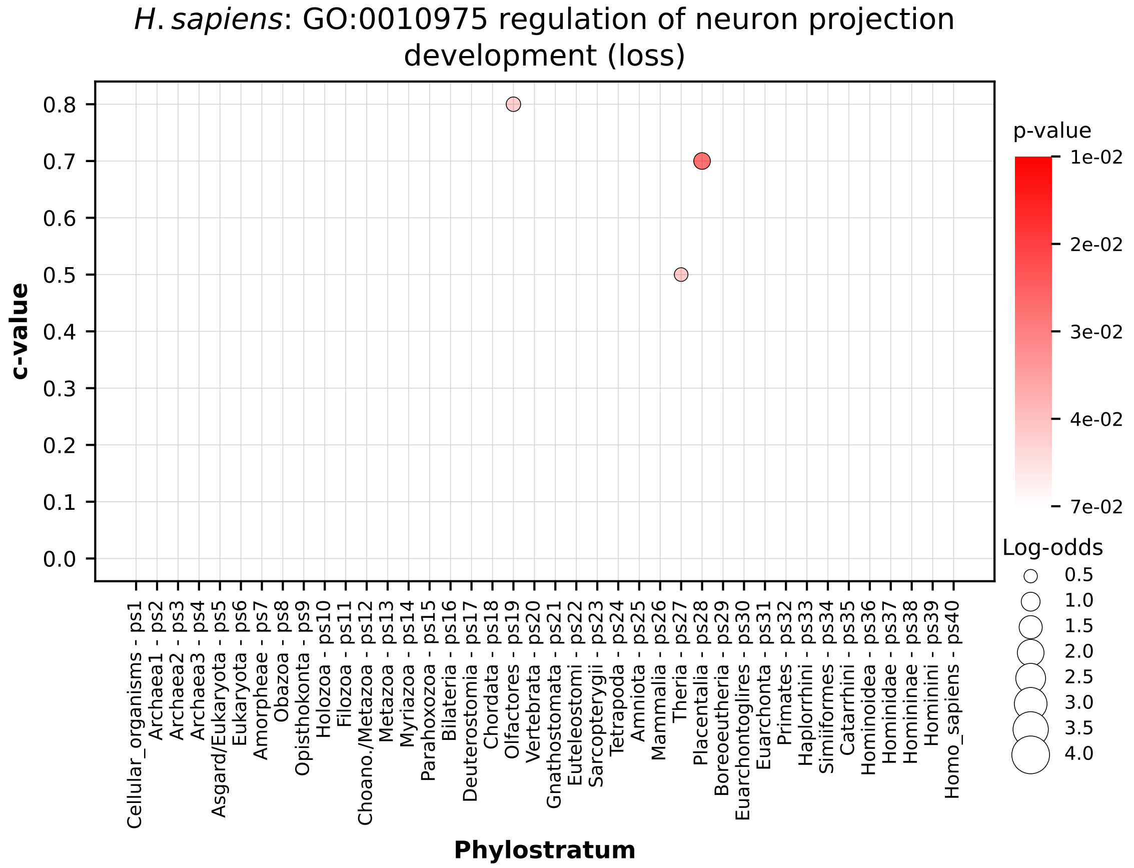

H. sapiens: GO:0010975 regulation of neuron projection development (loss)
c-value
0.0
0.1
0.2
0.3
0.4
0.5
0.6
0.7
0.8
Phylostratum
Cellular_organisms - ps1
Archaea1 - ps2
Archaea2 - ps3
Archaea3 - ps4
Asgard/Eukaryota - ps5
Eukaryota - ps6
Amorpheae - ps7
Obazoa - ps8
Opisthokonta - ps9
Holozoa - ps10
Filozoa - ps11
Choano./Metazoa - ps12
Metazoa - ps13
Myriazoa - ps14
Parahoxozoa - ps15
Bilateria - ps16
Deuterostomia - ps17
Chordata - ps18
Olfactores - ps19
Vertebrata - ps20
Gnathostomata - ps21
Euteleostomi - ps22
Sarcopterygii - ps23
Tetrapoda - ps24
Amniota - ps25
Mammalia - ps26
Theria - ps27
Placentalia - ps28
Boreoeutheria - ps29
Euarchontoglires - ps30
Euarchonta - ps31
Primates - ps32
Haplorrhini - ps33
Simiiformes - ps34
Catarrhini - ps35
Hominoidea - ps36
Hominidae - ps37
Homininae - ps38
Hominini - ps39
Homo_sapiens - ps40
p-value
1e-02
2e-02
3e-02
4e-02
7e-02
Log-odds
0.5
1.0
1.5
2.0
2.5
3.0
3.5
4.0

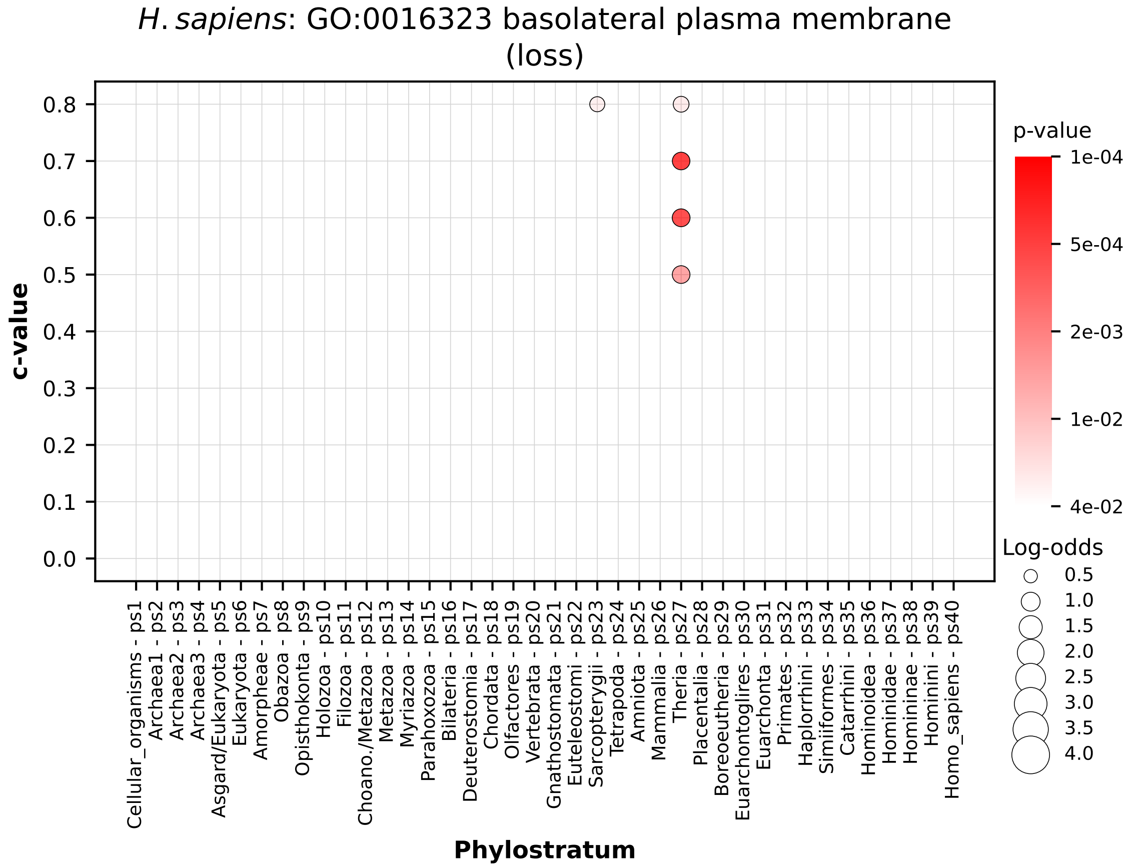

H. sapiens: GO:0016323 basolateral plasma membrane (loss)
c-value
Phylostratum
p-value
1e-04
5e-04
2e-03
1e-02
4e-02
Log-odds
0.5
1.0
1.5
2.0
2.5
3.0
3.5
4.0
Cellular_organisms - ps1
Archaea1 - ps2
Archaea2 - ps3
Archaea3 - ps4
Asgard/Eukaryota - ps5
Eukaryota - ps6
Amorpheae - ps7
Obazoa - ps8
Opisthokonta - ps9
Holozoa - ps10
Filozoa - ps11
Choano./Metazoa - ps12
Metazoa - ps13
Myriazoa - ps14
Parahoxozoa - ps15
Bilateria - ps16
Deuterostomia - ps17
Chordata - ps18
Olfactores - ps19
Vertebrata - ps20
Gnathostomata - ps21
Euteleostomi - ps22
Sarcopterygii - ps23
Tetrapoda - ps24
Amniota - ps25
Mammalia - ps26
Theria - ps27
Placentalia - ps28
Boreoeutheria - ps29
Euarchontoglires - ps30
Euarchonta - ps31
Primates - ps32
Haplorrhini - ps33
Simiiformes - ps34
Catarrhini - ps35
Hominoidea - ps36
Hominidae - ps37
Homininae - ps38
Hominini - ps39
Homo_sapiens - ps40

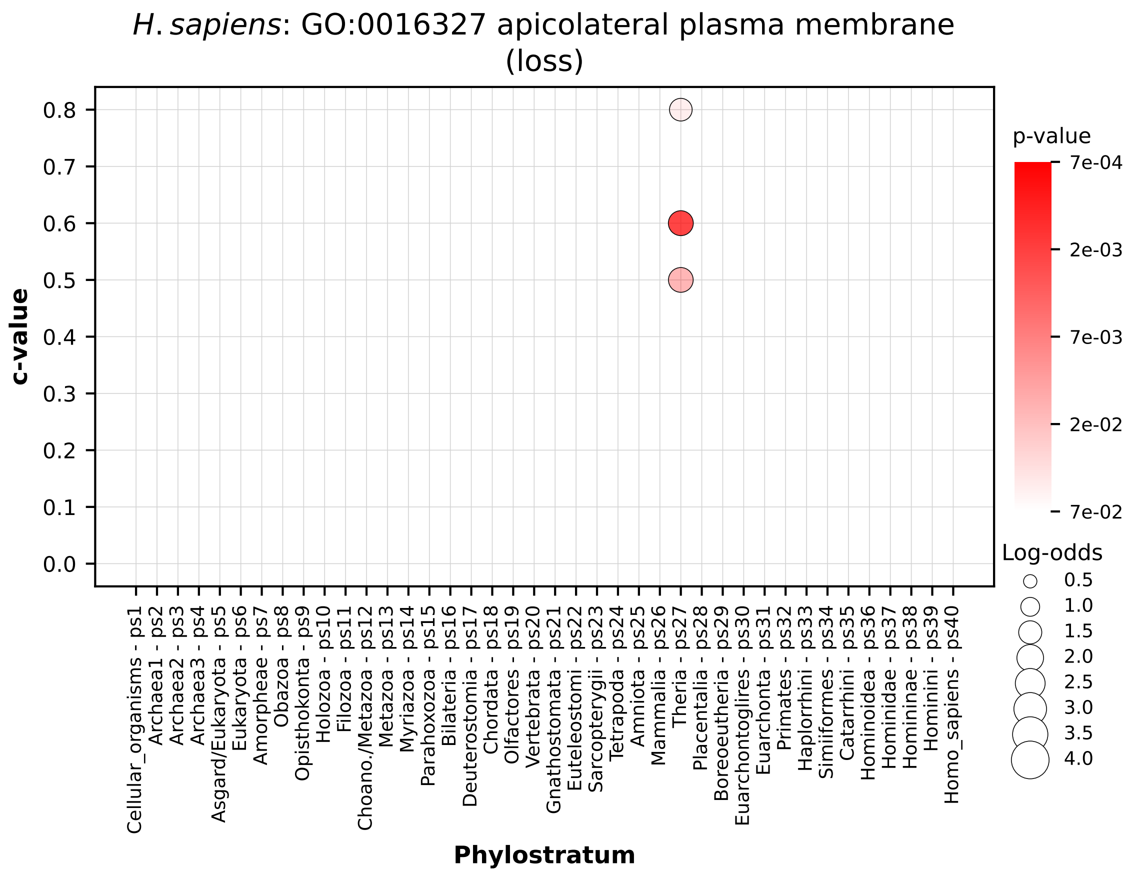

H. sapiens: GO:0016327 apicolateral plasma membrane (loss)
c-value
0.0
0.1
0.2
0.3
0.4
0.5
0.6
0.7
0.8
p-value
7e-04
2e-03
7e-03
2e-02
7e-02
Log-odds
0.5
1.0
1.5
2.0
2.5
3.0
3.5
4.0
Phylostratum
Cellular_organisms - ps1
Archaea1 - ps2
Archaea2 - ps3
Archaea3 - ps4
Asgard/Eukaryota - ps5
Eukaryota - ps6
Amorpheae - ps7
Obazoa - ps8
Opisthokonta - ps9
Holozoa - ps10
Filozoa - ps11
Choano./Metazoa - ps12
Metazoa - ps13
Myriazoa - ps14
Parahoxozoa - ps15
Bilateria - ps16
Deuterostomia - ps17
Chordata - ps18
Olfactores - ps19
Vertebrata - ps20
Gnathostomata - ps21
Euteleostomi - ps22
Sarcopterygii - ps23
Tetrapoda - ps24
Amniota - ps25
Mammalia - ps26
Theria - ps27
Placentalia - ps28
Boreoeutheria - ps29
Euarchontoglires - ps30
Euarchonta - ps31
Primates - ps32
Haplorrhini - ps33
Simiiformes - ps34
Catarrhini - ps35
Hominoidea - ps36
Hominidae - ps37
Homininae - ps38
Hominini - ps39
Homo_sapiens - ps40

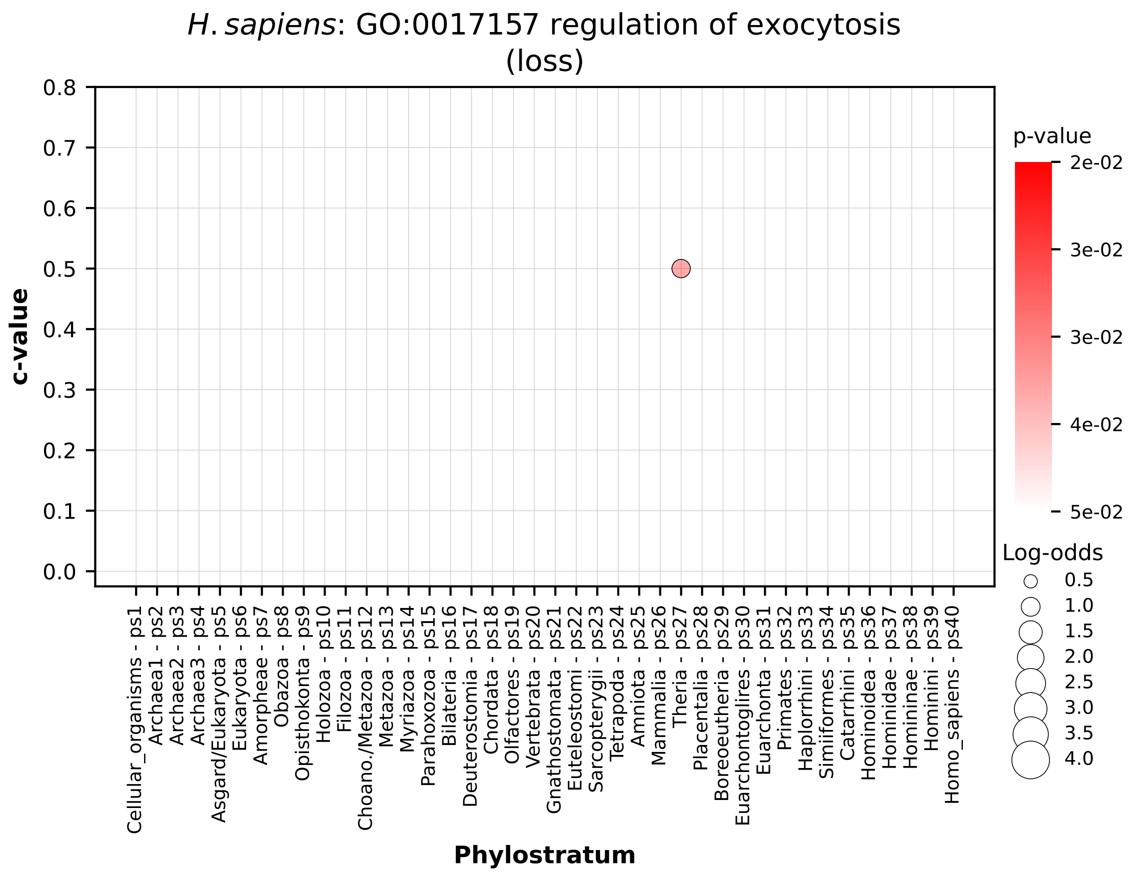
H. sapiens: GO:0017157 regulation of exocytosis
(loss)
c-value
0.8
0.7
0.6
0.5
0.4
0.3
0.2
0.1
0.0
p-value
2e-02
3e-02
3e-02
4e-02
5e-02
Log-odds
0.5
1.0
1.5
2.0
2.5
3.0
3.5
4.0
Cellular_organisms - ps1
Archaea1 - ps2
Archaea2 - ps3
Archaea3 - ps4
Asgard/Eukaryota - ps5
Eukaryota - ps6
Amorpheae - ps7
Obazoa - ps8
Opisthokonta - ps9
Holozoa - ps10
Filozoa - ps11
Choano./Metazoa - ps12
Metazoa - ps13
Myriazoa - ps14
Parahoxozoa - ps15
Bilateria - ps16
Deuterostomia - ps17
Chordata - ps18
Olfactores - ps19
Vertebrata - ps20
Gnathostomata - ps21
Euteleostomi - ps22
Sarcopterygii - ps23
Tetrapoda - ps24
Amniota - ps25
Mammalia - ps26
Theria - ps27
Placentalia - ps28
Boreoeutheria - ps29
Euarchontoglires - ps30
Euarchonta - ps31
Primates - ps32
Haplorrhini - ps33
Simiiformes - ps34
Catarrhini - ps35
Hominoidea - ps36
Hominidae - ps37
Homininae - ps38
Hominini - ps39
Homo_sapiens - ps40
Phylostratum

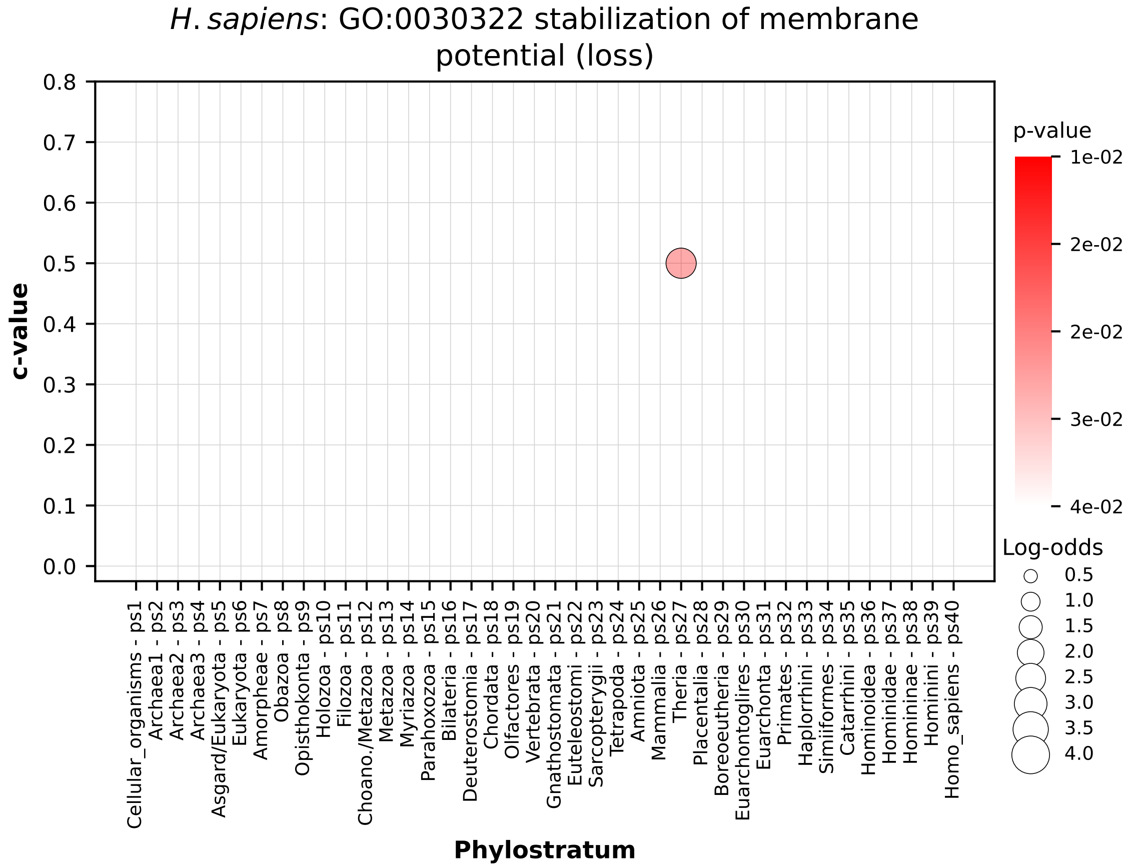

H. sapiens: GO:0030322 stabilization of membrane potential (loss)
c-value
0.8
0.7
0.6
0.5
0.4
0.3
0.2
0.1
0.0
p-value
1e-02
2e-02
2e-02
3e-02
4e-02
Log-odds
0.5
1.0
1.5
2.0
2.5
3.0
3.5
4.0
Cellular_organisms - ps1
Archaea1 - ps2
Archaea2 - ps3
Archaea3 - ps4
Asgard/Eukaryota - ps5
Eukaryota - ps6
Amorpheae - ps7
Obazoa - ps8
Opisthokonta - ps9
Holozoa - ps10
Filozoa - ps11
Choano./Metazoa - ps12
Metazoa - ps13
Myriazoa - ps14
Parahoxozoa - ps15
Bilateria - ps16
Deuterostomia - ps17
Chordata - ps18
Olfactores - ps19
Vertebrata - ps20
Gnathostomata - ps21
Euteleostomi - ps22
Sarcopterygii - ps23
Tetrapoda - ps24
Amniota - ps25
Mammalia - ps26
Theria - ps27
Placentalia - ps28
Boreoeutheria - ps29
Euarchontoglires - ps30
Euarchonta - ps31
Primates - ps32
Haplorrhini - ps33
Simiiformes - ps34
Catarrhini - ps35
Hominoidea - ps36
Hominidae - ps37
Homininae - ps38
Hominini - ps39
Homo_sapiens - ps40
Phylostratum

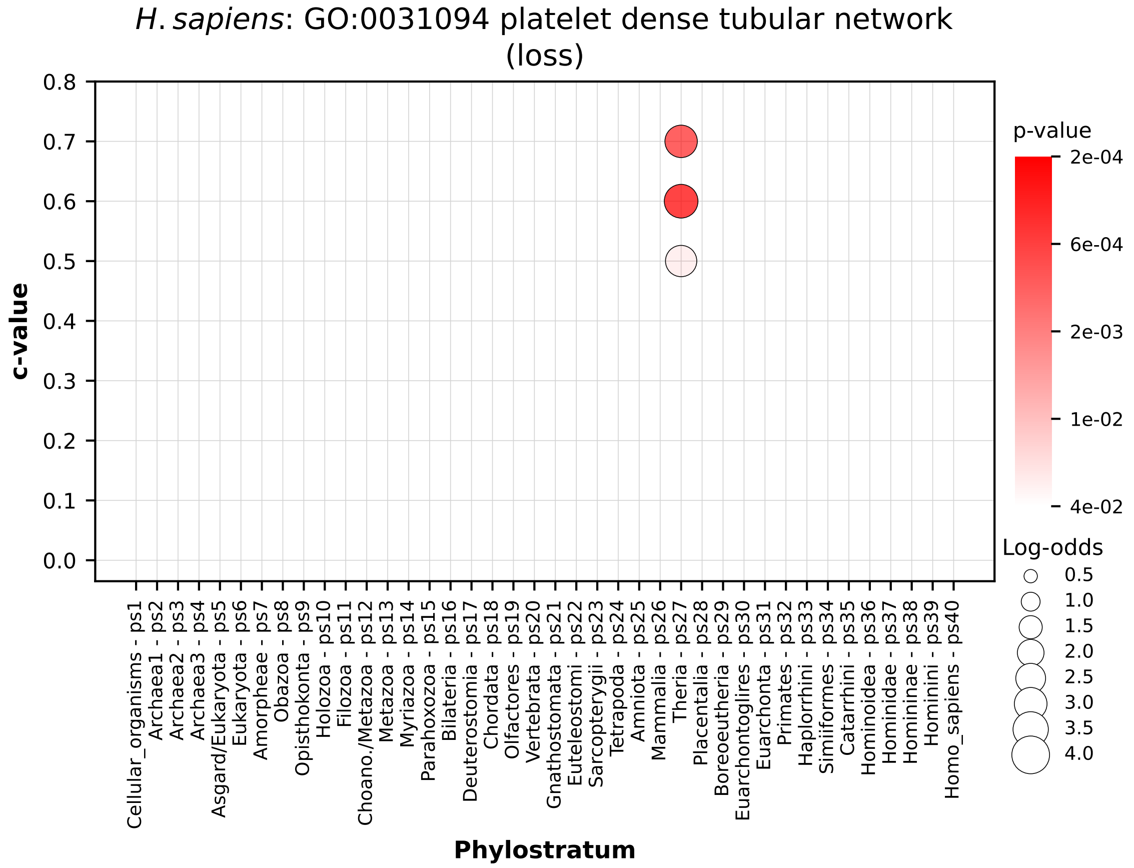
H. sapiens: GO:0031094 platelet dense tubular network
(loss)
c-value
0.8
0.7
0.6
0.5
0.4
0.3
0.2
0.1
0.0
p-value
2e-04
6e-04
2e-03
1e-02
4e-02
Log-odds
0.5
1.0
1.5
2.0
2.5
3.0
3.5
4.0
Cellular_organisms - ps1
Archaea1 - ps2
Archaea2 - ps3
Archaea3 - ps4
Asgard/Eukaryota - ps5
Eukaryota - ps6
Amorpheae - ps7
Obazoa - ps8
Opisthokonta - ps9
Holozoa - ps10
Filozoa - ps11
Choano./Metazoa - ps12
Metazoa - ps13
Myriazoa - ps14
Parahoxozoa - ps15
Bilateria - ps16
Deuterostomia - ps17
Chordata - ps18
Olfactores - ps19
Vertebrata - ps20
Gnathostomata - ps21
Euteleostomi - ps22
Sarcopterygii - ps23
Tetrapoda - ps24
Amniota - ps25
Mammalia - ps26
Theria - ps27
Placentalia - ps28
Boreoeutheria - ps29
Euarchontoglires - ps30
Euarchonta - ps31
Primates - ps32
Haplorrhini - ps33
Simiiformes - ps34
Catarrhini - ps35
Hominoidea - ps36
Hominidae - ps37
Homininae - ps38
Hominini - ps39
Homo_sapiens - ps40
Phylostratum

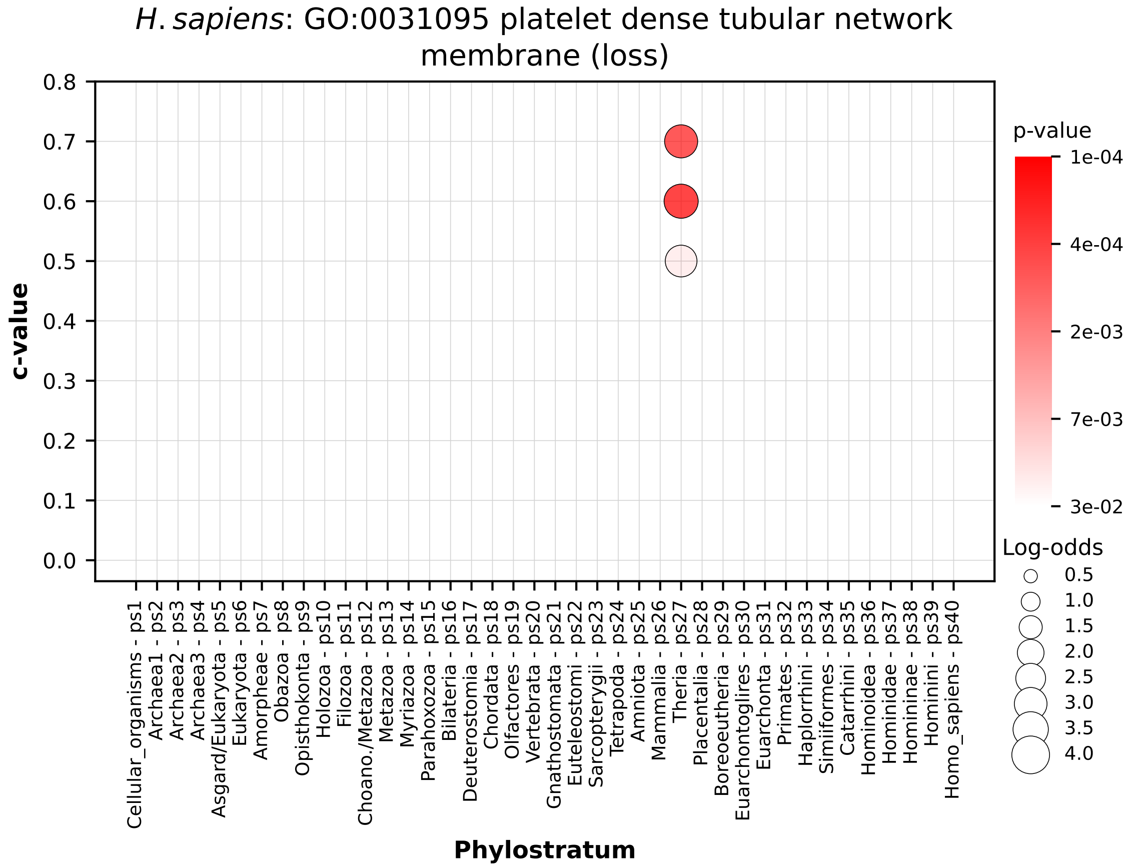

H. sapiens: GO:0031095 platelet dense tubular network membrane (loss)
c-value
0.8
0.7
0.6
0.5
0.4
0.3
0.2
0.1
0.0
p-value
1e-04
4e-04
2e-03
7e-03
3e-02
Log-odds
0.5
1.0
1.5
2.0
2.5
3.0
3.5
4.0
Cellular_organisms - ps1
Archaea1 - ps2
Archaea2 - ps3
Archaea3 - ps4
Asgard/Eukaryota - ps5
Eukaryota - ps6
Amorpheae - ps7
Obazoa - ps8
Opisthokonta - ps9
Holozoa - ps10
Filozoa - ps11
Choano./Metazoa - ps12
Metazoa - ps13
Myriazoa - ps14
Parahoxozoa - ps15
Bilateria - ps16
Deuterostomia - ps17
Chordata - ps18
Olfactores - ps19
Vertebrata - ps20
Gnathostomata - ps21
Euteleostomi - ps22
Sarcopterygii - ps23
Tetrapoda - ps24
Amniota - ps25
Mammalia - ps26
Theria - ps27
Placentalia - ps28
Boreoeutheria - ps29
Euarchontoglires - ps30
Euarchonta - ps31
Primates - ps32
Haplorrhini - ps33
Simiiformes - ps34
Catarrhini - ps35
Hominoidea - ps36
Hominidae - ps37
Homininae - ps38
Hominini - ps39
Homo_sapiens - ps40
Phylostratum

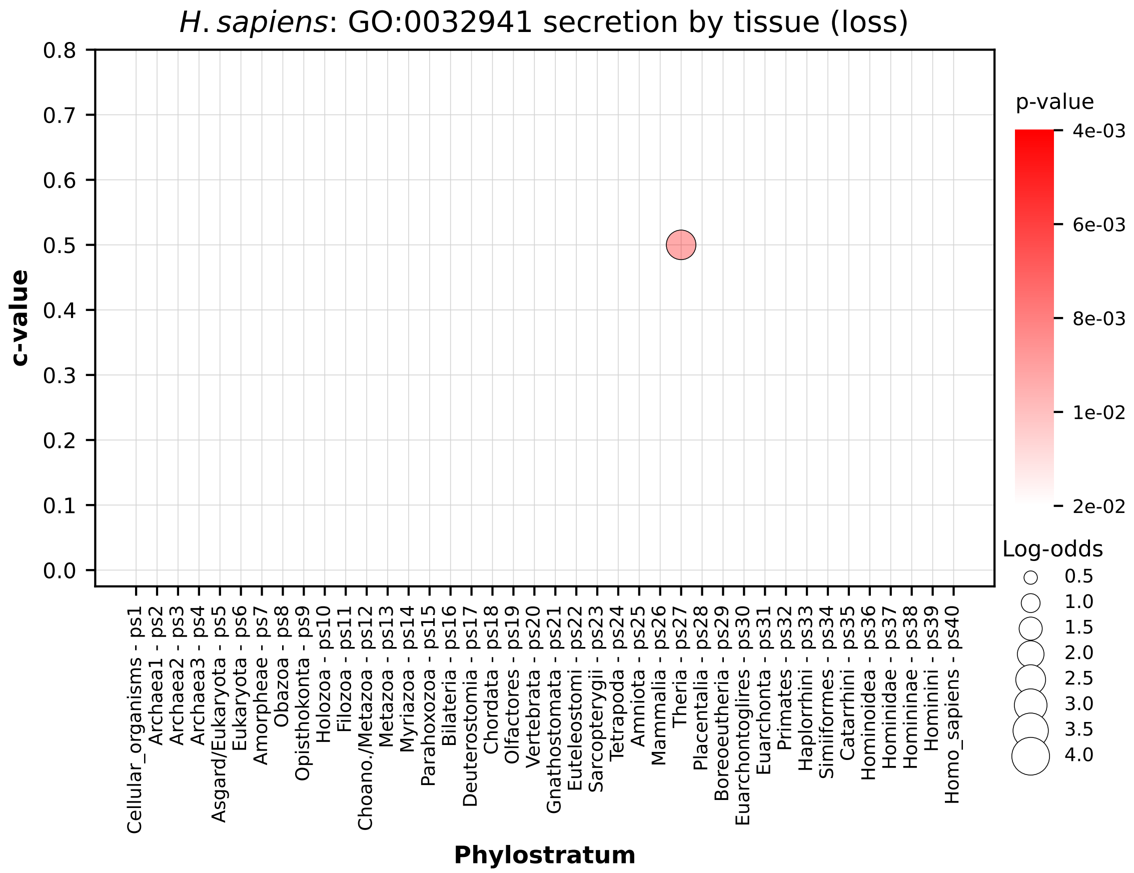

H. sapiens: GO:0032941 secretion by tissue (loss)
c-value
0.8
0.7
0.6
0.5
0.4
0.3
0.2
0.1
0.0
p-value
4e-03
6e-03
8e-03
1e-02
2e-02
Log-odds
0.5
1.0
1.5
2.0
2.5
3.0
3.5
4.0
Cellular_organisms - ps1
Archaea1 - ps2
Archaea2 - ps3
Archaea3 - ps4
Asgard/Eukaryota - ps5
Eukaryota - ps6
Amorpheae - ps7
Obazoa - ps8
Opisthokonta - ps9
Holozoa - ps10
Filozoa - ps11
Choano./Metazoa - ps12
Metazoa - ps13
Myriazoa - ps14
Parahoxozoa - ps15
Bilateria - ps16
Deuterostomia - ps17
Chordata - ps18
Olfactores - ps19
Vertebrata - ps20
Gnathostomata - ps21
Euteleostomi - ps22
Sarcopterygii - ps23
Tetrapoda - ps24
Amniota - ps25
Mammalia - ps26
Theria - ps27
Placentalia - ps28
Boreoeutheria - ps29
Euarchontoglires - ps30
Euarchonta - ps31
Primates - ps32
Haplorrhini - ps33
Simiiformes - ps34
Catarrhini - ps35
Hominoidea - ps36
Hominidae - ps37
Homininae - ps38
Hominini - ps39
Homo_sapiens - ps40
Phylostratum

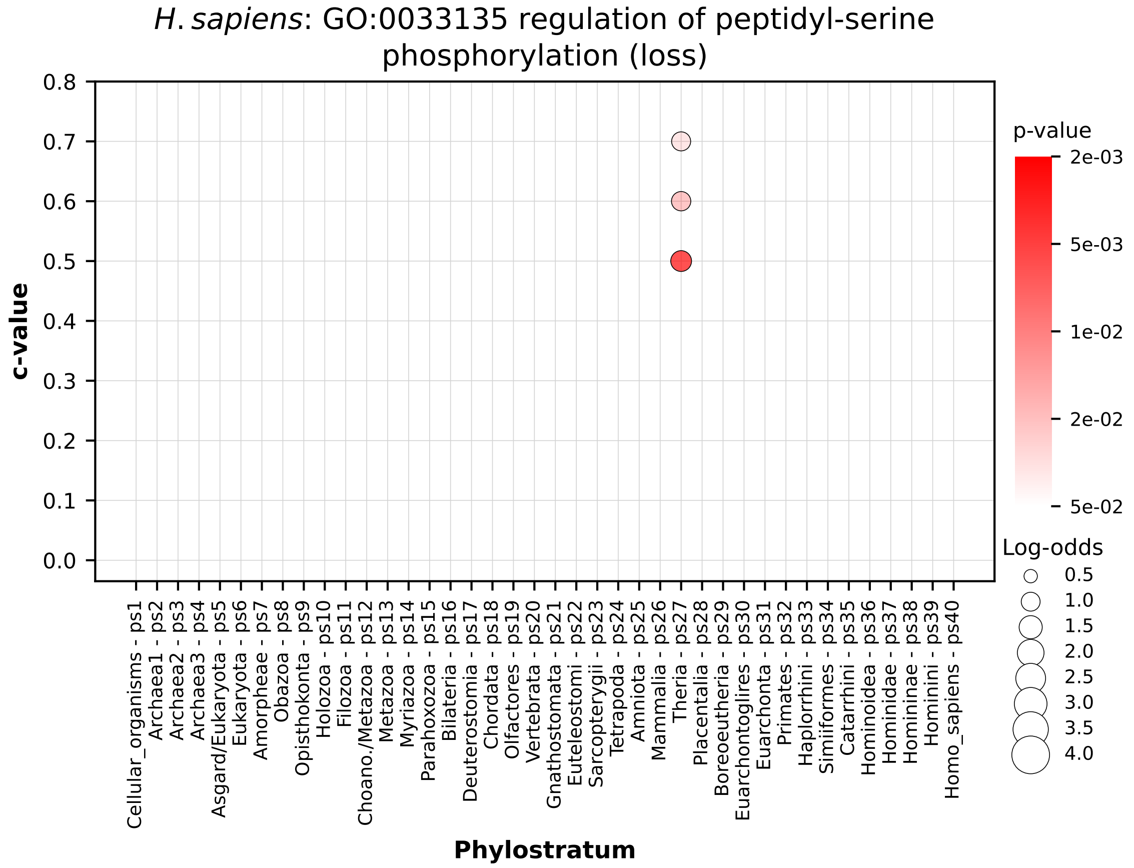

H. sapiens: GO:0033135 regulation of peptidyl-serine phosphorylation (loss)
c-value
0.8
0.7
0.6
0.5
0.4
0.3
0.2
0.1
0.0
Phylostratum
Cellular_organisms - ps1
Archaea1 - ps2
Archaea2 - ps3
Archaea3 - ps4
Asgard/Eukaryota - ps5
Eukaryota - ps6
Amorpheae - ps7
Obazoa - ps8
Opisthokonta - ps9
Holozoa - ps10
Filozoa - ps11
Choano./Metazoa - ps12
Metazoa - ps13
Myriazoa - ps14
Parahoxozoa - ps15
Bilateria - ps16
Deuterostomia - ps17
Chordata - ps18
Olfactores - ps19
Vertebrata - ps20
Gnathostomata - ps21
Euteleostomi - ps22
Sarcopterygii - ps23
Tetrapoda - ps24
Amniota - ps25
Mammalia - ps26
Theria - ps27
Placentalia - ps28
Boreoeutheria - ps29
Euarchontoglires - ps30
Euarchonta - ps31
Primates - ps32
Haplorrhini - ps33
Simiiformes - ps34
Catarrhini - ps35
Hominoidea - ps36
Hominidae - ps37
Homininae - ps38
Hominini - ps39
Homo_sapiens - ps40
p-value
2e-03
5e-03
1e-02
2e-02
5e-02
Log-odds
0.5
1.0
1.5
2.0
2.5
3.0
3.5
4.0

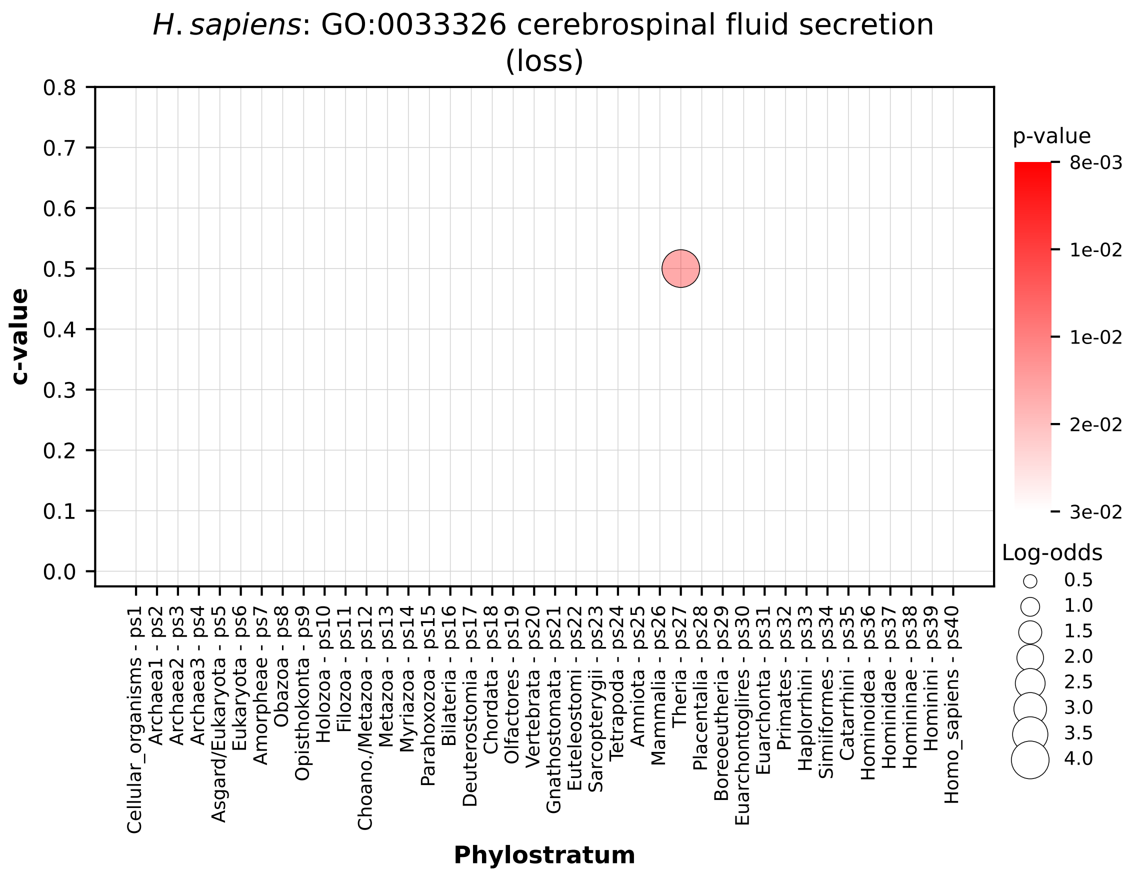

H. sapiens: GO:0033326 cerebrospinal fluid secretion (loss)
c-value
0.0
0.1
0.2
0.3
0.4
0.5
0.6
0.7
0.8
Phylostratum
Cellular_organisms - ps1
Archaea1 - ps2
Archaea2 - ps3
Archaea3 - ps4
Asgard/Eukaryota - ps5
Eukaryota - ps6
Amorpheae - ps7
Obazoa - ps8
Opisthokonta - ps9
Holozoa - ps10
Filozoa - ps11
Choano./Metazoa - ps12
Metazoa - ps13
Myriazoa - ps14
Parahoxozoa - ps15
Bilateria - ps16
Deuterostomia - ps17
Chordata - ps18
Olfactores - ps19
Vertebrata - ps20
Gnathostomata - ps21
Euteleostomi - ps22
Sarcopterygii - ps23
Tetrapoda - ps24
Amniota - ps25
Mammalia - ps26
Theria - ps27
Placentalia - ps28
Boreoeutheria - ps29
Euarchontoglires - ps30
Euarchonta - ps31
Primates - ps32
Haplorrhini - ps33
Simiiformes - ps34
Catarrhini - ps35
Hominoidea - ps36
Hominidae - ps37
Homininae - ps38
Hominini - ps39
Homo_sapiens - ps40
p-value
8e-03
1e-02
1e-02
2e-02
3e-02
Log-odds
0.5
1.0
1.5
2.0
2.5
3.0
3.5
4.0

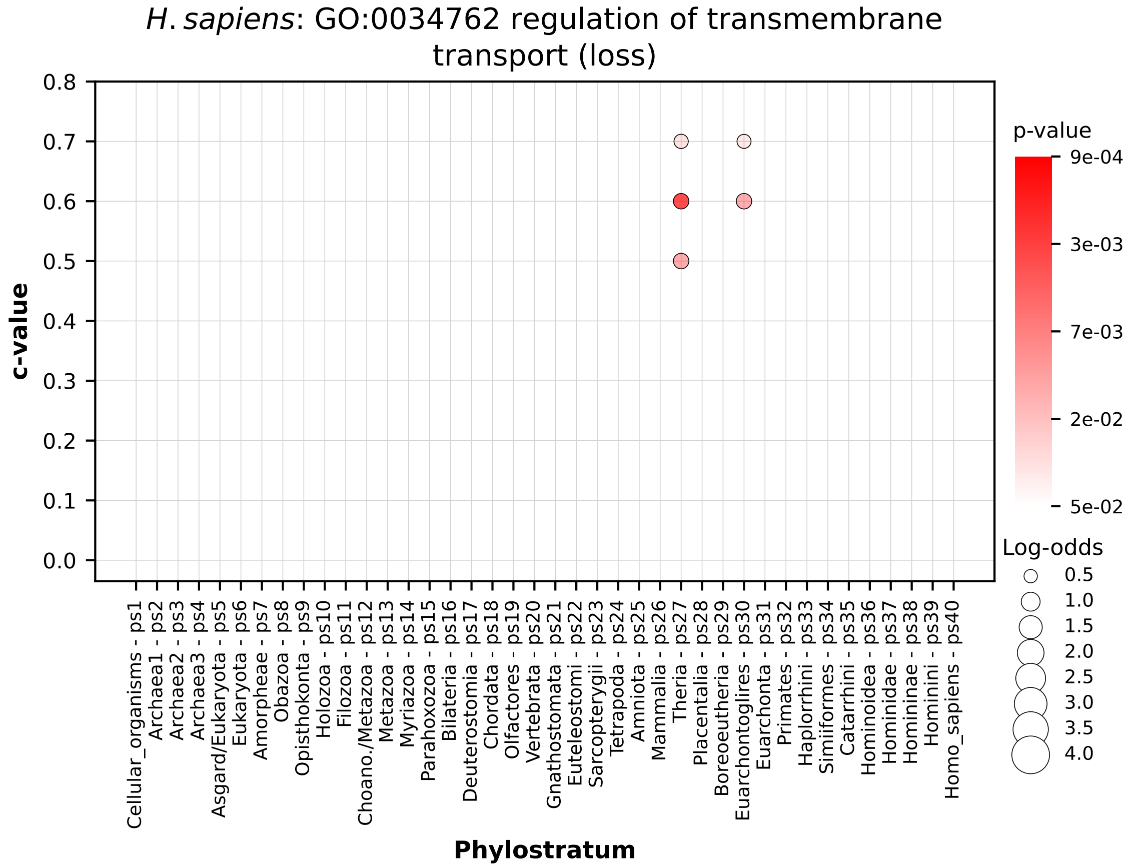

H. sapiens: GO:0034762 regulation of transmembrane transport (loss)
c-value
0.8
0.7
0.6
0.5
0.4
0.3
0.2
0.1
0.0
Phylostratum
Cellular_organisms - ps1
Archaea1 - ps2
Archaea2 - ps3
Archaea3 - ps4
Asgard/Eukaryota - ps5
Eukaryota - ps6
Amorpheae - ps7
Obazoa - ps8
Opisthokonta - ps9
Holozoa - ps10
Filozoa - ps11
Choano./Metazoa - ps12
Metazoa - ps13
Myriazoa - ps14
Parahoxozoa - ps15
Bilateria - ps16
Deuterostomia - ps17
Chordata - ps18
Olfactores - ps19
Vertebrata - ps20
Gnathostomata - ps21
Euteleostomi - ps22
Sarcopterygii - ps23
Tetrapoda - ps24
Amniota - ps25
Mammalia - ps26
Theria - ps27
Placentalia - ps28
Boreoeutheria - ps29
Euarchontoglires - ps30
Euarchonta - ps31
Primates - ps32
Haplorrhini - ps33
Simiiformes - ps34
Catarrhini - ps35
Hominoidea - ps36
Hominidae - ps37
Homininae - ps38
Hominini - ps39
Homo_sapiens - ps40
p-value
9e-04
3e-03
7e-03
2e-02
5e-02
Log-odds
0.5
1.0
1.5
2.0
2.5
3.0
3.5
4.0

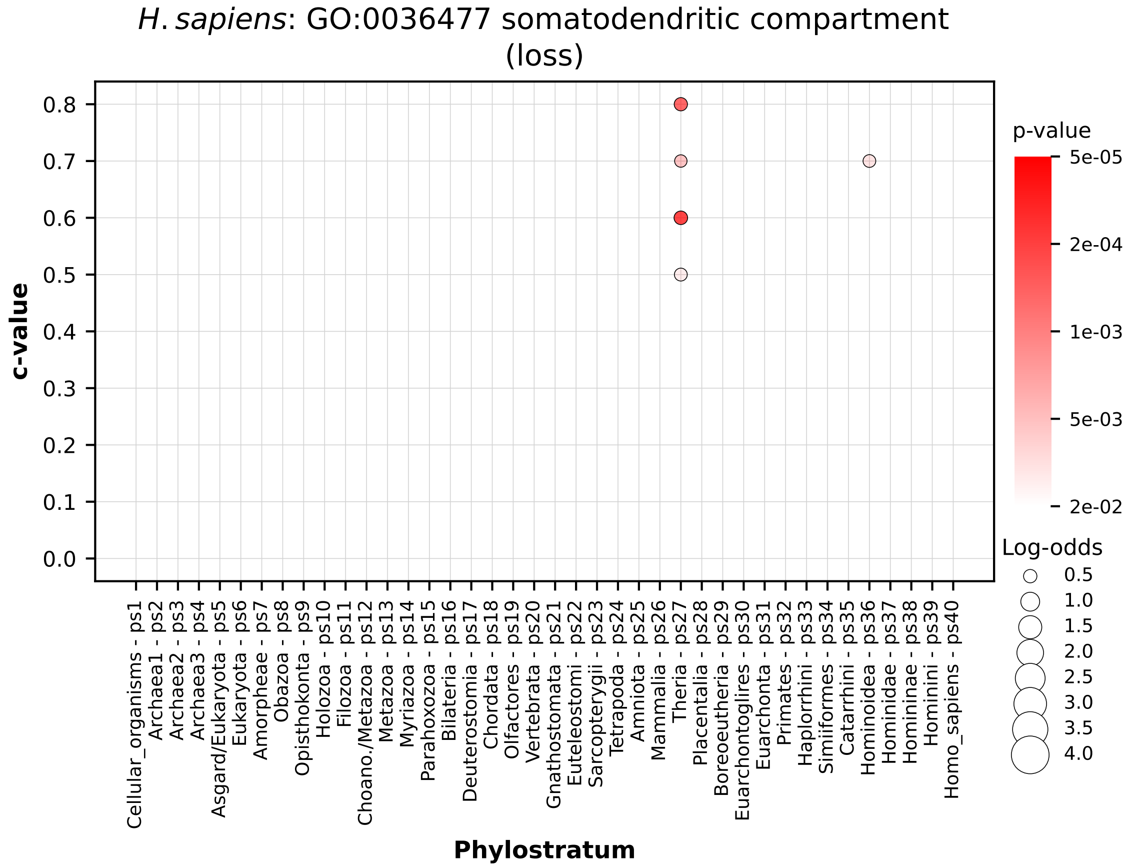
H. sapiens: GO:0036477 somatodendritic compartment
(loss)
c-value
0.0
0.1
0.2
0.3
0.4
0.5
0.6
0.7
0.8
Phylostratum
Cellular_organisms - ps1
Archaea1 - ps2
Archaea2 - ps3
Archaea3 - ps4
Asgard/Eukaryota - ps5
Eukaryota - ps6
Amorpheae - ps7
Obazoa - ps8
Opisthokonta - ps9
Holozoa - ps10
Filozoa - ps11
Choano./Metazoa - ps12
Metazoa - ps13
Myriazoa - ps14
Parahoxozoa - ps15
Bilateria - ps16
Deuterostomia - ps17
Chordata - ps18
Olfactores - ps19
Vertebrata - ps20
Gnathostomata - ps21
Euteleostomi - ps22
Sarcopterygii - ps23
Tetrapoda - ps24
Amniota - ps25
Mammalia - ps26
Theria - ps27
Placentalia - ps28
Boreoeutheria - ps29
Euarchontoglires - ps30
Euarchonta - ps31
Primates - ps32
Haplorrhini - ps33
Simiiformes - ps34
Catarrhini - ps35
Hominoidea - ps36
Hominidae - ps37
Homininae - ps38
Hominini - ps39
Homo_sapiens - ps40
p-value
5e-05
2e-04
1e-03
5e-03
2e-02
Log-odds
0.5
1.0
1.5
2.0
2.5
3.0
3.5
4.0

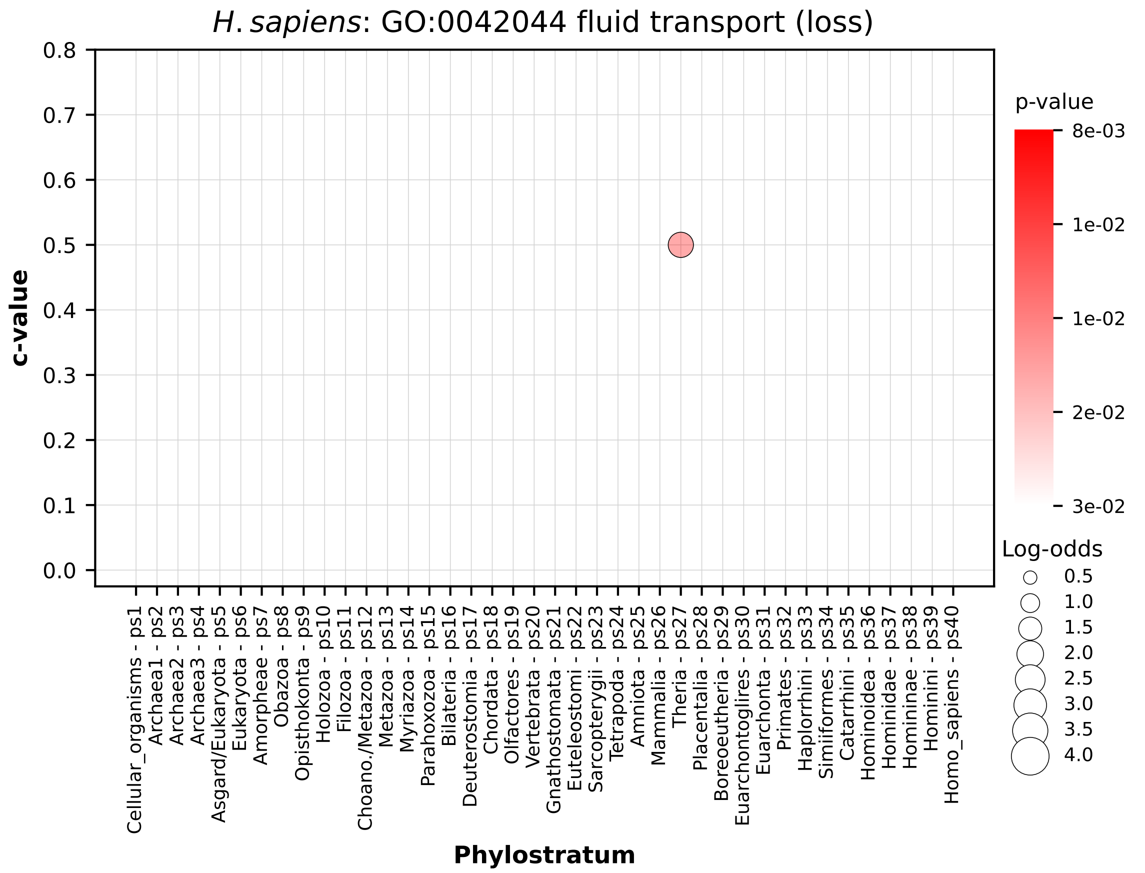
H. sapiens: GO:0042044 fluid transport (loss)
c-value
0.8
0.7
0.6
0.5
0.4
0.3
0.2
0.1
0.0
Phylostratum
Cellular_organisms - ps1
Archaea1 - ps2
Archaea2 - ps3
Archaea3 - ps4
Asgard/Eukaryota - ps5
Eukaryota - ps6
Amorpheae - ps7
Obazoa - ps8
Opisthokonta - ps9
Holozoa - ps10
Filozoa - ps11
Choano./Metazoa - ps12
Metazoa - ps13
Myriazoa - ps14
Parahoxozoa - ps15
Bilateria - ps16
Deuterostomia - ps17
Chordata - ps18
Olfactores - ps19
Vertebrata - ps20
Gnathostomata - ps21
Euteleostomi - ps22
Sarcopterygii - ps23
Tetrapoda - ps24
Amniota - ps25
Mammalia - ps26
Theria - ps27
Placentalia - ps28
Boreoeutheria - ps29
Euarchontoglires - ps30
Euarchonta - ps31
Primates - ps32
Haplorrhini - ps33
Simiiformes - ps34
Catarrhini - ps35
Hominoidea - ps36
Hominidae - ps37
Homininae - ps38
Hominini - ps39
Homo_sapiens - ps40
p-value
8e-03
1e-02
1e-02
2e-02
3e-02
Log-odds
0.5
1.0
1.5
2.0
2.5
3.0
3.5
4.0

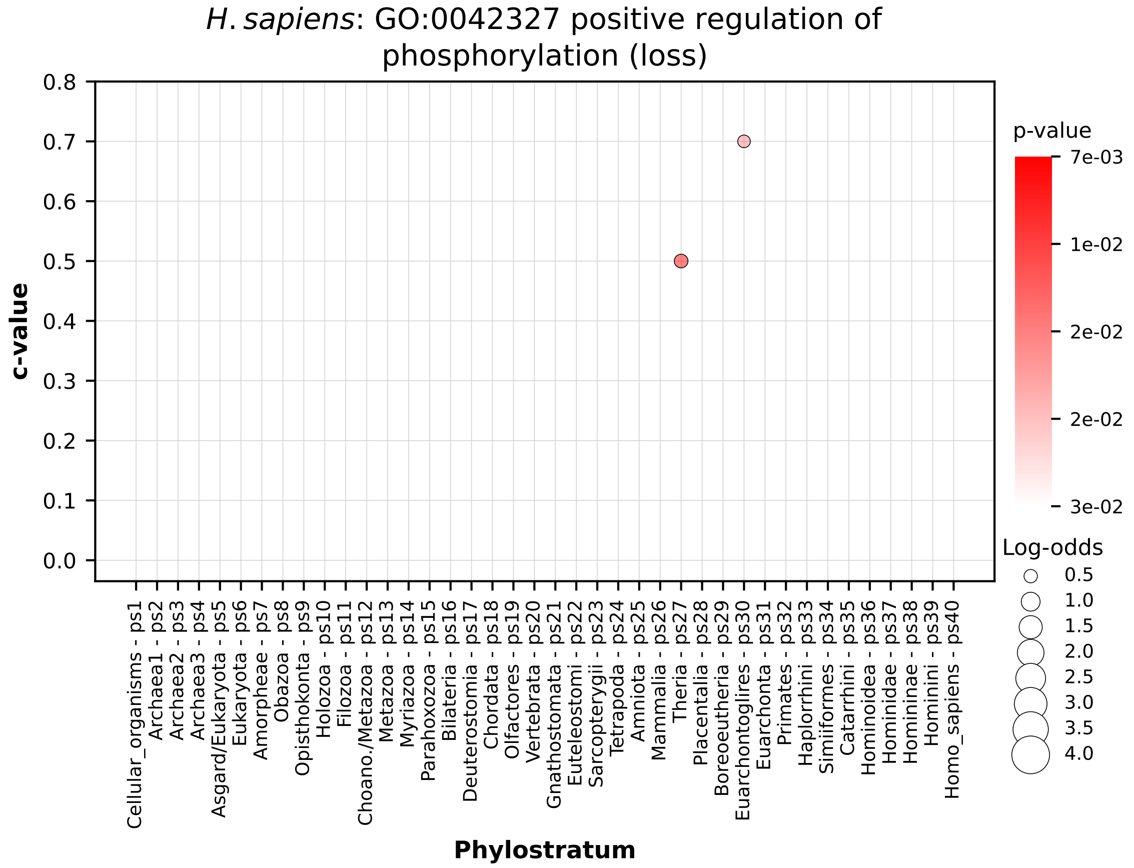

H. sapiens: GO:0042327 positive regulation of phosphorylation (loss)
c-value
0.8
0.7
0.6
0.5
0.4
0.3
0.2
0.1
0.0
p-value
7e-03
1e-02
2e-02
2e-02
3e-02
Log-odds
0.5
1.0
1.5
2.0
2.5
3.0
3.5
4.0
Cellular_organisms - ps1
Archaea1 - ps2
Archaea2 - ps3
Archaea3 - ps4
Asgard/Eukaryota - ps5
Eukaryota - ps6
Amorpheae - ps7
Obazoa - ps8
Opisthokonta - ps9
Holozoa - ps10
Filozoa - ps11
Choano./Metazoa - ps12
Metazoa - ps13
Myriazoa - ps14
Parahoxozoa - ps15
Bilateria - ps16
Deuterostomia - ps17
Chordata - ps18
Olfactores - ps19
Vertebrata - ps20
Gnathostomata - ps21
Euteleostomi - ps22
Sarcopterygii - ps23
Tetrapoda - ps24
Amniota - ps25
Mammalia - ps26
Theria - ps27
Placentalia - ps28
Boreoeutheria - ps29
Euarchontoglires - ps30
Euarchonta - ps31
Primates - ps32
Haplorrhini - ps33
Simiiformes - ps34
Catarrhini - ps35
Hominoidea - ps36
Hominidae - ps37
Homininae - ps38
Hominini - ps39
Homo_sapiens - ps40
Phylostratum

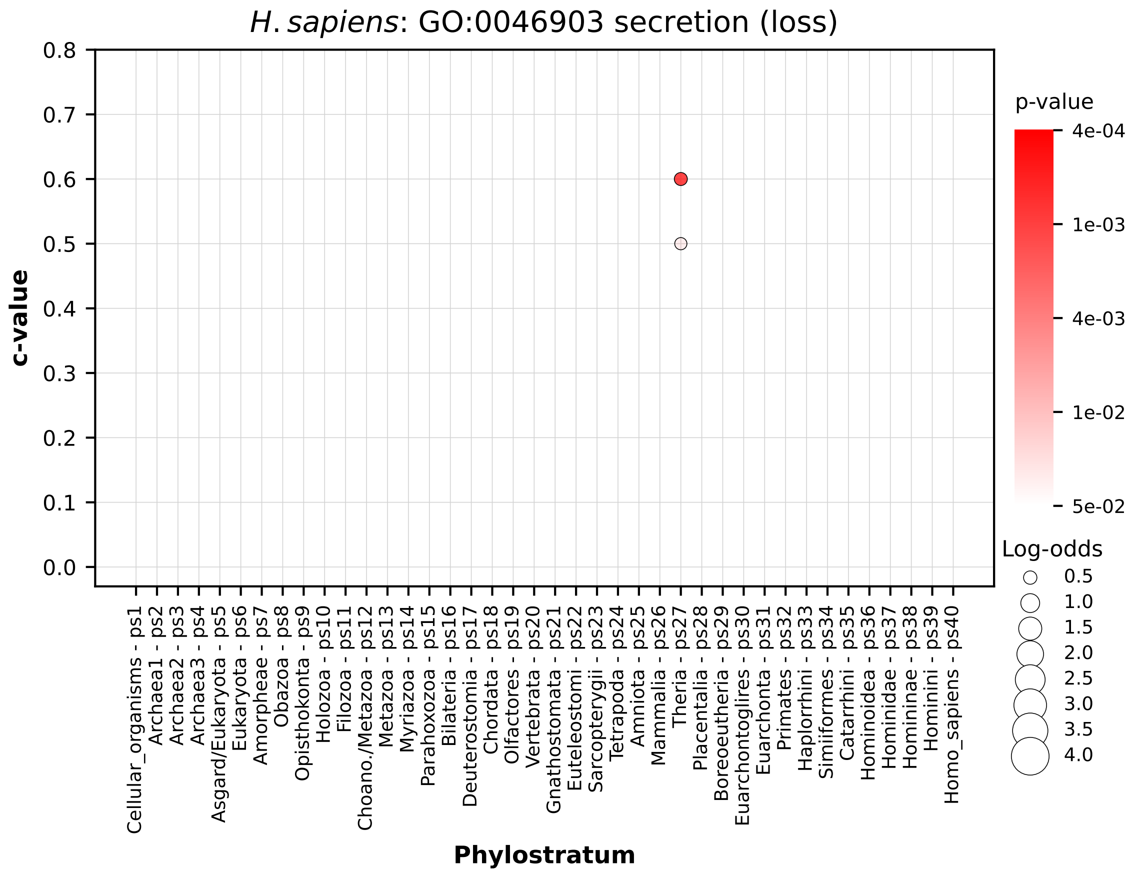

H. sapiens: GO:0046903 secretion (loss)
c-value
Phylostratum
p-value
4e-04
1e-03
4e-03
1e-02
5e-02
Log-odds
0.5
1.0
1.5
2.0
2.5
3.0
3.5
4.0
Cellular_organisms - ps1
Archaea1 - ps2
Archaea2 - ps3
Archaea3 - ps4
Asgard/Eukaryota - ps5
Eukaryota - ps6
Amorpheae - ps7
Obazoa - ps8
Opisthokonta - ps9
Holozoa - ps10
Filozoa - ps11
Choano./Metazoa - ps12
Metazoa - ps13
Myriazoa - ps14
Parahoxozoa - ps15
Bilateria - ps16
Deuterostomia - ps17
Chordata - ps18
Olfactores - ps19
Vertebrata - ps20
Gnathostomata - ps21
Euteleostomi - ps22
Sarcopterygii - ps23
Tetrapoda - ps24
Amniota - ps25
Mammalia - ps26
Theria - ps27
Placentalia - ps28
Boreoeutheria - ps29
Euarchontoglires - ps30
Euarchonta - ps31
Primates - ps32
Haplorrhini - ps33
Simiiformes - ps34
Catarrhini - ps35
Hominoidea - ps36
Hominidae - ps37
Homininae - ps38
Hominini - ps39
Homo_sapiens - ps40

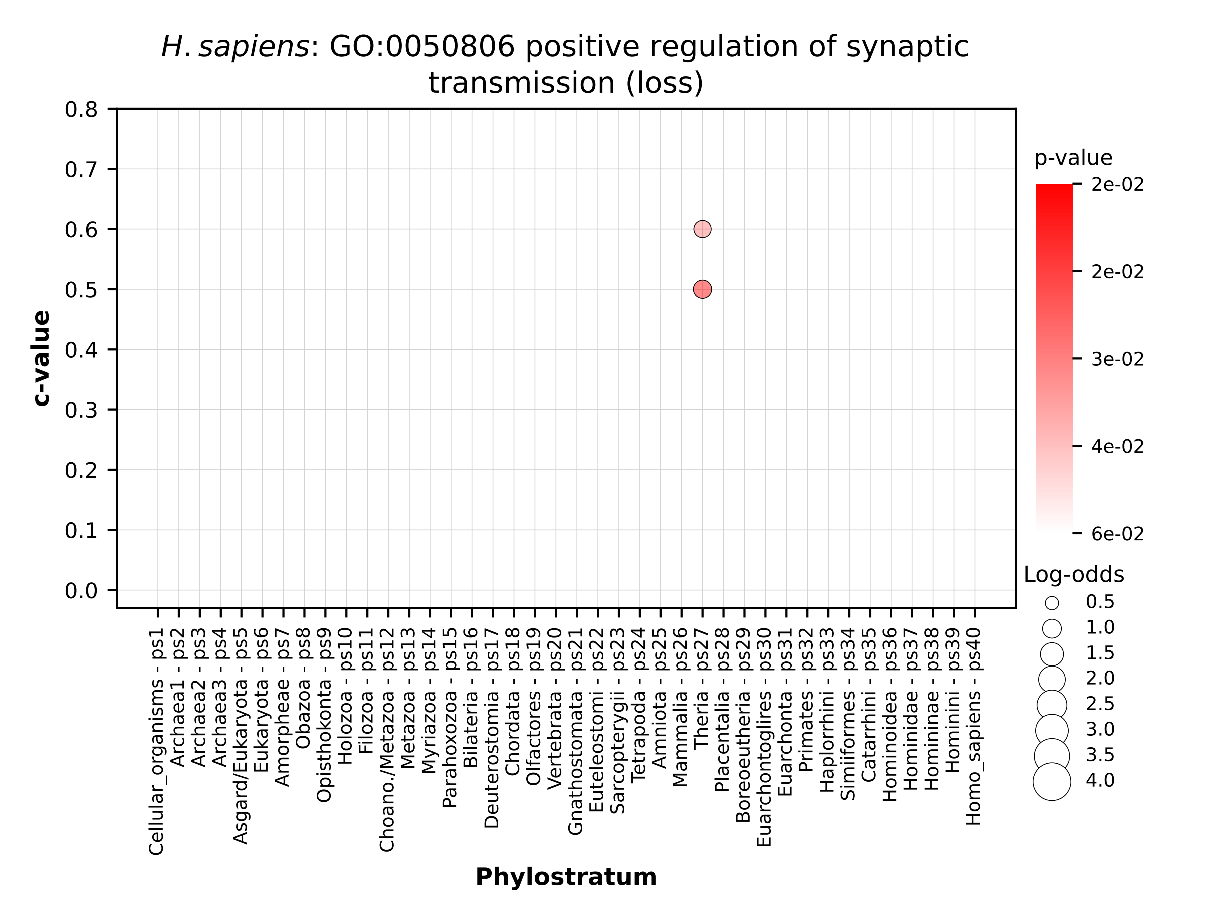

H. sapiens: GO:0050806 positive regulation of synaptic transmission (loss)
c-value
0.0
0.1
0.2
0.3
0.4
0.5
0.6
0.7
0.8
Phylostratum
Cellular_organisms - ps1
Archaea1 - ps2
Archaea2 - ps3
Archaea3 - ps4
Asgard/Eukaryota - ps5
Eukaryota - ps6
Amorpheae - ps7
Obazoa - ps8
Opisthokonta - ps9
Holozoa - ps10
Filozoa - ps11
Choano./Metazoa - ps12
Metazoa - ps13
Myriazoa - ps14
Parahoxozoa - ps15
Bilateria - ps16
Deuterostomia - ps17
Chordata - ps18
Olfactores - ps19
Vertebrata - ps20
Gnathostomata - ps21
Euteleostomi - ps22
Sarcopterygii - ps23
Tetrapoda - ps24
Amniota - ps25
Mammalia - ps26
Theria - ps27
Placentalia - ps28
Boreoeutheria - ps29
Euarchontoglires - ps30
Euarchonta - ps31
Primates - ps32
Haplorrhini - ps33
Simiiformes - ps34
Catarrhini - ps35
Hominoidea - ps36
Hominidae - ps37
Homininae - ps38
Hominini - ps39
Homo_sapiens - ps40
p-value
2e-02
2e-02
3e-02
4e-02
6e-02
Log-odds
0.5
1.0
1.5
2.0
2.5
3.0
3.5
4.0

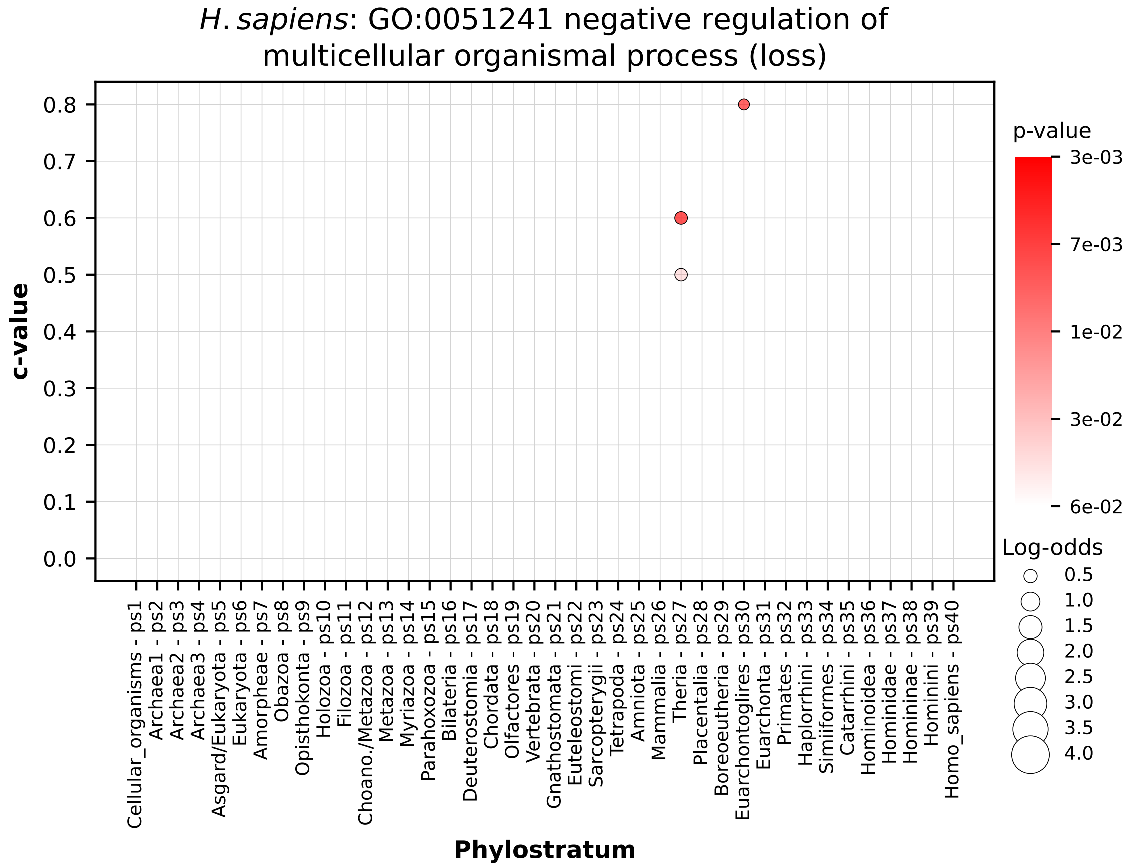

H. sapiens: GO:0051241 negative regulation of multicellular organismal process (loss)
c-value
0.0
0.1
0.2
0.3
0.4
0.5
0.6
0.7
0.8
Phylostratum
Cellular_organisms - ps1
Archaea1 - ps2
Archaea2 - ps3
Archaea3 - ps4
Asgard/Eukaryota - ps5
Eukaryota - ps6
Amorpheae - ps7
Obazoa - ps8
Opisthokonta - ps9
Holozoa - ps10
Filozoa - ps11
Choano./Metazoa - ps12
Metazoa - ps13
Myriazoa - ps14
Parahoxozoa - ps15
Bilateria - ps16
Deuterostomia - ps17
Chordata - ps18
Olfactores - ps19
Vertebrata - ps20
Gnathostomata - ps21
Euteleostomi - ps22
Sarcopterygii - ps23
Tetrapoda - ps24
Amniota - ps25
Mammalia - ps26
Theria - ps27
Placentalia - ps28
Boreoeutheria - ps29
Euarchontoglires - ps30
Euarchonta - ps31
Primates - ps32
Haplorrhini - ps33
Simiiformes - ps34
Catarrhini - ps35
Hominoidea - ps36
Hominidae - ps37
Homininae - ps38
Hominini - ps39
Homo_sapiens - ps40
p-value
3e-03
7e-03
1e-02
3e-02
6e-02
Log-odds
0.5
1.0
1.5
2.0
2.5
3.0
3.5
4.0

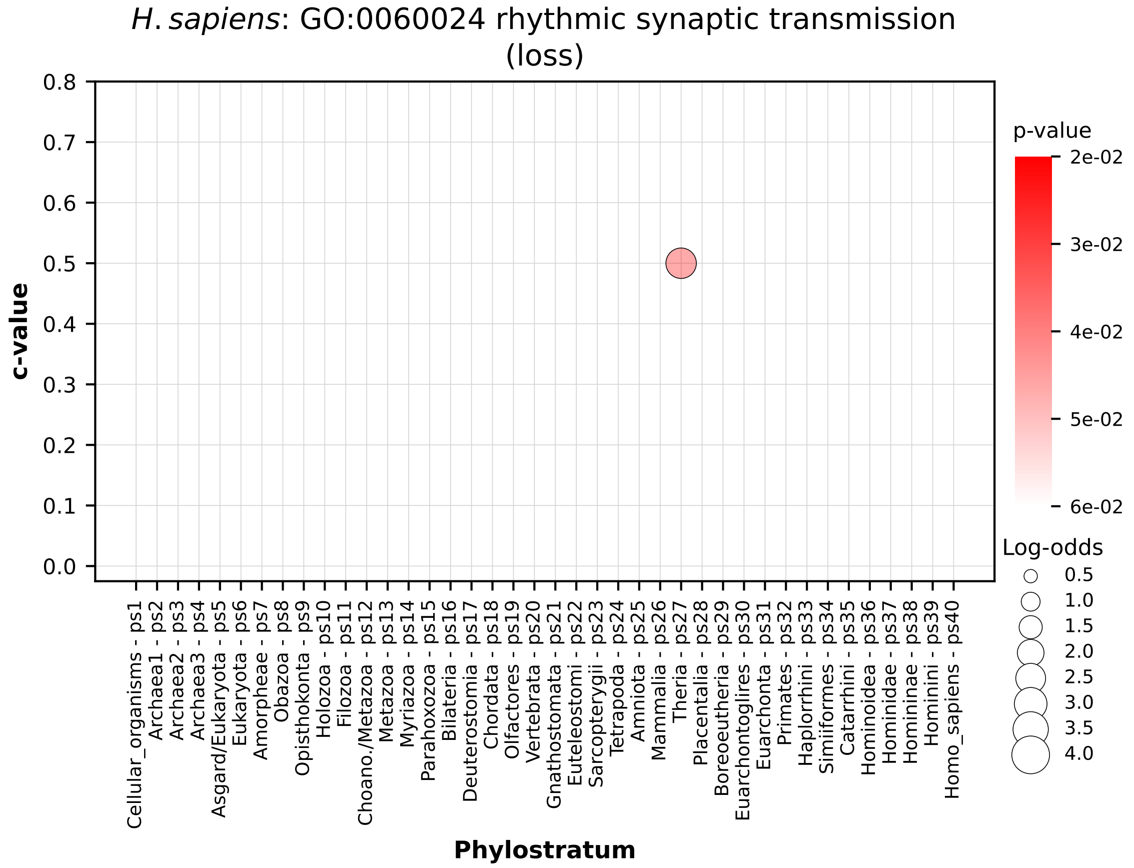

H. sapiens: GO:0060024 rhythmic synaptic transmission (loss)
c-value
0.8
0.7
0.6
0.5
0.4
0.3
0.2
0.1
0.0
Phylostratum
Cellular_organisms - ps1
Archaea1 - ps2
Archaea2 - ps3
Archaea3 - ps4
Asgard/Eukaryota - ps5
Eukaryota - ps6
Amorpheae - ps7
Obazoa - ps8
Opisthokonta - ps9
Holozoa - ps10
Filozoa - ps11
Choano./Metazoa - ps12
Metazoa - ps13
Myriazoa - ps14
Parahoxozoa - ps15
Bilateria - ps16
Deuterostomia - ps17
Chordata - ps18
Olfactores - ps19
Vertebrata - ps20
Gnathostomata - ps21
Euteleostomi - ps22
Sarcopterygii - ps23
Tetrapoda - ps24
Amniota - ps25
Mammalia - ps26
Theria - ps27
Placentalia - ps28
Boreoeutheria - ps29
Euarchontoglires - ps30
Euarchonta - ps31
Primates - ps32
Haplorrhini - ps33
Simiiformes - ps34
Catarrhini - ps35
Hominoidea - ps36
Hominidae - ps37
Homininae - ps38
Hominini - ps39
Homo_sapiens - ps40
p-value
2e-02
3e-02
4e-02
5e-02
6e-02
Log-odds
0.5
1.0
1.5
2.0
2.5
3.0
3.5
4.0

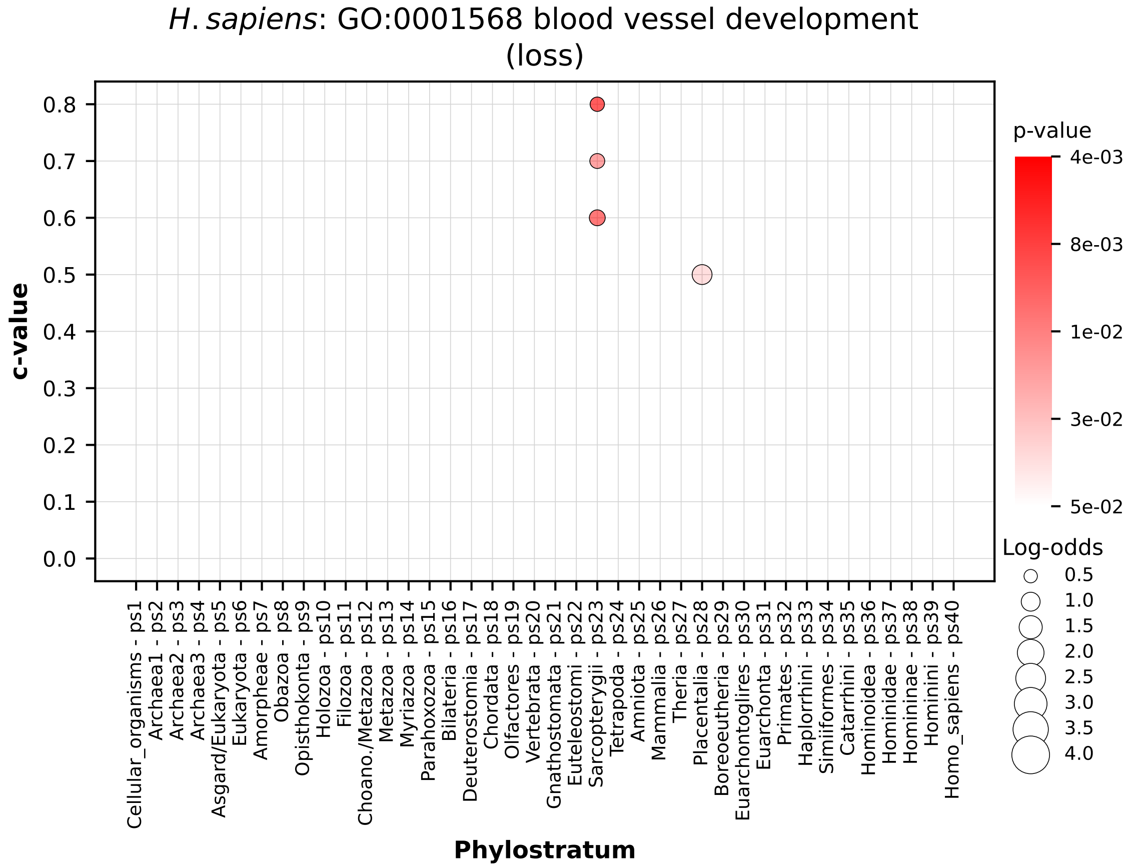

H. sapiens: GO:0001568 blood vessel development
(loss)
c-value
0.0
0.1
0.2
0.3
0.4
0.5
0.6
0.7
0.8
p-value
4e-03
8e-03
1e-02
3e-02
5e-02
Log-odds
0.5
1.0
1.5
2.0
2.5
3.0
3.5
4.0
Cellular_organisms - ps1
Archaea1 - ps2
Archaea2 - ps3
Archaea3 - ps4
Asgard/Eukaryota - ps5
Eukaryota - ps6
Amorpheae - ps7
Obazoa - ps8
Opisthokonta - ps9
Holozoa - ps10
Filozoa - ps11
Choano./Metazoa - ps12
Metazoa - ps13
Myriazoa - ps14
Parahoxozoa - ps15
Bilateria - ps16
Deuterostomia - ps17
Chordata - ps18
Olfactores - ps19
Vertebrata - ps20
Gnathostomata - ps21
Euteleostomi - ps22
Sarcopterygii - ps23
Tetrapoda - ps24
Amniota - ps25
Mammalia - ps26
Theria - ps27
Placentalia - ps28
Boreoeutheria - ps29
Euarchontoglires - ps30
Euarchonta - ps31
Primates - ps32
Haplorrhini - ps33
Simiiformes - ps34
Catarrhini - ps35
Hominoidea - ps36
Hominidae - ps37
Homininae - ps38
Hominini - ps39
Homo_sapiens - ps40
Phylostratum

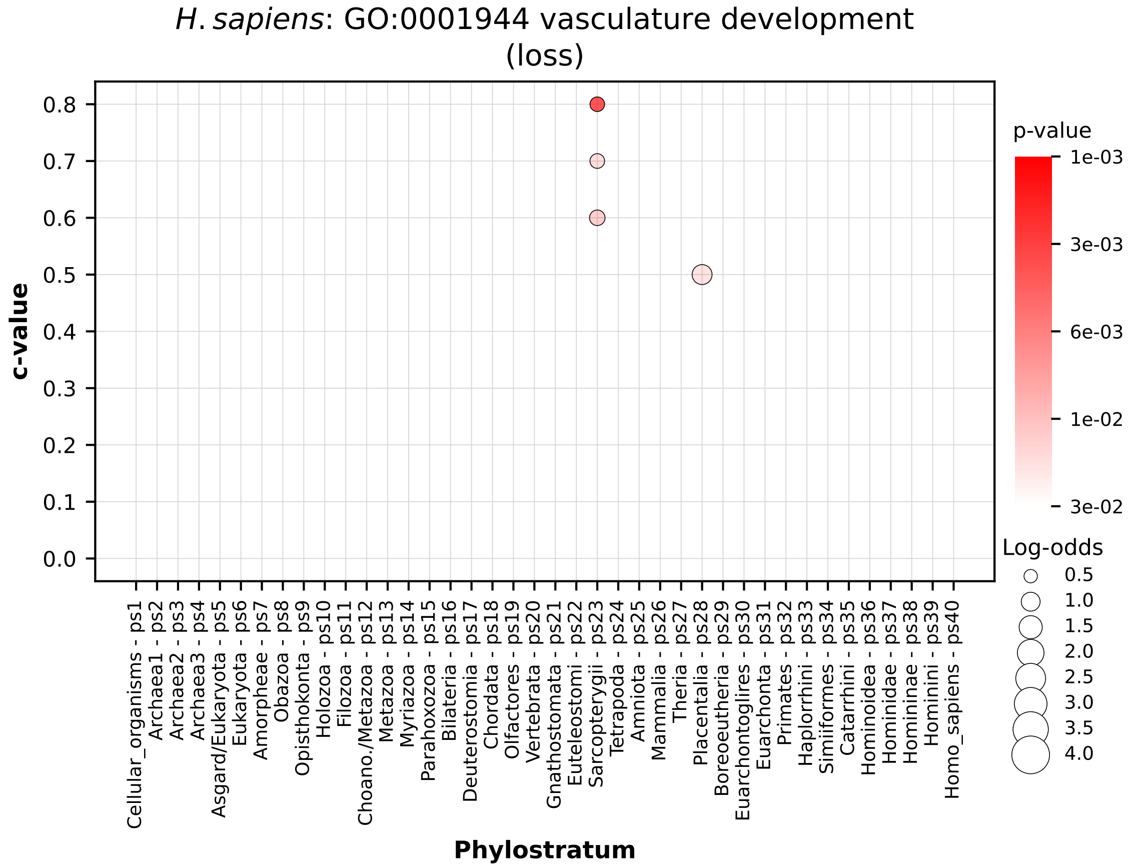

H. sapiens: GO:0001944 vasculature development (loss)
c-value
0.0
0.1
0.2
0.3
0.4
0.5
0.6
0.7
0.8
Phylostratum
Cellular_organisms - ps1
Archaea1 - ps2
Archaea2 - ps3
Archaea3 - ps4
Asgard/Eukaryota - ps5
Eukaryota - ps6
Amorpheae - ps7
Obazoa - ps8
Opisthokonta - ps9
Holozoa - ps10
Filozoa - ps11
Choano./Metazoa - ps12
Metazoa - ps13
Myriazoa - ps14
Parahoxozoa - ps15
Bilateria - ps16
Deuterostomia - ps17
Chordata - ps18
Olfactores - ps19
Vertebrata - ps20
Gnathostomata - ps21
Euteleostomi - ps22
Sarcopterygii - ps23
Tetrapoda - ps24
Amniota - ps25
Mammalia - ps26
Theria - ps27
Placentalia - ps28
Boreoeutheria - ps29
Euarchontoglires - ps30
Euarchonta - ps31
Primates - ps32
Haplorrhini - ps33
Simiiformes - ps34
Catarrhini - ps35
Hominoidea - ps36
Hominidae - ps37
Homininae - ps38
Hominini - ps39
Homo_sapiens - ps40
p-value
1e-03
3e-03
6e-03
1e-02
3e-02
Log-odds
0.5
1.0
1.5
2.0
2.5
3.0
3.5
4.0

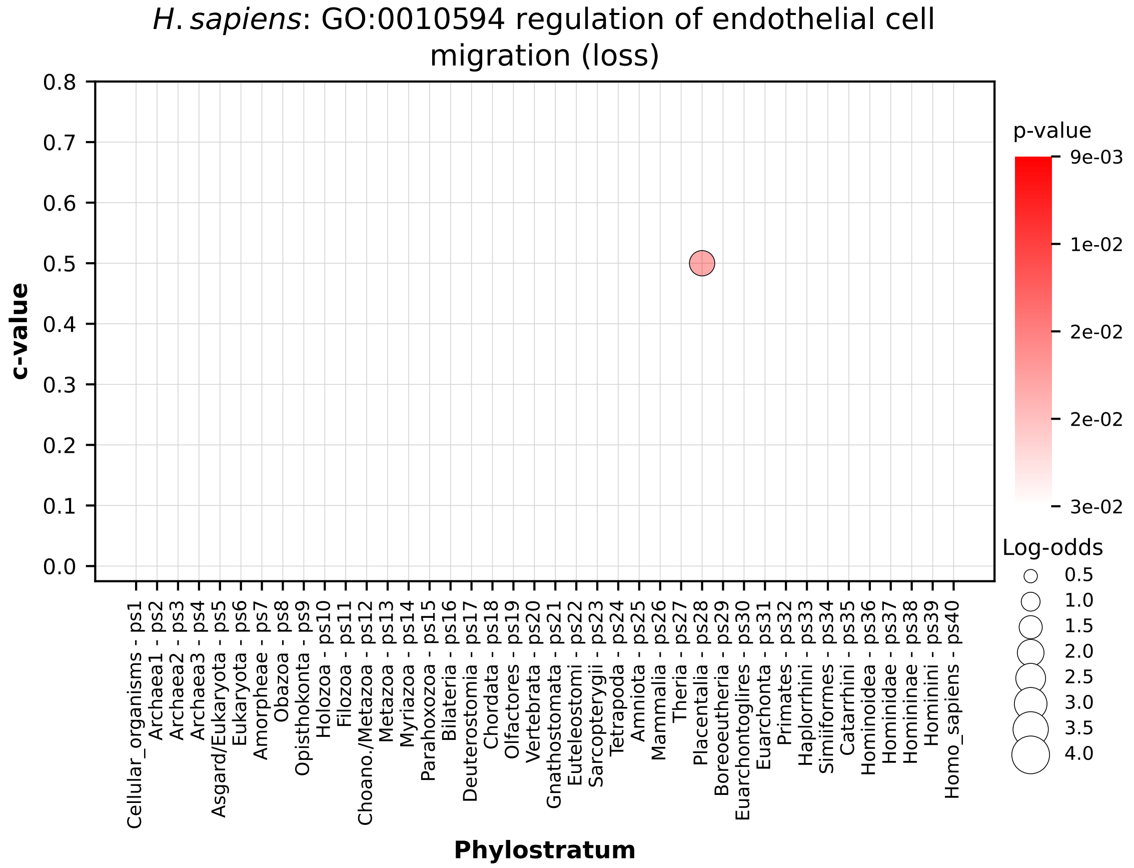

H. sapiens: GO:0010594 regulation of endothelial cell migration (loss)
c-value
0.0
0.1
0.2
0.3
0.4
0.5
0.6
0.7
0.8
Phylostratum
Cellular_organisms - ps1
Archaea1 - ps2
Archaea2 - ps3
Archaea3 - ps4
Asgard/Eukaryota - ps5
Eukaryota - ps6
Amorpheae - ps7
Obazoa - ps8
Opisthokonta - ps9
Holozoa - ps10
Filozoa - ps11
Choano./Metazoa - ps12
Metazoa - ps13
Myriazoa - ps14
Parahoxozoa - ps15
Bilateria - ps16
Deuterostomia - ps17
Chordata - ps18
Olfactores - ps19
Vertebrata - ps20
Gnathostomata - ps21
Euteleostomi - ps22
Sarcopterygii - ps23
Tetrapoda - ps24
Amniota - ps25
Mammalia - ps26
Theria - ps27
Placentalia - ps28
Boreoeutheria - ps29
Euarchontoglires - ps30
Euarchonta - ps31
Primates - ps32
Haplorrhini - ps33
Simiiformes - ps34
Catarrhini - ps35
Hominoidea - ps36
Hominidae - ps37
Homininae - ps38
Hominini - ps39
Homo_sapiens - ps40
p-value
9e-03
1e-02
2e-02
2e-02
3e-02
Log-odds
0.5
1.0
1.5
2.0
2.5
3.0
3.5
4.0

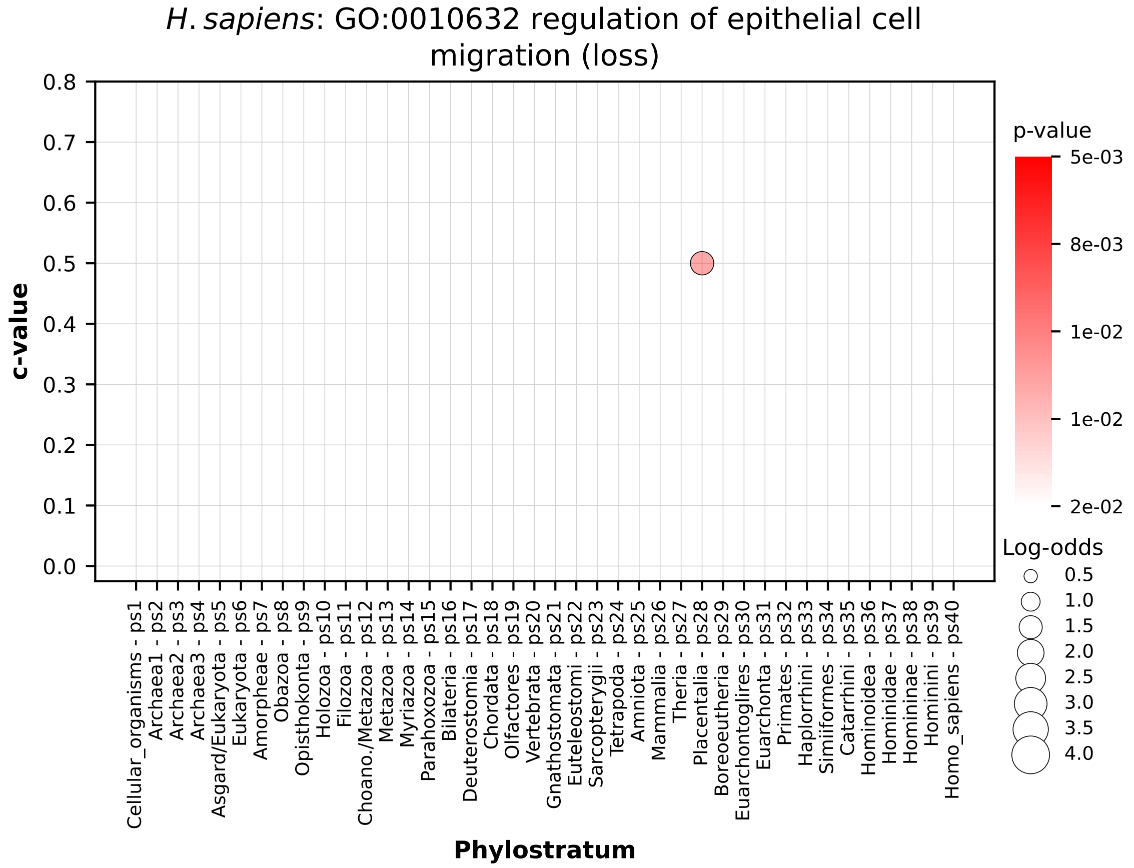

H. sapiens: GO:0010632 regulation of epithelial cell migration (loss)
c-value
0.8
0.7
0.6
0.5
0.4
0.3
0.2
0.1
0.0
Phylostratum
Cellular_organisms - ps1
Archaea1 - ps2
Archaea2 - ps3
Archaea3 - ps4
Asgard/Eukaryota - ps5
Eukaryota - ps6
Amorpheae - ps7
Obazoa - ps8
Opisthokonta - ps9
Holozoa - ps10
Filozoa - ps11
Choano./Metazoa - ps12
Metazoa - ps13
Myriazoa - ps14
Parahoxozoa - ps15
Bilateria - ps16
Deuterostomia - ps17
Chordata - ps18
Olfactores - ps19
Vertebrata - ps20
Gnathostomata - ps21
Euteleostomi - ps22
Sarcopterygii - ps23
Tetrapoda - ps24
Amniota - ps25
Mammalia - ps26
Theria - ps27
Placentalia - ps28
Boreoeutheria - ps29
Euarchontoglires - ps30
Euarchonta - ps31
Primates - ps32
Haplorrhini - ps33
Simiiformes - ps34
Catarrhini - ps35
Hominoidea - ps36
Hominidae - ps37
Homininae - ps38
Hominini - ps39
Homo_sapiens - ps40
p-value
5e-03
8e-03
1e-02
1e-02
2e-02
Log-odds
0.5
1.0
1.5
2.0
2.5
3.0
3.5
4.0

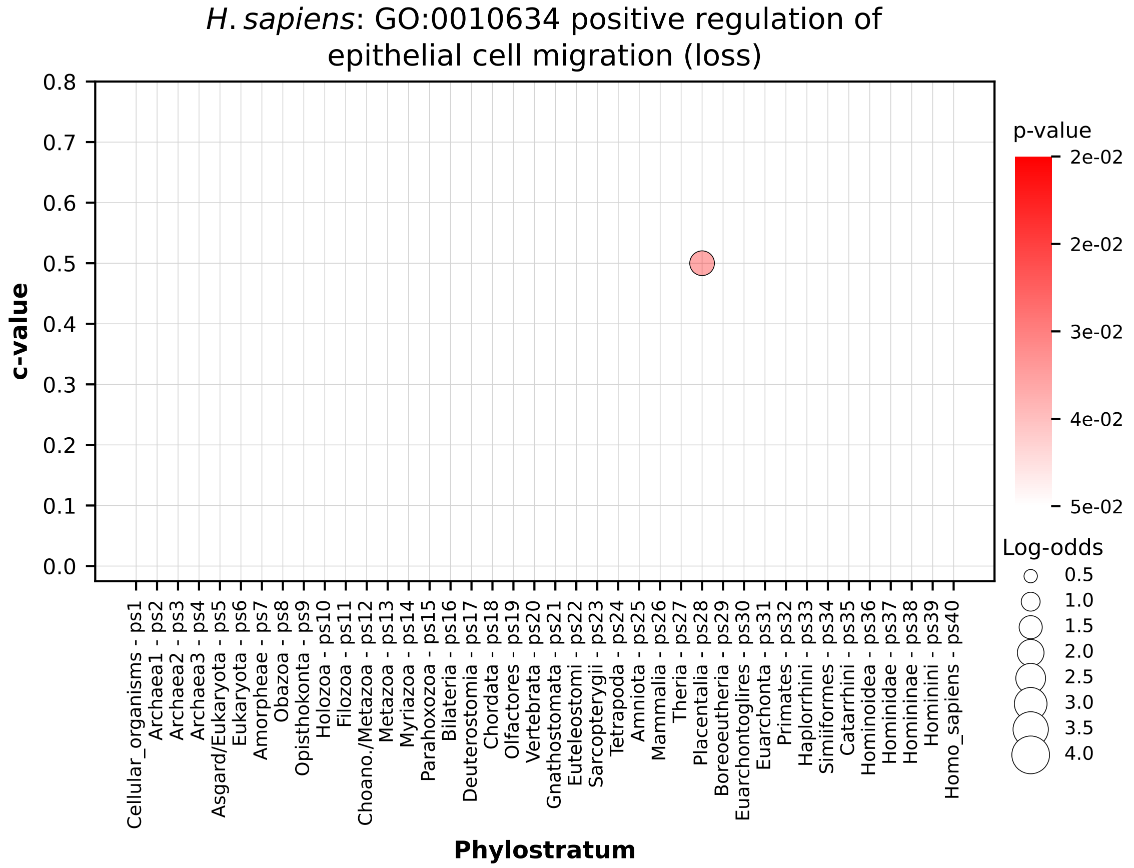
H. sapiens: GO:0010634 positive regulation of epithelial cell migration (loss)
c-value
0.8
0.7
0.6
0.5
0.4
0.3
0.2
0.1
0.0
Phylostratum
Cellular_organisms - ps1
Archaea1 - ps2
Archaea2 - ps3
Archaea3 - ps4
Asgard/Eukaryota - ps5
Eukaryota - ps6
Amorpheae - ps7
Obazoa - ps8
Opisthokonta - ps9
Holozoa - ps10
Filozoa - ps11
Choano./Metazoa - ps12
Metazoa - ps13
Myriazoa - ps14
Parahoxozoa - ps15
Bilateria - ps16
Deuterostomia - ps17
Chordata - ps18
Olfactores - ps19
Vertebrata - ps20
Gnathostomata - ps21
Euteleostomi - ps22
Sarcopterygii - ps23
Tetrapoda - ps24
Amniota - ps25
Mammalia - ps26
Theria - ps27
Placentalia - ps28
Boreoeutheria - ps29
Euarchontoglires - ps30
Euarchonta - ps31
Primates - ps32
Haplorrhini - ps33
Simiiformes - ps34
Catarrhini - ps35
Hominoidea - ps36
Hominidae - ps37
Homininae - ps38
Hominini - ps39
Homo_sapiens - ps40
p-value
2e-02
2e-02
3e-02
4e-02
5e-02
Log-odds
0.5
1.0
1.5
2.0
2.5
3.0
3.5
4.0

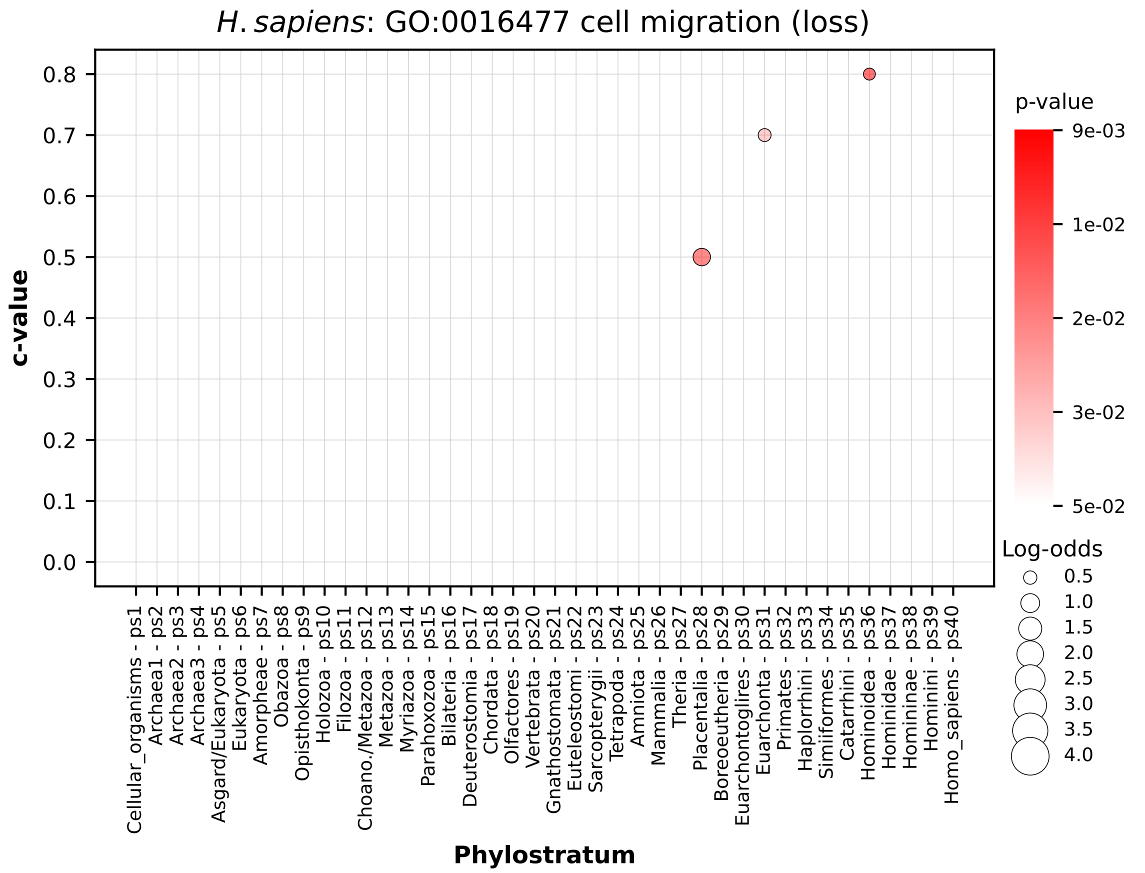
H. sapiens: GO:0016477 cell migration (loss)
c-value
0.0
0.1
0.2
0.3
0.4
0.5
0.6
0.7
0.8
Phylostratum
Cellular_organisms - ps1
Archaea1 - ps2
Archaea2 - ps3
Archaea3 - ps4
Asgard/Eukaryota - ps5
Eukaryota - ps6
Amorpheae - ps7
Obazoa - ps8
Opisthokonta - ps9
Holozoa - ps10
Filozoa - ps11
Choano./Metazoa - ps12
Metazoa - ps13
Myriazoa - ps14
Parahoxozoa - ps15
Bilateria - ps16
Deuterostomia - ps17
Chordata - ps18
Olfactores - ps19
Vertebrata - ps20
Gnathostomata - ps21
Euteleostomi - ps22
Sarcopterygii - ps23
Tetrapoda - ps24
Amniota - ps25
Mammalia - ps26
Theria - ps27
Placentalia - ps28
Boreoeutheria - ps29
Euarchontoglires - ps30
Euarchonta - ps31
Primates - ps32
Haplorrhini - ps33
Simiiformes - ps34
Catarrhini - ps35
Hominoidea - ps36
Hominidae - ps37
Homininae - ps38
Hominini - ps39
Homo_sapiens - ps40
p-value
9e-03
1e-02
2e-02
3e-02
5e-02
Log-odds
0.5
1.0
1.5
2.0
2.5
3.0
3.5
4.0

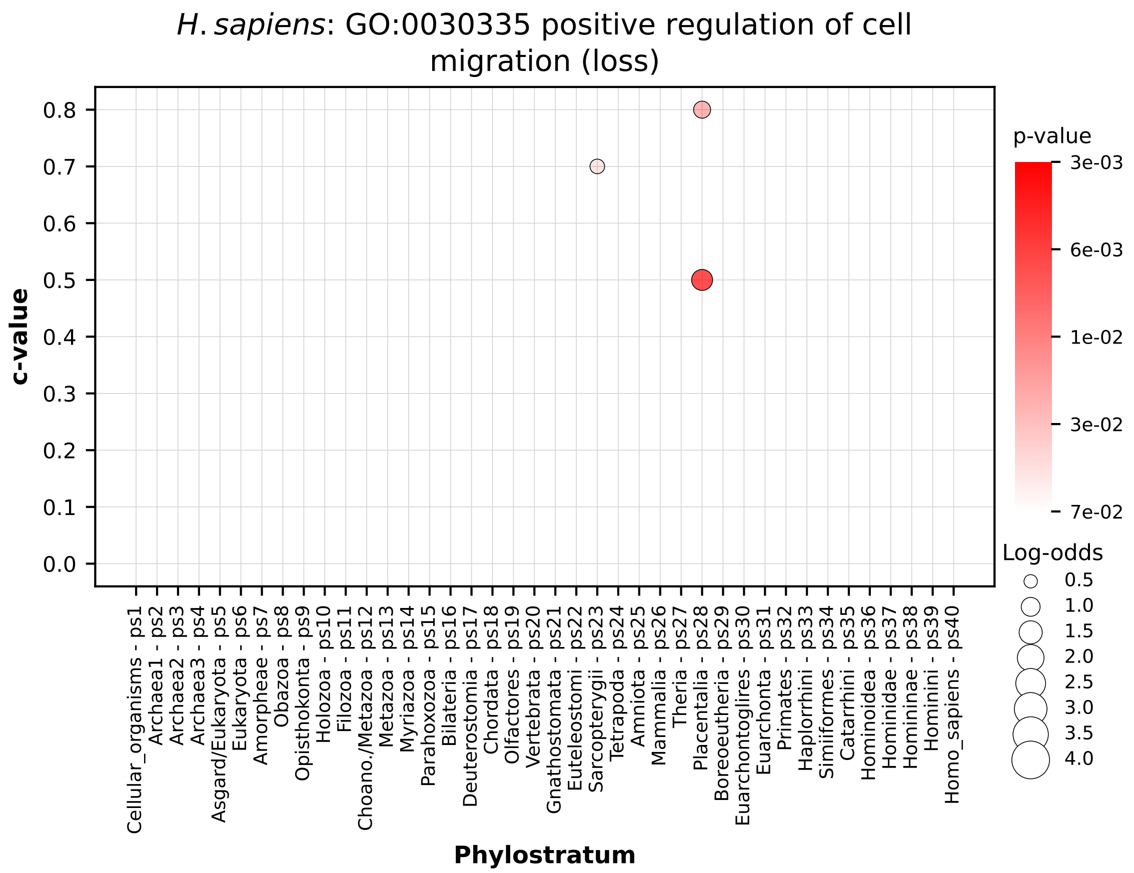

H. sapiens: GO:0030335 positive regulation of cell migration (loss)
c-value
0.8
0.7
0.6
0.5
0.4
0.3
0.2
0.1
0.0
p-value
3e-03
6e-03
1e-02
3e-02
7e-02
Log-odds
0.5
1.0
1.5
2.0
2.5
3.0
3.5
4.0
Cellular_organisms - ps1
Archaea1 - ps2
Archaea2 - ps3
Archaea3 - ps4
Asgard/Eukaryota - ps5
Eukaryota - ps6
Amorpheae - ps7
Obazoa - ps8
Opisthokonta - ps9
Holozoa - ps10
Filozoa - ps11
Choano./Metazoa - ps12
Metazoa - ps13
Myriazoa - ps14
Parahoxozoa - ps15
Bilateria - ps16
Deuterostomia - ps17
Chordata - ps18
Olfactores - ps19
Vertebrata - ps20
Gnathostomata - ps21
Euteleostomi - ps22
Sarcopterygii - ps23
Tetrapoda - ps24
Amniota - ps25
Mammalia - ps26
Theria - ps27
Placentalia - ps28
Boreoeutheria - ps29
Euarchontoglires - ps30
Euarchonta - ps31
Primates - ps32
Haplorrhini - ps33
Simiiformes - ps34
Catarrhini - ps35
Hominoidea - ps36
Hominidae - ps37
Homininae - ps38
Hominini - ps39
Homo_sapiens - ps40
Phylostratum

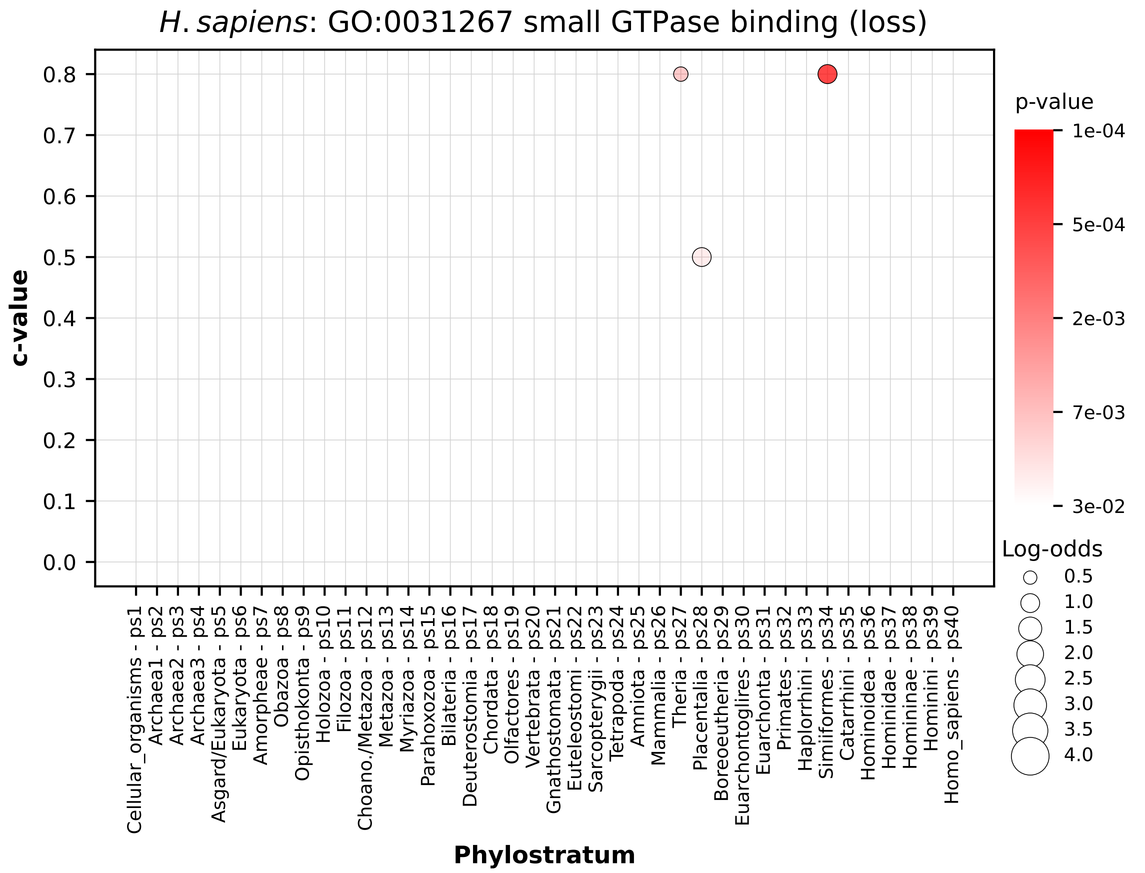

H. sapiens: GO:0031267 small GTPase binding (loss)
c-value
0.0
0.1
0.2
0.3
0.4
0.5
0.6
0.7
0.8
Phylostratum
Cellular_organisms - ps1
Archaea1 - ps2
Archaea2 - ps3
Archaea3 - ps4
Asgard/Eukaryota - ps5
Eukaryota - ps6
Amorpheae - ps7
Obazoa - ps8
Opisthokonta - ps9
Holozoa - ps10
Filozoa - ps11
Choano./Metazoa - ps12
Metazoa - ps13
Myriazoa - ps14
Parahoxozoa - ps15
Bilateria - ps16
Deuterostomia - ps17
Chordata - ps18
Olfactores - ps19
Vertebrata - ps20
Gnathostomata - ps21
Euteleostomi - ps22
Sarcopterygii - ps23
Tetrapoda - ps24
Amniota - ps25
Mammalia - ps26
Theria - ps27
Placentalia - ps28
Boreoeutheria - ps29
Euarchontoglires - ps30
Euarchonta - ps31
Primates - ps32
Haplorrhini - ps33
Simiiformes - ps34
Catarrhini - ps35
Hominoidea - ps36
Hominidae - ps37
Homininae - ps38
Hominini - ps39
Homo_sapiens - ps40
p-value
1e-04
5e-04
2e-03
7e-03
3e-02
Log-odds
0.5
1.0
1.5
2.0
2.5
3.0
3.5
4.0

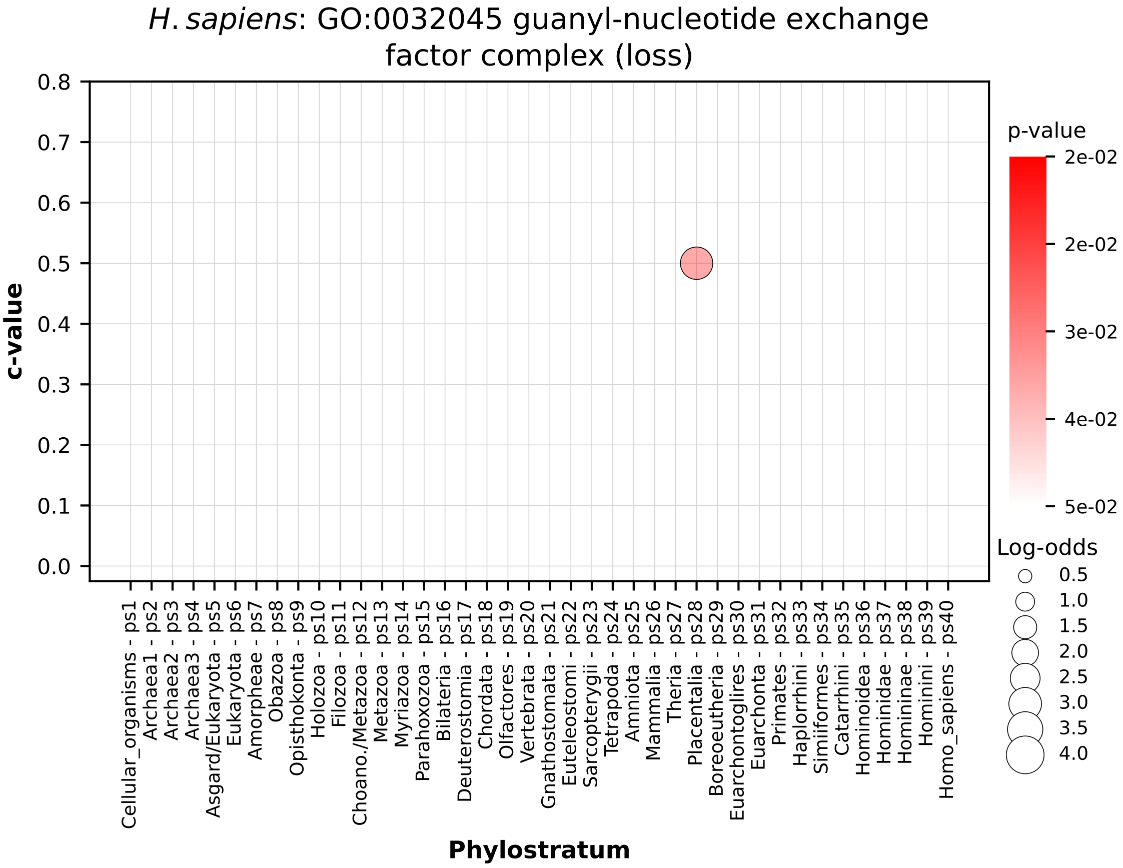

H. sapiens: GO:0032045 guanyl-nucleotide exchange factor complex (loss)
c-value
0.8
0.7
0.6
0.5
0.4
0.3
0.2
0.1
0.0
p-value
2e-02
2e-02
3e-02
4e-02
5e-02
Log-odds
0.5
1.0
1.5
2.0
2.5
3.0
3.5
4.0
Cellular_organisms - ps1
Archaea1 - ps2
Archaea2 - ps3
Archaea3 - ps4
Asgard/Eukaryota - ps5
Eukaryota - ps6
Amorpheae - ps7
Obazoa - ps8
Opisthokonta - ps9
Holozoa - ps10
Filozoa - ps11
Choano./Metazoa - ps12
Metazoa - ps13
Myriazoa - ps14
Parahoxozoa - ps15
Bilateria - ps16
Deuterostomia - ps17
Chordata - ps18
Olfactores - ps19
Vertebrata - ps20
Gnathostomata - ps21
Euteleostomi - ps22
Sarcopterygii - ps23
Tetrapoda - ps24
Amniota - ps25
Mammalia - ps26
Theria - ps27
Placentalia - ps28
Boreoeutheria - ps29
Euarchontoglires - ps30
Euarchonta - ps31
Primates - ps32
Haplorrhini - ps33
Simiiformes - ps34
Catarrhini - ps35
Hominoidea - ps36
Hominidae - ps37
Homininae - ps38
Hominini - ps39
Homo_sapiens - ps40
Phylostratum

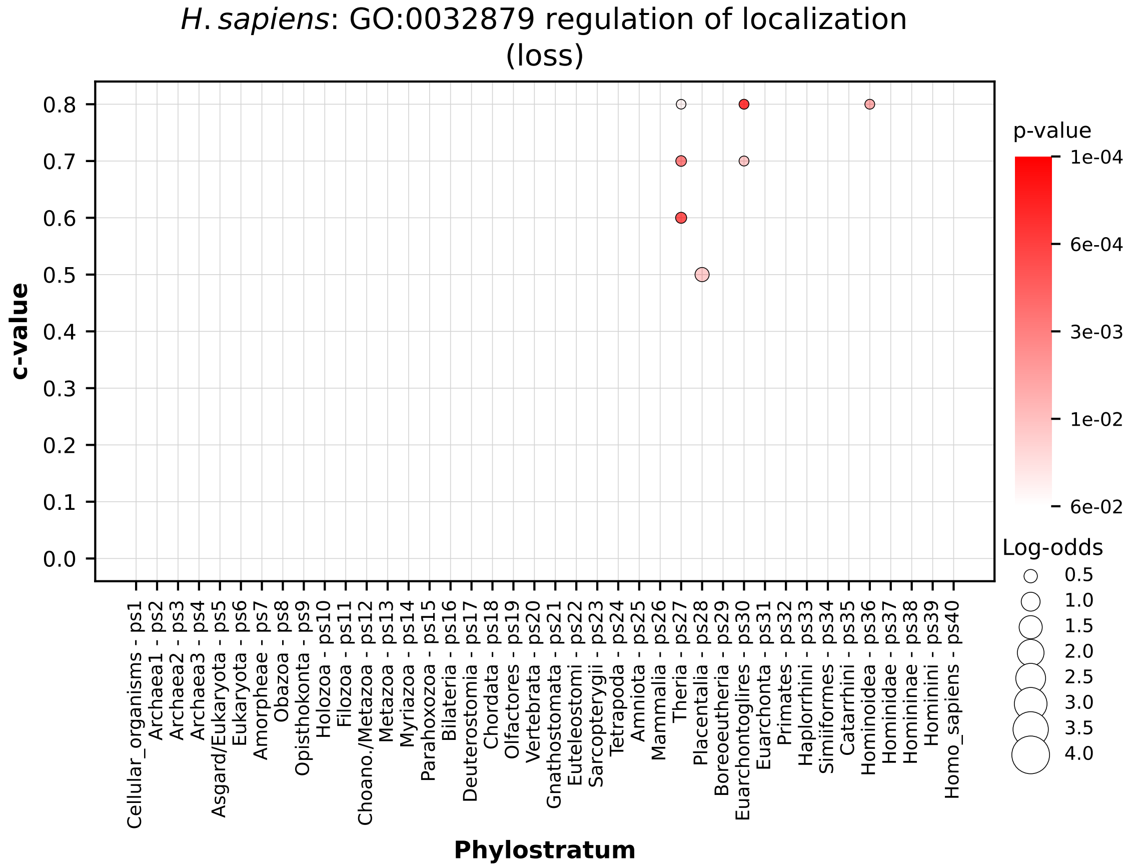

H. sapiens: GO:0032879 regulation of localization
(loss)
c-value
0.8
0.7
0.6
0.5
0.4
0.3
0.2
0.1
0.0
Phylostratum
Cellular_organisms - ps1
Archaea1 - ps2
Archaea2 - ps3
Archaea3 - ps4
Asgard/Eukaryota - ps5
Eukaryota - ps6
Amorpheae - ps7
Obazoa - ps8
Opisthokonta - ps9
Holozoa - ps10
Filozoa - ps11
Choano./Metazoa - ps12
Metazoa - ps13
Myriazoa - ps14
Parahoxozoa - ps15
Bilateria - ps16
Deuterostomia - ps17
Chordata - ps18
Olfactores - ps19
Vertebrata - ps20
Gnathostomata - ps21
Euteleostomi - ps22
Sarcopterygii - ps23
Tetrapoda - ps24
Amniota - ps25
Mammalia - ps26
Theria - ps27
Placentalia - ps28
Boreoeutheria - ps29
Euarchontoglires - ps30
Euarchonta - ps31
Primates - ps32
Haplorrhini - ps33
Simiiformes - ps34
Catarrhini - ps35
Hominoidea - ps36
Hominidae - ps37
Homininae - ps38
Hominini - ps39
Homo_sapiens - ps40
p-value
1e-04
6e-04
3e-03
1e-02
6e-02
Log-odds
0.5
1.0
1.5
2.0
2.5
3.0
3.5
4.0

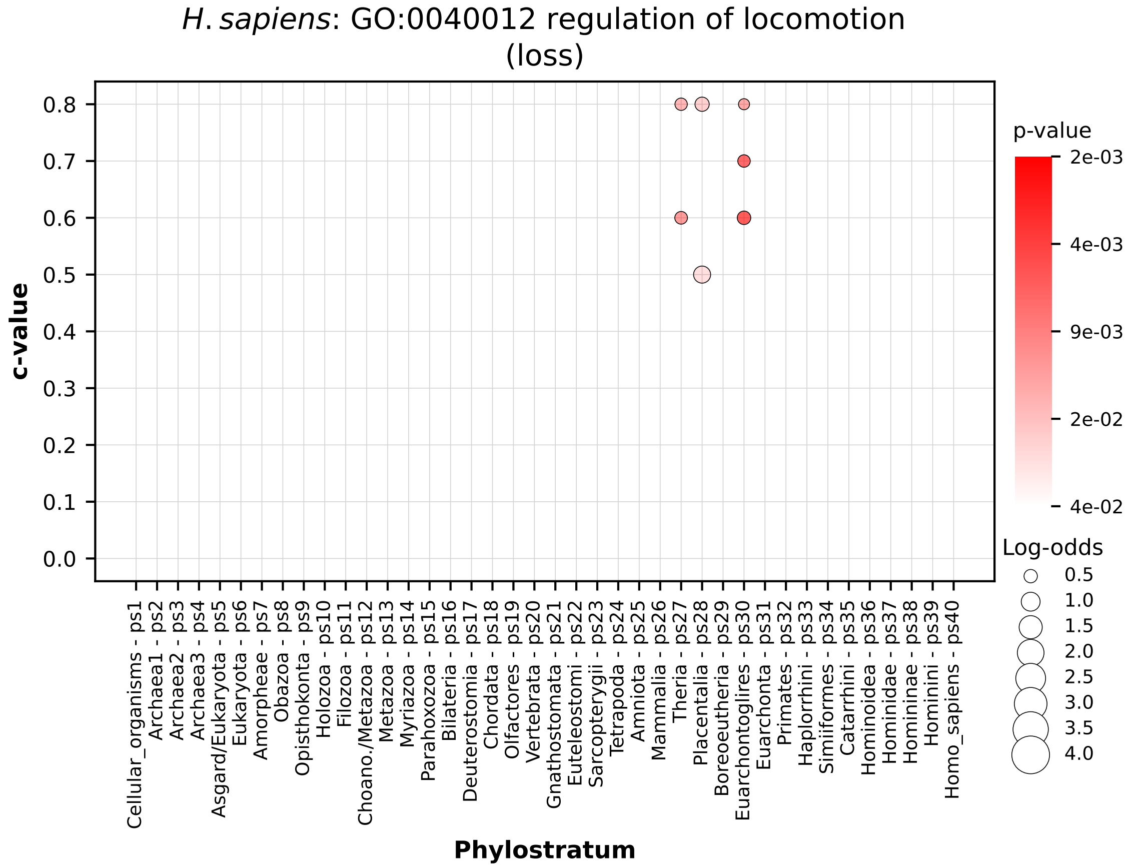

H. sapiens: GO:0040012 regulation of locomotion
(loss)
c-value
0.8
0.7
0.6
0.5
0.4
0.3
0.2
0.1
0.0
Phylostratum
Cellular_organisms - ps1
Archaea1 - ps2
Archaea2 - ps3
Archaea3 - ps4
Asgard/Eukaryota - ps5
Eukaryota - ps6
Amorpheae - ps7
Obazoa - ps8
Opisthokonta - ps9
Holozoa - ps10
Filozoa - ps11
Choano./Metazoa - ps12
Metazoa - ps13
Myriazoa - ps14
Parahoxozoa - ps15
Bilateria - ps16
Deuterostomia - ps17
Chordata - ps18
Olfactores - ps19
Vertebrata - ps20
Gnathostomata - ps21
Euteleostomi - ps22
Sarcopterygii - ps23
Tetrapoda - ps24
Amniota - ps25
Mammalia - ps26
Theria - ps27
Placentalia - ps28
Boreoeutheria - ps29
Euarchontoglires - ps30
Euarchonta - ps31
Primates - ps32
Haplorrhini - ps33
Simiiformes - ps34
Catarrhini - ps35
Hominoidea - ps36
Hominidae - ps37
Homininae - ps38
Hominini - ps39
Homo_sapiens - ps40
p-value
2e-03
4e-03
9e-03
2e-02
4e-02
Log-odds
0.5
1.0
1.5
2.0
2.5
3.0
3.5
4.0

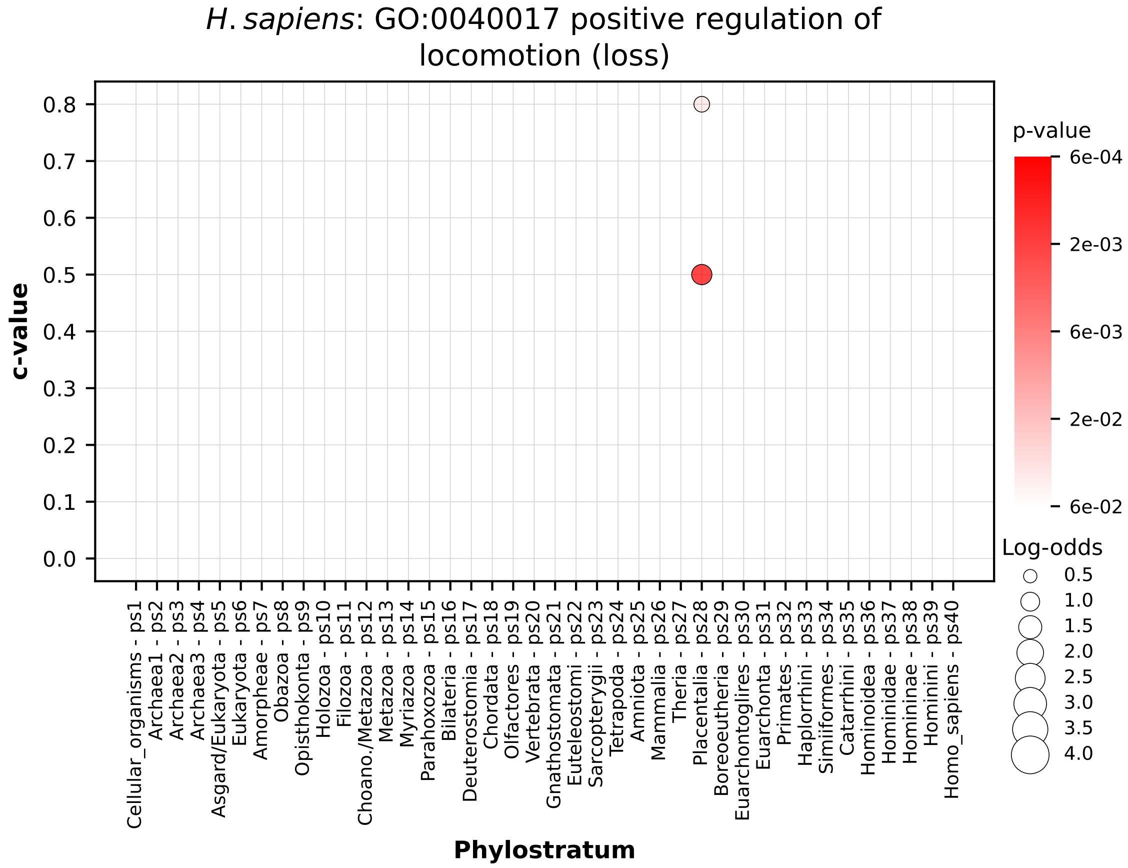

H. sapiens: GO:0040017 positive regulation of locomotion (loss)
c-value
0.0
0.1
0.2
0.3
0.4
0.5
0.6
0.7
0.8
Phylostratum
Cellular_organisms - ps1
Archaea1 - ps2
Archaea2 - ps3
Archaea3 - ps4
Asgard/Eukaryota - ps5
Eukaryota - ps6
Amorpheae - ps7
Obazoa - ps8
Opisthokonta - ps9
Holozoa - ps10
Filozoa - ps11
Choano./Metazoa - ps12
Metazoa - ps13
Myriazoa - ps14
Parahoxozoa - ps15
Bilateria - ps16
Deuterostomia - ps17
Chordata - ps18
Olfactores - ps19
Vertebrata - ps20
Gnathostomata - ps21
Euteleostomi - ps22
Sarcopterygii - ps23
Tetrapoda - ps24
Amniota - ps25
Mammalia - ps26
Theria - ps27
Placentalia - ps28
Boreoeutheria - ps29
Euarchontoglires - ps30
Euarchonta - ps31
Primates - ps32
Haplorrhini - ps33
Simiiformes - ps34
Catarrhini - ps35
Hominoidea - ps36
Hominidae - ps37
Homininae - ps38
Hominini - ps39
Homo_sapiens - ps40
p-value
6e-04
2e-03
6e-03
2e-02
6e-02
Log-odds
0.5
1.0
1.5
2.0
2.5
3.0
3.5
4.0

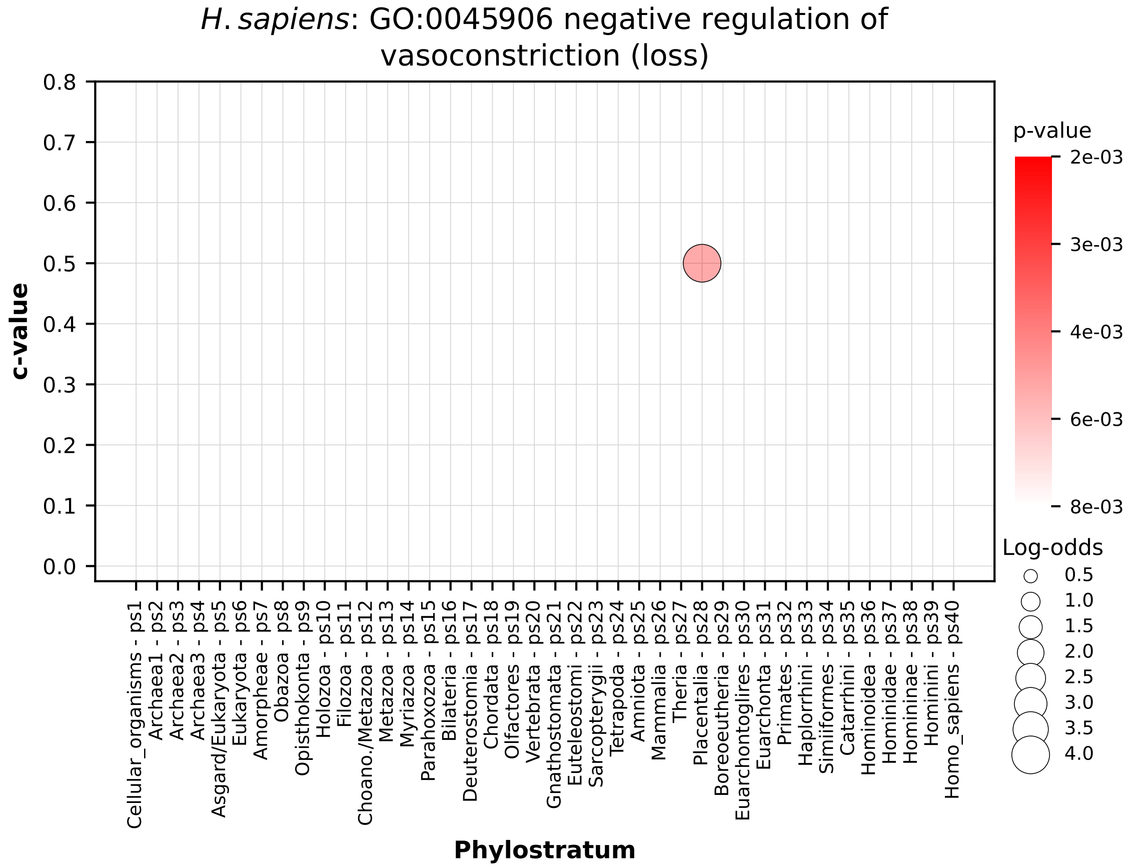

H. sapiens: GO:0045906 negative regulation of vasoconstriction (loss)
c-value
0.8
0.7
0.6
0.5
0.4
0.3
0.2
0.1
0.0
Phylostratum
Cellular_organisms - ps1
Archaea1 - ps2
Archaea2 - ps3
Archaea3 - ps4
Asgard/Eukaryota - ps5
Eukaryota - ps6
Amorpheae - ps7
Obazoa - ps8
Opisthokonta - ps9
Holozoa - ps10
Filozoa - ps11
Choano./Metazoa - ps12
Metazoa - ps13
Myriazoa - ps14
Parahoxozoa - ps15
Bilateria - ps16
Deuterostomia - ps17
Chordata - ps18
Olfactores - ps19
Vertebrata - ps20
Gnathostomata - ps21
Euteleostomi - ps22
Sarcopterygii - ps23
Tetrapoda - ps24
Amniota - ps25
Mammalia - ps26
Theria - ps27
Placentalia - ps28
Boreoeutheria - ps29
Euarchontoglires - ps30
Euarchonta - ps31
Primates - ps32
Haplorrhini - ps33
Simiiformes - ps34
Catarrhini - ps35
Hominoidea - ps36
Hominidae - ps37
Homininae - ps38
Hominini - ps39
Homo_sapiens - ps40
p-value
2e-03
3e-03
4e-03
6e-03
8e-03
Log-odds
0.5
1.0
1.5
2.0
2.5
3.0
3.5
4.0

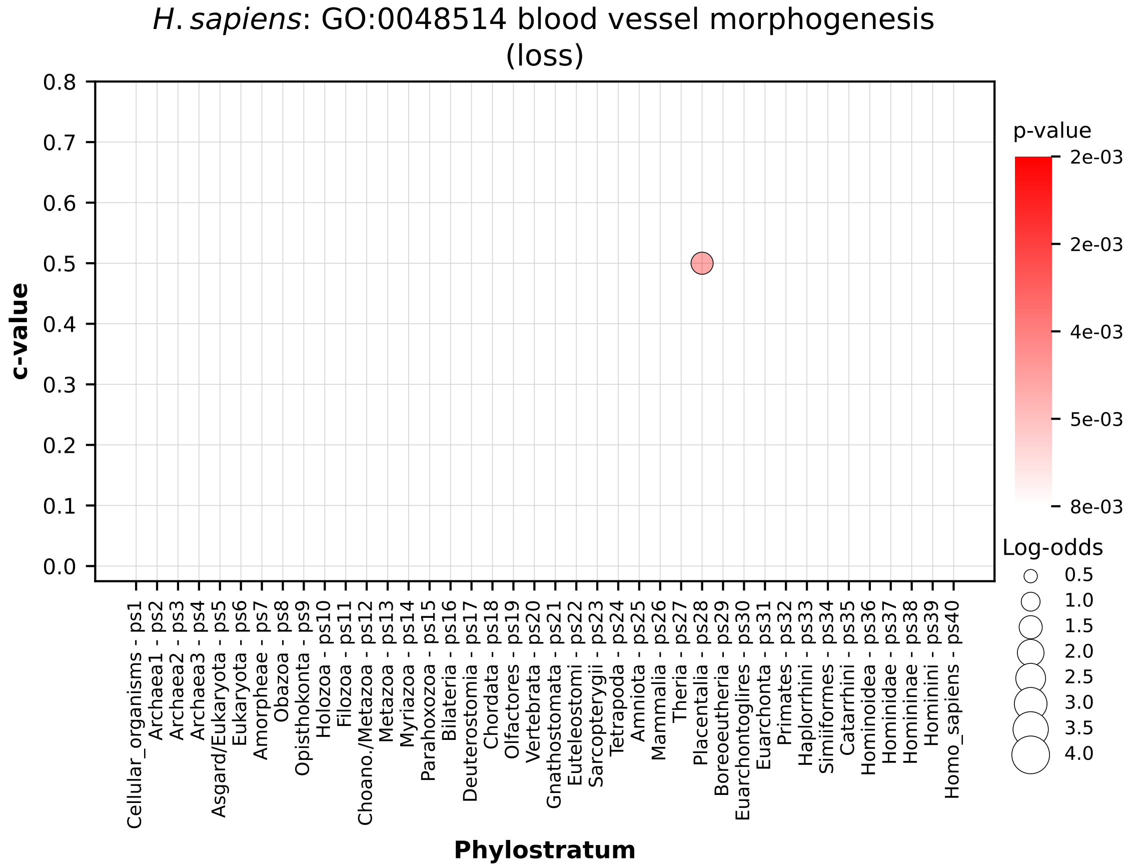

H. sapiens: GO:0048514 blood vessel morphogenesis
(loss)
c-value
0.8
0.7
0.6
0.5
0.4
0.3
0.2
0.1
0.0
Phylostratum
Cellular_organisms - ps1
Archaea1 - ps2
Archaea2 - ps3
Archaea3 - ps4
Asgard/Eukaryota - ps5
Eukaryota - ps6
Amorpheae - ps7
Obazoa - ps8
Opisthokonta - ps9
Holozoa - ps10
Filozoa - ps11
Choano./Metazoa - ps12
Metazoa - ps13
Myriazoa - ps14
Parahoxozoa - ps15
Bilateria - ps16
Deuterostomia - ps17
Chordata - ps18
Olfactores - ps19
Vertebrata - ps20
Gnathostomata - ps21
Euteleostomi - ps22
Sarcopterygii - ps23
Tetrapoda - ps24
Amniota - ps25
Mammalia - ps26
Theria - ps27
Placentalia - ps28
Boreoeutheria - ps29
Euarchontoglires - ps30
Euarchonta - ps31
Primates - ps32
Haplorrhini - ps33
Simiiformes - ps34
Catarrhini - ps35
Hominoidea - ps36
Hominidae - ps37
Homininae - ps38
Hominini - ps39
Homo_sapiens - ps40
p-value
2e-03
2e-03
4e-03
5e-03
8e-03
Log-odds
0.5
1.0
1.5
2.0
2.5
3.0
3.5
4.0

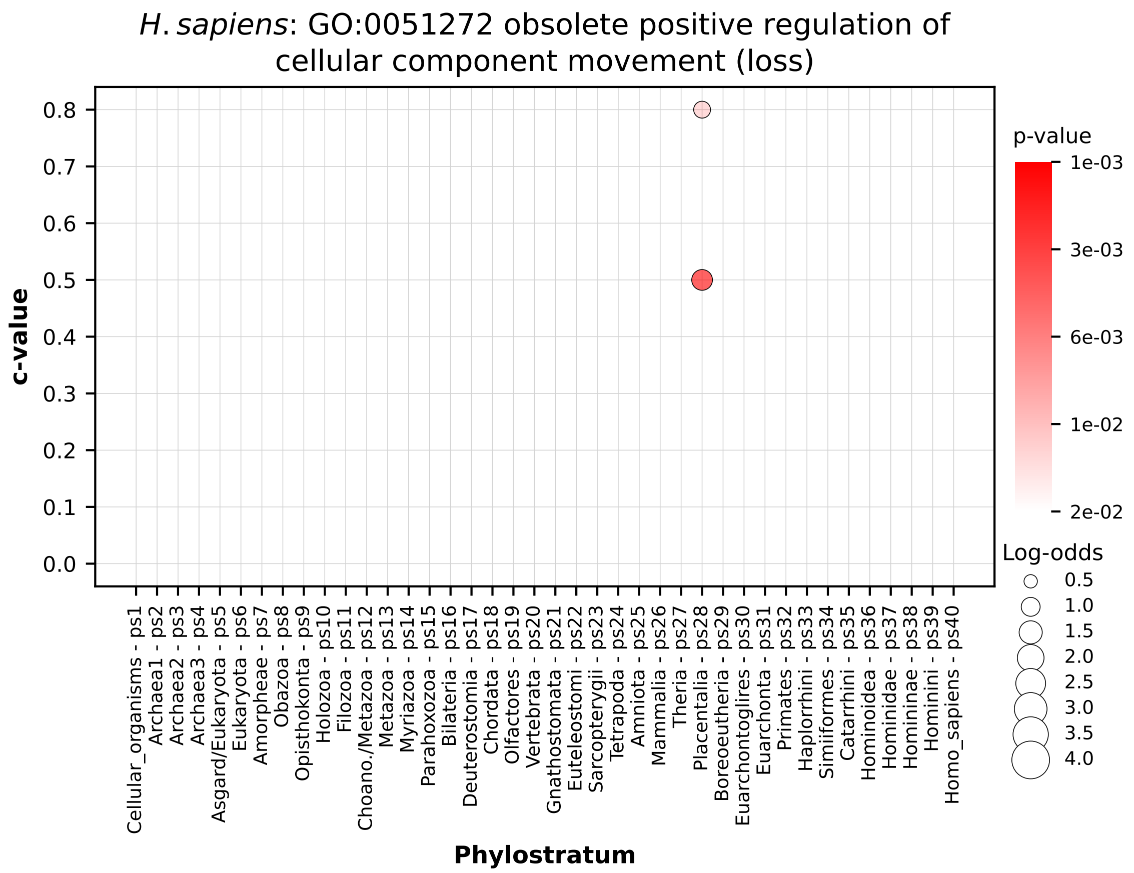
H. sapiens: GO:0051272 obsolete positive regulation of cellular component movement (loss)
c-value
Phylostratum
p-value
1e-03
3e-03
6e-03
1e-02
2e-02
Log-odds
0.5
1.0
1.5
2.0
2.5
3.0
3.5
4.0
0.0
0.1
0.2
0.3
0.4
0.5
0.6
0.7
0.8
Cellular_organisms - ps1
Archaea1 - ps2
Archaea2 - ps3
Archaea3 - ps4
Asgard/Eukaryota - ps5
Eukaryota - ps6
Amorpheae - ps7
Obazoa - ps8
Opisthokonta - ps9
Holozoa - ps10
Filozoa - ps11
Choano./Metazoa - ps12
Metazoa - ps13
Myriazoa - ps14
Parahoxozoa - ps15
Bilateria - ps16
Deuterostomia - ps17
Chordata - ps18
Olfactores - ps19
Vertebrata - ps20
Gnathostomata - ps21
Euteleostomi - ps22
Sarcopterygii - ps23
Tetrapoda - ps24
Amniota - ps25
Mammalia - ps26
Theria - ps27
Placentalia - ps28
Boreoeutheria - ps29
Euarchontoglires - ps30
Euarchonta - ps31
Primates - ps32
Haplorrhini - ps33
Simiiformes - ps34
Catarrhini - ps35
Hominoidea - ps36
Hominidae - ps37
Homininae - ps38
Hominini - ps39
Homo_sapiens - ps40

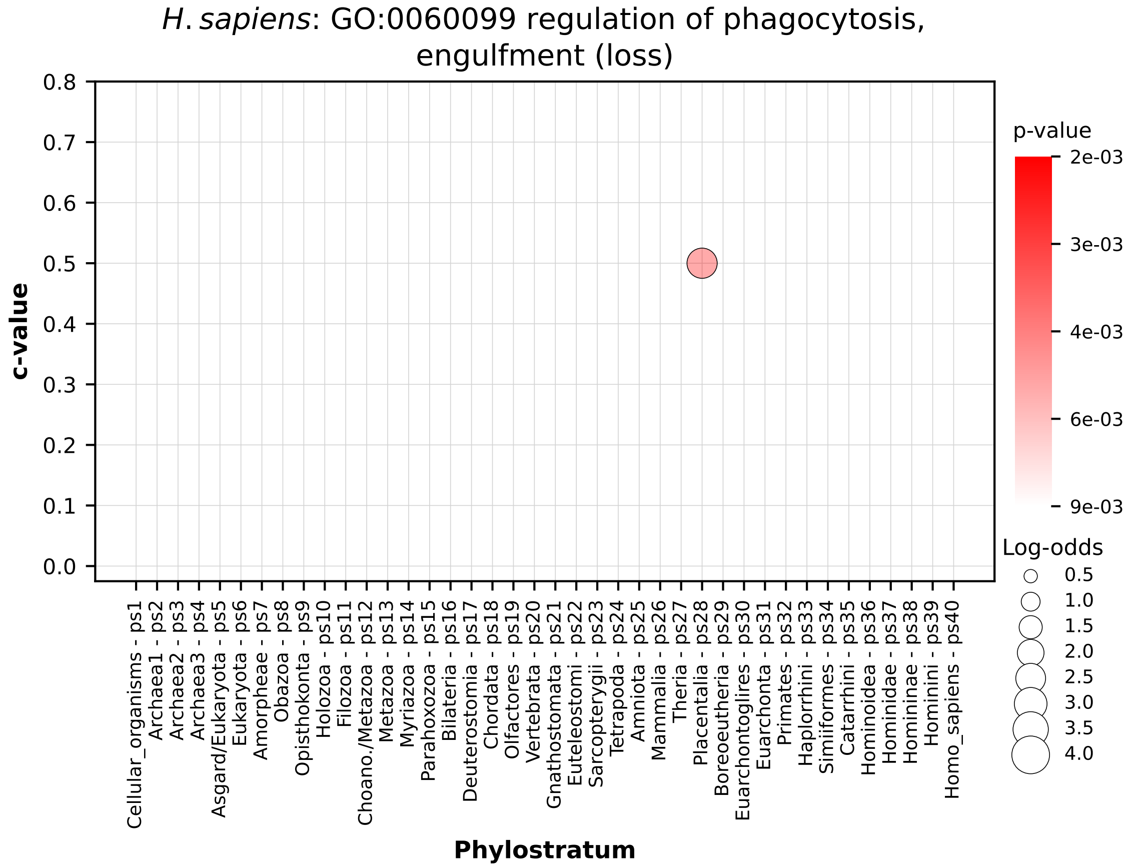

H. sapiens: GO:0060099 regulation of phagocytosis, engulfment (loss)
c-value
0.0
0.1
0.2
0.3
0.4
0.5
0.6
0.7
0.8
p-value
2e-03
3e-03
4e-03
6e-03
9e-03
Log-odds
0.5
1.0
1.5
2.0
2.5
3.0
3.5
4.0
Cellular_organisms - ps1
Archaea1 - ps2
Archaea2 - ps3
Archaea3 - ps4
Asgard/Eukaryota - ps5
Eukaryota - ps6
Amorpheae - ps7
Obazoa - ps8
Opisthokonta - ps9
Holozoa - ps10
Filozoa - ps11
Choano./Metazoa - ps12
Metazoa - ps13
Myriazoa - ps14
Parahoxozoa - ps15
Bilateria - ps16
Deuterostomia - ps17
Chordata - ps18
Olfactores - ps19
Vertebrata - ps20
Gnathostomata - ps21
Euteleostomi - ps22
Sarcopterygii - ps23
Tetrapoda - ps24
Amniota - ps25
Mammalia - ps26
Theria - ps27
Placentalia - ps28
Boreoeutheria - ps29
Euarchontoglires - ps30
Euarchonta - ps31
Primates - ps32
Haplorrhini - ps33
Simiiformes - ps34
Catarrhini - ps35
Hominoidea - ps36
Hominidae - ps37
Homininae - ps38
Hominini - ps39
Homo_sapiens - ps40
Phylostratum

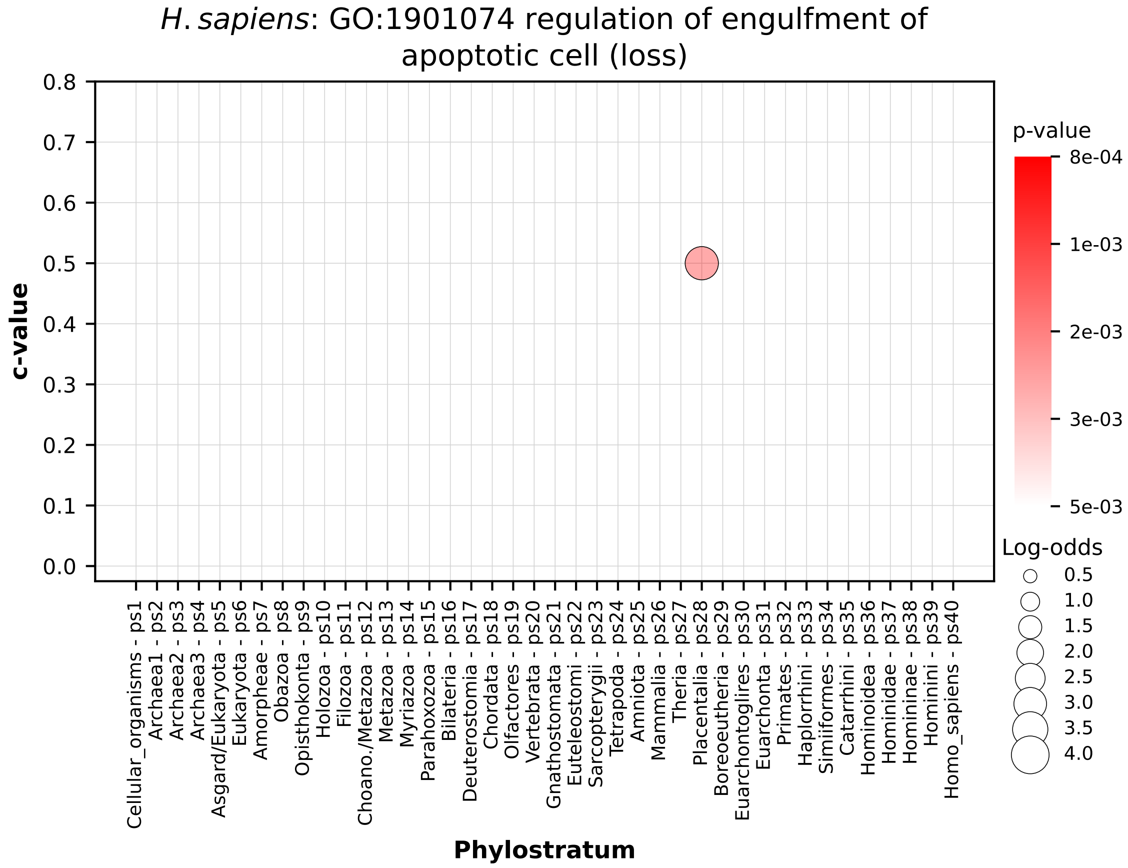

H. sapiens: GO:1901074 regulation of engulfment of apoptotic cell (loss)
c-value
0.8
0.7
0.6
0.5
0.4
0.3
0.2
0.1
0.0
p-value
8e-04
1e-03
2e-03
3e-03
5e-03
Log-odds
0.5
1.0
1.5
2.0
2.5
3.0
3.5
4.0
Cellular_organisms - ps1
Archaea1 - ps2
Archaea2 - ps3
Archaea3 - ps4
Asgard/Eukaryota - ps5
Eukaryota - ps6
Amorpheae - ps7
Obazoa - ps8
Opisthokonta - ps9
Holozoa - ps10
Filozoa - ps11
Choano./Metazoa - ps12
Metazoa - ps13
Myriazoa - ps14
Parahoxozoa - ps15
Bilateria - ps16
Deuterostomia - ps17
Chordata - ps18
Olfactores - ps19
Vertebrata - ps20
Gnathostomata - ps21
Euteleostomi - ps22
Sarcopterygii - ps23
Tetrapoda - ps24
Amniota - ps25
Mammalia - ps26
Theria - ps27
Placentalia - ps28
Boreoeutheria - ps29
Euarchontoglires - ps30
Euarchonta - ps31
Primates - ps32
Haplorrhini - ps33
Simiiformes - ps34
Catarrhini - ps35
Hominoidea - ps36
Hominidae - ps37
Homininae - ps38
Hominini - ps39
Homo_sapiens - ps40
Phylostratum

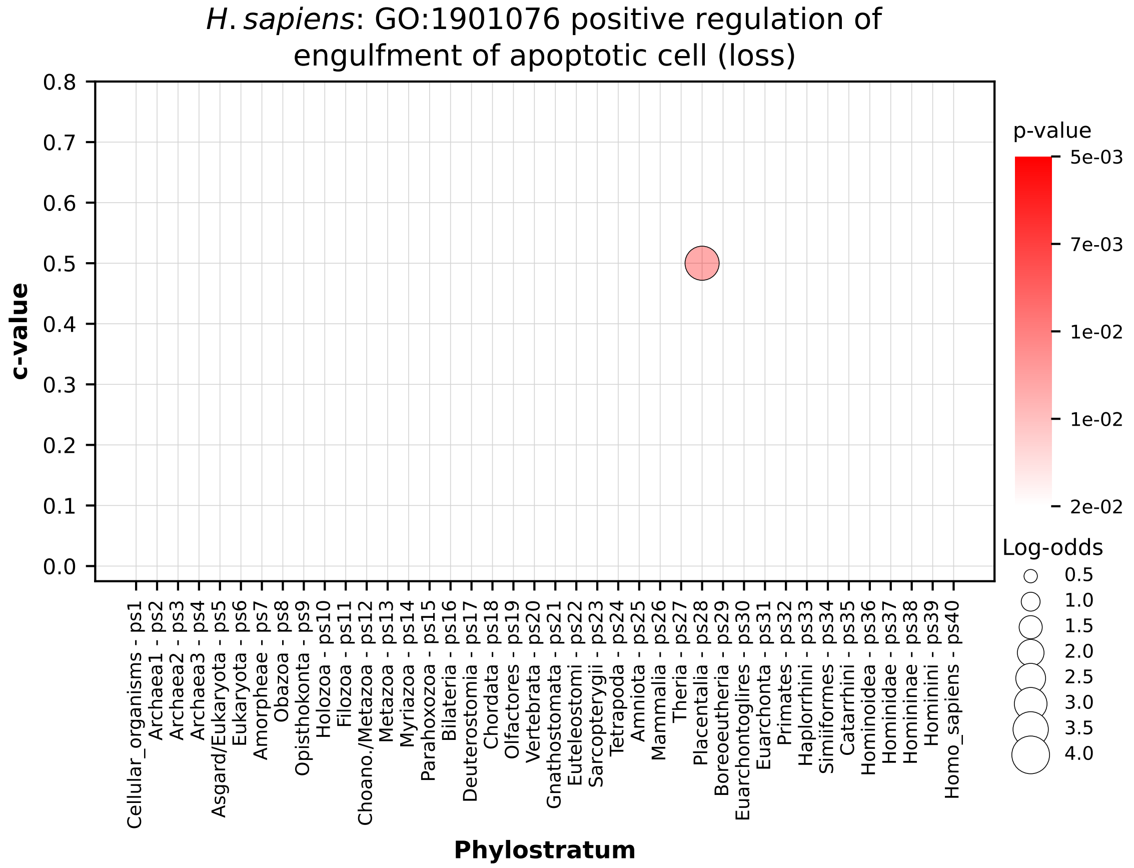
H. sapiens: GO:1901076 positive regulation of engulfment of apoptotic cell (loss)
c-value
0.8
0.7
0.6
0.5
0.4
0.3
0.2
0.1
0.0
Phylostratum
Cellular_organisms - ps1
Archaea1 - ps2
Archaea2 - ps3
Archaea3 - ps4
Asgard/Eukaryota - ps5
Eukaryota - ps6
Amorpheae - ps7
Obazoa - ps8
Opisthokonta - ps9
Holozoa - ps10
Filozoa - ps11
Choano./Metazoa - ps12
Metazoa - ps13
Myriazoa - ps14
Parahoxozoa - ps15
Bilateria - ps16
Deuterostomia - ps17
Chordata - ps18
Olfactores - ps19
Vertebrata - ps20
Gnathostomata - ps21
Euteleostomi - ps22
Sarcopterygii - ps23
Tetrapoda - ps24
Amniota - ps25
Mammalia - ps26
Theria - ps27
Placentalia - ps28
Boreoeutheria - ps29
Euarchontoglires - ps30
Euarchonta - ps31
Primates - ps32
Haplorrhini - ps33
Simiiformes - ps34
Catarrhini - ps35
Hominoidea - ps36
Hominidae - ps37
Homininae - ps38
Hominini - ps39
Homo_sapiens - ps40
p-value
5e-03
7e-03
1e-02
1e-02
2e-02
Log-odds
0.5
1.0
1.5
2.0
2.5
3.0
3.5
4.0

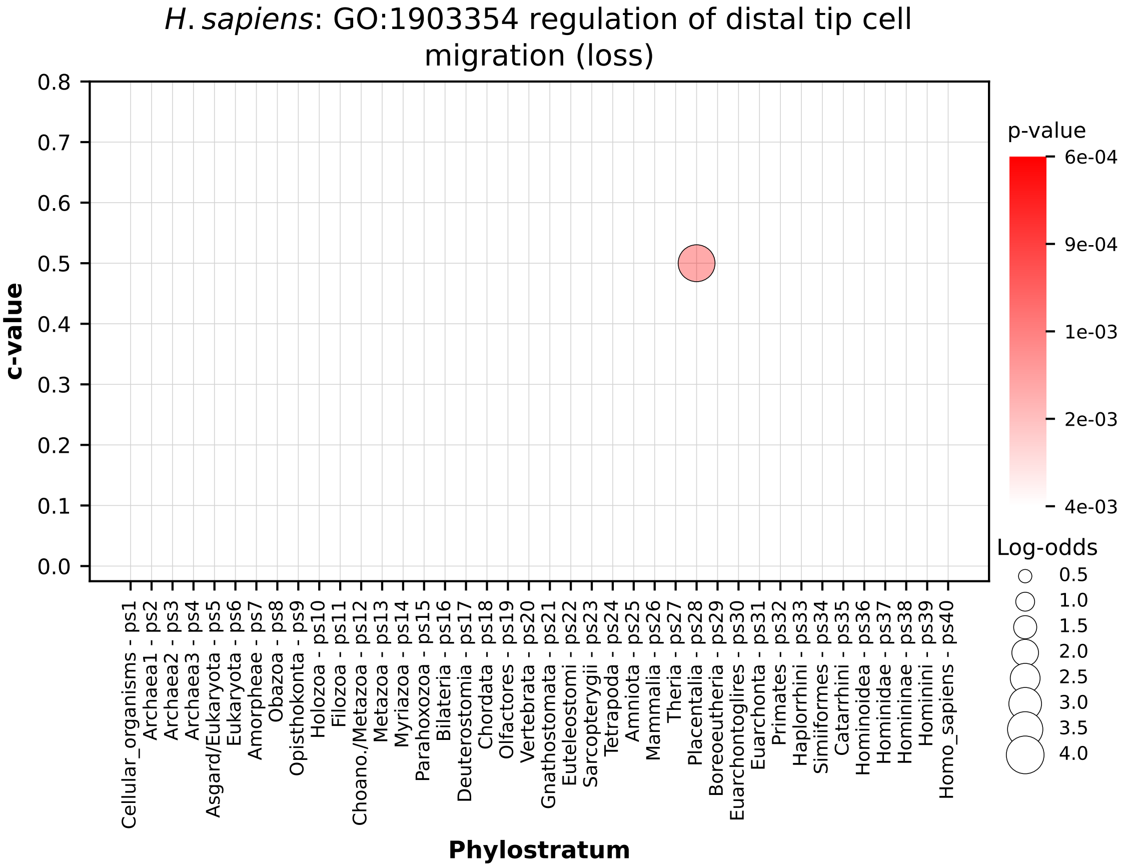

H. sapiens: GO:1903354 regulation of distal tip cell migration (loss)
c-value
0.8
0.7
0.6
0.5
0.4
0.3
0.2
0.1
0.0
Phylostratum
Cellular_organisms - ps1
Archaea1 - ps2
Archaea2 - ps3
Archaea3 - ps4
Asgard/Eukaryota - ps5
Eukaryota - ps6
Amorpheae - ps7
Obazoa - ps8
Opisthokonta - ps9
Holozoa - ps10
Filozoa - ps11
Choano./Metazoa - ps12
Metazoa - ps13
Myriazoa - ps14
Parahoxozoa - ps15
Bilateria - ps16
Deuterostomia - ps17
Chordata - ps18
Olfactores - ps19
Vertebrata - ps20
Gnathostomata - ps21
Euteleostomi - ps22
Sarcopterygii - ps23
Tetrapoda - ps24
Amniota - ps25
Mammalia - ps26
Theria - ps27
Placentalia - ps28
Boreoeutheria - ps29
Euarchontoglires - ps30
Euarchonta - ps31
Primates - ps32
Haplorrhini - ps33
Simiiformes - ps34
Catarrhini - ps35
Hominoidea - ps36
Hominidae - ps37
Homininae - ps38
Hominini - ps39
Homo_sapiens - ps40
p-value
6e-04
9e-04
1e-03
2e-03
4e-03
Log-odds
0.5
1.0
1.5
2.0
2.5
3.0
3.5
4.0

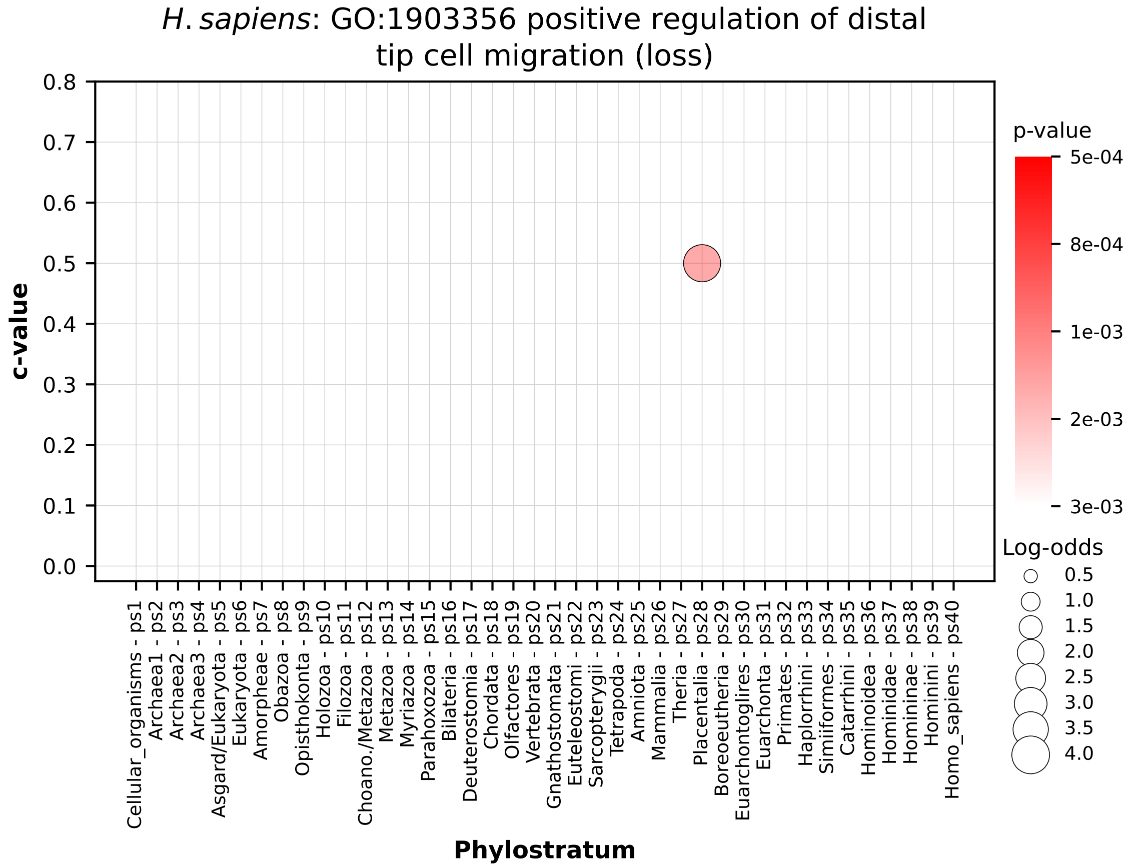

H. sapiens: GO:1903356 positive regulation of distal tip cell migration (loss)
c-value
0.0
0.1
0.2
0.3
0.4
0.5
0.6
0.7
0.8
Phylostratum
Cellular_organisms - ps1
Archaea1 - ps2
Archaea2 - ps3
Archaea3 - ps4
Asgard/Eukaryota - ps5
Eukaryota - ps6
Amorpheae - ps7
Obazoa - ps8
Opisthokonta - ps9
Holozoa - ps10
Filozoa - ps11
Choano./Metazoa - ps12
Metazoa - ps13
Myriazoa - ps14
Parahoxozoa - ps15
Bilateria - ps16
Deuterostomia - ps17
Chordata - ps18
Olfactores - ps19
Vertebrata - ps20
Gnathostomata - ps21
Euteleostomi - ps22
Sarcopterygii - ps23
Tetrapoda - ps24
Amniota - ps25
Mammalia - ps26
Theria - ps27
Placentalia - ps28
Boreoeutheria - ps29
Euarchontoglires - ps30
Euarchonta - ps31
Primates - ps32
Haplorrhini - ps33
Simiiformes - ps34
Catarrhini - ps35
Hominoidea - ps36
Hominidae - ps37
Homininae - ps38
Hominini - ps39
Homo_sapiens - ps40
p-value
5e-04
8e-04
1e-03
2e-03
3e-03
Log-odds
0.5
1.0
1.5
2.0
2.5
3.0
3.5
4.0

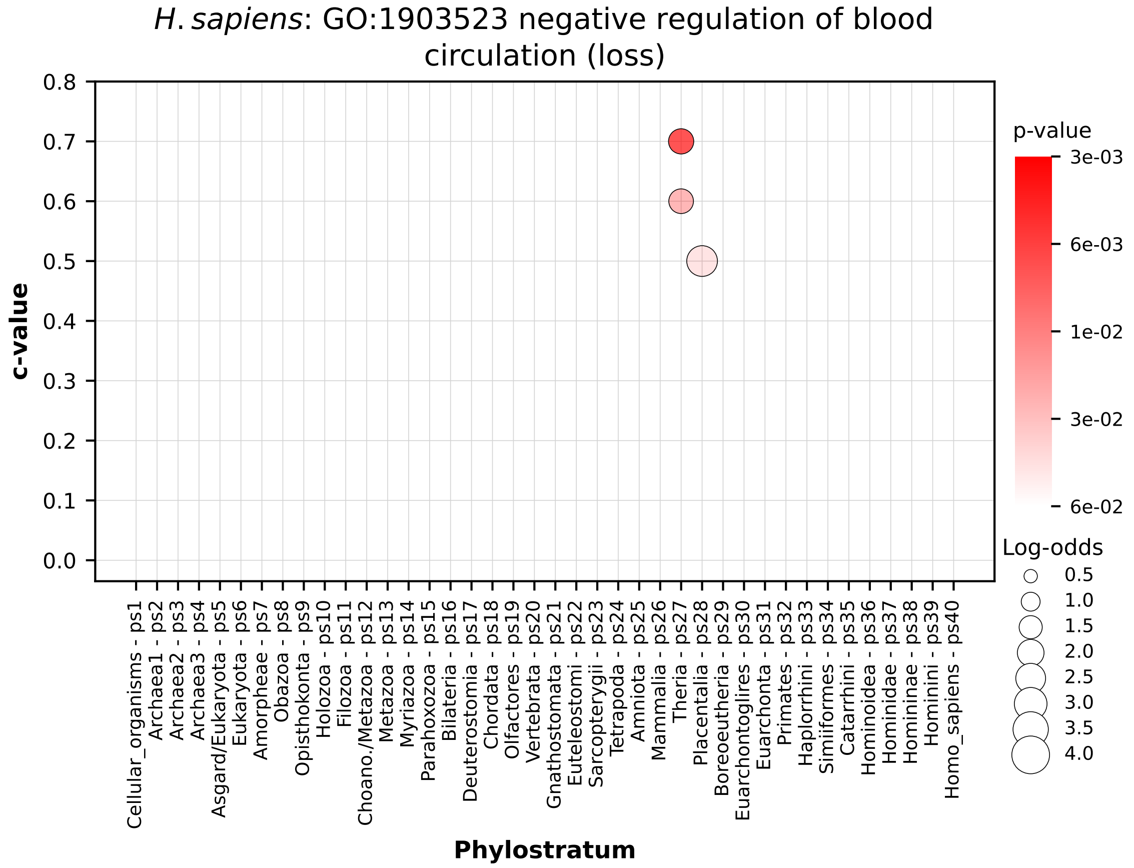
H. sapiens: GO:1903523 negative regulation of blood circulation (loss)
c-value
0.8
0.7
0.6
0.5
0.4
0.3
0.2
0.1
0.0
p-value
3e-03
6e-03
1e-02
3e-02
6e-02
Log-odds
0.5
1.0
1.5
2.0
2.5
3.0
3.5
4.0
Cellular_organisms - ps1
Archaea1 - ps2
Archaea2 - ps3
Archaea3 - ps4
Asgard/Eukaryota - ps5
Eukaryota - ps6
Amorpheae - ps7
Obazoa - ps8
Opisthokonta - ps9
Holozoa - ps10
Filozoa - ps11
Choano./Metazoa - ps12
Metazoa - ps13
Myriazoa - ps14
Parahoxozoa - ps15
Bilateria - ps16
Deuterostomia - ps17
Chordata - ps18
Olfactores - ps19
Vertebrata - ps20
Gnathostomata - ps21
Euteleostomi - ps22
Sarcopterygii - ps23
Tetrapoda - ps24
Amniota - ps25
Mammalia - ps26
Theria - ps27
Placentalia - ps28
Boreoeutheria - ps29
Euarchontoglires - ps30
Euarchonta - ps31
Primates - ps32
Haplorrhini - ps33
Simiiformes - ps34
Catarrhini - ps35
Hominoidea - ps36
Hominidae - ps37
Homininae - ps38
Hominini - ps39
Homo_sapiens - ps40
Phylostratum

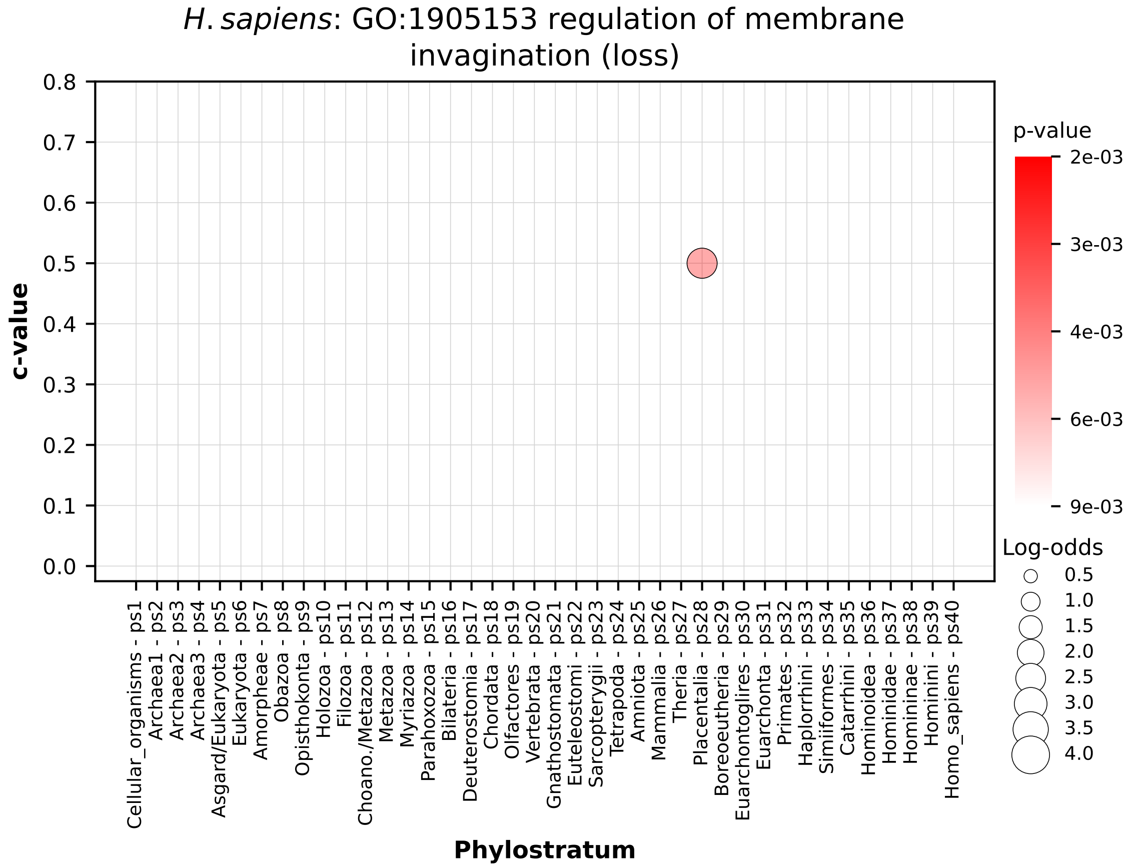

H. sapiens: GO:1905153 regulation of membrane invagination (loss)
c-value
0.8
0.7
0.6
0.5
0.4
0.3
0.2
0.1
0.0
Phylostratum
Cellular_organisms - ps1
Archaea1 - ps2
Archaea2 - ps3
Archaea3 - ps4
Asgard/Eukaryota - ps5
Eukaryota - ps6
Amorpheae - ps7
Obazoa - ps8
Opisthokonta - ps9
Holozoa - ps10
Filozoa - ps11
Choano./Metazoa - ps12
Metazoa - ps13
Myriazoa - ps14
Parahoxozoa - ps15
Bilateria - ps16
Deuterostomia - ps17
Chordata - ps18
Olfactores - ps19
Vertebrata - ps20
Gnathostomata - ps21
Euteleostomi - ps22
Sarcopterygii - ps23
Tetrapoda - ps24
Amniota - ps25
Mammalia - ps26
Theria - ps27
Placentalia - ps28
Boreoeutheria - ps29
Euarchontoglires - ps30
Euarchonta - ps31
Primates - ps32
Haplorrhini - ps33
Simiiformes - ps34
Catarrhini - ps35
Hominoidea - ps36
Hominidae - ps37
Homininae - ps38
Hominini - ps39
Homo_sapiens - ps40
p-value
2e-03
3e-03
4e-03
6e-03
9e-03
Log-odds
0.5
1.0
1.5
2.0
2.5
3.0
3.5
4.0

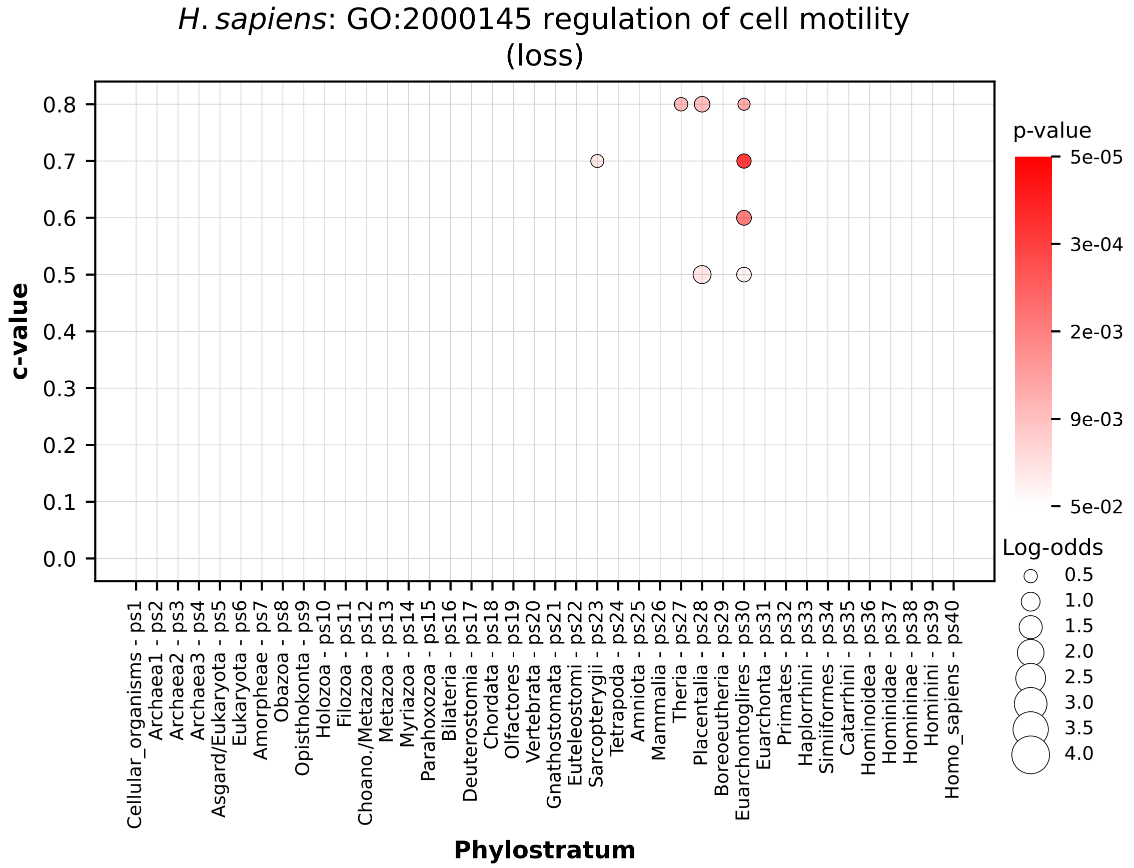

H. sapiens: GO:2000145 regulation of cell motility
(loss)
c-value
0.0
0.1
0.2
0.3
0.4
0.5
0.6
0.7
0.8
Phylostratum
Cellular_organisms - ps1
Archaea1 - ps2
Archaea2 - ps3
Archaea3 - ps4
Asgard/Eukaryota - ps5
Eukaryota - ps6
Amorpheae - ps7
Obazoa - ps8
Opisthokonta - ps9
Holozoa - ps10
Filozoa - ps11
Choano./Metazoa - ps12
Metazoa - ps13
Myriazoa - ps14
Parahoxozoa - ps15
Bilateria - ps16
Deuterostomia - ps17
Chordata - ps18
Olfactores - ps19
Vertebrata - ps20
Gnathostomata - ps21
Euteleostomi - ps22
Sarcopterygii - ps23
Tetrapoda - ps24
Amniota - ps25
Mammalia - ps26
Theria - ps27
Placentalia - ps28
Boreoeutheria - ps29
Euarchontoglires - ps30
Euarchonta - ps31
Primates - ps32
Haplorrhini - ps33
Simiiformes - ps34
Catarrhini - ps35
Hominoidea - ps36
Hominidae - ps37
Homininae - ps38
Hominini - ps39
Homo_sapiens - ps40
p-value
5e-05
3e-04
2e-03
9e-03
5e-02
Log-odds
0.5
1.0
1.5
2.0
2.5
3.0
3.5
4.0

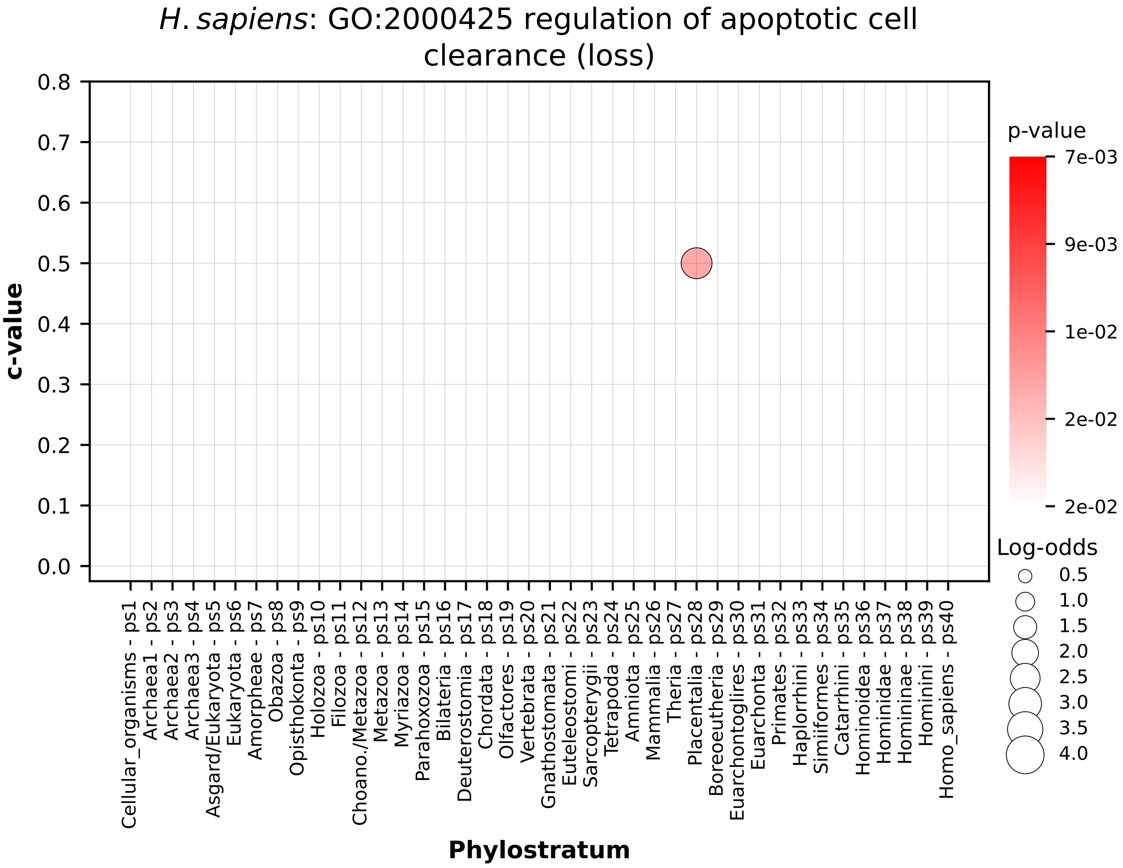

H. sapiens: GO:2000425 regulation of apoptotic cell clearance (loss)
c-value
0.8
0.7
0.6
0.5
0.4
0.3
0.2
0.1
0.0
Phylostratum
Cellular_organisms - ps1
Archaea1 - ps2
Archaea2 - ps3
Archaea3 - ps4
Asgard/Eukaryota - ps5
Eukaryota - ps6
Amorpheae - ps7
Obazoa - ps8
Opisthokonta - ps9
Holozoa - ps10
Filozoa - ps11
Choano./Metazoa - ps12
Metazoa - ps13
Myriazoa - ps14
Parahoxozoa - ps15
Bilateria - ps16
Deuterostomia - ps17
Chordata - ps18
Olfactores - ps19
Vertebrata - ps20
Gnathostomata - ps21
Euteleostomi - ps22
Sarcopterygii - ps23
Tetrapoda - ps24
Amniota - ps25
Mammalia - ps26
Theria - ps27
Placentalia - ps28
Boreoeutheria - ps29
Euarchontoglires - ps30
Euarchonta - ps31
Primates - ps32
Haplorrhini - ps33
Simiiformes - ps34
Catarrhini - ps35
Hominoidea - ps36
Hominidae - ps37
Homininae - ps38
Hominini - ps39
Homo_sapiens - ps40
p-value
7e-03
9e-03
1e-02
2e-02
2e-02
Log-odds
0.5
1.0
1.5
2.0
2.5
3.0
3.5
4.0

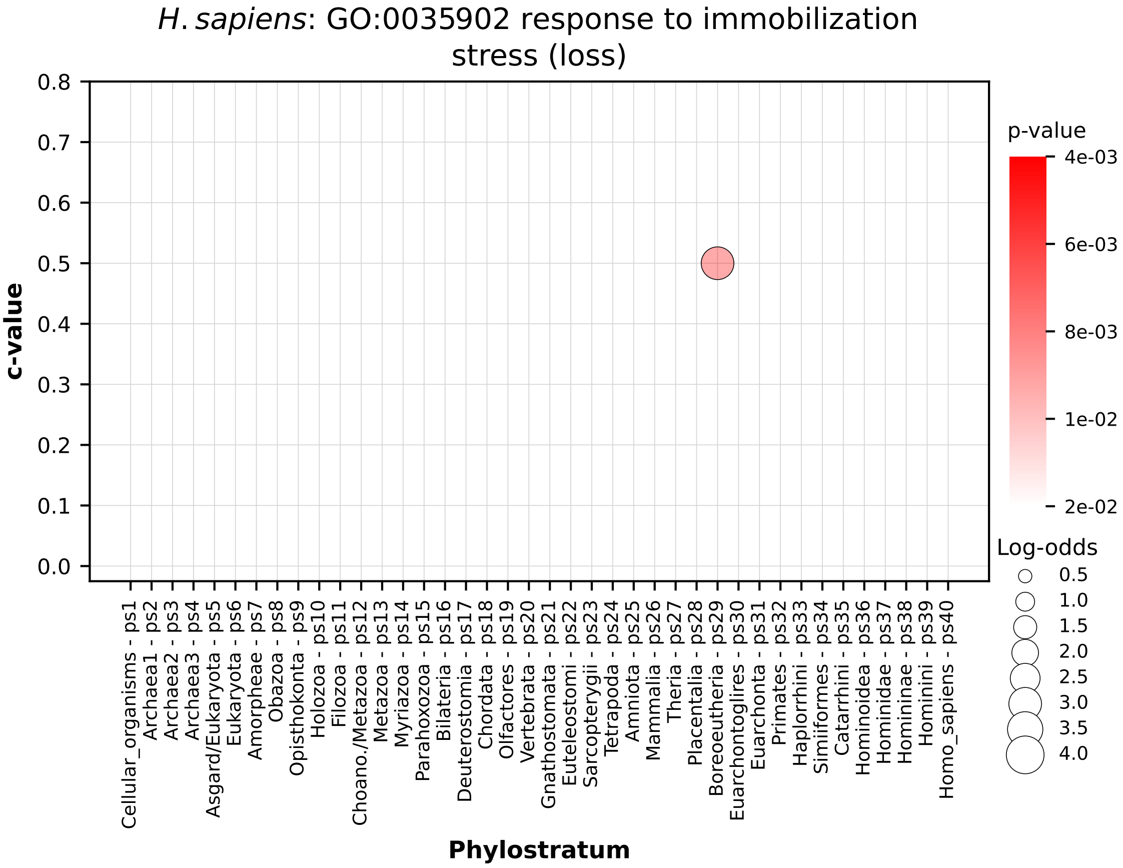

H. sapiens: GO:0035902 response to immobilization stress (loss)
c-value
0.0
0.1
0.2
0.3
0.4
0.5
0.6
0.7
0.8
Phylostratum
Cellular_organisms - ps1
Archaea1 - ps2
Archaea2 - ps3
Archaea3 - ps4
Asgard/Eukaryota - ps5
Eukaryota - ps6
Amorpheae - ps7
Obazoa - ps8
Opisthokonta - ps9
Holozoa - ps10
Filozoa - ps11
Choano./Metazoa - ps12
Metazoa - ps13
Myriazoa - ps14
Parahoxozoa - ps15
Bilateria - ps16
Deuterostomia - ps17
Chordata - ps18
Olfactores - ps19
Vertebrata - ps20
Gnathostomata - ps21
Euteleostomi - ps22
Sarcopterygii - ps23
Tetrapoda - ps24
Amniota - ps25
Mammalia - ps26
Theria - ps27
Placentalia - ps28
Boreoeutheria - ps29
Euarchontoglires - ps30
Euarchonta - ps31
Primates - ps32
Haplorrhini - ps33
Simiiformes - ps34
Catarrhini - ps35
Hominoidea - ps36
Hominidae - ps37
Homininae - ps38
Hominini - ps39
Homo_sapiens - ps40
p-value
4e-03
6e-03
8e-03
1e-02
2e-02
Log-odds
0.5
1.0
1.5
2.0
2.5
3.0
3.5
4.0

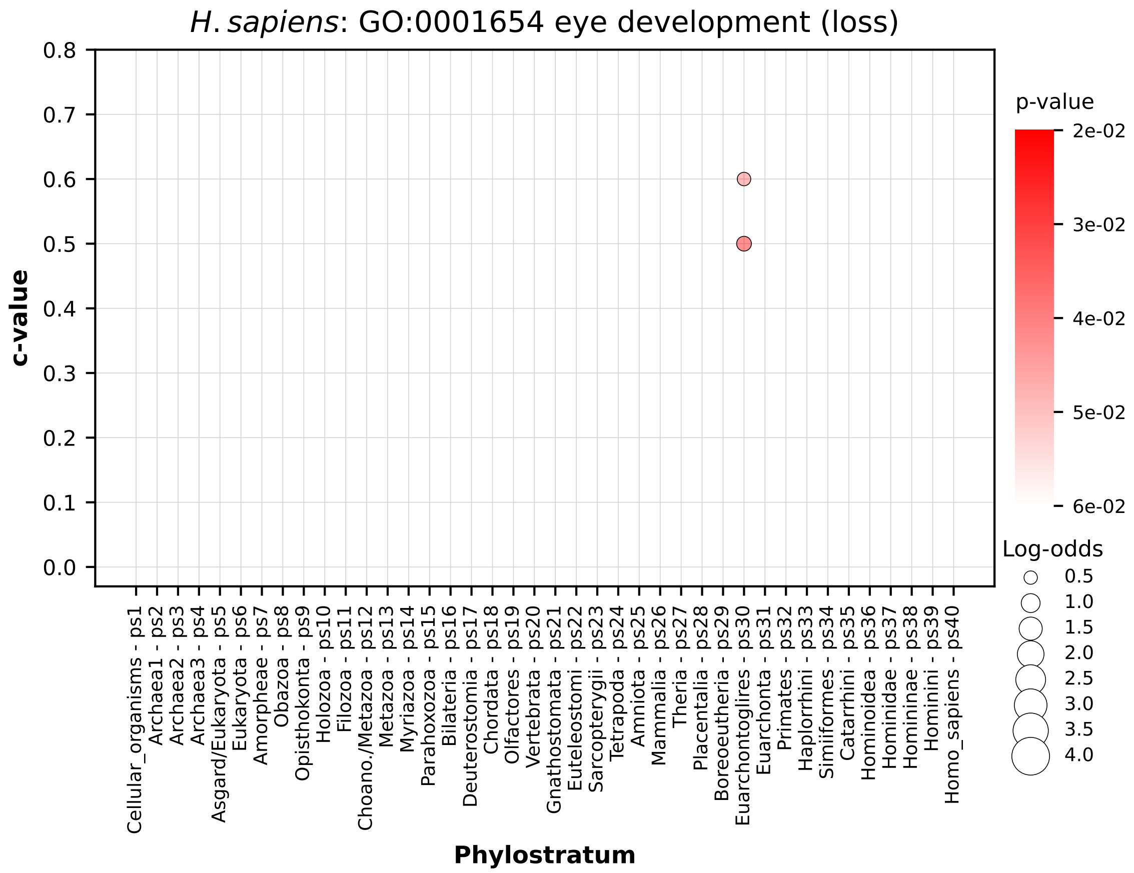

H. sapiens: GO:0001654 eye development (loss)
c-value
0.8
0.7
0.6
0.5
0.4
0.3
0.2
0.1
0.0
p-value
2e-02
3e-02
4e-02
5e-02
6e-02
Log-odds
0.5
1.0
1.5
2.0
2.5
3.0
3.5
4.0
Cellular_organisms - ps1
Archaea1 - ps2
Archaea2 - ps3
Archaea3 - ps4
Asgard/Eukaryota - ps5
Eukaryota - ps6
Amorpheae - ps7
Obazoa - ps8
Opisthokonta - ps9
Holozoa - ps10
Filozoa - ps11
Choano./Metazoa - ps12
Metazoa - ps13
Myriazoa - ps14
Parahoxozoa - ps15
Bilateria - ps16
Deuterostomia - ps17
Chordata - ps18
Olfactores - ps19
Vertebrata - ps20
Gnathostomata - ps21
Euteleostomi - ps22
Sarcopterygii - ps23
Tetrapoda - ps24
Amniota - ps25
Mammalia - ps26
Theria - ps27
Placentalia - ps28
Boreoeutheria - ps29
Euarchontoglires - ps30
Euarchonta - ps31
Primates - ps32
Haplorrhini - ps33
Simiiformes - ps34
Catarrhini - ps35
Hominoidea - ps36
Hominidae - ps37
Homininae - ps38
Hominini - ps39
Homo_sapiens - ps40
Phylostratum

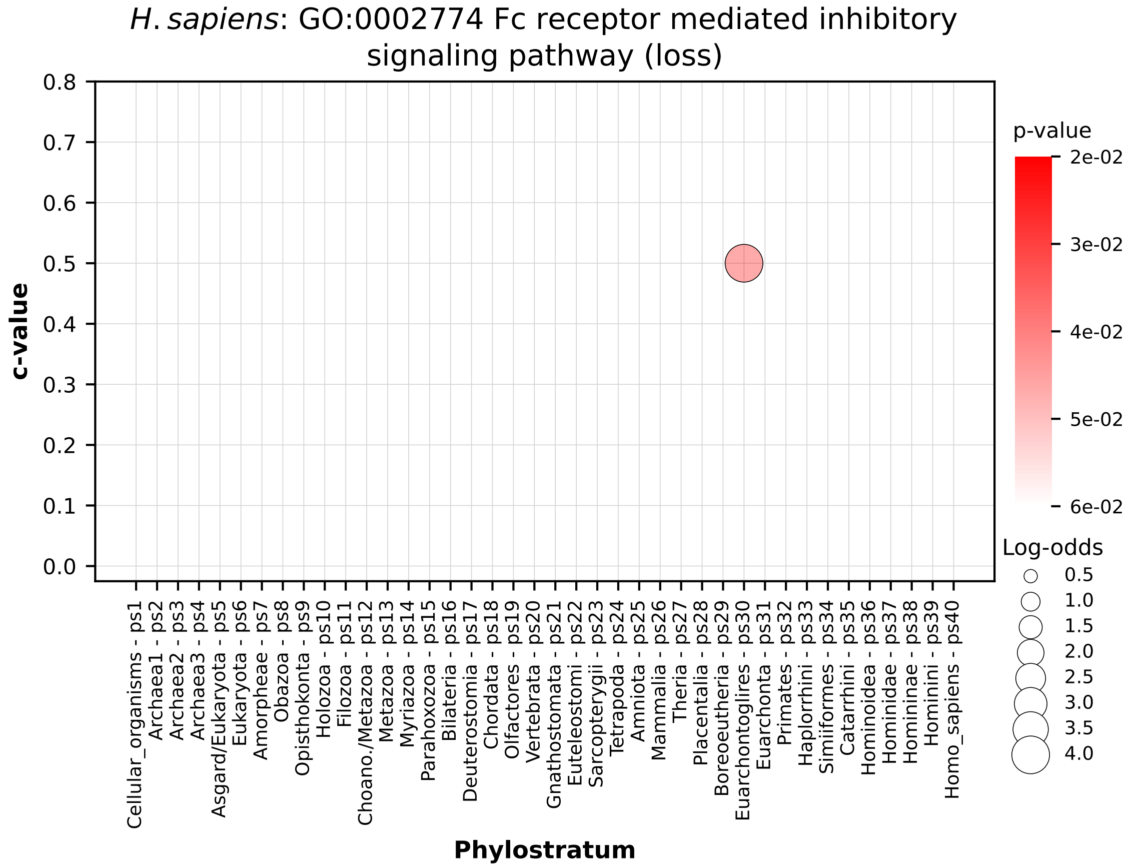

H. sapiens: GO:0002774 Fc receptor mediated inhibitory signaling pathway (loss)
c-value
0.8
0.7
0.6
0.5
0.4
0.3
0.2
0.1
0.0
Phylostratum
Cellular_organisms - ps1
Archaea1 - ps2
Archaea2 - ps3
Archaea3 - ps4
Asgard/Eukaryota - ps5
Eukaryota - ps6
Amorpheae - ps7
Obazoa - ps8
Opisthokonta - ps9
Holozoa - ps10
Filozoa - ps11
Choano./Metazoa - ps12
Metazoa - ps13
Myriazoa - ps14
Parahoxozoa - ps15
Bilateria - ps16
Deuterostomia - ps17
Chordata - ps18
Olfactores - ps19
Vertebrata - ps20
Gnathostomata - ps21
Euteleostomi - ps22
Sarcopterygii - ps23
Tetrapoda - ps24
Amniota - ps25
Mammalia - ps26
Theria - ps27
Placentalia - ps28
Boreoeutheria - ps29
Euarchontoglires - ps30
Euarchonta - ps31
Primates - ps32
Haplorrhini - ps33
Simiiformes - ps34
Catarrhini - ps35
Hominoidea - ps36
Hominidae - ps37
Homininae - ps38
Hominini - ps39
Homo_sapiens - ps40
p-value
2e-02
3e-02
4e-02
5e-02
6e-02
Log-odds
0.5
1.0
1.5
2.0
2.5
3.0
3.5
4.0

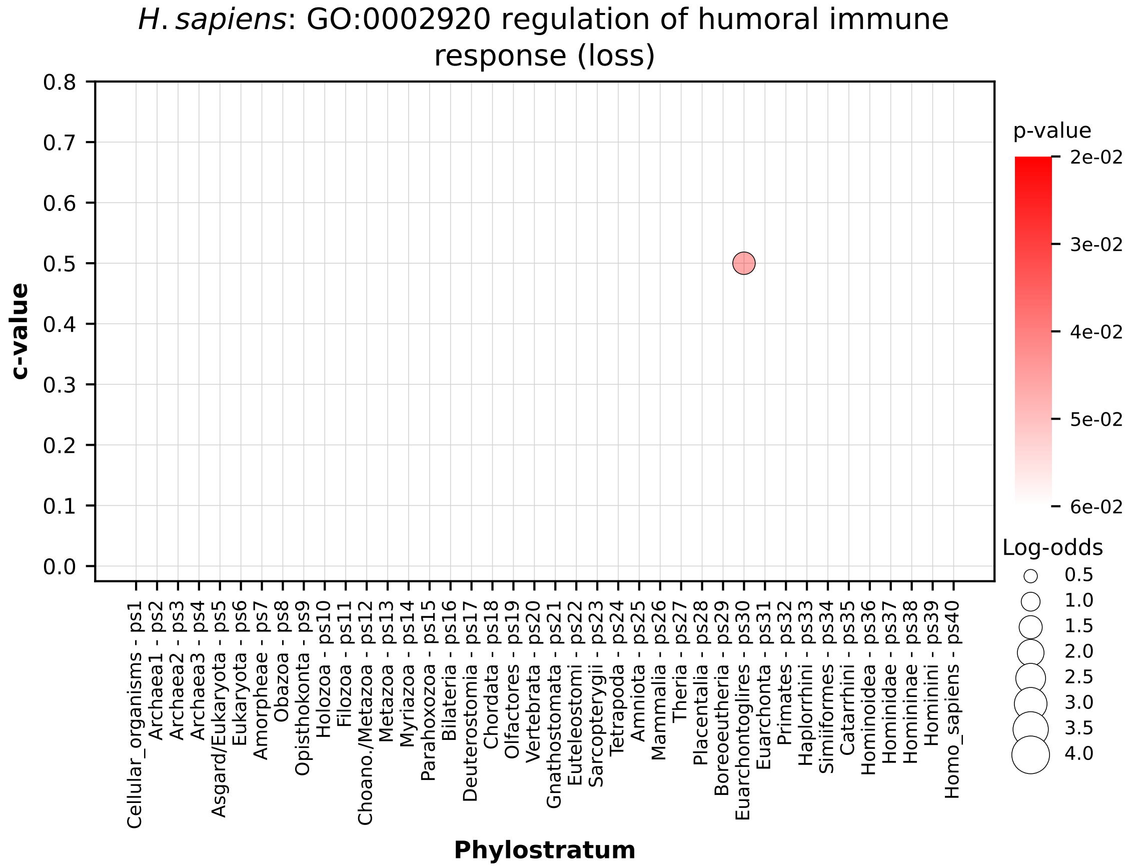

H. sapiens: GO:0002920 regulation of humoral immune response (loss)
c-value
0.0
0.1
0.2
0.3
0.4
0.5
0.6
0.7
0.8
Phylostratum
Cellular_organisms - ps1
Archaea1 - ps2
Archaea2 - ps3
Archaea3 - ps4
Asgard/Eukaryota - ps5
Eukaryota - ps6
Amorpheae - ps7
Obazoa - ps8
Opisthokonta - ps9
Holozoa - ps10
Filozoa - ps11
Choano./Metazoa - ps12
Metazoa - ps13
Myriazoa - ps14
Parahoxozoa - ps15
Bilateria - ps16
Deuterostomia - ps17
Chordata - ps18
Olfactores - ps19
Vertebrata - ps20
Gnathostomata - ps21
Euteleostomi - ps22
Sarcopterygii - ps23
Tetrapoda - ps24
Amniota - ps25
Mammalia - ps26
Theria - ps27
Placentalia - ps28
Boreoeutheria - ps29
Euarchontoglires - ps30
Euarchonta - ps31
Primates - ps32
Haplorrhini - ps33
Simiiformes - ps34
Catarrhini - ps35
Hominoidea - ps36
Hominidae - ps37
Homininae - ps38
Hominini - ps39
Homo_sapiens - ps40
p-value
2e-02
3e-02
4e-02
5e-02
6e-02
Log-odds
0.5
1.0
1.5
2.0
2.5
3.0
3.5
4.0

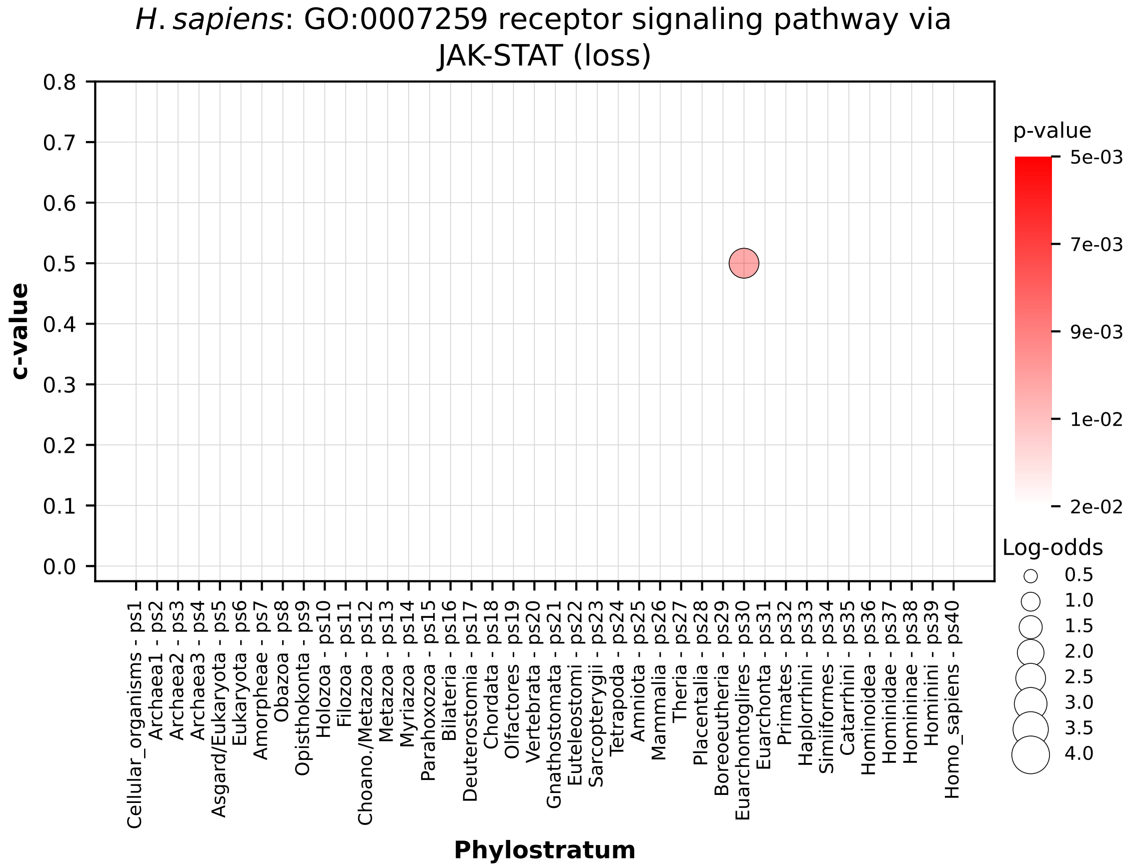
H. sapiens: GO:0007259 receptor signaling pathway via JAK-STAT (loss)
c-value
0.0
0.1
0.2
0.3
0.4
0.5
0.6
0.7
0.8
Phylostratum
Cellular_organisms - ps1
Archaea1 - ps2
Archaea2 - ps3
Archaea3 - ps4
Asgard/Eukaryota - ps5
Eukaryota - ps6
Amorpheae - ps7
Obazoa - ps8
Opisthokonta - ps9
Holozoa - ps10
Filozoa - ps11
Choano./Metazoa - ps12
Metazoa - ps13
Myriazoa - ps14
Parahoxozoa - ps15
Bilateria - ps16
Deuterostomia - ps17
Chordata - ps18
Olfactores - ps19
Vertebrata - ps20
Gnathostomata - ps21
Euteleostomi - ps22
Sarcopterygii - ps23
Tetrapoda - ps24
Amniota - ps25
Mammalia - ps26
Theria - ps27
Placentalia - ps28
Boreoeutheria - ps29
Euarchontoglires - ps30
Euarchonta - ps31
Primates - ps32
Haplorrhini - ps33
Simiiformes - ps34
Catarrhini - ps35
Hominoidea - ps36
Hominidae - ps37
Homininae - ps38
Hominini - ps39
Homo_sapiens - ps40
p-value
5e-03
7e-03
9e-03
1e-02
2e-02
Log-odds
0.5
1.0
1.5
2.0
2.5
3.0
3.5
4.0

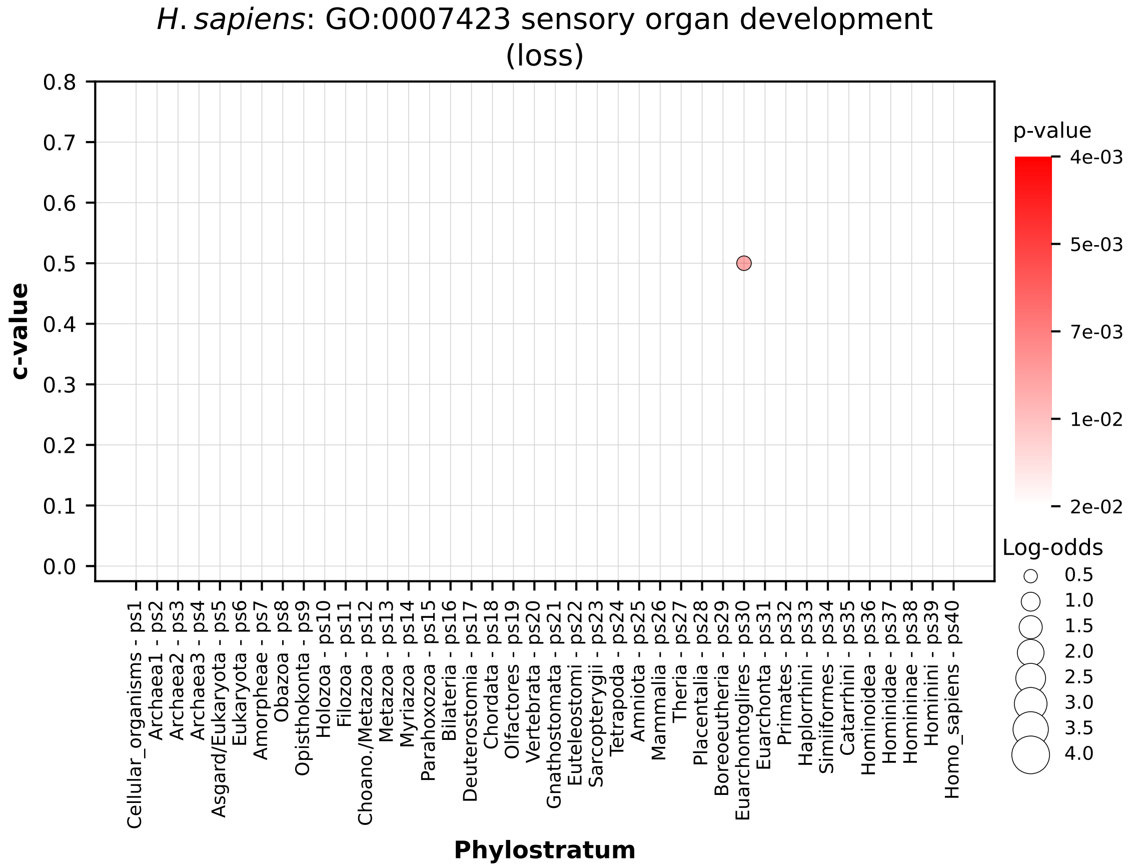

H. sapiens: GO:0007423 sensory organ development
(loss)
c-value
0.8
0.7
0.6
0.5
0.4
0.3
0.2
0.1
0.0
p-value
4e-03
5e-03
7e-03
1e-02
2e-02
Log-odds
0.5
1.0
1.5
2.0
2.5
3.0
3.5
4.0
Cellular_organisms - ps1
Archaea1 - ps2
Archaea2 - ps3
Archaea3 - ps4
Asgard/Eukaryota - ps5
Eukaryota - ps6
Amorpheae - ps7
Obazoa - ps8
Opisthokonta - ps9
Holozoa - ps10
Filozoa - ps11
Choano./Metazoa - ps12
Metazoa - ps13
Myriazoa - ps14
Parahoxozoa - ps15
Bilateria - ps16
Deuterostomia - ps17
Chordata - ps18
Olfactores - ps19
Vertebrata - ps20
Gnathostomata - ps21
Euteleostomi - ps22
Sarcopterygii - ps23
Tetrapoda - ps24
Amniota - ps25
Mammalia - ps26
Theria - ps27
Placentalia - ps28
Boreoeutheria - ps29
Euarchontoglires - ps30
Euarchonta - ps31
Primates - ps32
Haplorrhini - ps33
Simiiformes - ps34
Catarrhini - ps35
Hominoidea - ps36
Hominidae - ps37
Homininae - ps38
Hominini - ps39
Homo_sapiens - ps40
Phylostratum

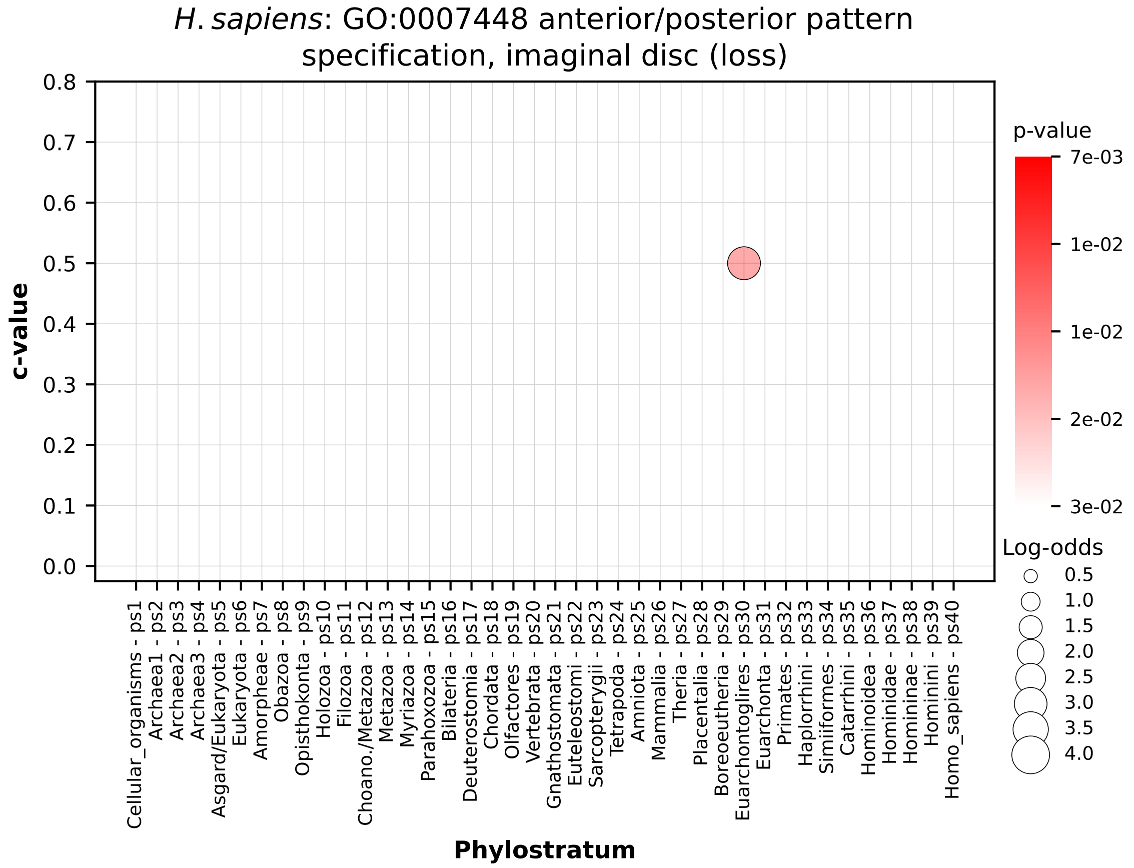

H. sapiens: GO:0007448 anterior/posterior pattern specification, imaginal disc (loss)
c-value
0.0
0.1
0.2
0.3
0.4
0.5
0.6
0.7
0.8
Phylostratum
Cellular_organisms - ps1
Archaea1 - ps2
Archaea2 - ps3
Archaea3 - ps4
Asgard/Eukaryota - ps5
Eukaryota - ps6
Amorpheae - ps7
Obazoa - ps8
Opisthokonta - ps9
Holozoa - ps10
Filozoa - ps11
Choano./Metazoa - ps12
Metazoa - ps13
Myriazoa - ps14
Parahoxozoa - ps15
Bilateria - ps16
Deuterostomia - ps17
Chordata - ps18
Olfactores - ps19
Vertebrata - ps20
Gnathostomata - ps21
Euteleostomi - ps22
Sarcopterygii - ps23
Tetrapoda - ps24
Amniota - ps25
Mammalia - ps26
Theria - ps27
Placentalia - ps28
Boreoeutheria - ps29
Euarchontoglires - ps30
Euarchonta - ps31
Primates - ps32
Haplorrhini - ps33
Simiiformes - ps34
Catarrhini - ps35
Hominoidea - ps36
Hominidae - ps37
Homininae - ps38
Hominini - ps39
Homo_sapiens - ps40
p-value
7e-03
1e-02
1e-02
2e-02
3e-02
Log-odds
0.5
1.0
1.5
2.0
2.5
3.0
3.5
4.0

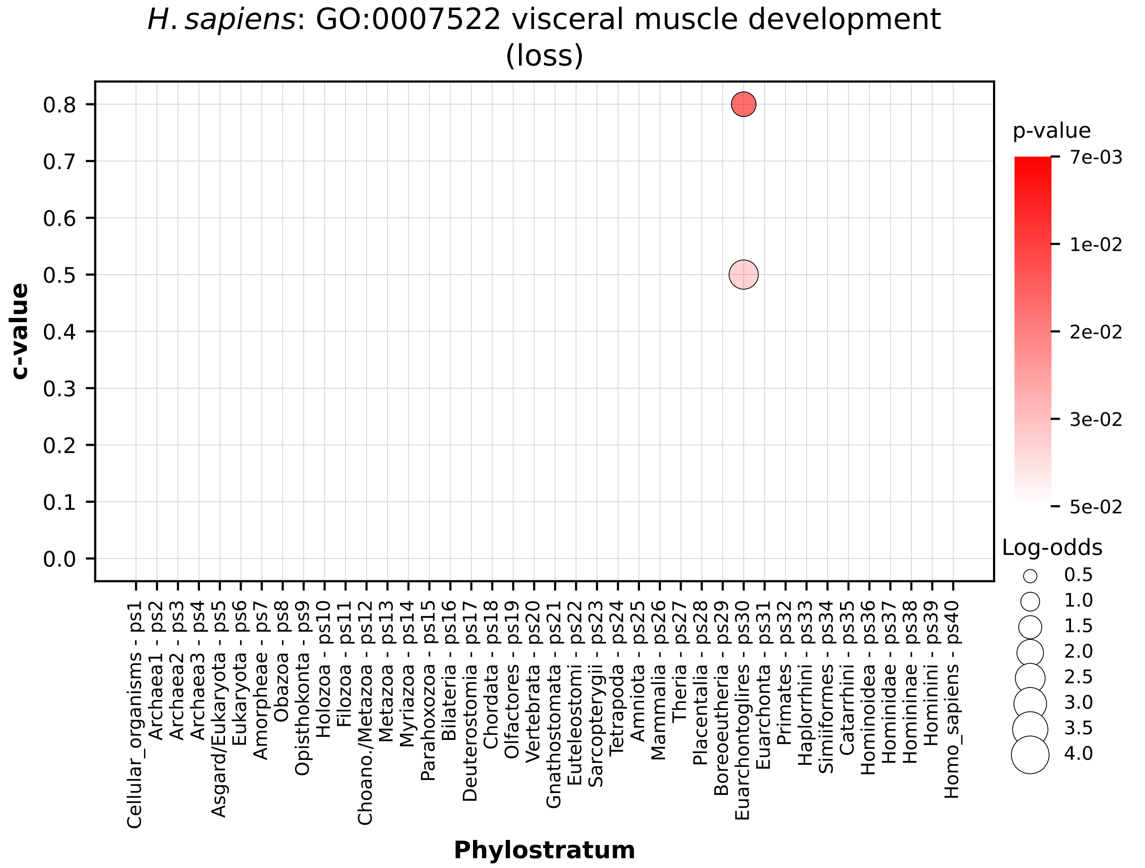

H. sapiens: GO:0007522 visceral muscle development (loss)
c-value
0.0
0.1
0.2
0.3
0.4
0.5
0.6
0.7
0.8
Phylostratum
Cellular_organisms - ps1
Archaea1 - ps2
Archaea2 - ps3
Archaea3 - ps4
Asgard/Eukaryota - ps5
Eukaryota - ps6
Amorpheae - ps7
Obazoa - ps8
Opisthokonta - ps9
Holozoa - ps10
Filozoa - ps11
Choano./Metazoa - ps12
Metazoa - ps13
Myriazoa - ps14
Parahoxozoa - ps15
Bilateria - ps16
Deuterostomia - ps17
Chordata - ps18
Olfactores - ps19
Vertebrata - ps20
Gnathostomata - ps21
Euteleostomi - ps22
Sarcopterygii - ps23
Tetrapoda - ps24
Amniota - ps25
Mammalia - ps26
Theria - ps27
Placentalia - ps28
Boreoeutheria - ps29
Euarchontoglires - ps30
Euarchonta - ps31
Primates - ps32
Haplorrhini - ps33
Simiiformes - ps34
Catarrhini - ps35
Hominoidea - ps36
Hominidae - ps37
Homininae - ps38
Hominini - ps39
Homo_sapiens - ps40
p-value
7e-03
1e-02
2e-02
3e-02
5e-02
Log-odds
0.5
1.0
1.5
2.0
2.5
3.0
3.5
4.0

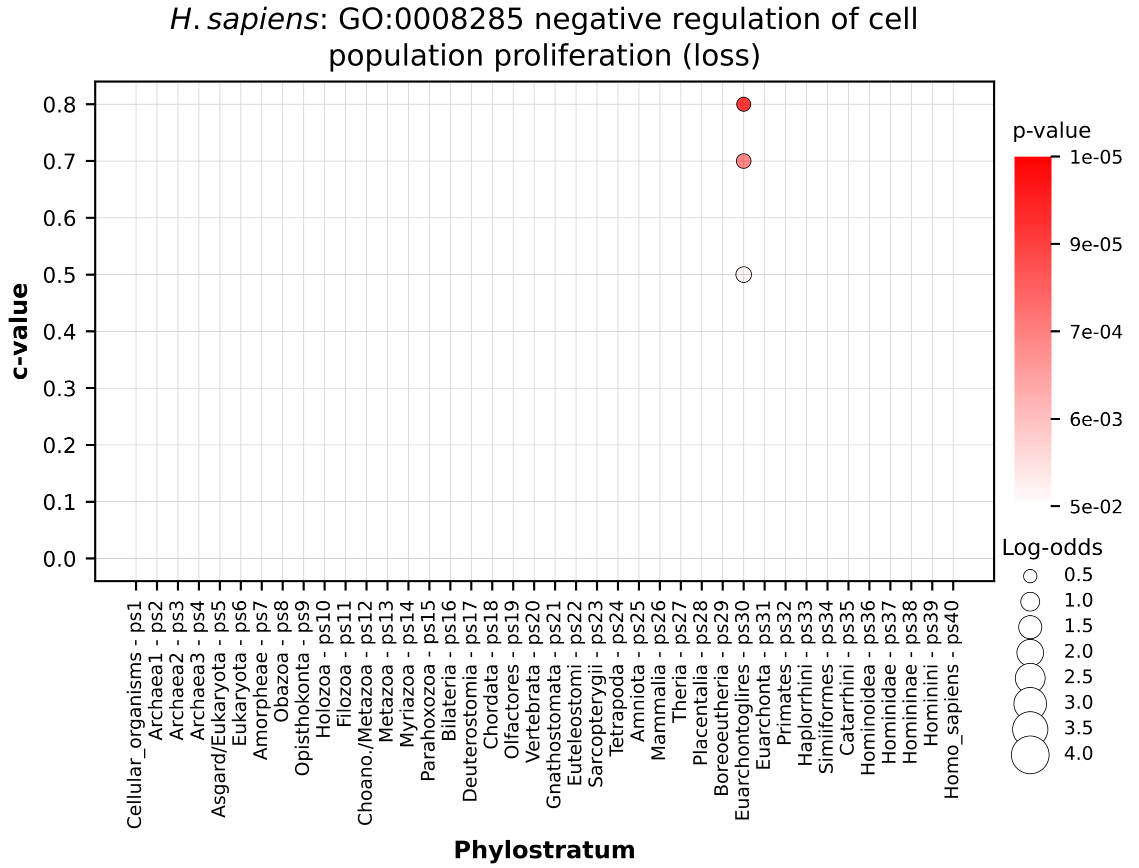
H. sapiens: GO:0008285 negative regulation of cell population proliferation (loss)
c-value
0.0
0.1
0.2
0.3
0.4
0.5
0.6
0.7
0.8
Phylostratum
Cellular_organisms - ps1
Archaea1 - ps2
Archaea2 - ps3
Archaea3 - ps4
Asgard/Eukaryota - ps5
Eukaryota - ps6
Amorpheae - ps7
Obazoa - ps8
Opisthokonta - ps9
Holozoa - ps10
Filozoa - ps11
Choano./Metazoa - ps12
Metazoa - ps13
Myriazoa - ps14
Parahoxozoa - ps15
Bilateria - ps16
Deuterostomia - ps17
Chordata - ps18
Olfactores - ps19
Vertebrata - ps20
Gnathostomata - ps21
Euteleostomi - ps22
Sarcopterygii - ps23
Tetrapoda - ps24
Amniota - ps25
Mammalia - ps26
Theria - ps27
Placentalia - ps28
Boreoeutheria - ps29
Euarchontoglires - ps30
Euarchonta - ps31
Primates - ps32
Haplorrhini - ps33
Simiiformes - ps34
Catarrhini - ps35
Hominoidea - ps36
Hominidae - ps37
Homininae - ps38
Hominini - ps39
Homo_sapiens - ps40
p-value
1e-05
9e-05
7e-04
6e-03
5e-02
Log-odds
0.5
1.0
1.5
2.0
2.5
3.0
3.5
4.0

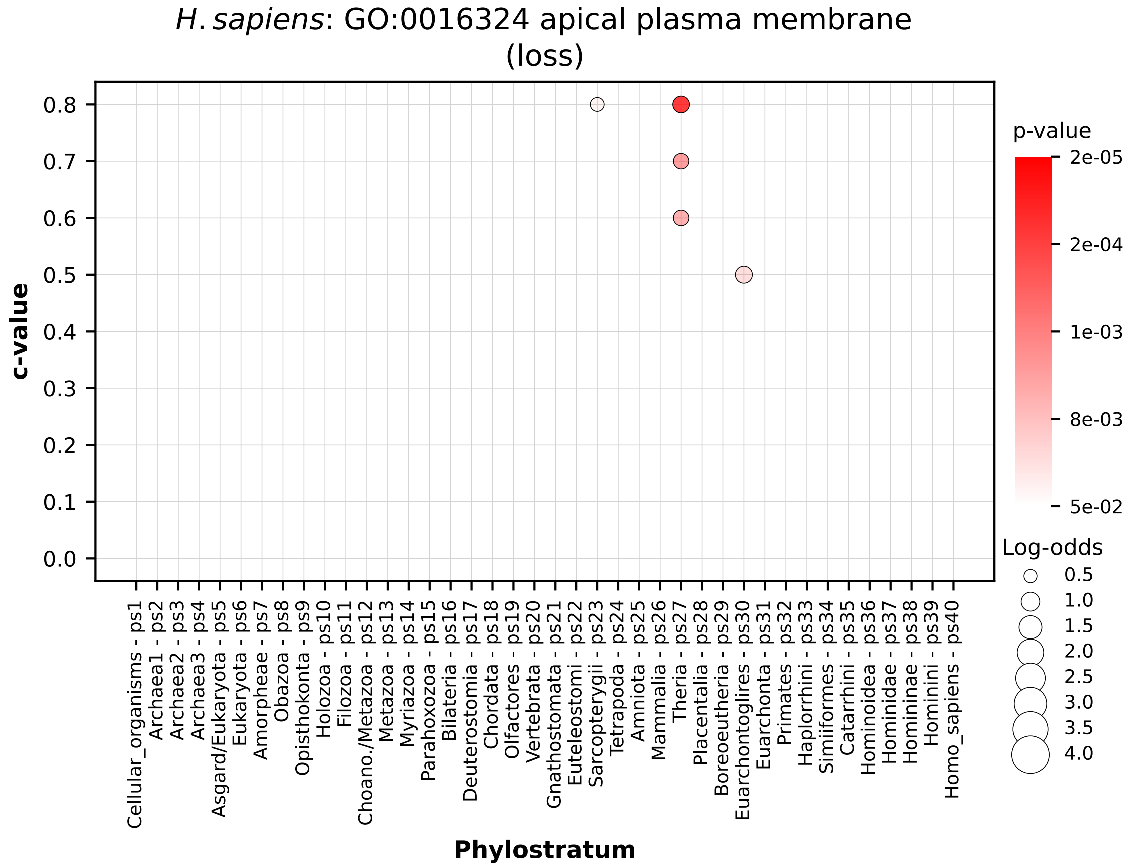
H. sapiens: GO:0016324 apical plasma membrane
(loss)
c-value
0.0
0.1
0.2
0.3
0.4
0.5
0.6
0.7
0.8
Phylostratum
Cellular_organisms - ps1
Archaea1 - ps2
Archaea2 - ps3
Archaea3 - ps4
Asgard/Eukaryota - ps5
Eukaryota - ps6
Amorpheae - ps7
Obazoa - ps8
Opisthokonta - ps9
Holozoa - ps10
Filozoa - ps11
Choano./Metazoa - ps12
Metazoa - ps13
Myriazoa - ps14
Parahoxozoa - ps15
Bilateria - ps16
Deuterostomia - ps17
Chordata - ps18
Olfactores - ps19
Vertebrata - ps20
Gnathostomata - ps21
Euteleostomi - ps22
Sarcopterygii - ps23
Tetrapoda - ps24
Amniota - ps25
Mammalia - ps26
Theria - ps27
Placentalia - ps28
Boreoeutheria - ps29
Euarchontoglires - ps30
Euarchonta - ps31
Primates - ps32
Haplorrhini - ps33
Simiiformes - ps34
Catarrhini - ps35
Hominoidea - ps36
Hominidae - ps37
Homininae - ps38
Hominini - ps39
Homo_sapiens - ps40
p-value
2e-05
2e-04
1e-03
8e-03
5e-02
Log-odds
0.5
1.0
1.5
2.0
2.5
3.0
3.5
4.0

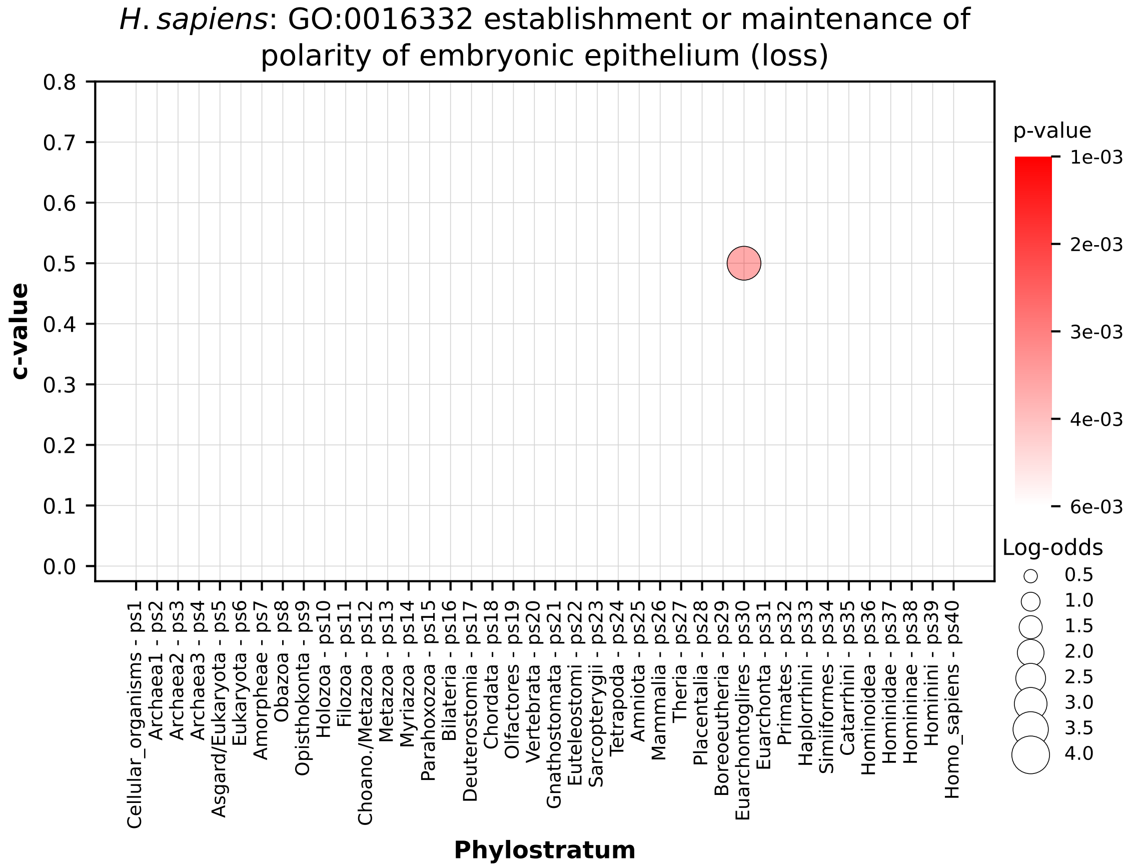

H. sapiens: GO:0016332 establishment or maintenance of polarity of embryonic epithelium (loss)
c-value
0.8
0.7
0.6
0.5
0.4
0.3
0.2
0.1
0.0
p-value
1e-03
2e-03
3e-03
4e-03
6e-03
Log-odds
0.5
1.0
1.5
2.0
2.5
3.0
3.5
4.0
Cellular_organisms - ps1
Archaea1 - ps2
Archaea2 - ps3
Archaea3 - ps4
Asgard/Eukaryota - ps5
Eukaryota - ps6
Amorpheae - ps7
Obazoa - ps8
Opisthokonta - ps9
Holozoa - ps10
Filozoa - ps11
Choano./Metazoa - ps12
Metazoa - ps13
Myriazoa - ps14
Parahoxozoa - ps15
Bilateria - ps16
Deuterostomia - ps17
Chordata - ps18
Olfactores - ps19
Vertebrata - ps20
Gnathostomata - ps21
Euteleostomi - ps22
Sarcopterygii - ps23
Tetrapoda - ps24
Amniota - ps25
Mammalia - ps26
Theria - ps27
Placentalia - ps28
Boreoeutheria - ps29
Euarchontoglires - ps30
Euarchonta - ps31
Primates - ps32
Haplorrhini - ps33
Simiiformes - ps34
Catarrhini - ps35
Hominoidea - ps36
Hominidae - ps37
Homininae - ps38
Hominini - ps39
Homo_sapiens - ps40
Phylostratum

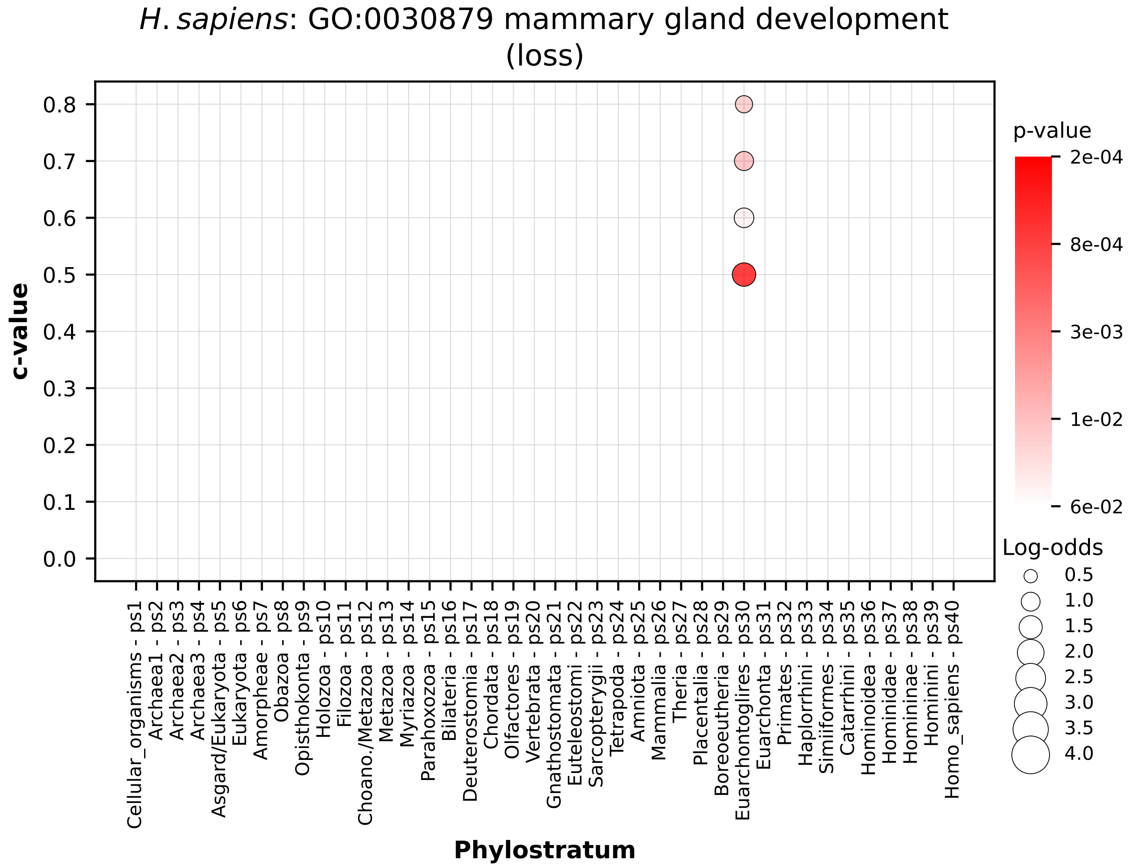

H. sapiens: GO:0030879 mammary gland development (loss)
c-value
0.0
0.1
0.2
0.3
0.4
0.5
0.6
0.7
0.8
Phylostratum
Cellular_organisms - ps1
Archaea1 - ps2
Archaea2 - ps3
Archaea3 - ps4
Asgard/Eukaryota - ps5
Eukaryota - ps6
Amorpheae - ps7
Obazoa - ps8
Opisthokonta - ps9
Holozoa - ps10
Filozoa - ps11
Choano./Metazoa - ps12
Metazoa - ps13
Myriazoa - ps14
Parahoxozoa - ps15
Bilateria - ps16
Deuterostomia - ps17
Chordata - ps18
Olfactores - ps19
Vertebrata - ps20
Gnathostomata - ps21
Euteleostomi - ps22
Sarcopterygii - ps23
Tetrapoda - ps24
Amniota - ps25
Mammalia - ps26
Theria - ps27
Placentalia - ps28
Boreoeutheria - ps29
Euarchontoglires - ps30
Euarchonta - ps31
Primates - ps32
Haplorrhini - ps33
Simiiformes - ps34
Catarrhini - ps35
Hominoidea - ps36
Hominidae - ps37
Homininae - ps38
Hominini - ps39
Homo_sapiens - ps40
p-value
2e-04
8e-04
3e-03
1e-02
6e-02
Log-odds
0.5
1.0
1.5
2.0
2.5
3.0
3.5
4.0

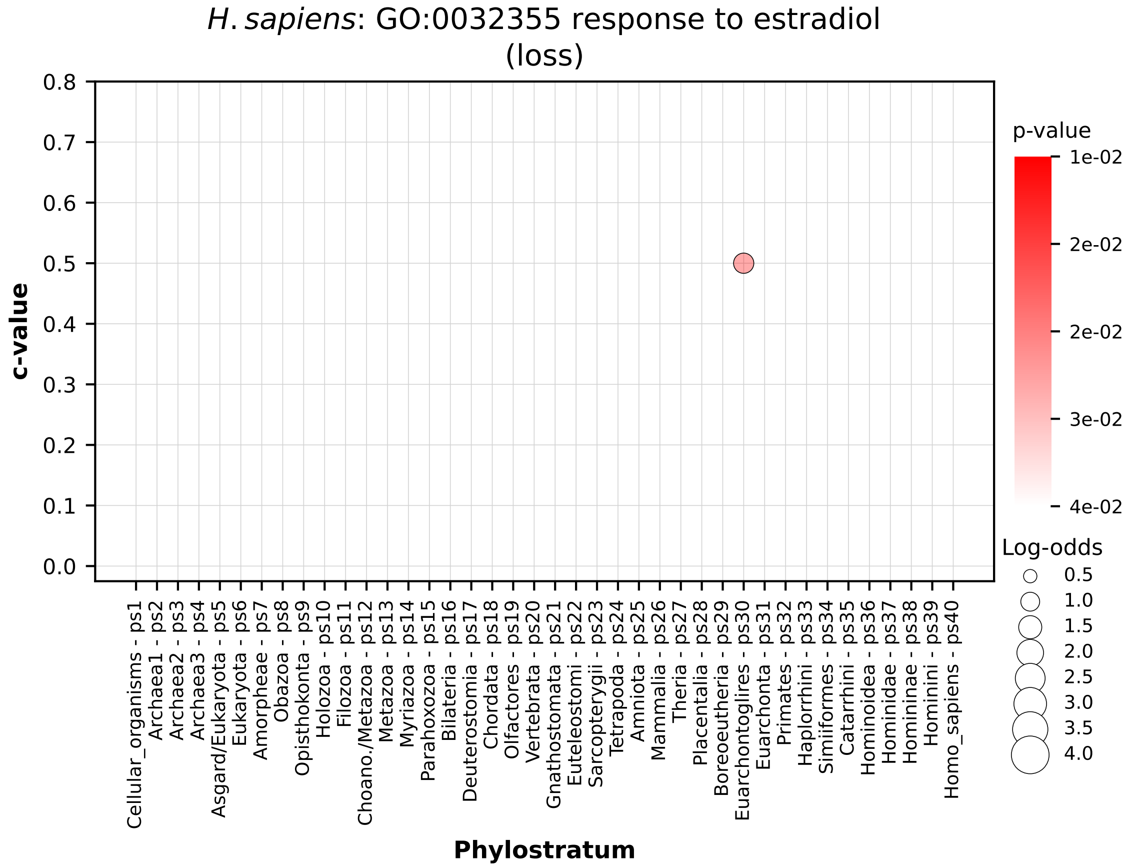
H. sapiens: GO:0032355 response to estradiol
(loss)
c-value
0.0
0.1
0.2
0.3
0.4
0.5
0.6
0.7
0.8
Phylostratum
Cellular_organisms - ps1
Archaea1 - ps2
Archaea2 - ps3
Archaea3 - ps4
Asgard/Eukaryota - ps5
Eukaryota - ps6
Amorpheae - ps7
Obazoa - ps8
Opisthokonta - ps9
Holozoa - ps10
Filozoa - ps11
Choano./Metazoa - ps12
Metazoa - ps13
Myriazoa - ps14
Parahoxozoa - ps15
Bilateria - ps16
Deuterostomia - ps17
Chordata - ps18
Olfactores - ps19
Vertebrata - ps20
Gnathostomata - ps21
Euteleostomi - ps22
Sarcopterygii - ps23
Tetrapoda - ps24
Amniota - ps25
Mammalia - ps26
Theria - ps27
Placentalia - ps28
Boreoeutheria - ps29
Euarchontoglires - ps30
Euarchonta - ps31
Primates - ps32
Haplorrhini - ps33
Simiiformes - ps34
Catarrhini - ps35
Hominoidea - ps36
Hominidae - ps37
Homininae - ps38
Hominini - ps39
Homo_sapiens - ps40
p-value
1e-02
2e-02
2e-02
3e-02
4e-02
Log-odds
0.5
1.0
1.5
2.0
2.5
3.0
3.5
4.0

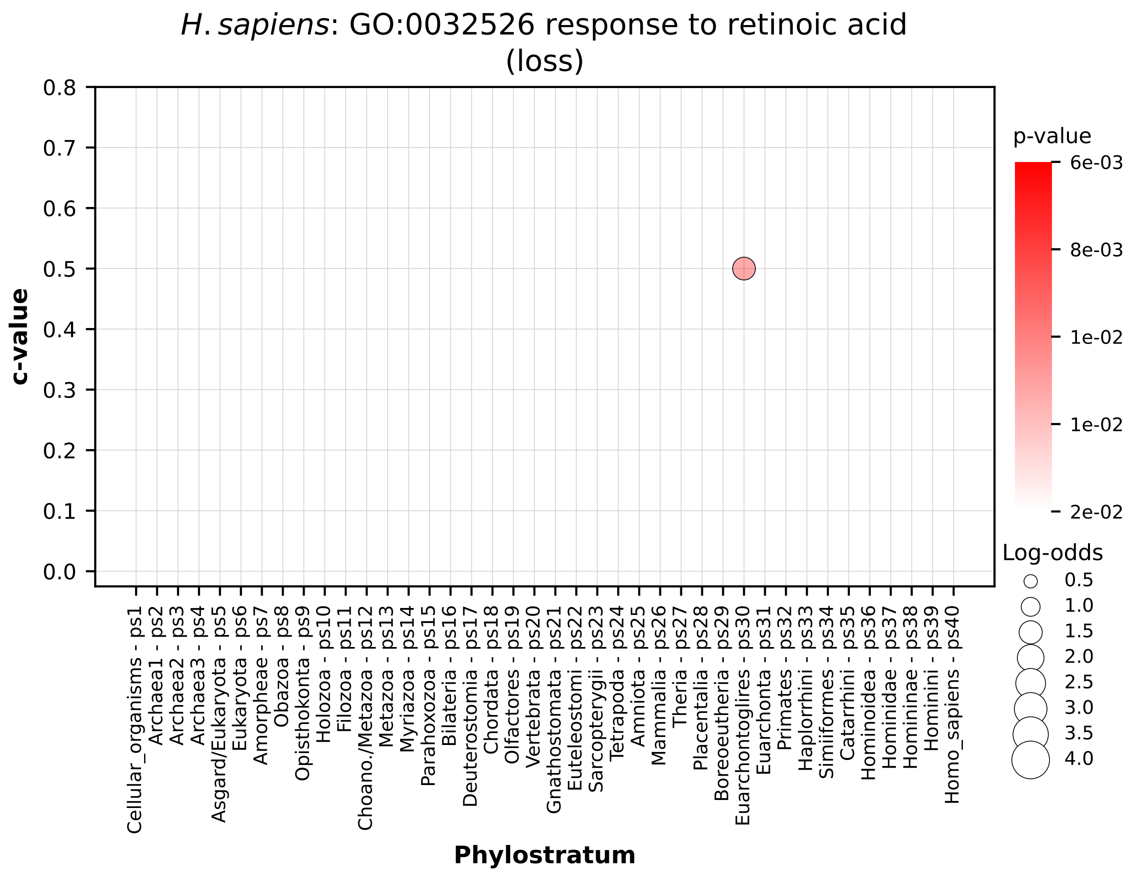
H. sapiens: GO:0032526 response to retinoic acid
(loss)
c-value
0.8
0.7
0.6
0.5
0.4
0.3
0.2
0.1
0.0
p-value
6e-03
8e-03
1e-02
1e-02
2e-02
Log-odds
0.5
1.0
1.5
2.0
2.5
3.0
3.5
4.0
Cellular_organisms - ps1
Archaea1 - ps2
Archaea2 - ps3
Archaea3 - ps4
Asgard/Eukaryota - ps5
Eukaryota - ps6
Amorpheae - ps7
Obazoa - ps8
Opisthokonta - ps9
Holozoa - ps10
Filozoa - ps11
Choano./Metazoa - ps12
Metazoa - ps13
Myriazoa - ps14
Parahoxozoa - ps15
Bilateria - ps16
Deuterostomia - ps17
Chordata - ps18
Olfactores - ps19
Vertebrata - ps20
Gnathostomata - ps21
Euteleostomi - ps22
Sarcopterygii - ps23
Tetrapoda - ps24
Amniota - ps25
Mammalia - ps26
Theria - ps27
Placentalia - ps28
Boreoeutheria - ps29
Euarchontoglires - ps30
Euarchonta - ps31
Primates - ps32
Haplorrhini - ps33
Simiiformes - ps34
Catarrhini - ps35
Hominoidea - ps36
Hominidae - ps37
Homininae - ps38
Hominini - ps39
Homo_sapiens - ps40
Phylostratum

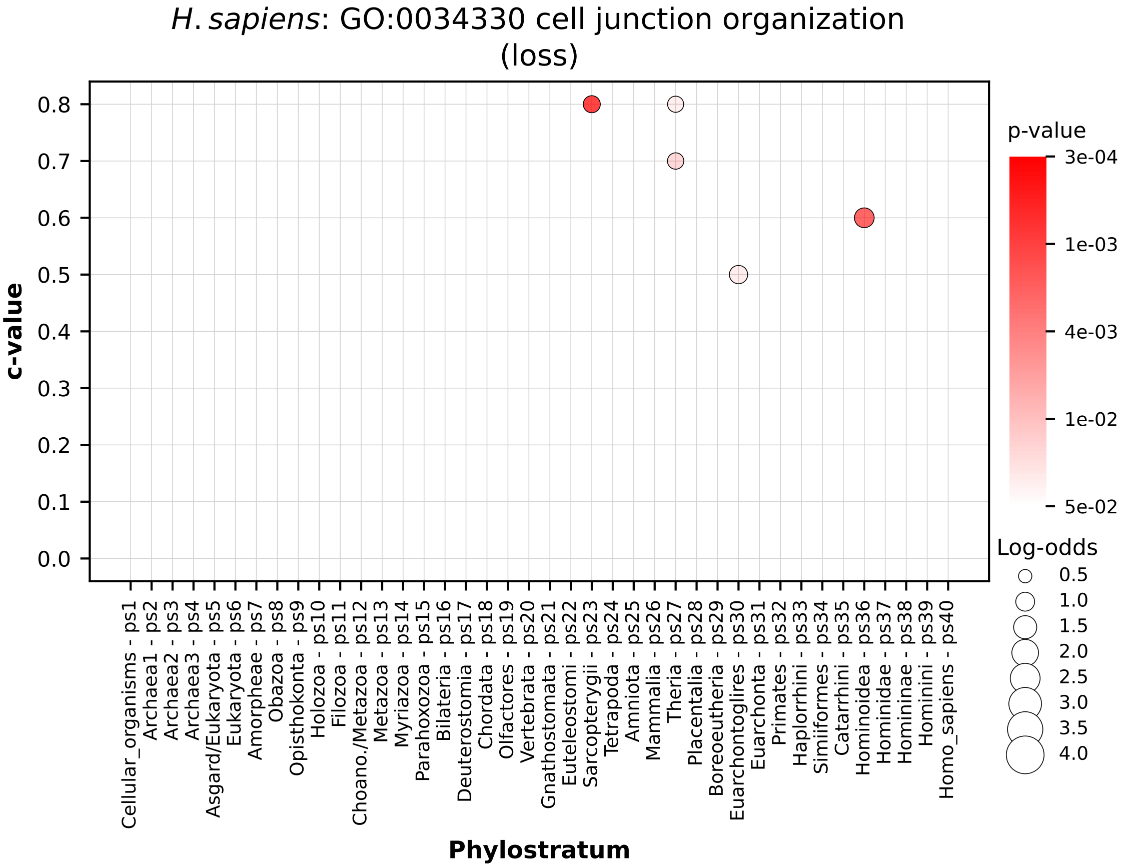

H. sapiens: GO:0034330 cell junction organization
(loss)
c-value
0.0
0.1
0.2
0.3
0.4
0.5
0.6
0.7
0.8
Phylostratum
Cellular_organisms - ps1
Archaea1 - ps2
Archaea2 - ps3
Archaea3 - ps4
Asgard/Eukaryota - ps5
Eukaryota - ps6
Amorpheae - ps7
Obazoa - ps8
Opisthokonta - ps9
Holozoa - ps10
Filozoa - ps11
Choano./Metazoa - ps12
Metazoa - ps13
Myriazoa - ps14
Parahoxozoa - ps15
Bilateria - ps16
Deuterostomia - ps17
Chordata - ps18
Olfactores - ps19
Vertebrata - ps20
Gnathostomata - ps21
Euteleostomi - ps22
Sarcopterygii - ps23
Tetrapoda - ps24
Amniota - ps25
Mammalia - ps26
Theria - ps27
Placentalia - ps28
Boreoeutheria - ps29
Euarchontoglires - ps30
Euarchonta - ps31
Primates - ps32
Haplorrhini - ps33
Simiiformes - ps34
Catarrhini - ps35
Hominoidea - ps36
Hominidae - ps37
Homininae - ps38
Hominini - ps39
Homo_sapiens - ps40
p-value
3e-04
1e-03
4e-03
1e-02
5e-02
Log-odds
0.5
1.0
1.5
2.0
2.5
3.0
3.5
4.0

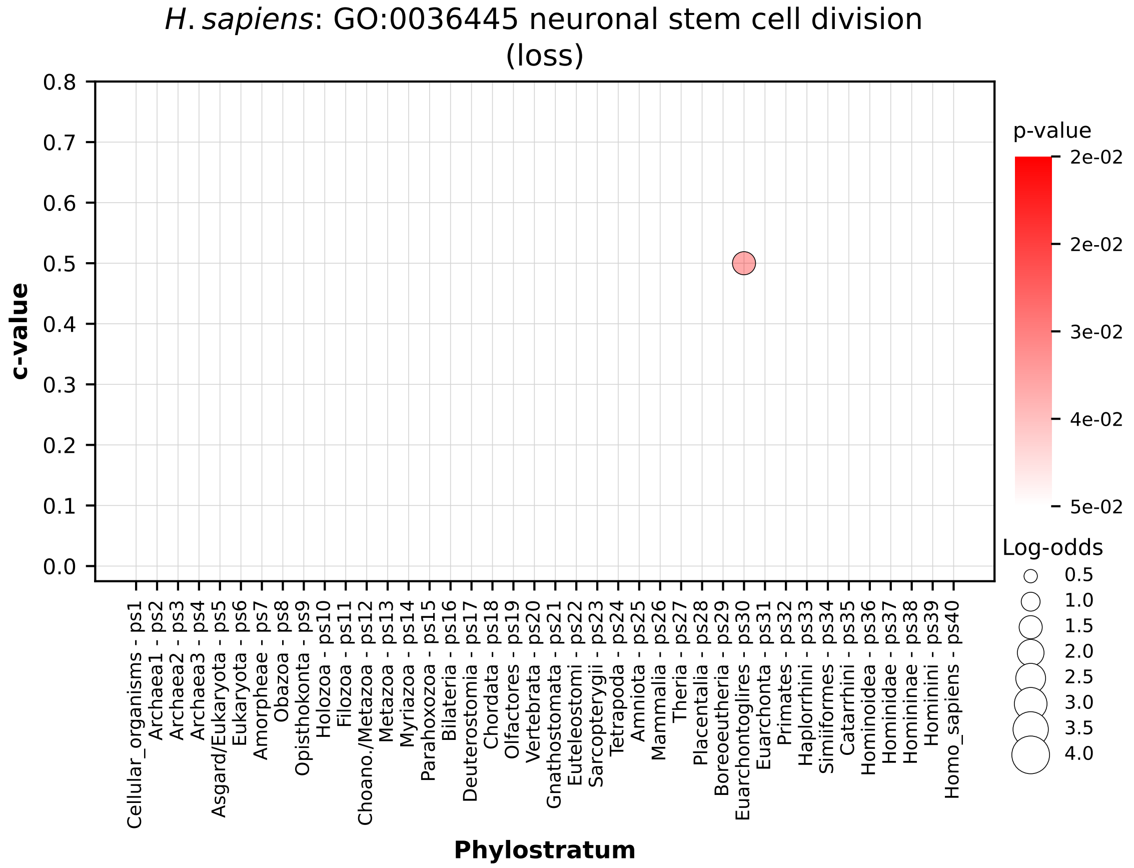

H. sapiens: GO:0036445 neuronal stem cell division
(loss)
c-value
0.8
0.7
0.6
0.5
0.4
0.3
0.2
0.1
0.0
Phylostratum
Cellular_organisms - ps1
Archaea1 - ps2
Archaea2 - ps3
Archaea3 - ps4
Asgard/Eukaryota - ps5
Eukaryota - ps6
Amorpheae - ps7
Obazoa - ps8
Opisthokonta - ps9
Holozoa - ps10
Filozoa - ps11
Choano./Metazoa - ps12
Metazoa - ps13
Myriazoa - ps14
Parahoxozoa - ps15
Bilateria - ps16
Deuterostomia - ps17
Chordata - ps18
Olfactores - ps19
Vertebrata - ps20
Gnathostomata - ps21
Euteleostomi - ps22
Sarcopterygii - ps23
Tetrapoda - ps24
Amniota - ps25
Mammalia - ps26
Theria - ps27
Placentalia - ps28
Boreoeutheria - ps29
Euarchontoglires - ps30
Euarchonta - ps31
Primates - ps32
Haplorrhini - ps33
Simiiformes - ps34
Catarrhini - ps35
Hominoidea - ps36
Hominidae - ps37
Homininae - ps38
Hominini - ps39
Homo_sapiens - ps40
p-value
2e-02
2e-02
3e-02
4e-02
5e-02
Log-odds
0.5
1.0
1.5
2.0
2.5
3.0
3.5
4.0

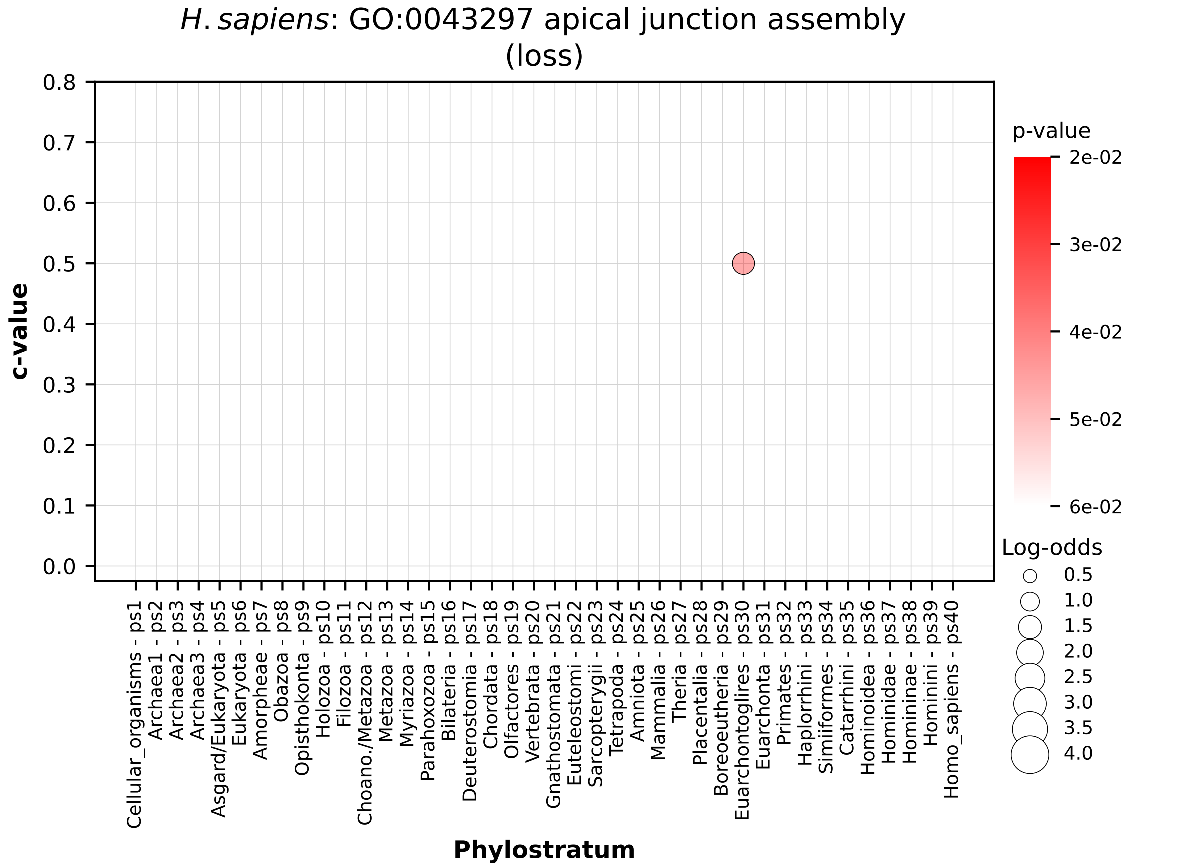
H. sapiens: GO:0043297 apical junction assembly
(loss)
c-value
0.0
0.1
0.2
0.3
0.4
0.5
0.6
0.7
0.8
Phylostratum
Cellular_organisms - ps1
Archaea1 - ps2
Archaea2 - ps3
Archaea3 - ps4
Asgard/Eukaryota - ps5
Eukaryota - ps6
Amorpheae - ps7
Obazoa - ps8
Opisthokonta - ps9
Holozoa - ps10
Filozoa - ps11
Choano./Metazoa - ps12
Metazoa - ps13
Myriazoa - ps14
Parahoxozoa - ps15
Bilateria - ps16
Deuterostomia - ps17
Chordata - ps18
Olfactores - ps19
Vertebrata - ps20
Gnathostomata - ps21
Euteleostomi - ps22
Sarcopterygii - ps23
Tetrapoda - ps24
Amniota - ps25
Mammalia - ps26
Theria - ps27
Placentalia - ps28
Boreoeutheria - ps29
Euarchontoglires - ps30
Euarchonta - ps31
Primates - ps32
Haplorrhini - ps33
Simiiformes - ps34
Catarrhini - ps35
Hominoidea - ps36
Hominidae - ps37
Homininae - ps38
Hominini - ps39
Homo_sapiens - ps40
p-value
2e-02
3e-02
4e-02
5e-02
6e-02
Log-odds
0.5
1.0
1.5
2.0
2.5
3.0
3.5
4.0

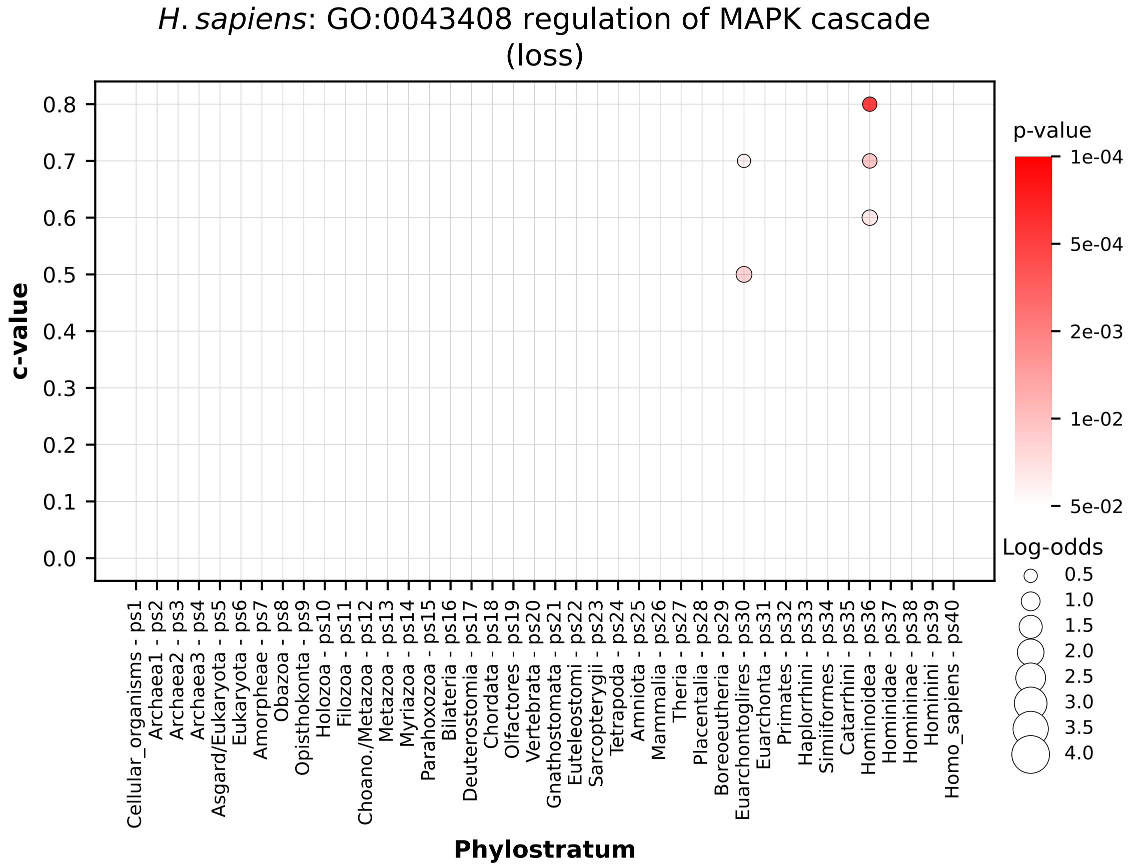

H. sapiens: GO:0043408 regulation of MAPK cascade (loss)
c-value
0.0
0.1
0.2
0.3
0.4
0.5
0.6
0.7
0.8
Phylostratum
Cellular_organisms - ps1
Archaea1 - ps2
Archaea2 - ps3
Archaea3 - ps4
Asgard/Eukaryota - ps5
Eukaryota - ps6
Amorpheae - ps7
Obazoa - ps8
Opisthokonta - ps9
Holozoa - ps10
Filozoa - ps11
Choano./Metazoa - ps12
Metazoa - ps13
Myriazoa - ps14
Parahoxozoa - ps15
Bilateria - ps16
Deuterostomia - ps17
Chordata - ps18
Olfactores - ps19
Vertebrata - ps20
Gnathostomata - ps21
Euteleostomi - ps22
Sarcopterygii - ps23
Tetrapoda - ps24
Amniota - ps25
Mammalia - ps26
Theria - ps27
Placentalia - ps28
Boreoeutheria - ps29
Euarchontoglires - ps30
Euarchonta - ps31
Primates - ps32
Haplorrhini - ps33
Simiiformes - ps34
Catarrhini - ps35
Hominoidea - ps36
Hominidae - ps37
Homininae - ps38
Hominini - ps39
Homo_sapiens - ps40
p-value
1e-04
5e-04
2e-03
1e-02
5e-02
Log-odds
0.5
1.0
1.5
2.0
2.5
3.0
3.5
4.0

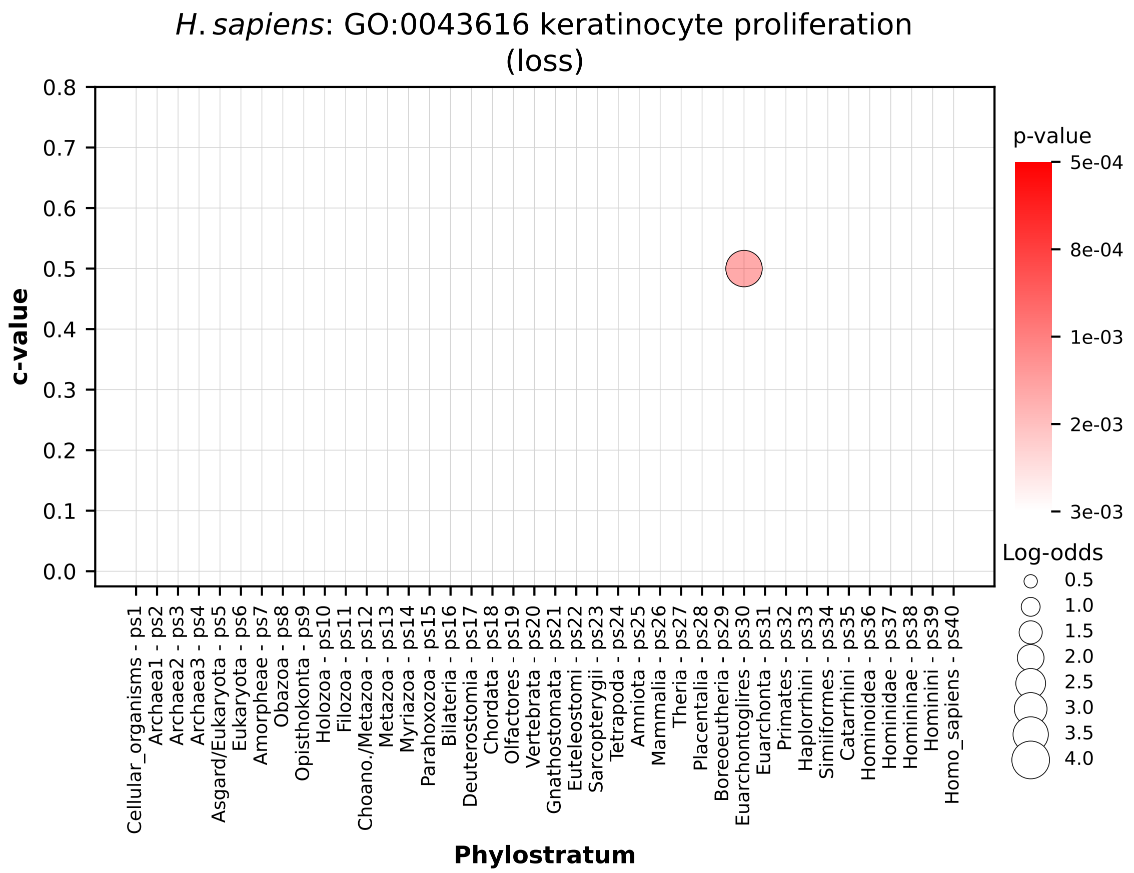

H. sapiens: GO:0043616 keratinocyte proliferation
(loss)
c-value
0.0
0.1
0.2
0.3
0.4
0.5
0.6
0.7
0.8
Phylostratum
Cellular_organisms - ps1
Archaea1 - ps2
Archaea2 - ps3
Archaea3 - ps4
Asgard/Eukaryota - ps5
Eukaryota - ps6
Amorpheae - ps7
Obazoa - ps8
Opisthokonta - ps9
Holozoa - ps10
Filozoa - ps11
Choano./Metazoa - ps12
Metazoa - ps13
Myriazoa - ps14
Parahoxozoa - ps15
Bilateria - ps16
Deuterostomia - ps17
Chordata - ps18
Olfactores - ps19
Vertebrata - ps20
Gnathostomata - ps21
Euteleostomi - ps22
Sarcopterygii - ps23
Tetrapoda - ps24
Amniota - ps25
Mammalia - ps26
Theria - ps27
Placentalia - ps28
Boreoeutheria - ps29
Euarchontoglires - ps30
Euarchonta - ps31
Primates - ps32
Haplorrhini - ps33
Simiiformes - ps34
Catarrhini - ps35
Hominoidea - ps36
Hominidae - ps37
Homininae - ps38
Hominini - ps39
Homo_sapiens - ps40
p-value
5e-04
8e-04
1e-03
2e-03
3e-03
Log-odds
0.5
1.0
1.5
2.0
2.5
3.0
3.5
4.0

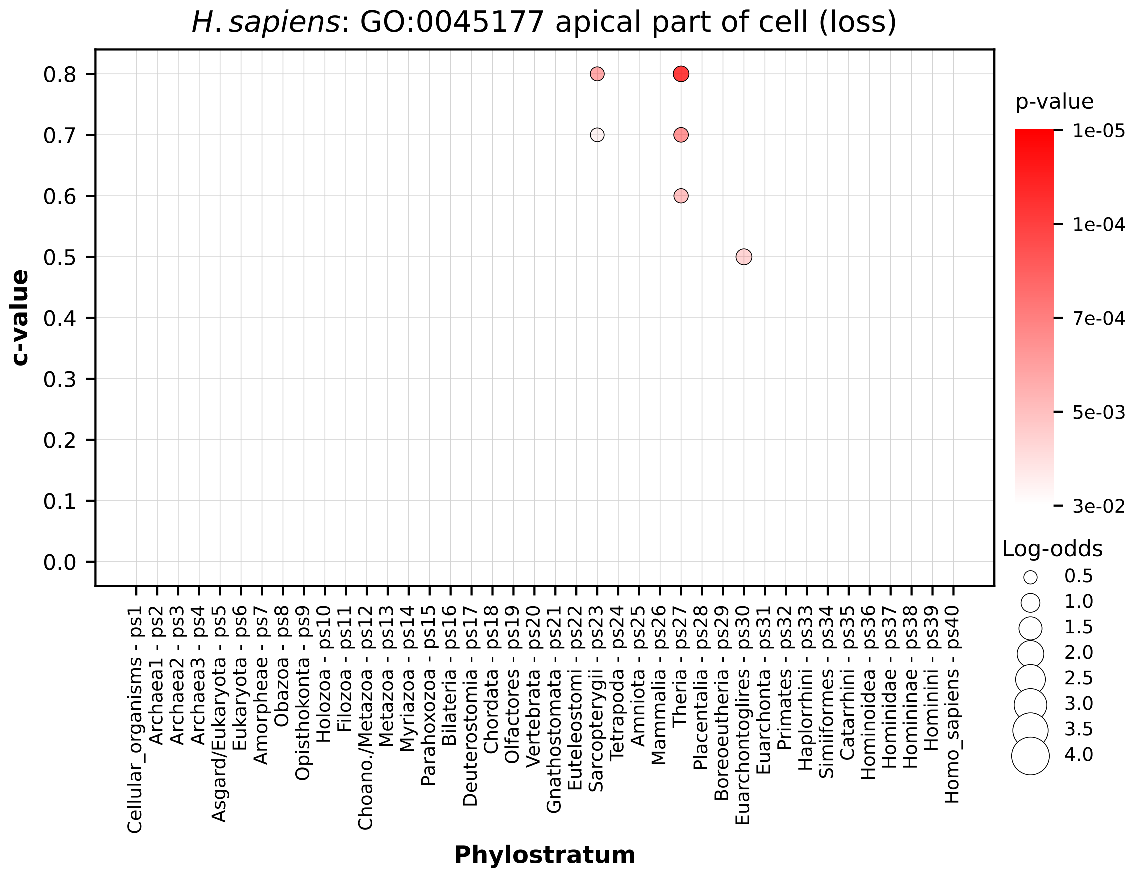
H. sapiens: GO:0045177 apical part of cell (loss)
c-value
0.0
0.1
0.2
0.3
0.4
0.5
0.6
0.7
0.8
p-value
1e-05
1e-04
7e-04
5e-03
3e-02
Log-odds
0.5
1.0
1.5
2.0
2.5
3.0
3.5
4.0
Cellular_organisms - ps1
Archaea1 - ps2
Archaea2 - ps3
Archaea3 - ps4
Asgard/Eukaryota - ps5
Eukaryota - ps6
Amorpheae - ps7
Obazoa - ps8
Opisthokonta - ps9
Holozoa - ps10
Filozoa - ps11
Choano./Metazoa - ps12
Metazoa - ps13
Myriazoa - ps14
Parahoxozoa - ps15
Bilateria - ps16
Deuterostomia - ps17
Chordata - ps18
Olfactores - ps19
Vertebrata - ps20
Gnathostomata - ps21
Euteleostomi - ps22
Sarcopterygii - ps23
Tetrapoda - ps24
Amniota - ps25
Mammalia - ps26
Theria - ps27
Placentalia - ps28
Boreoeutheria - ps29
Euarchontoglires - ps30
Euarchonta - ps31
Primates - ps32
Haplorrhini - ps33
Simiiformes - ps34
Catarrhini - ps35
Hominoidea - ps36
Hominidae - ps37
Homininae - ps38
Hominini - ps39
Homo_sapiens - ps40
Phylostratum

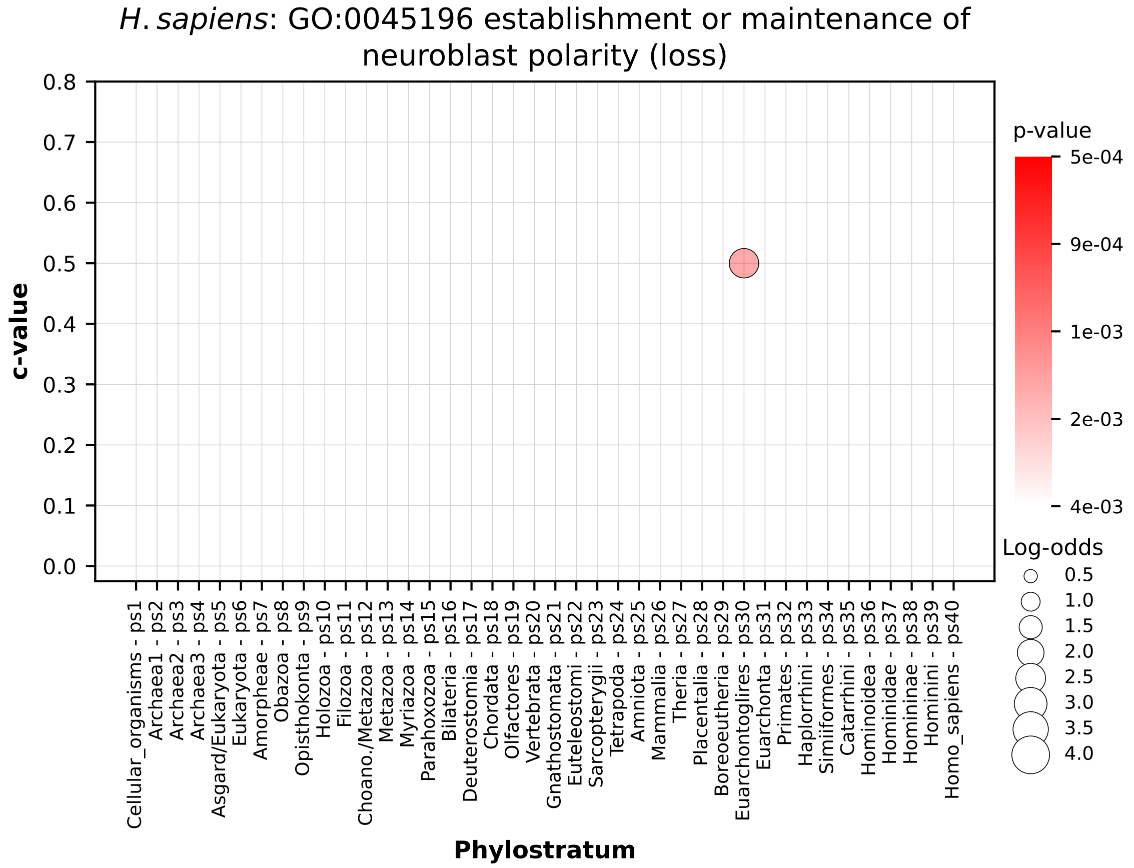

H. sapiens: GO:0045196 establishment or maintenance of neuroblast polarity (loss)
c-value
0.8
0.7
0.6
0.5
0.4
0.3
0.2
0.1
0.0
Phylostratum
Cellular_organisms - ps1
Archaea1 - ps2
Archaea2 - ps3
Archaea3 - ps4
Asgard/Eukaryota - ps5
Eukaryota - ps6
Amorpheae - ps7
Obazoa - ps8
Opisthokonta - ps9
Holozoa - ps10
Filozoa - ps11
Choano./Metazoa - ps12
Metazoa - ps13
Myriazoa - ps14
Parahoxozoa - ps15
Bilateria - ps16
Deuterostomia - ps17
Chordata - ps18
Olfactores - ps19
Vertebrata - ps20
Gnathostomata - ps21
Euteleostomi - ps22
Sarcopterygii - ps23
Tetrapoda - ps24
Amniota - ps25
Mammalia - ps26
Theria - ps27
Placentalia - ps28
Boreoeutheria - ps29
Euarchontoglires - ps30
Euarchonta - ps31
Primates - ps32
Haplorrhini - ps33
Simiiformes - ps34
Catarrhini - ps35
Hominoidea - ps36
Hominidae - ps37
Homininae - ps38
Hominini - ps39
Homo_sapiens - ps40
p-value
5e-04
9e-04
1e-03
2e-03
4e-03
Log-odds
0.5
1.0
1.5
2.0
2.5
3.0
3.5
4.0

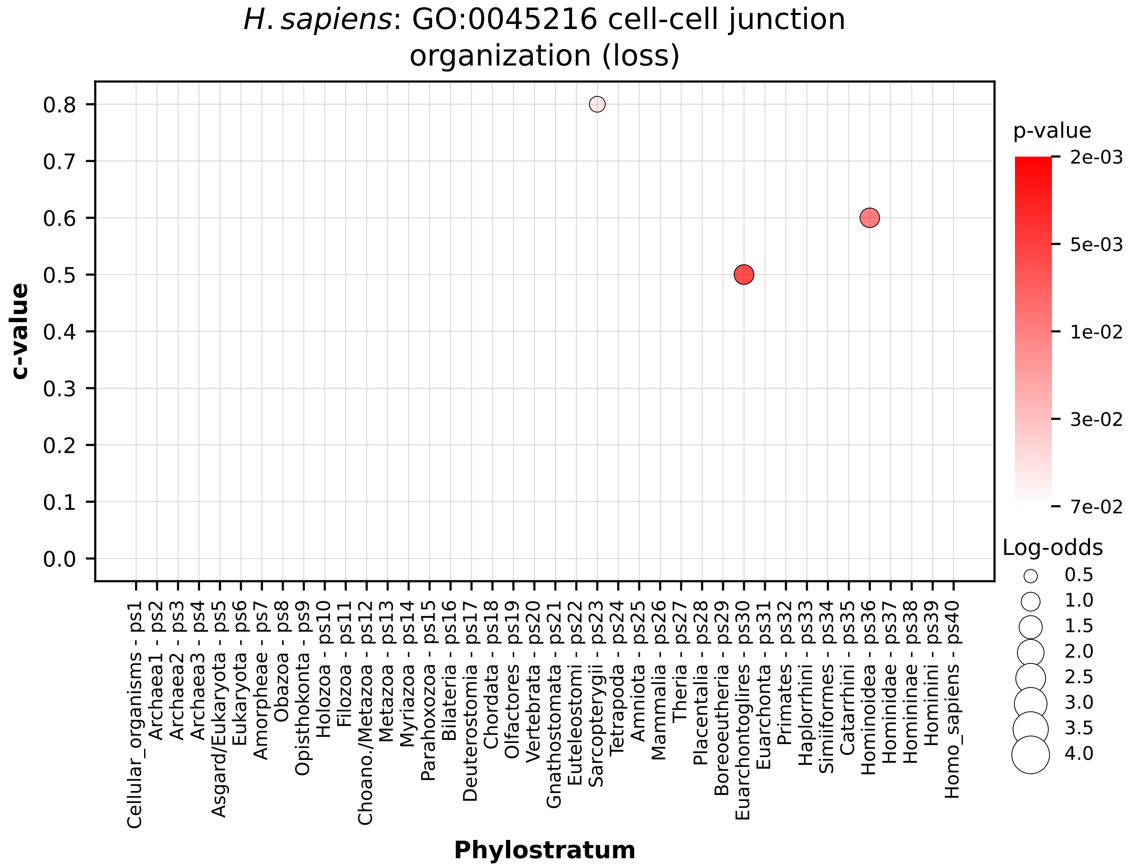

H. sapiens: GO:0045216 cell-cell junction organization (loss)
c-value
0.8
0.7
0.6
0.5
0.4
0.3
0.2
0.1
0.0
p-value
2e-03
5e-03
1e-02
3e-02
7e-02
Log-odds
0.5
1.0
1.5
2.0
2.5
3.0
3.5
4.0
Cellular_organisms - ps1
Archaea1 - ps2
Archaea2 - ps3
Archaea3 - ps4
Asgard/Eukaryota - ps5
Eukaryota - ps6
Amorpheae - ps7
Obazoa - ps8
Opisthokonta - ps9
Holozoa - ps10
Filozoa - ps11
Choano./Metazoa - ps12
Metazoa - ps13
Myriazoa - ps14
Parahoxozoa - ps15
Bilateria - ps16
Deuterostomia - ps17
Chordata - ps18
Olfactores - ps19
Vertebrata - ps20
Gnathostomata - ps21
Euteleostomi - ps22
Sarcopterygii - ps23
Tetrapoda - ps24
Amniota - ps25
Mammalia - ps26
Theria - ps27
Placentalia - ps28
Boreoeutheria - ps29
Euarchontoglires - ps30
Euarchonta - ps31
Primates - ps32
Haplorrhini - ps33
Simiiformes - ps34
Catarrhini - ps35
Hominoidea - ps36
Hominidae - ps37
Homininae - ps38
Hominini - ps39
Homo_sapiens - ps40
Phylostratum

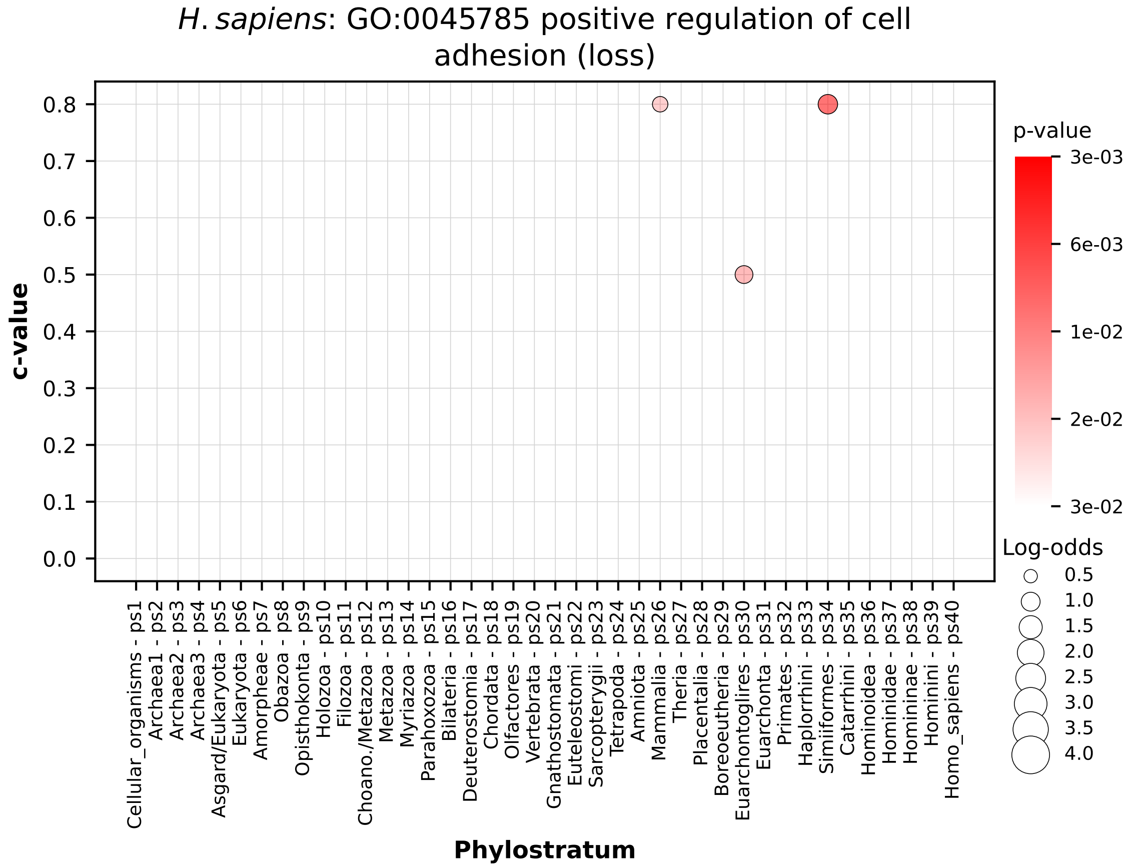

H. sapiens: GO:0045785 positive regulation of cell adhesion (loss)
c-value
0.8
0.7
0.6
0.5
0.4
0.3
0.2
0.1
0.0
p-value
3e-03
6e-03
1e-02
2e-02
3e-02
Log-odds
0.5
1.0
1.5
2.0
2.5
3.0
3.5
4.0
Cellular_organisms - ps1
Archaea1 - ps2
Archaea2 - ps3
Archaea3 - ps4
Asgard/Eukaryota - ps5
Eukaryota - ps6
Amorpheae - ps7
Obazoa - ps8
Opisthokonta - ps9
Holozoa - ps10
Filozoa - ps11
Choano./Metazoa - ps12
Metazoa - ps13
Myriazoa - ps14
Parahoxozoa - ps15
Bilateria - ps16
Deuterostomia - ps17
Chordata - ps18
Olfactores - ps19
Vertebrata - ps20
Gnathostomata - ps21
Euteleostomi - ps22
Sarcopterygii - ps23
Tetrapoda - ps24
Amniota - ps25
Mammalia - ps26
Theria - ps27
Placentalia - ps28
Boreoeutheria - ps29
Euarchontoglires - ps30
Euarchonta - ps31
Primates - ps32
Haplorrhini - ps33
Simiiformes - ps34
Catarrhini - ps35
Hominoidea - ps36
Hominidae - ps37
Homininae - ps38
Hominini - ps39
Homo_sapiens - ps40
Phylostratum

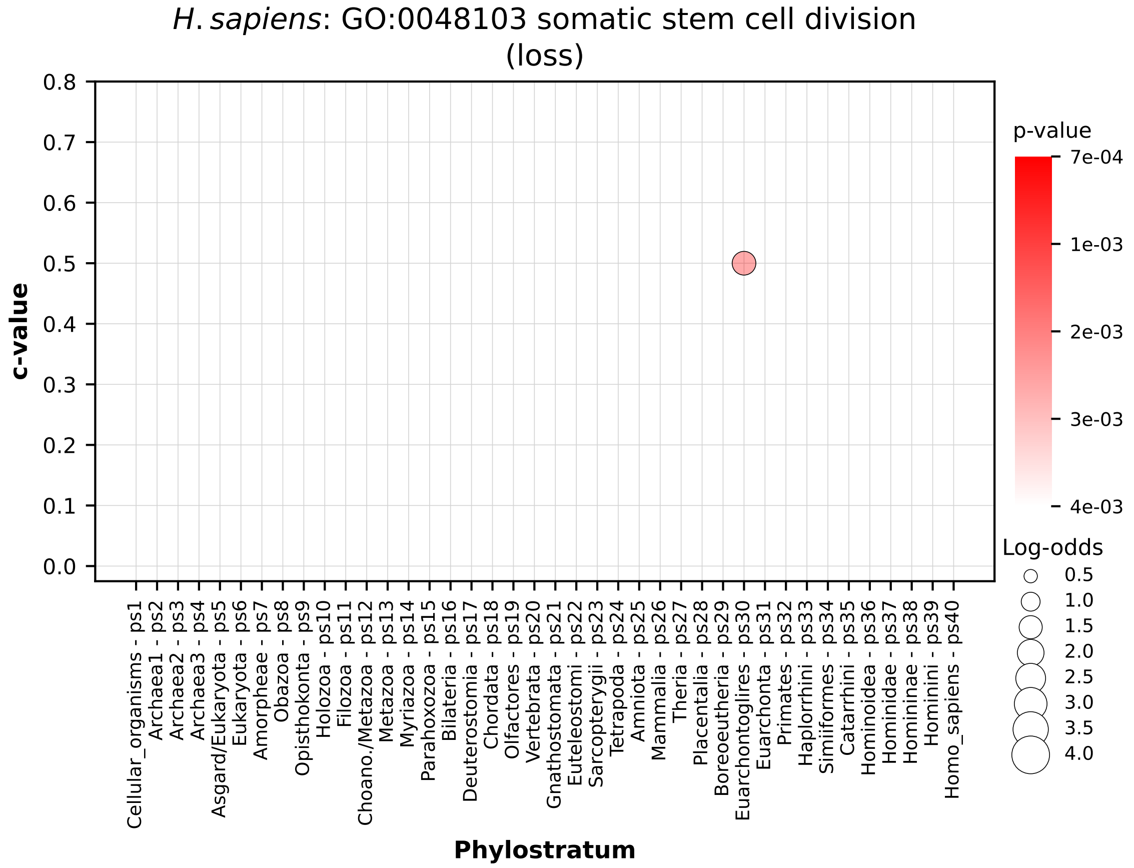

H. sapiens: GO:0048103 somatic stem cell division
(loss)
c-value
0.0
0.1
0.2
0.3
0.4
0.5
0.6
0.7
0.8
Phylostratum
Cellular_organisms - ps1
Archaea1 - ps2
Archaea2 - ps3
Archaea3 - ps4
Asgard/Eukaryota - ps5
Eukaryota - ps6
Amorpheae - ps7
Obazoa - ps8
Opisthokonta - ps9
Holozoa - ps10
Filozoa - ps11
Choano./Metazoa - ps12
Metazoa - ps13
Myriazoa - ps14
Parahoxozoa - ps15
Bilateria - ps16
Deuterostomia - ps17
Chordata - ps18
Olfactores - ps19
Vertebrata - ps20
Gnathostomata - ps21
Euteleostomi - ps22
Sarcopterygii - ps23
Tetrapoda - ps24
Amniota - ps25
Mammalia - ps26
Theria - ps27
Placentalia - ps28
Boreoeutheria - ps29
Euarchontoglires - ps30
Euarchonta - ps31
Primates - ps32
Haplorrhini - ps33
Simiiformes - ps34
Catarrhini - ps35
Hominoidea - ps36
Hominidae - ps37
Homininae - ps38
Hominini - ps39
Homo_sapiens - ps40
p-value
7e-04
1e-03
2e-03
3e-03
4e-03
Log-odds
0.5
1.0
1.5
2.0
2.5
3.0
3.5
4.0

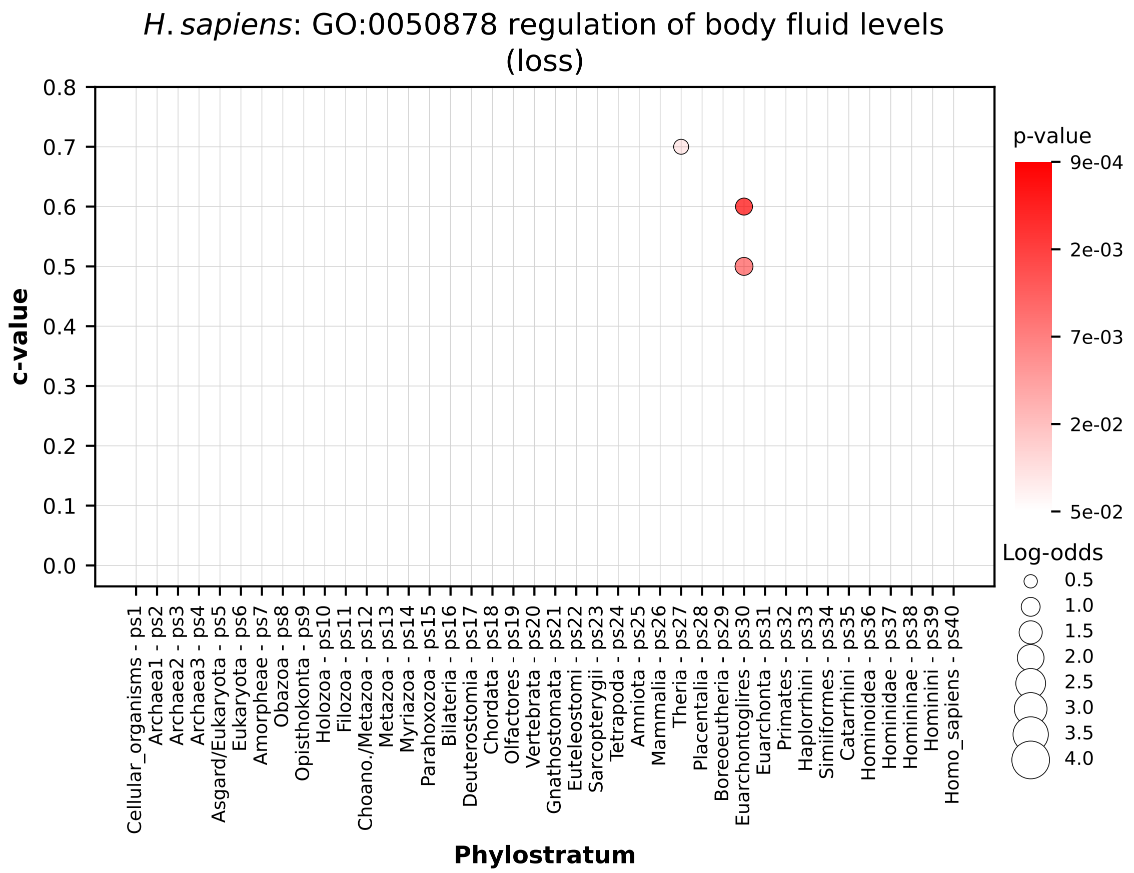

H. sapiens: GO:0050878 regulation of body fluid levels
(loss)
c-value
0.8
0.7
0.6
0.5
0.4
0.3
0.2
0.1
0.0
p-value
9e-04
2e-03
7e-03
2e-02
5e-02
Log-odds
0.5
1.0
1.5
2.0
2.5
3.0
3.5
4.0
Cellular_organisms - ps1
Archaea1 - ps2
Archaea2 - ps3
Archaea3 - ps4
Asgard/Eukaryota - ps5
Eukaryota - ps6
Amorpheae - ps7
Obazoa - ps8
Opisthokonta - ps9
Holozoa - ps10
Filozoa - ps11
Choano./Metazoa - ps12
Metazoa - ps13
Myriazoa - ps14
Parahoxozoa - ps15
Bilateria - ps16
Deuterostomia - ps17
Chordata - ps18
Olfactores - ps19
Vertebrata - ps20
Gnathostomata - ps21
Euteleostomi - ps22
Sarcopterygii - ps23
Tetrapoda - ps24
Amniota - ps25
Mammalia - ps26
Theria - ps27
Placentalia - ps28
Boreoeutheria - ps29
Euarchontoglires - ps30
Euarchonta - ps31
Primates - ps32
Haplorrhini - ps33
Simiiformes - ps34
Catarrhini - ps35
Hominoidea - ps36
Hominidae - ps37
Homininae - ps38
Hominini - ps39
Homo_sapiens - ps40
Phylostratum

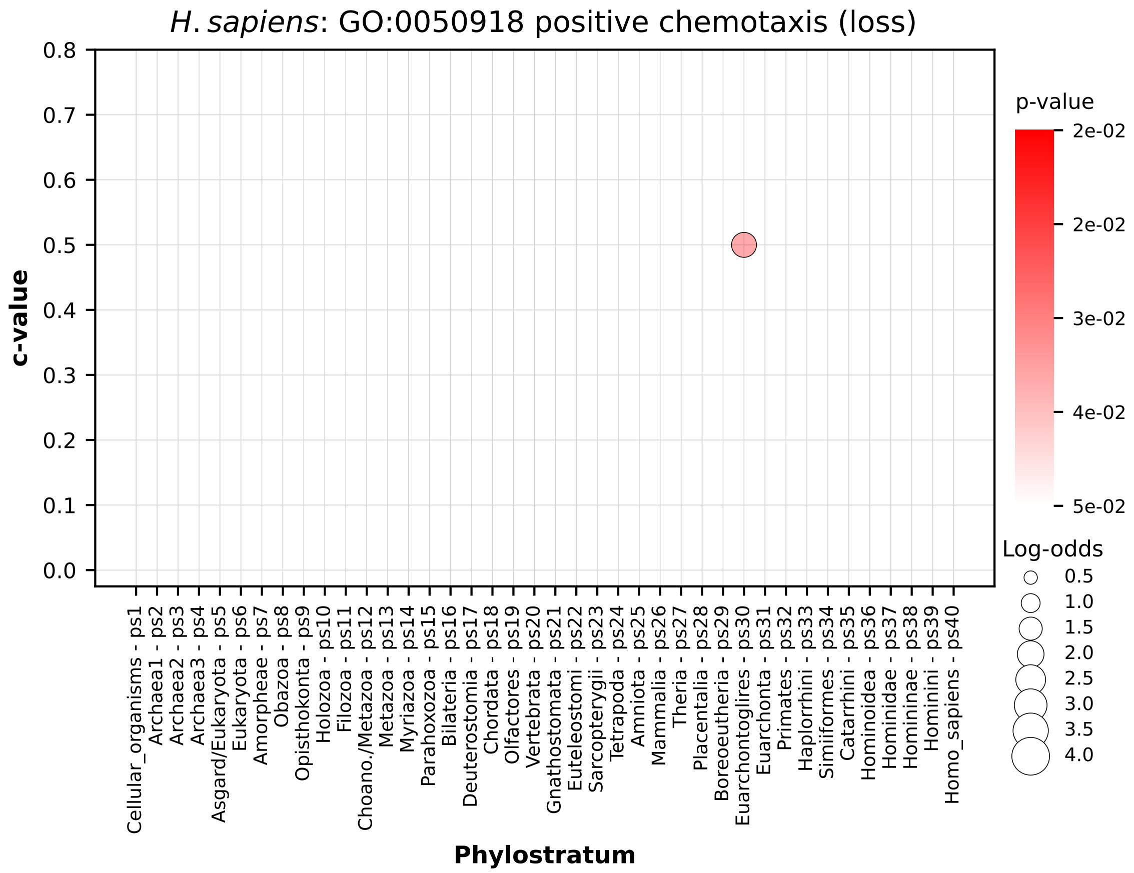

H. sapiens: GO:0050918 positive chemotaxis (loss)
c-value
0.8
0.7
0.6
0.5
0.4
0.3
0.2
0.1
0.0
Phylostratum
Cellular_organisms - ps1
Archaea1 - ps2
Archaea2 - ps3
Archaea3 - ps4
Asgard/Eukaryota - ps5
Eukaryota - ps6
Amorpheae - ps7
Obazoa - ps8
Opisthokonta - ps9
Holozoa - ps10
Filozoa - ps11
Choano./Metazoa - ps12
Metazoa - ps13
Myriazoa - ps14
Parahoxozoa - ps15
Bilateria - ps16
Deuterostomia - ps17
Chordata - ps18
Olfactores - ps19
Vertebrata - ps20
Gnathostomata - ps21
Euteleostomi - ps22
Sarcopterygii - ps23
Tetrapoda - ps24
Amniota - ps25
Mammalia - ps26
Theria - ps27
Placentalia - ps28
Boreoeutheria - ps29
Euarchontoglires - ps30
Euarchonta - ps31
Primates - ps32
Haplorrhini - ps33
Simiiformes - ps34
Catarrhini - ps35
Hominoidea - ps36
Hominidae - ps37
Homininae - ps38
Hominini - ps39
Homo_sapiens - ps40
p-value
2e-02
2e-02
3e-02
4e-02
5e-02
Log-odds
0.5
1.0
1.5
2.0
2.5
3.0
3.5
4.0

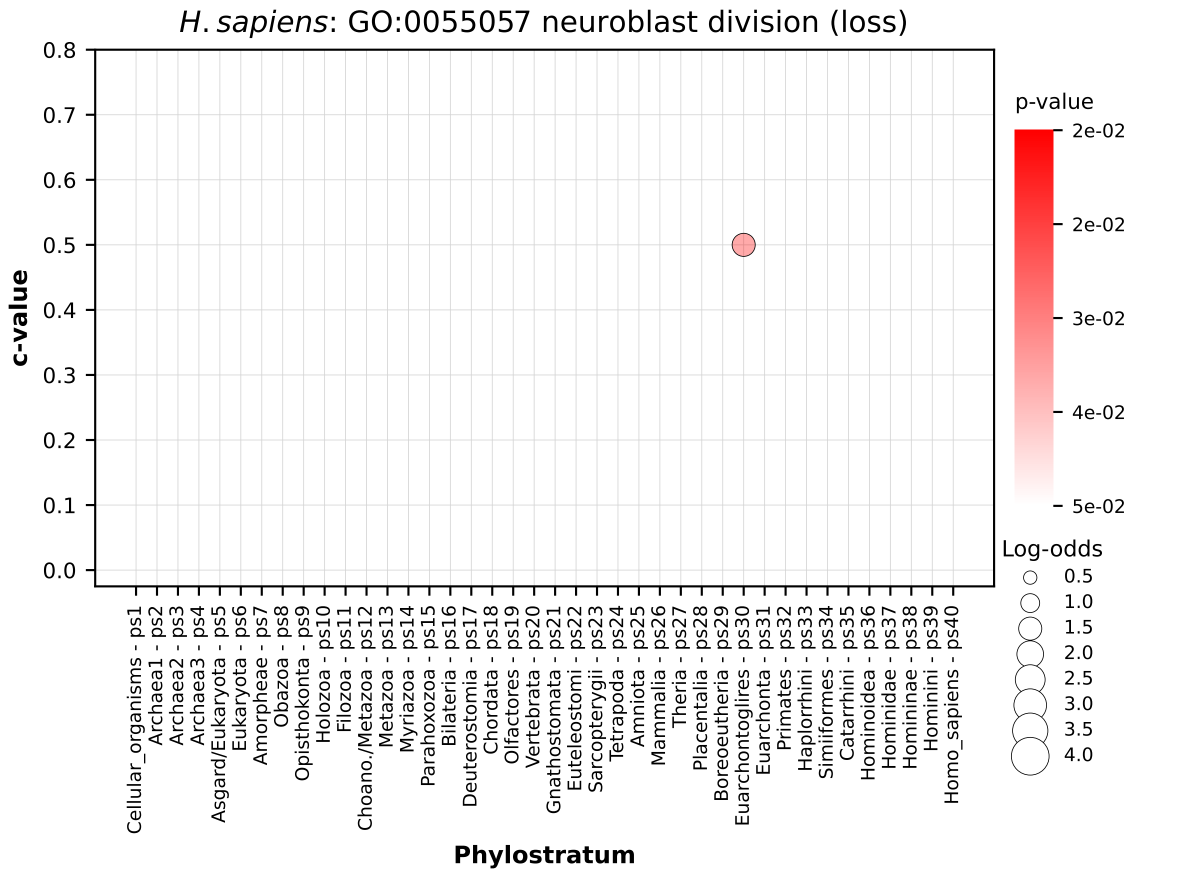

H. sapiens: GO:0055057 neuroblast division (loss)
c-value
0.0
0.1
0.2
0.3
0.4
0.5
0.6
0.7
0.8
Phylostratum
Cellular_organisms - ps1
Archaea1 - ps2
Archaea2 - ps3
Archaea3 - ps4
Asgard/Eukaryota - ps5
Eukaryota - ps6
Amorpheae - ps7
Obazoa - ps8
Opisthokonta - ps9
Holozoa - ps10
Filozoa - ps11
Choano./Metazoa - ps12
Metazoa - ps13
Myriazoa - ps14
Parahoxozoa - ps15
Bilateria - ps16
Deuterostomia - ps17
Chordata - ps18
Olfactores - ps19
Vertebrata - ps20
Gnathostomata - ps21
Euteleostomi - ps22
Sarcopterygii - ps23
Tetrapoda - ps24
Amniota - ps25
Mammalia - ps26
Theria - ps27
Placentalia - ps28
Boreoeutheria - ps29
Euarchontoglires - ps30
Euarchonta - ps31
Primates - ps32
Haplorrhini - ps33
Simiiformes - ps34
Catarrhini - ps35
Hominoidea - ps36
Hominidae - ps37
Homininae - ps38
Hominini - ps39
Homo_sapiens - ps40
p-value
2e-02
2e-02
3e-02
4e-02
5e-02
Log-odds
0.5
1.0
1.5
2.0
2.5
3.0
3.5
4.0

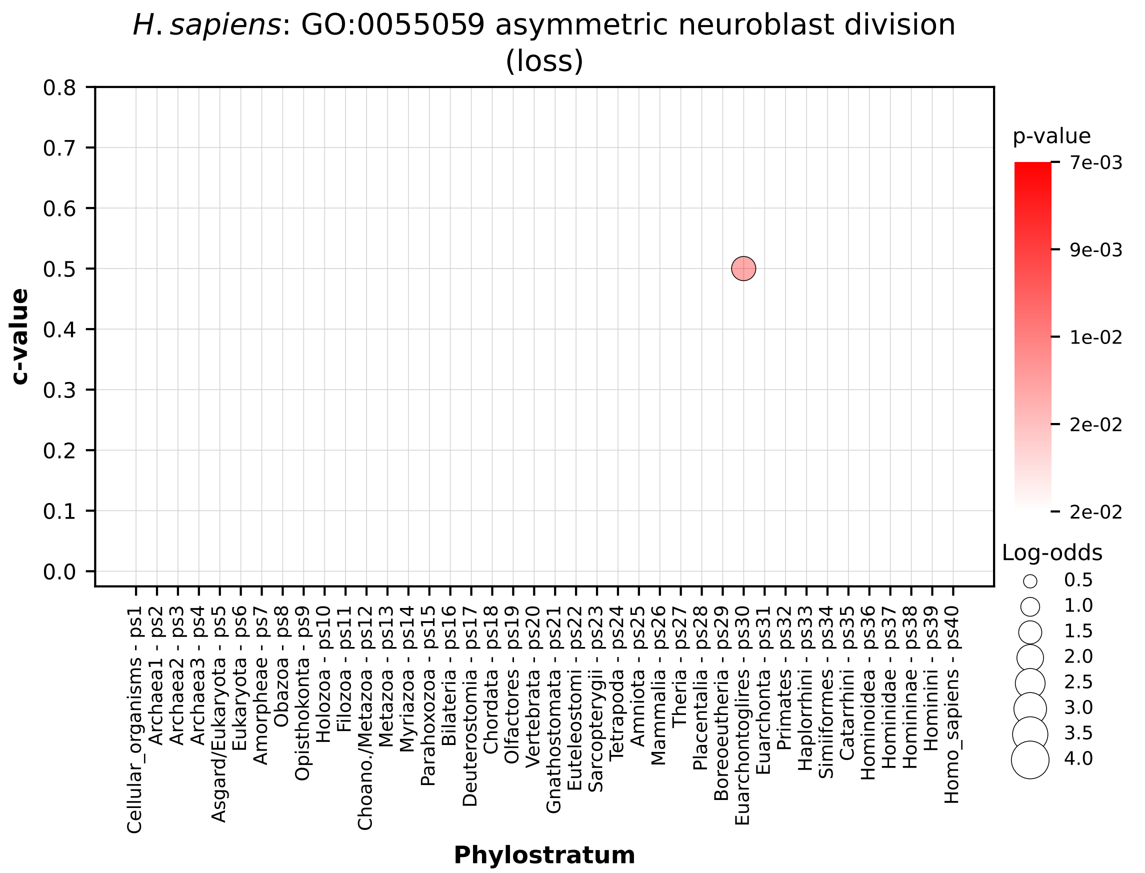

H. sapiens: GO:0055059 asymmetric neuroblast division (loss)
c-value
0.0
0.1
0.2
0.3
0.4
0.5
0.6
0.7
0.8
Phylostratum
Cellular_organisms - ps1
Archaea1 - ps2
Archaea2 - ps3
Archaea3 - ps4
Asgard/Eukaryota - ps5
Eukaryota - ps6
Amorpheae - ps7
Obazoa - ps8
Opisthokonta - ps9
Holozoa - ps10
Filozoa - ps11
Choano./Metazoa - ps12
Metazoa - ps13
Myriazoa - ps14
Parahoxozoa - ps15
Bilateria - ps16
Deuterostomia - ps17
Chordata - ps18
Olfactores - ps19
Vertebrata - ps20
Gnathostomata - ps21
Euteleostomi - ps22
Sarcopterygii - ps23
Tetrapoda - ps24
Amniota - ps25
Mammalia - ps26
Theria - ps27
Placentalia - ps28
Boreoeutheria - ps29
Euarchontoglires - ps30
Euarchonta - ps31
Primates - ps32
Haplorrhini - ps33
Simiiformes - ps34
Catarrhini - ps35
Hominoidea - ps36
Hominidae - ps37
Homininae - ps38
Hominini - ps39
Homo_sapiens - ps40
p-value
7e-03
9e-03
1e-02
2e-02
2e-02
Log-odds
0.5
1.0
1.5
2.0
2.5
3.0
3.5
4.0

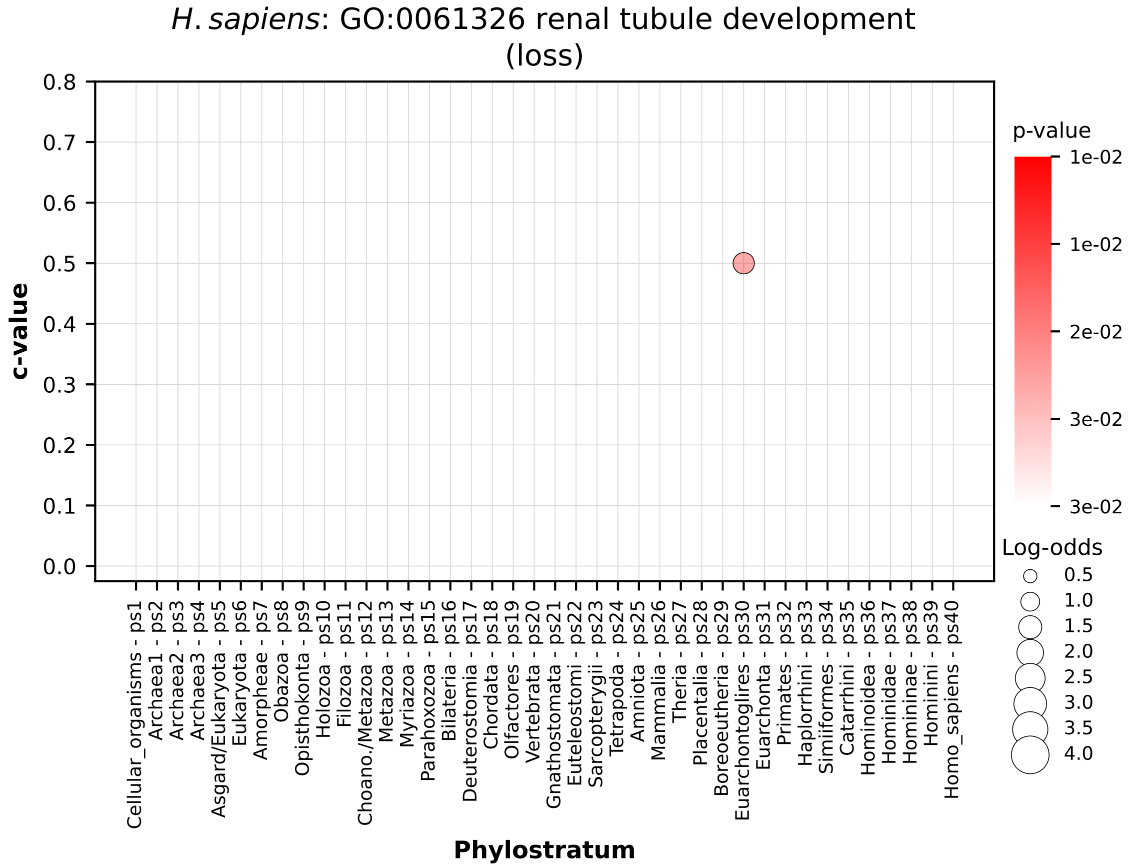
H. sapiens: GO:0061326 renal tubule development
(loss)
c-value
0.8
0.7
0.6
0.5
0.4
0.3
0.2
0.1
0.0
p-value
1e-02
1e-02
2e-02
3e-02
3e-02
Log-odds
0.5
1.0
1.5
2.0
2.5
3.0
3.5
4.0
Cellular_organisms - ps1
Archaea1 - ps2
Archaea2 - ps3
Archaea3 - ps4
Asgard/Eukaryota - ps5
Eukaryota - ps6
Amorpheae - ps7
Obazoa - ps8
Opisthokonta - ps9
Holozoa - ps10
Filozoa - ps11
Choano./Metazoa - ps12
Metazoa - ps13
Myriazoa - ps14
Parahoxozoa - ps15
Bilateria - ps16
Deuterostomia - ps17
Chordata - ps18
Olfactores - ps19
Vertebrata - ps20
Gnathostomata - ps21
Euteleostomi - ps22
Sarcopterygii - ps23
Tetrapoda - ps24
Amniota - ps25
Mammalia - ps26
Theria - ps27
Placentalia - ps28
Boreoeutheria - ps29
Euarchontoglires - ps30
Euarchonta - ps31
Primates - ps32
Haplorrhini - ps33
Simiiformes - ps34
Catarrhini - ps35
Hominoidea - ps36
Hominidae - ps37
Homininae - ps38
Hominini - ps39
Homo_sapiens - ps40
Phylostratum

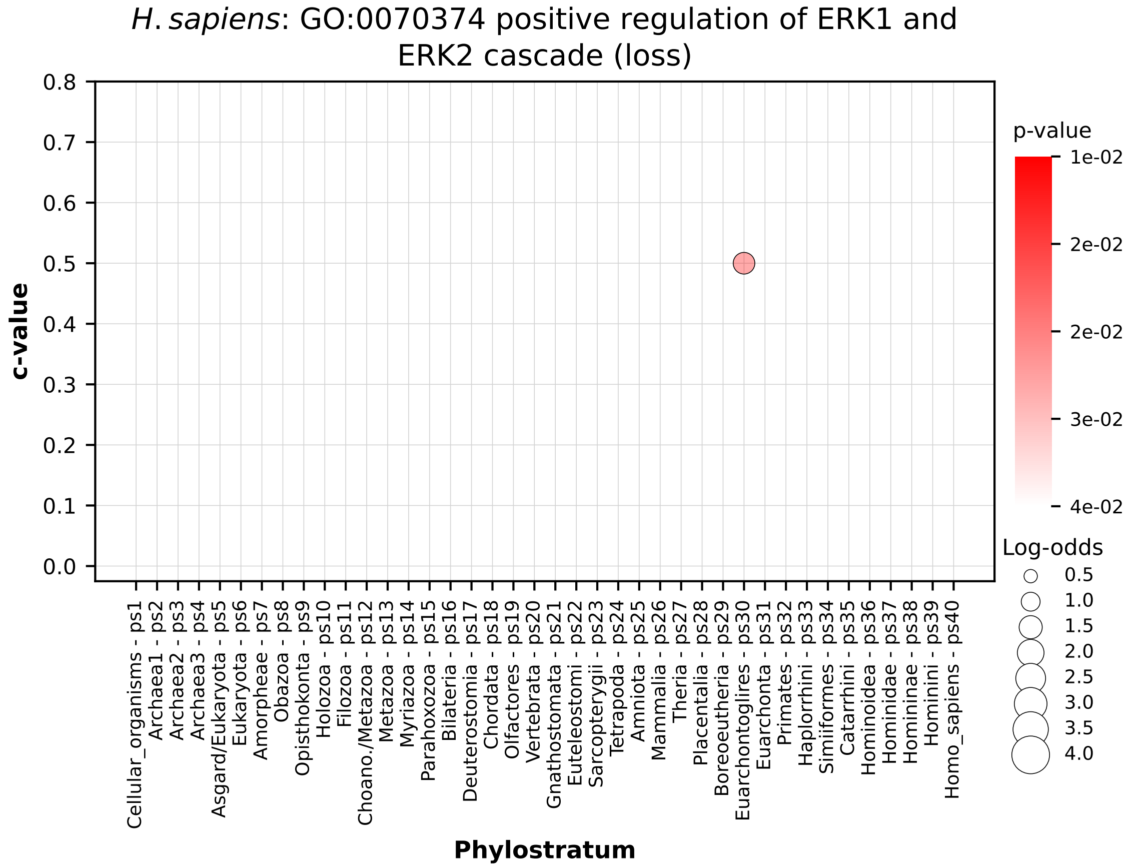

H. sapiens: GO:0070374 positive regulation of ERK1 and ERK2 cascade (loss)
c-value
0.0
0.1
0.2
0.3
0.4
0.5
0.6
0.7
0.8
Phylostratum
Cellular_organisms - ps1
Archaea1 - ps2
Archaea2 - ps3
Archaea3 - ps4
Asgard/Eukaryota - ps5
Eukaryota - ps6
Amorpheae - ps7
Obazoa - ps8
Opisthokonta - ps9
Holozoa - ps10
Filozoa - ps11
Choano./Metazoa - ps12
Metazoa - ps13
Myriazoa - ps14
Parahoxozoa - ps15
Bilateria - ps16
Deuterostomia - ps17
Chordata - ps18
Olfactores - ps19
Vertebrata - ps20
Gnathostomata - ps21
Euteleostomi - ps22
Sarcopterygii - ps23
Tetrapoda - ps24
Amniota - ps25
Mammalia - ps26
Theria - ps27
Placentalia - ps28
Boreoeutheria - ps29
Euarchontoglires - ps30
Euarchonta - ps31
Primates - ps32
Haplorrhini - ps33
Simiiformes - ps34
Catarrhini - ps35
Hominoidea - ps36
Hominidae - ps37
Homininae - ps38
Hominini - ps39
Homo_sapiens - ps40
p-value
1e-02
2e-02
2e-02
3e-02
4e-02
Log-odds
0.5
1.0
1.5
2.0
2.5
3.0
3.5
4.0

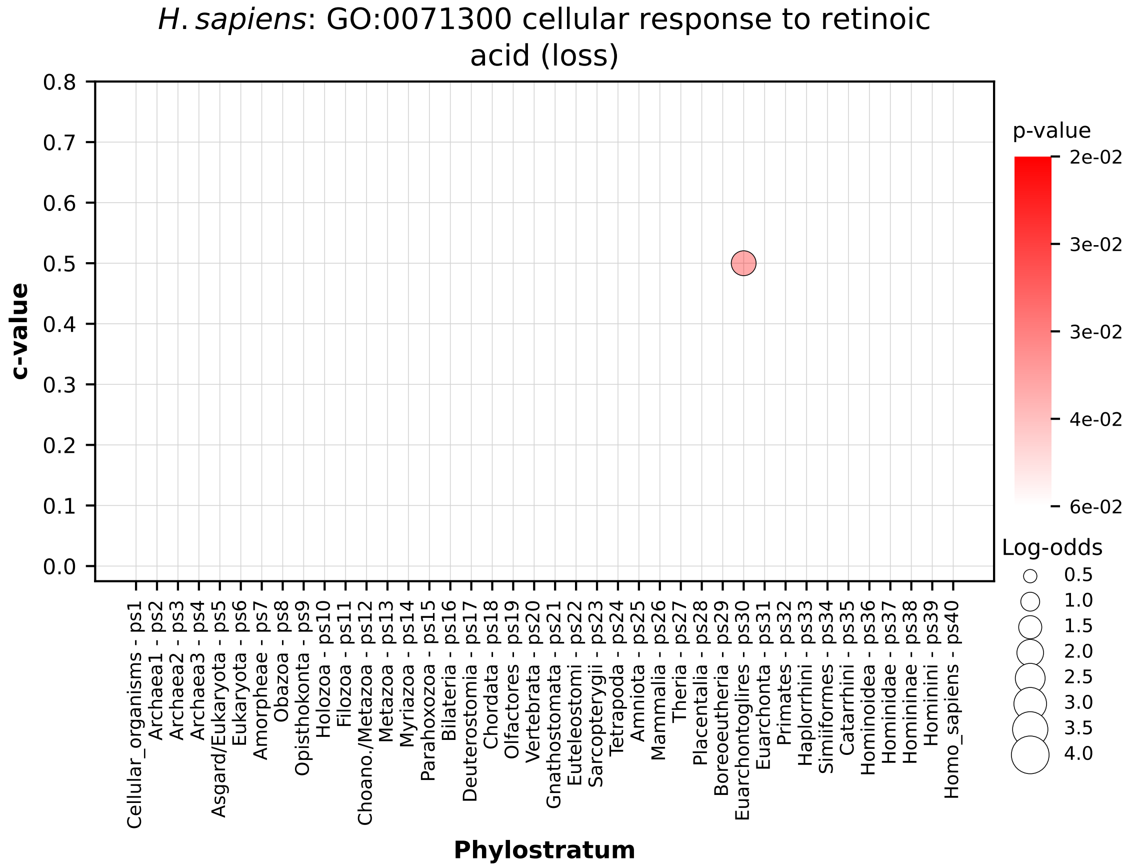

H. sapiens: GO:0071300 cellular response to retinoic acid (loss)
c-value
0.8
0.7
0.6
0.5
0.4
0.3
0.2
0.1
0.0
p-value
2e-02
3e-02
3e-02
4e-02
6e-02
Log-odds
0.5
1.0
1.5
2.0
2.5
3.0
3.5
4.0
Cellular_organisms - ps1
Archaea1 - ps2
Archaea2 - ps3
Archaea3 - ps4
Asgard/Eukaryota - ps5
Eukaryota - ps6
Amorpheae - ps7
Obazoa - ps8
Opisthokonta - ps9
Holozoa - ps10
Filozoa - ps11
Choano./Metazoa - ps12
Metazoa - ps13
Myriazoa - ps14
Parahoxozoa - ps15
Bilateria - ps16
Deuterostomia - ps17
Chordata - ps18
Olfactores - ps19
Vertebrata - ps20
Gnathostomata - ps21
Euteleostomi - ps22
Sarcopterygii - ps23
Tetrapoda - ps24
Amniota - ps25
Mammalia - ps26
Theria - ps27
Placentalia - ps28
Boreoeutheria - ps29
Euarchontoglires - ps30
Euarchonta - ps31
Primates - ps32
Haplorrhini - ps33
Simiiformes - ps34
Catarrhini - ps35
Hominoidea - ps36
Hominidae - ps37
Homininae - ps38
Hominini - ps39
Homo_sapiens - ps40
Phylostratum

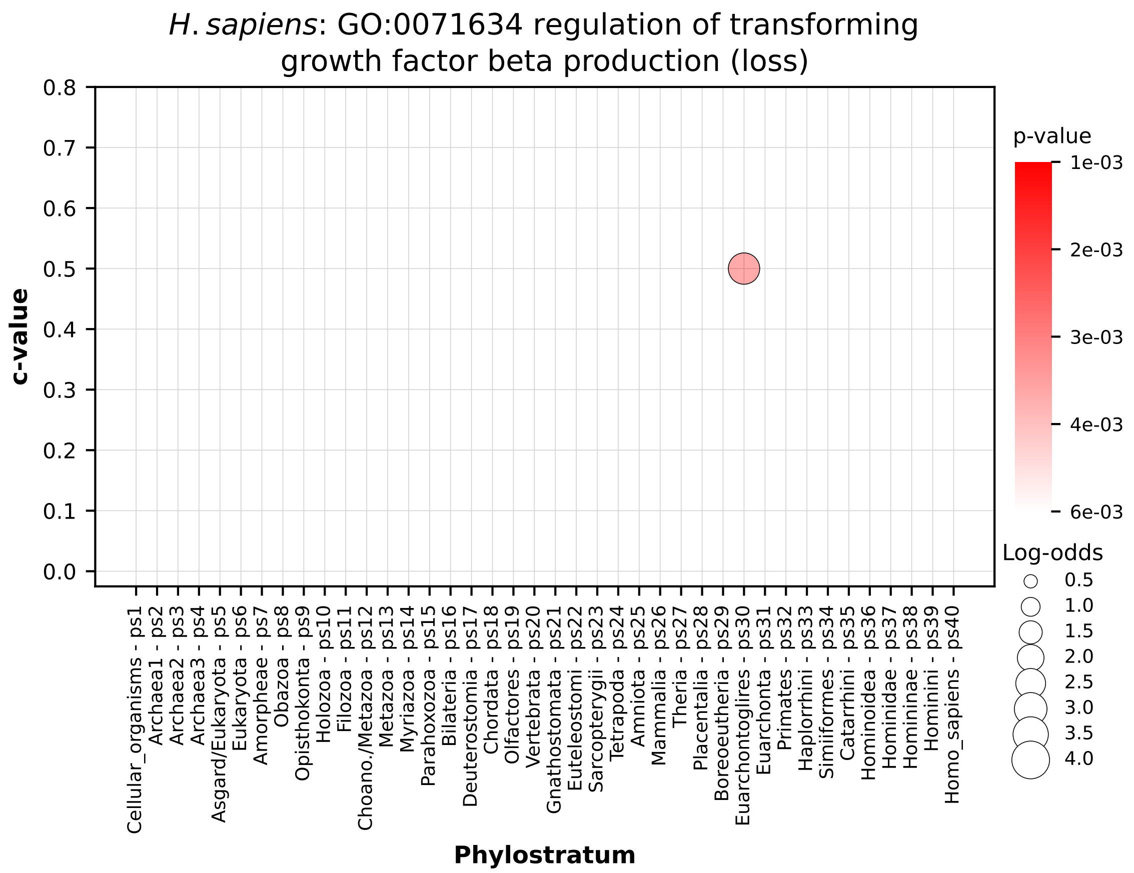
H. sapiens: GO:0071634 regulation of transforming growth factor beta production (loss)
c-value
0.8
0.7
0.6
0.5
0.4
0.3
0.2
0.1
0.0
Phylostratum
Cellular_organisms - ps1
Archaea1 - ps2
Archaea2 - ps3
Archaea3 - ps4
Asgard/Eukaryota - ps5
Eukaryota - ps6
Amorpheae - ps7
Obazoa - ps8
Opisthokonta - ps9
Holozoa - ps10
Filozoa - ps11
Choano./Metazoa - ps12
Metazoa - ps13
Myriazoa - ps14
Parahoxozoa - ps15
Bilateria - ps16
Deuterostomia - ps17
Chordata - ps18
Olfactores - ps19
Vertebrata - ps20
Gnathostomata - ps21
Euteleostomi - ps22
Sarcopterygii - ps23
Tetrapoda - ps24
Amniota - ps25
Mammalia - ps26
Theria - ps27
Placentalia - ps28
Boreoeutheria - ps29
Euarchontoglires - ps30
Euarchonta - ps31
Primates - ps32
Haplorrhini - ps33
Simiiformes - ps34
Catarrhini - ps35
Hominoidea - ps36
Hominidae - ps37
Homininae - ps38
Hominini - ps39
Homo_sapiens - ps40
p-value
1e-03
2e-03
3e-03
4e-03
6e-03
Log-odds
0.5
1.0
1.5
2.0
2.5
3.0
3.5
4.0

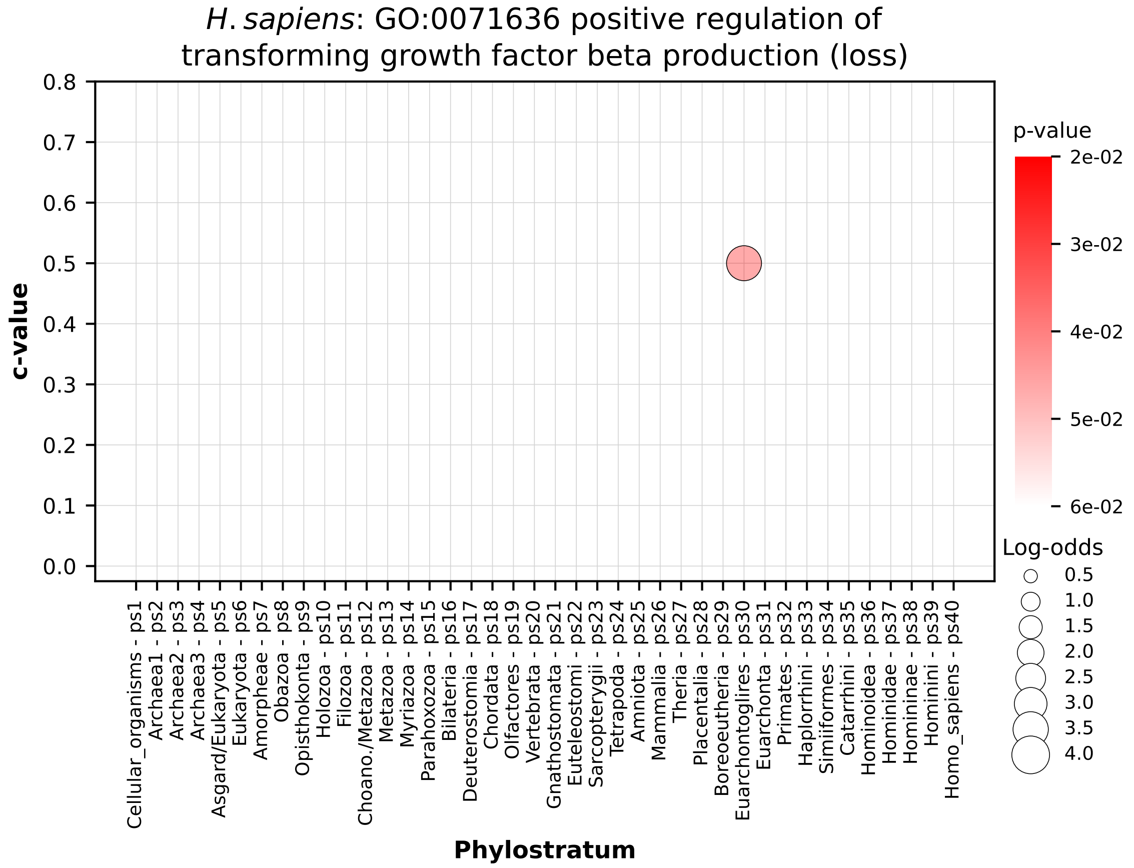

H. sapiens: GO:0071636 positive regulation of transforming growth factor beta production (loss)
c-value
0.8
0.7
0.6
0.5
0.4
0.3
0.2
0.1
0.0
p-value
2e-02
3e-02
4e-02
5e-02
6e-02
Log-odds
0.5
1.0
1.5
2.0
2.5
3.0
3.5
4.0
Cellular_organisms - ps1
Archaea1 - ps2
Archaea2 - ps3
Archaea3 - ps4
Asgard/Eukaryota - ps5
Eukaryota - ps6
Amorpheae - ps7
Obazoa - ps8
Opisthokonta - ps9
Holozoa - ps10
Filozoa - ps11
Choano./Metazoa - ps12
Metazoa - ps13
Myriazoa - ps14
Parahoxozoa - ps15
Bilateria - ps16
Deuterostomia - ps17
Chordata - ps18
Olfactores - ps19
Vertebrata - ps20
Gnathostomata - ps21
Euteleostomi - ps22
Sarcopterygii - ps23
Tetrapoda - ps24
Amniota - ps25
Mammalia - ps26
Theria - ps27
Placentalia - ps28
Boreoeutheria - ps29
Euarchontoglires - ps30
Euarchonta - ps31
Primates - ps32
Haplorrhini - ps33
Simiiformes - ps34
Catarrhini - ps35
Hominoidea - ps36
Hominidae - ps37
Homininae - ps38
Hominini - ps39
Homo_sapiens - ps40
Phylostratum

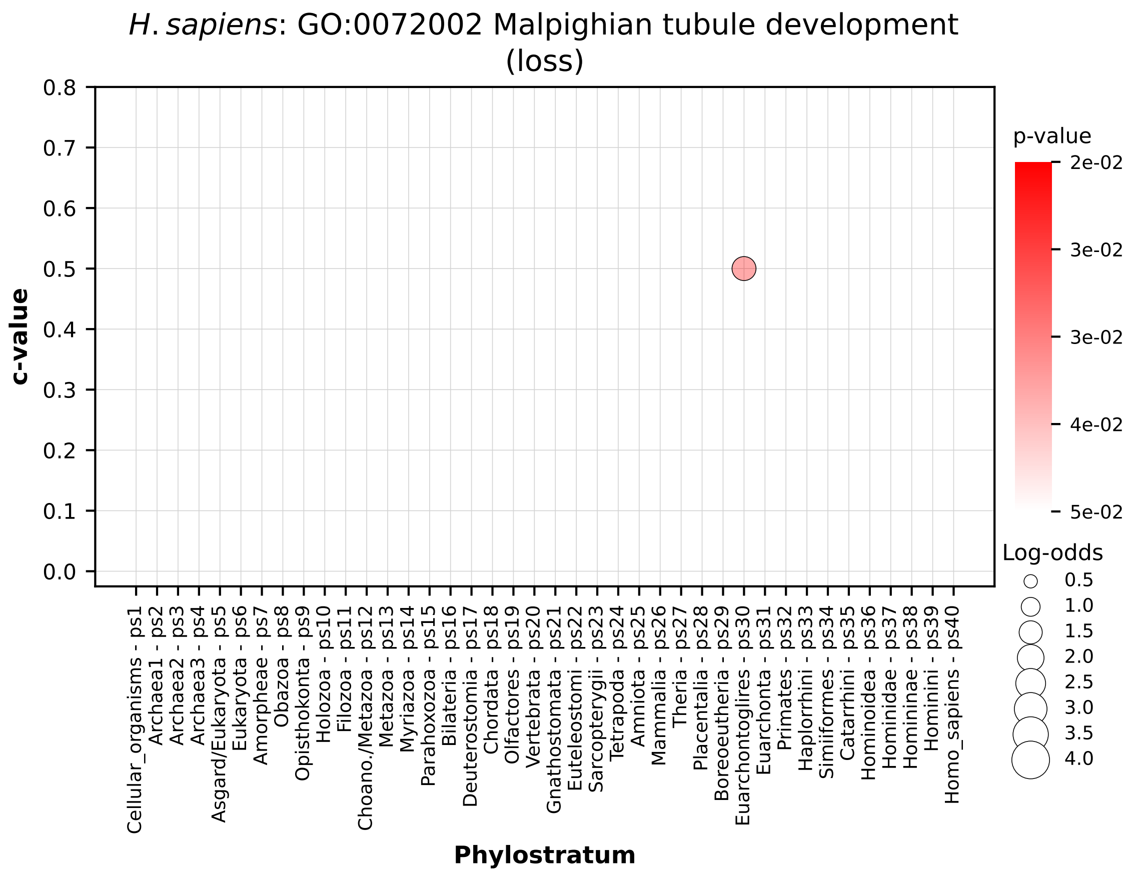

H. sapiens: GO:0072002 Malpighian tubule development (loss)
c-value
0.8
0.7
0.6
0.5
0.4
0.3
0.2
0.1
0.0
p-value
2e-02
3e-02
3e-02
4e-02
5e-02
Log-odds
0.5
1.0
1.5
2.0
2.5
3.0
3.5
4.0
Cellular_organisms - ps1
Archaea1 - ps2
Archaea2 - ps3
Archaea3 - ps4
Asgard/Eukaryota - ps5
Eukaryota - ps6
Amorpheae - ps7
Obazoa - ps8
Opisthokonta - ps9
Holozoa - ps10
Filozoa - ps11
Choano./Metazoa - ps12
Metazoa - ps13
Myriazoa - ps14
Parahoxozoa - ps15
Bilateria - ps16
Deuterostomia - ps17
Chordata - ps18
Olfactores - ps19
Vertebrata - ps20
Gnathostomata - ps21
Euteleostomi - ps22
Sarcopterygii - ps23
Tetrapoda - ps24
Amniota - ps25
Mammalia - ps26
Theria - ps27
Placentalia - ps28
Boreoeutheria - ps29
Euarchontoglires - ps30
Euarchonta - ps31
Primates - ps32
Haplorrhini - ps33
Simiiformes - ps34
Catarrhini - ps35
Hominoidea - ps36
Hominidae - ps37
Homininae - ps38
Hominini - ps39
Homo_sapiens - ps40
Phylostratum

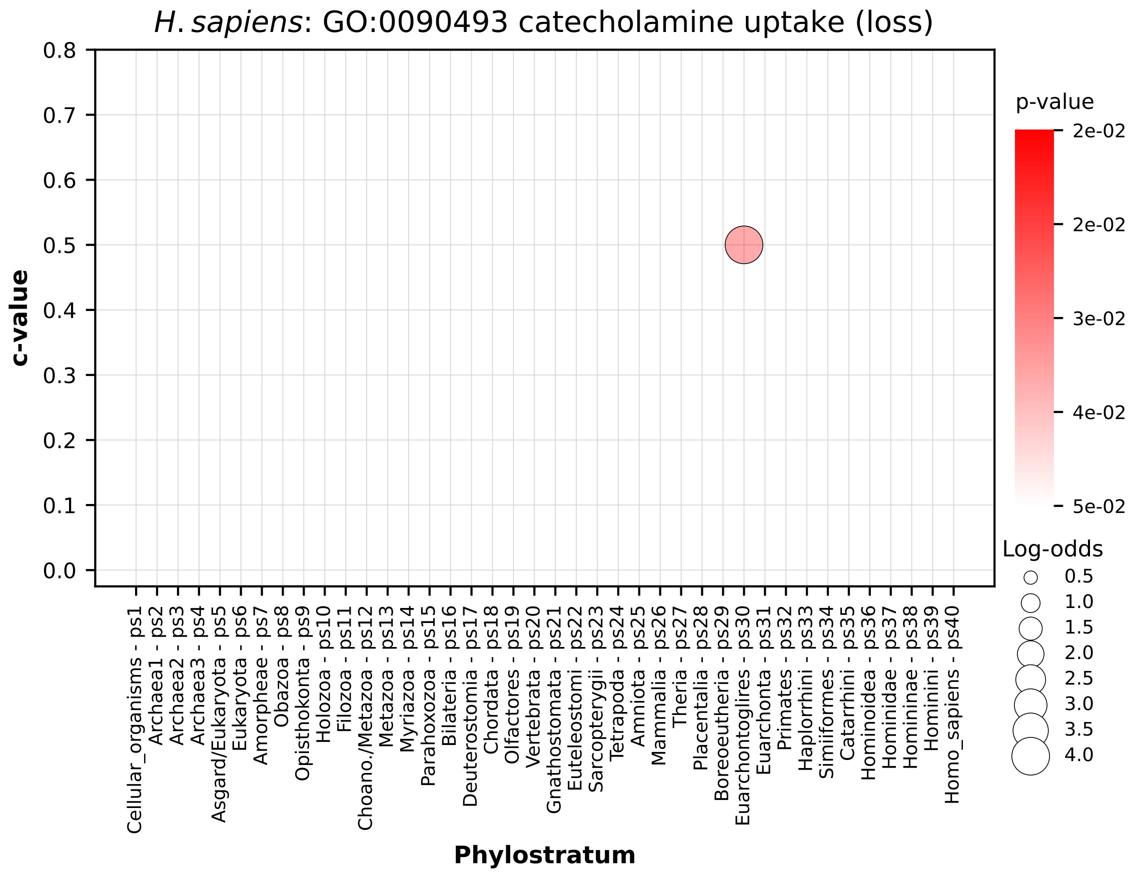

H. sapiens: GO:0090493 catecholamine uptake (loss)
c-value
0.8
0.7
0.6
0.5
0.4
0.3
0.2
0.1
0.0
Phylostratum
Cellular_organisms - ps1
Archaea1 - ps2
Archaea2 - ps3
Archaea3 - ps4
Asgard/Eukaryota - ps5
Eukaryota - ps6
Amorpheae - ps7
Obazoa - ps8
Opisthokonta - ps9
Holozoa - ps10
Filozoa - ps11
Choano./Metazoa - ps12
Metazoa - ps13
Myriazoa - ps14
Parahoxozoa - ps15
Bilateria - ps16
Deuterostomia - ps17
Chordata - ps18
Olfactores - ps19
Vertebrata - ps20
Gnathostomata - ps21
Euteleostomi - ps22
Sarcopterygii - ps23
Tetrapoda - ps24
Amniota - ps25
Mammalia - ps26
Theria - ps27
Placentalia - ps28
Boreoeutheria - ps29
Euarchontoglires - ps30
Euarchonta - ps31
Primates - ps32
Haplorrhini - ps33
Simiiformes - ps34
Catarrhini - ps35
Hominoidea - ps36
Hominidae - ps37
Homininae - ps38
Hominini - ps39
Homo_sapiens - ps40
p-value
2e-02
2e-02
3e-02
4e-02
5e-02
Log-odds
0.5
1.0
1.5
2.0
2.5
3.0
3.5
4.0

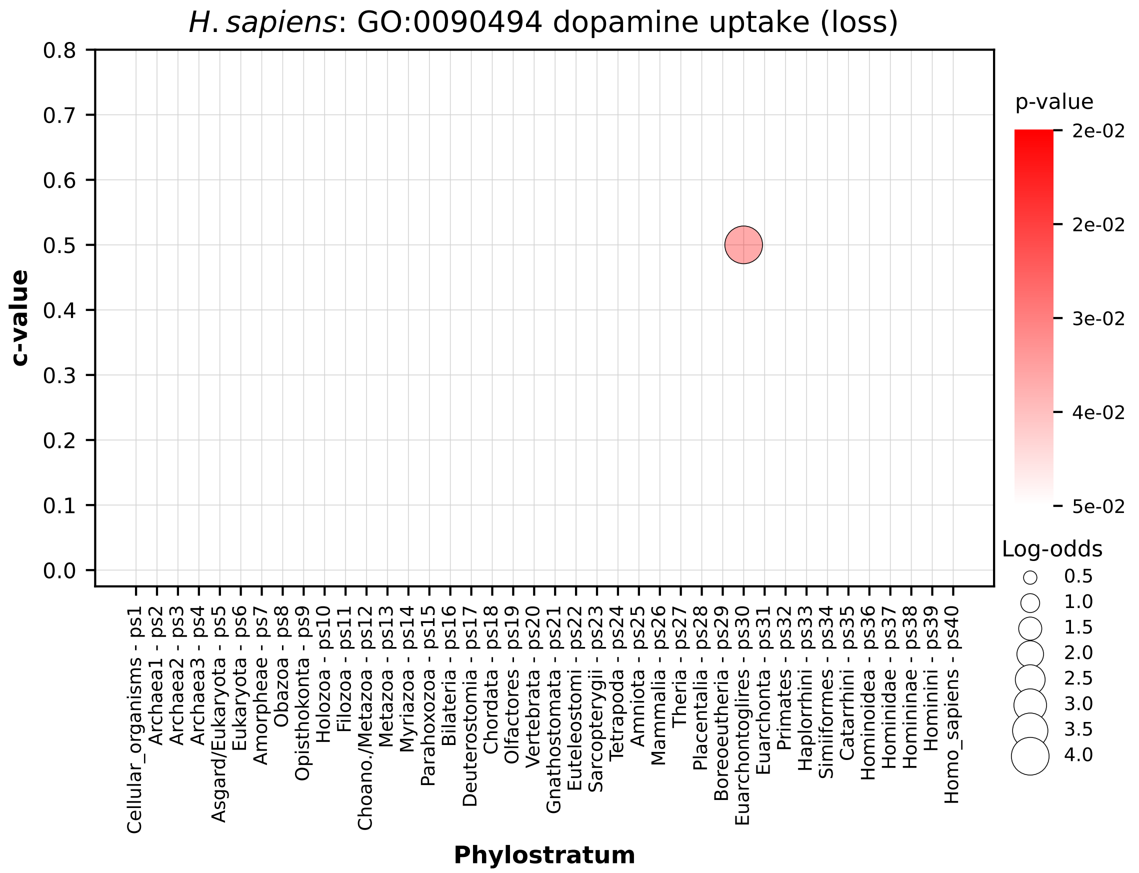

H. sapiens: GO:0090494 dopamine uptake (loss)
c-value
0.0
0.1
0.2
0.3
0.4
0.5
0.6
0.7
0.8
Phylostratum
Cellular_organisms - ps1
Archaea1 - ps2
Archaea2 - ps3
Archaea3 - ps4
Asgard/Eukaryota - ps5
Eukaryota - ps6
Amorpheae - ps7
Obazoa - ps8
Opisthokonta - ps9
Holozoa - ps10
Filozoa - ps11
Choano./Metazoa - ps12
Metazoa - ps13
Myriazoa - ps14
Parahoxozoa - ps15
Bilateria - ps16
Deuterostomia - ps17
Chordata - ps18
Olfactores - ps19
Vertebrata - ps20
Gnathostomata - ps21
Euteleostomi - ps22
Sarcopterygii - ps23
Tetrapoda - ps24
Amniota - ps25
Mammalia - ps26
Theria - ps27
Placentalia - ps28
Boreoeutheria - ps29
Euarchontoglires - ps30
Euarchonta - ps31
Primates - ps32
Haplorrhini - ps33
Simiiformes - ps34
Catarrhini - ps35
Hominoidea - ps36
Hominidae - ps37
Homininae - ps38
Hominini - ps39
Homo_sapiens - ps40
p-value
2e-02
2e-02
3e-02
4e-02
5e-02
Log-odds
0.5
1.0
1.5
2.0
2.5
3.0
3.5
4.0

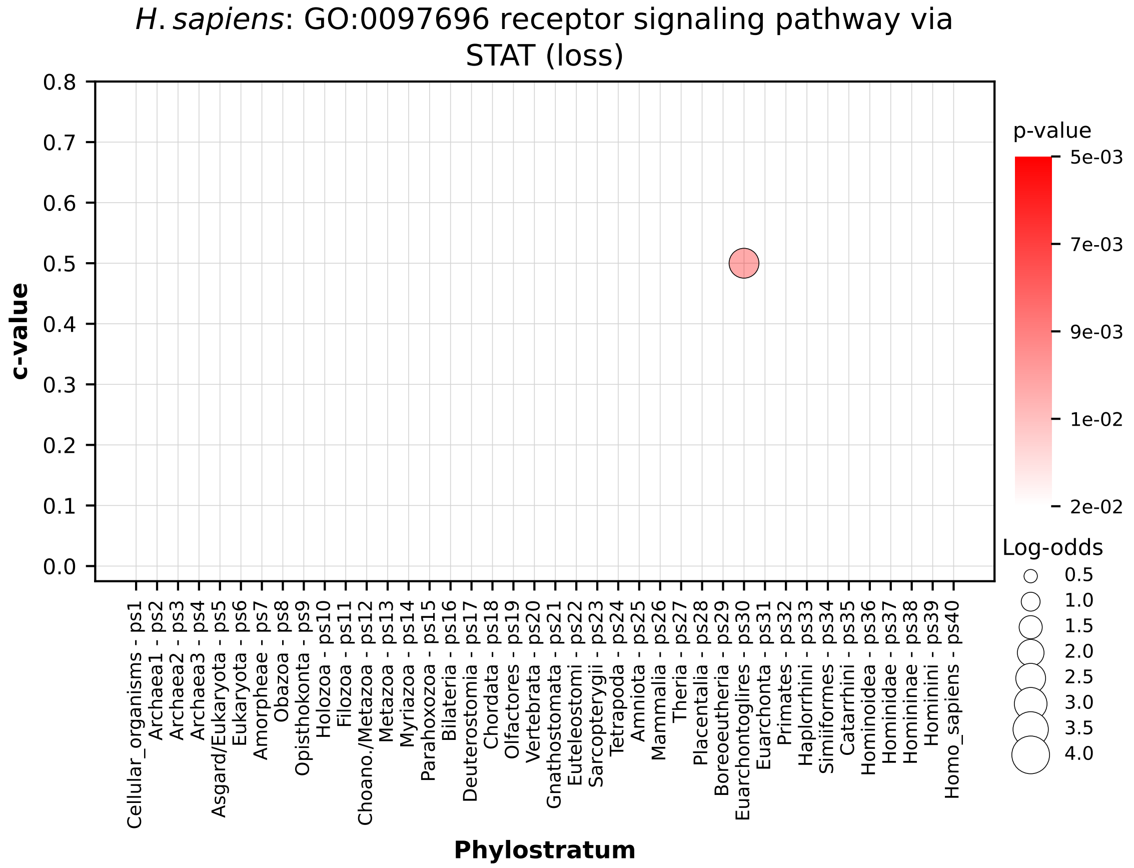

H. sapiens: GO:0097696 receptor signaling pathway via STAT (loss)
c-value
0.0
0.1
0.2
0.3
0.4
0.5
0.6
0.7
0.8
Phylostratum
Cellular_organisms - ps1
Archaea1 - ps2
Archaea2 - ps3
Archaea3 - ps4
Asgard/Eukaryota - ps5
Eukaryota - ps6
Amorpheae - ps7
Obazoa - ps8
Opisthokonta - ps9
Holozoa - ps10
Filozoa - ps11
Choano./Metazoa - ps12
Metazoa - ps13
Myriazoa - ps14
Parahoxozoa - ps15
Bilateria - ps16
Deuterostomia - ps17
Chordata - ps18
Olfactores - ps19
Vertebrata - ps20
Gnathostomata - ps21
Euteleostomi - ps22
Sarcopterygii - ps23
Tetrapoda - ps24
Amniota - ps25
Mammalia - ps26
Theria - ps27
Placentalia - ps28
Boreoeutheria - ps29
Euarchontoglires - ps30
Euarchonta - ps31
Primates - ps32
Haplorrhini - ps33
Simiiformes - ps34
Catarrhini - ps35
Hominoidea - ps36
Hominidae - ps37
Homininae - ps38
Hominini - ps39
Homo_sapiens - ps40
p-value
5e-03
7e-03
9e-03
1e-02
2e-02
Log-odds
0.5
1.0
1.5
2.0
2.5
3.0
3.5
4.0

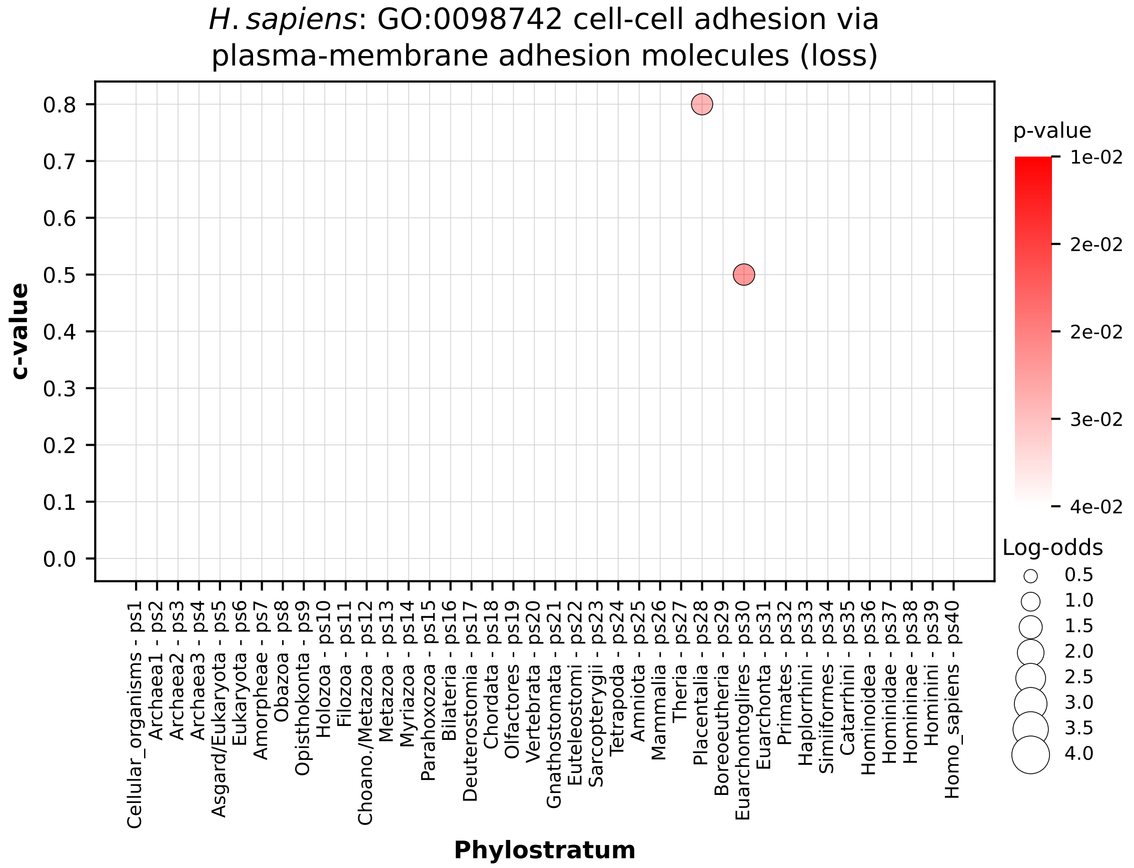

H. sapiens: GO:0098742 cell-cell adhesion via plasma-membrane adhesion molecules (loss)
c-value
0.0
0.1
0.2
0.3
0.4
0.5
0.6
0.7
0.8
Phylostratum
Cellular_organisms - ps1
Archaea1 - ps2
Archaea2 - ps3
Archaea3 - ps4
Asgard/Eukaryota - ps5
Eukaryota - ps6
Amorpheae - ps7
Obazoa - ps8
Opisthokonta - ps9
Holozoa - ps10
Filozoa - ps11
Choano./Metazoa - ps12
Metazoa - ps13
Myriazoa - ps14
Parahoxozoa - ps15
Bilateria - ps16
Deuterostomia - ps17
Chordata - ps18
Olfactores - ps19
Vertebrata - ps20
Gnathostomata - ps21
Euteleostomi - ps22
Sarcopterygii - ps23
Tetrapoda - ps24
Amniota - ps25
Mammalia - ps26
Theria - ps27
Placentalia - ps28
Boreoeutheria - ps29
Euarchontoglires - ps30
Euarchonta - ps31
Primates - ps32
Haplorrhini - ps33
Simiiformes - ps34
Catarrhini - ps35
Hominoidea - ps36
Hominidae - ps37
Homininae - ps38
Hominini - ps39
Homo_sapiens - ps40
p-value
1e-02
2e-02
2e-02
3e-02
4e-02
Log-odds
0.5
1.0
1.5
2.0
2.5
3.0
3.5
4.0

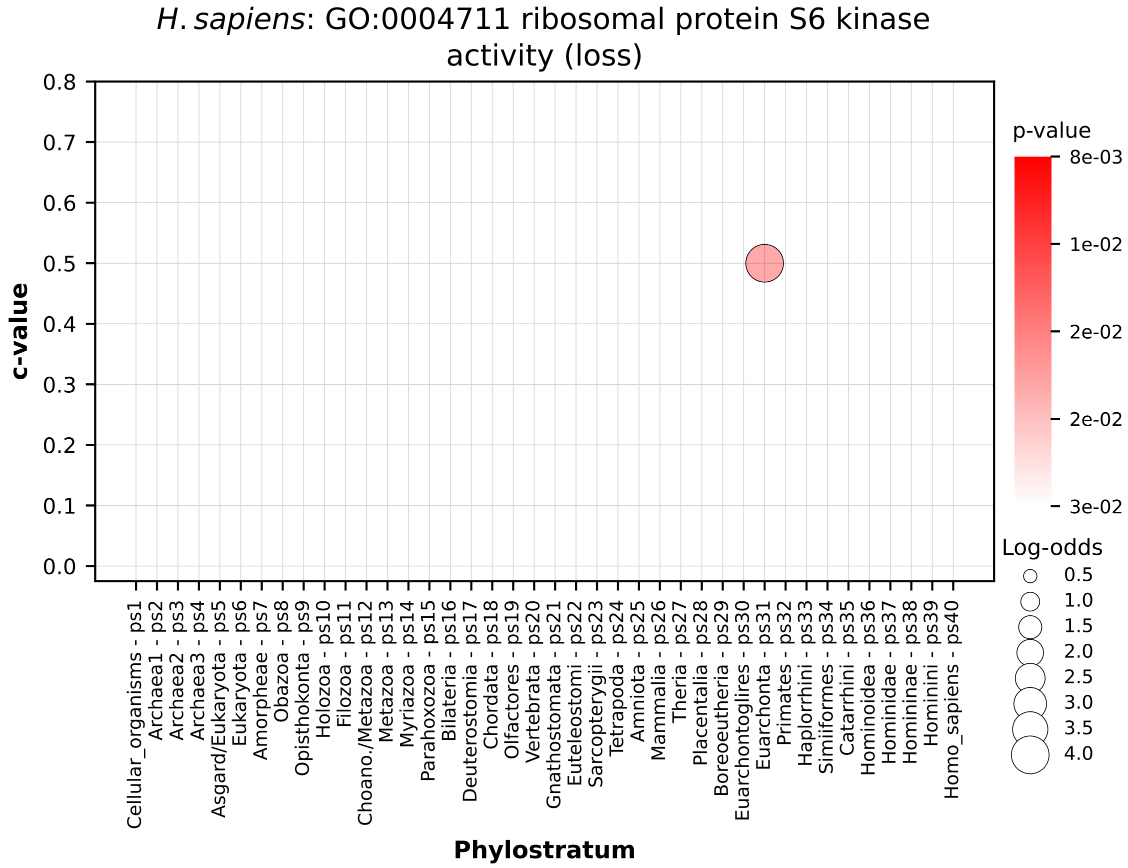

H. sapiens: GO:0004711 ribosomal protein S6 kinase activity (loss)
c-value
0.8
0.7
0.6
0.5
0.4
0.3
0.2
0.1
0.0
Phylostratum
Cellular_organisms - ps1
Archaea1 - ps2
Archaea2 - ps3
Archaea3 - ps4
Asgard/Eukaryota - ps5
Eukaryota - ps6
Amorpheae - ps7
Obazoa - ps8
Opisthokonta - ps9
Holozoa - ps10
Filozoa - ps11
Choano./Metazoa - ps12
Metazoa - ps13
Myriazoa - ps14
Parahoxozoa - ps15
Bilateria - ps16
Deuterostomia - ps17
Chordata - ps18
Olfactores - ps19
Vertebrata - ps20
Gnathostomata - ps21
Euteleostomi - ps22
Sarcopterygii - ps23
Tetrapoda - ps24
Amniota - ps25
Mammalia - ps26
Theria - ps27
Placentalia - ps28
Boreoeutheria - ps29
Euarchontoglires - ps30
Euarchonta - ps31
Primates - ps32
Haplorrhini - ps33
Simiiformes - ps34
Catarrhini - ps35
Hominoidea - ps36
Hominidae - ps37
Homininae - ps38
Hominini - ps39
Homo_sapiens - ps40
p-value
8e-03
1e-02
2e-02
2e-02
3e-02
Log-odds
0.5
1.0
1.5
2.0
2.5
3.0
3.5
4.0

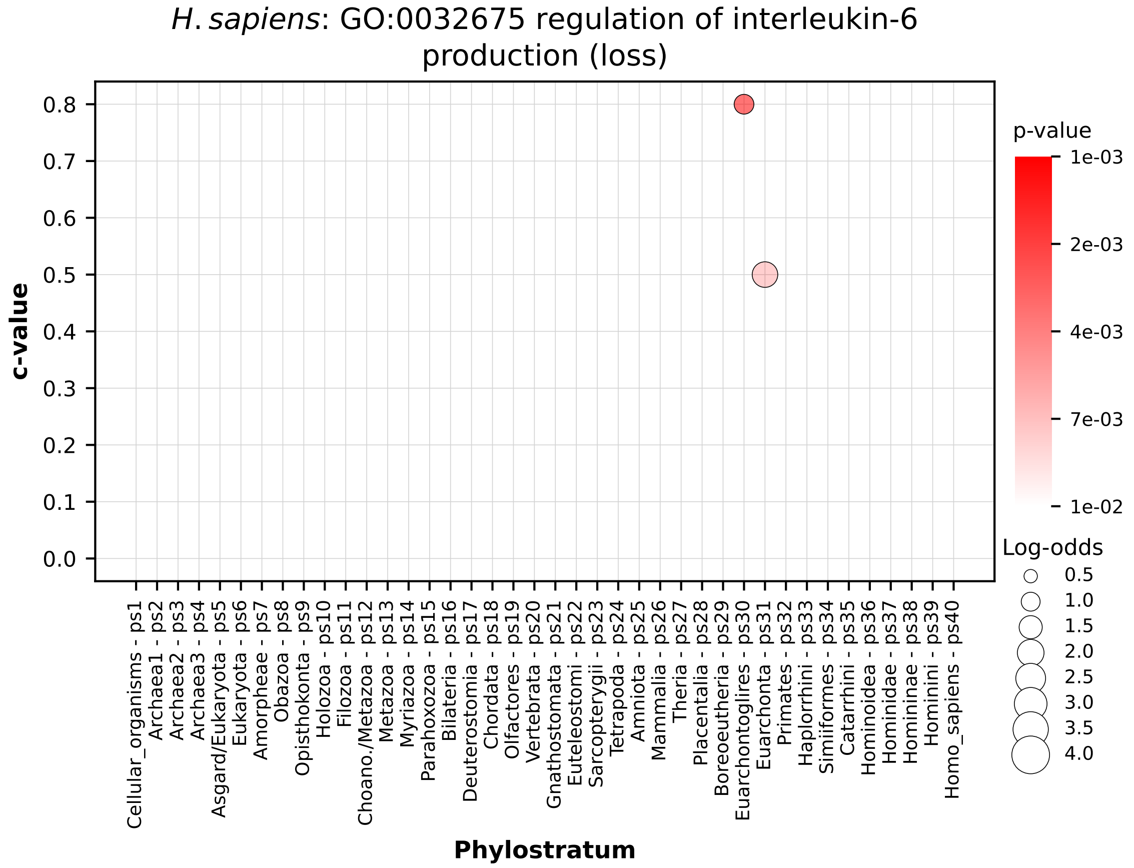
H. sapiens: GO:0032675 regulation of interleukin-6 production (loss)
c-value
0.8
0.7
0.6
0.5
0.4
0.3
0.2
0.1
0.0
Phylostratum
Cellular_organisms - ps1
Archaea1 - ps2
Archaea2 - ps3
Archaea3 - ps4
Asgard/Eukaryota - ps5
Eukaryota - ps6
Amorpheae - ps7
Obazoa - ps8
Opisthokonta - ps9
Holozoa - ps10
Filozoa - ps11
Choano./Metazoa - ps12
Metazoa - ps13
Myriazoa - ps14
Parahoxozoa - ps15
Bilateria - ps16
Deuterostomia - ps17
Chordata - ps18
Olfactores - ps19
Vertebrata - ps20
Gnathostomata - ps21
Euteleostomi - ps22
Sarcopterygii - ps23
Tetrapoda - ps24
Amniota - ps25
Mammalia - ps26
Theria - ps27
Placentalia - ps28
Boreoeutheria - ps29
Euarchontoglires - ps30
Euarchonta - ps31
Primates - ps32
Haplorrhini - ps33
Simiiformes - ps34
Catarrhini - ps35
Hominoidea - ps36
Hominidae - ps37
Homininae - ps38
Hominini - ps39
Homo_sapiens - ps40
p-value
1e-03
2e-03
4e-03
7e-03
1e-02
Log-odds
0.5
1.0
1.5
2.0
2.5
3.0
3.5
4.0

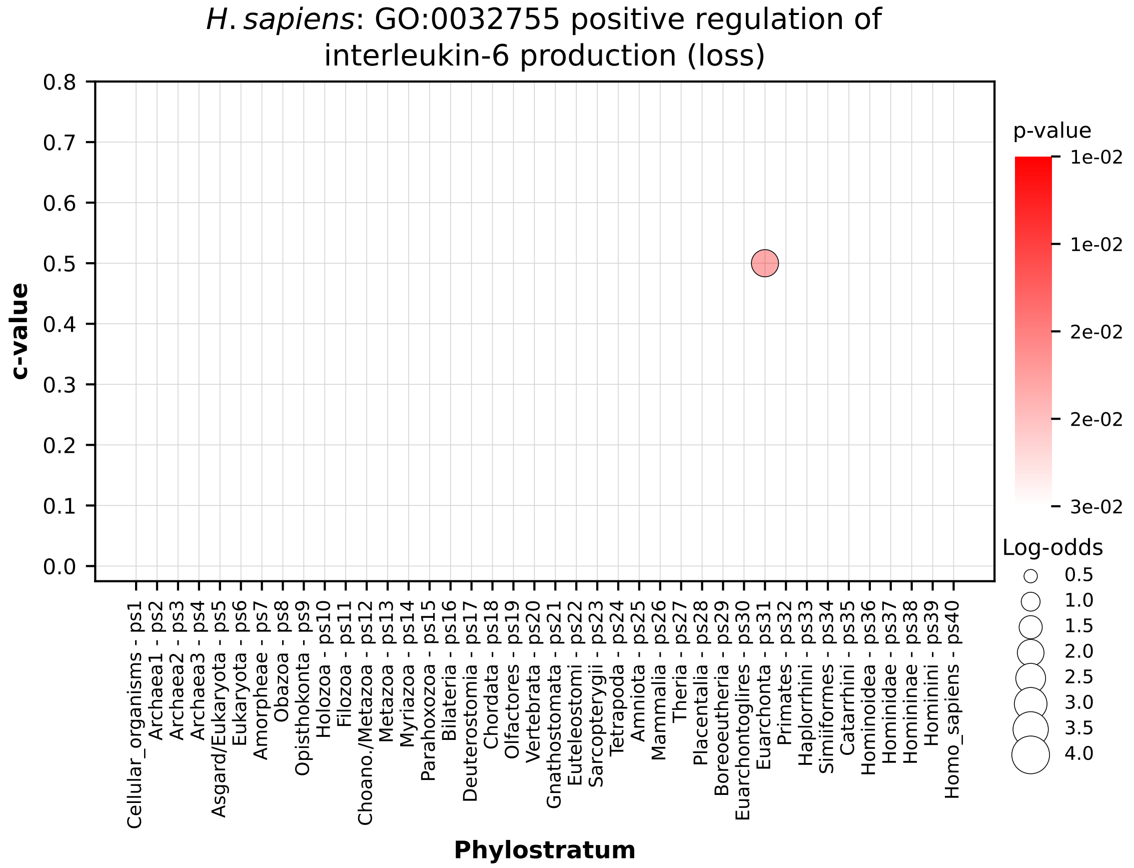
H. sapiens: GO:0032755 positive regulation of interleukin-6 production (loss)
c-value
0.8
0.7
0.6
0.5
0.4
0.3
0.2
0.1
0.0
p-value
1e-02
1e-02
2e-02
2e-02
3e-02
Log-odds
0.5
1.0
1.5
2.0
2.5
3.0
3.5
4.0
Cellular_organisms - ps1
Archaea1 - ps2
Archaea2 - ps3
Archaea3 - ps4
Asgard/Eukaryota - ps5
Eukaryota - ps6
Amorpheae - ps7
Obazoa - ps8
Opisthokonta - ps9
Holozoa - ps10
Filozoa - ps11
Choano./Metazoa - ps12
Metazoa - ps13
Myriazoa - ps14
Parahoxozoa - ps15
Bilateria - ps16
Deuterostomia - ps17
Chordata - ps18
Olfactores - ps19
Vertebrata - ps20
Gnathostomata - ps21
Euteleostomi - ps22
Sarcopterygii - ps23
Tetrapoda - ps24
Amniota - ps25
Mammalia - ps26
Theria - ps27
Placentalia - ps28
Boreoeutheria - ps29
Euarchontoglires - ps30
Euarchonta - ps31
Primates - ps32
Haplorrhini - ps33
Simiiformes - ps34
Catarrhini - ps35
Hominoidea - ps36
Hominidae - ps37
Homininae - ps38
Hominini - ps39
Homo_sapiens - ps40
Phylostratum

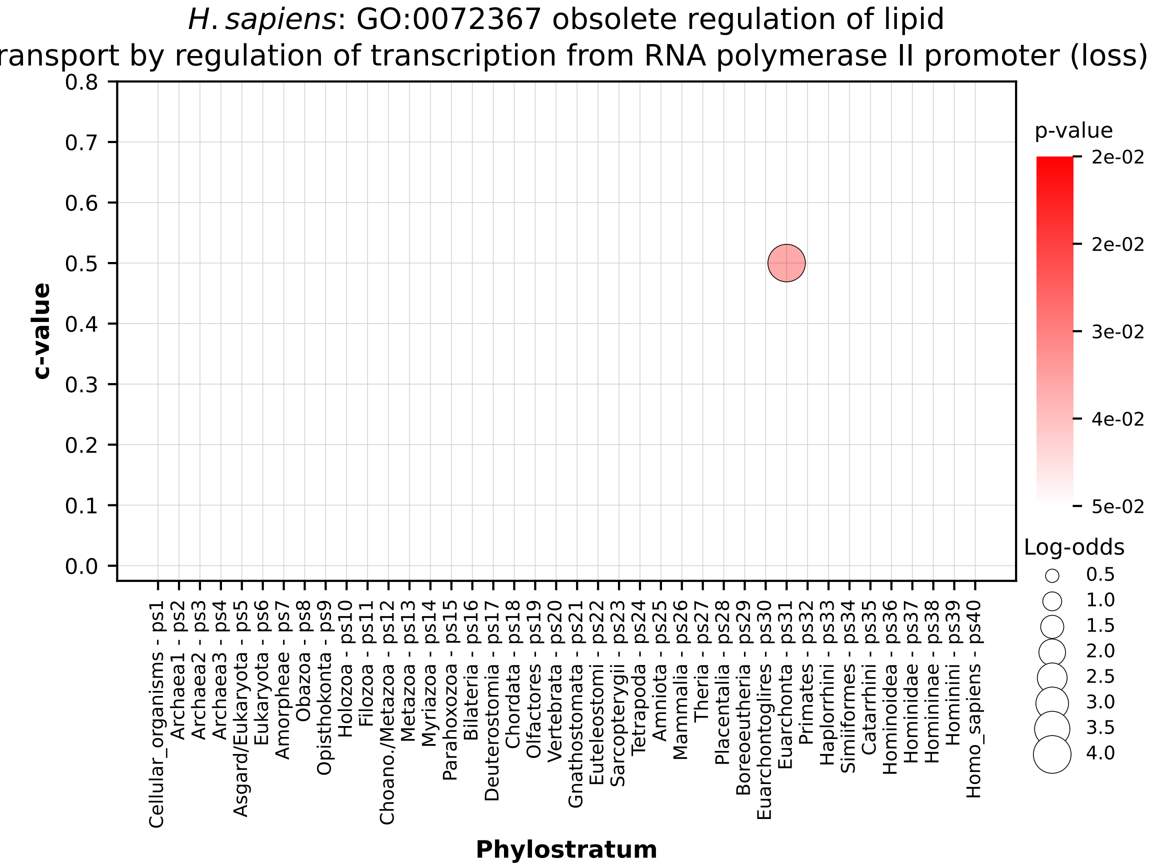

H. sapiens: GO:0072367 obsolete regulation of lipid
transport by regulation of transcription from RNA polymerase II promoter (loss)
c-value
0.8
0.7
0.6
0.5
0.4
0.3
0.2
0.1
0.0
Phylostratum
Cellular_organisms - ps1
Archaea1 - ps2
Archaea2 - ps3
Archaea3 - ps4
Asgard/Eukaryota - ps5
Eukaryota - ps6
Amorpheae - ps7
Obazoa - ps8
Opisthokonta - ps9
Holozoa - ps10
Filozoa - ps11
Choano./Metazoa - ps12
Metazoa - ps13
Myriazoa - ps14
Parahoxozoa - ps15
Bilateria - ps16
Deuterostomia - ps17
Chordata - ps18
Olfactores - ps19
Vertebrata - ps20
Gnathostomata - ps21
Euteleostomi - ps22
Sarcopterygii - ps23
Tetrapoda - ps24
Amniota - ps25
Mammalia - ps26
Theria - ps27
Placentalia - ps28
Boreoeutheria - ps29
Euarchontoglires - ps30
Euarchonta - ps31
Primates - ps32
Haplorrhini - ps33
Simiiformes - ps34
Catarrhini - ps35
Hominoidea - ps36
Hominidae - ps37
Homininae - ps38
Hominini - ps39
Homo_sapiens - ps40
p-value
2e-02
2e-02
3e-02
4e-02
5e-02
Log-odds
0.5
1.0
1.5
2.0
2.5
3.0
3.5
4.0

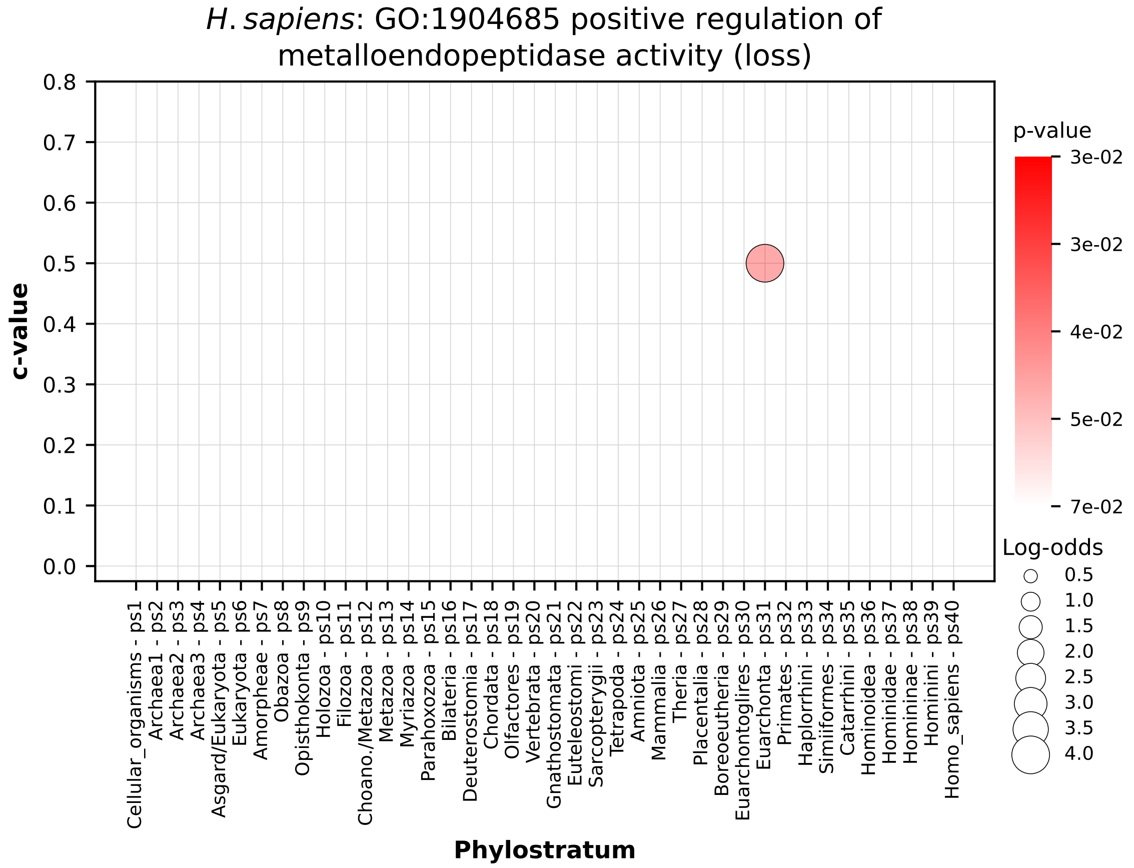
H. sapiens: GO:1904685 positive regulation of metalloendopeptidase activity (loss)
c-value
0.8
0.7
0.6
0.5
0.4
0.3
0.2
0.1
0.0
p-value
3e-02
3e-02
4e-02
5e-02
7e-02
Log-odds
0.5
1.0
1.5
2.0
2.5
3.0
3.5
4.0
Cellular_organisms - ps1
Archaea1 - ps2
Archaea2 - ps3
Archaea3 - ps4
Asgard/Eukaryota - ps5
Eukaryota - ps6
Amorpheae - ps7
Obazoa - ps8
Opisthokonta - ps9
Holozoa - ps10
Filozoa - ps11
Choano./Metazoa - ps12
Metazoa - ps13
Myriazoa - ps14
Parahoxozoa - ps15
Bilateria - ps16
Deuterostomia - ps17
Chordata - ps18
Olfactores - ps19
Vertebrata - ps20
Gnathostomata - ps21
Euteleostomi - ps22
Sarcopterygii - ps23
Tetrapoda - ps24
Amniota - ps25
Mammalia - ps26
Theria - ps27
Placentalia - ps28
Boreoeutheria - ps29
Euarchontoglires - ps30
Euarchonta - ps31
Primates - ps32
Haplorrhini - ps33
Simiiformes - ps34
Catarrhini - ps35
Hominoidea - ps36
Hominidae - ps37
Homininae - ps38
Hominini - ps39
Homo_sapiens - ps40
Phylostratum

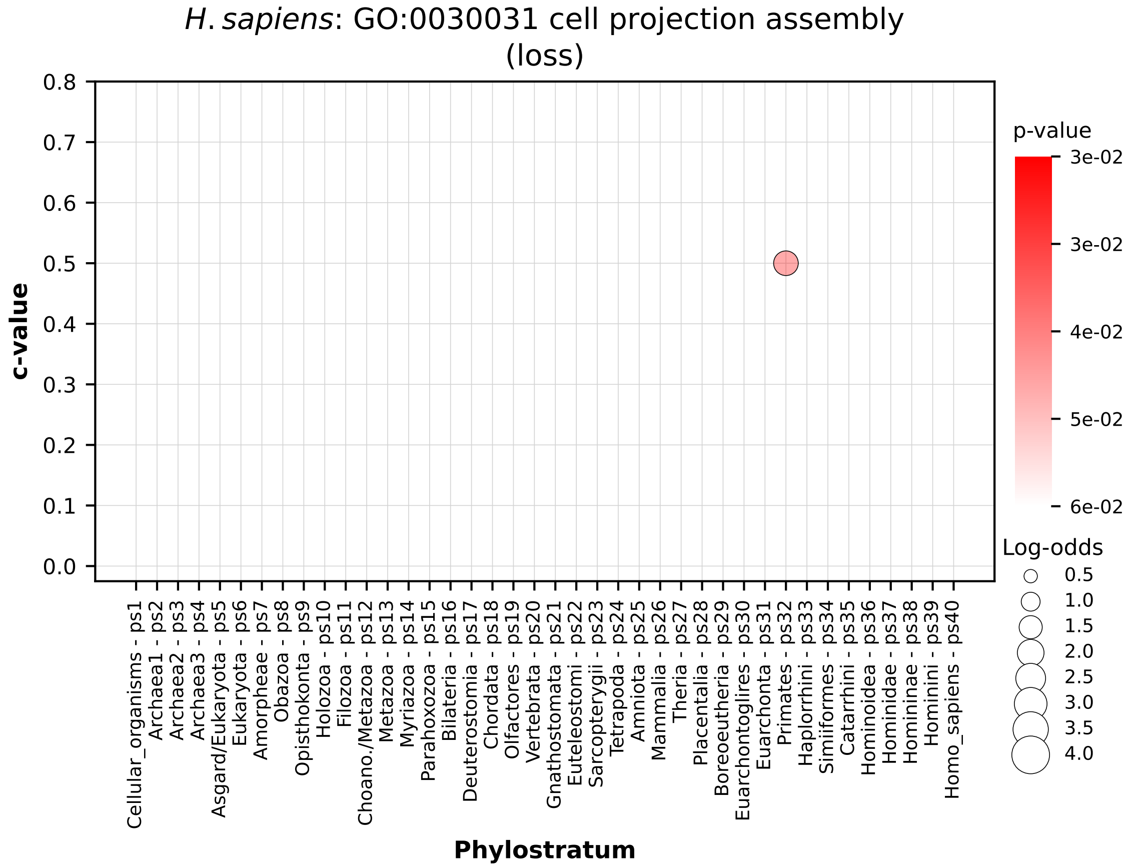

H. sapiens: GO:0030031 cell projection assembly
(loss)
c-value
0.8
0.7
0.6
0.5
0.4
0.3
0.2
0.1
0.0
Phylostratum
Cellular_organisms - ps1
Archaea1 - ps2
Archaea2 - ps3
Archaea3 - ps4
Asgard/Eukaryota - ps5
Eukaryota - ps6
Amorpheae - ps7
Obazoa - ps8
Opisthokonta - ps9
Holozoa - ps10
Filozoa - ps11
Choano./Metazoa - ps12
Metazoa - ps13
Myriazoa - ps14
Parahoxozoa - ps15
Bilateria - ps16
Deuterostomia - ps17
Chordata - ps18
Olfactores - ps19
Vertebrata - ps20
Gnathostomata - ps21
Euteleostomi - ps22
Sarcopterygii - ps23
Tetrapoda - ps24
Amniota - ps25
Mammalia - ps26
Theria - ps27
Placentalia - ps28
Boreoeutheria - ps29
Euarchontoglires - ps30
Euarchonta - ps31
Primates - ps32
Haplorrhini - ps33
Simiiformes - ps34
Catarrhini - ps35
Hominoidea - ps36
Hominidae - ps37
Homininae - ps38
Hominini - ps39
Homo_sapiens - ps40
p-value
3e-02
3e-02
4e-02
5e-02
6e-02
Log-odds
0.5
1.0
1.5
2.0
2.5
3.0
3.5
4.0

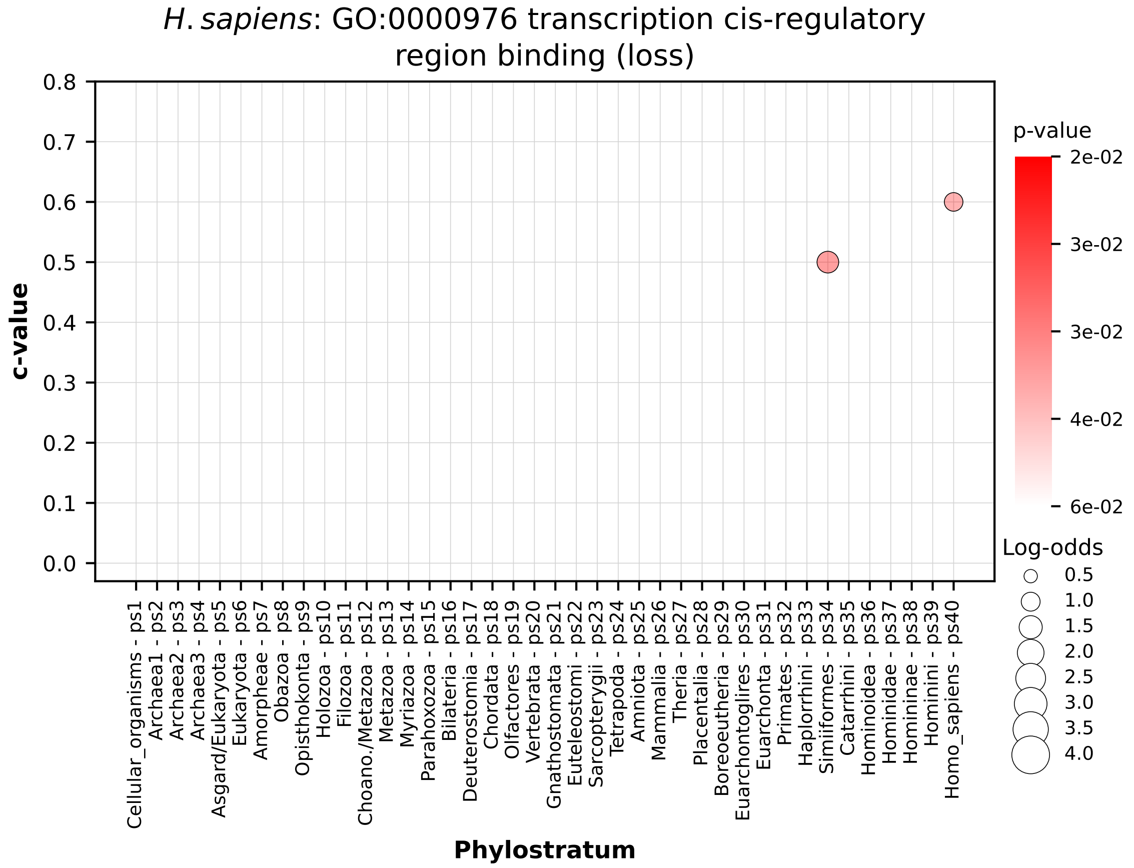

H. sapiens: GO:0000976 transcription cis-regulatory region binding (loss)
c-value
0.8
0.7
0.6
0.5
0.4
0.3
0.2
0.1
0.0
p-value
2e-02
3e-02
3e-02
4e-02
6e-02
Log-odds
0.5
1.0
1.5
2.0
2.5
3.0
3.5
4.0
Cellular_organisms - ps1
Archaea1 - ps2
Archaea2 - ps3
Archaea3 - ps4
Asgard/Eukaryota - ps5
Eukaryota - ps6
Amorpheae - ps7
Obazoa - ps8
Opisthokonta - ps9
Holozoa - ps10
Filozoa - ps11
Choano./Metazoa - ps12
Metazoa - ps13
Myriazoa - ps14
Parahoxozoa - ps15
Bilateria - ps16
Deuterostomia - ps17
Chordata - ps18
Olfactores - ps19
Vertebrata - ps20
Gnathostomata - ps21
Euteleostomi - ps22
Sarcopterygii - ps23
Tetrapoda - ps24
Amniota - ps25
Mammalia - ps26
Theria - ps27
Placentalia - ps28
Boreoeutheria - ps29
Euarchontoglires - ps30
Euarchonta - ps31
Primates - ps32
Haplorrhini - ps33
Simiiformes - ps34
Catarrhini - ps35
Hominoidea - ps36
Hominidae - ps37
Homininae - ps38
Hominini - ps39
Homo_sapiens - ps40
Phylostratum

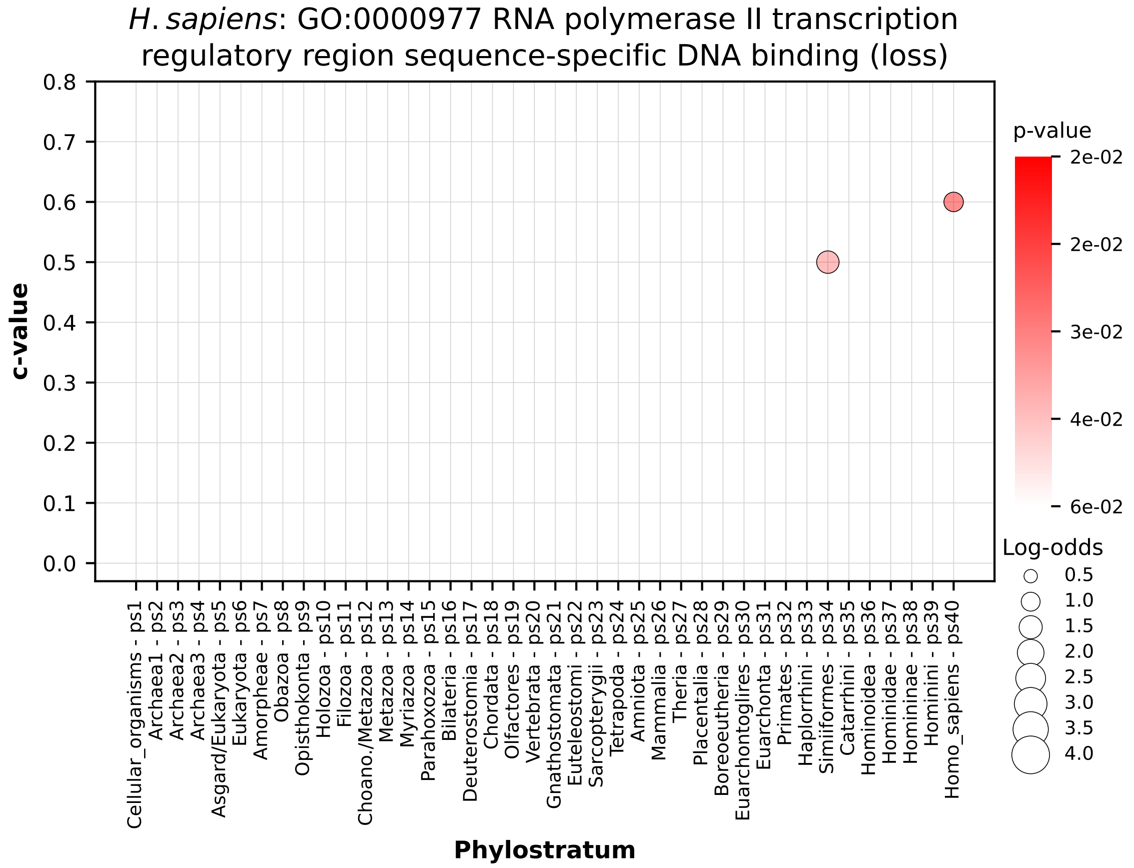

H. sapiens: GO:0000977 RNA polymerase II transcription regulatory region sequence-specific DNA binding (loss)
c-value
0.8
0.7
0.6
0.5
0.4
0.3
0.2
0.1
0.0
Phylostratum
Cellular_organisms - ps1
Archaea1 - ps2
Archaea2 - ps3
Archaea3 - ps4
Asgard/Eukaryota - ps5
Eukaryota - ps6
Amorpheae - ps7
Obazoa - ps8
Opisthokonta - ps9
Holozoa - ps10
Filozoa - ps11
Choano./Metazoa - ps12
Metazoa - ps13
Myriazoa - ps14
Parahoxozoa - ps15
Bilateria - ps16
Deuterostomia - ps17
Chordata - ps18
Olfactores - ps19
Vertebrata - ps20
Gnathostomata - ps21
Euteleostomi - ps22
Sarcopterygii - ps23
Tetrapoda - ps24
Amniota - ps25
Mammalia - ps26
Theria - ps27
Placentalia - ps28
Boreoeutheria - ps29
Euarchontoglires - ps30
Euarchonta - ps31
Primates - ps32
Haplorrhini - ps33
Simiiformes - ps34
Catarrhini - ps35
Hominoidea - ps36
Hominidae - ps37
Homininae - ps38
Hominini - ps39
Homo_sapiens - ps40
p-value
2e-02
2e-02
3e-02
4e-02
6e-02
Log-odds
0.5
1.0
1.5
2.0
2.5
3.0
3.5
4.0

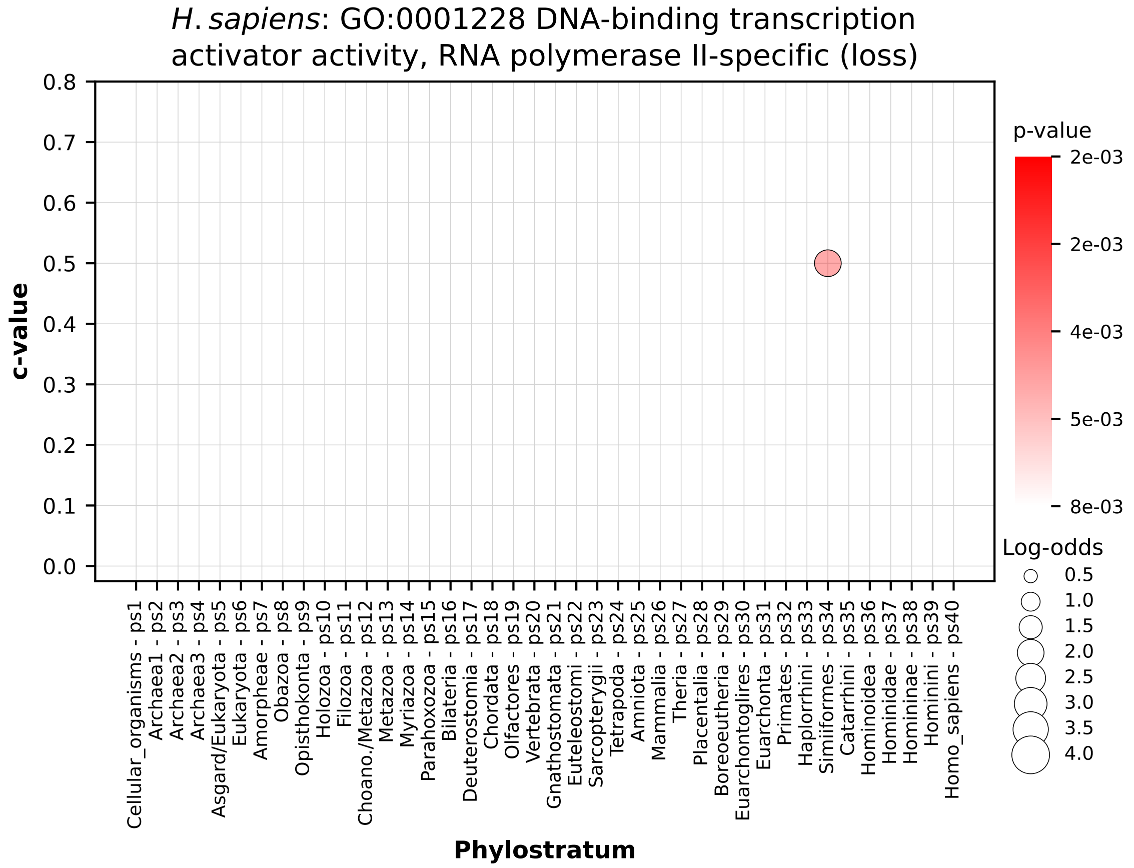

H. sapiens: GO:0001228 DNA-binding transcription activator activity, RNA polymerase II-specific (loss)
c-value
0.8
0.7
0.6
0.5
0.4
0.3
0.2
0.1
0.0
Phylostratum
Cellular_organisms - ps1
Archaea1 - ps2
Archaea2 - ps3
Archaea3 - ps4
Asgard/Eukaryota - ps5
Eukaryota - ps6
Amorpheae - ps7
Obazoa - ps8
Opisthokonta - ps9
Holozoa - ps10
Filozoa - ps11
Choano./Metazoa - ps12
Metazoa - ps13
Myriazoa - ps14
Parahoxozoa - ps15
Bilateria - ps16
Deuterostomia - ps17
Chordata - ps18
Olfactores - ps19
Vertebrata - ps20
Gnathostomata - ps21
Euteleostomi - ps22
Sarcopterygii - ps23
Tetrapoda - ps24
Amniota - ps25
Mammalia - ps26
Theria - ps27
Placentalia - ps28
Boreoeutheria - ps29
Euarchontoglires - ps30
Euarchonta - ps31
Primates - ps32
Haplorrhini - ps33
Simiiformes - ps34
Catarrhini - ps35
Hominoidea - ps36
Hominidae - ps37
Homininae - ps38
Hominini - ps39
Homo_sapiens - ps40
p-value
2e-03
2e-03
4e-03
5e-03
8e-03
Log-odds
0.5
1.0
1.5
2.0
2.5
3.0
3.5
4.0

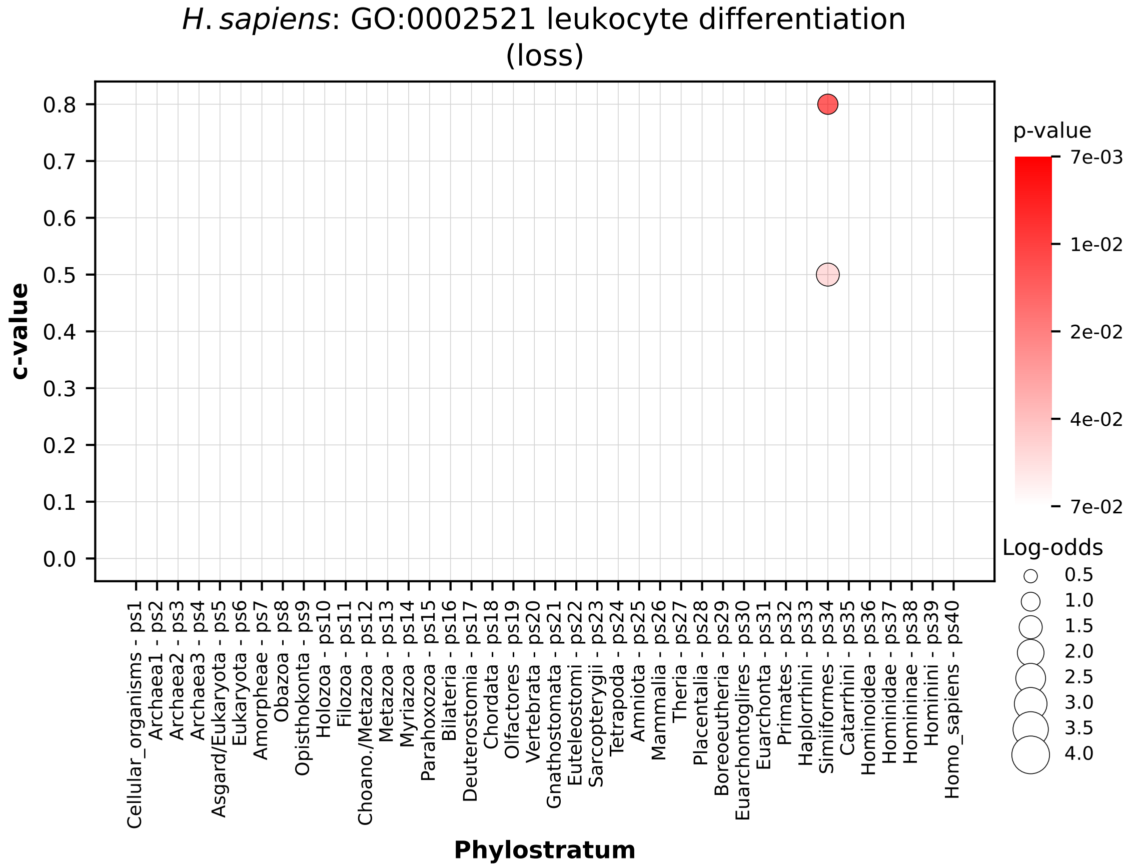

H. sapiens: GO:0002521 leukocyte differentiation
(loss)
c-value
0.0
0.1
0.2
0.3
0.4
0.5
0.6
0.7
0.8
Phylostratum
Cellular_organisms - ps1
Archaea1 - ps2
Archaea2 - ps3
Archaea3 - ps4
Asgard/Eukaryota - ps5
Eukaryota - ps6
Amorpheae - ps7
Obazoa - ps8
Opisthokonta - ps9
Holozoa - ps10
Filozoa - ps11
Choano./Metazoa - ps12
Metazoa - ps13
Myriazoa - ps14
Parahoxozoa - ps15
Bilateria - ps16
Deuterostomia - ps17
Chordata - ps18
Olfactores - ps19
Vertebrata - ps20
Gnathostomata - ps21
Euteleostomi - ps22
Sarcopterygii - ps23
Tetrapoda - ps24
Amniota - ps25
Mammalia - ps26
Theria - ps27
Placentalia - ps28
Boreoeutheria - ps29
Euarchontoglires - ps30
Euarchonta - ps31
Primates - ps32
Haplorrhini - ps33
Simiiformes - ps34
Catarrhini - ps35
Hominoidea - ps36
Hominidae - ps37
Homininae - ps38
Hominini - ps39
Homo_sapiens - ps40
p-value
7e-03
1e-02
2e-02
4e-02
7e-02
Log-odds
0.5
1.0
1.5
2.0
2.5
3.0
3.5
4.0

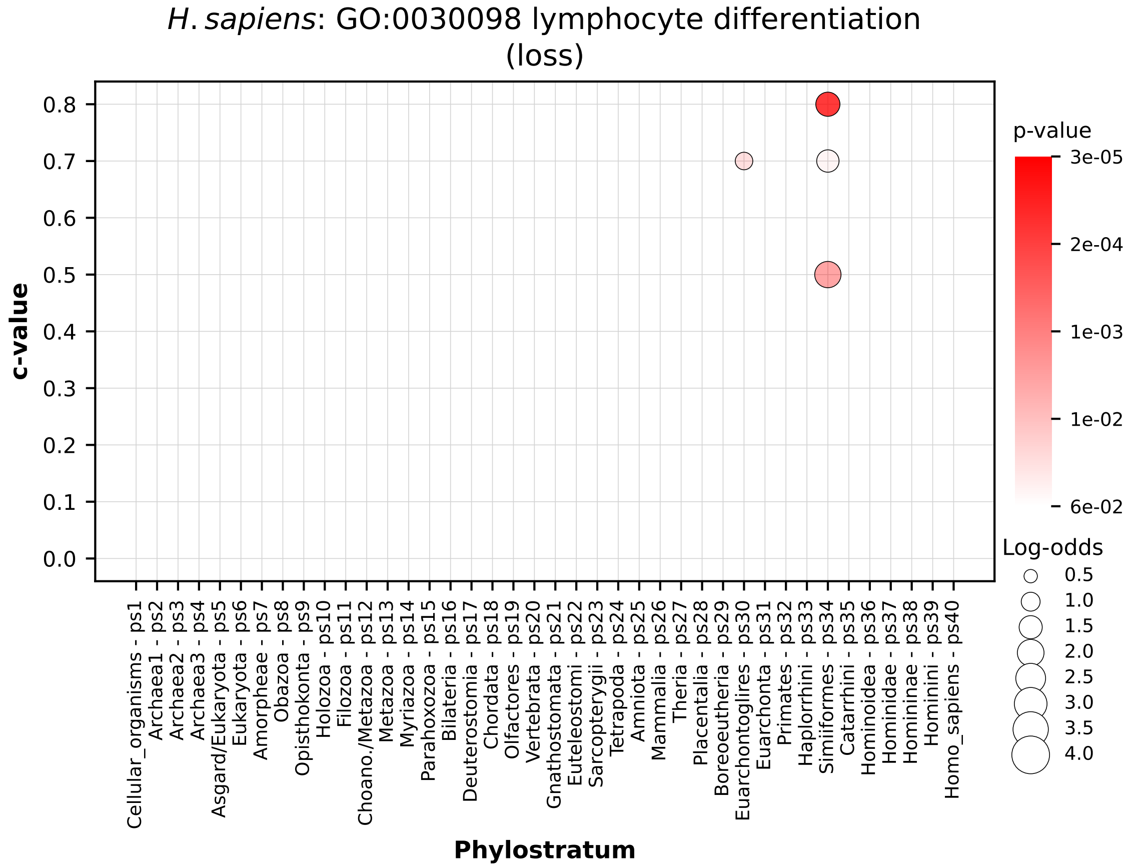
H. sapiens: GO:0030098 lymphocyte differentiation
(loss)
c-value
0.8
0.7
0.6
0.5
0.4
0.3
0.2
0.1
0.0
p-value
3e-05
2e-04
1e-03
1e-02
6e-02
Log-odds
0.5
1.0
1.5
2.0
2.5
3.0
3.5
4.0
Cellular_organisms - ps1
Archaea1 - ps2
Archaea2 - ps3
Archaea3 - ps4
Asgard/Eukaryota - ps5
Eukaryota - ps6
Amorpheae - ps7
Obazoa - ps8
Opisthokonta - ps9
Holozoa - ps10
Filozoa - ps11
Choano./Metazoa - ps12
Metazoa - ps13
Myriazoa - ps14
Parahoxozoa - ps15
Bilateria - ps16
Deuterostomia - ps17
Chordata - ps18
Olfactores - ps19
Vertebrata - ps20
Gnathostomata - ps21
Euteleostomi - ps22
Sarcopterygii - ps23
Tetrapoda - ps24
Amniota - ps25
Mammalia - ps26
Theria - ps27
Placentalia - ps28
Boreoeutheria - ps29
Euarchontoglires - ps30
Euarchonta - ps31
Primates - ps32
Haplorrhini - ps33
Simiiformes - ps34
Catarrhini - ps35
Hominoidea - ps36
Hominidae - ps37
Homininae - ps38
Hominini - ps39
Homo_sapiens - ps40
Phylostratum

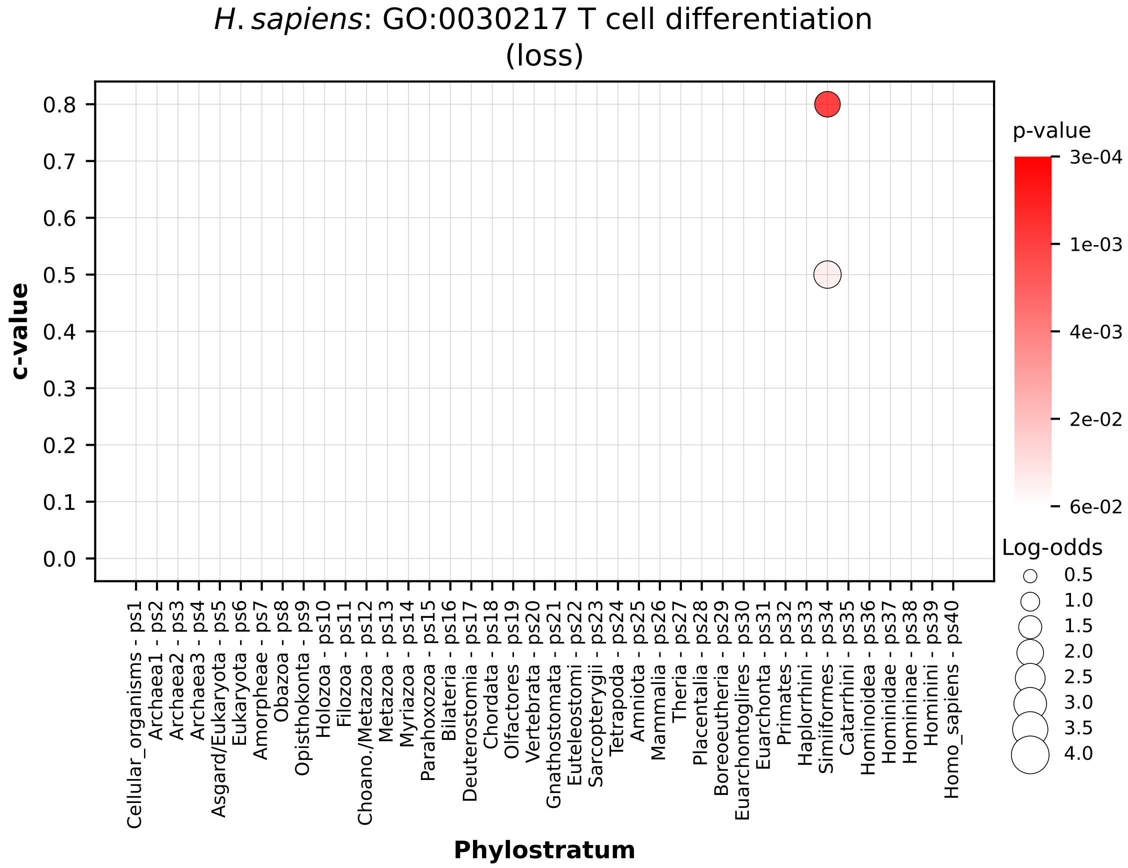
H. sapiens: GO:0030217 T cell differentiation
(loss)
c-value
0.8
0.7
0.6
0.5
0.4
0.3
0.2
0.1
0.0
p-value
3e-04
1e-03
4e-03
2e-02
6e-02
Log-odds
0.5
1.0
1.5
2.0
2.5
3.0
3.5
4.0
Cellular_organisms - ps1
Archaea1 - ps2
Archaea2 - ps3
Archaea3 - ps4
Asgard/Eukaryota - ps5
Eukaryota - ps6
Amorpheae - ps7
Obazoa - ps8
Opisthokonta - ps9
Holozoa - ps10
Filozoa - ps11
Choano./Metazoa - ps12
Metazoa - ps13
Myriazoa - ps14
Parahoxozoa - ps15
Bilateria - ps16
Deuterostomia - ps17
Chordata - ps18
Olfactores - ps19
Vertebrata - ps20
Gnathostomata - ps21
Euteleostomi - ps22
Sarcopterygii - ps23
Tetrapoda - ps24
Amniota - ps25
Mammalia - ps26
Theria - ps27
Placentalia - ps28
Boreoeutheria - ps29
Euarchontoglires - ps30
Euarchonta - ps31
Primates - ps32
Haplorrhini - ps33
Simiiformes - ps34
Catarrhini - ps35
Hominoidea - ps36
Hominidae - ps37
Homininae - ps38
Hominini - ps39
Homo_sapiens - ps40
Phylostratum

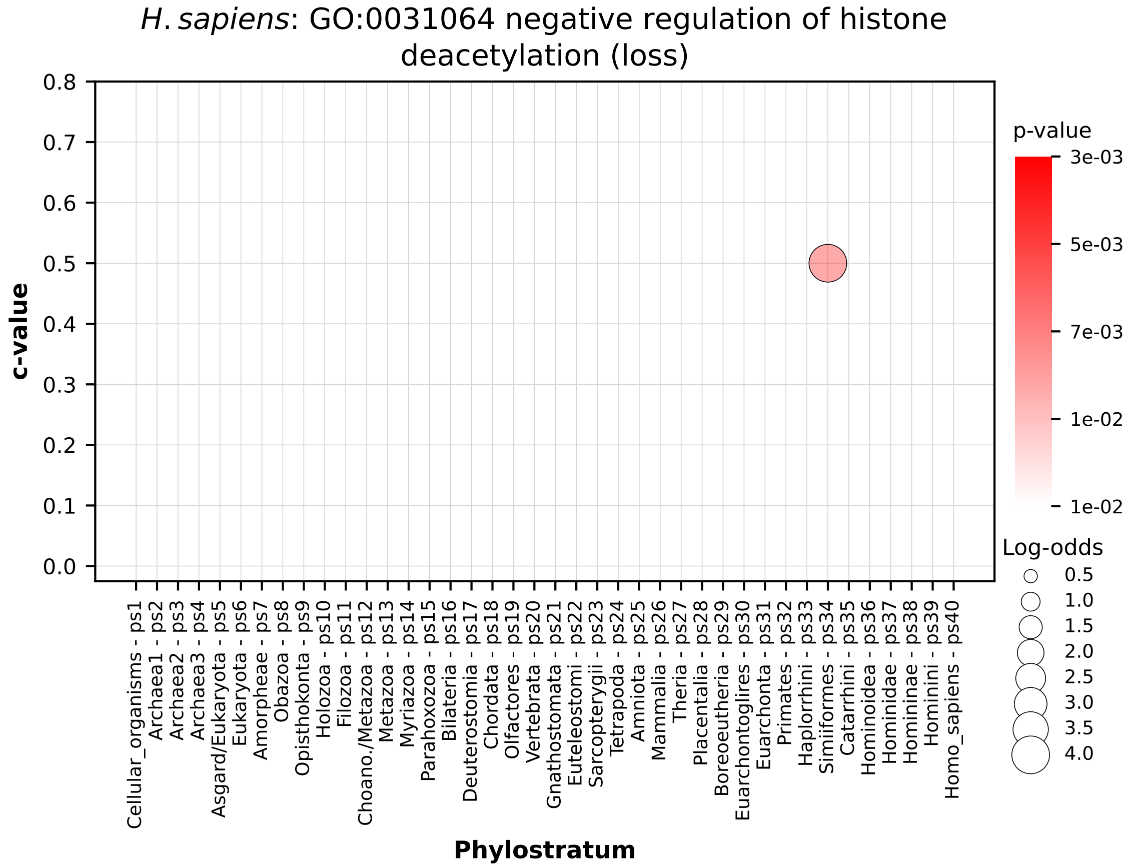

H. sapiens: GO:0031064 negative regulation of histone deacetylation (loss)
c-value
Phylostratum
p-value
3e-03
5e-03
7e-03
1e-02
1e-02
Log-odds
0.5
1.0
1.5
2.0
2.5
3.0
3.5
4.0
0.8
0.7
0.6
0.5
0.4
0.3
0.2
0.1
0.0
Cellular_organisms - ps1
Archaea1 - ps2
Archaea2 - ps3
Archaea3 - ps4
Asgard/Eukaryota - ps5
Eukaryota - ps6
Amorpheae - ps7
Obazoa - ps8
Opisthokonta - ps9
Holozoa - ps10
Filozoa - ps11
Choano./Metazoa - ps12
Metazoa - ps13
Myriazoa - ps14
Parahoxozoa - ps15
Bilateria - ps16
Deuterostomia - ps17
Chordata - ps18
Olfactores - ps19
Vertebrata - ps20
Gnathostomata - ps21
Euteleostomi - ps22
Sarcopterygii - ps23
Tetrapoda - ps24
Amniota - ps25
Mammalia - ps26
Theria - ps27
Placentalia - ps28
Boreoeutheria - ps29
Euarchontoglires - ps30
Euarchonta - ps31
Primates - ps32
Haplorrhini - ps33
Simiiformes - ps34
Catarrhini - ps35
Hominoidea - ps36
Hominidae - ps37
Homininae - ps38
Hominini - ps39
Homo_sapiens - ps40

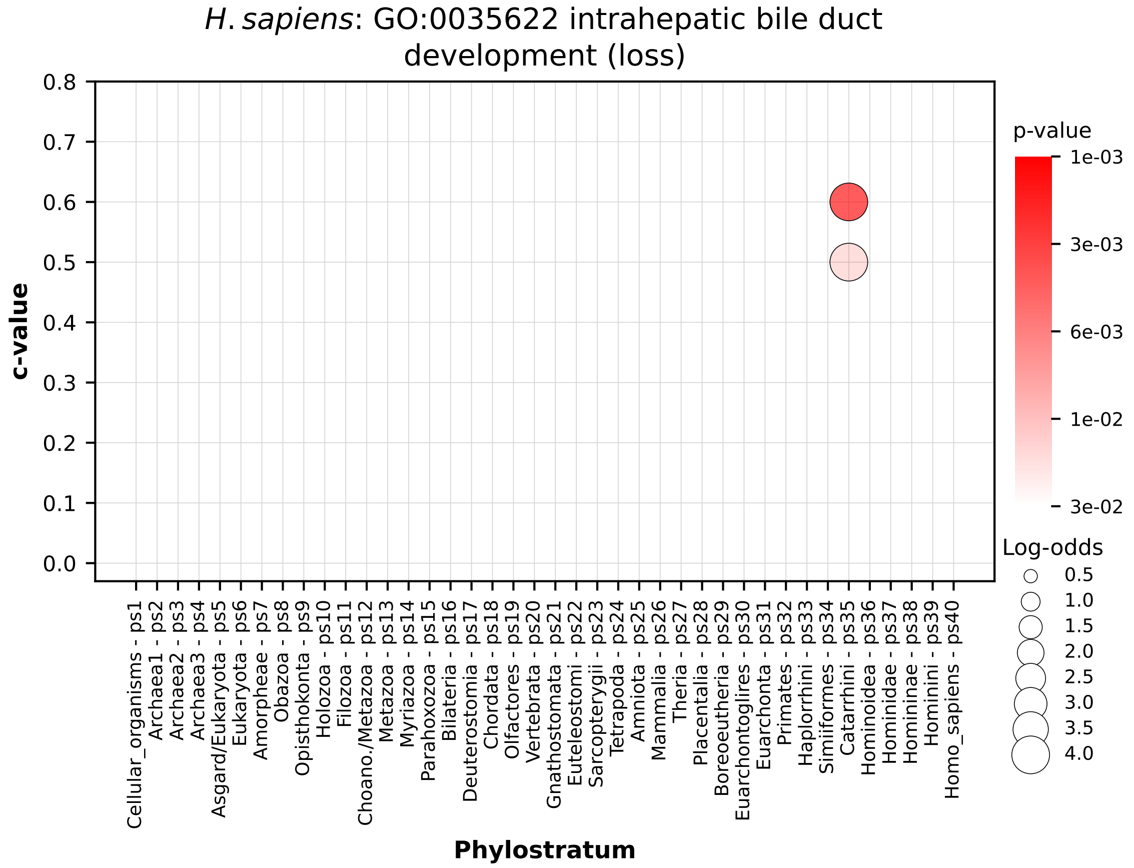

H. sapiens: GO:0035622 intrahepatic bile duct development (loss)
c-value
0.8
0.7
0.6
0.5
0.4
0.3
0.2
0.1
0.0
p-value
1e-03
3e-03
6e-03
1e-02
3e-02
Log-odds
0.5
1.0
1.5
2.0
2.5
3.0
3.5
4.0
Cellular_organisms - ps1
Archaea1 - ps2
Archaea2 - ps3
Archaea3 - ps4
Asgard/Eukaryota - ps5
Eukaryota - ps6
Amorpheae - ps7
Obazoa - ps8
Opisthokonta - ps9
Holozoa - ps10
Filozoa - ps11
Choano./Metazoa - ps12
Metazoa - ps13
Myriazoa - ps14
Parahoxozoa - ps15
Bilateria - ps16
Deuterostomia - ps17
Chordata - ps18
Olfactores - ps19
Vertebrata - ps20
Gnathostomata - ps21
Euteleostomi - ps22
Sarcopterygii - ps23
Tetrapoda - ps24
Amniota - ps25
Mammalia - ps26
Theria - ps27
Placentalia - ps28
Boreoeutheria - ps29
Euarchontoglires - ps30
Euarchonta - ps31
Primates - ps32
Haplorrhini - ps33
Simiiformes - ps34
Catarrhini - ps35
Hominoidea - ps36
Hominidae - ps37
Homininae - ps38
Hominini - ps39
Homo_sapiens - ps40
Phylostratum

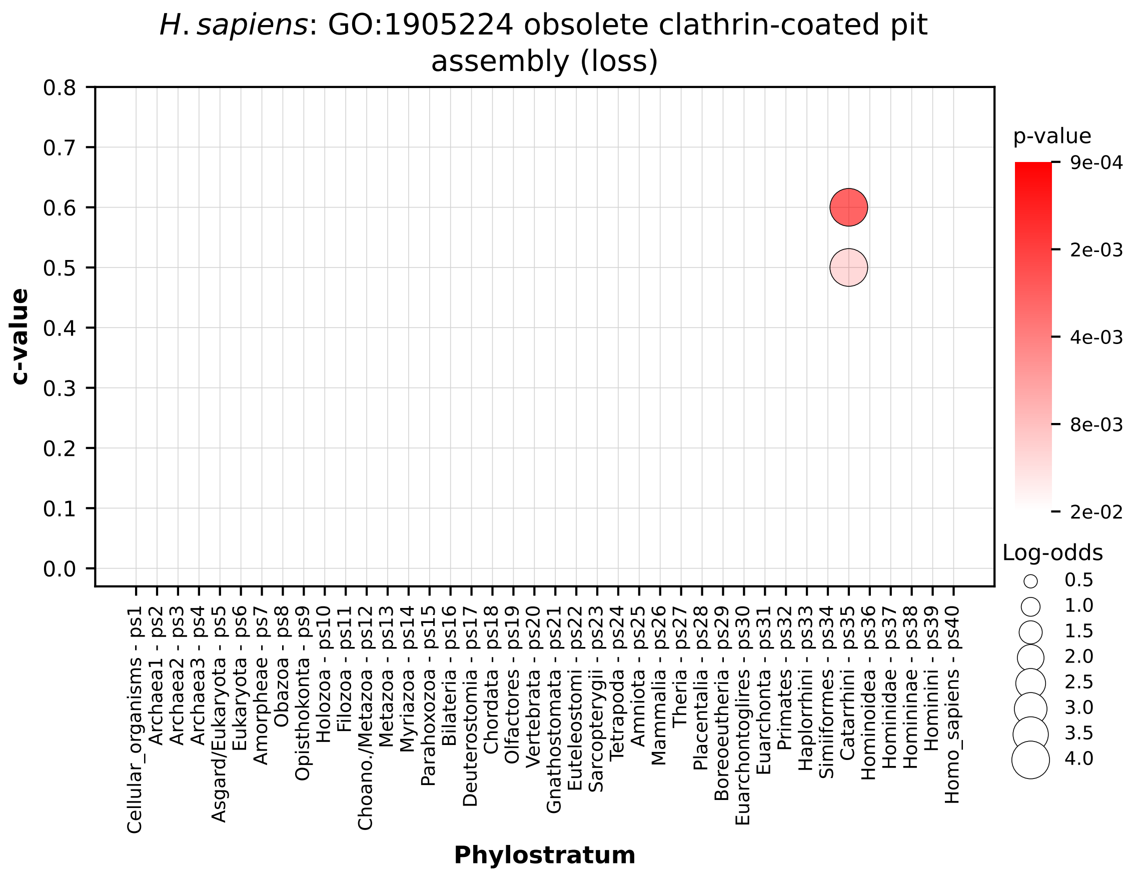

H. sapiens: GO:1905224 obsolete clathrin-coated pit assembly (loss)
c-value
0.8
0.7
0.6
0.5
0.4
0.3
0.2
0.1
0.0
p-value
9e-04
2e-03
4e-03
8e-03
2e-02
Log-odds
0.5
1.0
1.5
2.0
2.5
3.0
3.5
4.0
Cellular_organisms - ps1
Archaea1 - ps2
Archaea2 - ps3
Archaea3 - ps4
Asgard/Eukaryota - ps5
Eukaryota - ps6
Amorpheae - ps7
Obazoa - ps8
Opisthokonta - ps9
Holozoa - ps10
Filozoa - ps11
Choano./Metazoa - ps12
Metazoa - ps13
Myriazoa - ps14
Parahoxozoa - ps15
Bilateria - ps16
Deuterostomia - ps17
Chordata - ps18
Olfactores - ps19
Vertebrata - ps20
Gnathostomata - ps21
Euteleostomi - ps22
Sarcopterygii - ps23
Tetrapoda - ps24
Amniota - ps25
Mammalia - ps26
Theria - ps27
Placentalia - ps28
Boreoeutheria - ps29
Euarchontoglires - ps30
Euarchonta - ps31
Primates - ps32
Haplorrhini - ps33
Simiiformes - ps34
Catarrhini - ps35
Hominoidea - ps36
Hominidae - ps37
Homininae - ps38
Hominini - ps39
Homo_sapiens - ps40
Phylostratum

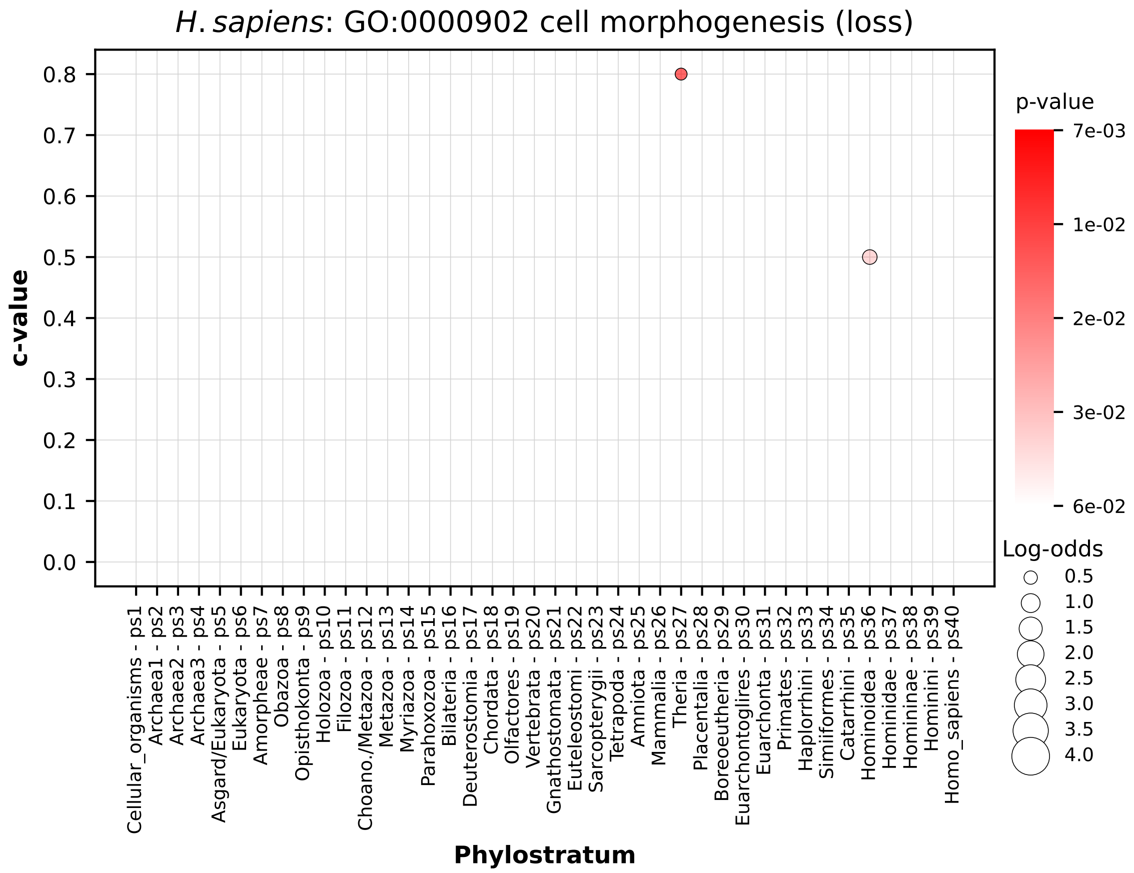

H. sapiens: GO:0000902 cell morphogenesis (loss)
c-value
0.0
0.1
0.2
0.3
0.4
0.5
0.6
0.7
0.8
p-value
7e-03
1e-02
2e-02
3e-02
6e-02
Log-odds
0.5
1.0
1.5
2.0
2.5
3.0
3.5
4.0
Cellular_organisms - ps1
Archaea1 - ps2
Archaea2 - ps3
Archaea3 - ps4
Asgard/Eukaryota - ps5
Eukaryota - ps6
Amorpheae - ps7
Obazoa - ps8
Opisthokonta - ps9
Holozoa - ps10
Filozoa - ps11
Choano./Metazoa - ps12
Metazoa - ps13
Myriazoa - ps14
Parahoxozoa - ps15
Bilateria - ps16
Deuterostomia - ps17
Chordata - ps18
Olfactores - ps19
Vertebrata - ps20
Gnathostomata - ps21
Euteleostomi - ps22
Sarcopterygii - ps23
Tetrapoda - ps24
Amniota - ps25
Mammalia - ps26
Theria - ps27
Placentalia - ps28
Boreoeutheria - ps29
Euarchontoglires - ps30
Euarchonta - ps31
Primates - ps32
Haplorrhini - ps33
Simiiformes - ps34
Catarrhini - ps35
Hominoidea - ps36
Hominidae - ps37
Homininae - ps38
Hominini - ps39
Homo_sapiens - ps40
Phylostratum

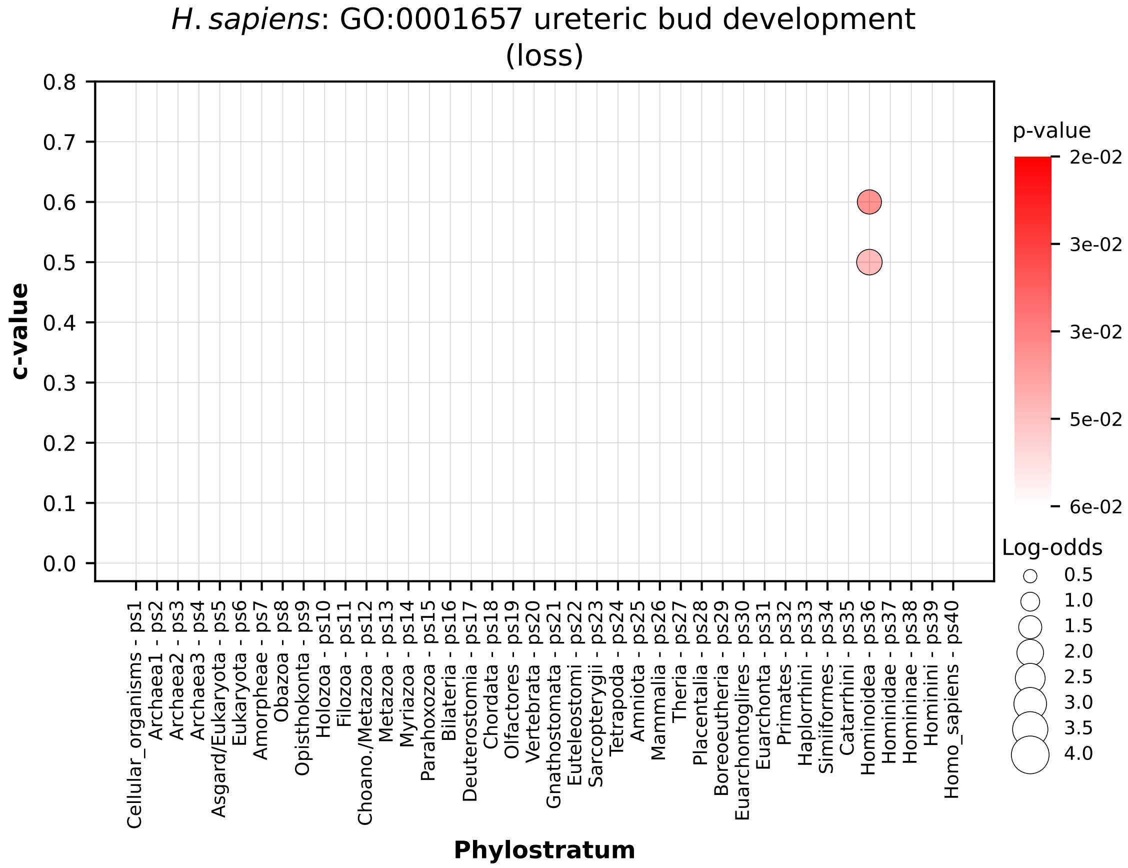

H. sapiens: GO:0001657 ureteric bud development (loss)
c-value
0.8
0.7
0.6
0.5
0.4
0.3
0.2
0.1
0.0
Phylostratum
Cellular_organisms - ps1
Archaea1 - ps2
Archaea2 - ps3
Archaea3 - ps4
Asgard/Eukaryota - ps5
Eukaryota - ps6
Amorpheae - ps7
Obazoa - ps8
Opisthokonta - ps9
Holozoa - ps10
Filozoa - ps11
Choano./Metazoa - ps12
Metazoa - ps13
Myriazoa - ps14
Parahoxozoa - ps15
Bilateria - ps16
Deuterostomia - ps17
Chordata - ps18
Olfactores - ps19
Vertebrata - ps20
Gnathostomata - ps21
Euteleostomi - ps22
Sarcopterygii - ps23
Tetrapoda - ps24
Amniota - ps25
Mammalia - ps26
Theria - ps27
Placentalia - ps28
Boreoeutheria - ps29
Euarchontoglires - ps30
Euarchonta - ps31
Primates - ps32
Haplorrhini - ps33
Simiiformes - ps34
Catarrhini - ps35
Hominoidea - ps36
Hominidae - ps37
Homininae - ps38
Hominini - ps39
Homo_sapiens - ps40
p-value
2e-02
3e-02
3e-02
5e-02
6e-02
Log-odds
0.5
1.0
1.5
2.0
2.5
3.0
3.5
4.0

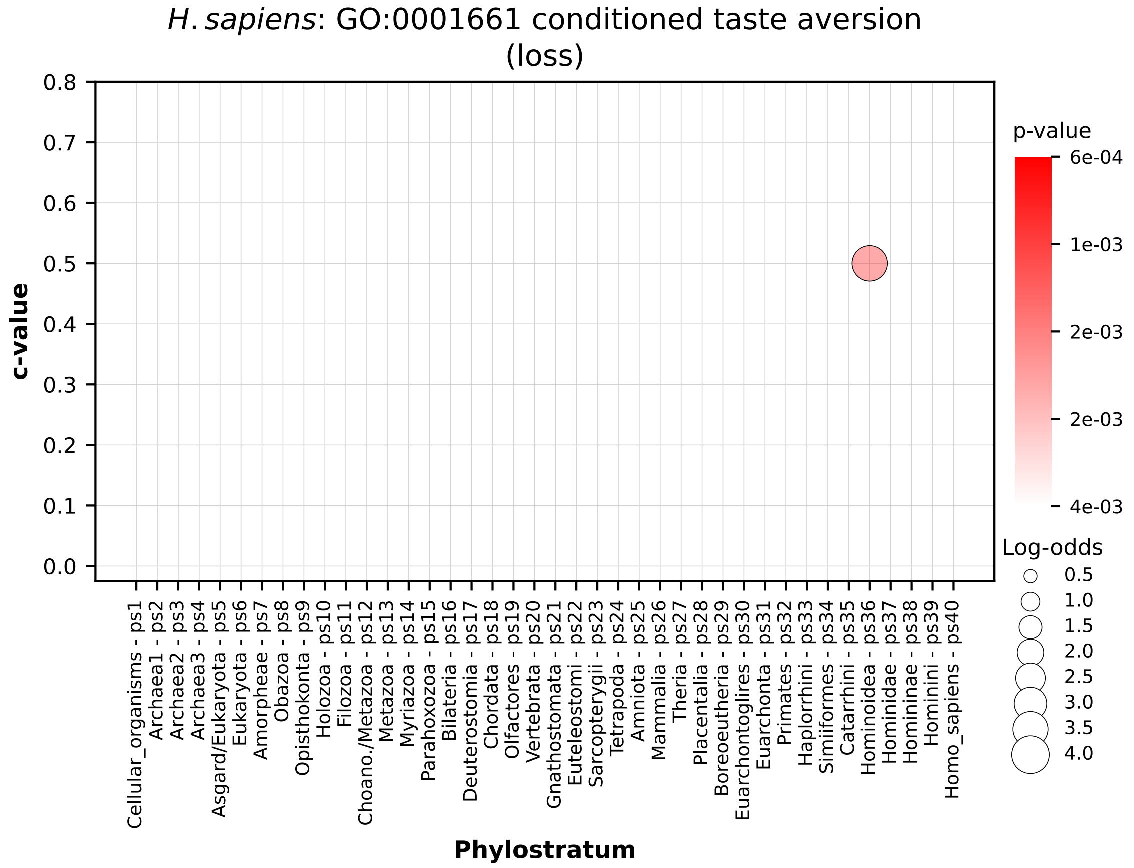

H. sapiens: GO:0001661 conditioned taste aversion
(loss)
c-value
0.8
0.7
0.6
0.5
0.4
0.3
0.2
0.1
0.0
p-value
6e-04
1e-03
2e-03
2e-03
4e-03
Log-odds
0.5
1.0
1.5
2.0
2.5
3.0
3.5
4.0
Cellular_organisms - ps1
Archaea1 - ps2
Archaea2 - ps3
Archaea3 - ps4
Asgard/Eukaryota - ps5
Eukaryota - ps6
Amorpheae - ps7
Obazoa - ps8
Opisthokonta - ps9
Holozoa - ps10
Filozoa - ps11
Choano./Metazoa - ps12
Metazoa - ps13
Myriazoa - ps14
Parahoxozoa - ps15
Bilateria - ps16
Deuterostomia - ps17
Chordata - ps18
Olfactores - ps19
Vertebrata - ps20
Gnathostomata - ps21
Euteleostomi - ps22
Sarcopterygii - ps23
Tetrapoda - ps24
Amniota - ps25
Mammalia - ps26
Theria - ps27
Placentalia - ps28
Boreoeutheria - ps29
Euarchontoglires - ps30
Euarchonta - ps31
Primates - ps32
Haplorrhini - ps33
Simiiformes - ps34
Catarrhini - ps35
Hominoidea - ps36
Hominidae - ps37
Homininae - ps38
Hominini - ps39
Homo_sapiens - ps40
Phylostratum

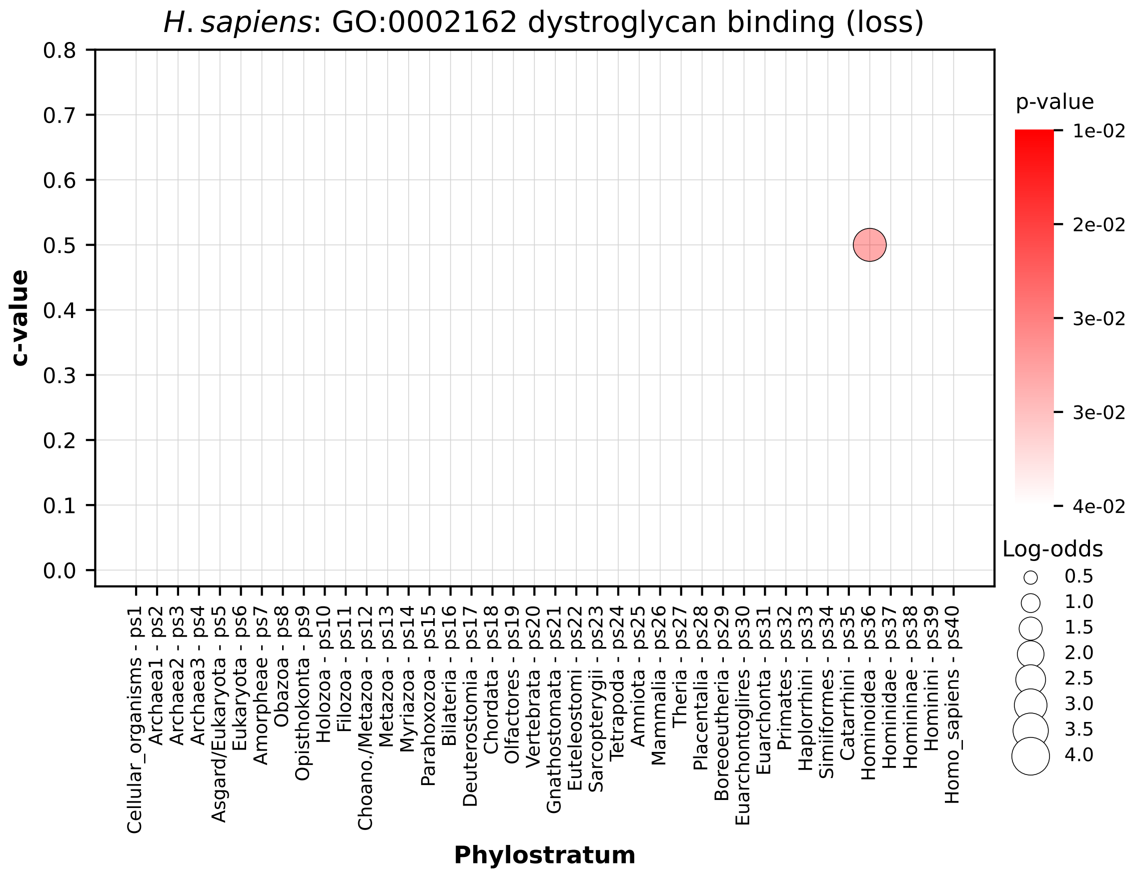

H. sapiens: GO:0002162 dystroglycan binding (loss)
c-value
0.0
0.1
0.2
0.3
0.4
0.5
0.6
0.7
0.8
Phylostratum
Cellular_organisms - ps1
Archaea1 - ps2
Archaea2 - ps3
Archaea3 - ps4
Asgard/Eukaryota - ps5
Eukaryota - ps6
Amorpheae - ps7
Obazoa - ps8
Opisthokonta - ps9
Holozoa - ps10
Filozoa - ps11
Choano./Metazoa - ps12
Metazoa - ps13
Myriazoa - ps14
Parahoxozoa - ps15
Bilateria - ps16
Deuterostomia - ps17
Chordata - ps18
Olfactores - ps19
Vertebrata - ps20
Gnathostomata - ps21
Euteleostomi - ps22
Sarcopterygii - ps23
Tetrapoda - ps24
Amniota - ps25
Mammalia - ps26
Theria - ps27
Placentalia - ps28
Boreoeutheria - ps29
Euarchontoglires - ps30
Euarchonta - ps31
Primates - ps32
Haplorrhini - ps33
Simiiformes - ps34
Catarrhini - ps35
Hominoidea - ps36
Hominidae - ps37
Homininae - ps38
Hominini - ps39
Homo_sapiens - ps40
p-value
1e-02
2e-02
3e-02
3e-02
4e-02
Log-odds
0.5
1.0
1.5
2.0
2.5
3.0
3.5
4.0

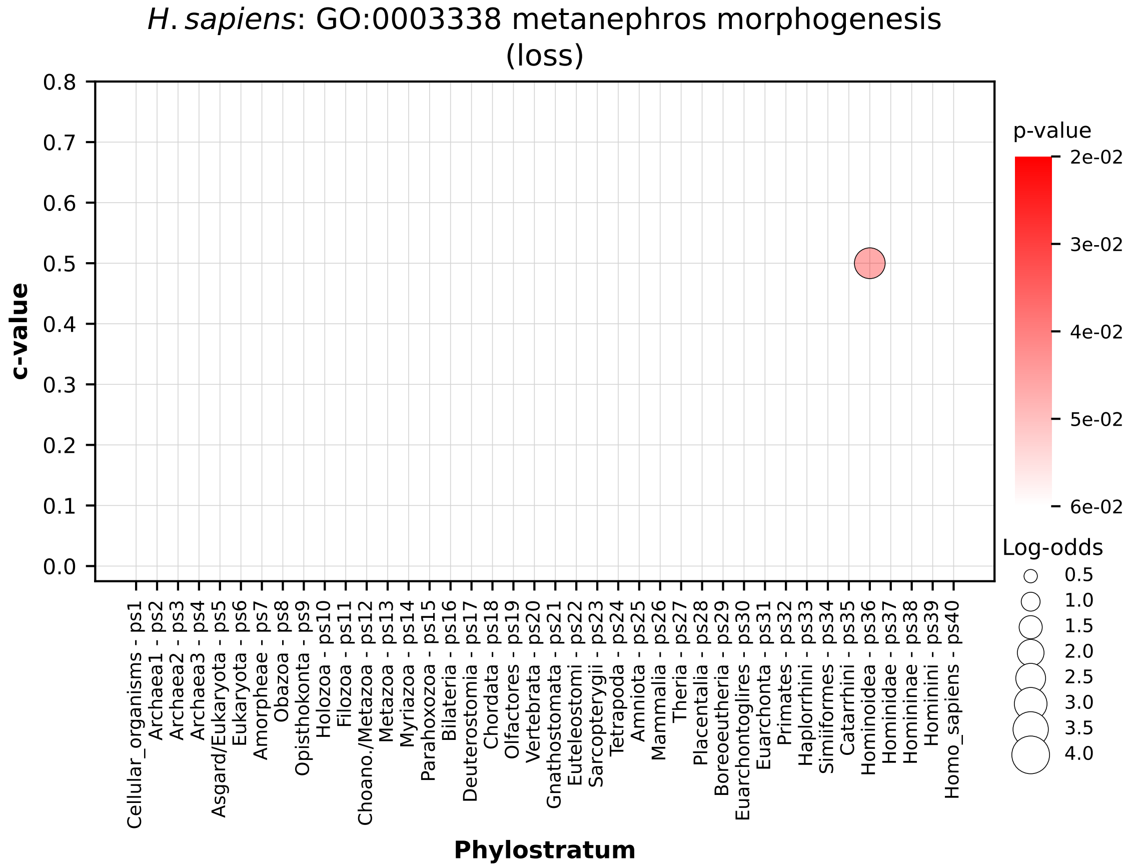

H. sapiens: GO:0003338 metanephros morphogenesis (loss)
c-value
0.8
0.7
0.6
0.5
0.4
0.3
0.2
0.1
0.0
p-value
2e-02
3e-02
4e-02
5e-02
6e-02
Log-odds
0.5
1.0
1.5
2.0
2.5
3.0
3.5
4.0
Cellular_organisms - ps1
Archaea1 - ps2
Archaea2 - ps3
Archaea3 - ps4
Asgard/Eukaryota - ps5
Eukaryota - ps6
Amorpheae - ps7
Obazoa - ps8
Opisthokonta - ps9
Holozoa - ps10
Filozoa - ps11
Choano./Metazoa - ps12
Metazoa - ps13
Myriazoa - ps14
Parahoxozoa - ps15
Bilateria - ps16
Deuterostomia - ps17
Chordata - ps18
Olfactores - ps19
Vertebrata - ps20
Gnathostomata - ps21
Euteleostomi - ps22
Sarcopterygii - ps23
Tetrapoda - ps24
Amniota - ps25
Mammalia - ps26
Theria - ps27
Placentalia - ps28
Boreoeutheria - ps29
Euarchontoglires - ps30
Euarchonta - ps31
Primates - ps32
Haplorrhini - ps33
Simiiformes - ps34
Catarrhini - ps35
Hominoidea - ps36
Hominidae - ps37
Homininae - ps38
Hominini - ps39
Homo_sapiens - ps40
Phylostratum

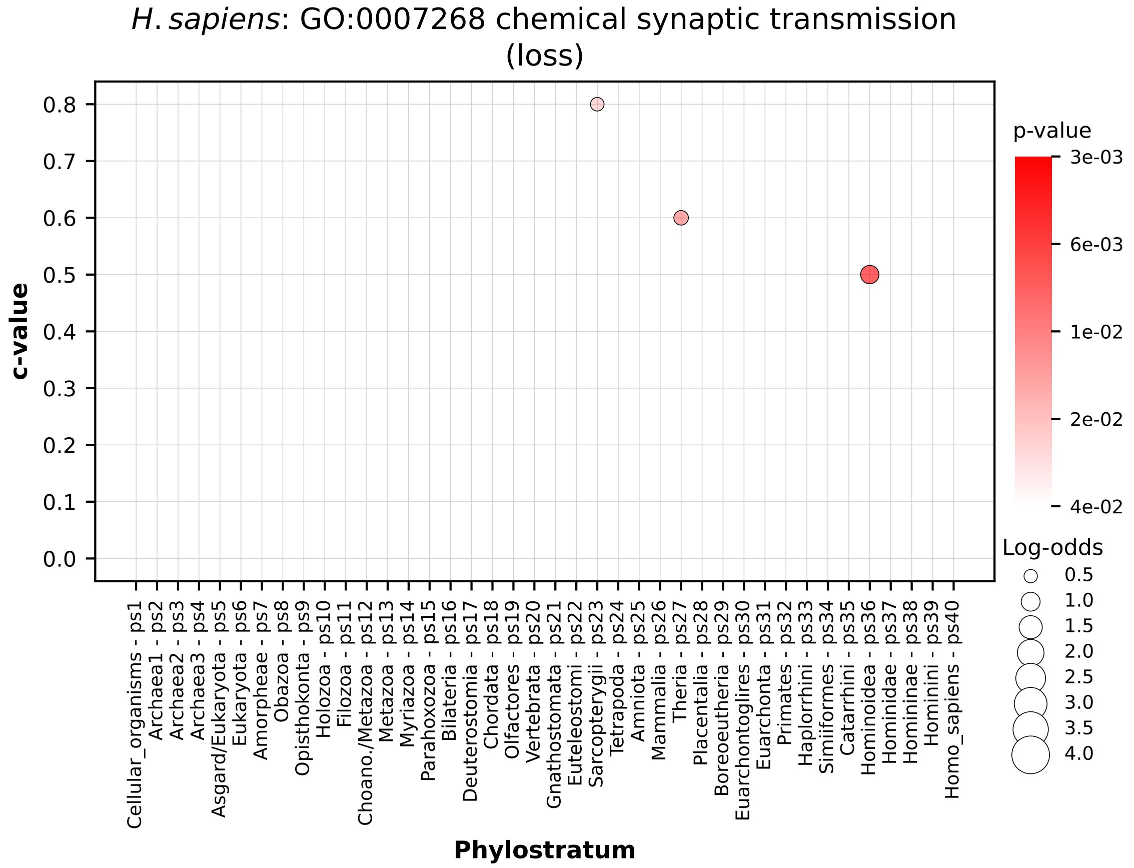
H. sapiens: GO:0007268 chemical synaptic transmission (loss)
c-value
0.0
0.1
0.2
0.3
0.4
0.5
0.6
0.7
0.8
Phylostratum
Cellular_organisms - ps1
Archaea1 - ps2
Archaea2 - ps3
Archaea3 - ps4
Asgard/Eukaryota - ps5
Eukaryota - ps6
Amorpheae - ps7
Obazoa - ps8
Opisthokonta - ps9
Holozoa - ps10
Filozoa - ps11
Choano./Metazoa - ps12
Metazoa - ps13
Myriazoa - ps14
Parahoxozoa - ps15
Bilateria - ps16
Deuterostomia - ps17
Chordata - ps18
Olfactores - ps19
Vertebrata - ps20
Gnathostomata - ps21
Euteleostomi - ps22
Sarcopterygii - ps23
Tetrapoda - ps24
Amniota - ps25
Mammalia - ps26
Theria - ps27
Placentalia - ps28
Boreoeutheria - ps29
Euarchontoglires - ps30
Euarchonta - ps31
Primates - ps32
Haplorrhini - ps33
Simiiformes - ps34
Catarrhini - ps35
Hominoidea - ps36
Hominidae - ps37
Homininae - ps38
Hominini - ps39
Homo_sapiens - ps40
p-value
3e-03
6e-03
1e-02
2e-02
4e-02
Log-odds
0.5
1.0
1.5
2.0
2.5
3.0
3.5
4.0

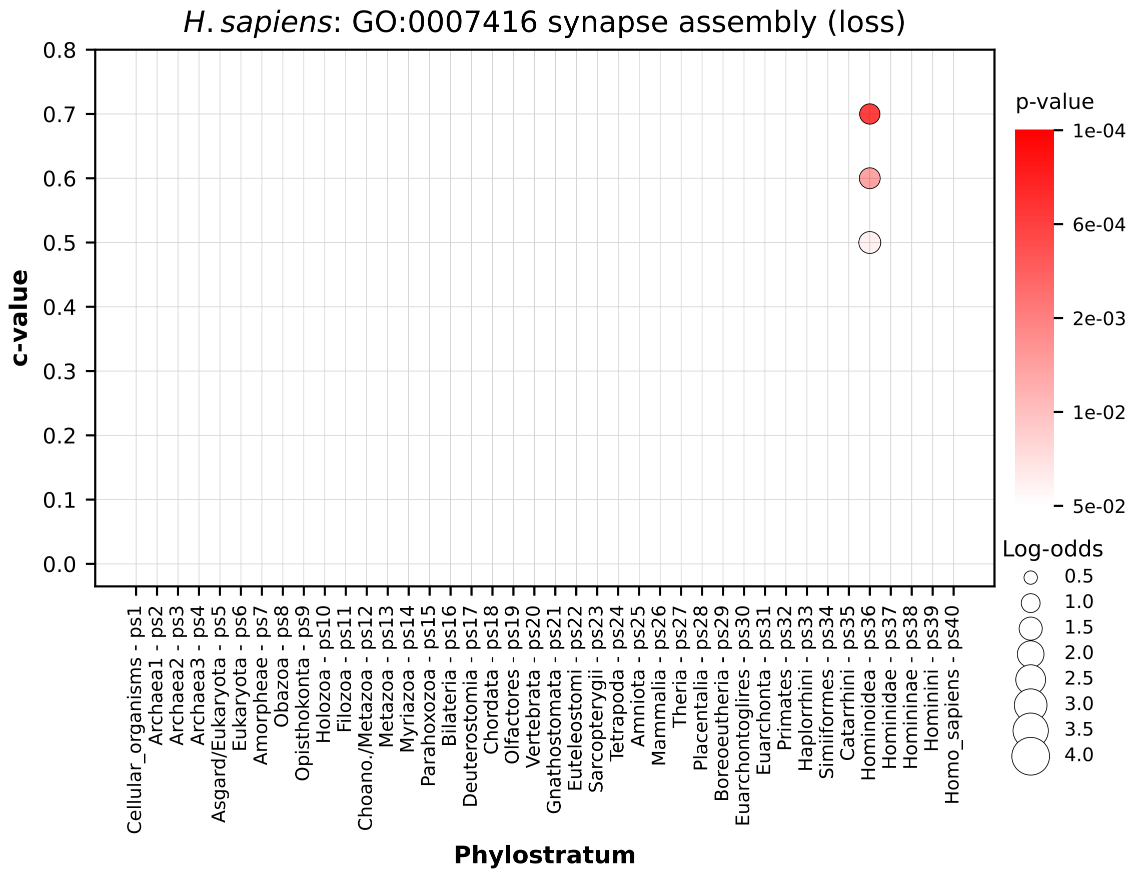
H. sapiens: GO:0007416 synapse assembly (loss)
c-value
0.8
0.7
0.6
0.5
0.4
0.3
0.2
0.1
0.0
p-value
1e-04
6e-04
2e-03
1e-02
5e-02
Log-odds
0.5
1.0
1.5
2.0
2.5
3.0
3.5
4.0
Cellular_organisms - ps1
Archaea1 - ps2
Archaea2 - ps3
Archaea3 - ps4
Asgard/Eukaryota - ps5
Eukaryota - ps6
Amorpheae - ps7
Obazoa - ps8
Opisthokonta - ps9
Holozoa - ps10
Filozoa - ps11
Choano./Metazoa - ps12
Metazoa - ps13
Myriazoa - ps14
Parahoxozoa - ps15
Bilateria - ps16
Deuterostomia - ps17
Chordata - ps18
Olfactores - ps19
Vertebrata - ps20
Gnathostomata - ps21
Euteleostomi - ps22
Sarcopterygii - ps23
Tetrapoda - ps24
Amniota - ps25
Mammalia - ps26
Theria - ps27
Placentalia - ps28
Boreoeutheria - ps29
Euarchontoglires - ps30
Euarchonta - ps31
Primates - ps32
Haplorrhini - ps33
Simiiformes - ps34
Catarrhini - ps35
Hominoidea - ps36
Hominidae - ps37
Homininae - ps38
Hominini - ps39
Homo_sapiens - ps40
Phylostratum

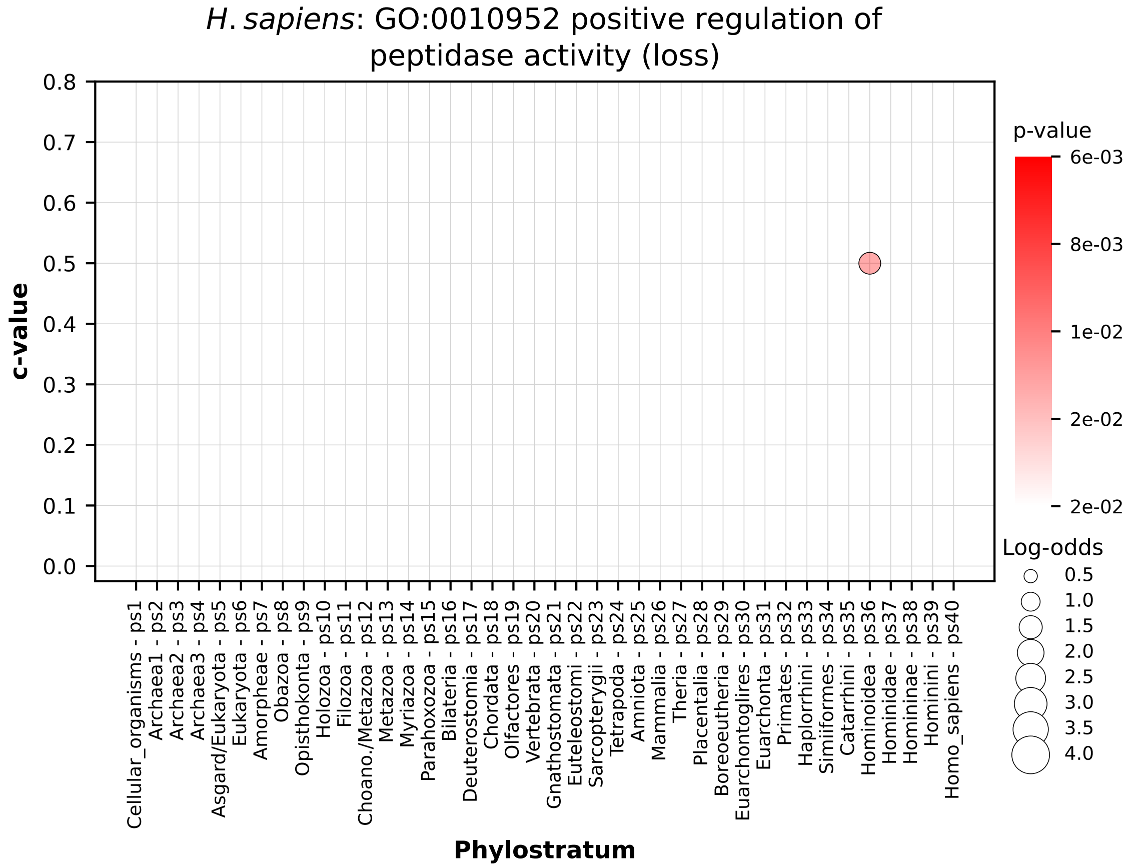

H. sapiens: GO:0010952 positive regulation of peptidase activity (loss)
c-value
0.0
0.1
0.2
0.3
0.4
0.5
0.6
0.7
0.8
p-value
6e-03
8e-03
1e-02
2e-02
2e-02
Log-odds
0.5
1.0
1.5
2.0
2.5
3.0
3.5
4.0
Cellular_organisms - ps1
Archaea1 - ps2
Archaea2 - ps3
Archaea3 - ps4
Asgard/Eukaryota - ps5
Eukaryota - ps6
Amorpheae - ps7
Obazoa - ps8
Opisthokonta - ps9
Holozoa - ps10
Filozoa - ps11
Choano./Metazoa - ps12
Metazoa - ps13
Myriazoa - ps14
Parahoxozoa - ps15
Bilateria - ps16
Deuterostomia - ps17
Chordata - ps18
Olfactores - ps19
Vertebrata - ps20
Gnathostomata - ps21
Euteleostomi - ps22
Sarcopterygii - ps23
Tetrapoda - ps24
Amniota - ps25
Mammalia - ps26
Theria - ps27
Placentalia - ps28
Boreoeutheria - ps29
Euarchontoglires - ps30
Euarchonta - ps31
Primates - ps32
Haplorrhini - ps33
Simiiformes - ps34
Catarrhini - ps35
Hominoidea - ps36
Hominidae - ps37
Homininae - ps38
Hominini - ps39
Homo_sapiens - ps40
Phylostratum

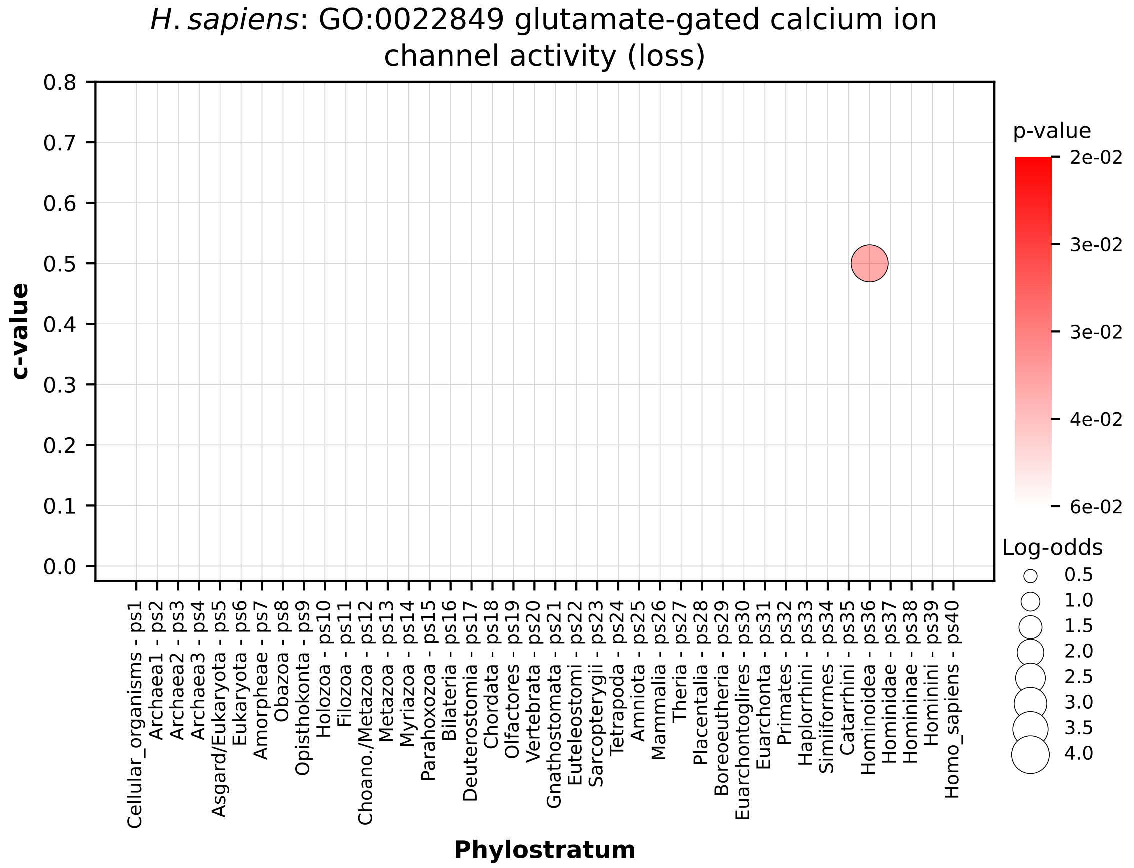
H. sapiens: GO:0022849 glutamate-gated calcium ion channel activity (loss)
c-value
0.0
0.1
0.2
0.3
0.4
0.5
0.6
0.7
0.8
Phylostratum
Cellular_organisms - ps1
Archaea1 - ps2
Archaea2 - ps3
Archaea3 - ps4
Asgard/Eukaryota - ps5
Eukaryota - ps6
Amorpheae - ps7
Obazoa - ps8
Opisthokonta - ps9
Holozoa - ps10
Filozoa - ps11
Choano./Metazoa - ps12
Metazoa - ps13
Myriazoa - ps14
Parahoxozoa - ps15
Bilateria - ps16
Deuterostomia - ps17
Chordata - ps18
Olfactores - ps19
Vertebrata - ps20
Gnathostomata - ps21
Euteleostomi - ps22
Sarcopterygii - ps23
Tetrapoda - ps24
Amniota - ps25
Mammalia - ps26
Theria - ps27
Placentalia - ps28
Boreoeutheria - ps29
Euarchontoglires - ps30
Euarchonta - ps31
Primates - ps32
Haplorrhini - ps33
Simiiformes - ps34
Catarrhini - ps35
Hominoidea - ps36
Hominidae - ps37
Homininae - ps38
Hominini - ps39
Homo_sapiens - ps40
p-value
2e-02
3e-02
3e-02
4e-02
6e-02
Log-odds
0.5
1.0
1.5
2.0
2.5
3.0
3.5
4.0

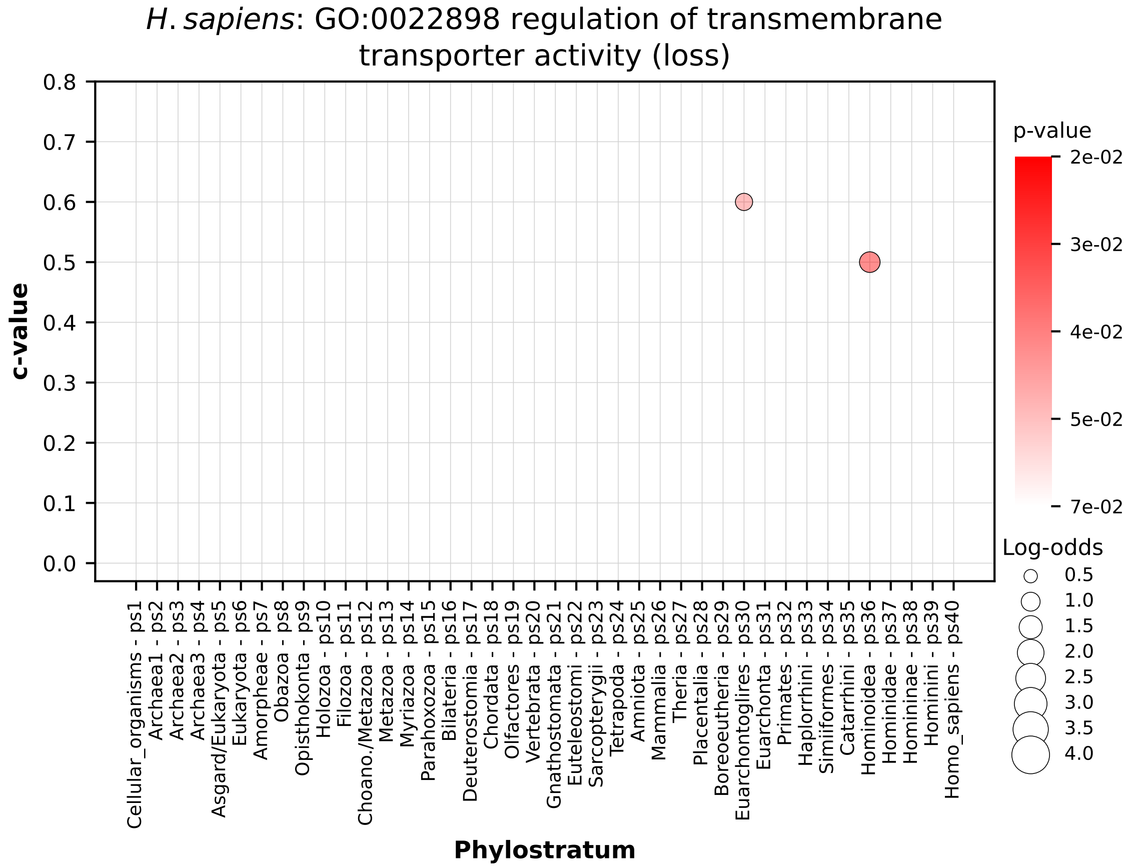

H. sapiens: GO:0022898 regulation of transmembrane transporter activity (loss)
c-value
0.8
0.7
0.6
0.5
0.4
0.3
0.2
0.1
0.0
Phylostratum
Cellular_organisms - ps1
Archaea1 - ps2
Archaea2 - ps3
Archaea3 - ps4
Asgard/Eukaryota - ps5
Eukaryota - ps6
Amorpheae - ps7
Obazoa - ps8
Opisthokonta - ps9
Holozoa - ps10
Filozoa - ps11
Choano./Metazoa - ps12
Metazoa - ps13
Myriazoa - ps14
Parahoxozoa - ps15
Bilateria - ps16
Deuterostomia - ps17
Chordata - ps18
Olfactores - ps19
Vertebrata - ps20
Gnathostomata - ps21
Euteleostomi - ps22
Sarcopterygii - ps23
Tetrapoda - ps24
Amniota - ps25
Mammalia - ps26
Theria - ps27
Placentalia - ps28
Boreoeutheria - ps29
Euarchontoglires - ps30
Euarchonta - ps31
Primates - ps32
Haplorrhini - ps33
Simiiformes - ps34
Catarrhini - ps35
Hominoidea - ps36
Hominidae - ps37
Homininae - ps38
Hominini - ps39
Homo_sapiens - ps40
p-value
2e-02
3e-02
4e-02
5e-02
7e-02
Log-odds
0.5
1.0
1.5
2.0
2.5
3.0
3.5
4.0

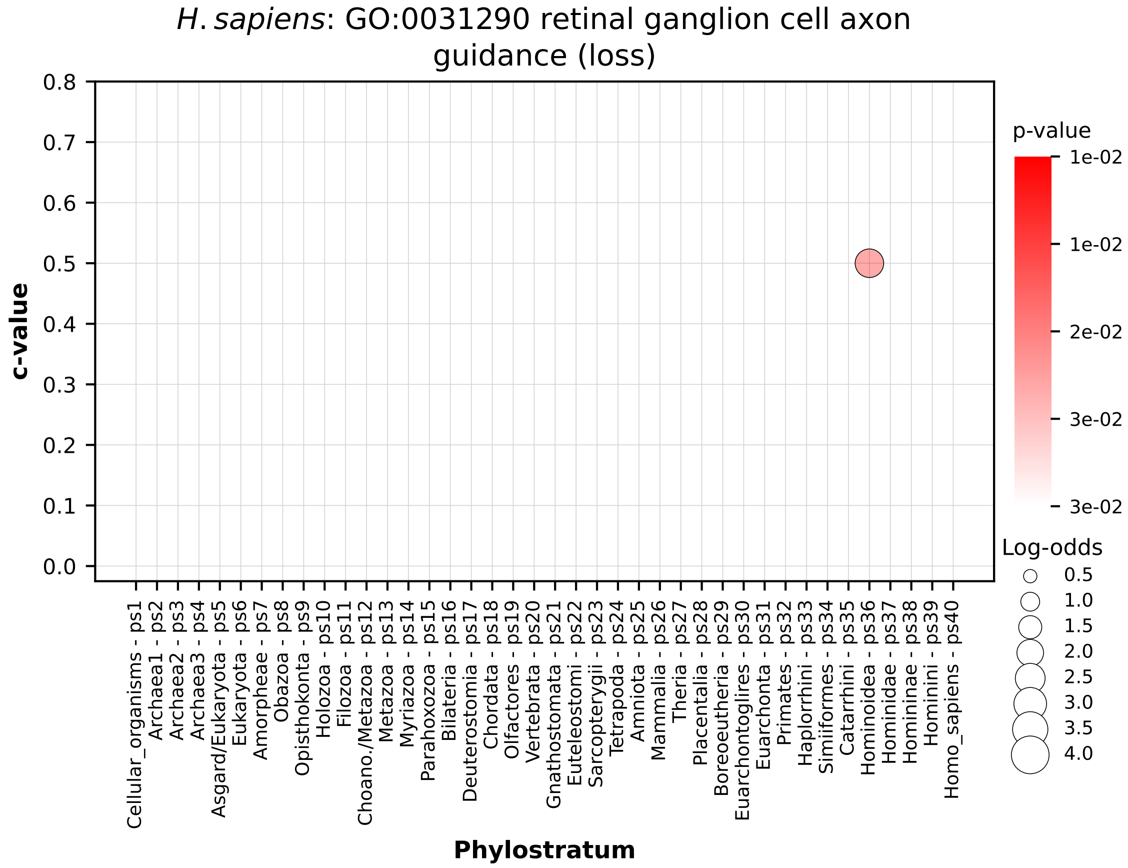

H. sapiens: GO:0031290 retinal ganglion cell axon guidance (loss)
c-value
0.8
0.7
0.6
0.5
0.4
0.3
0.2
0.1
0.0
Phylostratum
Cellular_organisms - ps1
Archaea1 - ps2
Archaea2 - ps3
Archaea3 - ps4
Asgard/Eukaryota - ps5
Eukaryota - ps6
Amorpheae - ps7
Obazoa - ps8
Opisthokonta - ps9
Holozoa - ps10
Filozoa - ps11
Choano./Metazoa - ps12
Metazoa - ps13
Myriazoa - ps14
Parahoxozoa - ps15
Bilateria - ps16
Deuterostomia - ps17
Chordata - ps18
Olfactores - ps19
Vertebrata - ps20
Gnathostomata - ps21
Euteleostomi - ps22
Sarcopterygii - ps23
Tetrapoda - ps24
Amniota - ps25
Mammalia - ps26
Theria - ps27
Placentalia - ps28
Boreoeutheria - ps29
Euarchontoglires - ps30
Euarchonta - ps31
Primates - ps32
Haplorrhini - ps33
Simiiformes - ps34
Catarrhini - ps35
Hominoidea - ps36
Hominidae - ps37
Homininae - ps38
Hominini - ps39
Homo_sapiens - ps40
p-value
1e-02
1e-02
2e-02
3e-02
3e-02
Log-odds
0.5
1.0
1.5
2.0
2.5
3.0
3.5
4.0

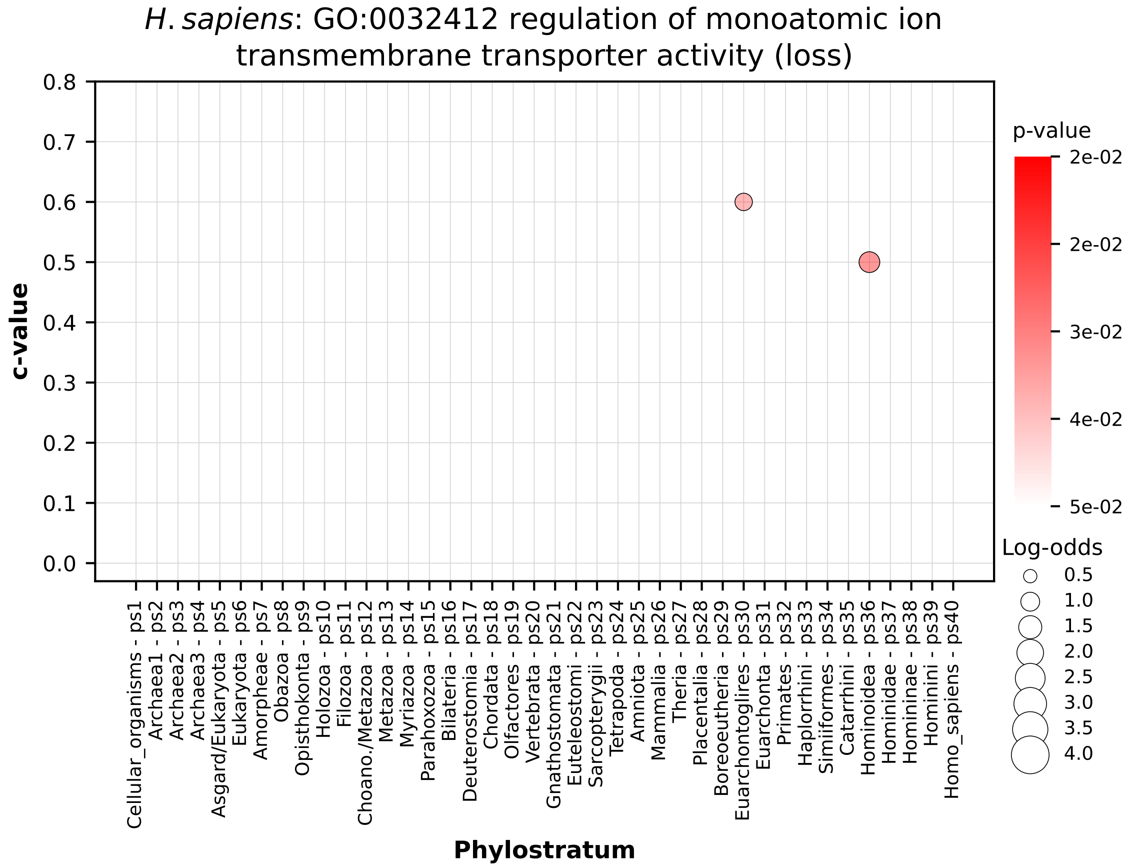

H. sapiens: GO:0032412 regulation of monoatomic ion transmembrane transporter activity (loss)
c-value
0.0
0.1
0.2
0.3
0.4
0.5
0.6
0.7
0.8
p-value
2e-02
2e-02
3e-02
4e-02
5e-02
Log-odds
0.5
1.0
1.5
2.0
2.5
3.0
3.5
4.0
Cellular_organisms - ps1
Archaea1 - ps2
Archaea2 - ps3
Archaea3 - ps4
Asgard/Eukaryota - ps5
Eukaryota - ps6
Amorpheae - ps7
Obazoa - ps8
Opisthokonta - ps9
Holozoa - ps10
Filozoa - ps11
Choano./Metazoa - ps12
Metazoa - ps13
Myriazoa - ps14
Parahoxozoa - ps15
Bilateria - ps16
Deuterostomia - ps17
Chordata - ps18
Olfactores - ps19
Vertebrata - ps20
Gnathostomata - ps21
Euteleostomi - ps22
Sarcopterygii - ps23
Tetrapoda - ps24
Amniota - ps25
Mammalia - ps26
Theria - ps27
Placentalia - ps28
Boreoeutheria - ps29
Euarchontoglires - ps30
Euarchonta - ps31
Primates - ps32
Haplorrhini - ps33
Simiiformes - ps34
Catarrhini - ps35
Hominoidea - ps36
Hominidae - ps37
Homininae - ps38
Hominini - ps39
Homo_sapiens - ps40
Phylostratum

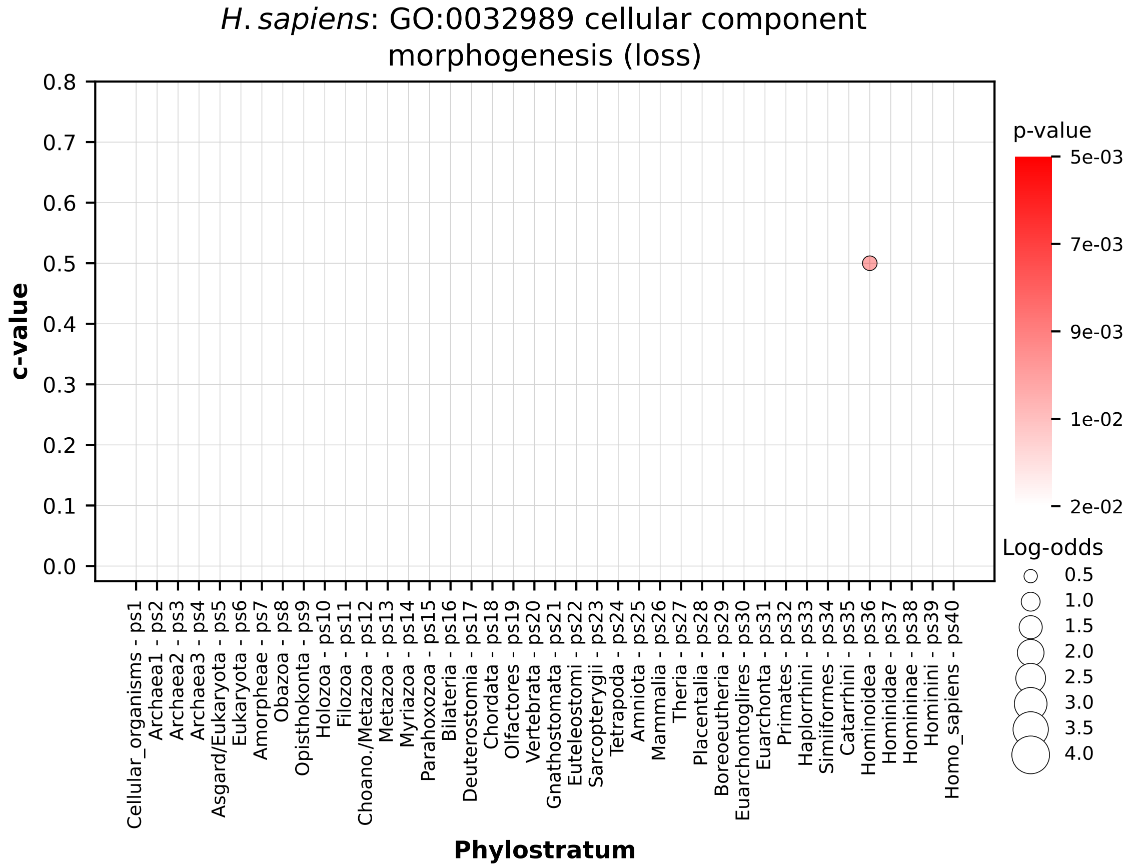

H. sapiens: GO:0032989 cellular component morphogenesis (loss)
c-value
0.8
0.7
0.6
0.5
0.4
0.3
0.2
0.1
0.0
p-value
5e-03
7e-03
9e-03
1e-02
2e-02
Log-odds
0.5
1.0
1.5
2.0
2.5
3.0
3.5
4.0
Cellular_organisms - ps1
Archaea1 - ps2
Archaea2 - ps3
Archaea3 - ps4
Asgard/Eukaryota - ps5
Eukaryota - ps6
Amorpheae - ps7
Obazoa - ps8
Opisthokonta - ps9
Holozoa - ps10
Filozoa - ps11
Choano./Metazoa - ps12
Metazoa - ps13
Myriazoa - ps14
Parahoxozoa - ps15
Bilateria - ps16
Deuterostomia - ps17
Chordata - ps18
Olfactores - ps19
Vertebrata - ps20
Gnathostomata - ps21
Euteleostomi - ps22
Sarcopterygii - ps23
Tetrapoda - ps24
Amniota - ps25
Mammalia - ps26
Theria - ps27
Placentalia - ps28
Boreoeutheria - ps29
Euarchontoglires - ps30
Euarchonta - ps31
Primates - ps32
Haplorrhini - ps33
Simiiformes - ps34
Catarrhini - ps35
Hominoidea - ps36
Hominidae - ps37
Homininae - ps38
Hominini - ps39
Homo_sapiens - ps40
Phylostratum

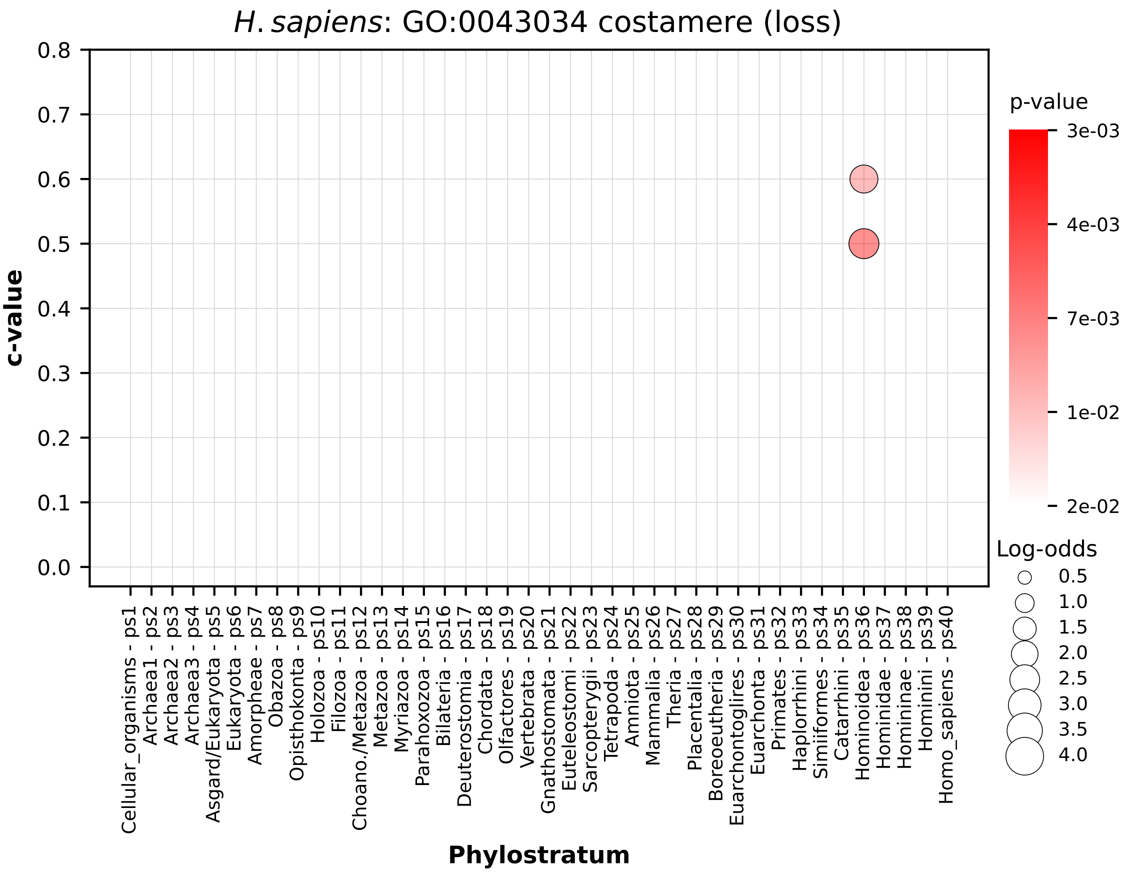

H. sapiens: GO:0043034 costamere (loss)
c-value
0.8
0.7
0.6
0.5
0.4
0.3
0.2
0.1
0.0
p-value
3e-03
4e-03
7e-03
1e-02
2e-02
Log-odds
0.5
1.0
1.5
2.0
2.5
3.0
3.5
4.0
Cellular_organisms - ps1
Archaea1 - ps2
Archaea2 - ps3
Archaea3 - ps4
Asgard/Eukaryota - ps5
Eukaryota - ps6
Amorpheae - ps7
Obazoa - ps8
Opisthokonta - ps9
Holozoa - ps10
Filozoa - ps11
Choano./Metazoa - ps12
Metazoa - ps13
Myriazoa - ps14
Parahoxozoa - ps15
Bilateria - ps16
Deuterostomia - ps17
Chordata - ps18
Olfactores - ps19
Vertebrata - ps20
Gnathostomata - ps21
Euteleostomi - ps22
Sarcopterygii - ps23
Tetrapoda - ps24
Amniota - ps25
Mammalia - ps26
Theria - ps27
Placentalia - ps28
Boreoeutheria - ps29
Euarchontoglires - ps30
Euarchonta - ps31
Primates - ps32
Haplorrhini - ps33
Simiiformes - ps34
Catarrhini - ps35
Hominoidea - ps36
Hominidae - ps37
Homininae - ps38
Hominini - ps39
Homo_sapiens - ps40
Phylostratum

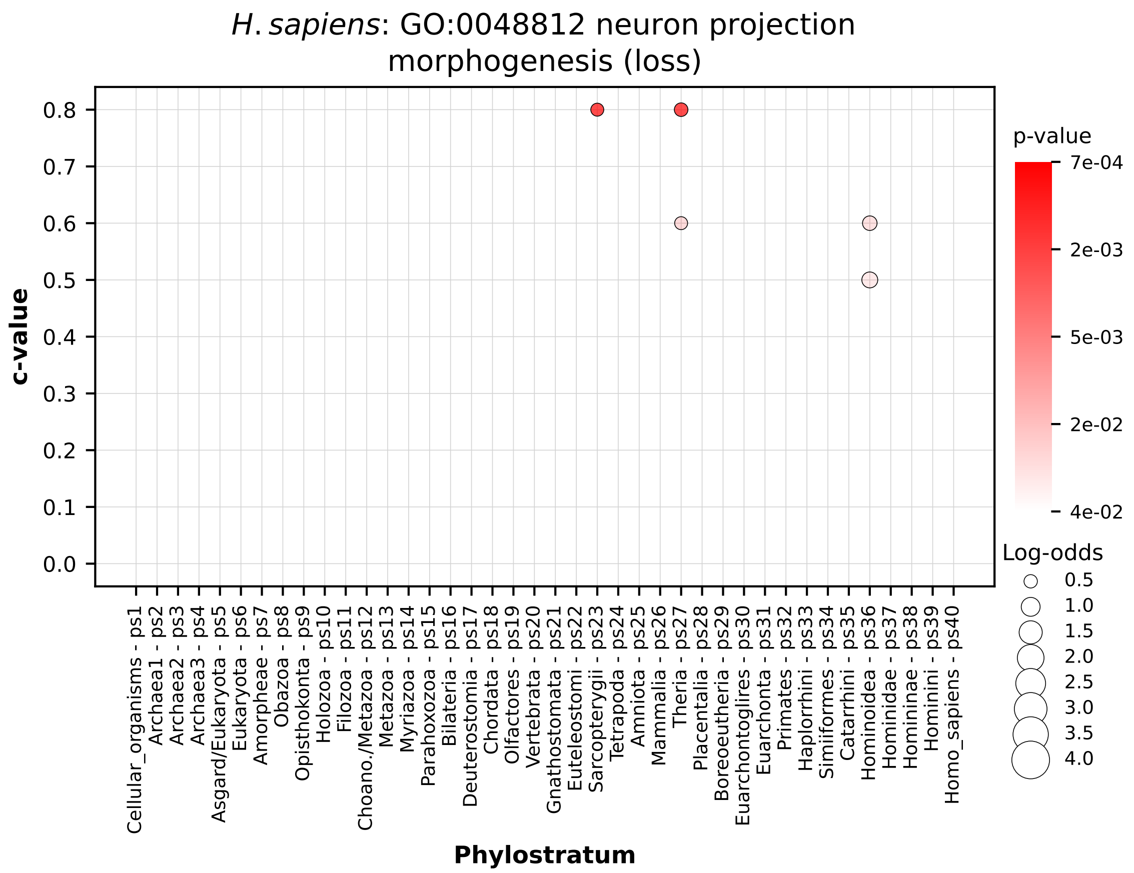

H. sapiens: GO:0048812 neuron projection morphogenesis (loss)
c-value
0.8
0.7
0.6
0.5
0.4
0.3
0.2
0.1
0.0
p-value
7e-04
2e-03
5e-03
2e-02
4e-02
Log-odds
0.5
1.0
1.5
2.0
2.5
3.0
3.5
4.0
Cellular_organisms - ps1
Archaea1 - ps2
Archaea2 - ps3
Archaea3 - ps4
Asgard/Eukaryota - ps5
Eukaryota - ps6
Amorpheae - ps7
Obazoa - ps8
Opisthokonta - ps9
Holozoa - ps10
Filozoa - ps11
Choano./Metazoa - ps12
Metazoa - ps13
Myriazoa - ps14
Parahoxozoa - ps15
Bilateria - ps16
Deuterostomia - ps17
Chordata - ps18
Olfactores - ps19
Vertebrata - ps20
Gnathostomata - ps21
Euteleostomi - ps22
Sarcopterygii - ps23
Tetrapoda - ps24
Amniota - ps25
Mammalia - ps26
Theria - ps27
Placentalia - ps28
Boreoeutheria - ps29
Euarchontoglires - ps30
Euarchonta - ps31
Primates - ps32
Haplorrhini - ps33
Simiiformes - ps34
Catarrhini - ps35
Hominoidea - ps36
Hominidae - ps37
Homininae - ps38
Hominini - ps39
Homo_sapiens - ps40
Phylostratum

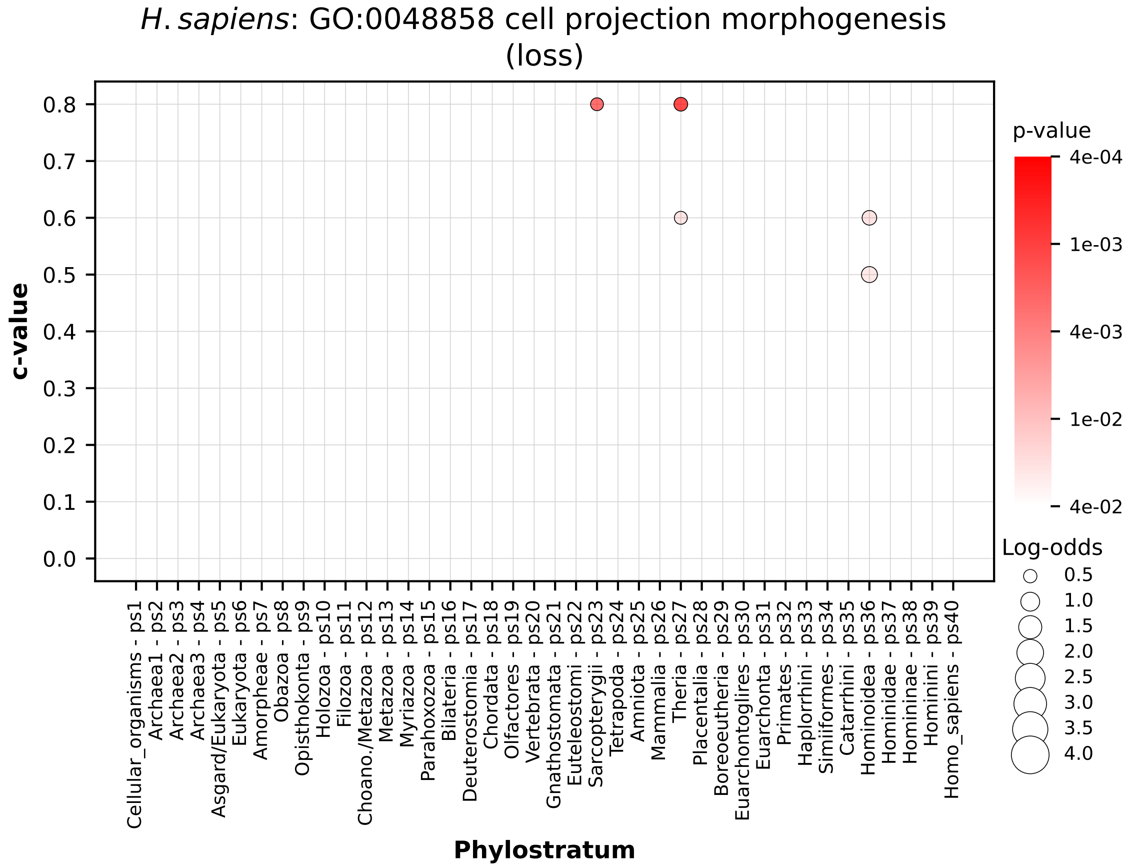

H. sapiens: GO:0048858 cell projection morphogenesis
(loss)
c-value
0.8
0.7
0.6
0.5
0.4
0.3
0.2
0.1
0.0
p-value
4e-04
1e-03
4e-03
1e-02
4e-02
Log-odds
0.5
1.0
1.5
2.0
2.5
3.0
3.5
4.0
Cellular_organisms - ps1
Archaea1 - ps2
Archaea2 - ps3
Archaea3 - ps4
Asgard/Eukaryota - ps5
Eukaryota - ps6
Amorpheae - ps7
Obazoa - ps8
Opisthokonta - ps9
Holozoa - ps10
Filozoa - ps11
Choano./Metazoa - ps12
Metazoa - ps13
Myriazoa - ps14
Parahoxozoa - ps15
Bilateria - ps16
Deuterostomia - ps17
Chordata - ps18
Olfactores - ps19
Vertebrata - ps20
Gnathostomata - ps21
Euteleostomi - ps22
Sarcopterygii - ps23
Tetrapoda - ps24
Amniota - ps25
Mammalia - ps26
Theria - ps27
Placentalia - ps28
Boreoeutheria - ps29
Euarchontoglires - ps30
Euarchonta - ps31
Primates - ps32
Haplorrhini - ps33
Simiiformes - ps34
Catarrhini - ps35
Hominoidea - ps36
Hominidae - ps37
Homininae - ps38
Hominini - ps39
Homo_sapiens - ps40
Phylostratum

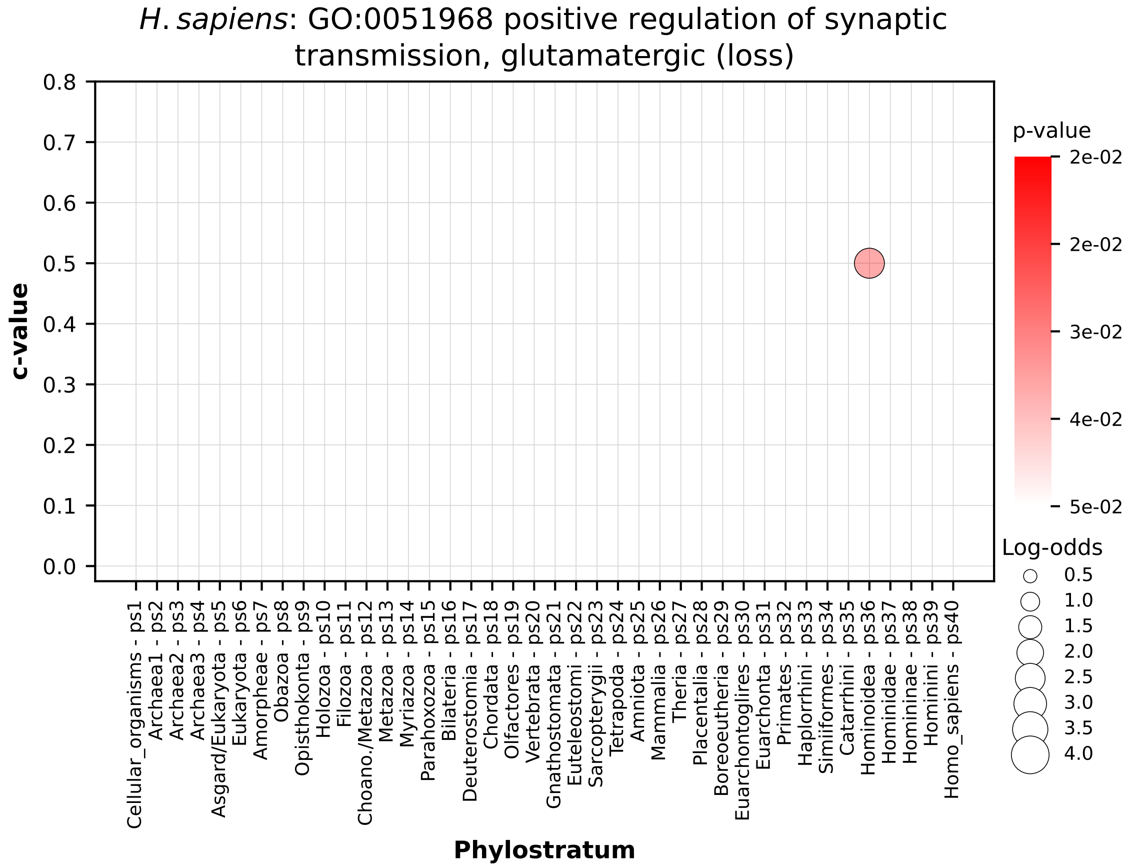

H. sapiens: GO:0051968 positive regulation of synaptic transmission, glutamatergic (loss)
c-value
0.0
0.1
0.2
0.3
0.4
0.5
0.6
0.7
0.8
Phylostratum
Cellular_organisms - ps1
Archaea1 - ps2
Archaea2 - ps3
Archaea3 - ps4
Asgard/Eukaryota - ps5
Eukaryota - ps6
Amorpheae - ps7
Obazoa - ps8
Opisthokonta - ps9
Holozoa - ps10
Filozoa - ps11
Choano./Metazoa - ps12
Metazoa - ps13
Myriazoa - ps14
Parahoxozoa - ps15
Bilateria - ps16
Deuterostomia - ps17
Chordata - ps18
Olfactores - ps19
Vertebrata - ps20
Gnathostomata - ps21
Euteleostomi - ps22
Sarcopterygii - ps23
Tetrapoda - ps24
Amniota - ps25
Mammalia - ps26
Theria - ps27
Placentalia - ps28
Boreoeutheria - ps29
Euarchontoglires - ps30
Euarchonta - ps31
Primates - ps32
Haplorrhini - ps33
Simiiformes - ps34
Catarrhini - ps35
Hominoidea - ps36
Hominidae - ps37
Homininae - ps38
Hominini - ps39
Homo_sapiens - ps40
p-value
2e-02
2e-02
3e-02
4e-02
5e-02
Log-odds
0.5
1.0
1.5
2.0
2.5
3.0
3.5
4.0

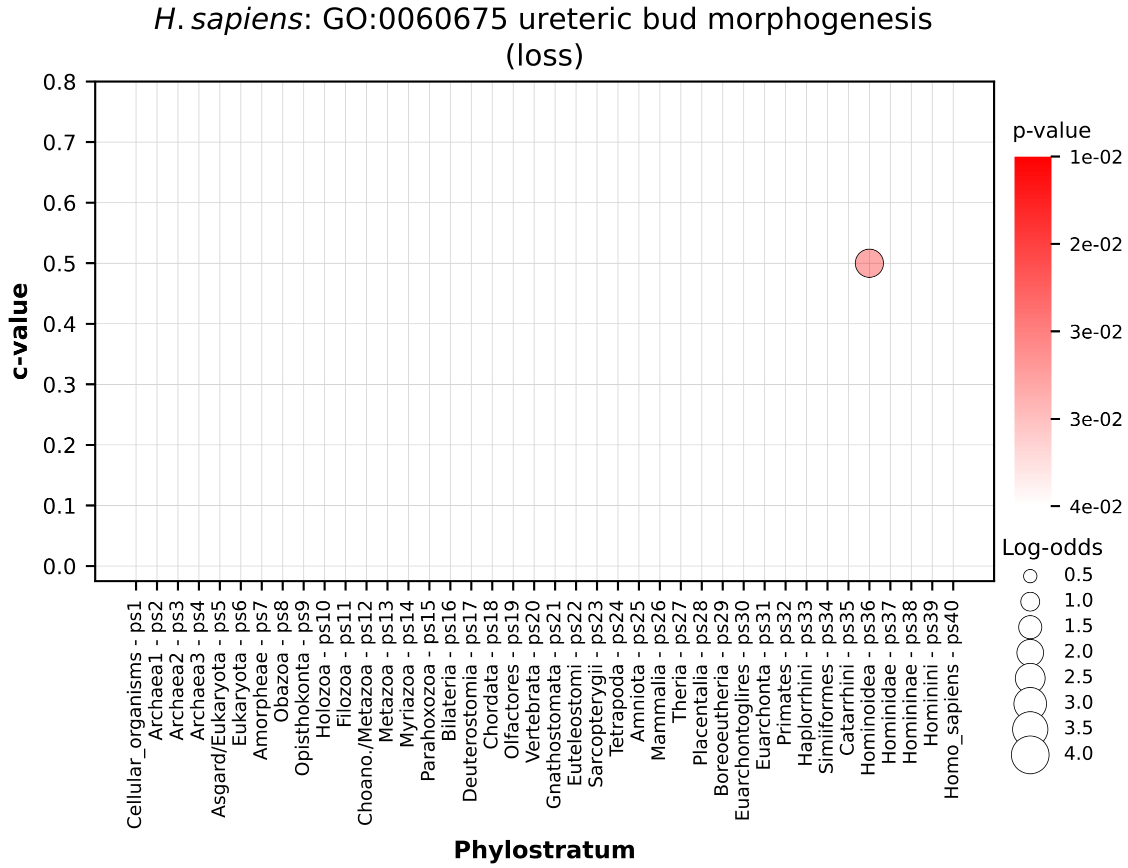
H. sapiens: GO:0060675 ureteric bud morphogenesis (loss)
c-value
Phylostratum
p-value
Log-odds
Cellular_organisms - ps1
Archaea1 - ps2
Archaea2 - ps3
Archaea3 - ps4
Asgard/Eukaryota - ps5
Eukaryota - ps6
Amorpheae - ps7
Obazoa - ps8
Opisthokonta - ps9
Holozoa - ps10
Filozoa - ps11
Choano./Metazoa - ps12
Metazoa - ps13
Myriazoa - ps14
Parahoxozoa - ps15
Bilateria - ps16
Deuterostomia - ps17
Chordata - ps18
Olfactores - ps19
Vertebrata - ps20
Gnathostomata - ps21
Euteleostomi - ps22
Sarcopterygii - ps23
Tetrapoda - ps24
Amniota - ps25
Mammalia - ps26
Theria - ps27
Placentalia - ps28
Boreoeutheria - ps29
Euarchontoglires - ps30
Euarchonta - ps31
Primates - ps32
Haplorrhini - ps33
Simiiformes - ps34
Catarrhini - ps35
Hominoidea - ps36
Hominidae - ps37
Homininae - ps38
Hominini - ps39
Homo_sapiens - ps40

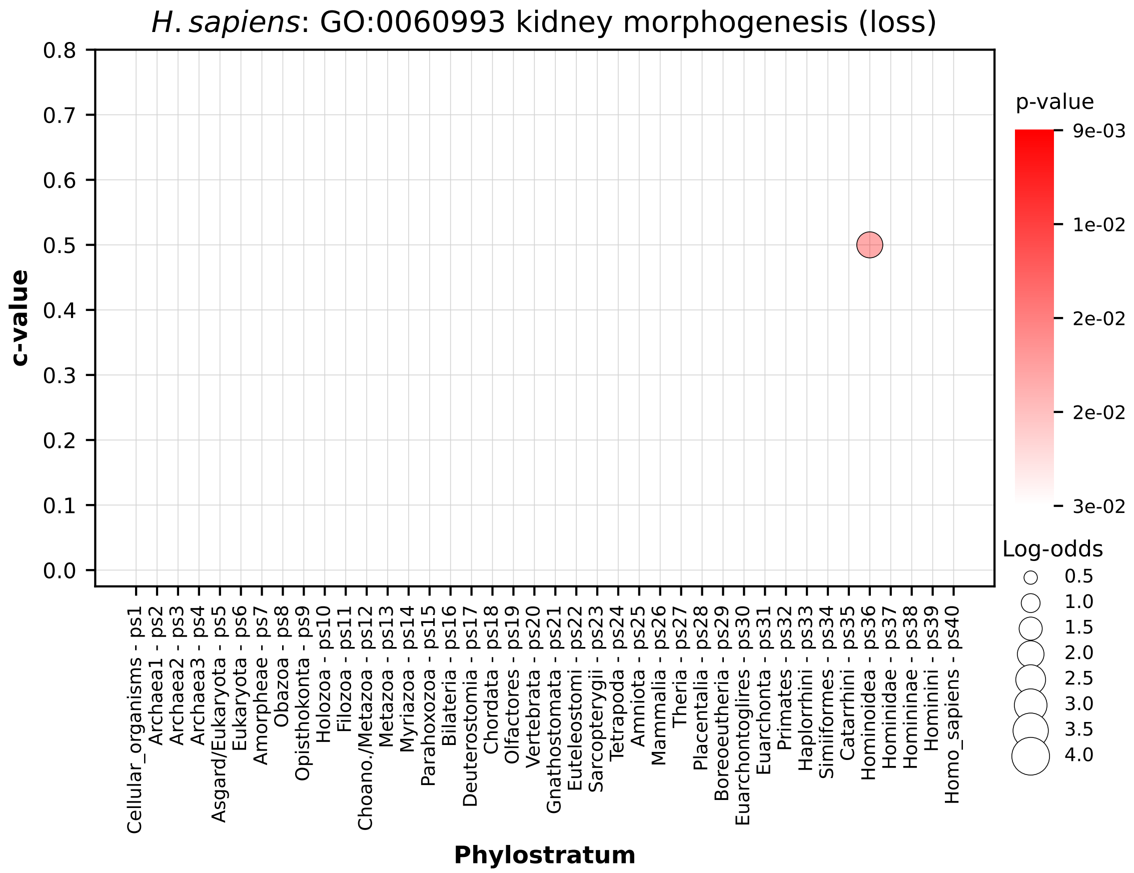
H. sapiens: GO:0060993 kidney morphogenesis (loss)
c-value
0.0
0.1
0.2
0.3
0.4
0.5
0.6
0.7
0.8
Phylostratum
Cellular_organisms - ps1
Archaea1 - ps2
Archaea2 - ps3
Archaea3 - ps4
Asgard/Eukaryota - ps5
Eukaryota - ps6
Amorpheae - ps7
Obazoa - ps8
Opisthokonta - ps9
Holozoa - ps10
Filozoa - ps11
Choano./Metazoa - ps12
Metazoa - ps13
Myriazoa - ps14
Parahoxozoa - ps15
Bilateria - ps16
Deuterostomia - ps17
Chordata - ps18
Olfactores - ps19
Vertebrata - ps20
Gnathostomata - ps21
Euteleostomi - ps22
Sarcopterygii - ps23
Tetrapoda - ps24
Amniota - ps25
Mammalia - ps26
Theria - ps27
Placentalia - ps28
Boreoeutheria - ps29
Euarchontoglires - ps30
Euarchonta - ps31
Primates - ps32
Haplorrhini - ps33
Simiiformes - ps34
Catarrhini - ps35
Hominoidea - ps36
Hominidae - ps37
Homininae - ps38
Hominini - ps39
Homo_sapiens - ps40
p-value
9e-03
1e-02
2e-02
2e-02
3e-02
Log-odds
0.5
1.0
1.5
2.0
2.5
3.0
3.5
4.0

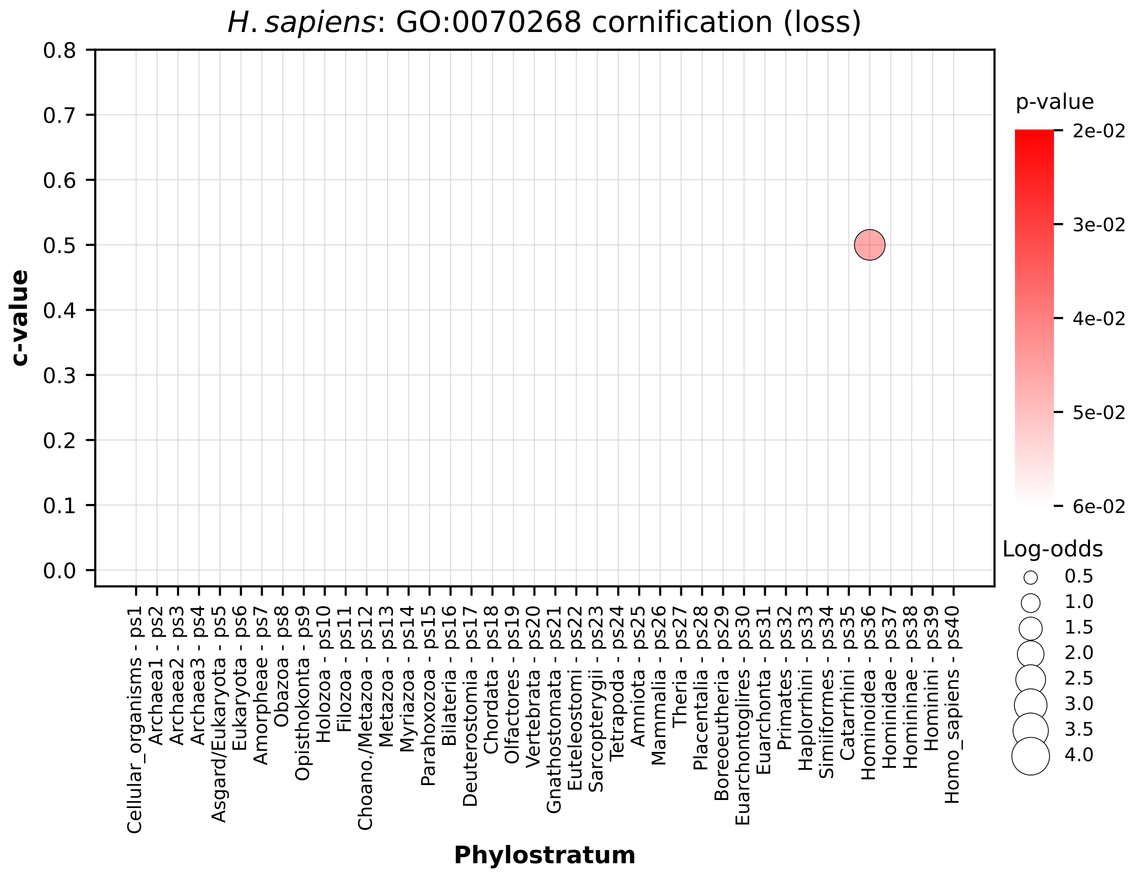

H. sapiens: GO:0070268 cornification (loss)
c-value
0.8
0.7
0.6
0.5
0.4
0.3
0.2
0.1
0.0
p-value
2e-02
3e-02
4e-02
5e-02
6e-02
Log-odds
0.5
1.0
1.5
2.0
2.5
3.0
3.5
4.0
Cellular_organisms - ps1
Archaea1 - ps2
Archaea2 - ps3
Archaea3 - ps4
Asgard/Eukaryota - ps5
Eukaryota - ps6
Amorpheae - ps7
Obazoa - ps8
Opisthokonta - ps9
Holozoa - ps10
Filozoa - ps11
Choano./Metazoa - ps12
Metazoa - ps13
Myriazoa - ps14
Parahoxozoa - ps15
Bilateria - ps16
Deuterostomia - ps17
Chordata - ps18
Olfactores - ps19
Vertebrata - ps20
Gnathostomata - ps21
Euteleostomi - ps22
Sarcopterygii - ps23
Tetrapoda - ps24
Amniota - ps25
Mammalia - ps26
Theria - ps27
Placentalia - ps28
Boreoeutheria - ps29
Euarchontoglires - ps30
Euarchonta - ps31
Primates - ps32
Haplorrhini - ps33
Simiiformes - ps34
Catarrhini - ps35
Hominoidea - ps36
Hominidae - ps37
Homininae - ps38
Hominini - ps39
Homo_sapiens - ps40
Phylostratum

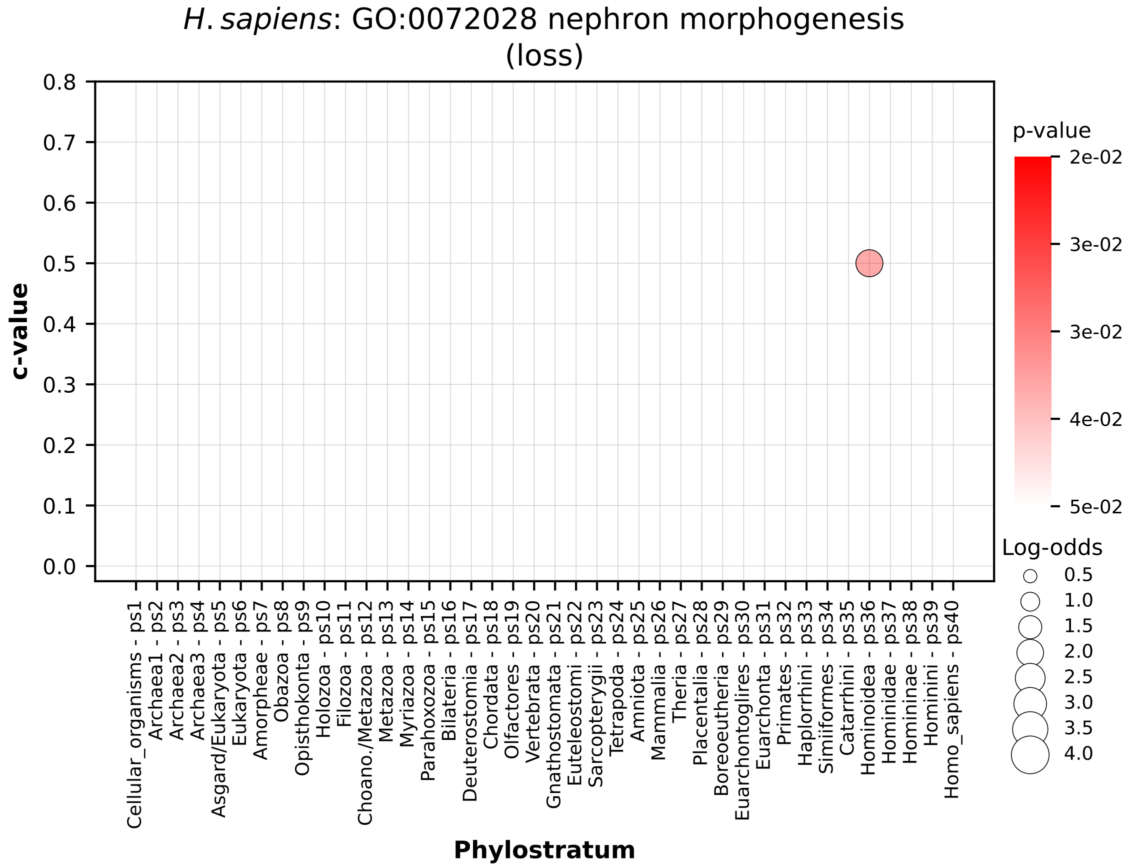

H. sapiens: GO:0072028 nephron morphogenesis
(loss)
c-value
0.8
0.7
0.6
0.5
0.4
0.3
0.2
0.1
0.0
p-value
2e-02
3e-02
3e-02
4e-02
5e-02
Log-odds
0.5
1.0
1.5
2.0
2.5
3.0
3.5
4.0
Cellular_organisms - ps1
Archaea1 - ps2
Archaea2 - ps3
Archaea3 - ps4
Asgard/Eukaryota - ps5
Eukaryota - ps6
Amorpheae - ps7
Obazoa - ps8
Opisthokonta - ps9
Holozoa - ps10
Filozoa - ps11
Choano./Metazoa - ps12
Metazoa - ps13
Myriazoa - ps14
Parahoxozoa - ps15
Bilateria - ps16
Deuterostomia - ps17
Chordata - ps18
Olfactores - ps19
Vertebrata - ps20
Gnathostomata - ps21
Euteleostomi - ps22
Sarcopterygii - ps23
Tetrapoda - ps24
Amniota - ps25
Mammalia - ps26
Theria - ps27
Placentalia - ps28
Boreoeutheria - ps29
Euarchontoglires - ps30
Euarchonta - ps31
Primates - ps32
Haplorrhini - ps33
Simiiformes - ps34
Catarrhini - ps35
Hominoidea - ps36
Hominidae - ps37
Homininae - ps38
Hominini - ps39
Homo_sapiens - ps40
Phylostratum

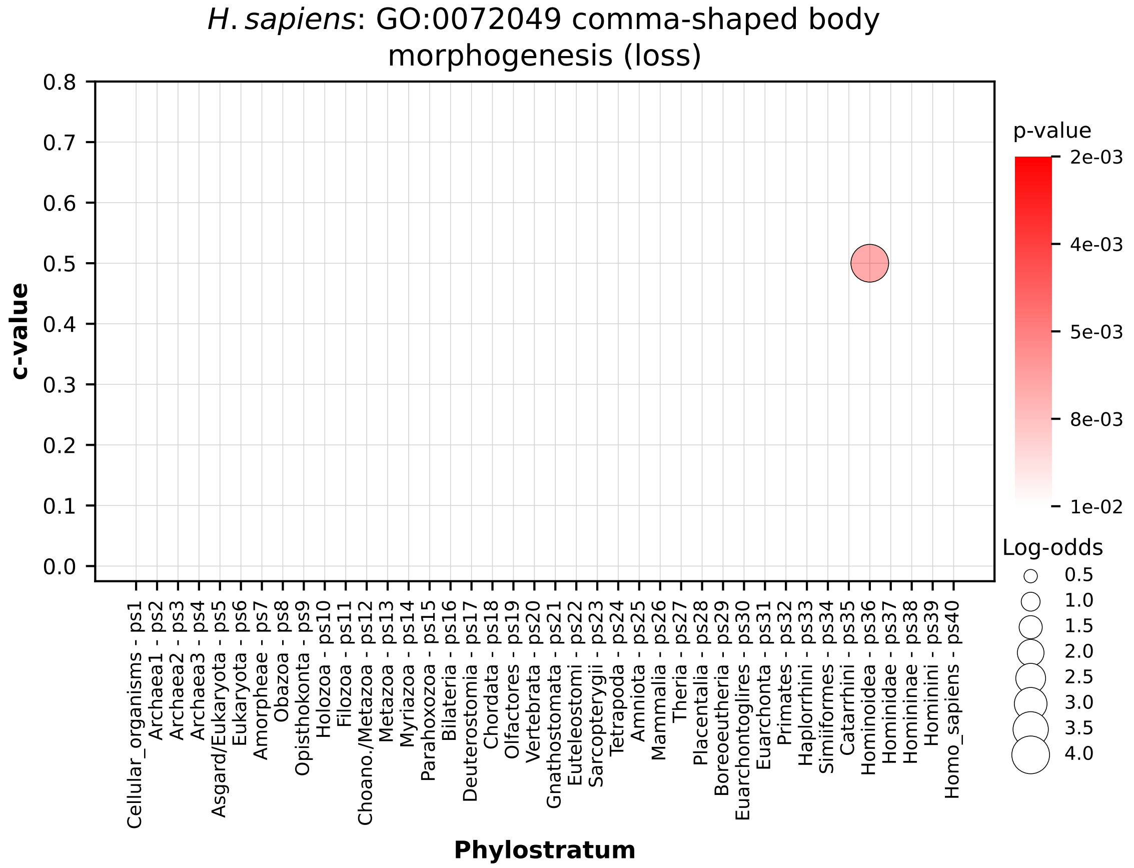

H. sapiens: GO:0072049 comma-shaped body morphogenesis (loss)
c-value
0.8
0.7
0.6
0.5
0.4
0.3
0.2
0.1
0.0
Phylostratum
Cellular_organisms - ps1
Archaea1 - ps2
Archaea2 - ps3
Archaea3 - ps4
Asgard/Eukaryota - ps5
Eukaryota - ps6
Amorpheae - ps7
Obazoa - ps8
Opisthokonta - ps9
Holozoa - ps10
Filozoa - ps11
Choano./Metazoa - ps12
Metazoa - ps13
Myriazoa - ps14
Parahoxozoa - ps15
Bilateria - ps16
Deuterostomia - ps17
Chordata - ps18
Olfactores - ps19
Vertebrata - ps20
Gnathostomata - ps21
Euteleostomi - ps22
Sarcopterygii - ps23
Tetrapoda - ps24
Amniota - ps25
Mammalia - ps26
Theria - ps27
Placentalia - ps28
Boreoeutheria - ps29
Euarchontoglires - ps30
Euarchonta - ps31
Primates - ps32
Haplorrhini - ps33
Simiiformes - ps34
Catarrhini - ps35
Hominoidea - ps36
Hominidae - ps37
Homininae - ps38
Hominini - ps39
Homo_sapiens - ps40
p-value
2e-03
4e-03
5e-03
8e-03
1e-02
Log-odds
0.5
1.0
1.5
2.0
2.5
3.0
3.5
4.0

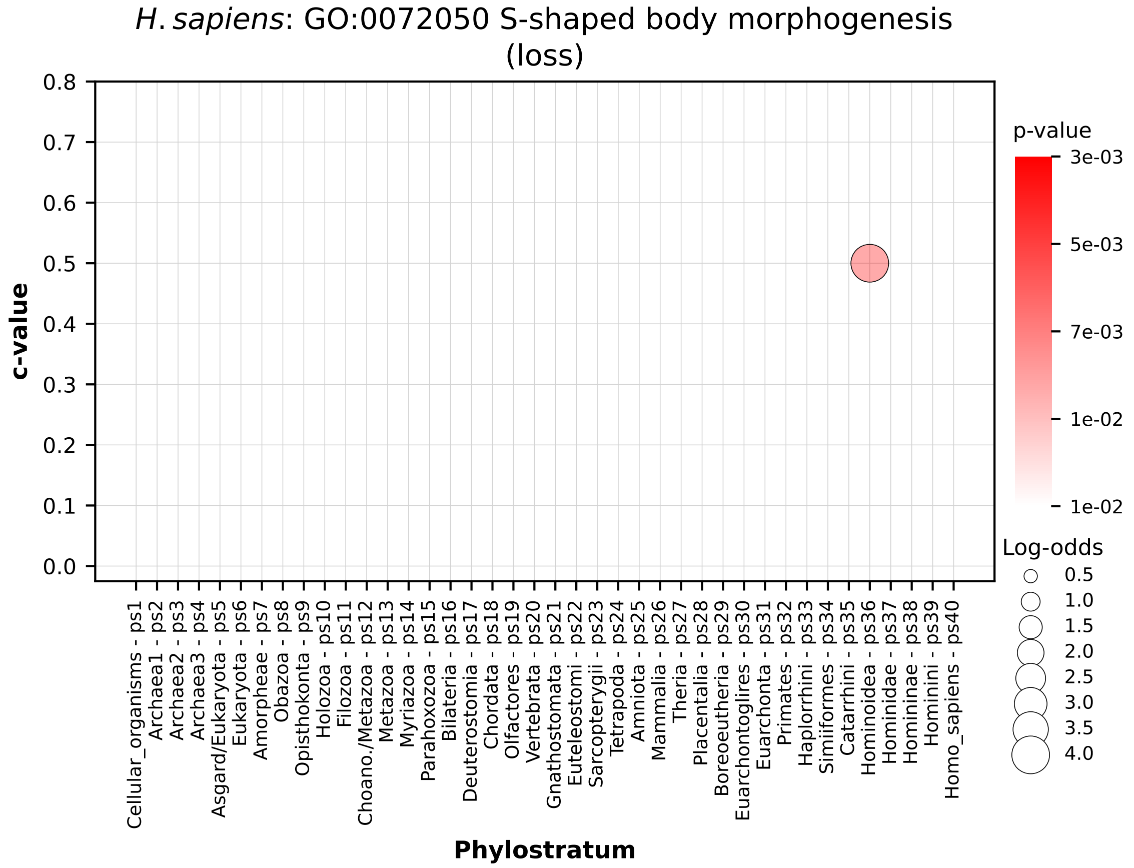
H. sapiens: GO:0072050 S-shaped body morphogenesis
(loss)
c-value
0.8
0.7
0.6
0.5
0.4
0.3
0.2
0.1
0.0
p-value
3e-03
5e-03
7e-03
1e-02
1e-02
Log-odds
0.5
1.0
1.5
2.0
2.5
3.0
3.5
4.0
Cellular_organisms - ps1
Archaea1 - ps2
Archaea2 - ps3
Archaea3 - ps4
Asgard/Eukaryota - ps5
Eukaryota - ps6
Amorpheae - ps7
Obazoa - ps8
Opisthokonta - ps9
Holozoa - ps10
Filozoa - ps11
Choano./Metazoa - ps12
Metazoa - ps13
Myriazoa - ps14
Parahoxozoa - ps15
Bilateria - ps16
Deuterostomia - ps17
Chordata - ps18
Olfactores - ps19
Vertebrata - ps20
Gnathostomata - ps21
Euteleostomi - ps22
Sarcopterygii - ps23
Tetrapoda - ps24
Amniota - ps25
Mammalia - ps26
Theria - ps27
Placentalia - ps28
Boreoeutheria - ps29
Euarchontoglires - ps30
Euarchonta - ps31
Primates - ps32
Haplorrhini - ps33
Simiiformes - ps34
Catarrhini - ps35
Hominoidea - ps36
Hominidae - ps37
Homininae - ps38
Hominini - ps39
Homo_sapiens - ps40
Phylostratum

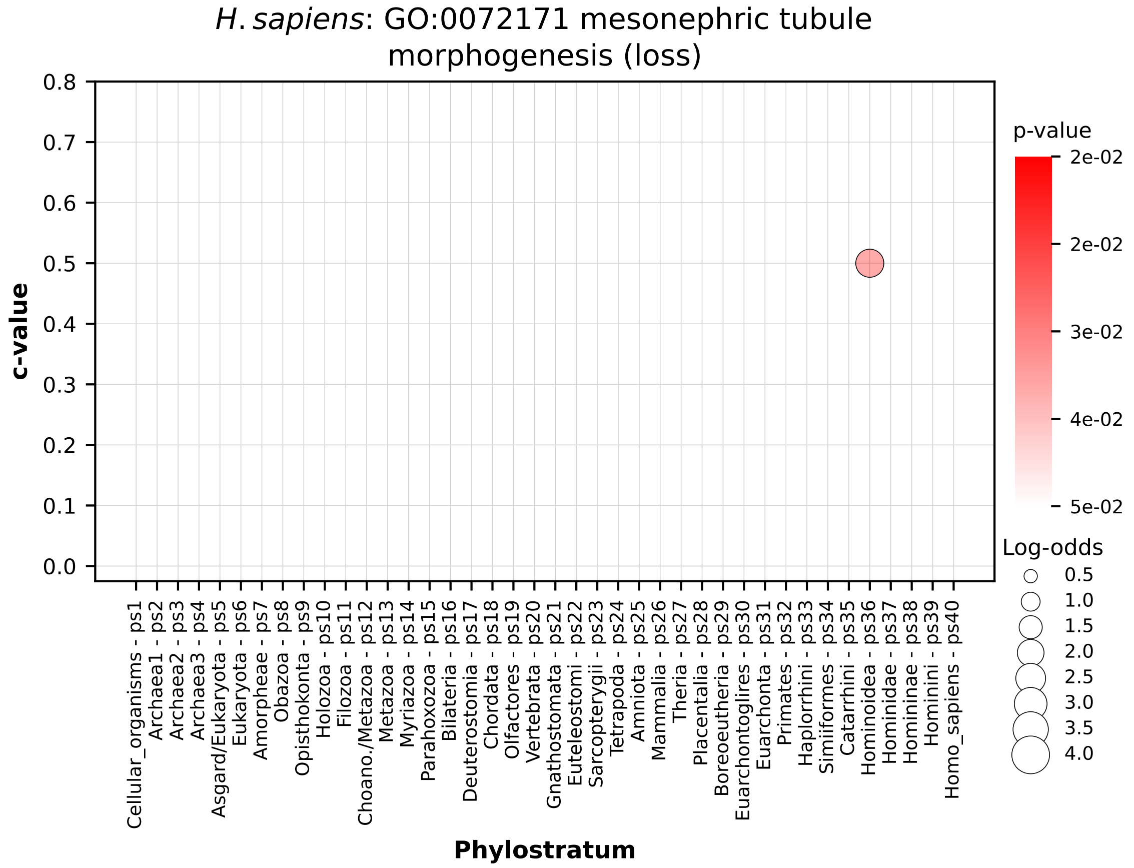

H. sapiens: GO:0072171 mesonephric tubule morphogenesis (loss)
c-value
0.0
0.1
0.2
0.3
0.4
0.5
0.6
0.7
0.8
Phylostratum
Cellular_organisms - ps1
Archaea1 - ps2
Archaea2 - ps3
Archaea3 - ps4
Asgard/Eukaryota - ps5
Eukaryota - ps6
Amorpheae - ps7
Obazoa - ps8
Opisthokonta - ps9
Holozoa - ps10
Filozoa - ps11
Choano./Metazoa - ps12
Metazoa - ps13
Myriazoa - ps14
Parahoxozoa - ps15
Bilateria - ps16
Deuterostomia - ps17
Chordata - ps18
Olfactores - ps19
Vertebrata - ps20
Gnathostomata - ps21
Euteleostomi - ps22
Sarcopterygii - ps23
Tetrapoda - ps24
Amniota - ps25
Mammalia - ps26
Theria - ps27
Placentalia - ps28
Boreoeutheria - ps29
Euarchontoglires - ps30
Euarchonta - ps31
Primates - ps32
Haplorrhini - ps33
Simiiformes - ps34
Catarrhini - ps35
Hominoidea - ps36
Hominidae - ps37
Homininae - ps38
Hominini - ps39
Homo_sapiens - ps40
p-value
2e-02
2e-02
3e-02
4e-02
5e-02
Log-odds
0.5
1.0
1.5
2.0
2.5
3.0
3.5
4.0

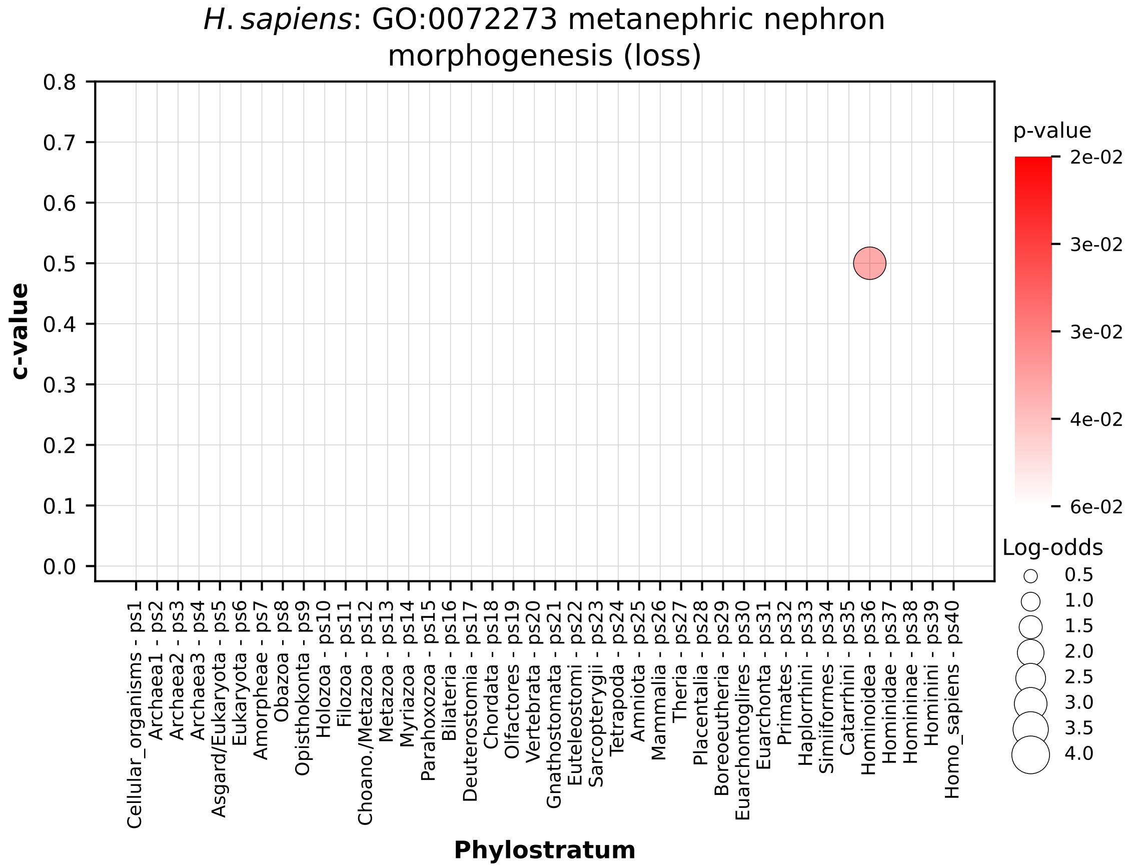
H. sapiens: GO:0072273 metanephric nephron morphogenesis (loss)
c-value
0.0
0.1
0.2
0.3
0.4
0.5
0.6
0.7
0.8
Phylostratum
Cellular_organisms - ps1
Archaea1 - ps2
Archaea2 - ps3
Archaea3 - ps4
Asgard/Eukaryota - ps5
Eukaryota - ps6
Amorpheae - ps7
Obazoa - ps8
Opisthokonta - ps9
Holozoa - ps10
Filozoa - ps11
Choano./Metazoa - ps12
Metazoa - ps13
Myriazoa - ps14
Parahoxozoa - ps15
Bilateria - ps16
Deuterostomia - ps17
Chordata - ps18
Olfactores - ps19
Vertebrata - ps20
Gnathostomata - ps21
Euteleostomi - ps22
Sarcopterygii - ps23
Tetrapoda - ps24
Amniota - ps25
Mammalia - ps26
Theria - ps27
Placentalia - ps28
Boreoeutheria - ps29
Euarchontoglires - ps30
Euarchonta - ps31
Primates - ps32
Haplorrhini - ps33
Simiiformes - ps34
Catarrhini - ps35
Hominoidea - ps36
Hominidae - ps37
Homininae - ps38
Hominini - ps39
Homo_sapiens - ps40
p-value
2e-02
3e-02
3e-02
4e-02
6e-02
Log-odds
0.5
1.0
1.5
2.0
2.5
3.0
3.5
4.0

H. sapiens: GO:0072278 metanephric comma-shaped body morphogenesis (loss)

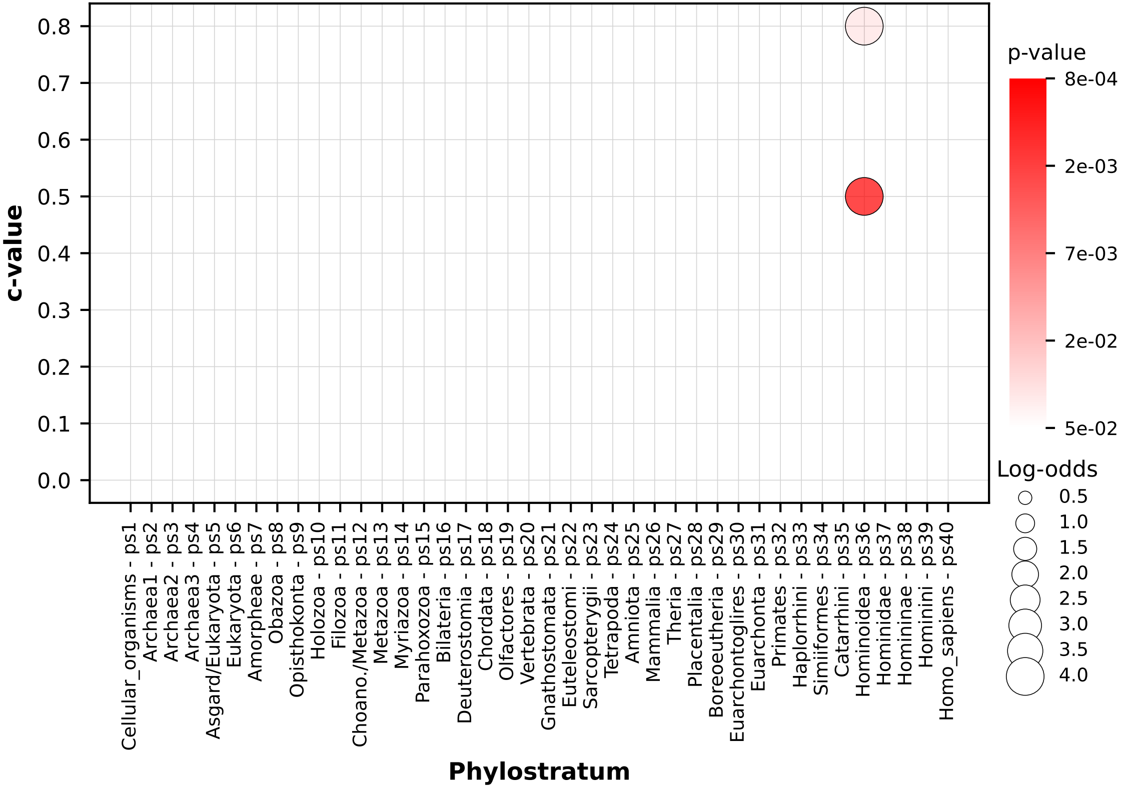

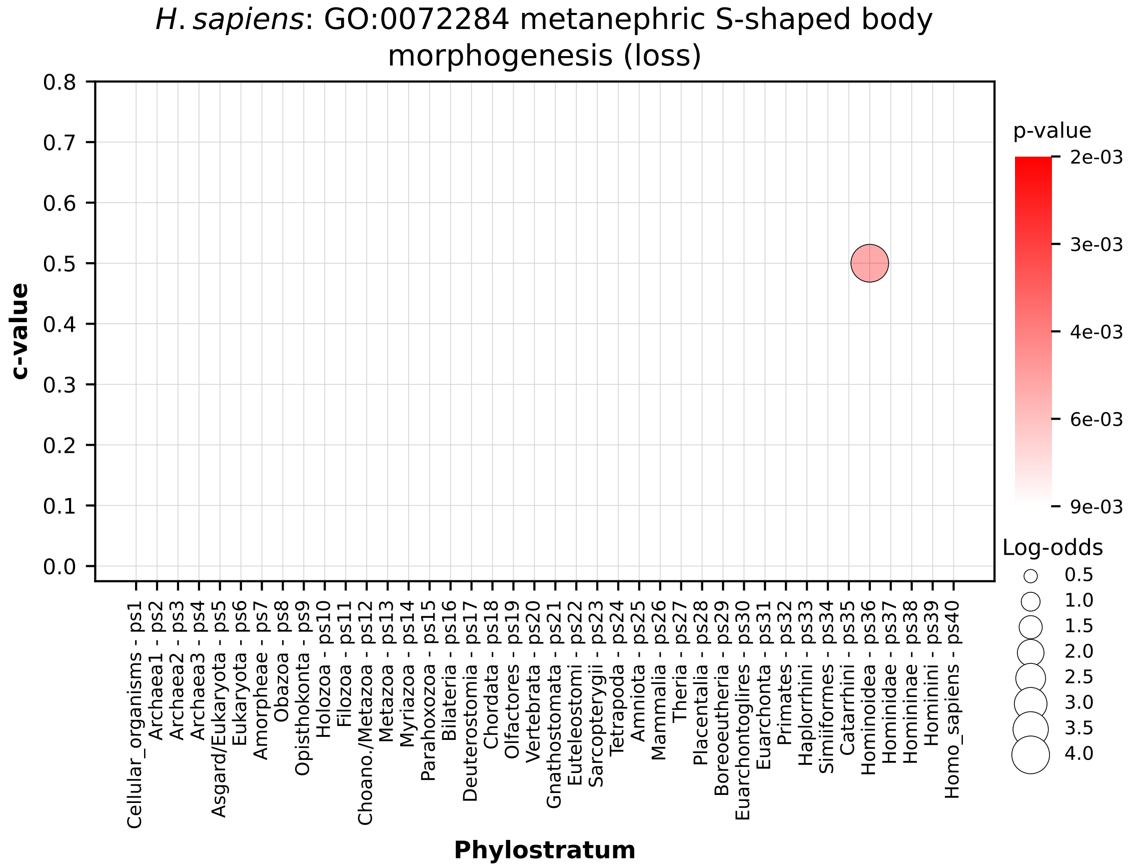
H. sapiens: GO:0072284 metanephric S-shaped body morphogenesis (loss)
c-value
Phylostratum
p-value
2e-03
3e-03
4e-03
6e-03
9e-03
Log-odds
0.5
1.0
1.5
2.0
2.5
3.0
3.5
4.0
0.0
0.1
0.2
0.3
0.4
0.5
0.6
0.7
0.8
Cellular_organisms - ps1
Archaea1 - ps2
Archaea2 - ps3
Archaea3 - ps4
Asgard/Eukaryota - ps5
Eukaryota - ps6
Amorpheae - ps7
Obazoa - ps8
Opisthokonta - ps9
Holozoa - ps10
Filozoa - ps11
Choano./Metazoa - ps12
Metazoa - ps13
Myriazoa - ps14
Parahoxozoa - ps15
Bilateria - ps16
Deuterostomia - ps17
Chordata - ps18
Olfactores - ps19
Vertebrata - ps20
Gnathostomata - ps21
Euteleostomi - ps22
Sarcopterygii - ps23
Tetrapoda - ps24
Amniota - ps25
Mammalia - ps26
Theria - ps27
Placentalia - ps28
Boreoeutheria - ps29
Euarchontoglires - ps30
Euarchonta - ps31
Primates - ps32
Haplorrhini - ps33
Simiiformes - ps34
Catarrhini - ps35
Hominoidea - ps36
Hominidae - ps37
Homininae - ps38
Hominini - ps39
Homo_sapiens - ps40

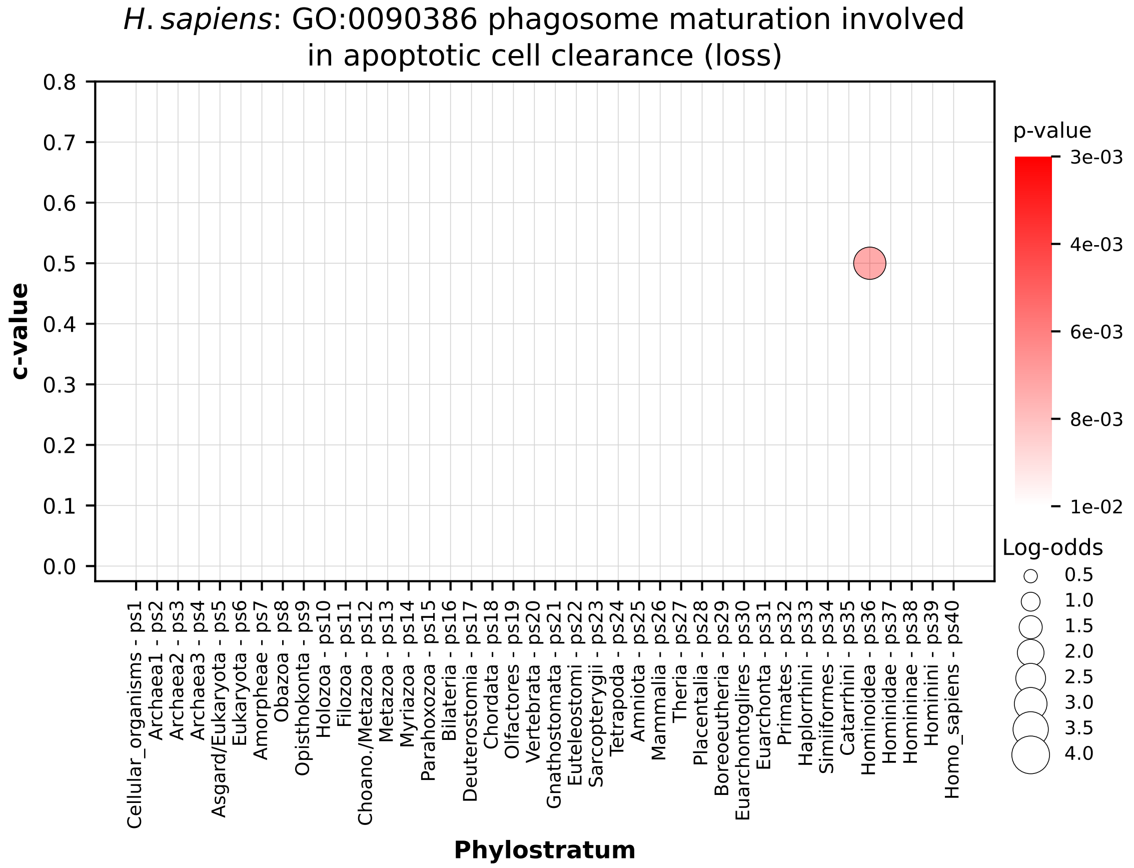
H. sapiens: GO:0090386 phagosome maturation involved in apoptotic cell clearance (loss)
c-value
0.0
0.1
0.2
0.3
0.4
0.5
0.6
0.7
0.8
Phylostratum
Cellular_organisms - ps1
Archaea1 - ps2
Archaea2 - ps3
Archaea3 - ps4
Asgard/Eukaryota - ps5
Eukaryota - ps6
Amorpheae - ps7
Obazoa - ps8
Opisthokonta - ps9
Holozoa - ps10
Filozoa - ps11
Choano./Metazoa - ps12
Metazoa - ps13
Myriazoa - ps14
Parahoxozoa - ps15
Bilateria - ps16
Deuterostomia - ps17
Chordata - ps18
Olfactores - ps19
Vertebrata - ps20
Gnathostomata - ps21
Euteleostomi - ps22
Sarcopterygii - ps23
Tetrapoda - ps24
Amniota - ps25
Mammalia - ps26
Theria - ps27
Placentalia - ps28
Boreoeutheria - ps29
Euarchontoglires - ps30
Euarchonta - ps31
Primates - ps32
Haplorrhini - ps33
Simiiformes - ps34
Catarrhini - ps35
Hominoidea - ps36
Hominidae - ps37
Homininae - ps38
Hominini - ps39
Homo_sapiens - ps40
p-value
3e-03
4e-03
6e-03
8e-03
1e-02
Log-odds
0.5
1.0
1.5
2.0
2.5
3.0
3.5
4.0

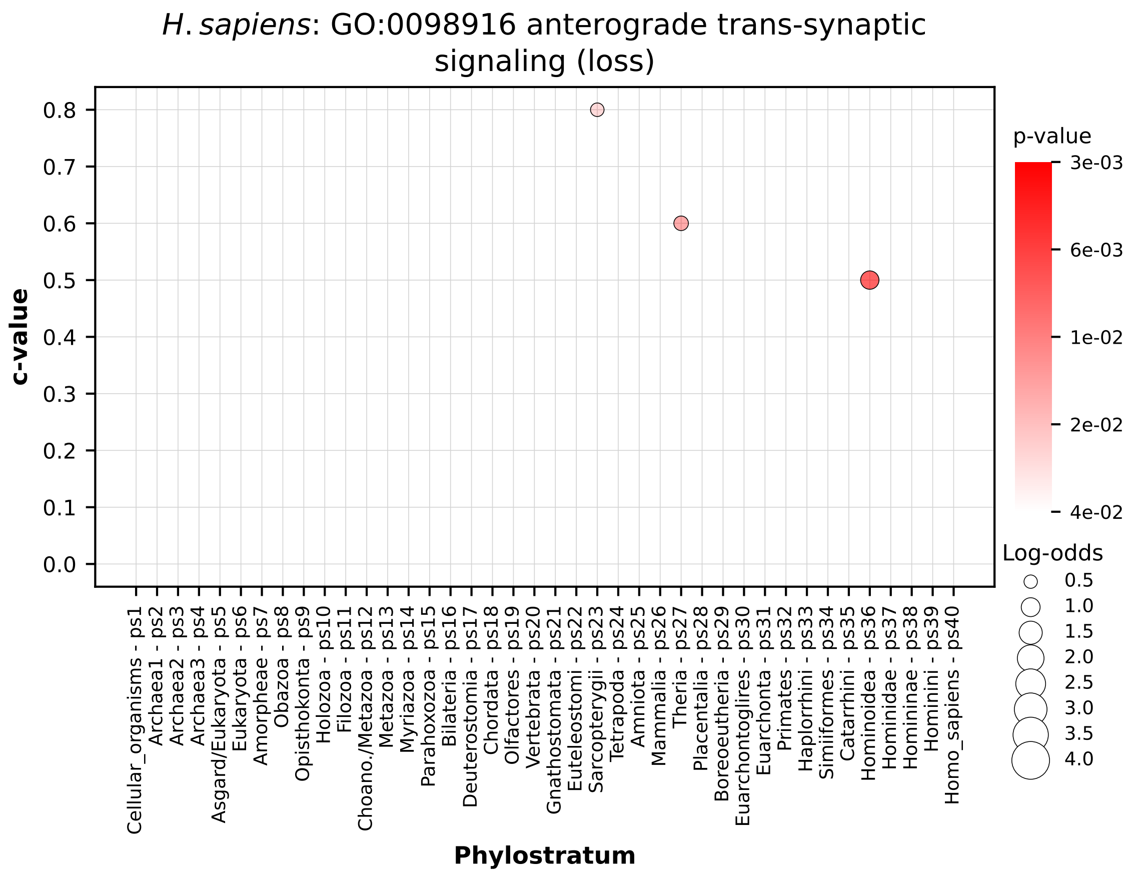

H. sapiens: GO:0098916 anterograde trans-synaptic signaling (loss)
c-value
0.0
0.1
0.2
0.3
0.4
0.5
0.6
0.7
0.8
p-value
3e-03
6e-03
1e-02
2e-02
4e-02
Log-odds
0.5
1.0
1.5
2.0
2.5
3.0
3.5
4.0
Cellular_organisms - ps1
Archaea1 - ps2
Archaea2 - ps3
Archaea3 - ps4
Asgard/Eukaryota - ps5
Eukaryota - ps6
Amorpheae - ps7
Obazoa - ps8
Opisthokonta - ps9
Holozoa - ps10
Filozoa - ps11
Choano./Metazoa - ps12
Metazoa - ps13
Myriazoa - ps14
Parahoxozoa - ps15
Bilateria - ps16
Deuterostomia - ps17
Chordata - ps18
Olfactores - ps19
Vertebrata - ps20
Gnathostomata - ps21
Euteleostomi - ps22
Sarcopterygii - ps23
Tetrapoda - ps24
Amniota - ps25
Mammalia - ps26
Theria - ps27
Placentalia - ps28
Boreoeutheria - ps29
Euarchontoglires - ps30
Euarchonta - ps31
Primates - ps32
Haplorrhini - ps33
Simiiformes - ps34
Catarrhini - ps35
Hominoidea - ps36
Hominidae - ps37
Homininae - ps38
Hominini - ps39
Homo_sapiens - ps40
Phylostratum

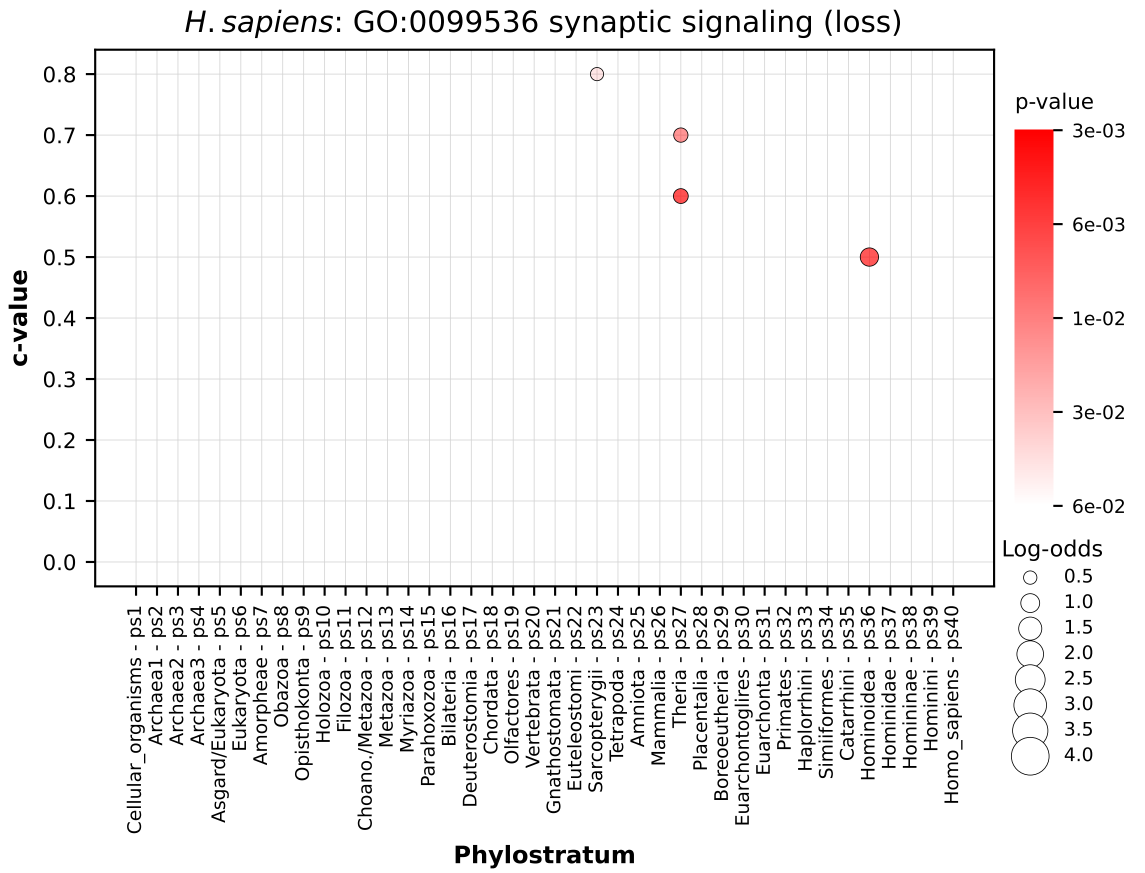

H. sapiens: GO:0099536 synaptic signaling (loss)
c-value
0.0
0.1
0.2
0.3
0.4
0.5
0.6
0.7
0.8
p-value
3e-03
6e-03
1e-02
3e-02
6e-02
Log-odds
0.5
1.0
1.5
2.0
2.5
3.0
3.5
4.0
Cellular_organisms - ps1
Archaea1 - ps2
Archaea2 - ps3
Archaea3 - ps4
Asgard/Eukaryota - ps5
Eukaryota - ps6
Amorpheae - ps7
Obazoa - ps8
Opisthokonta - ps9
Holozoa - ps10
Filozoa - ps11
Choano./Metazoa - ps12
Metazoa - ps13
Myriazoa - ps14
Parahoxozoa - ps15
Bilateria - ps16
Deuterostomia - ps17
Chordata - ps18
Olfactores - ps19
Vertebrata - ps20
Gnathostomata - ps21
Euteleostomi - ps22
Sarcopterygii - ps23
Tetrapoda - ps24
Amniota - ps25
Mammalia - ps26
Theria - ps27
Placentalia - ps28
Boreoeutheria - ps29
Euarchontoglires - ps30
Euarchonta - ps31
Primates - ps32
Haplorrhini - ps33
Simiiformes - ps34
Catarrhini - ps35
Hominoidea - ps36
Hominidae - ps37
Homininae - ps38
Hominini - ps39
Homo_sapiens - ps40
Phylostratum

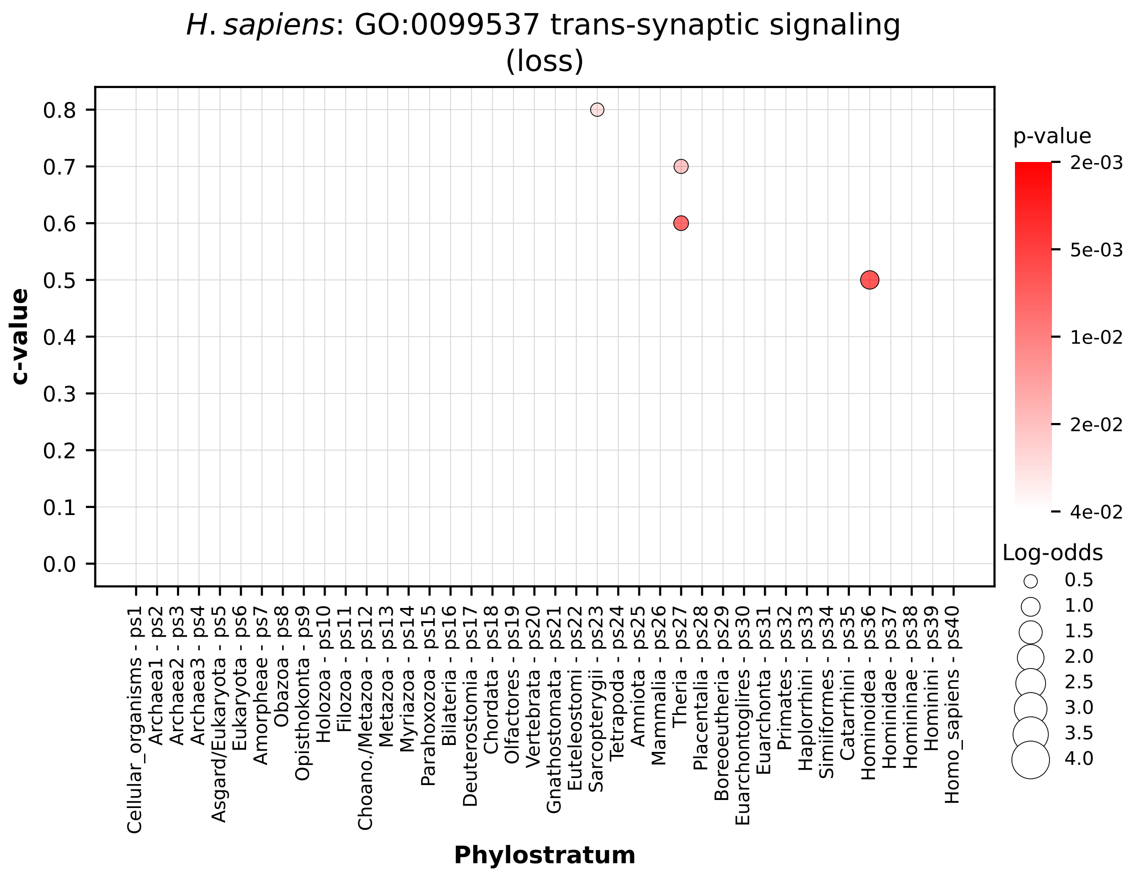

H. sapiens: GO:0099537 trans-synaptic signaling (loss)
c-value
0.0
0.1
0.2
0.3
0.4
0.5
0.6
0.7
0.8
Phylostratum
Cellular_organisms - ps1
Archaea1 - ps2
Archaea2 - ps3
Archaea3 - ps4
Asgard/Eukaryota - ps5
Eukaryota - ps6
Amorpheae - ps7
Obazoa - ps8
Opisthokonta - ps9
Holozoa - ps10
Filozoa - ps11
Choano./Metazoa - ps12
Metazoa - ps13
Myriazoa - ps14
Parahoxozoa - ps15
Bilateria - ps16
Deuterostomia - ps17
Chordata - ps18
Olfactores - ps19
Vertebrata - ps20
Gnathostomata - ps21
Euteleostomi - ps22
Sarcopterygii - ps23
Tetrapoda - ps24
Amniota - ps25
Mammalia - ps26
Theria - ps27
Placentalia - ps28
Boreoeutheria - ps29
Euarchontoglires - ps30
Euarchonta - ps31
Primates - ps32
Haplorrhini - ps33
Simiiformes - ps34
Catarrhini - ps35
Hominoidea - ps36
Hominidae - ps37
Homininae - ps38
Hominini - ps39
Homo_sapiens - ps40
p-value
2e-03
5e-03
1e-02
2e-02
4e-02
Log-odds
0.5
1.0
1.5
2.0
2.5
3.0
3.5
4.0

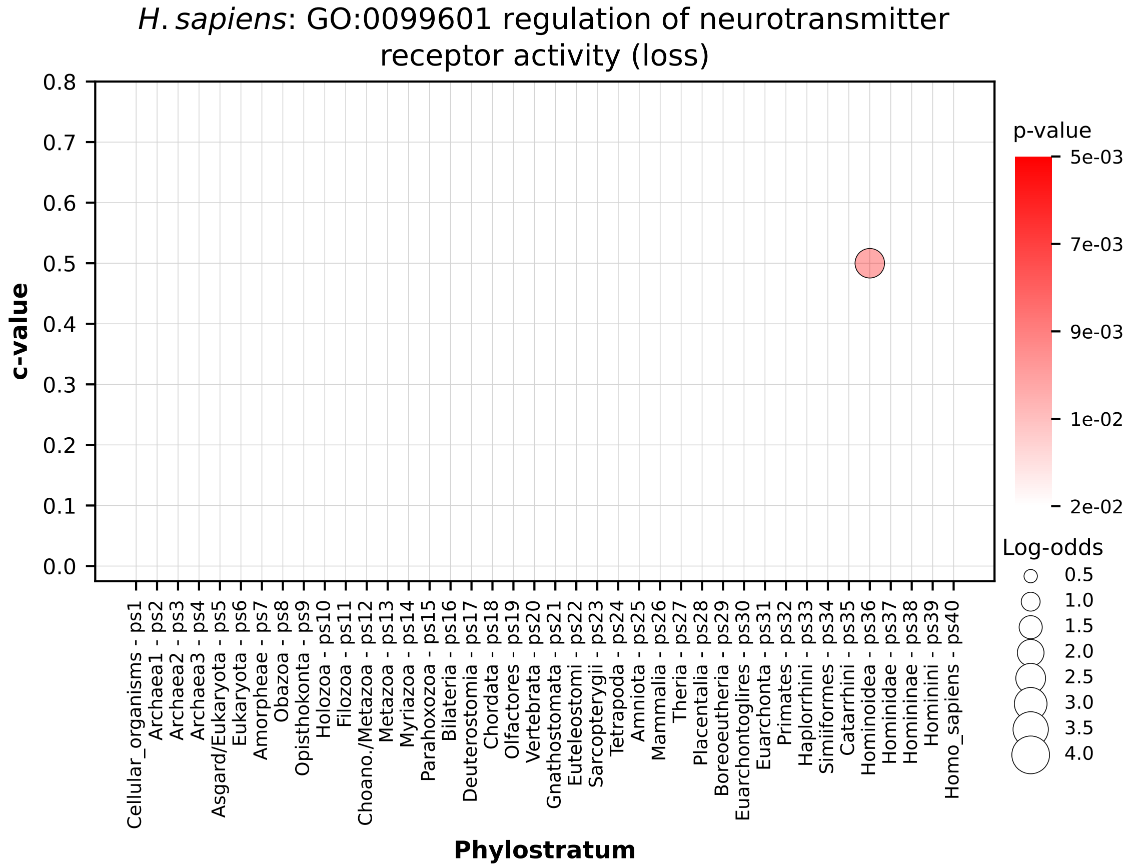
H. sapiens: GO:0099601 regulation of neurotransmitter receptor activity (loss)
c-value
0.8
0.7
0.6
0.5
0.4
0.3
0.2
0.1
0.0
Phylostratum
Cellular_organisms - ps1
Archaea1 - ps2
Archaea2 - ps3
Archaea3 - ps4
Asgard/Eukaryota - ps5
Eukaryota - ps6
Amorpheae - ps7
Obazoa - ps8
Opisthokonta - ps9
Holozoa - ps10
Filozoa - ps11
Choano./Metazoa - ps12
Metazoa - ps13
Myriazoa - ps14
Parahoxozoa - ps15
Bilateria - ps16
Deuterostomia - ps17
Chordata - ps18
Olfactores - ps19
Vertebrata - ps20
Gnathostomata - ps21
Euteleostomi - ps22
Sarcopterygii - ps23
Tetrapoda - ps24
Amniota - ps25
Mammalia - ps26
Theria - ps27
Placentalia - ps28
Boreoeutheria - ps29
Euarchontoglires - ps30
Euarchonta - ps31
Primates - ps32
Haplorrhini - ps33
Simiiformes - ps34
Catarrhini - ps35
Hominoidea - ps36
Hominidae - ps37
Homininae - ps38
Hominini - ps39
Homo_sapiens - ps40
p-value
5e-03
7e-03
9e-03
1e-02
2e-02
Log-odds
0.5
1.0
1.5
2.0
2.5
3.0
3.5
4.0

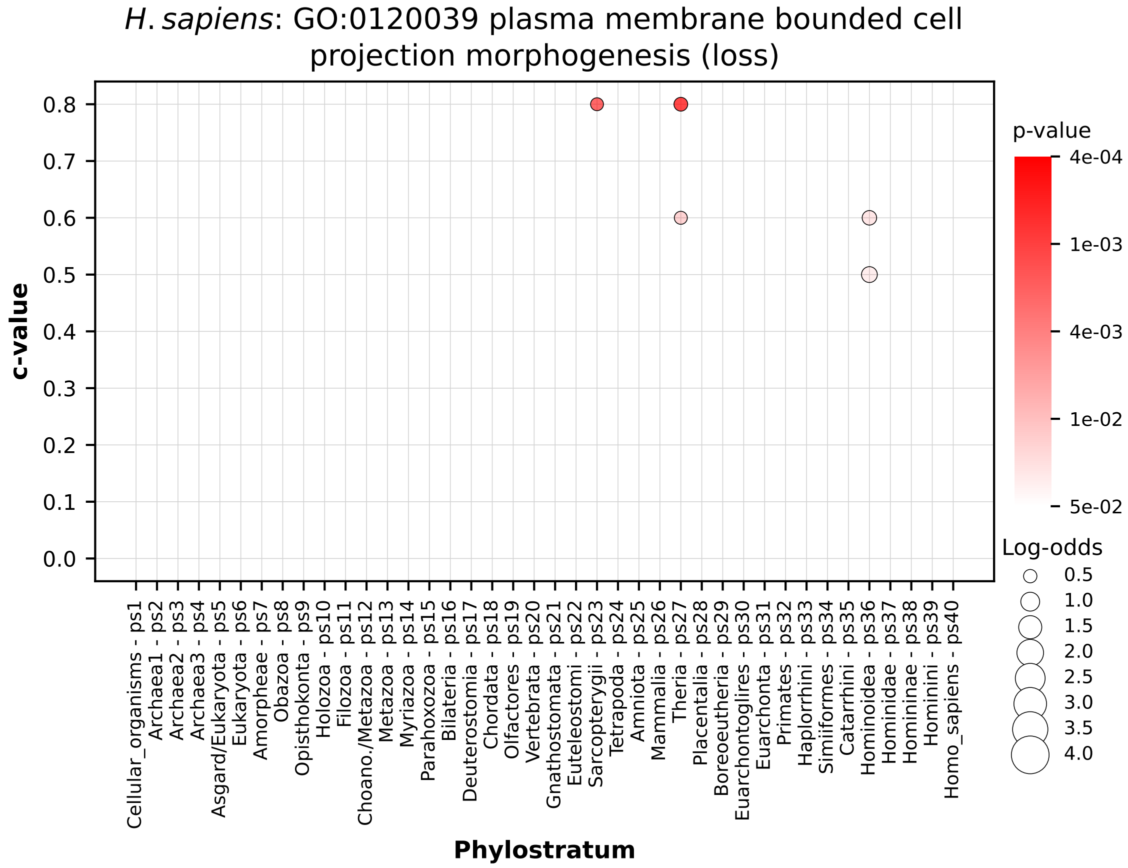
H. sapiens: GO:0120039 plasma membrane bounded cell projection morphogenesis (loss)
c-value
Phylostratum
p-value
4e-04
1e-03
4e-03
1e-02
5e-02
Log-odds
0.5
1.0
1.5
2.0
2.5
3.0
3.5
4.0
Cellular_organisms - ps1
Archaea1 - ps2
Archaea2 - ps3
Archaea3 - ps4
Asgard/Eukaryota - ps5
Eukaryota - ps6
Amorpheae - ps7
Obazoa - ps8
Opisthokonta - ps9
Holozoa - ps10
Filozoa - ps11
Choano./Metazoa - ps12
Metazoa - ps13
Myriazoa - ps14
Parahoxozoa - ps15
Bilateria - ps16
Deuterostomia - ps17
Chordata - ps18
Olfactores - ps19
Vertebrata - ps20
Gnathostomata - ps21
Euteleostomi - ps22
Sarcopterygii - ps23
Tetrapoda - ps24
Amniota - ps25
Mammalia - ps26
Theria - ps27
Placentalia - ps28
Boreoeutheria - ps29
Euarchontoglires - ps30
Euarchonta - ps31
Primates - ps32
Haplorrhini - ps33
Simiiformes - ps34
Catarrhini - ps35
Hominoidea - ps36
Hominidae - ps37
Homininae - ps38
Hominini - ps39
Homo_sapiens - ps40

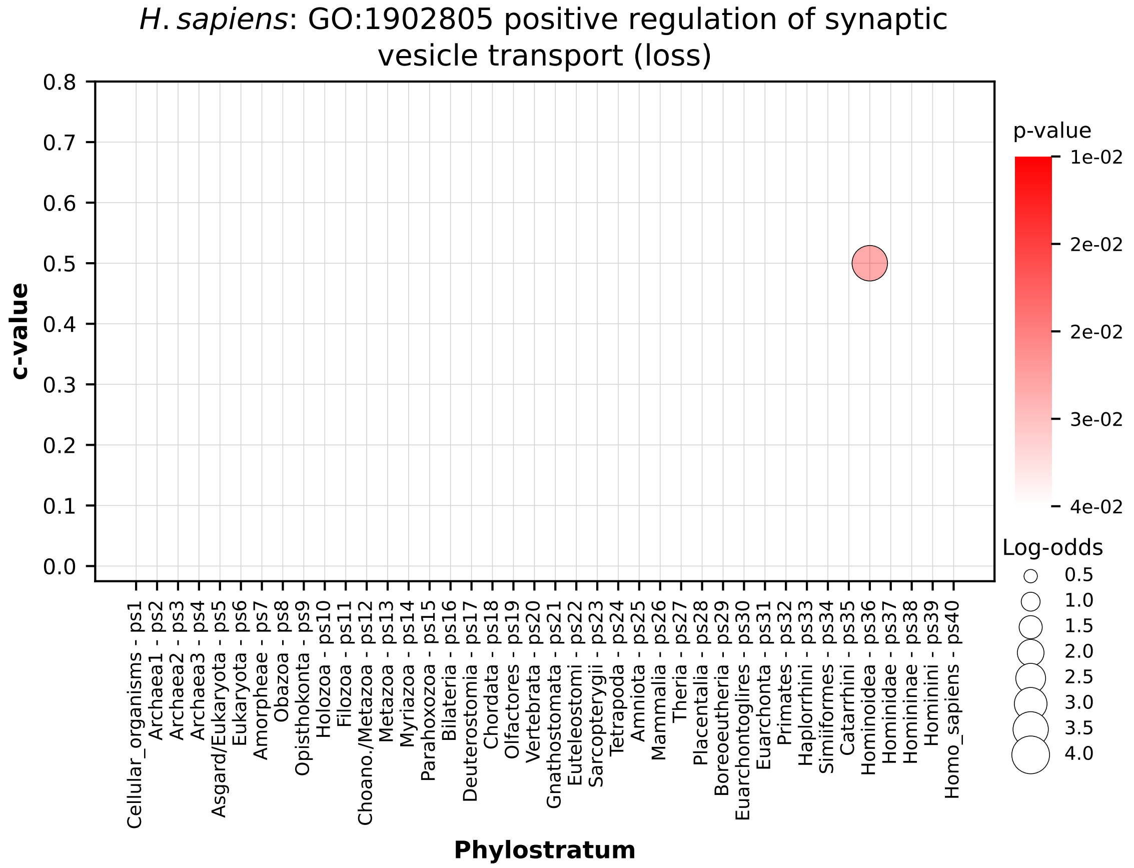
H. sapiens: GO:1902805 positive regulation of synaptic vesicle transport (loss)
c-value
Phylostratum
p-value
1e-02
2e-02
2e-02
3e-02
4e-02
Log-odds
0.5
1.0
1.5
2.0
2.5
3.0
3.5
4.0
Cellular_organisms - ps1
Archaea1 - ps2
Archaea2 - ps3
Archaea3 - ps4
Asgard/Eukaryota - ps5
Eukaryota - ps6
Amorpheae - ps7
Obazoa - ps8
Opisthokonta - ps9
Holozoa - ps10
Filozoa - ps11
Choano./Metazoa - ps12
Metazoa - ps13
Myriazoa - ps14
Parahoxozoa - ps15
Bilateria - ps16
Deuterostomia - ps17
Chordata - ps18
Olfactores - ps19
Vertebrata - ps20
Gnathostomata - ps21
Euteleostomi - ps22
Sarcopterygii - ps23
Tetrapoda - ps24
Amniota - ps25
Mammalia - ps26
Theria - ps27
Placentalia - ps28
Boreoeutheria - ps29
Euarchontoglires - ps30
Euarchonta - ps31
Primates - ps32
Haplorrhini - ps33
Simiiformes - ps34
Catarrhini - ps35
Hominoidea - ps36
Hominidae - ps37
Homininae - ps38
Hominini - ps39
Homo_sapiens - ps40

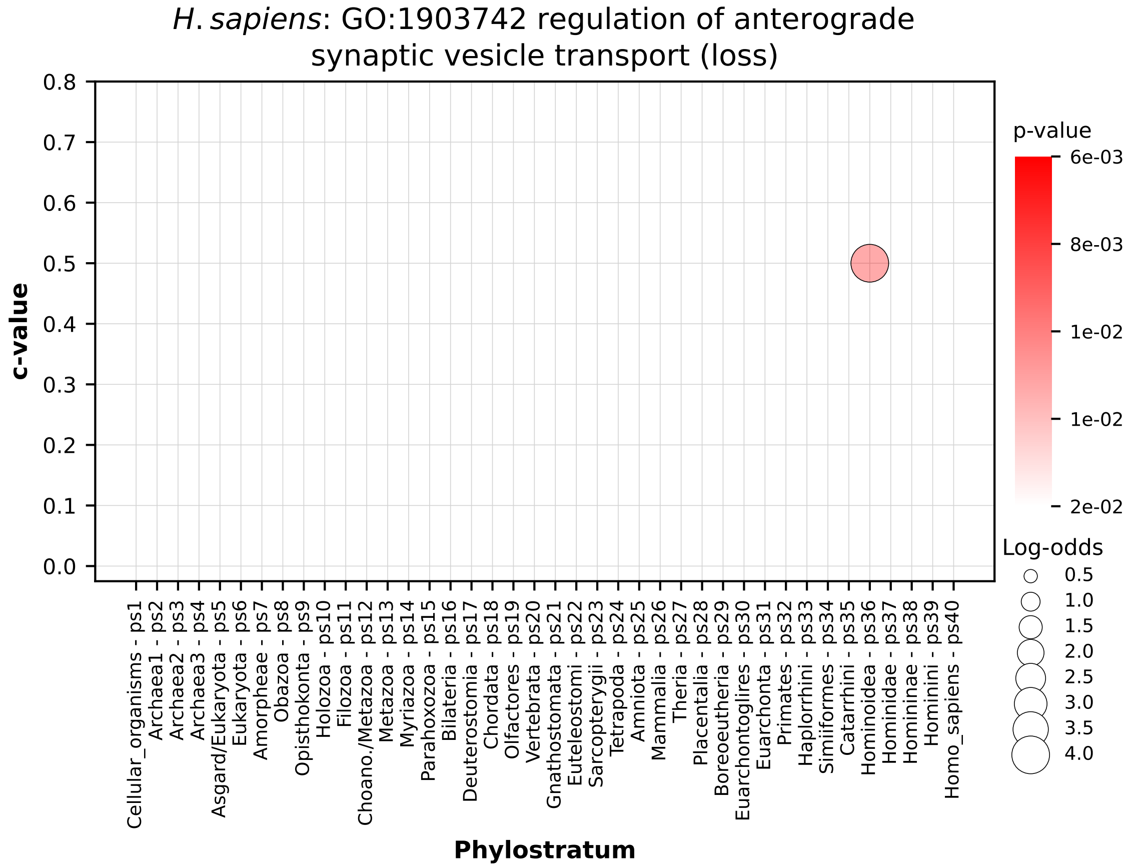

H. sapiens: GO:1903742 regulation of anterograde synaptic vesicle transport (loss)
c-value
0.0
0.1
0.2
0.3
0.4
0.5
0.6
0.7
0.8
Phylostratum
Cellular_organisms - ps1
Archaea1 - ps2
Archaea2 - ps3
Archaea3 - ps4
Asgard/Eukaryota - ps5
Eukaryota - ps6
Amorpheae - ps7
Obazoa - ps8
Opisthokonta - ps9
Holozoa - ps10
Filozoa - ps11
Choano./Metazoa - ps12
Metazoa - ps13
Myriazoa - ps14
Parahoxozoa - ps15
Bilateria - ps16
Deuterostomia - ps17
Chordata - ps18
Olfactores - ps19
Vertebrata - ps20
Gnathostomata - ps21
Euteleostomi - ps22
Sarcopterygii - ps23
Tetrapoda - ps24
Amniota - ps25
Mammalia - ps26
Theria - ps27
Placentalia - ps28
Boreoeutheria - ps29
Euarchontoglires - ps30
Euarchonta - ps31
Primates - ps32
Haplorrhini - ps33
Simiiformes - ps34
Catarrhini - ps35
Hominoidea - ps36
Hominidae - ps37
Homininae - ps38
Hominini - ps39
Homo_sapiens - ps40
p-value
6e-03
8e-03
1e-02
1e-02
2e-02
Log-odds
0.5
1.0
1.5
2.0
2.5
3.0
3.5
4.0

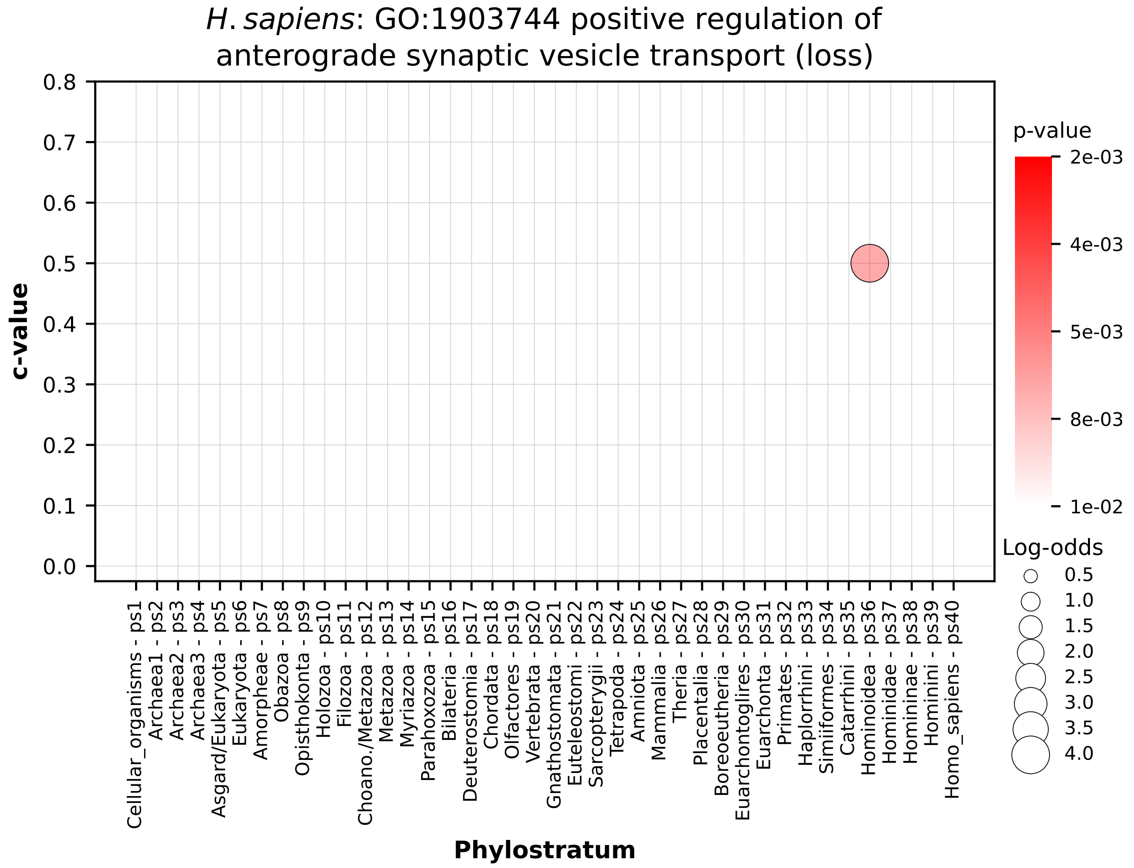

H. sapiens: GO:1903744 positive regulation of anterograde synaptic vesicle transport (loss)
c-value
0.0
0.1
0.2
0.3
0.4
0.5
0.6
0.7
0.8
p-value
2e-03
4e-03
5e-03
8e-03
1e-02
Log-odds
0.5
1.0
1.5
2.0
2.5
3.0
3.5
4.0
Cellular_organisms - ps1
Archaea1 - ps2
Archaea2 - ps3
Archaea3 - ps4
Asgard/Eukaryota - ps5
Eukaryota - ps6
Amorpheae - ps7
Obazoa - ps8
Opisthokonta - ps9
Holozoa - ps10
Filozoa - ps11
Choano./Metazoa - ps12
Metazoa - ps13
Myriazoa - ps14
Parahoxozoa - ps15
Bilateria - ps16
Deuterostomia - ps17
Chordata - ps18
Olfactores - ps19
Vertebrata - ps20
Gnathostomata - ps21
Euteleostomi - ps22
Sarcopterygii - ps23
Tetrapoda - ps24
Amniota - ps25
Mammalia - ps26
Theria - ps27
Placentalia - ps28
Boreoeutheria - ps29
Euarchontoglires - ps30
Euarchonta - ps31
Primates - ps32
Haplorrhini - ps33
Simiiformes - ps34
Catarrhini - ps35
Hominoidea - ps36
Hominidae - ps37
Homininae - ps38
Hominini - ps39
Homo_sapiens - ps40
Phylostratum

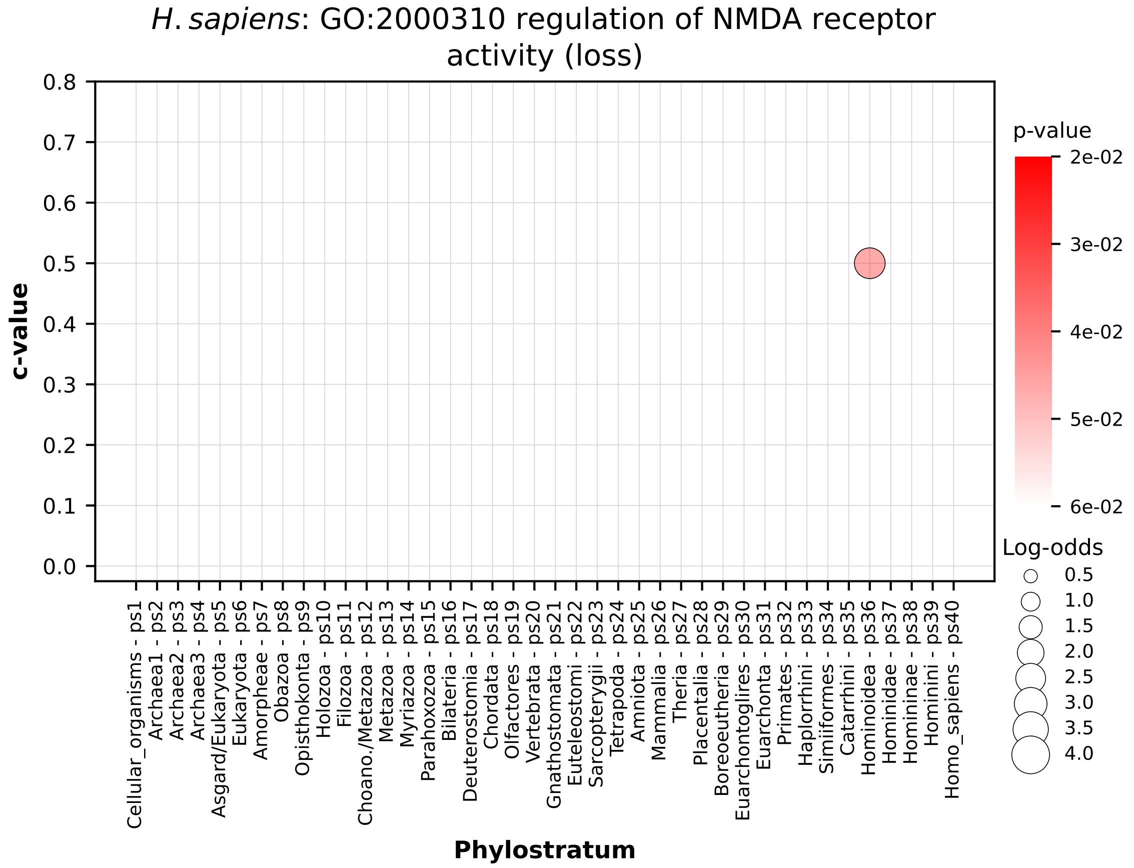
H. sapiens: GO:2000310 regulation of NMDA receptor activity (loss)
c-value
0.0
0.1
0.2
0.3
0.4
0.5
0.6
0.7
0.8
Phylostratum
Cellular_organisms - ps1
Archaea1 - ps2
Archaea2 - ps3
Archaea3 - ps4
Asgard/Eukaryota - ps5
Eukaryota - ps6
Amorpheae - ps7
Obazoa - ps8
Opisthokonta - ps9
Holozoa - ps10
Filozoa - ps11
Choano./Metazoa - ps12
Metazoa - ps13
Myriazoa - ps14
Parahoxozoa - ps15
Bilateria - ps16
Deuterostomia - ps17
Chordata - ps18
Olfactores - ps19
Vertebrata - ps20
Gnathostomata - ps21
Euteleostomi - ps22
Sarcopterygii - ps23
Tetrapoda - ps24
Amniota - ps25
Mammalia - ps26
Theria - ps27
Placentalia - ps28
Boreoeutheria - ps29
Euarchontoglires - ps30
Euarchonta - ps31
Primates - ps32
Haplorrhini - ps33
Simiiformes - ps34
Catarrhini - ps35
Hominoidea - ps36
Hominidae - ps37
Homininae - ps38
Hominini - ps39
Homo_sapiens - ps40
p-value
2e-02
3e-02
4e-02
5e-02
6e-02
Log-odds
0.5
1.0
1.5
2.0
2.5
3.0
3.5
4.0

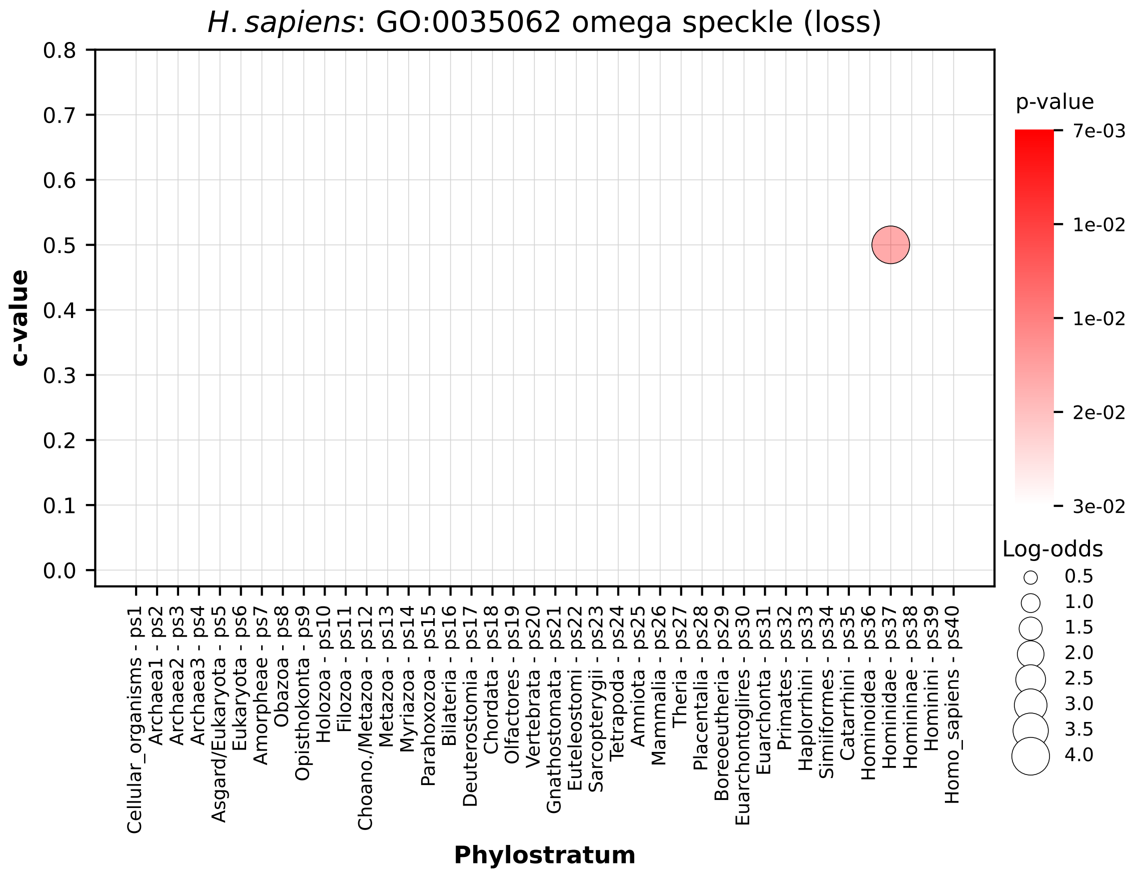

H. sapiens: GO:0035062 omega speckle (loss)
c-value
0.0
0.1
0.2
0.3
0.4
0.5
0.6
0.7
0.8
Phylostratum
Cellular_organisms - ps1
Archaea1 - ps2
Archaea2 - ps3
Archaea3 - ps4
Asgard/Eukaryota - ps5
Eukaryota - ps6
Amorpheae - ps7
Obazoa - ps8
Opisthokonta - ps9
Holozoa - ps10
Filozoa - ps11
Choano./Metazoa - ps12
Metazoa - ps13
Myriazoa - ps14
Parahoxozoa - ps15
Bilateria - ps16
Deuterostomia - ps17
Chordata - ps18
Olfactores - ps19
Vertebrata - ps20
Gnathostomata - ps21
Euteleostomi - ps22
Sarcopterygii - ps23
Tetrapoda - ps24
Amniota - ps25
Mammalia - ps26
Theria - ps27
Placentalia - ps28
Boreoeutheria - ps29
Euarchontoglires - ps30
Euarchonta - ps31
Primates - ps32
Haplorrhini - ps33
Simiiformes - ps34
Catarrhini - ps35
Hominoidea - ps36
Hominidae - ps37
Homininae - ps38
Hominini - ps39
Homo_sapiens - ps40
p-value
7e-03
1e-02
1e-02
2e-02
3e-02
Log-odds
0.5
1.0
1.5
2.0
2.5
3.0
3.5
4.0

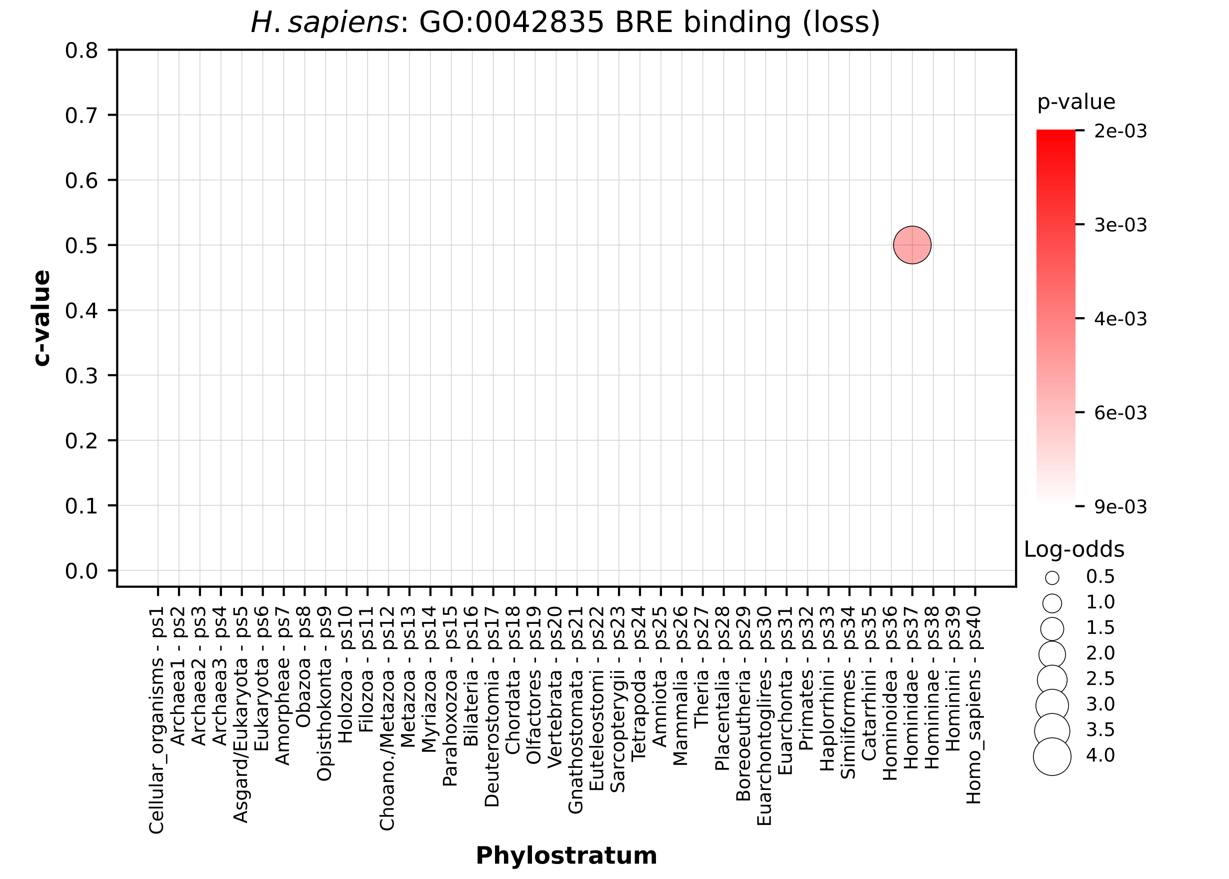
H. sapiens: GO:0042835 BRE binding (loss)
c-value
0.8
0.7
0.6
0.5
0.4
0.3
0.2
0.1
0.0
p-value
2e-03
3e-03
4e-03
6e-03
9e-03
Log-odds
0.5
1.0
1.5
2.0
2.5
3.0
3.5
4.0
Cellular_organisms - ps1
Archaea1 - ps2
Archaea2 - ps3
Archaea3 - ps4
Asgard/Eukaryota - ps5
Eukaryota - ps6
Amorpheae - ps7
Obazoa - ps8
Opisthokonta - ps9
Holozoa - ps10
Filozoa - ps11
Choano./Metazoa - ps12
Metazoa - ps13
Myriazoa - ps14
Parahoxozoa - ps15
Bilateria - ps16
Deuterostomia - ps17
Chordata - ps18
Olfactores - ps19
Vertebrata - ps20
Gnathostomata - ps21
Euteleostomi - ps22
Sarcopterygii - ps23
Tetrapoda - ps24
Amniota - ps25
Mammalia - ps26
Theria - ps27
Placentalia - ps28
Boreoeutheria - ps29
Euarchontoglires - ps30
Euarchonta - ps31
Primates - ps32
Haplorrhini - ps33
Simiiformes - ps34
Catarrhini - ps35
Hominoidea - ps36
Hominidae - ps37
Homininae - ps38
Hominini - ps39
Homo_sapiens - ps40
Phylostratum

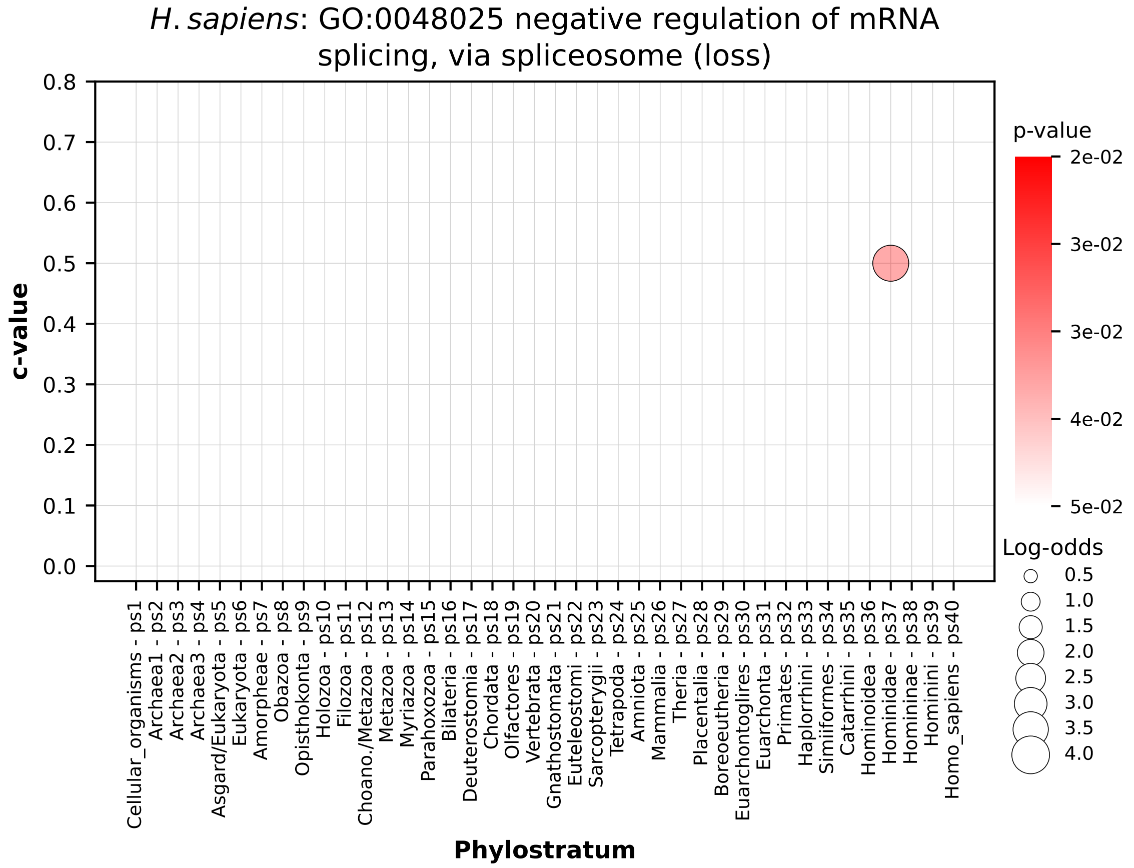
H. sapiens: GO:0048025 negative regulation of mRNA splicing, via spliceosome (loss)
c-value
0.8
0.7
0.6
0.5
0.4
0.3
0.2
0.1
0.0
p-value
2e-02
3e-02
3e-02
4e-02
5e-02
Log-odds
0.5
1.0
1.5
2.0
2.5
3.0
3.5
4.0
Cellular_organisms - ps1
Archaea1 - ps2
Archaea2 - ps3
Archaea3 - ps4
Asgard/Eukaryota - ps5
Eukaryota - ps6
Amorpheae - ps7
Obazoa - ps8
Opisthokonta - ps9
Holozoa - ps10
Filozoa - ps11
Choano./Metazoa - ps12
Metazoa - ps13
Myriazoa - ps14
Parahoxozoa - ps15
Bilateria - ps16
Deuterostomia - ps17
Chordata - ps18
Olfactores - ps19
Vertebrata - ps20
Gnathostomata - ps21
Euteleostomi - ps22
Sarcopterygii - ps23
Tetrapoda - ps24
Amniota - ps25
Mammalia - ps26
Theria - ps27
Placentalia - ps28
Boreoeutheria - ps29
Euarchontoglires - ps30
Euarchonta - ps31
Primates - ps32
Haplorrhini - ps33
Simiiformes - ps34
Catarrhini - ps35
Hominoidea - ps36
Hominidae - ps37
Homininae - ps38
Hominini - ps39
Homo_sapiens - ps40
Phylostratum

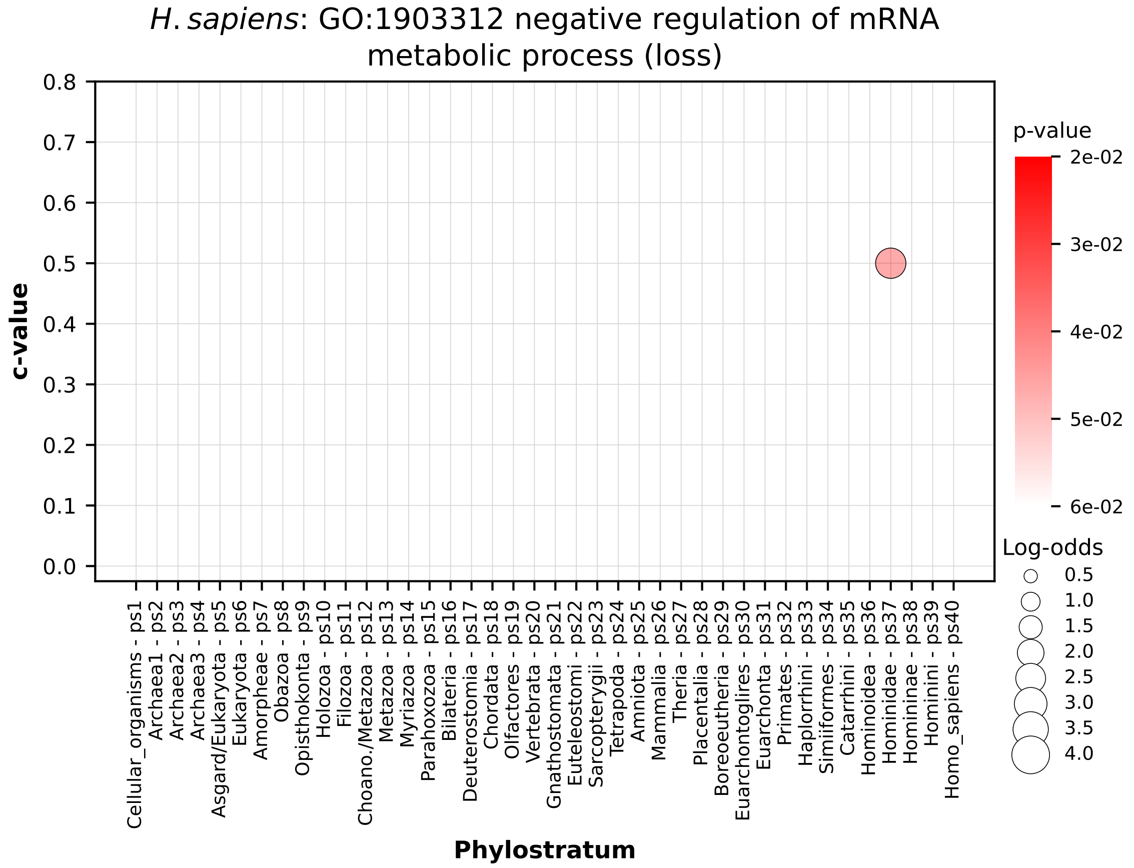
H. sapiens: GO:1903312 negative regulation of mRNA metabolic process (loss)
c-value
0.8
0.7
0.6
0.5
0.4
0.3
0.2
0.1
0.0
Phylostratum
Cellular_organisms - ps1
Archaea1 - ps2
Archaea2 - ps3
Archaea3 - ps4
Asgard/Eukaryota - ps5
Eukaryota - ps6
Amorpheae - ps7
Obazoa - ps8
Opisthokonta - ps9
Holozoa - ps10
Filozoa - ps11
Choano./Metazoa - ps12
Metazoa - ps13
Myriazoa - ps14
Parahoxozoa - ps15
Bilateria - ps16
Deuterostomia - ps17
Chordata - ps18
Olfactores - ps19
Vertebrata - ps20
Gnathostomata - ps21
Euteleostomi - ps22
Sarcopterygii - ps23
Tetrapoda - ps24
Amniota - ps25
Mammalia - ps26
Theria - ps27
Placentalia - ps28
Boreoeutheria - ps29
Euarchontoglires - ps30
Euarchonta - ps31
Primates - ps32
Haplorrhini - ps33
Simiiformes - ps34
Catarrhini - ps35
Hominoidea - ps36
Hominidae - ps37
Homininae - ps38
Hominini - ps39
Homo_sapiens - ps40
p-value
2e-02
3e-02
4e-02
5e-02
6e-02
Log-odds
0.5
1.0
1.5
2.0
2.5
3.0
3.5
4.0

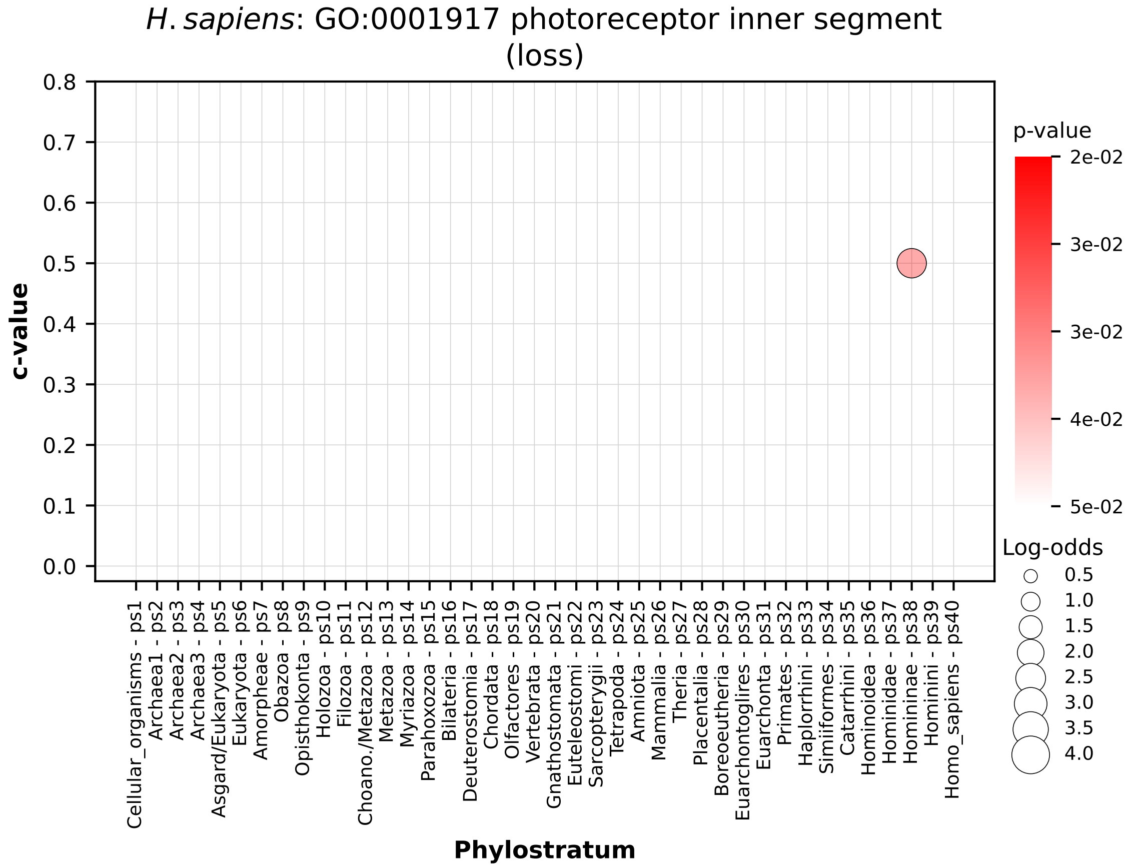

H. sapiens: GO:0001917 photoreceptor inner segment (loss)
c-value
0.8
0.7
0.6
0.5
0.4
0.3
0.2
0.1
0.0
Phylostratum
Cellular_organisms - ps1
Archaea1 - ps2
Archaea2 - ps3
Archaea3 - ps4
Asgard/Eukaryota - ps5
Eukaryota - ps6
Amorpheae - ps7
Obazoa - ps8
Opisthokonta - ps9
Holozoa - ps10
Filozoa - ps11
Choano./Metazoa - ps12
Metazoa - ps13
Myriazoa - ps14
Parahoxozoa - ps15
Bilateria - ps16
Deuterostomia - ps17
Chordata - ps18
Olfactores - ps19
Vertebrata - ps20
Gnathostomata - ps21
Euteleostomi - ps22
Sarcopterygii - ps23
Tetrapoda - ps24
Amniota - ps25
Mammalia - ps26
Theria - ps27
Placentalia - ps28
Boreoeutheria - ps29
Euarchontoglires - ps30
Euarchonta - ps31
Primates - ps32
Haplorrhini - ps33
Simiiformes - ps34
Catarrhini - ps35
Hominoidea - ps36
Hominidae - ps37
Homininae - ps38
Hominini - ps39
Homo_sapiens - ps40
p-value
2e-02
3e-02
3e-02
4e-02
5e-02
Log-odds
0.5
1.0
1.5
2.0
2.5
3.0
3.5
4.0

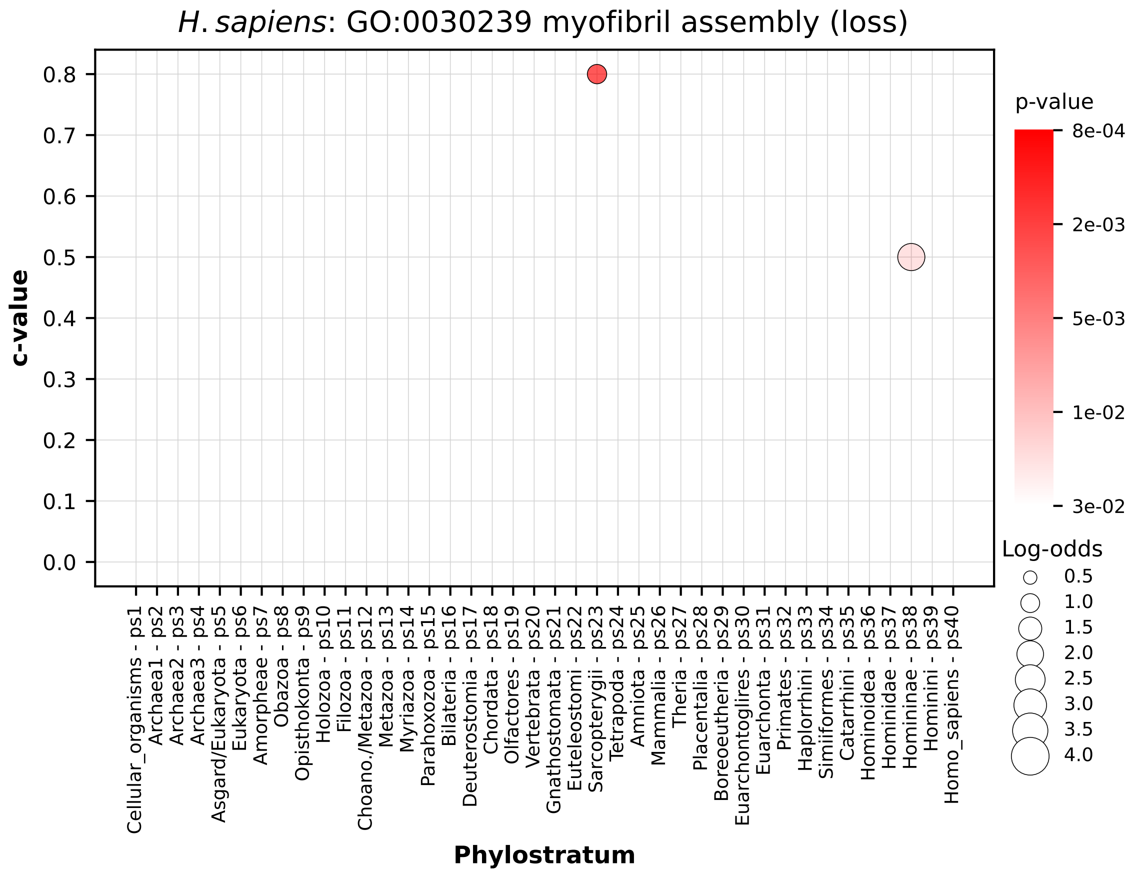

H. sapiens: GO:0030239 myofibril assembly (loss)
c-value
0.0
0.1
0.2
0.3
0.4
0.5
0.6
0.7
0.8
Phylostratum
Cellular_organisms - ps1
Archaea1 - ps2
Archaea2 - ps3
Archaea3 - ps4
Asgard/Eukaryota - ps5
Eukaryota - ps6
Amorpheae - ps7
Obazoa - ps8
Opisthokonta - ps9
Holozoa - ps10
Filozoa - ps11
Choano./Metazoa - ps12
Metazoa - ps13
Myriazoa - ps14
Parahoxozoa - ps15
Bilateria - ps16
Deuterostomia - ps17
Chordata - ps18
Olfactores - ps19
Vertebrata - ps20
Gnathostomata - ps21
Euteleostomi - ps22
Sarcopterygii - ps23
Tetrapoda - ps24
Amniota - ps25
Mammalia - ps26
Theria - ps27
Placentalia - ps28
Boreoeutheria - ps29
Euarchontoglires - ps30
Euarchonta - ps31
Primates - ps32
Haplorrhini - ps33
Simiiformes - ps34
Catarrhini - ps35
Hominoidea - ps36
Hominidae - ps37
Homininae - ps38
Hominini - ps39
Homo_sapiens - ps40
p-value
8e-04
2e-03
5e-03
1e-02
3e-02
Log-odds
0.5
1.0
1.5
2.0
2.5
3.0
3.5
4.0

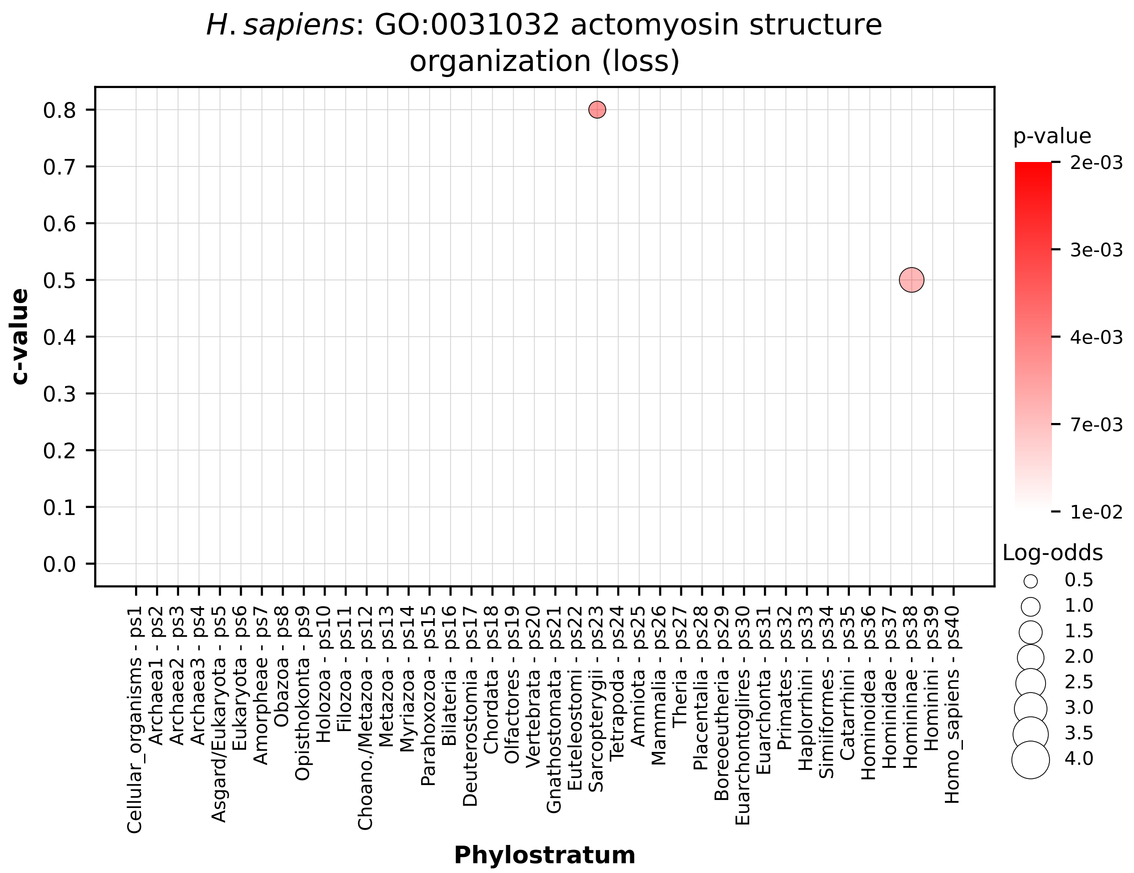

H. sapiens: GO:0031032 actomyosin structure organization (loss)
c-value
0.0
0.1
0.2
0.3
0.4
0.5
0.6
0.7
0.8
Phylostratum
Cellular_organisms - ps1
Archaea1 - ps2
Archaea2 - ps3
Archaea3 - ps4
Asgard/Eukaryota - ps5
Eukaryota - ps6
Amorpheae - ps7
Obazoa - ps8
Opisthokonta - ps9
Holozoa - ps10
Filozoa - ps11
Choano./Metazoa - ps12
Metazoa - ps13
Myriazoa - ps14
Parahoxozoa - ps15
Bilateria - ps16
Deuterostomia - ps17
Chordata - ps18
Olfactores - ps19
Vertebrata - ps20
Gnathostomata - ps21
Euteleostomi - ps22
Sarcopterygii - ps23
Tetrapoda - ps24
Amniota - ps25
Mammalia - ps26
Theria - ps27
Placentalia - ps28
Boreoeutheria - ps29
Euarchontoglires - ps30
Euarchonta - ps31
Primates - ps32
Haplorrhini - ps33
Simiiformes - ps34
Catarrhini - ps35
Hominoidea - ps36
Hominidae - ps37
Homininae - ps38
Hominini - ps39
Homo_sapiens - ps40
p-value
2e-03
3e-03
4e-03
7e-03
1e-02
Log-odds
0.5
1.0
1.5
2.0
2.5
3.0
3.5
4.0

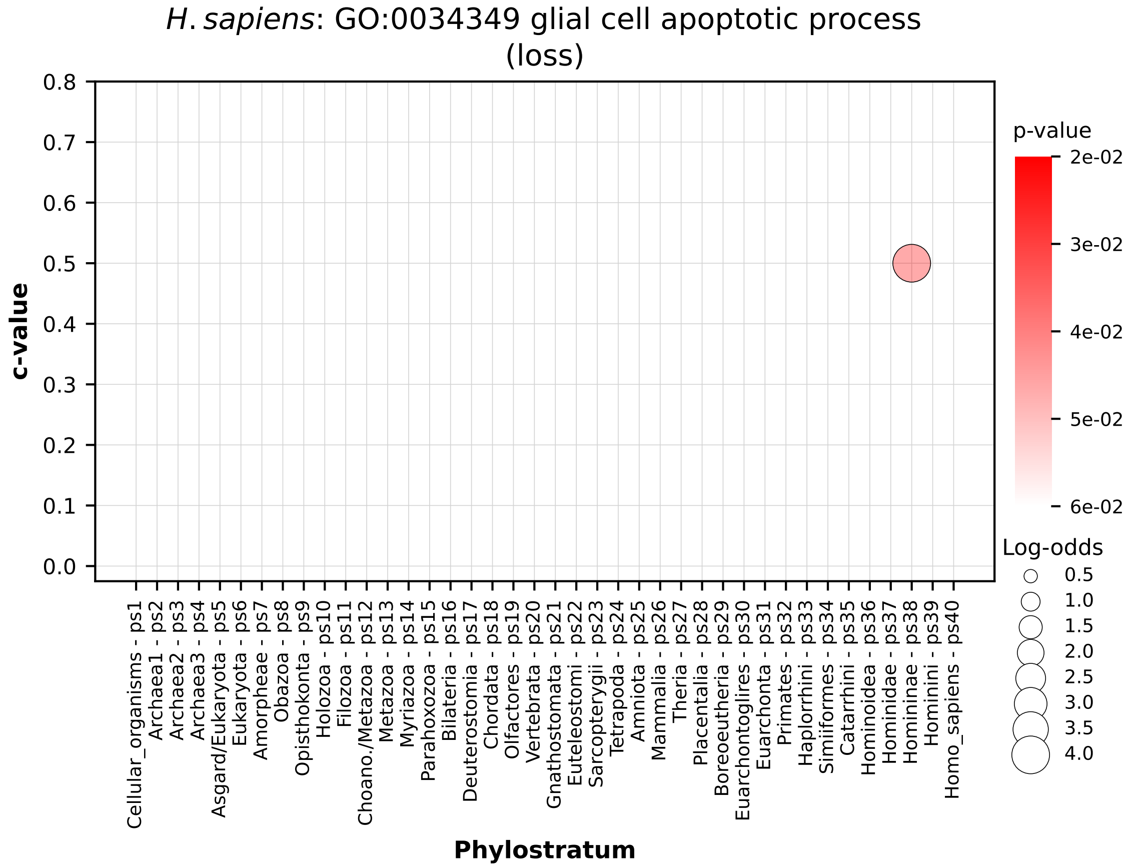

H. sapiens: GO:0034349 glial cell apoptotic process (loss)
c-value
0.0
0.1
0.2
0.3
0.4
0.5
0.6
0.7
0.8
p-value
2e-02
3e-02
4e-02
5e-02
6e-02
Log-odds
0.5
1.0
1.5
2.0
2.5
3.0
3.5
4.0
Cellular_organisms - ps1
Archaea1 - ps2
Archaea2 - ps3
Archaea3 - ps4
Asgard/Eukaryota - ps5
Eukaryota - ps6
Amorpheae - ps7
Obazoa - ps8
Opisthokonta - ps9
Holozoa - ps10
Filozoa - ps11
Choano./Metazoa - ps12
Metazoa - ps13
Myriazoa - ps14
Parahoxozoa - ps15
Bilateria - ps16
Deuterostomia - ps17
Chordata - ps18
Olfactores - ps19
Vertebrata - ps20
Gnathostomata - ps21
Euteleostomi - ps22
Sarcopterygii - ps23
Tetrapoda - ps24
Amniota - ps25
Mammalia - ps26
Theria - ps27
Placentalia - ps28
Boreoeutheria - ps29
Euarchontoglires - ps30
Euarchonta - ps31
Primates - ps32
Haplorrhini - ps33
Simiiformes - ps34
Catarrhini - ps35
Hominoidea - ps36
Hominidae - ps37
Homininae - ps38
Hominini - ps39
Homo_sapiens - ps40
Phylostratum

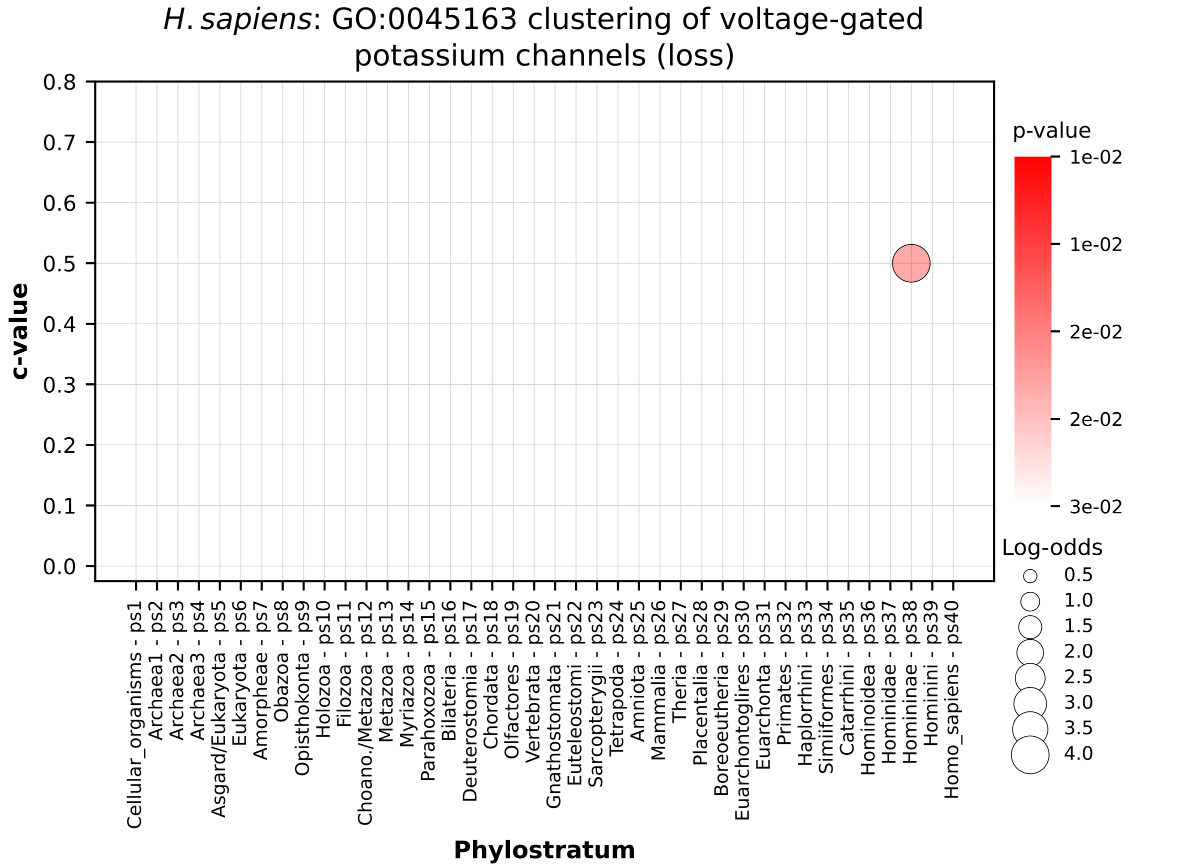
H. sapiens: GO:0045163 clustering of voltage-gated potassium channels (loss)
c-value
0.8
0.7
0.6
0.5
0.4
0.3
0.2
0.1
0.0
p-value
1e-02
1e-02
2e-02
2e-02
3e-02
Log-odds
0.5
1.0
1.5
2.0
2.5
3.0
3.5
4.0
Cellular_organisms - ps1
Archaea1 - ps2
Archaea2 - ps3
Archaea3 - ps4
Asgard/Eukaryota - ps5
Eukaryota - ps6
Amorpheae - ps7
Obazoa - ps8
Opisthokonta - ps9
Holozoa - ps10
Filozoa - ps11
Choano./Metazoa - ps12
Metazoa - ps13
Myriazoa - ps14
Parahoxozoa - ps15
Bilateria - ps16
Deuterostomia - ps17
Chordata - ps18
Olfactores - ps19
Vertebrata - ps20
Gnathostomata - ps21
Euteleostomi - ps22
Sarcopterygii - ps23
Tetrapoda - ps24
Amniota - ps25
Mammalia - ps26
Theria - ps27
Placentalia - ps28
Boreoeutheria - ps29
Euarchontoglires - ps30
Euarchonta - ps31
Primates - ps32
Haplorrhini - ps33
Simiiformes - ps34
Catarrhini - ps35
Hominoidea - ps36
Hominidae - ps37
Homininae - ps38
Hominini - ps39
Homo_sapiens - ps40
Phylostratum

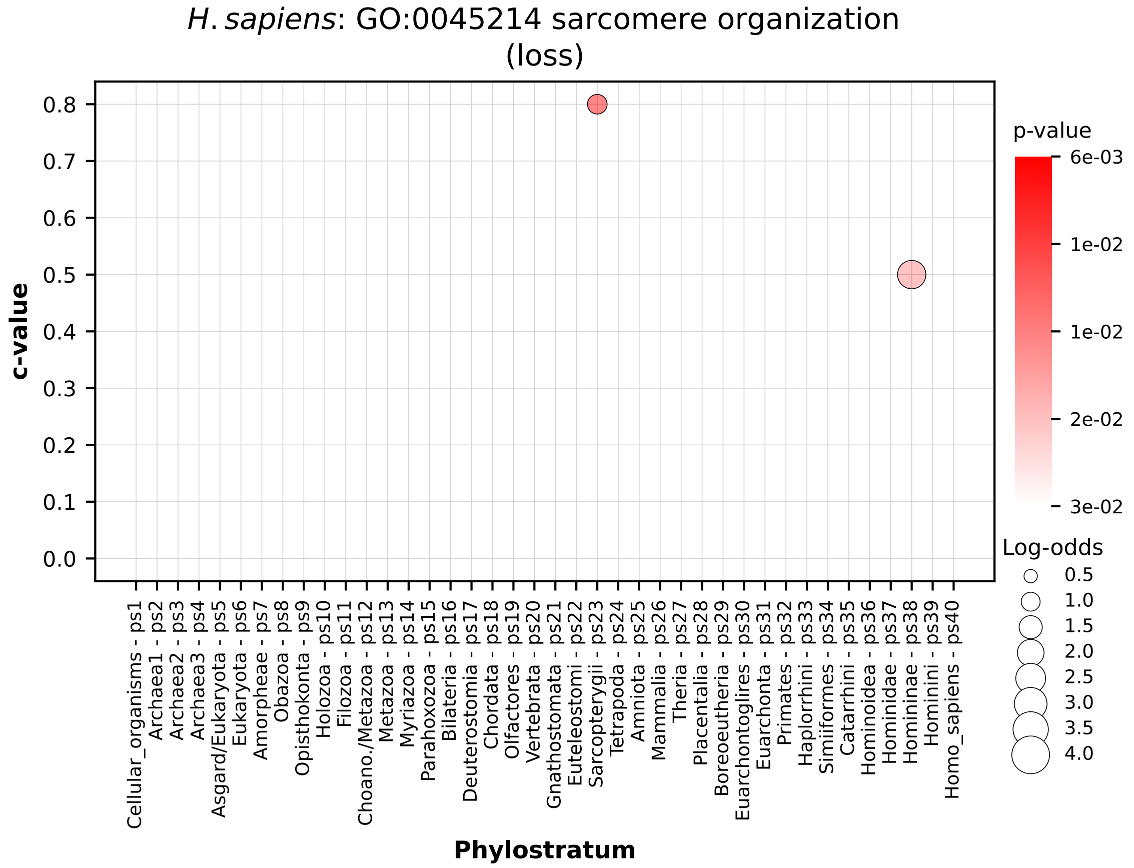

H. sapiens: GO:0045214 sarcomere organization
(loss)
c-value
0.8
0.7
0.6
0.5
0.4
0.3
0.2
0.1
0.0
p-value
6e-03
1e-02
1e-02
2e-02
3e-02
Log-odds
0.5
1.0
1.5
2.0
2.5
3.0
3.5
4.0
Cellular_organisms - ps1
Archaea1 - ps2
Archaea2 - ps3
Archaea3 - ps4
Asgard/Eukaryota - ps5
Eukaryota - ps6
Amorpheae - ps7
Obazoa - ps8
Opisthokonta - ps9
Holozoa - ps10
Filozoa - ps11
Choano./Metazoa - ps12
Metazoa - ps13
Myriazoa - ps14
Parahoxozoa - ps15
Bilateria - ps16
Deuterostomia - ps17
Chordata - ps18
Olfactores - ps19
Vertebrata - ps20
Gnathostomata - ps21
Euteleostomi - ps22
Sarcopterygii - ps23
Tetrapoda - ps24
Amniota - ps25
Mammalia - ps26
Theria - ps27
Placentalia - ps28
Boreoeutheria - ps29
Euarchontoglires - ps30
Euarchonta - ps31
Primates - ps32
Haplorrhini - ps33
Simiiformes - ps34
Catarrhini - ps35
Hominoidea - ps36
Hominidae - ps37
Homininae - ps38
Hominini - ps39
Homo_sapiens - ps40
Phylostratum

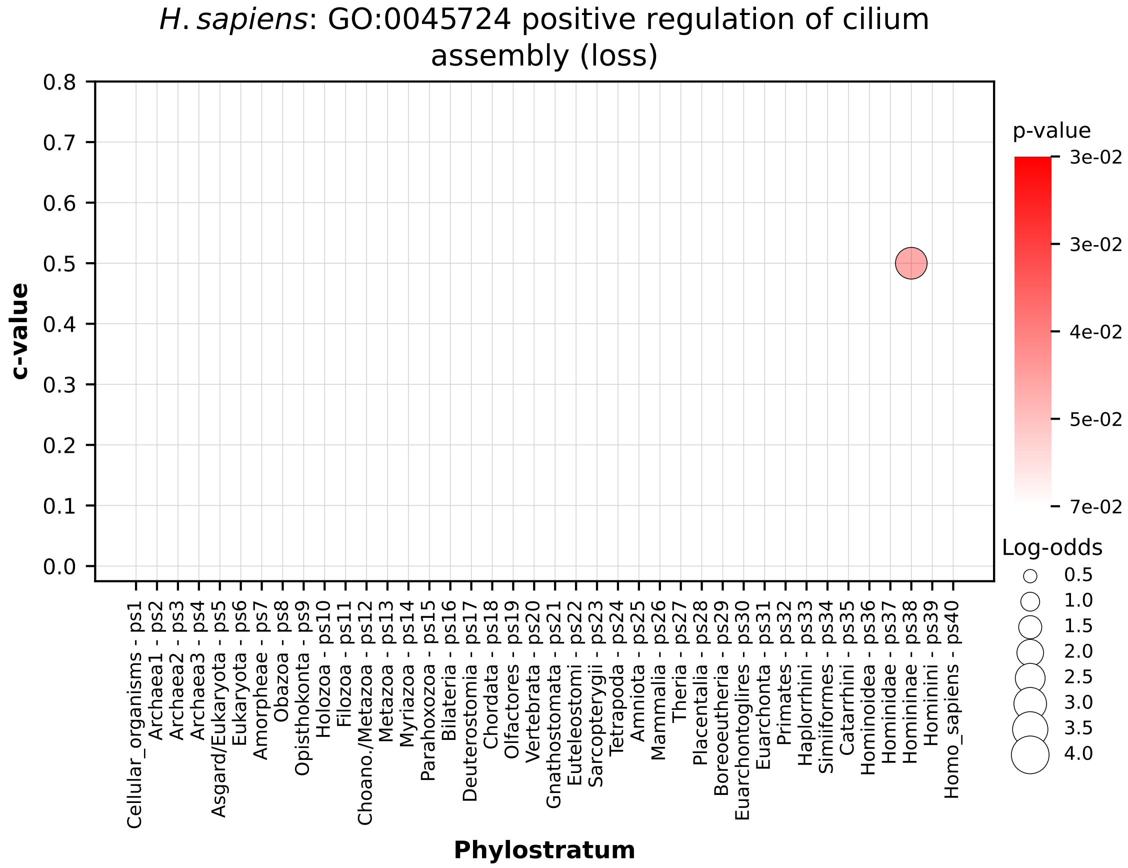
H. sapiens: GO:0045724 positive regulation of cilium assembly (loss)
c-value
0.8
0.7
0.6
0.5
0.4
0.3
0.2
0.1
0.0
p-value
3e-02
3e-02
4e-02
5e-02
7e-02
Log-odds
0.5
1.0
1.5
2.0
2.5
3.0
3.5
4.0
Cellular_organisms - ps1
Archaea1 - ps2
Archaea2 - ps3
Archaea3 - ps4
Asgard/Eukaryota - ps5
Eukaryota - ps6
Amorpheae - ps7
Obazoa - ps8
Opisthokonta - ps9
Holozoa - ps10
Filozoa - ps11
Choano./Metazoa - ps12
Metazoa - ps13
Myriazoa - ps14
Parahoxozoa - ps15
Bilateria - ps16
Deuterostomia - ps17
Chordata - ps18
Olfactores - ps19
Vertebrata - ps20
Gnathostomata - ps21
Euteleostomi - ps22
Sarcopterygii - ps23
Tetrapoda - ps24
Amniota - ps25
Mammalia - ps26
Theria - ps27
Placentalia - ps28
Boreoeutheria - ps29
Euarchontoglires - ps30
Euarchonta - ps31
Primates - ps32
Haplorrhini - ps33
Similformes - ps34
Catarrhini - ps35
Hominoidea - ps36
Hominidae - ps37
Homininae - ps38
Hominini - ps39
Homo_sapiens - ps40
Phylostratum

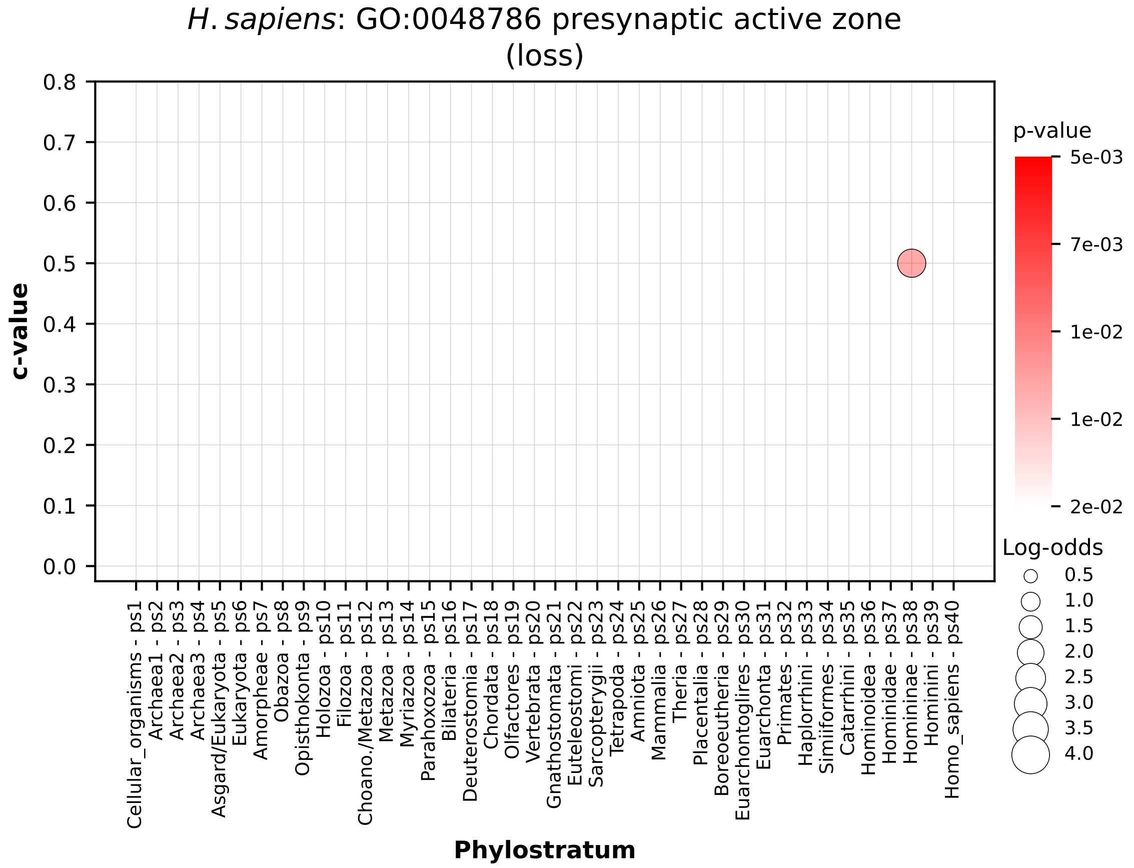
H. sapiens: GO:0048786 presynaptic active zone
(loss)
c-value
0.8
0.7
0.6
0.5
0.4
0.3
0.2
0.1
0.0
p-value
5e-03
7e-03
1e-02
1e-02
2e-02
Log-odds
0.5
1.0
1.5
2.0
2.5
3.0
3.5
4.0
Cellular_organisms - ps1
Archaea1 - ps2
Archaea2 - ps3
Archaea3 - ps4
Asgard/Eukaryota - ps5
Eukaryota - ps6
Amorpheae - ps7
Obazoa - ps8
Opisthokonta - ps9
Holozoa - ps10
Filozoa - ps11
Choano./Metazoa - ps12
Metazoa - ps13
Myriazoa - ps14
Parahoxozoa - ps15
Bilateria - ps16
Deuterostomia - ps17
Chordata - ps18
Olfactores - ps19
Vertebrata - ps20
Gnathostomata - ps21
Euteleostomi - ps22
Sarcopterygii - ps23
Tetrapoda - ps24
Amniota - ps25
Mammalia - ps26
Theria - ps27
Placentalia - ps28
Boreoeutheria - ps29
Euarchontoglires - ps30
Euarchonta - ps31
Primates - ps32
Haplorrhini - ps33
Simiiformes - ps34
Catarrhini - ps35
Hominoidea - ps36
Hominidae - ps37
Homininae - ps38
Hominini - ps39
Homo_sapiens - ps40
Phylostratum

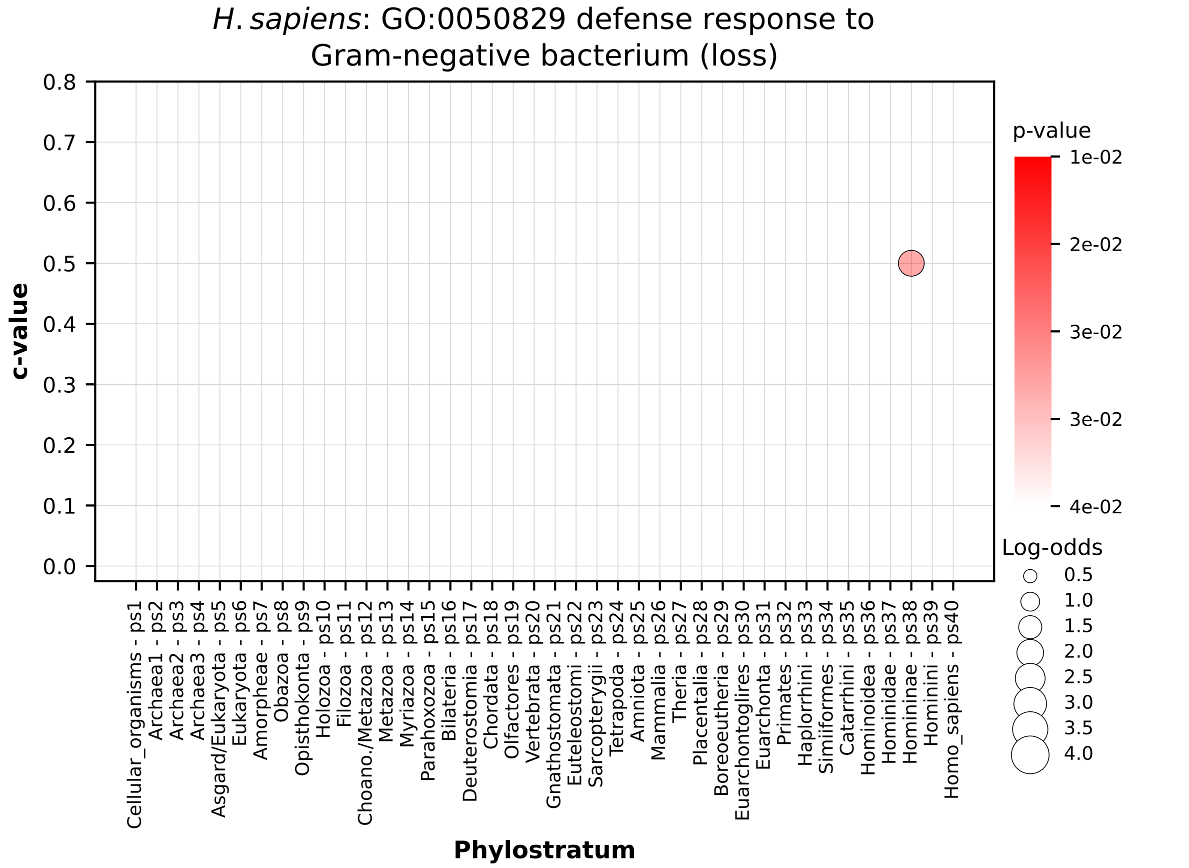

H. sapiens: GO:0050829 defense response to Gram-negative bacterium (loss)
c-value
0.8
0.7
0.6
0.5
0.4
0.3
0.2
0.1
0.0
p-value
1e-02
2e-02
3e-02
3e-02
4e-02
Log-odds
0.5
1.0
1.5
2.0
2.5
3.0
3.5
4.0
Cellular_organisms - ps1
Archaea1 - ps2
Archaea2 - ps3
Archaea3 - ps4
Asgard/Eukaryota - ps5
Eukaryota - ps6
Amorpheae - ps7
Obazoa - ps8
Opisthokonta - ps9
Holozoa - ps10
Filozoa - ps11
Choano./Metazoa - ps12
Metazoa - ps13
Myriazoa - ps14
Parahoxozoa - ps15
Bilateria - ps16
Deuterostomia - ps17
Chordata - ps18
Olfactores - ps19
Vertebrata - ps20
Gnathostomata - ps21
Euteleostomi - ps22
Sarcopterygii - ps23
Tetrapoda - ps24
Amniota - ps25
Mammalia - ps26
Theria - ps27
Placentalia - ps28
Boreoeutheria - ps29
Euarchontoglires - ps30
Euarchonta - ps31
Primates - ps32
Haplorrhini - ps33
Simiiformes - ps34
Catarrhini - ps35
Hominoidea - ps36
Hominidae - ps37
Homininae - ps38
Hominini - ps39
Homo_sapiens - ps40
Phylostratum

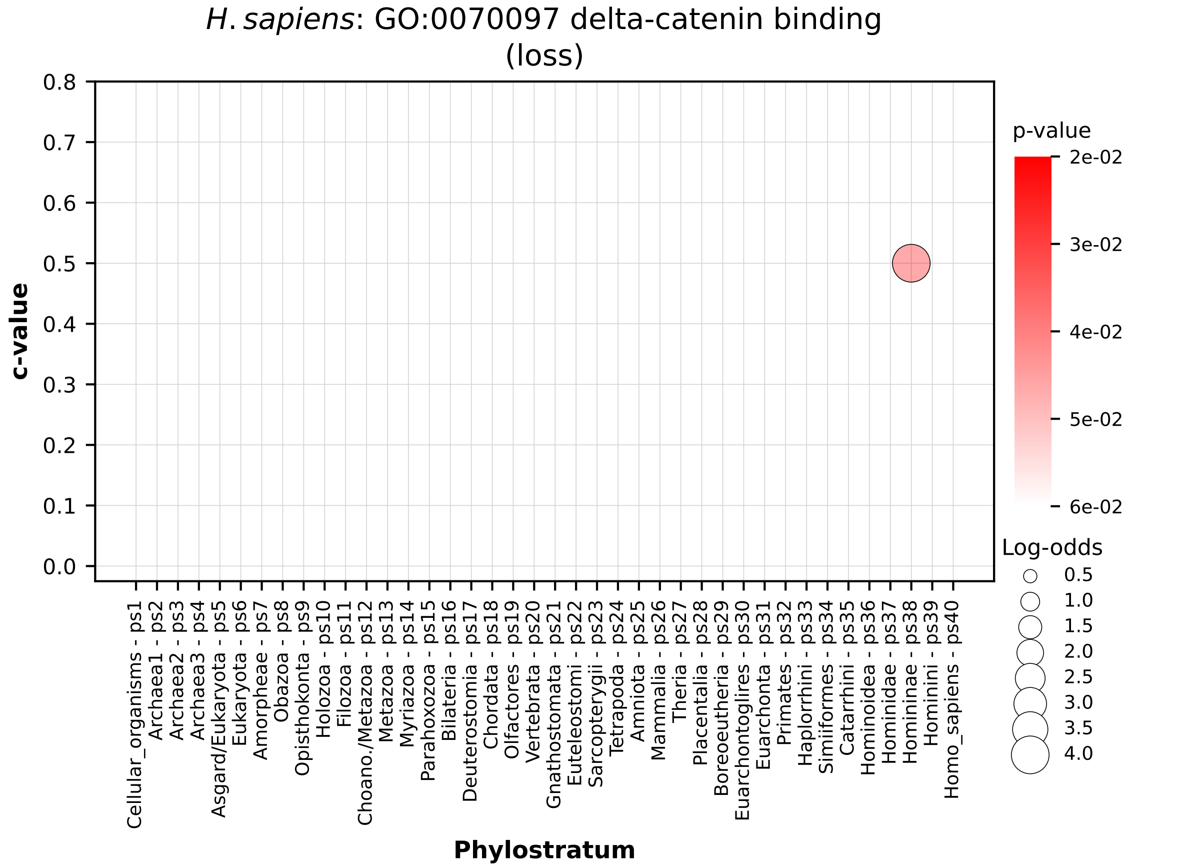
H. sapiens: GO:0070097 delta-catenin binding
(loss)
c-value
0.8
0.7
0.6
0.5
0.4
0.3
0.2
0.1
0.0
Phylostratum
Cellular_organisms - ps1
Archaea1 - ps2
Archaea2 - ps3
Archaea3 - ps4
Asgard/Eukaryota - ps5
Eukaryota - ps6
Amorpheae - ps7
Obazoa - ps8
Opisthokonta - ps9
Holozoa - ps10
Filozoa - ps11
Choano./Metazoa - ps12
Metazoa - ps13
Myriazoa - ps14
Parahoxozoa - ps15
Bilateria - ps16
Deuterostomia - ps17
Chordata - ps18
Olfactores - ps19
Vertebrata - ps20
Gnathostomata - ps21
Euteleostomi - ps22
Sarcopterygii - ps23
Tetrapoda - ps24
Amniota - ps25
Mammalia - ps26
Theria - ps27
Placentalia - ps28
Boreoeutheria - ps29
Euarchontoglires - ps30
Euarchonta - ps31
Primates - ps32
Haplorrhini - ps33
Simiiformes - ps34
Catarrhini - ps35
Hominoidea - ps36
Hominidae - ps37
Homininae - ps38
Hominini - ps39
Homo_sapiens - ps40
p-value
2e-02
3e-02
4e-02
5e-02
6e-02
Log-odds
0.5
1.0
1.5
2.0
2.5
3.0
3.5
4.0

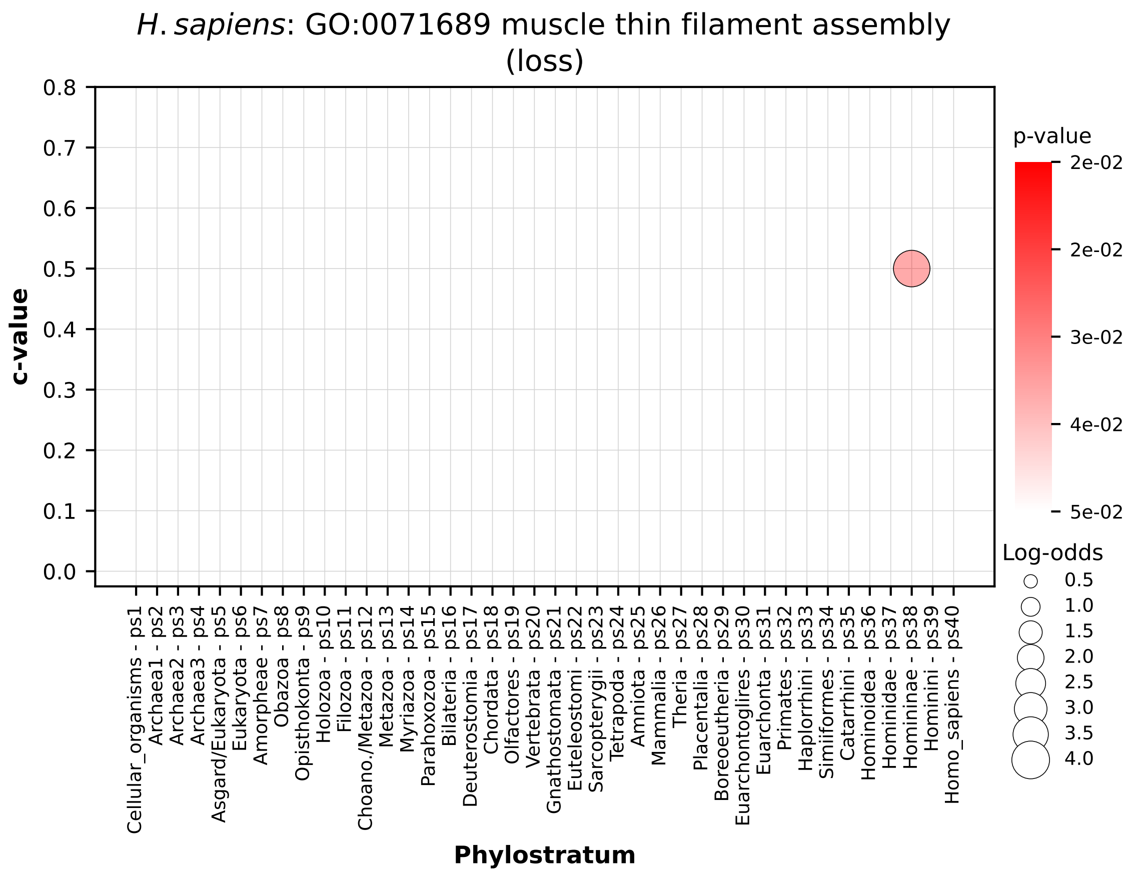

H. sapiens: GO:0071689 muscle thin filament assembly
(loss)
c-value
0.8
0.7
0.6
0.5
0.4
0.3
0.2
0.1
0.0
Phylostratum
Cellular_organisms - ps1
Archaea1 - ps2
Archaea2 - ps3
Archaea3 - ps4
Asgard/Eukaryota - ps5
Eukaryota - ps6
Amorpheae - ps7
Obazoa - ps8
Opisthokonta - ps9
Holozoa - ps10
Filozoa - ps11
Choano./Metazoa - ps12
Metazoa - ps13
Myriazoa - ps14
Parahoxozoa - ps15
Bilateria - ps16
Deuterostomia - ps17
Chordata - ps18
Olfactores - ps19
Vertebrata - ps20
Gnathostomata - ps21
Euteleostomi - ps22
Sarcopterygii - ps23
Tetrapoda - ps24
Amniota - ps25
Mammalia - ps26
Theria - ps27
Placentalia - ps28
Boreoeutheria - ps29
Euarchontoglires - ps30
Euarchonta - ps31
Primates - ps32
Haplorrhini - ps33
Simiiformes - ps34
Catarrhini - ps35
Hominoidea - ps36
Hominidae - ps37
Homininae - ps38
Hominini - ps39
Homo_sapiens - ps40
p-value
2e-02
2e-02
3e-02
4e-02
5e-02
Log-odds
0.5
1.0
1.5
2.0
2.5
3.0
3.5
4.0

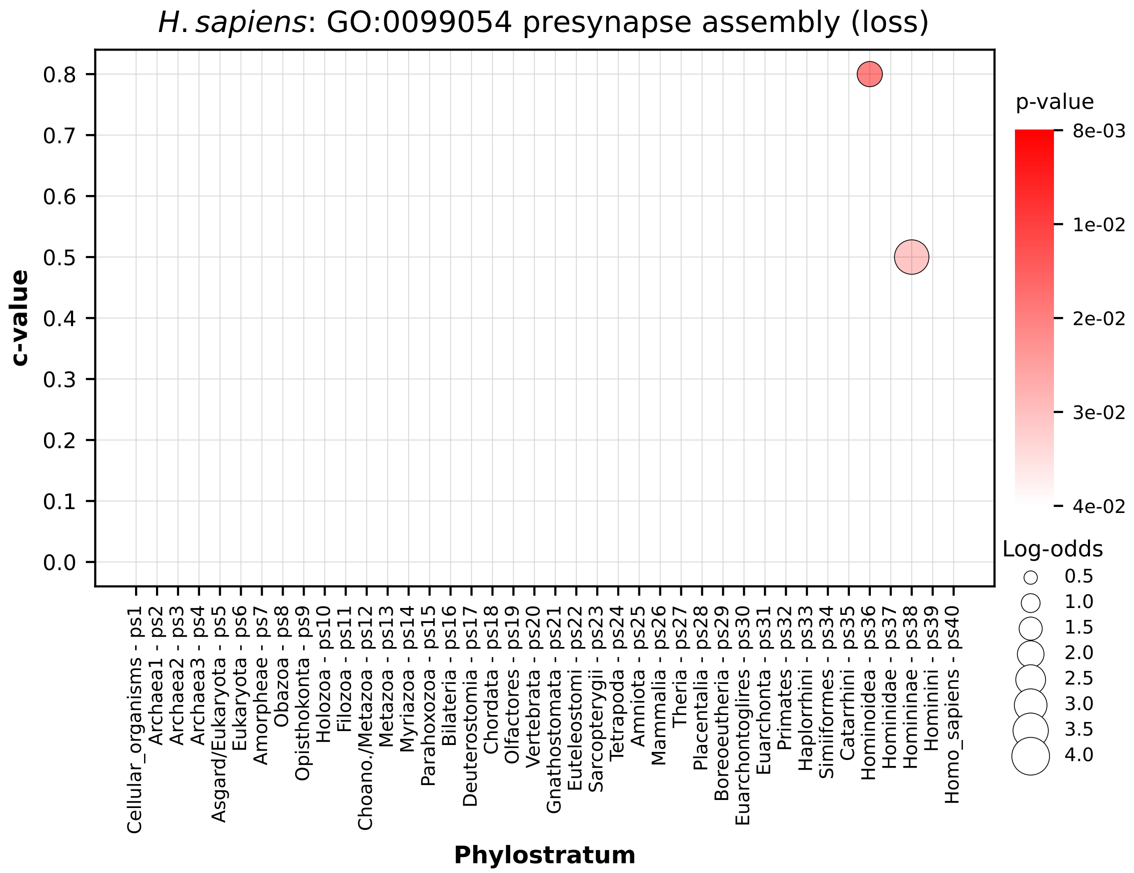

H. sapiens: GO:0099054 presynapse assembly (loss)
c-value
Phylostratum
p-value
8e-03
1e-02
2e-02
3e-02
4e-02
Log-odds
0.5
1.0
1.5
2.0
2.5
3.0
3.5
4.0
Cellular_organisms - ps1
Archaea1 - ps2
Archaea2 - ps3
Archaea3 - ps4
Asgard/Eukaryota - ps5
Eukaryota - ps6
Amorpheae - ps7
Obazoa - ps8
Opisthokonta - ps9
Holozoa - ps10
Filozoa - ps11
Choano./Metazoa - ps12
Metazoa - ps13
Myriazoa - ps14
Parahoxozoa - ps15
Bilateria - ps16
Deuterostomia - ps17
Chordata - ps18
Olfactores - ps19
Vertebrata - ps20
Gnathostomata - ps21
Euteleostomi - ps22
Sarcopterygii - ps23
Tetrapoda - ps24
Amniota - ps25
Mammalia - ps26
Theria - ps27
Placentalia - ps28
Boreoeutheria - ps29
Euarchontoglires - ps30
Euarchonta - ps31
Primates - ps32
Haplorrhini - ps33
Simiiformes - ps34
Catarrhini - ps35
Hominoidea - ps36
Hominidae - ps37
Homininae - ps38
Hominini - ps39
Homo_sapiens - ps40

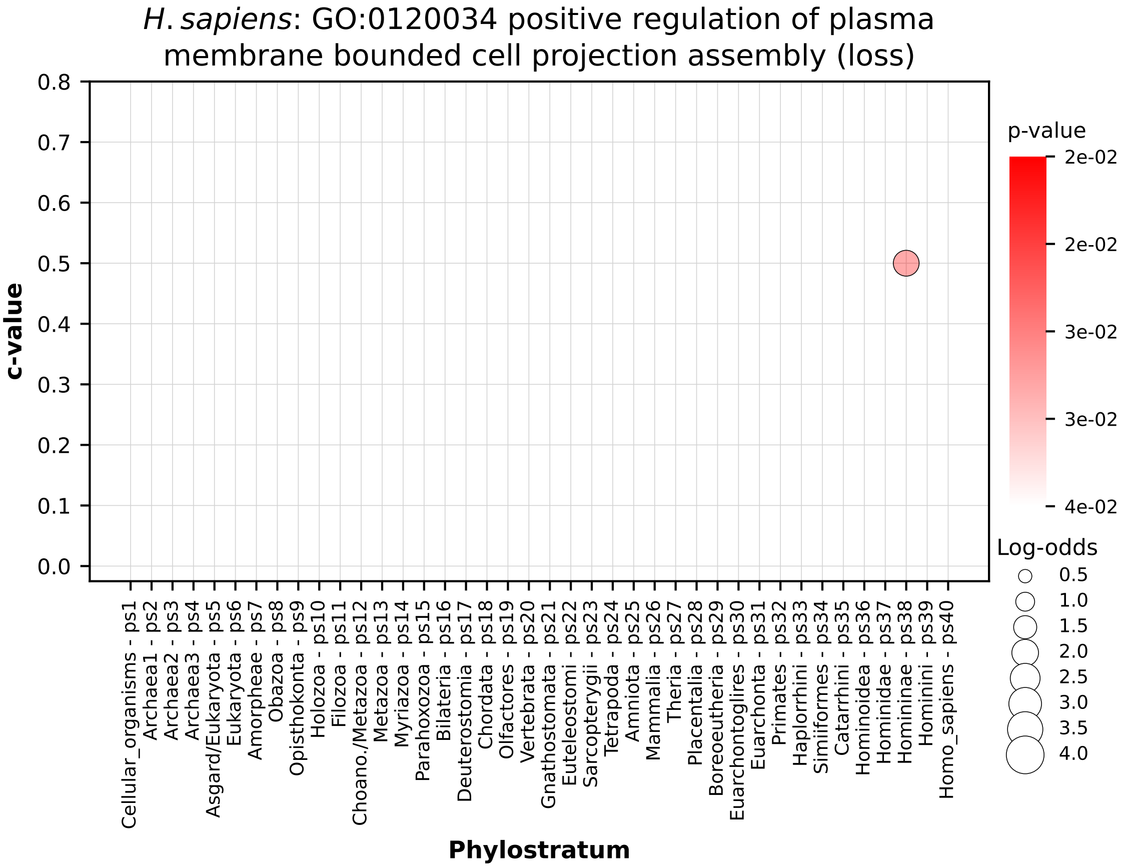

H. sapiens: GO:0120034 positive regulation of plasma membrane bounded cell projection assembly (loss)
c-value
0.8
0.7
0.6
0.5
0.4
0.3
0.2
0.1
0.0
Phylostratum
Cellular_organisms - ps1
Archaea1 - ps2
Archaea2 - ps3
Archaea3 - ps4
Asgard/Eukaryota - ps5
Eukaryota - ps6
Amorpheae - ps7
Obazoa - ps8
Opisthokonta - ps9
Holozoa - ps10
Filozoa - ps11
Choano./Metazoa - ps12
Metazoa - ps13
Myriazoa - ps14
Parahoxozoa - ps15
Bilateria - ps16
Deuterostomia - ps17
Chordata - ps18
Olfactores - ps19
Vertebrata - ps20
Gnathostomata - ps21
Euteleostomi - ps22
Sarcopterygii - ps23
Tetrapoda - ps24
Amniota - ps25
Mammalia - ps26
Theria - ps27
Placentalia - ps28
Boreoeutheria - ps29
Euarchontoglires - ps30
Euarchonta - ps31
Primates - ps32
Haplorrhini - ps33
Simiiformes - ps34
Catarrhini - ps35
Hominoidea - ps36
Hominidae - ps37
Homininae - ps38
Hominini - ps39
Homo_sapiens - ps40
p-value
2e-02
2e-02
3e-02
3e-02
4e-02
Log-odds
0.5
1.0
1.5
2.0
2.5
3.0
3.5
4.0

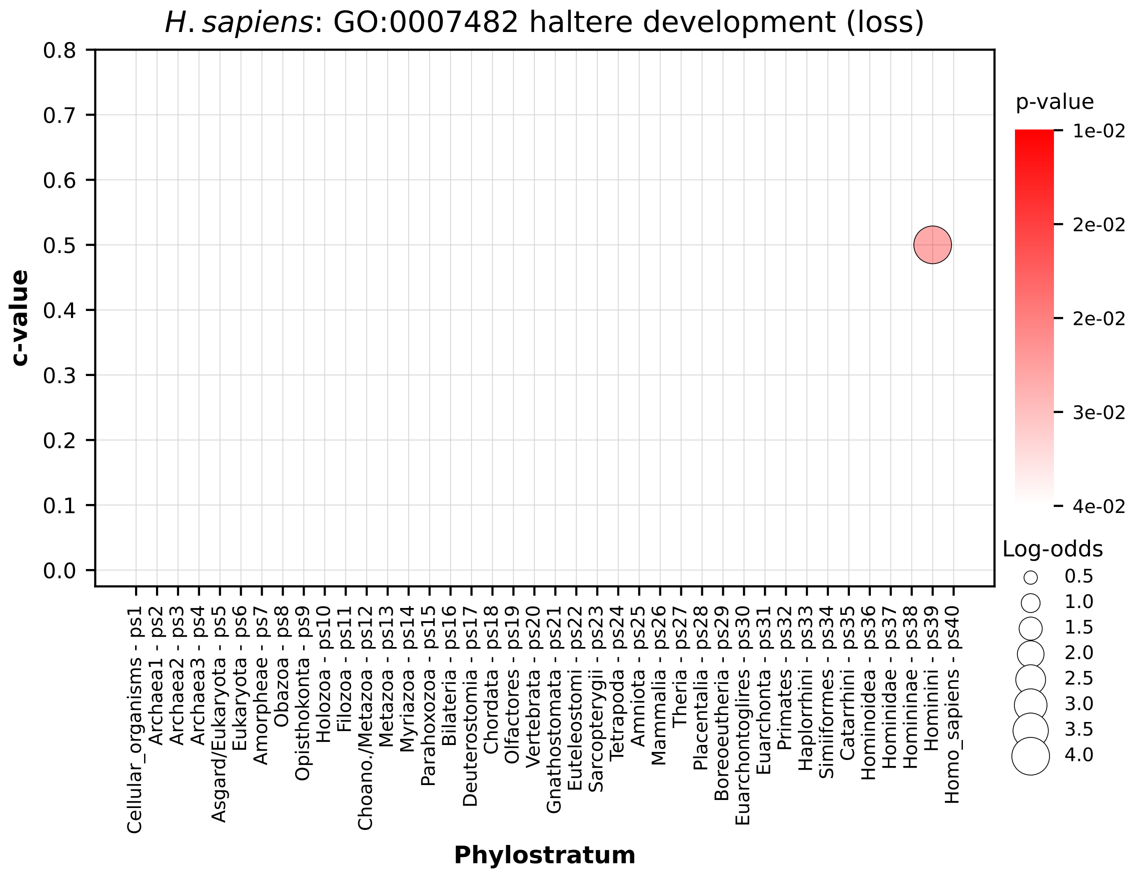

H. sapiens: GO:0007482 haltere development (loss)
c-value
0.8
0.7
0.6
0.5
0.4
0.3
0.2
0.1
0.0
p-value
1e-02
2e-02
2e-02
3e-02
4e-02
Log-odds
0.5
1.0
1.5
2.0
2.5
3.0
3.5
4.0
Cellular_organisms - ps1
Archaea1 - ps2
Archaea2 - ps3
Archaea3 - ps4
Asgard/Eukaryota - ps5
Eukaryota - ps6
Amorpheae - ps7
Obazoa - ps8
Opisthokonta - ps9
Holozoa - ps10
Filozoa - ps11
Choano./Metazoa - ps12
Metazoa - ps13
Myriazoa - ps14
Parahoxozoa - ps15
Bilateria - ps16
Deuterostomia - ps17
Chordata - ps18
Olfactores - ps19
Vertebrata - ps20
Gnathostomata - ps21
Euteleostomi - ps22
Sarcopterygii - ps23
Tetrapoda - ps24
Amniota - ps25
Mammalia - ps26
Theria - ps27
Placentalia - ps28
Boreoeutheria - ps29
Euarchontoglires - ps30
Euarchonta - ps31
Primates - ps32
Haplorrhini - ps33
Simiiformes - ps34
Catarrhini - ps35
Hominoidea - ps36
Hominidae - ps37
Homininae - ps38
Hominini - ps39
Homo_sapiens - ps40
Phylostratum

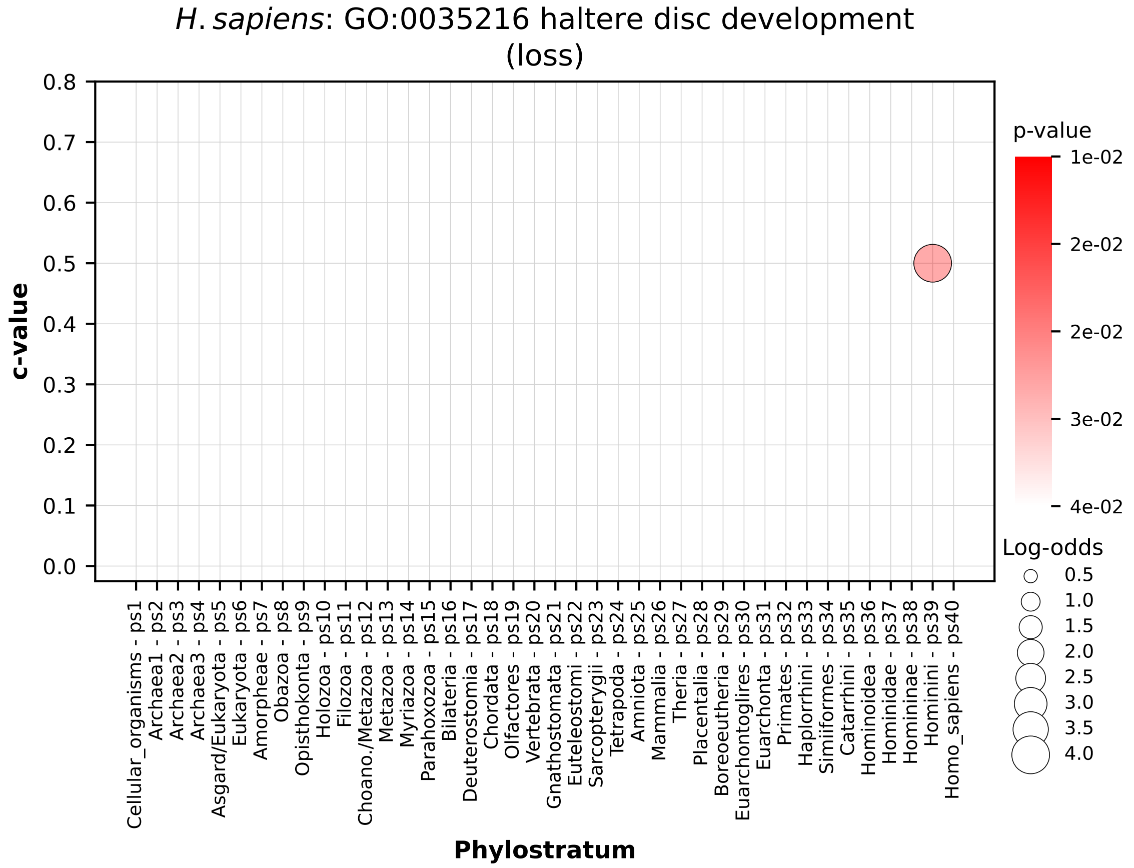
H. sapiens: GO:0035216 haltere disc development
(loss)
c-value
0.8
0.7
0.6
0.5
0.4
0.3
0.2
0.1
0.0
p-value
1e-02
2e-02
2e-02
3e-02
4e-02
Log-odds
0.5
1.0
1.5
2.0
2.5
3.0
3.5
4.0
Cellular_organisms - ps1
Archaea1 - ps2
Archaea2 - ps3
Archaea3 - ps4
Asgard/Eukaryota - ps5
Eukaryota - ps6
Amorpheae - ps7
Obazoa - ps8
Opisthokonta - ps9
Holozoa - ps10
Filozoa - ps11
Choano./Metazoa - ps12
Metazoa - ps13
Myriazoa - ps14
Parahoxozoa - ps15
Bilateria - ps16
Deuterostomia - ps17
Chordata - ps18
Olfactores - ps19
Vertebrata - ps20
Gnathostomata - ps21
Euteleostomi - ps22
Sarcopterygii - ps23
Tetrapoda - ps24
Amniota - ps25
Mammalia - ps26
Theria - ps27
Placentalia - ps28
Boreoeutheria - ps29
Euarchontoglires - ps30
Euarchonta - ps31
Primates - ps32
Haplorrhini - ps33
Simiiformes - ps34
Catarrhini - ps35
Hominoidea - ps36
Hominidae - ps37
Homininae - ps38
Hominini - ps39
Homo_sapiens - ps40
Phylostratum

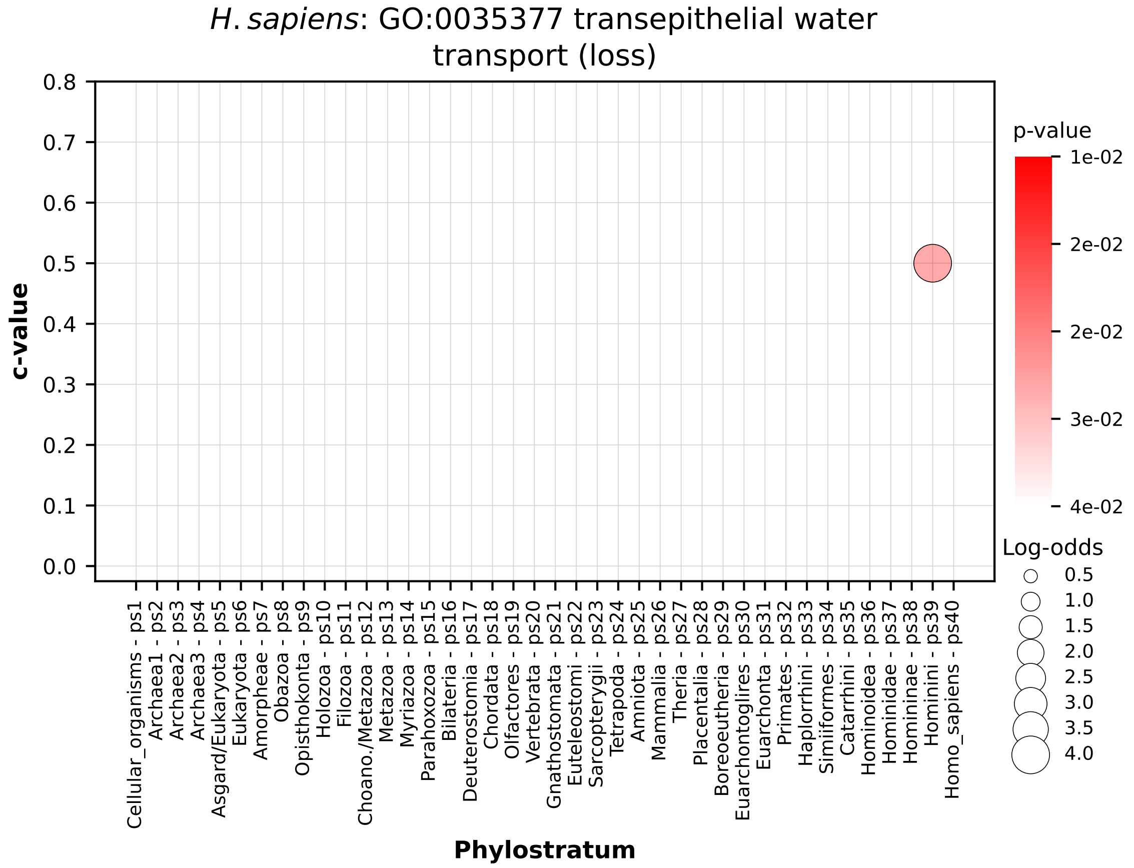

H. sapiens: GO:0035377 transepithelial water transport (loss)
c-value
0.8
0.7
0.6
0.5
0.4
0.3
0.2
0.1
0.0
Phylostratum
Cellular_organisms - ps1
Archaea1 - ps2
Archaea2 - ps3
Archaea3 - ps4
Asgard/Eukaryota - ps5
Eukaryota - ps6
Amorpheae - ps7
Obazoa - ps8
Opisthokonta - ps9
Holozoa - ps10
Filozoa - ps11
Choano./Metazoa - ps12
Metazoa - ps13
Myriazoa - ps14
Parahoxozoa - ps15
Bilateria - ps16
Deuterostomia - ps17
Chordata - ps18
Olfactores - ps19
Vertebrata - ps20
Gnathostomata - ps21
Euteleostomi - ps22
Sarcopterygii - ps23
Tetrapoda - ps24
Amniota - ps25
Mammalia - ps26
Theria - ps27
Placentalia - ps28
Boreoeutheria - ps29
Euarchontoglires - ps30
Euarchonta - ps31
Primates - ps32
Haplorrhini - ps33
Simiiformes - ps34
Catarrhini - ps35
Hominoidea - ps36
Hominidae - ps37
Homininae - ps38
Hominini - ps39
Homo_sapiens - ps40
p-value
1e-02
2e-02
2e-02
3e-02
4e-02
Log-odds
0.5
1.0
1.5
2.0
2.5
3.0
3.5
4.0

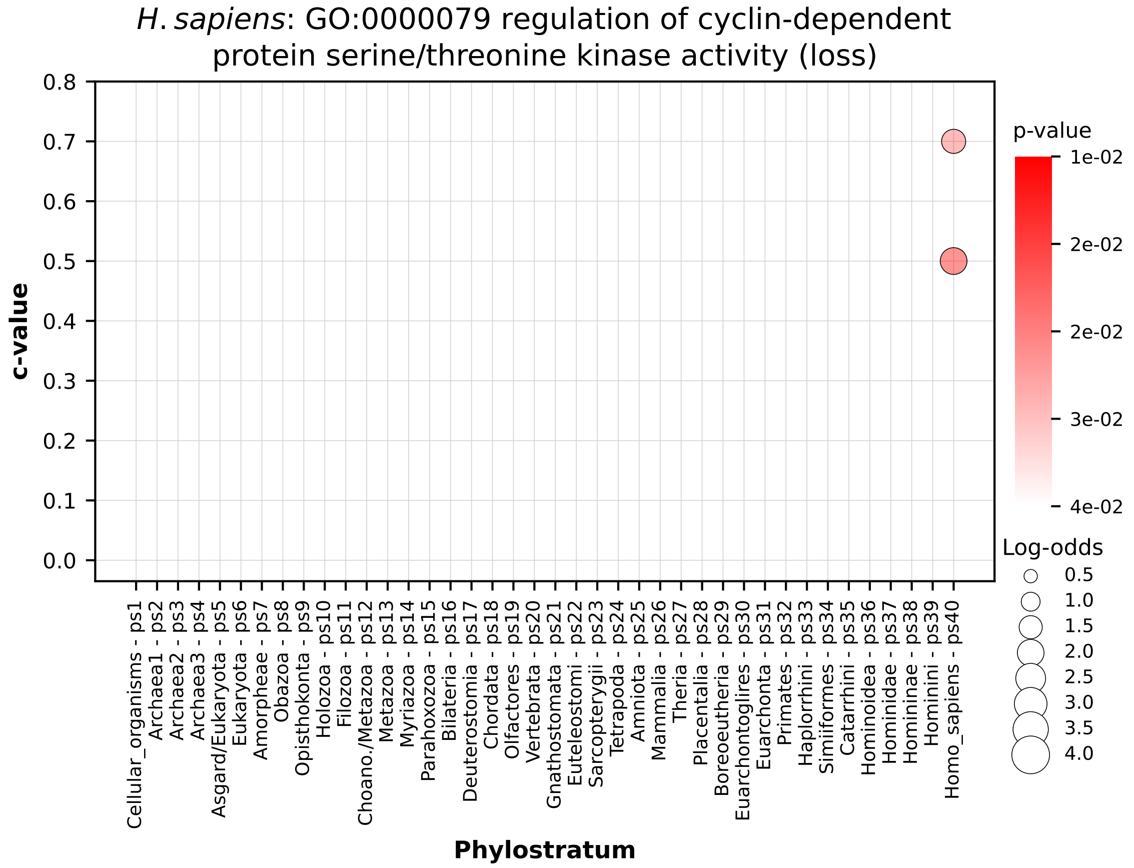

H. sapiens: GO:0000079 regulation of cyclin-dependent protein serine/threonine kinase activity (loss)
c-value
0.8
0.7
0.6
0.5
0.4
0.3
0.2
0.1
0.0
p-value
1e-02
2e-02
2e-02
3e-02
4e-02
Log-odds
0.5
1.0
1.5
2.0
2.5
3.0
3.5
4.0
Cellular_organisms - ps1
Archaea1 - ps2
Archaea2 - ps3
Archaea3 - ps4
Asgard/Eukaryota - ps5
Eukaryota - ps6
Amorpheae - ps7
Obazoa - ps8
Opisthokonta - ps9
Holozoa - ps10
Filozoa - ps11
Choano./Metazoa - ps12
Metazoa - ps13
Myriazoa - ps14
Parahoxozoa - ps15
Bilateria - ps16
Deuterostomia - ps17
Chordata - ps18
Olfactores - ps19
Vertebrata - ps20
Gnathostomata - ps21
Euteleostomi - ps22
Sarcopterygii - ps23
Tetrapoda - ps24
Amniota - ps25
Mammalia - ps26
Theria - ps27
Placentalia - ps28
Boreoeutheria - ps29
Euarchontoglires - ps30
Euarchonta - ps31
Primates - ps32
Haplorrhini - ps33
Simiiformes - ps34
Catarrhini - ps35
Hominoidea - ps36
Hominidae - ps37
Homininae - ps38
Hominini - ps39
Homo_sapiens - ps40
Phylostratum

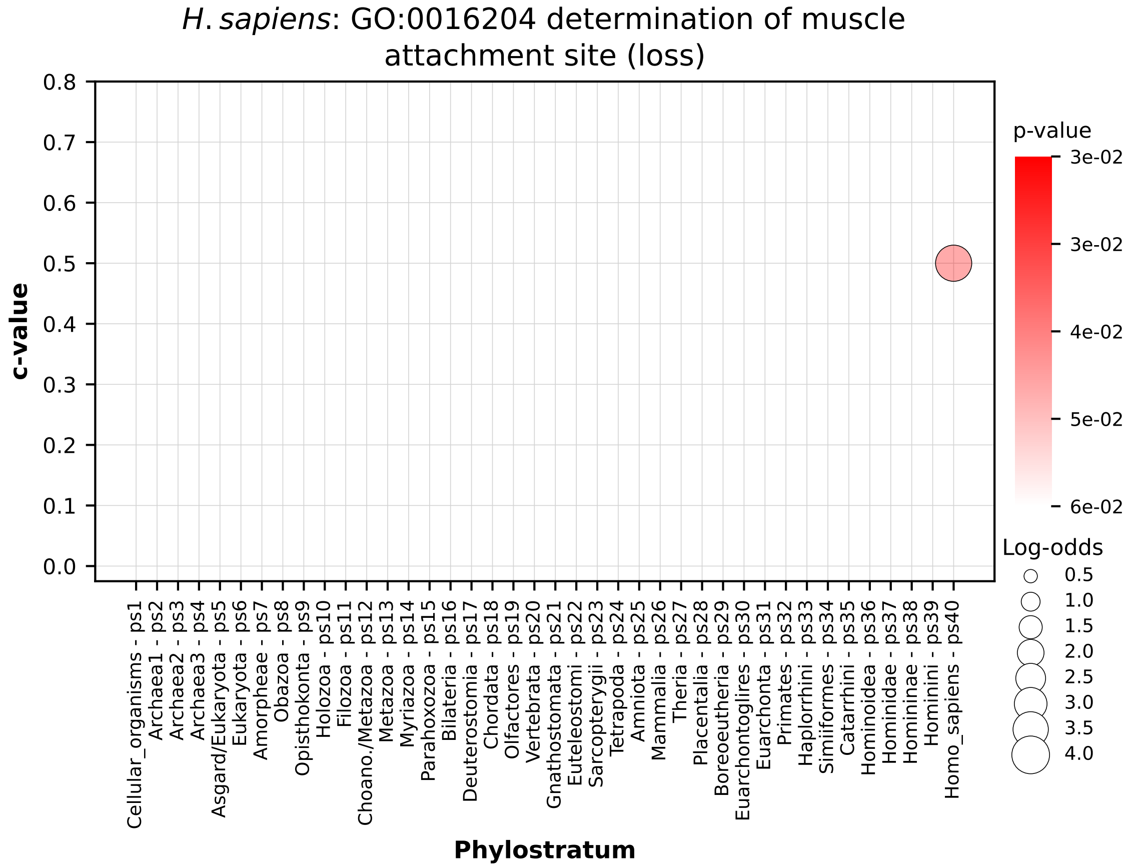

H. sapiens: GO:0016204 determination of muscle attachment site (loss)
c-value
0.8
0.7
0.6
0.5
0.4
0.3
0.2
0.1
0.0
p-value
3e-02
3e-02
4e-02
5e-02
6e-02
Log-odds
0.5
1.0
1.5
2.0
2.5
3.0
3.5
4.0
Cellular_organisms - ps1
Archaea1 - ps2
Archaea2 - ps3
Archaea3 - ps4
Asgard/Eukaryota - ps5
Eukaryota - ps6
Amorpheae - ps7
Obazoa - ps8
Opisthokonta - ps9
Holozoa - ps10
Filozoa - ps11
Choano./Metazoa - ps12
Metazoa - ps13
Myriazoa - ps14
Parahoxozoa - ps15
Bilateria - ps16
Deuterostomia - ps17
Chordata - ps18
Olfactores - ps19
Vertebrata - ps20
Gnathostomata - ps21
Euteleostomi - ps22
Sarcopterygii - ps23
Tetrapoda - ps24
Amniota - ps25
Mammalia - ps26
Theria - ps27
Placentalia - ps28
Boreoeutheria - ps29
Euarchontoglires - ps30
Euarchonta - ps31
Primates - ps32
Haplorrhini - ps33
Simiiformes - ps34
Catarrhini - ps35
Hominoidea - ps36
Hominidae - ps37
Homininae - ps38
Hominini - ps39
Homo_sapiens - ps40
Phylostratum

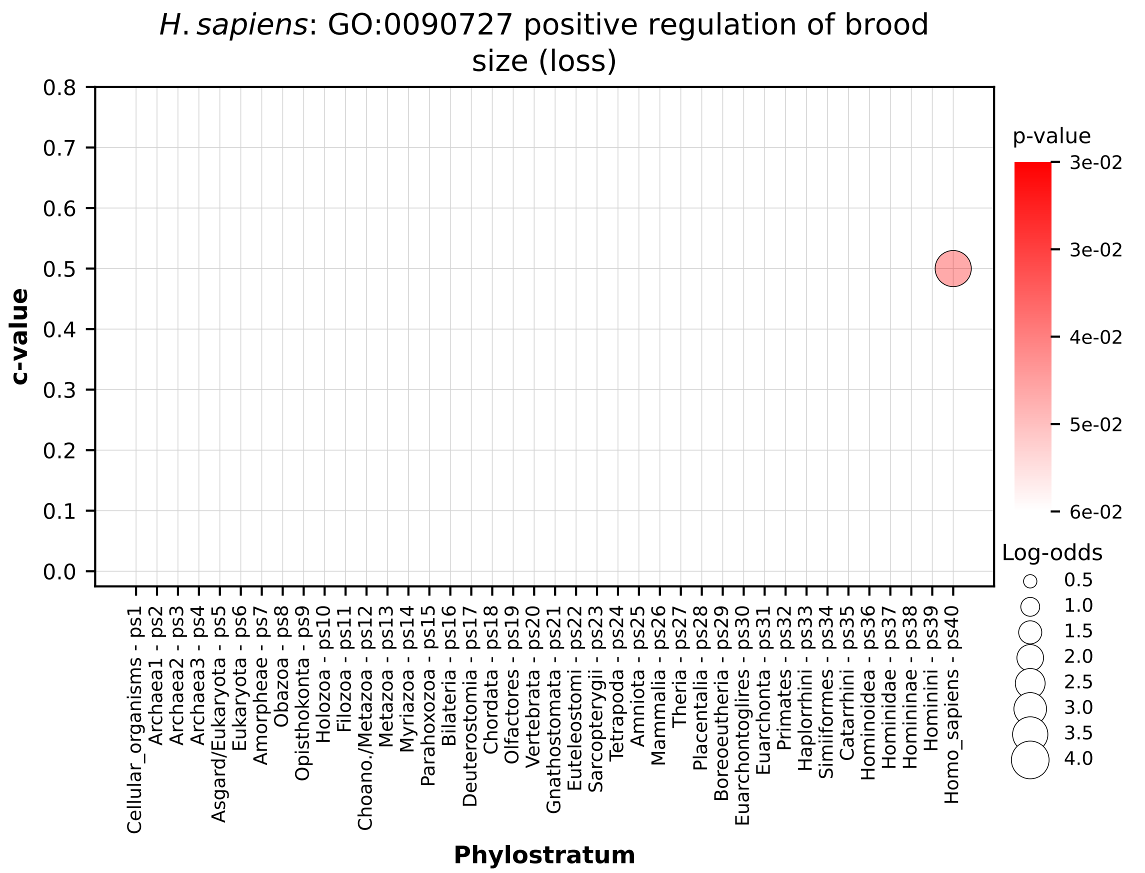
H. sapiens: GO:0090727 positive regulation of brood size (loss)
c-value
0.8
0.7
0.6
0.5
0.4
0.3
0.2
0.1
0.0
p-value
3e-02
3e-02
4e-02
5e-02
6e-02
Log-odds
0.5
1.0
1.5
2.0
2.5
3.0
3.5
4.0
Cellular_organisms - ps1
Archaea1 - ps2
Archaea2 - ps3
Archaea3 - ps4
Asgard/Eukaryota - ps5
Eukaryota - ps6
Amorpheae - ps7
Obazoa - ps8
Opisthokonta - ps9
Holozoa - ps10
Filozoa - ps11
Choano./Metazoa - ps12
Metazoa - ps13
Myriazoa - ps14
Parahoxozoa - ps15
Bilateria - ps16
Deuterostomia - ps17
Chordata - ps18
Olfactores - ps19
Vertebrata - ps20
Gnathostomata - ps21
Euteleostomi - ps22
Sarcopterygii - ps23
Tetrapoda - ps24
Amniota - ps25
Mammalia - ps26
Theria - ps27
Placentalia - ps28
Boreoeutheria - ps29
Euarchontoglires - ps30
Euarchonta - ps31
Primates - ps32
Haplorrhini - ps33
Simiiformes - ps34
Catarrhini - ps35
Hominoidea - ps36
Hominidae - ps37
Homininae - ps38
Hominini - ps39
Homo_sapiens - ps40
Phylostratum

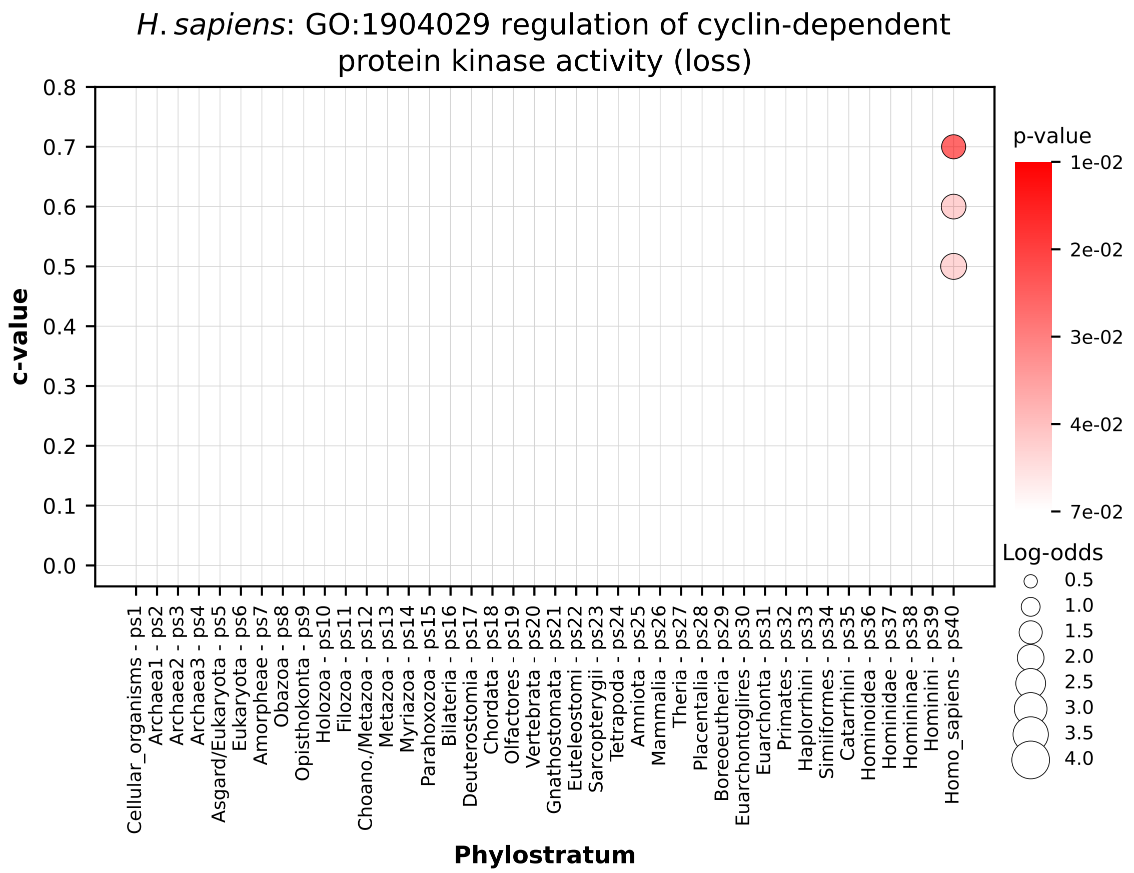

H. sapiens: GO:1904029 regulation of cyclin-dependent protein kinase activity (loss)
c-value
0.0
0.1
0.2
0.3
0.4
0.5
0.6
0.7
0.8
Phylostratum
Cellular_organisms - ps1
Archaea1 - ps2
Archaea2 - ps3
Archaea3 - ps4
Asgard/Eukaryota - ps5
Eukaryota - ps6
Amorpheae - ps7
Obazoa - ps8
Opisthokonta - ps9
Holozoa - ps10
Filozoa - ps11
Choano./Metazoa - ps12
Metazoa - ps13
Myriazoa - ps14
Parahoxozoa - ps15
Bilateria - ps16
Deuterostomia - ps17
Chordata - ps18
Olfactores - ps19
Vertebrata - ps20
Gnathostomata - ps21
Euteleostomi - ps22
Sarcopterygii - ps23
Tetrapoda - ps24
Amniota - ps25
Mammalia - ps26
Theria - ps27
Placentalia - ps28
Boreoeutheria - ps29
Euarchontoglires - ps30
Euarchonta - ps31
Primates - ps32
Haplorrhini - ps33
Simiiformes - ps34
Catarrhini - ps35
Hominoidea - ps36
Hominidae - ps37
Homininae - ps38
Hominini - ps39
Homo_sapiens - ps40
p-value
1e-02
2e-02
3e-02
4e-02
7e-02
Log-odds
0.5
1.0
1.5
2.0
2.5
3.0
3.5
4.0

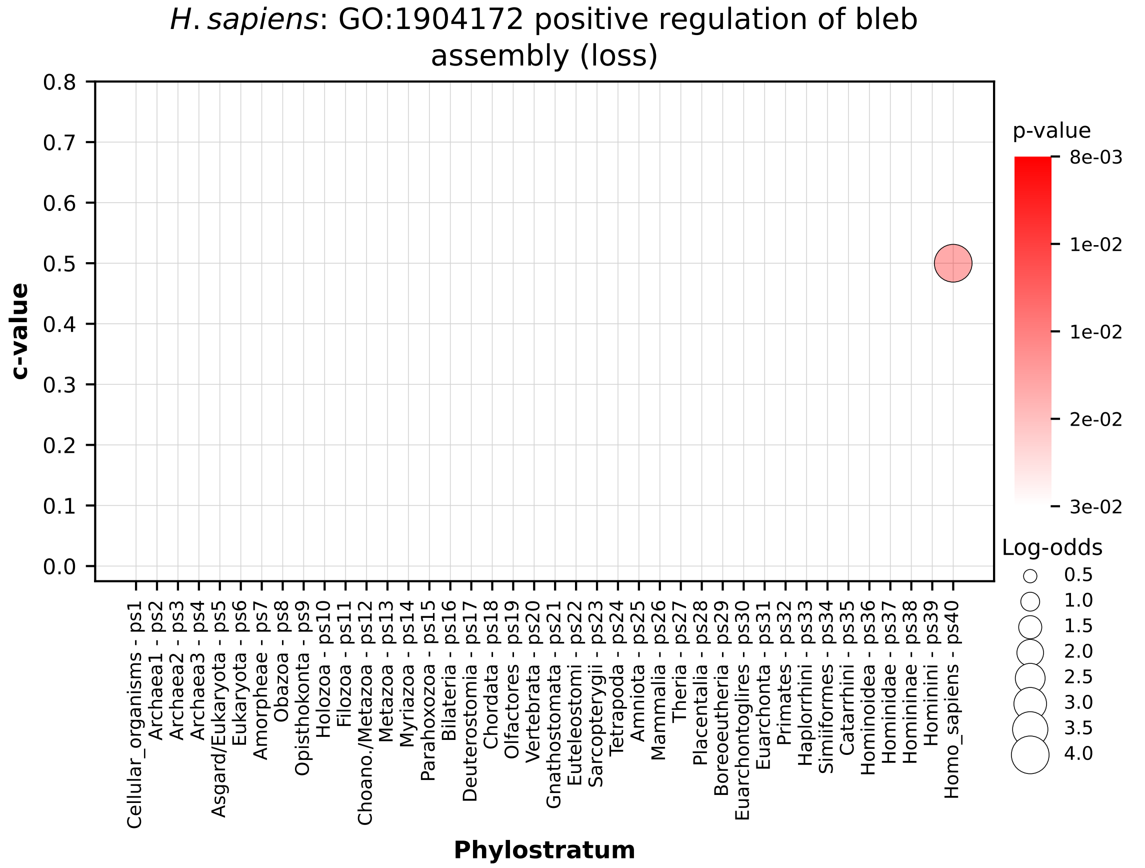

H. sapiens: GO:1904172 positive regulation of bleb assembly (loss)
c-value
0.8
0.7
0.6
0.5
0.4
0.3
0.2
0.1
0.0
Phylostratum
Cellular_organisms - ps1
Archaea1 - ps2
Archaea2 - ps3
Archaea3 - ps4
Asgard/Eukaryota - ps5
Eukaryota - ps6
Amorpheae - ps7
Obazoa - ps8
Opisthokonta - ps9
Holozoa - ps10
Filozoa - ps11
Choano./Metazoa - ps12
Metazoa - ps13
Myriazoa - ps14
Parahoxozoa - ps15
Bilateria - ps16
Deuterostomia - ps17
Chordata - ps18
Olfactores - ps19
Vertebrata - ps20
Gnathostomata - ps21
Euteleostomi - ps22
Sarcopterygii - ps23
Tetrapoda - ps24
Amniota - ps25
Mammalia - ps26
Theria - ps27
Placentalia - ps28
Boreoeutheria - ps29
Euarchontoglires - ps30
Euarchonta - ps31
Primates - ps32
Haplorrhini - ps33
Simiiformes - ps34
Catarrhini - ps35
Hominoidea - ps36
Hominidae - ps37
Homininae - ps38
Hominini - ps39
Homo_sapiens - ps40
p-value
8e-03
1e-02
1e-02
2e-02
3e-02
Log-odds
0.5
1.0
1.5
2.0
2.5
3.0
3.5
4.0

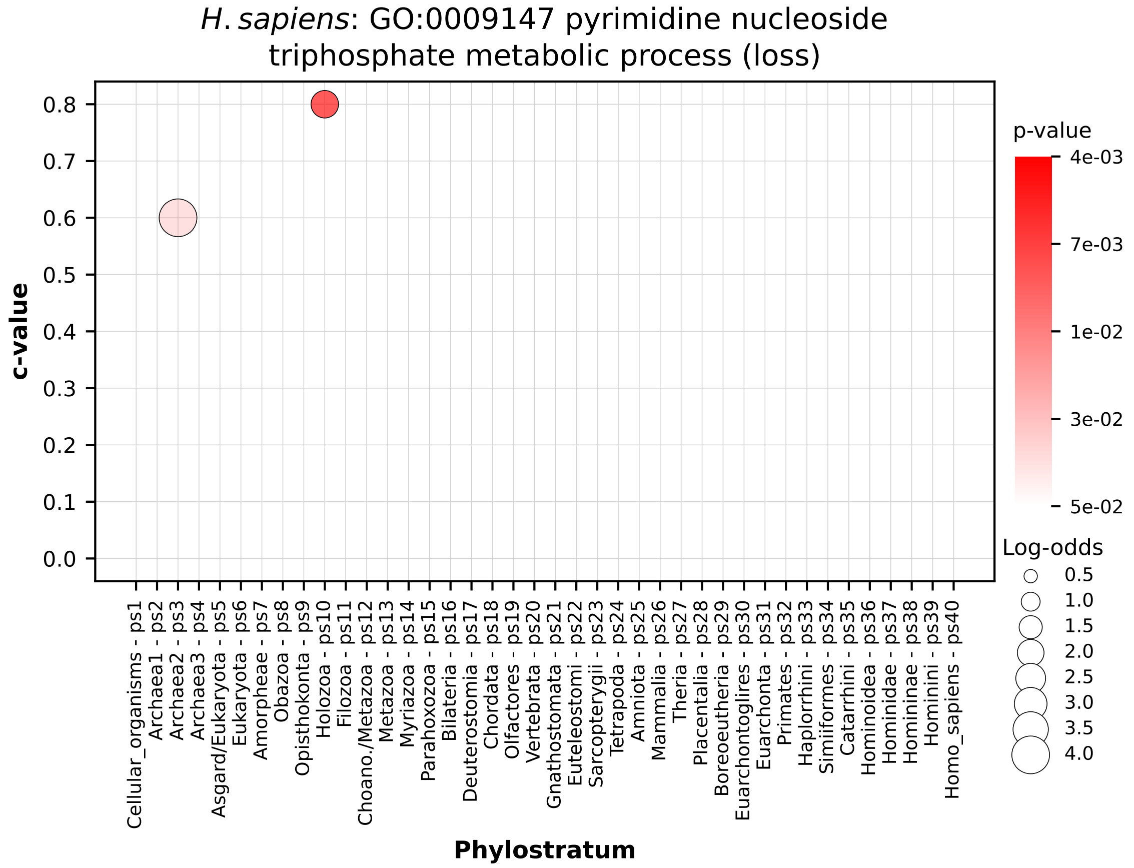

H. sapiens: GO:0009147 pyrimidine nucleoside triphosphate metabolic process (loss)
c-value
0.0
0.1
0.2
0.3
0.4
0.5
0.6
0.7
0.8
Phylostratum
Cellular_organisms - ps1
Archaea1 - ps2
Archaea2 - ps3
Archaea3 - ps4
Asgard/Eukaryota - ps5
Eukaryota - ps6
Amorpheae - ps7
Obazoa - ps8
Opisthokonta - ps9
Holozoa - ps10
Filozoa - ps11
Choano./Metazoa - ps12
Metazoa - ps13
Myriazoa - ps14
Parahoxozoa - ps15
Bilateria - ps16
Deuterostomia - ps17
Chordata - ps18
Olfactores - ps19
Vertebrata - ps20
Gnathostomata - ps21
Euteleostomi - ps22
Sarcopterygii - ps23
Tetrapoda - ps24
Amniota - ps25
Mammalia - ps26
Theria - ps27
Placentalia - ps28
Boreoeutheria - ps29
Euarchontoglires - ps30
Euarchonta - ps31
Primates - ps32
Haplorrhini - ps33
Simiiformes - ps34
Catarrhini - ps35
Hominoidea - ps36
Hominidae - ps37
Homininae - ps38
Hominini - ps39
Homo_sapiens - ps40
p-value
4e-03
7e-03
1e-02
3e-02
5e-02
Log-odds
0.5
1.0
1.5
2.0
2.5
3.0
3.5
4.0

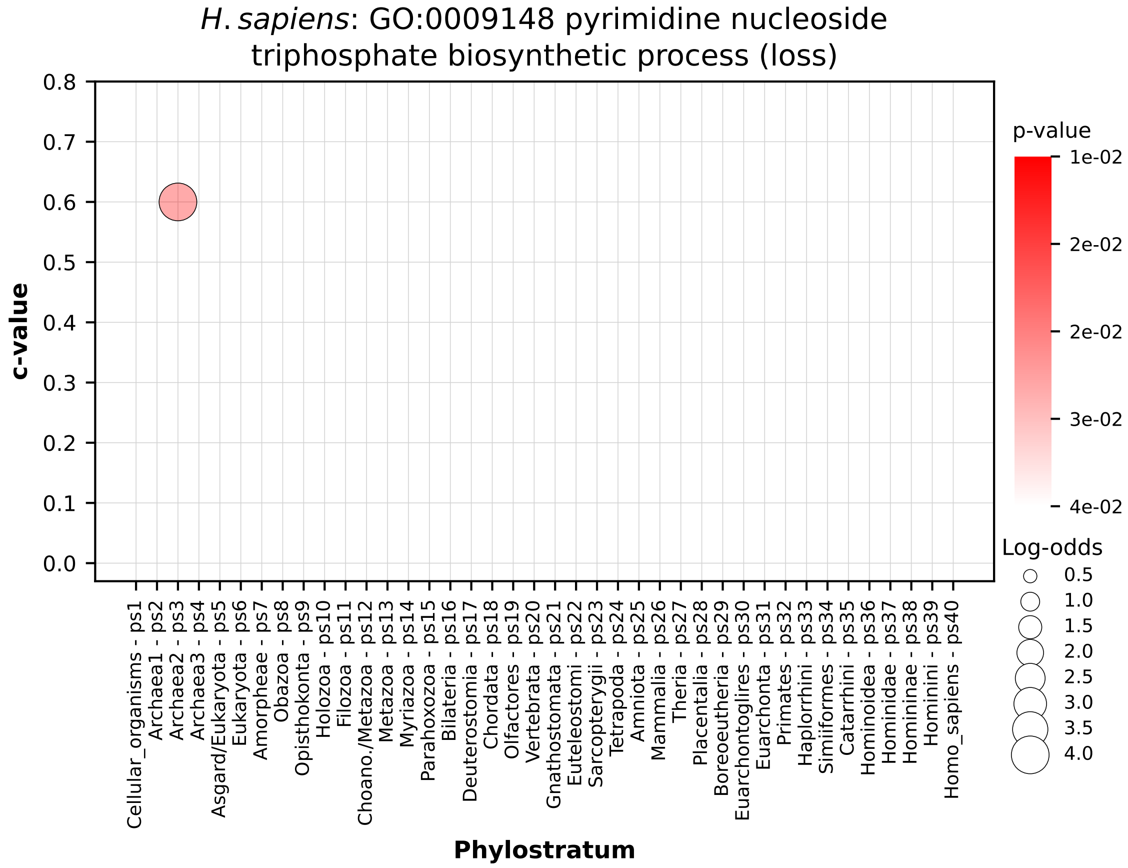

H. sapiens: GO:0009148 pyrimidine nucleoside triphosphate biosynthetic process (loss)
c-value
0.8
0.7
0.6
0.5
0.4
0.3
0.2
0.1
0.0
p-value
1e-02
2e-02
2e-02
3e-02
4e-02
Log-odds
0.5
1.0
1.5
2.0
2.5
3.0
3.5
4.0
Cellular_organisms - ps1
Archaea1 - ps2
Archaea2 - ps3
Archaea3 - ps4
Asgard/Eukaryota - ps5
Eukaryota - ps6
Amorpheae - ps7
Obazoa - ps8
Opisthokonta - ps9
Holozoa - ps10
Filozoa - ps11
Choano./Metazoa - ps12
Metazoa - ps13
Myriazoa - ps14
Parahoxozoa - ps15
Bilateria - ps16
Deuterostomia - ps17
Chordata - ps18
Olfactores - ps19
Vertebrata - ps20
Gnathostomata - ps21
Euteleostomi - ps22
Sarcopterygii - ps23
Tetrapoda - ps24
Amniota - ps25
Mammalia - ps26
Theria - ps27
Placentalia - ps28
Boreoeutheria - ps29
Euarchontoglires - ps30
Euarchonta - ps31
Primates - ps32
Haplorrhini - ps33
Simiiformes - ps34
Catarrhini - ps35
Hominoidea - ps36
Hominidae - ps37
Homininae - ps38
Hominini - ps39
Homo_sapiens - ps40
Phylostratum

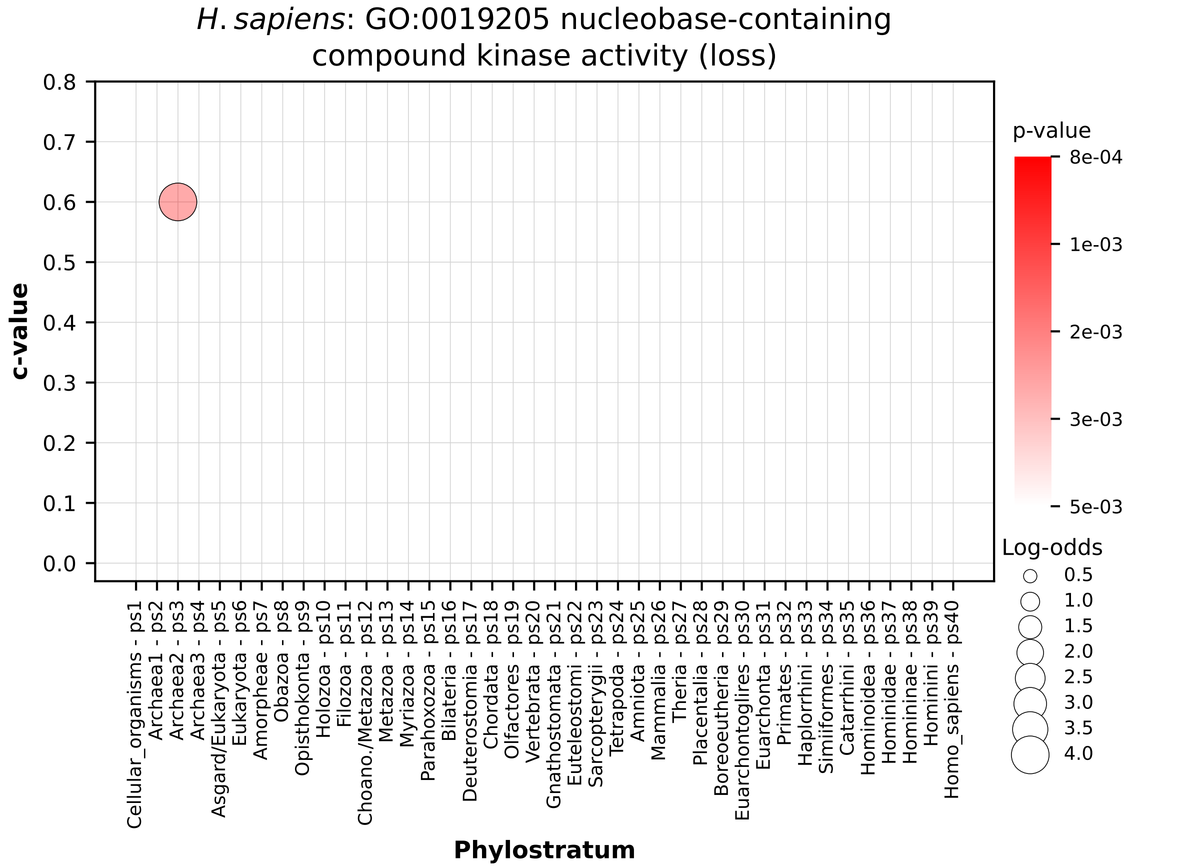
H. sapiens: GO:0019205 nucleobase-containing compound kinase activity (loss)
c-value
0.8
0.7
0.6
0.5
0.4
0.3
0.2
0.1
0.0
Phylostratum
Cellular_organisms - ps1
Archaea1 - ps2
Archaea2 - ps3
Archaea3 - ps4
Asgard/Eukaryota - ps5
Eukaryota - ps6
Amorpheae - ps7
Obazoa - ps8
Opisthokonta - ps9
Holozoa - ps10
Filozoa - ps11
Choano./Metazoa - ps12
Metazoa - ps13
Myriazoa - ps14
Parahoxozoa - ps15
Bilateria - ps16
Deuterostomia - ps17
Chordata - ps18
Olfactores - ps19
Vertebrata - ps20
Gnathostomata - ps21
Euteleostomi - ps22
Sarcopterygii - ps23
Tetrapoda - ps24
Amniota - ps25
Mammalia - ps26
Theria - ps27
Placentalia - ps28
Boreoeutheria - ps29
Euarchontoglires - ps30
Euarchonta - ps31
Primates - ps32
Haplorrhini - ps33
Simiiformes - ps34
Catarrhini - ps35
Hominoidea - ps36
Hominidae - ps37
Homininae - ps38
Hominini - ps39
Homo_sapiens - ps40
p-value
8e-04
1e-03
2e-03
3e-03
5e-03
Log-odds
0.5
1.0
1.5
2.0
2.5
3.0
3.5
4.0

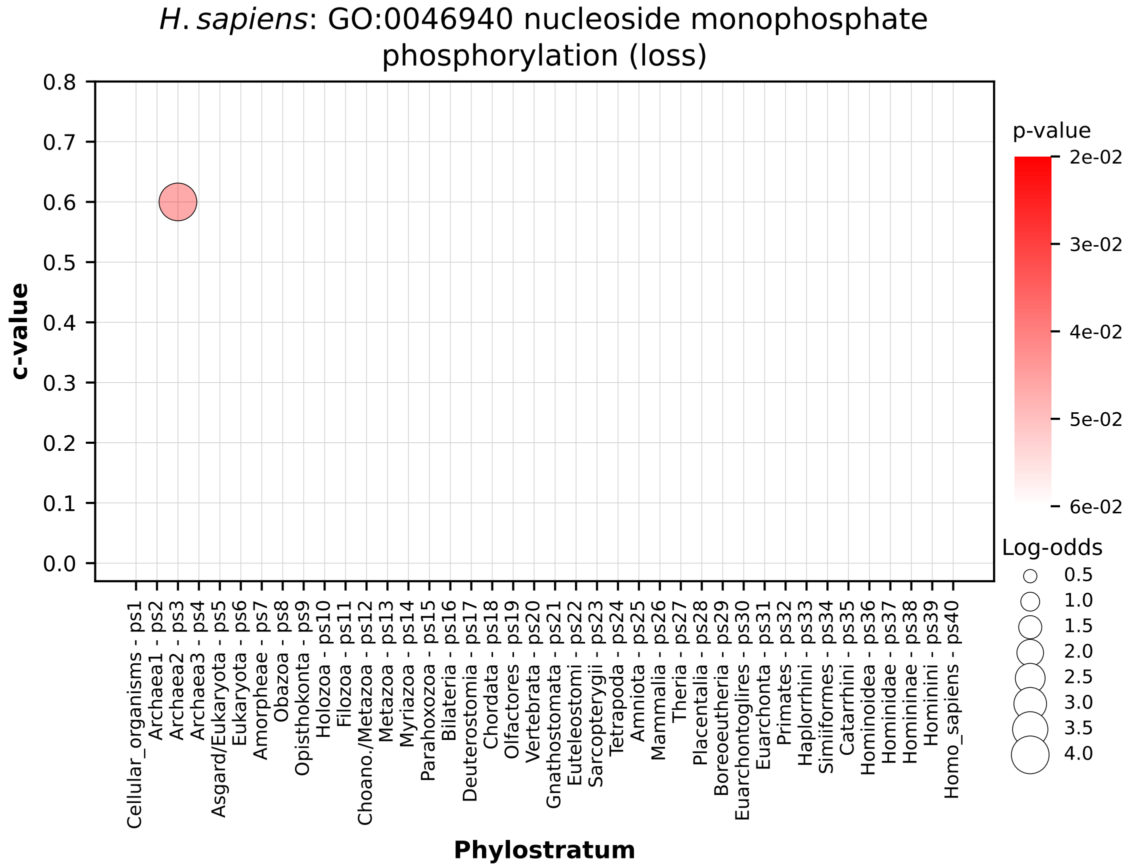

H. sapiens: GO:0046940 nucleoside monophosphate phosphorylation (loss)
c-value
0.0
0.1
0.2
0.3
0.4
0.5
0.6
0.7
0.8
Phylostratum
Cellular_organisms - ps1
Archaea1 - ps2
Archaea2 - ps3
Archaea3 - ps4
Asgard/Eukaryota - ps5
Eukaryota - ps6
Amorpheae - ps7
Obazoa - ps8
Opisthokonta - ps9
Holozoa - ps10
Filozoa - ps11
Choano./Metazoa - ps12
Metazoa - ps13
Myriazoa - ps14
Parahoxozoa - ps15
Bilateria - ps16
Deuterostomia - ps17
Chordata - ps18
Olfactores - ps19
Vertebrata - ps20
Gnathostomata - ps21
Euteleostomi - ps22
Sarcopterygii - ps23
Tetrapoda - ps24
Amniota - ps25
Mammalia - ps26
Theria - ps27
Placentalia - ps28
Boreoeutheria - ps29
Euarchontoglires - ps30
Euarchonta - ps31
Primates - ps32
Haplorrhini - ps33
Simiiformes - ps34
Catarrhini - ps35
Hominoidea - ps36
Hominidae - ps37
Homininae - ps38
Hominini - ps39
Homo_sapiens - ps40
p-value
2e-02
3e-02
4e-02
5e-02
6e-02
Log-odds
0.5
1.0
1.5
2.0
2.5
3.0
3.5
4.0

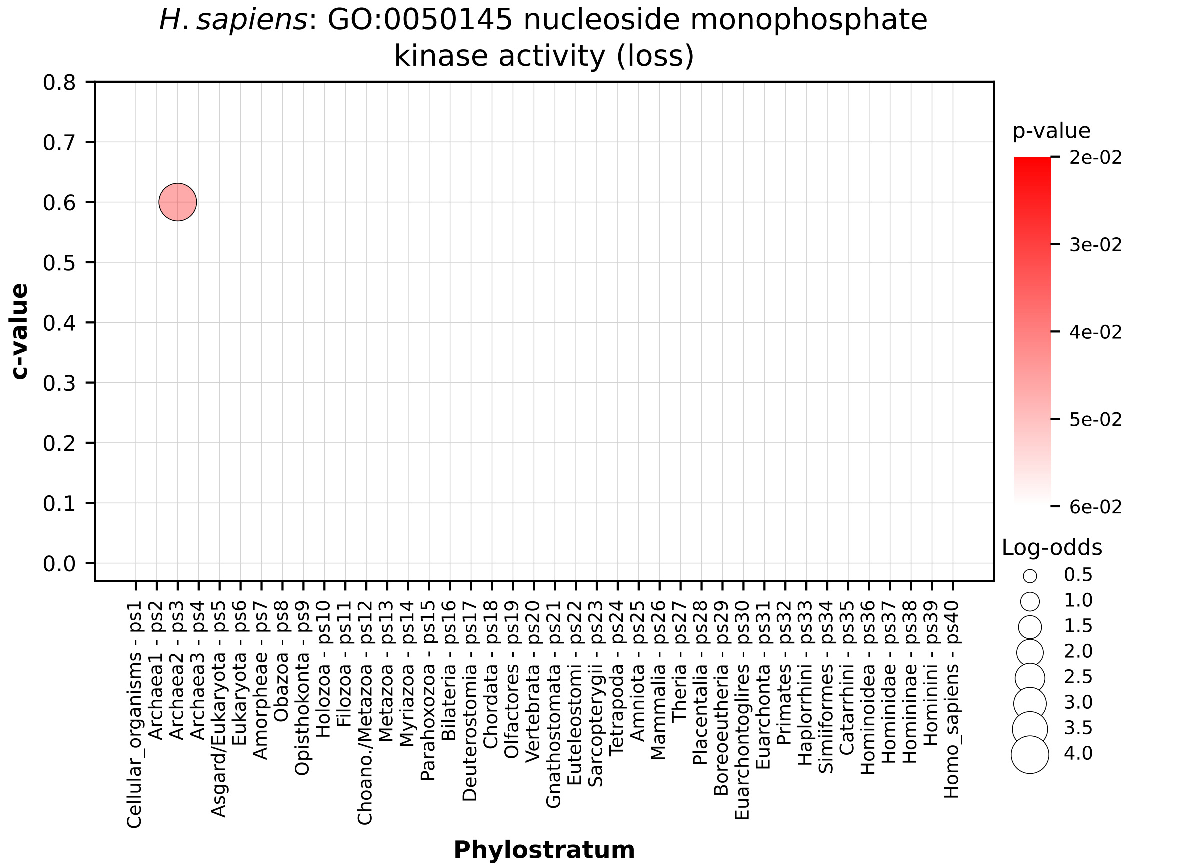

H. sapiens: GO:0050145 nucleoside monophosphate kinase activity (loss)
c-value
0.8
0.7
0.6
0.5
0.4
0.3
0.2
0.1
0.0
Phylostratum
Cellular_organisms - ps1
Archaea1 - ps2
Archaea2 - ps3
Archaea3 - ps4
Asgard/Eukaryota - ps5
Eukaryota - ps6
Amorpheae - ps7
Obazoa - ps8
Opisthokonta - ps9
Holozoa - ps10
Filozoa - ps11
Choano./Metazoa - ps12
Metazoa - ps13
Myriazoa - ps14
Parahoxozoa - ps15
Bilateria - ps16
Deuterostomia - ps17
Chordata - ps18
Olfactores - ps19
Vertebrata - ps20
Gnathostomata - ps21
Euteleostomi - ps22
Sarcopterygii - ps23
Tetrapoda - ps24
Amniota - ps25
Mammalia - ps26
Theria - ps27
Placentalia - ps28
Boreoeutheria - ps29
Euarchontoglires - ps30
Euarchonta - ps31
Primates - ps32
Haplorrhini - ps33
Simiiformes - ps34
Catarrhini - ps35
Hominoidea - ps36
Hominidae - ps37
Homininae - ps38
Hominini - ps39
Homo_sapiens - ps40
p-value
2e-02
3e-02
4e-02
5e-02
6e-02
Log-odds
0.5
1.0
1.5
2.0
2.5
3.0
3.5
4.0

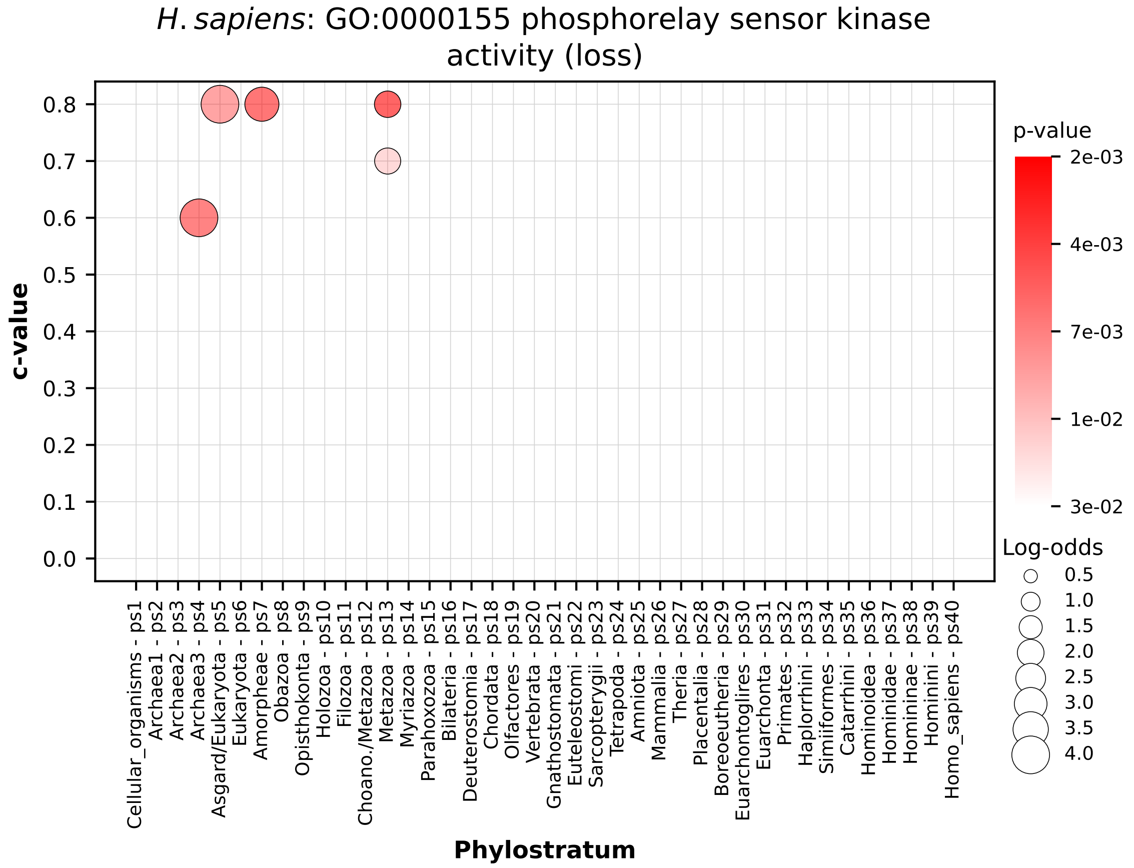
H. sapiens: GO:0000155 phosphorelay sensor kinase activity (loss)
c-value
0.0
0.1
0.2
0.3
0.4
0.5
0.6
0.7
0.8
Phylostratum
Cellular_organisms - ps1
Archaea1 - ps2
Archaea2 - ps3
Archaea3 - ps4
Asgard/Eukaryota - ps5
Eukaryota - ps6
Amorpheae - ps7
Obazoa - ps8
Opisthokonta - ps9
Holozoa - ps10
Filozoa - ps11
Choano./Metazoa - ps12
Metazoa - ps13
Myriazoa - ps14
Parahoxozoa - ps15
Bilateria - ps16
Deuterostomia - ps17
Chordata - ps18
Olfactores - ps19
Vertebrata - ps20
Gnathostomata - ps21
Euteleostomi - ps22
Sarcopterygii - ps23
Tetrapoda - ps24
Amniota - ps25
Mammalia - ps26
Theria - ps27
Placentalia - ps28
Boreoeutheria - ps29
Euarchontoglires - ps30
Euarchonta - ps31
Primates - ps32
Haplorrhini - ps33
Simiiformes - ps34
Catarrhini - ps35
Hominoidea - ps36
Hominidae - ps37
Homininae - ps38
Hominini - ps39
Homo_sapiens - ps40
p-value
2e-03
4e-03
7e-03
1e-02
3e-02
Log-odds
0.5
1.0
1.5
2.0
2.5
3.0
3.5
4.0

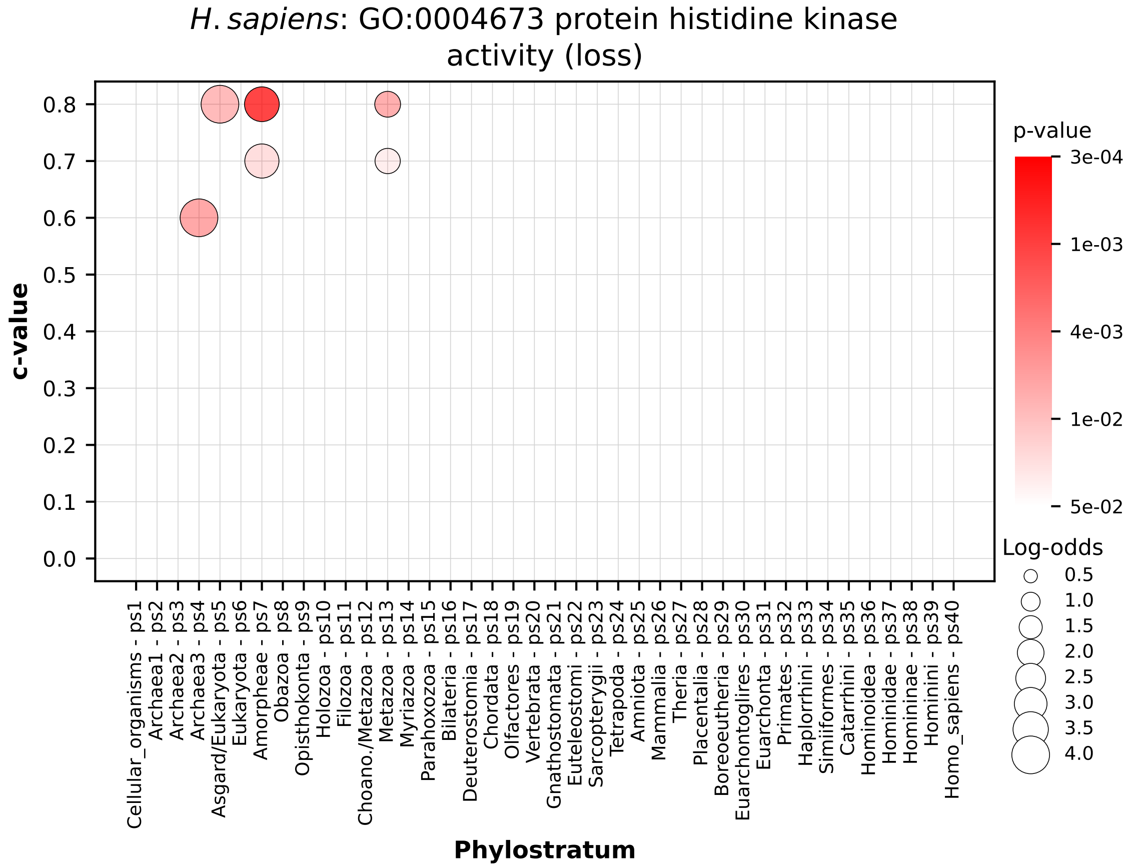

H. sapiens: GO:0004673 protein histidine kinase activity (loss)
c-value
0.0
0.1
0.2
0.3
0.4
0.5
0.6
0.7
0.8
p-value
3e-04
1e-03
4e-03
1e-02
5e-02
Log-odds
0.5
1.0
1.5
2.0
2.5
3.0
3.5
4.0
Cellular_organisms - ps1
Archaea1 - ps2
Archaea2 - ps3
Archaea3 - ps4
Asgard/Eukaryota - ps5
Eukaryota - ps6
Amorpheae - ps7
Obazoa - ps8
Opisthokonta - ps9
Holozoa - ps10
Filozoa - ps11
Choano./Metazoa - ps12
Metazoa - ps13
Myriazoa - ps14
Parahoxozoa - ps15
Bilateria - ps16
Deuterostomia - ps17
Chordata - ps18
Olfactores - ps19
Vertebrata - ps20
Gnathostomata - ps21
Euteleostomi - ps22
Sarcopterygii - ps23
Tetrapoda - ps24
Amniota - ps25
Mammalia - ps26
Theria - ps27
Placentalia - ps28
Boreoeutheria - ps29
Euarchontoglires - ps30
Euarchonta - ps31
Primates - ps32
Haplorrhini - ps33
Simiiformes - ps34
Catarrhini - ps35
Hominoidea - ps36
Hominidae - ps37
Homininae - ps38
Hominini - ps39
Homo_sapiens - ps40
Phylostratum

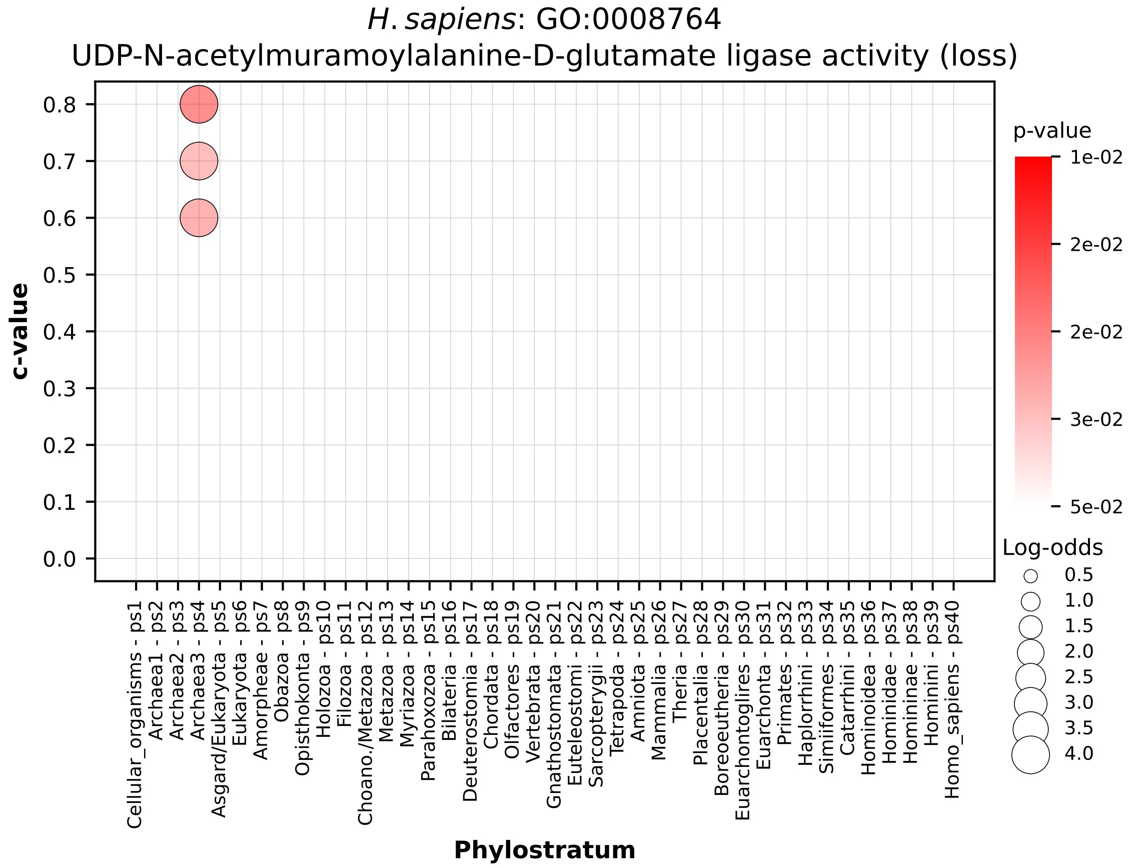
H. sapiens: GO:0008764
UDP-N-acetylmuramoylalanine-D-glutamate ligase activity (loss)
c-value
0.0
0.1
0.2
0.3
0.4
0.5
0.6
0.7
0.8
p-value
1e-02
2e-02
2e-02
3e-02
5e-02
Log-odds
0.5
1.0
1.5
2.0
2.5
3.0
3.5
4.0
Cellular_organisms - ps1
Archaea1 - ps2
Archaea2 - ps3
Archaea3 - ps4
Asgard/Eukaryota - ps5
Eukaryota - ps6
Amorpheae - ps7
Obazoa - ps8
Opisthokonta - ps9
Holozoa - ps10
Filozoa - ps11
Choano./Metazoa - ps12
Metazoa - ps13
Myriazoa - ps14
Parahoxozoa - ps15
Bilateria - ps16
Deuterostomia - ps17
Chordata - ps18
Olfactores - ps19
Vertebrata - ps20
Gnathostomata - ps21
Euteleostomi - ps22
Sarcopterygii - ps23
Tetrapoda - ps24
Amniota - ps25
Mammalia - ps26
Theria - ps27
Placentalia - ps28
Boreoeutheria - ps29
Euarchontoglires - ps30
Euarchonta - ps31
Primates - ps32
Haplorrhini - ps33
Simiiformes - ps34
Catarrhini - ps35
Hominoidea - ps36
Hominidae - ps37
Homininae - ps38
Hominini - ps39
Homo_sapiens - ps40
Phylostratum

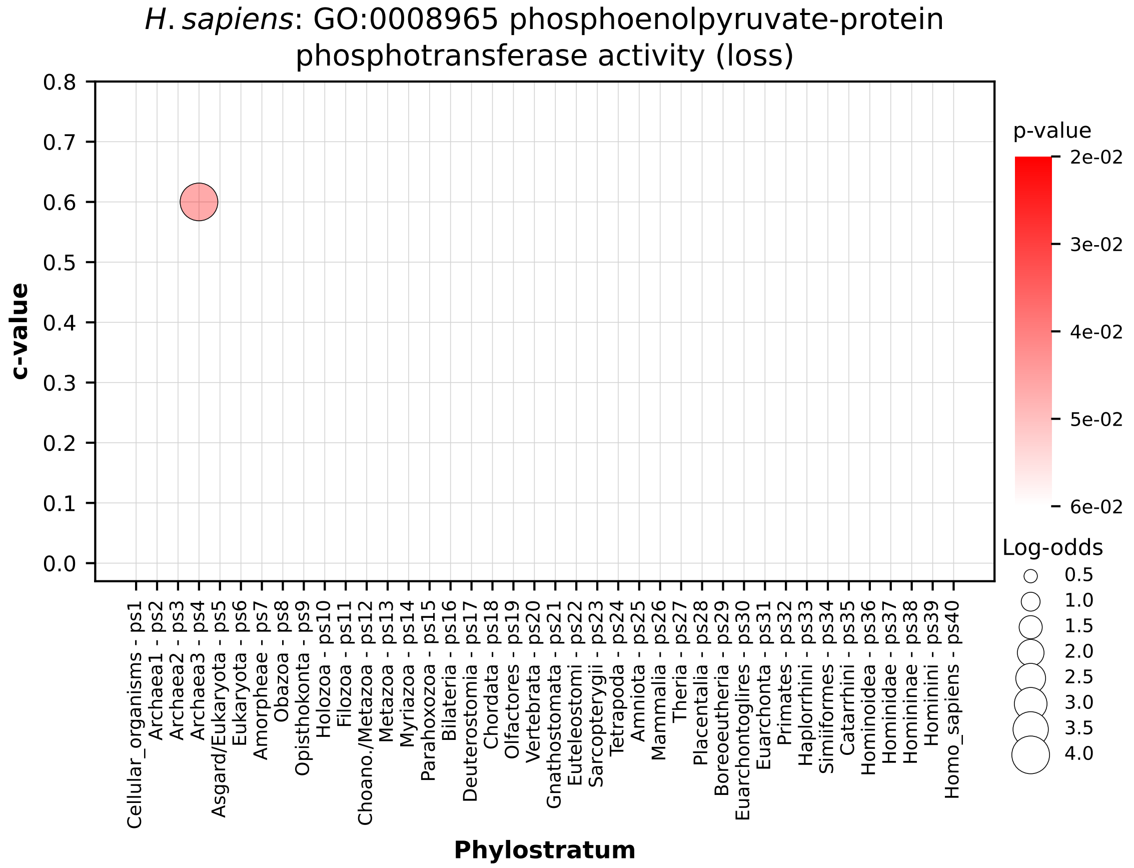

H. sapiens: GO:0008965 phosphoenolpyruvate-protein phosphotransferase activity (loss)
c-value
0.8
0.7
0.6
0.5
0.4
0.3
0.2
0.1
0.0
p-value
2e-02
3e-02
4e-02
5e-02
6e-02
Log-odds
0.5
1.0
1.5
2.0
2.5
3.0
3.5
4.0
Cellular_organisms - ps1
Archaea1 - ps2
Archaea2 - ps3
Archaea3 - ps4
Asgard/Eukaryota - ps5
Eukaryota - ps6
Amorpheae - ps7
Obazoa - ps8
Opisthokonta - ps9
Holozoa - ps10
Filozoa - ps11
Choano./Metazoa - ps12
Metazoa - ps13
Myriazoa - ps14
Parahoxozoa - ps15
Bilateria - ps16
Deuterostomia - ps17
Chordata - ps18
Olfactores - ps19
Vertebrata - ps20
Gnathostomata - ps21
Euteleostomi - ps22
Sarcopterygii - ps23
Tetrapoda - ps24
Amniota - ps25
Mammalia - ps26
Theria - ps27
Placentalia - ps28
Boreoeutheria - ps29
Euarchontoglires - ps30
Euarchonta - ps31
Primates - ps32
Haplorrhini - ps33
Simiiformes - ps34
Catarrhini - ps35
Hominoidea - ps36
Hominidae - ps37
Homininae - ps38
Hominini - ps39
Homo_sapiens - ps40
Phylostratum

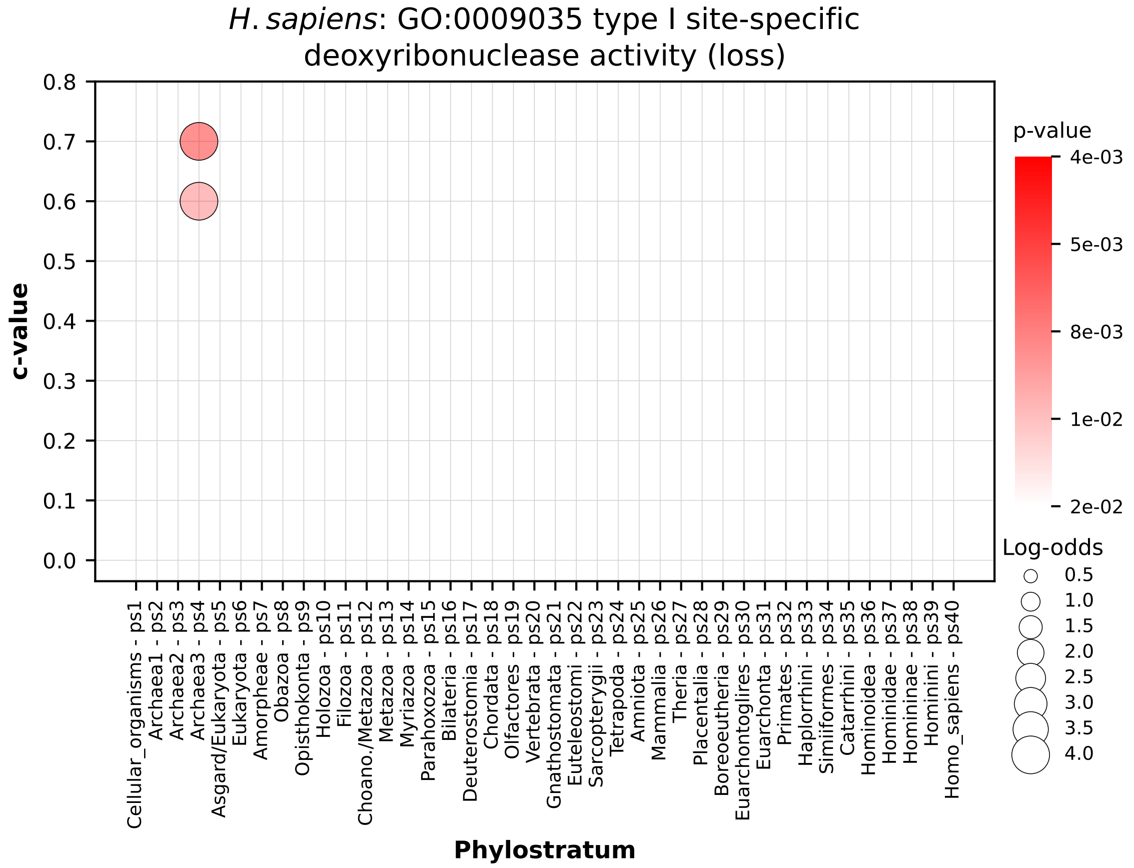
H. sapiens: GO:0009035 type I site-specific deoxyribonuclease activity (loss)
c-value
0.8
0.7
0.6
0.5
0.4
0.3
0.2
0.1
0.0
p-value
4e-03
5e-03
8e-03
1e-02
2e-02
Log-odds
0.5
1.0
1.5
2.0
2.5
3.0
3.5
4.0
Cellular_organisms - ps1
Archaea1 - ps2
Archaea2 - ps3
Archaea3 - ps4
Asgard/Eukaryota - ps5
Eukaryota - ps6
Amorpheae - ps7
Obazoa - ps8
Opisthokonta - ps9
Holozoa - ps10
Filozoa - ps11
Choano./Metazoa - ps12
Metazoa - ps13
Myriazoa - ps14
Parahoxozoa - ps15
Bilateria - ps16
Deuterostomia - ps17
Chordata - ps18
Olfactores - ps19
Vertebrata - ps20
Gnathostomata - ps21
Euteleostomi - ps22
Sarcopterygii - ps23
Tetrapoda - ps24
Amniota - ps25
Mammalia - ps26
Theria - ps27
Placentalia - ps28
Boreoeutheria - ps29
Euarchontoglires - ps30
Euarchonta - ps31
Primates - ps32
Haplorrhini - ps33
Simiiformes - ps34
Catarrhini - ps35
Hominoidea - ps36
Hominidae - ps37
Homininae - ps38
Hominini - ps39
Homo_sapiens - ps40
Phylostratum

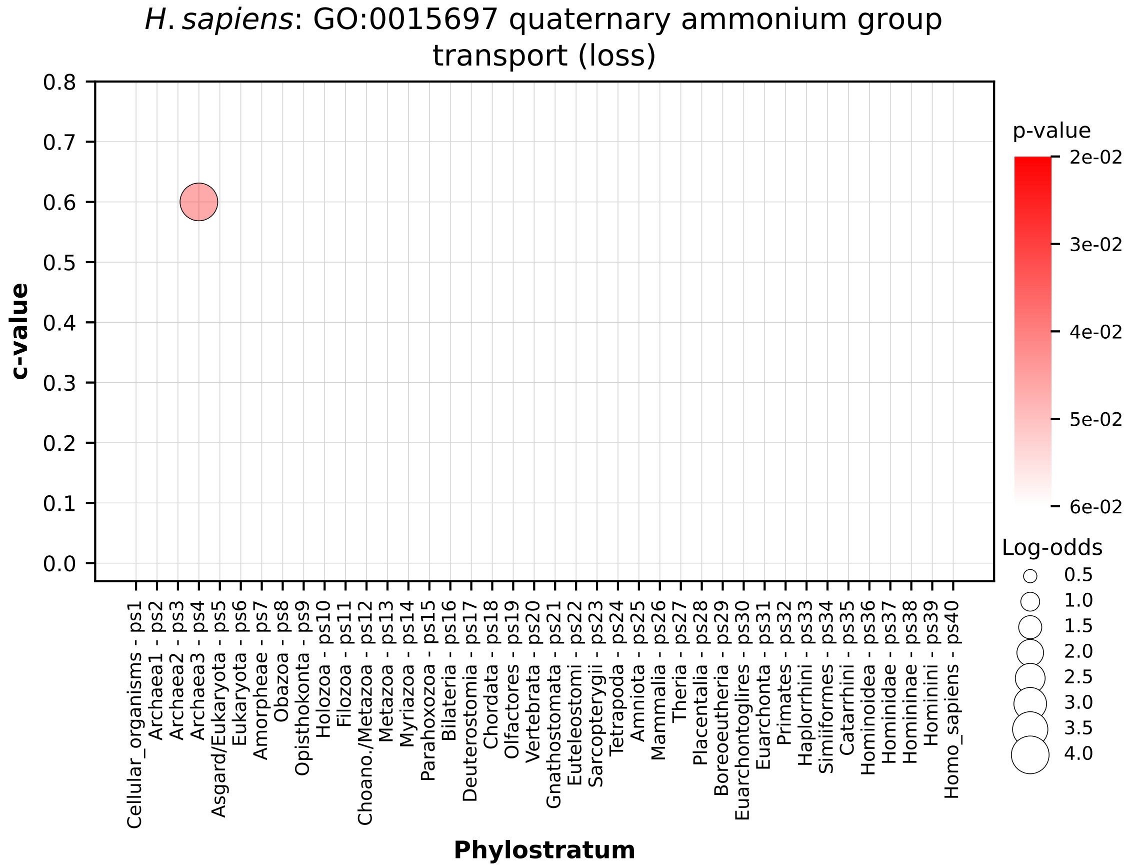

H. sapiens: GO:0015697 quaternary ammonium group transport (loss)
c-value
0.8
0.7
0.6
0.5
0.4
0.3
0.2
0.1
0.0
p-value
2e-02
3e-02
4e-02
5e-02
6e-02
Log-odds
0.5
1.0
1.5
2.0
2.5
3.0
3.5
4.0
Cellular_organisms - ps1
Archaea1 - ps2
Archaea2 - ps3
Archaea3 - ps4
Asgard/Eukaryota - ps5
Eukaryota - ps6
Amorpheae - ps7
Obazoa - ps8
Opisthokonta - ps9
Holozoa - ps10
Filozoa - ps11
Choano./Metazoa - ps12
Metazoa - ps13
Myriazoa - ps14
Parahoxozoa - ps15
Bilateria - ps16
Deuterostomia - ps17
Chordata - ps18
Olfactores - ps19
Vertebrata - ps20
Gnathostomata - ps21
Euteleostomi - ps22
Sarcopterygii - ps23
Tetrapoda - ps24
Amniota - ps25
Mammalia - ps26
Theria - ps27
Placentalia - ps28
Boreoeutheria - ps29
Euarchontoglires - ps30
Euarchonta - ps31
Primates - ps32
Haplorrhini - ps33
Simiiformes - ps34
Catarrhini - ps35
Hominoidea - ps36
Hominidae - ps37
Homininae - ps38
Hominini - ps39
Homo_sapiens - ps40
Phylostratum

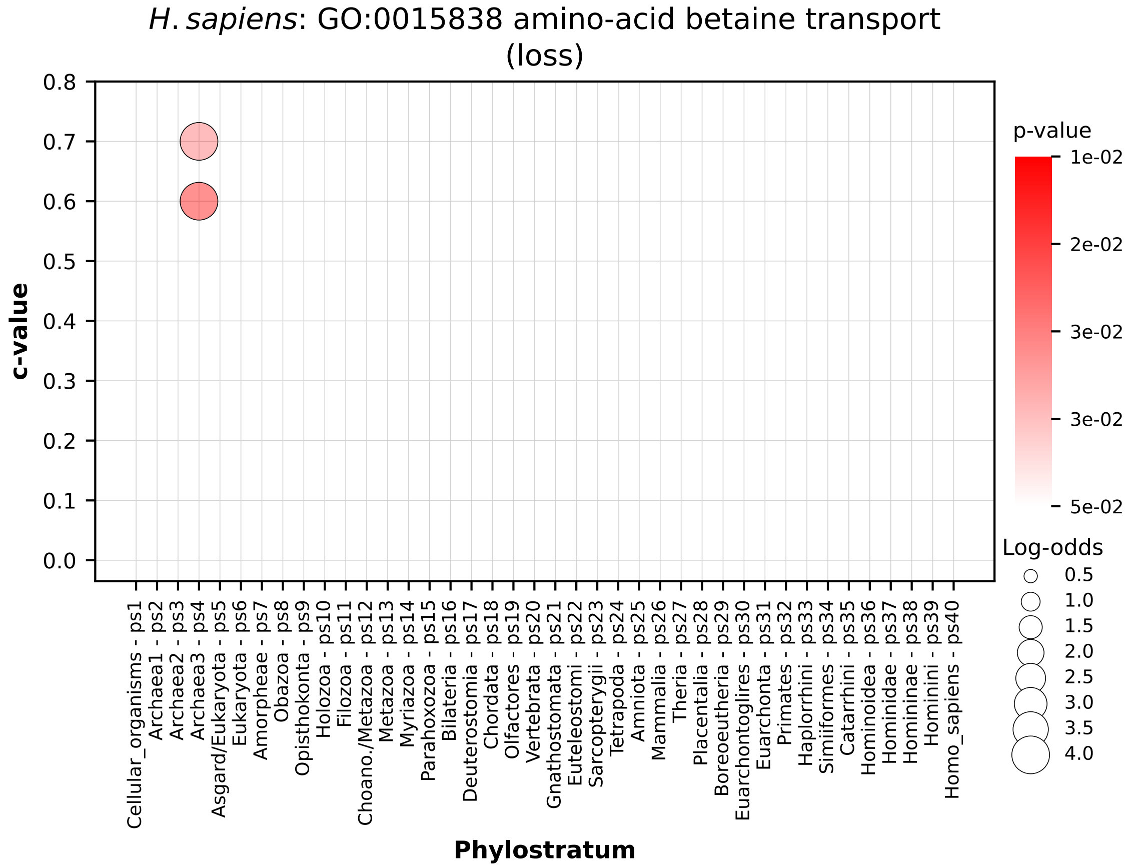

H. sapiens: GO:0015838 amino-acid betaine transport (loss)
c-value
0.8
0.7
0.6
0.5
0.4
0.3
0.2
0.1
0.0
p-value
1e-02
2e-02
3e-02
3e-02
5e-02
Log-odds
0.5
1.0
1.5
2.0
2.5
3.0
3.5
4.0
Cellular_organisms - ps1
Archaea1 - ps2
Archaea2 - ps3
Archaea3 - ps4
Asgard/Eukaryota - ps5
Eukaryota - ps6
Amorpheae - ps7
Obazoa - ps8
Opisthokonta - ps9
Holozoa - ps10
Filozoa - ps11
Choano./Metazoa - ps12
Metazoa - ps13
Myriazoa - ps14
Parahoxozoa - ps15
Bilateria - ps16
Deuterostomia - ps17
Chordata - ps18
Olfactores - ps19
Vertebrata - ps20
Gnathostomata - ps21
Euteleostomi - ps22
Sarcopterygii - ps23
Tetrapoda - ps24
Amniota - ps25
Mammalia - ps26
Theria - ps27
Placentalia - ps28
Boreoeutheria - ps29
Euarchontoglires - ps30
Euarchonta - ps31
Primates - ps32
Haplorrhini - ps33
Simiiformes - ps34
Catarrhini - ps35
Hominoidea - ps36
Hominidae - ps37
Homininae - ps38
Hominini - ps39
Homo_sapiens - ps40
Phylostratum

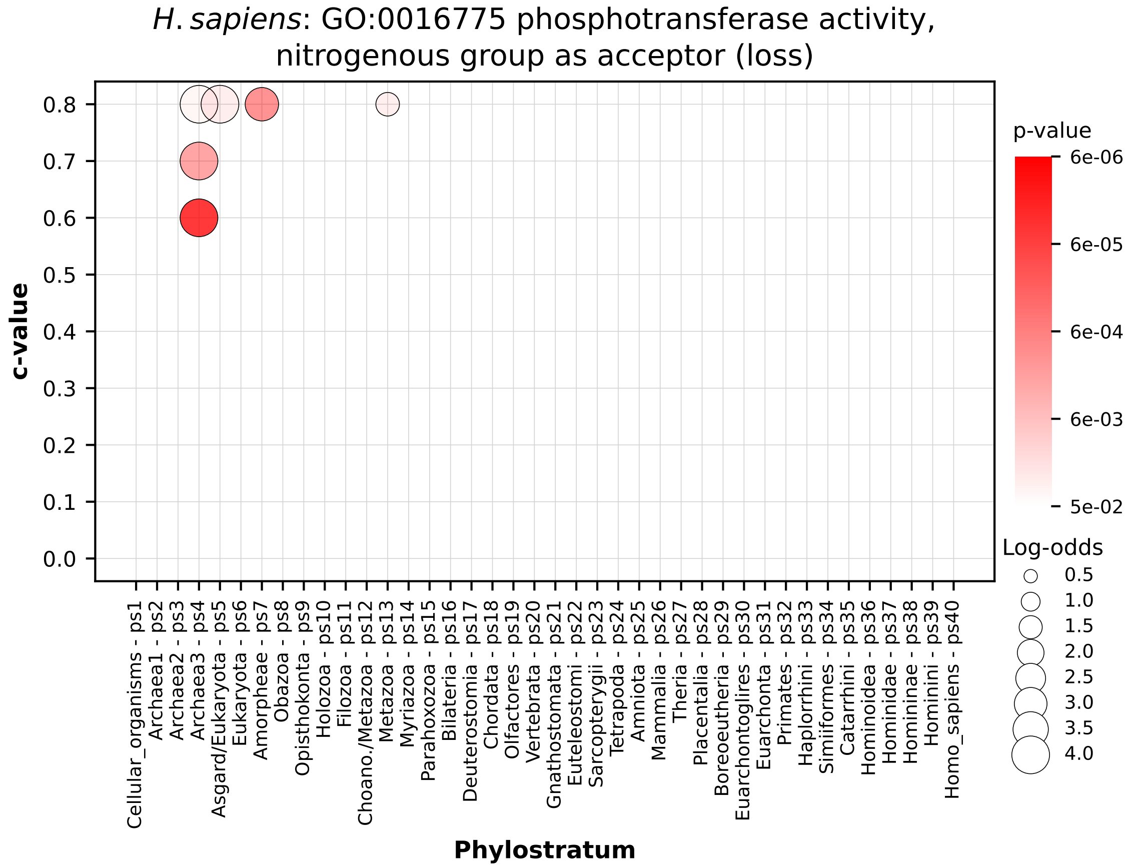
H. sapiens: GO:0016775 phosphotransferase activity, nitrogenous group as acceptor (loss)
c-value
0.0
0.1
0.2
0.3
0.4
0.5
0.6
0.7
0.8
p-value
6e-06
6e-05
6e-04
6e-03
5e-02
Log-odds
0.5
1.0
1.5
2.0
2.5
3.0
3.5
4.0
Cellular_organisms - ps1
Archaea1 - ps2
Archaea2 - ps3
Archaea3 - ps4
Asgard/Eukaryota - ps5
Eukaryota - ps6
Amorpheae - ps7
Obazoa - ps8
Opisthokonta - ps9
Holozoa - ps10
Filozoa - ps11
Choano./Metazoa - ps12
Metazoa - ps13
Myriazoa - ps14
Parahoxozoa - ps15
Bilateria - ps16
Deuterostomia - ps17
Chordata - ps18
Olfactores - ps19
Vertebrata - ps20
Gnathostomata - ps21
Euteleostomi - ps22
Sarcopterygii - ps23
Tetrapoda - ps24
Amniota - ps25
Mammalia - ps26
Theria - ps27
Placentalia - ps28
Boreoeutheria - ps29
Euarchontoglires - ps30
Euarchonta - ps31
Primates - ps32
Haplorrhini - ps33
Simiiformes - ps34
Catarrhini - ps35
Hominoidea - ps36
Hominidae - ps37
Homininae - ps38
Hominini - ps39
Homo_sapiens - ps40
Phylostratum

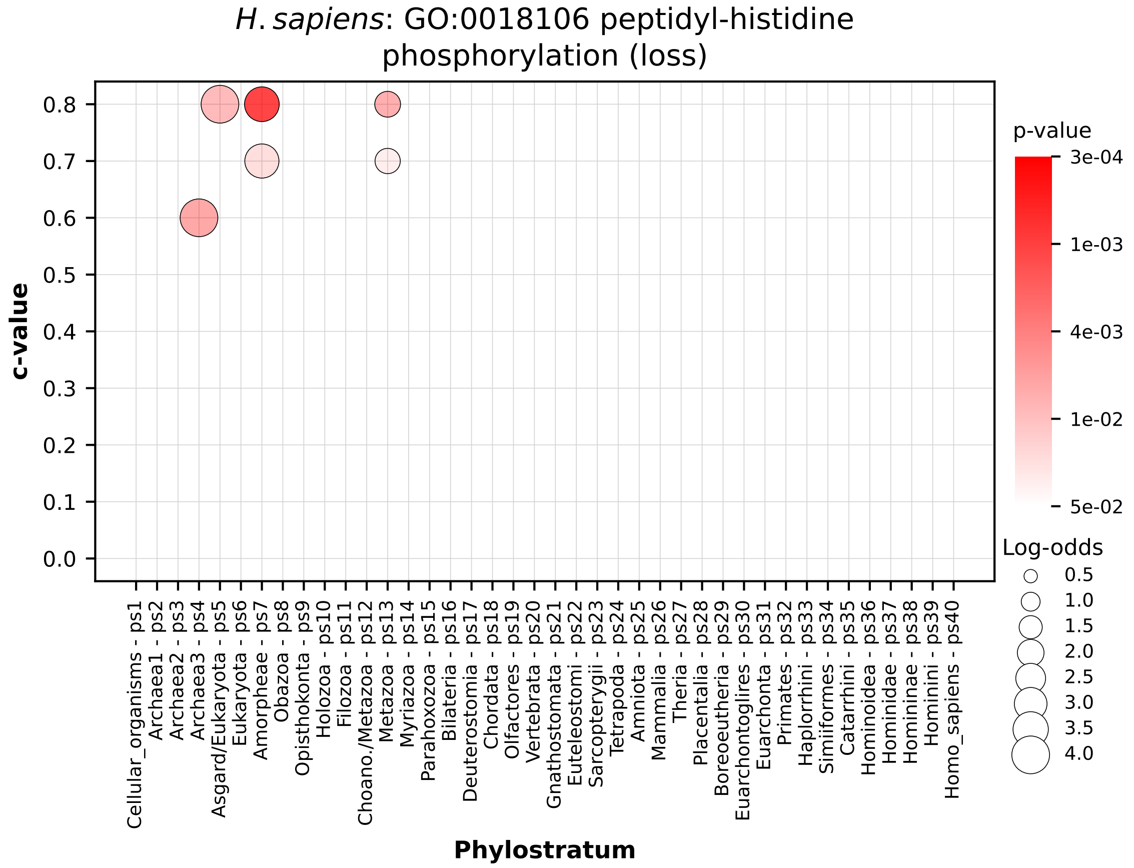

H. sapiens: GO:0018106 peptidyl-histidine phosphorylation (loss)
c-value
0.0
0.1
0.2
0.3
0.4
0.5
0.6
0.7
0.8
Phylostratum
Cellular_organisms - ps1
Archaea1 - ps2
Archaea2 - ps3
Archaea3 - ps4
Asgard/Eukaryota - ps5
Eukaryota - ps6
Amorpheae - ps7
Obazoa - ps8
Opisthokonta - ps9
Holozoa - ps10
Filozoa - ps11
Choano./Metazoa - ps12
Metazoa - ps13
Myriazoa - ps14
Parahoxozoa - ps15
Bilateria - ps16
Deuterostomia - ps17
Chordata - ps18
Olfactores - ps19
Vertebrata - ps20
Gnathostomata - ps21
Euteleostomi - ps22
Sarcopterygii - ps23
Tetrapoda - ps24
Amniota - ps25
Mammalia - ps26
Theria - ps27
Placentalia - ps28
Boreoeutheria - ps29
Euarchontoglires - ps30
Euarchonta - ps31
Primates - ps32
Haplorrhini - ps33
Simiiformes - ps34
Catarrhini - ps35
Hominoidea - ps36
Hominidae - ps37
Homininae - ps38
Hominini - ps39
Homo_sapiens - ps40
p-value
3e-04
1e-03
4e-03
1e-02
5e-02
Log-odds
0.5
1.0
1.5
2.0
2.5
3.0
3.5
4.0

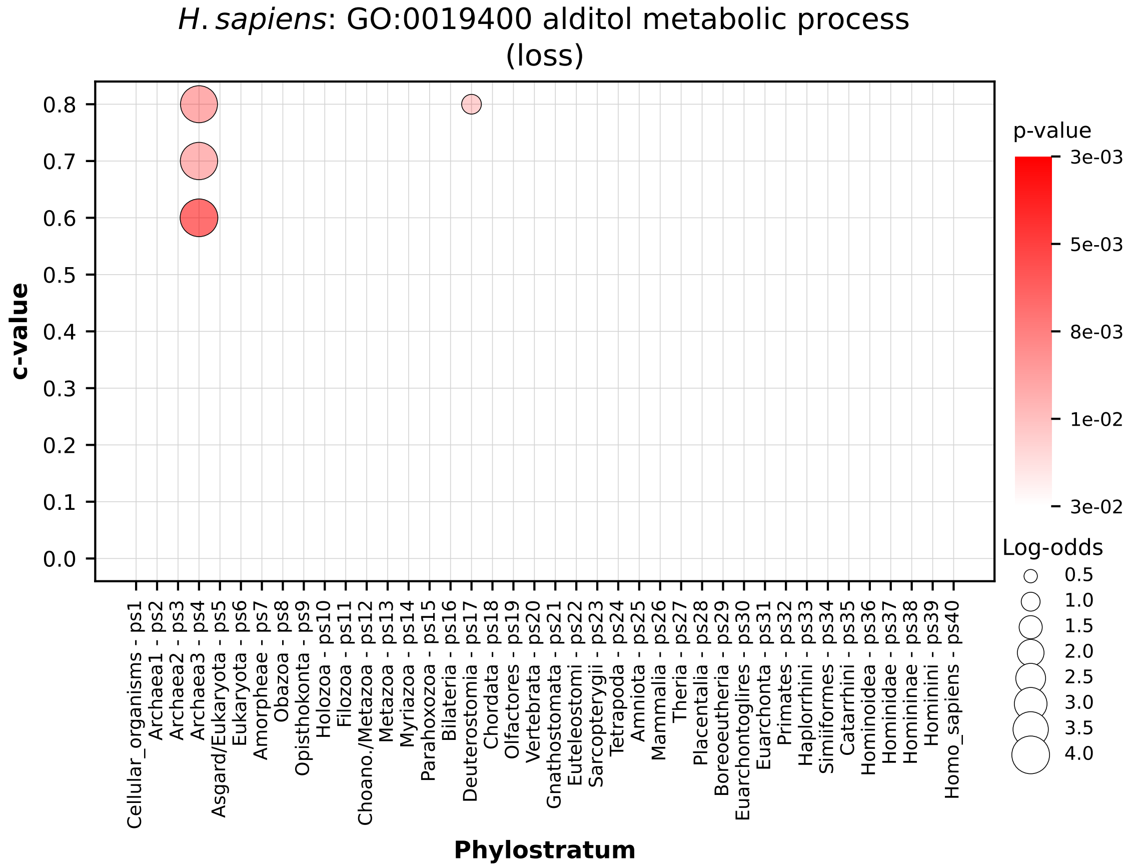
H. sapiens: GO:0019400 alditol metabolic process
(loss)
c-value
0.8
0.7
0.6
0.5
0.4
0.3
0.2
0.1
0.0
p-value
3e-03
5e-03
8e-03
1e-02
3e-02
Log-odds
0.5
1.0
1.5
2.0
2.5
3.0
3.5
4.0
Cellular_organisms - ps1
Archaea1 - ps2
Archaea2 - ps3
Archaea3 - ps4
Asgard/Eukaryota - ps5
Eukaryota - ps6
Amorpheae - ps7
Obazoa - ps8
Opisthokonta - ps9
Holozoa - ps10
Filozoa - ps11
Choano./Metazoa - ps12
Metazoa - ps13
Myriazoa - ps14
Parahoxozoa - ps15
Bilateria - ps16
Deuterostomia - ps17
Chordata - ps18
Olfactores - ps19
Vertebrata - ps20
Gnathostomata - ps21
Euteleostomi - ps22
Sarcopterygii - ps23
Tetrapoda - ps24
Amniota - ps25
Mammalia - ps26
Theria - ps27
Placentalia - ps28
Boreoeutheria - ps29
Euarchontoglires - ps30
Euarchonta - ps31
Primates - ps32
Haplorrhini - ps33
Simiiformes - ps34
Catarrhini - ps35
Hominoidea - ps36
Hominidae - ps37
Homininae - ps38
Hominini - ps39
Homo_sapiens - ps40
Phylostratum

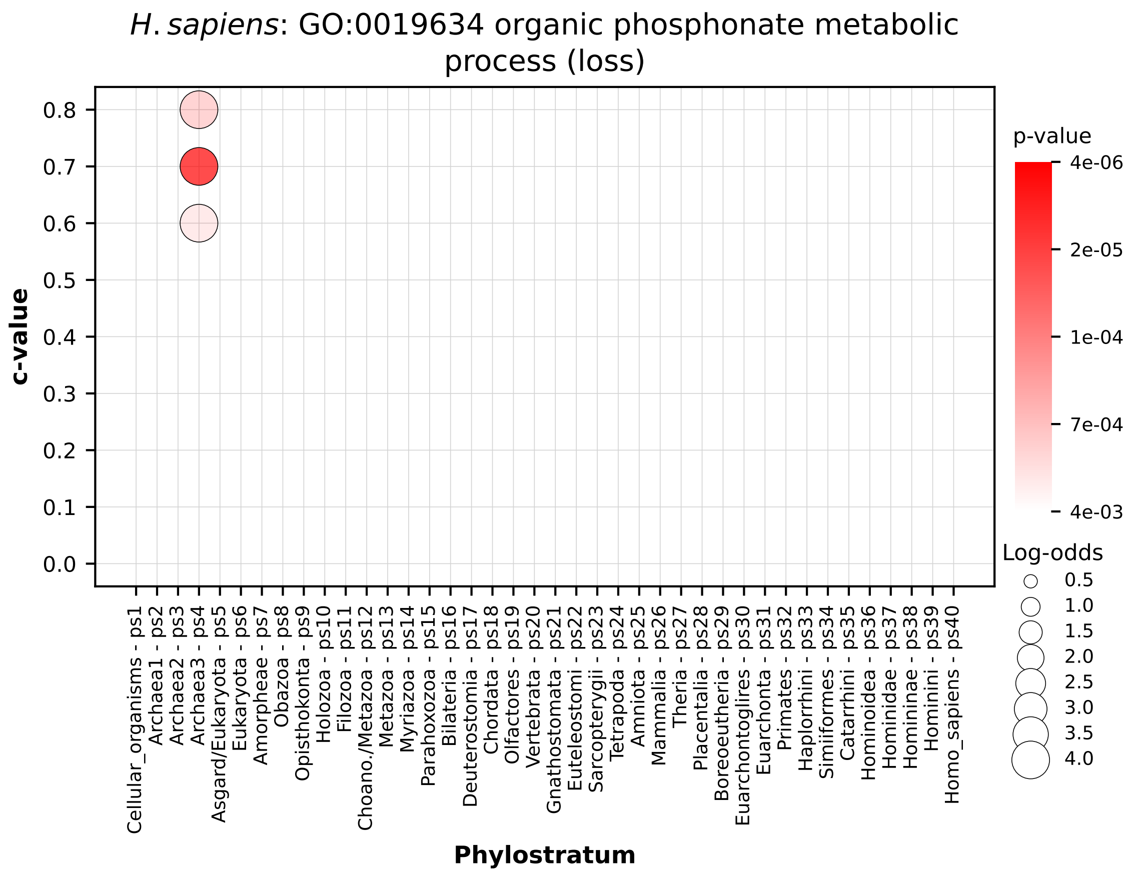
$H. sapiens$: GO:0019634 organic phosphonate metabolic process (loss)
c-value
0.8
0.7
0.6
0.5
0.4
0.3
0.2
0.1
0.0
p-value
4e-06
2e-05
1e-04
7e-04
4e-03
Log-odds
0.5
1.0
1.5
2.0
2.5
3.0
3.5
4.0
Cellular_organisms - ps1
Archaea1 - ps2
Archaea2 - ps3
Archaea3 - ps4
Asgard/Eukaryota - ps5
Eukaryota - ps6
Amorpheae - ps7
Obazoa - ps8
Opisthokonta - ps9
Holozoa - ps10
Filozoa - ps11
Choano./Metazoa - ps12
Metazoa - ps13
Myriazoa - ps14
Parahoxozoa - ps15
Bilateria - ps16
Deuterostomia - ps17
Chordata - ps18
Olfactores - ps19
Vertebrata - ps20
Gnathostomata - ps21
Euteleostomi - ps22
Sarcopterygii - ps23
Tetrapoda - ps24
Amniota - ps25
Mammalia - ps26
Theria - ps27
Placentalia - ps28
Boreoeutheria - ps29
Euarchontoglires - ps30
Euarchonta - ps31
Primates - ps32
Haplorrhini - ps33
Simiiformes - ps34
Catarrhini - ps35
Hominoidea - ps36
Hominidae - ps37
Homininae - ps38
Hominini - ps39
Homo_sapiens - ps40
Phylostratum

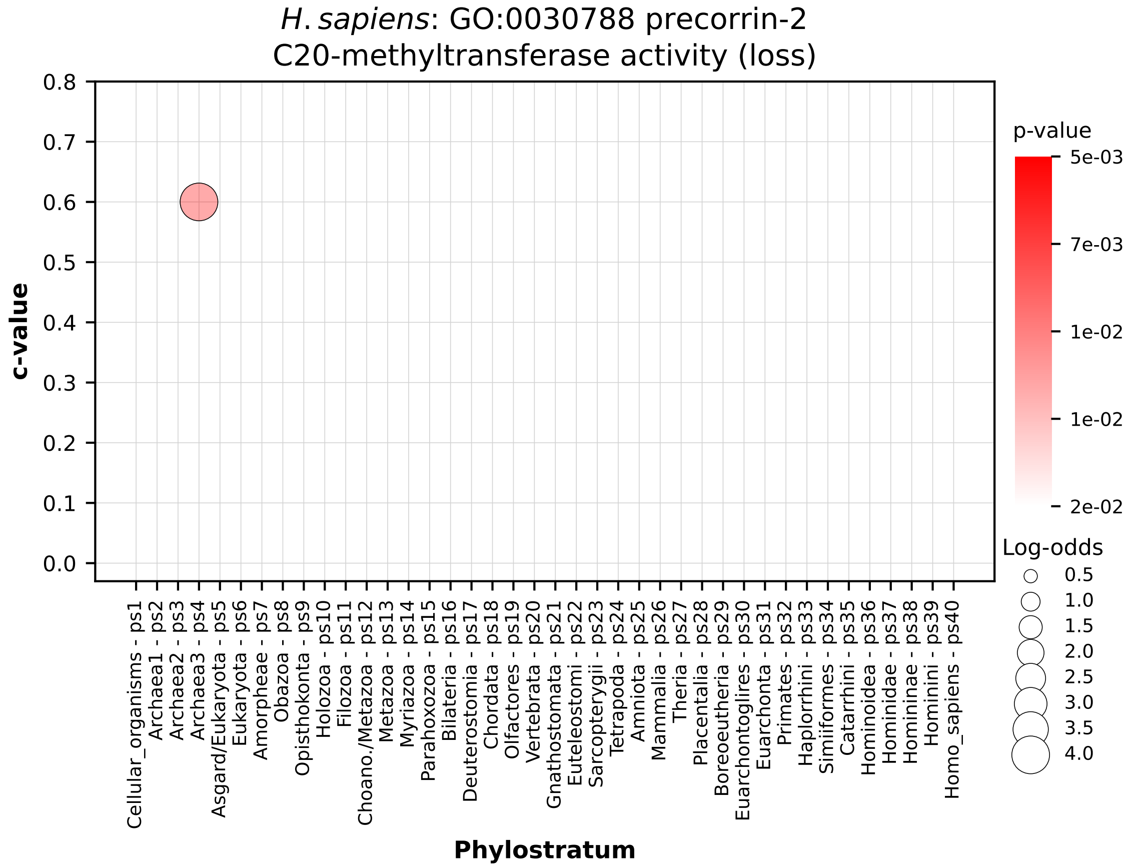

H. sapiens: GO:0030788 precorrin-2 C20-methyltransferase activity (loss)
c-value
0.8
0.7
0.6
0.5
0.4
0.3
0.2
0.1
0.0
p-value
5e-03
7e-03
1e-02
1e-02
2e-02
Log-odds
0.5
1.0
1.5
2.0
2.5
3.0
3.5
4.0
Cellular_organisms - ps1
Archaea1 - ps2
Archaea2 - ps3
Archaea3 - ps4
Asgard/Eukaryota - ps5
Eukaryota - ps6
Amorpheae - ps7
Obazoa - ps8
Opisthokonta - ps9
Holozoa - ps10
Filozoa - ps11
Choano./Metazoa - ps12
Metazoa - ps13
Myriazoa - ps14
Parahoxozoa - ps15
Bilateria - ps16
Deuterostomia - ps17
Chordata - ps18
Olfactores - ps19
Vertebrata - ps20
Gnathostomata - ps21
Euteleostomi - ps22
Sarcopterygii - ps23
Tetrapoda - ps24
Amniota - ps25
Mammalia - ps26
Theria - ps27
Placentalia - ps28
Boreoeutheria - ps29
Euarchontoglires - ps30
Euarchonta - ps31
Primates - ps32
Haplorrhini - ps33
Simiiformes - ps34
Catarrhini - ps35
Hominoidea - ps36
Hominidae - ps37
Homininae - ps38
Hominini - ps39
Homo_sapiens - ps40
Phylostratum

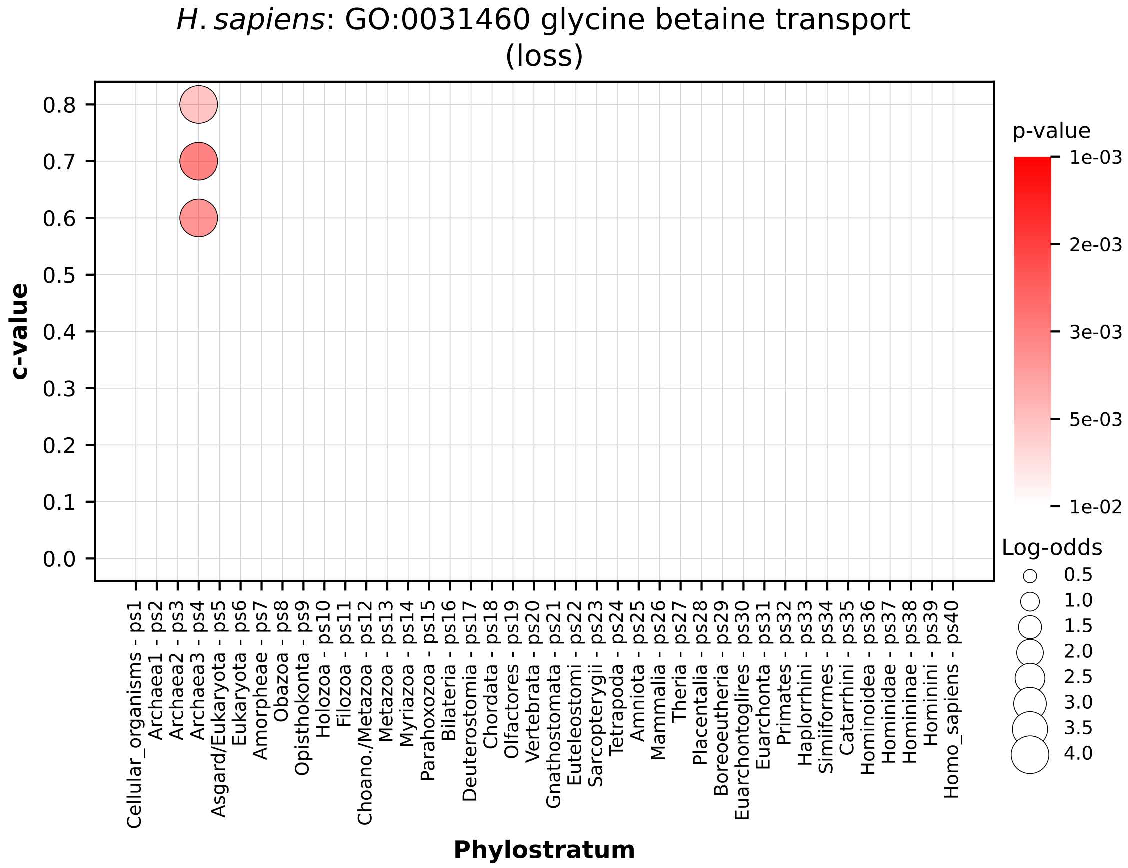
H. sapiens: GO:0031460 glycine betaine transport
(loss)
c-value
0.0
0.1
0.2
0.3
0.4
0.5
0.6
0.7
0.8
p-value
1e-03
2e-03
3e-03
5e-03
1e-02
Log-odds
0.5
1.0
1.5
2.0
2.5
3.0
3.5
4.0
Cellular_organisms - ps1
Archaea1 - ps2
Archaea2 - ps3
Archaea3 - ps4
Asgard/Eukaryota - ps5
Eukaryota - ps6
Amorpheae - ps7
Obazoa - ps8
Opisthokonta - ps9
Holozoa - ps10
Filozoa - ps11
Choano./Metazoa - ps12
Metazoa - ps13
Myriazoa - ps14
Parahoxozoa - ps15
Bilateria - ps16
Deuterostomia - ps17
Chordata - ps18
Olfactores - ps19
Vertebrata - ps20
Gnathostomata - ps21
Euteleostomi - ps22
Sarcopterygii - ps23
Tetrapoda - ps24
Amniota - ps25
Mammalia - ps26
Theria - ps27
Placentalia - ps28
Boreoeutheria - ps29
Euarchontoglires - ps30
Euarchonta - ps31
Primates - ps32
Haplorrhini - ps33
Simiiformes - ps34
Catarrhini - ps35
Hominoidea - ps36
Hominidae - ps37
Homininae - ps38
Hominini - ps39
Homo_sapiens - ps40
Phylostratum

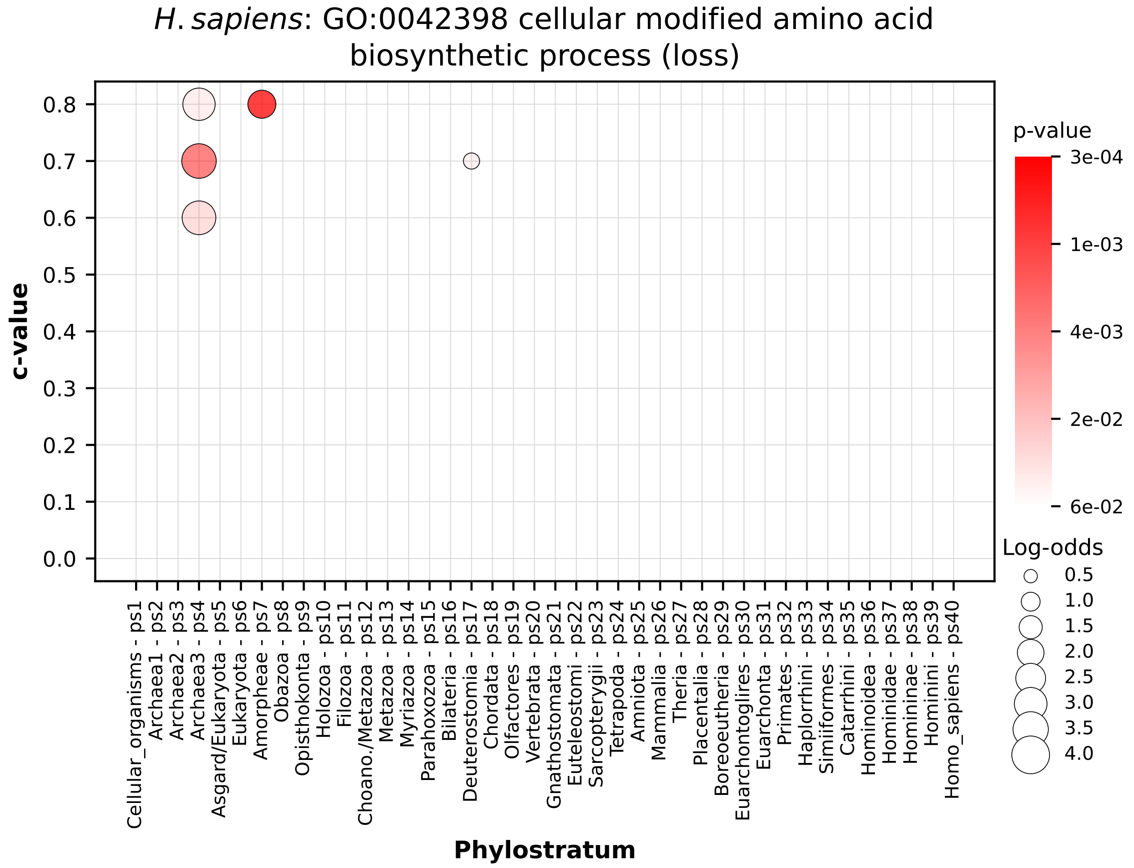

H. sapiens: GO:0042398 cellular modified amino acid biosynthetic process (loss)
c-value
0.8
0.7
0.6
0.5
0.4
0.3
0.2
0.1
0.0
p-value
3e-04
1e-03
4e-03
2e-02
6e-02
Log-odds
0.5
1.0
1.5
2.0
2.5
3.0
3.5
4.0
Cellular_organisms - ps1
Archaea1 - ps2
Archaea2 - ps3
Archaea3 - ps4
Asgard/Eukaryota - ps5
Eukaryota - ps6
Amorpheae - ps7
Obazoa - ps8
Opisthokonta - ps9
Holozoa - ps10
Filozoa - ps11
Choano./Metazoa - ps12
Metazoa - ps13
Myriazoa - ps14
Parahoxozoa - ps15
Bilateria - ps16
Deuterostomia - ps17
Chordata - ps18
Olfactores - ps19
Vertebrata - ps20
Gnathostomata - ps21
Euteleostomi - ps22
Sarcopterygii - ps23
Tetrapoda - ps24
Amniota - ps25
Mammalia - ps26
Theria - ps27
Placentalia - ps28
Boreoeutheria - ps29
Euarchontoglires - ps30
Euarchonta - ps31
Primates - ps32
Haplorrhini - ps33
Simiiformes - ps34
Catarrhini - ps35
Hominoidea - ps36
Hominidae - ps37
Homininae - ps38
Hominini - ps39
Homo_sapiens - ps40
Phylostratum

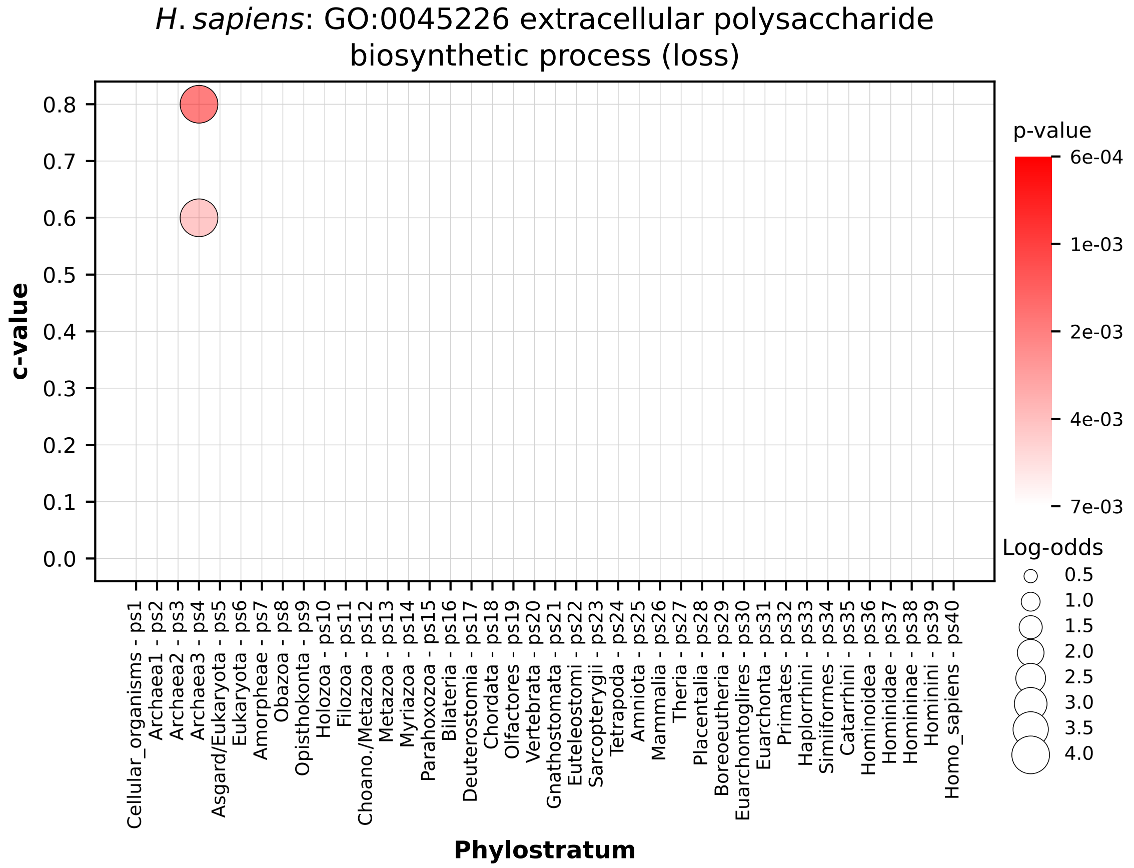

H. sapiens: GO:0045226 extracellular polysaccharide biosynthetic process (loss)
c-value
0.8
0.7
0.6
0.5
0.4
0.3
0.2
0.1
0.0
Phylostratum
Cellular_organisms - ps1
Archaea1 - ps2
Archaea2 - ps3
Archaea3 - ps4
Asgard/Eukaryota - ps5
Eukaryota - ps6
Amorpheae - ps7
Obazoa - ps8
Opisthokonta - ps9
Holozoa - ps10
Filozoa - ps11
Choano./Metazoa - ps12
Metazoa - ps13
Myriazoa - ps14
Parahoxozoa - ps15
Bilateria - ps16
Deuterostomia - ps17
Chordata - ps18
Olfactores - ps19
Vertebrata - ps20
Gnathostomata - ps21
Euteleostomi - ps22
Sarcopterygii - ps23
Tetrapoda - ps24
Amniota - ps25
Mammalia - ps26
Theria - ps27
Placentalia - ps28
Boreoeutheria - ps29
Euarchontoglires - ps30
Euarchonta - ps31
Primates - ps32
Haplorrhini - ps33
Simiiformes - ps34
Catarrhini - ps35
Hominoidea - ps36
Hominidae - ps37
Homininae - ps38
Hominini - ps39
Homo_sapiens - ps40
p-value
6e-04
1e-03
2e-03
4e-03
7e-03
Log-odds
0.5
1.0
1.5
2.0
2.5
3.0
3.5
4.0

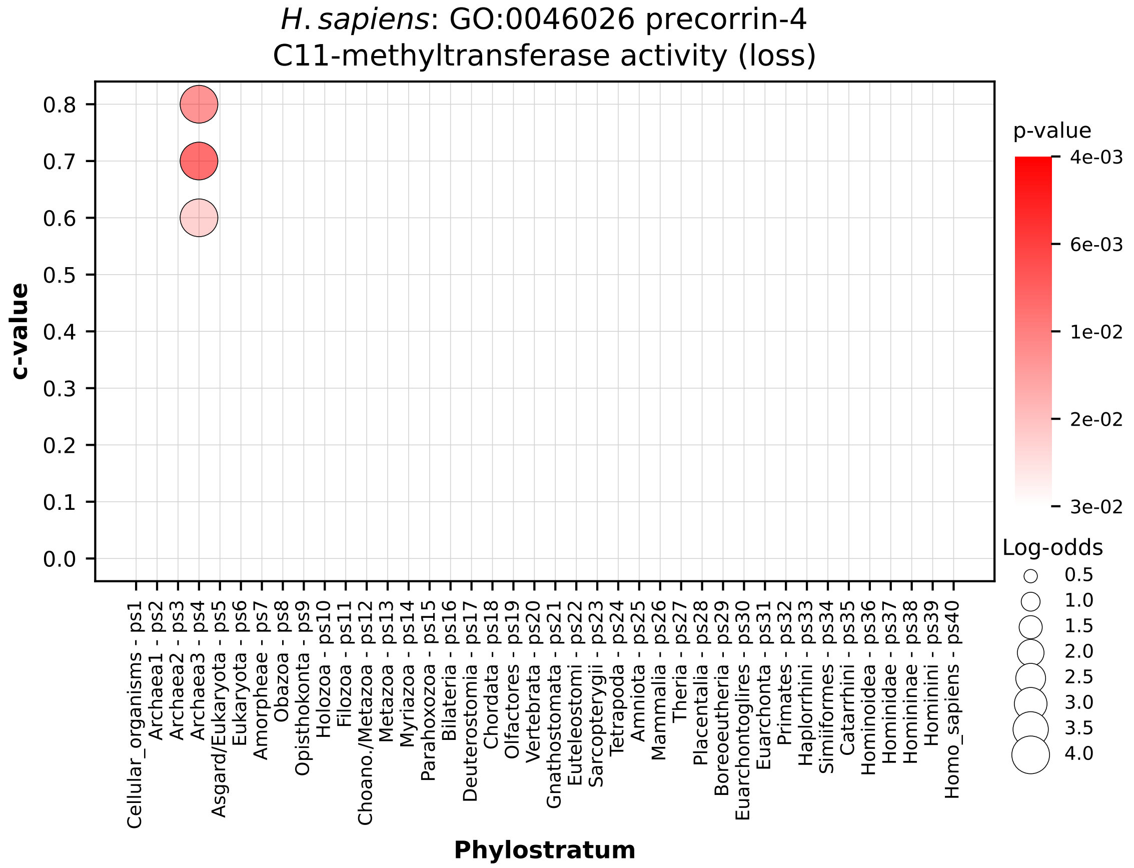

H. sapiens: GO:0046026 precorrin-4 C11-methyltransferase activity (loss)
c-value
0.8
0.7
0.6
0.5
0.4
0.3
0.2
0.1
0.0
p-value
4e-03
6e-03
1e-02
2e-02
3e-02
Log-odds
0.5
1.0
1.5
2.0
2.5
3.0
3.5
4.0
Cellular_organisms - ps1
Archaea1 - ps2
Archaea2 - ps3
Archaea3 - ps4
Asgard/Eukaryota - ps5
Eukaryota - ps6
Amorpheae - ps7
Obazoa - ps8
Opisthokonta - ps9
Holozoa - ps10
Filozoa - ps11
Choano./Metazoa - ps12
Metazoa - ps13
Myriazoa - ps14
Parahoxozoa - ps15
Bilateria - ps16
Deuterostomia - ps17
Chordata - ps18
Olfactores - ps19
Vertebrata - ps20
Gnathostomata - ps21
Euteleostomi - ps22
Sarcopterygii - ps23
Tetrapoda - ps24
Amniota - ps25
Mammalia - ps26
Theria - ps27
Placentalia - ps28
Boreoeutheria - ps29
Euarchontoglires - ps30
Euarchonta - ps31
Primates - ps32
Haplorrhini - ps33
Simiiformes - ps34
Catarrhini - ps35
Hominoidea - ps36
Hominidae - ps37
Homininae - ps38
Hominini - ps39
Homo_sapiens - ps40
Phylostratum

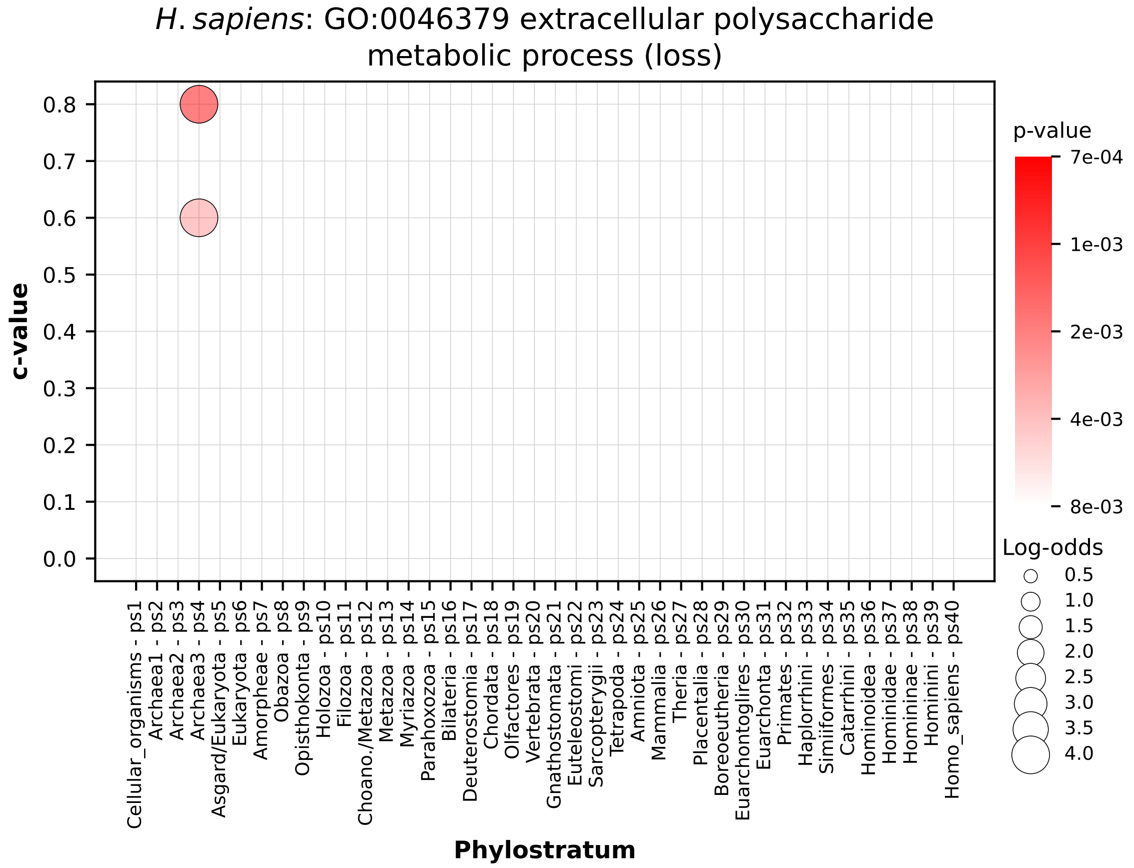

H. sapiens: GO:0046379 extracellular polysaccharide metabolic process (loss)
c-value
0.0
0.1
0.2
0.3
0.4
0.5
0.6
0.7
0.8
p-value
7e-04
1e-03
2e-03
4e-03
8e-03
Log-odds
0.5
1.0
1.5
2.0
2.5
3.0
3.5
4.0
Cellular_organisms - ps1
Archaea1 - ps2
Archaea2 - ps3
Archaea3 - ps4
Asgard/Eukaryota - ps5
Eukaryota - ps6
Amorpheae - ps7
Obazoa - ps8
Opisthokonta - ps9
Holozoa - ps10
Filozoa - ps11
Choano./Metazoa - ps12
Metazoa - ps13
Myriazoa - ps14
Parahoxozoa - ps15
Bilateria - ps16
Deuterostomia - ps17
Chordata - ps18
Olfactores - ps19
Vertebrata - ps20
Gnathostomata - ps21
Euteleostomi - ps22
Sarcopterygii - ps23
Tetrapoda - ps24
Amniota - ps25
Mammalia - ps26
Theria - ps27
Placentalia - ps28
Boreoeutheria - ps29
Euarchontoglires - ps30
Euarchonta - ps31
Primates - ps32
Haplorrhini - ps33
Simiiformes - ps34
Catarrhini - ps35
Hominoidea - ps36
Hominidae - ps37
Homininae - ps38
Hominini - ps39
Homo_sapiens - ps40
Phylostratum

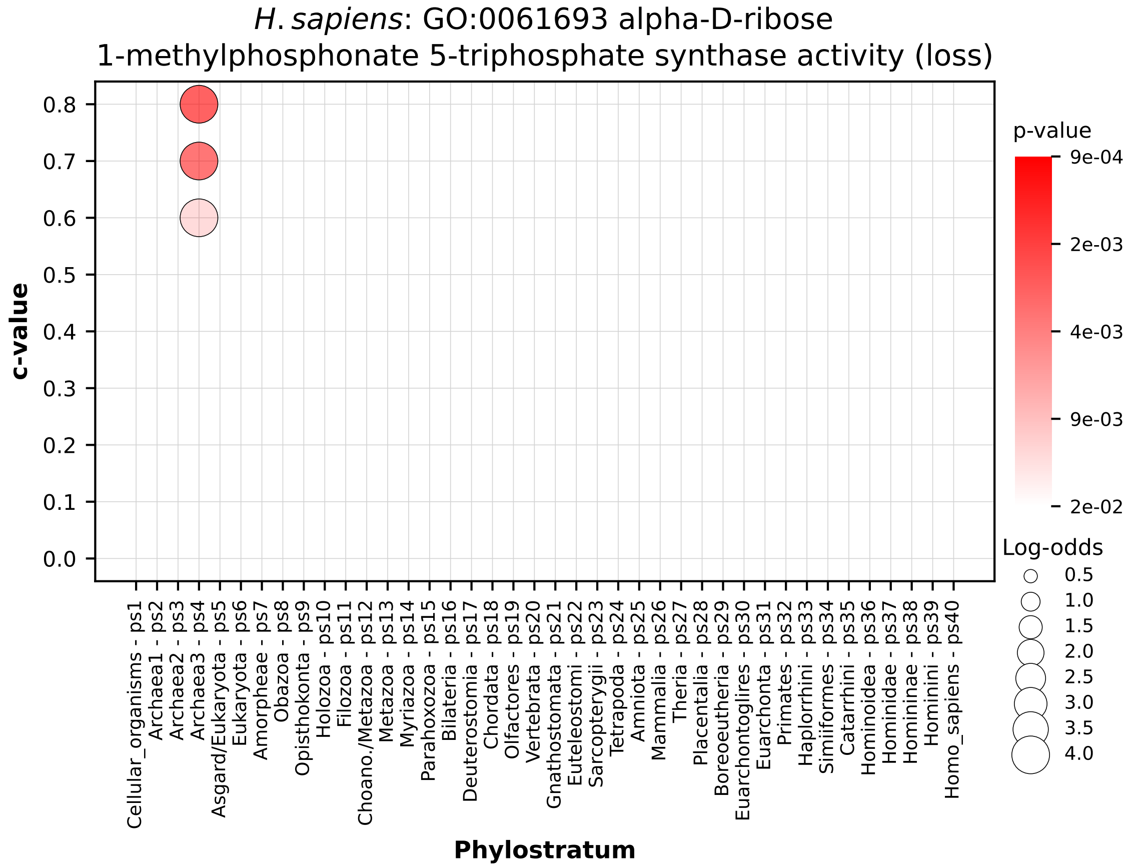

H. sapiens: GO:0061693 alpha-D-ribose
1-methylphosphonate 5-triphosphate synthase activity (loss)
c-value
0.0
0.1
0.2
0.3
0.4
0.5
0.6
0.7
0.8
Phylostratum
Cellular_organisms - ps1
Archaea1 - ps2
Archaea2 - ps3
Archaea3 - ps4
Asgard/Eukaryota - ps5
Eukaryota - ps6
Amorpheae - ps7
Obazoa - ps8
Opisthokonta - ps9
Holozoa - ps10
Filozoa - ps11
Choano./Metazoa - ps12
Metazoa - ps13
Myriazoa - ps14
Parahoxozoa - ps15
Bilateria - ps16
Deuterostomia - ps17
Chordata - ps18
Olfactores - ps19
Vertebrata - ps20
Gnathostomata - ps21
Euteleostomi - ps22
Sarcopterygii - ps23
Tetrapoda - ps24
Amniota - ps25
Mammalia - ps26
Theria - ps27
Placentalia - ps28
Boreoeutheria - ps29
Euarchontoglires - ps30
Euarchonta - ps31
Primates - ps32
Haplorrhini - ps33
Simiiformes - ps34
Catarrhini - ps35
Hominoidea - ps36
Hominidae - ps37
Homininae - ps38
Hominini - ps39
Homo_sapiens - ps40
p-value
9e-04
2e-03
4e-03
9e-03
2e-02
Log-odds
0.5
1.0
1.5
2.0
2.5
3.0
3.5
4.0

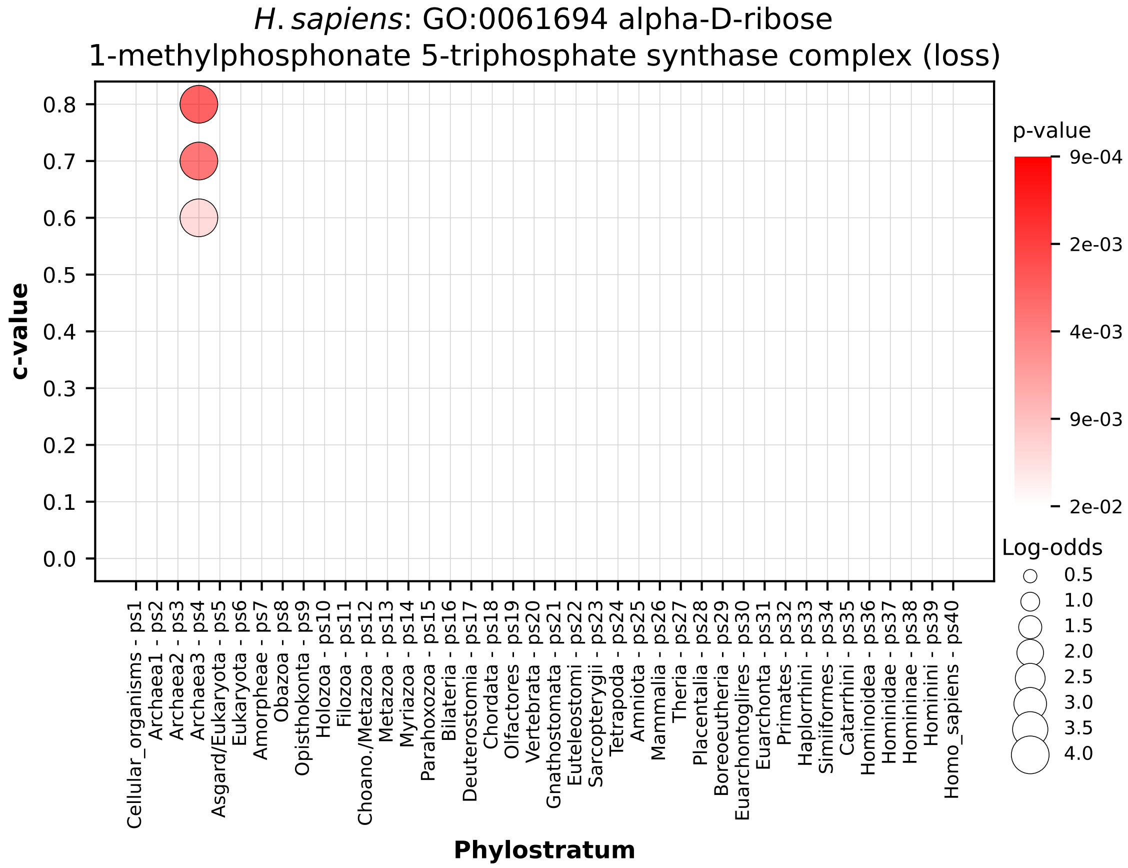

H. sapiens: GO:0061694 alpha-D-ribose
1-methylphosphonate 5-triphosphate synthase complex (loss)
c-value
0.0
0.1
0.2
0.3
0.4
0.5
0.6
0.7
0.8
Phylostratum
Cellular_organisms - ps1
Archaea1 - ps2
Archaea2 - ps3
Archaea3 - ps4
Asgard/Eukaryota - ps5
Eukaryota - ps6
Amorpheae - ps7
Obazoa - ps8
Opisthokonta - ps9
Holozoa - ps10
Filozoa - ps11
Choano./Metazoa - ps12
Metazoa - ps13
Myriazoa - ps14
Parahoxozoa - ps15
Bilateria - ps16
Deuterostomia - ps17
Chordata - ps18
Olfactores - ps19
Vertebrata - ps20
Gnathostomata - ps21
Euteleostomi - ps22
Sarcopterygii - ps23
Tetrapoda - ps24
Amniota - ps25
Mammalia - ps26
Theria - ps27
Placentalia - ps28
Boreoeutheria - ps29
Euarchontoglires - ps30
Euarchonta - ps31
Primates - ps32
Haplorrhini - ps33
Simiiformes - ps34
Catarrhini - ps35
Hominoidea - ps36
Hominidae - ps37
Homininae - ps38
Hominini - ps39
Homo_sapiens - ps40
p-value
9e-04
2e-03
4e-03
9e-03
2e-02
Log-odds
0.5
1.0
1.5
2.0
2.5
3.0
3.5
4.0

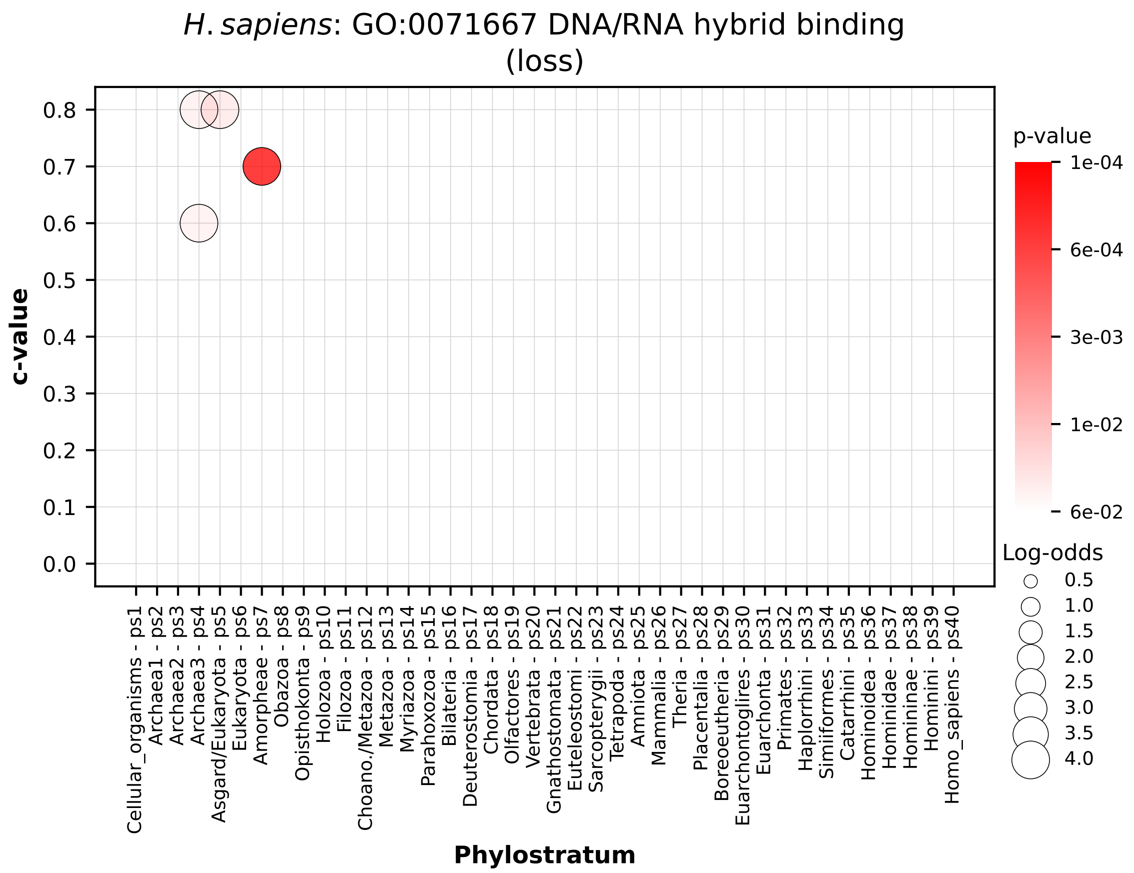

H. sapiens: GO:0071667 DNA/RNA hybrid binding
(loss)
c-value
0.0
0.1
0.2
0.3
0.4
0.5
0.6
0.7
0.8
Phylostratum
Cellular_organisms - ps1
Archaea1 - ps2
Archaea2 - ps3
Archaea3 - ps4
Asgard/Eukaryota - ps5
Eukaryota - ps6
Amorpheae - ps7
Obazoa - ps8
Opisthokonta - ps9
Holozoa - ps10
Filozoa - ps11
Choano./Metazoa - ps12
Metazoa - ps13
Myriazoa - ps14
Parahoxozoa - ps15
Bilateria - ps16
Deuterostomia - ps17
Chordata - ps18
Olfactores - ps19
Vertebrata - ps20
Gnathostomata - ps21
Euteleostomi - ps22
Sarcopterygii - ps23
Tetrapoda - ps24
Amniota - ps25
Mammalia - ps26
Theria - ps27
Placentalia - ps28
Boreoeutheria - ps29
Euarchontoglires - ps30
Euarchonta - ps31
Primates - ps32
Haplorrhini - ps33
Simiiformes - ps34
Catarrhini - ps35
Hominoidea - ps36
Hominidae - ps37
Homininae - ps38
Hominini - ps39
Homo_sapiens - ps40
p-value
1e-04
6e-04
3e-03
1e-02
6e-02
Log-odds
0.5
1.0
1.5
2.0
2.5
3.0
3.5
4.0

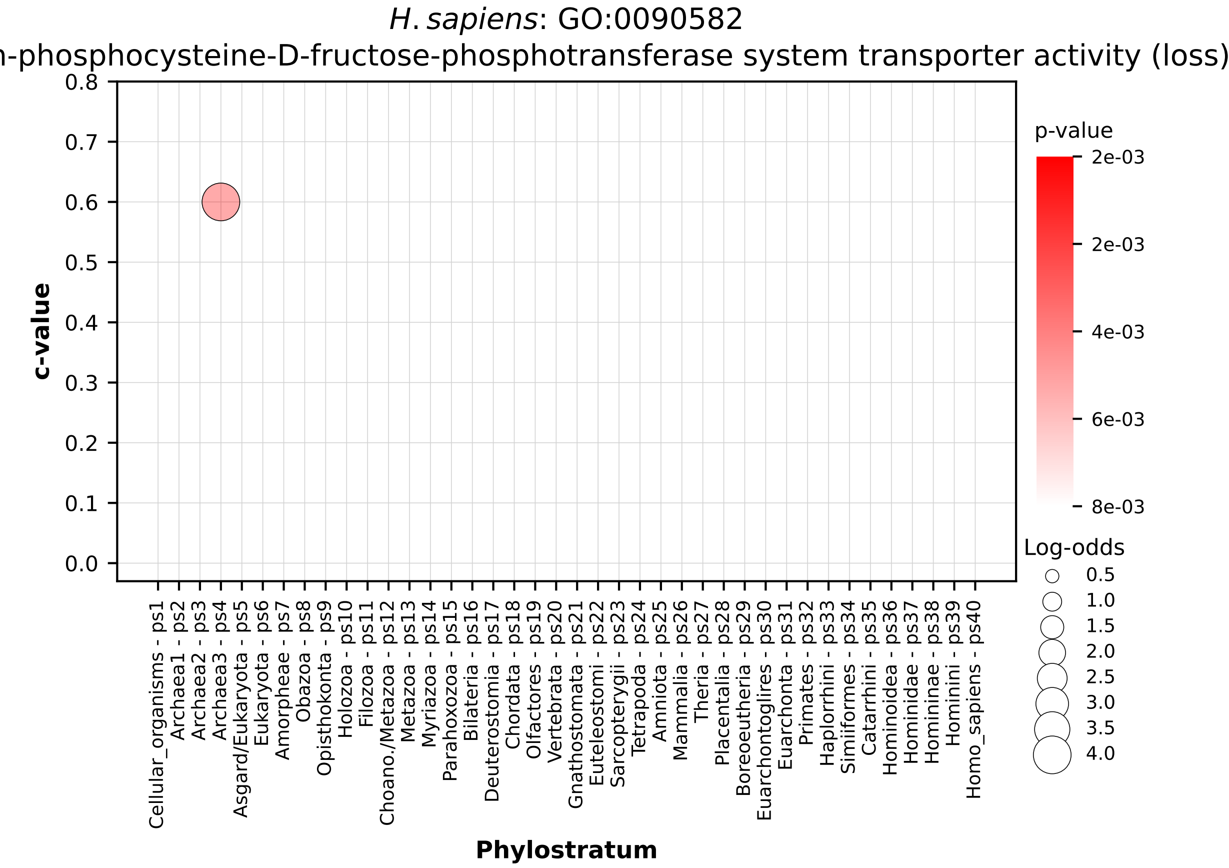

H. sapiens: GO:0090582
n-phosphocysteine-D-fructose-phosphotransferase system transporter activity (loss)
c-value
0.8
0.7
0.6
0.5
0.4
0.3
0.2
0.1
0.0
p-value
2e-03
2e-03
4e-03
6e-03
8e-03
Log-odds
0.5
1.0
1.5
2.0
2.5
3.0
3.5
4.0
Cellular_organisms - ps1
Archaea1 - ps2
Archaea2 - ps3
Archaea3 - ps4
Asgard/Eukaryota - ps5
Eukaryota - ps6
Amorpheae - ps7
Obazoa - ps8
Opisthokonta - ps9
Holozoa - ps10
Filozoa - ps11
Choano./Metazoa - ps12
Metazoa - ps13
Myriazoa - ps14
Parahoxozoa - ps15
Bilateria - ps16
Deuterostomia - ps17
Chordata - ps18
Olfactores - ps19
Vertebrata - ps20
Gnathostomata - ps21
Euteleostomi - ps22
Sarcopterygii - ps23
Tetrapoda - ps24
Amniota - ps25
Mammalia - ps26
Theria - ps27
Placentalia - ps28
Boreoeutheria - ps29
Euarchontoglires - ps30
Euarchonta - ps31
Primates - ps32
Haplorrhini - ps33
Simiiformes - ps34
Catarrhini - ps35
Hominoidea - ps36
Hominidae - ps37
Homininae - ps38
Hominini - ps39
Homo_sapiens - ps40
Phylostratum

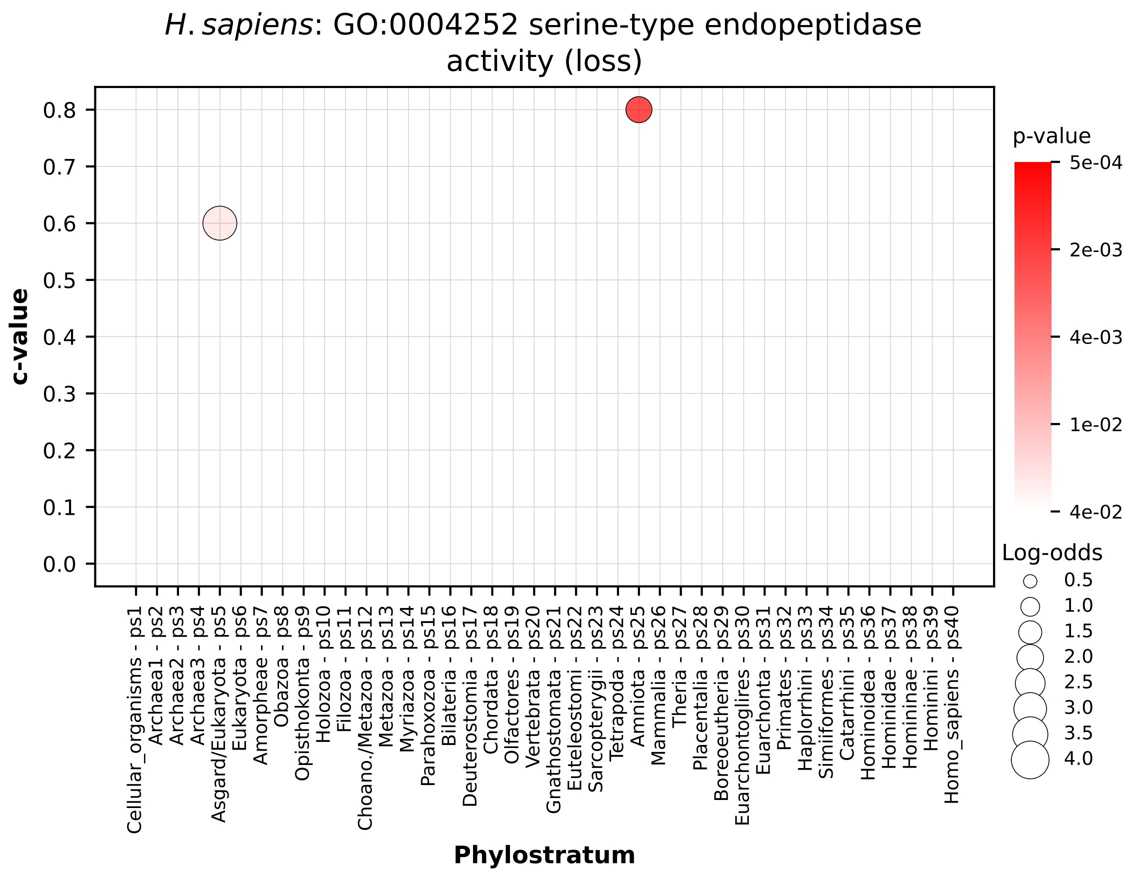

H. sapiens: GO:0004252 serine-type endopeptidase activity (loss)
c-value
0.0
0.1
0.2
0.3
0.4
0.5
0.6
0.7
0.8
Phylostratum
Cellular_organisms - ps1
Archaea1 - ps2
Archaea2 - ps3
Archaea3 - ps4
Asgard/Eukaryota - ps5
Eukaryota - ps6
Amorpheae - ps7
Obazoa - ps8
Opisthokonta - ps9
Holozoa - ps10
Filozoa - ps11
Choano./Metazoa - ps12
Metazoa - ps13
Myriazoa - ps14
Parahoxozoa - ps15
Bilateria - ps16
Deuterostomia - ps17
Chordata - ps18
Olfactores - ps19
Vertebrata - ps20
Gnathostomata - ps21
Euteleostomi - ps22
Sarcopterygii - ps23
Tetrapoda - ps24
Amniota - ps25
Mammalia - ps26
Theria - ps27
Placentalia - ps28
Boreoeutheria - ps29
Euarchontoglires - ps30
Euarchonta - ps31
Primates - ps32
Haplorrhini - ps33
Simiiformes - ps34
Catarrhini - ps35
Hominoidea - ps36
Hominidae - ps37
Homininae - ps38
Hominini - ps39
Homo_sapiens - ps40
p-value
5e-04
2e-03
4e-03
1e-02
4e-02
Log-odds
0.5
1.0
1.5
2.0
2.5
3.0
3.5
4.0

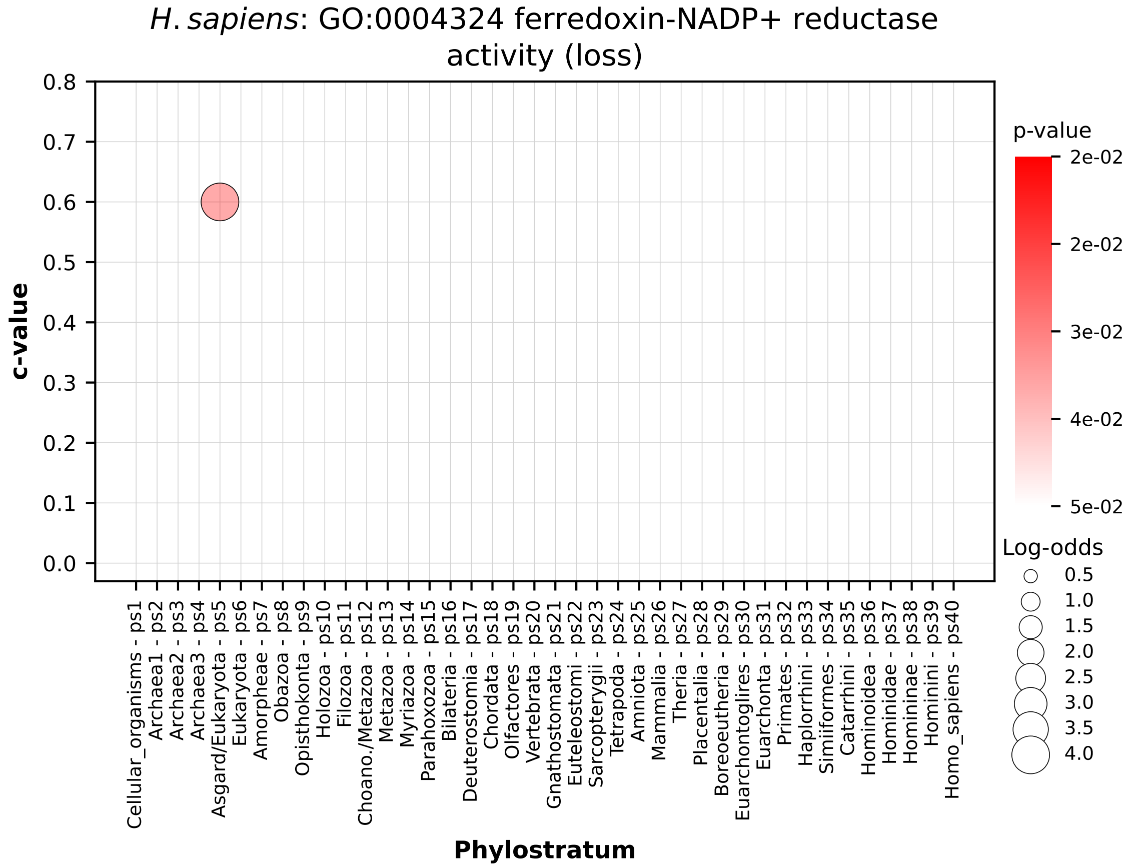

H. sapiens: GO:0004324 ferredoxin-NADP+ reductase activity (loss)
c-value
0.8
0.7
0.6
0.5
0.4
0.3
0.2
0.1
0.0
Phylostratum
Cellular_organisms - ps1
Archaea1 - ps2
Archaea2 - ps3
Archaea3 - ps4
Asgard/Eukaryota - ps5
Eukaryota - ps6
Amorpheae - ps7
Obazoa - ps8
Opisthokonta - ps9
Holozoa - ps10
Filozoa - ps11
Choano./Metazoa - ps12
Metazoa - ps13
Myriazoa - ps14
Parahoxozoa - ps15
Bilateria - ps16
Deuterostomia - ps17
Chordata - ps18
Olfactores - ps19
Vertebrata - ps20
Gnathostomata - ps21
Euteleostomi - ps22
Sarcopterygii - ps23
Tetrapoda - ps24
Amniota - ps25
Mammalia - ps26
Theria - ps27
Placentalia - ps28
Boreoeutheria - ps29
Euarchontoglires - ps30
Euarchonta - ps31
Primates - ps32
Haplorrhini - ps33
Simiiformes - ps34
Catarrhini - ps35
Hominoidea - ps36
Hominidae - ps37
Homininae - ps38
Hominini - ps39
Homo_sapiens - ps40
p-value
2e-02
2e-02
3e-02
4e-02
5e-02
Log-odds
0.5
1.0
1.5
2.0
2.5
3.0
3.5
4.0

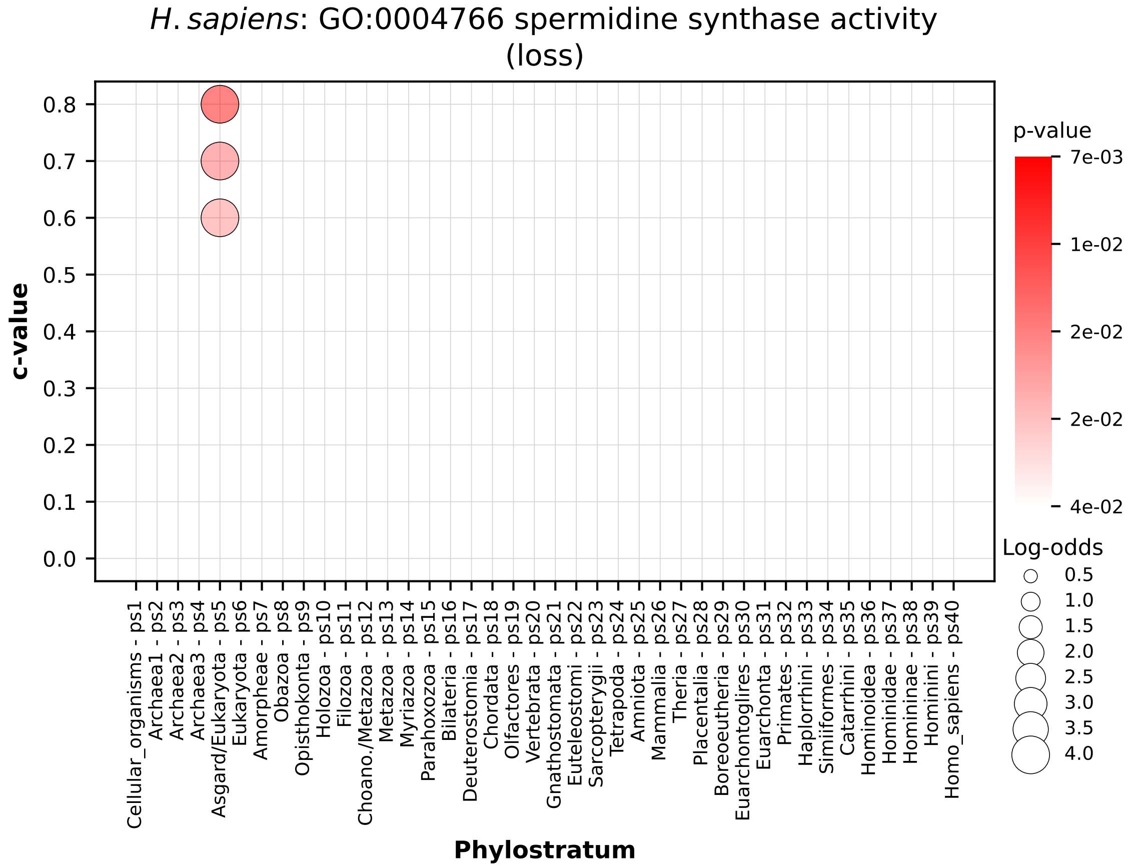

H. sapiens: GO:0004766 spermidine synthase activity
(loss)
c-value
0.8
0.7
0.6
0.5
0.4
0.3
0.2
0.1
0.0
p-value
7e-03
1e-02
2e-02
2e-02
4e-02
Log-odds
0.5
1.0
1.5
2.0
2.5
3.0
3.5
4.0
Cellular_organisms - ps1
Archaea1 - ps2
Archaea2 - ps3
Archaea3 - ps4
Asgard/Eukaryota - ps5
Eukaryota - ps6
Amorpheae - ps7
Obazoa - ps8
Opisthokonta - ps9
Holozoa - ps10
Filozoa - ps11
Choano./Metazoa - ps12
Metazoa - ps13
Myriazoa - ps14
Parahoxozoa - ps15
Bilateria - ps16
Deuterostomia - ps17
Chordata - ps18
Olfactores - ps19
Vertebrata - ps20
Gnathostomata - ps21
Euteleostomi - ps22
Sarcopterygii - ps23
Tetrapoda - ps24
Amniota - ps25
Mammalia - ps26
Theria - ps27
Placentalia - ps28
Boreoeutheria - ps29
Euarchontoglires - ps30
Euarchonta - ps31
Primates - ps32
Haplorrhini - ps33
Simiiformes - ps34
Catarrhini - ps35
Hominoidea - ps36
Hominidae - ps37
Homininae - ps38
Hominini - ps39
Homo_sapiens - ps40
Phylostratum

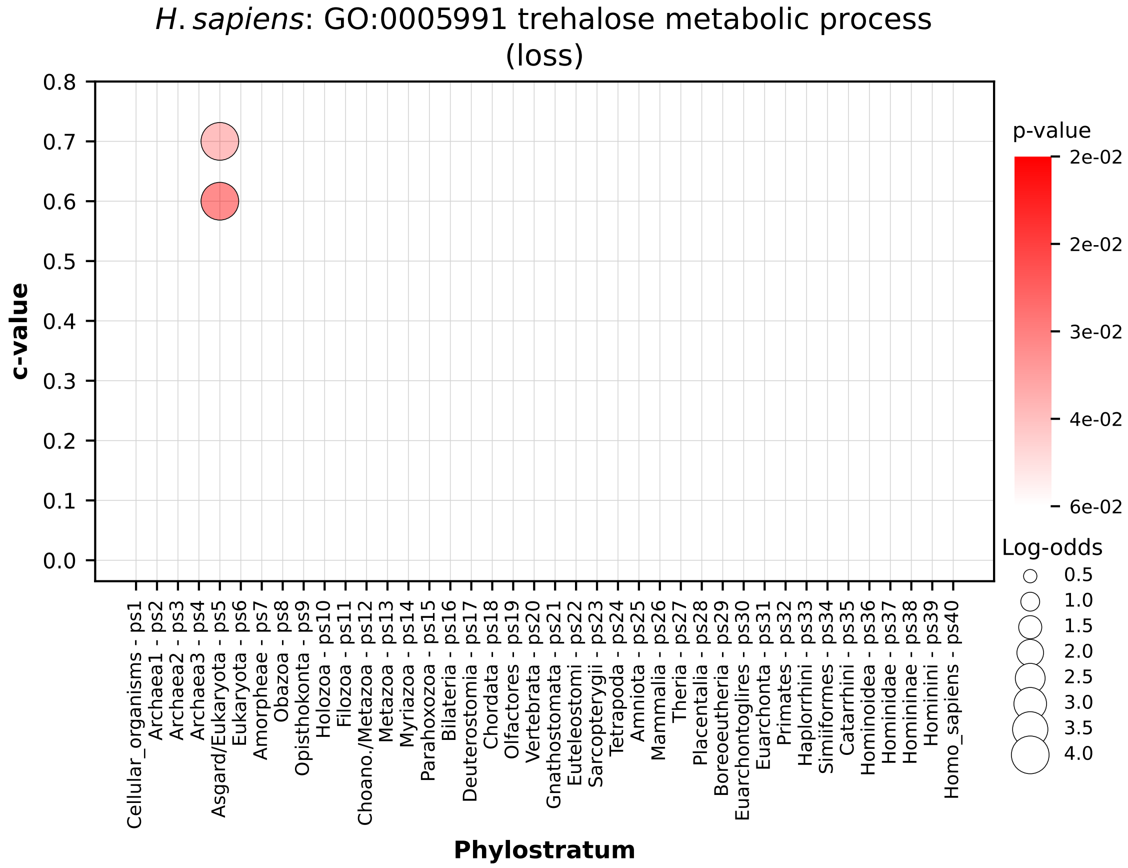

H. sapiens: GO:0005991 trehalose metabolic process (loss)
c-value
0.8
0.7
0.6
0.5
0.4
0.3
0.2
0.1
0.0
Phylostratum
Cellular_organisms - ps1
Archaea1 - ps2
Archaea2 - ps3
Archaea3 - ps4
Asgard/Eukaryota - ps5
Eukaryota - ps6
Amorpheae - ps7
Obazoa - ps8
Opisthokonta - ps9
Holozoa - ps10
Filozoa - ps11
Choano./Metazoa - ps12
Metazoa - ps13
Myriazoa - ps14
Parahoxozoa - ps15
Bilateria - ps16
Deuterostomia - ps17
Chordata - ps18
Olfactores - ps19
Vertebrata - ps20
Gnathostomata - ps21
Euteleostomi - ps22
Sarcopterygii - ps23
Tetrapoda - ps24
Amniota - ps25
Mammalia - ps26
Theria - ps27
Placentalia - ps28
Boreoeutheria - ps29
Euarchontoglires - ps30
Euarchonta - ps31
Primates - ps32
Haplorrhini - ps33
Simiiformes - ps34
Catarrhini - ps35
Hominoidea - ps36
Hominidae - ps37
Homininae - ps38
Hominini - ps39
Homo_sapiens - ps40
p-value
2e-02
2e-02
3e-02
4e-02
6e-02
Log-odds
0.5
1.0
1.5
2.0
2.5
3.0
3.5
4.0

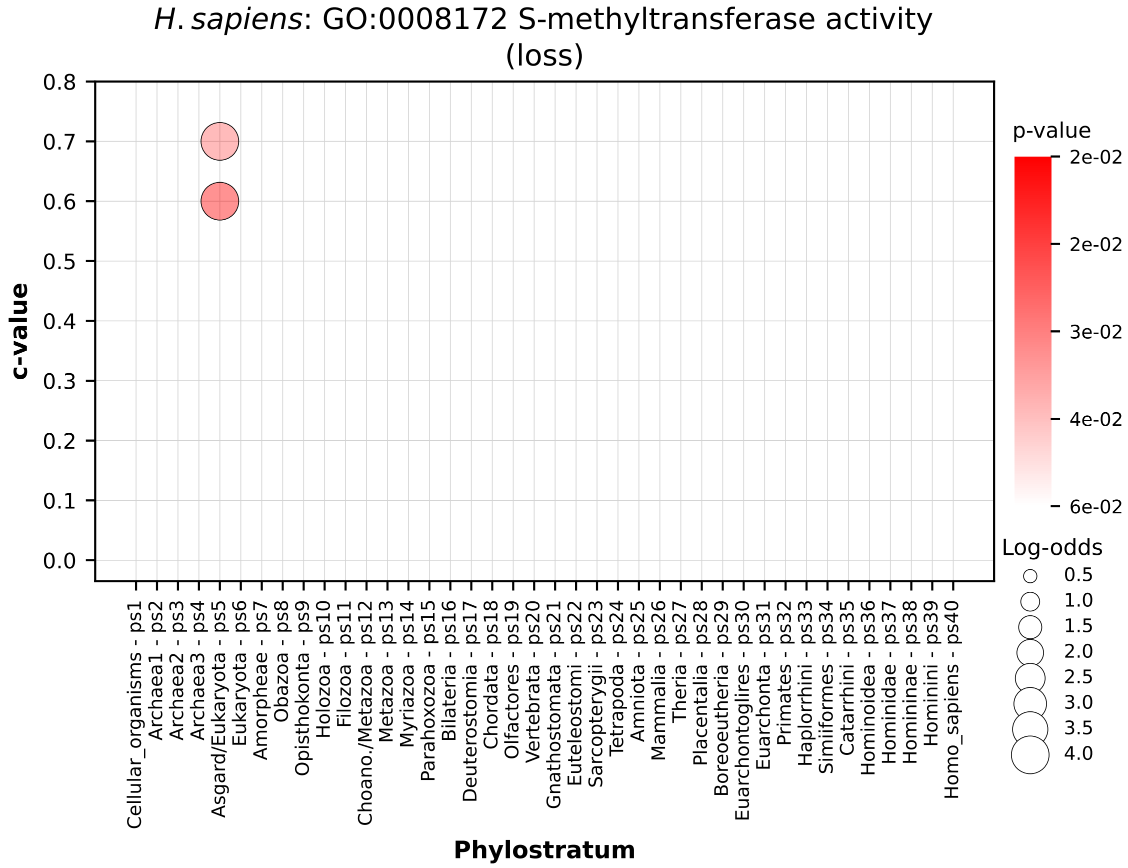

H. sapiens: GO:0008172 S-methyltransferase activity
(loss)
c-value
0.8
0.7
0.6
0.5
0.4
0.3
0.2
0.1
0.0
p-value
2e-02
2e-02
3e-02
4e-02
6e-02
Log-odds
0.5
1.0
1.5
2.0
2.5
3.0
3.5
4.0
Cellular_organisms - ps1
Archaea1 - ps2
Archaea2 - ps3
Archaea3 - ps4
Asgard/Eukaryota - ps5
Eukaryota - ps6
Amorpheae - ps7
Obazoa - ps8
Opisthokonta - ps9
Holozoa - ps10
Filozoa - ps11
Choano./Metazoa - ps12
Metazoa - ps13
Myriazoa - ps14
Parahoxozoa - ps15
Bilateria - ps16
Deuterostomia - ps17
Chordata - ps18
Olfactores - ps19
Vertebrata - ps20
Gnathostomata - ps21
Euteleostomi - ps22
Sarcopterygii - ps23
Tetrapoda - ps24
Amniota - ps25
Mammalia - ps26
Theria - ps27
Placentalia - ps28
Boreoeutheria - ps29
Euarchontoglires - ps30
Euarchonta - ps31
Primates - ps32
Haplorrhini - ps33
Simiiformes - ps34
Catarrhini - ps35
Hominoidea - ps36
Hominidae - ps37
Homininae - ps38
Hominini - ps39
Homo_sapiens - ps40
Phylostratum

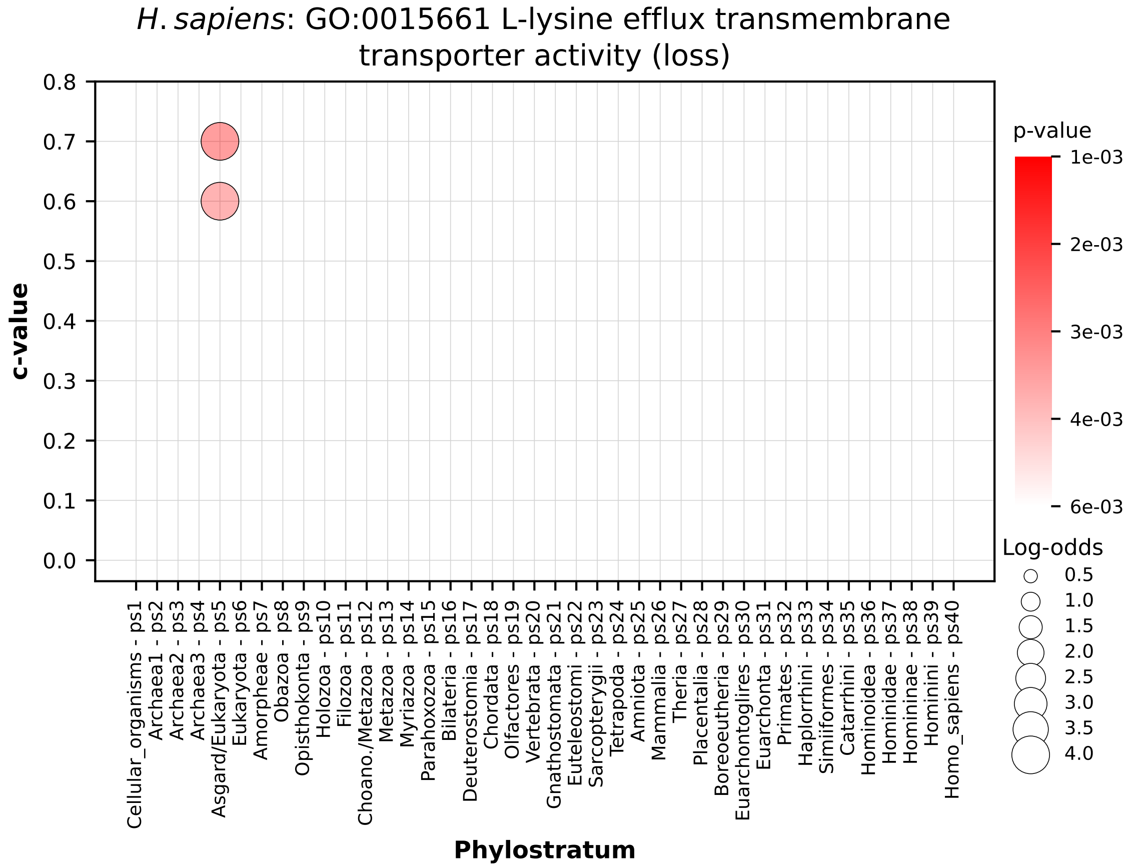

H. sapiens: GO:0015661 L-lysine efflux transmembrane transporter activity (loss)
c-value
0.8
0.7
0.6
0.5
0.4
0.3
0.2
0.1
0.0
Phylostratum
Cellular_organisms - ps1
Archaea1 - ps2
Archaea2 - ps3
Archaea3 - ps4
Asgard/Eukaryota - ps5
Eukaryota - ps6
Amorpheae - ps7
Obazoa - ps8
Opisthokonta - ps9
Holozoa - ps10
Filozoa - ps11
Choano./Metazoa - ps12
Metazoa - ps13
Myriazoa - ps14
Parahoxozoa - ps15
Bilateria - ps16
Deuterostomia - ps17
Chordata - ps18
Olfactores - ps19
Vertebrata - ps20
Gnathostomata - ps21
Euteleostomi - ps22
Sarcopterygii - ps23
Tetrapoda - ps24
Amniota - ps25
Mammalia - ps26
Theria - ps27
Placentalia - ps28
Boreoeutheria - ps29
Euarchontoglires - ps30
Euarchonta - ps31
Primates - ps32
Haplorrhini - ps33
Simiiformes - ps34
Catarrhini - ps35
Hominoidea - ps36
Hominidae - ps37
Homininae - ps38
Hominini - ps39
Homo_sapiens - ps40
p-value
1e-03
2e-03
3e-03
4e-03
6e-03
Log-odds
0.5
1.0
1.5
2.0
2.5
3.0
3.5
4.0

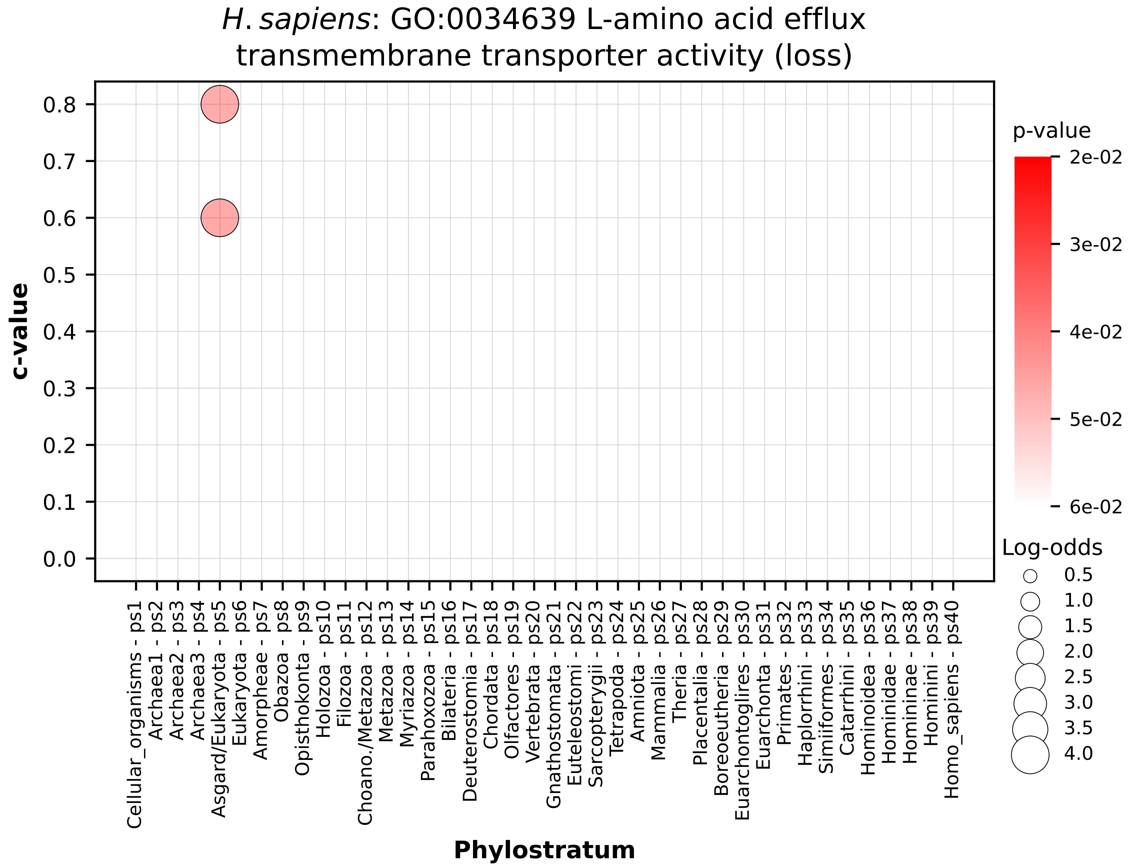

H. sapiens: GO:0034639 L-amino acid efflux transmembrane transporter activity (loss)
c-value
0.8
0.7
0.6
0.5
0.4
0.3
0.2
0.1
0.0
p-value
2e-02
3e-02
4e-02
5e-02
6e-02
Log-odds
0.5
1.0
1.5
2.0
2.5
3.0
3.5
4.0
Cellular_organisms - ps1
Archaea1 - ps2
Archaea2 - ps3
Archaea3 - ps4
Asgard/Eukaryota - ps5
Eukaryota - ps6
Amorpheae - ps7
Obazoa - ps8
Opisthokonta - ps9
Holozoa - ps10
Filozoa - ps11
Choano./Metazoa - ps12
Metazoa - ps13
Myriazoa - ps14
Parahoxozoa - ps15
Bilateria - ps16
Deuterostomia - ps17
Chordata - ps18
Olfactores - ps19
Vertebrata - ps20
Gnathostomata - ps21
Euteleostomi - ps22
Sarcopterygii - ps23
Tetrapoda - ps24
Amniota - ps25
Mammalia - ps26
Theria - ps27
Placentalia - ps28
Boreoeutheria - ps29
Euarchontoglires - ps30
Euarchonta - ps31
Primates - ps32
Haplorrhini - ps33
Simiiformes - ps34
Catarrhini - ps35
Hominoidea - ps36
Hominidae - ps37
Homininae - ps38
Hominini - ps39
Homo_sapiens - ps40
Phylostratum

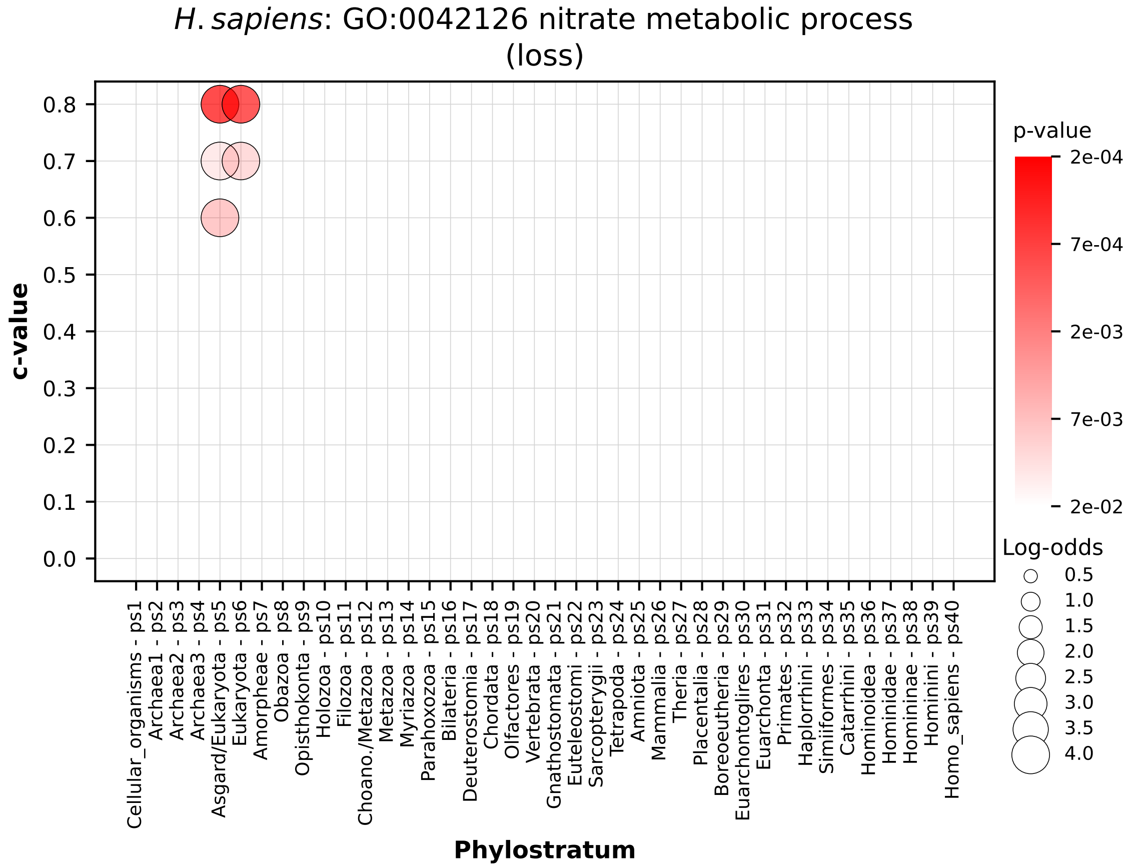

H. sapiens: GO:0042126 nitrate metabolic process
(loss)
c-value
0.0
0.1
0.2
0.3
0.4
0.5
0.6
0.7
0.8
p-value
2e-04
7e-04
2e-03
7e-03
2e-02
Log-odds
0.5
1.0
1.5
2.0
2.5
3.0
3.5
4.0
Cellular_organisms - ps1
Archaea1 - ps2
Archaea2 - ps3
Archaea3 - ps4
Asgard/Eukaryota - ps5
Eukaryota - ps6
Amorpheae - ps7
Obazoa - ps8
Opisthokonta - ps9
Holozoa - ps10
Filozoa - ps11
Choano./Metazoa - ps12
Metazoa - ps13
Myriazoa - ps14
Parahoxozoa - ps15
Bilateria - ps16
Deuterostomia - ps17
Chordata - ps18
Olfactores - ps19
Vertebrata - ps20
Gnathostomata - ps21
Euteleostomi - ps22
Sarcopterygii - ps23
Tetrapoda - ps24
Amniota - ps25
Mammalia - ps26
Theria - ps27
Placentalia - ps28
Boreoeutheria - ps29
Euarchontoglires - ps30
Euarchonta - ps31
Primates - ps32
Haplorrhini - ps33
Simiiformes - ps34
Catarrhini - ps35
Hominoidea - ps36
Hominidae - ps37
Homininae - ps38
Hominini - ps39
Homo_sapiens - ps40
Phylostratum

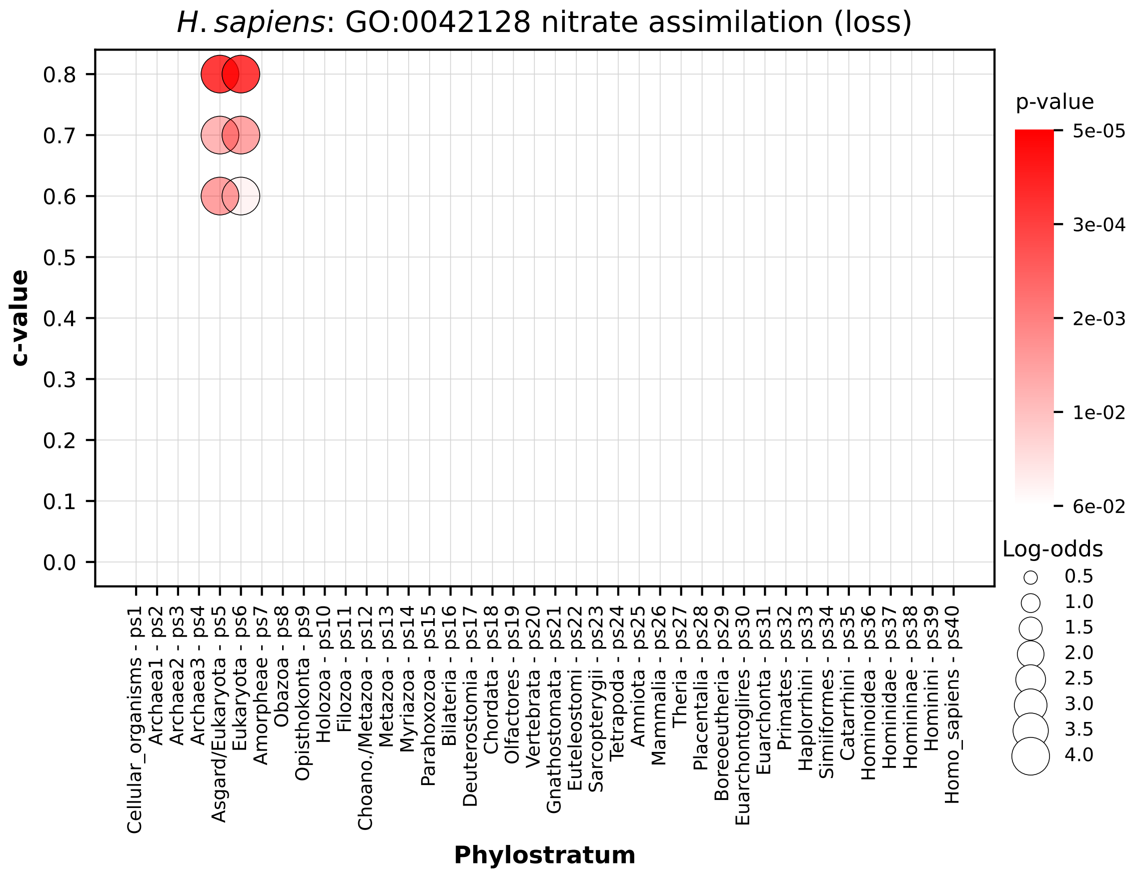

H. sapiens: GO:0042128 nitrate assimilation (loss)
c-value
0.0
0.1
0.2
0.3
0.4
0.5
0.6
0.7
0.8
p-value
5e-05
3e-04
2e-03
1e-02
6e-02
Log-odds
0.5
1.0
1.5
2.0
2.5
3.0
3.5
4.0
Cellular_organisms - ps1
Archaea1 - ps2
Archaea2 - ps3
Archaea3 - ps4
Asgard/Eukaryota - ps5
Eukaryota - ps6
Amorpheae - ps7
Obazoa - ps8
Opisthokonta - ps9
Holozoa - ps10
Filozoa - ps11
Choano./Metazoa - ps12
Metazoa - ps13
Myriazoa - ps14
Parahoxozoa - ps15
Bilateria - ps16
Deuterostomia - ps17
Chordata - ps18
Olfactores - ps19
Vertebrata - ps20
Gnathostomata - ps21
Euteleostomi - ps22
Sarcopterygii - ps23
Tetrapoda - ps24
Amniota - ps25
Mammalia - ps26
Theria - ps27
Placentalia - ps28
Boreoeutheria - ps29
Euarchontoglires - ps30
Euarchonta - ps31
Primates - ps32
Haplorrhini - ps33
Simiiformes - ps34
Catarrhini - ps35
Hominoidea - ps36
Hominidae - ps37
Homininae - ps38
Hominini - ps39
Homo_sapiens - ps40
Phylostratum

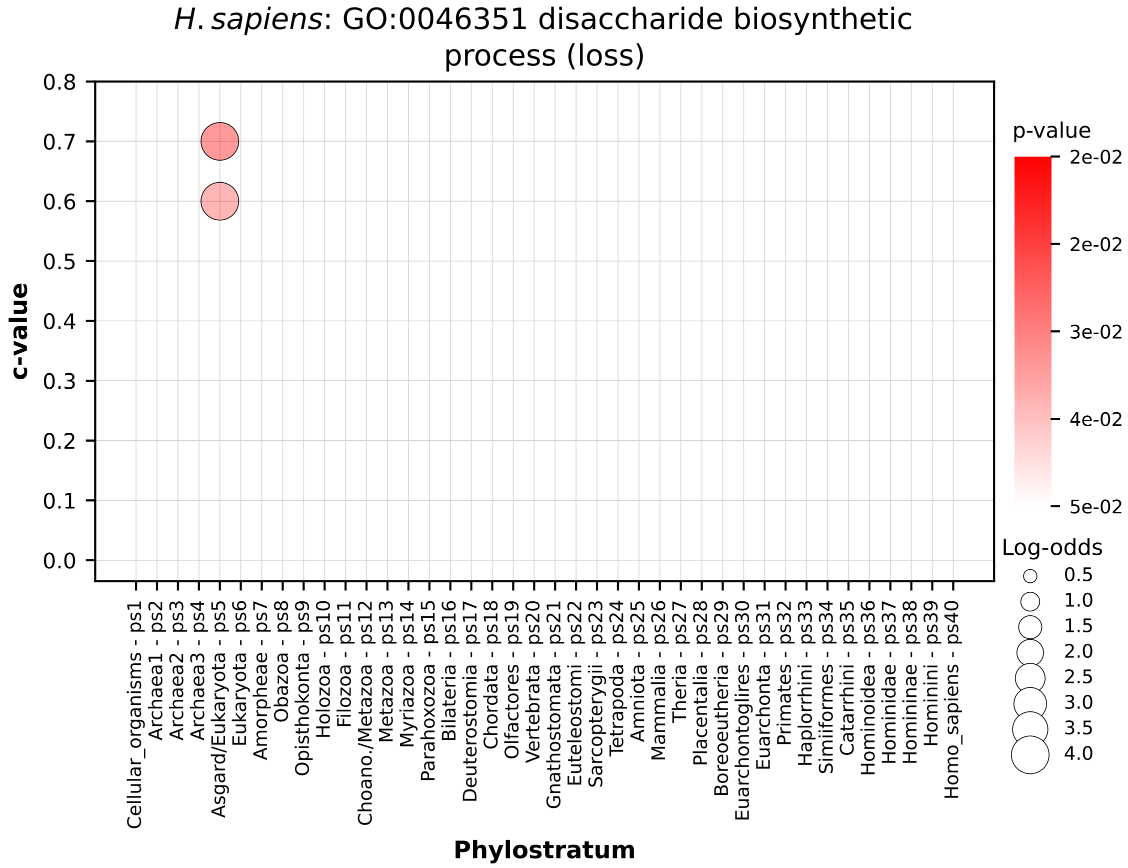

H. sapiens: GO:0046351 disaccharide biosynthetic process (loss)
c-value
0.8
0.7
0.6
0.5
0.4
0.3
0.2
0.1
0.0
Phylostratum
Cellular_organisms - ps1
Archaea1 - ps2
Archaea2 - ps3
Archaea3 - ps4
Asgard/Eukaryota - ps5
Eukaryota - ps6
Amorpheae - ps7
Obazoa - ps8
Opisthokonta - ps9
Holozoa - ps10
Filozoa - ps11
Choano./Metazoa - ps12
Metazoa - ps13
Myriazoa - ps14
Parahoxozoa - ps15
Bilateria - ps16
Deuterostomia - ps17
Chordata - ps18
Olfactores - ps19
Vertebrata - ps20
Gnathostomata - ps21
Euteleostomi - ps22
Sarcopterygii - ps23
Tetrapoda - ps24
Amniota - ps25
Mammalia - ps26
Theria - ps27
Placentalia - ps28
Boreoeutheria - ps29
Euarchontoglires - ps30
Euarchonta - ps31
Primates - ps32
Haplorrhini - ps33
Simiiformes - ps34
Catarrhini - ps35
Hominoidea - ps36
Hominidae - ps37
Homininae - ps38
Hominini - ps39
Homo_sapiens - ps40
p-value
2e-02
2e-02
3e-02
4e-02
5e-02
Log-odds
0.5
1.0
1.5
2.0
2.5
3.0
3.5
4.0

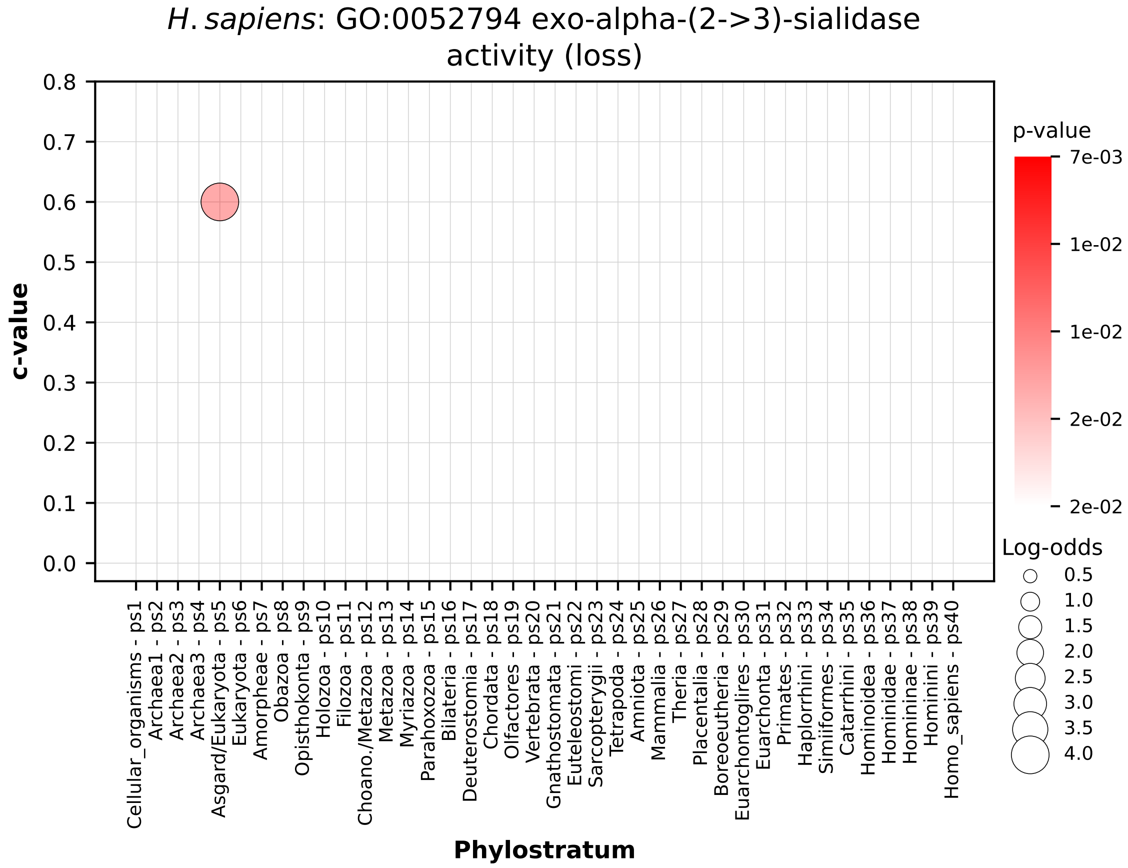

H. sapiens: GO:0052794 exo-alpha-(2->3)-sialidase activity (loss)
c-value
0.8
0.7
0.6
0.5
0.4
0.3
0.2
0.1
0.0
p-value
7e-03
1e-02
1e-02
2e-02
2e-02
[truncated: 517,695 more chars]
